# Supplementary material for: A new perspective on selenium’s impact on renal function: European population-based analysis of plasma proteome-mediated Mendelian randomization study
Source: Front Endocrinol (Lausanne). 2024 Sep 12;15:1410463. doi: 10.3389/fendo.2024.1410463 (PMC11424436; doi:10.3389/fendo.2024.1410463)
Supplement: Supplementary Figure 3 — Leave-one-out plots of all the mendelian randomization analyses in this study. [file DataSheet3.pdf]

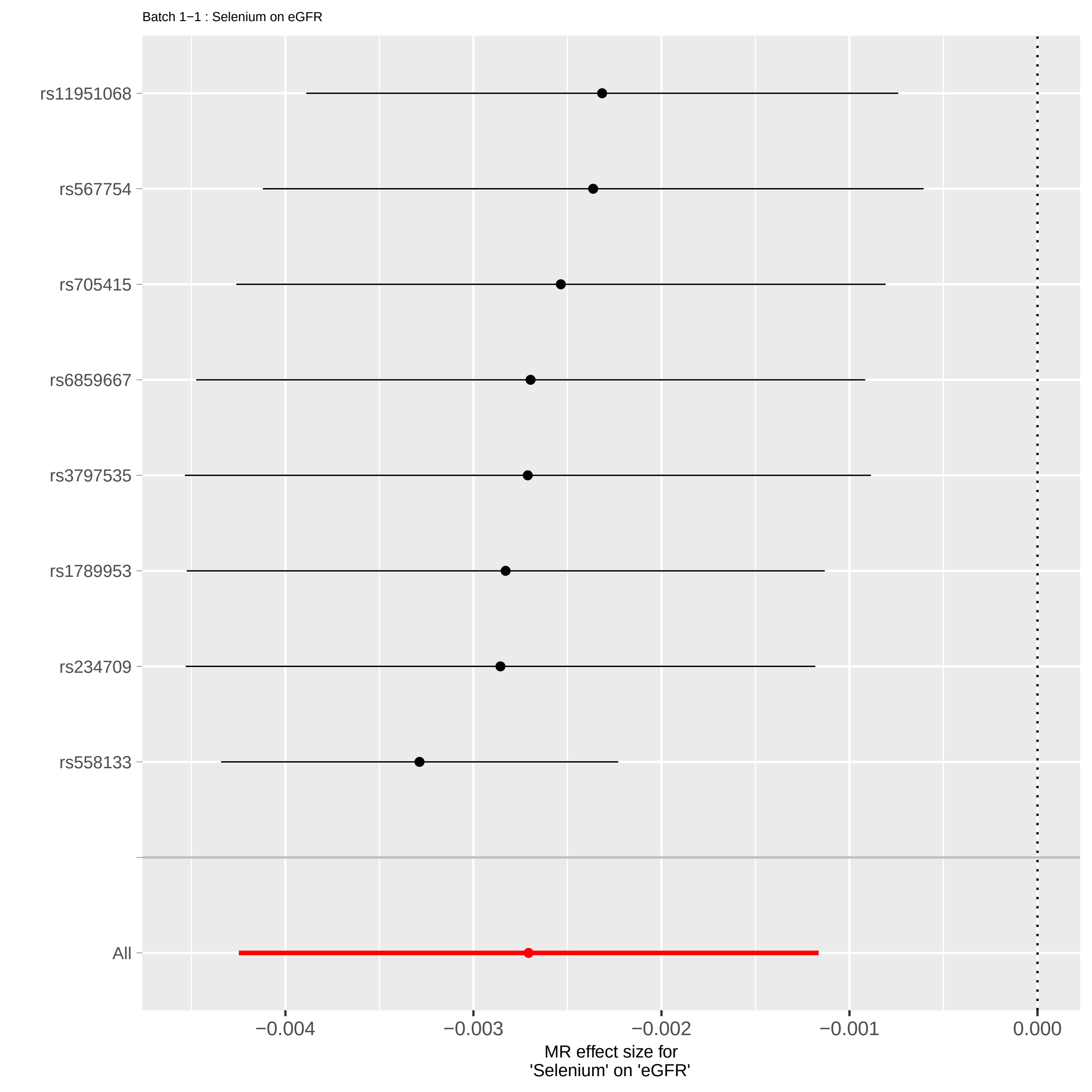

Batch 1−1 : Selenium on eGFR
rs11951068
rs567754
rs705415
rs6859667
rs3797535
rs1789953
rs234709
rs558133
All
−0.004
−0.003
−0.002
−0.001
0.000
MR effect size for
'Selenium' on 'eGFR'

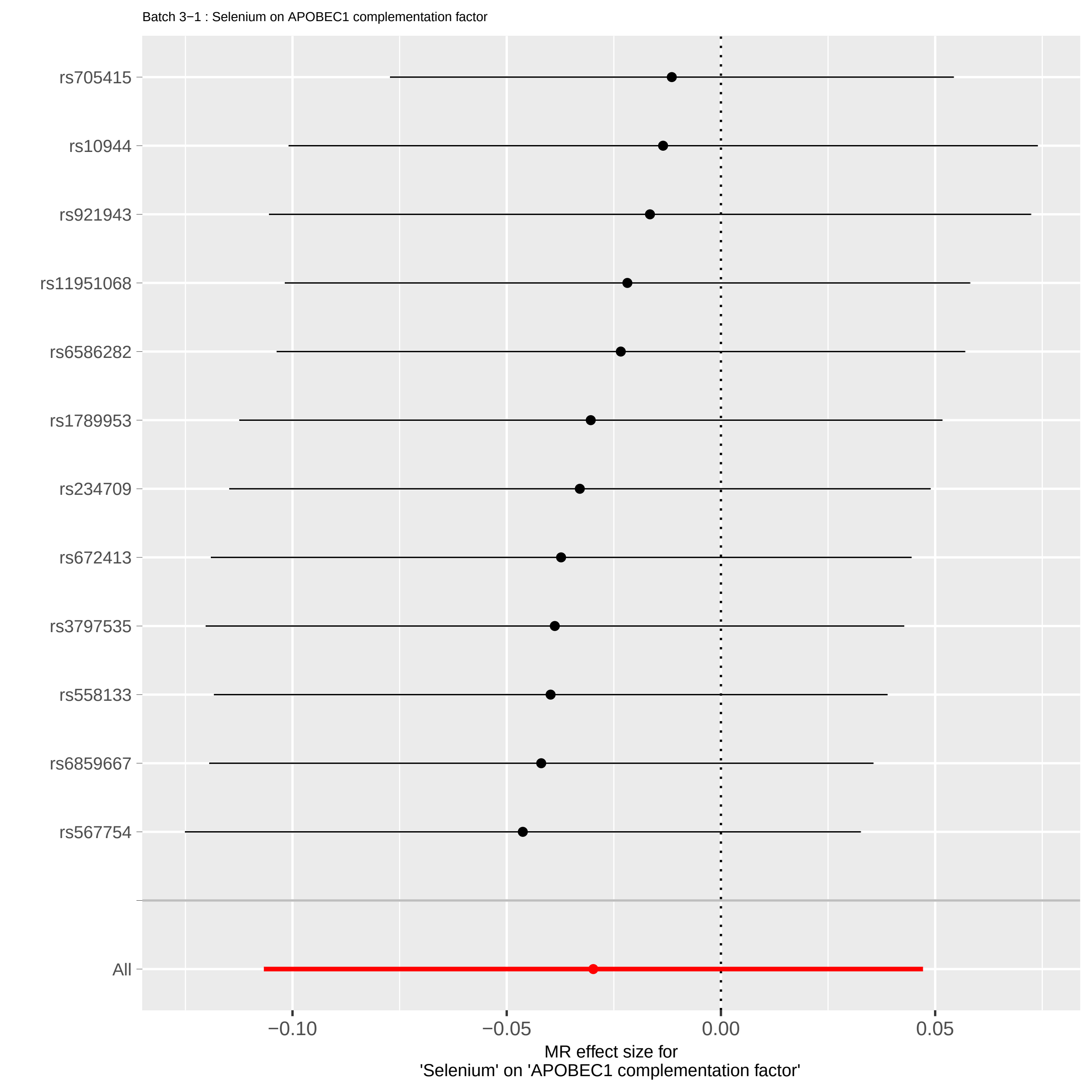

Batch 3−1 : Selenium on APOBEC1 complementation factor
rs705415
rs10944
rs921943
rs11951068
rs6586282
rs1789953
rs234709
rs672413
rs3797535
rs558133
rs6859667
rs567754
All
−0.10
−0.05
0.00
0.05
MR effect size for
'Selenium' on 'APOBEC1 complementation factor'

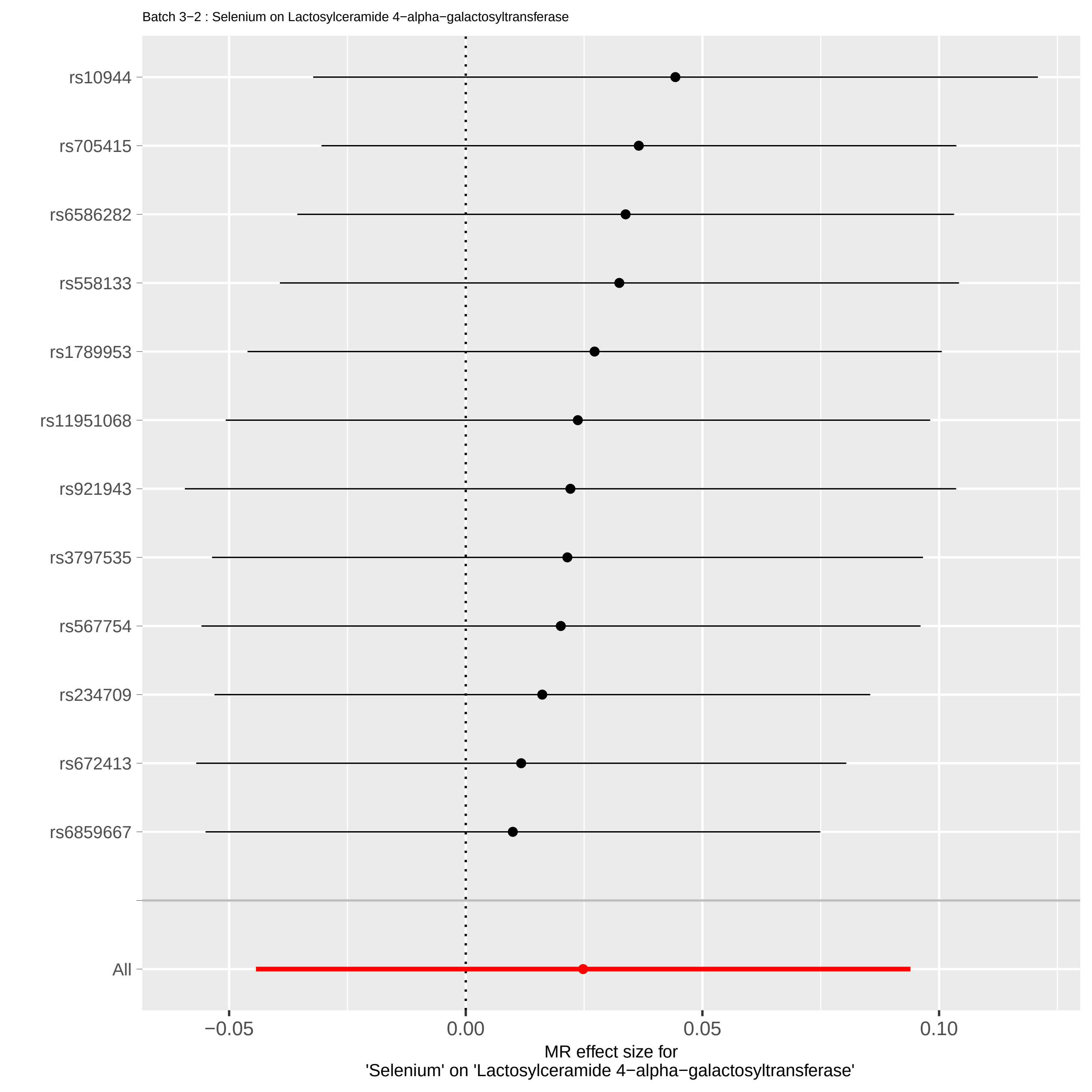

Batch 3−2 : Selenium on Lactosylceramide 4−alpha−galactosyltransferase
rs10944
rs705415
rs6586282
rs558133
rs1789953
rs11951068
rs921943
rs3797535
rs567754
rs234709
rs672413
rs6859667
All
−0.05
0.00
0.05
0.10
MR effect size for
'Selenium' on 'Lactosylceramide 4−alpha−galactosyltransferase'

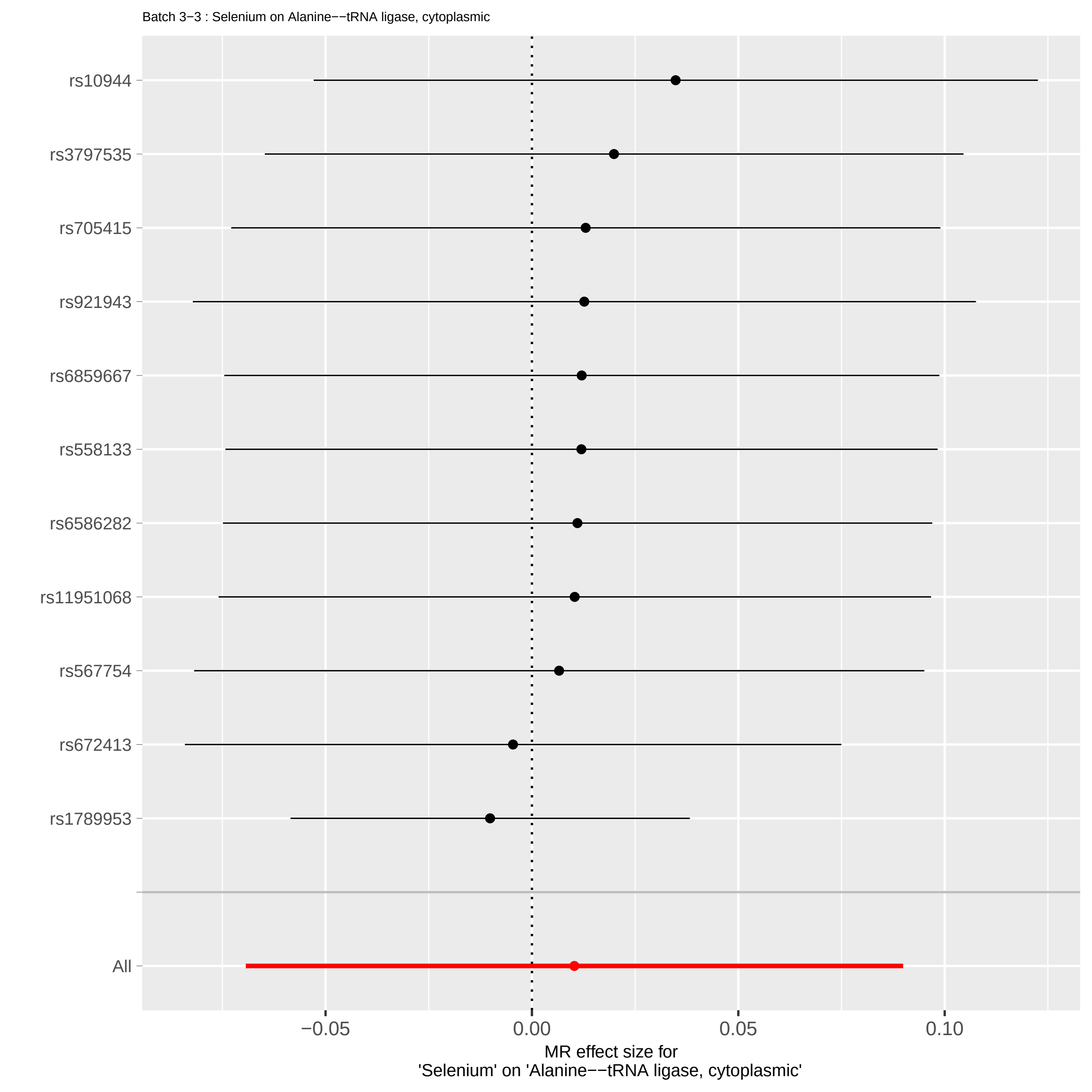
Batch 3−3 : Selenium on Alanine−−tRNA ligase, cytoplasmic
rs10944
rs3797535
rs705415
rs921943
rs6859667
rs558133
rs6586282
rs11951068
rs567754
rs672413
rs1789953
All
−0.05
0.00
0.05
0.10
MR effect size for
'Selenium' on 'Alanine−−tRNA ligase, cytoplasmic'

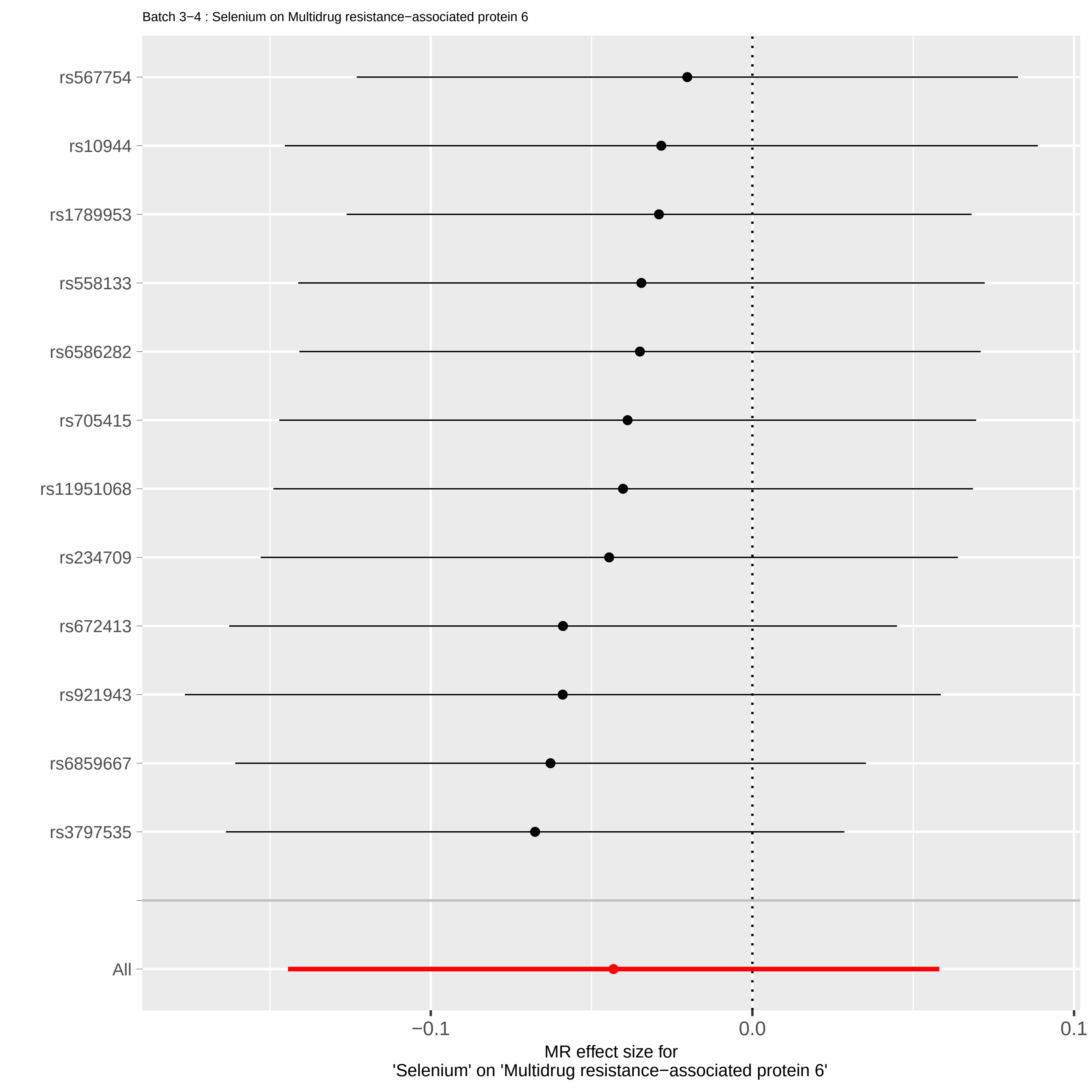

Batch 3−4 : Selenium on Multidrug resistance−associated protein 6
rs567754
rs10944
rs1789953
rs558133
rs6586282
rs705415
rs11951068
rs234709
rs672413
rs921943
rs6859667
rs3797535
All
−0.1
0.0
0.1
MR effect size for
'Selenium' on 'Multidrug resistance−associated protein 6'

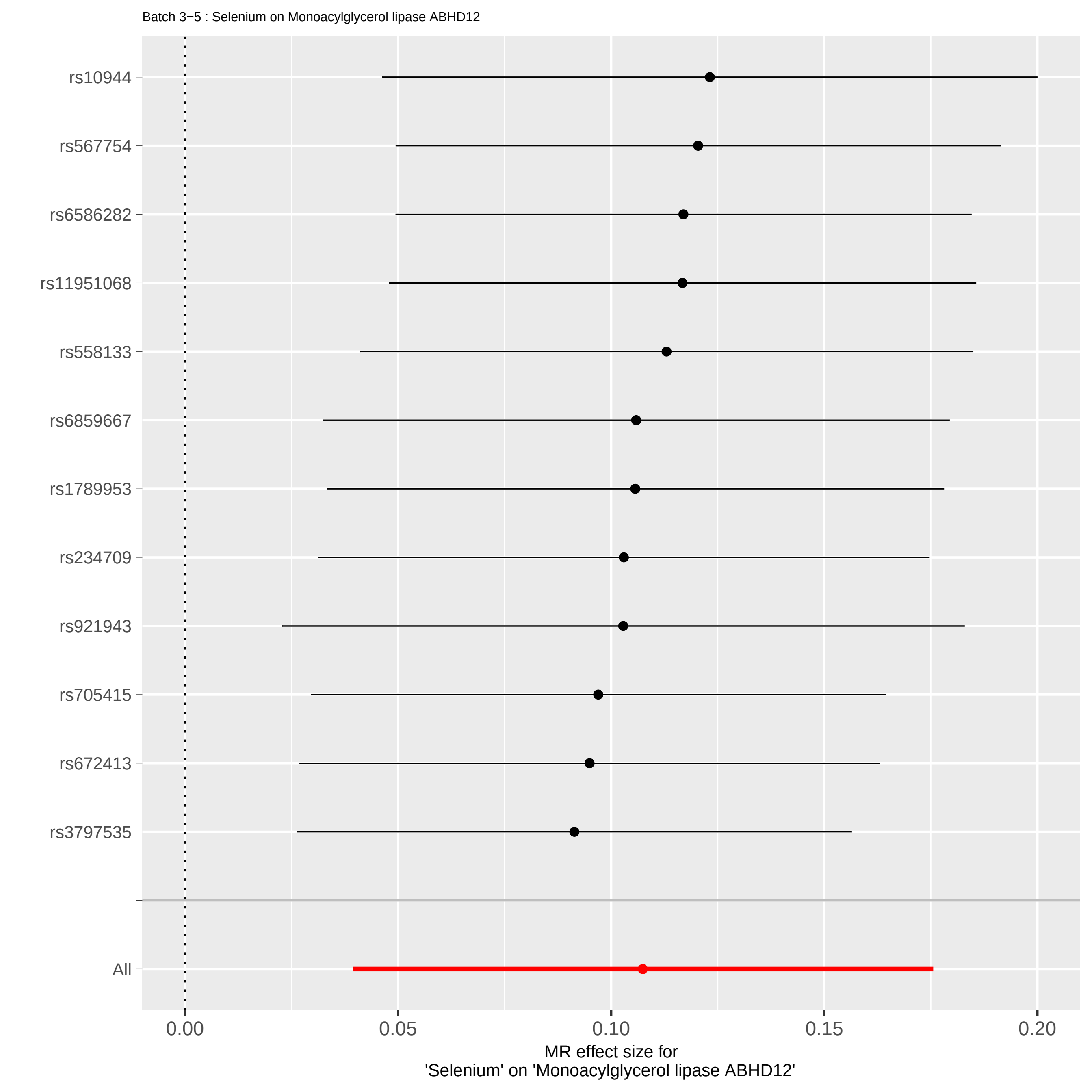

Batch 3−5 : Selenium on Monoacylglycerol lipase ABHD12
rs10944
rs567754
rs6586282
rs11951068
rs558133
rs6859667
rs1789953
rs234709
rs921943
rs705415
rs672413
rs3797535
All
0.00
0.05
0.10
0.15
0.20
MR effect size for
'Selenium' on 'Monoacylglycerol lipase ABHD12'

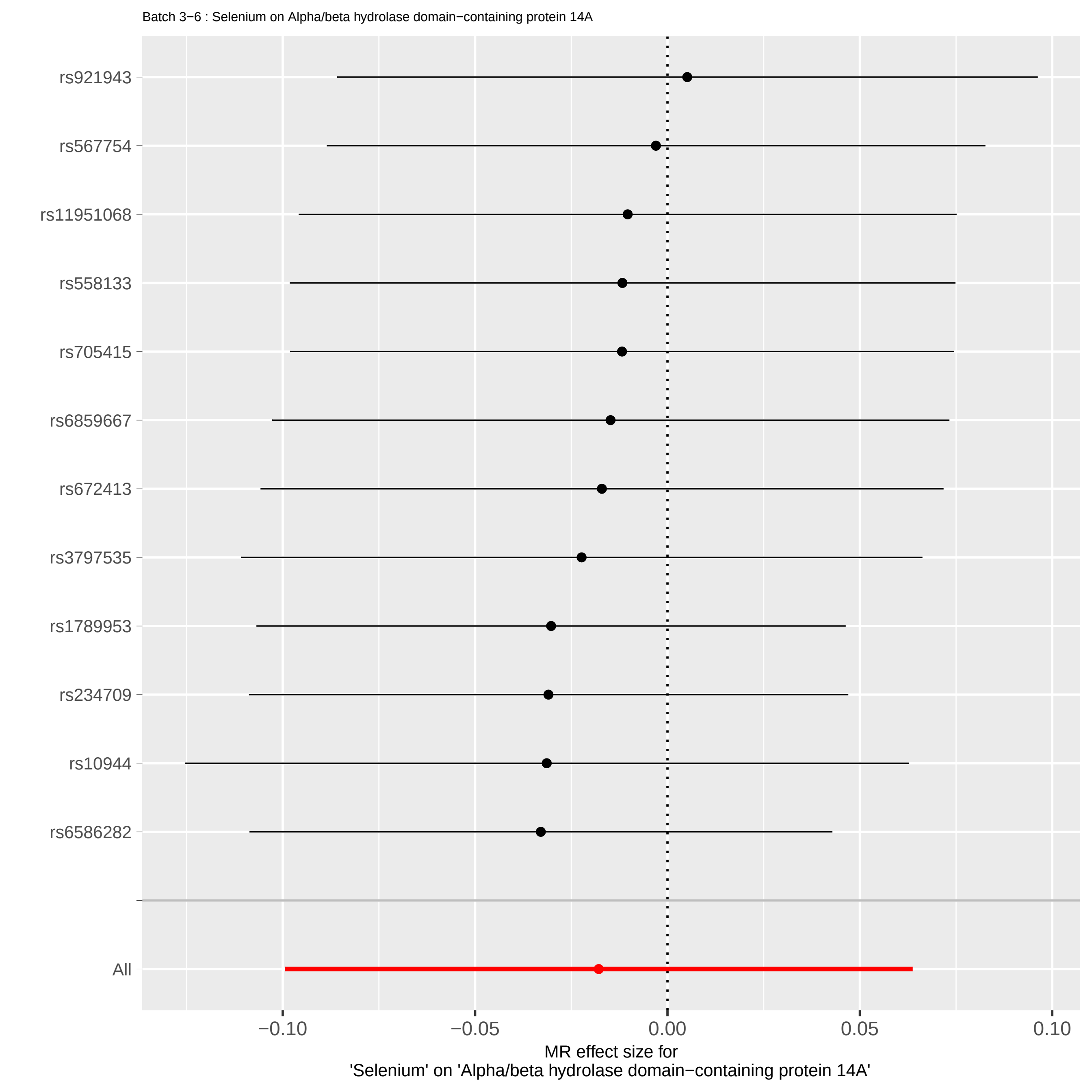

Batch 3−6 : Selenium on Alpha/beta hydrolase domain−containing protein 14A
rs921943
rs567754
rs11951068
rs558133
rs705415
rs6859667
rs672413
rs3797535
rs1789953
rs234709
rs10944
rs6586282
All
−0.10
−0.05
0.00
0.05
0.10
MR effect size for
'Selenium' on 'Alpha/beta hydrolase domain−containing protein 14A'

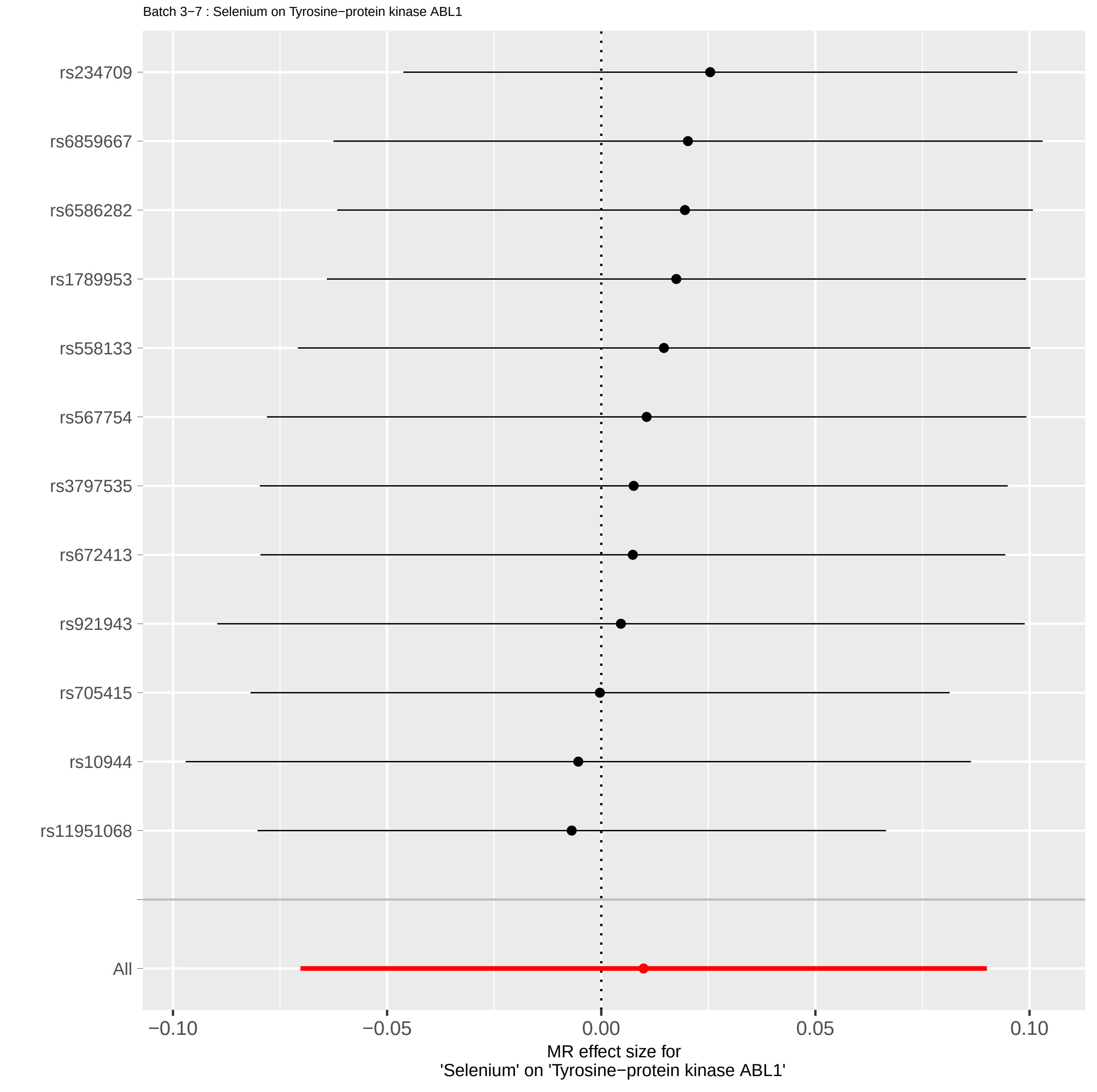
Batch 3−7 : Selenium on Tyrosine−protein kinase ABL1
rs234709
rs6859667
rs6586282
rs1789953
rs558133
rs567754
rs3797535
rs672413
rs921943
rs705415
rs10944
rs11951068
All
−0.10
−0.05
0.00
0.05
0.10
MR effect size for
'Selenium' on 'Tyrosine−protein kinase ABL1'

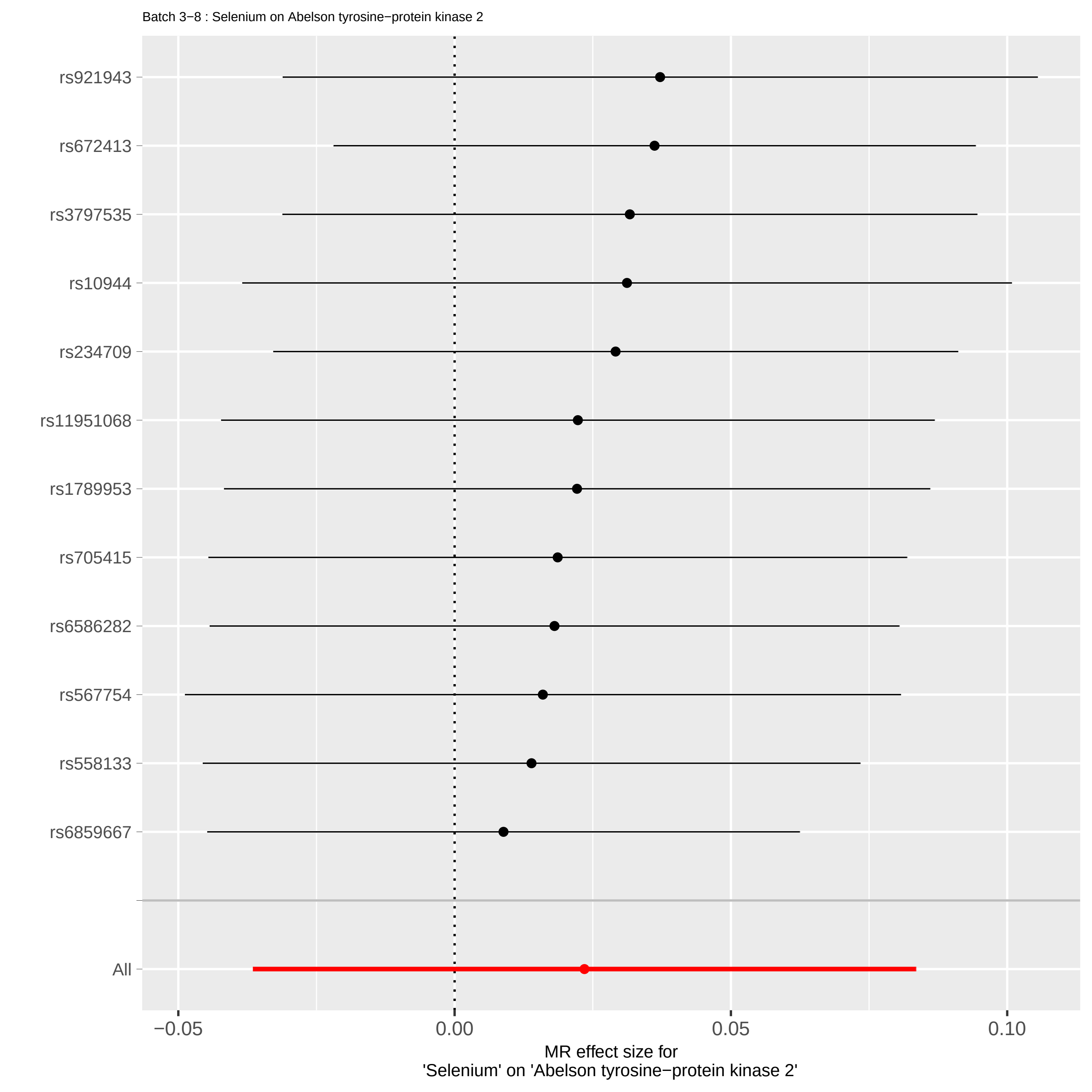
Batch 3−8 : Selenium on Abelson tyrosine−protein kinase 2
rs921943
rs672413
rs3797535
rs10944
rs234709
rs11951068
rs1789953
rs705415
rs6586282
rs567754
rs558133
rs6859667
All
−0.05
0.00
0.05
0.10
MR effect size for
'Selenium' on 'Abelson tyrosine−protein kinase 2'

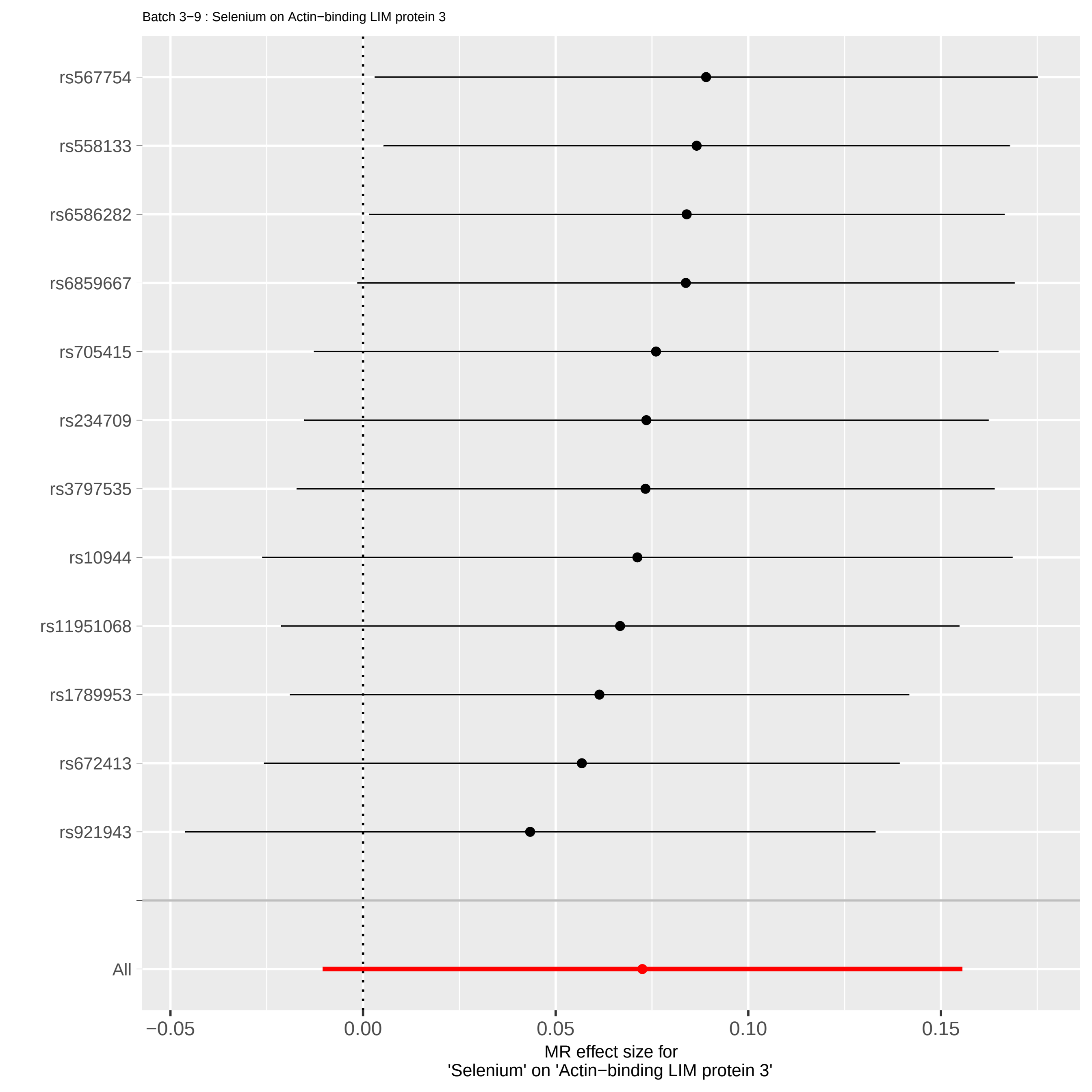

Batch 3−9 : Selenium on Actin−binding LIM protein 3
rs567754
rs558133
rs6586282
rs6859667
rs705415
rs234709
rs3797535
rs10944
rs11951068
rs1789953
rs672413
rs921943
All
−0.05
0.00
0.05
0.10
0.15
MR effect size for
'Selenium' on 'Actin−binding LIM protein 3'

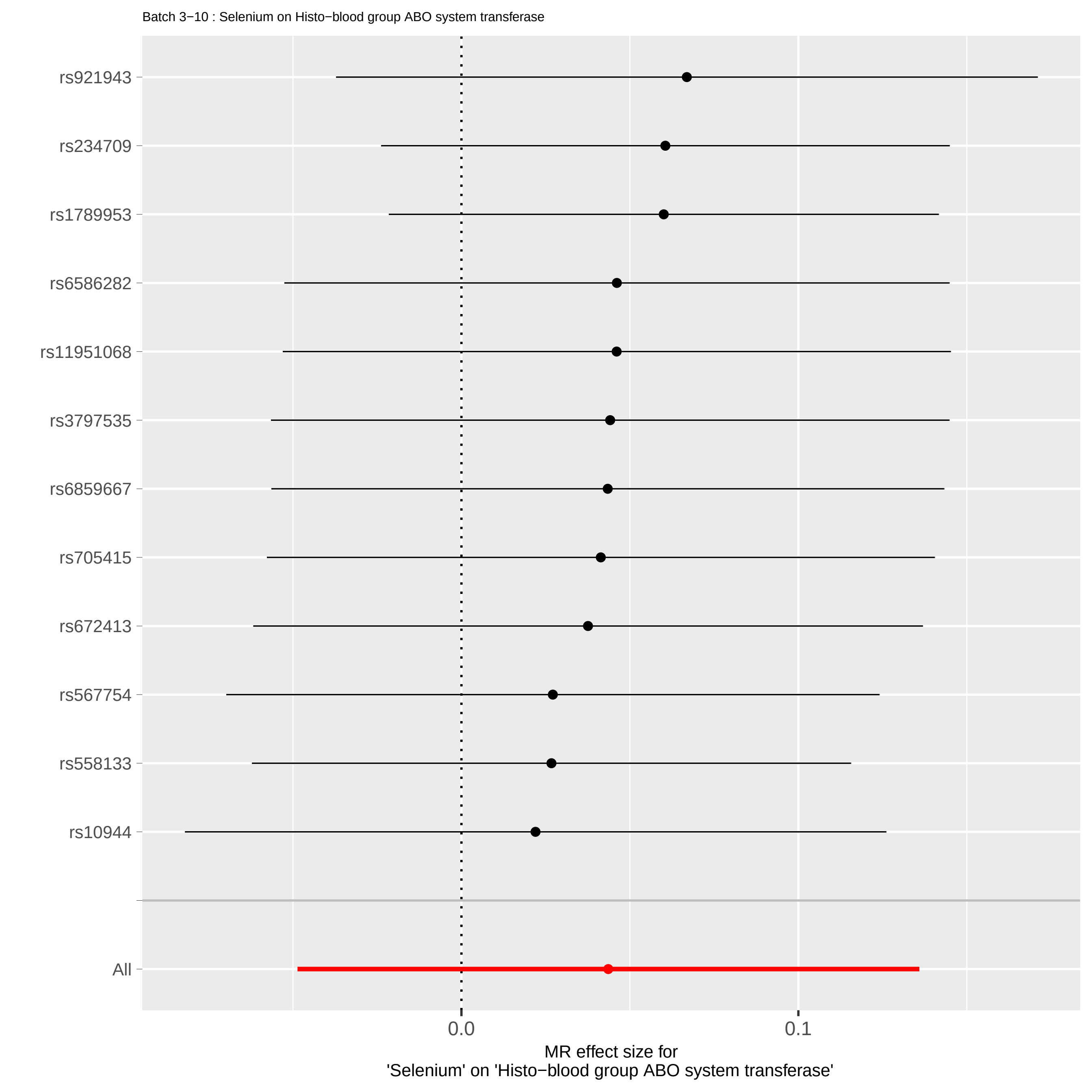
Batch 3−10 : Selenium on Histo−blood group ABO system transferase
rs921943
rs234709
rs1789953
rs6586282
rs11951068
rs3797535
rs6859667
rs705415
rs672413
rs567754
rs558133
rs10944
All
0.0
0.1
MR effect size for
'Selenium' on 'Histo−blood group ABO system transferase'

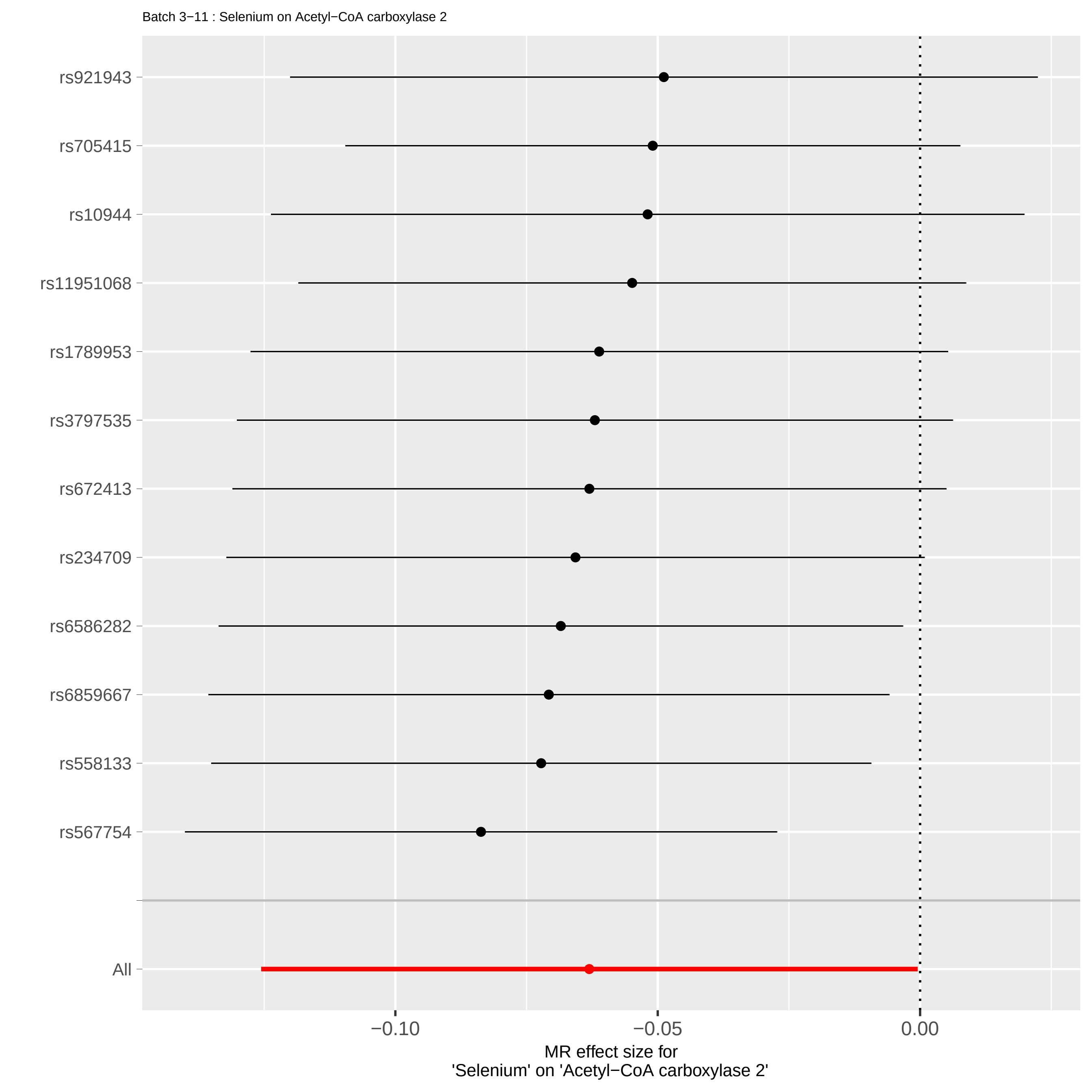
Batch 3−11 : Selenium on Acetyl−CoA carboxylase 2
rs921943
rs705415
rs10944
rs11951068
rs1789953
rs3797535
rs672413
rs234709
rs6586282
rs6859667
rs558133
rs567754
All
−0.10
−0.05
0.00
MR effect size for
'Selenium' on 'Acetyl−CoA carboxylase 2'

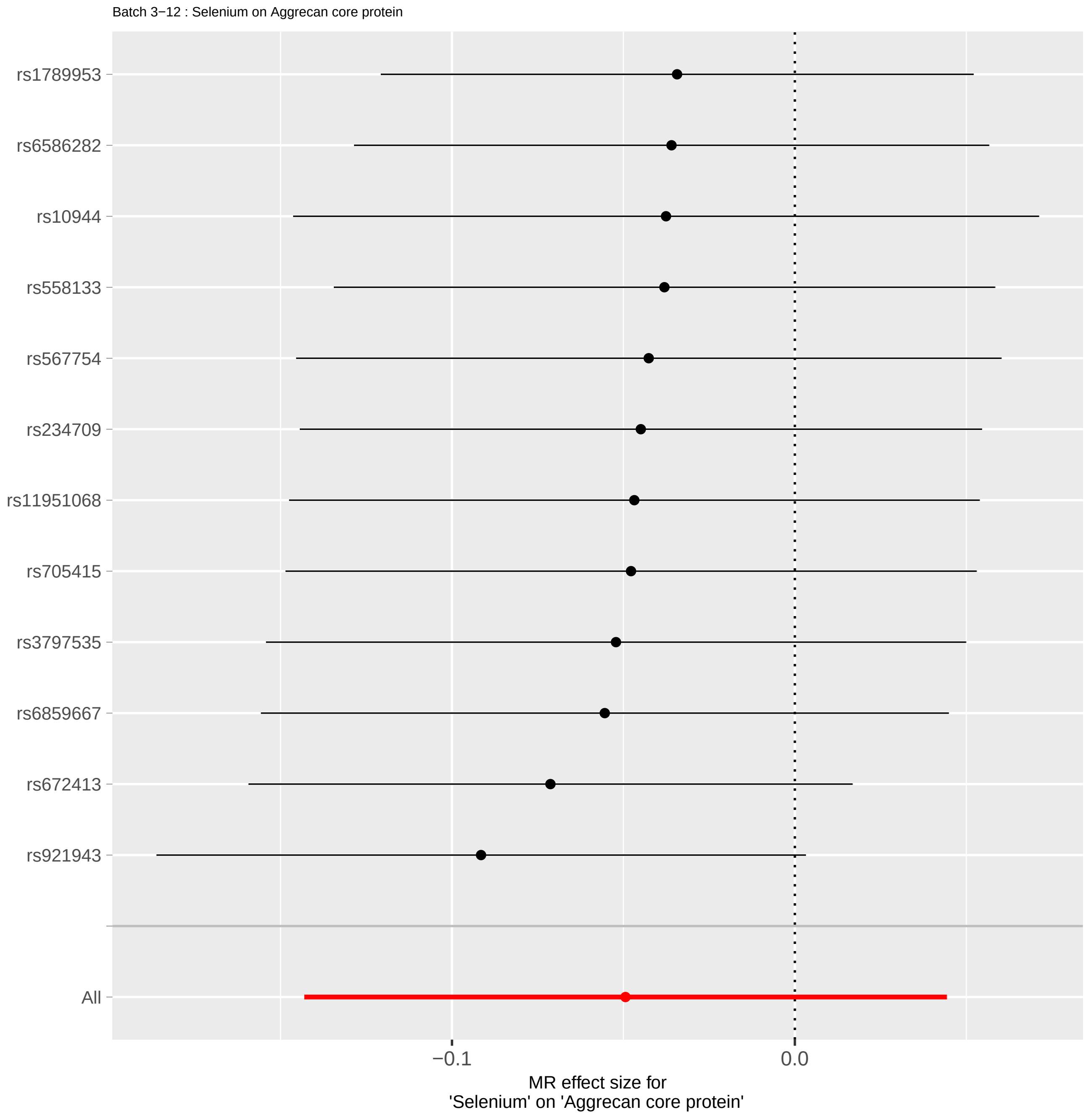

Batch 3−12 : Selenium on Aggrecan core protein
rs1789953
rs6586282
rs10944
rs558133
rs567754
rs234709
rs11951068
rs705415
rs3797535
rs6859667
rs672413
rs921943
All
−0.1
0.0
MR effect size for
'Selenium' on 'Aggrecan core protein'

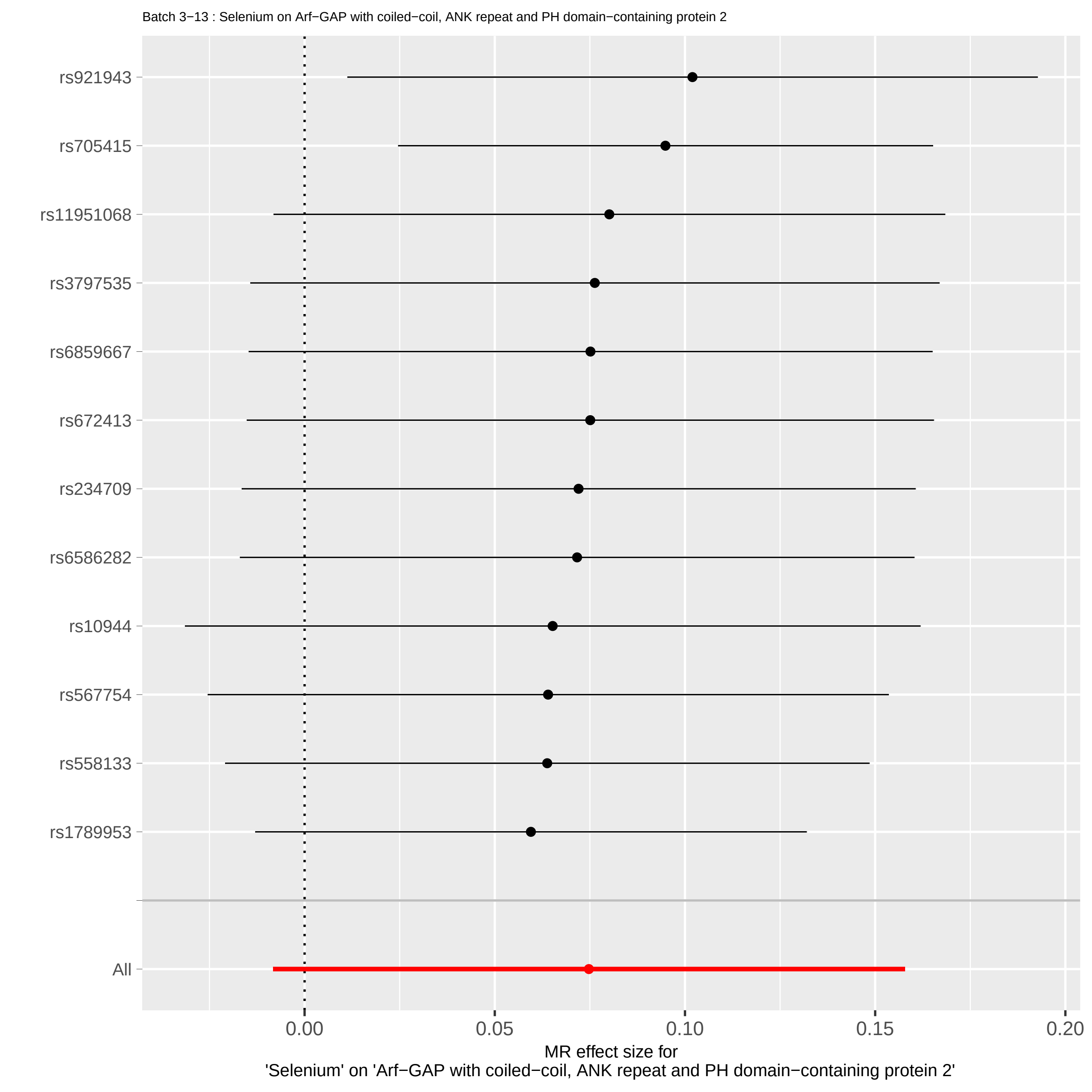

Batch 3−13 : Selenium on Arf−GAP with coiled−coil, ANK repeat and PH domain−containing protein 2
rs921943
rs705415
rs11951068
rs3797535
rs6859667
rs672413
rs234709
rs6586282
rs10944
rs567754
rs558133
rs1789953
All
0.00
0.05
0.10
0.15
0.20
MR effect size for
'Selenium' on 'Arf−GAP with coiled−coil, ANK repeat and PH domain−containing protein 2'

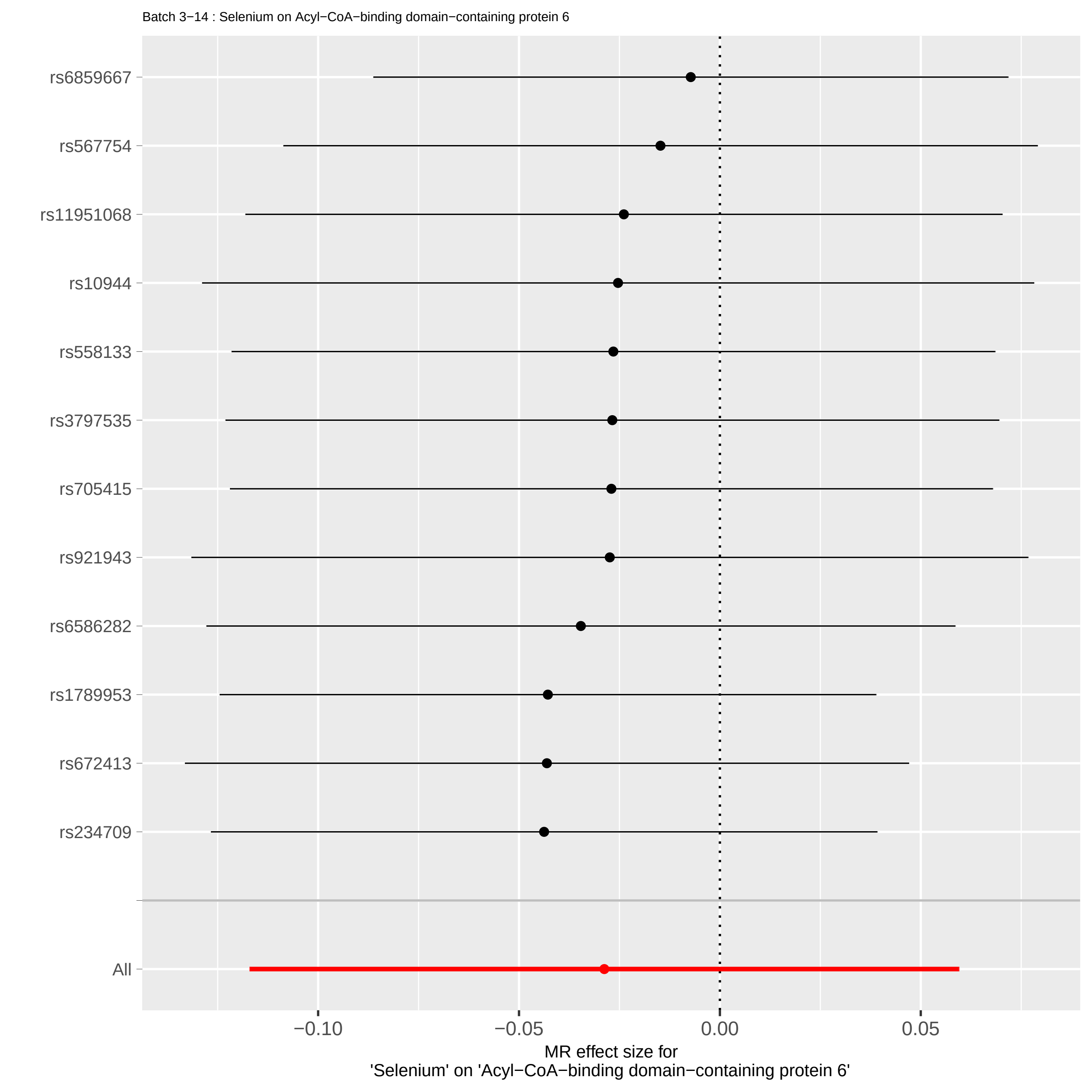

Batch 3−14 : Selenium on Acyl−CoA−binding domain−containing protein 6
rs6859667
rs567754
rs11951068
rs10944
rs558133
rs3797535
rs705415
rs921943
rs6586282
rs1789953
rs672413
rs234709
All
−0.10
−0.05
0.00
0.05
MR effect size for
'Selenium' on 'Acyl−CoA−binding domain−containing protein 6'

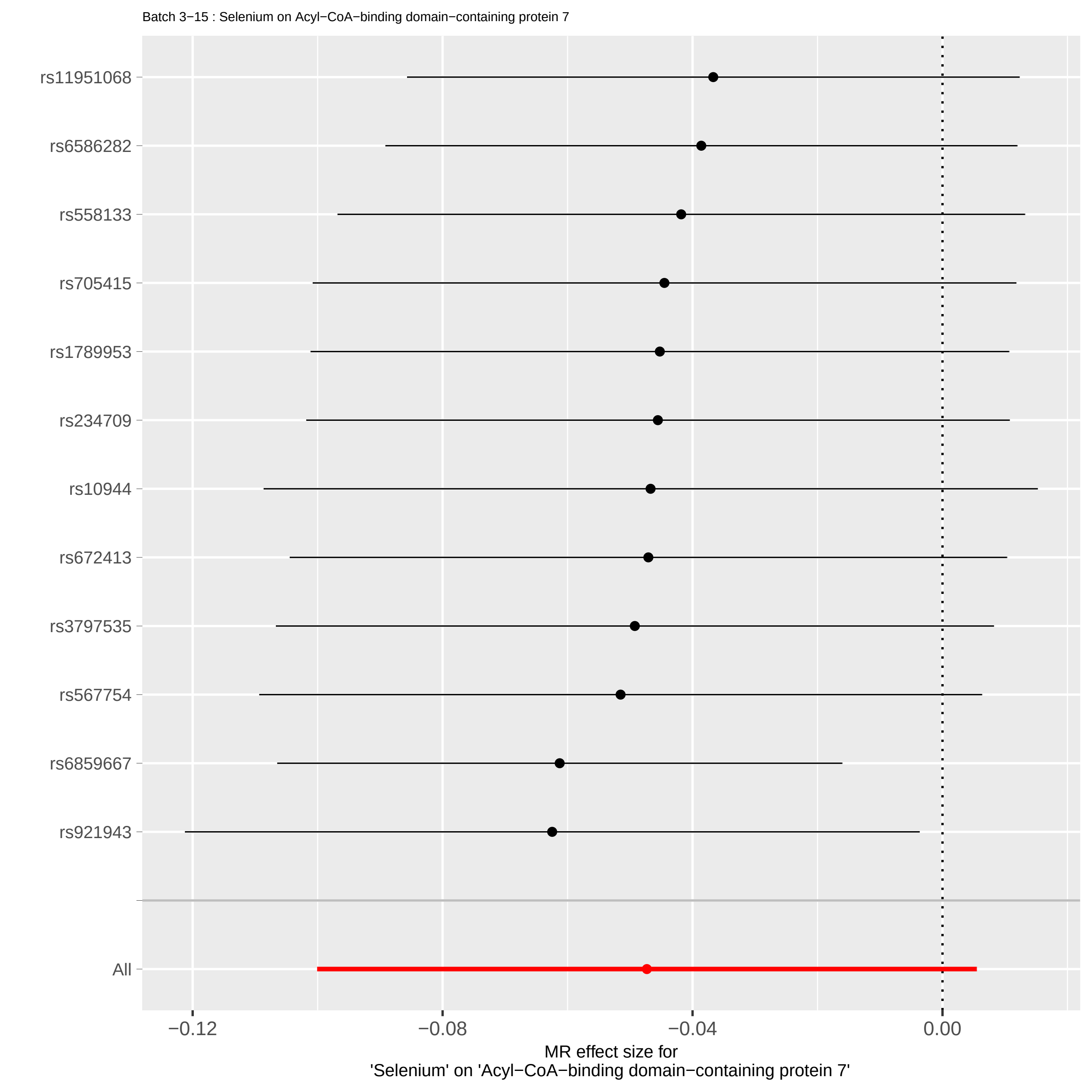
Batch 3−15 : Selenium on Acyl−CoA−binding domain−containing protein 7
rs11951068
rs6586282
rs558133
rs705415
rs1789953
rs234709
rs10944
rs672413
rs3797535
rs567754
rs6859667
rs921943
All
−0.12
−0.08
−0.04
0.00
MR effect size for
'Selenium' on 'Acyl−CoA−binding domain−containing protein 7'

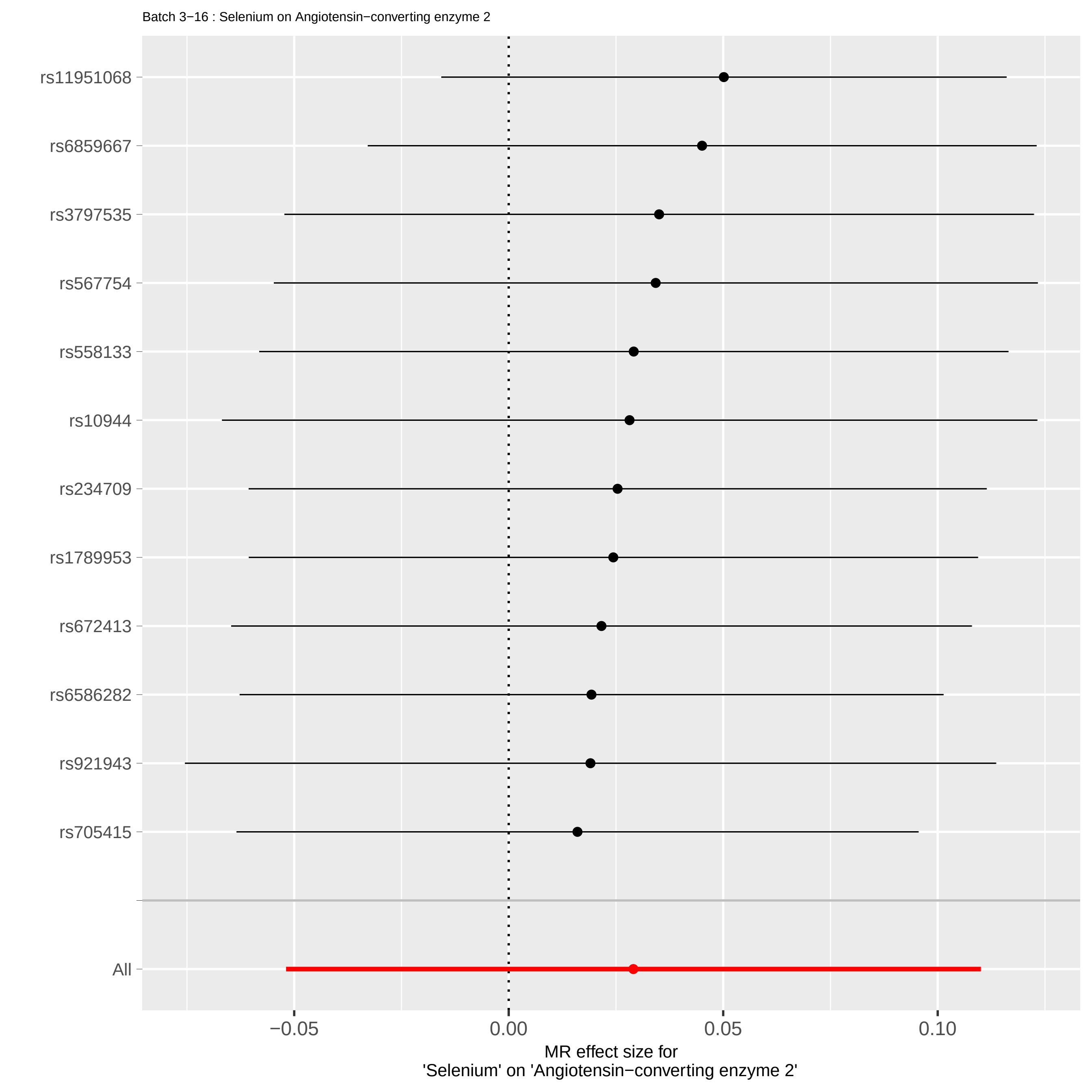
Batch 3−16 : Selenium on Angiotensin−converting enzyme 2
rs11951068
rs6859667
rs3797535
rs567754
rs558133
rs10944
rs234709
rs1789953
rs672413
rs6586282
rs921943
rs705415
All
−0.05
0.00
0.05
0.10
MR effect size for
'Selenium' on 'Angiotensin−converting enzyme 2'

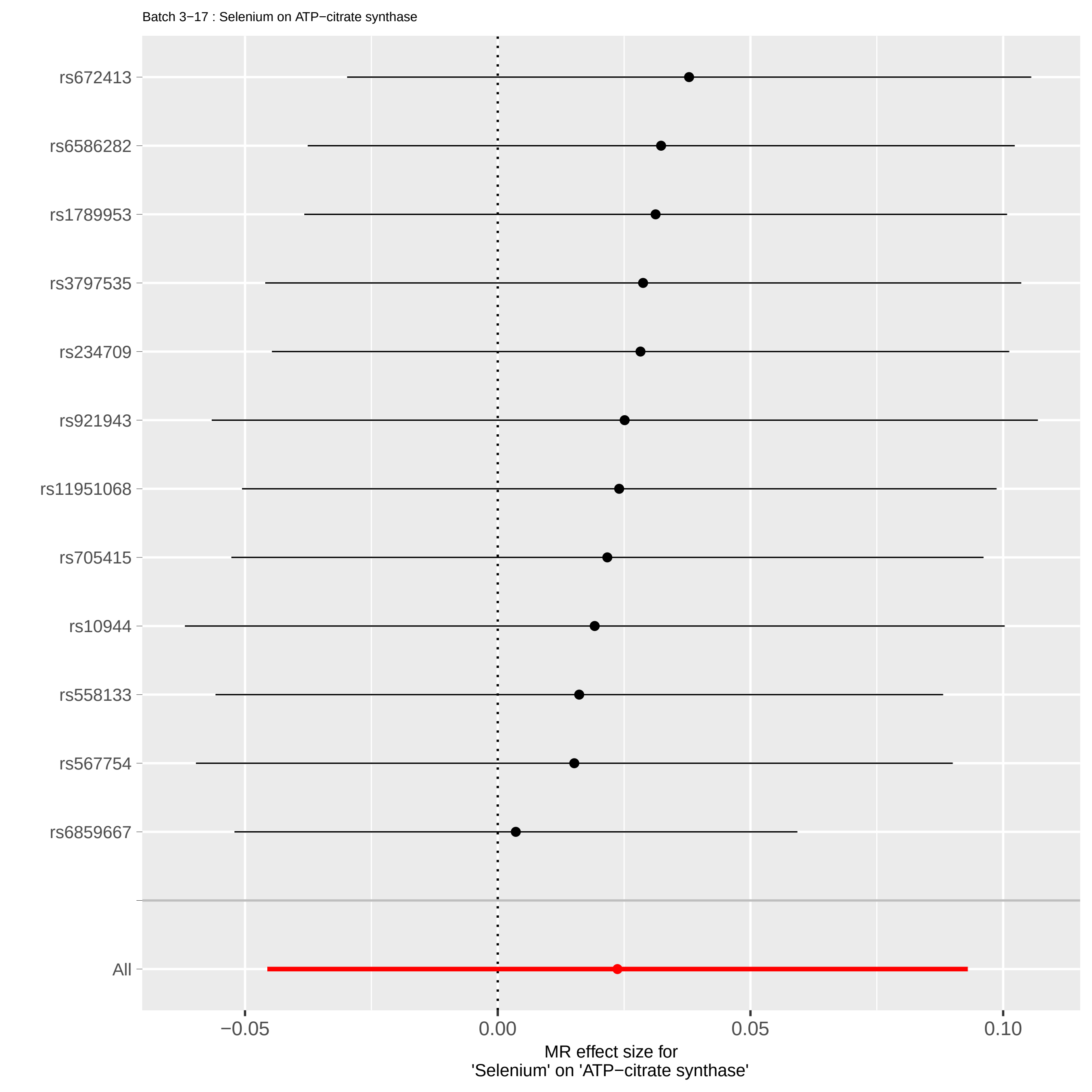

Batch 3−17 : Selenium on ATP−citrate synthase
rs672413
rs6586282
rs1789953
rs3797535
rs234709
rs921943
rs11951068
rs705415
rs10944
rs558133
rs567754
rs6859667
All
−0.05
0.00
0.05
0.10
MR effect size for
'Selenium' on 'ATP−citrate synthase'

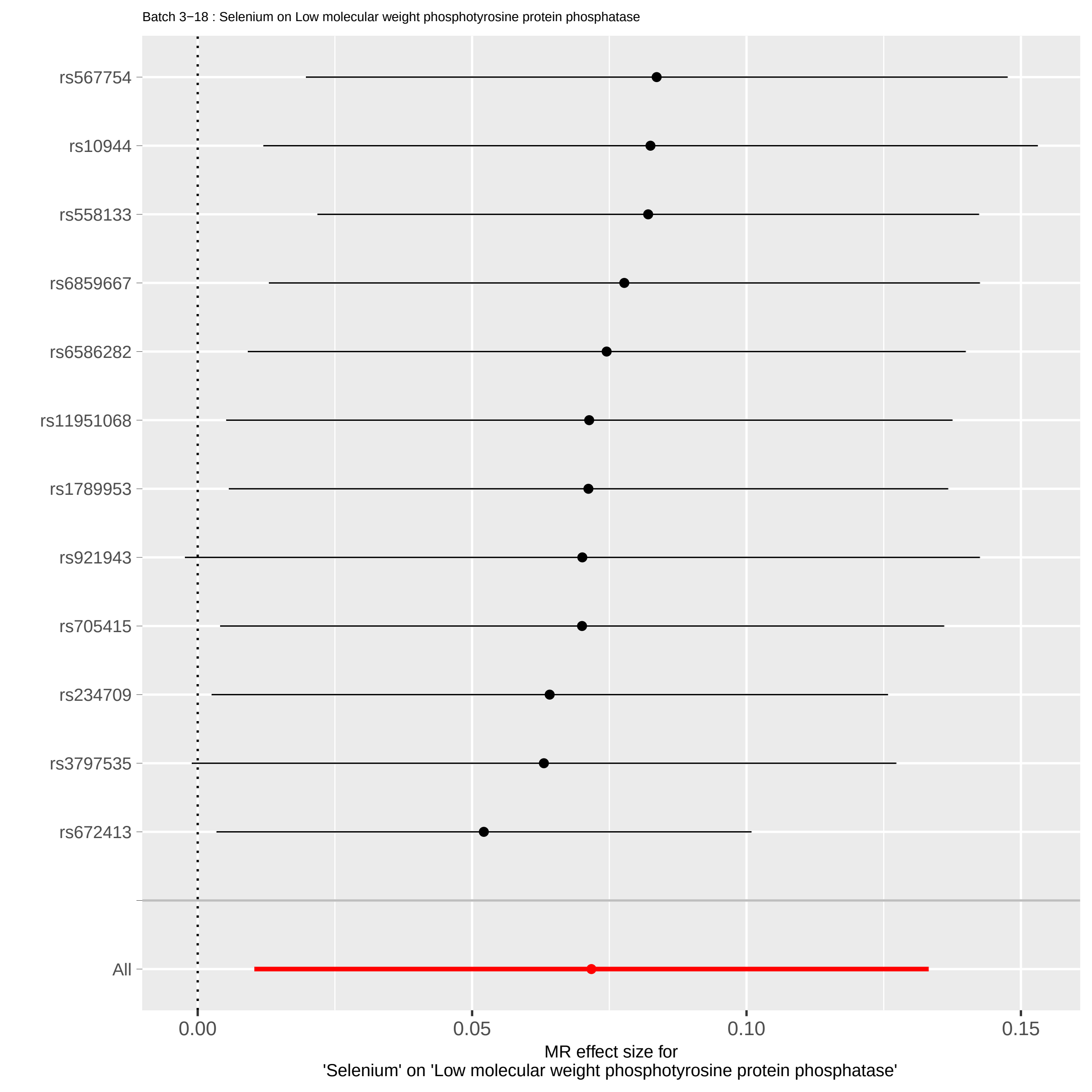
Batch 3−18 : Selenium on Low molecular weight phosphotyrosine protein phosphatase
rs567754
rs10944
rs558133
rs6859667
rs6586282
rs11951068
rs1789953
rs921943
rs705415
rs234709
rs3797535
rs672413
All
0.00
0.05
0.10
0.15
MR effect size for
'Selenium' on 'Low molecular weight phosphotyrosine protein phosphatase'

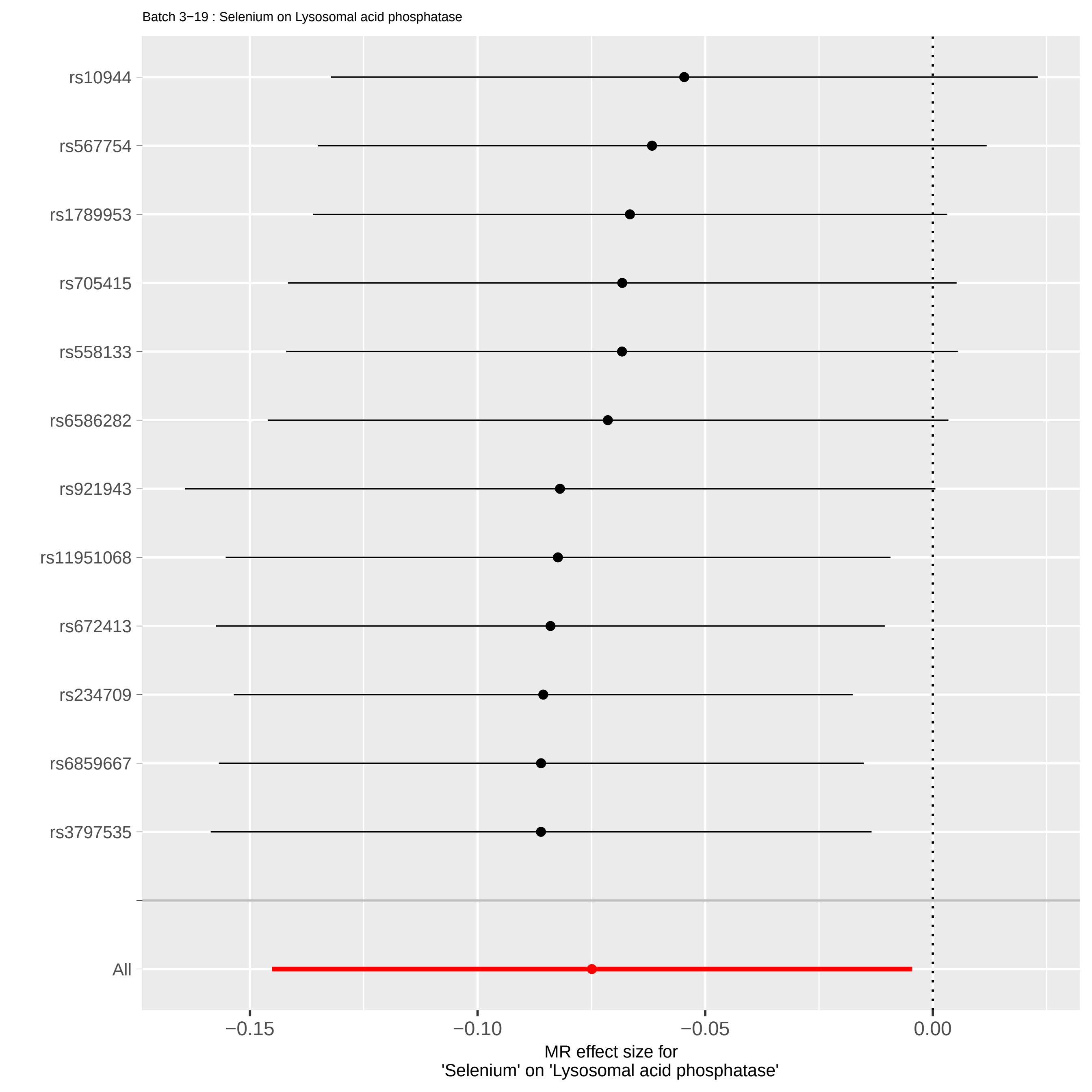

Batch 3−19 : Selenium on Lysosomal acid phosphatase
rs10944
rs567754
rs1789953
rs705415
rs558133
rs6586282
rs921943
rs11951068
rs672413
rs234709
rs6859667
rs3797535
All
−0.15
−0.10
−0.05
0.00
MR effect size for
'Selenium' on 'Lysosomal acid phosphatase'

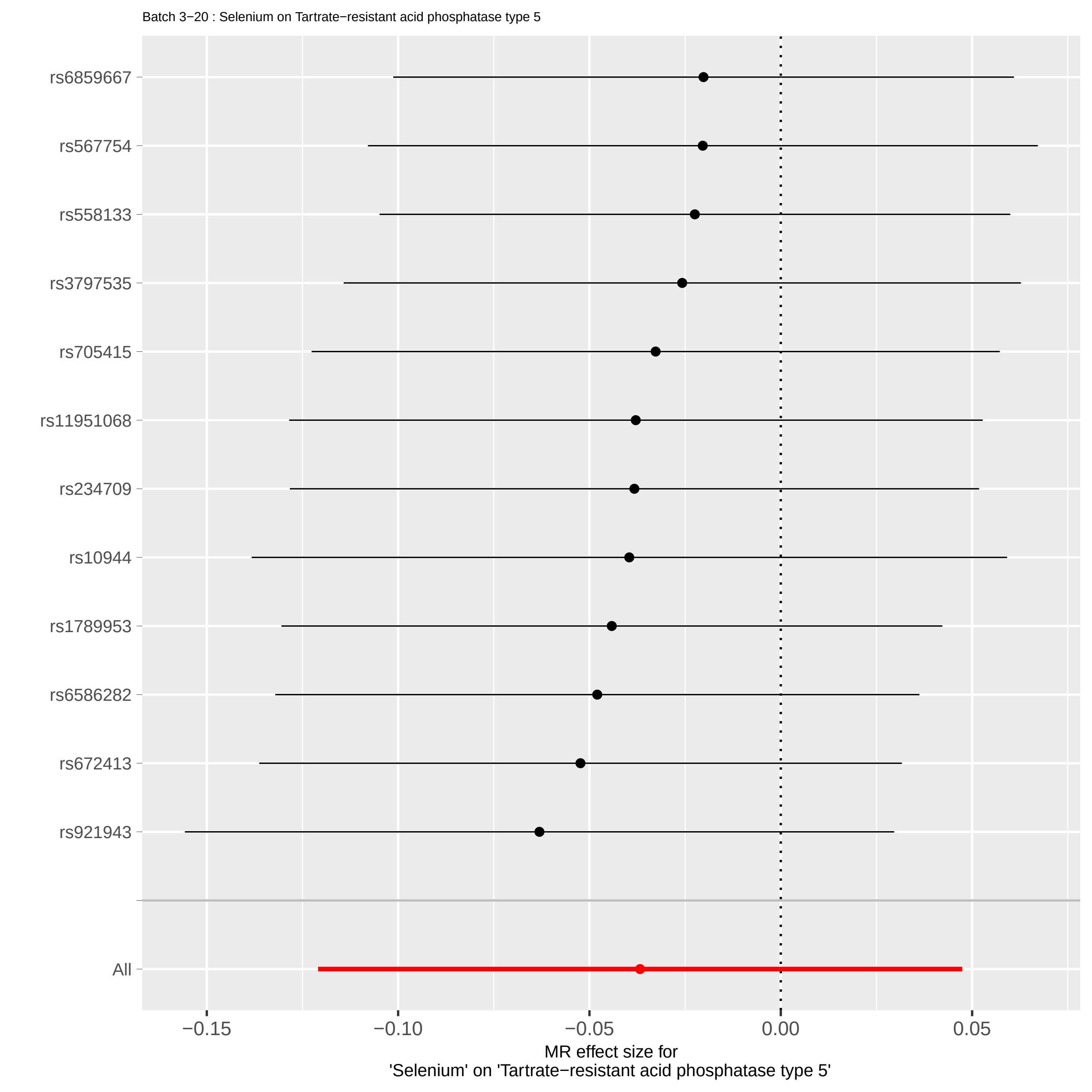

Batch 3−20 : Selenium on Tartrate−resistant acid phosphatase type 5
rs6859667
rs567754
rs558133
rs3797535
rs705415
rs11951068
rs234709
rs10944
rs1789953
rs6586282
rs672413
rs921943
All
−0.15
−0.10
−0.05
0.00
0.05
MR effect size for
'Selenium' on 'Tartrate−resistant acid phosphatase type 5'

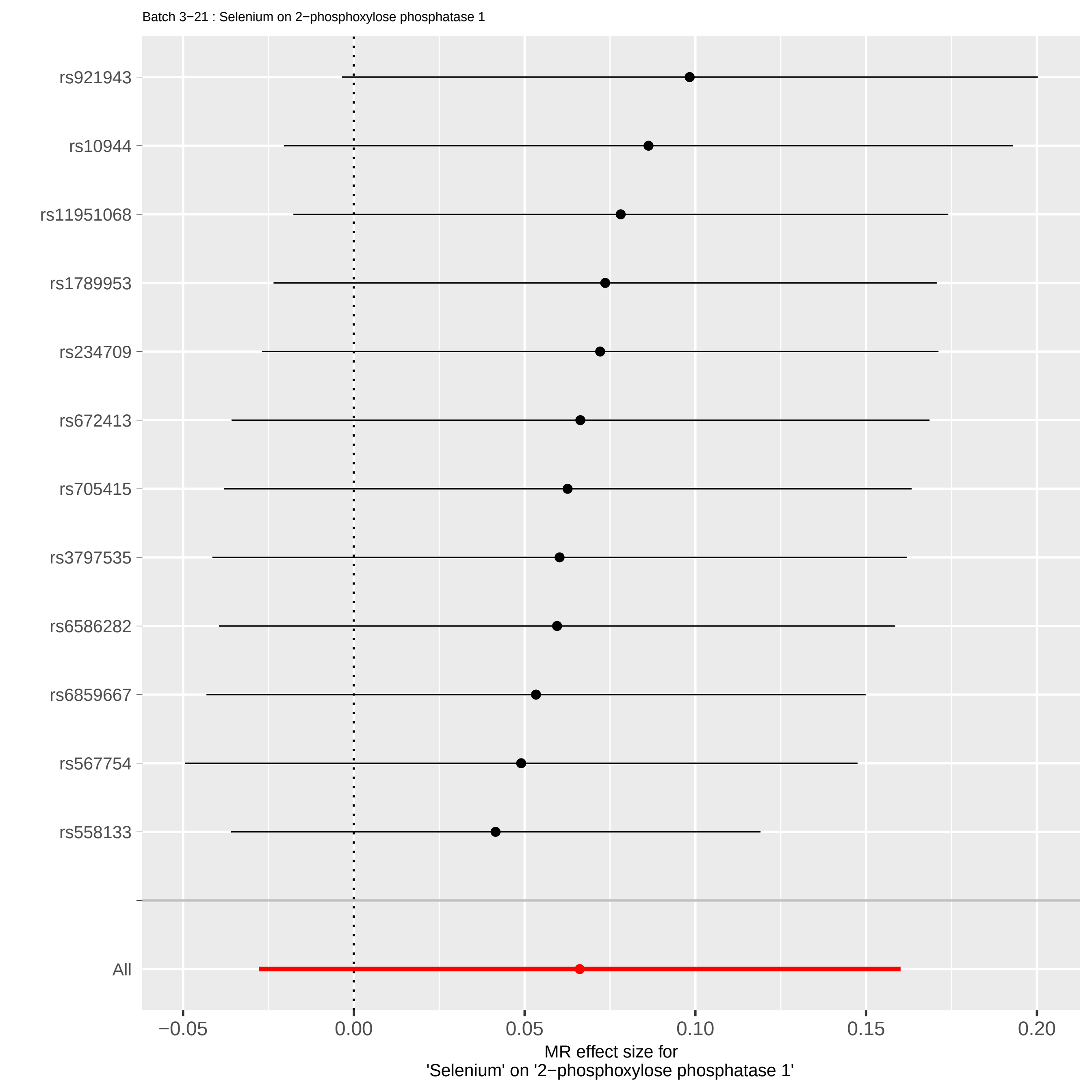
Batch 3−21 : Selenium on 2−phosphoxylose phosphatase 1
rs921943
rs10944
rs11951068
rs1789953
rs234709
rs672413
rs705415
rs3797535
rs6586282
rs6859667
rs567754
rs558133
All
−0.05
0.00
0.05
0.10
0.15
0.20
MR effect size for
'Selenium' on '2−phosphoxylose phosphatase 1'

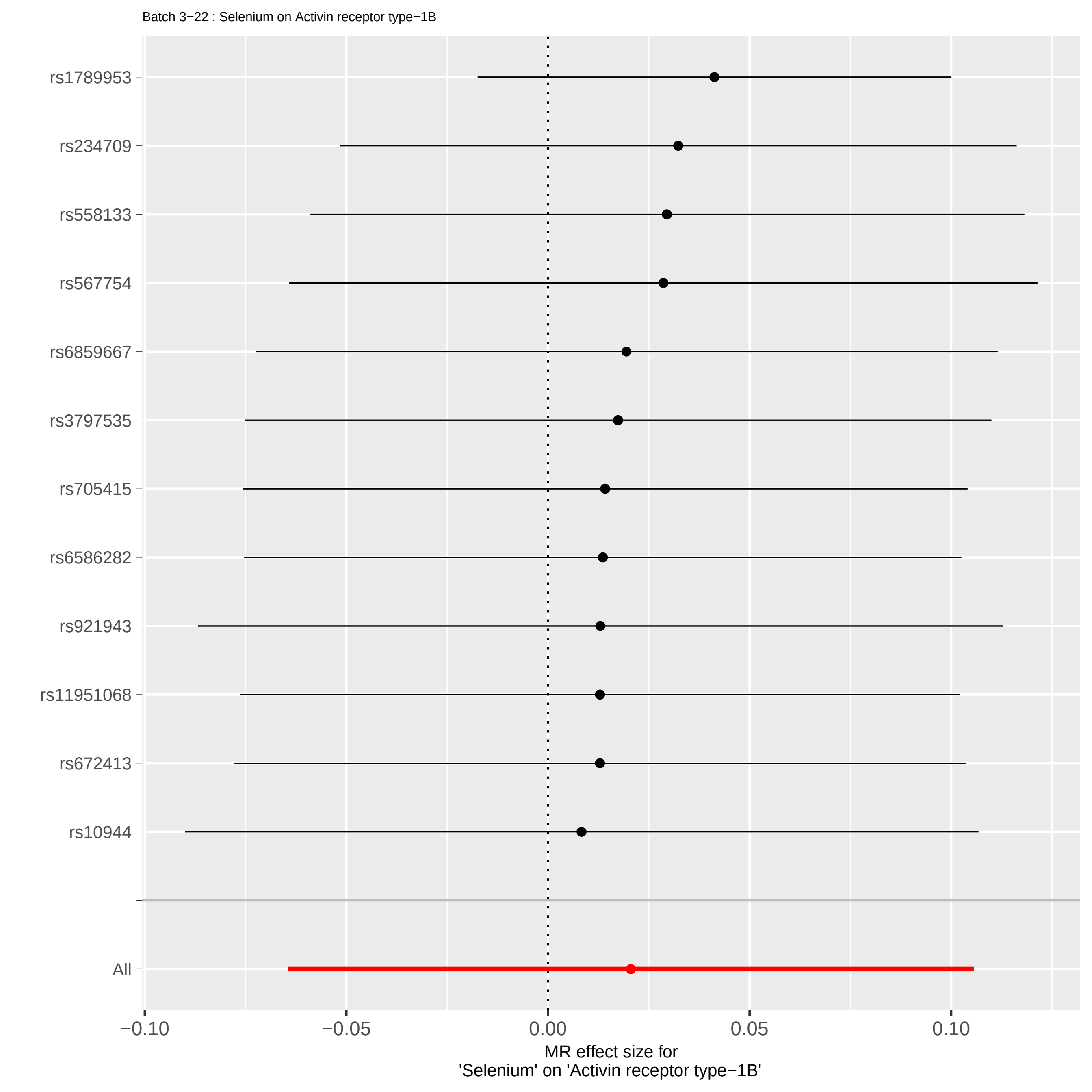

Batch 3−22 : Selenium on Activin receptor type−1B
rs1789953
rs234709
rs558133
rs567754
rs6859667
rs3797535
rs705415
rs6586282
rs921943
rs11951068
rs672413
rs10944
All
−0.10
−0.05
0.00
0.05
0.10
MR effect size for
'Selenium' on 'Activin receptor type−1B'

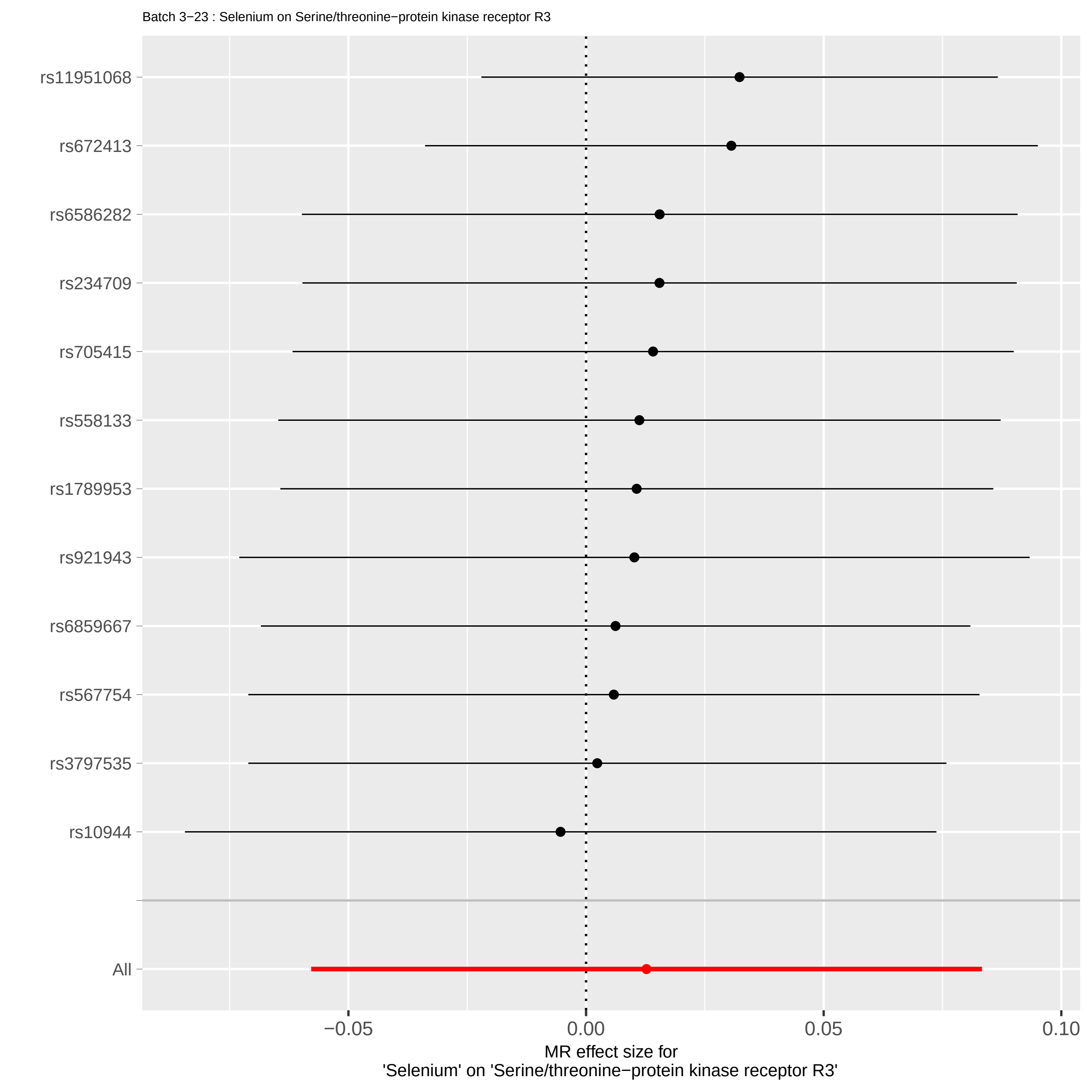

Batch 3−23 : Selenium on Serine/threonine−protein kinase receptor R3
rs11951068
rs672413
rs6586282
rs234709
rs705415
rs558133
rs1789953
rs921943
rs6859667
rs567754
rs3797535
rs10944
All
−0.05
0.00
0.05
0.10
MR effect size for
'Selenium' on 'Serine/threonine−protein kinase receptor R3'

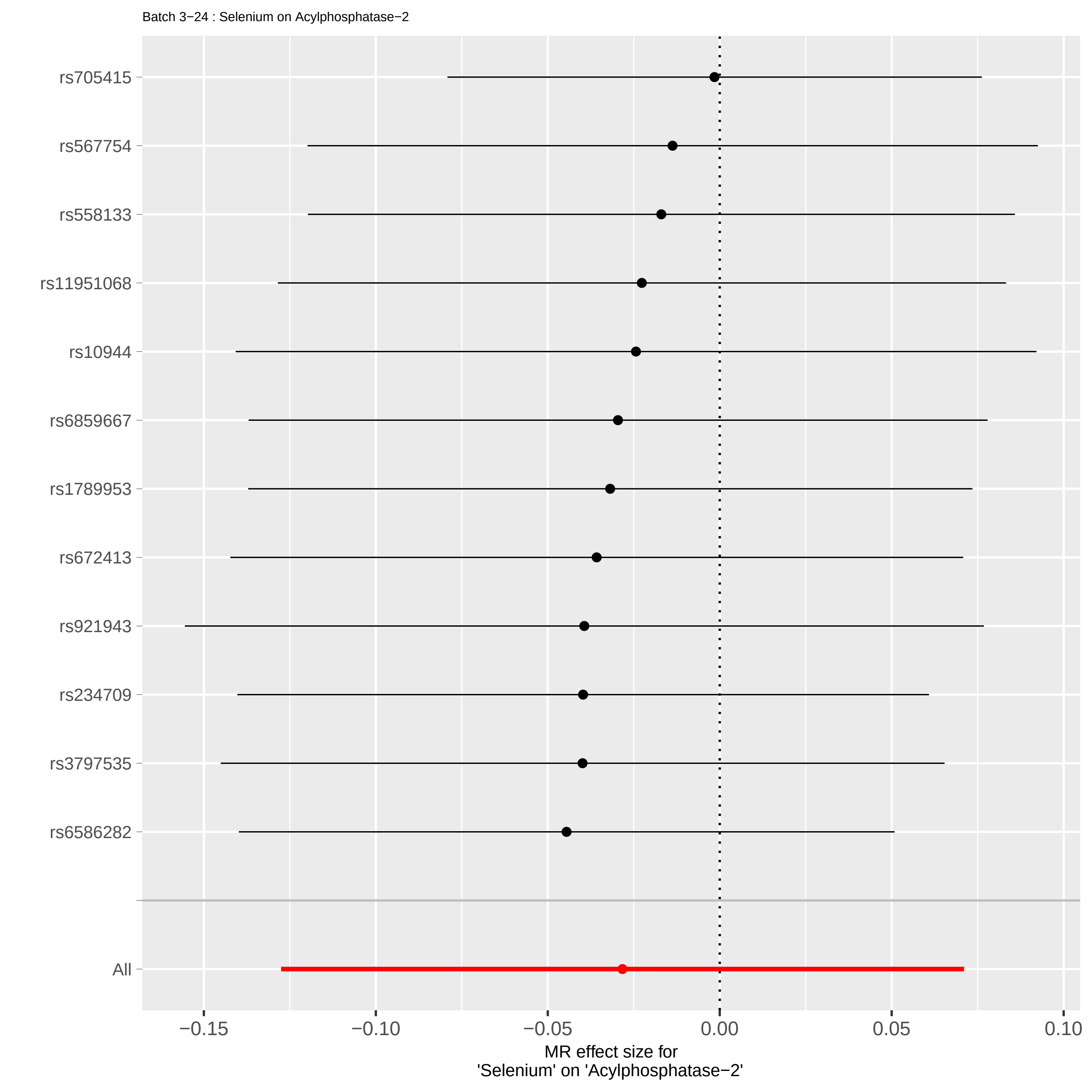

Batch 3−24 : Selenium on Acylphosphatase−2
rs705415
rs567754
rs558133
rs11951068
rs10944
rs6859667
rs1789953
rs672413
rs921943
rs234709
rs3797535
rs6586282
All
−0.15
−0.10
−0.05
0.00
0.05
0.10
MR effect size for
'Selenium' on 'Acylphosphatase−2'

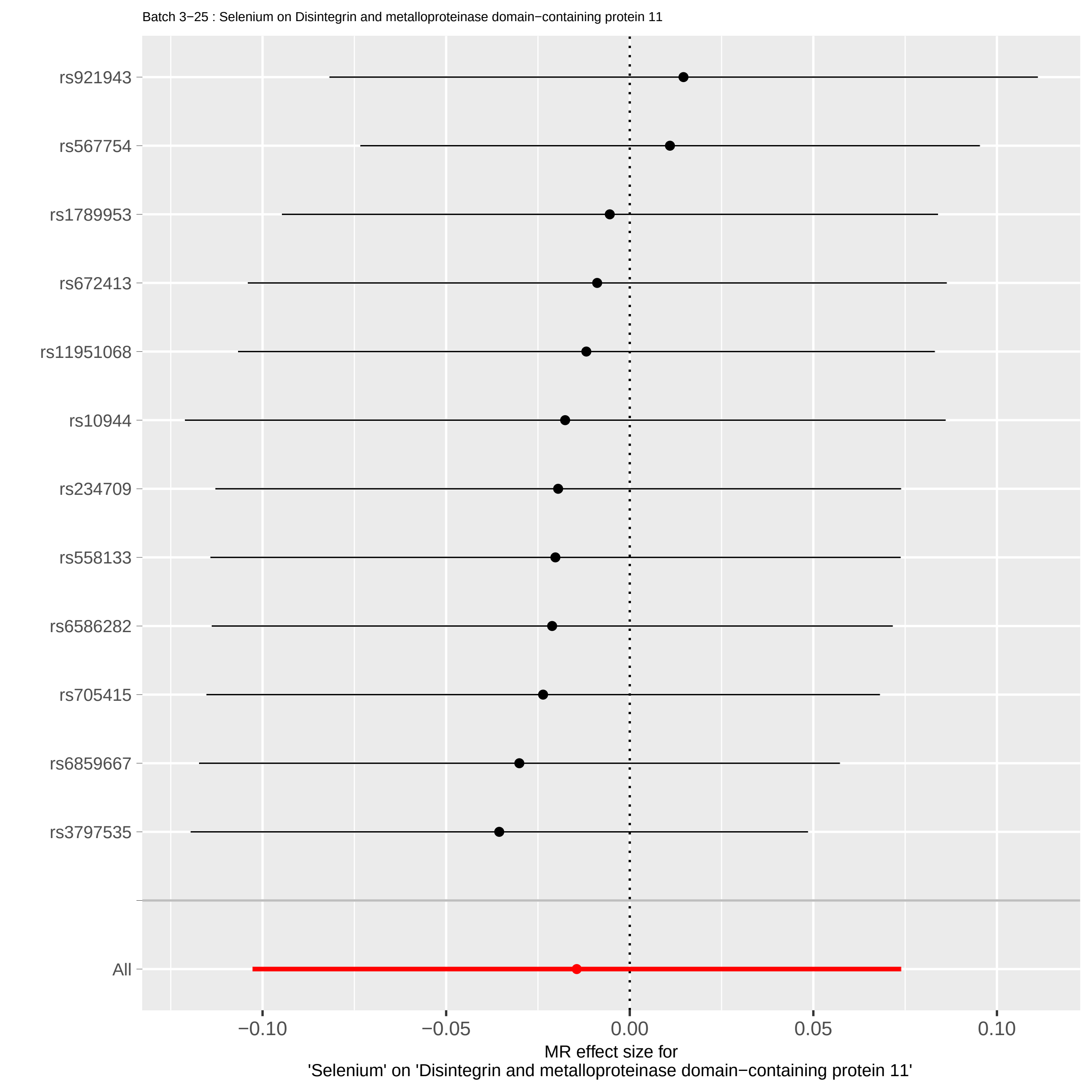
Batch 3−25 : Selenium on Disintegrin and metalloproteinase domain−containing protein 11
rs921943
rs567754
rs1789953
rs672413
rs11951068
rs10944
rs234709
rs558133
rs6586282
rs705415
rs6859667
rs3797535
All
−0.10
−0.05
0.00
0.05
0.10
MR effect size for
'Selenium' on 'Disintegrin and metalloproteinase domain−containing protein 11'

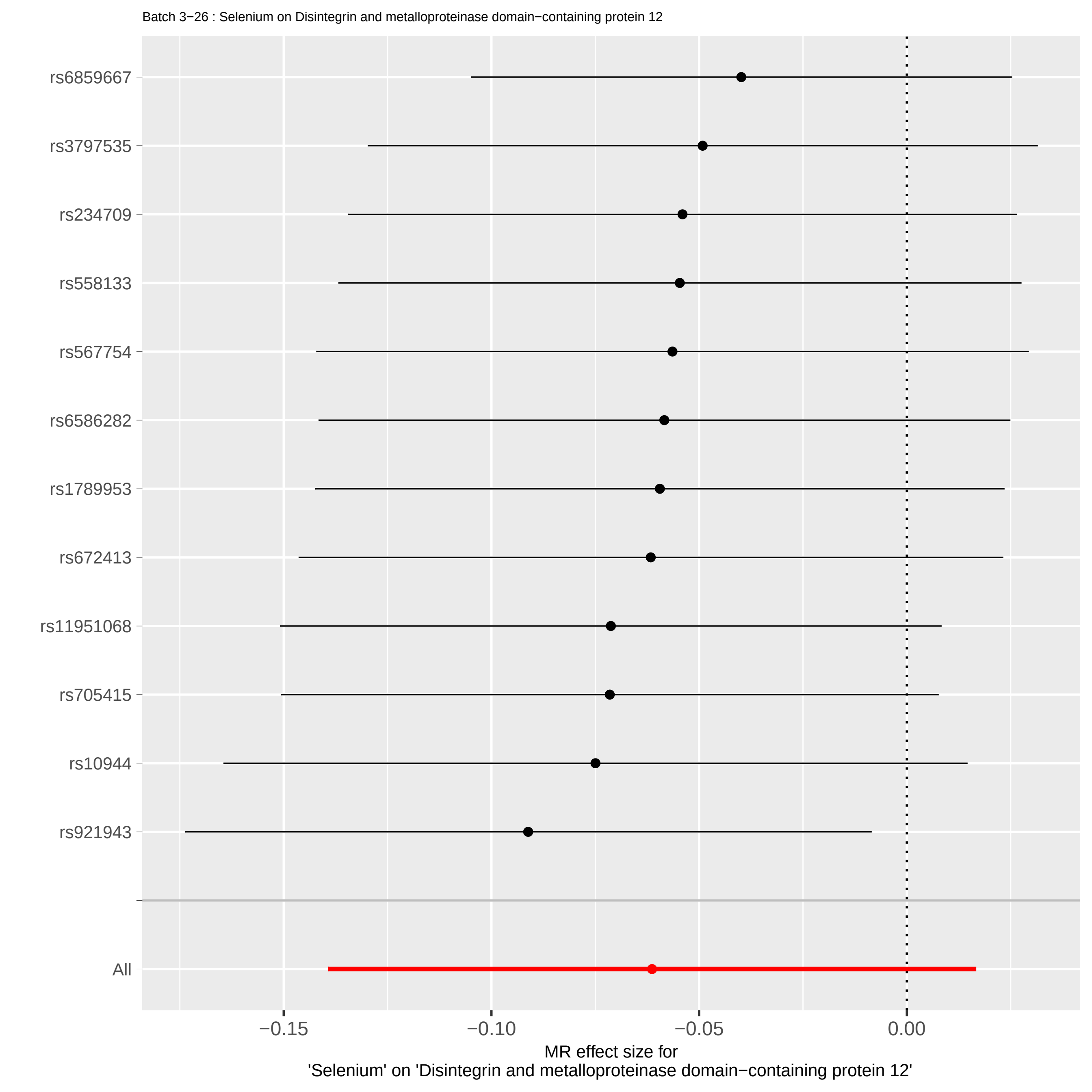

Batch 3−26 : Selenium on Disintegrin and metalloproteinase domain−containing protein 12
rs6859667
rs3797535
rs234709
rs558133
rs567754
rs6586282
rs1789953
rs672413
rs11951068
rs705415
rs10944
rs921943
All
−0.15
−0.10
−0.05
0.00
MR effect size for
'Selenium' on 'Disintegrin and metalloproteinase domain−containing protein 12'

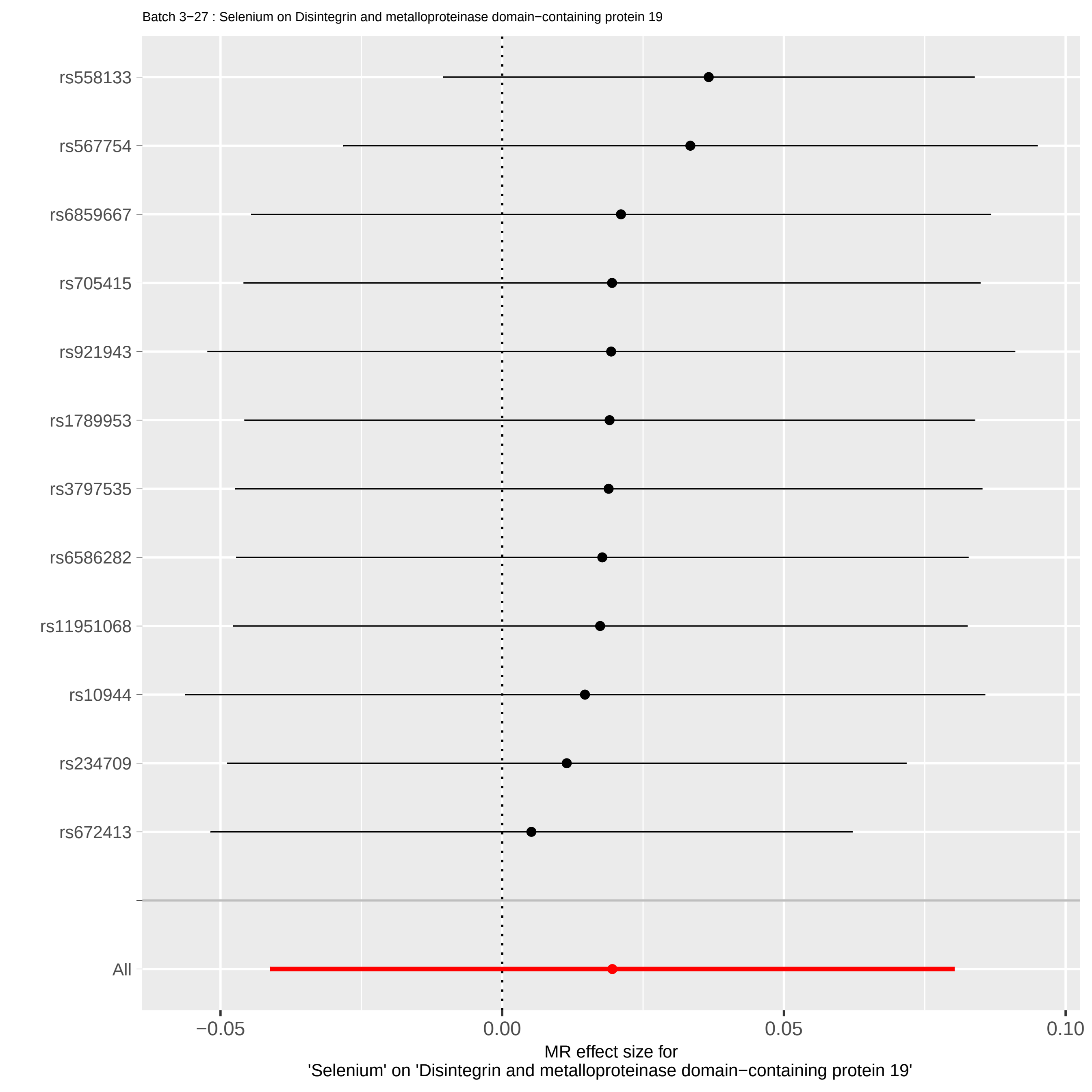

Batch 3−27 : Selenium on Disintegrin and metalloproteinase domain−containing protein 19
rs558133
rs567754
rs6859667
rs705415
rs921943
rs1789953
rs3797535
rs6586282
rs11951068
rs10944
rs234709
rs672413
All
−0.05
0.00
0.05
0.10
MR effect size for
'Selenium' on 'Disintegrin and metalloproteinase domain−containing protein 19'

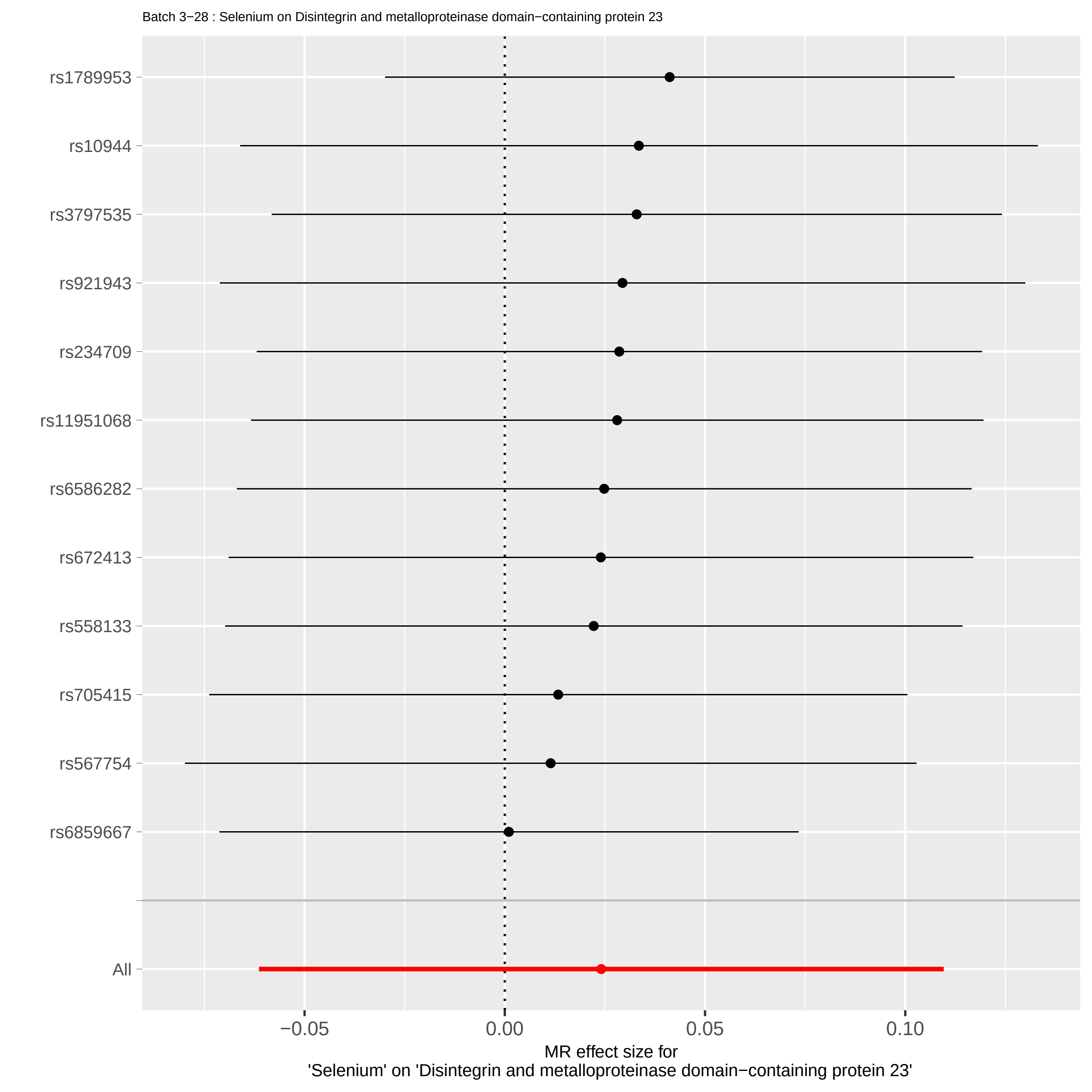

Batch 3−28 : Selenium on Disintegrin and metalloproteinase domain−containing protein 23
rs1789953
rs10944
rs3797535
rs921943
rs234709
rs11951068
rs6586282
rs672413
rs558133
rs705415
rs567754
rs6859667
All
−0.05
0.00
0.05
0.10
MR effect size for
'Selenium' on 'Disintegrin and metalloproteinase domain−containing protein 23'

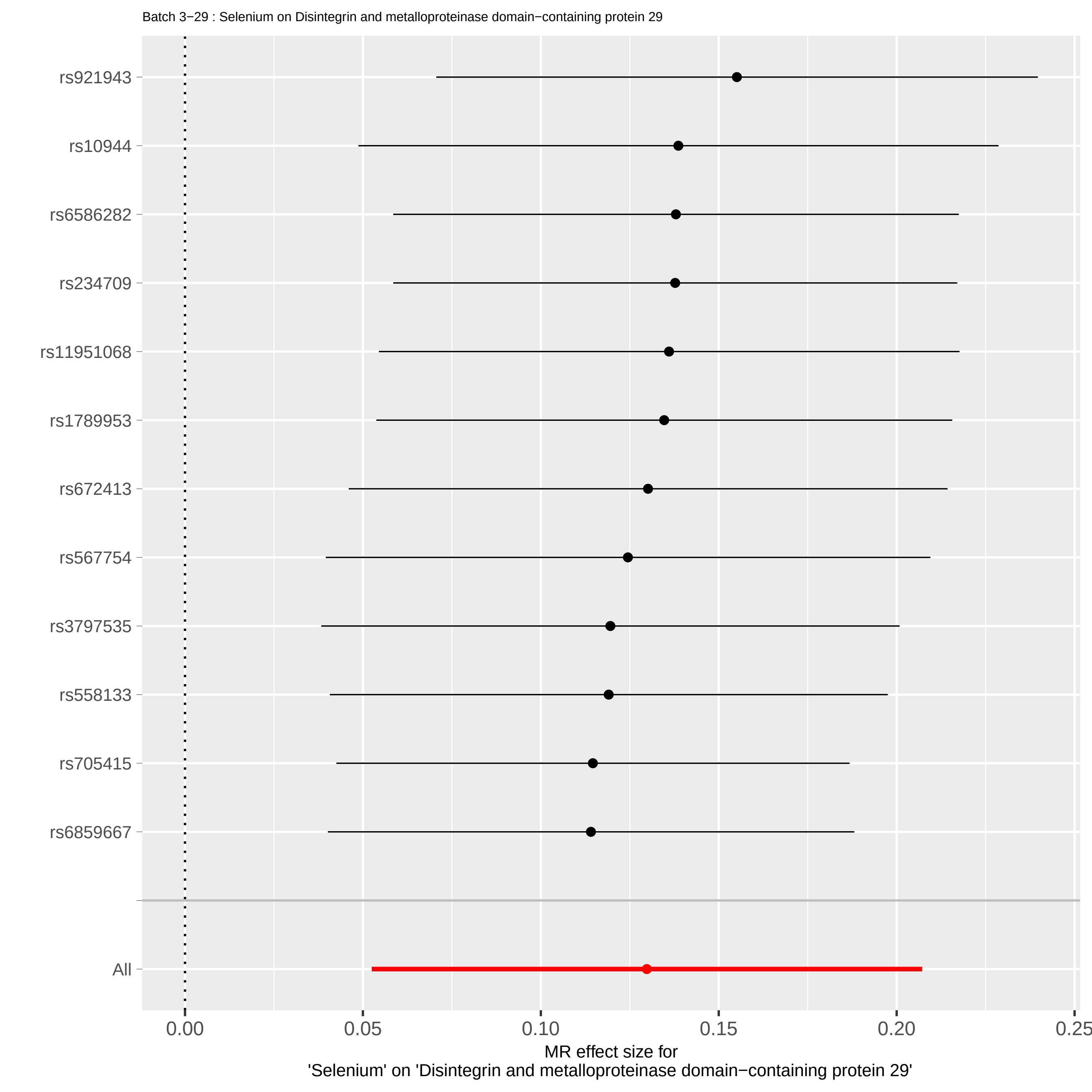

Batch 3−29 : Selenium on Disintegrin and metalloproteinase domain−containing protein 29
rs921943
rs10944
rs6586282
rs234709
rs11951068
rs1789953
rs672413
rs567754
rs3797535
rs558133
rs705415
rs6859667
All
0.00
0.05
0.10
0.15
0.20
0.25
MR effect size for
'Selenium' on 'Disintegrin and metalloproteinase domain−containing protein 29'

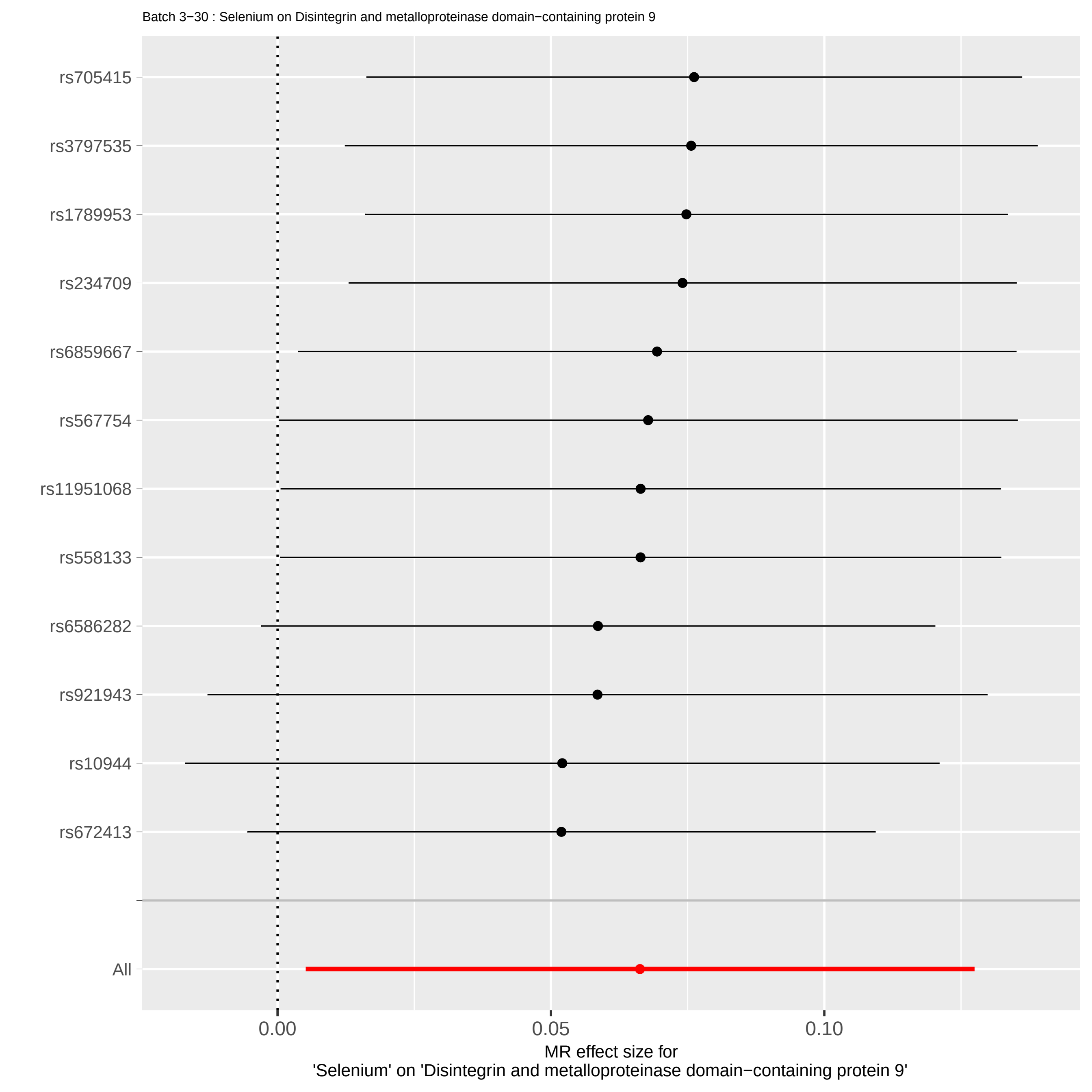

Batch 3−30 : Selenium on Disintegrin and metalloproteinase domain−containing protein 9
rs705415
rs3797535
rs1789953
rs234709
rs6859667
rs567754
rs11951068
rs558133
rs6586282
rs921943
rs10944
rs672413
All
0.00
0.05
0.10
MR effect size for
'Selenium' on 'Disintegrin and metalloproteinase domain−containing protein 9'

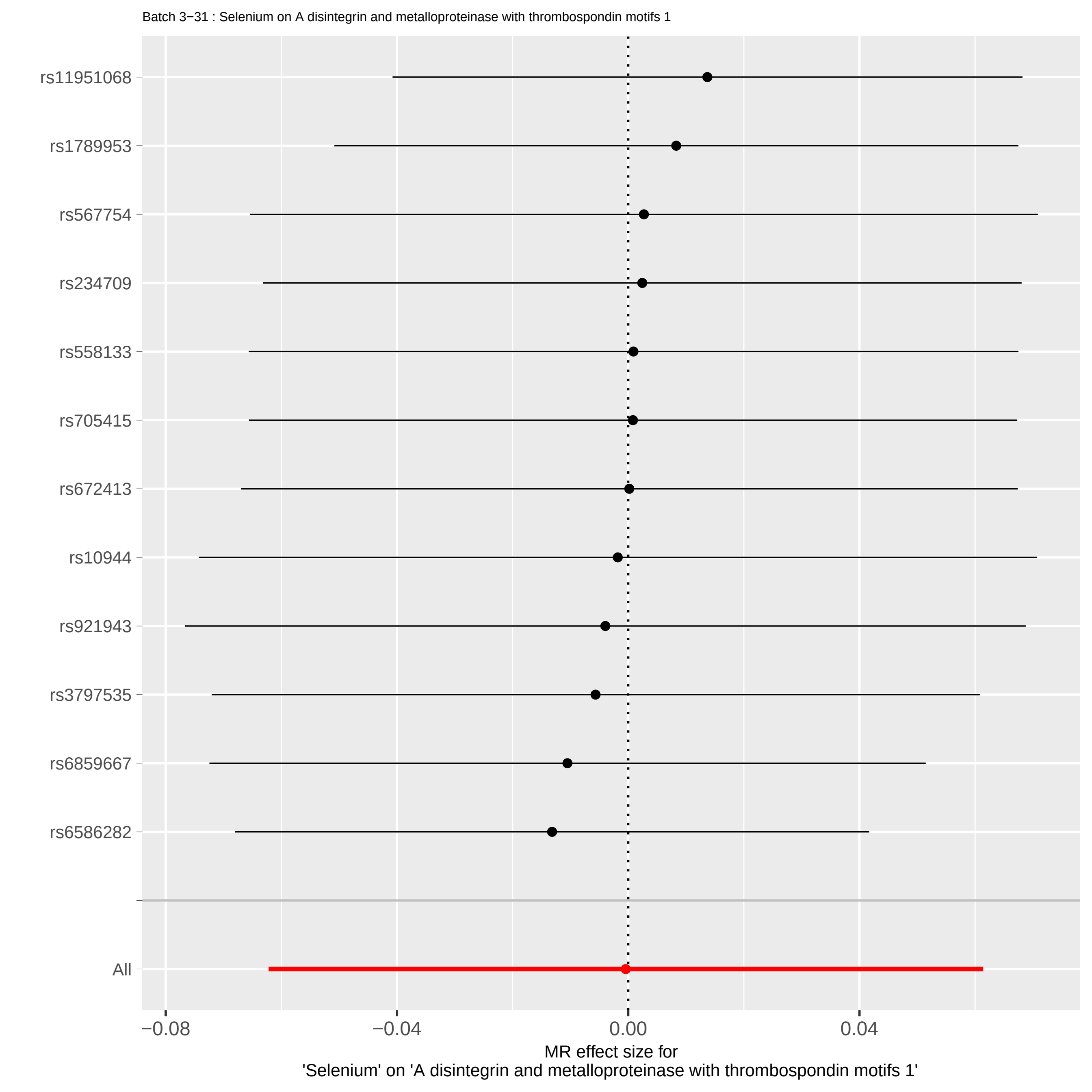
Batch 3−31 : Selenium on A disintegrin and metalloproteinase with thrombospondin motifs 1
rs11951068
rs1789953
rs567754
rs234709
rs558133
rs705415
rs672413
rs10944
rs921943
rs3797535
rs6859667
rs6586282
All
−0.08
−0.04
0.00
0.04
MR effect size for
'Selenium' on 'A disintegrin and metalloproteinase with thrombospondin motifs 1'

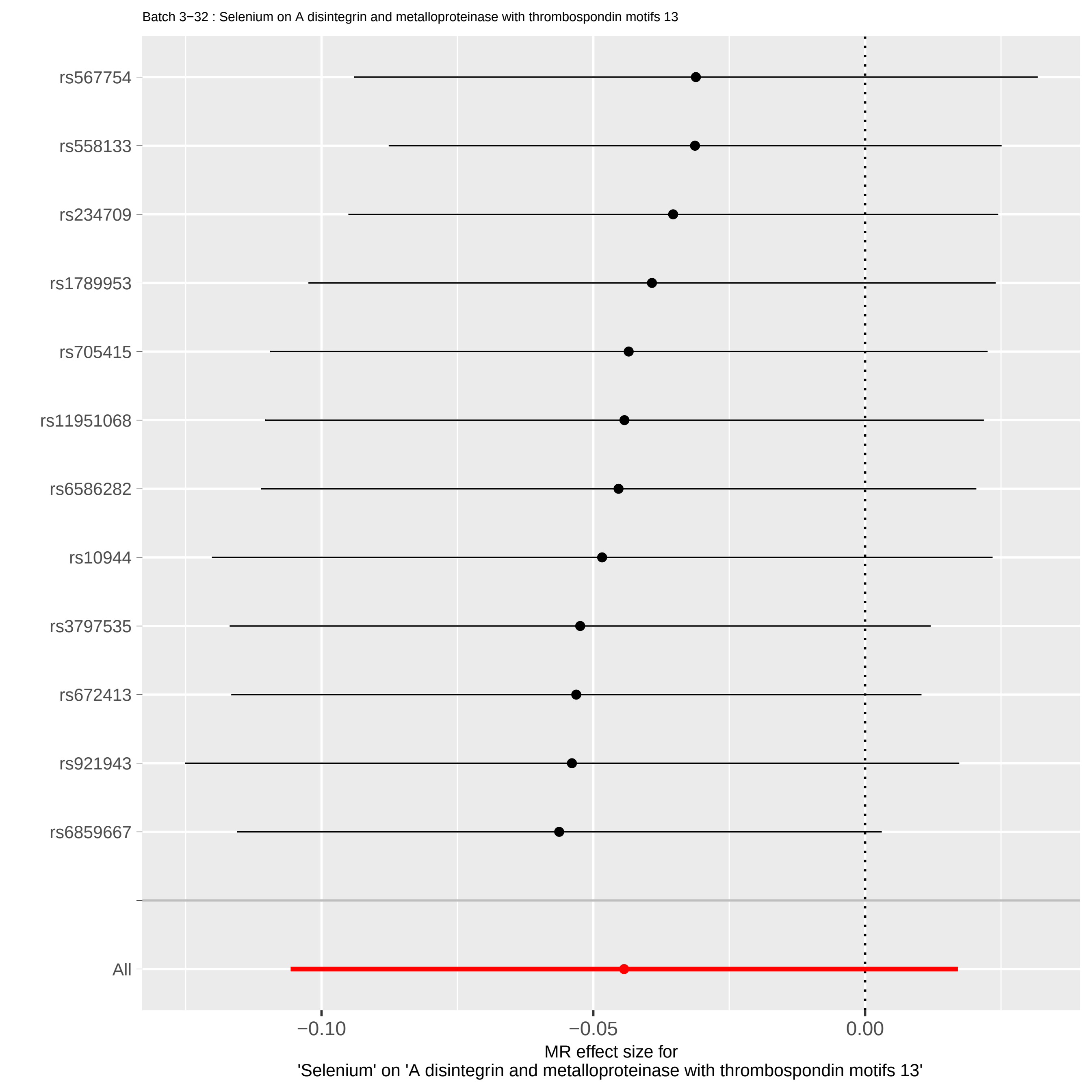

Batch 3−32 : Selenium on A disintegrin and metalloproteinase with thrombospondin motifs 13
rs567754
rs558133
rs234709
rs1789953
rs705415
rs11951068
rs6586282
rs10944
rs3797535
rs672413
rs921943
rs6859667
All
−0.10
−0.05
0.00
MR effect size for
'Selenium' on 'A disintegrin and metalloproteinase with thrombospondin motifs 13'

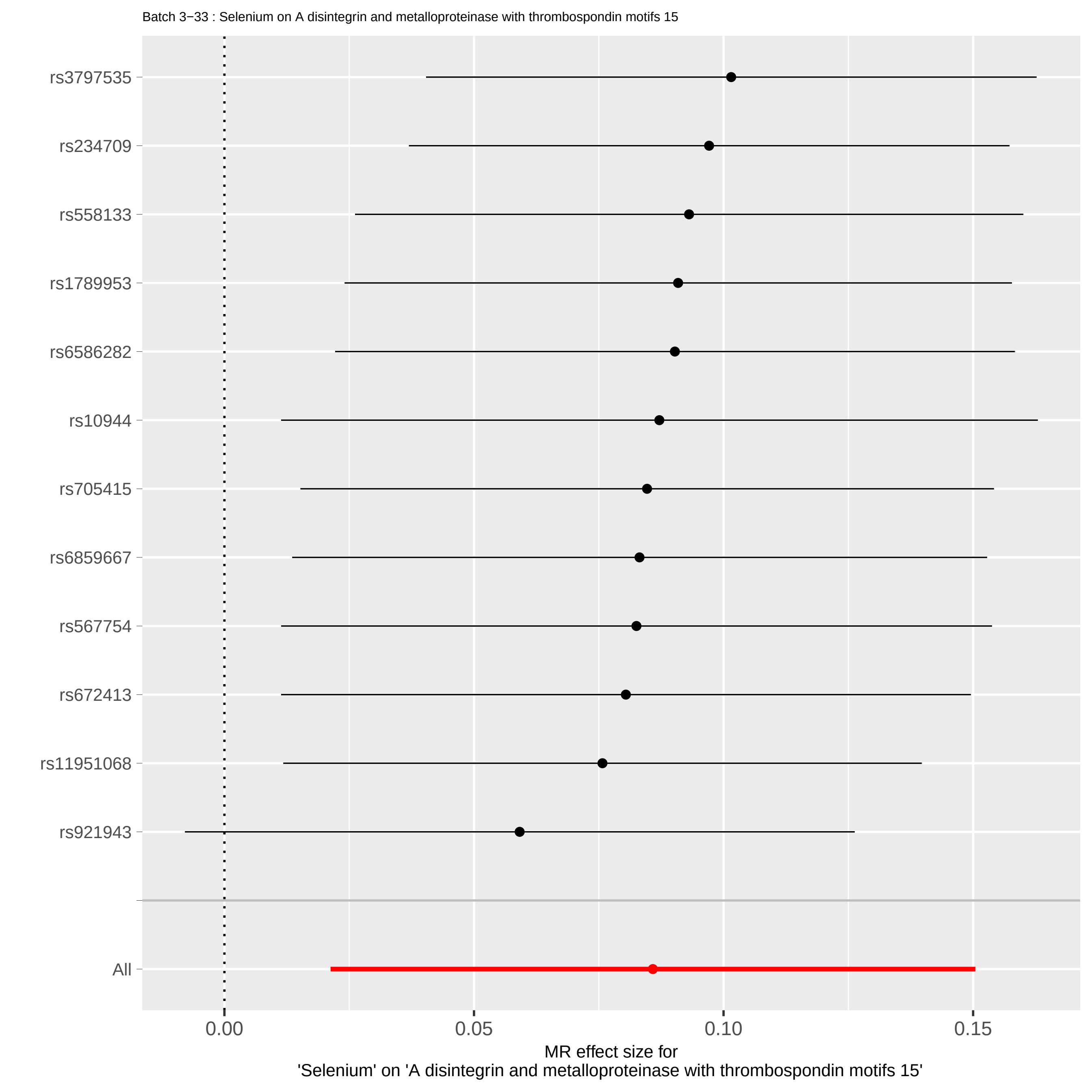
Batch 3−33 : Selenium on A disintegrin and metalloproteinase with thrombospondin motifs 15
rs3797535
rs234709
rs558133
rs1789953
rs6586282
rs10944
rs705415
rs6859667
rs567754
rs672413
rs11951068
rs921943
All
0.00
0.05
0.10
0.15
MR effect size for
'Selenium' on 'A disintegrin and metalloproteinase with thrombospondin motifs 15'

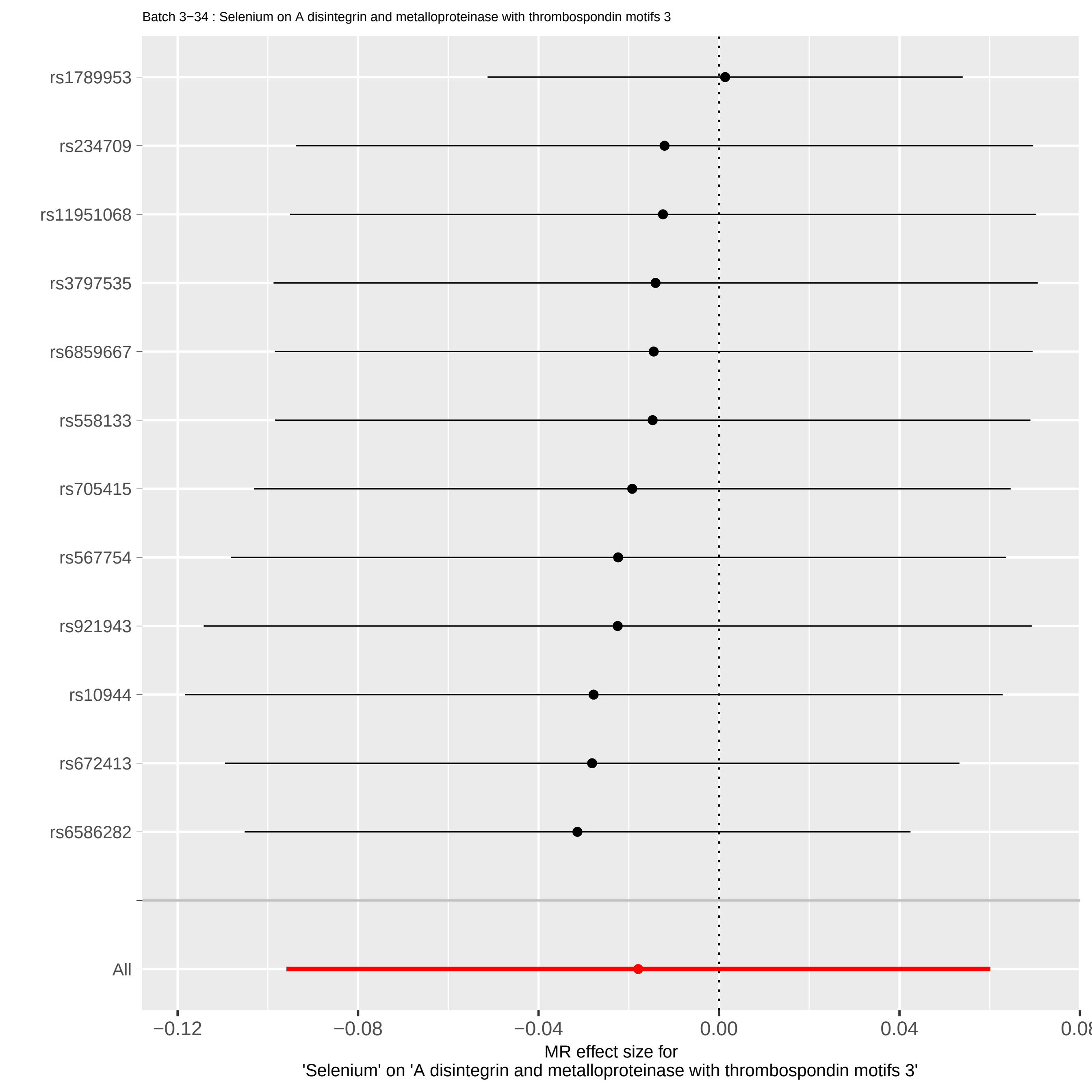

Batch 3−34 : Selenium on A disintegrin and metalloproteinase with thrombospondin motifs 3
rs1789953
rs234709
rs11951068
rs3797535
rs6859667
rs558133
rs705415
rs567754
rs921943
rs10944
rs672413
rs6586282
All
−0.12
−0.08
−0.04
0.00
0.04
0.08
MR effect size for
'Selenium' on 'A disintegrin and metalloproteinase with thrombospondin motifs 3'

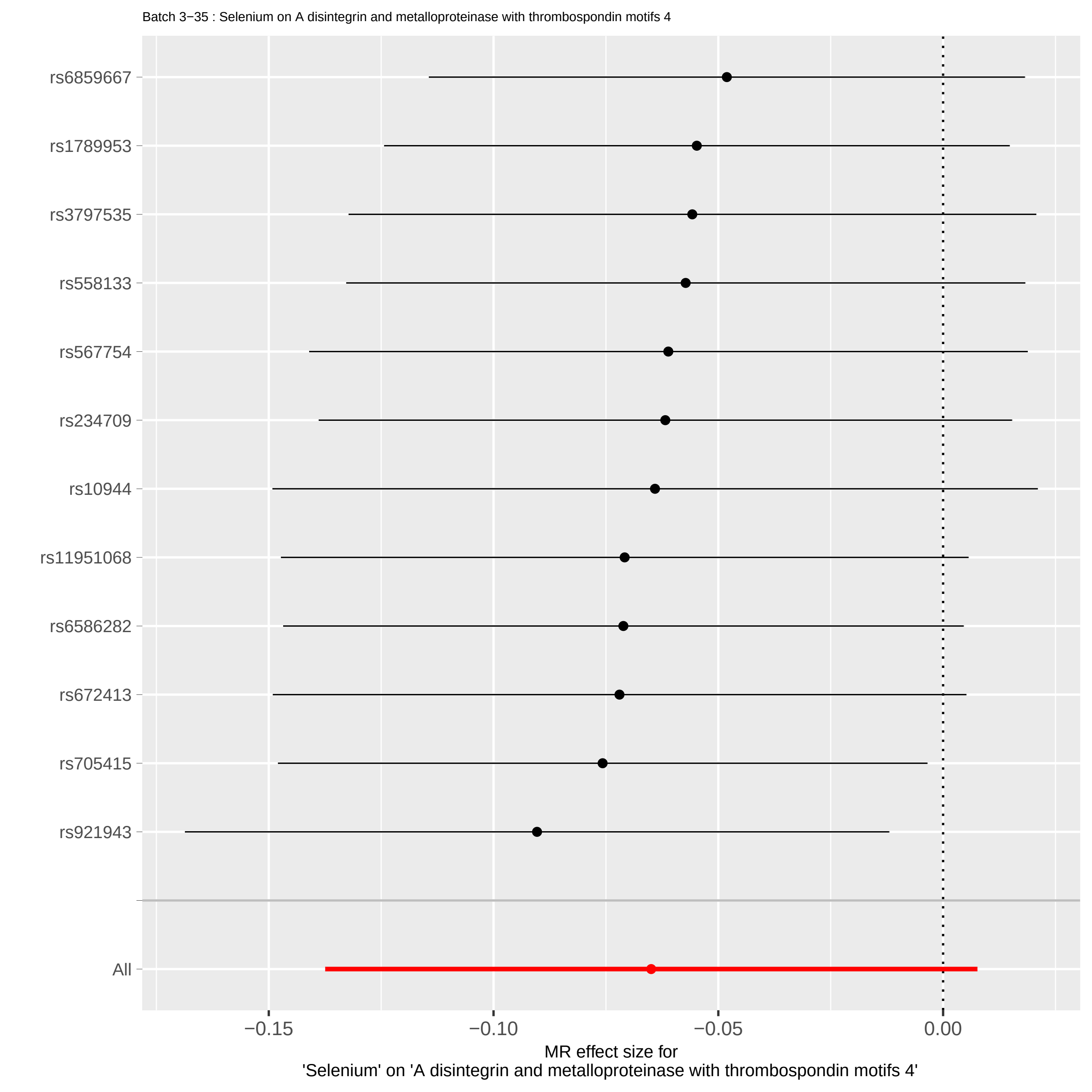

Batch 3−35 : Selenium on A disintegrin and metalloproteinase with thrombospondin motifs 4
rs6859667
rs1789953
rs3797535
rs558133
rs567754
rs234709
rs10944
rs11951068
rs6586282
rs672413
rs705415
rs921943
All
−0.15
−0.10
−0.05
0.00
MR effect size for
'Selenium' on 'A disintegrin and metalloproteinase with thrombospondin motifs 4'

Batch 3−36 : Selenium on A disintegrin and metalloproteinase with thrombospondin motifs 5

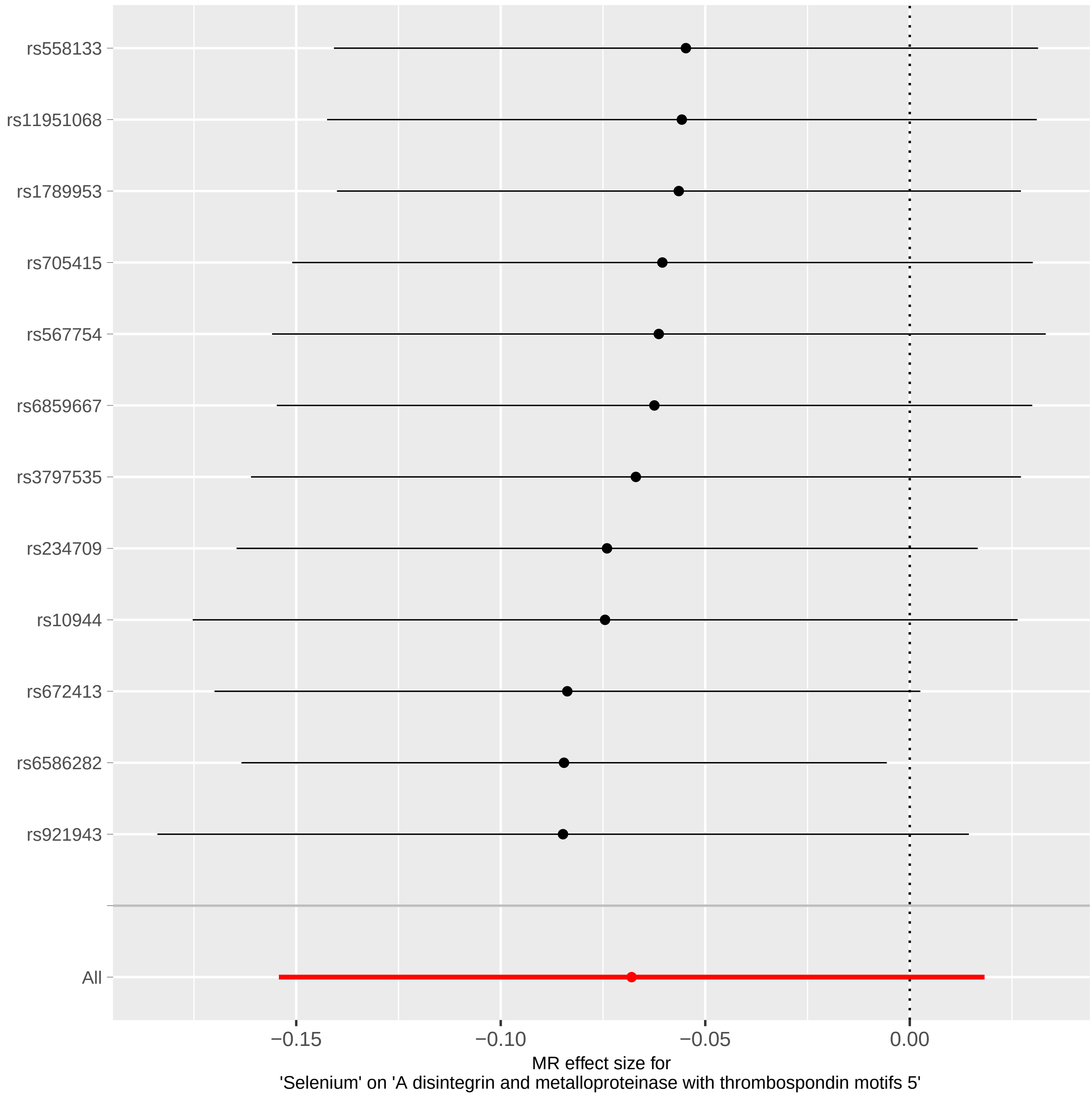

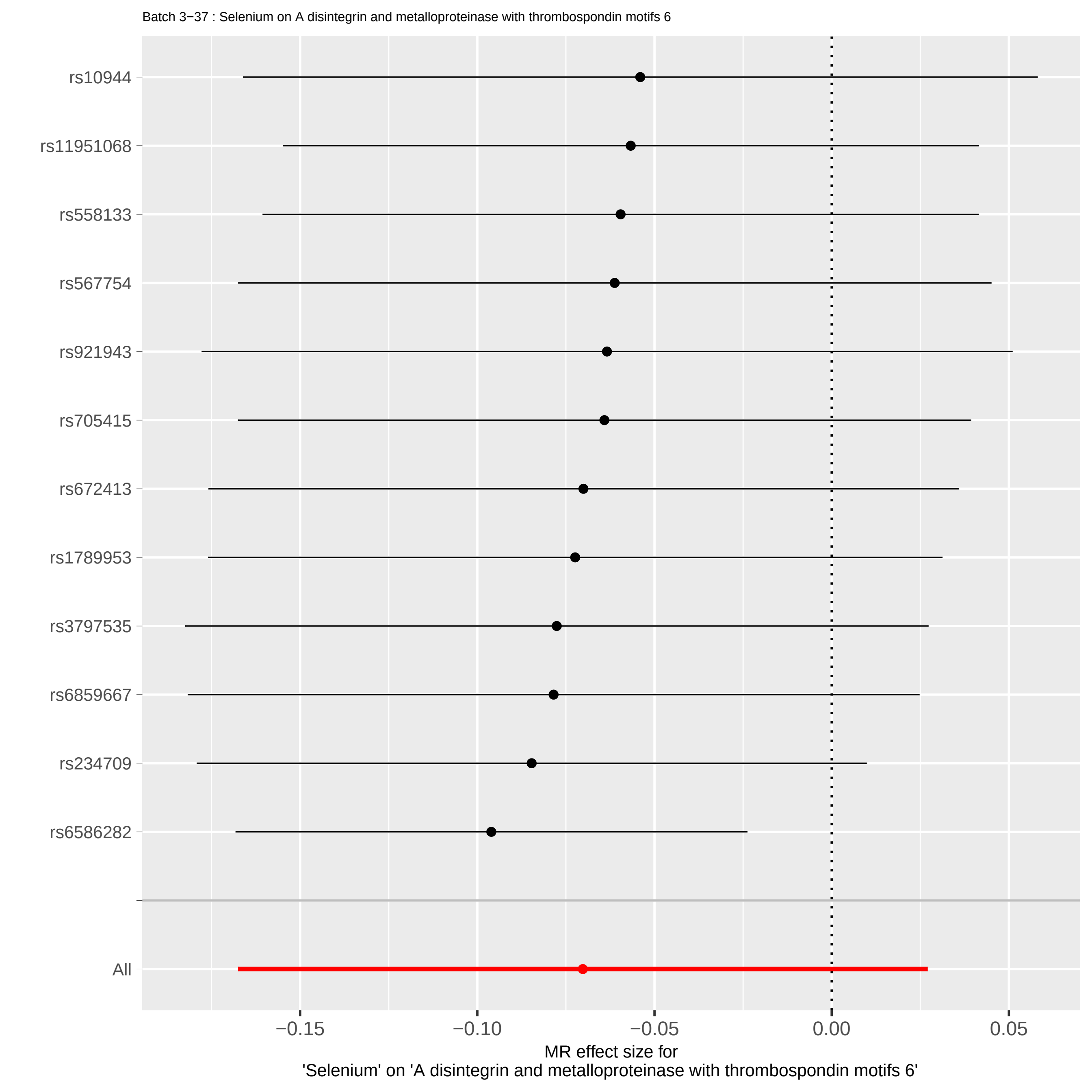

Batch 3−37 : Selenium on A disintegrin and metalloproteinase with thrombospondin motifs 6
rs10944
rs11951068
rs558133
rs567754
rs921943
rs705415
rs672413
rs1789953
rs3797535
rs6859667
rs234709
rs6586282
All
−0.15
−0.10
−0.05
0.00
0.05
MR effect size for
'Selenium' on 'A disintegrin and metalloproteinase with thrombospondin motifs 6'

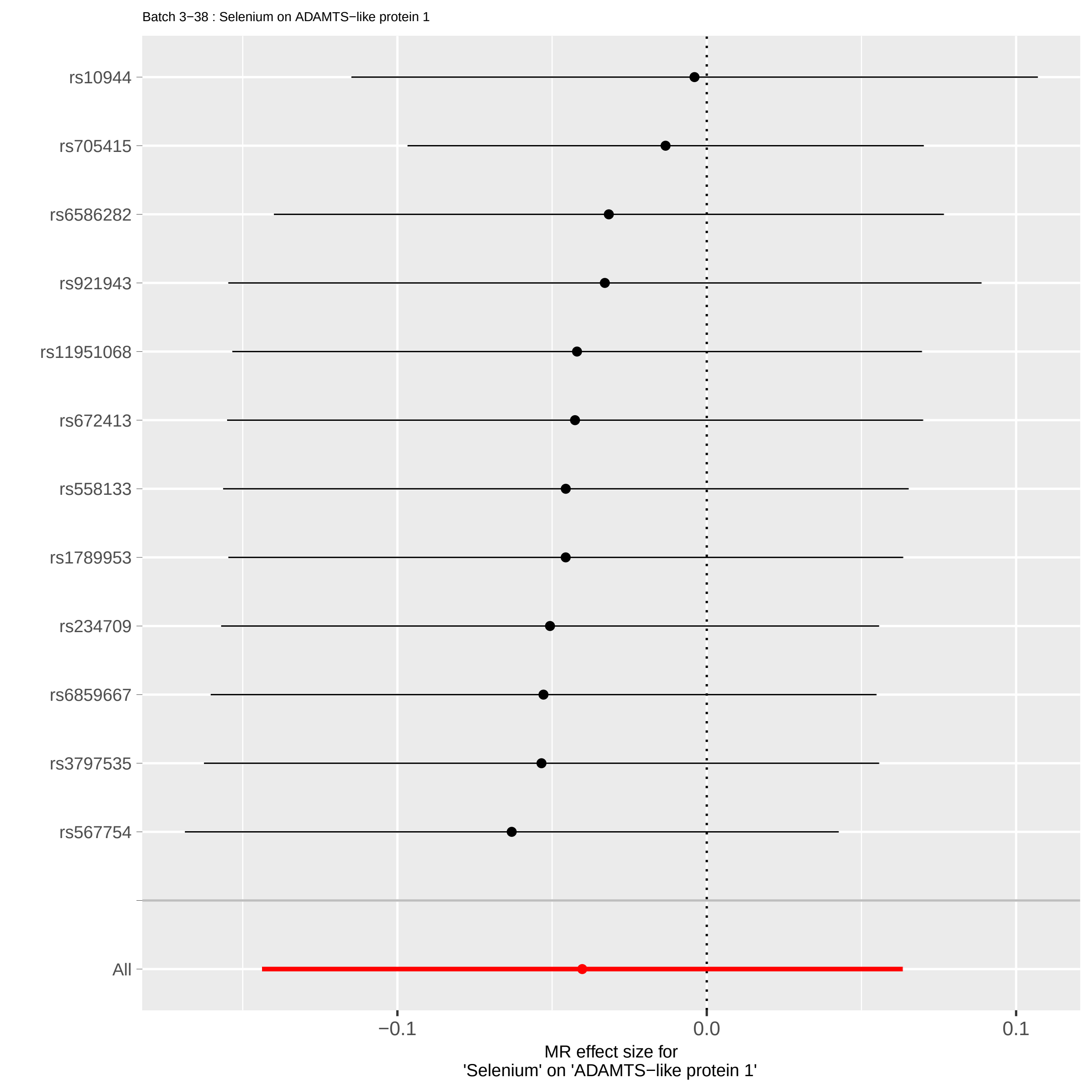

Batch 3−38 : Selenium on ADAMTS−like protein 1
rs10944
rs705415
rs6586282
rs921943
rs11951068
rs672413
rs558133
rs1789953
rs234709
rs6859667
rs3797535
rs567754
All
−0.1
0.0
0.1
MR effect size for
'Selenium' on 'ADAMTS−like protein 1'

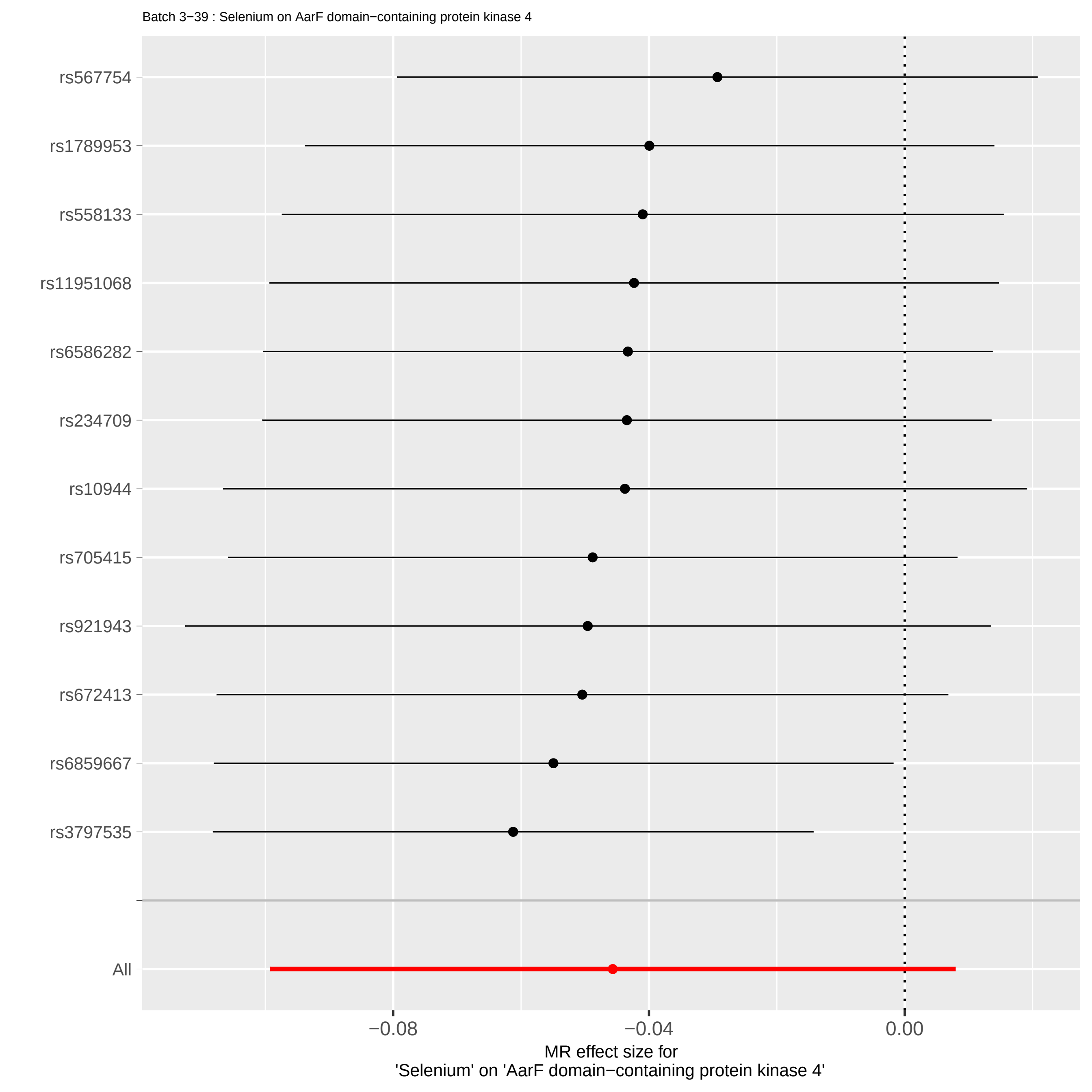

Batch 3−39 : Selenium on AarF domain−containing protein kinase 4
rs567754
rs1789953
rs558133
rs11951068
rs6586282
rs234709
rs10944
rs705415
rs921943
rs672413
rs6859667
rs3797535
All
−0.08
−0.04
0.00
MR effect size for
'Selenium' on 'AarF domain−containing protein kinase 4'

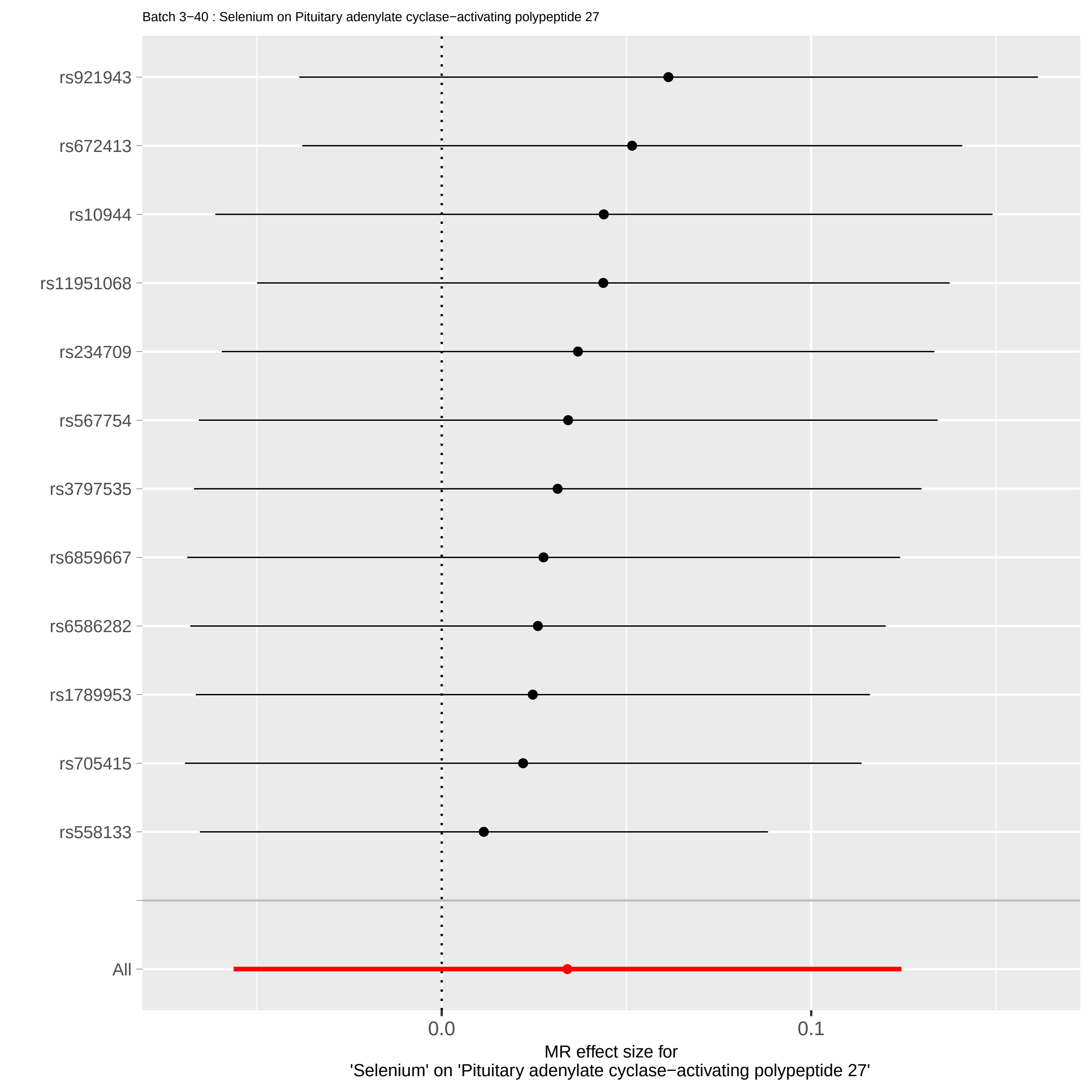

Batch 3−40 : Selenium on Pituitary adenylate cyclase−activating polypeptide 27
rs921943
rs672413
rs10944
rs11951068
rs234709
rs567754
rs3797535
rs6859667
rs6586282
rs1789953
rs705415
rs558133
All
0.0
0.1
MR effect size for
'Selenium' on 'Pituitary adenylate cyclase−activating polypeptide 27'

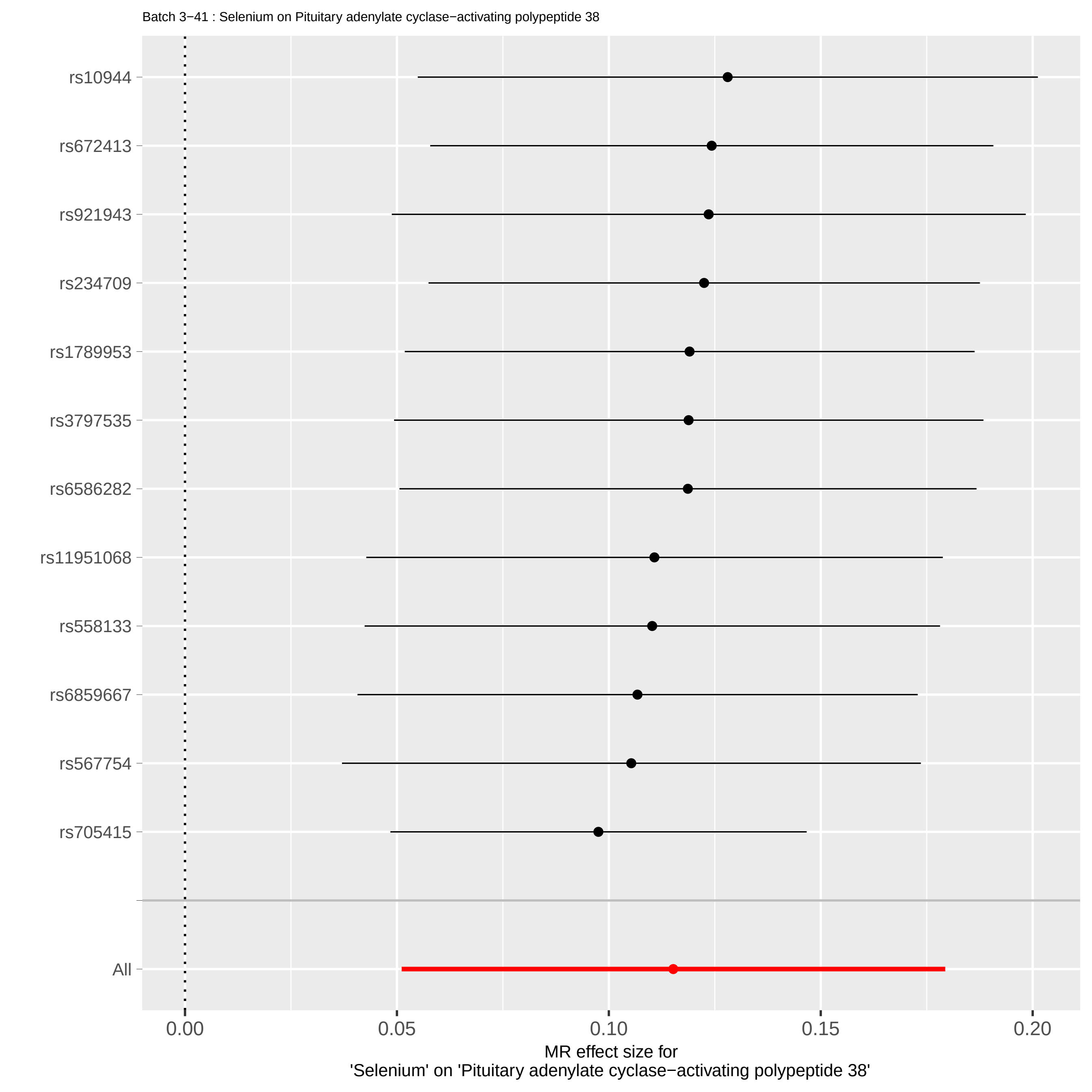
Batch 3−41 : Selenium on Pituitary adenylate cyclase−activating polypeptide 38
rs10944
rs672413
rs921943
rs234709
rs1789953
rs3797535
rs6586282
rs11951068
rs558133
rs6859667
rs567754
rs705415
All
0.00
0.05
0.10
0.15
0.20
MR effect size for
'Selenium' on 'Pituitary adenylate cyclase−activating polypeptide 38'

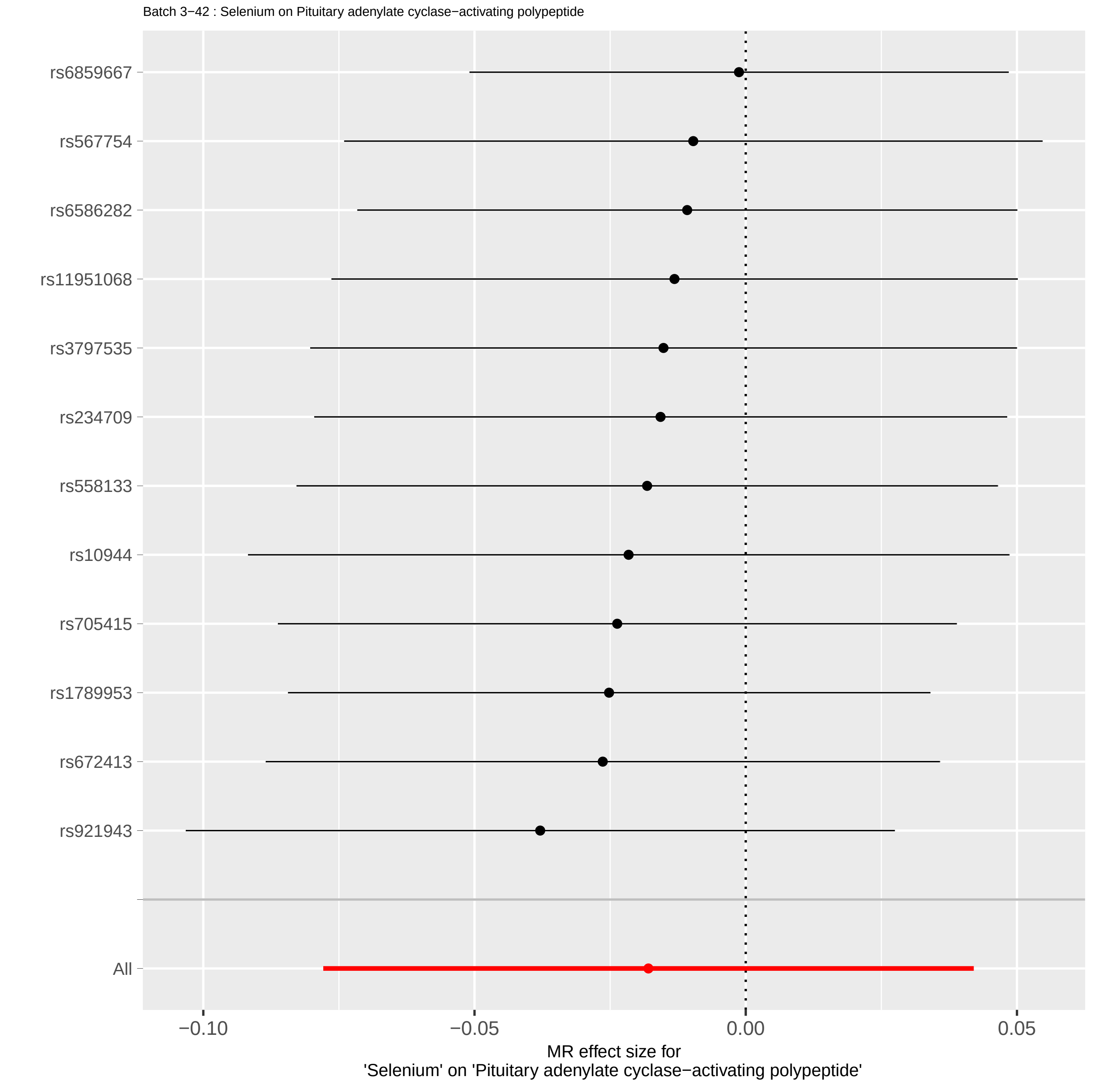
Batch 3−42 : Selenium on Pituitary adenylate cyclase−activating polypeptide
rs6859667
rs567754
rs6586282
rs11951068
rs3797535
rs234709
rs558133
rs10944
rs705415
rs1789953
rs672413
rs921943
All
−0.10
−0.05
0.00
0.05
MR effect size for
'Selenium' on 'Pituitary adenylate cyclase−activating polypeptide'

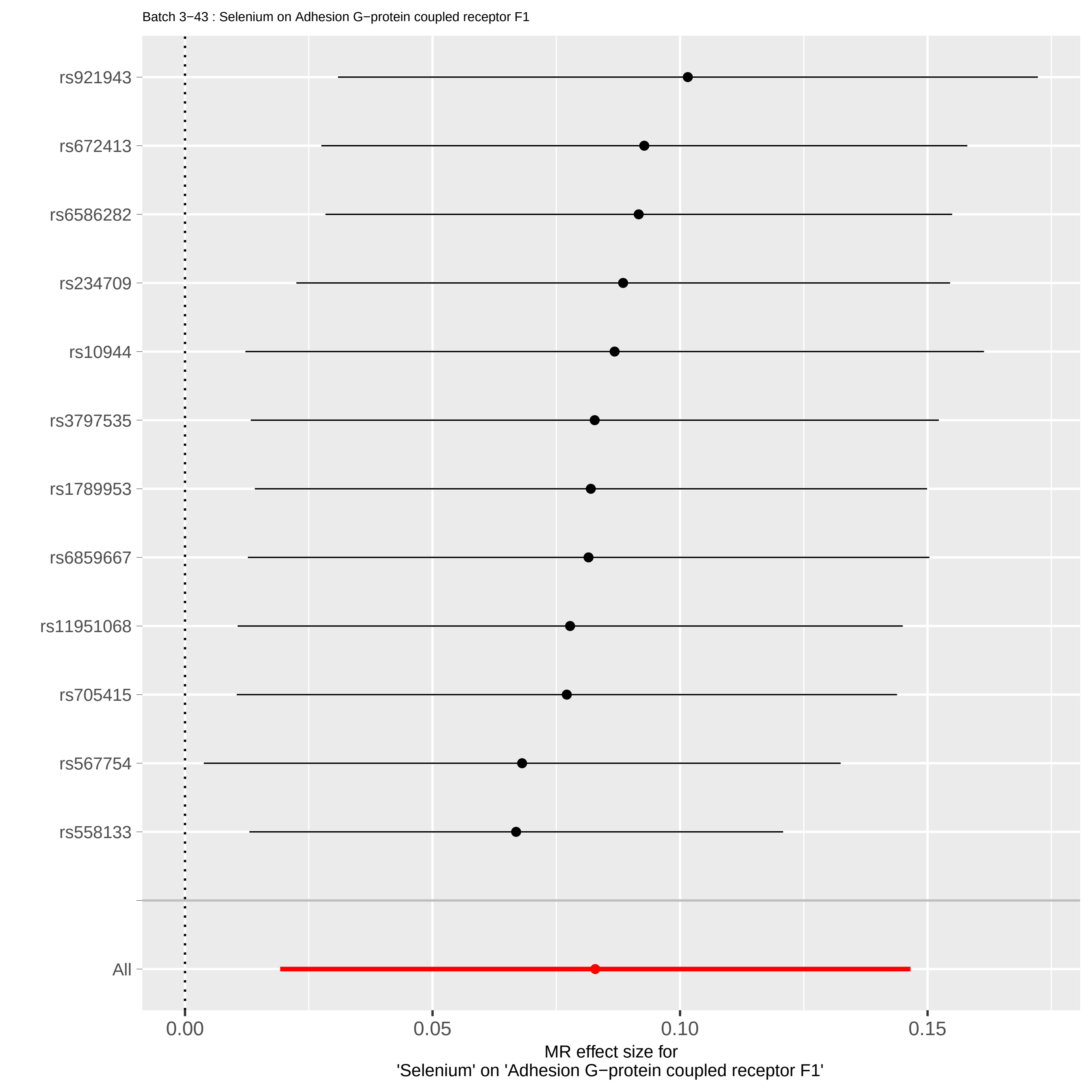

Batch 3−43 : Selenium on Adhesion G−protein coupled receptor F1
rs921943
rs672413
rs6586282
rs234709
rs10944
rs3797535
rs1789953
rs6859667
rs11951068
rs705415
rs567754
rs558133
All
0.00
0.05
0.10
0.15
MR effect size for
'Selenium' on 'Adhesion G−protein coupled receptor F1'

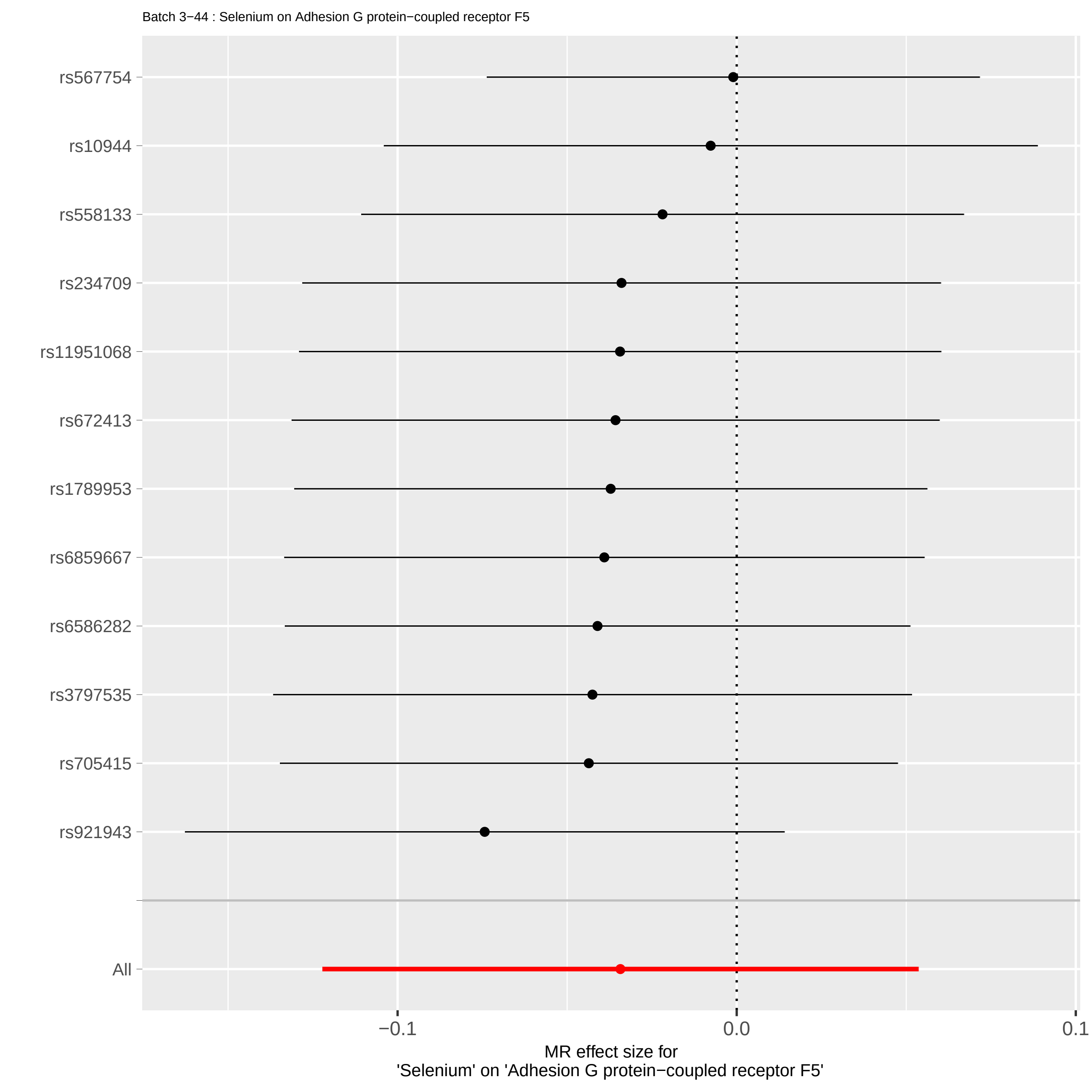

Batch 3−44 : Selenium on Adhesion G protein−coupled receptor F5
rs567754
rs10944
rs558133
rs234709
rs11951068
rs672413
rs1789953
rs6859667
rs6586282
rs3797535
rs705415
rs921943
All
−0.1
0.0
0.1
MR effect size for
'Selenium' on 'Adhesion G protein−coupled receptor F5'

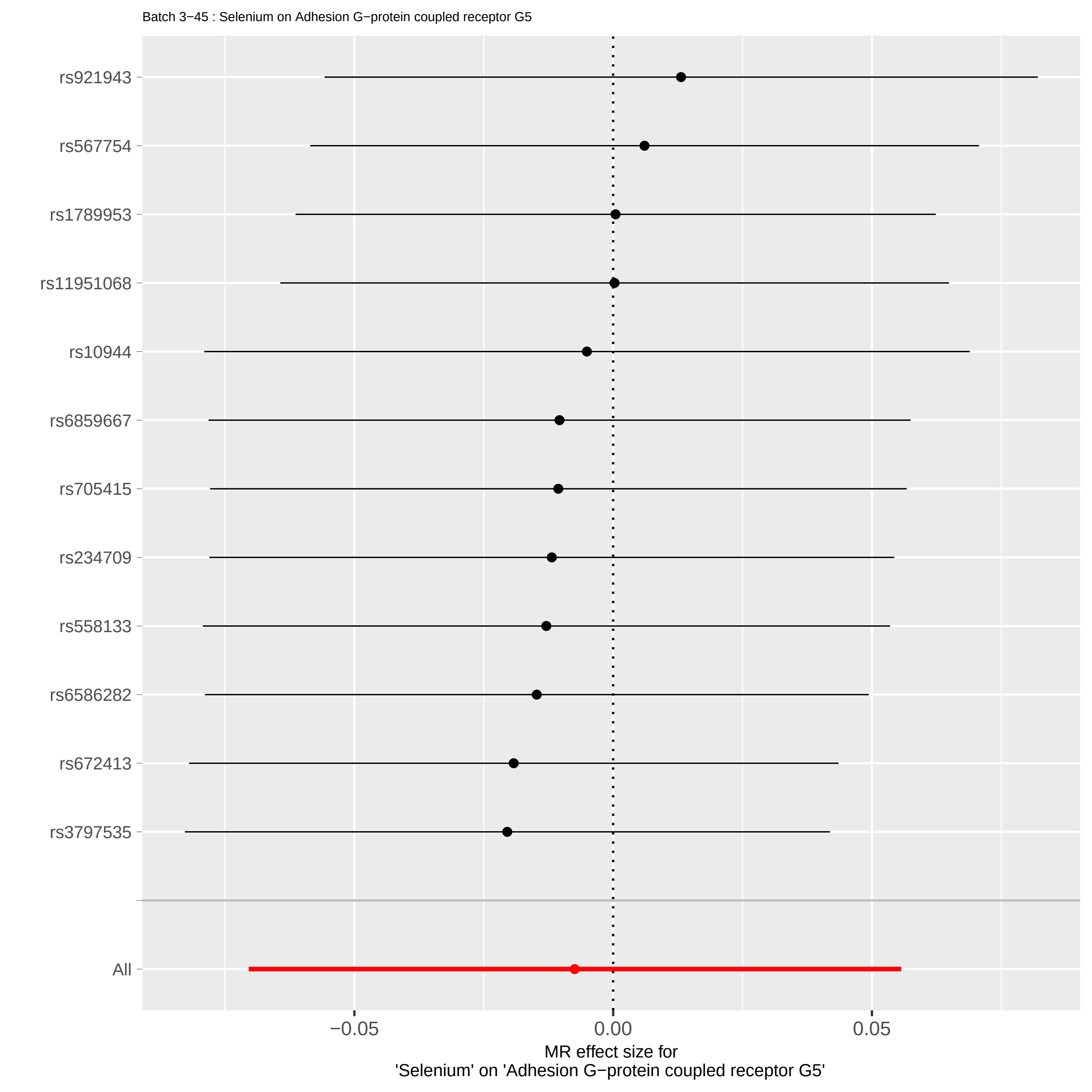

Batch 3−45 : Selenium on Adhesion G−protein coupled receptor G5
rs921943
rs567754
rs1789953
rs11951068
rs10944
rs6859667
rs705415
rs234709
rs558133
rs6586282
rs672413
rs3797535
All
−0.05
0.00
0.05
MR effect size for
'Selenium' on 'Adhesion G−protein coupled receptor G5'

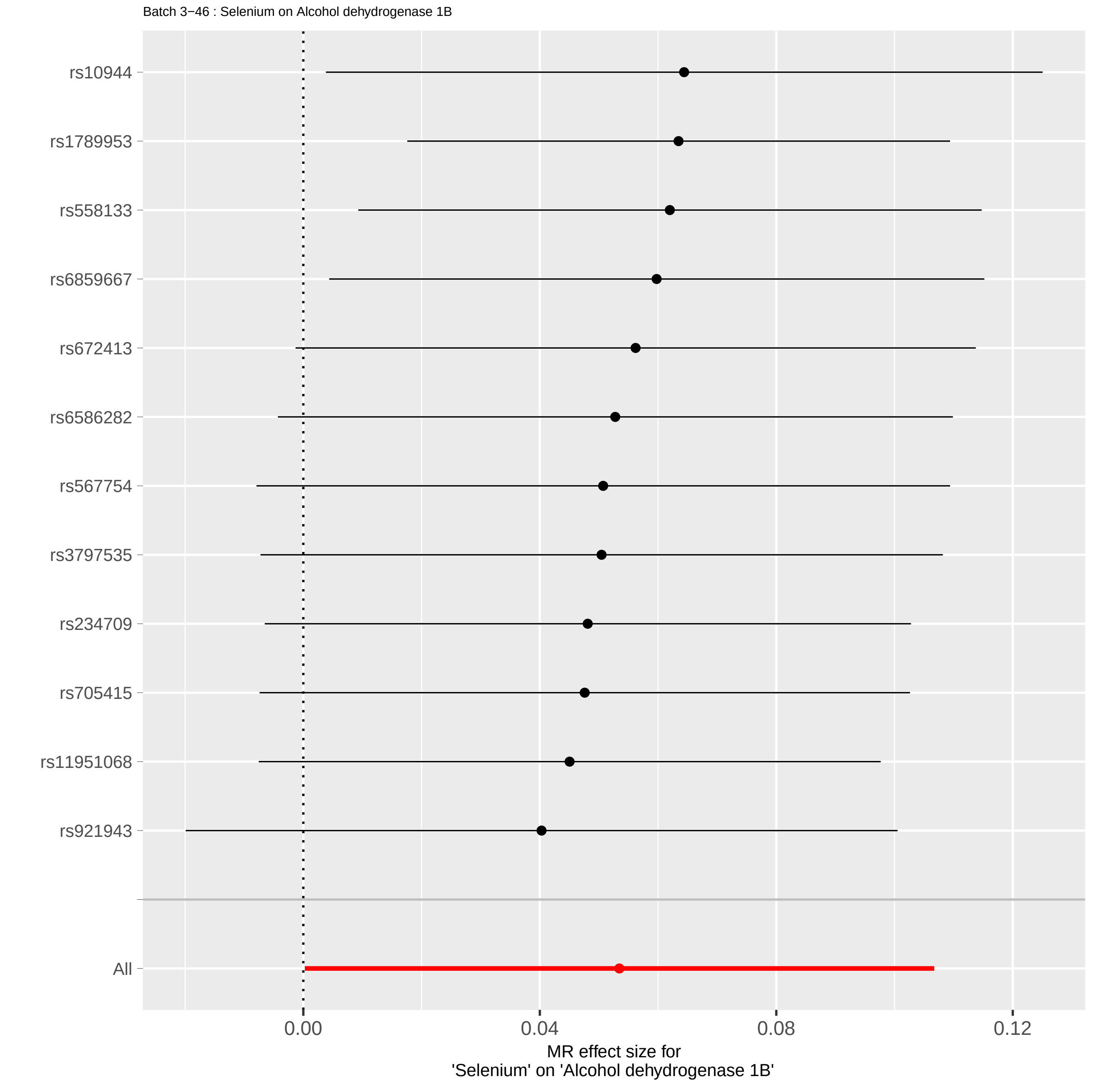
Batch 3−46 : Selenium on Alcohol dehydrogenase 1B
rs10944
rs1789953
rs558133
rs6859667
rs672413
rs6586282
rs567754
rs3797535
rs234709
rs705415
rs11951068
rs921943
All
0.00
0.04
0.08
0.12
MR effect size for
'Selenium' on 'Alcohol dehydrogenase 1B'

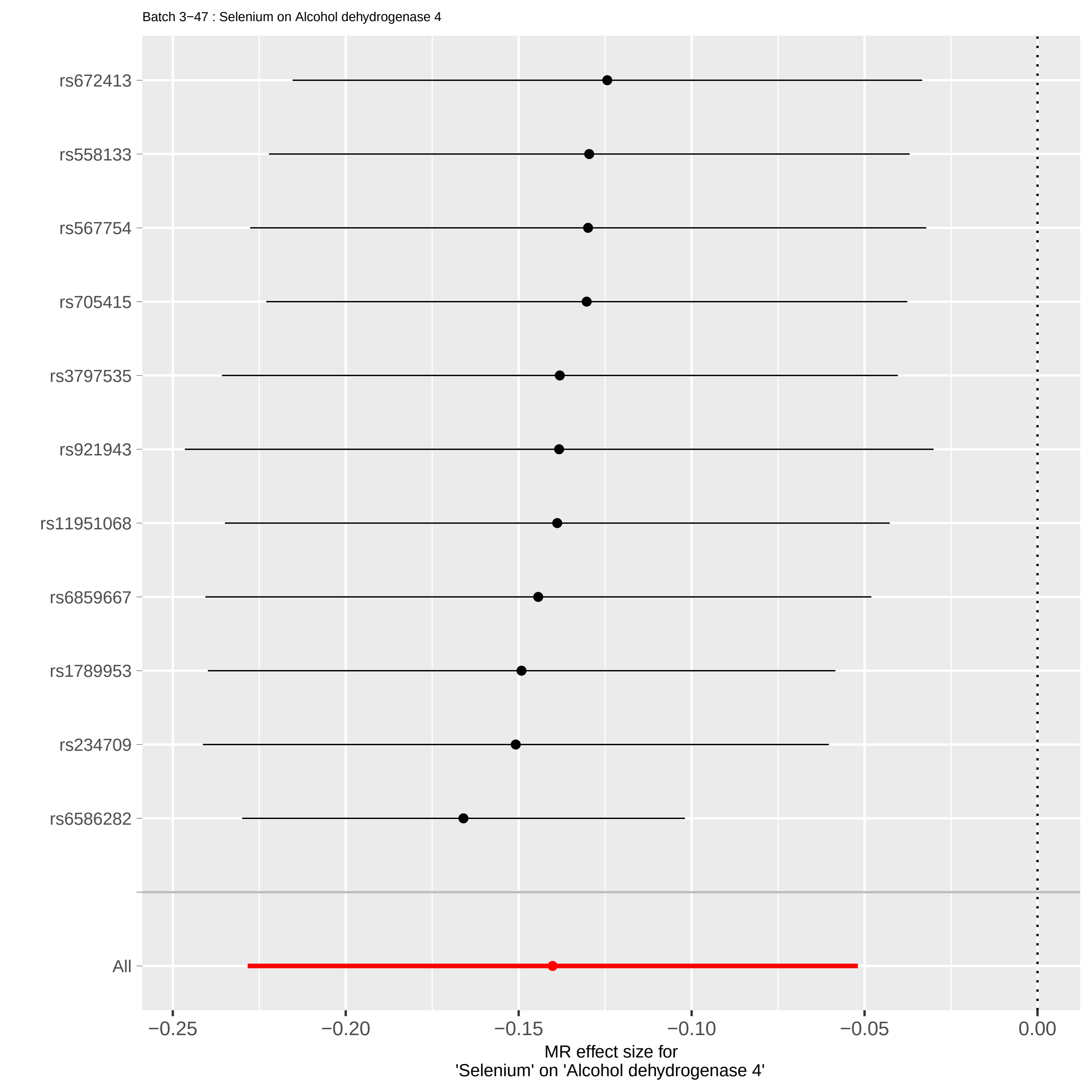

Batch 3−47 : Selenium on Alcohol dehydrogenase 4
rs672413
rs558133
rs567754
rs705415
rs3797535
rs921943
rs11951068
rs6859667
rs1789953
rs234709
rs6586282
All
−0.25
−0.20
−0.15
−0.10
−0.05
0.00
MR effect size for
'Selenium' on 'Alcohol dehydrogenase 4'

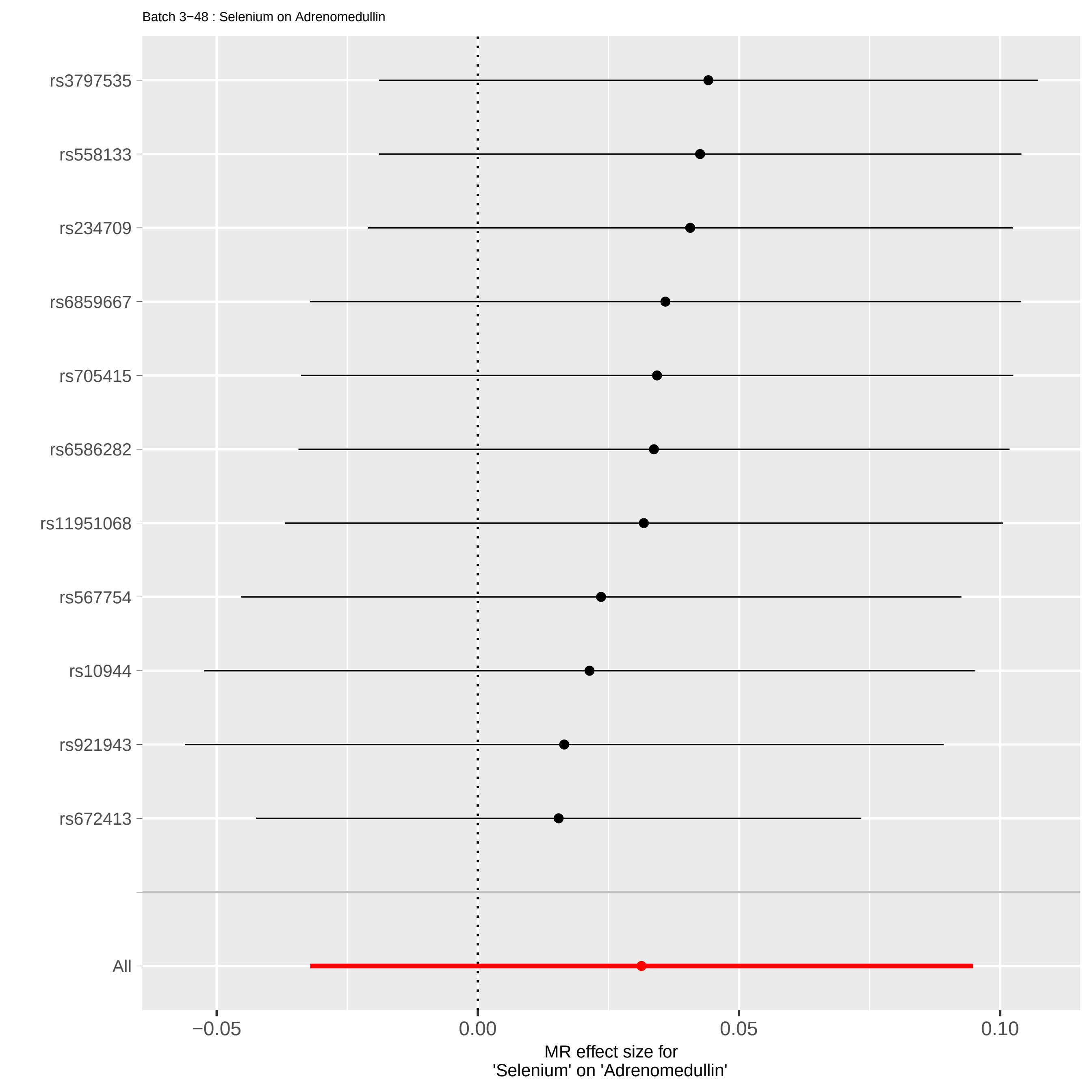

Batch 3−48 : Selenium on Adrenomedullin
rs3797535
rs558133
rs234709
rs6859667
rs705415
rs6586282
rs11951068
rs567754
rs10944
rs921943
rs672413
All
−0.05
0.00
0.05
0.10
MR effect size for
'Selenium' on 'Adrenomedullin'

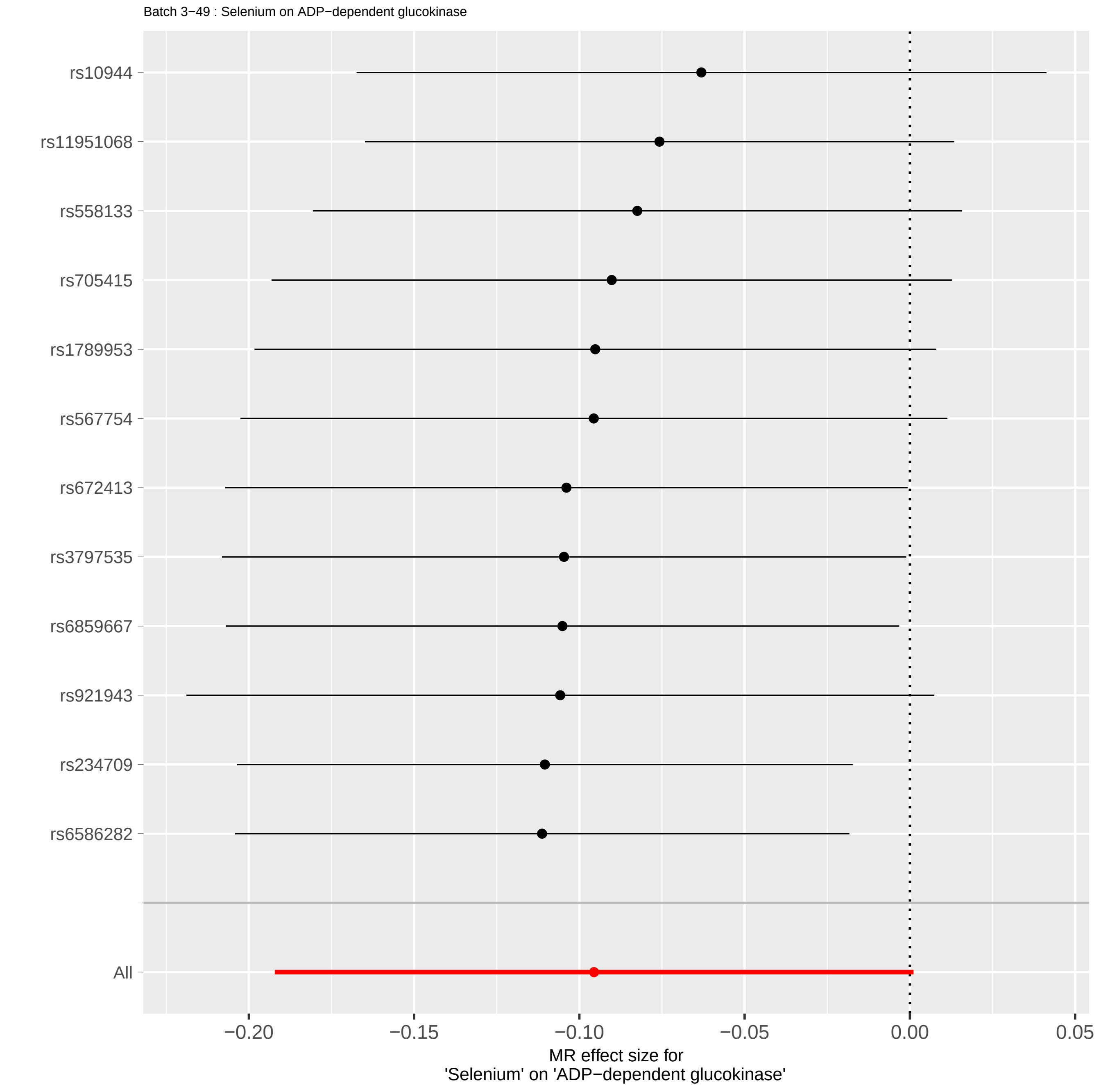

Batch 3−49 : Selenium on ADP−dependent glucokinase
rs10944
rs11951068
rs558133
rs705415
rs1789953
rs567754
rs672413
rs3797535
rs6859667
rs921943
rs234709
rs6586282
All
−0.20
−0.15
−0.10
−0.05
0.00
0.05
MR effect size for
'Selenium' on 'ADP−dependent glucokinase'

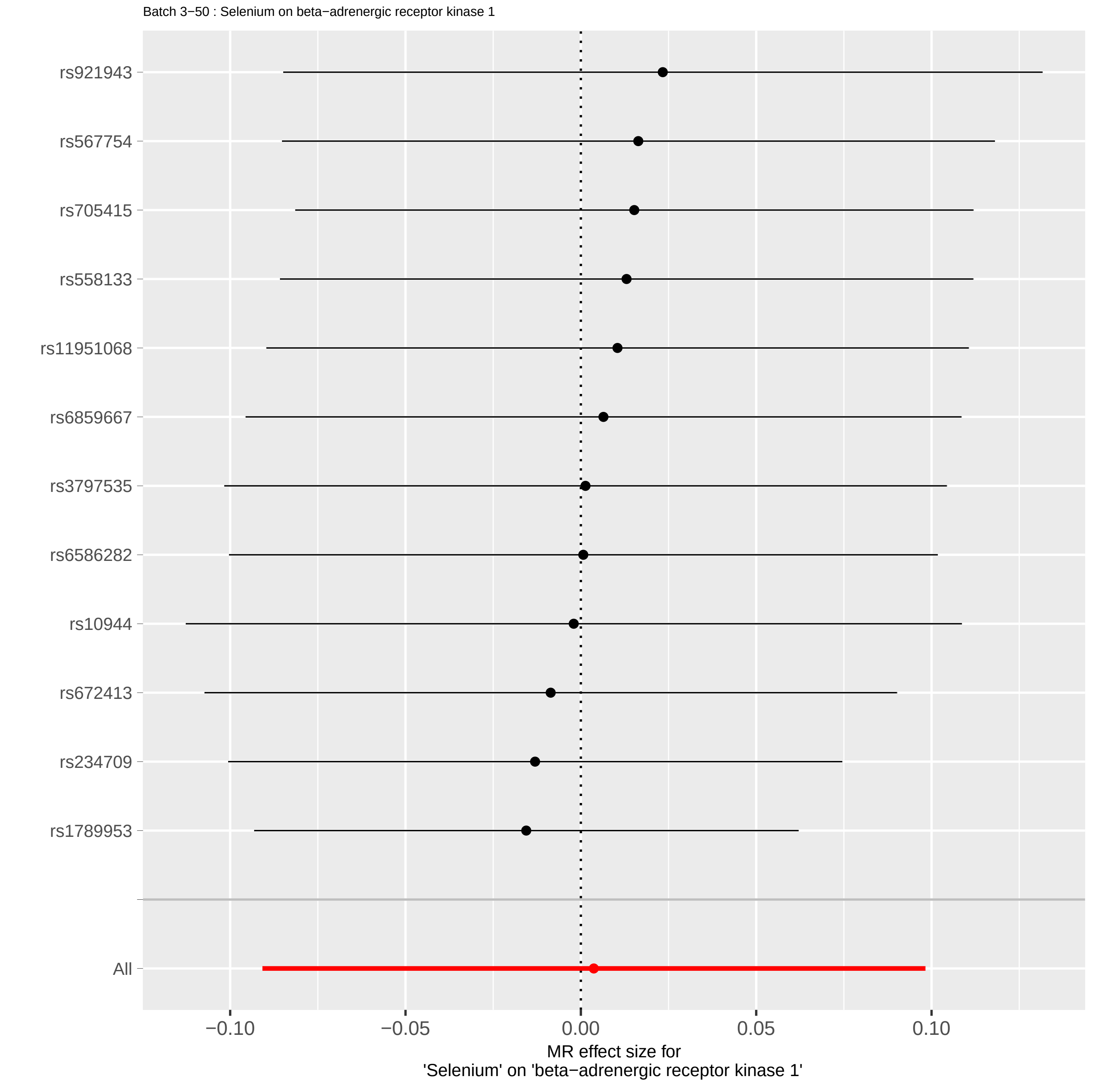

Batch 3−50 : Selenium on beta−adrenergic receptor kinase 1
rs921943
rs567754
rs705415
rs558133
rs11951068
rs6859667
rs3797535
rs6586282
rs10944
rs672413
rs234709
rs1789953
All
−0.10
−0.05
0.00
0.05
0.10
MR effect size for
'Selenium' on 'beta−adrenergic receptor kinase 1'

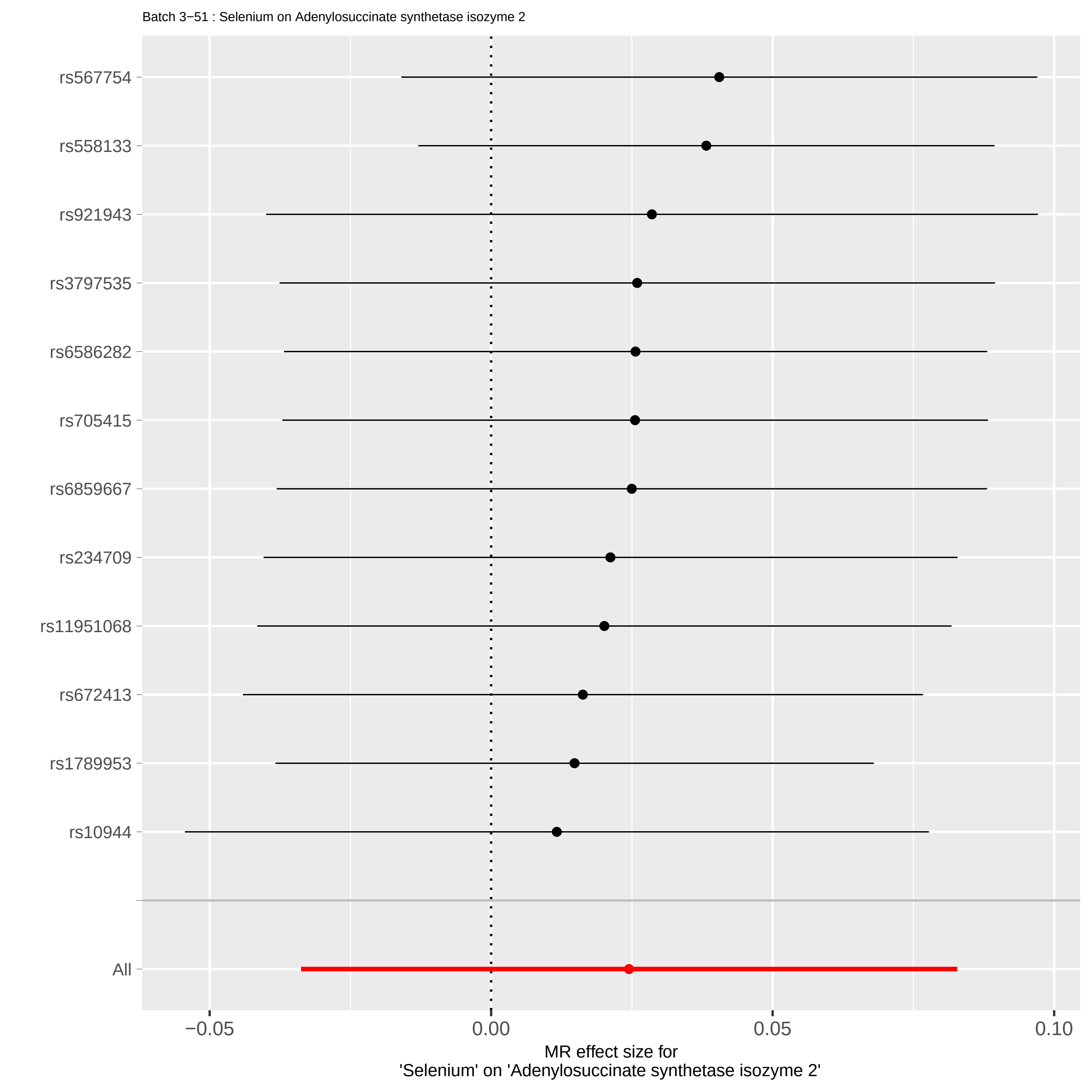

Batch 3−51 : Selenium on Adenylosuccinate synthetase isozyme 2
rs567754
rs558133
rs921943
rs3797535
rs6586282
rs705415
rs6859667
rs234709
rs11951068
rs672413
rs1789953
rs10944
All
−0.05
0.00
0.05
0.10
MR effect size for
'Selenium' on 'Adenylosuccinate synthetase isozyme 2'

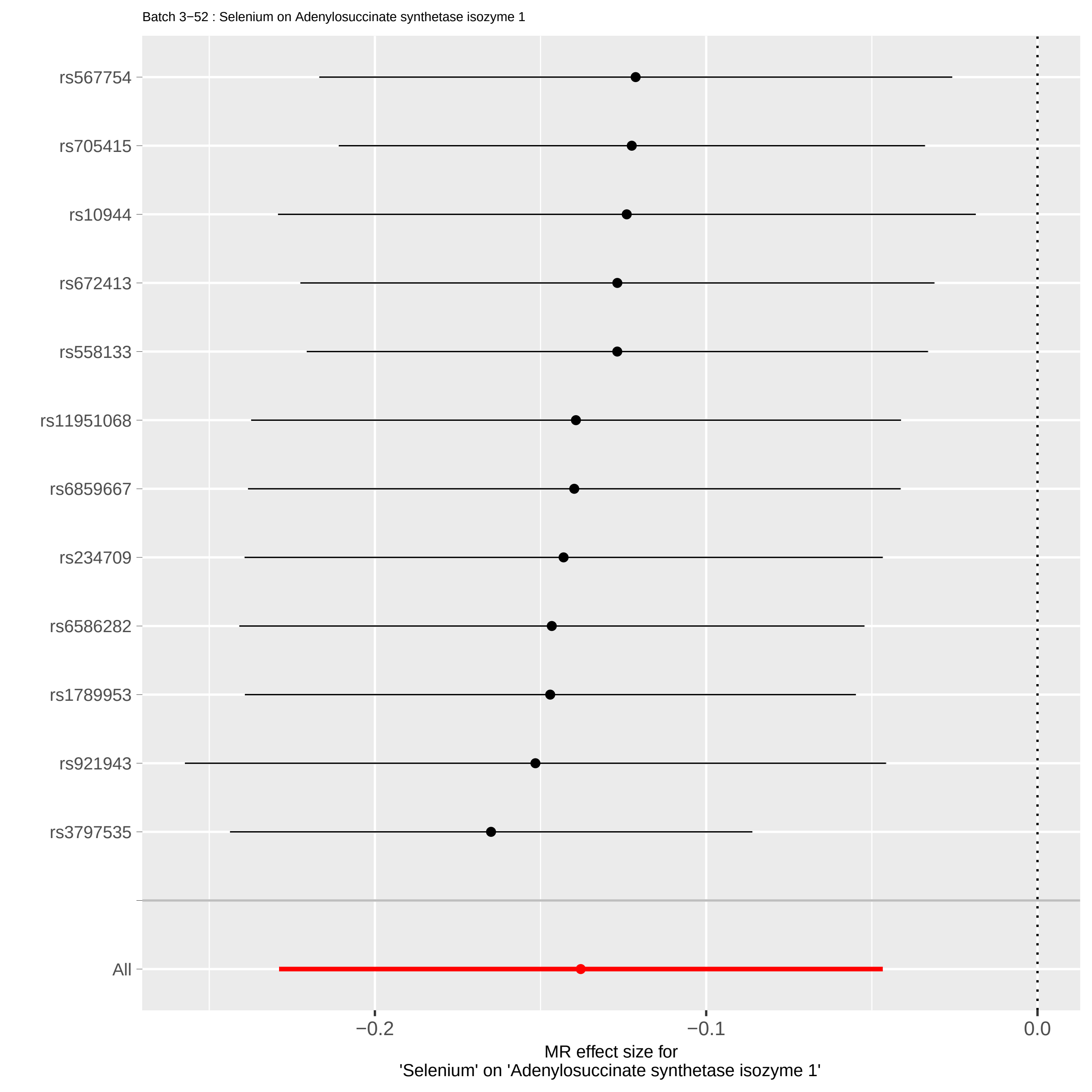

Batch 3−52 : Selenium on Adenylosuccinate synthetase isozyme 1
rs567754
rs705415
rs10944
rs672413
rs558133
rs11951068
rs6859667
rs234709
rs6586282
rs1789953
rs921943
rs3797535
All
−0.2
−0.1
0.0
MR effect size for
'Selenium' on 'Adenylosuccinate synthetase isozyme 1'

Batch 3−53 : Selenium on alpha−Fetoprotein

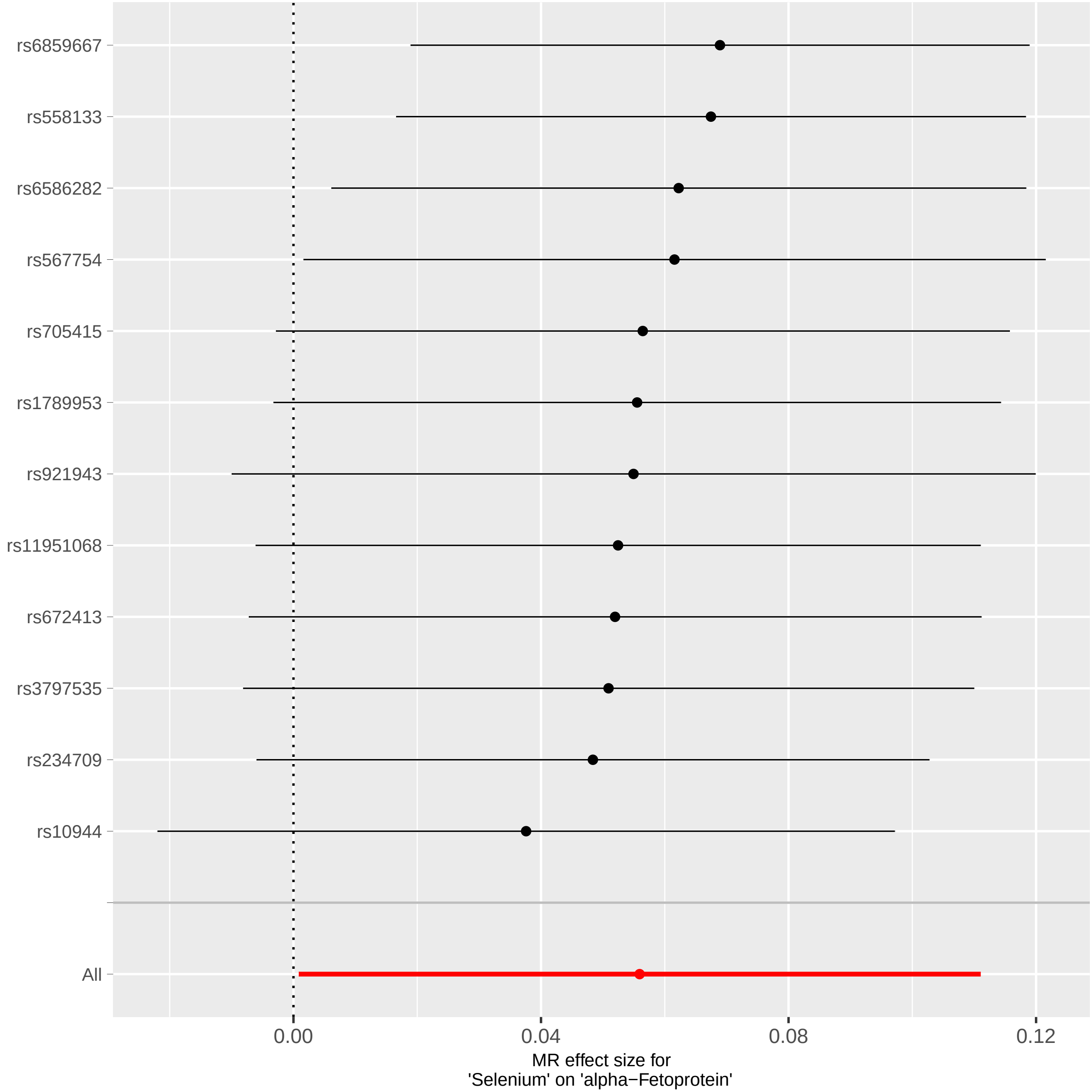

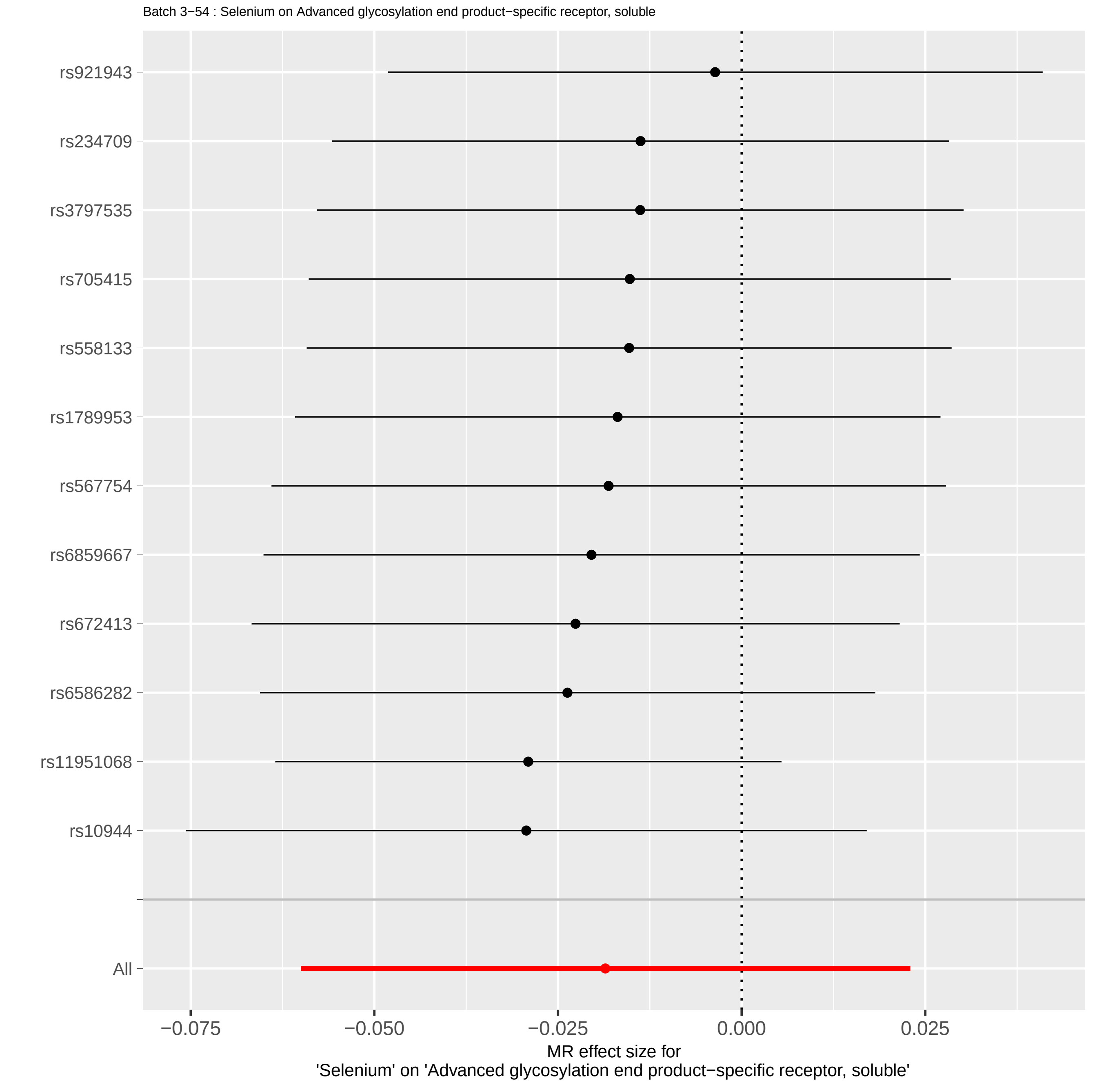

Batch 3−54 : Selenium on Advanced glycosylation end product−specific receptor, soluble
rs921943
rs234709
rs3797535
rs705415
rs558133
rs1789953
rs567754
rs6859667
rs672413
rs6586282
rs11951068
rs10944
All
−0.075
−0.050
−0.025
0.000
0.025
MR effect size for
'Selenium' on 'Advanced glycosylation end product−specific receptor, soluble'

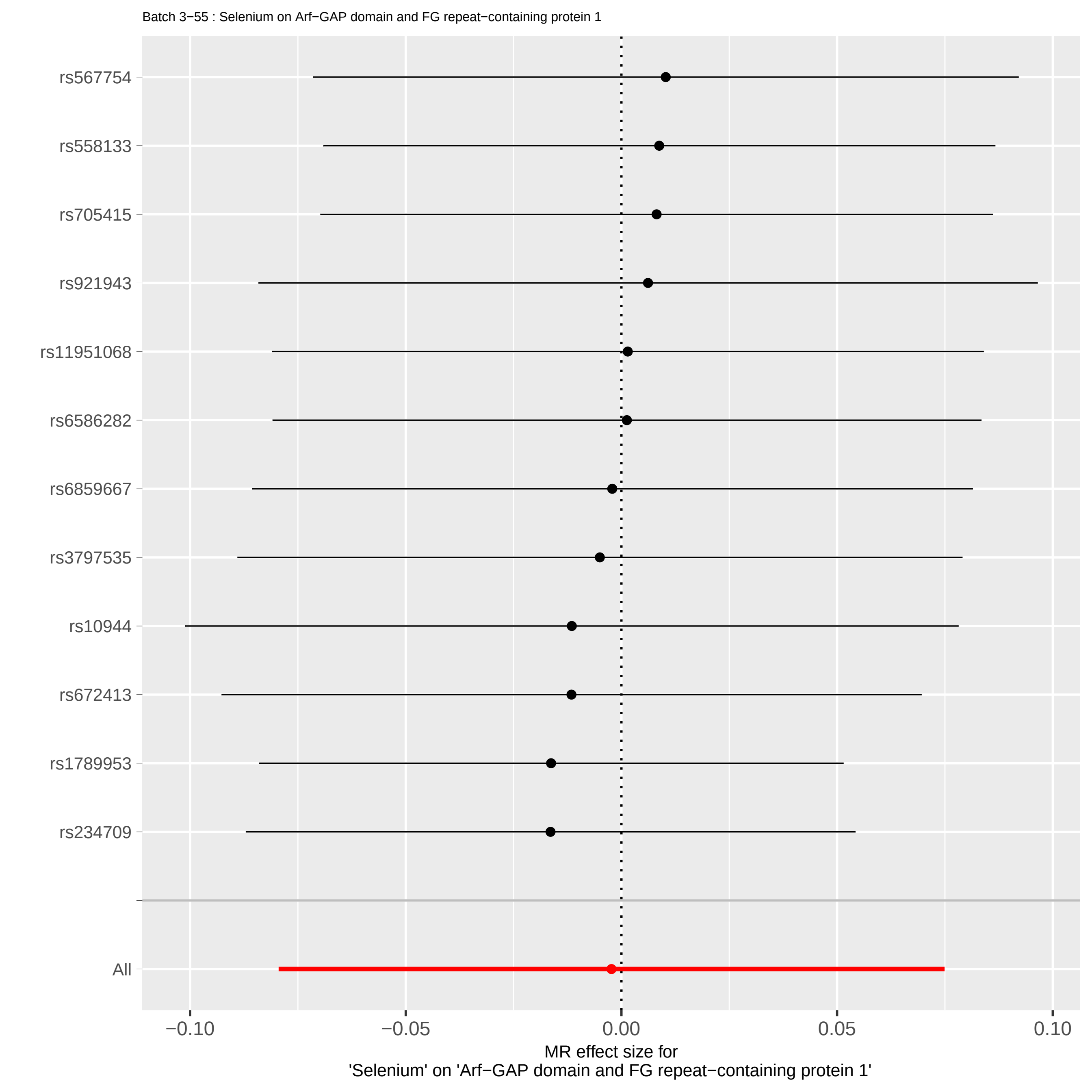

Batch 3−55 : Selenium on Arf−GAP domain and FG repeat−containing protein 1
rs567754
rs558133
rs705415
rs921943
rs11951068
rs6586282
rs6859667
rs3797535
rs10944
rs672413
rs1789953
rs234709
All
−0.10
−0.05
0.00
0.05
0.10
MR effect size for
'Selenium' on 'Arf−GAP domain and FG repeat−containing protein 1'

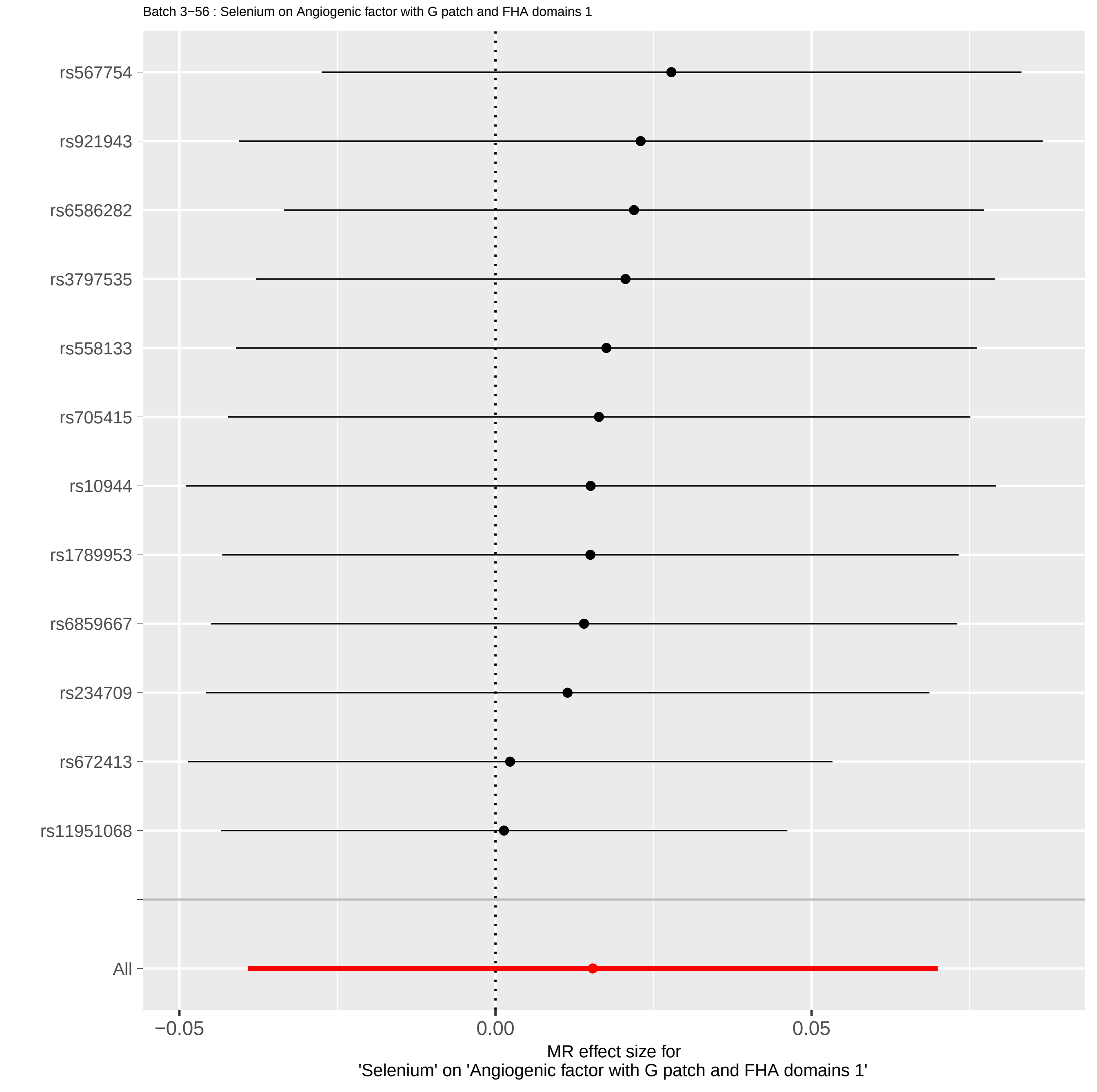

Batch 3−56 : Selenium on Angiogenic factor with G patch and FHA domains 1
rs567754
rs921943
rs6586282
rs3797535
rs558133
rs705415
rs10944
rs1789953
rs6859667
rs234709
rs672413
rs11951068
All
−0.05
0.00
0.05
MR effect size for
'Selenium' on 'Angiogenic factor with G patch and FHA domains 1'

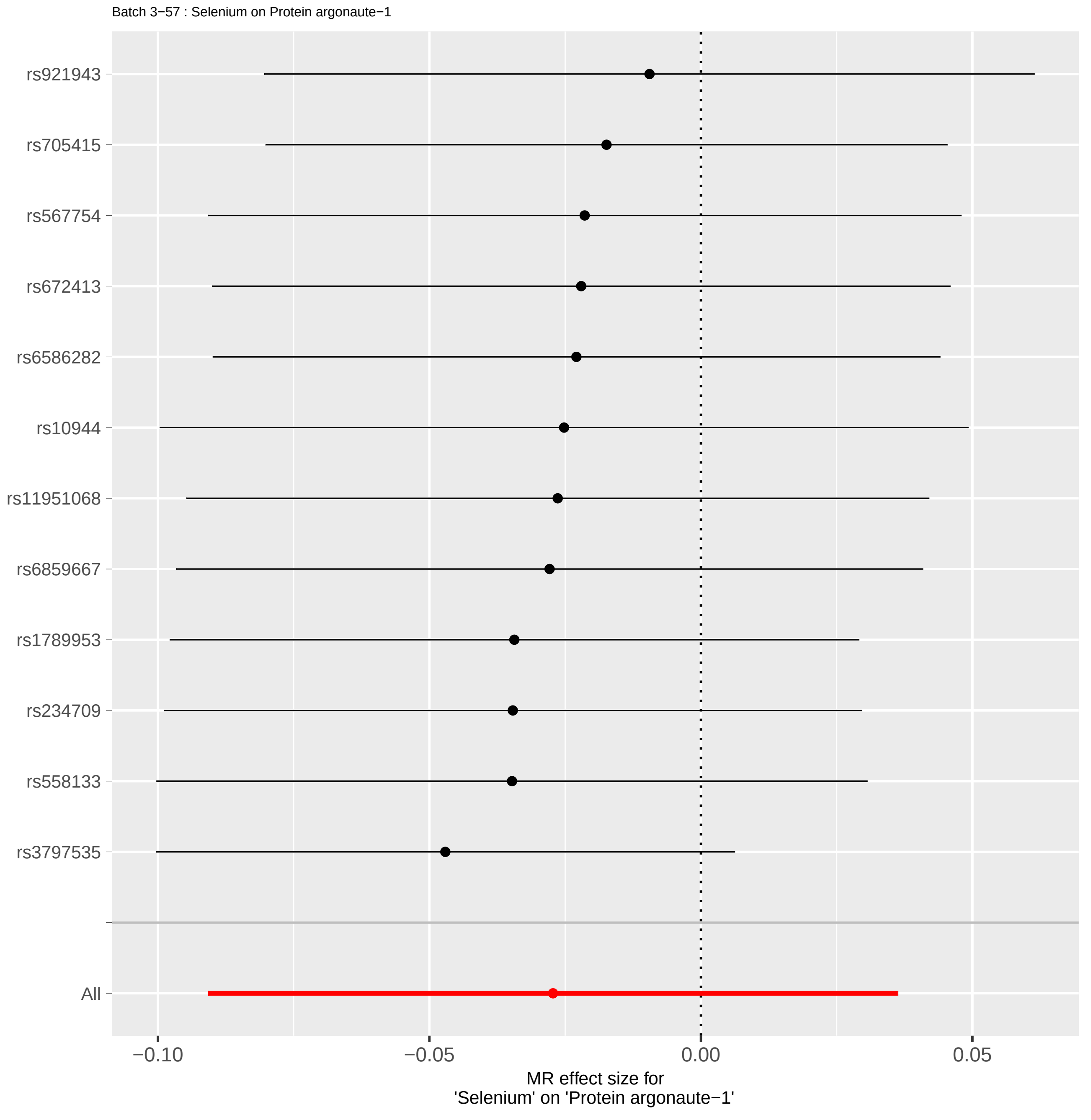

Batch 3−57 : Selenium on Protein argonaute−1
rs921943
rs705415
rs567754
rs672413
rs6586282
rs10944
rs11951068
rs6859667
rs1789953
rs234709
rs558133
rs3797535
All
−0.10
−0.05
0.00
0.05
MR effect size for
'Selenium' on 'Protein argonaute−1'

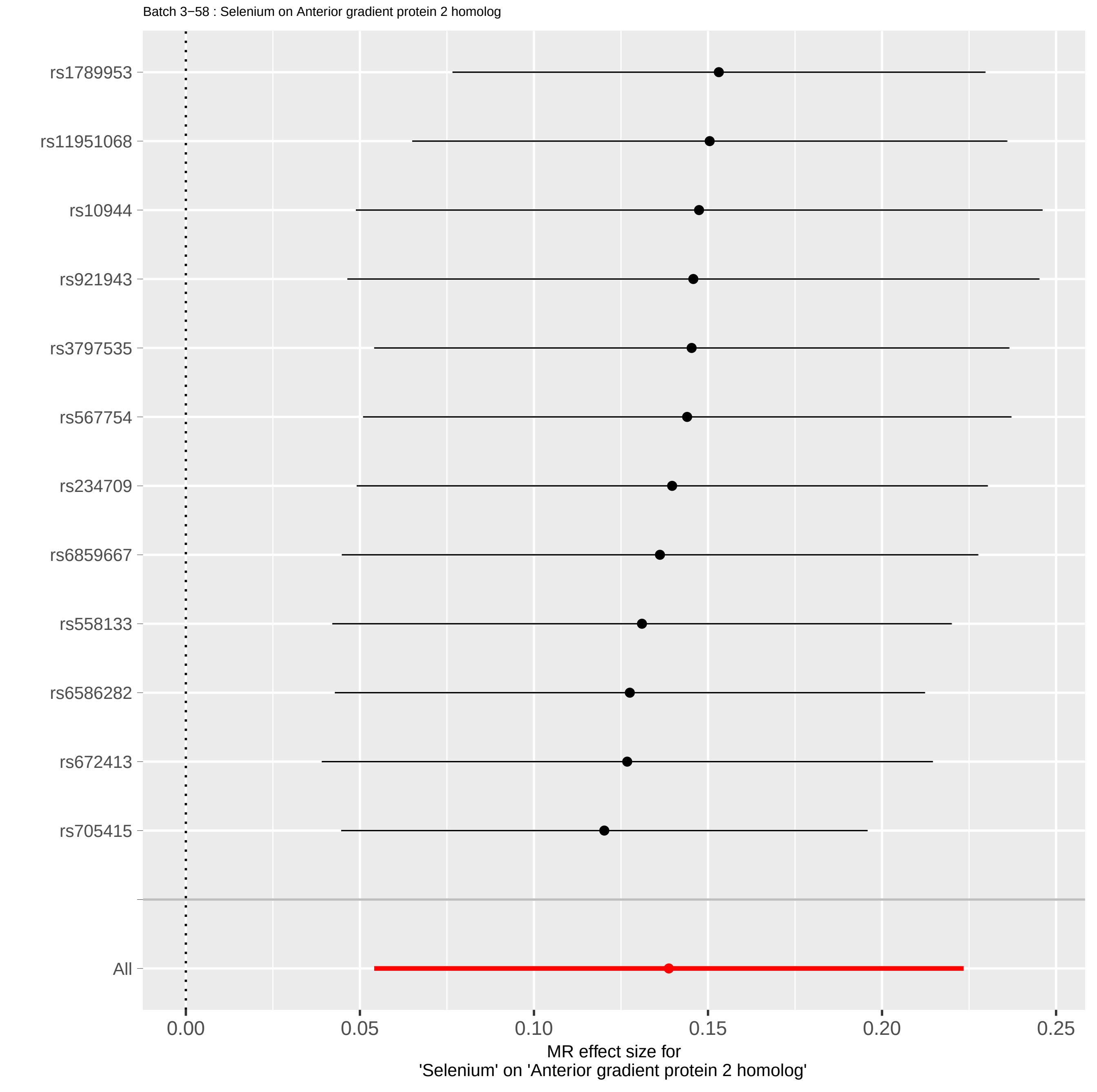

Batch 3−58 : Selenium on Anterior gradient protein 2 homolog
rs1789953
rs11951068
rs10944
rs921943
rs3797535
rs567754
rs234709
rs6859667
rs558133
rs6586282
rs672413
rs705415
All
0.00
0.05
0.10
0.15
0.20
0.25
MR effect size for
'Selenium' on 'Anterior gradient protein 2 homolog'

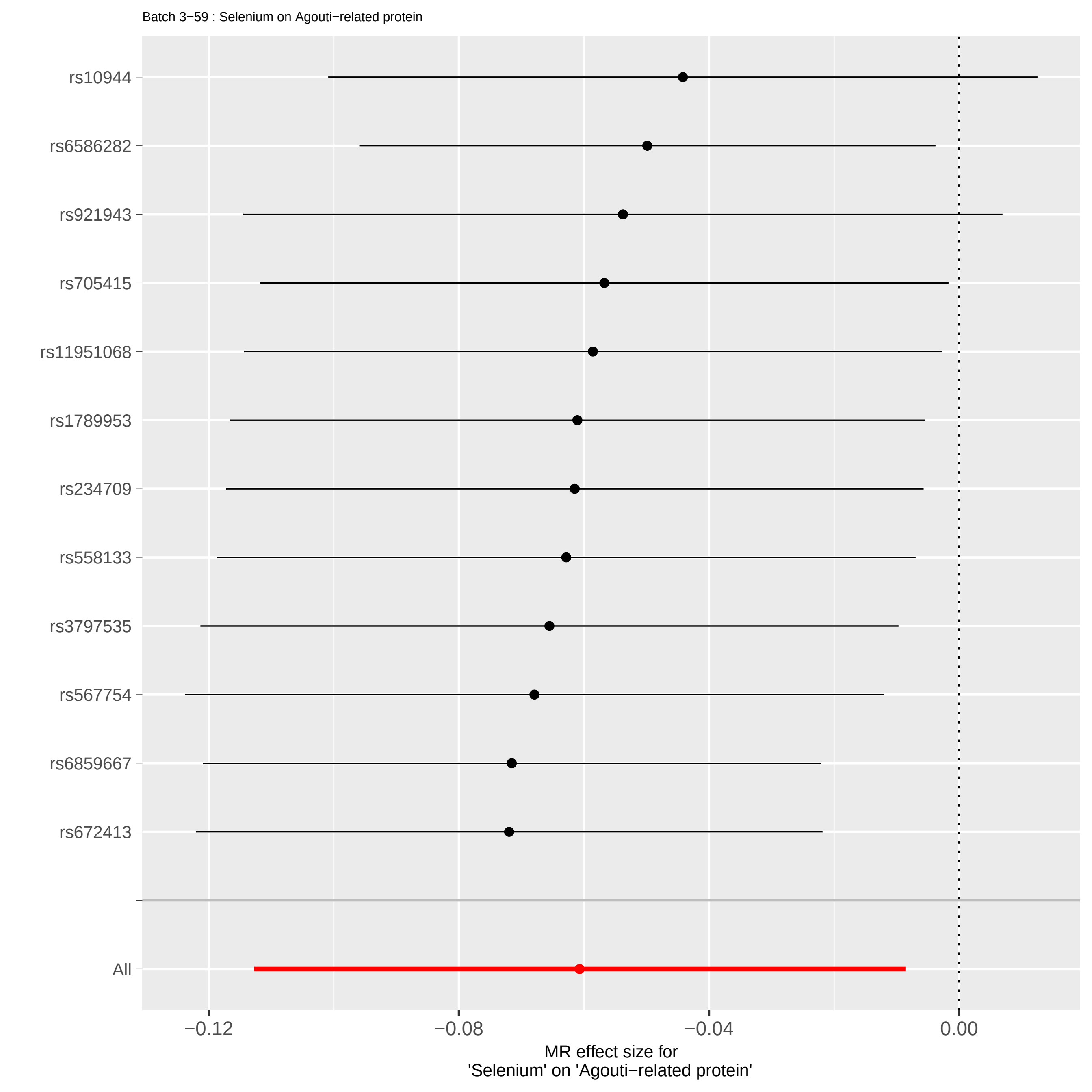

Batch 3−59 : Selenium on Agouti−related protein
rs10944
rs6586282
rs921943
rs705415
rs11951068
rs1789953
rs234709
rs558133
rs3797535
rs567754
rs6859667
rs672413
All
−0.12
−0.08
−0.04
0.00
MR effect size for
'Selenium' on 'Agouti−related protein'

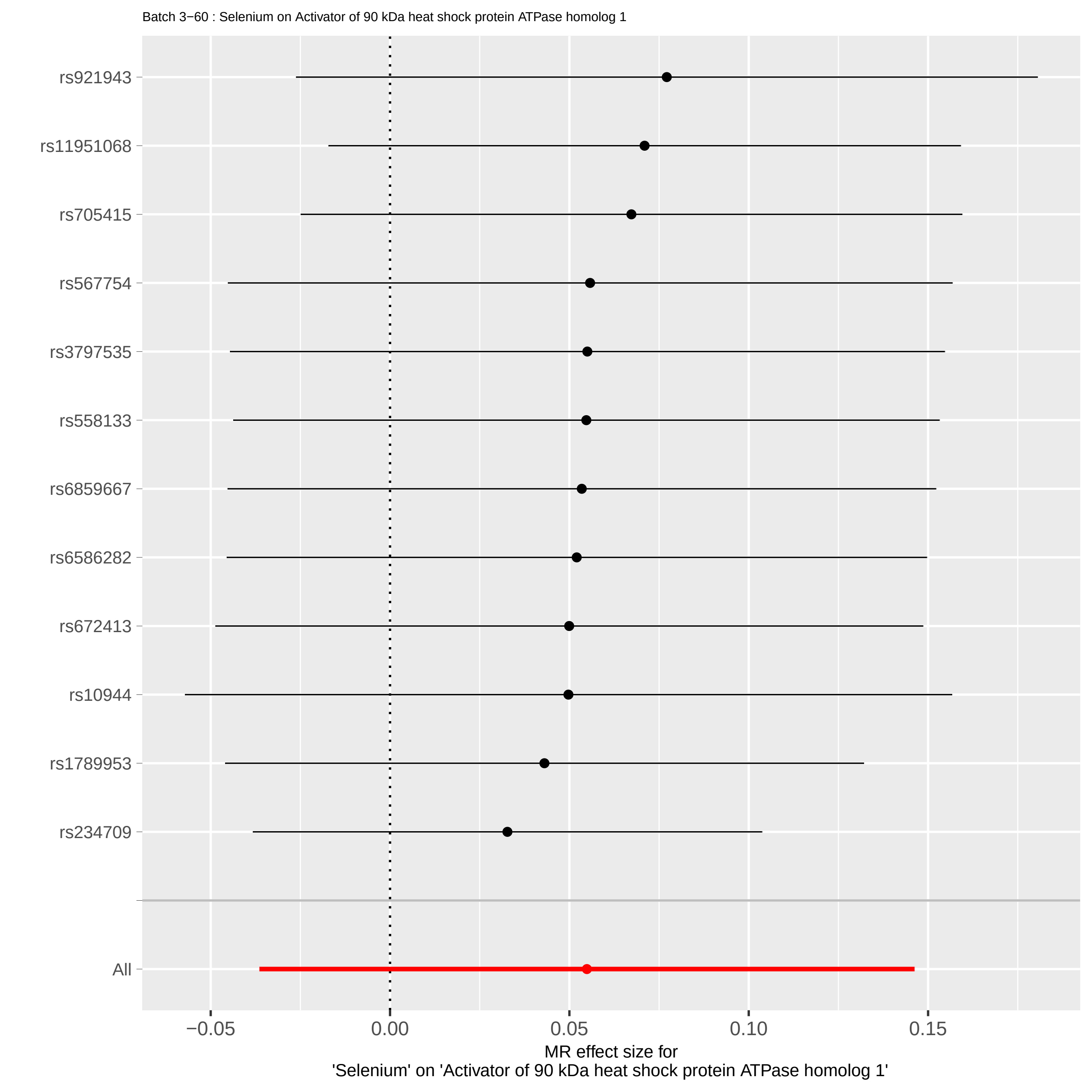

Batch 3−60 : Selenium on Activator of 90 kDa heat shock protein ATPase homolog 1
rs921943
rs11951068
rs705415
rs567754
rs3797535
rs558133
rs6859667
rs6586282
rs672413
rs10944
rs1789953
rs234709
All
−0.05
0.00
0.05
0.10
0.15
MR effect size for
'Selenium' on 'Activator of 90 kDa heat shock protein ATPase homolog 1'

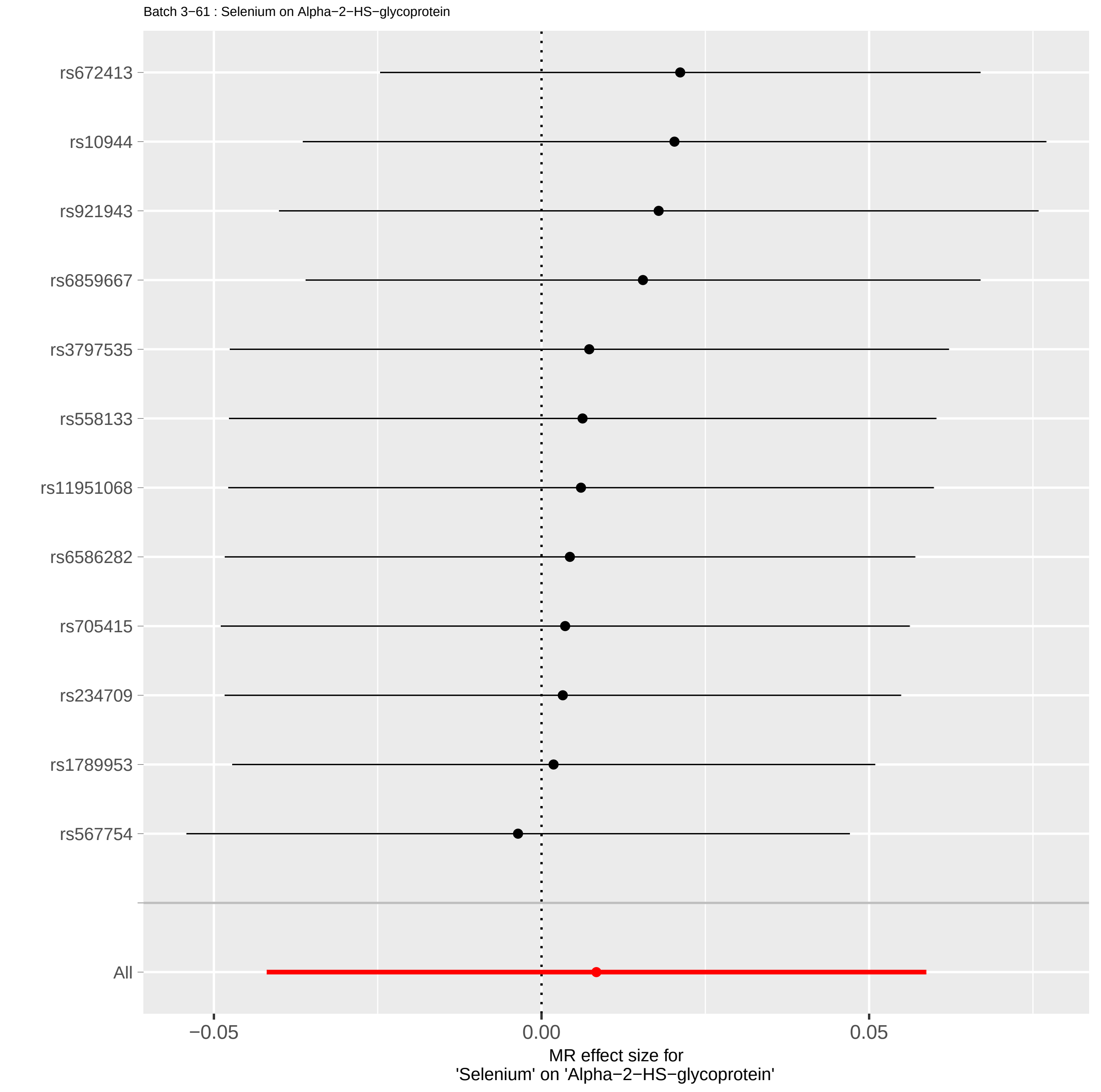

Batch 3−61 : Selenium on Alpha−2−HS−glycoprotein
rs672413
rs10944
rs921943
rs6859667
rs3797535
rs558133
rs11951068
rs6586282
rs705415
rs234709
rs1789953
rs567754
All
−0.05
0.00
0.05
MR effect size for
'Selenium' on 'Alpha−2−HS−glycoprotein'

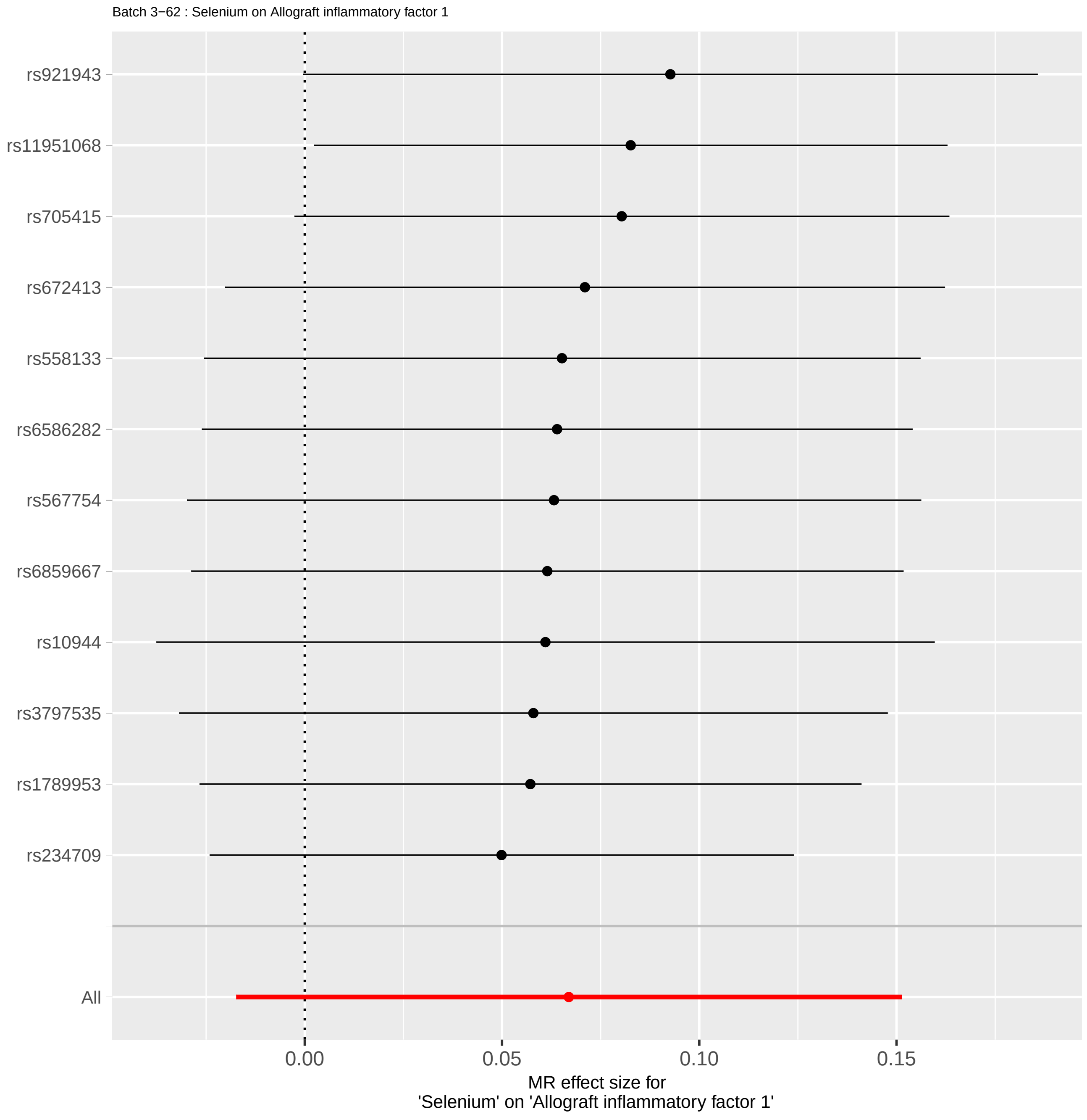

Batch 3−62 : Selenium on Allograft inflammatory factor 1
rs921943
rs11951068
rs705415
rs672413
rs558133
rs6586282
rs567754
rs6859667
rs10944
rs3797535
rs1789953
rs234709
All
0.00
0.05
0.10
0.15
MR effect size for
'Selenium' on 'Allograft inflammatory factor 1'

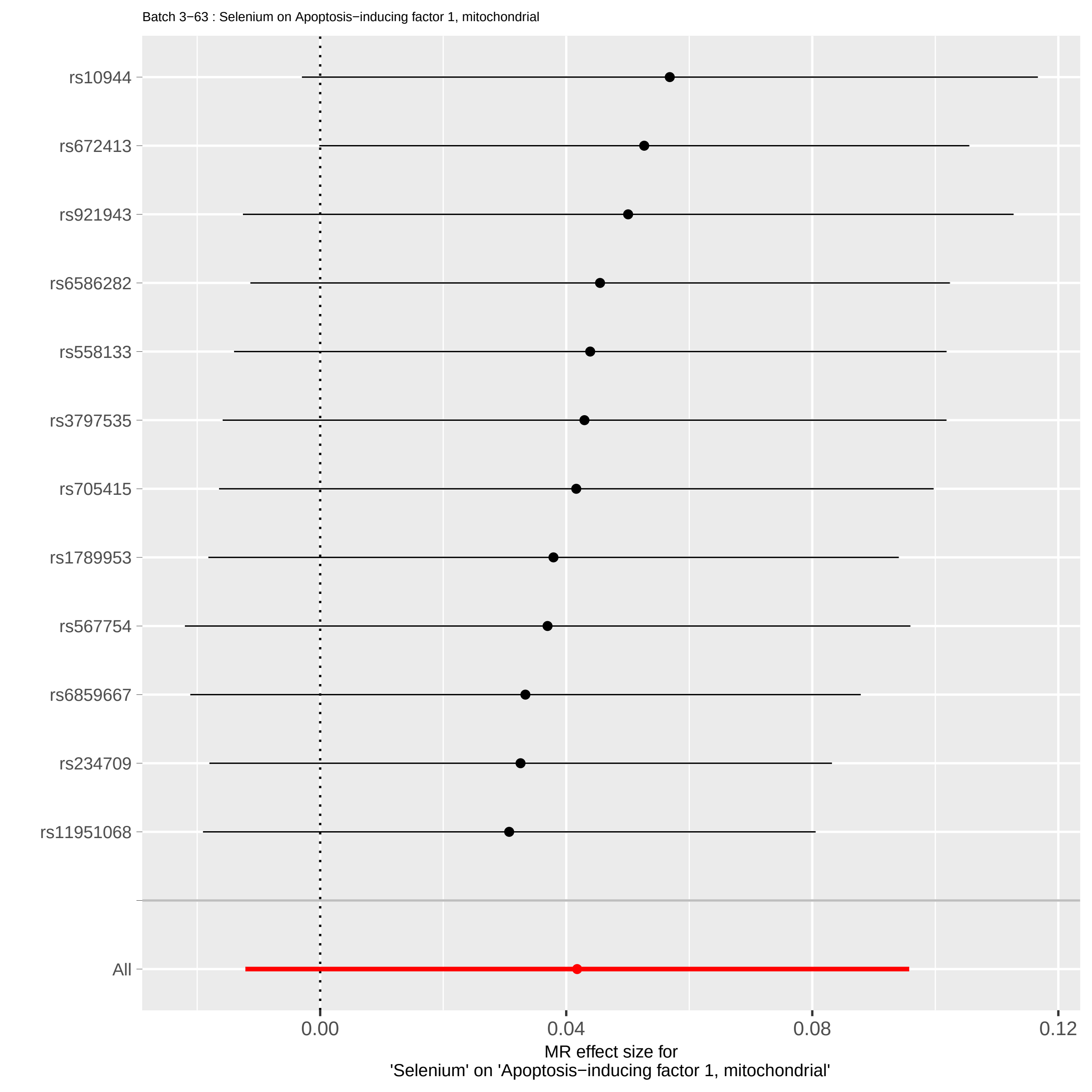

Batch 3−63 : Selenium on Apoptosis−inducing factor 1, mitochondrial
rs10944
rs672413
rs921943
rs6586282
rs558133
rs3797535
rs705415
rs1789953
rs567754
rs6859667
rs234709
rs11951068
All
0.00
0.04
0.08
0.12
MR effect size for
'Selenium' on 'Apoptosis−inducing factor 1, mitochondrial'

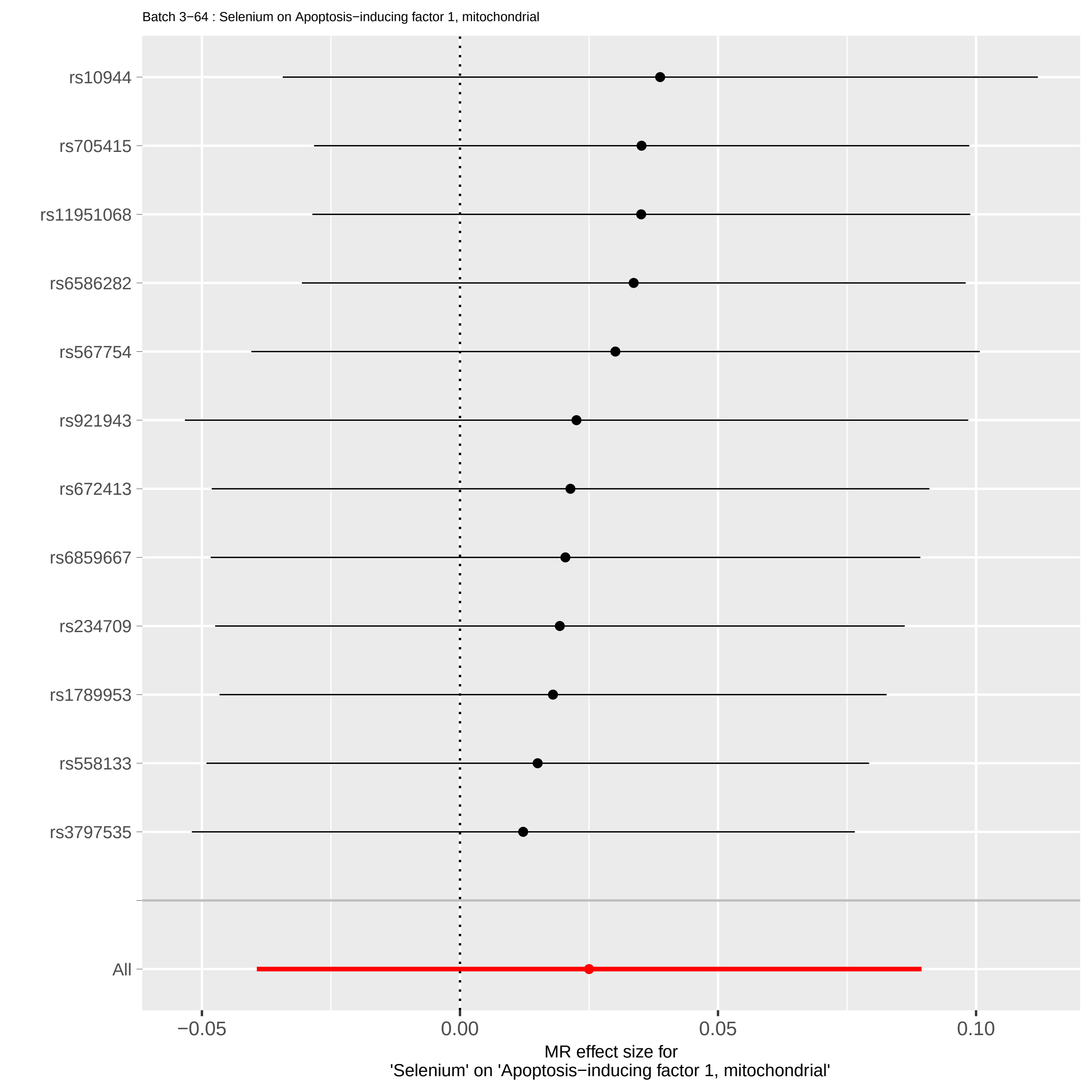

Batch 3−64 : Selenium on Apoptosis−inducing factor 1, mitochondrial
rs10944
rs705415
rs11951068
rs6586282
rs567754
rs921943
rs672413
rs6859667
rs234709
rs1789953
rs558133
rs3797535
All
−0.05
0.00
0.05
0.10
MR effect size for
'Selenium' on 'Apoptosis−inducing factor 1, mitochondrial'

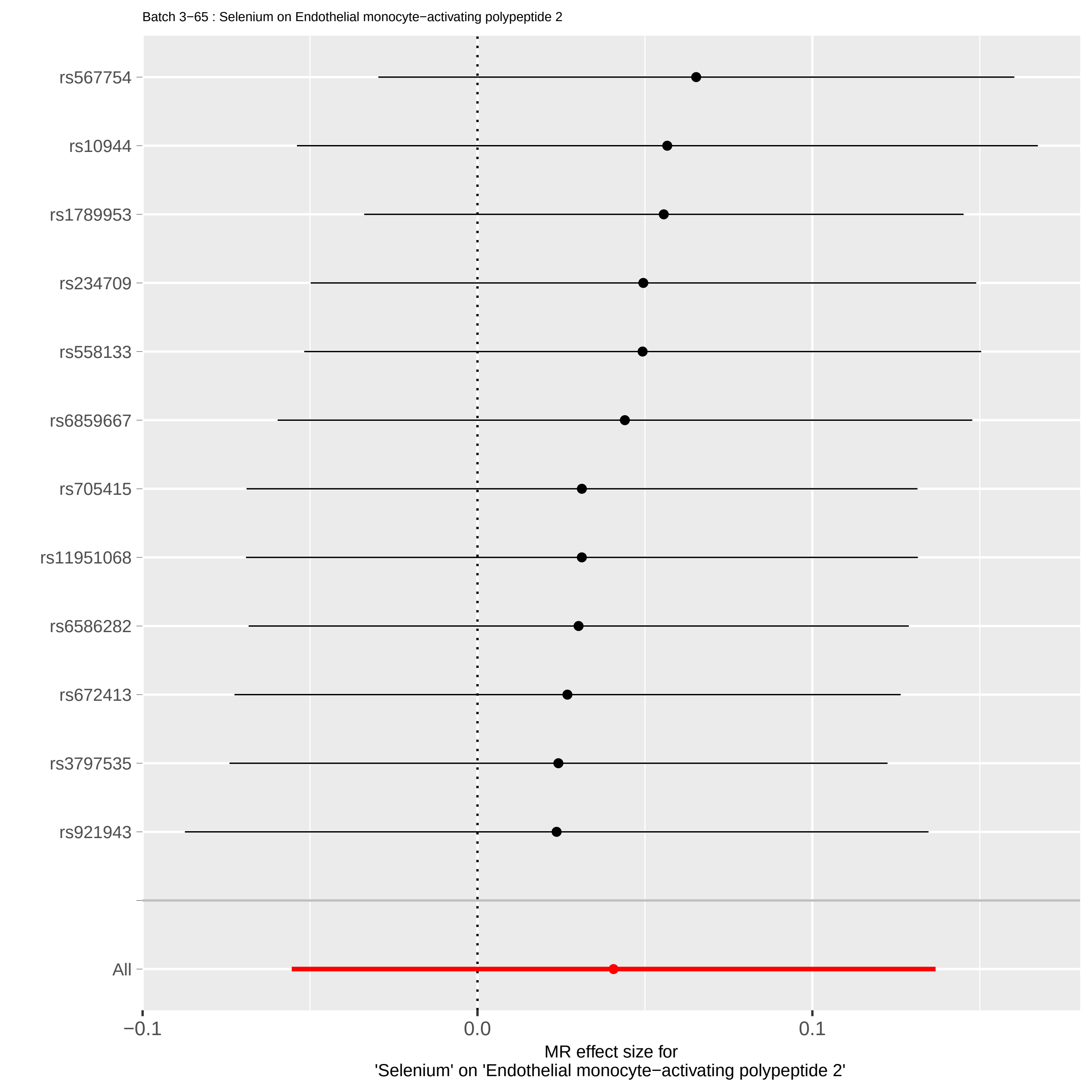

Batch 3−65 : Selenium on Endothelial monocyte−activating polypeptide 2
rs567754
rs10944
rs1789953
rs234709
rs558133
rs6859667
rs705415
rs11951068
rs6586282
rs672413
rs3797535
rs921943
All
−0.1
0.0
0.1
MR effect size for
'Selenium' on 'Endothelial monocyte−activating polypeptide 2'

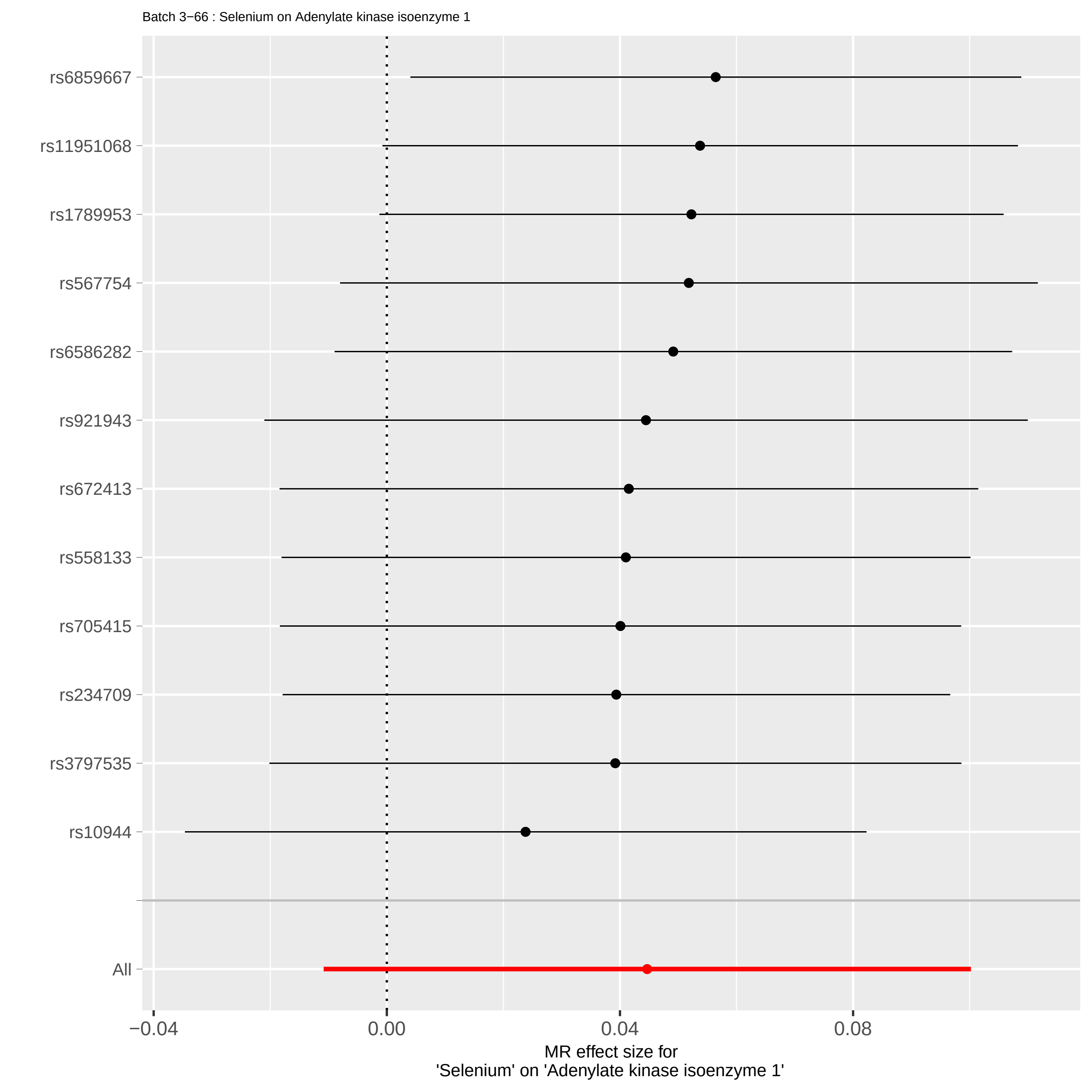

Batch 3−66 : Selenium on Adenylate kinase isoenzyme 1
rs6859667
rs11951068
rs1789953
rs567754
rs6586282
rs921943
rs672413
rs558133
rs705415
rs234709
rs3797535
rs10944
All
−0.04
0.00
0.04
0.08
MR effect size for
'Selenium' on 'Adenylate kinase isoenzyme 1'

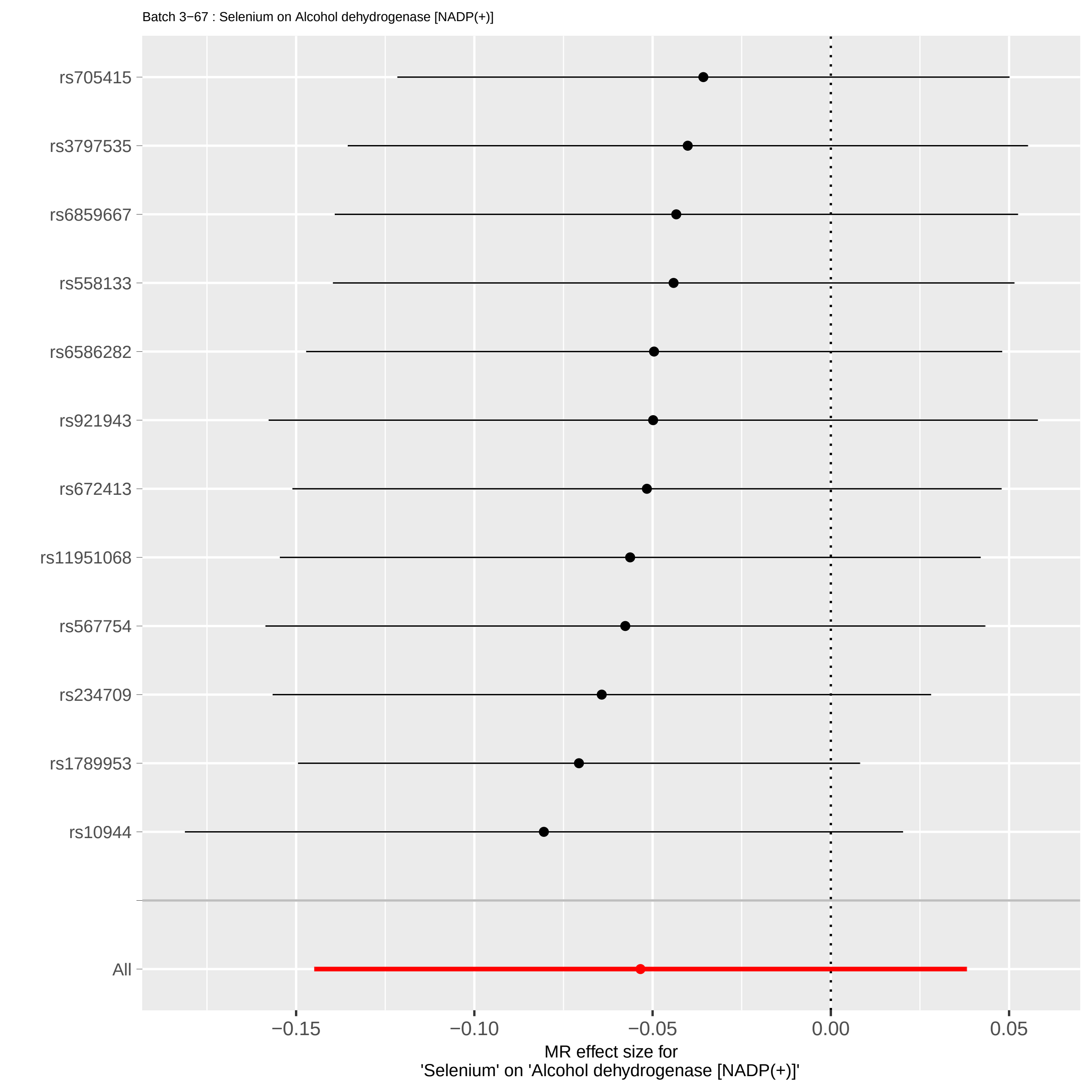

Batch 3−67 : Selenium on Alcohol dehydrogenase [NADP(+)]
rs705415
rs3797535
rs6859667
rs558133
rs6586282
rs921943
rs672413
rs11951068
rs567754
rs234709
rs1789953
rs10944
All
−0.15
−0.10
−0.05
0.00
0.05
MR effect size for
'Selenium' on 'Alcohol dehydrogenase [NADP(+)]'

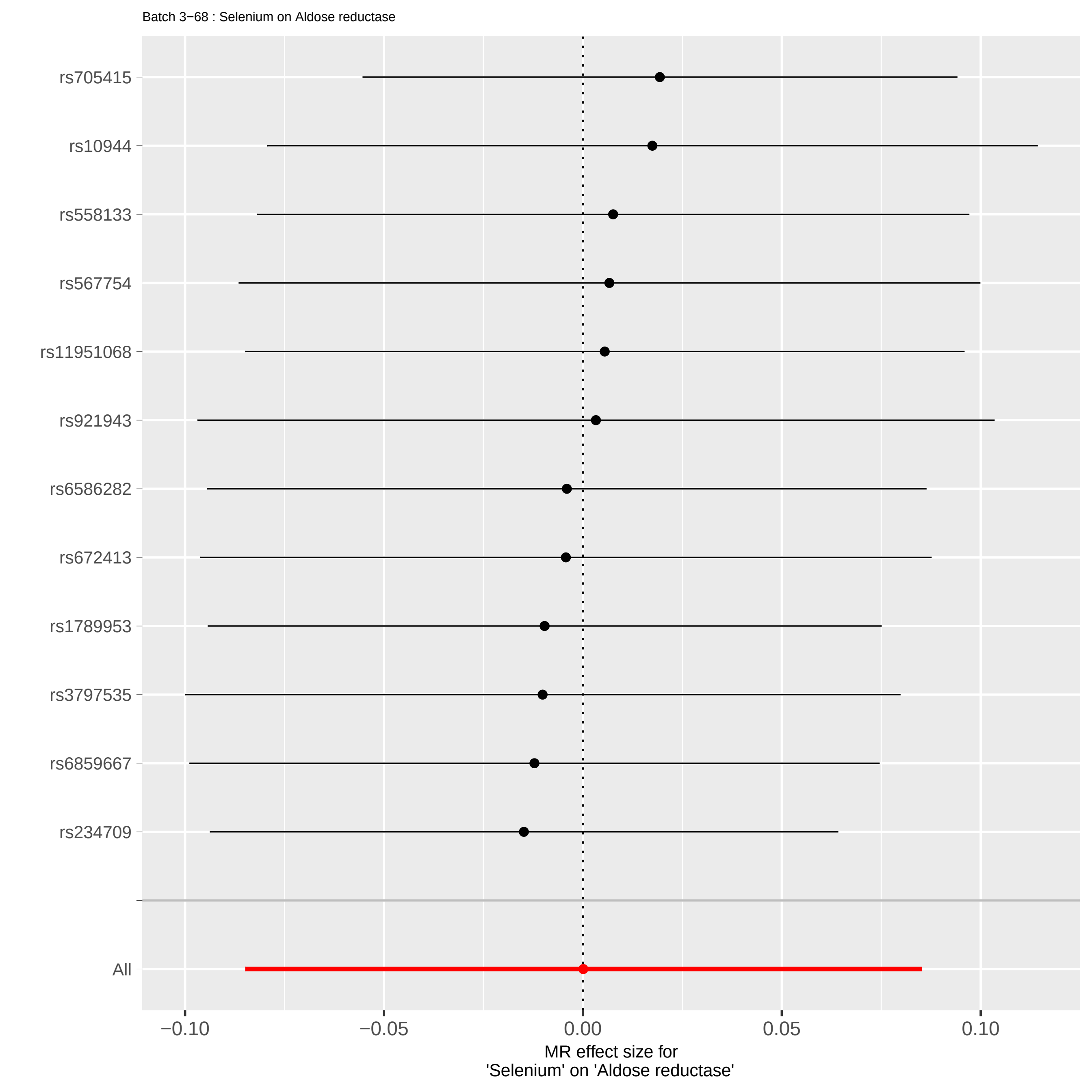

Batch 3−68 : Selenium on Aldose reductase
rs705415
rs10944
rs558133
rs567754
rs11951068
rs921943
rs6586282
rs672413
rs1789953
rs3797535
rs6859667
rs234709
All
−0.10
−0.05
0.00
0.05
0.10
MR effect size for
'Selenium' on 'Aldose reductase'

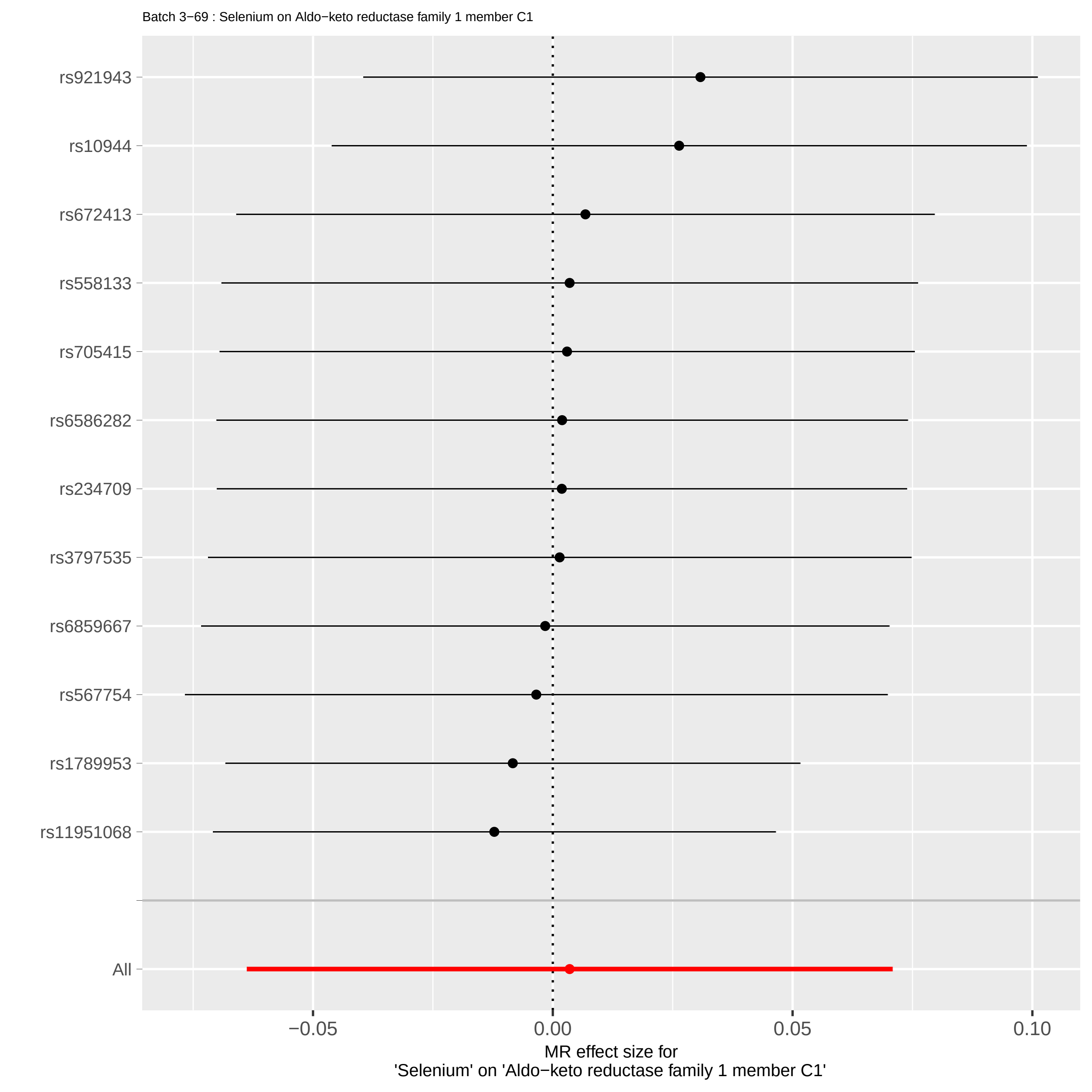

Batch 3−69 : Selenium on Aldo−keto reductase family 1 member C1
rs921943
rs10944
rs672413
rs558133
rs705415
rs6586282
rs234709
rs3797535
rs6859667
rs567754
rs1789953
rs11951068
All
−0.05
0.00
0.05
0.10
MR effect size for
'Selenium' on 'Aldo−keto reductase family 1 member C1'

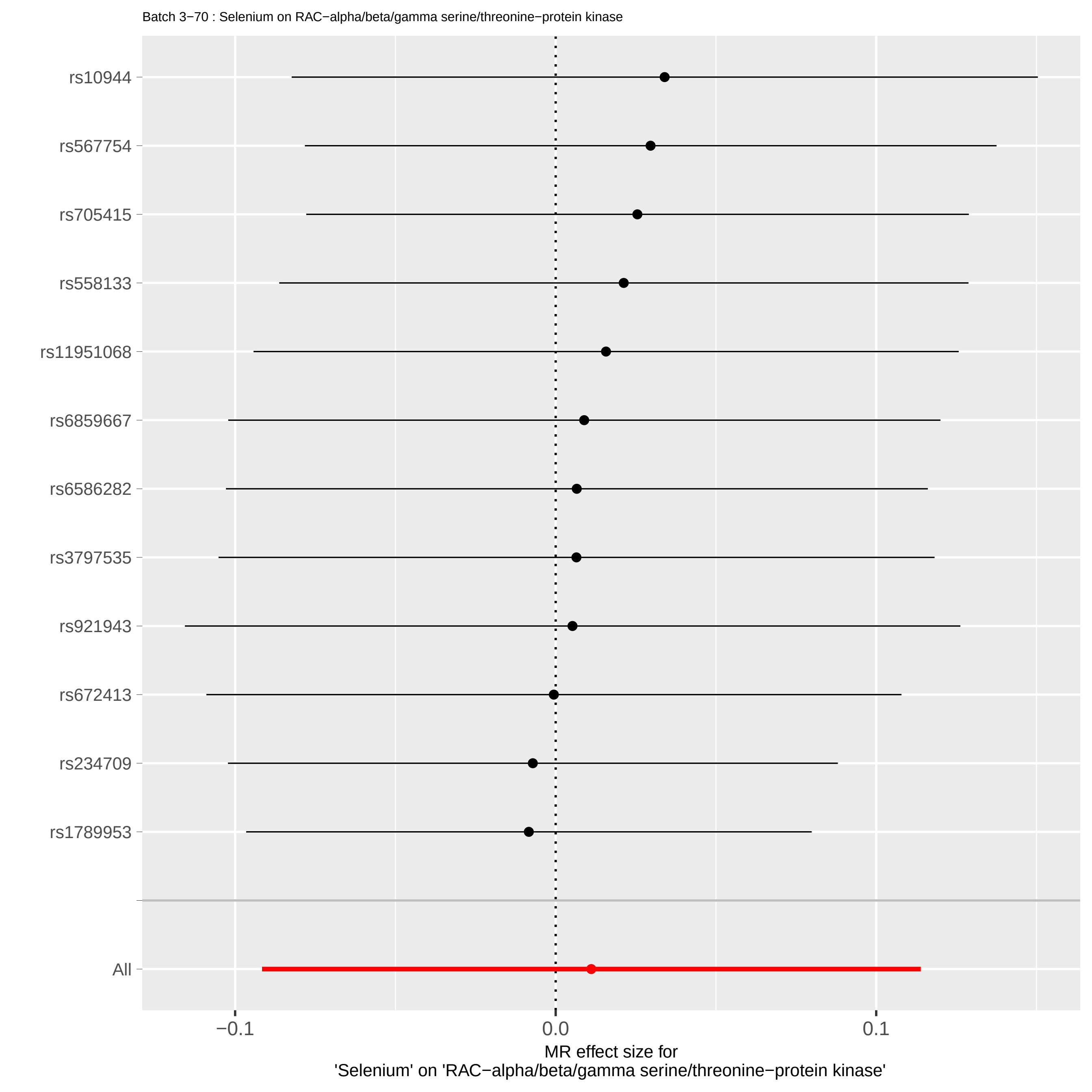

Batch 3−70 : Selenium on RAC−alpha/beta/gamma serine/threonine−protein kinase
rs10944
rs567754
rs705415
rs558133
rs11951068
rs6859667
rs6586282
rs3797535
rs921943
rs672413
rs234709
rs1789953
All
−0.1
0.0
0.1
MR effect size for
'Selenium' on 'RAC−alpha/beta/gamma serine/threonine−protein kinase'

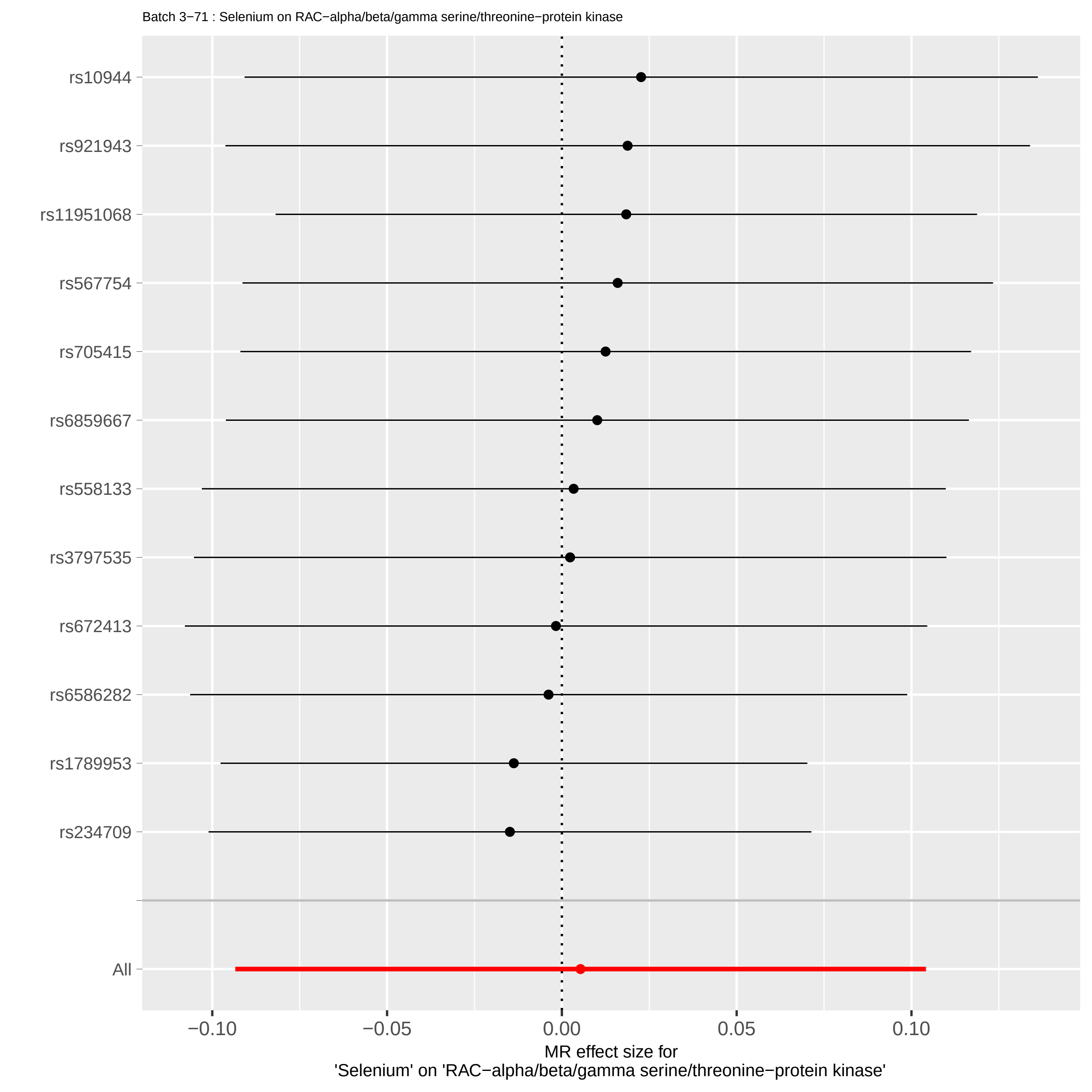

Batch 3−71 : Selenium on RAC−alpha/beta/gamma serine/threonine−protein kinase
rs10944
rs921943
rs11951068
rs567754
rs705415
rs6859667
rs558133
rs3797535
rs672413
rs6586282
rs1789953
rs234709
All
−0.10
−0.05
0.00
0.05
0.10
MR effect size for
'Selenium' on 'RAC−alpha/beta/gamma serine/threonine−protein kinase'

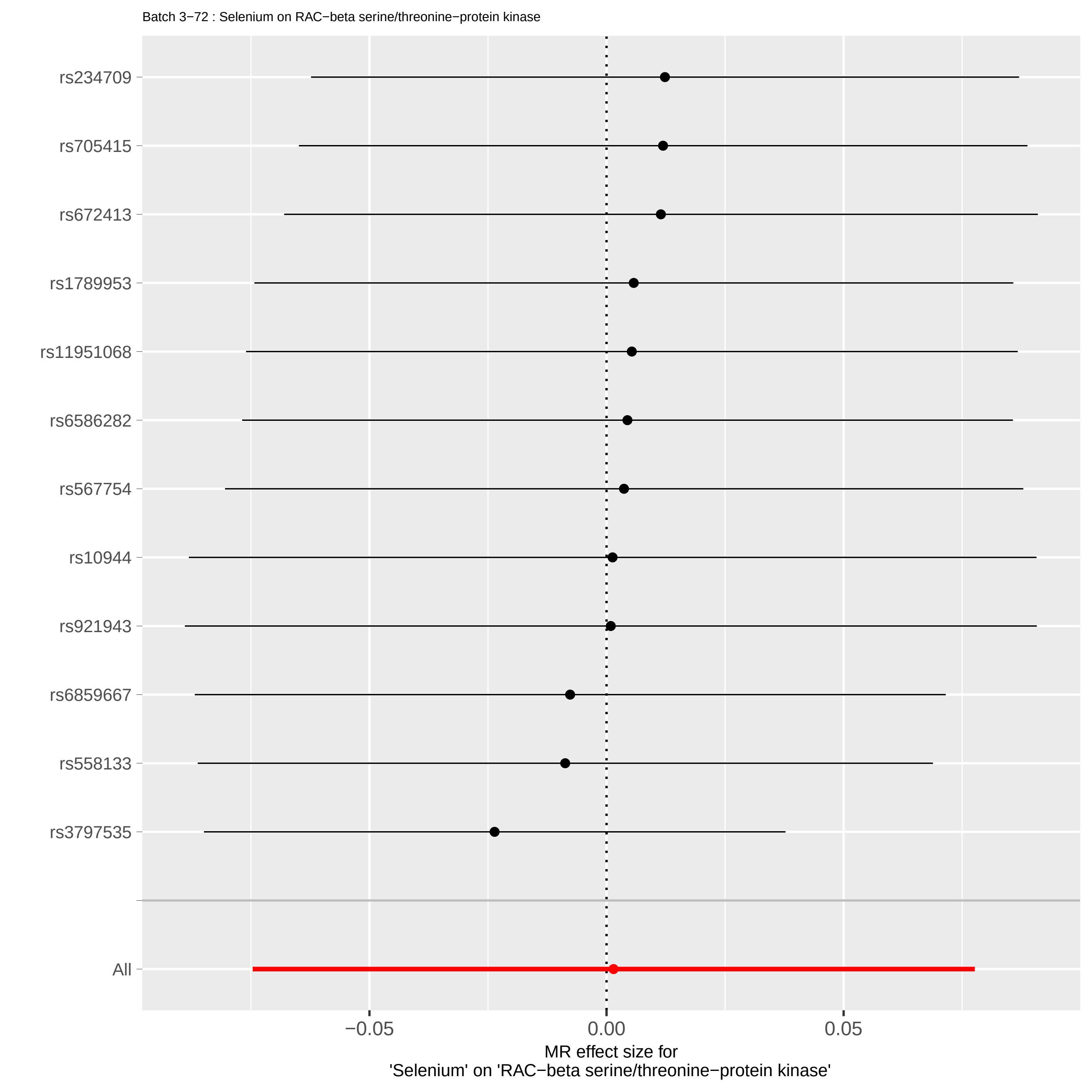

Batch 3−72 : Selenium on RAC−beta serine/threonine−protein kinase
rs234709
rs705415
rs672413
rs1789953
rs11951068
rs6586282
rs567754
rs10944
rs921943
rs6859667
rs558133
rs3797535
All
−0.05
0.00
0.05
MR effect size for
'Selenium' on 'RAC−beta serine/threonine−protein kinase'

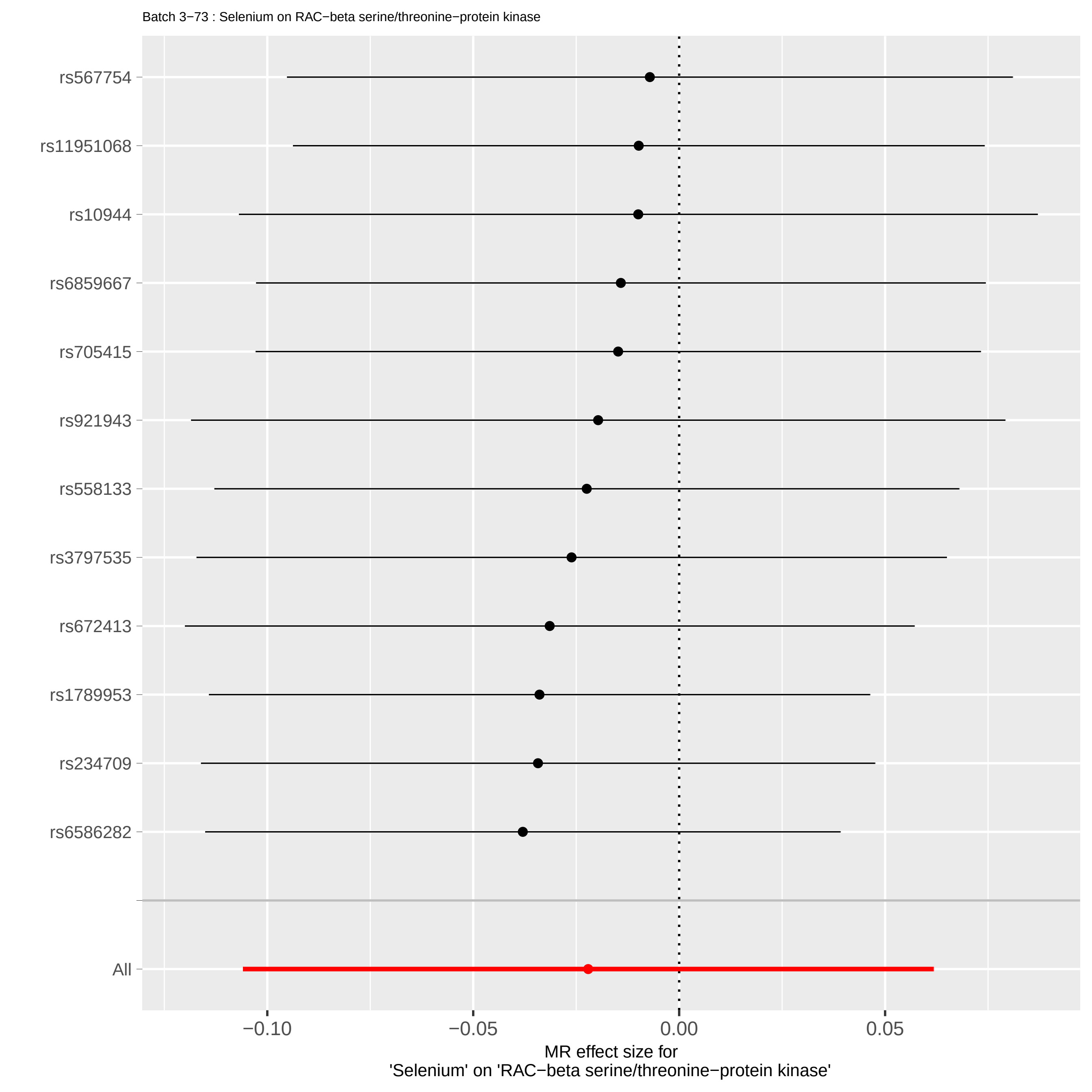
Batch 3−73 : Selenium on RAC−beta serine/threonine−protein kinase
rs567754
rs11951068
rs10944
rs6859667
rs705415
rs921943
rs558133
rs3797535
rs672413
rs1789953
rs234709
rs6586282
All
−0.10
−0.05
0.00
0.05
MR effect size for
'Selenium' on 'RAC−beta serine/threonine−protein kinase'

Batch 3−74 : Selenium on CD166 antigen

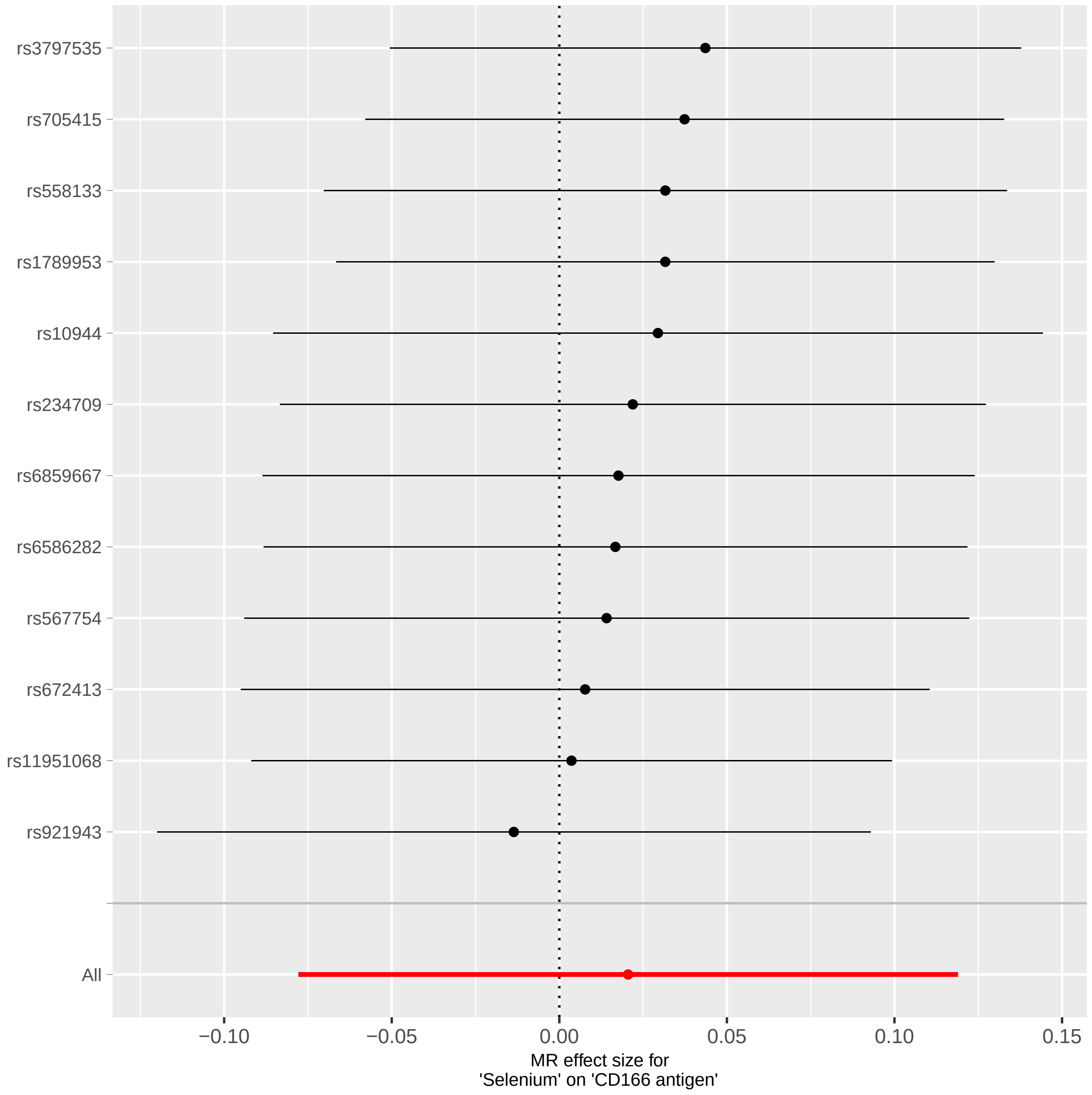

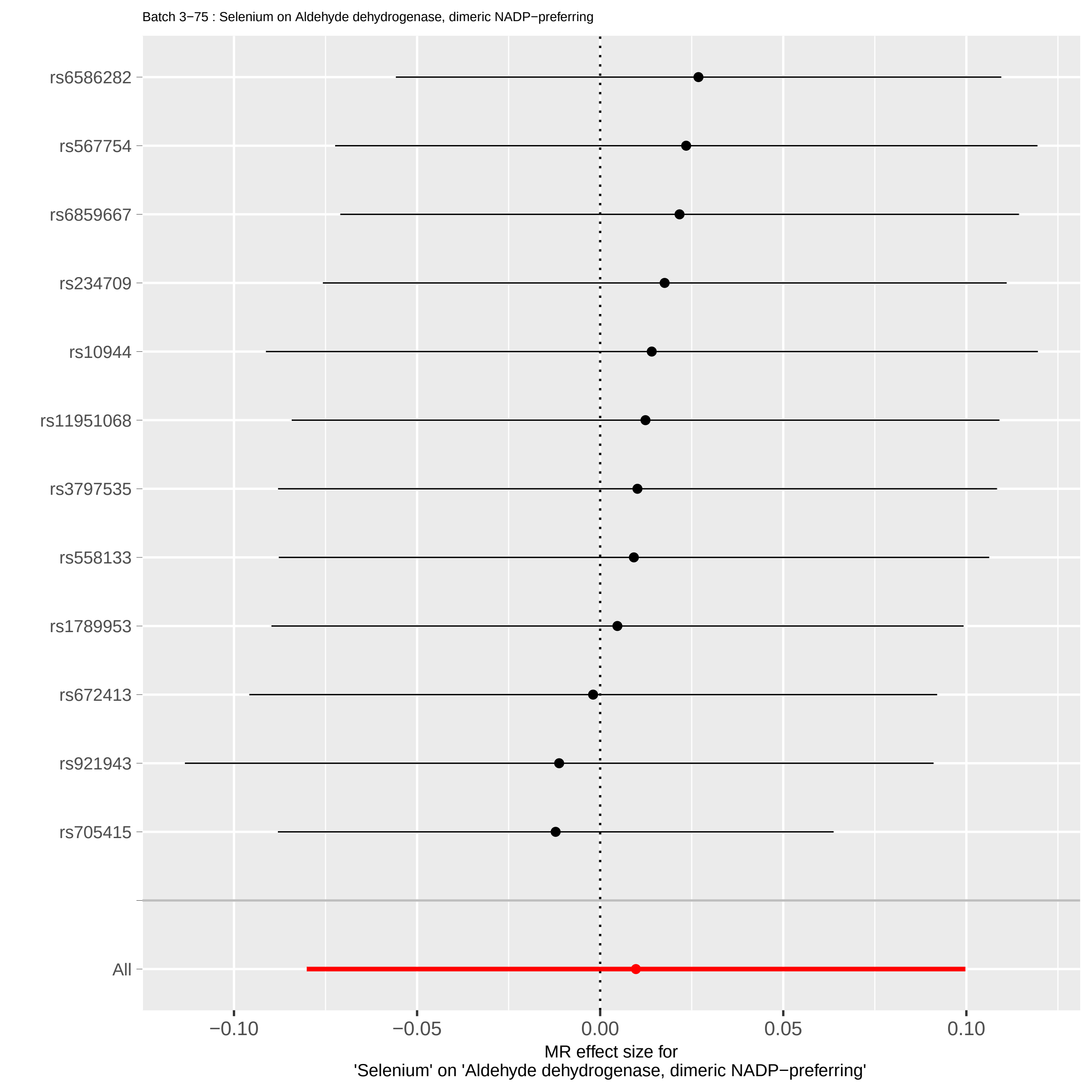
Batch 3−75 : Selenium on Aldehyde dehydrogenase, dimeric NADP−preferring
rs6586282
rs567754
rs6859667
rs234709
rs10944
rs11951068
rs3797535
rs558133
rs1789953
rs672413
rs921943
rs705415
All
−0.10
−0.05
0.00
0.05
0.10
MR effect size for
'Selenium' on 'Aldehyde dehydrogenase, dimeric NADP−preferring'

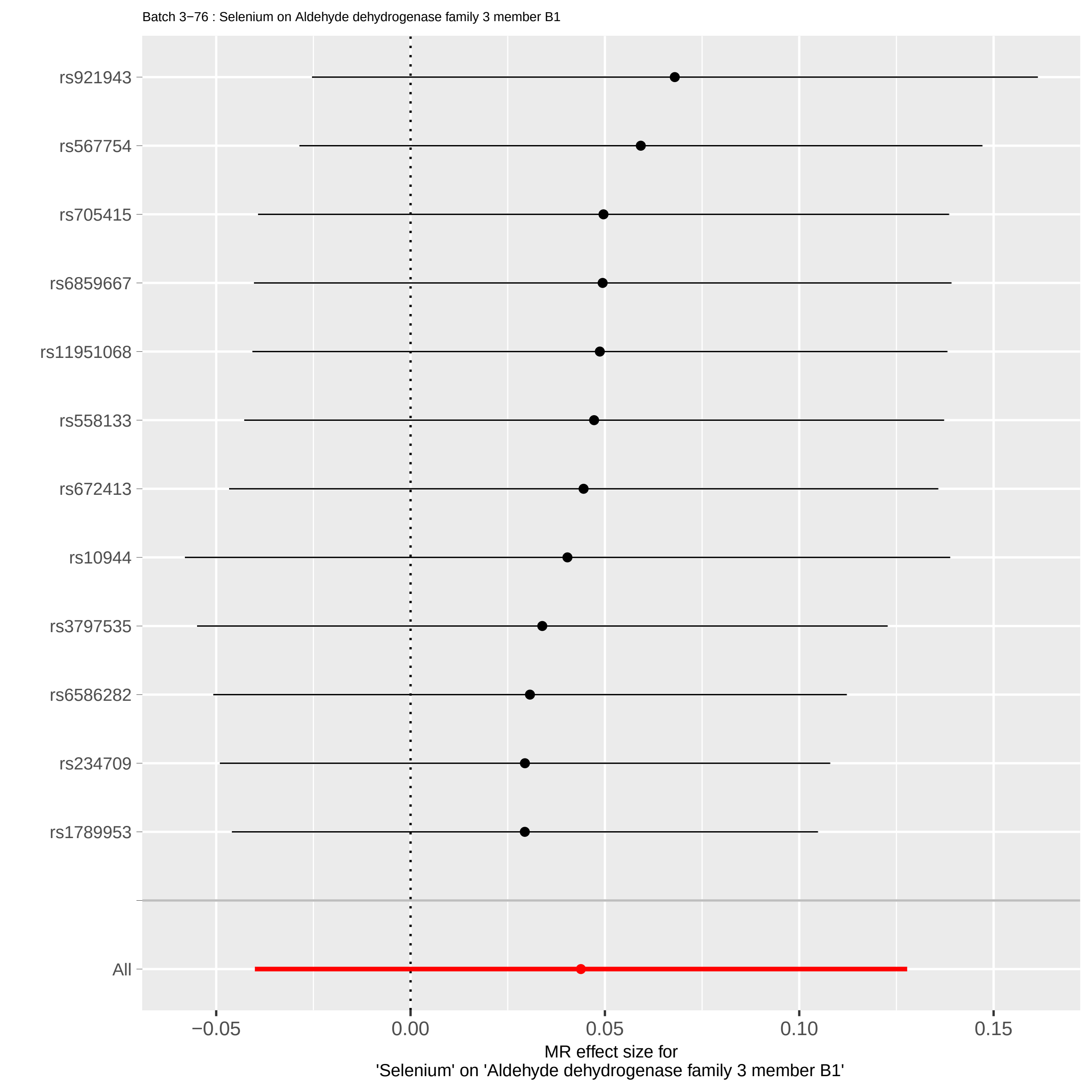

Batch 3−76 : Selenium on Aldehyde dehydrogenase family 3 member B1
rs921943
rs567754
rs705415
rs6859667
rs11951068
rs558133
rs672413
rs10944
rs3797535
rs6586282
rs234709
rs1789953
All
−0.05
0.00
0.05
0.10
0.15
MR effect size for
'Selenium' on 'Aldehyde dehydrogenase family 3 member B1'

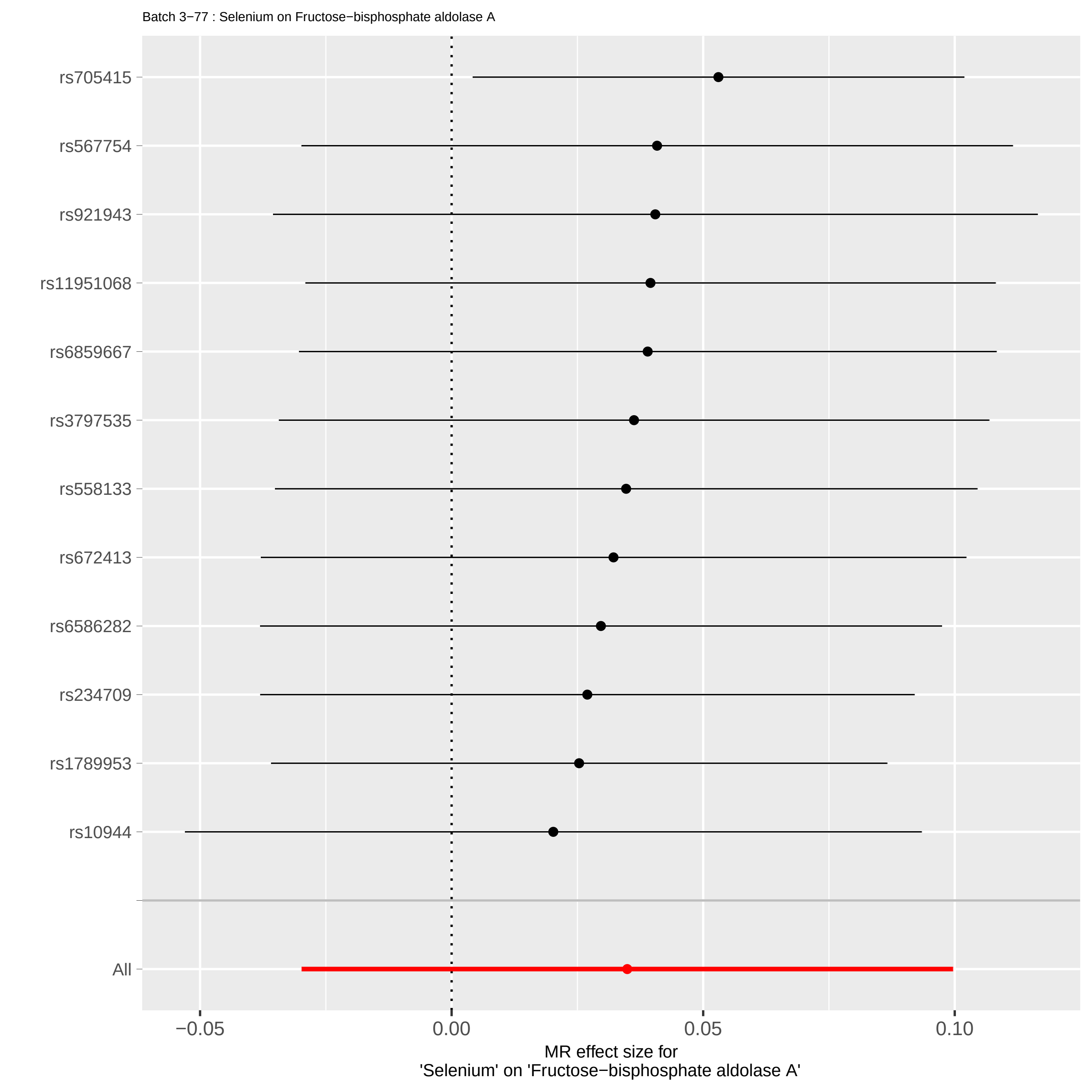

Batch 3−77 : Selenium on Fructose−bisphosphate aldolase A
rs705415
rs567754
rs921943
rs11951068
rs6859667
rs3797535
rs558133
rs672413
rs6586282
rs234709
rs1789953
rs10944
All
−0.05
0.00
0.05
0.10
MR effect size for
'Selenium' on 'Fructose−bisphosphate aldolase A'

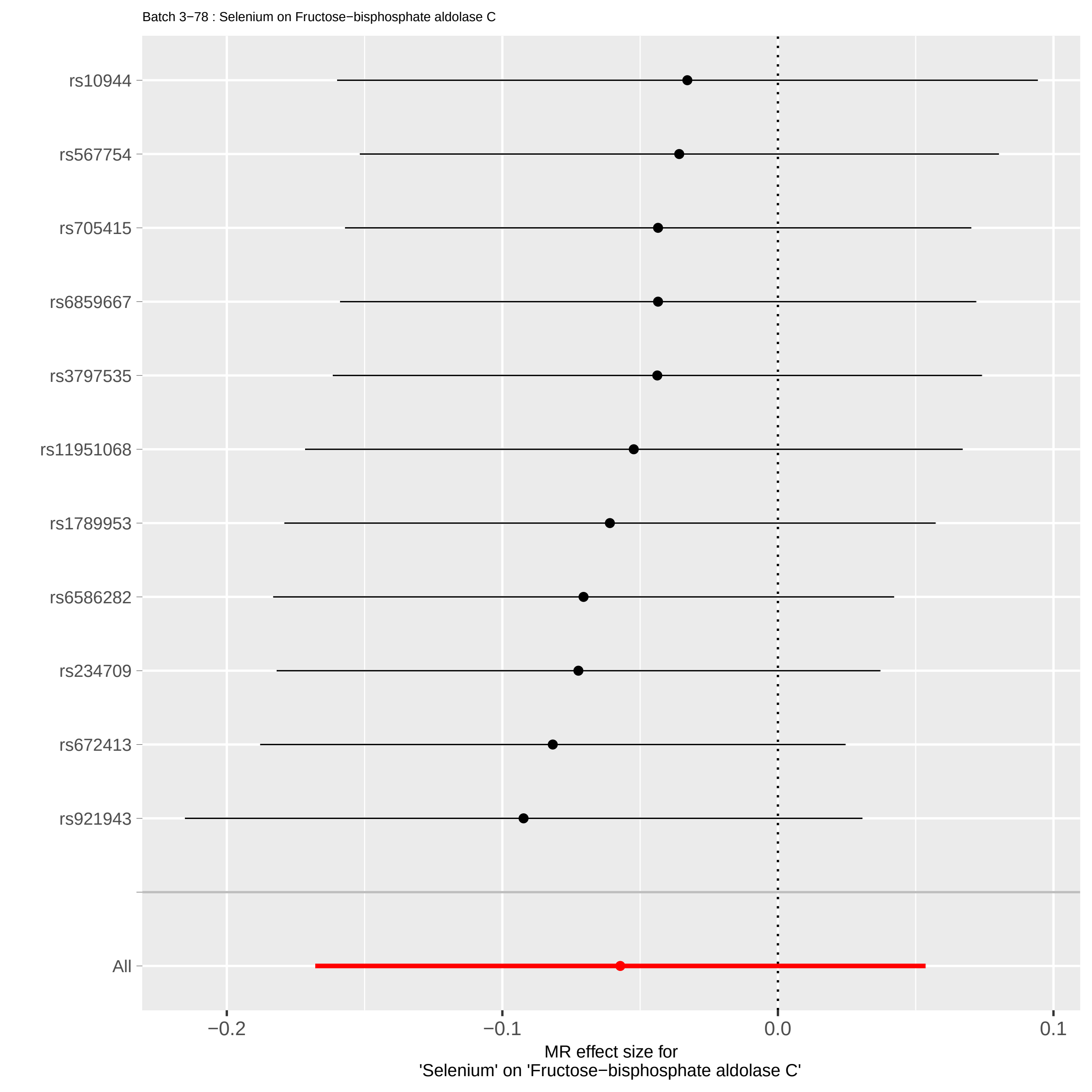

Batch 3−78 : Selenium on Fructose−bisphosphate aldolase C
rs10944
rs567754
rs705415
rs6859667
rs3797535
rs11951068
rs1789953
rs6586282
rs234709
rs672413
rs921943
All
−0.2
−0.1
0.0
0.1
MR effect size for
'Selenium' on 'Fructose−bisphosphate aldolase C'

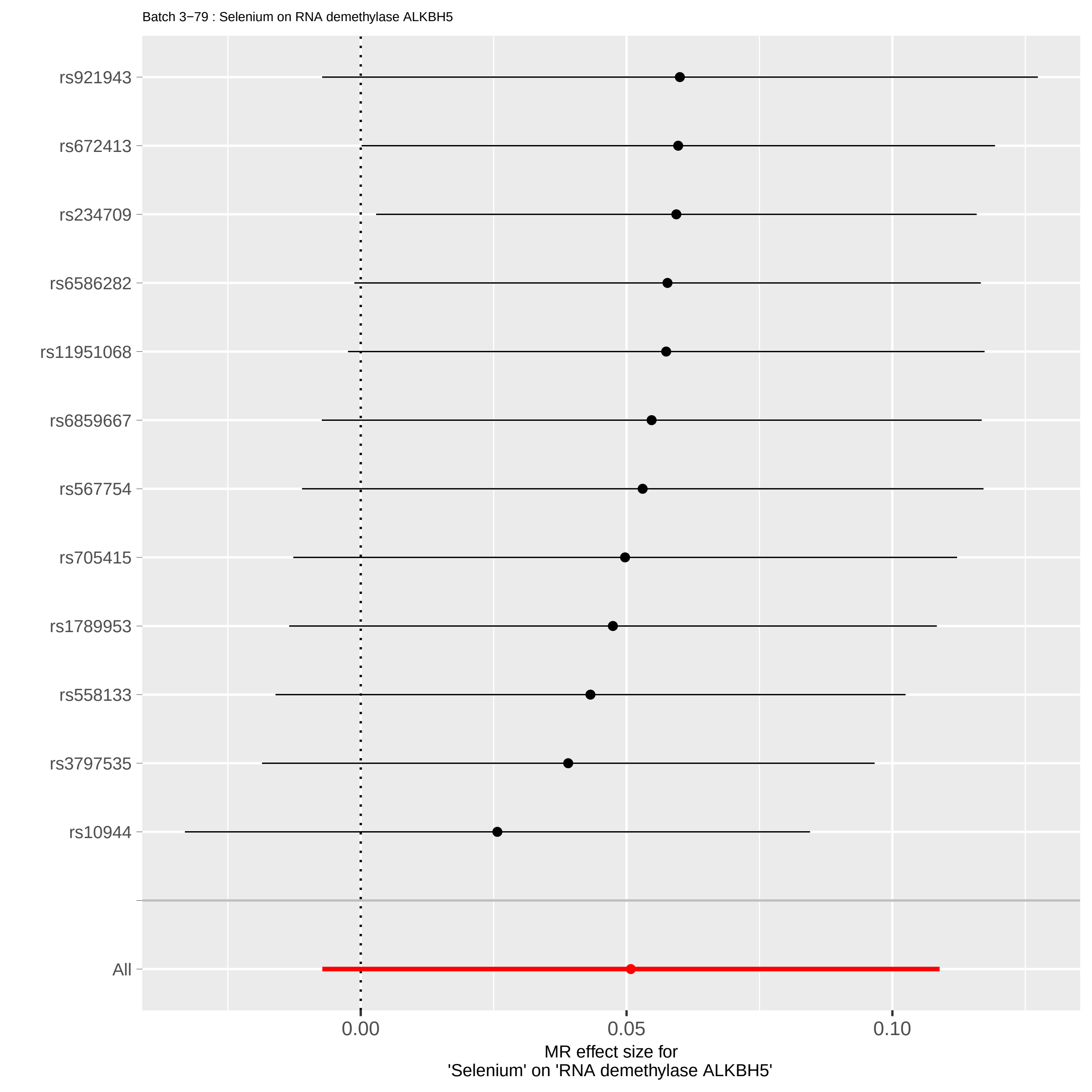
Batch 3−79 : Selenium on RNA demethylase ALKBH5
rs921943
rs672413
rs234709
rs6586282
rs11951068
rs6859667
rs567754
rs705415
rs1789953
rs558133
rs3797535
rs10944
All
0.00
0.05
0.10
MR effect size for
'Selenium' on 'RNA demethylase ALKBH5'

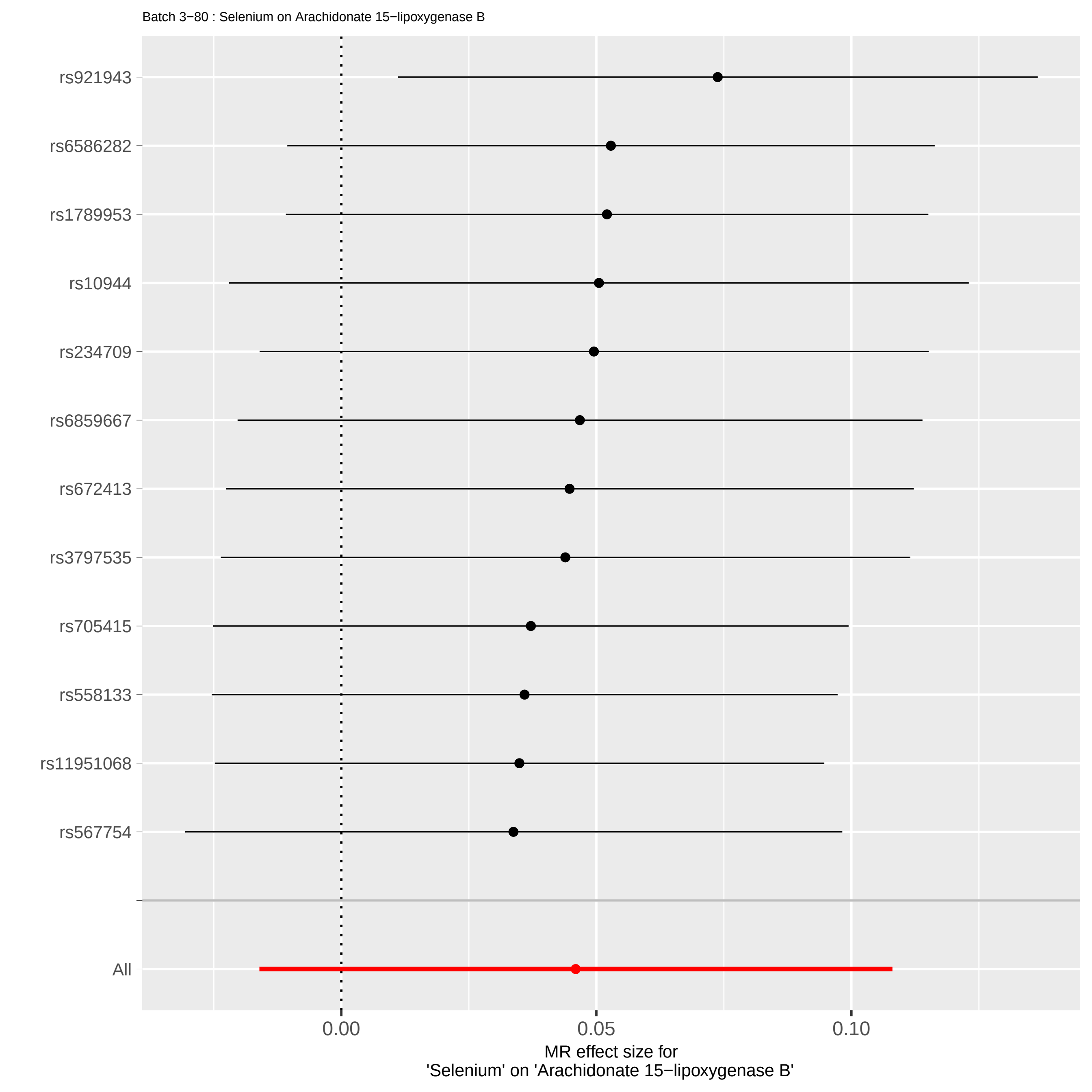
Batch 3−80 : Selenium on Arachidonate 15−lipoxygenase B
rs921943
rs6586282
rs1789953
rs10944
rs234709
rs6859667
rs672413
rs3797535
rs705415
rs558133
rs11951068
rs567754
All
0.00
0.05
0.10
MR effect size for
'Selenium' on 'Arachidonate 15−lipoxygenase B'

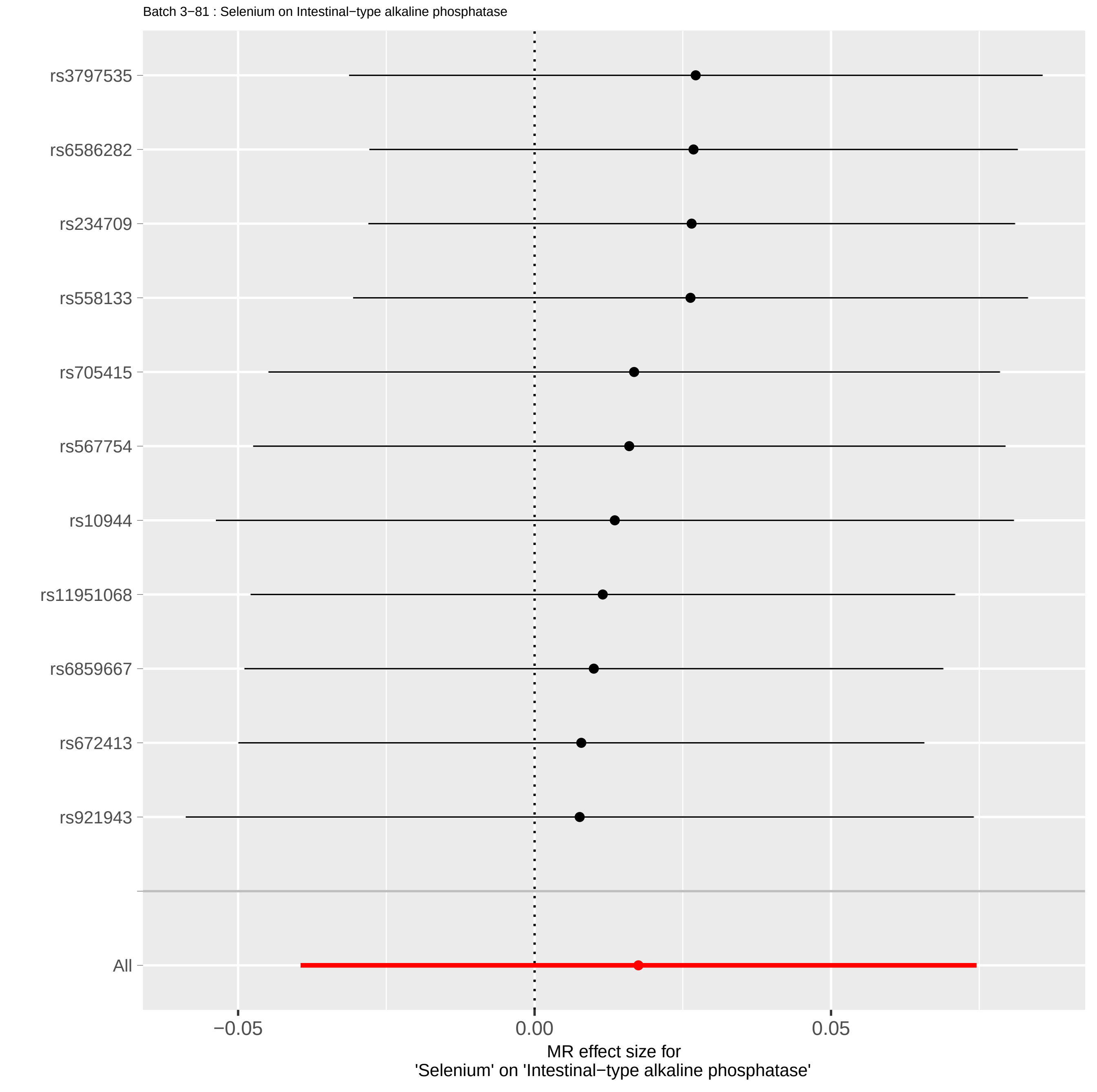

Batch 3−81 : Selenium on Intestinal−type alkaline phosphatase
rs3797535
rs6586282
rs234709
rs558133
rs705415
rs567754
rs10944
rs11951068
rs6859667
rs672413
rs921943
All
−0.05
0.00
0.05
MR effect size for
'Selenium' on 'Intestinal−type alkaline phosphatase'

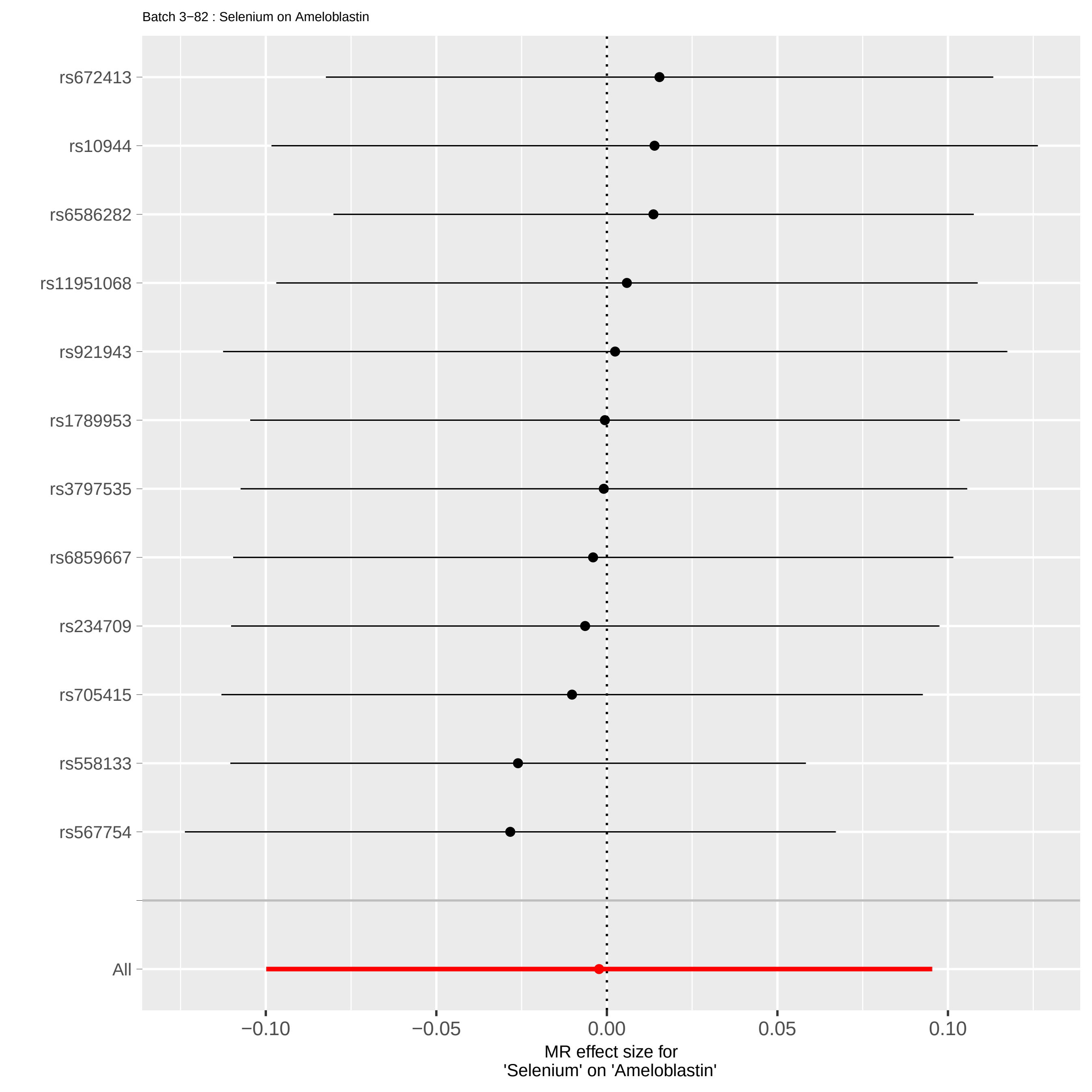

Batch 3−82 : Selenium on Ameloblastin
rs672413
rs10944
rs6586282
rs11951068
rs921943
rs1789953
rs3797535
rs6859667
rs234709
rs705415
rs558133
rs567754
All
−0.10
−0.05
0.00
0.05
0.10
MR effect size for
'Selenium' on 'Ameloblastin'

Batch 3−83 : Selenium on Amelogenin, X isoform

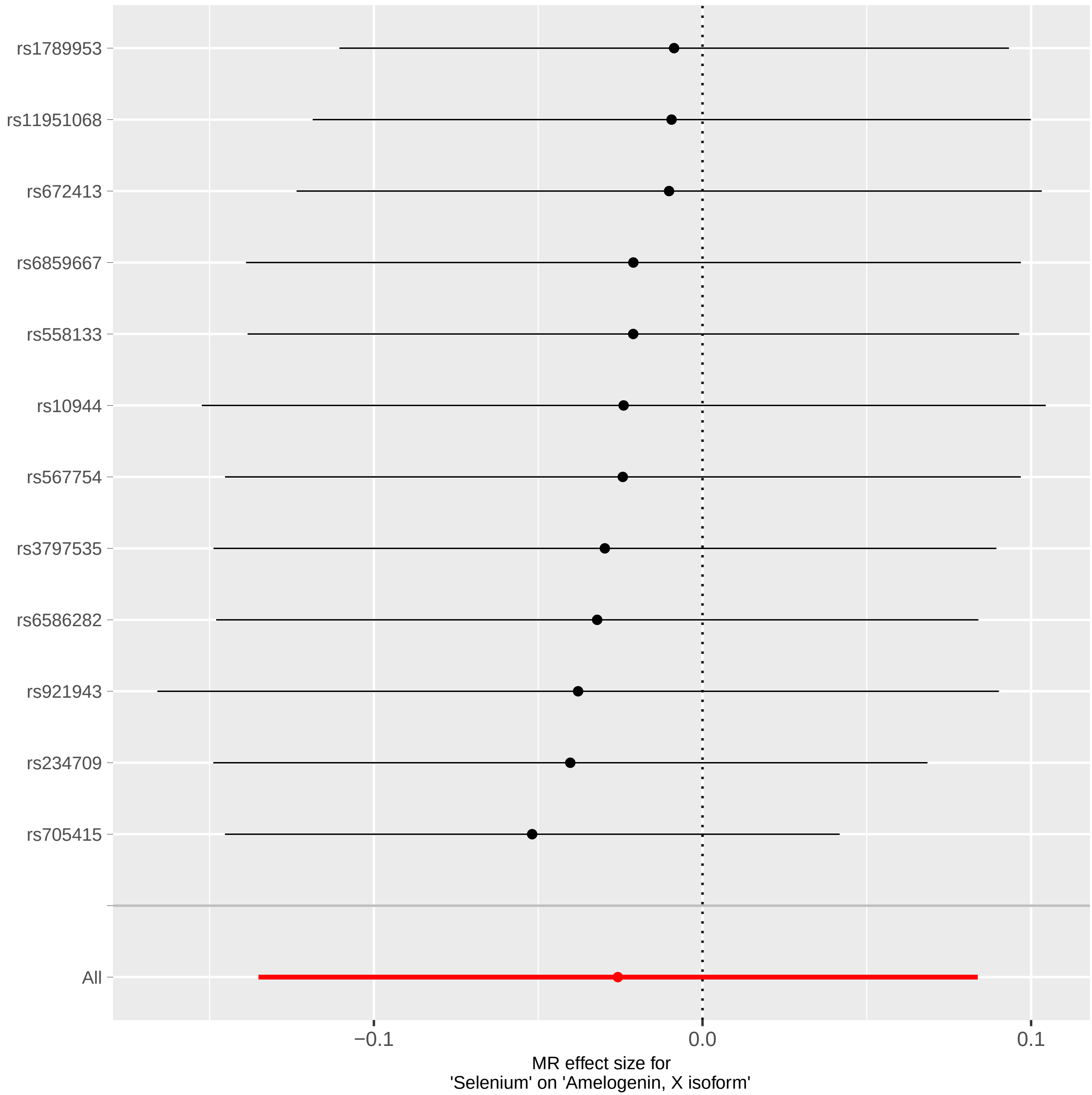

Batch 3−84 : Selenium on Muellerian−inhibiting factor

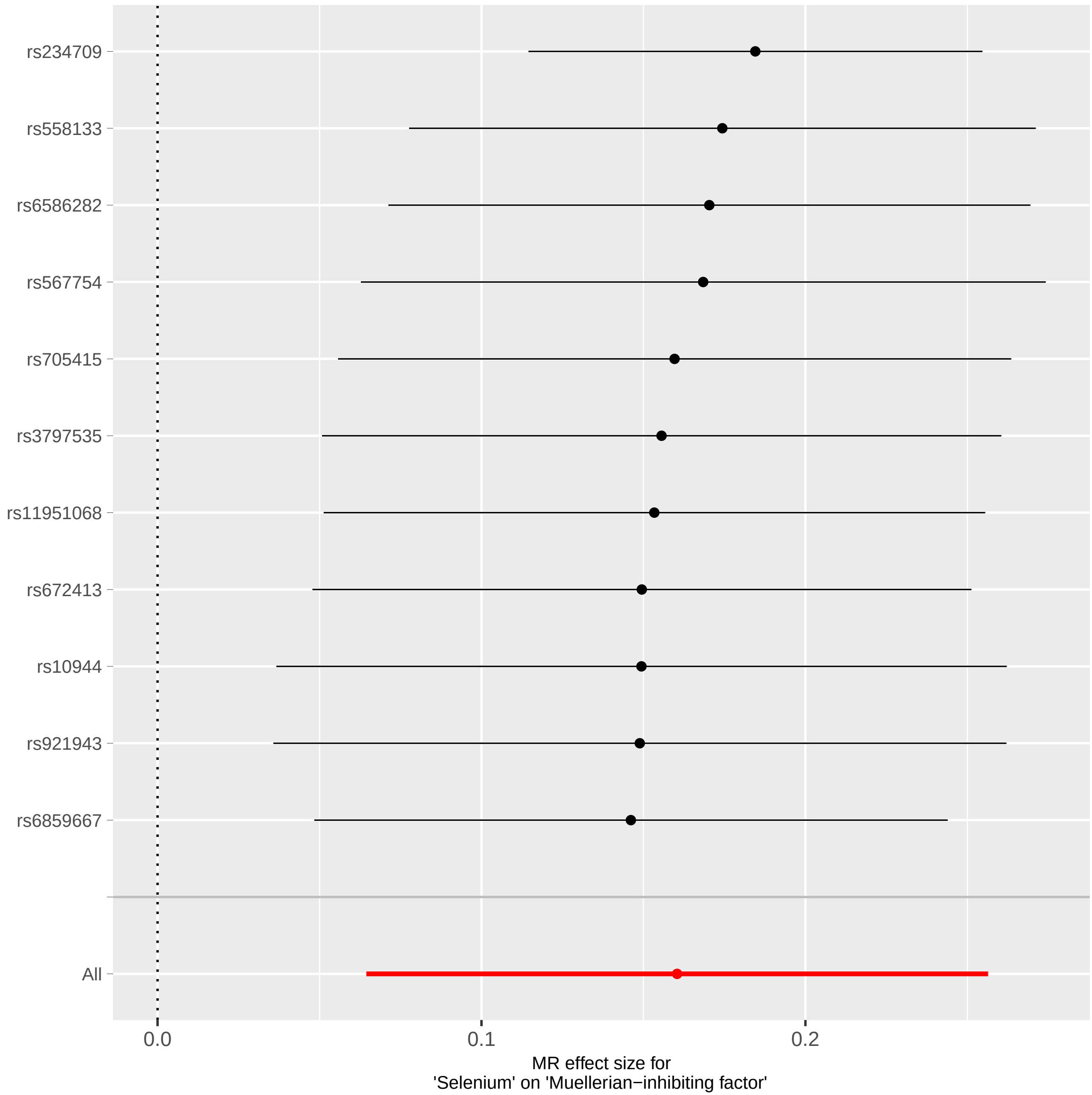

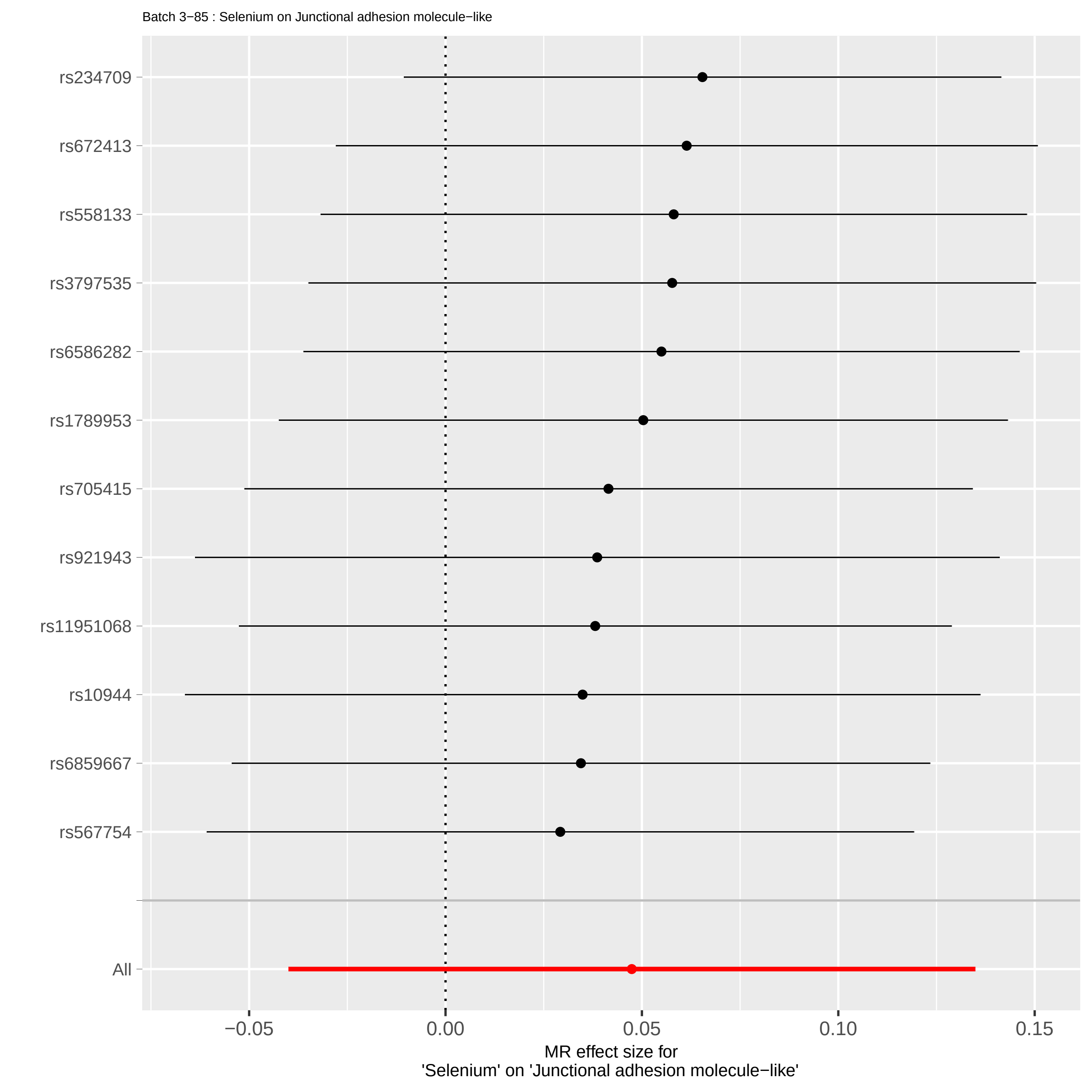

Batch 3−85 : Selenium on Junctional adhesion molecule−like
rs234709
rs672413
rs558133
rs3797535
rs6586282
rs1789953
rs705415
rs921943
rs11951068
rs10944
rs6859667
rs567754
All
−0.05
0.00
0.05
0.10
0.15
MR effect size for
'Selenium' on 'Junctional adhesion molecule−like'

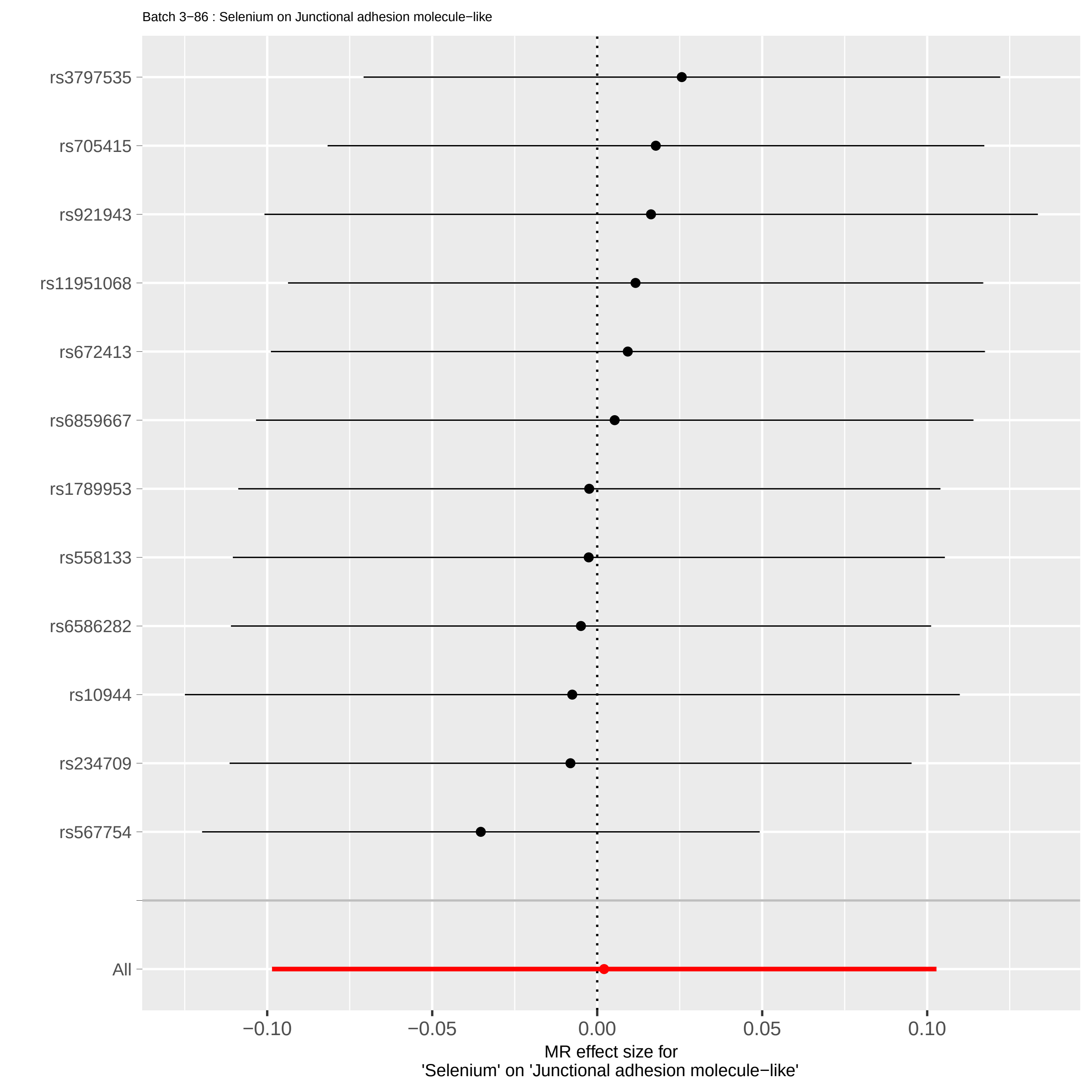
Batch 3−86 : Selenium on Junctional adhesion molecule−like
rs3797535
rs705415
rs921943
rs11951068
rs672413
rs6859667
rs1789953
rs558133
rs6586282
rs10944
rs234709
rs567754
All
−0.10
−0.05
0.00
0.05
0.10
MR effect size for
'Selenium' on 'Junctional adhesion molecule−like'

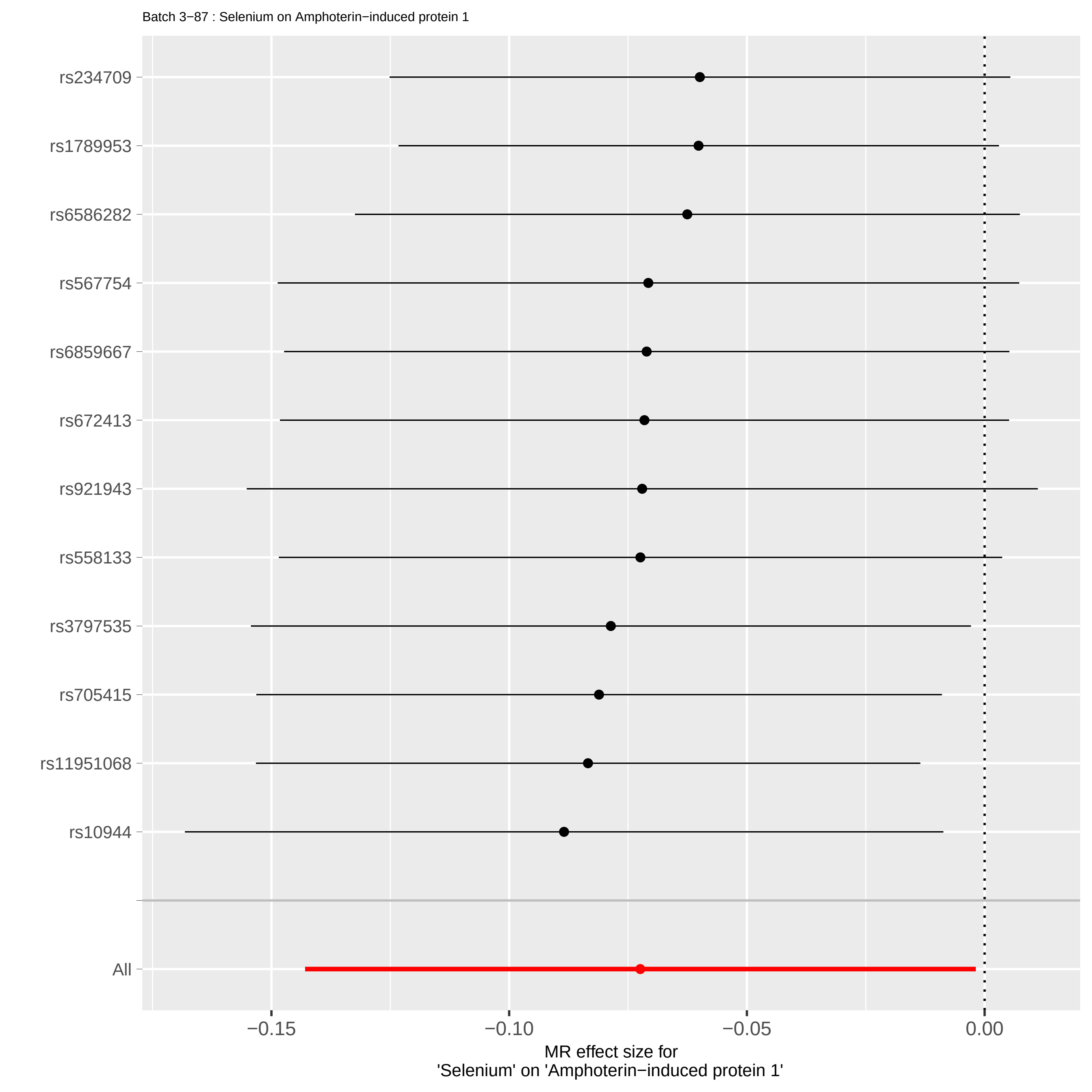

Batch 3−87 : Selenium on Amphoterin−induced protein 1
rs234709
rs1789953
rs6586282
rs567754
rs6859667
rs672413
rs921943
rs558133
rs3797535
rs705415
rs11951068
rs10944
All
−0.15
−0.10
−0.05
0.00
MR effect size for
'Selenium' on 'Amphoterin−induced protein 1'

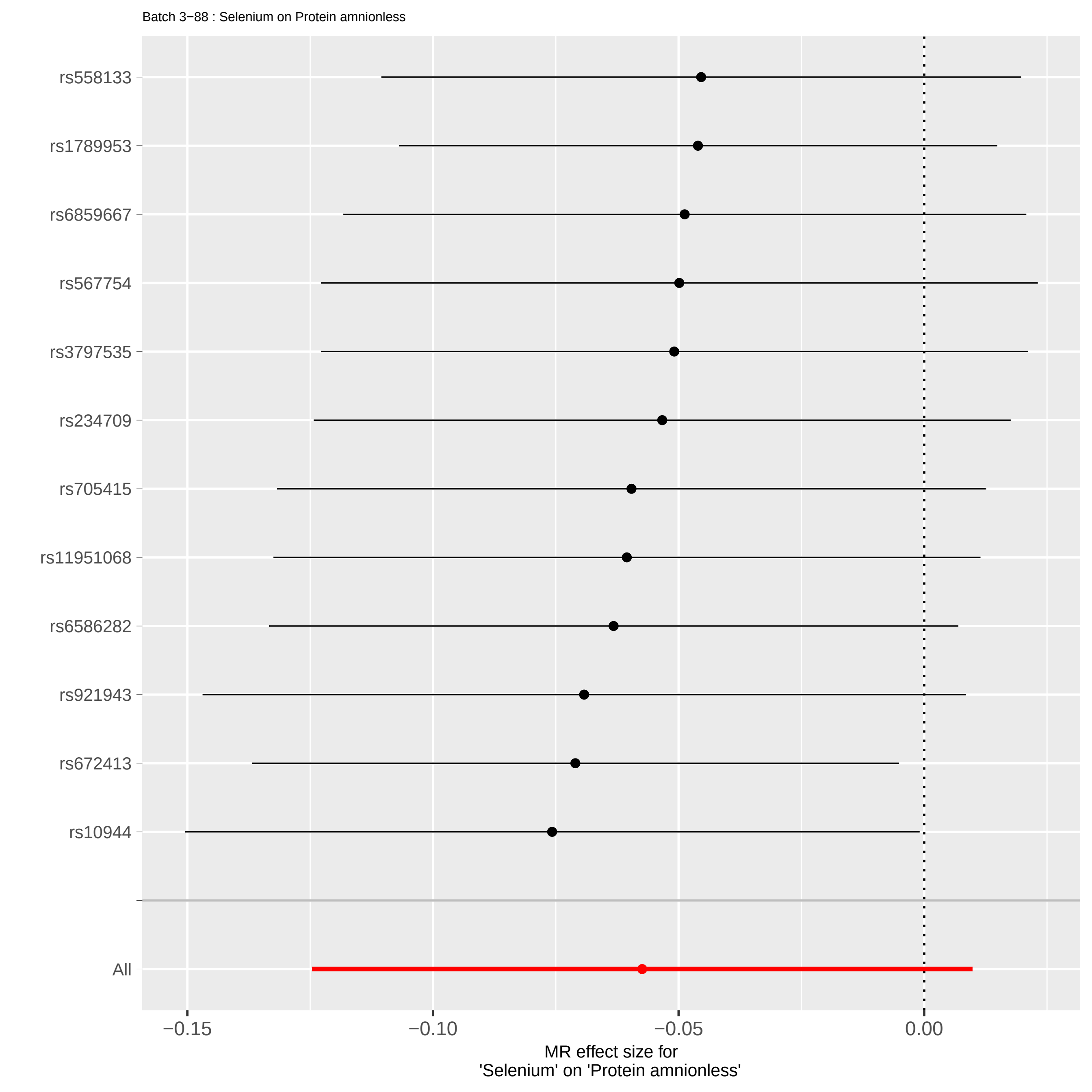

Batch 3−88 : Selenium on Protein amnionless
rs558133
rs1789953
rs6859667
rs567754
rs3797535
rs234709
rs705415
rs11951068
rs6586282
rs921943
rs672413
rs10944
All
−0.15
−0.10
−0.05
0.00
MR effect size for
'Selenium' on 'Protein amnionless'

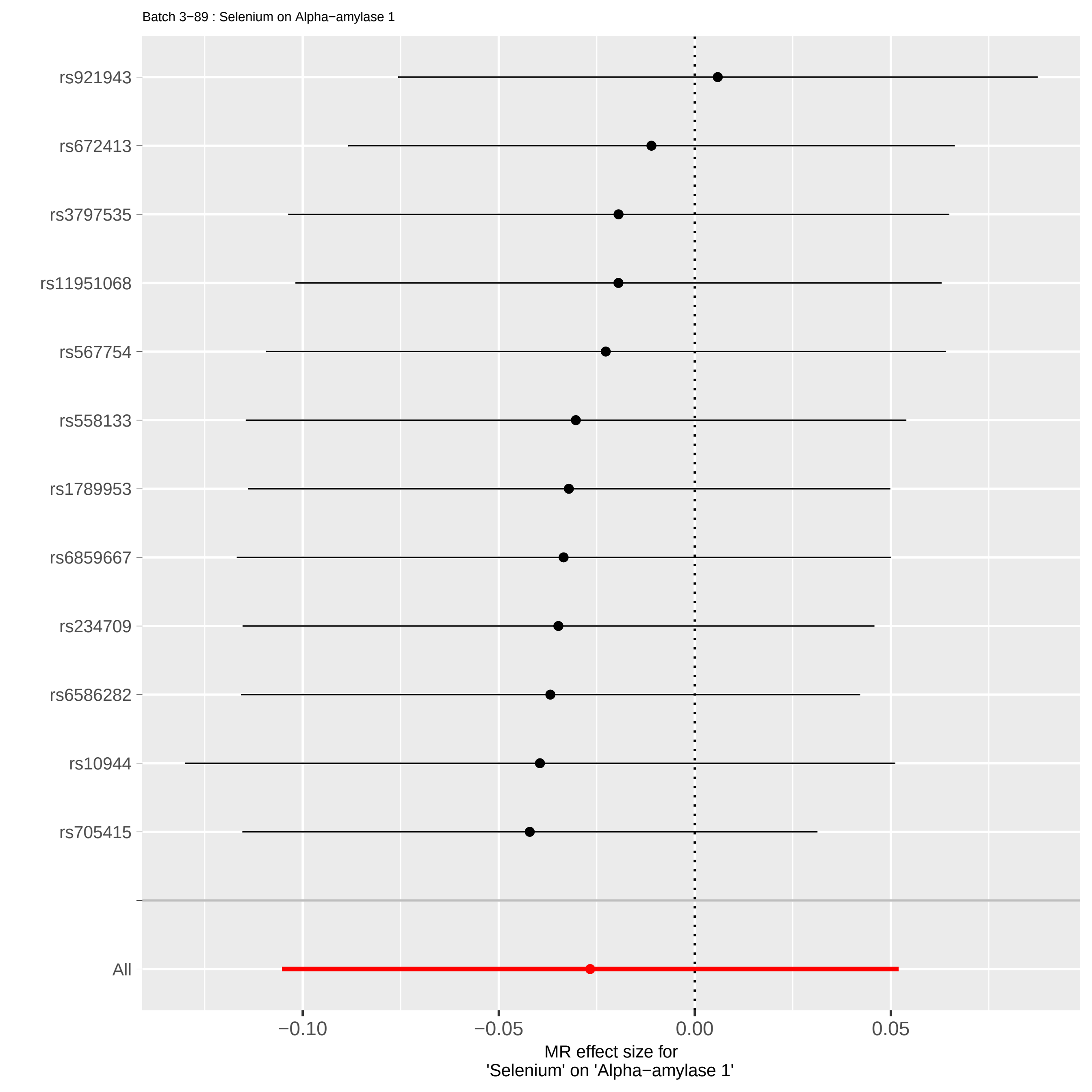

Batch 3−89 : Selenium on Alpha−amylase 1
rs921943
rs672413
rs3797535
rs11951068
rs567754
rs558133
rs1789953
rs6859667
rs234709
rs6586282
rs10944
rs705415
All
−0.10
−0.05
0.00
0.05
MR effect size for
'Selenium' on 'Alpha−amylase 1'

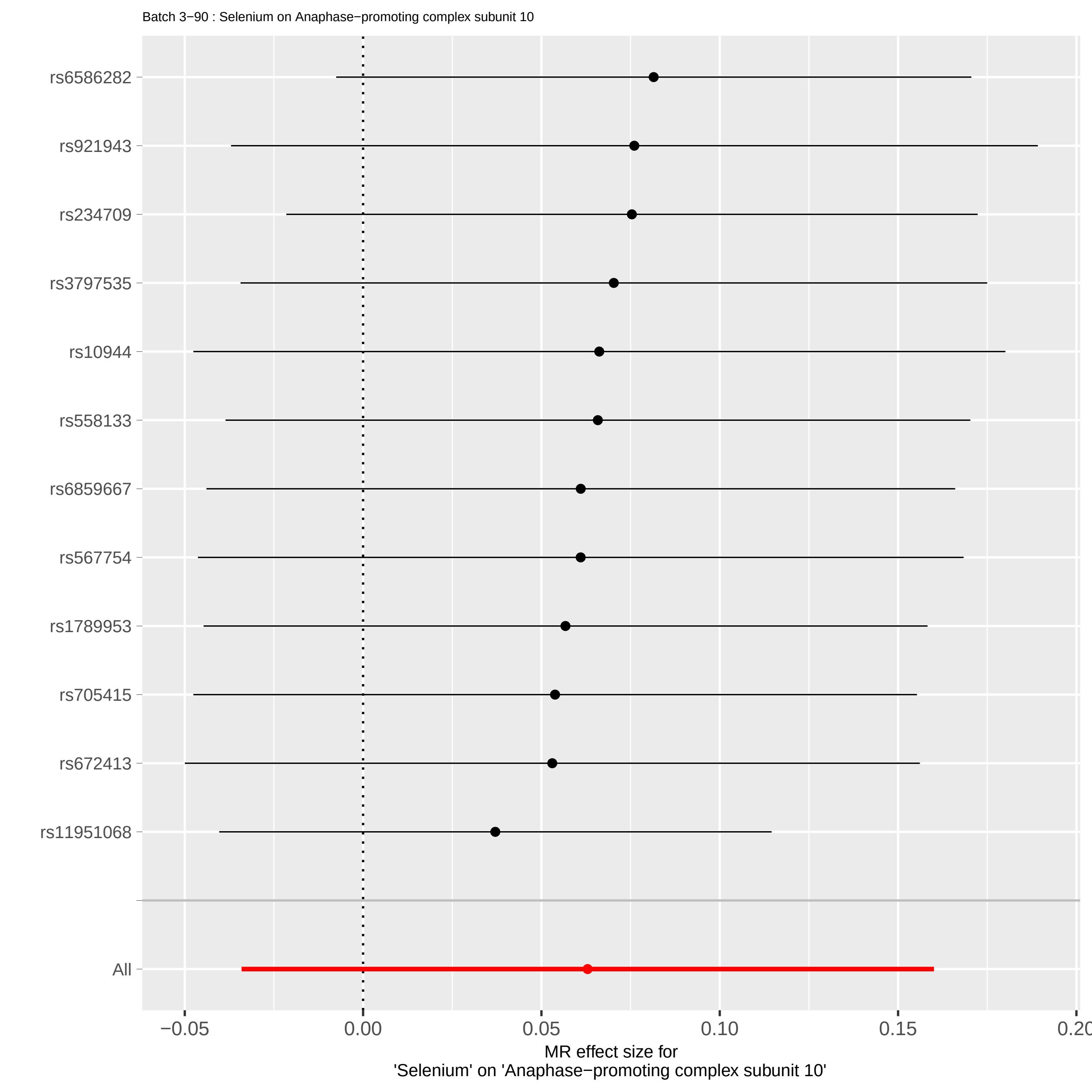

Batch 3−90 : Selenium on Anaphase−promoting complex subunit 10
rs6586282
rs921943
rs234709
rs3797535
rs10944
rs558133
rs6859667
rs567754
rs1789953
rs705415
rs672413
rs11951068
All
−0.05
0.00
0.05
0.10
0.15
0.20
MR effect size for
'Selenium' on 'Anaphase−promoting complex subunit 10'

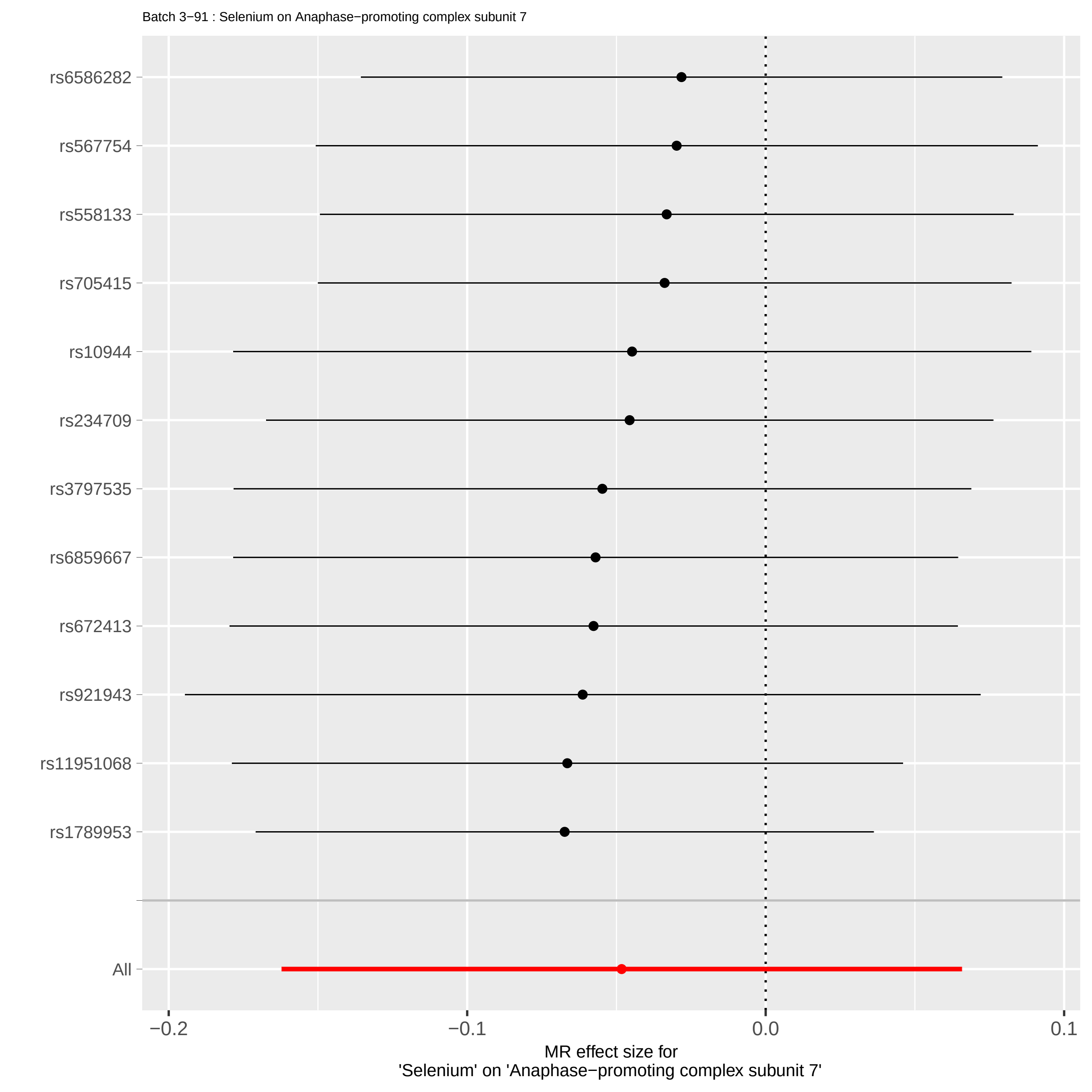
Batch 3−91 : Selenium on Anaphase−promoting complex subunit 7
rs6586282
rs567754
rs558133
rs705415
rs10944
rs234709
rs3797535
rs6859667
rs672413
rs921943
rs11951068
rs1789953
All
−0.2
−0.1
0.0
0.1
MR effect size for
'Selenium' on 'Anaphase−promoting complex subunit 7'

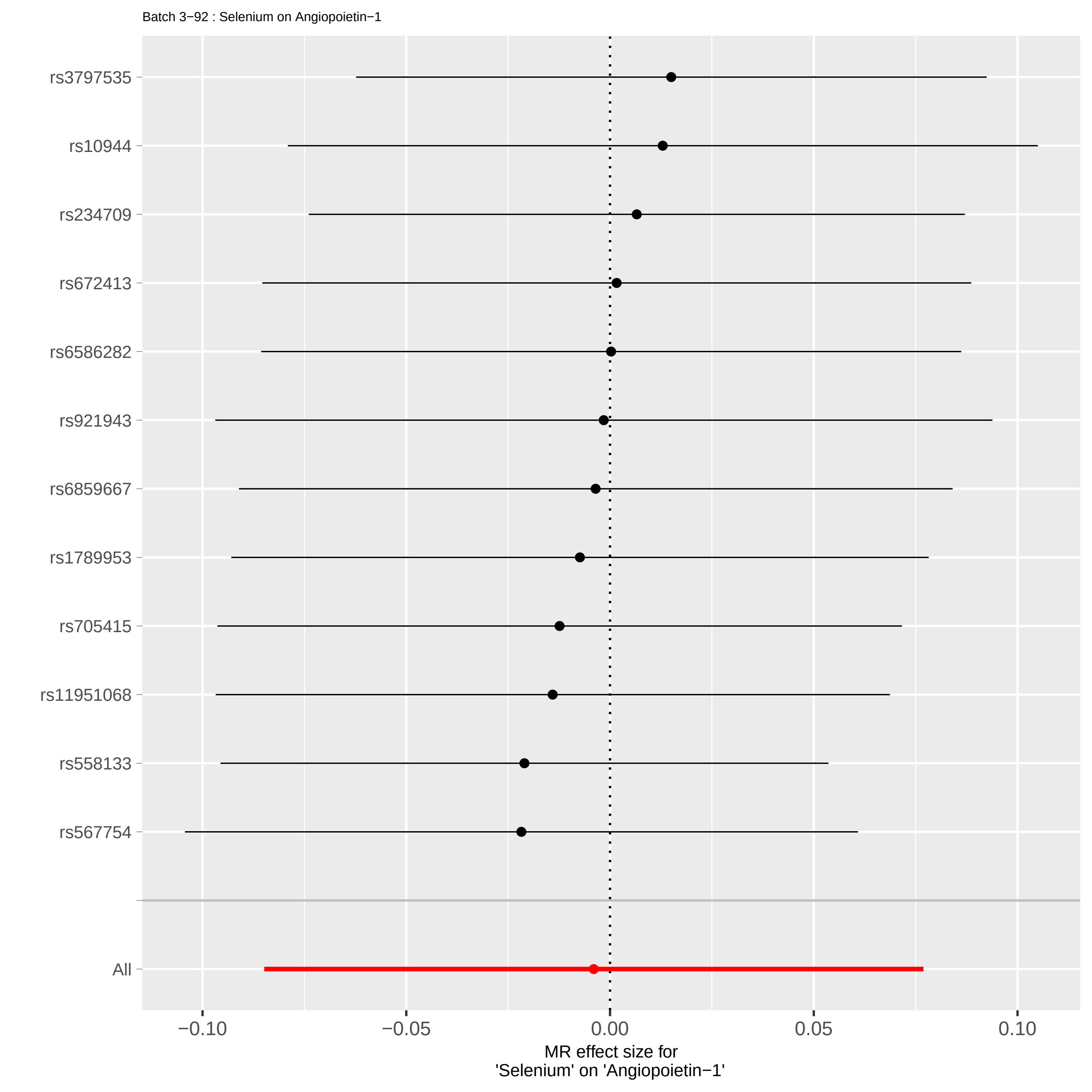

Batch 3−92 : Selenium on Angiopoietin−1
rs3797535
rs10944
rs234709
rs672413
rs6586282
rs921943
rs6859667
rs1789953
rs705415
rs11951068
rs558133
rs567754
All
−0.10
−0.05
0.00
0.05
0.10
MR effect size for
'Selenium' on 'Angiopoietin−1'

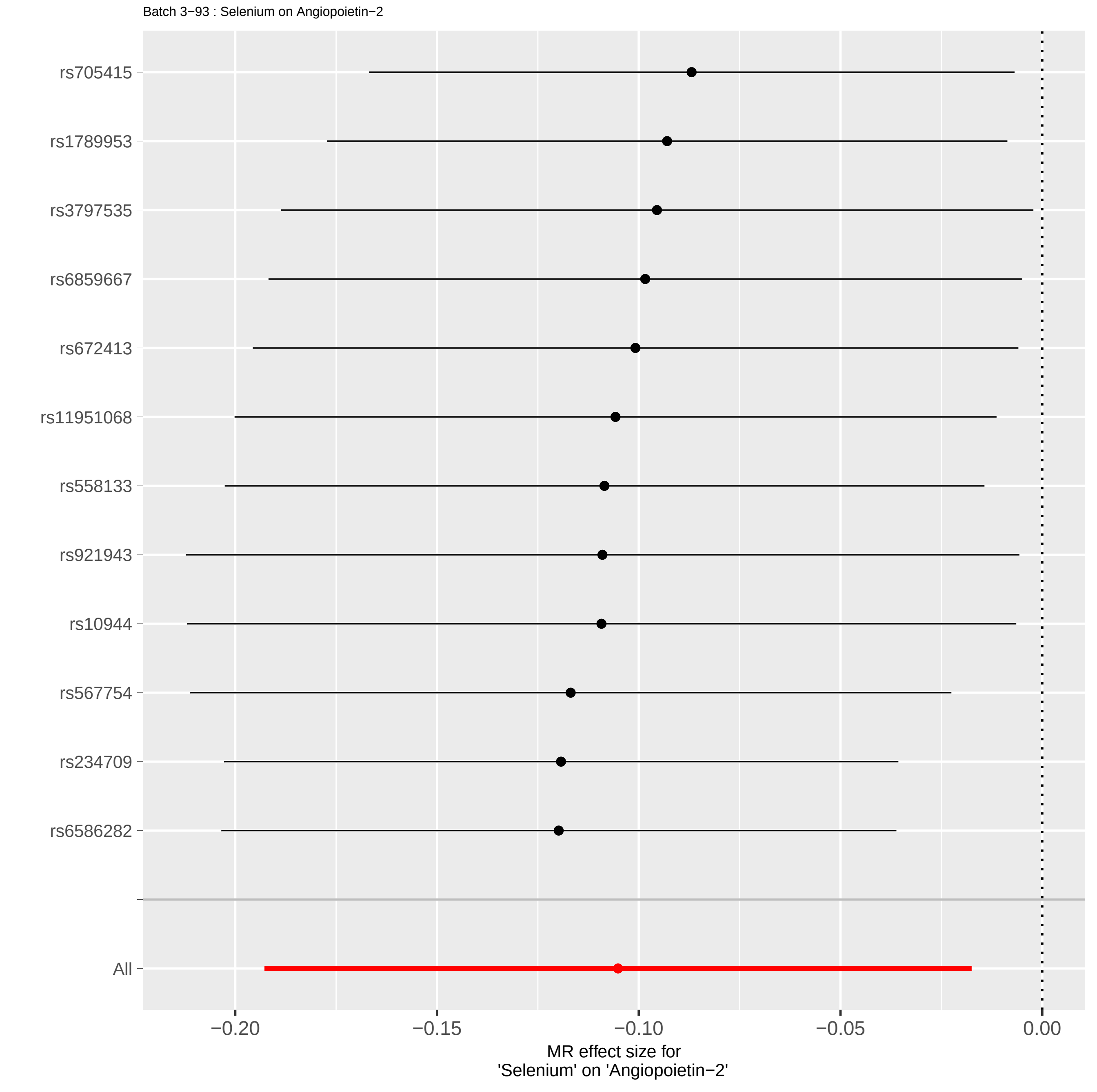

Batch 3−93 : Selenium on Angiopoietin−2
rs705415
rs1789953
rs3797535
rs6859667
rs672413
rs11951068
rs558133
rs921943
rs10944
rs567754
rs234709
rs6586282
All
−0.20
−0.15
−0.10
−0.05
0.00
MR effect size for
'Selenium' on 'Angiopoietin−2'

Batch 3−94 : Selenium on Angiopoietin−2

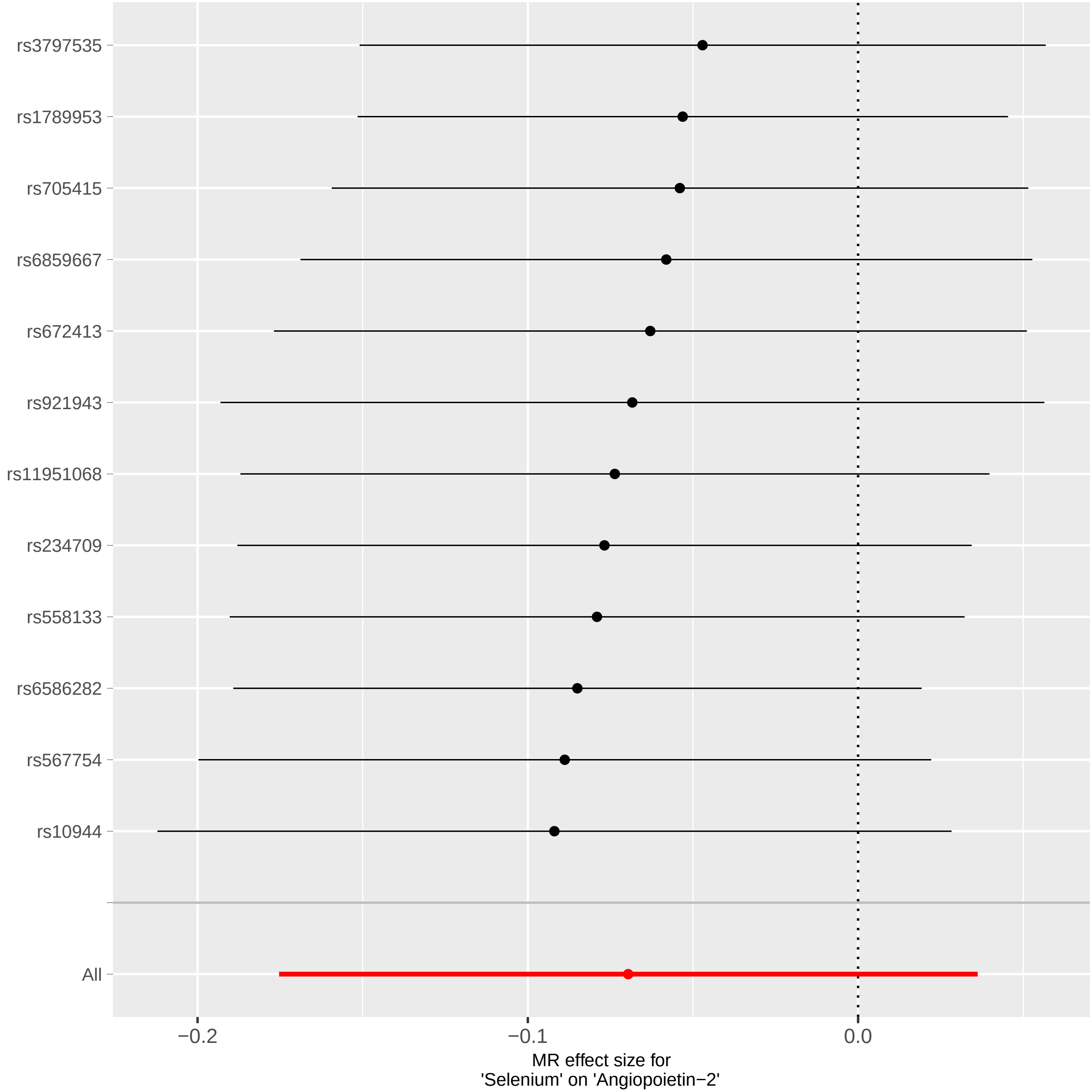

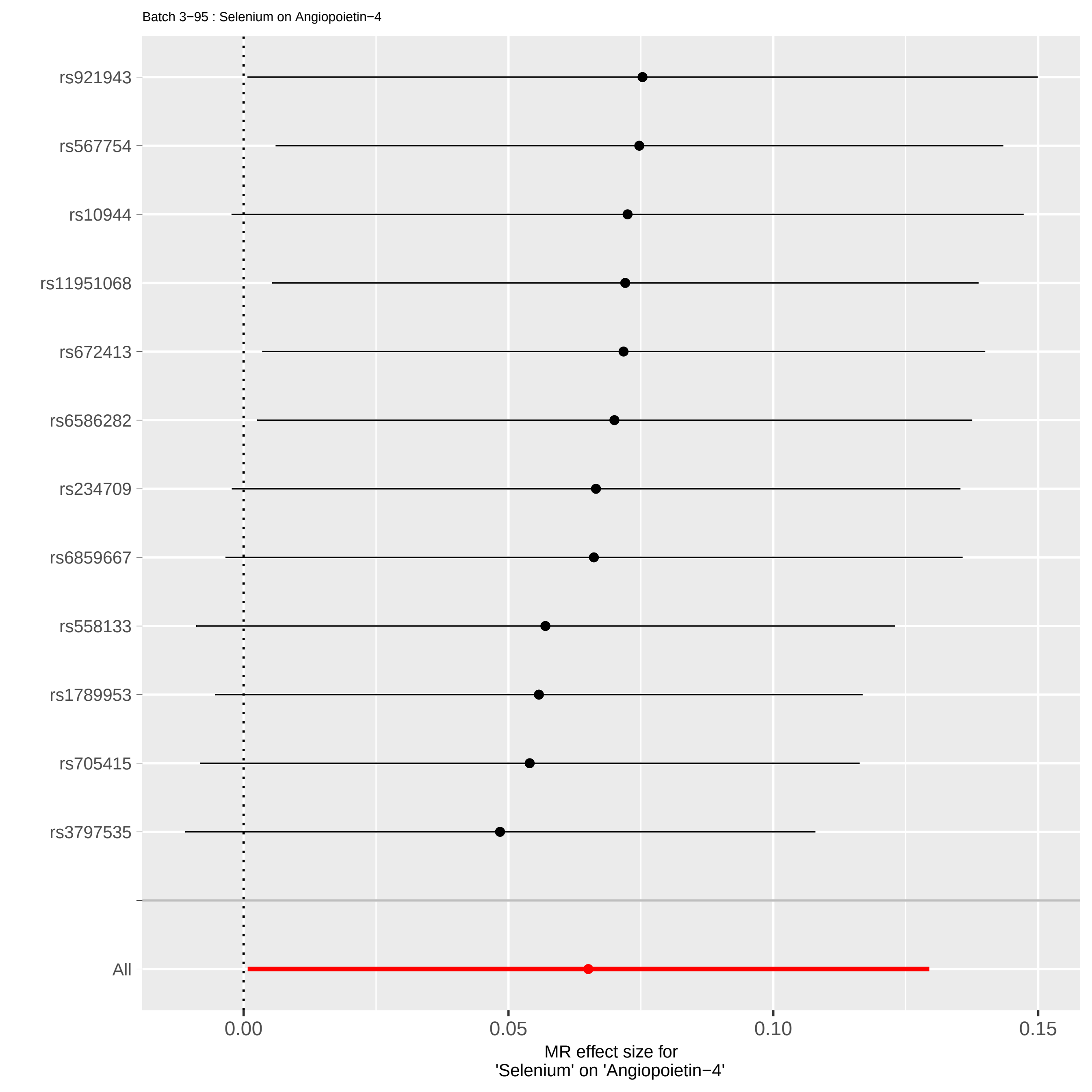

Batch 3−95 : Selenium on Angiopoietin−4
rs921943
rs567754
rs10944
rs11951068
rs672413
rs6586282
rs234709
rs6859667
rs558133
rs1789953
rs705415
rs3797535
All
0.00
0.05
0.10
0.15
MR effect size for
'Selenium' on 'Angiopoietin−4'

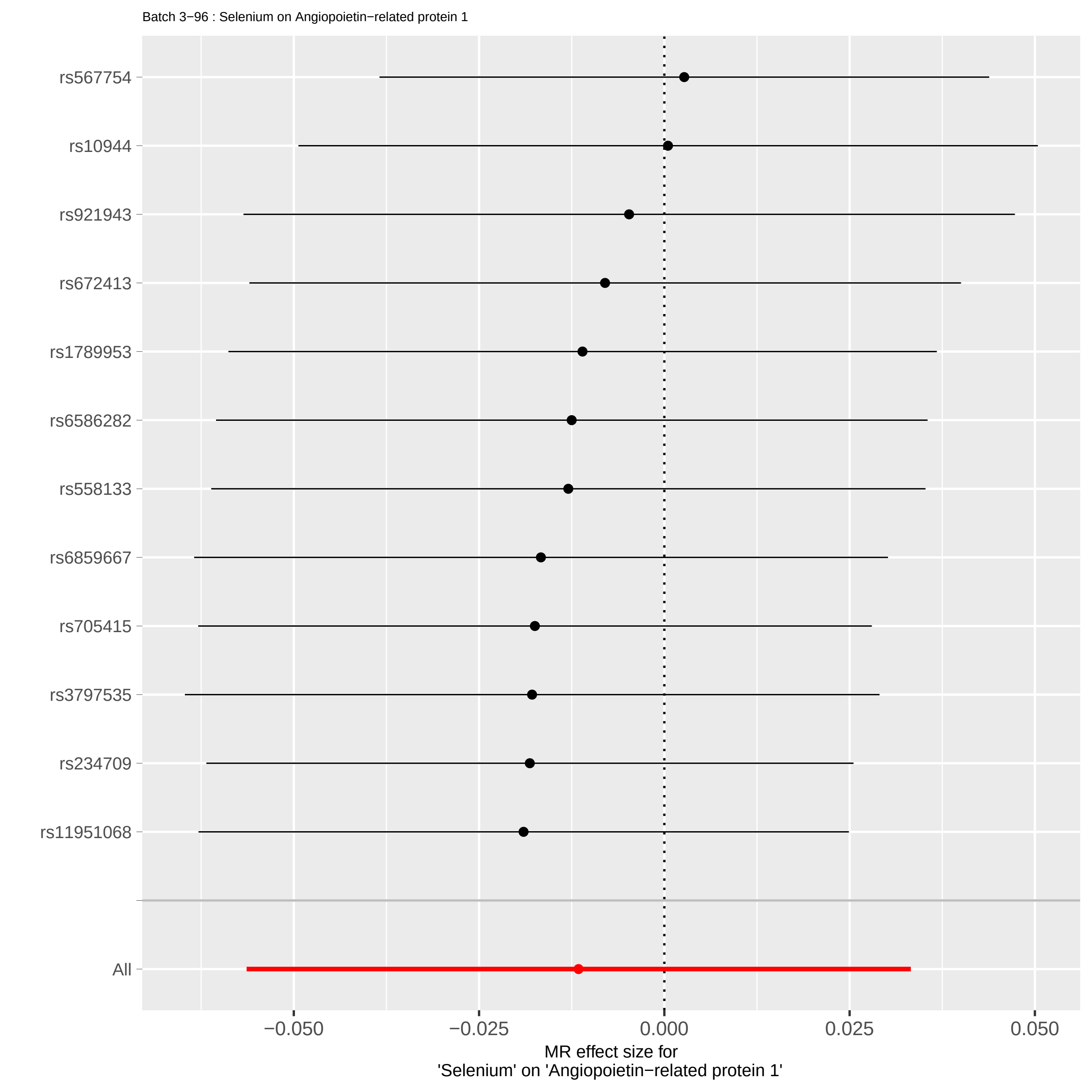
Batch 3−96 : Selenium on Angiopoietin−related protein 1
rs567754
rs10944
rs921943
rs672413
rs1789953
rs6586282
rs558133
rs6859667
rs705415
rs3797535
rs234709
rs11951068
All
−0.050
−0.025
0.000
0.025
0.050
MR effect size for
'Selenium' on 'Angiopoietin−related protein 1'

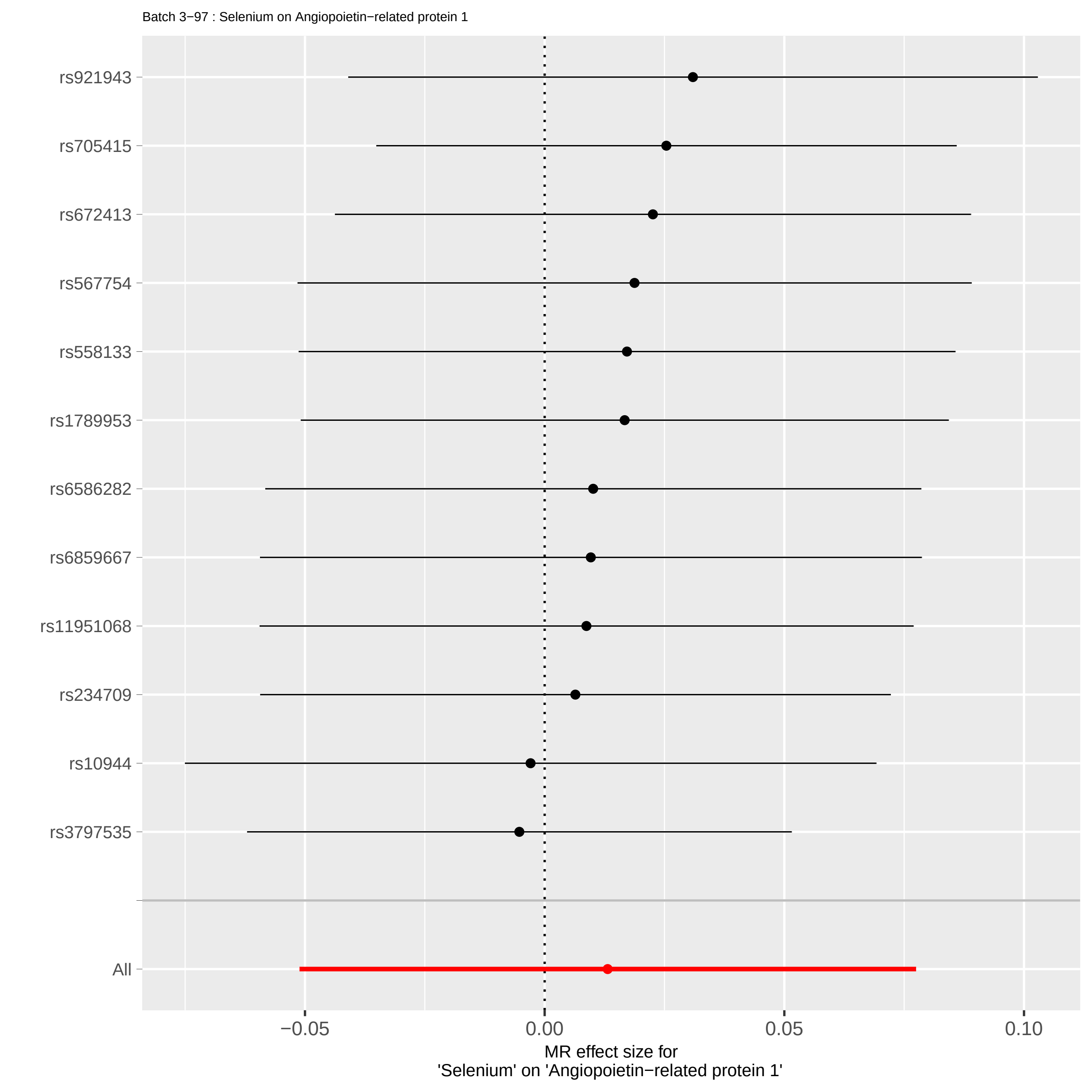

Batch 3−97 : Selenium on Angiopoietin−related protein 1
rs921943
rs705415
rs672413
rs567754
rs558133
rs1789953
rs6586282
rs6859667
rs11951068
rs234709
rs10944
rs3797535
All
−0.05
0.00
0.05
0.10
MR effect size for
'Selenium' on 'Angiopoietin−related protein 1'

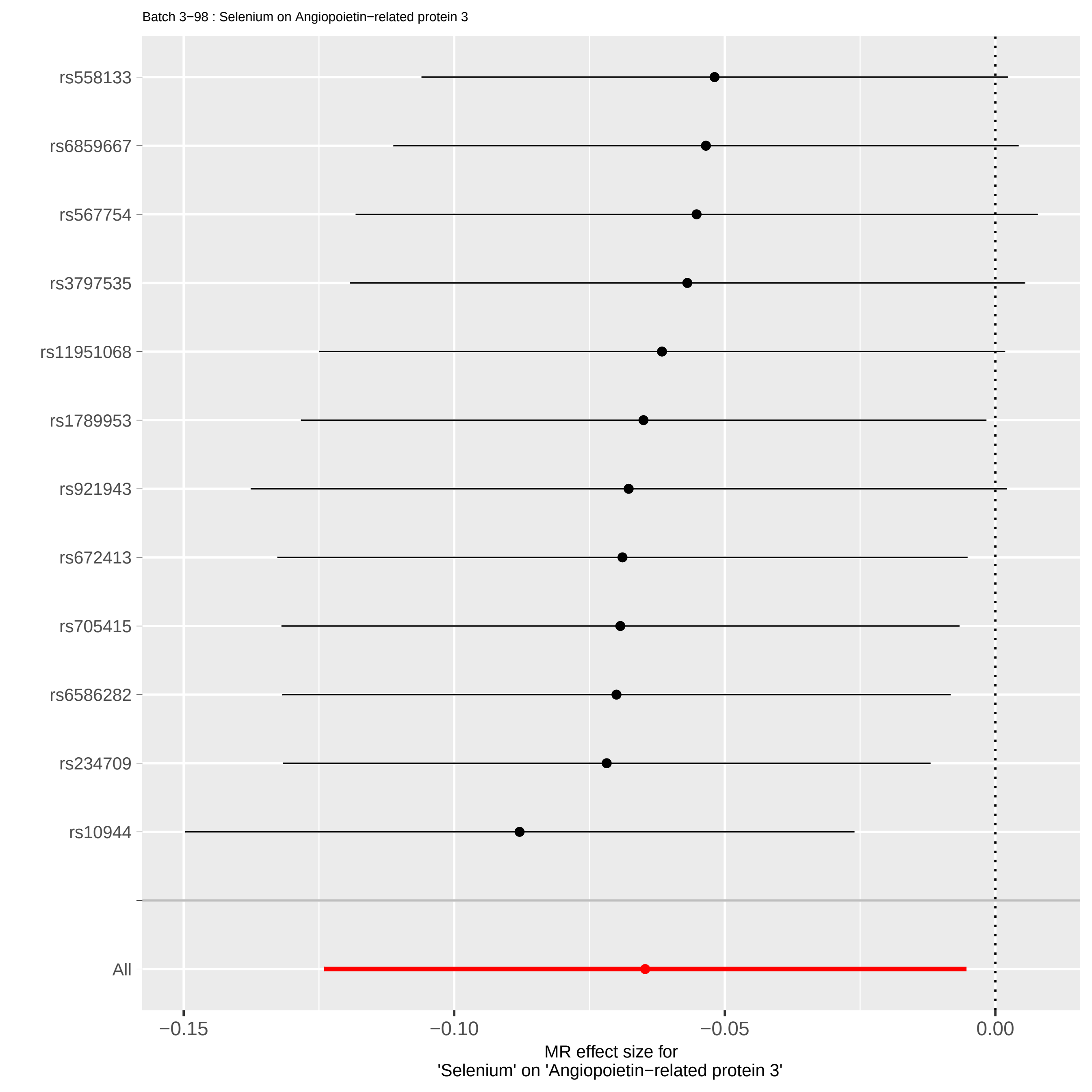

Batch 3−98 : Selenium on Angiopoietin−related protein 3
rs558133
rs6859667
rs567754
rs3797535
rs11951068
rs1789953
rs921943
rs672413
rs705415
rs6586282
rs234709
rs10944
All
−0.15
−0.10
−0.05
0.00
MR effect size for
'Selenium' on 'Angiopoietin−related protein 3'

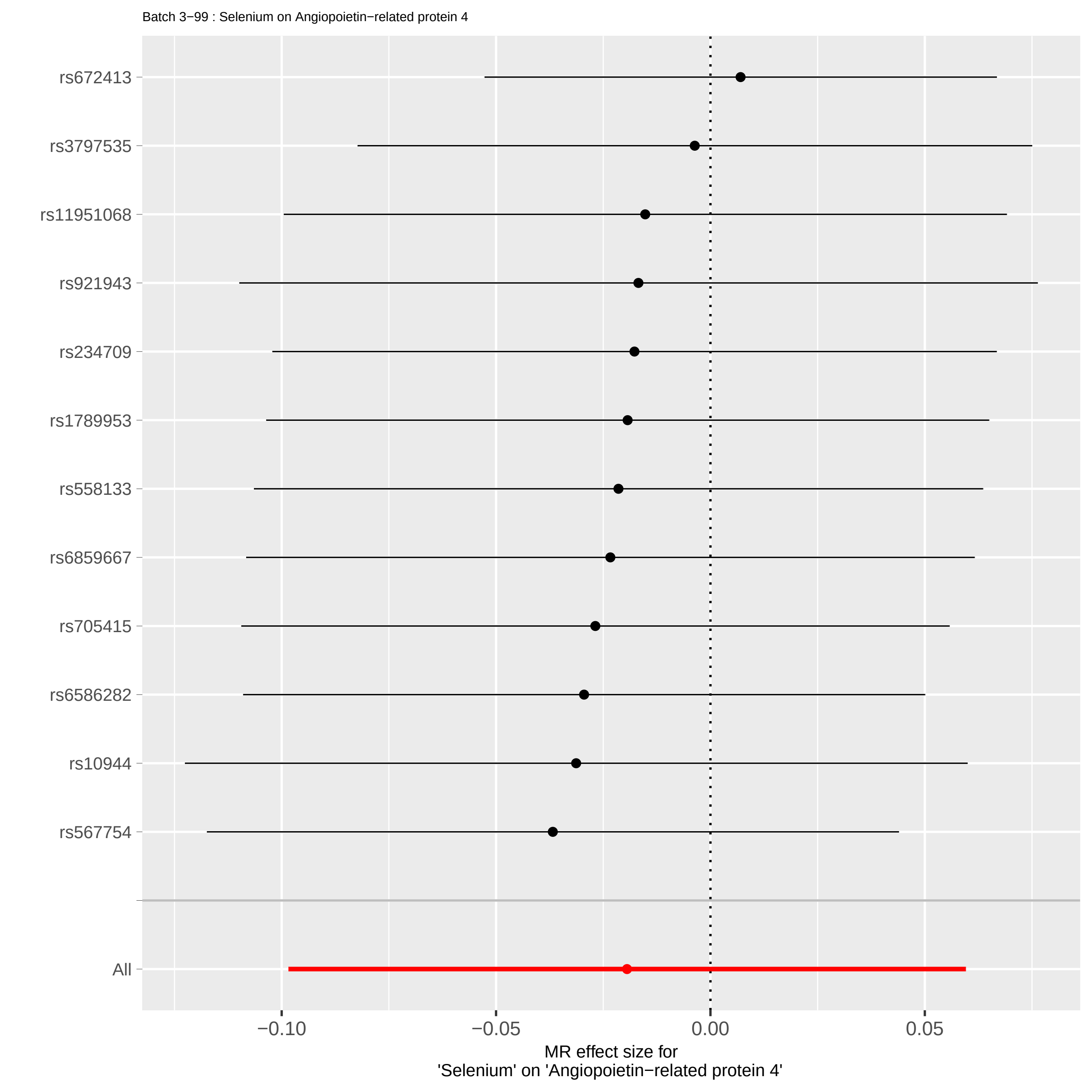
Batch 3−99 : Selenium on Angiopoietin−related protein 4
rs672413
rs3797535
rs11951068
rs921943
rs234709
rs1789953
rs558133
rs6859667
rs705415
rs6586282
rs10944
rs567754
All
−0.10
−0.05
0.00
0.05
MR effect size for
'Selenium' on 'Angiopoietin−related protein 4'

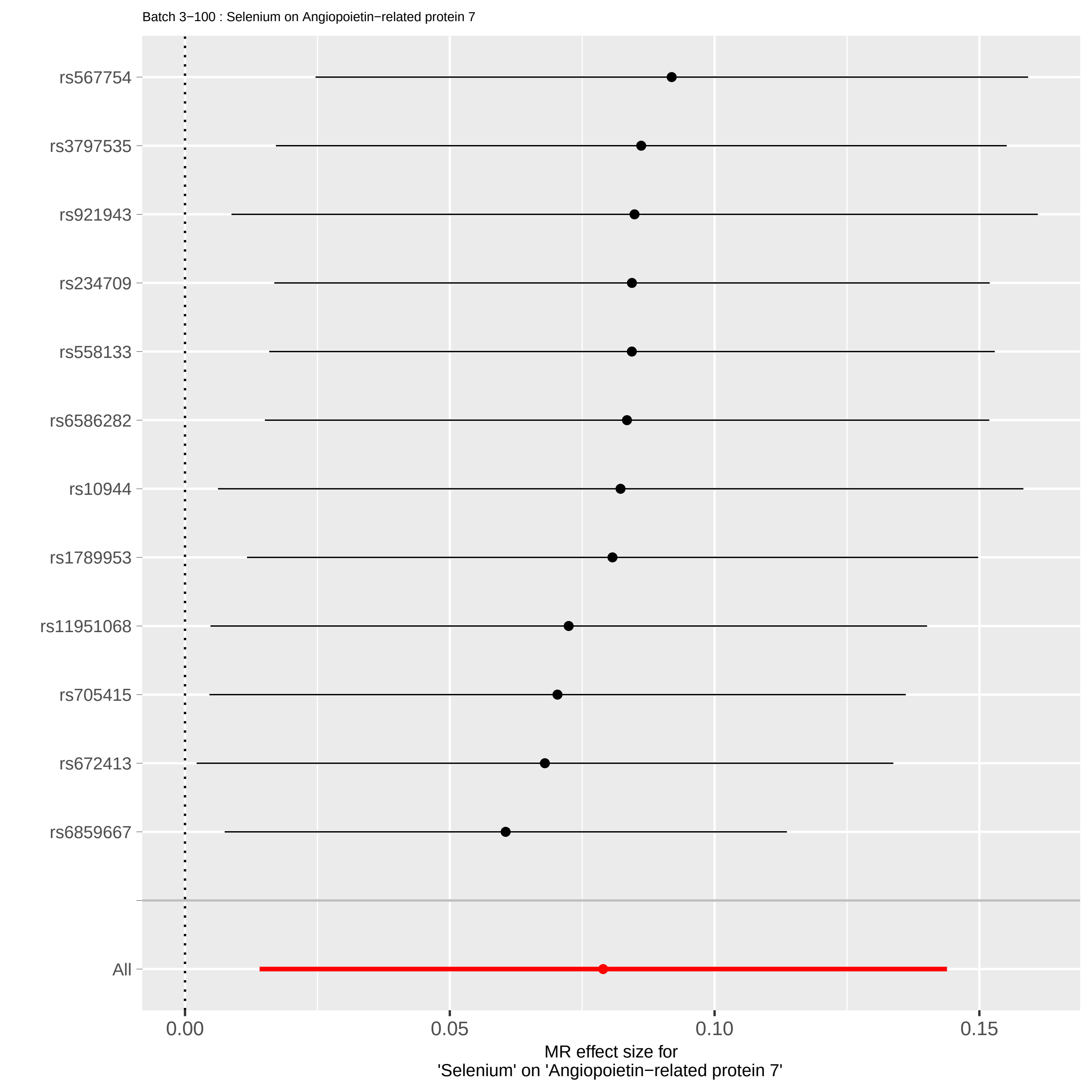

Batch 3−100 : Selenium on Angiopoietin−related protein 7
rs567754
rs3797535
rs921943
rs234709
rs558133
rs6586282
rs10944
rs1789953
rs11951068
rs705415
rs672413
rs6859667
All
0.00
0.05
0.10
0.15
MR effect size for
'Selenium' on 'Angiopoietin−related protein 7'

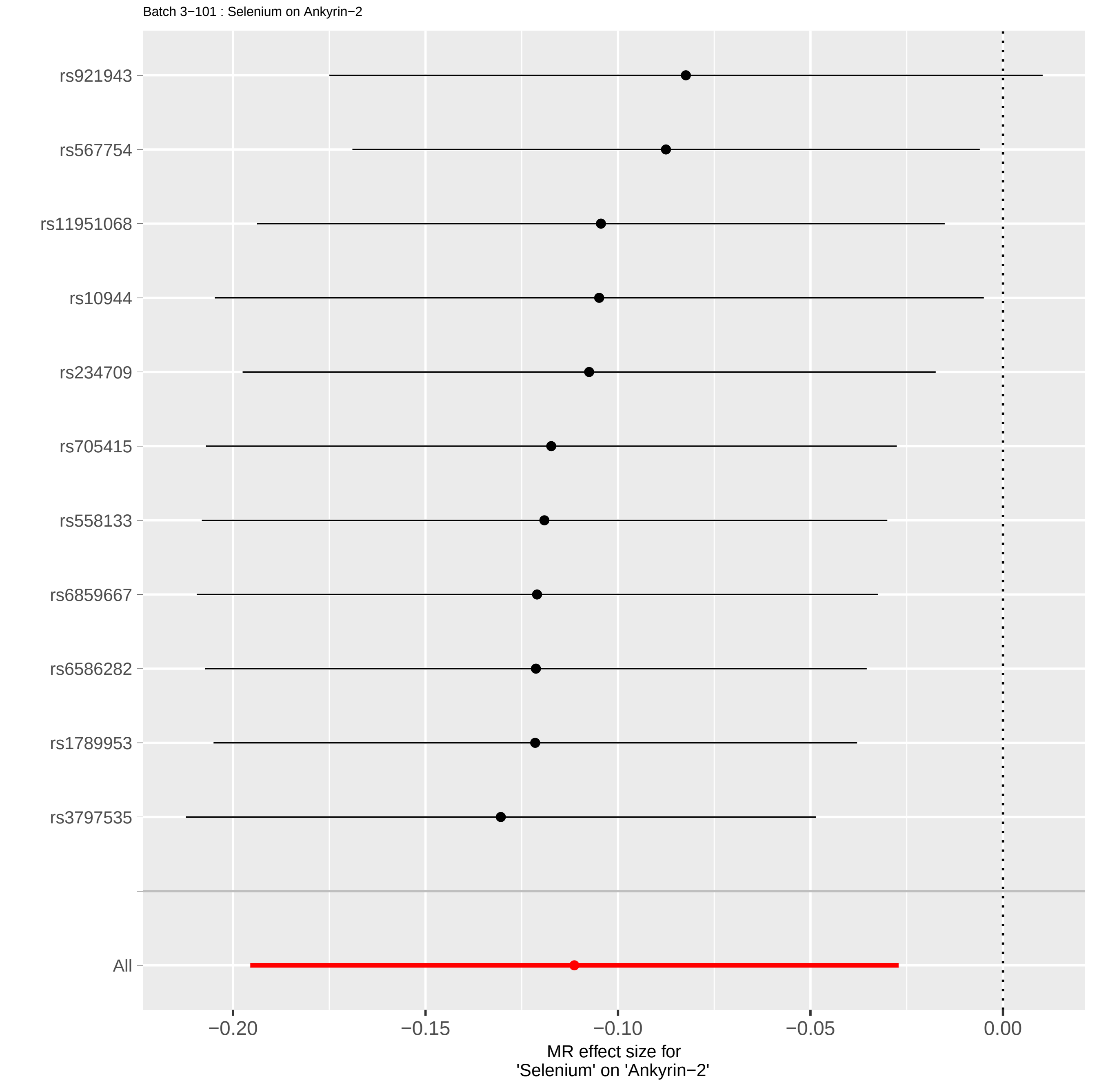

Batch 3−101 : Selenium on Ankyrin−2
rs921943
rs567754
rs11951068
rs10944
rs234709
rs705415
rs558133
rs6859667
rs6586282
rs1789953
rs3797535
All
−0.20
−0.15
−0.10
−0.05
0.00
MR effect size for
'Selenium' on 'Ankyrin−2'

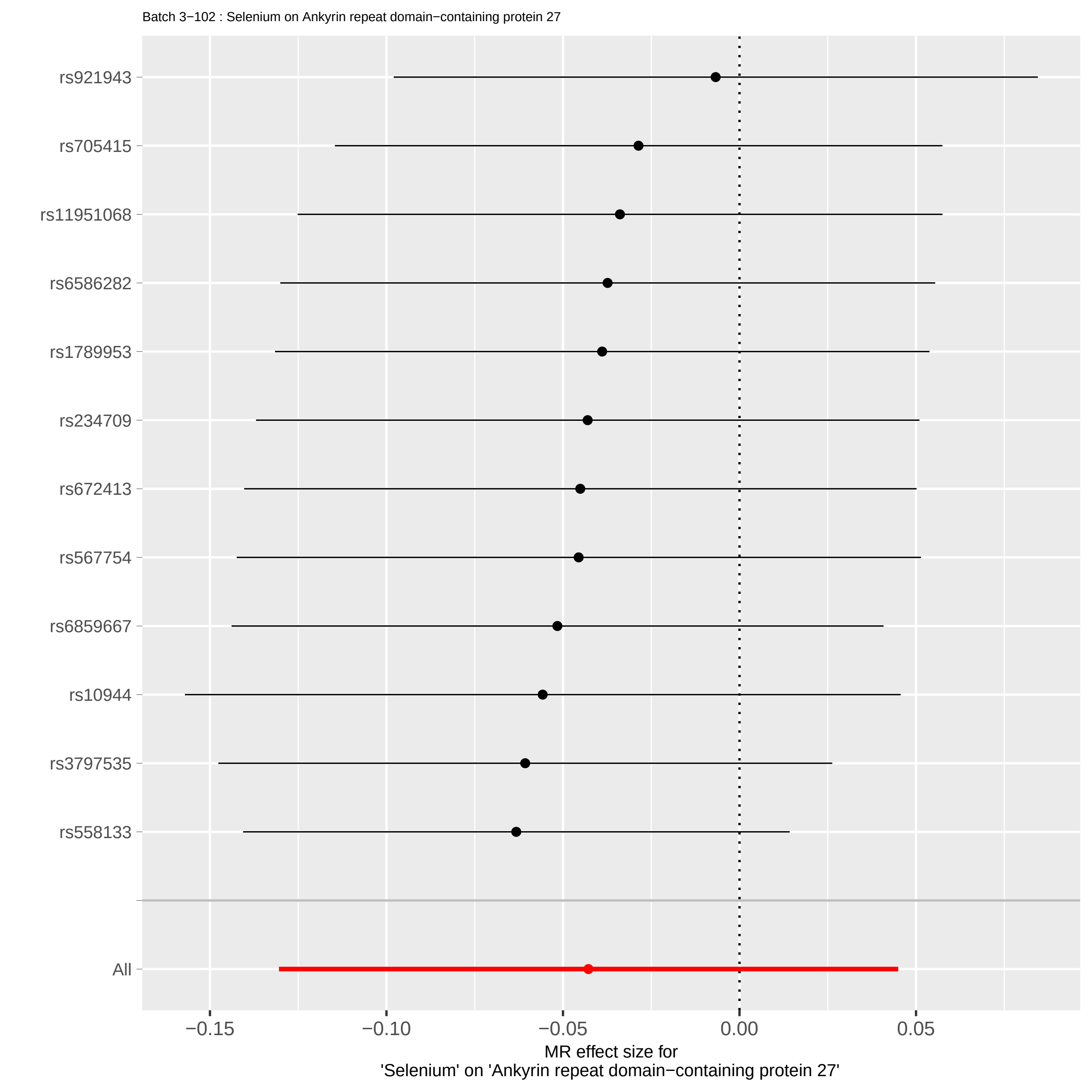

Batch 3−102 : Selenium on Ankyrin repeat domain−containing protein 27
rs921943
rs705415
rs11951068
rs6586282
rs1789953
rs234709
rs672413
rs567754
rs6859667
rs10944
rs3797535
rs558133
All
−0.15
−0.10
−0.05
0.00
0.05
MR effect size for
'Selenium' on 'Ankyrin repeat domain−containing protein 27'

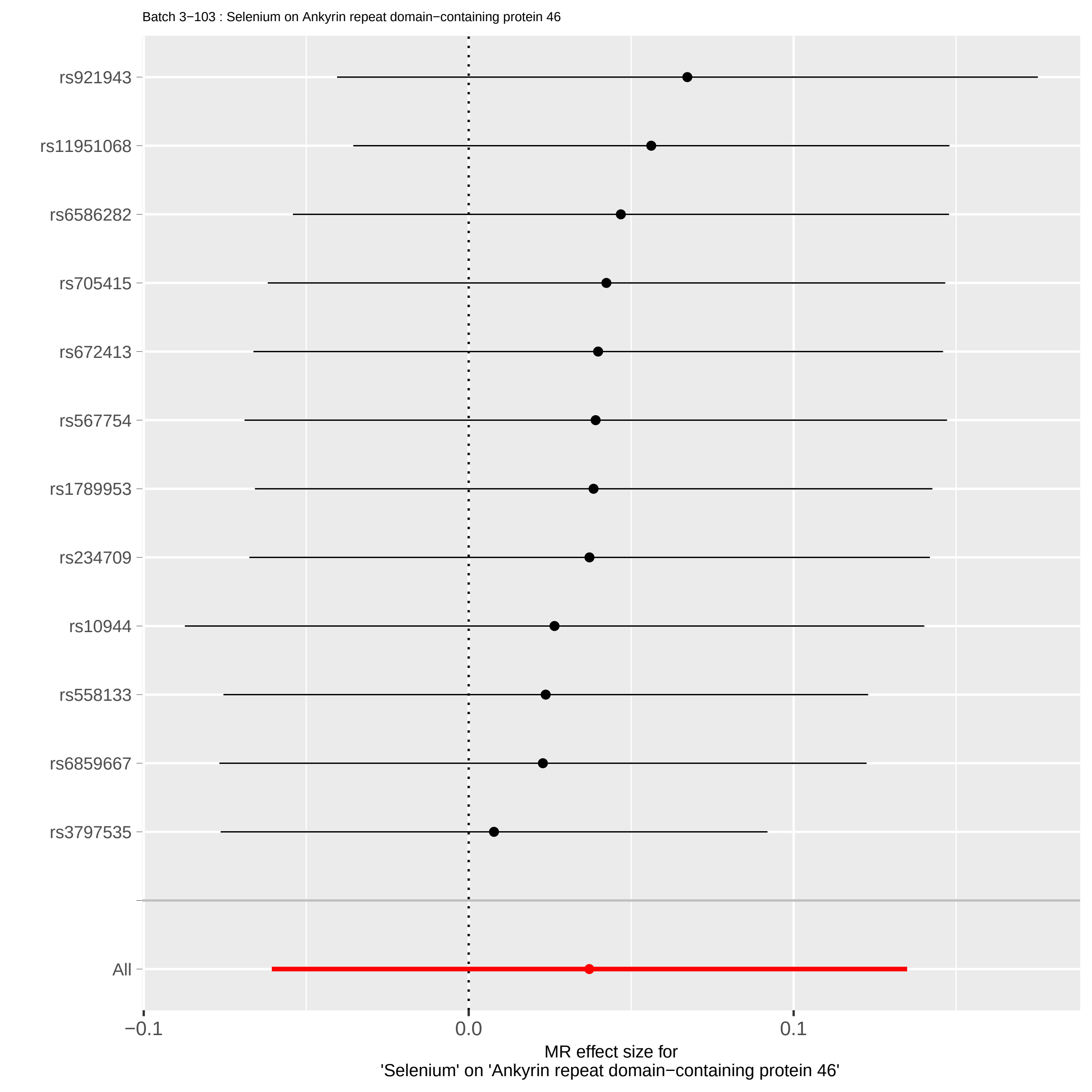

Batch 3−103 : Selenium on Ankyrin repeat domain−containing protein 46
rs921943
rs11951068
rs6586282
rs705415
rs672413
rs567754
rs1789953
rs234709
rs10944
rs558133
rs6859667
rs3797535
All
−0.1
0.0
0.1
MR effect size for
'Selenium' on 'Ankyrin repeat domain−containing protein 46'

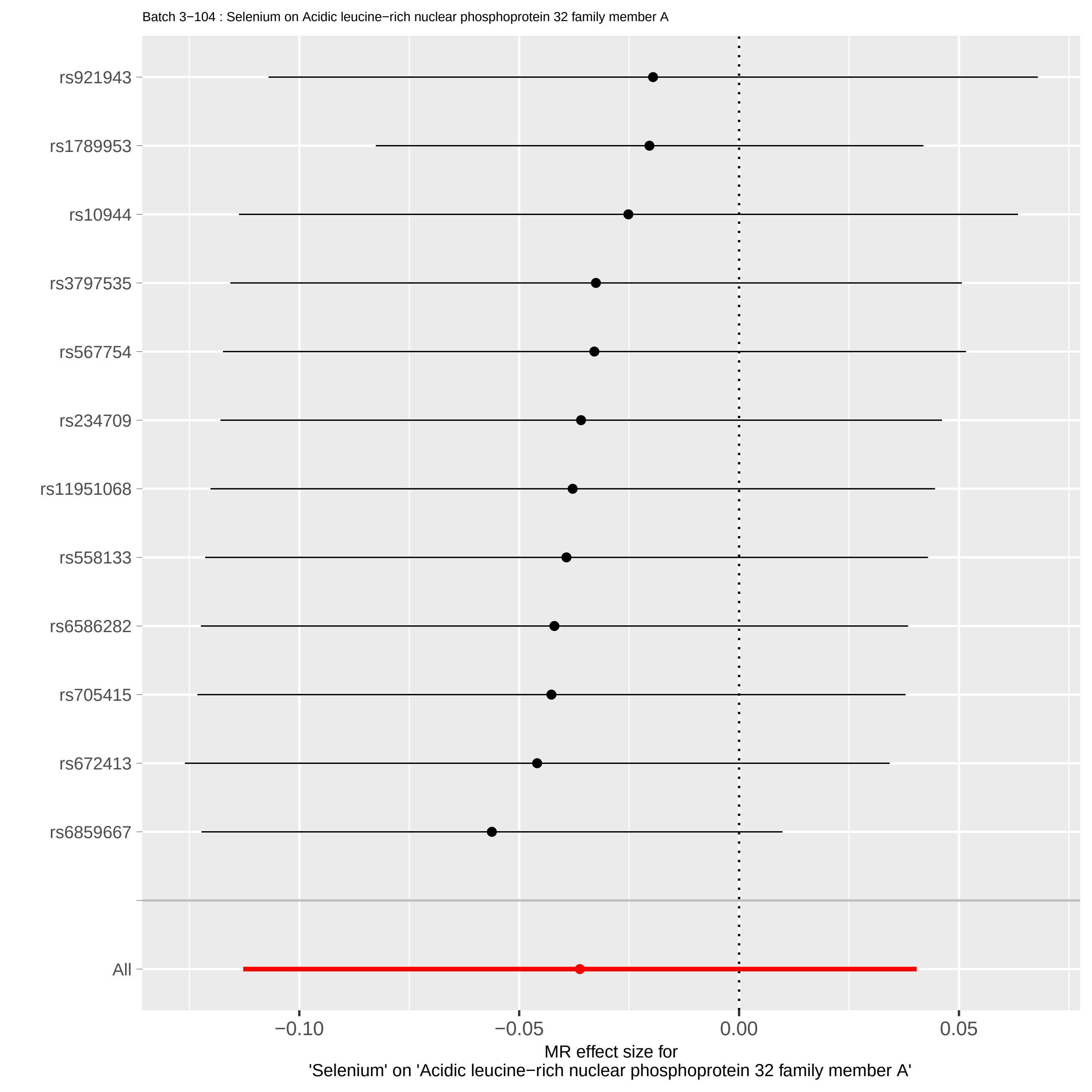

Batch 3−104 : Selenium on Acidic leucine−rich nuclear phosphoprotein 32 family member A
rs921943
rs1789953
rs10944
rs3797535
rs567754
rs234709
rs11951068
rs558133
rs6586282
rs705415
rs672413
rs6859667
All
−0.10
−0.05
0.00
0.05
MR effect size for
'Selenium' on 'Acidic leucine−rich nuclear phosphoprotein 32 family member A'

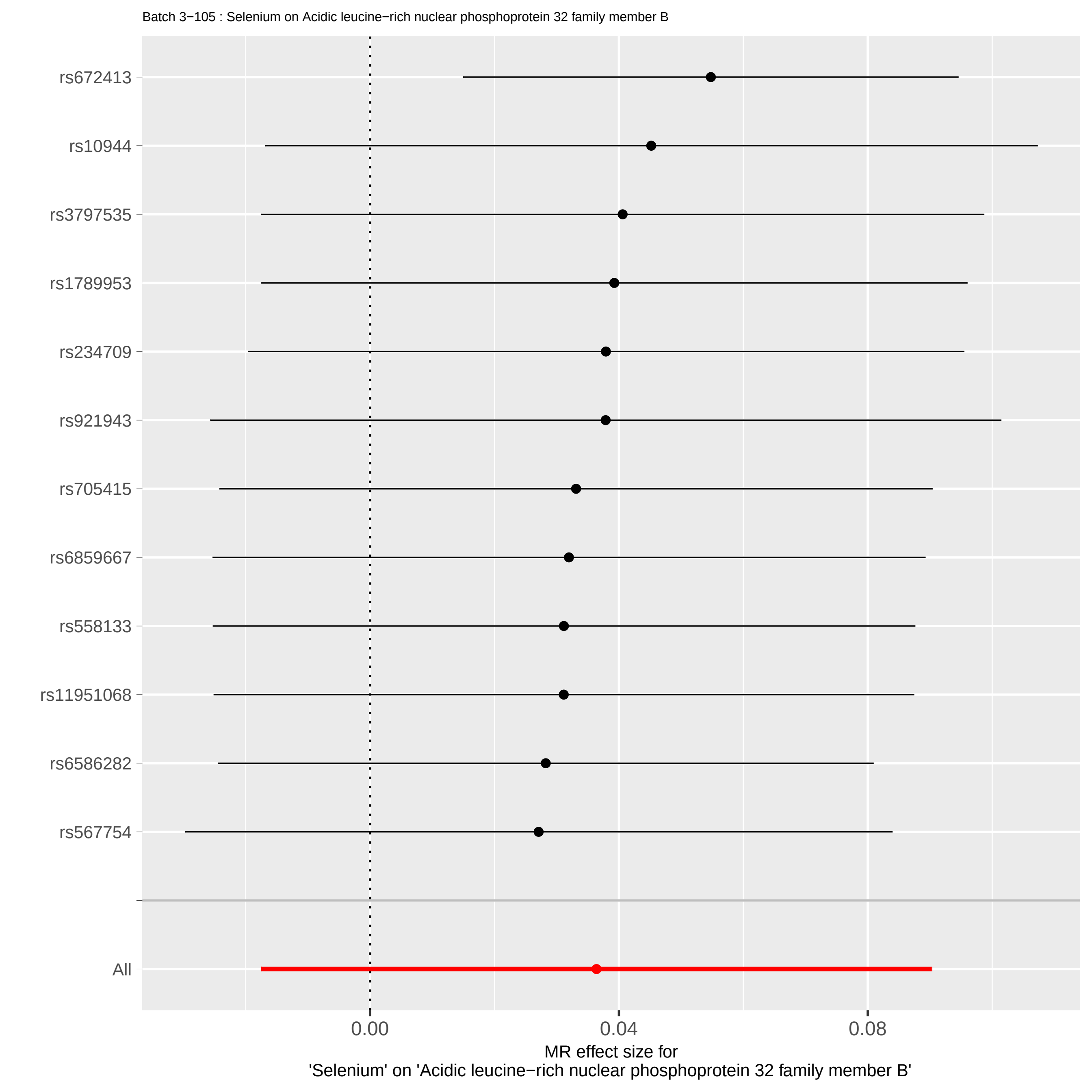

Batch 3−105 : Selenium on Acidic leucine−rich nuclear phosphoprotein 32 family member B
rs672413
rs10944
rs3797535
rs1789953
rs234709
rs921943
rs705415
rs6859667
rs558133
rs11951068
rs6586282
rs567754
All
0.00
0.04
0.08
MR effect size for
'Selenium' on 'Acidic leucine−rich nuclear phosphoprotein 32 family member B'

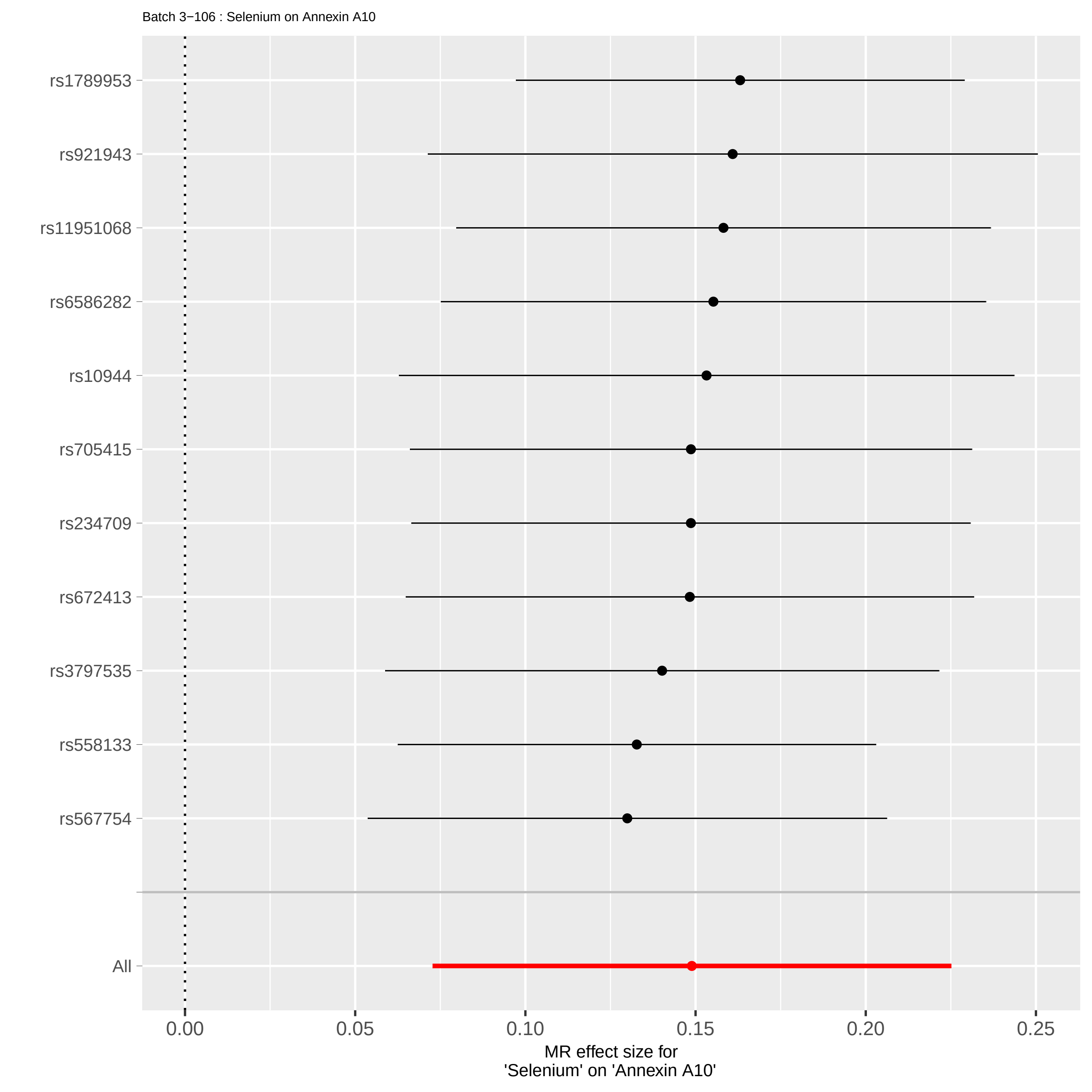

Batch 3−106 : Selenium on Annexin A10
rs1789953
rs921943
rs11951068
rs6586282
rs10944
rs705415
rs234709
rs672413
rs3797535
rs558133
rs567754
All
0.00
0.05
0.10
0.15
0.20
0.25
MR effect size for
'Selenium' on 'Annexin A10'

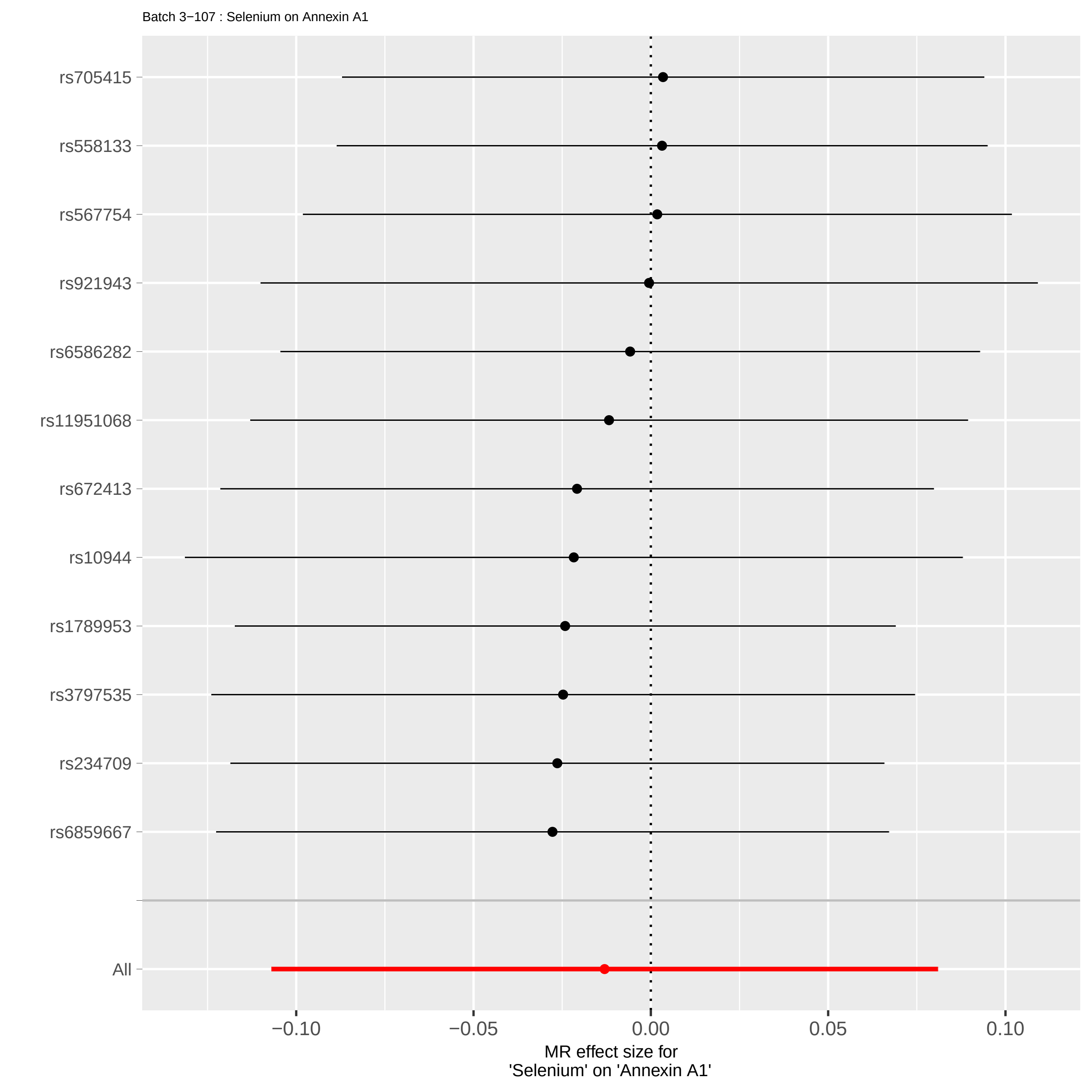
Batch 3−107 : Selenium on Annexin A1
rs705415
rs558133
rs567754
rs921943
rs6586282
rs11951068
rs672413
rs10944
rs1789953
rs3797535
rs234709
rs6859667
All
−0.10
−0.05
0.00
0.05
0.10
MR effect size for
'Selenium' on 'Annexin A1'

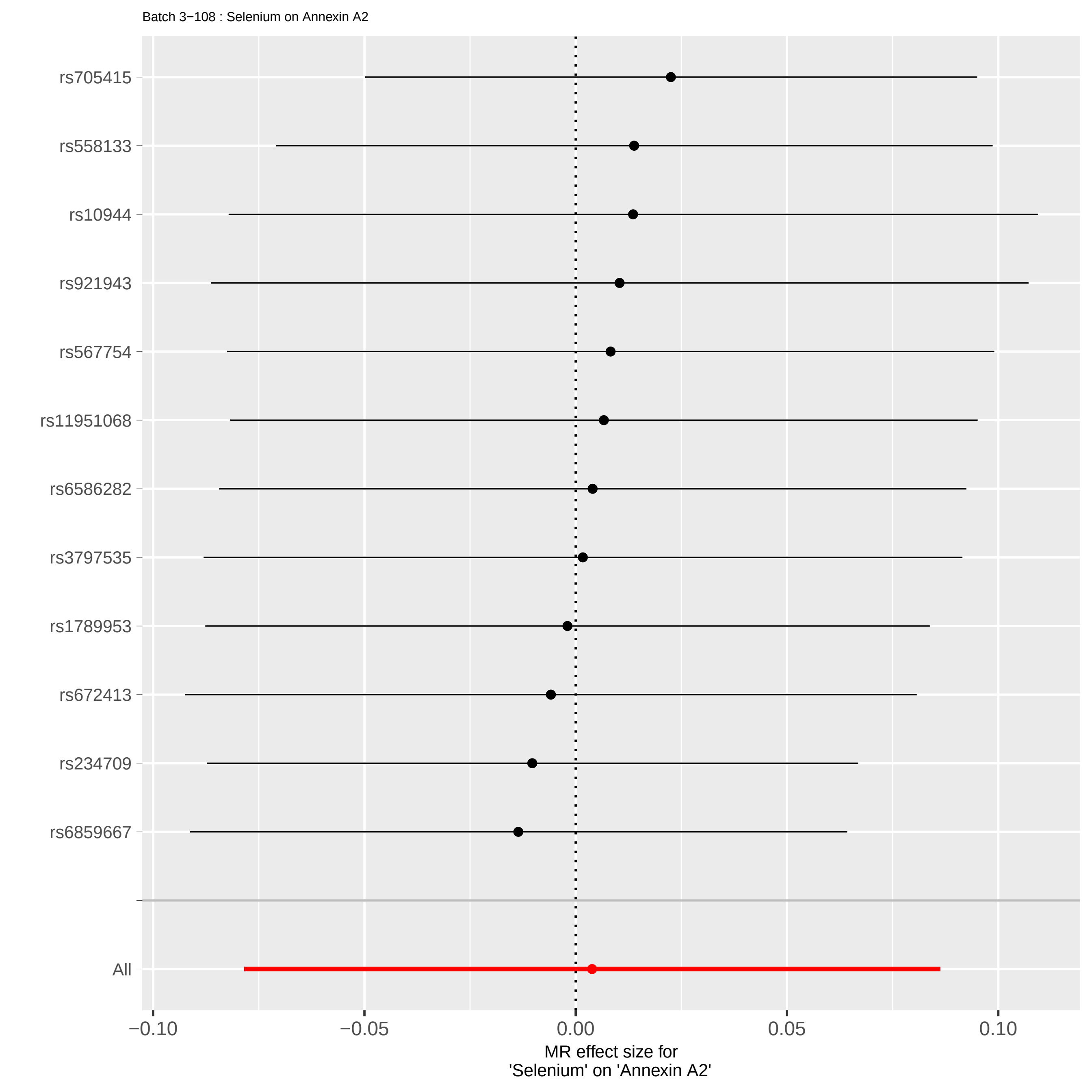
Batch 3−108 : Selenium on Annexin A2
rs705415
rs558133
rs10944
rs921943
rs567754
rs11951068
rs6586282
rs3797535
rs1789953
rs672413
rs234709
rs6859667
All
−0.10
−0.05
0.00
0.05
0.10
MR effect size for
'Selenium' on 'Annexin A2'

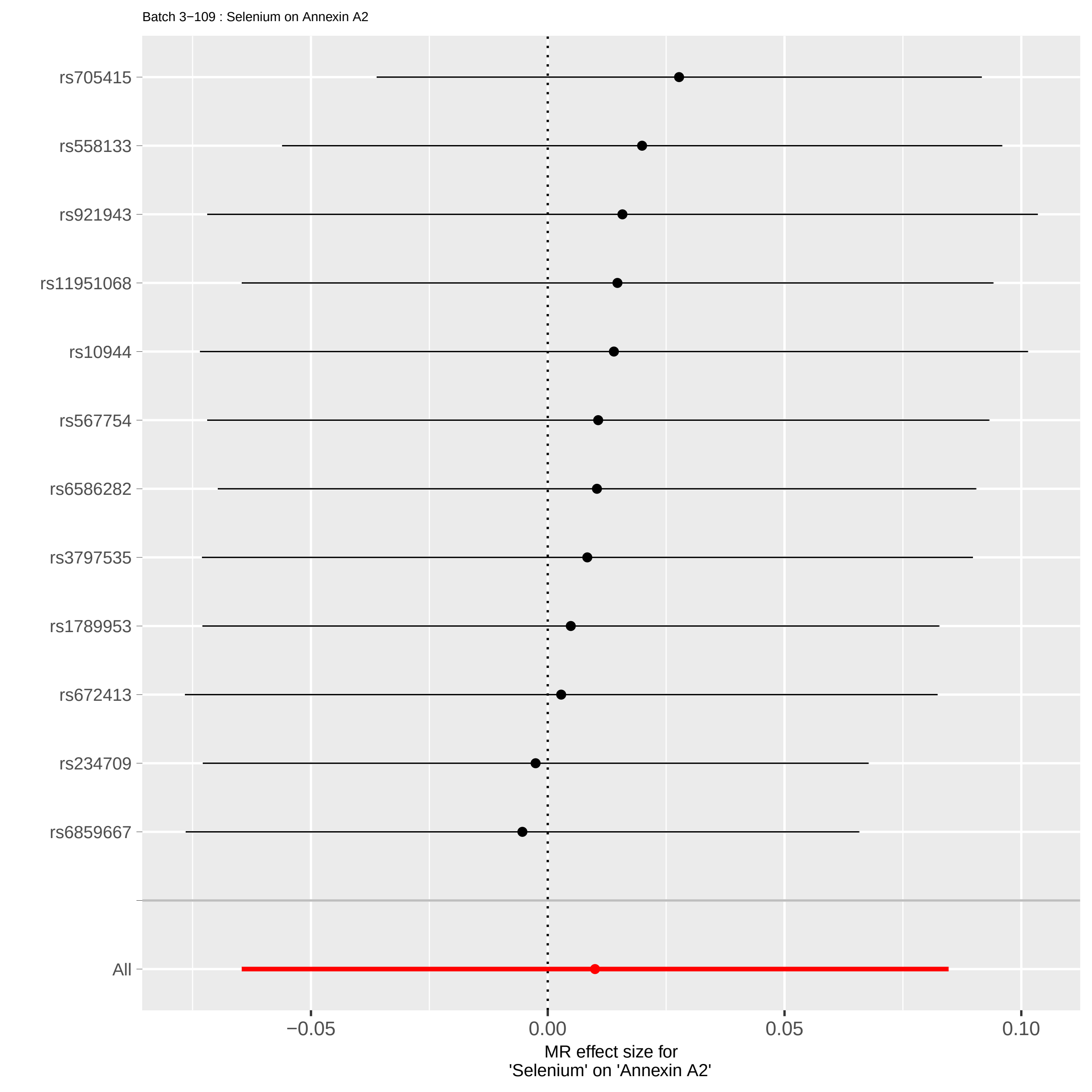
Batch 3−109 : Selenium on Annexin A2
rs705415
rs558133
rs921943
rs11951068
rs10944
rs567754
rs6586282
rs3797535
rs1789953
rs672413
rs234709
rs6859667
All
−0.05
0.00
0.05
0.10
MR effect size for
'Selenium' on 'Annexin A2'

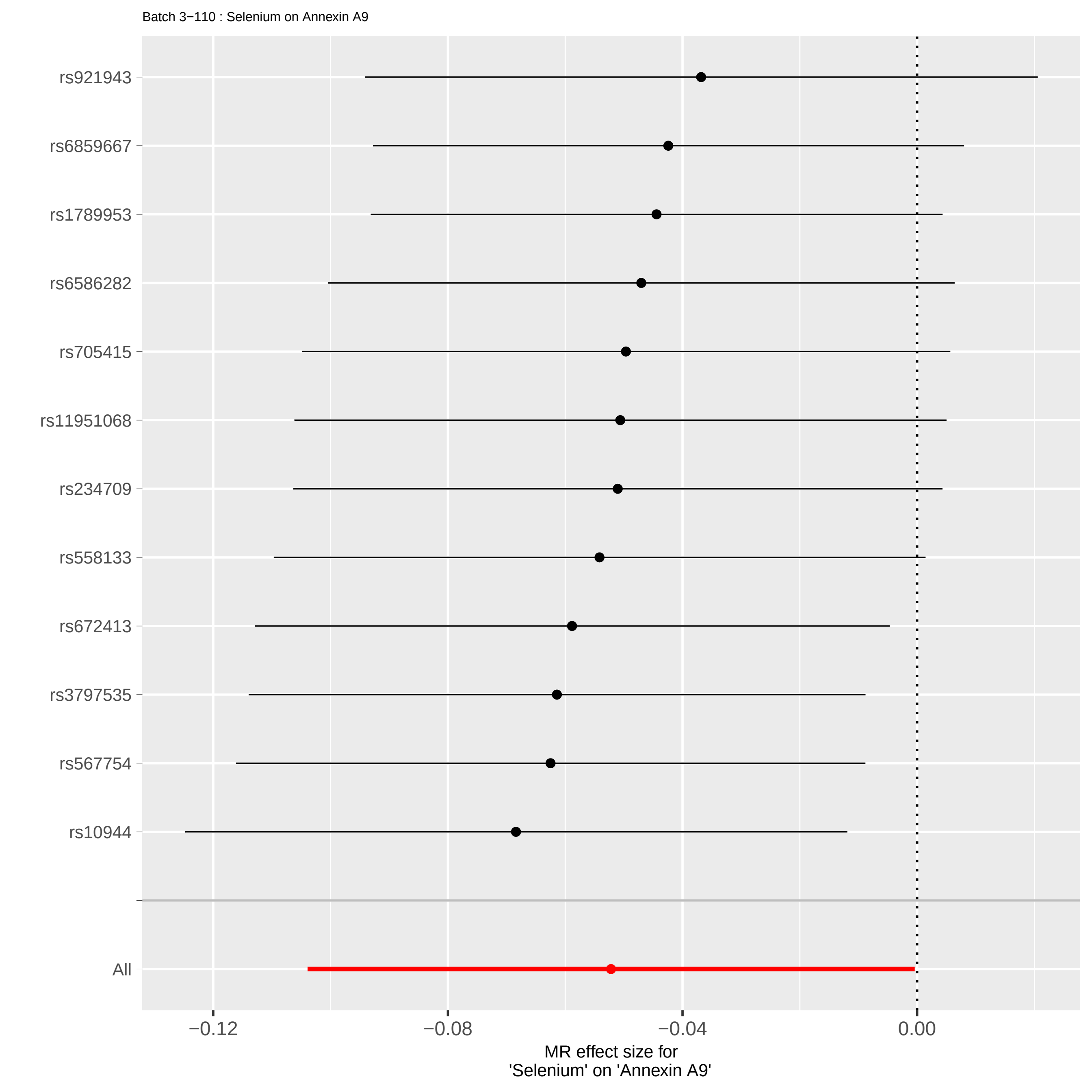

Batch 3−110 : Selenium on Annexin A9
rs921943
rs6859667
rs1789953
rs6586282
rs705415
rs11951068
rs234709
rs558133
rs672413
rs3797535
rs567754
rs10944
All
−0.12
−0.08
−0.04
0.00
MR effect size for
'Selenium' on 'Annexin A9'

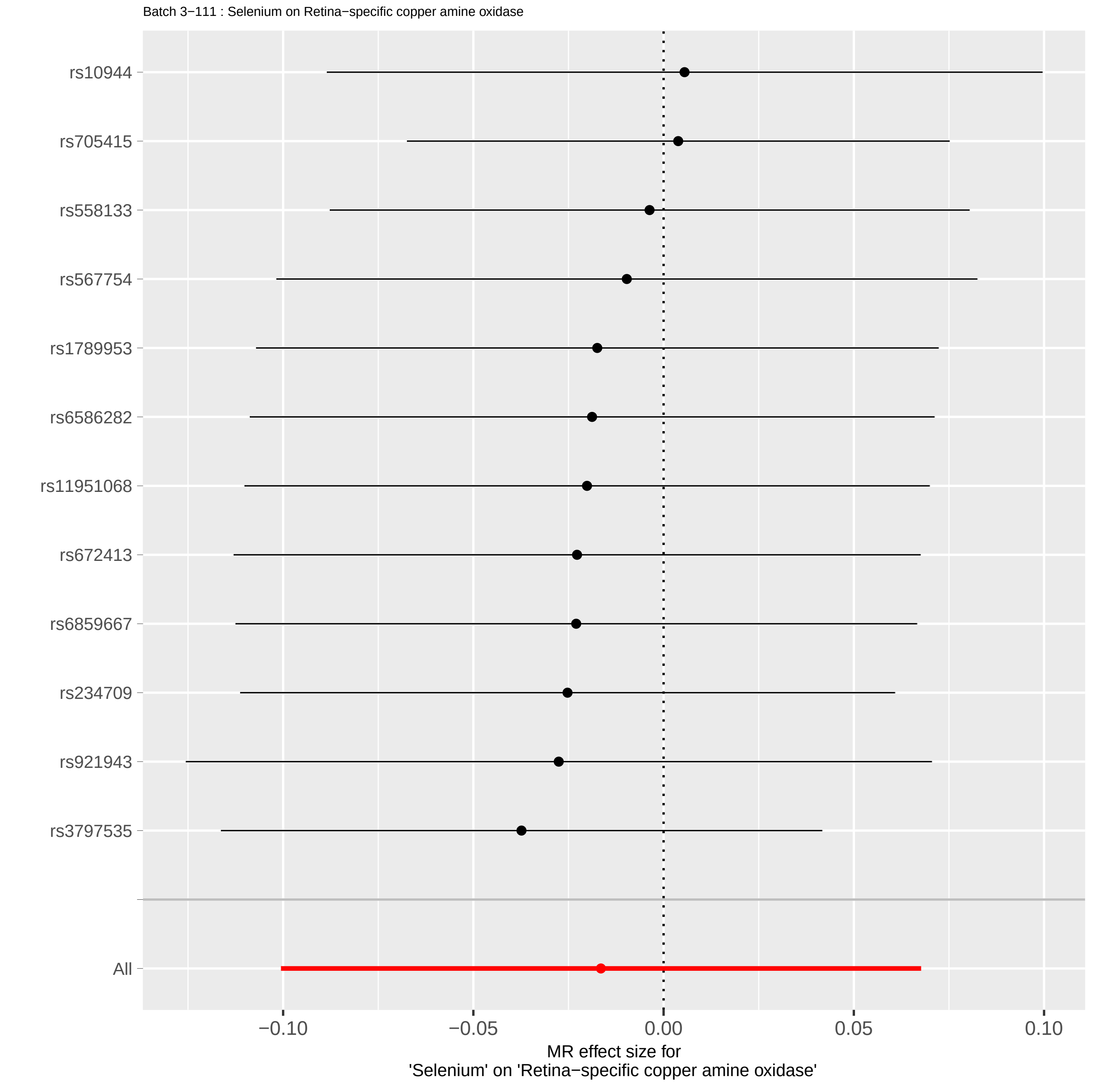
Batch 3−111 : Selenium on Retina−specific copper amine oxidase
rs10944
rs705415
rs558133
rs567754
rs1789953
rs6586282
rs11951068
rs672413
rs6859667
rs234709
rs921943
rs3797535
All
−0.10
−0.05
0.00
0.05
0.10
MR effect size for
'Selenium' on 'Retina−specific copper amine oxidase'

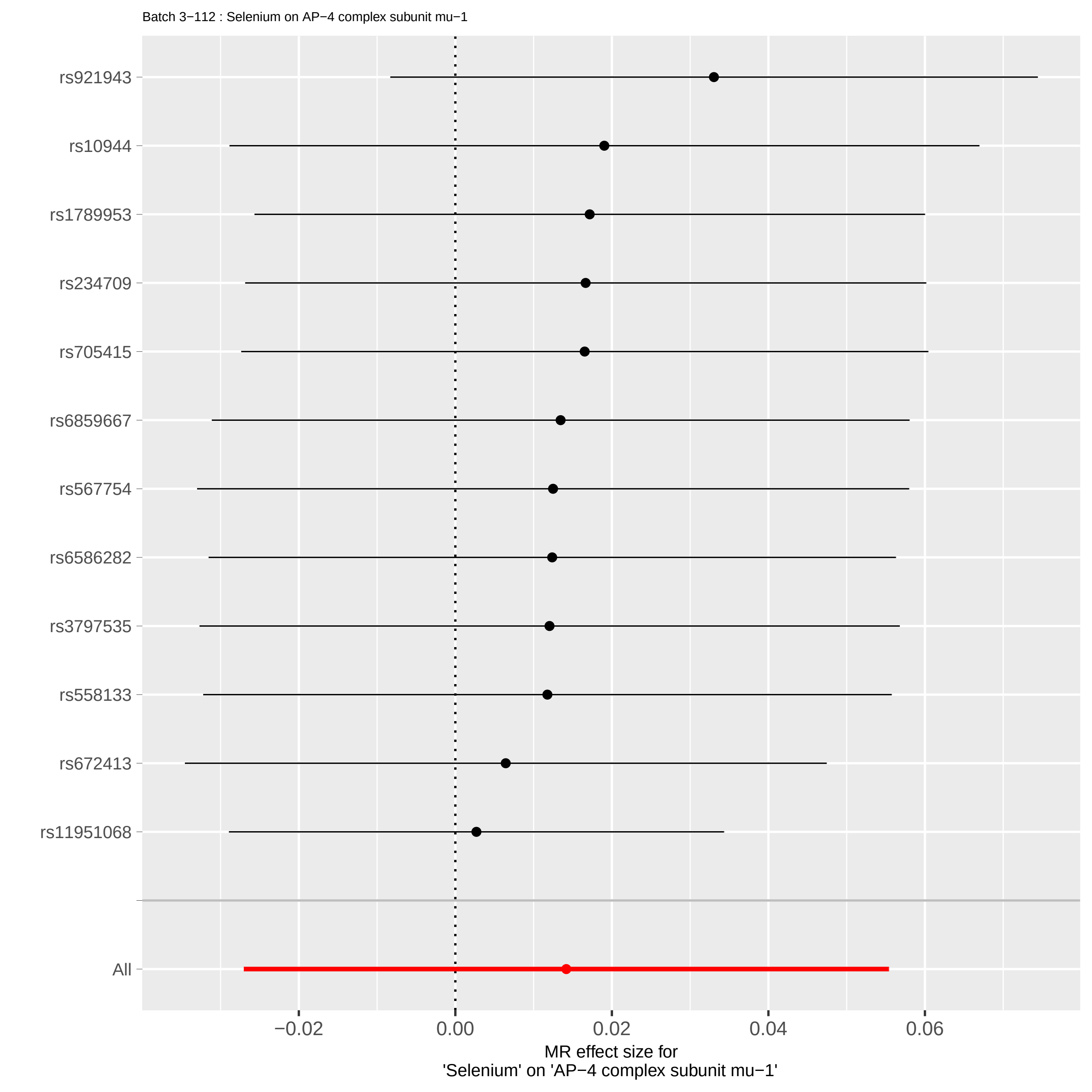

Batch 3−112 : Selenium on AP−4 complex subunit mu−1
rs921943
rs10944
rs1789953
rs234709
rs705415
rs6859667
rs567754
rs6586282
rs3797535
rs558133
rs672413
rs11951068
All
−0.02
0.00
0.02
0.04
0.06
MR effect size for
'Selenium' on 'AP−4 complex subunit mu−1'

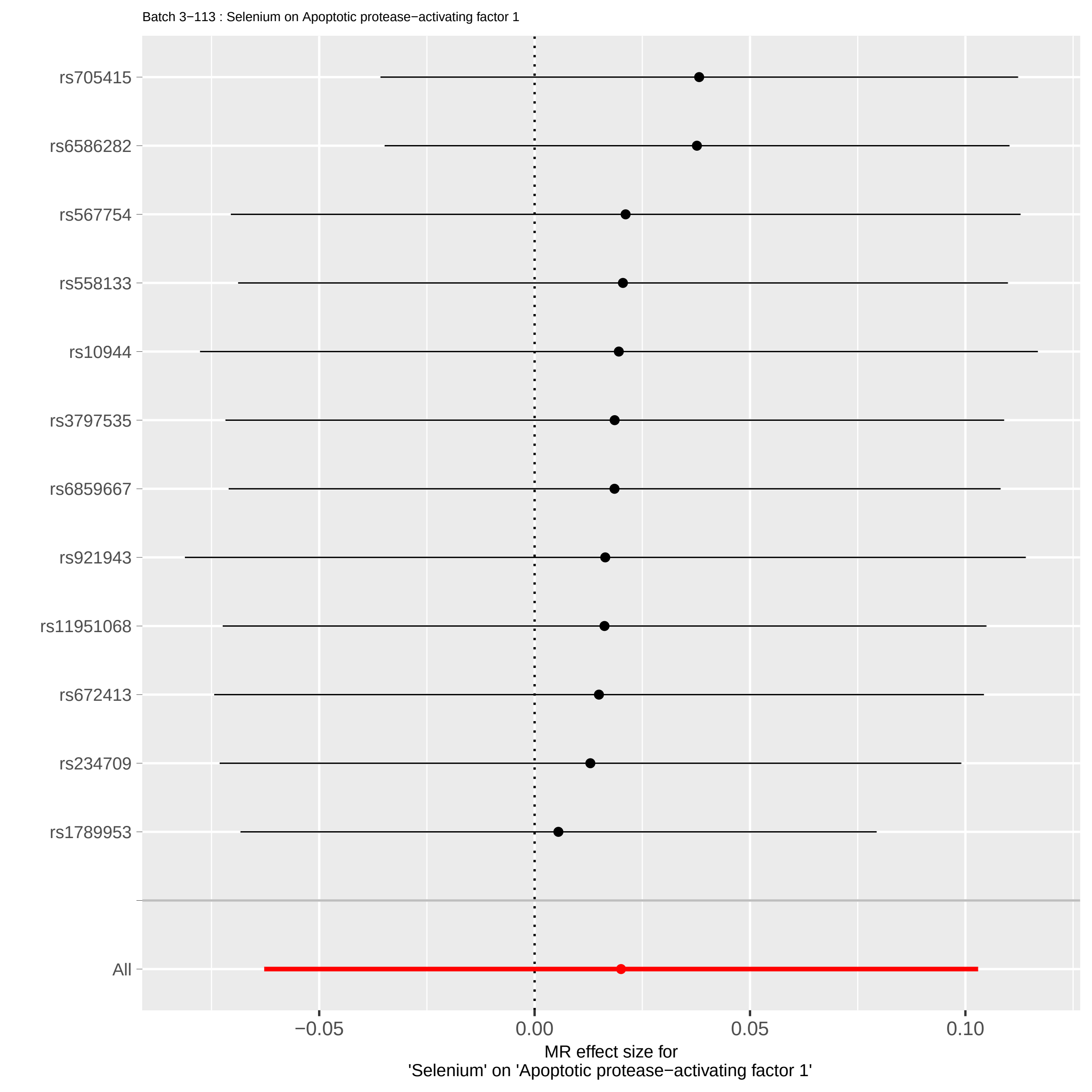

Batch 3−113 : Selenium on Apoptotic protease−activating factor 1
rs705415
rs6586282
rs567754
rs558133
rs10944
rs3797535
rs6859667
rs921943
rs11951068
rs672413
rs234709
rs1789953
All
−0.05
0.00
0.05
0.10
MR effect size for
'Selenium' on 'Apoptotic protease−activating factor 1'

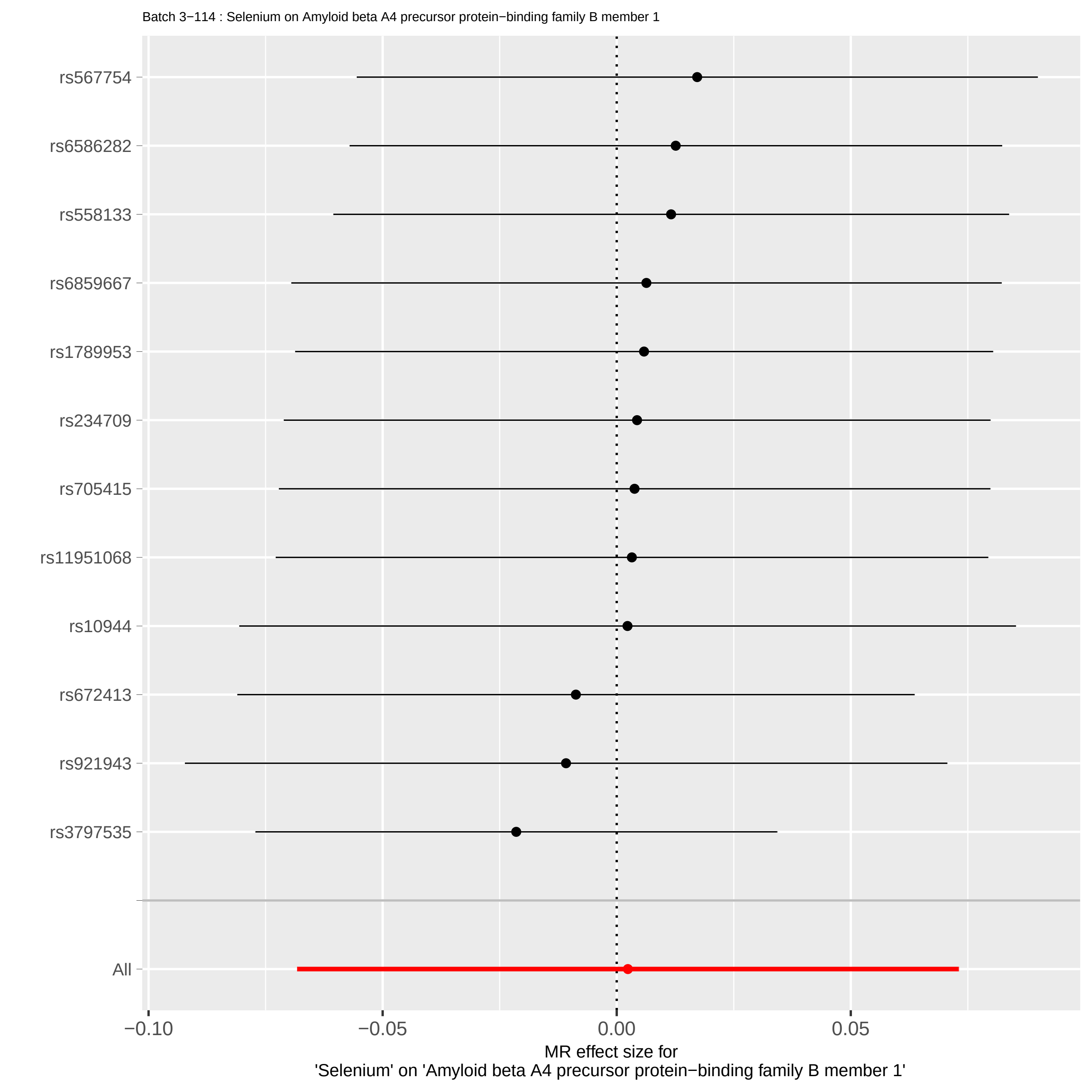
Batch 3−114 : Selenium on Amyloid beta A4 precursor protein−binding family B member 1
rs567754
rs6586282
rs558133
rs6859667
rs1789953
rs234709
rs705415
rs11951068
rs10944
rs672413
rs921943
rs3797535
All
−0.10
−0.05
0.00
0.05
MR effect size for
'Selenium' on 'Amyloid beta A4 precursor protein−binding family B member 1'

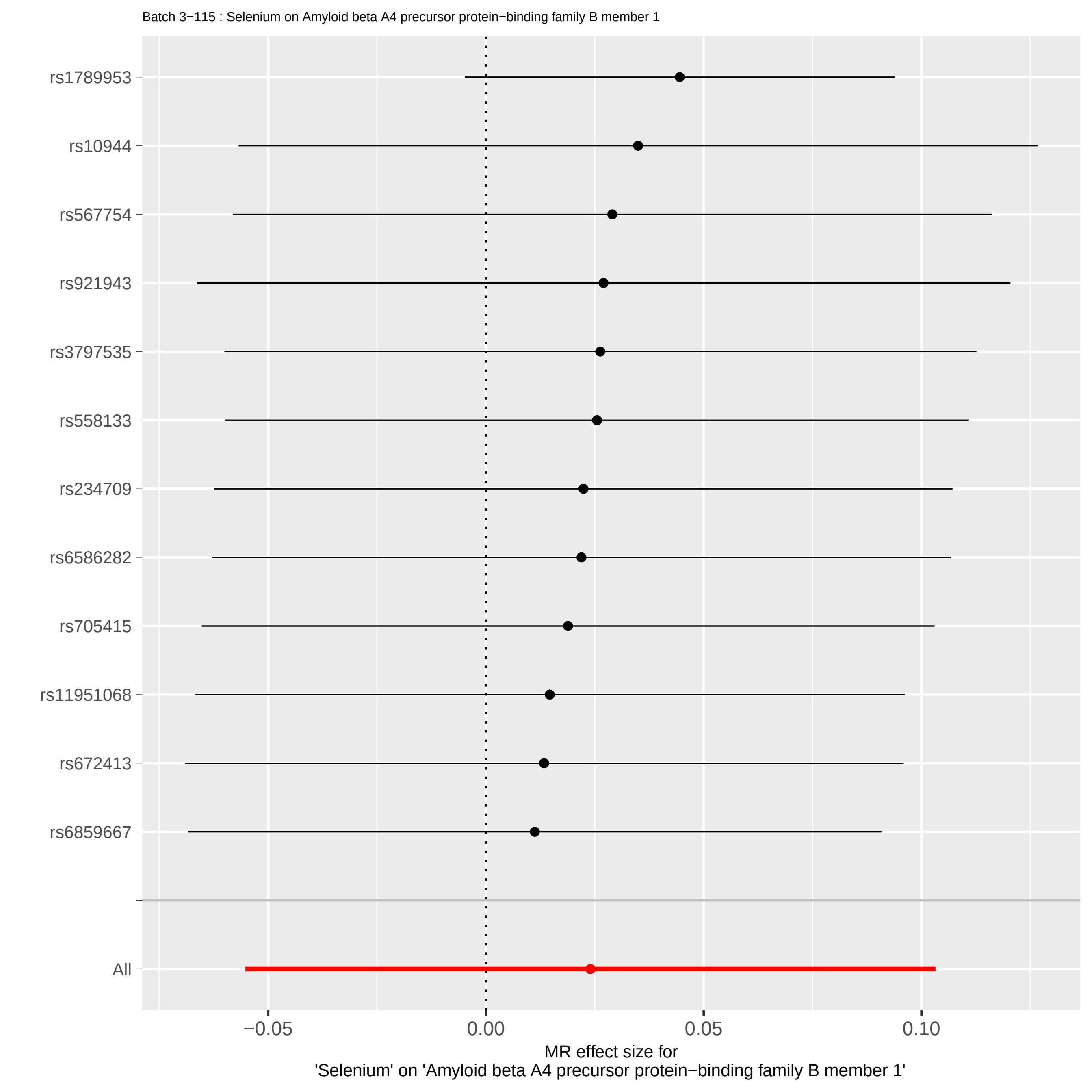

Batch 3−115 : Selenium on Amyloid beta A4 precursor protein−binding family B member 1
rs1789953
rs10944
rs567754
rs921943
rs3797535
rs558133
rs234709
rs6586282
rs705415
rs11951068
rs672413
rs6859667
All
−0.05
0.00
0.05
0.10
MR effect size for
'Selenium' on 'Amyloid beta A4 precursor protein−binding family B member 1'

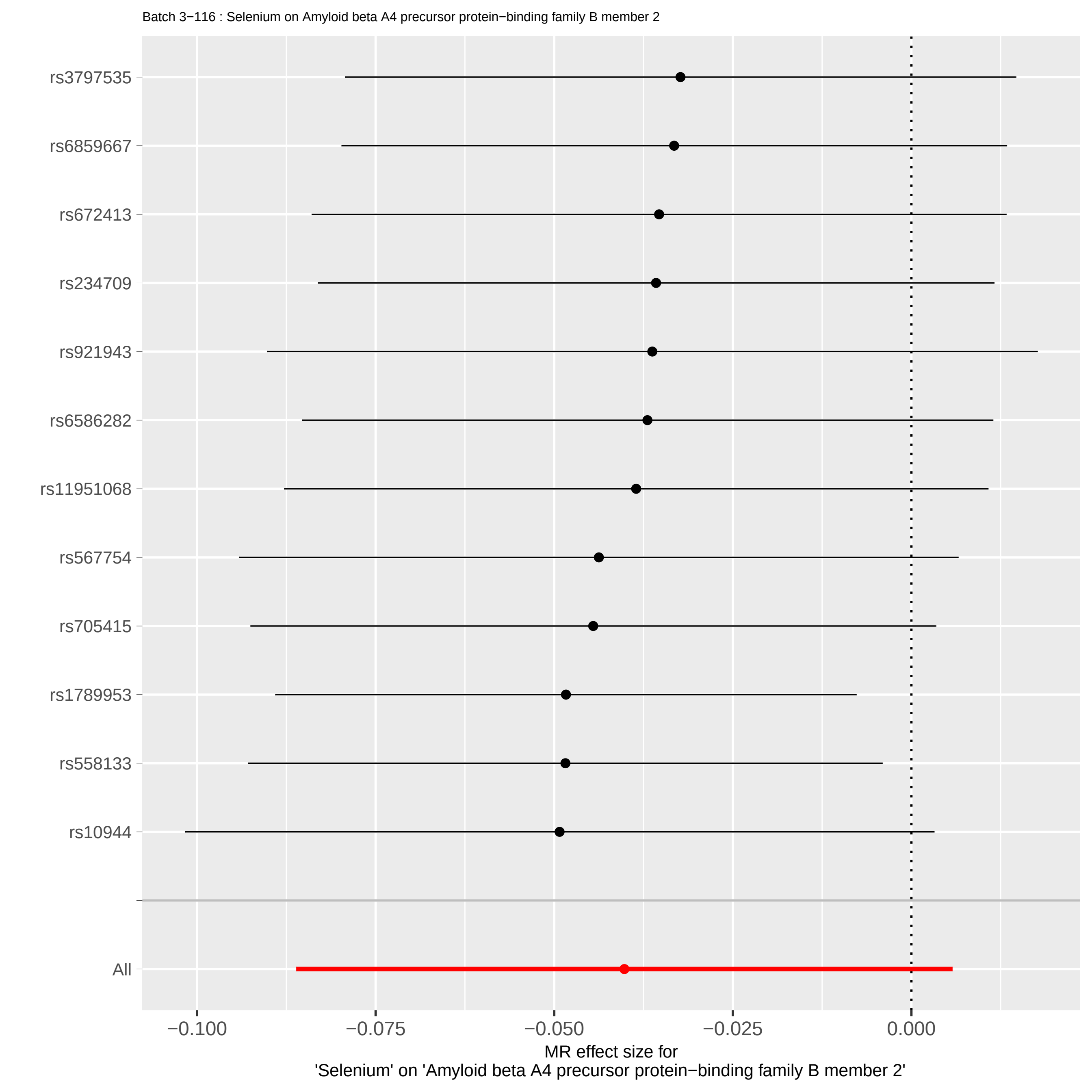
Batch 3−116 : Selenium on Amyloid beta A4 precursor protein−binding family B member 2
rs3797535
rs6859667
rs672413
rs234709
rs921943
rs6586282
rs11951068
rs567754
rs705415
rs1789953
rs558133
rs10944
All
−0.100
−0.075
−0.050
−0.025
0.000
MR effect size for
'Selenium' on 'Amyloid beta A4 precursor protein−binding family B member 2'

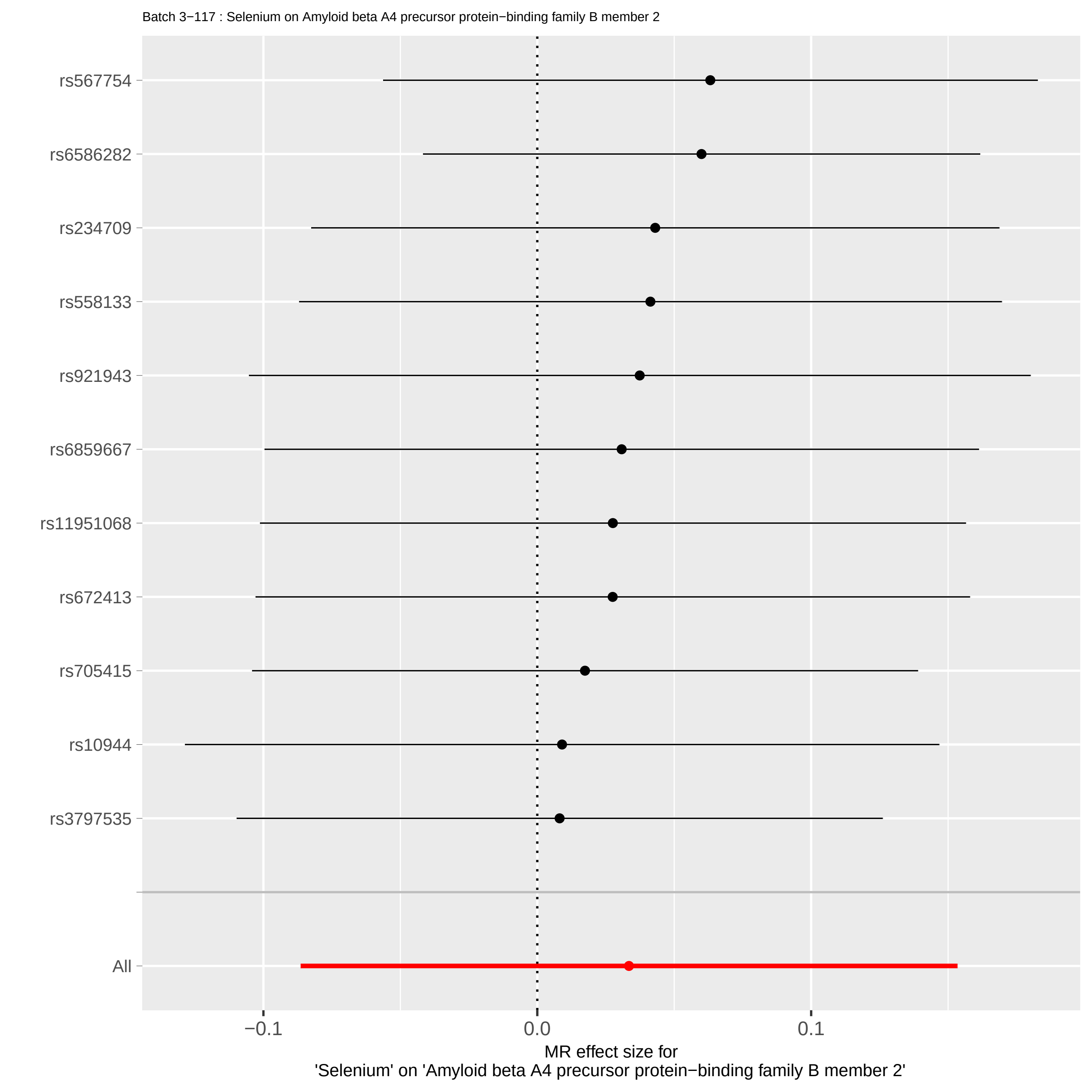

Batch 3−117 : Selenium on Amyloid beta A4 precursor protein−binding family B member 2
rs567754
rs6586282
rs234709
rs558133
rs921943
rs6859667
rs11951068
rs672413
rs705415
rs10944
rs3797535
All
−0.1
0.0
0.1
MR effect size for
'Selenium' on 'Amyloid beta A4 precursor protein−binding family B member 2'

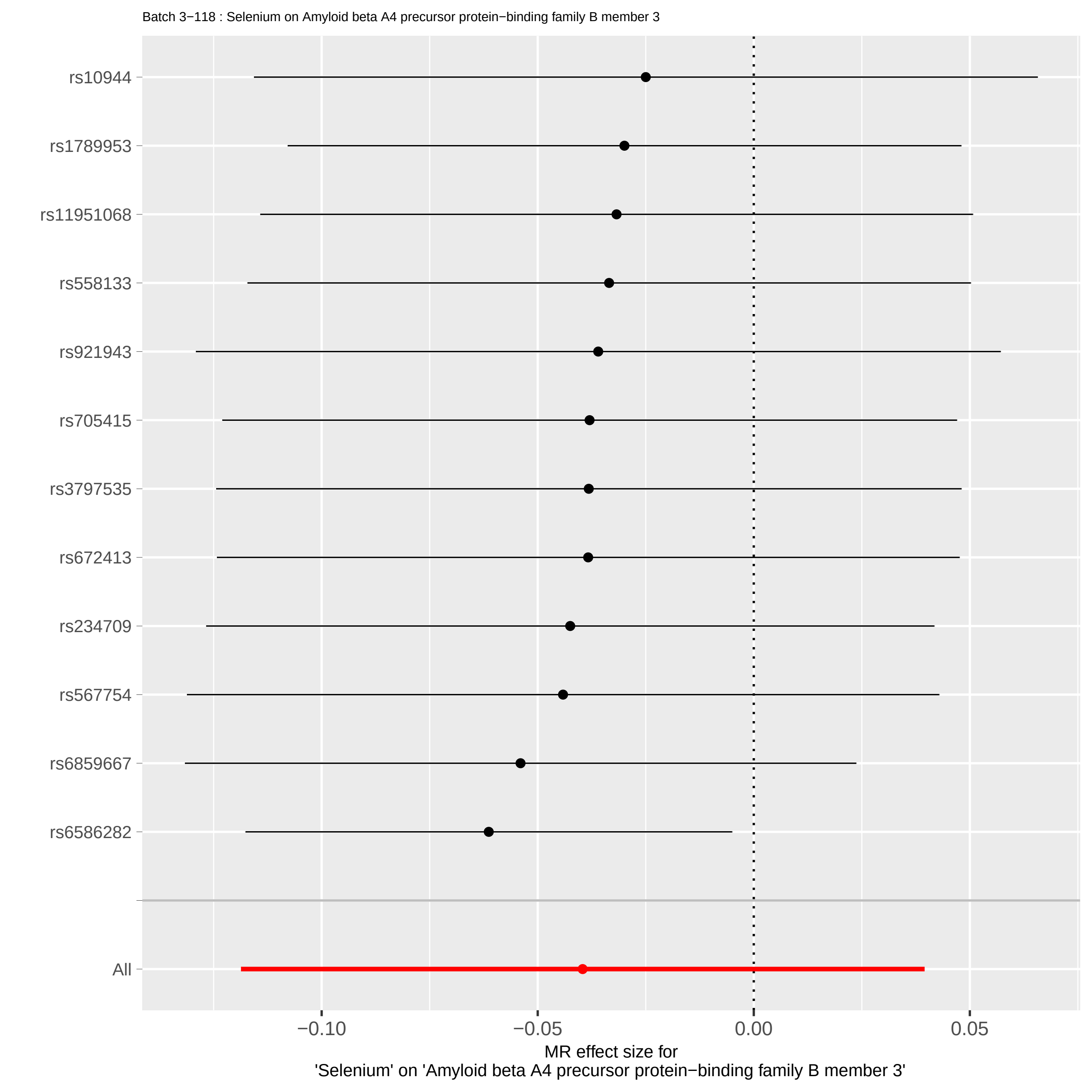

Batch 3−118 : Selenium on Amyloid beta A4 precursor protein−binding family B member 3
rs10944
rs1789953
rs11951068
rs558133
rs921943
rs705415
rs3797535
rs672413
rs234709
rs567754
rs6859667
rs6586282
All
−0.10
−0.05
0.00
0.05
MR effect size for
'Selenium' on 'Amyloid beta A4 precursor protein−binding family B member 3'

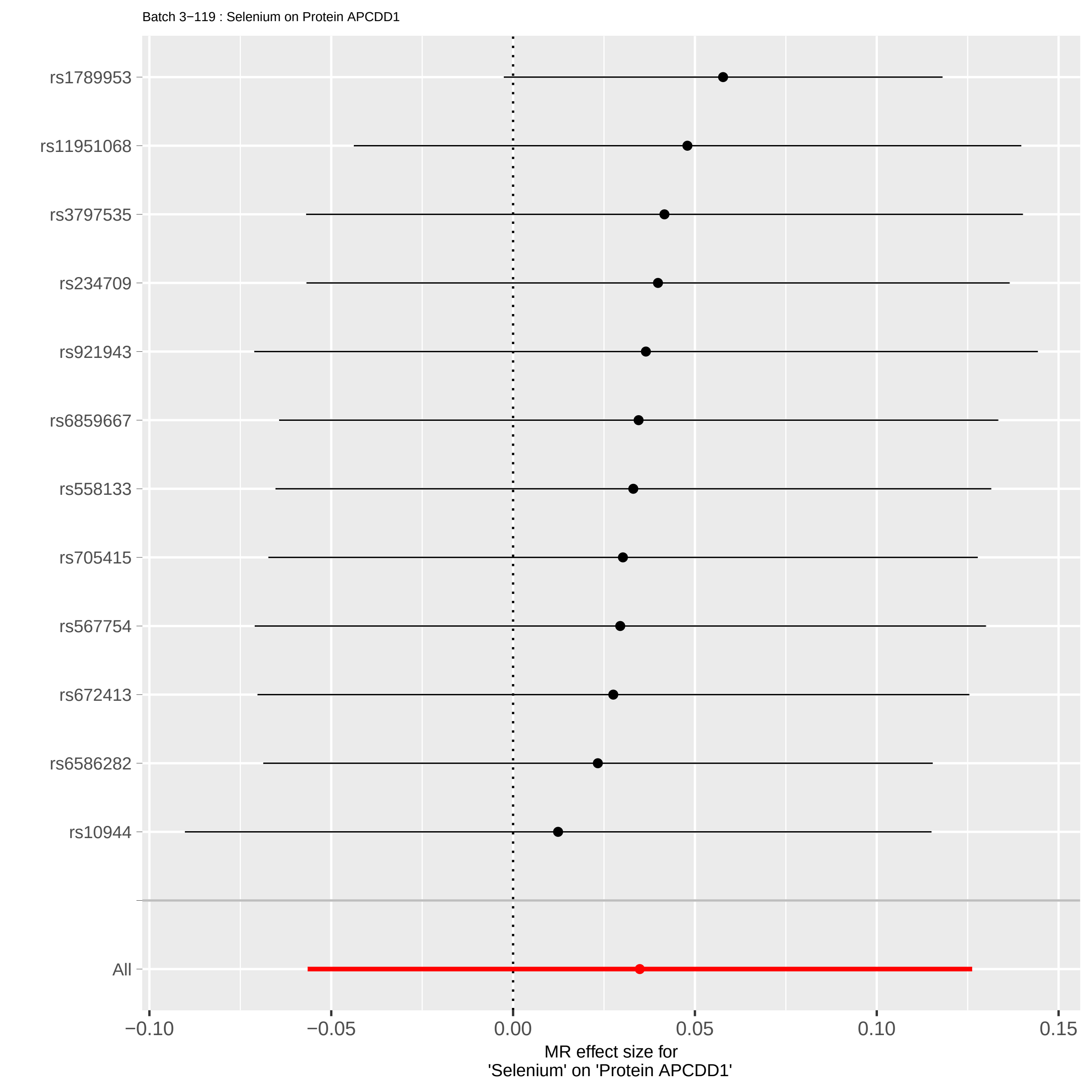
Batch 3−119 : Selenium on Protein APCDD1
rs1789953
rs11951068
rs3797535
rs234709
rs921943
rs6859667
rs558133
rs705415
rs567754
rs672413
rs6586282
rs10944
All
−0.10
−0.05
0.00
0.05
0.10
0.15
MR effect size for
'Selenium' on 'Protein APCDD1'

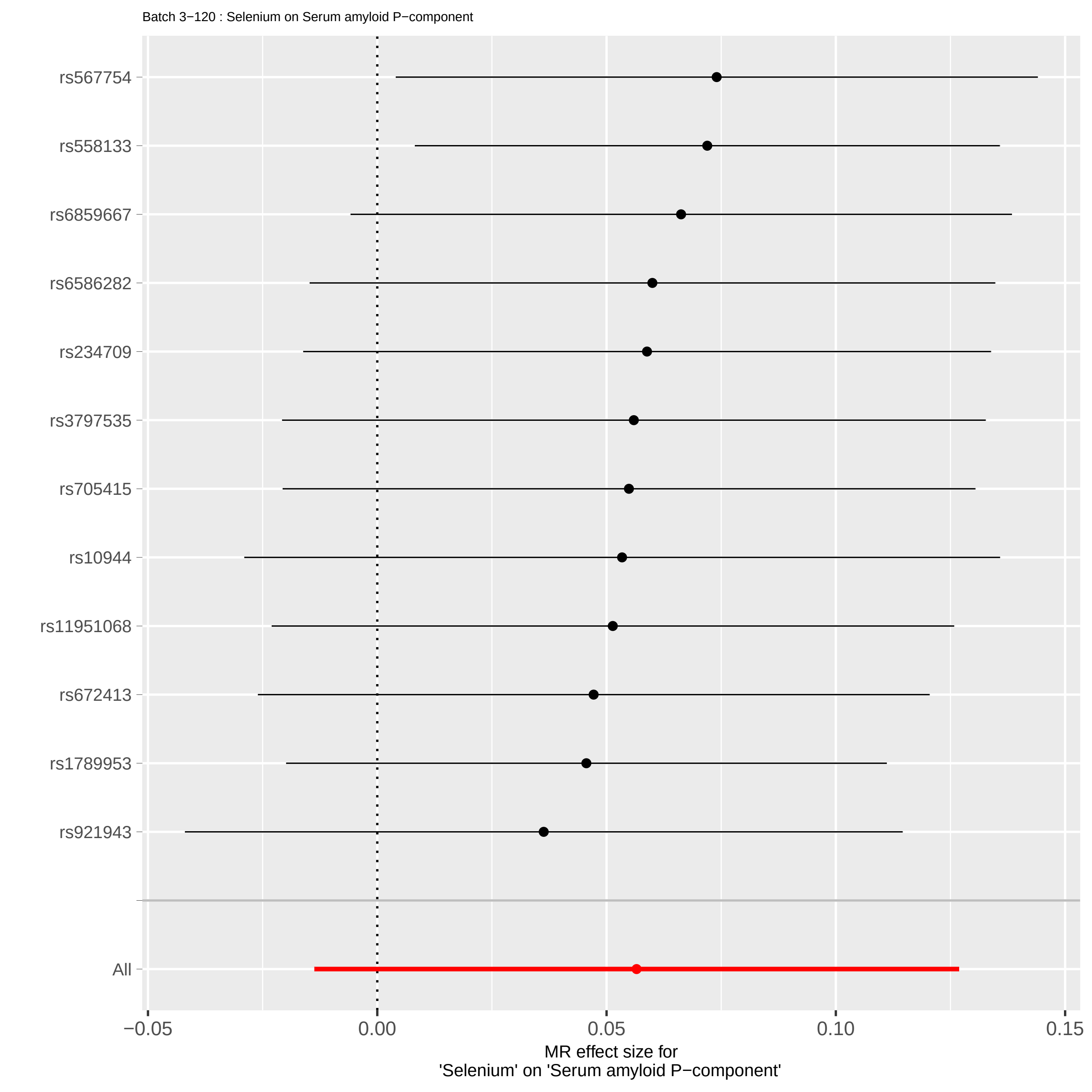

Batch 3−120 : Selenium on Serum amyloid P−component
rs567754
rs558133
rs6859667
rs6586282
rs234709
rs3797535
rs705415
rs10944
rs11951068
rs672413
rs1789953
rs921943
All
−0.05
0.00
0.05
0.10
0.15
MR effect size for
'Selenium' on 'Serum amyloid P−component'

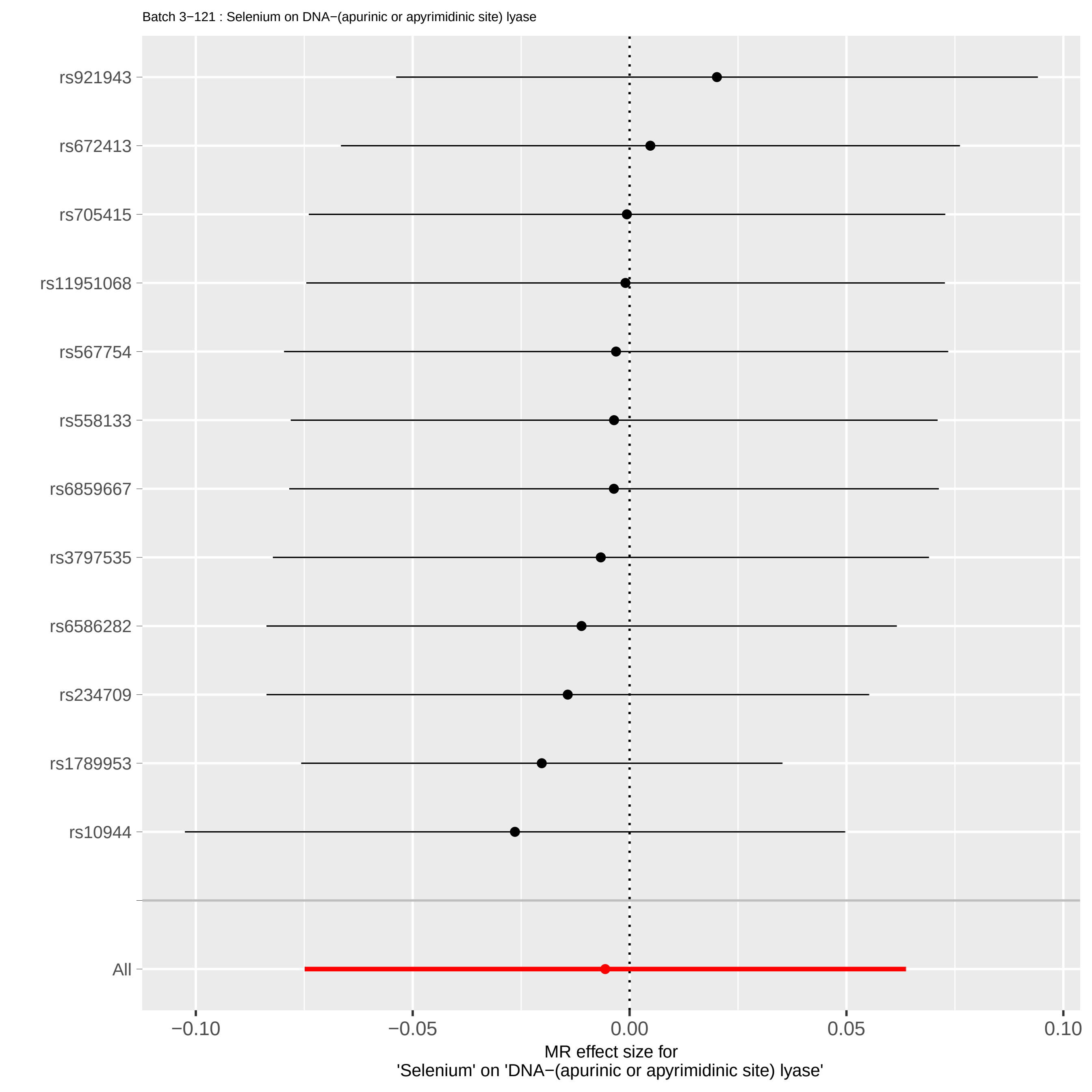

Batch 3−121 : Selenium on DNA−(apurinic or apyrimidinic site) lyase
rs921943
rs672413
rs705415
rs11951068
rs567754
rs558133
rs6859667
rs3797535
rs6586282
rs234709
rs1789953
rs10944
All
−0.10
−0.05
0.00
0.05
0.10
MR effect size for
'Selenium' on 'DNA−(apurinic or apyrimidinic site) lyase'

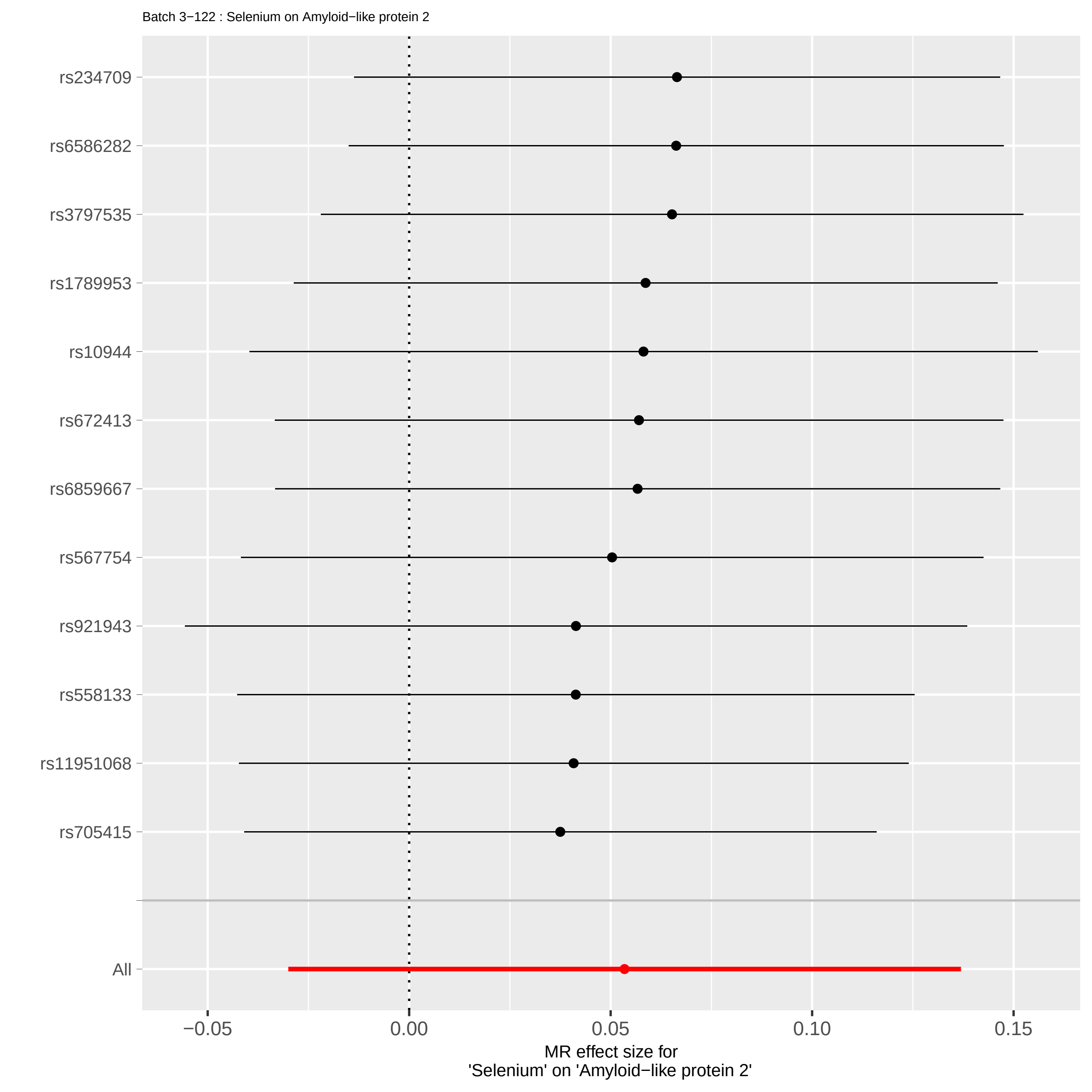

Batch 3−122 : Selenium on Amyloid−like protein 2
rs234709
rs6586282
rs3797535
rs1789953
rs10944
rs672413
rs6859667
rs567754
rs921943
rs558133
rs11951068
rs705415
All
−0.05
0.00
0.05
0.10
0.15
MR effect size for
'Selenium' on 'Amyloid−like protein 2'

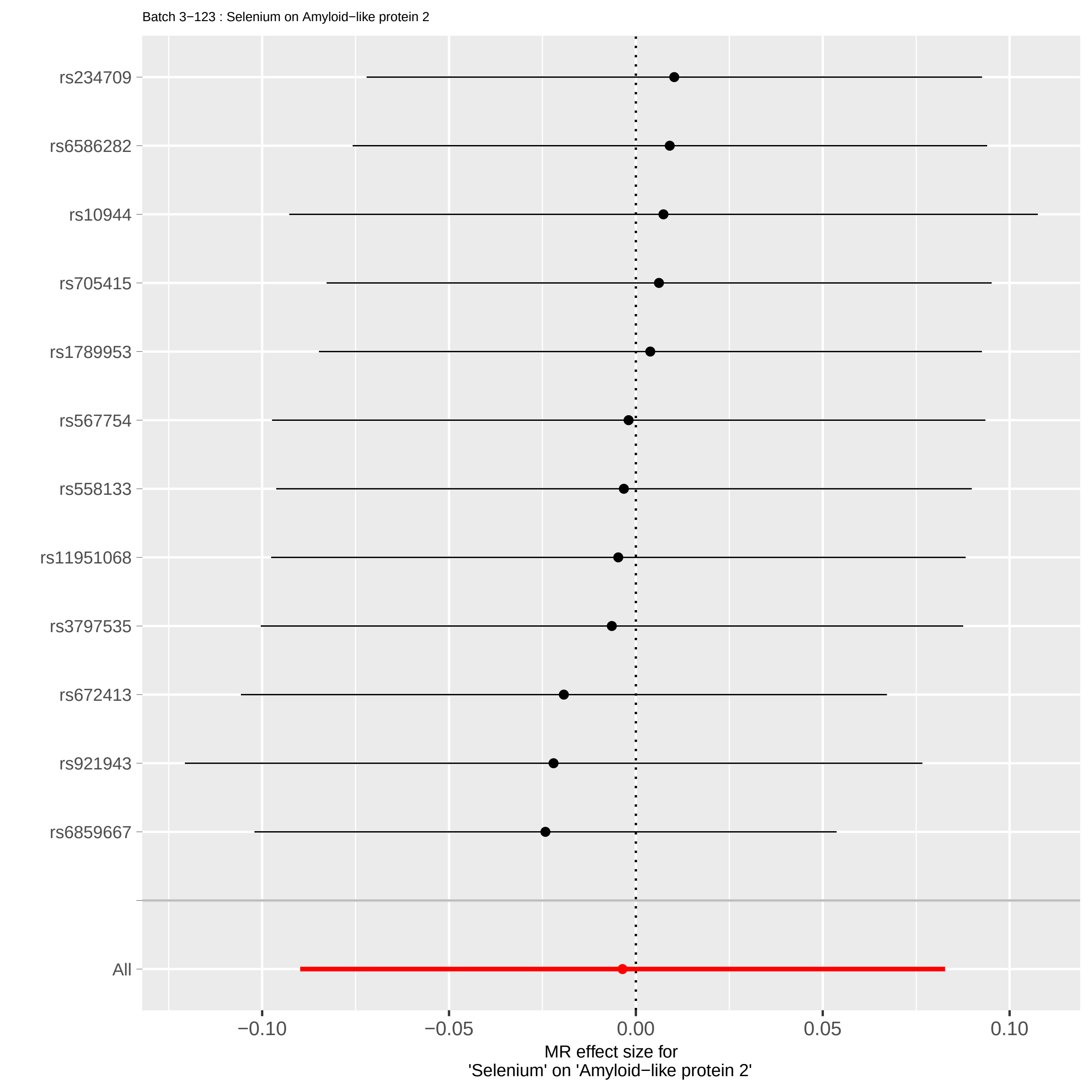

Batch 3−123 : Selenium on Amyloid−like protein 2
rs234709
rs6586282
rs10944
rs705415
rs1789953
rs567754
rs558133
rs11951068
rs3797535
rs672413
rs921943
rs6859667
All
−0.10
−0.05
0.00
0.05
0.10
MR effect size for
'Selenium' on 'Amyloid−like protein 2'

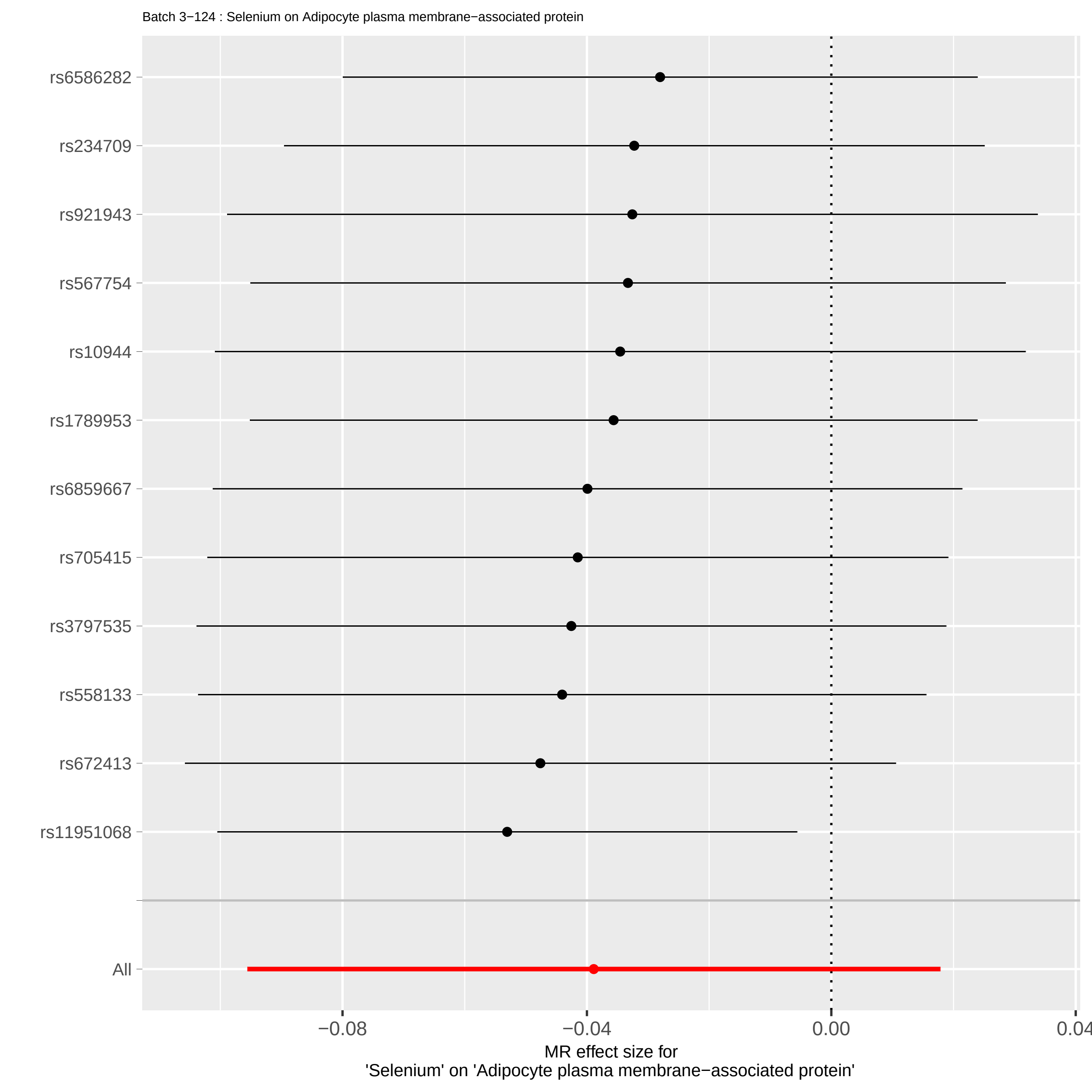

Batch 3−124 : Selenium on Adipocyte plasma membrane−associated protein
rs6586282
rs234709
rs921943
rs567754
rs10944
rs1789953
rs6859667
rs705415
rs3797535
rs558133
rs672413
rs11951068
All
−0.08
−0.04
0.00
0.04
MR effect size for
'Selenium' on 'Adipocyte plasma membrane−associated protein'

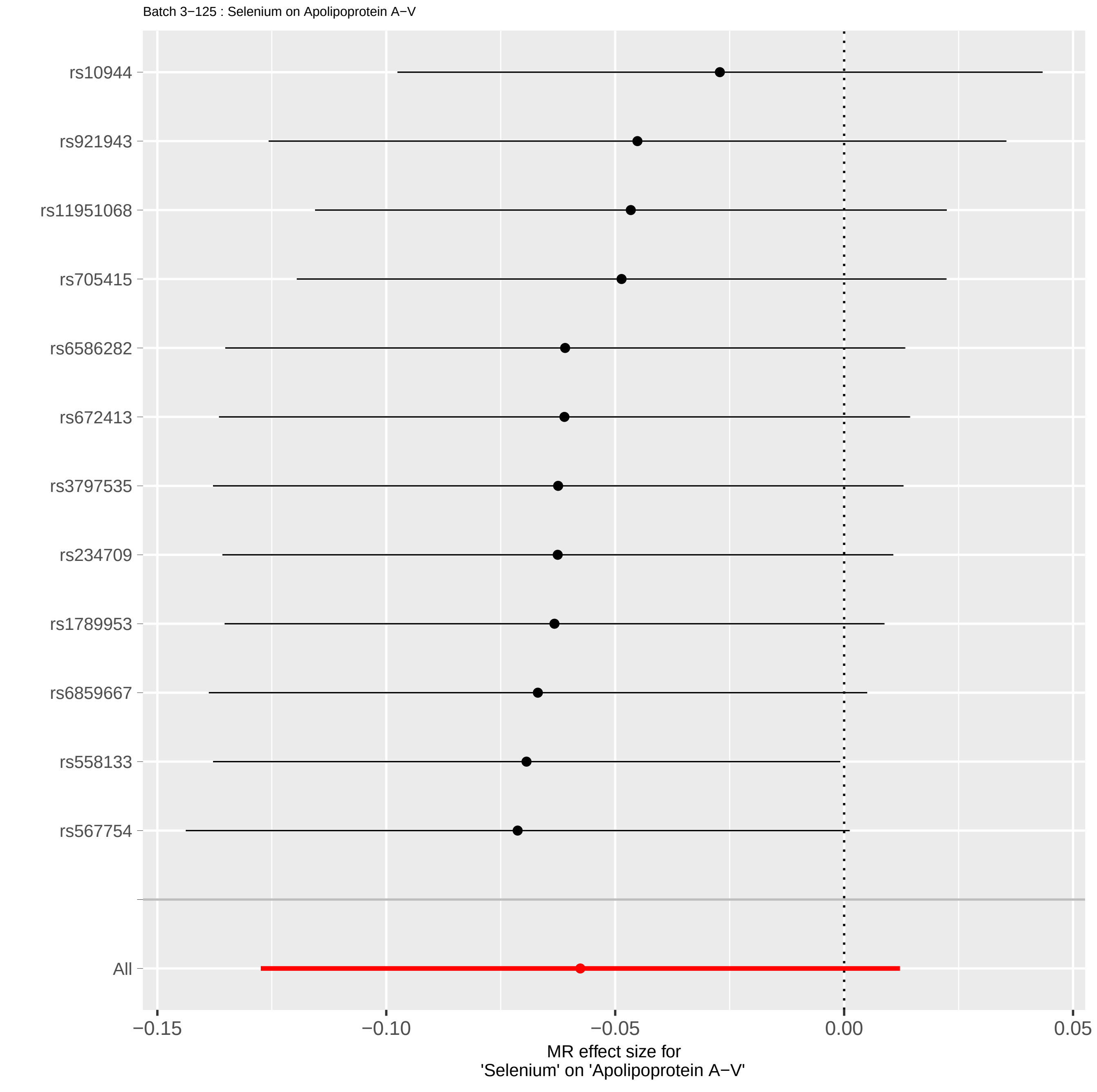

Batch 3−125 : Selenium on Apolipoprotein A−V
rs10944
rs921943
rs11951068
rs705415
rs6586282
rs672413
rs3797535
rs234709
rs1789953
rs6859667
rs558133
rs567754
All
−0.15
−0.10
−0.05
0.00
0.05
MR effect size for
'Selenium' on 'Apolipoprotein A−V'

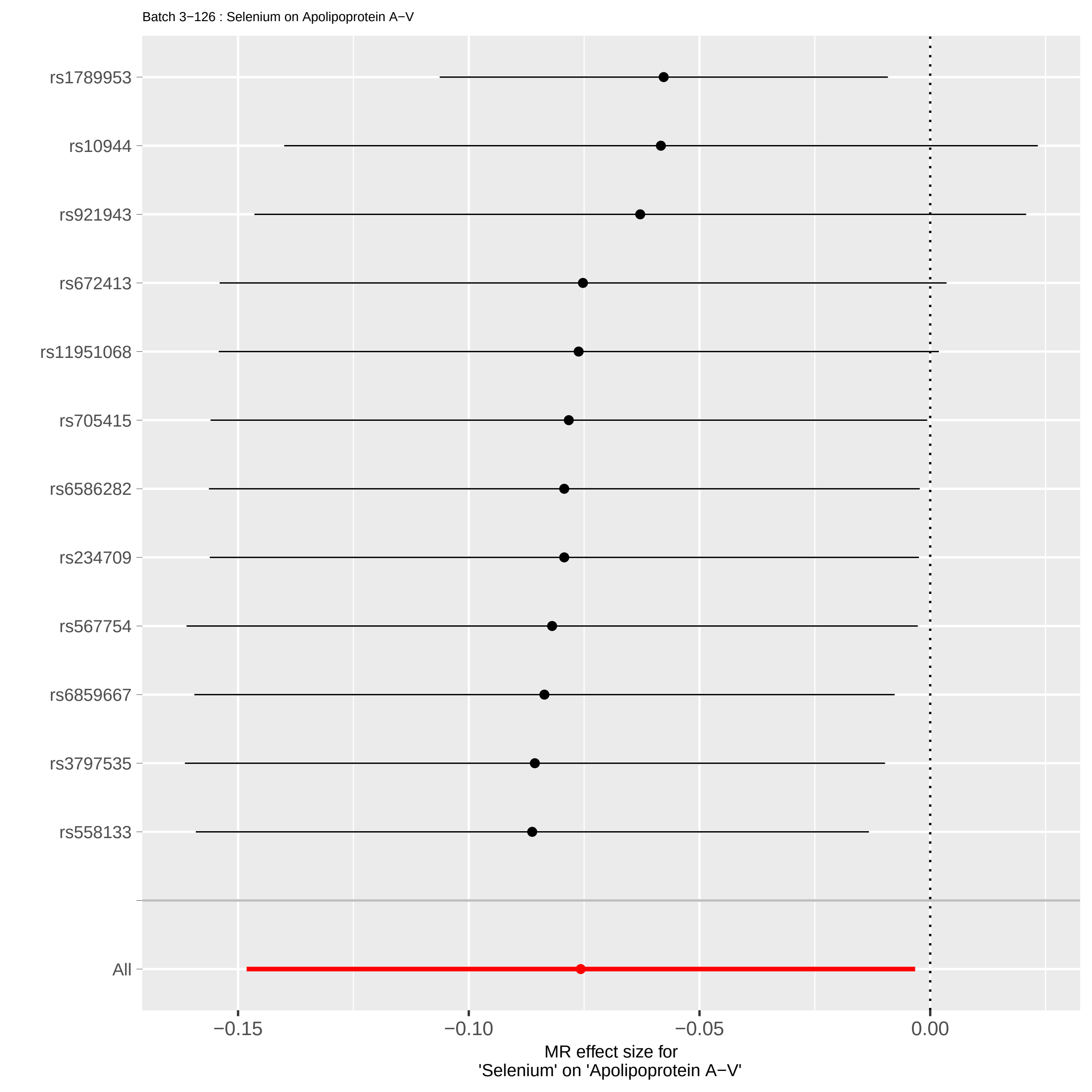

Batch 3−126 : Selenium on Apolipoprotein A−V
rs1789953
rs10944
rs921943
rs672413
rs11951068
rs705415
rs6586282
rs234709
rs567754
rs6859667
rs3797535
rs558133
All
−0.15
−0.10
−0.05
0.00
MR effect size for
'Selenium' on 'Apolipoprotein A−V'

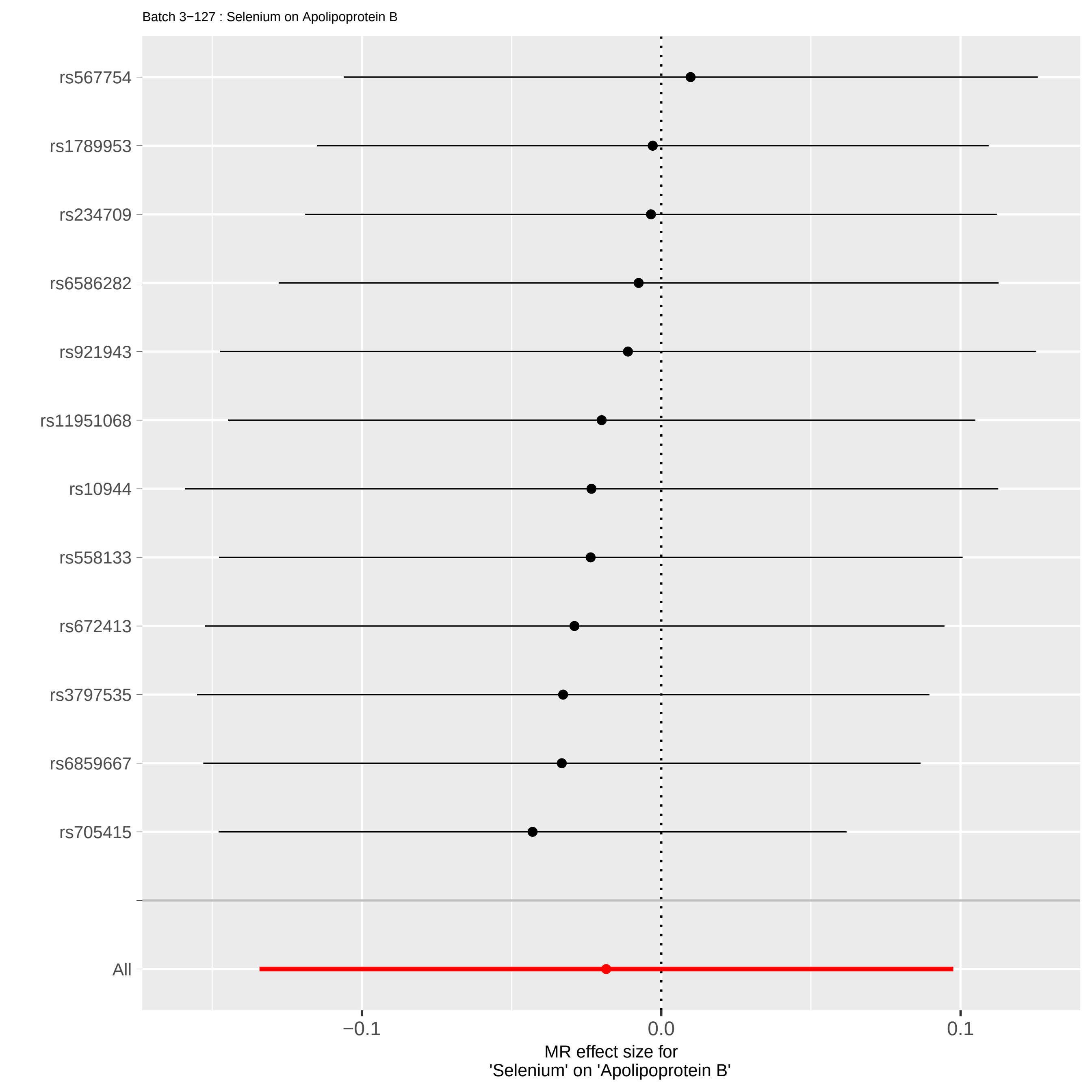
Batch 3−127 : Selenium on Apolipoprotein B
rs567754
rs1789953
rs234709
rs6586282
rs921943
rs11951068
rs10944
rs558133
rs672413
rs3797535
rs6859667
rs705415
All
−0.1
0.0
0.1
MR effect size for
'Selenium' on 'Apolipoprotein B'

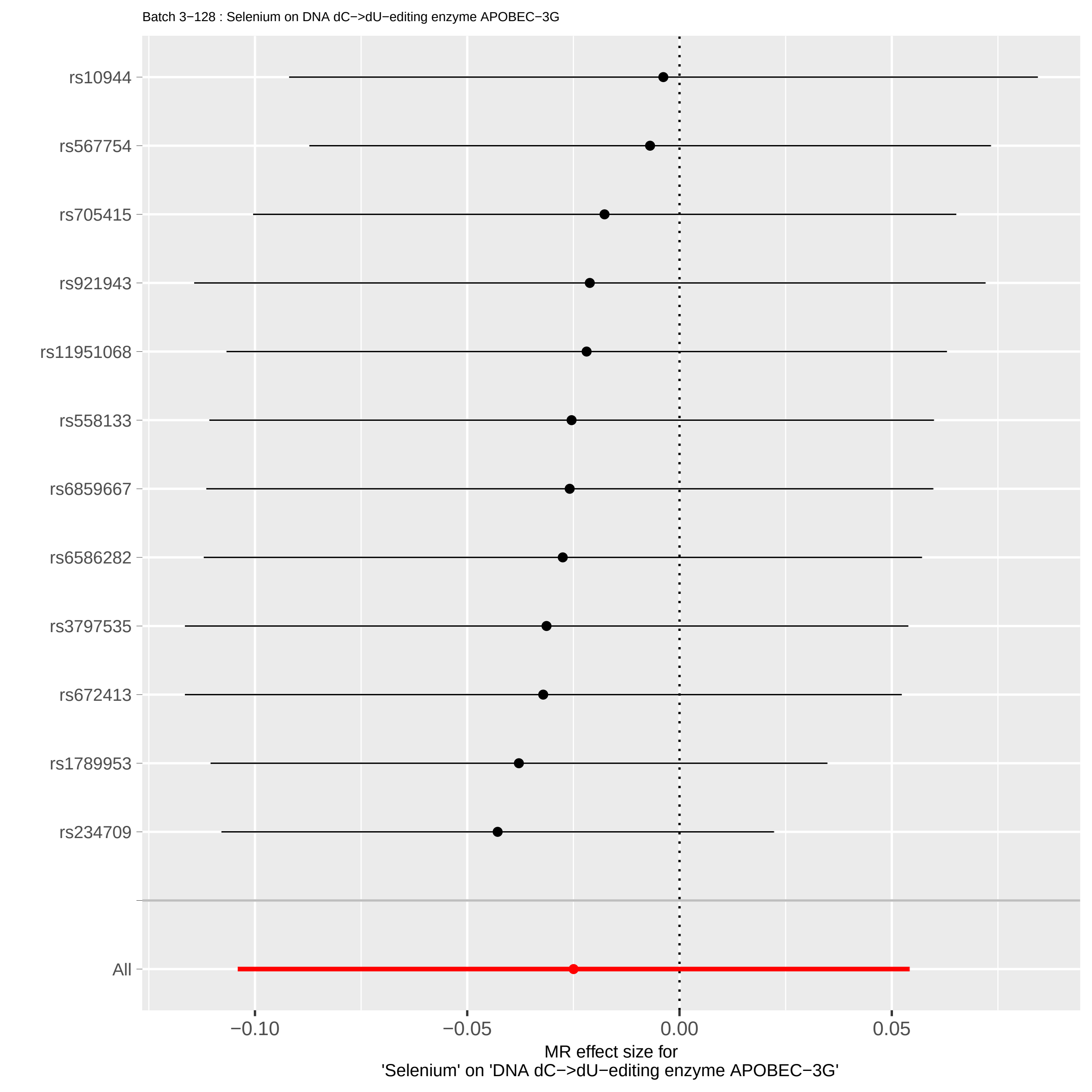
Batch 3−128 : Selenium on DNA dC−>dU−editing enzyme APOBEC−3G
rs10944
rs567754
rs705415
rs921943
rs11951068
rs558133
rs6859667
rs6586282
rs3797535
rs672413
rs1789953
rs234709
All
−0.10
−0.05
0.00
0.05
MR effect size for
'Selenium' on 'DNA dC−>dU−editing enzyme APOBEC−3G'

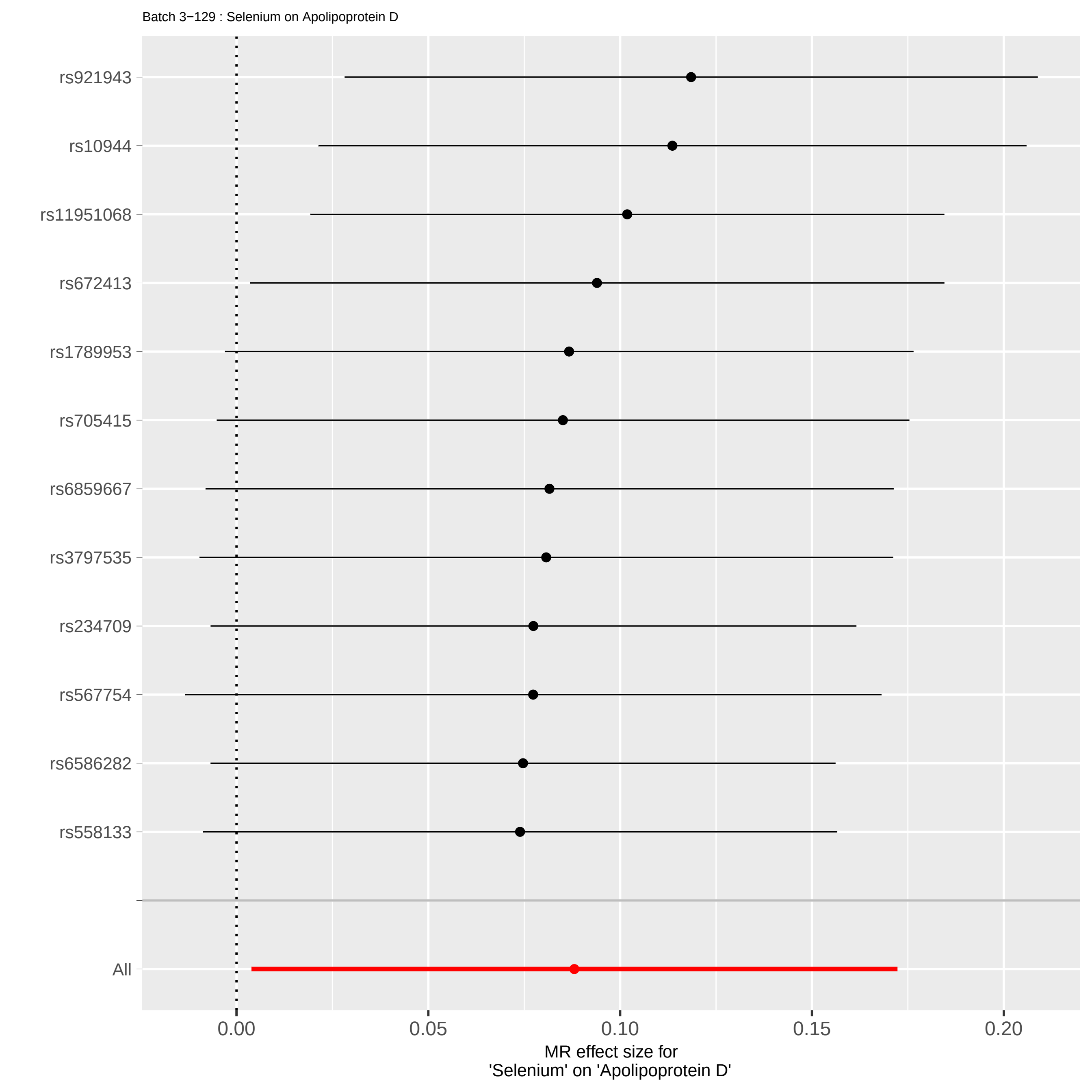

Batch 3−129 : Selenium on Apolipoprotein D
rs921943
rs10944
rs11951068
rs672413
rs1789953
rs705415
rs6859667
rs3797535
rs234709
rs567754
rs6586282
rs558133
All
0.00
0.05
0.10
0.15
0.20
MR effect size for
'Selenium' on 'Apolipoprotein D'

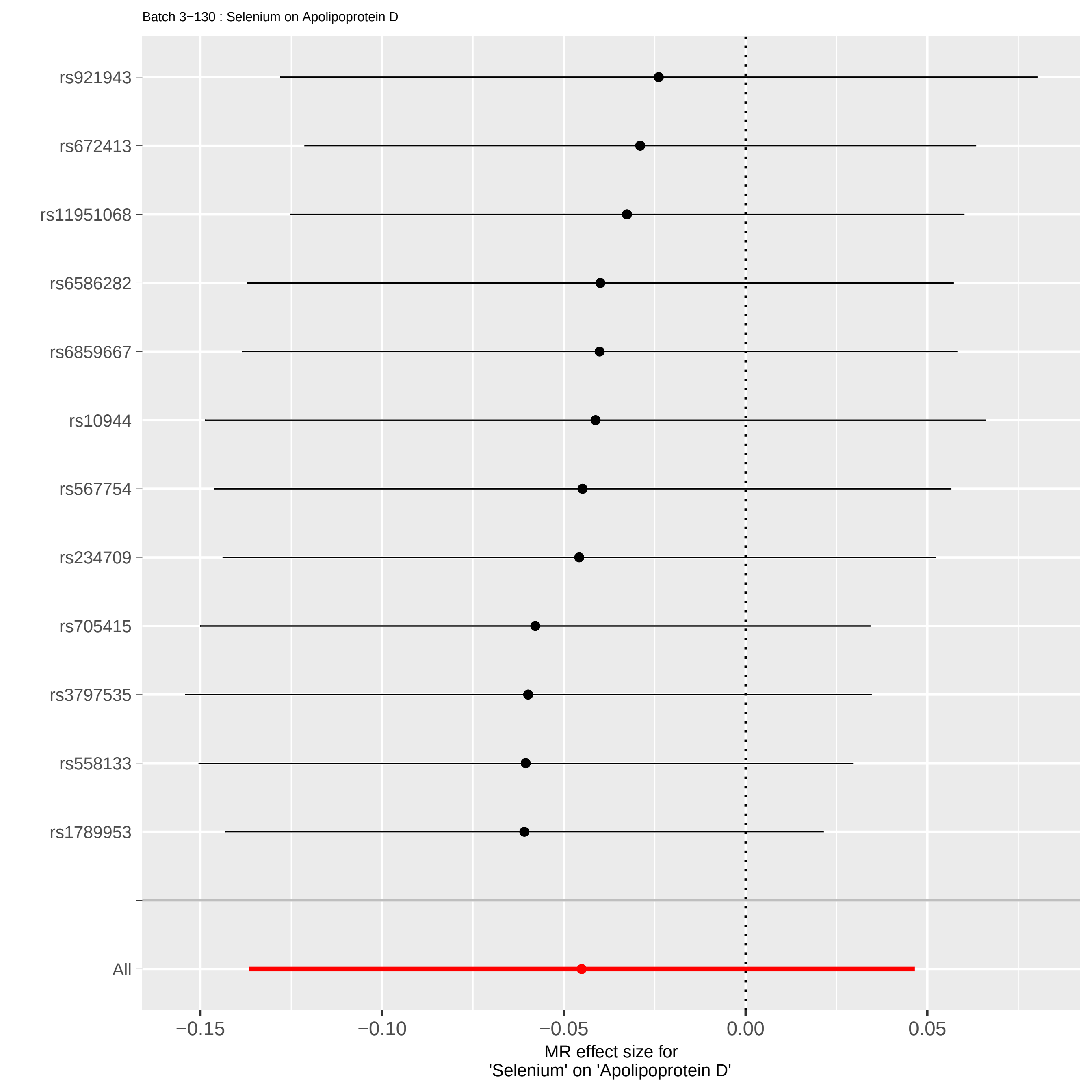

Batch 3−130 : Selenium on Apolipoprotein D
rs921943
rs672413
rs11951068
rs6586282
rs6859667
rs10944
rs567754
rs234709
rs705415
rs3797535
rs558133
rs1789953
All
−0.15
−0.10
−0.05
0.00
0.05
MR effect size for
'Selenium' on 'Apolipoprotein D'

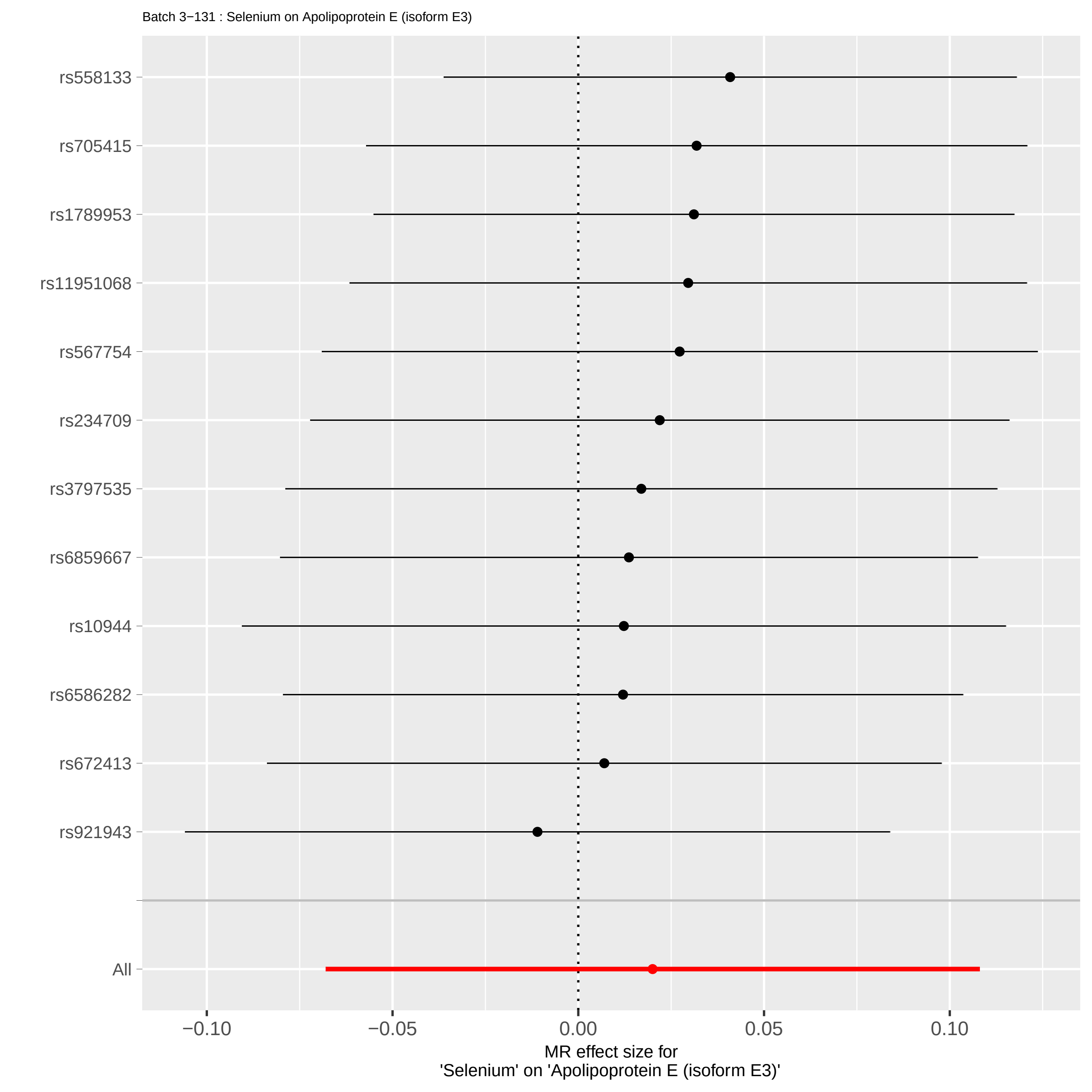

Batch 3−131 : Selenium on Apolipoprotein E (isoform E3)
rs558133
rs705415
rs1789953
rs11951068
rs567754
rs234709
rs3797535
rs6859667
rs10944
rs6586282
rs672413
rs921943
All
−0.10
−0.05
0.00
0.05
0.10
MR effect size for
'Selenium' on 'Apolipoprotein E (isoform E3)'

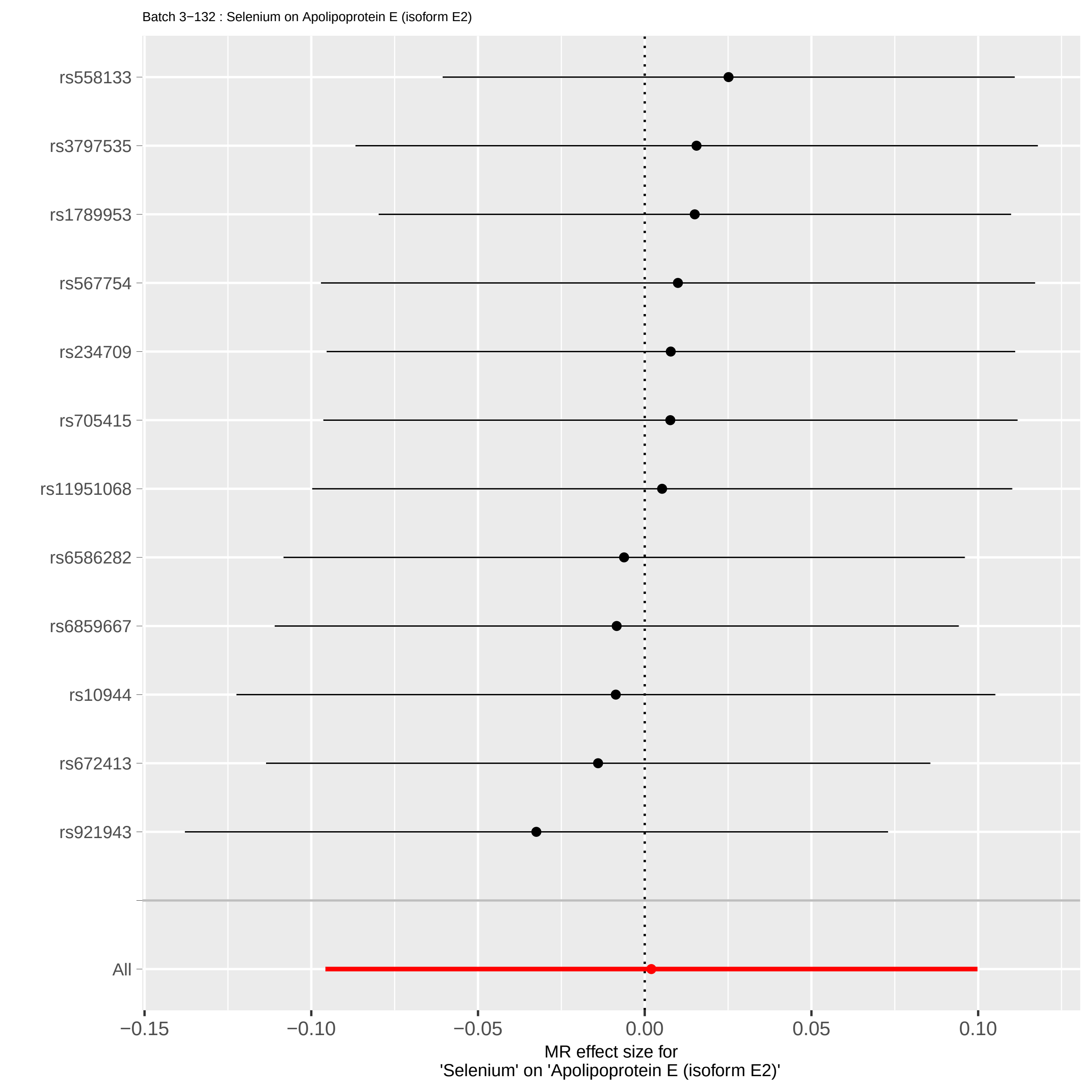
Batch 3−132 : Selenium on Apolipoprotein E (isoform E2)
rs558133
rs3797535
rs1789953
rs567754
rs234709
rs705415
rs11951068
rs6586282
rs6859667
rs10944
rs672413
rs921943
All
−0.15
−0.10
−0.05
0.00
0.05
0.10
MR effect size for
'Selenium' on 'Apolipoprotein E (isoform E2)'

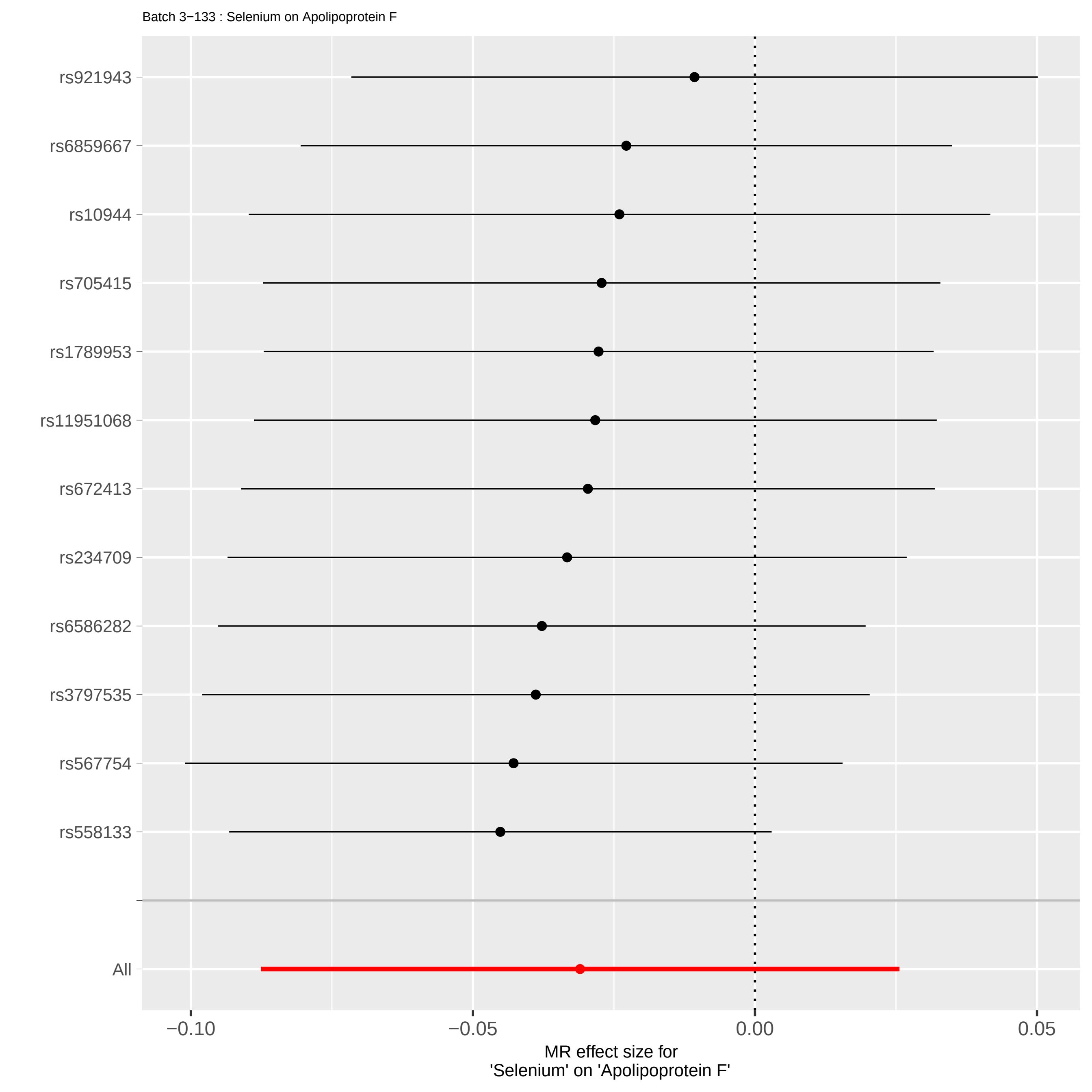

Batch 3−133 : Selenium on Apolipoprotein F
rs921943
rs6859667
rs10944
rs705415
rs1789953
rs11951068
rs672413
rs234709
rs6586282
rs3797535
rs567754
rs558133
All
−0.10
−0.05
0.00
0.05
MR effect size for
'Selenium' on 'Apolipoprotein F'

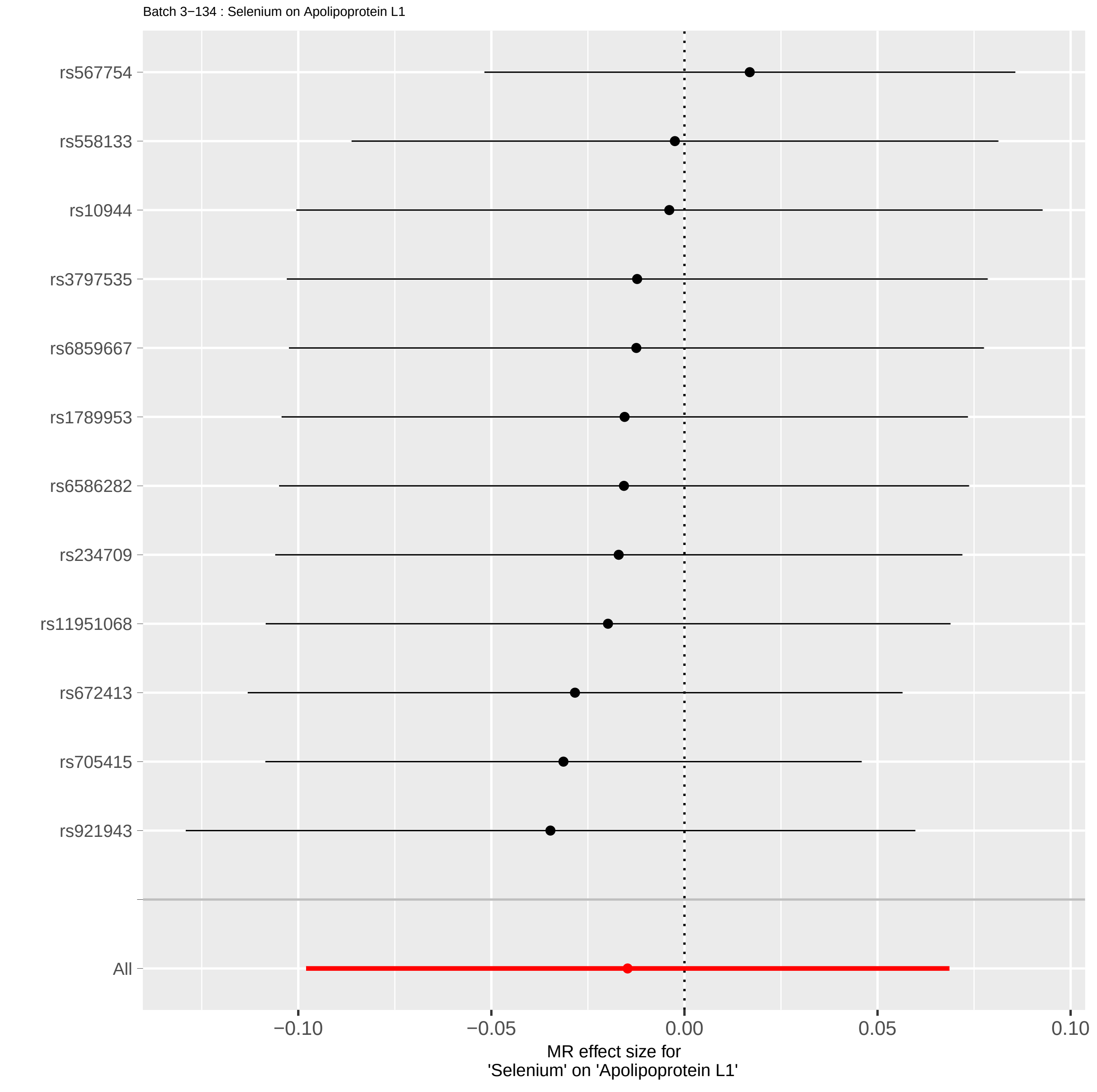
Batch 3−134 : Selenium on Apolipoprotein L1
rs567754
rs558133
rs10944
rs3797535
rs6859667
rs1789953
rs6586282
rs234709
rs11951068
rs672413
rs705415
rs921943
All
−0.10
−0.05
0.00
0.05
0.10
MR effect size for
'Selenium' on 'Apolipoprotein L1'

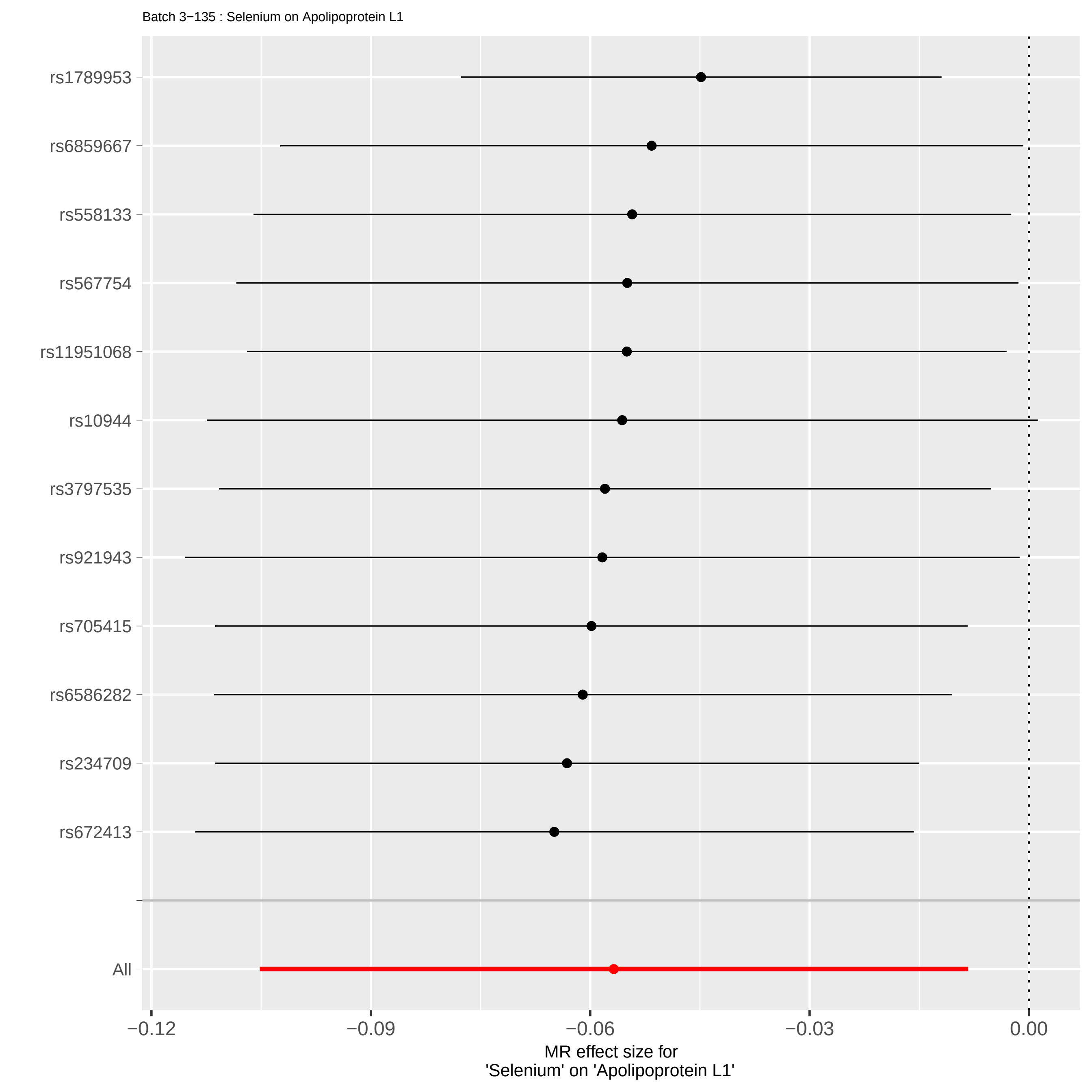

Batch 3−135 : Selenium on Apolipoprotein L1
rs1789953
rs6859667
rs558133
rs567754
rs11951068
rs10944
rs3797535
rs921943
rs705415
rs6586282
rs234709
rs672413
All
−0.12
−0.09
−0.06
−0.03
0.00
MR effect size for
'Selenium' on 'Apolipoprotein L1'

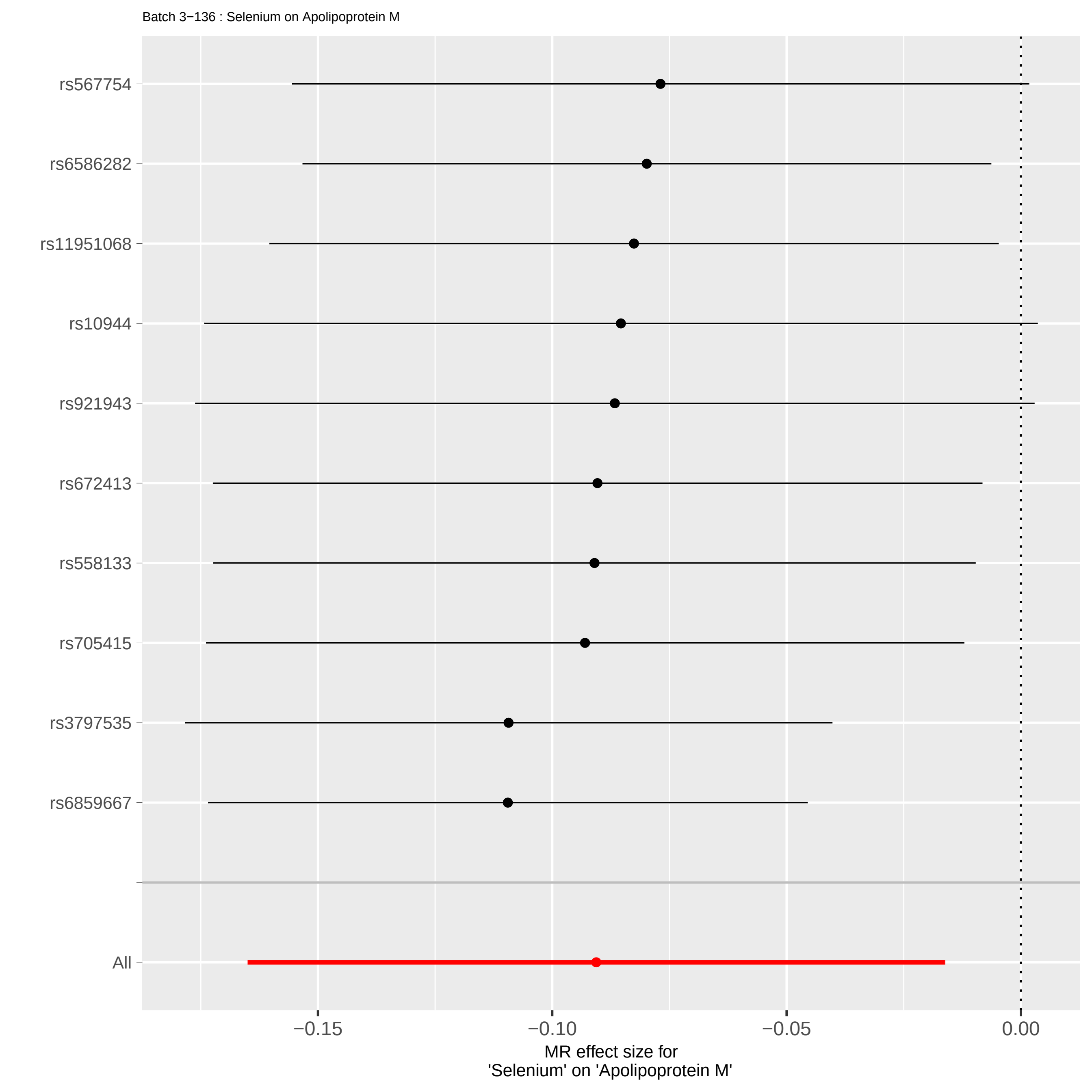
Batch 3−136 : Selenium on Apolipoprotein M
rs567754
rs6586282
rs11951068
rs10944
rs921943
rs672413
rs558133
rs705415
rs3797535
rs6859667
All
−0.15
−0.10
−0.05
0.00
MR effect size for
'Selenium' on 'Apolipoprotein M'

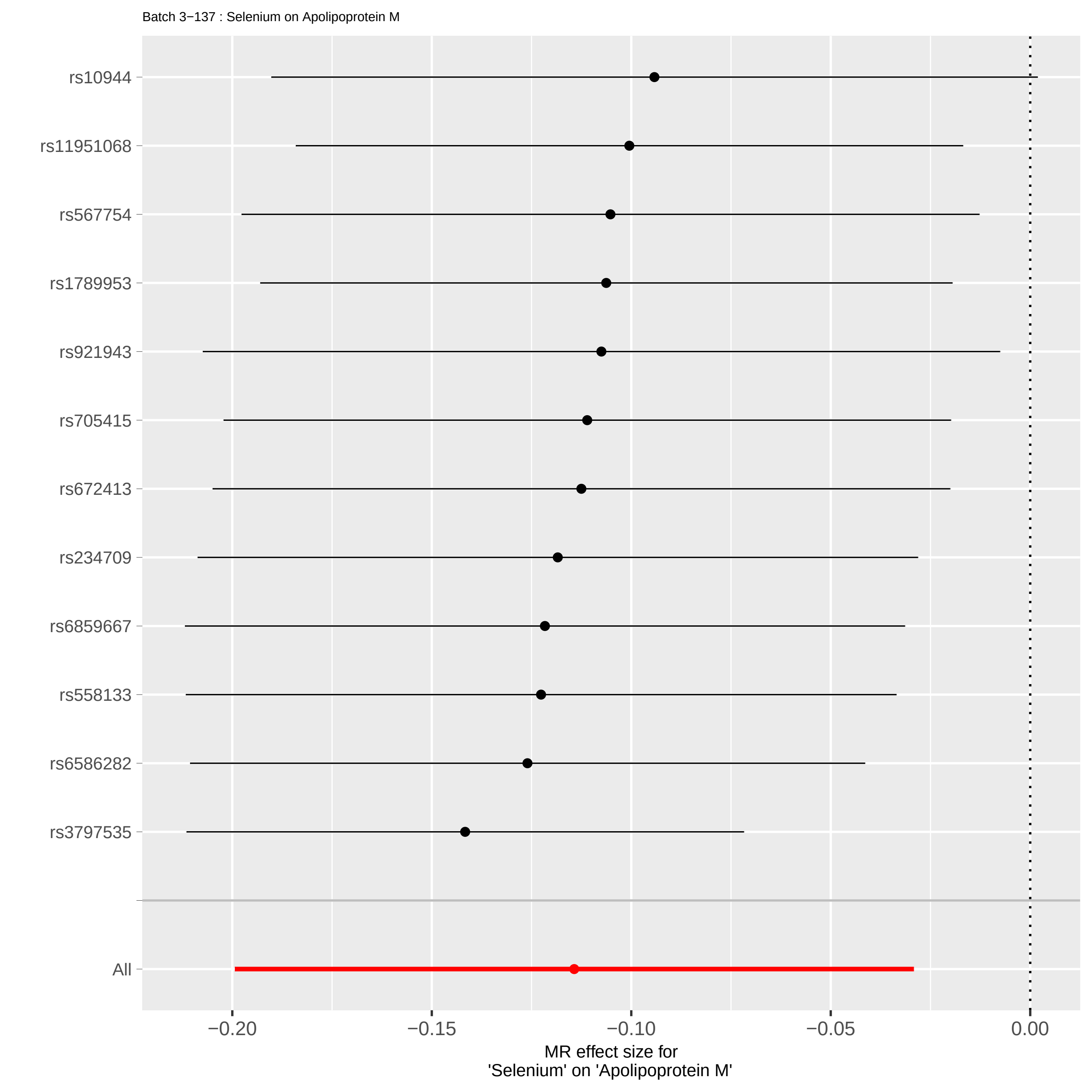

Batch 3−137 : Selenium on Apolipoprotein M
rs10944
rs11951068
rs567754
rs1789953
rs921943
rs705415
rs672413
rs234709
rs6859667
rs558133
rs6586282
rs3797535
All
−0.20
−0.15
−0.10
−0.05
0.00
MR effect size for
'Selenium' on 'Apolipoprotein M'

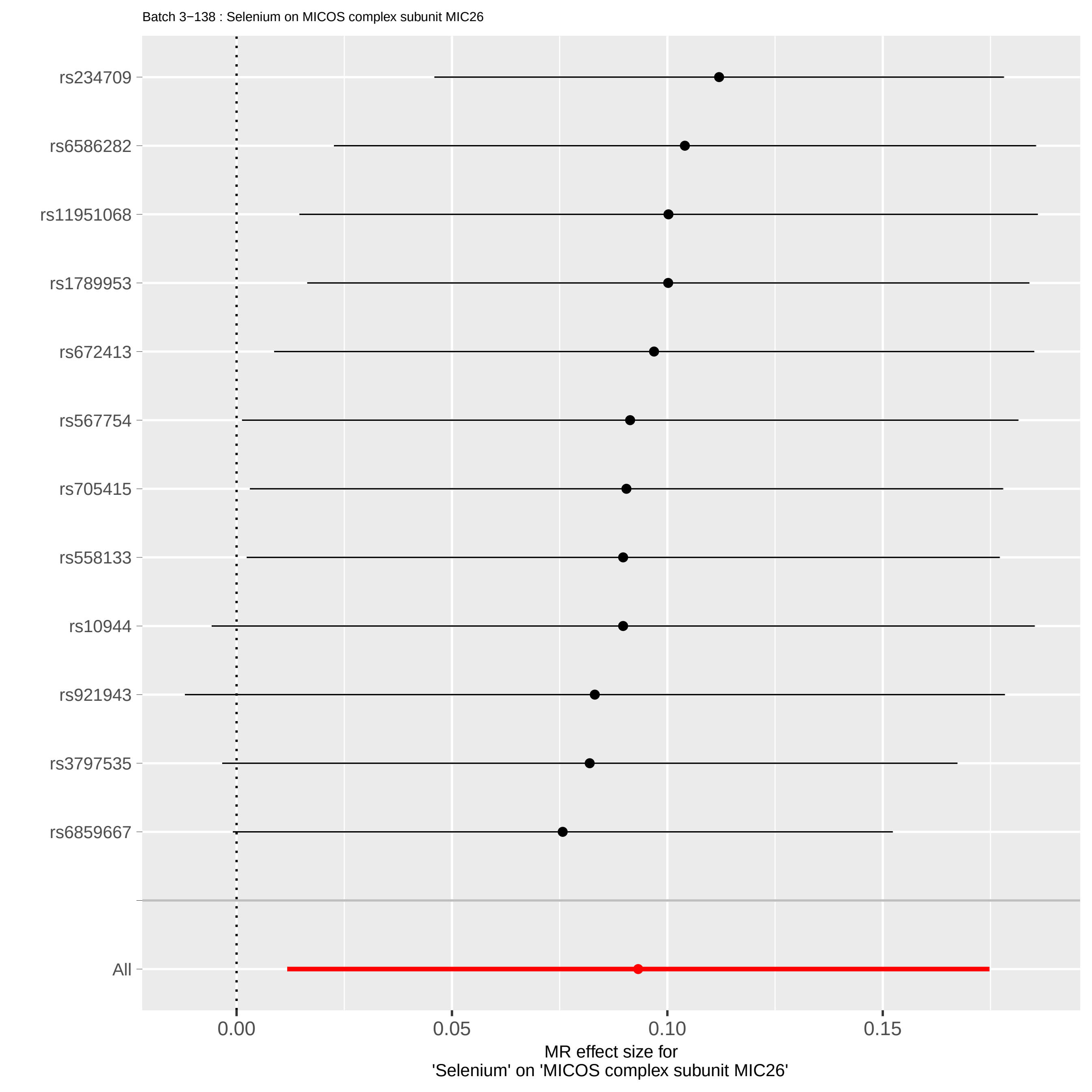
Batch 3−138 : Selenium on MICOS complex subunit MIC26
rs234709
rs6586282
rs11951068
rs1789953
rs672413
rs567754
rs705415
rs558133
rs10944
rs921943
rs3797535
rs6859667
All
0.00
0.05
0.10
0.15
MR effect size for
'Selenium' on 'MICOS complex subunit MIC26'

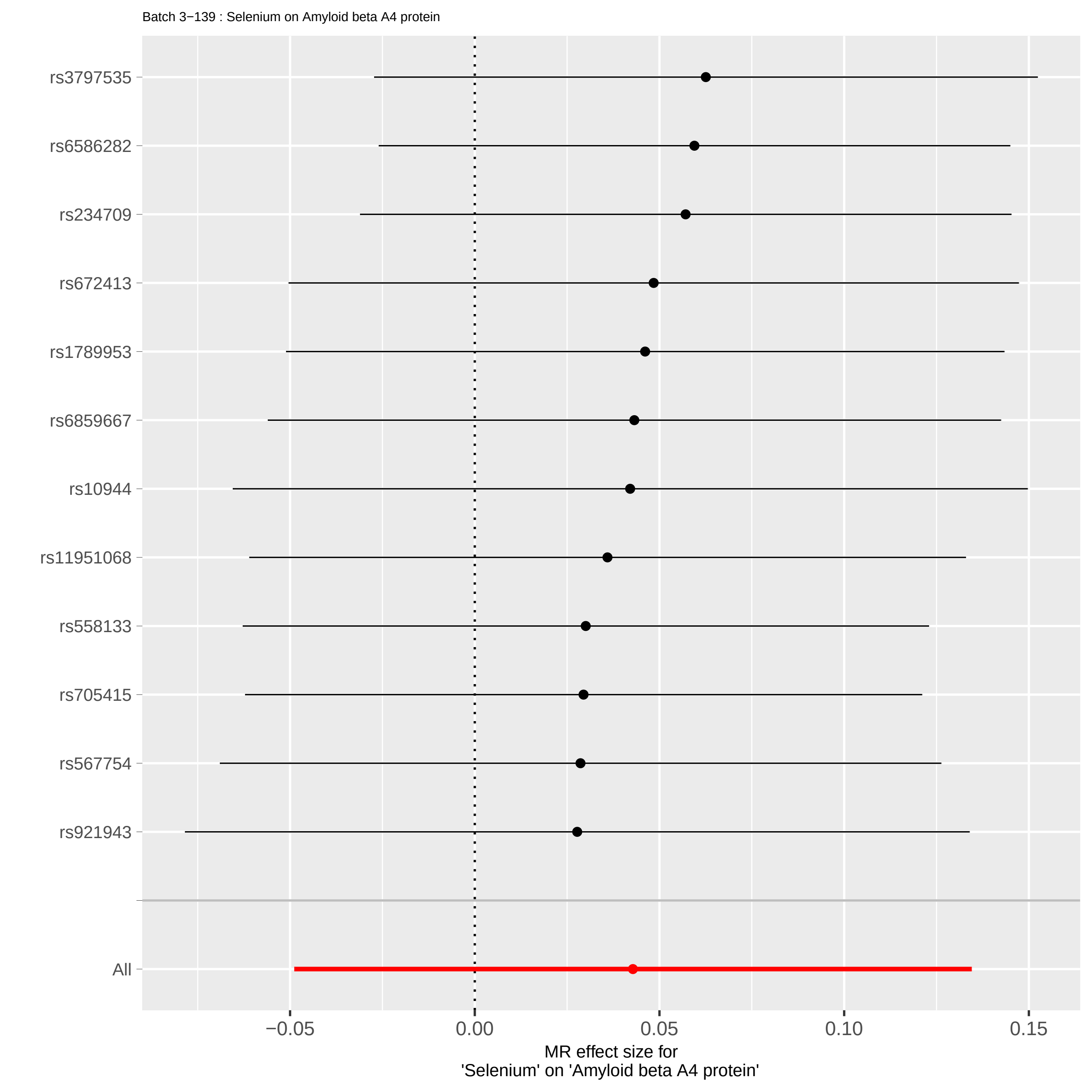

Batch 3−139 : Selenium on Amyloid beta A4 protein
rs3797535
rs6586282
rs234709
rs672413
rs1789953
rs6859667
rs10944
rs11951068
rs558133
rs705415
rs567754
rs921943
All
−0.05
0.00
0.05
0.10
0.15
MR effect size for
'Selenium' on 'Amyloid beta A4 protein'

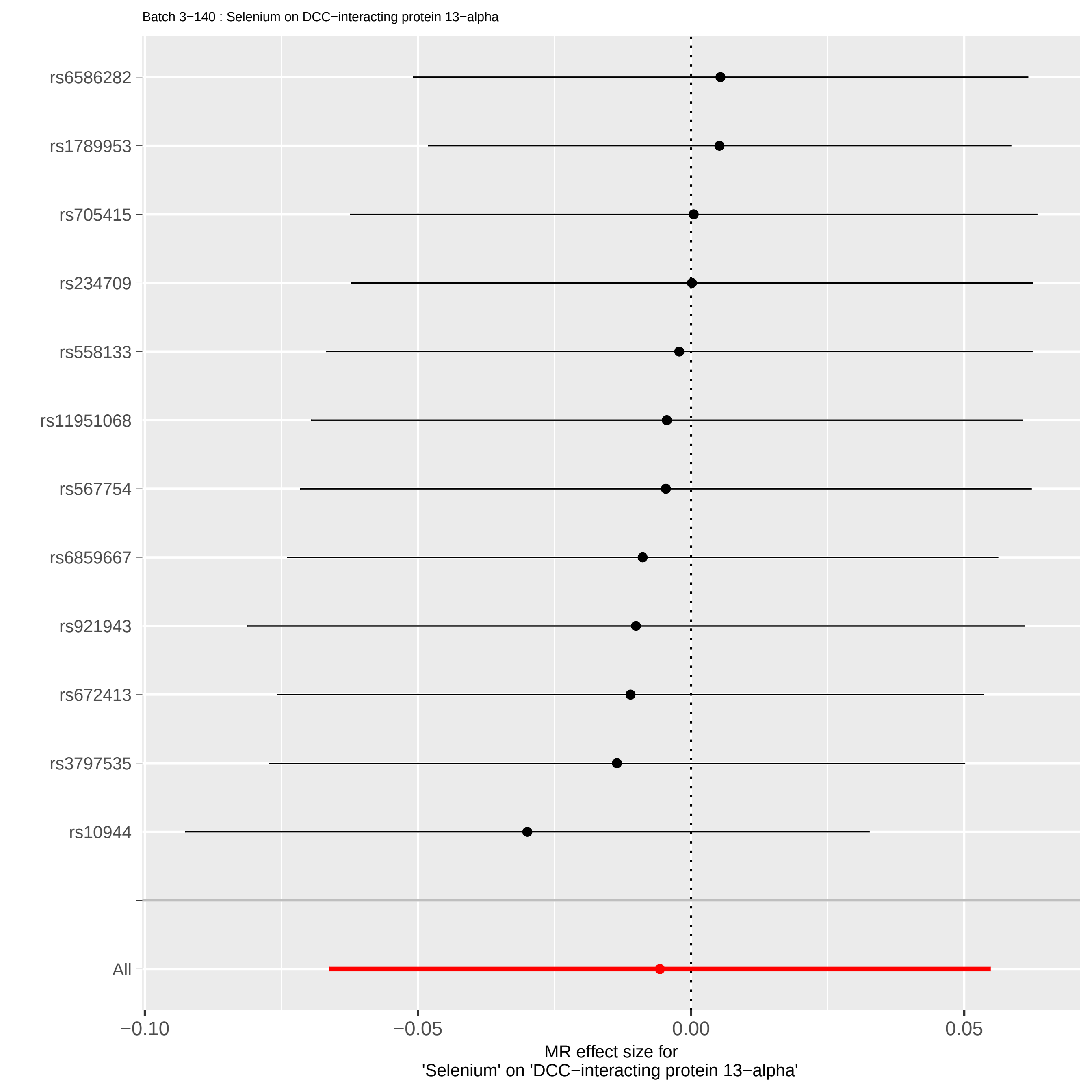

Batch 3−140 : Selenium on DCC−interacting protein 13−alpha
rs6586282
rs1789953
rs705415
rs234709
rs558133
rs11951068
rs567754
rs6859667
rs921943
rs672413
rs3797535
rs10944
All
−0.10
−0.05
0.00
0.05
MR effect size for
'Selenium' on 'DCC−interacting protein 13−alpha'

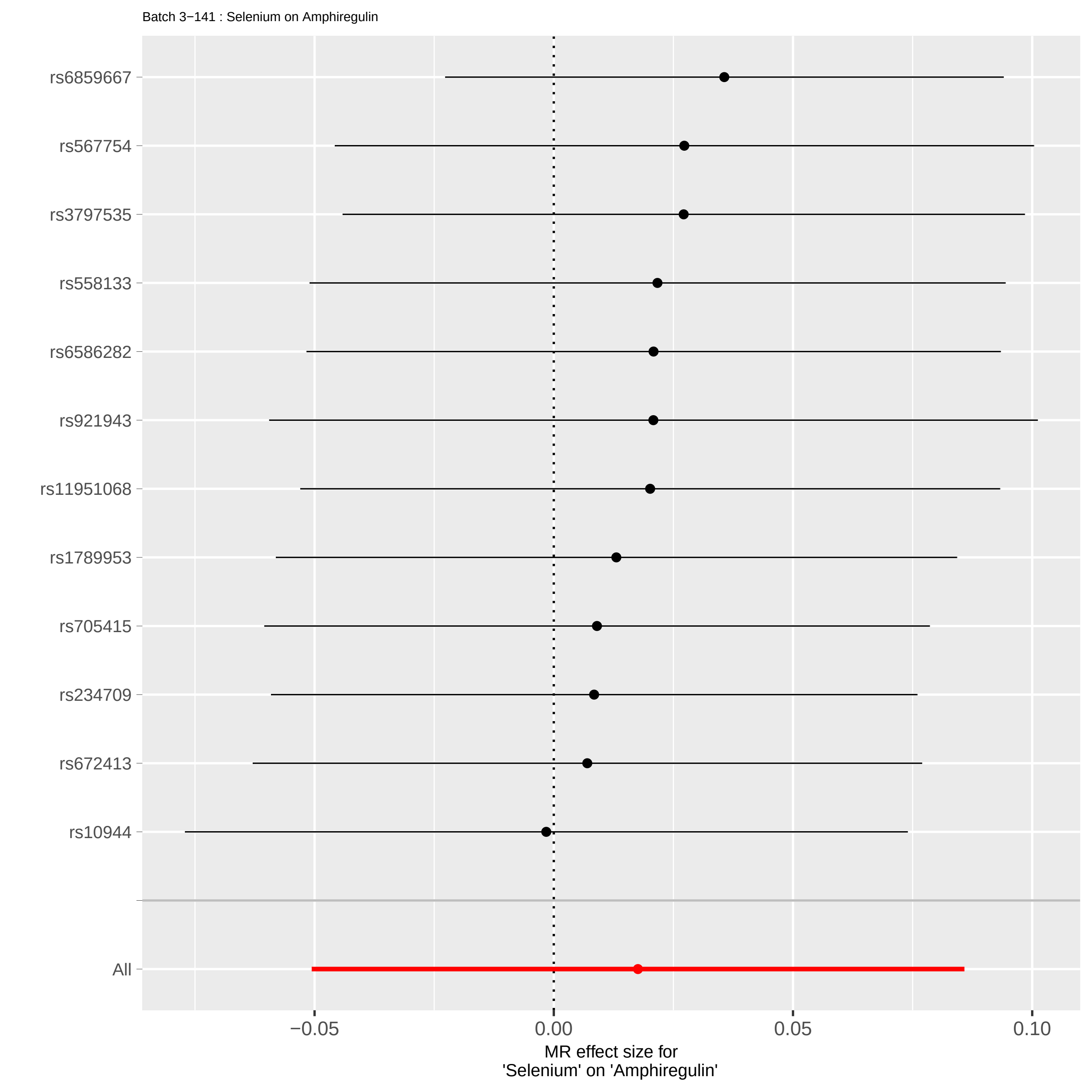
Batch 3−141 : Selenium on Amphiregulin
rs6859667
rs567754
rs3797535
rs558133
rs6586282
rs921943
rs11951068
rs1789953
rs705415
rs234709
rs672413
rs10944
All
−0.05
0.00
0.05
0.10
MR effect size for
'Selenium' on 'Amphiregulin'

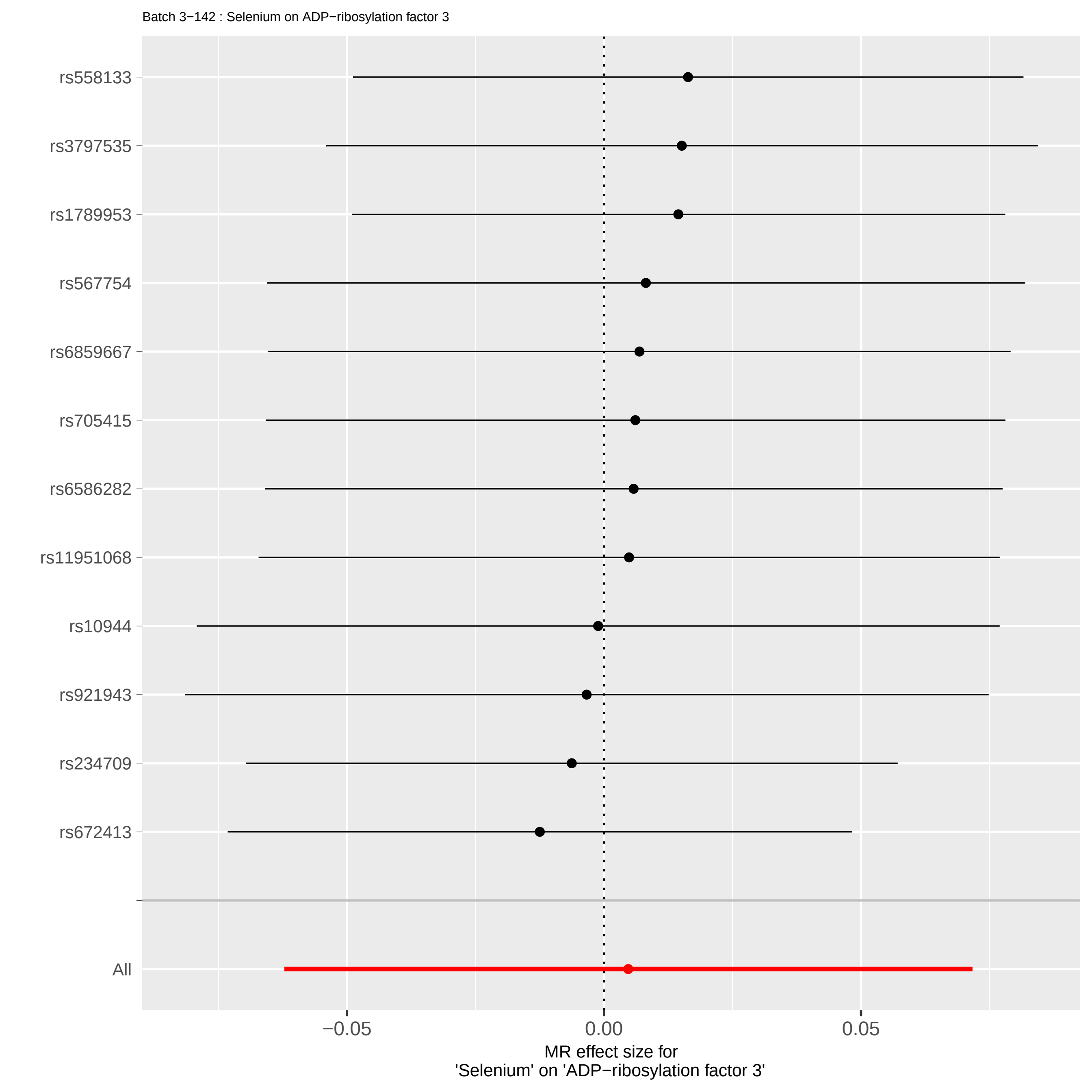

Batch 3−142 : Selenium on ADP−ribosylation factor 3
rs558133
rs3797535
rs1789953
rs567754
rs6859667
rs705415
rs6586282
rs11951068
rs10944
rs921943
rs234709
rs672413
All
−0.05
0.00
0.05
MR effect size for
'Selenium' on 'ADP−ribosylation factor 3'

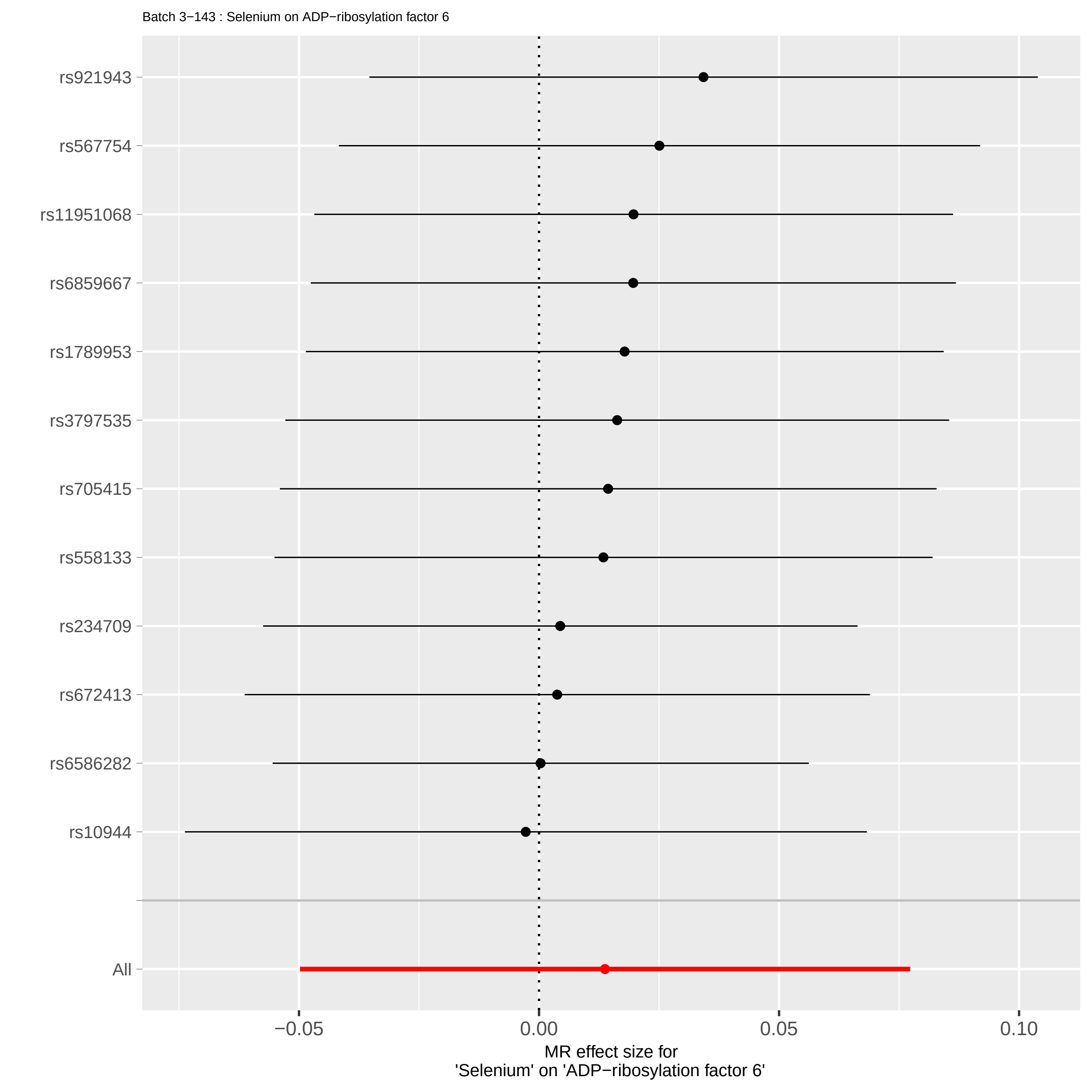

Batch 3−143 : Selenium on ADP−ribosylation factor 6
rs921943
rs567754
rs11951068
rs6859667
rs1789953
rs3797535
rs705415
rs558133
rs234709
rs672413
rs6586282
rs10944
All
−0.05
0.00
0.05
0.10
MR effect size for
'Selenium' on 'ADP−ribosylation factor 6'

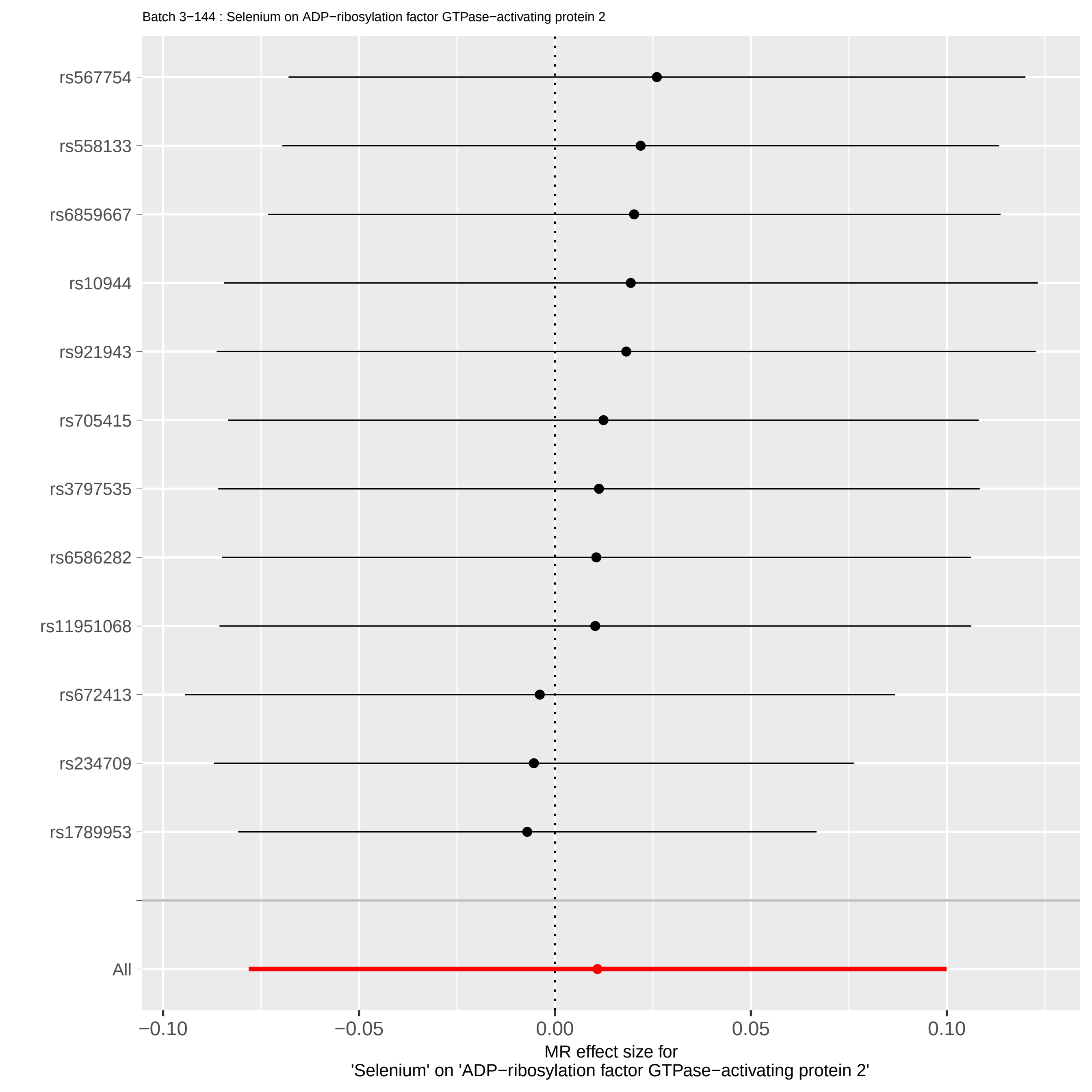

Batch 3−144 : Selenium on ADP−ribosylation factor GTPase−activating protein 2
rs567754
rs558133
rs6859667
rs10944
rs921943
rs705415
rs3797535
rs6586282
rs11951068
rs672413
rs234709
rs1789953
All
−0.10
−0.05
0.00
0.05
0.10
MR effect size for
'Selenium' on 'ADP−ribosylation factor GTPase−activating protein 2'

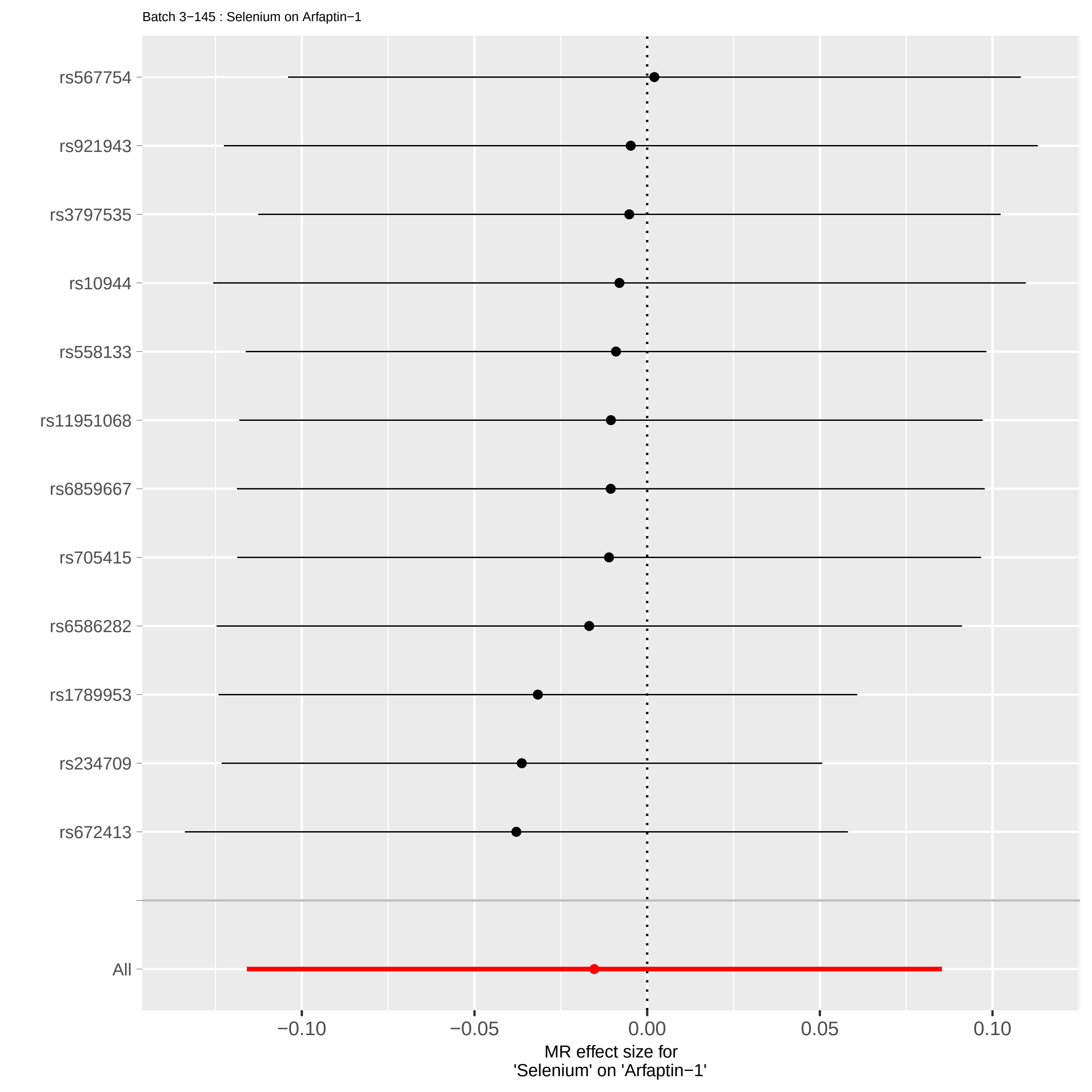

Batch 3−145 : Selenium on Arfaptin−1
rs567754
rs921943
rs3797535
rs10944
rs558133
rs11951068
rs6859667
rs705415
rs6586282
rs1789953
rs234709
rs672413
All
−0.10
−0.05
0.00
0.05
0.10
MR effect size for
'Selenium' on 'Arfaptin−1'

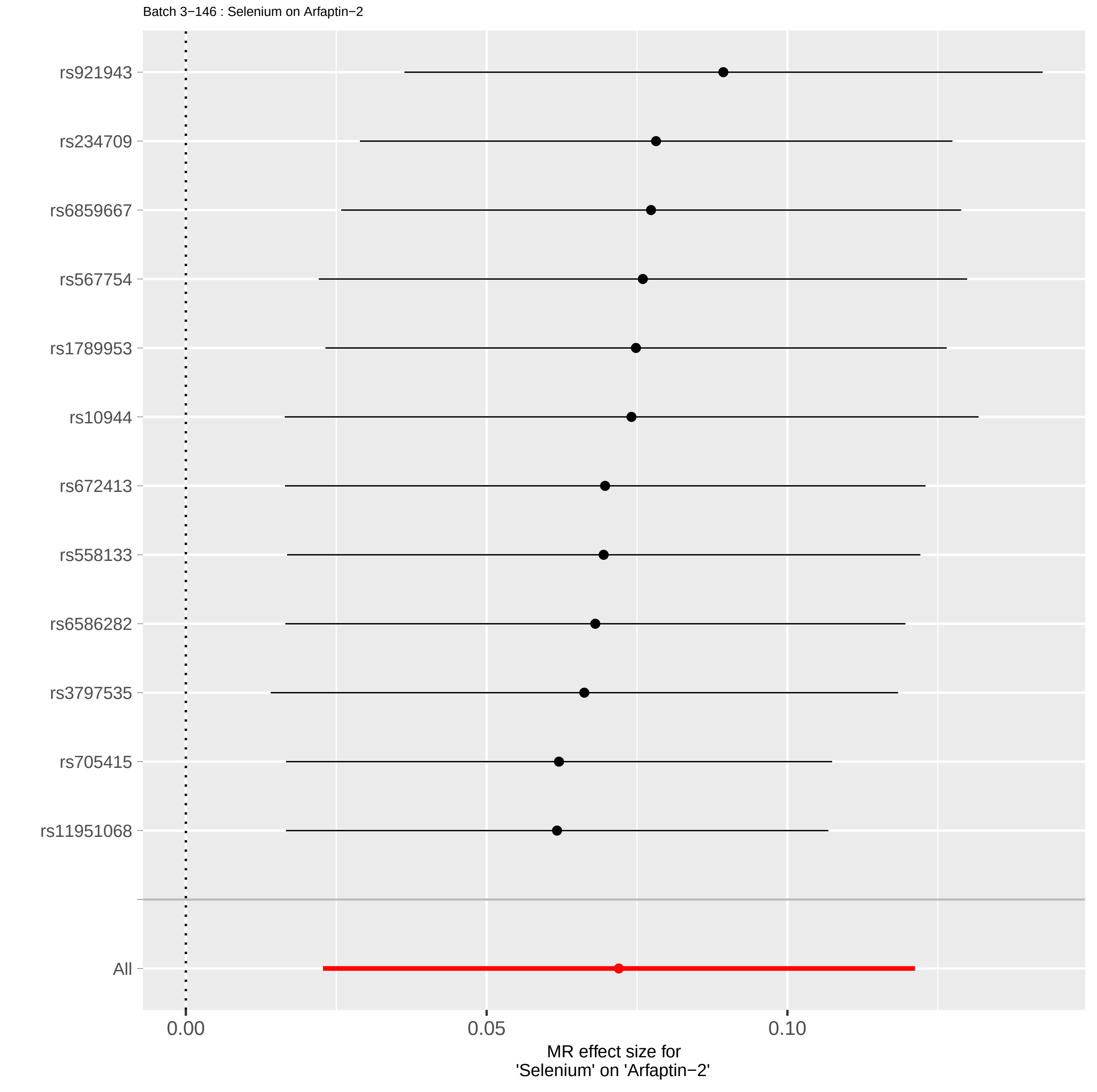

Batch 3−146 : Selenium on Arfaptin−2
rs921943
rs234709
rs6859667
rs567754
rs1789953
rs10944
rs672413
rs558133
rs6586282
rs3797535
rs705415
rs11951068
All
0.00
0.05
0.10
MR effect size for
'Selenium' on 'Arfaptin−2'

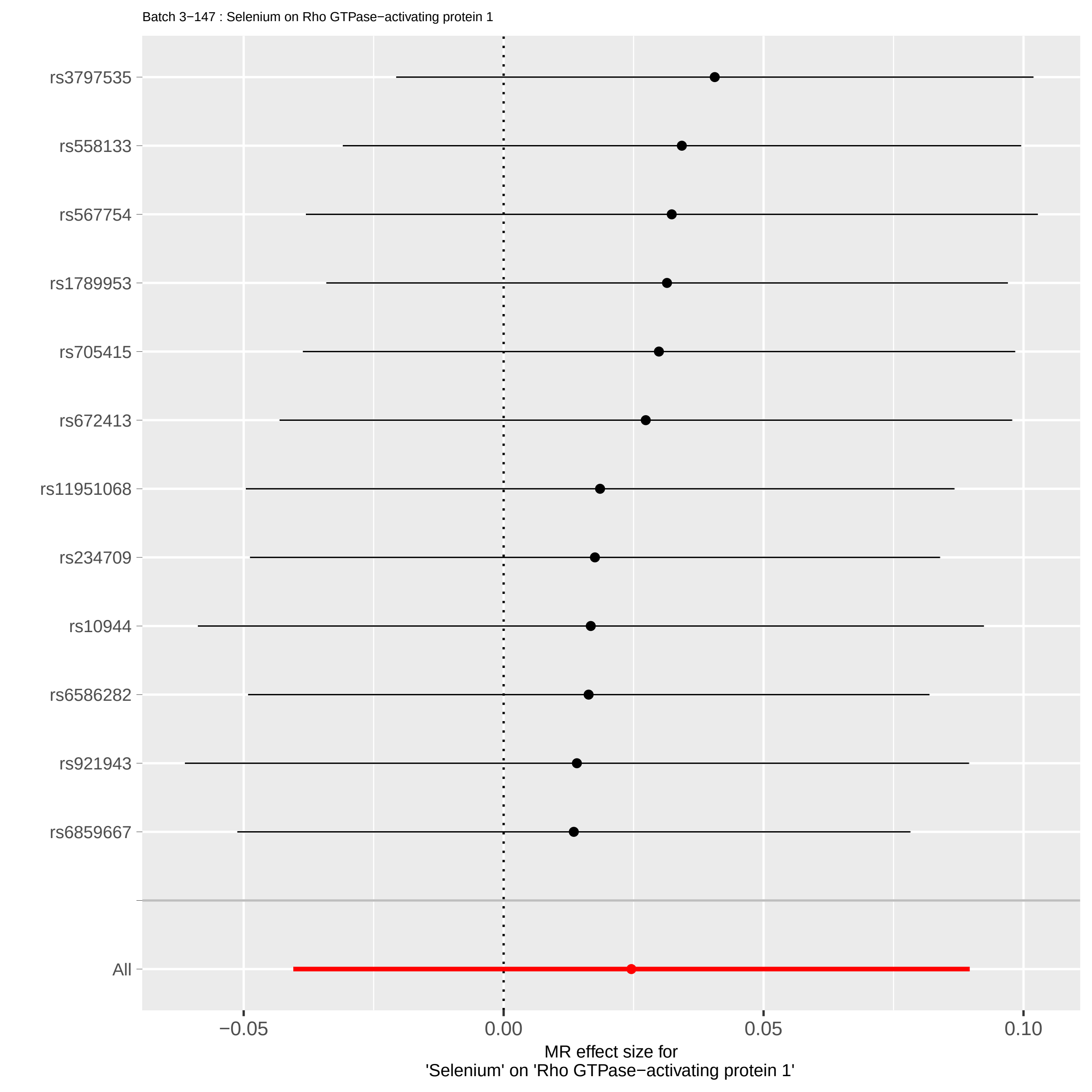

Batch 3−147 : Selenium on Rho GTPase−activating protein 1
rs3797535
rs558133
rs567754
rs1789953
rs705415
rs672413
rs11951068
rs234709
rs10944
rs6586282
rs921943
rs6859667
All
−0.05
0.00
0.05
0.10
MR effect size for
'Selenium' on 'Rho GTPase−activating protein 1'

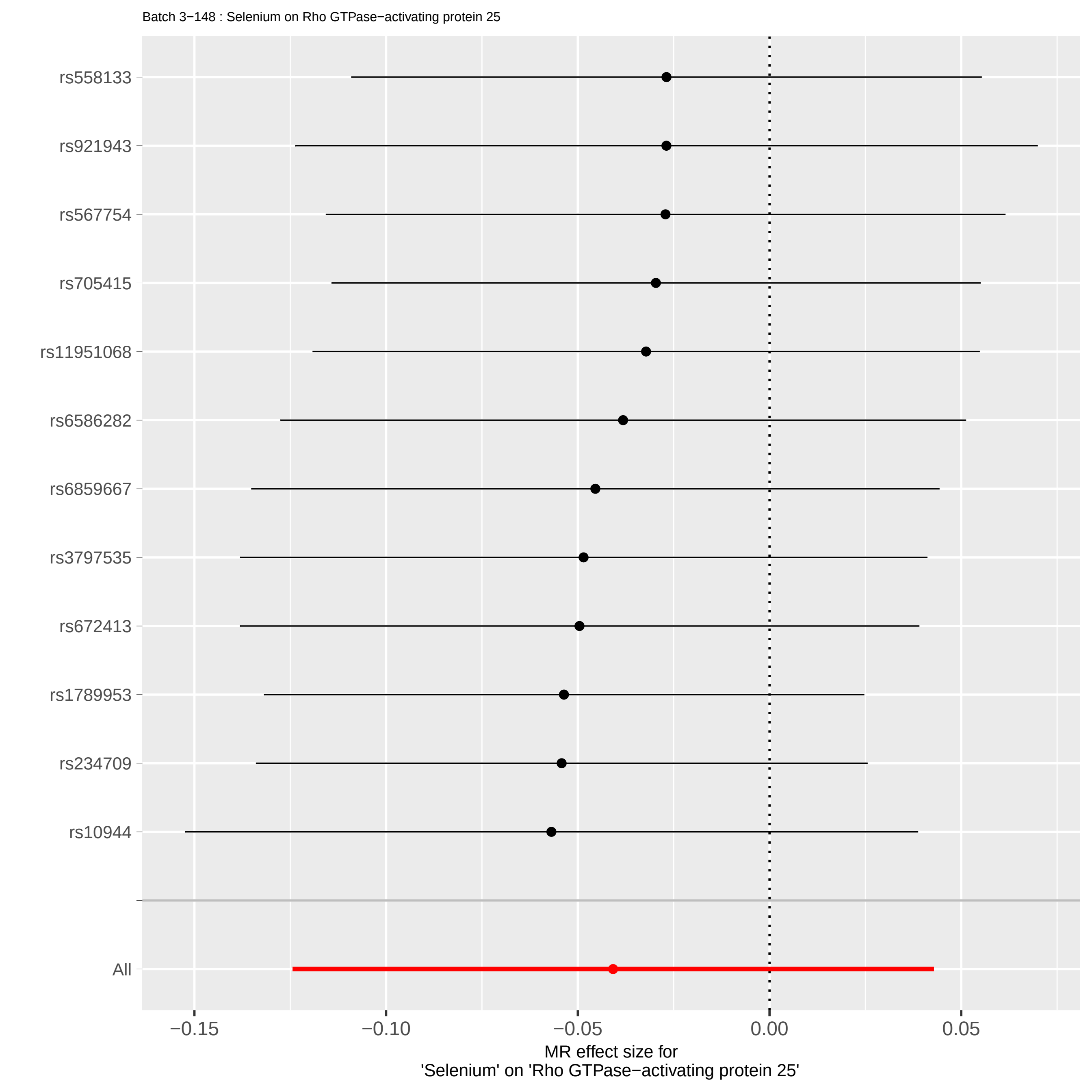
Batch 3−148 : Selenium on Rho GTPase−activating protein 25
rs558133
rs921943
rs567754
rs705415
rs11951068
rs6586282
rs6859667
rs3797535
rs672413
rs1789953
rs234709
rs10944
All
−0.15
−0.10
−0.05
0.00
0.05
MR effect size for
'Selenium' on 'Rho GTPase−activating protein 25'

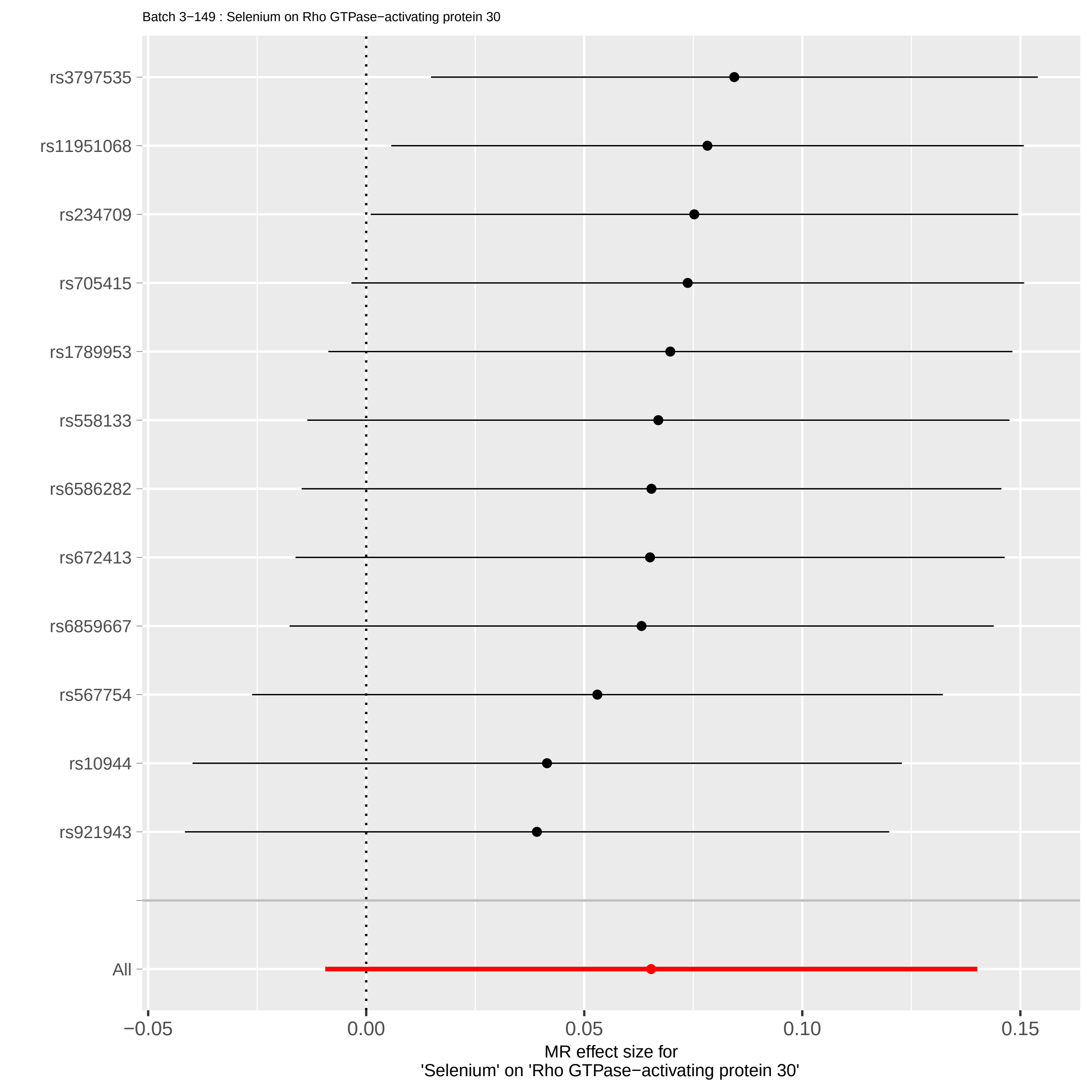

Batch 3−149 : Selenium on Rho GTPase−activating protein 30
rs3797535
rs11951068
rs234709
rs705415
rs1789953
rs558133
rs6586282
rs672413
rs6859667
rs567754
rs10944
rs921943
All
−0.05
0.00
0.05
0.10
0.15
MR effect size for
'Selenium' on 'Rho GTPase−activating protein 30'

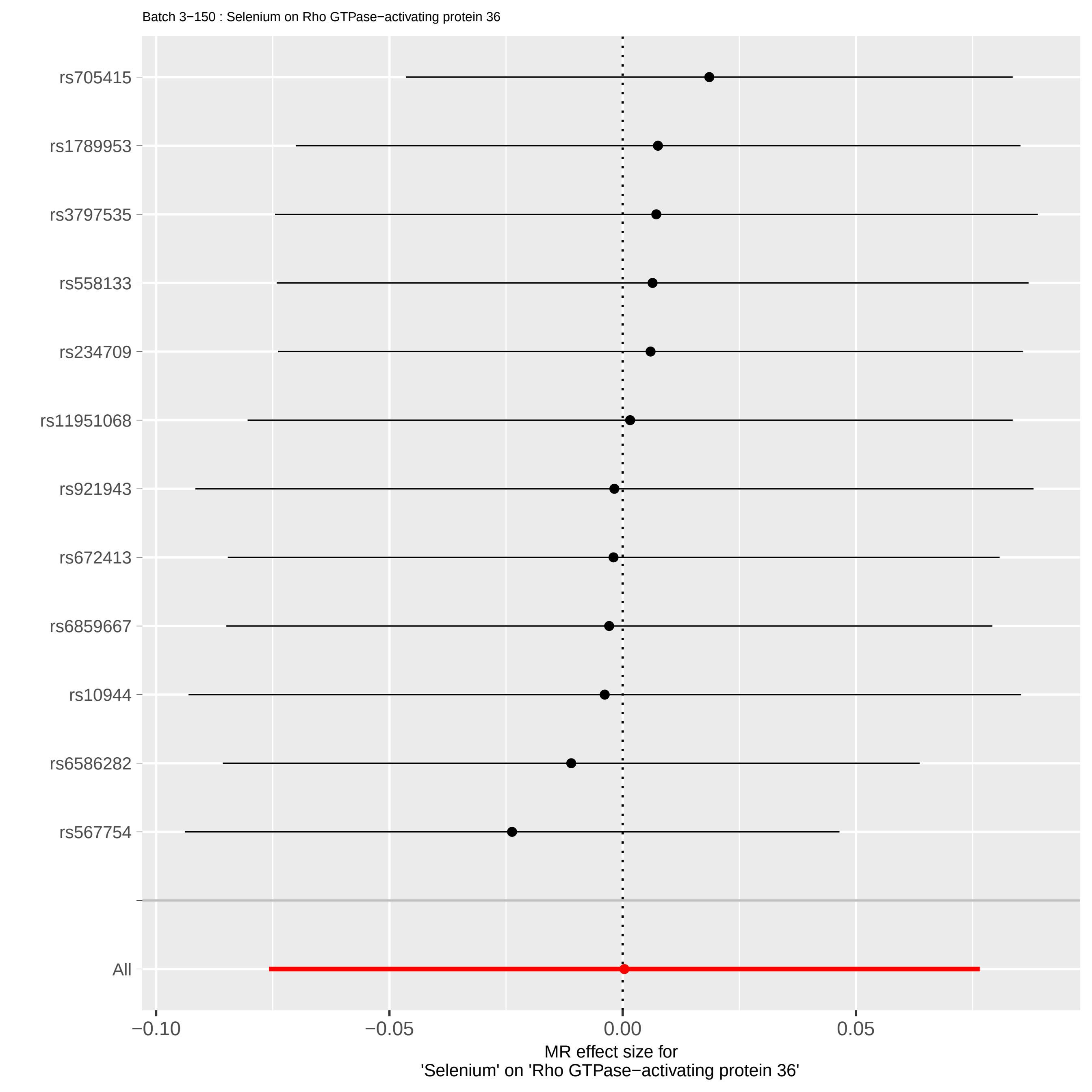

Batch 3−150 : Selenium on Rho GTPase−activating protein 36
rs705415
rs1789953
rs3797535
rs558133
rs234709
rs11951068
rs921943
rs672413
rs6859667
rs10944
rs6586282
rs567754
All
−0.10
−0.05
0.00
0.05
MR effect size for
'Selenium' on 'Rho GTPase−activating protein 36'

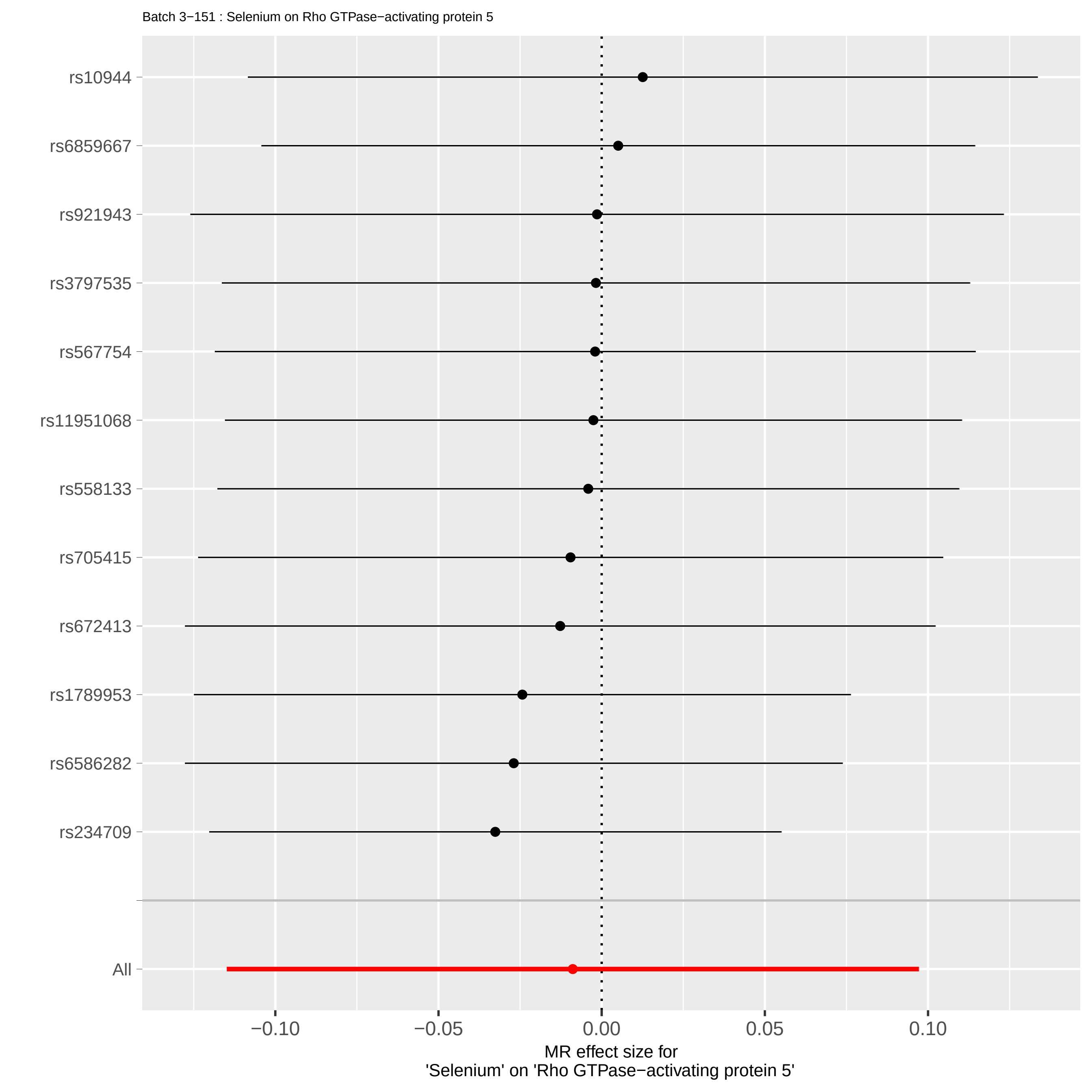

Batch 3−151 : Selenium on Rho GTPase−activating protein 5
rs10944
rs6859667
rs921943
rs3797535
rs567754
rs11951068
rs558133
rs705415
rs672413
rs1789953
rs6586282
rs234709
All
−0.10
−0.05
0.00
0.05
0.10
MR effect size for
'Selenium' on 'Rho GTPase−activating protein 5'

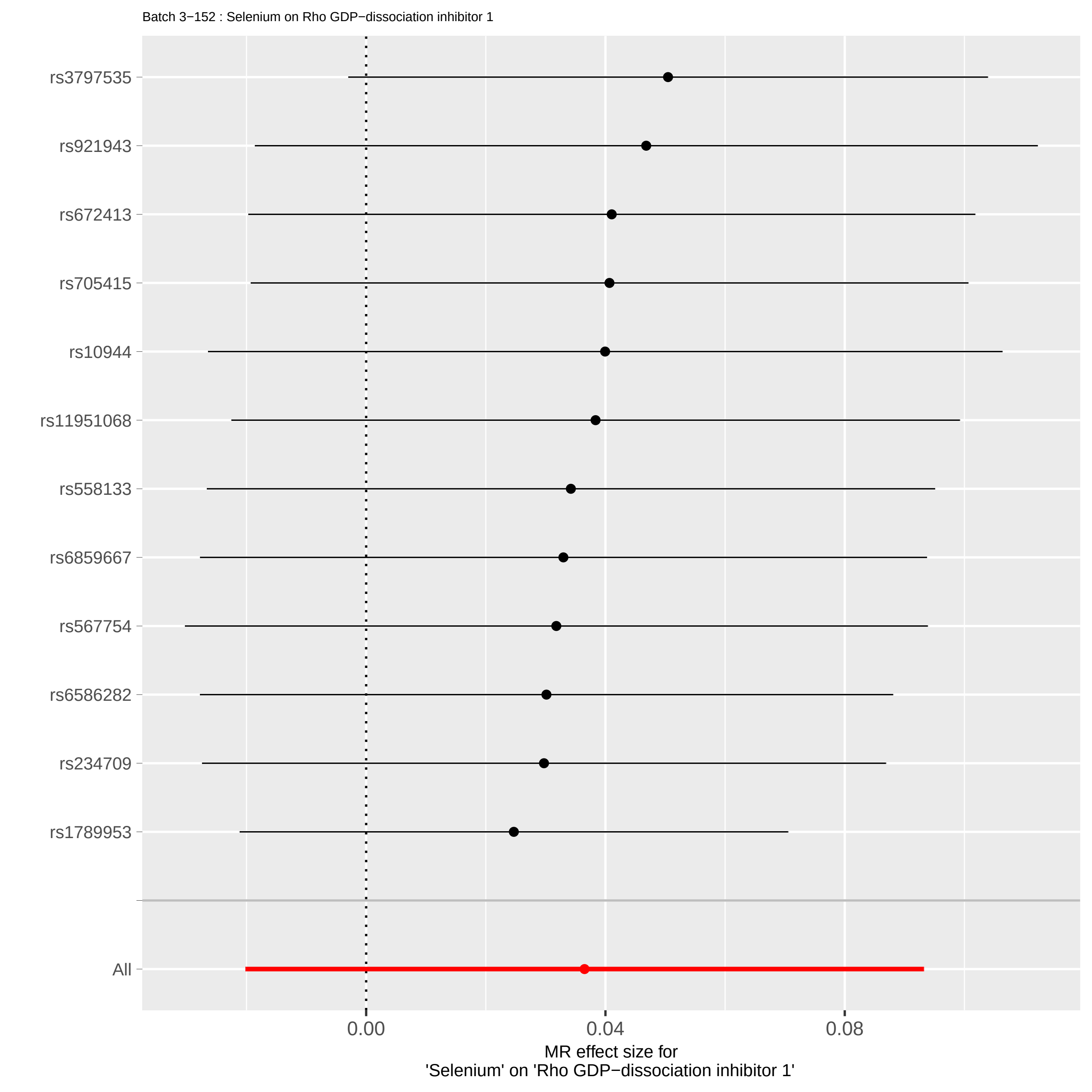

Batch 3−152 : Selenium on Rho GDP−dissociation inhibitor 1
rs3797535
rs921943
rs672413
rs705415
rs10944
rs11951068
rs558133
rs6859667
rs567754
rs6586282
rs234709
rs1789953
All
0.00
0.04
0.08
MR effect size for
'Selenium' on 'Rho GDP−dissociation inhibitor 1'

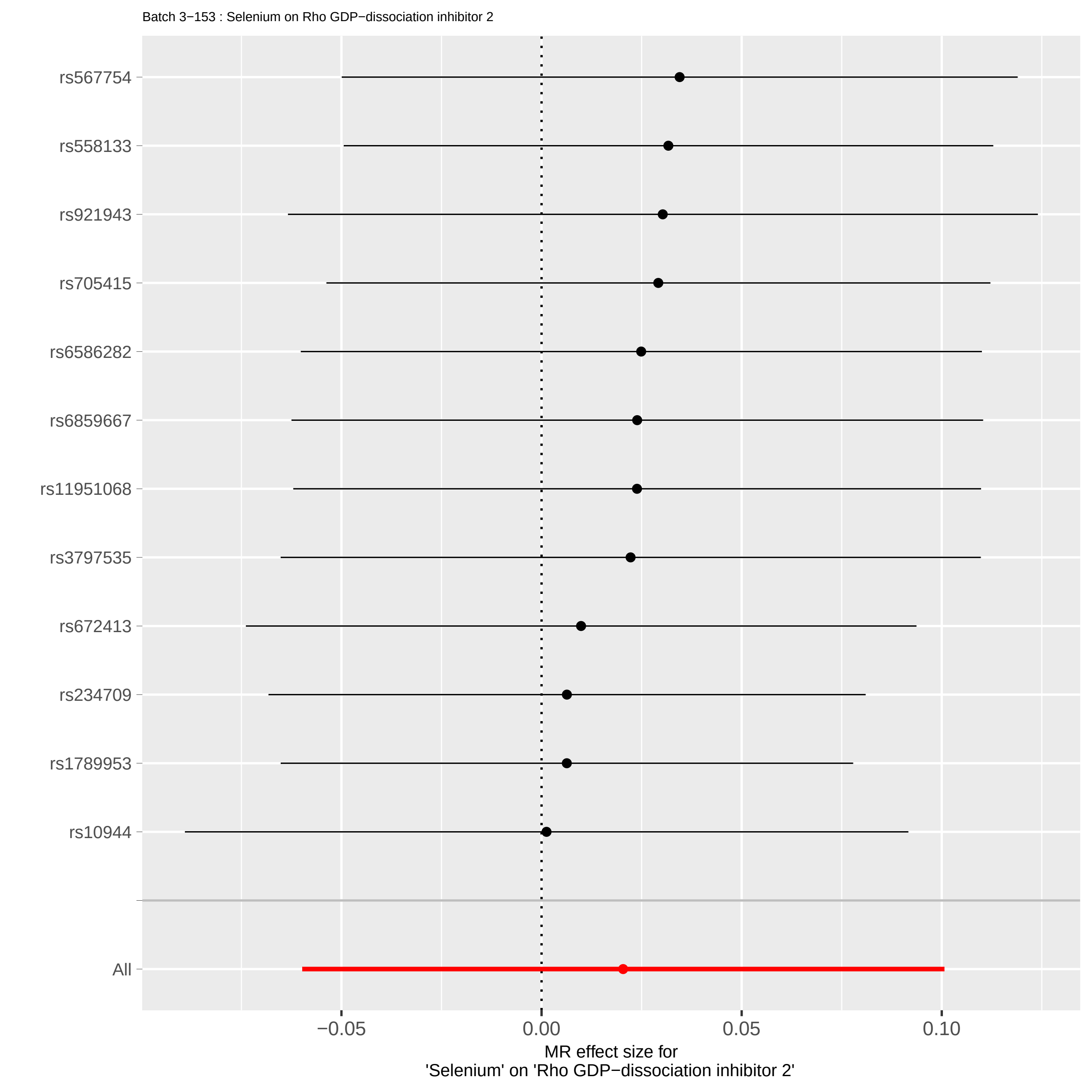

Batch 3−153 : Selenium on Rho GDP−dissociation inhibitor 2
rs567754
rs558133
rs921943
rs705415
rs6586282
rs6859667
rs11951068
rs3797535
rs672413
rs234709
rs1789953
rs10944
All
−0.05
0.00
0.05
0.10
MR effect size for
'Selenium' on 'Rho GDP−dissociation inhibitor 2'

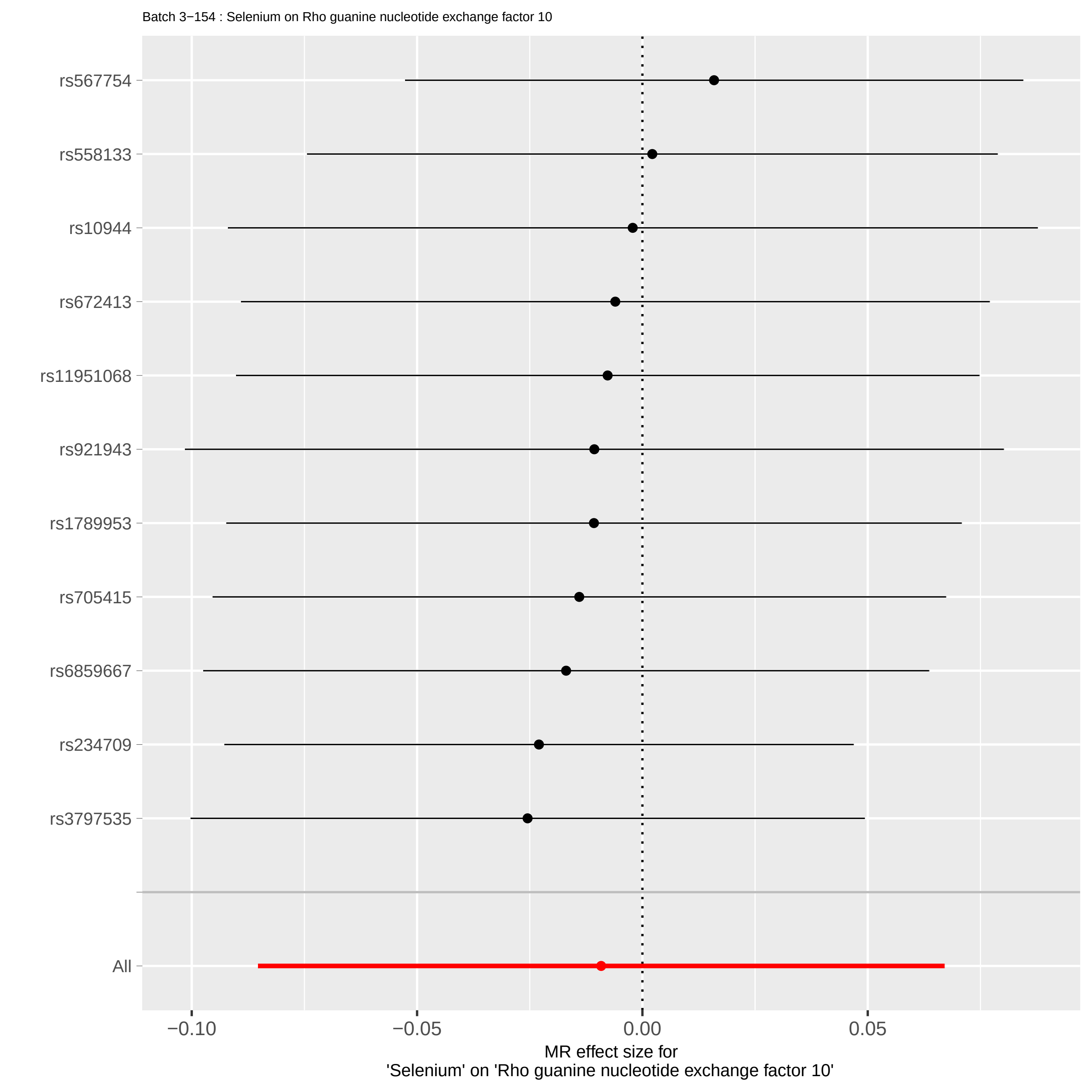

Batch 3−154 : Selenium on Rho guanine nucleotide exchange factor 10
rs567754
rs558133
rs10944
rs672413
rs11951068
rs921943
rs1789953
rs705415
rs6859667
rs234709
rs3797535
All
−0.10
−0.05
0.00
0.05
MR effect size for
'Selenium' on 'Rho guanine nucleotide exchange factor 10'

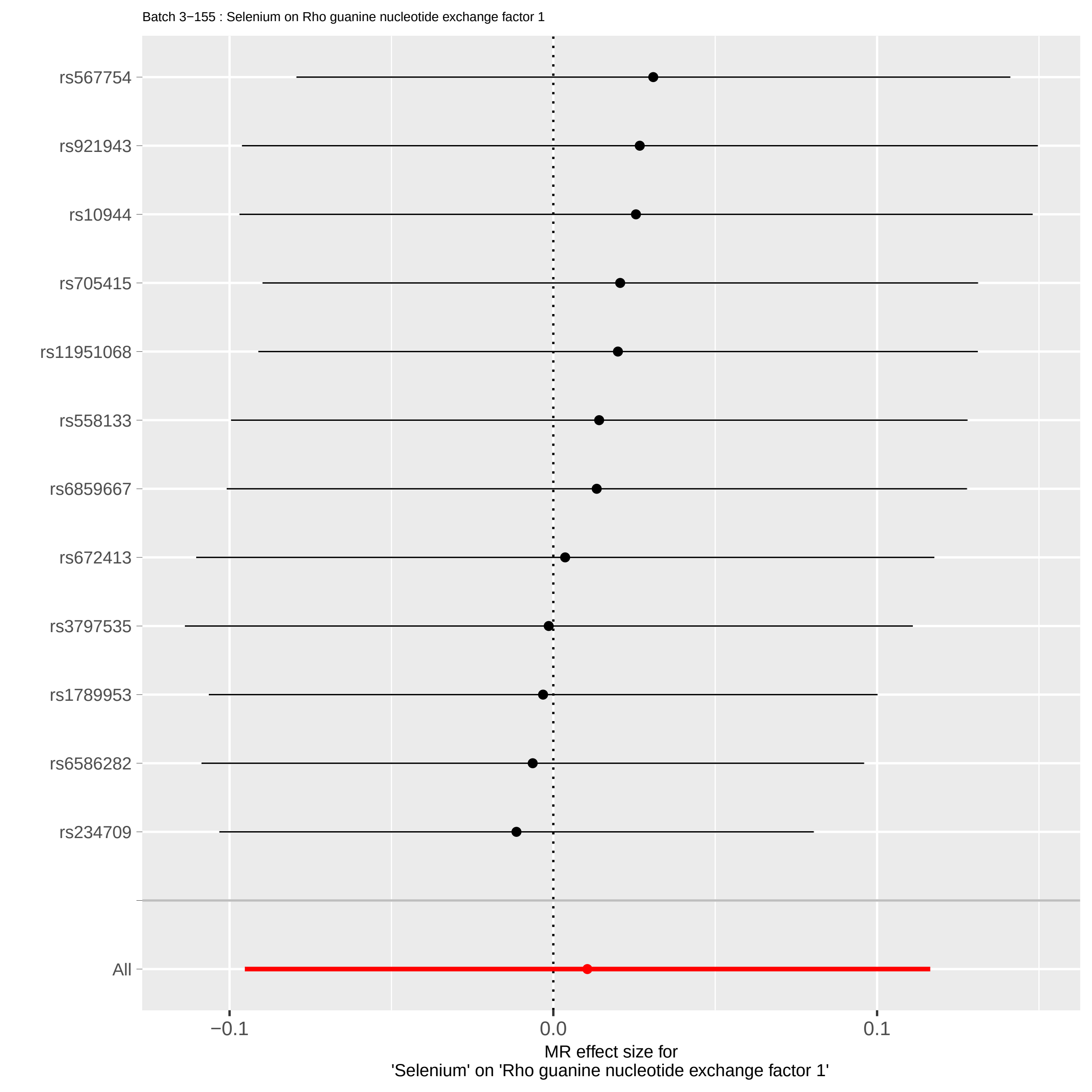

Batch 3−155 : Selenium on Rho guanine nucleotide exchange factor 1
rs567754
rs921943
rs10944
rs705415
rs11951068
rs558133
rs6859667
rs672413
rs3797535
rs1789953
rs6586282
rs234709
All
−0.1
0.0
0.1
MR effect size for
'Selenium' on 'Rho guanine nucleotide exchange factor 1'

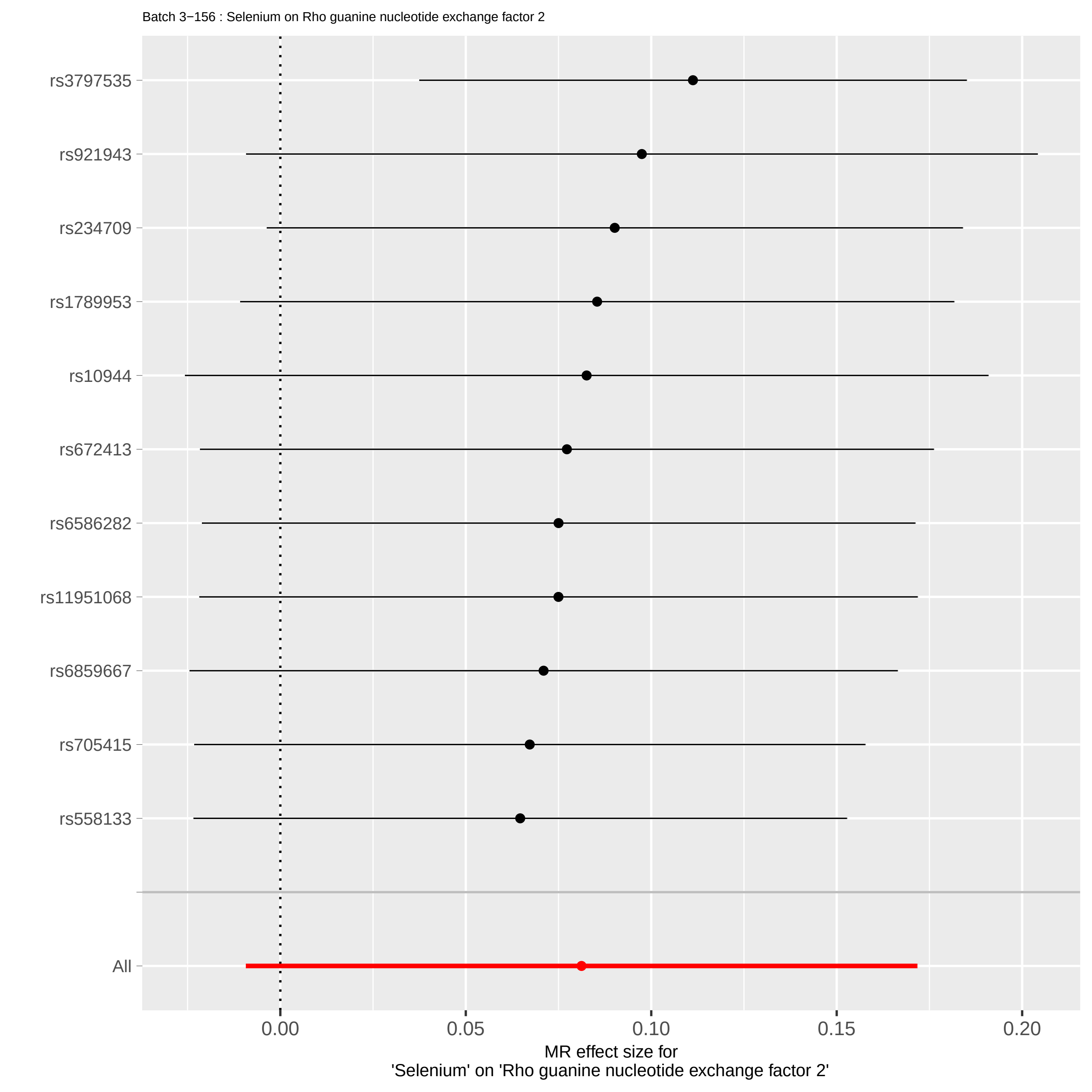

Batch 3−156 : Selenium on Rho guanine nucleotide exchange factor 2
rs3797535
rs921943
rs234709
rs1789953
rs10944
rs672413
rs6586282
rs11951068
rs6859667
rs705415
rs558133
All
0.00
0.05
0.10
0.15
0.20
MR effect size for
'Selenium' on 'Rho guanine nucleotide exchange factor 2'

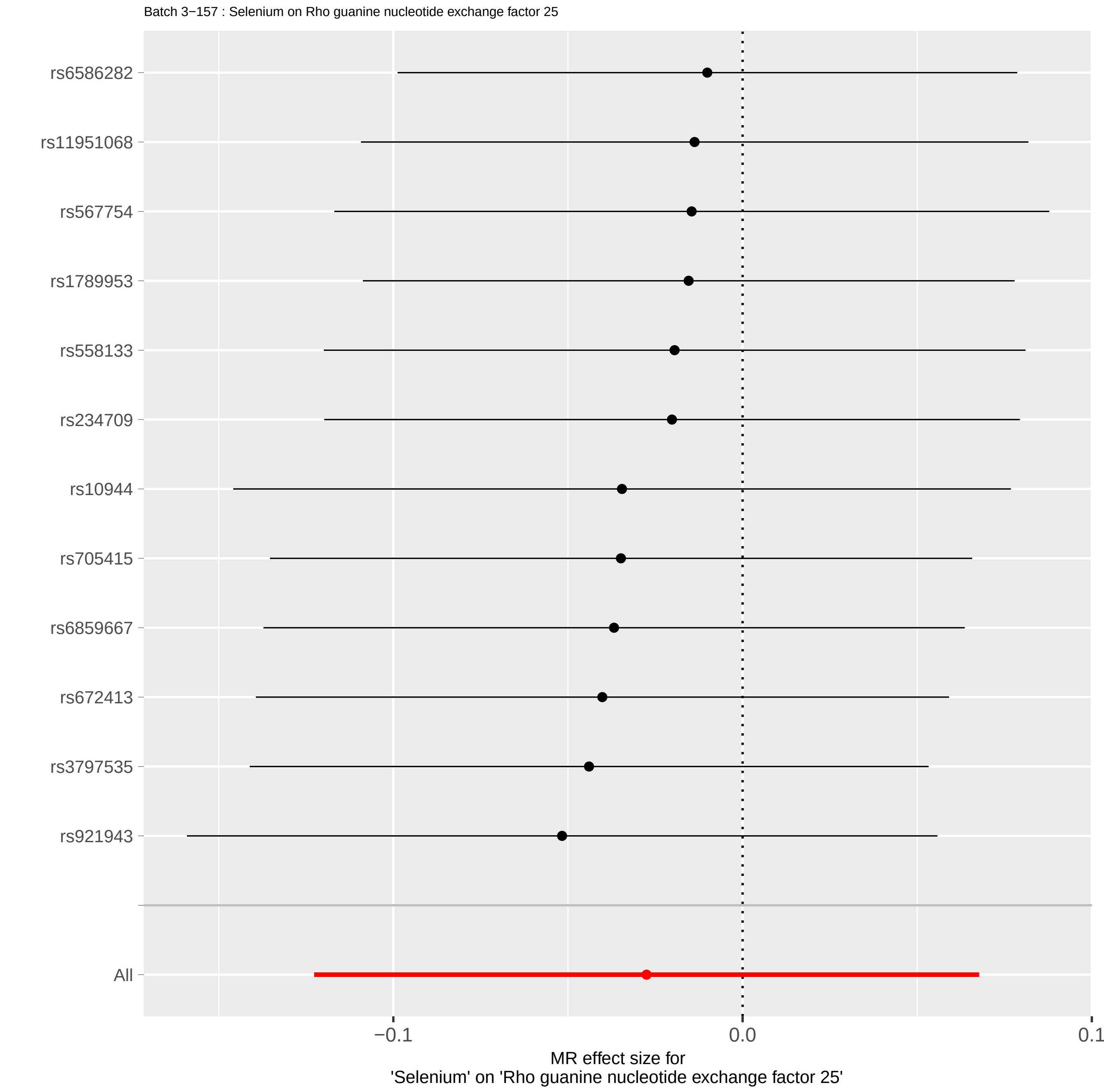
Batch 3−157 : Selenium on Rho guanine nucleotide exchange factor 25
rs6586282
rs11951068
rs567754
rs1789953
rs558133
rs234709
rs10944
rs705415
rs6859667
rs672413
rs3797535
rs921943
All
−0.1
0.0
0.1
MR effect size for
'Selenium' on 'Rho guanine nucleotide exchange factor 25'

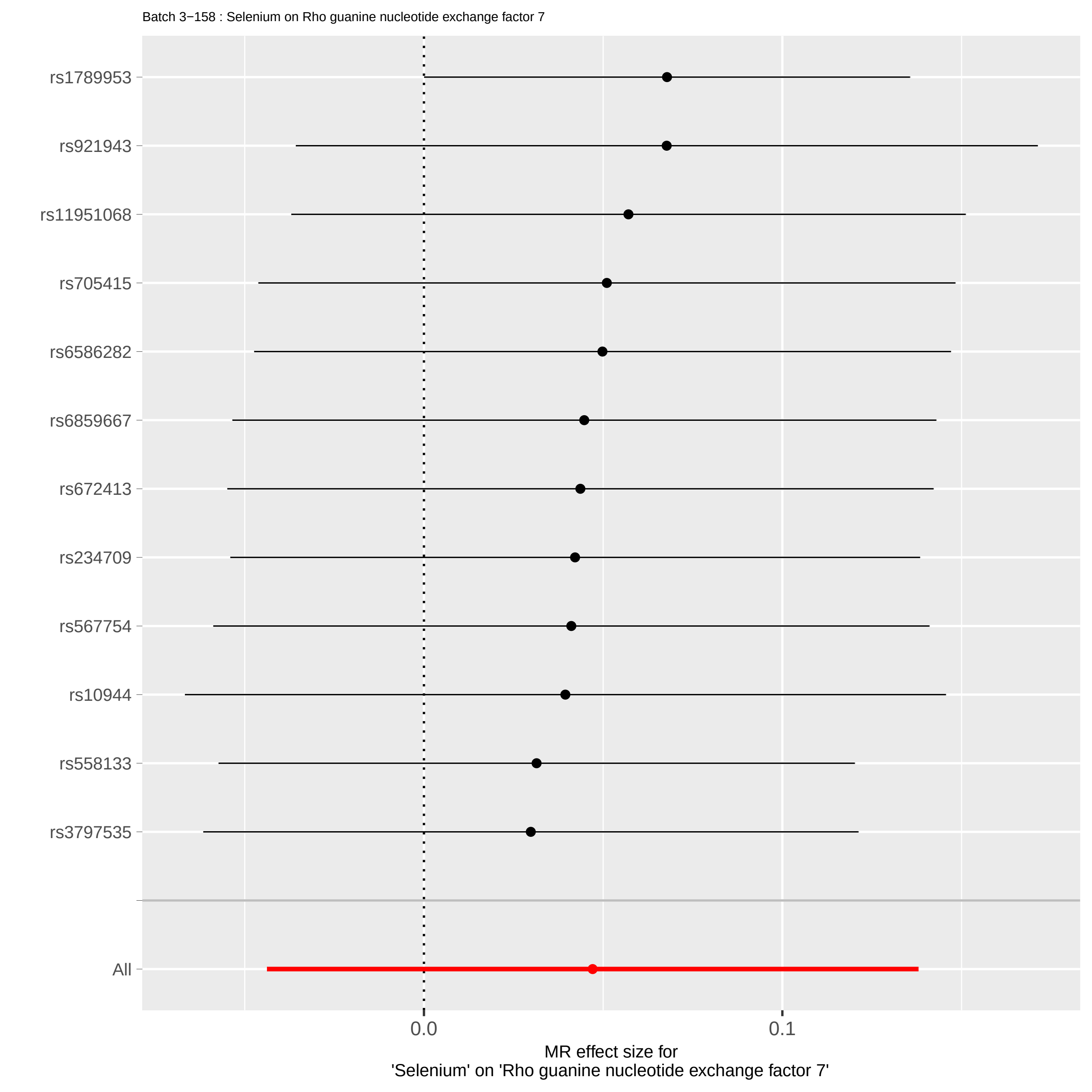

Batch 3−158 : Selenium on Rho guanine nucleotide exchange factor 7
rs1789953
rs921943
rs11951068
rs705415
rs6586282
rs6859667
rs672413
rs234709
rs567754
rs10944
rs558133
rs3797535
All
0.0
0.1
MR effect size for
'Selenium' on 'Rho guanine nucleotide exchange factor 7'

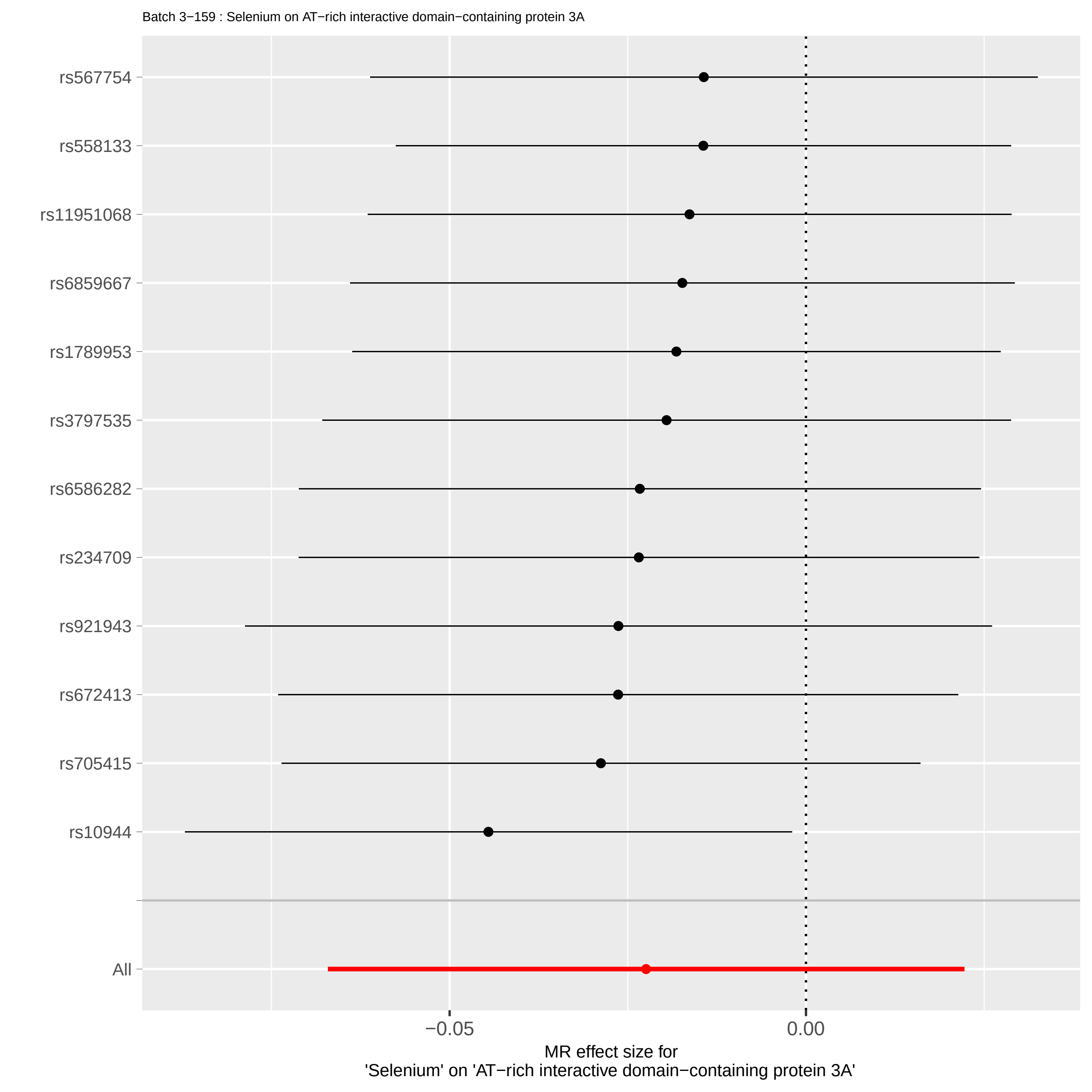
Batch 3−159 : Selenium on AT−rich interactive domain−containing protein 3A
rs567754
rs558133
rs11951068
rs6859667
rs1789953
rs3797535
rs6586282
rs234709
rs921943
rs672413
rs705415
rs10944
All
−0.05
0.00
MR effect size for
'Selenium' on 'AT−rich interactive domain−containing protein 3A'

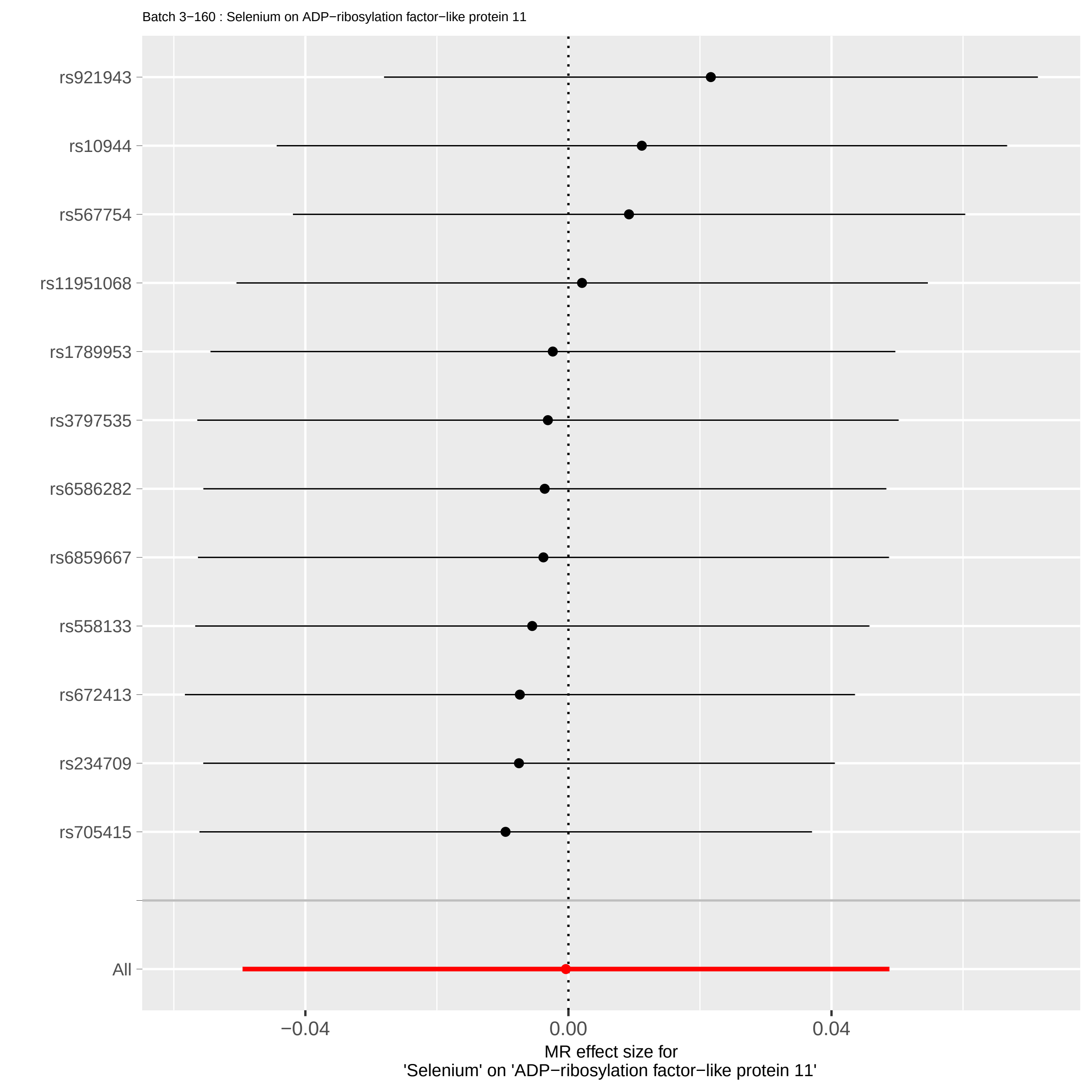

Batch 3−160 : Selenium on ADP−ribosylation factor−like protein 11
rs921943
rs10944
rs567754
rs11951068
rs1789953
rs3797535
rs6586282
rs6859667
rs558133
rs672413
rs234709
rs705415
All
−0.04
0.00
0.04
MR effect size for
'Selenium' on 'ADP−ribosylation factor−like protein 11'

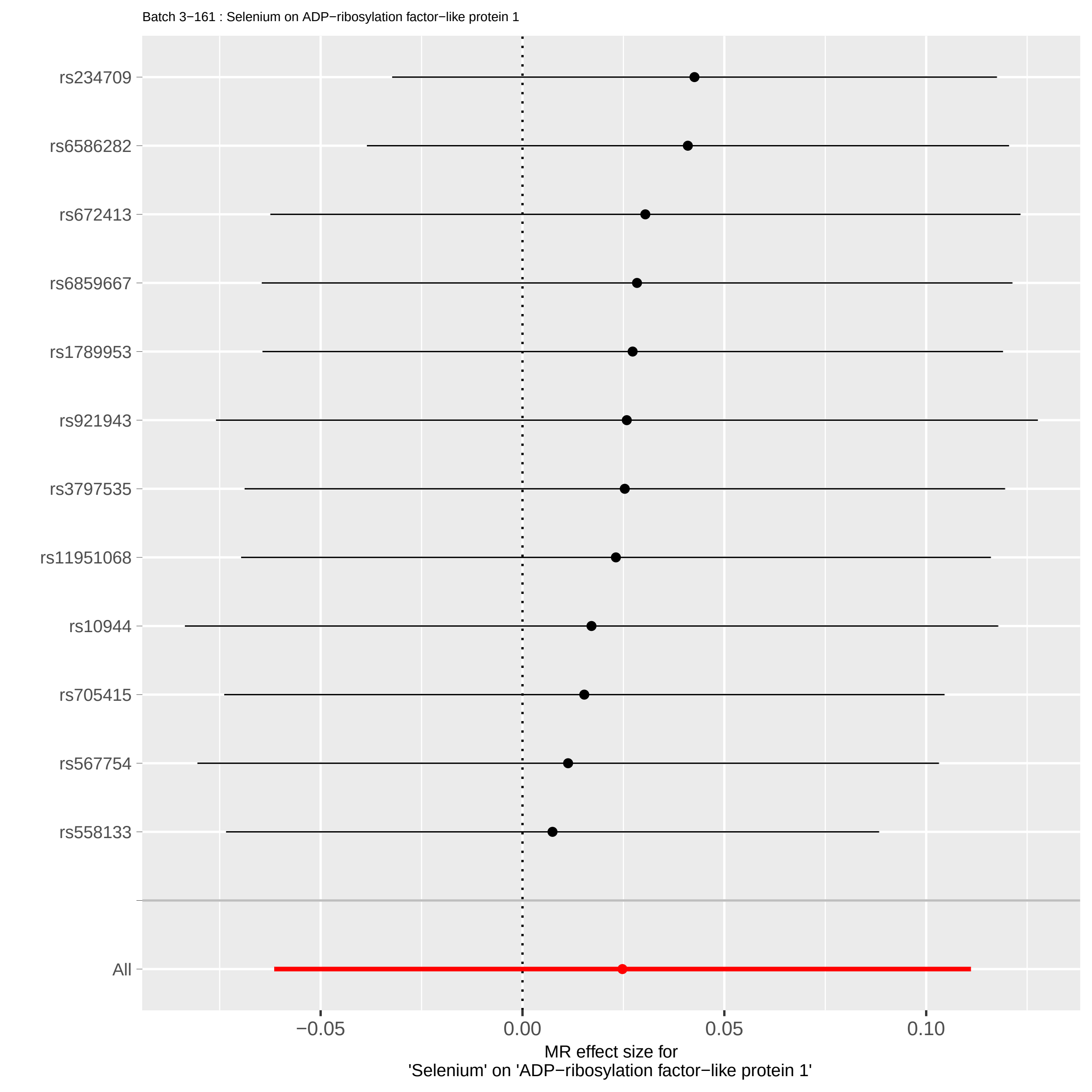

Batch 3−161 : Selenium on ADP−ribosylation factor−like protein 1
rs234709
rs6586282
rs672413
rs6859667
rs1789953
rs921943
rs3797535
rs11951068
rs10944
rs705415
rs567754
rs558133
All
−0.05
0.00
0.05
0.10
MR effect size for
'Selenium' on 'ADP−ribosylation factor−like protein 1'

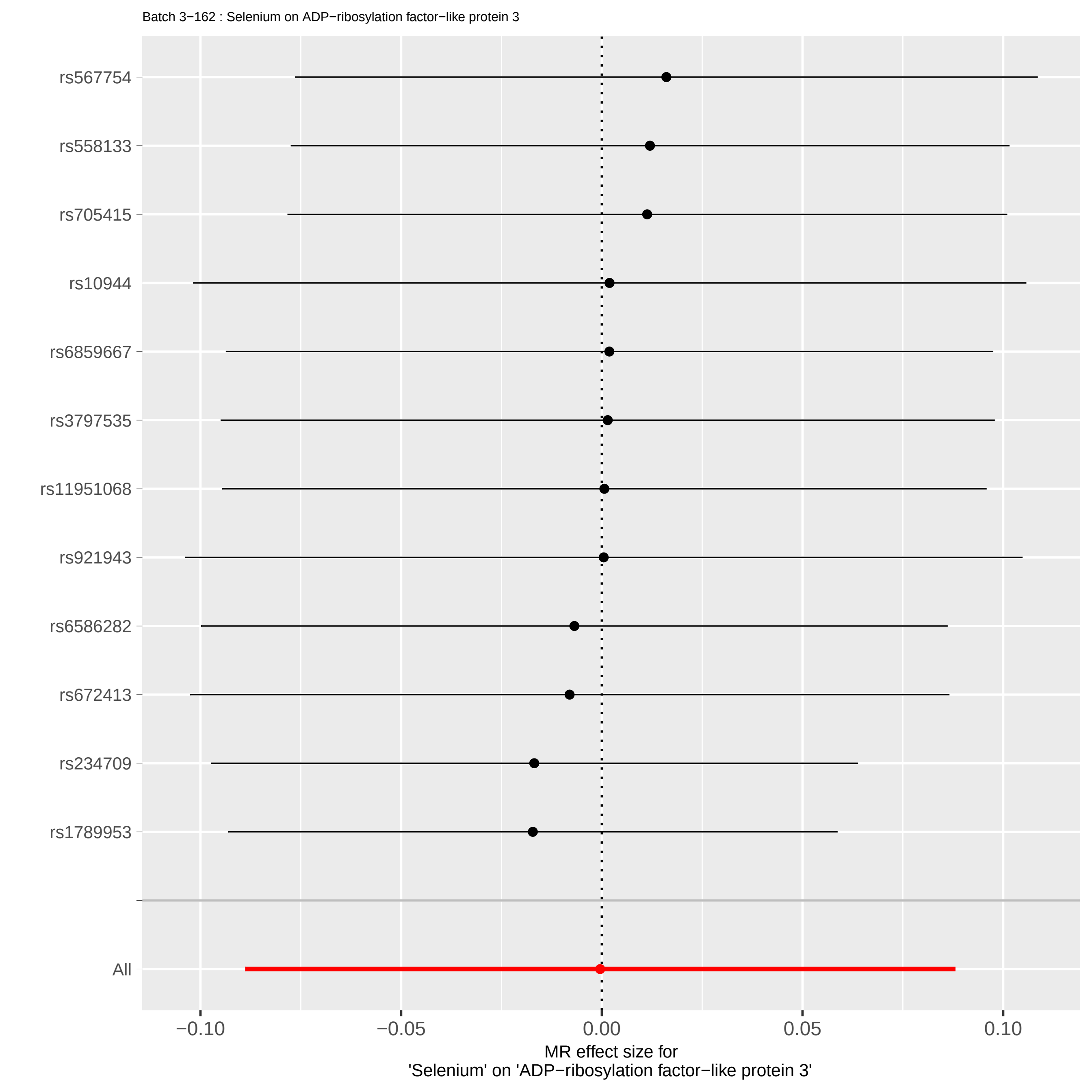

Batch 3−162 : Selenium on ADP−ribosylation factor−like protein 3
rs567754
rs558133
rs705415
rs10944
rs6859667
rs3797535
rs11951068
rs921943
rs6586282
rs672413
rs234709
rs1789953
All
−0.10
−0.05
0.00
0.05
0.10
MR effect size for
'Selenium' on 'ADP−ribosylation factor−like protein 3'

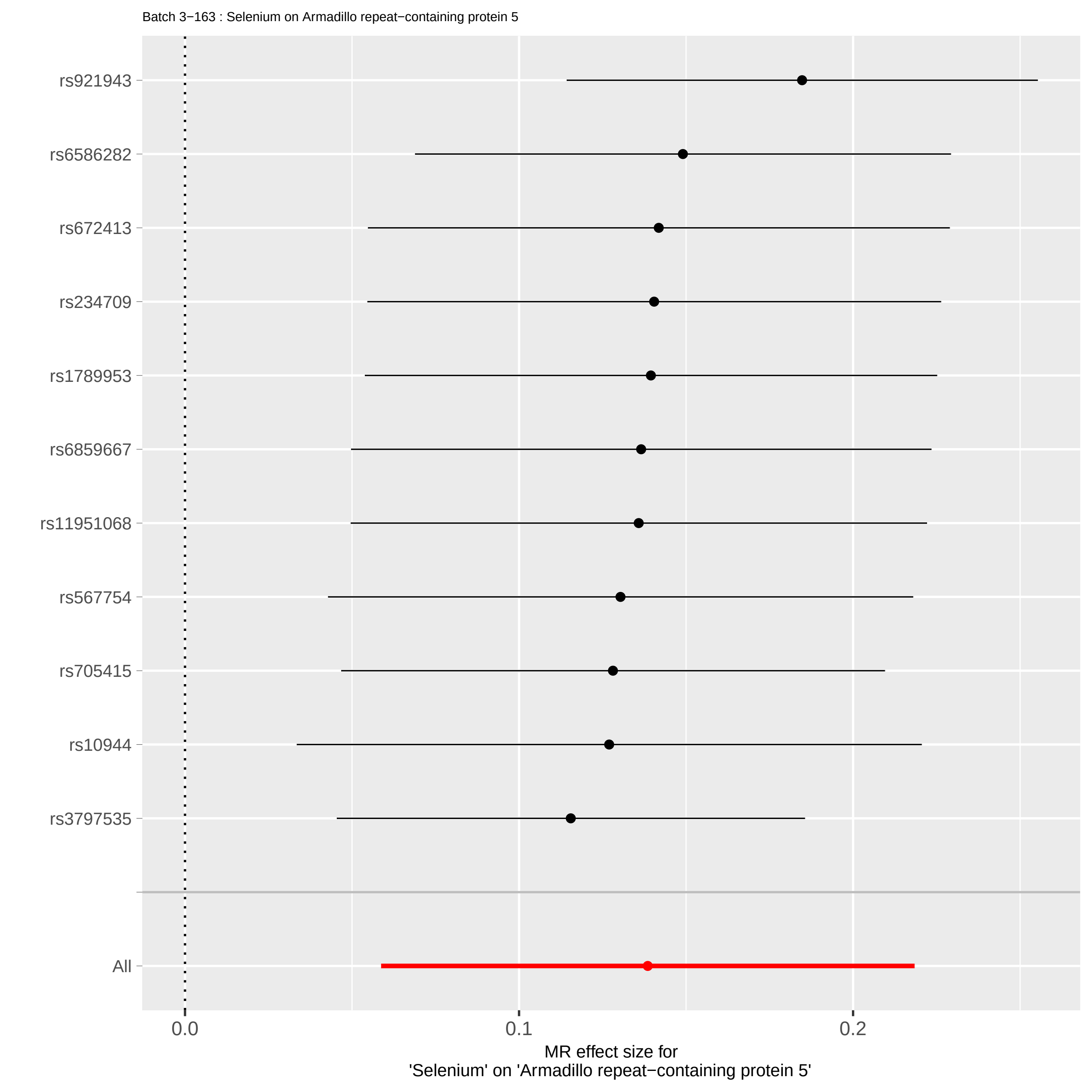

Batch 3−163 : Selenium on Armadillo repeat−containing protein 5
rs921943
rs6586282
rs672413
rs234709
rs1789953
rs6859667
rs11951068
rs567754
rs705415
rs10944
rs3797535
All
0.0
0.1
0.2
MR effect size for
'Selenium' on 'Armadillo repeat−containing protein 5'

Batch 3−164 : Selenium on Armadillo repeat−containing protein 5

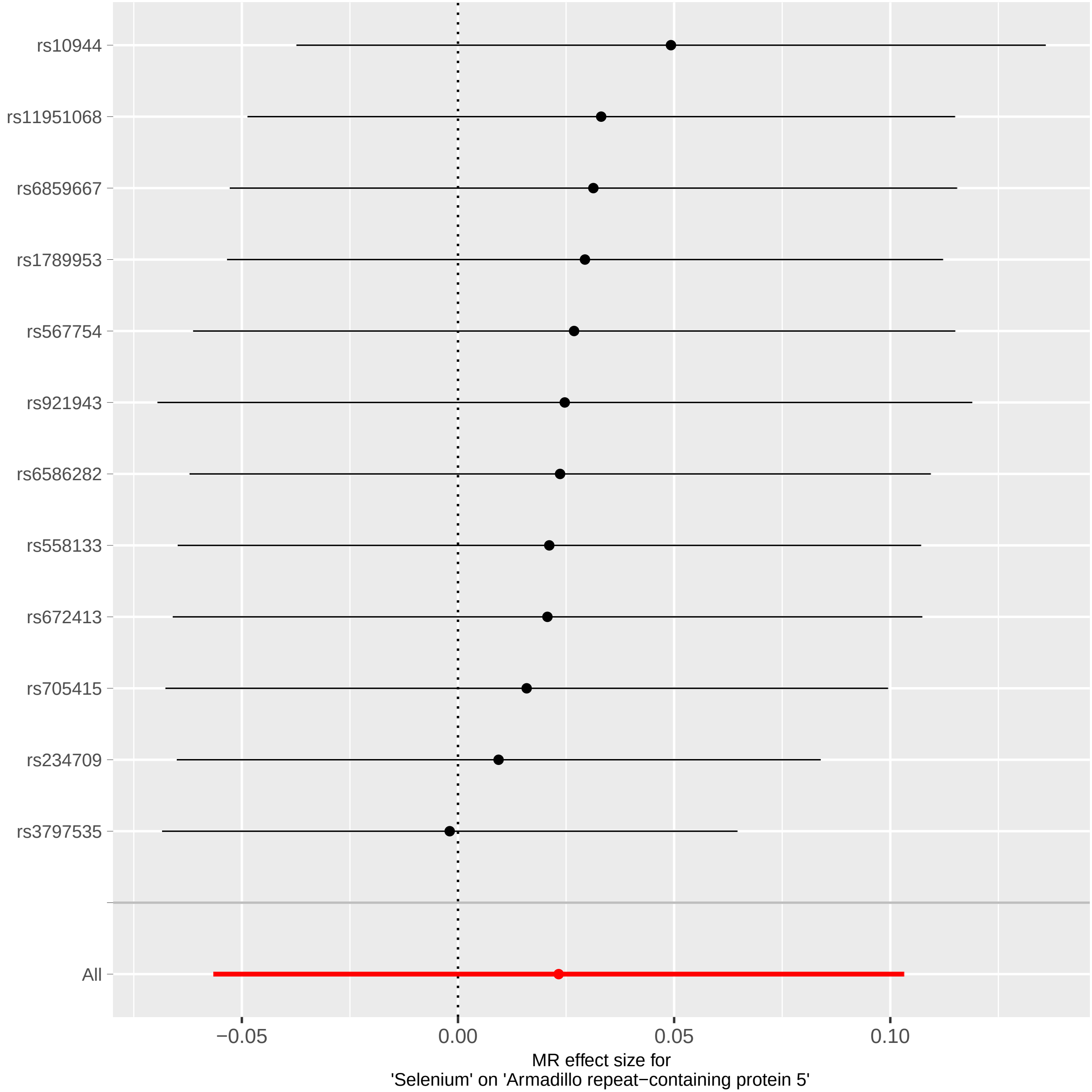

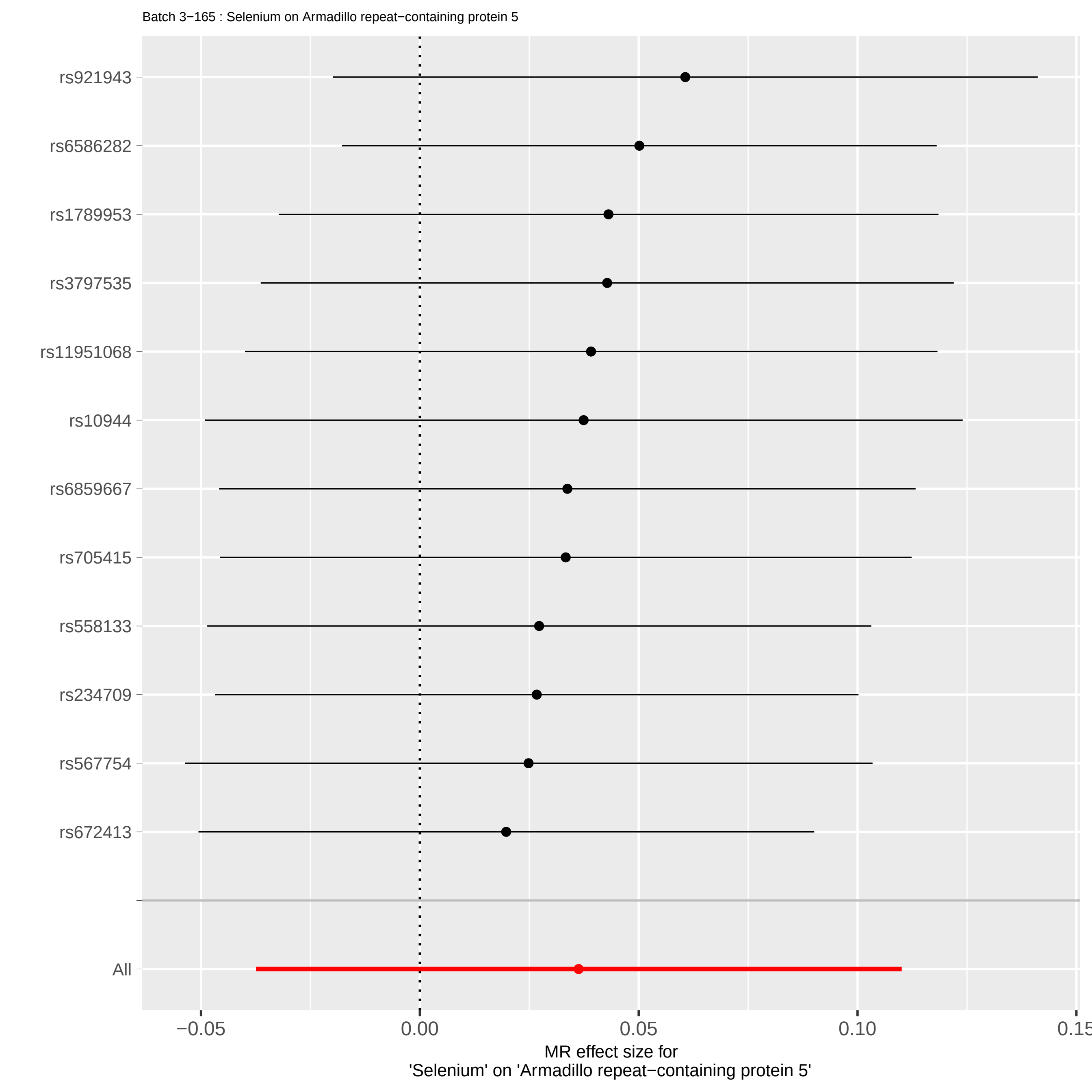
Batch 3−165 : Selenium on Armadillo repeat−containing protein 5
rs921943
rs6586282
rs1789953
rs3797535
rs11951068
rs10944
rs6859667
rs705415
rs558133
rs234709
rs567754
rs672413
All
−0.05
0.00
0.05
0.10
0.15
MR effect size for
'Selenium' on 'Armadillo repeat−containing protein 5'

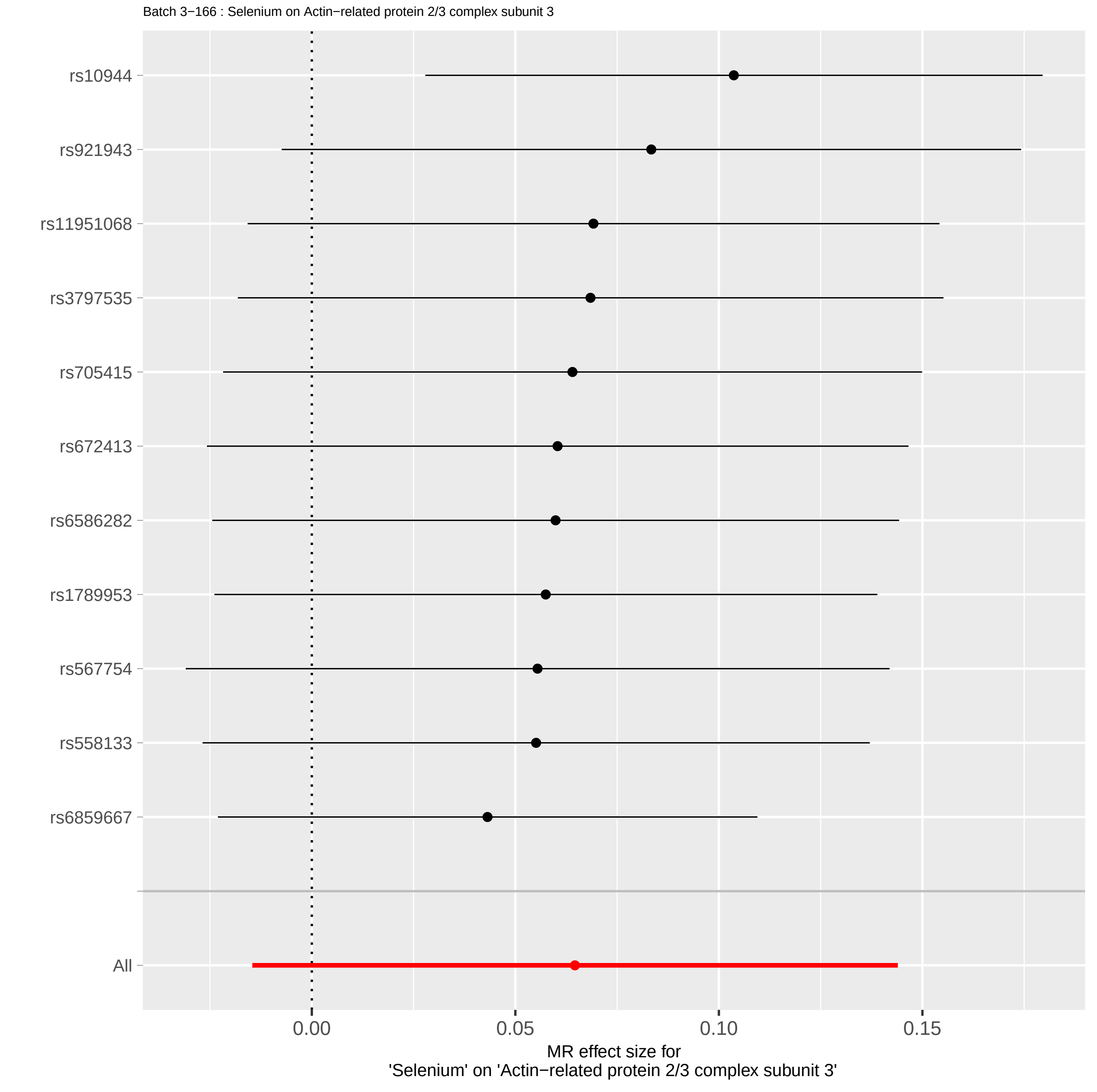
Batch 3−166 : Selenium on Actin−related protein 2/3 complex subunit 3
rs10944
rs921943
rs11951068
rs3797535
rs705415
rs672413
rs6586282
rs1789953
rs567754
rs558133
rs6859667
All
0.00
0.05
0.10
0.15
MR effect size for
'Selenium' on 'Actin−related protein 2/3 complex subunit 3'

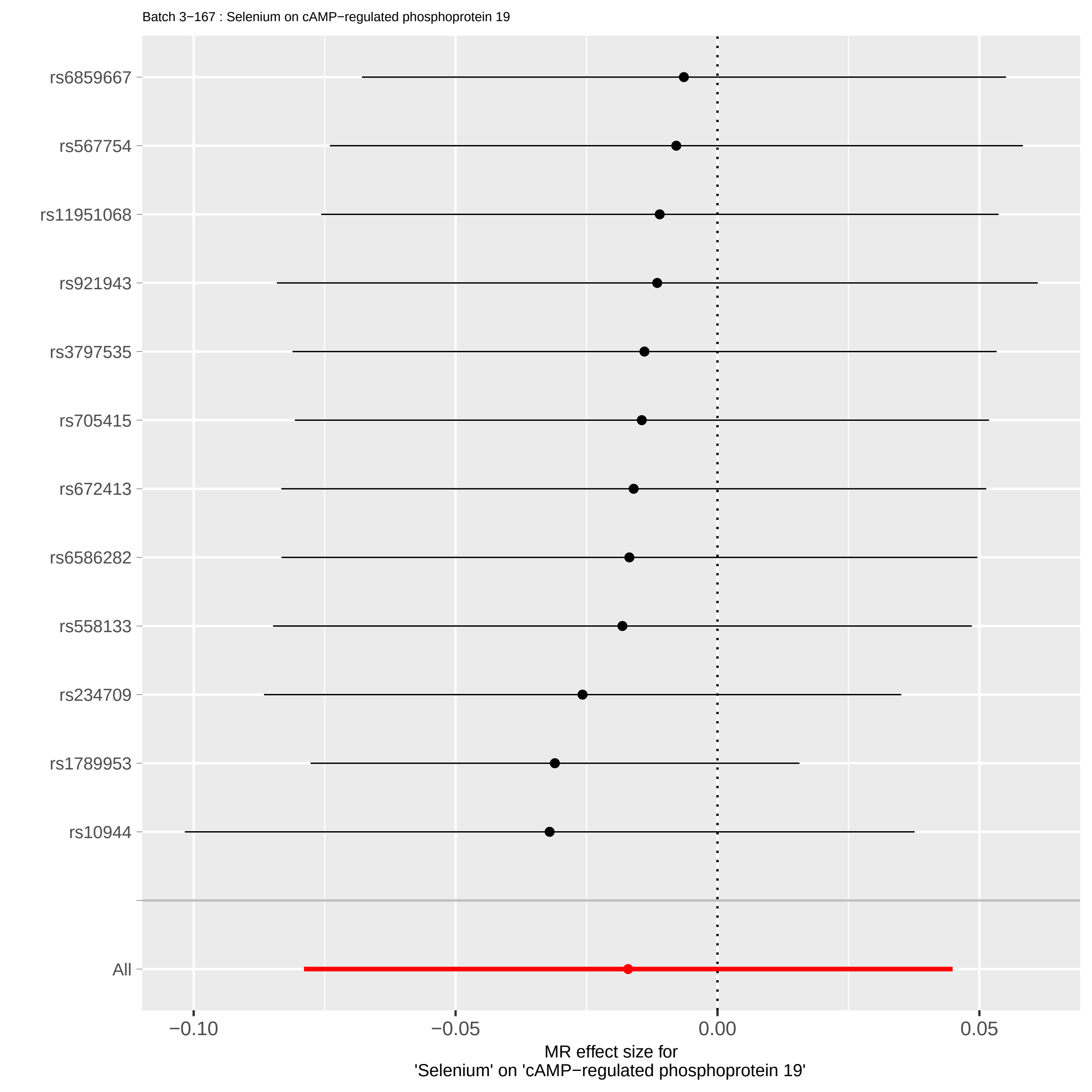

Batch 3−167 : Selenium on cAMP−regulated phosphoprotein 19
rs6859667
rs567754
rs11951068
rs921943
rs3797535
rs705415
rs672413
rs6586282
rs558133
rs234709
rs1789953
rs10944
All
−0.10
−0.05
0.00
0.05
MR effect size for
'Selenium' on 'cAMP−regulated phosphoprotein 19'

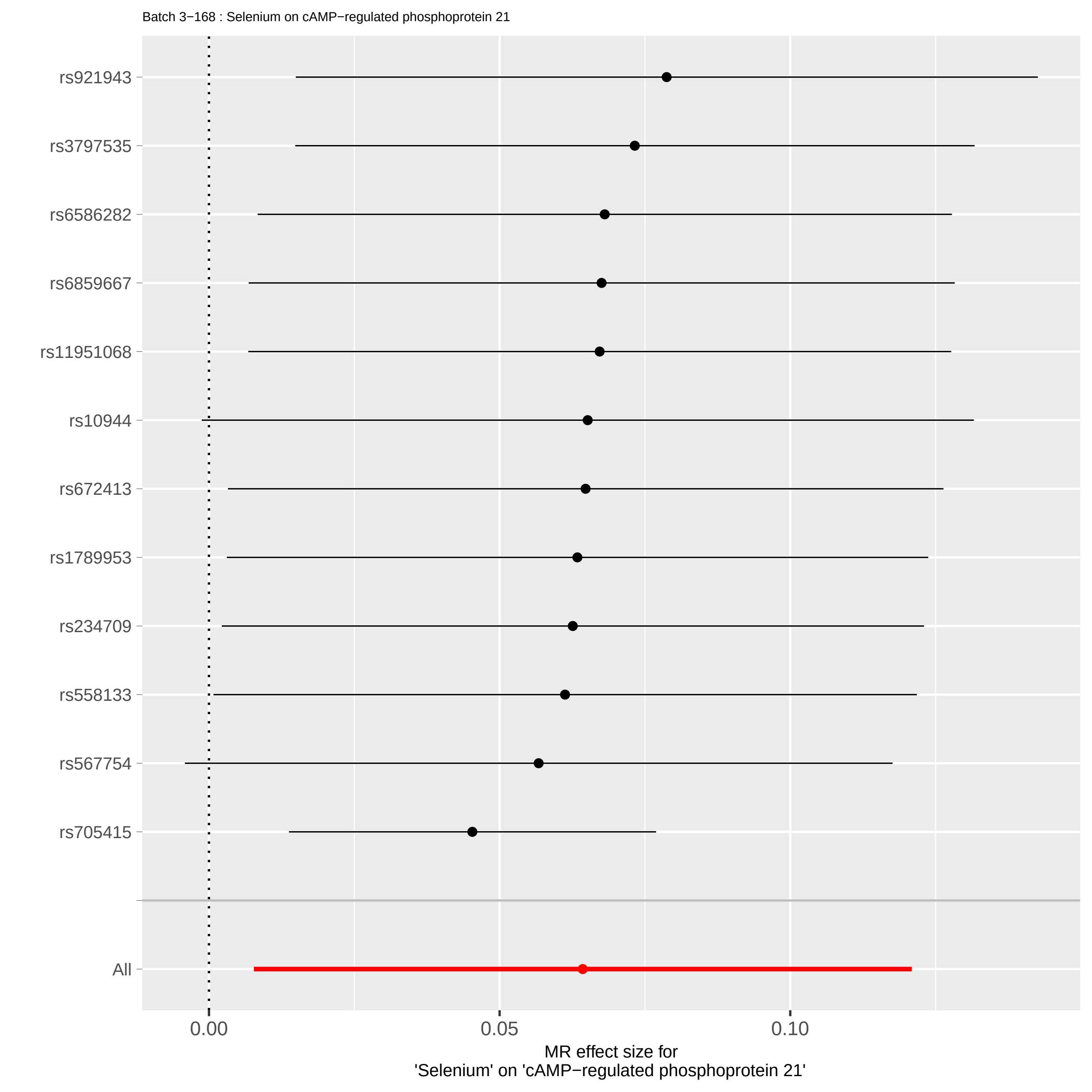

Batch 3−168 : Selenium on cAMP−regulated phosphoprotein 21
rs921943
rs3797535
rs6586282
rs6859667
rs11951068
rs10944
rs672413
rs1789953
rs234709
rs558133
rs567754
rs705415
All
0.00
0.05
0.10
MR effect size for
'Selenium' on 'cAMP−regulated phosphoprotein 21'

Batch 3−169 : Selenium on Beta−arrestin−1

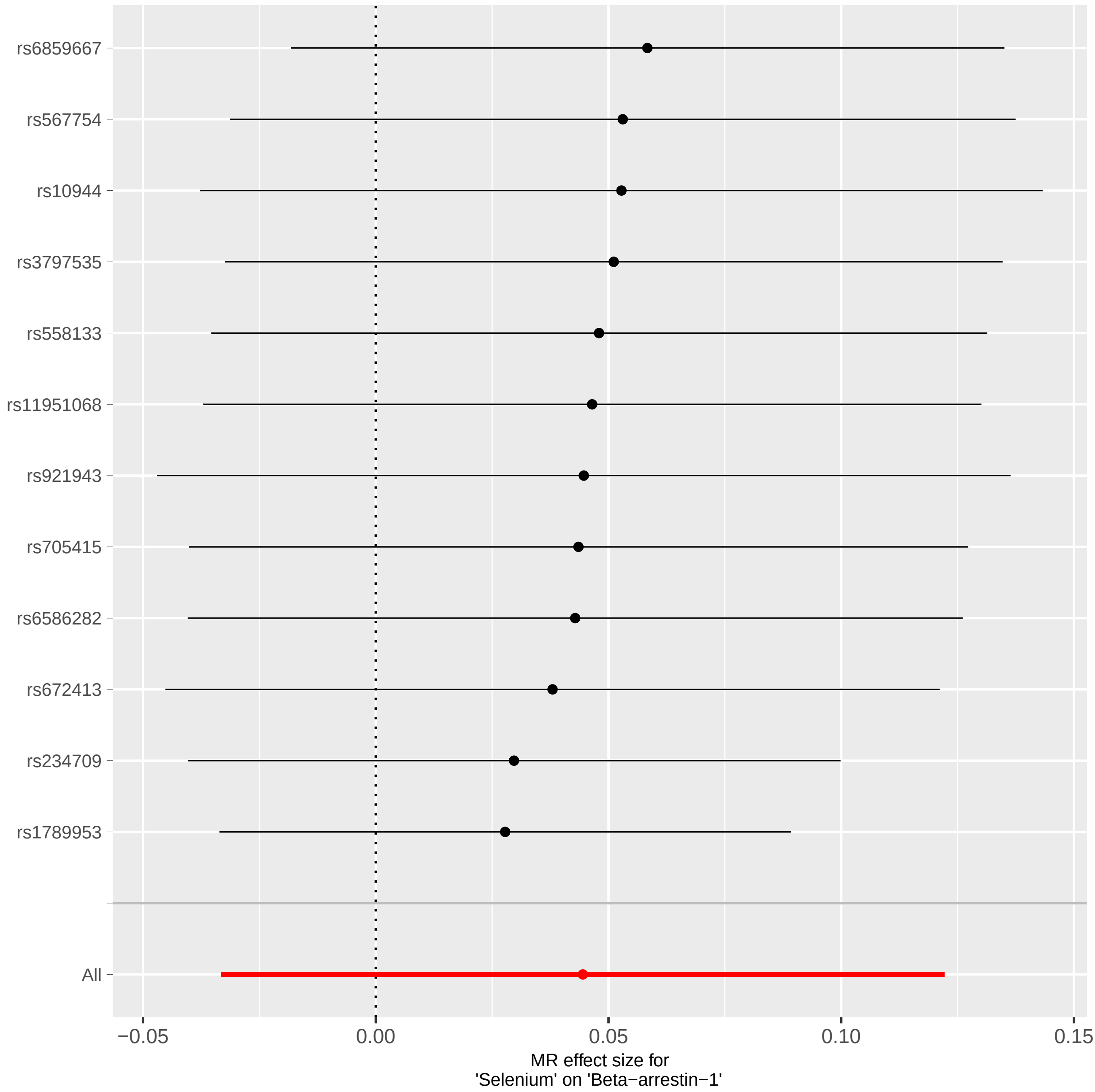

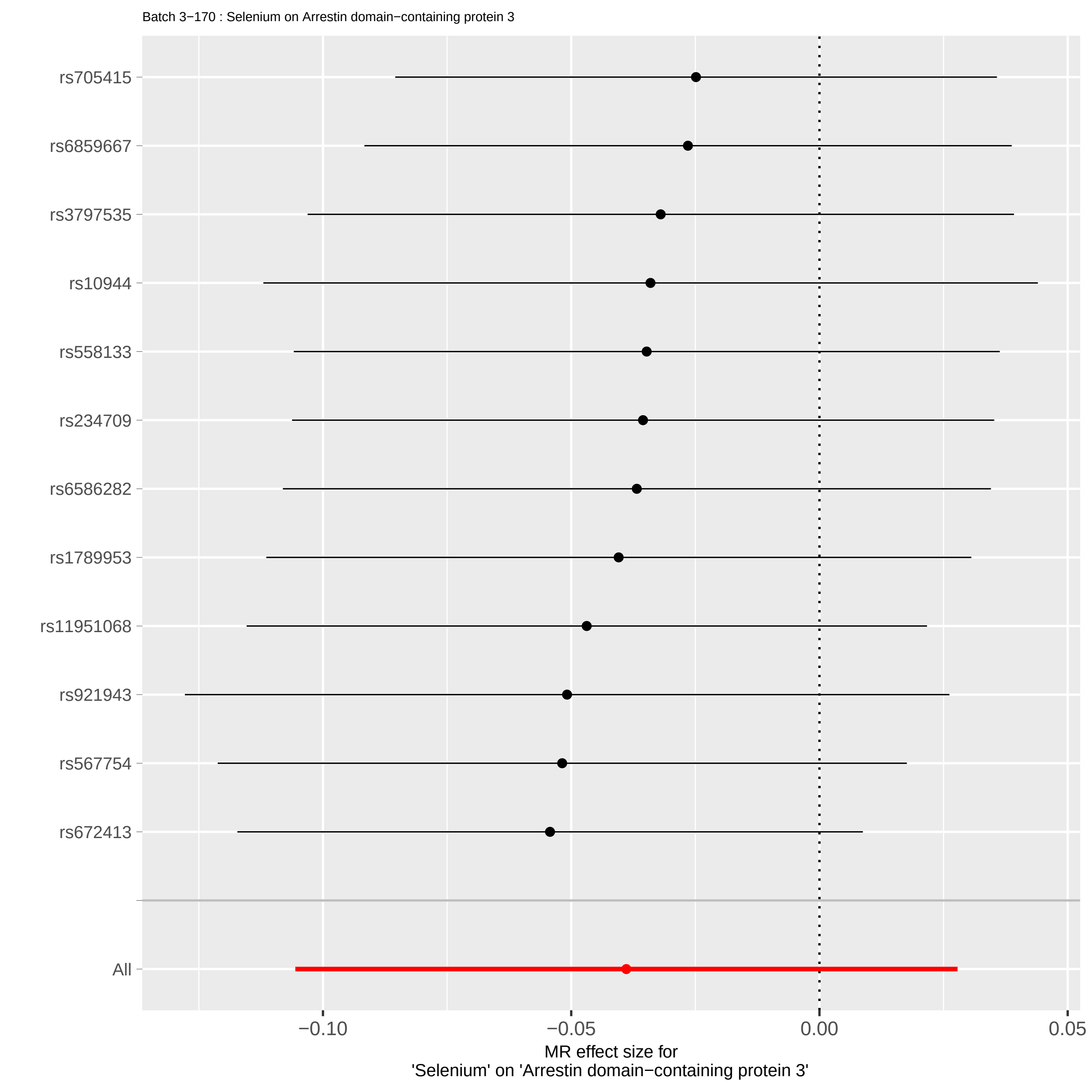

Batch 3−170 : Selenium on Arrestin domain−containing protein 3
rs705415
rs6859667
rs3797535
rs10944
rs558133
rs234709
rs6586282
rs1789953
rs11951068
rs921943
rs567754
rs672413
All
−0.10
−0.05
0.00
0.05
MR effect size for
'Selenium' on 'Arrestin domain−containing protein 3'

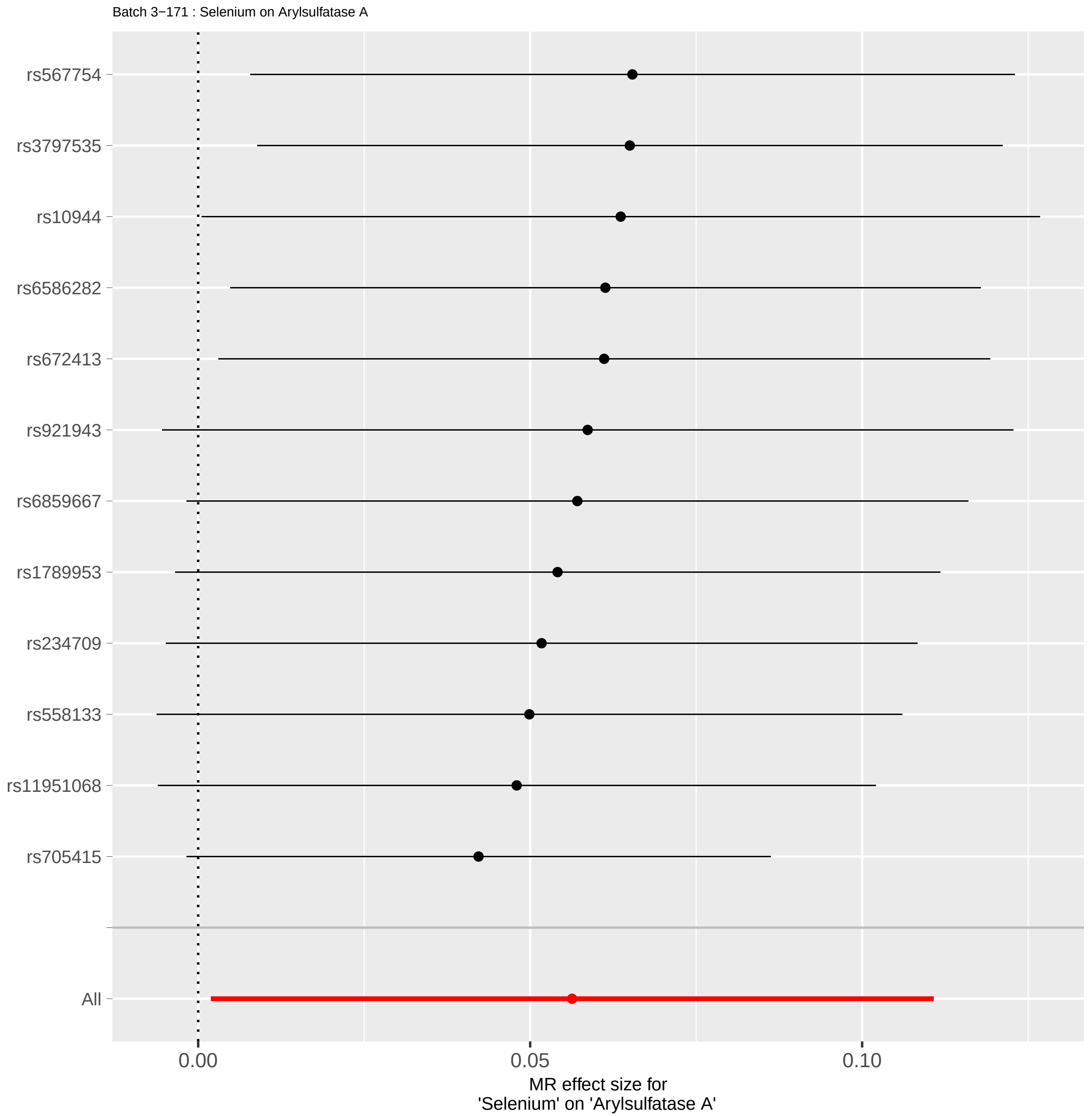
Batch 3−171 : Selenium on Arylsulfatase A
rs567754
rs3797535
rs10944
rs6586282
rs672413
rs921943
rs6859667
rs1789953
rs234709
rs558133
rs11951068
rs705415
All
0.00
0.05
0.10
MR effect size for
'Selenium' on 'Arylsulfatase A'

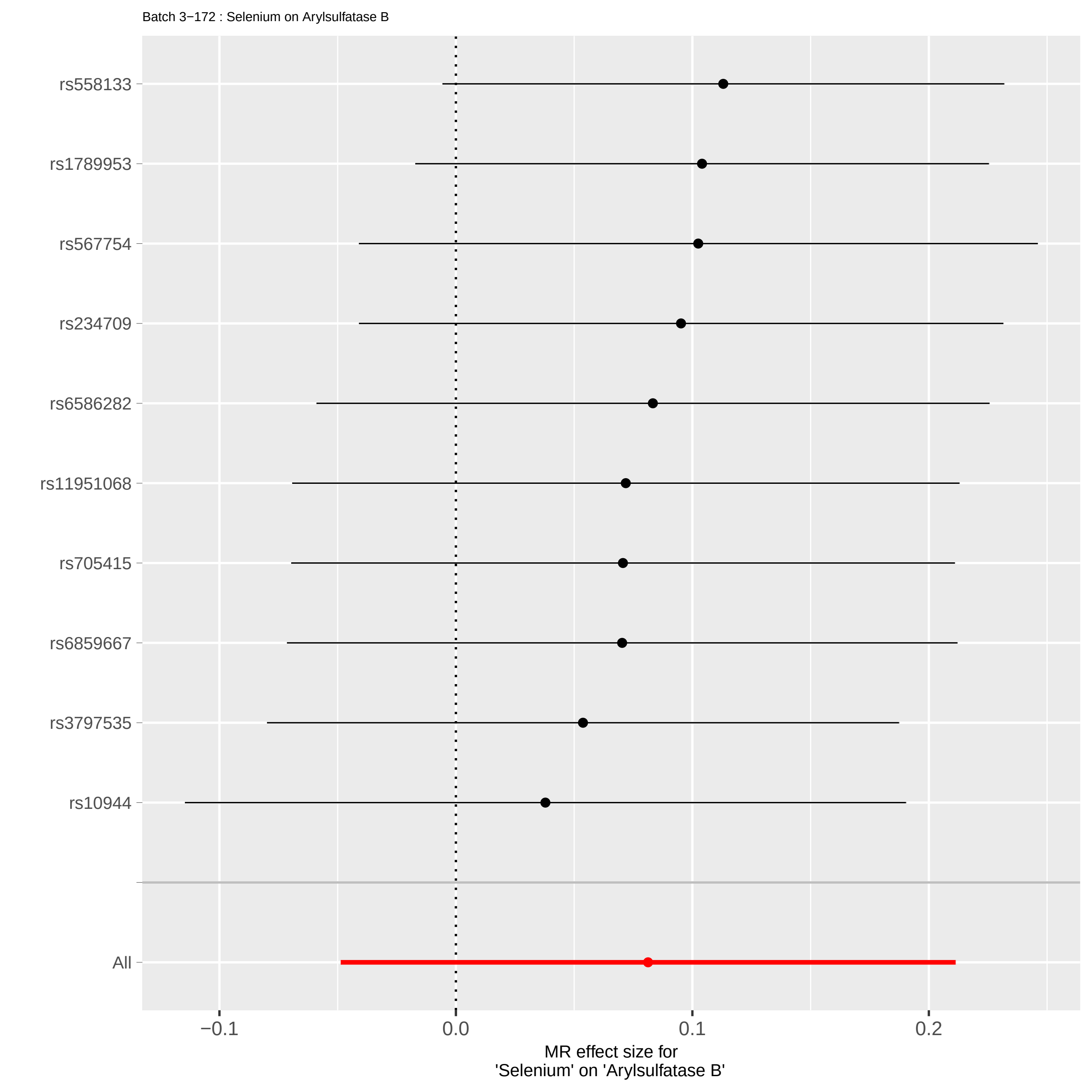
Batch 3−172 : Selenium on Arylsulfatase B
rs558133
rs1789953
rs567754
rs234709
rs6586282
rs11951068
rs705415
rs6859667
rs3797535
rs10944
All
−0.1
0.0
0.1
0.2
MR effect size for
'Selenium' on 'Arylsulfatase B'

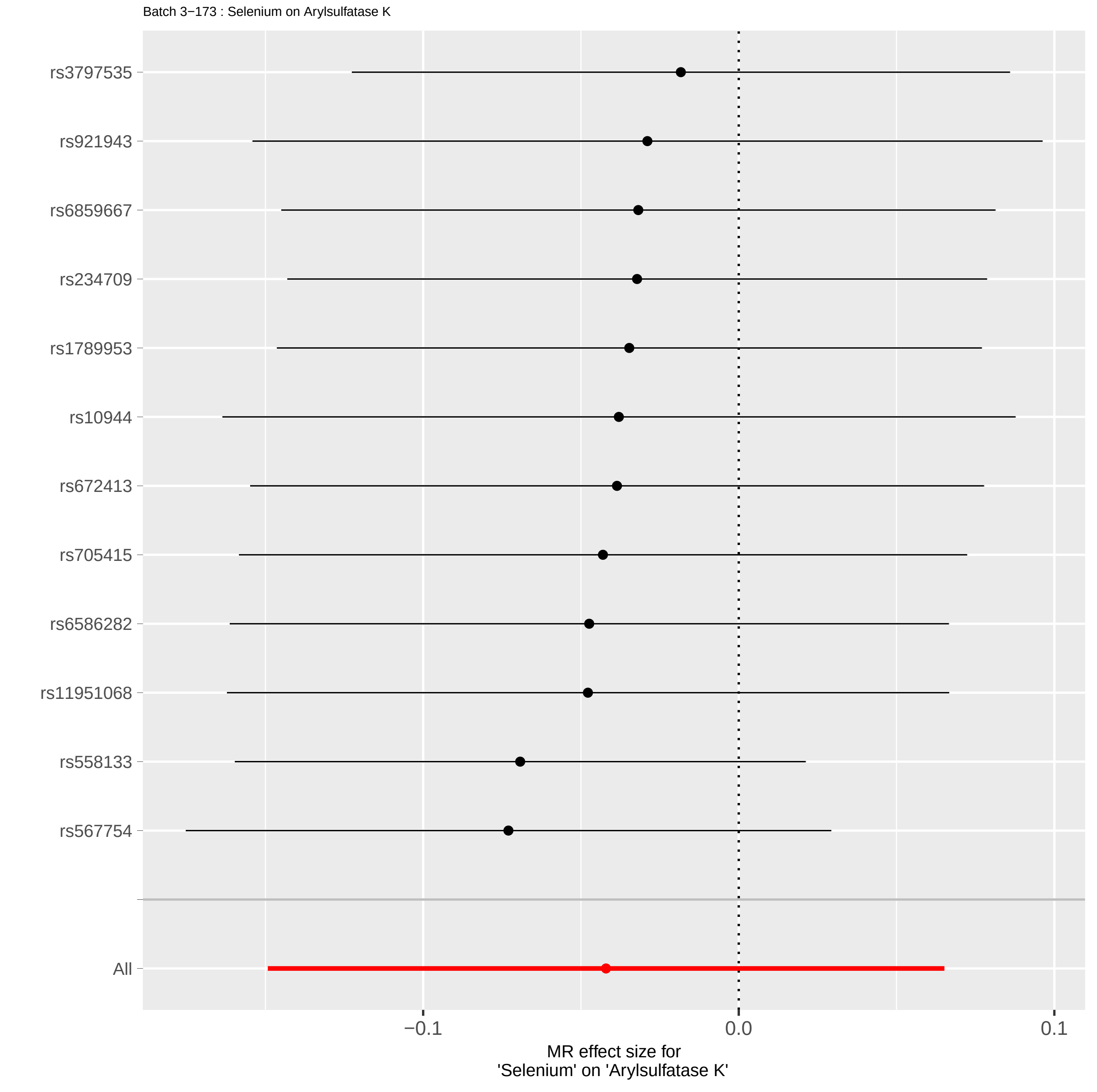

Batch 3−173 : Selenium on Arylsulfatase K
rs3797535
rs921943
rs6859667
rs234709
rs1789953
rs10944
rs672413
rs705415
rs6586282
rs11951068
rs558133
rs567754
All
−0.1
0.0
0.1
MR effect size for
'Selenium' on 'Arylsulfatase K'

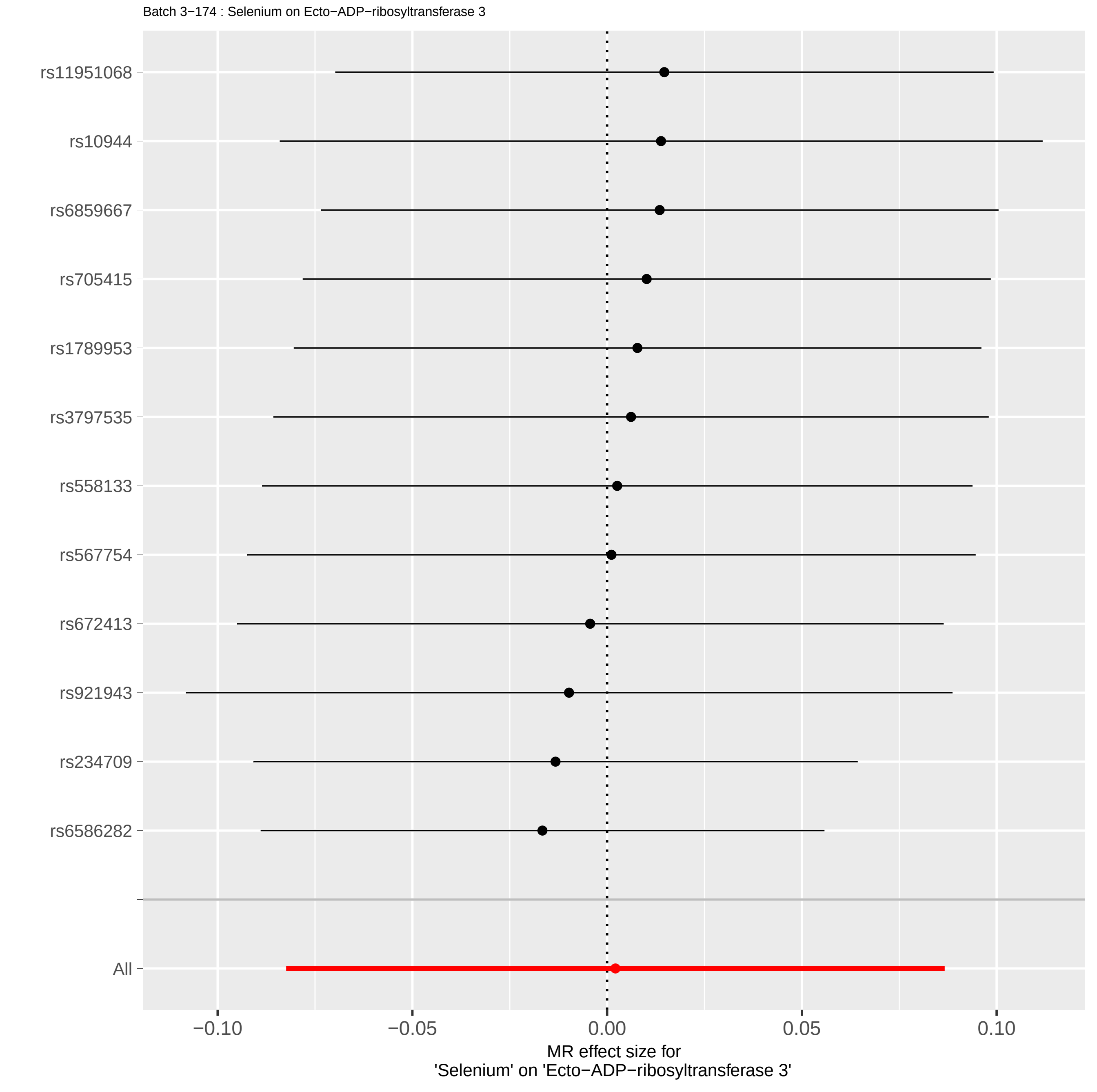
Batch 3−174 : Selenium on Ecto−ADP−ribosyltransferase 3
rs11951068
rs10944
rs6859667
rs705415
rs1789953
rs3797535
rs558133
rs567754
rs672413
rs921943
rs234709
rs6586282
All
−0.10
−0.05
0.00
0.05
0.10
MR effect size for
'Selenium' on 'Ecto−ADP−ribosyltransferase 3'

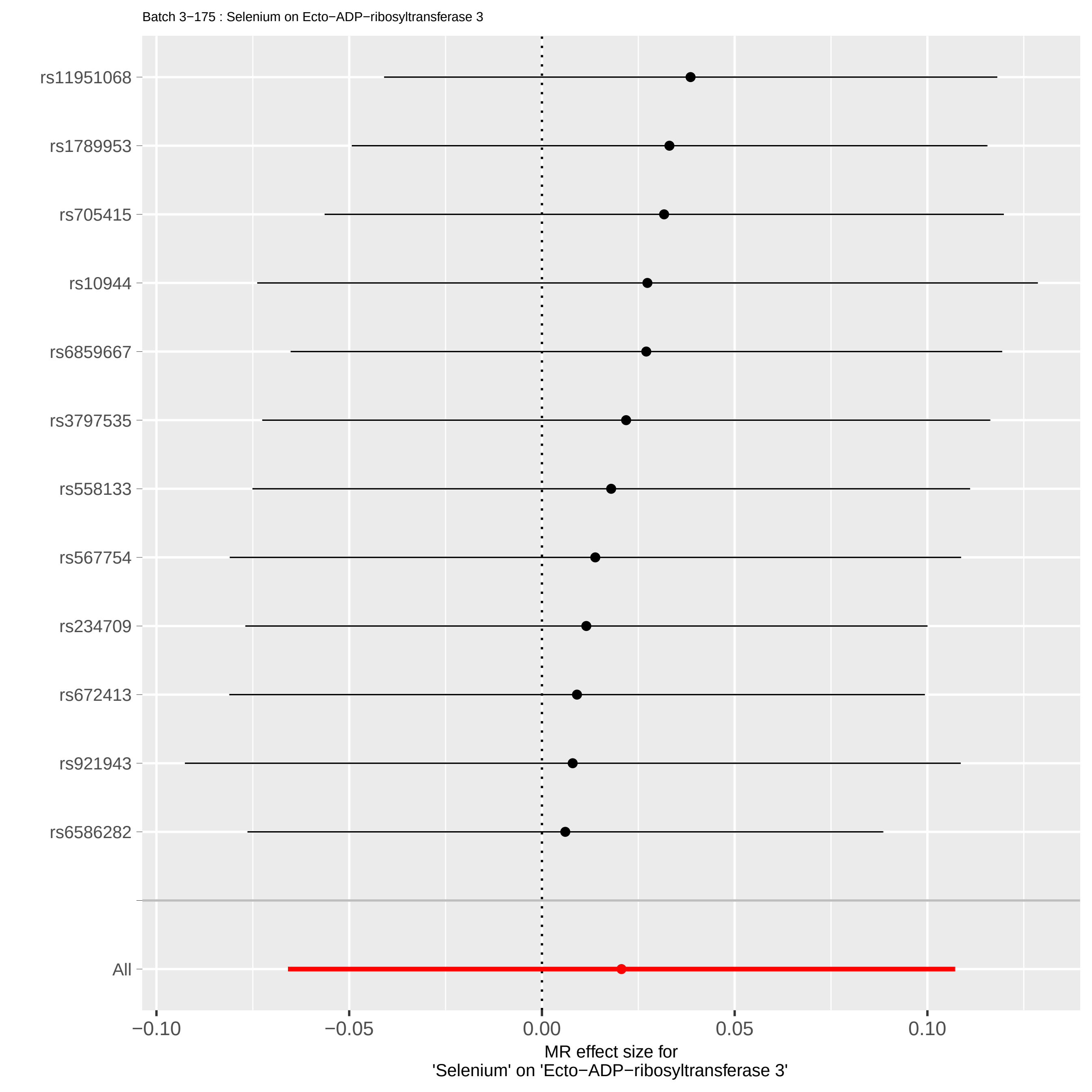

Batch 3−175 : Selenium on Ecto−ADP−ribosyltransferase 3
rs11951068
rs1789953
rs705415
rs10944
rs6859667
rs3797535
rs558133
rs567754
rs234709
rs672413
rs921943
rs6586282
All
−0.10
−0.05
0.00
0.05
0.10
MR effect size for
'Selenium' on 'Ecto−ADP−ribosyltransferase 3'

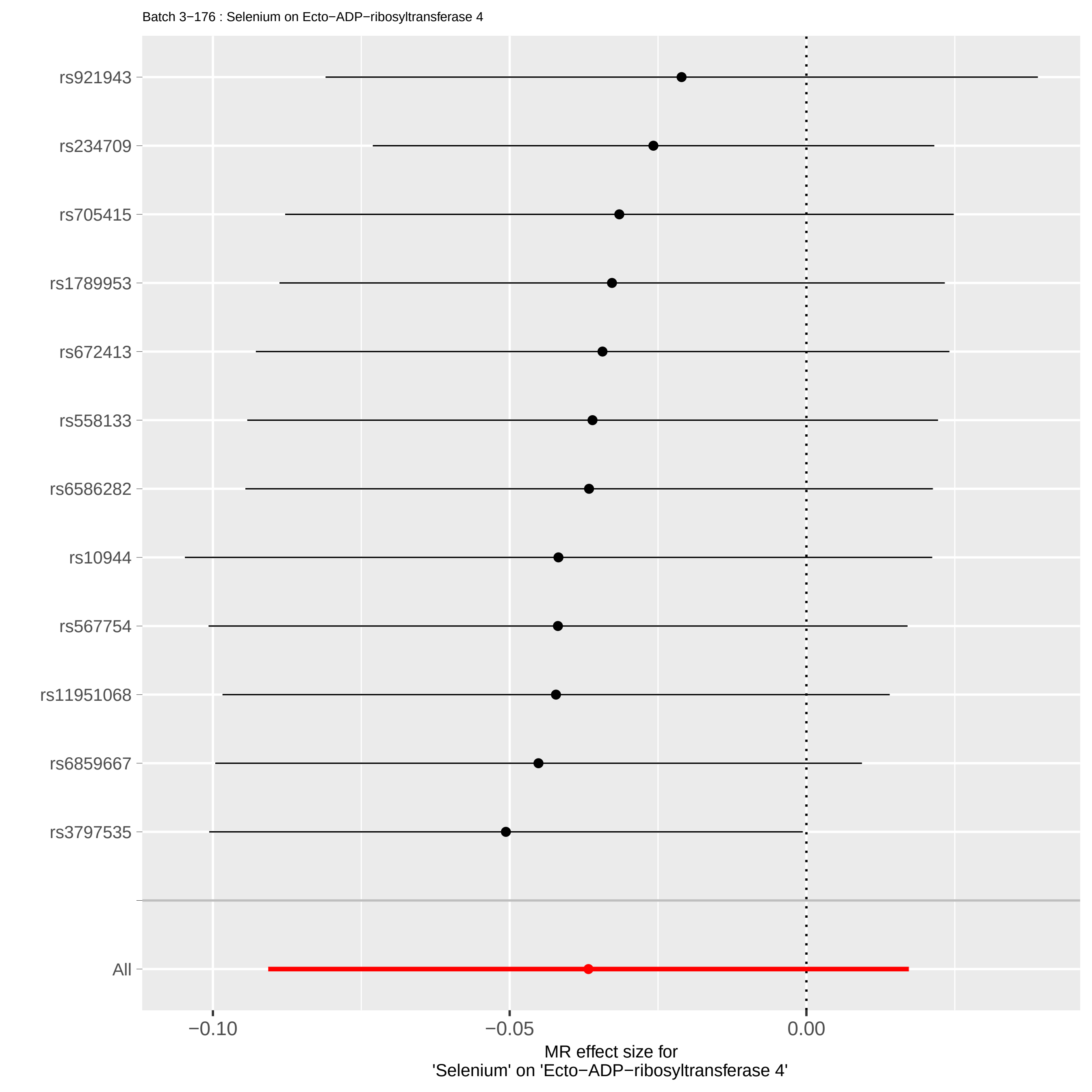

Batch 3−176 : Selenium on Ecto−ADP−ribosyltransferase 4
rs921943
rs234709
rs705415
rs1789953
rs672413
rs558133
rs6586282
rs10944
rs567754
rs11951068
rs6859667
rs3797535
All
−0.10
−0.05
0.00
MR effect size for
'Selenium' on 'Ecto−ADP−ribosyltransferase 4'

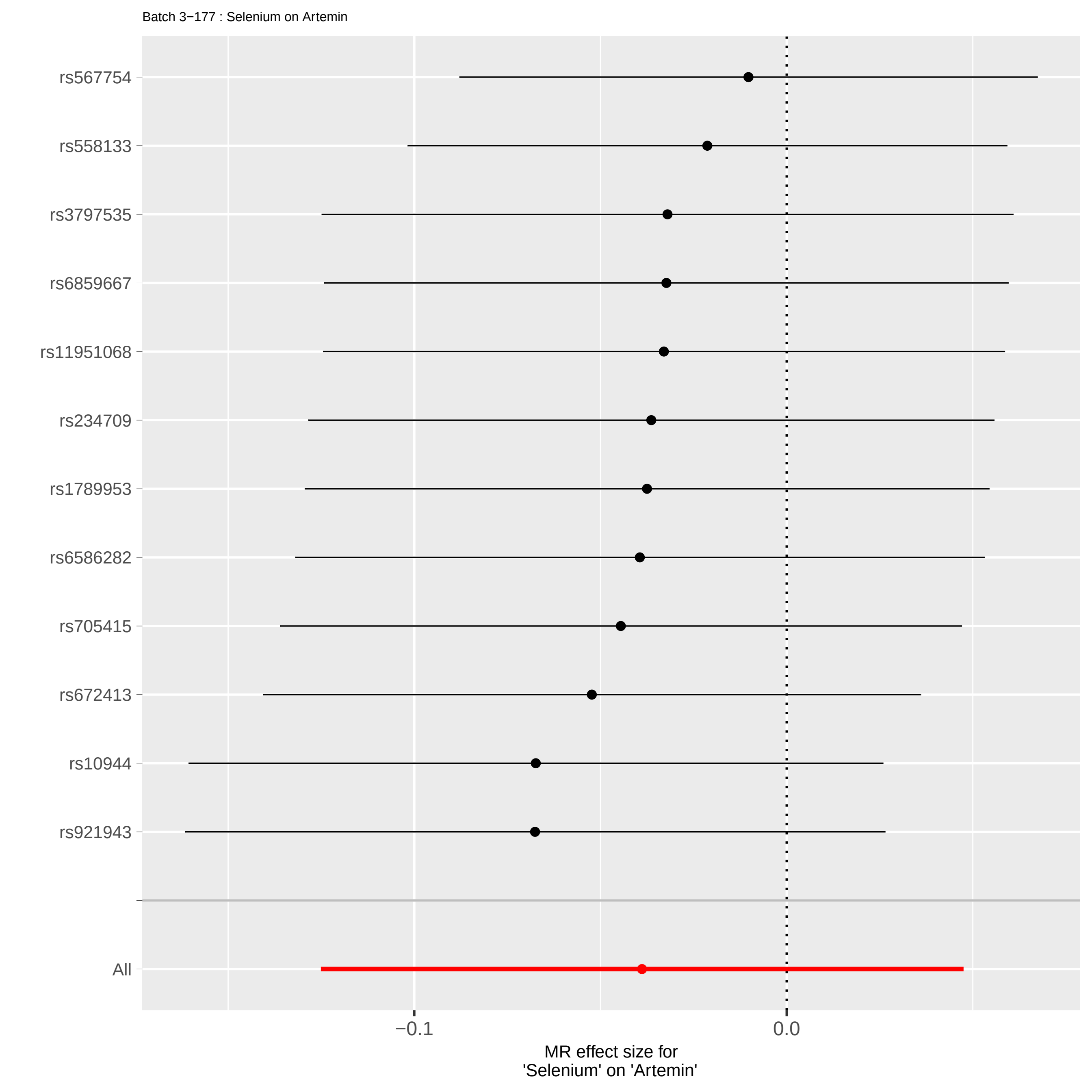
Batch 3−177 : Selenium on Artemin
rs567754
rs558133
rs3797535
rs6859667
rs11951068
rs234709
rs1789953
rs6586282
rs705415
rs672413
rs10944
rs921943
All
−0.1
0.0
MR effect size for
'Selenium' on 'Artemin'

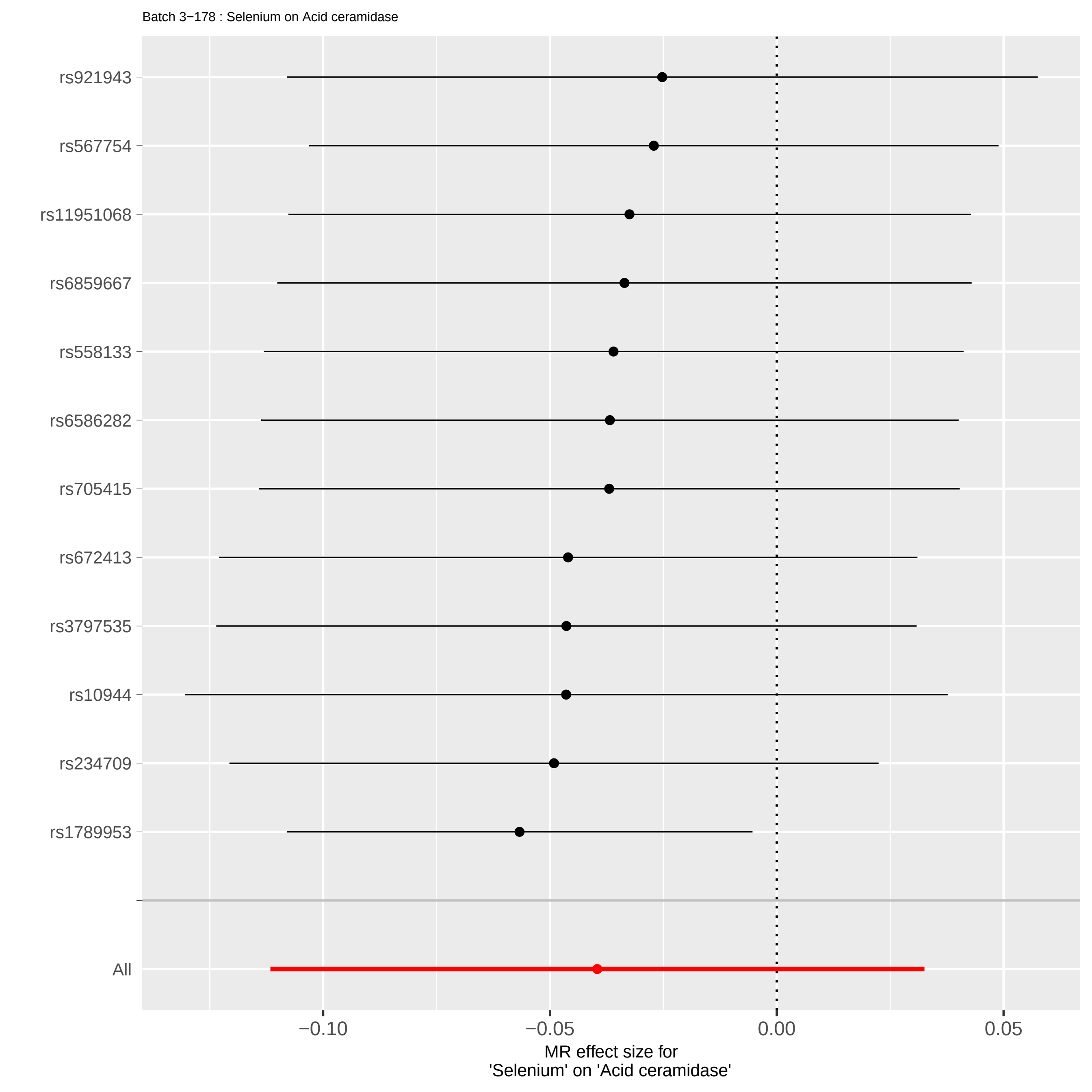

Batch 3−178 : Selenium on Acid ceramidase
rs921943
rs567754
rs11951068
rs6859667
rs558133
rs6586282
rs705415
rs672413
rs3797535
rs10944
rs234709
rs1789953
All
−0.10
−0.05
0.00
0.05
MR effect size for
'Selenium' on 'Acid ceramidase'

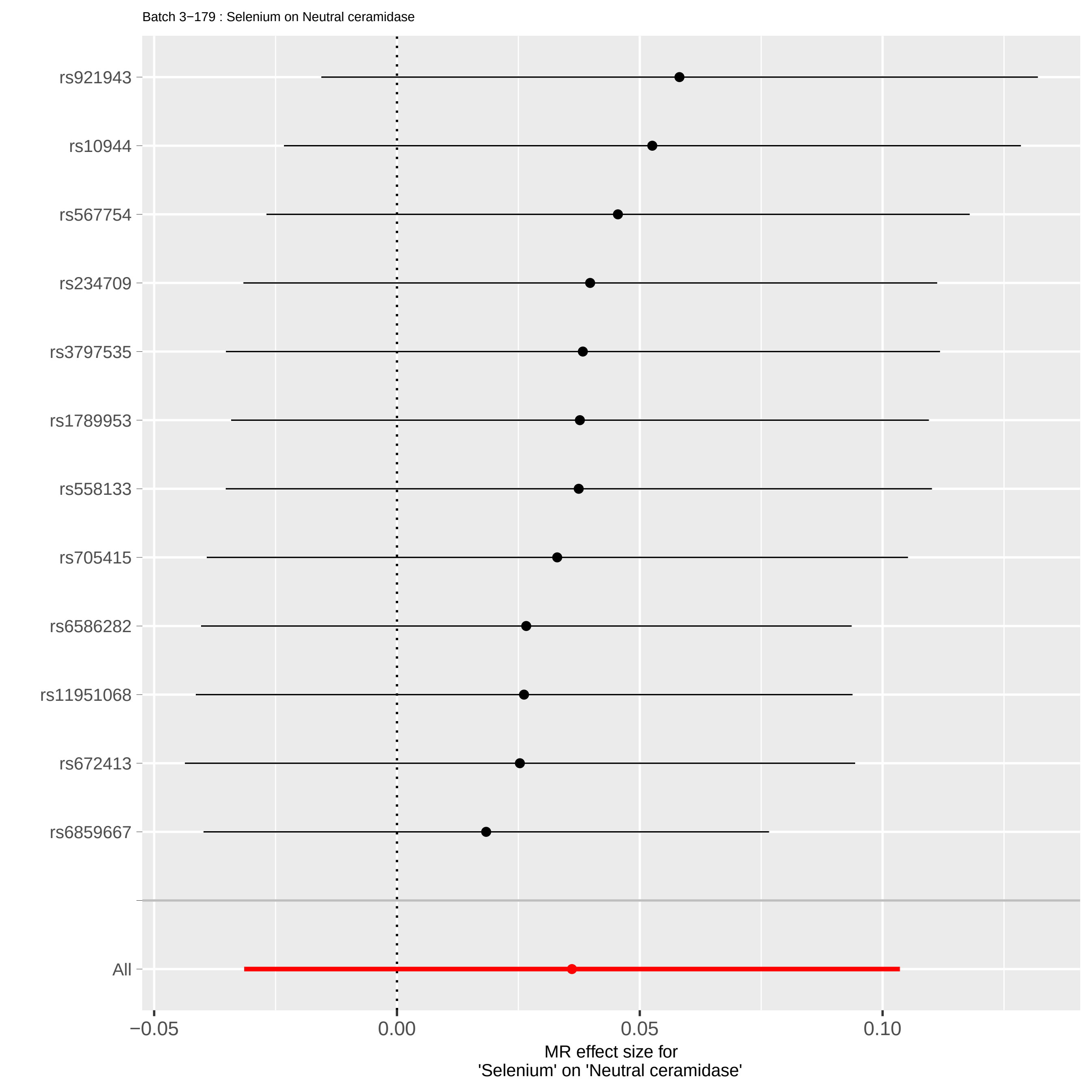

Batch 3−179 : Selenium on Neutral ceramidase
rs921943
rs10944
rs567754
rs234709
rs3797535
rs1789953
rs558133
rs705415
rs6586282
rs11951068
rs672413
rs6859667
All
−0.05
0.00
0.05
0.10
MR effect size for
'Selenium' on 'Neutral ceramidase'

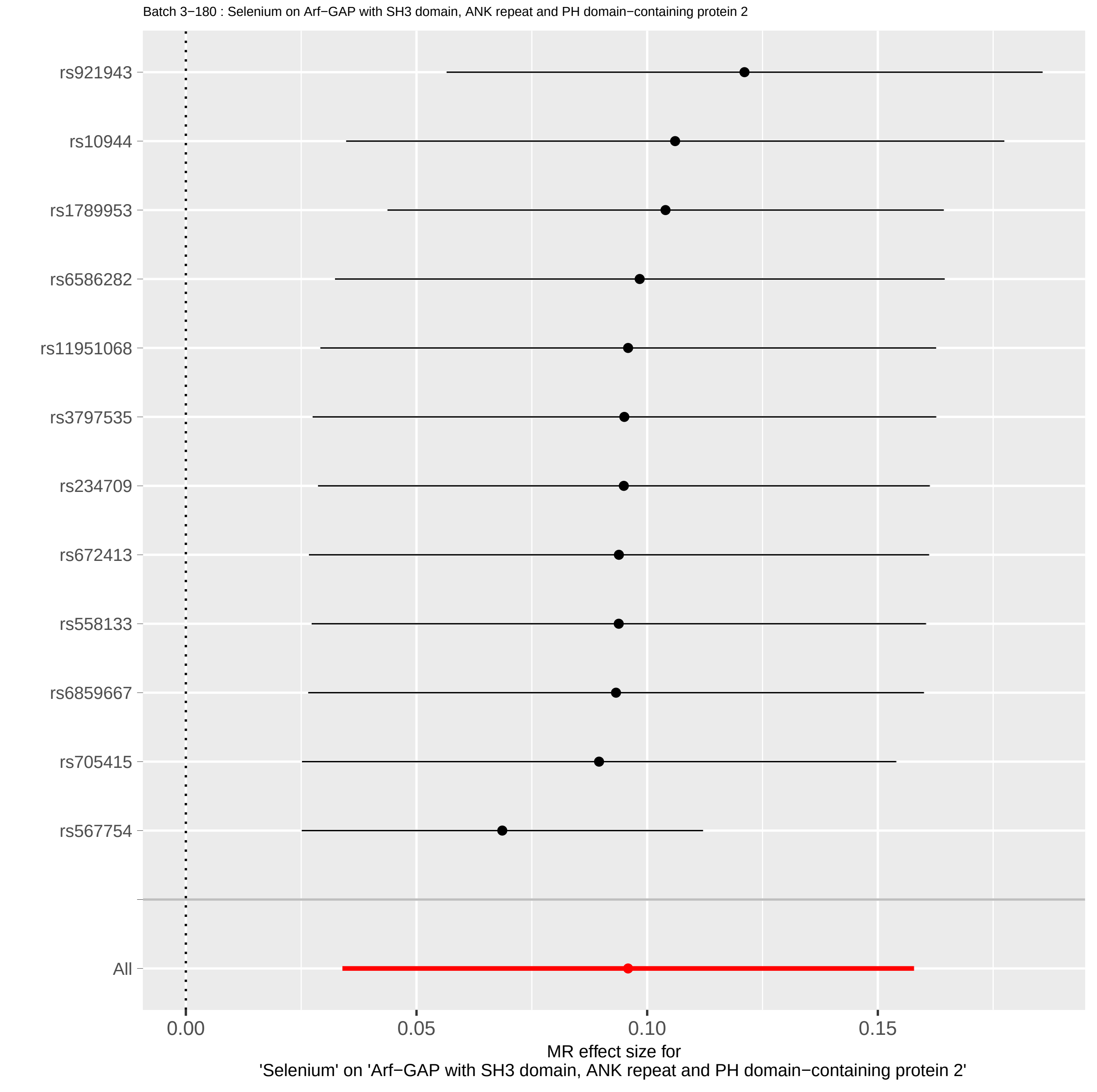
Batch 3−180 : Selenium on Arf−GAP with SH3 domain, ANK repeat and PH domain−containing protein 2
rs921943
rs10944
rs1789953
rs6586282
rs11951068
rs3797535
rs234709
rs672413
rs558133
rs6859667
rs705415
rs567754
All
0.00
0.05
0.10
0.15
MR effect size for
'Selenium' on 'Arf−GAP with SH3 domain, ANK repeat and PH domain−containing protein 2'

Batch 3−181 : Selenium on Activating signal cointegrator 1 complex subunit 1

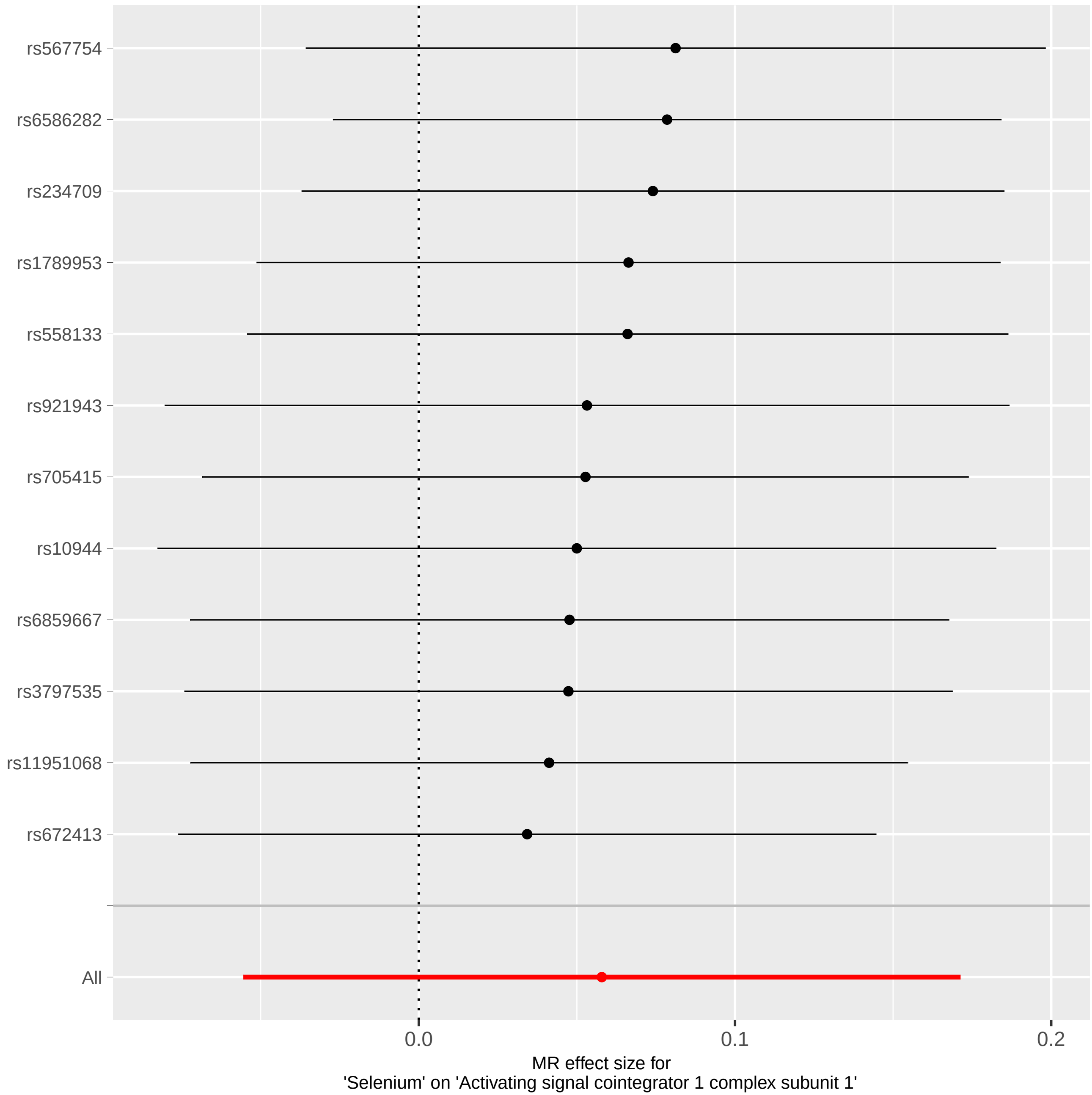

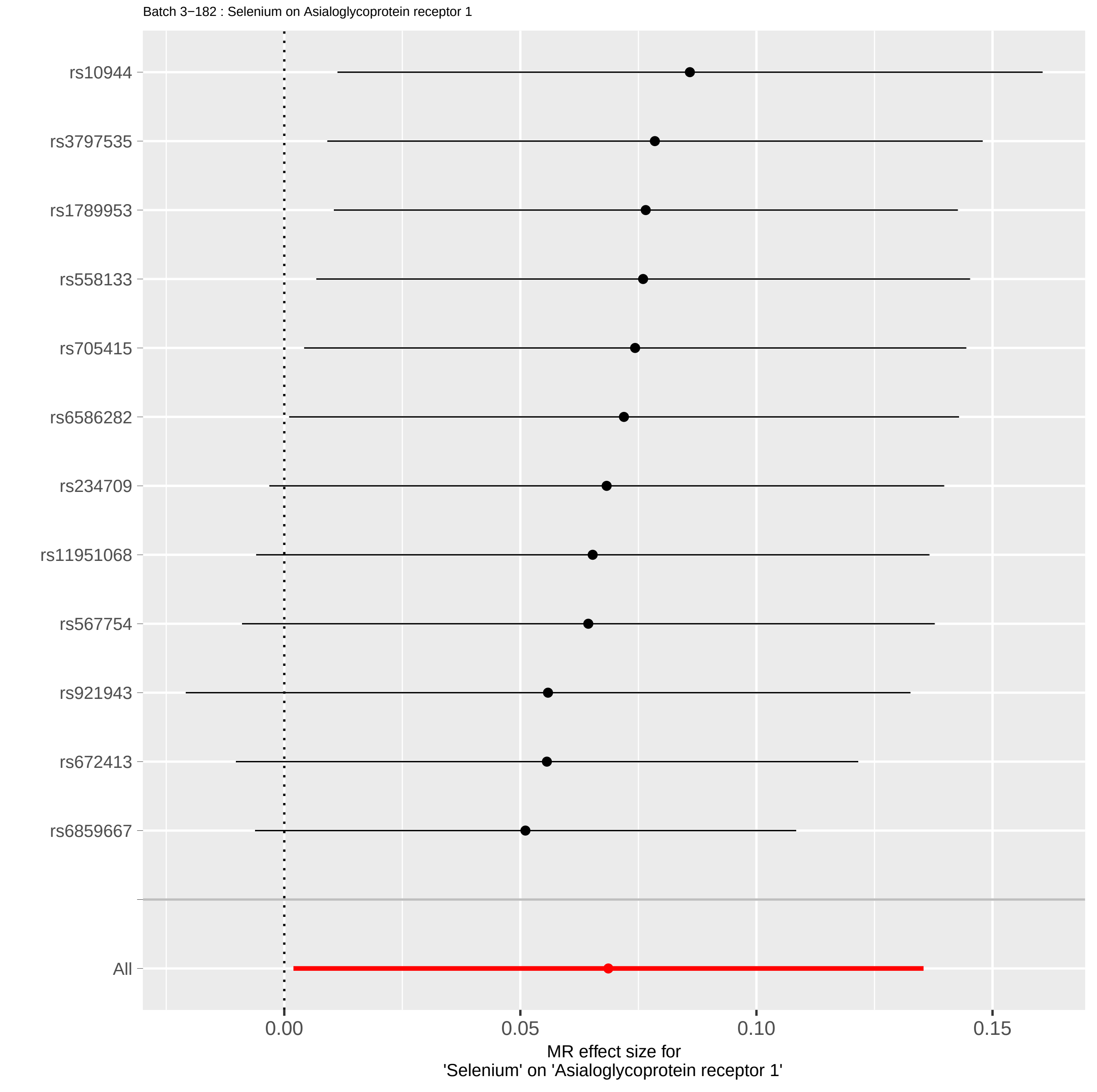
Batch 3−182 : Selenium on Asialoglycoprotein receptor 1
rs10944
rs3797535
rs1789953
rs558133
rs705415
rs6586282
rs234709
rs11951068
rs567754
rs921943
rs672413
rs6859667
All
0.00
0.05
0.10
0.15
MR effect size for
'Selenium' on 'Asialoglycoprotein receptor 1'

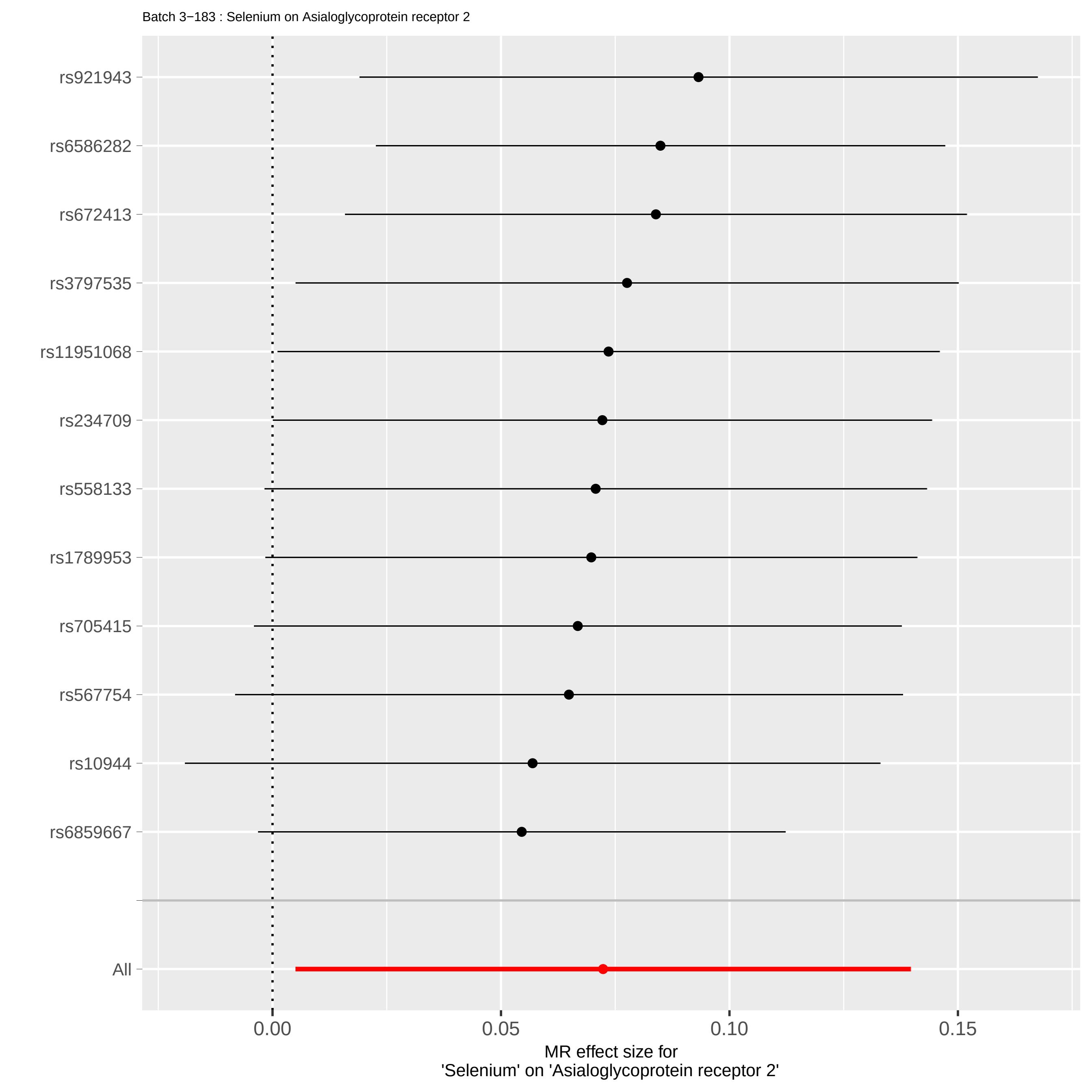

Batch 3−183 : Selenium on Asialoglycoprotein receptor 2
rs921943
rs6586282
rs672413
rs3797535
rs11951068
rs234709
rs558133
rs1789953
rs705415
rs567754
rs10944
rs6859667
All
0.00
0.05
0.10
0.15
MR effect size for
'Selenium' on 'Asialoglycoprotein receptor 2'

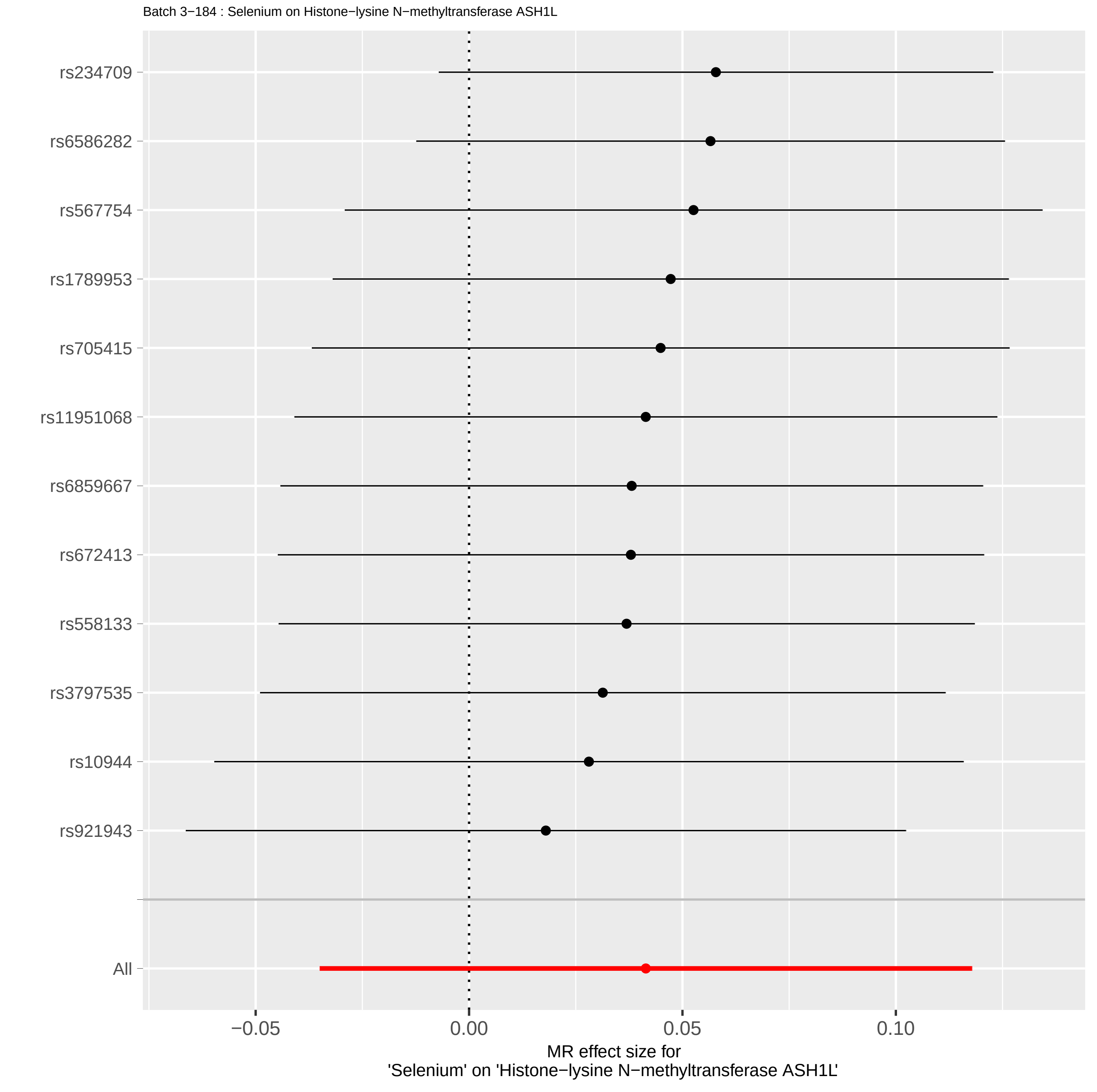
Batch 3−184 : Selenium on Histone−lysine N−methyltransferase ASH1L
rs234709
rs6586282
rs567754
rs1789953
rs705415
rs11951068
rs6859667
rs672413
rs558133
rs3797535
rs10944
rs921943
All
−0.05
0.00
0.05
0.10
MR effect size for
'Selenium' on 'Histone−lysine N−methyltransferase ASH1L'

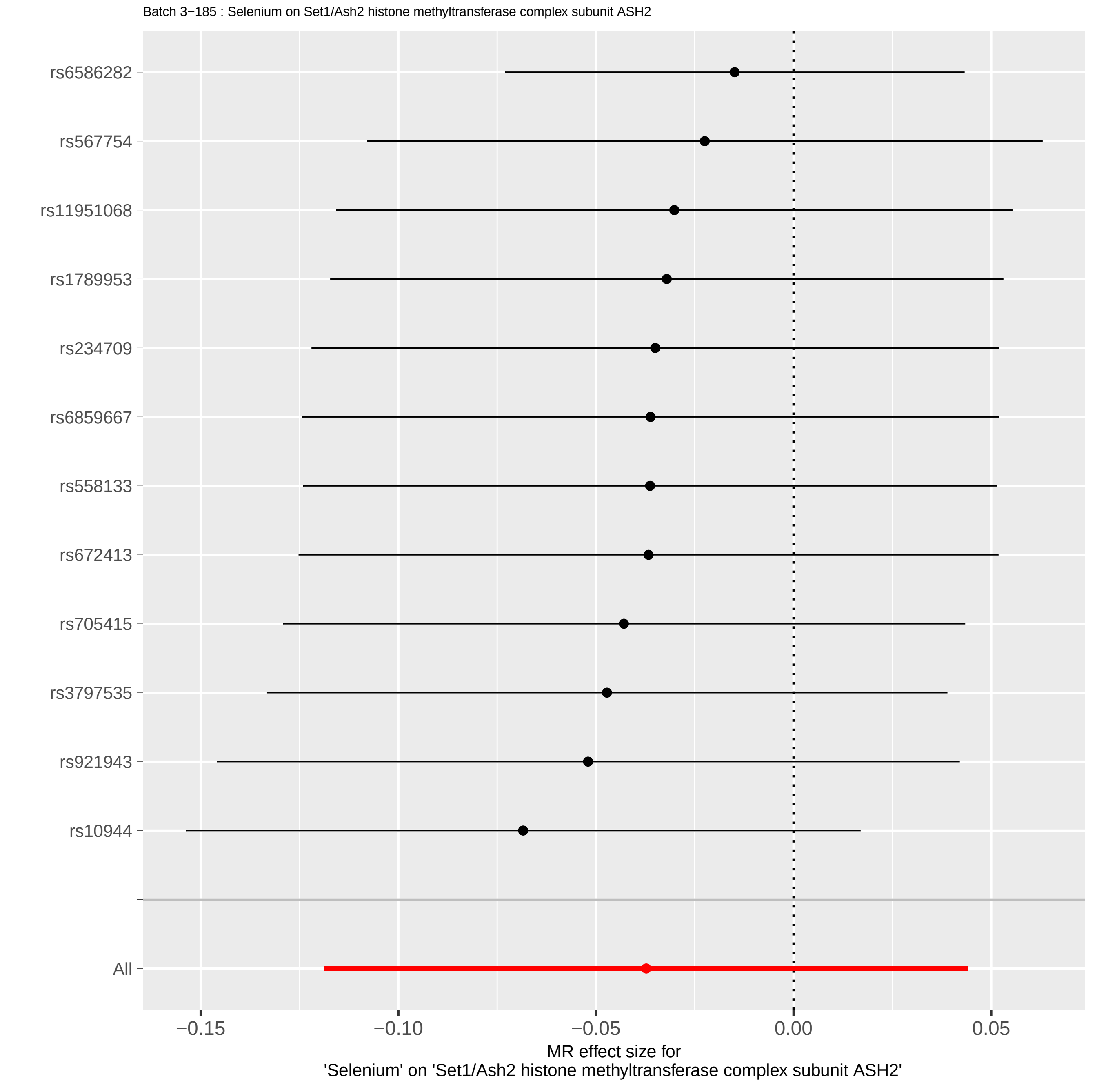

Batch 3−185 : Selenium on Set1/Ash2 histone methyltransferase complex subunit ASH2
rs6586282
rs567754
rs11951068
rs1789953
rs234709
rs6859667
rs558133
rs672413
rs705415
rs3797535
rs921943
rs10944
All
−0.15
−0.10
−0.05
0.00
0.05
MR effect size for
'Selenium' on 'Set1/Ash2 histone methyltransferase complex subunit ASH2'

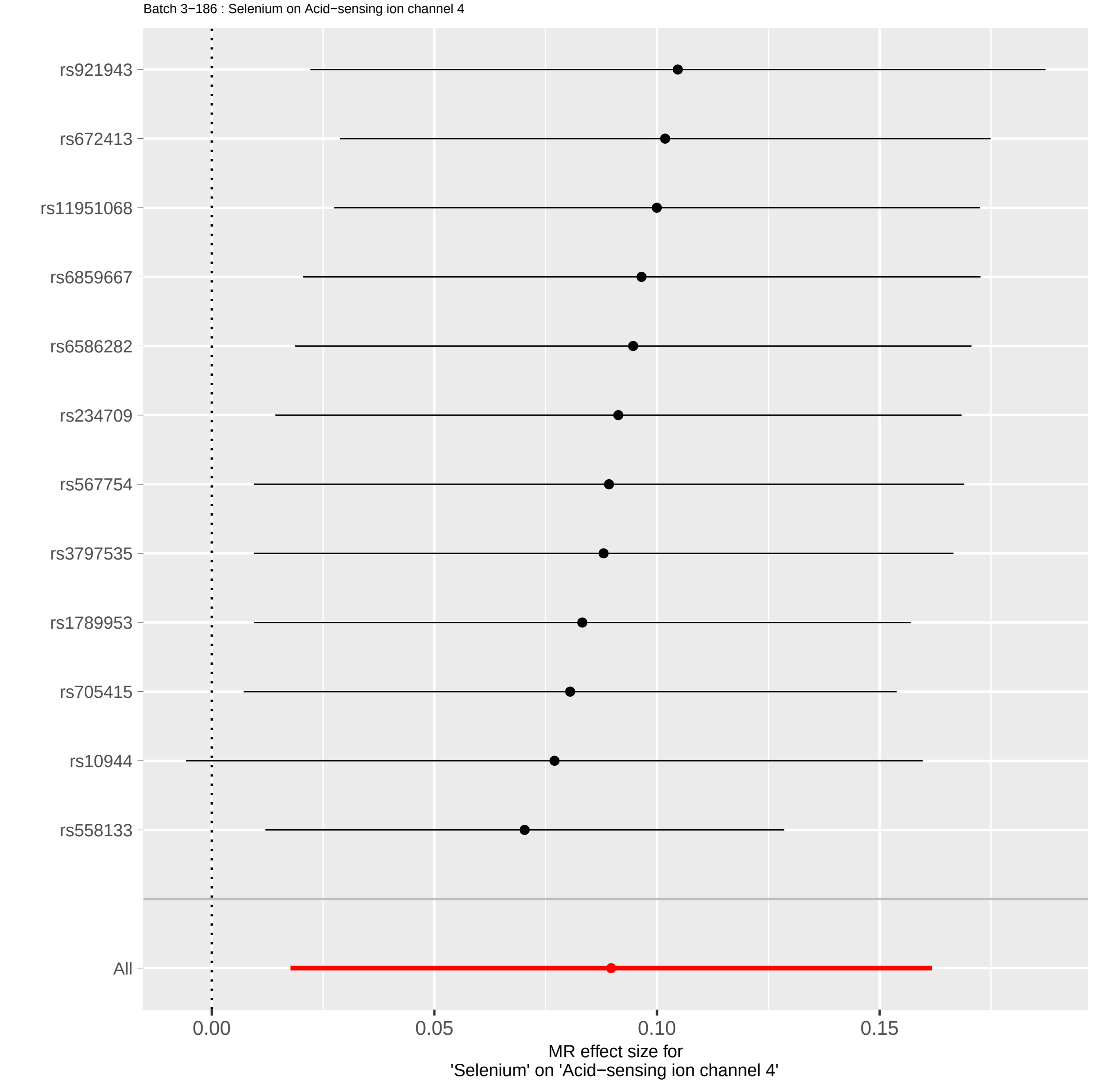
Batch 3−186 : Selenium on Acid−sensing ion channel 4
rs921943
rs672413
rs11951068
rs6859667
rs6586282
rs234709
rs567754
rs3797535
rs1789953
rs705415
rs10944
rs558133
All
0.00
0.05
0.10
0.15
MR effect size for
'Selenium' on 'Acid−sensing ion channel 4'

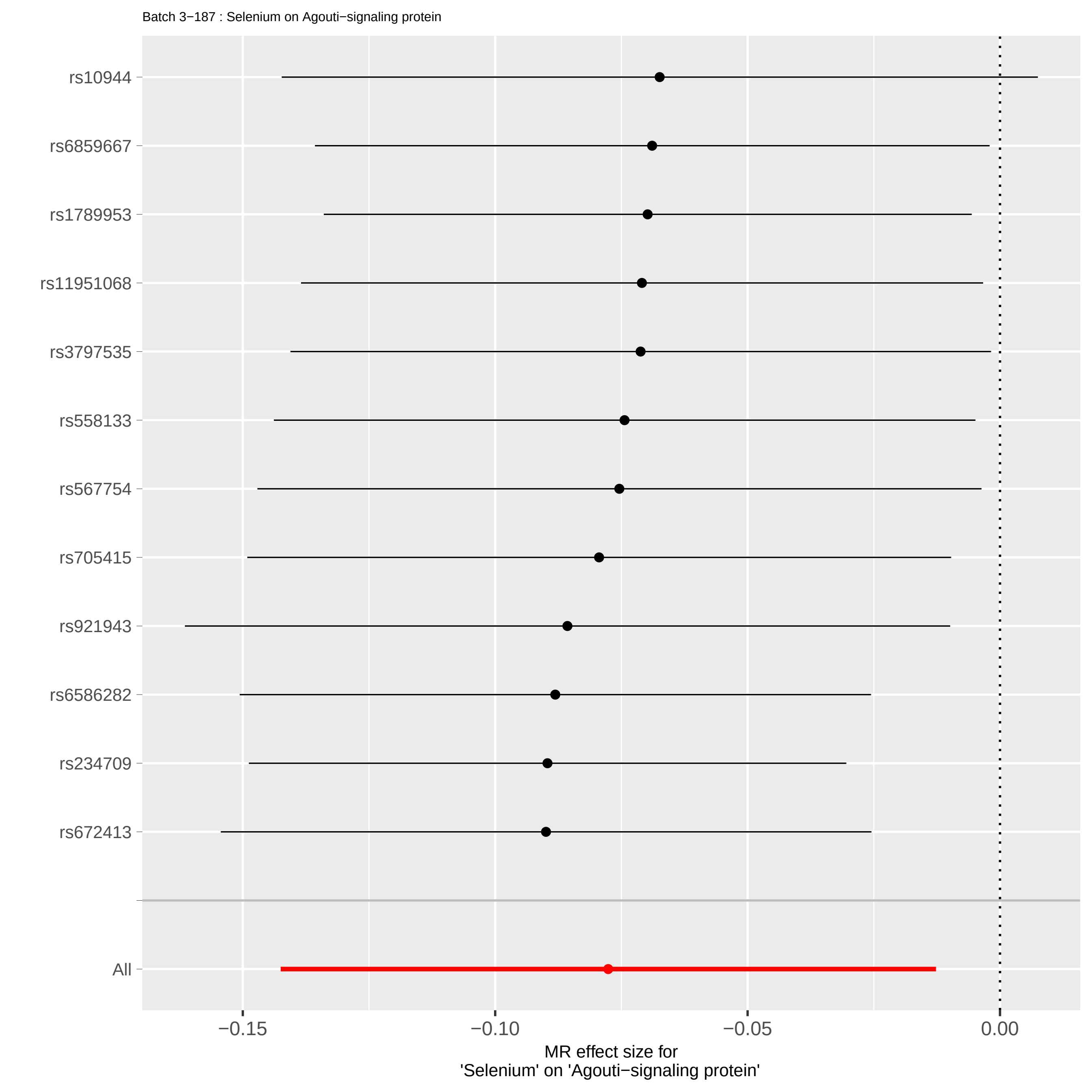

Batch 3−187 : Selenium on Agouti−signaling protein
rs10944
rs6859667
rs1789953
rs11951068
rs3797535
rs558133
rs567754
rs705415
rs921943
rs6586282
rs234709
rs672413
All
−0.15
−0.10
−0.05
0.00
MR effect size for
'Selenium' on 'Agouti−signaling protein'

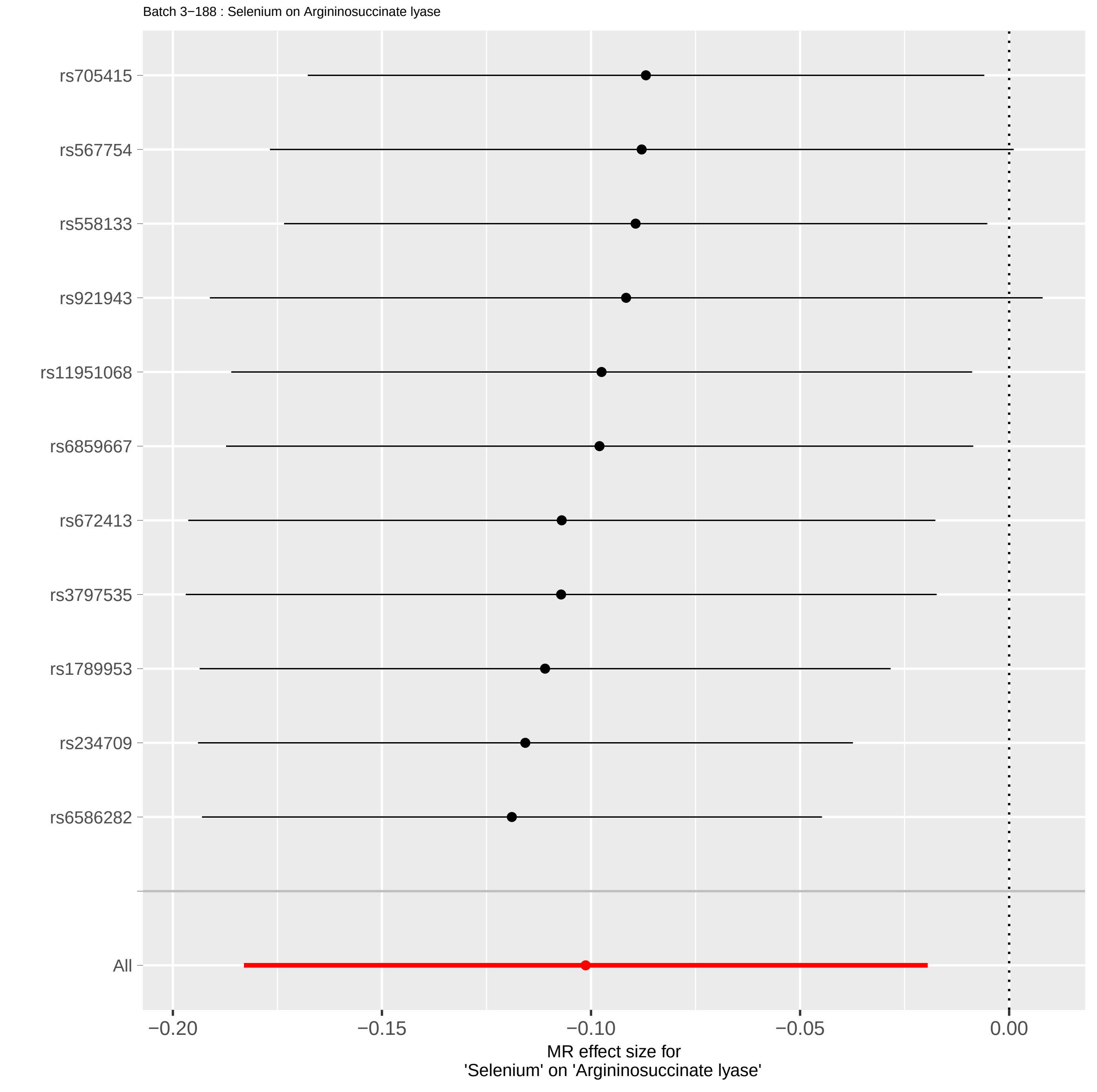
Batch 3−188 : Selenium on Argininosuccinate lyase
rs705415
rs567754
rs558133
rs921943
rs11951068
rs6859667
rs672413
rs3797535
rs1789953
rs234709
rs6586282
All
−0.20
−0.15
−0.10
−0.05
0.00
MR effect size for
'Selenium' on 'Argininosuccinate lyase'

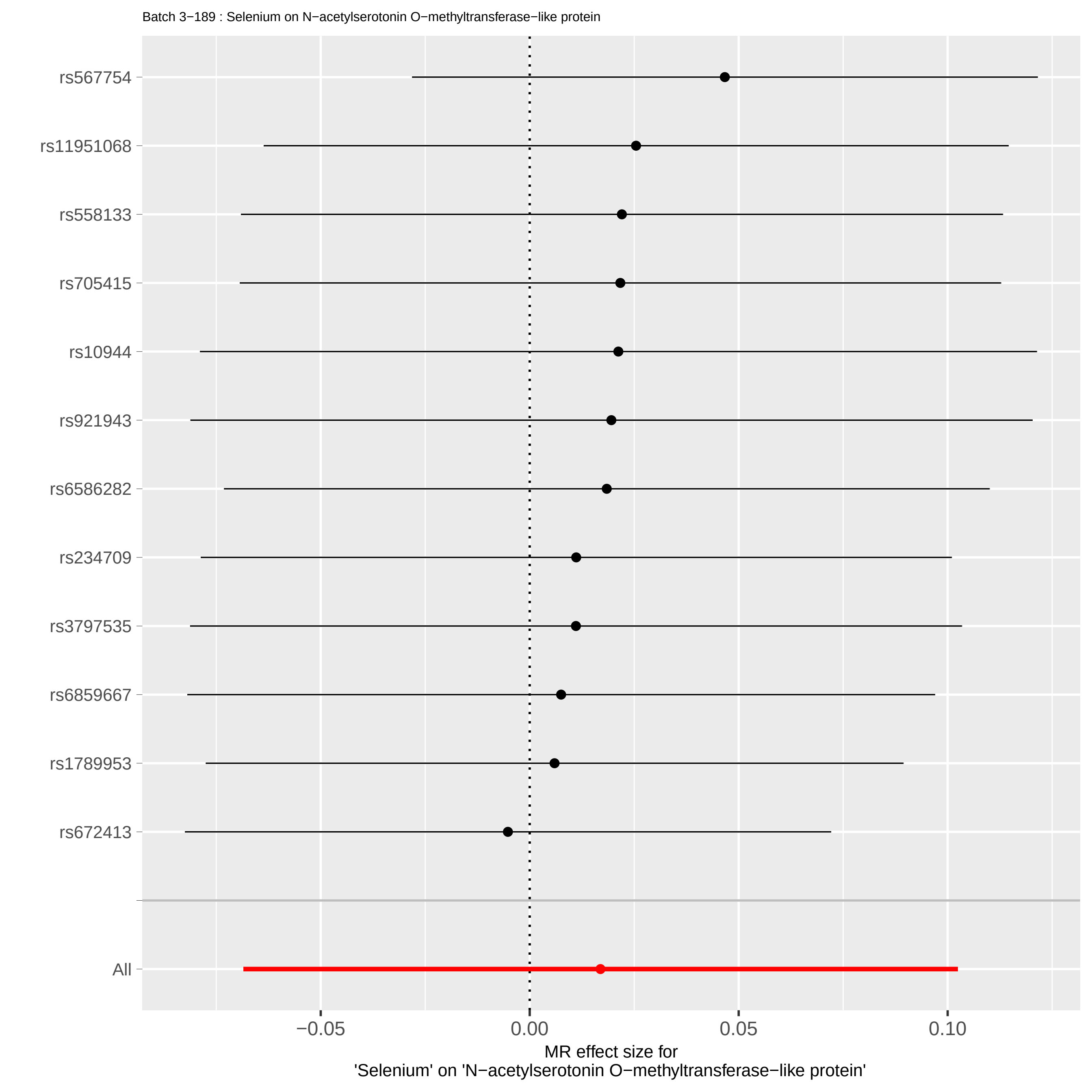
Batch 3−189 : Selenium on N−acetylserotonin O−methyltransferase−like protein
rs567754
rs11951068
rs558133
rs705415
rs10944
rs921943
rs6586282
rs234709
rs3797535
rs6859667
rs1789953
rs672413
All
−0.05
0.00
0.05
0.10
MR effect size for
'Selenium' on 'N−acetylserotonin O−methyltransferase−like protein'

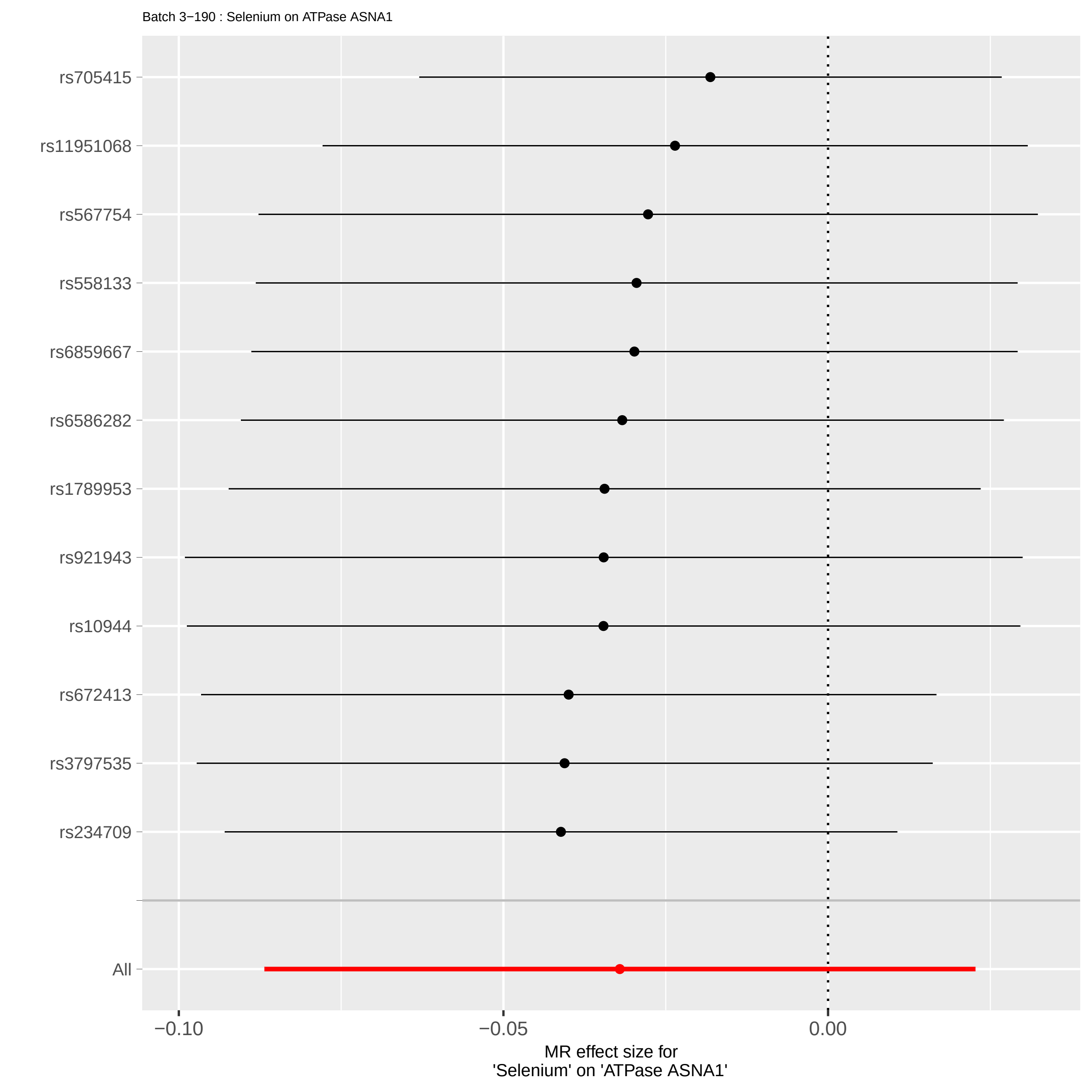

Batch 3−190 : Selenium on ATPase ASNA1
rs705415
rs11951068
rs567754
rs558133
rs6859667
rs6586282
rs1789953
rs921943
rs10944
rs672413
rs3797535
rs234709
All
−0.10
−0.05
0.00
MR effect size for
'Selenium' on 'ATPase ASNA1'

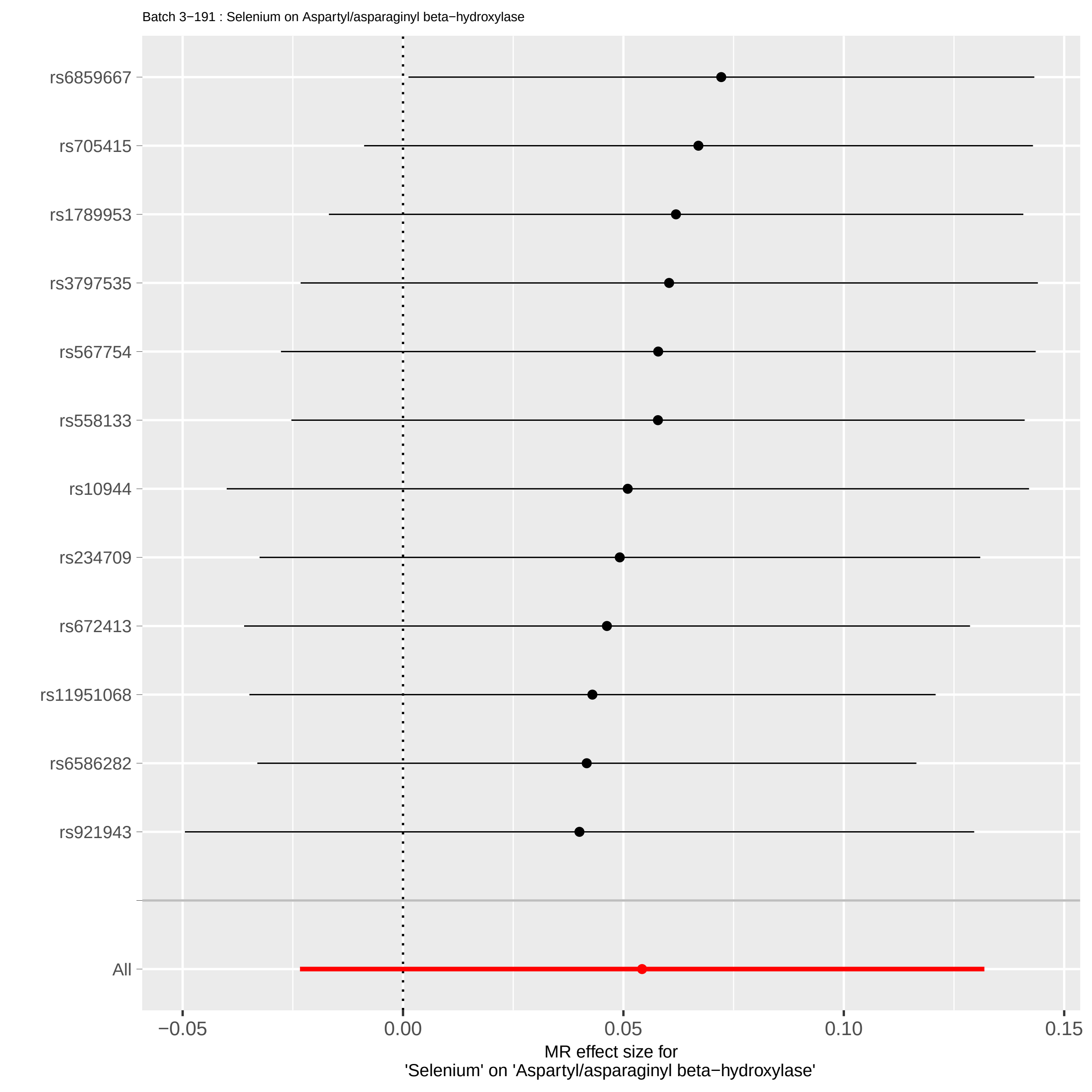
Batch 3−191 : Selenium on Aspartyl/asparaginyl beta−hydroxylase
rs6859667
rs705415
rs1789953
rs3797535
rs567754
rs558133
rs10944
rs234709
rs672413
rs11951068
rs6586282
rs921943
All
−0.05
0.00
0.05
0.10
0.15
MR effect size for
'Selenium' on 'Aspartyl/asparaginyl beta−hydroxylase'

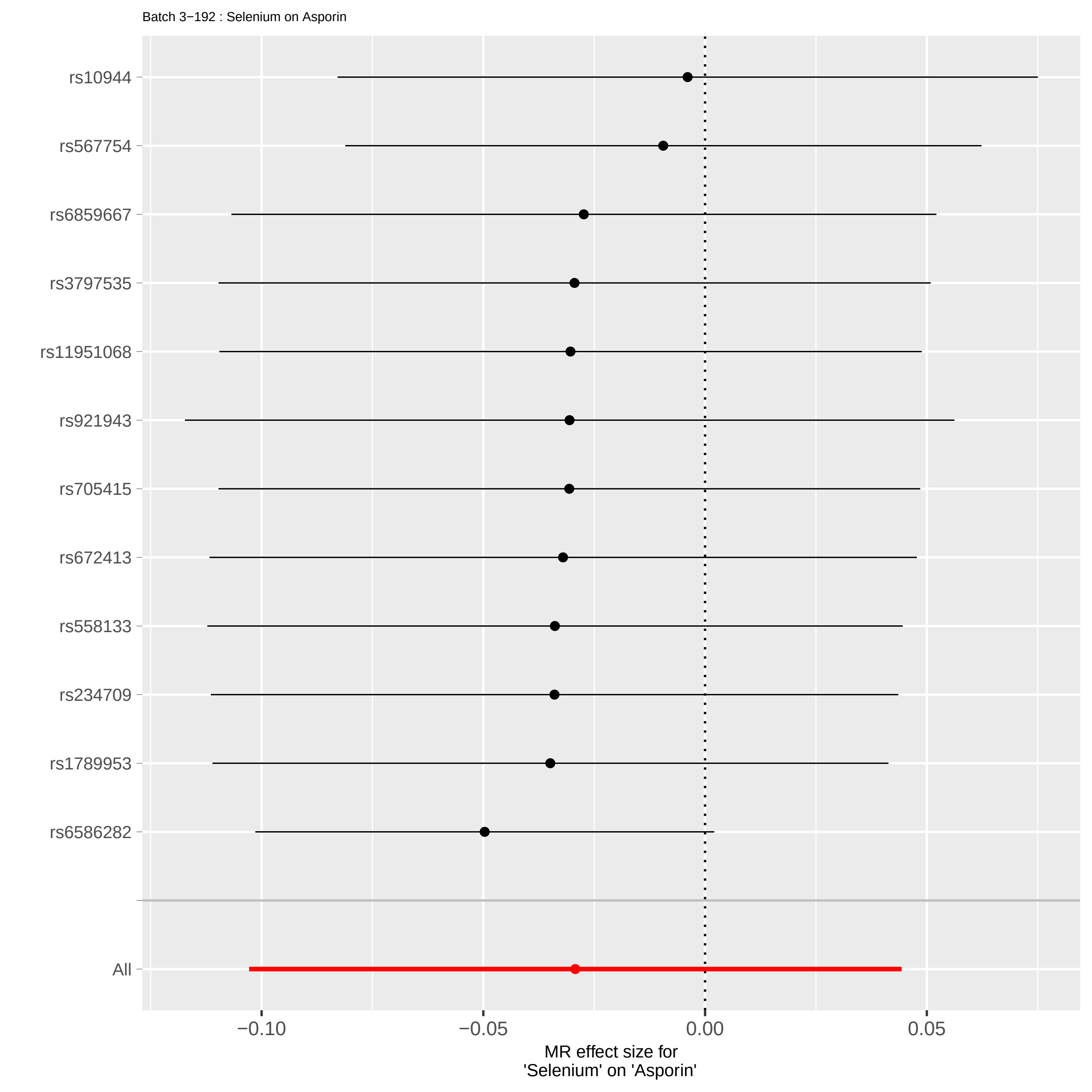

Batch 3−192 : Selenium on Asporin
rs10944
rs567754
rs6859667
rs3797535
rs11951068
rs921943
rs705415
rs672413
rs558133
rs234709
rs1789953
rs6586282
All
−0.10
−0.05
0.00
0.05
MR effect size for
'Selenium' on 'Asporin'

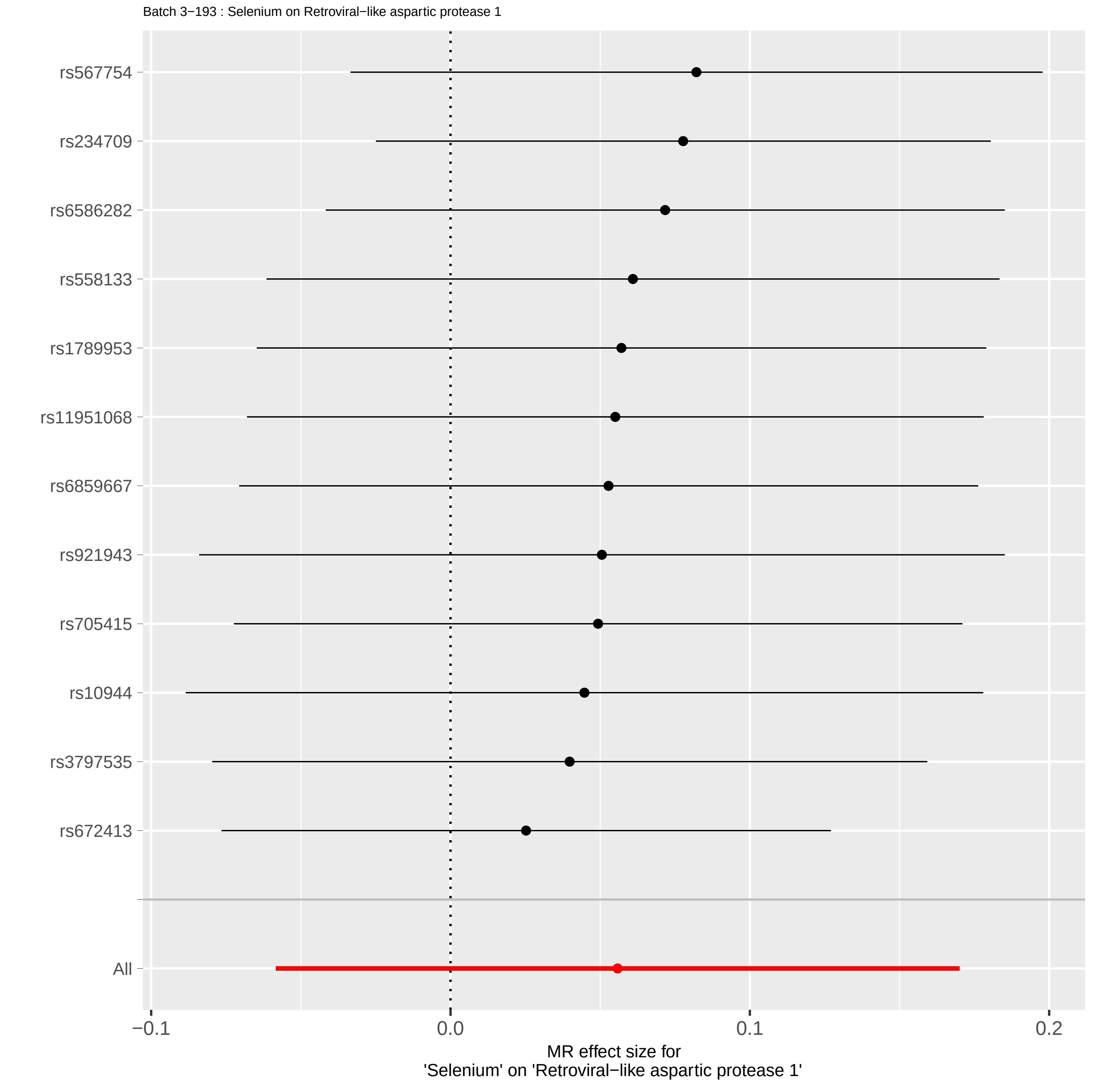
Batch 3−193 : Selenium on Retroviral−like aspartic protease 1
rs567754
rs234709
rs6586282
rs558133
rs1789953
rs11951068
rs6859667
rs921943
rs705415
rs10944
rs3797535
rs672413
All
−0.1
0.0
0.1
0.2
MR effect size for
'Selenium' on 'Retroviral−like aspartic protease 1'

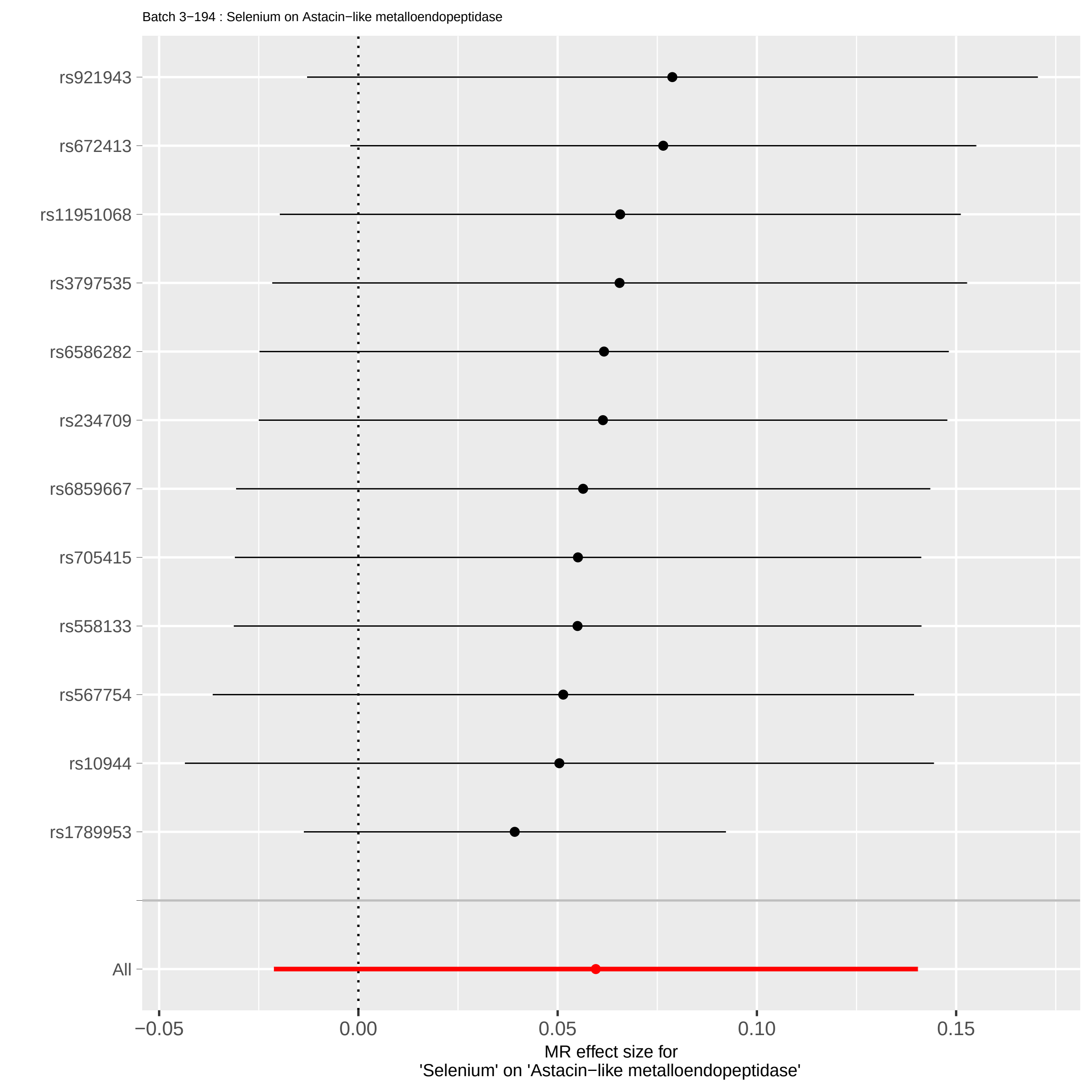
Batch 3−194 : Selenium on Astacin−like metalloendopeptidase
rs921943
rs672413
rs11951068
rs3797535
rs6586282
rs234709
rs6859667
rs705415
rs558133
rs567754
rs10944
rs1789953
All
−0.05
0.00
0.05
0.10
0.15
MR effect size for
'Selenium' on 'Astacin−like metalloendopeptidase'

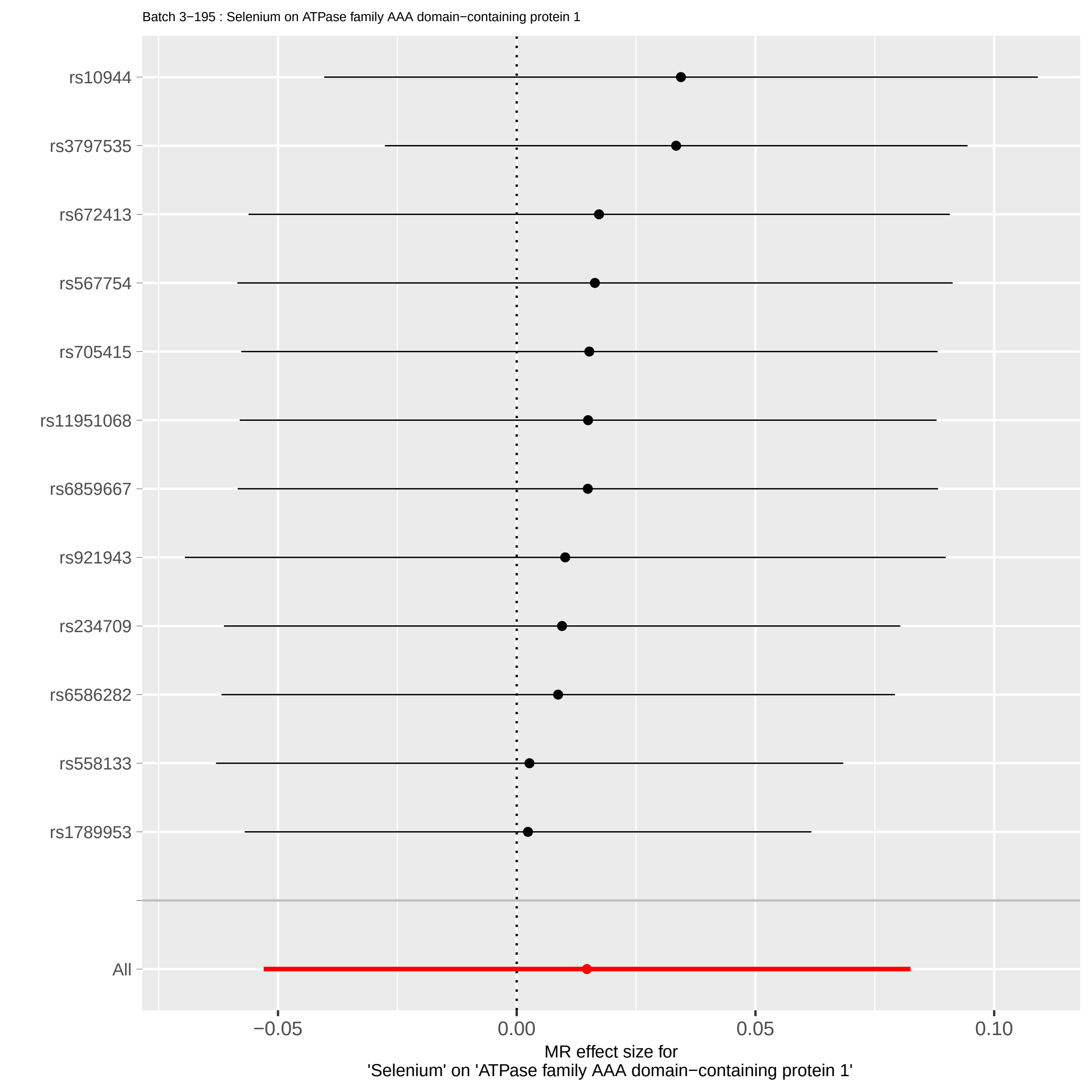

Batch 3−195 : Selenium on ATPase family AAA domain−containing protein 1
rs10944
rs3797535
rs672413
rs567754
rs705415
rs11951068
rs6859667
rs921943
rs234709
rs6586282
rs558133
rs1789953
All
−0.05
0.00
0.05
0.10
MR effect size for
'Selenium' on 'ATPase family AAA domain−containing protein 1'

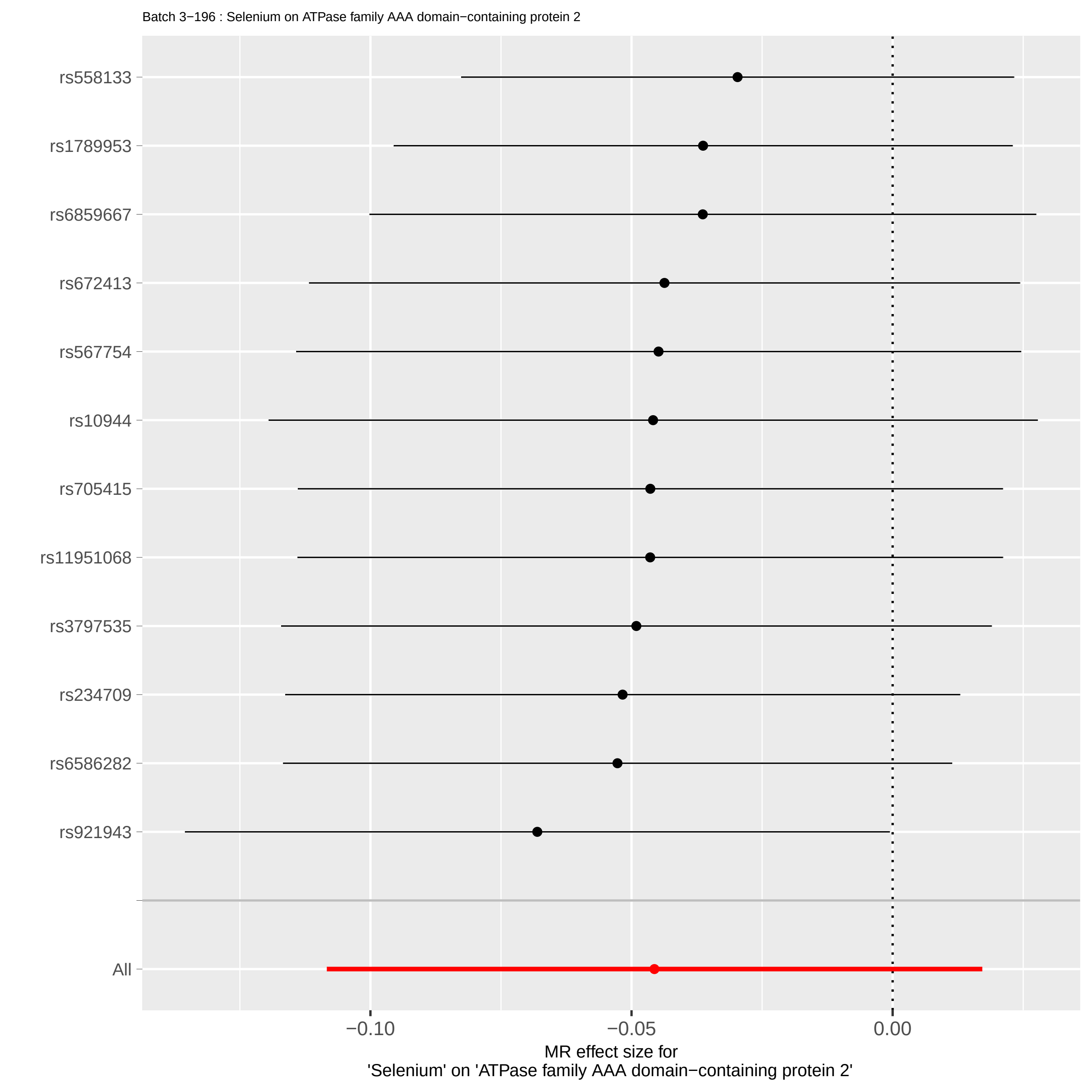

Batch 3−196 : Selenium on ATPase family AAA domain−containing protein 2
rs558133
rs1789953
rs6859667
rs672413
rs567754
rs10944
rs705415
rs11951068
rs3797535
rs234709
rs6586282
rs921943
All
−0.10
−0.05
0.00
MR effect size for
'Selenium' on 'ATPase family AAA domain−containing protein 2'

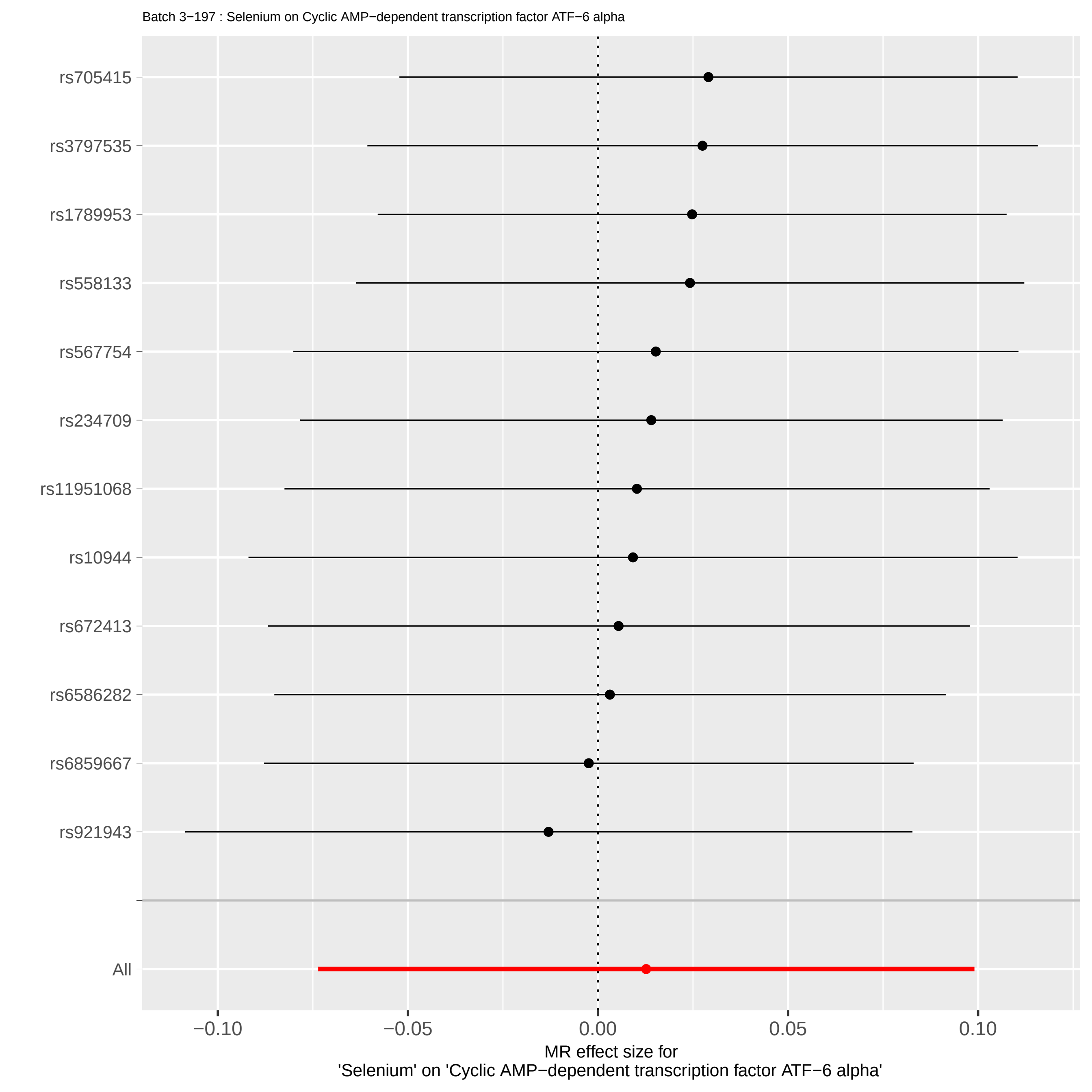

Batch 3−197 : Selenium on Cyclic AMP−dependent transcription factor ATF−6 alpha
rs705415
rs3797535
rs1789953
rs558133
rs567754
rs234709
rs11951068
rs10944
rs672413
rs6586282
rs6859667
rs921943
All
−0.10
−0.05
0.00
0.05
0.10
MR effect size for
'Selenium' on 'Cyclic AMP−dependent transcription factor ATF−6 alpha'

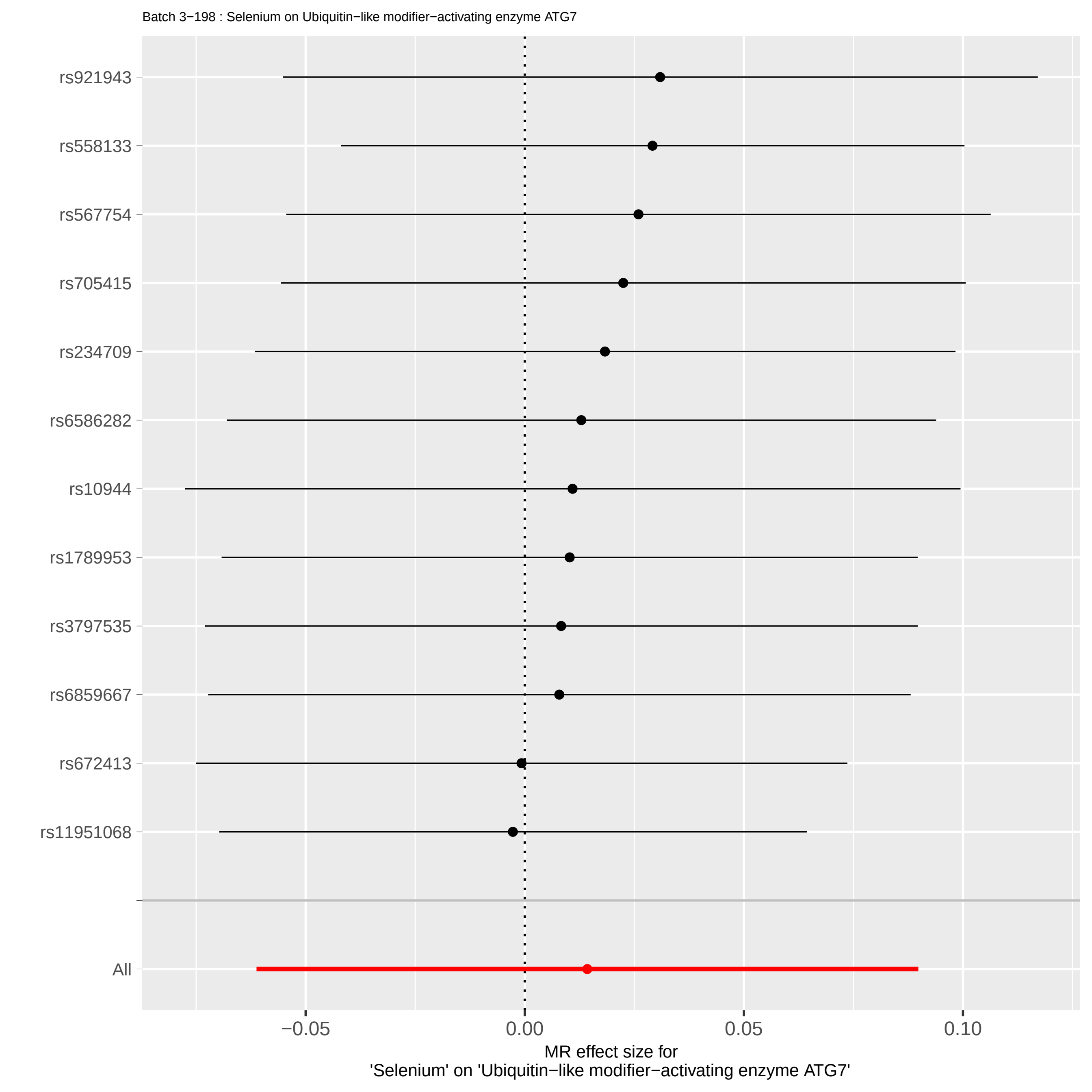

Batch 3−198 : Selenium on Ubiquitin−like modifier−activating enzyme ATG7
rs921943
rs558133
rs567754
rs705415
rs234709
rs6586282
rs10944
rs1789953
rs3797535
rs6859667
rs672413
rs11951068
All
−0.05
0.00
0.05
0.10
MR effect size for
'Selenium' on 'Ubiquitin−like modifier−activating enzyme ATG7'

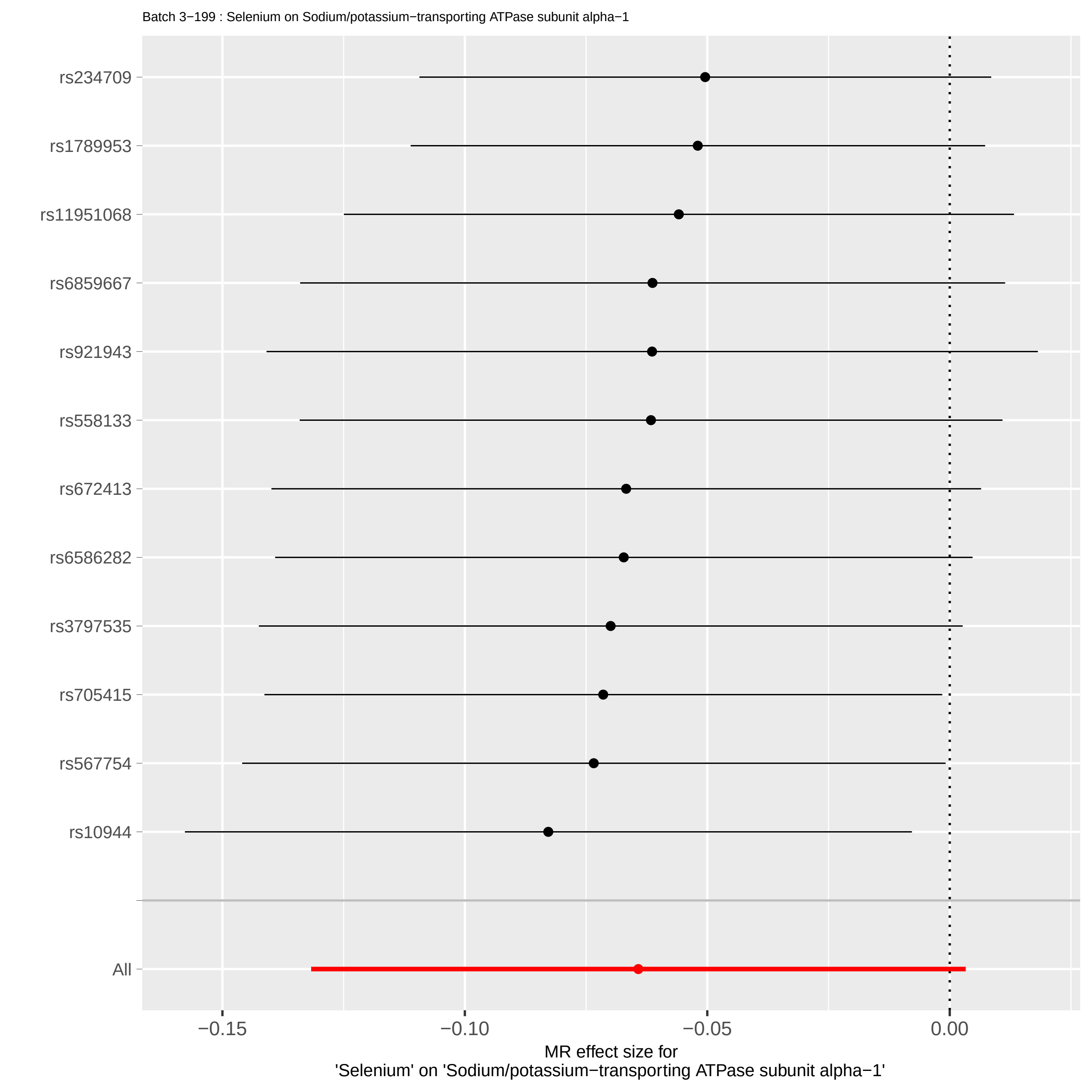
Batch 3−199 : Selenium on Sodium/potassium−transporting ATPase subunit alpha−1
rs234709
rs1789953
rs11951068
rs6859667
rs921943
rs558133
rs672413
rs6586282
rs3797535
rs705415
rs567754
rs10944
All
−0.15
−0.10
−0.05
0.00
MR effect size for
'Selenium' on 'Sodium/potassium−transporting ATPase subunit alpha−1'

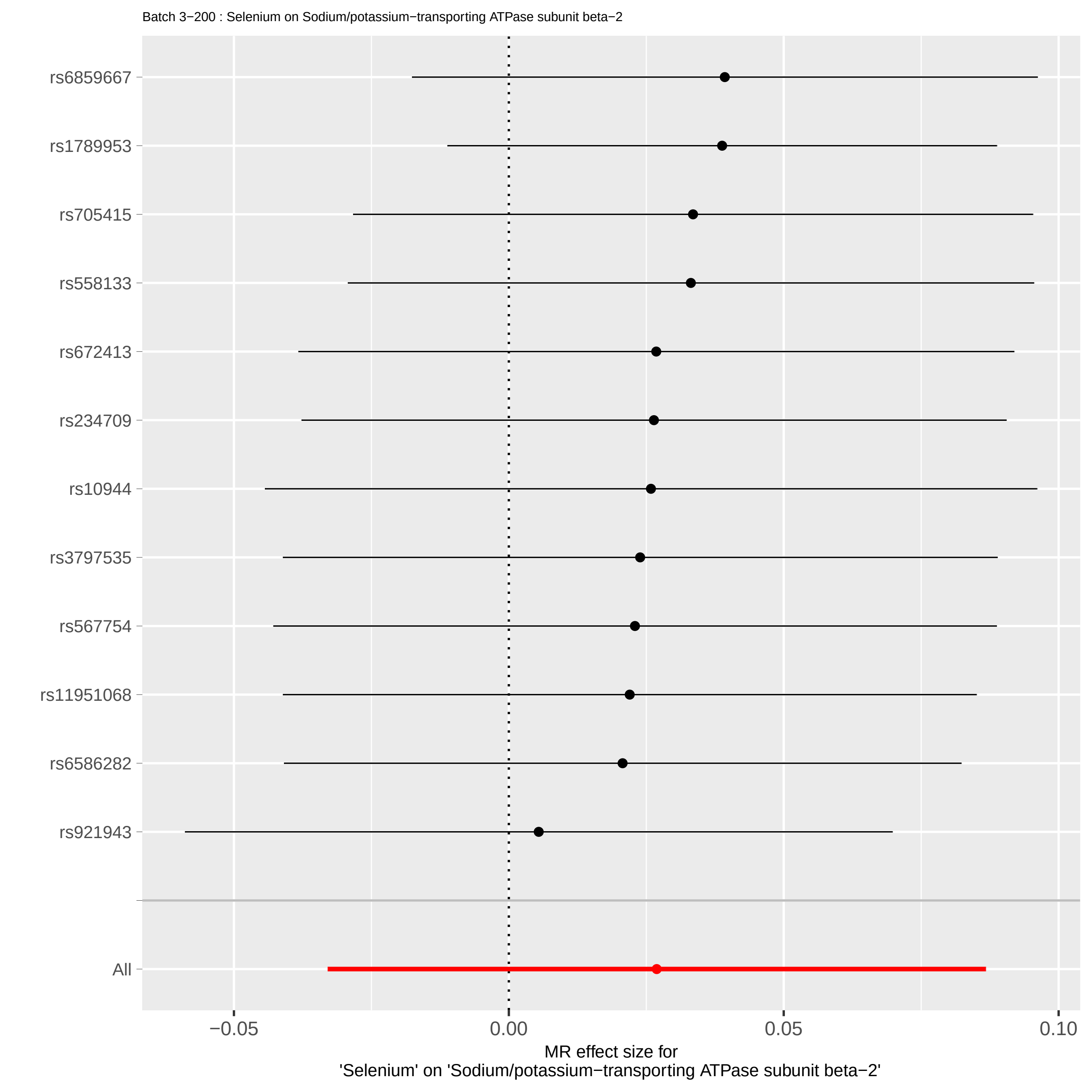

Batch 3−200 : Selenium on Sodium/potassium−transporting ATPase subunit beta−2
rs6859667
rs1789953
rs705415
rs558133
rs672413
rs234709
rs10944
rs3797535
rs567754
rs11951068
rs6586282
rs921943
All
−0.05
0.00
0.05
0.10
MR effect size for
'Selenium' on 'Sodium/potassium−transporting ATPase subunit beta−2'

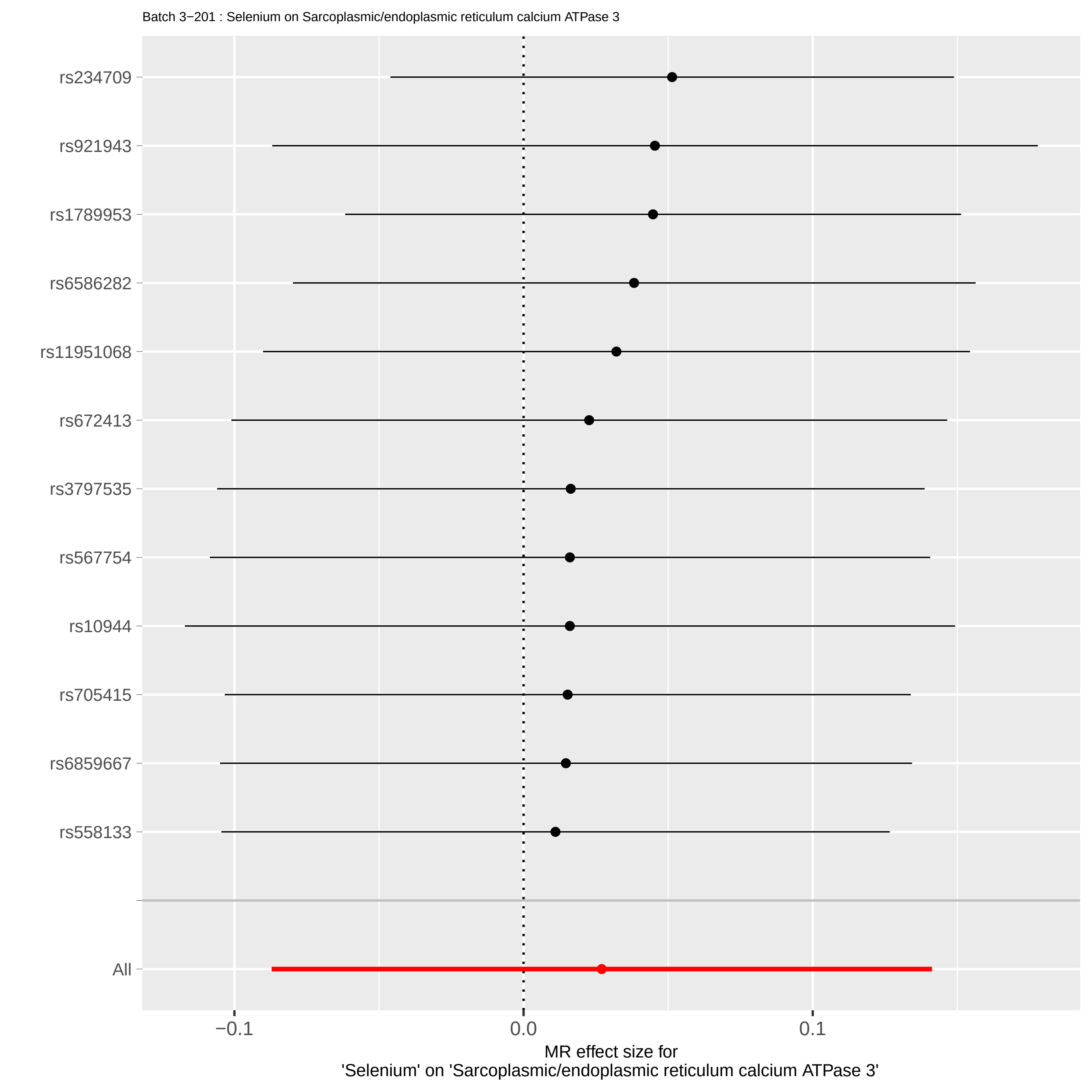

Batch 3−201 : Selenium on Sarcoplasmic/endoplasmic reticulum calcium ATPase 3
rs234709
rs921943
rs1789953
rs6586282
rs11951068
rs672413
rs3797535
rs567754
rs10944
rs705415
rs6859667
rs558133
All
−0.1
0.0
0.1
MR effect size for
'Selenium' on 'Sarcoplasmic/endoplasmic reticulum calcium ATPase 3'

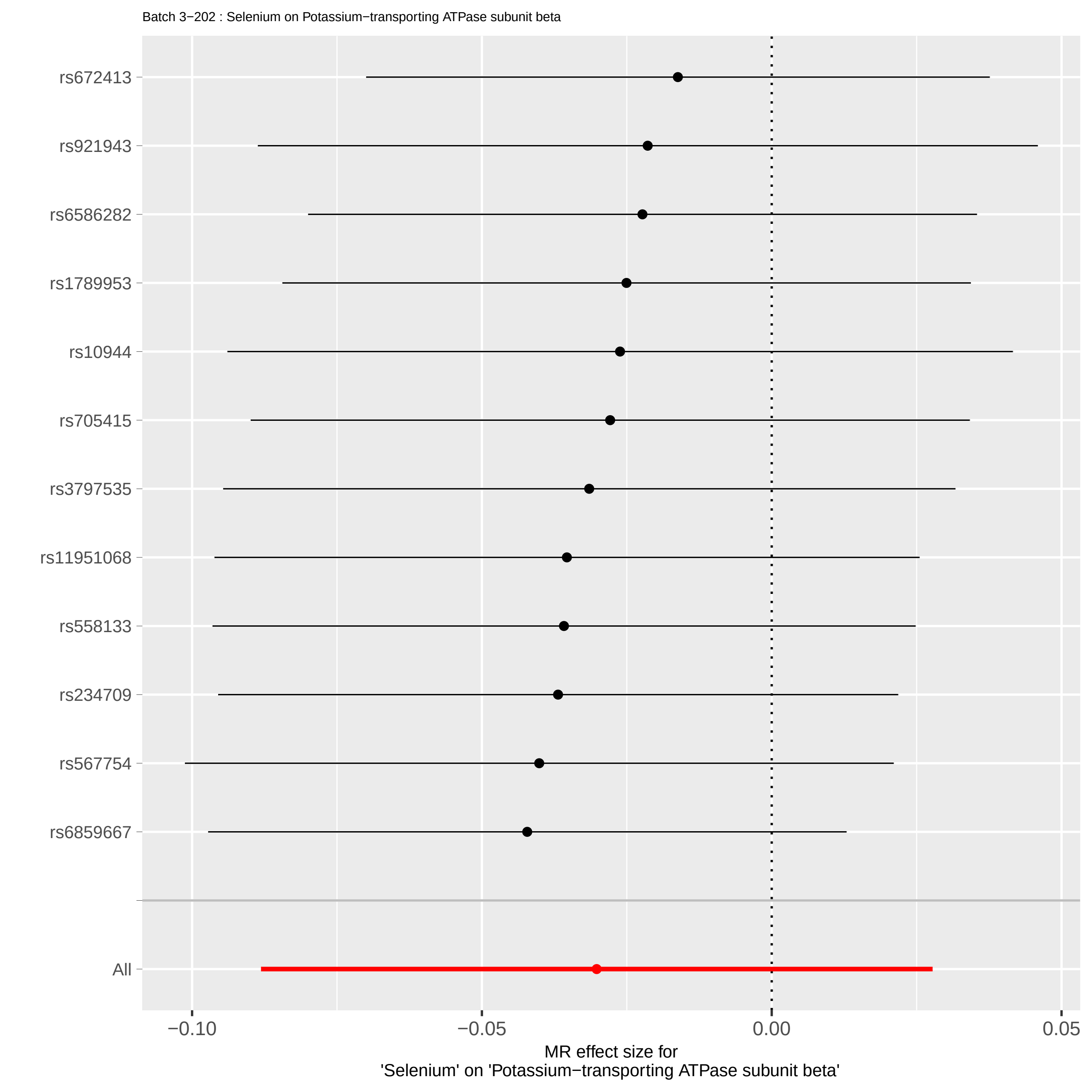
Batch 3−202 : Selenium on Potassium−transporting ATPase subunit beta
rs672413
rs921943
rs6586282
rs1789953
rs10944
rs705415
rs3797535
rs11951068
rs558133
rs234709
rs567754
rs6859667
All
−0.10
−0.05
0.00
0.05
MR effect size for
'Selenium' on 'Potassium−transporting ATPase subunit beta'

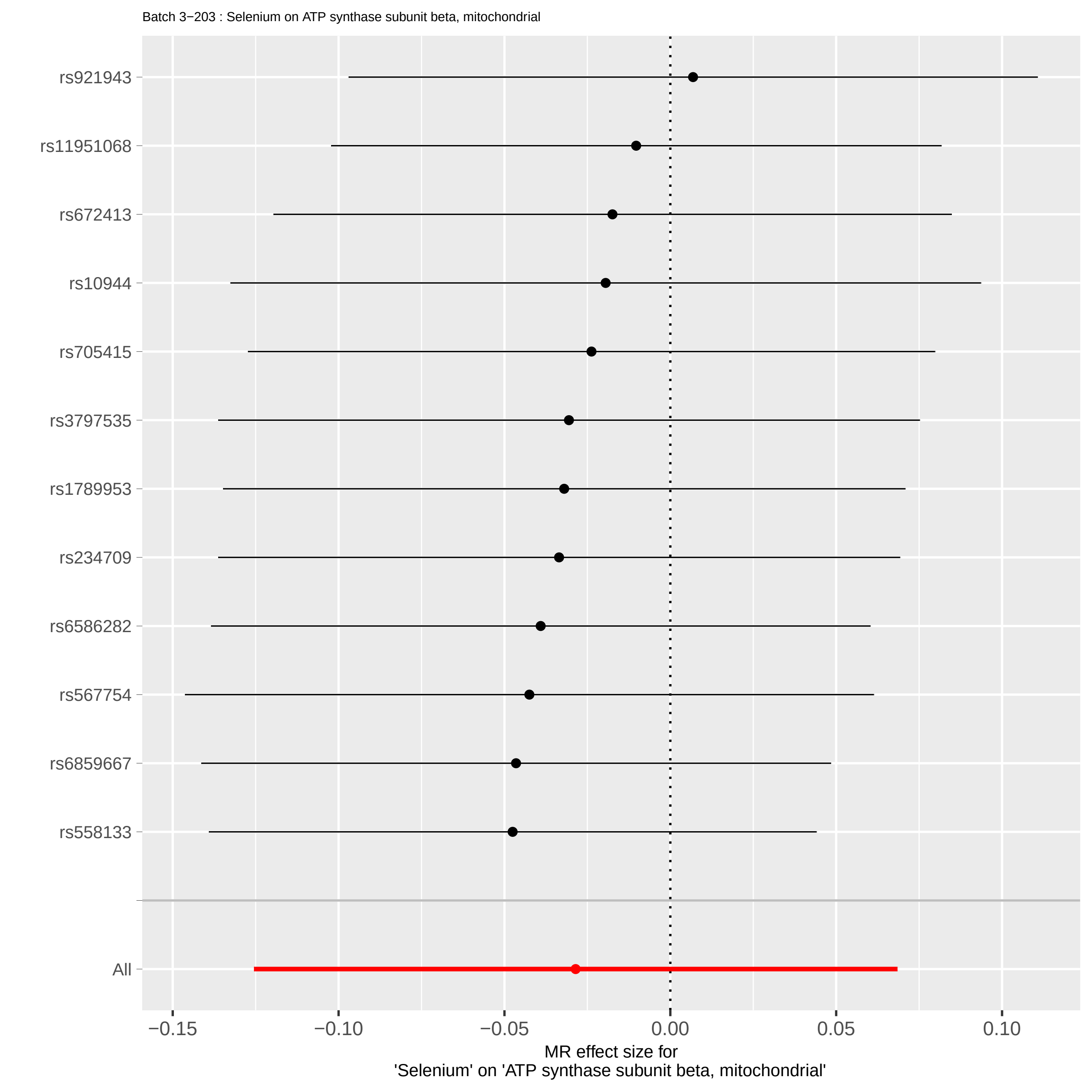

Batch 3−203 : Selenium on ATP synthase subunit beta, mitochondrial
rs921943
rs11951068
rs672413
rs10944
rs705415
rs3797535
rs1789953
rs234709
rs6586282
rs567754
rs6859667
rs558133
All
−0.15
−0.10
−0.05
0.00
0.05
0.10
MR effect size for
'Selenium' on 'ATP synthase subunit beta, mitochondrial'

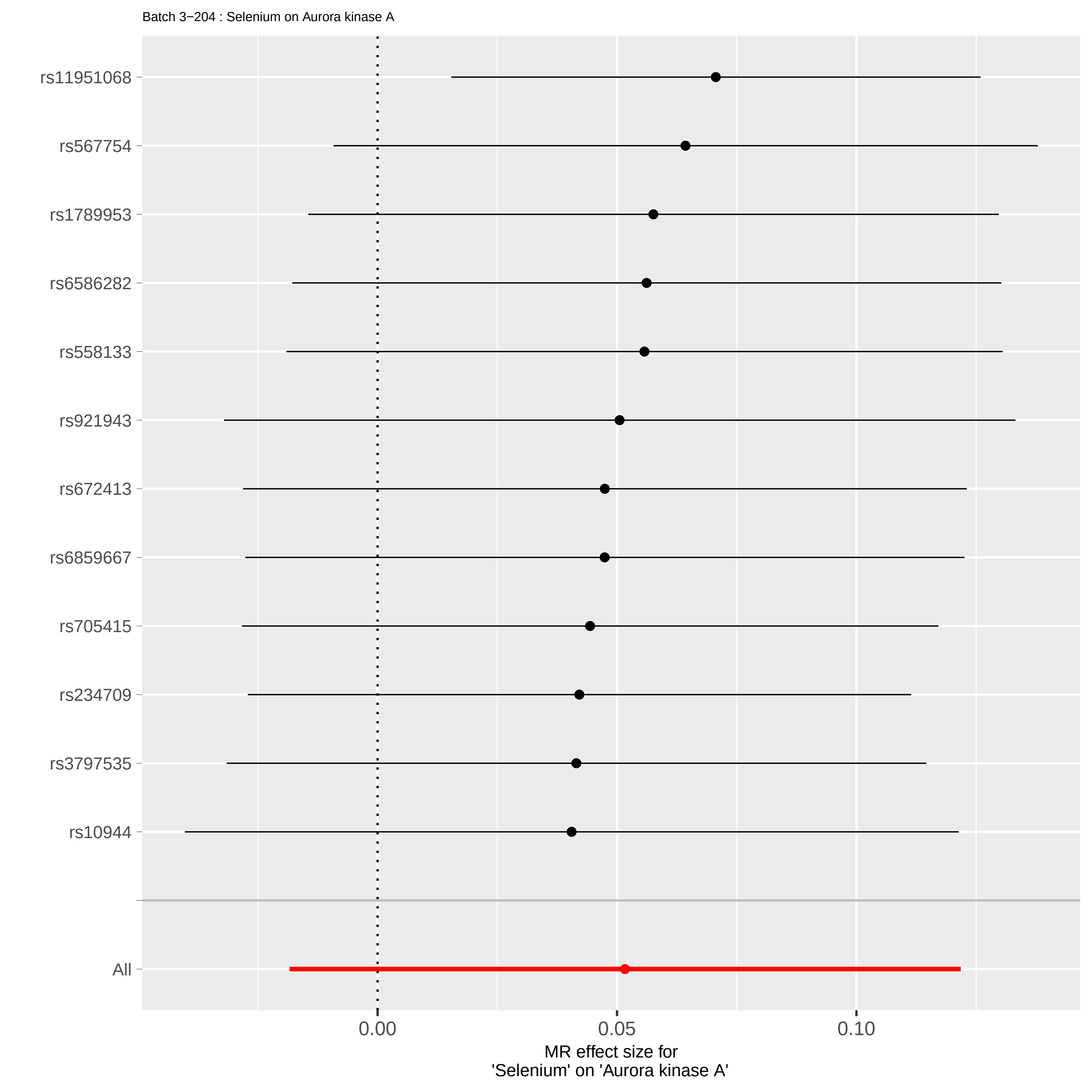

Batch 3−204 : Selenium on Aurora kinase A
rs11951068
rs567754
rs1789953
rs6586282
rs558133
rs921943
rs672413
rs6859667
rs705415
rs234709
rs3797535
rs10944
All
0.00
0.05
0.10
MR effect size for
'Selenium' on 'Aurora kinase A'

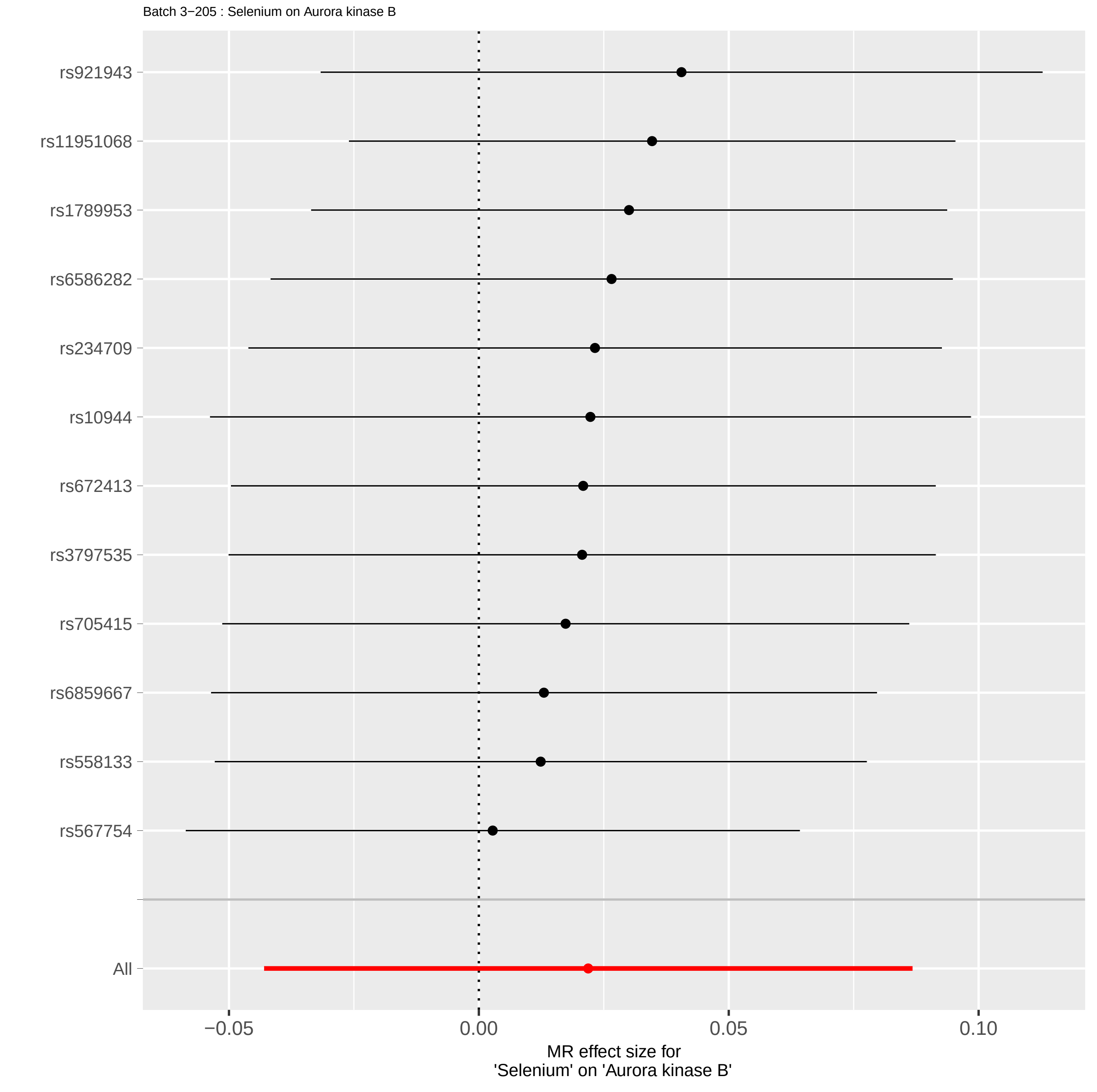
Batch 3−205 : Selenium on Aurora kinase B
rs921943
rs11951068
rs1789953
rs6586282
rs234709
rs10944
rs672413
rs3797535
rs705415
rs6859667
rs558133
rs567754
All
−0.05
0.00
0.05
0.10
MR effect size for
'Selenium' on 'Aurora kinase B'

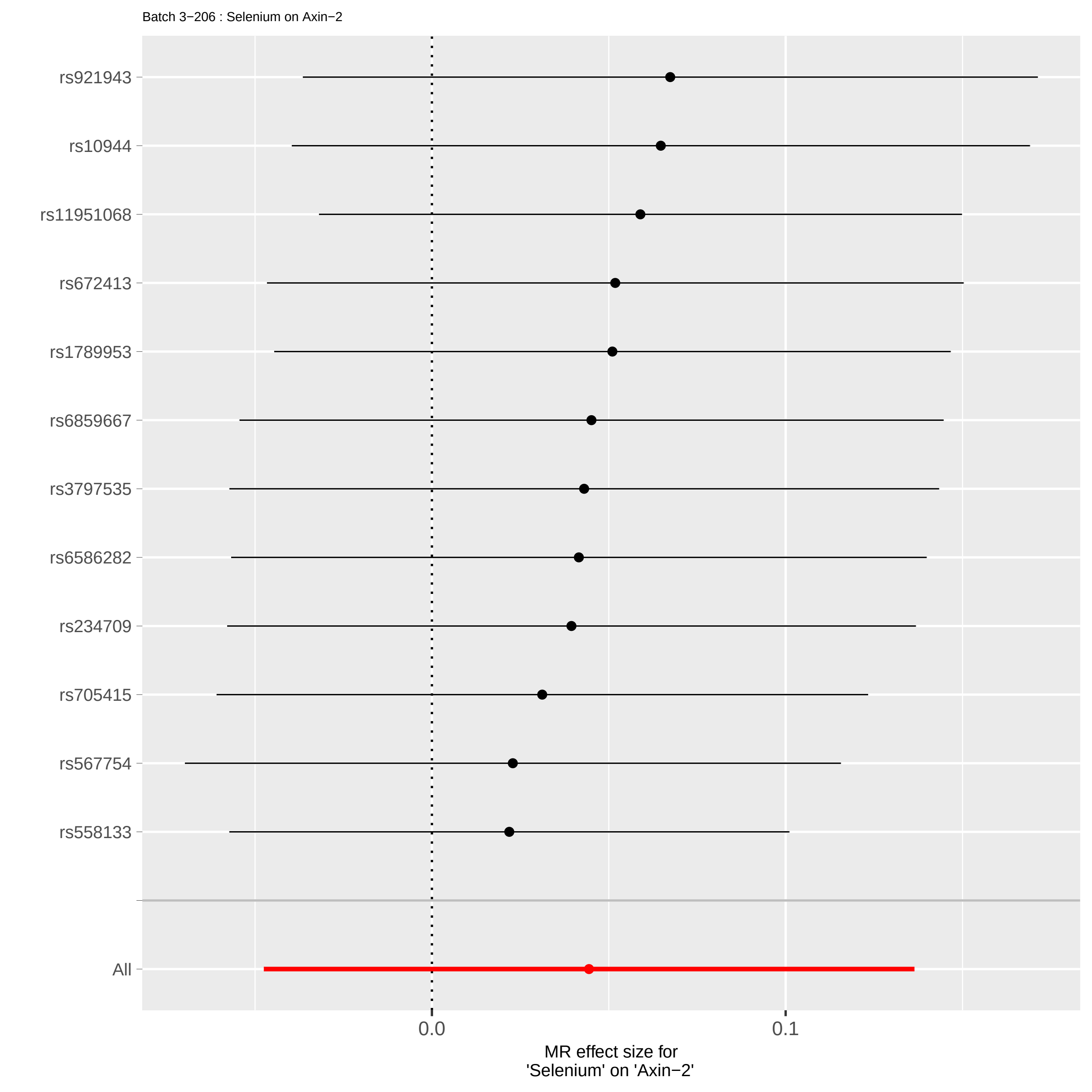
Batch 3−206 : Selenium on Axin−2
rs921943
rs10944
rs11951068
rs672413
rs1789953
rs6859667
rs3797535
rs6586282
rs234709
rs705415
rs567754
rs558133
All
0.0
0.1
MR effect size for
'Selenium' on 'Axin−2'

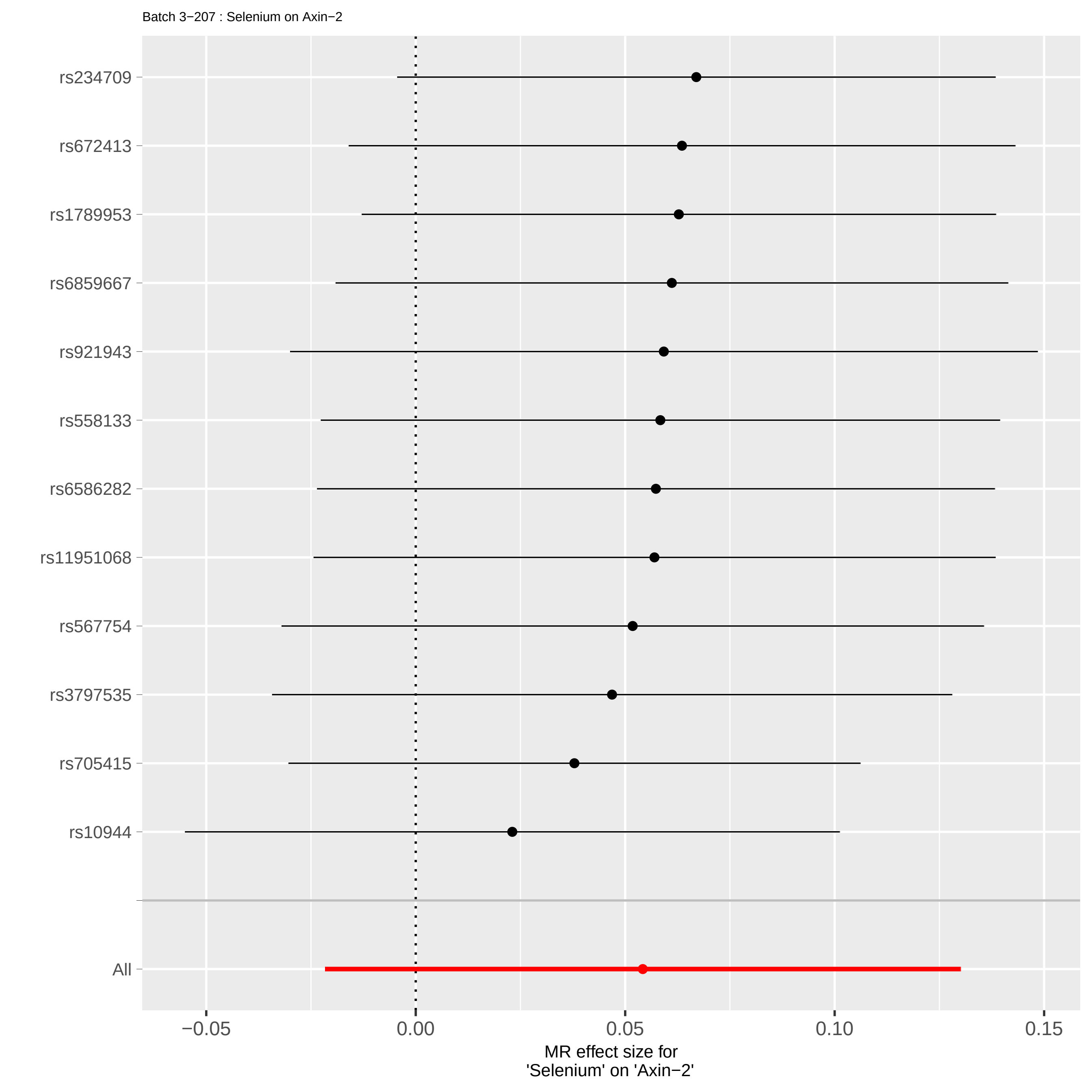

Batch 3−207 : Selenium on Axin−2
rs234709
rs672413
rs1789953
rs6859667
rs921943
rs558133
rs6586282
rs11951068
rs567754
rs3797535
rs705415
rs10944
All
−0.05
0.00
0.05
0.10
0.15
MR effect size for
'Selenium' on 'Axin−2'

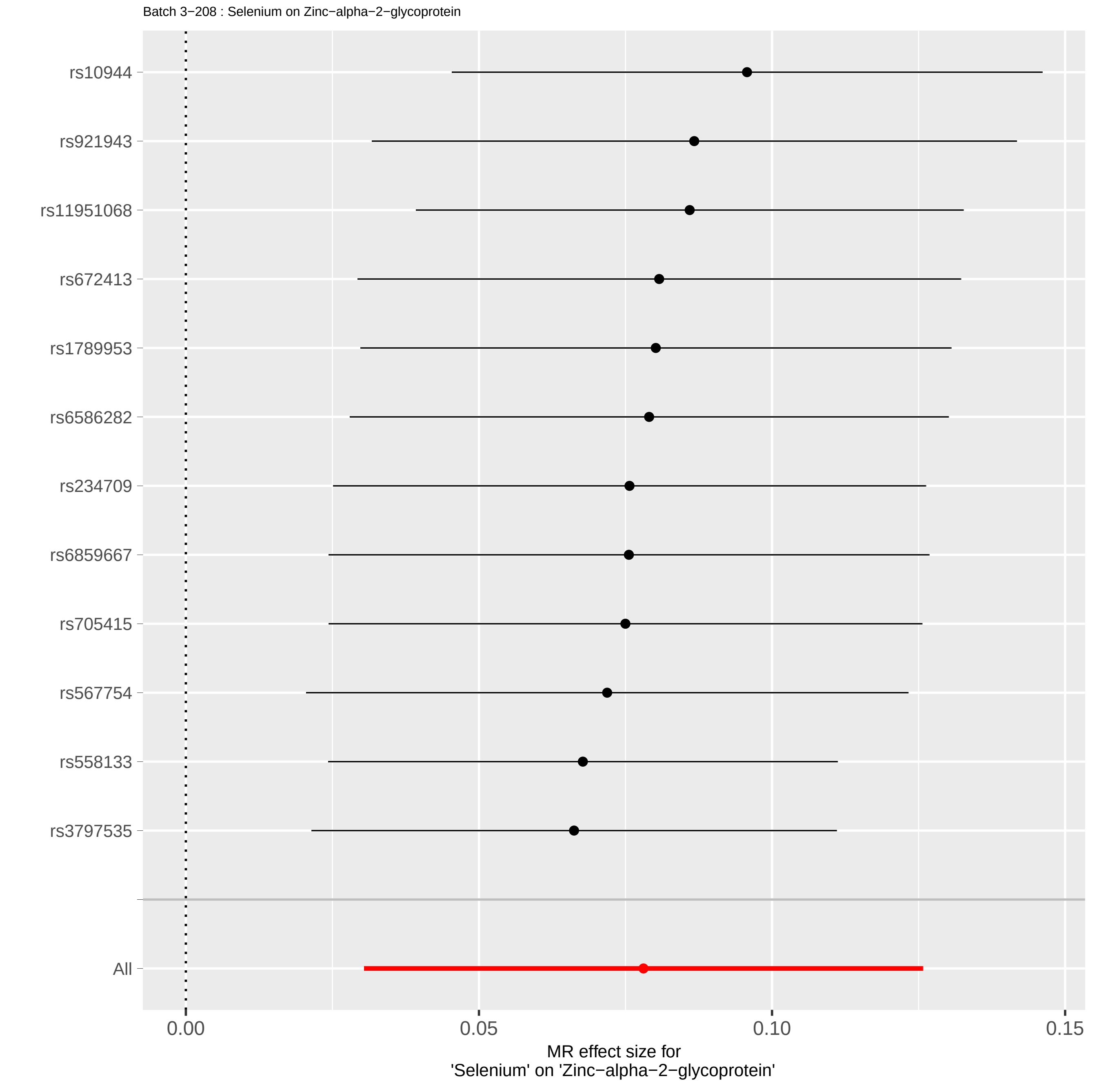

Batch 3−208 : Selenium on Zinc−alpha−2−glycoprotein
rs10944
rs921943
rs11951068
rs672413
rs1789953
rs6586282
rs234709
rs6859667
rs705415
rs567754
rs558133
rs3797535
All
0.00
0.05
0.10
0.15
MR effect size for
'Selenium' on 'Zinc−alpha−2−glycoprotein'

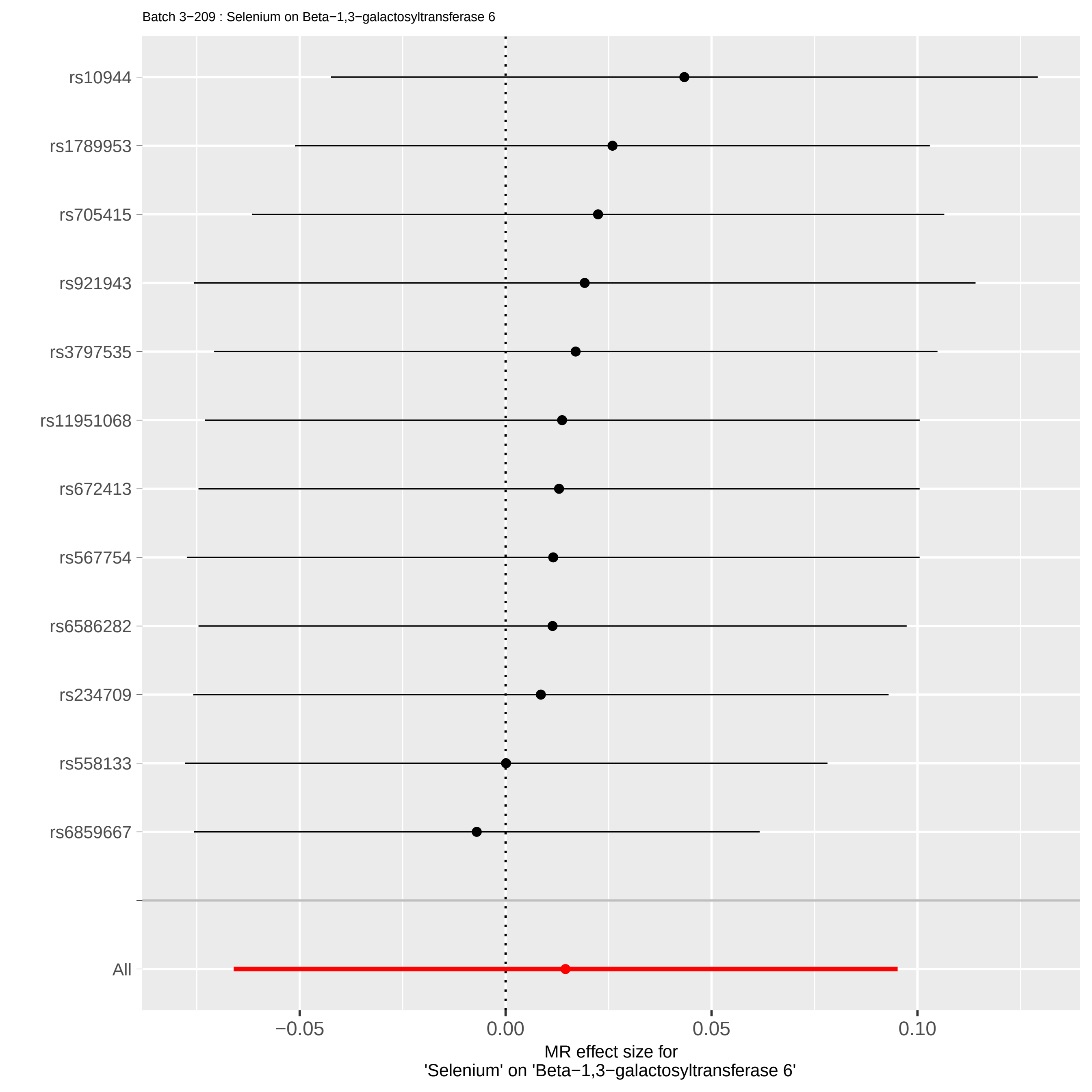

Batch 3−209 : Selenium on Beta−1,3−galactosyltransferase 6
rs10944
rs1789953
rs705415
rs921943
rs3797535
rs11951068
rs672413
rs567754
rs6586282
rs234709
rs558133
rs6859667
All
−0.05
0.00
0.05
0.10
MR effect size for
'Selenium' on 'Beta−1,3−galactosyltransferase 6'

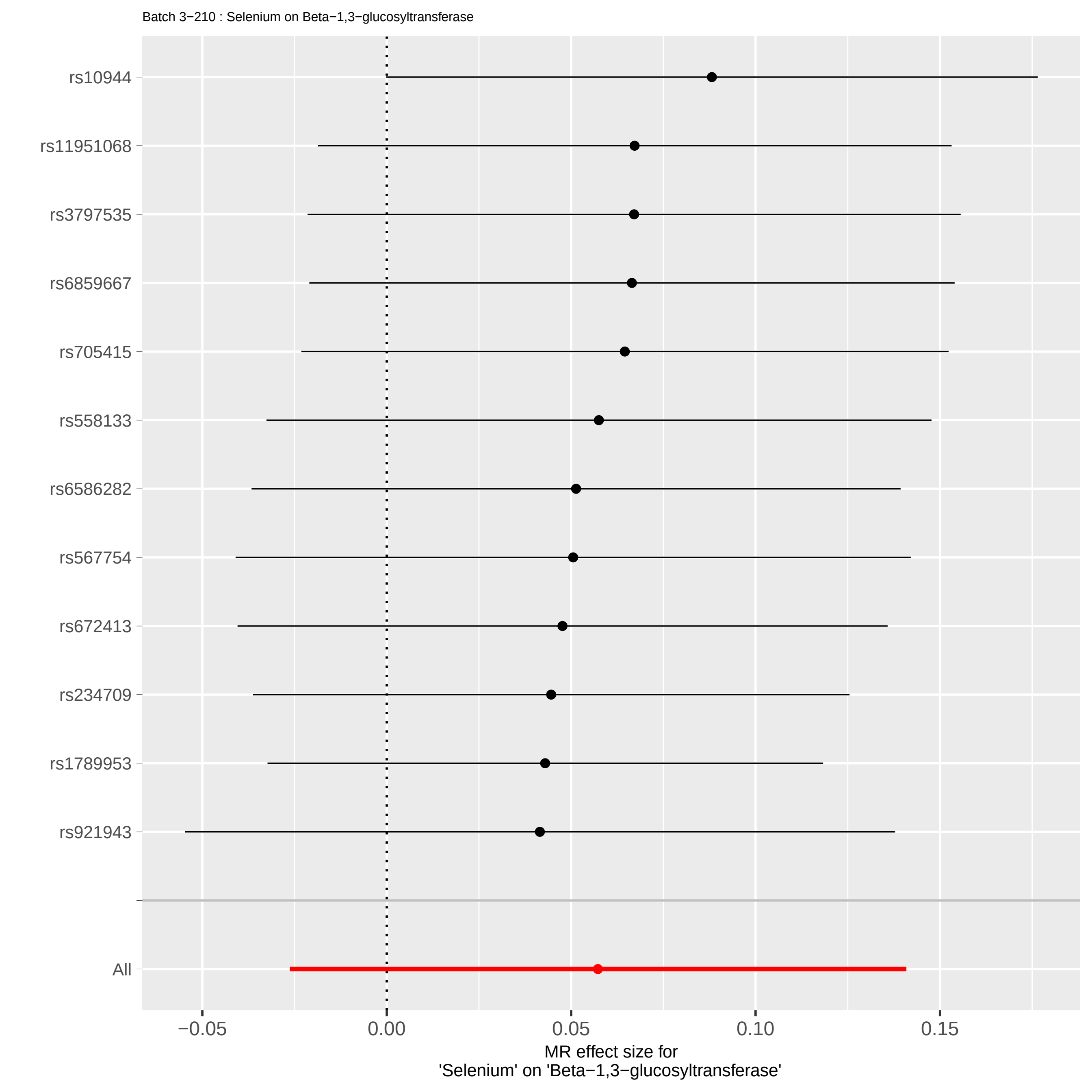
Batch 3−210 : Selenium on Beta−1,3−glucosyltransferase
rs10944
rs11951068
rs3797535
rs6859667
rs705415
rs558133
rs6586282
rs567754
rs672413
rs234709
rs1789953
rs921943
All
−0.05
0.00
0.05
0.10
0.15
MR effect size for
'Selenium' on 'Beta−1,3−glucosyltransferase'

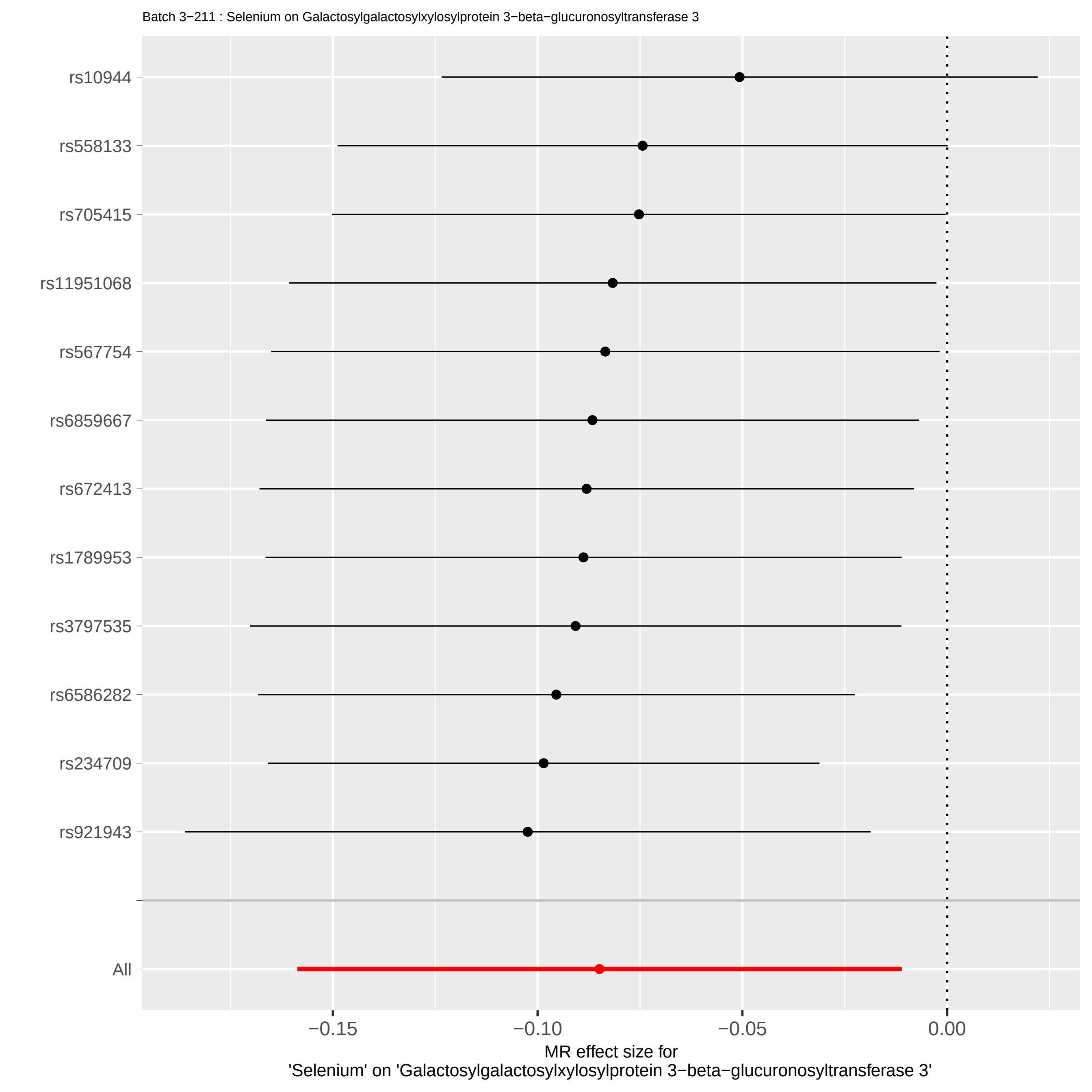

Batch 3−211 : Selenium on Galactosylgalactosylxylosylprotein 3−beta−glucuronosyltransferase 3
rs10944
rs558133
rs705415
rs11951068
rs567754
rs6859667
rs672413
rs1789953
rs3797535
rs6586282
rs234709
rs921943
All
−0.15
−0.10
−0.05
0.00
MR effect size for
'Selenium' on 'Galactosylgalactosylxylosylprotein 3−beta−glucuronosyltransferase 3'

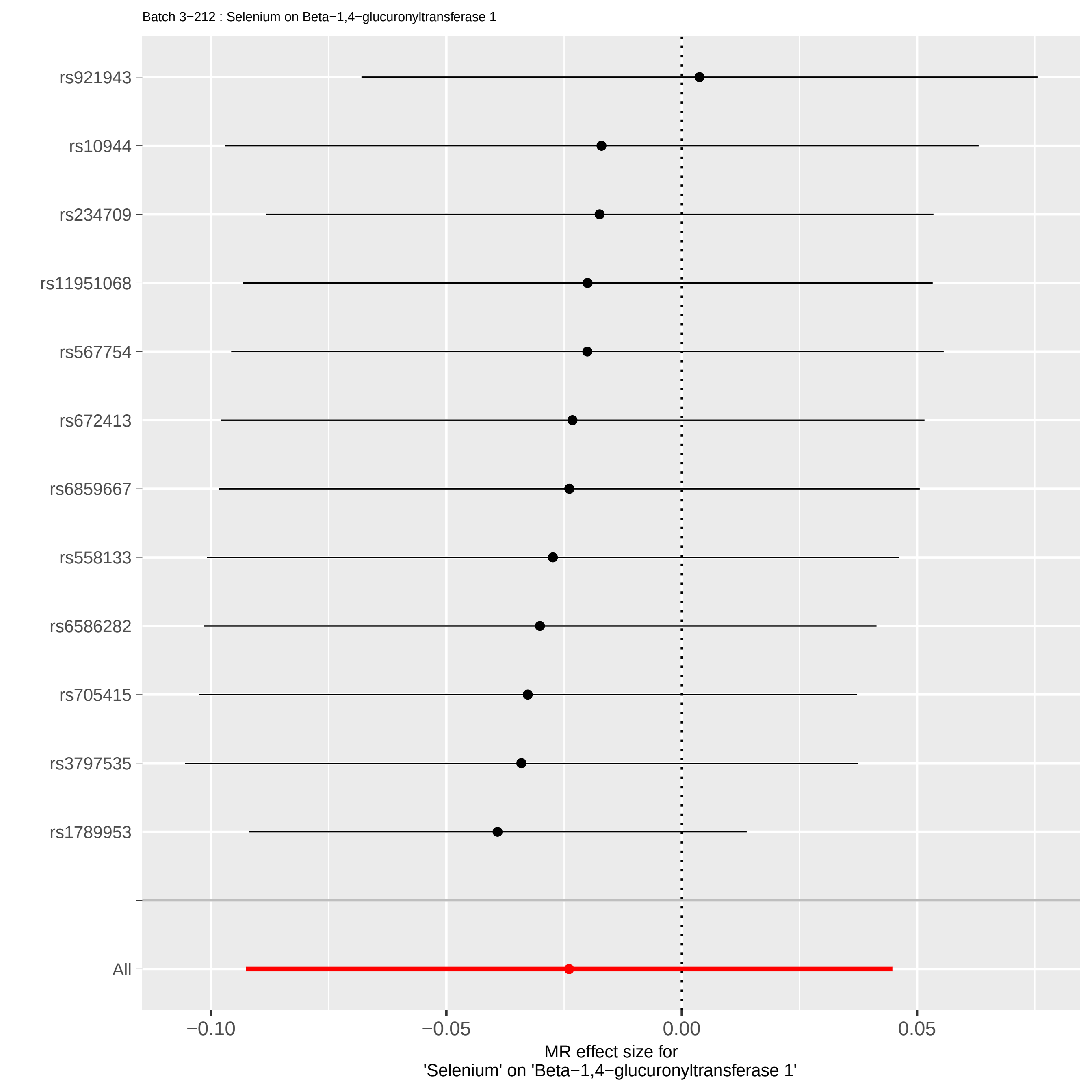

Batch 3−212 : Selenium on Beta−1,4−glucuronyltransferase 1
rs921943
rs10944
rs234709
rs11951068
rs567754
rs672413
rs6859667
rs558133
rs6586282
rs705415
rs3797535
rs1789953
All
−0.10
−0.05
0.00
0.05
MR effect size for
'Selenium' on 'Beta−1,4−glucuronyltransferase 1'

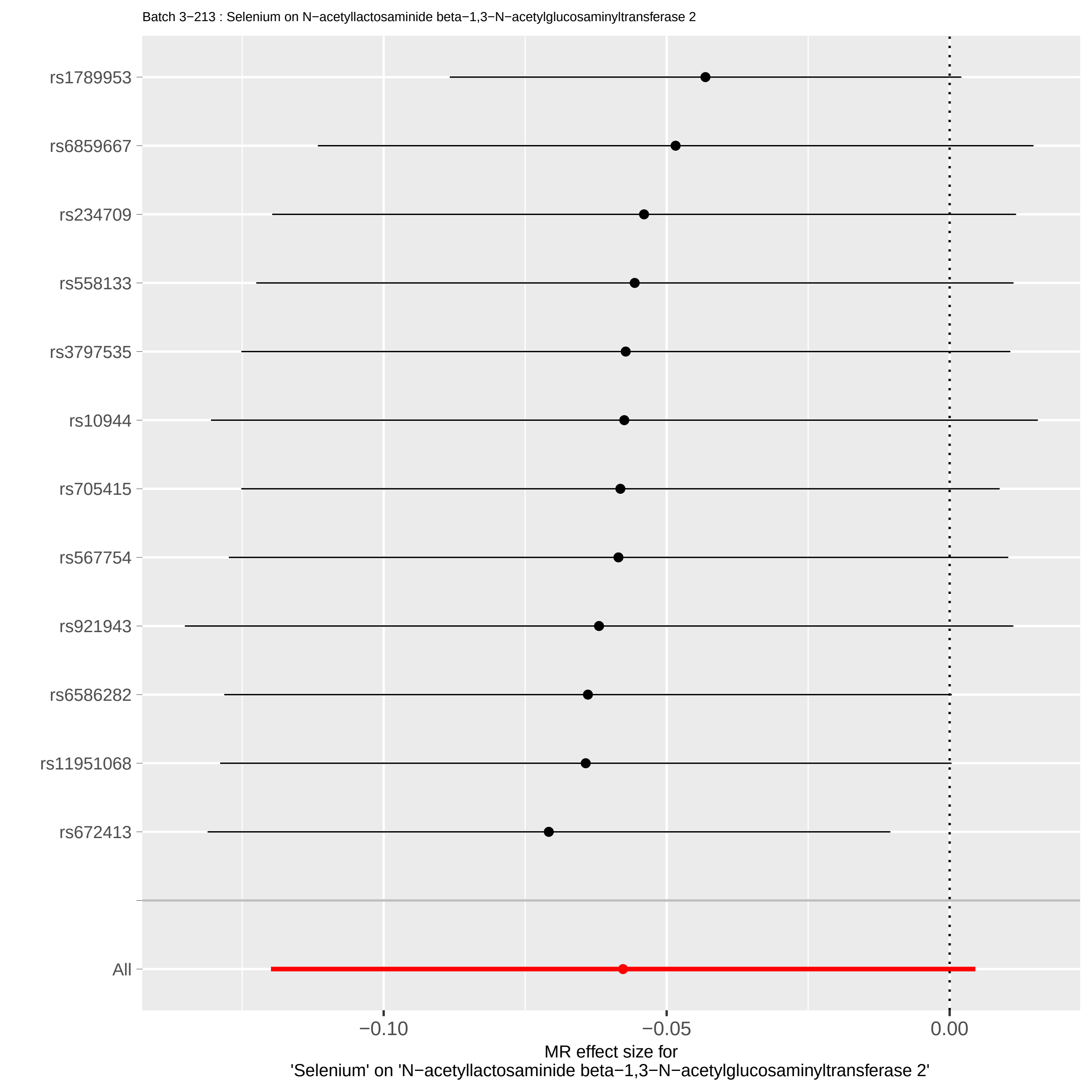

Batch 3−213 : Selenium on N−acetyllactosaminide beta−1,3−N−acetylglucosaminyltransferase 2
rs1789953
rs6859667
rs234709
rs558133
rs3797535
rs10944
rs705415
rs567754
rs921943
rs6586282
rs11951068
rs672413
All
−0.10
−0.05
0.00
MR effect size for
'Selenium' on 'N−acetyllactosaminide beta−1,3−N−acetylglucosaminyltransferase 2'

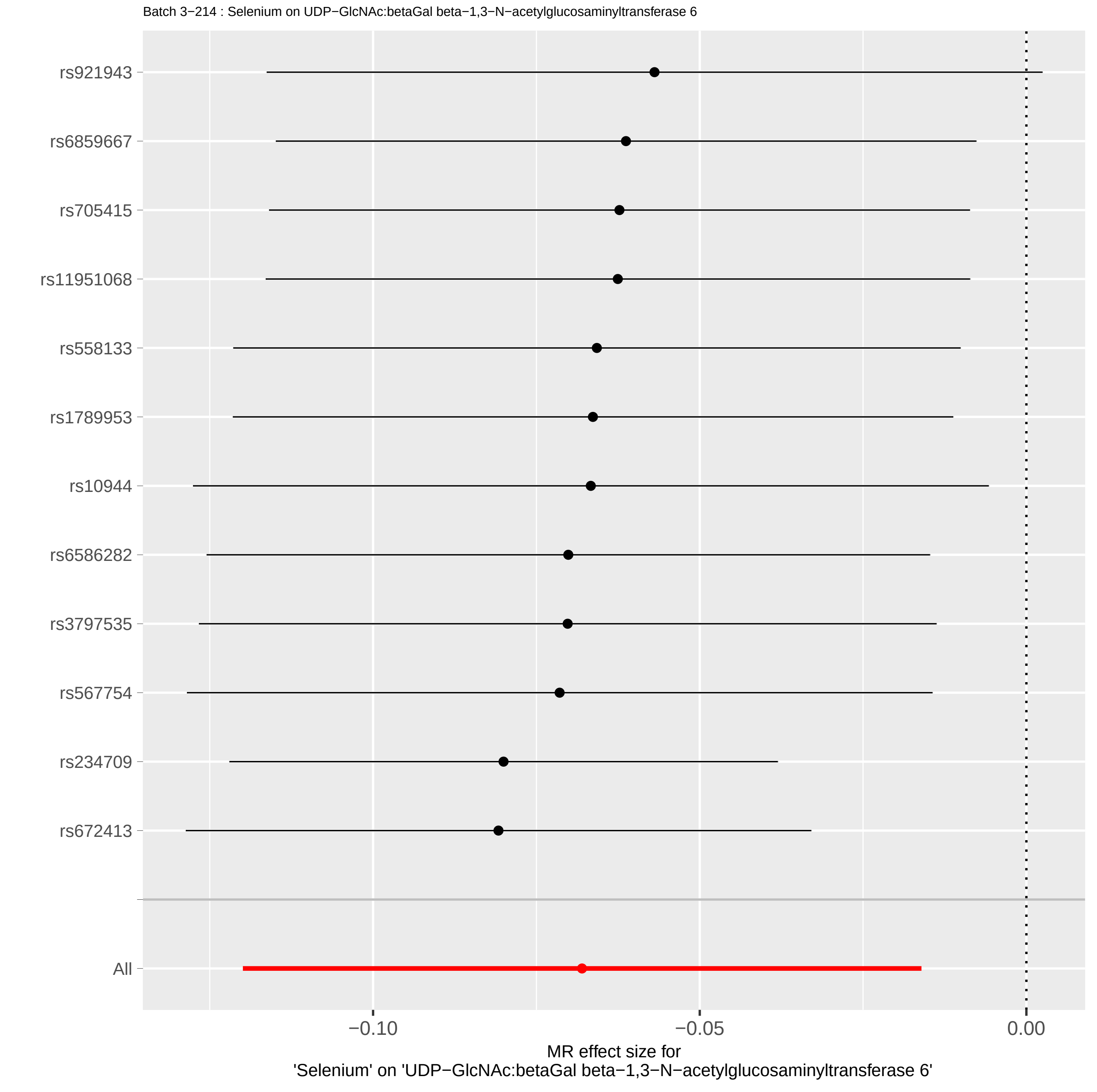

Batch 3−214 : Selenium on UDP−GlcNAc:betaGal beta−1,3−N−acetylglucosaminyltransferase 6
rs921943
rs6859667
rs705415
rs11951068
rs558133
rs1789953
rs10944
rs6586282
rs3797535
rs567754
rs234709
rs672413
All
−0.10
−0.05
0.00
MR effect size for
'Selenium' on 'UDP−GlcNAc:betaGal beta−1,3−N−acetylglucosaminyltransferase 6'

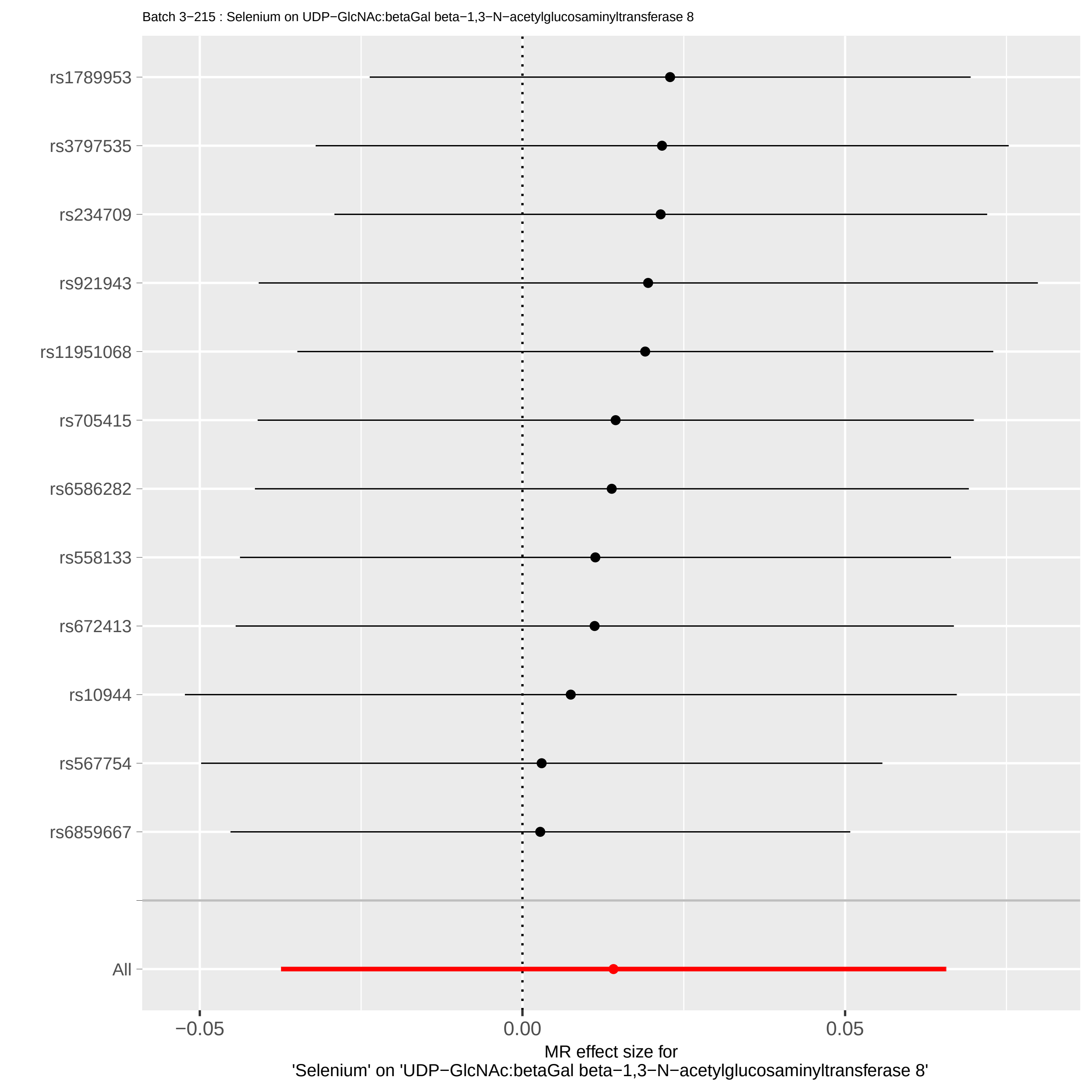

Batch 3−215 : Selenium on UDP−GlcNAc:betaGal beta−1,3−N−acetylglucosaminyltransferase 8
rs1789953
rs3797535
rs234709
rs921943
rs11951068
rs705415
rs6586282
rs558133
rs672413
rs10944
rs567754
rs6859667
All
−0.05
0.00
0.05
MR effect size for
'Selenium' on 'UDP−GlcNAc:betaGal beta−1,3−N−acetylglucosaminyltransferase 8'

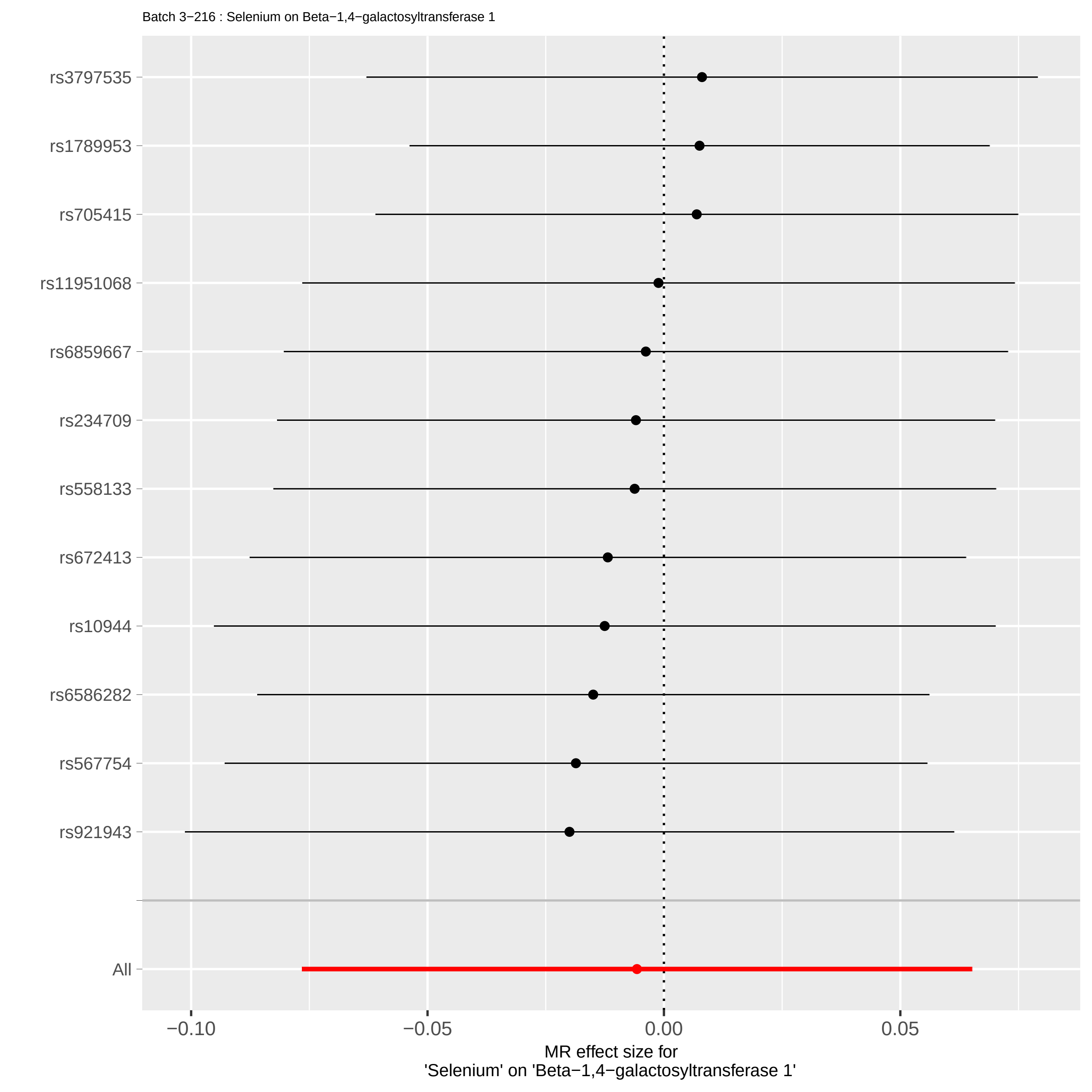

Batch 3−216 : Selenium on Beta−1,4−galactosyltransferase 1
rs3797535
rs1789953
rs705415
rs11951068
rs6859667
rs234709
rs558133
rs672413
rs10944
rs6586282
rs567754
rs921943
All
−0.10
−0.05
0.00
0.05
MR effect size for
'Selenium' on 'Beta−1,4−galactosyltransferase 1'

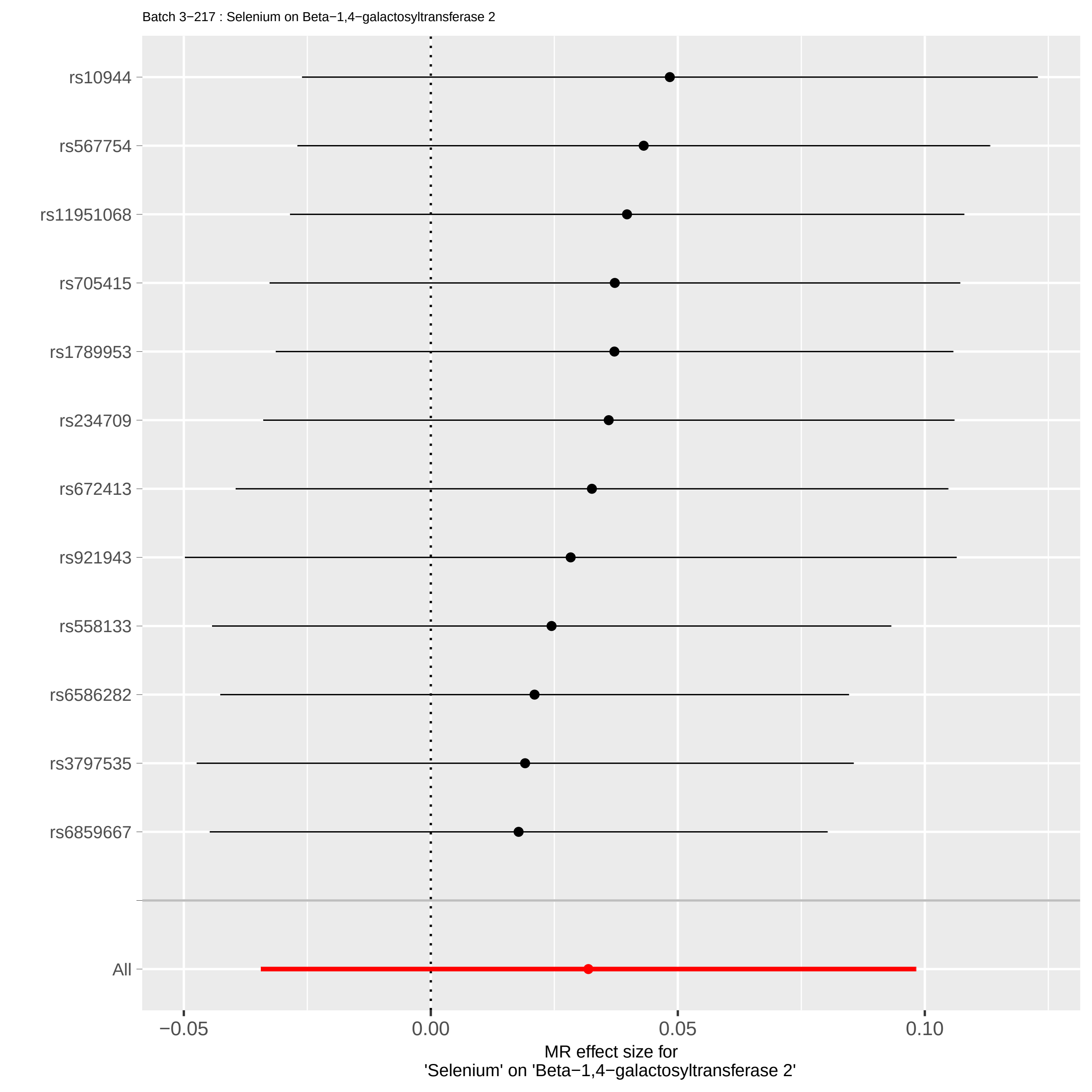

Batch 3−217 : Selenium on Beta−1,4−galactosyltransferase 2
rs10944
rs567754
rs11951068
rs705415
rs1789953
rs234709
rs672413
rs921943
rs558133
rs6586282
rs3797535
rs6859667
All
−0.05
0.00
0.05
0.10
MR effect size for
'Selenium' on 'Beta−1,4−galactosyltransferase 2'

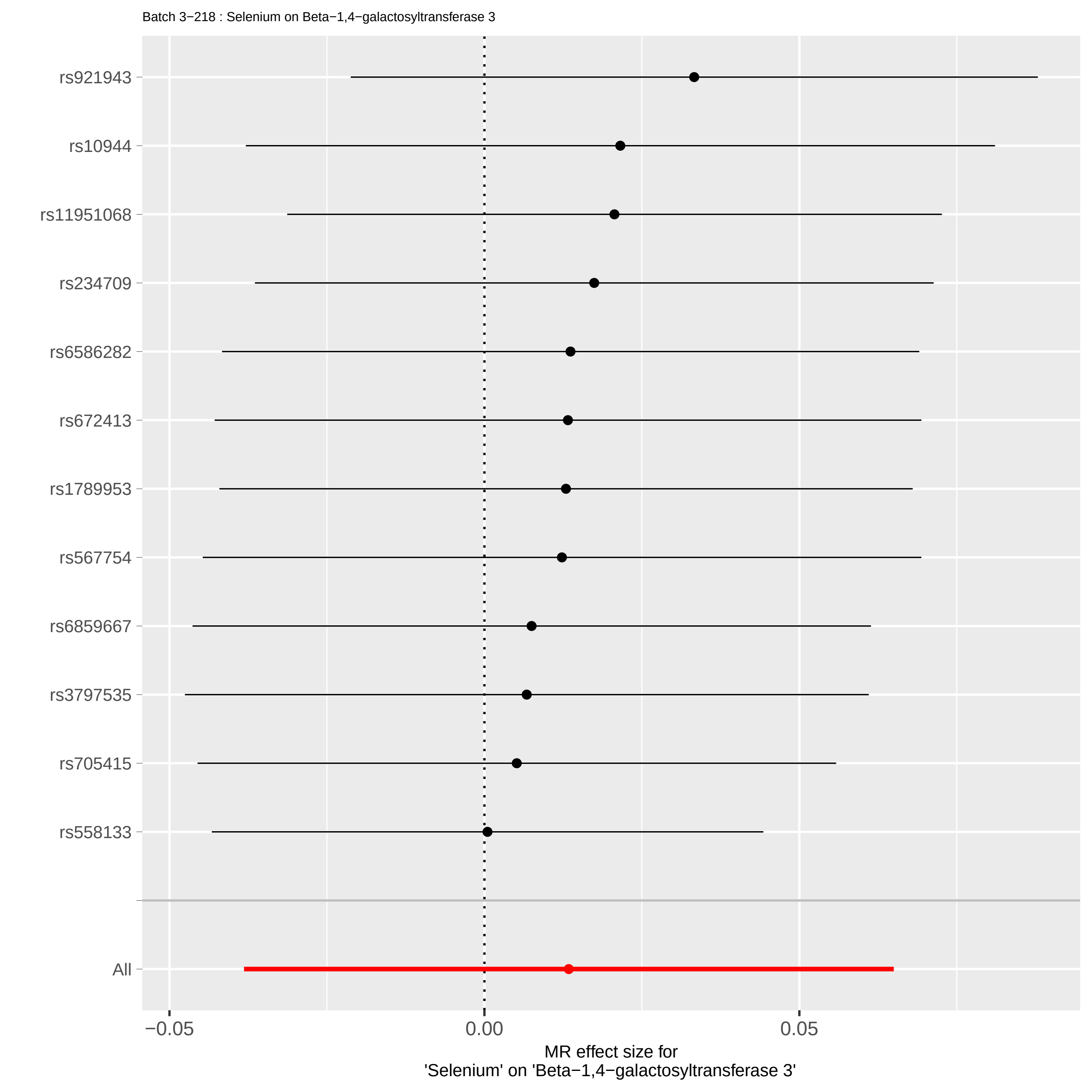

Batch 3−218 : Selenium on Beta−1,4−galactosyltransferase 3
rs921943
rs10944
rs11951068
rs234709
rs6586282
rs672413
rs1789953
rs567754
rs6859667
rs3797535
rs705415
rs558133
All
−0.05
0.00
0.05
MR effect size for
'Selenium' on 'Beta−1,4−galactosyltransferase 3'

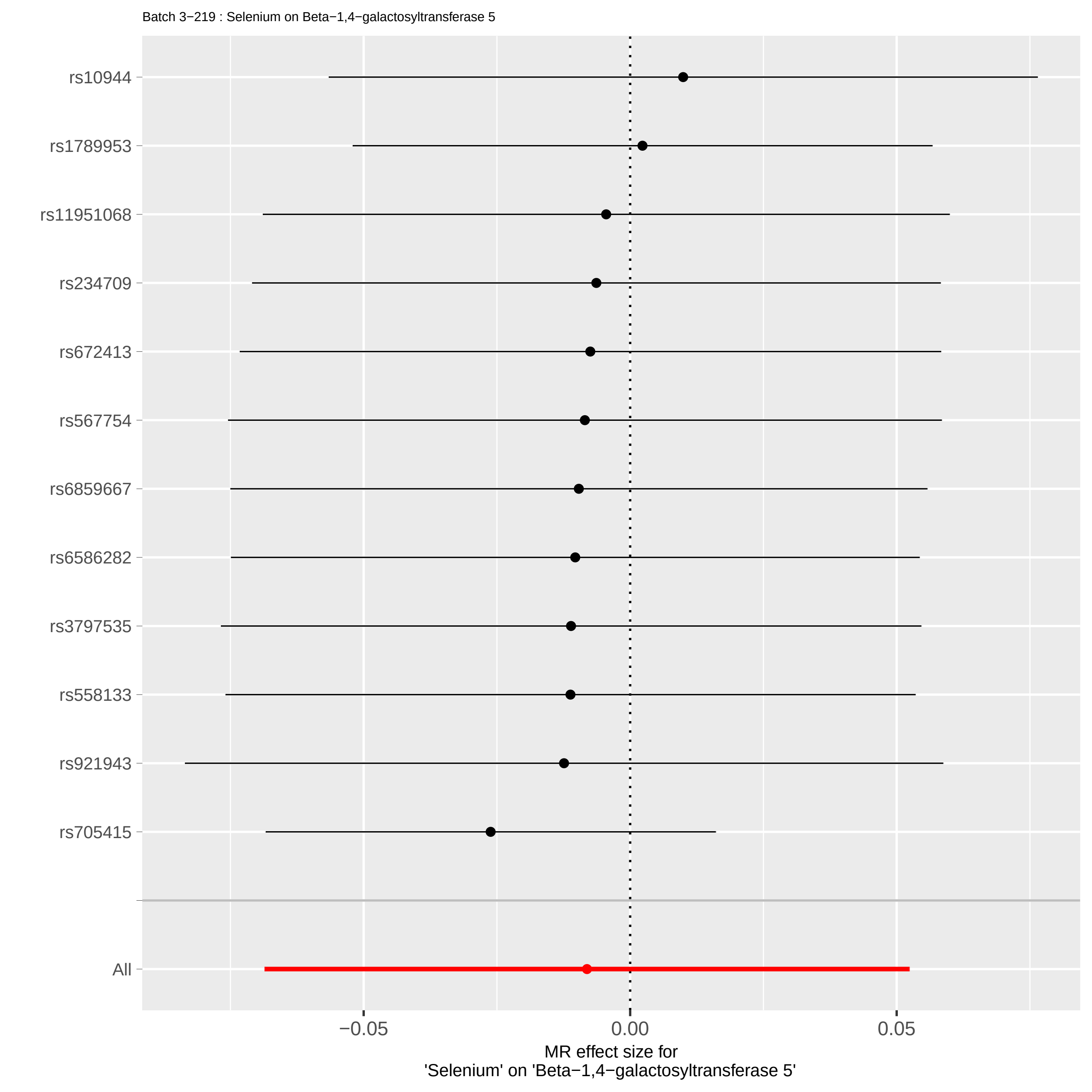

Batch 3−219 : Selenium on Beta−1,4−galactosyltransferase 5
rs10944
rs1789953
rs11951068
rs234709
rs672413
rs567754
rs6859667
rs6586282
rs3797535
rs558133
rs921943
rs705415
All
−0.05
0.00
0.05
MR effect size for
'Selenium' on 'Beta−1,4−galactosyltransferase 5'

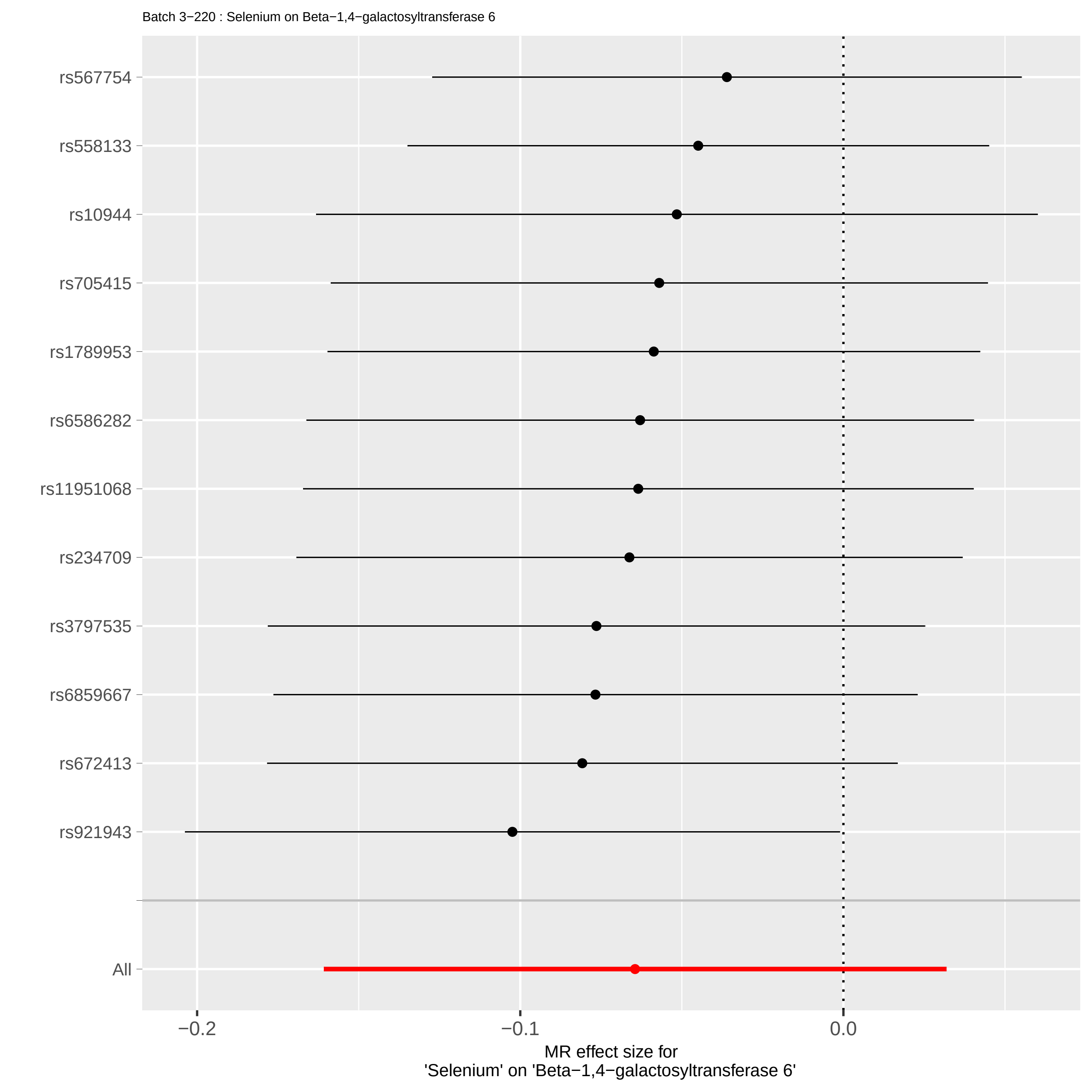

Batch 3−220 : Selenium on Beta−1,4−galactosyltransferase 6
rs567754
rs558133
rs10944
rs705415
rs1789953
rs6586282
rs11951068
rs234709
rs3797535
rs6859667
rs672413
rs921943
All
−0.2
−0.1
0.0
MR effect size for
'Selenium' on 'Beta−1,4−galactosyltransferase 6'

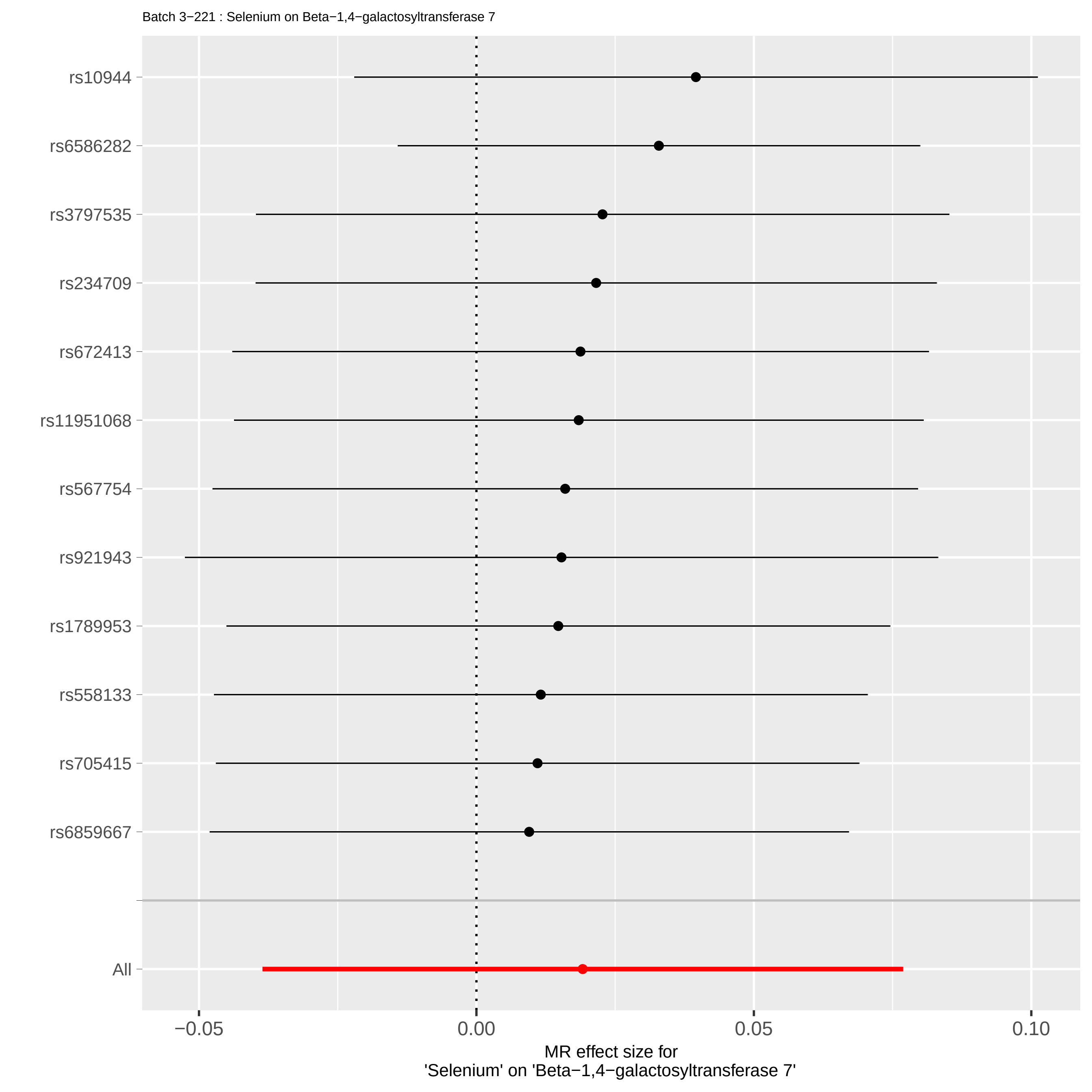

Batch 3−221 : Selenium on Beta−1,4−galactosyltransferase 7
rs10944
rs6586282
rs3797535
rs234709
rs672413
rs11951068
rs567754
rs921943
rs1789953
rs558133
rs705415
rs6859667
All
−0.05
0.00
0.05
0.10
MR effect size for
'Selenium' on 'Beta−1,4−galactosyltransferase 7'

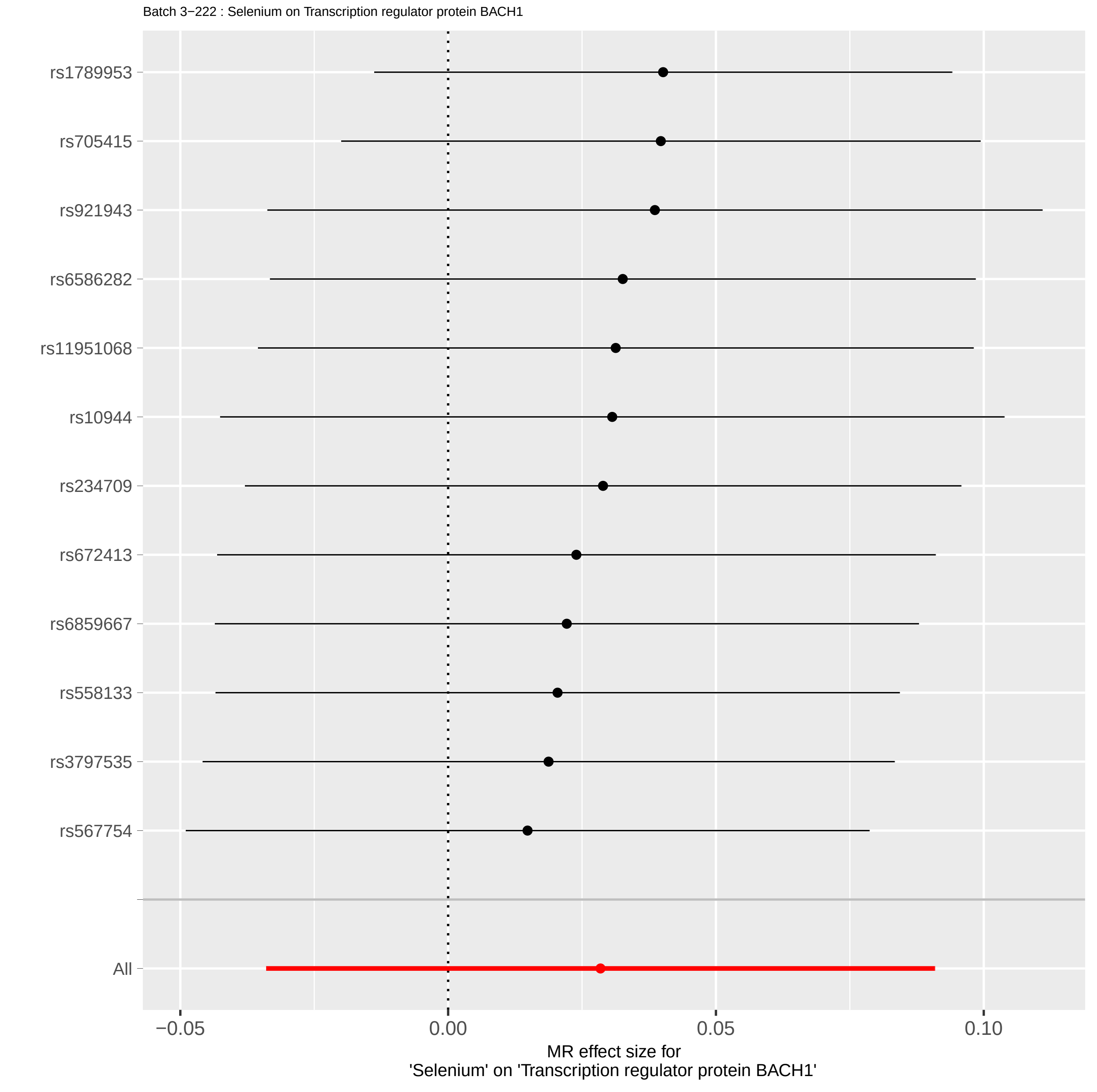

Batch 3−222 : Selenium on Transcription regulator protein BACH1
rs1789953
rs705415
rs921943
rs6586282
rs11951068
rs10944
rs234709
rs672413
rs6859667
rs558133
rs3797535
rs567754
All
−0.05
0.00
0.05
0.10
MR effect size for
'Selenium' on 'Transcription regulator protein BACH1'

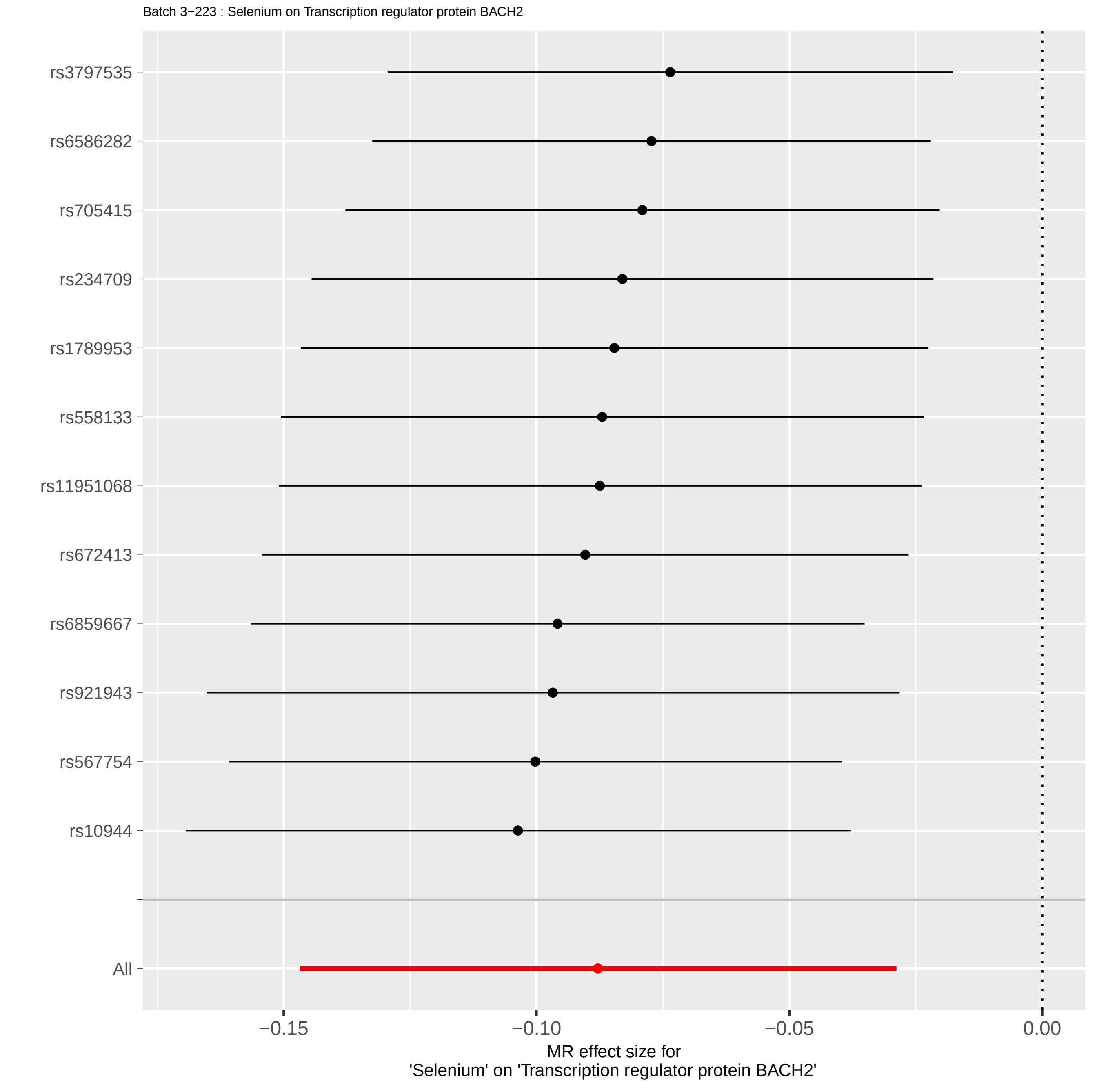

Batch 3−223 : Selenium on Transcription regulator protein BACH2
rs3797535
rs6586282
rs705415
rs234709
rs1789953
rs558133
rs11951068
rs672413
rs6859667
rs921943
rs567754
rs10944
All
−0.15
−0.10
−0.05
0.00
MR effect size for
'Selenium' on 'Transcription regulator protein BACH2'

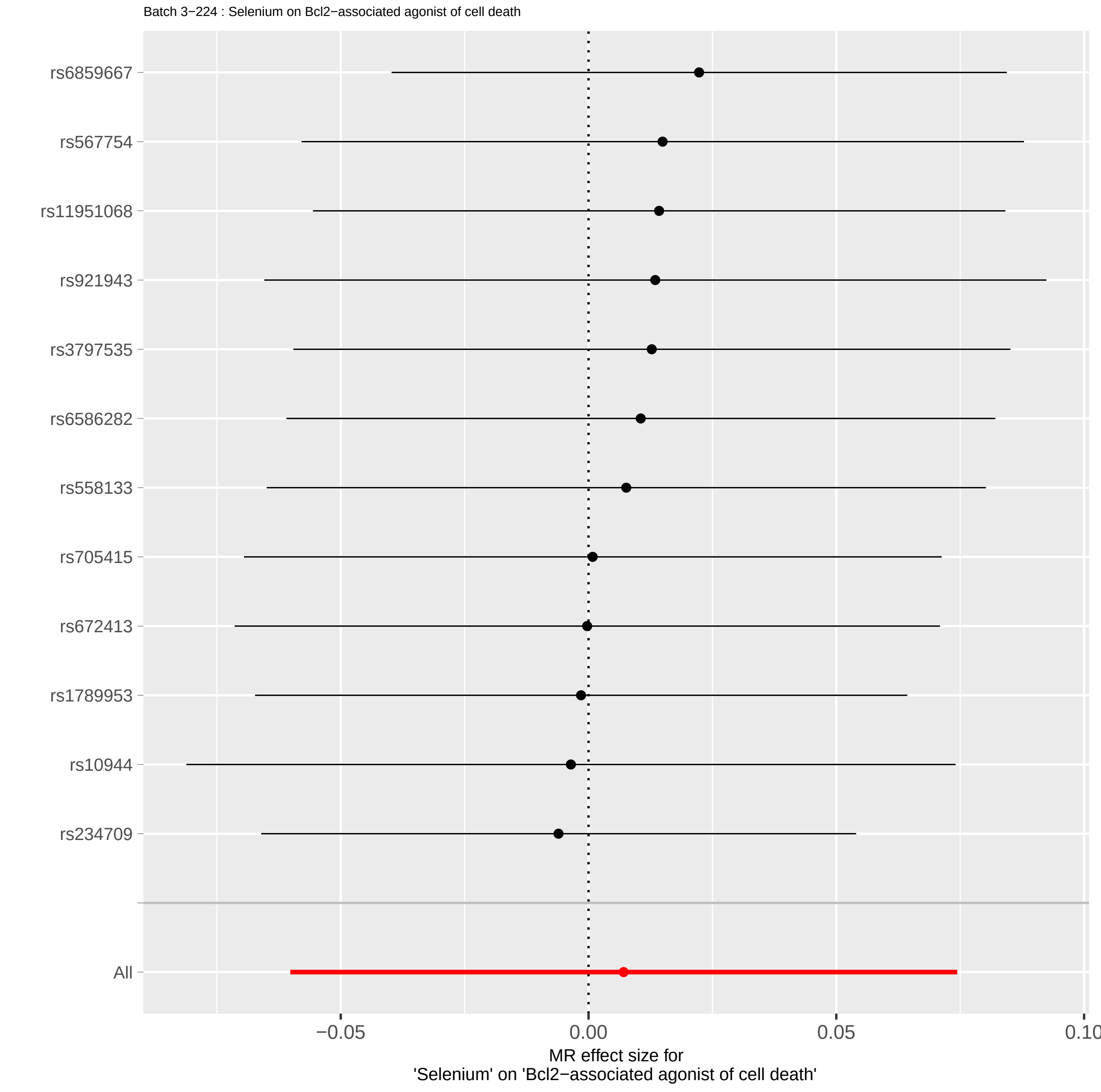

Batch 3−224 : Selenium on Bcl2−associated agonist of cell death
rs6859667
rs567754
rs11951068
rs921943
rs3797535
rs6586282
rs558133
rs705415
rs672413
rs1789953
rs10944
rs234709
All
−0.05
0.00
0.05
0.10
MR effect size for
'Selenium' on 'Bcl2−associated agonist of cell death'

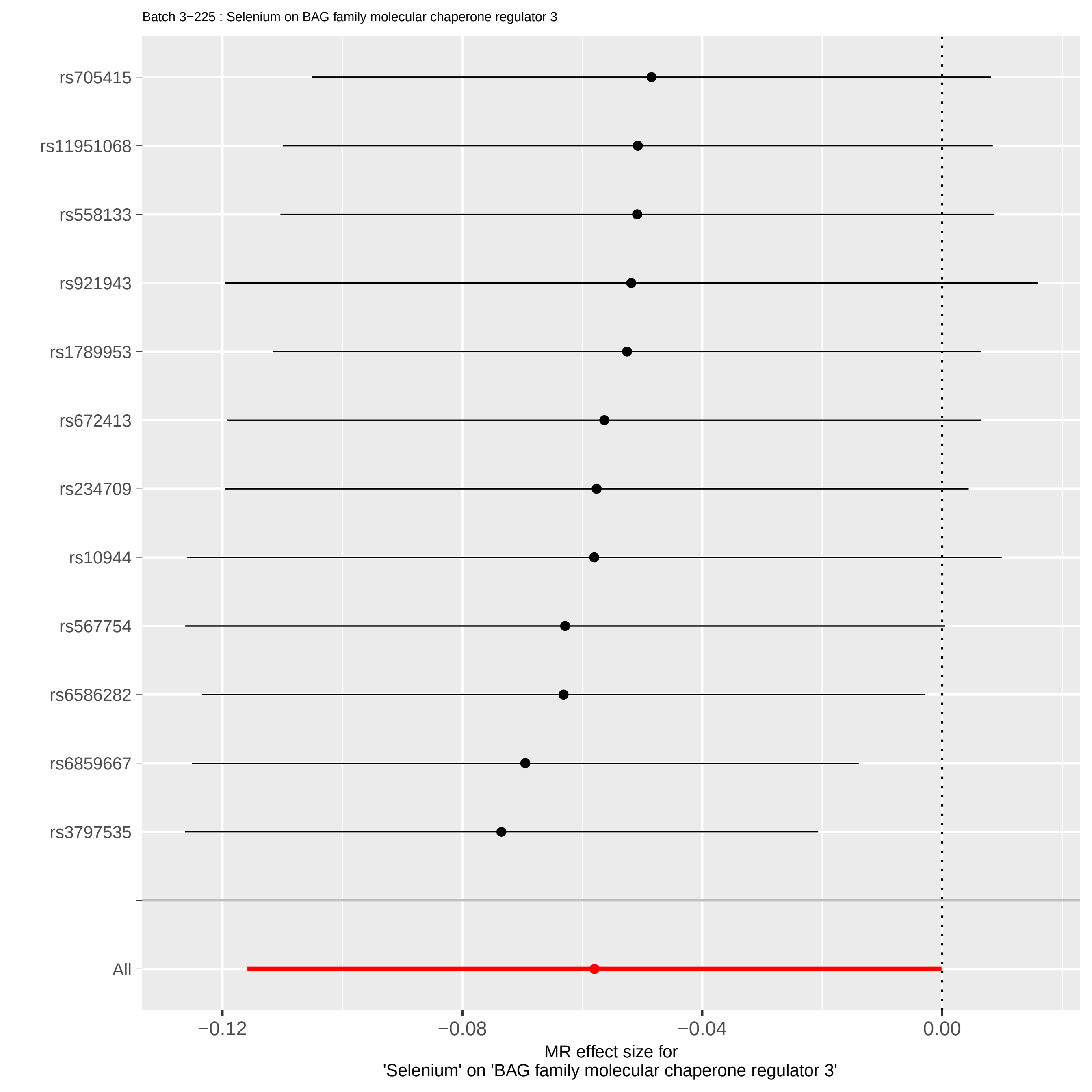
Batch 3−225 : Selenium on BAG family molecular chaperone regulator 3
rs705415
rs11951068
rs558133
rs921943
rs1789953
rs672413
rs234709
rs10944
rs567754
rs6586282
rs6859667
rs3797535
All
−0.12
−0.08
−0.04
0.00
MR effect size for
'Selenium' on 'BAG family molecular chaperone regulator 3'

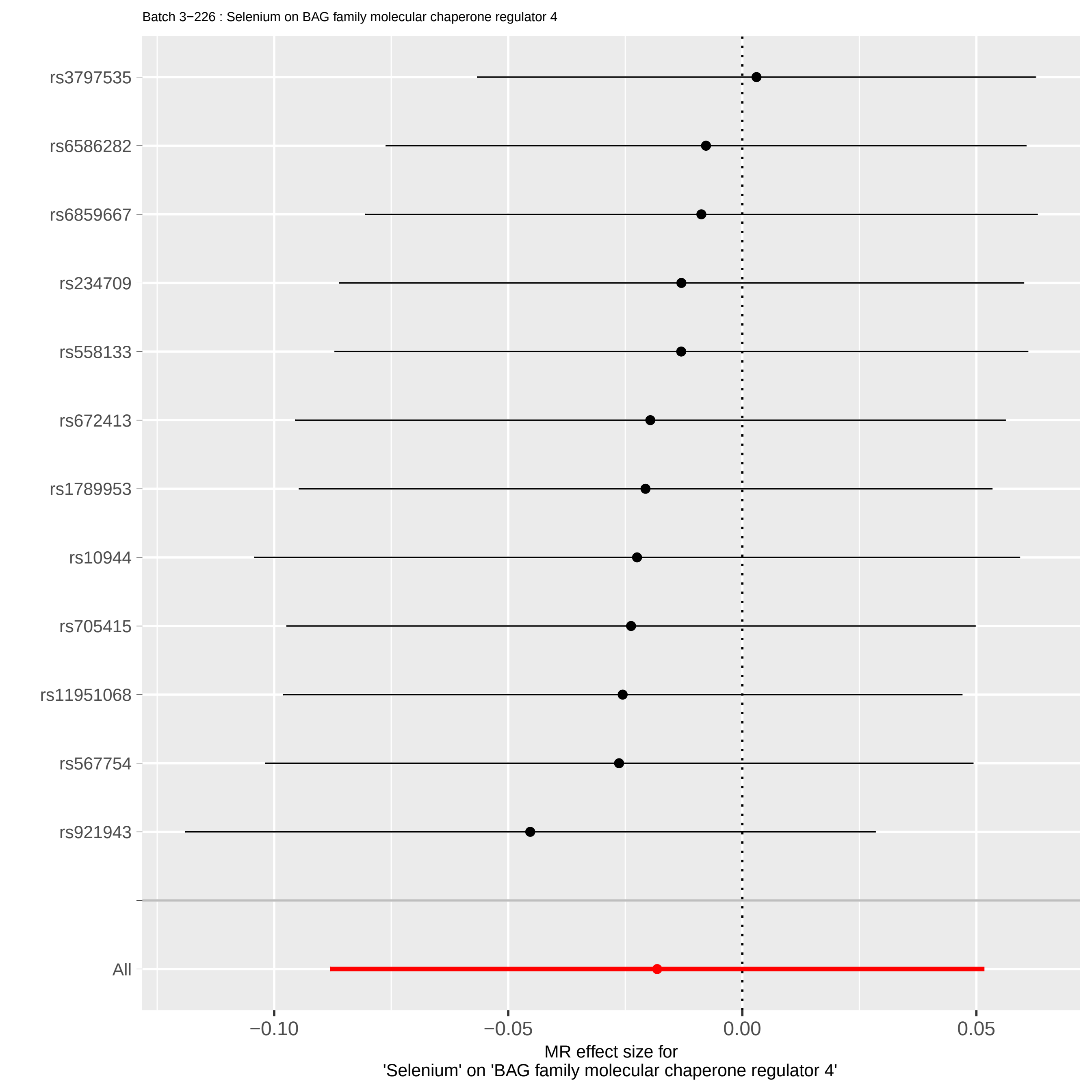

Batch 3−226 : Selenium on BAG family molecular chaperone regulator 4
rs3797535
rs6586282
rs6859667
rs234709
rs558133
rs672413
rs1789953
rs10944
rs705415
rs11951068
rs567754
rs921943
All
−0.10
−0.05
0.00
0.05
MR effect size for
'Selenium' on 'BAG family molecular chaperone regulator 4'

Batch 3−227 : Selenium on B melanoma antigen 2

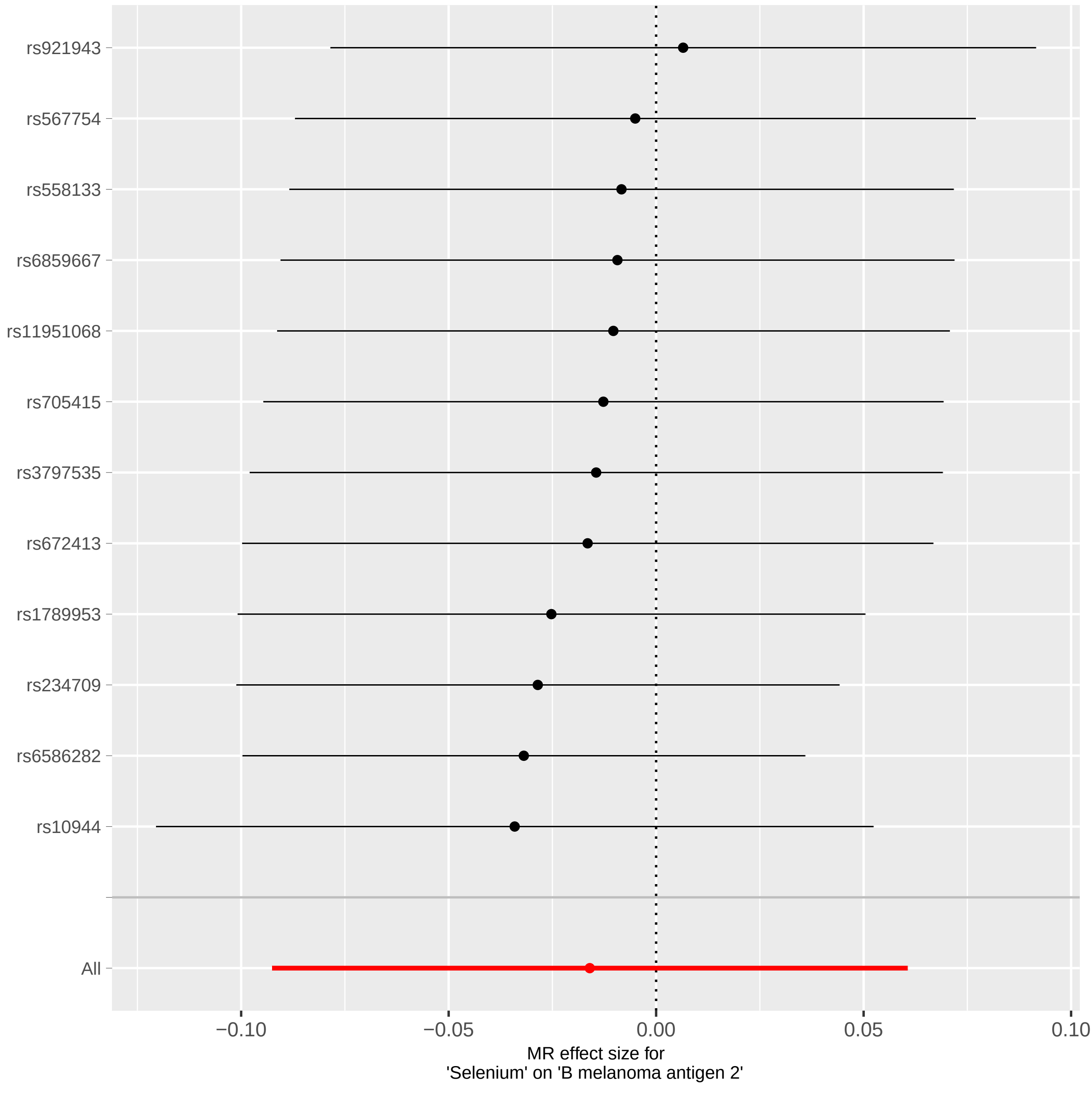

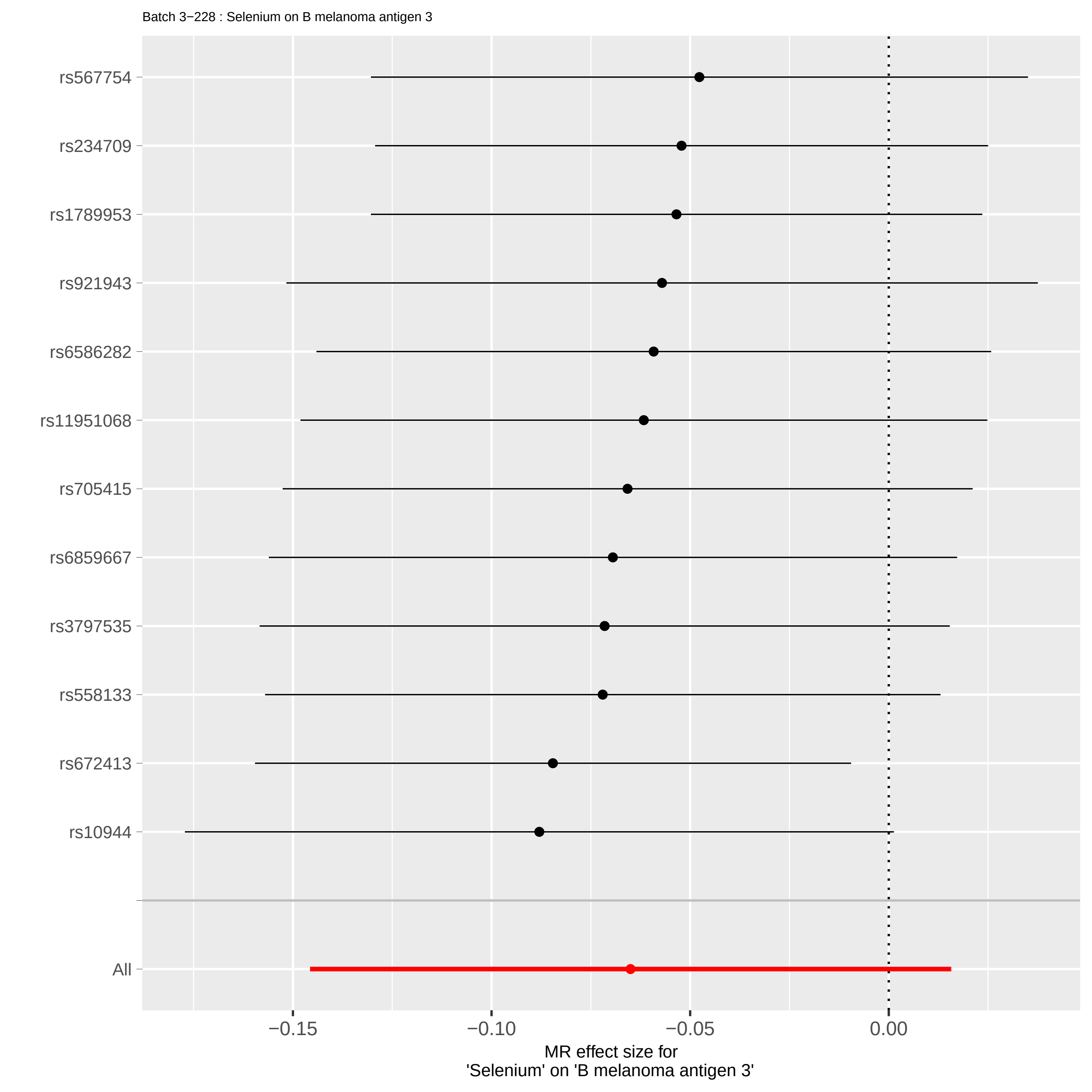

Batch 3−228 : Selenium on B melanoma antigen 3
rs567754
rs234709
rs1789953
rs921943
rs6586282
rs11951068
rs705415
rs6859667
rs3797535
rs558133
rs672413
rs10944
All
−0.15
−0.10
−0.05
0.00
MR effect size for
'Selenium' on 'B melanoma antigen 3'

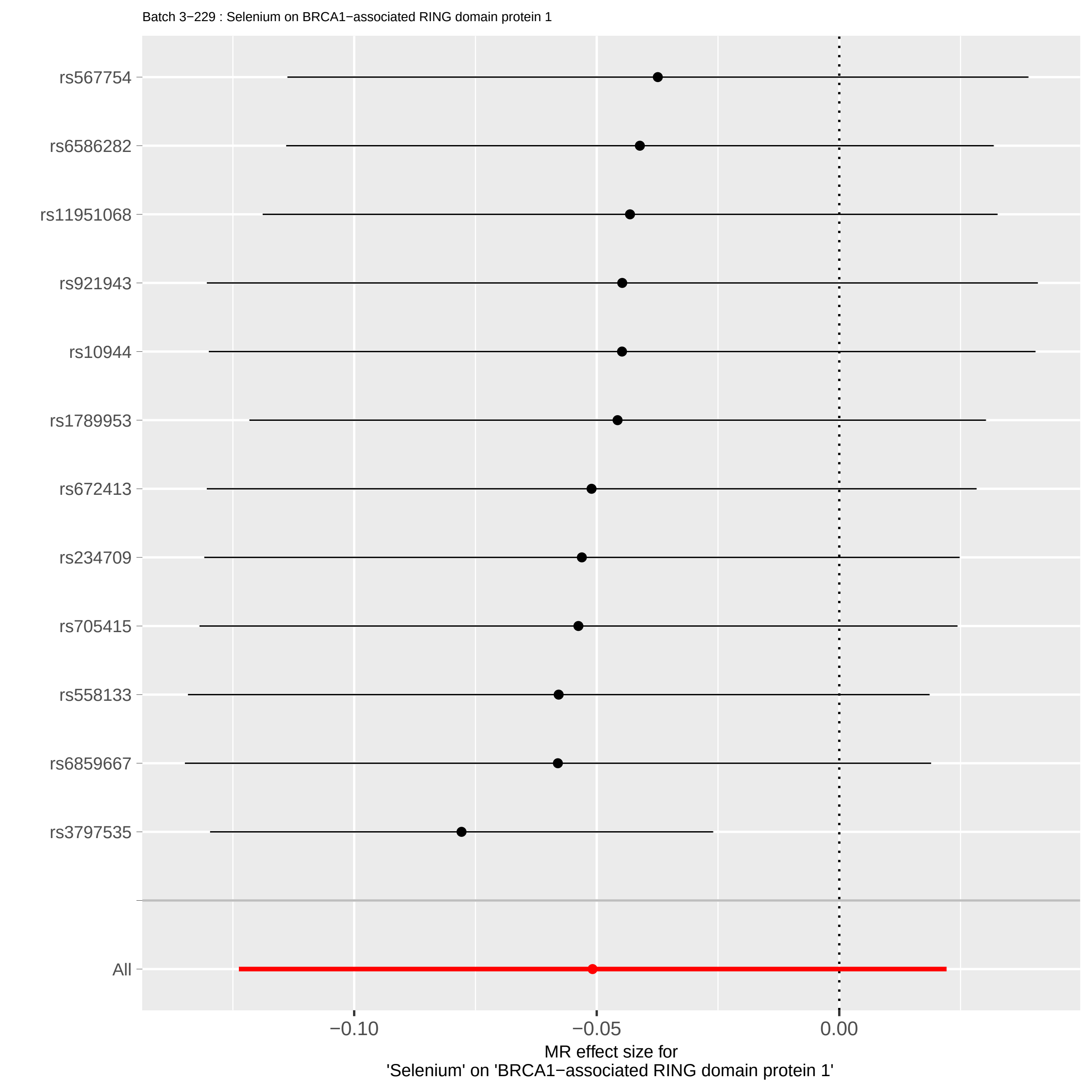

Batch 3−229 : Selenium on BRCA1−associated RING domain protein 1
rs567754
rs6586282
rs11951068
rs921943
rs10944
rs1789953
rs672413
rs234709
rs705415
rs558133
rs6859667
rs3797535
All
−0.10
−0.05
0.00
MR effect size for
'Selenium' on 'BRCA1−associated RING domain protein 1'

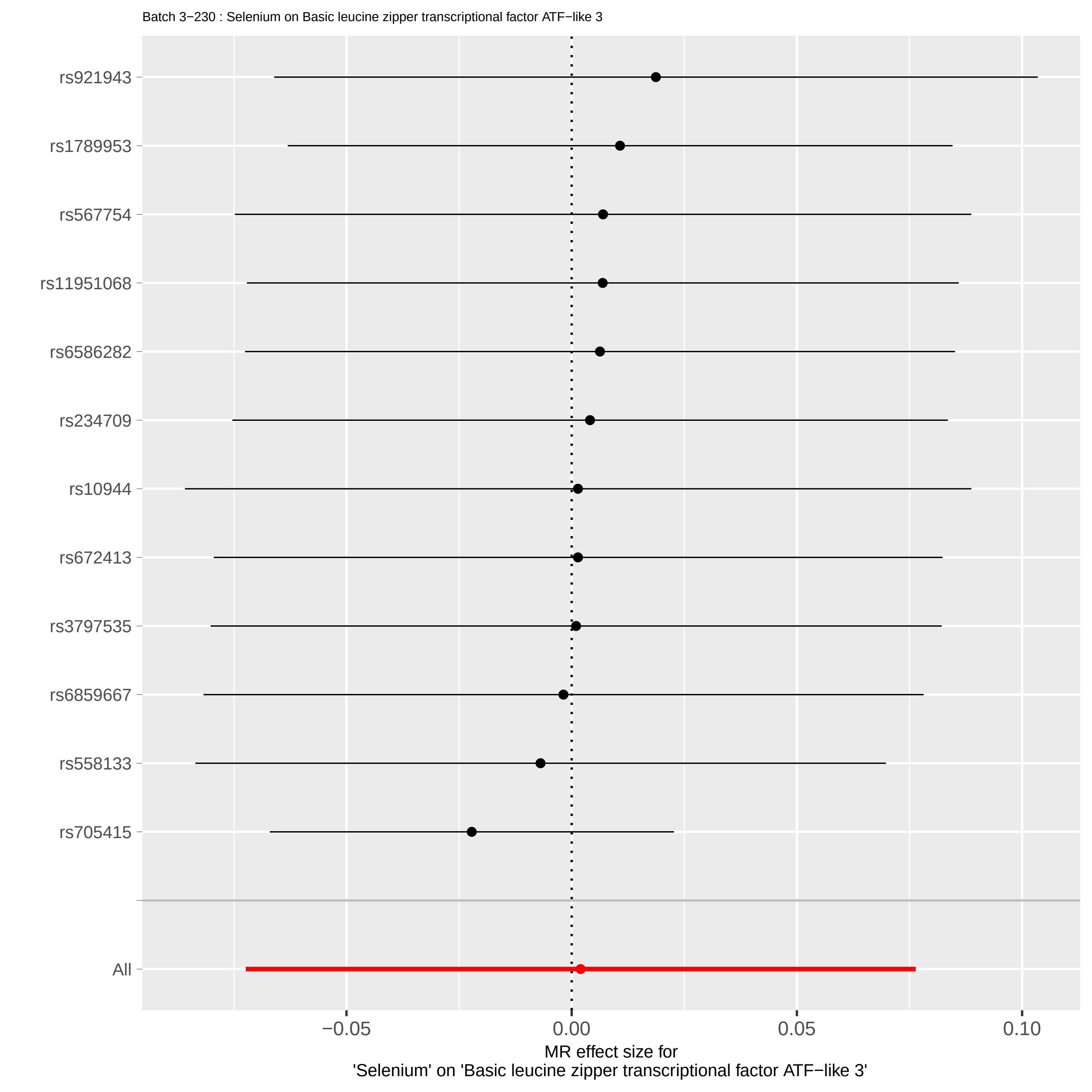

Batch 3−230 : Selenium on Basic leucine zipper transcriptional factor ATF−like 3
rs921943
rs1789953
rs567754
rs11951068
rs6586282
rs234709
rs10944
rs672413
rs3797535
rs6859667
rs558133
rs705415
All
−0.05
0.00
0.05
0.10
MR effect size for
'Selenium' on 'Basic leucine zipper transcriptional factor ATF−like 3'

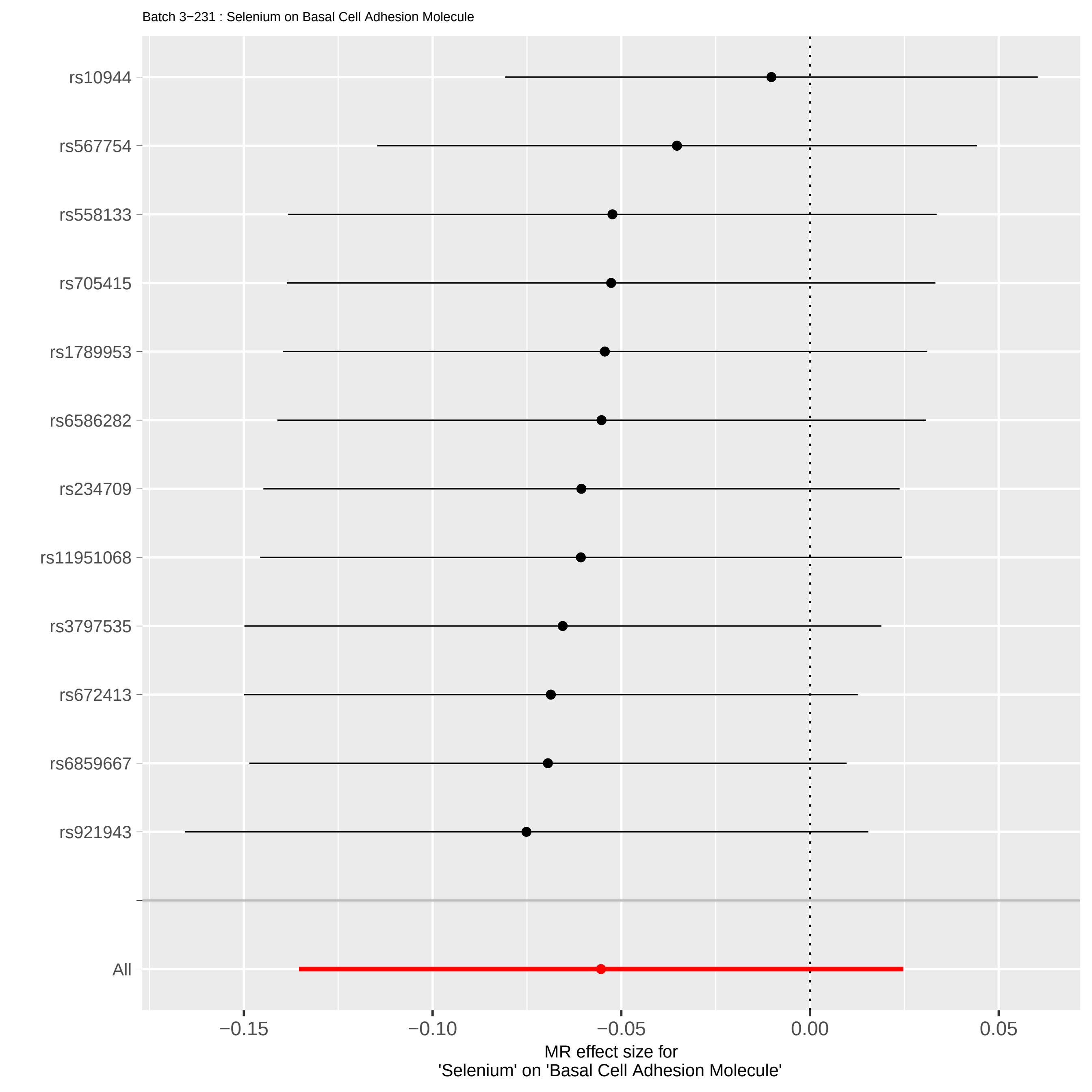

Batch 3−231 : Selenium on Basal Cell Adhesion Molecule
rs10944
rs567754
rs558133
rs705415
rs1789953
rs6586282
rs234709
rs11951068
rs3797535
rs672413
rs6859667
rs921943
All
−0.15
−0.10
−0.05
0.00
0.05
MR effect size for
'Selenium' on 'Basal Cell Adhesion Molecule'

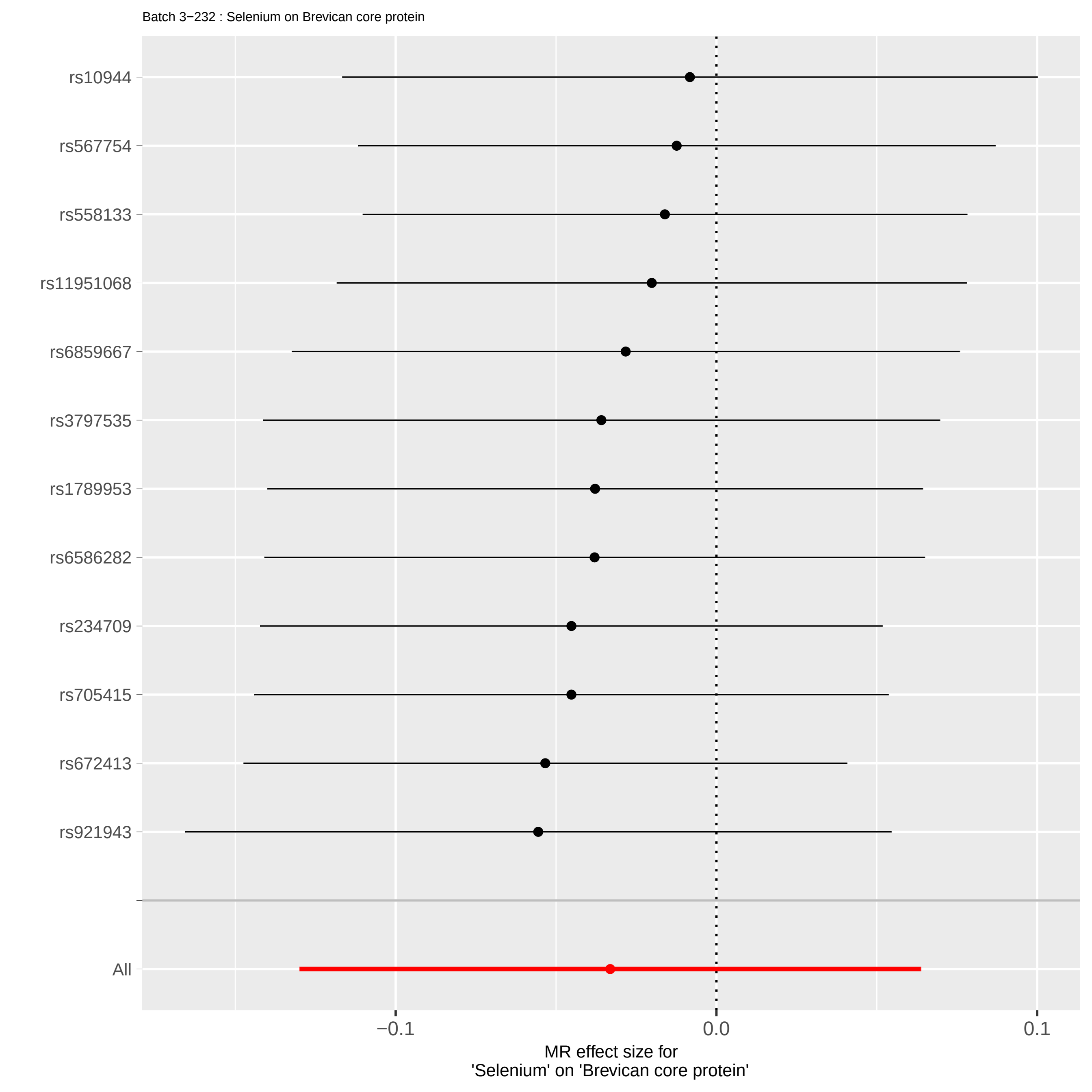
Batch 3−232 : Selenium on Brevican core protein
rs10944
rs567754
rs558133
rs11951068
rs6859667
rs3797535
rs1789953
rs6586282
rs234709
rs705415
rs672413
rs921943
All
−0.1
0.0
0.1
MR effect size for
'Selenium' on 'Brevican core protein'

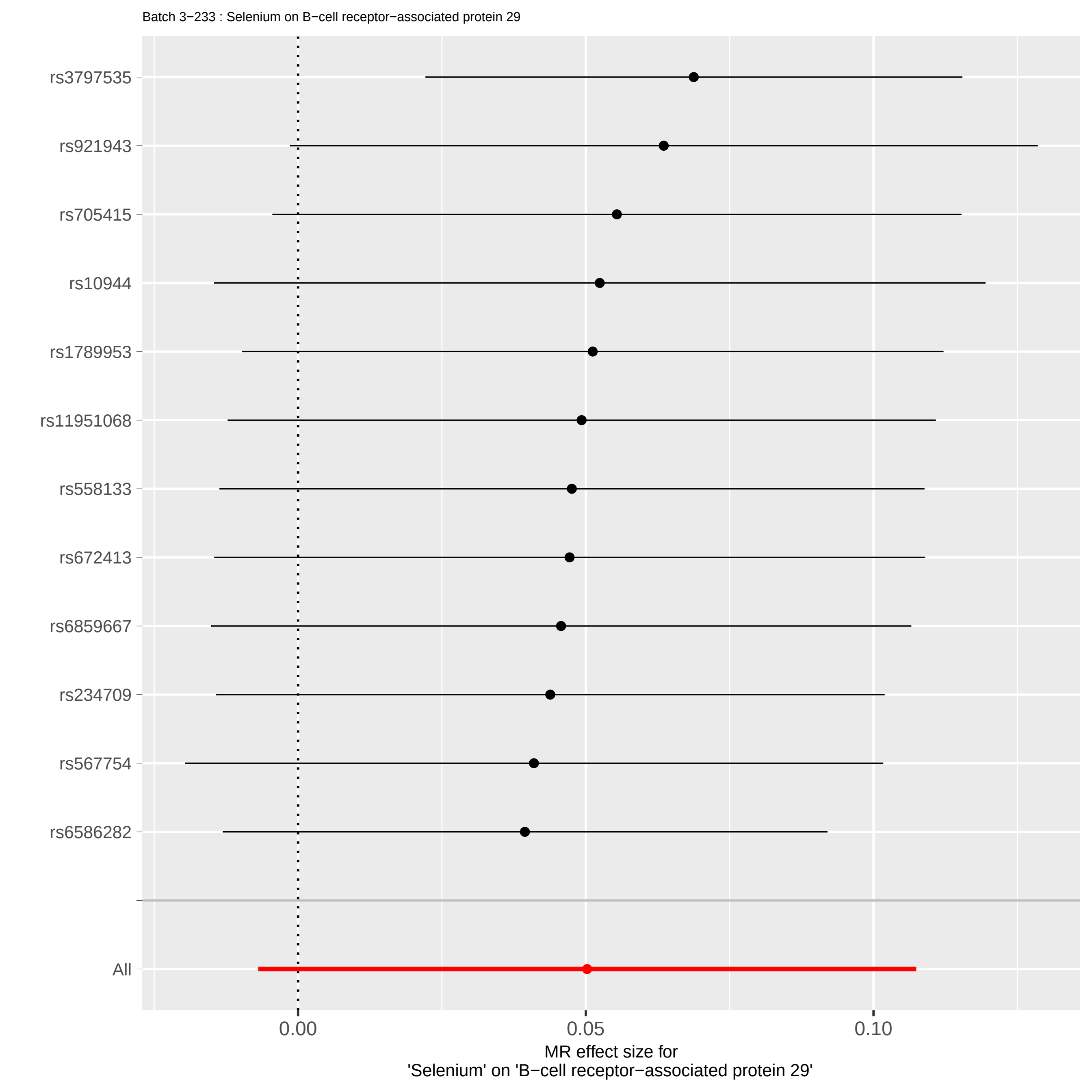
Batch 3−233 : Selenium on B−cell receptor−associated protein 29
rs3797535
rs921943
rs705415
rs10944
rs1789953
rs11951068
rs558133
rs672413
rs6859667
rs234709
rs567754
rs6586282
All
0.00
0.05
0.10
MR effect size for
'Selenium' on 'B−cell receptor−associated protein 29'

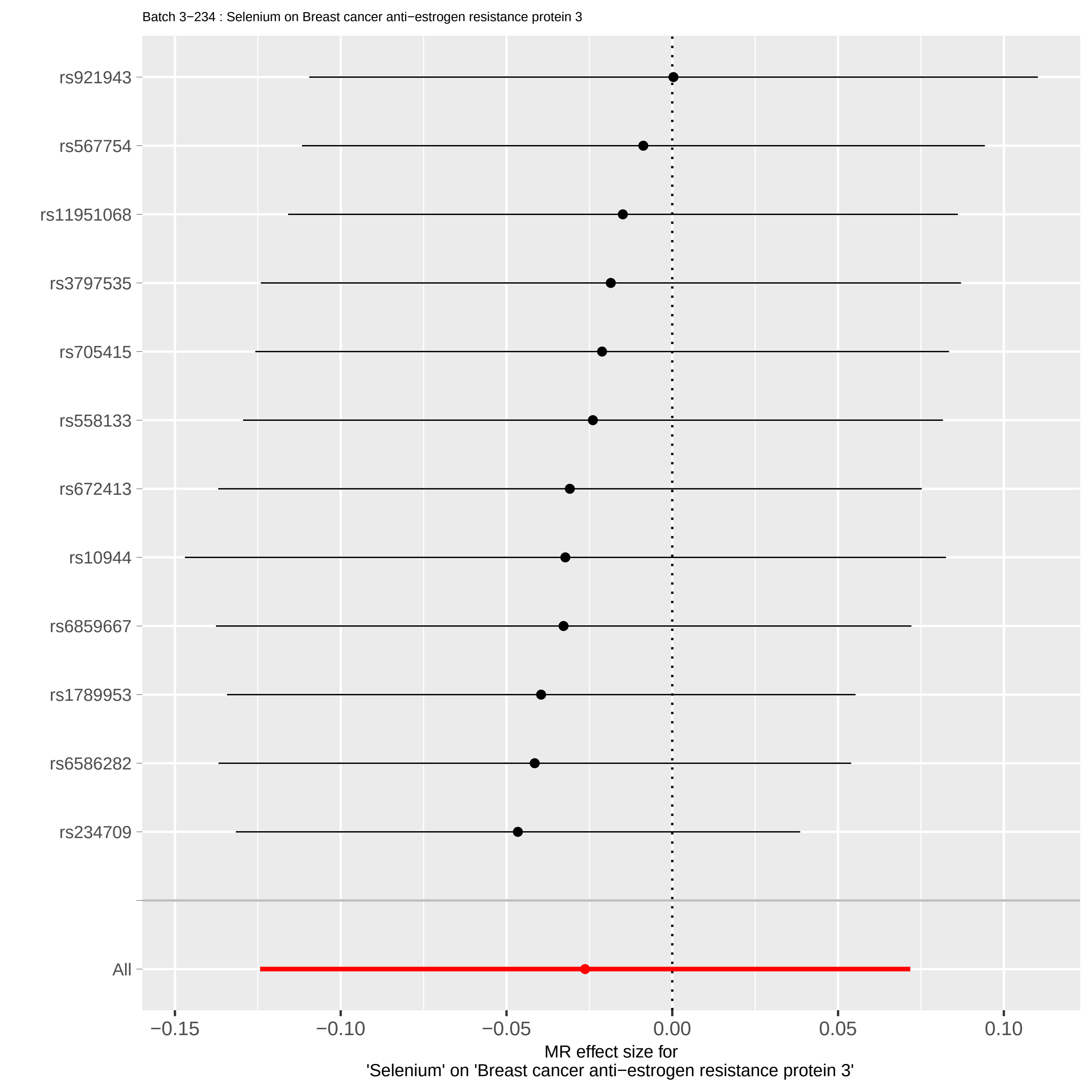

Batch 3−234 : Selenium on Breast cancer anti−estrogen resistance protein 3
rs921943
rs567754
rs11951068
rs3797535
rs705415
rs558133
rs672413
rs10944
rs6859667
rs1789953
rs6586282
rs234709
All
−0.15
−0.10
−0.05
0.00
0.05
0.10
MR effect size for
'Selenium' on 'Breast cancer anti−estrogen resistance protein 3'

Batch 3−235 : Selenium on B−cell lymphoma/leukemia 10

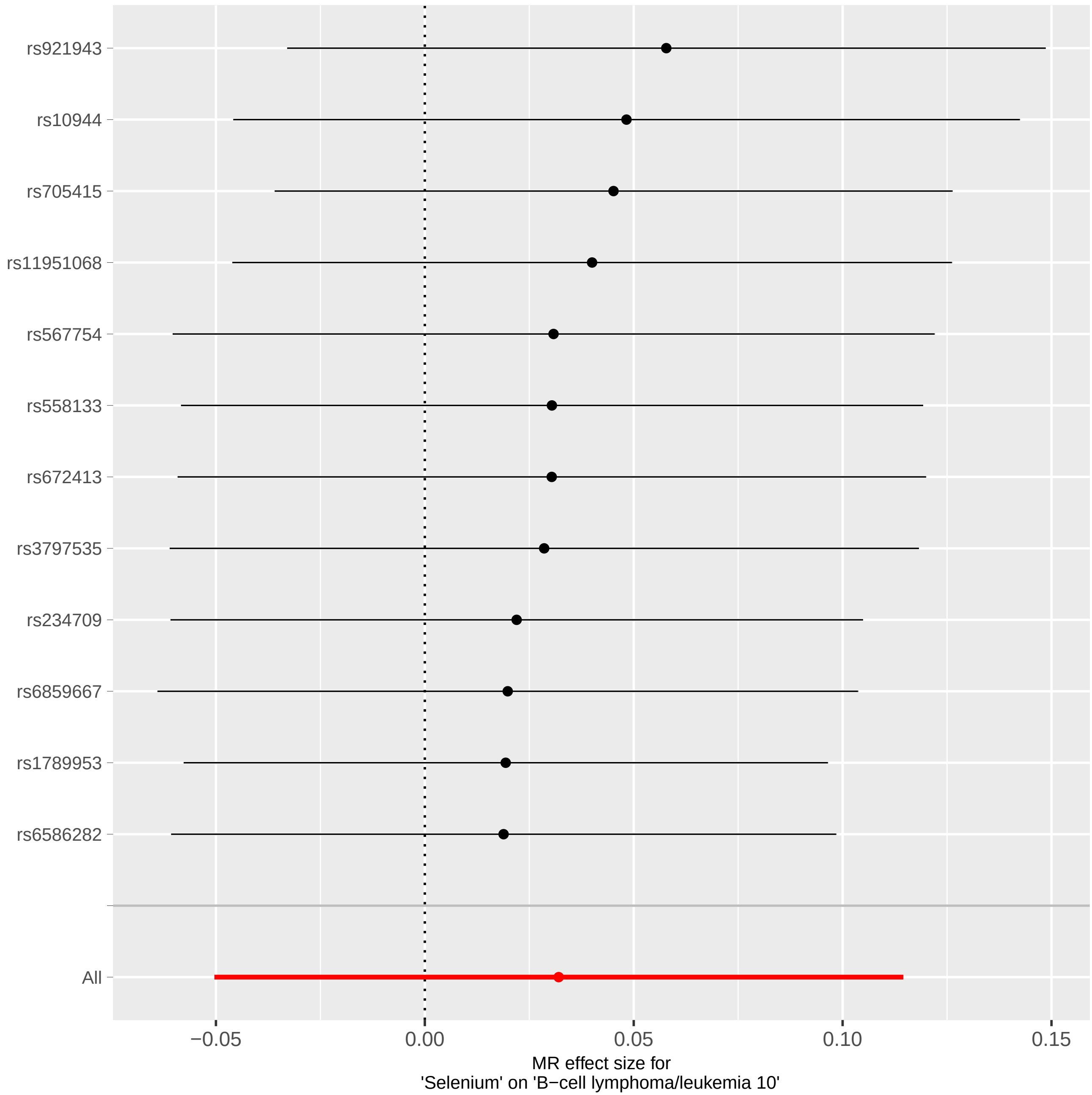

Batch 3−236 : Selenium on Apoptosis regulator Bcl−2

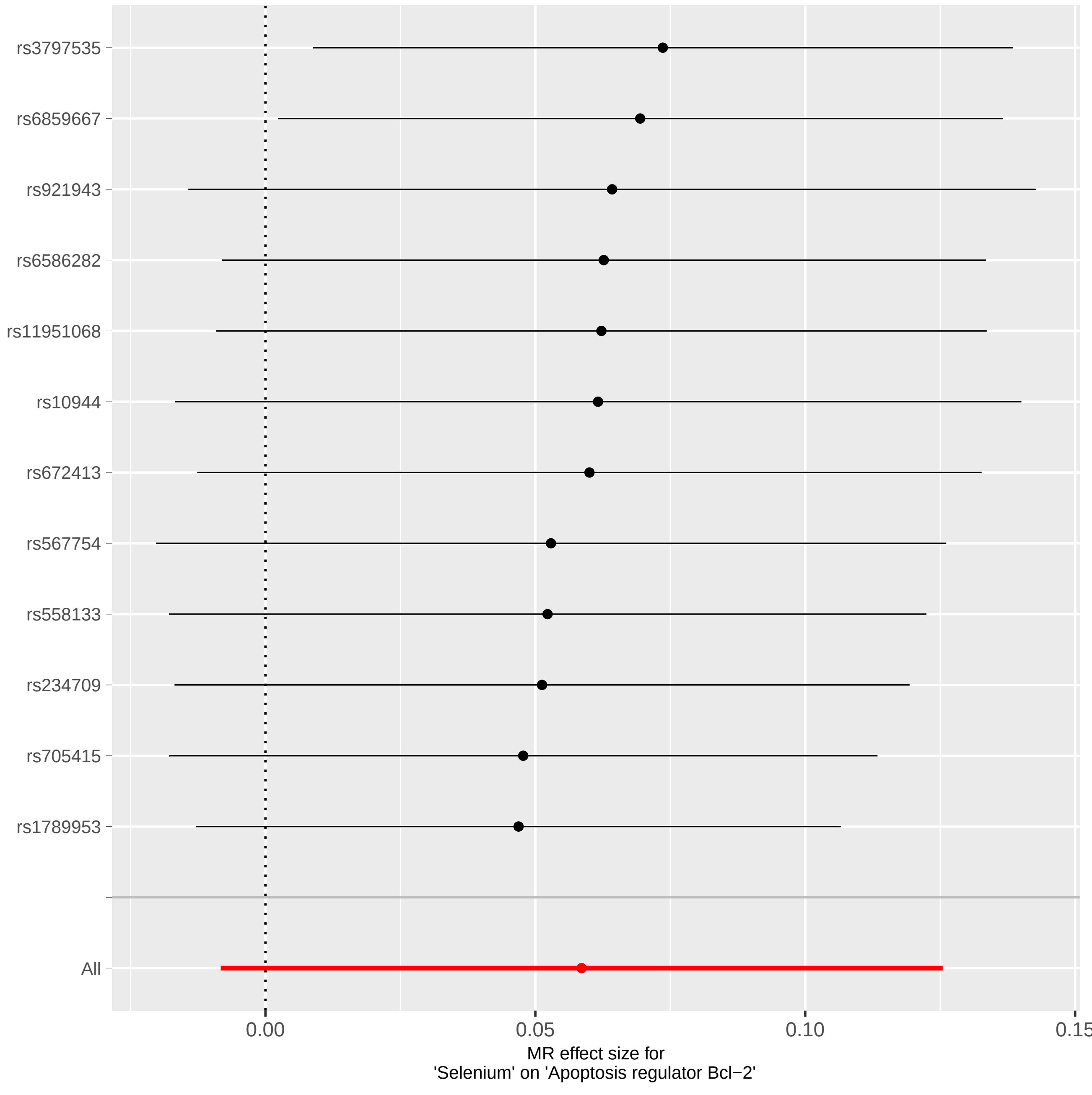

Batch 3−237 : Selenium on Bcl−2−related protein A1

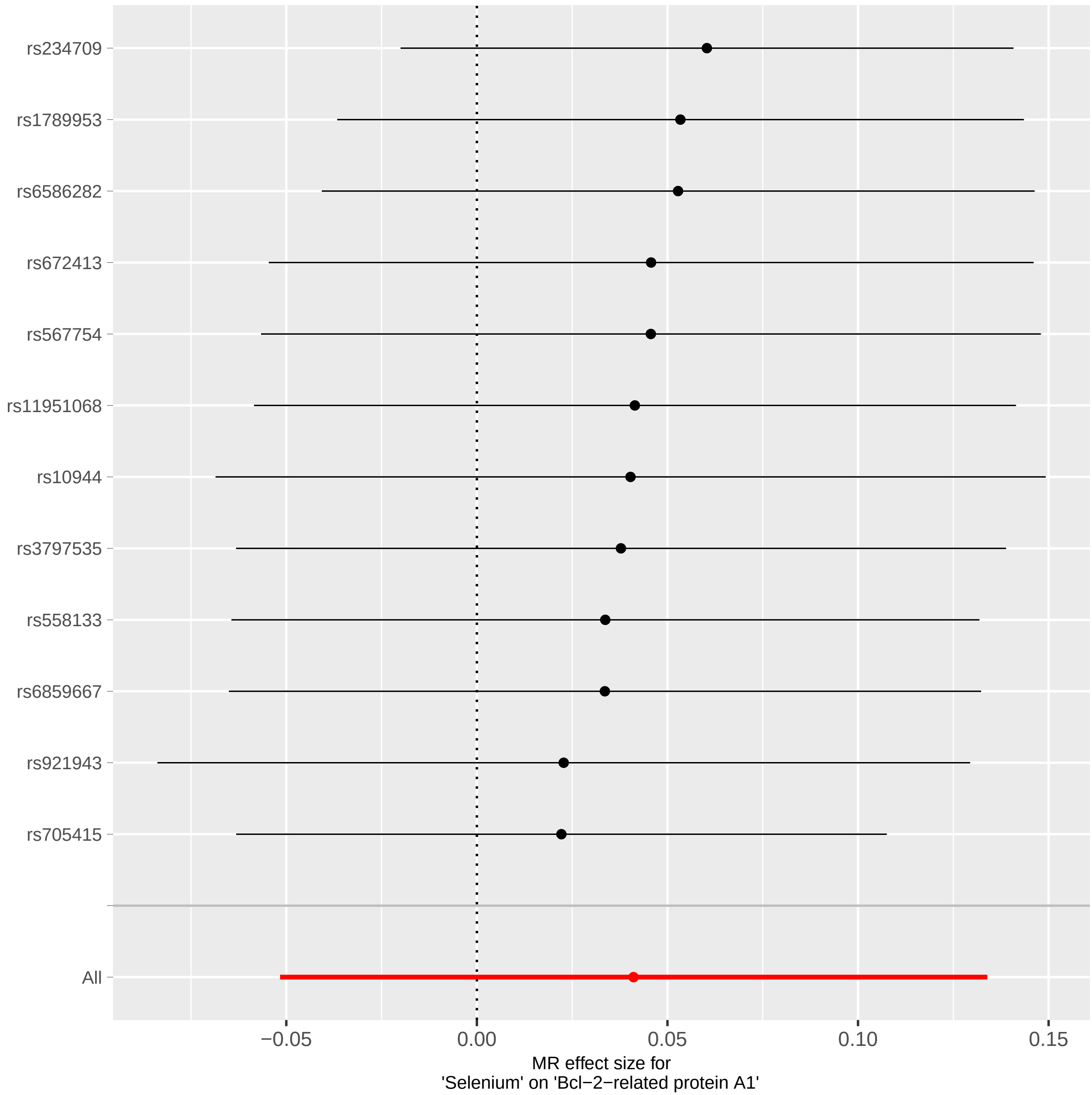

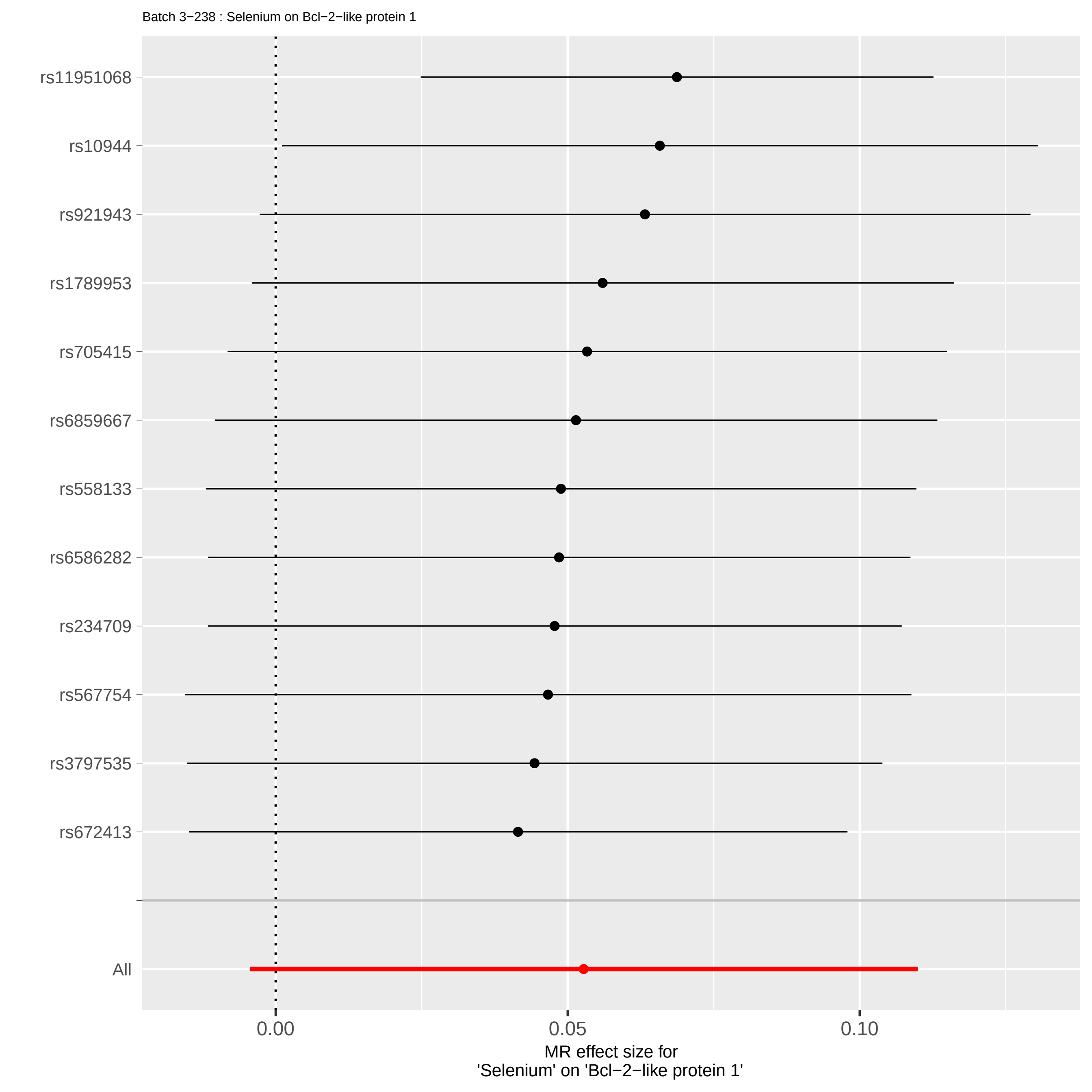

Batch 3−238 : Selenium on Bcl−2−like protein 1
rs11951068
rs10944
rs921943
rs1789953
rs705415
rs6859667
rs558133
rs6586282
rs234709
rs567754
rs3797535
rs672413
All
0.00
0.05
0.10
MR effect size for
'Selenium' on 'Bcl−2−like protein 1'

Batch 3−239 : Selenium on Bcl−2−like protein 2

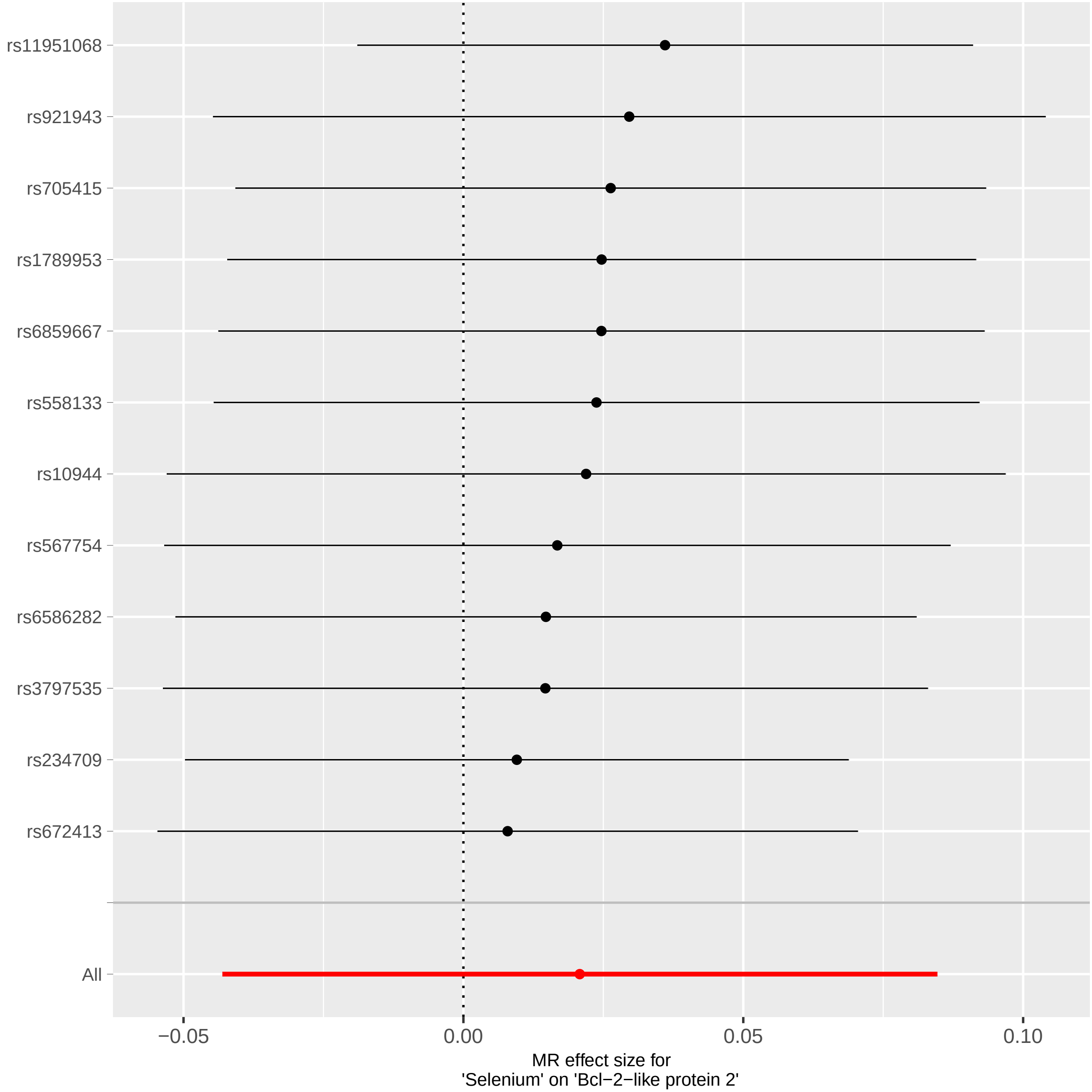

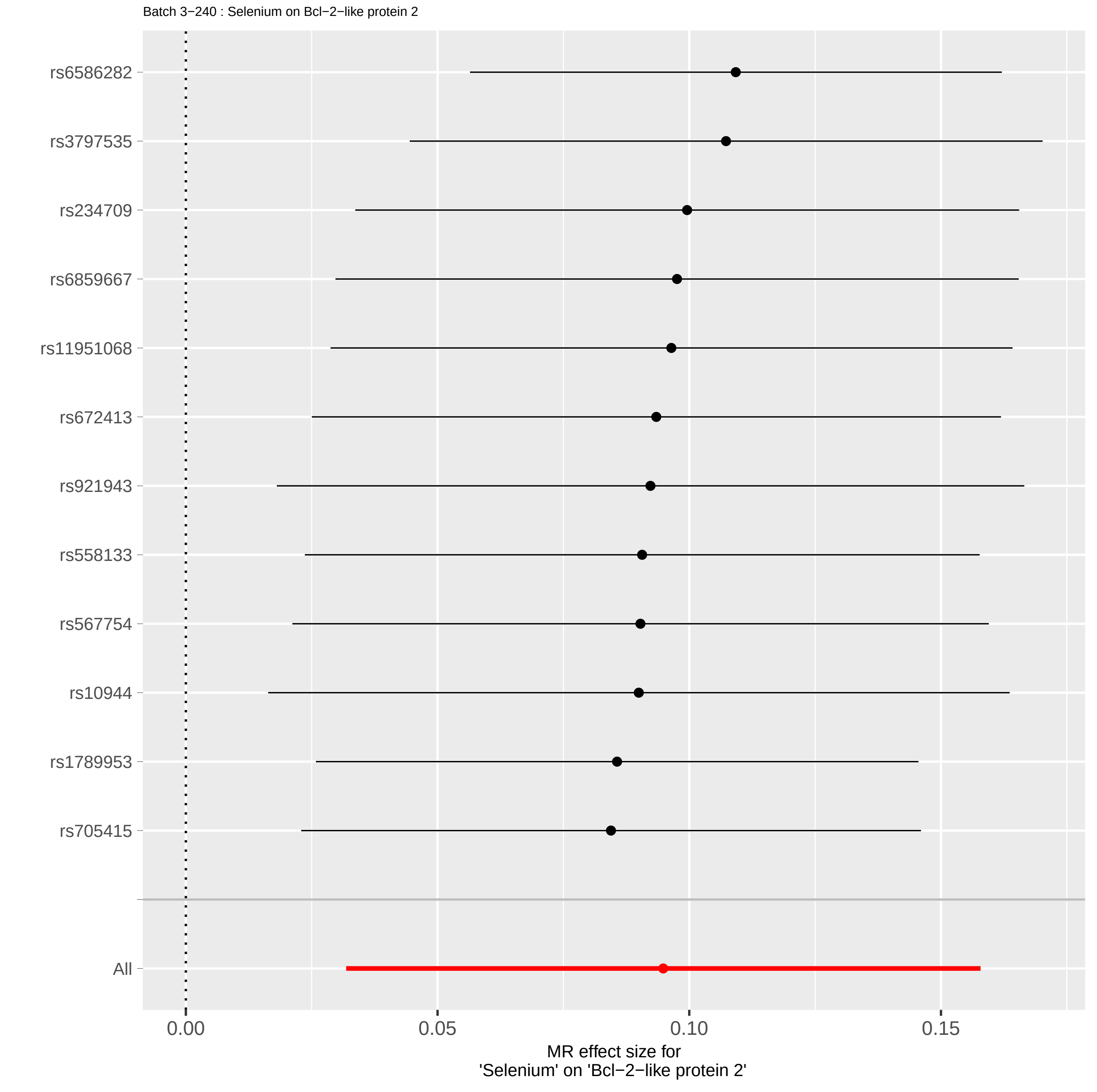
Batch 3−240 : Selenium on Bcl−2−like protein 2
rs6586282
rs3797535
rs234709
rs6859667
rs11951068
rs672413
rs921943
rs558133
rs567754
rs10944
rs1789953
rs705415
All
0.00
0.05
0.10
0.15
MR effect size for
'Selenium' on 'Bcl−2−like protein 2'

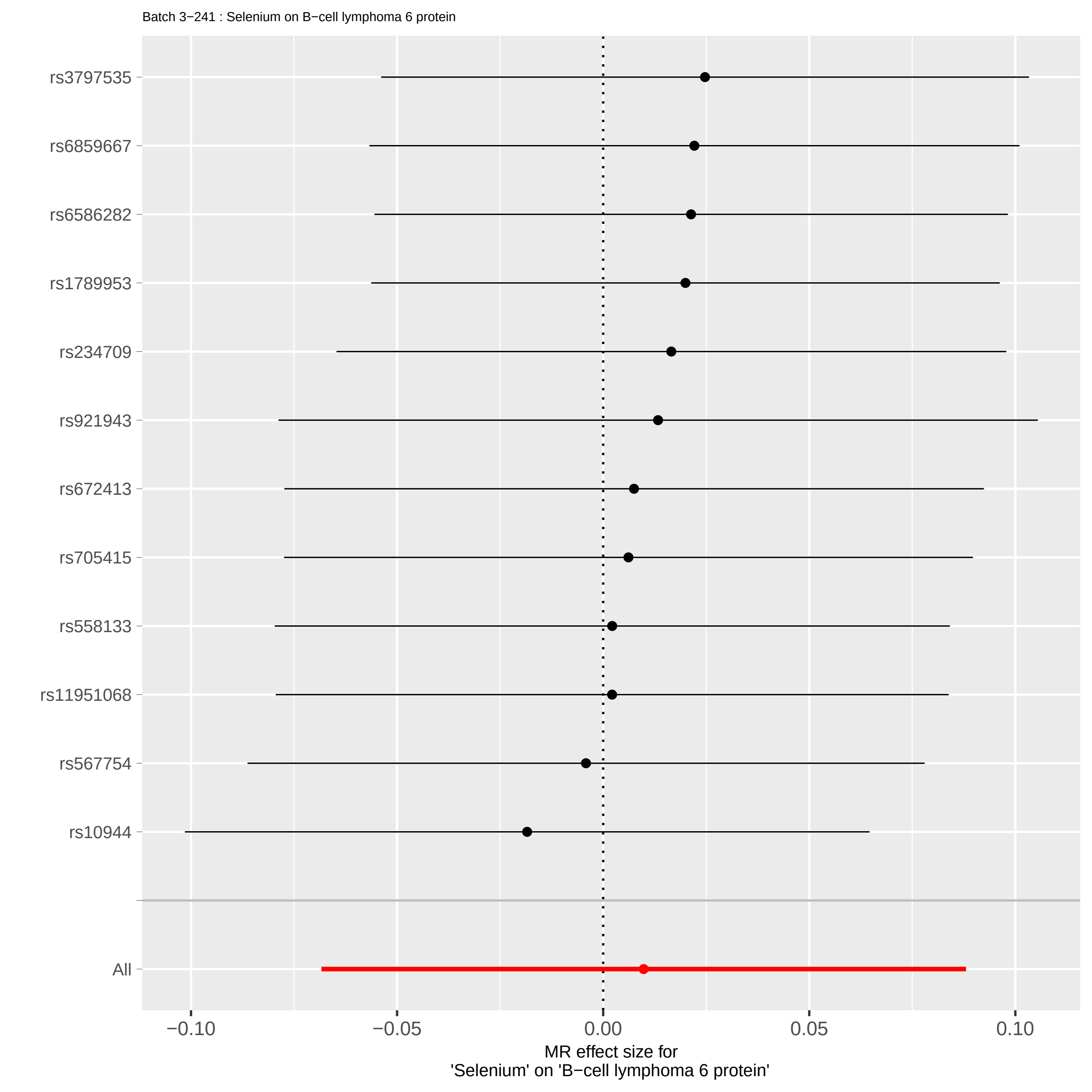

Batch 3−241 : Selenium on B−cell lymphoma 6 protein
rs3797535
rs6859667
rs6586282
rs1789953
rs234709
rs921943
rs672413
rs705415
rs558133
rs11951068
rs567754
rs10944
All
−0.10
−0.05
0.00
0.05
0.10
MR effect size for
'Selenium' on 'B−cell lymphoma 6 protein'

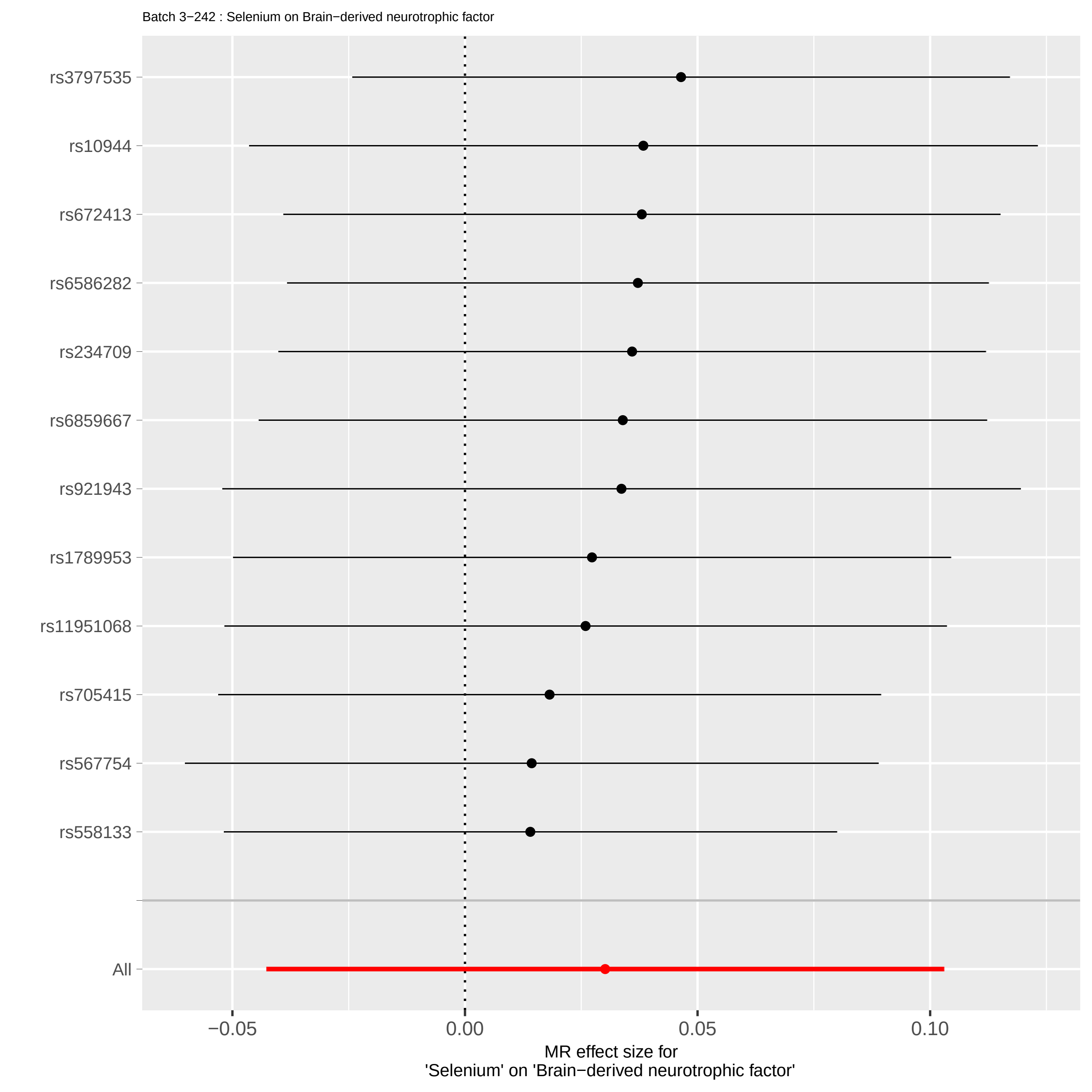
Batch 3−242 : Selenium on Brain−derived neurotrophic factor
rs3797535
rs10944
rs672413
rs6586282
rs234709
rs6859667
rs921943
rs1789953
rs11951068
rs705415
rs567754
rs558133
All
−0.05
0.00
0.05
0.10
MR effect size for
'Selenium' on 'Brain−derived neurotrophic factor'

Batch 3−243 : Selenium on Transcription factor TFIIIB component B" homolog

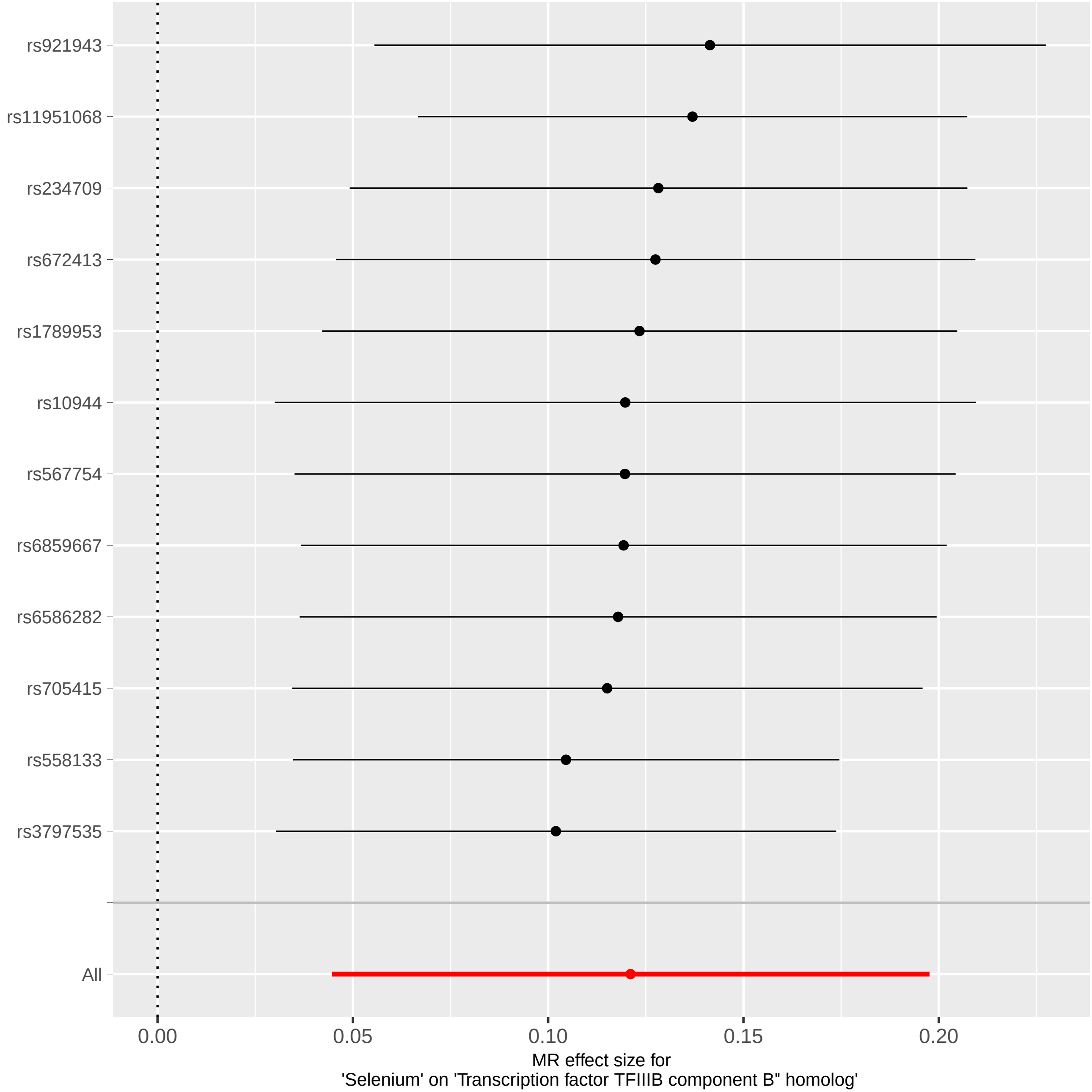

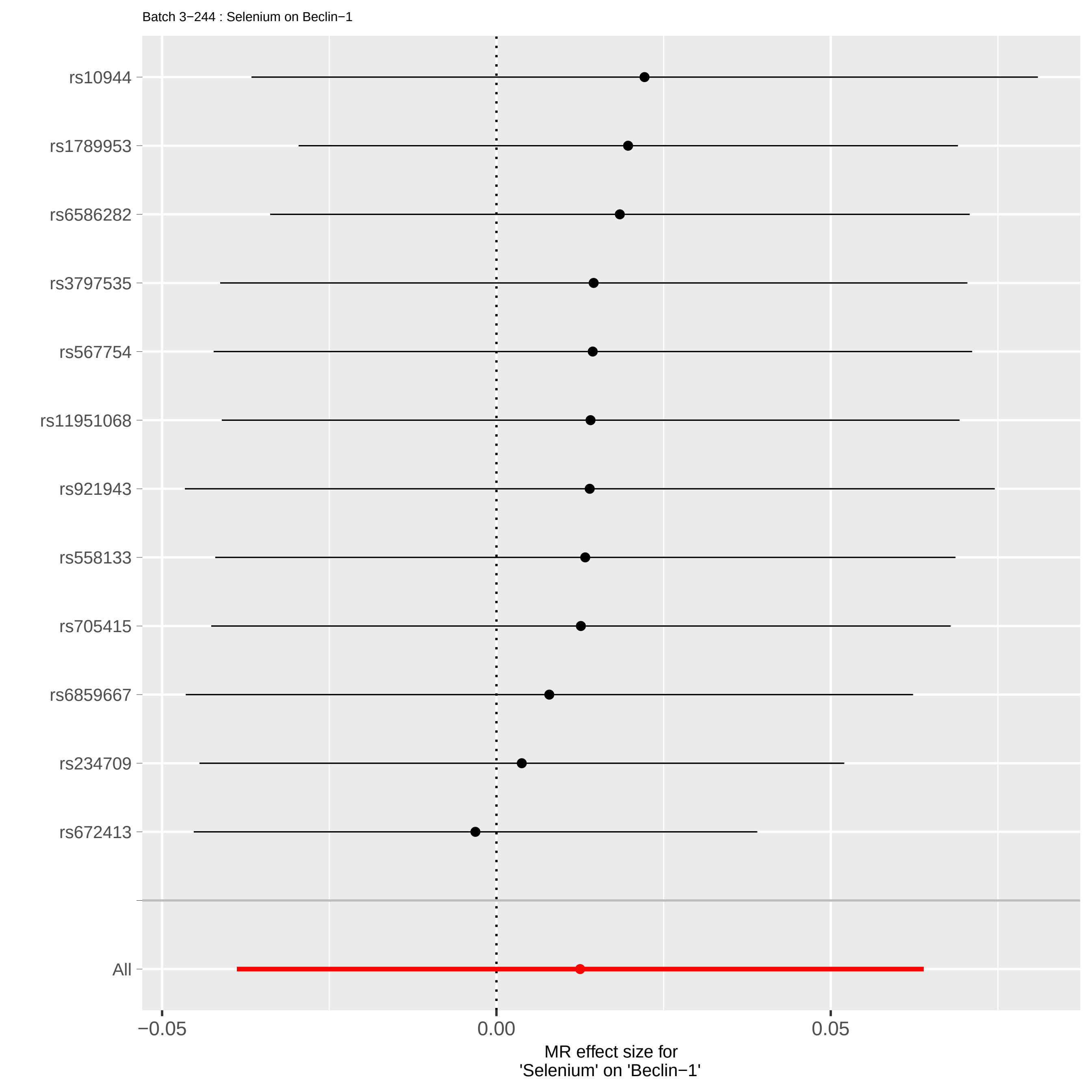

Batch 3−244 : Selenium on Beclin−1
rs10944
rs1789953
rs6586282
rs3797535
rs567754
rs11951068
rs921943
rs558133
rs705415
rs6859667
rs234709
rs672413
All
−0.05
0.00
0.05
MR effect size for
'Selenium' on 'Beclin−1'

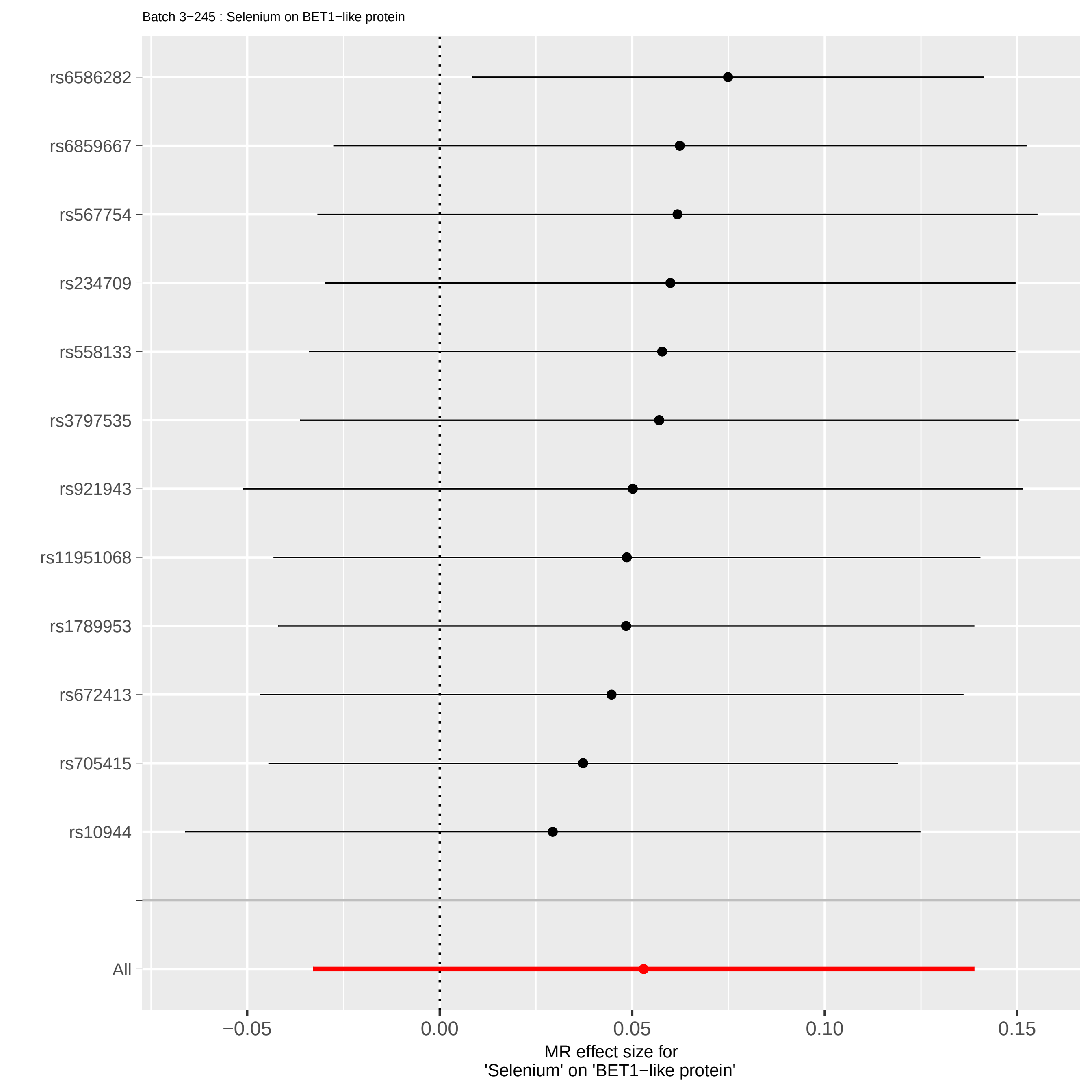

Batch 3−245 : Selenium on BET1−like protein
rs6586282
rs6859667
rs567754
rs234709
rs558133
rs3797535
rs921943
rs11951068
rs1789953
rs672413
rs705415
rs10944
All
−0.05
0.00
0.05
0.10
0.15
MR effect size for
'Selenium' on 'BET1−like protein'

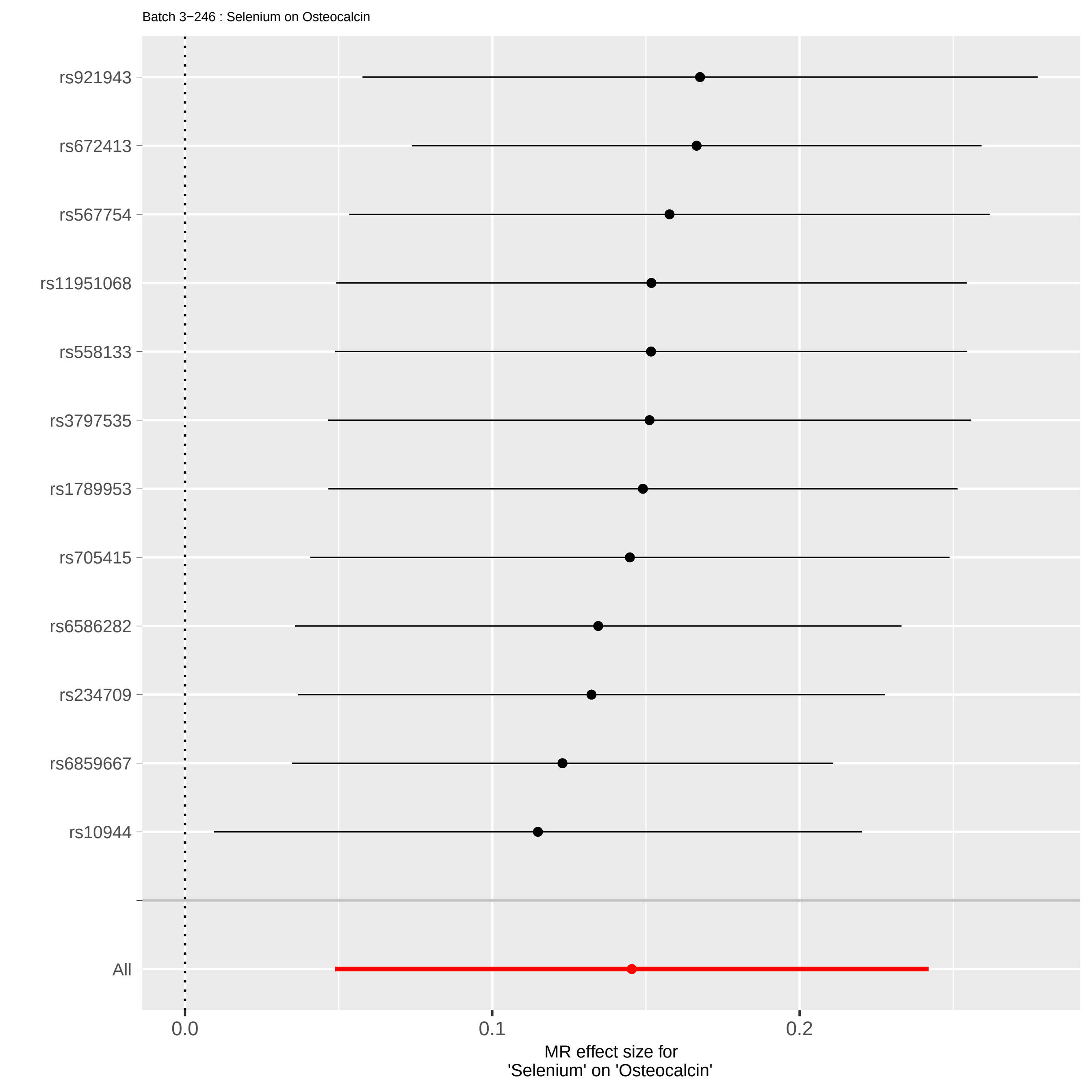

Batch 3−246 : Selenium on Osteocalcin
rs921943
rs672413
rs567754
rs11951068
rs558133
rs3797535
rs1789953
rs705415
rs6586282
rs234709
rs6859667
rs10944
All
0.0
0.1
0.2
MR effect size for
'Selenium' on 'Osteocalcin'

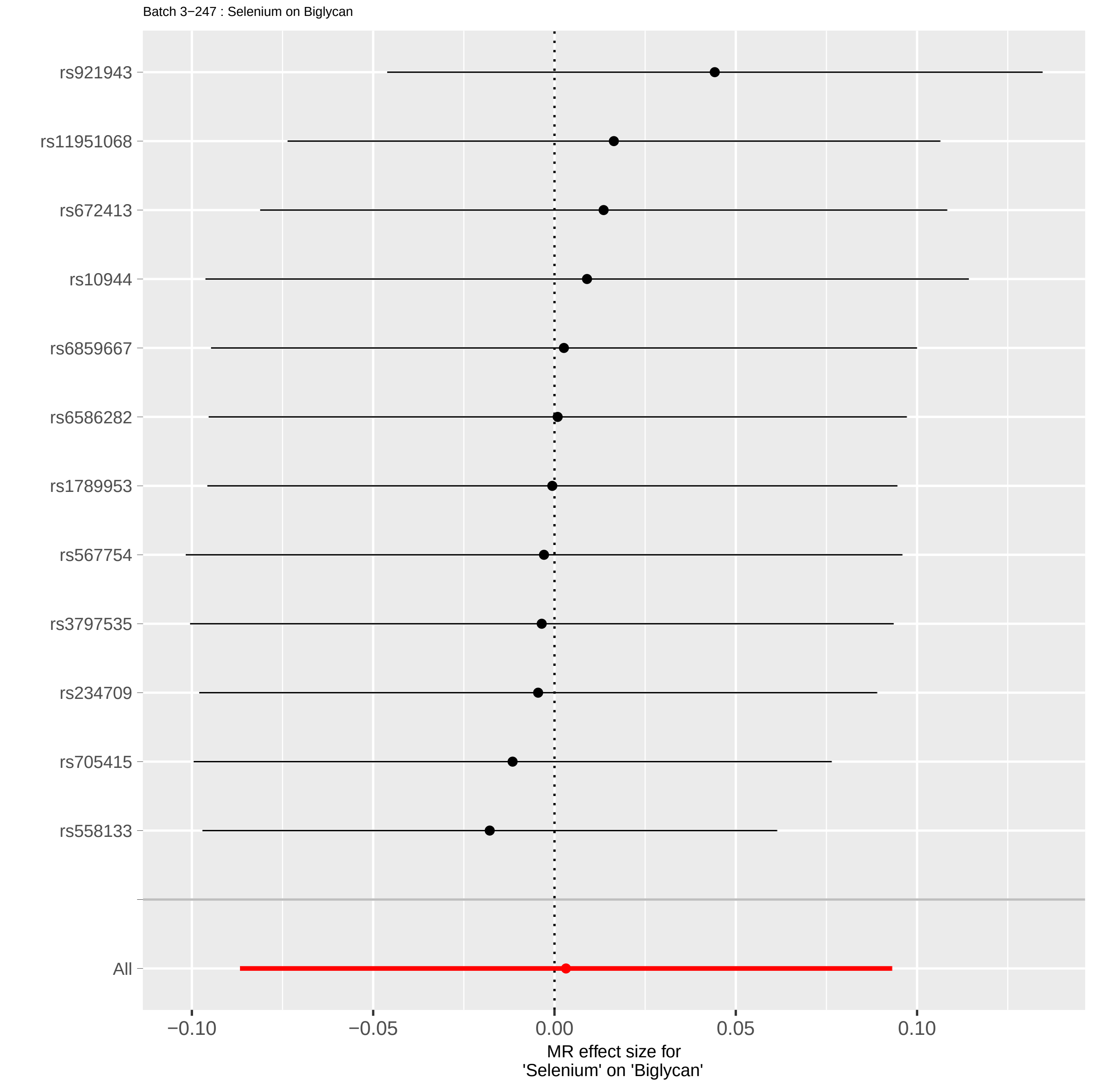
Batch 3−247 : Selenium on Biglycan
rs921943
rs11951068
rs672413
rs10944
rs6859667
rs6586282
rs1789953
rs567754
rs3797535
rs234709
rs705415
rs558133
All
−0.10
−0.05
0.00
0.05
0.10
MR effect size for
'Selenium' on 'Biglycan'

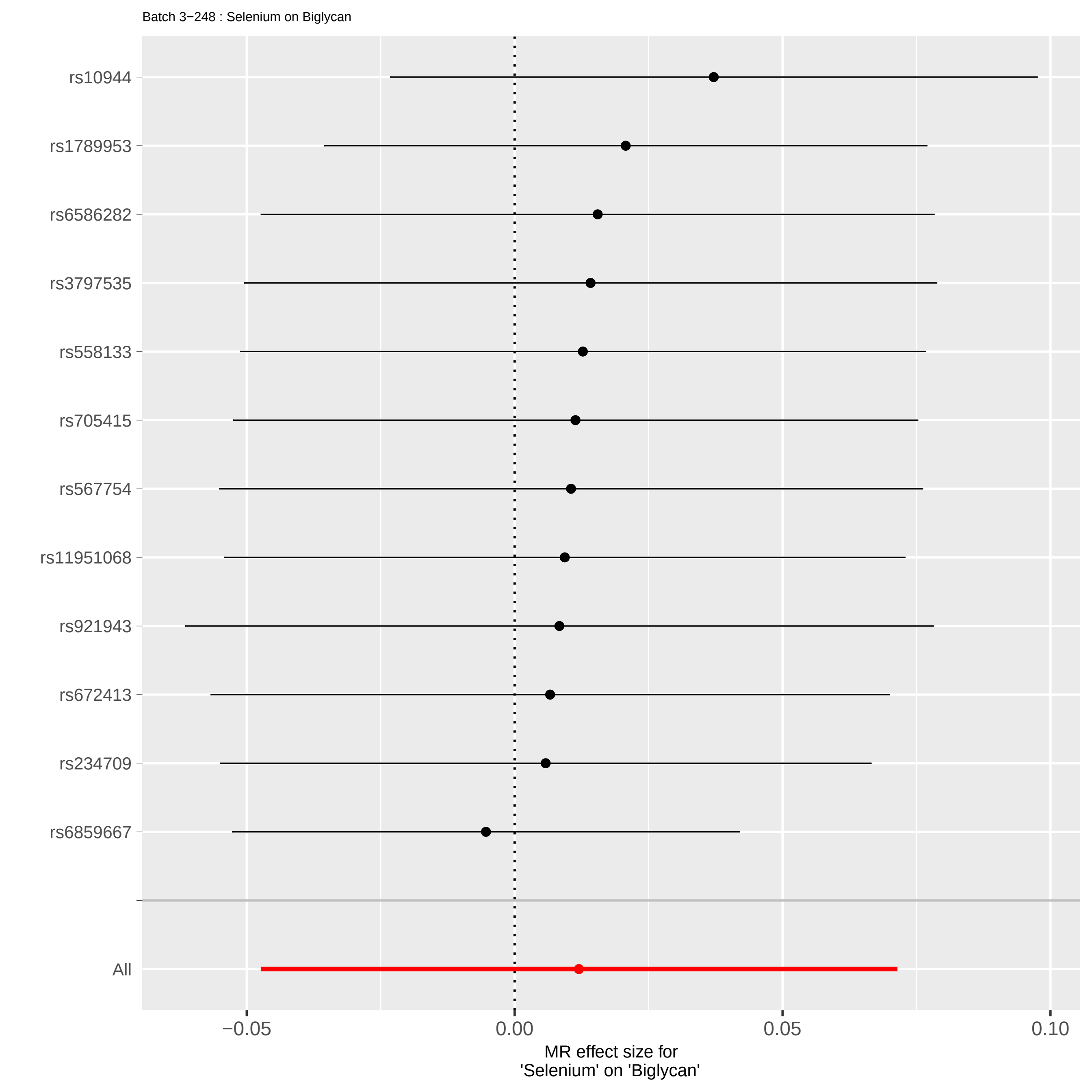
Batch 3−248 : Selenium on Biglycan
rs10944
rs1789953
rs6586282
rs3797535
rs558133
rs705415
rs567754
rs11951068
rs921943
rs672413
rs234709
rs6859667
All
−0.05
0.00
0.05
0.10
MR effect size for
'Selenium' on 'Biglycan'

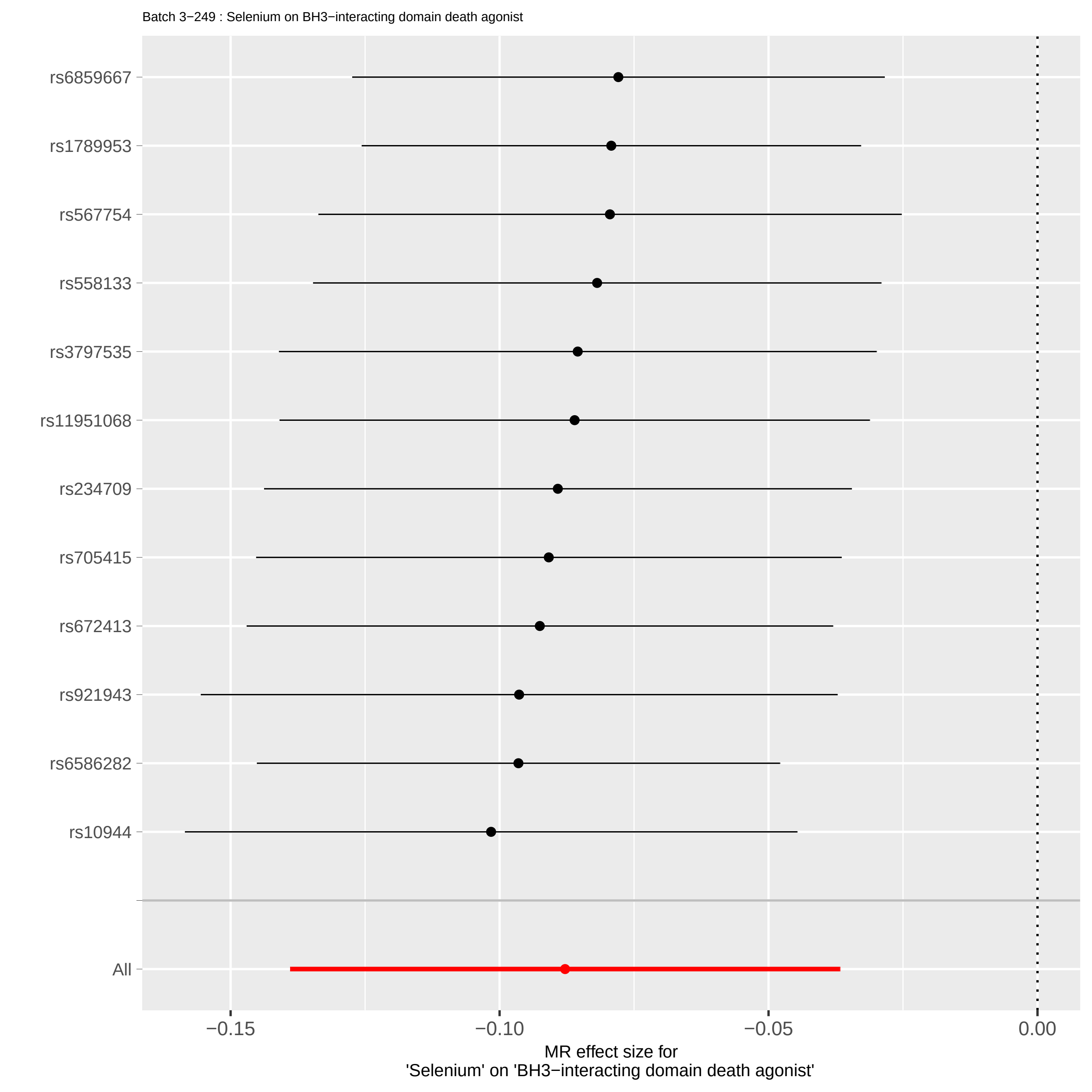

Batch 3−249 : Selenium on BH3−interacting domain death agonist
rs6859667
rs1789953
rs567754
rs558133
rs3797535
rs11951068
rs234709
rs705415
rs672413
rs921943
rs6586282
rs10944
All
−0.15
−0.10
−0.05
0.00
MR effect size for
'Selenium' on 'BH3−interacting domain death agonist'

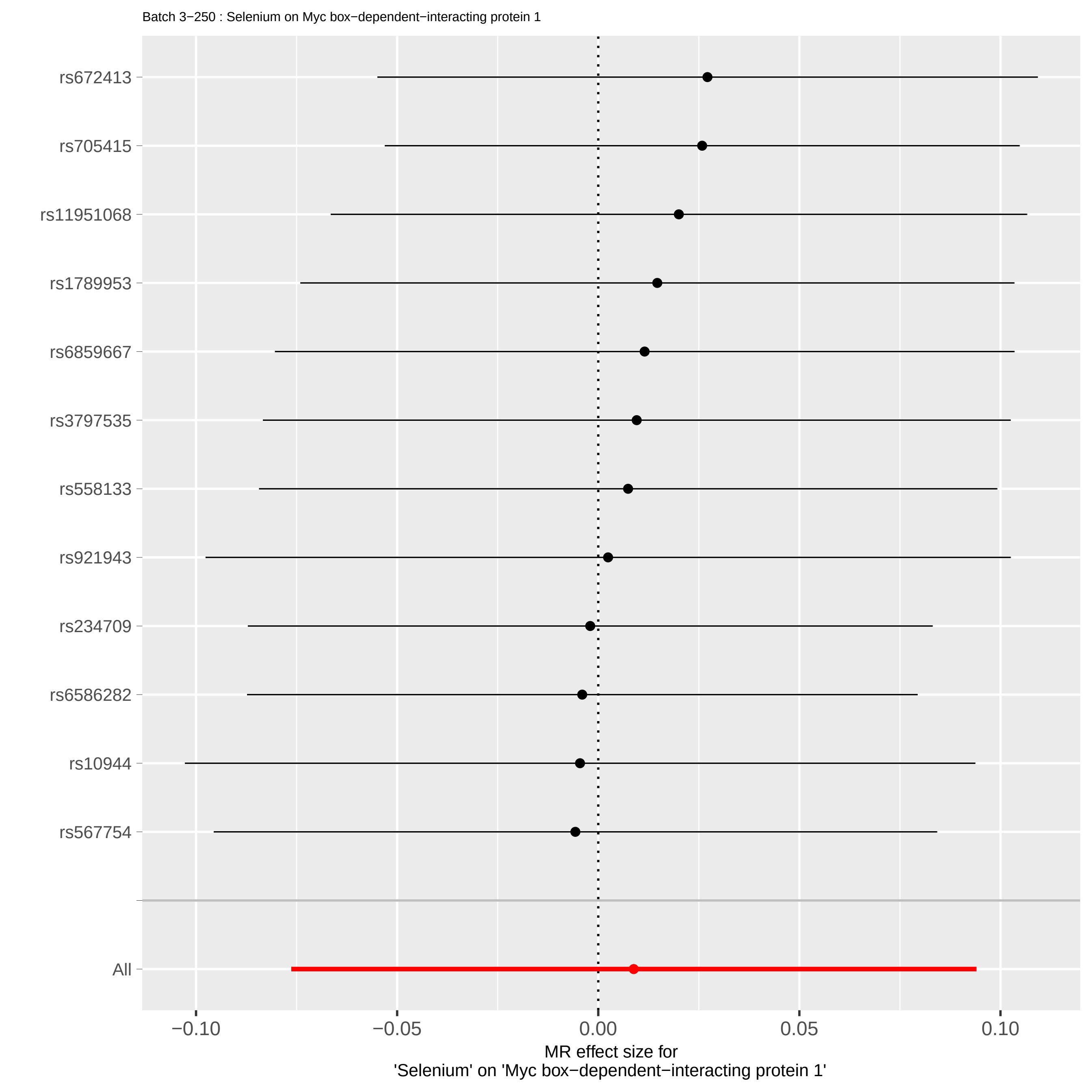

Batch 3−250 : Selenium on Myc box−dependent−interacting protein 1
rs672413
rs705415
rs11951068
rs1789953
rs6859667
rs3797535
rs558133
rs921943
rs234709
rs6586282
rs10944
rs567754
All
−0.10
−0.05
0.00
0.05
0.10
MR effect size for
'Selenium' on 'Myc box−dependent−interacting protein 1'

Batch 3−251 : Selenium on Baculoviral IAP repeat−containing protein 3

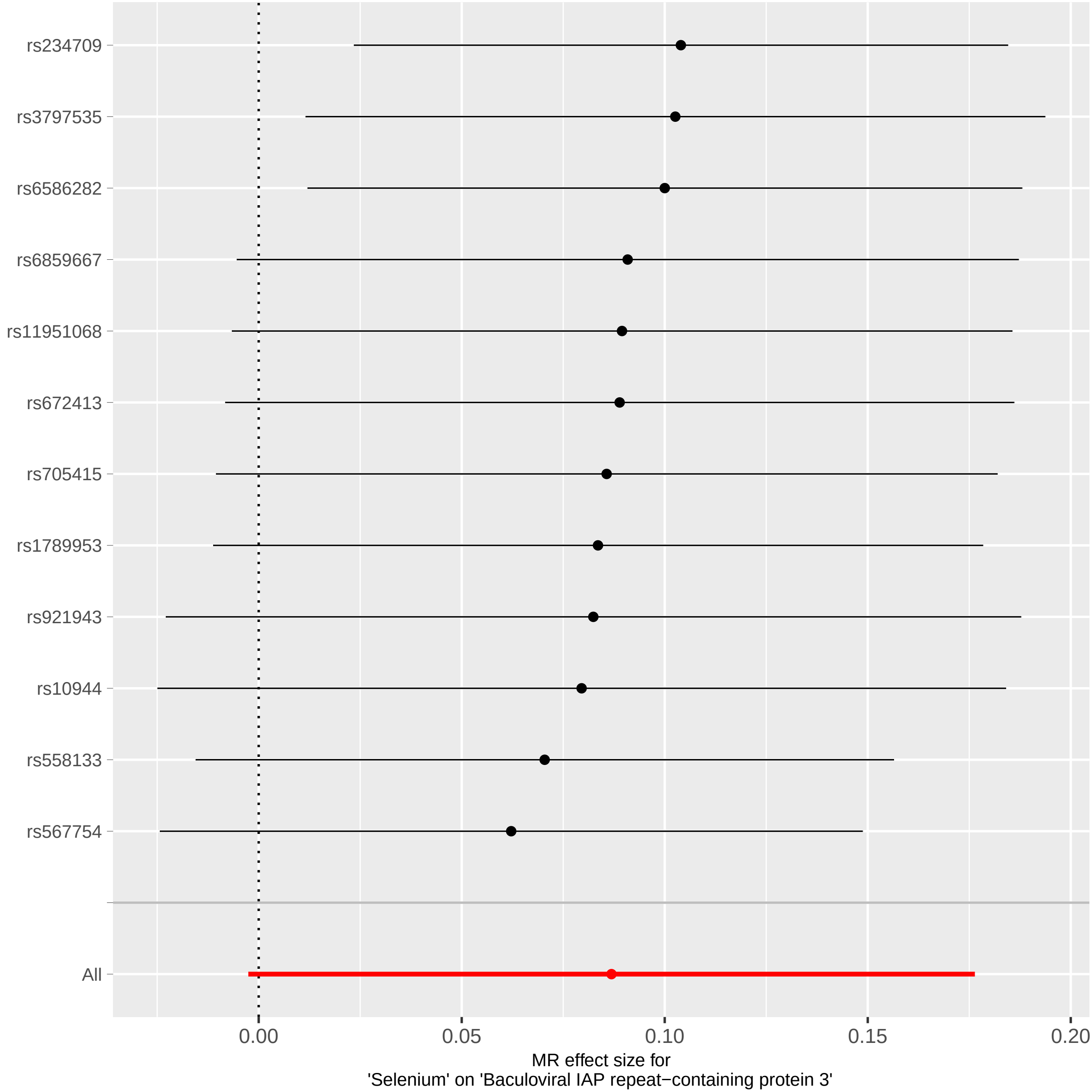

Batch 3−252 : Selenium on Baculoviral IAP repeat−containing protein 5

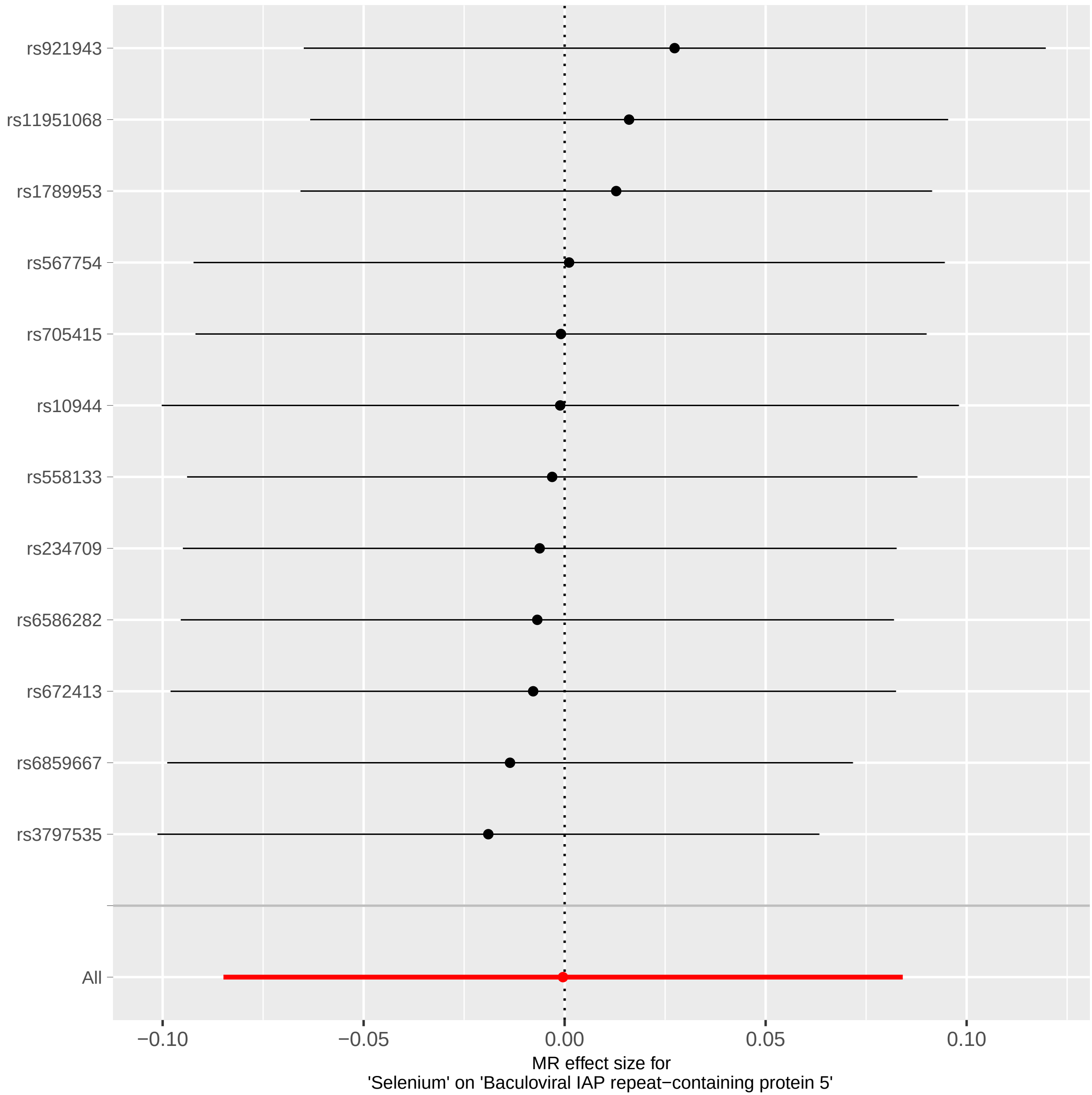

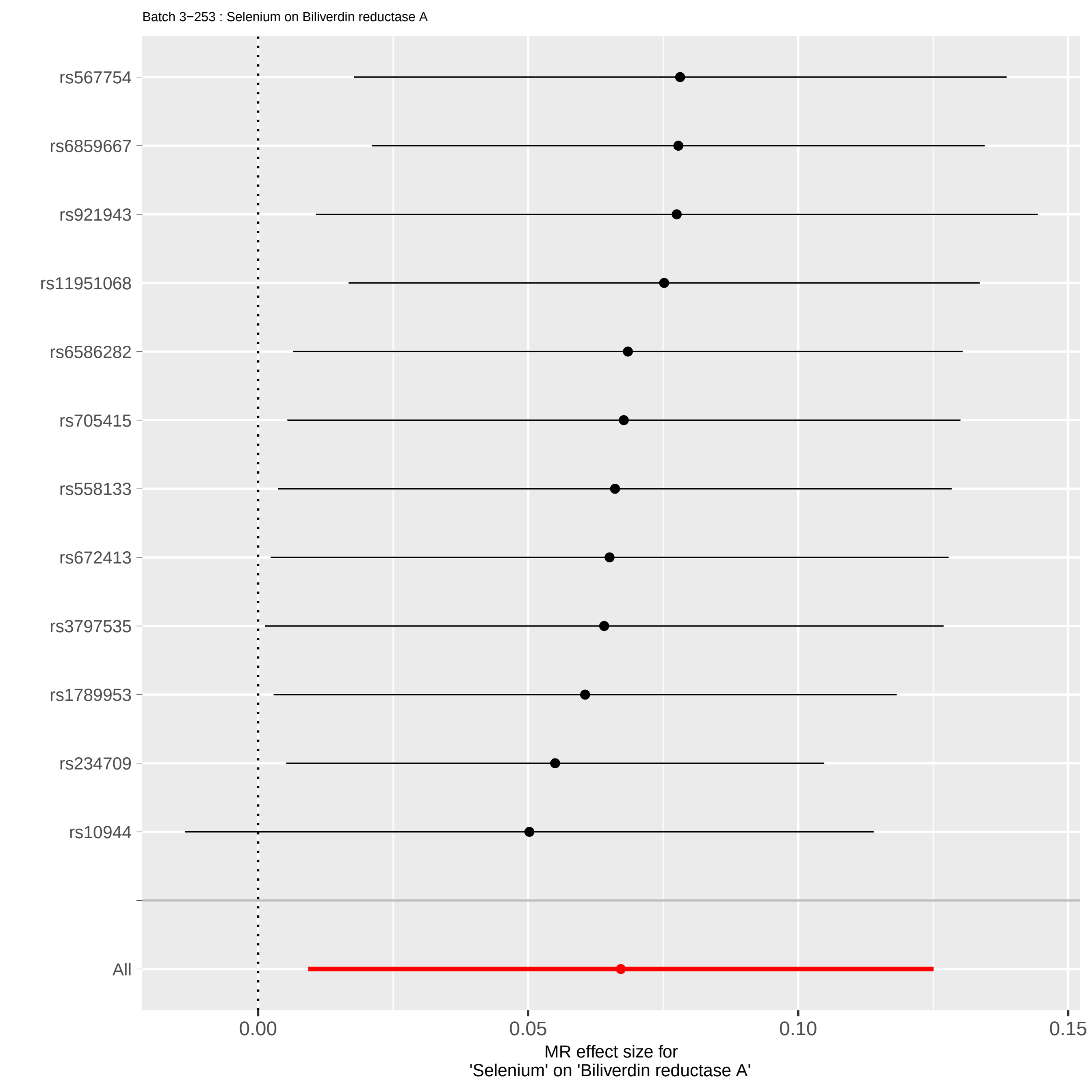

Batch 3−253 : Selenium on Biliverdin reductase A
rs567754
rs6859667
rs921943
rs11951068
rs6586282
rs705415
rs558133
rs672413
rs3797535
rs1789953
rs234709
rs10944
All
0.00
0.05
0.10
0.15
MR effect size for
'Selenium' on 'Biliverdin reductase A'

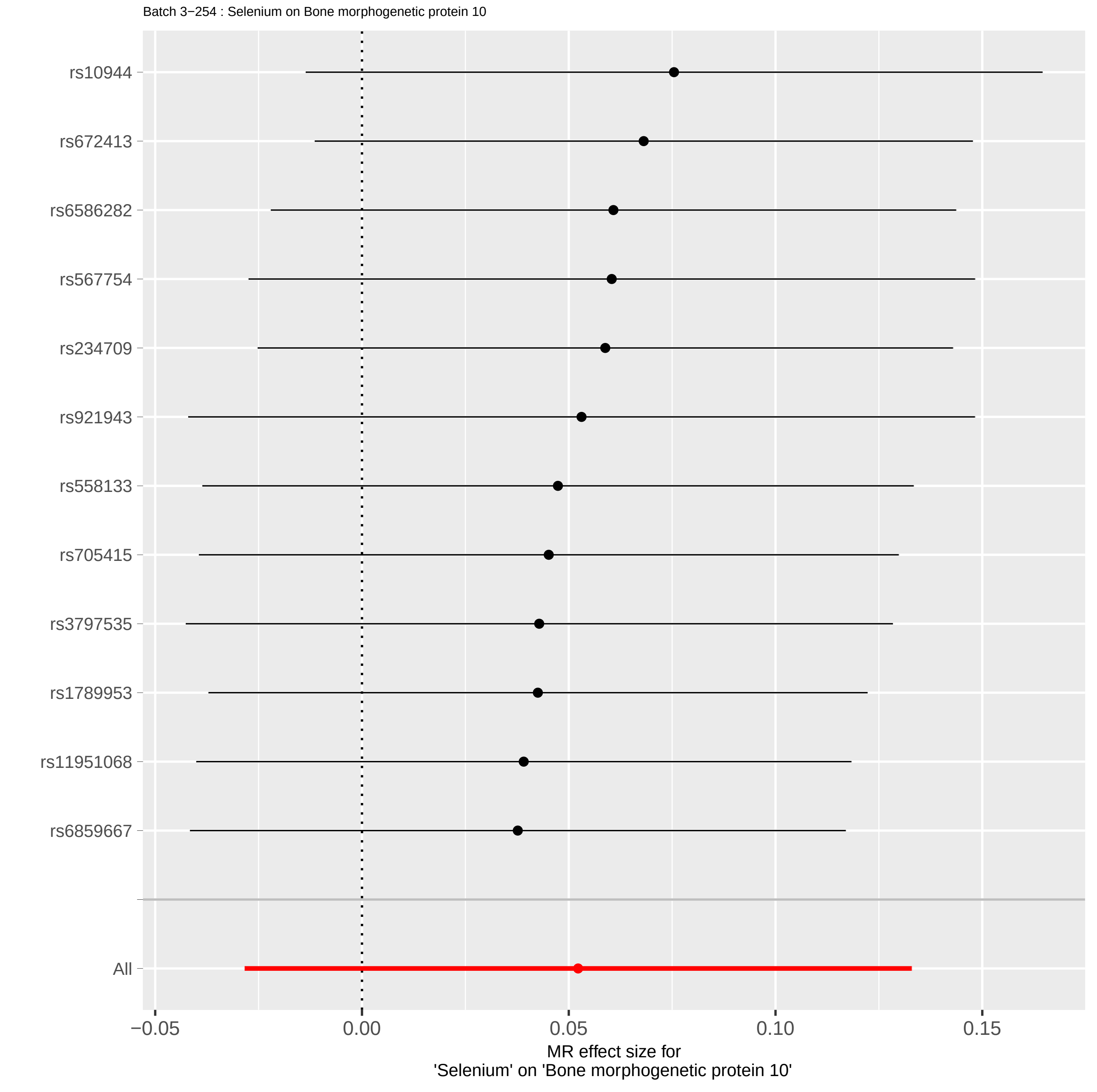

Batch 3−254 : Selenium on Bone morphogenetic protein 10
rs10944
rs672413
rs6586282
rs567754
rs234709
rs921943
rs558133
rs705415
rs3797535
rs1789953
rs11951068
rs6859667
All
−0.05
0.00
0.05
0.10
0.15
MR effect size for
'Selenium' on 'Bone morphogenetic protein 10'

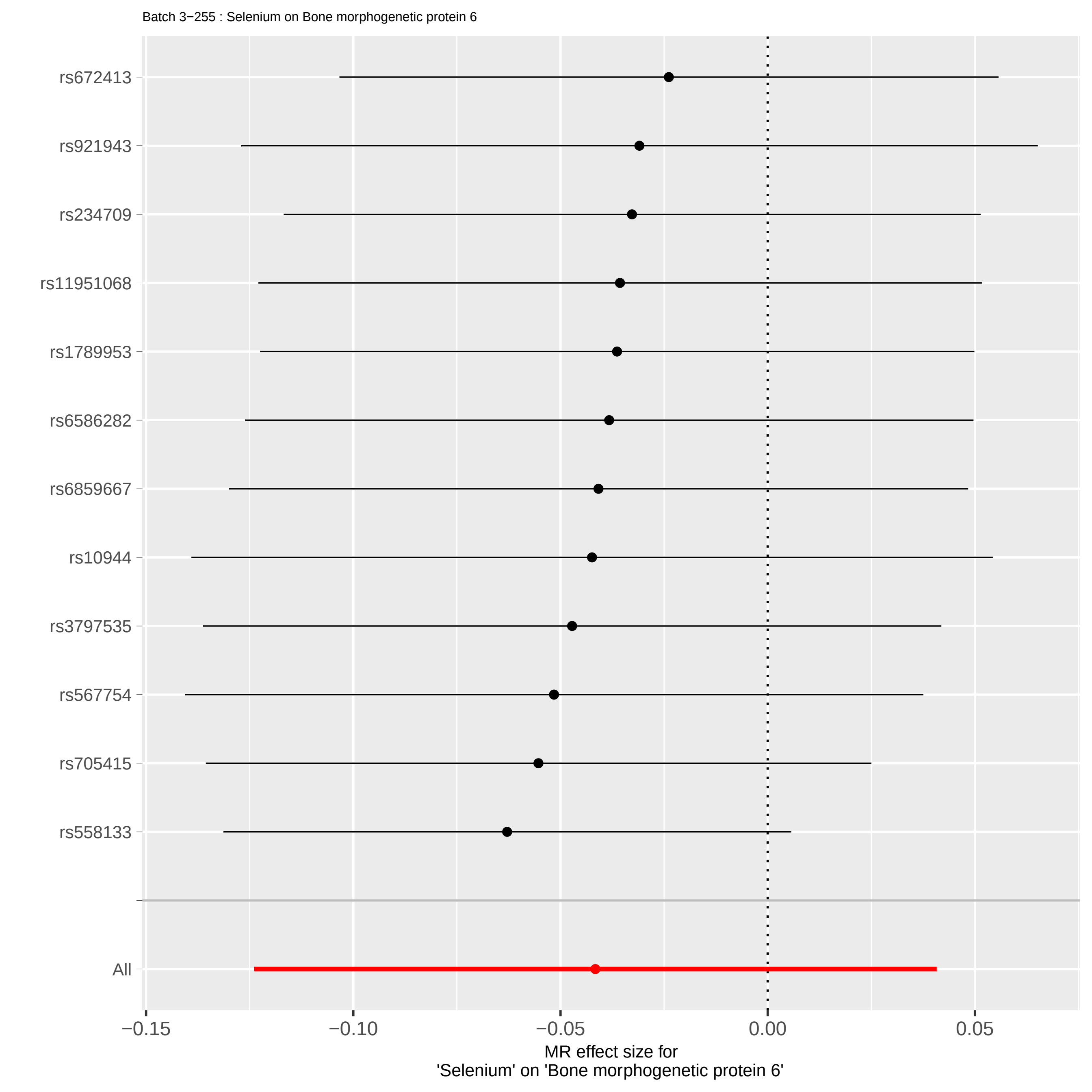

Batch 3−255 : Selenium on Bone morphogenetic protein 6
rs672413
rs921943
rs234709
rs11951068
rs1789953
rs6586282
rs6859667
rs10944
rs3797535
rs567754
rs705415
rs558133
All
−0.15
−0.10
−0.05
0.00
0.05
MR effect size for
'Selenium' on 'Bone morphogenetic protein 6'

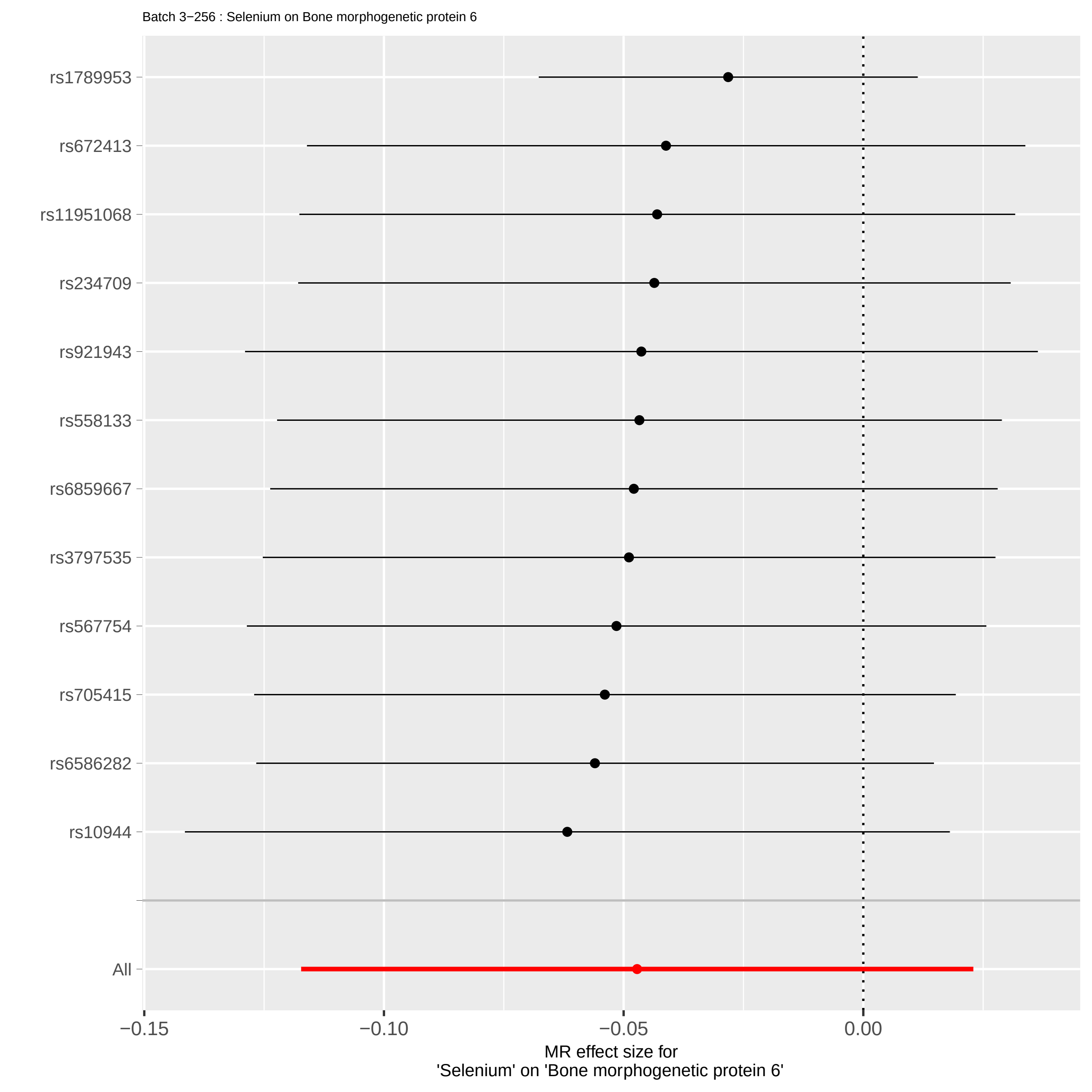

Batch 3−256 : Selenium on Bone morphogenetic protein 6
rs1789953
rs672413
rs11951068
rs234709
rs921943
rs558133
rs6859667
rs3797535
rs567754
rs705415
rs6586282
rs10944
All
−0.15
−0.10
−0.05
0.00
MR effect size for
'Selenium' on 'Bone morphogenetic protein 6'

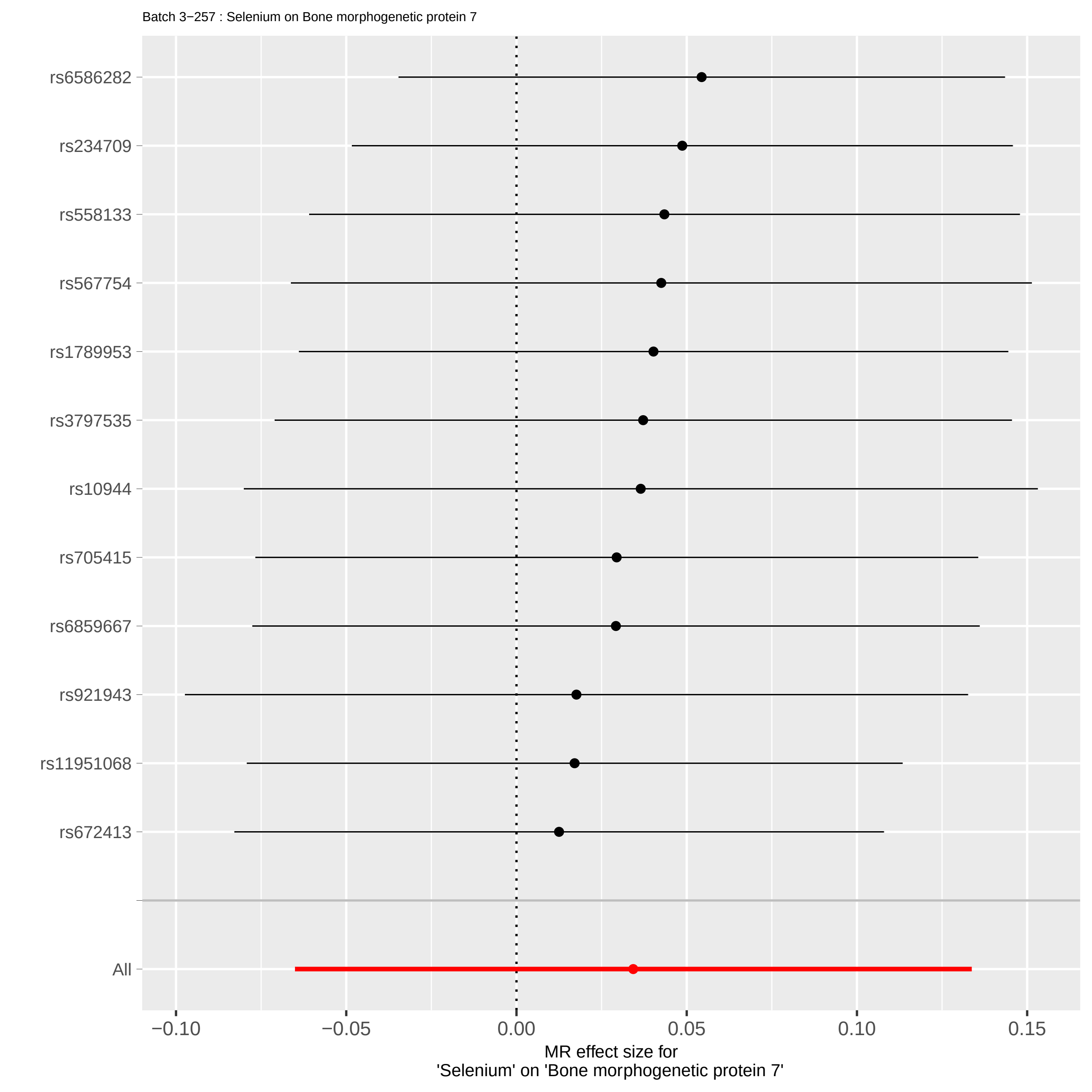

Batch 3−257 : Selenium on Bone morphogenetic protein 7
rs6586282
rs234709
rs558133
rs567754
rs1789953
rs3797535
rs10944
rs705415
rs6859667
rs921943
rs11951068
rs672413
All
−0.10
−0.05
0.00
0.05
0.10
0.15
MR effect size for
'Selenium' on 'Bone morphogenetic protein 7'

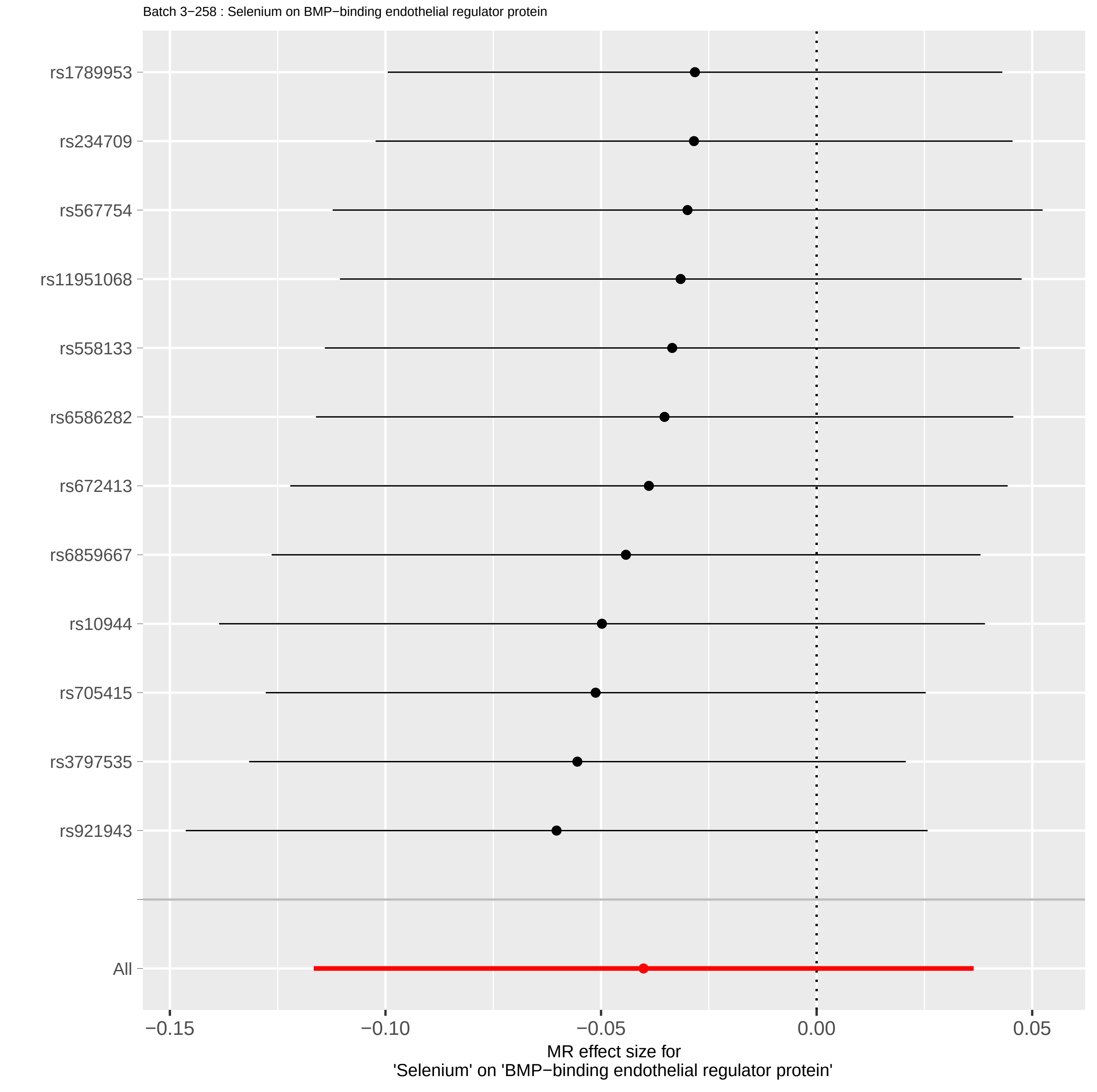

Batch 3−258 : Selenium on BMP−binding endothelial regulator protein
rs1789953
rs234709
rs567754
rs11951068
rs558133
rs6586282
rs672413
rs6859667
rs10944
rs705415
rs3797535
rs921943
All
−0.15
−0.10
−0.05
0.00
0.05
MR effect size for
'Selenium' on 'BMP−binding endothelial regulator protein'

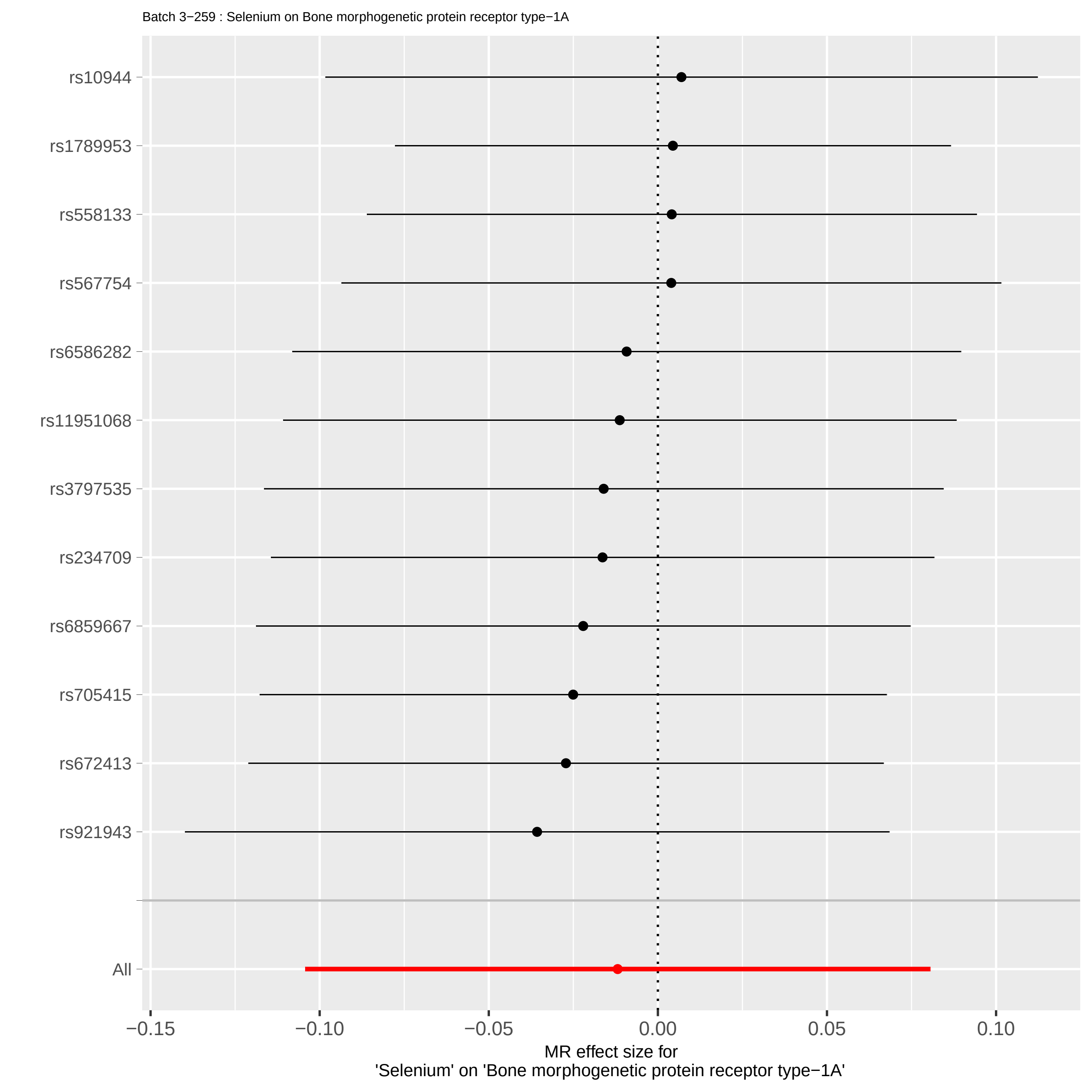

Batch 3−259 : Selenium on Bone morphogenetic protein receptor type−1A
rs10944
rs1789953
rs558133
rs567754
rs6586282
rs11951068
rs3797535
rs234709
rs6859667
rs705415
rs672413
rs921943
All
−0.15
−0.10
−0.05
0.00
0.05
0.10
MR effect size for
'Selenium' on 'Bone morphogenetic protein receptor type−1A'

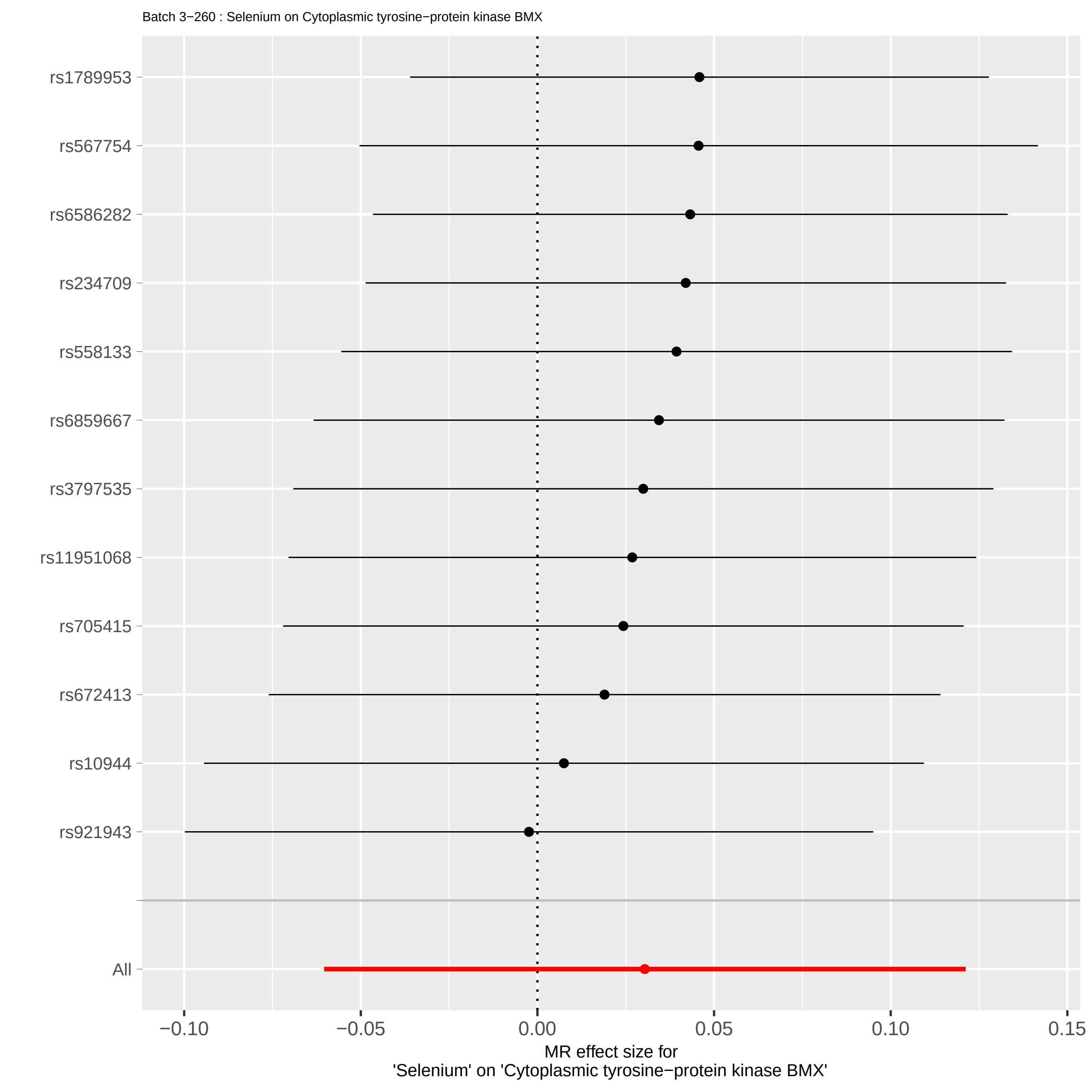

Batch 3−260 : Selenium on Cytoplasmic tyrosine−protein kinase BMX
rs1789953
rs567754
rs6586282
rs234709
rs558133
rs6859667
rs3797535
rs11951068
rs705415
rs672413
rs10944
rs921943
All
−0.10
−0.05
0.00
0.05
0.10
0.15
MR effect size for
'Selenium' on 'Cytoplasmic tyrosine−protein kinase BMX'

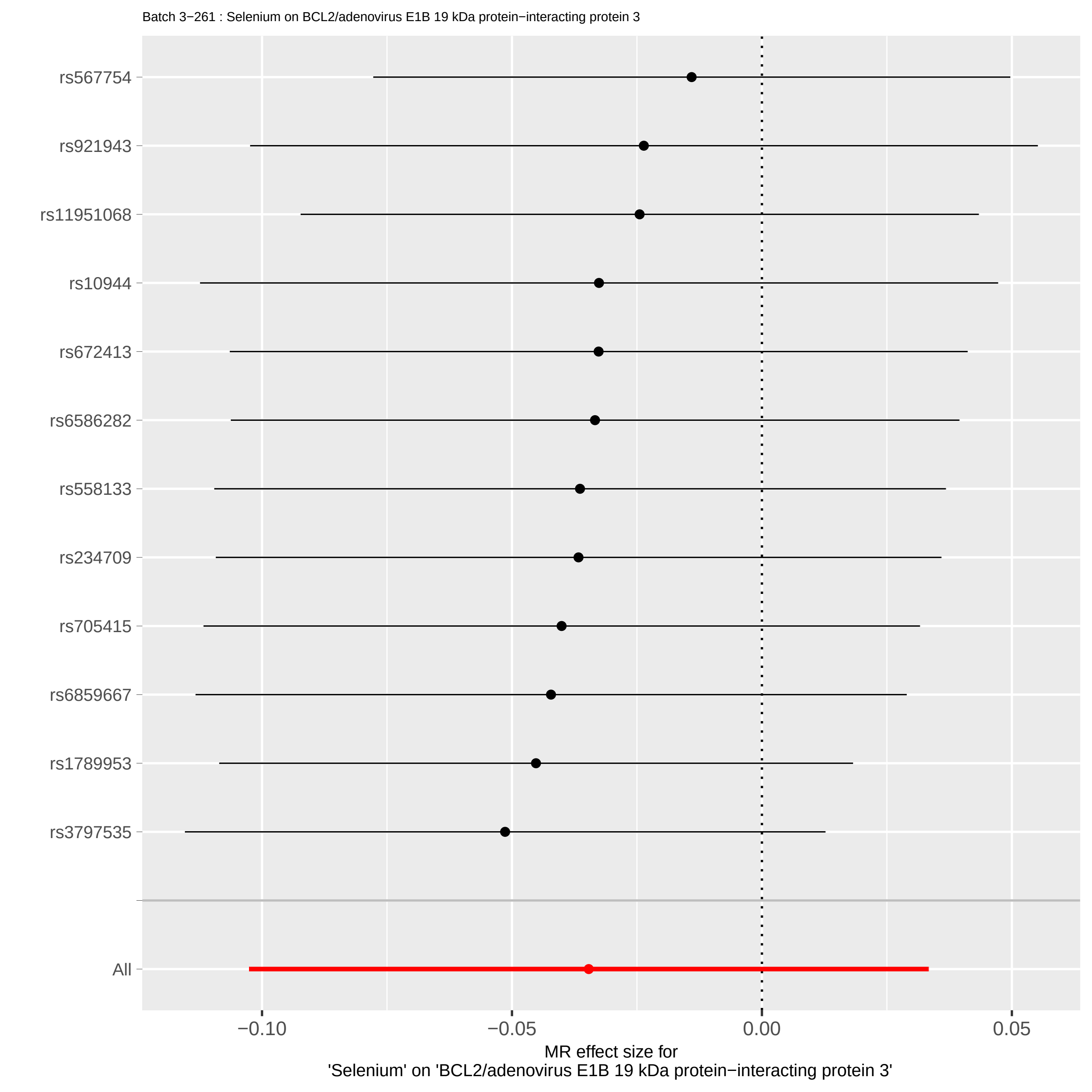

Batch 3−261 : Selenium on BCL2/adenovirus E1B 19 kDa protein−interacting protein 3
rs567754
rs921943
rs11951068
rs10944
rs672413
rs6586282
rs558133
rs234709
rs705415
rs6859667
rs1789953
rs3797535
All
−0.10
−0.05
0.00
0.05
MR effect size for
'Selenium' on 'BCL2/adenovirus E1B 19 kDa protein−interacting protein 3'

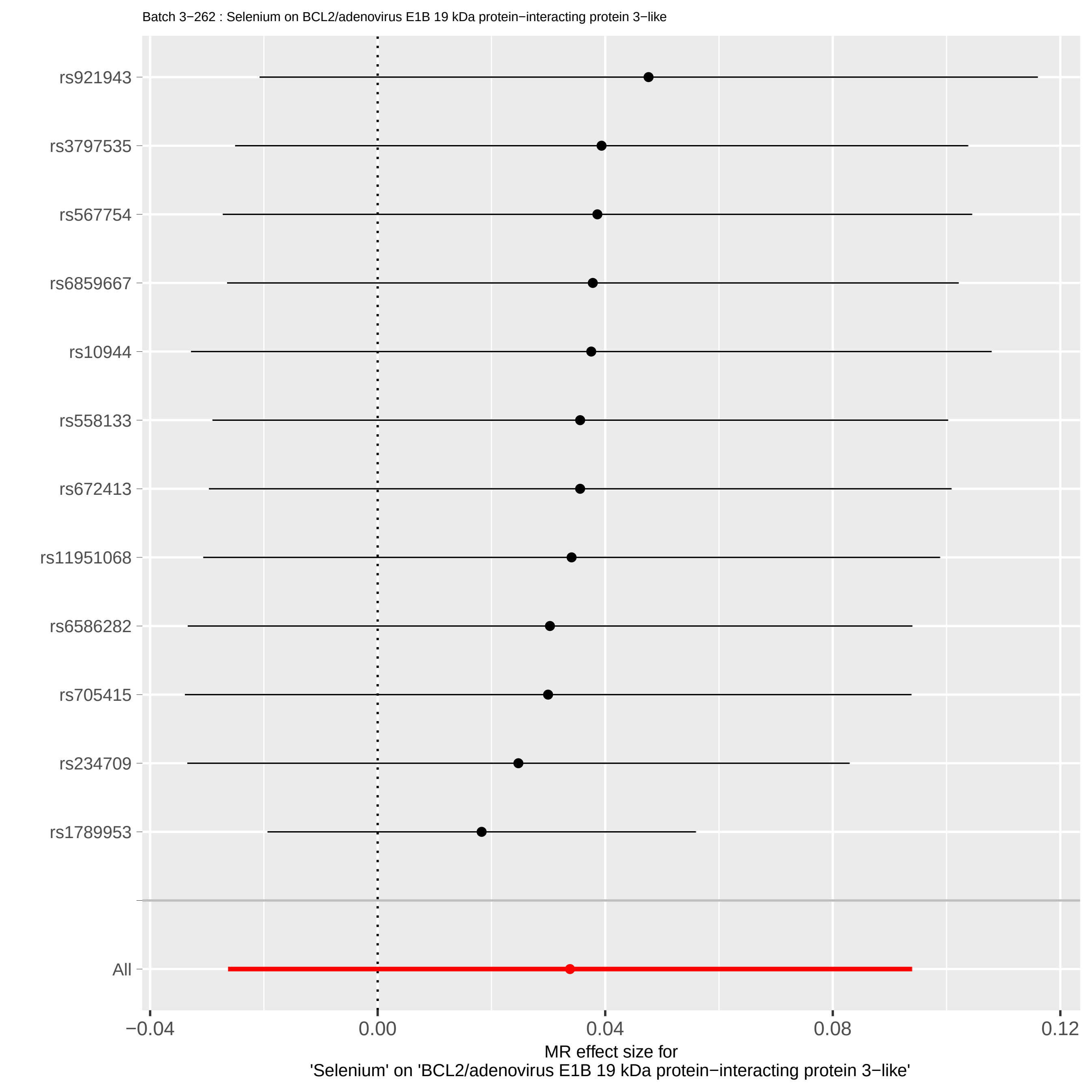
Batch 3−262 : Selenium on BCL2/adenovirus E1B 19 kDa protein−interacting protein 3−like
rs921943
rs3797535
rs567754
rs6859667
rs10944
rs558133
rs672413
rs11951068
rs6586282
rs705415
rs234709
rs1789953
All
−0.04
0.00
0.04
0.08
0.12
MR effect size for
'Selenium' on 'BCL2/adenovirus E1B 19 kDa protein−interacting protein 3−like'

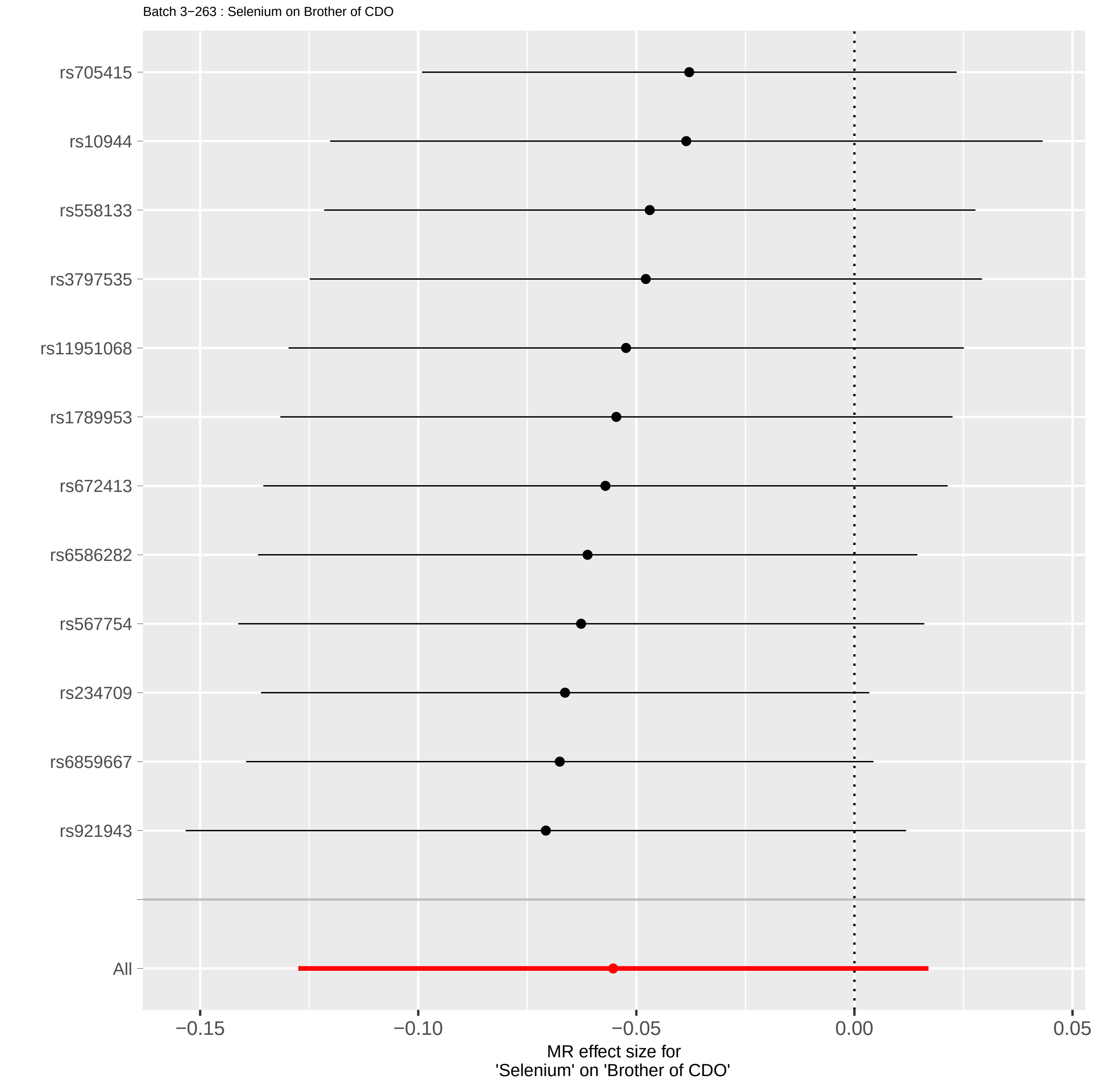

Batch 3−263 : Selenium on Brother of CDO
rs705415
rs10944
rs558133
rs3797535
rs11951068
rs1789953
rs672413
rs6586282
rs567754
rs234709
rs6859667
rs921943
All
−0.15
−0.10
−0.05
0.00
0.05
MR effect size for
'Selenium' on 'Brother of CDO'

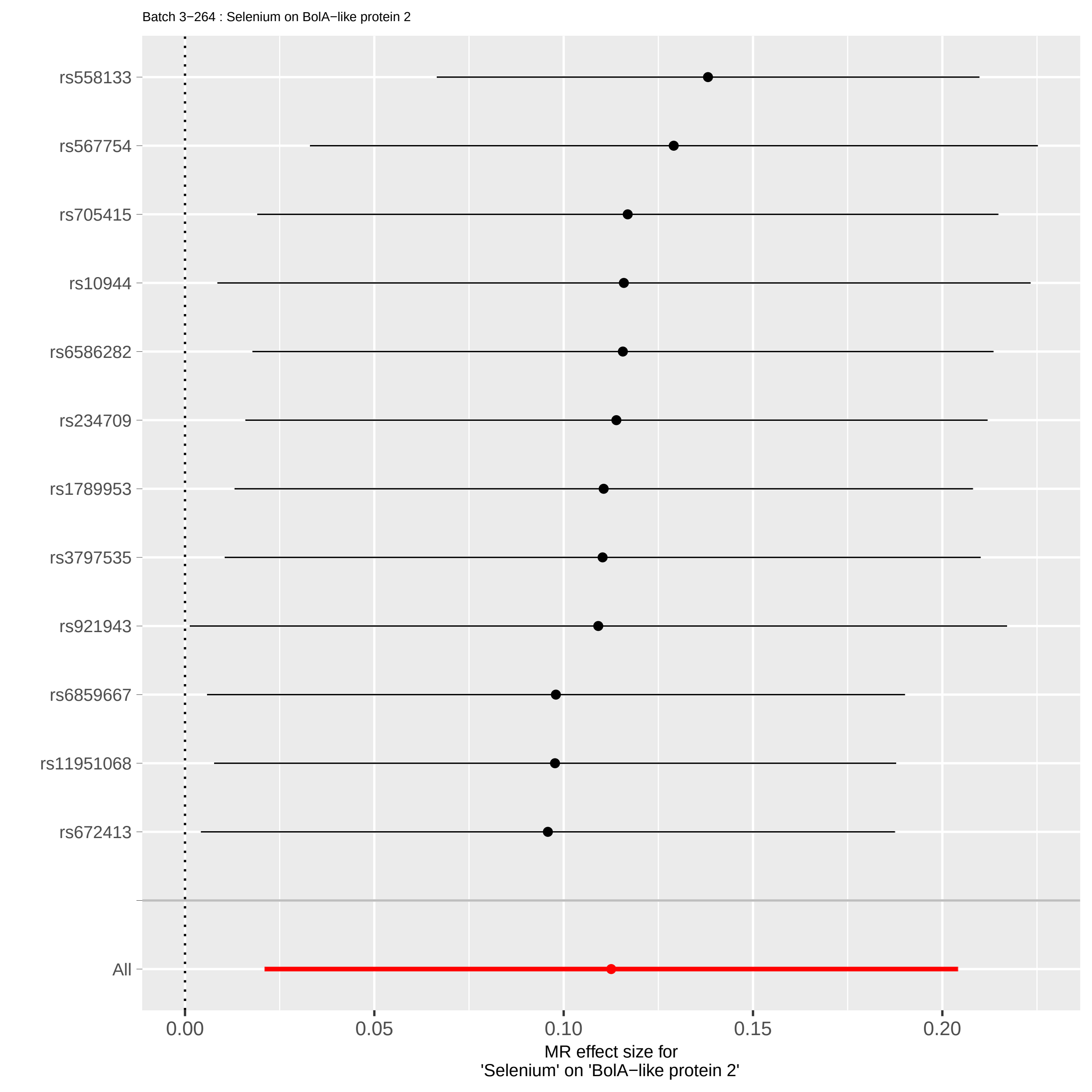

Batch 3−264 : Selenium on BolA−like protein 2
rs558133
rs567754
rs705415
rs10944
rs6586282
rs234709
rs1789953
rs3797535
rs921943
rs6859667
rs11951068
rs672413
All
0.00
0.05
0.10
0.15
0.20
MR effect size for
'Selenium' on 'BolA−like protein 2'

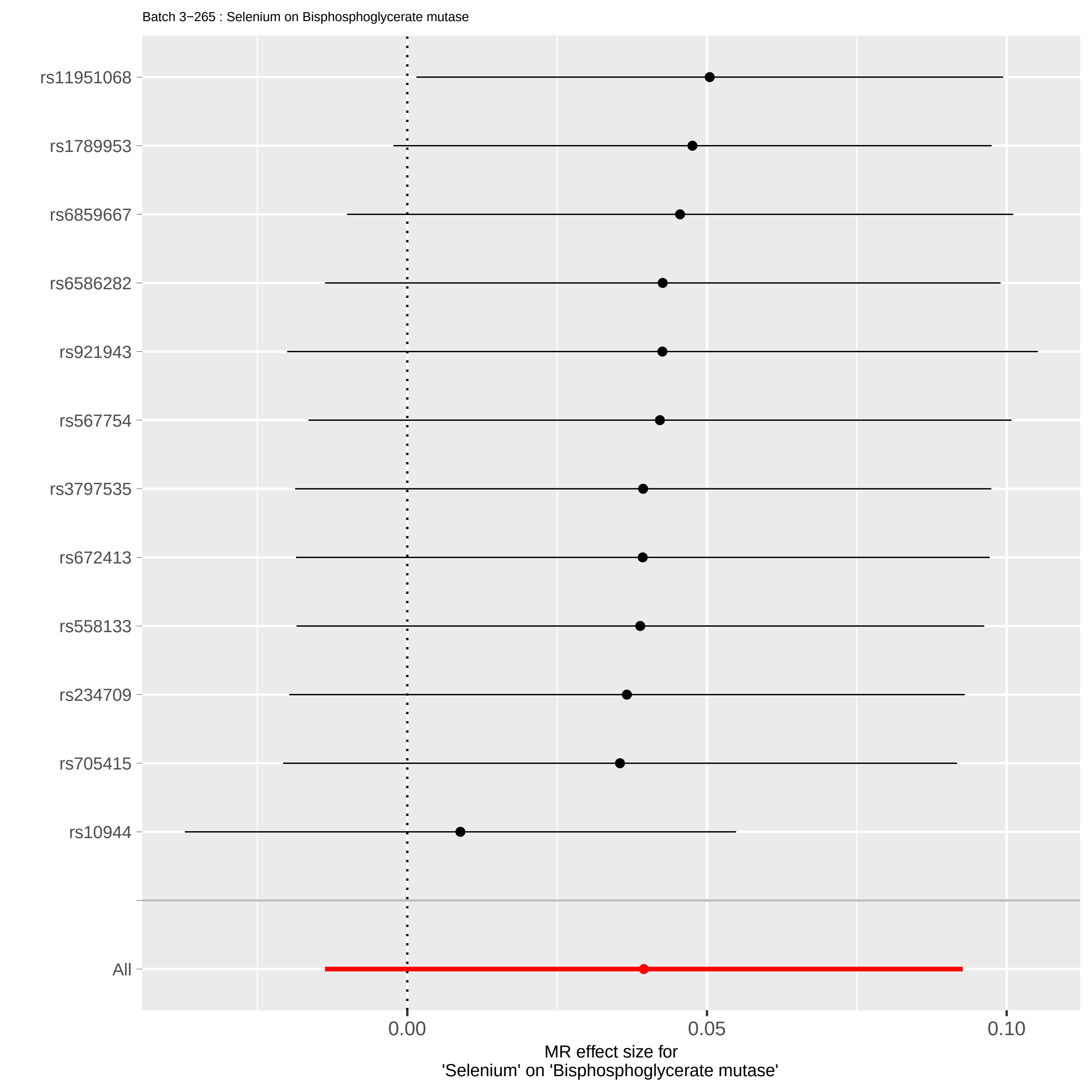
Batch 3−265 : Selenium on Bisphosphoglycerate mutase
rs11951068
rs1789953
rs6859667
rs6586282
rs921943
rs567754
rs3797535
rs672413
rs558133
rs234709
rs705415
rs10944
All
0.00
0.05
0.10
MR effect size for
'Selenium' on 'Bisphosphoglycerate mutase'

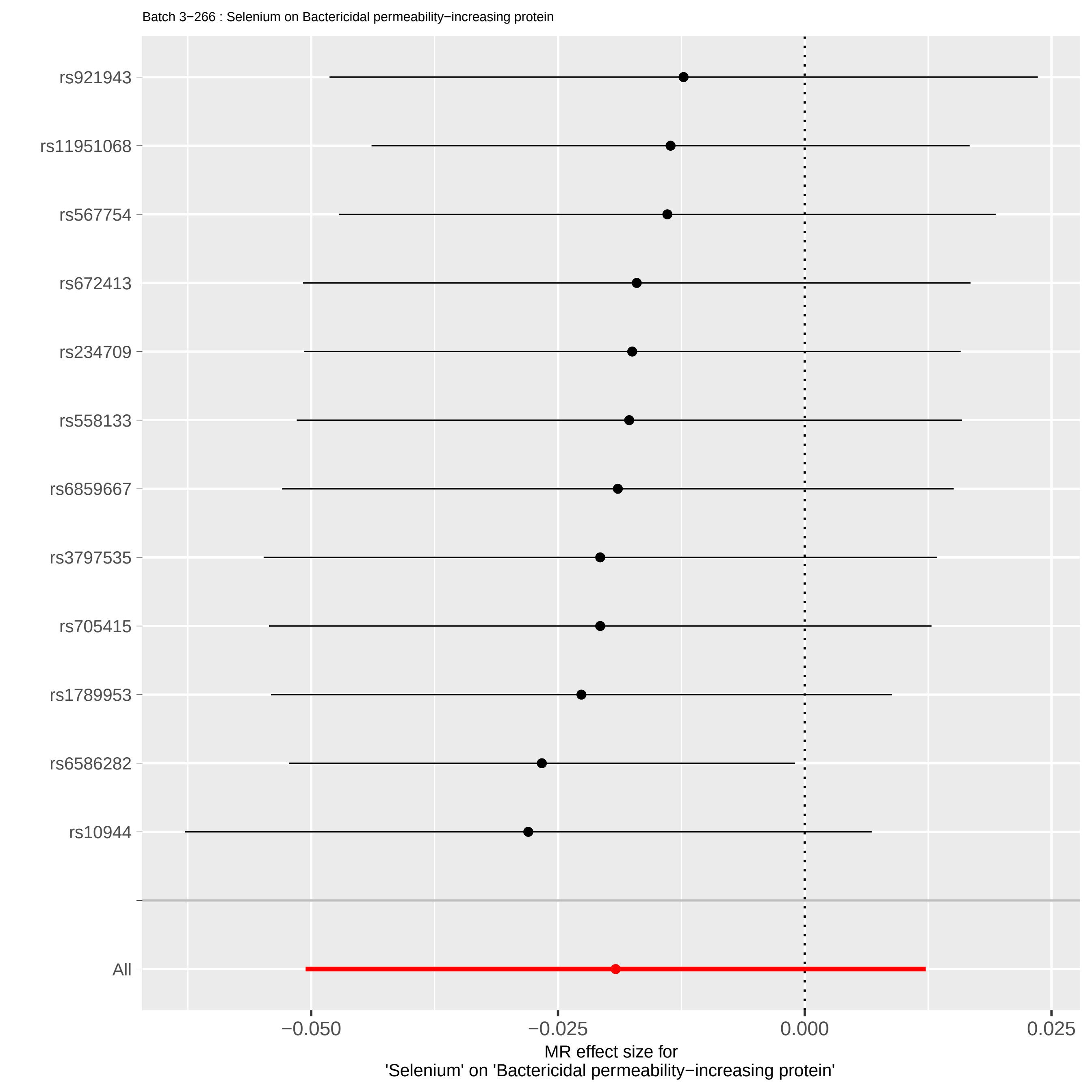
Batch 3−266 : Selenium on Bactericidal permeability−increasing protein
rs921943
rs11951068
rs567754
rs672413
rs234709
rs558133
rs6859667
rs3797535
rs705415
rs1789953
rs6586282
rs10944
All
−0.050
−0.025
0.000
0.025
MR effect size for
'Selenium' on 'Bactericidal permeability−increasing protein'

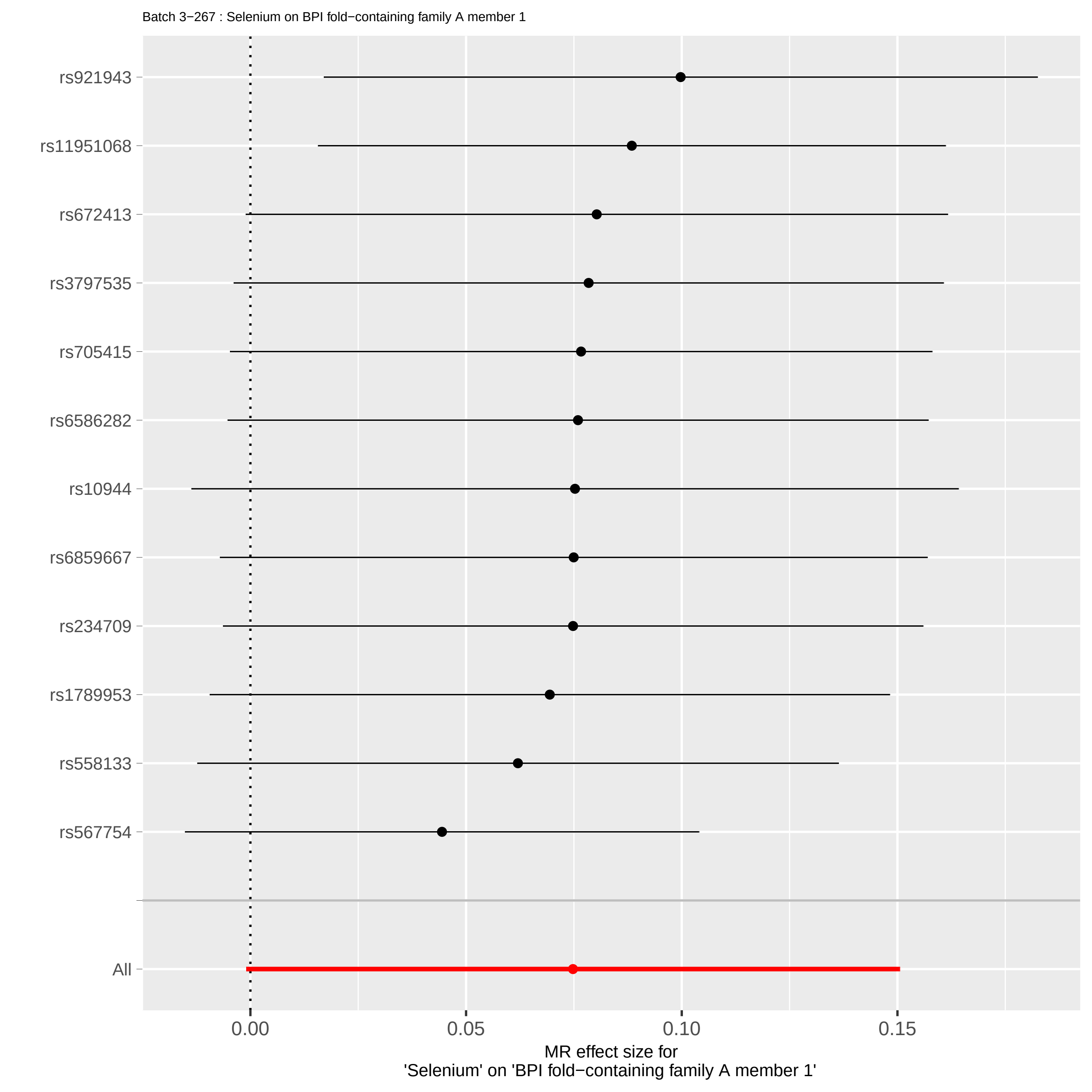
Batch 3−267 : Selenium on BPI fold−containing family A member 1
rs921943
rs11951068
rs672413
rs3797535
rs705415
rs6586282
rs10944
rs6859667
rs234709
rs1789953
rs558133
rs567754
All
0.00
0.05
0.10
0.15
MR effect size for
'Selenium' on 'BPI fold−containing family A member 1'

Batch 3−268 : Selenium on BPI fold−containing family B member 1

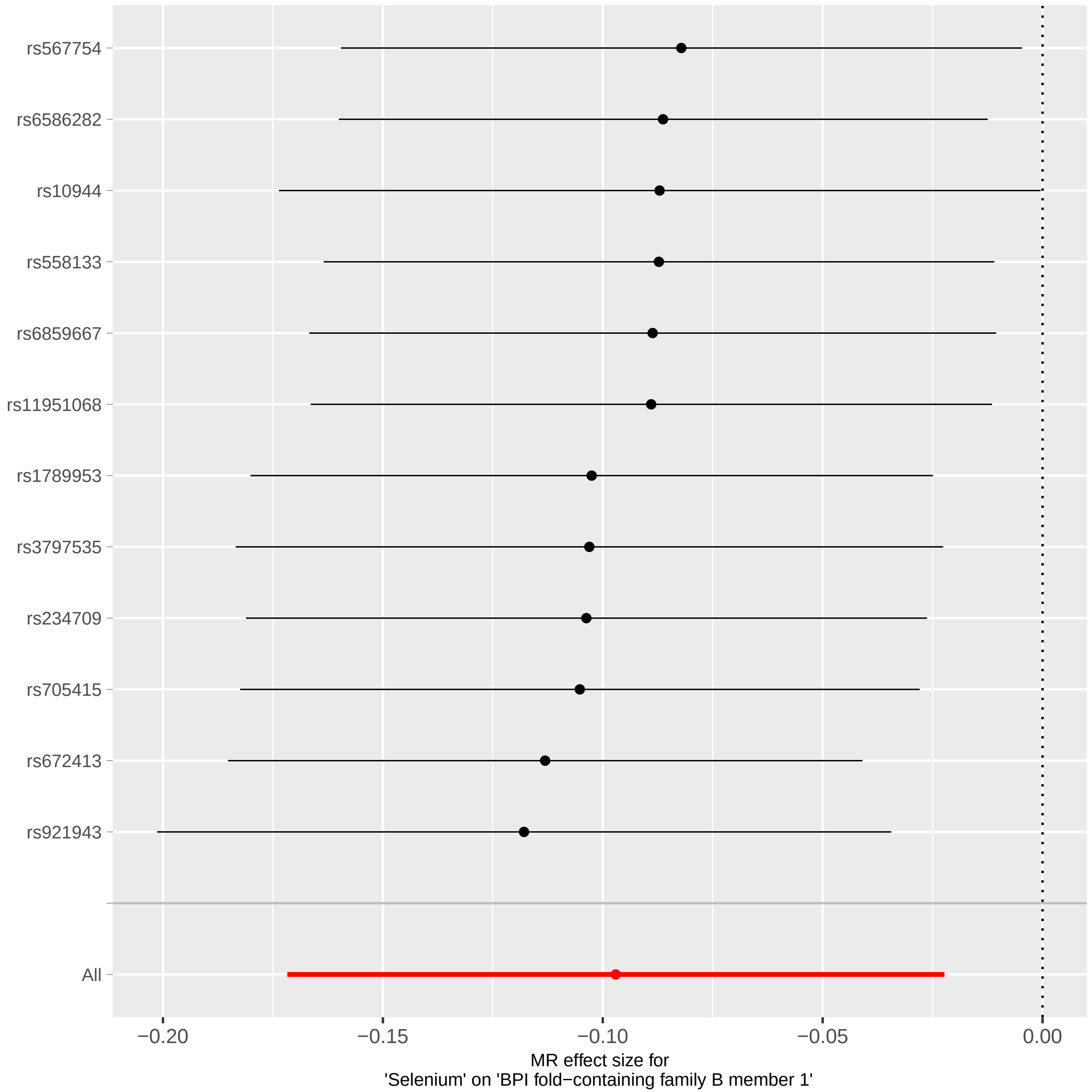

Batch 3−269 : Selenium on Bromodomain−containing protein 1

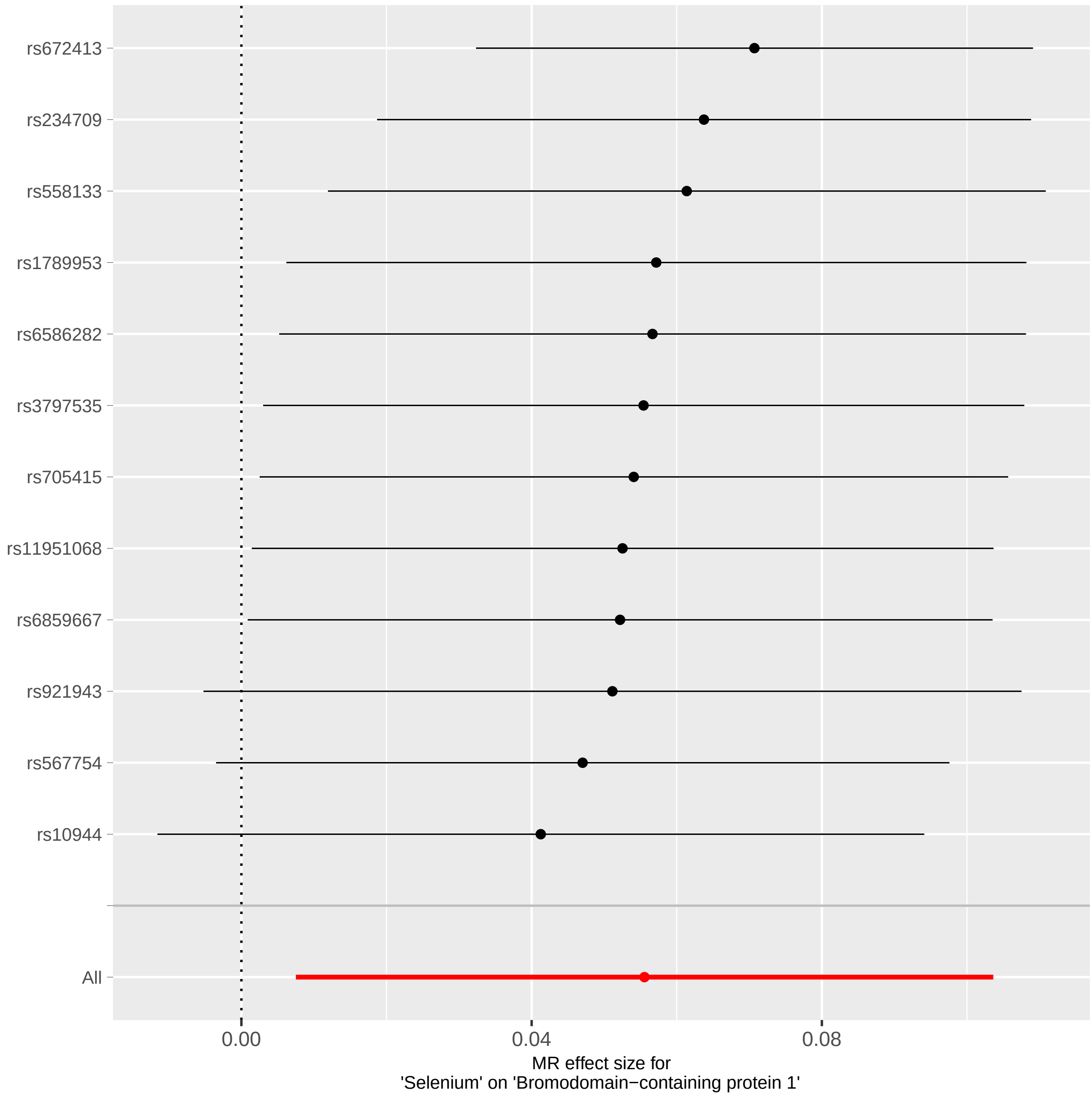

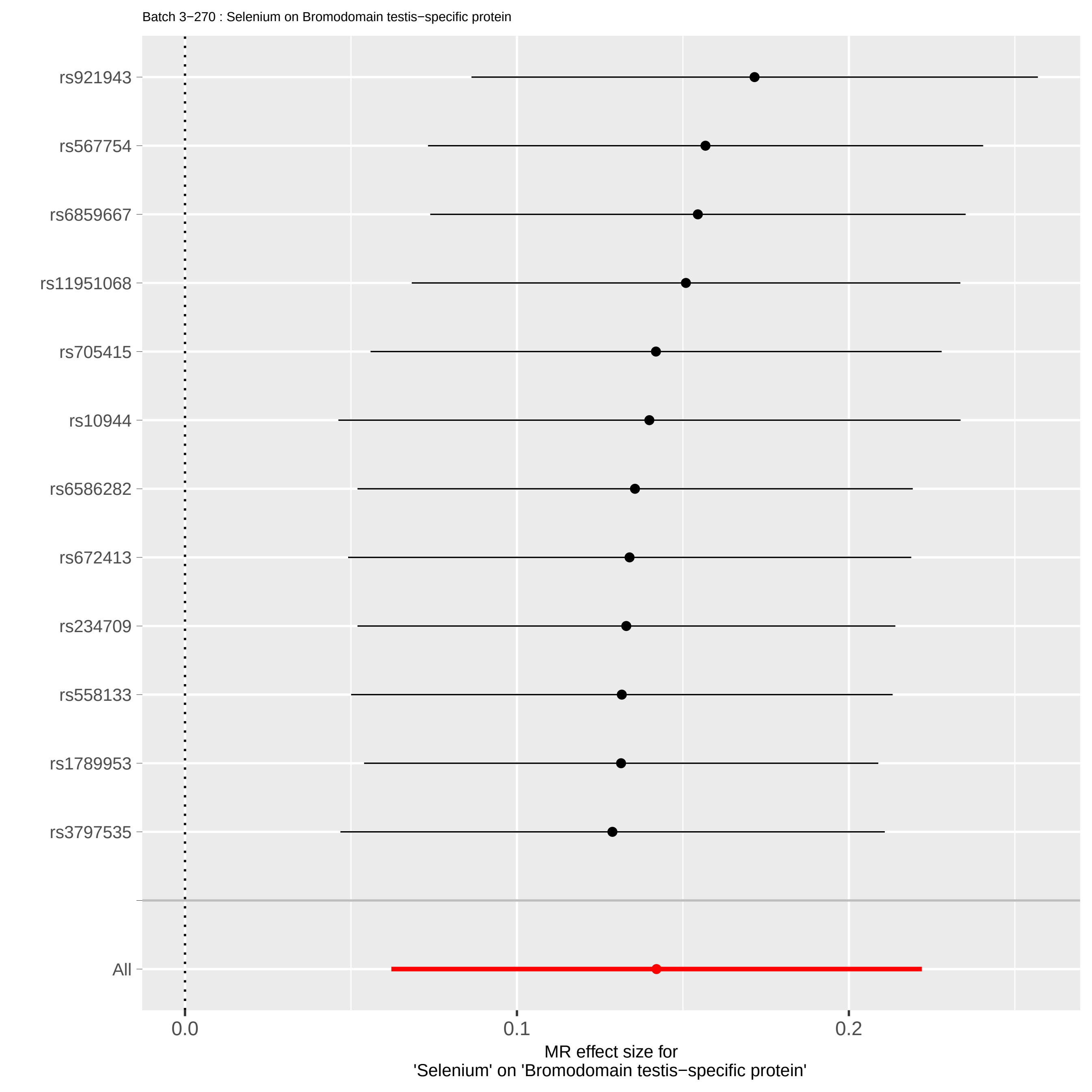

Batch 3−270 : Selenium on Bromodomain testis−specific protein
rs921943
rs567754
rs6859667
rs11951068
rs705415
rs10944
rs6586282
rs672413
rs234709
rs558133
rs1789953
rs3797535
All
0.0
0.1
0.2
MR effect size for
'Selenium' on 'Bromodomain testis−specific protein'

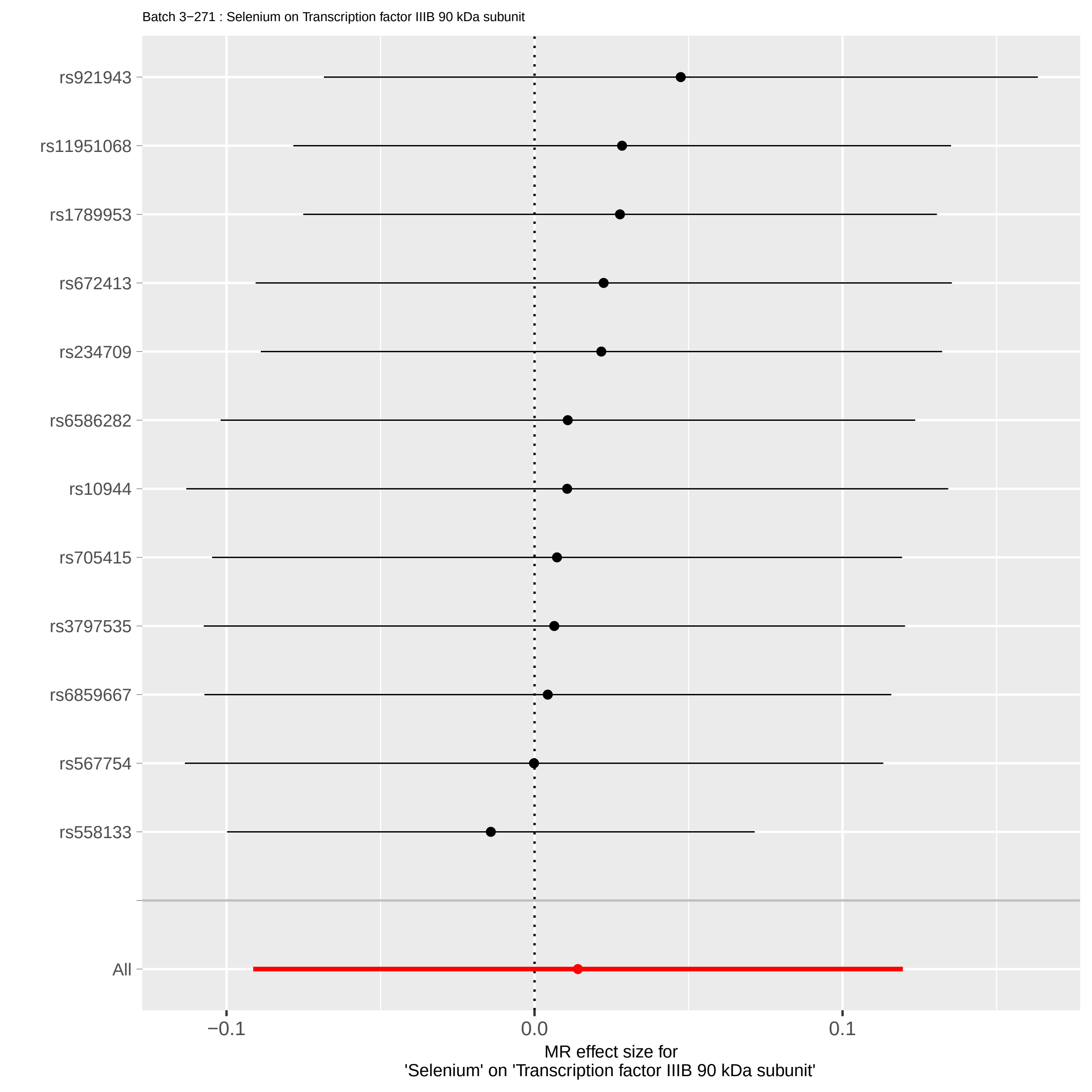

Batch 3−271 : Selenium on Transcription factor IIIB 90 kDa subunit
rs921943
rs11951068
rs1789953
rs672413
rs234709
rs6586282
rs10944
rs705415
rs3797535
rs6859667
rs567754
rs558133
All
−0.1
0.0
0.1
MR effect size for
'Selenium' on 'Transcription factor IIIB 90 kDa subunit'

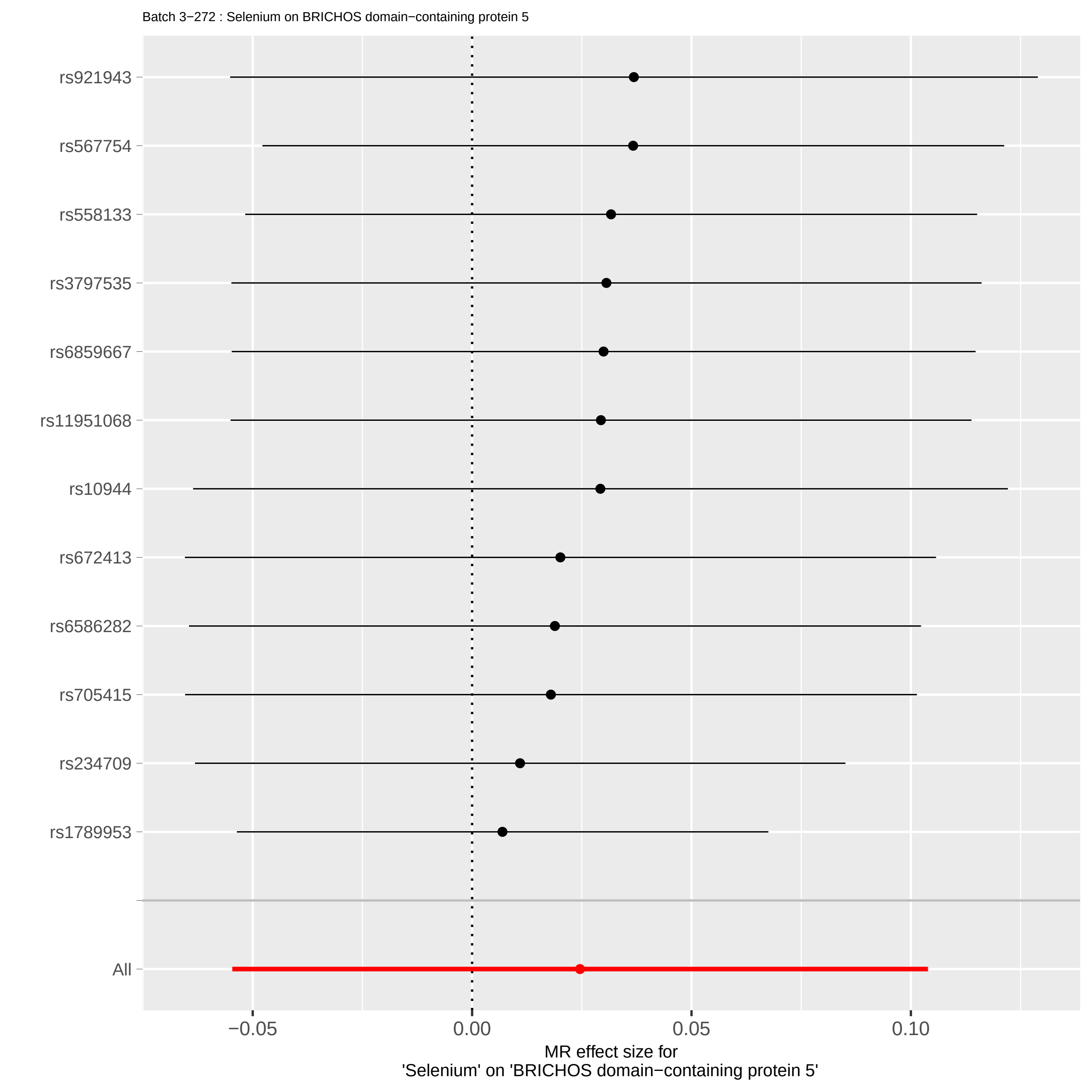

Batch 3−272 : Selenium on BRICHOS domain−containing protein 5
rs921943
rs567754
rs558133
rs3797535
rs6859667
rs11951068
rs10944
rs672413
rs6586282
rs705415
rs234709
rs1789953
All
−0.05
0.00
0.05
0.10
MR effect size for
'Selenium' on 'BRICHOS domain−containing protein 5'

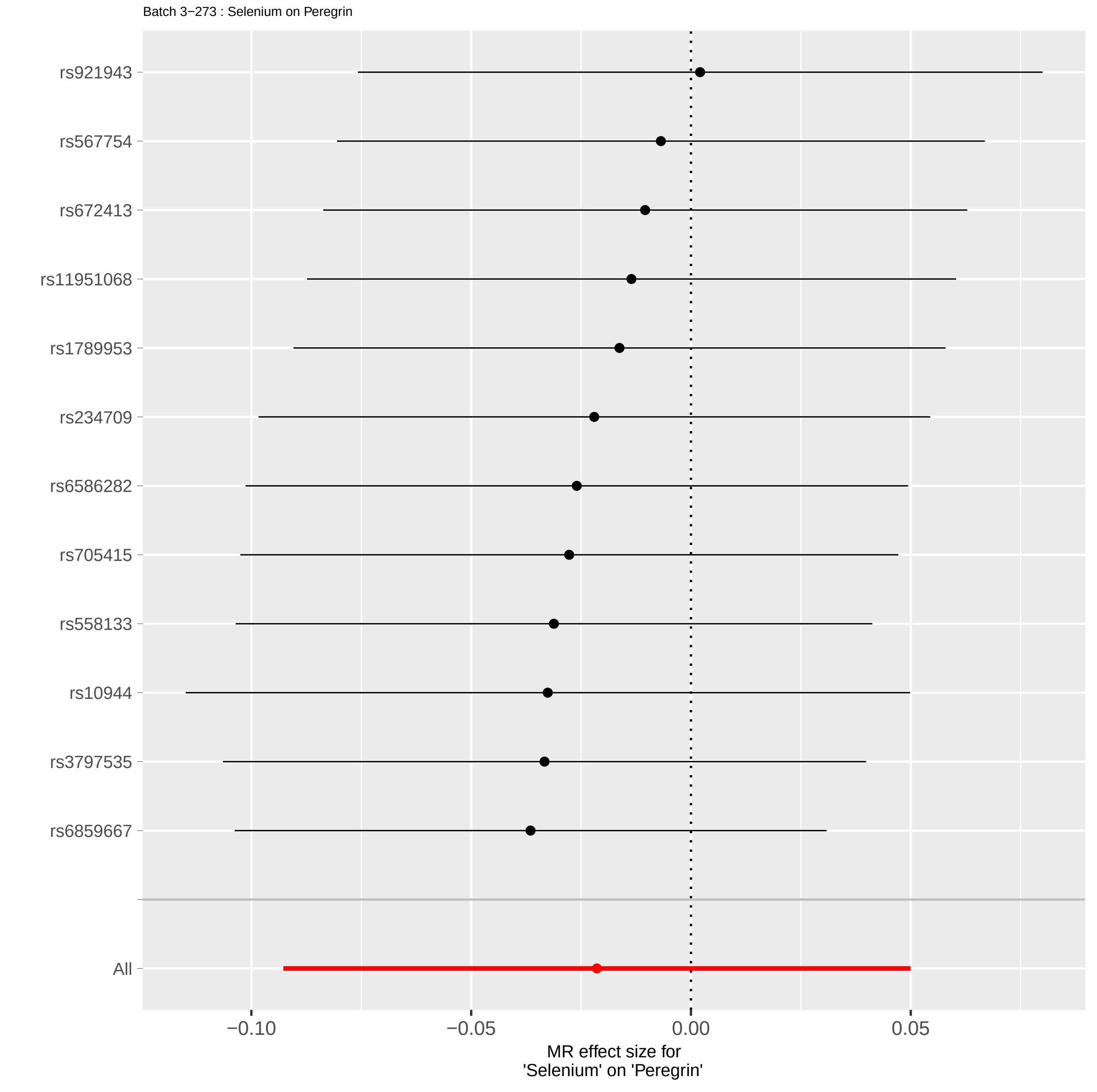
Batch 3−273 : Selenium on Peregrin
rs921943
rs567754
rs672413
rs11951068
rs1789953
rs234709
rs6586282
rs705415
rs558133
rs10944
rs3797535
rs6859667
All
−0.10
−0.05
0.00
0.05
MR effect size for
'Selenium' on 'Peregrin'

Batch 3−274 : Selenium on Serine/threonine−protein kinase BRSK2

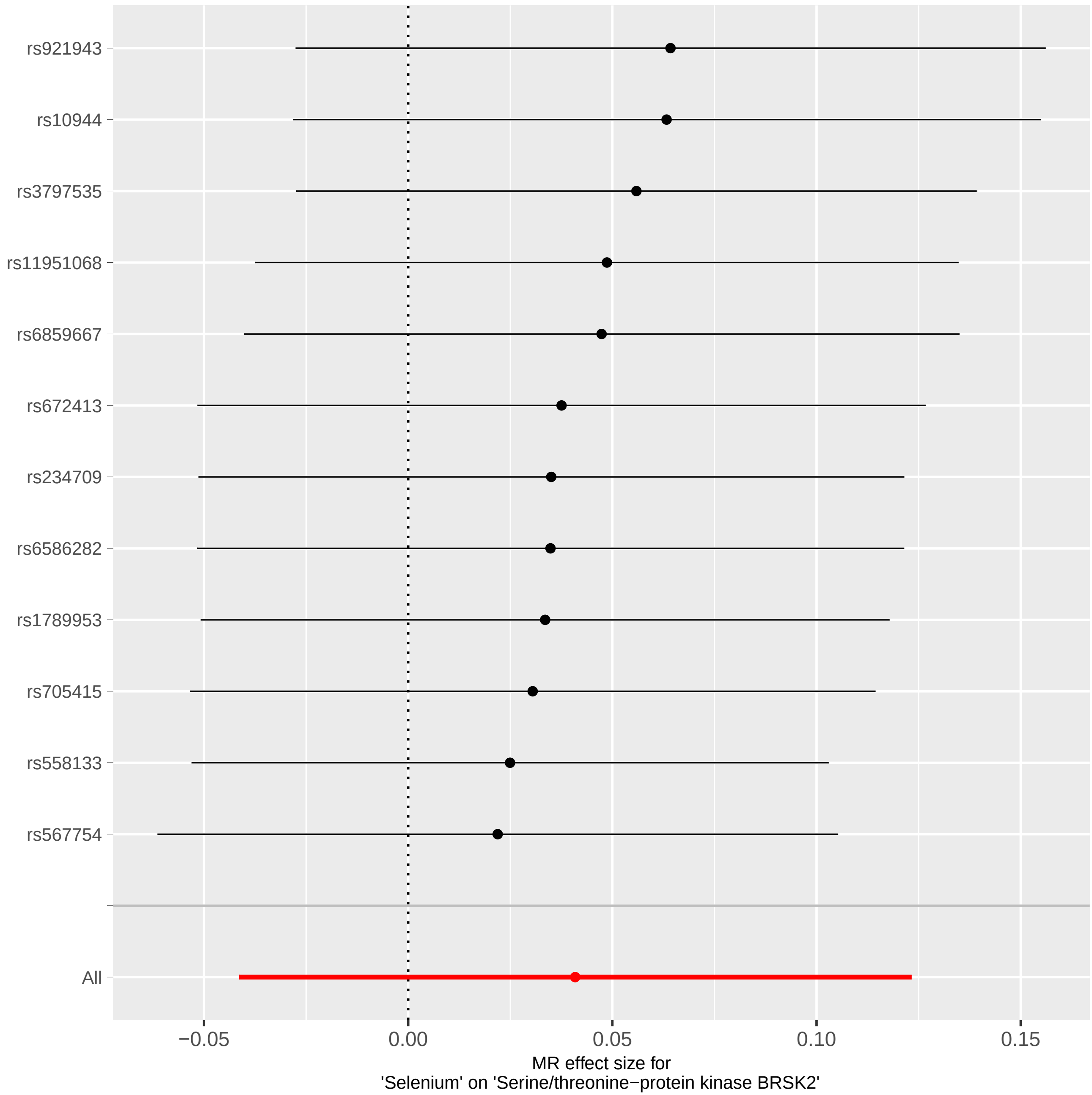

Batch 3−275 : Selenium on Basigin

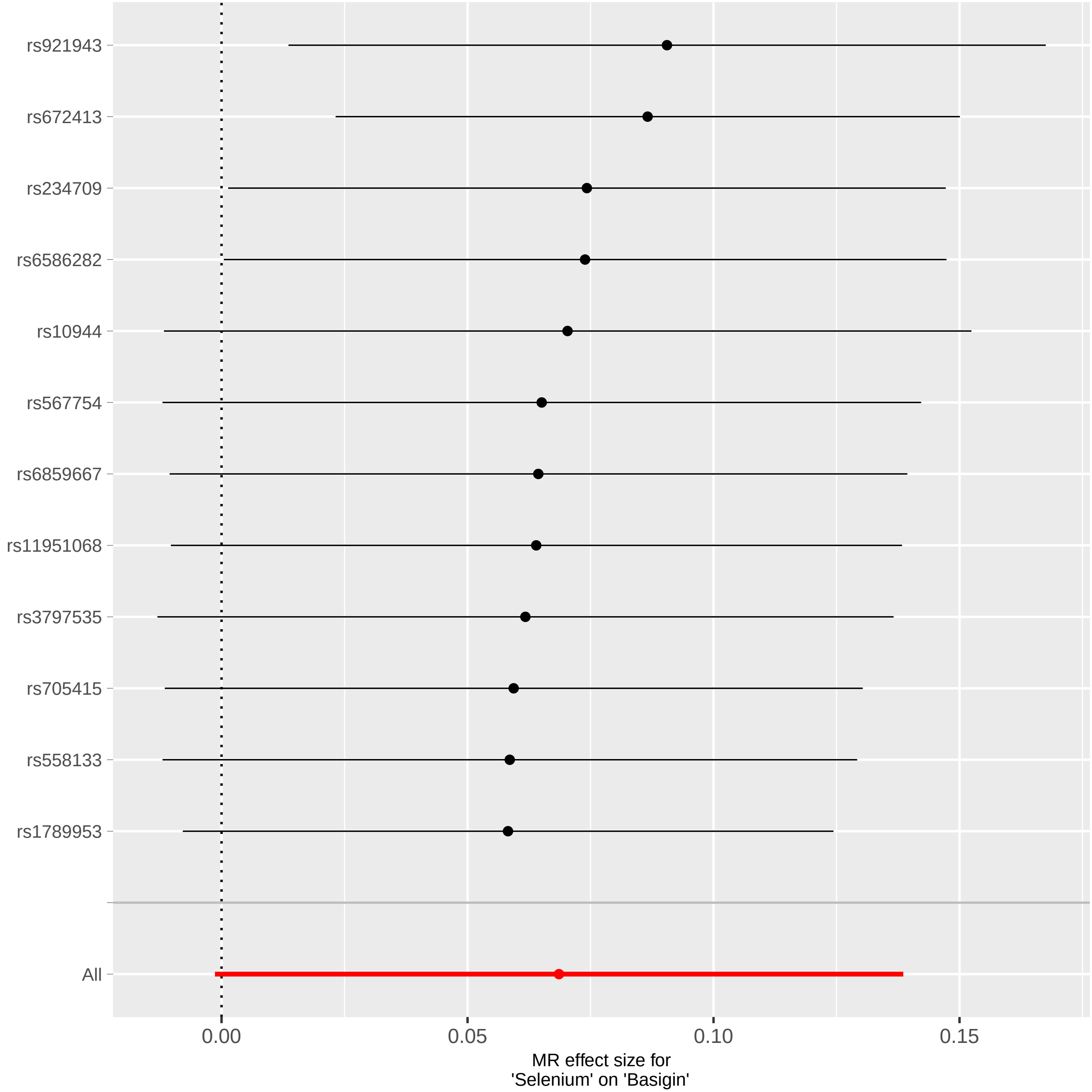

Batch 3−276 : Selenium on ADP−ribosyl cyclase/cyclic ADP−ribose hydrolase 2

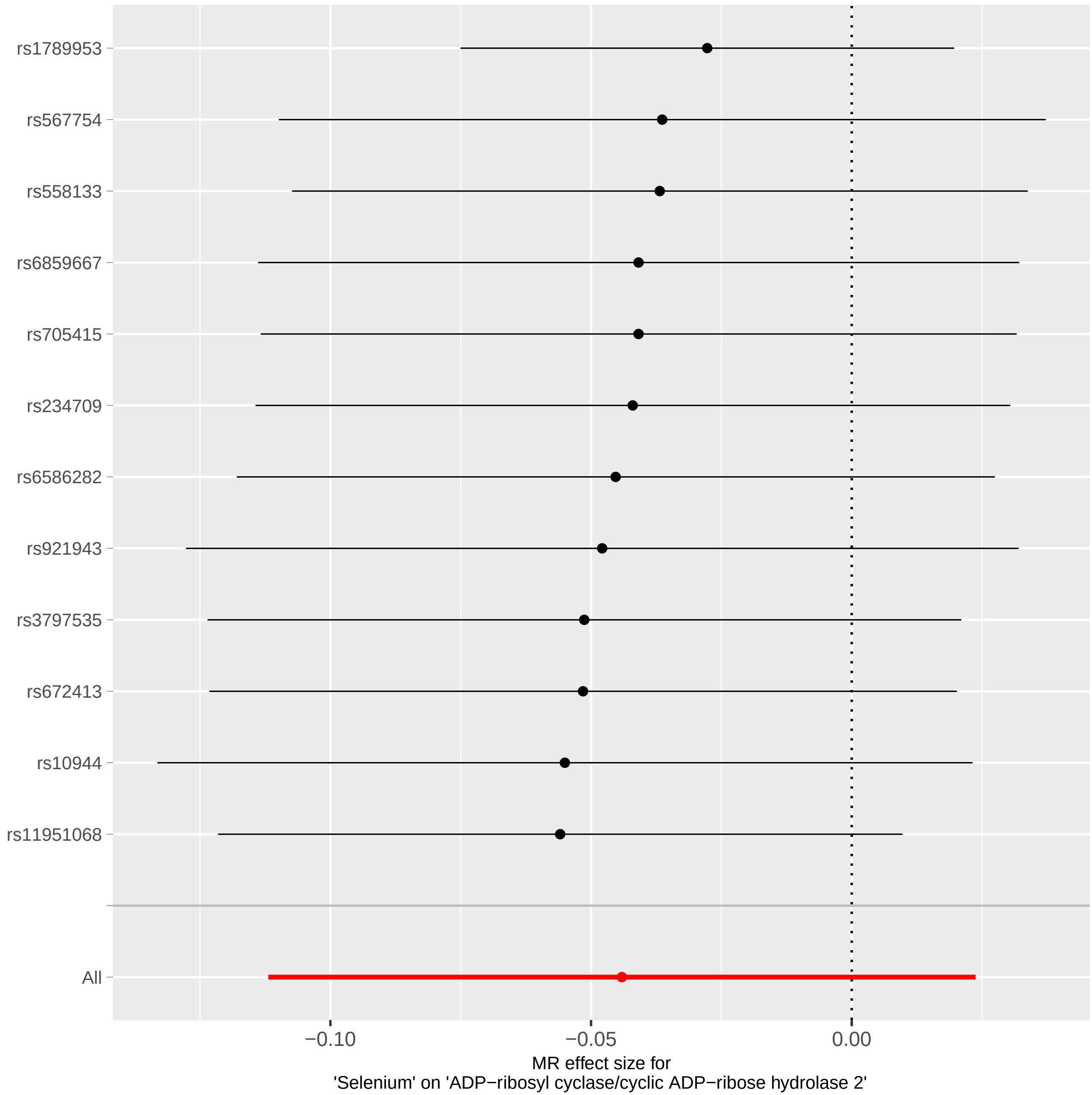

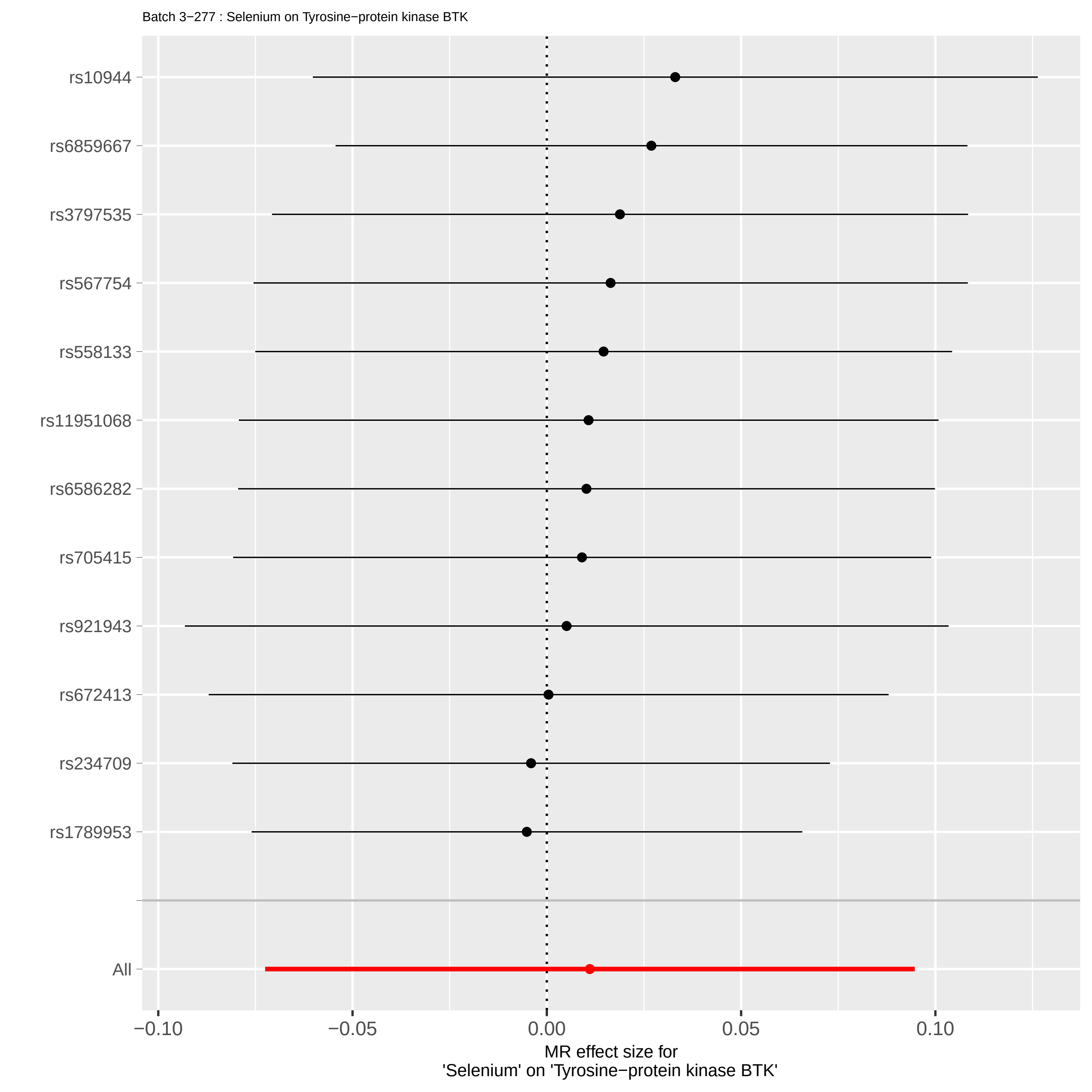
Batch 3−277 : Selenium on Tyrosine−protein kinase BTK
rs10944
rs6859667
rs3797535
rs567754
rs558133
rs11951068
rs6586282
rs705415
rs921943
rs672413
rs234709
rs1789953
All
−0.10
−0.05
0.00
0.05
0.10
MR effect size for
'Selenium' on 'Tyrosine−protein kinase BTK'

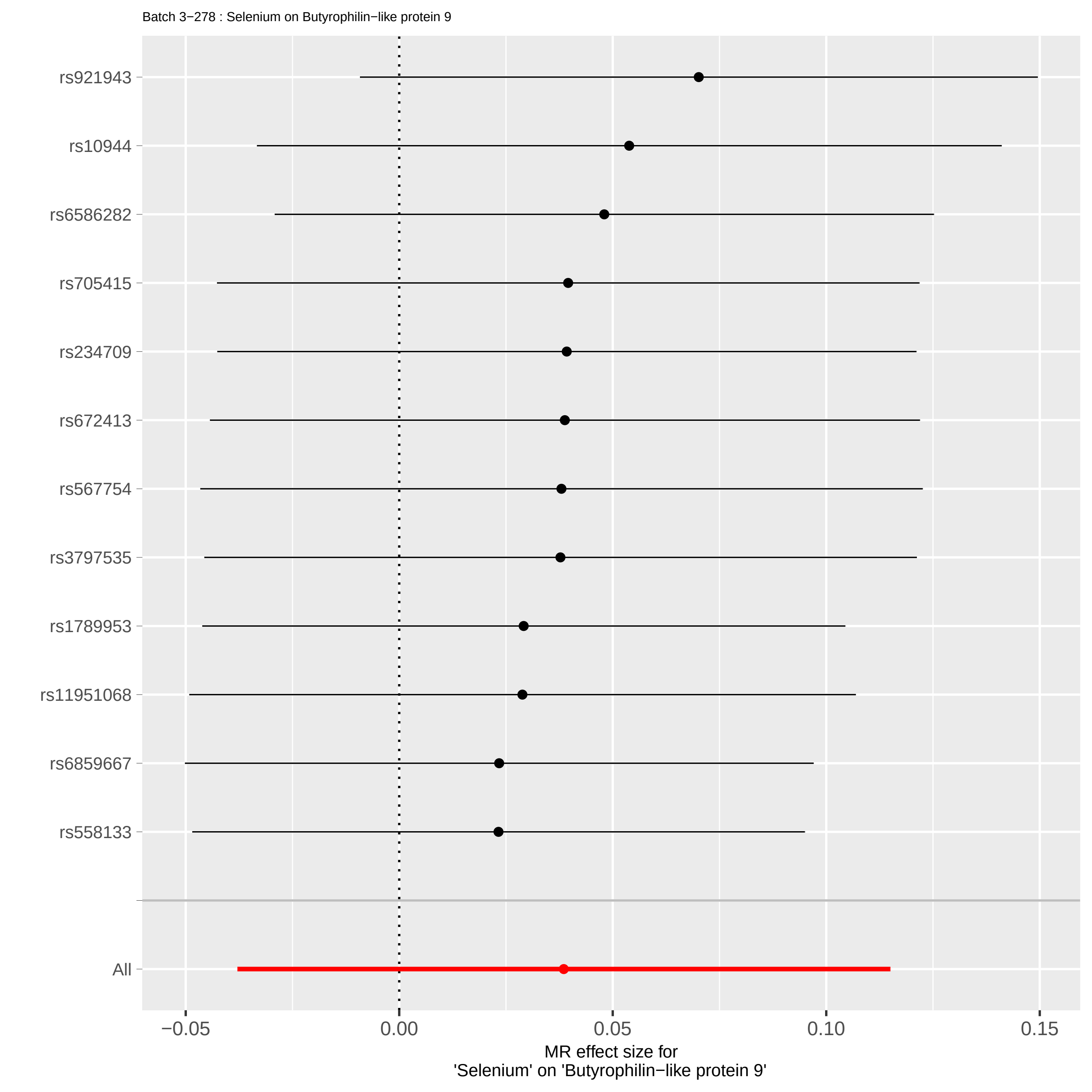

Batch 3−278 : Selenium on Butyrophilin−like protein 9
rs921943
rs10944
rs6586282
rs705415
rs234709
rs672413
rs567754
rs3797535
rs1789953
rs11951068
rs6859667
rs558133
All
−0.05
0.00
0.05
0.10
0.15
MR effect size for
'Selenium' on 'Butyrophilin−like protein 9'

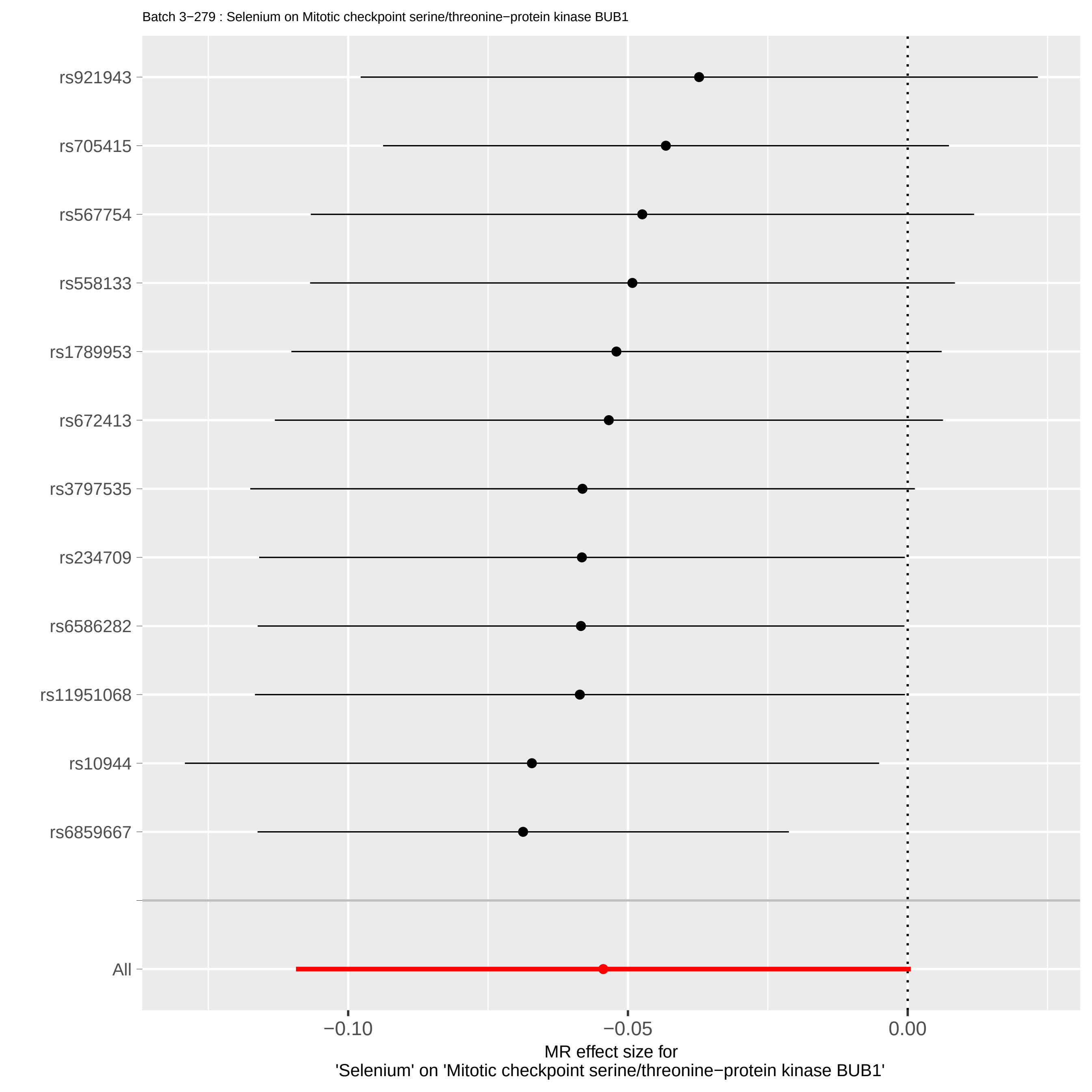

Batch 3−279 : Selenium on Mitotic checkpoint serine/threonine−protein kinase BUB1
rs921943
rs705415
rs567754
rs558133
rs1789953
rs672413
rs3797535
rs234709
rs6586282
rs11951068
rs10944
rs6859667
All
−0.10
−0.05
0.00
MR effect size for
'Selenium' on 'Mitotic checkpoint serine/threonine−protein kinase BUB1'

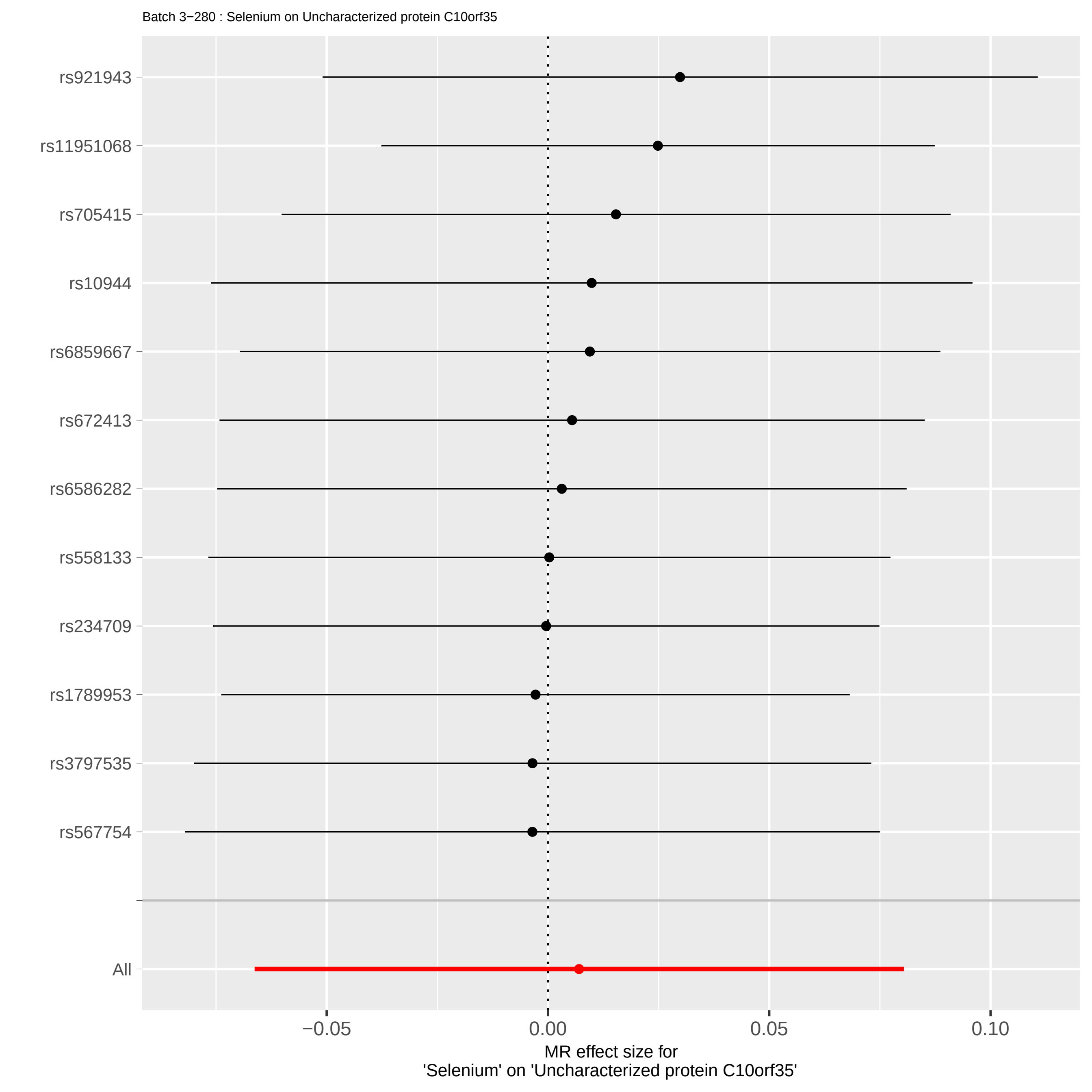

Batch 3−280 : Selenium on Uncharacterized protein C10orf35
rs921943
rs11951068
rs705415
rs10944
rs6859667
rs672413
rs6586282
rs558133
rs234709
rs1789953
rs3797535
rs567754
All
−0.05
0.00
0.05
0.10
MR effect size for
'Selenium' on 'Uncharacterized protein C10orf35'

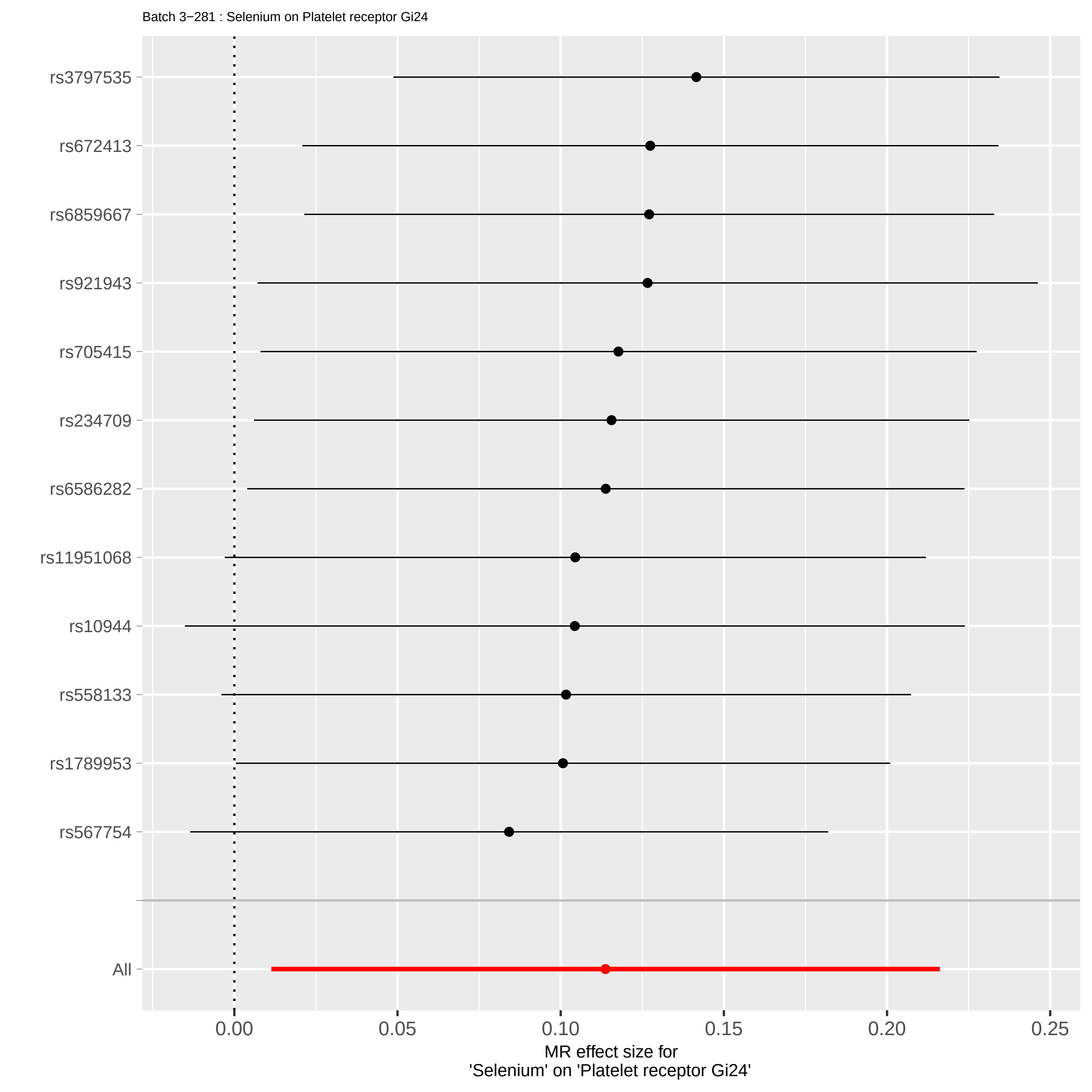

Batch 3−281 : Selenium on Platelet receptor Gi24
rs3797535
rs672413
rs6859667
rs921943
rs705415
rs234709
rs6586282
rs11951068
rs10944
rs558133
rs1789953
rs567754
All
0.00
0.05
0.10
0.15
0.20
0.25
MR effect size for
'Selenium' on 'Platelet receptor Gi24'

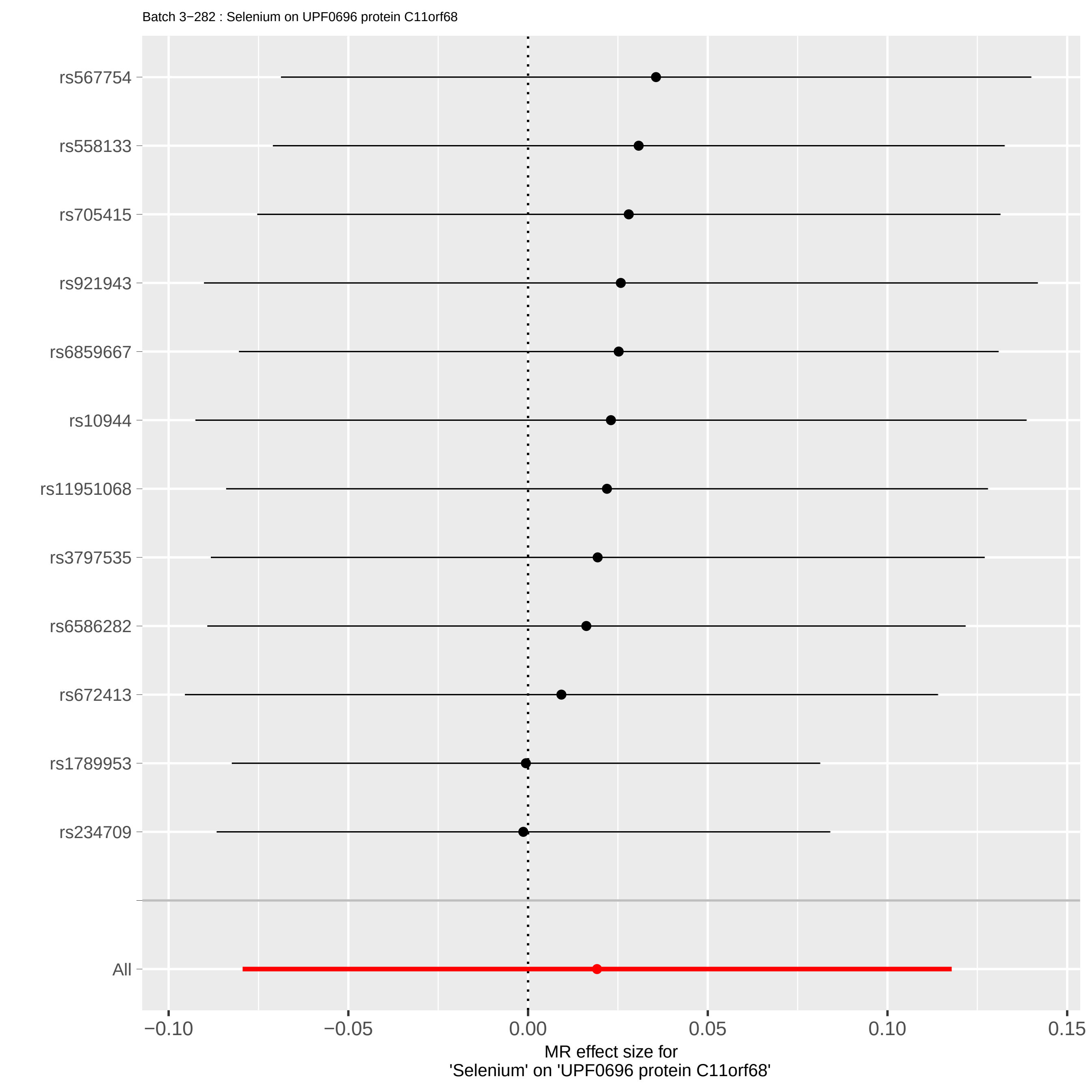
Batch 3−282 : Selenium on UPF0696 protein C11orf68
rs567754
rs558133
rs705415
rs921943
rs6859667
rs10944
rs11951068
rs3797535
rs6586282
rs672413
rs1789953
rs234709
All
−0.10
−0.05
0.00
0.05
0.10
0.15
MR effect size for
'Selenium' on 'UPF0696 protein C11orf68'

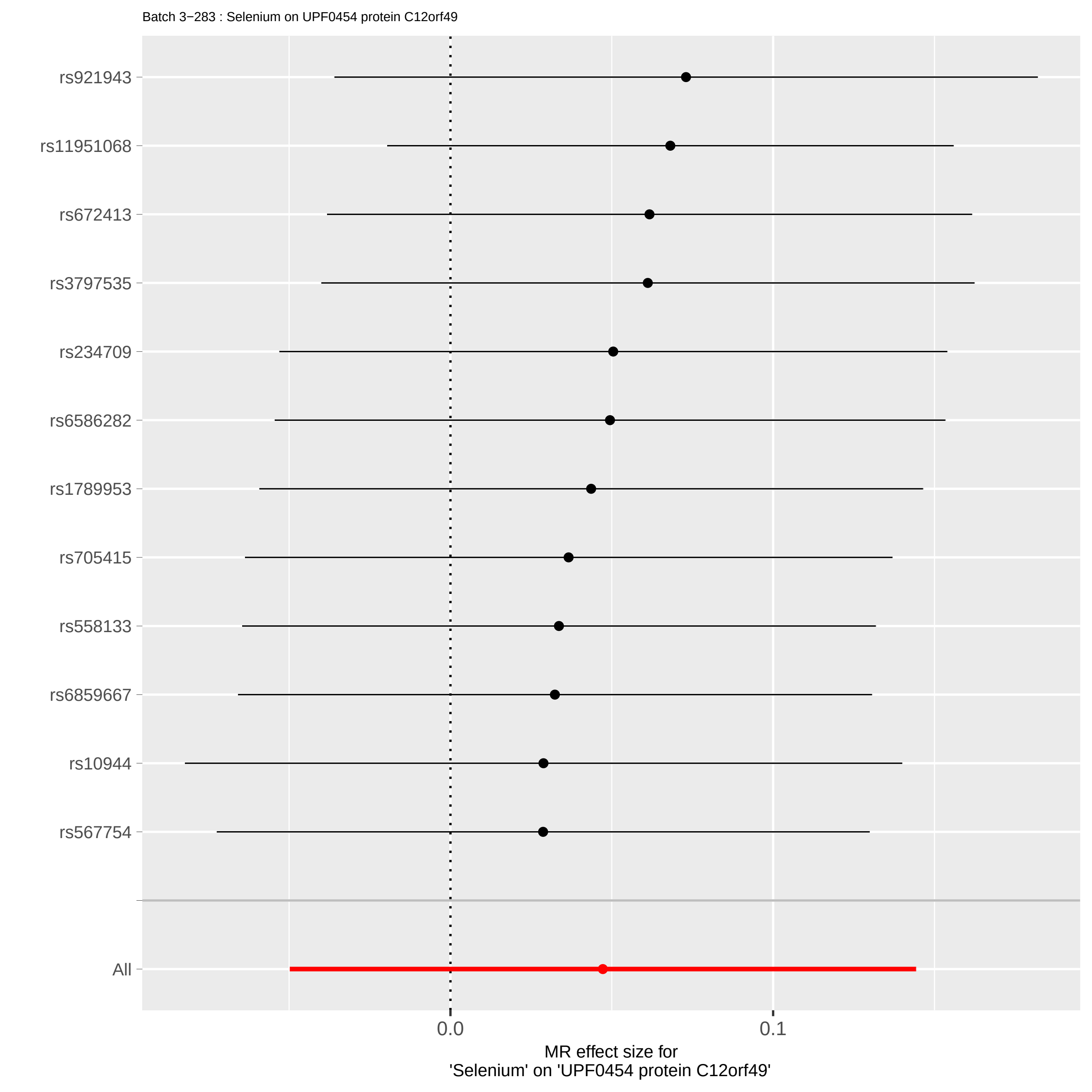

Batch 3−283 : Selenium on UPF0454 protein C12orf49
rs921943
rs11951068
rs672413
rs3797535
rs234709
rs6586282
rs1789953
rs705415
rs558133
rs6859667
rs10944
rs567754
All
0.0
0.1
MR effect size for
'Selenium' on 'UPF0454 protein C12orf49'

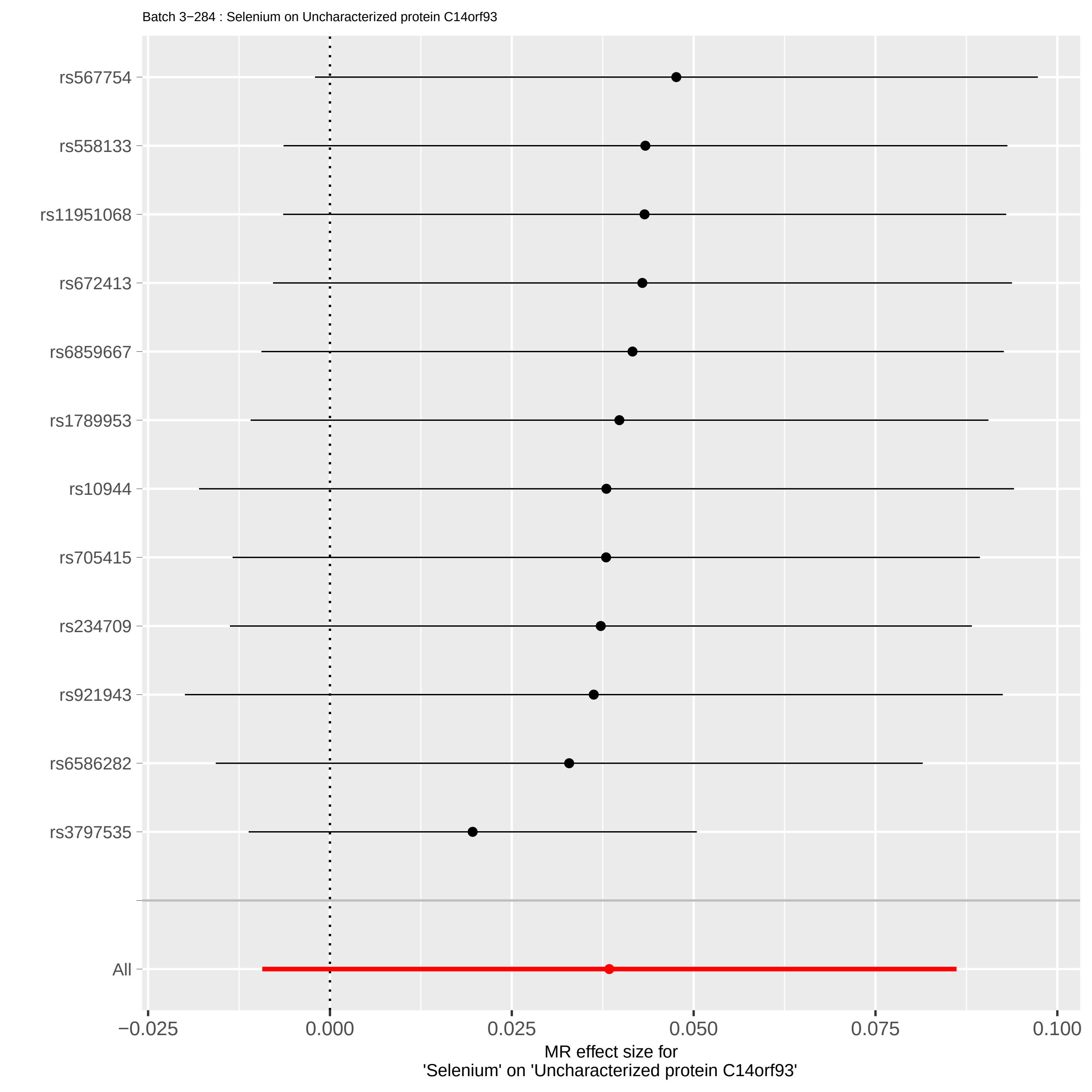
Batch 3−284 : Selenium on Uncharacterized protein C14orf93
rs567754
rs558133
rs11951068
rs672413
rs6859667
rs1789953
rs10944
rs705415
rs234709
rs921943
rs6586282
rs3797535
All
−0.025
0.000
0.025
0.050
0.075
0.100
MR effect size for
'Selenium' on 'Uncharacterized protein C14orf93'

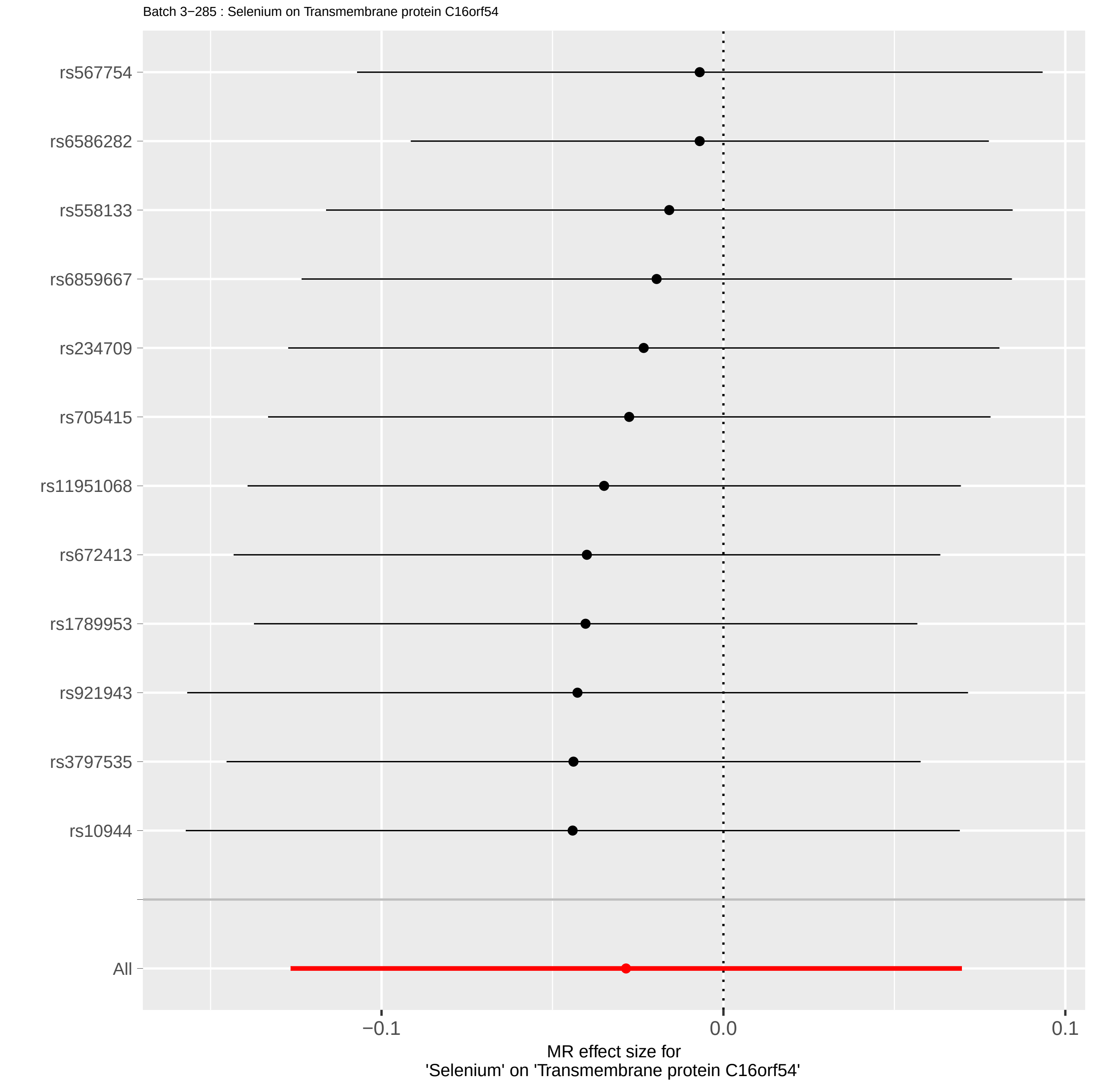

Batch 3−285 : Selenium on Transmembrane protein C16orf54
rs567754
rs6586282
rs558133
rs6859667
rs234709
rs705415
rs11951068
rs672413
rs1789953
rs921943
rs3797535
rs10944
All
−0.1
0.0
0.1
MR effect size for
'Selenium' on 'Transmembrane protein C16orf54'

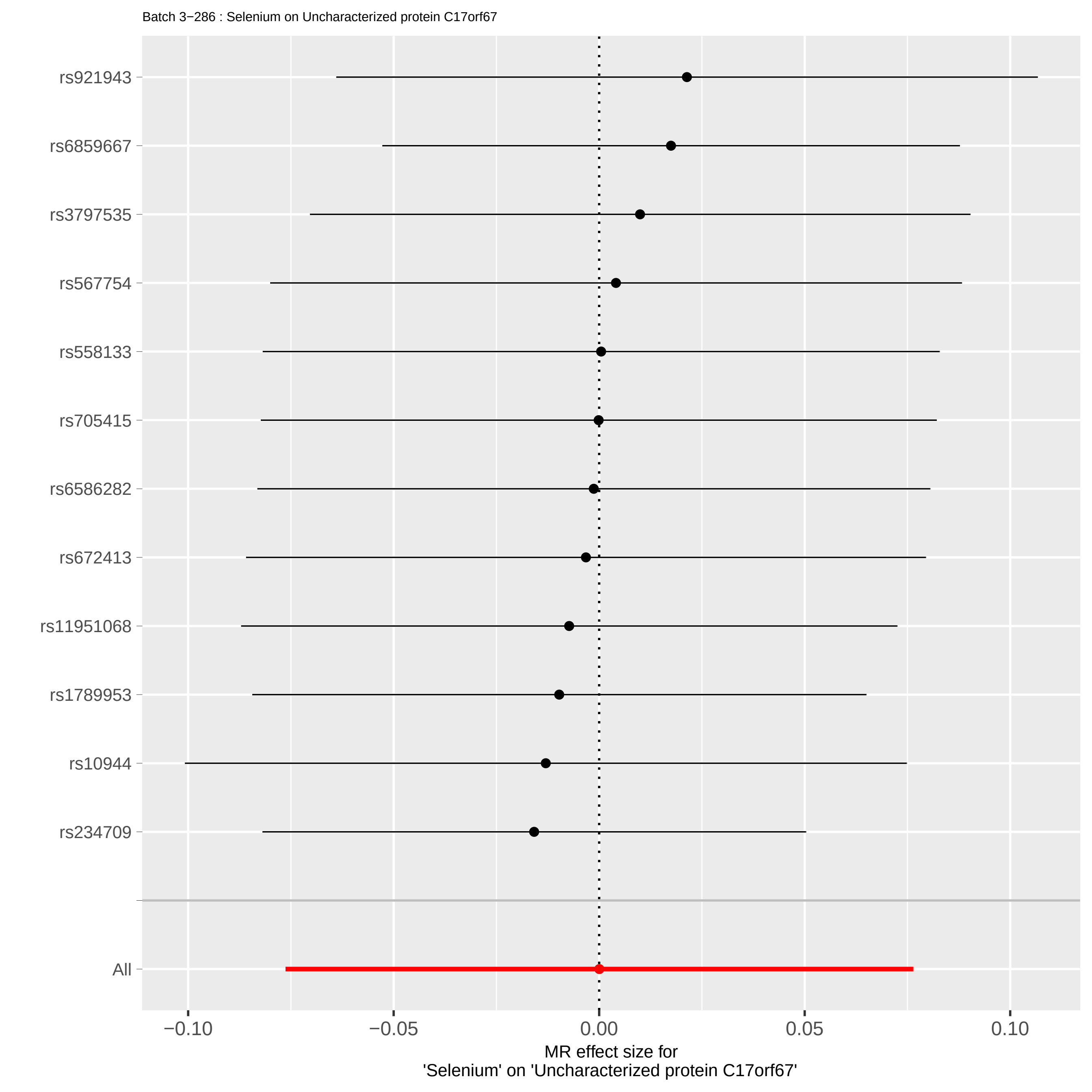

Batch 3−286 : Selenium on Uncharacterized protein C17orf67
rs921943
rs6859667
rs3797535
rs567754
rs558133
rs705415
rs6586282
rs672413
rs11951068
rs1789953
rs10944
rs234709
All
−0.10
−0.05
0.00
0.05
0.10
MR effect size for
'Selenium' on 'Uncharacterized protein C17orf67'

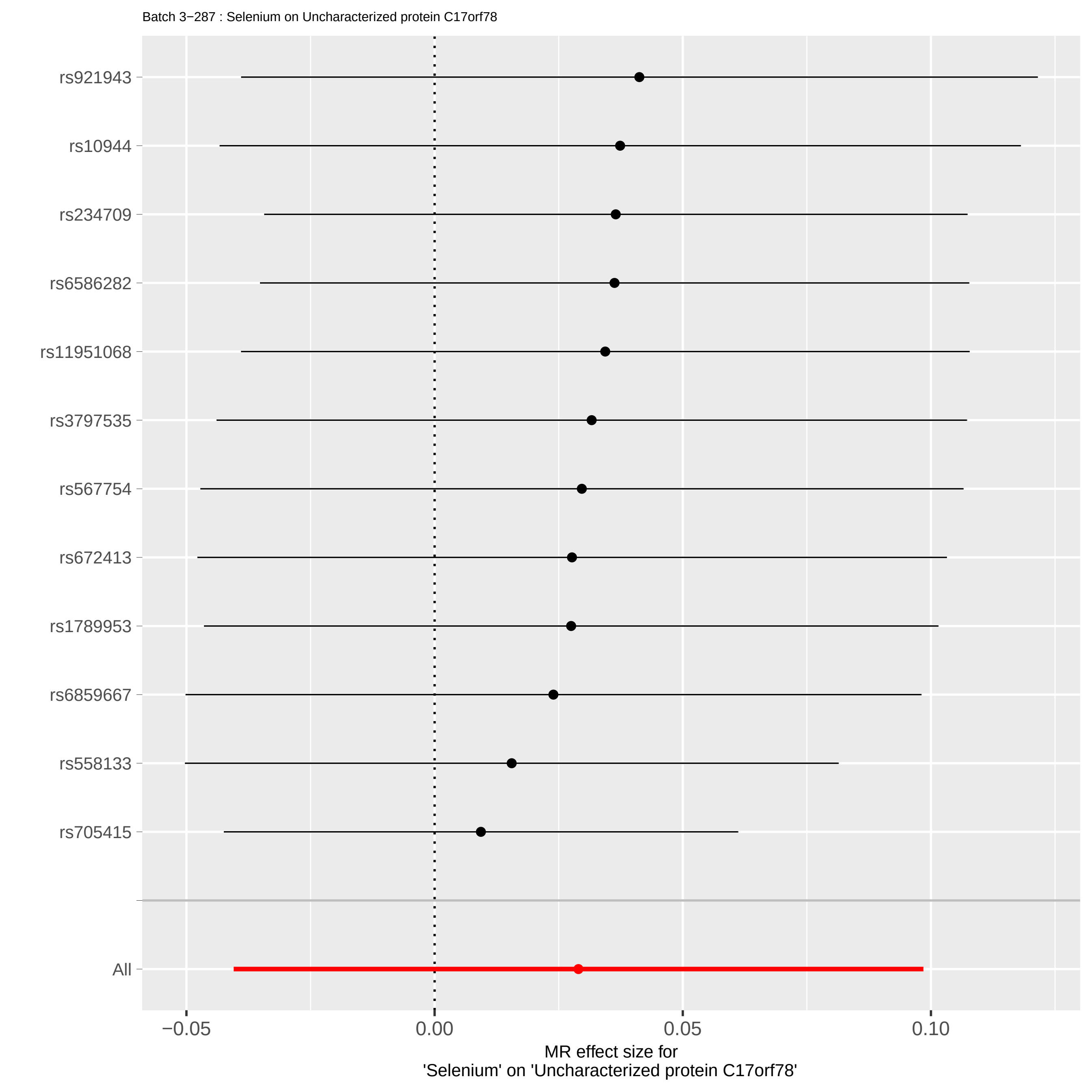
Batch 3−287 : Selenium on Uncharacterized protein C17orf78
rs921943
rs10944
rs234709
rs6586282
rs11951068
rs3797535
rs567754
rs672413
rs1789953
rs6859667
rs558133
rs705415
All
−0.05
0.00
0.05
0.10
MR effect size for
'Selenium' on 'Uncharacterized protein C17orf78'

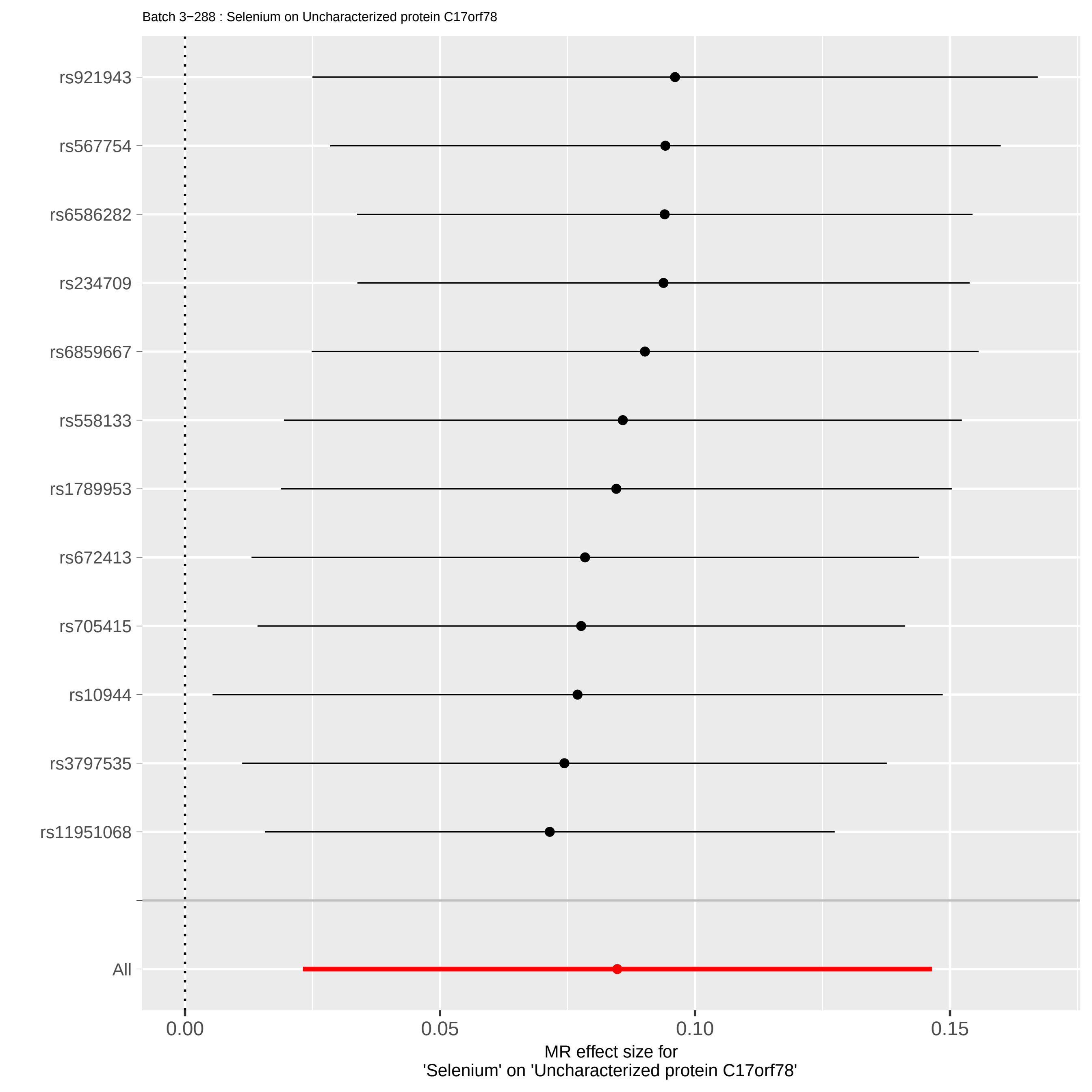

Batch 3−288 : Selenium on Uncharacterized protein C17orf78
rs921943
rs567754
rs6586282
rs234709
rs6859667
rs558133
rs1789953
rs672413
rs705415
rs10944
rs3797535
rs11951068
All
0.00
0.05
0.10
0.15
MR effect size for
'Selenium' on 'Uncharacterized protein C17orf78'

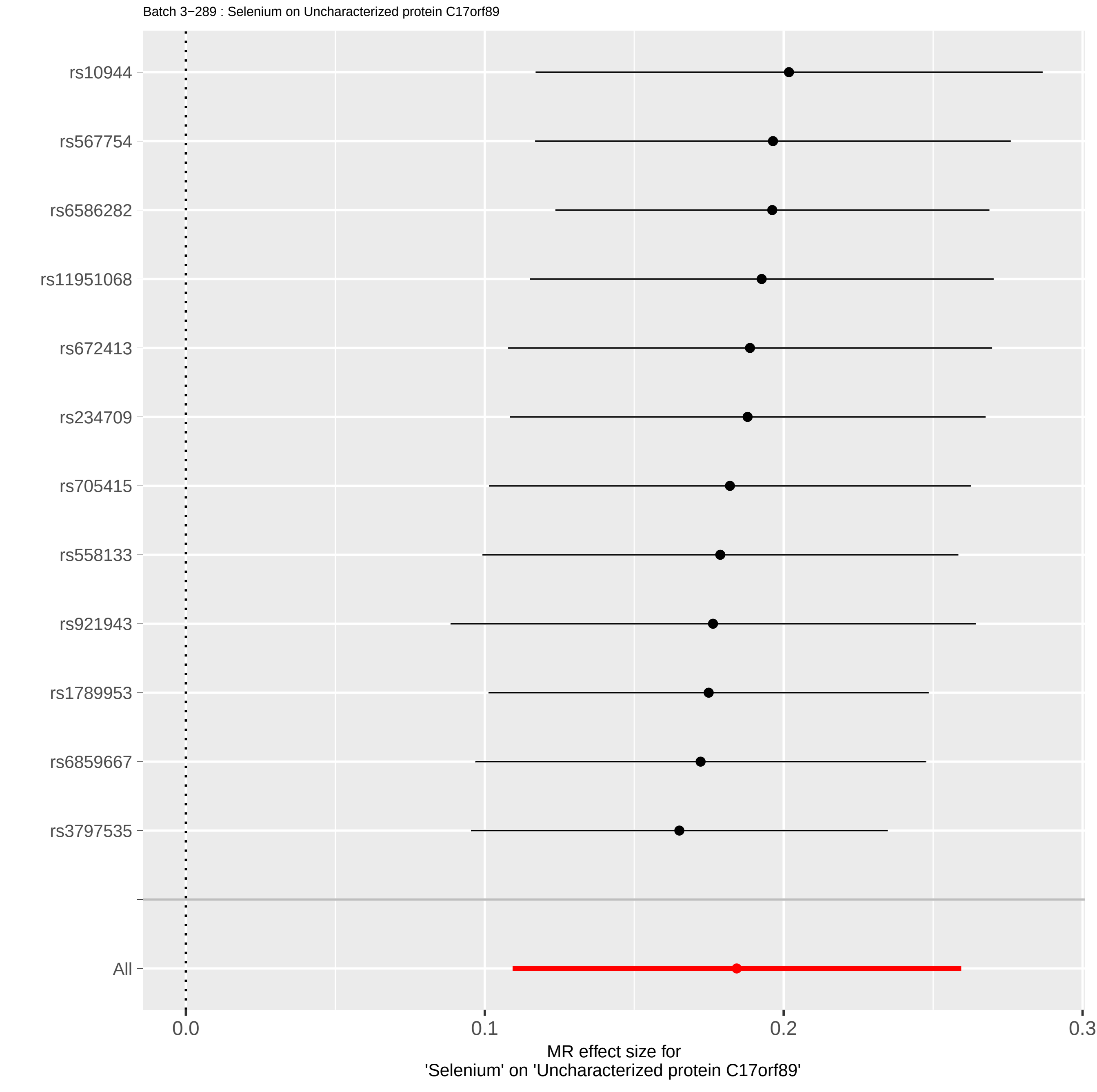

Batch 3−289 : Selenium on Uncharacterized protein C17orf89
rs10944
rs567754
rs6586282
rs11951068
rs672413
rs234709
rs705415
rs558133
rs921943
rs1789953
rs6859667
rs3797535
All
0.0
0.1
0.2
0.3
MR effect size for
'Selenium' on 'Uncharacterized protein C17orf89'

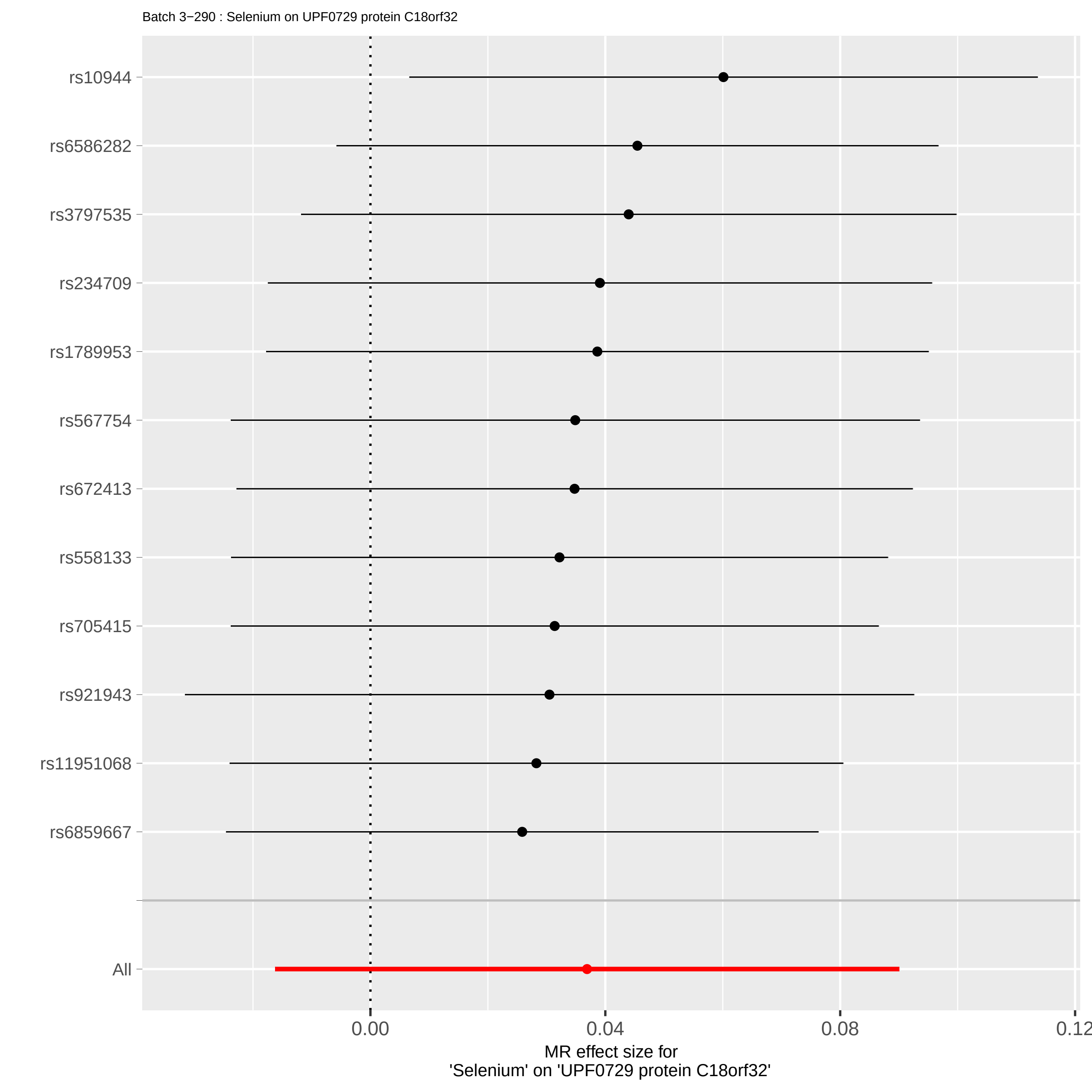

Batch 3−290 : Selenium on UPF0729 protein C18orf32
rs10944
rs6586282
rs3797535
rs234709
rs1789953
rs567754
rs672413
rs558133
rs705415
rs921943
rs11951068
rs6859667
All
0.00
0.04
0.08
0.12
MR effect size for
'Selenium' on 'UPF0729 protein C18orf32'

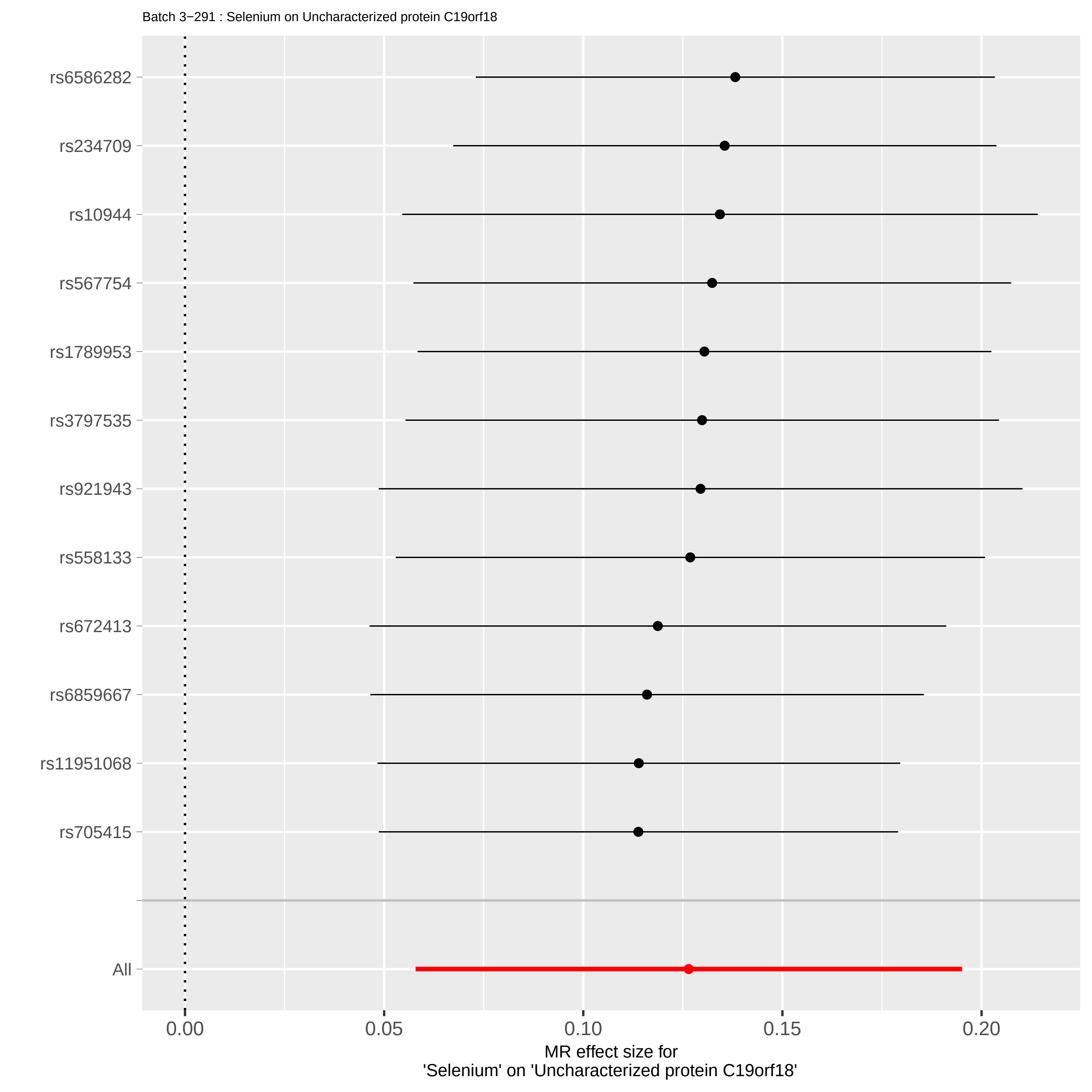

Batch 3−291 : Selenium on Uncharacterized protein C19orf18
rs6586282
rs234709
rs10944
rs567754
rs1789953
rs3797535
rs921943
rs558133
rs672413
rs6859667
rs11951068
rs705415
All
0.00
0.05
0.10
0.15
0.20
MR effect size for
'Selenium' on 'Uncharacterized protein C19orf18'

Batch 3−292 : Selenium on Angiopoietin−like protein 8

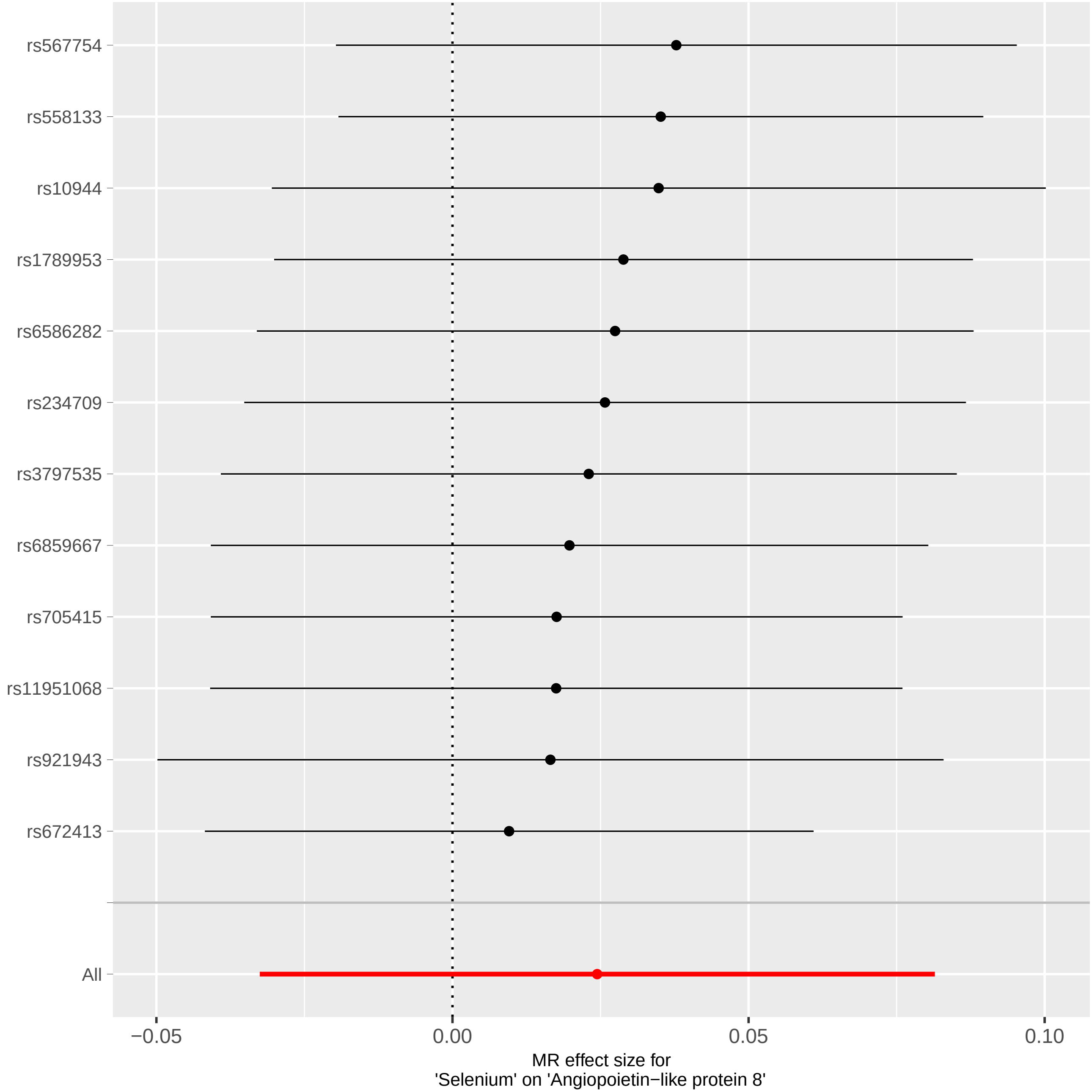

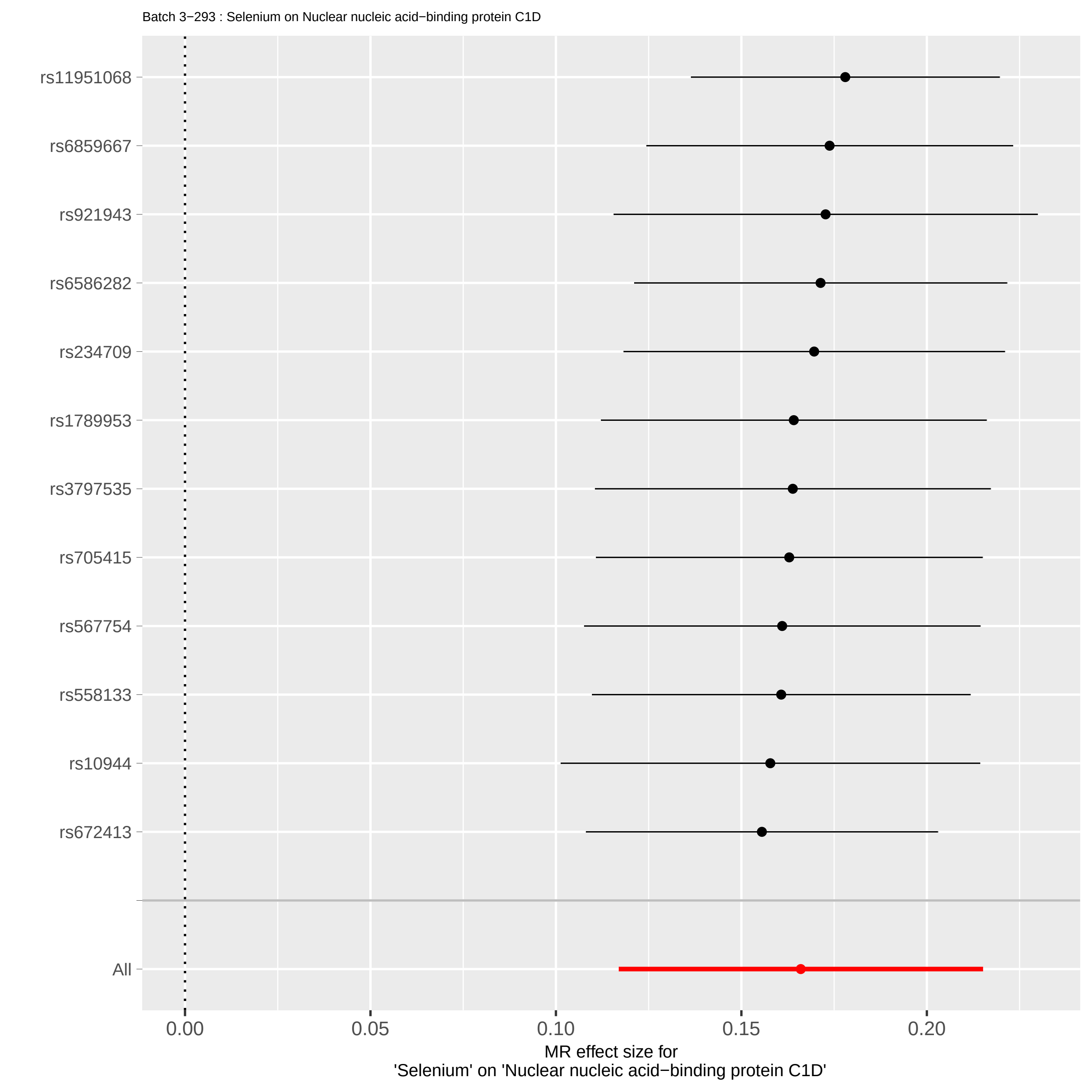

Batch 3−293 : Selenium on Nuclear nucleic acid−binding protein C1D
rs11951068
rs6859667
rs921943
rs6586282
rs234709
rs1789953
rs3797535
rs705415
rs567754
rs558133
rs10944
rs672413
All
0.00
0.05
0.10
0.15
0.20
MR effect size for
'Selenium' on 'Nuclear nucleic acid−binding protein C1D'

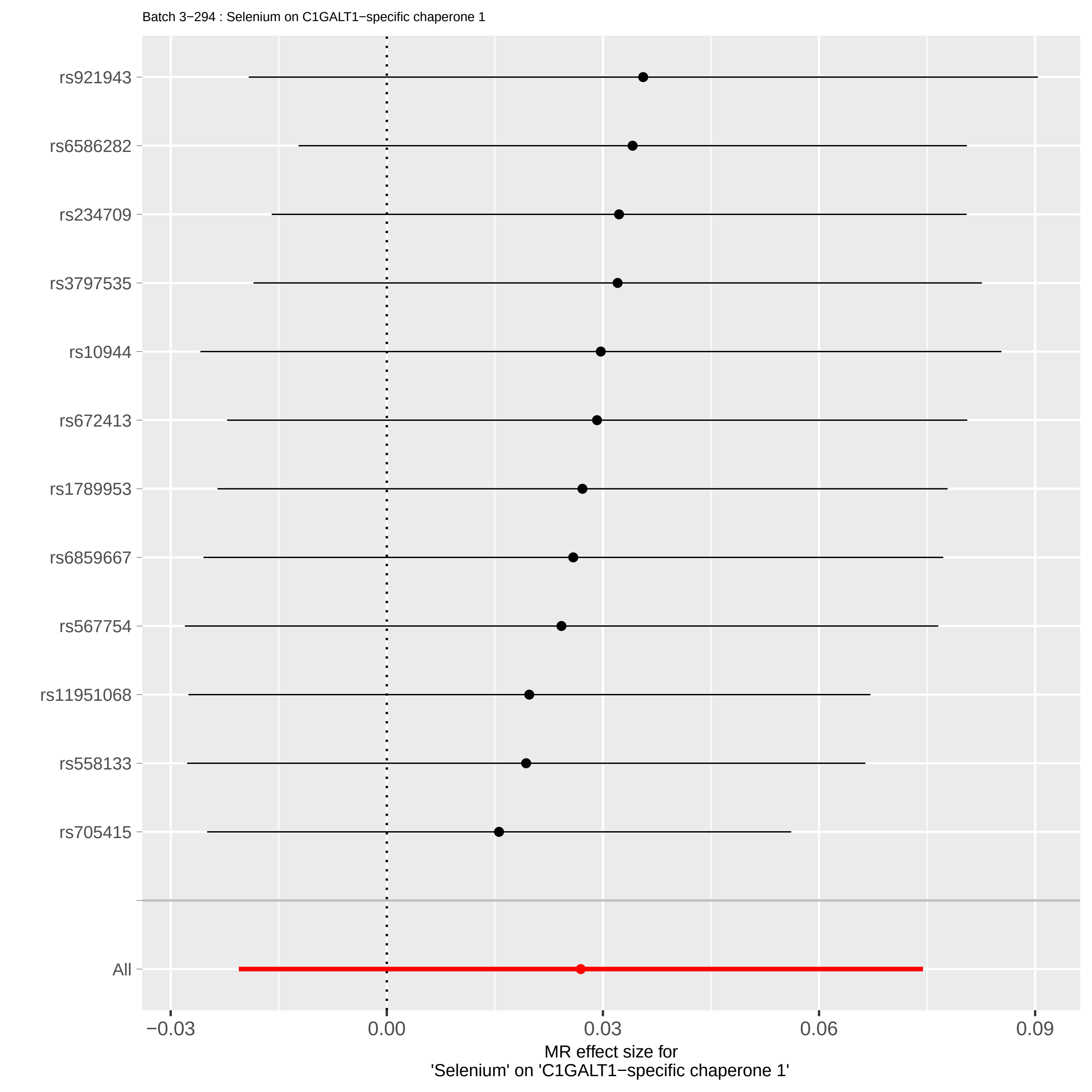
Batch 3−294 : Selenium on C1GALT1−specific chaperone 1
rs921943
rs6586282
rs234709
rs3797535
rs10944
rs672413
rs1789953
rs6859667
rs567754
rs11951068
rs558133
rs705415
All
−0.03
0.00
0.03
0.06
0.09
MR effect size for
'Selenium' on 'C1GALT1−specific chaperone 1'

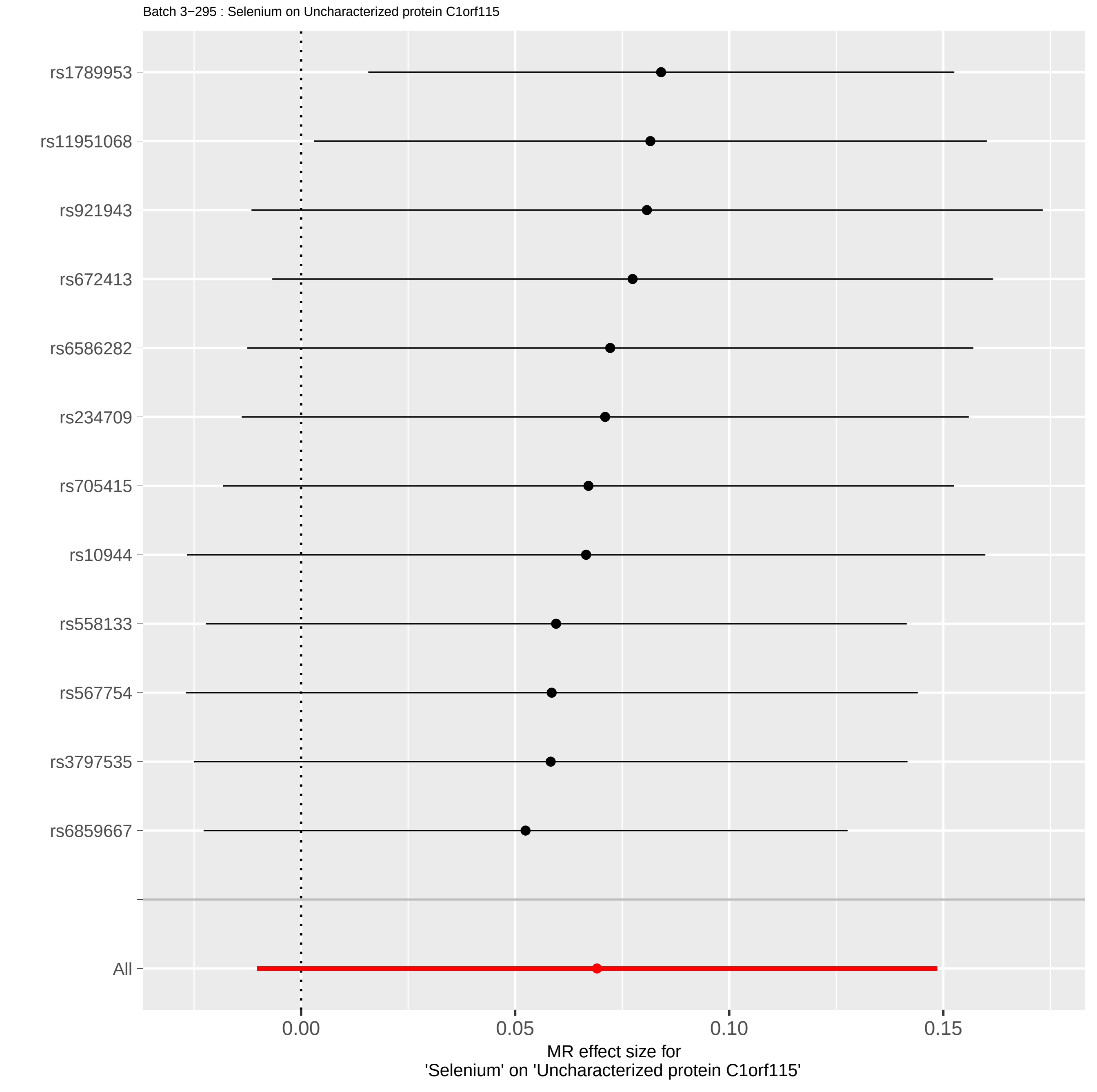

Batch 3−295 : Selenium on Uncharacterized protein C1orf115
rs1789953
rs11951068
rs921943
rs672413
rs6586282
rs234709
rs705415
rs10944
rs558133
rs567754
rs3797535
rs6859667
All
0.00
0.05
0.10
0.15
MR effect size for
'Selenium' on 'Uncharacterized protein C1orf115'

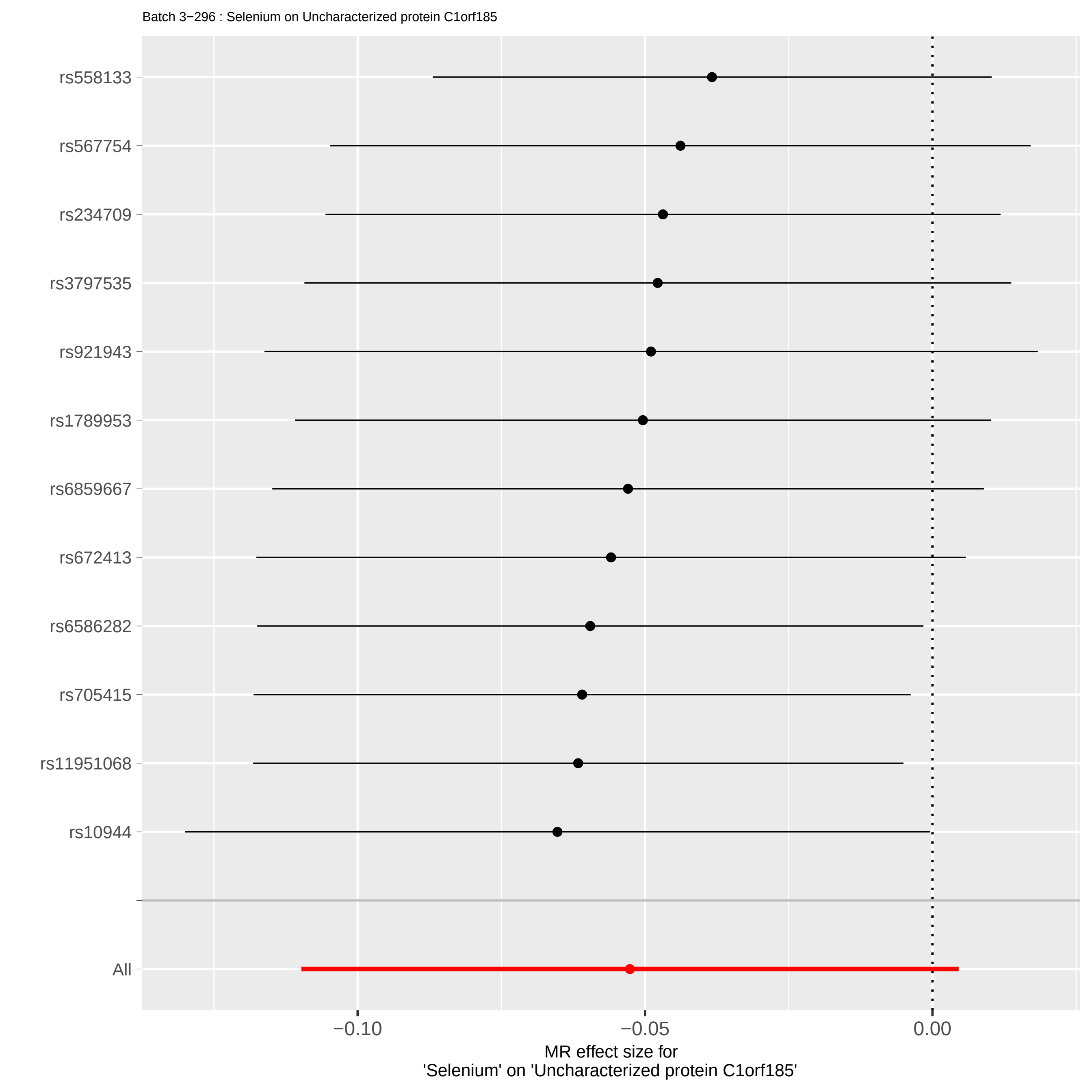
Batch 3−296 : Selenium on Uncharacterized protein C1orf185
rs558133
rs567754
rs234709
rs3797535
rs921943
rs1789953
rs6859667
rs672413
rs6586282
rs705415
rs11951068
rs10944
All
−0.10
−0.05
0.00
MR effect size for
'Selenium' on 'Uncharacterized protein C1orf185'

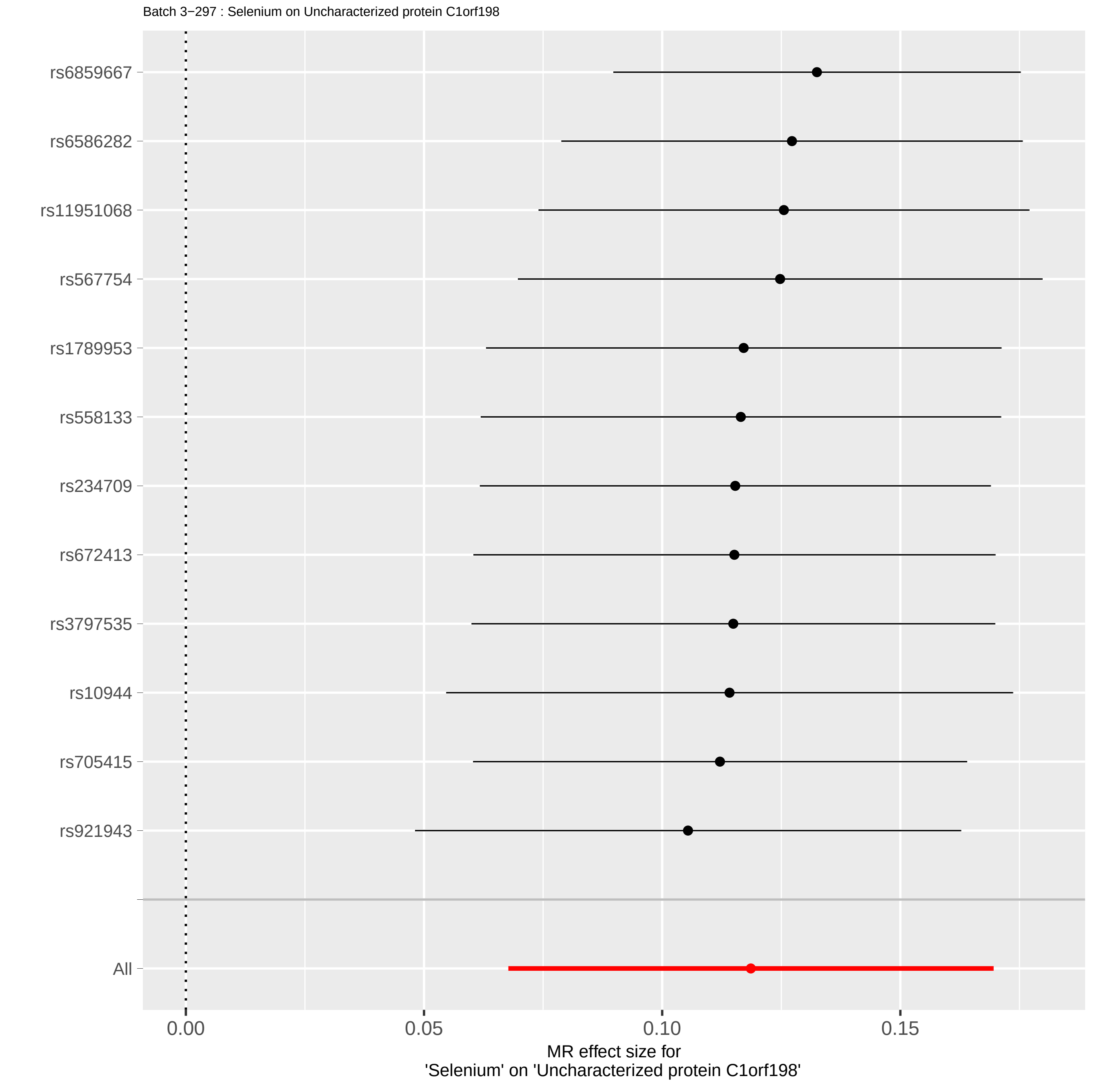
Batch 3−297 : Selenium on Uncharacterized protein C1orf198
rs6859667
rs6586282
rs11951068
rs567754
rs1789953
rs558133
rs234709
rs672413
rs3797535
rs10944
rs705415
rs921943
All
0.00
0.05
0.10
0.15
MR effect size for
'Selenium' on 'Uncharacterized protein C1orf198'

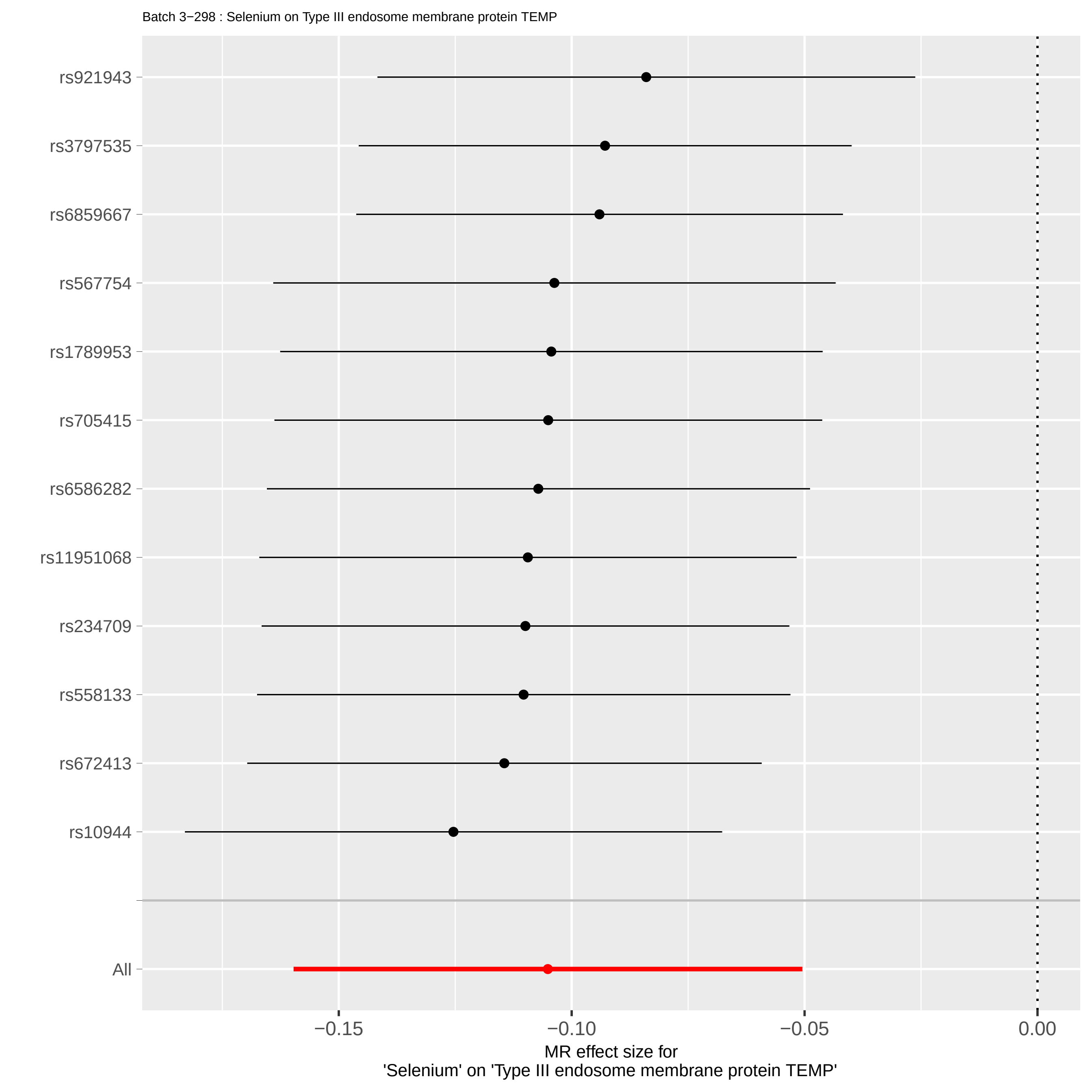

Batch 3−298 : Selenium on Type III endosome membrane protein TEMP
rs921943
rs3797535
rs6859667
rs567754
rs1789953
rs705415
rs6586282
rs11951068
rs234709
rs558133
rs672413
rs10944
All
−0.15
−0.10
−0.05
0.00
MR effect size for
'Selenium' on 'Type III endosome membrane protein TEMP'

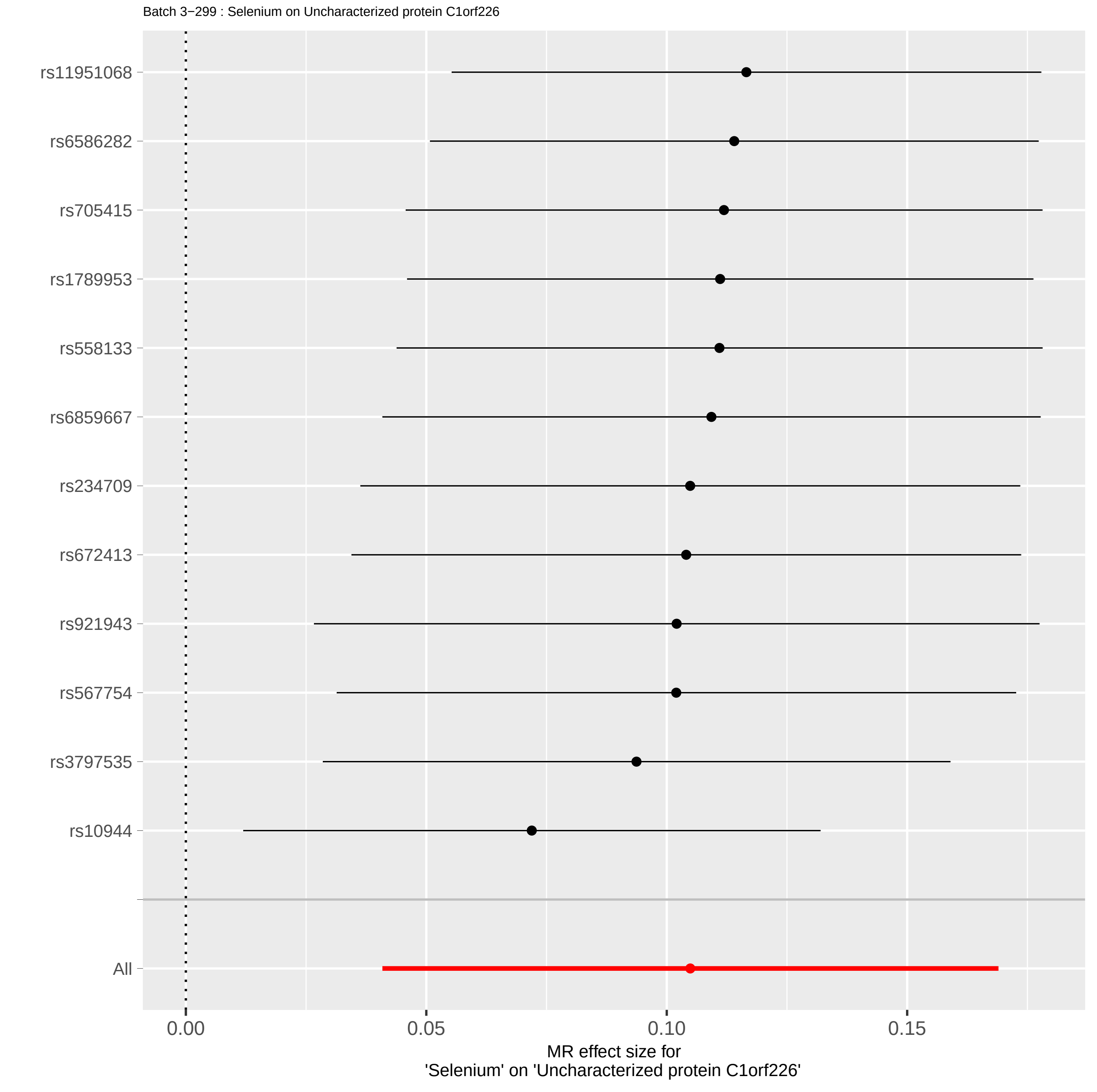

Batch 3−299 : Selenium on Uncharacterized protein C1orf226
rs11951068
rs6586282
rs705415
rs1789953
rs558133
rs6859667
rs234709
rs672413
rs921943
rs567754
rs3797535
rs10944
All
0.00
0.05
0.10
0.15
MR effect size for
'Selenium' on 'Uncharacterized protein C1orf226'

Batch 3−300 : Selenium on Complement component 1 Q subcomponent−binding protein, mitochondrial

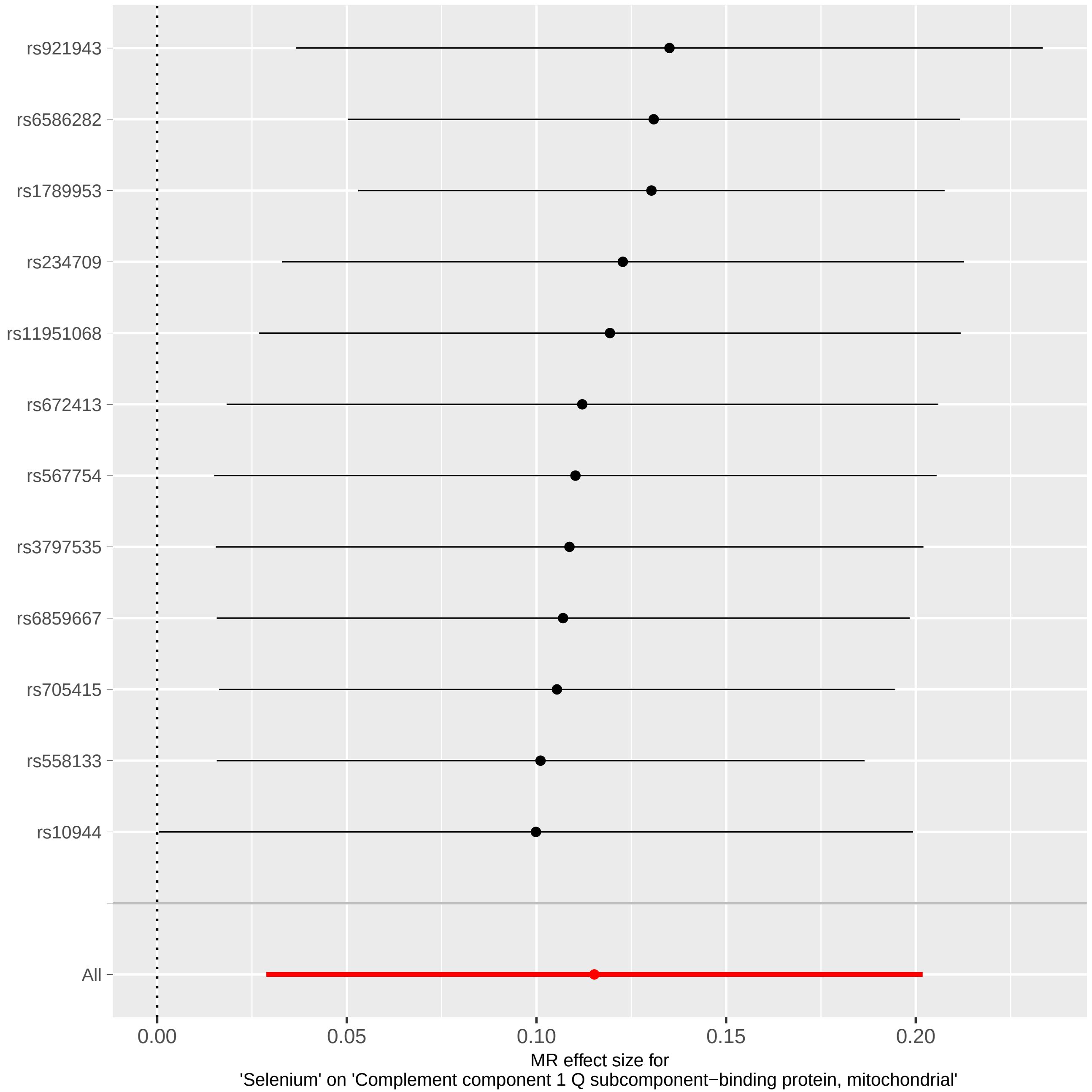

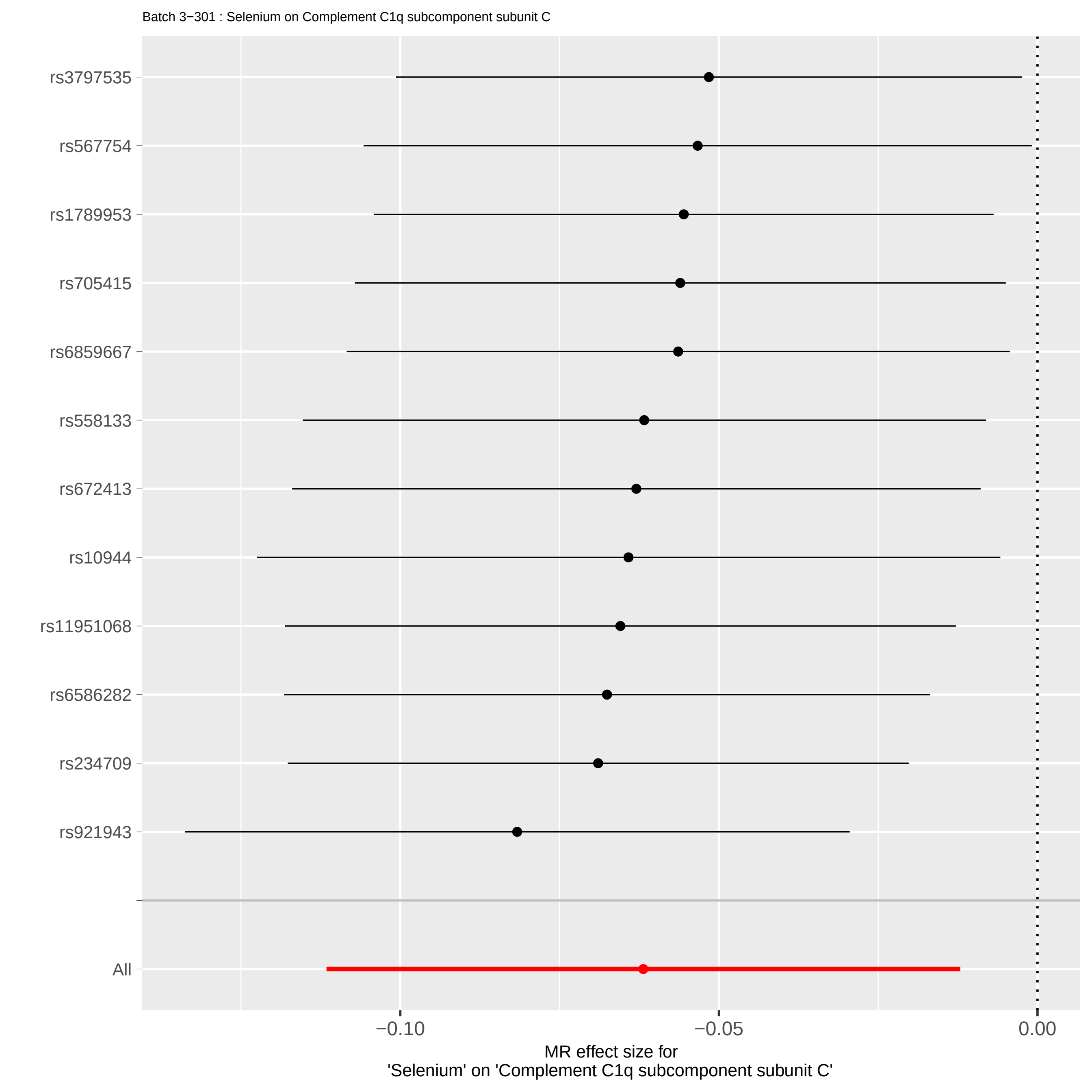
Batch 3−301 : Selenium on Complement C1q subcomponent subunit C
rs3797535
rs567754
rs1789953
rs705415
rs6859667
rs558133
rs672413
rs10944
rs11951068
rs6586282
rs234709
rs921943
All
−0.10
−0.05
0.00
MR effect size for
'Selenium' on 'Complement C1q subcomponent subunit C'

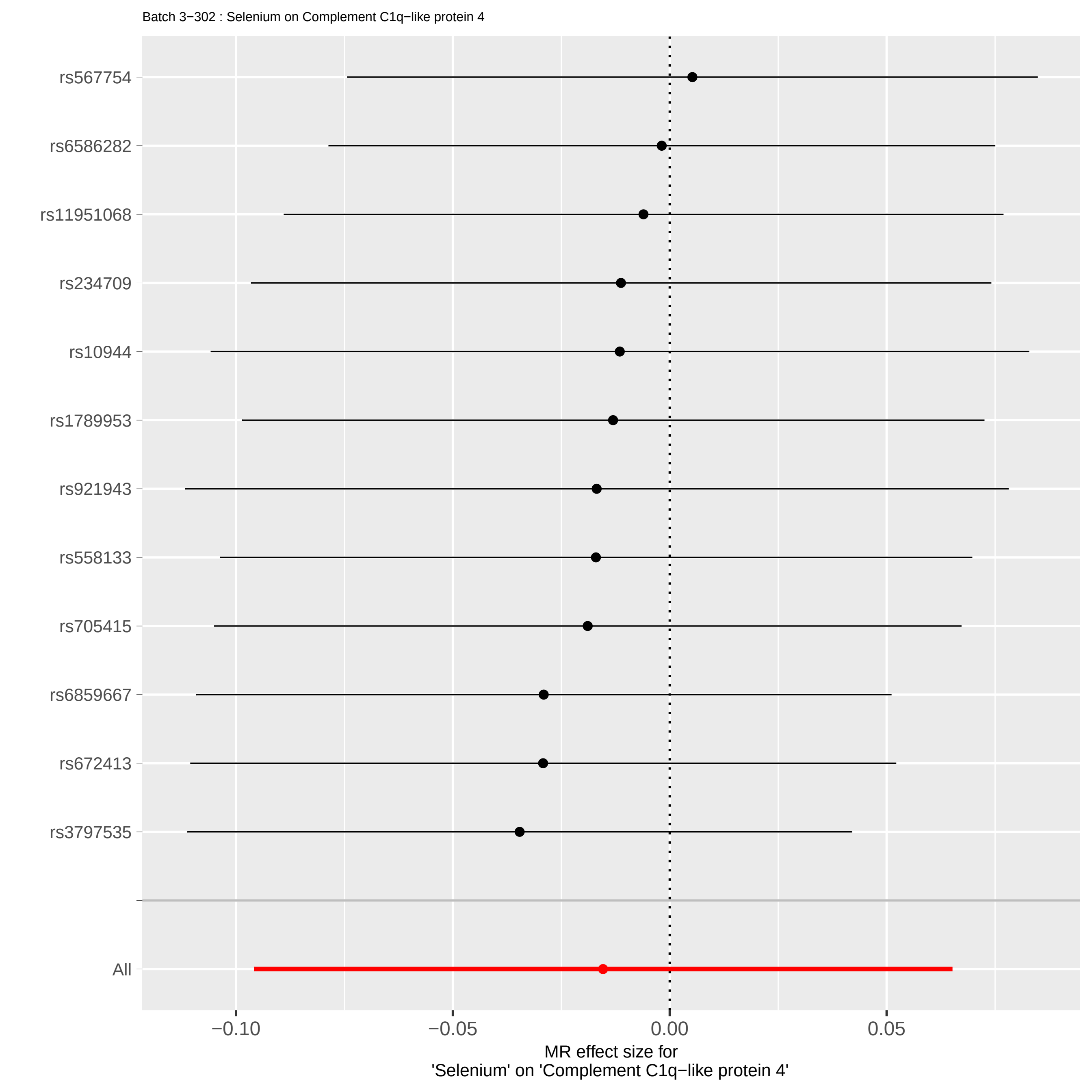

Batch 3−302 : Selenium on Complement C1q−like protein 4
rs567754
rs6586282
rs11951068
rs234709
rs10944
rs1789953
rs921943
rs558133
rs705415
rs6859667
rs672413
rs3797535
All
−0.10
−0.05
0.00
0.05
MR effect size for
'Selenium' on 'Complement C1q−like protein 4'

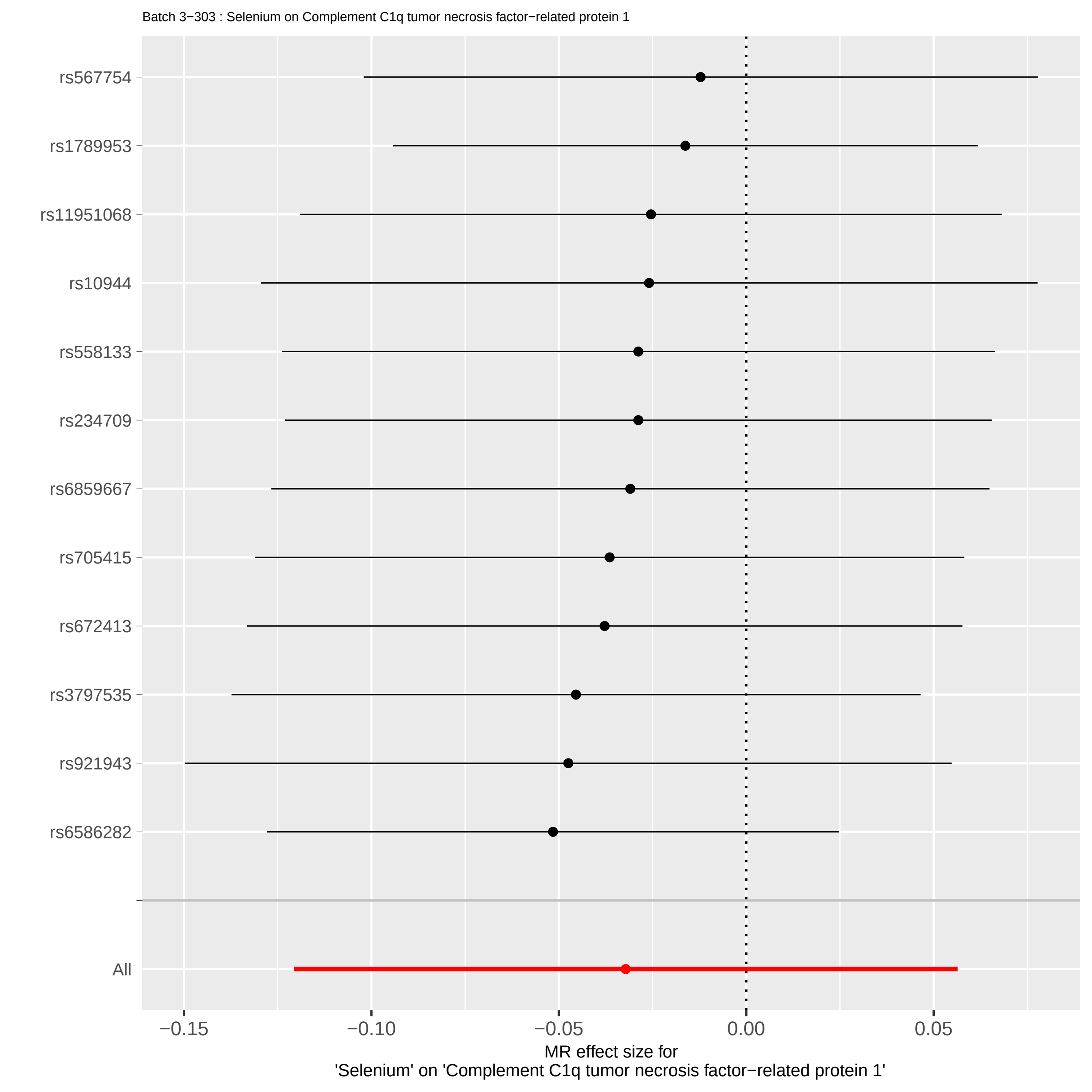
Batch 3−303 : Selenium on Complement C1q tumor necrosis factor−related protein 1
rs567754
rs1789953
rs11951068
rs10944
rs558133
rs234709
rs6859667
rs705415
rs672413
rs3797535
rs921943
rs6586282
All
−0.15
−0.10
−0.05
0.00
0.05
MR effect size for
'Selenium' on 'Complement C1q tumor necrosis factor−related protein 1'

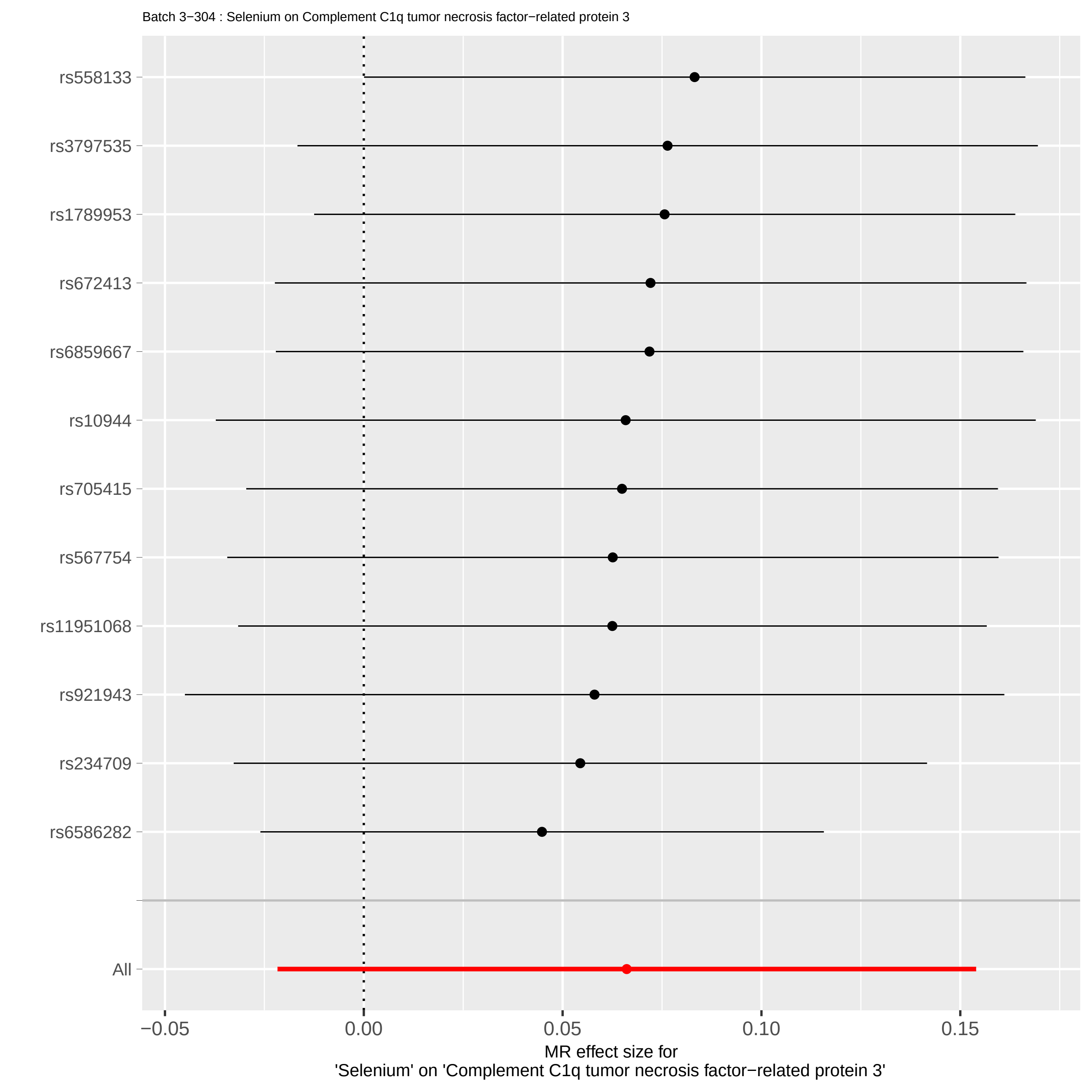
Batch 3−304 : Selenium on Complement C1q tumor necrosis factor−related protein 3
rs558133
rs3797535
rs1789953
rs672413
rs6859667
rs10944
rs705415
rs567754
rs11951068
rs921943
rs234709
rs6586282
All
−0.05
0.00
0.05
0.10
0.15
MR effect size for
'Selenium' on 'Complement C1q tumor necrosis factor−related protein 3'

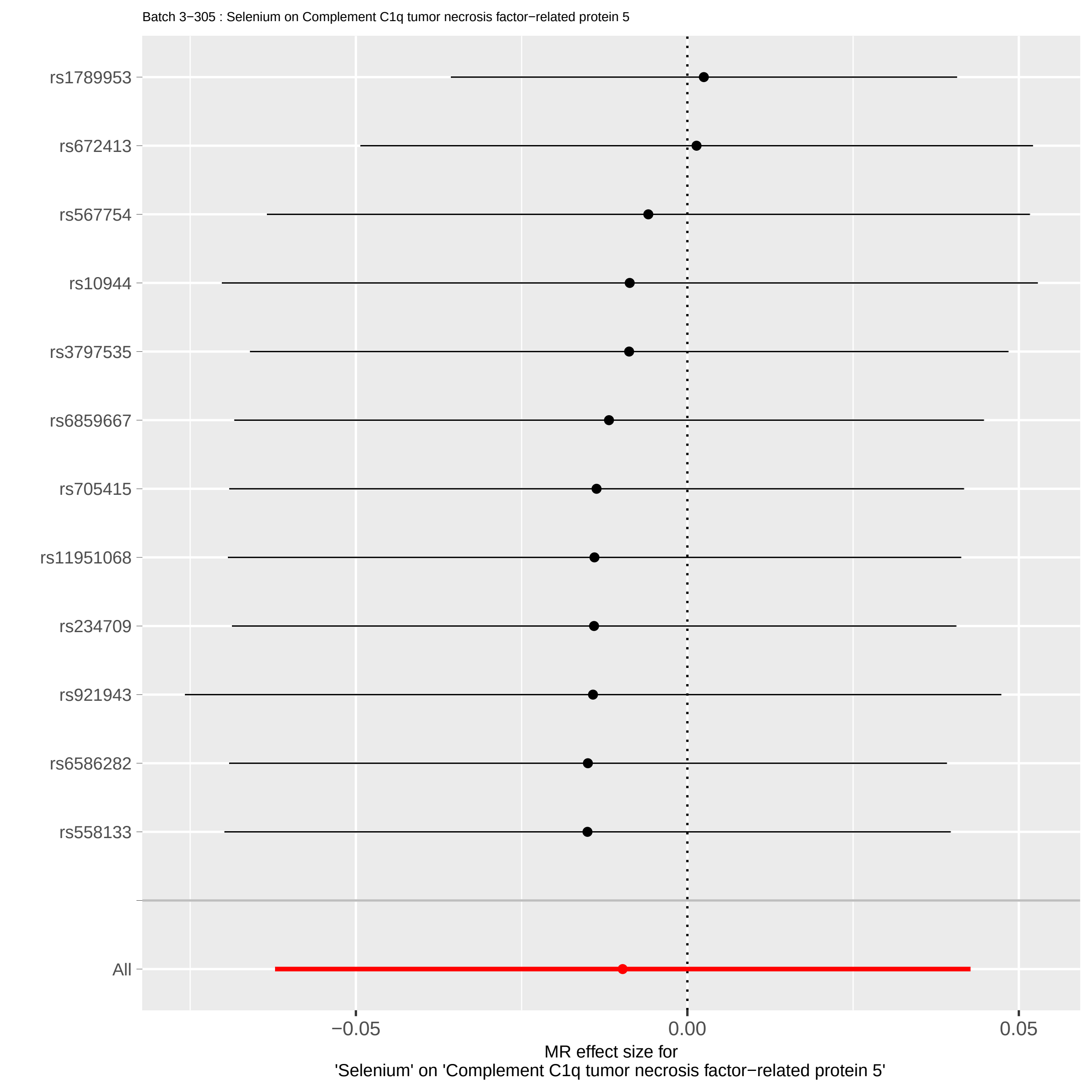
Batch 3−305 : Selenium on Complement C1q tumor necrosis factor−related protein 5
rs1789953
rs672413
rs567754
rs10944
rs3797535
rs6859667
rs705415
rs11951068
rs234709
rs921943
rs6586282
rs558133
All
−0.05
0.00
0.05
MR effect size for
'Selenium' on 'Complement C1q tumor necrosis factor−related protein 5'

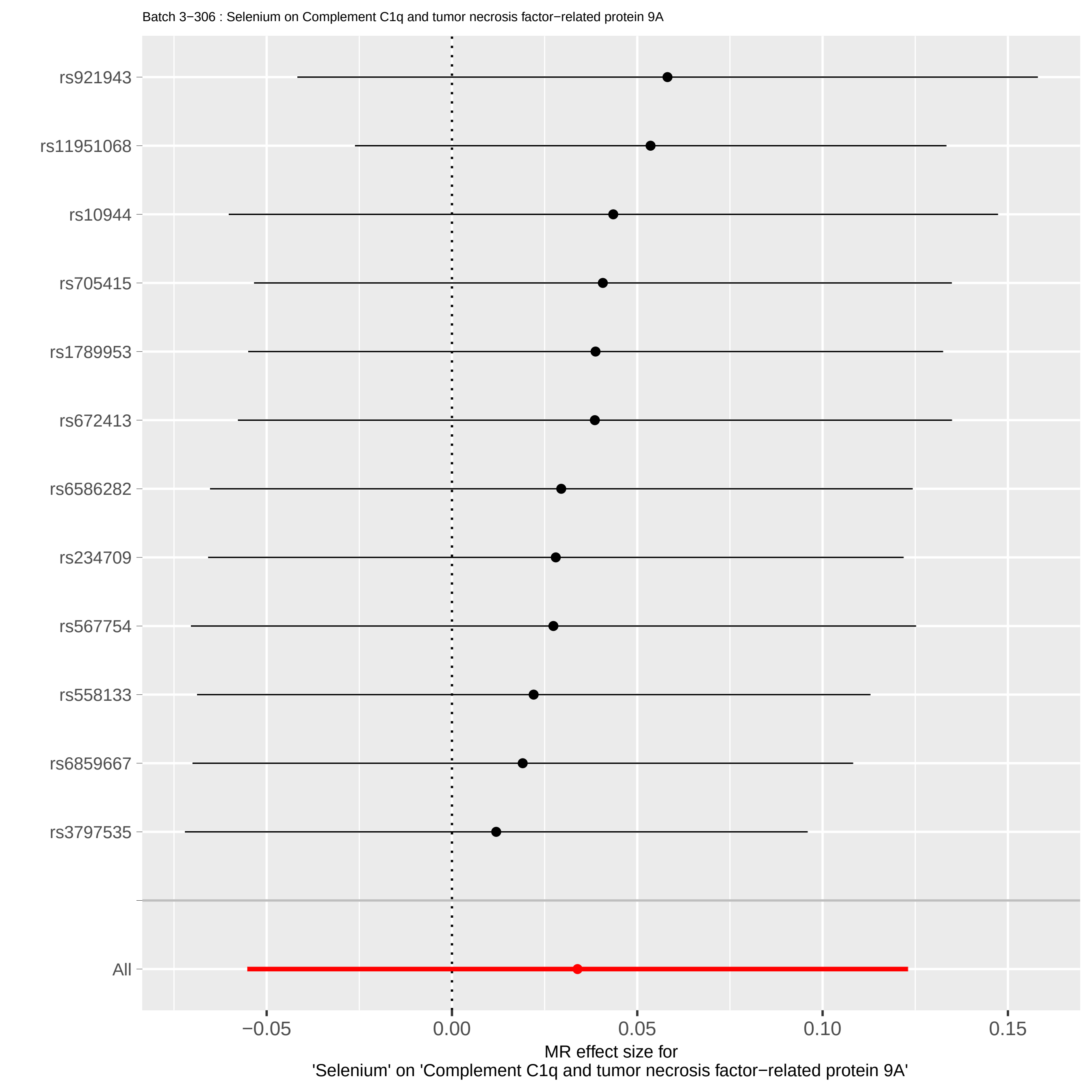
Batch 3−306 : Selenium on Complement C1q and tumor necrosis factor−related protein 9A
rs921943
rs11951068
rs10944
rs705415
rs1789953
rs672413
rs6586282
rs234709
rs567754
rs558133
rs6859667
rs3797535
All
−0.05
0.00
0.05
0.10
0.15
MR effect size for
'Selenium' on 'Complement C1q and tumor necrosis factor−related protein 9A'

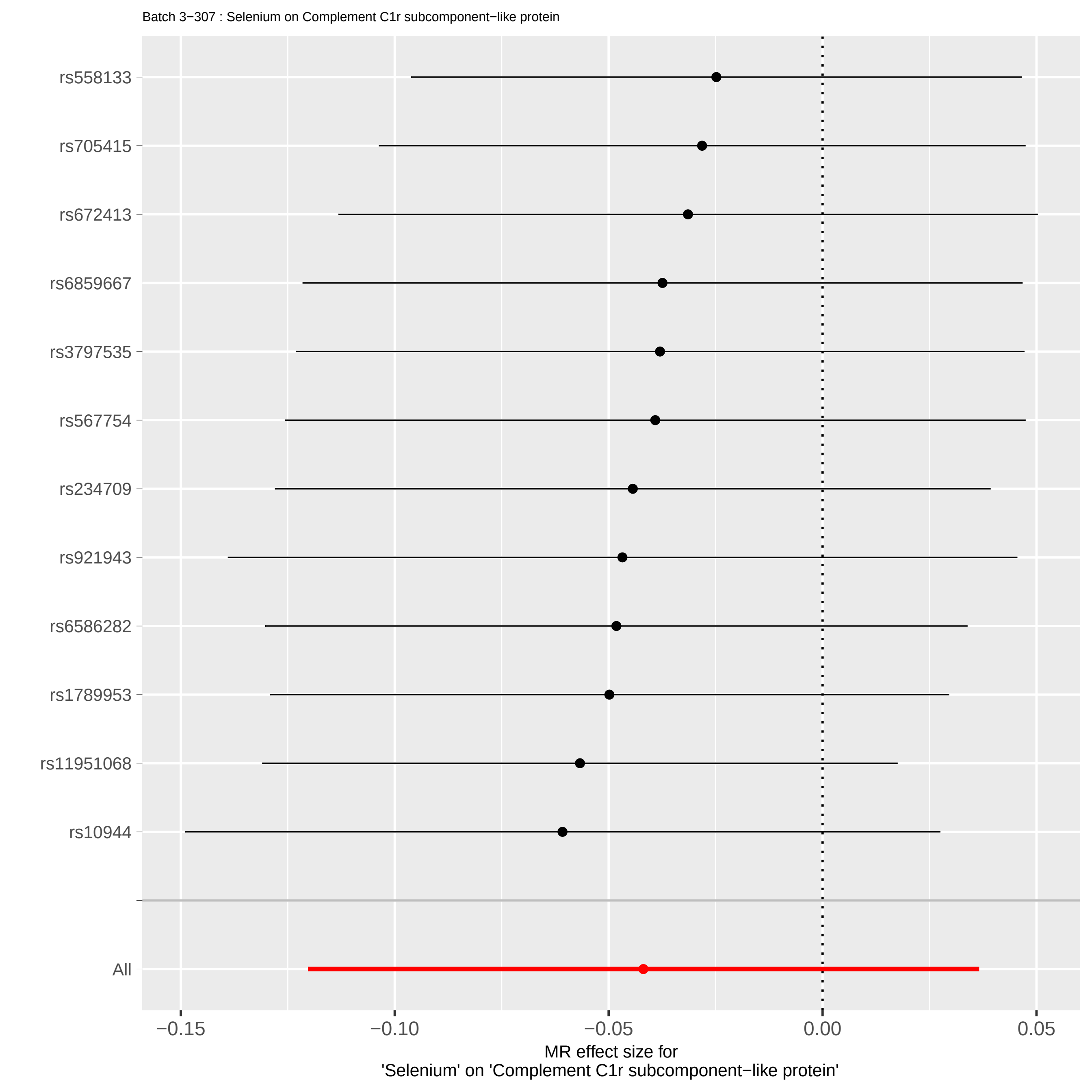

Batch 3−307 : Selenium on Complement C1r subcomponent−like protein
rs558133
rs705415
rs672413
rs6859667
rs3797535
rs567754
rs234709
rs921943
rs6586282
rs1789953
rs11951068
rs10944
All
−0.15
−0.10
−0.05
0.00
0.05
MR effect size for
'Selenium' on 'Complement C1r subcomponent−like protein'

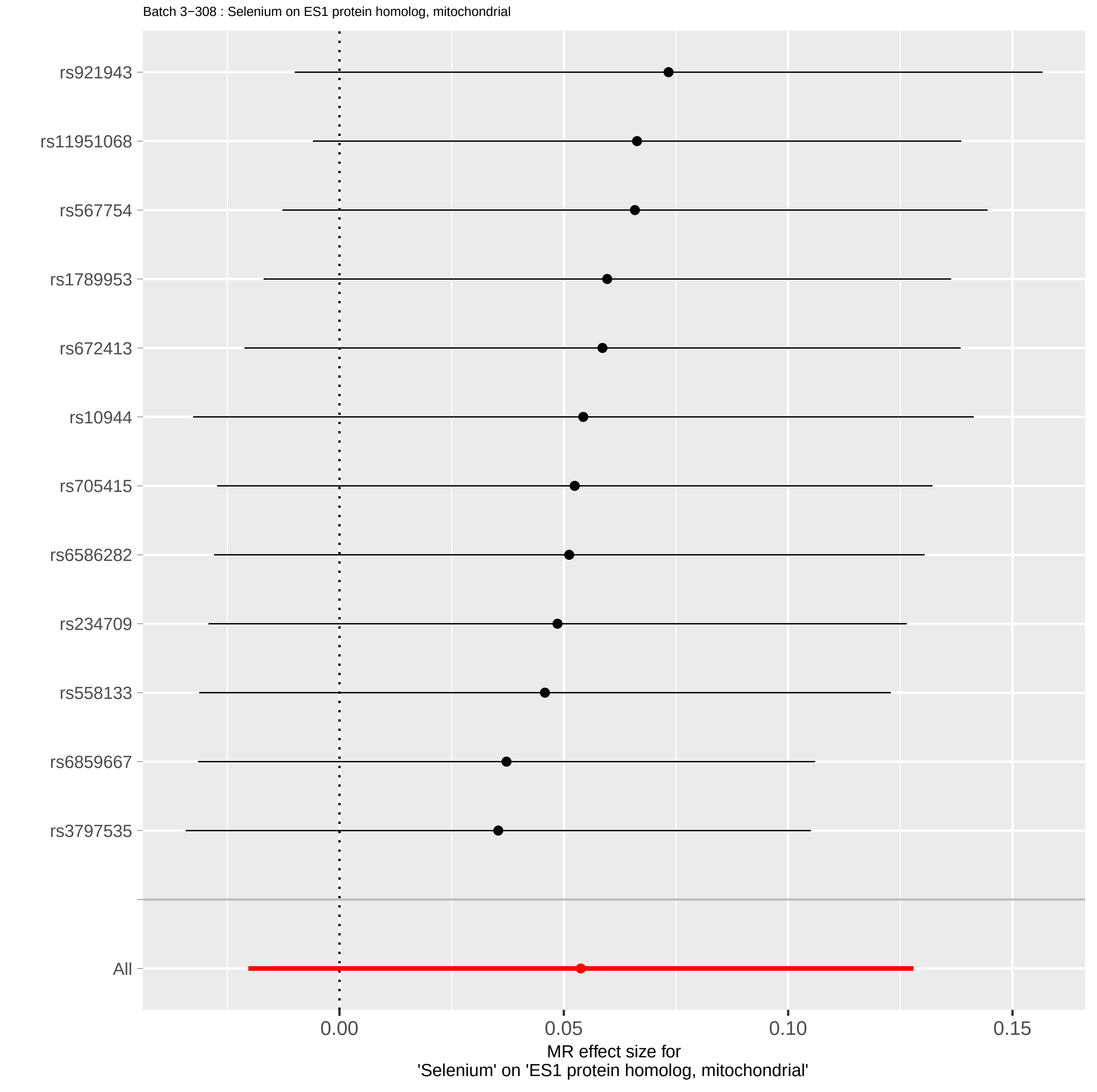

Batch 3−308 : Selenium on ES1 protein homolog, mitochondrial
rs921943
rs11951068
rs567754
rs1789953
rs672413
rs10944
rs705415
rs6586282
rs234709
rs558133
rs6859667
rs3797535
All
0.00
0.05
0.10
0.15
MR effect size for
'Selenium' on 'ES1 protein homolog, mitochondrial'

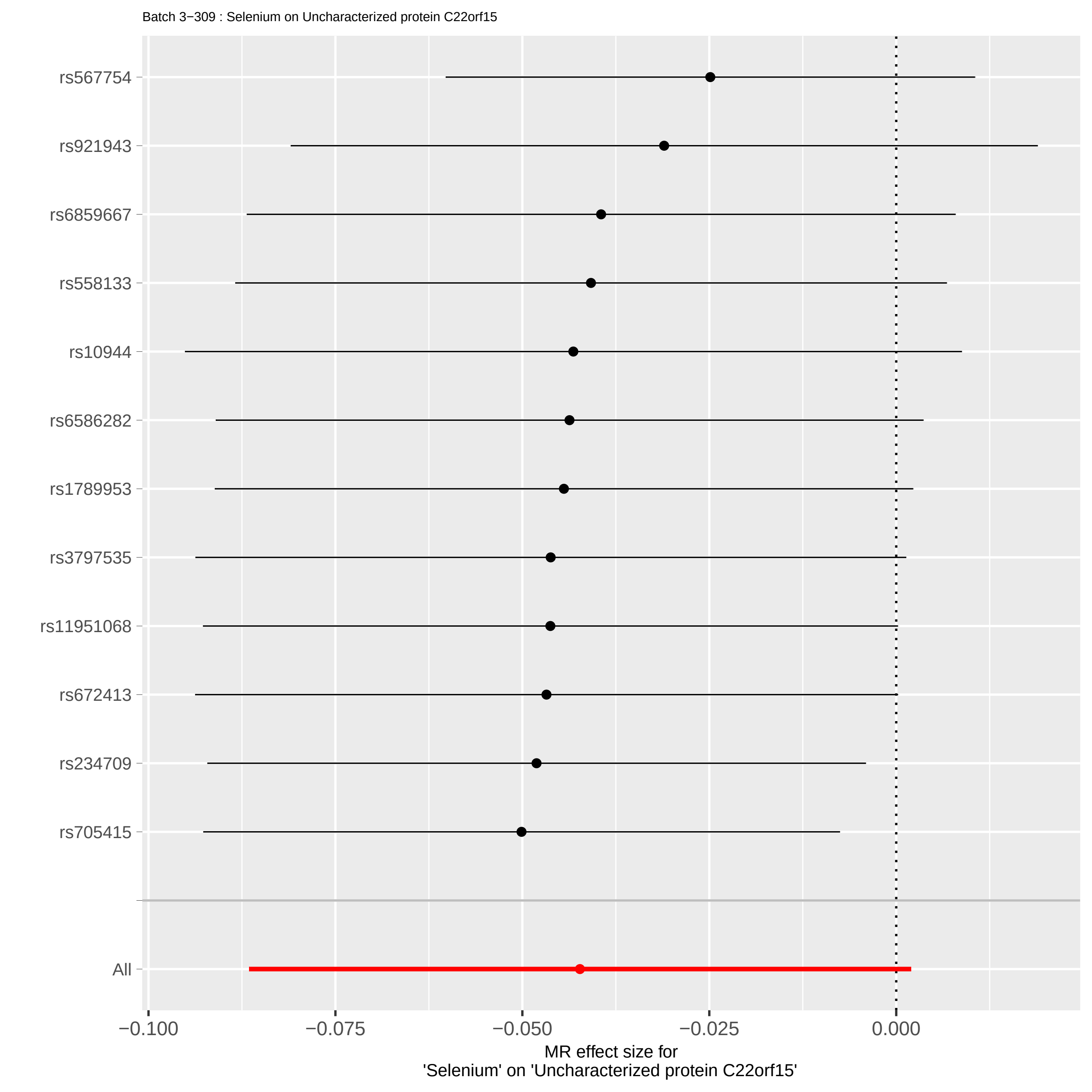

Batch 3−309 : Selenium on Uncharacterized protein C22orf15
rs567754
rs921943
rs6859667
rs558133
rs10944
rs6586282
rs1789953
rs3797535
rs11951068
rs672413
rs234709
rs705415
All
−0.100
−0.075
−0.050
−0.025
0.000
MR effect size for
'Selenium' on 'Uncharacterized protein C22orf15'

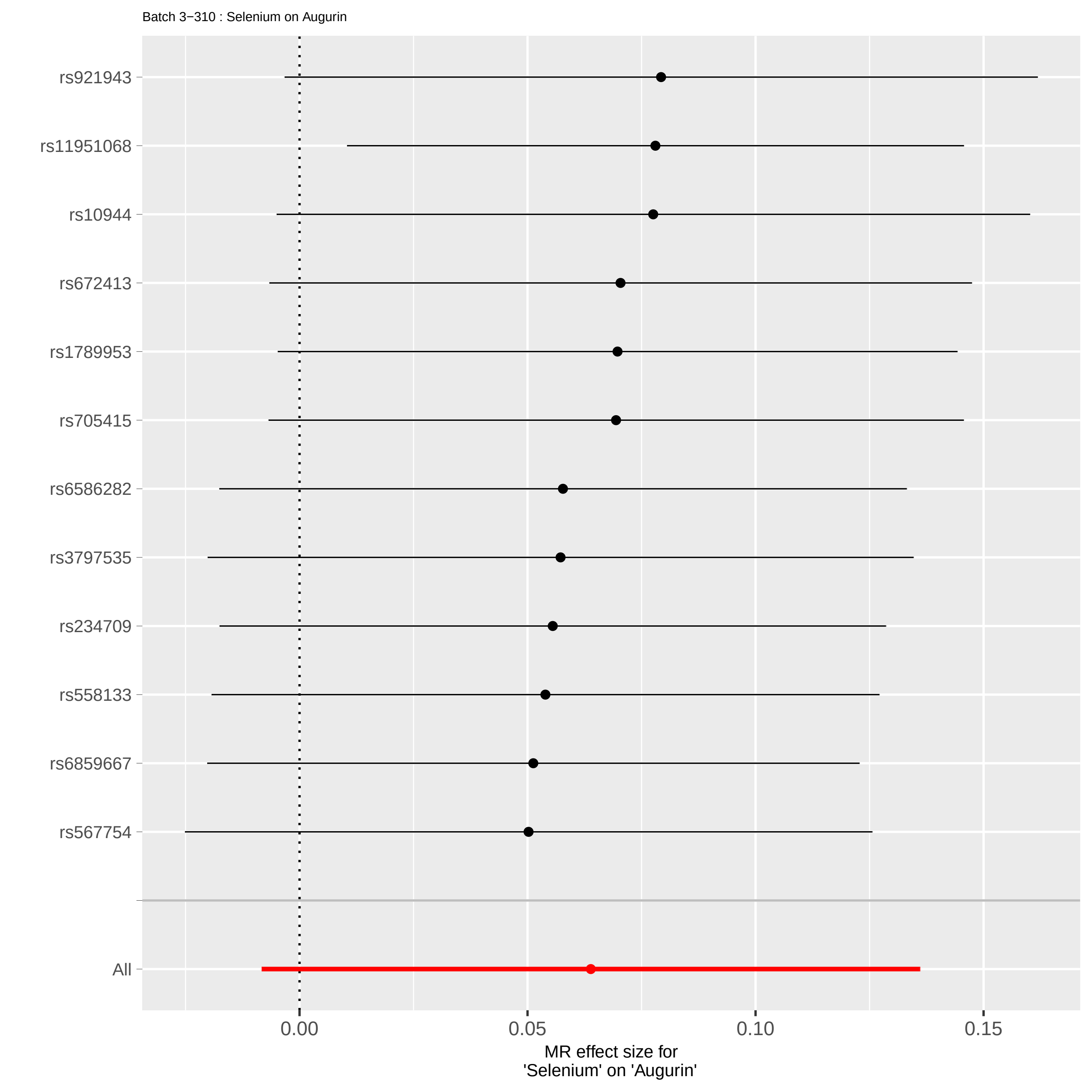

Batch 3−310 : Selenium on Augurin
rs921943
rs11951068
rs10944
rs672413
rs1789953
rs705415
rs6586282
rs3797535
rs234709
rs558133
rs6859667
rs567754
All
0.00
0.05
0.10
0.15
MR effect size for
'Selenium' on 'Augurin'

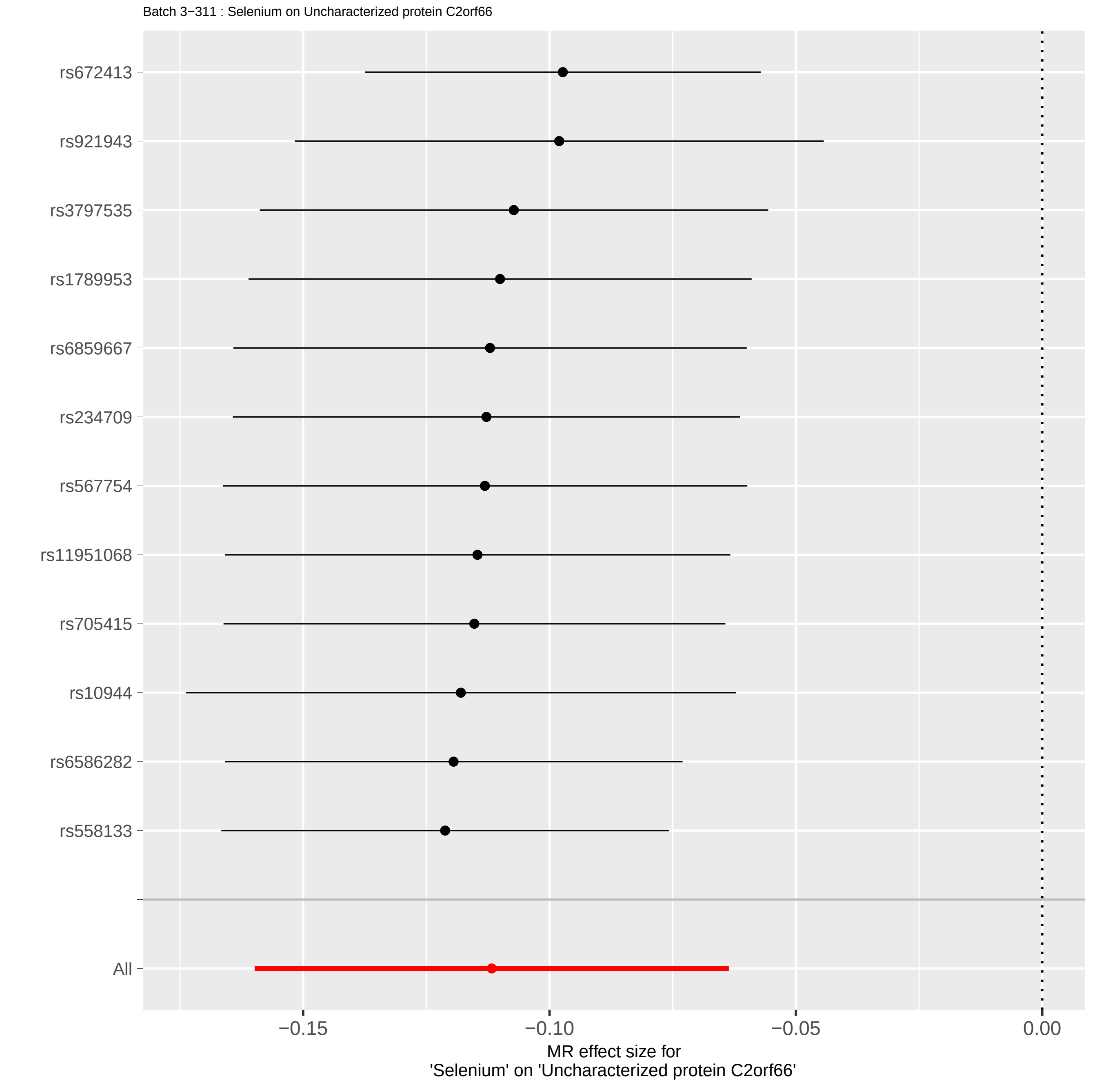

Batch 3−311 : Selenium on Uncharacterized protein C2orf66
rs672413
rs921943
rs3797535
rs1789953
rs6859667
rs234709
rs567754
rs11951068
rs705415
rs10944
rs6586282
rs558133
All
−0.15
−0.10
−0.05
0.00
MR effect size for
'Selenium' on 'Uncharacterized protein C2orf66'

Batch 3−312 : Selenium on Uncharacterized protein C2orf82

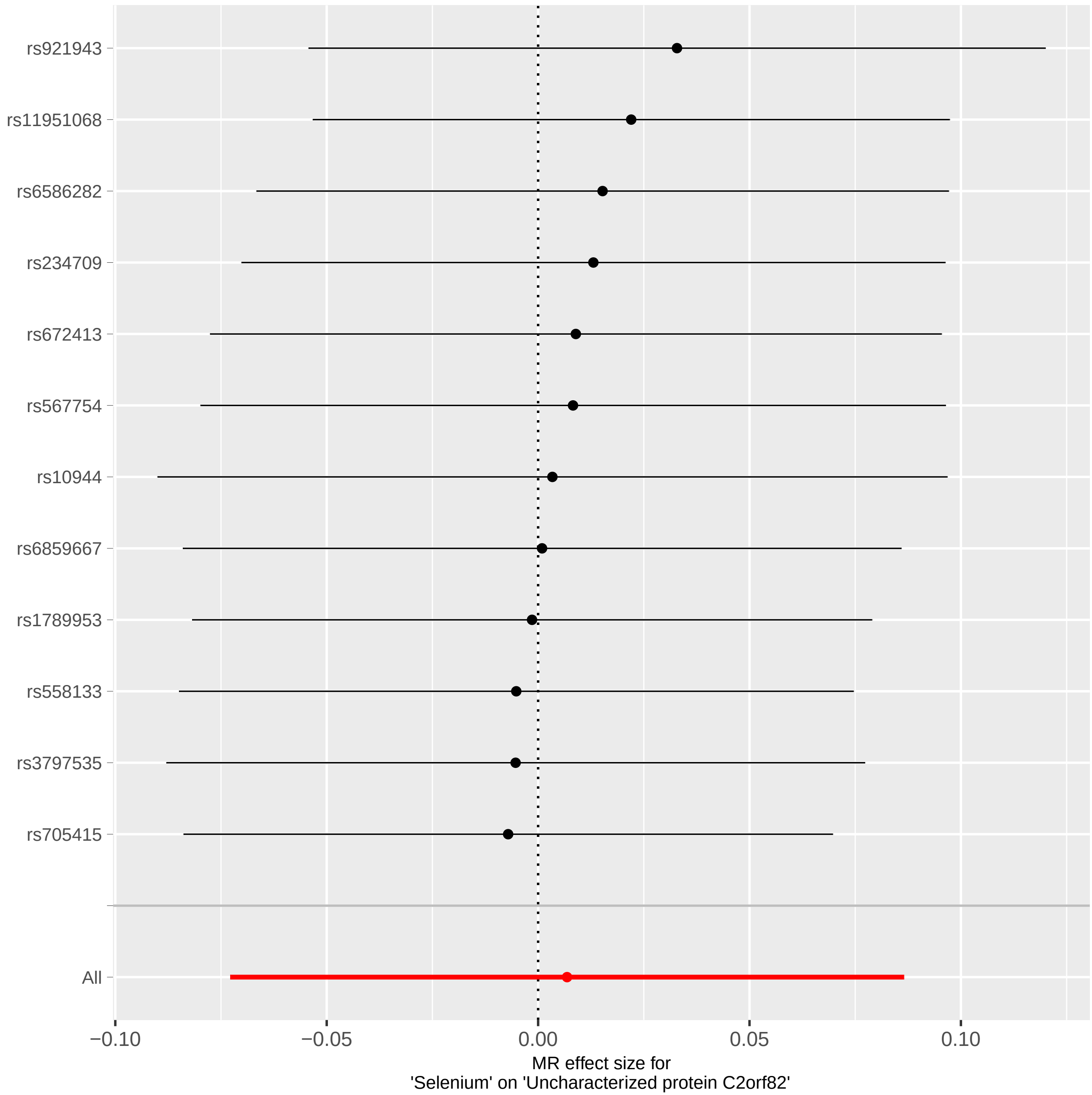

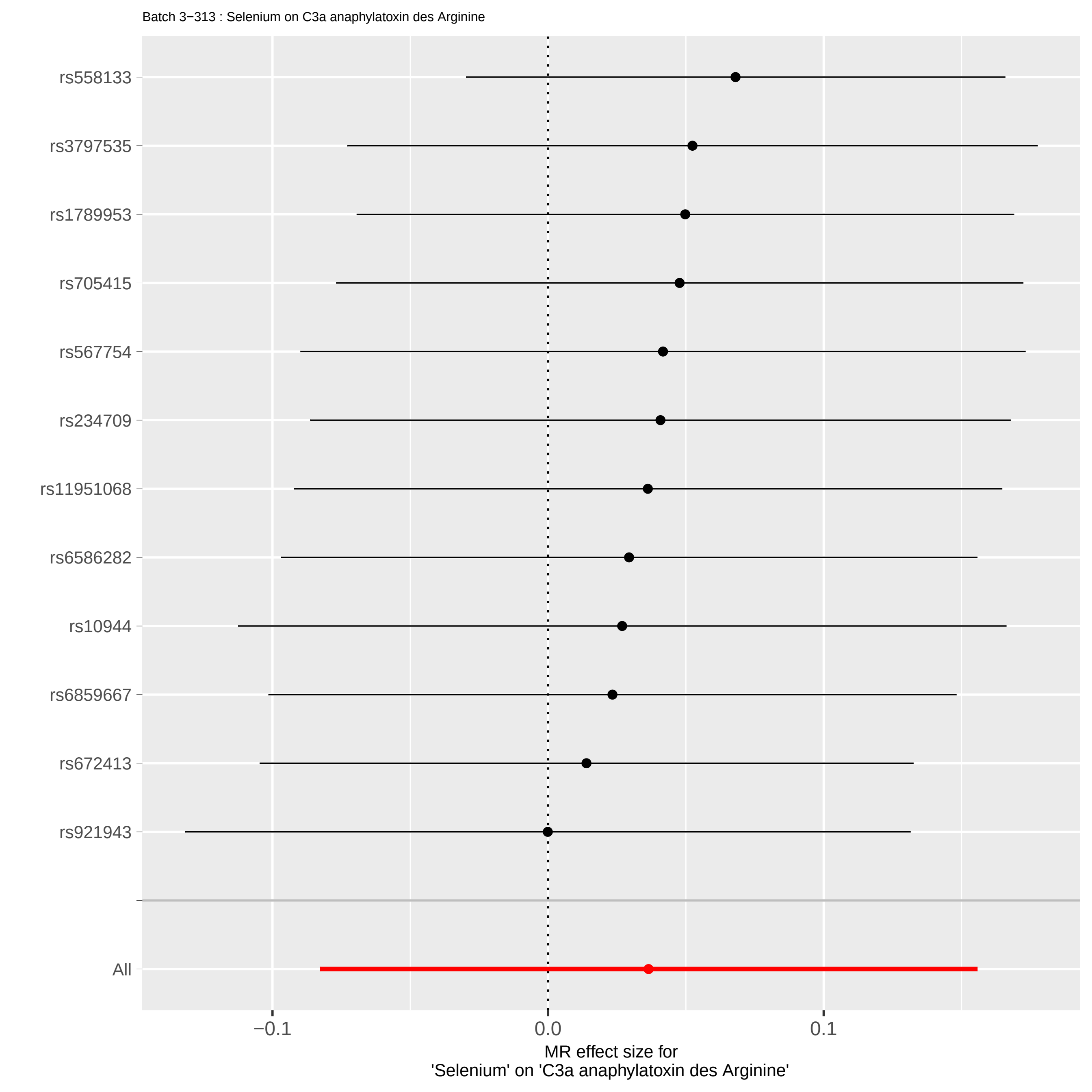

Batch 3−313 : Selenium on C3a anaphylatoxin des Arginine
rs558133
rs3797535
rs1789953
rs705415
rs567754
rs234709
rs11951068
rs6586282
rs10944
rs6859667
rs672413
rs921943
All
−0.1
0.0
0.1
MR effect size for
'Selenium' on 'C3a anaphylatoxin des Arginine'

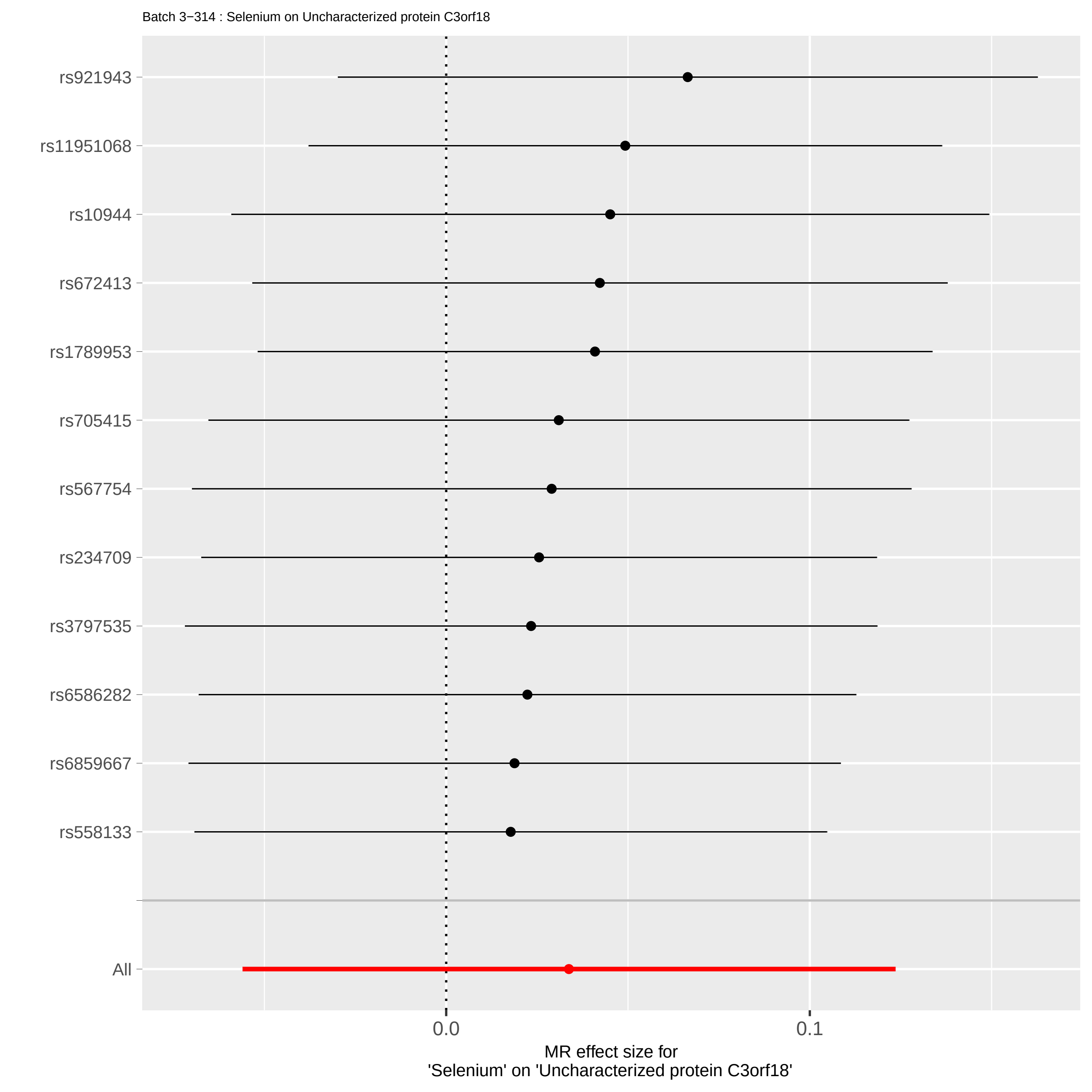

Batch 3−314 : Selenium on Uncharacterized protein C3orf18
rs921943
rs11951068
rs10944
rs672413
rs1789953
rs705415
rs567754
rs234709
rs3797535
rs6586282
rs6859667
rs558133
All
0.0
0.1
MR effect size for
'Selenium' on 'Uncharacterized protein C3orf18'

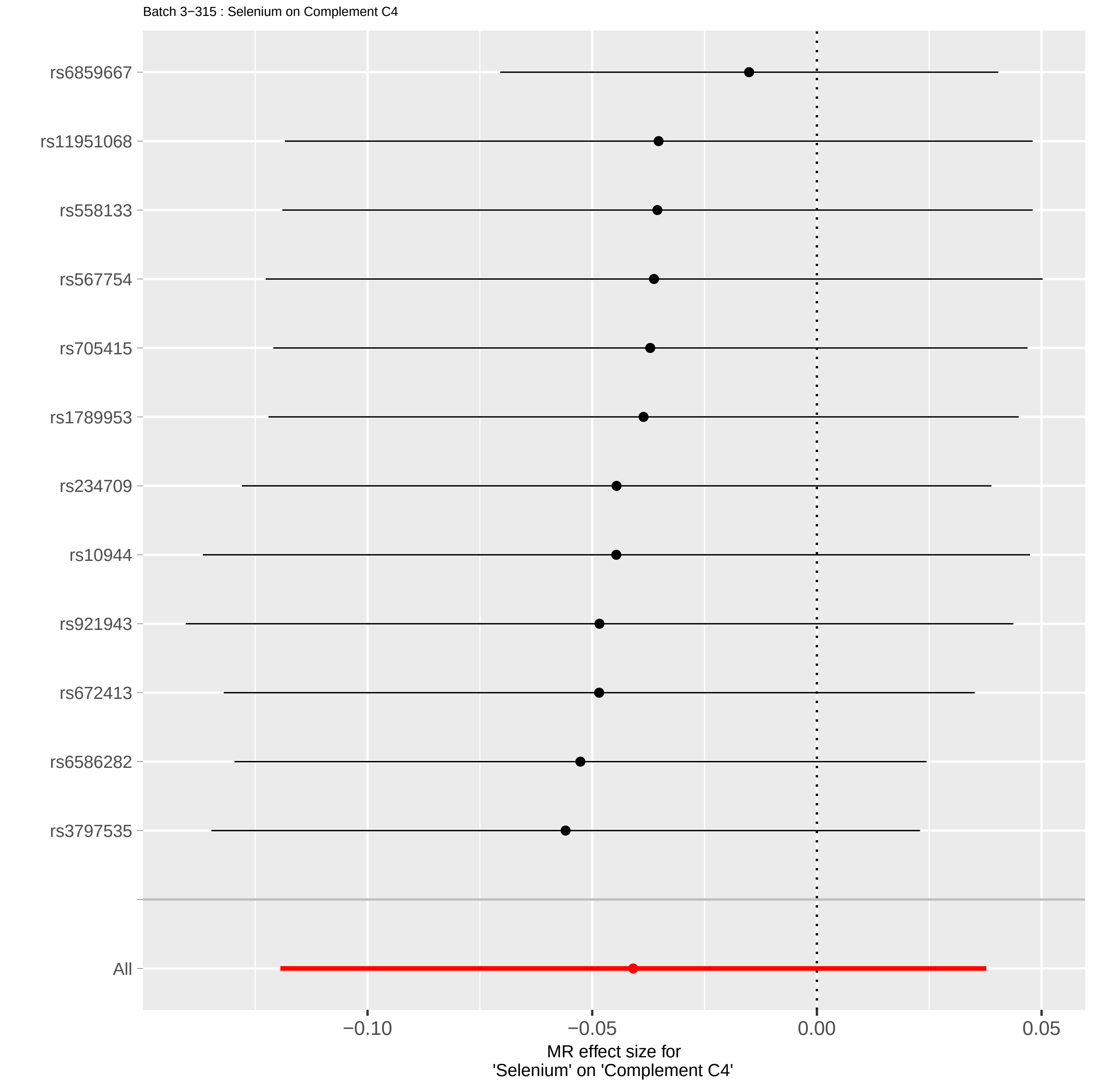

Batch 3−315 : Selenium on Complement C4
rs6859667
rs11951068
rs558133
rs567754
rs705415
rs1789953
rs234709
rs10944
rs921943
rs672413
rs6586282
rs3797535
All
−0.10
−0.05
0.00
0.05
MR effect size for
'Selenium' on 'Complement C4'

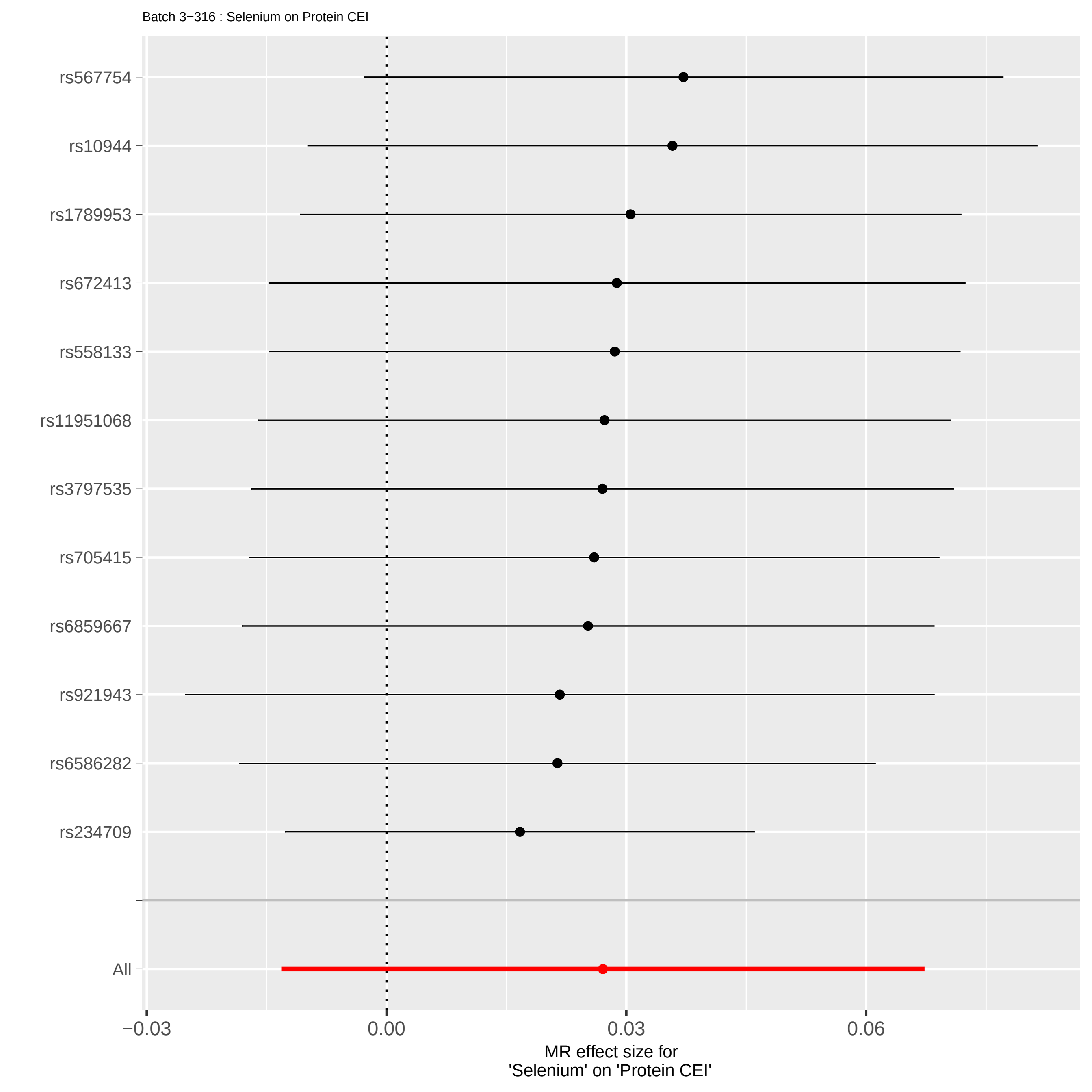

Batch 3−316 : Selenium on Protein CEI
rs567754
rs10944
rs1789953
rs672413
rs558133
rs11951068
rs3797535
rs705415
rs6859667
rs921943
rs6586282
rs234709
All
−0.03
0.00
0.03
0.06
MR effect size for
'Selenium' on 'Protein CEI'

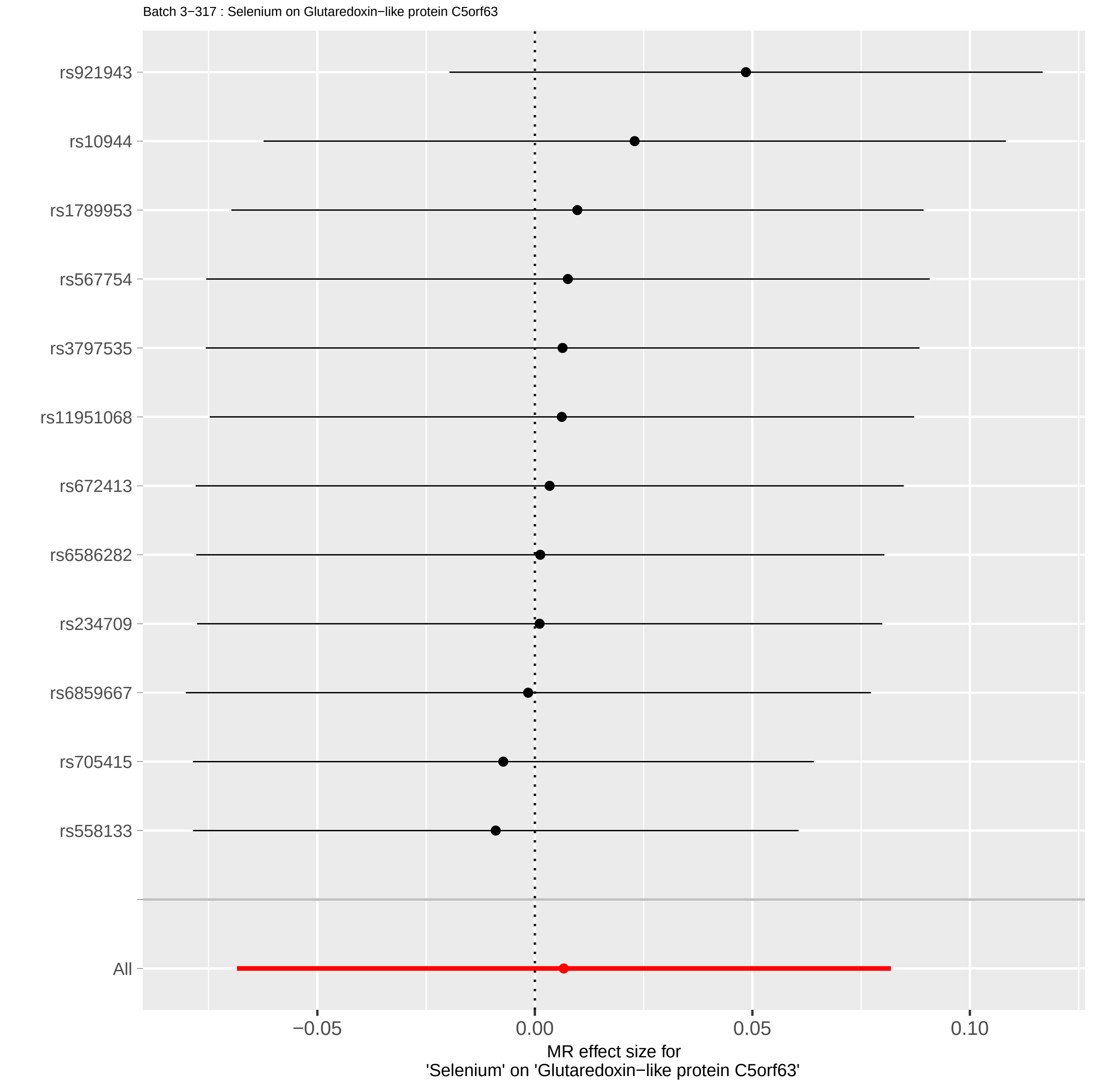

Batch 3−317 : Selenium on Glutaredoxin−like protein C5orf63
rs921943
rs10944
rs1789953
rs567754
rs3797535
rs11951068
rs672413
rs6586282
rs234709
rs6859667
rs705415
rs558133
All
−0.05
0.00
0.05
0.10
MR effect size for
'Selenium' on 'Glutaredoxin−like protein C5orf63'

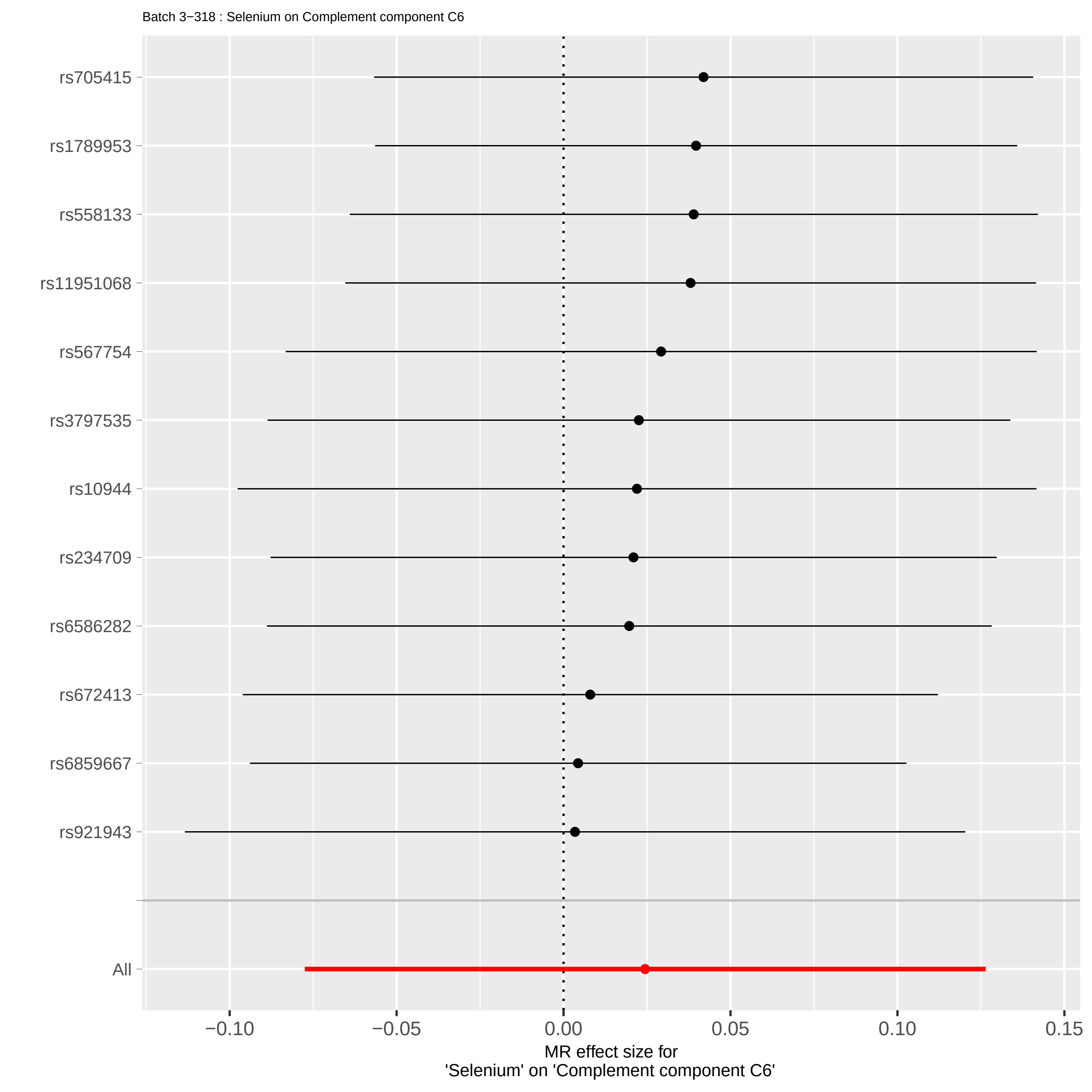

Batch 3−318 : Selenium on Complement component C6
rs705415
rs1789953
rs558133
rs11951068
rs567754
rs3797535
rs10944
rs234709
rs6586282
rs672413
rs6859667
rs921943
All
−0.10
−0.05
0.00
0.05
0.10
0.15
MR effect size for
'Selenium' on 'Complement component C6'

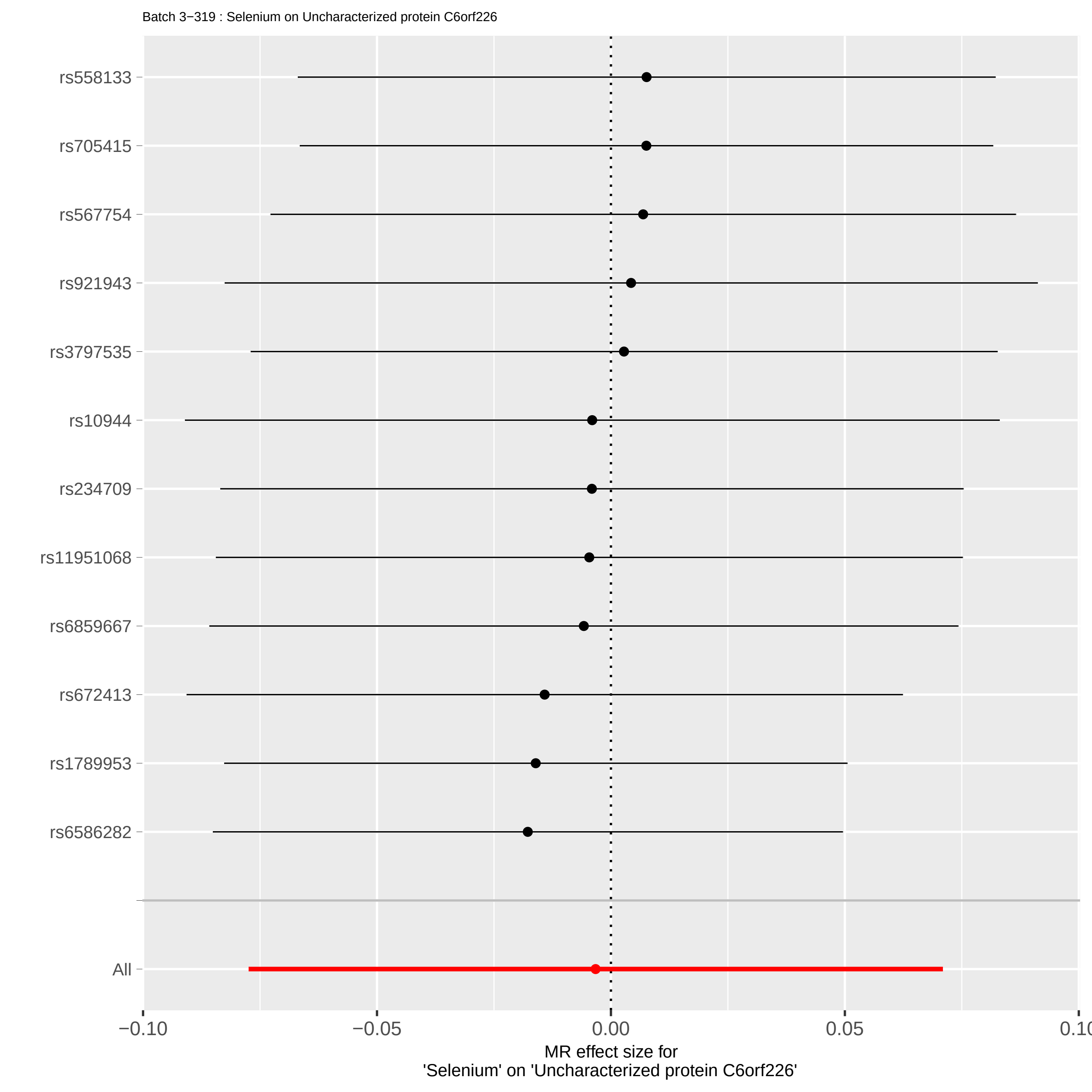

Batch 3−319 : Selenium on Uncharacterized protein C6orf226
rs558133
rs705415
rs567754
rs921943
rs3797535
rs10944
rs234709
rs11951068
rs6859667
rs672413
rs1789953
rs6586282
All
−0.10
−0.05
0.00
0.05
0.10
MR effect size for
'Selenium' on 'Uncharacterized protein C6orf226'

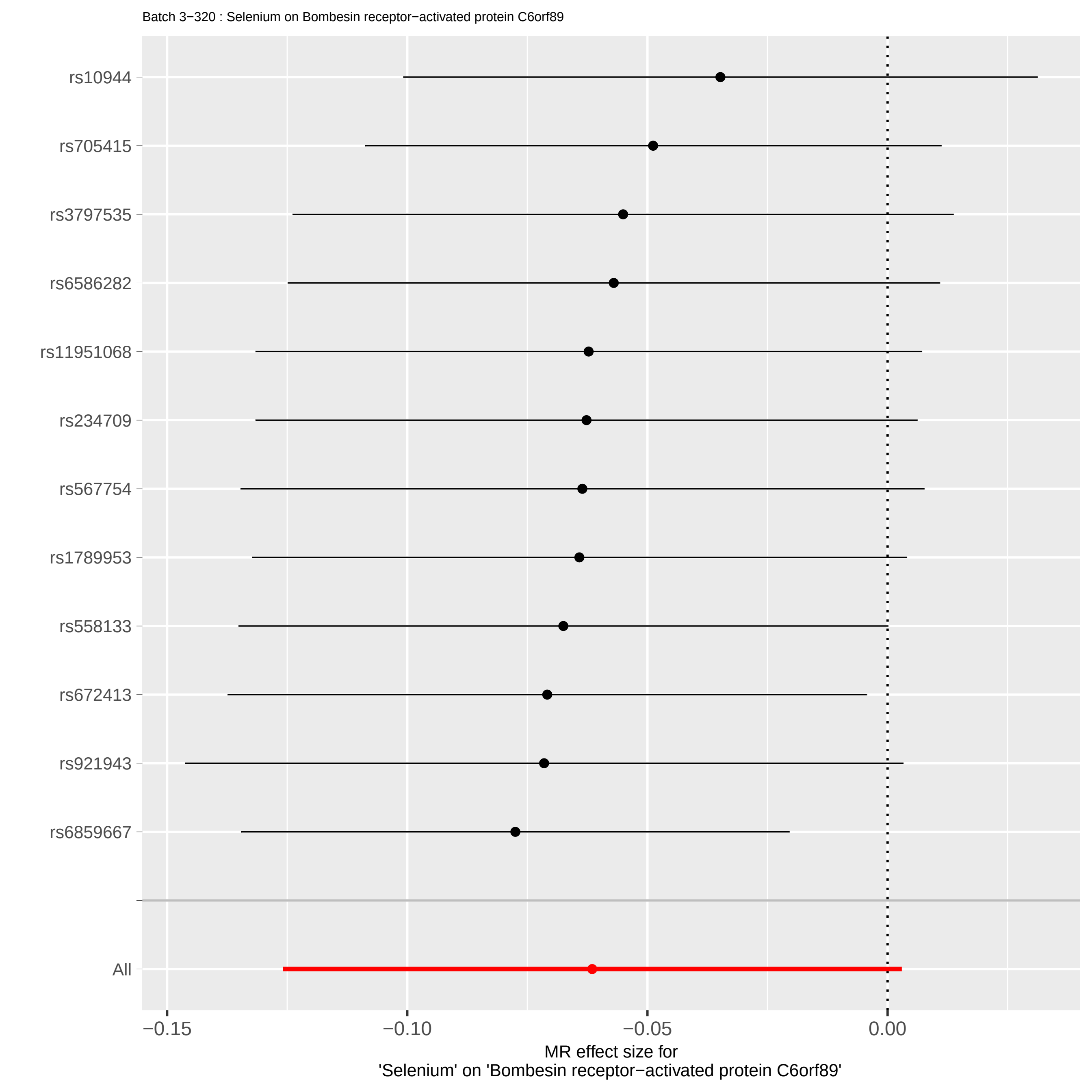

Batch 3−320 : Selenium on Bombesin receptor−activated protein C6orf89
rs10944
rs705415
rs3797535
rs6586282
rs11951068
rs234709
rs567754
rs1789953
rs558133
rs672413
rs921943
rs6859667
All
−0.15
−0.10
−0.05
0.00
MR effect size for
'Selenium' on 'Bombesin receptor−activated protein C6orf89'

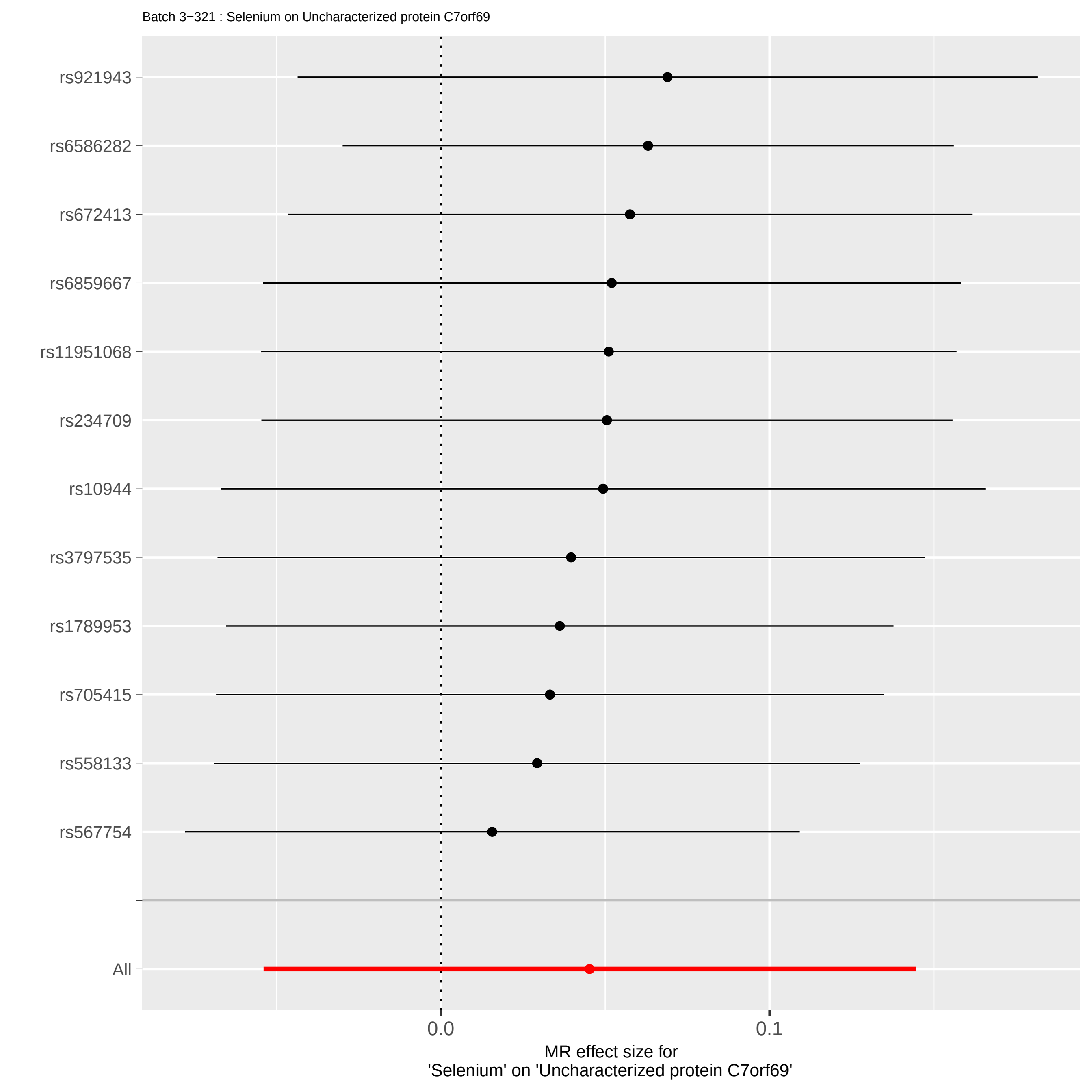

Batch 3−321 : Selenium on Uncharacterized protein C7orf69
rs921943
rs6586282
rs672413
rs6859667
rs11951068
rs234709
rs10944
rs3797535
rs1789953
rs705415
rs558133
rs567754
All
0.0
0.1
MR effect size for
'Selenium' on 'Uncharacterized protein C7orf69'

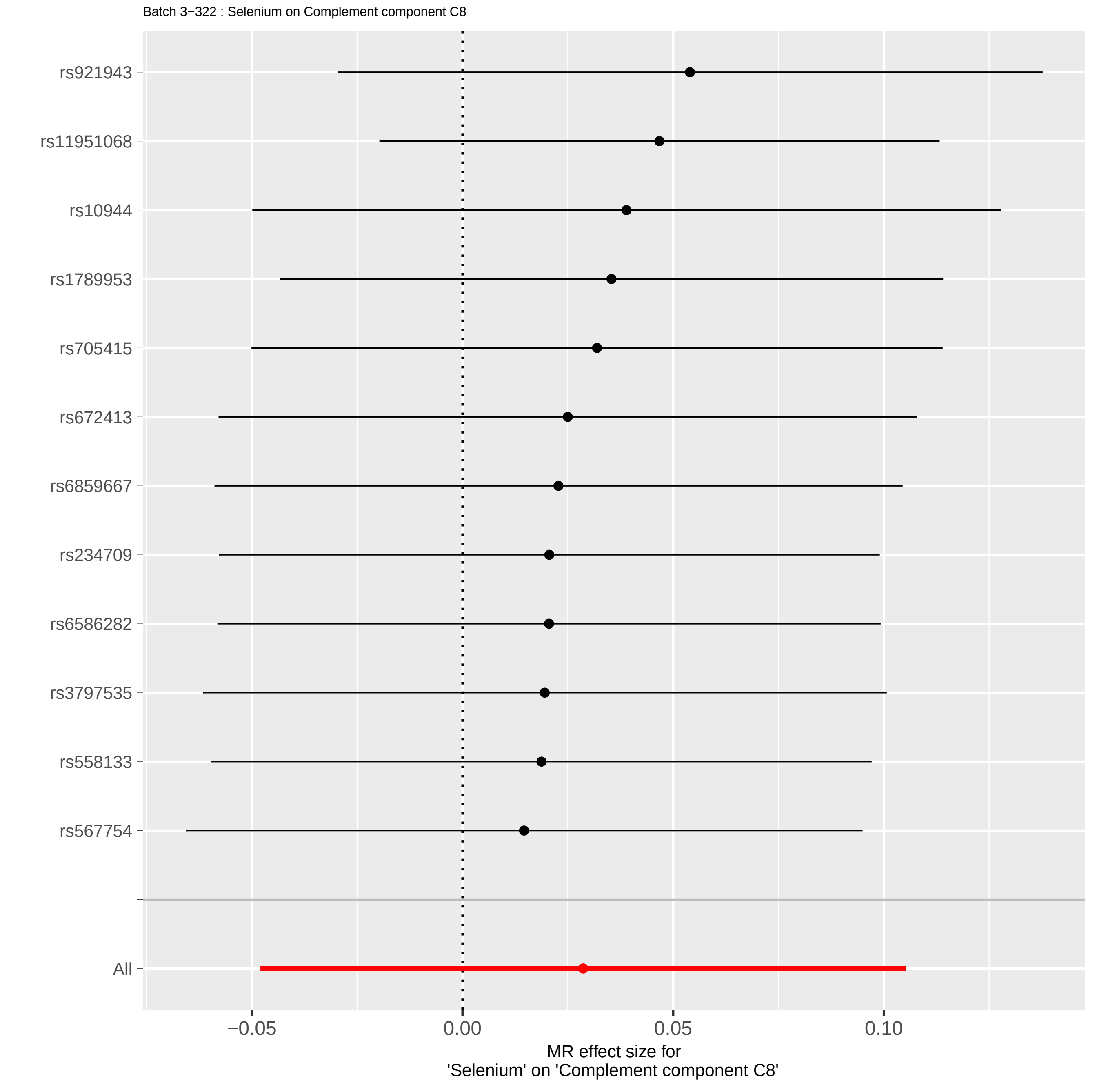
Batch 3−322 : Selenium on Complement component C8
rs921943
rs11951068
rs10944
rs1789953
rs705415
rs672413
rs6859667
rs234709
rs6586282
rs3797535
rs558133
rs567754
All
−0.05
0.00
0.05
0.10
MR effect size for
'Selenium' on 'Complement component C8'

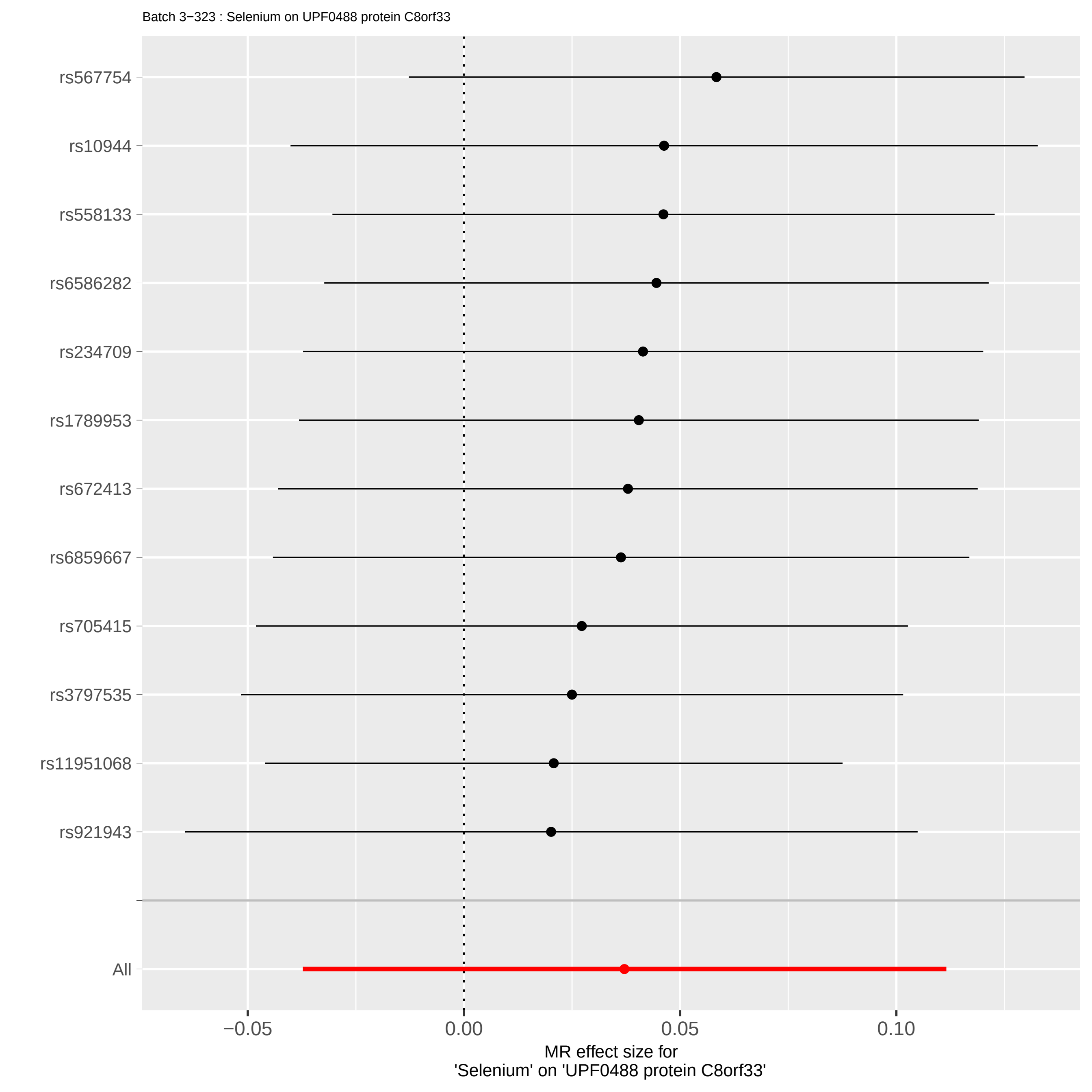

Batch 3−323 : Selenium on UPF0488 protein C8orf33
rs567754
rs10944
rs558133
rs6586282
rs234709
rs1789953
rs672413
rs6859667
rs705415
rs3797535
rs11951068
rs921943
All
−0.05
0.00
0.05
0.10
MR effect size for
'Selenium' on 'UPF0488 protein C8orf33'

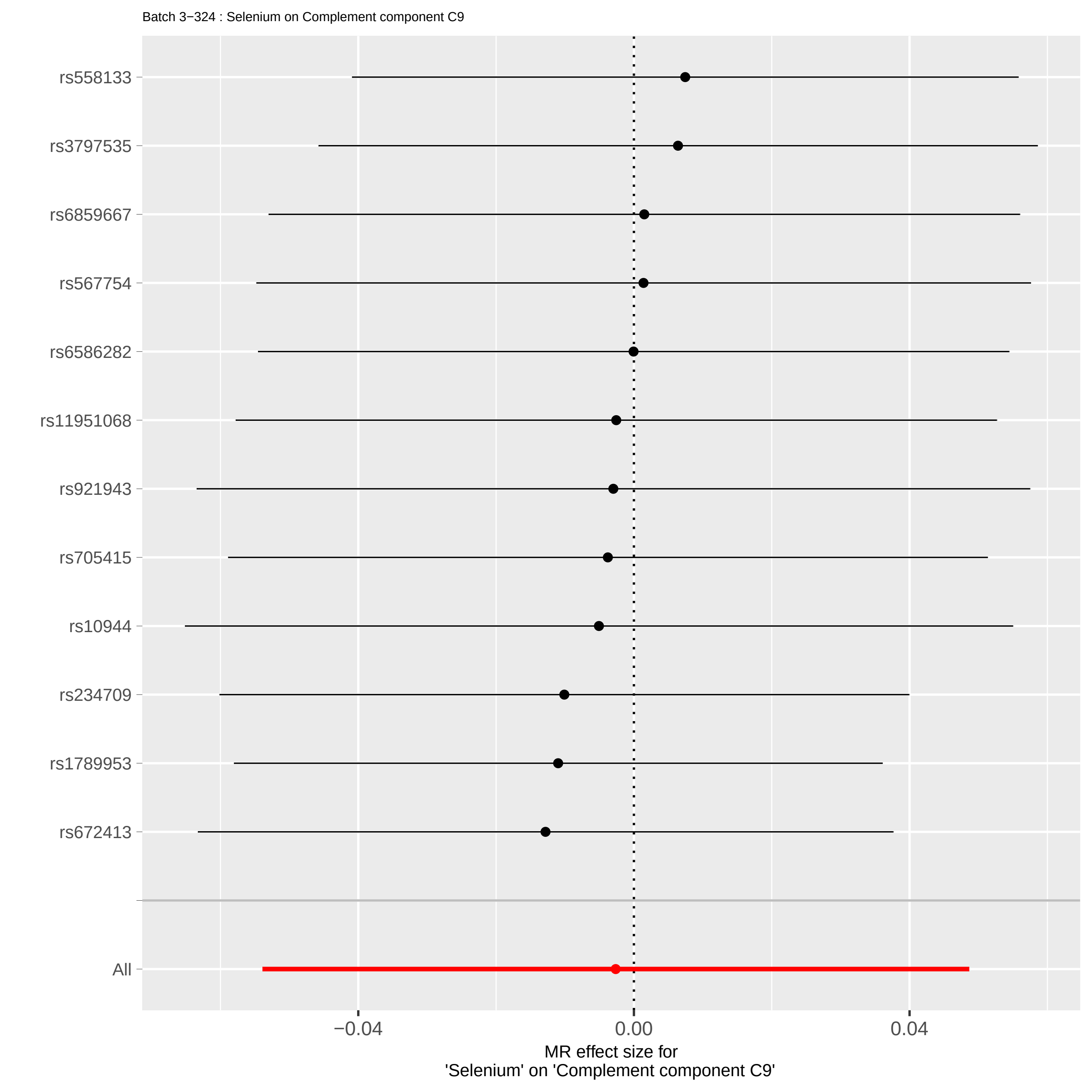

Batch 3−324 : Selenium on Complement component C9
rs558133
rs3797535
rs6859667
rs567754
rs6586282
rs11951068
rs921943
rs705415
rs10944
rs234709
rs1789953
rs672413
All
−0.04
0.00
0.04
MR effect size for
'Selenium' on 'Complement component C9'

Batch 3−325 : Selenium on Bcl10−interacting CARD protein

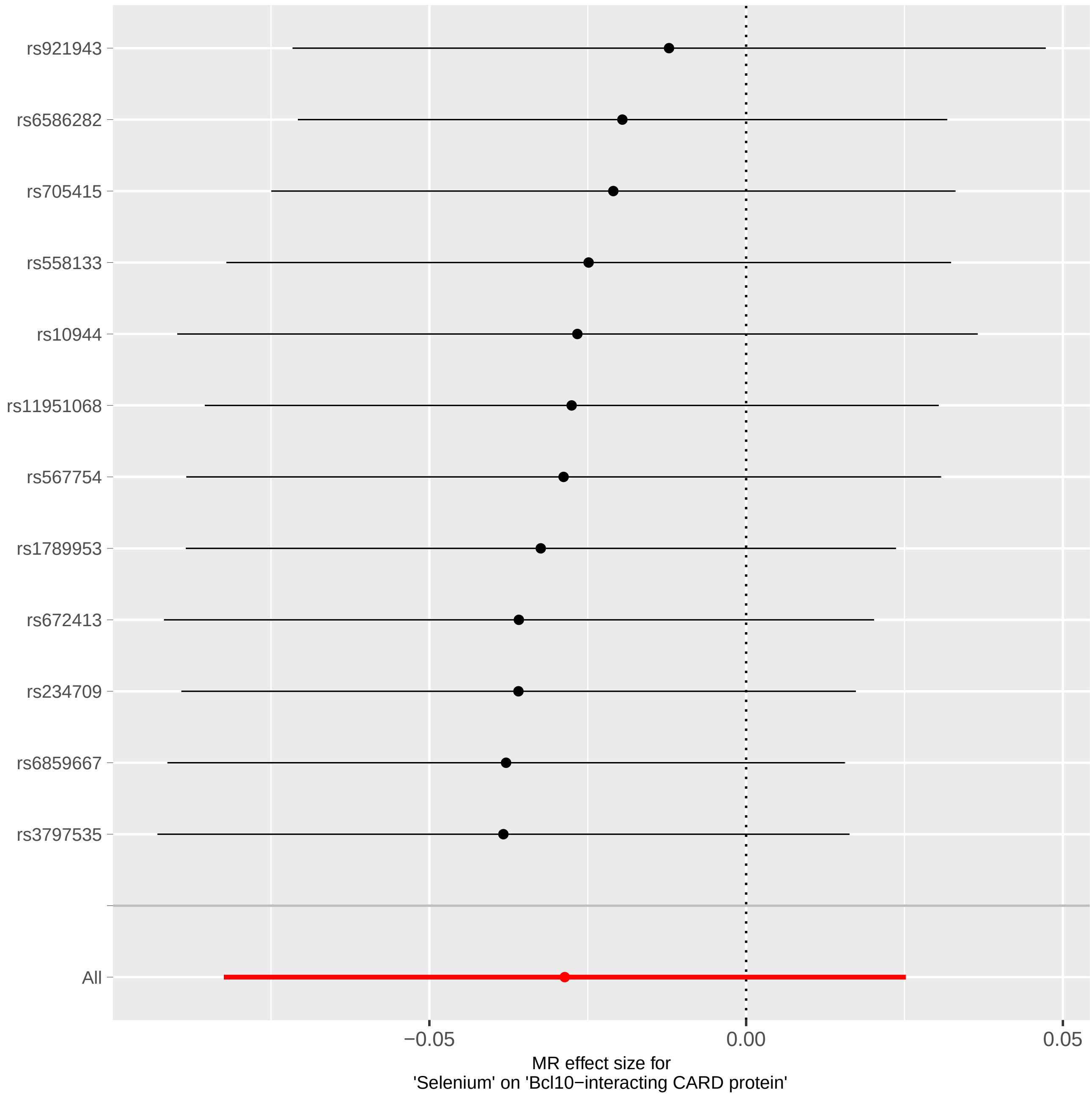

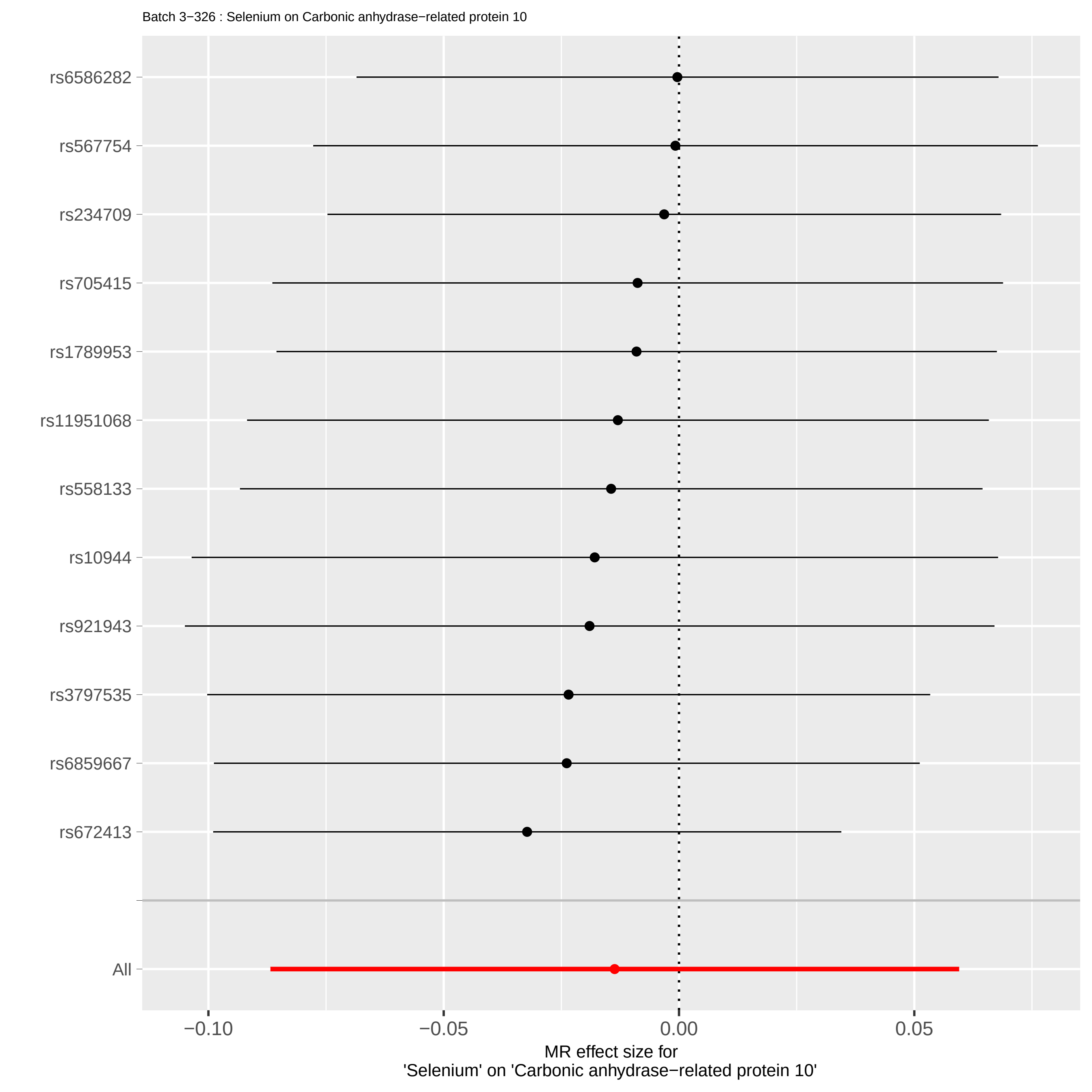

Batch 3−326 : Selenium on Carbonic anhydrase−related protein 10
rs6586282
rs567754
rs234709
rs705415
rs1789953
rs11951068
rs558133
rs10944
rs921943
rs3797535
rs6859667
rs672413
All
−0.10
−0.05
0.00
0.05
MR effect size for
'Selenium' on 'Carbonic anhydrase−related protein 10'

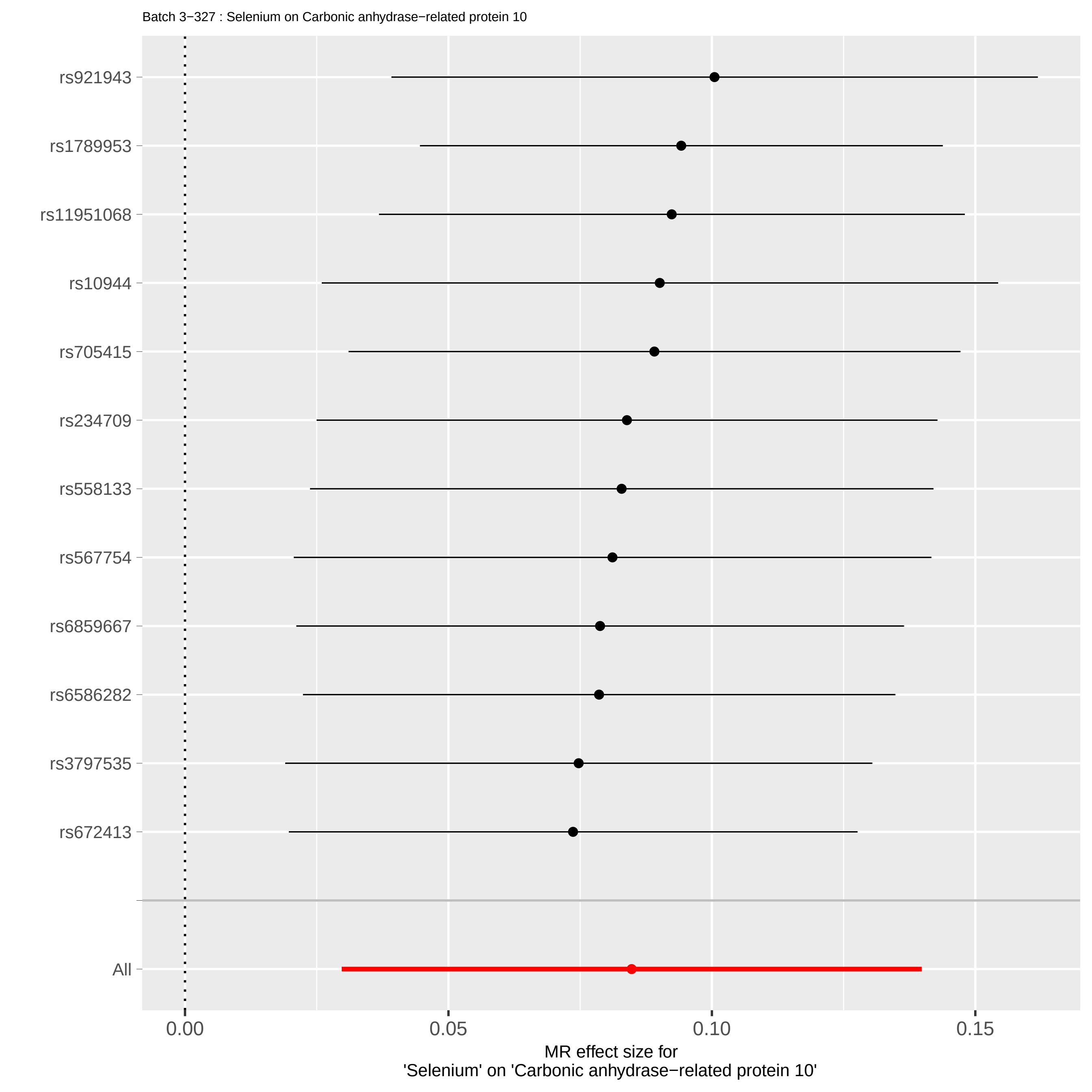
Batch 3−327 : Selenium on Carbonic anhydrase−related protein 10
rs921943
rs1789953
rs11951068
rs10944
rs705415
rs234709
rs558133
rs567754
rs6859667
rs6586282
rs3797535
rs672413
All
0.00
0.05
0.10
0.15
MR effect size for
'Selenium' on 'Carbonic anhydrase−related protein 10'

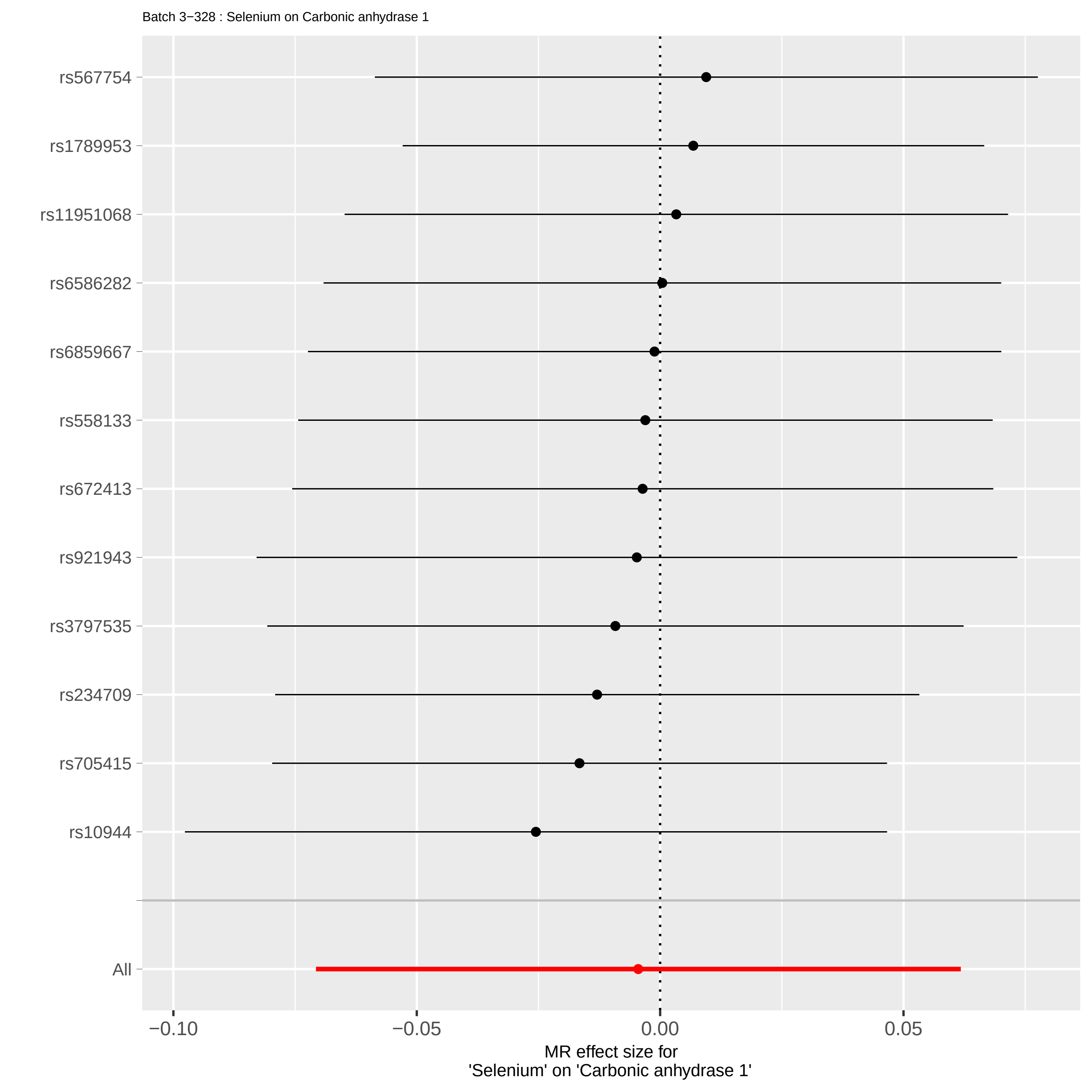
Batch 3−328 : Selenium on Carbonic anhydrase 1
rs567754
rs1789953
rs11951068
rs6586282
rs6859667
rs558133
rs672413
rs921943
rs3797535
rs234709
rs705415
rs10944
All
−0.10
−0.05
0.00
0.05
MR effect size for
'Selenium' on 'Carbonic anhydrase 1'

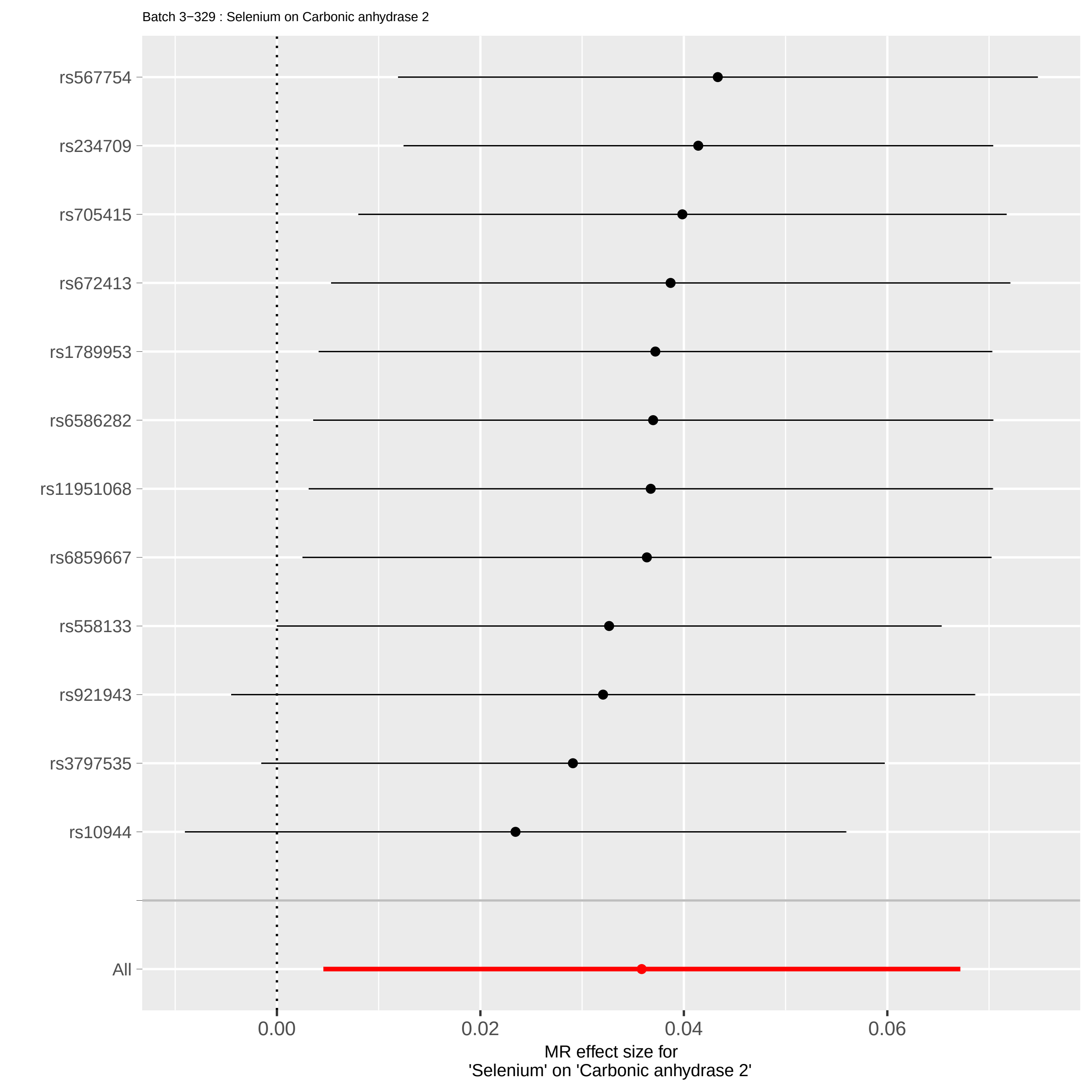
Batch 3−329 : Selenium on Carbonic anhydrase 2
rs567754
rs234709
rs705415
rs672413
rs1789953
rs6586282
rs11951068
rs6859667
rs558133
rs921943
rs3797535
rs10944
All
0.00
0.02
0.04
0.06
MR effect size for
'Selenium' on 'Carbonic anhydrase 2'

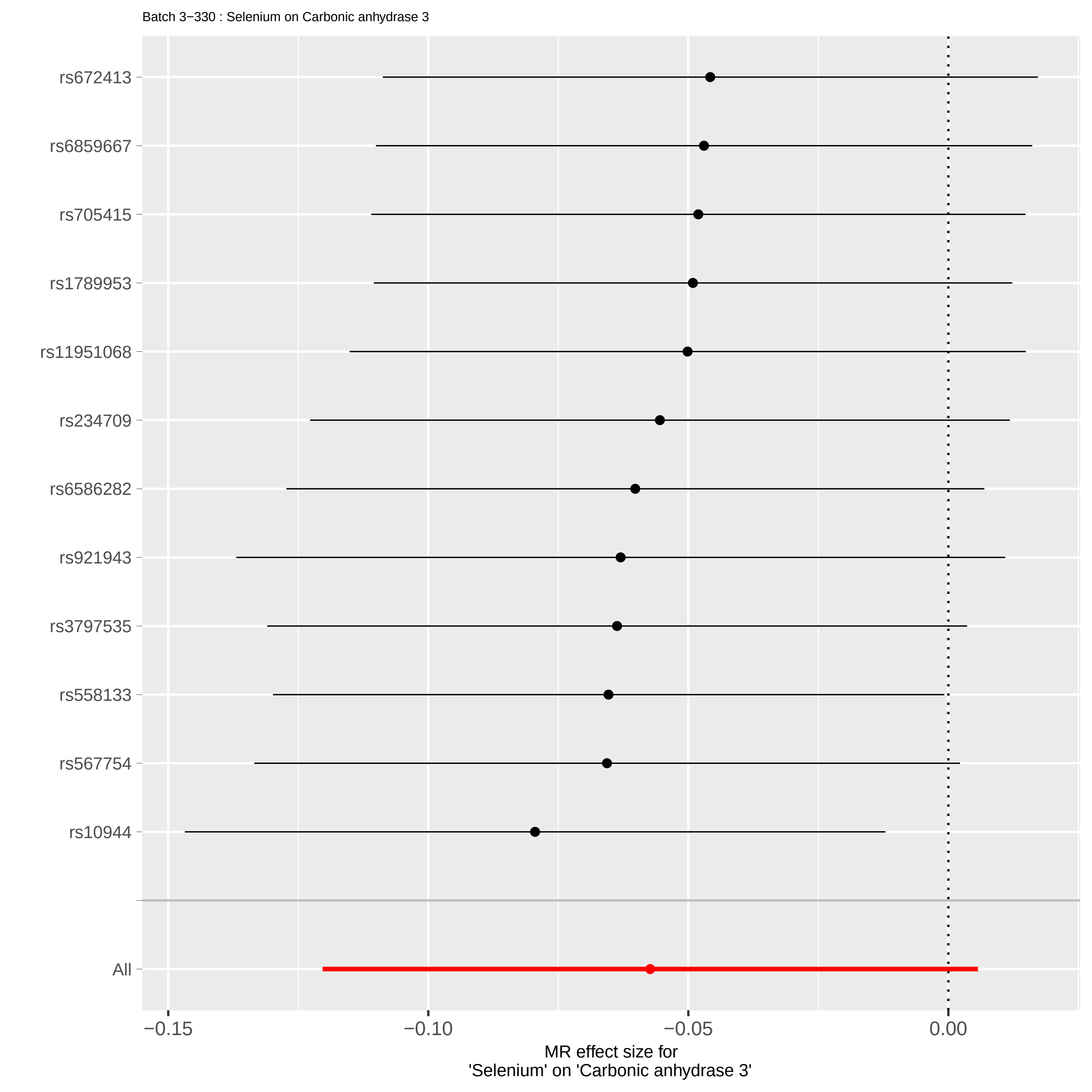
Batch 3−330 : Selenium on Carbonic anhydrase 3
rs672413
rs6859667
rs705415
rs1789953
rs11951068
rs234709
rs6586282
rs921943
rs3797535
rs558133
rs567754
rs10944
All
−0.15
−0.10
−0.05
0.00
MR effect size for
'Selenium' on 'Carbonic anhydrase 3'

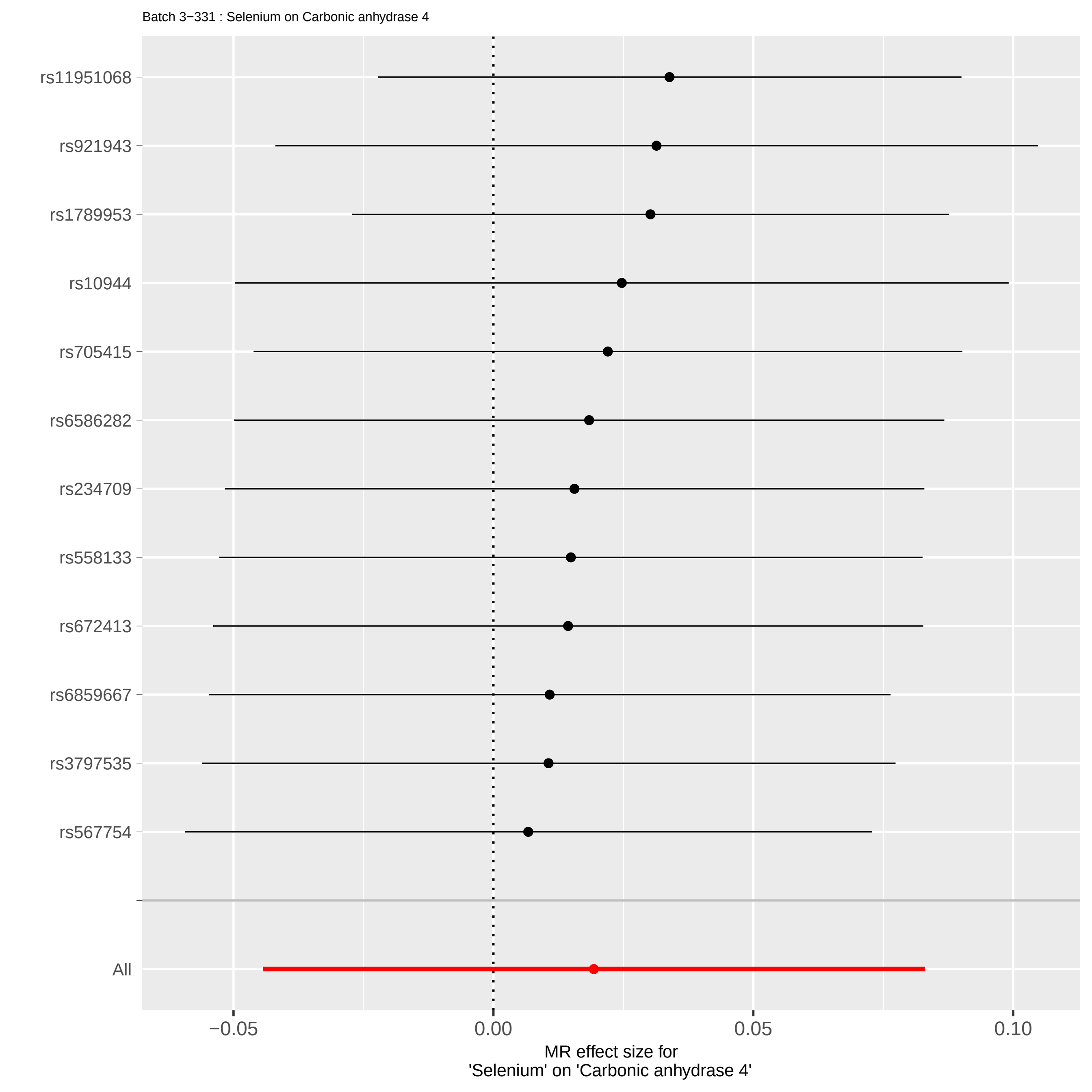

Batch 3−331 : Selenium on Carbonic anhydrase 4
rs11951068
rs921943
rs1789953
rs10944
rs705415
rs6586282
rs234709
rs558133
rs672413
rs6859667
rs3797535
rs567754
All
−0.05
0.00
0.05
0.10
MR effect size for
'Selenium' on 'Carbonic anhydrase 4'

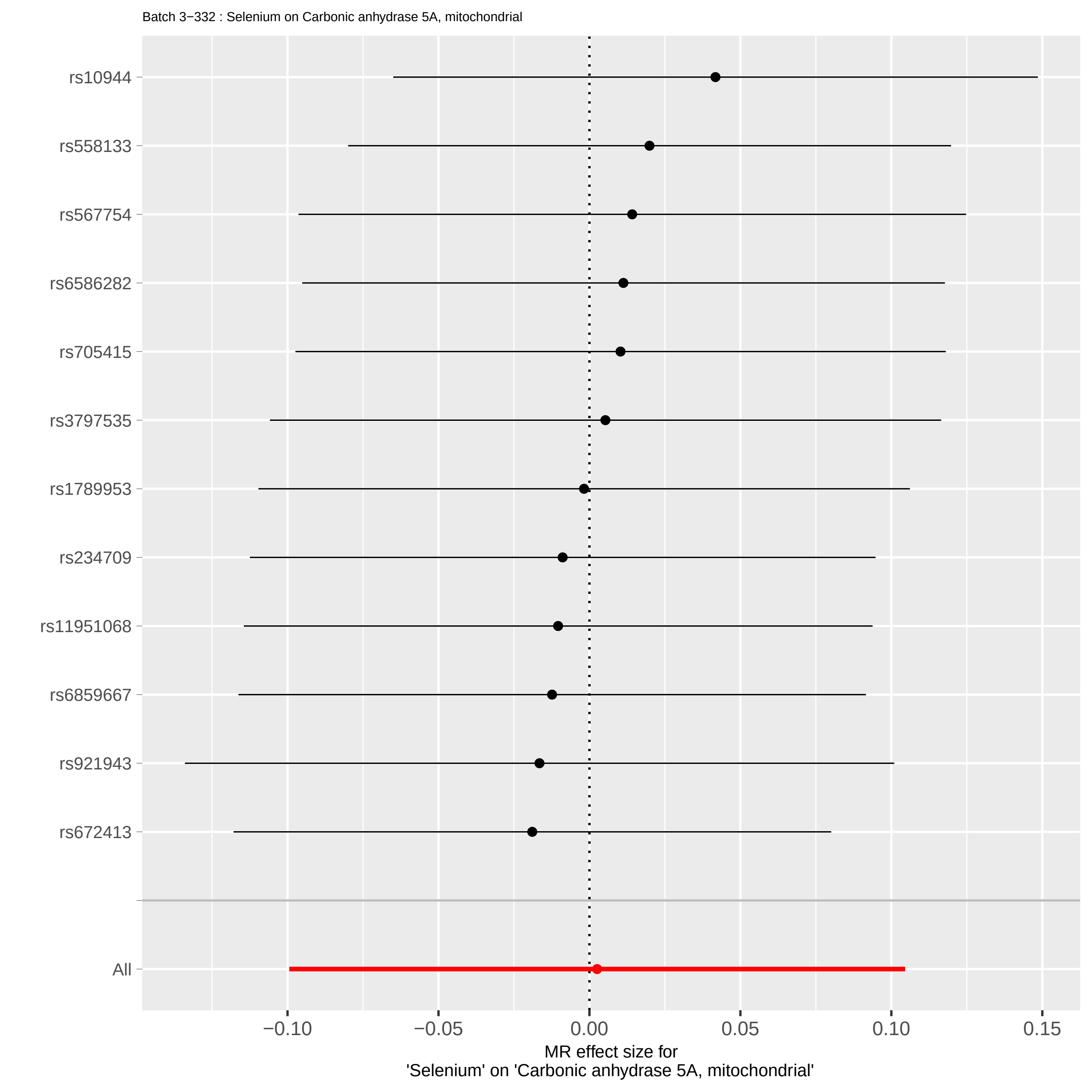
Batch 3−332 : Selenium on Carbonic anhydrase 5A, mitochondrial
rs10944
rs558133
rs567754
rs6586282
rs705415
rs3797535
rs1789953
rs234709
rs11951068
rs6859667
rs921943
rs672413
All
−0.10
−0.05
0.00
0.05
0.10
0.15
MR effect size for
'Selenium' on 'Carbonic anhydrase 5A, mitochondrial'

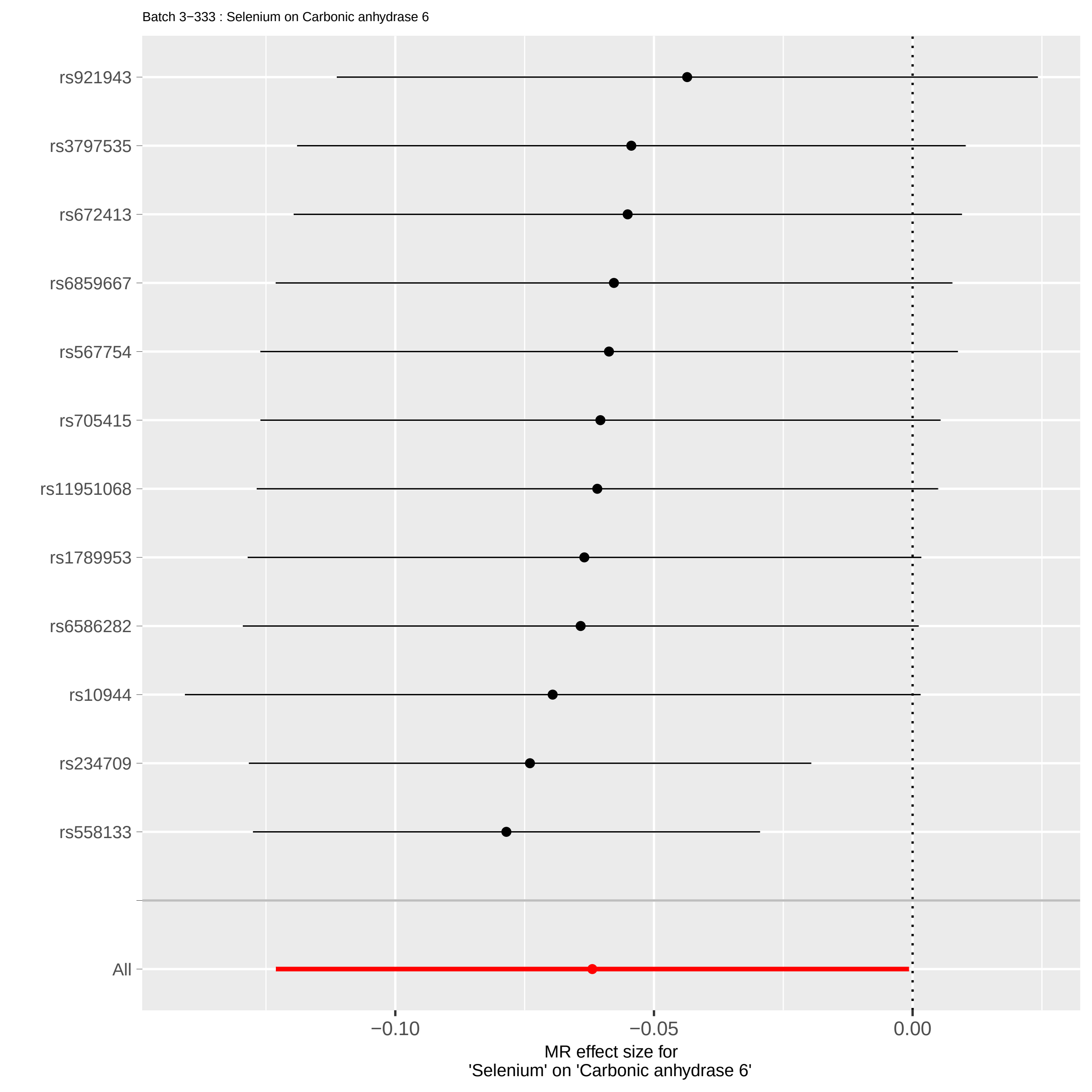
Batch 3−333 : Selenium on Carbonic anhydrase 6
rs921943
rs3797535
rs672413
rs6859667
rs567754
rs705415
rs11951068
rs1789953
rs6586282
rs10944
rs234709
rs558133
All
−0.10
−0.05
0.00
MR effect size for
'Selenium' on 'Carbonic anhydrase 6'

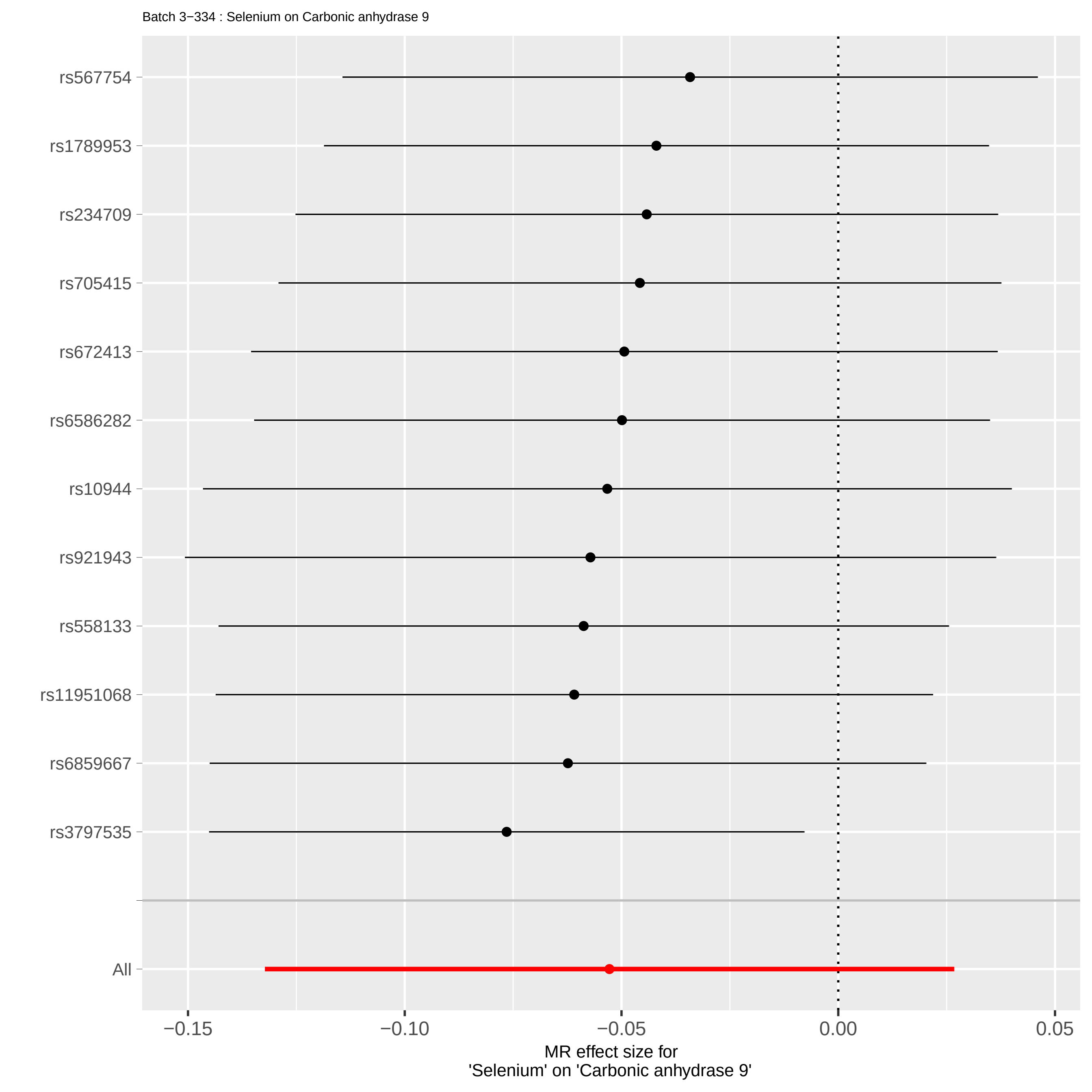

Batch 3−334 : Selenium on Carbonic anhydrase 9
rs567754
rs1789953
rs234709
rs705415
rs672413
rs6586282
rs10944
rs921943
rs558133
rs11951068
rs6859667
rs3797535
All
−0.15
−0.10
−0.05
0.00
0.05
MR effect size for
'Selenium' on 'Carbonic anhydrase 9'

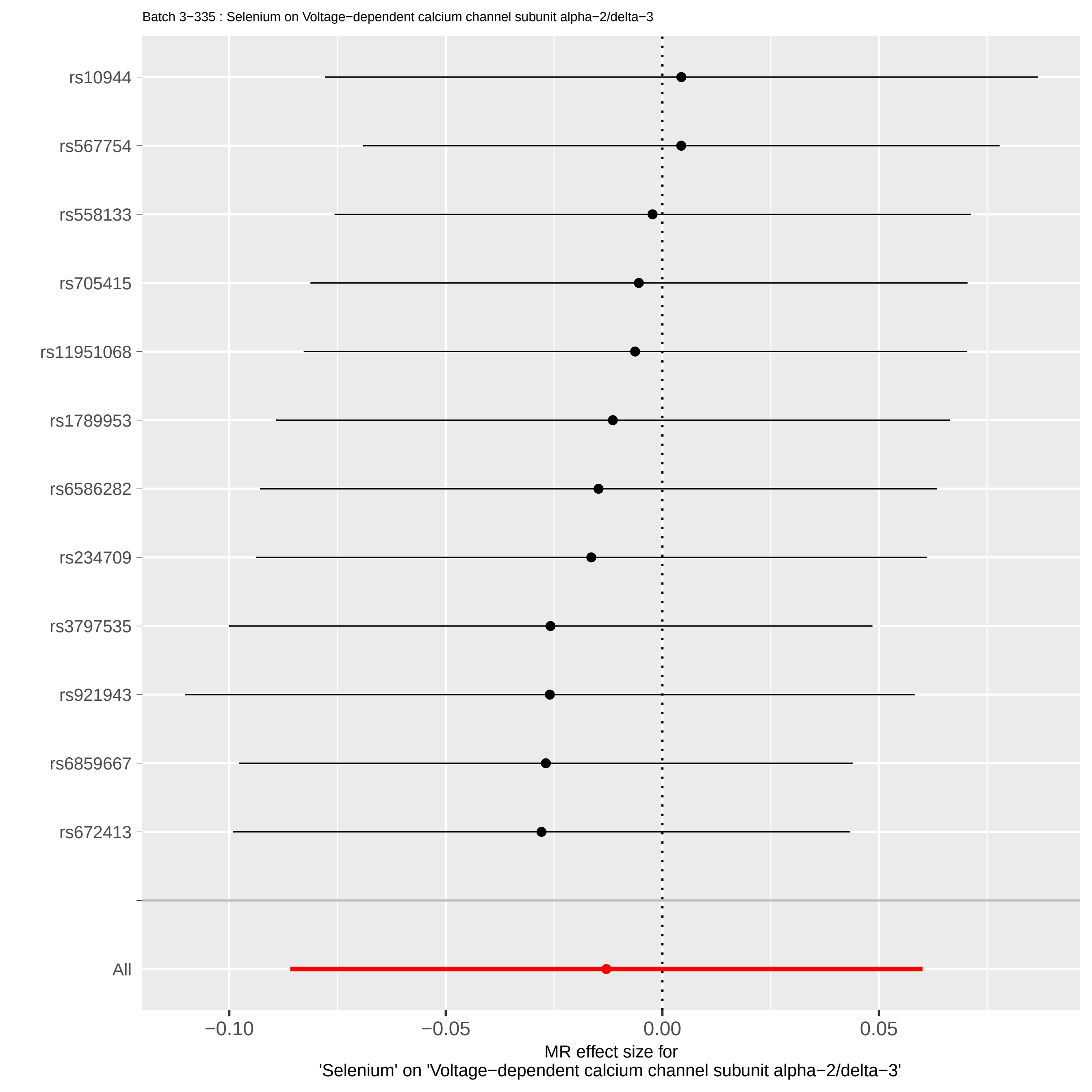

Batch 3−335 : Selenium on Voltage−dependent calcium channel subunit alpha−2/delta−3
rs10944
rs567754
rs558133
rs705415
rs11951068
rs1789953
rs6586282
rs234709
rs3797535
rs921943
rs6859667
rs672413
All
−0.10
−0.05
0.00
0.05
MR effect size for
'Selenium' on 'Voltage−dependent calcium channel subunit alpha−2/delta−3'

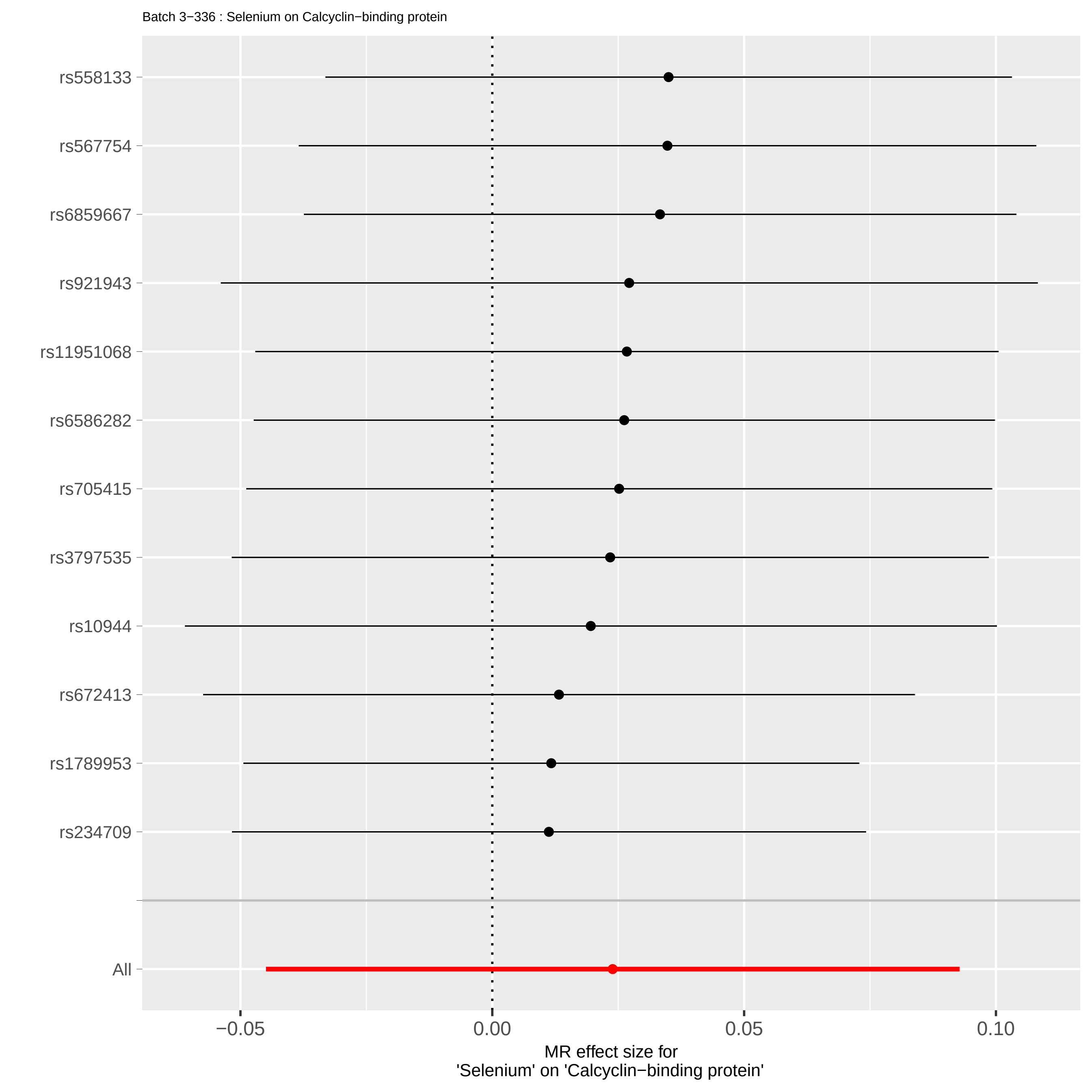

Batch 3−336 : Selenium on Calcyclin−binding protein
rs558133
rs567754
rs6859667
rs921943
rs11951068
rs6586282
rs705415
rs3797535
rs10944
rs672413
rs1789953
rs234709
All
−0.05
0.00
0.05
0.10
MR effect size for
'Selenium' on 'Calcyclin−binding protein'

Batch 3−337 : Selenium on Cell adhesion molecule 1

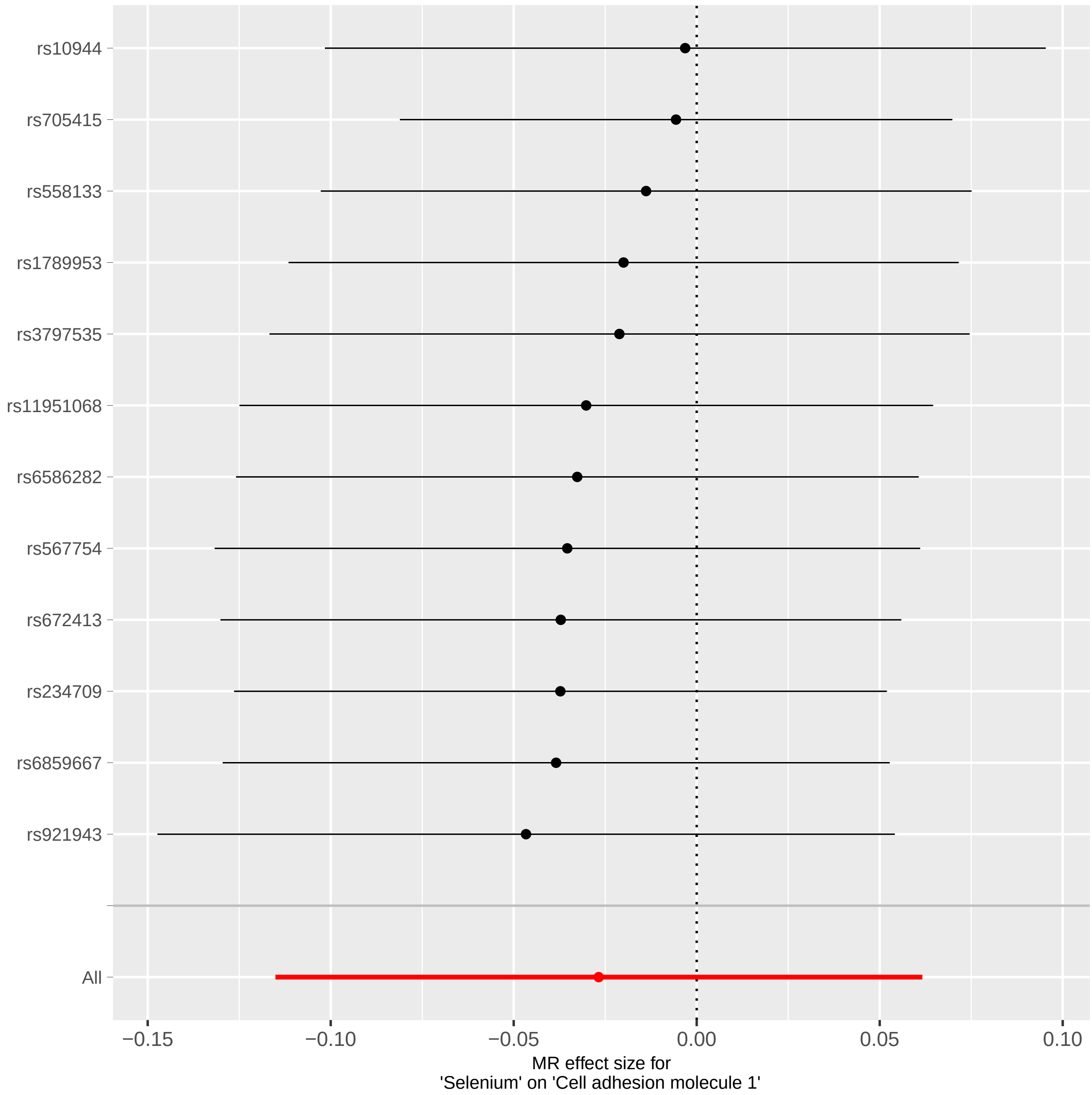

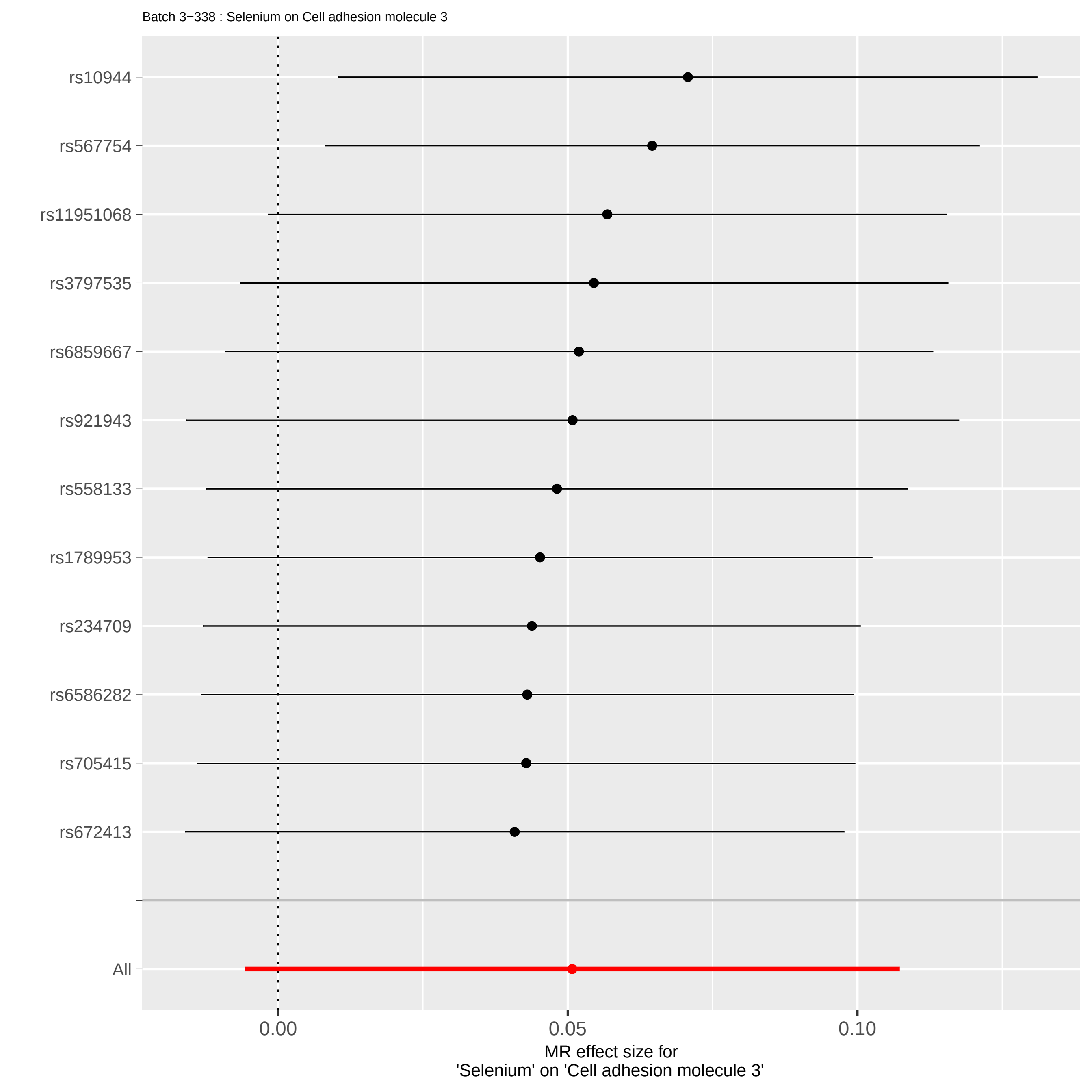
Batch 3−338 : Selenium on Cell adhesion molecule 3
rs10944
rs567754
rs11951068
rs3797535
rs6859667
rs921943
rs558133
rs1789953
rs234709
rs6586282
rs705415
rs672413
All
0.00
0.05
0.10
MR effect size for
'Selenium' on 'Cell adhesion molecule 3'

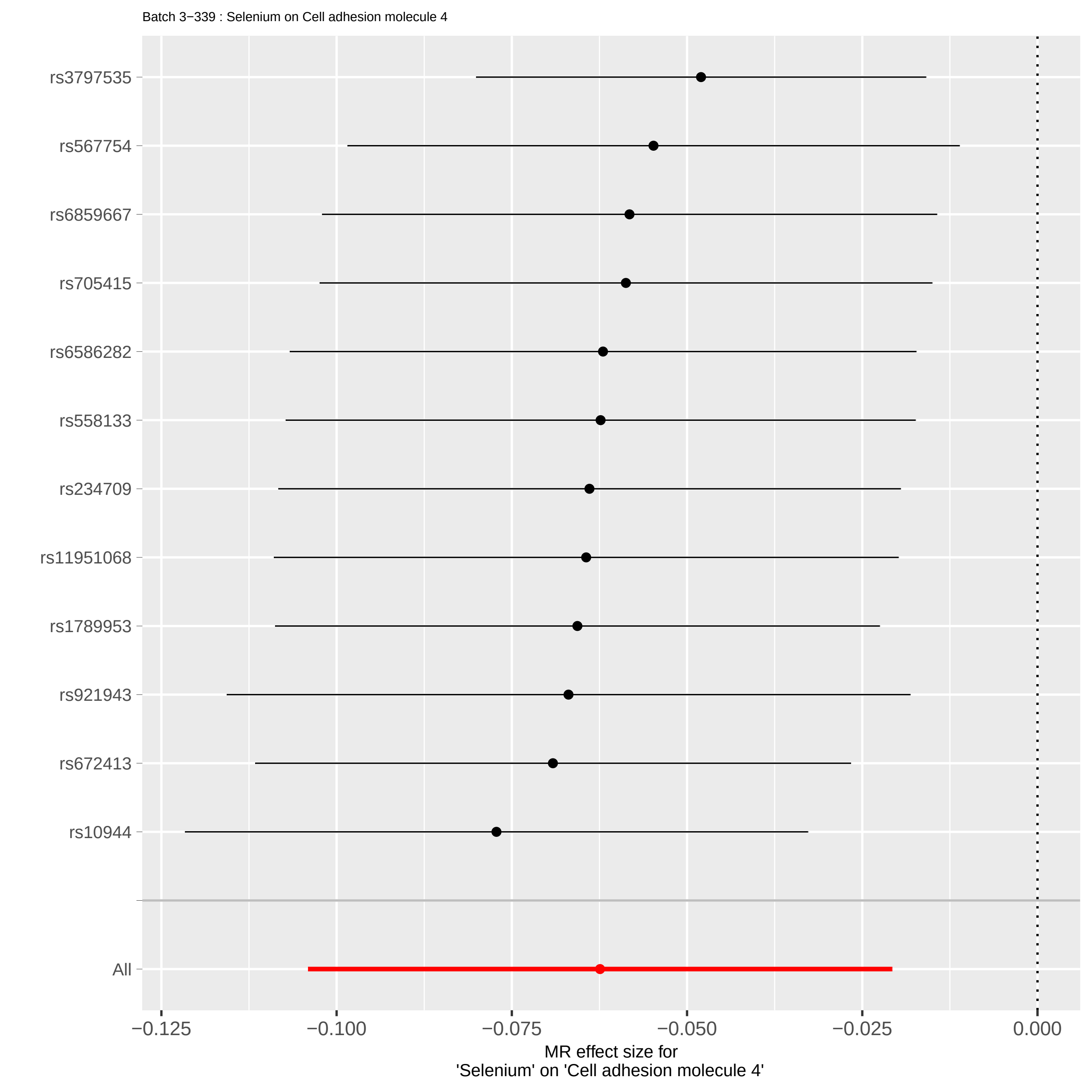
Batch 3−339 : Selenium on Cell adhesion molecule 4
rs3797535
rs567754
rs6859667
rs705415
rs6586282
rs558133
rs234709
rs11951068
rs1789953
rs921943
rs672413
rs10944
All
−0.125
−0.100
−0.075
−0.050
−0.025
0.000
MR effect size for
'Selenium' on 'Cell adhesion molecule 4'

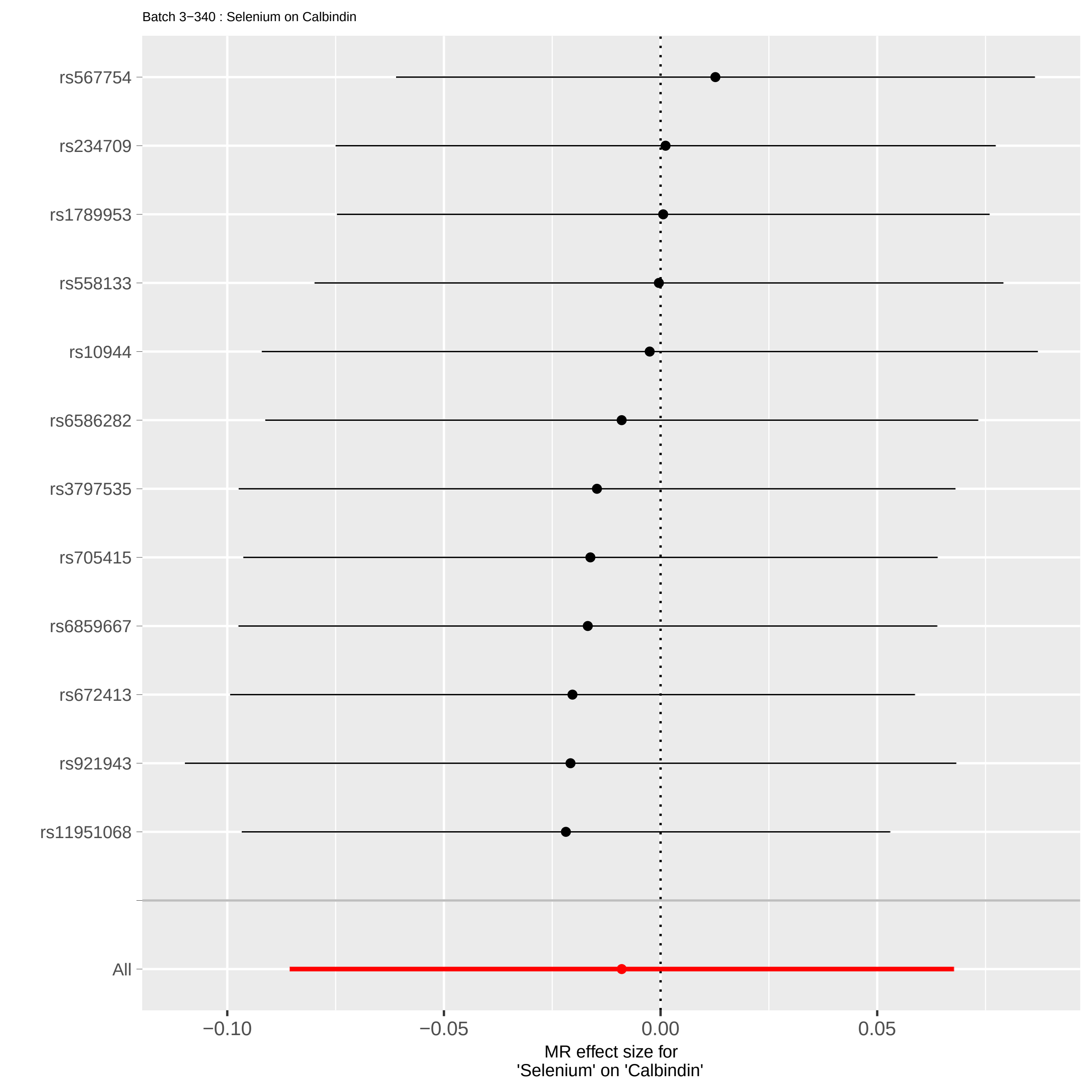

Batch 3−340 : Selenium on Calbindin
rs567754
rs234709
rs1789953
rs558133
rs10944
rs6586282
rs3797535
rs705415
rs6859667
rs672413
rs921943
rs11951068
All
−0.10
−0.05
0.00
0.05
MR effect size for
'Selenium' on 'Calbindin'

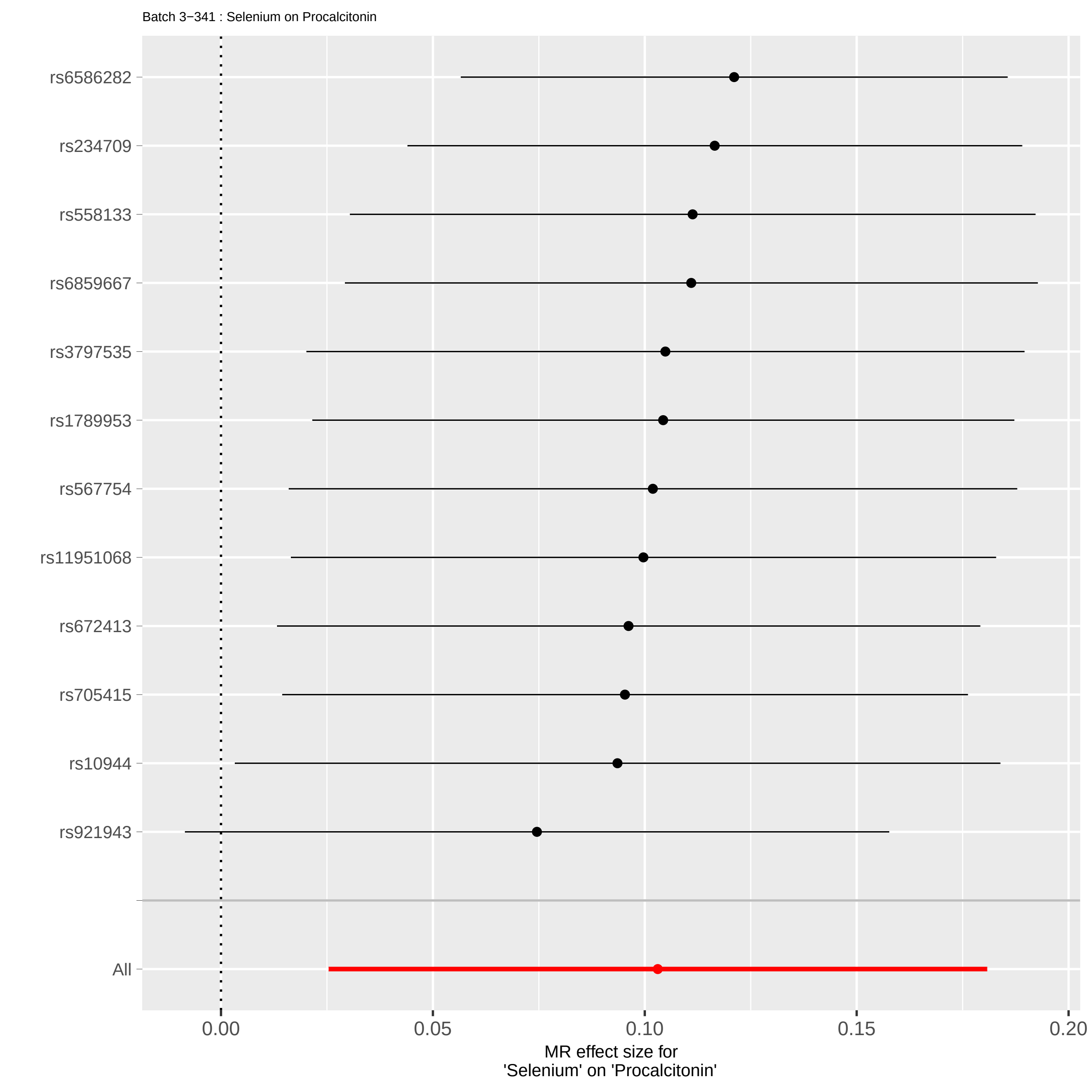

Batch 3−341 : Selenium on Procalcitonin
rs6586282
rs234709
rs558133
rs6859667
rs3797535
rs1789953
rs567754
rs11951068
rs672413
rs705415
rs10944
rs921943
All
0.00
0.05
0.10
0.15
0.20
MR effect size for
'Selenium' on 'Procalcitonin'

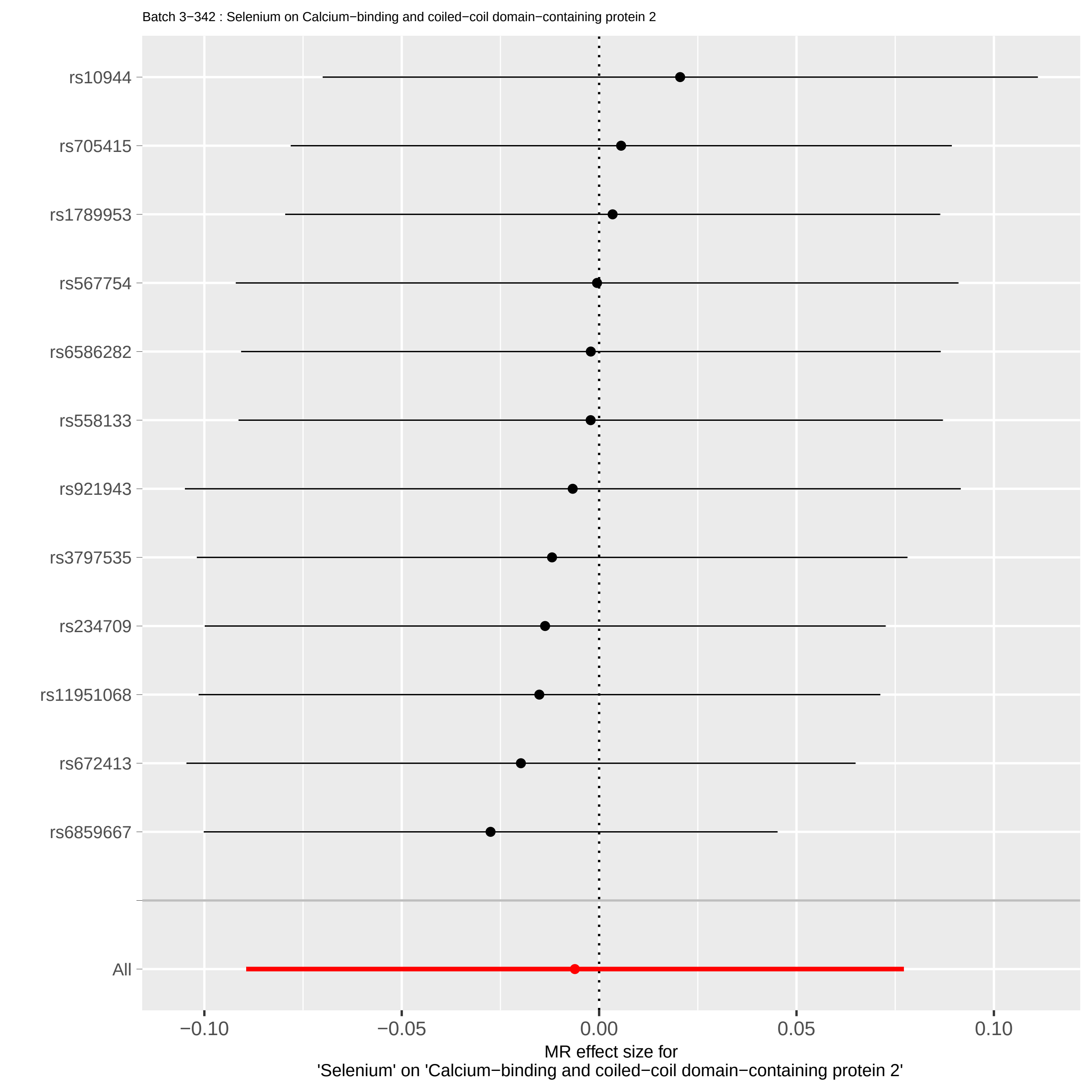

Batch 3−342 : Selenium on Calcium−binding and coiled−coil domain−containing protein 2
rs10944
rs705415
rs1789953
rs567754
rs6586282
rs558133
rs921943
rs3797535
rs234709
rs11951068
rs672413
rs6859667
All
−0.10
−0.05
0.00
0.05
0.10
MR effect size for
'Selenium' on 'Calcium−binding and coiled−coil domain−containing protein 2'

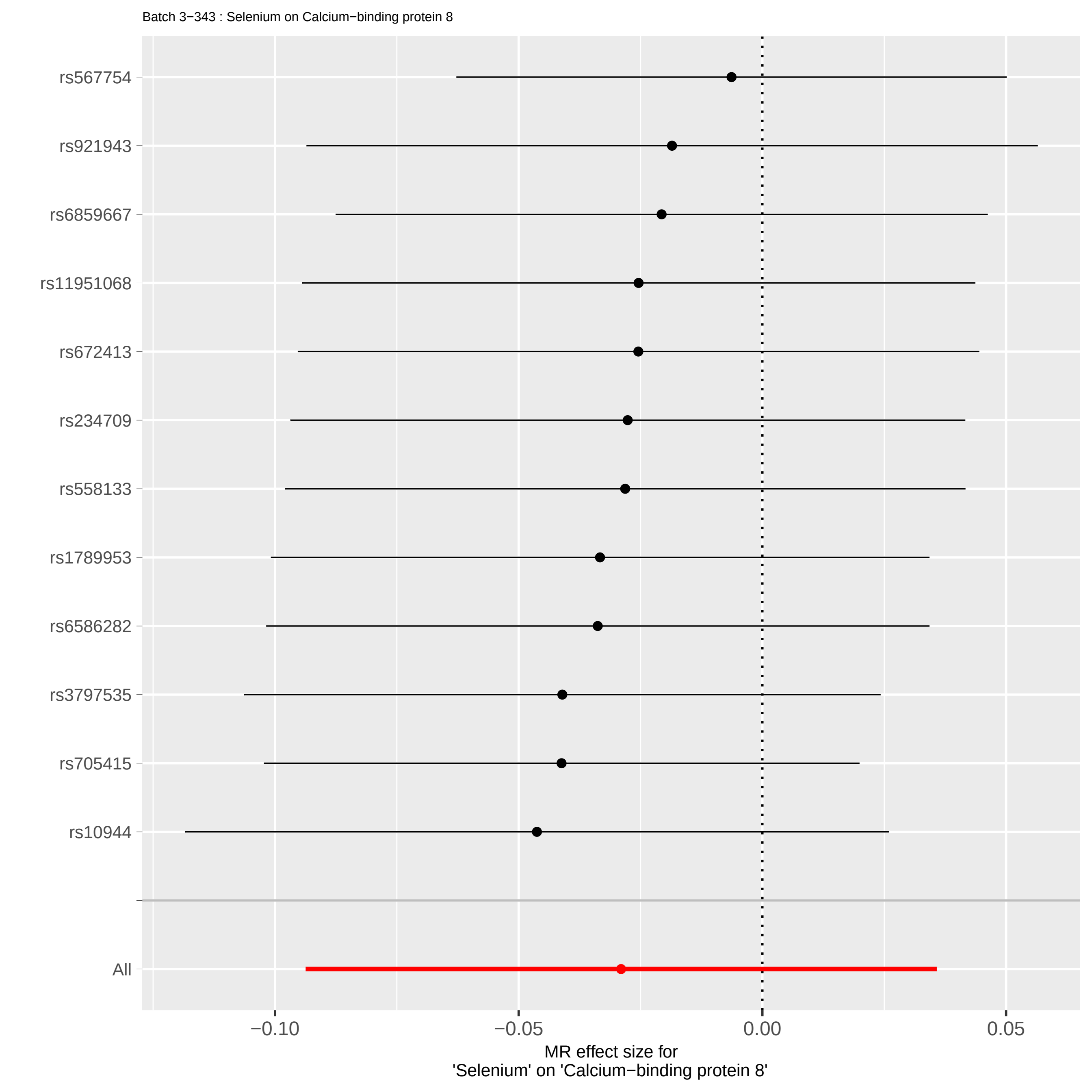

Batch 3−343 : Selenium on Calcium−binding protein 8
rs567754
rs921943
rs6859667
rs11951068
rs672413
rs234709
rs558133
rs1789953
rs6586282
rs3797535
rs705415
rs10944
All
−0.10
−0.05
0.00
0.05
MR effect size for
'Selenium' on 'Calcium−binding protein 8'

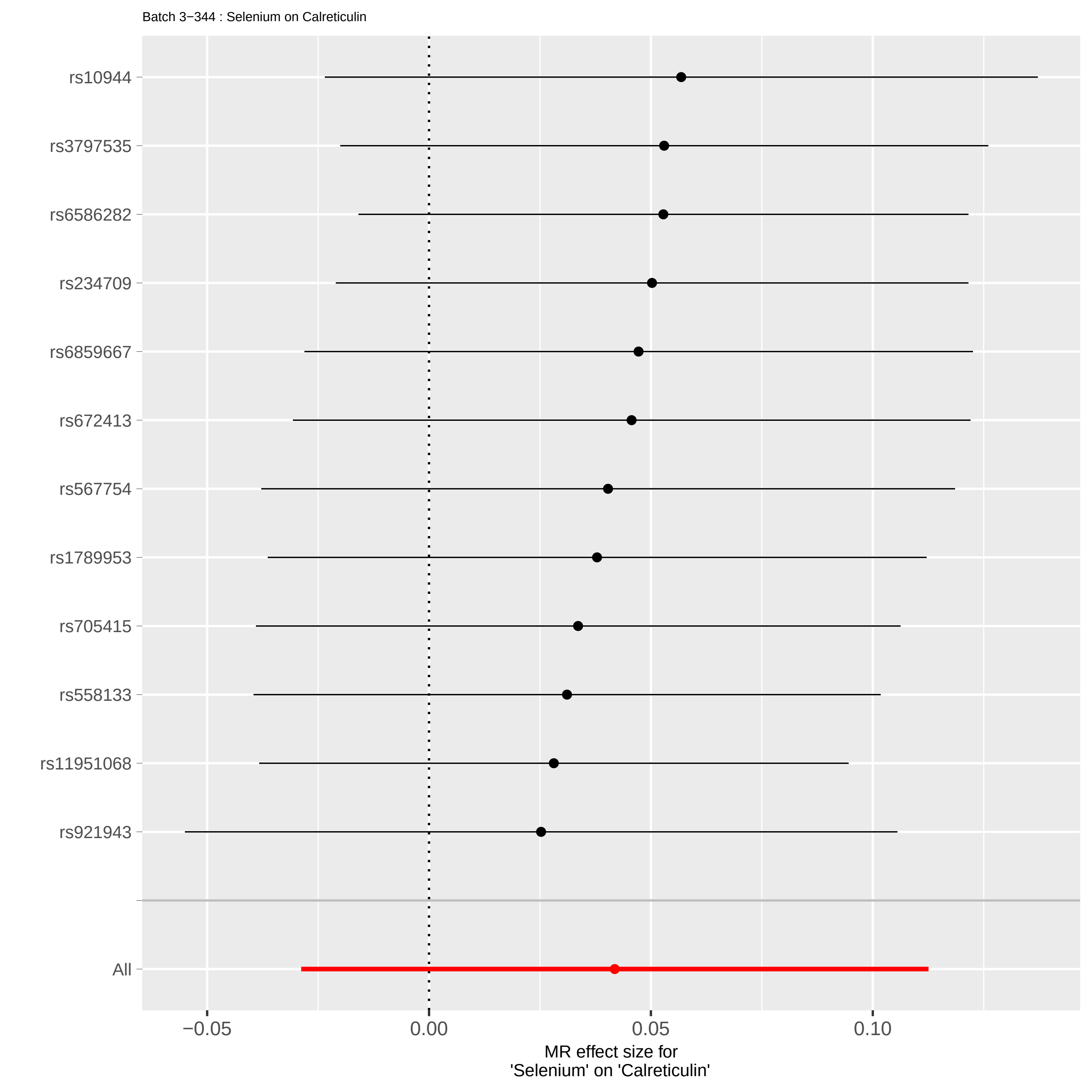

Batch 3−344 : Selenium on Calreticulin
rs10944
rs3797535
rs6586282
rs234709
rs6859667
rs672413
rs567754
rs1789953
rs705415
rs558133
rs11951068
rs921943
All
−0.05
0.00
0.05
0.10
MR effect size for
'Selenium' on 'Calreticulin'

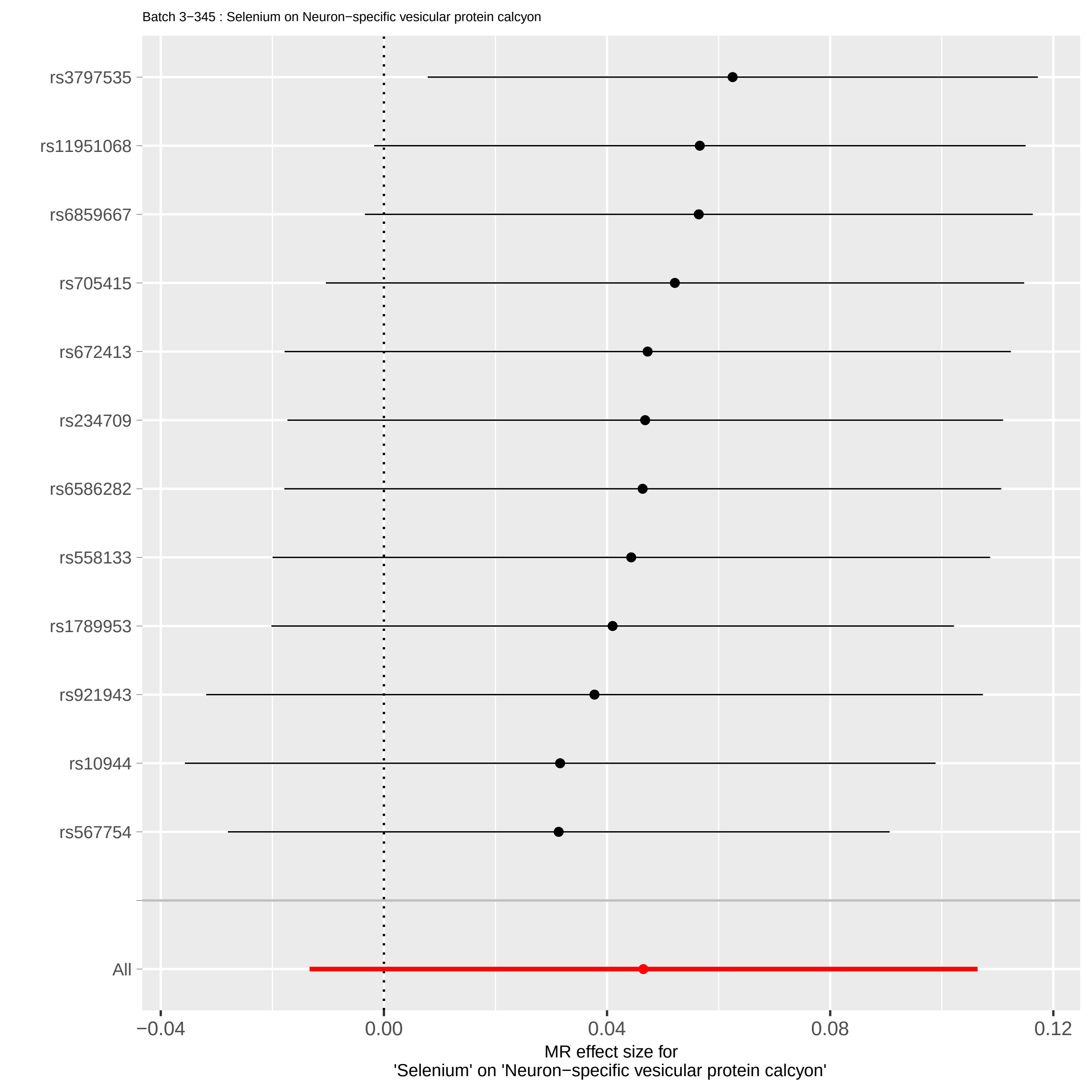

Batch 3−345 : Selenium on Neuron−specific vesicular protein calcyon
rs3797535
rs11951068
rs6859667
rs705415
rs672413
rs234709
rs6586282
rs558133
rs1789953
rs921943
rs10944
rs567754
All
−0.04
0.00
0.04
0.08
0.12
MR effect size for
'Selenium' on 'Neuron−specific vesicular protein calcyon'

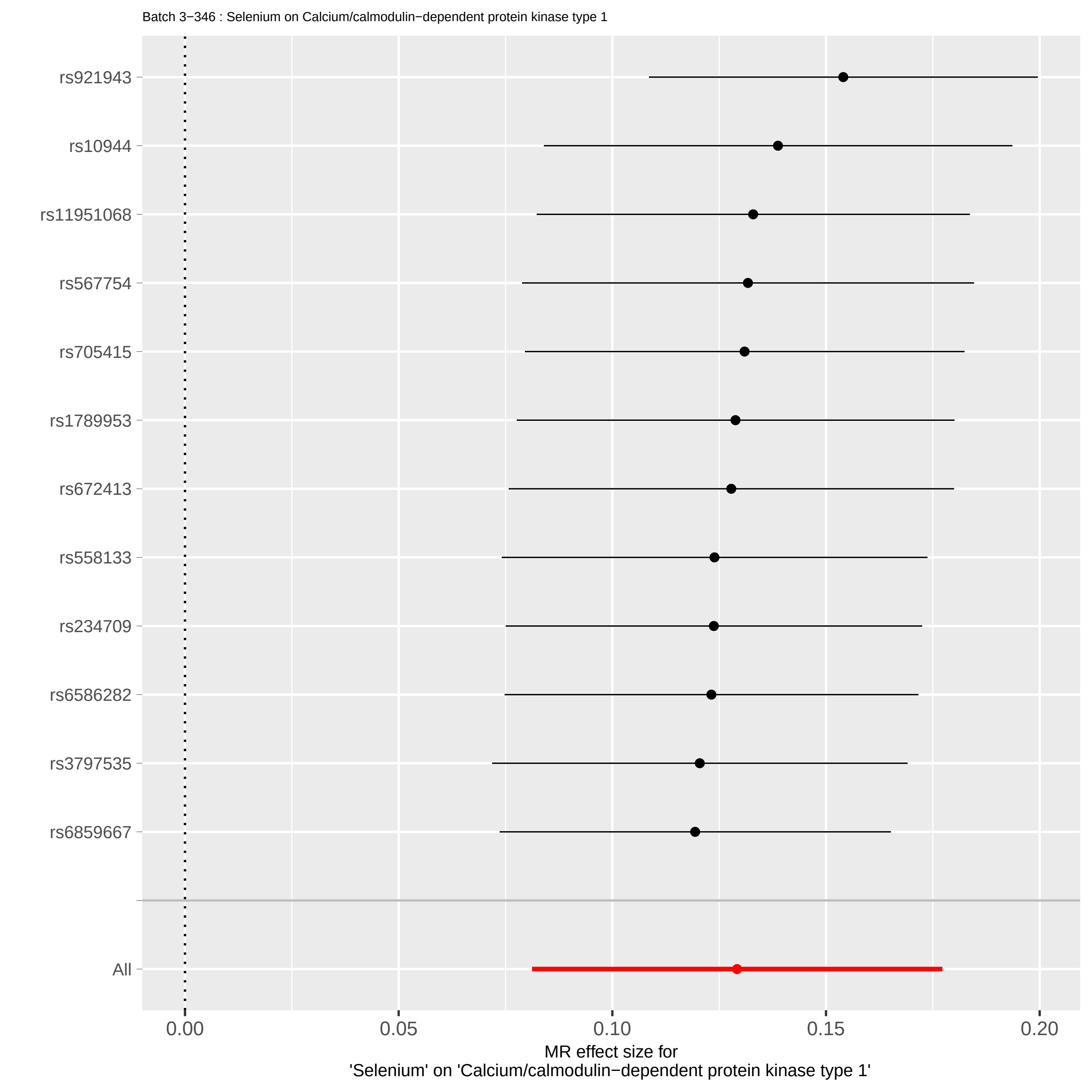

Batch 3−346 : Selenium on Calcium/calmodulin−dependent protein kinase type 1
rs921943
rs10944
rs11951068
rs567754
rs705415
rs1789953
rs672413
rs558133
rs234709
rs6586282
rs3797535
rs6859667
All
0.00
0.05
0.10
0.15
0.20
MR effect size for
'Selenium' on 'Calcium/calmodulin−dependent protein kinase type 1'

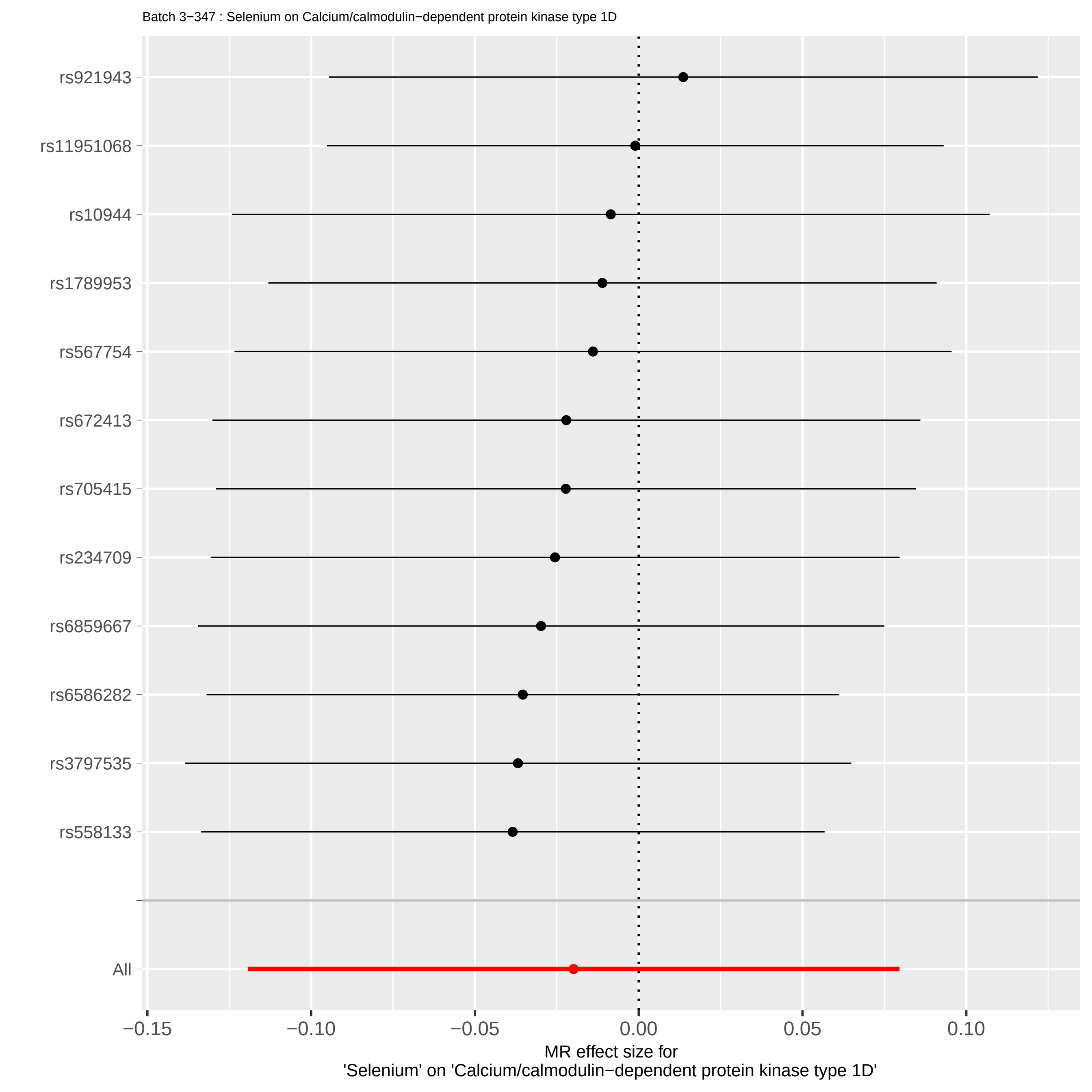

Batch 3−347 : Selenium on Calcium/calmodulin−dependent protein kinase type 1D
rs921943
rs11951068
rs10944
rs1789953
rs567754
rs672413
rs705415
rs234709
rs6859667
rs6586282
rs3797535
rs558133
All
−0.15
−0.10
−0.05
0.00
0.05
0.10
MR effect size for
'Selenium' on 'Calcium/calmodulin−dependent protein kinase type 1D'

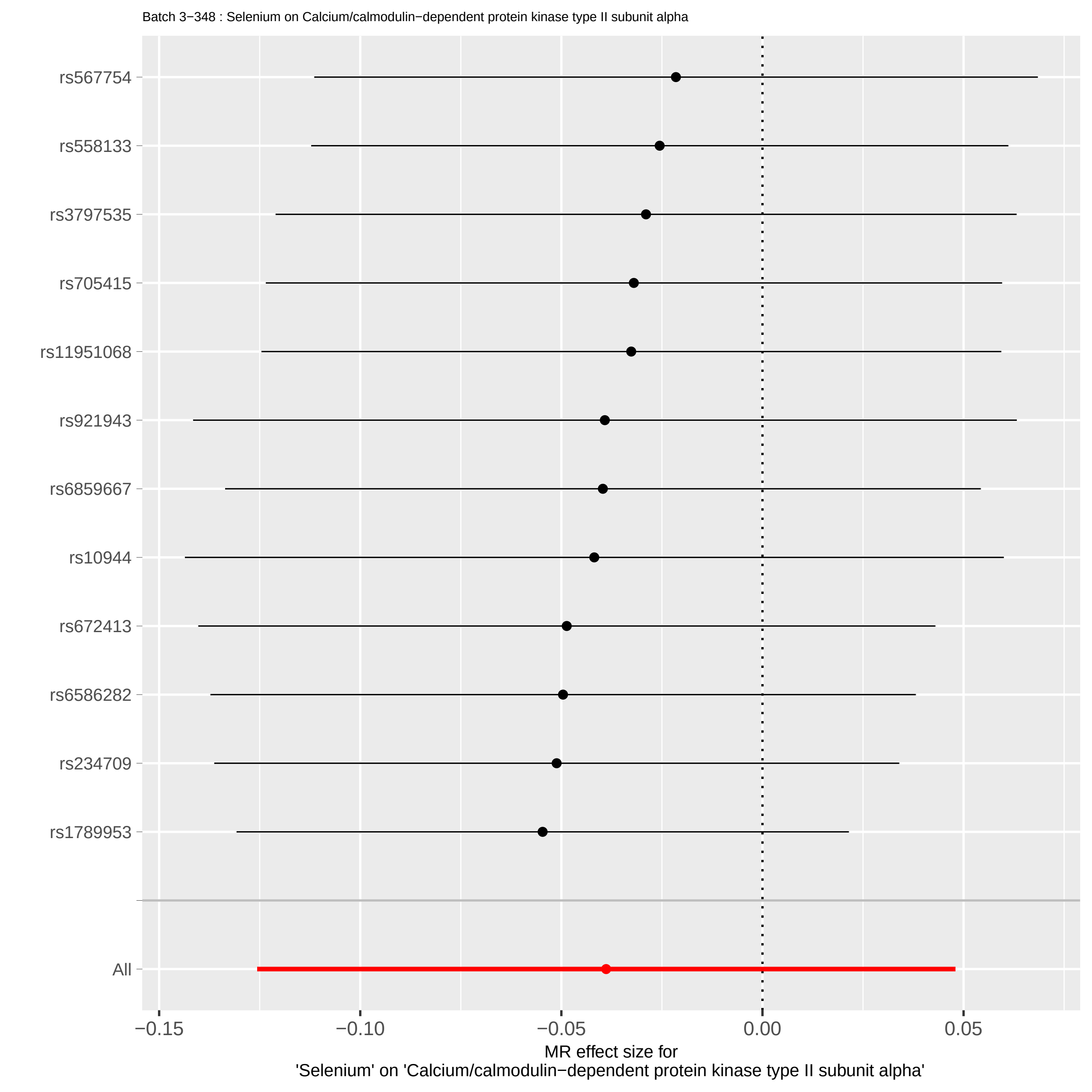

Batch 3−348 : Selenium on Calcium/calmodulin−dependent protein kinase type II subunit alpha
rs567754
rs558133
rs3797535
rs705415
rs11951068
rs921943
rs6859667
rs10944
rs672413
rs6586282
rs234709
rs1789953
All
−0.15
−0.10
−0.05
0.00
0.05
MR effect size for
'Selenium' on 'Calcium/calmodulin−dependent protein kinase type II subunit alpha'

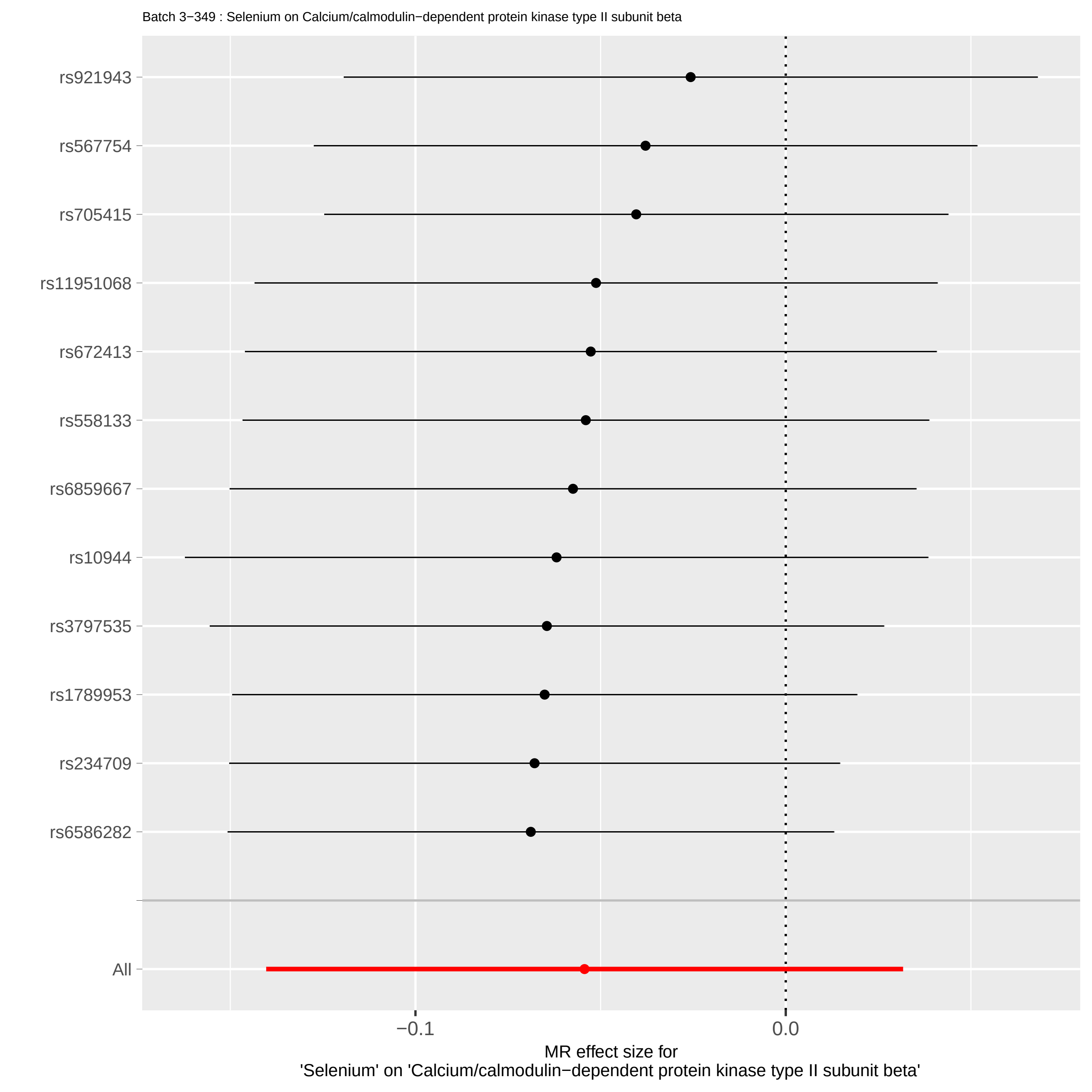

Batch 3−349 : Selenium on Calcium/calmodulin−dependent protein kinase type II subunit beta
rs921943
rs567754
rs705415
rs11951068
rs672413
rs558133
rs6859667
rs10944
rs3797535
rs1789953
rs234709
rs6586282
All
−0.1
0.0
MR effect size for
'Selenium' on 'Calcium/calmodulin−dependent protein kinase type II subunit beta'

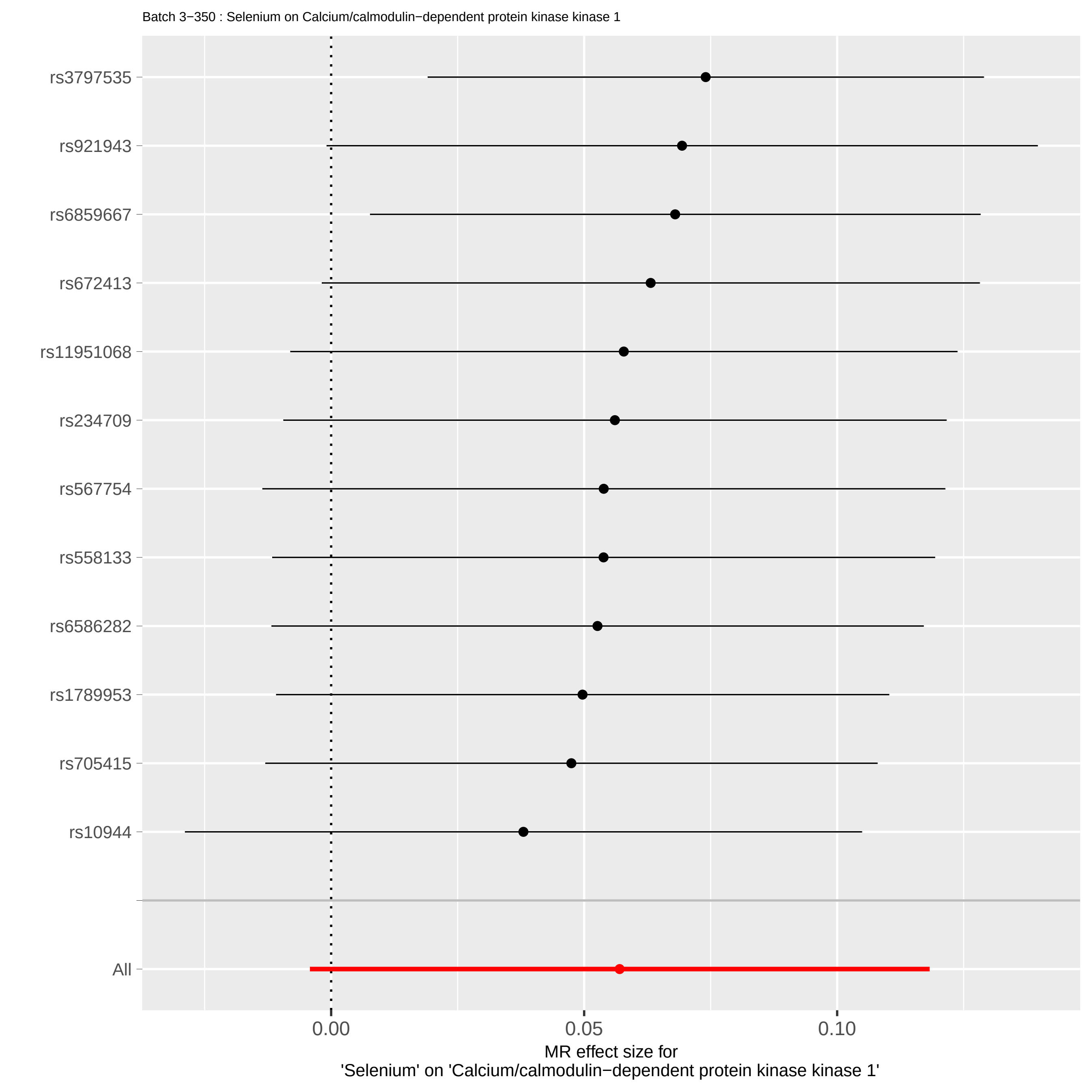
Batch 3−350 : Selenium on Calcium/calmodulin−dependent protein kinase kinase 1
rs3797535
rs921943
rs6859667
rs672413
rs11951068
rs234709
rs567754
rs558133
rs6586282
rs1789953
rs705415
rs10944
All
0.00
0.05
0.10
MR effect size for
'Selenium' on 'Calcium/calmodulin−dependent protein kinase kinase 1'

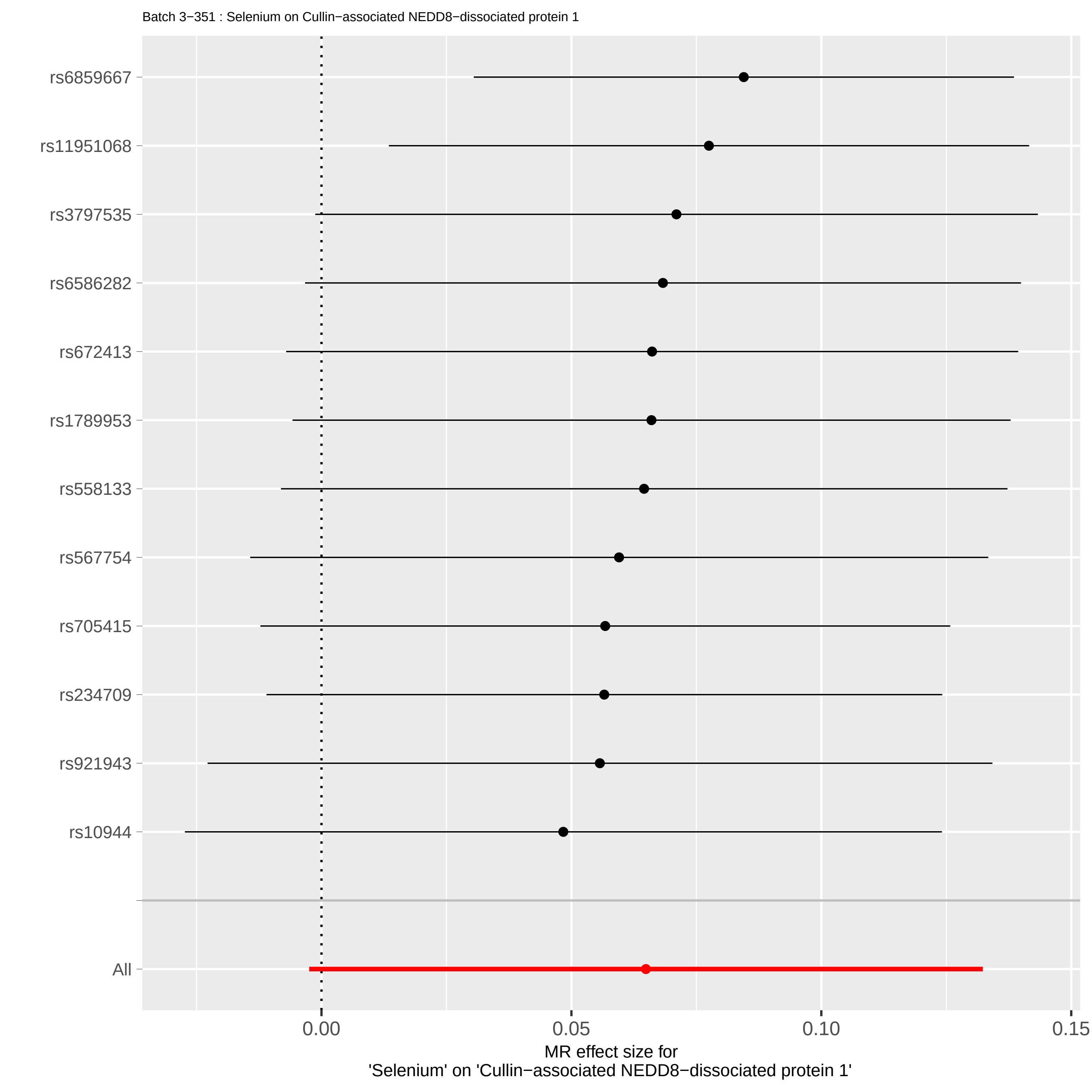

Batch 3−351 : Selenium on Cullin−associated NEDD8−dissociated protein 1
rs6859667
rs11951068
rs3797535
rs6586282
rs672413
rs1789953
rs558133
rs567754
rs705415
rs234709
rs921943
rs10944
All
0.00
0.05
0.10
0.15
MR effect size for
'Selenium' on 'Cullin−associated NEDD8−dissociated protein 1'

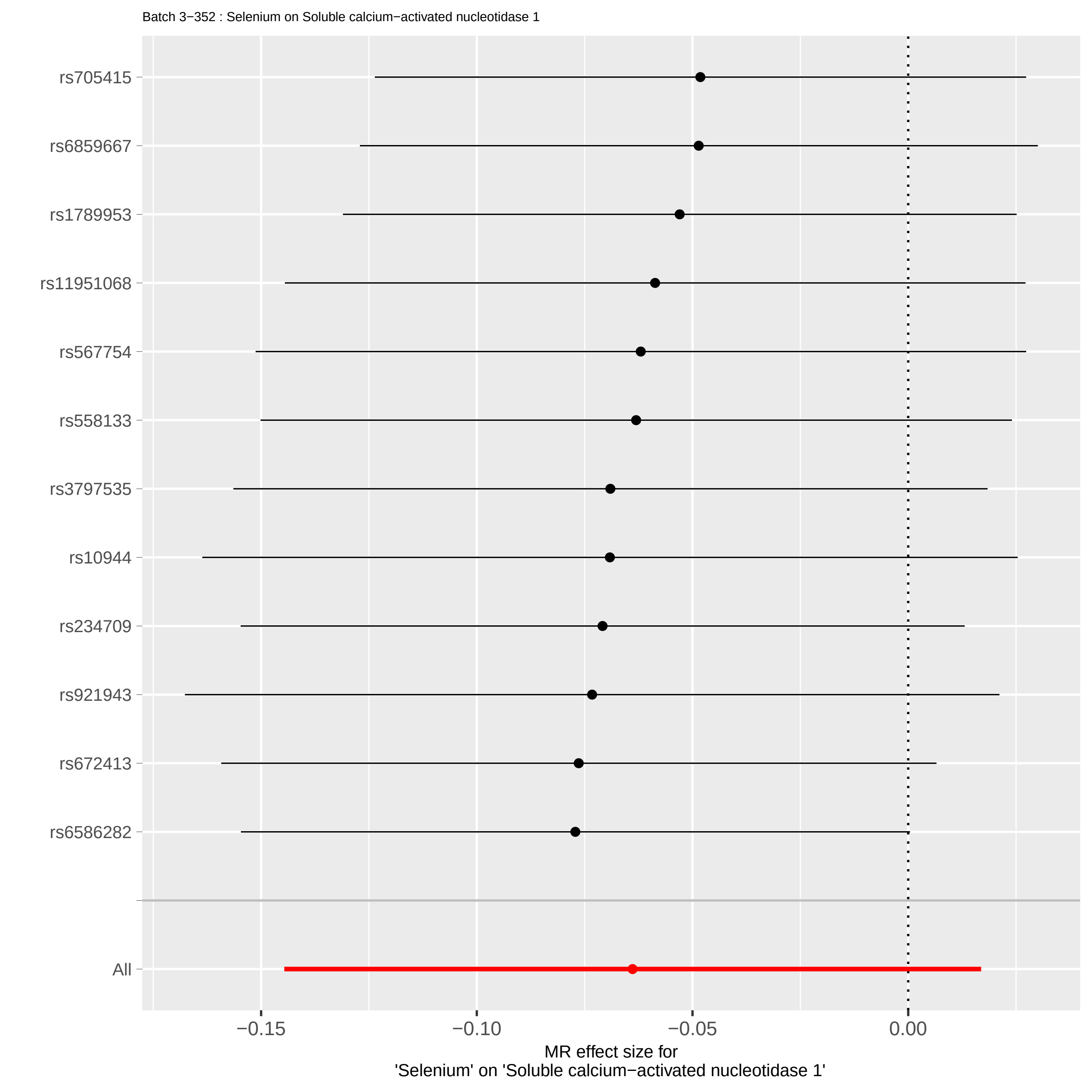

Batch 3−352 : Selenium on Soluble calcium−activated nucleotidase 1
rs705415
rs6859667
rs1789953
rs11951068
rs567754
rs558133
rs3797535
rs10944
rs234709
rs921943
rs672413
rs6586282
All
−0.15
−0.10
−0.05
0.00
MR effect size for
'Selenium' on 'Soluble calcium−activated nucleotidase 1'

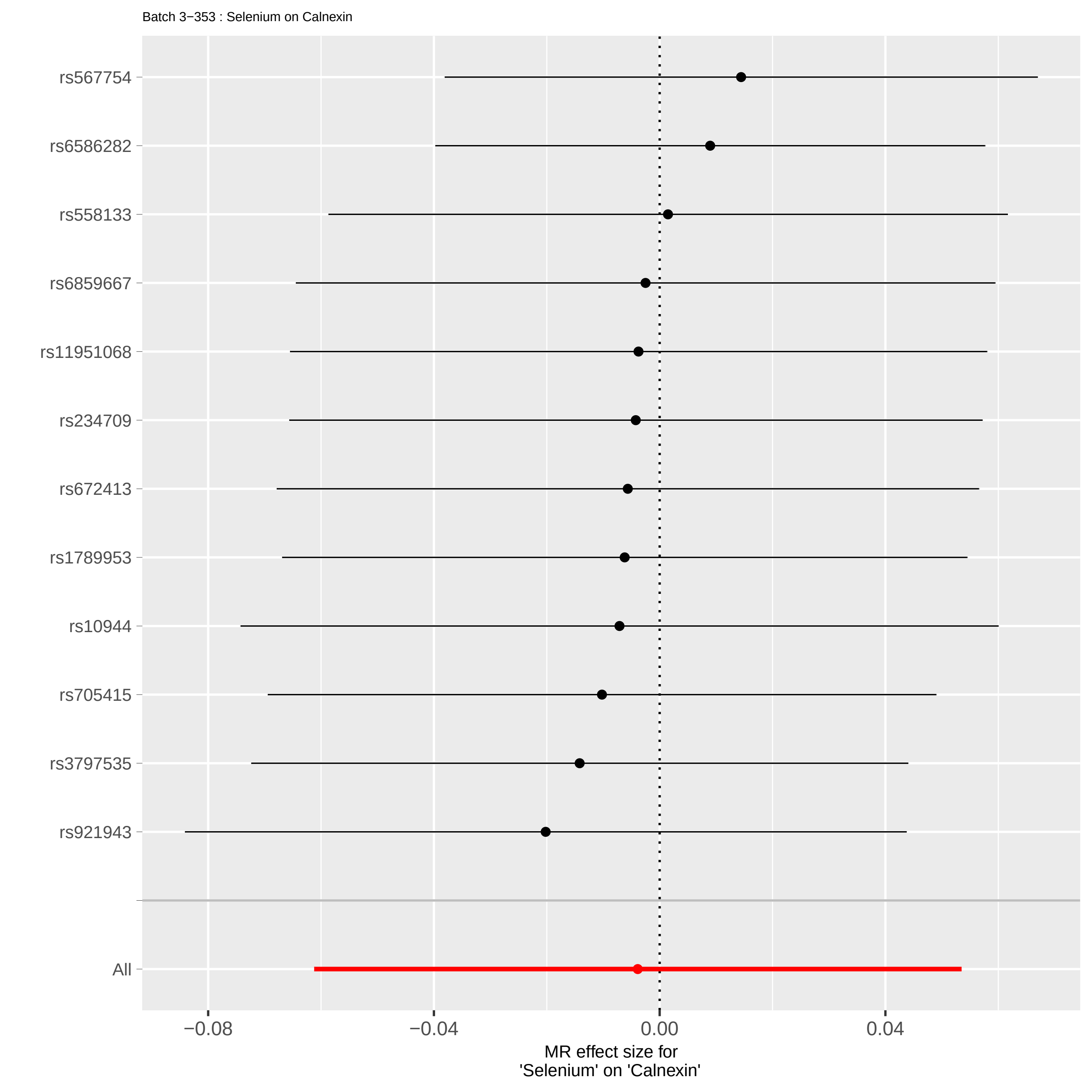

Batch 3−353 : Selenium on Calnexin
rs567754
rs6586282
rs558133
rs6859667
rs11951068
rs234709
rs672413
rs1789953
rs10944
rs705415
rs3797535
rs921943
All
−0.08
−0.04
0.00
0.04
MR effect size for
'Selenium' on 'Calnexin'

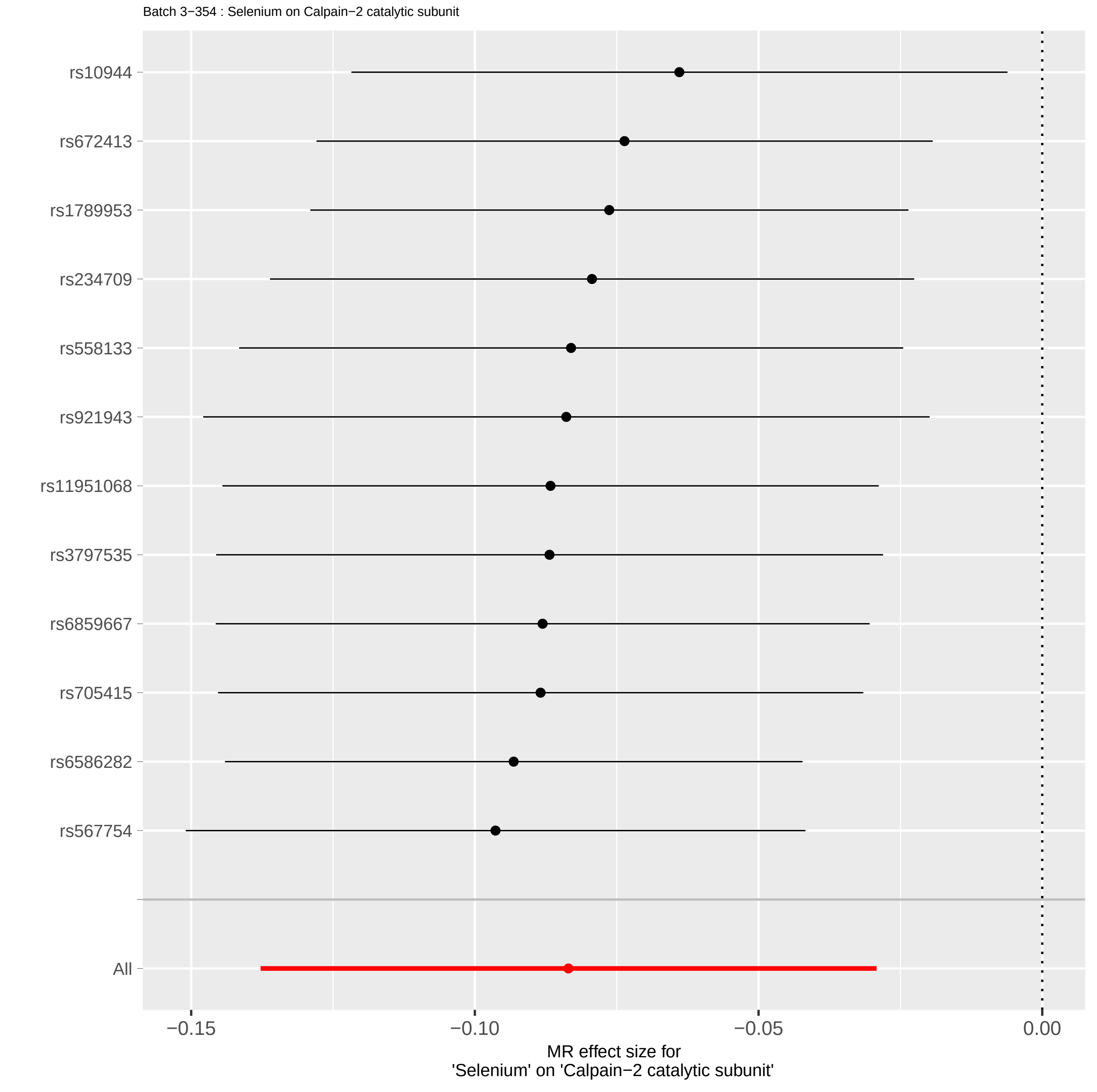

Batch 3−354 : Selenium on Calpain−2 catalytic subunit
rs10944
rs672413
rs1789953
rs234709
rs558133
rs921943
rs11951068
rs3797535
rs6859667
rs705415
rs6586282
rs567754
All
−0.15
−0.10
−0.05
0.00
MR effect size for
'Selenium' on 'Calpain−2 catalytic subunit'

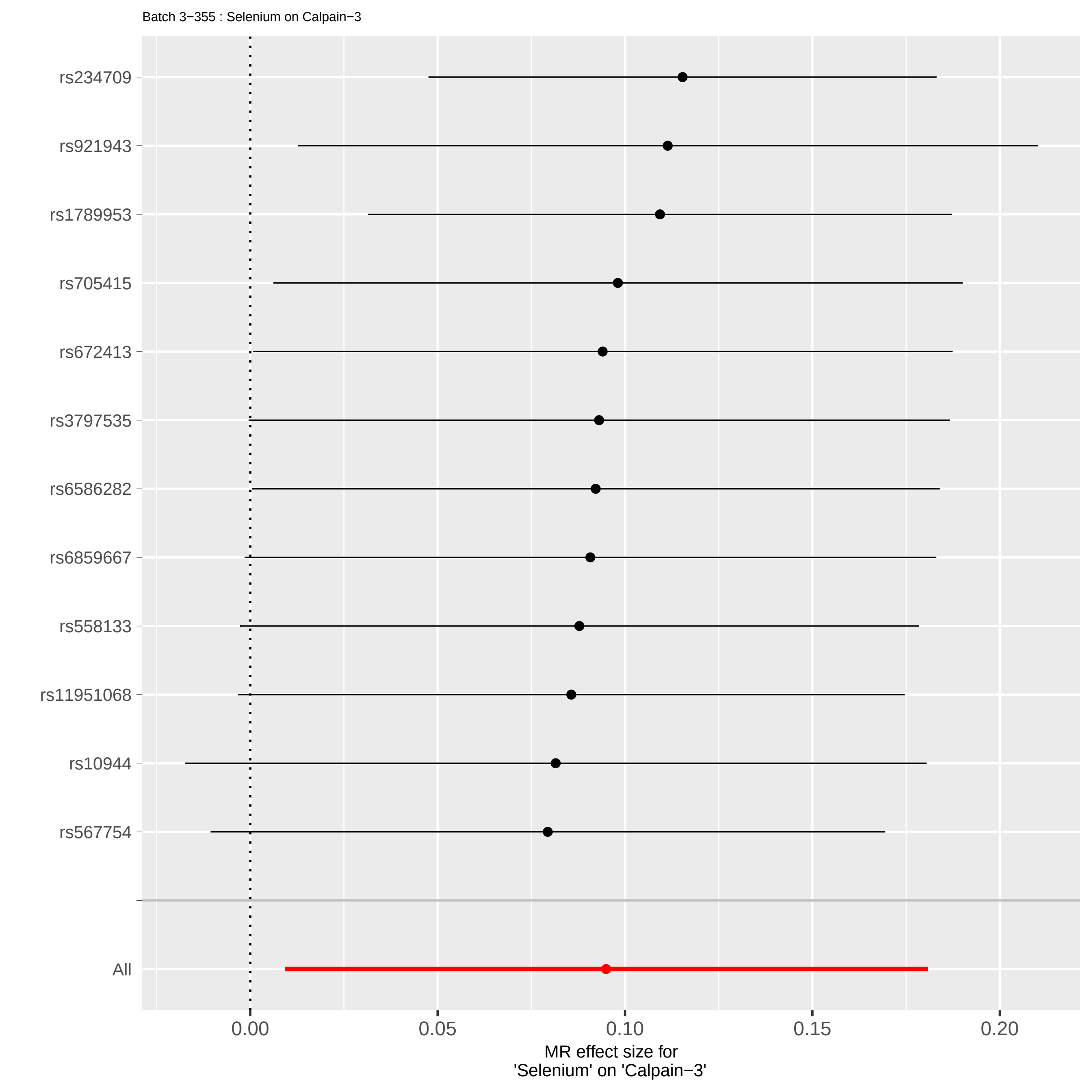

Batch 3−355 : Selenium on Calpain−3
rs234709
rs921943
rs1789953
rs705415
rs672413
rs3797535
rs6586282
rs6859667
rs558133
rs11951068
rs10944
rs567754
All
0.00
0.05
0.10
0.15
0.20
MR effect size for
'Selenium' on 'Calpain−3'

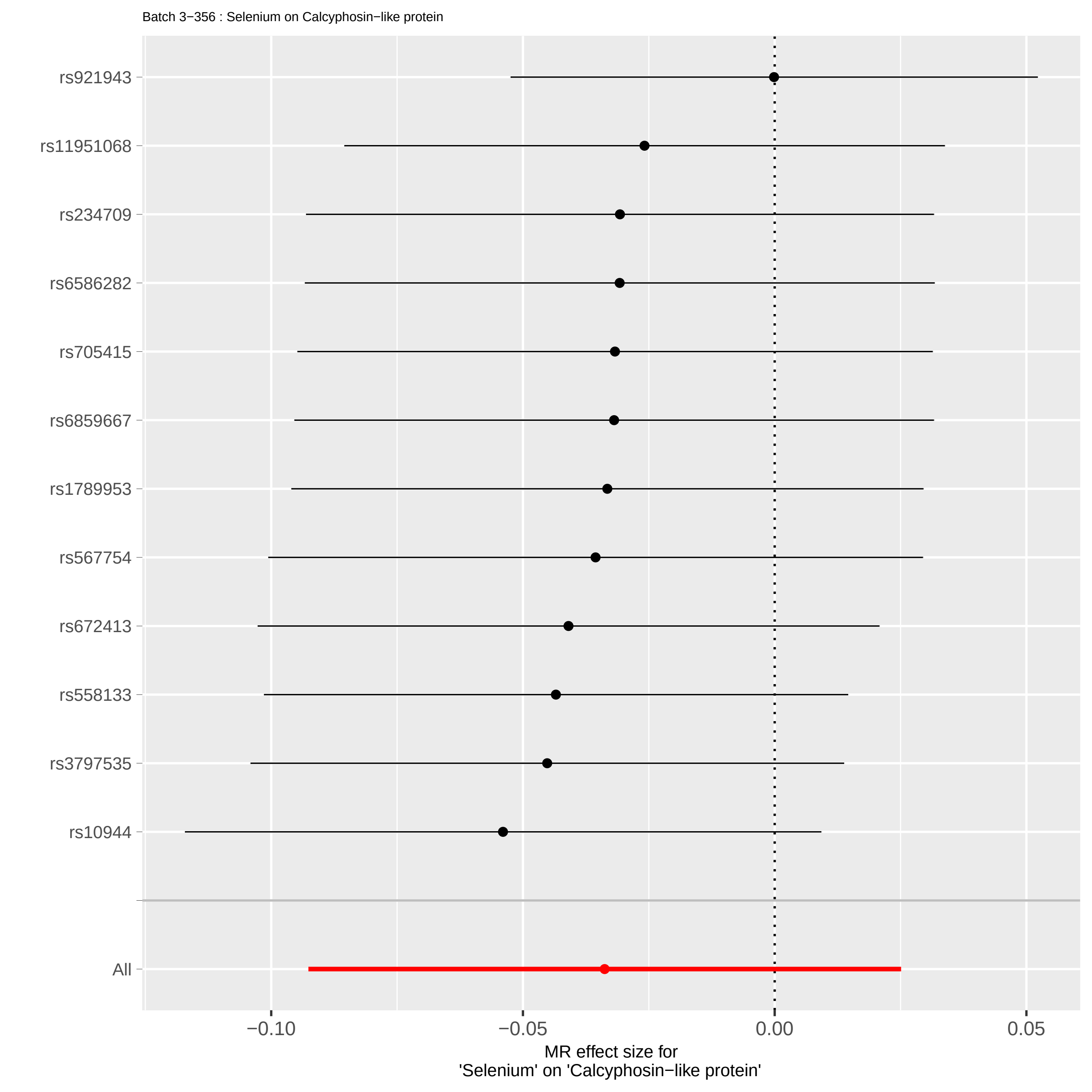

Batch 3−356 : Selenium on Calcyphosin−like protein
rs921943
rs11951068
rs234709
rs6586282
rs705415
rs6859667
rs1789953
rs567754
rs672413
rs558133
rs3797535
rs10944
All
−0.10
−0.05
0.00
0.05
MR effect size for
'Selenium' on 'Calcyphosin−like protein'

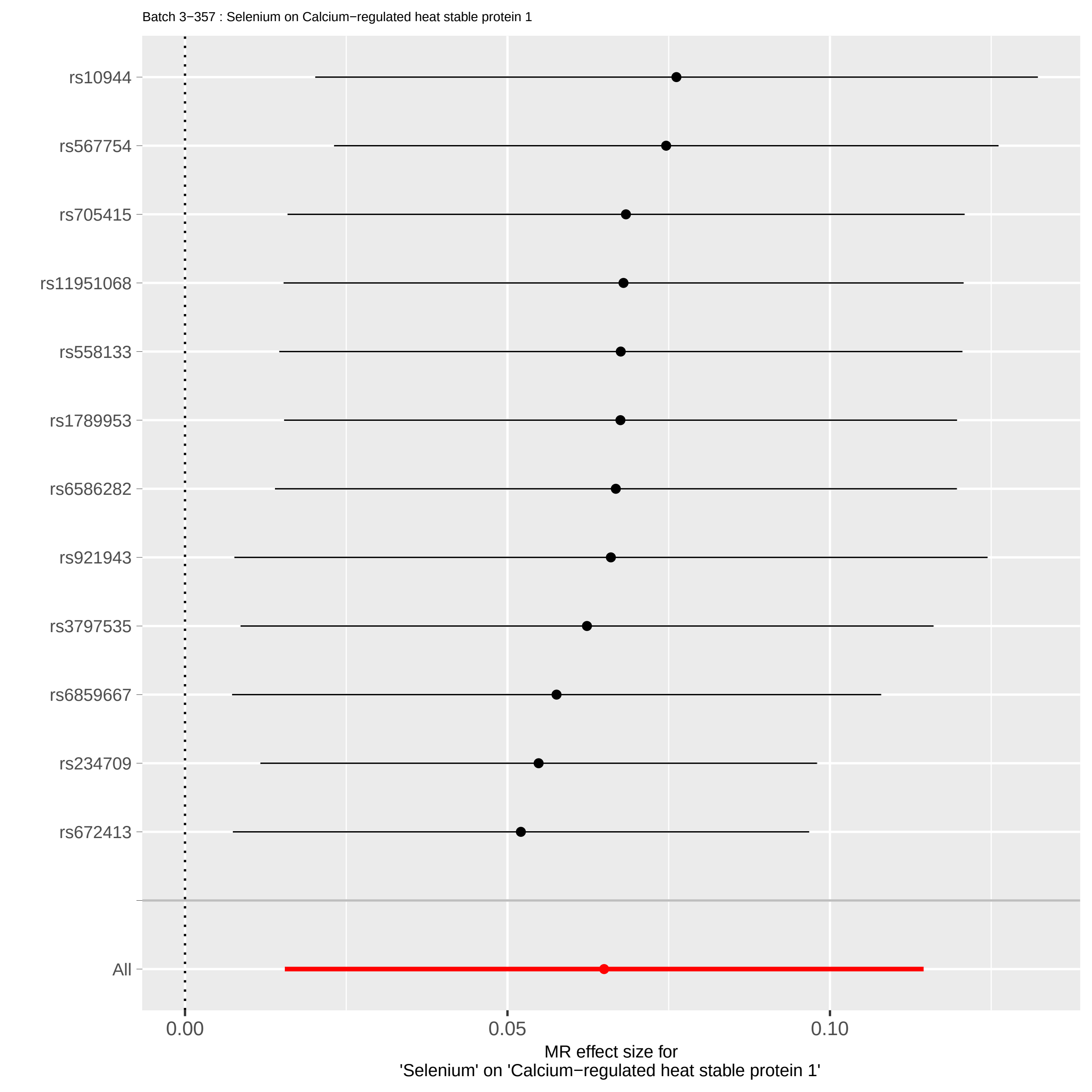
Batch 3−357 : Selenium on Calcium−regulated heat stable protein 1
rs10944
rs567754
rs705415
rs11951068
rs558133
rs1789953
rs6586282
rs921943
rs3797535
rs6859667
rs234709
rs672413
All
0.00
0.05
0.10
MR effect size for
'Selenium' on 'Calcium−regulated heat stable protein 1'

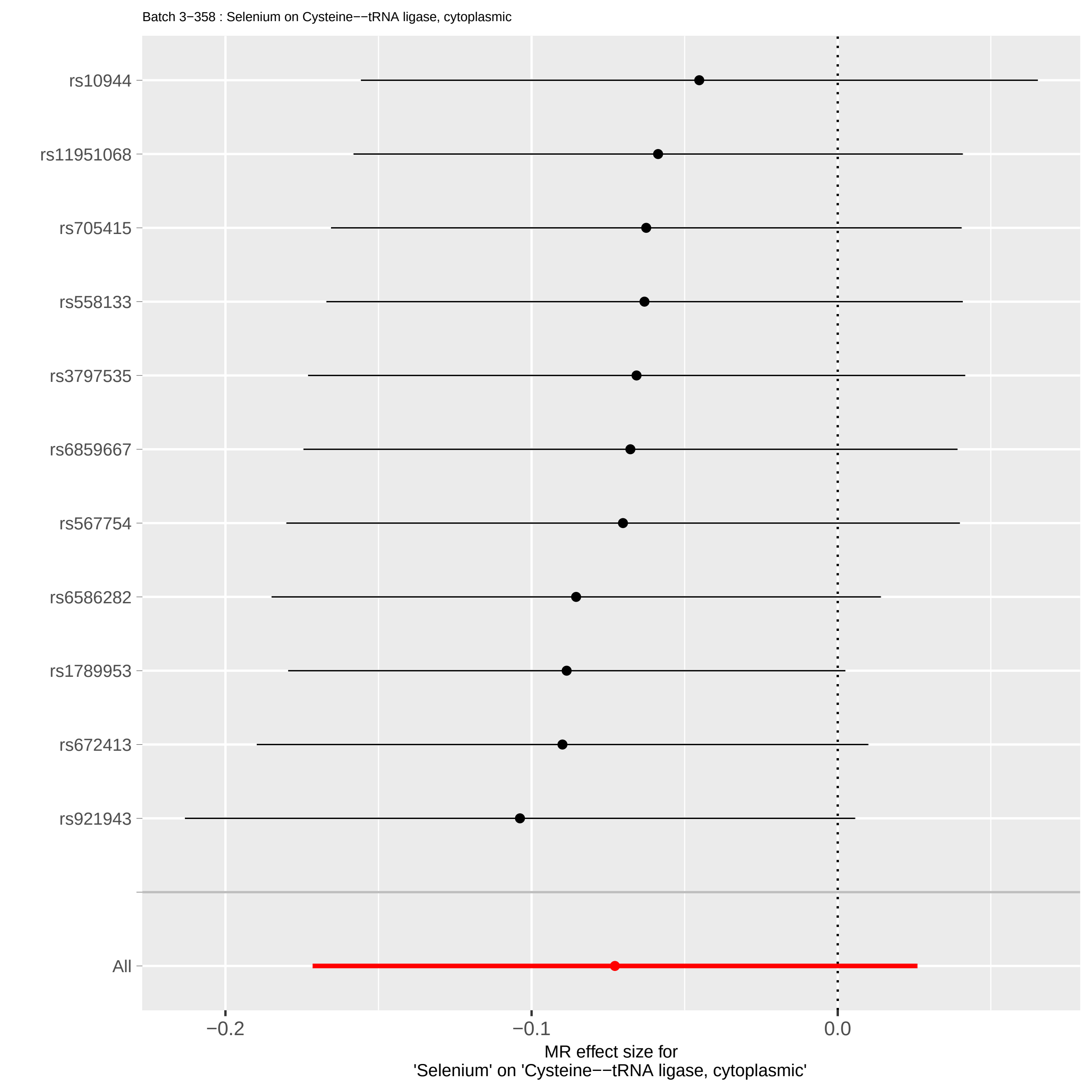
Batch 3−358 : Selenium on Cysteine−−tRNA ligase, cytoplasmic
rs10944
rs11951068
rs705415
rs558133
rs3797535
rs6859667
rs567754
rs6586282
rs1789953
rs672413
rs921943
All
−0.2
−0.1
0.0
MR effect size for
'Selenium' on 'Cysteine−−tRNA ligase, cytoplasmic'

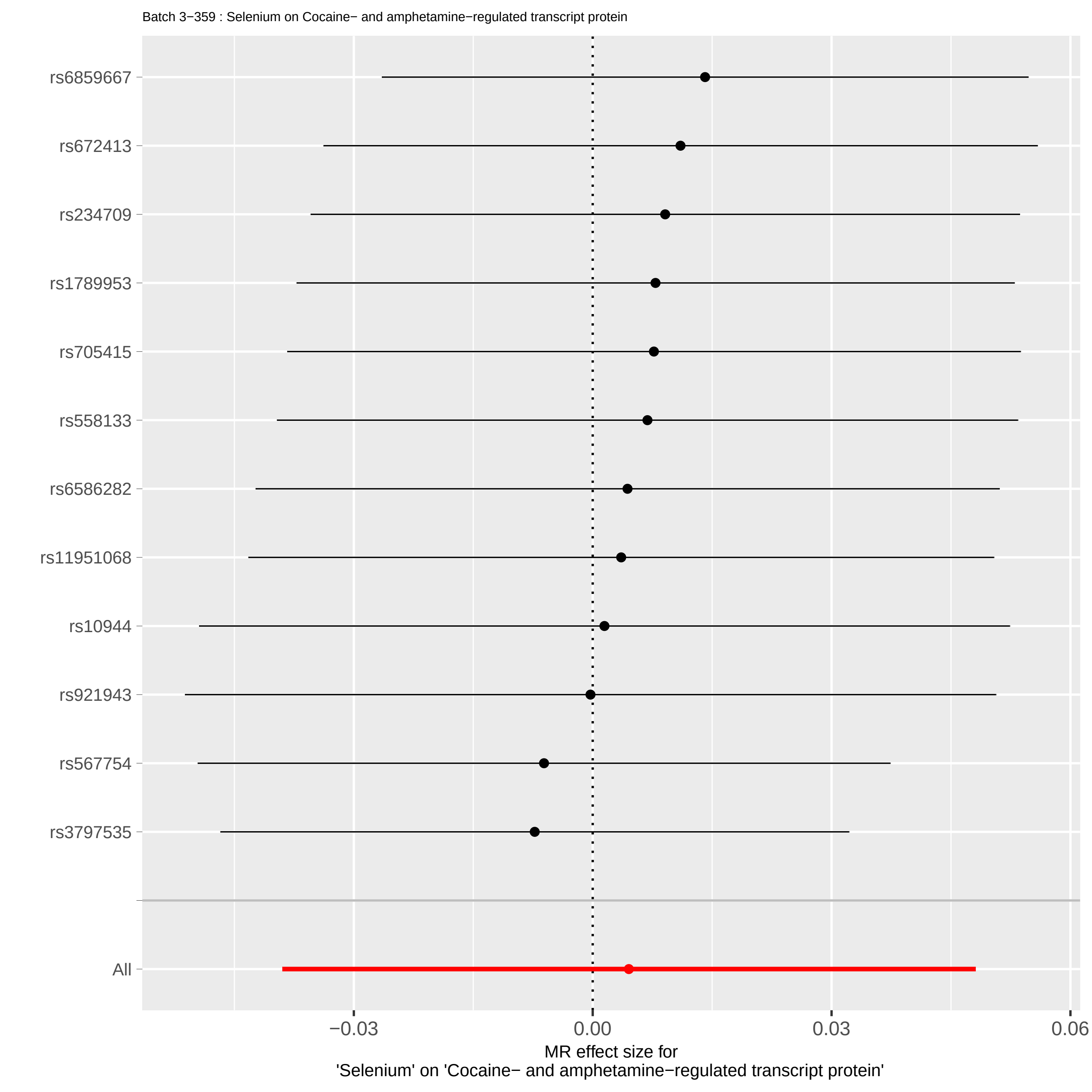

Batch 3−359 : Selenium on Cocaine− and amphetamine−regulated transcript protein
rs6859667
rs672413
rs234709
rs1789953
rs705415
rs558133
rs6586282
rs11951068
rs10944
rs921943
rs567754
rs3797535
All
−0.03
0.00
0.03
0.06
MR effect size for
'Selenium' on 'Cocaine− and amphetamine−regulated transcript protein'

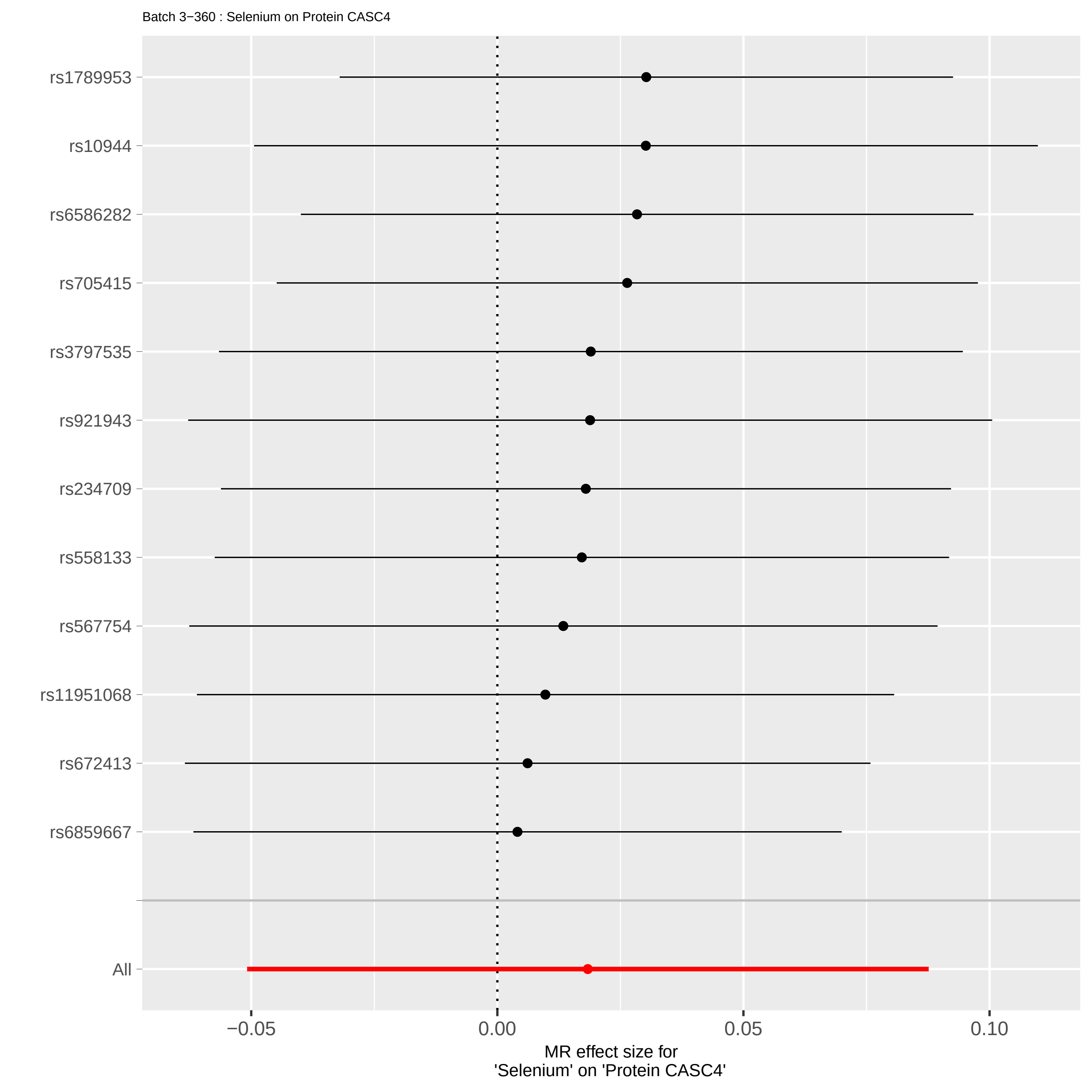
Batch 3−360 : Selenium on Protein CASC4
rs1789953
rs10944
rs6586282
rs705415
rs3797535
rs921943
rs234709
rs558133
rs567754
rs11951068
rs672413
rs6859667
All
−0.05
0.00
0.05
0.10
MR effect size for
'Selenium' on 'Protein CASC4'

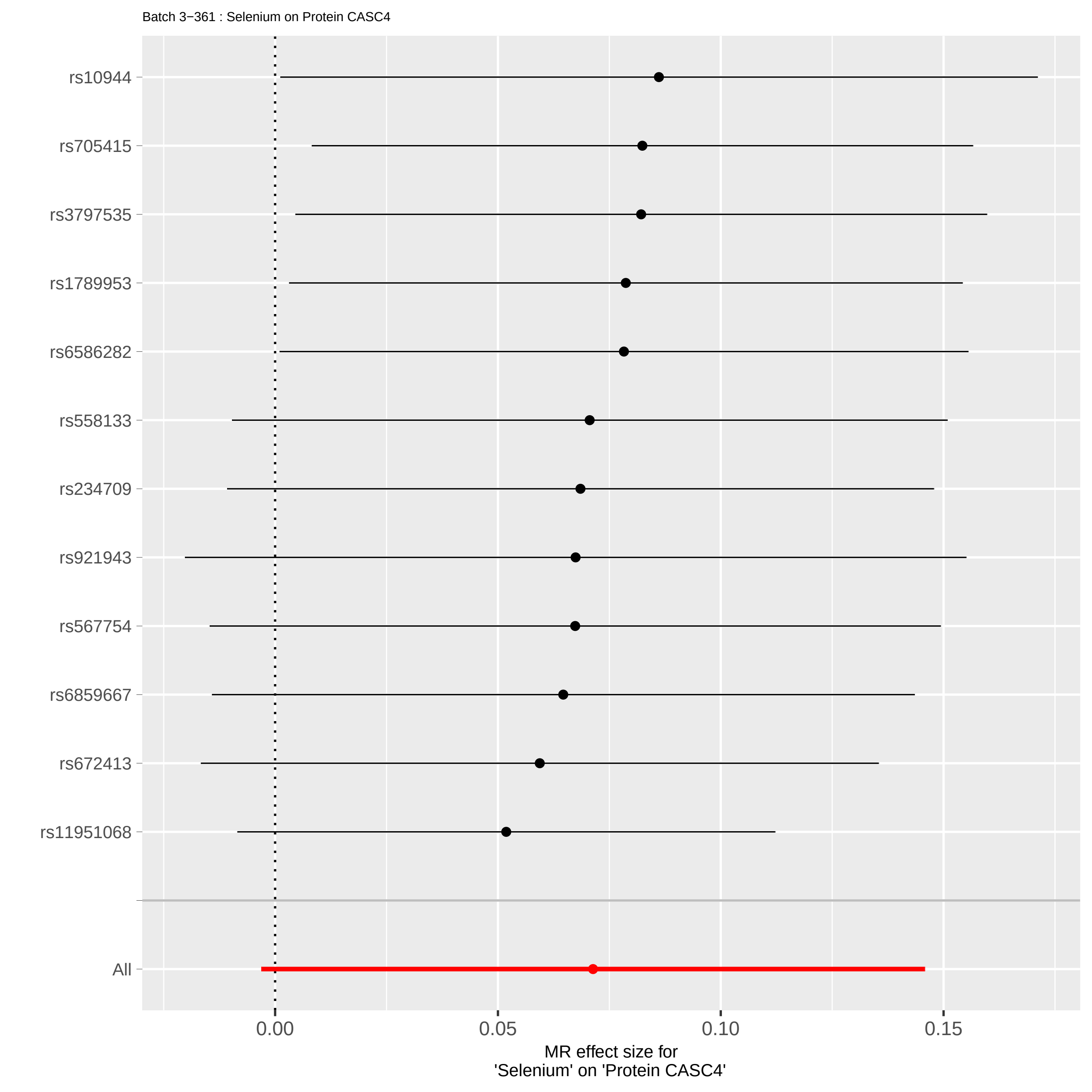

Batch 3−361 : Selenium on Protein CASC4
rs10944
rs705415
rs3797535
rs1789953
rs6586282
rs558133
rs234709
rs921943
rs567754
rs6859667
rs672413
rs11951068
All
0.00
0.05
0.10
0.15
MR effect size for
'Selenium' on 'Protein CASC4'

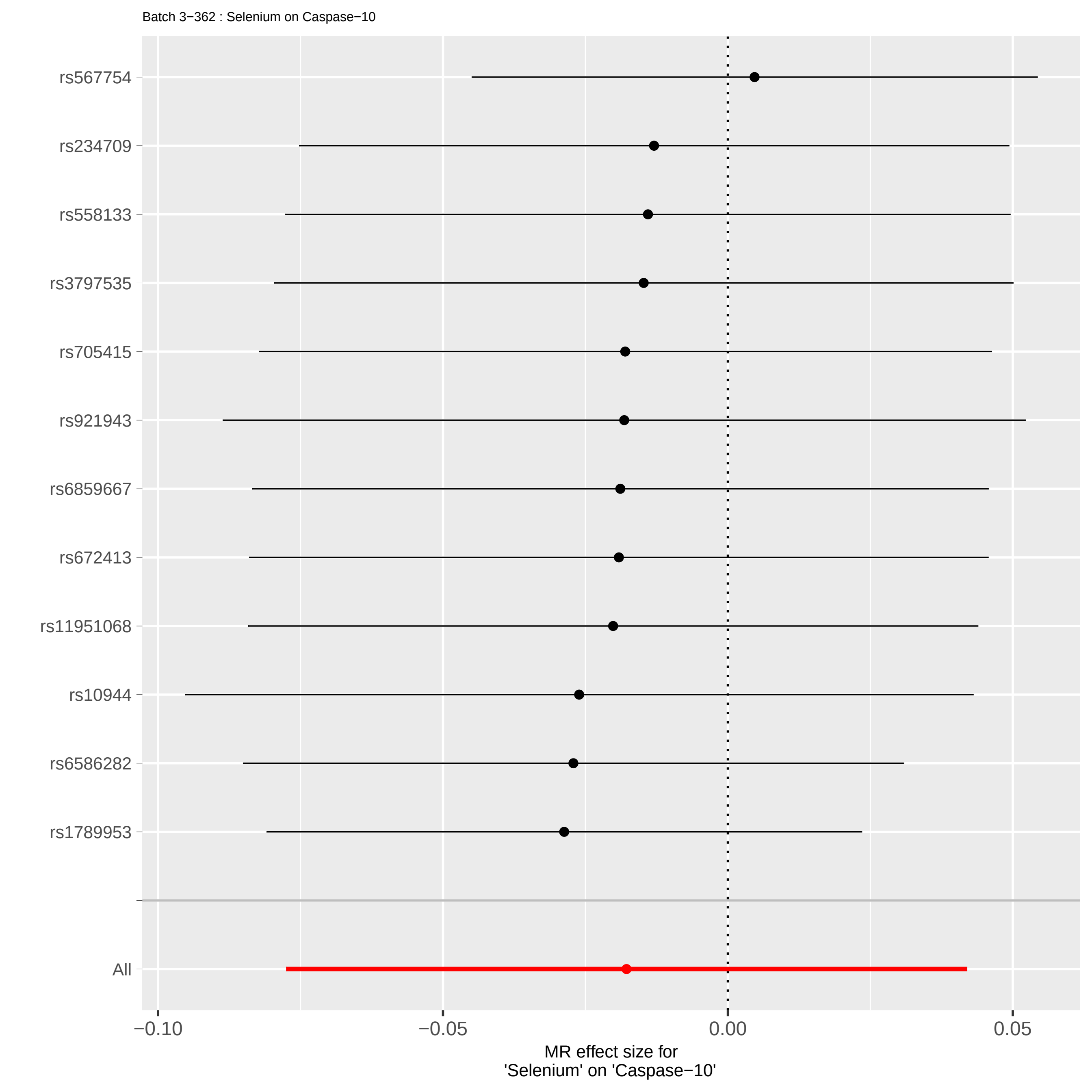

Batch 3−362 : Selenium on Caspase−10
rs567754
rs234709
rs558133
rs3797535
rs705415
rs921943
rs6859667
rs672413
rs11951068
rs10944
rs6586282
rs1789953
All
−0.10
−0.05
0.00
0.05
MR effect size for
'Selenium' on 'Caspase−10'

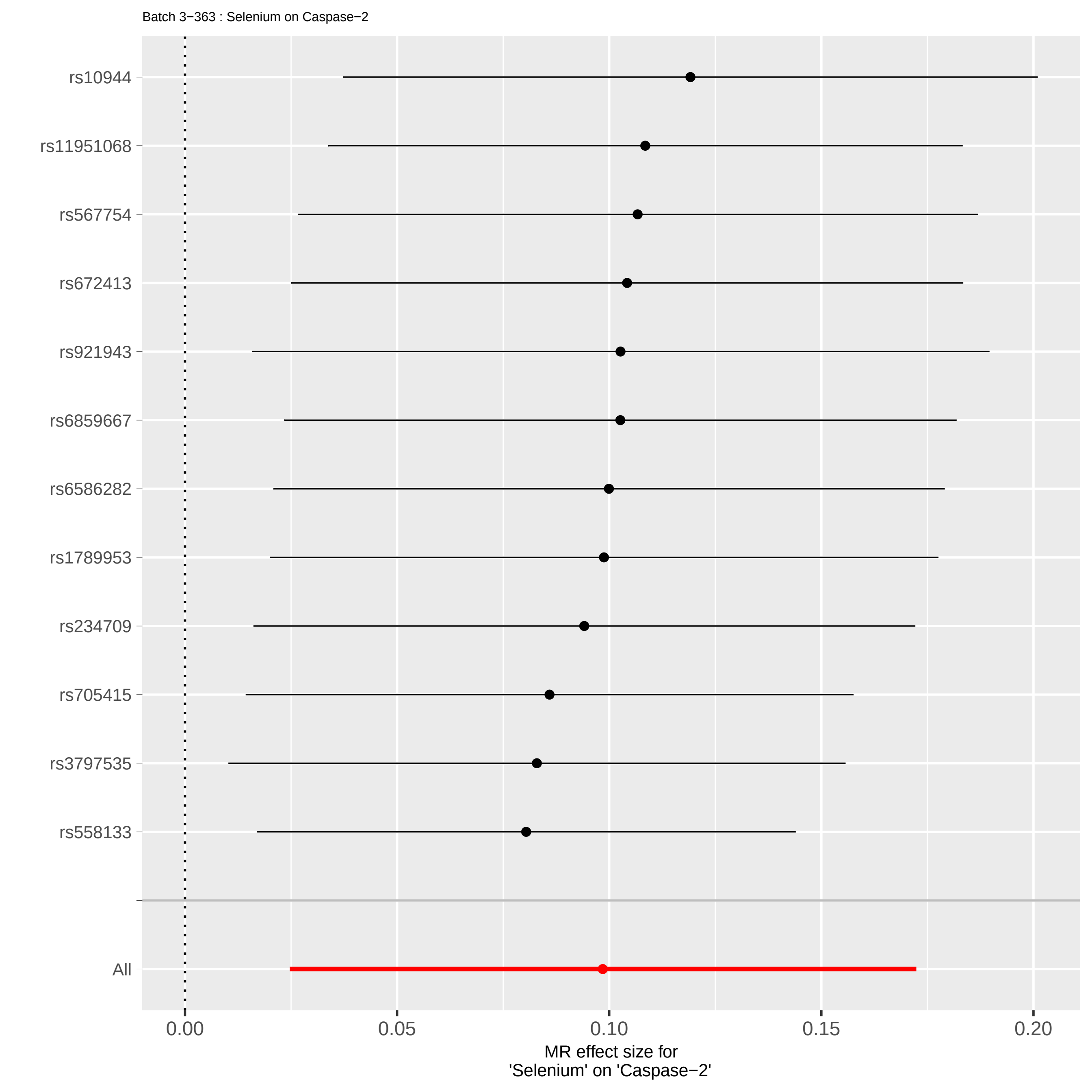

Batch 3−363 : Selenium on Caspase−2
rs10944
rs11951068
rs567754
rs672413
rs921943
rs6859667
rs6586282
rs1789953
rs234709
rs705415
rs3797535
rs558133
All
0.00
0.05
0.10
0.15
0.20
MR effect size for
'Selenium' on 'Caspase−2'

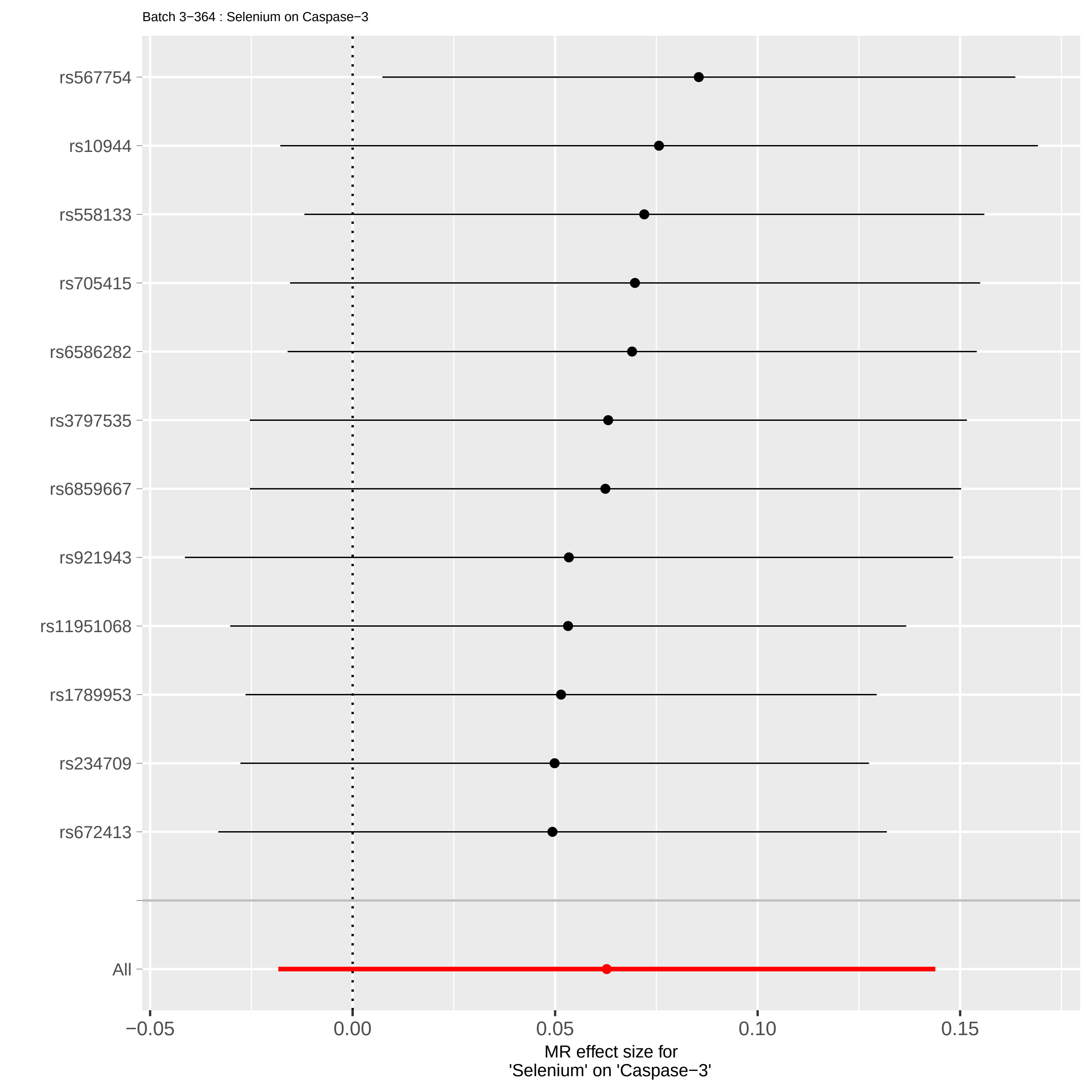

Batch 3−364 : Selenium on Caspase−3
rs567754
rs10944
rs558133
rs705415
rs6586282
rs3797535
rs6859667
rs921943
rs11951068
rs1789953
rs234709
rs672413
All
−0.05
0.00
0.05
0.10
0.15
MR effect size for
'Selenium' on 'Caspase−3'

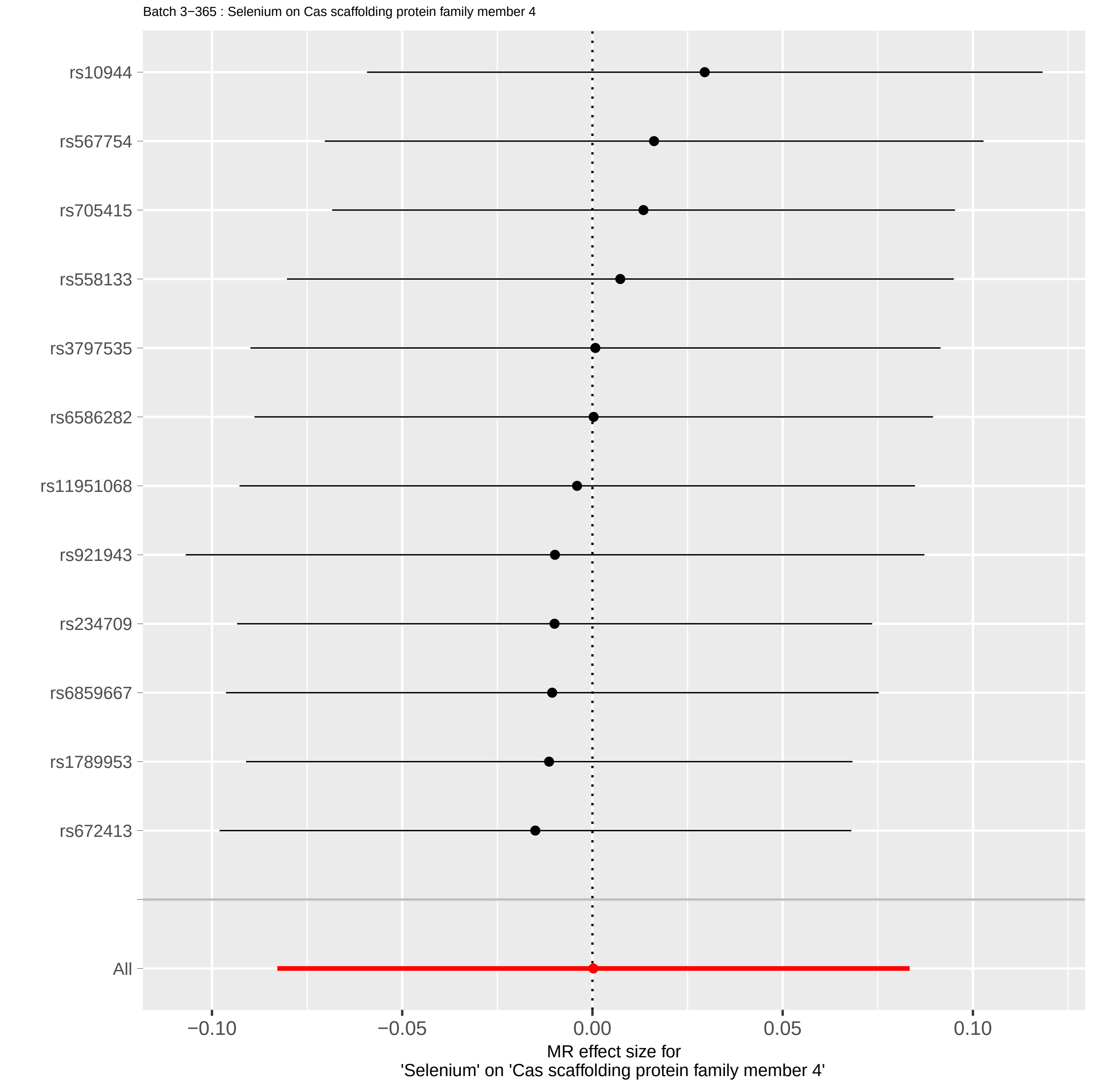

Batch 3−365 : Selenium on Cas scaffolding protein family member 4
rs10944
rs567754
rs705415
rs558133
rs3797535
rs6586282
rs11951068
rs921943
rs234709
rs6859667
rs1789953
rs672413
All
−0.10
−0.05
0.00
0.05
0.10
MR effect size for
'Selenium' on 'Cas scaffolding protein family member 4'

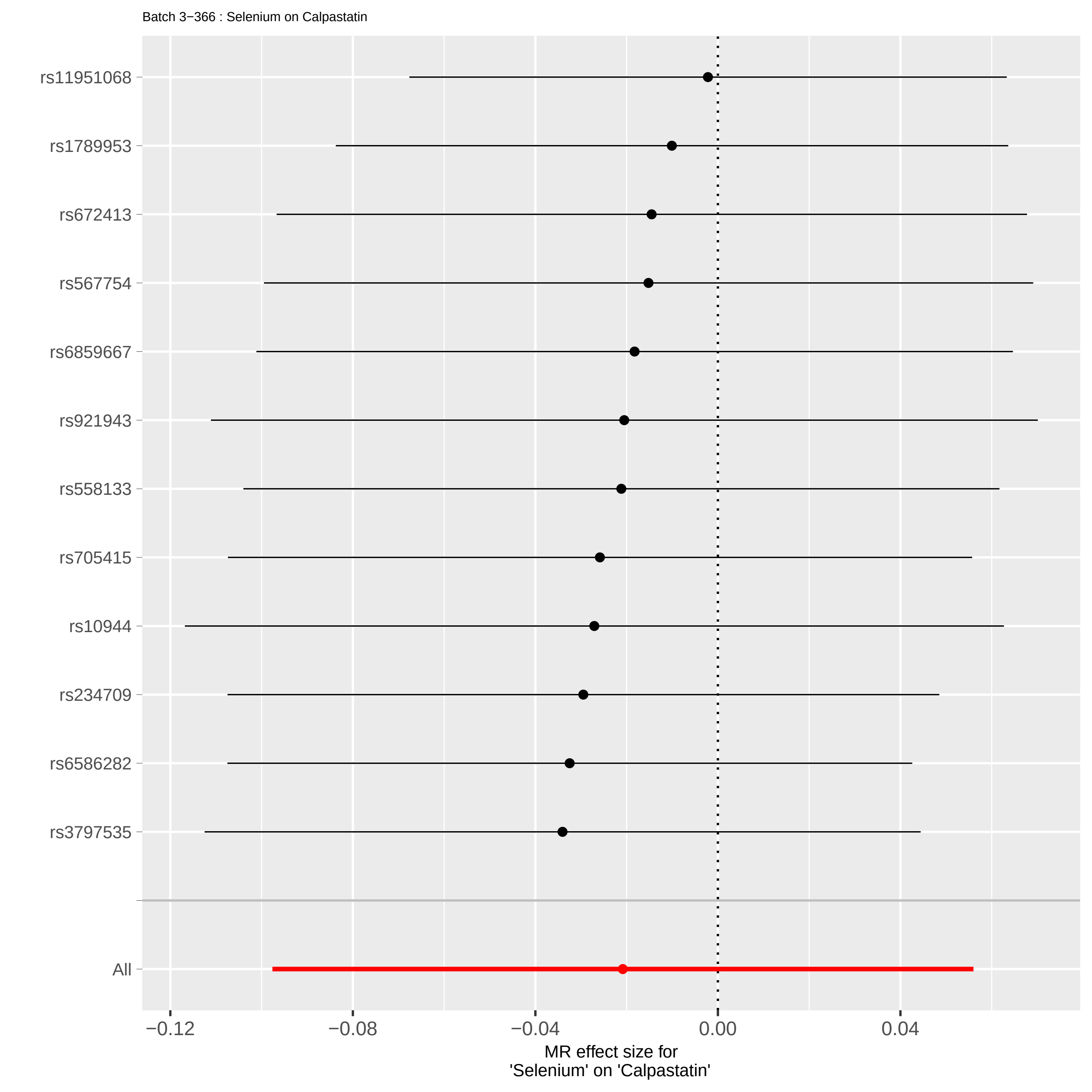

Batch 3−366 : Selenium on Calpastatin
rs11951068
rs1789953
rs672413
rs567754
rs6859667
rs921943
rs558133
rs705415
rs10944
rs234709
rs6586282
rs3797535
All
−0.12
−0.08
−0.04
0.00
0.04
MR effect size for
'Selenium' on 'Calpastatin'

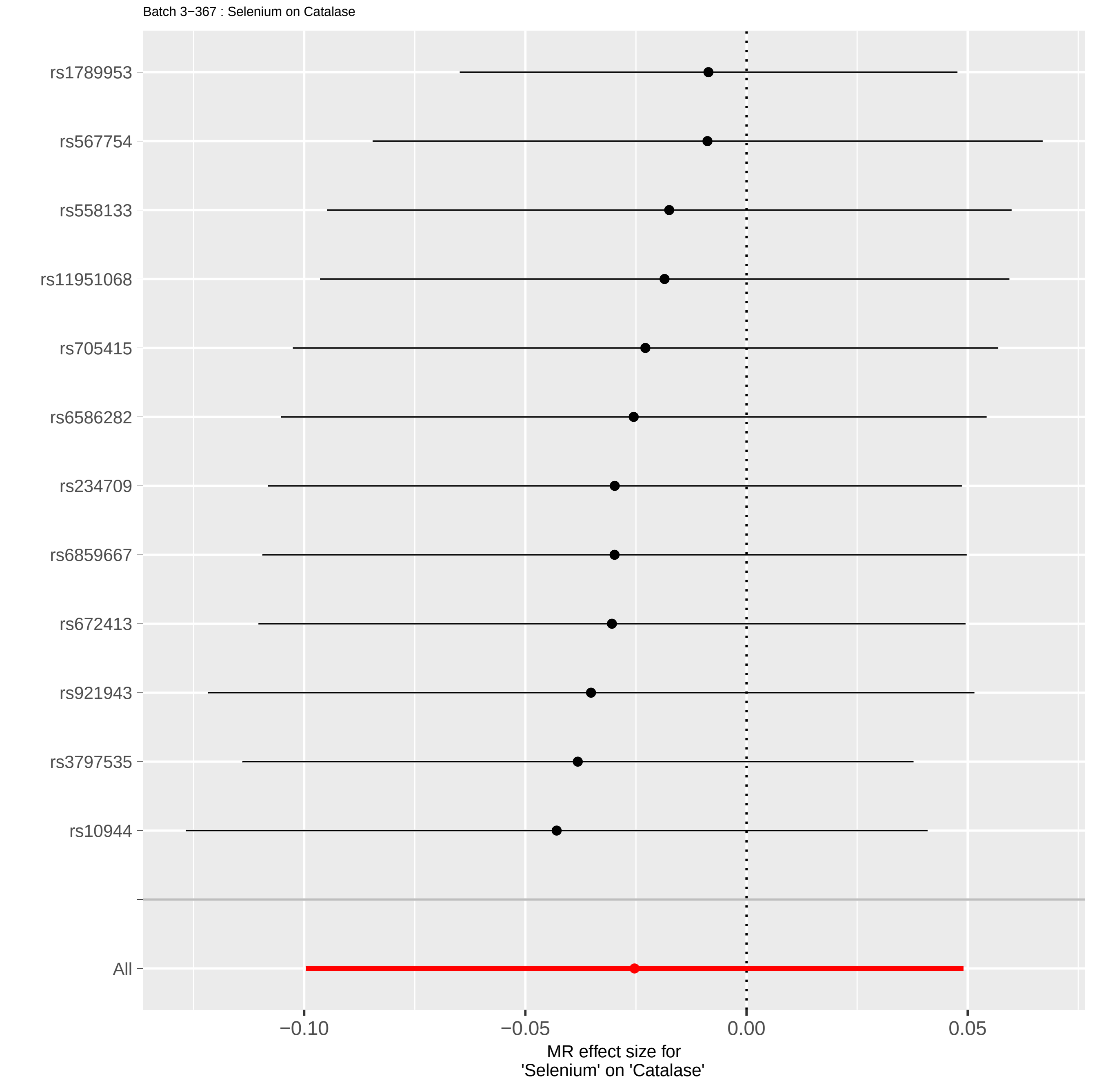
Batch 3−367 : Selenium on Catalase
rs1789953
rs567754
rs558133
rs11951068
rs705415
rs6586282
rs234709
rs6859667
rs672413
rs921943
rs3797535
rs10944
All
−0.10
−0.05
0.00
0.05
MR effect size for
'Selenium' on 'Catalase'

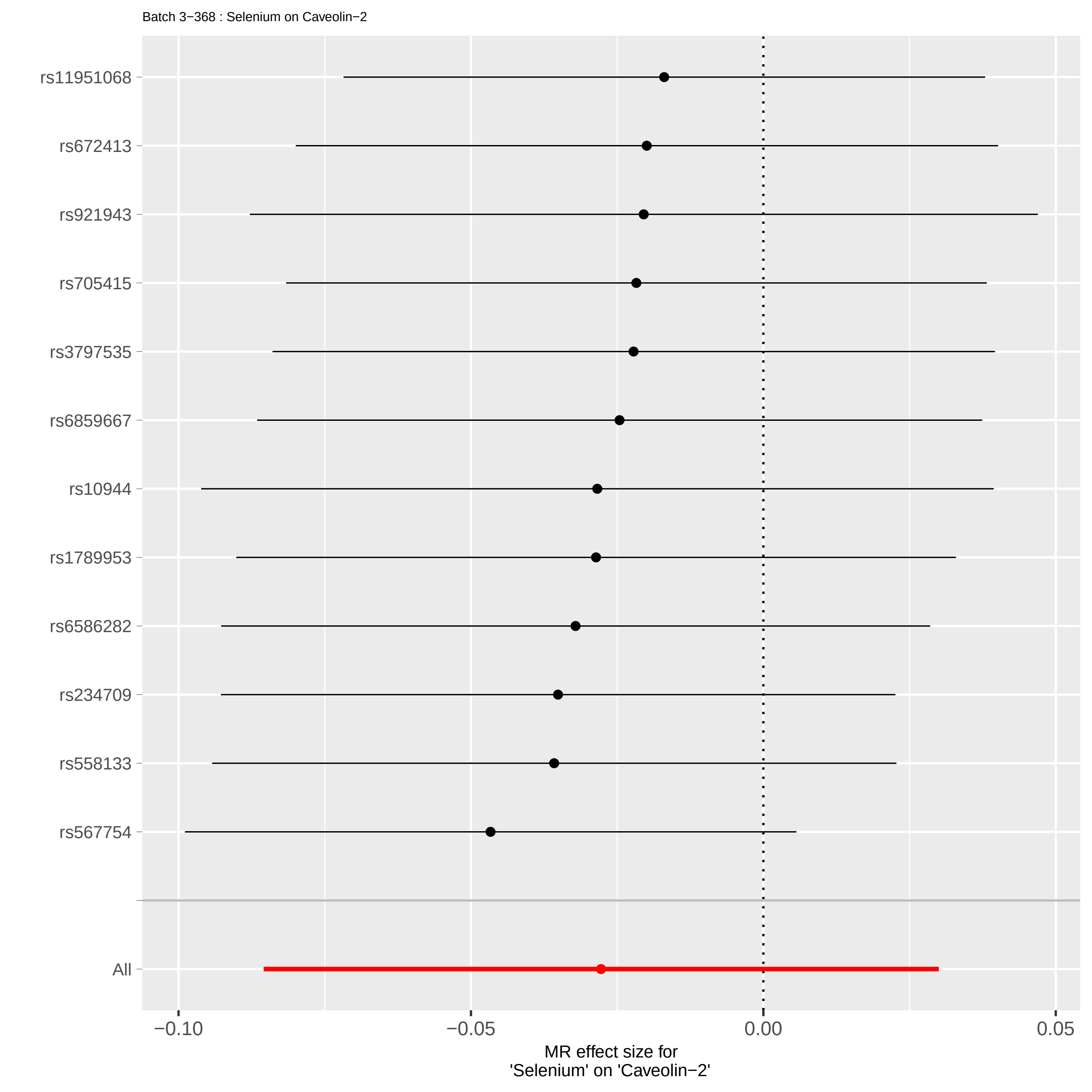
Batch 3−368 : Selenium on Caveolin−2
rs11951068
rs672413
rs921943
rs705415
rs3797535
rs6859667
rs10944
rs1789953
rs6586282
rs234709
rs558133
rs567754
All
−0.10
−0.05
0.00
0.05
MR effect size for
'Selenium' on 'Caveolin−2'

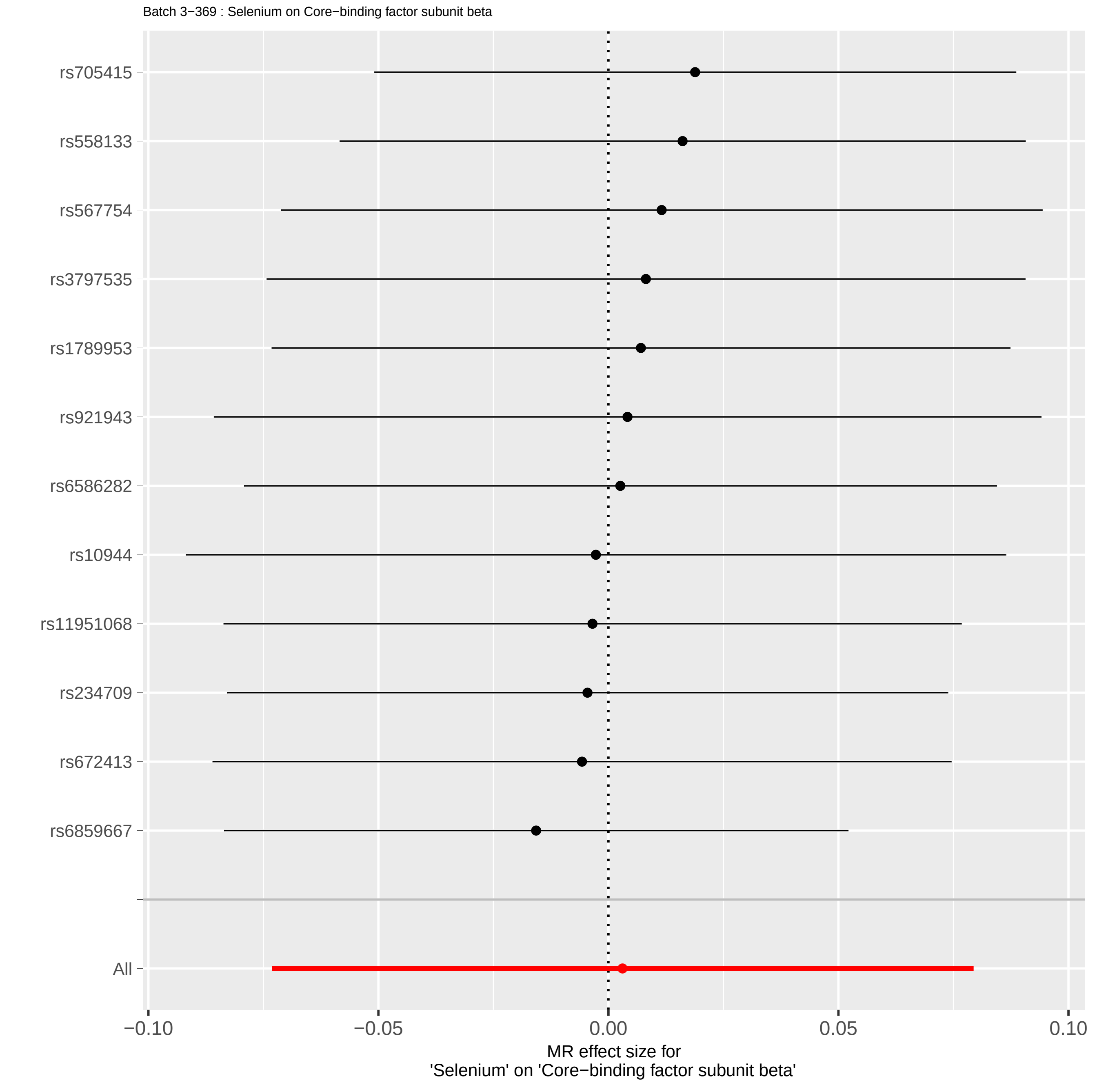

Batch 3−369 : Selenium on Core−binding factor subunit beta
rs705415
rs558133
rs567754
rs3797535
rs1789953
rs921943
rs6586282
rs10944
rs11951068
rs234709
rs672413
rs6859667
All
−0.10
−0.05
0.00
0.05
0.10
MR effect size for
'Selenium' on 'Core−binding factor subunit beta'

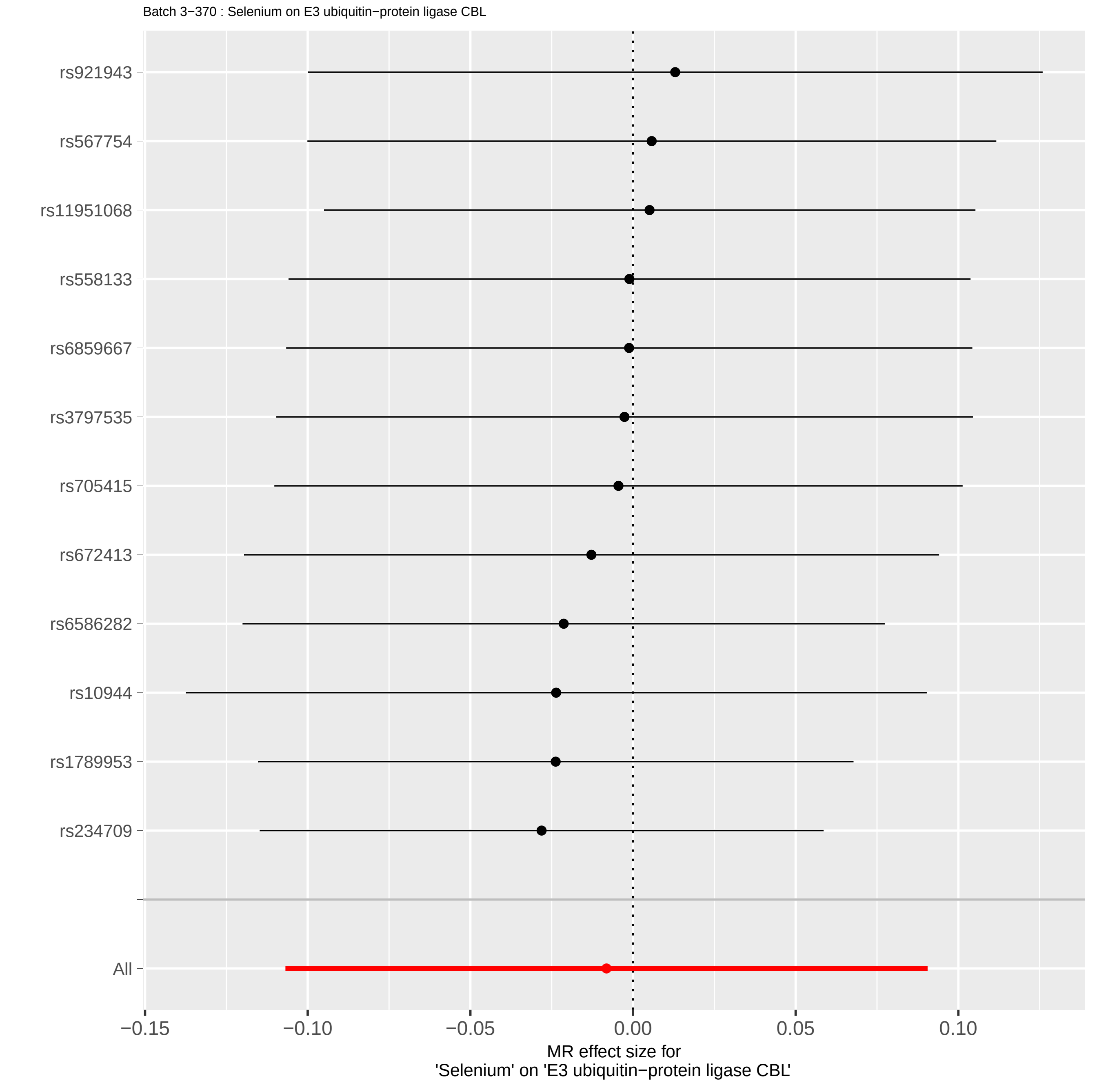

Batch 3−370 : Selenium on E3 ubiquitin−protein ligase CBL
rs921943
rs567754
rs11951068
rs558133
rs6859667
rs3797535
rs705415
rs672413
rs6586282
rs10944
rs1789953
rs234709
All
−0.15
−0.10
−0.05
0.00
0.05
0.10
MR effect size for
'Selenium' on 'E3 ubiquitin−protein ligase CBL'

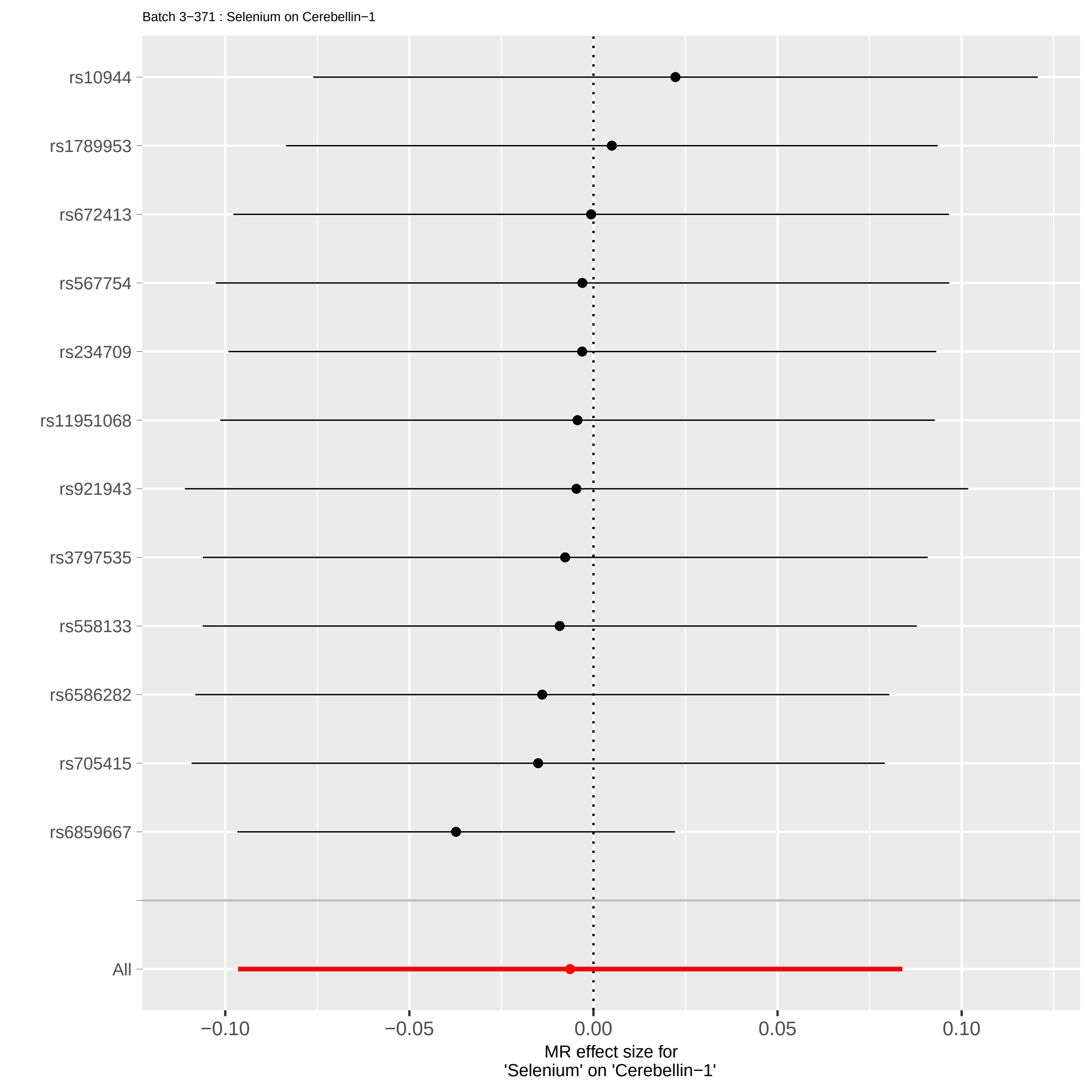

Batch 3−371 : Selenium on Cerebellin−1
rs10944
rs1789953
rs672413
rs567754
rs234709
rs11951068
rs921943
rs3797535
rs558133
rs6586282
rs705415
rs6859667
All
−0.10
−0.05
0.00
0.05
0.10
MR effect size for
'Selenium' on 'Cerebellin−1'

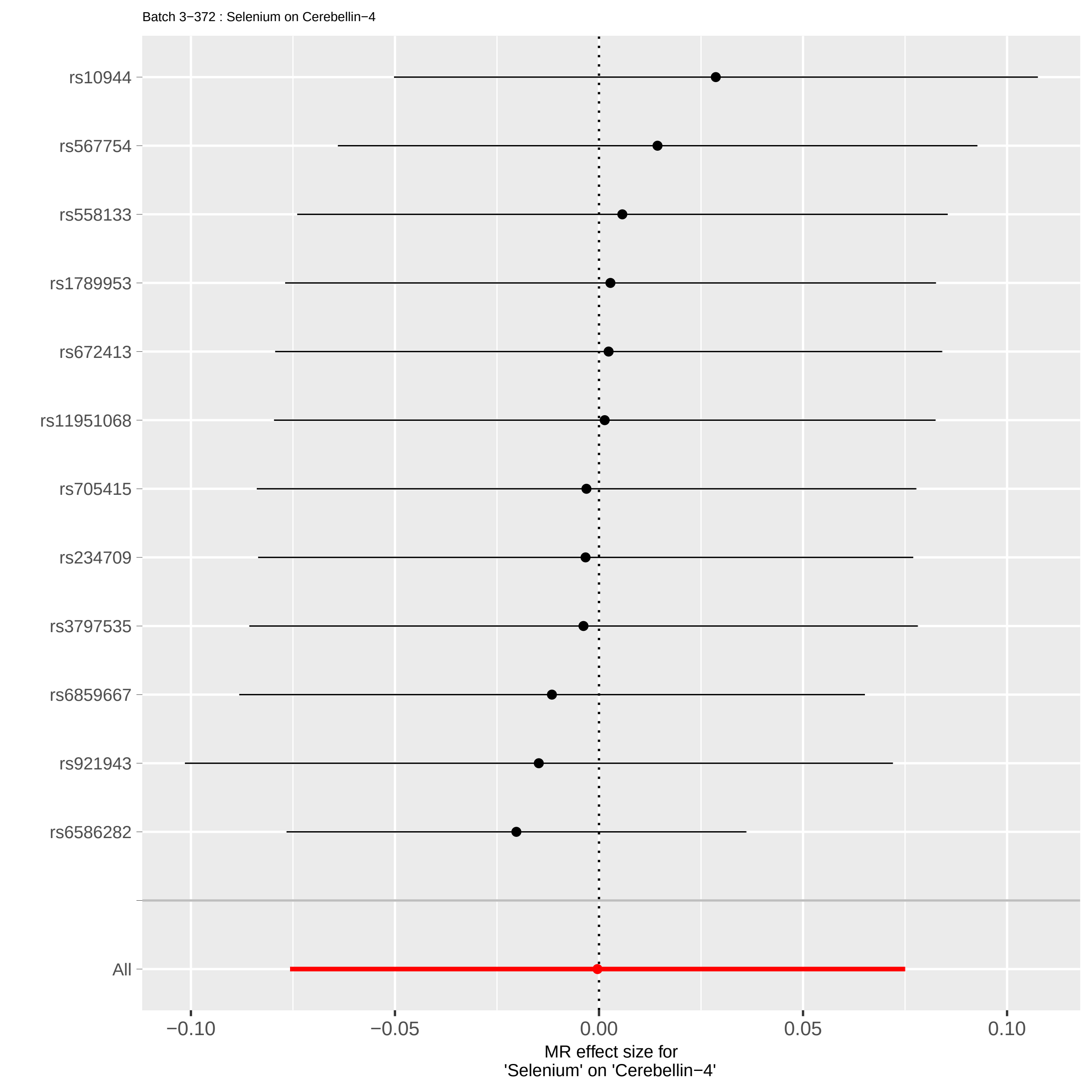

Batch 3−372 : Selenium on Cerebellin−4
rs10944
rs567754
rs558133
rs1789953
rs672413
rs11951068
rs705415
rs234709
rs3797535
rs6859667
rs921943
rs6586282
All
−0.10
−0.05
0.00
0.05
0.10
MR effect size for
'Selenium' on 'Cerebellin−4'

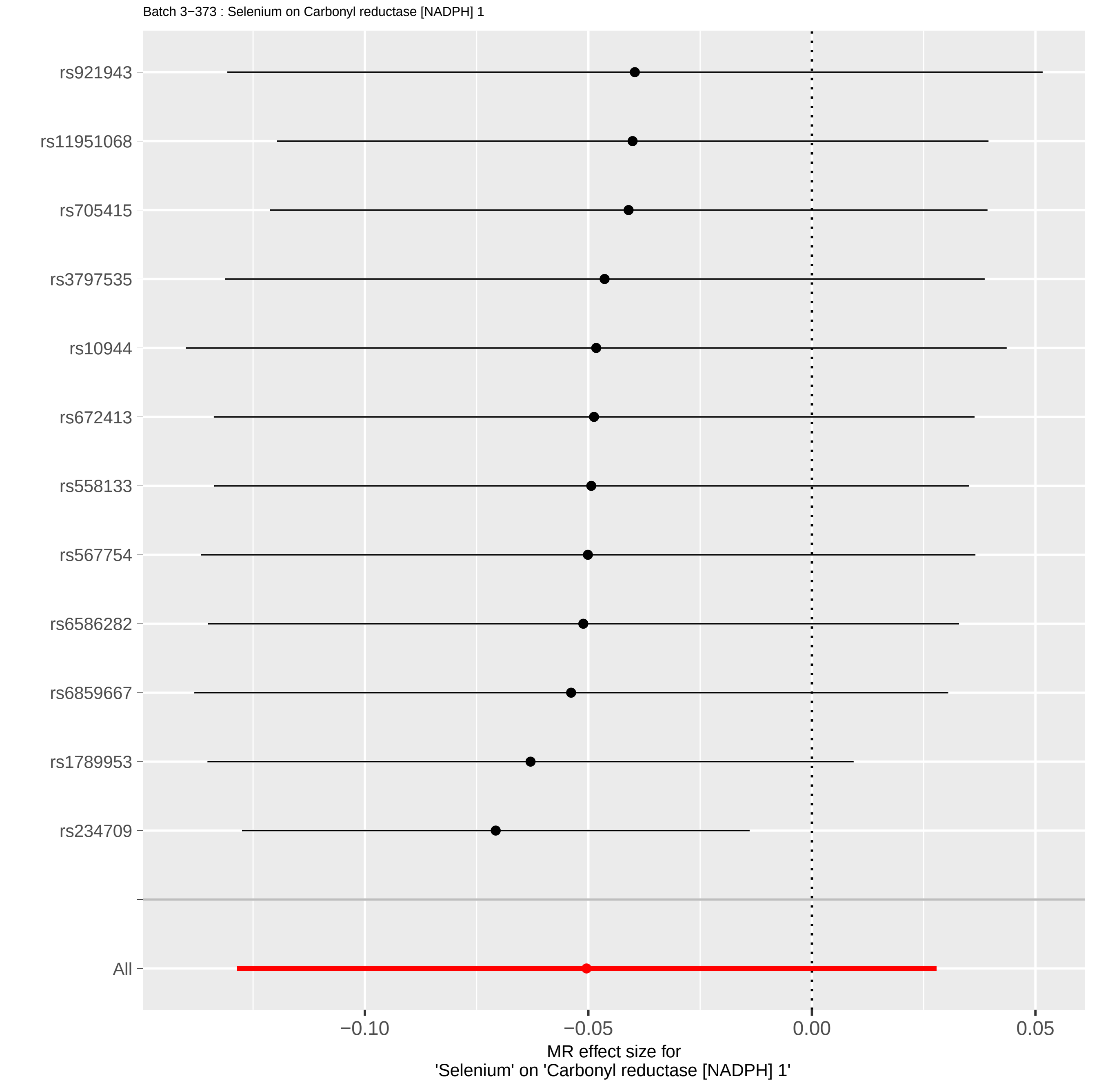

Batch 3−373 : Selenium on Carbonyl reductase [NADPH] 1
rs921943
rs11951068
rs705415
rs3797535
rs10944
rs672413
rs558133
rs567754
rs6586282
rs6859667
rs1789953
rs234709
All
−0.10
−0.05
0.00
0.05
MR effect size for
'Selenium' on 'Carbonyl reductase [NADPH] 1'

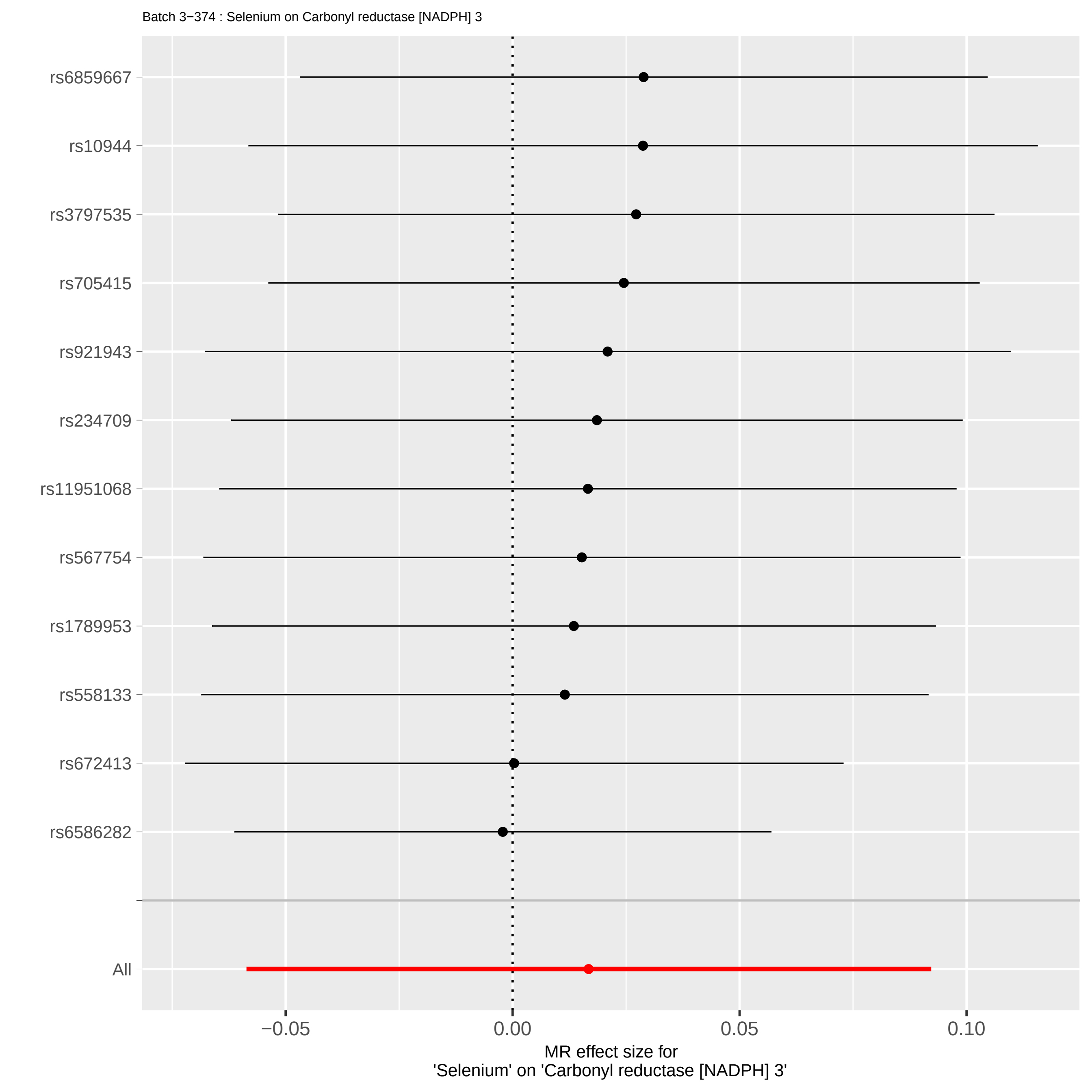

Batch 3−374 : Selenium on Carbonyl reductase [NADPH] 3
rs6859667
rs10944
rs3797535
rs705415
rs921943
rs234709
rs11951068
rs567754
rs1789953
rs558133
rs672413
rs6586282
All
−0.05
0.00
0.05
0.10
MR effect size for
'Selenium' on 'Carbonyl reductase [NADPH] 3'

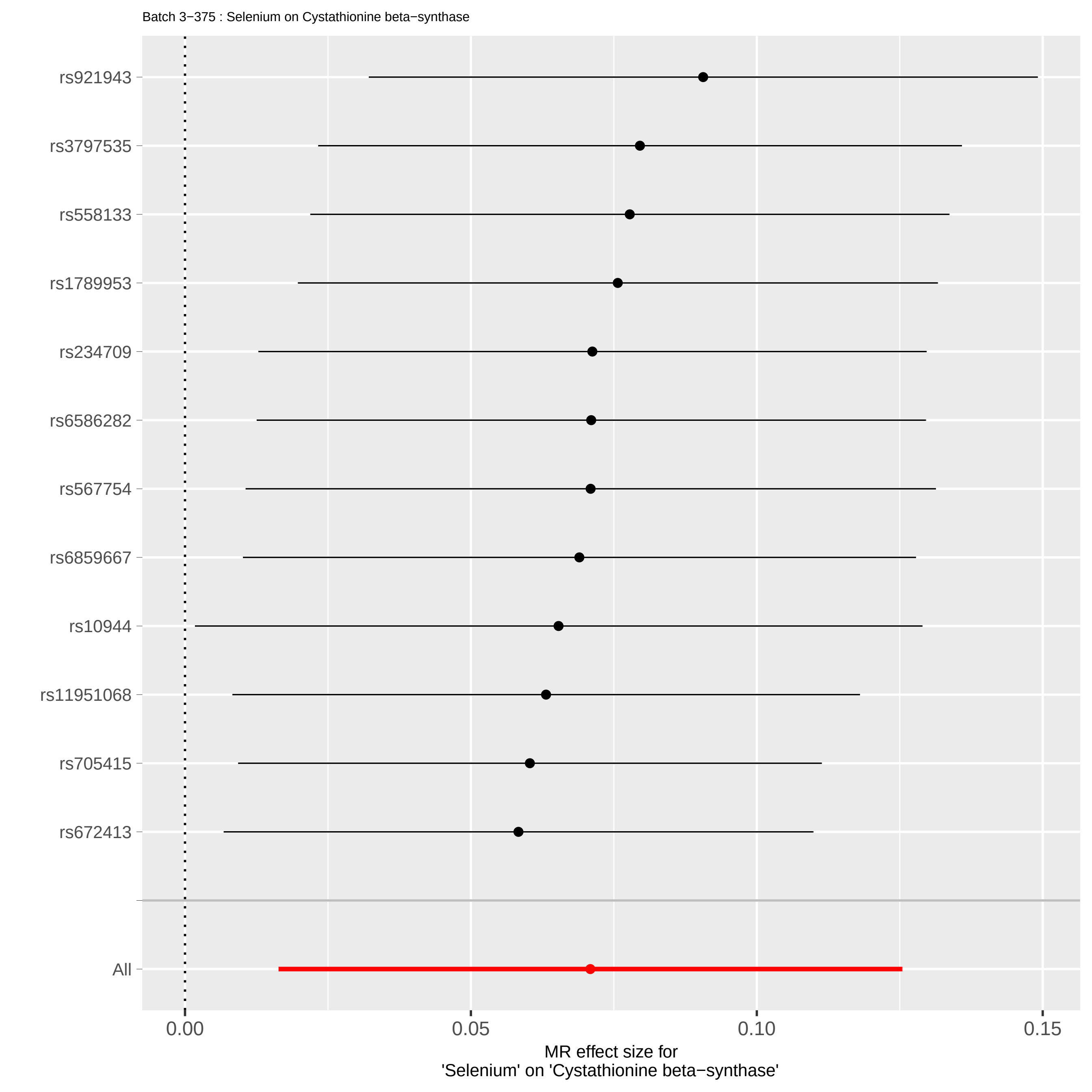

Batch 3−375 : Selenium on Cystathionine beta−synthase
rs921943
rs3797535
rs558133
rs1789953
rs234709
rs6586282
rs567754
rs6859667
rs10944
rs11951068
rs705415
rs672413
All
0.00
0.05
0.10
0.15
MR effect size for
'Selenium' on 'Cystathionine beta−synthase'

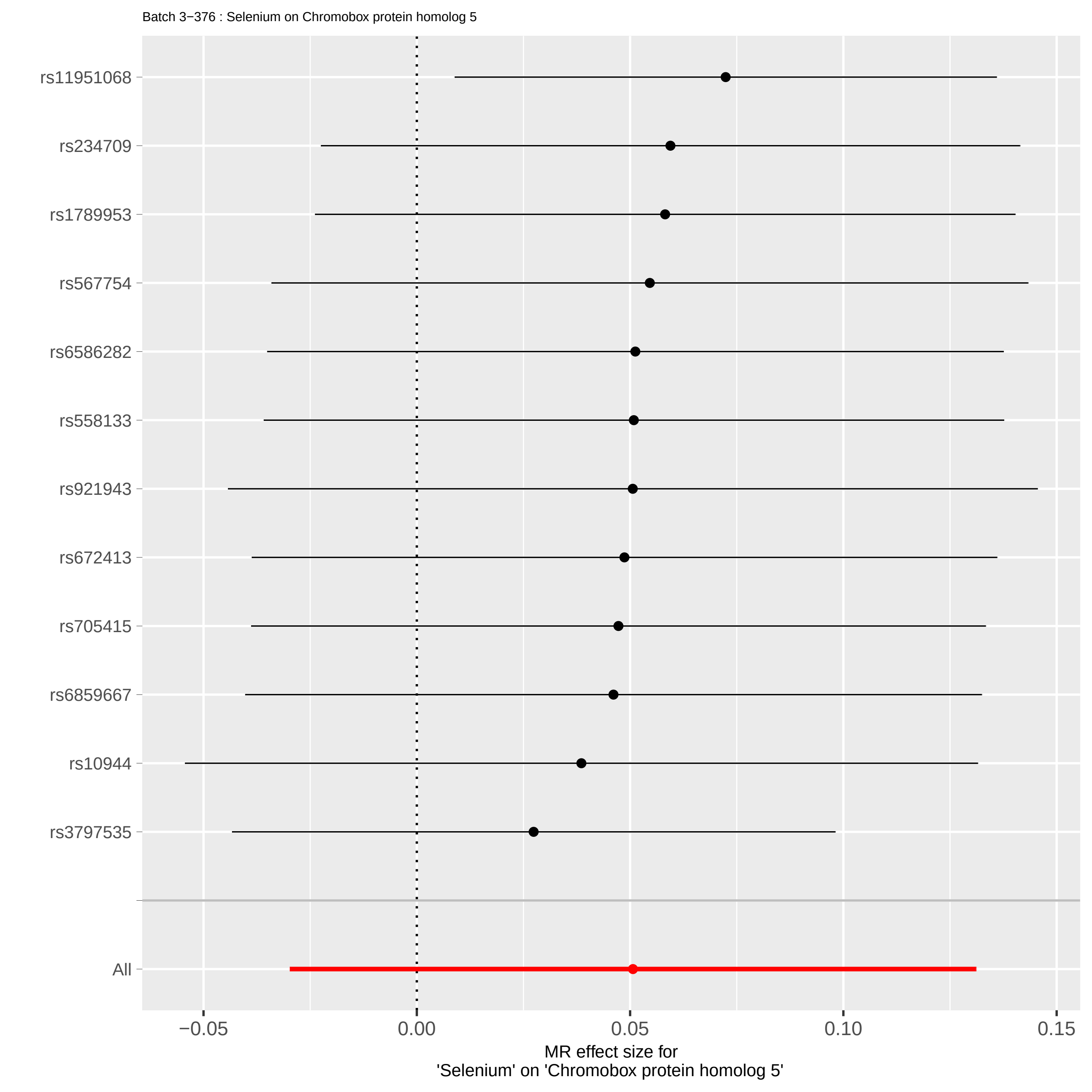
Batch 3−376 : Selenium on Chromobox protein homolog 5
rs11951068
rs234709
rs1789953
rs567754
rs6586282
rs558133
rs921943
rs672413
rs705415
rs6859667
rs10944
rs3797535
All
−0.05
0.00
0.05
0.10
0.15
MR effect size for
'Selenium' on 'Chromobox protein homolog 5'

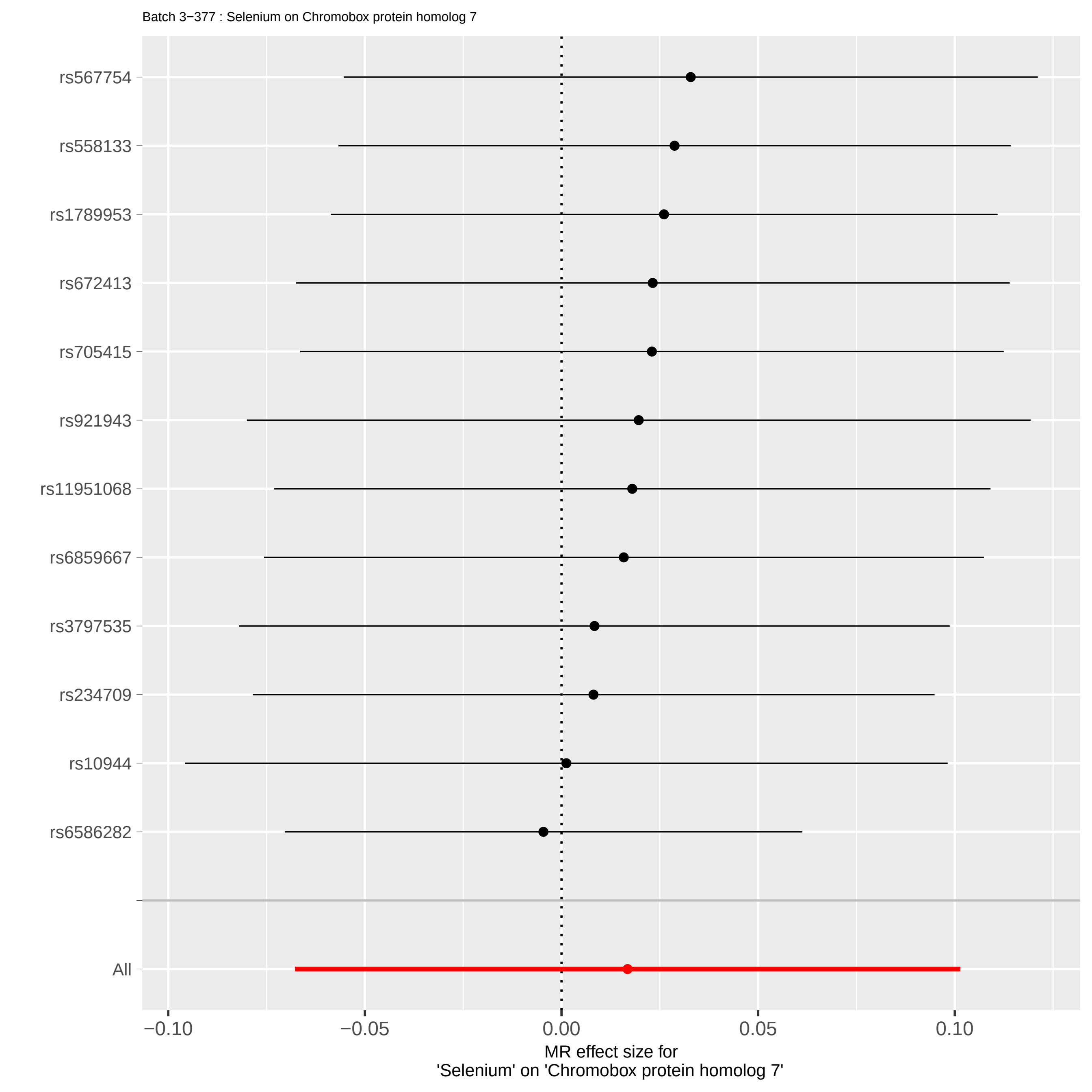

Batch 3−377 : Selenium on Chromobox protein homolog 7
rs567754
rs558133
rs1789953
rs672413
rs705415
rs921943
rs11951068
rs6859667
rs3797535
rs234709
rs10944
rs6586282
All
−0.10
−0.05
0.00
0.05
0.10
MR effect size for
'Selenium' on 'Chromobox protein homolog 7'

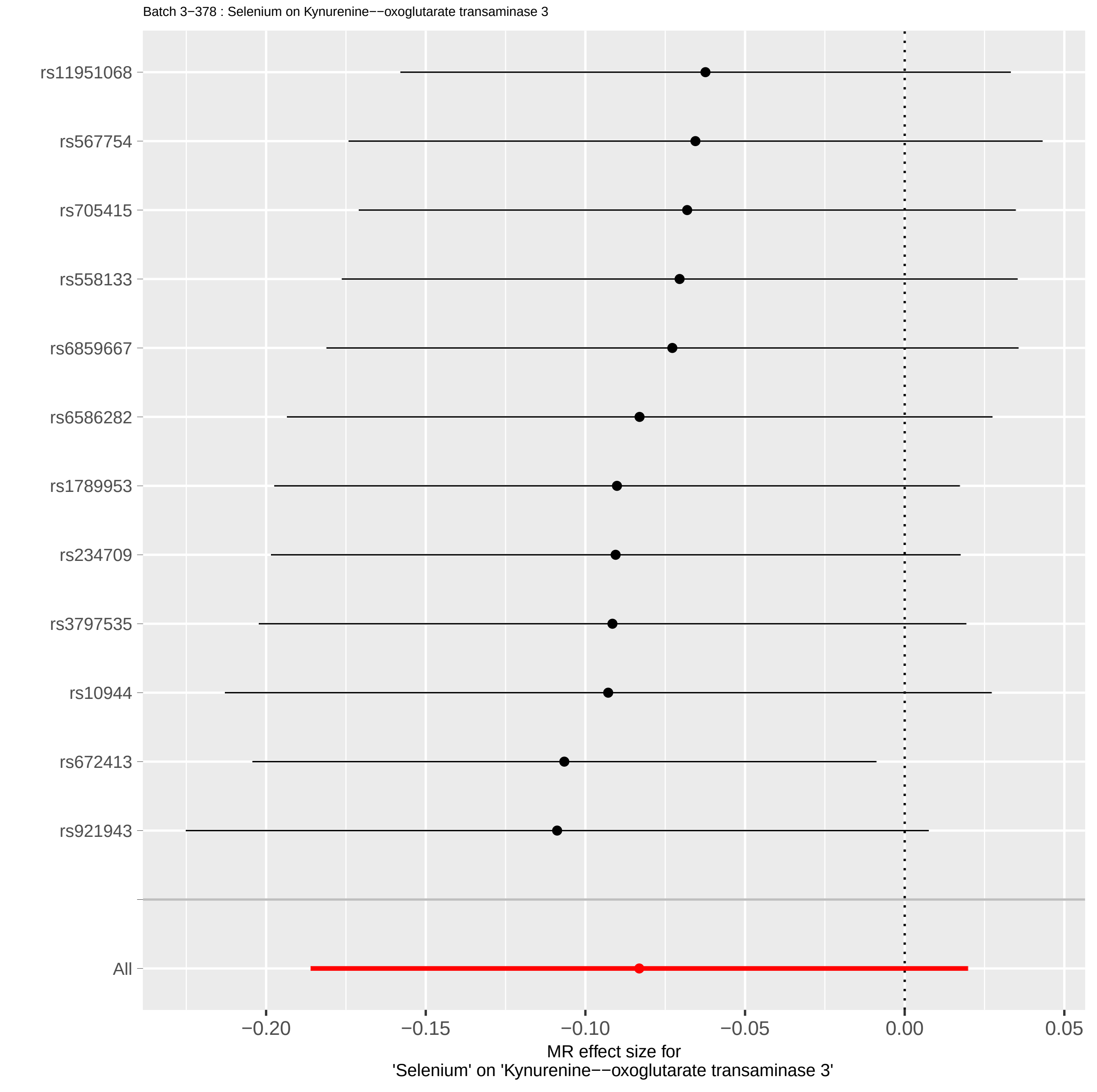

Batch 3−378 : Selenium on Kynurenine−−oxoglutarate transaminase 3
rs11951068
rs567754
rs705415
rs558133
rs6859667
rs6586282
rs1789953
rs234709
rs3797535
rs10944
rs672413
rs921943
All
−0.20
−0.15
−0.10
−0.05
0.00
0.05
MR effect size for
'Selenium' on 'Kynurenine−−oxoglutarate transaminase 3'

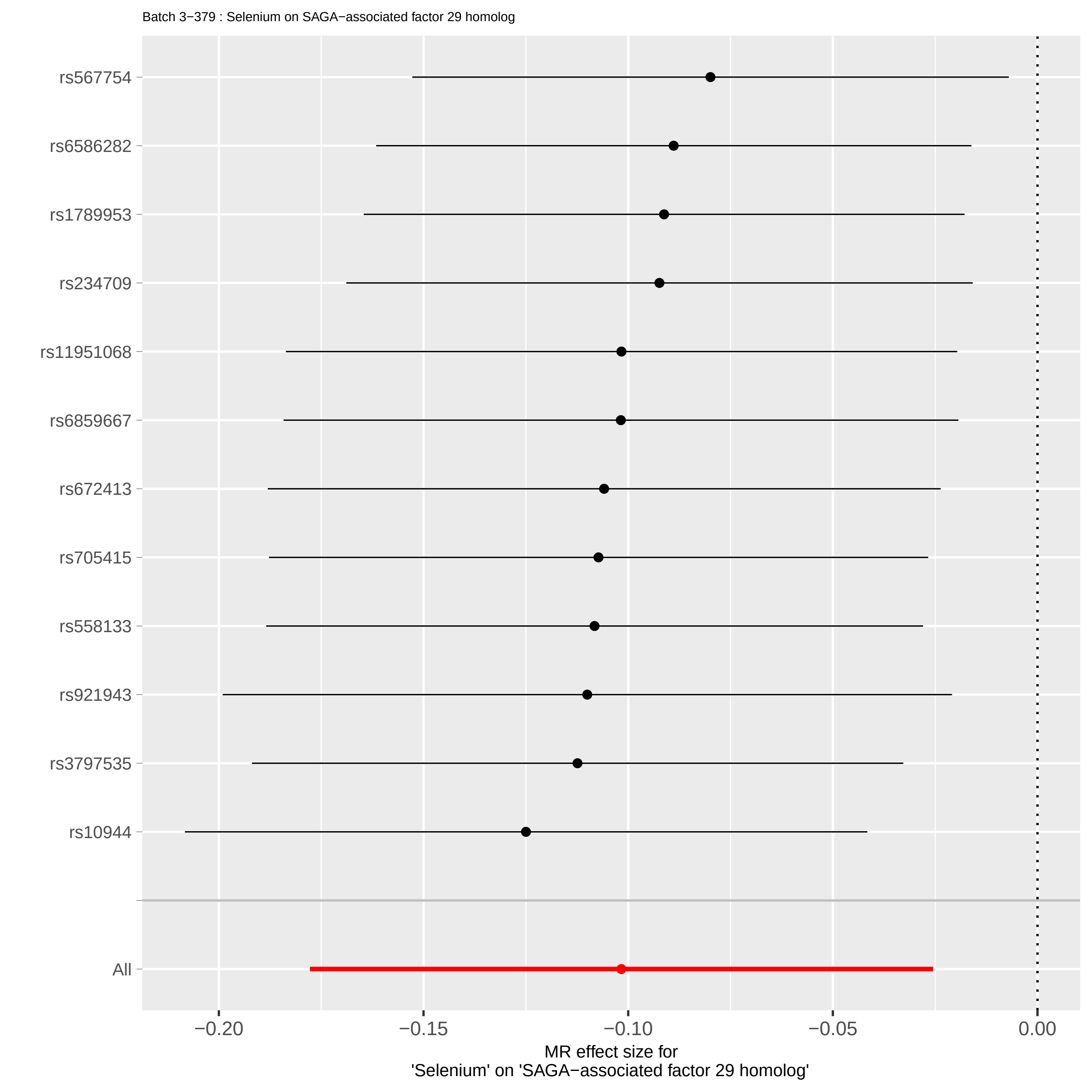
Batch 3−379 : Selenium on SAGA−associated factor 29 homolog
rs567754
rs6586282
rs1789953
rs234709
rs11951068
rs6859667
rs672413
rs705415
rs558133
rs921943
rs3797535
rs10944
All
−0.20
−0.15
−0.10
−0.05
0.00
MR effect size for
'Selenium' on 'SAGA−associated factor 29 homolog'

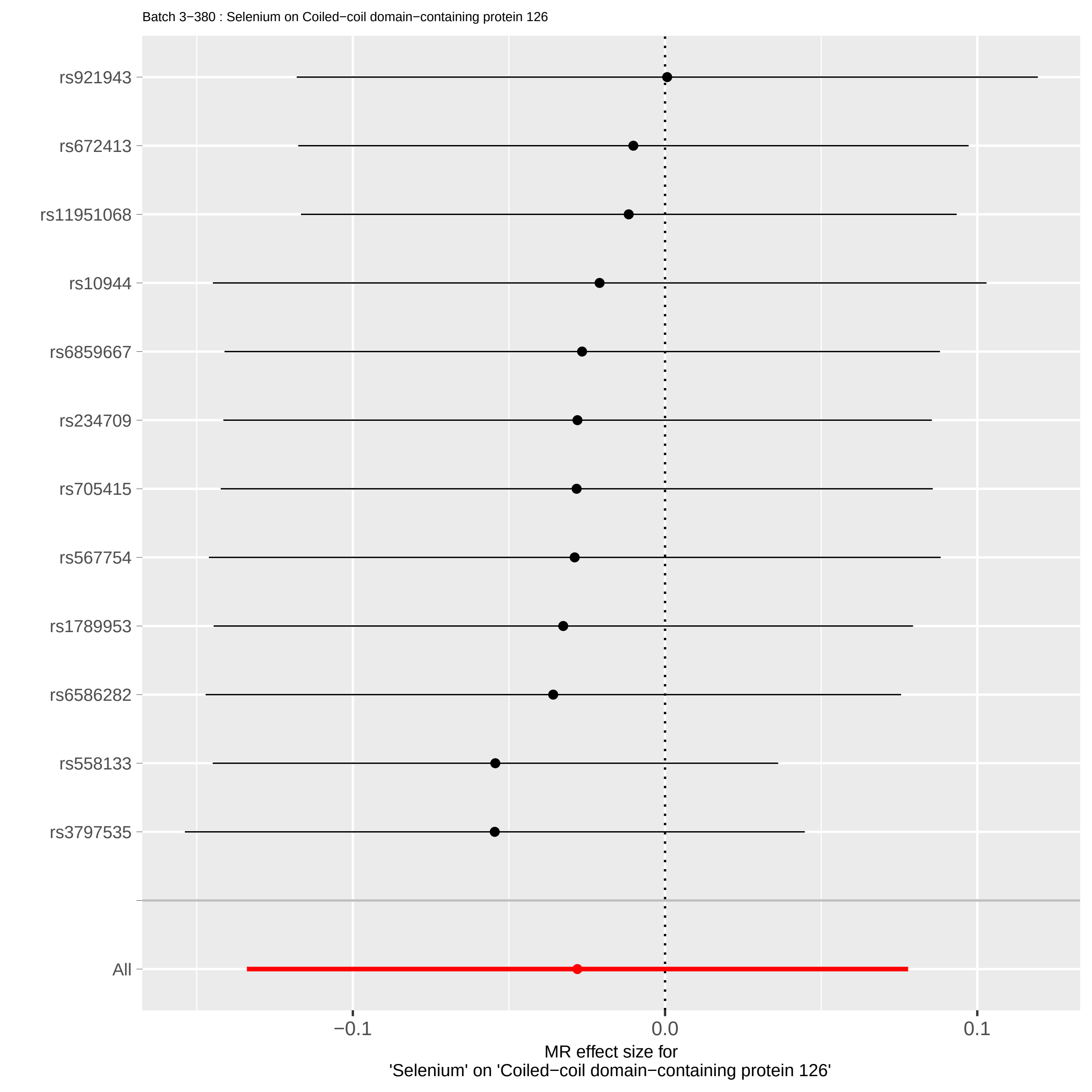

Batch 3−380 : Selenium on Coiled−coil domain−containing protein 126
rs921943
rs672413
rs11951068
rs10944
rs6859667
rs234709
rs705415
rs567754
rs1789953
rs6586282
rs558133
rs3797535
All
−0.1
0.0
0.1
MR effect size for
'Selenium' on 'Coiled−coil domain−containing protein 126'

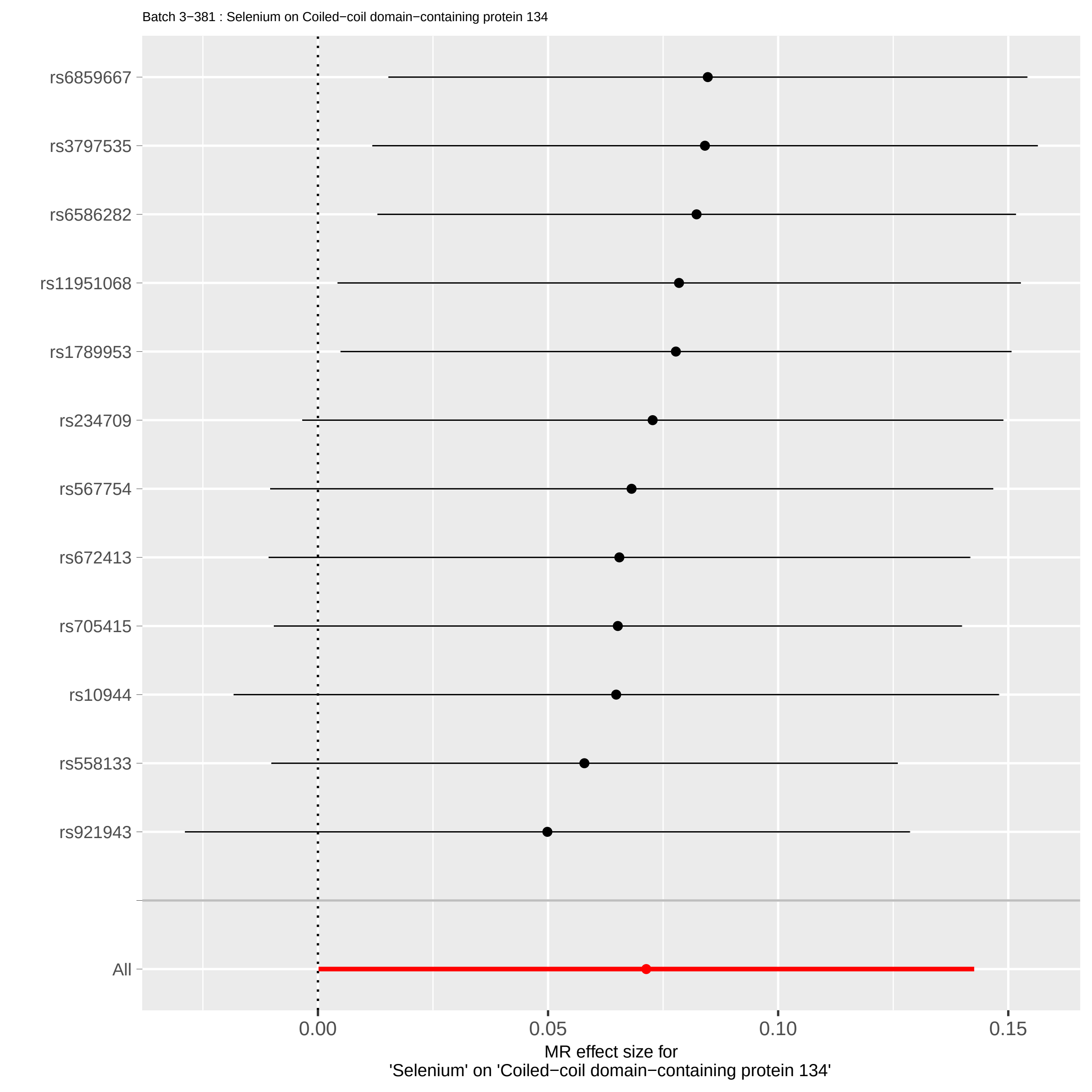
Batch 3−381 : Selenium on Coiled−coil domain−containing protein 134
rs6859667
rs3797535
rs6586282
rs11951068
rs1789953
rs234709
rs567754
rs672413
rs705415
rs10944
rs558133
rs921943
All
0.00
0.05
0.10
0.15
MR effect size for
'Selenium' on 'Coiled−coil domain−containing protein 134'

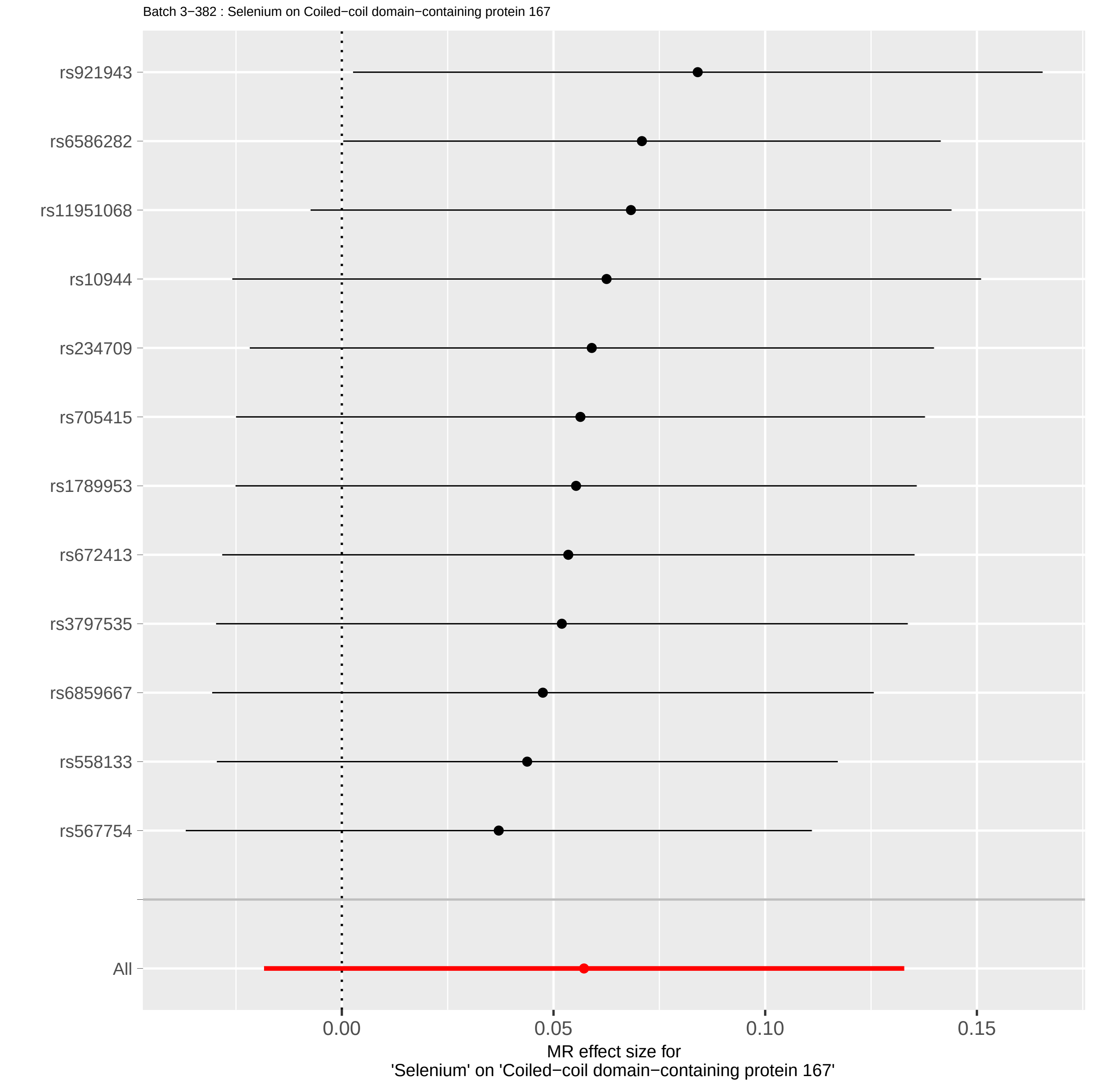

Batch 3−382 : Selenium on Coiled−coil domain−containing protein 167
rs921943
rs6586282
rs11951068
rs10944
rs234709
rs705415
rs1789953
rs672413
rs3797535
rs6859667
rs558133
rs567754
All
0.00
0.05
0.10
0.15
MR effect size for
'Selenium' on 'Coiled−coil domain−containing protein 167'

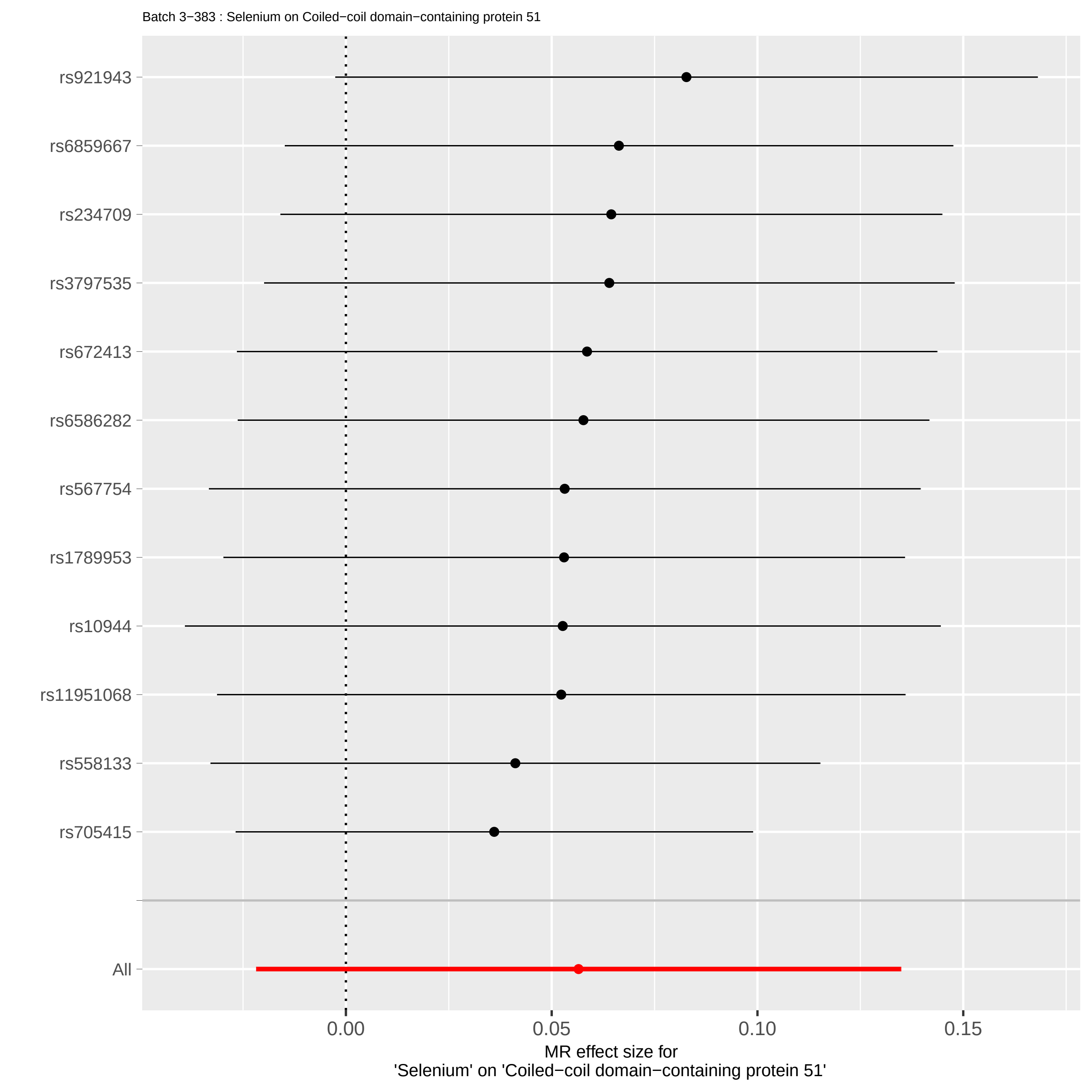

Batch 3−383 : Selenium on Coiled−coil domain−containing protein 51
rs921943
rs6859667
rs234709
rs3797535
rs672413
rs6586282
rs567754
rs1789953
rs10944
rs11951068
rs558133
rs705415
All
0.00
0.05
0.10
0.15
MR effect size for
'Selenium' on 'Coiled−coil domain−containing protein 51'

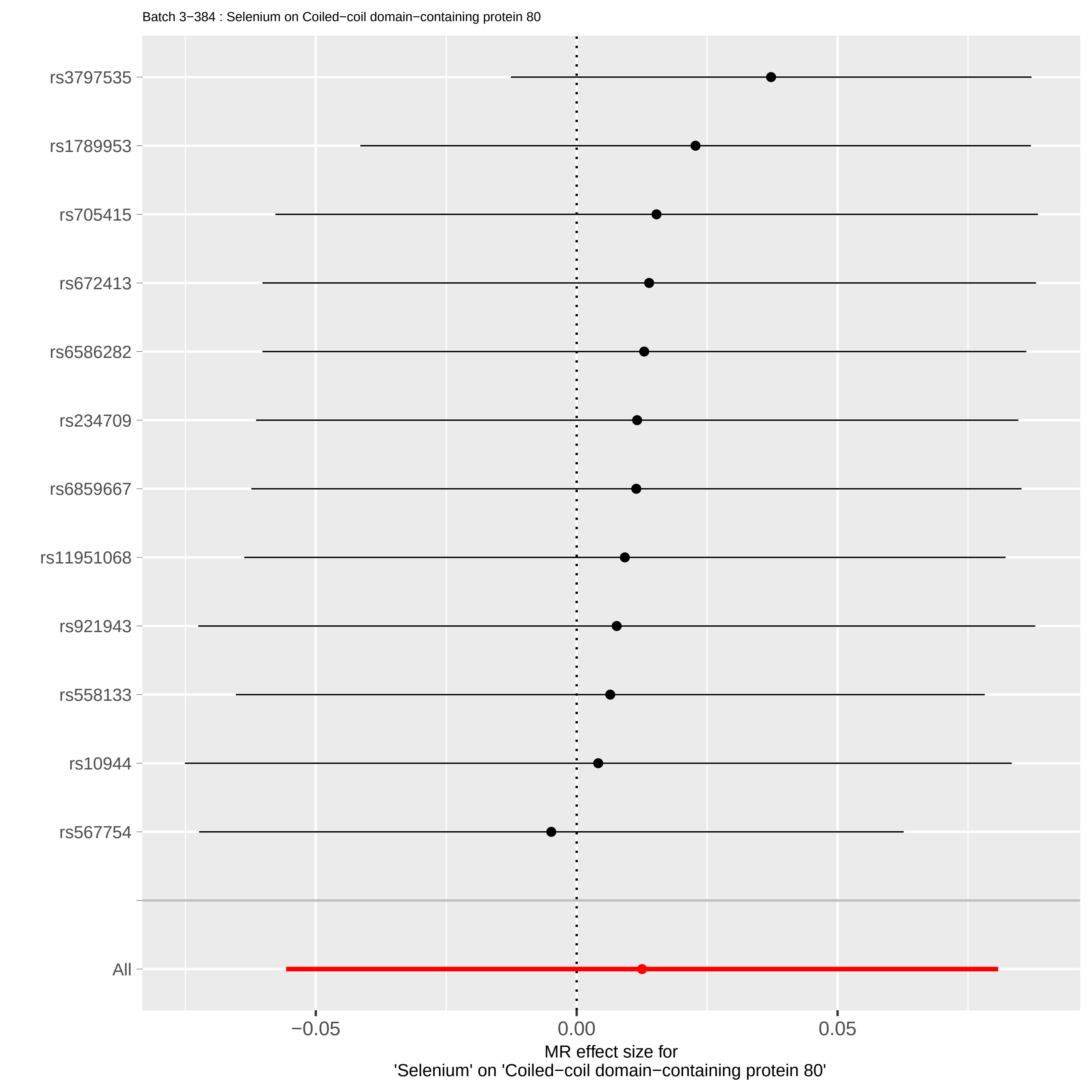

Batch 3−384 : Selenium on Coiled−coil domain−containing protein 80
rs3797535
rs1789953
rs705415
rs672413
rs6586282
rs234709
rs6859667
rs11951068
rs921943
rs558133
rs10944
rs567754
All
−0.05
0.00
0.05
MR effect size for
'Selenium' on 'Coiled−coil domain−containing protein 80'

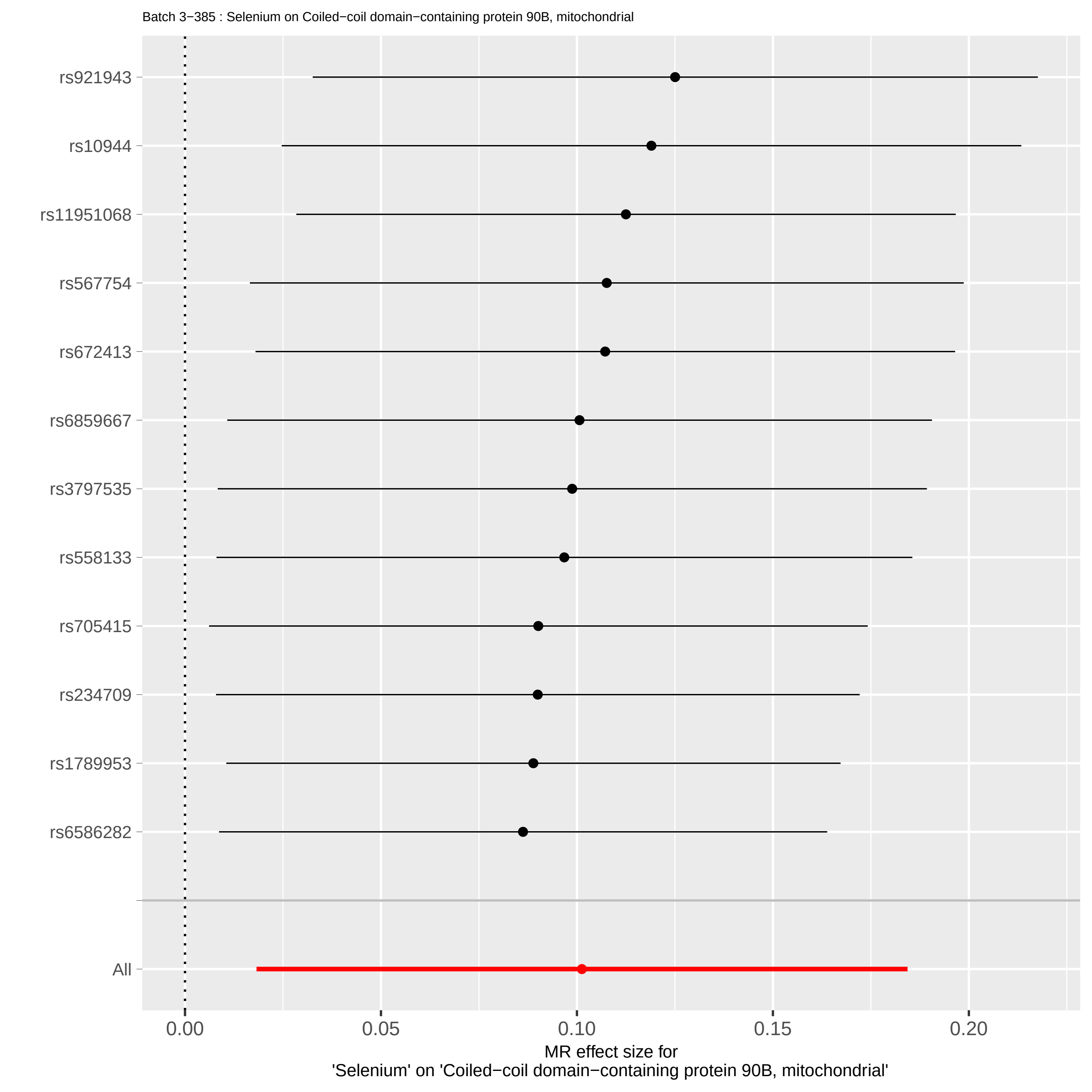

Batch 3−385 : Selenium on Coiled−coil domain−containing protein 90B, mitochondrial
rs921943
rs10944
rs11951068
rs567754
rs672413
rs6859667
rs3797535
rs558133
rs705415
rs234709
rs1789953
rs6586282
All
0.00
0.05
0.10
0.15
0.20
MR effect size for
'Selenium' on 'Coiled−coil domain−containing protein 90B, mitochondrial'

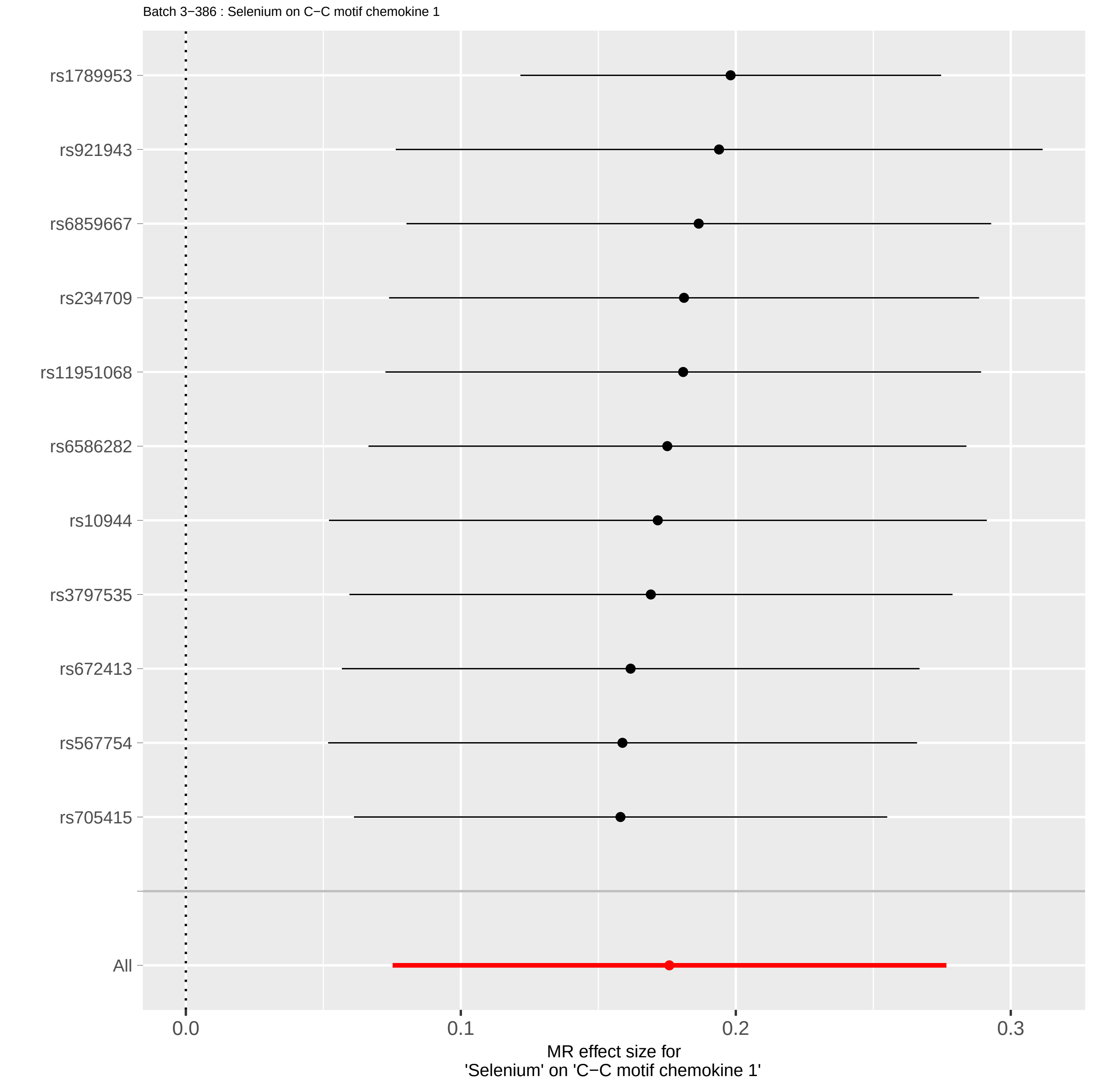

Batch 3−386 : Selenium on C−C motif chemokine 1
rs1789953
rs921943
rs6859667
rs234709
rs11951068
rs6586282
rs10944
rs3797535
rs672413
rs567754
rs705415
All
0.0
0.1
0.2
0.3
MR effect size for
'Selenium' on 'C−C motif chemokine 1'

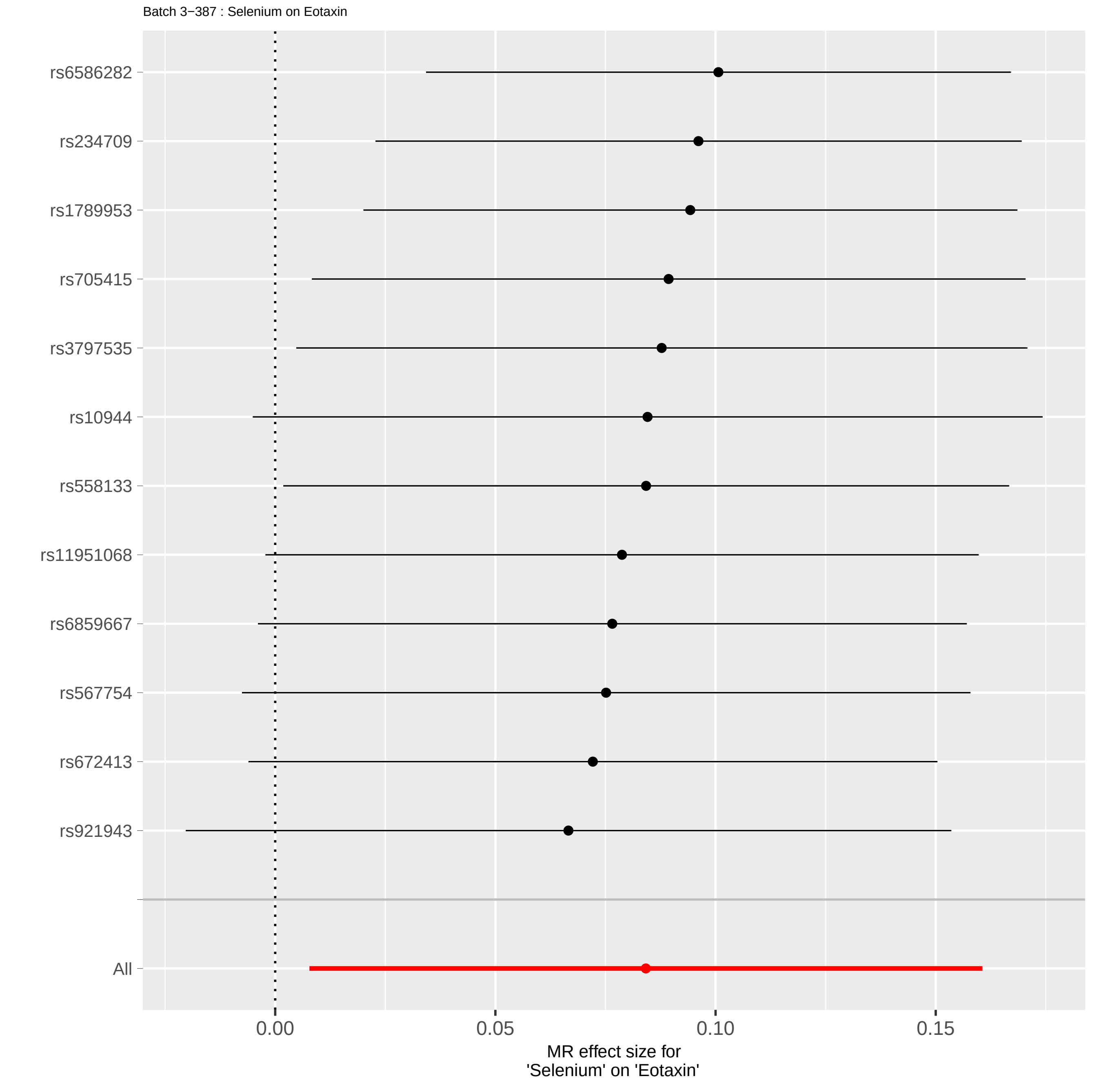

Batch 3−387 : Selenium on Eotaxin
rs6586282
rs234709
rs1789953
rs705415
rs3797535
rs10944
rs558133
rs11951068
rs6859667
rs567754
rs672413
rs921943
All
0.00
0.05
0.10
0.15
MR effect size for
'Selenium' on 'Eotaxin'

Batch 3−388 : Selenium on C−C motif chemokine 1

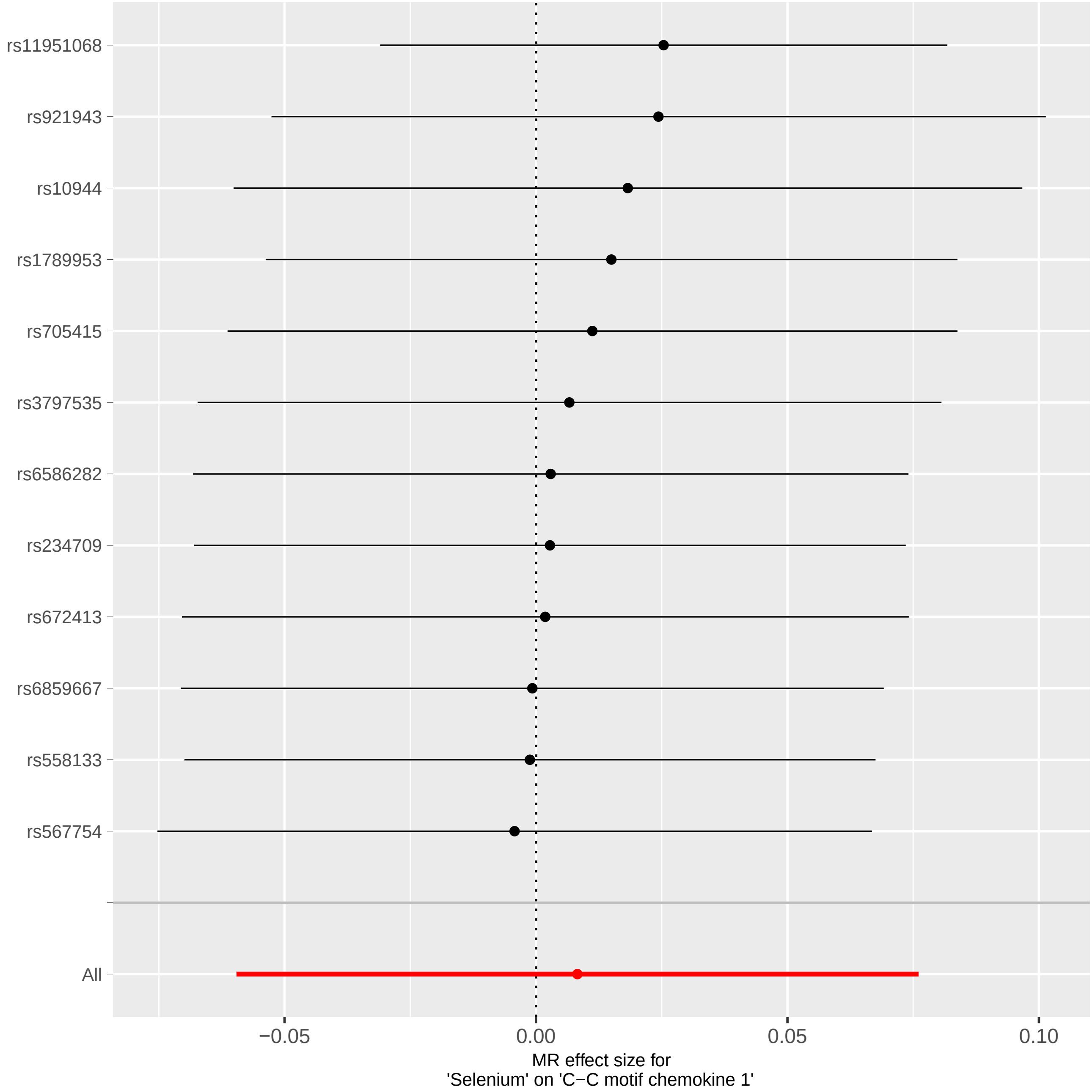

Batch 3−389 : Selenium on C−C motif chemokine 13

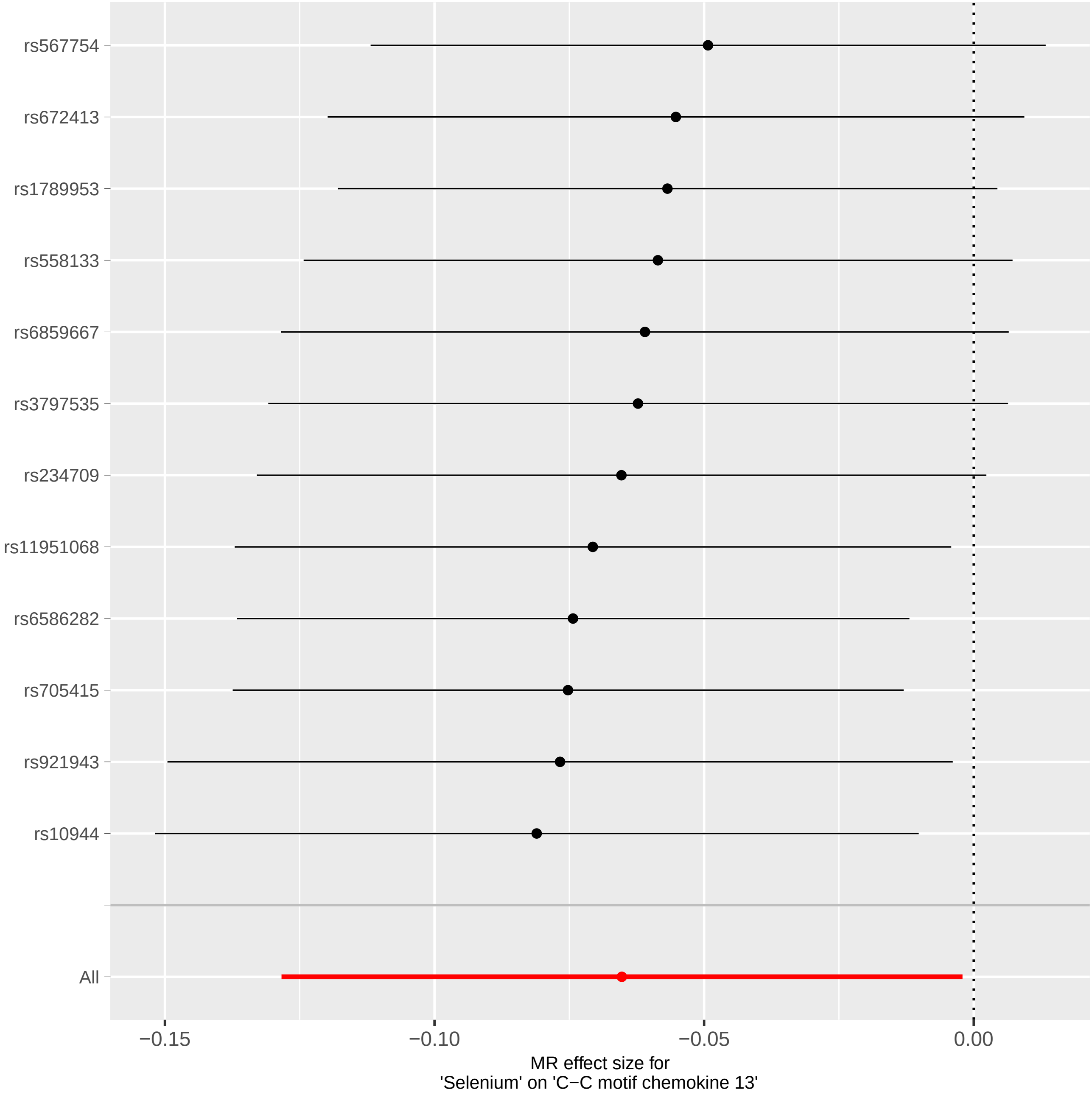

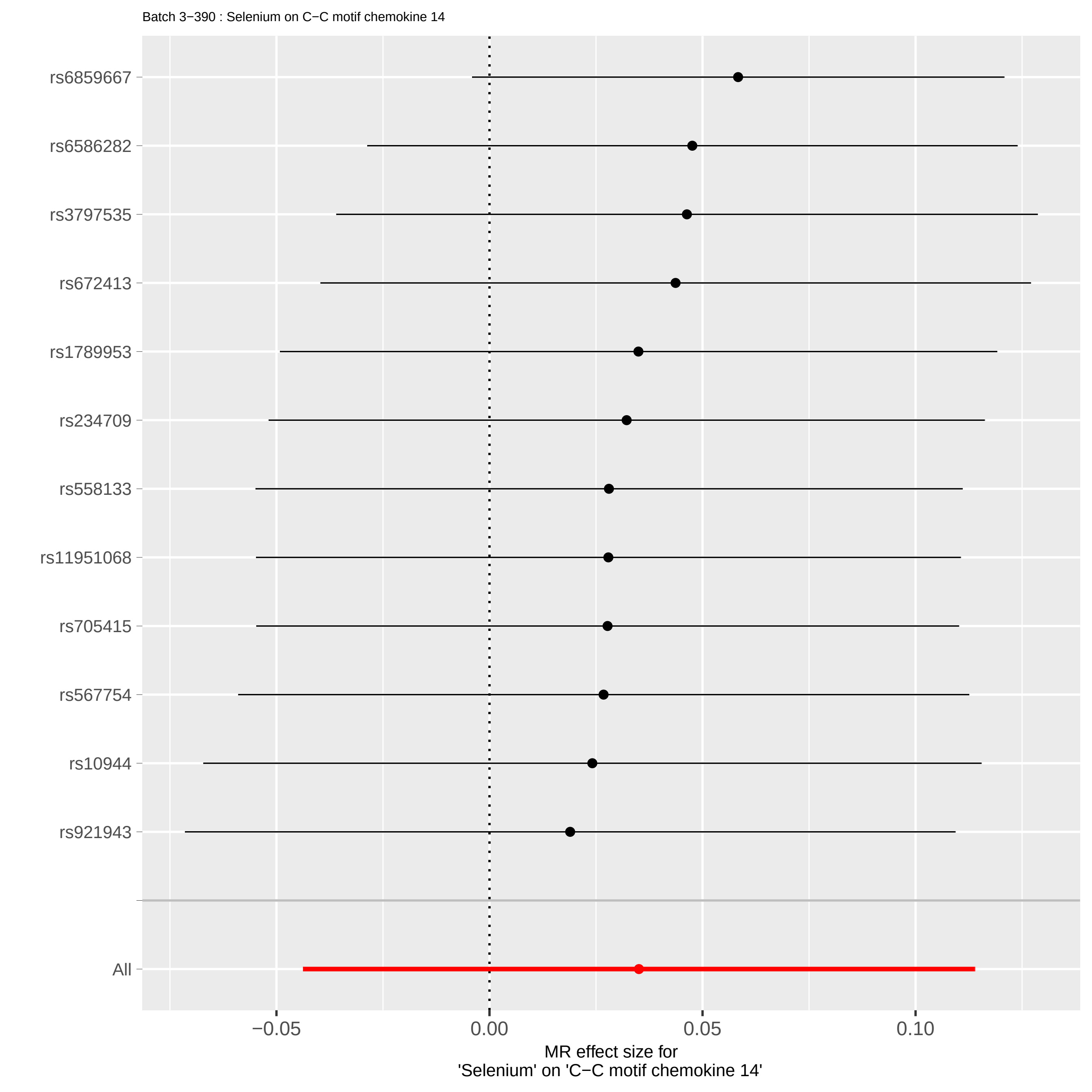
Batch 3−390 : Selenium on C−C motif chemokine 14
rs6859667
rs6586282
rs3797535
rs672413
rs1789953
rs234709
rs558133
rs11951068
rs705415
rs567754
rs10944
rs921943
All
−0.05
0.00
0.05
0.10
MR effect size for
'Selenium' on 'C−C motif chemokine 14'

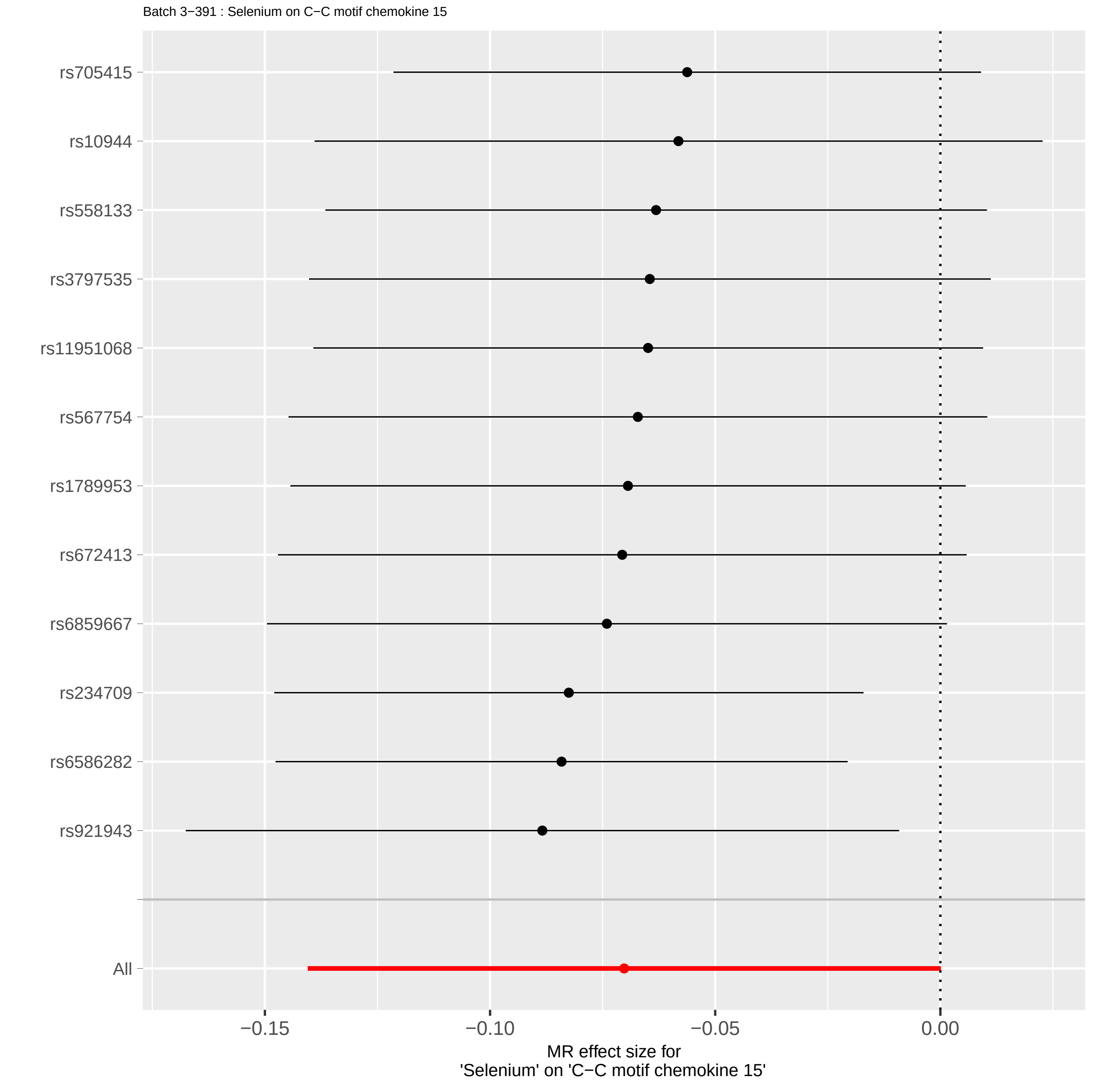

Batch 3−391 : Selenium on C−C motif chemokine 15
rs705415
rs10944
rs558133
rs3797535
rs11951068
rs567754
rs1789953
rs672413
rs6859667
rs234709
rs6586282
rs921943
All
−0.15
−0.10
−0.05
0.00
MR effect size for
'Selenium' on 'C−C motif chemokine 15'

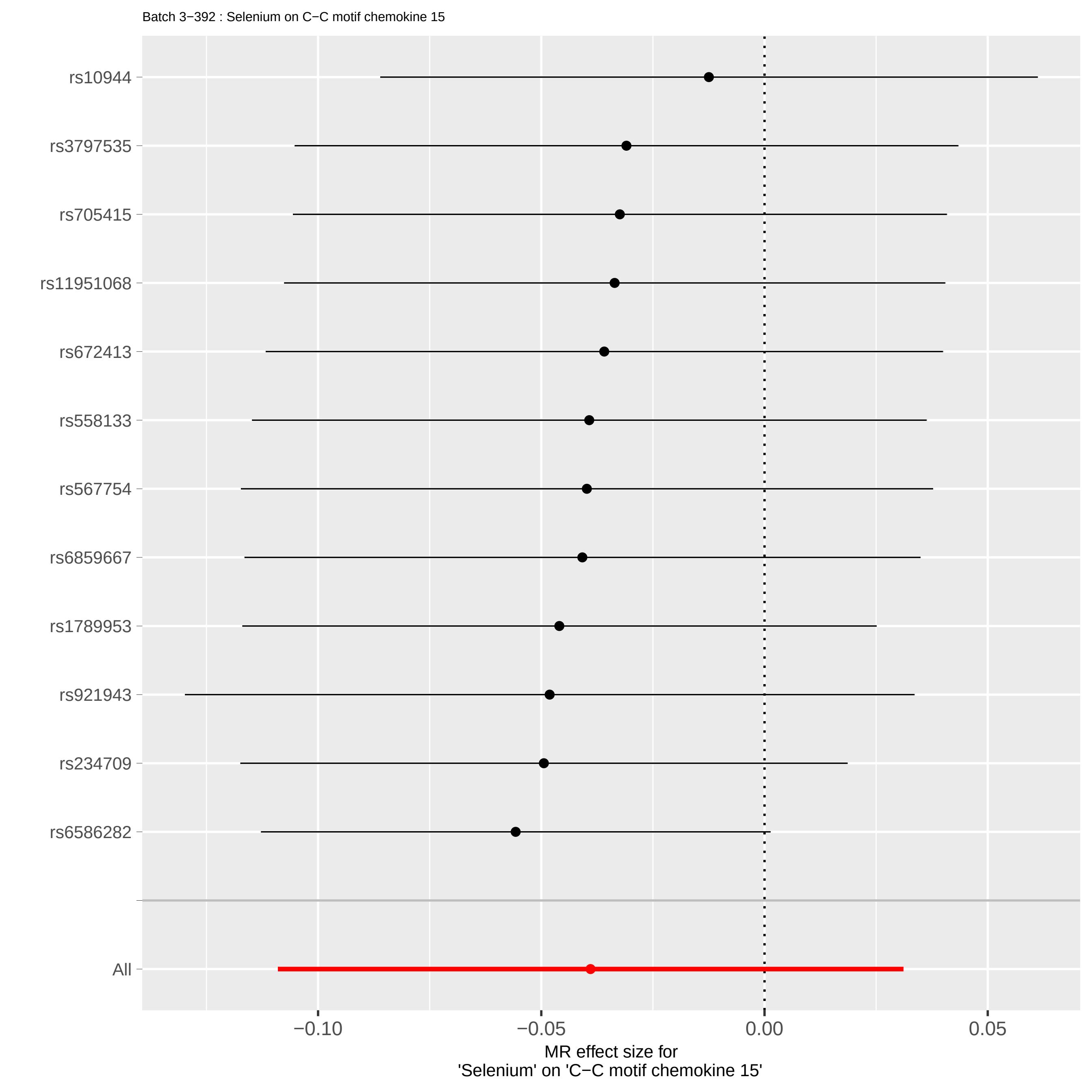
Batch 3−392 : Selenium on C−C motif chemokine 15
rs10944
rs3797535
rs705415
rs11951068
rs672413
rs558133
rs567754
rs6859667
rs1789953
rs921943
rs234709
rs6586282
All
−0.10
−0.05
0.00
0.05
MR effect size for
'Selenium' on 'C−C motif chemokine 15'

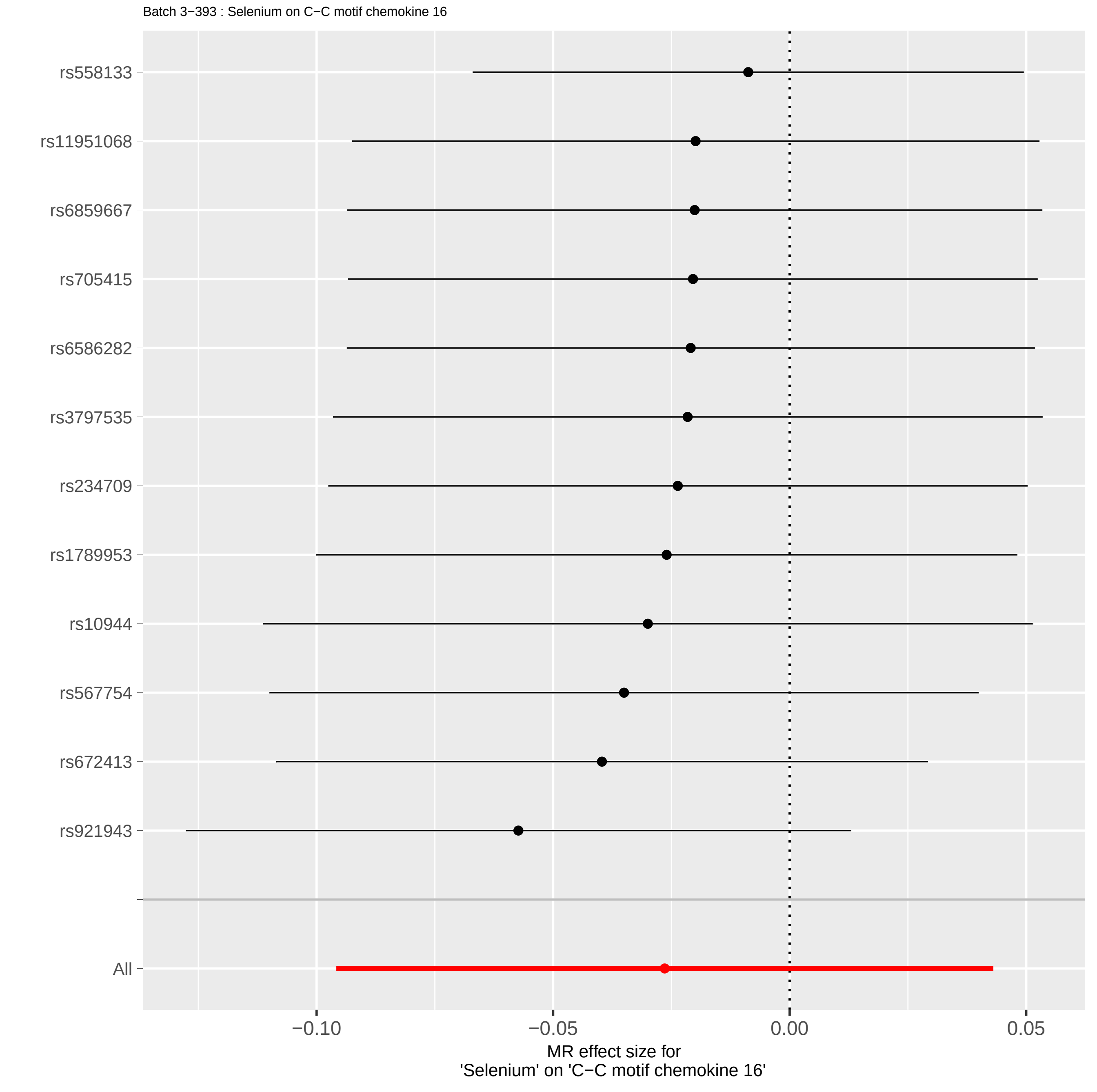

Batch 3−393 : Selenium on C−C motif chemokine 16
rs558133
rs11951068
rs6859667
rs705415
rs6586282
rs3797535
rs234709
rs1789953
rs10944
rs567754
rs672413
rs921943
All
−0.10
−0.05
0.00
0.05
MR effect size for
'Selenium' on 'C−C motif chemokine 16'

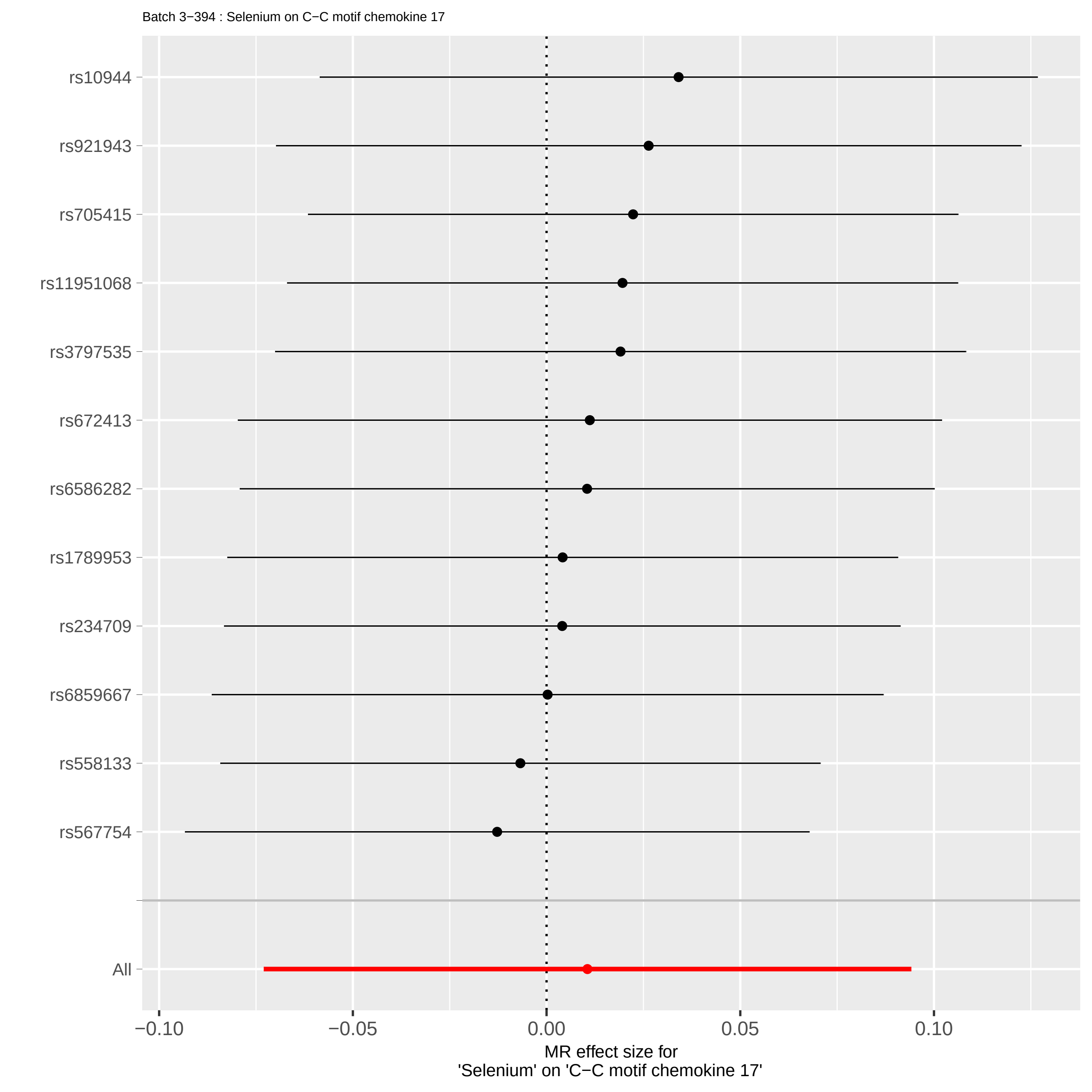

Batch 3−394 : Selenium on C−C motif chemokine 17
rs10944
rs921943
rs705415
rs11951068
rs3797535
rs672413
rs6586282
rs1789953
rs234709
rs6859667
rs558133
rs567754
All
−0.10
−0.05
0.00
0.05
0.10
MR effect size for
'Selenium' on 'C−C motif chemokine 17'

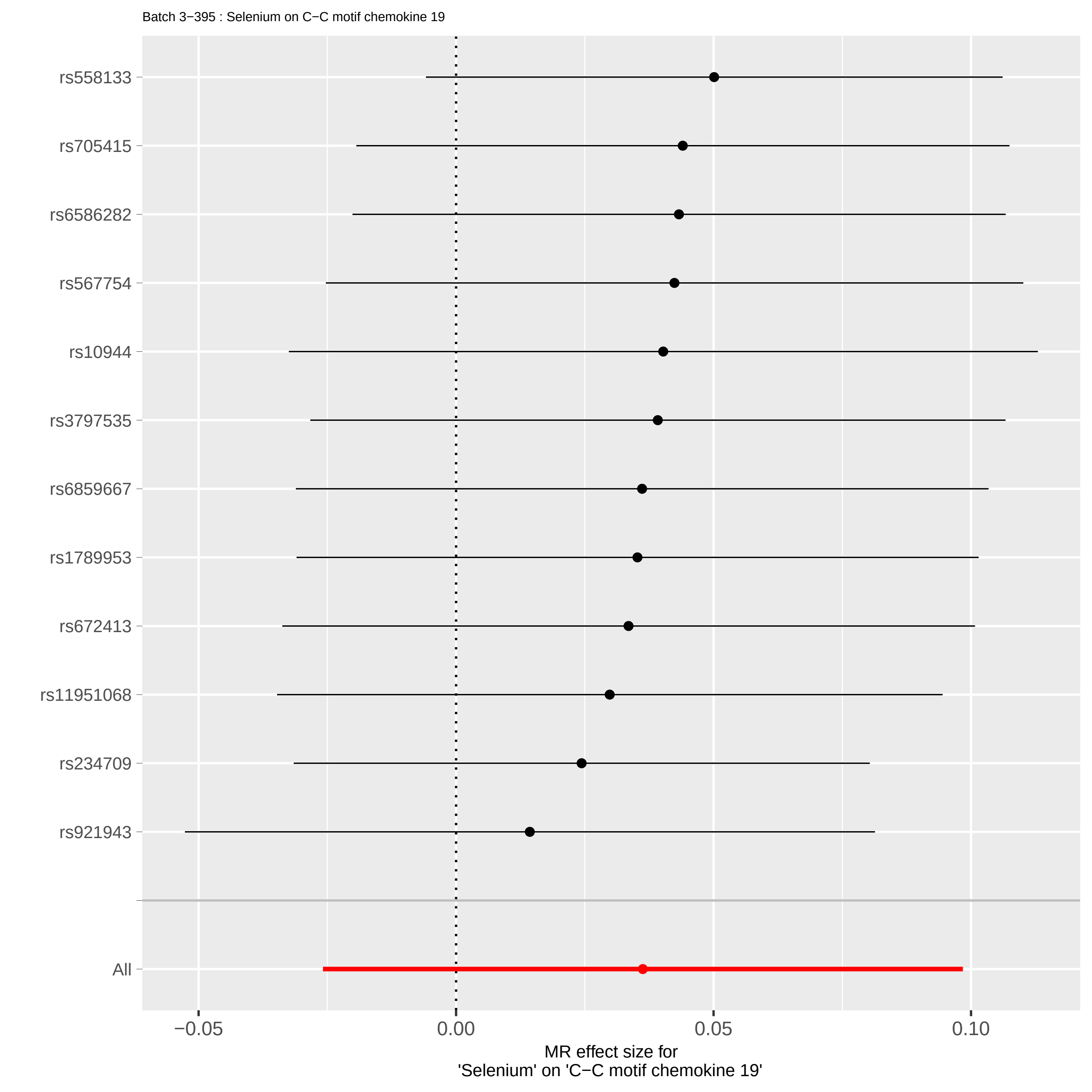

Batch 3−395 : Selenium on C−C motif chemokine 19
rs558133
rs705415
rs6586282
rs567754
rs10944
rs3797535
rs6859667
rs1789953
rs672413
rs11951068
rs234709
rs921943
All
−0.05
0.00
0.05
0.10
MR effect size for
'Selenium' on 'C−C motif chemokine 19'

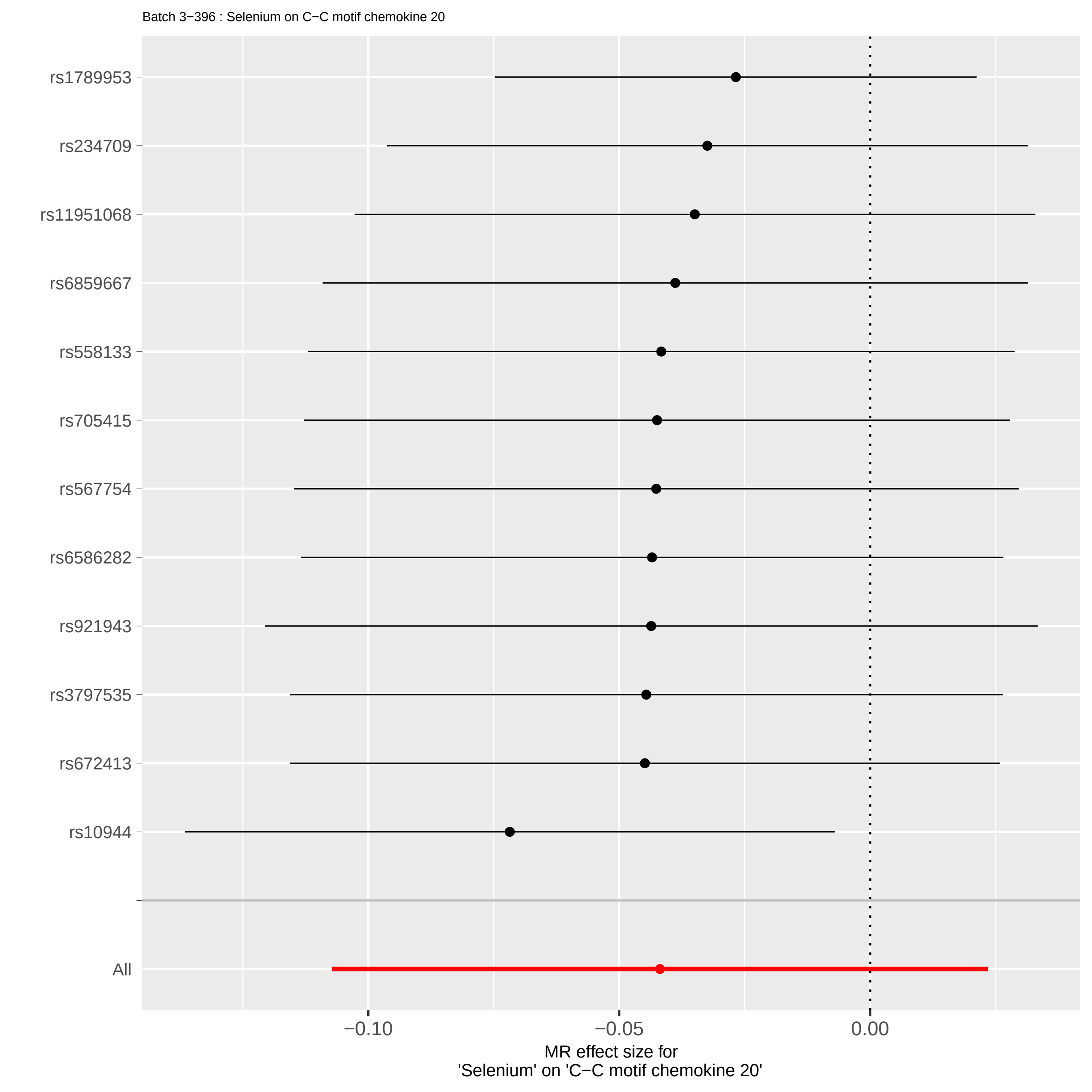
Batch 3−396 : Selenium on C−C motif chemokine 20
rs1789953
rs234709
rs11951068
rs6859667
rs558133
rs705415
rs567754
rs6586282
rs921943
rs3797535
rs672413
rs10944
All
−0.10
−0.05
0.00
MR effect size for
'Selenium' on 'C−C motif chemokine 20'

Batch 3−397 : Selenium on C−C motif chemokine 21

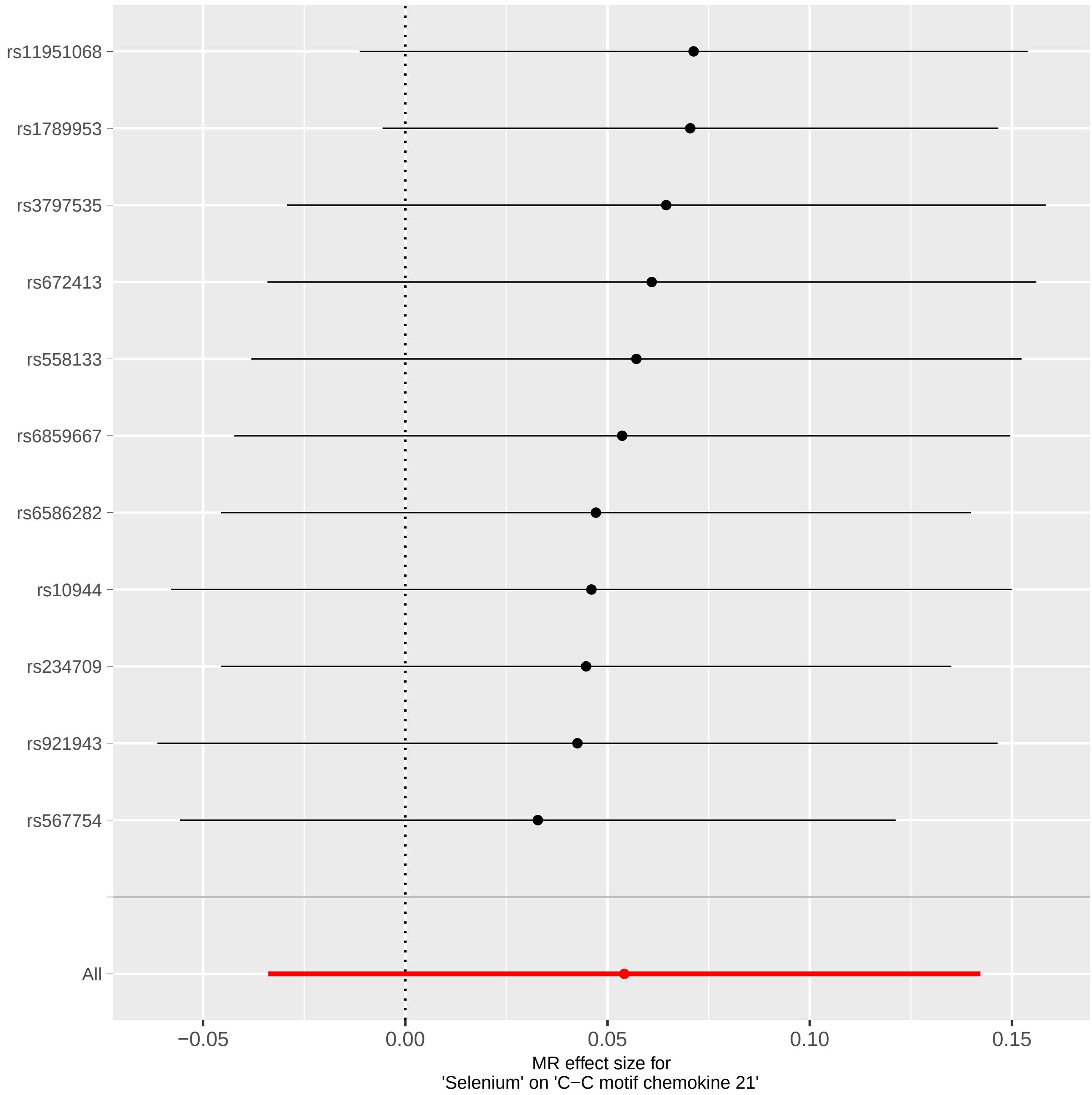

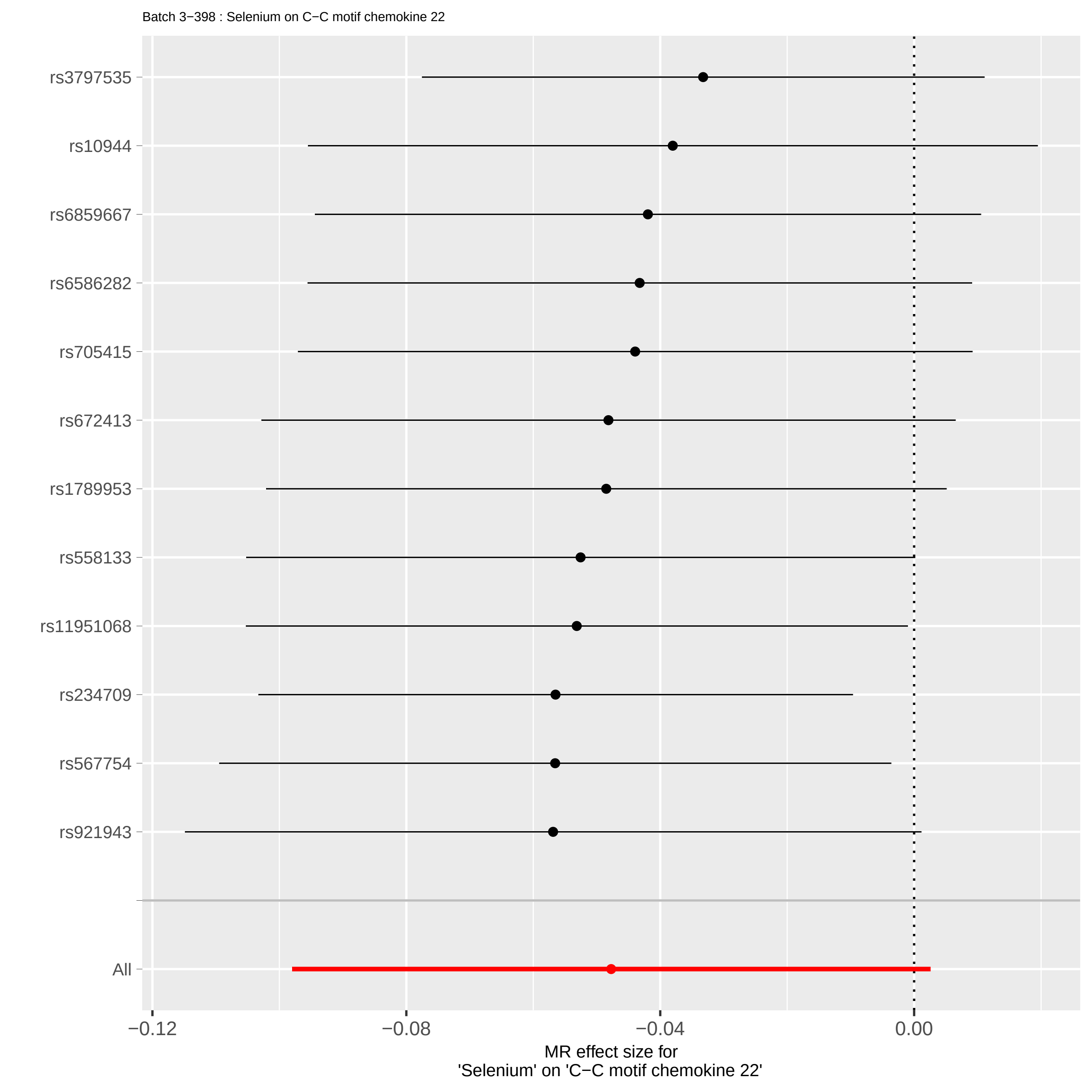
Batch 3−398 : Selenium on C−C motif chemokine 22
rs3797535
rs10944
rs6859667
rs6586282
rs705415
rs672413
rs1789953
rs558133
rs11951068
rs234709
rs567754
rs921943
All
−0.12
−0.08
−0.04
0.00
MR effect size for
'Selenium' on 'C−C motif chemokine 22'

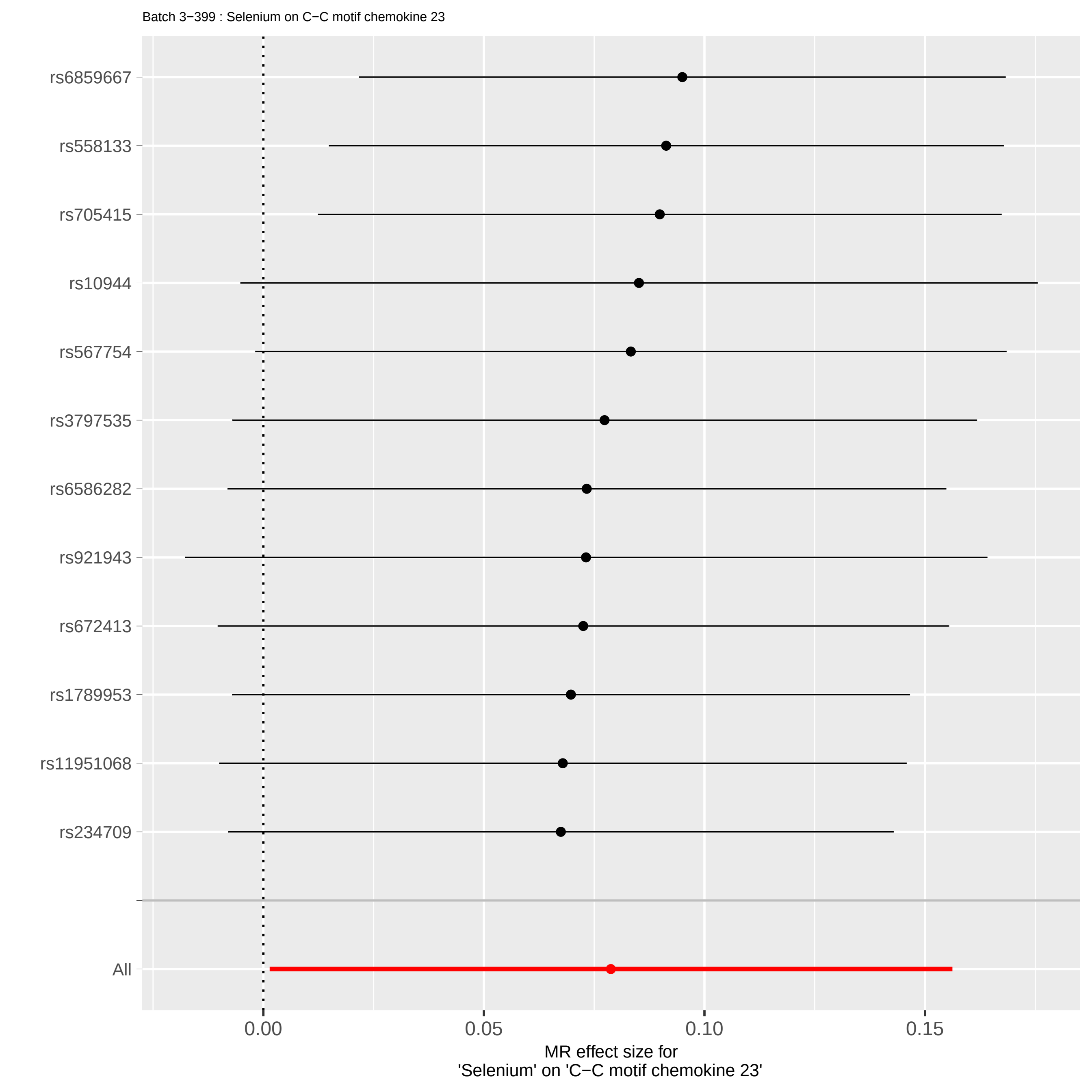

Batch 3−399 : Selenium on C−C motif chemokine 23
rs6859667
rs558133
rs705415
rs10944
rs567754
rs3797535
rs6586282
rs921943
rs672413
rs1789953
rs11951068
rs234709
All
0.00
0.05
0.10
0.15
MR effect size for
'Selenium' on 'C−C motif chemokine 23'

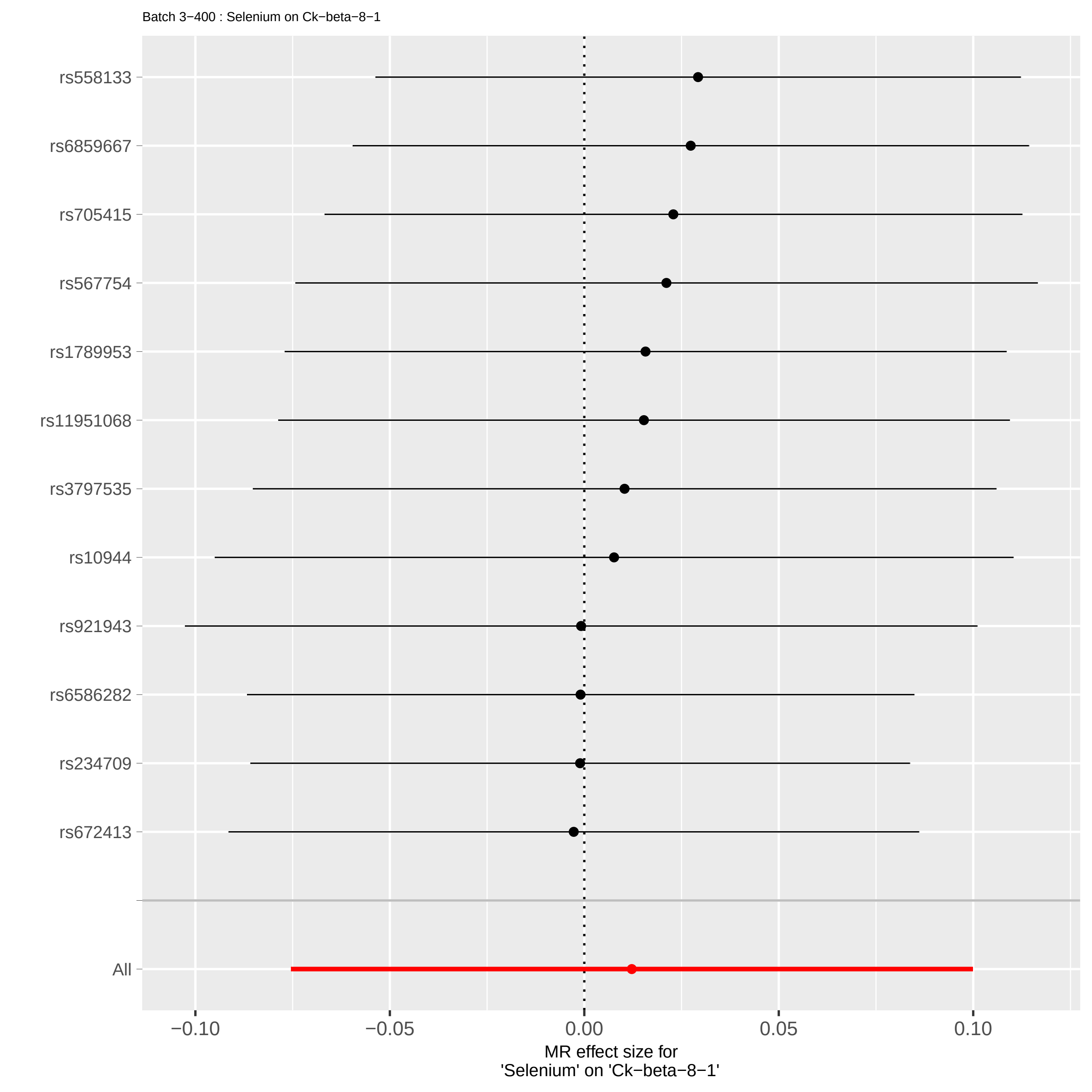

Batch 3−400 : Selenium on Ck−beta−8−1
rs558133
rs6859667
rs705415
rs567754
rs1789953
rs11951068
rs3797535
rs10944
rs921943
rs6586282
rs234709
rs672413
All
−0.10
−0.05
0.00
0.05
0.10
MR effect size for
'Selenium' on 'Ck−beta−8−1'

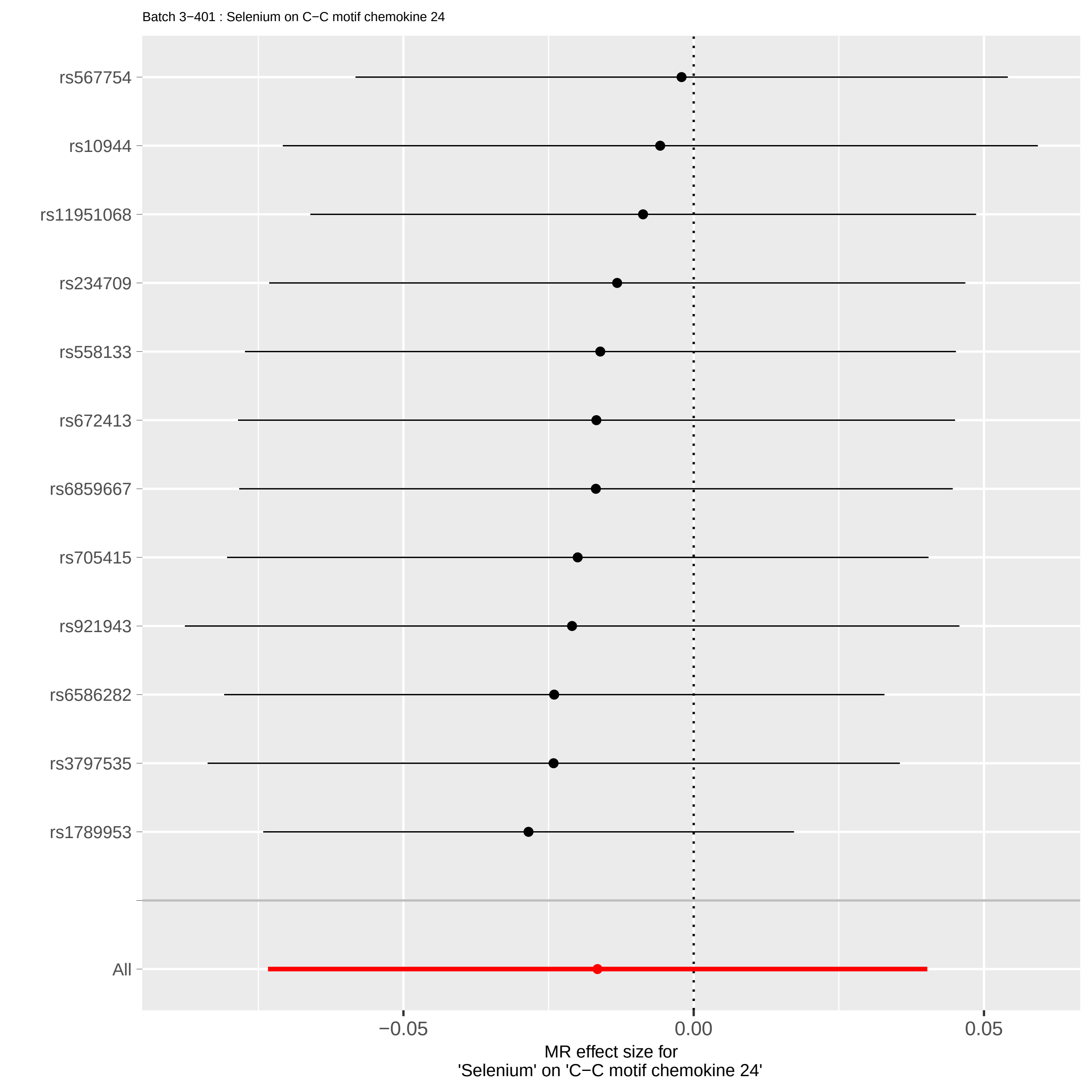
Batch 3−401 : Selenium on C−C motif chemokine 24
rs567754
rs10944
rs11951068
rs234709
rs558133
rs672413
rs6859667
rs705415
rs921943
rs6586282
rs3797535
rs1789953
All
−0.05
0.00
0.05
MR effect size for
'Selenium' on 'C−C motif chemokine 24'

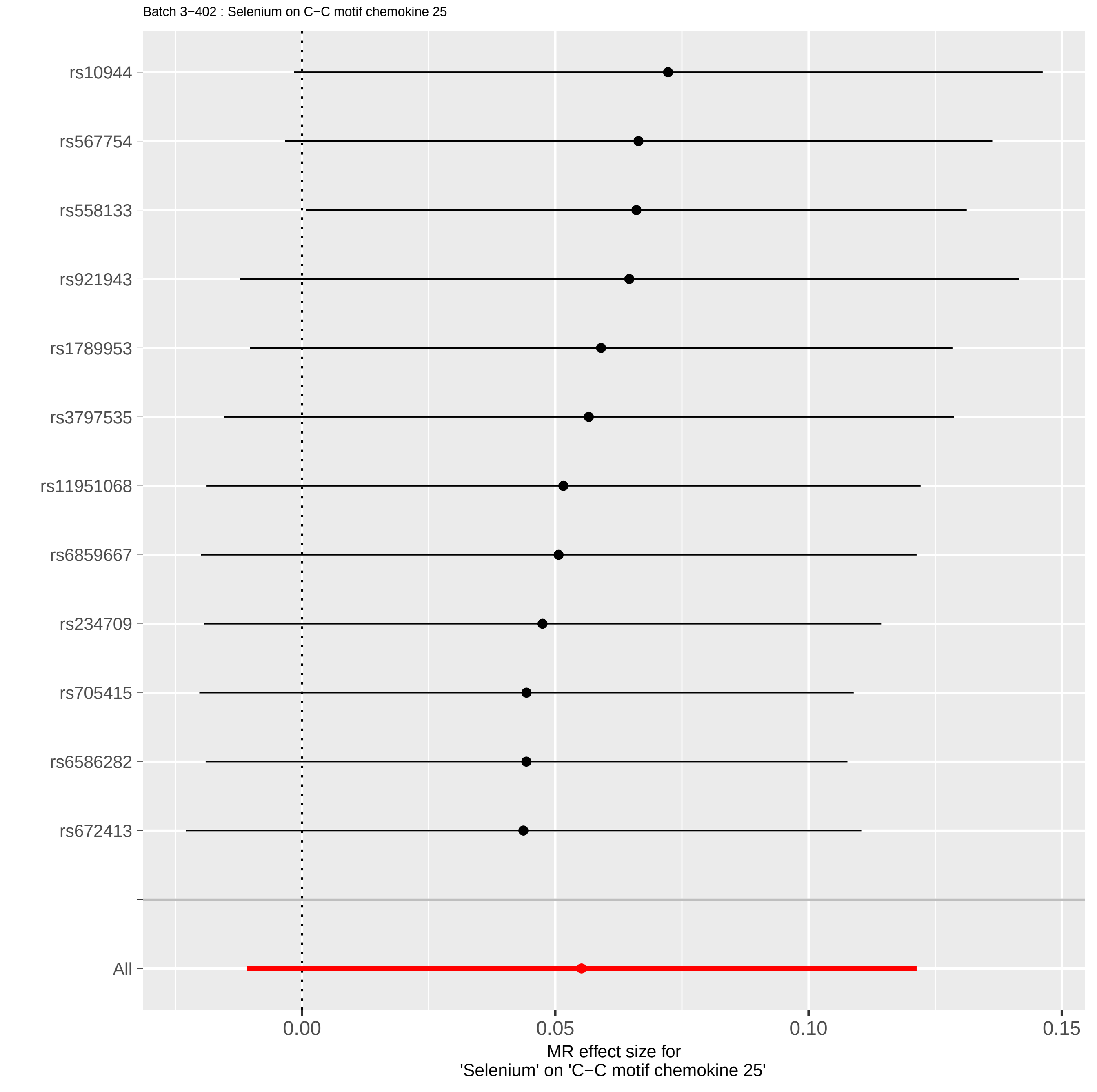

Batch 3−402 : Selenium on C−C motif chemokine 25
rs10944
rs567754
rs558133
rs921943
rs1789953
rs3797535
rs11951068
rs6859667
rs234709
rs705415
rs6586282
rs672413
All
0.00
0.05
0.10
0.15
MR effect size for
'Selenium' on 'C−C motif chemokine 25'

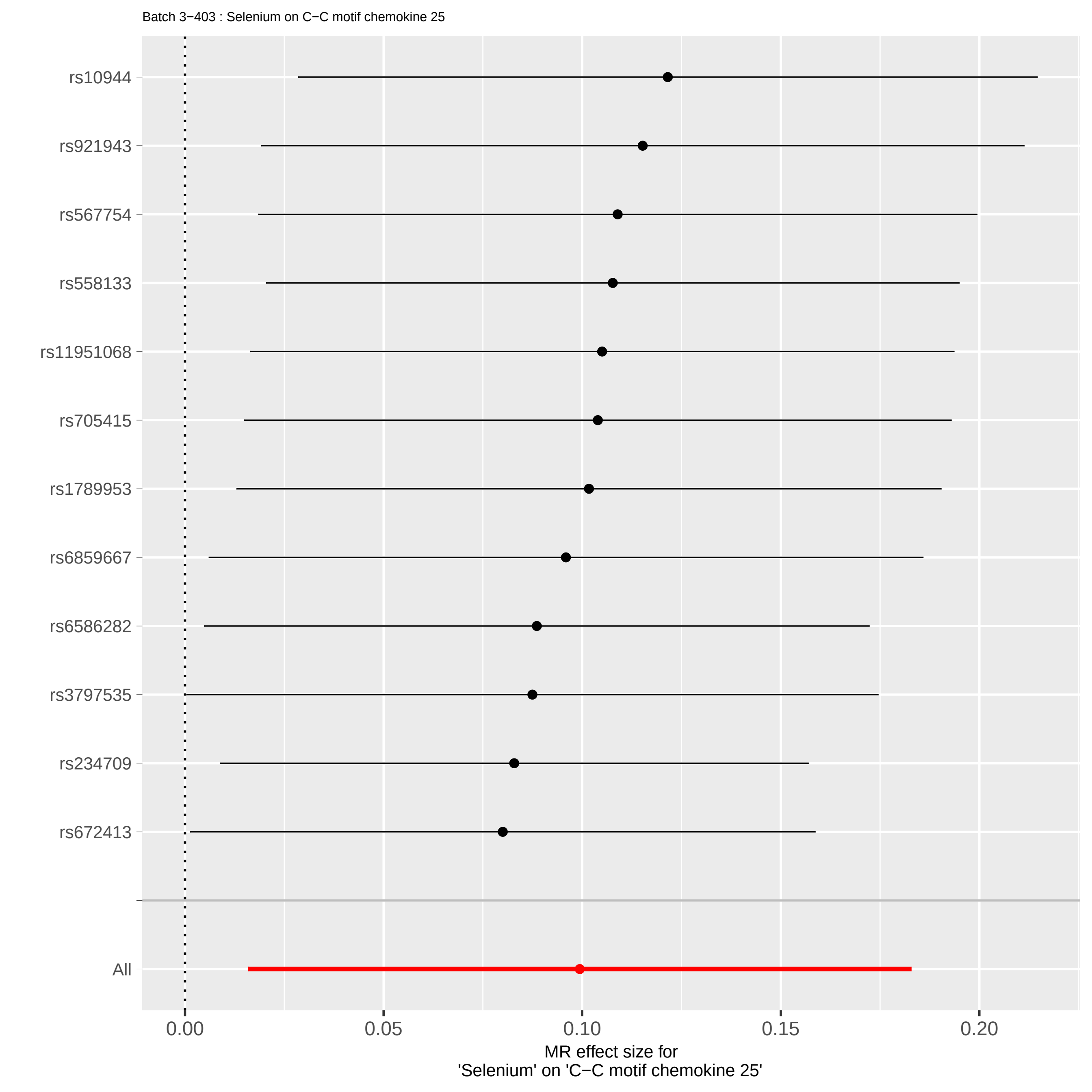

Batch 3−403 : Selenium on C−C motif chemokine 25
rs10944
rs921943
rs567754
rs558133
rs11951068
rs705415
rs1789953
rs6859667
rs6586282
rs3797535
rs234709
rs672413
All
0.00
0.05
0.10
0.15
0.20
MR effect size for
'Selenium' on 'C−C motif chemokine 25'

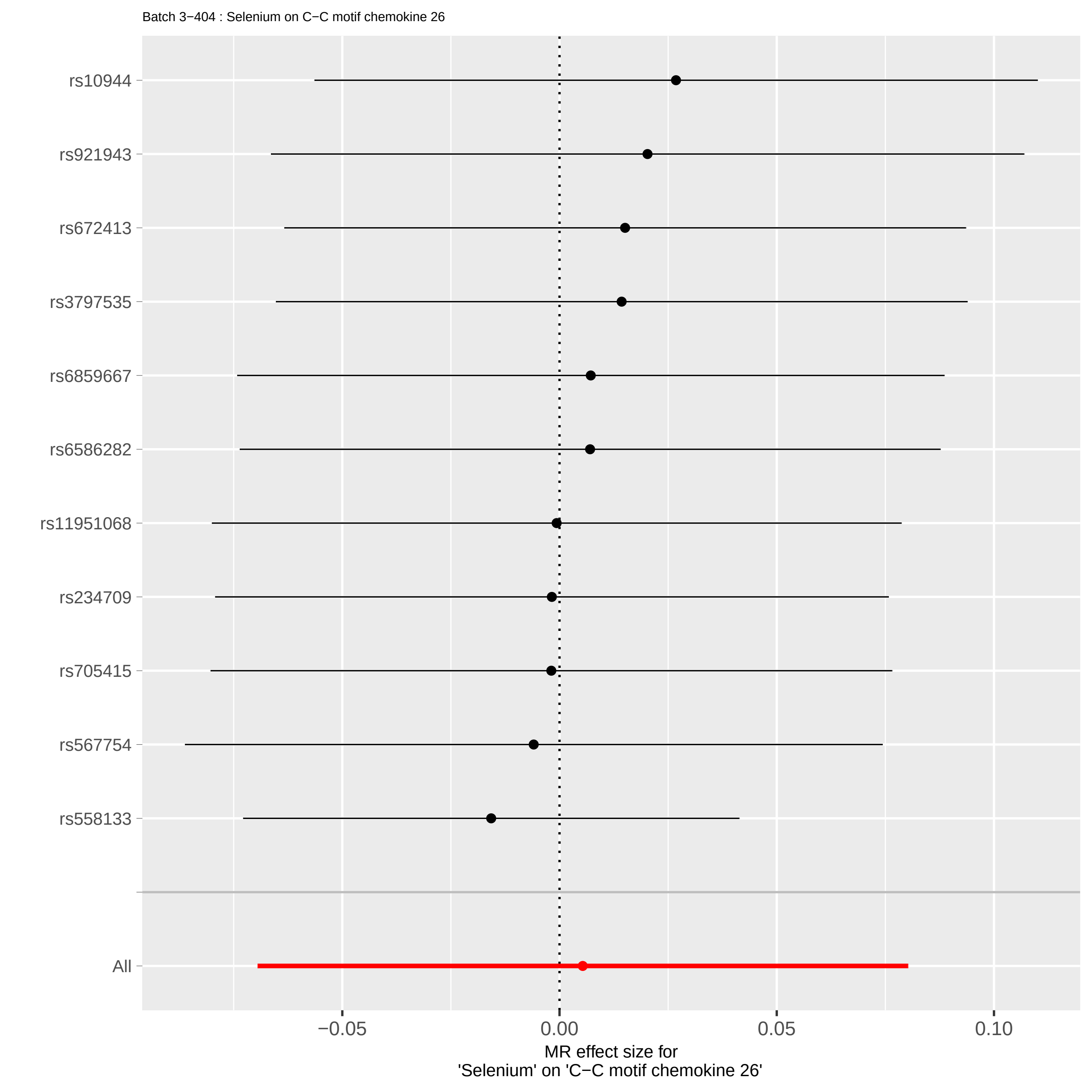

Batch 3−404 : Selenium on C−C motif chemokine 26
rs10944
rs921943
rs672413
rs3797535
rs6859667
rs6586282
rs11951068
rs234709
rs705415
rs567754
rs558133
All
−0.05
0.00
0.05
0.10
MR effect size for
'Selenium' on 'C−C motif chemokine 26'

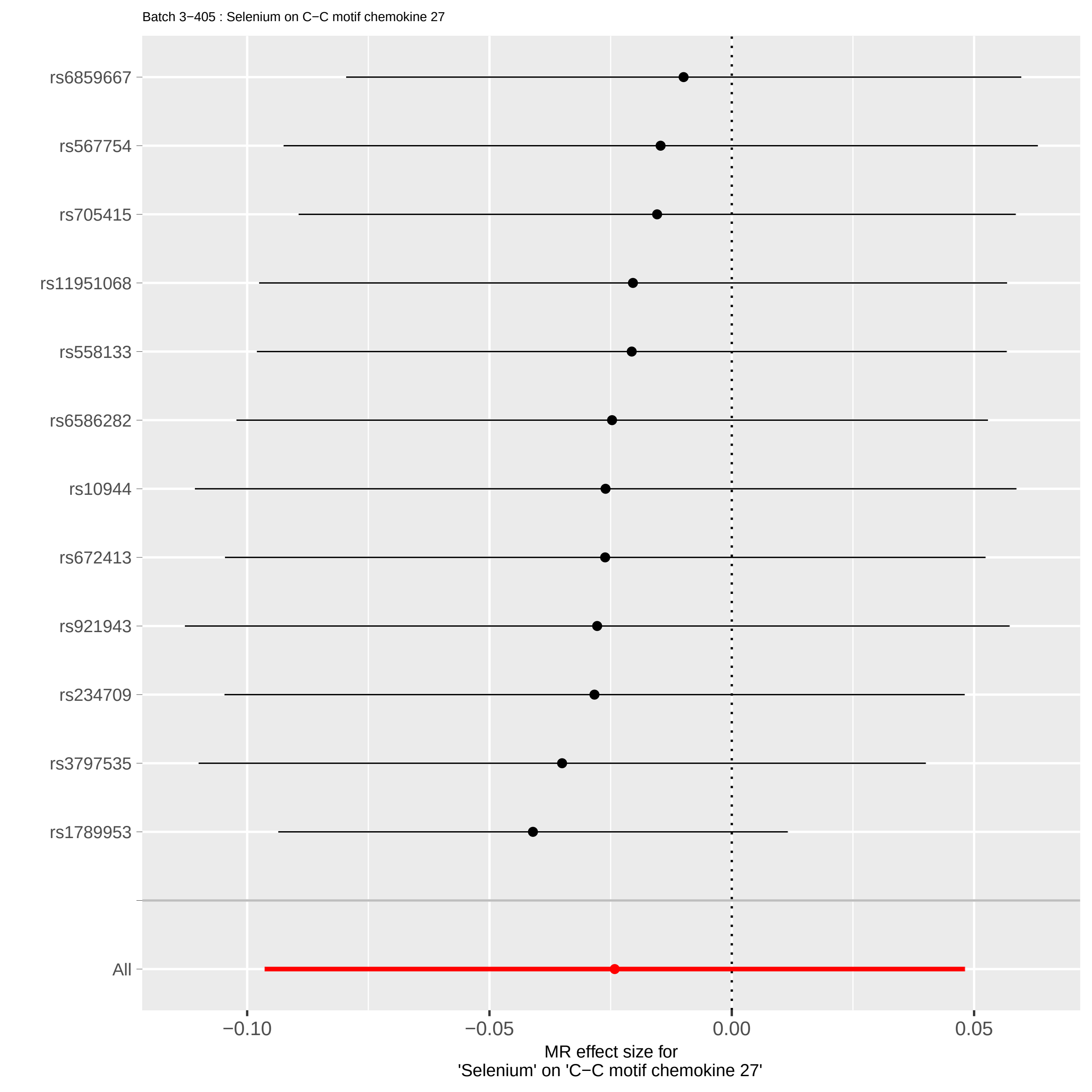
Batch 3−405 : Selenium on C−C motif chemokine 27
rs6859667
rs567754
rs705415
rs11951068
rs558133
rs6586282
rs10944
rs672413
rs921943
rs234709
rs3797535
rs1789953
All
−0.10
−0.05
0.00
0.05
MR effect size for
'Selenium' on 'C−C motif chemokine 27'

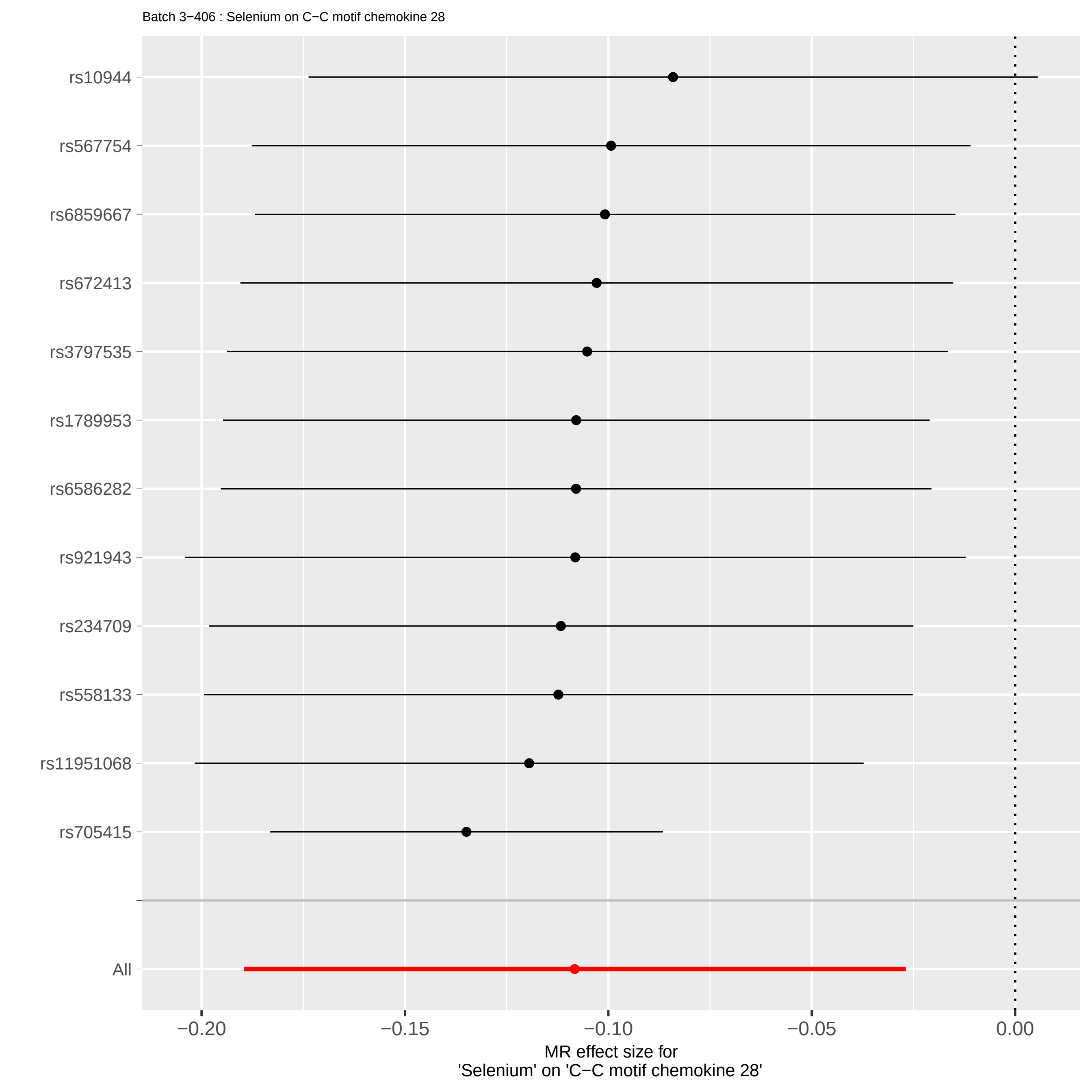

Batch 3−406 : Selenium on C−C motif chemokine 28
rs10944
rs567754
rs6859667
rs672413
rs3797535
rs1789953
rs6586282
rs921943
rs234709
rs558133
rs11951068
rs705415
All
−0.20
−0.15
−0.10
−0.05
0.00
MR effect size for
'Selenium' on 'C−C motif chemokine 28'

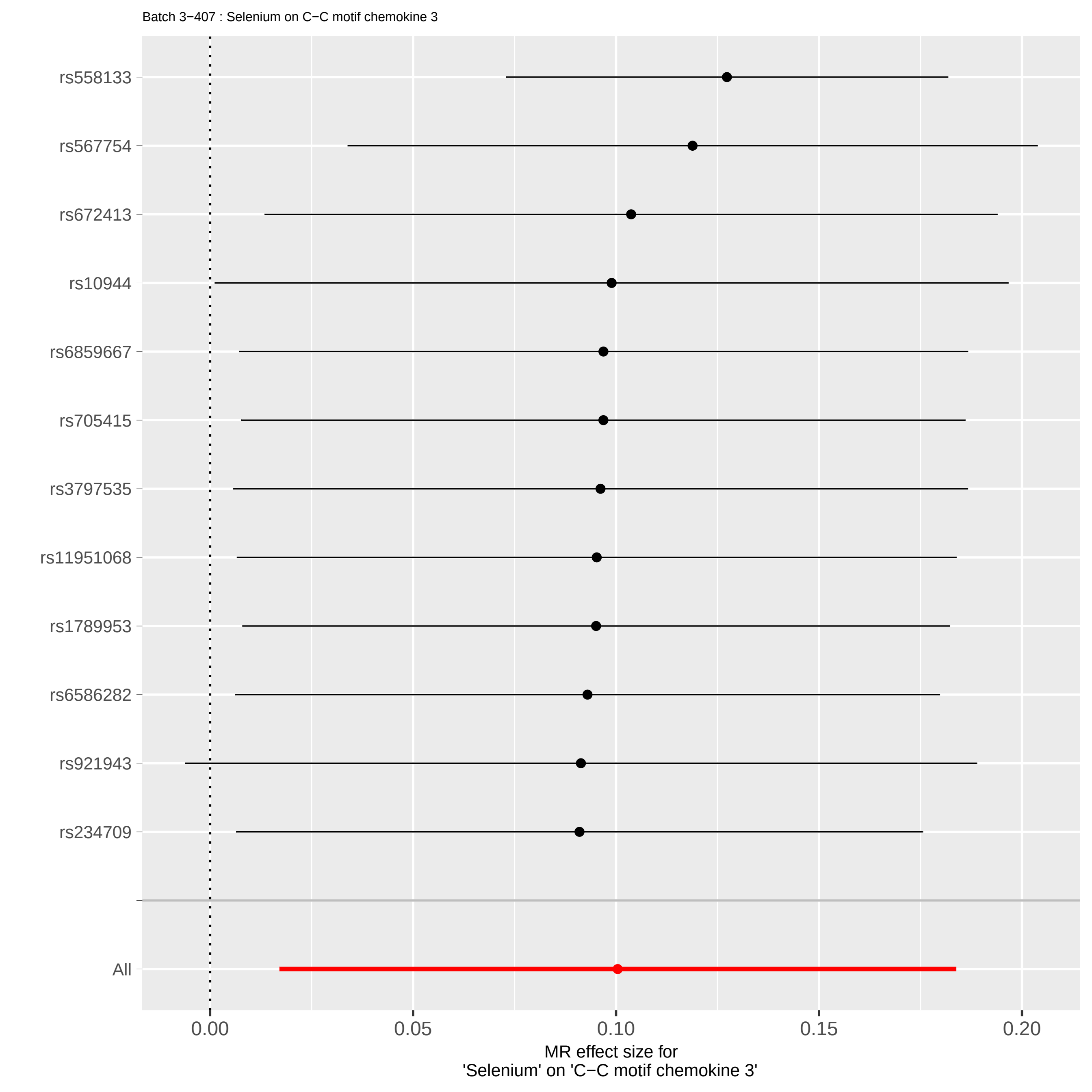

Batch 3−407 : Selenium on C−C motif chemokine 3
rs558133
rs567754
rs672413
rs10944
rs6859667
rs705415
rs3797535
rs11951068
rs1789953
rs6586282
rs921943
rs234709
All
0.00
0.05
0.10
0.15
0.20
MR effect size for
'Selenium' on 'C−C motif chemokine 3'

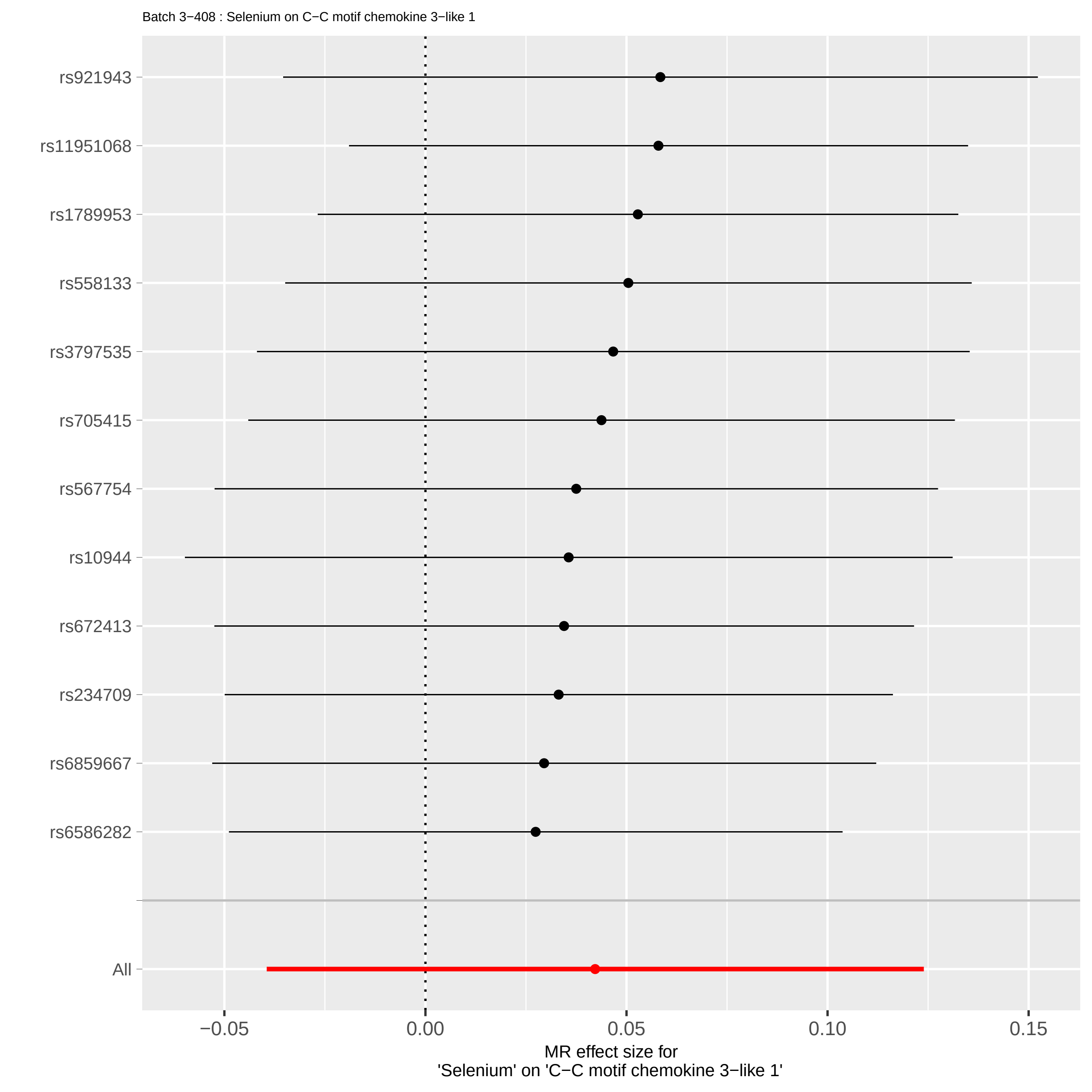
Batch 3−408 : Selenium on C−C motif chemokine 3−like 1
rs921943
rs11951068
rs1789953
rs558133
rs3797535
rs705415
rs567754
rs10944
rs672413
rs234709
rs6859667
rs6586282
All
−0.05
0.00
0.05
0.10
0.15
MR effect size for
'Selenium' on 'C−C motif chemokine 3−like 1'

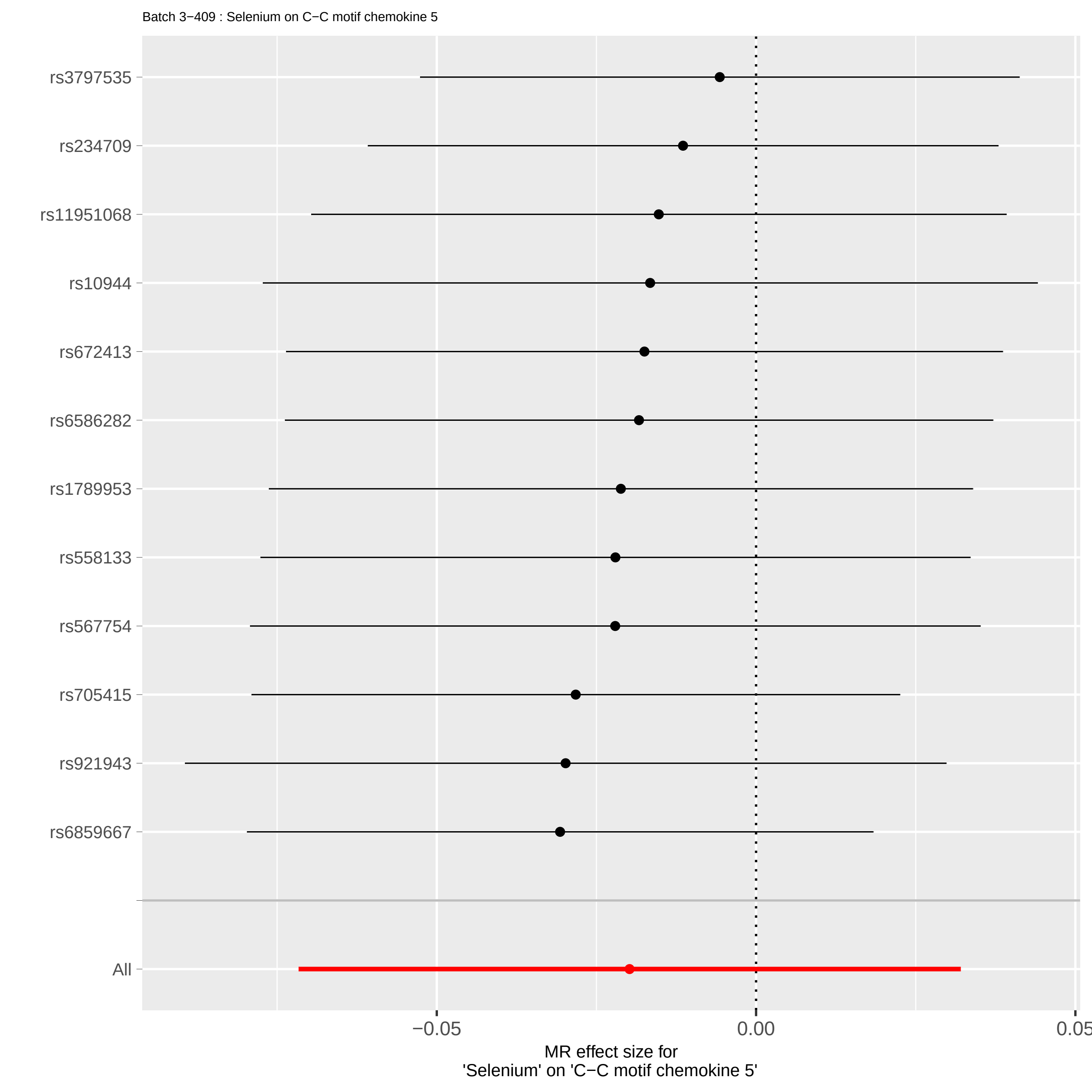

Batch 3−409 : Selenium on C−C motif chemokine 5
rs3797535
rs234709
rs11951068
rs10944
rs672413
rs6586282
rs1789953
rs558133
rs567754
rs705415
rs921943
rs6859667
All
−0.05
0.00
0.05
MR effect size for
'Selenium' on 'C−C motif chemokine 5'

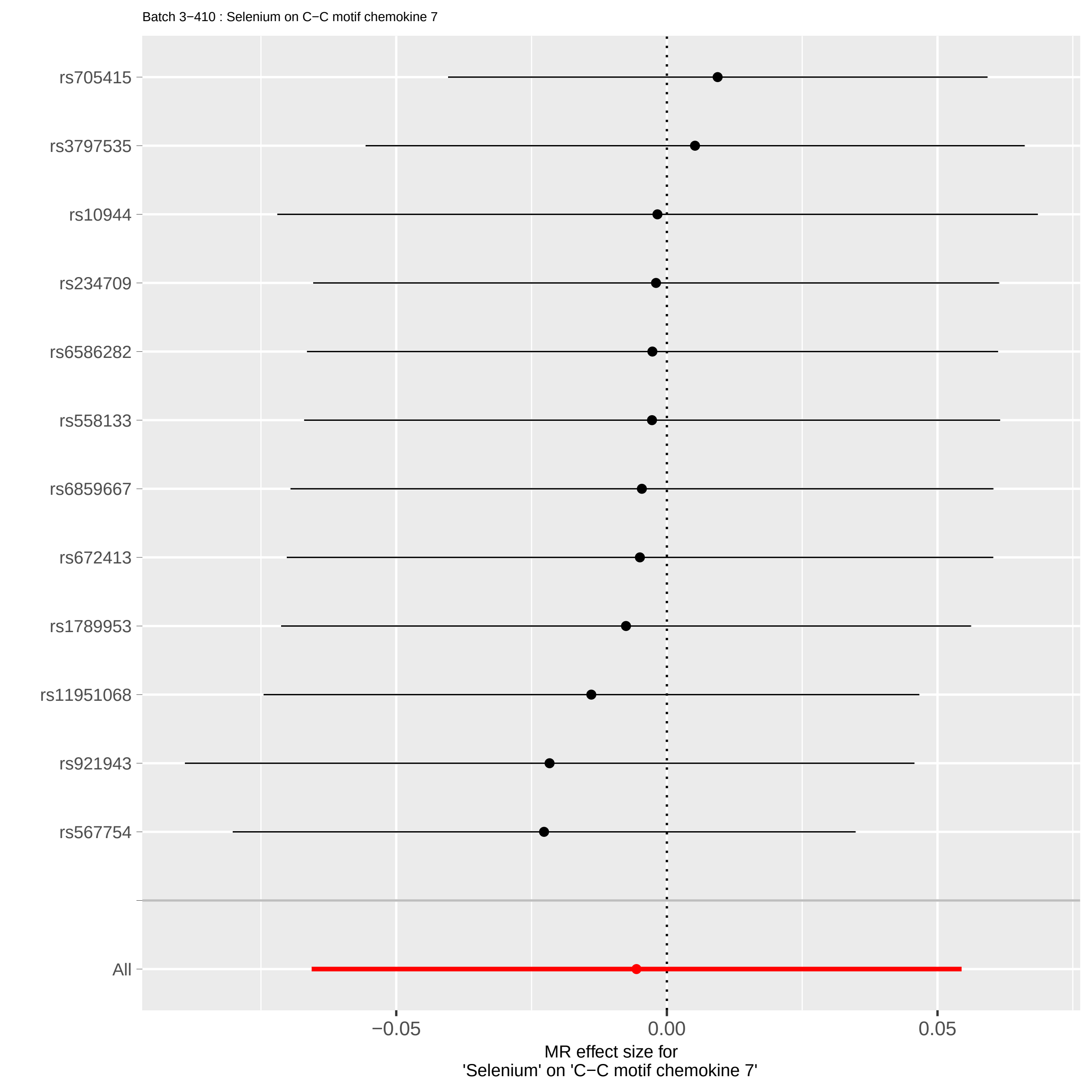

Batch 3−410 : Selenium on C−C motif chemokine 7
rs705415
rs3797535
rs10944
rs234709
rs6586282
rs558133
rs6859667
rs672413
rs1789953
rs11951068
rs921943
rs567754
All
−0.05
0.00
0.05
MR effect size for
'Selenium' on 'C−C motif chemokine 7'

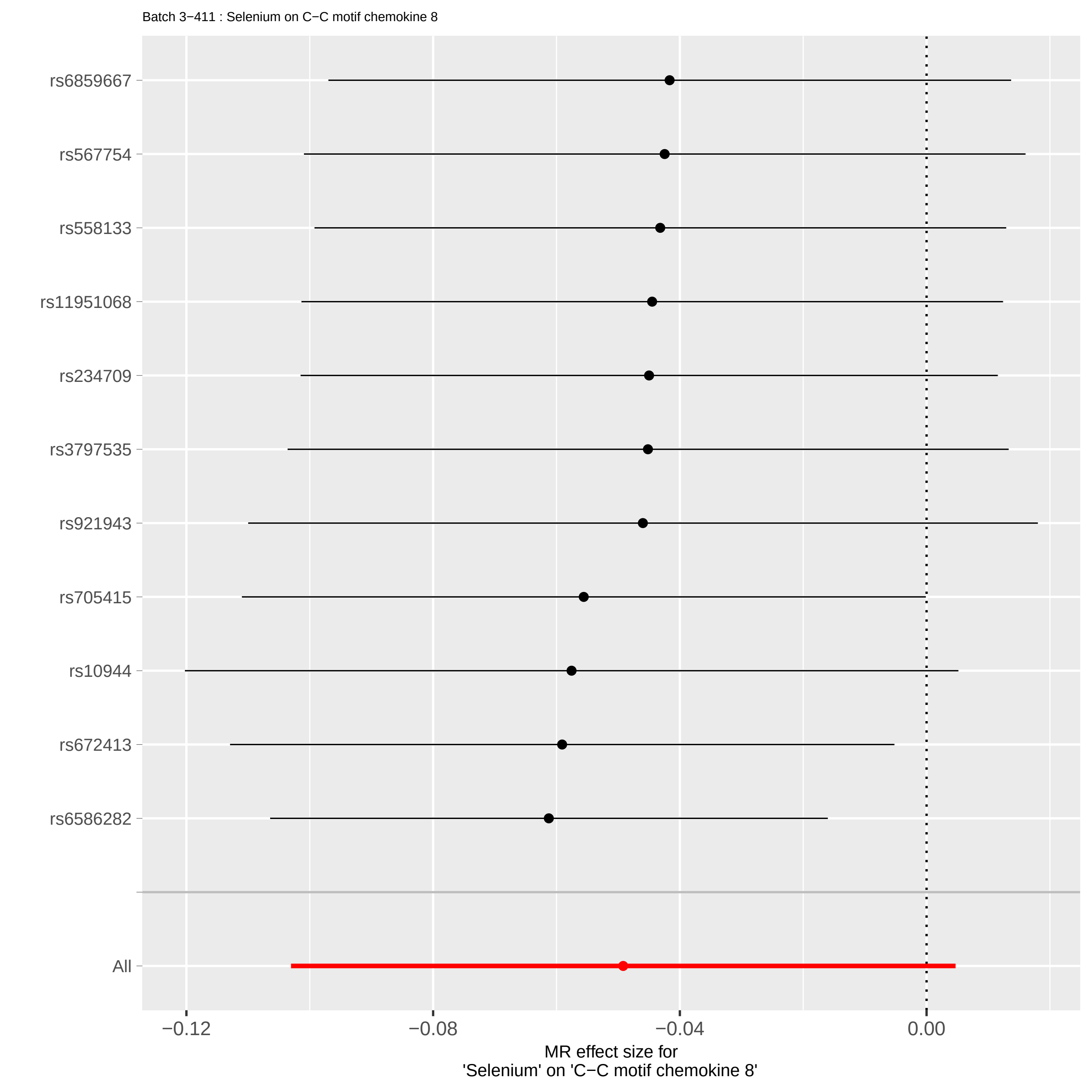

Batch 3−411 : Selenium on C−C motif chemokine 8
rs6859667
rs567754
rs558133
rs11951068
rs234709
rs3797535
rs921943
rs705415
rs10944
rs672413
rs6586282
All
−0.12
−0.08
−0.04
0.00
MR effect size for
'Selenium' on 'C−C motif chemokine 8'

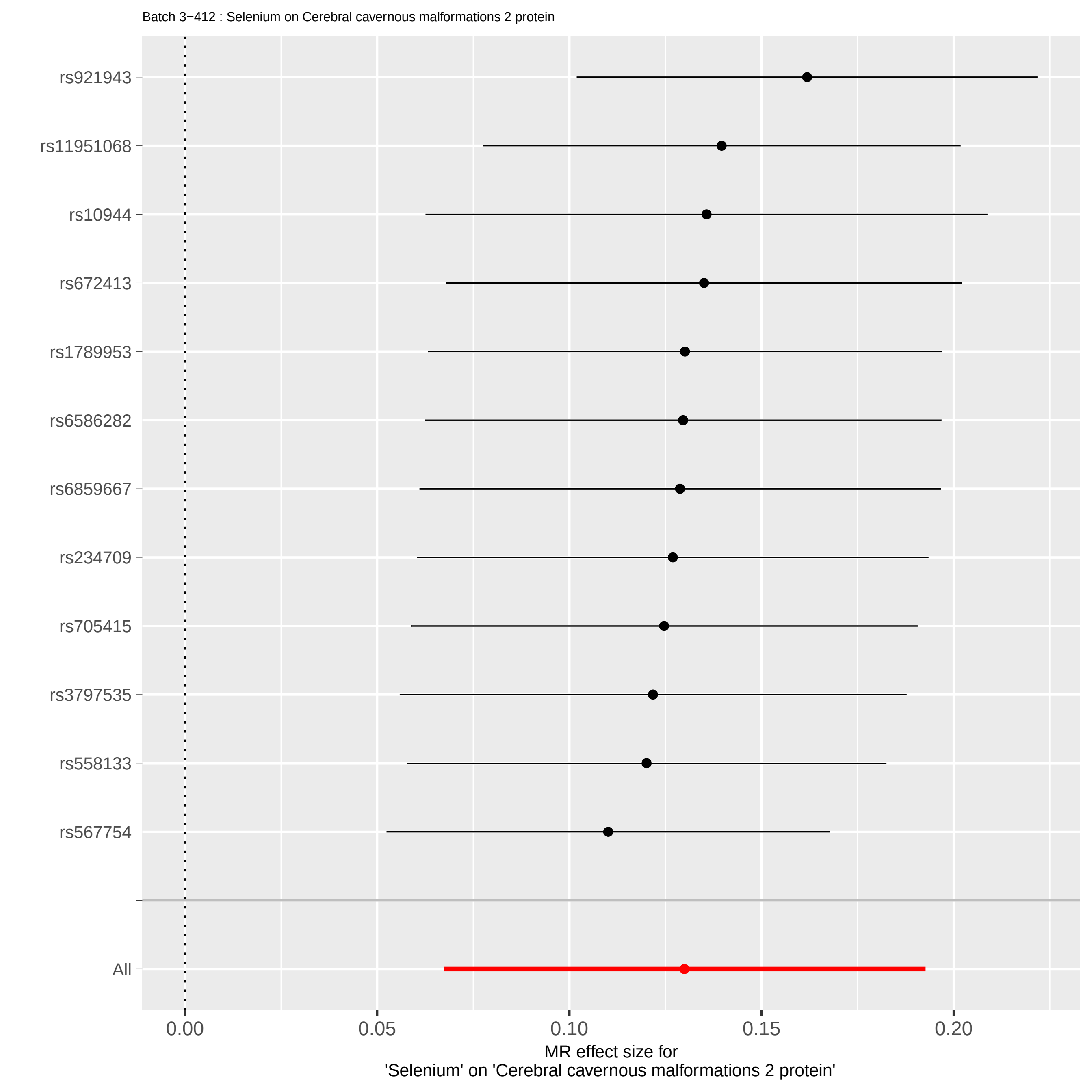

Batch 3−412 : Selenium on Cerebral cavernous malformations 2 protein
rs921943
rs11951068
rs10944
rs672413
rs1789953
rs6586282
rs6859667
rs234709
rs705415
rs3797535
rs558133
rs567754
All
0.00
0.05
0.10
0.15
0.20
MR effect size for
'Selenium' on 'Cerebral cavernous malformations 2 protein'

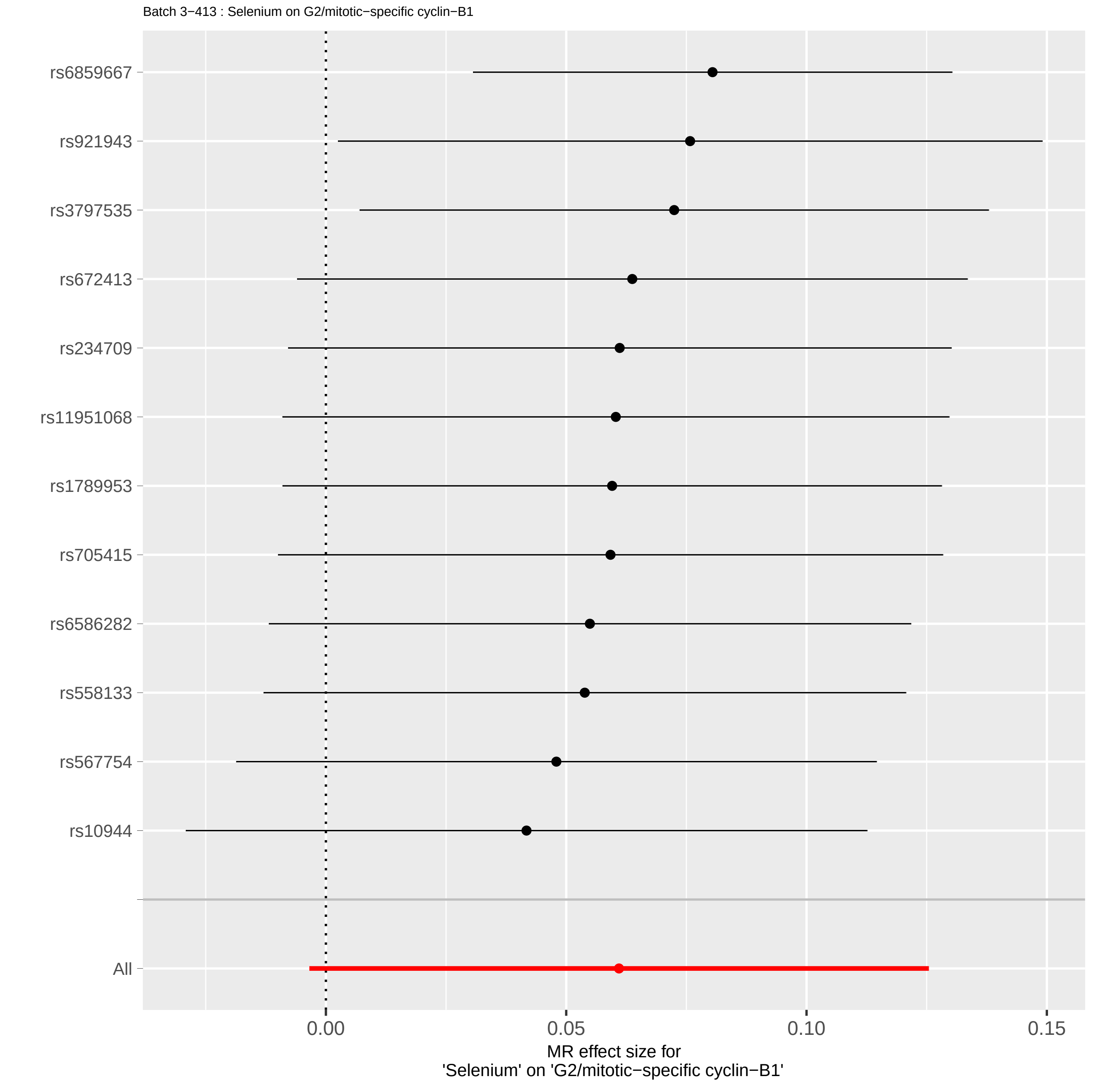
Batch 3−413 : Selenium on G2/mitotic−specific cyclin−B1
rs6859667
rs921943
rs3797535
rs672413
rs234709
rs11951068
rs1789953
rs705415
rs6586282
rs558133
rs567754
rs10944
All
0.00
0.05
0.10
0.15
MR effect size for
'Selenium' on 'G2/mitotic−specific cyclin−B1'

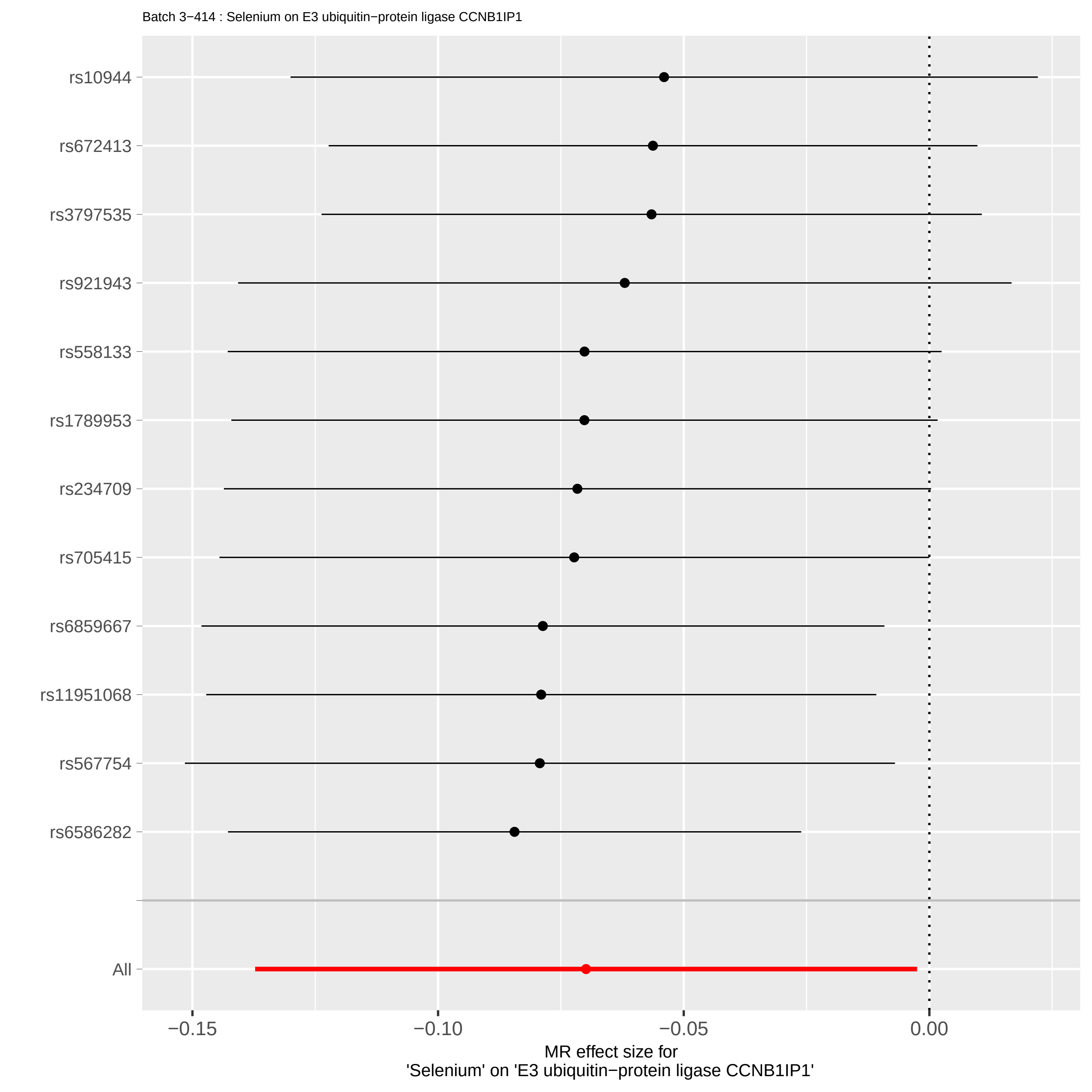
Batch 3−414 : Selenium on E3 ubiquitin−protein ligase CCNB1IP1
rs10944
rs672413
rs3797535
rs921943
rs558133
rs1789953
rs234709
rs705415
rs6859667
rs11951068
rs567754
rs6586282
All
−0.15
−0.10
−0.05
0.00
MR effect size for
'Selenium' on 'E3 ubiquitin−protein ligase CCNB1IP1'

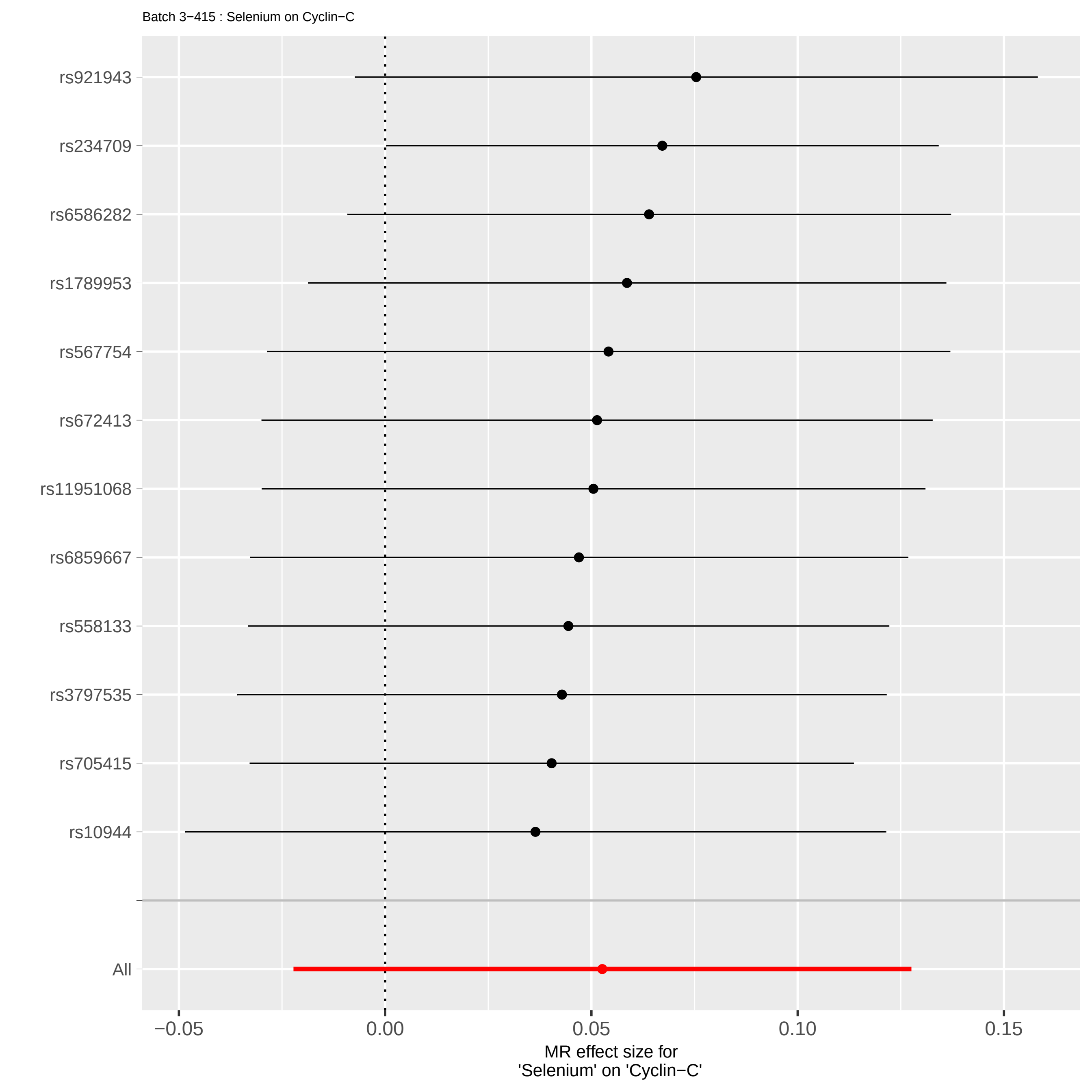

Batch 3−415 : Selenium on Cyclin−C
rs921943
rs234709
rs6586282
rs1789953
rs567754
rs672413
rs11951068
rs6859667
rs558133
rs3797535
rs705415
rs10944
All
−0.05
0.00
0.05
0.10
0.15
MR effect size for
'Selenium' on 'Cyclin−C'

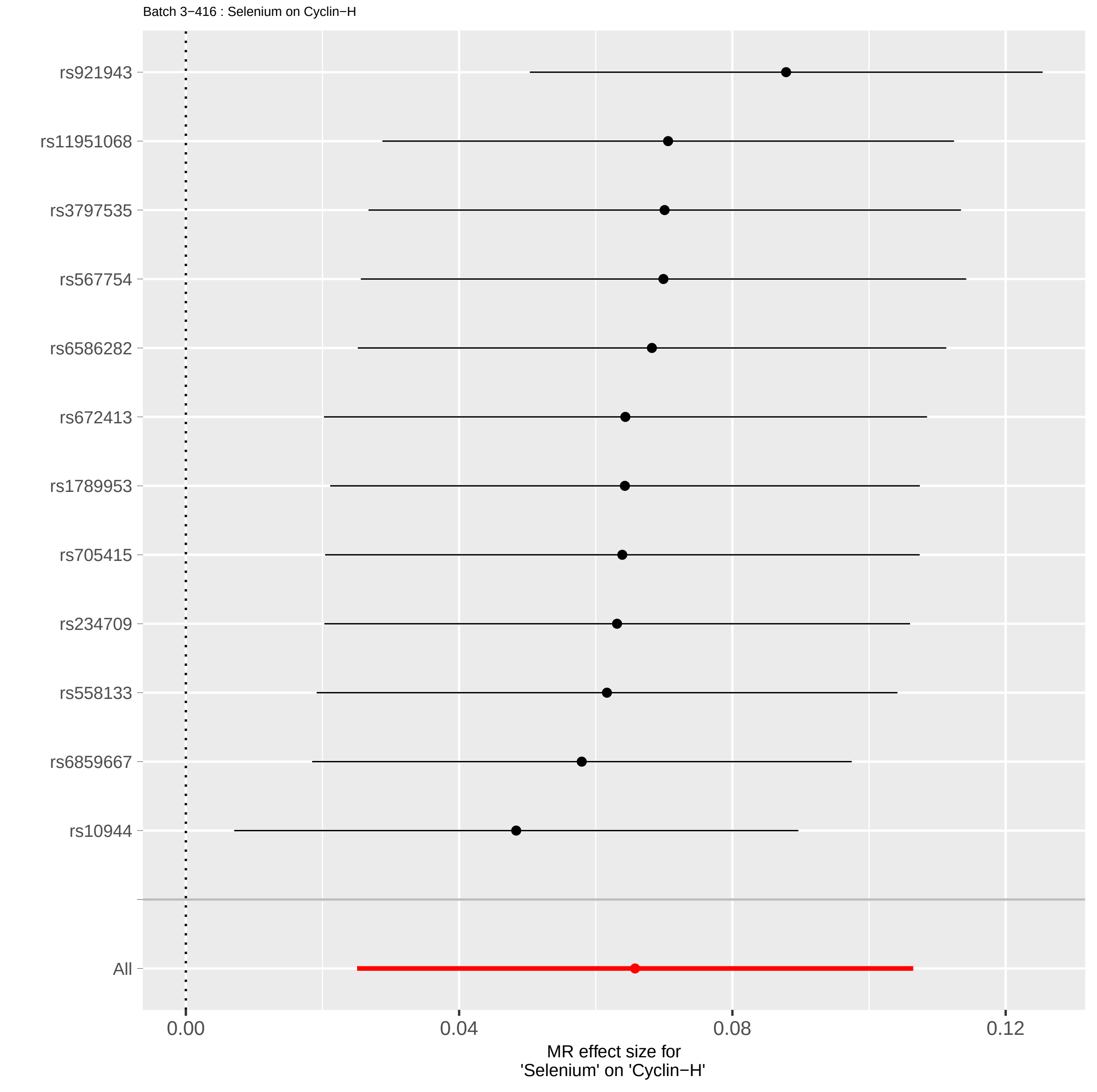
Batch 3−416 : Selenium on Cyclin−H
rs921943
rs11951068
rs3797535
rs567754
rs6586282
rs672413
rs1789953
rs705415
rs234709
rs558133
rs6859667
rs10944
All
0.00
0.04
0.08
0.12
MR effect size for
'Selenium' on 'Cyclin−H'

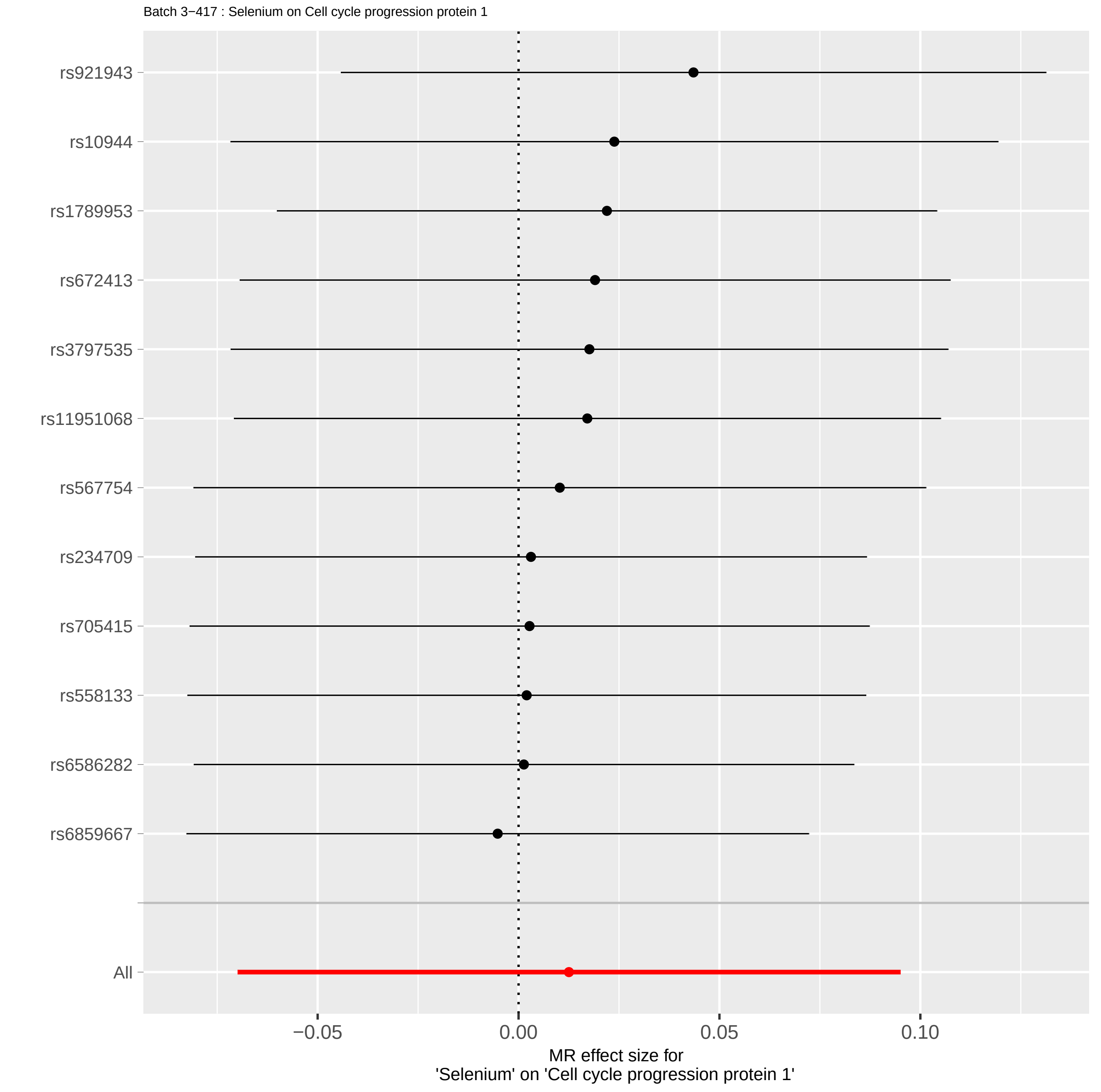
Batch 3−417 : Selenium on Cell cycle progression protein 1
rs921943
rs10944
rs1789953
rs672413
rs3797535
rs11951068
rs567754
rs234709
rs705415
rs558133
rs6586282
rs6859667
All
−0.05
0.00
0.05
0.10
MR effect size for
'Selenium' on 'Cell cycle progression protein 1'

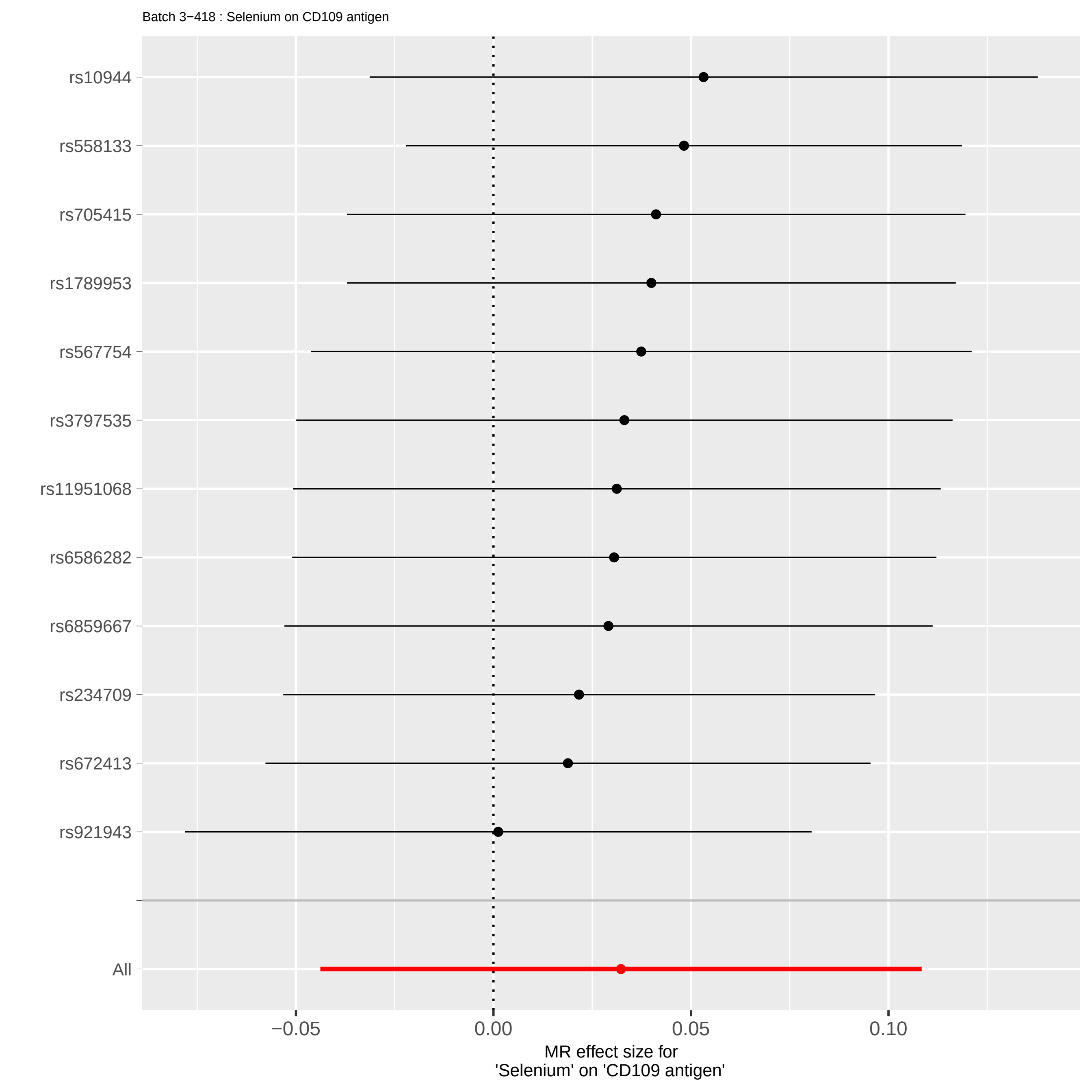

Batch 3−418 : Selenium on CD109 antigen
rs10944
rs558133
rs705415
rs1789953
rs567754
rs3797535
rs11951068
rs6586282
rs6859667
rs234709
rs672413
rs921943
All
−0.05
0.00
0.05
0.10
MR effect size for
'Selenium' on 'CD109 antigen'

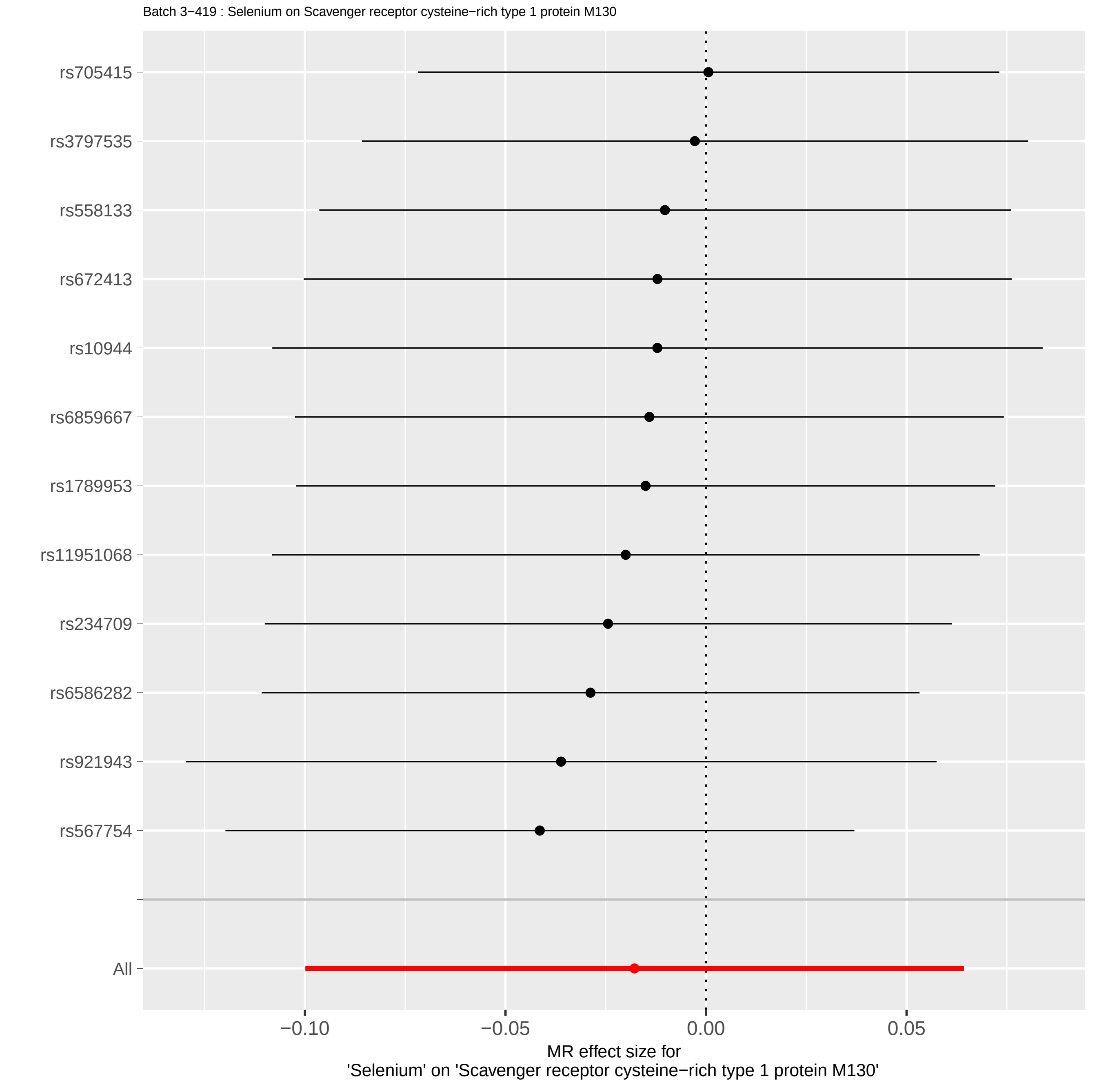

Batch 3−419 : Selenium on Scavenger receptor cysteine−rich type 1 protein M130
rs705415
rs3797535
rs558133
rs672413
rs10944
rs6859667
rs1789953
rs11951068
rs234709
rs6586282
rs921943
rs567754
All
−0.10
−0.05
0.00
0.05
MR effect size for
'Selenium' on 'Scavenger receptor cysteine−rich type 1 protein M130'

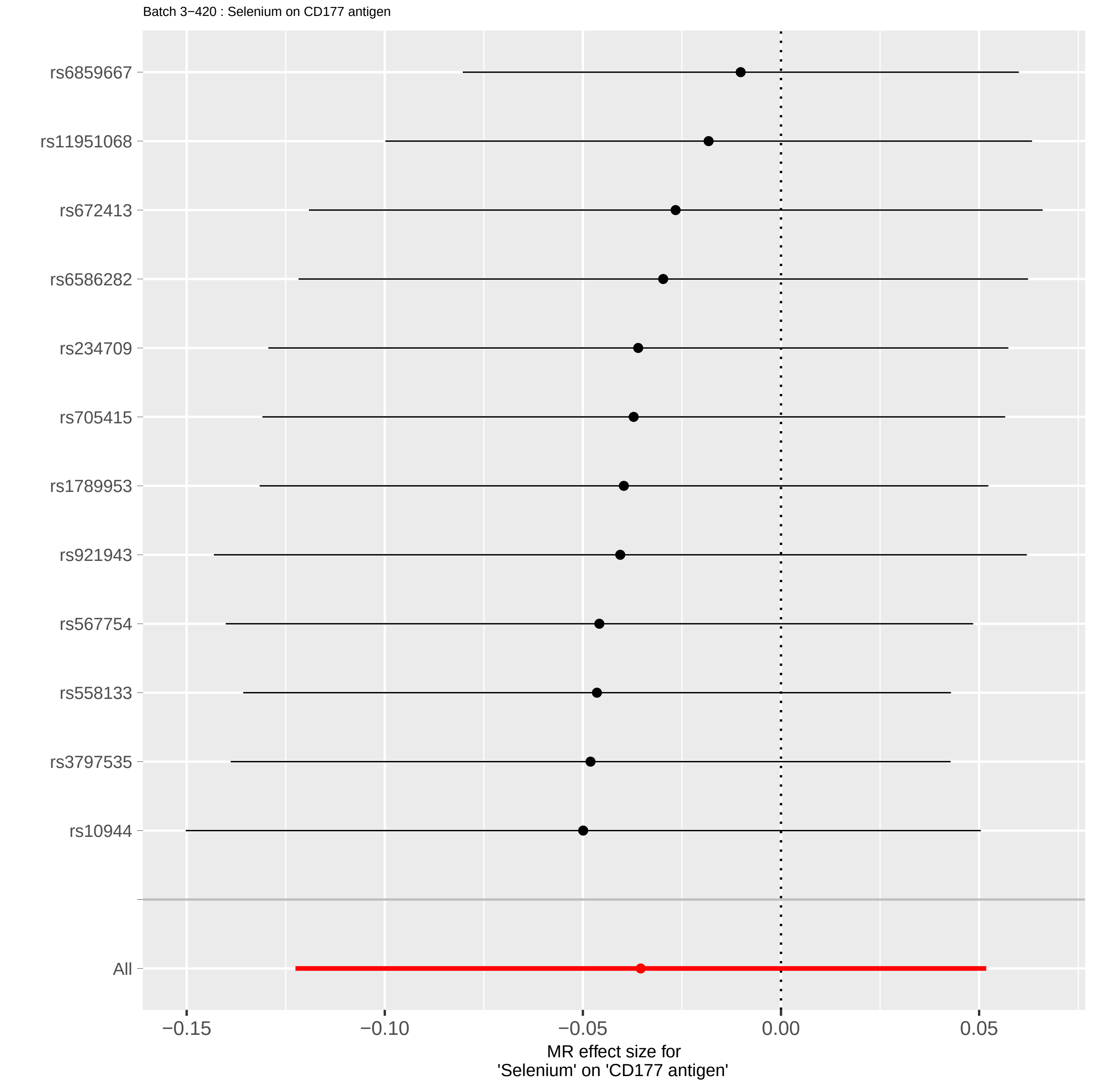

Batch 3−420 : Selenium on CD177 antigen
rs6859667
rs11951068
rs672413
rs6586282
rs234709
rs705415
rs1789953
rs921943
rs567754
rs558133
rs3797535
rs10944
All
−0.15
−0.10
−0.05
0.00
0.05
MR effect size for
'Selenium' on 'CD177 antigen'

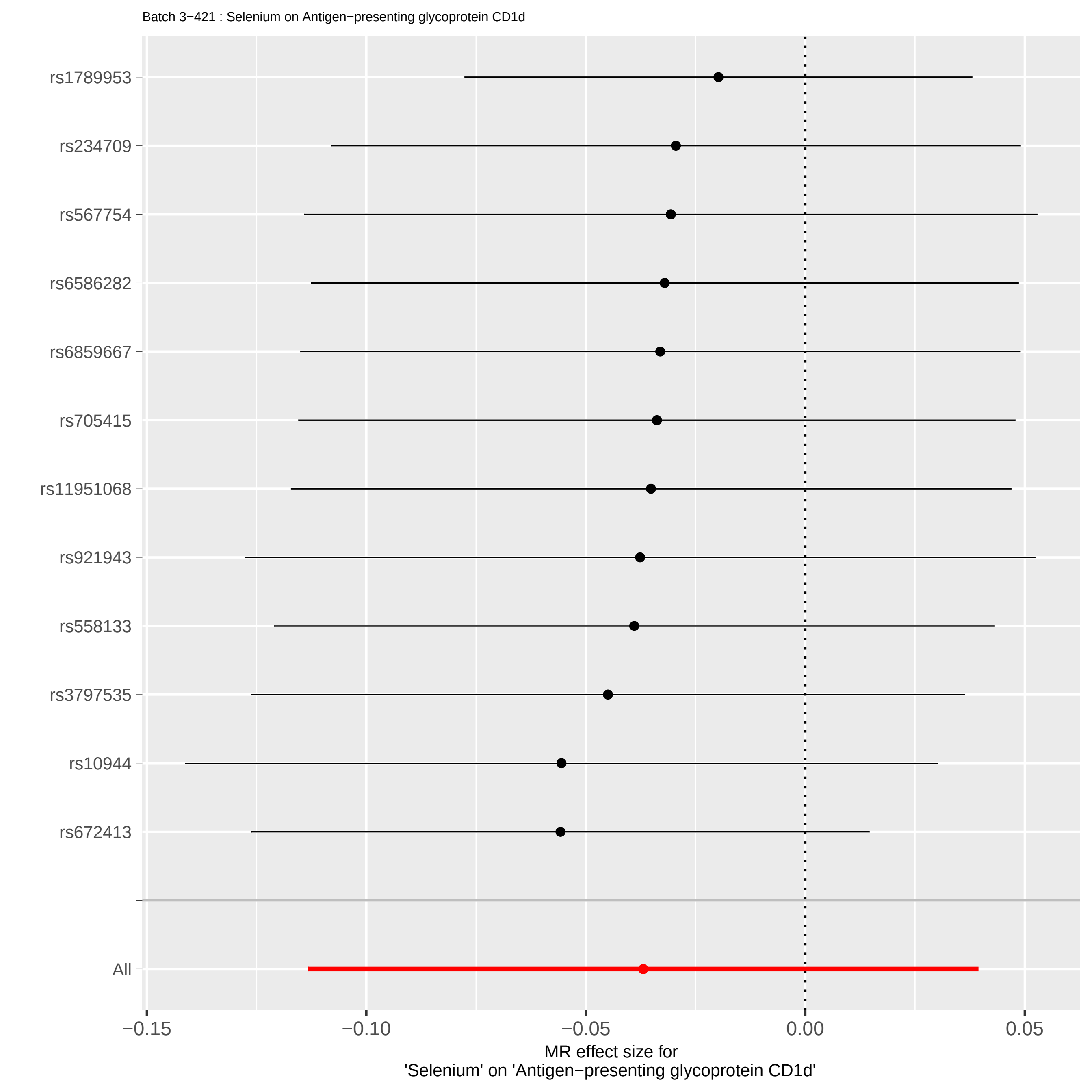

Batch 3−421 : Selenium on Antigen−presenting glycoprotein CD1d
rs1789953
rs234709
rs567754
rs6586282
rs6859667
rs705415
rs11951068
rs921943
rs558133
rs3797535
rs10944
rs672413
All
−0.15
−0.10
−0.05
0.00
0.05
MR effect size for
'Selenium' on 'Antigen−presenting glycoprotein CD1d'

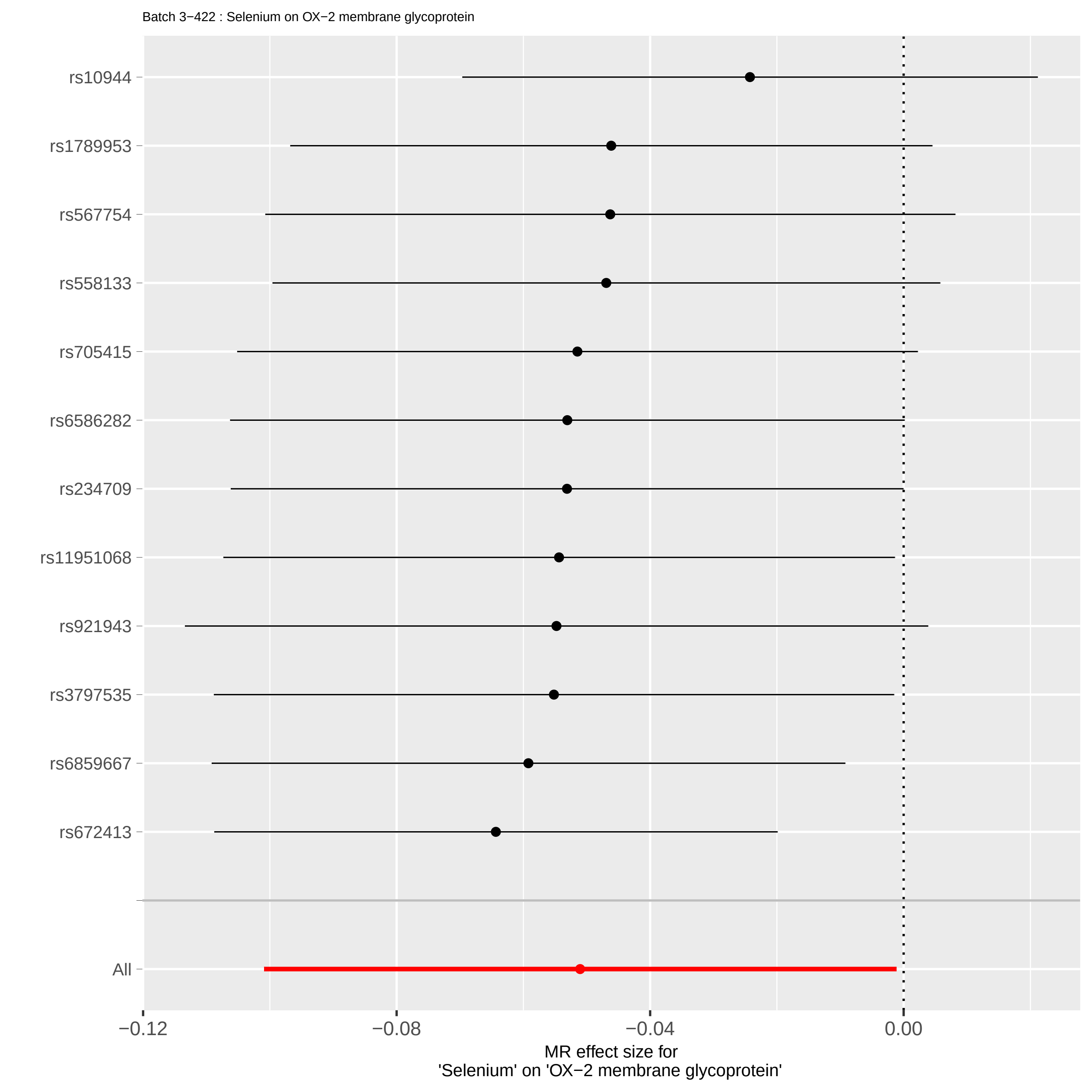

Batch 3−422 : Selenium on OX−2 membrane glycoprotein
rs10944
rs1789953
rs567754
rs558133
rs705415
rs6586282
rs234709
rs11951068
rs921943
rs3797535
rs6859667
rs672413
All
−0.12
−0.08
−0.04
0.00
MR effect size for
'Selenium' on 'OX−2 membrane glycoprotein'

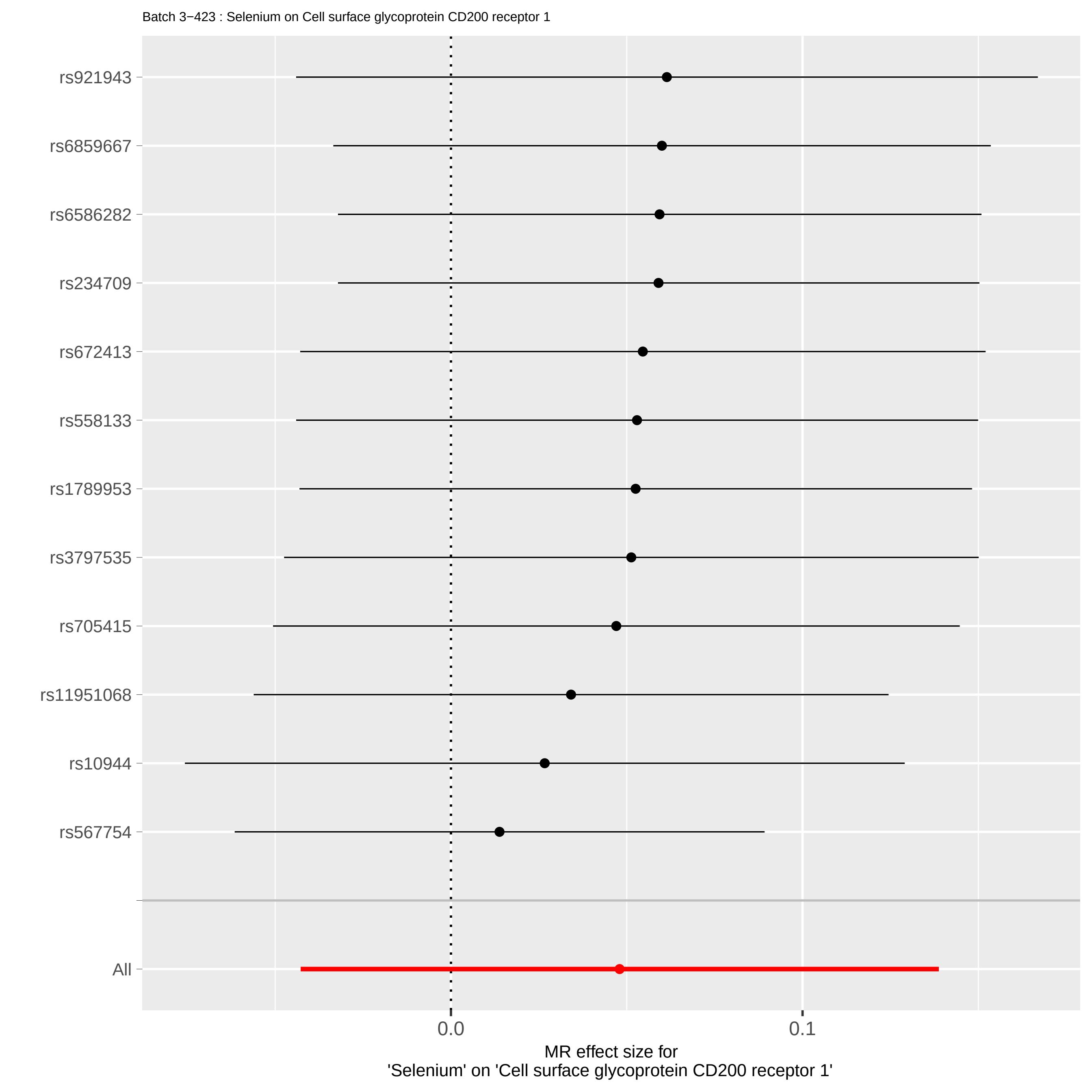
Batch 3−423 : Selenium on Cell surface glycoprotein CD200 receptor 1
rs921943
rs6859667
rs6586282
rs234709
rs672413
rs558133
rs1789953
rs3797535
rs705415
rs11951068
rs10944
rs567754
All
0.0
0.1
MR effect size for
'Selenium' on 'Cell surface glycoprotein CD200 receptor 1'

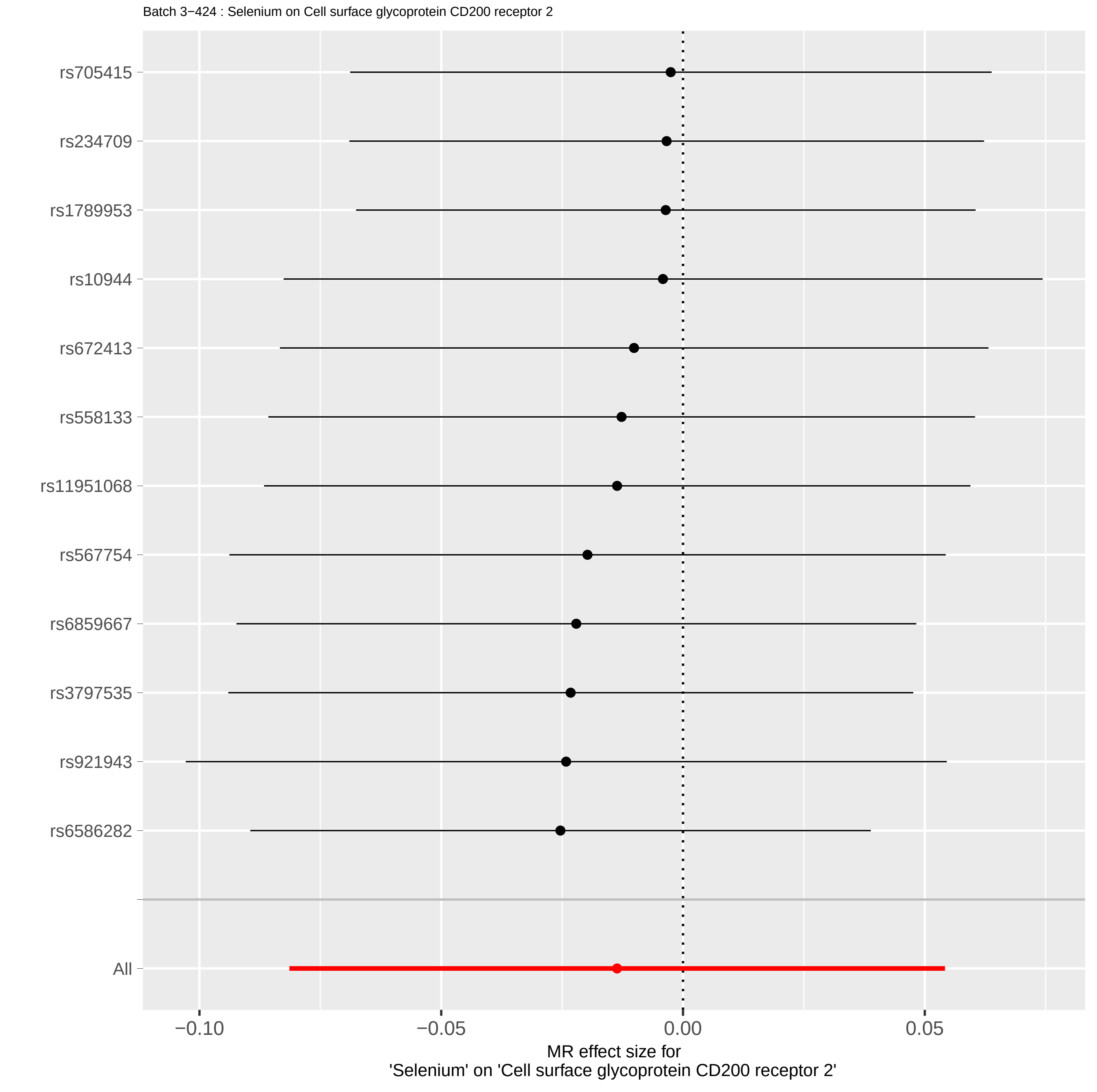

Batch 3−424 : Selenium on Cell surface glycoprotein CD200 receptor 2
rs705415
rs234709
rs1789953
rs10944
rs672413
rs558133
rs11951068
rs567754
rs6859667
rs3797535
rs921943
rs6586282
All
−0.10
−0.05
0.00
0.05
MR effect size for
'Selenium' on 'Cell surface glycoprotein CD200 receptor 2'

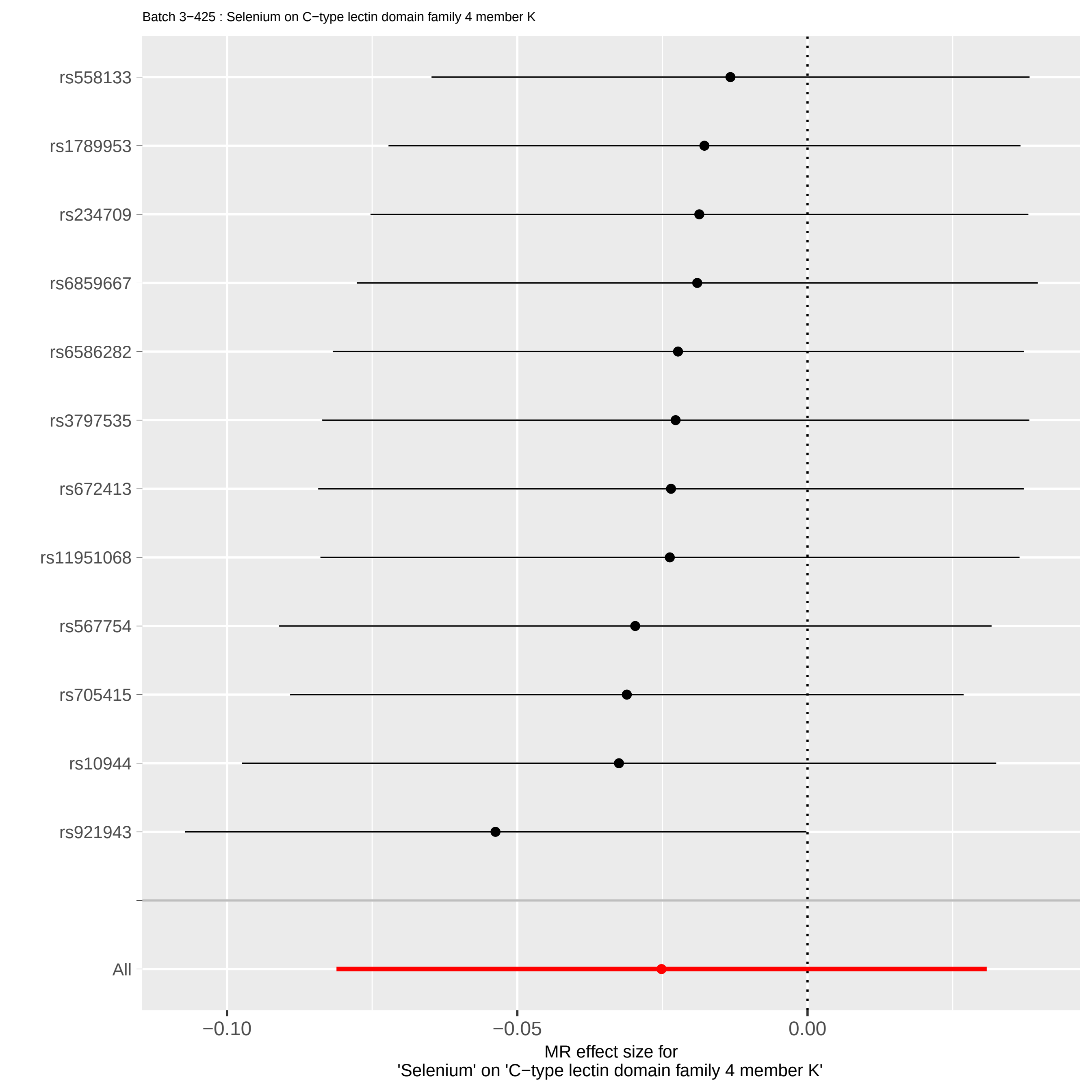

Batch 3−425 : Selenium on C−type lectin domain family 4 member K
rs558133
rs1789953
rs234709
rs6859667
rs6586282
rs3797535
rs672413
rs11951068
rs567754
rs705415
rs10944
rs921943
All
−0.10
−0.05
0.00
MR effect size for
'Selenium' on 'C−type lectin domain family 4 member K'

Batch 3−426 : Selenium on CD209 antigen

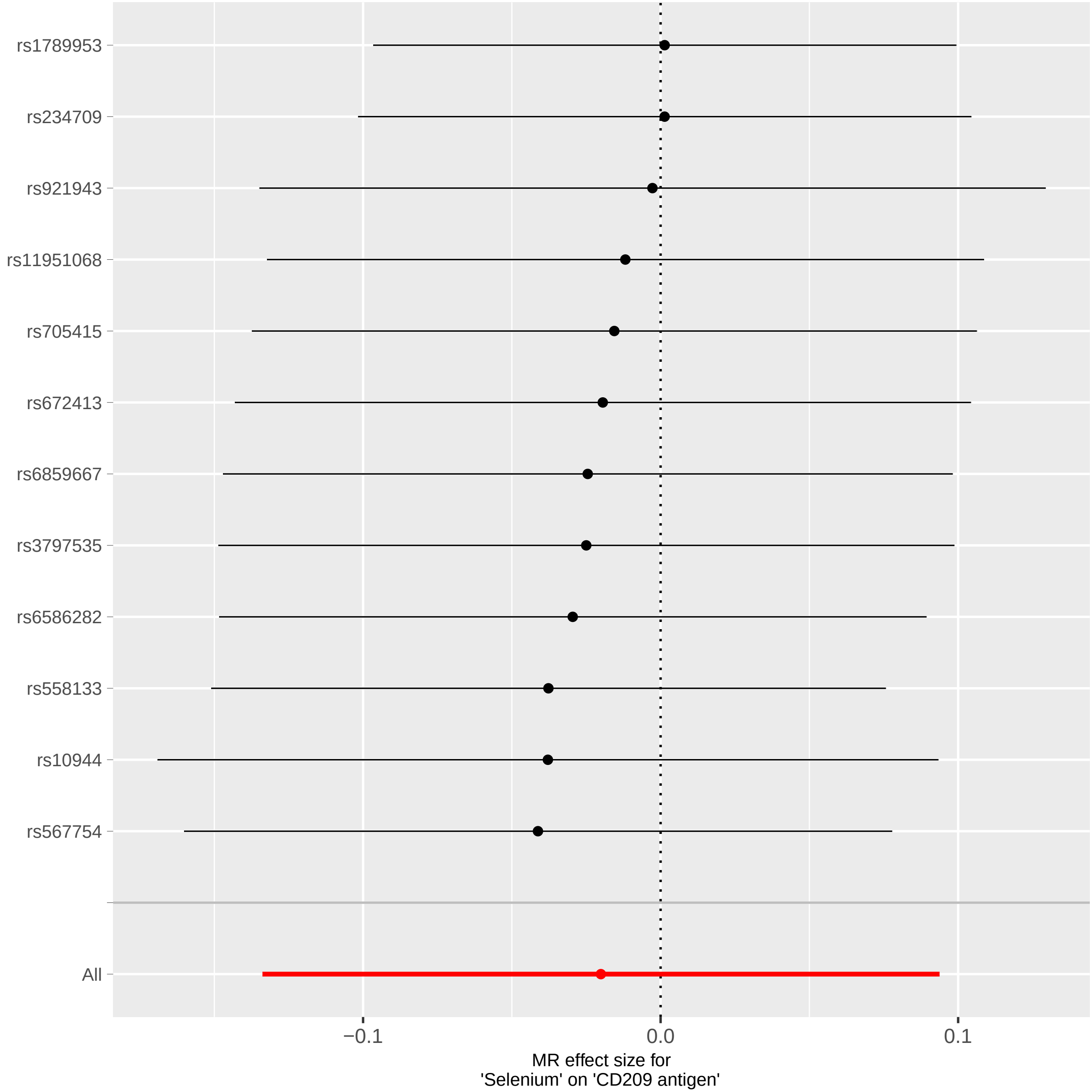

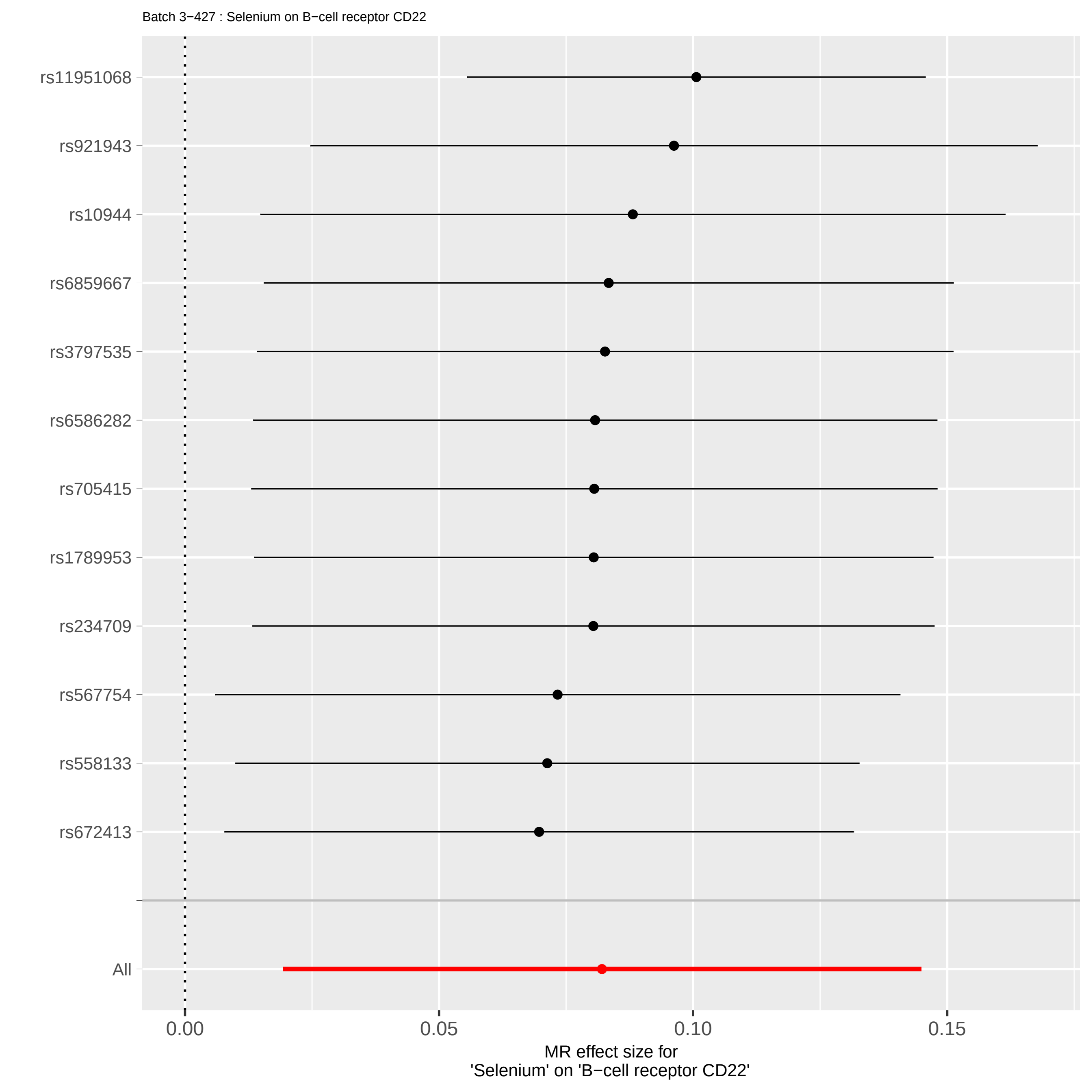
Batch 3−427 : Selenium on B−cell receptor CD22
rs11951068
rs921943
rs10944
rs6859667
rs3797535
rs6586282
rs705415
rs1789953
rs234709
rs567754
rs558133
rs672413
All
0.00
0.05
0.10
0.15
MR effect size for
'Selenium' on 'B−cell receptor CD22'

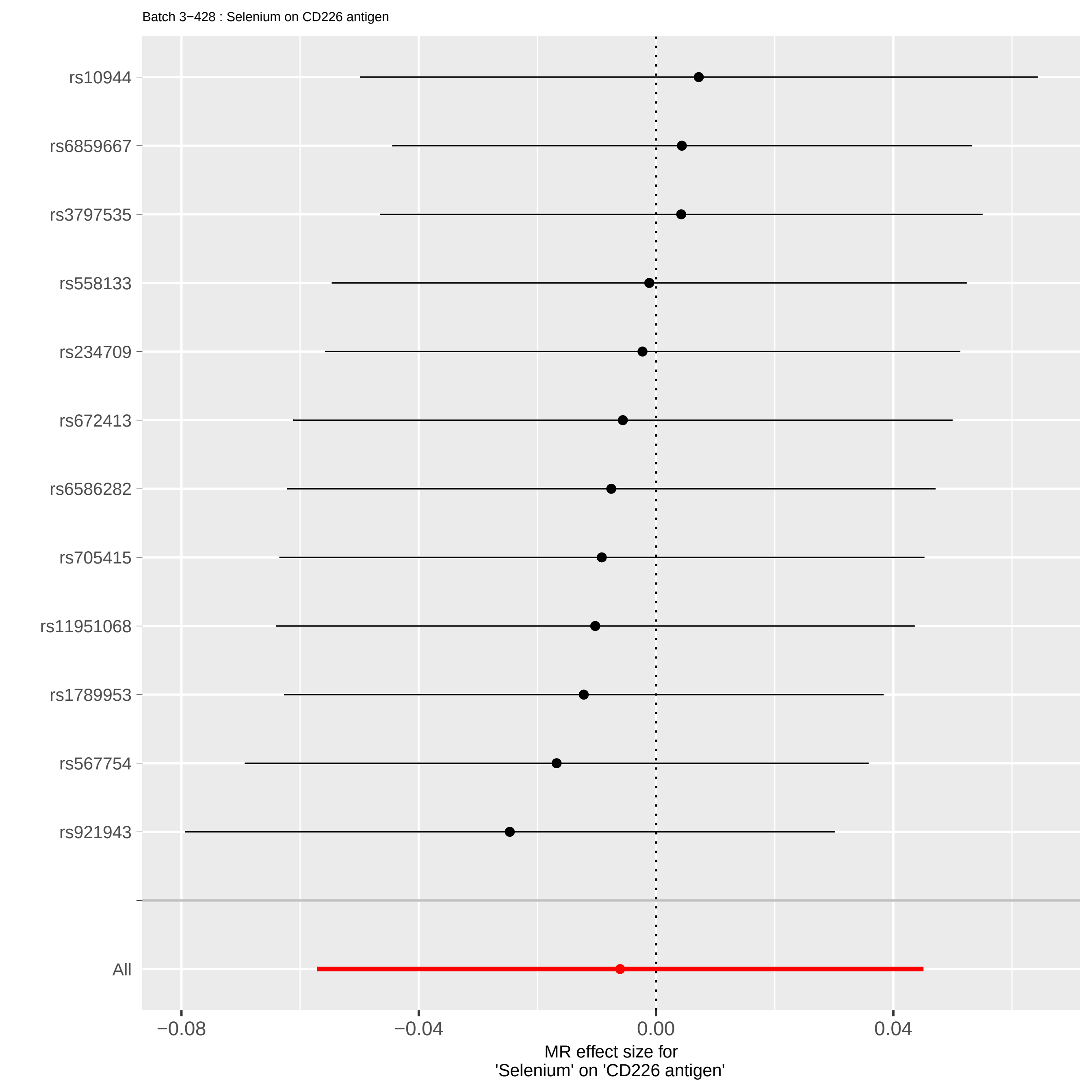

Batch 3−428 : Selenium on CD226 antigen
rs10944
rs6859667
rs3797535
rs558133
rs234709
rs672413
rs6586282
rs705415
rs11951068
rs1789953
rs567754
rs921943
All
−0.08
−0.04
0.00
0.04
MR effect size for
'Selenium' on 'CD226 antigen'

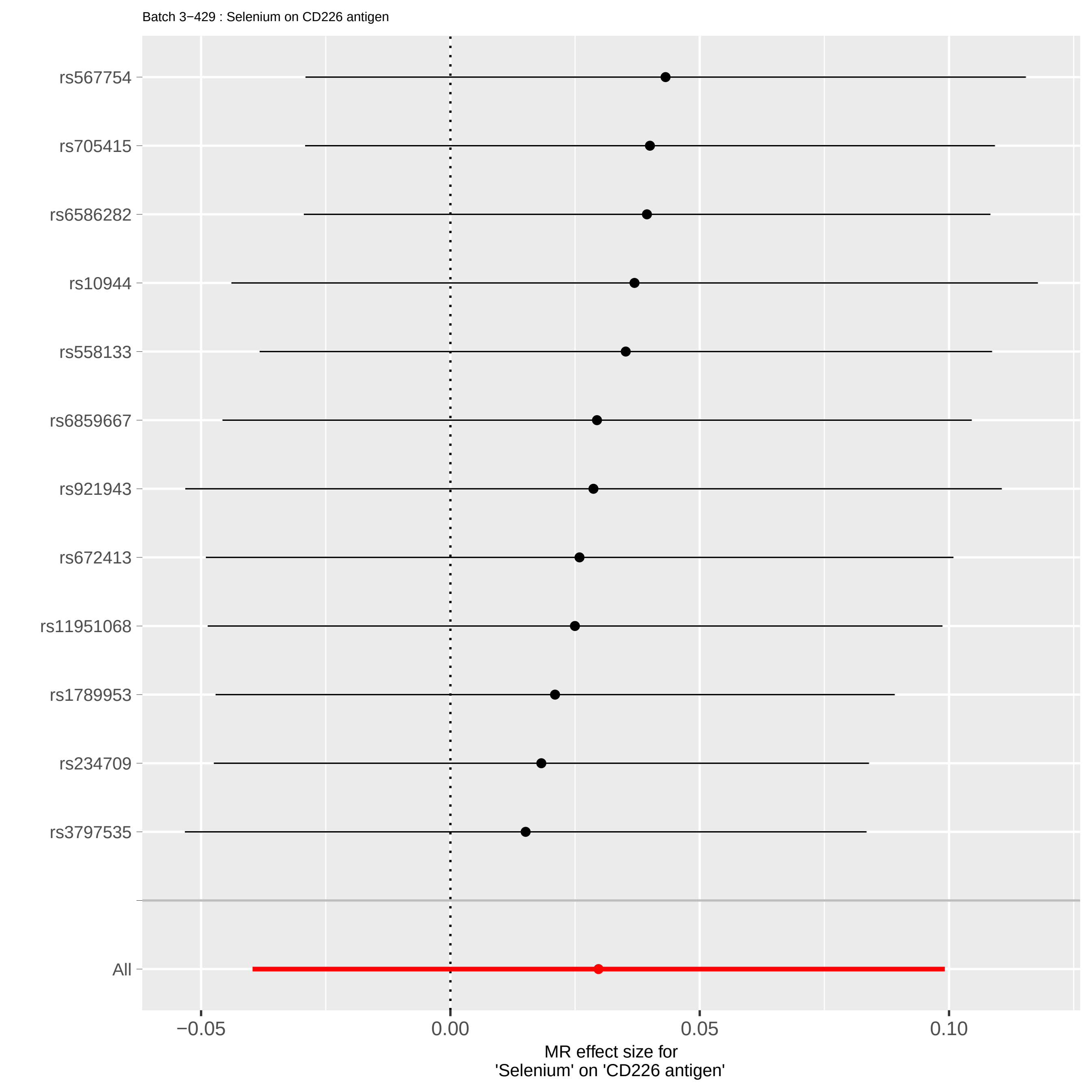
Batch 3−429 : Selenium on CD226 antigen
rs567754
rs705415
rs6586282
rs10944
rs558133
rs6859667
rs921943
rs672413
rs11951068
rs1789953
rs234709
rs3797535
All
−0.05
0.00
0.05
0.10
MR effect size for
'Selenium' on 'CD226 antigen'

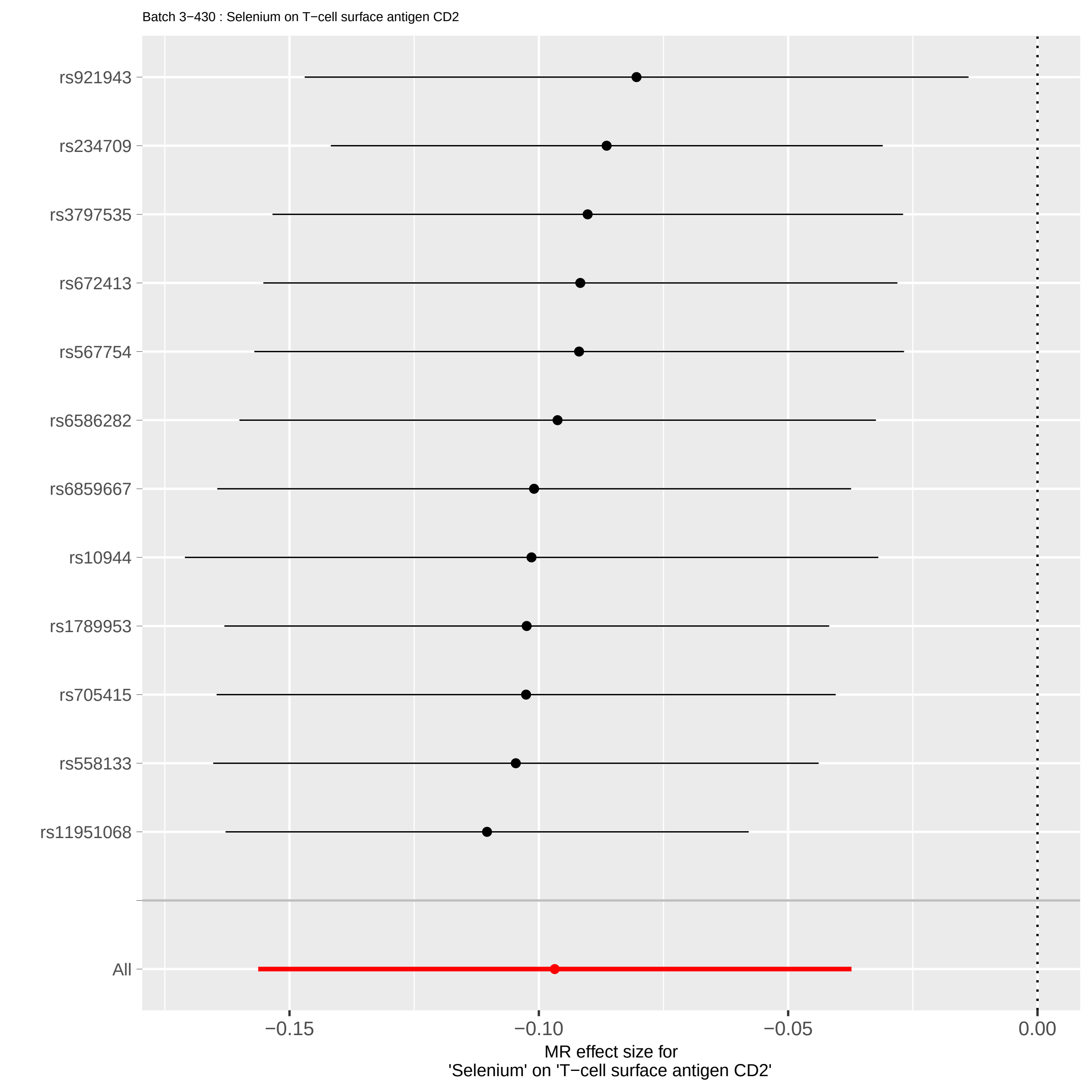
Batch 3−430 : Selenium on T−cell surface antigen CD2
rs921943
rs234709
rs3797535
rs672413
rs567754
rs6586282
rs6859667
rs10944
rs1789953
rs705415
rs558133
rs11951068
All
−0.15
−0.10
−0.05
0.00
MR effect size for
'Selenium' on 'T−cell surface antigen CD2'

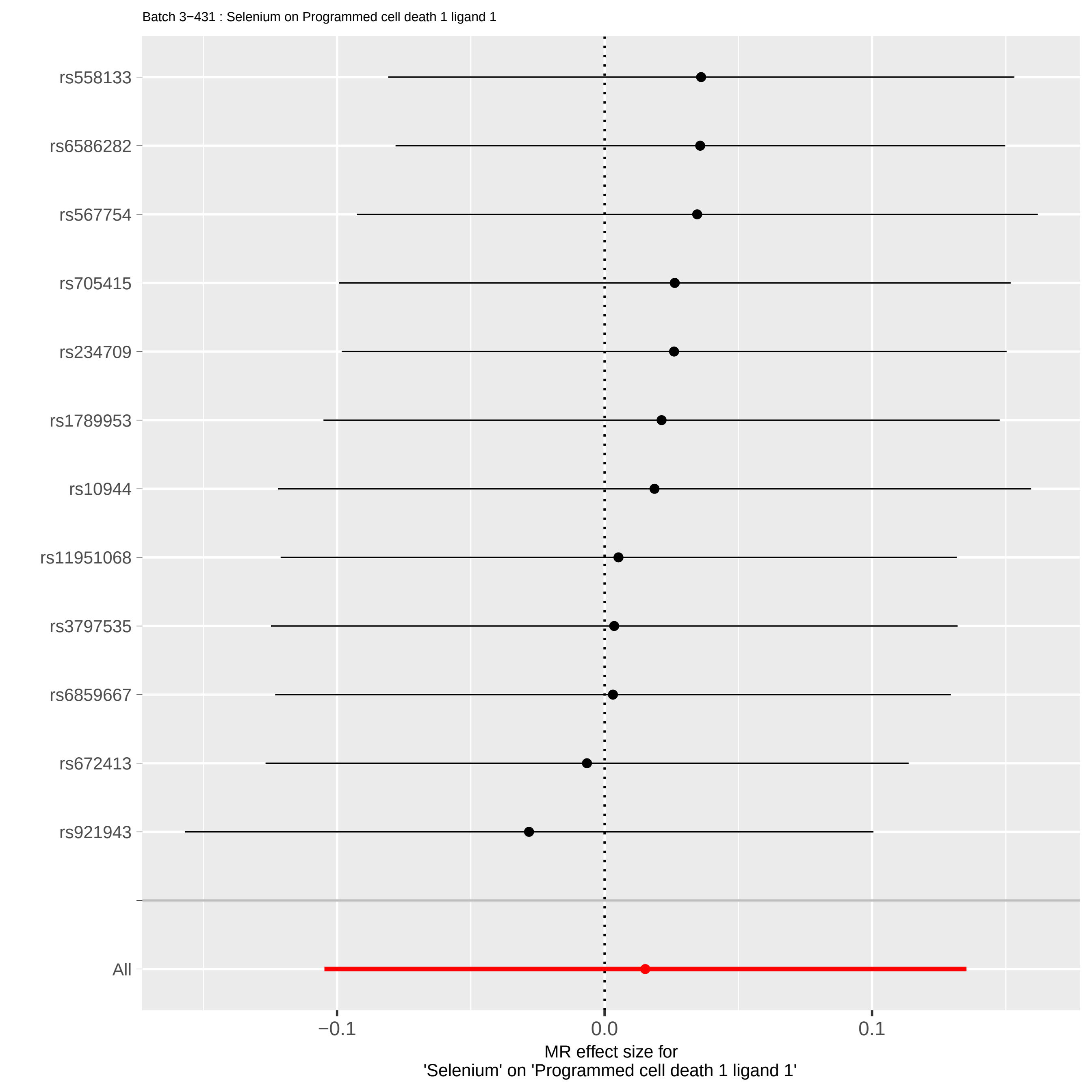

Batch 3−431 : Selenium on Programmed cell death 1 ligand 1
rs558133
rs6586282
rs567754
rs705415
rs234709
rs1789953
rs10944
rs11951068
rs3797535
rs6859667
rs672413
rs921943
All
−0.1
0.0
0.1
MR effect size for
'Selenium' on 'Programmed cell death 1 ligand 1'

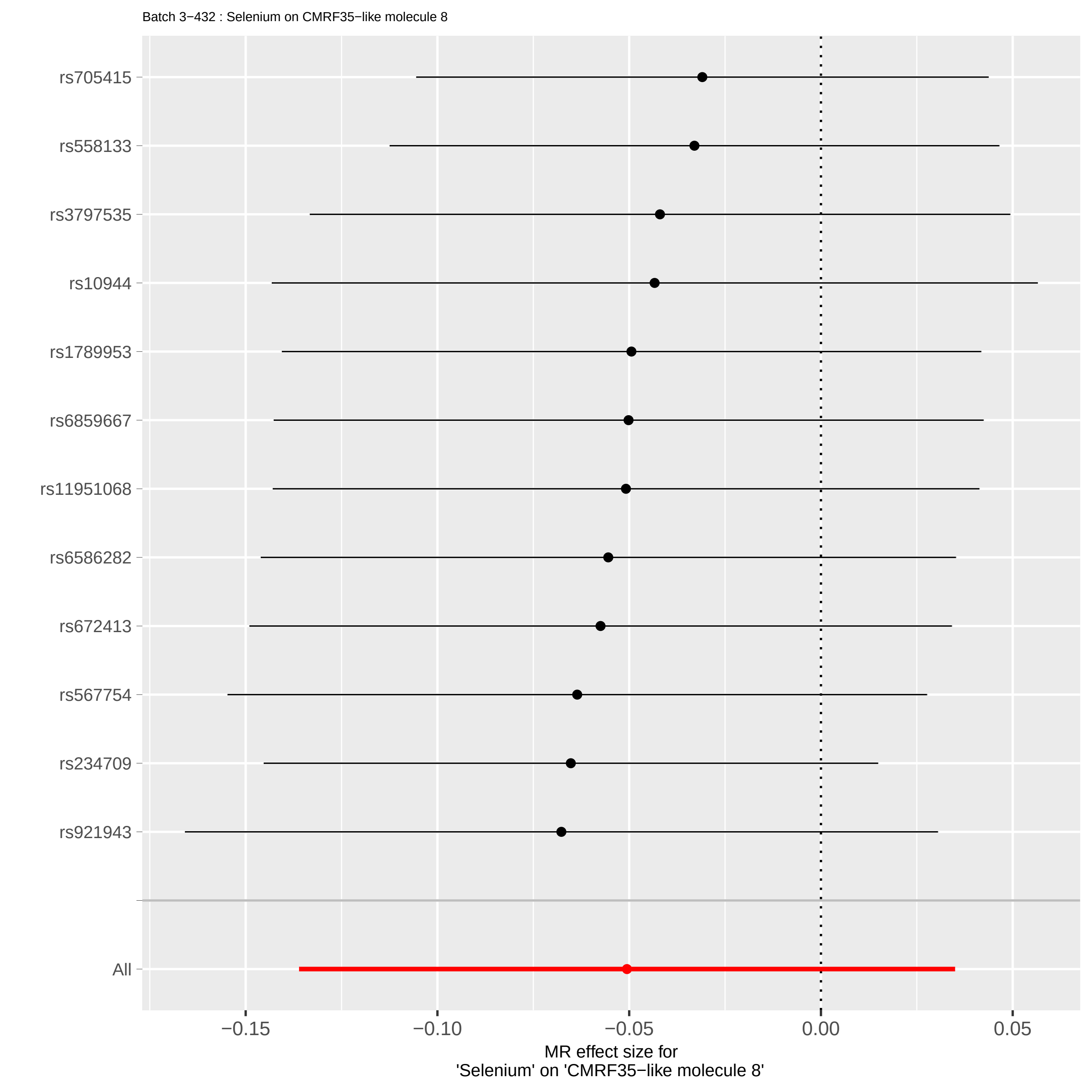
Batch 3−432 : Selenium on CMRF35−like molecule 8
rs705415
rs558133
rs3797535
rs10944
rs1789953
rs6859667
rs11951068
rs6586282
rs672413
rs567754
rs234709
rs921943
All
−0.15
−0.10
−0.05
0.00
0.05
MR effect size for
'Selenium' on 'CMRF35−like molecule 8'

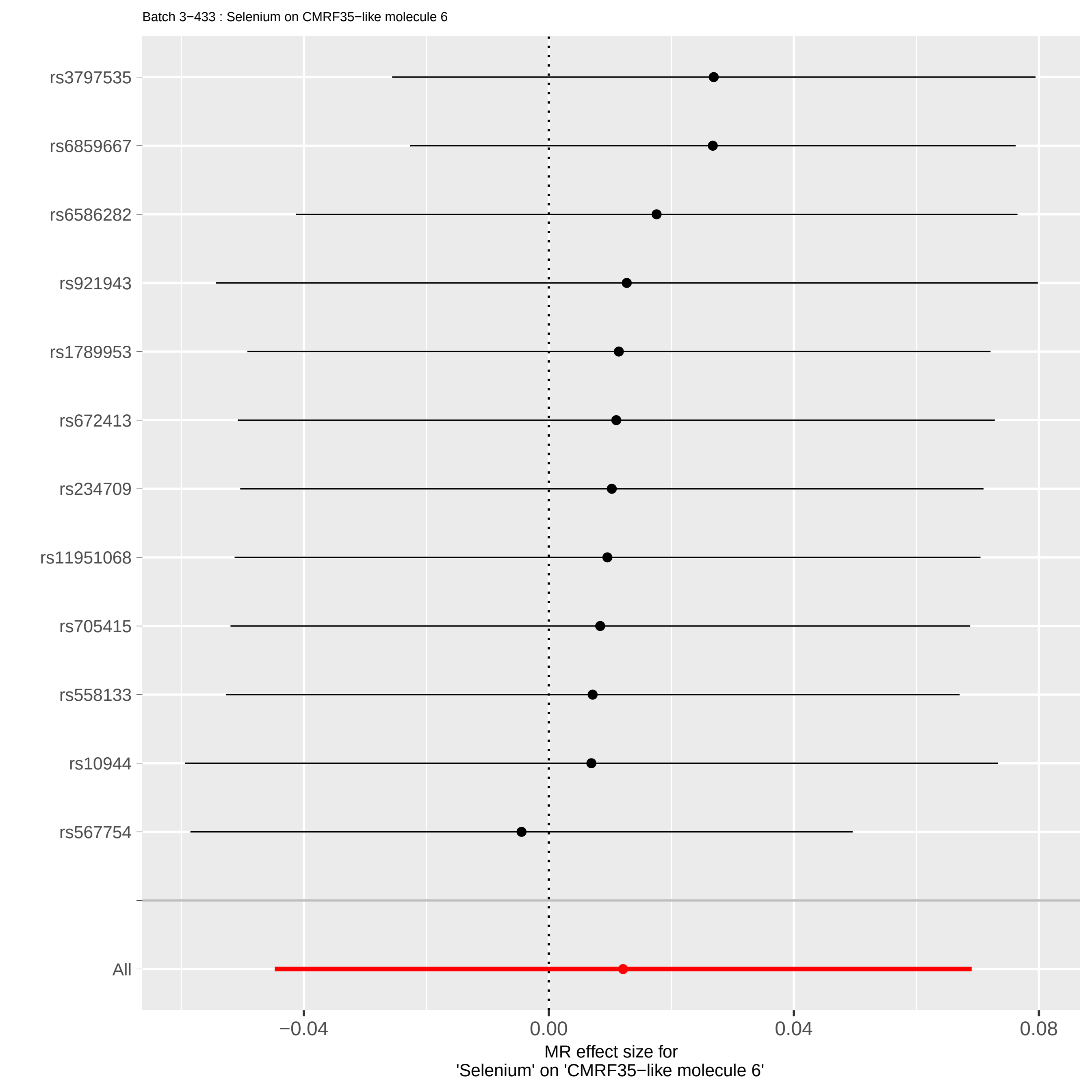
Batch 3−433 : Selenium on CMRF35−like molecule 6
rs3797535
rs6859667
rs6586282
rs921943
rs1789953
rs672413
rs234709
rs11951068
rs705415
rs558133
rs10944
rs567754
All
−0.04
0.00
0.04
0.08
MR effect size for
'Selenium' on 'CMRF35−like molecule 6'

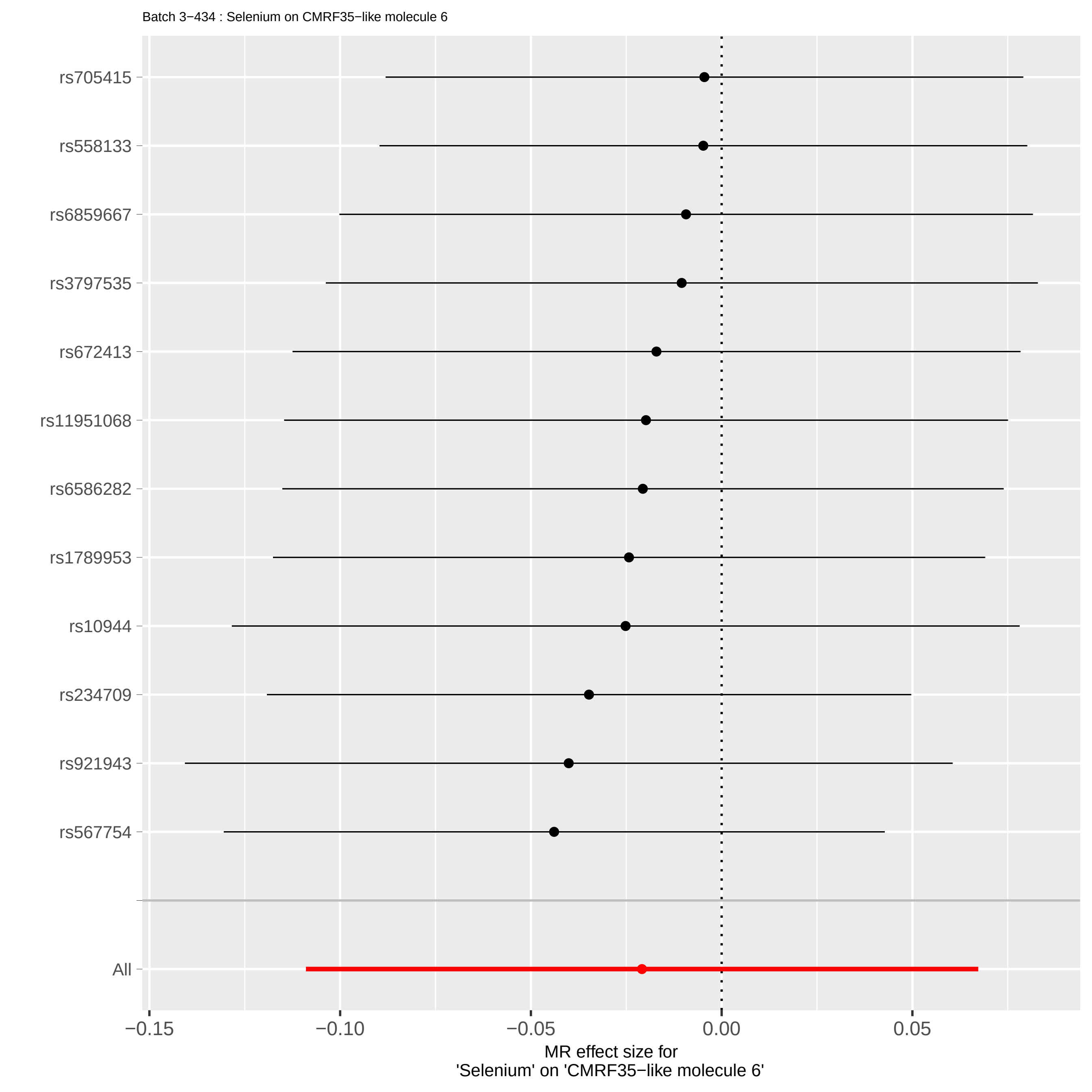
Batch 3−434 : Selenium on CMRF35−like molecule 6
rs705415
rs558133
rs6859667
rs3797535
rs672413
rs11951068
rs6586282
rs1789953
rs10944
rs234709
rs921943
rs567754
All
−0.15
−0.10
−0.05
0.00
0.05
MR effect size for
'Selenium' on 'CMRF35−like molecule 6'

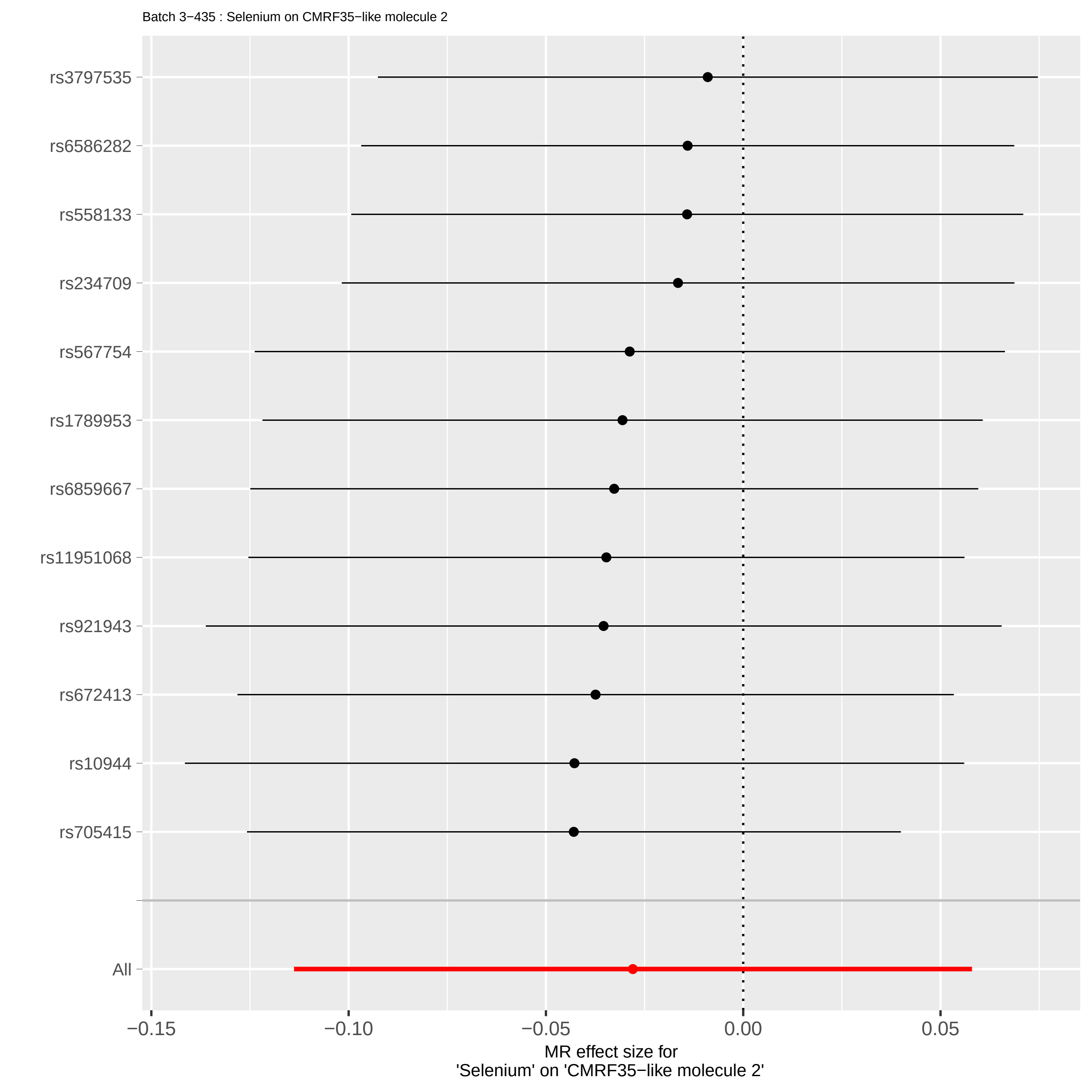

Batch 3−435 : Selenium on CMRF35−like molecule 2
rs3797535
rs6586282
rs558133
rs234709
rs567754
rs1789953
rs6859667
rs11951068
rs921943
rs672413
rs10944
rs705415
All
−0.15
−0.10
−0.05
0.00
0.05
MR effect size for
'Selenium' on 'CMRF35−like molecule 2'

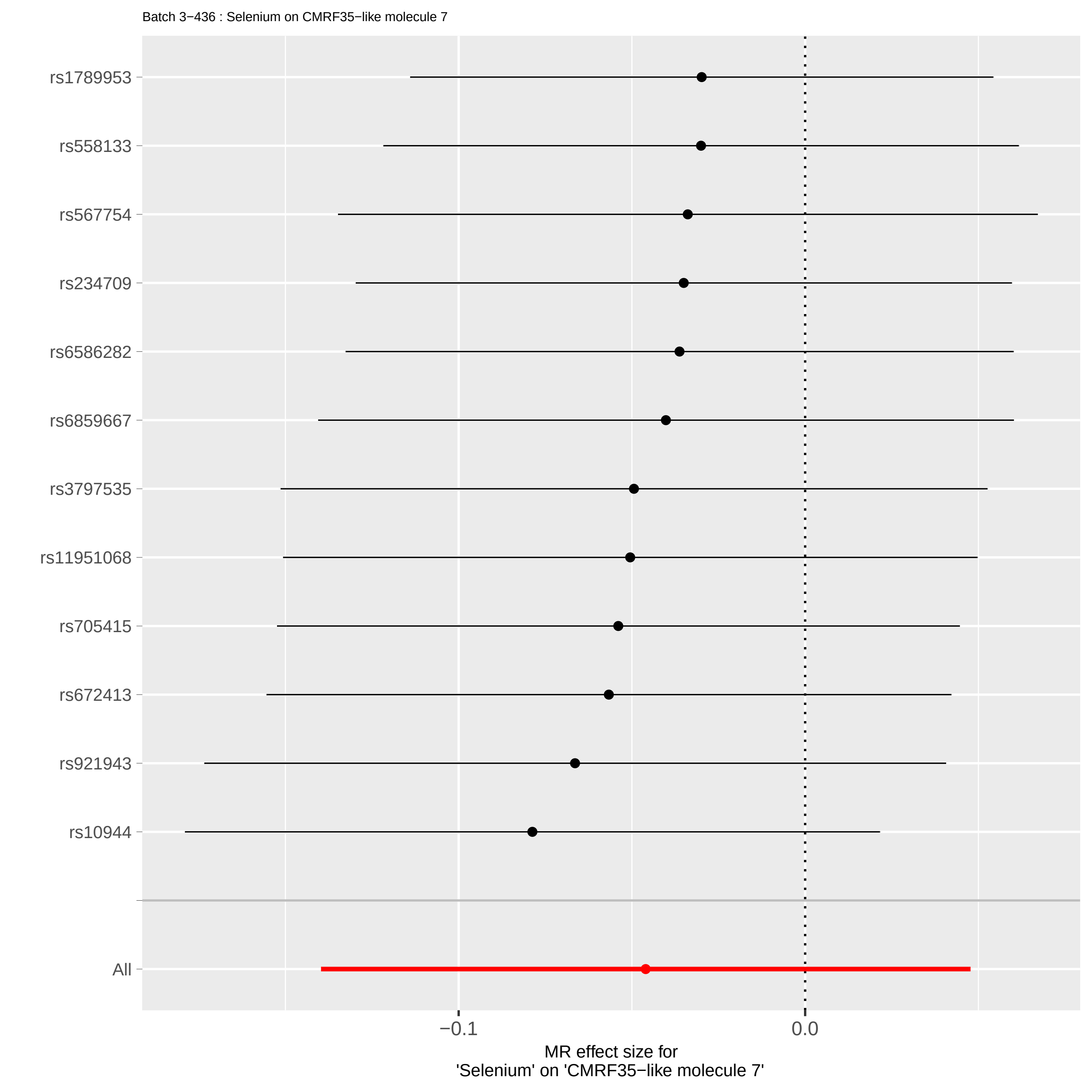
Batch 3−436 : Selenium on CMRF35−like molecule 7
rs1789953
rs558133
rs567754
rs234709
rs6586282
rs6859667
rs3797535
rs11951068
rs705415
rs672413
rs921943
rs10944
All
−0.1
0.0
MR effect size for
'Selenium' on 'CMRF35−like molecule 7'

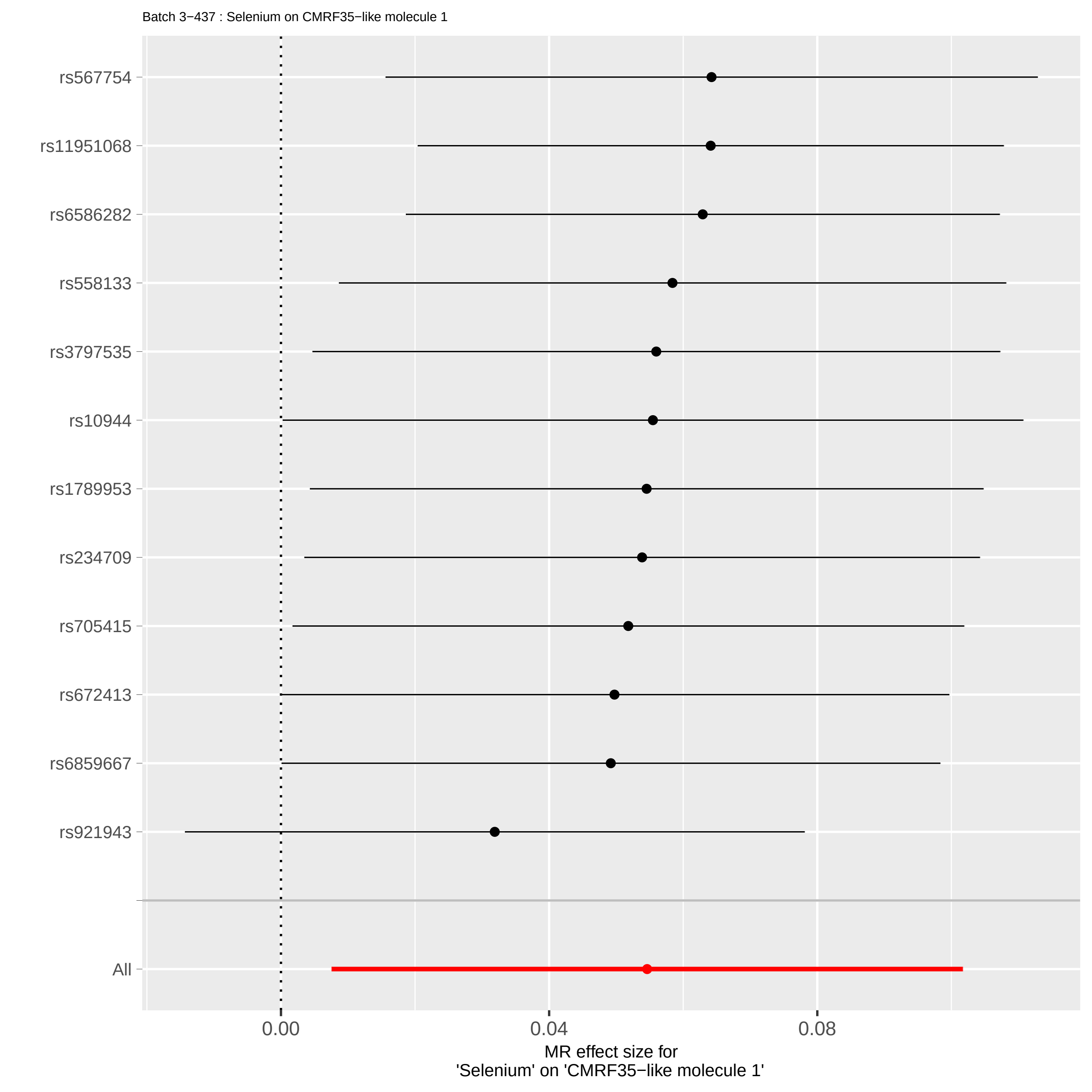

Batch 3−437 : Selenium on CMRF35−like molecule 1
rs567754
rs11951068
rs6586282
rs558133
rs3797535
rs10944
rs1789953
rs234709
rs705415
rs672413
rs6859667
rs921943
All
0.00
0.04
0.08
MR effect size for
'Selenium' on 'CMRF35−like molecule 1'

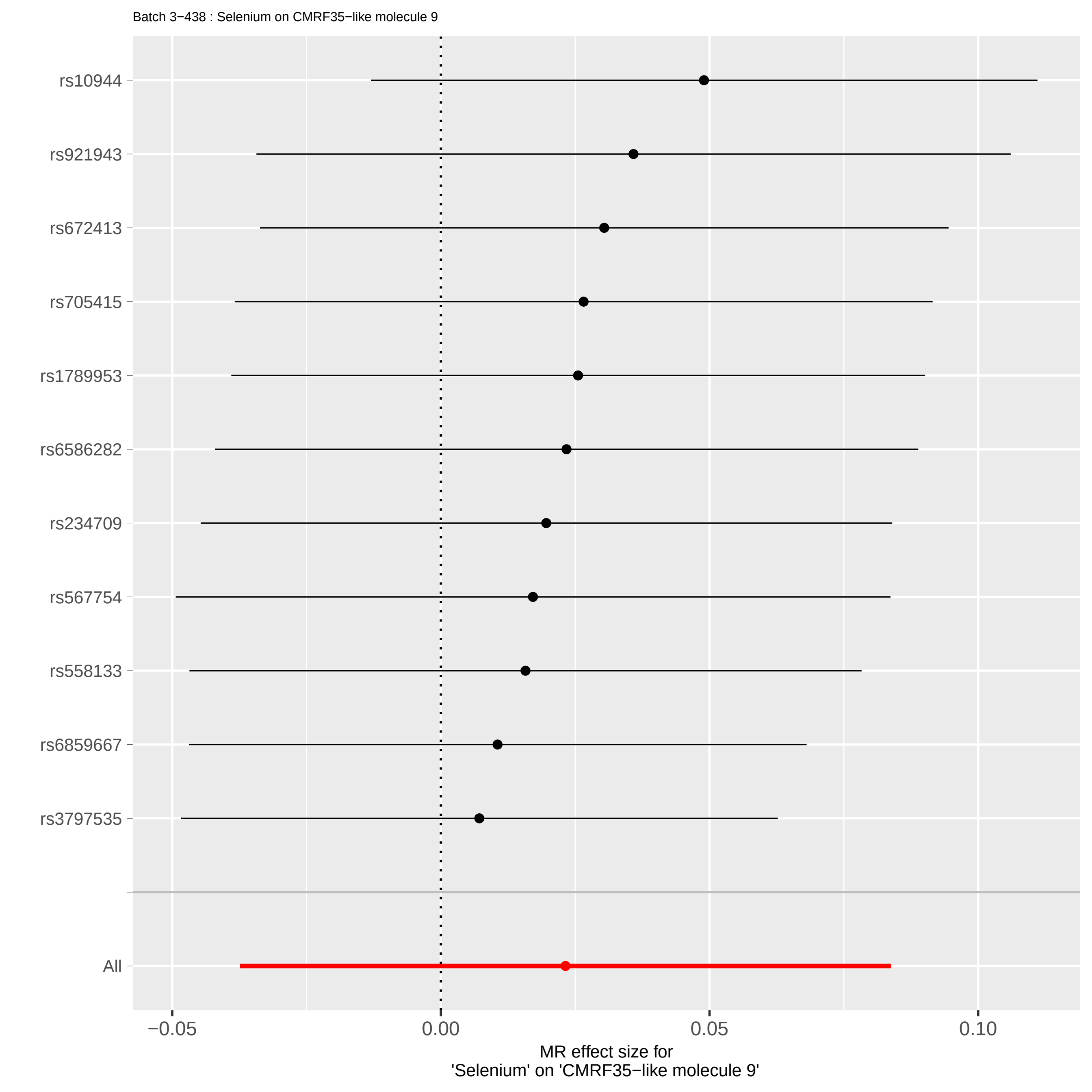

Batch 3−438 : Selenium on CMRF35−like molecule 9
rs10944
rs921943
rs672413
rs705415
rs1789953
rs6586282
rs234709
rs567754
rs558133
rs6859667
rs3797535
All
−0.05
0.00
0.05
0.10
MR effect size for
'Selenium' on 'CMRF35−like molecule 9'

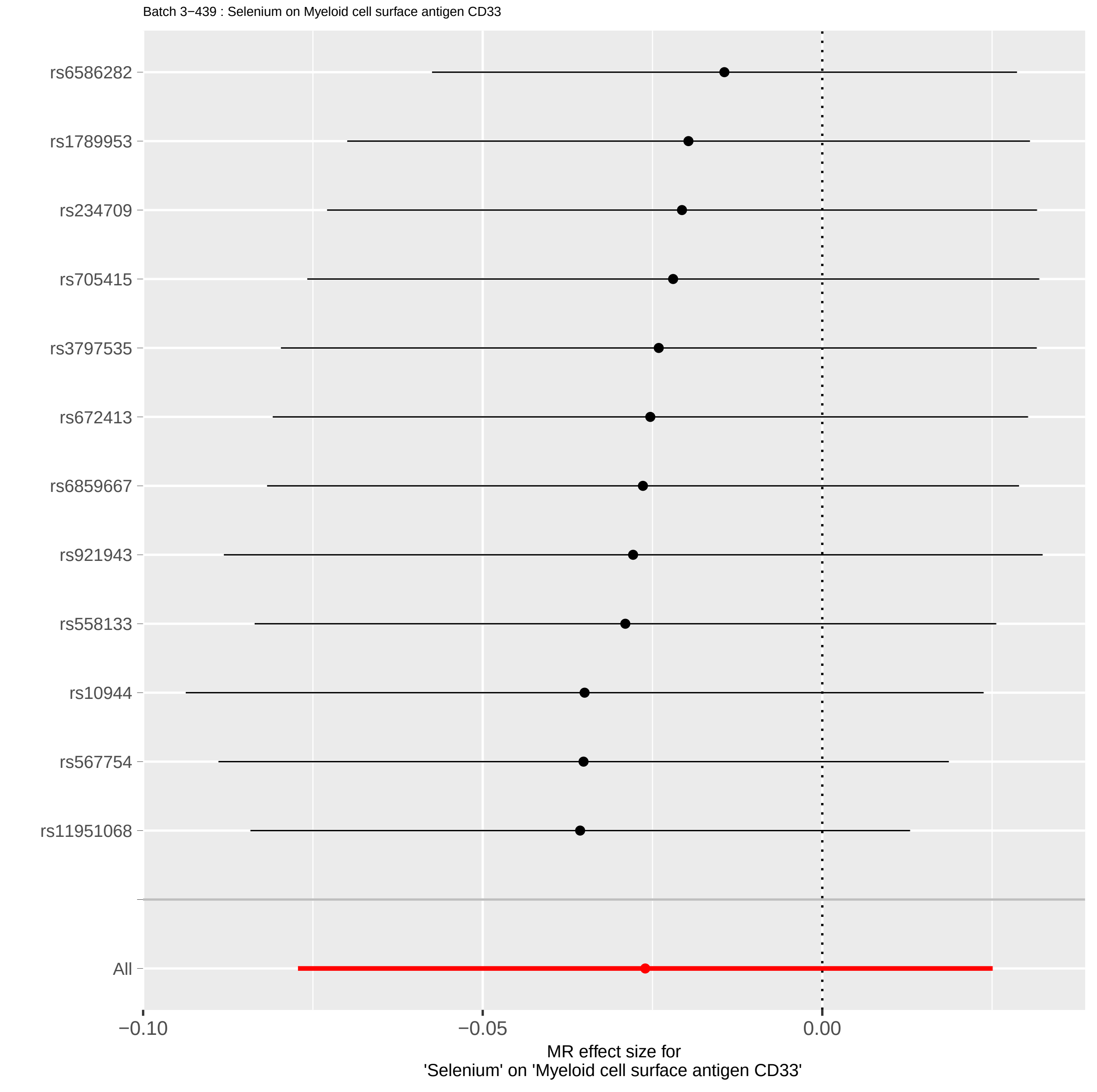

Batch 3−439 : Selenium on Myeloid cell surface antigen CD33
rs6586282
rs1789953
rs234709
rs705415
rs3797535
rs672413
rs6859667
rs921943
rs558133
rs10944
rs567754
rs11951068
All
−0.10
−0.05
0.00
MR effect size for
'Selenium' on 'Myeloid cell surface antigen CD33'

Batch 3−440 : Selenium on Hematopoietic progenitor cell antigen CD34

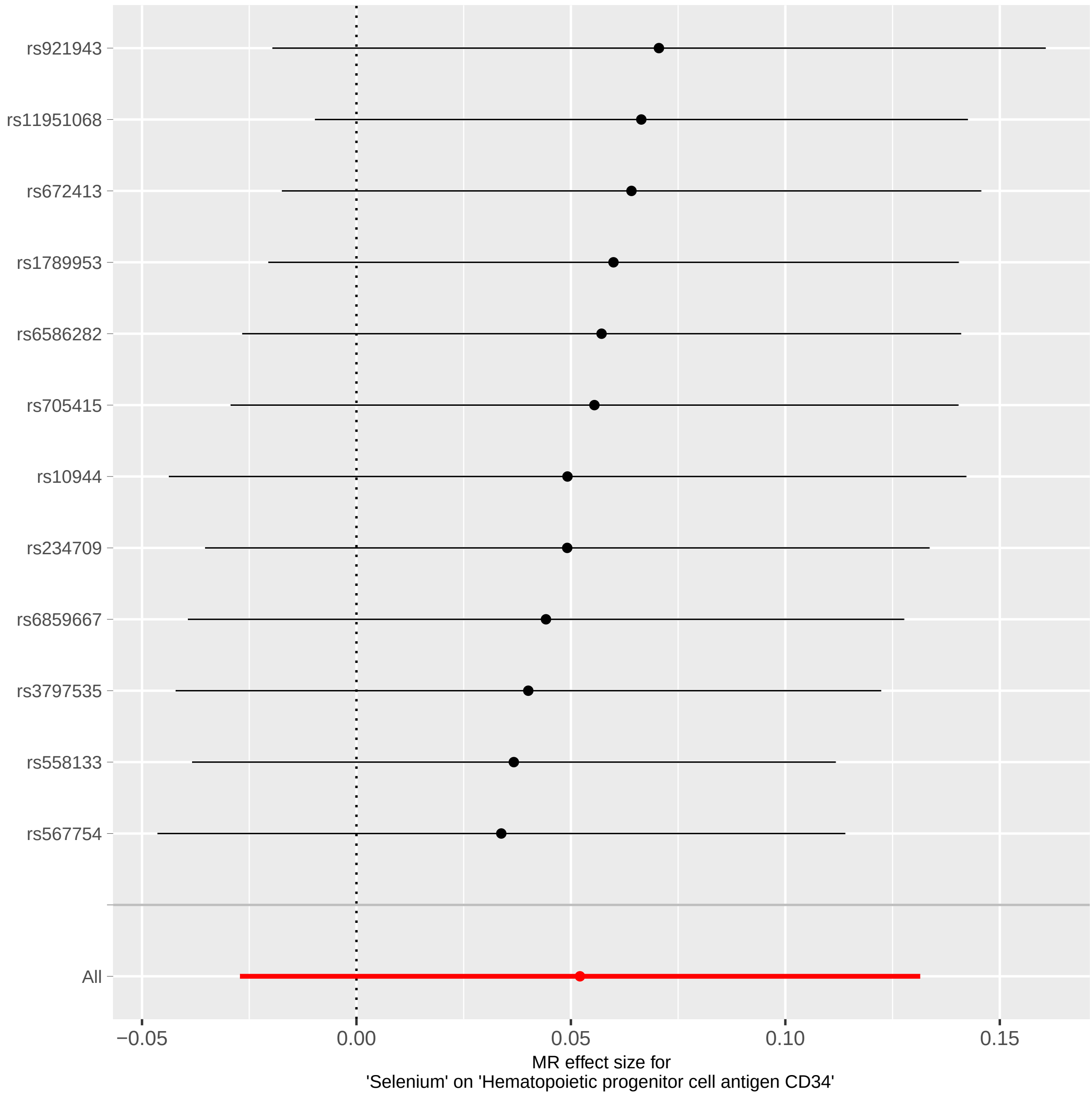

Batch 3−441 : Selenium on Hematopoietic progenitor cell antigen CD34

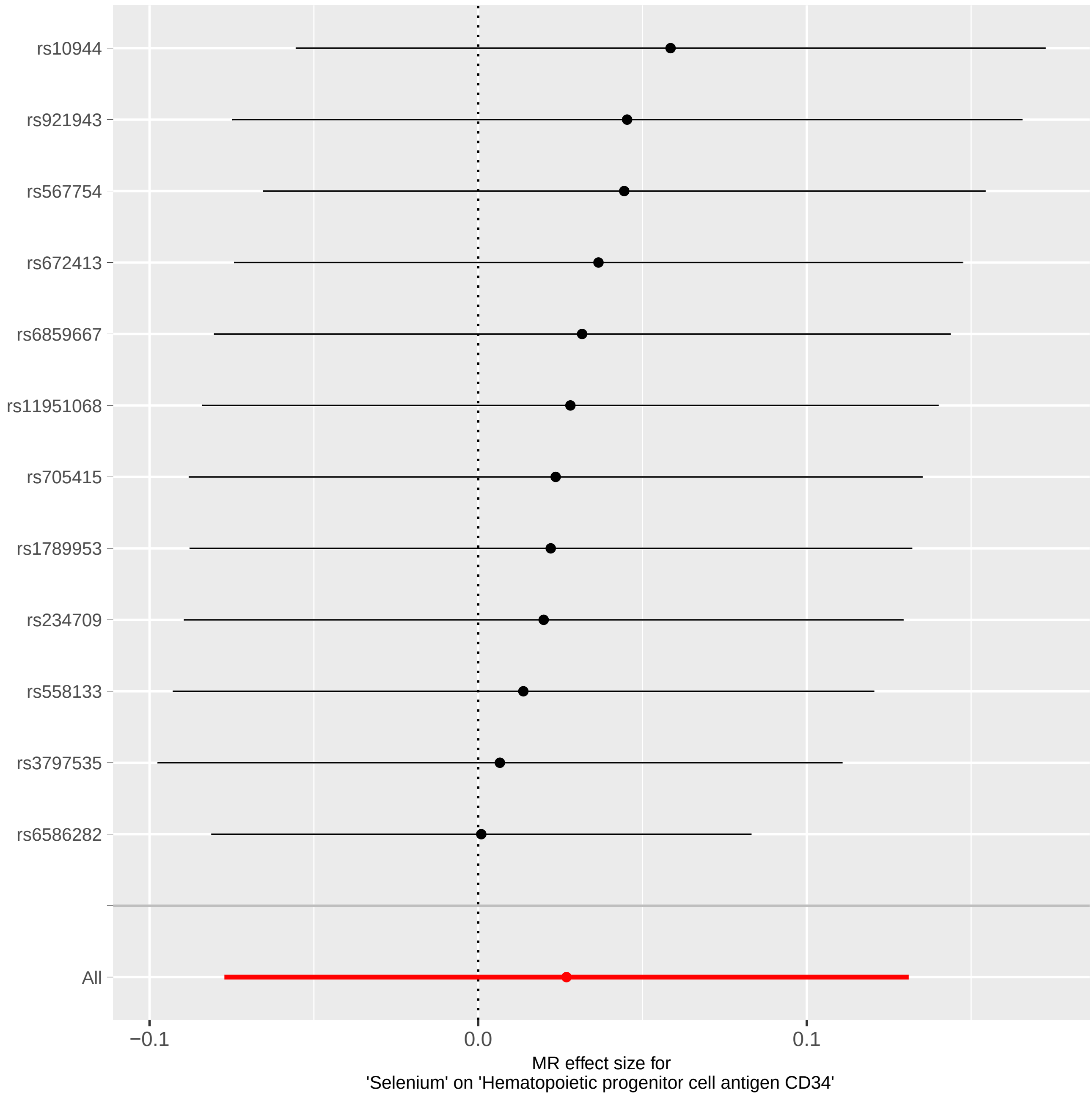

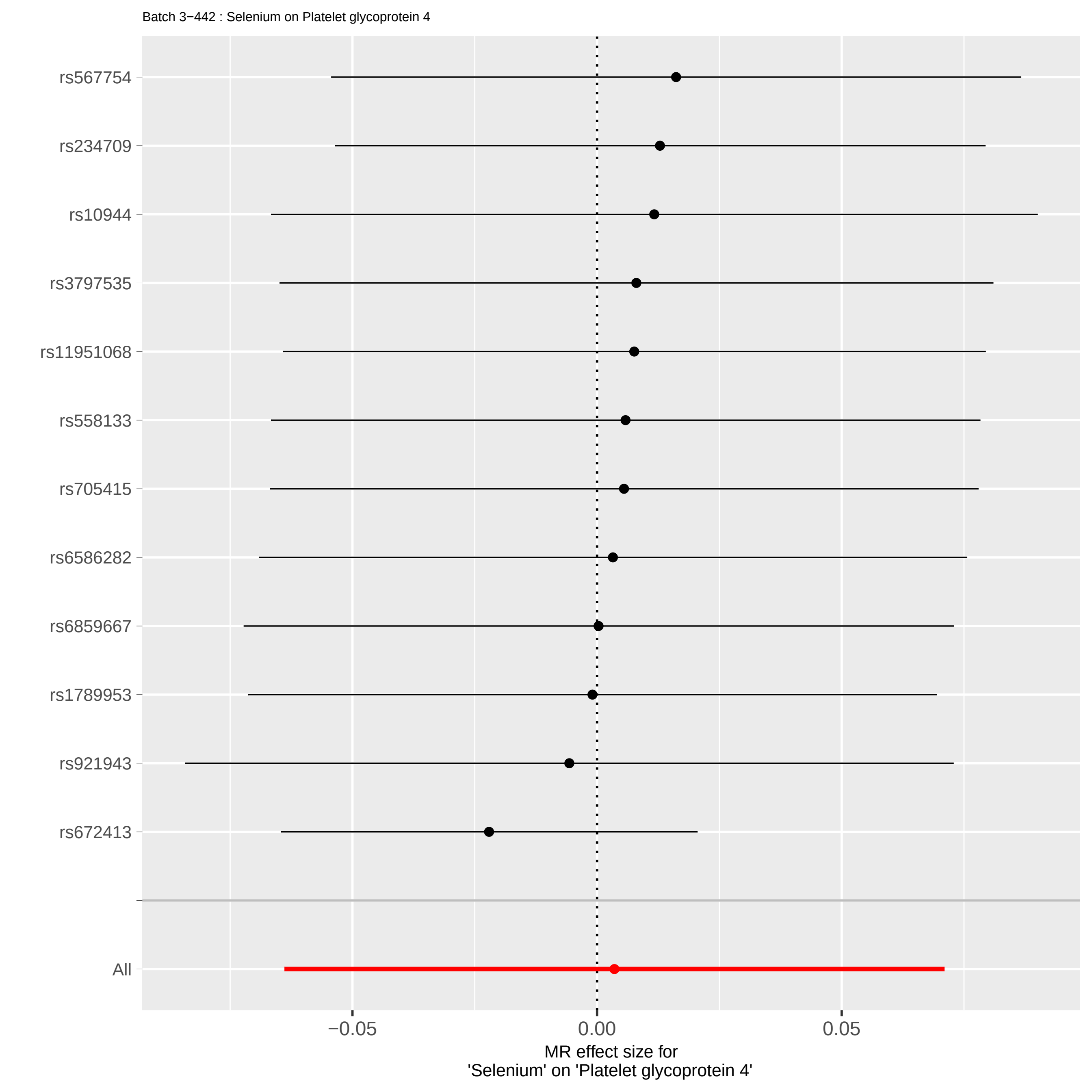

Batch 3−442 : Selenium on Platelet glycoprotein 4
rs567754
rs234709
rs10944
rs3797535
rs11951068
rs558133
rs705415
rs6586282
rs6859667
rs1789953
rs921943
rs672413
All
−0.05
0.00
0.05
MR effect size for
'Selenium' on 'Platelet glycoprotein 4'

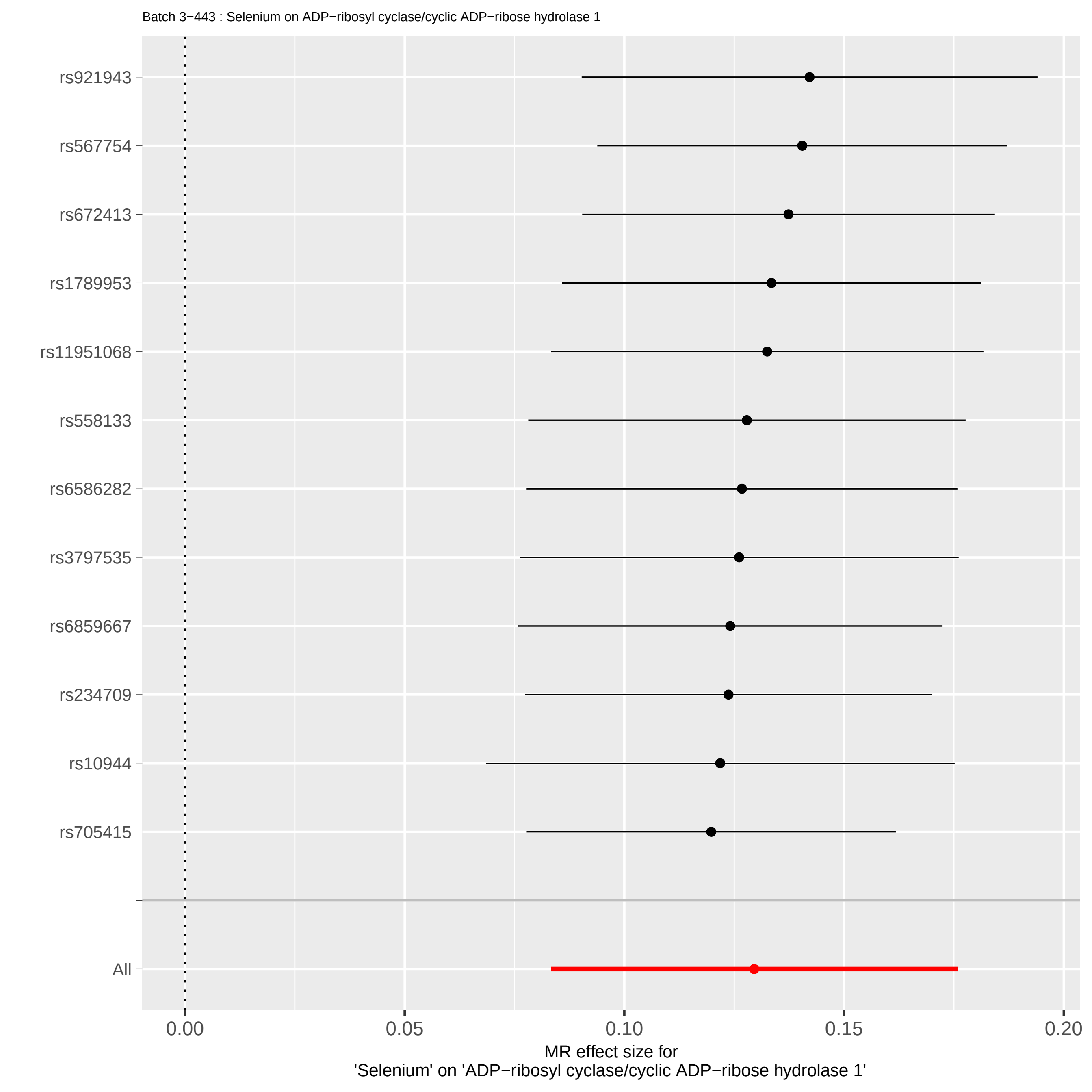
Batch 3−443 : Selenium on ADP−ribosyl cyclase/cyclic ADP−ribose hydrolase 1
rs921943
rs567754
rs672413
rs1789953
rs11951068
rs558133
rs6586282
rs3797535
rs6859667
rs234709
rs10944
rs705415
All
0.00
0.05
0.10
0.15
0.20
MR effect size for
'Selenium' on 'ADP−ribosyl cyclase/cyclic ADP−ribose hydrolase 1'

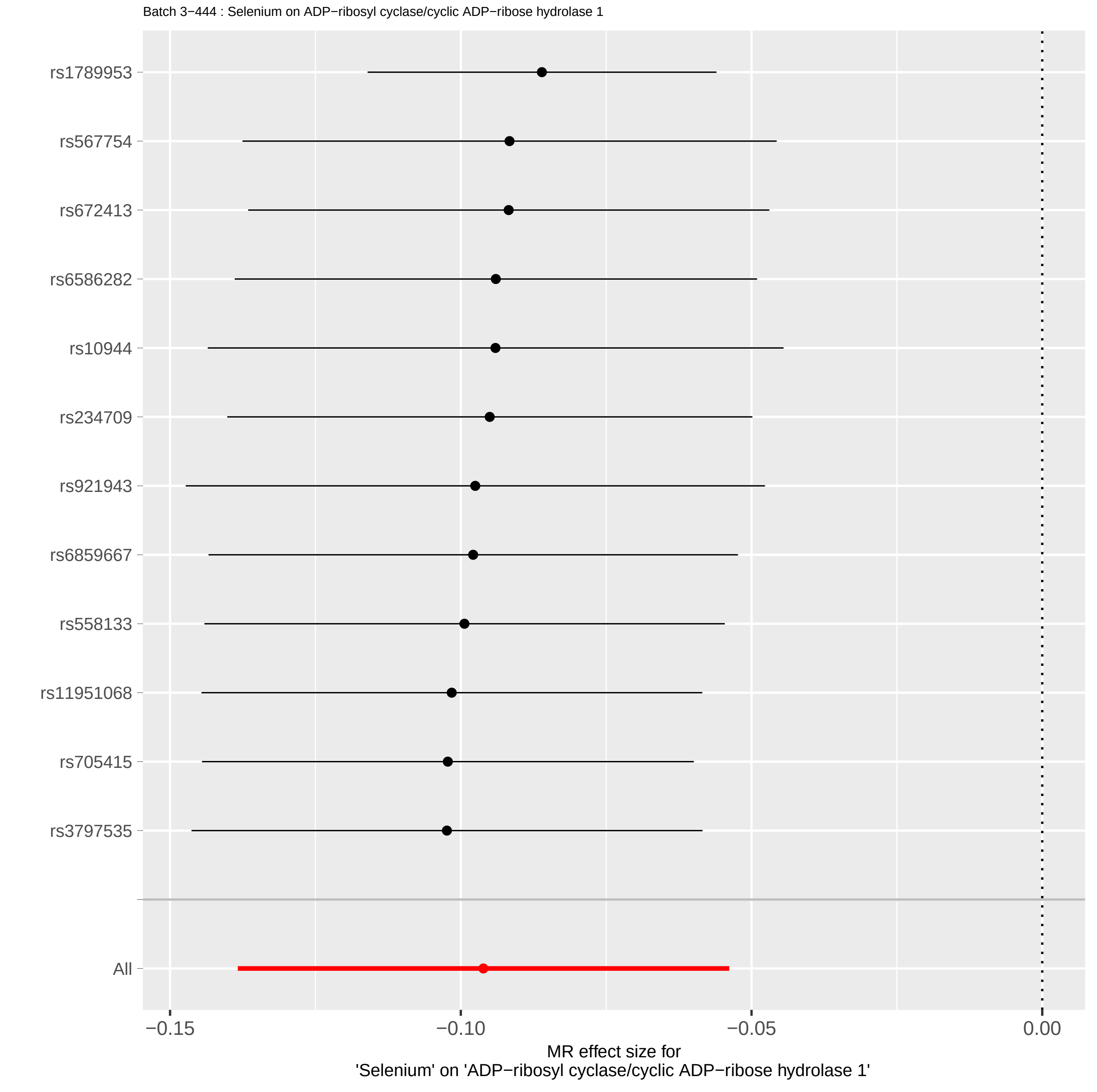

Batch 3−444 : Selenium on ADP−ribosyl cyclase/cyclic ADP−ribose hydrolase 1
rs1789953
rs567754
rs672413
rs6586282
rs10944
rs234709
rs921943
rs6859667
rs558133
rs11951068
rs705415
rs3797535
All
−0.15
−0.10
−0.05
0.00
MR effect size for
'Selenium' on 'ADP−ribosyl cyclase/cyclic ADP−ribose hydrolase 1'

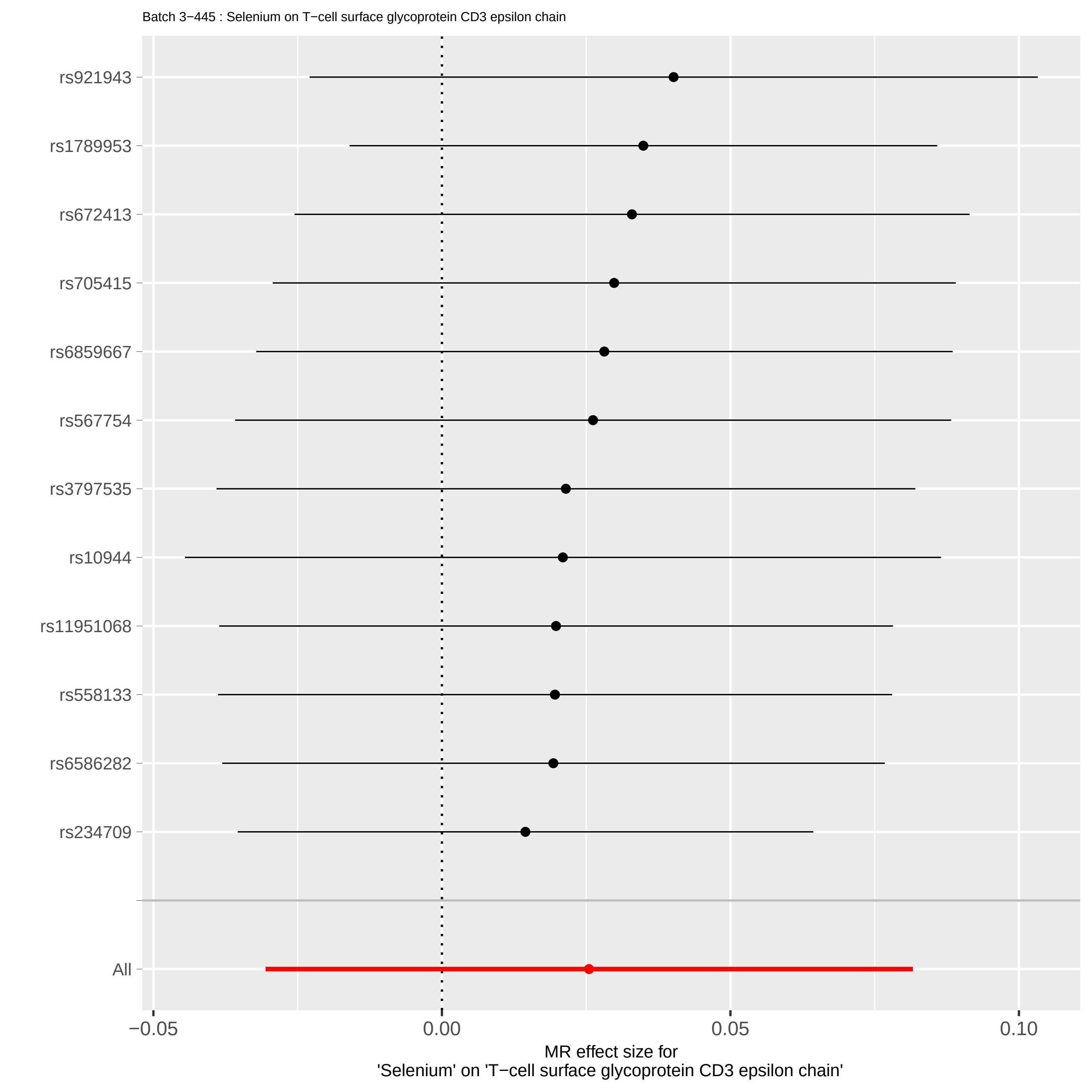

Batch 3−445 : Selenium on T−cell surface glycoprotein CD3 epsilon chain
rs921943
rs1789953
rs672413
rs705415
rs6859667
rs567754
rs3797535
rs10944
rs11951068
rs558133
rs6586282
rs234709
All
−0.05
0.00
0.05
0.10
MR effect size for
'Selenium' on 'T−cell surface glycoprotein CD3 epsilon chain'

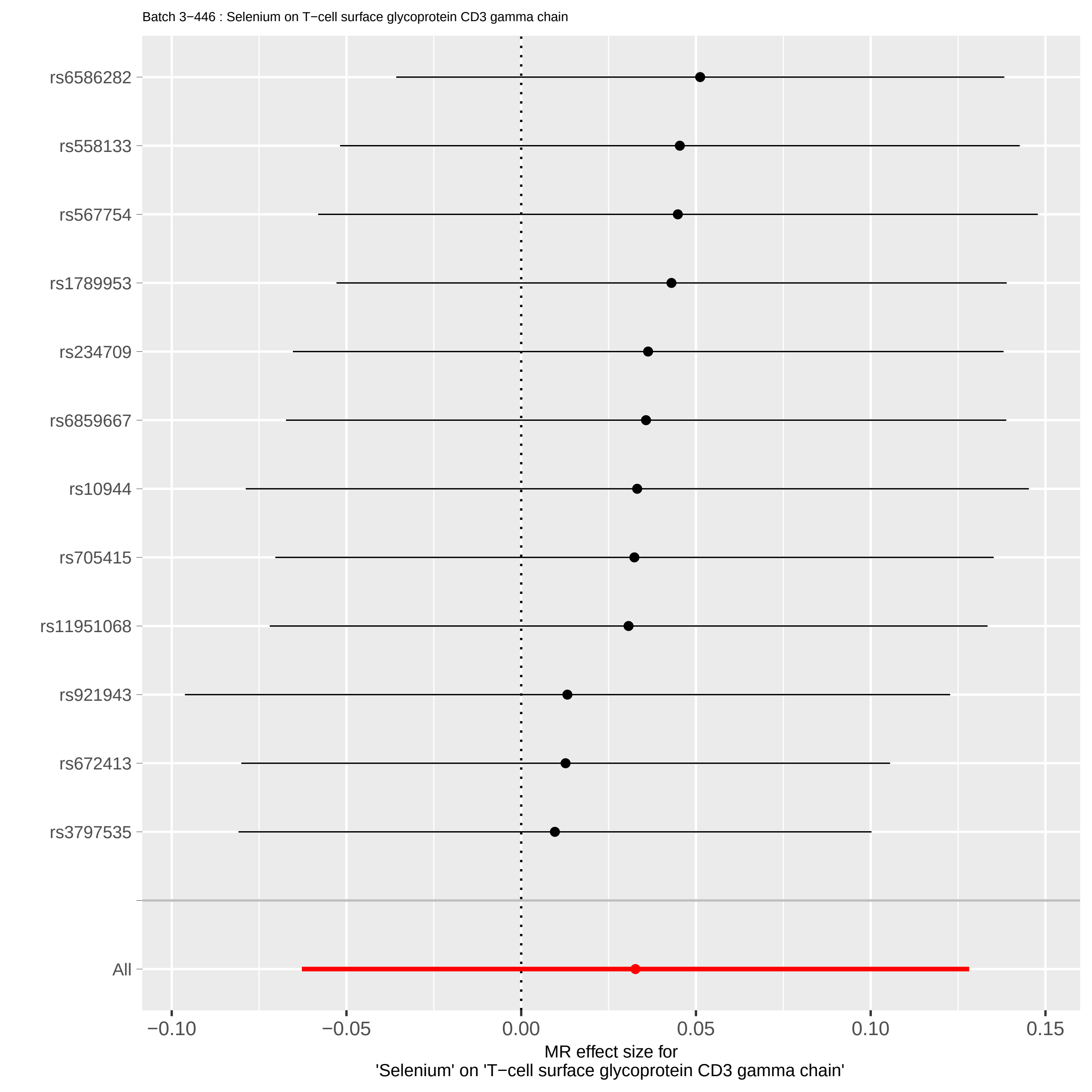

Batch 3−446 : Selenium on T−cell surface glycoprotein CD3 gamma chain
rs6586282
rs558133
rs567754
rs1789953
rs234709
rs6859667
rs10944
rs705415
rs11951068
rs921943
rs672413
rs3797535
All
−0.10
−0.05
0.00
0.05
0.10
0.15
MR effect size for
'Selenium' on 'T−cell surface glycoprotein CD3 gamma chain'

Batch 3−447 : Selenium on CD40 ligand

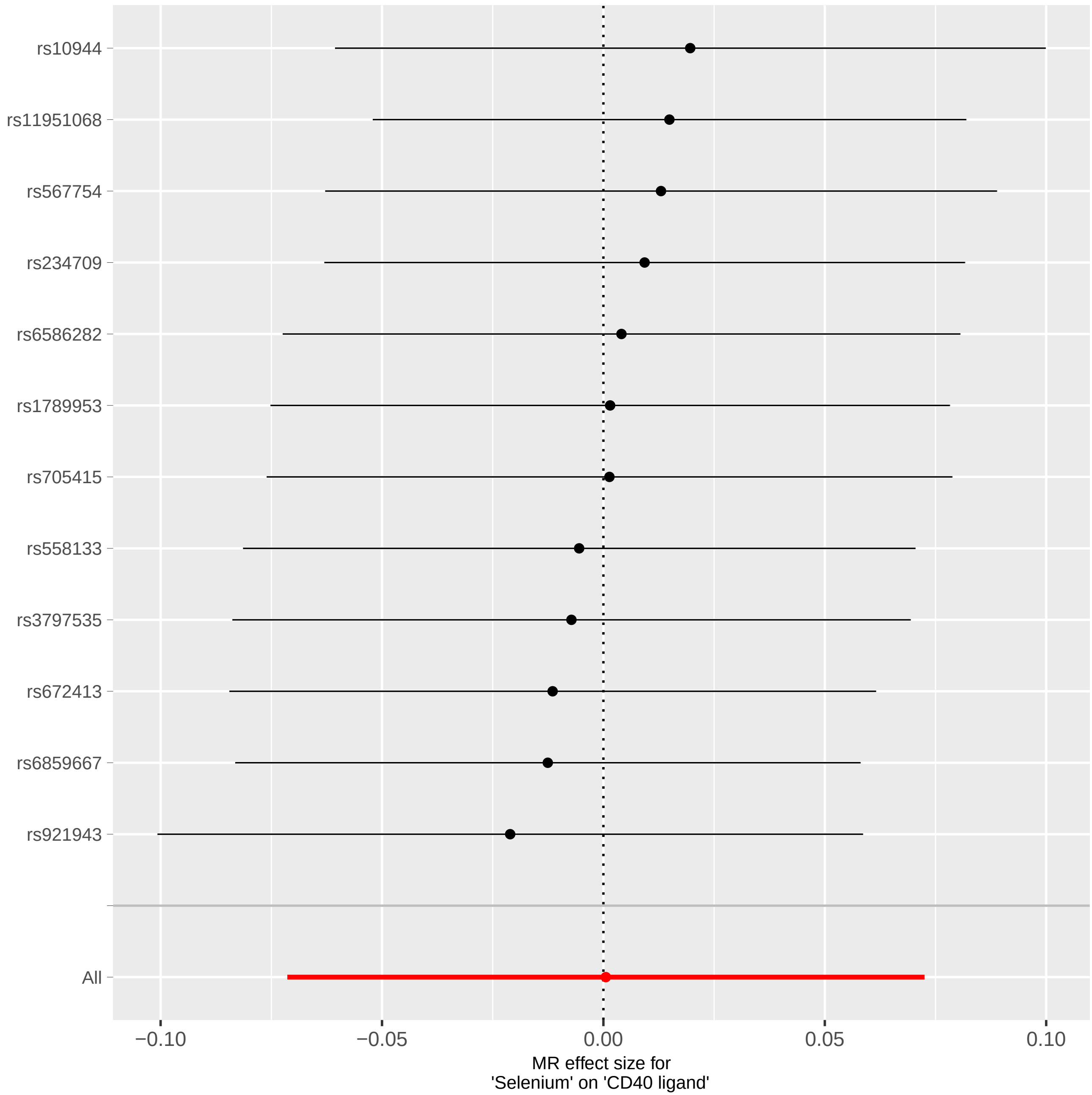

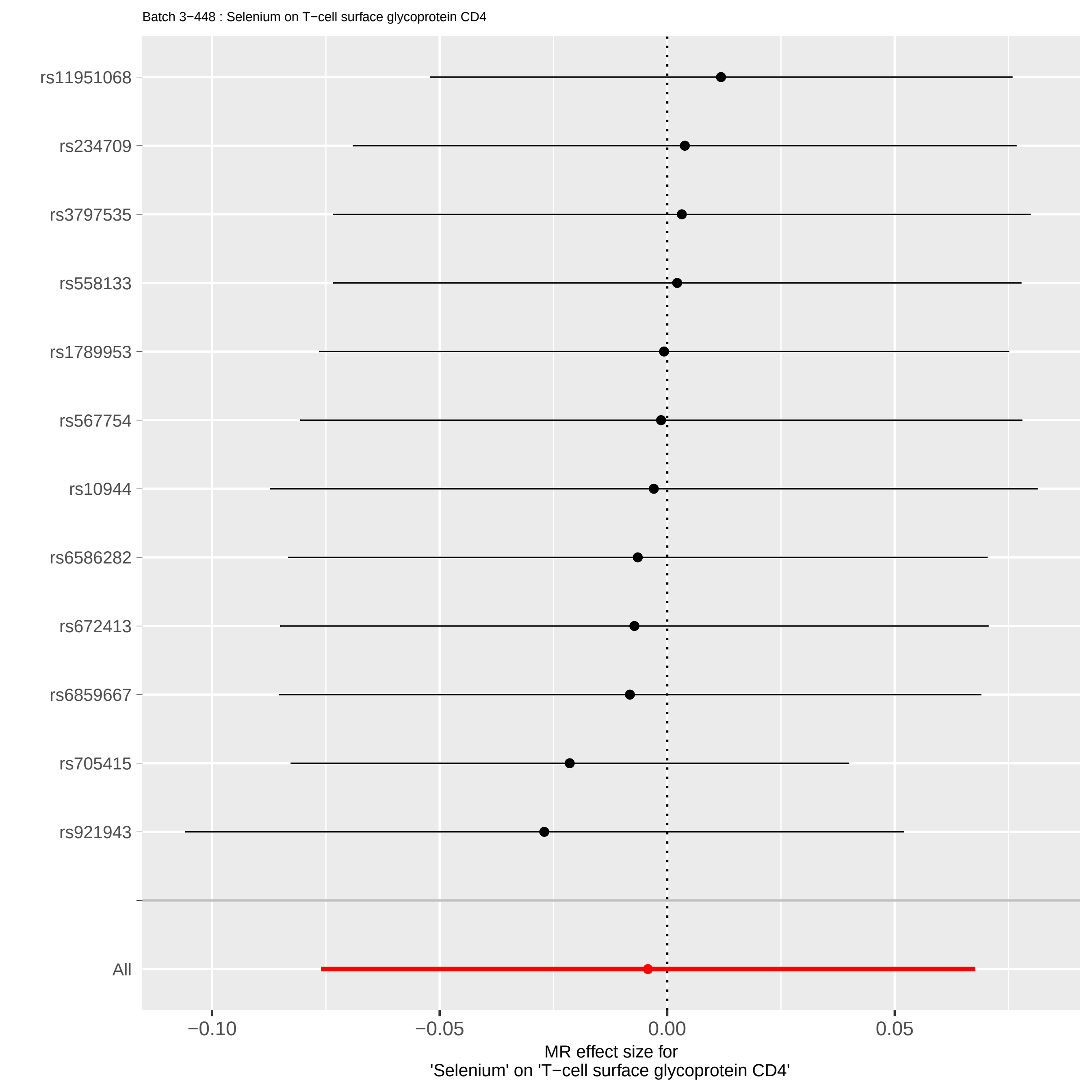
Batch 3−448 : Selenium on T−cell surface glycoprotein CD4
rs11951068
rs234709
rs3797535
rs558133
rs1789953
rs567754
rs10944
rs6586282
rs672413
rs6859667
rs705415
rs921943
All
−0.10
−0.05
0.00
0.05
MR effect size for
'Selenium' on 'T−cell surface glycoprotein CD4'

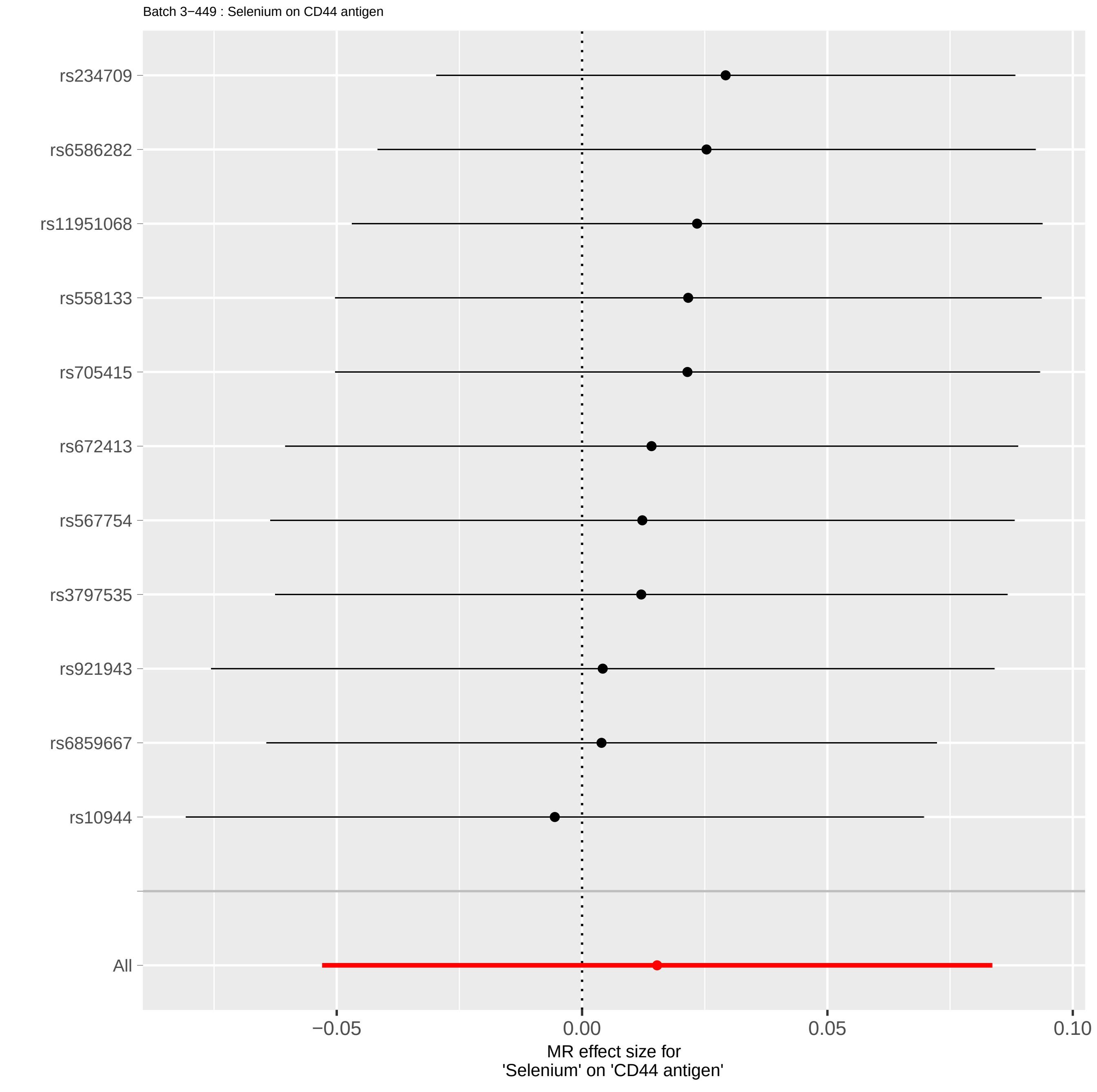

Batch 3−449 : Selenium on CD44 antigen
rs234709
rs6586282
rs11951068
rs558133
rs705415
rs672413
rs567754
rs3797535
rs921943
rs6859667
rs10944
All
−0.05
0.00
0.05
0.10
MR effect size for
'Selenium' on 'CD44 antigen'

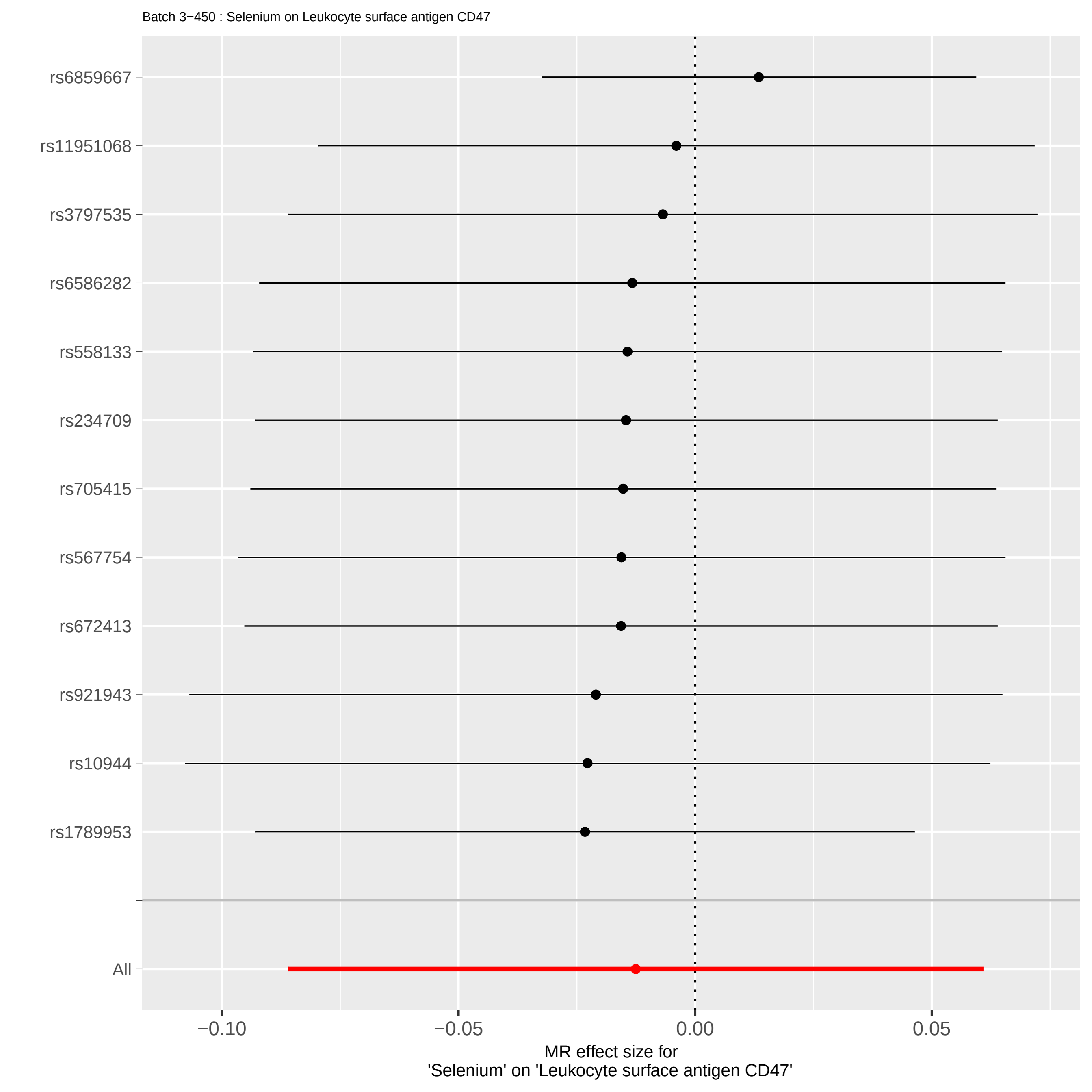
Batch 3−450 : Selenium on Leukocyte surface antigen CD47
rs6859667
rs11951068
rs3797535
rs6586282
rs558133
rs234709
rs705415
rs567754
rs672413
rs921943
rs10944
rs1789953
All
−0.10
−0.05
0.00
0.05
MR effect size for
'Selenium' on 'Leukocyte surface antigen CD47'

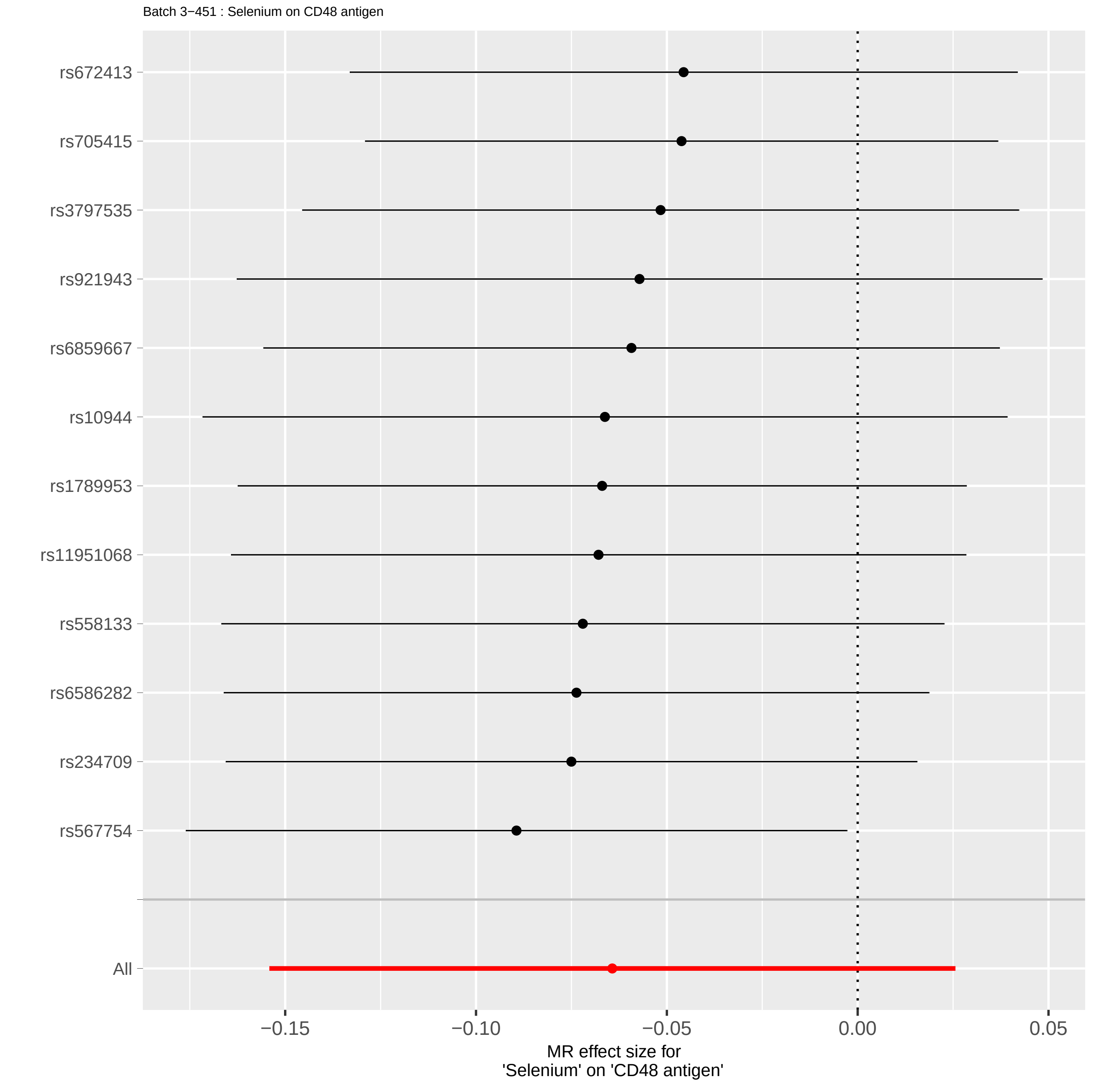

Batch 3−451 : Selenium on CD48 antigen
rs672413
rs705415
rs3797535
rs921943
rs6859667
rs10944
rs1789953
rs11951068
rs558133
rs6586282
rs234709
rs567754
All
−0.15
−0.10
−0.05
0.00
0.05
MR effect size for
'Selenium' on 'CD48 antigen'

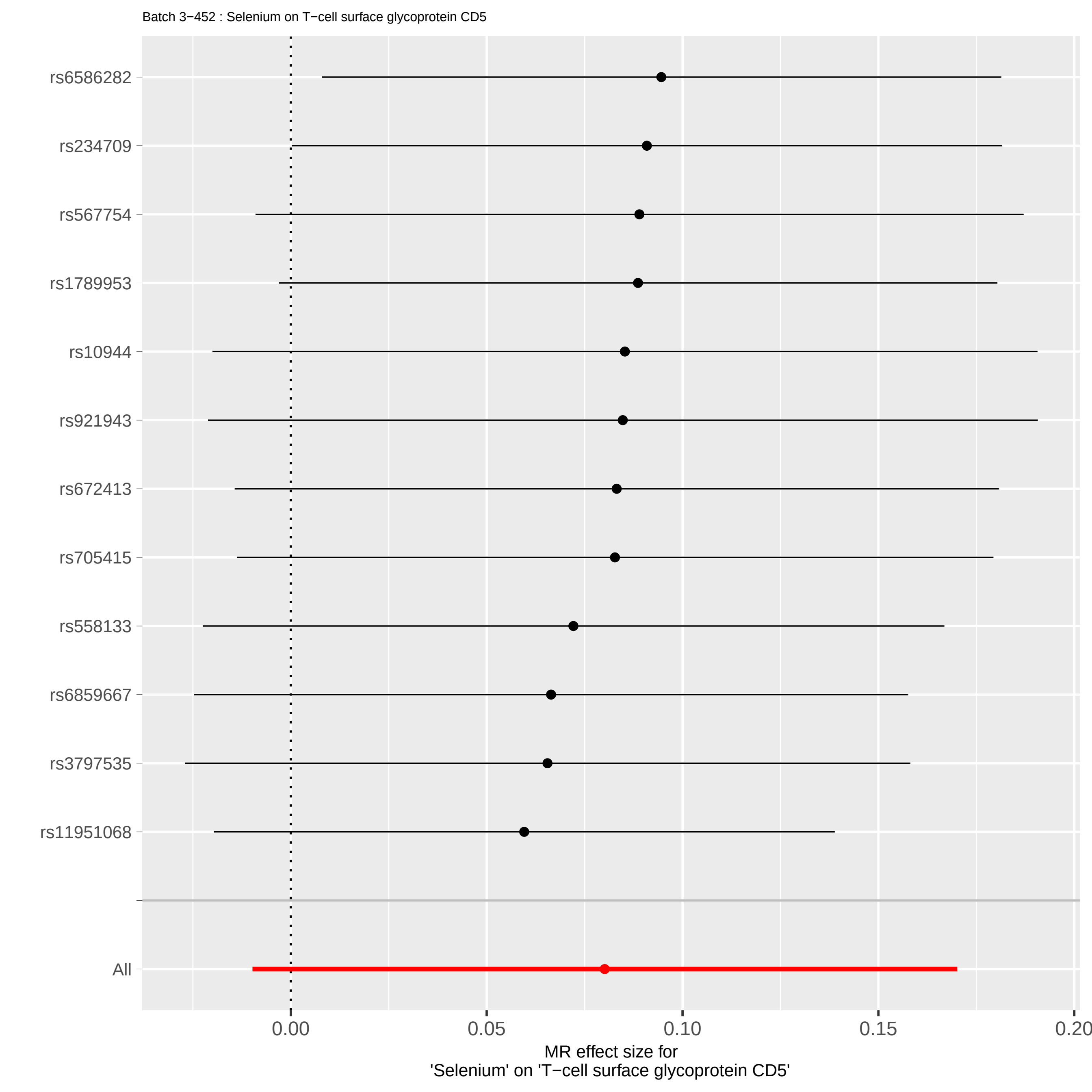

Batch 3−452 : Selenium on T−cell surface glycoprotein CD5
rs6586282
rs234709
rs567754
rs1789953
rs10944
rs921943
rs672413
rs705415
rs558133
rs6859667
rs3797535
rs11951068
All
0.00
0.05
0.10
0.15
0.20
MR effect size for
'Selenium' on 'T−cell surface glycoprotein CD5'

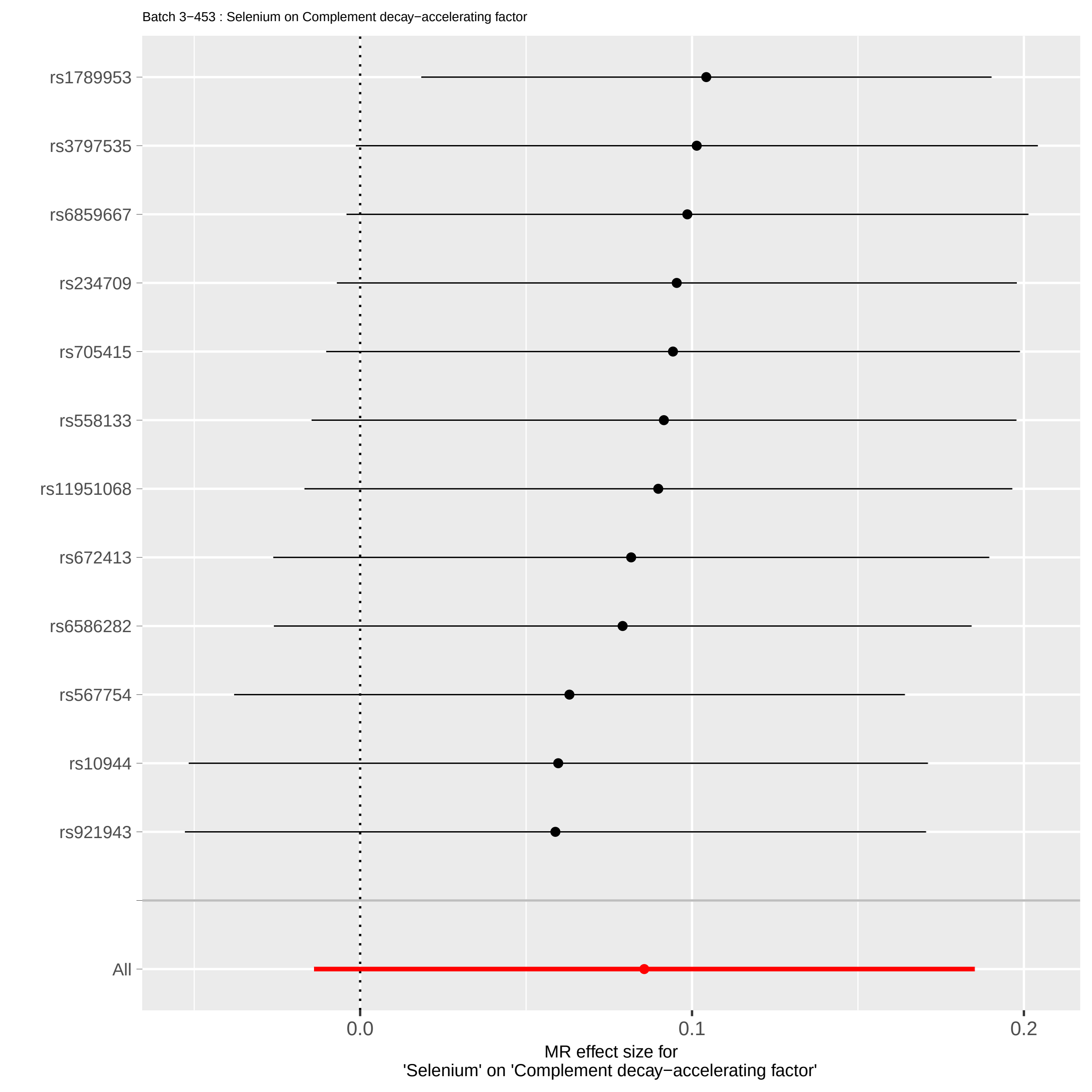
Batch 3−453 : Selenium on Complement decay−accelerating factor
rs1789953
rs3797535
rs6859667
rs234709
rs705415
rs558133
rs11951068
rs672413
rs6586282
rs567754
rs10944
rs921943
All
0.0
0.1
0.2
MR effect size for
'Selenium' on 'Complement decay−accelerating factor'

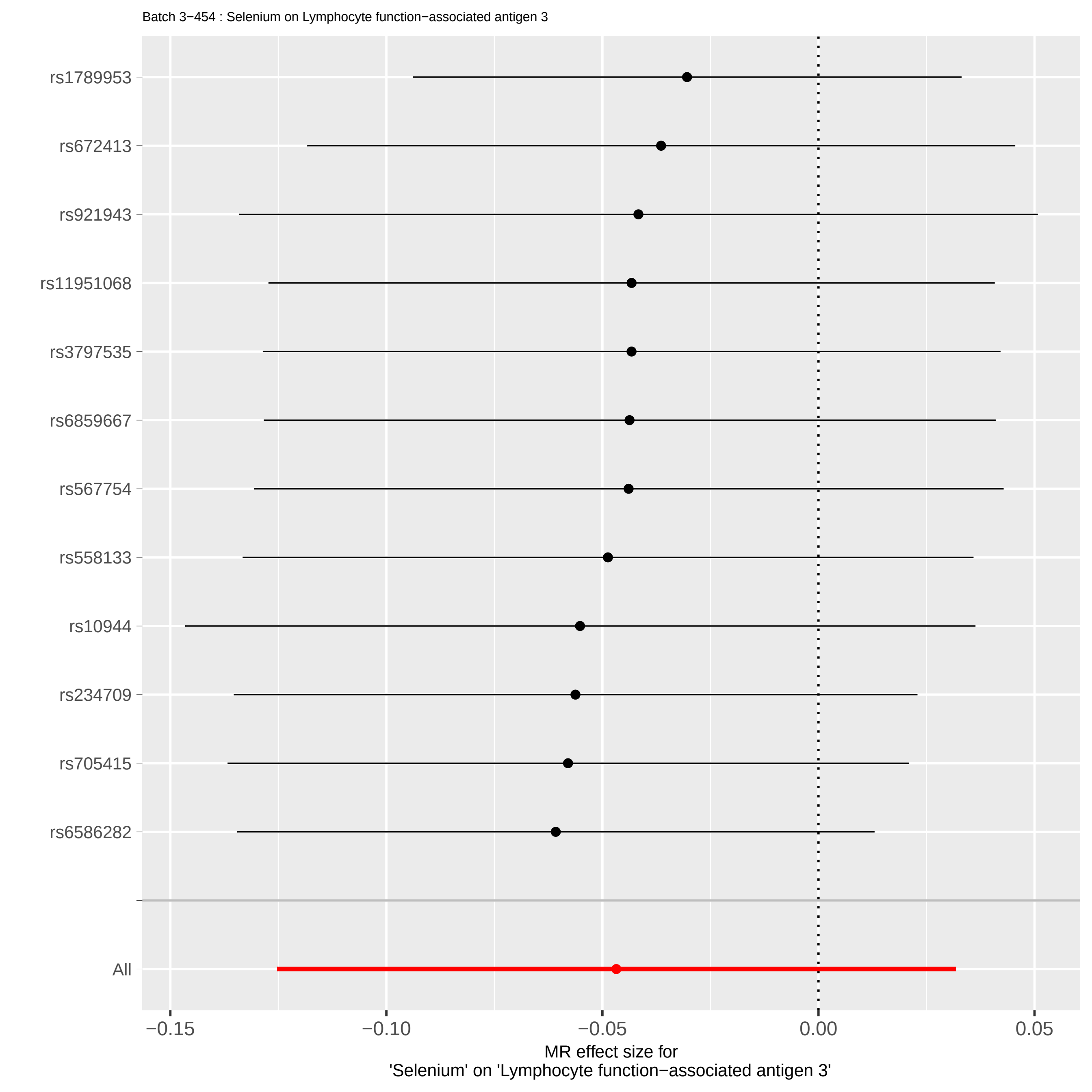
Batch 3−454 : Selenium on Lymphocyte function−associated antigen 3
rs1789953
rs672413
rs921943
rs11951068
rs3797535
rs6859667
rs567754
rs558133
rs10944
rs234709
rs705415
rs6586282
All
−0.15
−0.10
−0.05
0.00
0.05
MR effect size for
'Selenium' on 'Lymphocyte function−associated antigen 3'

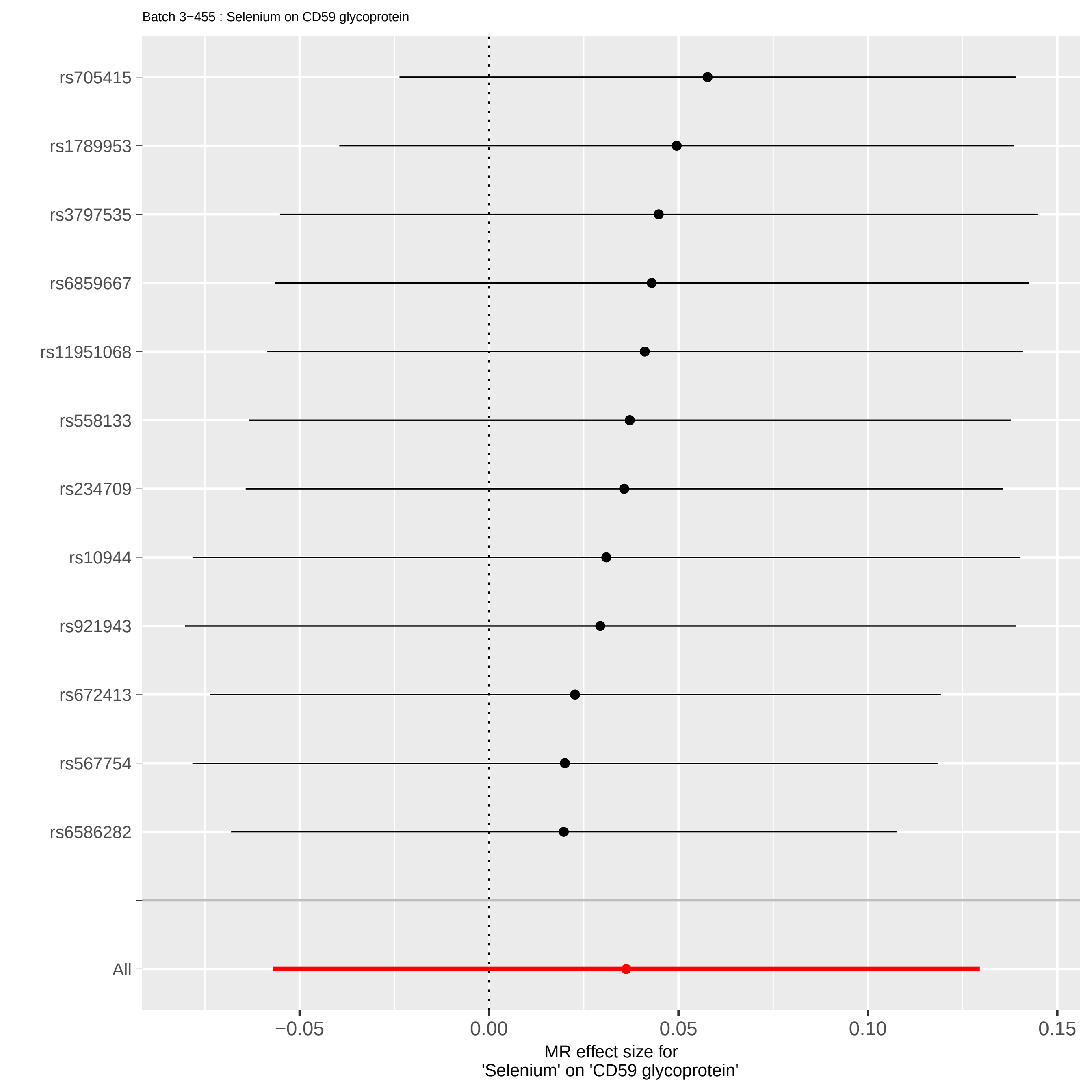

Batch 3−455 : Selenium on CD59 glycoprotein
rs705415
rs1789953
rs3797535
rs6859667
rs11951068
rs558133
rs234709
rs10944
rs921943
rs672413
rs567754
rs6586282
All
−0.05
0.00
0.05
0.10
0.15
MR effect size for
'Selenium' on 'CD59 glycoprotein'

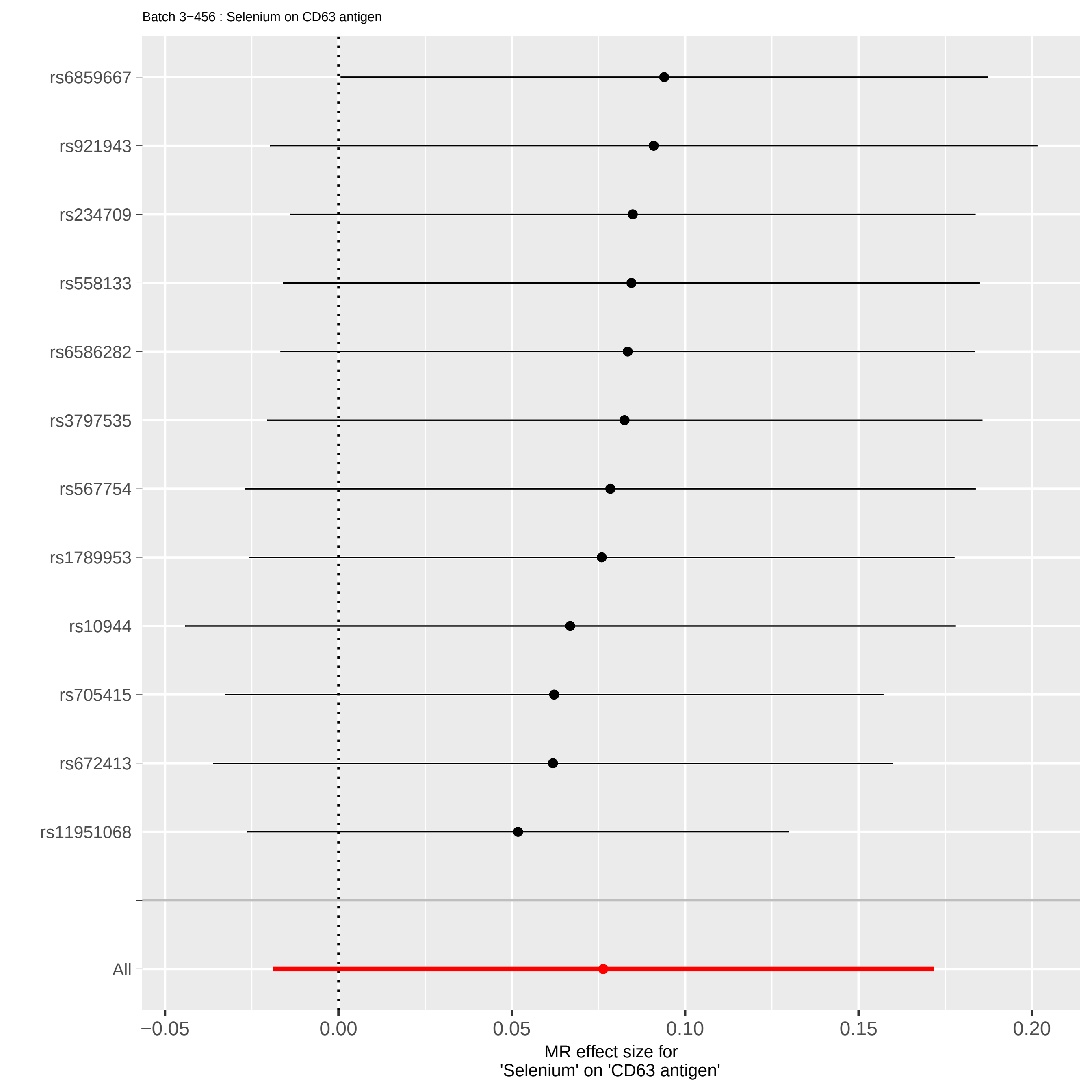

Batch 3−456 : Selenium on CD63 antigen
rs6859667
rs921943
rs234709
rs558133
rs6586282
rs3797535
rs567754
rs1789953
rs10944
rs705415
rs672413
rs11951068
All
−0.05
0.00
0.05
0.10
0.15
0.20
MR effect size for
'Selenium' on 'CD63 antigen'

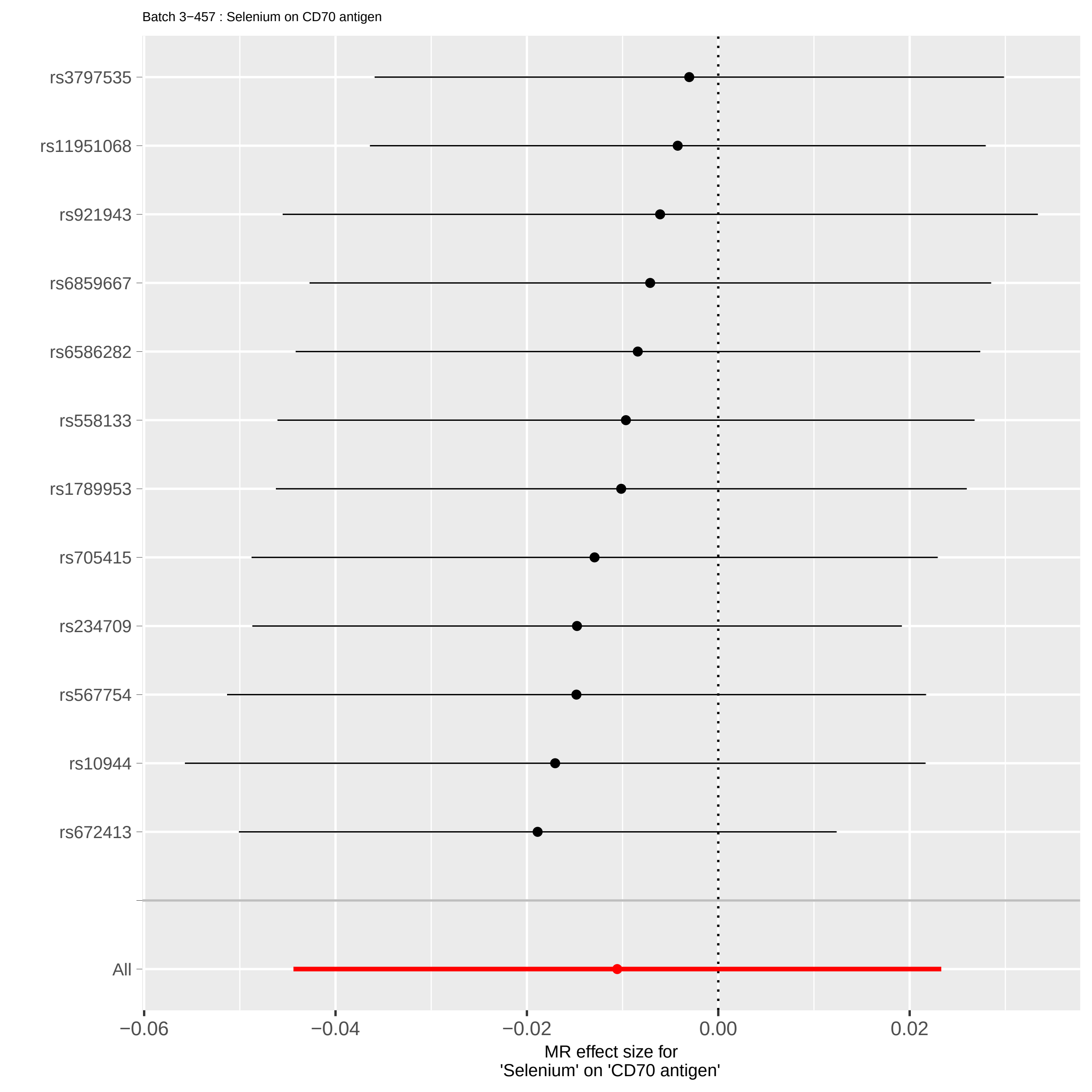

Batch 3−457 : Selenium on CD70 antigen
rs3797535
rs11951068
rs921943
rs6859667
rs6586282
rs558133
rs1789953
rs705415
rs234709
rs567754
rs10944
rs672413
All
−0.06
−0.04
−0.02
0.00
0.02
MR effect size for
'Selenium' on 'CD70 antigen'

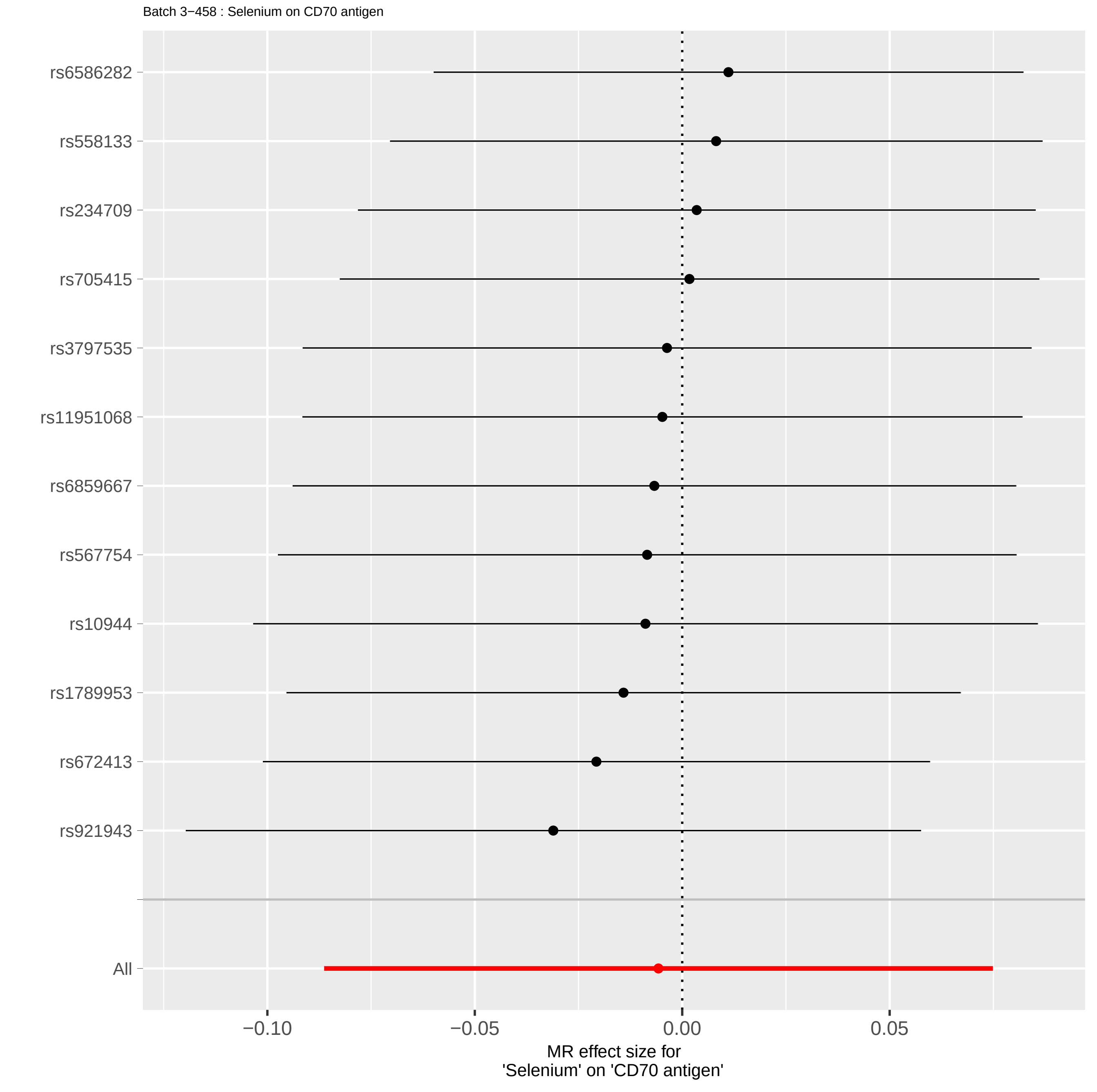

Batch 3−458 : Selenium on CD70 antigen
rs6586282
rs558133
rs234709
rs705415
rs3797535
rs11951068
rs6859667
rs567754
rs10944
rs1789953
rs672413
rs921943
All
−0.10
−0.05
0.00
0.05
MR effect size for
'Selenium' on 'CD70 antigen'

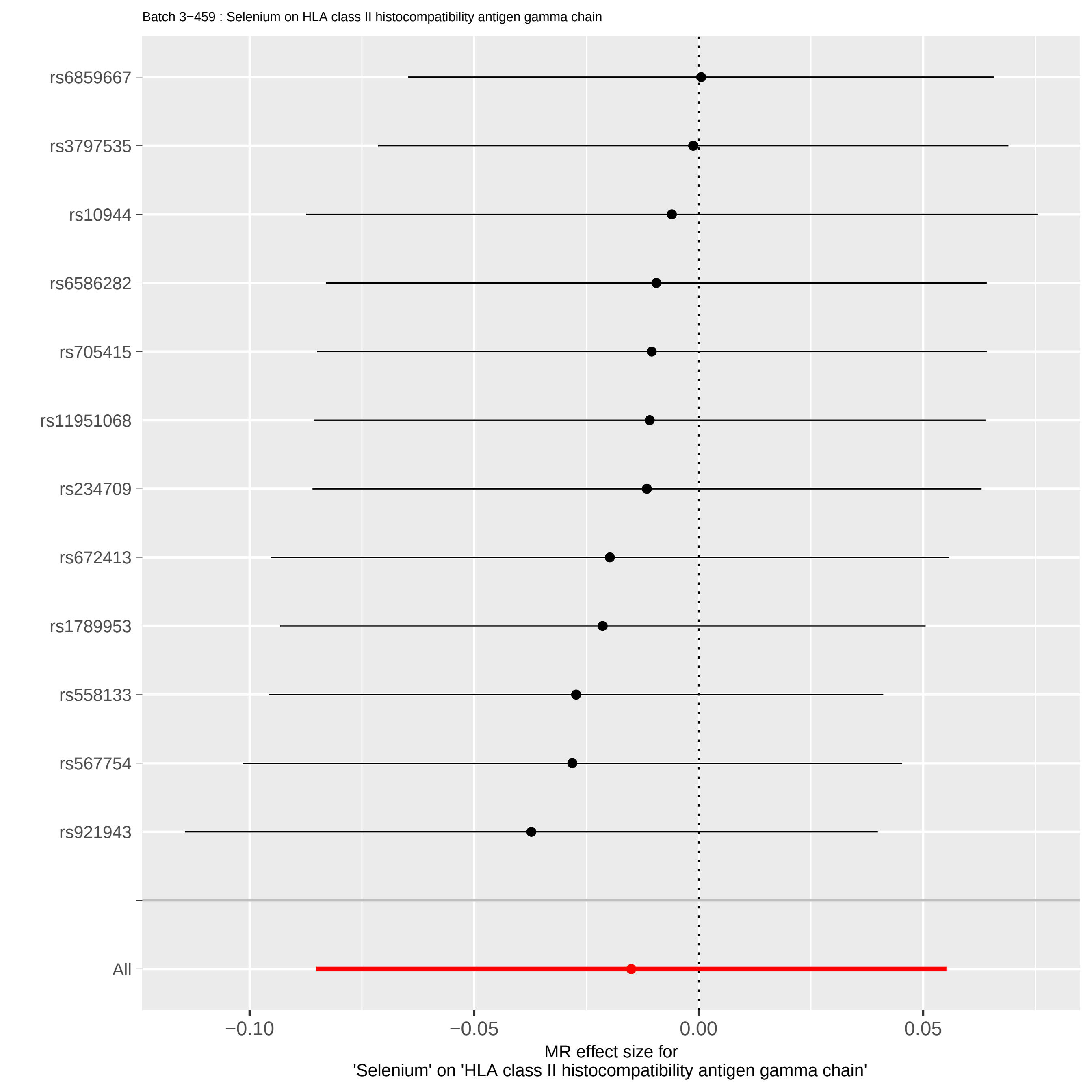

Batch 3−459 : Selenium on HLA class II histocompatibility antigen gamma chain
rs6859667
rs3797535
rs10944
rs6586282
rs705415
rs11951068
rs234709
rs672413
rs1789953
rs558133
rs567754
rs921943
All
−0.10
−0.05
0.00
0.05
MR effect size for
'Selenium' on 'HLA class II histocompatibility antigen gamma chain'

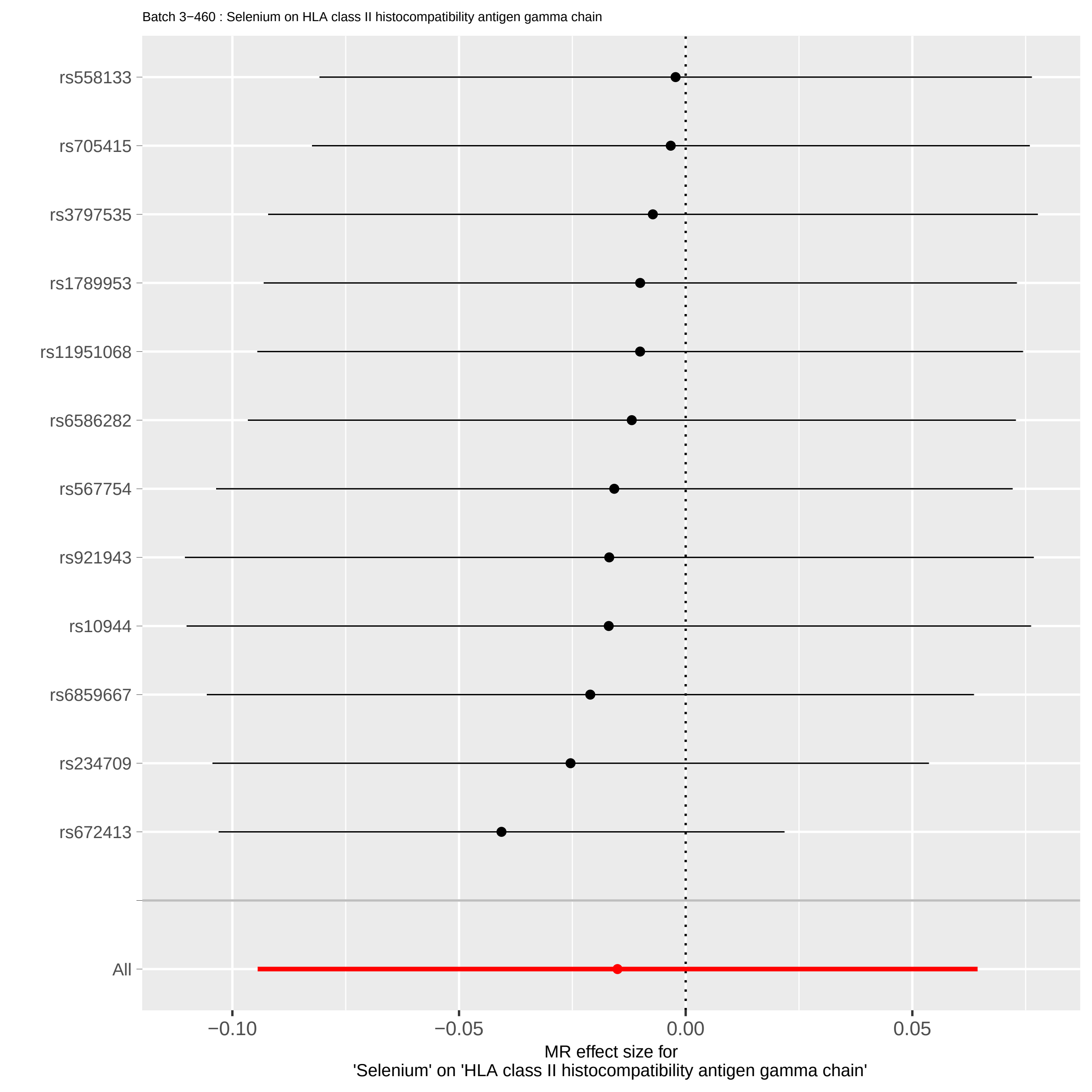
Batch 3−460 : Selenium on HLA class II histocompatibility antigen gamma chain
rs558133
rs705415
rs3797535
rs1789953
rs11951068
rs6586282
rs567754
rs921943
rs10944
rs6859667
rs234709
rs672413
All
−0.10
−0.05
0.00
0.05
MR effect size for
'Selenium' on 'HLA class II histocompatibility antigen gamma chain'

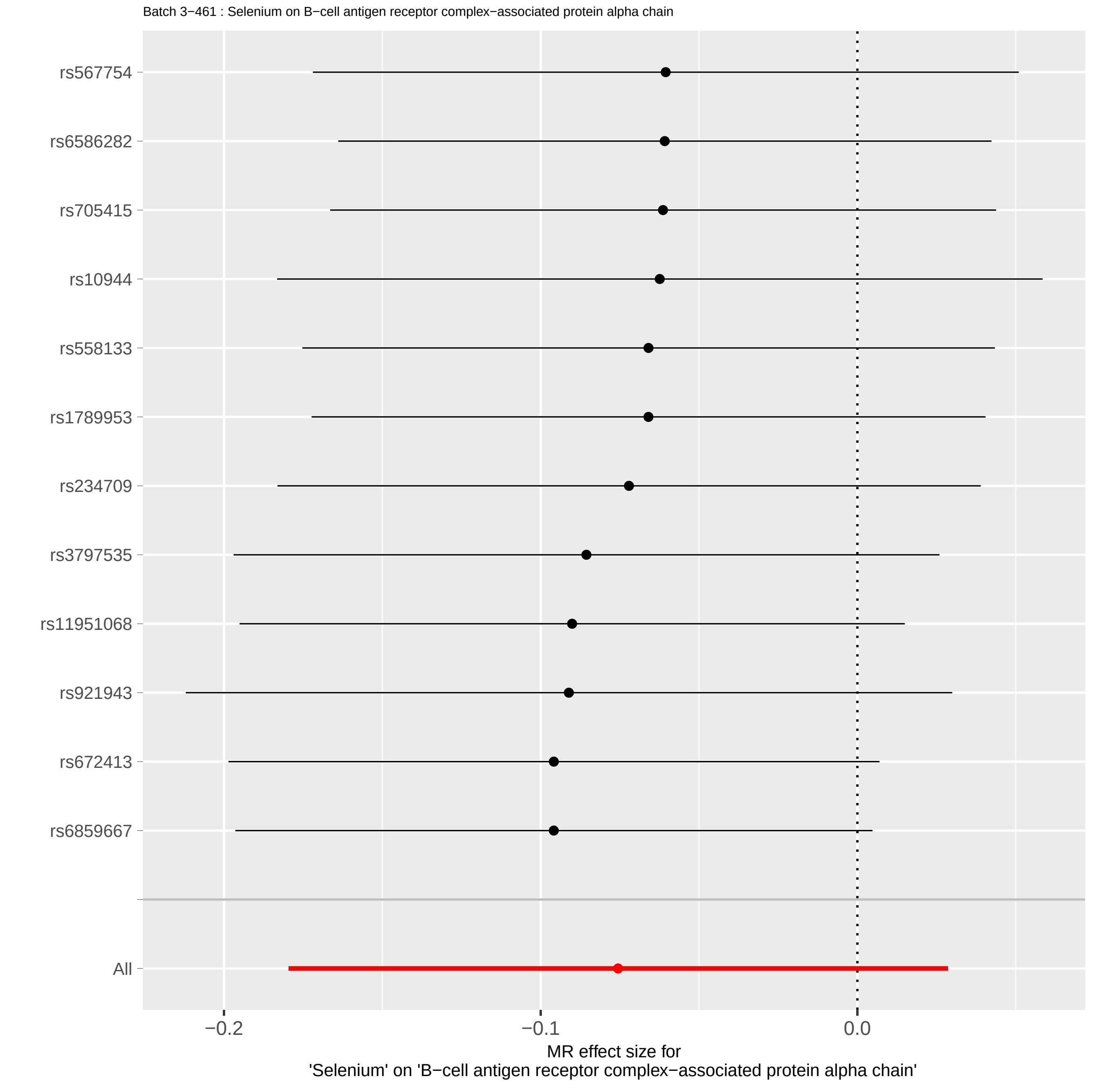

Batch 3−461 : Selenium on B−cell antigen receptor complex−associated protein alpha chain
rs567754
rs6586282
rs705415
rs10944
rs558133
rs1789953
rs234709
rs3797535
rs11951068
rs921943
rs672413
rs6859667
All
−0.2
−0.1
0.0
MR effect size for
'Selenium' on 'B−cell antigen receptor complex−associated protein alpha chain'

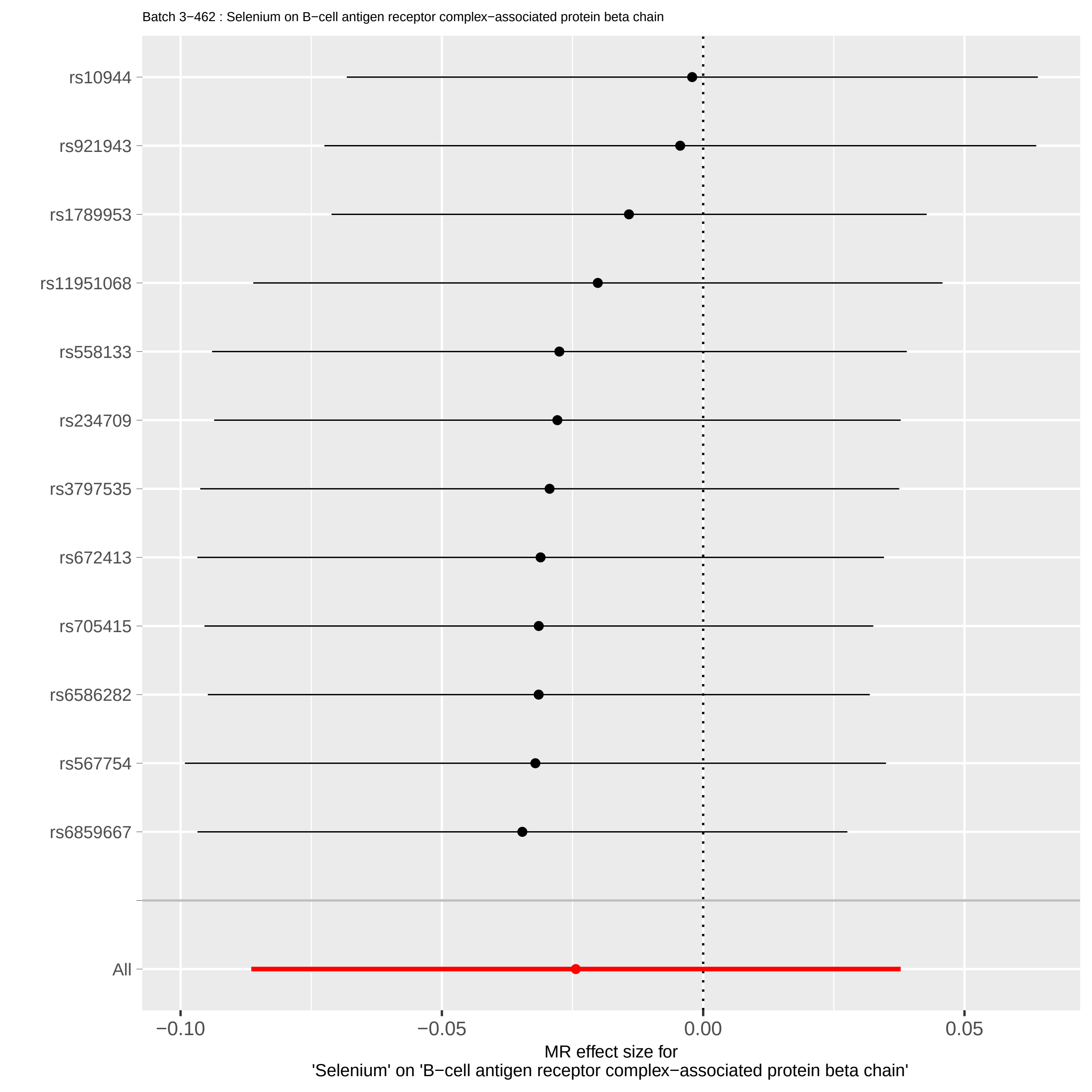

Batch 3−462 : Selenium on B−cell antigen receptor complex−associated protein beta chain
rs10944
rs921943
rs1789953
rs11951068
rs558133
rs234709
rs3797535
rs672413
rs705415
rs6586282
rs567754
rs6859667
All
−0.10
−0.05
0.00
0.05
MR effect size for
'Selenium' on 'B−cell antigen receptor complex−associated protein beta chain'

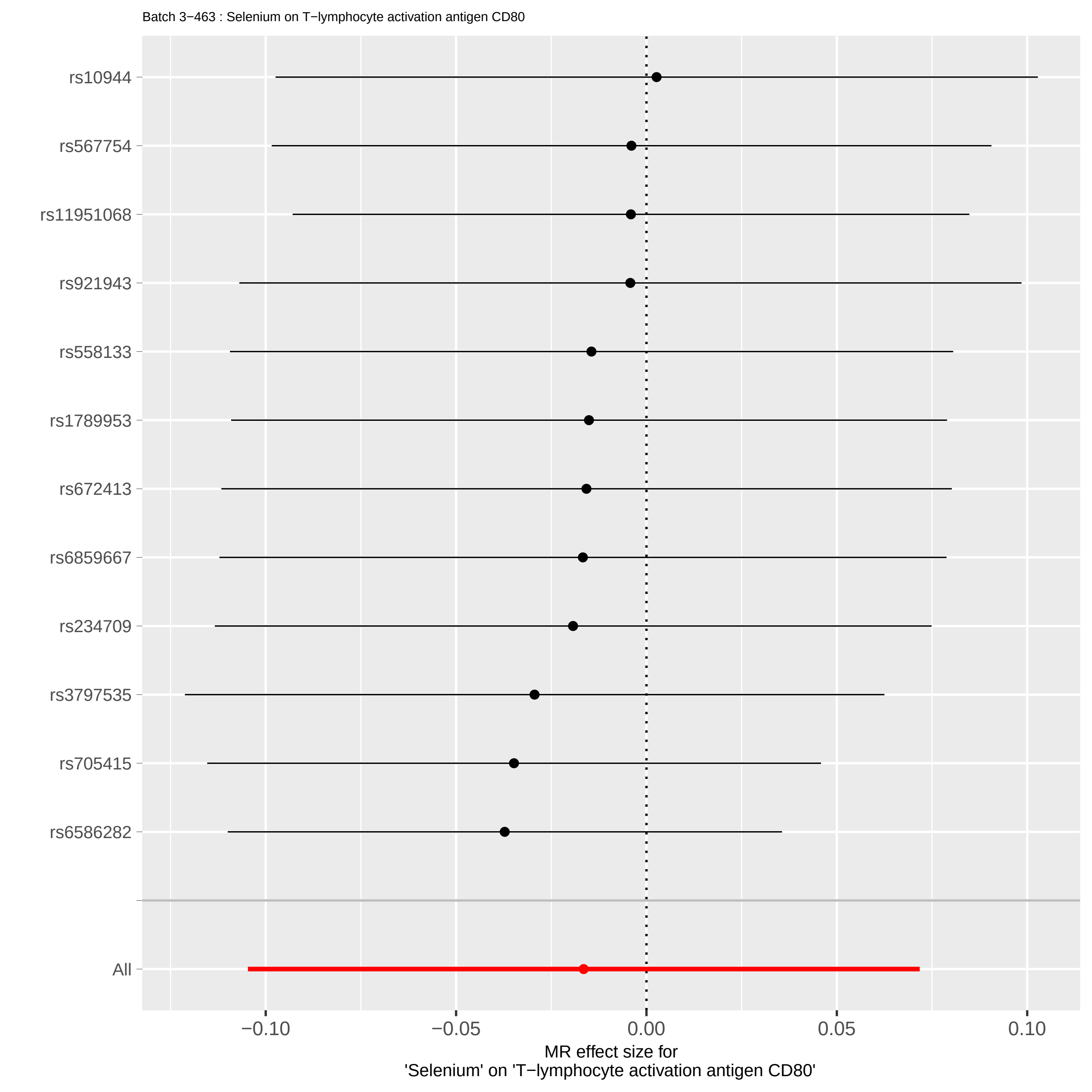

Batch 3−463 : Selenium on T−lymphocyte activation antigen CD80
rs10944
rs567754
rs11951068
rs921943
rs558133
rs1789953
rs672413
rs6859667
rs234709
rs3797535
rs705415
rs6586282
All
−0.10
−0.05
0.00
0.05
0.10
MR effect size for
'Selenium' on 'T−lymphocyte activation antigen CD80'

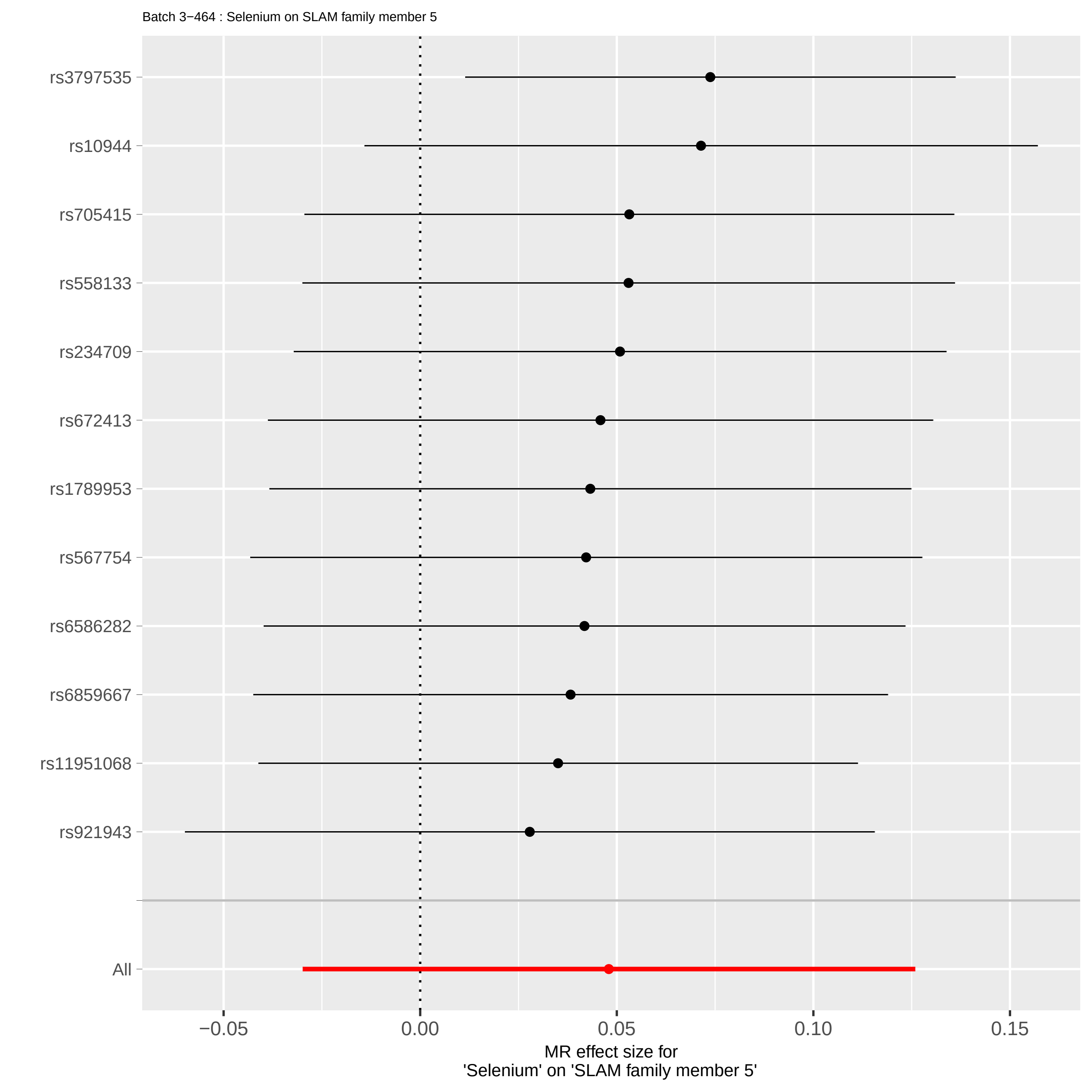
Batch 3−464 : Selenium on SLAM family member 5
rs3797535
rs10944
rs705415
rs558133
rs234709
rs672413
rs1789953
rs567754
rs6586282
rs6859667
rs11951068
rs921943
All
−0.05
0.00
0.05
0.10
0.15
MR effect size for
'Selenium' on 'SLAM family member 5'

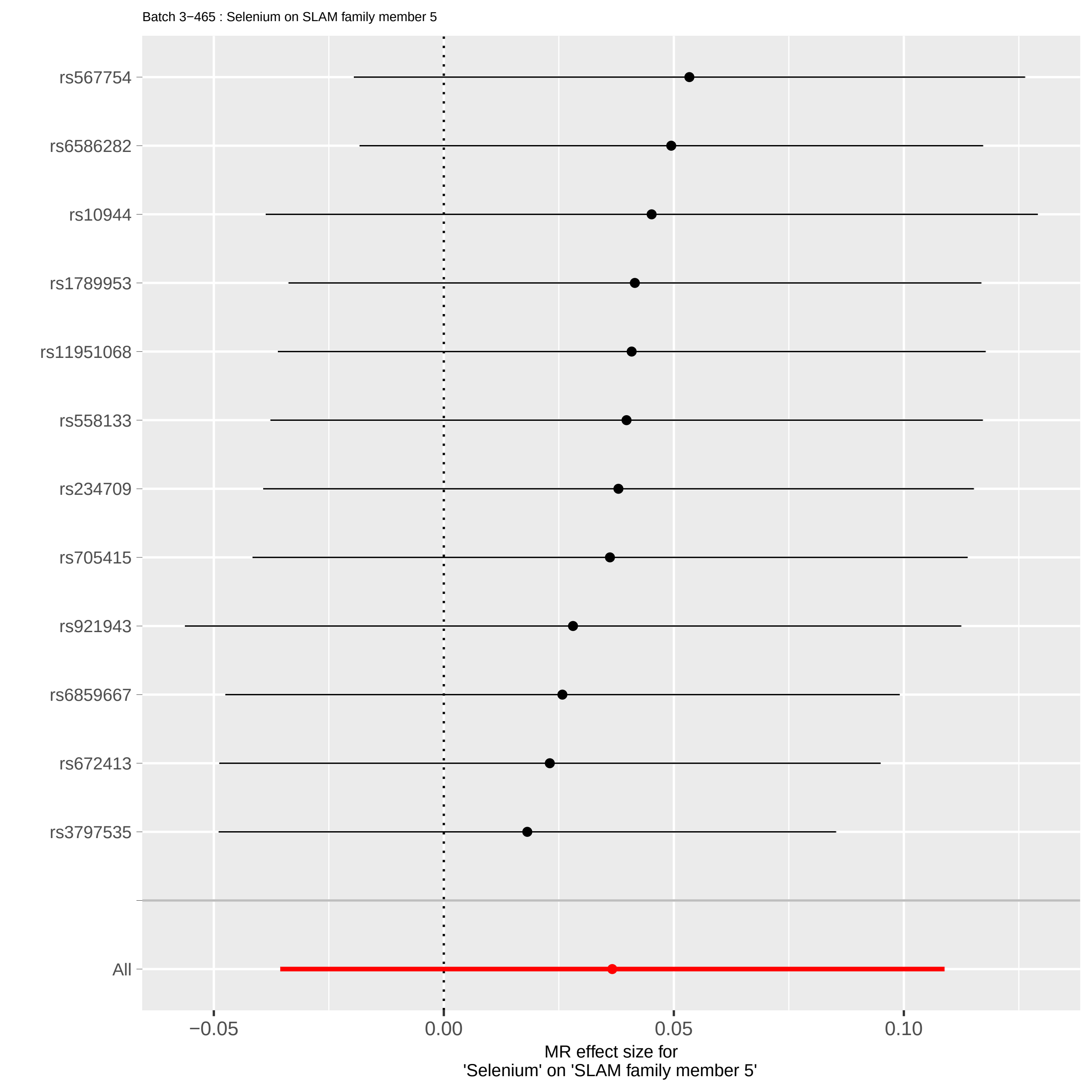
Batch 3−465 : Selenium on SLAM family member 5
rs567754
rs6586282
rs10944
rs1789953
rs11951068
rs558133
rs234709
rs705415
rs921943
rs6859667
rs672413
rs3797535
All
−0.05
0.00
0.05
0.10
MR effect size for
'Selenium' on 'SLAM family member 5'

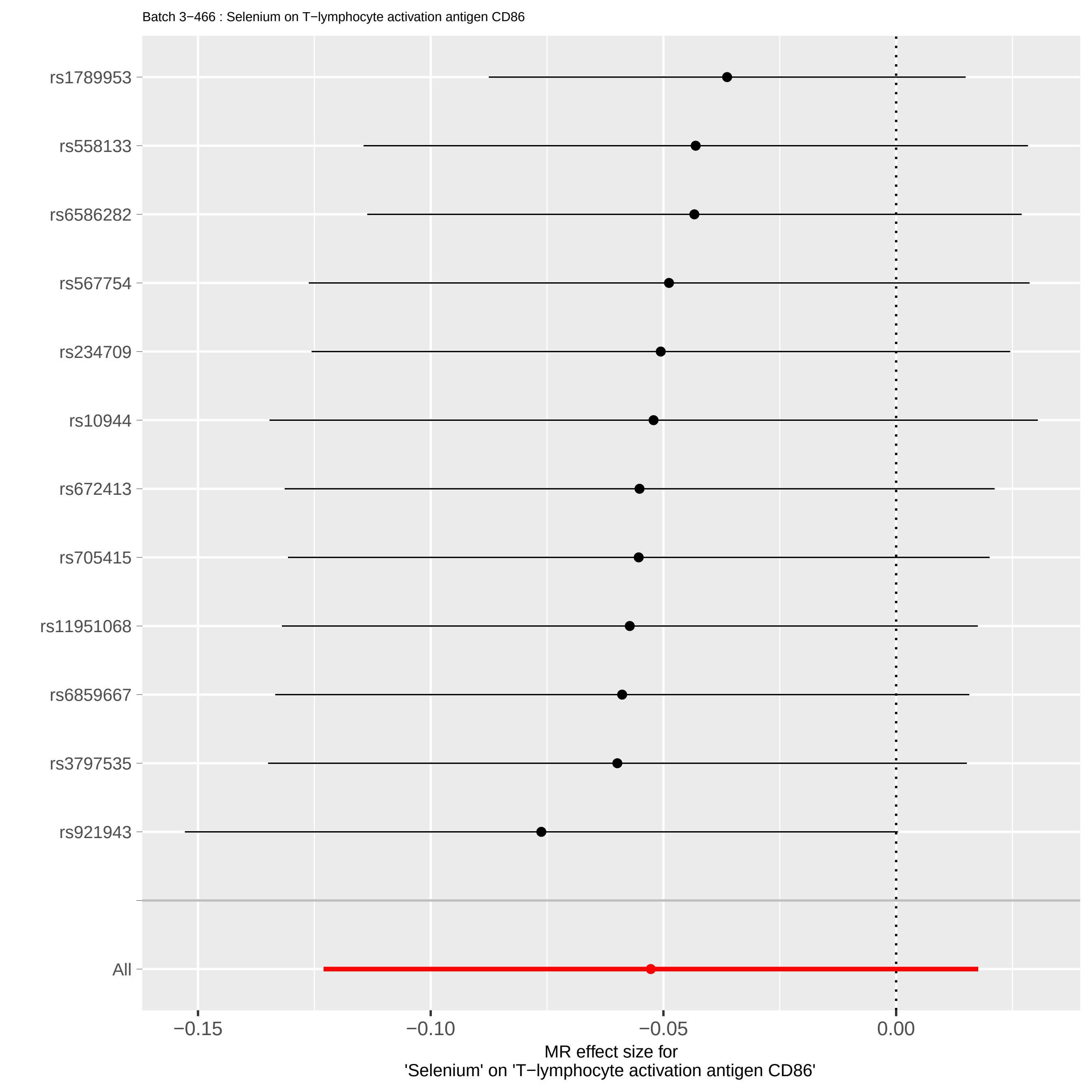

Batch 3−466 : Selenium on T−lymphocyte activation antigen CD86
rs1789953
rs558133
rs6586282
rs567754
rs234709
rs10944
rs672413
rs705415
rs11951068
rs6859667
rs3797535
rs921943
All
−0.15
−0.10
−0.05
0.00
MR effect size for
'Selenium' on 'T−lymphocyte activation antigen CD86'

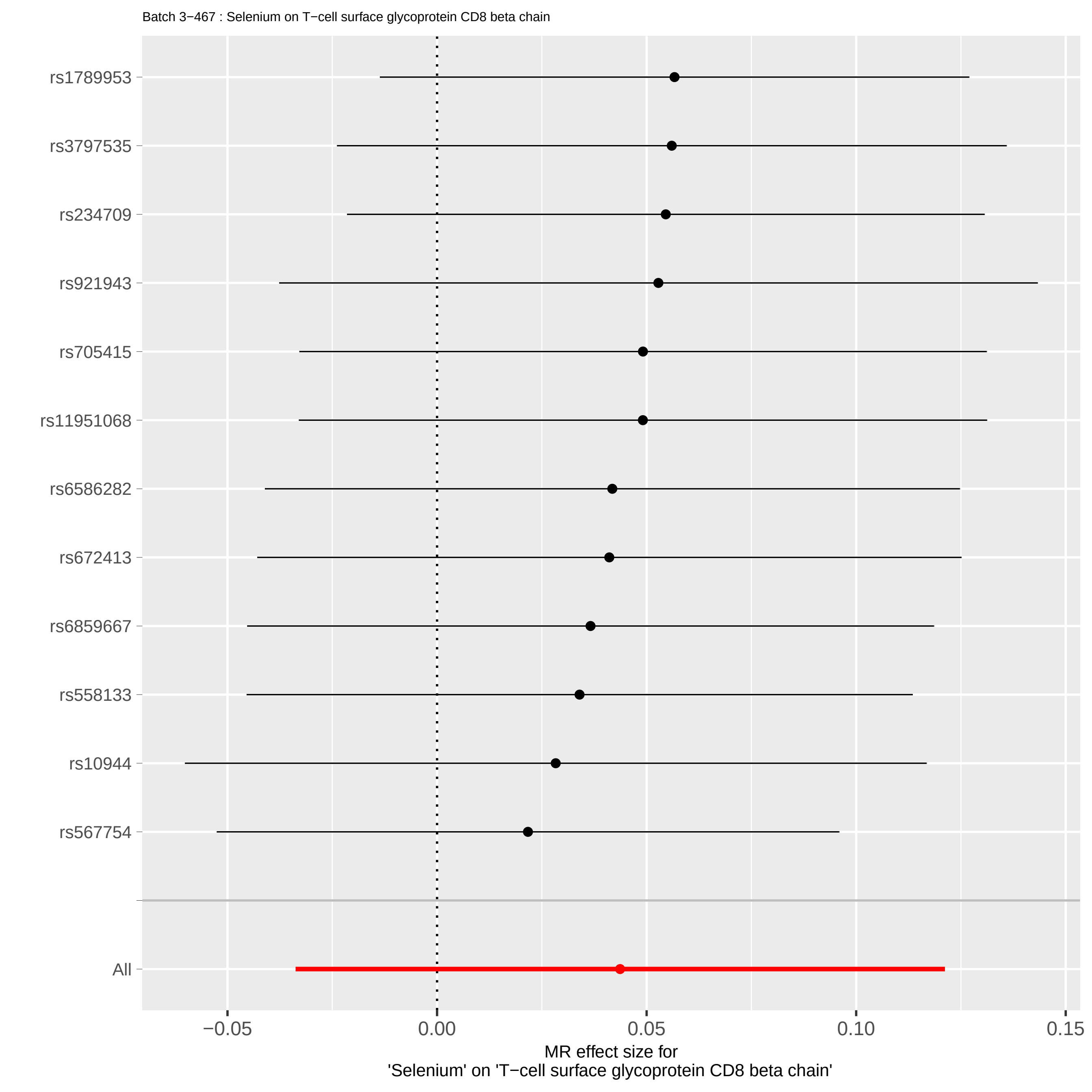

Batch 3−467 : Selenium on T−cell surface glycoprotein CD8 beta chain
rs1789953
rs3797535
rs234709
rs921943
rs705415
rs11951068
rs6586282
rs672413
rs6859667
rs558133
rs10944
rs567754
All
−0.05
0.00
0.05
0.10
0.15
MR effect size for
'Selenium' on 'T−cell surface glycoprotein CD8 beta chain'

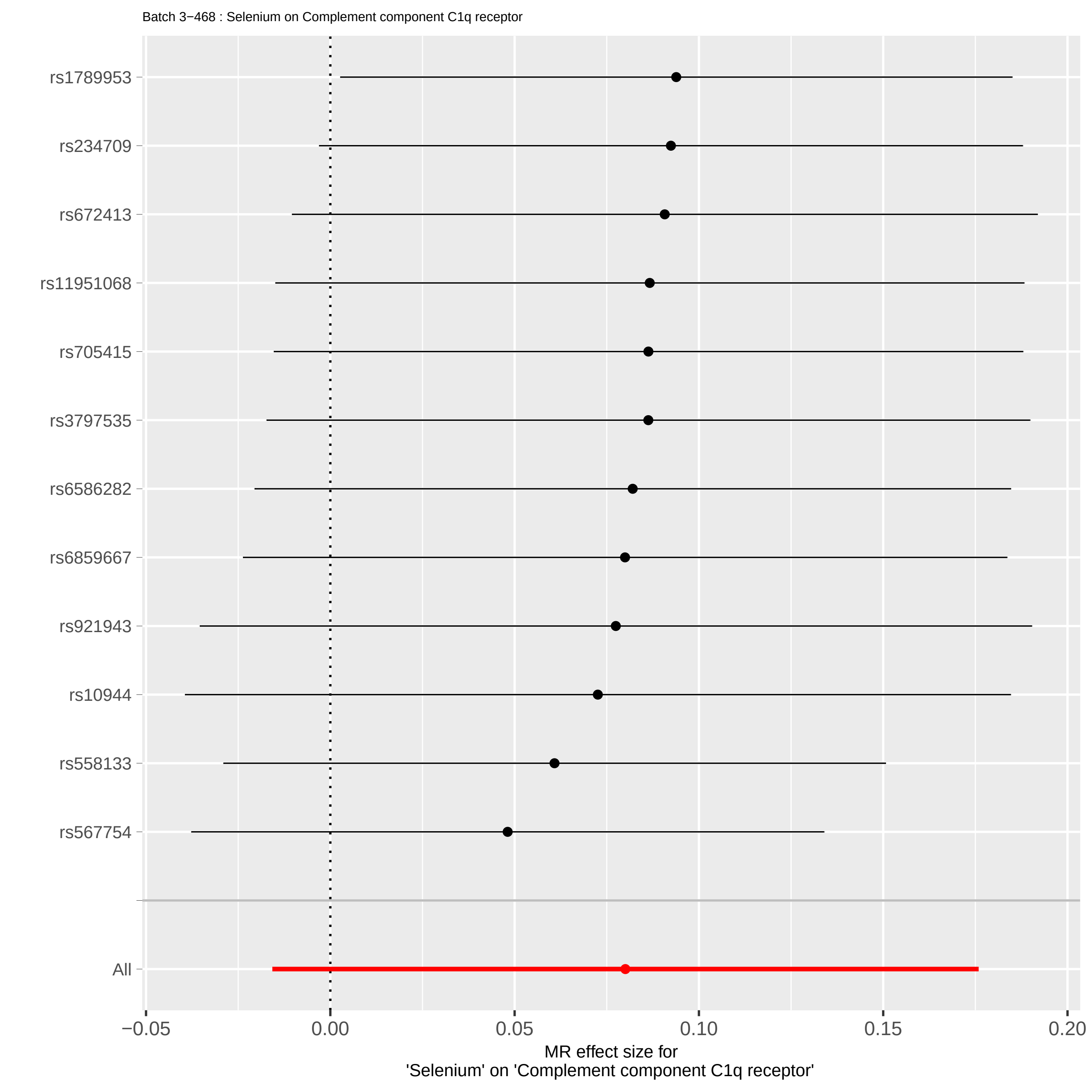

Batch 3−468 : Selenium on Complement component C1q receptor
rs1789953
rs234709
rs672413
rs11951068
rs705415
rs3797535
rs6586282
rs6859667
rs921943
rs10944
rs558133
rs567754
All
−0.05
0.00
0.05
0.10
0.15
0.20
MR effect size for
'Selenium' on 'Complement component C1q receptor'

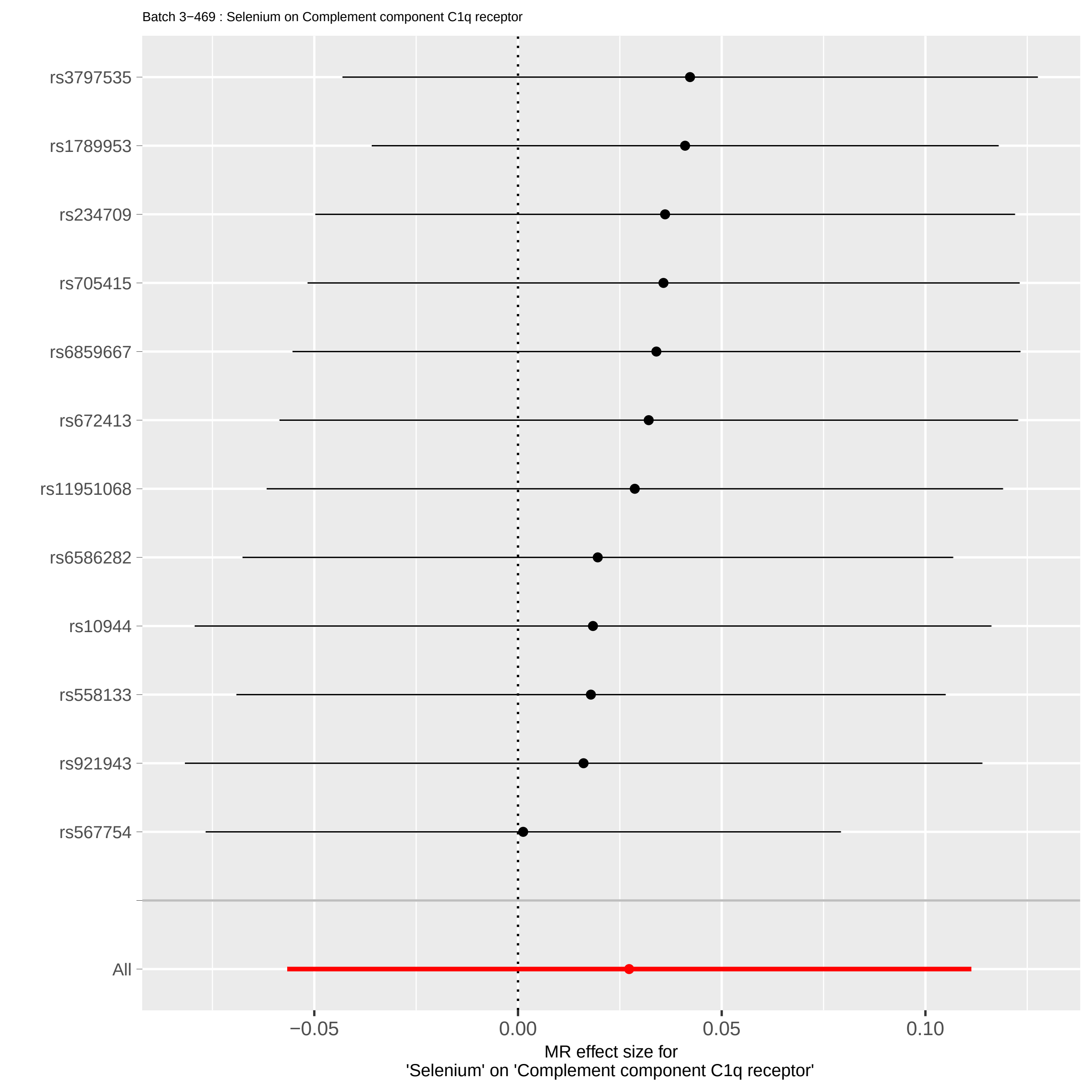

Batch 3−469 : Selenium on Complement component C1q receptor
rs3797535
rs1789953
rs234709
rs705415
rs6859667
rs672413
rs11951068
rs6586282
rs10944
rs558133
rs921943
rs567754
All
−0.05
0.00
0.05
0.10
MR effect size for
'Selenium' on 'Complement component C1q receptor'

Batch 3−470 : Selenium on T−cell surface protein tactile

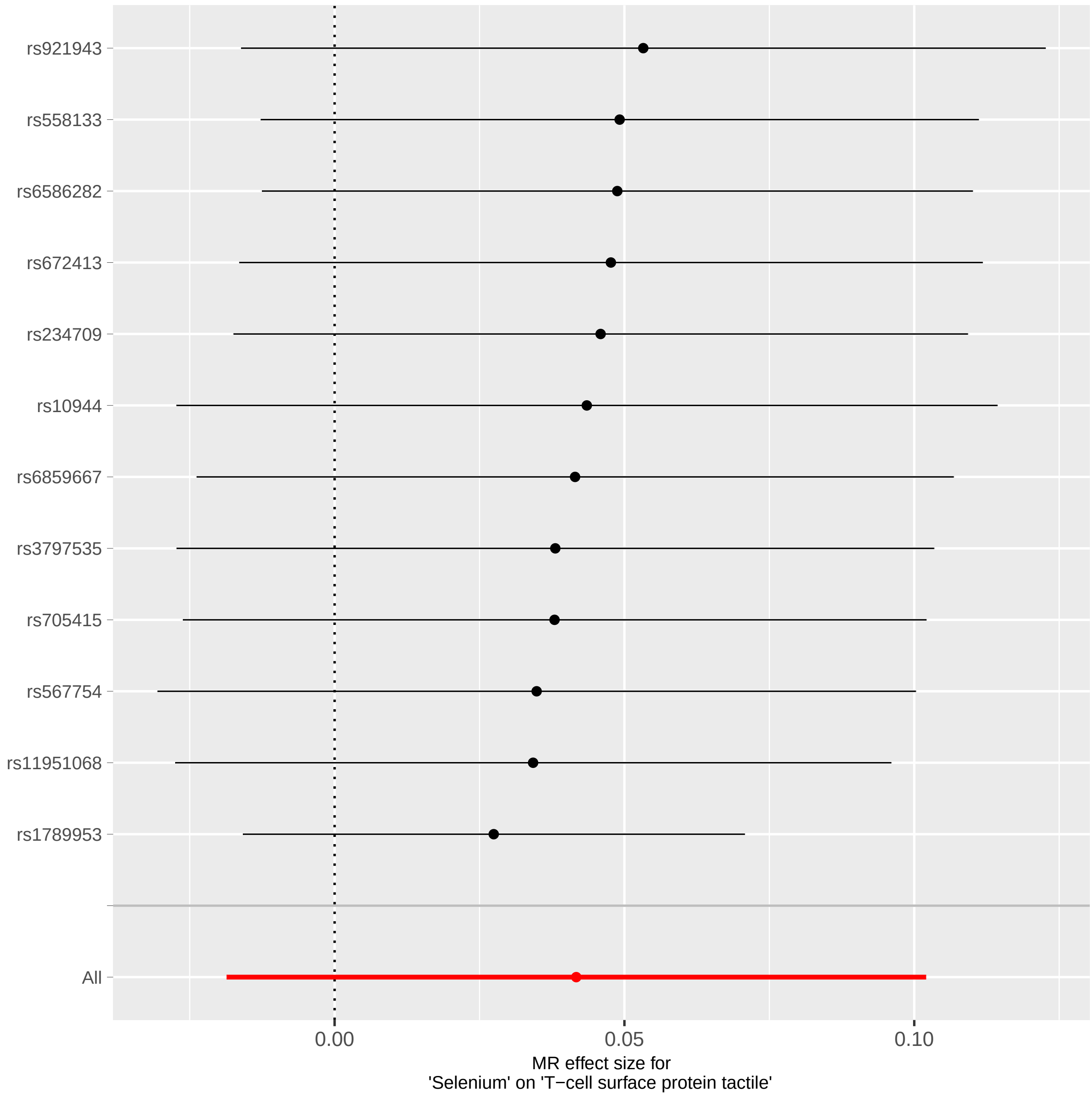

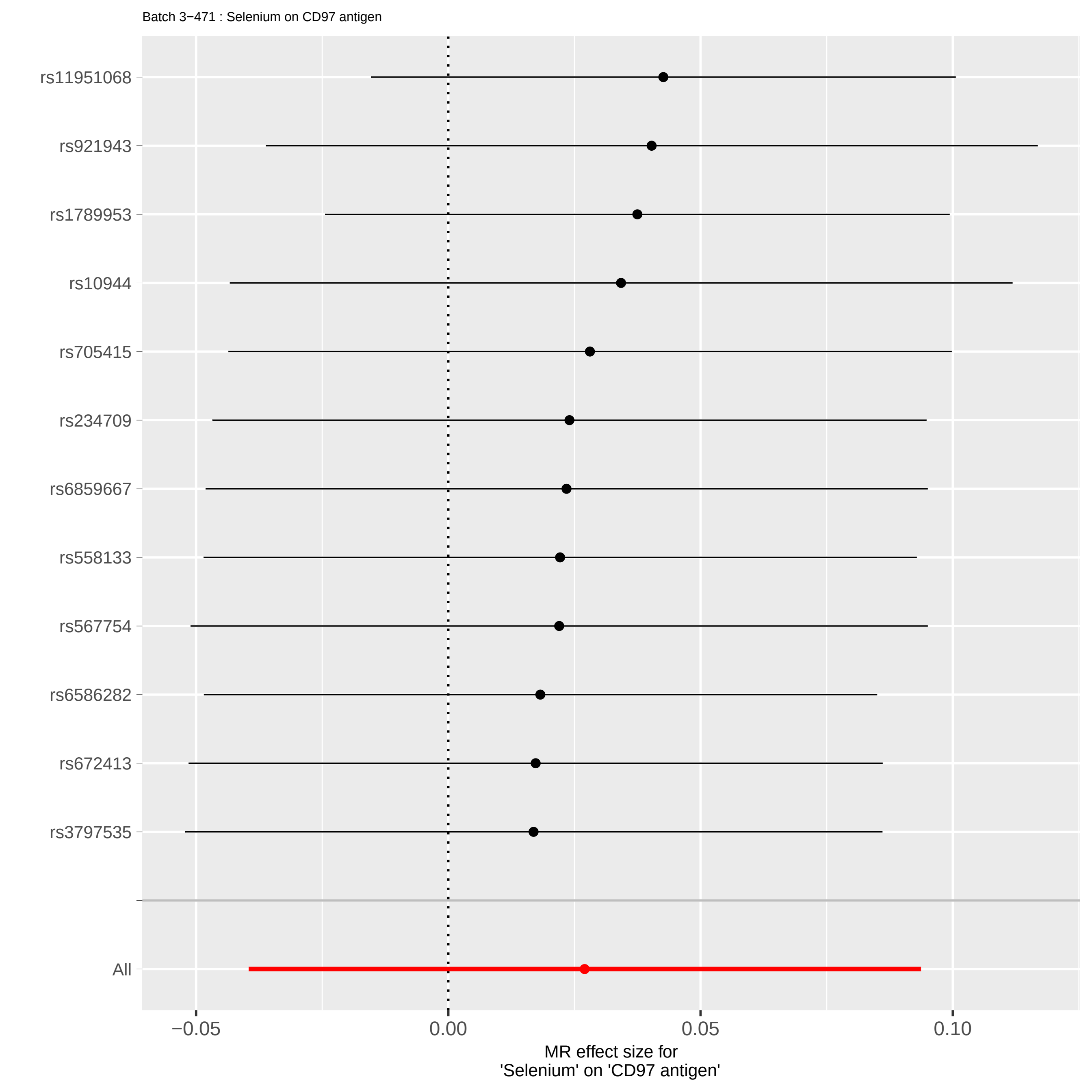

Batch 3−471 : Selenium on CD97 antigen
rs11951068
rs921943
rs1789953
rs10944
rs705415
rs234709
rs6859667
rs558133
rs567754
rs6586282
rs672413
rs3797535
All
−0.05
0.00
0.05
0.10
MR effect size for
'Selenium' on 'CD97 antigen'

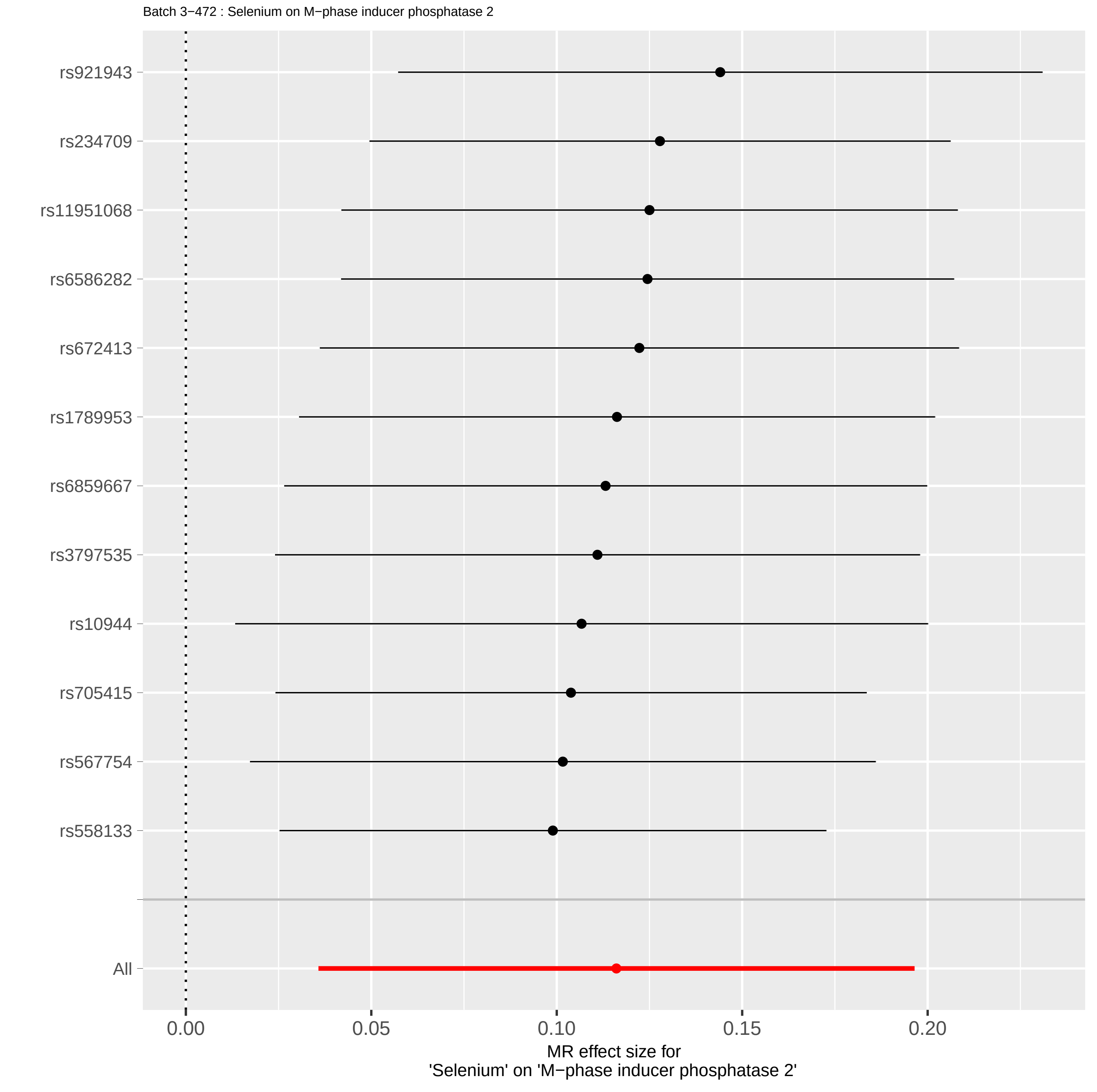
Batch 3−472 : Selenium on M−phase inducer phosphatase 2
rs921943
rs234709
rs11951068
rs6586282
rs672413
rs1789953
rs6859667
rs3797535
rs10944
rs705415
rs567754
rs558133
All
0.00
0.05
0.10
0.15
0.20
MR effect size for
'Selenium' on 'M−phase inducer phosphatase 2'

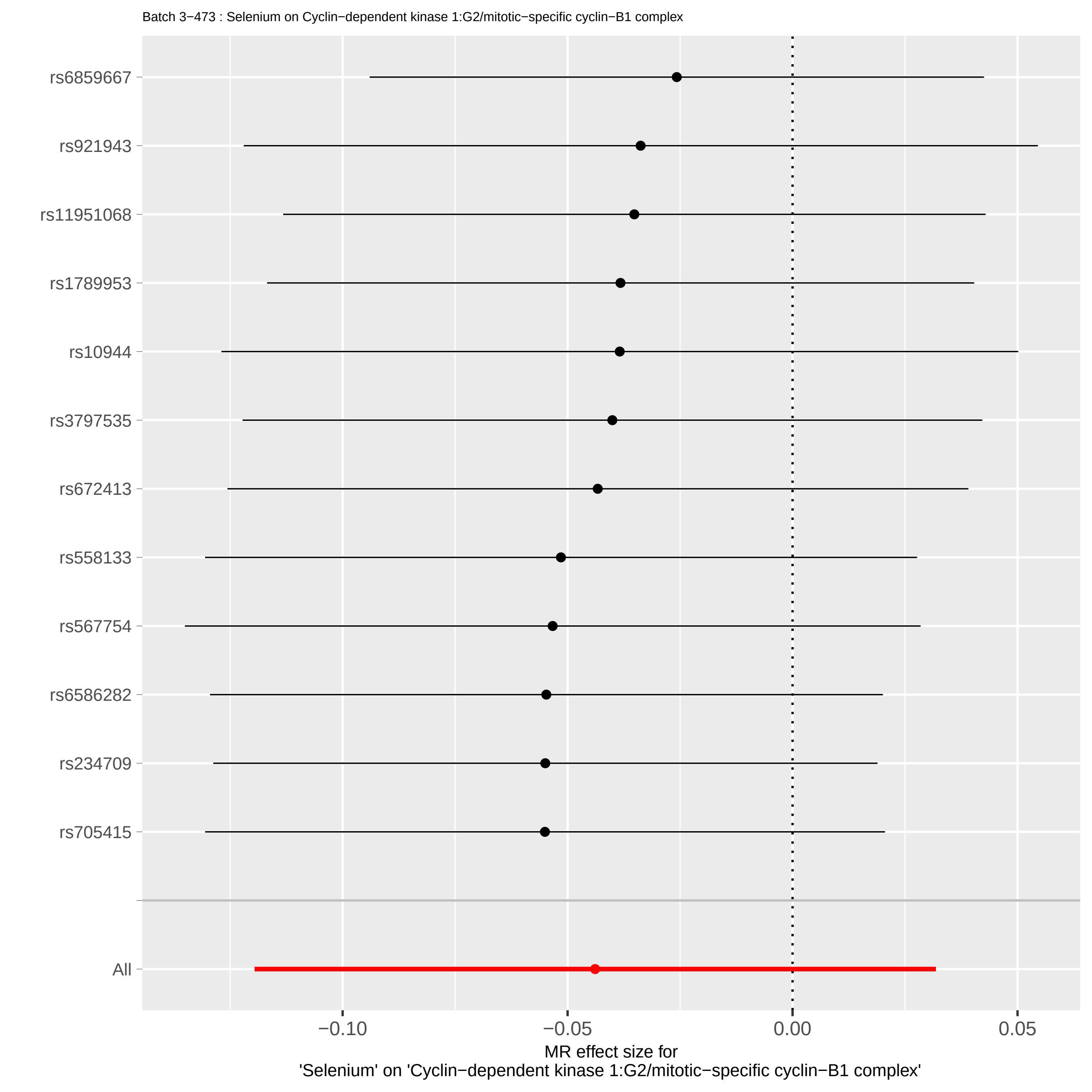

Batch 3−473 : Selenium on Cyclin−dependent kinase 1:G2/mitotic−specific cyclin−B1 complex
rs6859667
rs921943
rs11951068
rs1789953
rs10944
rs3797535
rs672413
rs558133
rs567754
rs6586282
rs234709
rs705415
All
−0.10
−0.05
0.00
0.05
MR effect size for
'Selenium' on 'Cyclin−dependent kinase 1:G2/mitotic−specific cyclin−B1 complex'

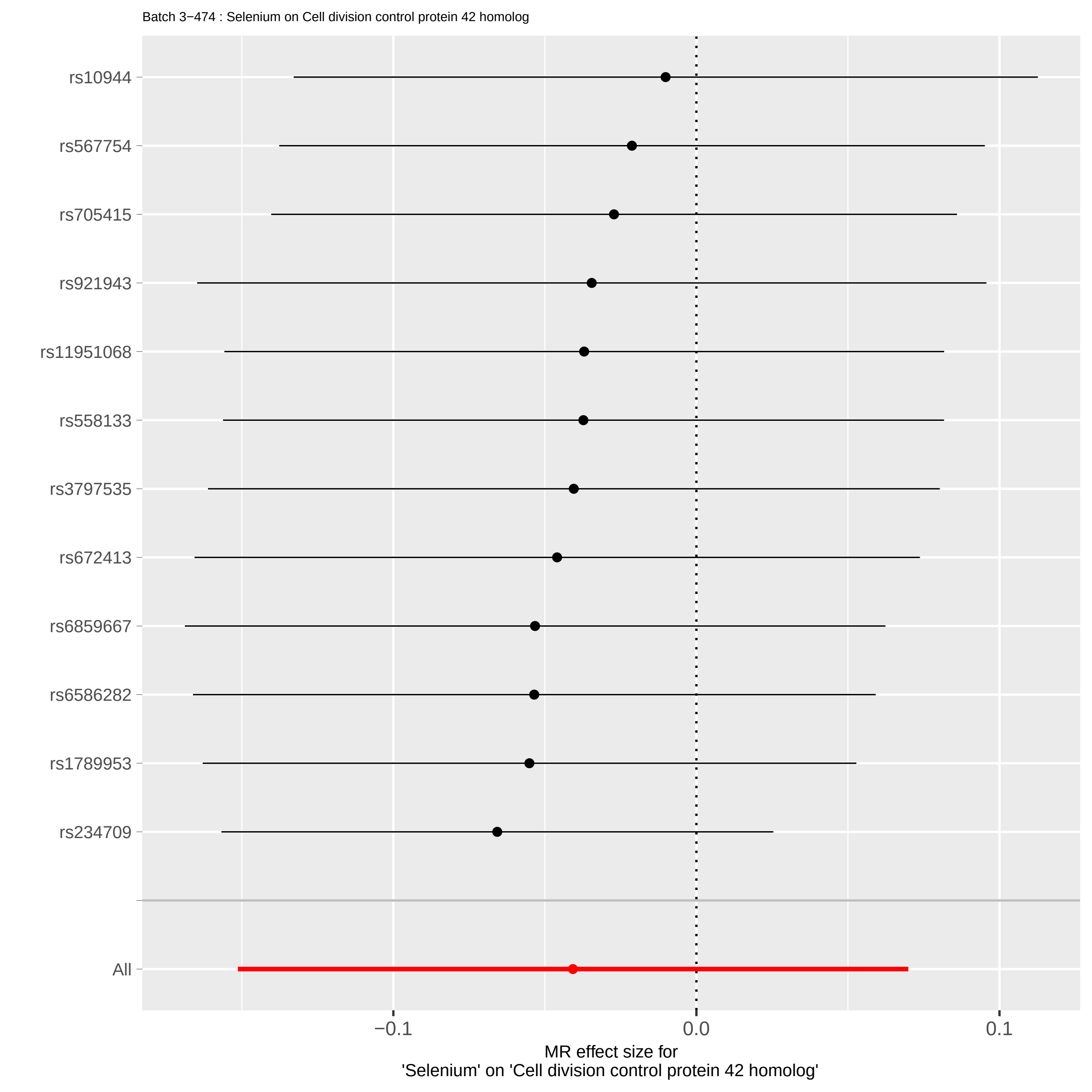

Batch 3−474 : Selenium on Cell division control protein 42 homolog
rs10944
rs567754
rs705415
rs921943
rs11951068
rs558133
rs3797535
rs672413
rs6859667
rs6586282
rs1789953
rs234709
All
−0.1
0.0
0.1
MR effect size for
'Selenium' on 'Cell division control protein 42 homolog'

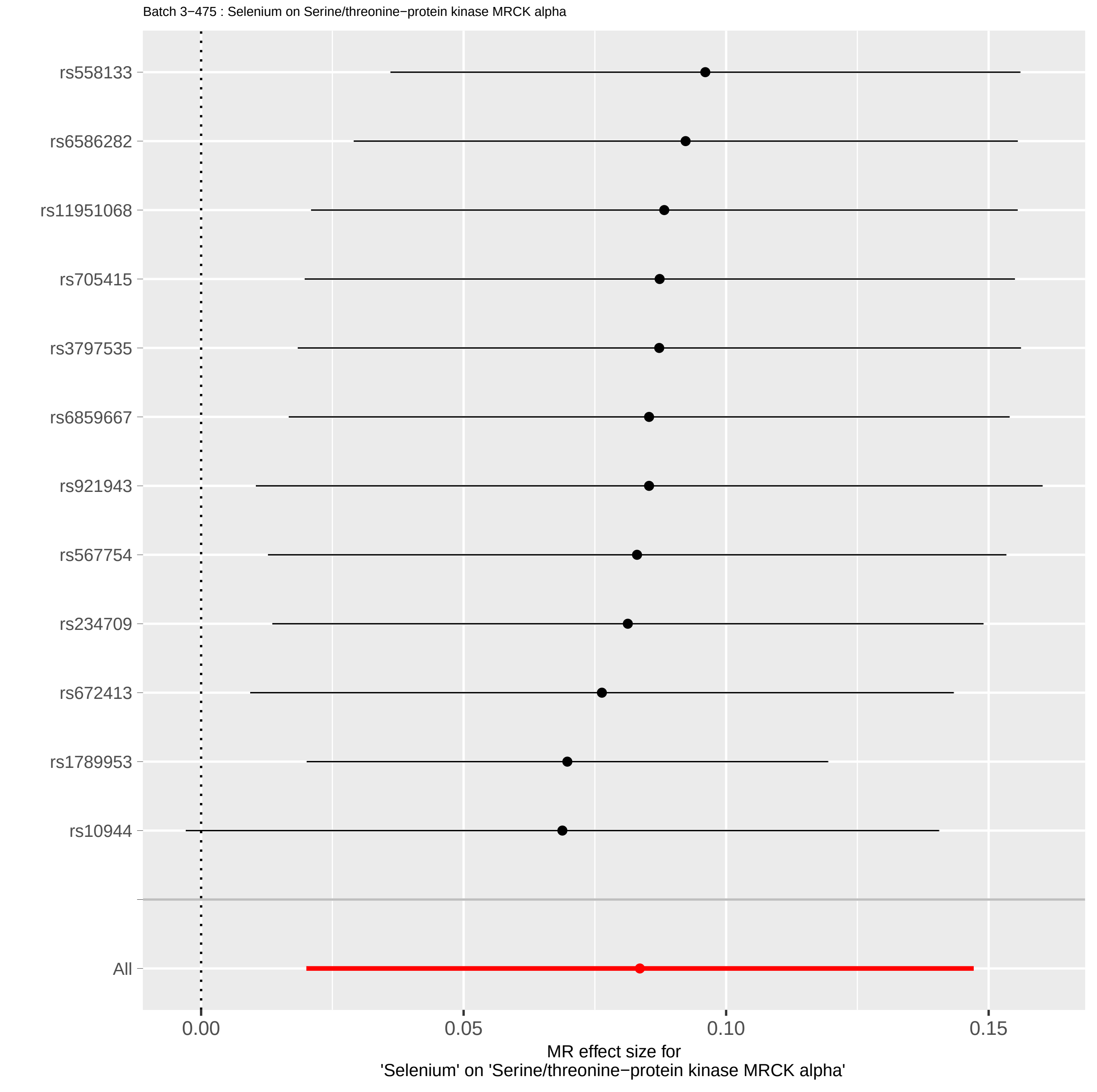
Batch 3−475 : Selenium on Serine/threonine−protein kinase MRCK alpha
rs558133
rs6586282
rs11951068
rs705415
rs3797535
rs6859667
rs921943
rs567754
rs234709
rs672413
rs1789953
rs10944
All
0.00
0.05
0.10
0.15
MR effect size for
'Selenium' on 'Serine/threonine−protein kinase MRCK alpha'

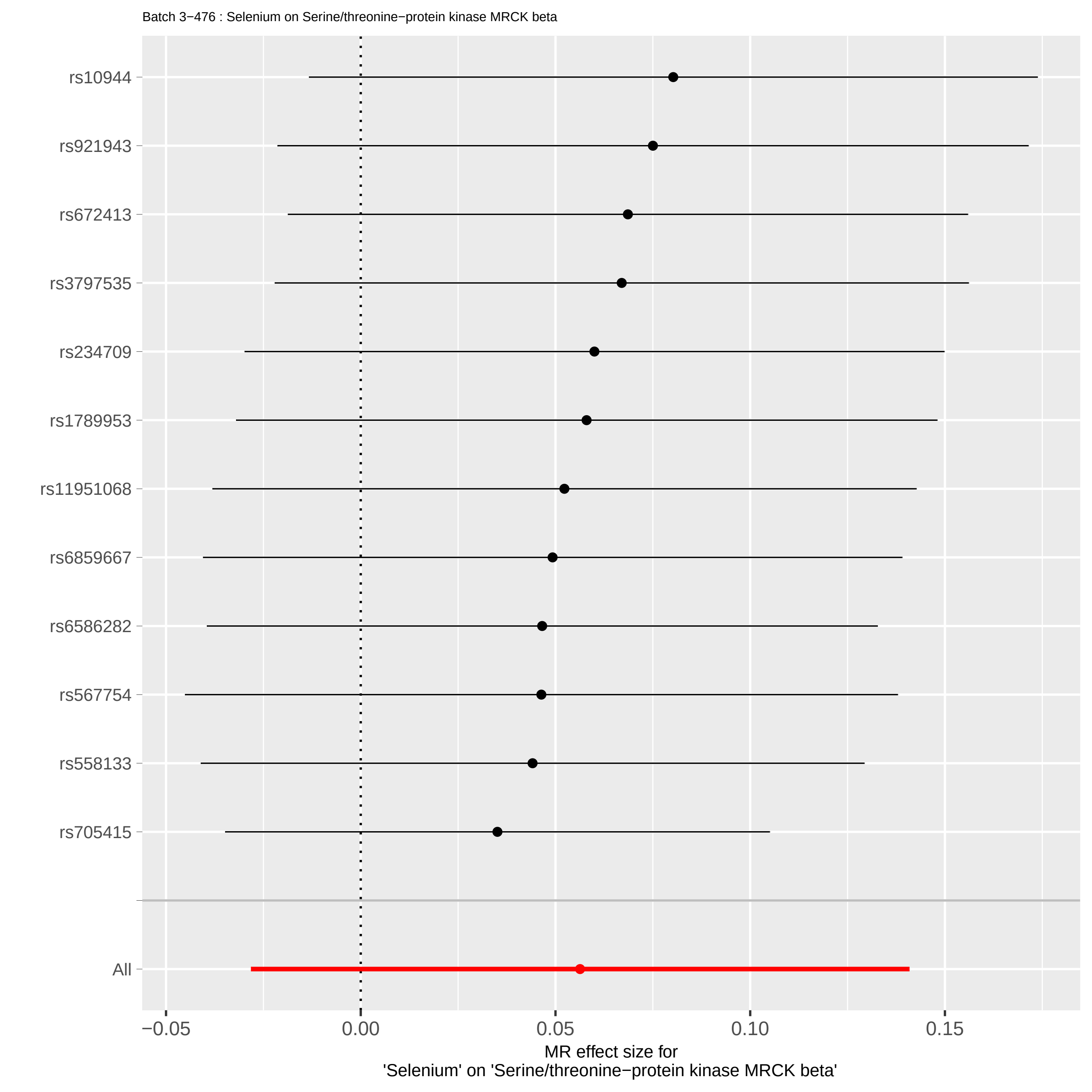
Batch 3−476 : Selenium on Serine/threonine−protein kinase MRCK beta
rs10944
rs921943
rs672413
rs3797535
rs234709
rs1789953
rs11951068
rs6859667
rs6586282
rs567754
rs558133
rs705415
All
−0.05
0.00
0.05
0.10
0.15
MR effect size for
'Selenium' on 'Serine/threonine−protein kinase MRCK beta'

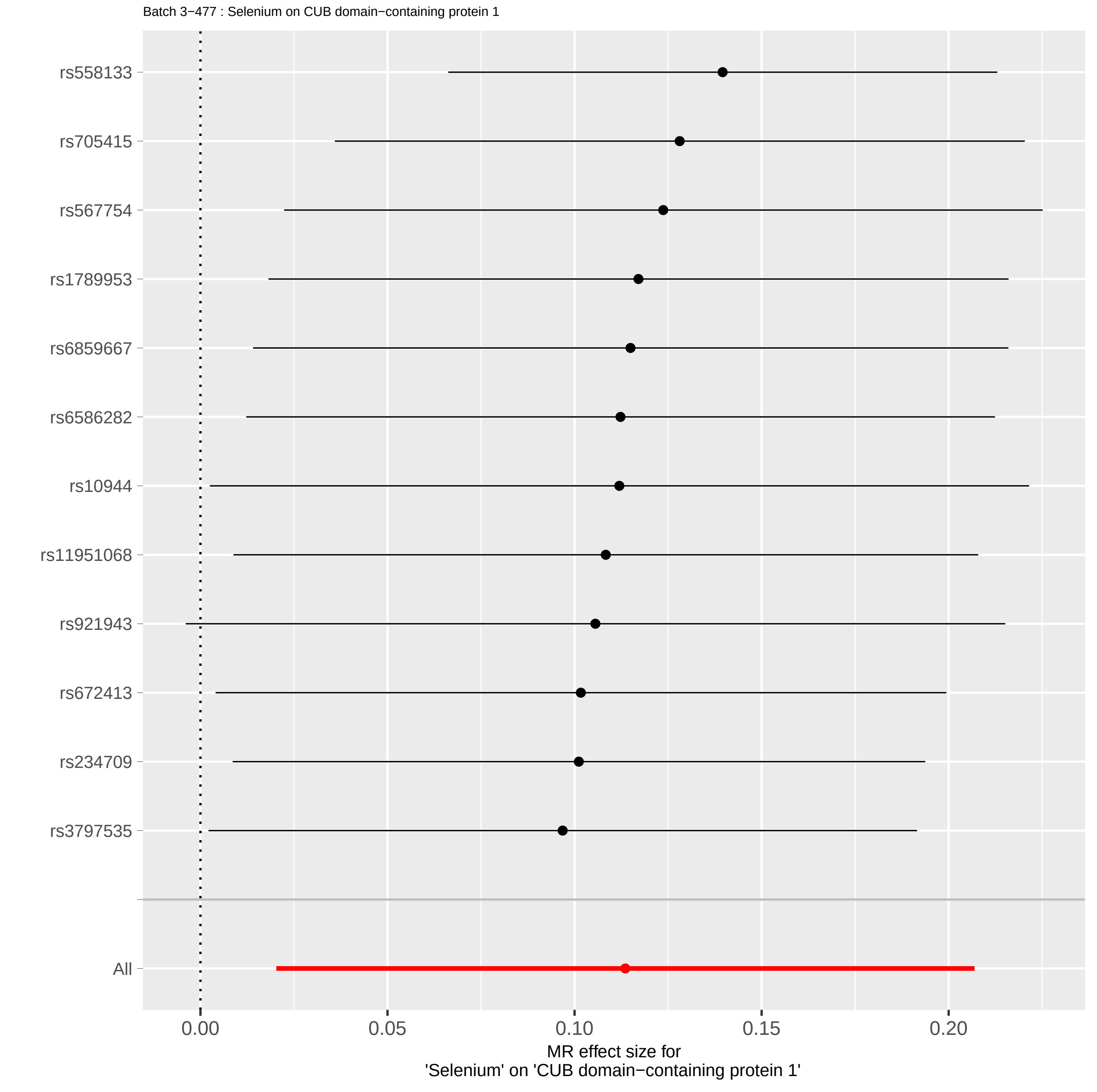

Batch 3−477 : Selenium on CUB domain−containing protein 1
rs558133
rs705415
rs567754
rs1789953
rs6859667
rs6586282
rs10944
rs11951068
rs921943
rs672413
rs234709
rs3797535
All
0.00
0.05
0.10
0.15
0.20
MR effect size for
'Selenium' on 'CUB domain−containing protein 1'

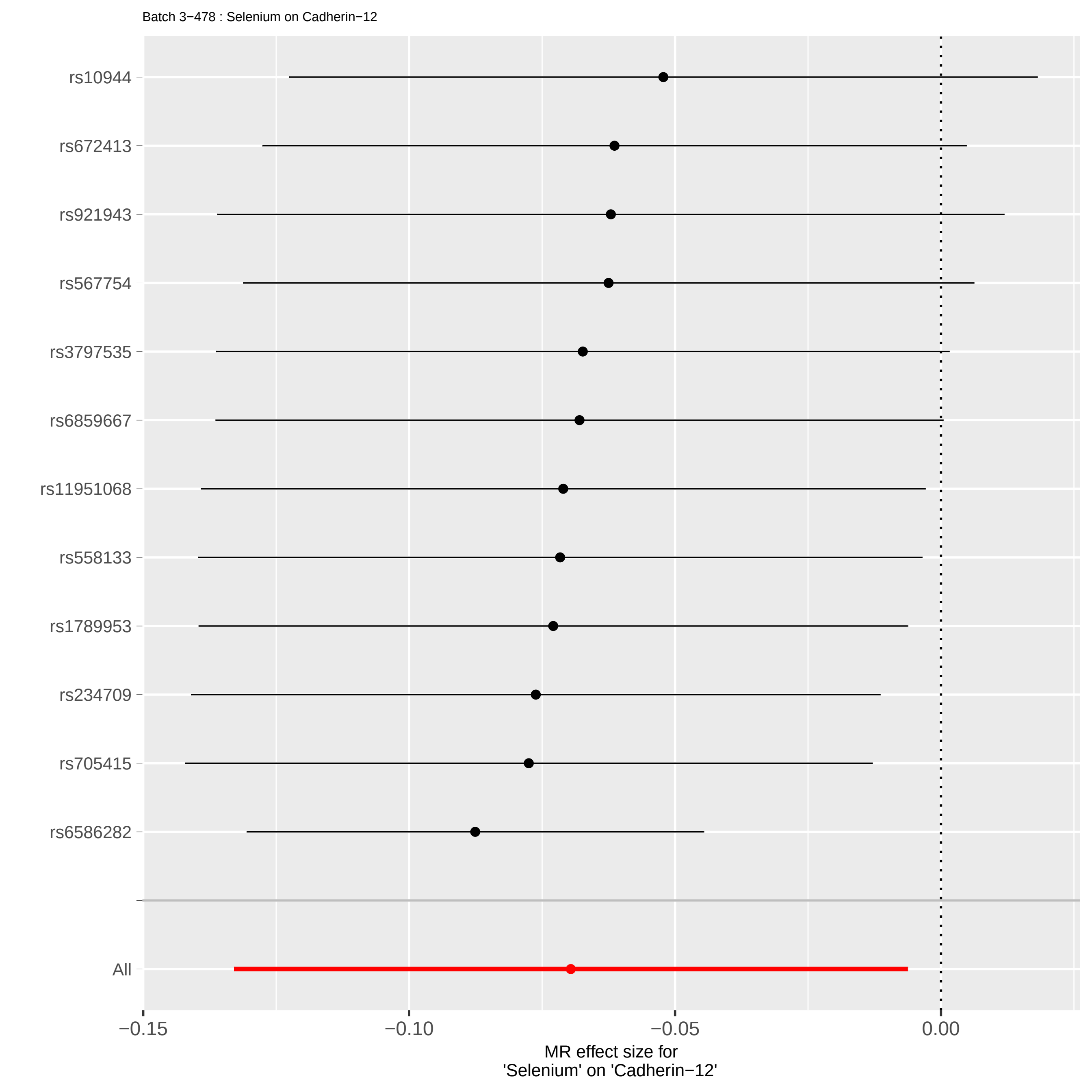

Batch 3−478 : Selenium on Cadherin−12
rs10944
rs672413
rs921943
rs567754
rs3797535
rs6859667
rs11951068
rs558133
rs1789953
rs234709
rs705415
rs6586282
All
−0.15
−0.10
−0.05
0.00
MR effect size for
'Selenium' on 'Cadherin−12'

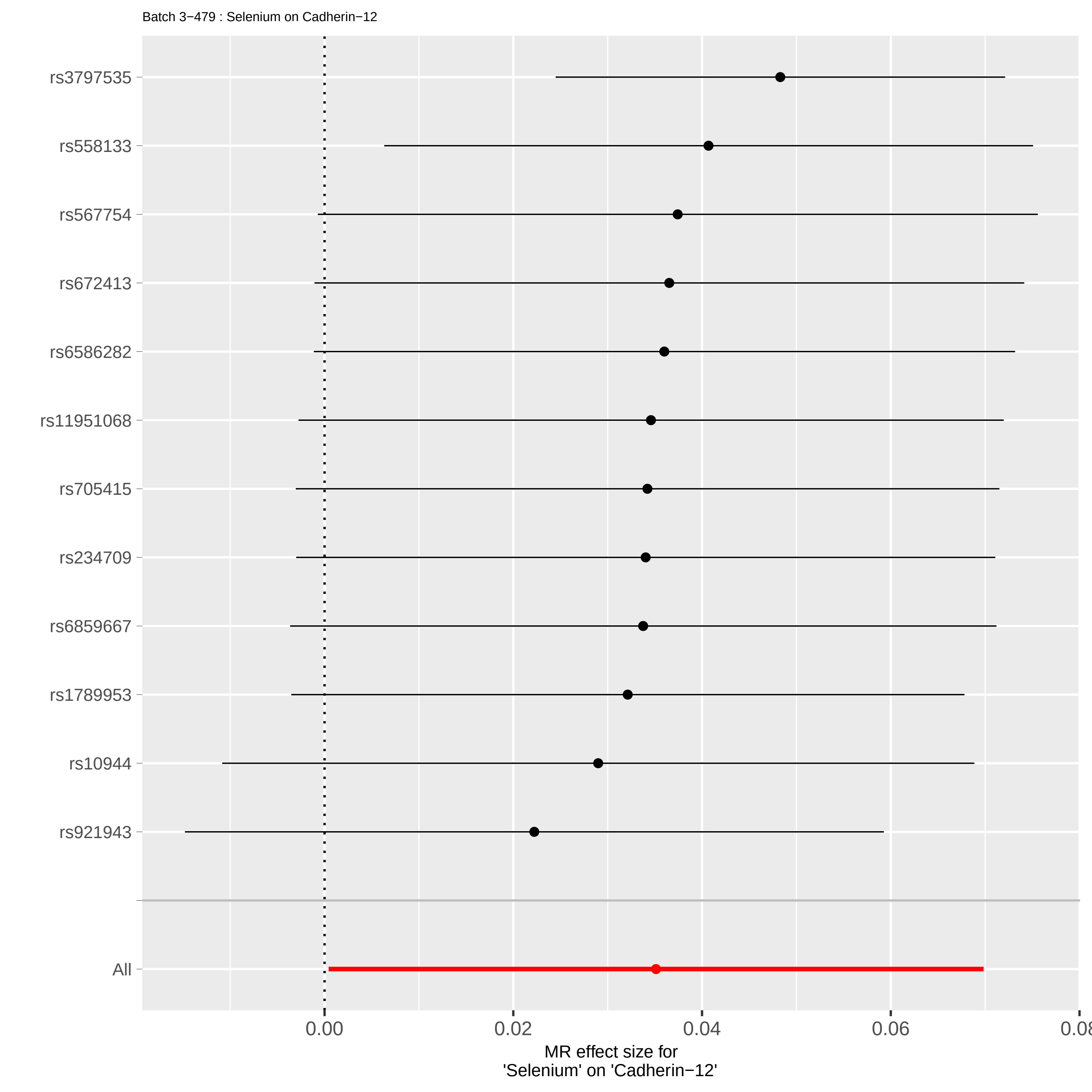

Batch 3−479 : Selenium on Cadherin−12
rs3797535
rs558133
rs567754
rs672413
rs6586282
rs11951068
rs705415
rs234709
rs6859667
rs1789953
rs10944
rs921943
All
0.00
0.02
0.04
0.06
0.08
MR effect size for
'Selenium' on 'Cadherin−12'

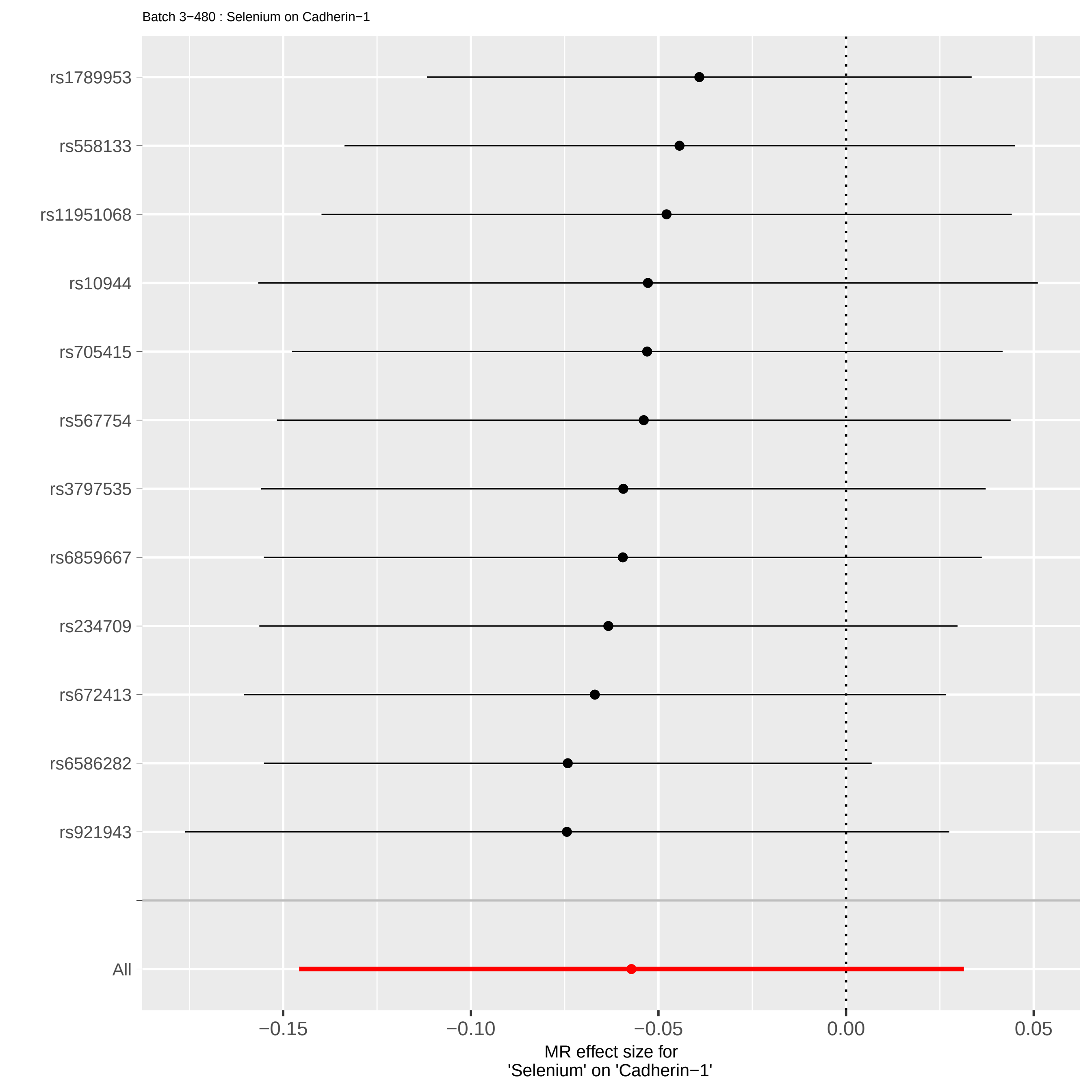

Batch 3−480 : Selenium on Cadherin−1
rs1789953
rs558133
rs11951068
rs10944
rs705415
rs567754
rs3797535
rs6859667
rs234709
rs672413
rs6586282
rs921943
All
−0.15
−0.10
−0.05
0.00
0.05
MR effect size for
'Selenium' on 'Cadherin−1'

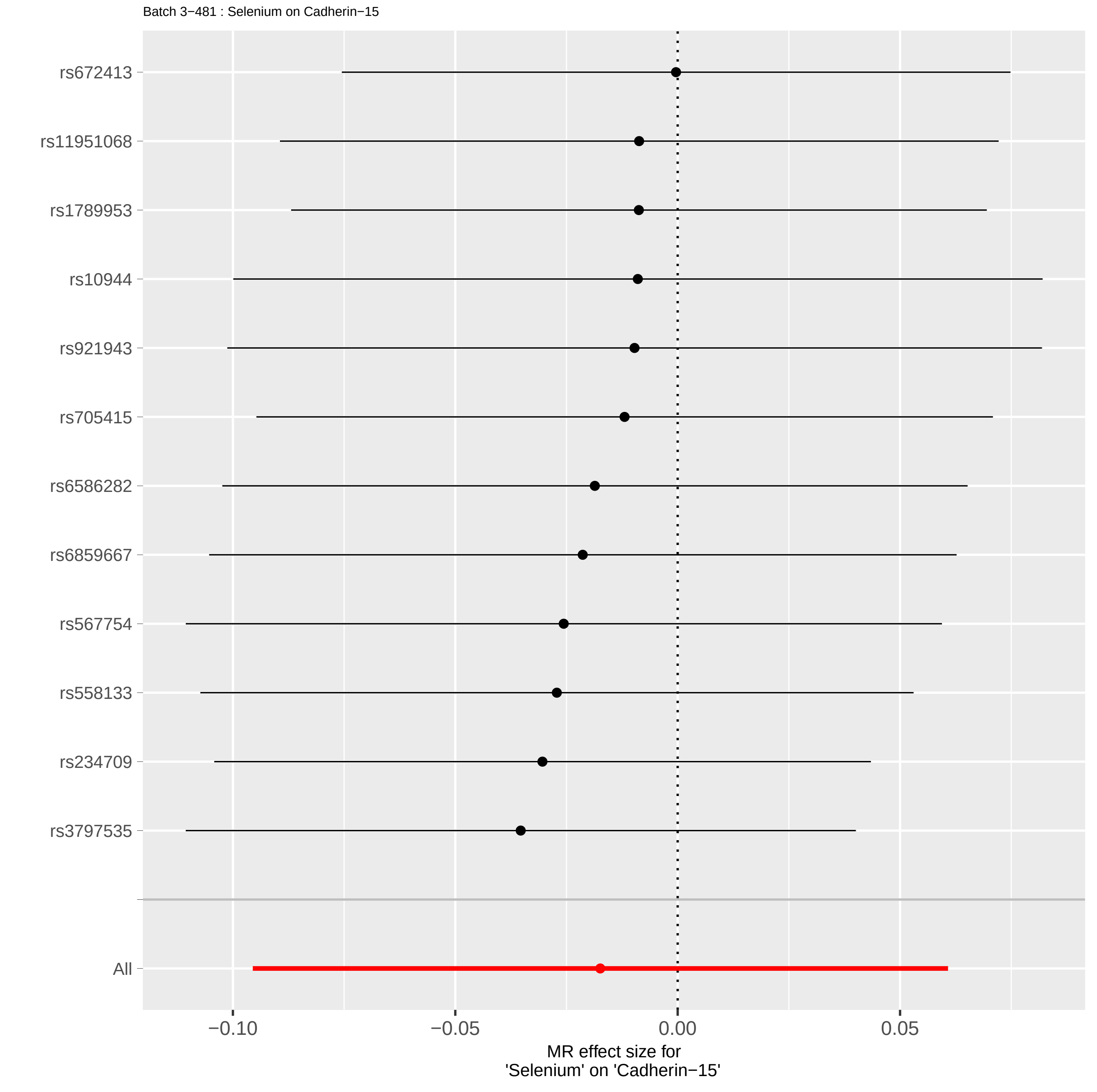

Batch 3−481 : Selenium on Cadherin−15
rs672413
rs11951068
rs1789953
rs10944
rs921943
rs705415
rs6586282
rs6859667
rs567754
rs558133
rs234709
rs3797535
All
−0.10
−0.05
0.00
0.05
MR effect size for
'Selenium' on 'Cadherin−15'

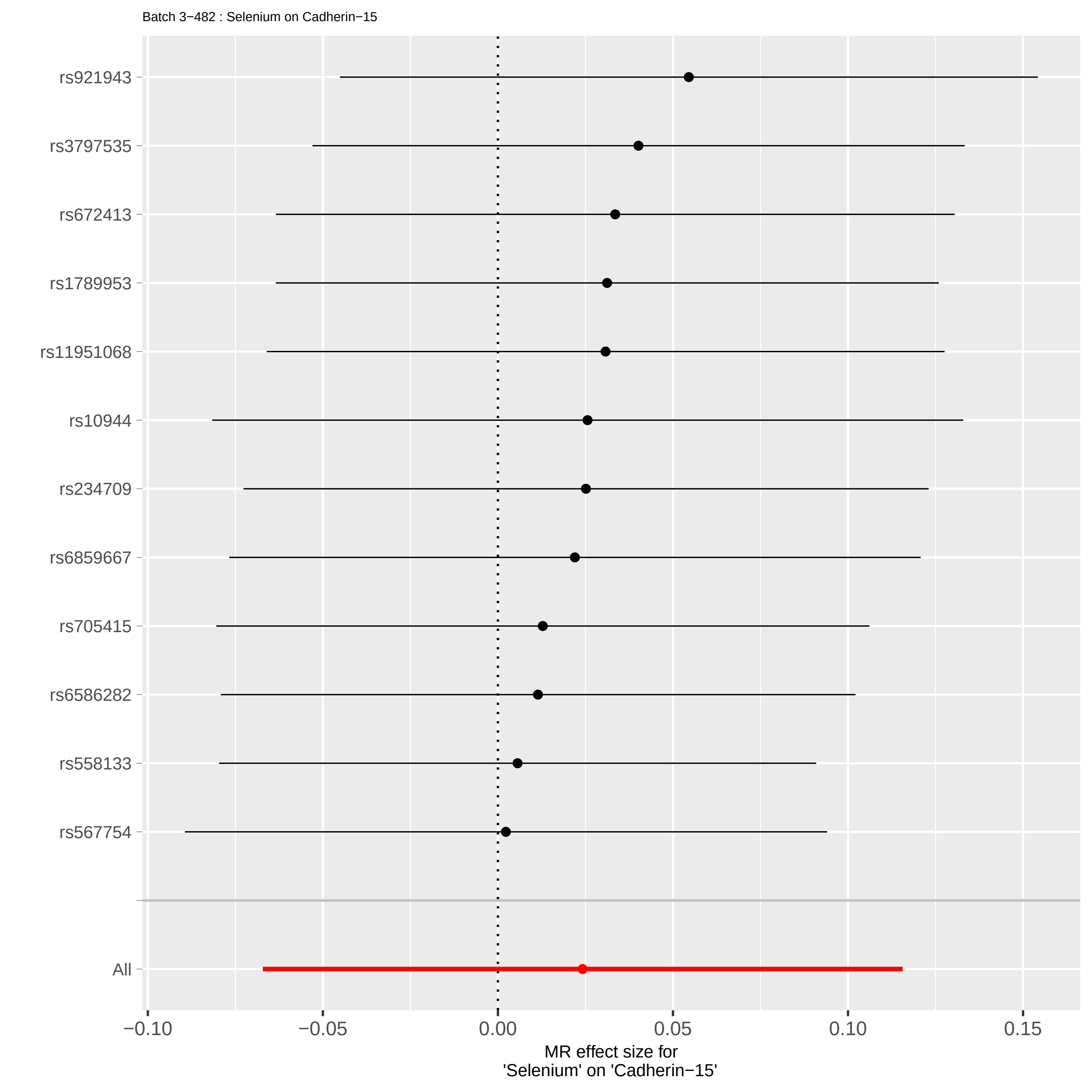

Batch 3−482 : Selenium on Cadherin−15
rs921943
rs3797535
rs672413
rs1789953
rs11951068
rs10944
rs234709
rs6859667
rs705415
rs6586282
rs558133
rs567754
All
−0.10
−0.05
0.00
0.05
0.10
0.15
MR effect size for
'Selenium' on 'Cadherin−15'

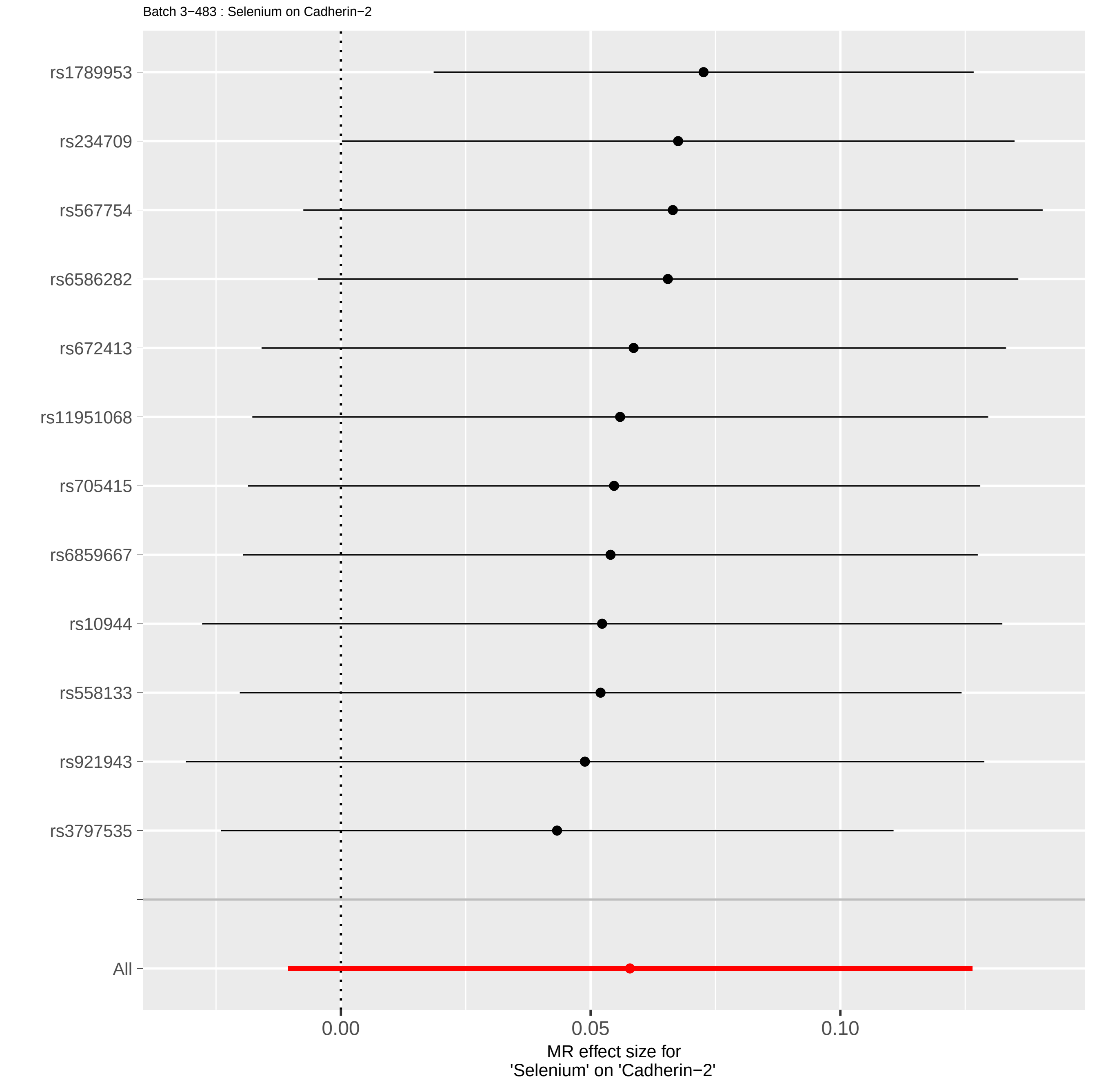

Batch 3−483 : Selenium on Cadherin−2
rs1789953
rs234709
rs567754
rs6586282
rs672413
rs11951068
rs705415
rs6859667
rs10944
rs558133
rs921943
rs3797535
All
0.00
0.05
0.10
MR effect size for
'Selenium' on 'Cadherin−2'

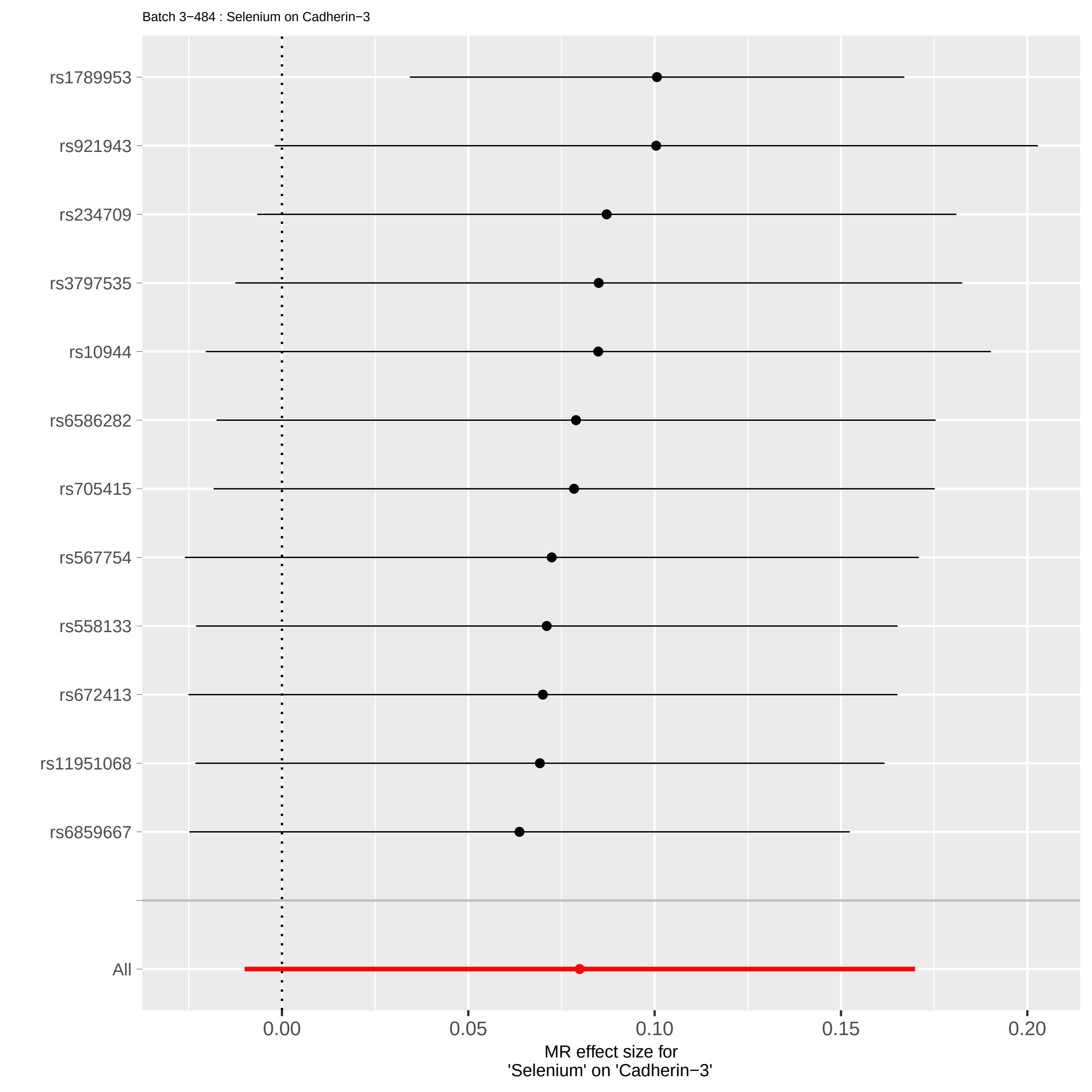
Batch 3−484 : Selenium on Cadherin−3
rs1789953
rs921943
rs234709
rs3797535
rs10944
rs6586282
rs705415
rs567754
rs558133
rs672413
rs11951068
rs6859667
All
0.00
0.05
0.10
0.15
0.20
MR effect size for
'Selenium' on 'Cadherin−3'

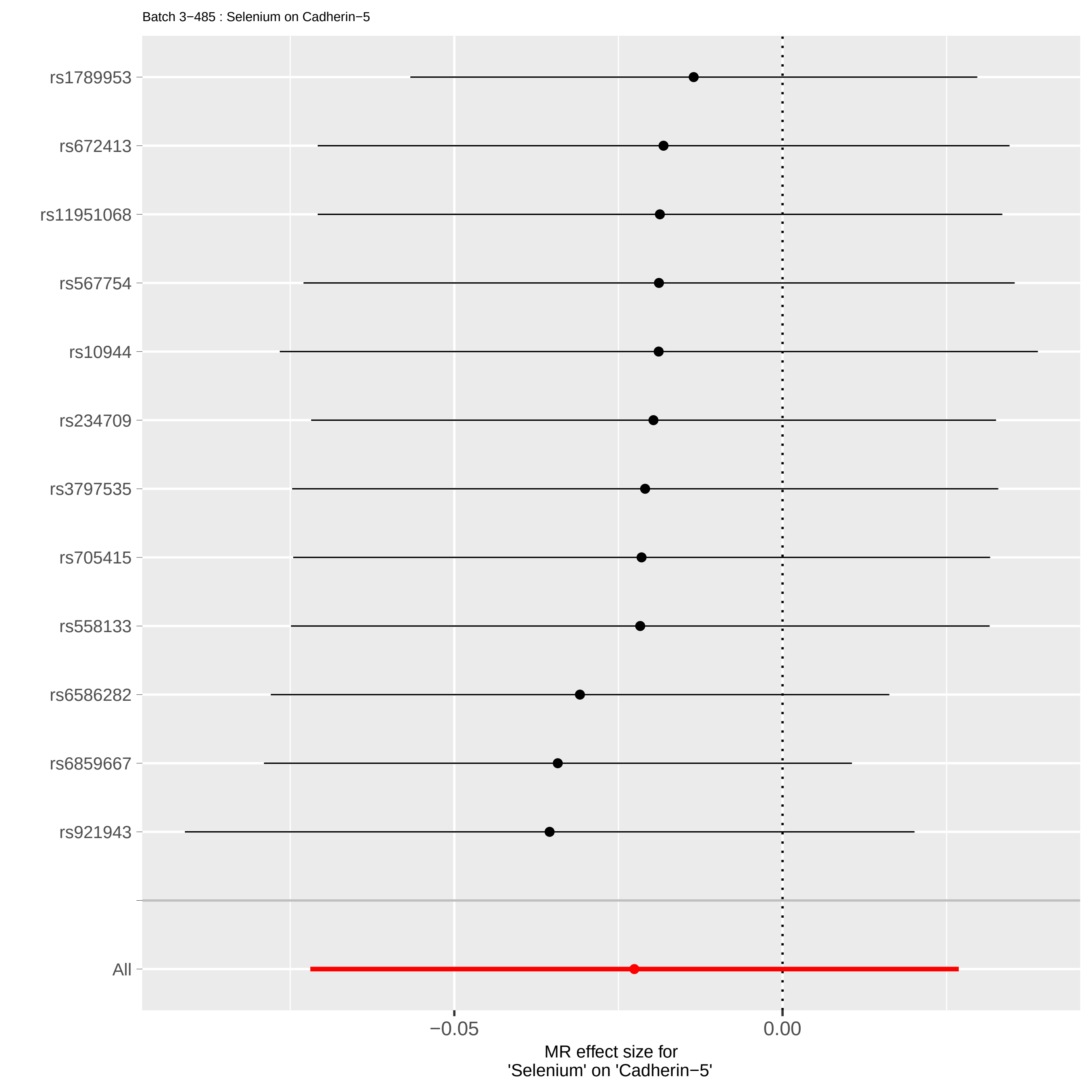
Batch 3−485 : Selenium on Cadherin−5
rs1789953
rs672413
rs11951068
rs567754
rs10944
rs234709
rs3797535
rs705415
rs558133
rs6586282
rs6859667
rs921943
All
−0.05
0.00
MR effect size for
'Selenium' on 'Cadherin−5'

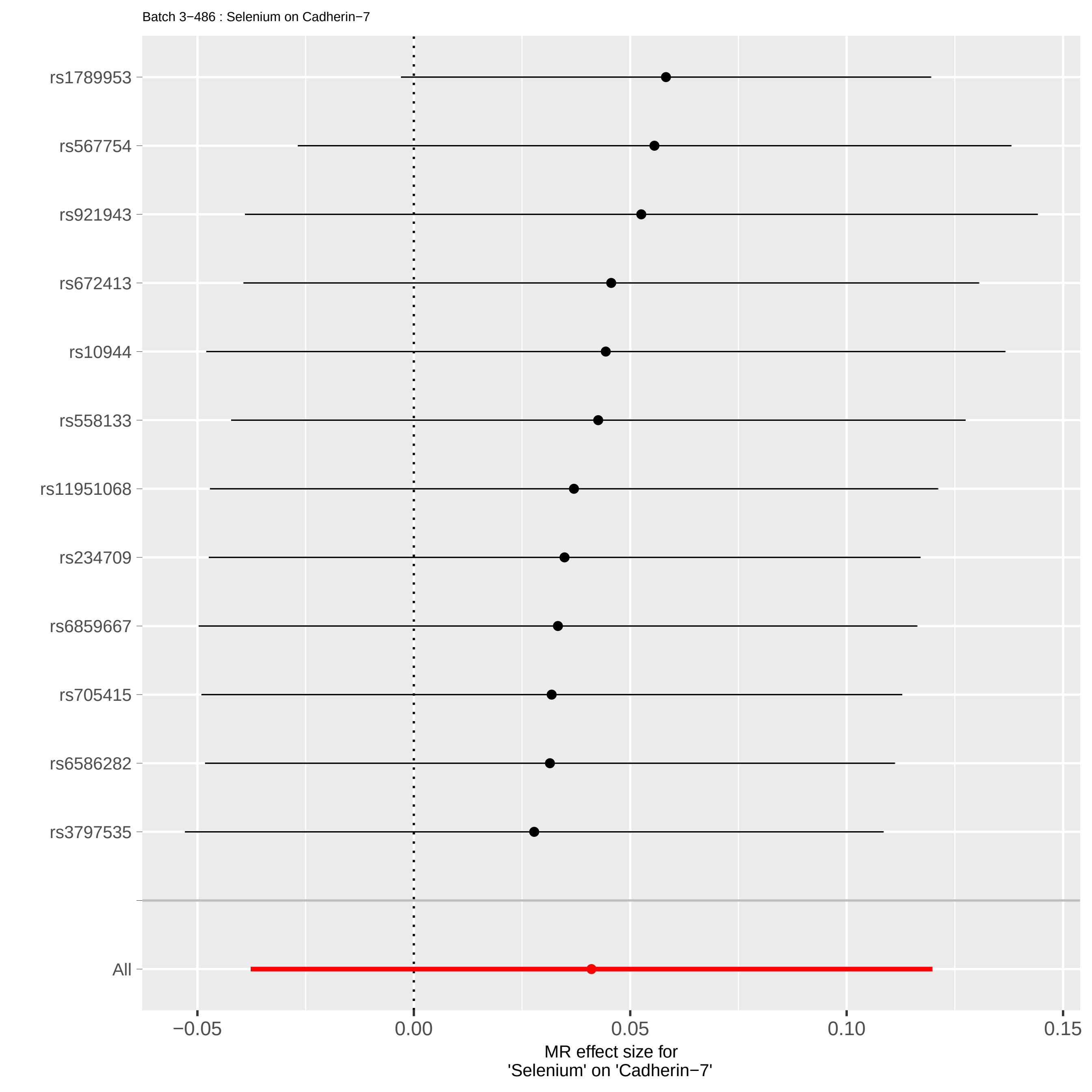

Batch 3−486 : Selenium on Cadherin−7
rs1789953
rs567754
rs921943
rs672413
rs10944
rs558133
rs11951068
rs234709
rs6859667
rs705415
rs6586282
rs3797535
All
−0.05
0.00
0.05
0.10
0.15
MR effect size for
'Selenium' on 'Cadherin−7'

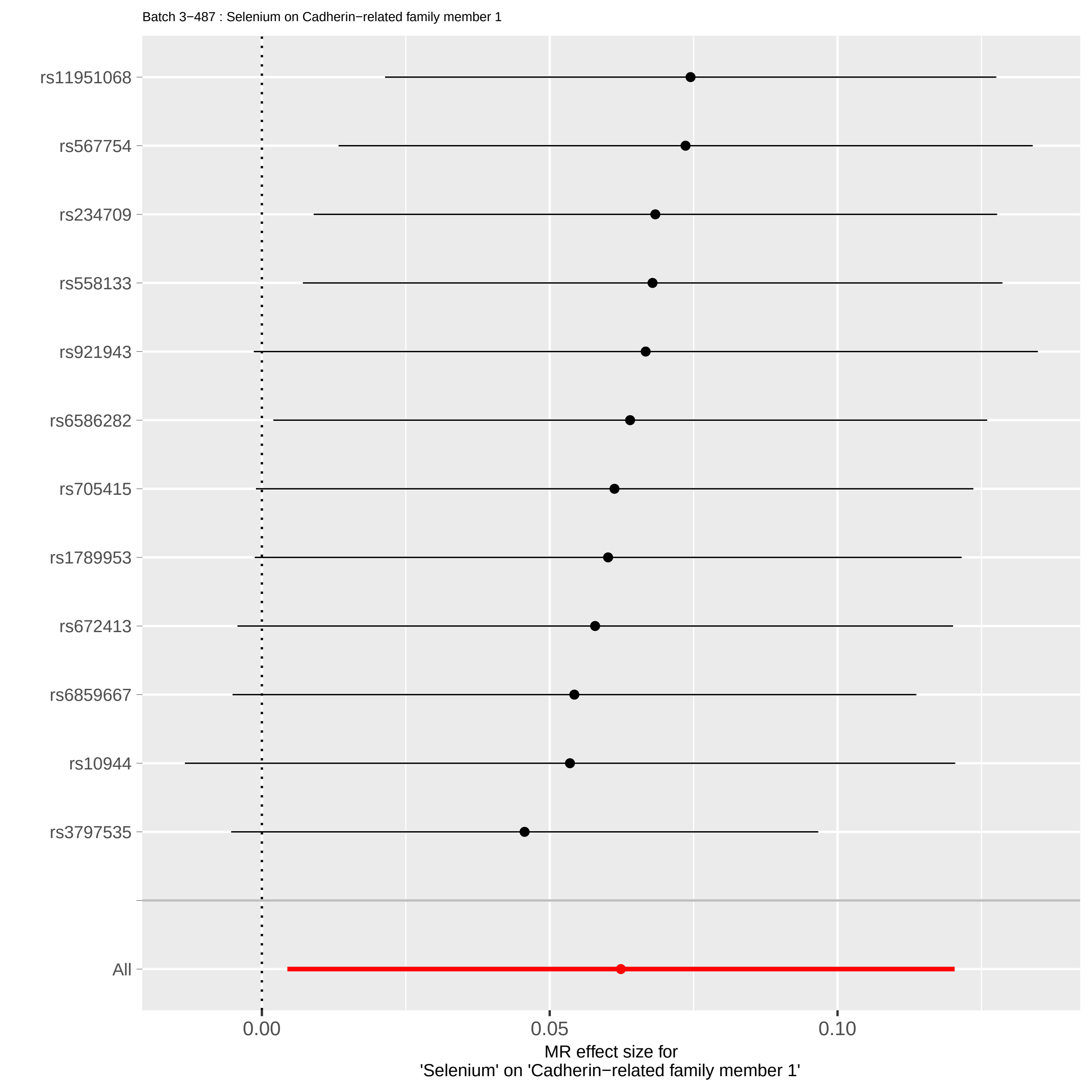

Batch 3−487 : Selenium on Cadherin−related family member 1
rs11951068
rs567754
rs234709
rs558133
rs921943
rs6586282
rs705415
rs1789953
rs672413
rs6859667
rs10944
rs3797535
All
0.00
0.05
0.10
MR effect size for
'Selenium' on 'Cadherin−related family member 1'

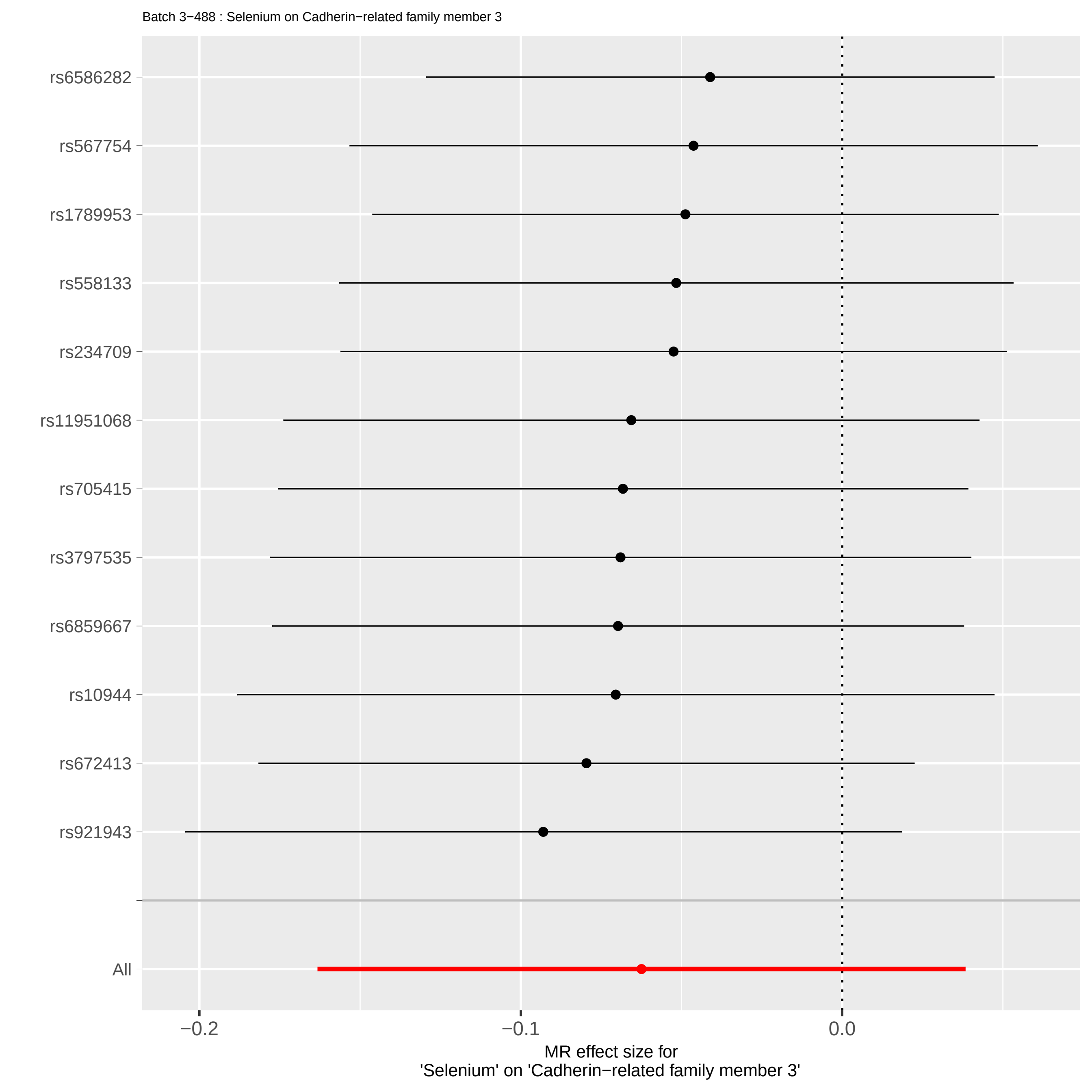

Batch 3−488 : Selenium on Cadherin−related family member 3
rs6586282
rs567754
rs1789953
rs558133
rs234709
rs11951068
rs705415
rs3797535
rs6859667
rs10944
rs672413
rs921943
All
−0.2
−0.1
0.0
MR effect size for
'Selenium' on 'Cadherin−related family member 3'

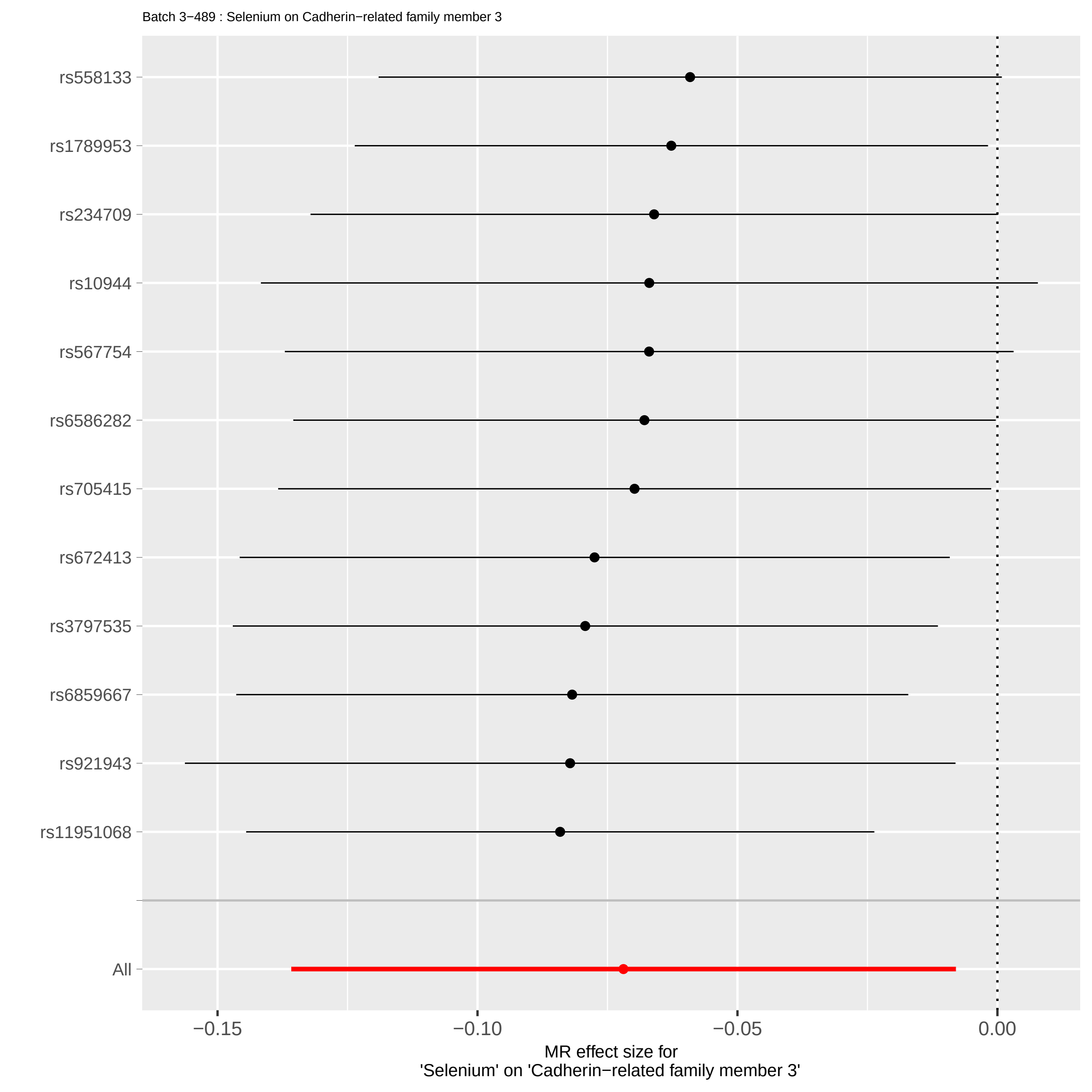

Batch 3−489 : Selenium on Cadherin−related family member 3
rs558133
rs1789953
rs234709
rs10944
rs567754
rs6586282
rs705415
rs672413
rs3797535
rs6859667
rs921943
rs11951068
All
−0.15
−0.10
−0.05
0.00
MR effect size for
'Selenium' on 'Cadherin−related family member 3'

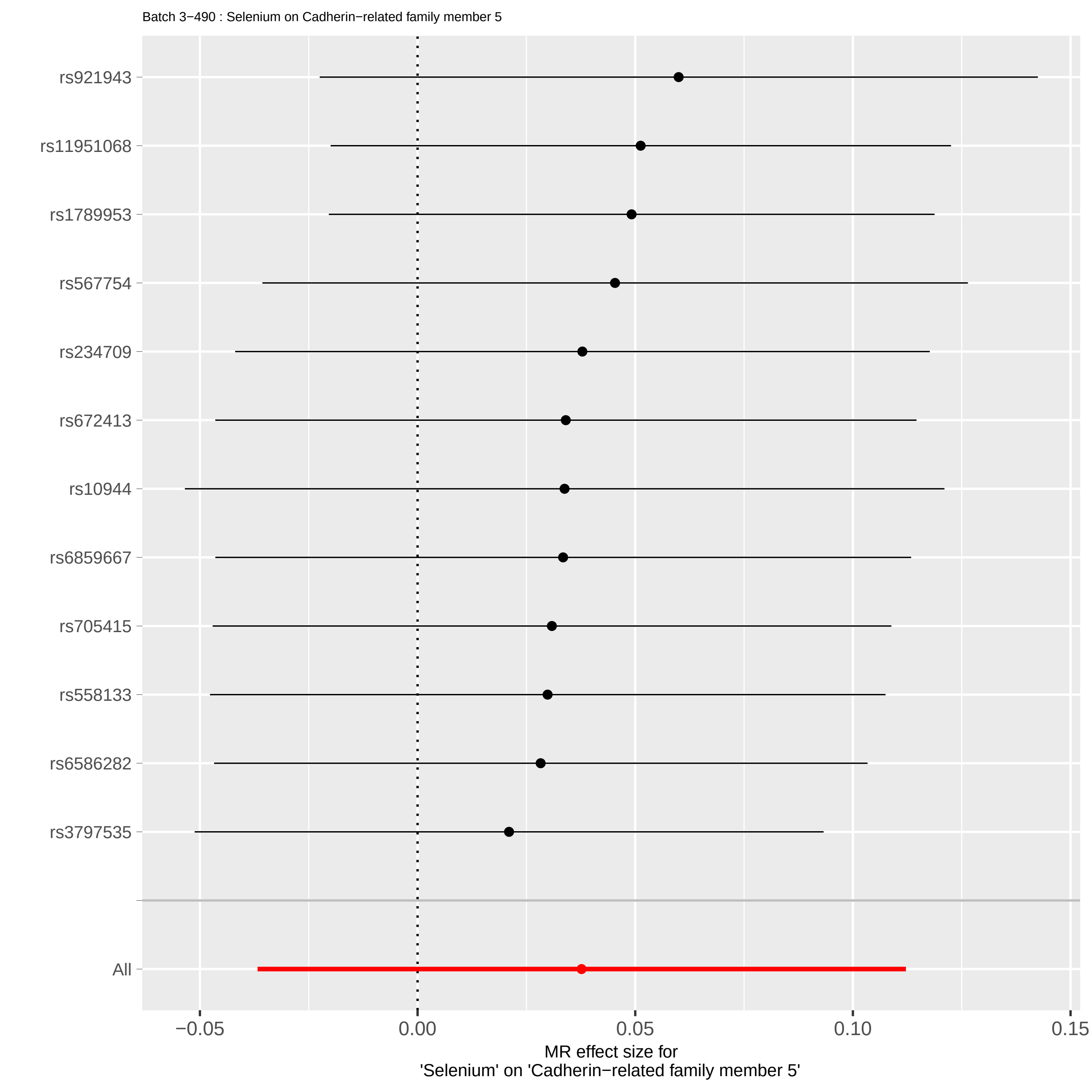
Batch 3−490 : Selenium on Cadherin−related family member 5
rs921943
rs11951068
rs1789953
rs567754
rs234709
rs672413
rs10944
rs6859667
rs705415
rs558133
rs6586282
rs3797535
All
−0.05
0.00
0.05
0.10
0.15
MR effect size for
'Selenium' on 'Cadherin−related family member 5'

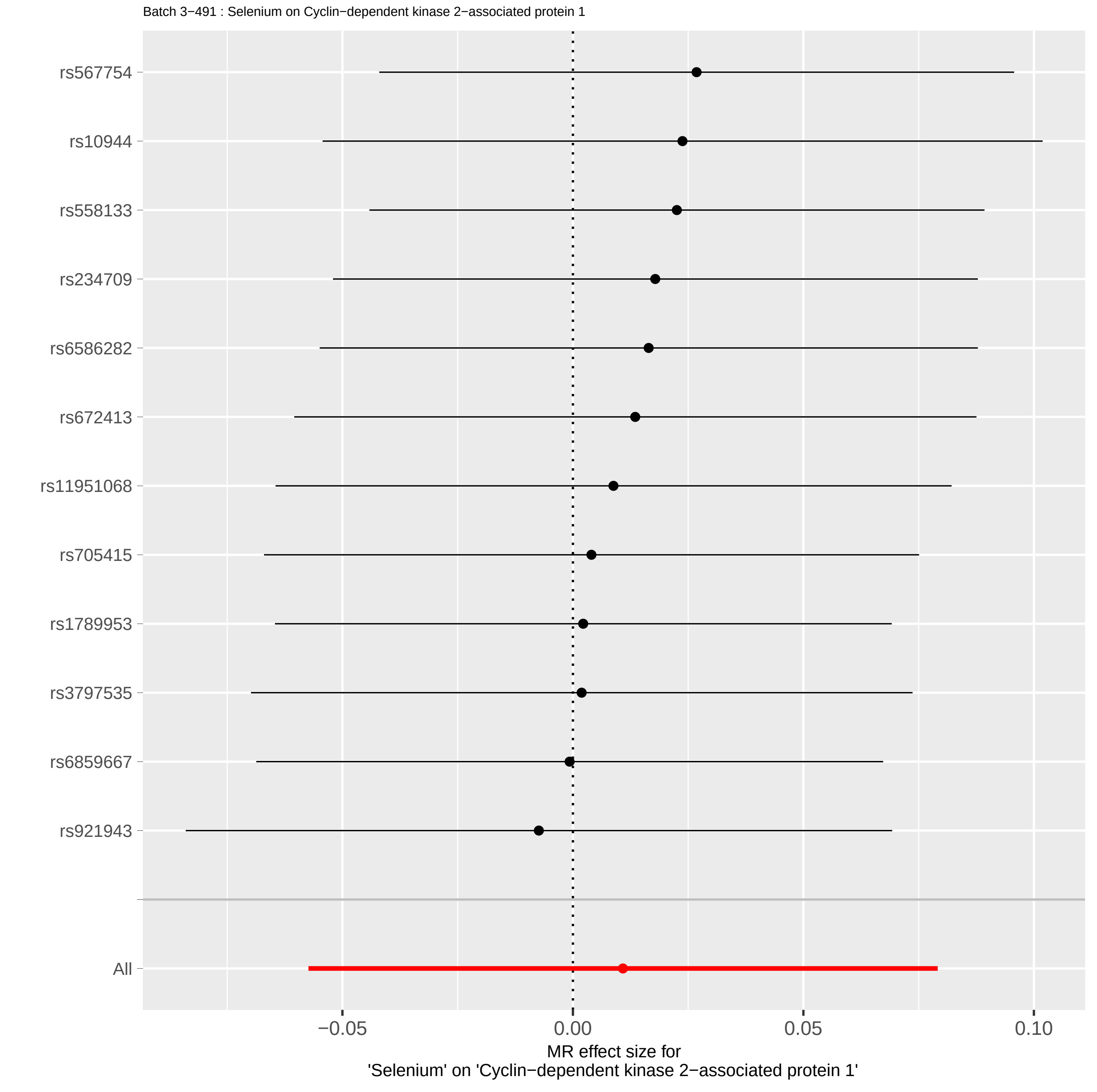

Batch 3−491 : Selenium on Cyclin−dependent kinase 2−associated protein 1
rs567754
rs10944
rs558133
rs234709
rs6586282
rs672413
rs11951068
rs705415
rs1789953
rs3797535
rs6859667
rs921943
All
−0.05
0.00
0.05
0.10
MR effect size for
'Selenium' on 'Cyclin−dependent kinase 2−associated protein 1'

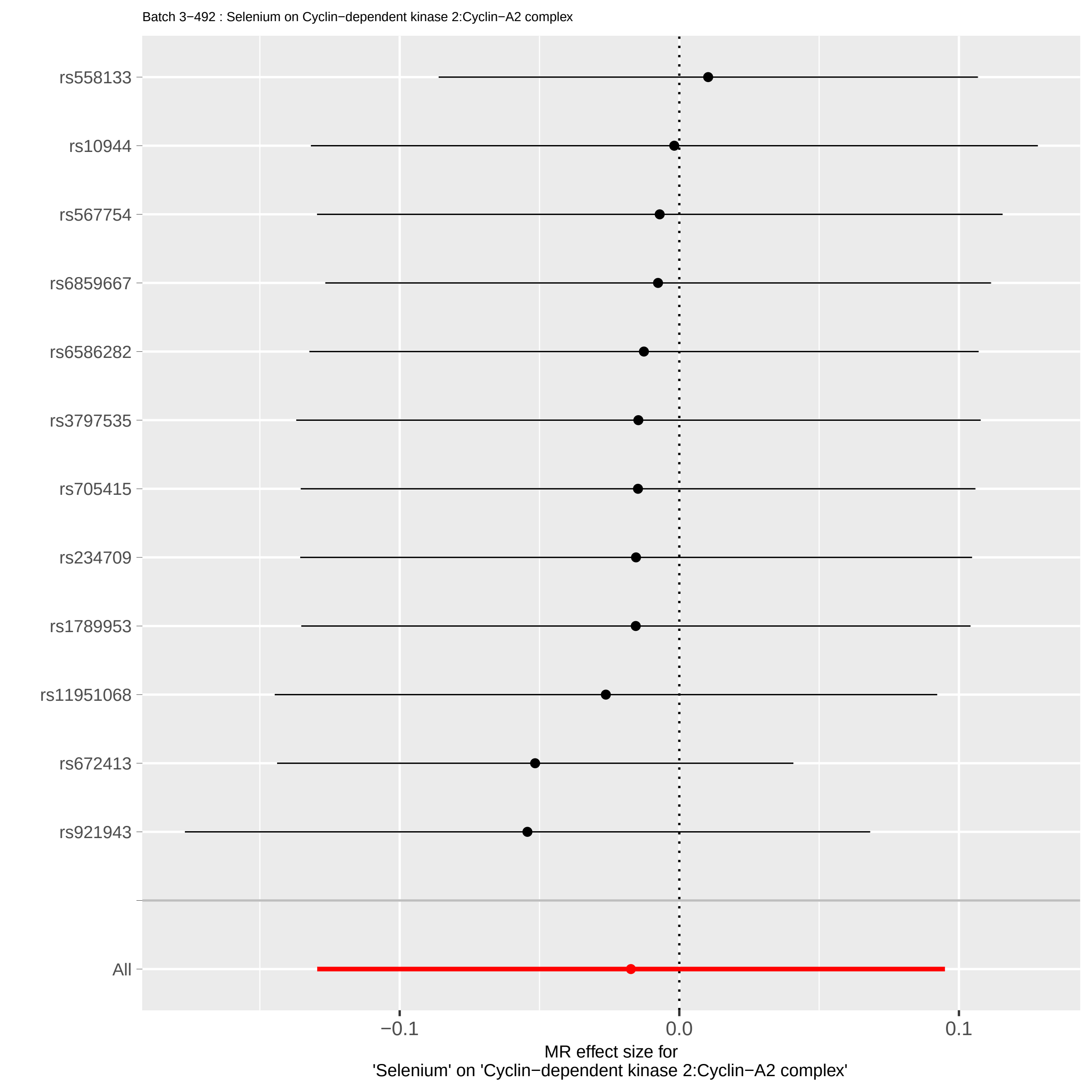

Batch 3−492 : Selenium on Cyclin−dependent kinase 2:Cyclin−A2 complex
rs558133
rs10944
rs567754
rs6859667
rs6586282
rs3797535
rs705415
rs234709
rs1789953
rs11951068
rs672413
rs921943
All
−0.1
0.0
0.1
MR effect size for
'Selenium' on 'Cyclin−dependent kinase 2:Cyclin−A2 complex'

Batch 3−493 : Selenium on Cyclin−dependent kinase 5:Cyclin−dependent kinase 5 activator 1 complex

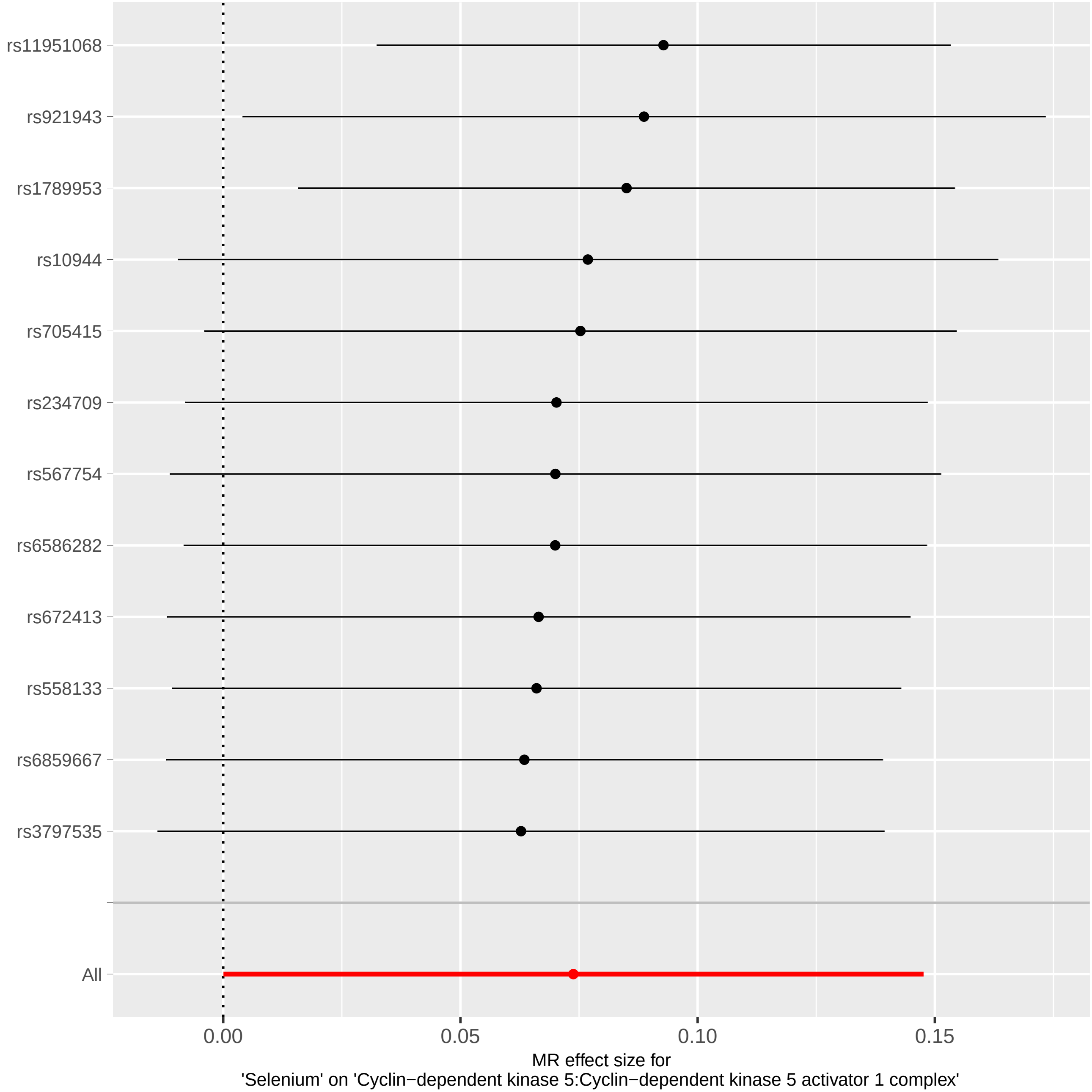

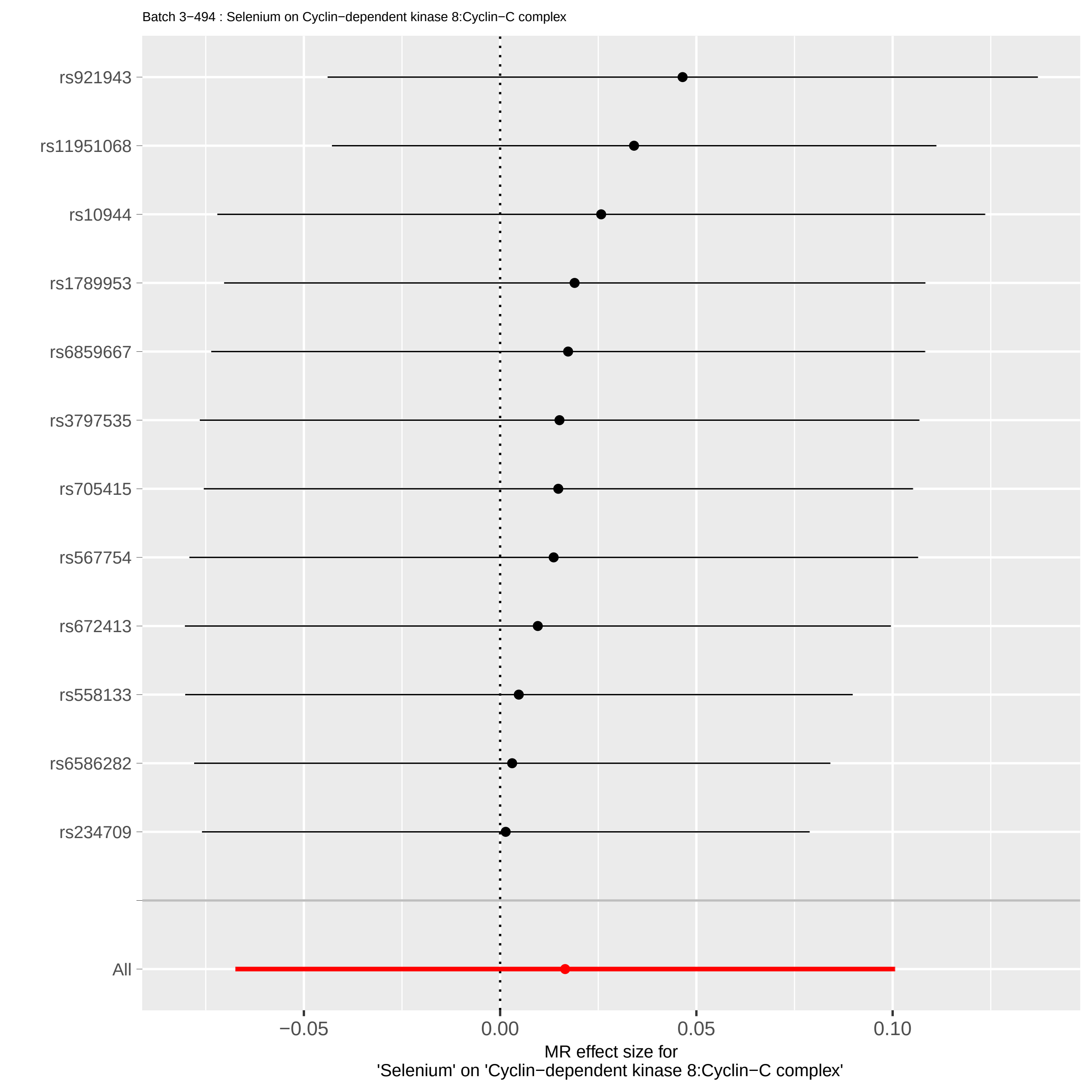

Batch 3−494 : Selenium on Cyclin−dependent kinase 8:Cyclin−C complex
rs921943
rs11951068
rs10944
rs1789953
rs6859667
rs3797535
rs705415
rs567754
rs672413
rs558133
rs6586282
rs234709
All
−0.05
0.00
0.05
0.10
MR effect size for
'Selenium' on 'Cyclin−dependent kinase 8:Cyclin−C complex'

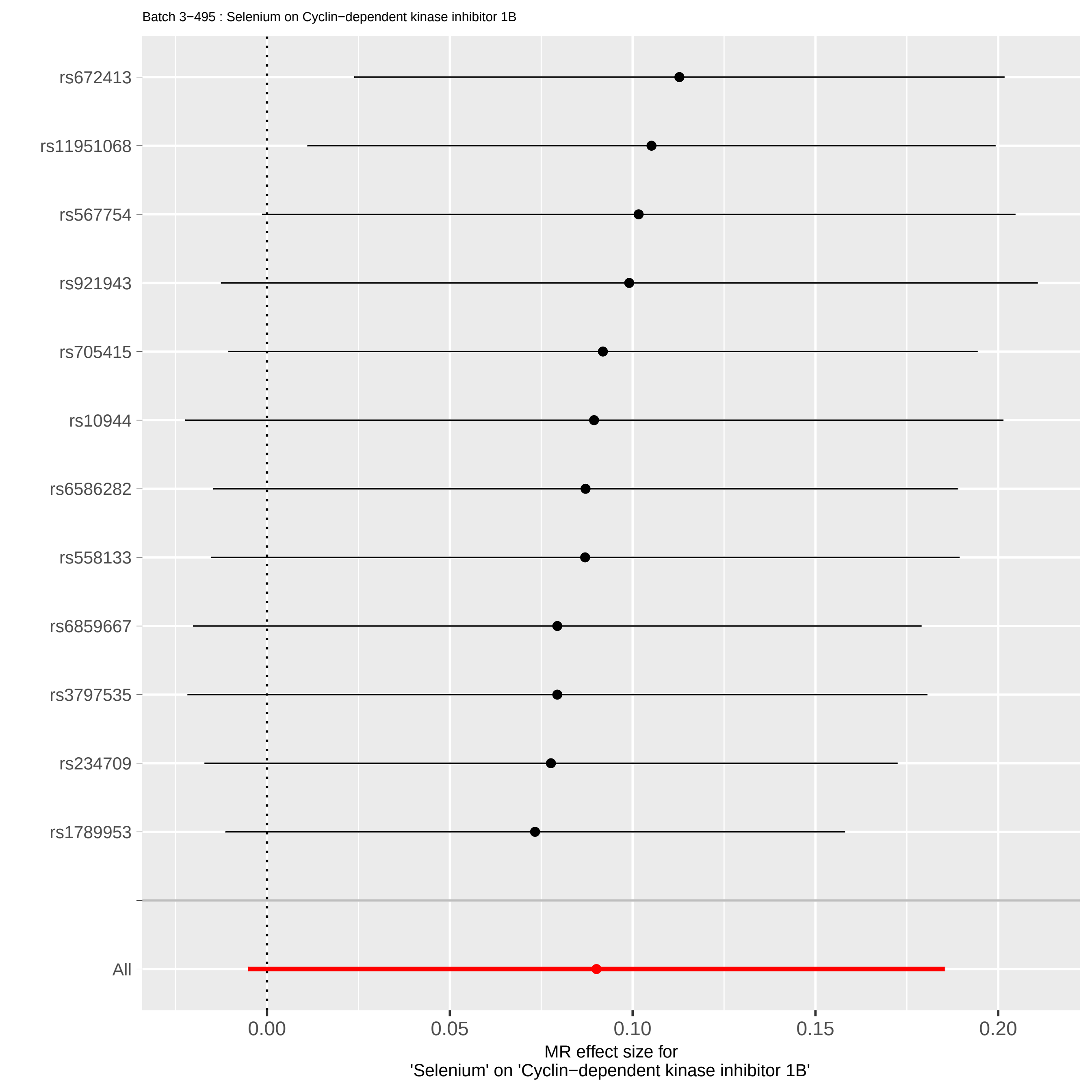

Batch 3−495 : Selenium on Cyclin−dependent kinase inhibitor 1B
rs672413
rs11951068
rs567754
rs921943
rs705415
rs10944
rs6586282
rs558133
rs6859667
rs3797535
rs234709
rs1789953
All
0.00
0.05
0.10
0.15
0.20
MR effect size for
'Selenium' on 'Cyclin−dependent kinase inhibitor 1B'

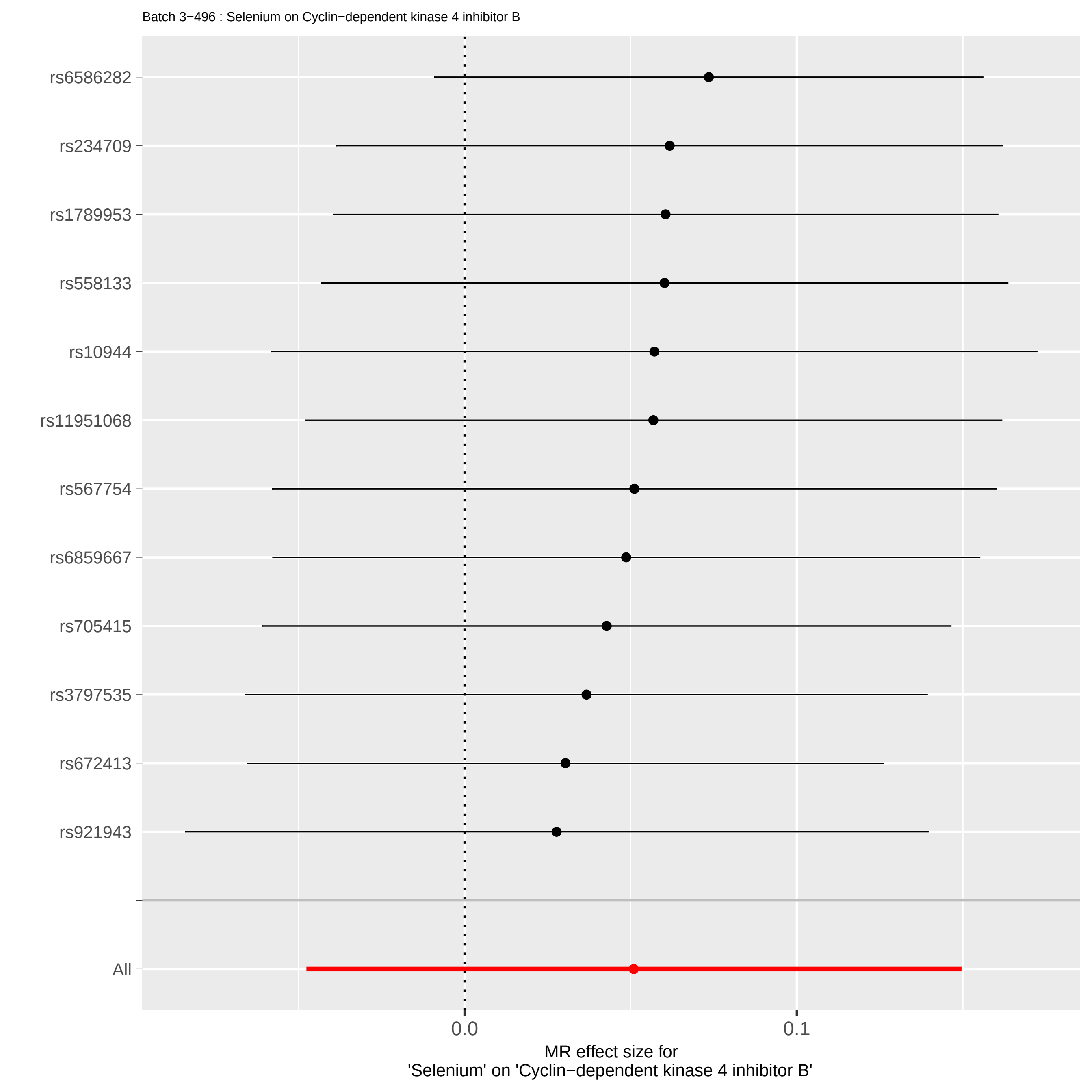
Batch 3−496 : Selenium on Cyclin−dependent kinase 4 inhibitor B
rs6586282
rs234709
rs1789953
rs558133
rs10944
rs11951068
rs567754
rs6859667
rs705415
rs3797535
rs672413
rs921943
All
0.0
0.1
MR effect size for
'Selenium' on 'Cyclin−dependent kinase 4 inhibitor B'

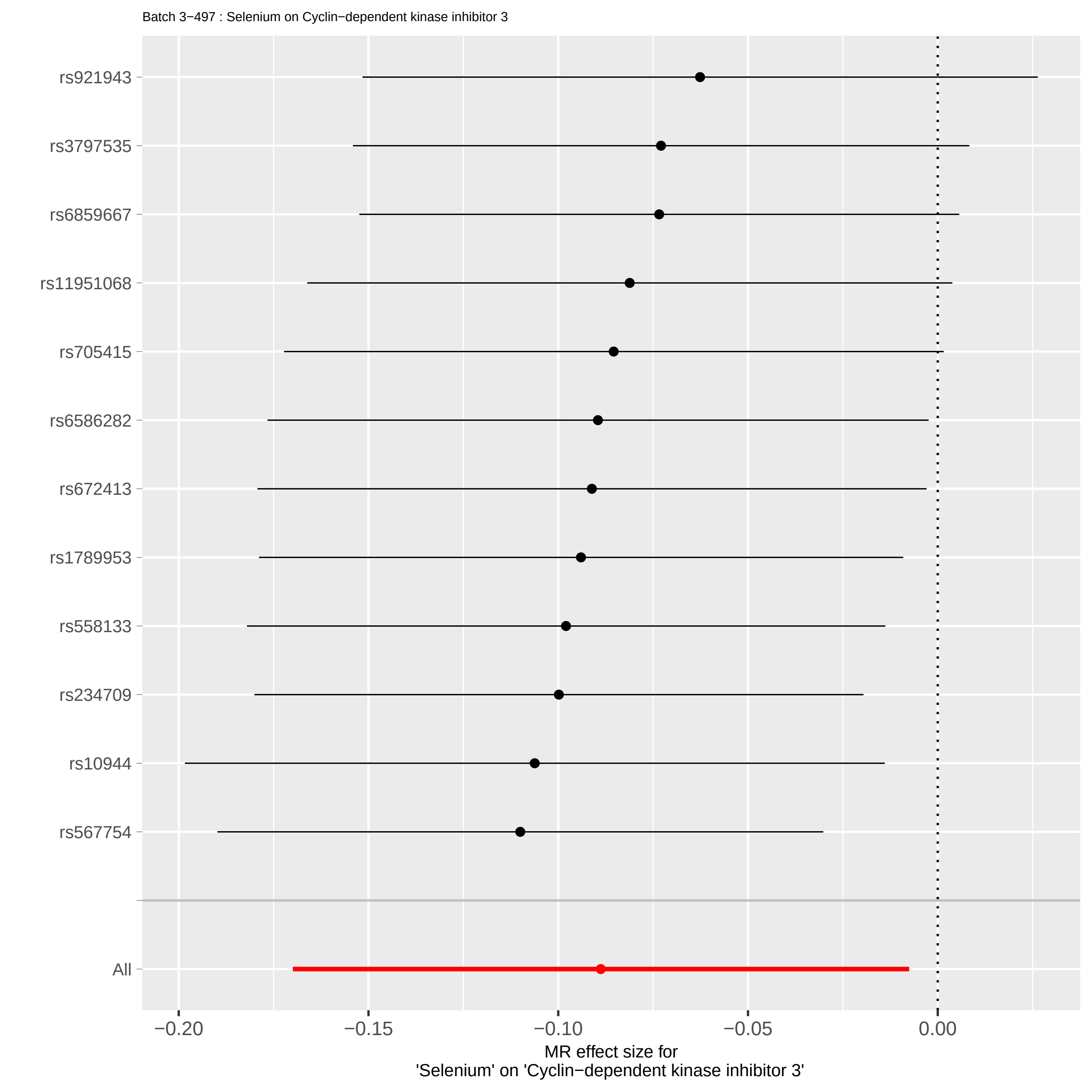

Batch 3−497 : Selenium on Cyclin−dependent kinase inhibitor 3
rs921943
rs3797535
rs6859667
rs11951068
rs705415
rs6586282
rs672413
rs1789953
rs558133
rs234709
rs10944
rs567754
All
−0.20
−0.15
−0.10
−0.05
0.00
MR effect size for
'Selenium' on 'Cyclin−dependent kinase inhibitor 3'

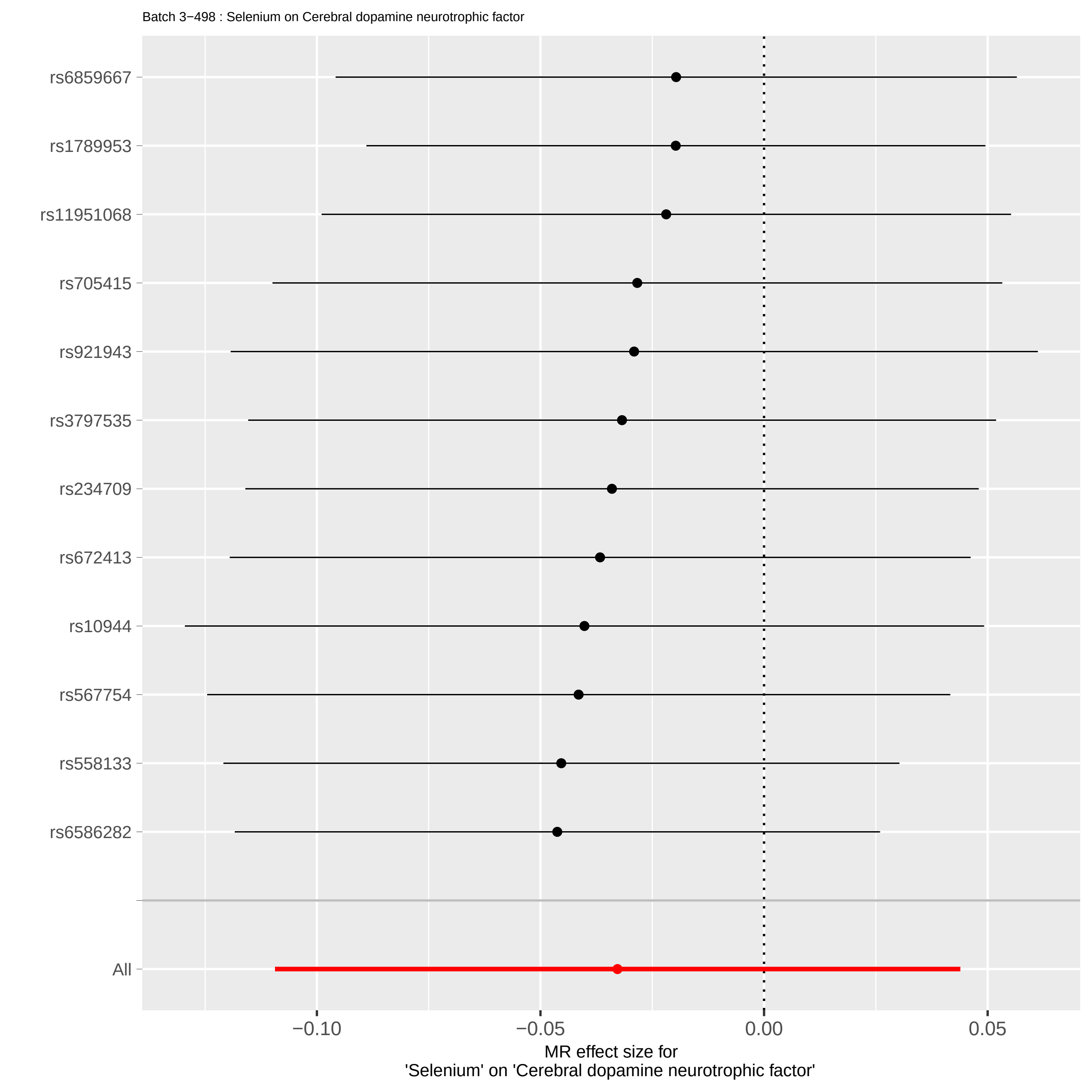

Batch 3−498 : Selenium on Cerebral dopamine neurotrophic factor
rs6859667
rs1789953
rs11951068
rs705415
rs921943
rs3797535
rs234709
rs672413
rs10944
rs567754
rs558133
rs6586282
All
−0.10
−0.05
0.00
0.05
MR effect size for
'Selenium' on 'Cerebral dopamine neurotrophic factor'

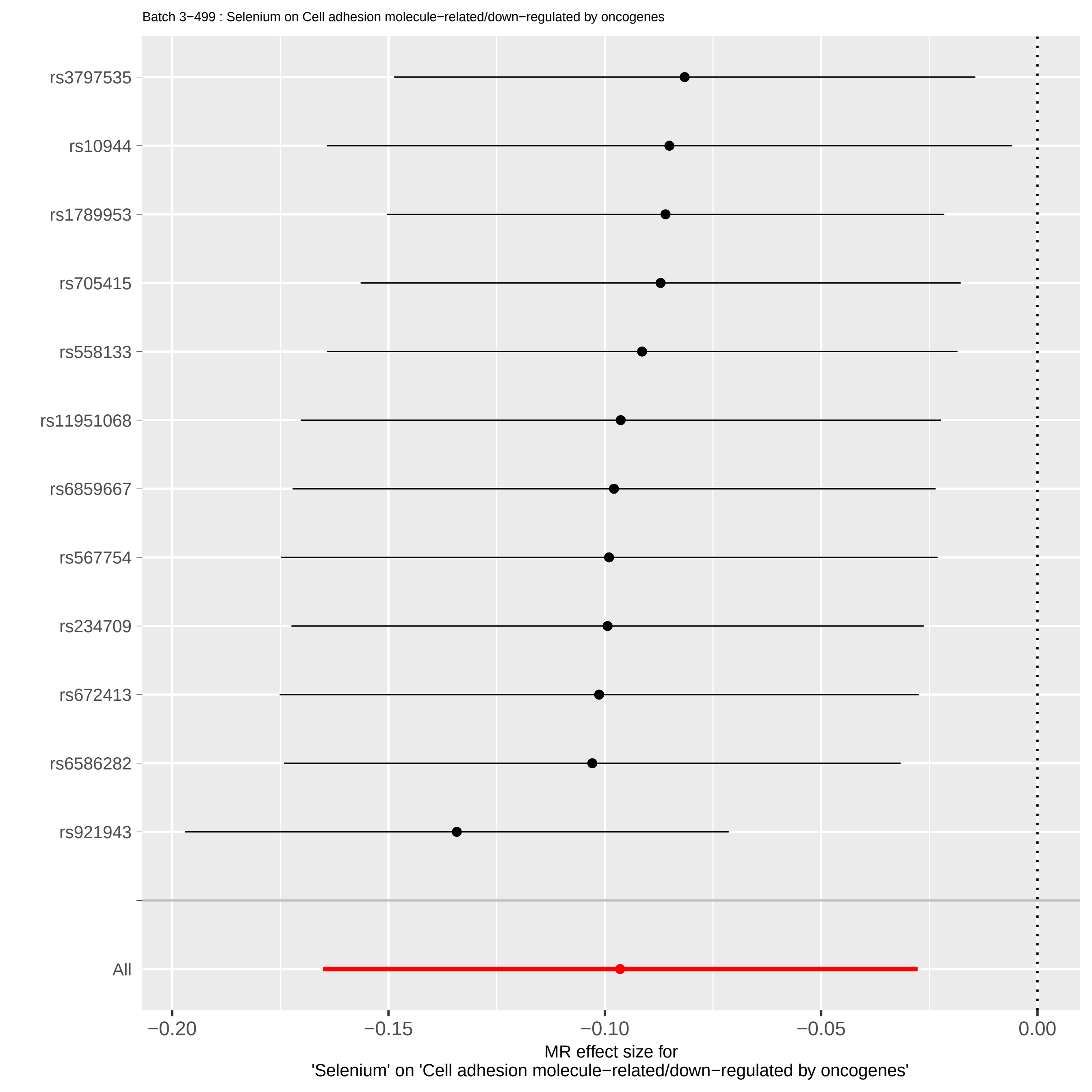

Batch 3−499 : Selenium on Cell adhesion molecule−related/down−regulated by oncogenes
rs3797535
rs10944
rs1789953
rs705415
rs558133
rs11951068
rs6859667
rs567754
rs234709
rs672413
rs6586282
rs921943
All
−0.20
−0.15
−0.10
−0.05
0.00
MR effect size for
'Selenium' on 'Cell adhesion molecule−related/down−regulated by oncogenes'

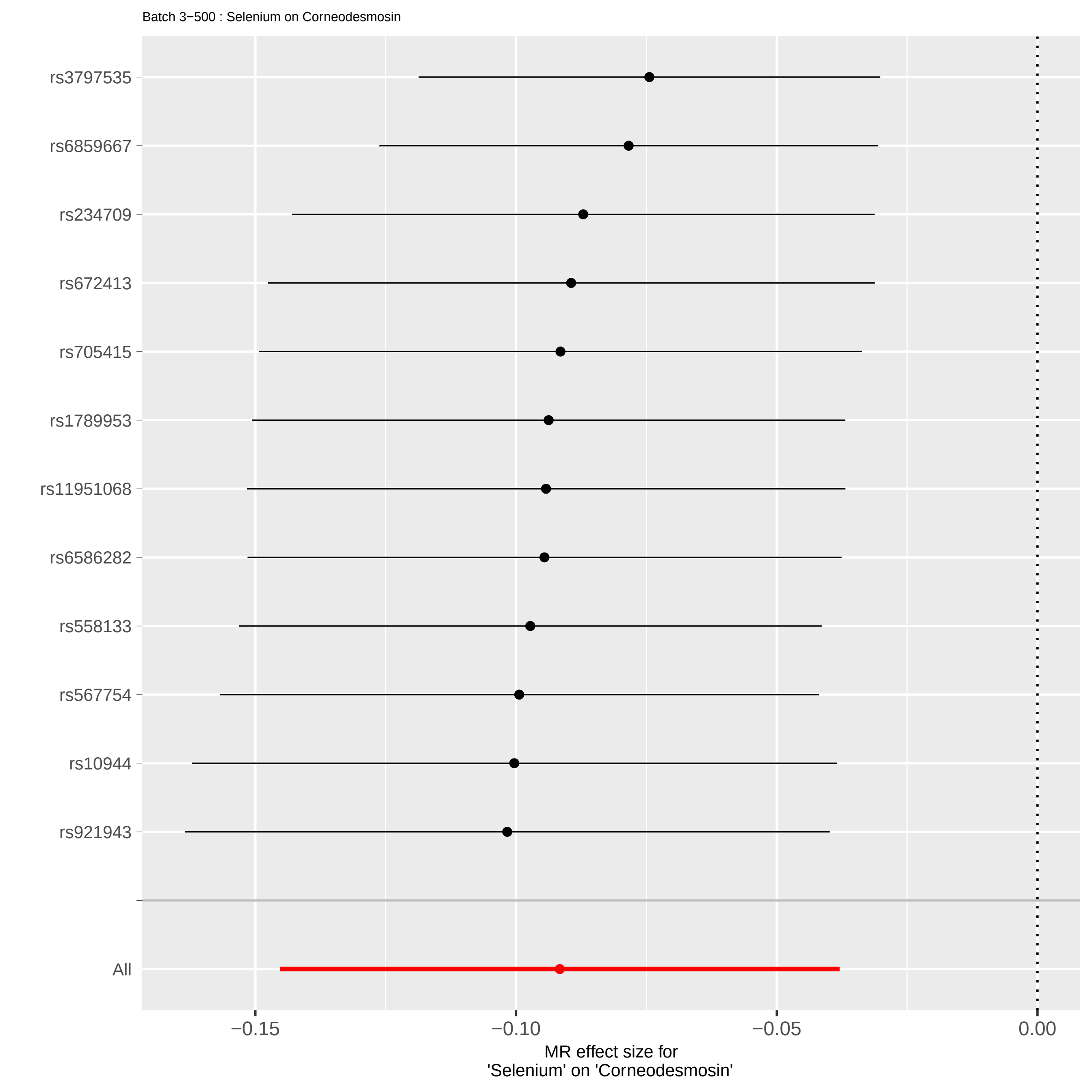
Batch 3−500 : Selenium on Corneodesmosin
rs3797535
rs6859667
rs234709
rs672413
rs705415
rs1789953
rs11951068
rs6586282
rs558133
rs567754
rs10944
rs921943
All
−0.15
−0.10
−0.05
0.00
MR effect size for
'Selenium' on 'Corneodesmosin'

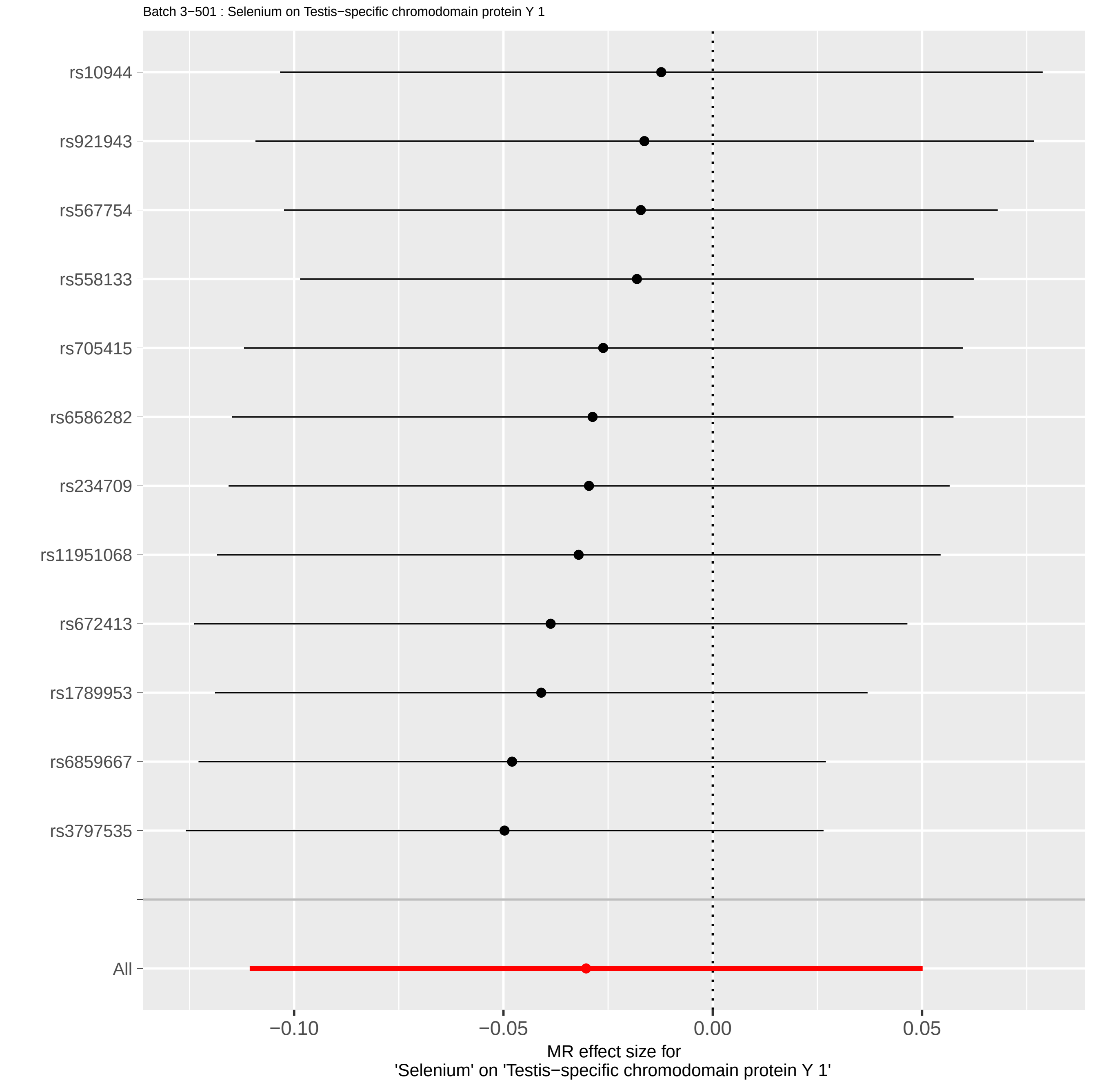

Batch 3−501 : Selenium on Testis−specific chromodomain protein Y 1
rs10944
rs921943
rs567754
rs558133
rs705415
rs6586282
rs234709
rs11951068
rs672413
rs1789953
rs6859667
rs3797535
All
−0.10
−0.05
0.00
0.05
MR effect size for
'Selenium' on 'Testis−specific chromodomain protein Y 1'

Batch 3−502 : Selenium on Carcinoembryonic antigen−related cell adhesion molecule 1

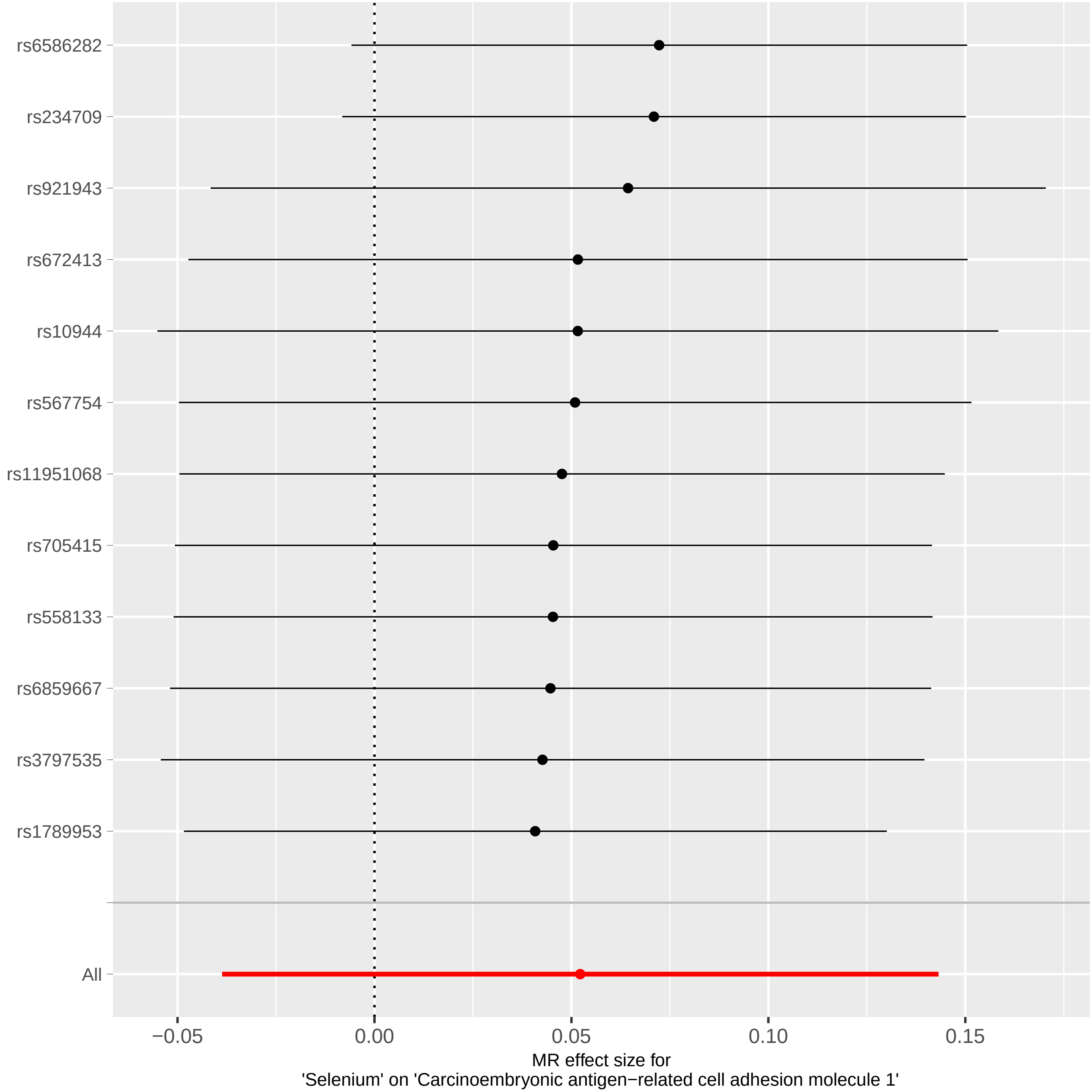

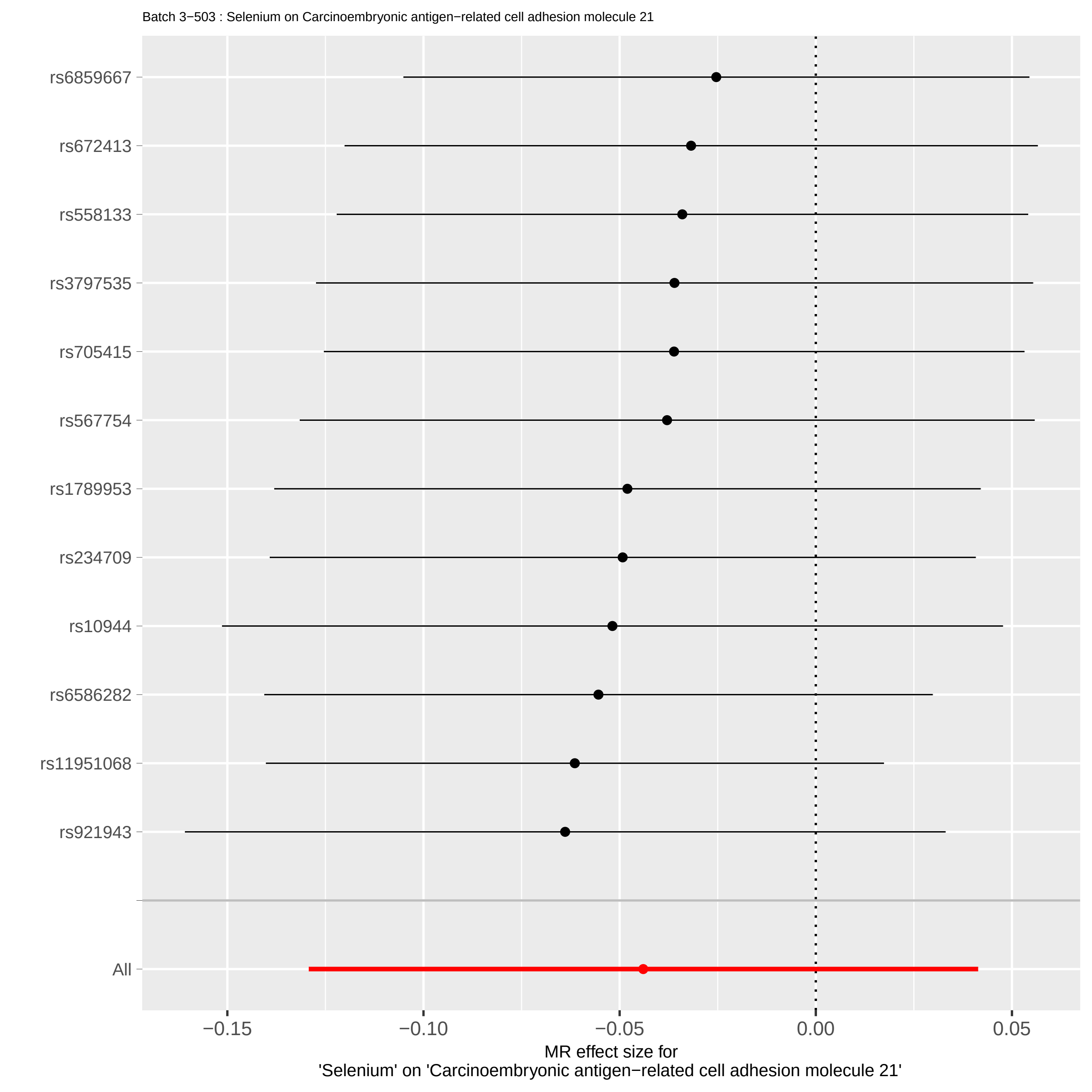

Batch 3−503 : Selenium on Carcinoembryonic antigen−related cell adhesion molecule 21
rs6859667
rs672413
rs558133
rs3797535
rs705415
rs567754
rs1789953
rs234709
rs10944
rs6586282
rs11951068
rs921943
All
−0.15
−0.10
−0.05
0.00
0.05
MR effect size for
'Selenium' on 'Carcinoembryonic antigen−related cell adhesion molecule 21'

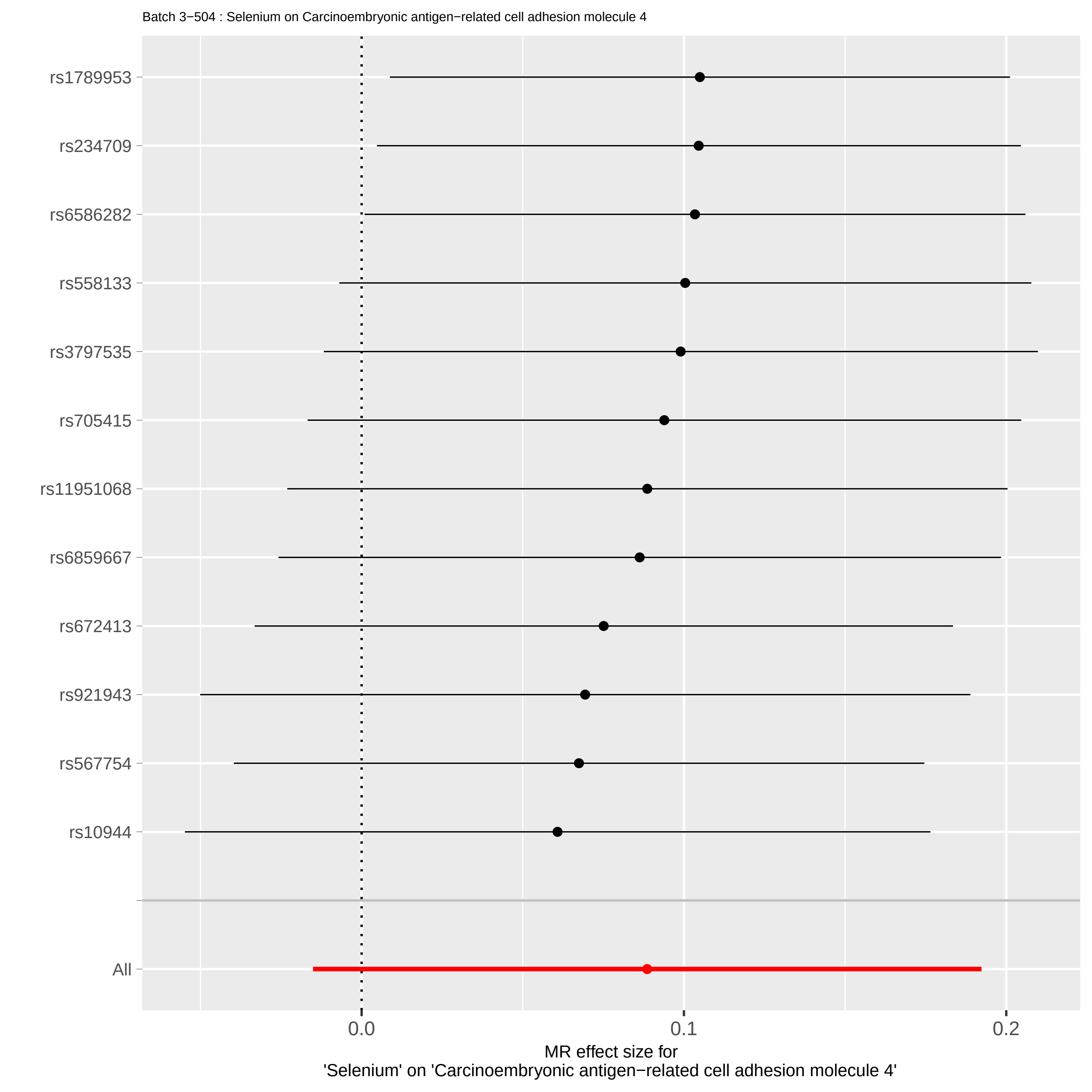

Batch 3−504 : Selenium on Carcinoembryonic antigen−related cell adhesion molecule 4
rs1789953
rs234709
rs6586282
rs558133
rs3797535
rs705415
rs11951068
rs6859667
rs672413
rs921943
rs567754
rs10944
All
0.0
0.1
0.2
MR effect size for
'Selenium' on 'Carcinoembryonic antigen−related cell adhesion molecule 4'

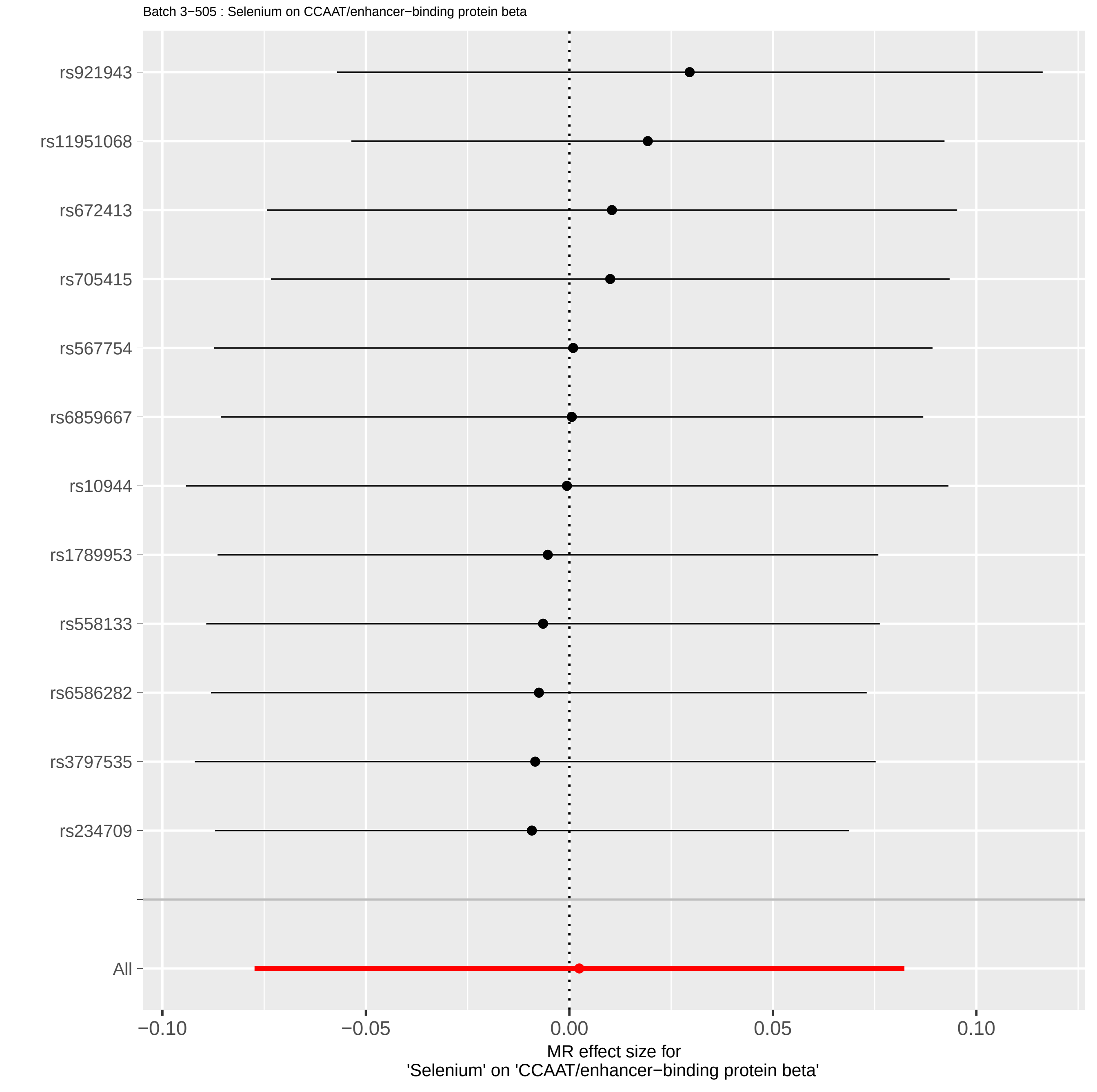
Batch 3−505 : Selenium on CCAAT/enhancer−binding protein beta
rs921943
rs11951068
rs672413
rs705415
rs567754
rs6859667
rs10944
rs1789953
rs558133
rs6586282
rs3797535
rs234709
All
−0.10
−0.05
0.00
0.05
0.10
MR effect size for
'Selenium' on 'CCAAT/enhancer−binding protein beta'

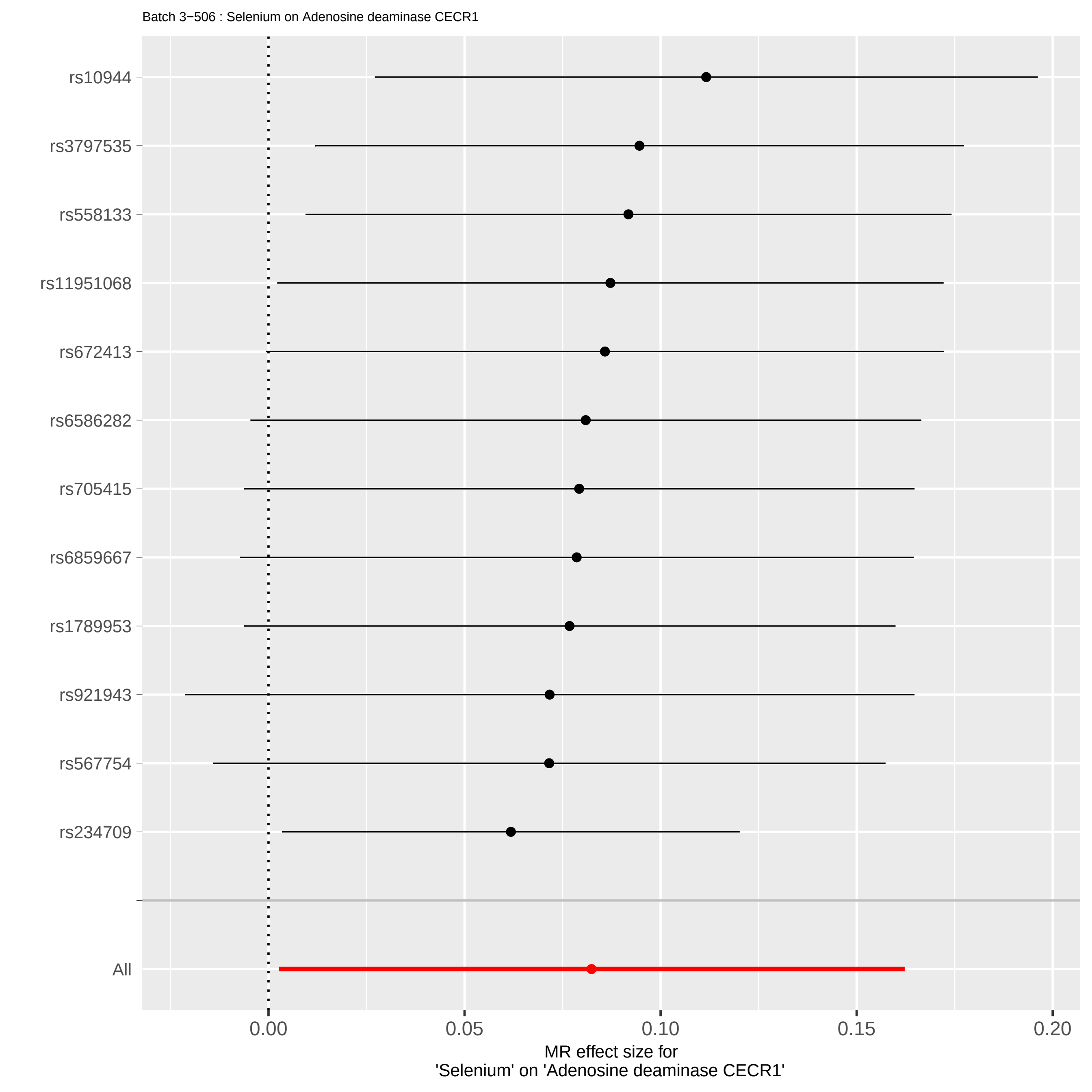
Batch 3−506 : Selenium on Adenosine deaminase CECR1
rs10944
rs3797535
rs558133
rs11951068
rs672413
rs6586282
rs705415
rs6859667
rs1789953
rs921943
rs567754
rs234709
All
0.00
0.05
0.10
0.15
0.20
MR effect size for
'Selenium' on 'Adenosine deaminase CECR1'

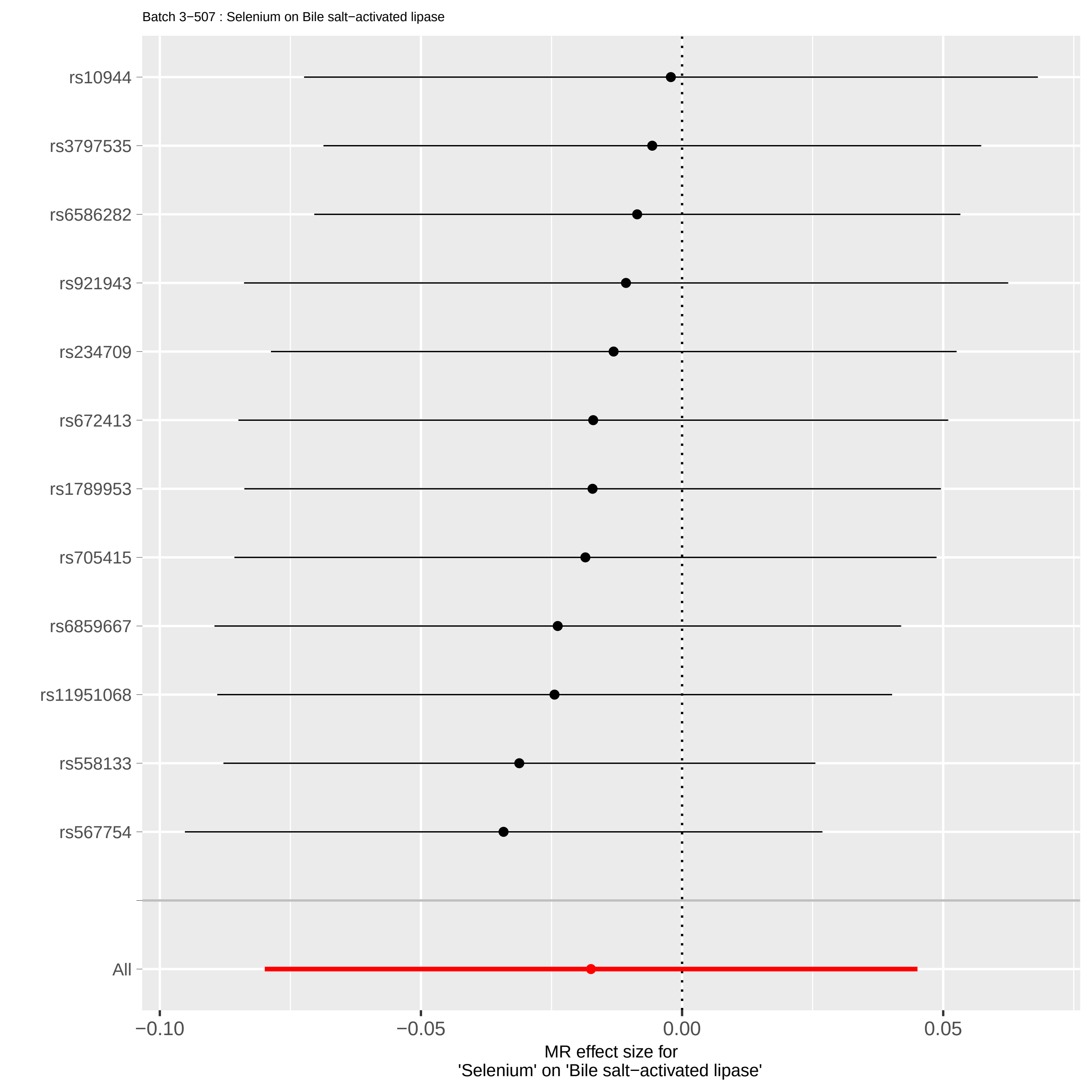

Batch 3−507 : Selenium on Bile salt−activated lipase
rs10944
rs3797535
rs6586282
rs921943
rs234709
rs672413
rs1789953
rs705415
rs6859667
rs11951068
rs558133
rs567754
All
−0.10
−0.05
0.00
0.05
MR effect size for
'Selenium' on 'Bile salt−activated lipase'

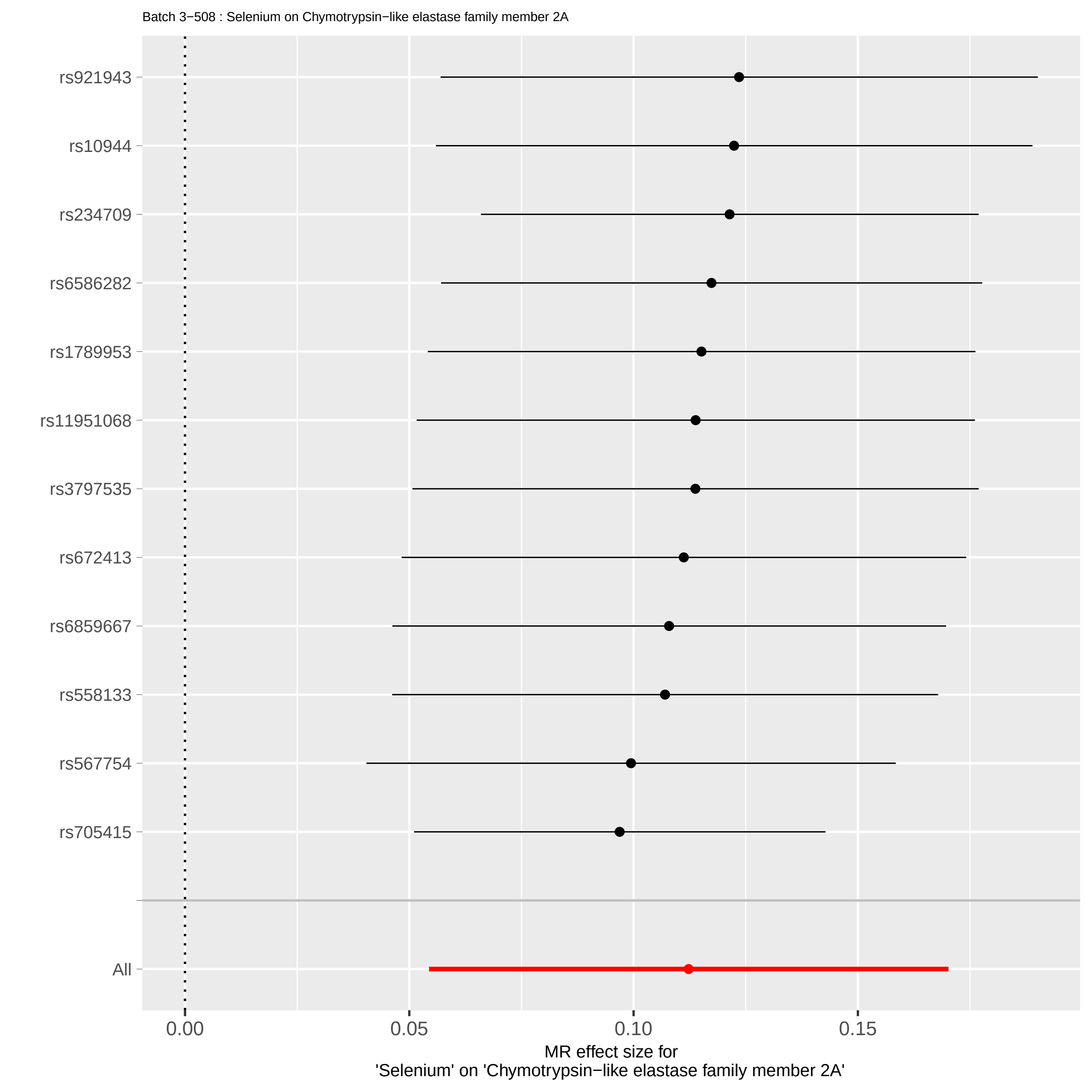

Batch 3−508 : Selenium on Chymotrypsin−like elastase family member 2A
rs921943
rs10944
rs234709
rs6586282
rs1789953
rs11951068
rs3797535
rs672413
rs6859667
rs558133
rs567754
rs705415
All
0.00
0.05
0.10
0.15
MR effect size for
'Selenium' on 'Chymotrypsin−like elastase family member 2A'

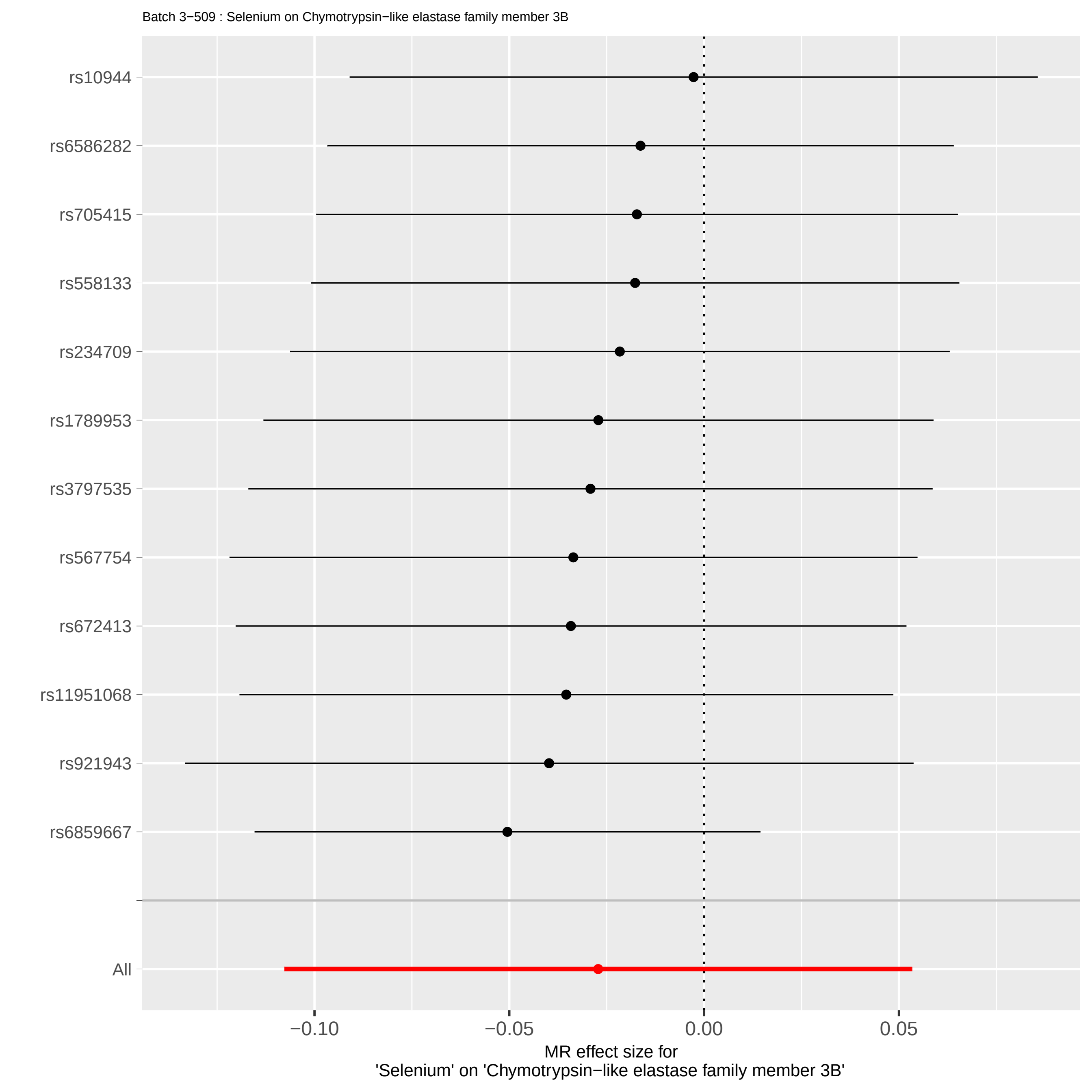
Batch 3−509 : Selenium on Chymotrypsin−like elastase family member 3B
rs10944
rs6586282
rs705415
rs558133
rs234709
rs1789953
rs3797535
rs567754
rs672413
rs11951068
rs921943
rs6859667
All
−0.10
−0.05
0.00
0.05
MR effect size for
'Selenium' on 'Chymotrypsin−like elastase family member 3B'

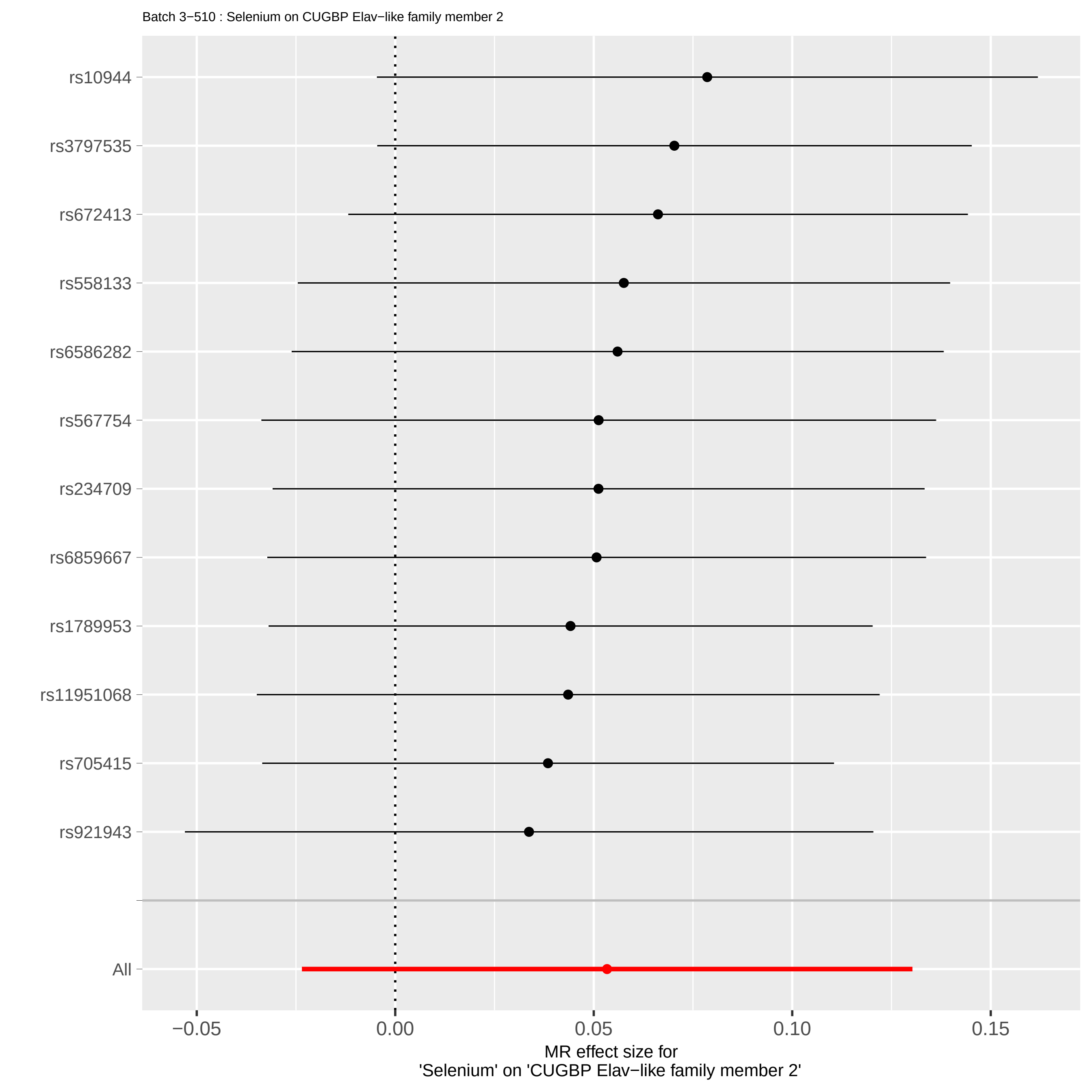
Batch 3−510 : Selenium on CUGBP Elav−like family member 2
rs10944
rs3797535
rs672413
rs558133
rs6586282
rs567754
rs234709
rs6859667
rs1789953
rs11951068
rs705415
rs921943
All
−0.05
0.00
0.05
0.10
0.15
MR effect size for
'Selenium' on 'CUGBP Elav−like family member 2'

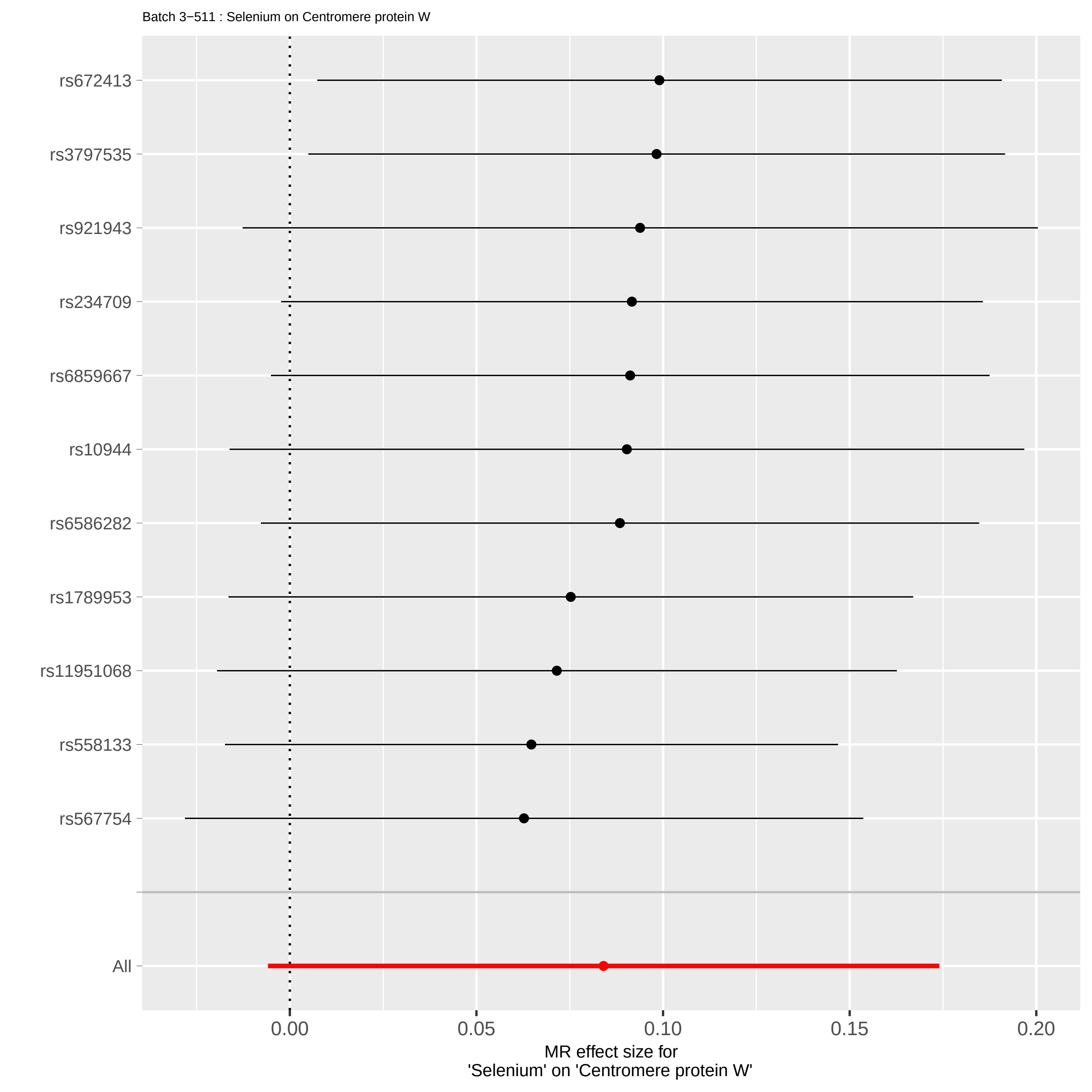

Batch 3−511 : Selenium on Centromere protein W
rs672413
rs3797535
rs921943
rs234709
rs6859667
rs10944
rs6586282
rs1789953
rs11951068
rs558133
rs567754
All
0.00
0.05
0.10
0.15
0.20
MR effect size for
'Selenium' on 'Centromere protein W'

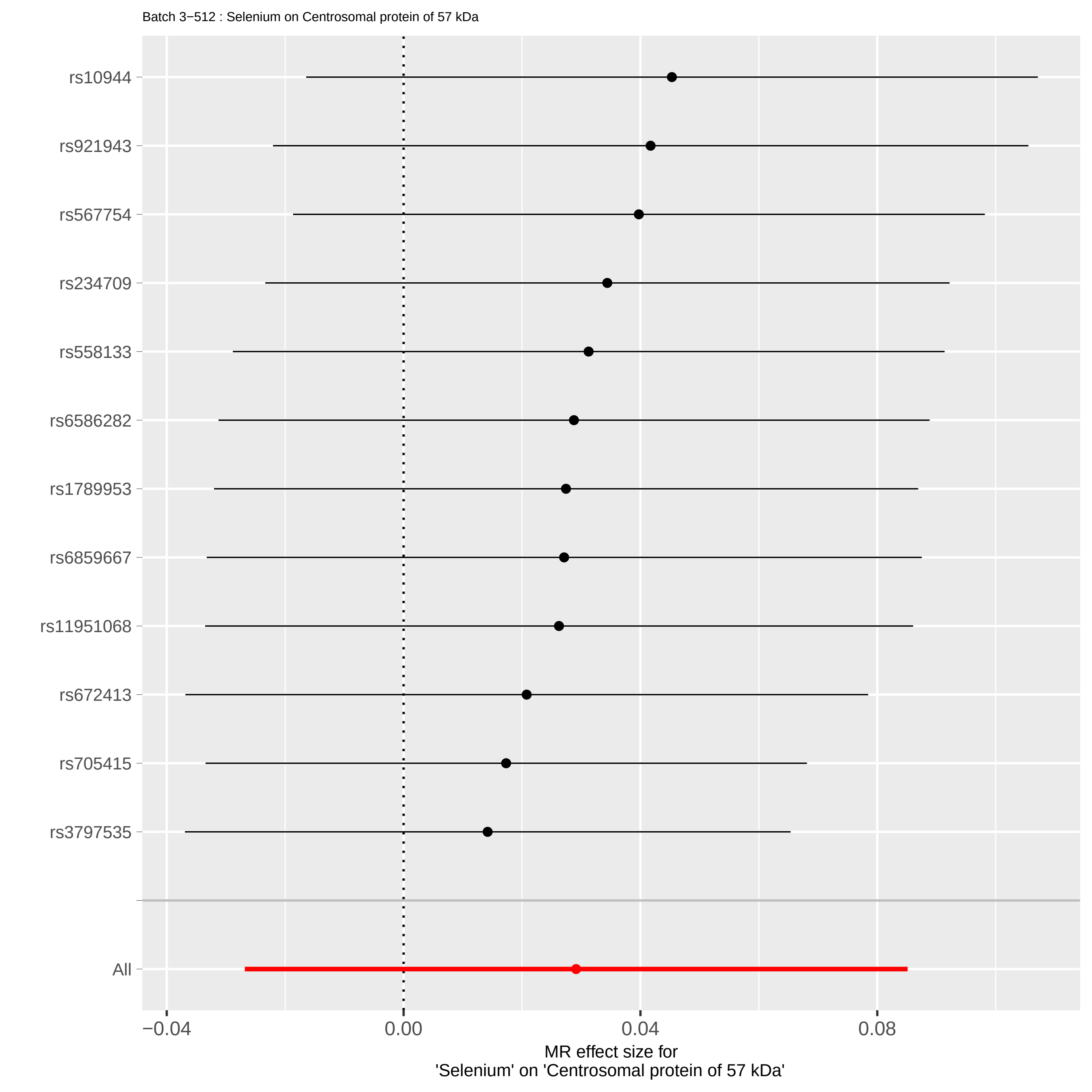

Batch 3−512 : Selenium on Centrosomal protein of 57 kDa
rs10944
rs921943
rs567754
rs234709
rs558133
rs6586282
rs1789953
rs6859667
rs11951068
rs672413
rs705415
rs3797535
All
−0.04
0.00
0.04
0.08
MR effect size for
'Selenium' on 'Centrosomal protein of 57 kDa'

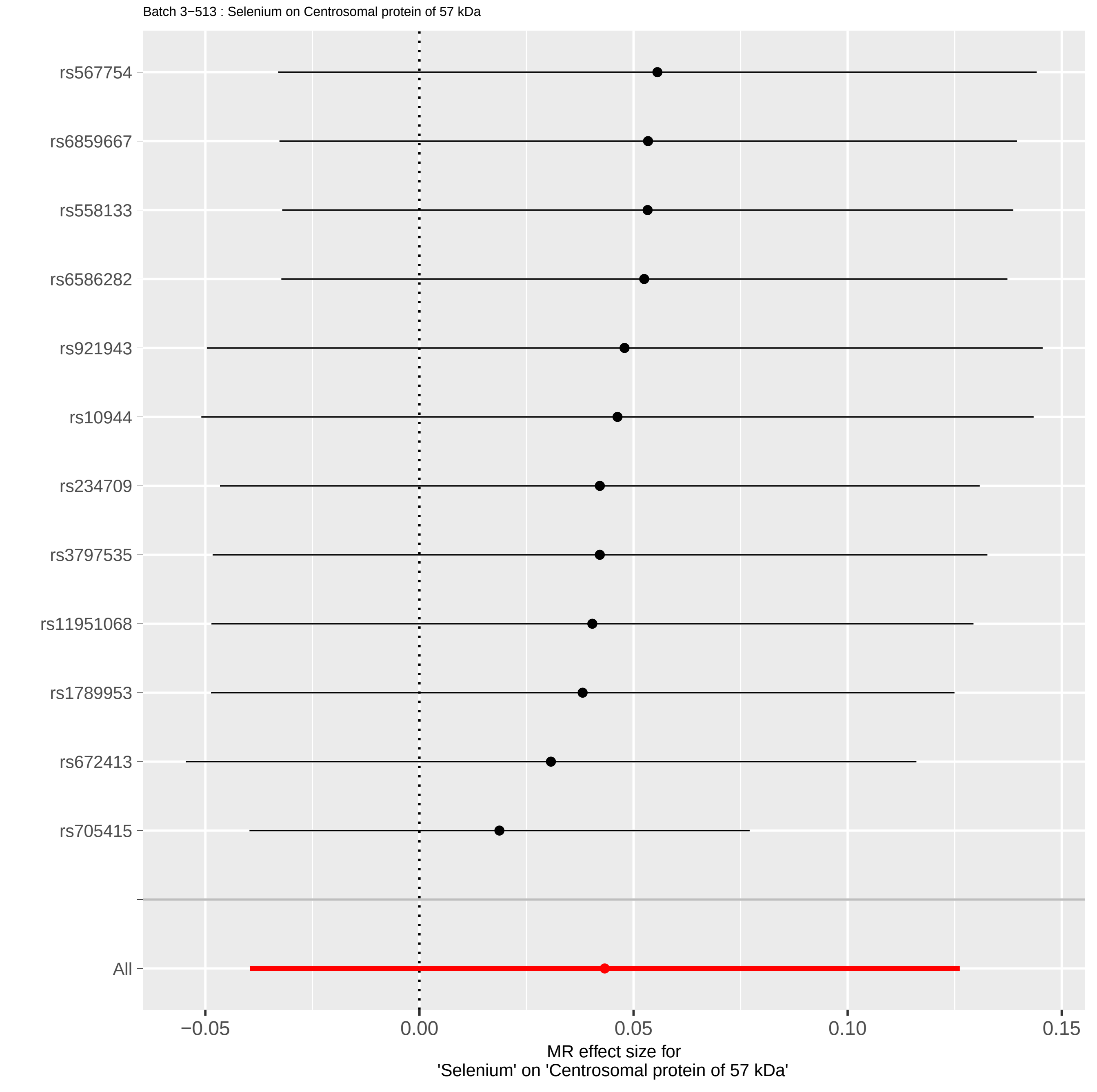

Batch 3−513 : Selenium on Centrosomal protein of 57 kDa
rs567754
rs6859667
rs558133
rs6586282
rs921943
rs10944
rs234709
rs3797535
rs11951068
rs1789953
rs672413
rs705415
All
−0.05
0.00
0.05
0.10
0.15
MR effect size for
'Selenium' on 'Centrosomal protein of 57 kDa'

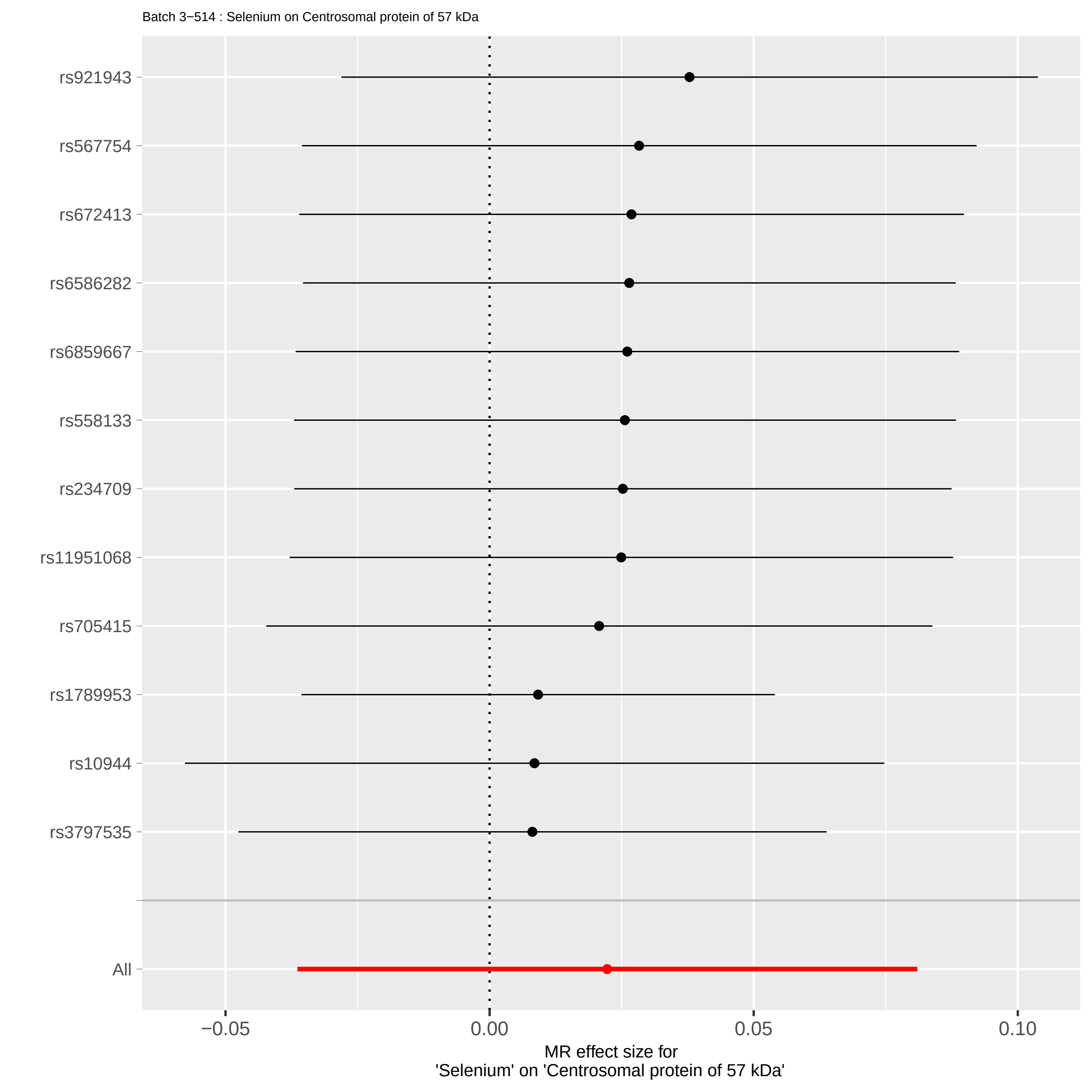
Batch 3−514 : Selenium on Centrosomal protein of 57 kDa
rs921943
rs567754
rs672413
rs6586282
rs6859667
rs558133
rs234709
rs11951068
rs705415
rs1789953
rs10944
rs3797535
All
−0.05
0.00
0.05
0.10
MR effect size for
'Selenium' on 'Centrosomal protein of 57 kDa'

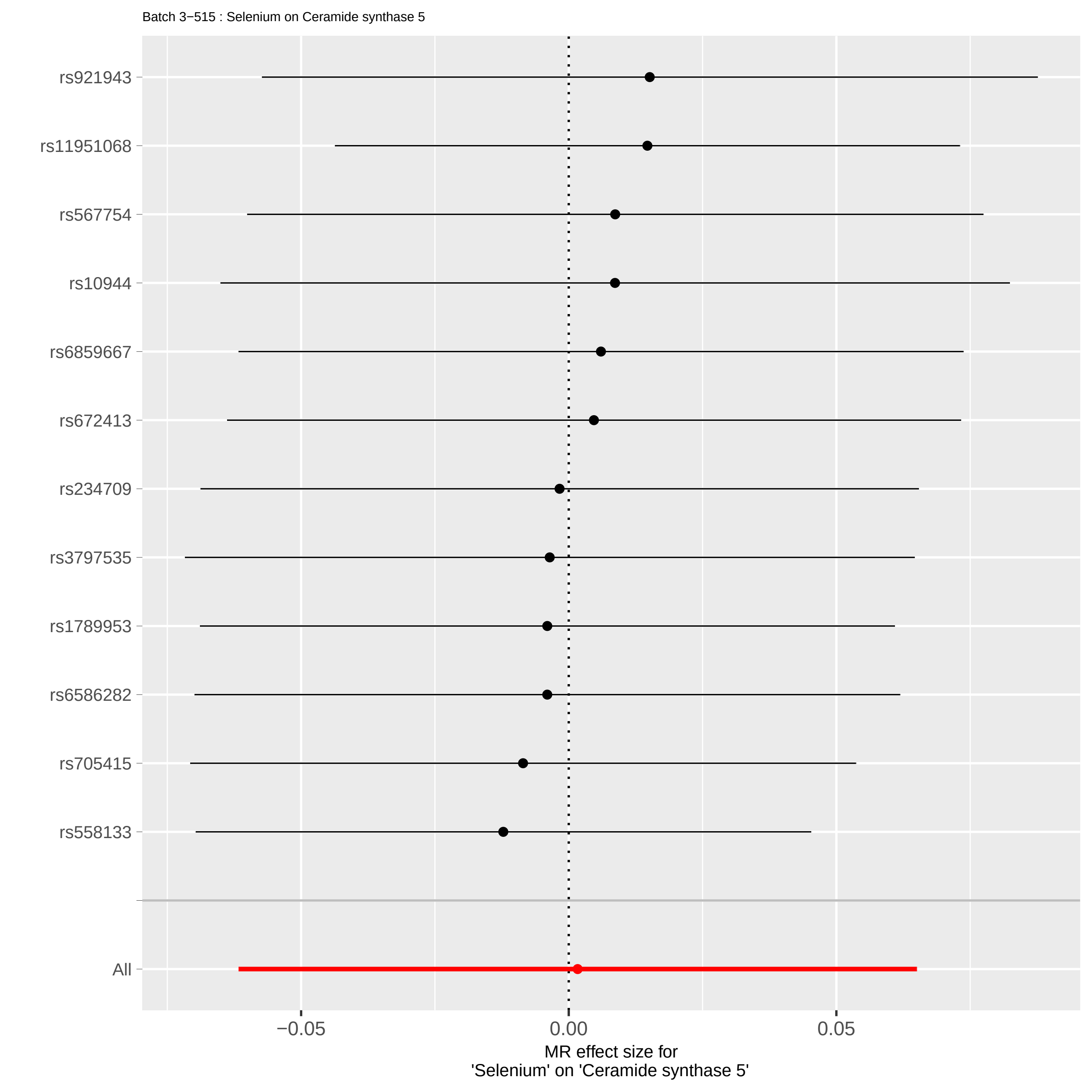

Batch 3−515 : Selenium on Ceramide synthase 5
rs921943
rs11951068
rs567754
rs10944
rs6859667
rs672413
rs234709
rs3797535
rs1789953
rs6586282
rs705415
rs558133
All
−0.05
0.00
0.05
MR effect size for
'Selenium' on 'Ceramide synthase 5'

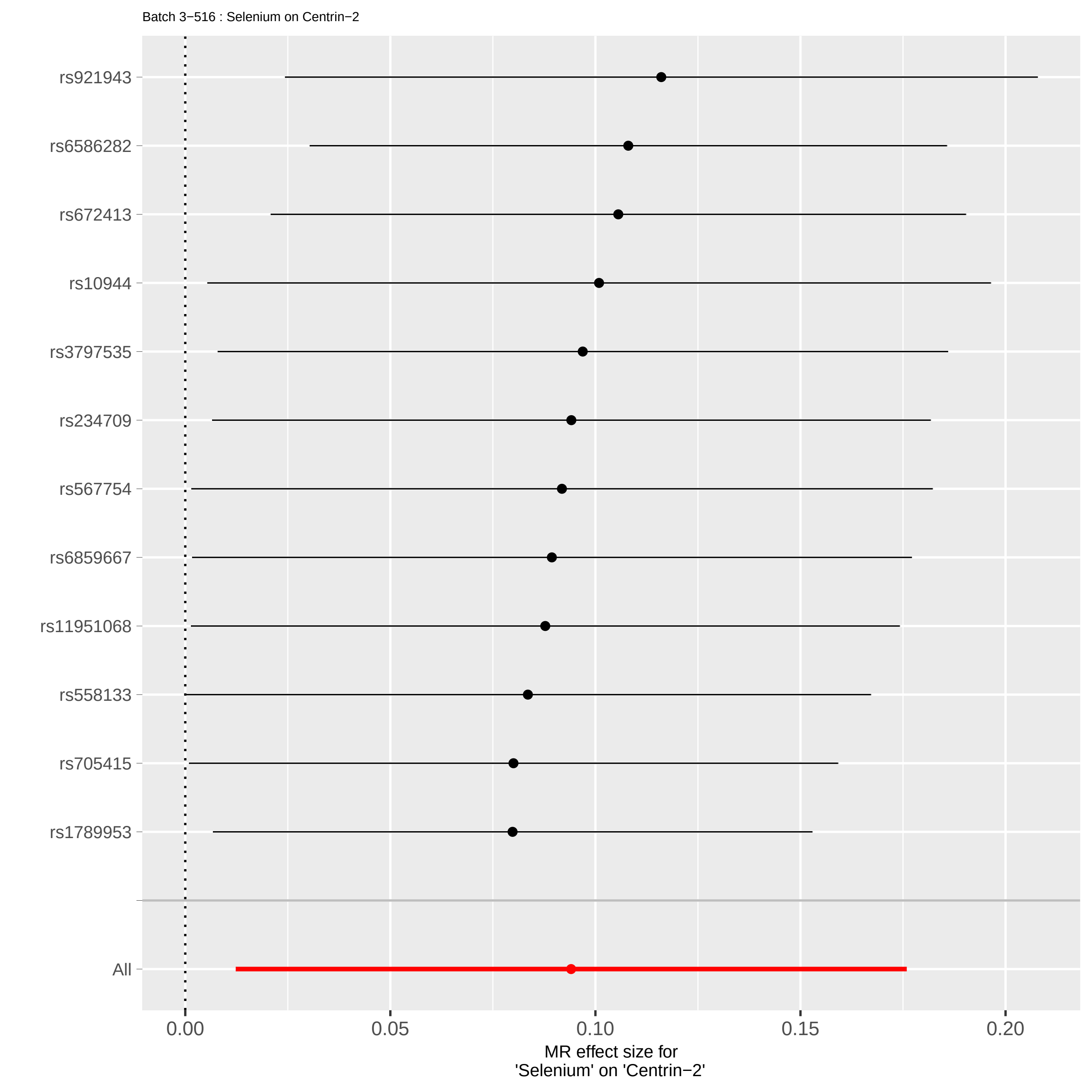

Batch 3−516 : Selenium on Centrin−2
rs921943
rs6586282
rs672413
rs10944
rs3797535
rs234709
rs567754
rs6859667
rs11951068
rs558133
rs705415
rs1789953
All
0.00
0.05
0.10
0.15
0.20
MR effect size for
'Selenium' on 'Centrin−2'

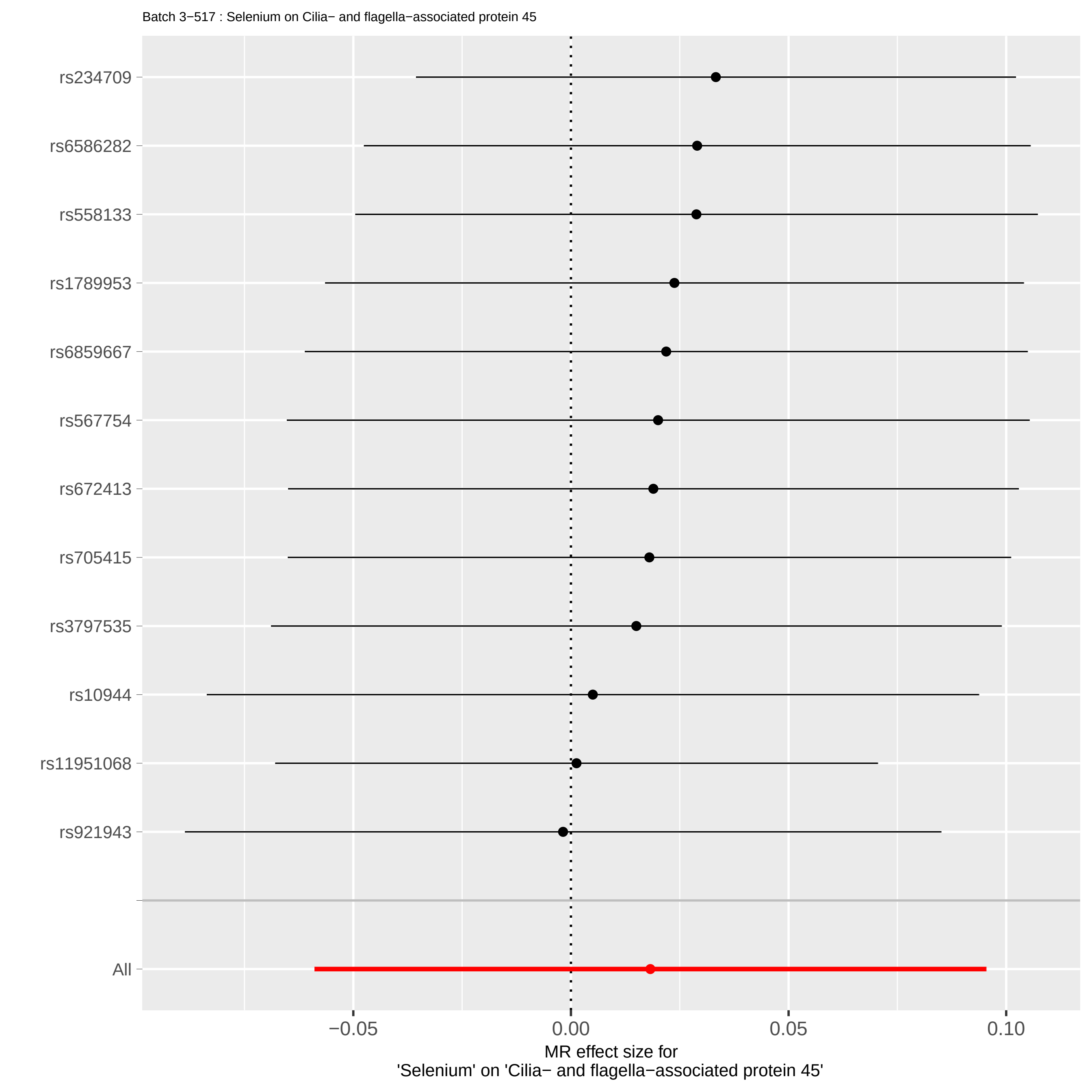

Batch 3−517 : Selenium on Cilia− and flagella−associated protein 45
rs234709
rs6586282
rs558133
rs1789953
rs6859667
rs567754
rs672413
rs705415
rs3797535
rs10944
rs11951068
rs921943
All
−0.05
0.00
0.05
0.10
MR effect size for
'Selenium' on 'Cilia− and flagella−associated protein 45'

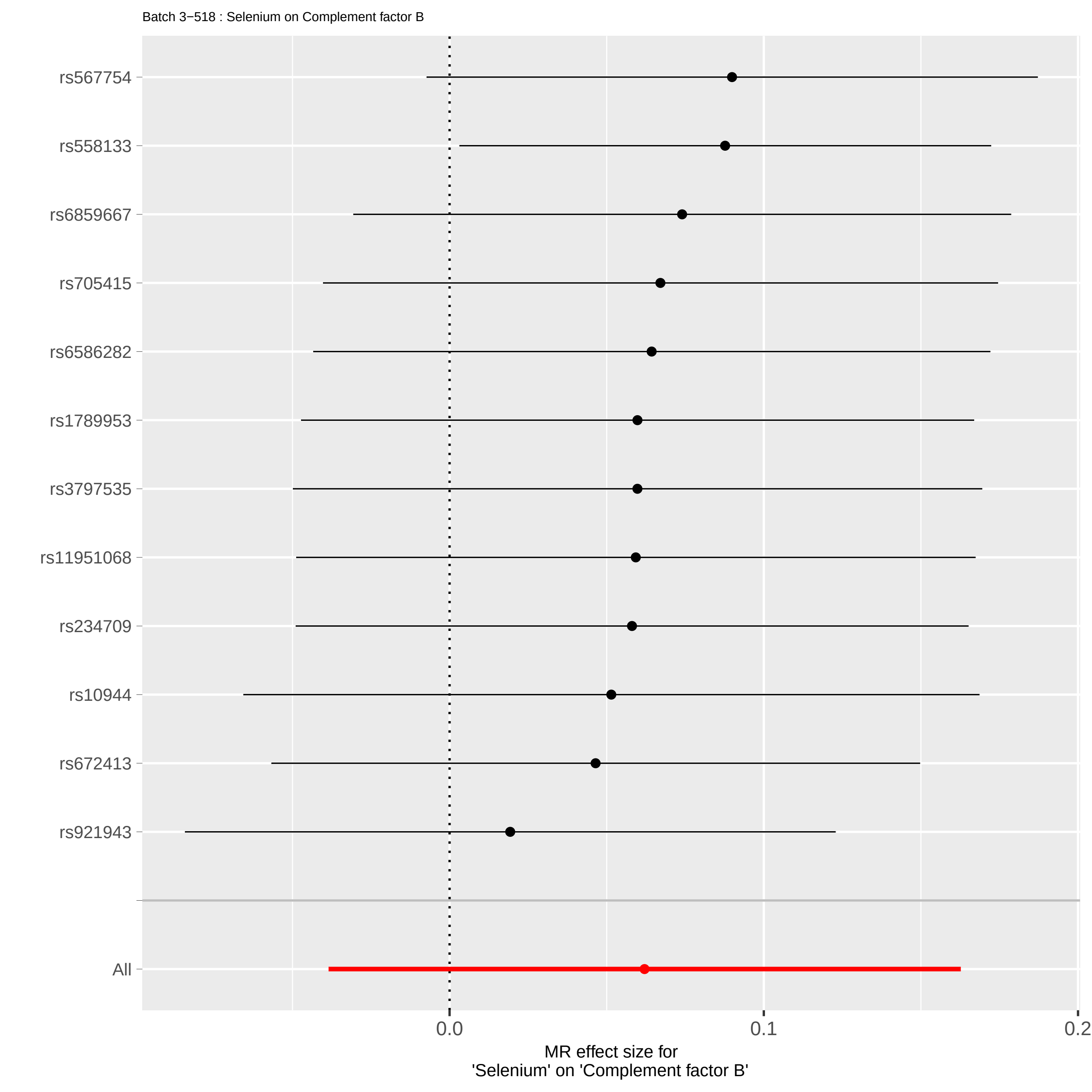

Batch 3−518 : Selenium on Complement factor B
rs567754
rs558133
rs6859667
rs705415
rs6586282
rs1789953
rs3797535
rs11951068
rs234709
rs10944
rs672413
rs921943
All
0.0
0.1
0.2
MR effect size for
'Selenium' on 'Complement factor B'

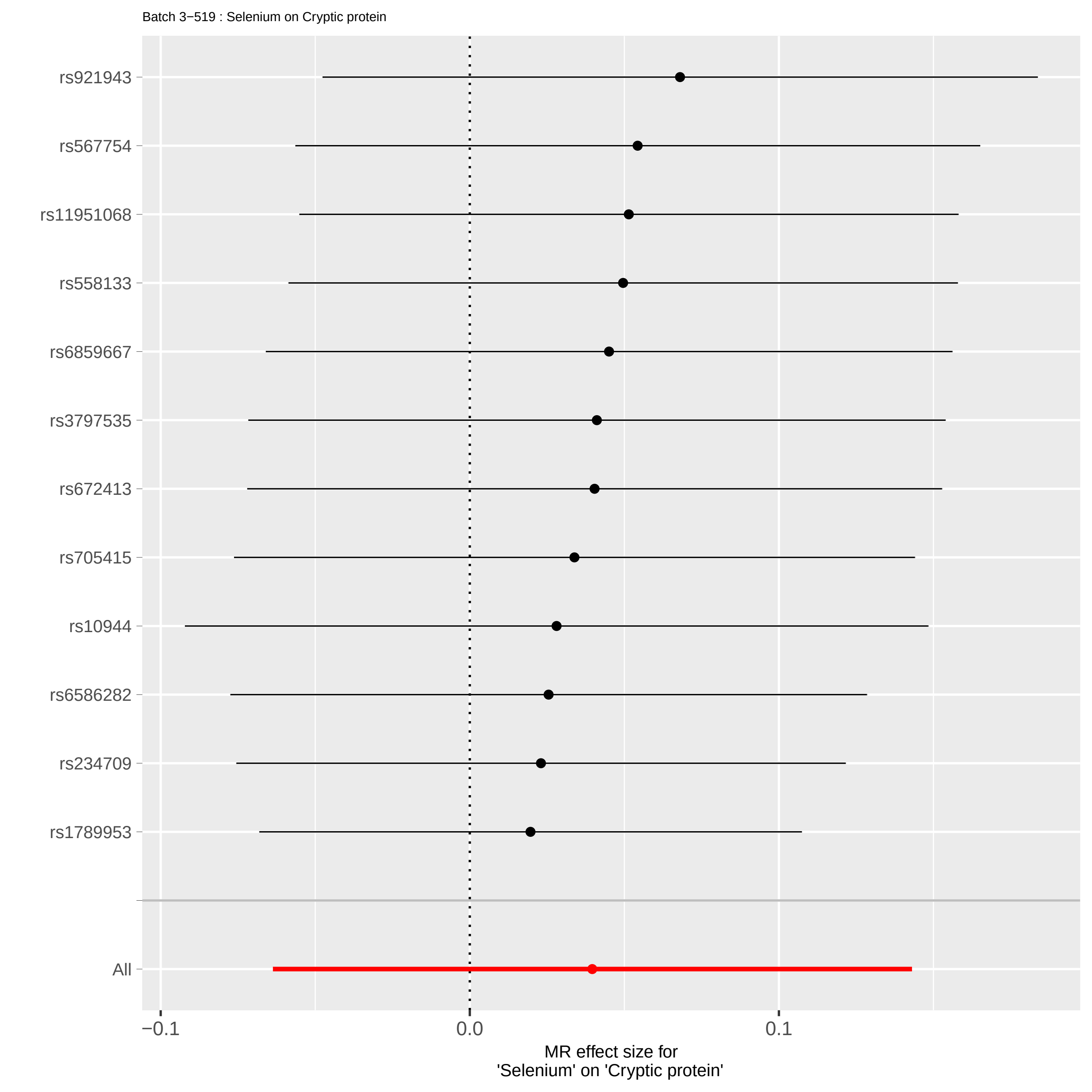

Batch 3−519 : Selenium on Cryptic protein
rs921943
rs567754
rs11951068
rs558133
rs6859667
rs3797535
rs672413
rs705415
rs10944
rs6586282
rs234709
rs1789953
All
−0.1
0.0
0.1
MR effect size for
'Selenium' on 'Cryptic protein'

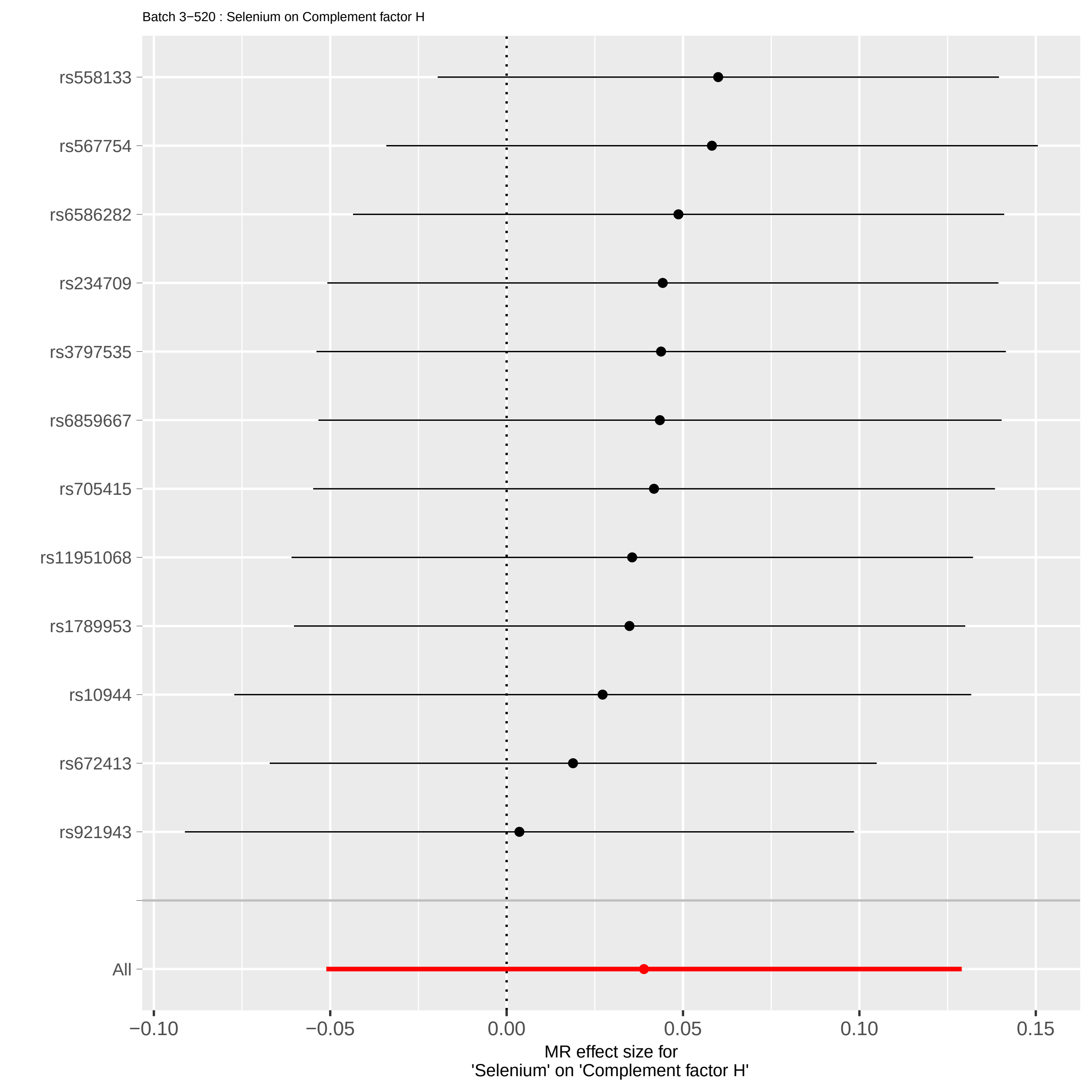

Batch 3−520 : Selenium on Complement factor H
rs558133
rs567754
rs6586282
rs234709
rs3797535
rs6859667
rs705415
rs11951068
rs1789953
rs10944
rs672413
rs921943
All
−0.10
−0.05
0.00
0.05
0.10
0.15
MR effect size for
'Selenium' on 'Complement factor H'

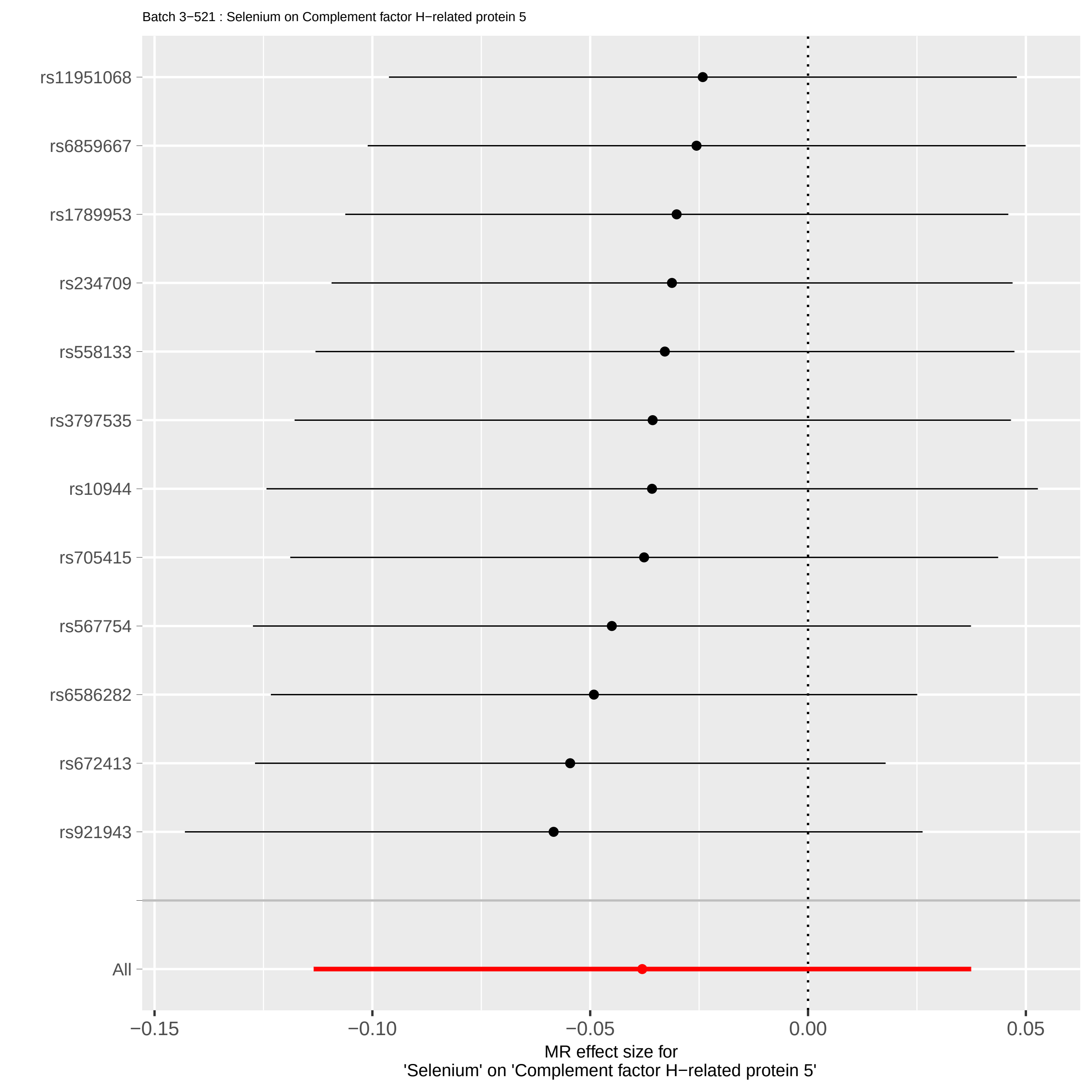

Batch 3−521 : Selenium on Complement factor H−related protein 5
rs11951068
rs6859667
rs1789953
rs234709
rs558133
rs3797535
rs10944
rs705415
rs567754
rs6586282
rs672413
rs921943
All
−0.15
−0.10
−0.05
0.00
0.05
MR effect size for
'Selenium' on 'Complement factor H−related protein 5'

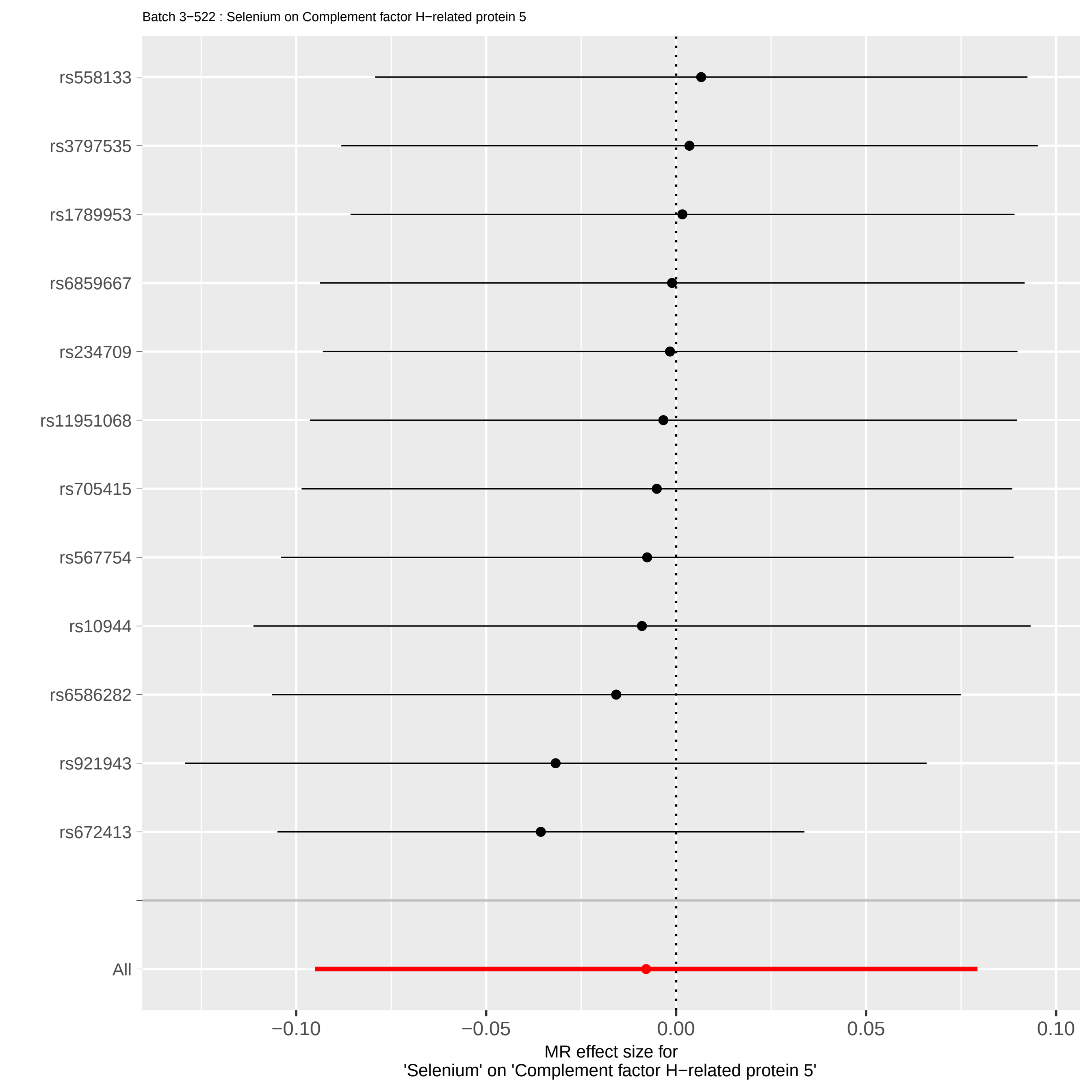

Batch 3−522 : Selenium on Complement factor H−related protein 5
rs558133
rs3797535
rs1789953
rs6859667
rs234709
rs11951068
rs705415
rs567754
rs10944
rs6586282
rs921943
rs672413
All
−0.10
−0.05
0.00
0.05
0.10
MR effect size for
'Selenium' on 'Complement factor H−related protein 5'

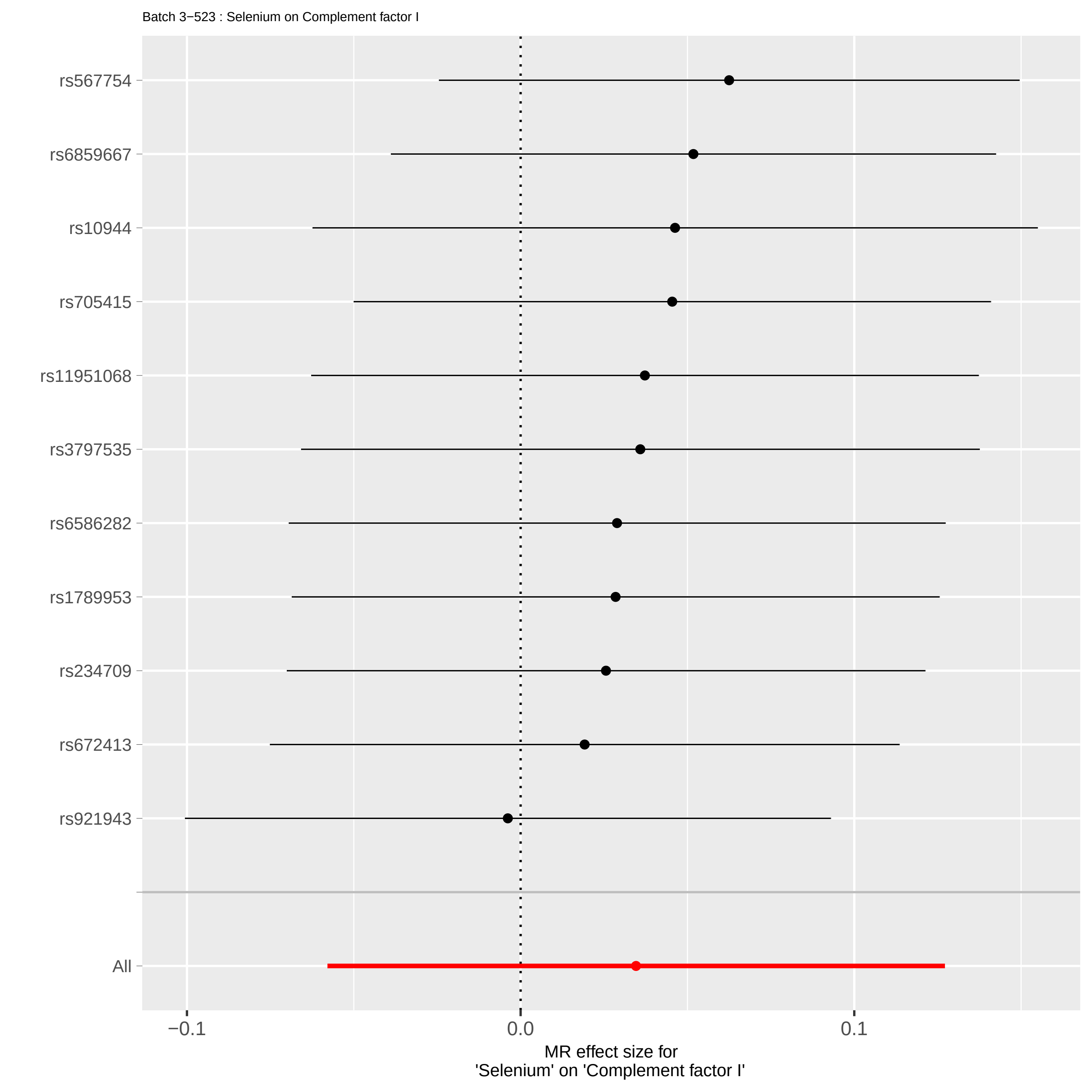
Batch 3−523 : Selenium on Complement factor I
rs567754
rs6859667
rs10944
rs705415
rs11951068
rs3797535
rs6586282
rs1789953
rs234709
rs672413
rs921943
All
−0.1
0.0
0.1
MR effect size for
'Selenium' on 'Complement factor I'

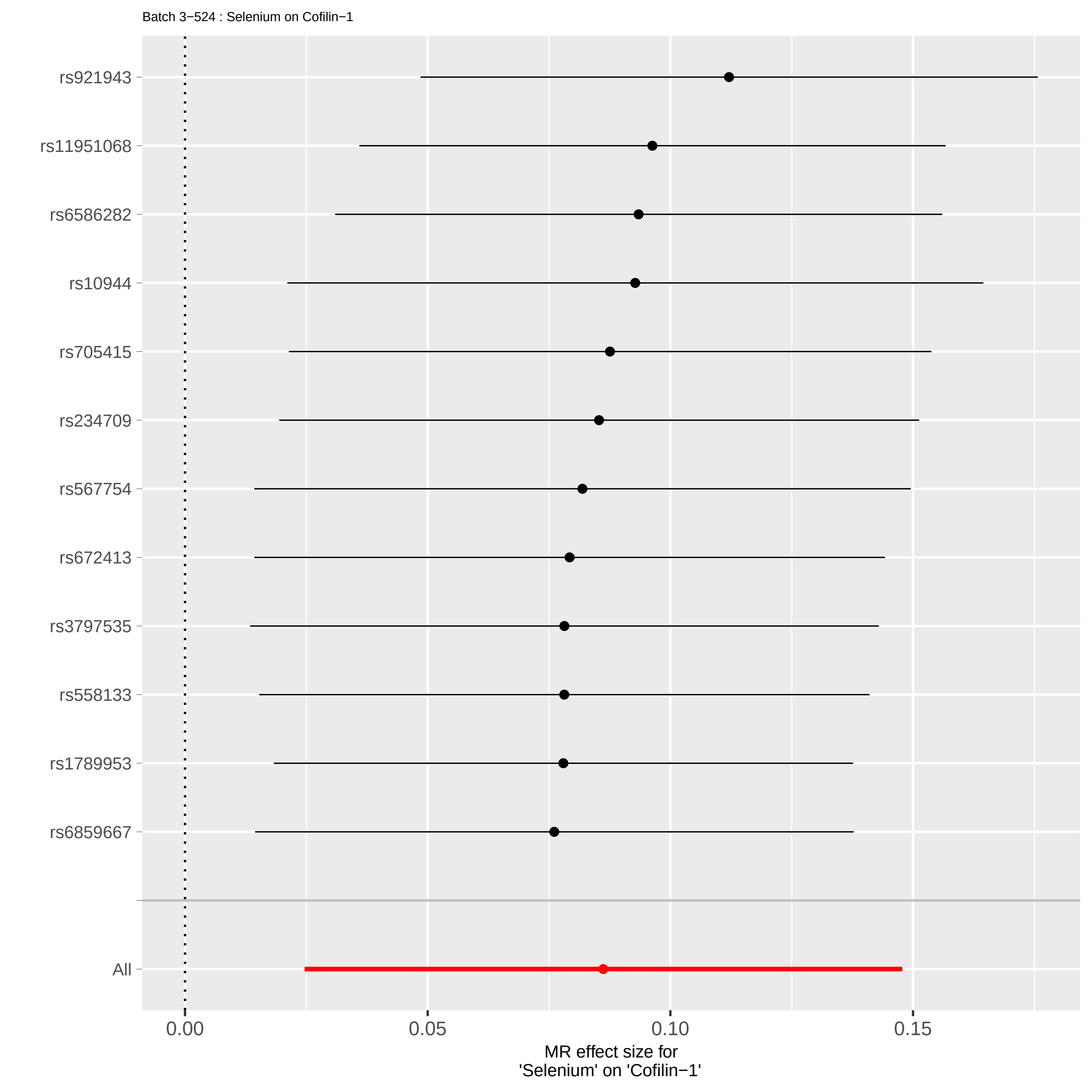

Batch 3−524 : Selenium on Cofilin−1
rs921943
rs11951068
rs6586282
rs10944
rs705415
rs234709
rs567754
rs672413
rs3797535
rs558133
rs1789953
rs6859667
All
0.00
0.05
0.10
0.15
MR effect size for
'Selenium' on 'Cofilin−1'

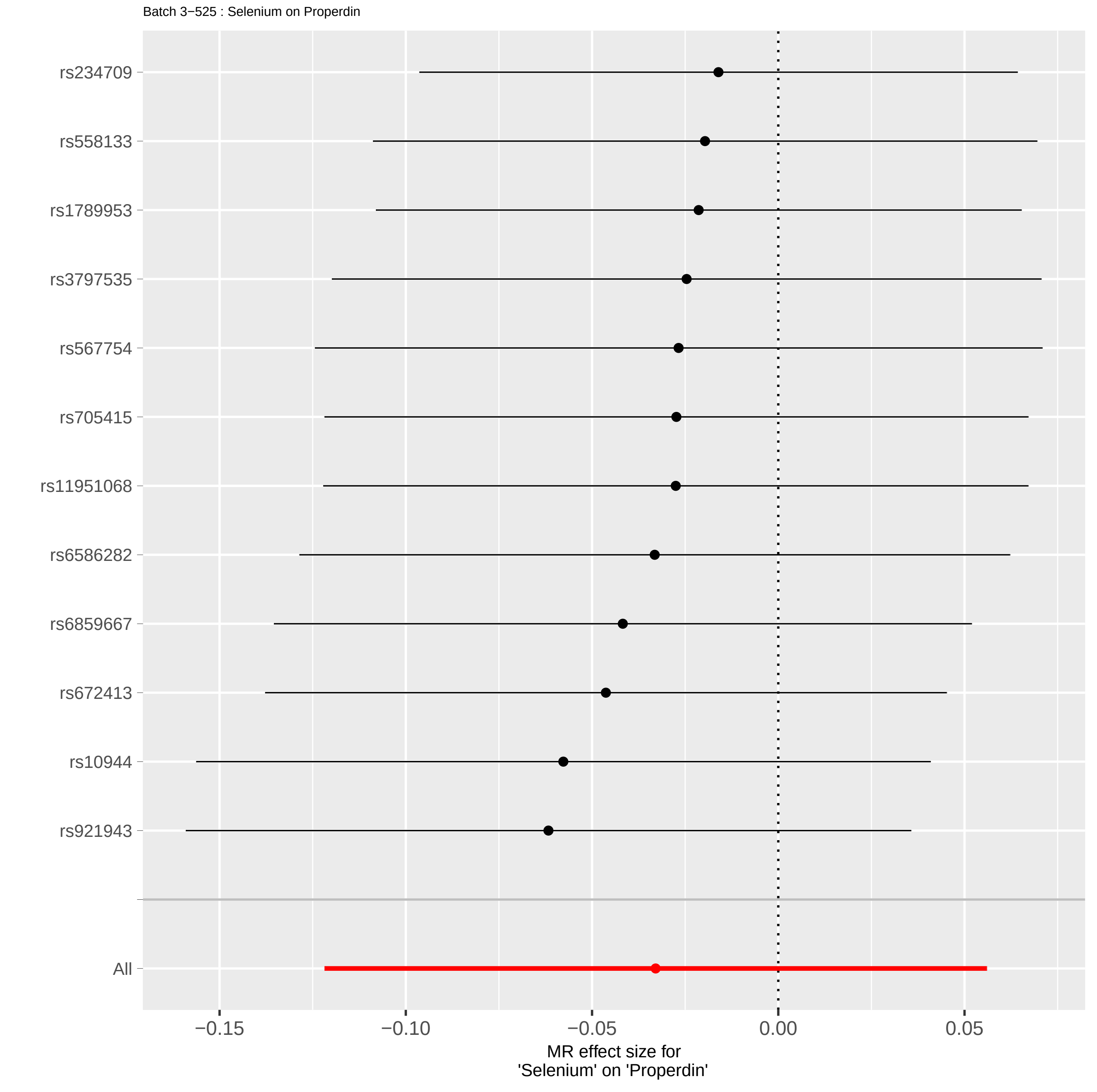

Batch 3−525 : Selenium on Properdin
rs234709
rs558133
rs1789953
rs3797535
rs567754
rs705415
rs11951068
rs6586282
rs6859667
rs672413
rs10944
rs921943
All
−0.15
−0.10
−0.05
0.00
0.05
MR effect size for
'Selenium' on 'Properdin'

Batch 3−526 : Selenium on Glycoprotein hormones alpha chain

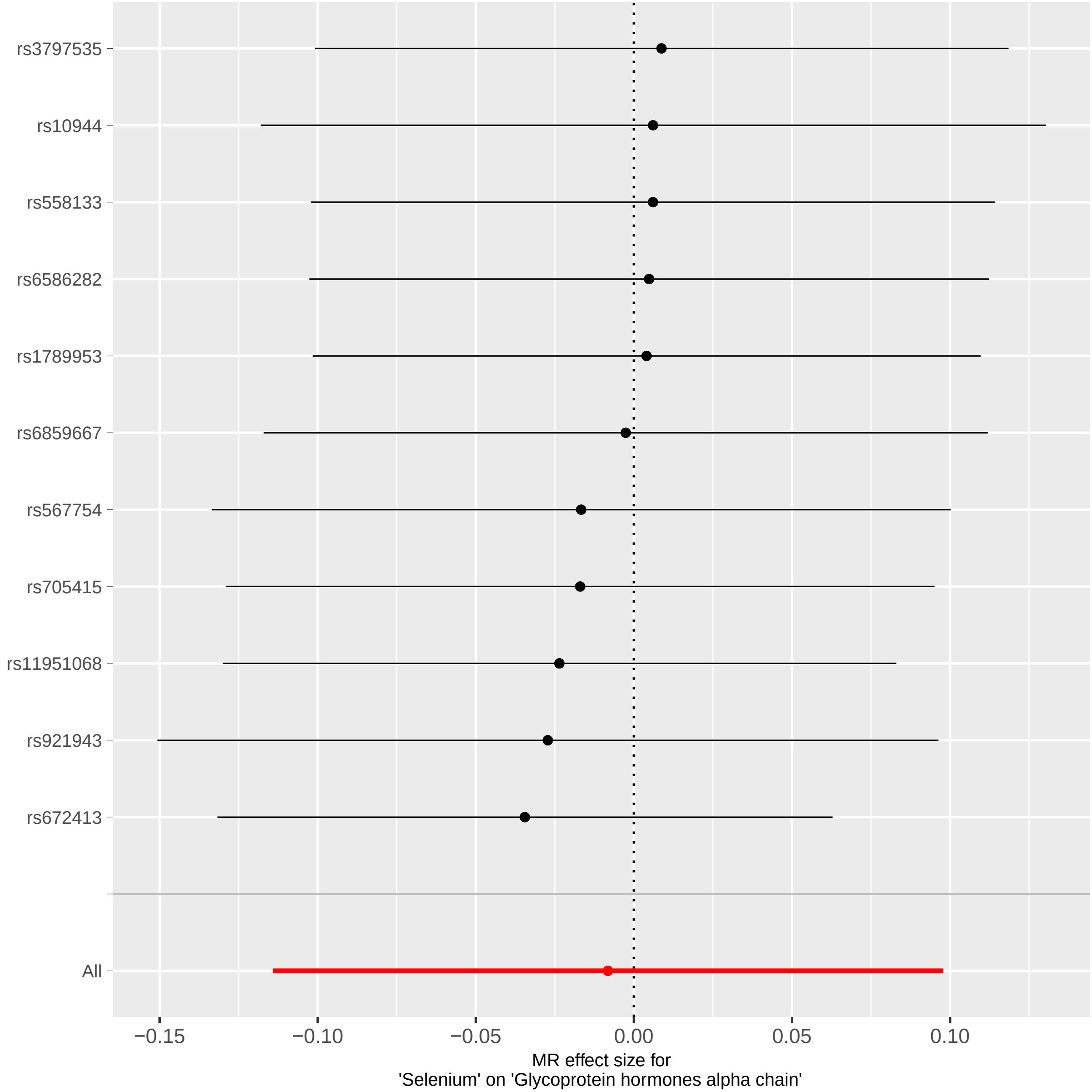

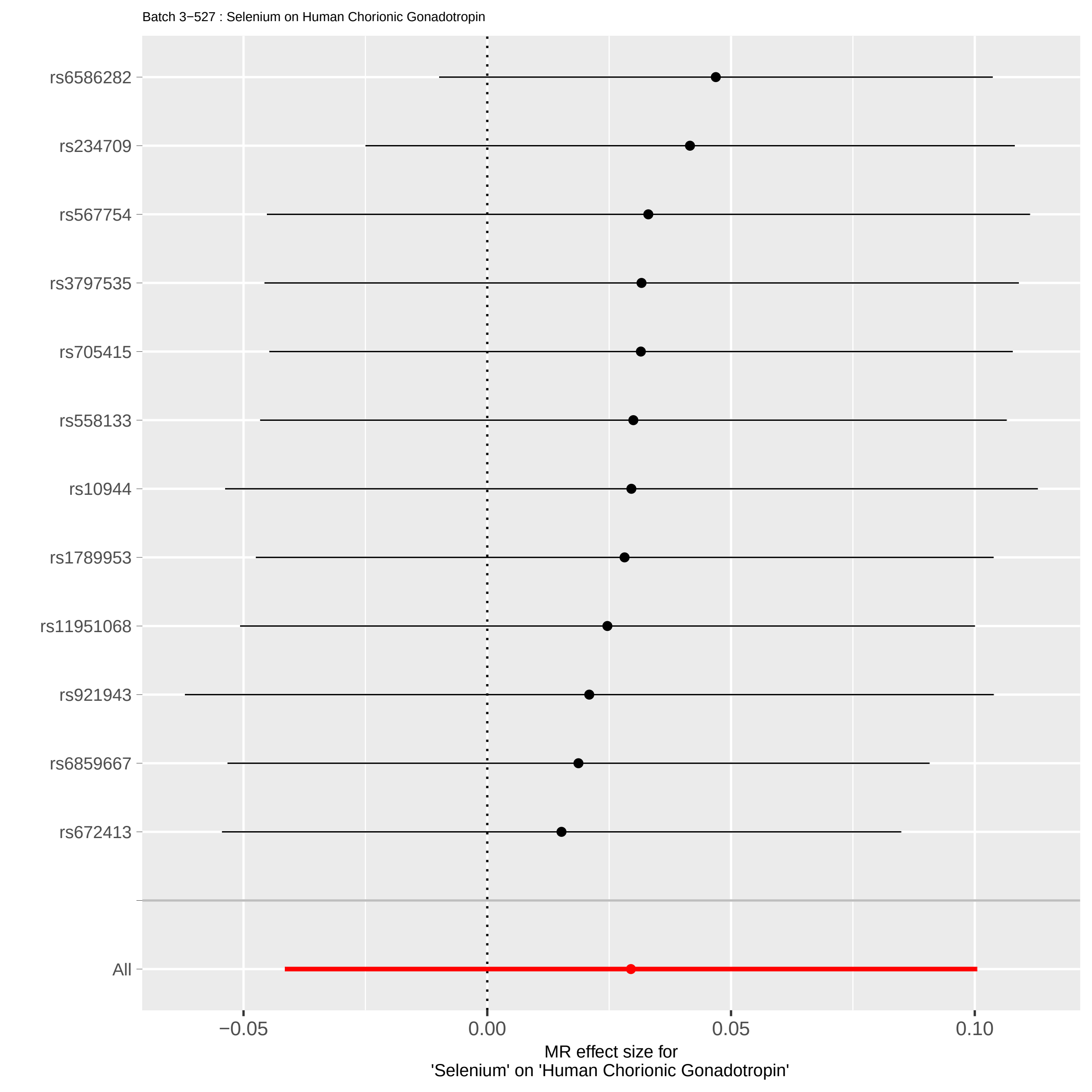

Batch 3−527 : Selenium on Human Chorionic Gonadotropin
rs6586282
rs234709
rs567754
rs3797535
rs705415
rs558133
rs10944
rs1789953
rs11951068
rs921943
rs6859667
rs672413
All
−0.05
0.00
0.05
0.10
MR effect size for
'Selenium' on 'Human Chorionic Gonadotropin'

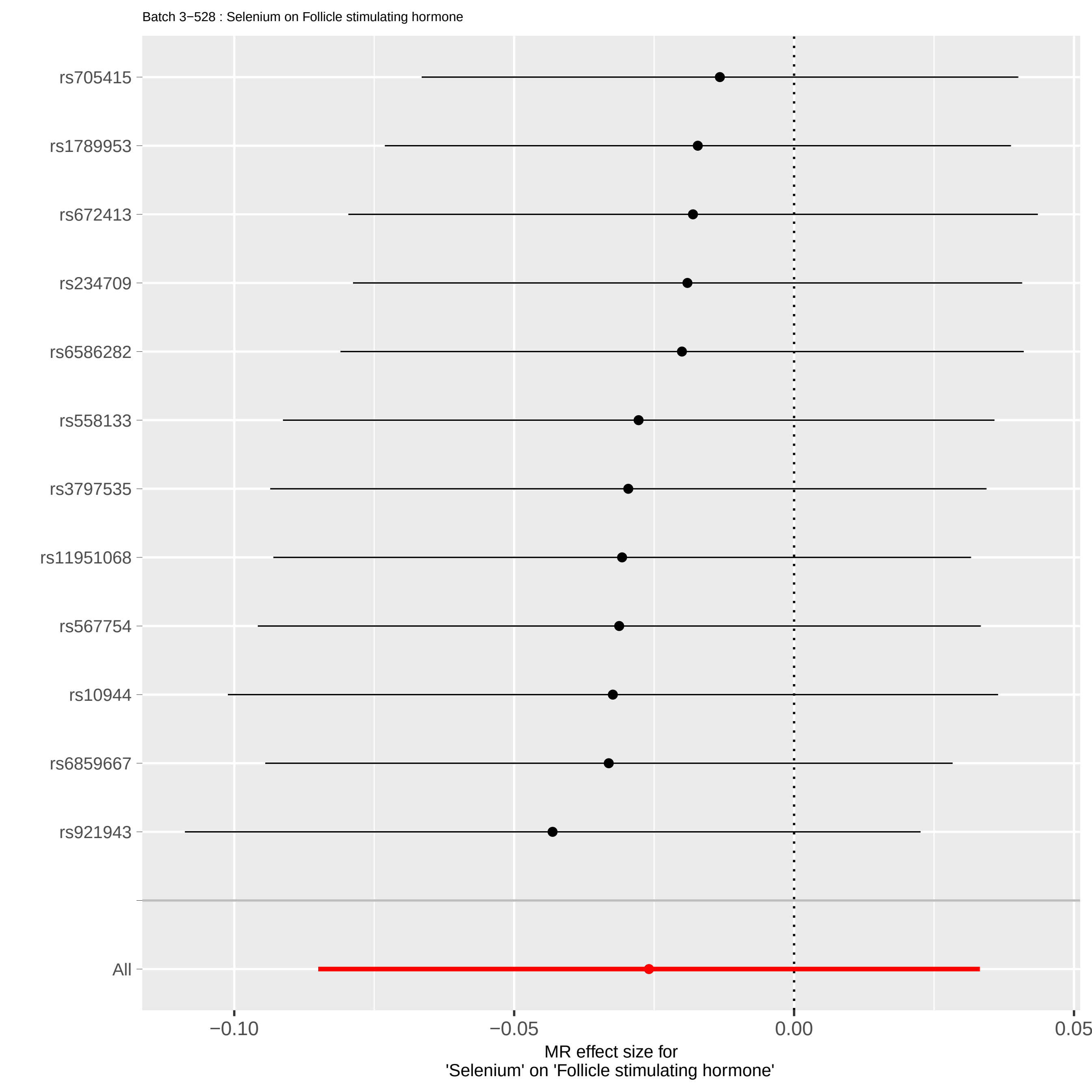
Batch 3−528 : Selenium on Follicle stimulating hormone
rs705415
rs1789953
rs672413
rs234709
rs6586282
rs558133
rs3797535
rs11951068
rs567754
rs10944
rs6859667
rs921943
All
−0.10
−0.05
0.00
0.05
MR effect size for
'Selenium' on 'Follicle stimulating hormone'

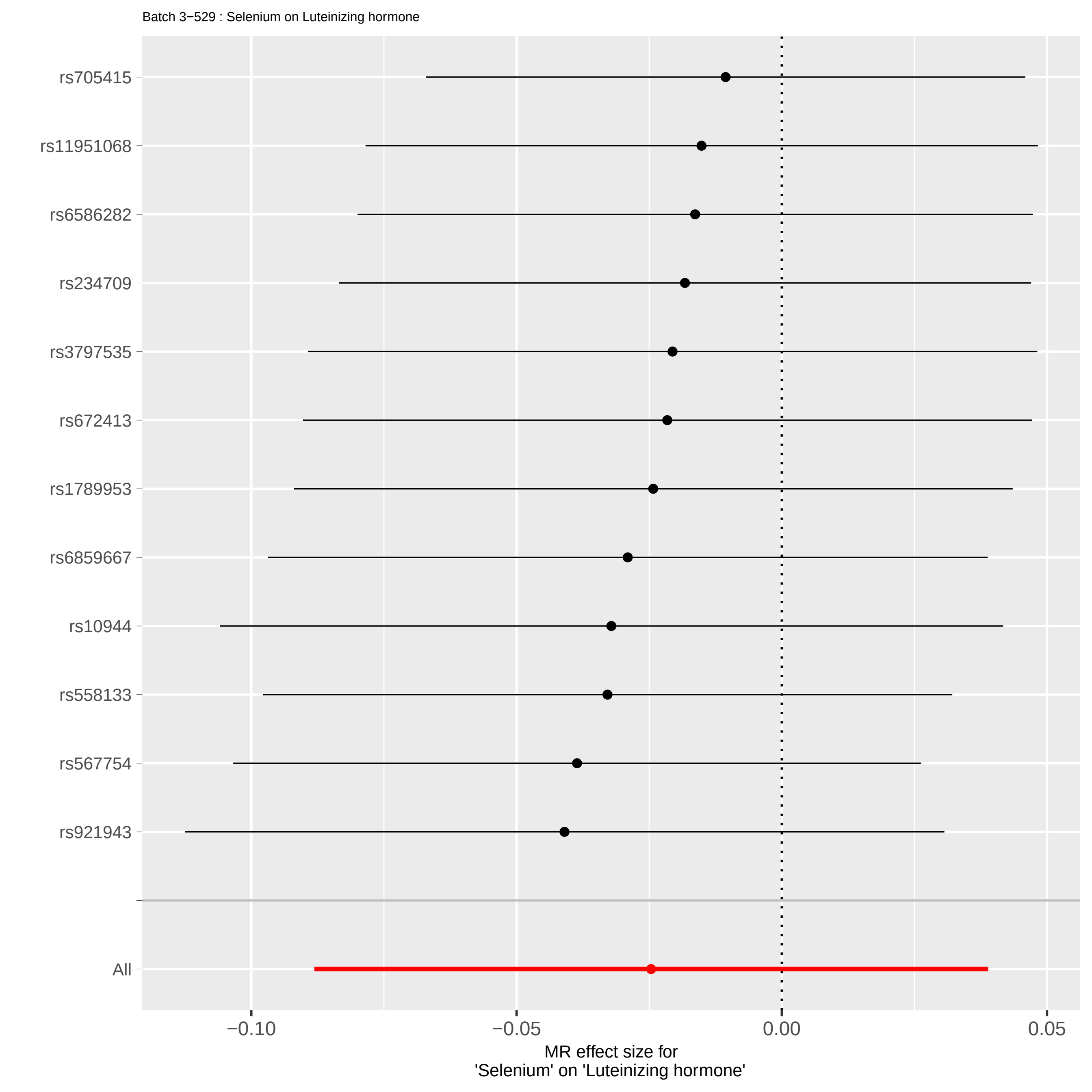

Batch 3−529 : Selenium on Luteinizing hormone
rs705415
rs11951068
rs6586282
rs234709
rs3797535
rs672413
rs1789953
rs6859667
rs10944
rs558133
rs567754
rs921943
All
−0.10
−0.05
0.00
0.05
MR effect size for
'Selenium' on 'Luteinizing hormone'

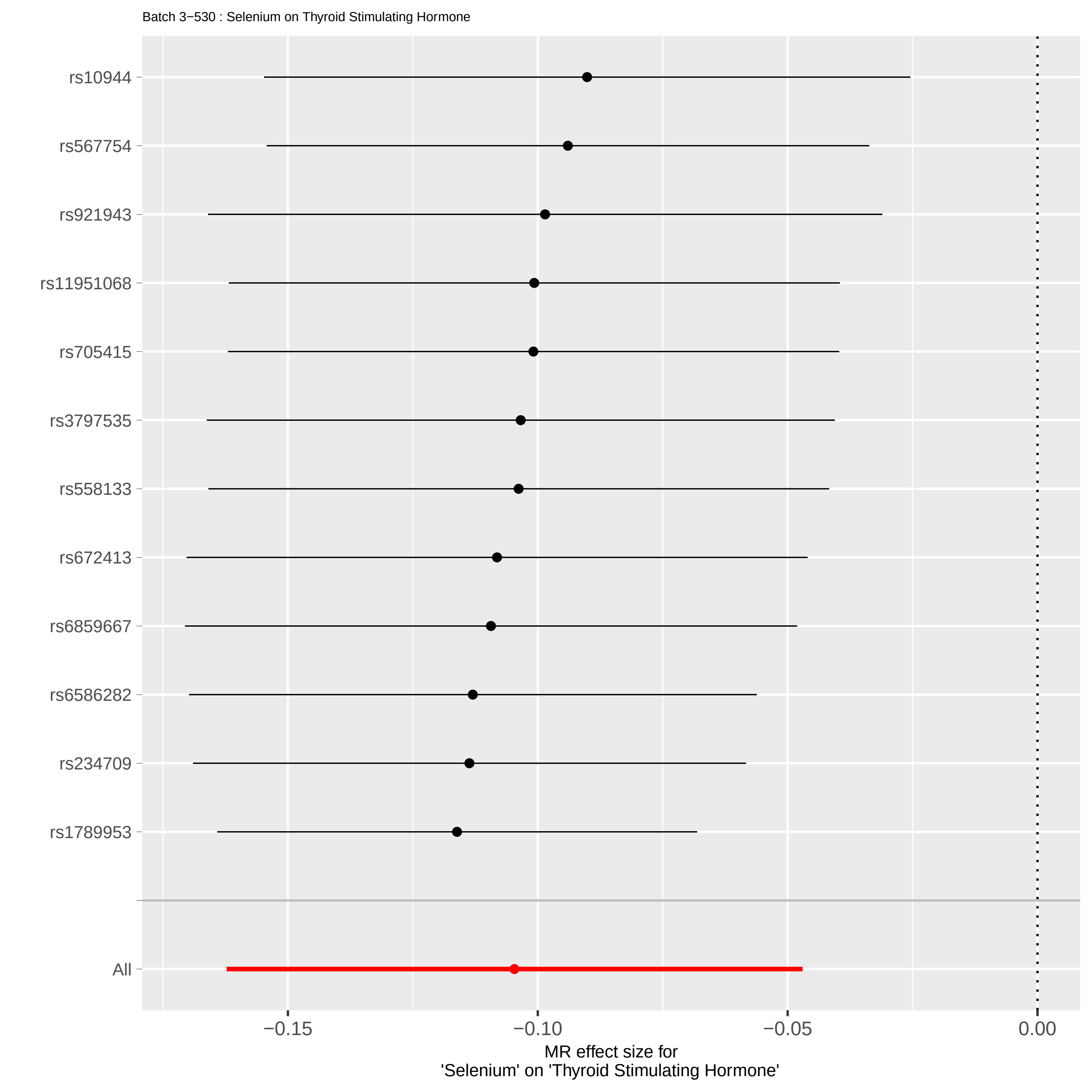
Batch 3−530 : Selenium on Thyroid Stimulating Hormone
rs10944
rs567754
rs921943
rs11951068
rs705415
rs3797535
rs558133
rs672413
rs6859667
rs6586282
rs234709
rs1789953
All
−0.15
−0.10
−0.05
0.00
MR effect size for
'Selenium' on 'Thyroid Stimulating Hormone'

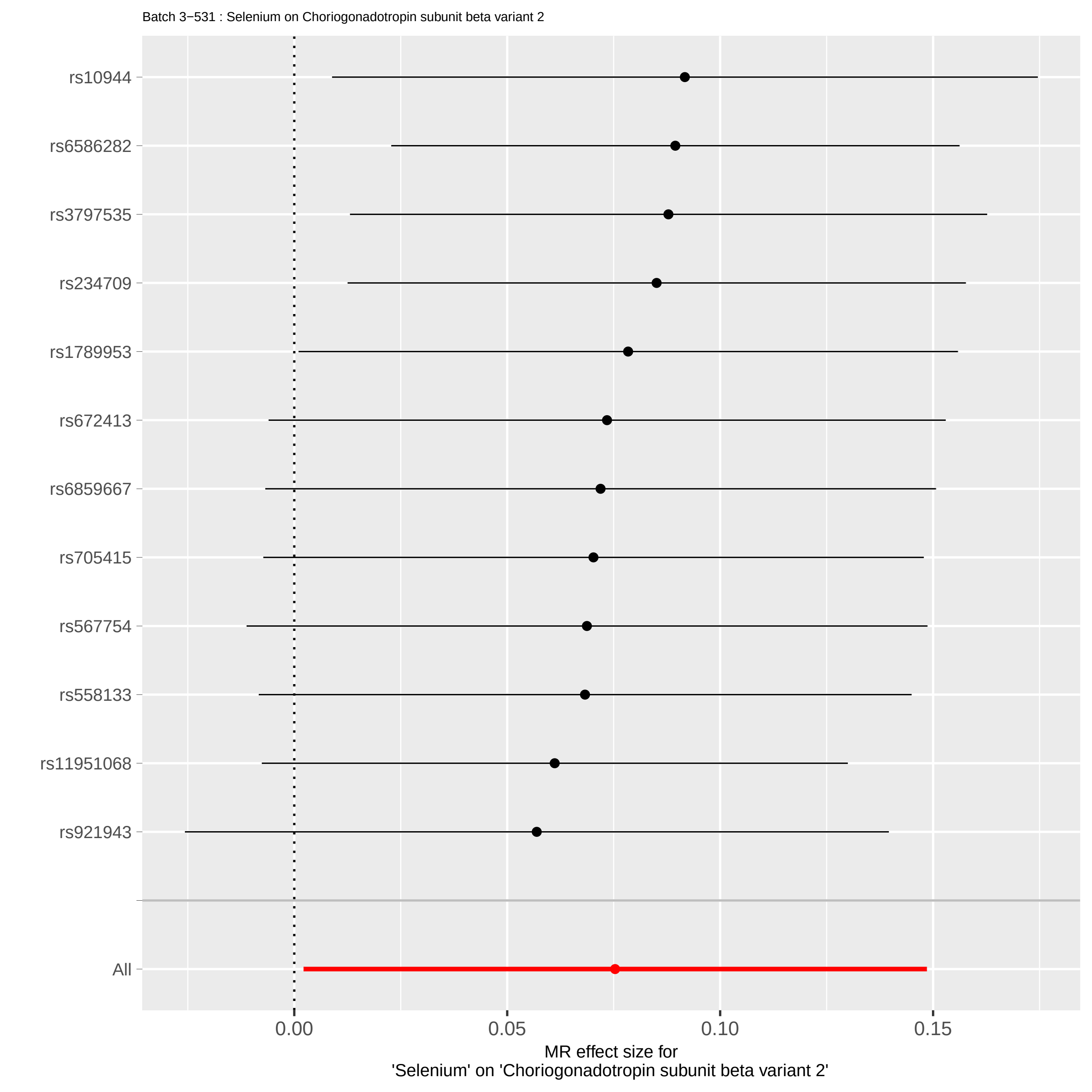

Batch 3−531 : Selenium on Choriogonadotropin subunit beta variant 2
rs10944
rs6586282
rs3797535
rs234709
rs1789953
rs672413
rs6859667
rs705415
rs567754
rs558133
rs11951068
rs921943
All
0.00
0.05
0.10
0.15
MR effect size for
'Selenium' on 'Choriogonadotropin subunit beta variant 2'

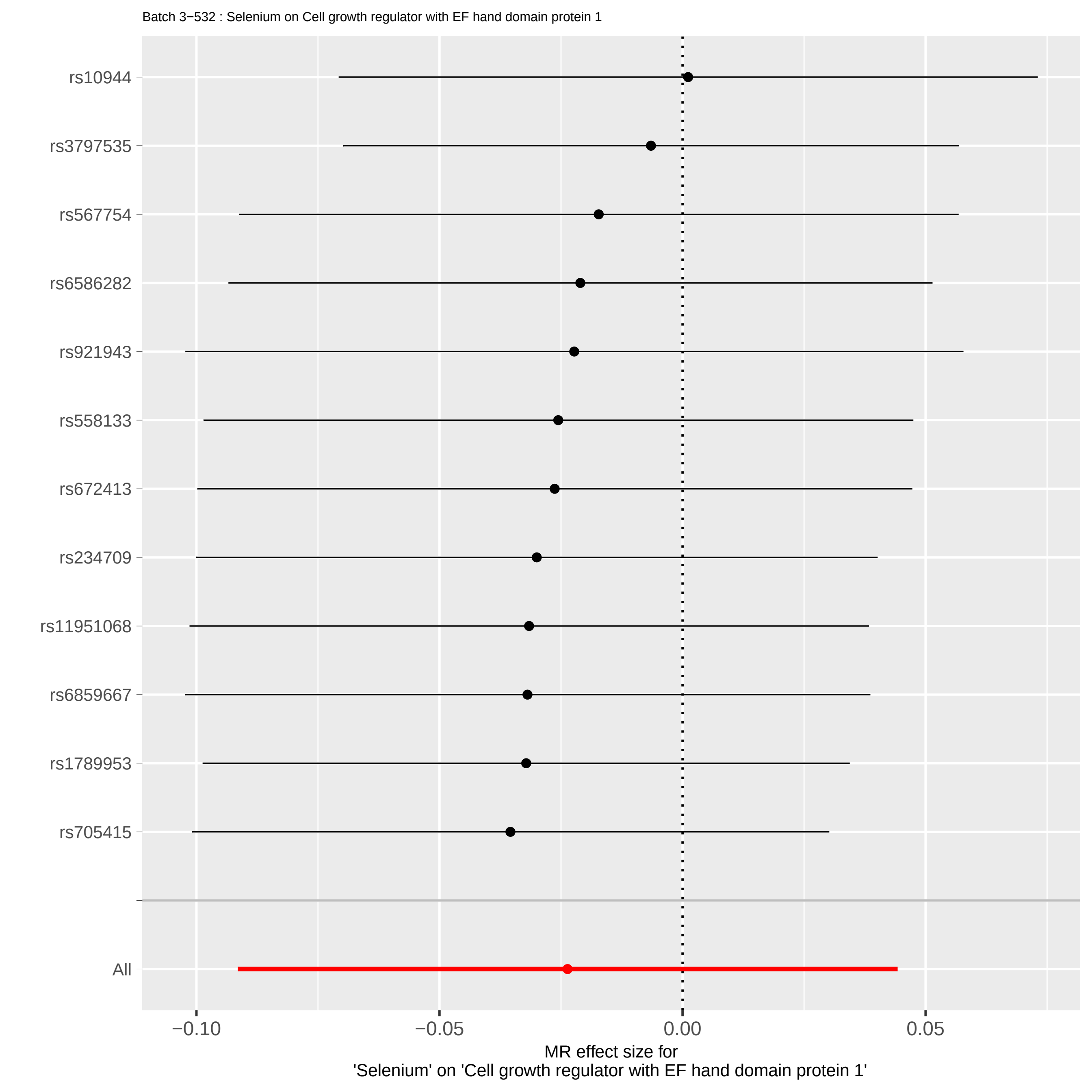

Batch 3−532 : Selenium on Cell growth regulator with EF hand domain protein 1
rs10944
rs3797535
rs567754
rs6586282
rs921943
rs558133
rs672413
rs234709
rs11951068
rs6859667
rs1789953
rs705415
All
−0.10
−0.05
0.00
0.05
MR effect size for
'Selenium' on 'Cell growth regulator with EF hand domain protein 1'

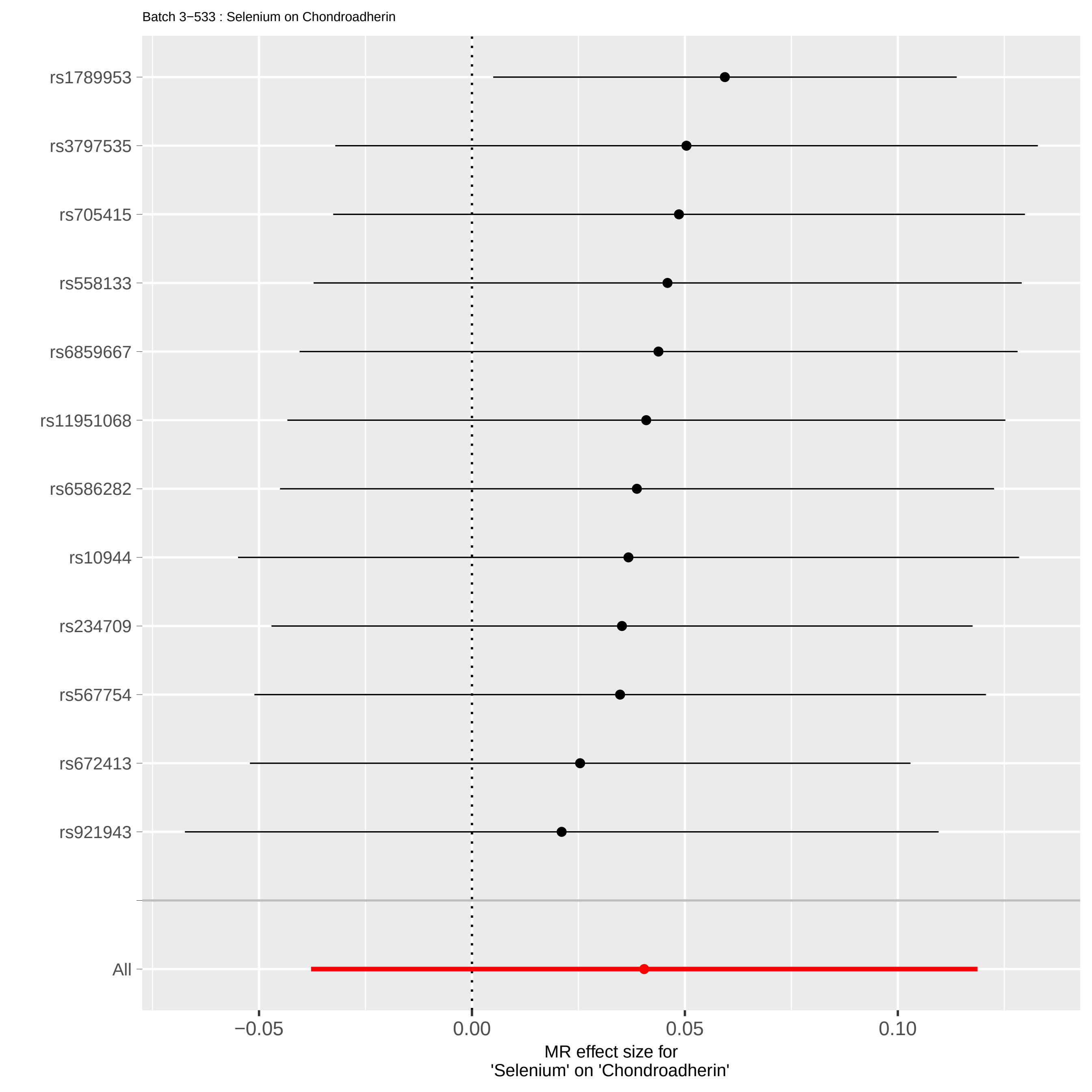

Batch 3−533 : Selenium on Chondroadherin
rs1789953
rs3797535
rs705415
rs558133
rs6859667
rs11951068
rs6586282
rs10944
rs234709
rs567754
rs672413
rs921943
All
−0.05
0.00
0.05
0.10
MR effect size for
'Selenium' on 'Chondroadherin'

Batch 3−534 : Selenium on Coiled−coil−helix−coiled−coil−helix domain−containing protein 10, mitochondrial

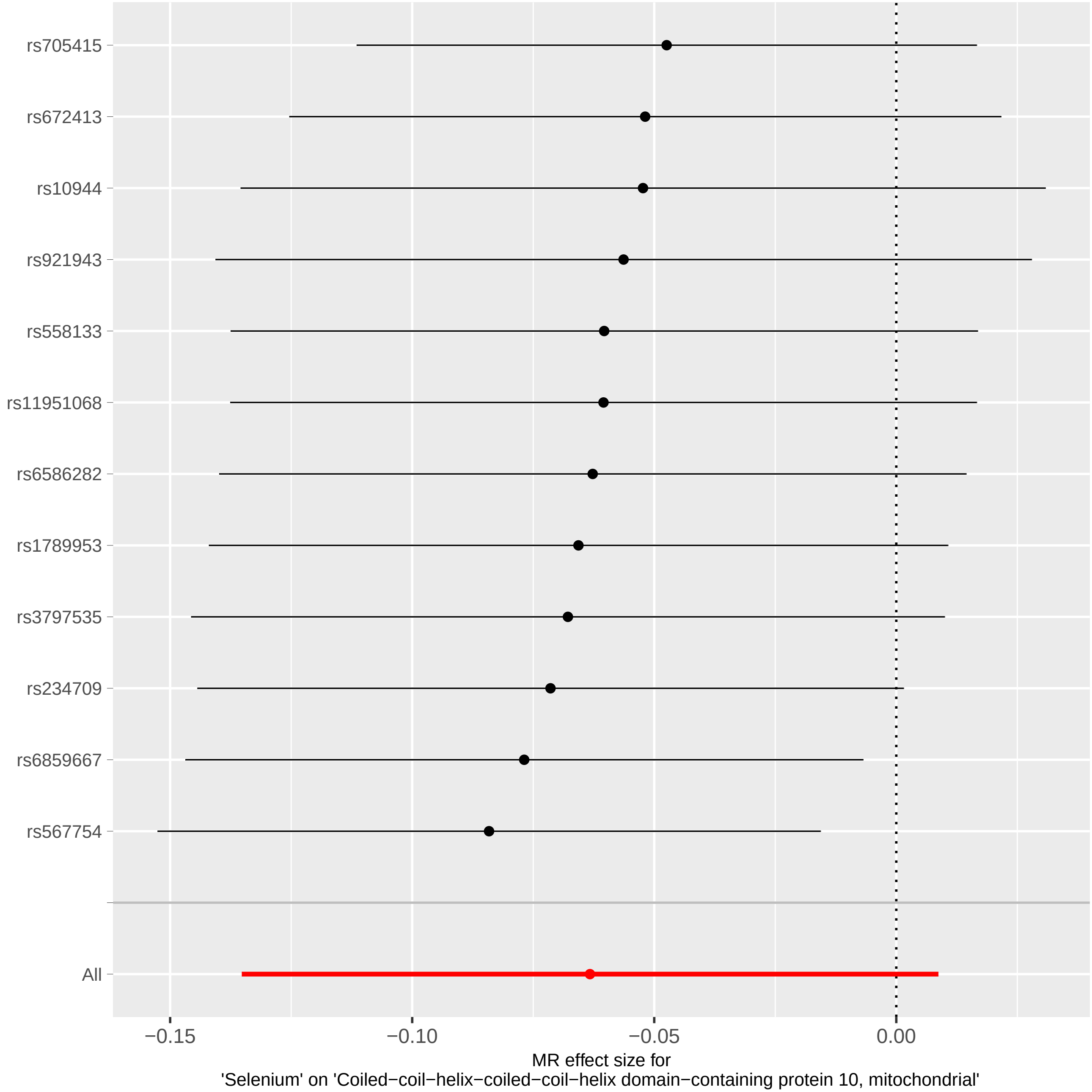

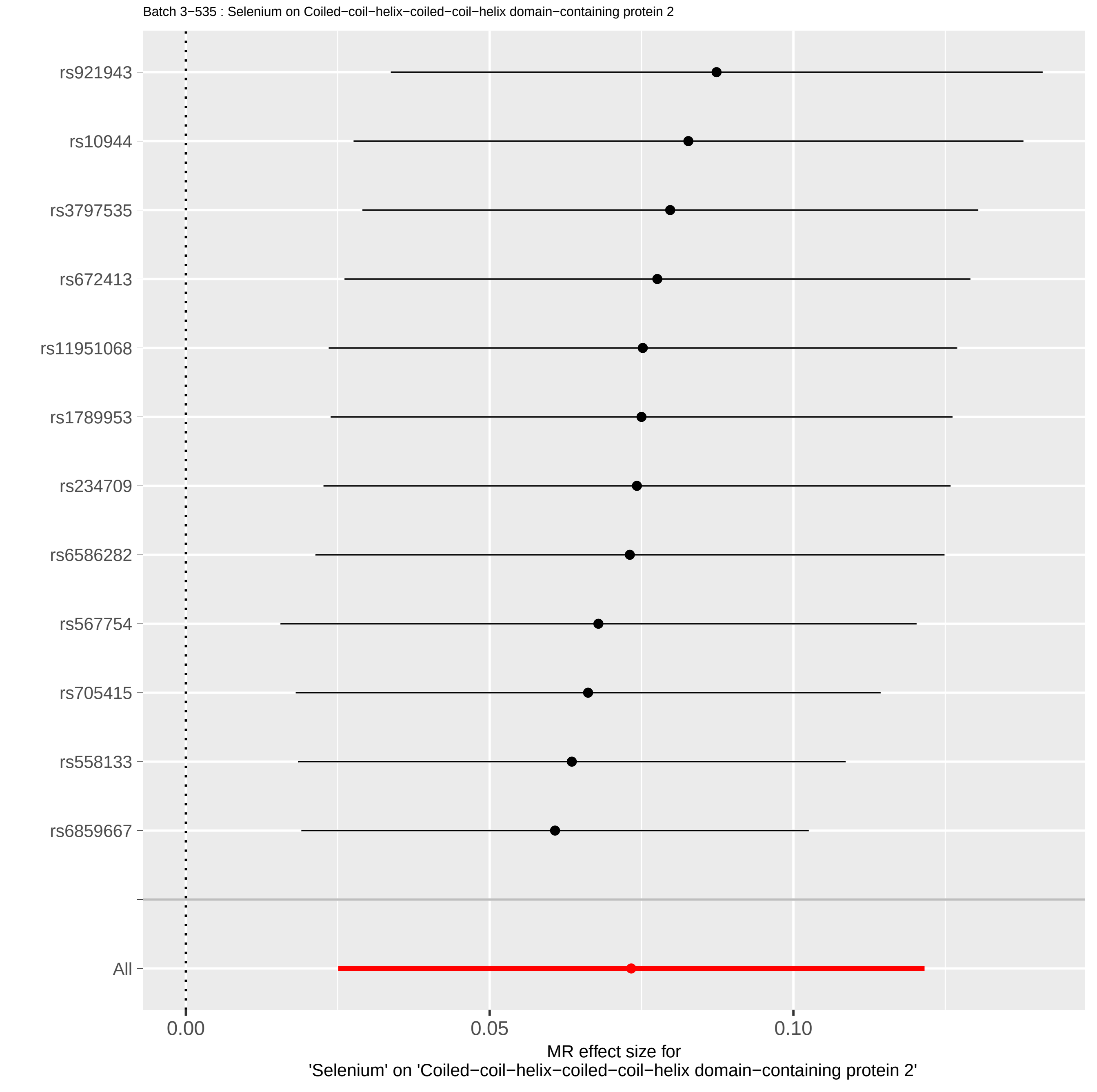

Batch 3−535 : Selenium on Coiled−coil−helix−coiled−coil−helix domain−containing protein 2
rs921943
rs10944
rs3797535
rs672413
rs11951068
rs1789953
rs234709
rs6586282
rs567754
rs705415
rs558133
rs6859667
All
0.00
0.05
0.10
MR effect size for
'Selenium' on 'Coiled−coil−helix−coiled−coil−helix domain−containing protein 2'

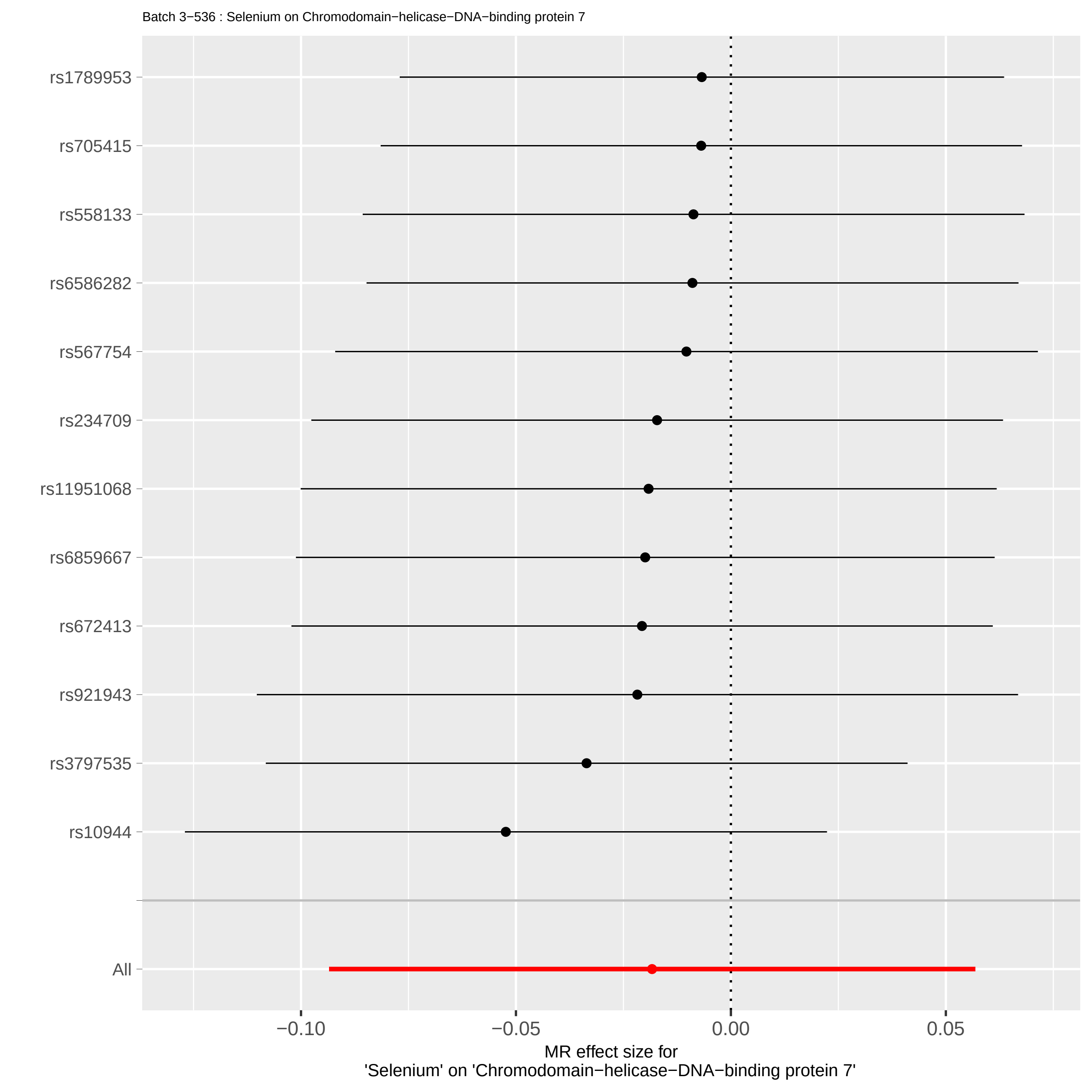

Batch 3−536 : Selenium on Chromodomain−helicase−DNA−binding protein 7
rs1789953
rs705415
rs558133
rs6586282
rs567754
rs234709
rs11951068
rs6859667
rs672413
rs921943
rs3797535
rs10944
All
−0.10
−0.05
0.00
0.05
MR effect size for
'Selenium' on 'Chromodomain−helicase−DNA−binding protein 7'

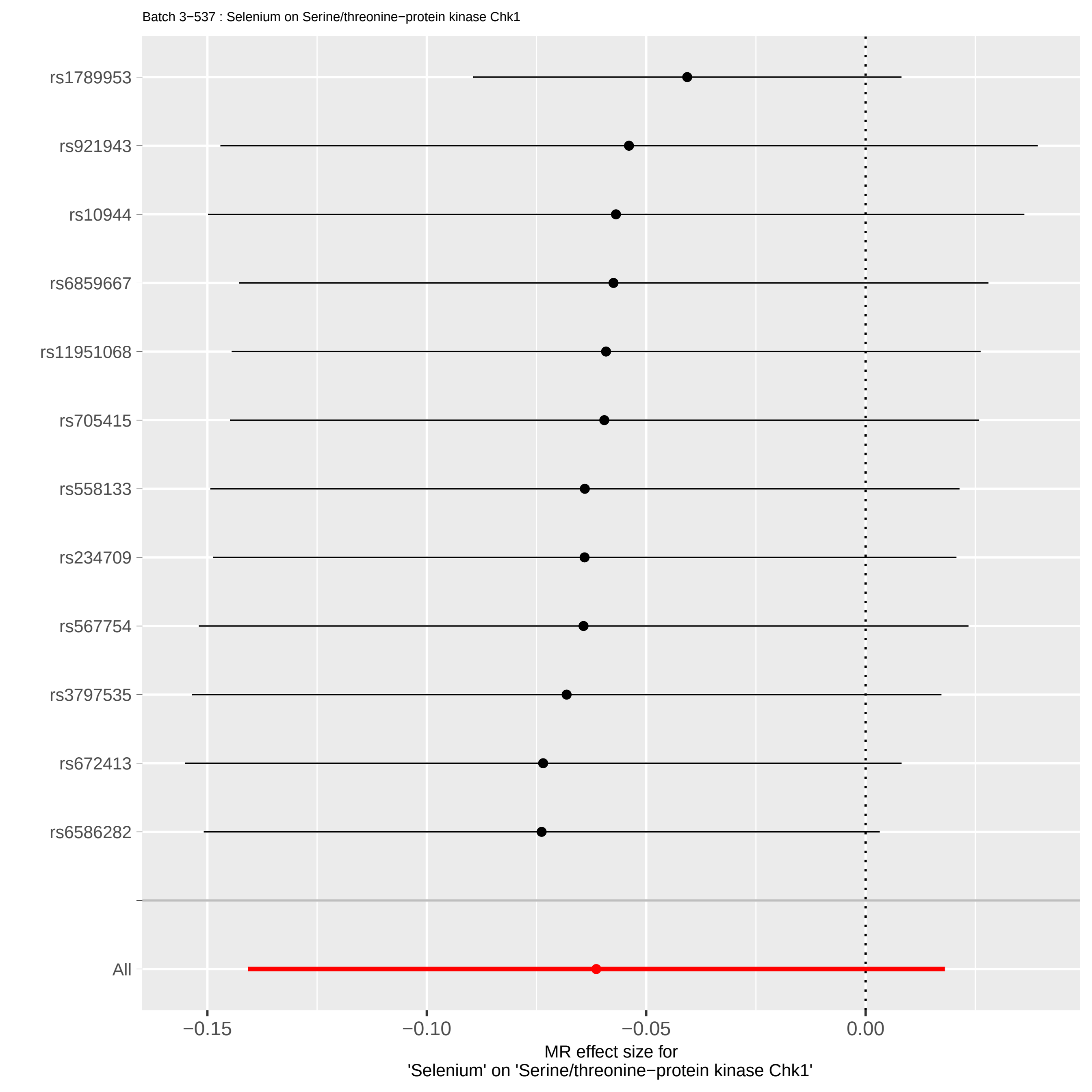

Batch 3−537 : Selenium on Serine/threonine−protein kinase Chk1
rs1789953
rs921943
rs10944
rs6859667
rs11951068
rs705415
rs558133
rs234709
rs567754
rs3797535
rs672413
rs6586282
All
−0.15
−0.10
−0.05
0.00
MR effect size for
'Selenium' on 'Serine/threonine−protein kinase Chk1'

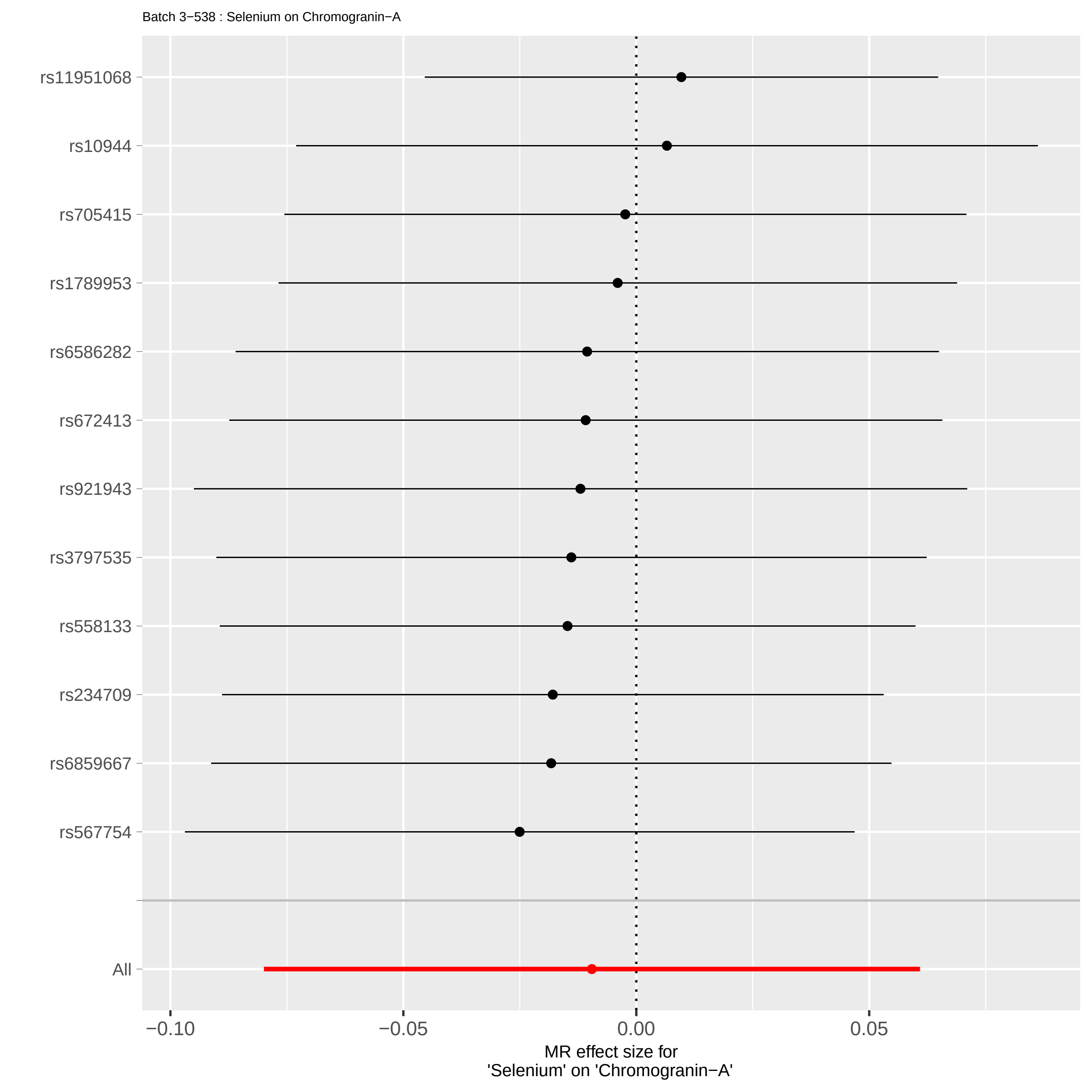

Batch 3−538 : Selenium on Chromogranin−A
rs11951068
rs10944
rs705415
rs1789953
rs6586282
rs672413
rs921943
rs3797535
rs558133
rs234709
rs6859667
rs567754
All
−0.10
−0.05
0.00
0.05
MR effect size for
'Selenium' on 'Chromogranin−A'

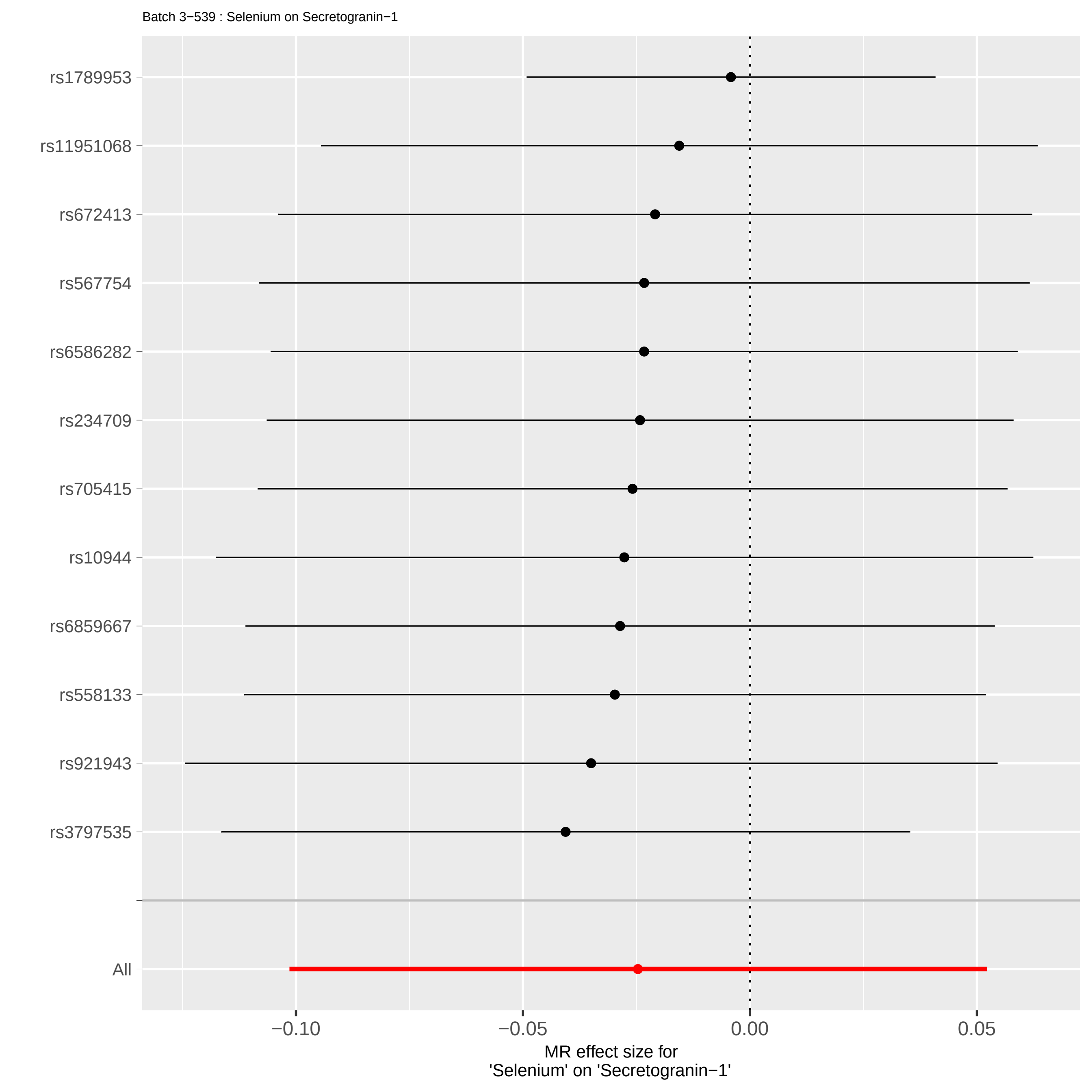

Batch 3−539 : Selenium on Secretogranin−1
rs1789953
rs11951068
rs672413
rs567754
rs6586282
rs234709
rs705415
rs10944
rs6859667
rs558133
rs921943
rs3797535
All
−0.10
−0.05
0.00
0.05
MR effect size for
'Selenium' on 'Secretogranin−1'

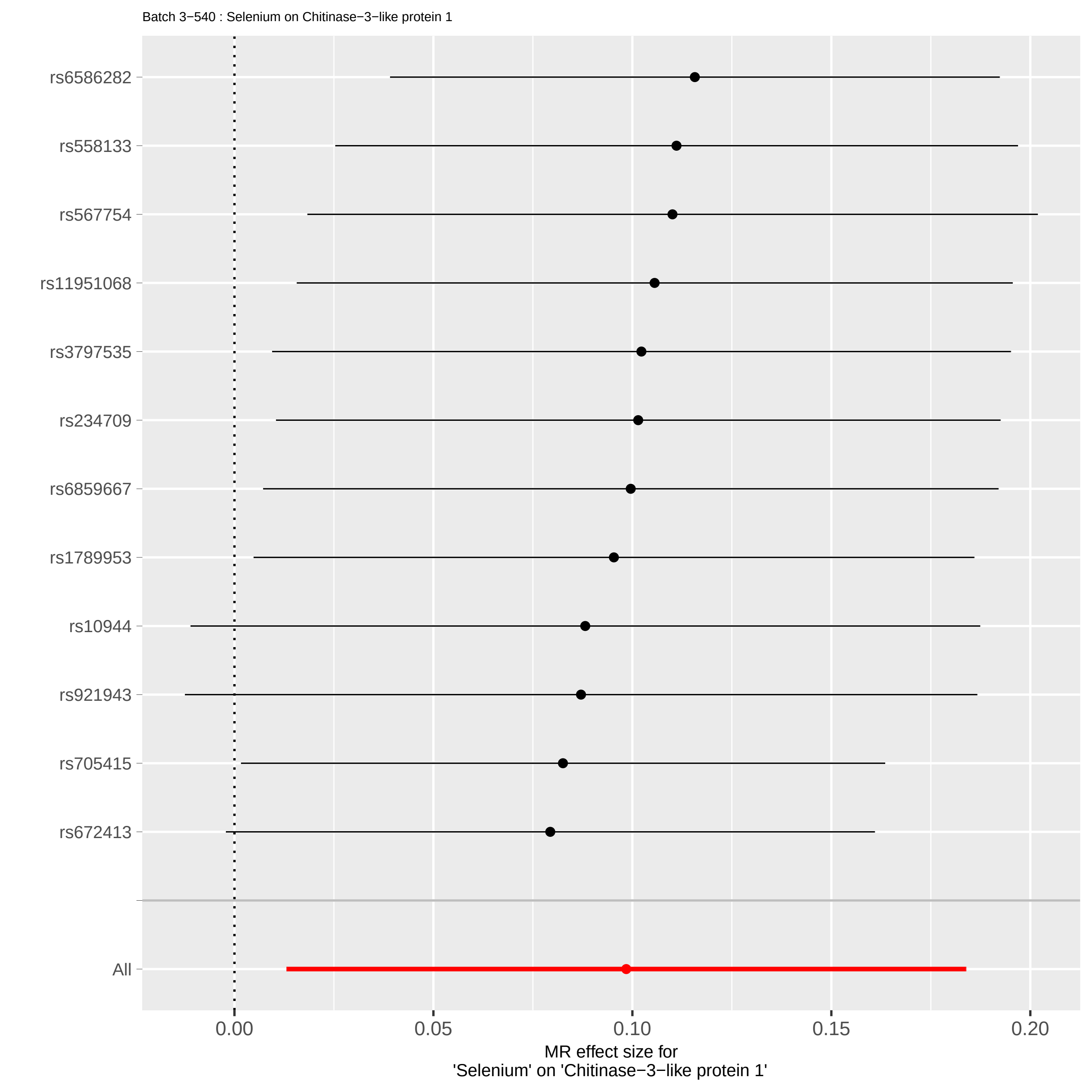

Batch 3−540 : Selenium on Chitinase−3−like protein 1
rs6586282
rs558133
rs567754
rs11951068
rs3797535
rs234709
rs6859667
rs1789953
rs10944
rs921943
rs705415
rs672413
All
0.00
0.05
0.10
0.15
0.20
MR effect size for
'Selenium' on 'Chitinase−3−like protein 1'

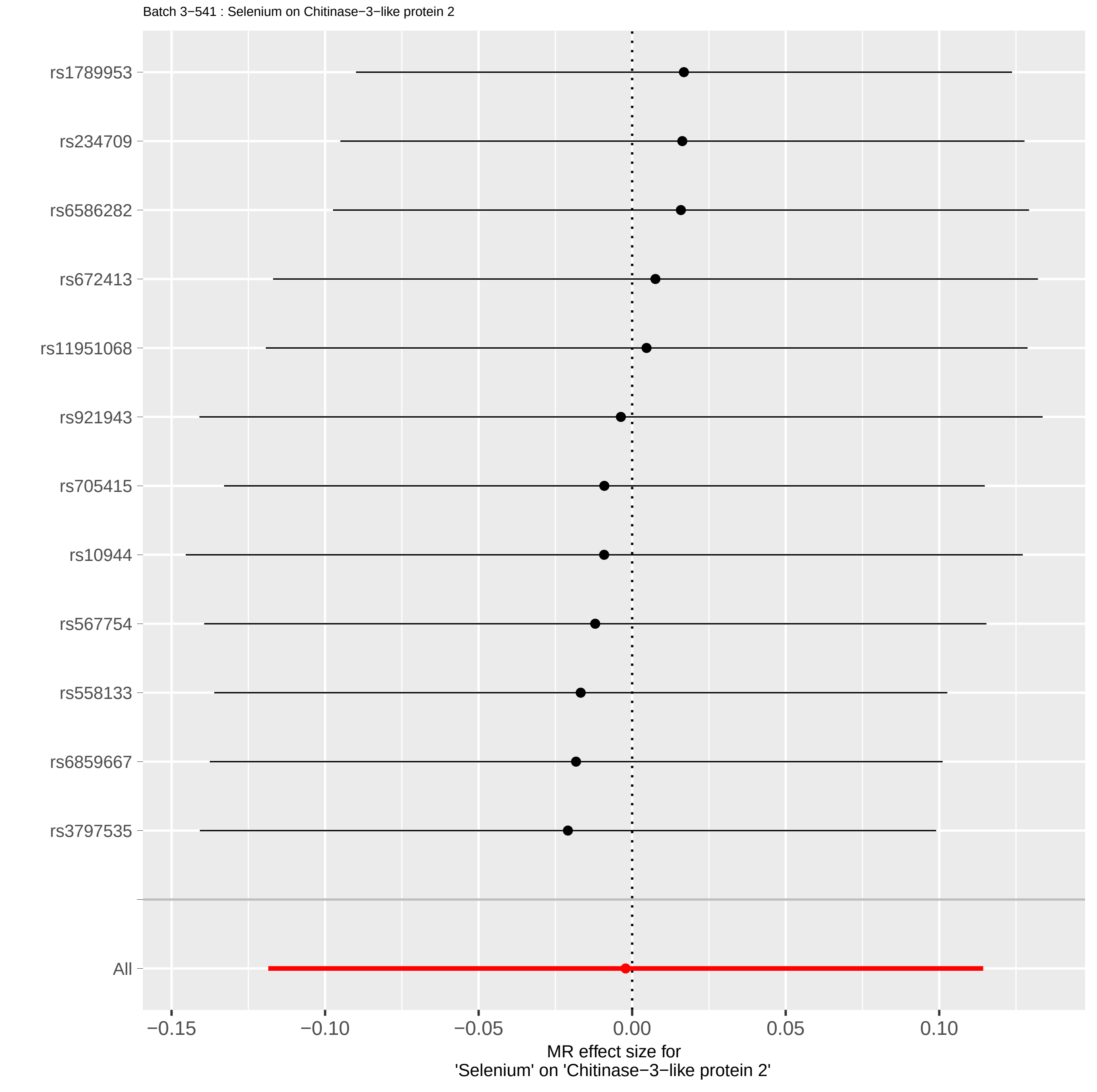

Batch 3−541 : Selenium on Chitinase−3−like protein 2
rs1789953
rs234709
rs6586282
rs672413
rs11951068
rs921943
rs705415
rs10944
rs567754
rs558133
rs6859667
rs3797535
All
−0.15
−0.10
−0.05
0.00
0.05
0.10
MR effect size for
'Selenium' on 'Chitinase−3−like protein 2'

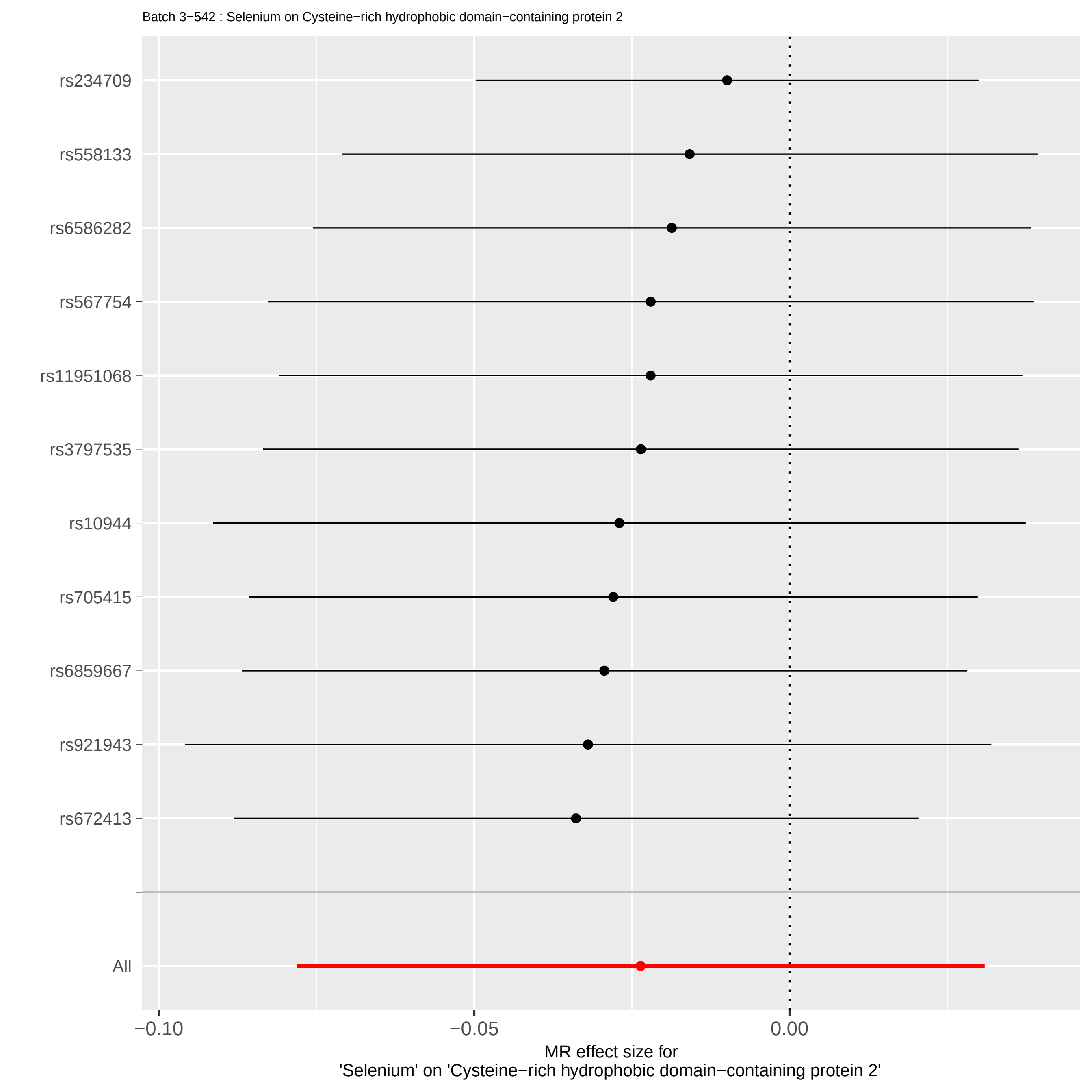

Batch 3−542 : Selenium on Cysteine−rich hydrophobic domain−containing protein 2
rs234709
rs558133
rs6586282
rs567754
rs11951068
rs3797535
rs10944
rs705415
rs6859667
rs921943
rs672413
All
−0.10
−0.05
0.00
MR effect size for
'Selenium' on 'Cysteine−rich hydrophobic domain−containing protein 2'

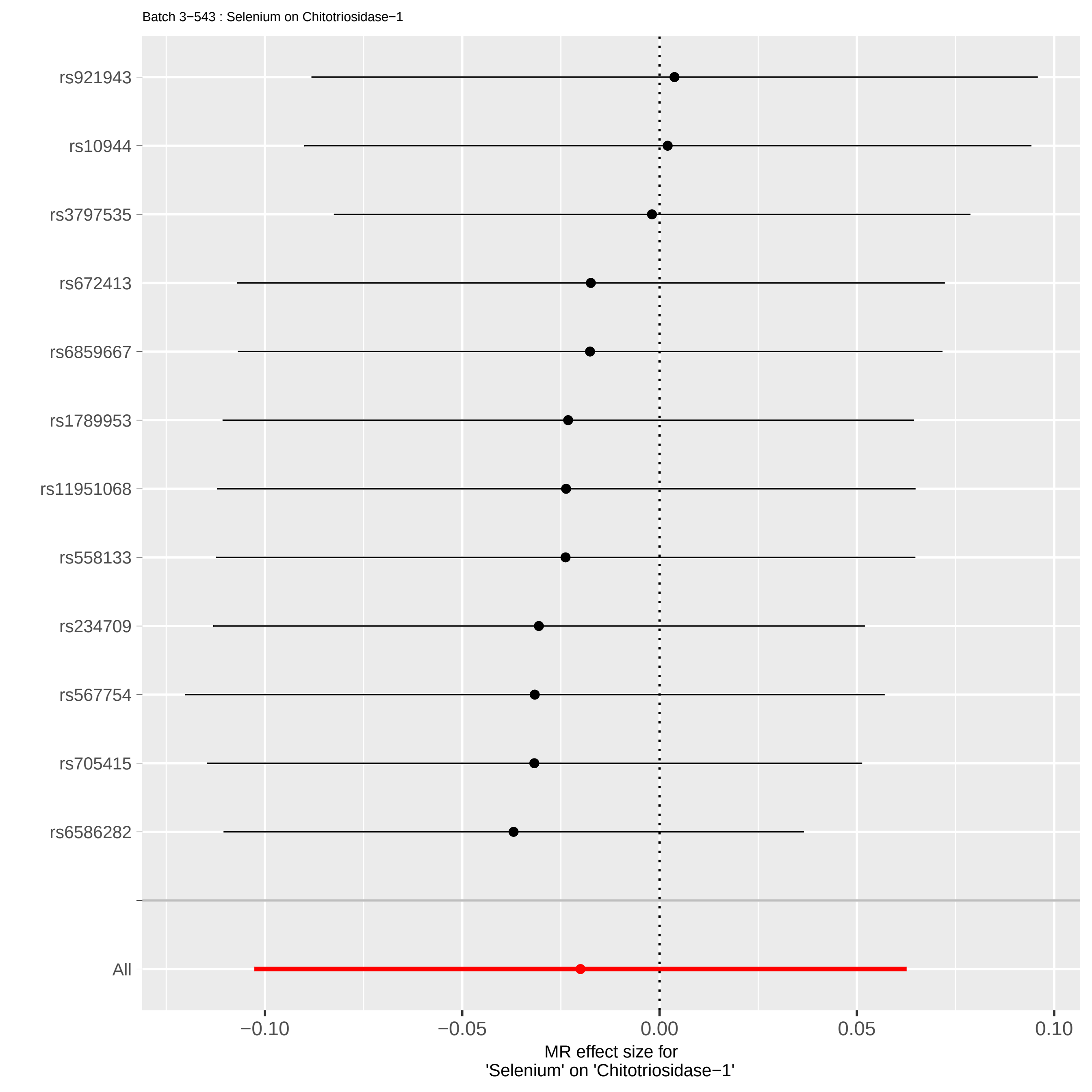

Batch 3−543 : Selenium on Chitotriosidase−1
rs921943
rs10944
rs3797535
rs672413
rs6859667
rs1789953
rs11951068
rs558133
rs234709
rs567754
rs705415
rs6586282
All
−0.10
−0.05
0.00
0.05
0.10
MR effect size for
'Selenium' on 'Chitotriosidase−1'

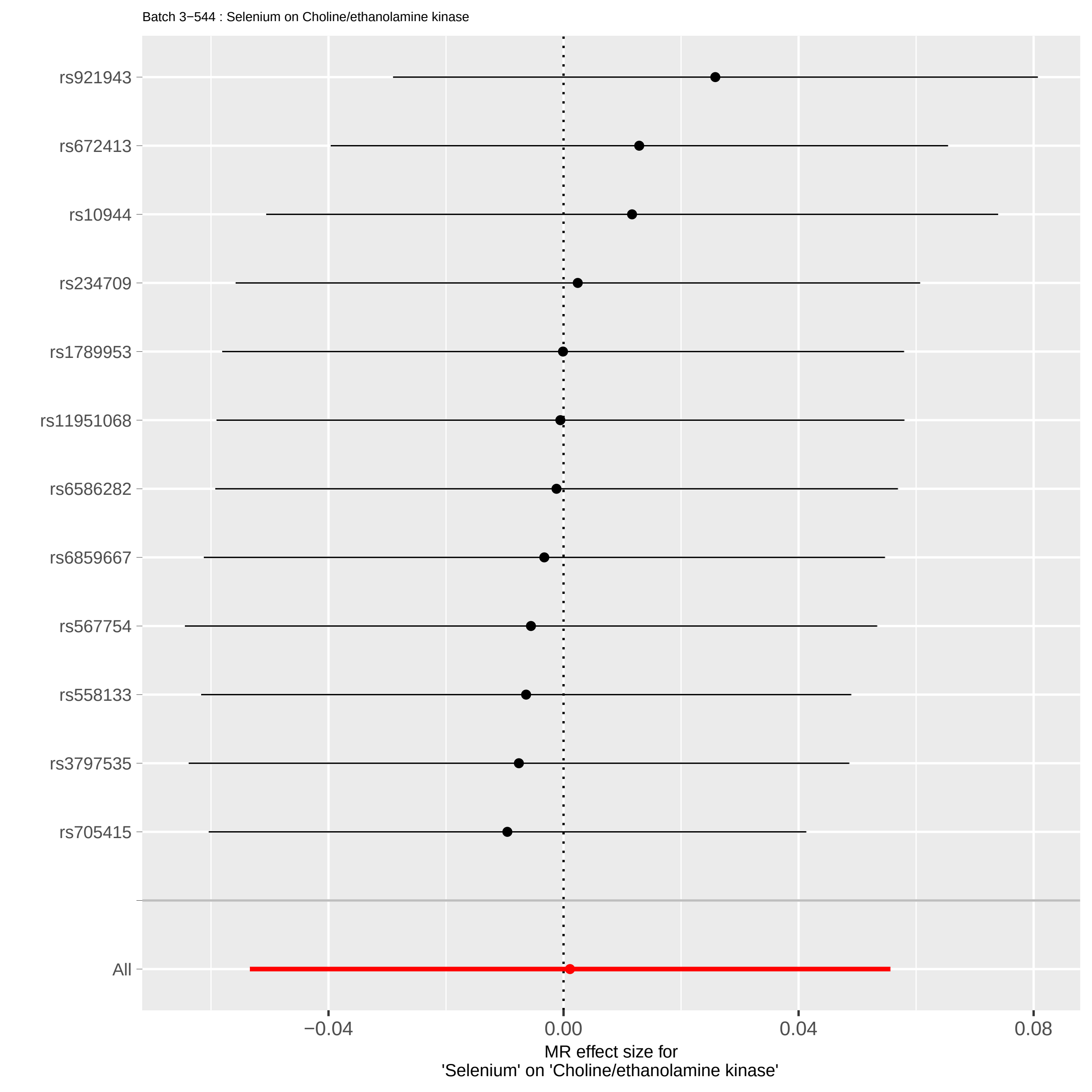

Batch 3−544 : Selenium on Choline/ethanolamine kinase
rs921943
rs672413
rs10944
rs234709
rs1789953
rs11951068
rs6586282
rs6859667
rs567754
rs558133
rs3797535
rs705415
All
−0.04
0.00
0.04
0.08
MR effect size for
'Selenium' on 'Choline/ethanolamine kinase'

Batch 3−545 : Selenium on Neural cell adhesion molecule L1−like protein

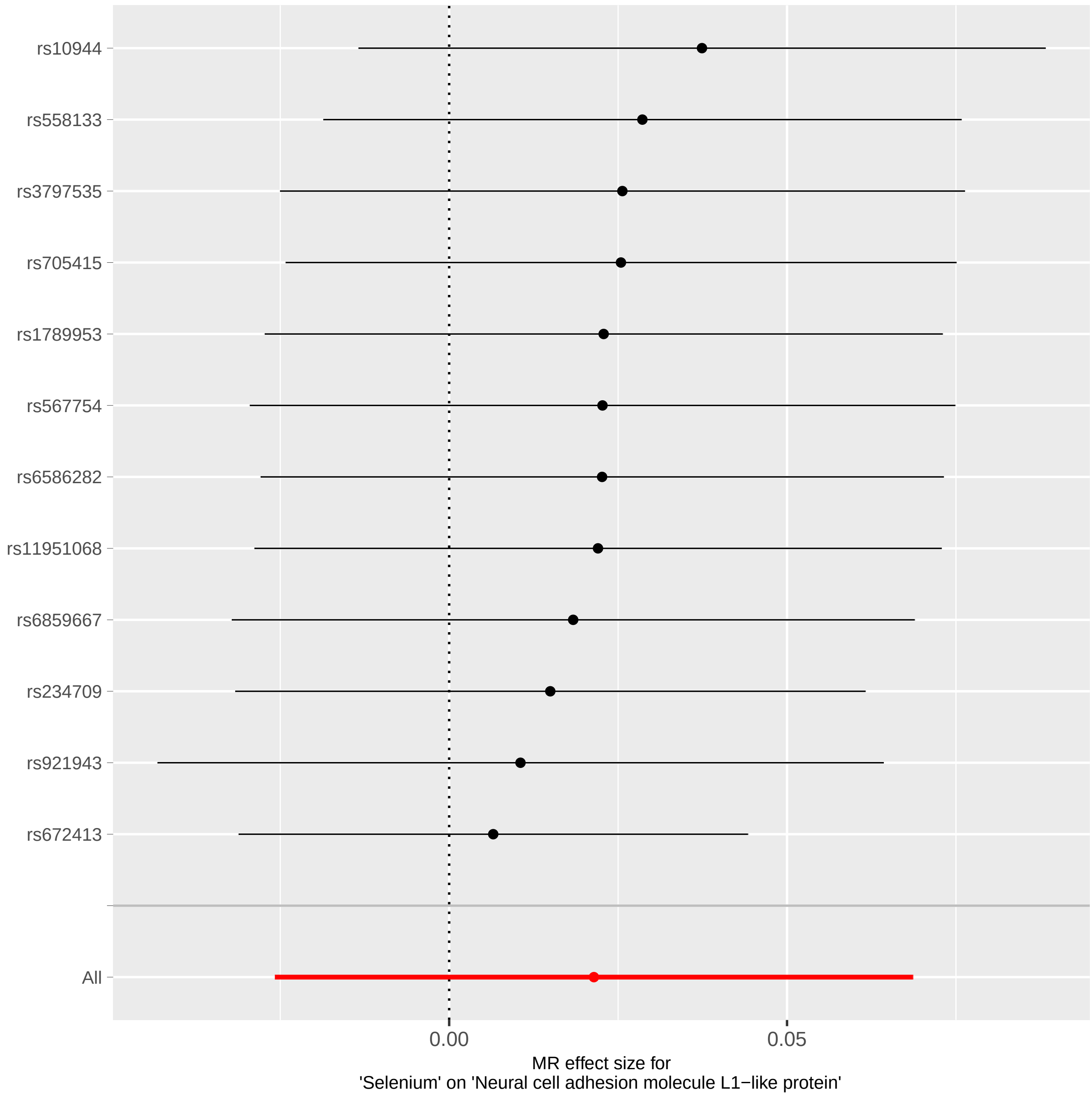

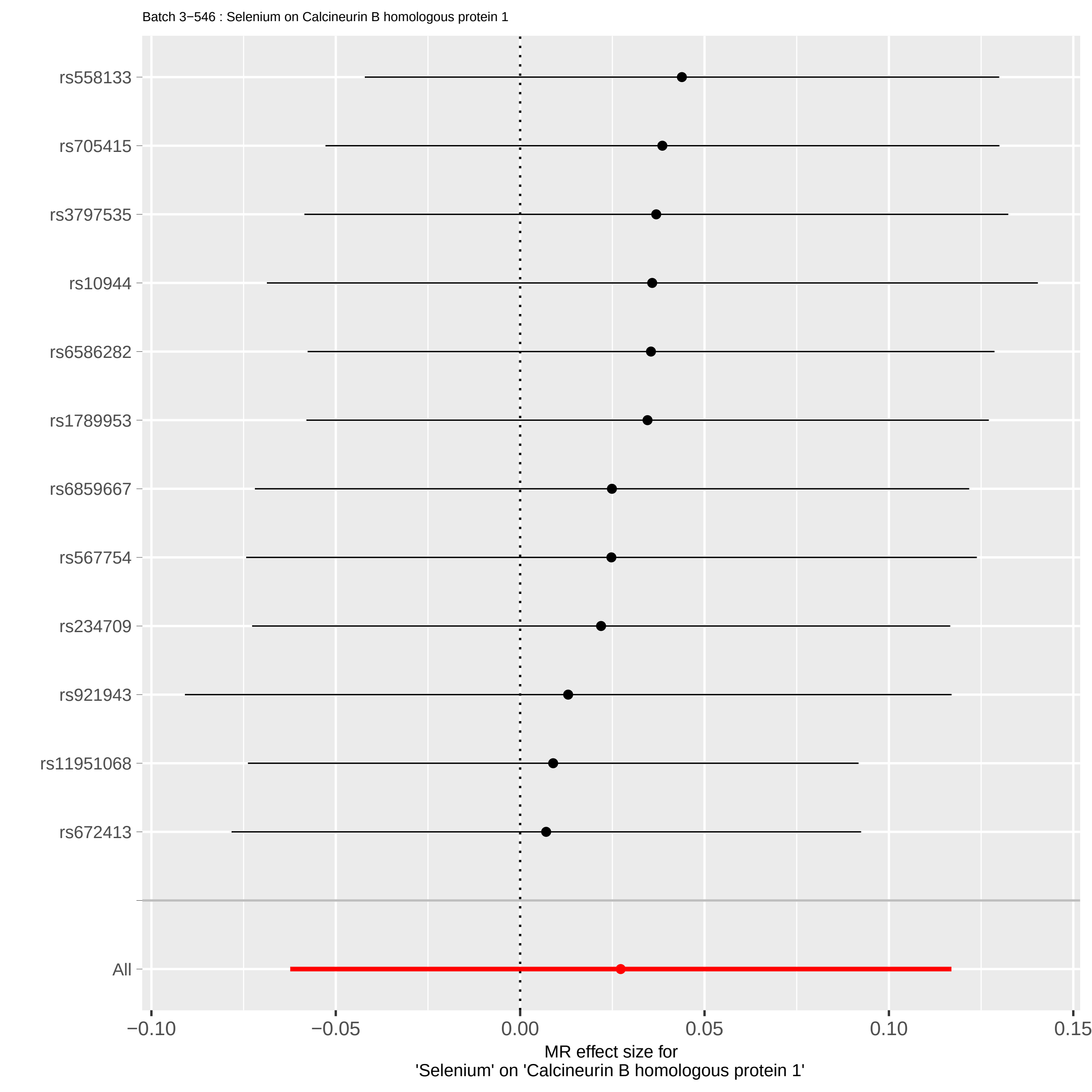

Batch 3−546 : Selenium on Calcineurin B homologous protein 1
rs558133
rs705415
rs3797535
rs10944
rs6586282
rs1789953
rs6859667
rs567754
rs234709
rs921943
rs11951068
rs672413
All
−0.10
−0.05
0.00
0.05
0.10
0.15
MR effect size for
'Selenium' on 'Calcineurin B homologous protein 1'

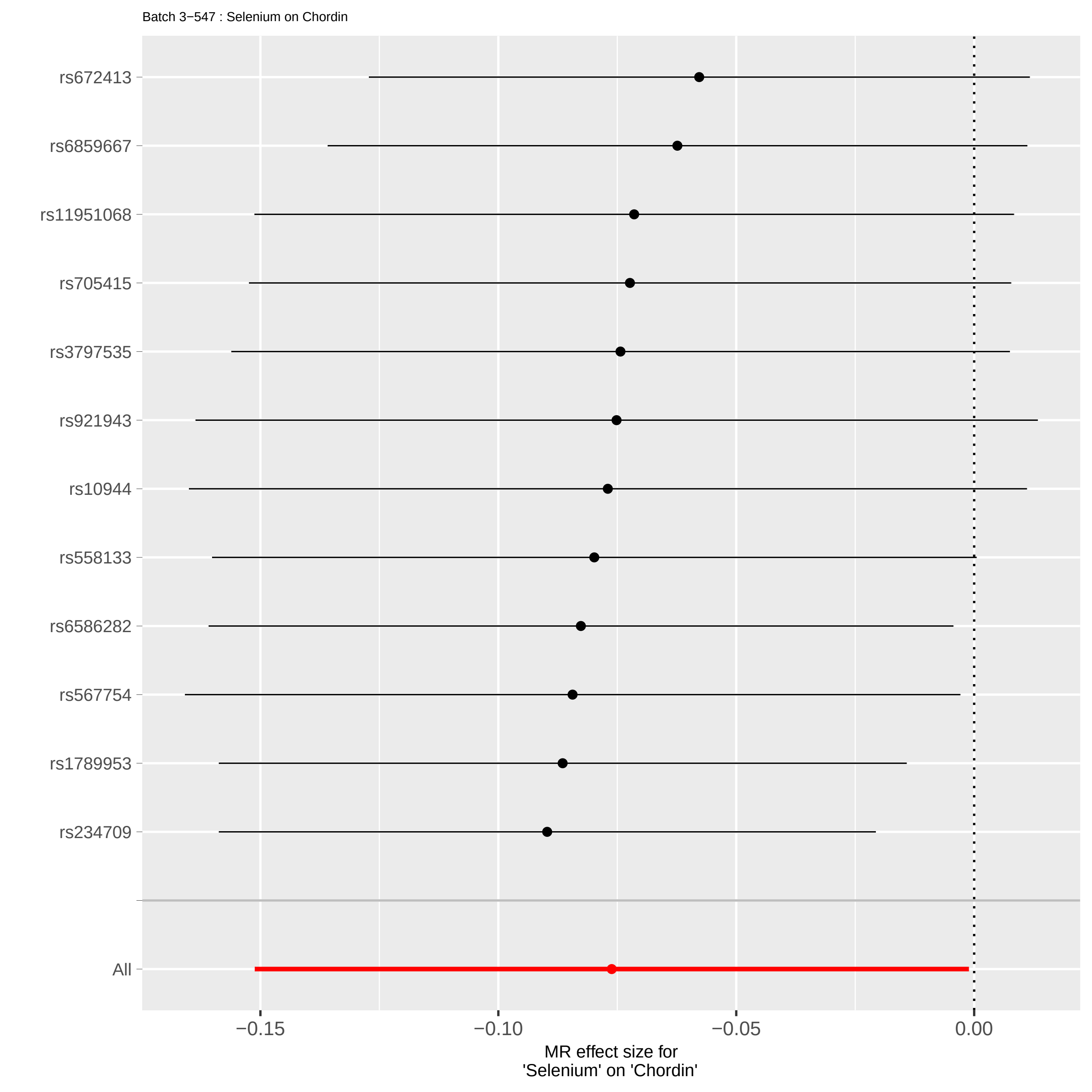

Batch 3−547 : Selenium on Chordin
rs672413
rs6859667
rs11951068
rs705415
rs3797535
rs921943
rs10944
rs558133
rs6586282
rs567754
rs1789953
rs234709
All
−0.15
−0.10
−0.05
0.00
MR effect size for
'Selenium' on 'Chordin'

Batch 3−548 : Selenium on Chordin−like protein 1

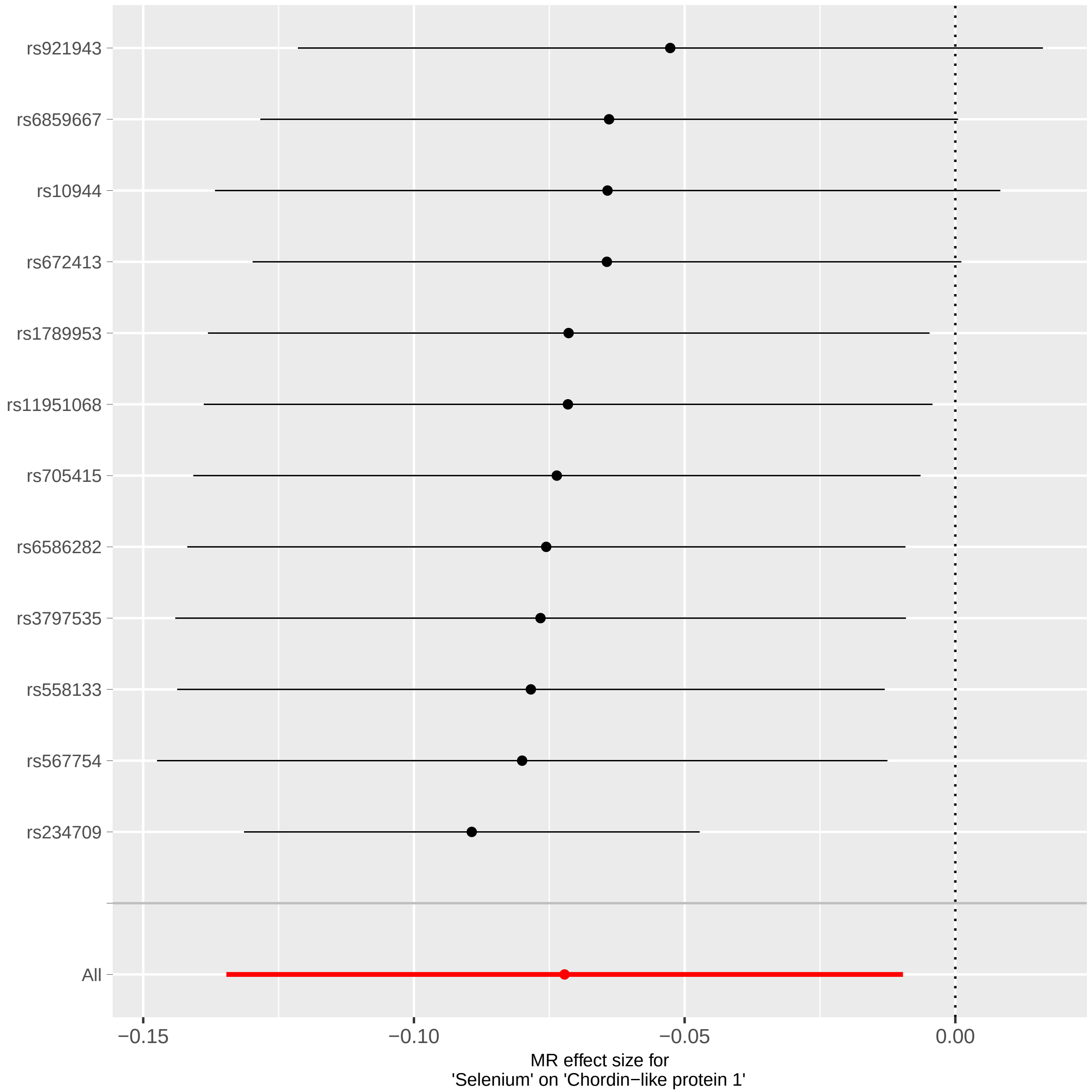

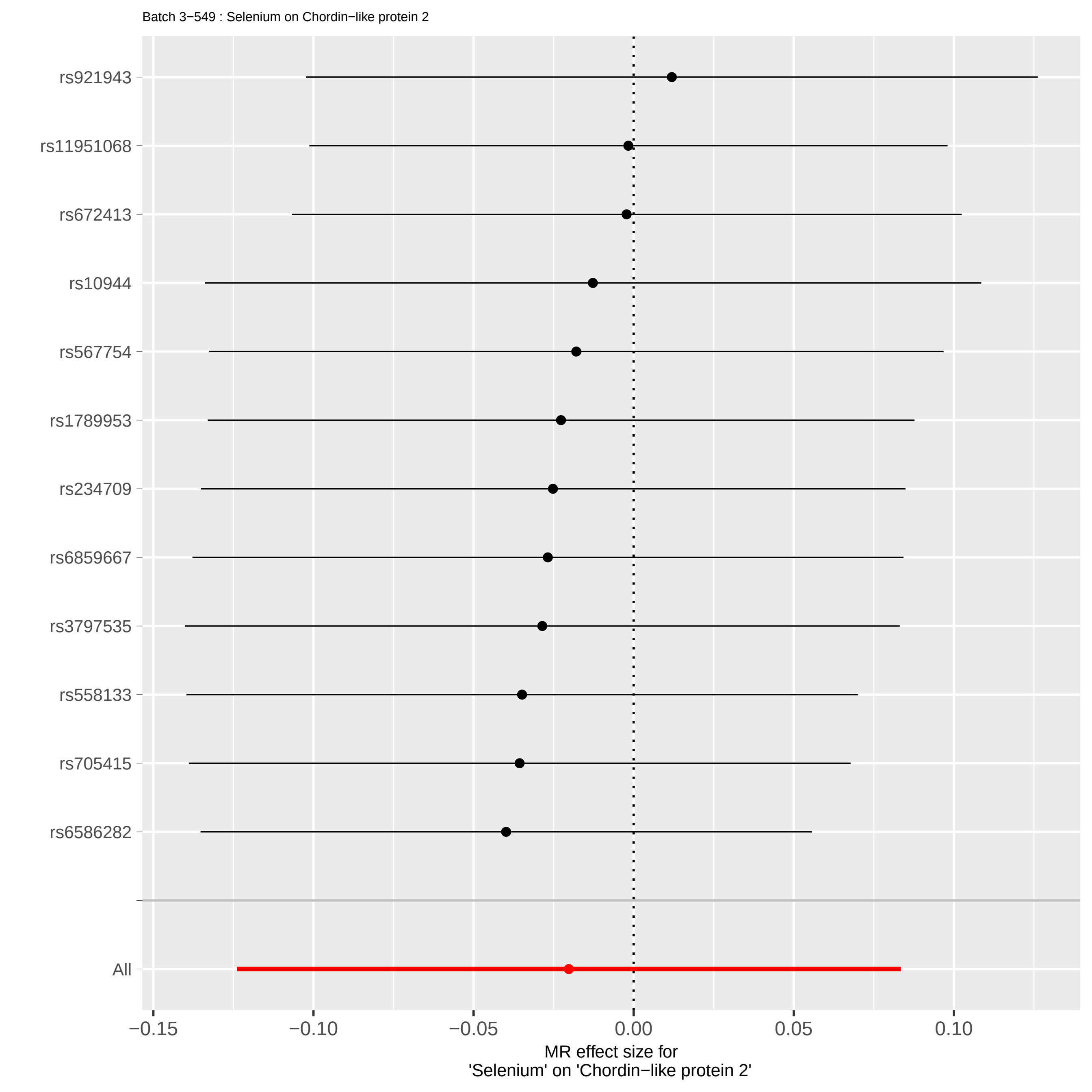
Batch 3−549 : Selenium on Chordin−like protein 2
rs921943
rs11951068
rs672413
rs10944
rs567754
rs1789953
rs234709
rs6859667
rs3797535
rs558133
rs705415
rs6586282
All
−0.15
−0.10
−0.05
0.00
0.05
0.10
MR effect size for
'Selenium' on 'Chordin−like protein 2'

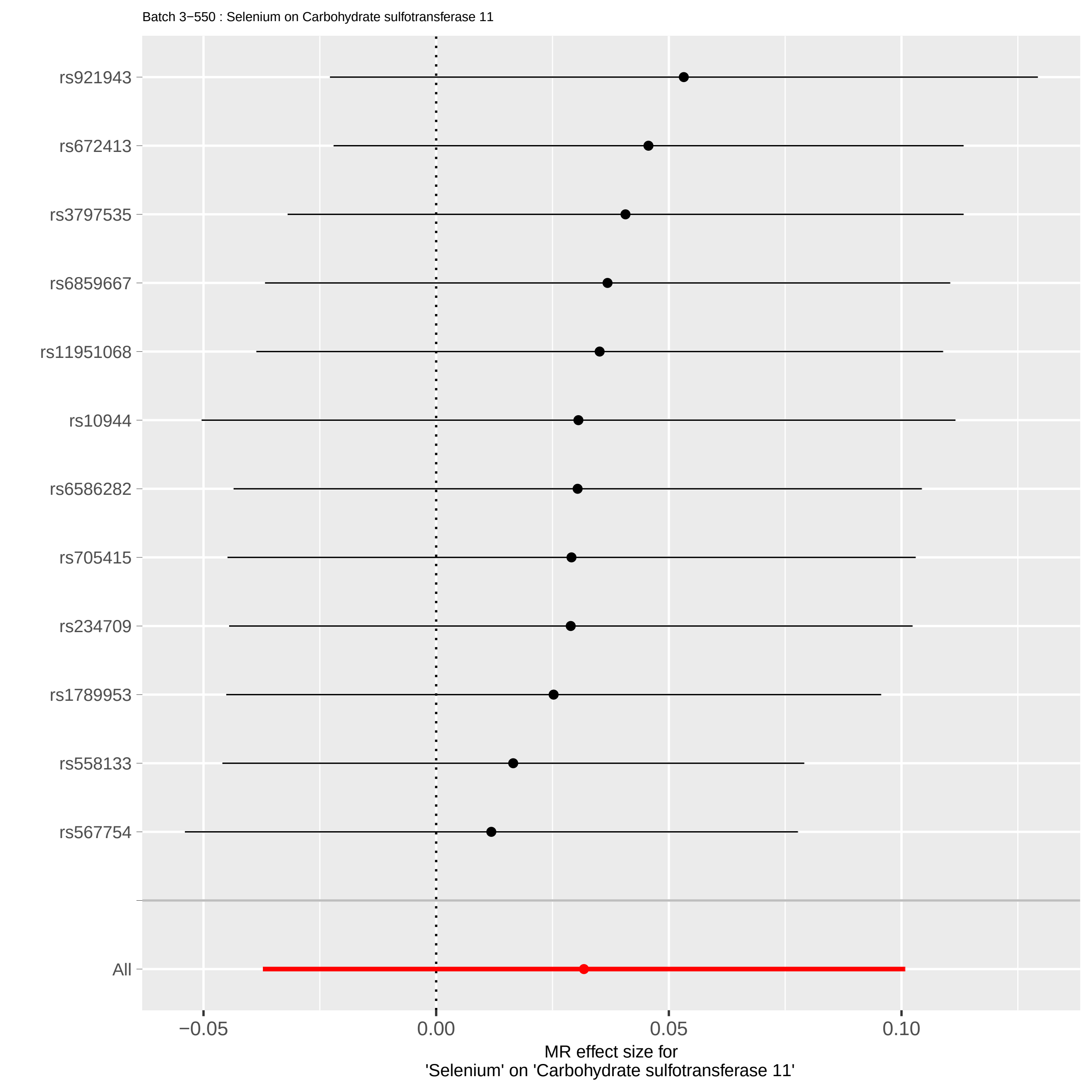

Batch 3−550 : Selenium on Carbohydrate sulfotransferase 11
rs921943
rs672413
rs3797535
rs6859667
rs11951068
rs10944
rs6586282
rs705415
rs234709
rs1789953
rs558133
rs567754
All
−0.05
0.00
0.05
0.10
MR effect size for
'Selenium' on 'Carbohydrate sulfotransferase 11'

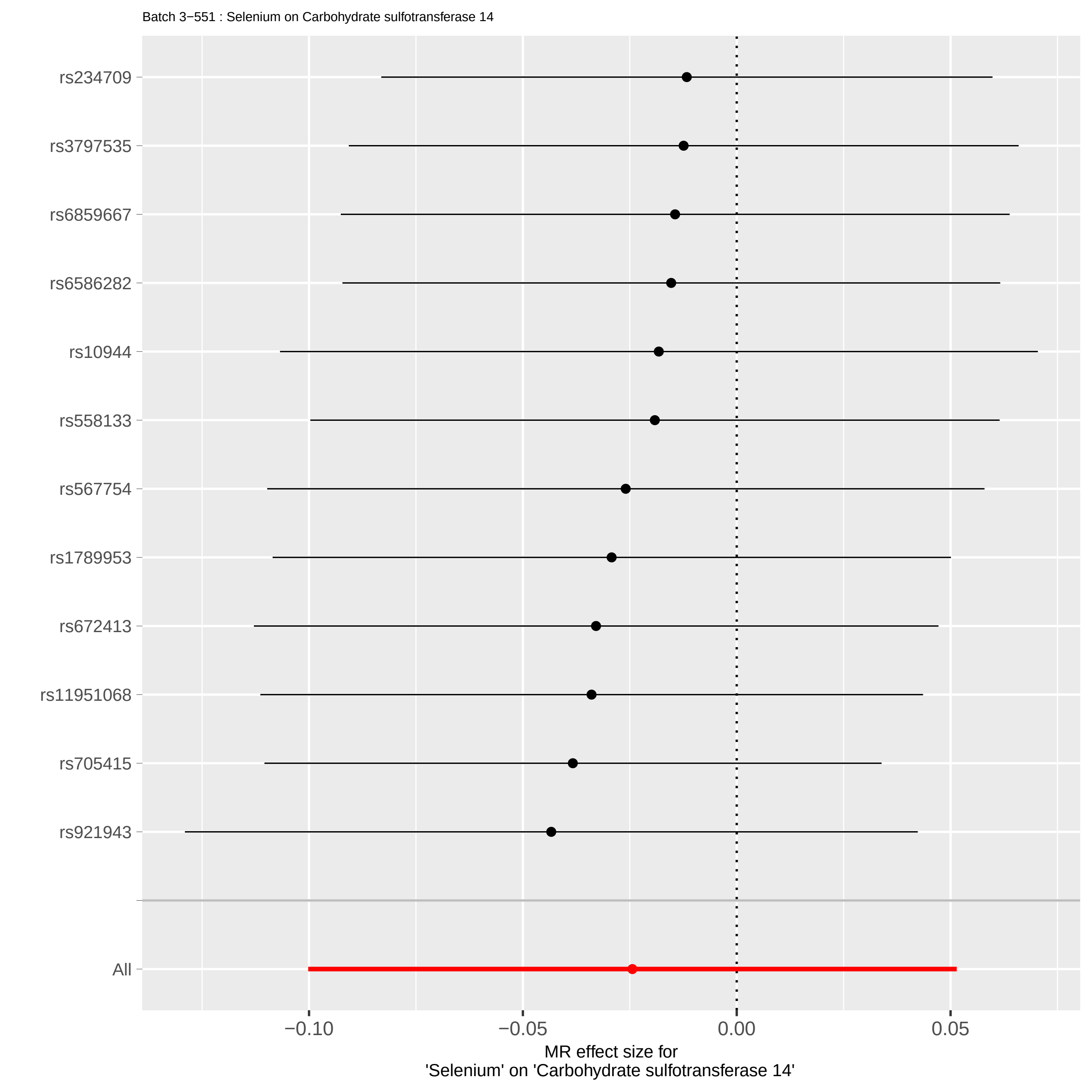

Batch 3−551 : Selenium on Carbohydrate sulfotransferase 14
rs234709
rs3797535
rs6859667
rs6586282
rs10944
rs558133
rs567754
rs1789953
rs672413
rs11951068
rs705415
rs921943
All
−0.10
−0.05
0.00
0.05
MR effect size for
'Selenium' on 'Carbohydrate sulfotransferase 14'

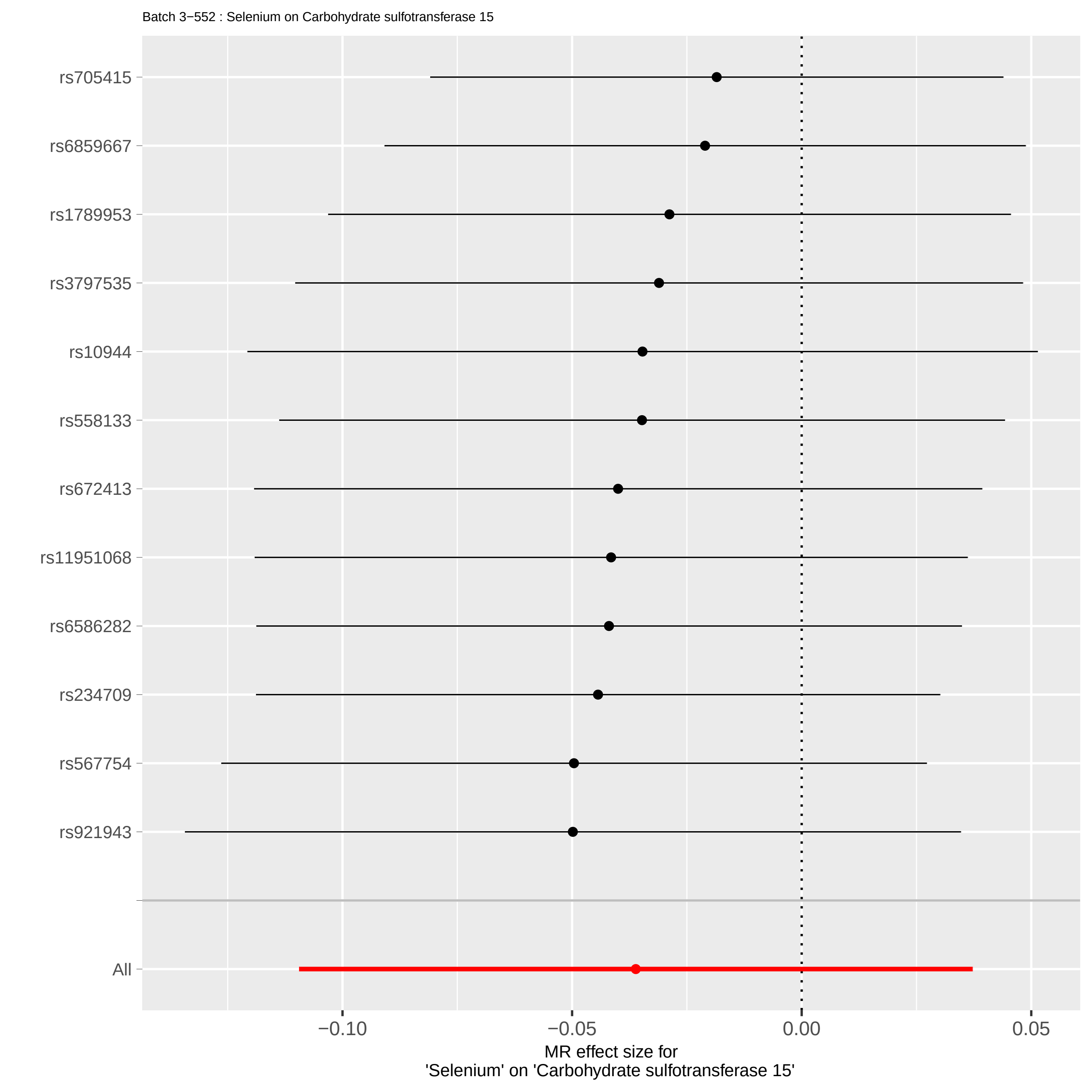

Batch 3−552 : Selenium on Carbohydrate sulfotransferase 15
rs705415
rs6859667
rs1789953
rs3797535
rs10944
rs558133
rs672413
rs11951068
rs6586282
rs234709
rs567754
rs921943
All
−0.10
−0.05
0.00
0.05
MR effect size for
'Selenium' on 'Carbohydrate sulfotransferase 15'

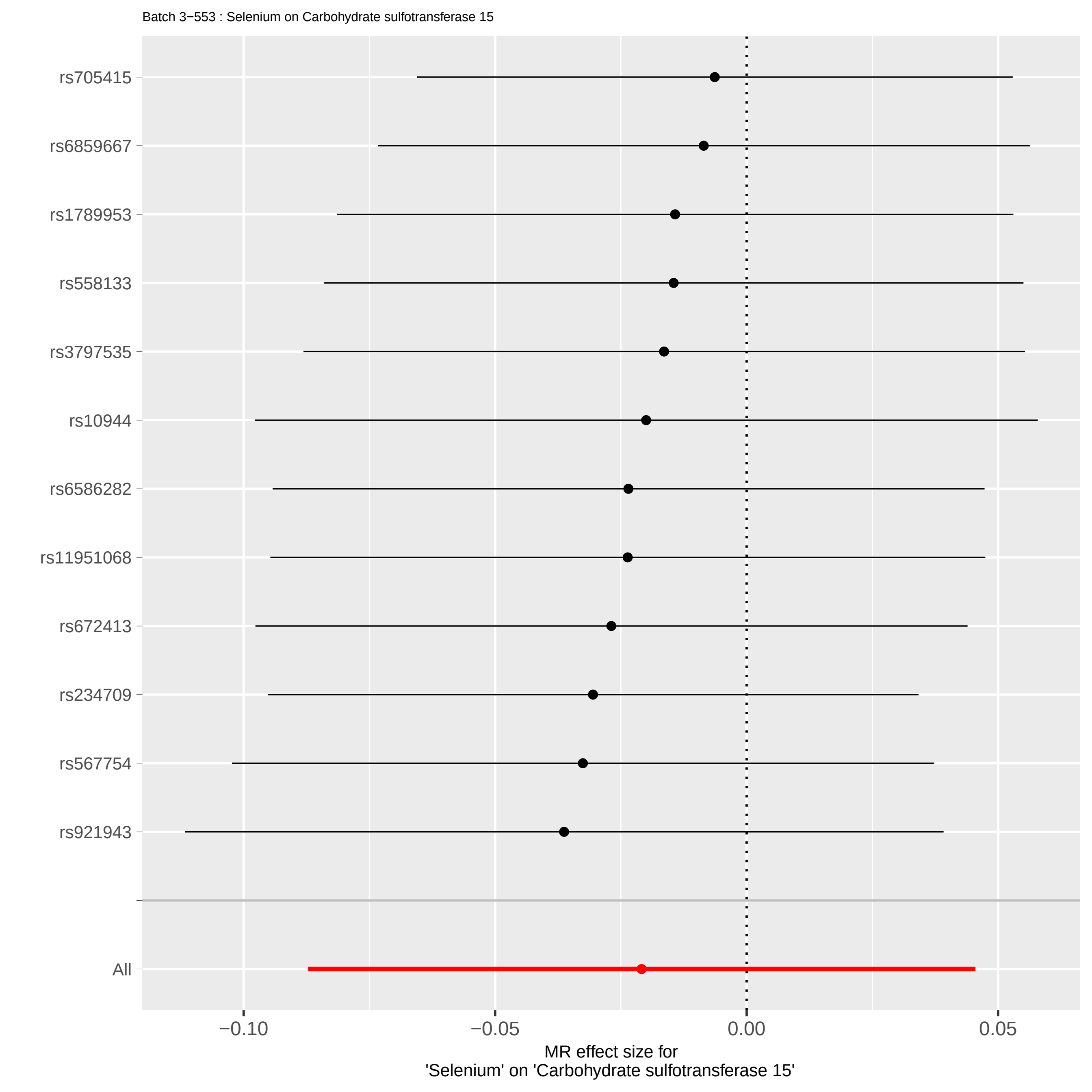

Batch 3−553 : Selenium on Carbohydrate sulfotransferase 15
rs705415
rs6859667
rs1789953
rs558133
rs3797535
rs10944
rs6586282
rs11951068
rs672413
rs234709
rs567754
rs921943
All
−0.10
−0.05
0.00
0.05
MR effect size for
'Selenium' on 'Carbohydrate sulfotransferase 15'

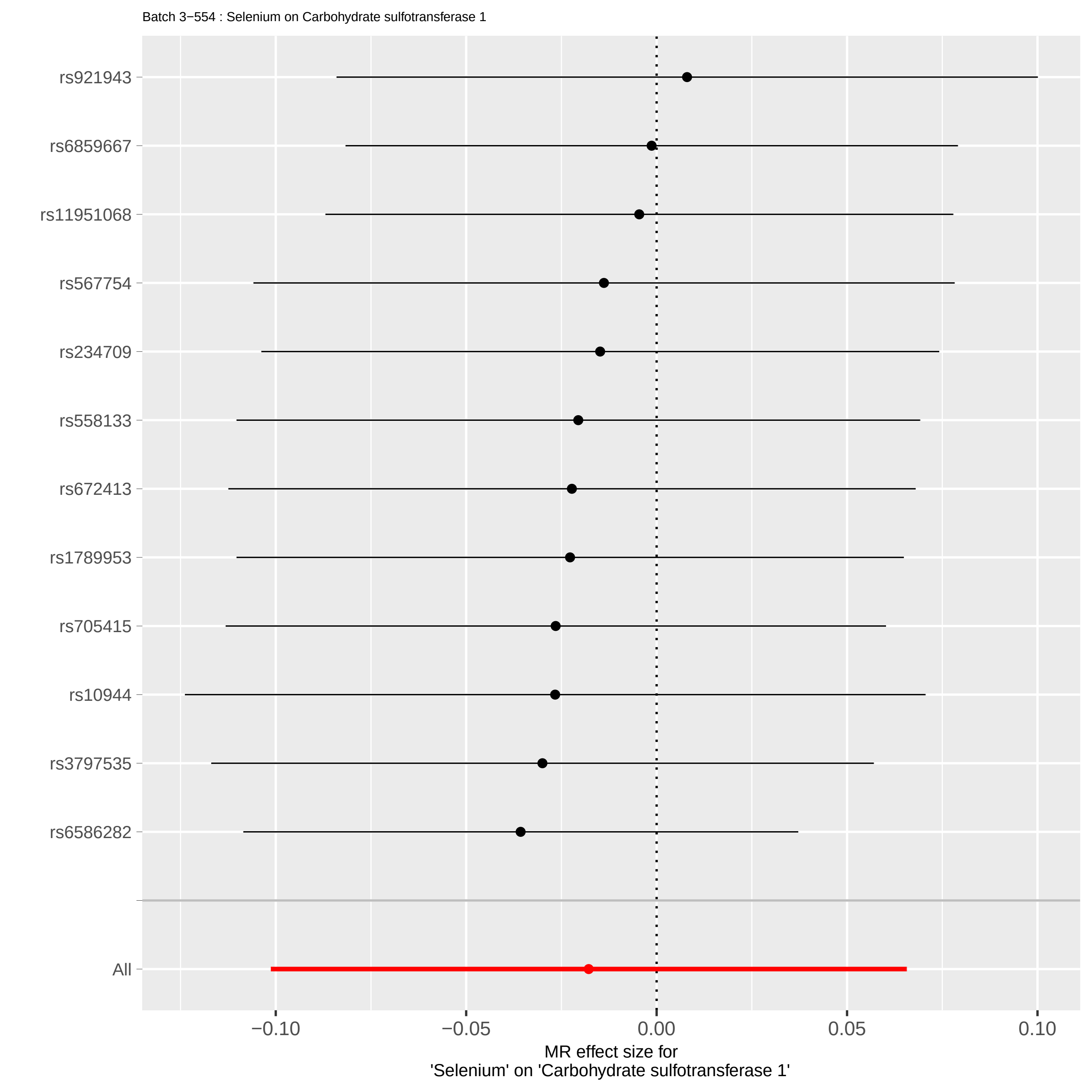

Batch 3−554 : Selenium on Carbohydrate sulfotransferase 1
rs921943
rs6859667
rs11951068
rs567754
rs234709
rs558133
rs672413
rs1789953
rs705415
rs10944
rs3797535
rs6586282
All
−0.10
−0.05
0.00
0.05
0.10
MR effect size for
'Selenium' on 'Carbohydrate sulfotransferase 1'

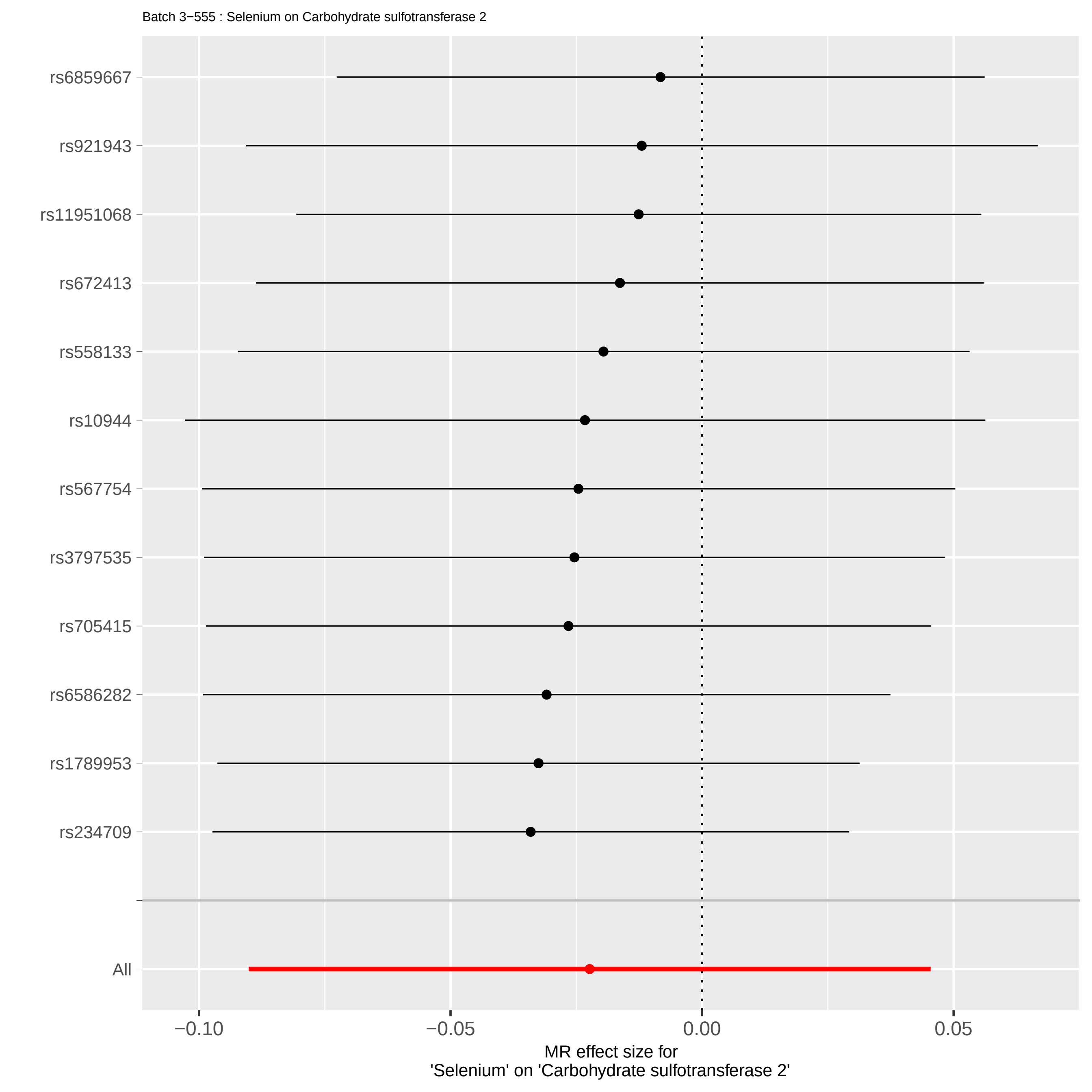

Batch 3−555 : Selenium on Carbohydrate sulfotransferase 2
rs6859667
rs921943
rs11951068
rs672413
rs558133
rs10944
rs567754
rs3797535
rs705415
rs6586282
rs1789953
rs234709
All
−0.10
−0.05
0.00
0.05
MR effect size for
'Selenium' on 'Carbohydrate sulfotransferase 2'

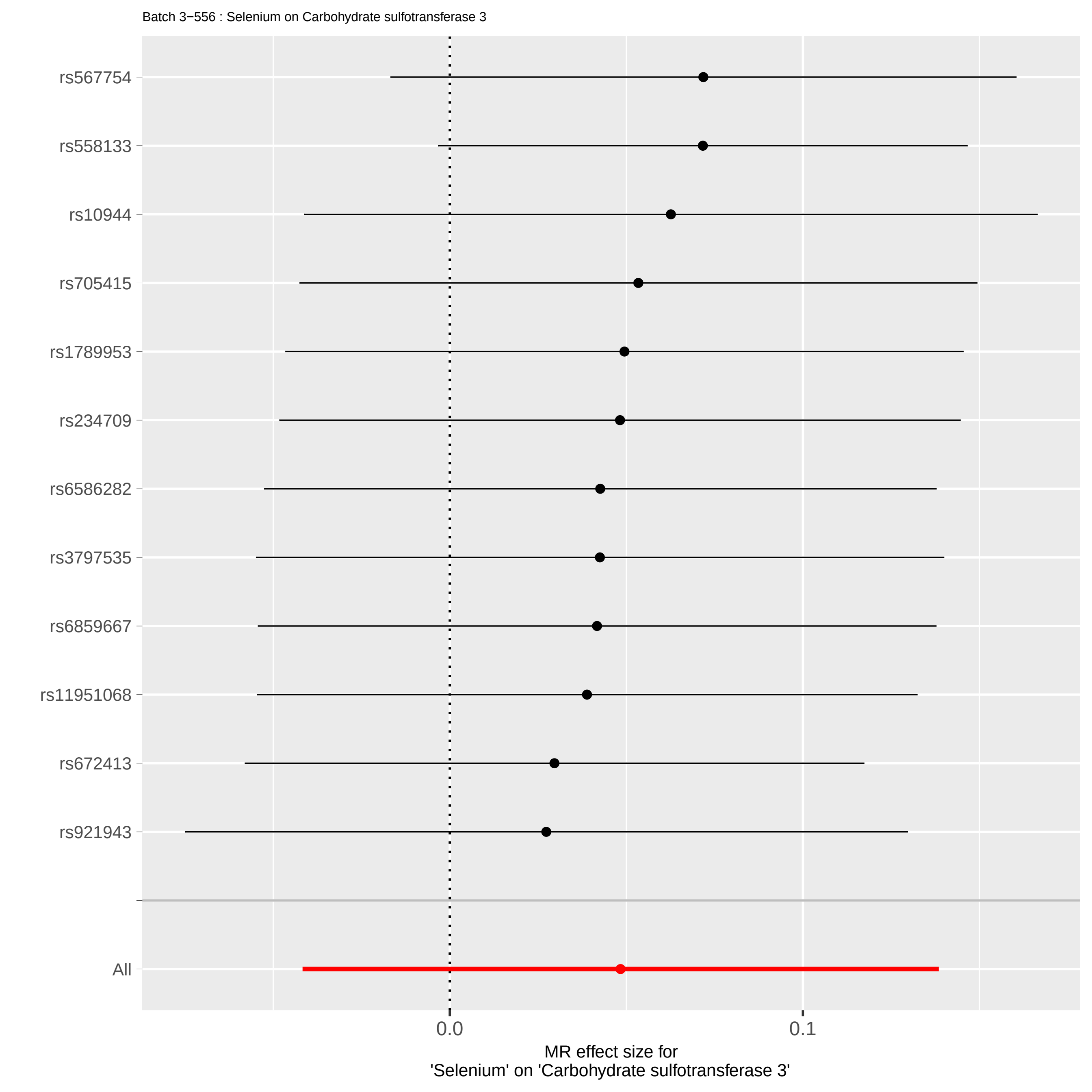

Batch 3−556 : Selenium on Carbohydrate sulfotransferase 3
rs567754
rs558133
rs10944
rs705415
rs1789953
rs234709
rs6586282
rs3797535
rs6859667
rs11951068
rs672413
rs921943
All
0.0
0.1
MR effect size for
'Selenium' on 'Carbohydrate sulfotransferase 3'

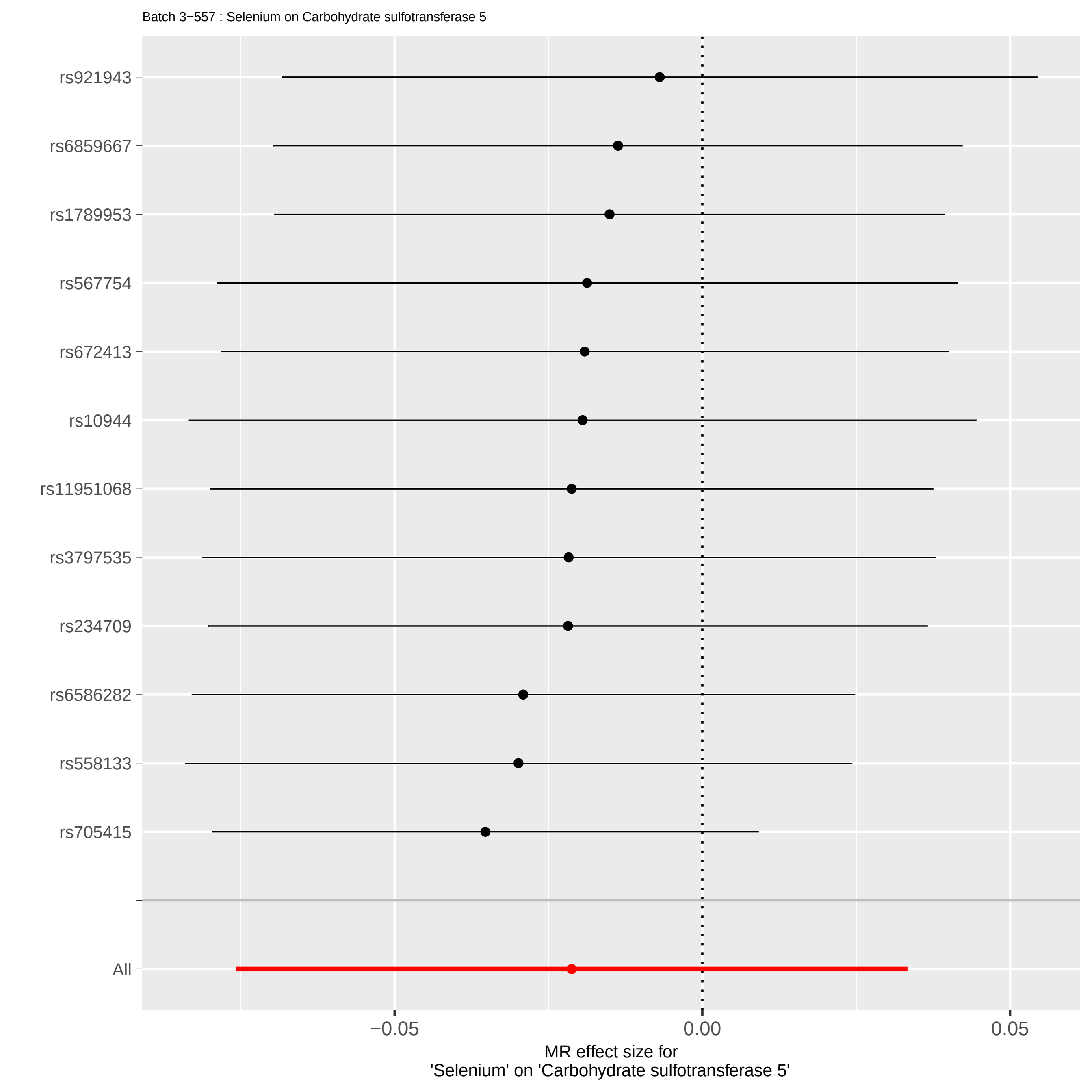
Batch 3−557 : Selenium on Carbohydrate sulfotransferase 5
rs921943
rs6859667
rs1789953
rs567754
rs672413
rs10944
rs11951068
rs3797535
rs234709
rs6586282
rs558133
rs705415
All
−0.05
0.00
0.05
MR effect size for
'Selenium' on 'Carbohydrate sulfotransferase 5'

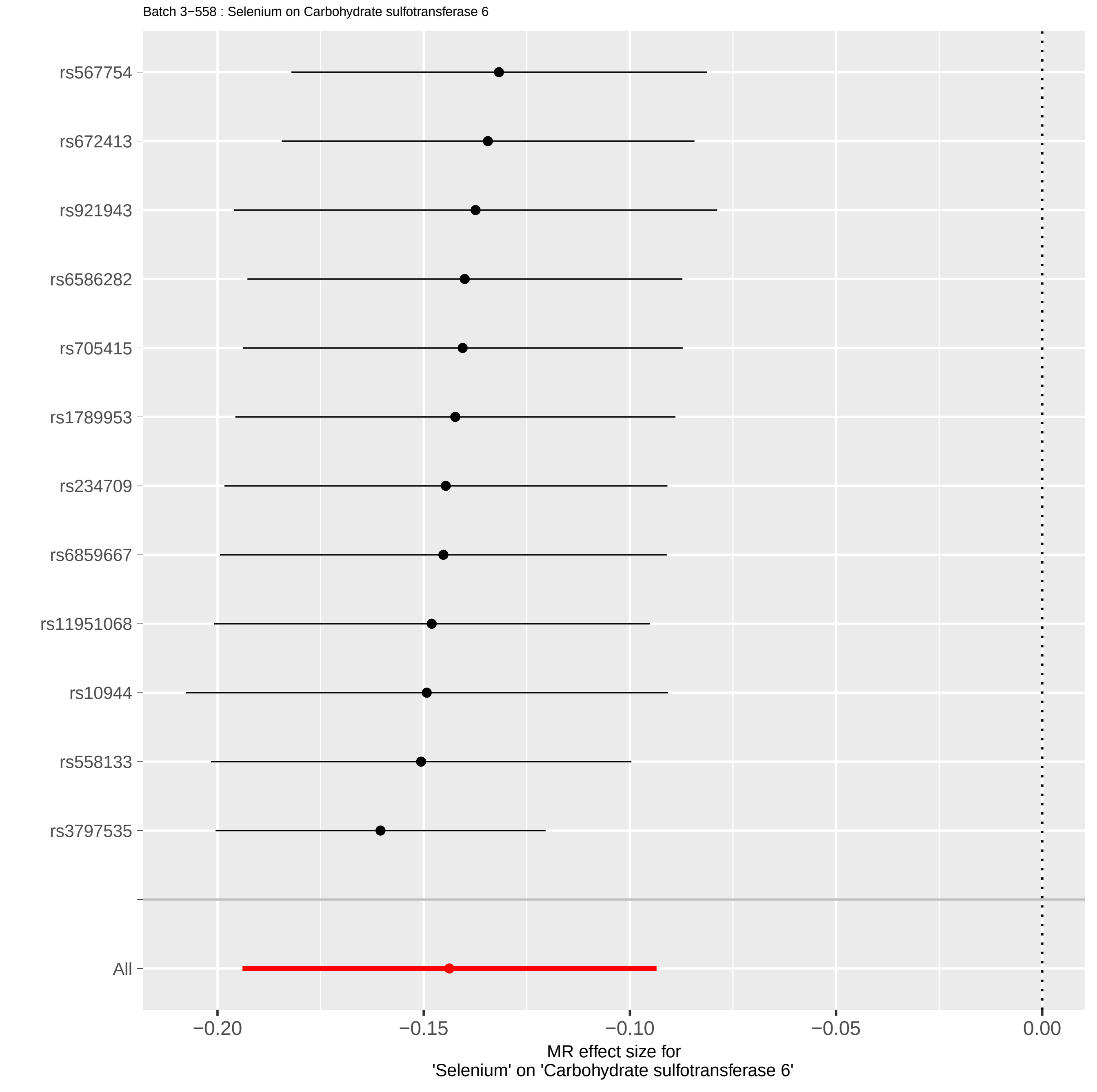
Batch 3−558 : Selenium on Carbohydrate sulfotransferase 6
rs567754
rs672413
rs921943
rs6586282
rs705415
rs1789953
rs234709
rs6859667
rs11951068
rs10944
rs558133
rs3797535
All
−0.20
−0.15
−0.10
−0.05
0.00
MR effect size for
'Selenium' on 'Carbohydrate sulfotransferase 6'

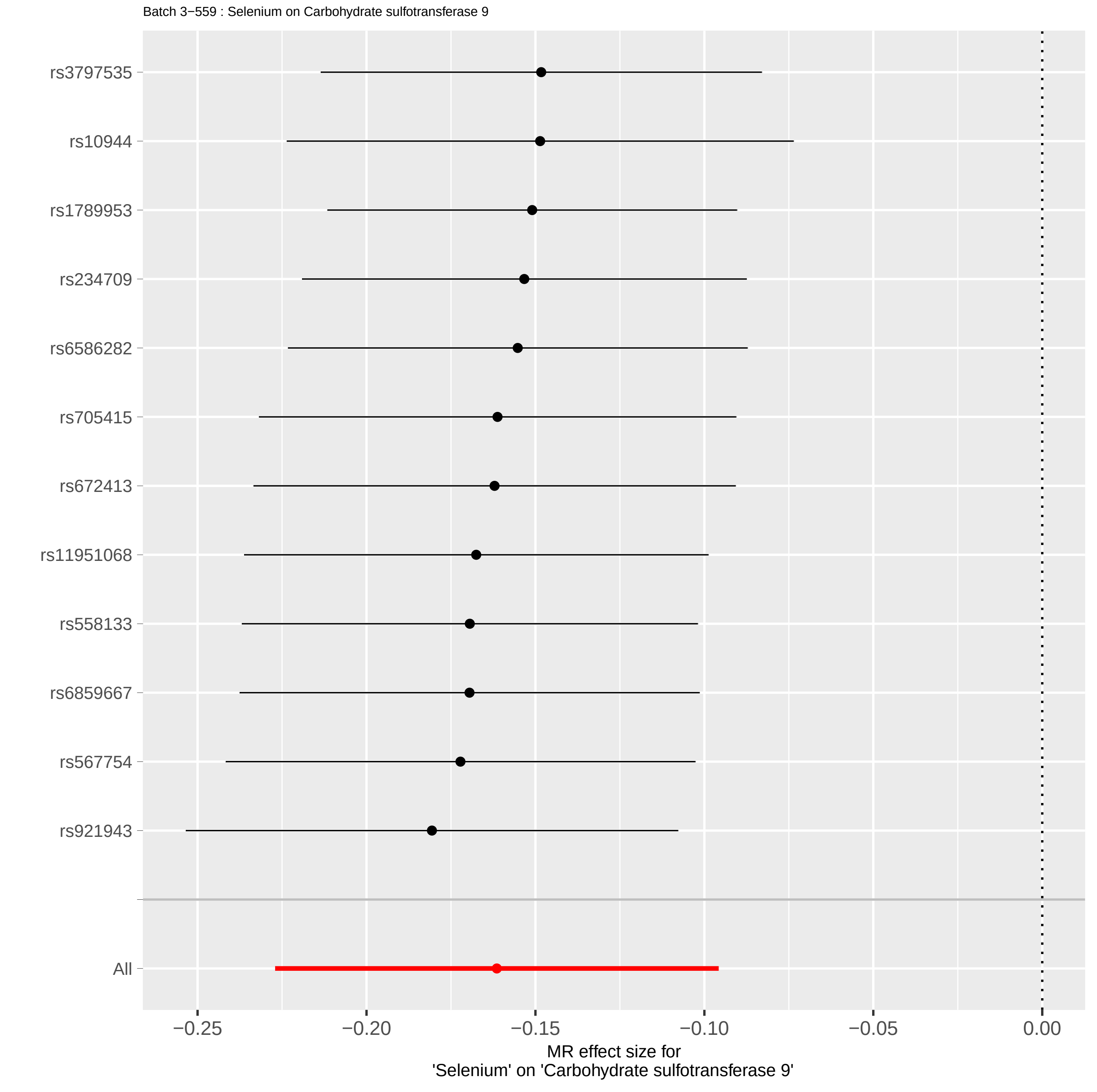

Batch 3−559 : Selenium on Carbohydrate sulfotransferase 9
rs3797535
rs10944
rs1789953
rs234709
rs6586282
rs705415
rs672413
rs11951068
rs558133
rs6859667
rs567754
rs921943
All
−0.25
−0.20
−0.15
−0.10
−0.05
0.00
MR effect size for
'Selenium' on 'Carbohydrate sulfotransferase 9'

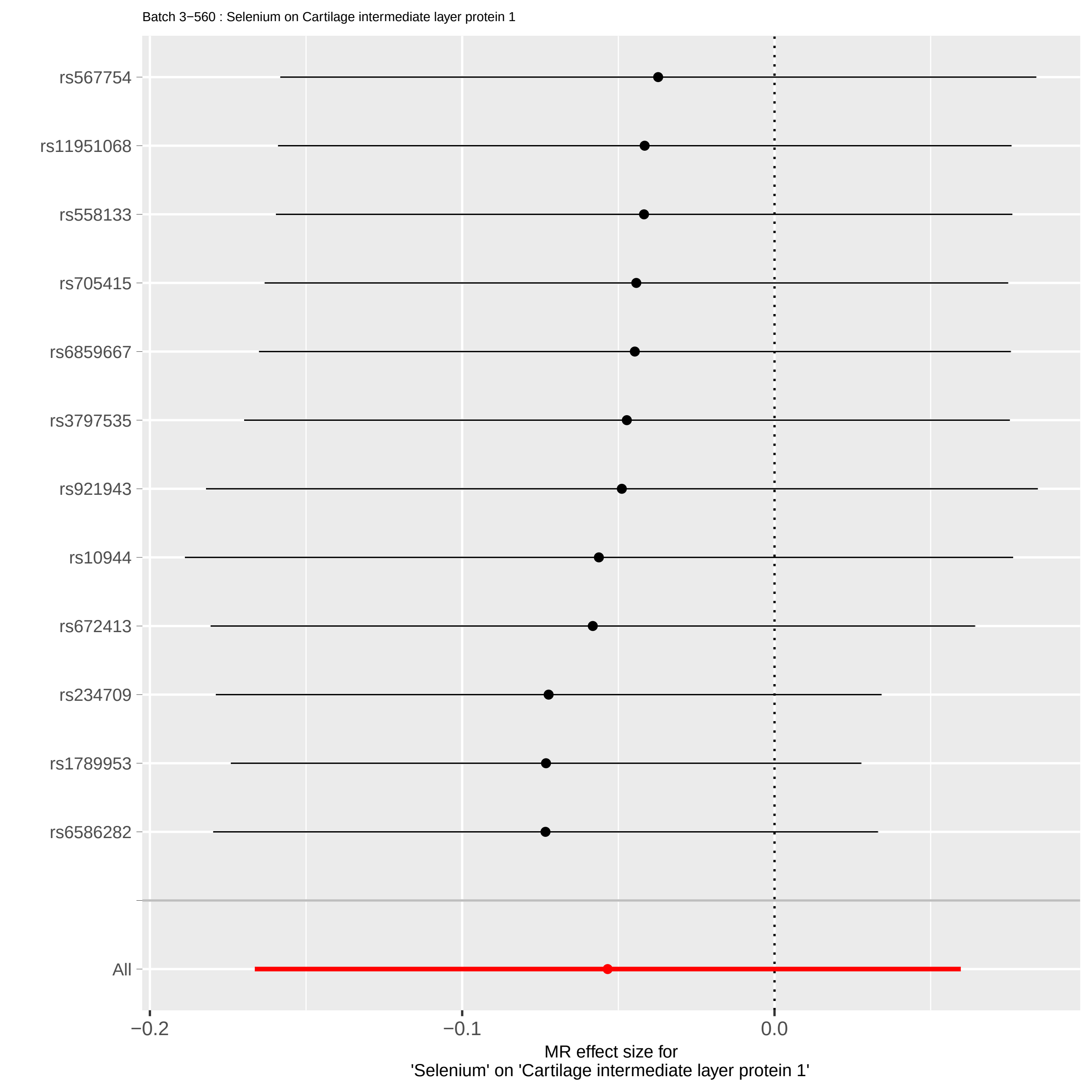
Batch 3−560 : Selenium on Cartilage intermediate layer protein 1
rs567754
rs11951068
rs558133
rs705415
rs6859667
rs3797535
rs921943
rs10944
rs672413
rs234709
rs1789953
rs6586282
All
−0.2
−0.1
0.0
MR effect size for
'Selenium' on 'Cartilage intermediate layer protein 1'

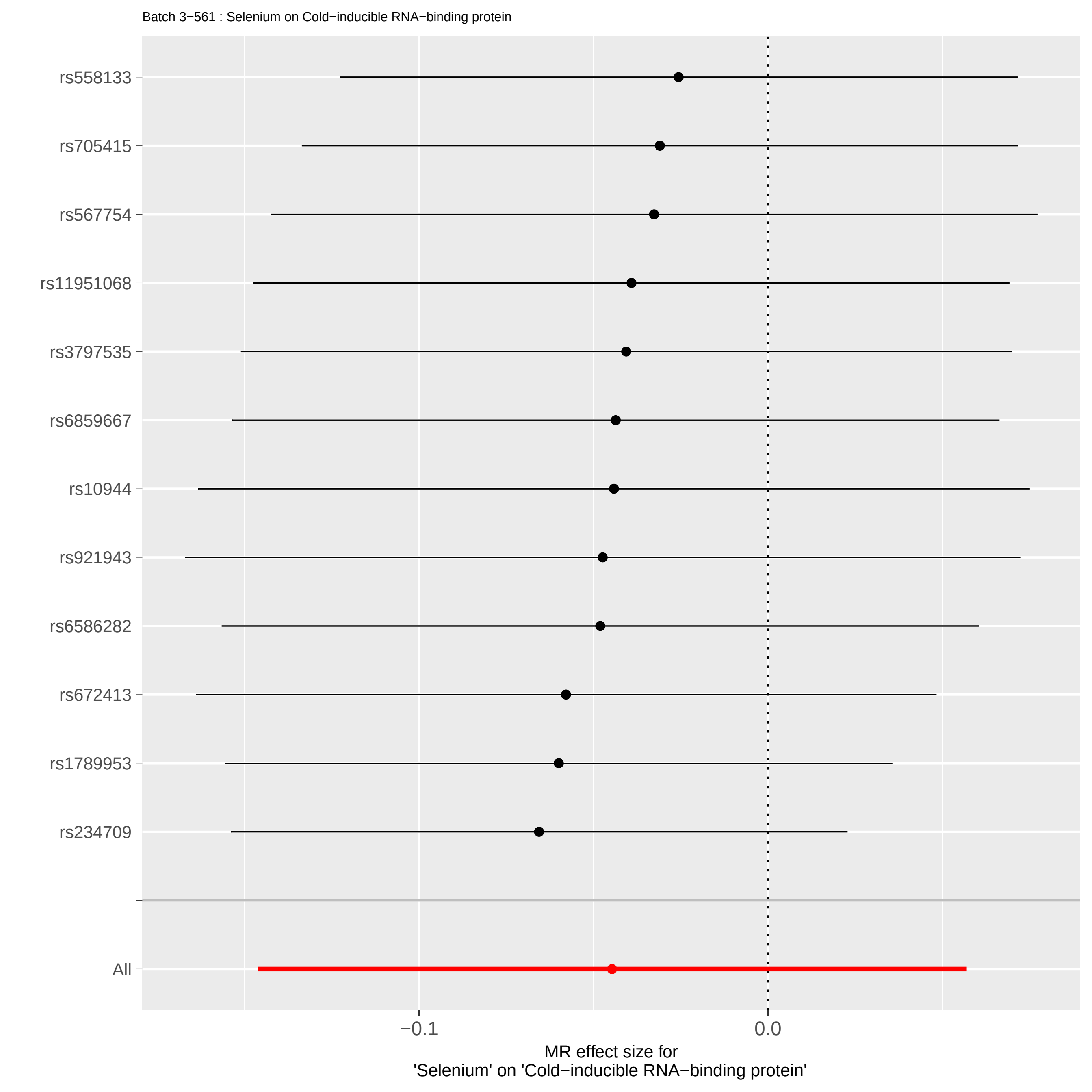

Batch 3−561 : Selenium on Cold−inducible RNA−binding protein
rs558133
rs705415
rs567754
rs11951068
rs3797535
rs6859667
rs10944
rs921943
rs6586282
rs672413
rs1789953
rs234709
All
−0.1
0.0
MR effect size for
'Selenium' on 'Cold−inducible RNA−binding protein'

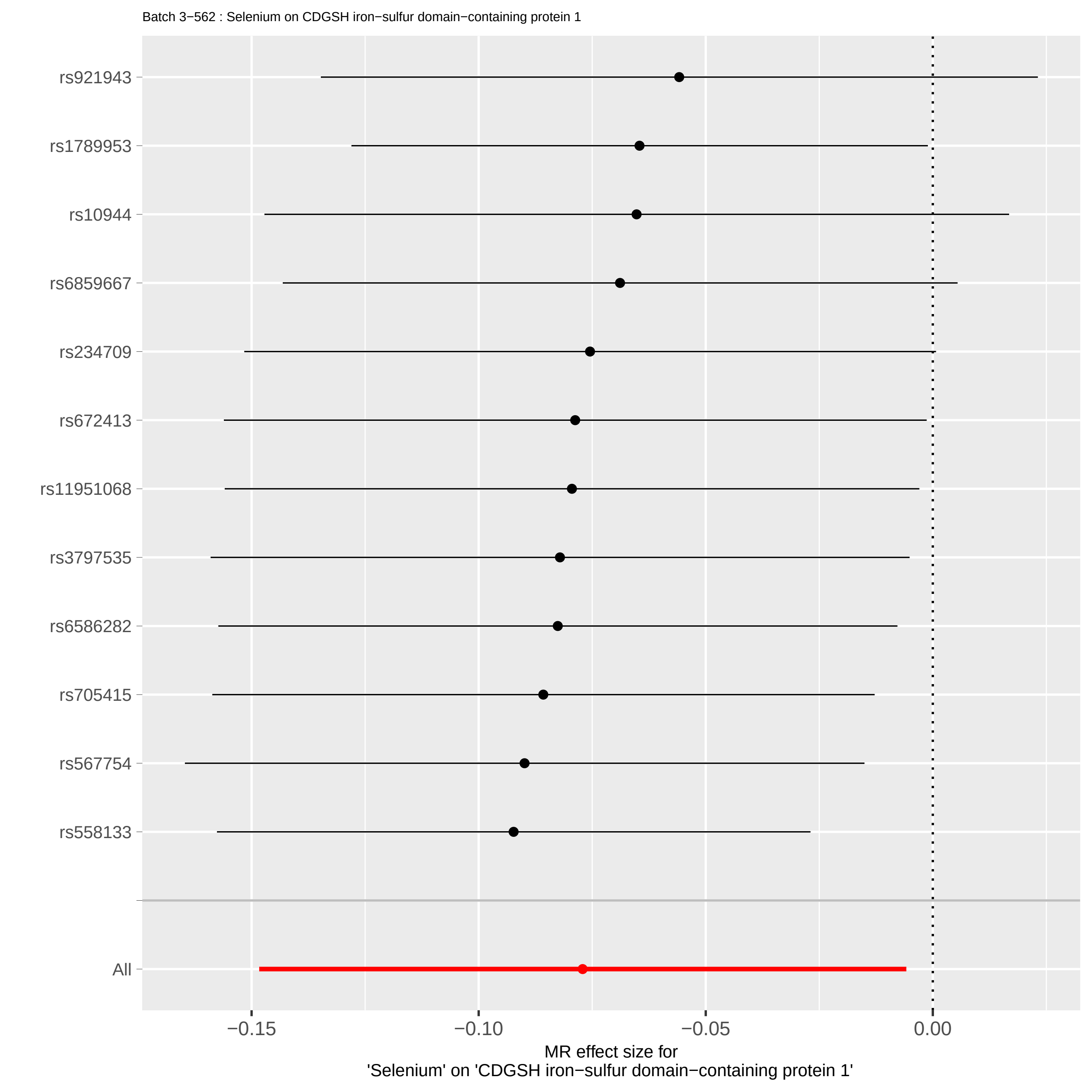
Batch 3−562 : Selenium on CDGSH iron−sulfur domain−containing protein 1
rs921943
rs1789953
rs10944
rs6859667
rs234709
rs672413
rs11951068
rs3797535
rs6586282
rs705415
rs567754
rs558133
All
−0.15
−0.10
−0.05
0.00
MR effect size for
'Selenium' on 'CDGSH iron−sulfur domain−containing protein 1'

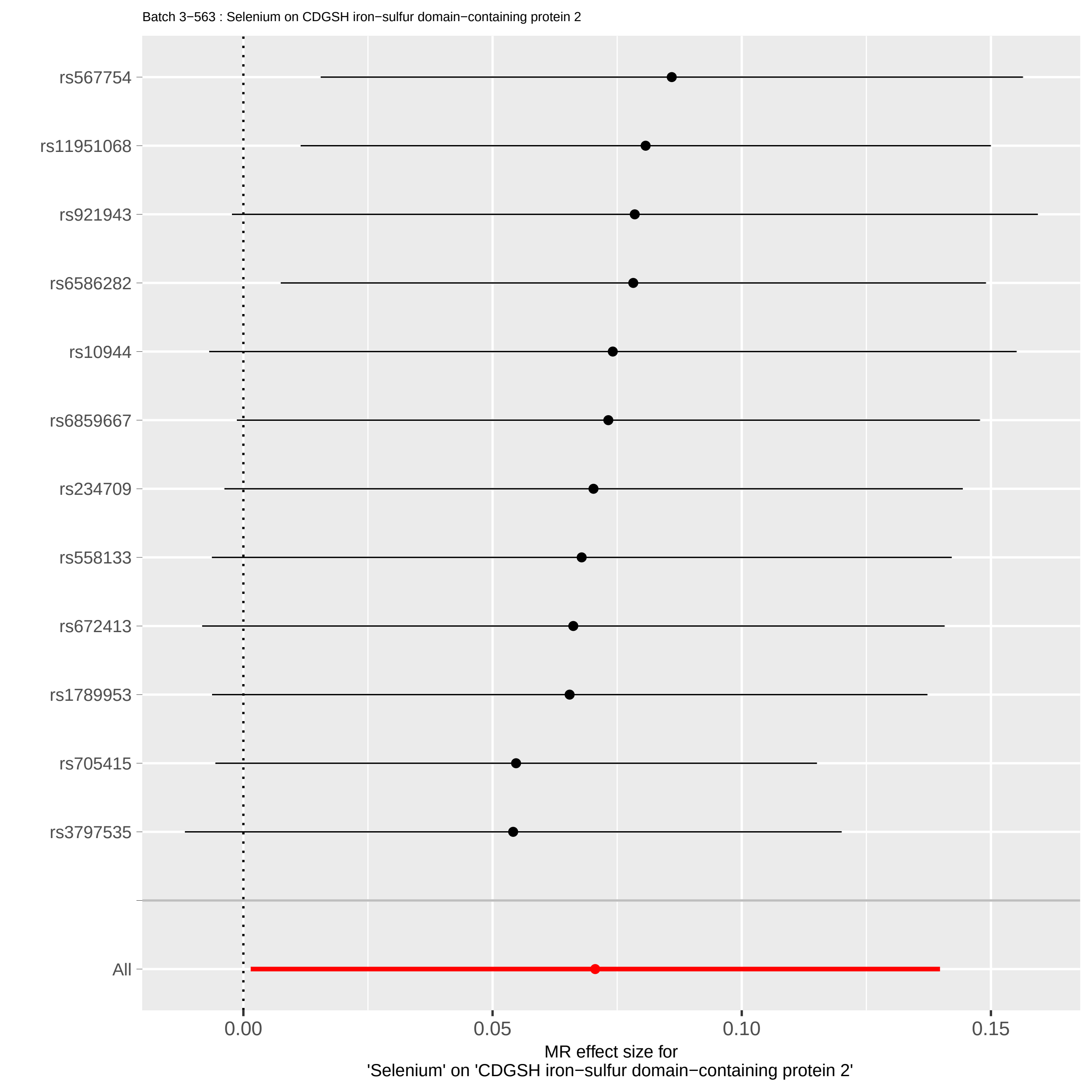

Batch 3−563 : Selenium on CDGSH iron−sulfur domain−containing protein 2
rs567754
rs11951068
rs921943
rs6586282
rs10944
rs6859667
rs234709
rs558133
rs672413
rs1789953
rs705415
rs3797535
All
0.00
0.05
0.10
0.15
MR effect size for
'Selenium' on 'CDGSH iron−sulfur domain−containing protein 2'

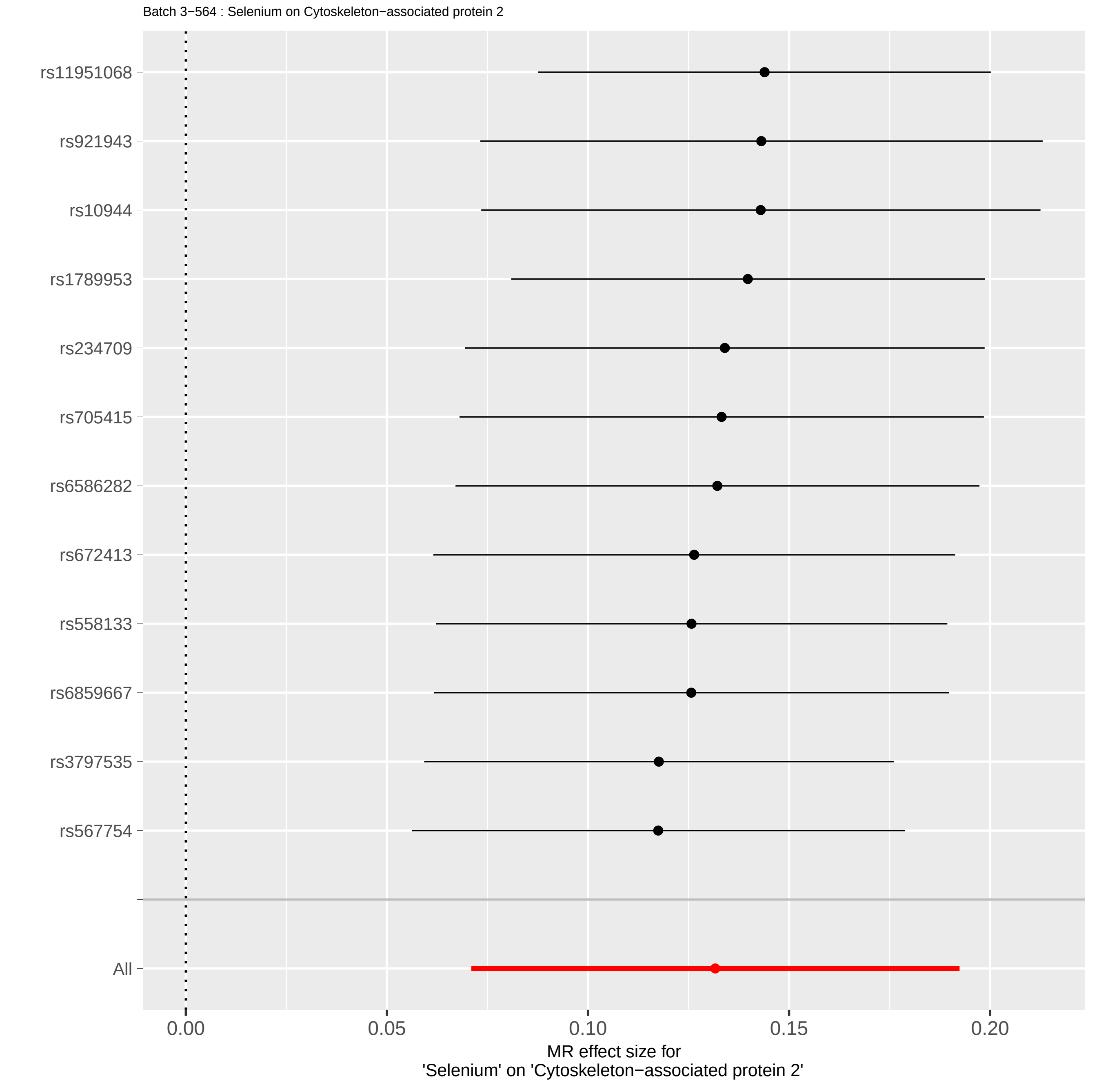

Batch 3−564 : Selenium on Cytoskeleton−associated protein 2
rs11951068
rs921943
rs10944
rs1789953
rs234709
rs705415
rs6586282
rs672413
rs558133
rs6859667
rs3797535
rs567754
All
0.00
0.05
0.10
0.15
0.20
MR effect size for
'Selenium' on 'Cytoskeleton−associated protein 2'

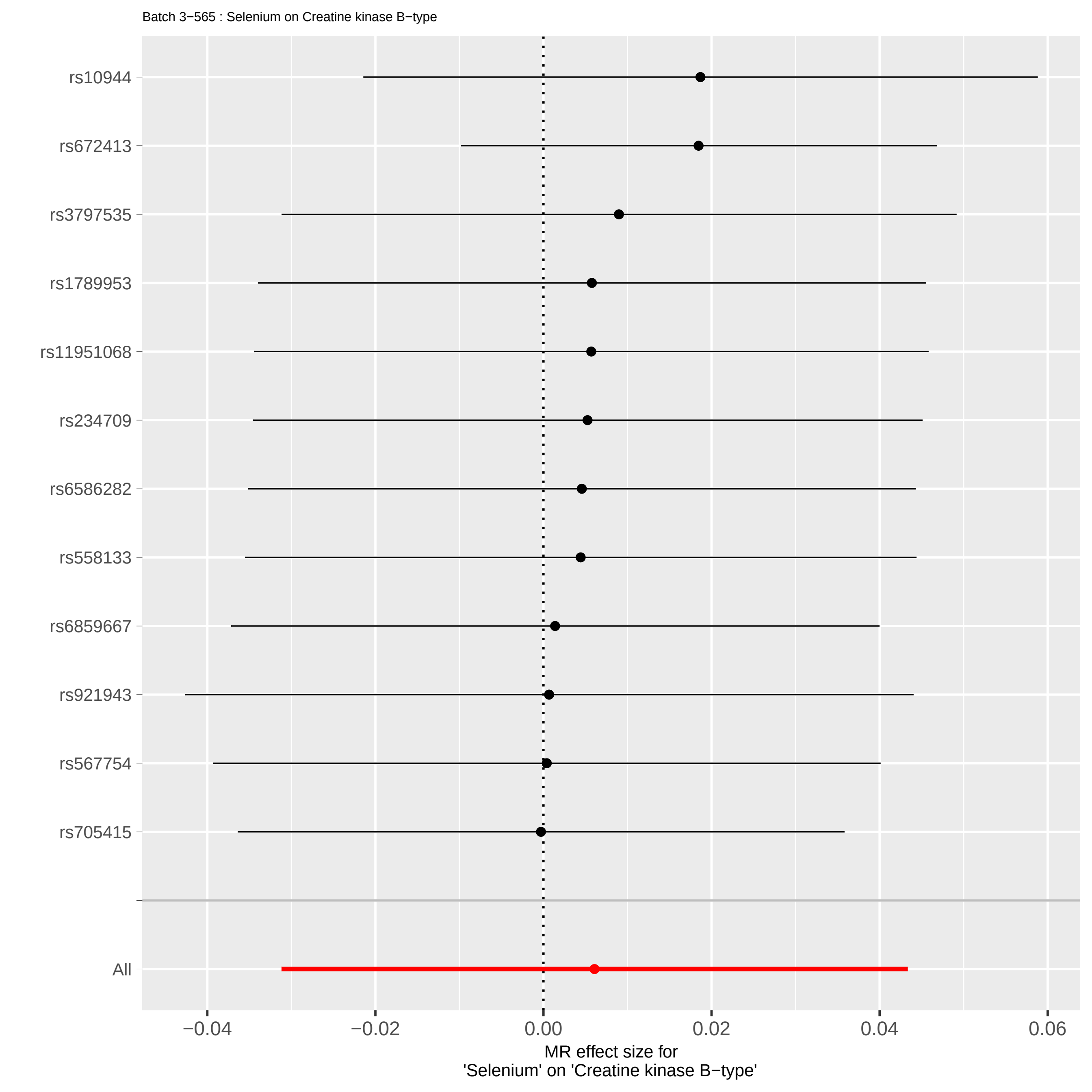

Batch 3−565 : Selenium on Creatine kinase B−type
rs10944
rs672413
rs3797535
rs1789953
rs11951068
rs234709
rs6586282
rs558133
rs6859667
rs921943
rs567754
rs705415
All
−0.04
−0.02
0.00
0.02
0.04
0.06
MR effect size for
'Selenium' on 'Creatine kinase B−type'

Batch 3−566 : Selenium on Creatine kinase M−type

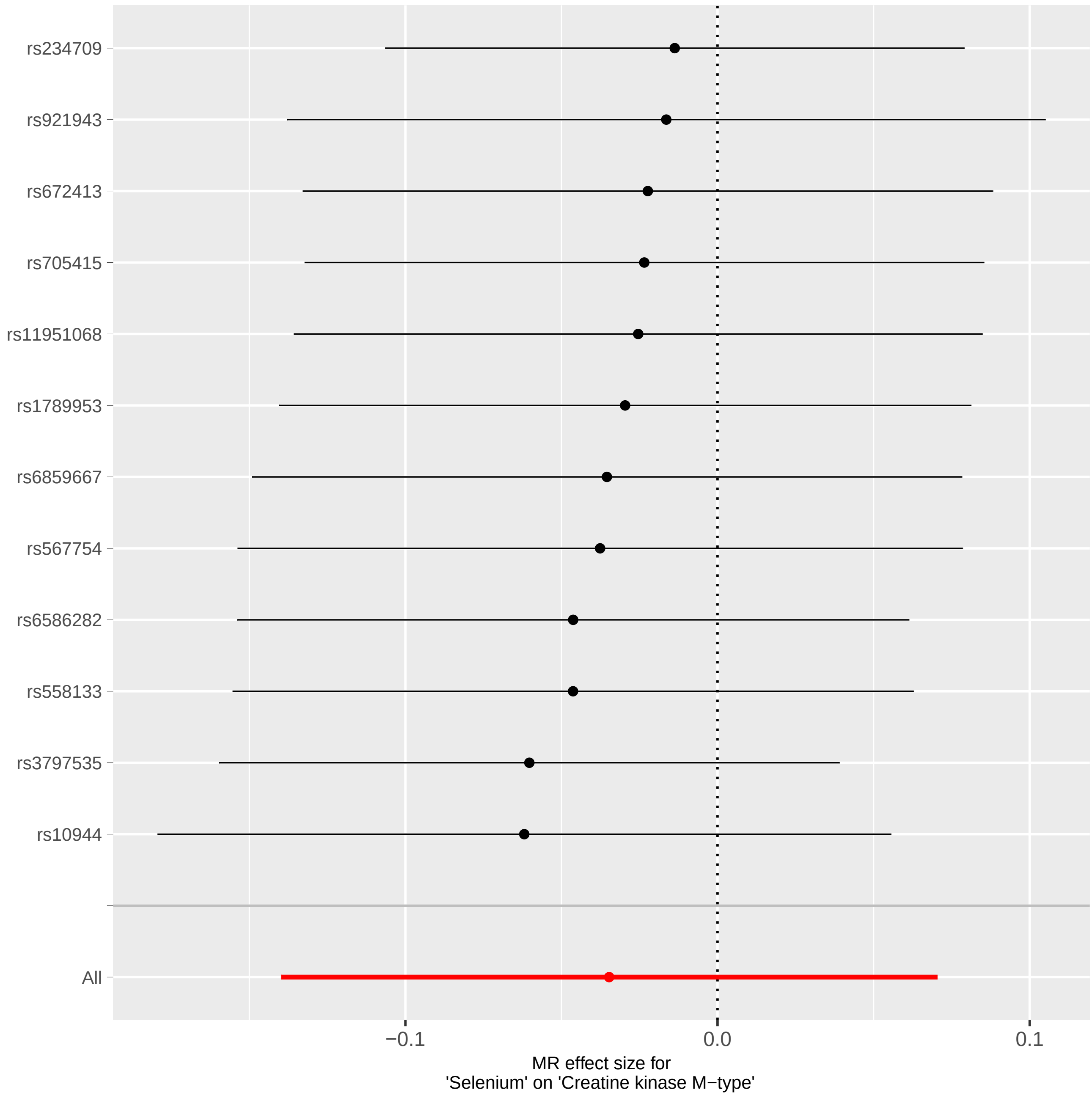

Batch 3−567 : Selenium on Galectin−10

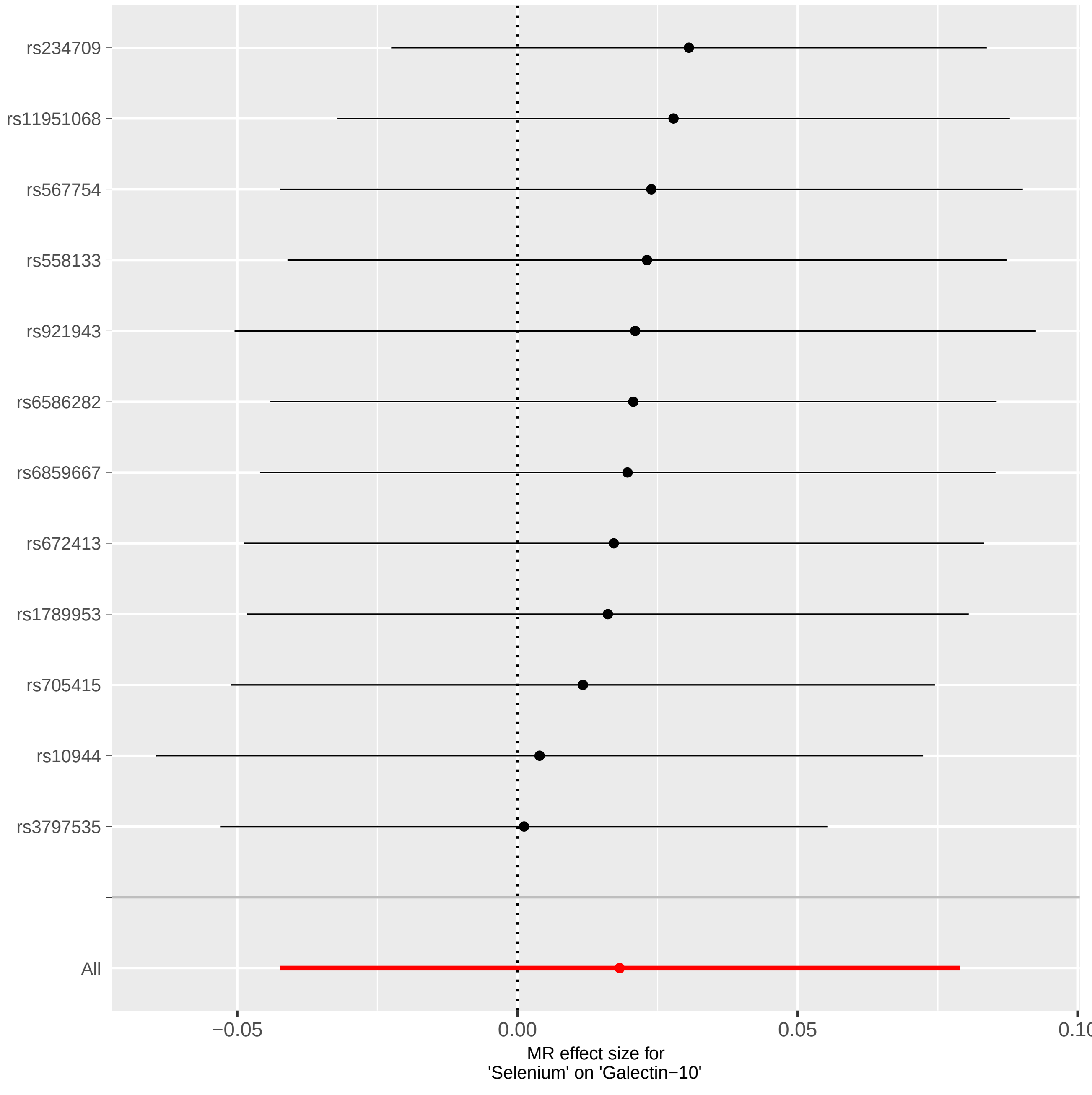

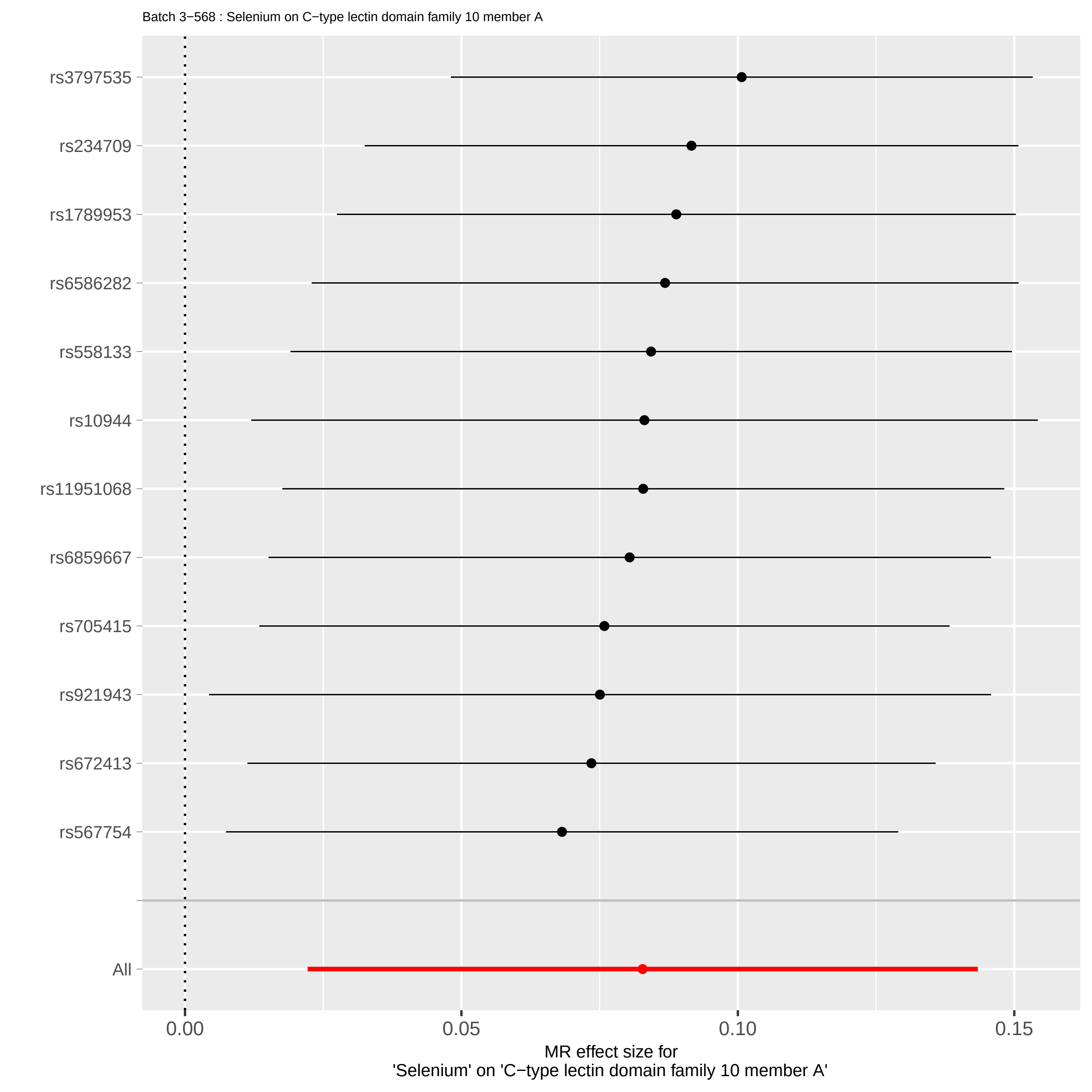

Batch 3−568 : Selenium on C−type lectin domain family 10 member A
rs3797535
rs234709
rs1789953
rs6586282
rs558133
rs10944
rs11951068
rs6859667
rs705415
rs921943
rs672413
rs567754
All
0.00
0.05
0.10
0.15
MR effect size for
'Selenium' on 'C−type lectin domain family 10 member A'

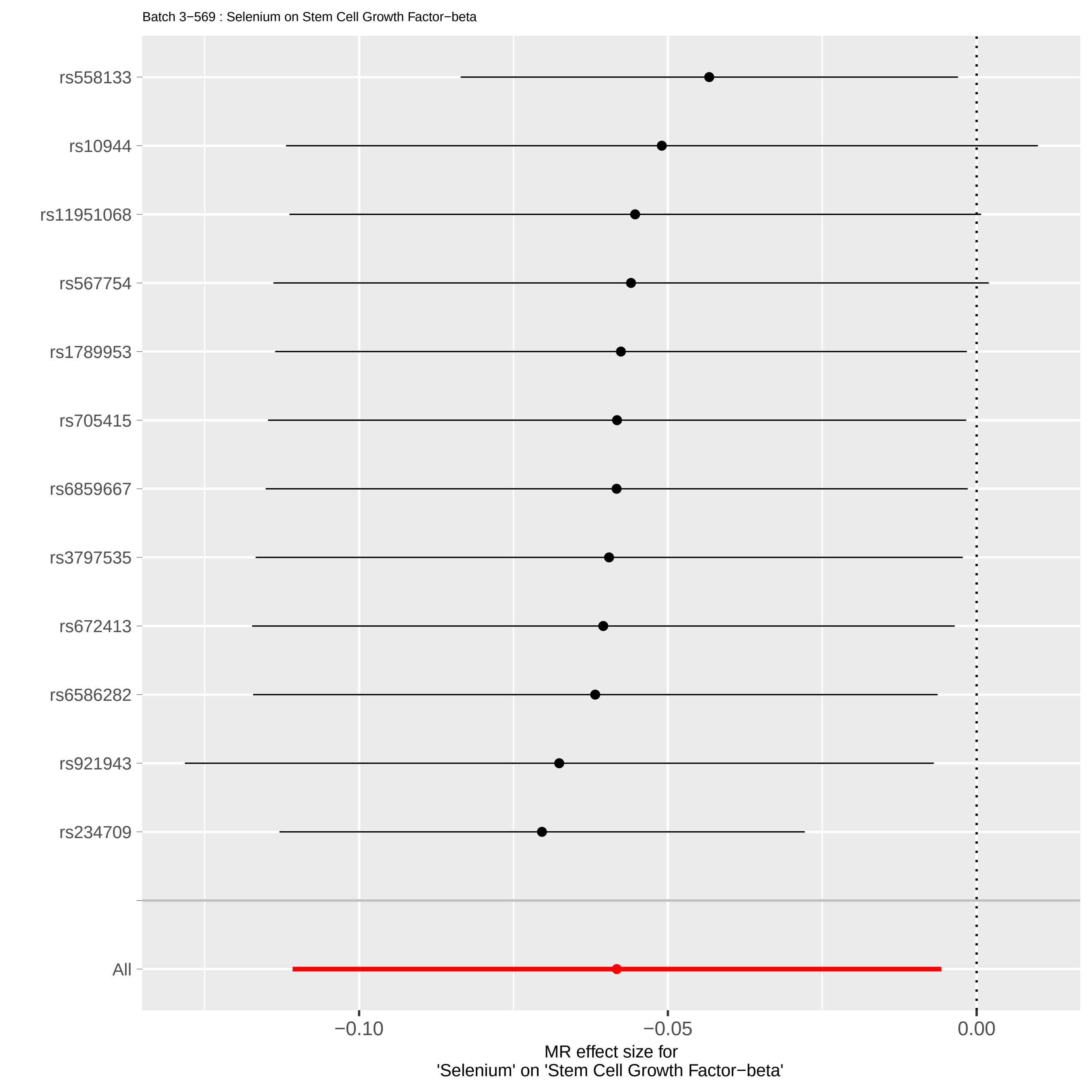

Batch 3−569 : Selenium on Stem Cell Growth Factor−beta
rs558133
rs10944
rs11951068
rs567754
rs1789953
rs705415
rs6859667
rs3797535
rs672413
rs6586282
rs921943
rs234709
All
−0.10
−0.05
0.00
MR effect size for
'Selenium' on 'Stem Cell Growth Factor−beta'

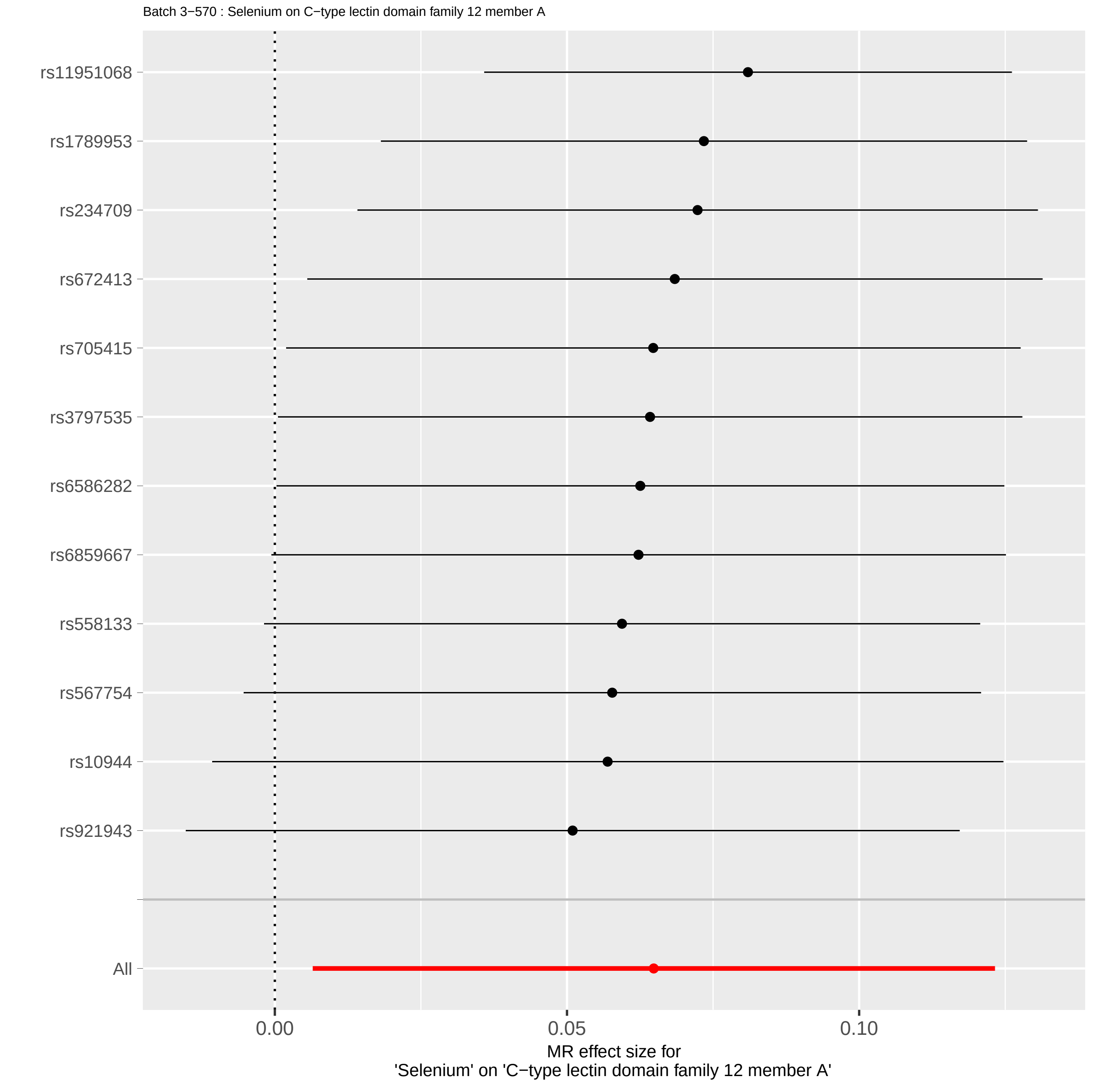
Batch 3−570 : Selenium on C−type lectin domain family 12 member A
rs11951068
rs1789953
rs234709
rs672413
rs705415
rs3797535
rs6586282
rs6859667
rs558133
rs567754
rs10944
rs921943
All
0.00
0.05
0.10
MR effect size for
'Selenium' on 'C−type lectin domain family 12 member A'

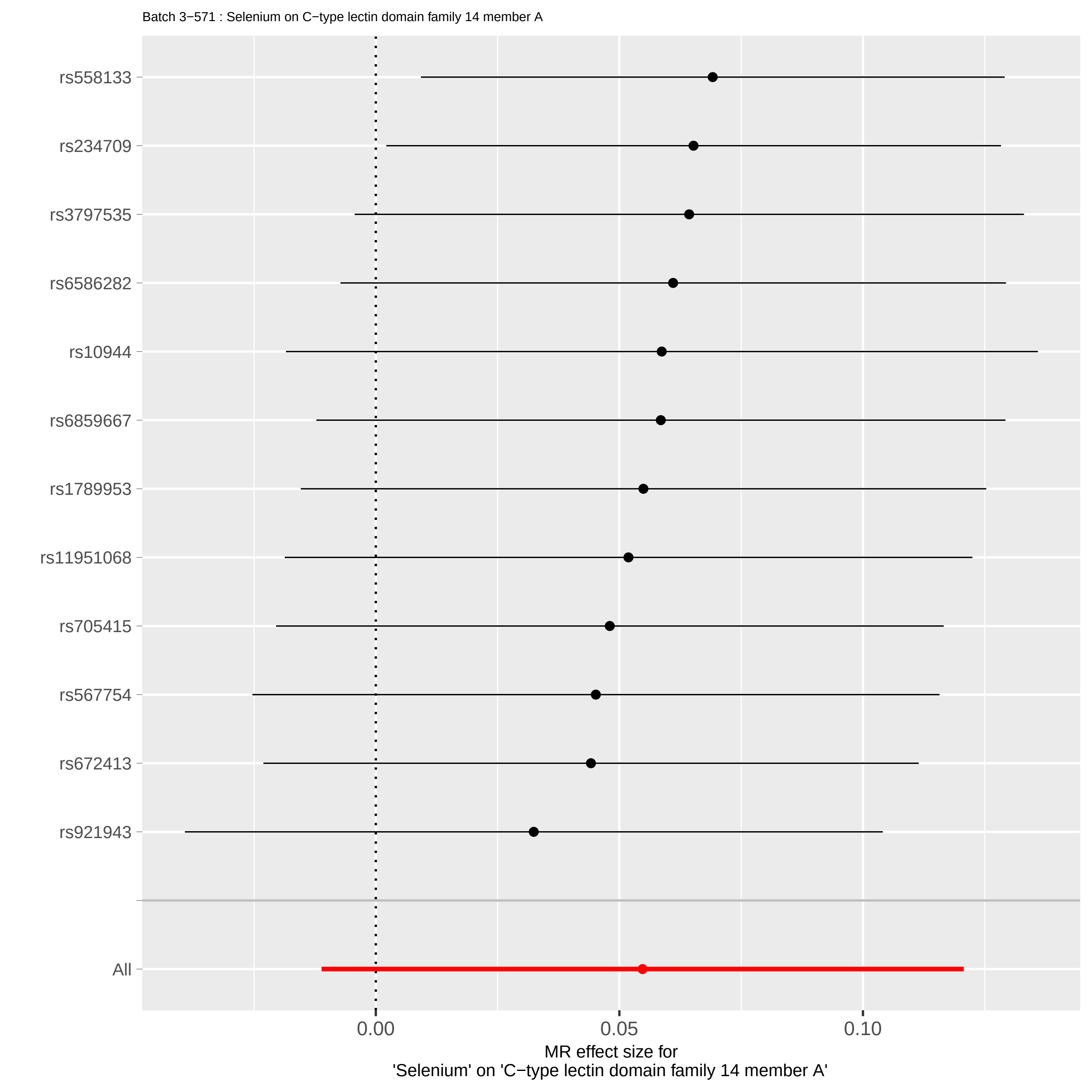

Batch 3−571 : Selenium on C−type lectin domain family 14 member A
rs558133
rs234709
rs3797535
rs6586282
rs10944
rs6859667
rs1789953
rs11951068
rs705415
rs567754
rs672413
rs921943
All
0.00
0.05
0.10
MR effect size for
'Selenium' on 'C−type lectin domain family 14 member A'

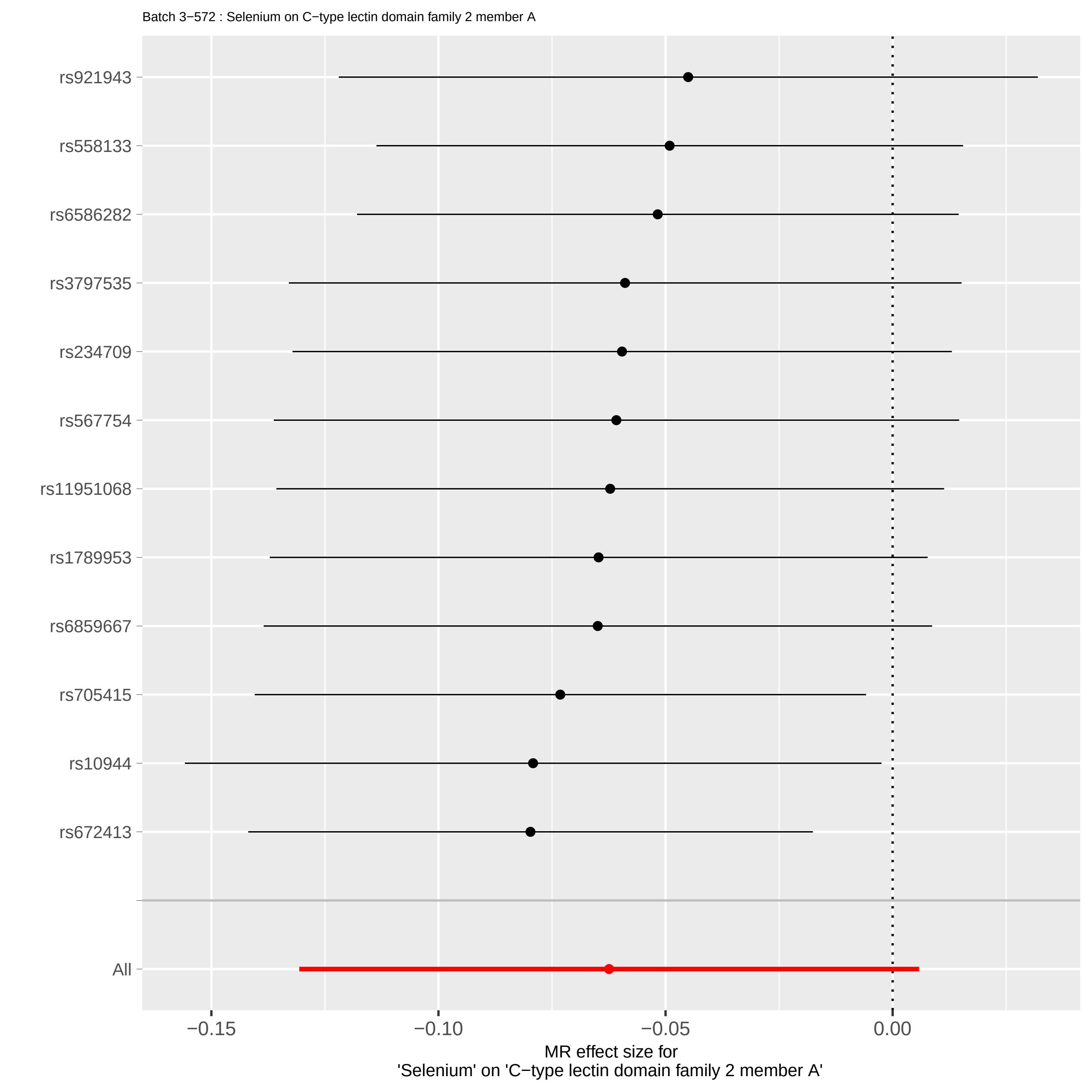

Batch 3−572 : Selenium on C−type lectin domain family 2 member A
rs921943
rs558133
rs6586282
rs3797535
rs234709
rs567754
rs11951068
rs1789953
rs6859667
rs705415
rs10944
rs672413
All
−0.15
−0.10
−0.05
0.00
MR effect size for
'Selenium' on 'C−type lectin domain family 2 member A'

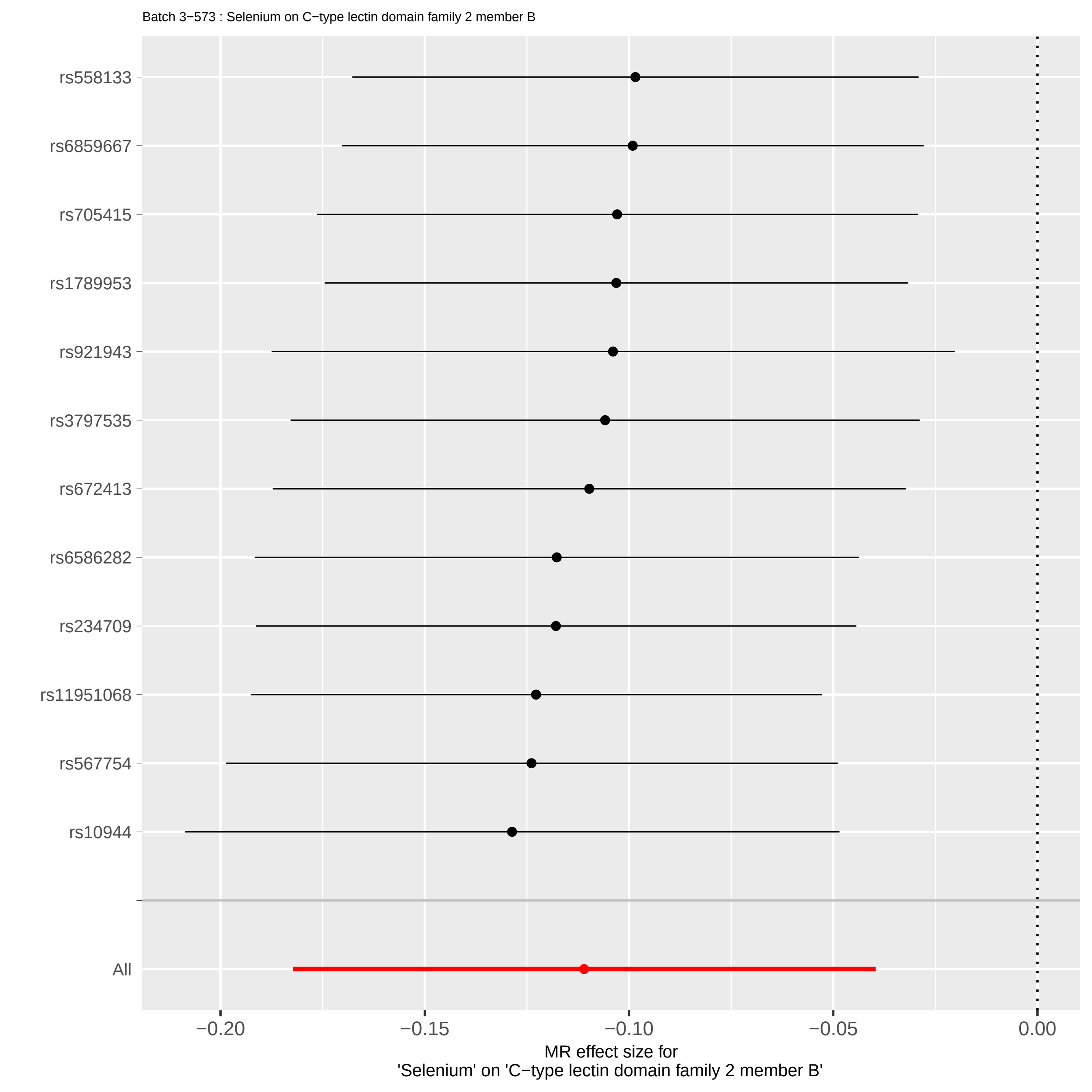
Batch 3−573 : Selenium on C−type lectin domain family 2 member B
rs558133
rs6859667
rs705415
rs1789953
rs921943
rs3797535
rs672413
rs6586282
rs234709
rs11951068
rs567754
rs10944
All
−0.20
−0.15
−0.10
−0.05
0.00
MR effect size for
'Selenium' on 'C−type lectin domain family 2 member B'

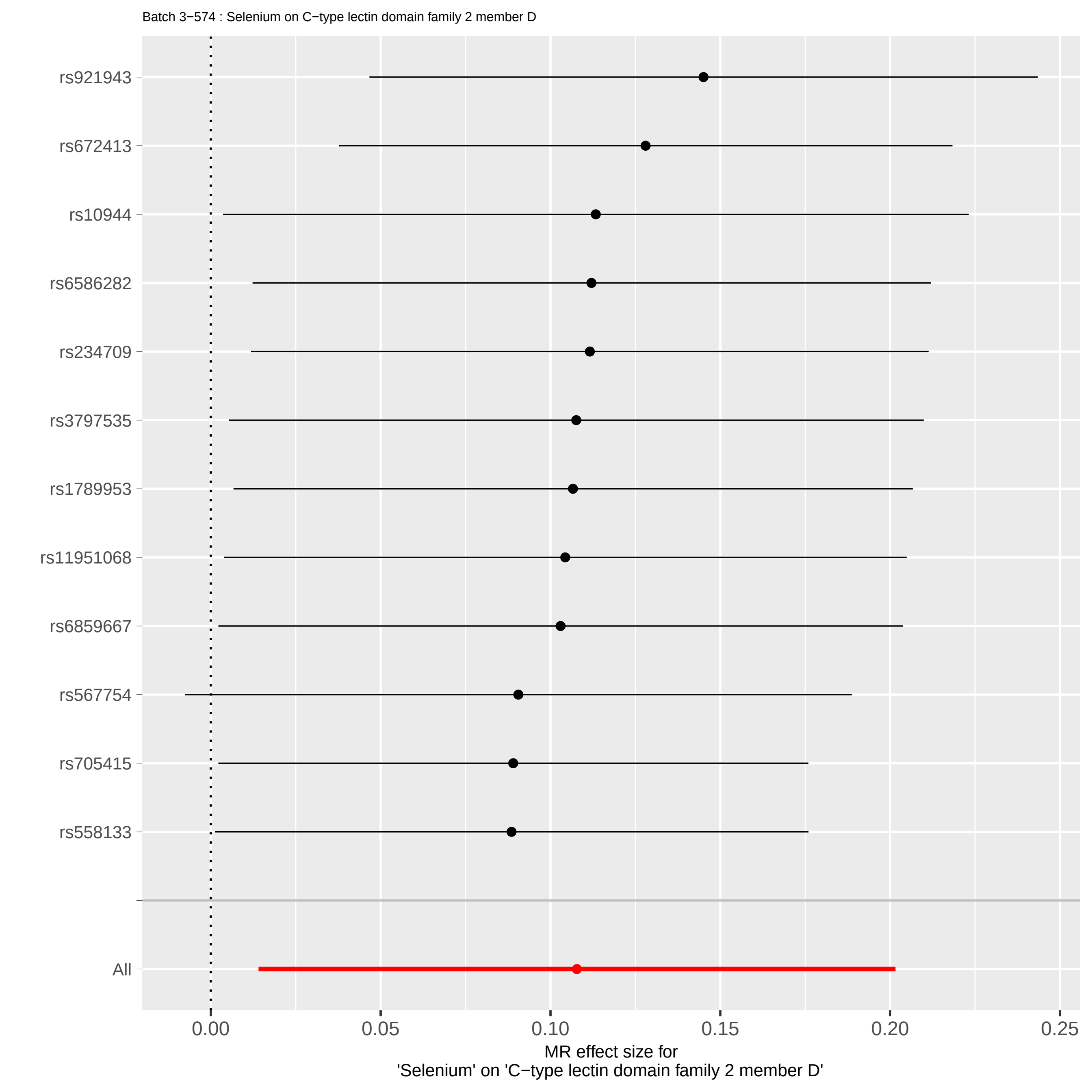

Batch 3−574 : Selenium on C−type lectin domain family 2 member D
rs921943
rs672413
rs10944
rs6586282
rs234709
rs3797535
rs1789953
rs11951068
rs6859667
rs567754
rs705415
rs558133
All
0.00
0.05
0.10
0.15
0.20
0.25
MR effect size for
'Selenium' on 'C−type lectin domain family 2 member D'

Batch 3−575 : Selenium on C−type lectin domain family 2 member L

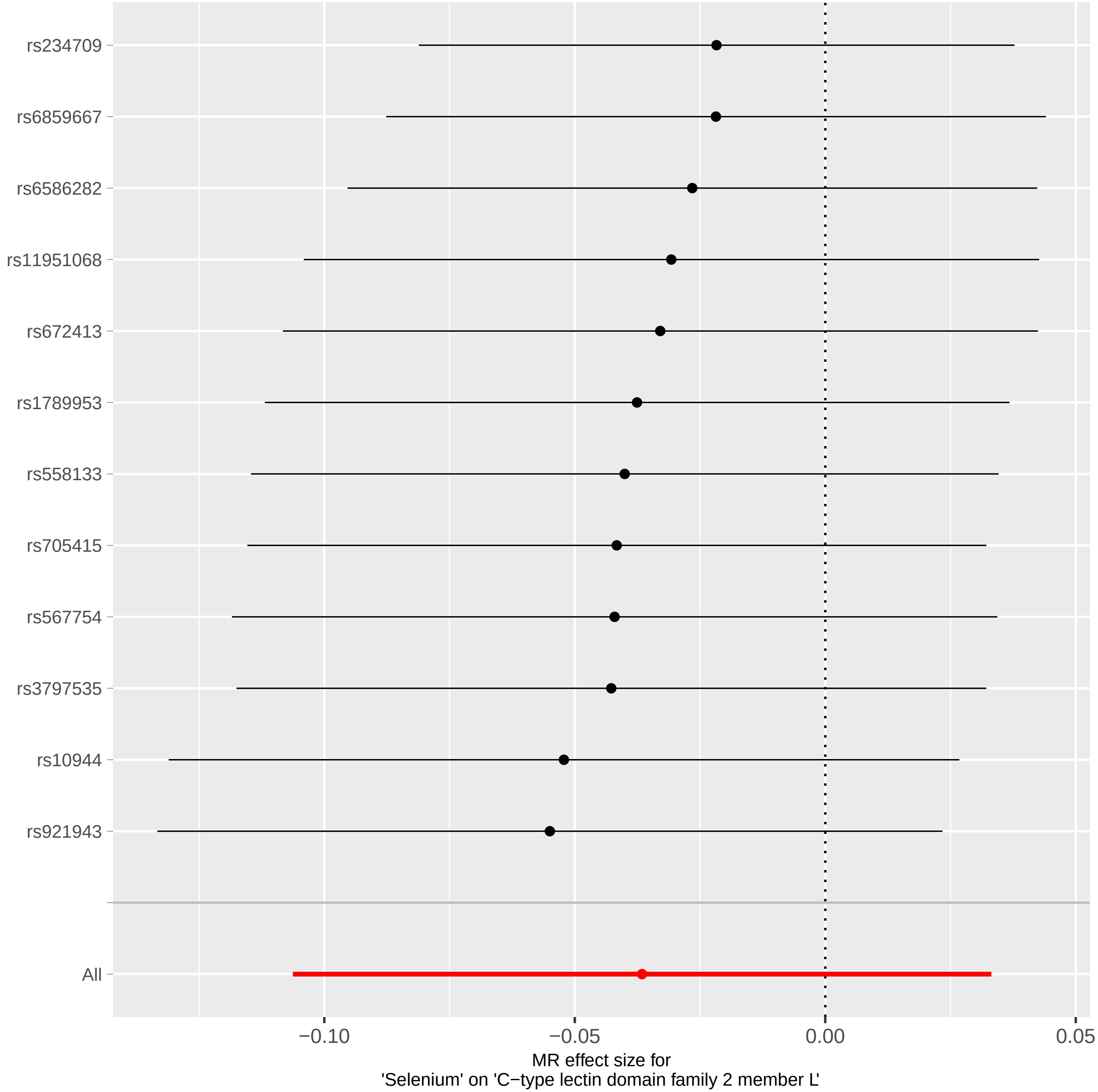

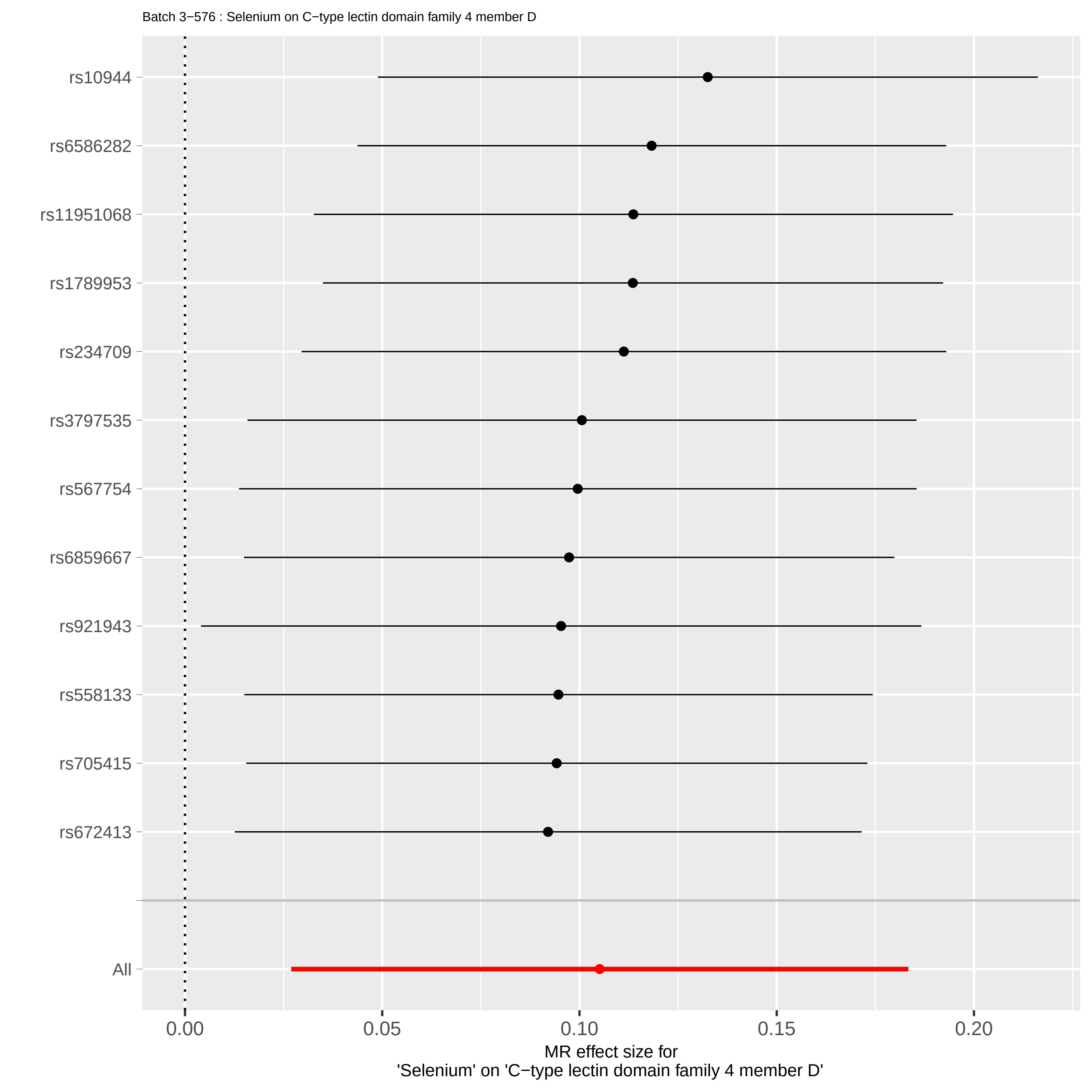

Batch 3−576 : Selenium on C−type lectin domain family 4 member D
rs10944
rs6586282
rs11951068
rs1789953
rs234709
rs3797535
rs567754
rs6859667
rs921943
rs558133
rs705415
rs672413
All
0.00
0.05
0.10
0.15
0.20
MR effect size for
'Selenium' on 'C−type lectin domain family 4 member D'

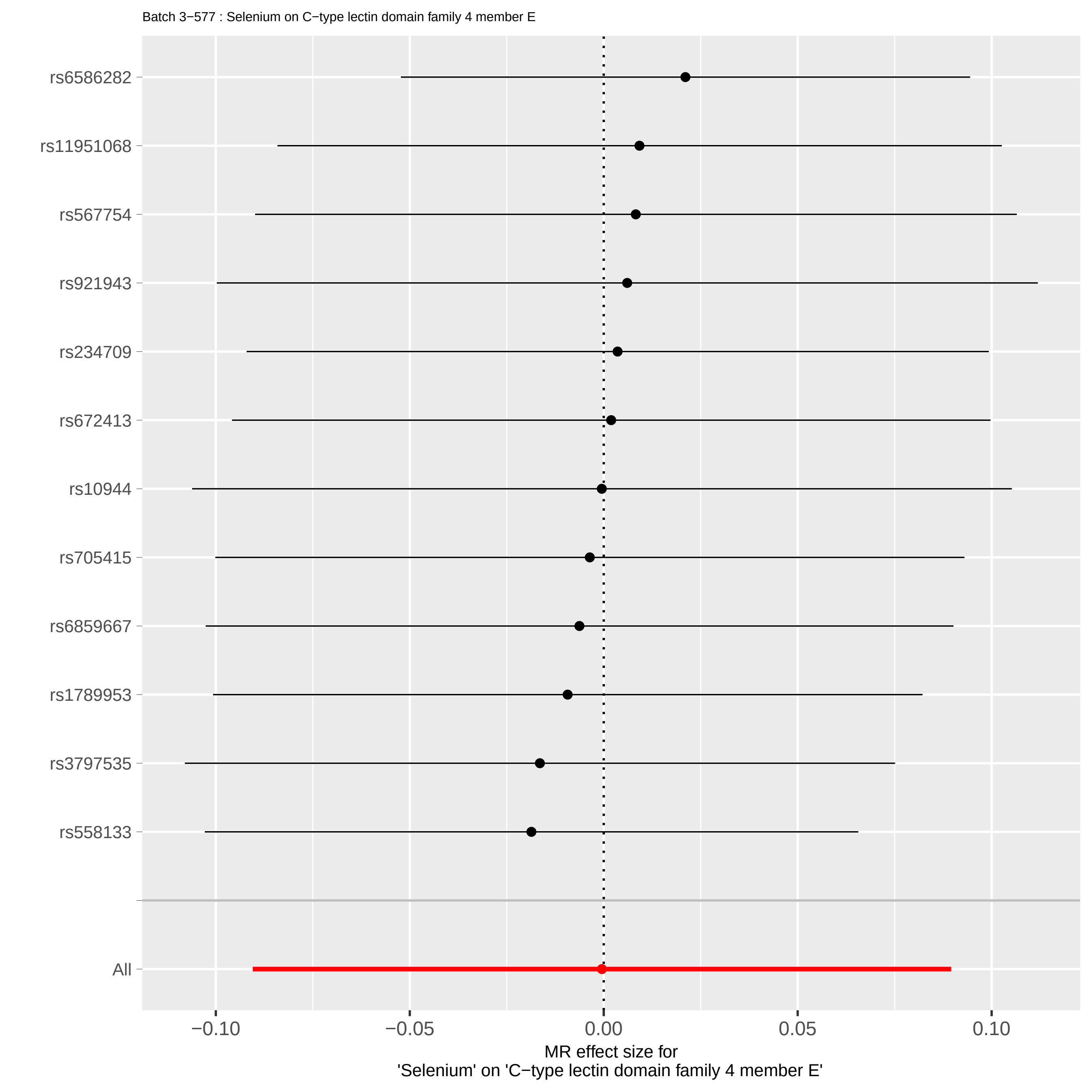
Batch 3−577 : Selenium on C−type lectin domain family 4 member E
rs6586282
rs11951068
rs567754
rs921943
rs234709
rs672413
rs10944
rs705415
rs6859667
rs1789953
rs3797535
rs558133
All
−0.10
−0.05
0.00
0.05
0.10
MR effect size for
'Selenium' on 'C−type lectin domain family 4 member E'

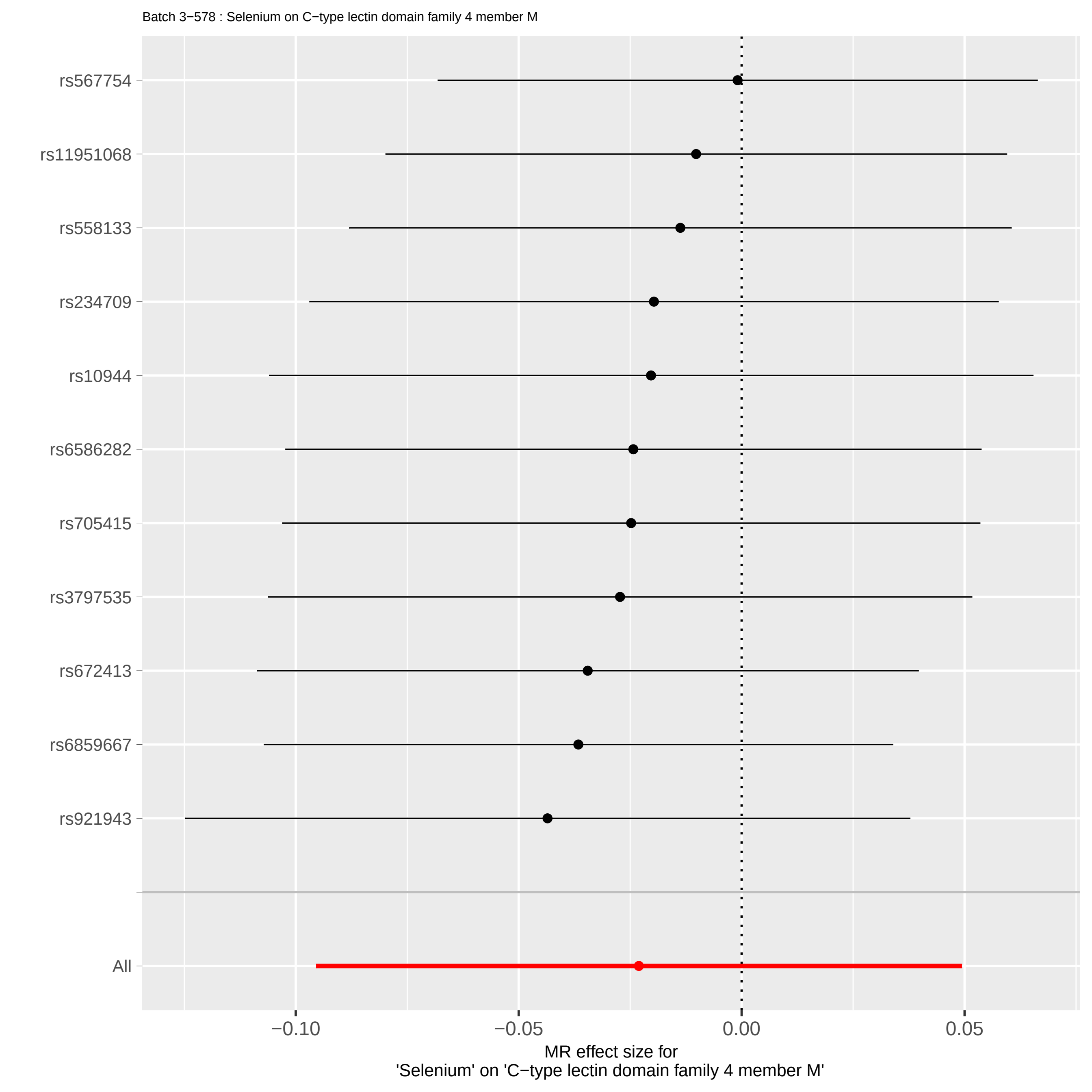

Batch 3−578 : Selenium on C−type lectin domain family 4 member M
rs567754
rs11951068
rs558133
rs234709
rs10944
rs6586282
rs705415
rs3797535
rs672413
rs6859667
rs921943
All
−0.10
−0.05
0.00
0.05
MR effect size for
'Selenium' on 'C−type lectin domain family 4 member M'

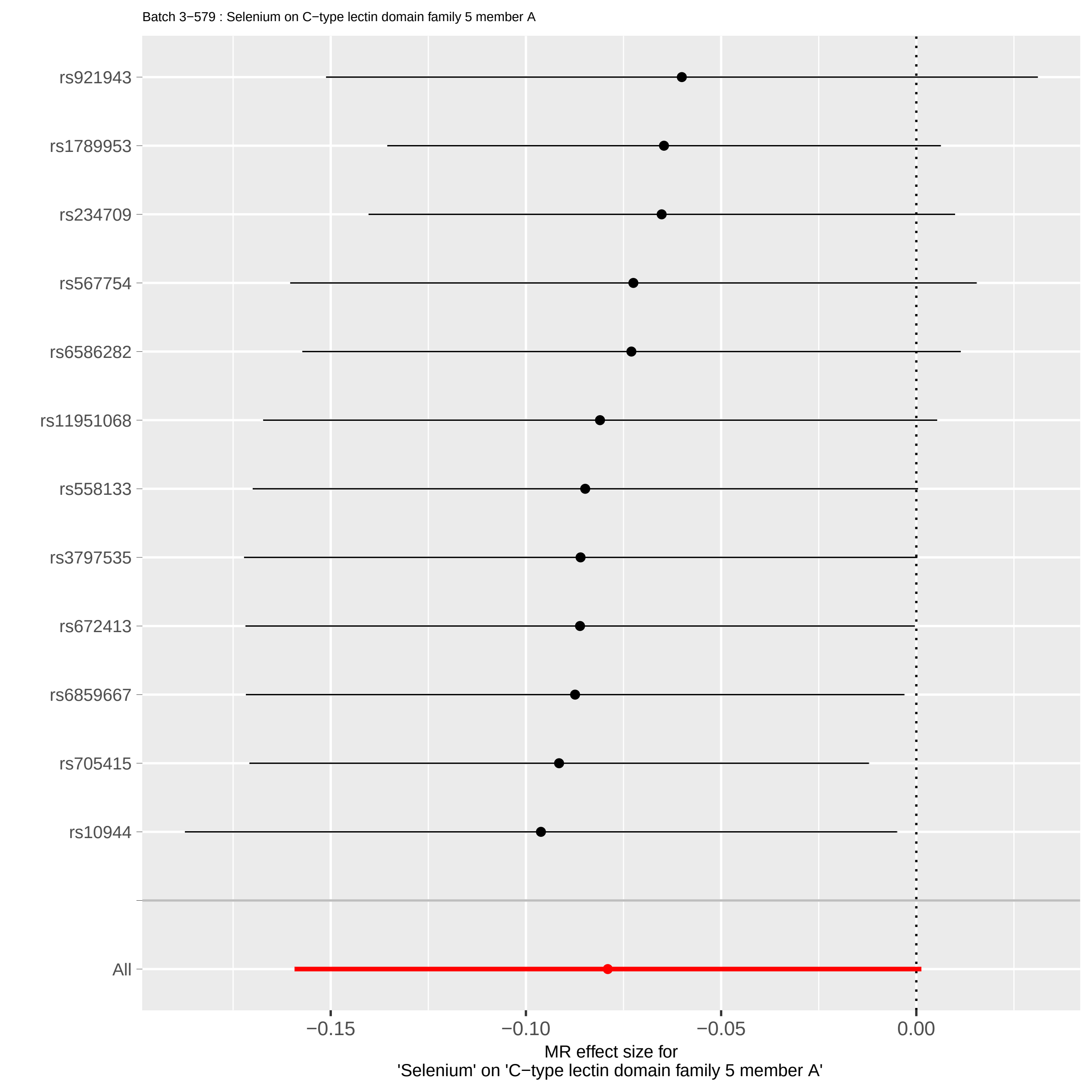

Batch 3−579 : Selenium on C−type lectin domain family 5 member A
rs921943
rs1789953
rs234709
rs567754
rs6586282
rs11951068
rs558133
rs3797535
rs672413
rs6859667
rs705415
rs10944
All
−0.15
−0.10
−0.05
0.00
MR effect size for
'Selenium' on 'C−type lectin domain family 5 member A'

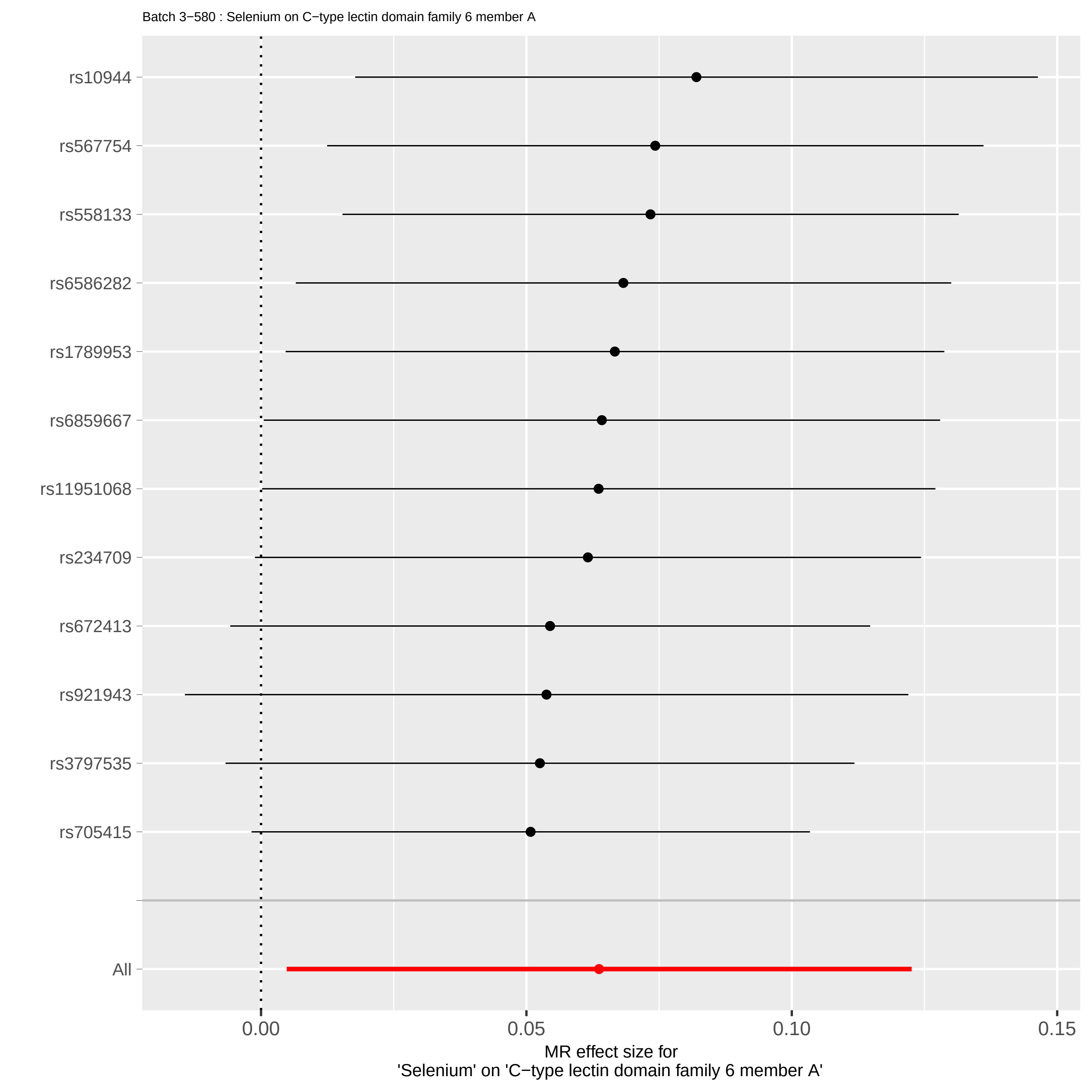
Batch 3−580 : Selenium on C−type lectin domain family 6 member A
rs10944
rs567754
rs558133
rs6586282
rs1789953
rs6859667
rs11951068
rs234709
rs672413
rs921943
rs3797535
rs705415
All
0.00
0.05
0.10
0.15
MR effect size for
'Selenium' on 'C−type lectin domain family 6 member A'

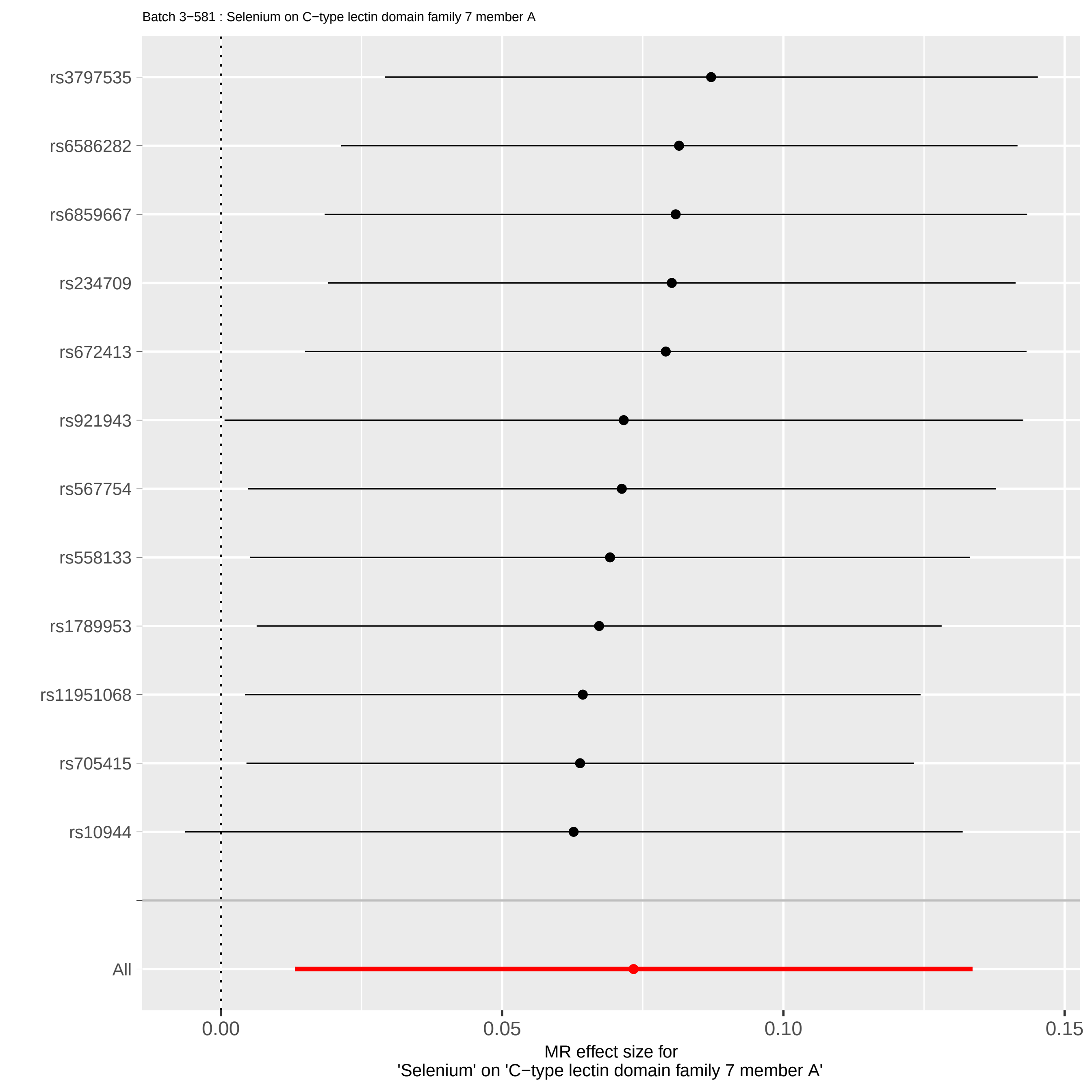

Batch 3−581 : Selenium on C−type lectin domain family 7 member A
rs3797535
rs6586282
rs6859667
rs234709
rs672413
rs921943
rs567754
rs558133
rs1789953
rs11951068
rs705415
rs10944
All
0.00
0.05
0.10
0.15
MR effect size for
'Selenium' on 'C−type lectin domain family 7 member A'

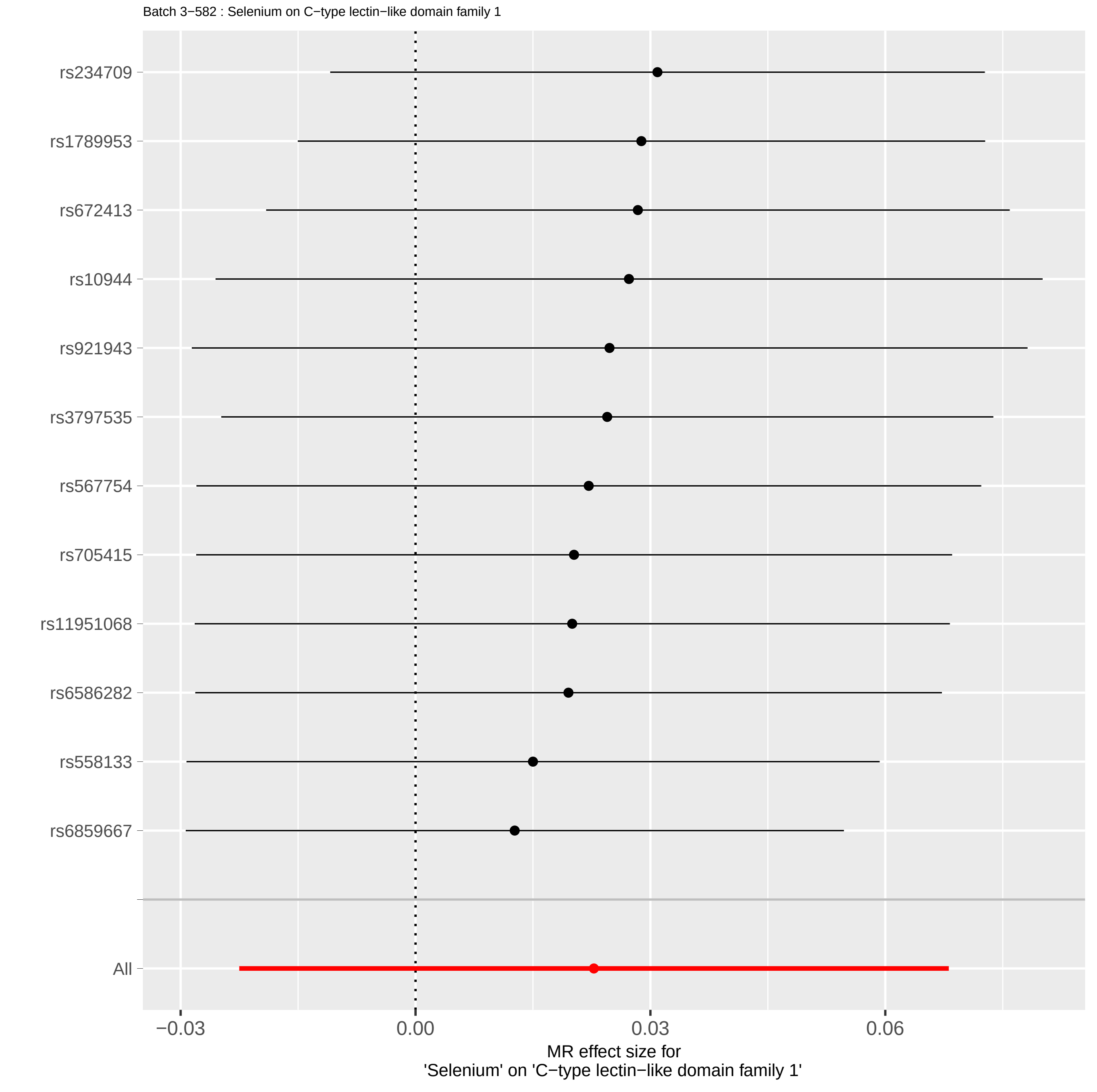

Batch 3−582 : Selenium on C−type lectin−like domain family 1
rs234709
rs1789953
rs672413
rs10944
rs921943
rs3797535
rs567754
rs705415
rs11951068
rs6586282
rs558133
rs6859667
All
−0.03
0.00
0.03
0.06
MR effect size for
'Selenium' on 'C−type lectin−like domain family 1'

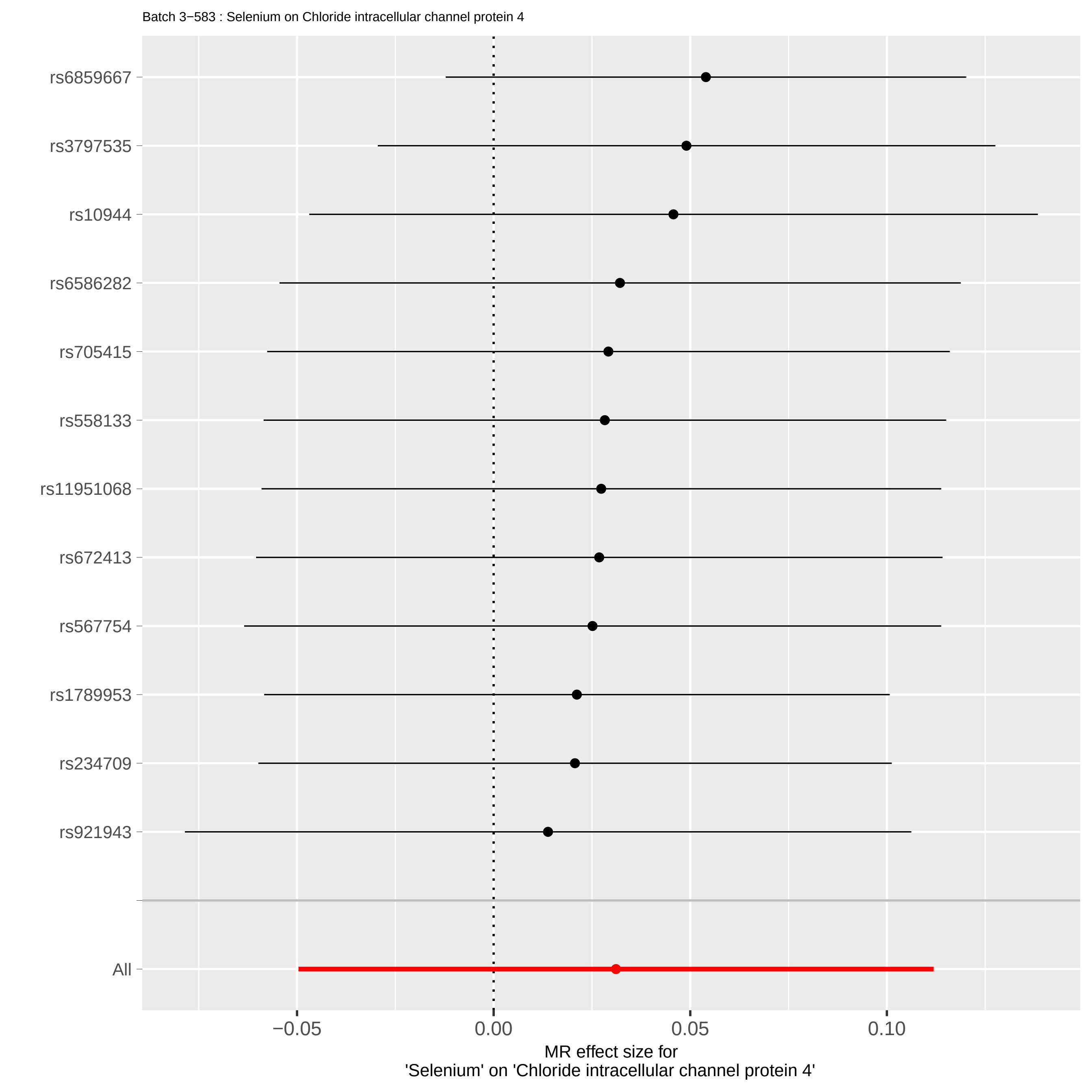
Batch 3−583 : Selenium on Chloride intracellular channel protein 4
rs6859667
rs3797535
rs10944
rs6586282
rs705415
rs558133
rs11951068
rs672413
rs567754
rs1789953
rs234709
rs921943
All
−0.05
0.00
0.05
0.10
MR effect size for
'Selenium' on 'Chloride intracellular channel protein 4'

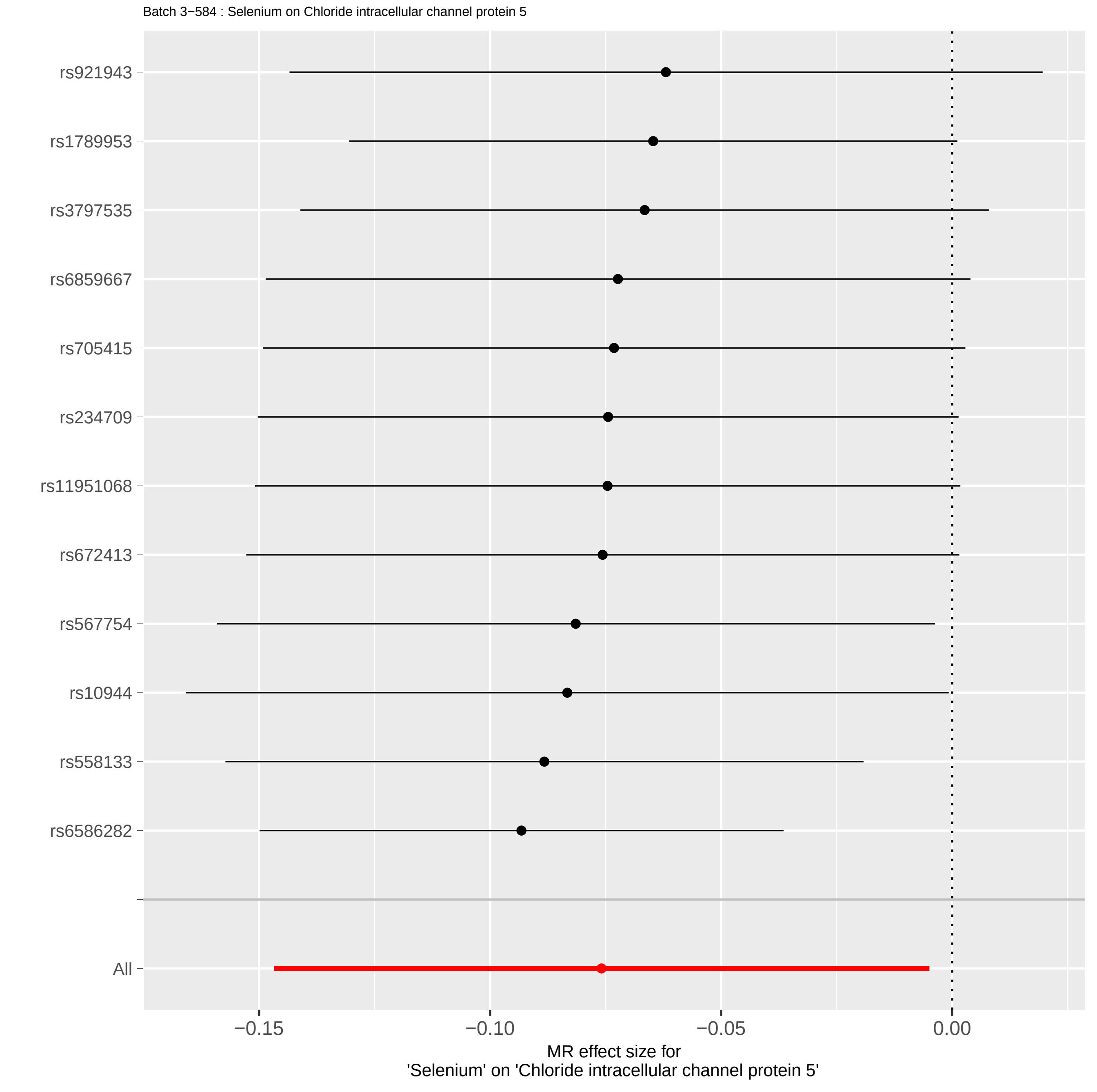

Batch 3−584 : Selenium on Chloride intracellular channel protein 5
rs921943
rs1789953
rs3797535
rs6859667
rs705415
rs234709
rs11951068
rs672413
rs567754
rs10944
rs558133
rs6586282
All
−0.15
−0.10
−0.05
0.00
MR effect size for
'Selenium' on 'Chloride intracellular channel protein 5'

Batch 3−585 : Selenium on Clathrin interactor 1

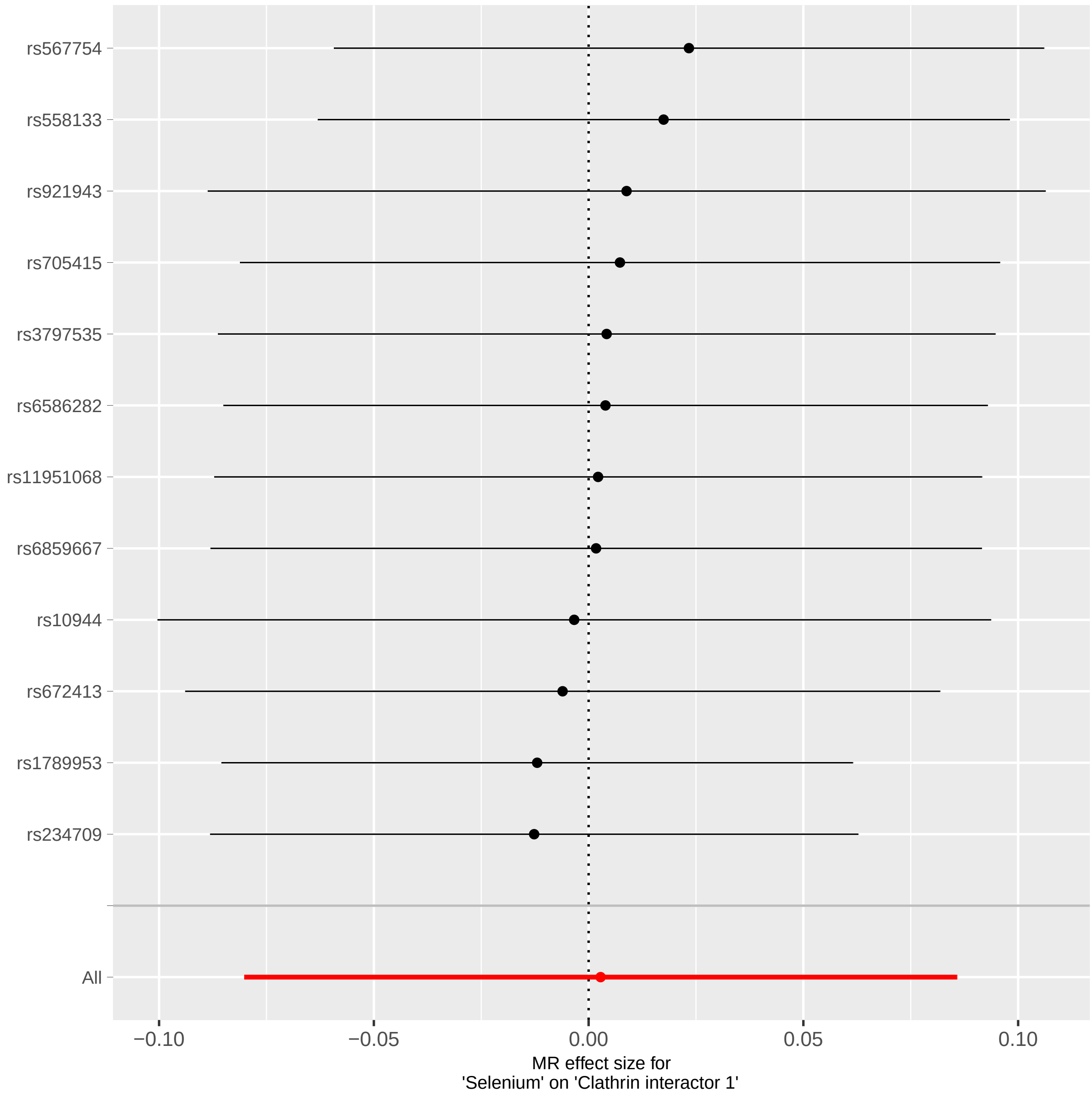

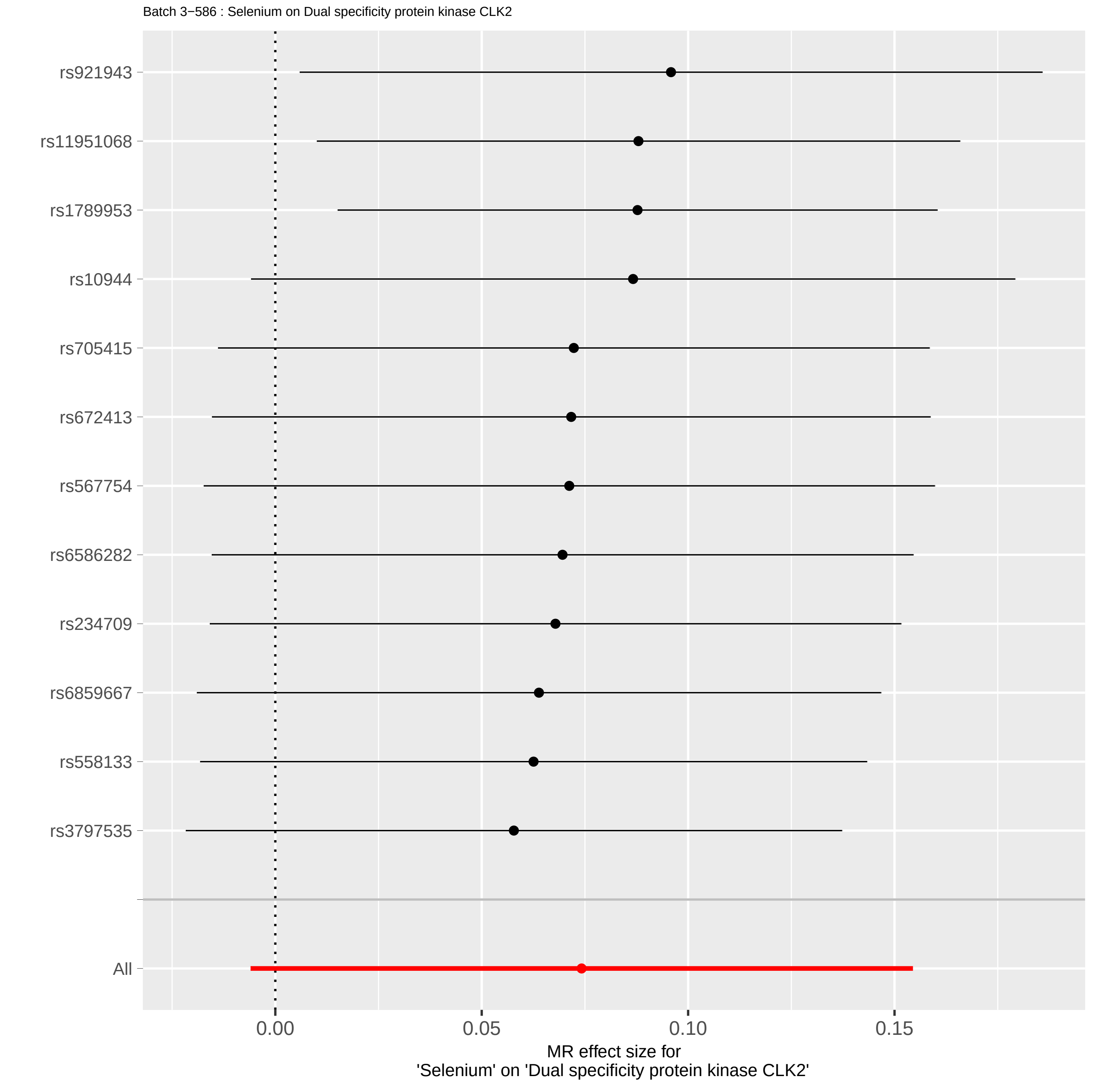

Batch 3−586 : Selenium on Dual specificity protein kinase CLK2
rs921943
rs11951068
rs1789953
rs10944
rs705415
rs672413
rs567754
rs6586282
rs234709
rs6859667
rs558133
rs3797535
All
0.00
0.05
0.10
0.15
MR effect size for
'Selenium' on 'Dual specificity protein kinase CLK2'

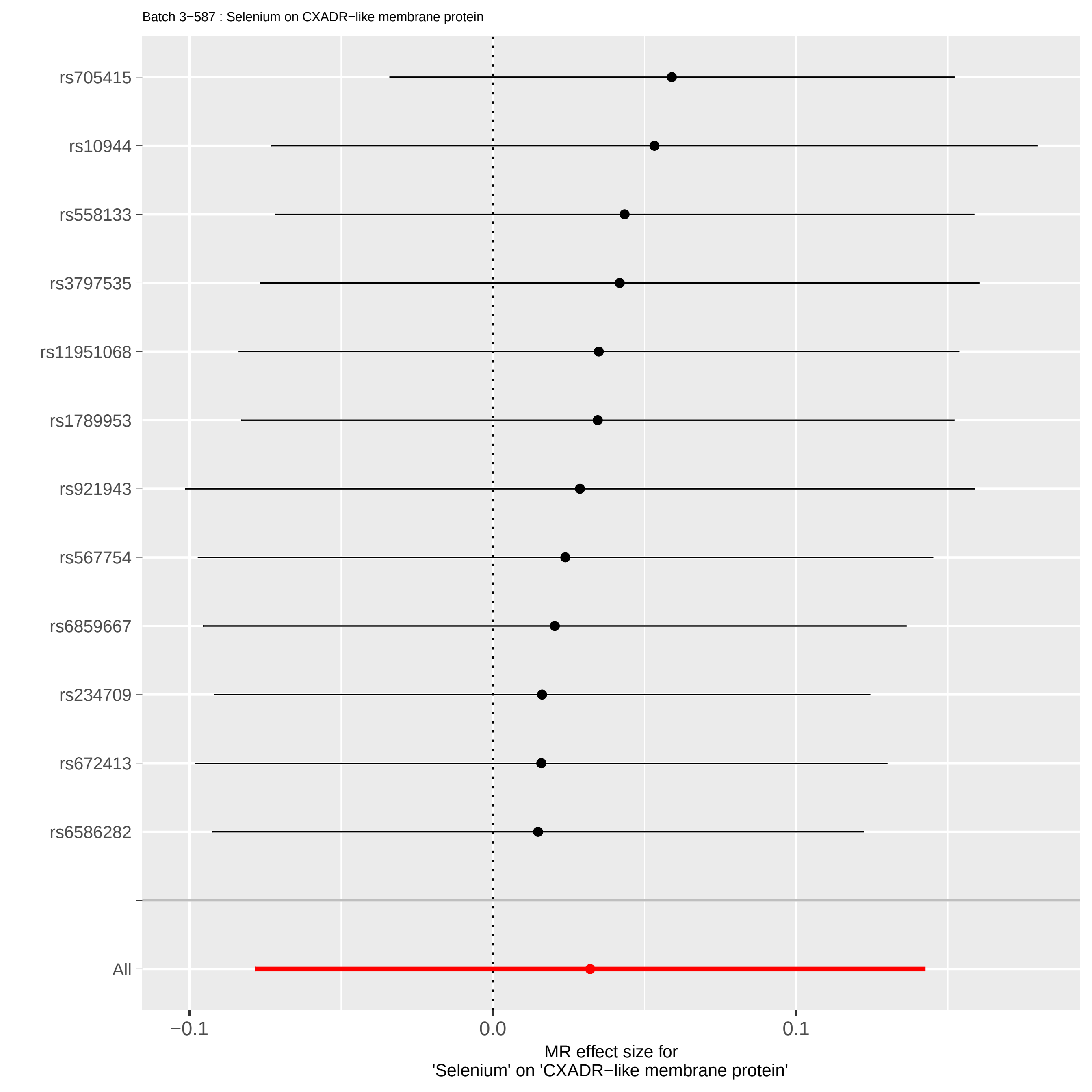

Batch 3−587 : Selenium on CXADR−like membrane protein
rs705415
rs10944
rs558133
rs3797535
rs11951068
rs1789953
rs921943
rs567754
rs6859667
rs234709
rs672413
rs6586282
All
−0.1
0.0
0.1
MR effect size for
'Selenium' on 'CXADR−like membrane protein'

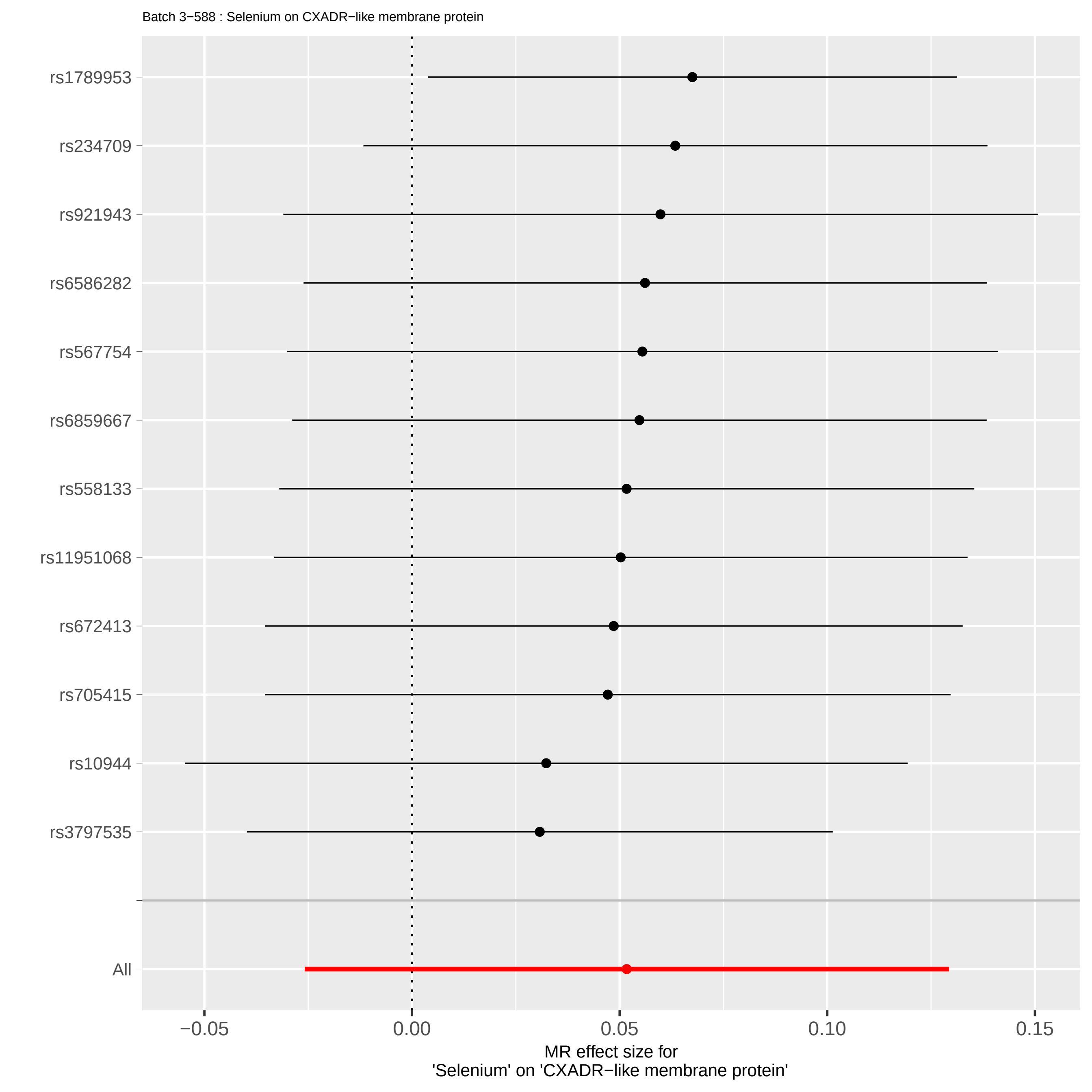

Batch 3−588 : Selenium on CXADR−like membrane protein
rs1789953
rs234709
rs921943
rs6586282
rs567754
rs6859667
rs558133
rs11951068
rs672413
rs705415
rs10944
rs3797535
All
−0.05
0.00
0.05
0.10
0.15
MR effect size for
'Selenium' on 'CXADR−like membrane protein'

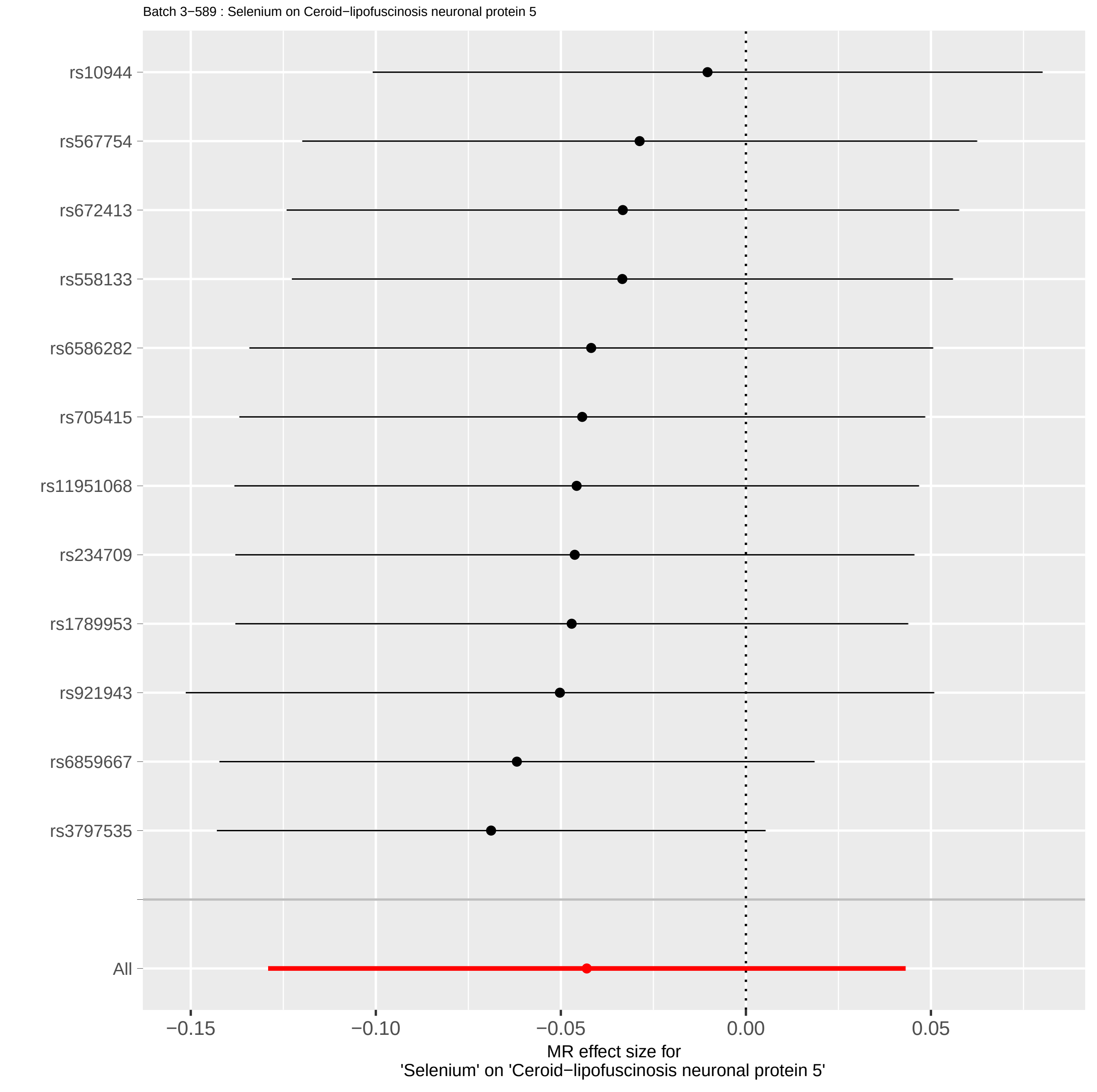

Batch 3−589 : Selenium on Ceroid−lipofuscinosis neuronal protein 5
rs10944
rs567754
rs672413
rs558133
rs6586282
rs705415
rs11951068
rs234709
rs1789953
rs921943
rs6859667
rs3797535
All
−0.15
−0.10
−0.05
0.00
0.05
MR effect size for
'Selenium' on 'Ceroid−lipofuscinosis neuronal protein 5'

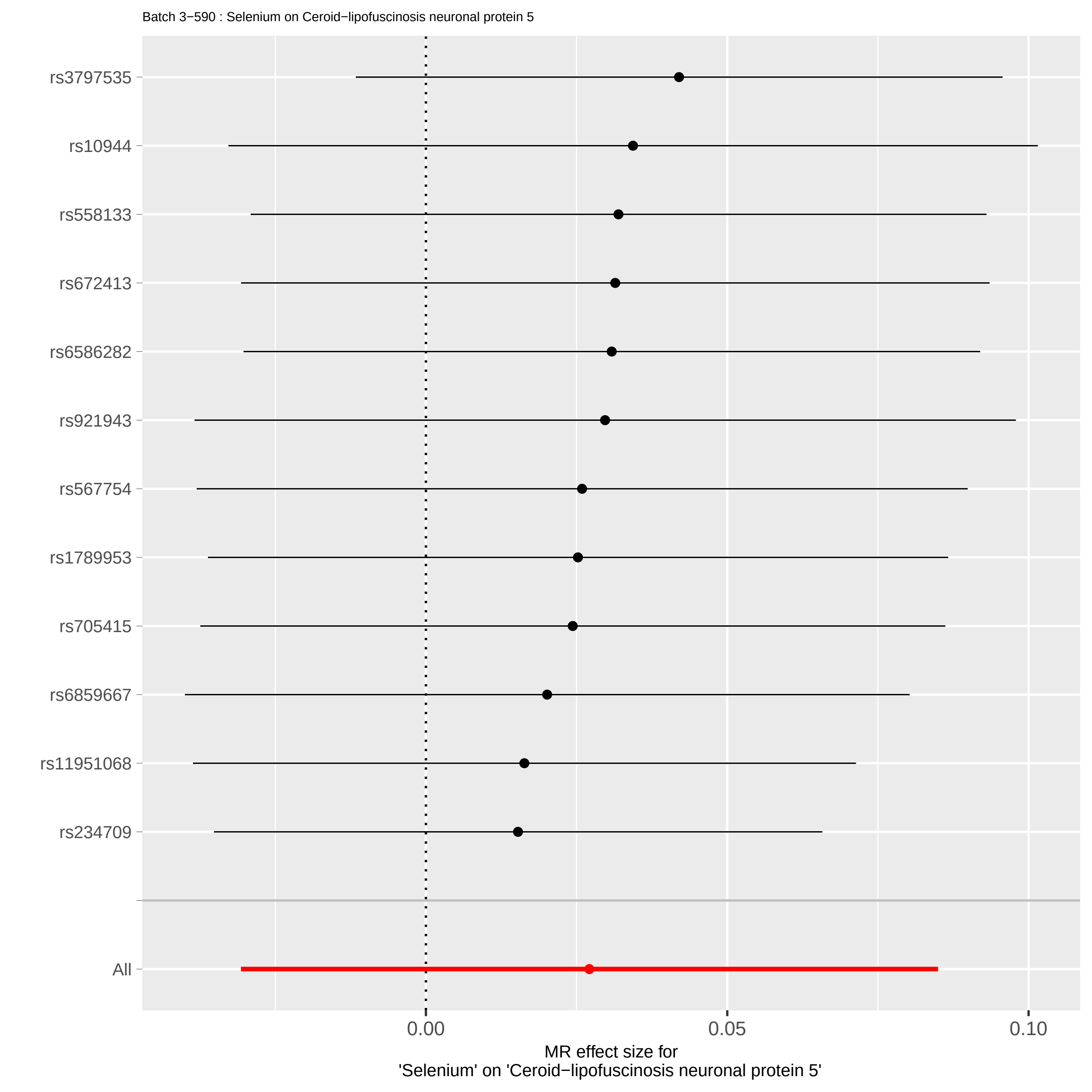
Batch 3−590 : Selenium on Ceroid−lipofuscinosis neuronal protein 5
rs3797535
rs10944
rs558133
rs672413
rs6586282
rs921943
rs567754
rs1789953
rs705415
rs6859667
rs11951068
rs234709
All
0.00
0.05
0.10
MR effect size for
'Selenium' on 'Ceroid−lipofuscinosis neuronal protein 5'

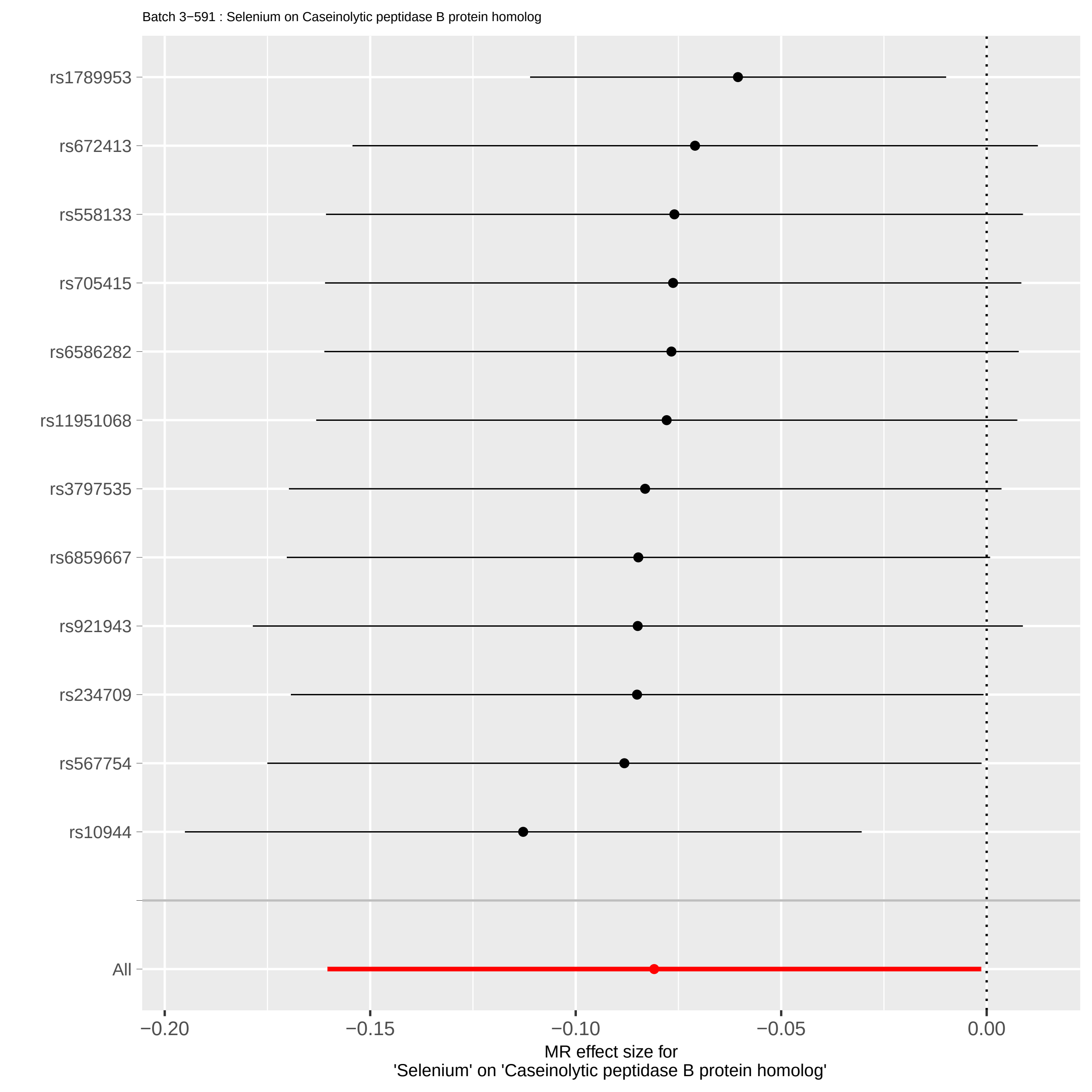

Batch 3−591 : Selenium on Caseinolytic peptidase B protein homolog
rs1789953
rs672413
rs558133
rs705415
rs6586282
rs11951068
rs3797535
rs6859667
rs921943
rs234709
rs567754
rs10944
All
−0.20
−0.15
−0.10
−0.05
0.00
MR effect size for
'Selenium' on 'Caseinolytic peptidase B protein homolog'

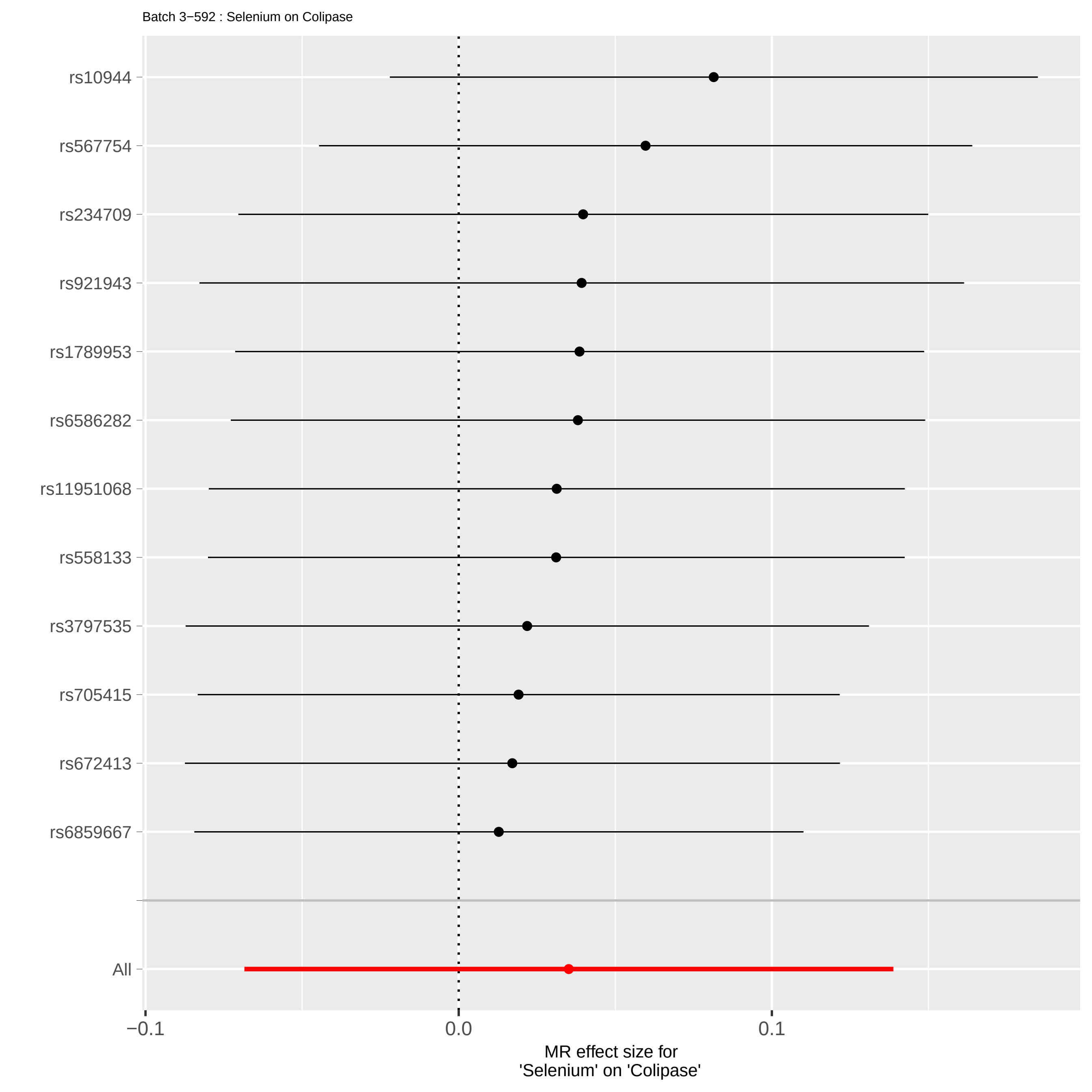

Batch 3−592 : Selenium on Colipase
rs10944
rs567754
rs234709
rs921943
rs1789953
rs6586282
rs11951068
rs558133
rs3797535
rs705415
rs672413
rs6859667
All
−0.1
0.0
0.1
MR effect size for
'Selenium' on 'Colipase'

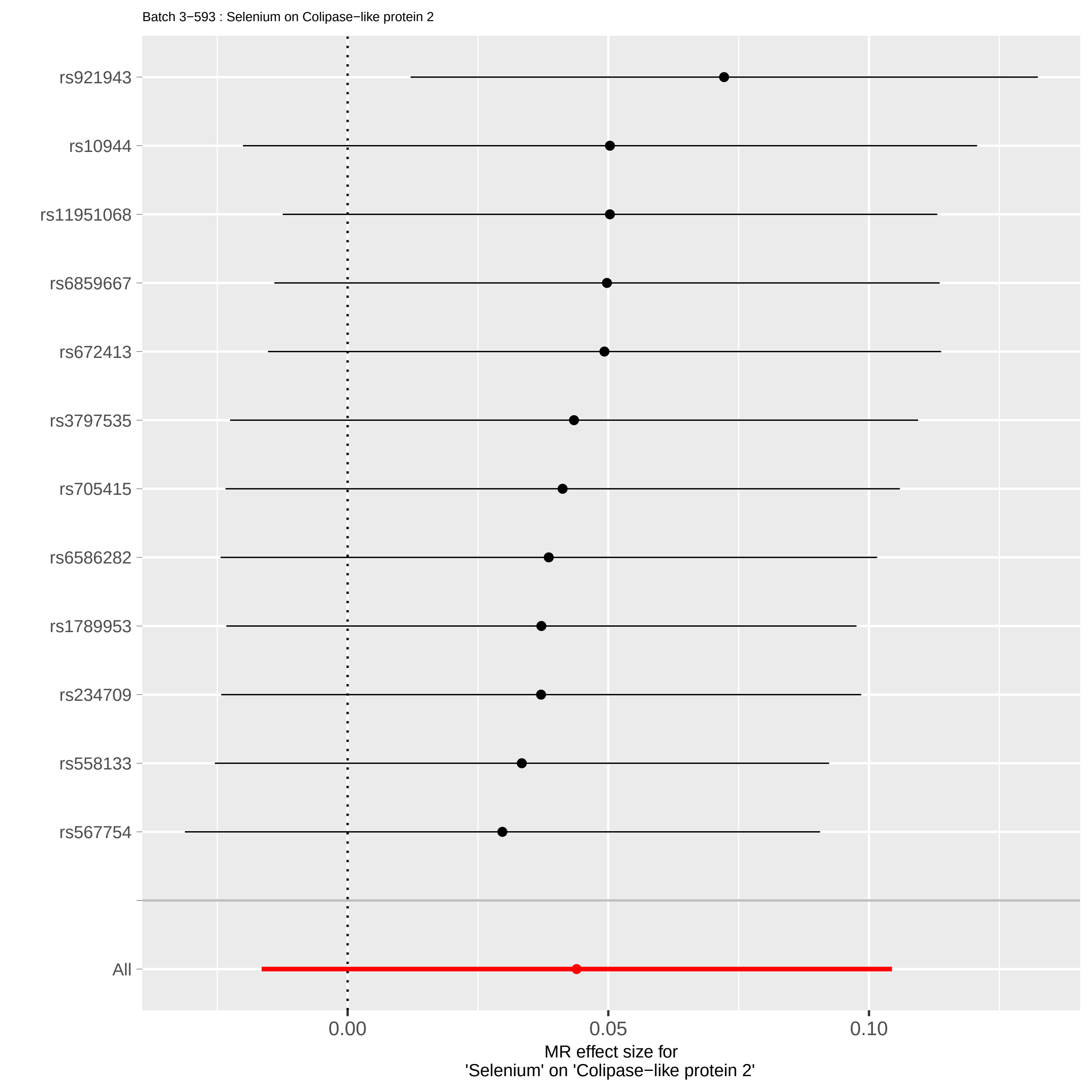
Batch 3−593 : Selenium on Colipase−like protein 2
rs921943
rs10944
rs11951068
rs6859667
rs672413
rs3797535
rs705415
rs6586282
rs1789953
rs234709
rs558133
rs567754
All
0.00
0.05
0.10
MR effect size for
'Selenium' on 'Colipase−like protein 2'

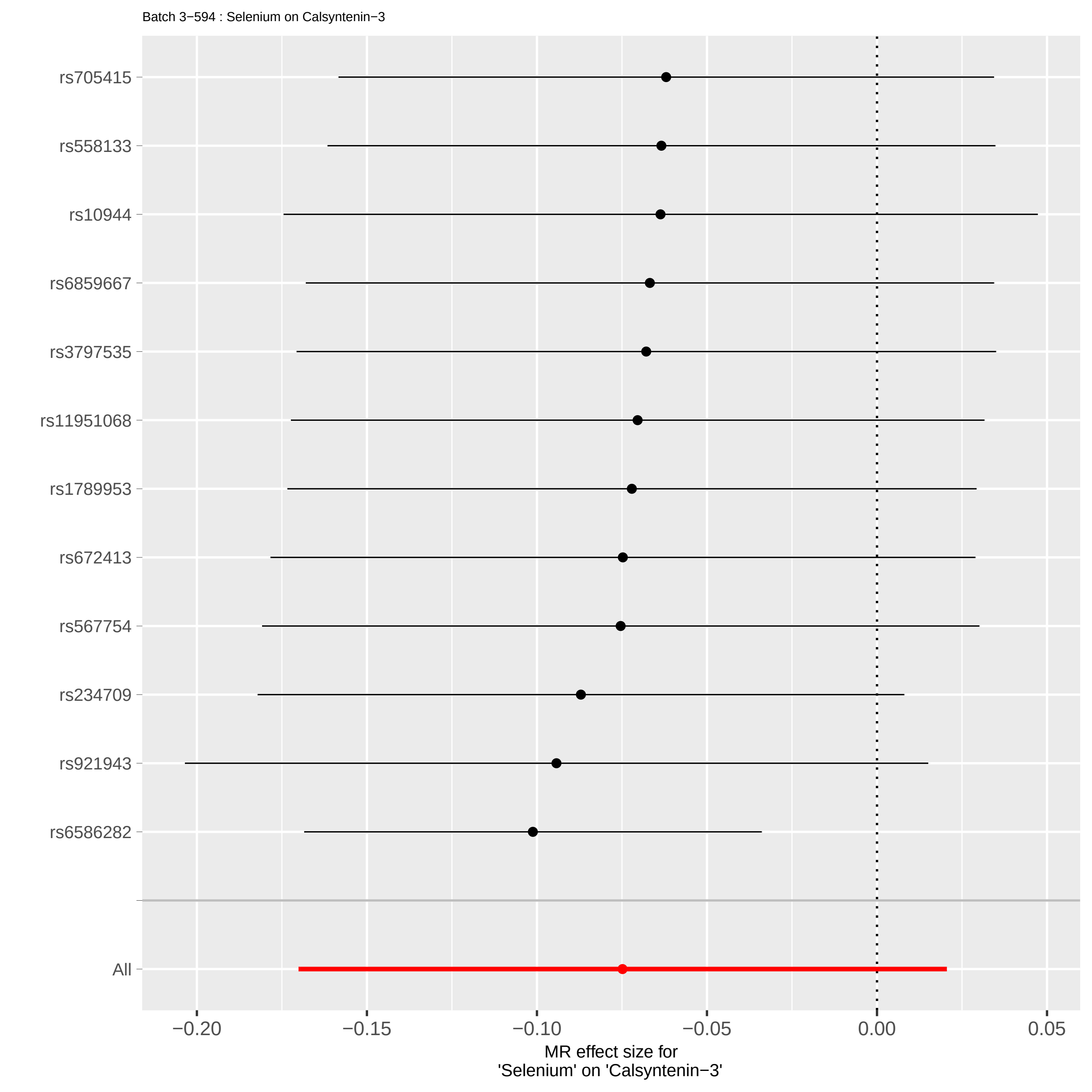

Batch 3−594 : Selenium on Calsyntenin−3
rs705415
rs558133
rs10944
rs6859667
rs3797535
rs11951068
rs1789953
rs672413
rs567754
rs234709
rs921943
rs6586282
All
−0.20
−0.15
−0.10
−0.05
0.00
0.05
MR effect size for
'Selenium' on 'Calsyntenin−3'

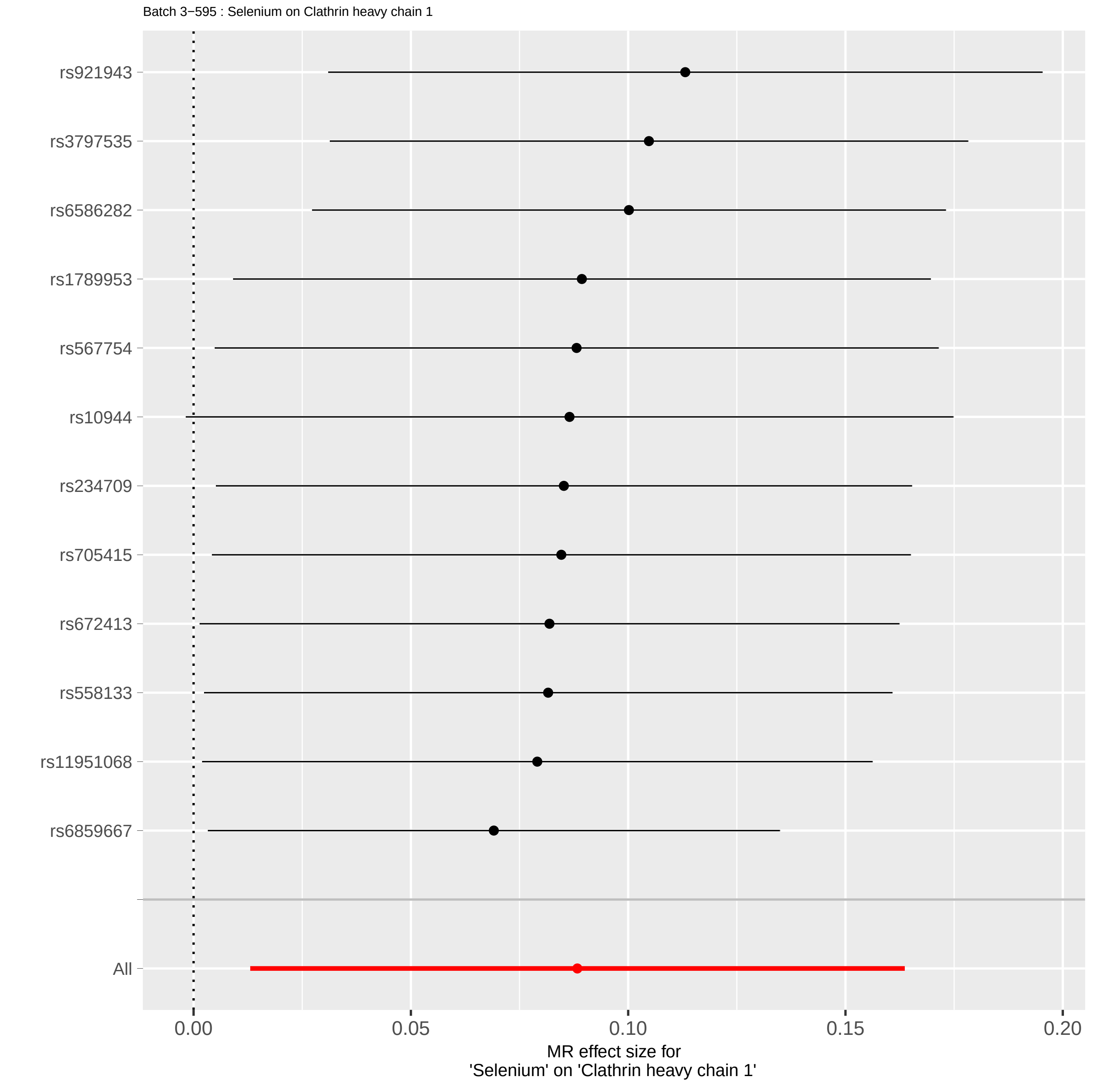
Batch 3−595 : Selenium on Clathrin heavy chain 1
rs921943
rs3797535
rs6586282
rs1789953
rs567754
rs10944
rs234709
rs705415
rs672413
rs558133
rs11951068
rs6859667
All
0.00
0.05
0.10
0.15
0.20
MR effect size for
'Selenium' on 'Clathrin heavy chain 1'

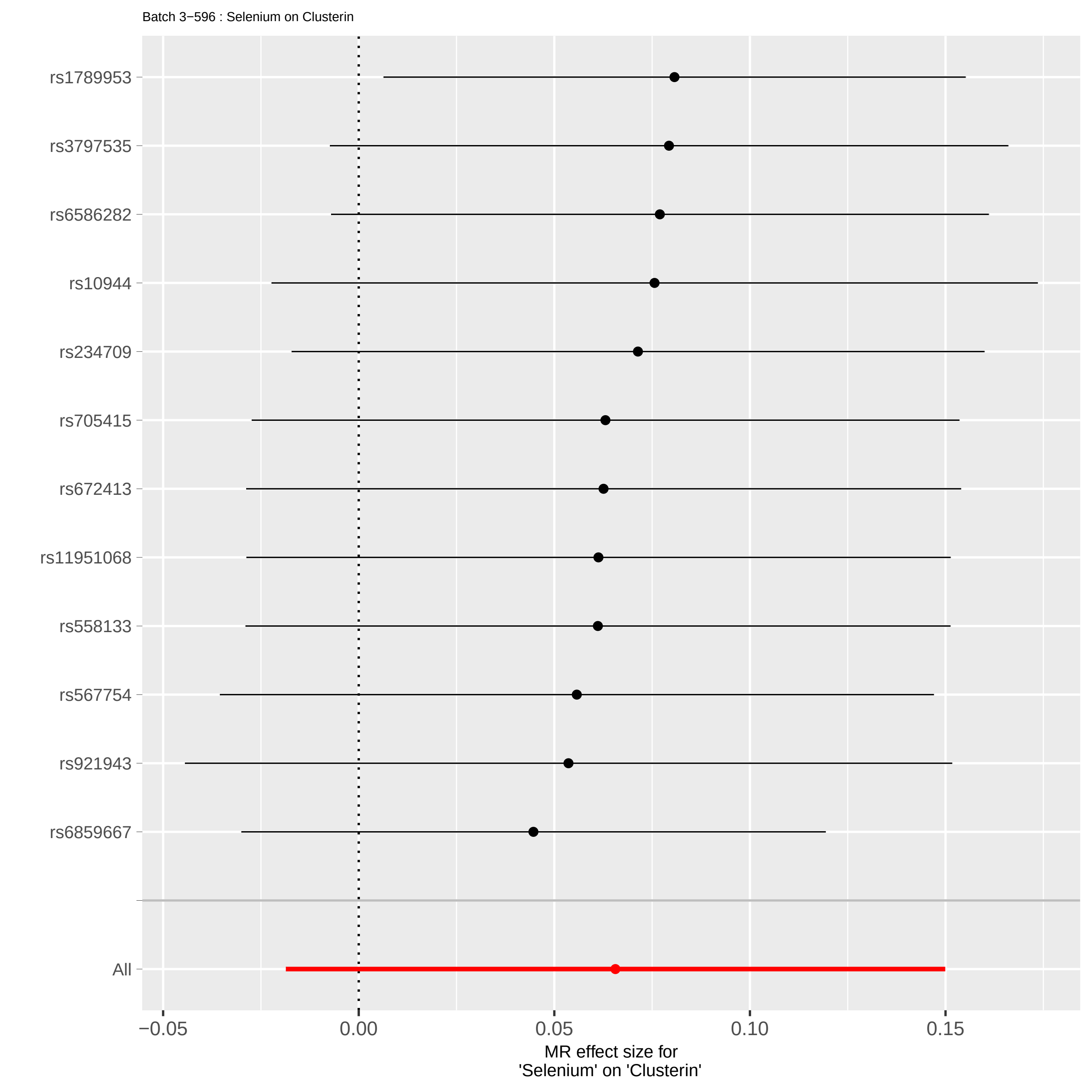
Batch 3−596 : Selenium on Clusterin
rs1789953
rs3797535
rs6586282
rs10944
rs234709
rs705415
rs672413
rs11951068
rs558133
rs567754
rs921943
rs6859667
All
−0.05
0.00
0.05
0.10
0.15
MR effect size for
'Selenium' on 'Clusterin'

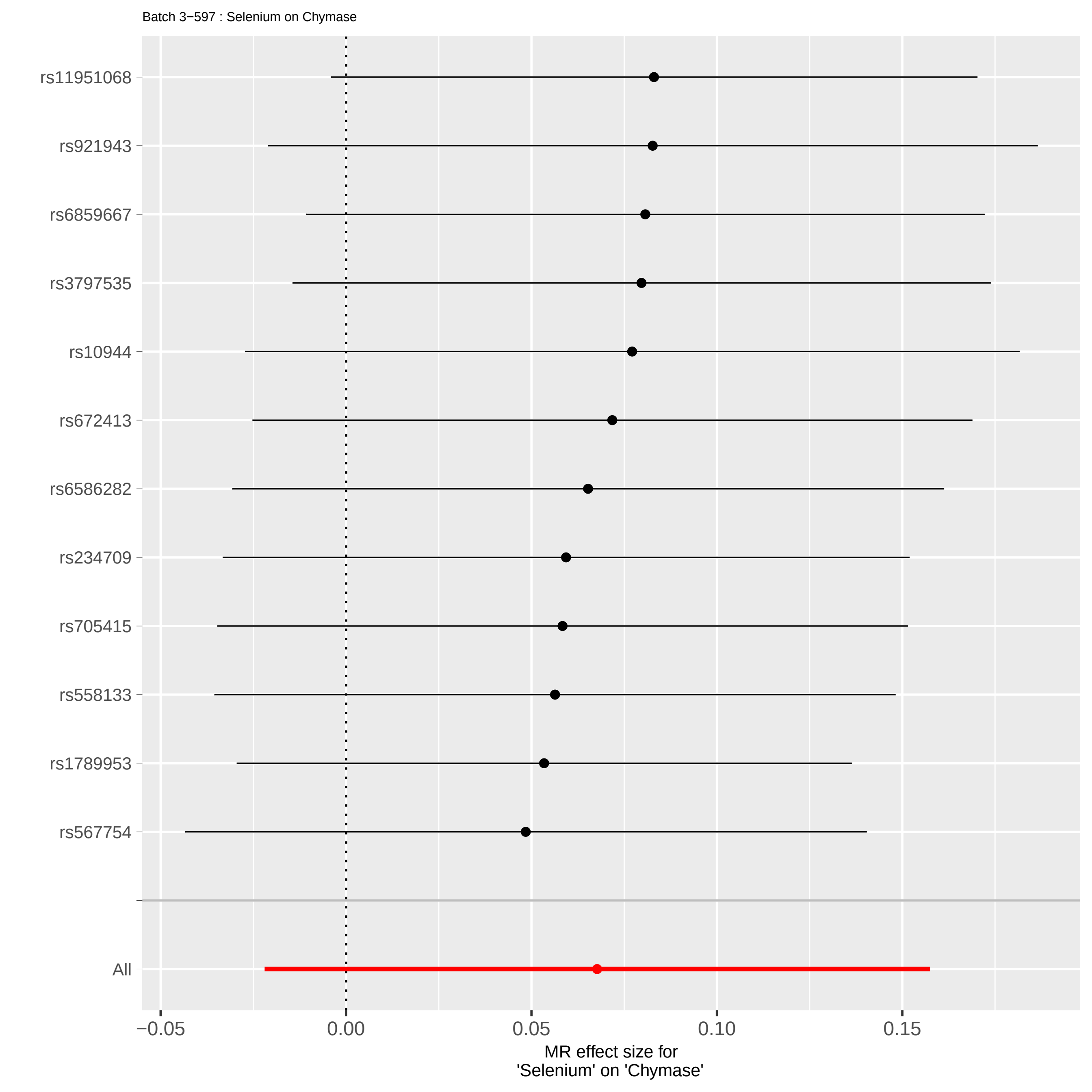

Batch 3−597 : Selenium on Chymase
rs11951068
rs921943
rs6859667
rs3797535
rs10944
rs672413
rs6586282
rs234709
rs705415
rs558133
rs1789953
rs567754
All
−0.05
0.00
0.05
0.10
0.15
MR effect size for
'Selenium' on 'Chymase'

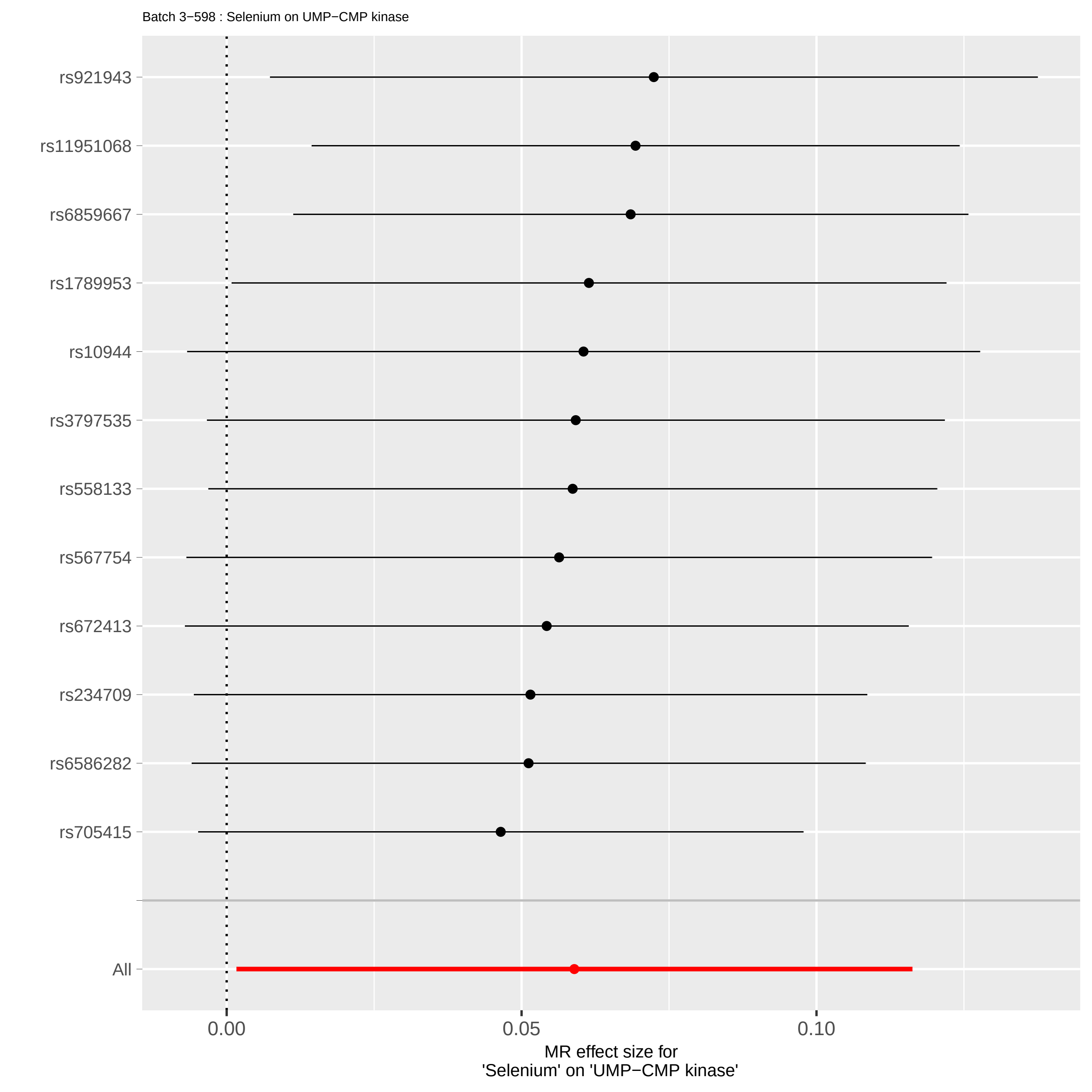

Batch 3−598 : Selenium on UMP−CMP kinase
rs921943
rs11951068
rs6859667
rs1789953
rs10944
rs3797535
rs558133
rs567754
rs672413
rs234709
rs6586282
rs705415
All
0.00
0.05
0.10
MR effect size for
'Selenium' on 'UMP−CMP kinase'

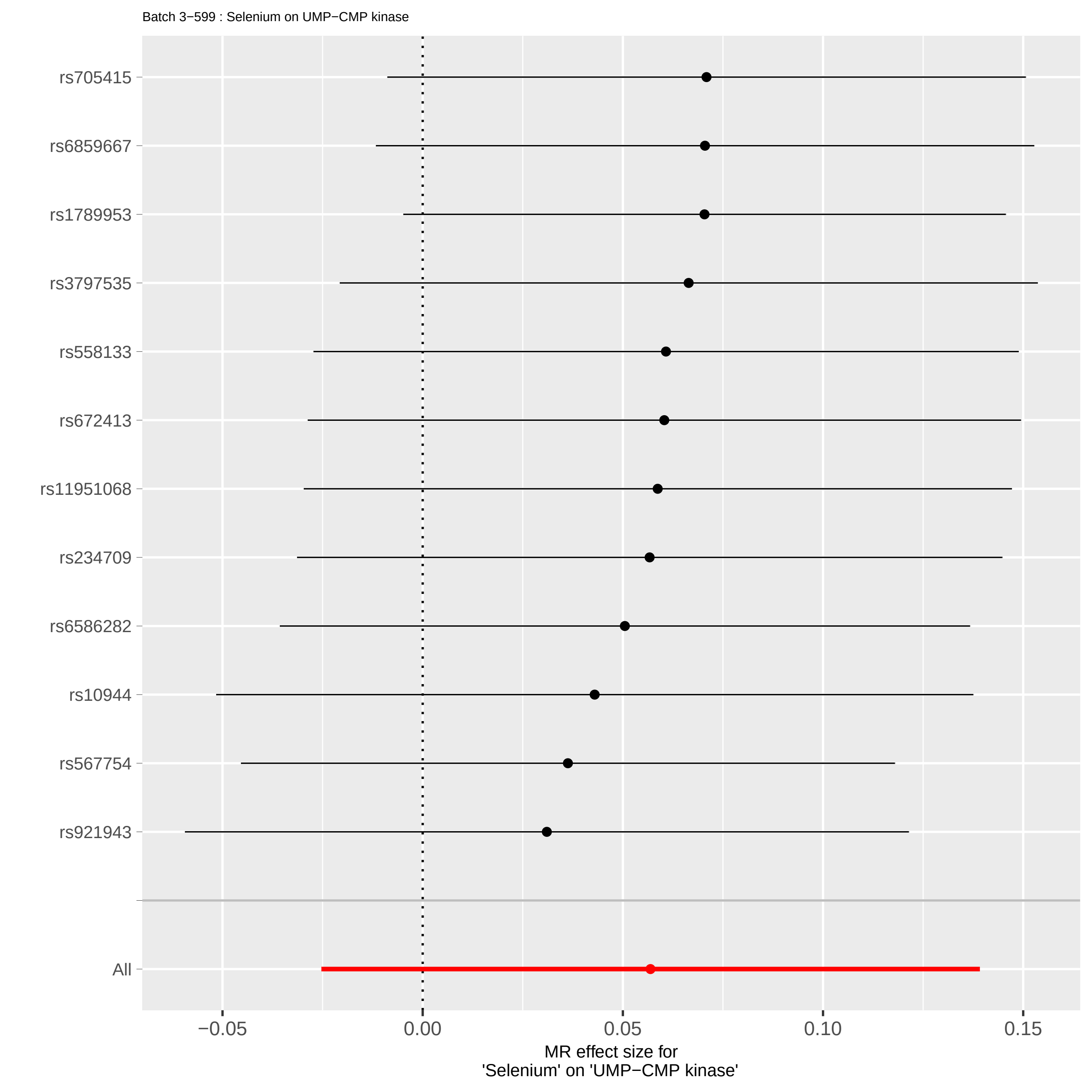

Batch 3−599 : Selenium on UMP−CMP kinase
rs705415
rs6859667
rs1789953
rs3797535
rs558133
rs672413
rs11951068
rs234709
rs6586282
rs10944
rs567754
rs921943
All
−0.05
0.00
0.05
0.10
0.15
MR effect size for
'Selenium' on 'UMP−CMP kinase'

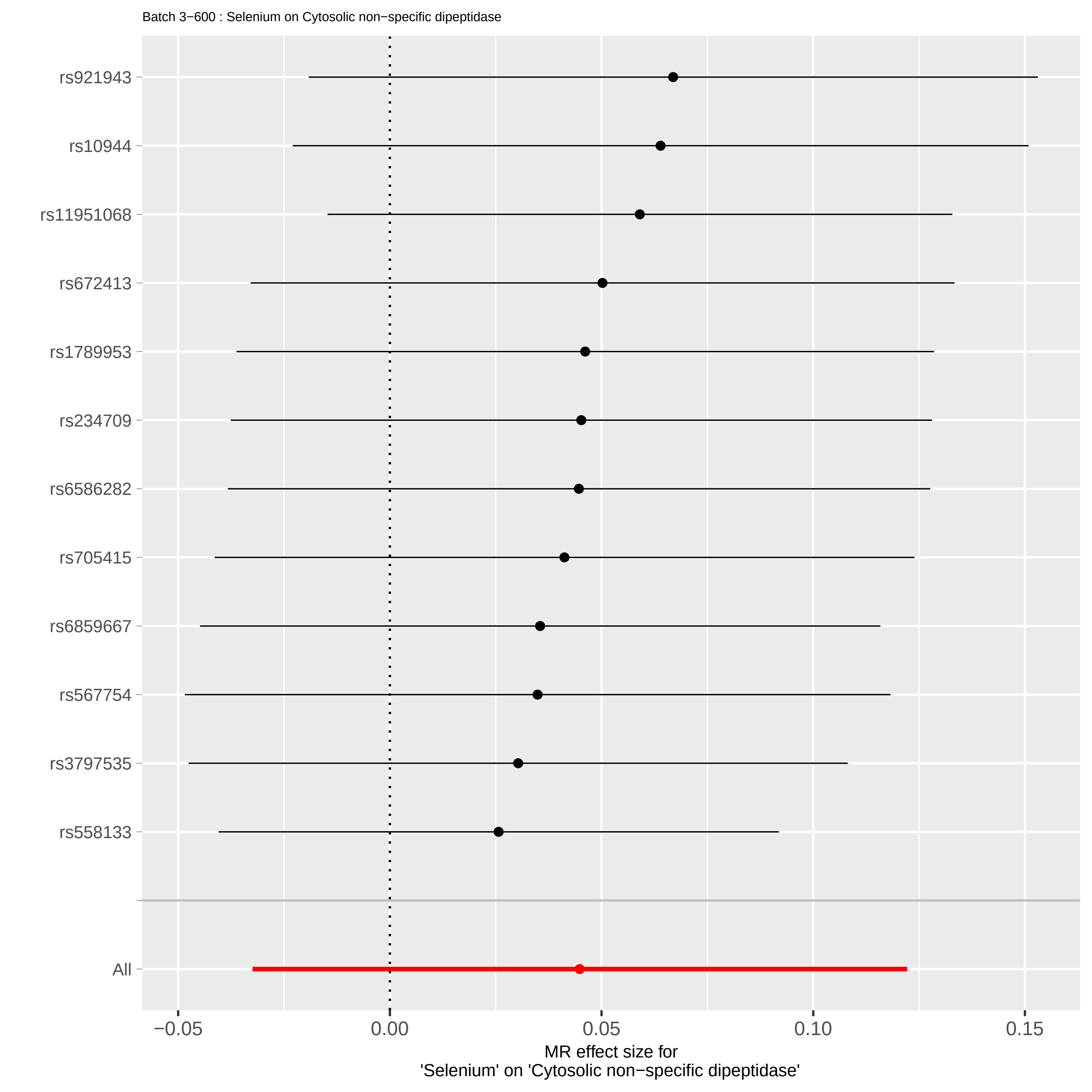

Batch 3−600 : Selenium on Cytosolic non−specific dipeptidase
rs921943
rs10944
rs11951068
rs672413
rs1789953
rs234709
rs6586282
rs705415
rs6859667
rs567754
rs3797535
rs558133
All
−0.05
0.00
0.05
0.10
0.15
MR effect size for
'Selenium' on 'Cytosolic non−specific dipeptidase'

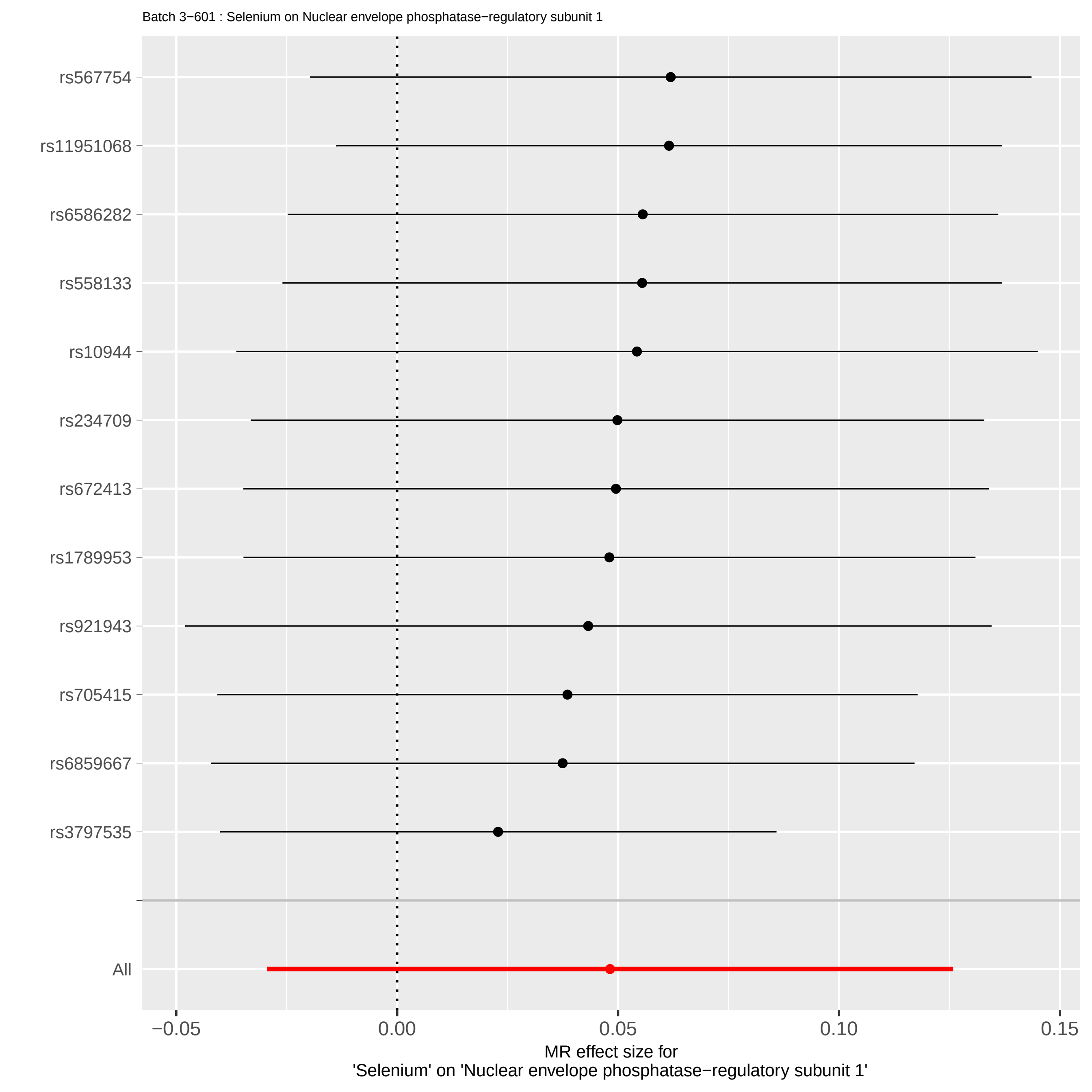

Batch 3−601 : Selenium on Nuclear envelope phosphatase−regulatory subunit 1
rs567754
rs11951068
rs6586282
rs558133
rs10944
rs234709
rs672413
rs1789953
rs921943
rs705415
rs6859667
rs3797535
All
−0.05
0.00
0.05
0.10
0.15
MR effect size for
'Selenium' on 'Nuclear envelope phosphatase−regulatory subunit 1'

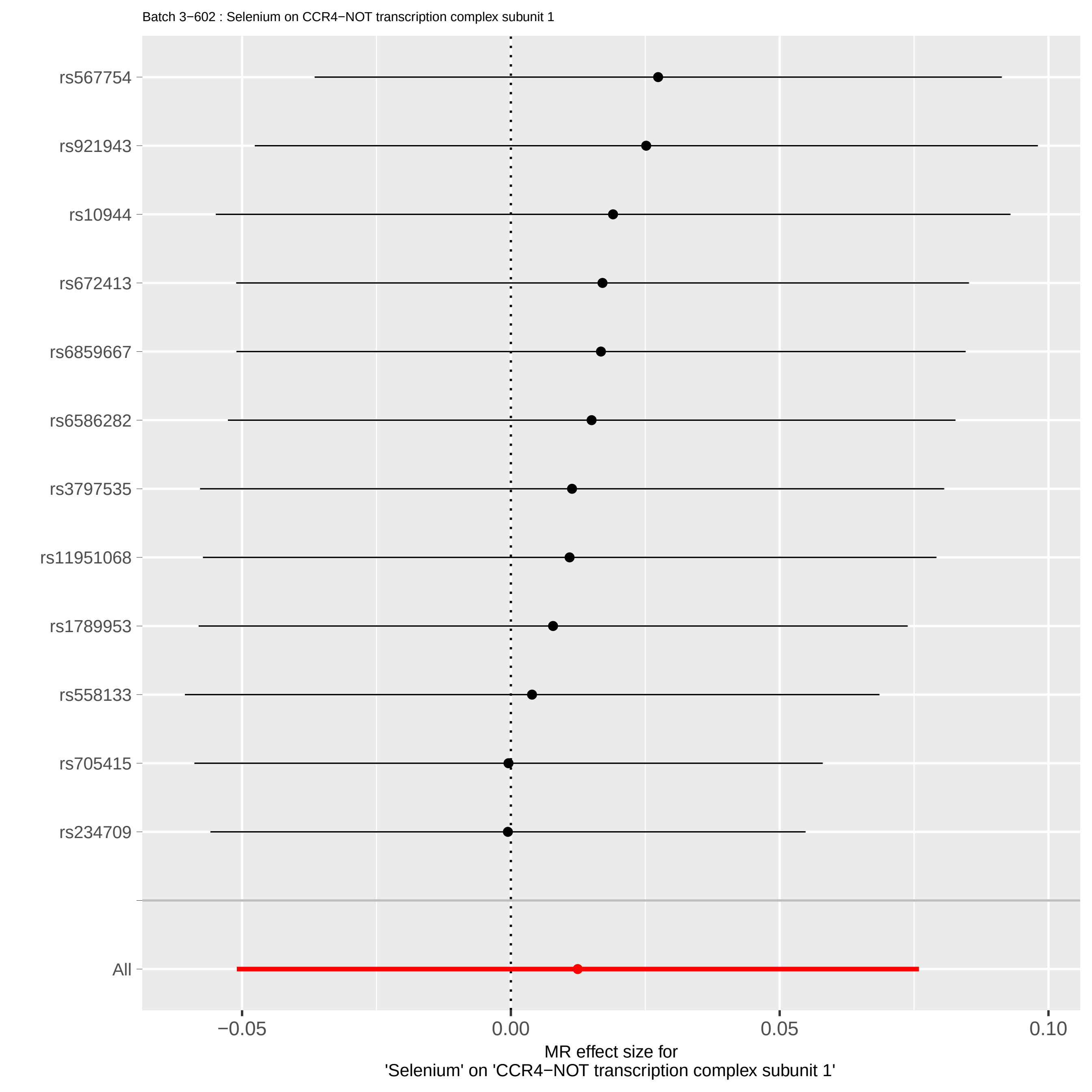

Batch 3−602 : Selenium on CCR4−NOT transcription complex subunit 1
rs567754
rs921943
rs10944
rs672413
rs6859667
rs6586282
rs3797535
rs11951068
rs1789953
rs558133
rs705415
rs234709
All
−0.05
0.00
0.05
0.10
MR effect size for
'Selenium' on 'CCR4−NOT transcription complex subunit 1'

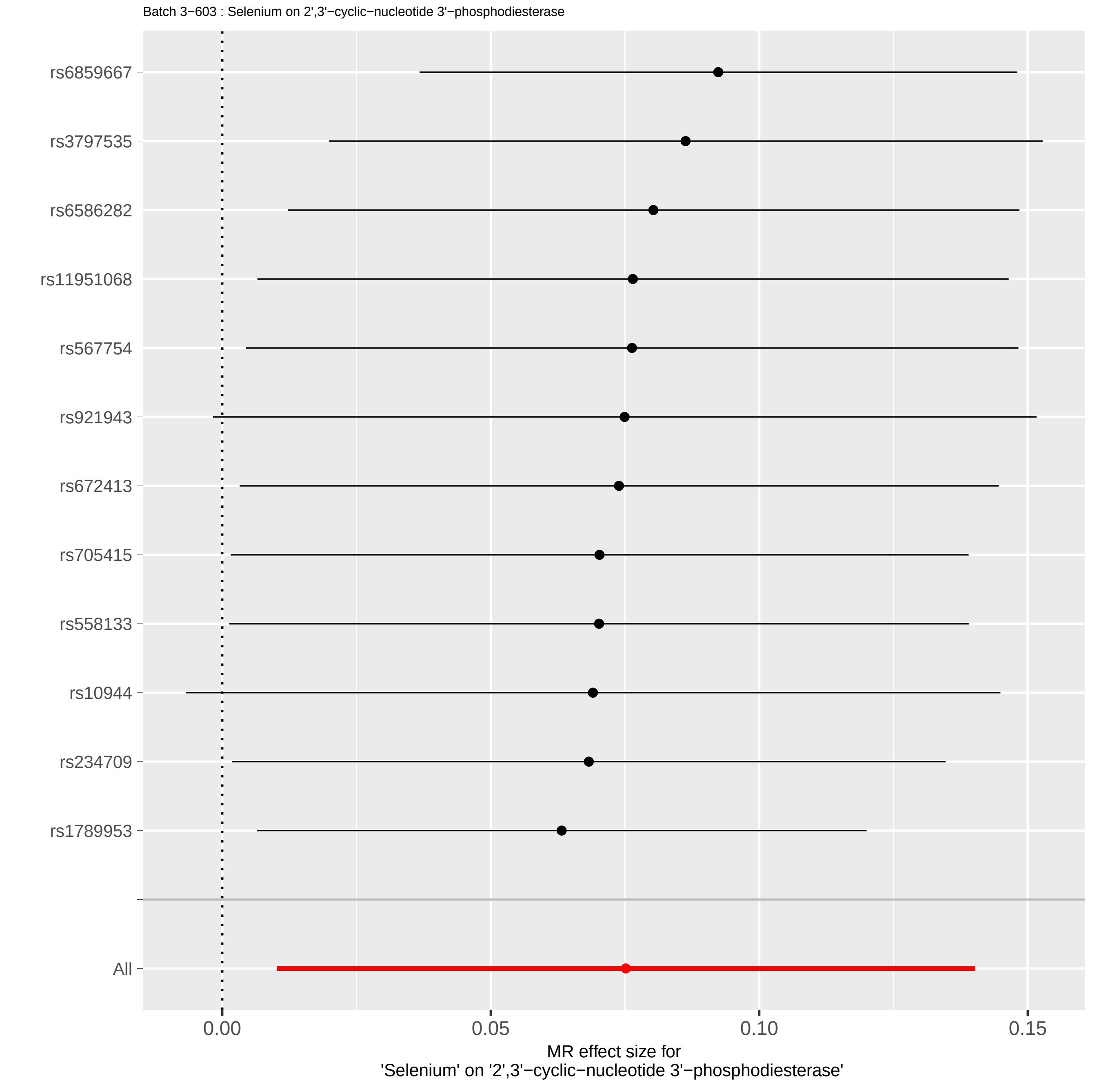

Batch 3−603 : Selenium on 2',3'−cyclic−nucleotide 3'−phosphodiesterase
rs6859667
rs3797535
rs6586282
rs11951068
rs567754
rs921943
rs672413
rs705415
rs558133
rs10944
rs234709
rs1789953
All
0.00
0.05
0.10
0.15
MR effect size for
'Selenium' on '2',3'−cyclic−nucleotide 3'−phosphodiesterase'

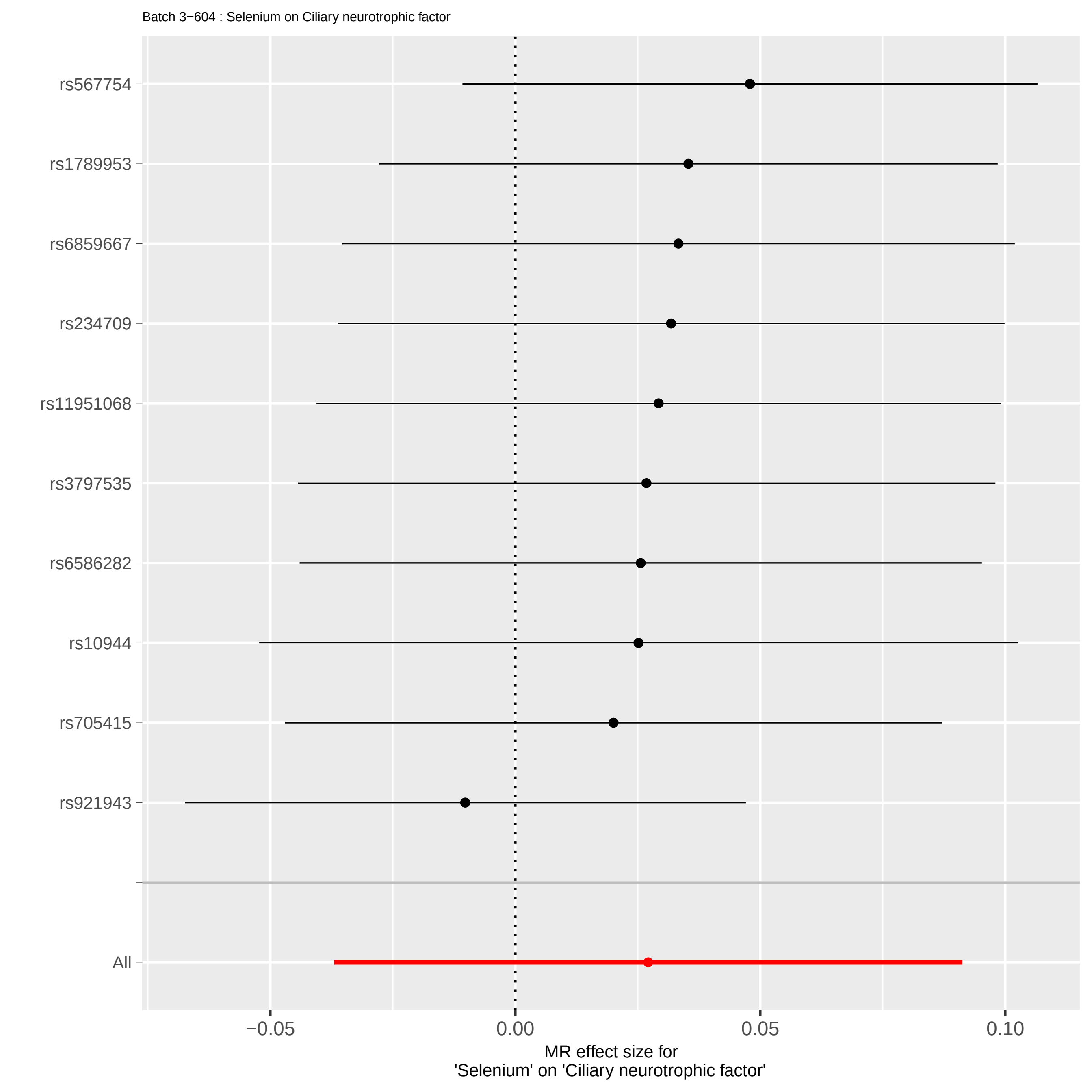
Batch 3−604 : Selenium on Ciliary neurotrophic factor
rs567754
rs1789953
rs6859667
rs234709
rs11951068
rs3797535
rs6586282
rs10944
rs705415
rs921943
All
−0.05
0.00
0.05
0.10
MR effect size for
'Selenium' on 'Ciliary neurotrophic factor'

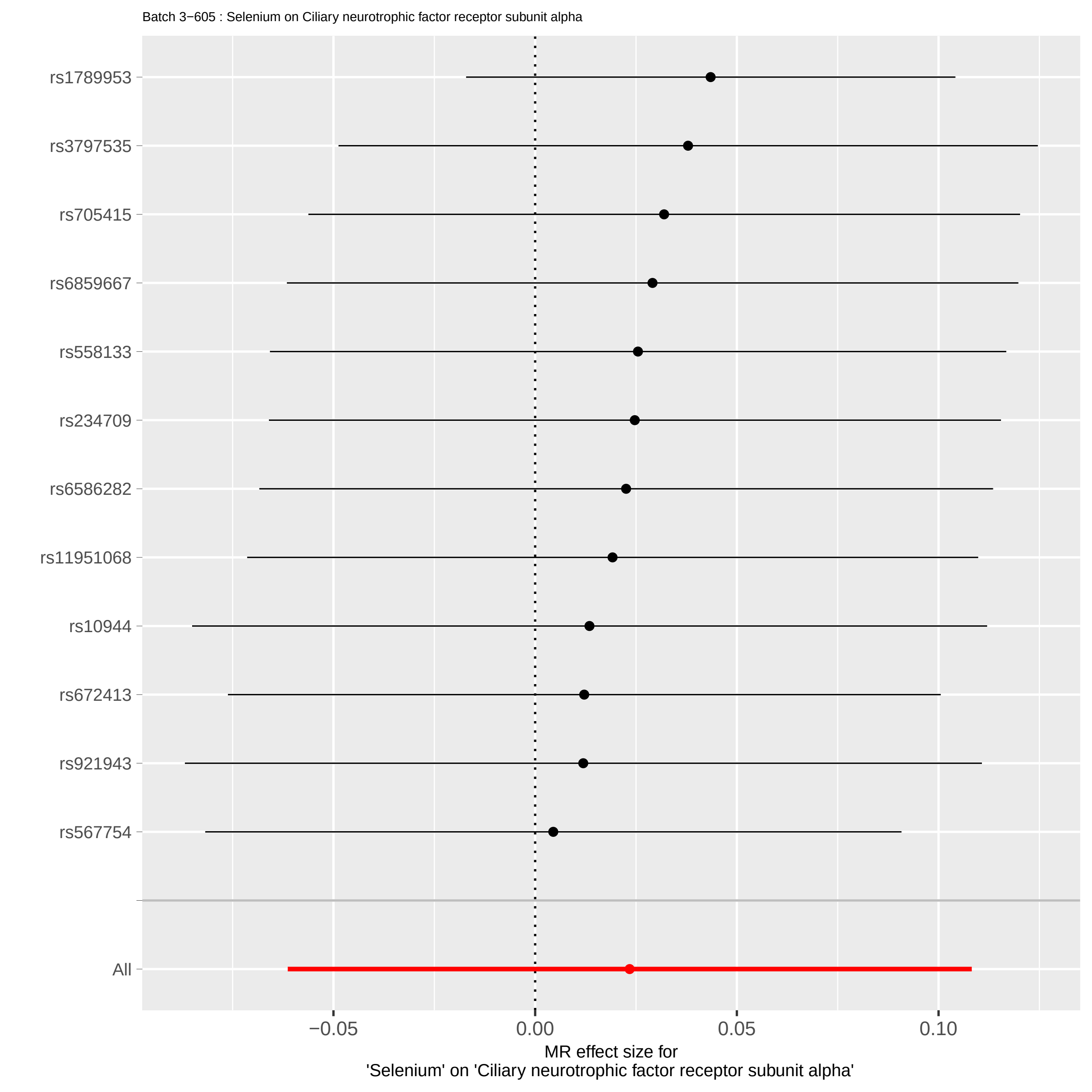

Batch 3−605 : Selenium on Ciliary neurotrophic factor receptor subunit alpha
rs1789953
rs3797535
rs705415
rs6859667
rs558133
rs234709
rs6586282
rs11951068
rs10944
rs672413
rs921943
rs567754
All
−0.05
0.00
0.05
0.10
MR effect size for
'Selenium' on 'Ciliary neurotrophic factor receptor subunit alpha'

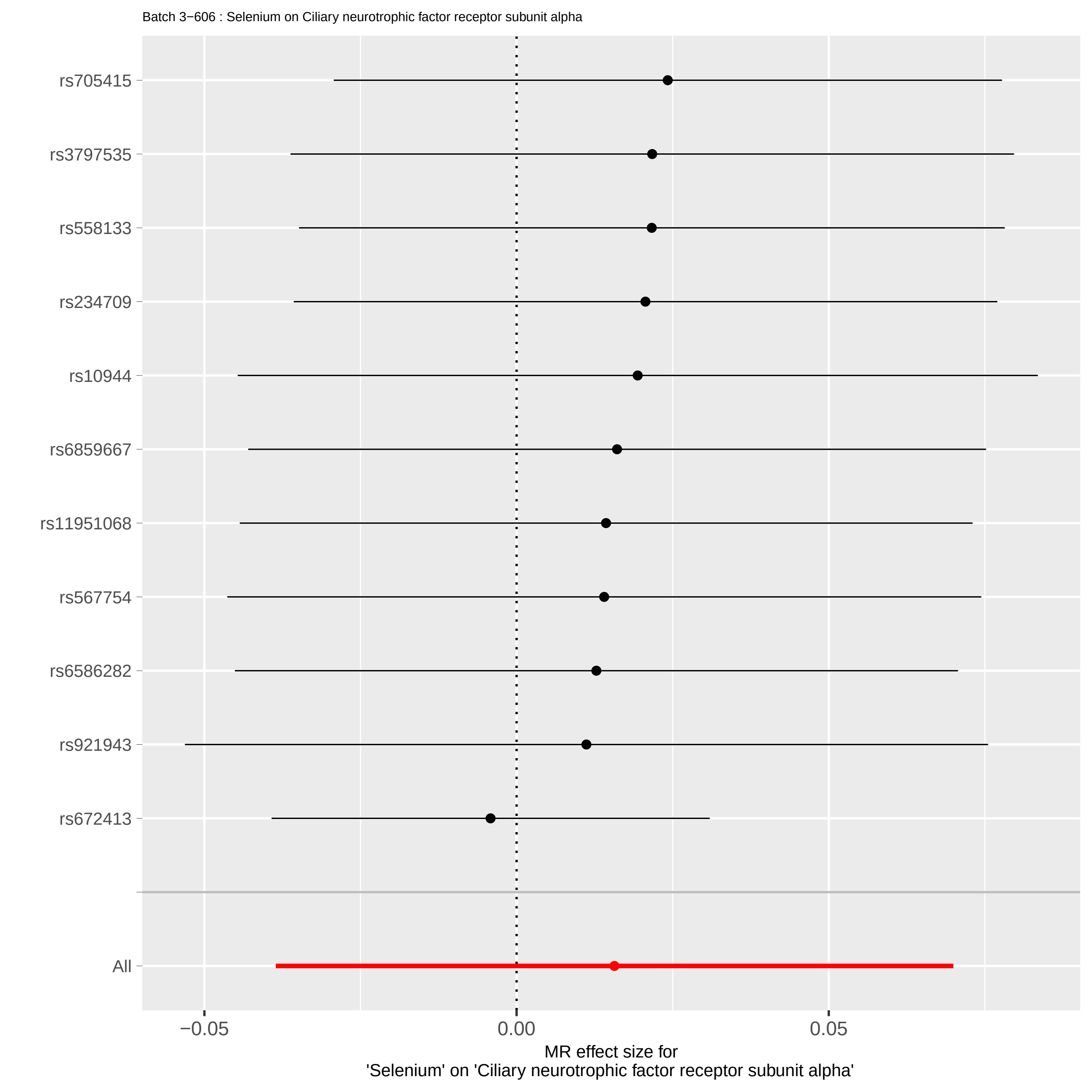
Batch 3−606 : Selenium on Ciliary neurotrophic factor receptor subunit alpha
rs705415
rs3797535
rs558133
rs234709
rs10944
rs6859667
rs11951068
rs567754
rs6586282
rs921943
rs672413
All
−0.05
0.00
0.05
MR effect size for
'Selenium' on 'Ciliary neurotrophic factor receptor subunit alpha'

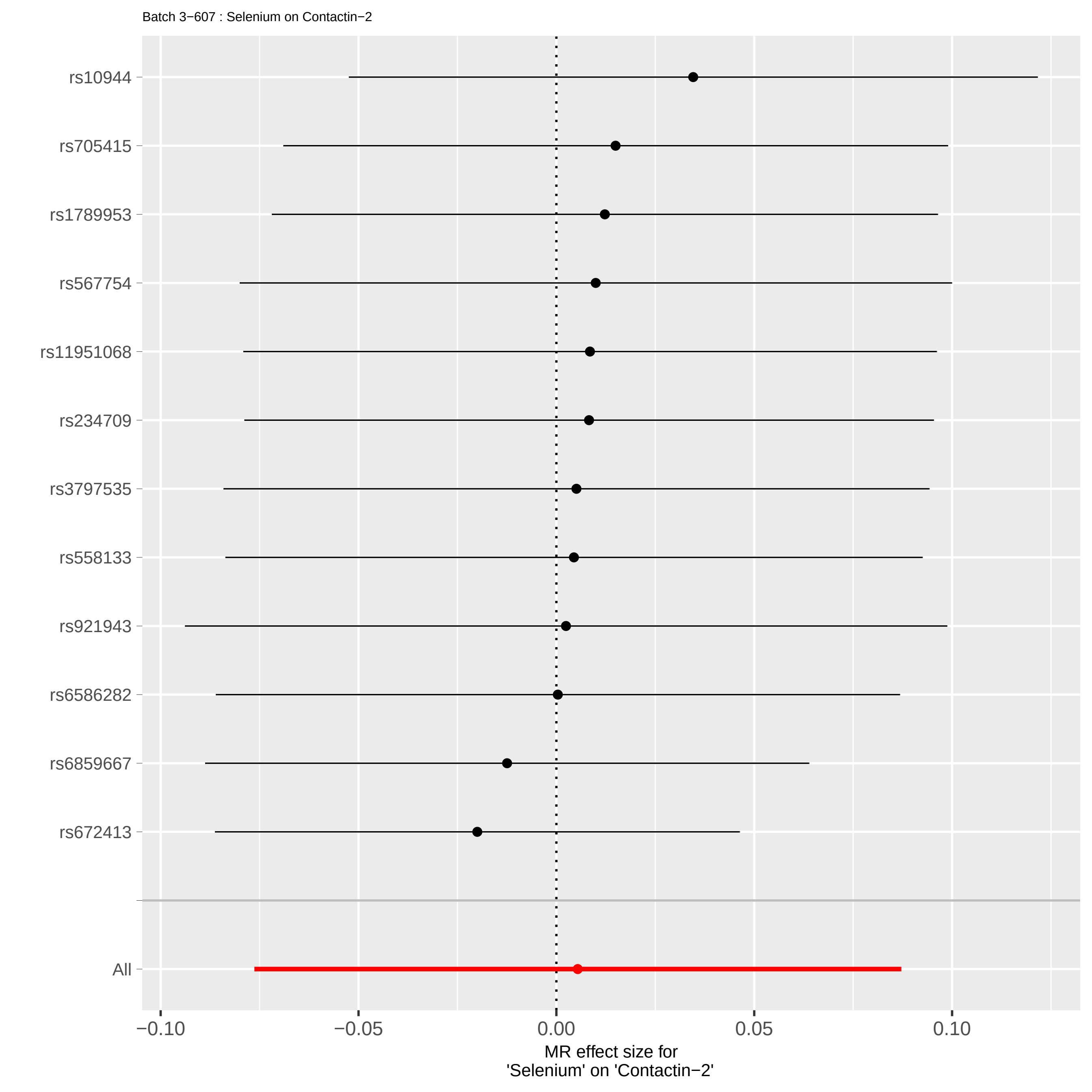
Batch 3−607 : Selenium on Contactin−2
rs10944
rs705415
rs1789953
rs567754
rs11951068
rs234709
rs3797535
rs558133
rs921943
rs6586282
rs6859667
rs672413
All
−0.10
−0.05
0.00
0.05
0.10
MR effect size for
'Selenium' on 'Contactin−2'

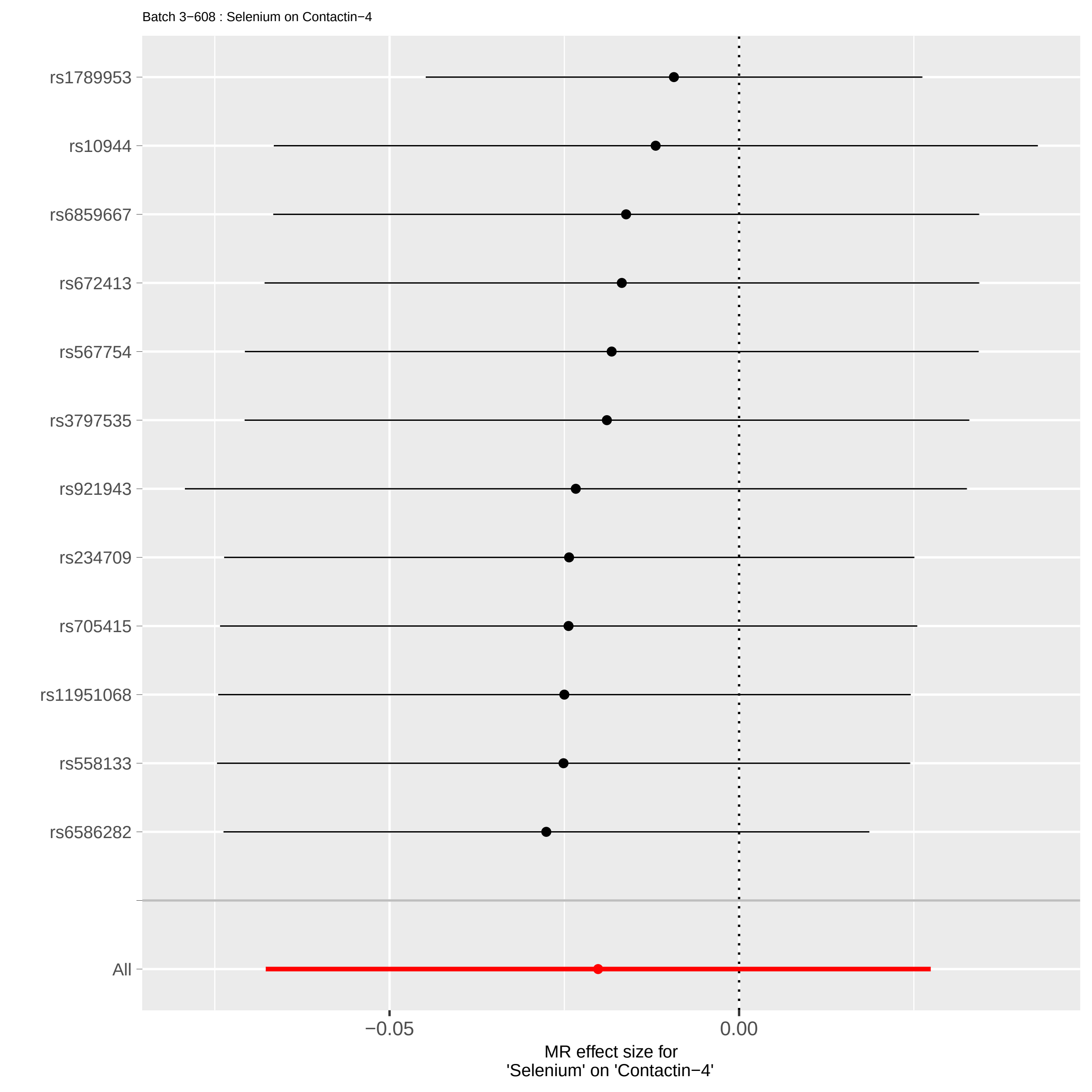

Batch 3−608 : Selenium on Contactin−4
rs1789953
rs10944
rs6859667
rs672413
rs567754
rs3797535
rs921943
rs234709
rs705415
rs11951068
rs558133
rs6586282
All
−0.05
0.00
MR effect size for
'Selenium' on 'Contactin−4'

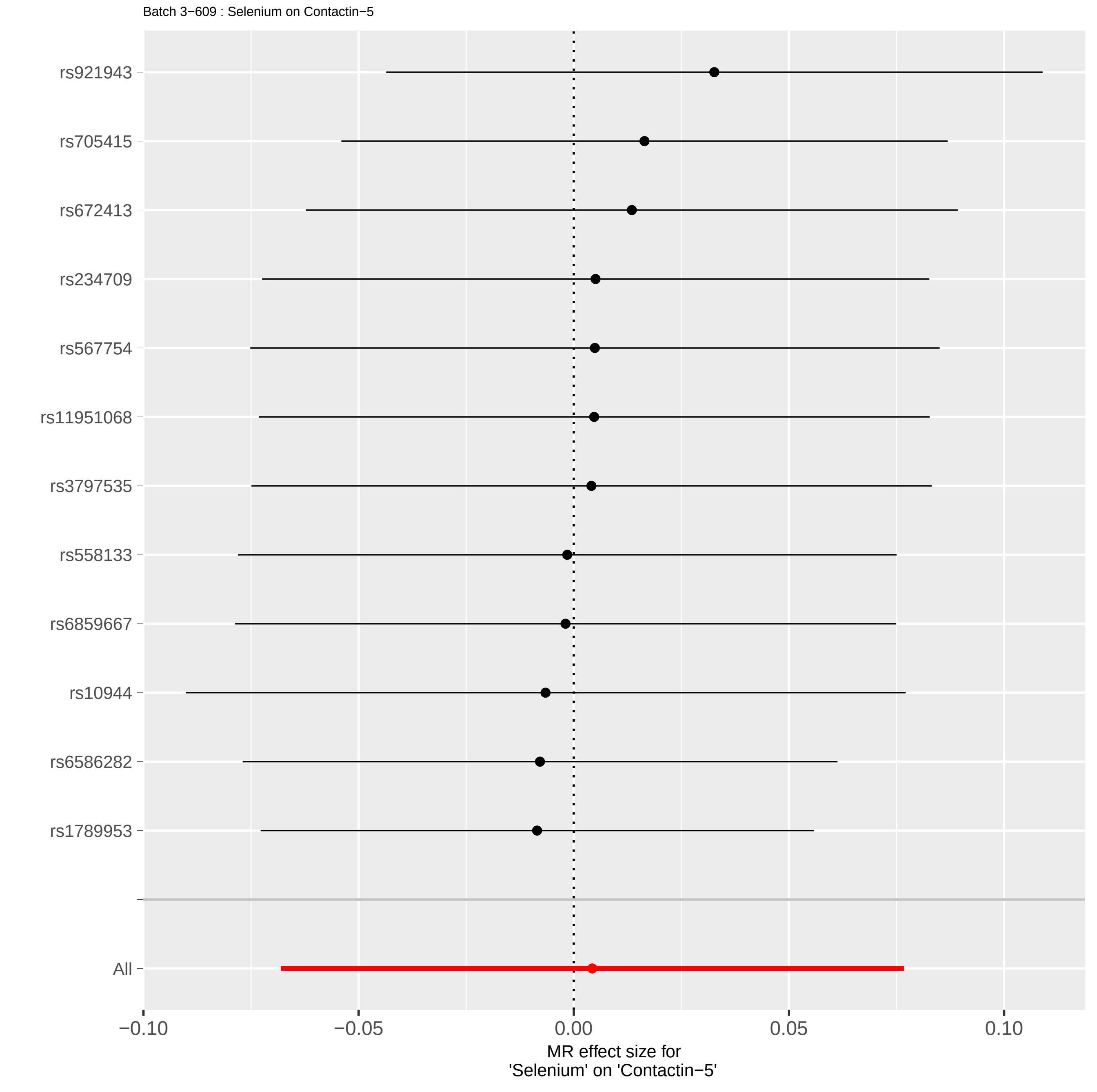

Batch 3−609 : Selenium on Contactin−5
rs921943
rs705415
rs672413
rs234709
rs567754
rs11951068
rs3797535
rs558133
rs6859667
rs10944
rs6586282
rs1789953
All
−0.10
−0.05
0.00
0.05
0.10
MR effect size for
'Selenium' on 'Contactin−5'

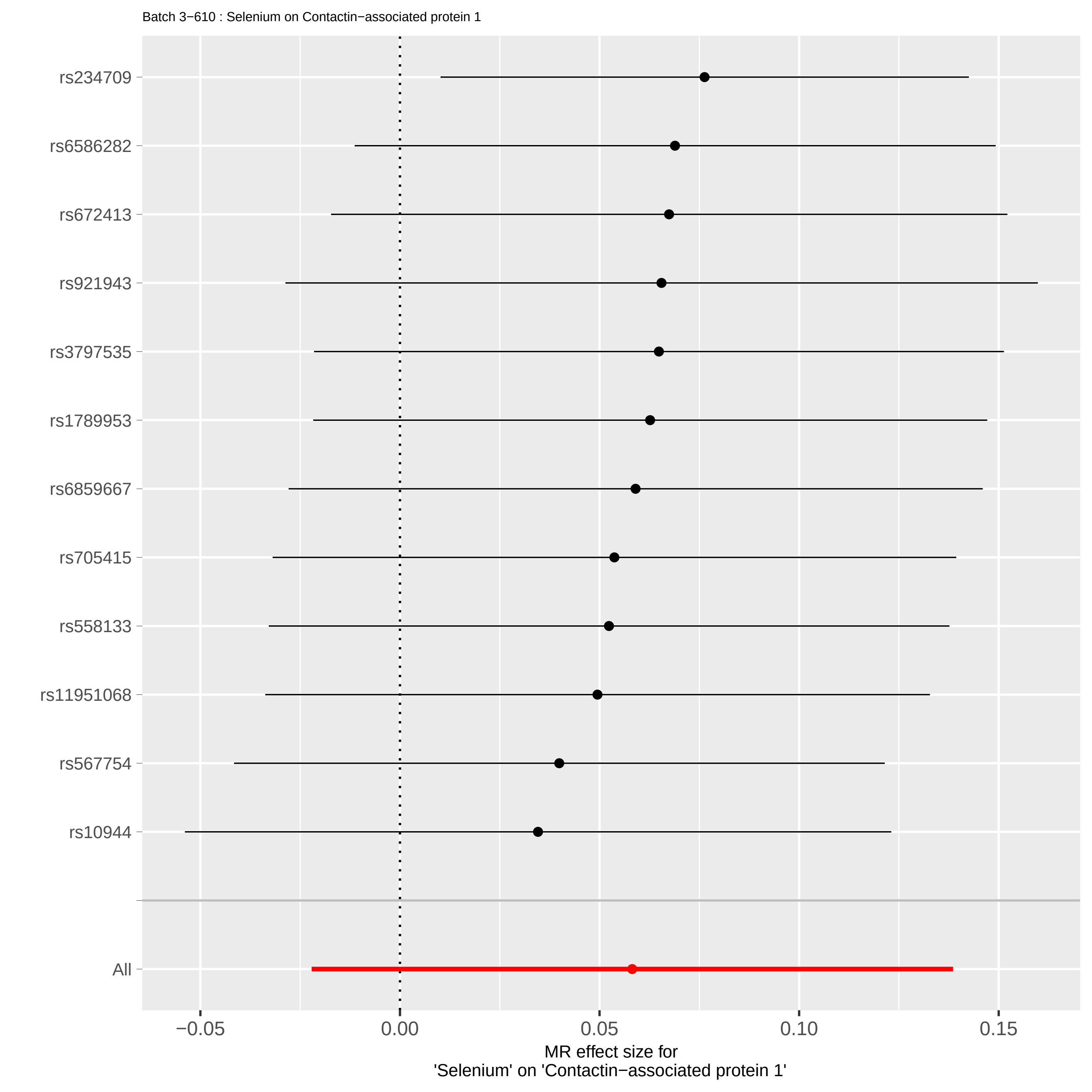

Batch 3−610 : Selenium on Contactin−associated protein 1
rs234709
rs6586282
rs672413
rs921943
rs3797535
rs1789953
rs6859667
rs705415
rs558133
rs11951068
rs567754
rs10944
All
−0.05
0.00
0.05
0.10
0.15
MR effect size for
'Selenium' on 'Contactin−associated protein 1'

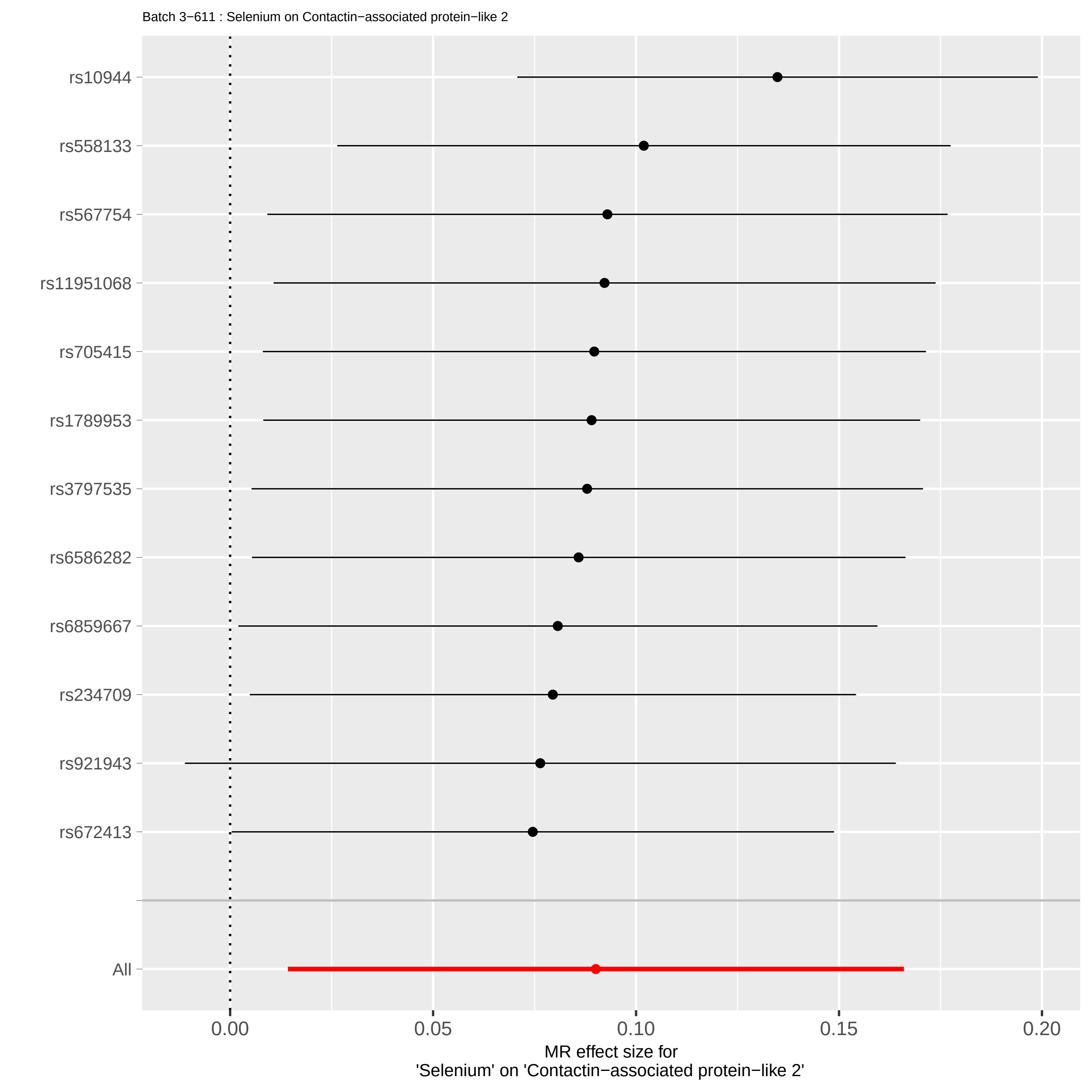

Batch 3−611 : Selenium on Contactin−associated protein−like 2
rs10944
rs558133
rs567754
rs11951068
rs705415
rs1789953
rs3797535
rs6586282
rs6859667
rs234709
rs921943
rs672413
All
0.00
0.05
0.10
0.15
0.20
MR effect size for
'Selenium' on 'Contactin−associated protein−like 2'

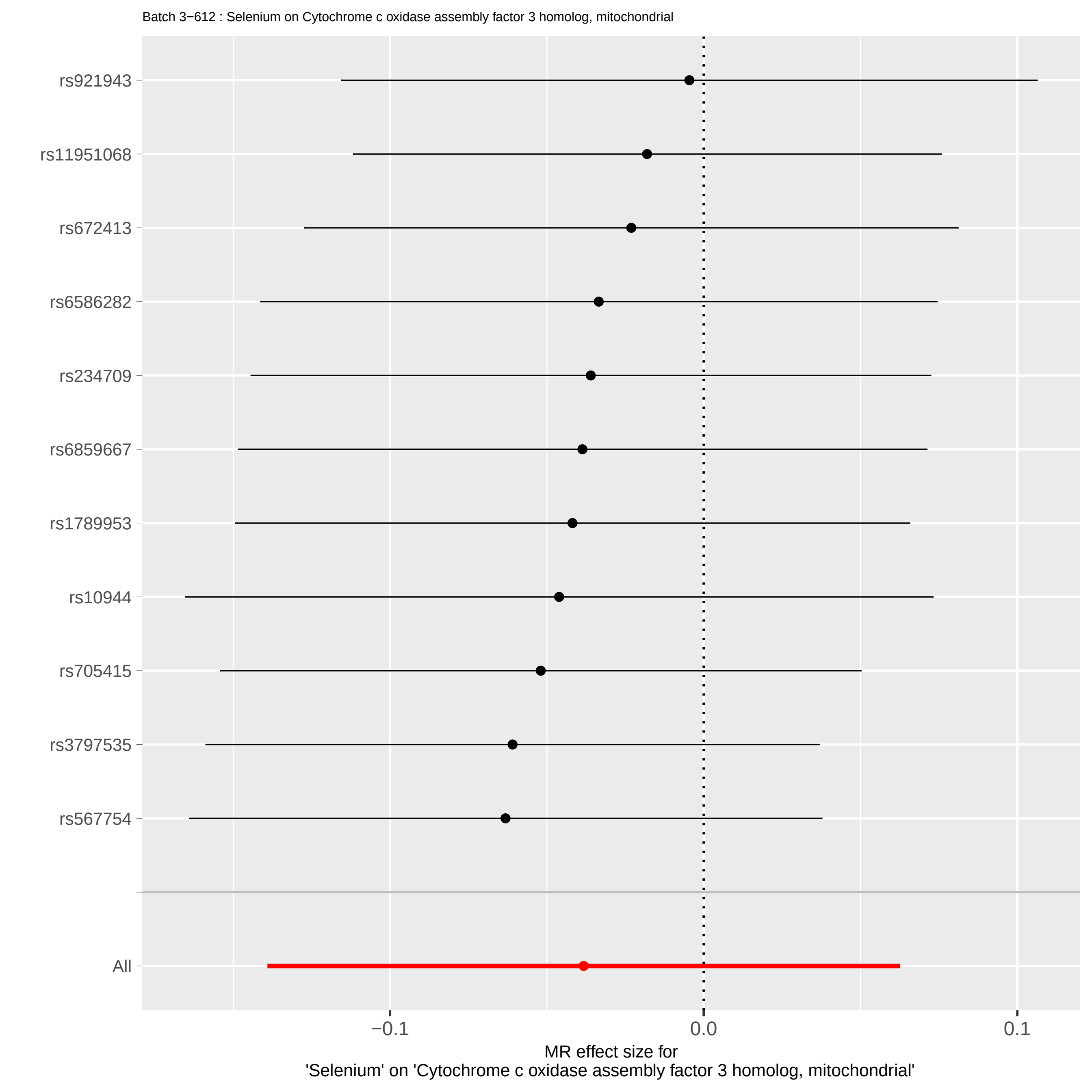
Batch 3−612 : Selenium on Cytochrome c oxidase assembly factor 3 homolog, mitochondrial
rs921943
rs11951068
rs672413
rs6586282
rs234709
rs6859667
rs1789953
rs10944
rs705415
rs3797535
rs567754
All
−0.1
0.0
0.1
MR effect size for
'Selenium' on 'Cytochrome c oxidase assembly factor 3 homolog, mitochondrial'

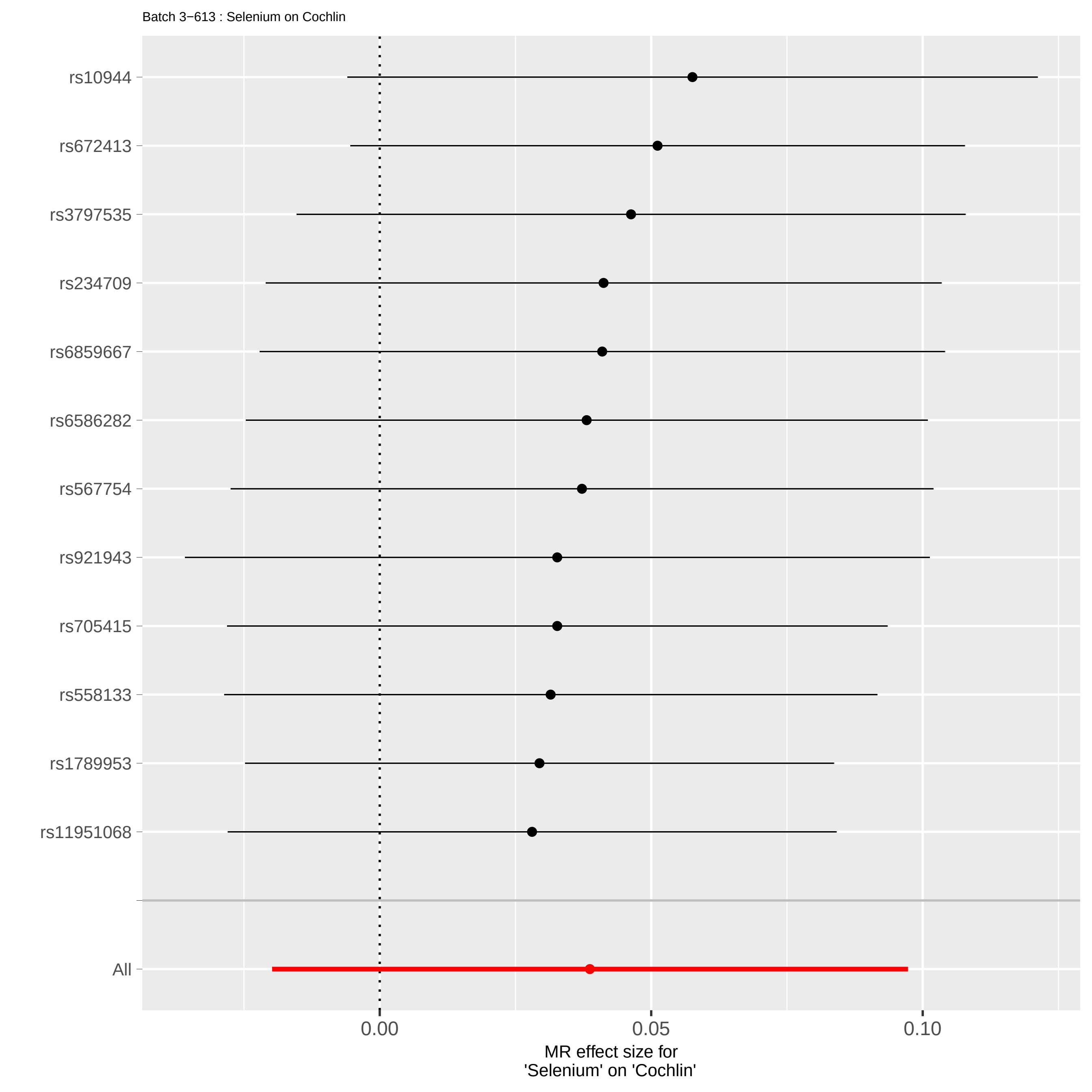

Batch 3−613 : Selenium on Cochlin
rs10944
rs672413
rs3797535
rs234709
rs6859667
rs6586282
rs567754
rs921943
rs705415
rs558133
rs1789953
rs11951068
All
0.00
0.05
0.10
MR effect size for
'Selenium' on 'Cochlin'

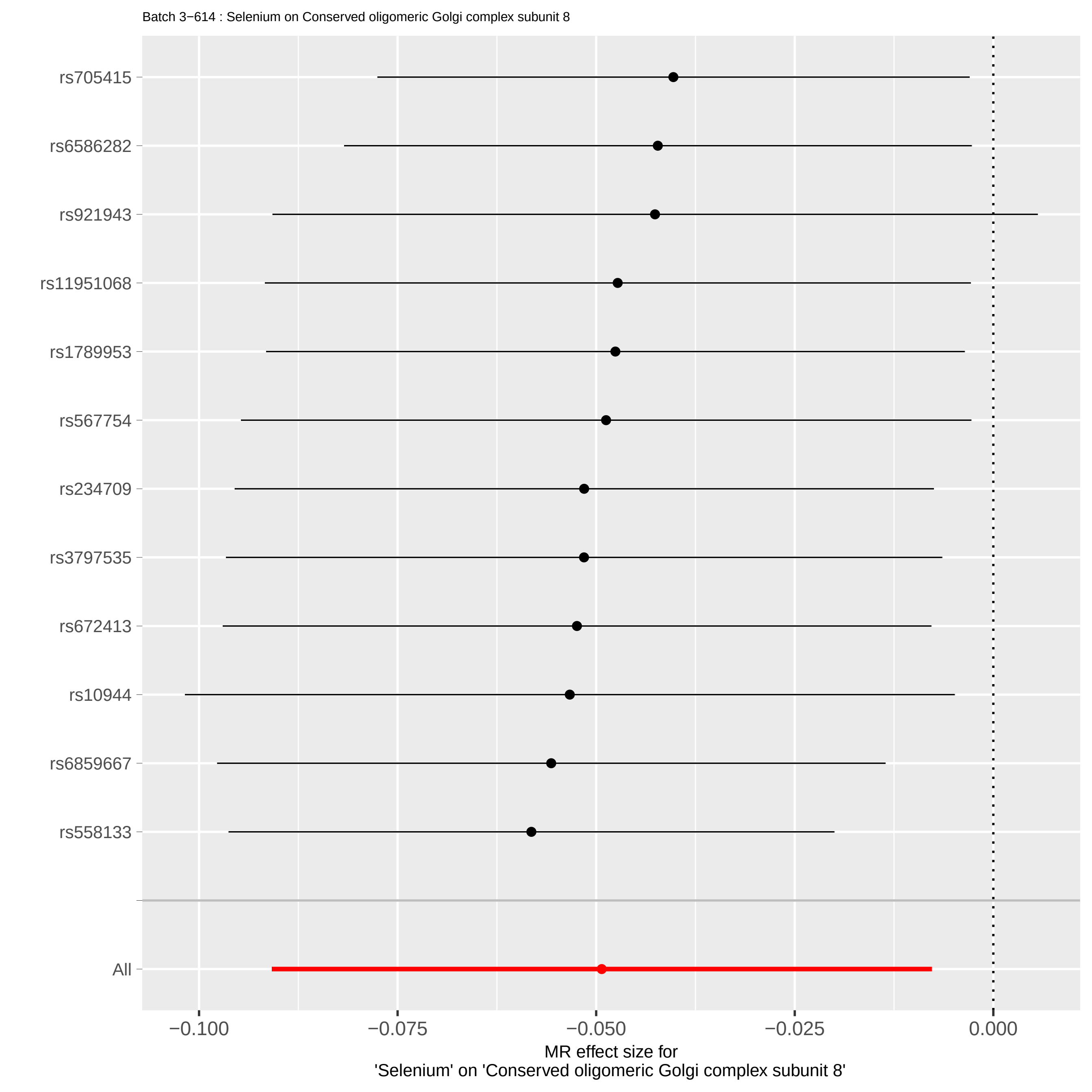
Batch 3−614 : Selenium on Conserved oligomeric Golgi complex subunit 8
rs705415
rs6586282
rs921943
rs11951068
rs1789953
rs567754
rs234709
rs3797535
rs672413
rs10944
rs6859667
rs558133
All
−0.100
−0.075
−0.050
−0.025
0.000
MR effect size for
'Selenium' on 'Conserved oligomeric Golgi complex subunit 8'

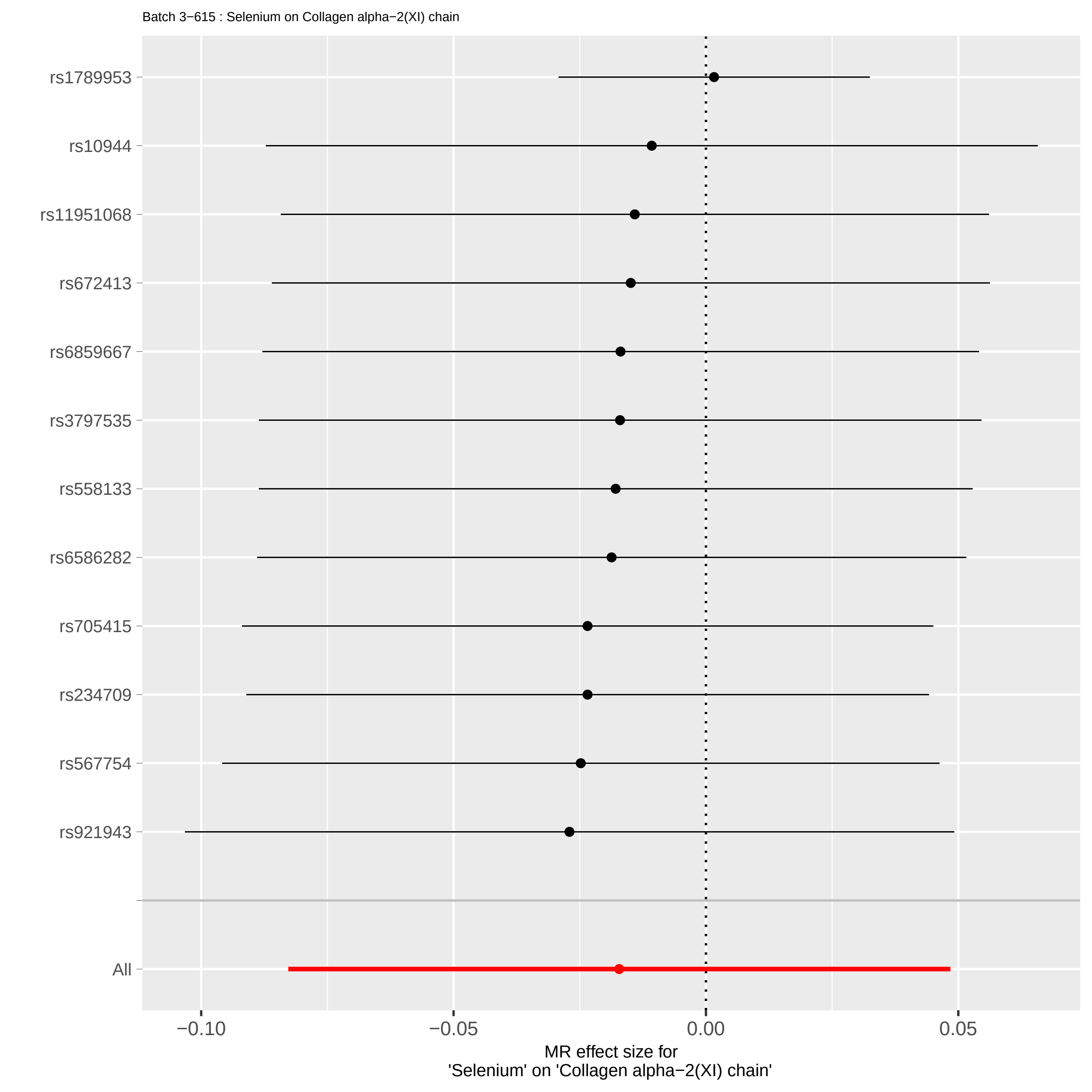

Batch 3−615 : Selenium on Collagen alpha−2(XI) chain
rs1789953
rs10944
rs11951068
rs672413
rs6859667
rs3797535
rs558133
rs6586282
rs705415
rs234709
rs567754
rs921943
All
−0.10
−0.05
0.00
0.05
MR effect size for
'Selenium' on 'Collagen alpha−2(XI) chain'

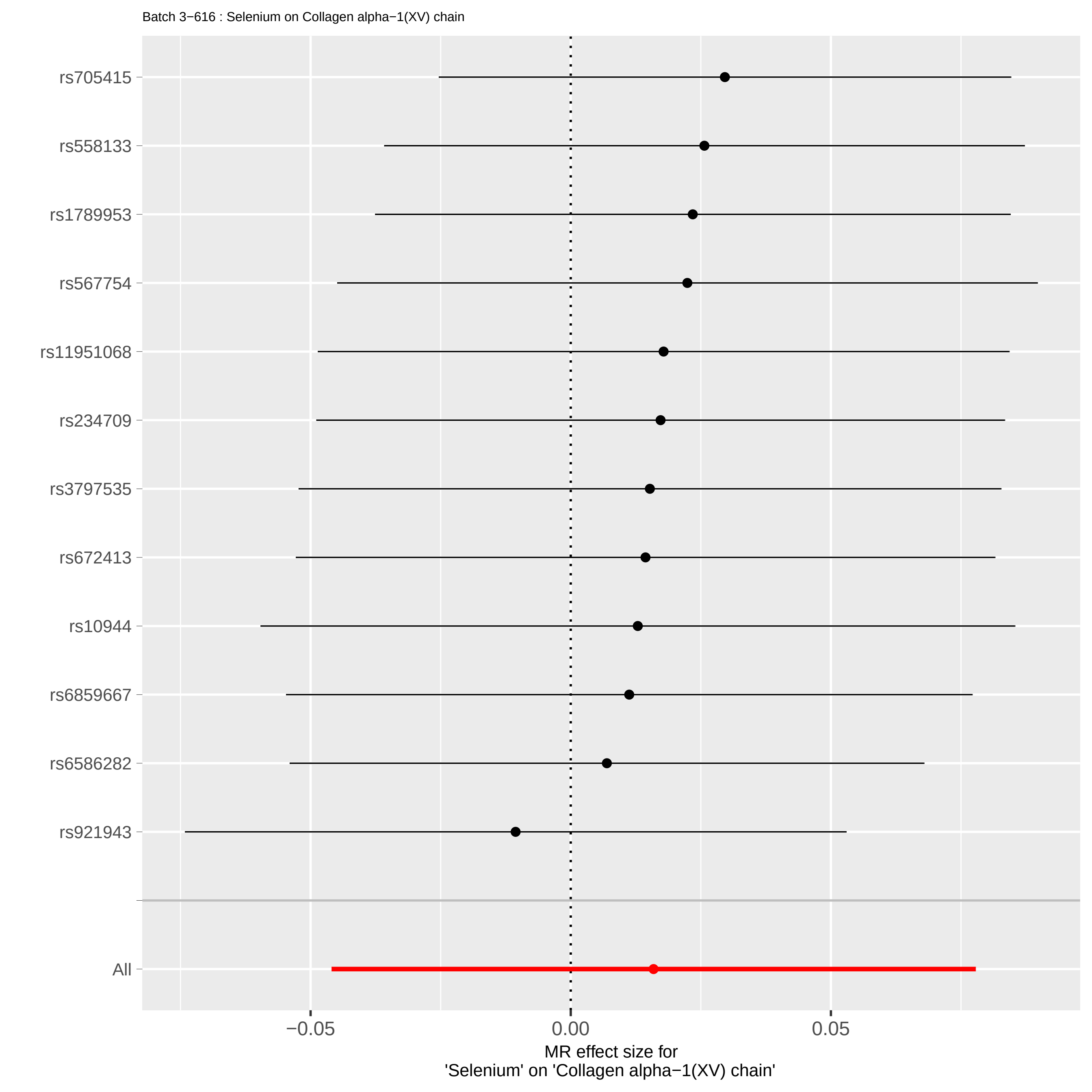

Batch 3−616 : Selenium on Collagen alpha−1(XV) chain
rs705415
rs558133
rs1789953
rs567754
rs11951068
rs234709
rs3797535
rs672413
rs10944
rs6859667
rs6586282
rs921943
All
−0.05
0.00
0.05
MR effect size for
'Selenium' on 'Collagen alpha−1(XV) chain'

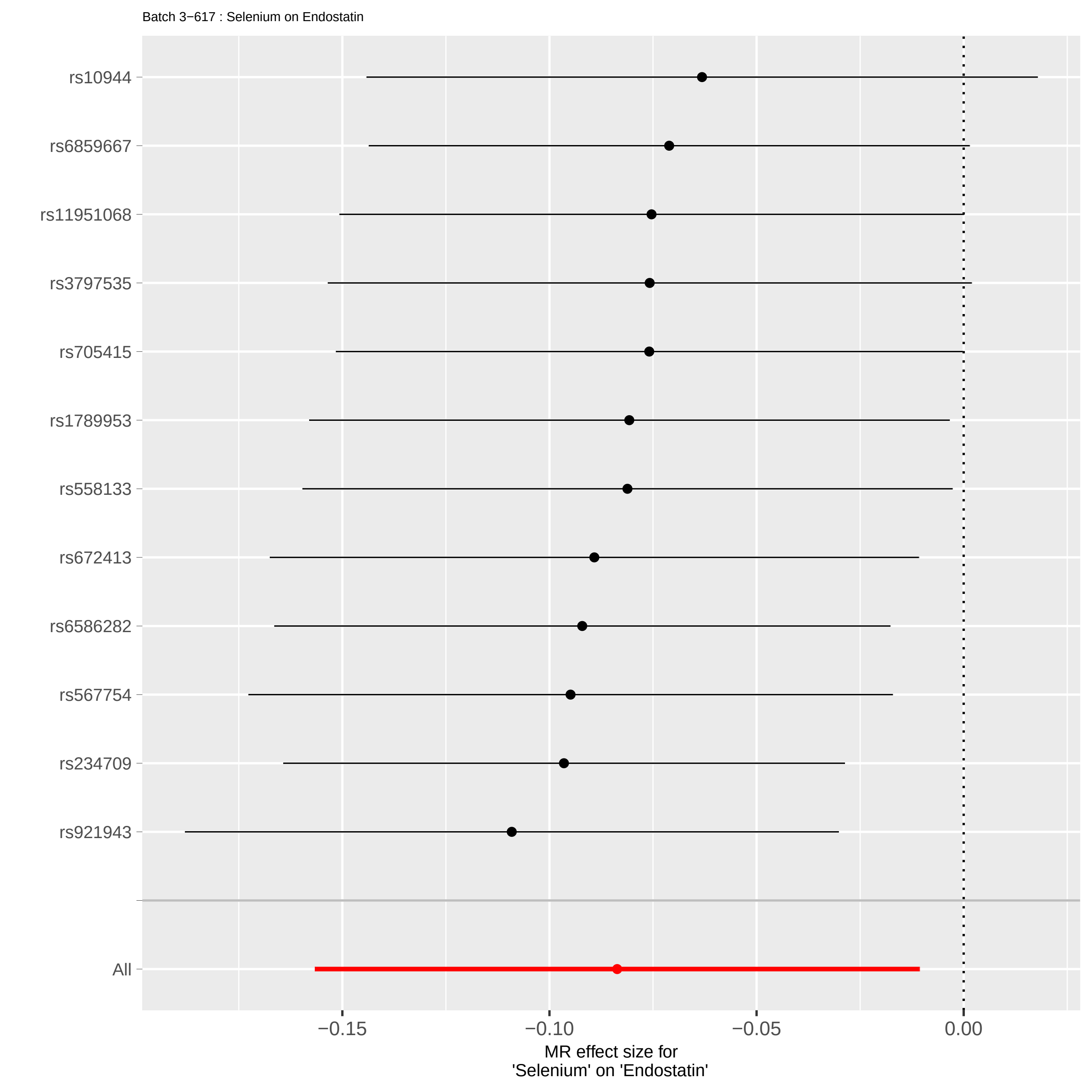

Batch 3−617 : Selenium on Endostatin
rs10944
rs6859667
rs11951068
rs3797535
rs705415
rs1789953
rs558133
rs672413
rs6586282
rs567754
rs234709
rs921943
All
−0.15
−0.10
−0.05
0.00
MR effect size for
'Selenium' on 'Endostatin'

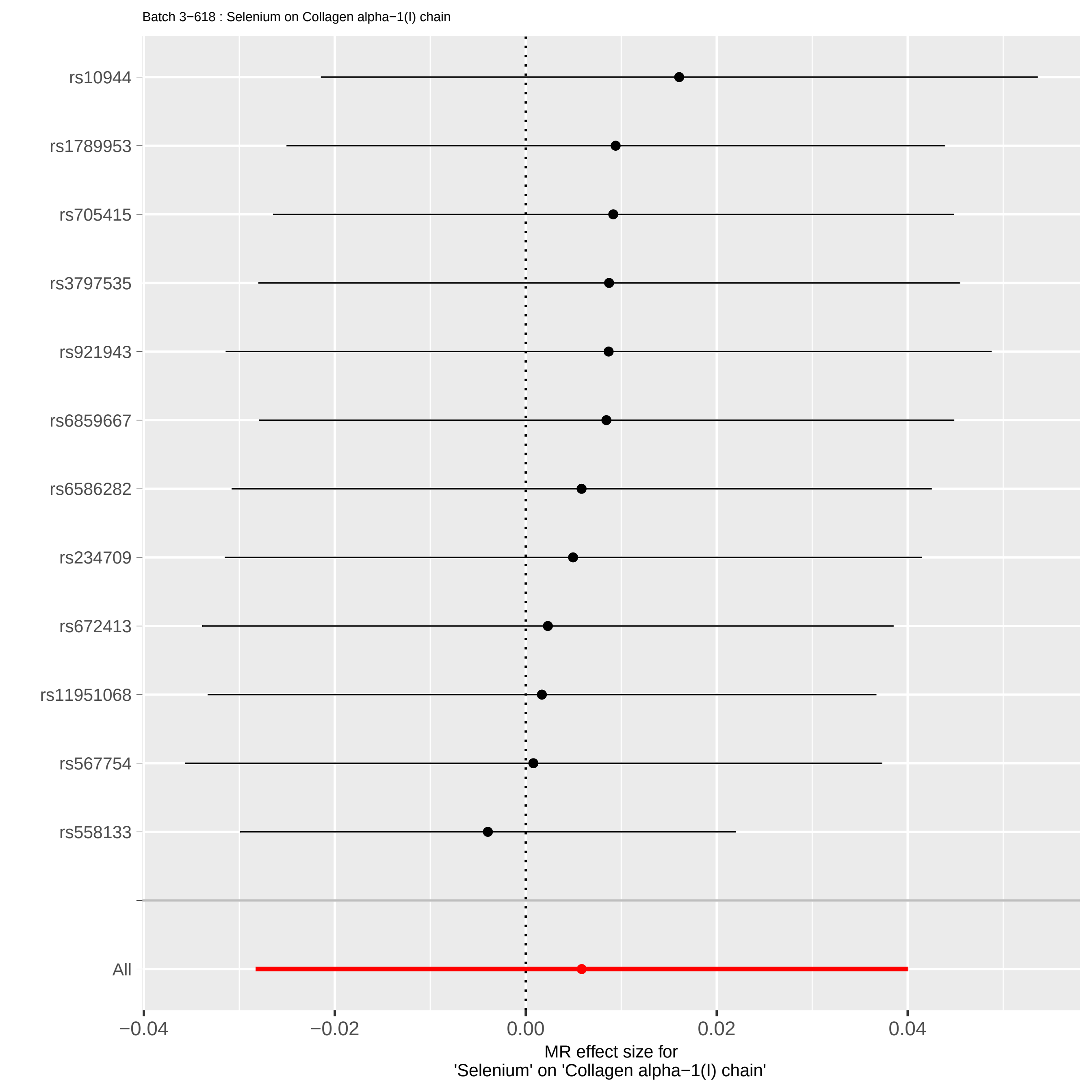
Batch 3−618 : Selenium on Collagen alpha−1(I) chain
rs10944
rs1789953
rs705415
rs3797535
rs921943
rs6859667
rs6586282
rs234709
rs672413
rs11951068
rs567754
rs558133
All
−0.04
−0.02
0.00
0.02
0.04
MR effect size for
'Selenium' on 'Collagen alpha−1(I) chain'

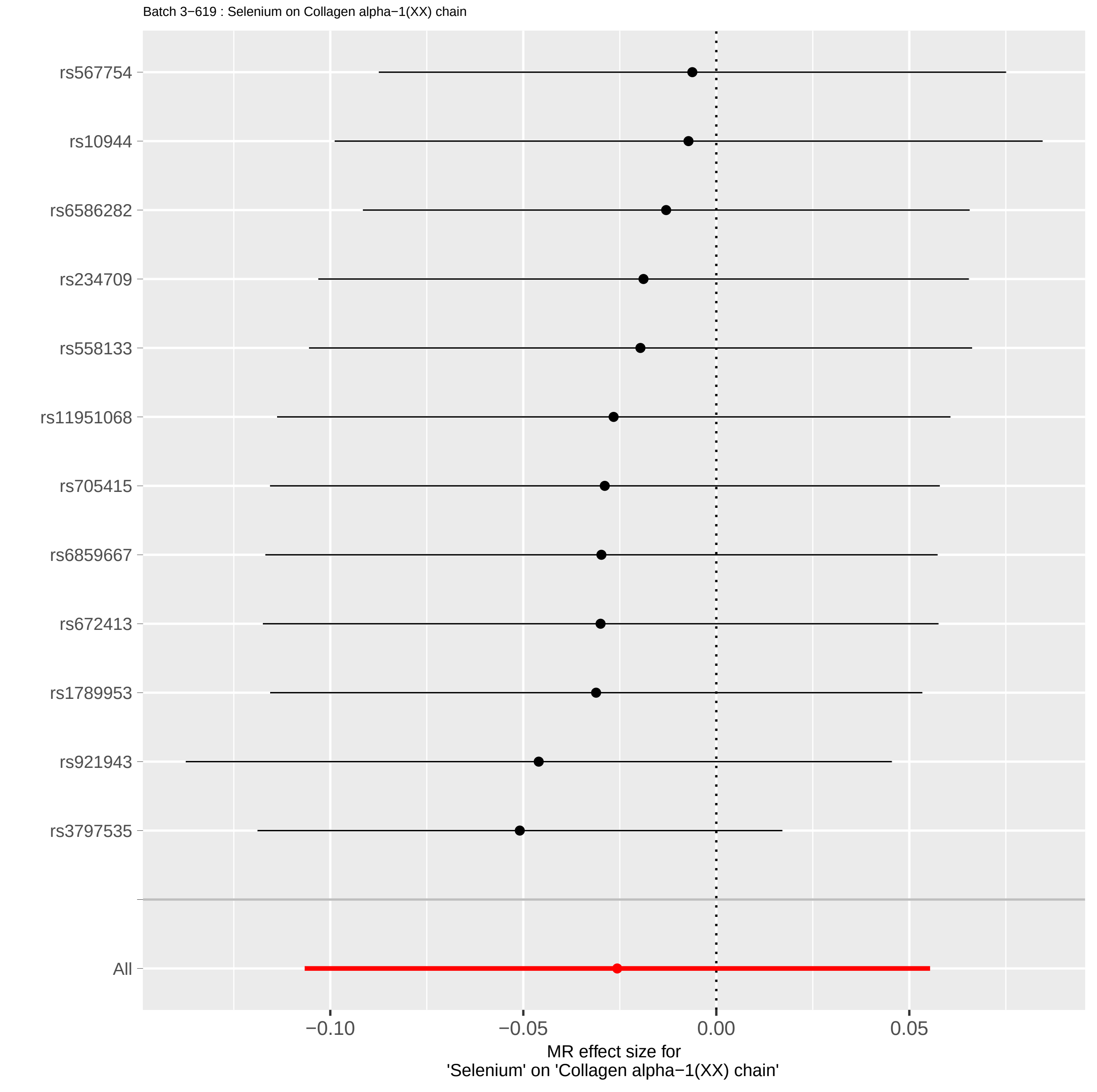
Batch 3−619 : Selenium on Collagen alpha−1(XX) chain
rs567754
rs10944
rs6586282
rs234709
rs558133
rs11951068
rs705415
rs6859667
rs672413
rs1789953
rs921943
rs3797535
All
−0.10
−0.05
0.00
0.05
MR effect size for
'Selenium' on 'Collagen alpha−1(XX) chain'

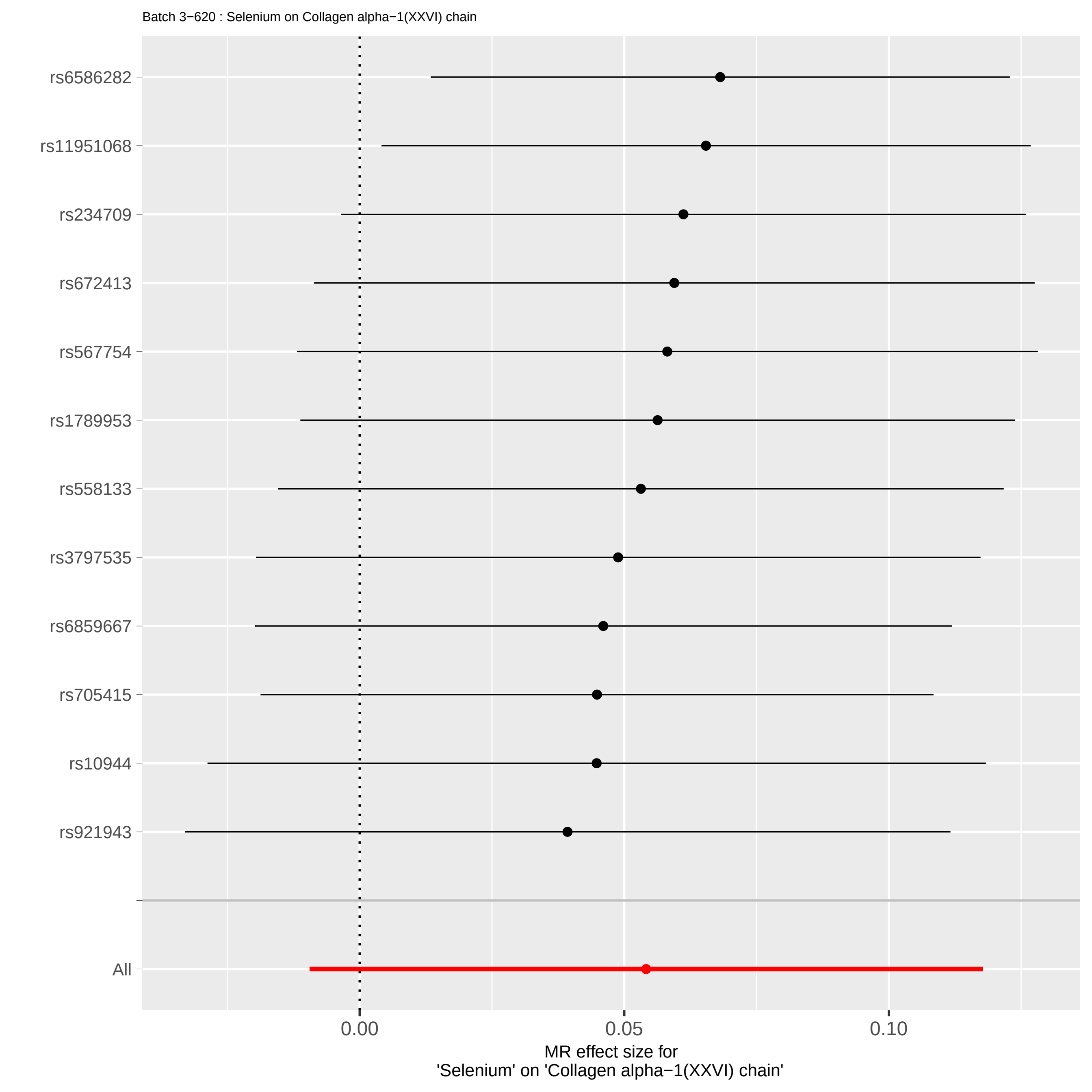
Batch 3−620 : Selenium on Collagen alpha−1(XXVI) chain
rs6586282
rs11951068
rs234709
rs672413
rs567754
rs1789953
rs558133
rs3797535
rs6859667
rs705415
rs10944
rs921943
All
0.00
0.05
0.10
MR effect size for
'Selenium' on 'Collagen alpha−1(XXVI) chain'

Batch 3−621 : Selenium on Collagen alpha−1(XXVIII) chain

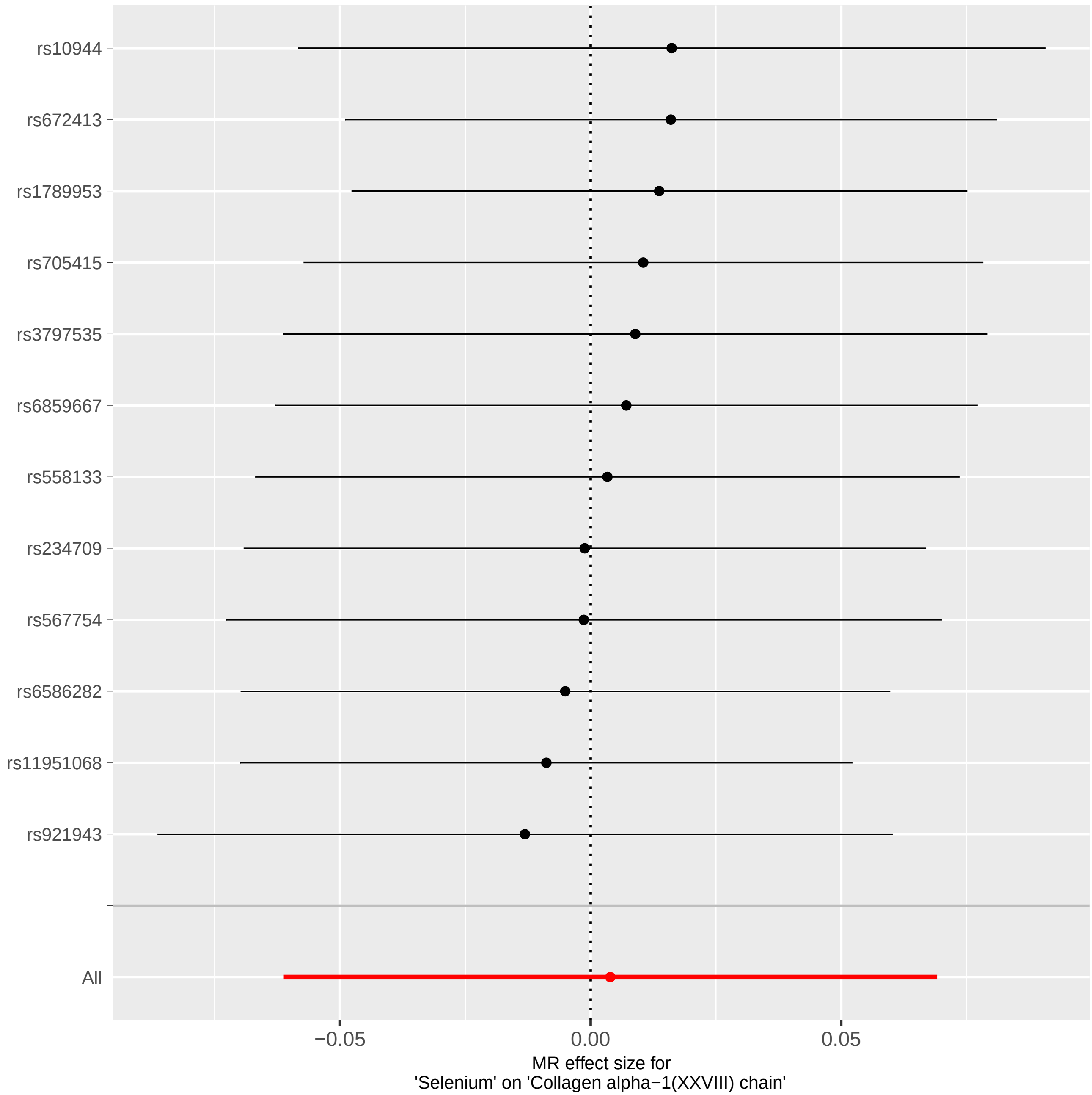

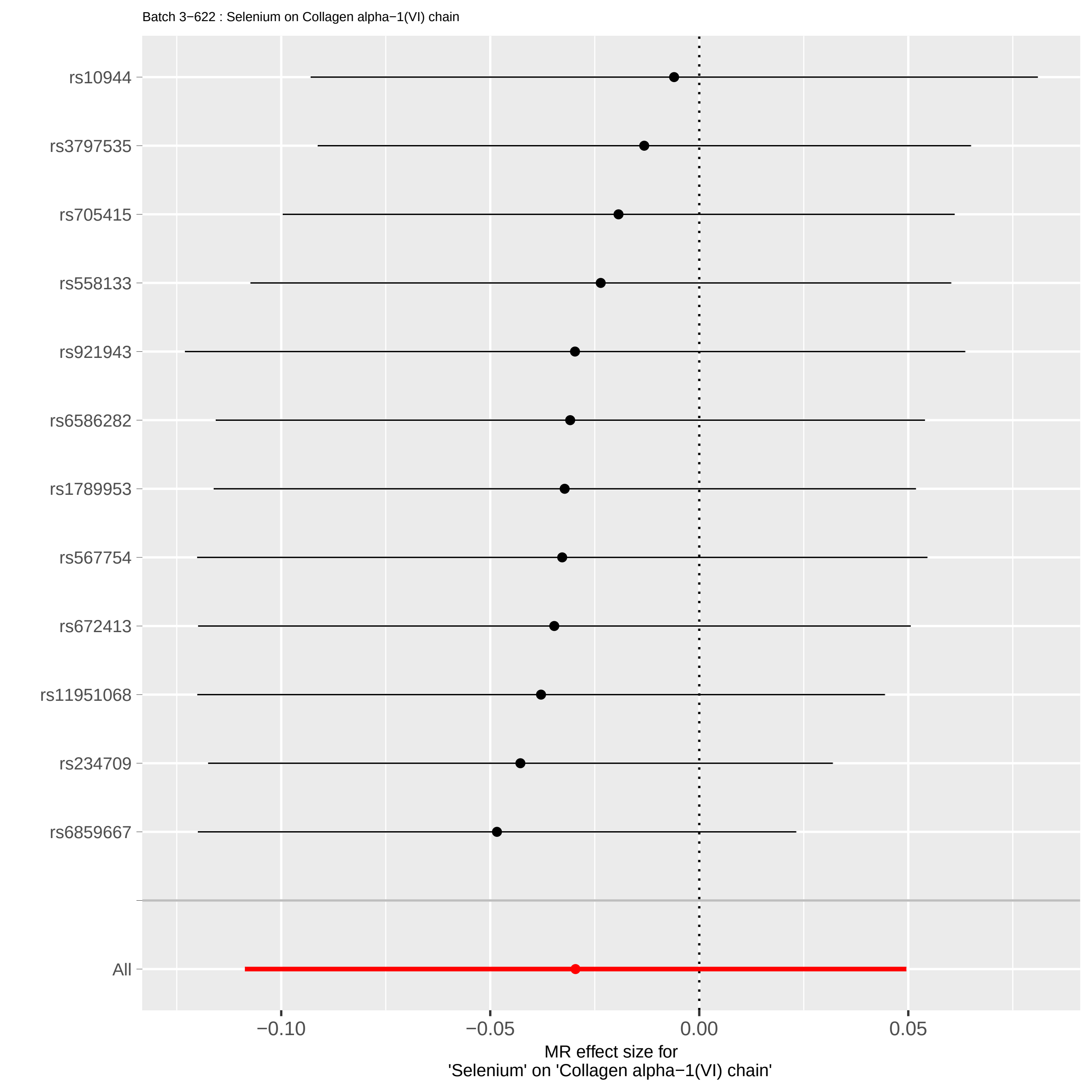
Batch 3−622 : Selenium on Collagen alpha−1(VI) chain
rs10944
rs3797535
rs705415
rs558133
rs921943
rs6586282
rs1789953
rs567754
rs672413
rs11951068
rs234709
rs6859667
All
−0.10
−0.05
0.00
0.05
MR effect size for
'Selenium' on 'Collagen alpha−1(VI) chain'

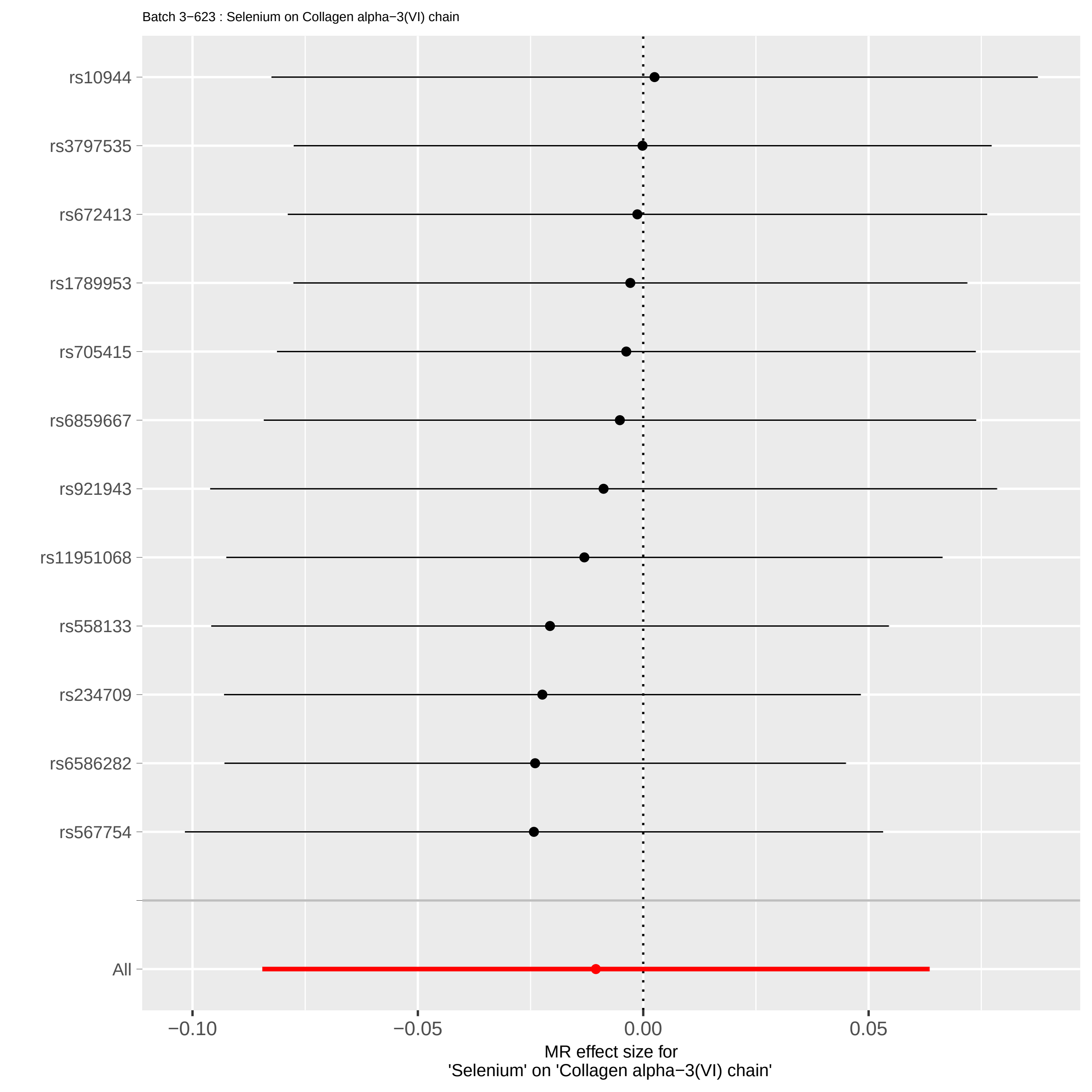

Batch 3−623 : Selenium on Collagen alpha−3(VI) chain
rs10944
rs3797535
rs672413
rs1789953
rs705415
rs6859667
rs921943
rs11951068
rs558133
rs234709
rs6586282
rs567754
All
−0.10
−0.05
0.00
0.05
MR effect size for
'Selenium' on 'Collagen alpha−3(VI) chain'

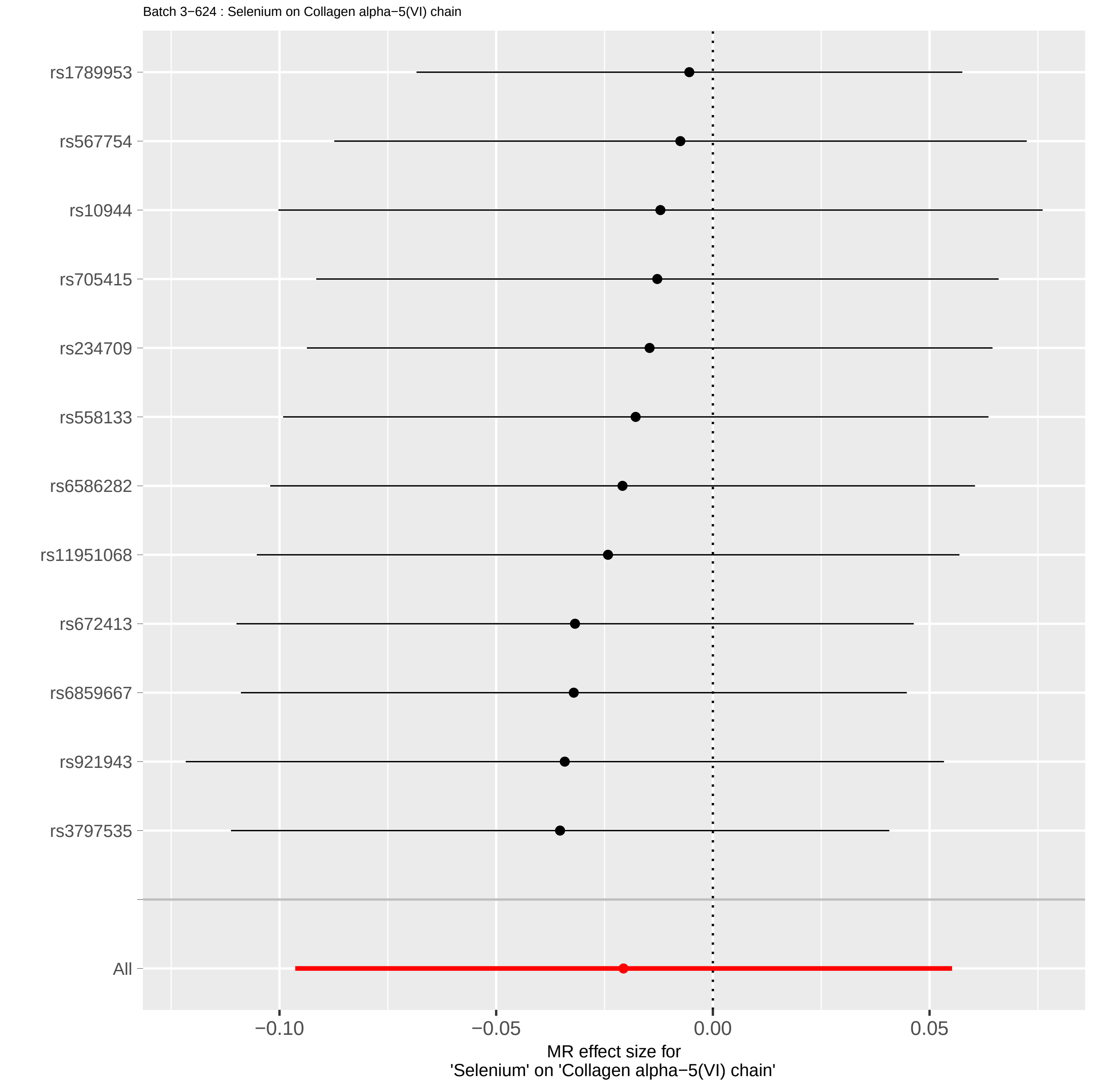

Batch 3−624 : Selenium on Collagen alpha−5(VI) chain
rs1789953
rs567754
rs10944
rs705415
rs234709
rs558133
rs6586282
rs11951068
rs672413
rs6859667
rs921943
rs3797535
All
−0.10
−0.05
0.00
0.05
MR effect size for
'Selenium' on 'Collagen alpha−5(VI) chain'

Batch 3−625 : Selenium on Collagen alpha−1(VIII) chain

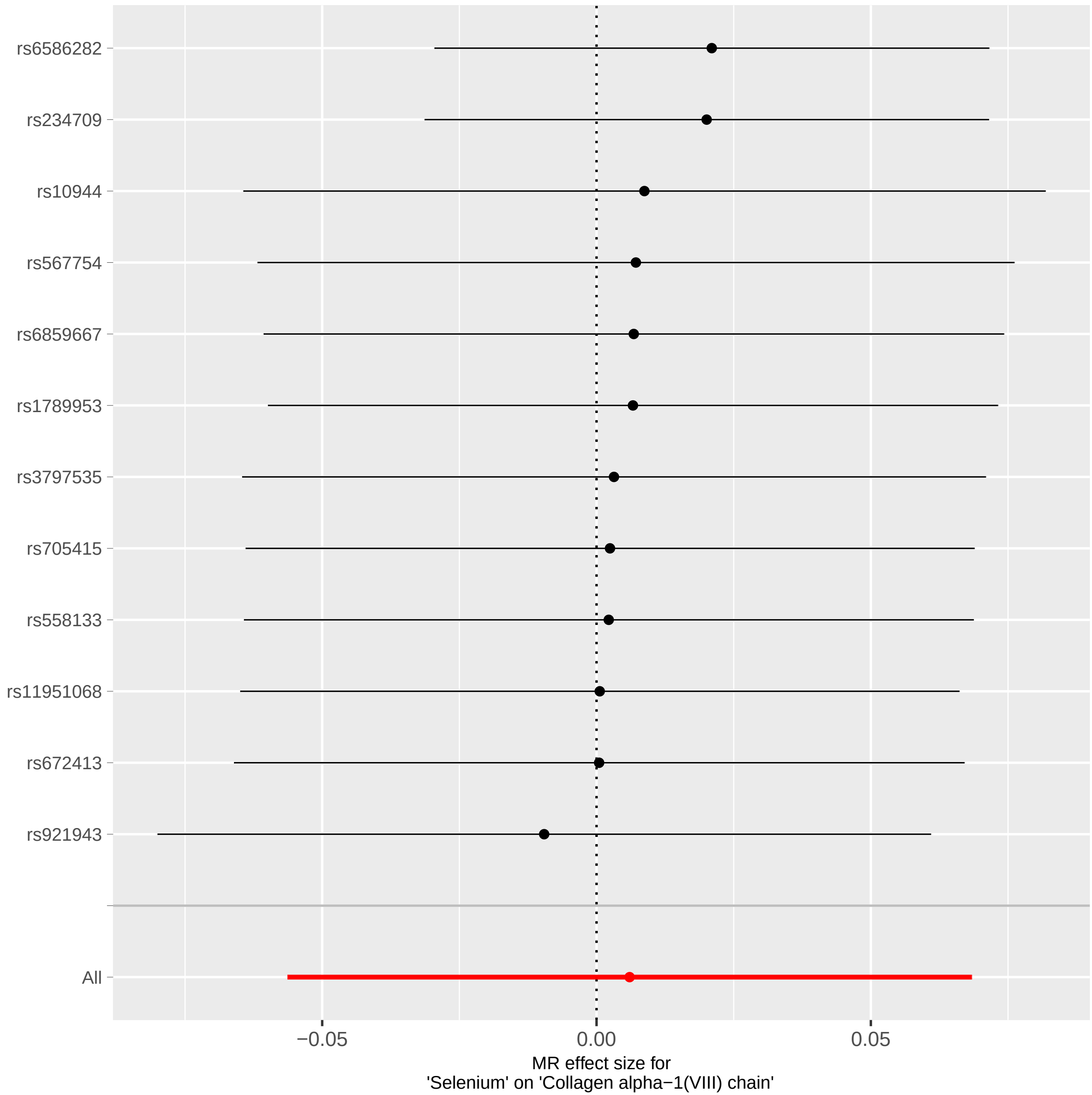

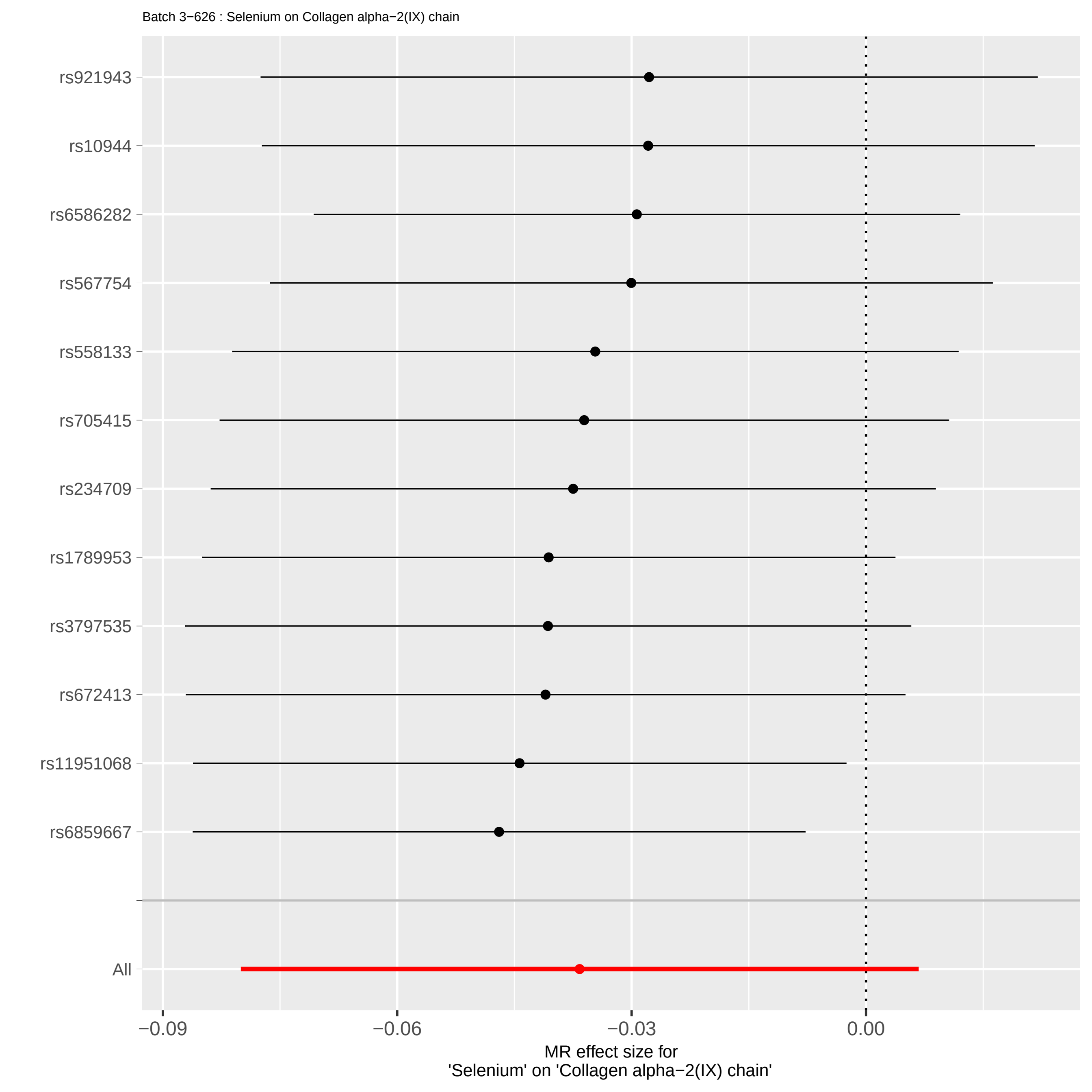

Batch 3−626 : Selenium on Collagen alpha−2(IX) chain
rs921943
rs10944
rs6586282
rs567754
rs558133
rs705415
rs234709
rs1789953
rs3797535
rs672413
rs11951068
rs6859667
All
−0.09
−0.06
−0.03
0.00
MR effect size for
'Selenium' on 'Collagen alpha−2(IX) chain'

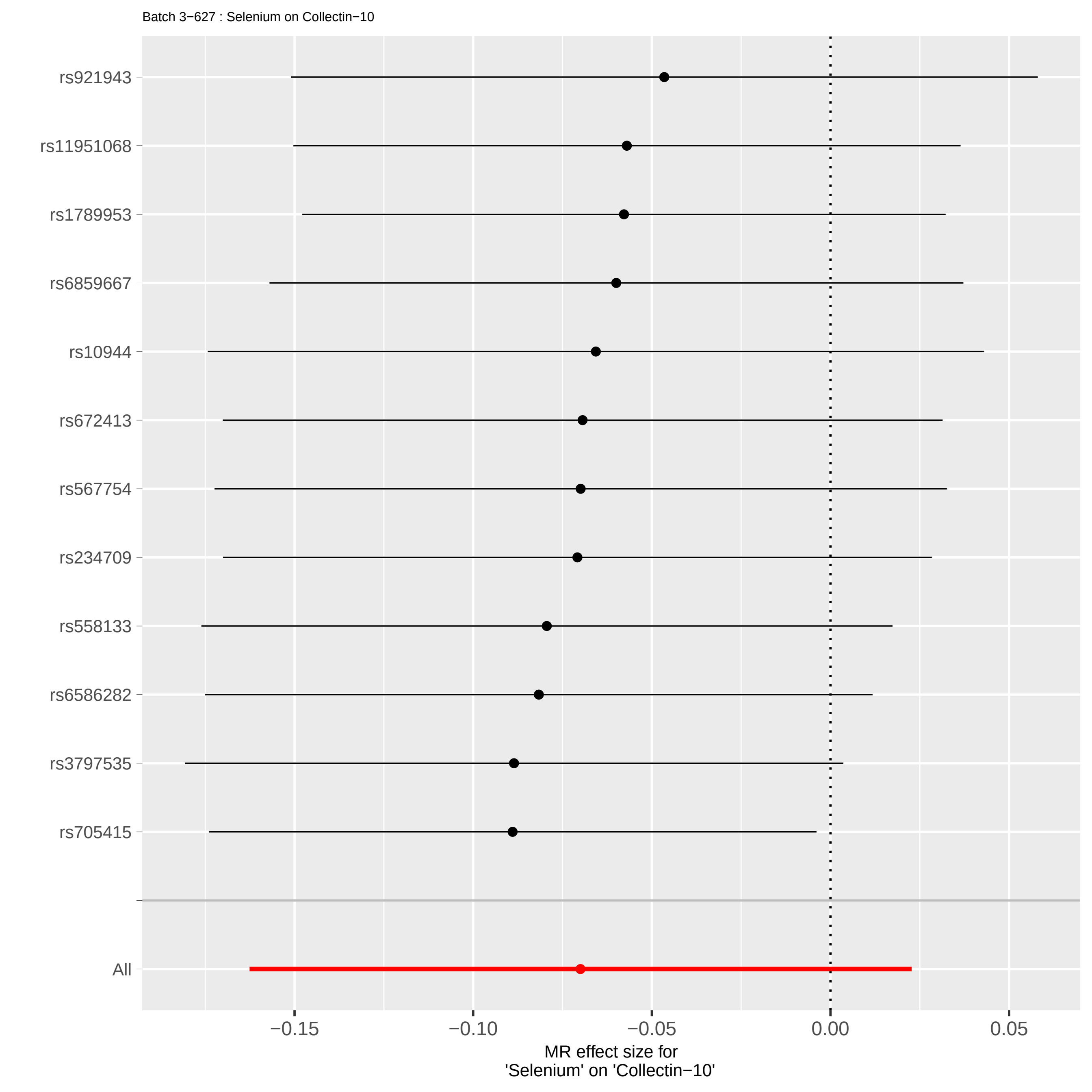

Batch 3−627 : Selenium on Collectin−10
rs921943
rs11951068
rs1789953
rs6859667
rs10944
rs672413
rs567754
rs234709
rs558133
rs6586282
rs3797535
rs705415
All
−0.15
−0.10
−0.05
0.00
0.05
MR effect size for
'Selenium' on 'Collectin−10'

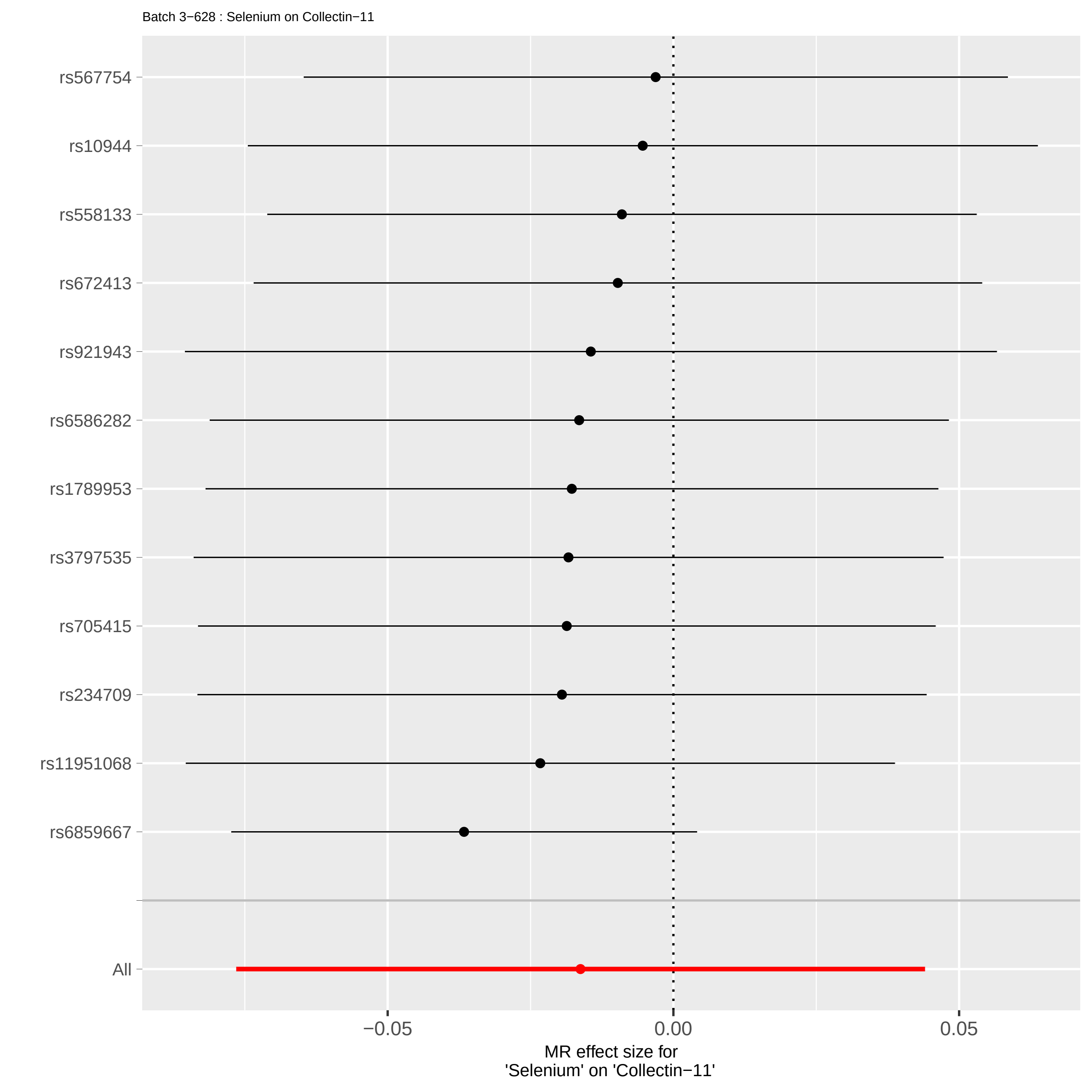
Batch 3−628 : Selenium on Collectin−11
rs567754
rs10944
rs558133
rs672413
rs921943
rs6586282
rs1789953
rs3797535
rs705415
rs234709
rs11951068
rs6859667
All
−0.05
0.00
0.05
MR effect size for
'Selenium' on 'Collectin−11'

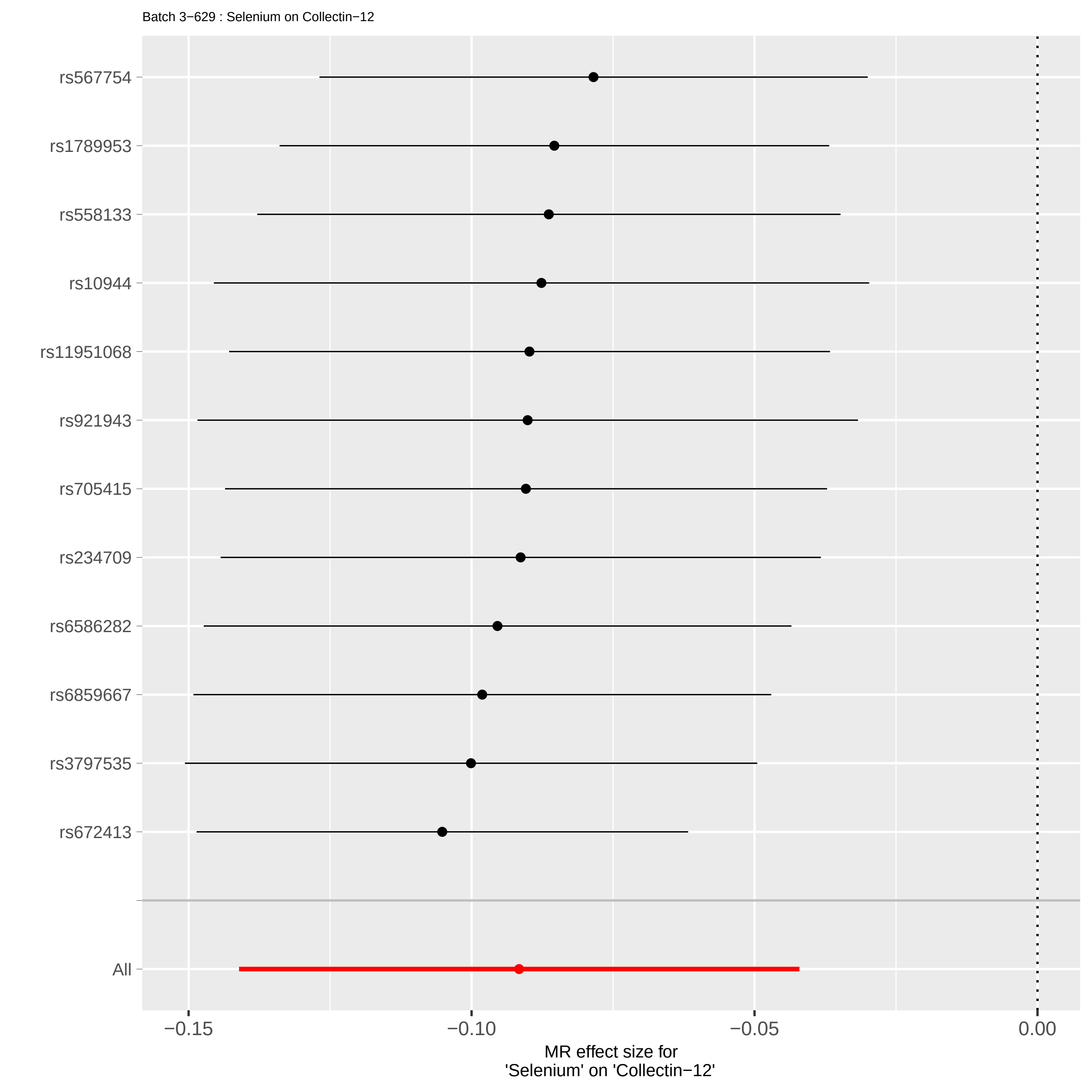
Batch 3−629 : Selenium on Collectin−12
rs567754
rs1789953
rs558133
rs10944
rs11951068
rs921943
rs705415
rs234709
rs6586282
rs6859667
rs3797535
rs672413
All
−0.15
−0.10
−0.05
0.00
MR effect size for
'Selenium' on 'Collectin−12'

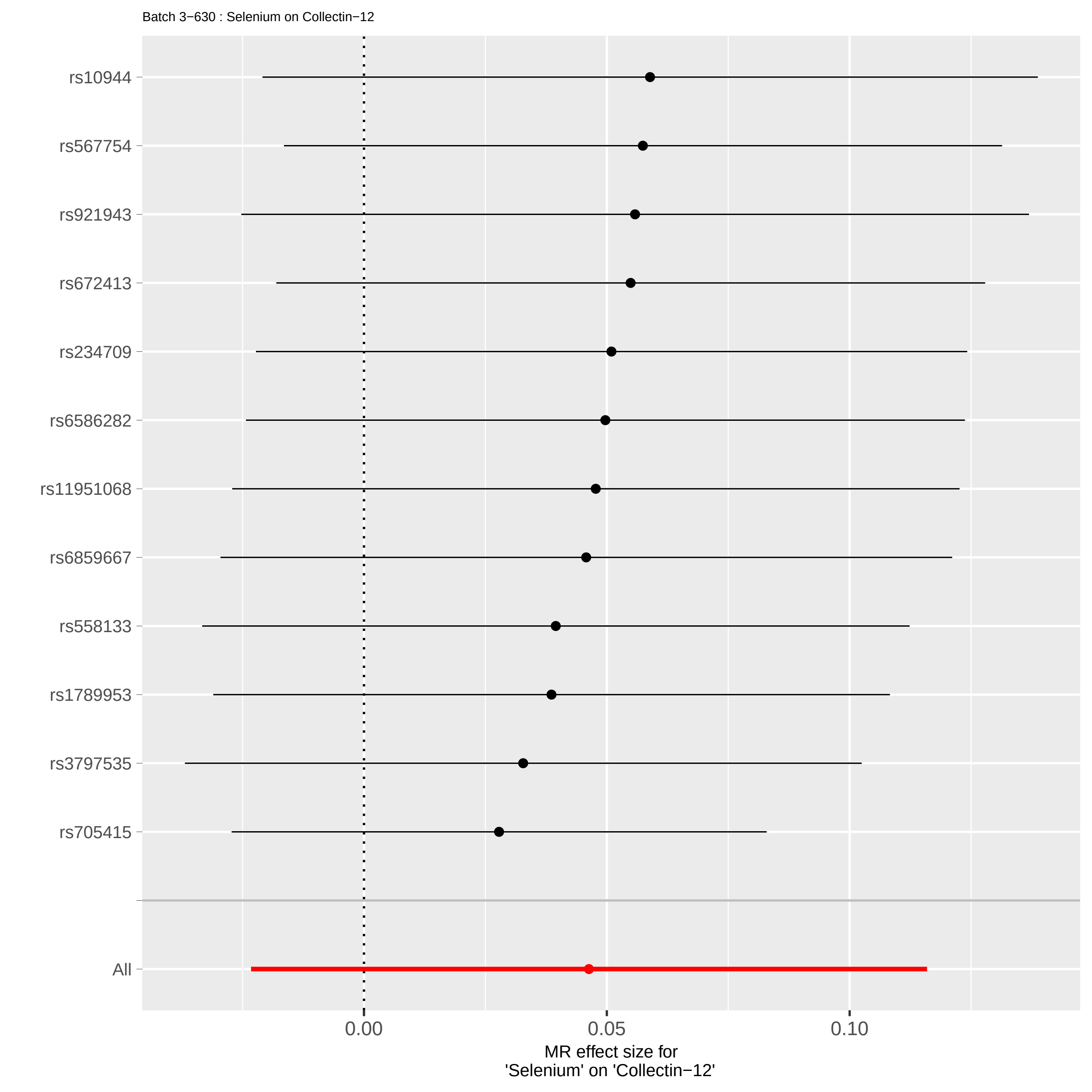

Batch 3−630 : Selenium on Collectin−12
rs10944
rs567754
rs921943
rs672413
rs234709
rs6586282
rs11951068
rs6859667
rs558133
rs1789953
rs3797535
rs705415
All
0.00
0.05
0.10
MR effect size for
'Selenium' on 'Collectin−12'

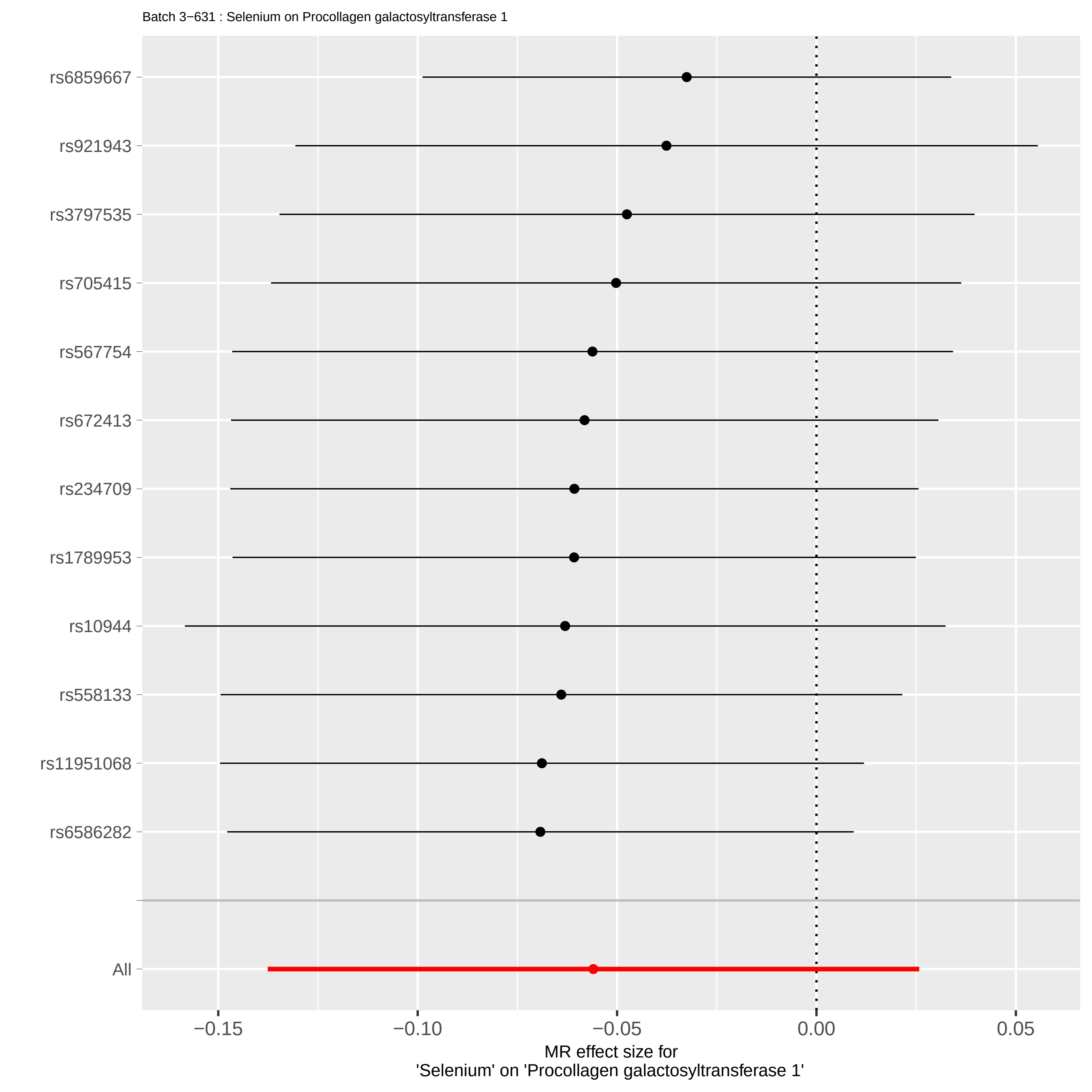

Batch 3−631 : Selenium on Procollagen galactosyltransferase 1
rs6859667
rs921943
rs3797535
rs705415
rs567754
rs672413
rs234709
rs1789953
rs10944
rs558133
rs11951068
rs6586282
All
−0.15
−0.10
−0.05
0.00
0.05
MR effect size for
'Selenium' on 'Procollagen galactosyltransferase 1'

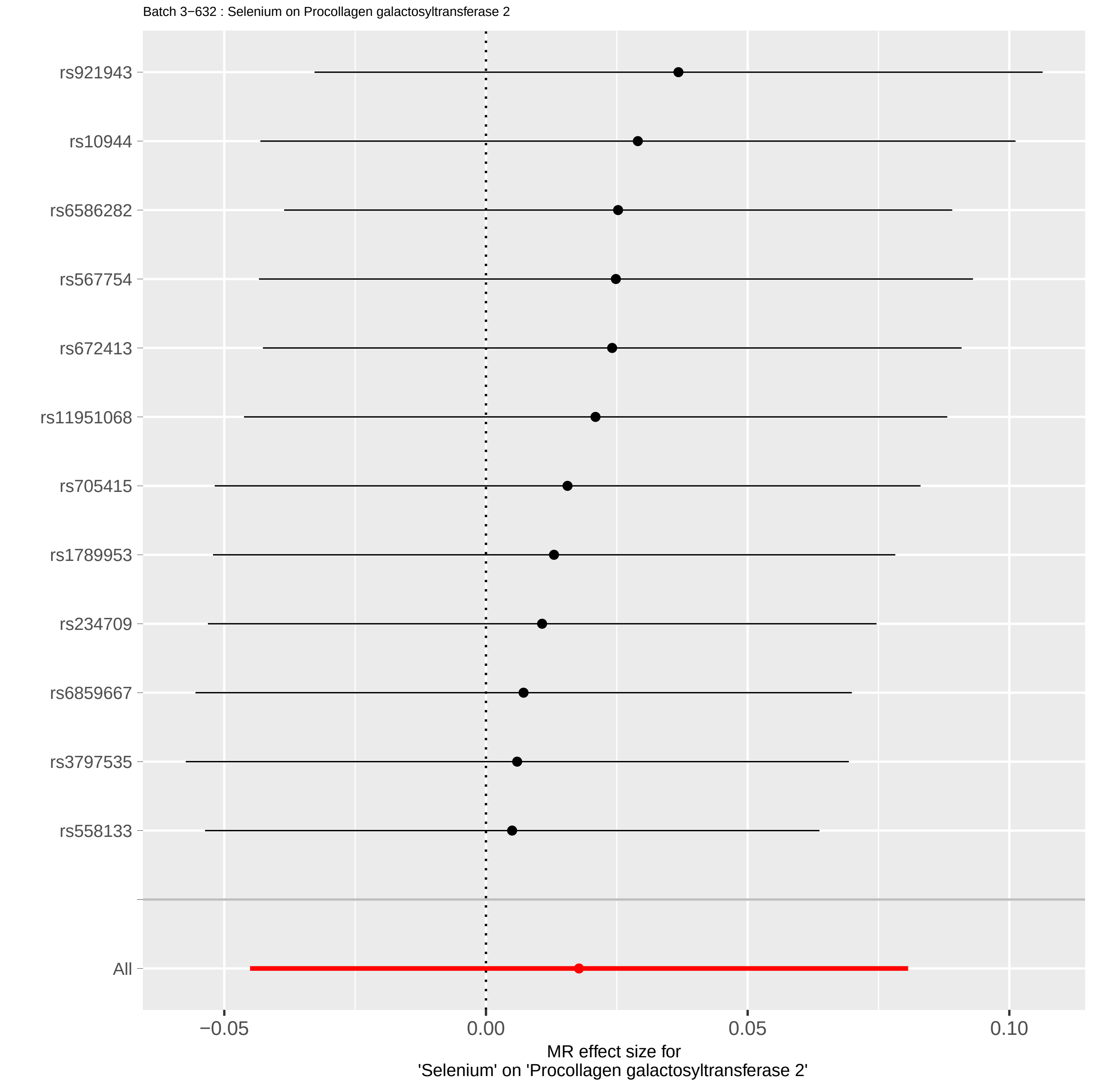
Batch 3−632 : Selenium on Procollagen galactosyltransferase 2
rs921943
rs10944
rs6586282
rs567754
rs672413
rs11951068
rs705415
rs1789953
rs234709
rs6859667
rs3797535
rs558133
All
−0.05
0.00
0.05
0.10
MR effect size for
'Selenium' on 'Procollagen galactosyltransferase 2'

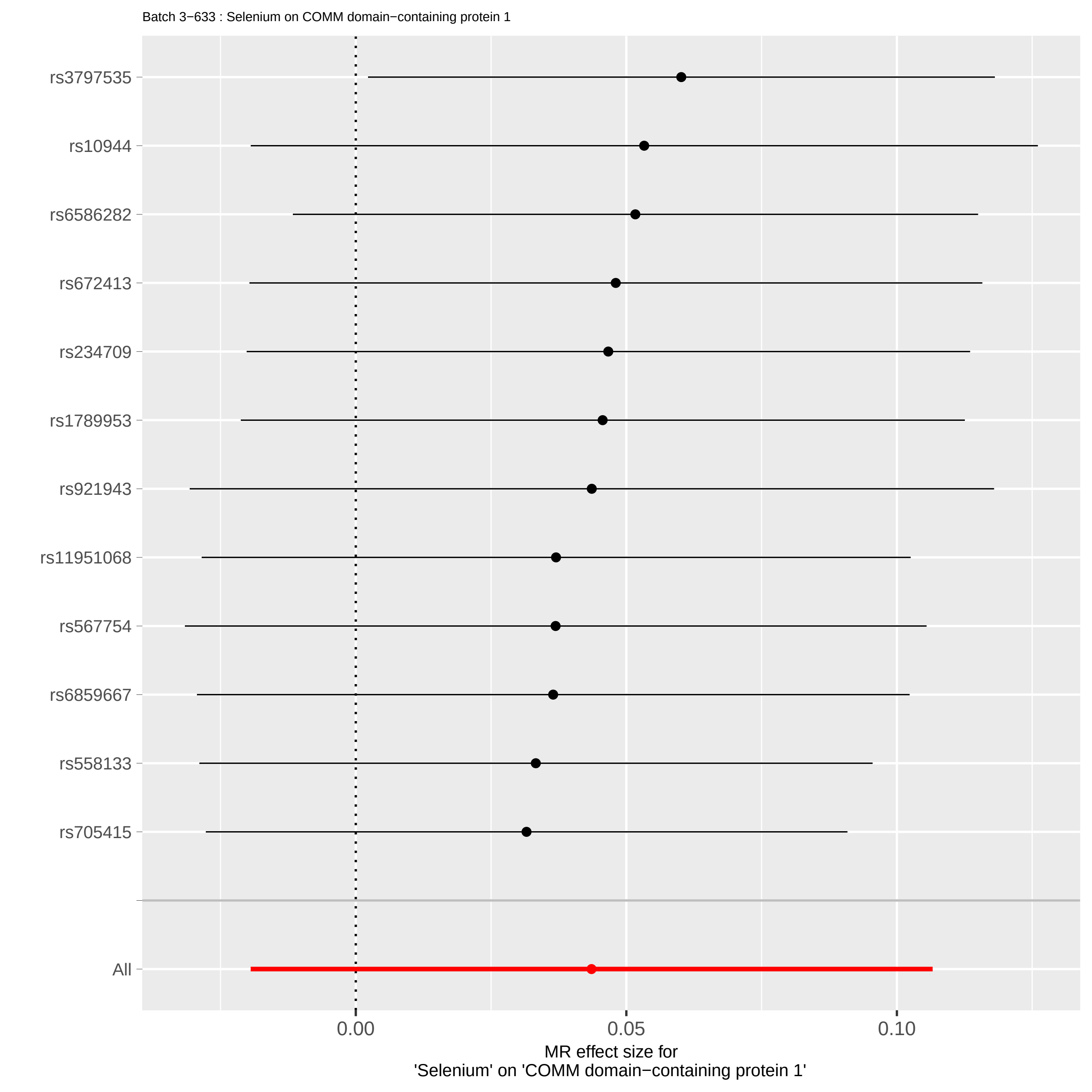

Batch 3−633 : Selenium on COMM domain−containing protein 1
rs3797535
rs10944
rs6586282
rs672413
rs234709
rs1789953
rs921943
rs11951068
rs567754
rs6859667
rs558133
rs705415
All
0.00
0.05
0.10
MR effect size for
'Selenium' on 'COMM domain−containing protein 1'

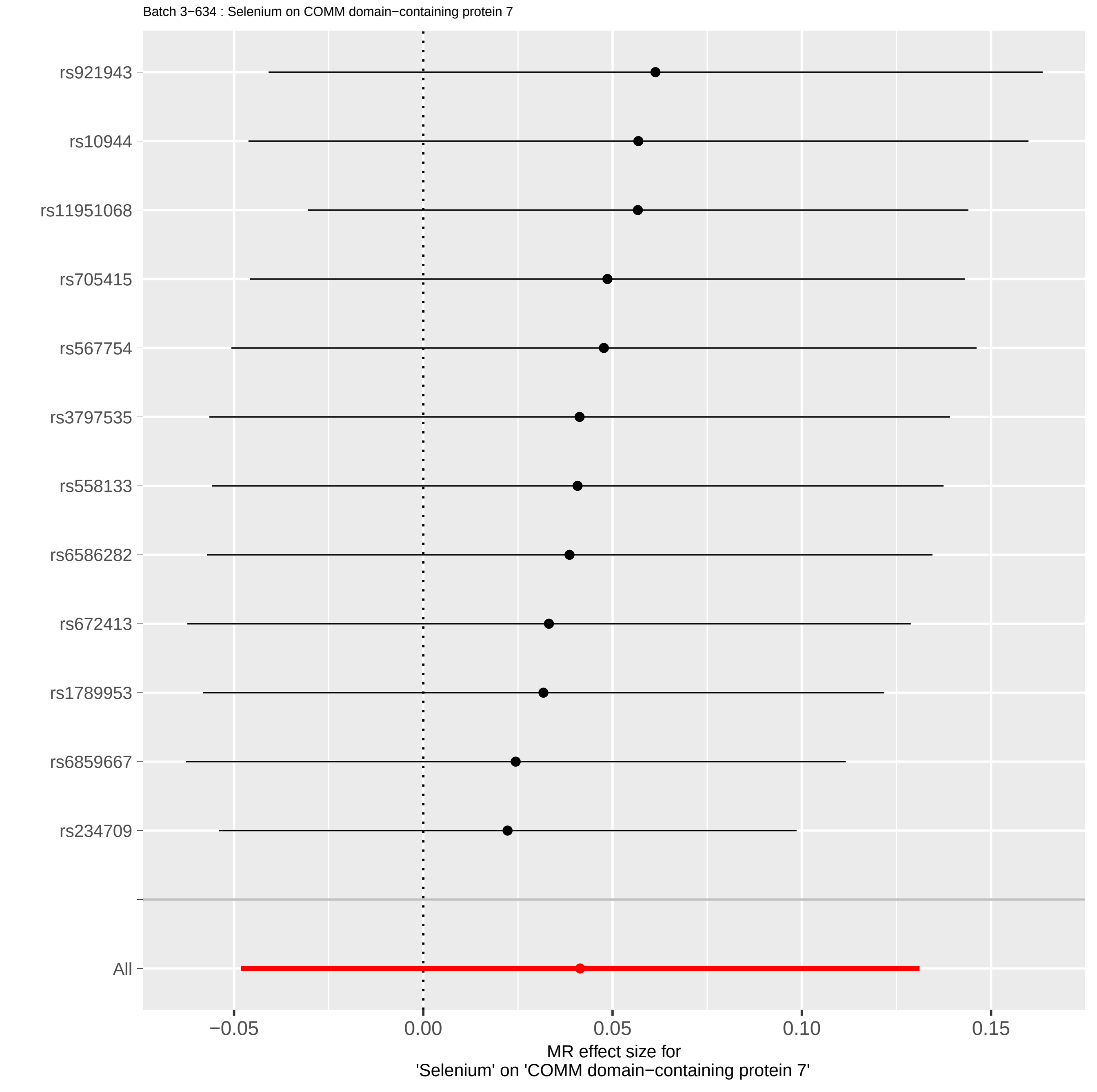

Batch 3−634 : Selenium on COMM domain−containing protein 7
rs921943
rs10944
rs11951068
rs705415
rs567754
rs3797535
rs558133
rs6586282
rs672413
rs1789953
rs6859667
rs234709
All
−0.05
0.00
0.05
0.10
0.15
MR effect size for
'Selenium' on 'COMM domain−containing protein 7'

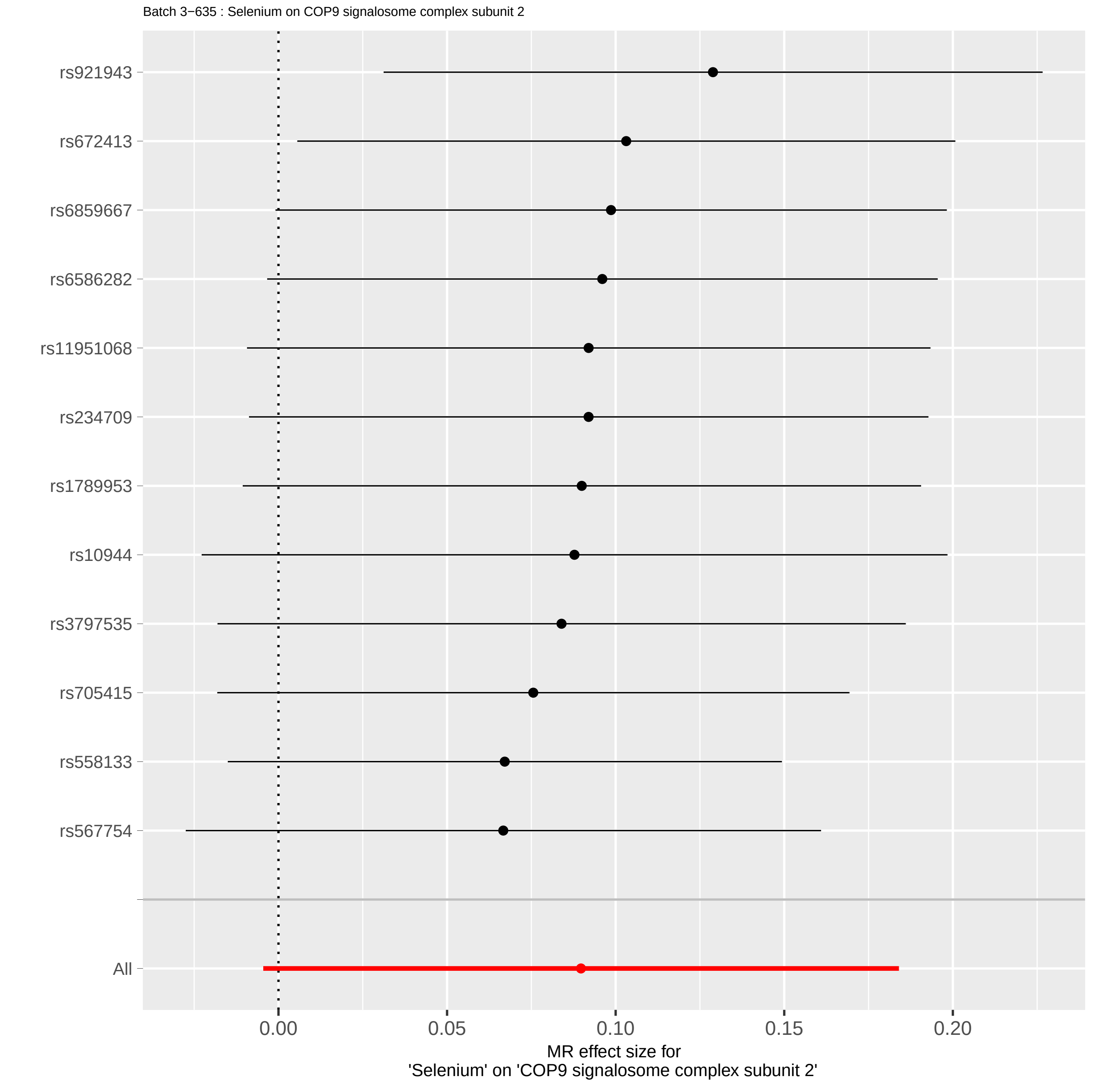

Batch 3−635 : Selenium on COP9 signalosome complex subunit 2
rs921943
rs672413
rs6859667
rs6586282
rs11951068
rs234709
rs1789953
rs10944
rs3797535
rs705415
rs558133
rs567754
All
0.00
0.05
0.10
0.15
0.20
MR effect size for
'Selenium' on 'COP9 signalosome complex subunit 2'

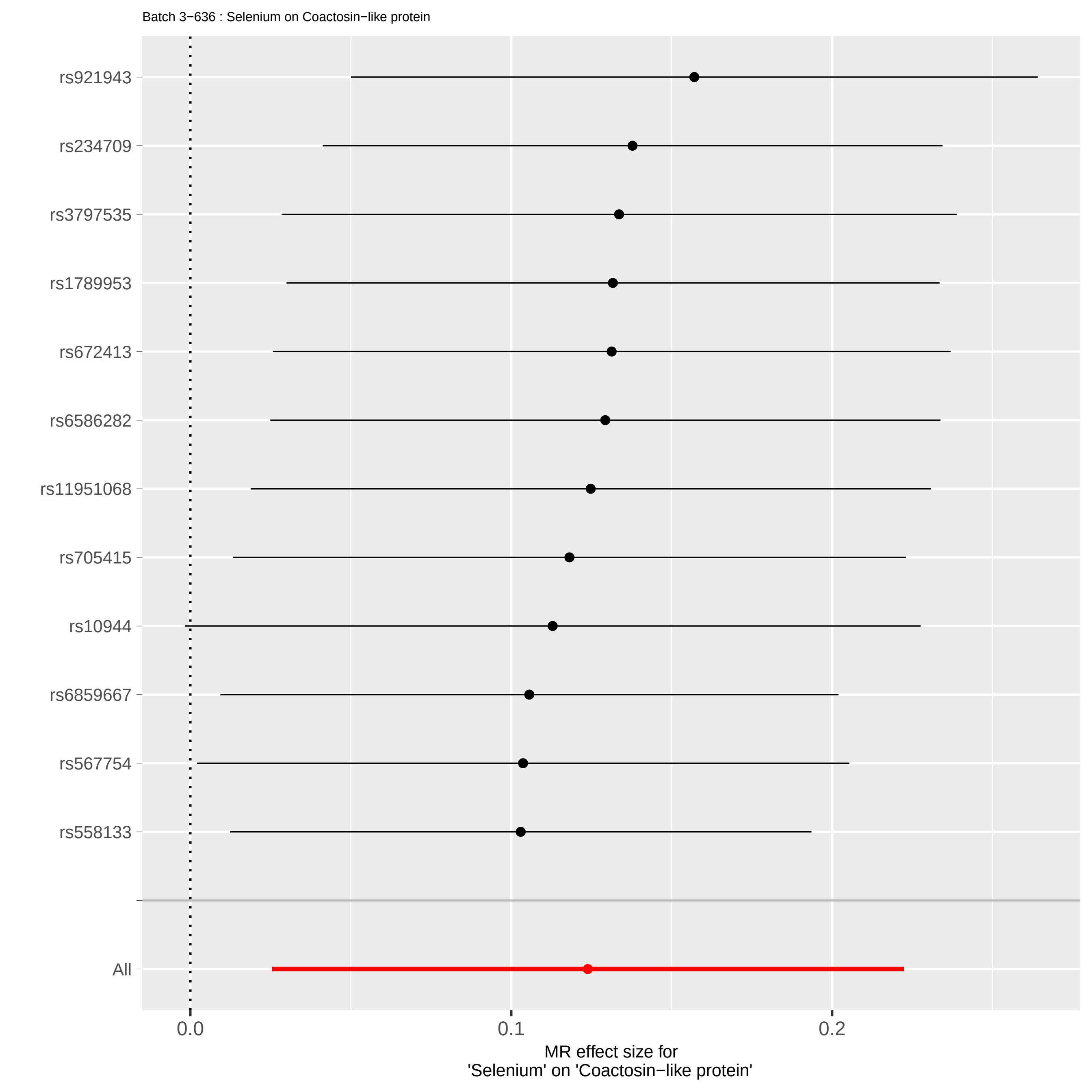

Batch 3−636 : Selenium on Coactosin−like protein
rs921943
rs234709
rs3797535
rs1789953
rs672413
rs6586282
rs11951068
rs705415
rs10944
rs6859667
rs567754
rs558133
All
0.0
0.1
0.2
MR effect size for
'Selenium' on 'Coactosin−like protein'

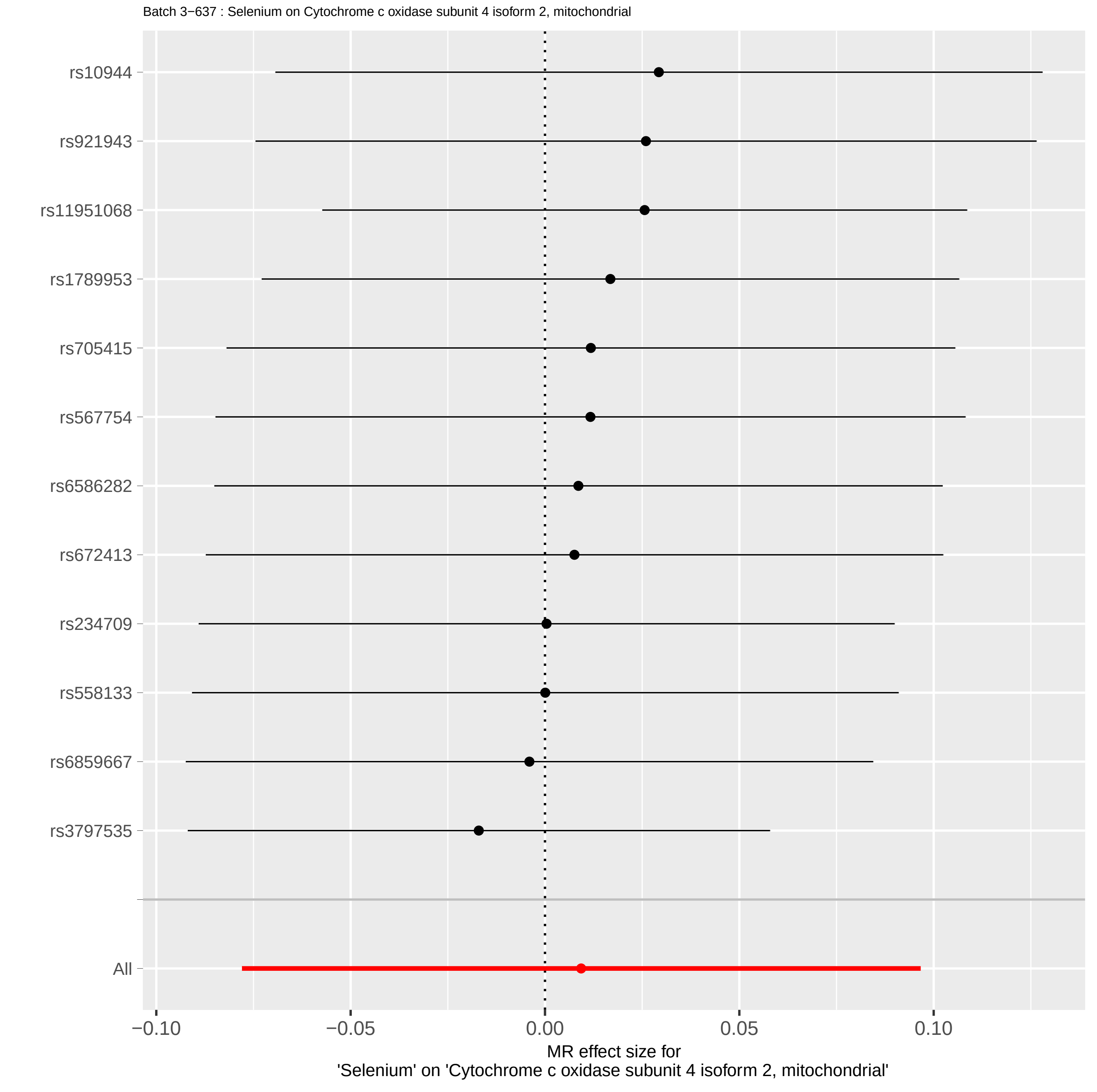

Batch 3−637 : Selenium on Cytochrome c oxidase subunit 4 isoform 2, mitochondrial
rs10944
rs921943
rs11951068
rs1789953
rs705415
rs567754
rs6586282
rs672413
rs234709
rs558133
rs6859667
rs3797535
All
−0.10
−0.05
0.00
0.05
0.10
MR effect size for
'Selenium' on 'Cytochrome c oxidase subunit 4 isoform 2, mitochondrial'

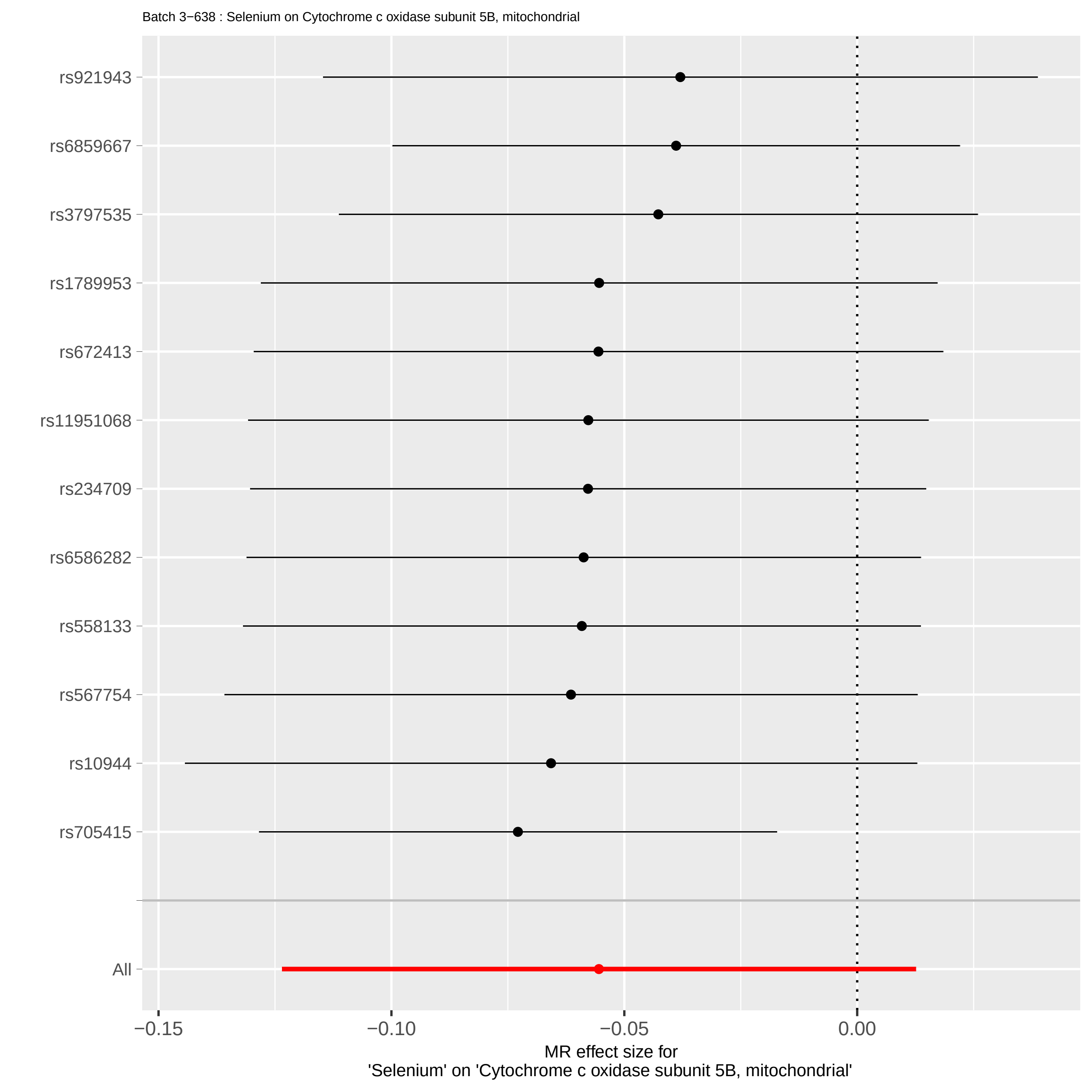

Batch 3−638 : Selenium on Cytochrome c oxidase subunit 5B, mitochondrial
rs921943
rs6859667
rs3797535
rs1789953
rs672413
rs11951068
rs234709
rs6586282
rs558133
rs567754
rs10944
rs705415
All
−0.15
−0.10
−0.05
0.00
MR effect size for
'Selenium' on 'Cytochrome c oxidase subunit 5B, mitochondrial'

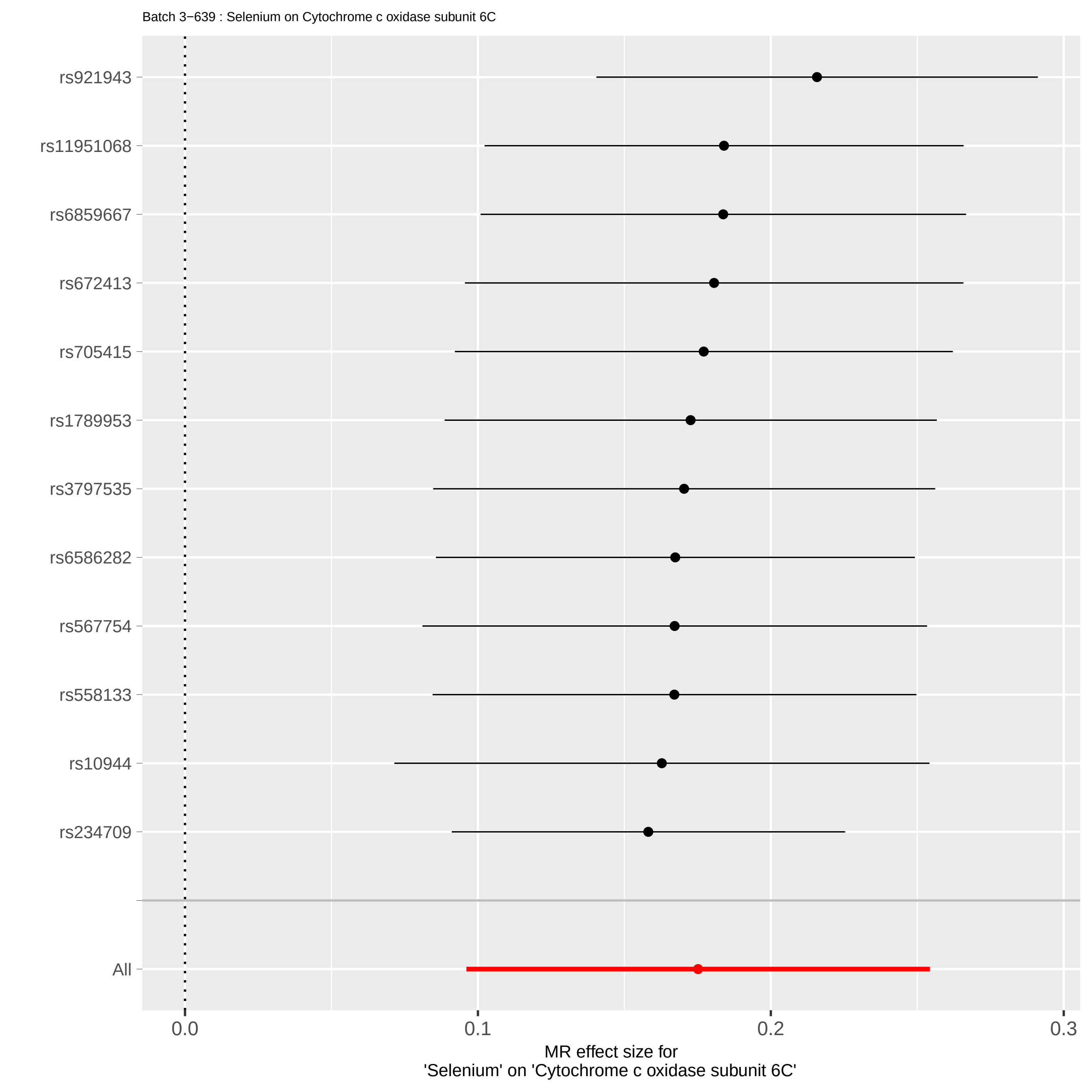

Batch 3−639 : Selenium on Cytochrome c oxidase subunit 6C
rs921943
rs11951068
rs6859667
rs672413
rs705415
rs1789953
rs3797535
rs6586282
rs567754
rs558133
rs10944
rs234709
All
0.0
0.1
0.2
0.3
MR effect size for
'Selenium' on 'Cytochrome c oxidase subunit 6C'

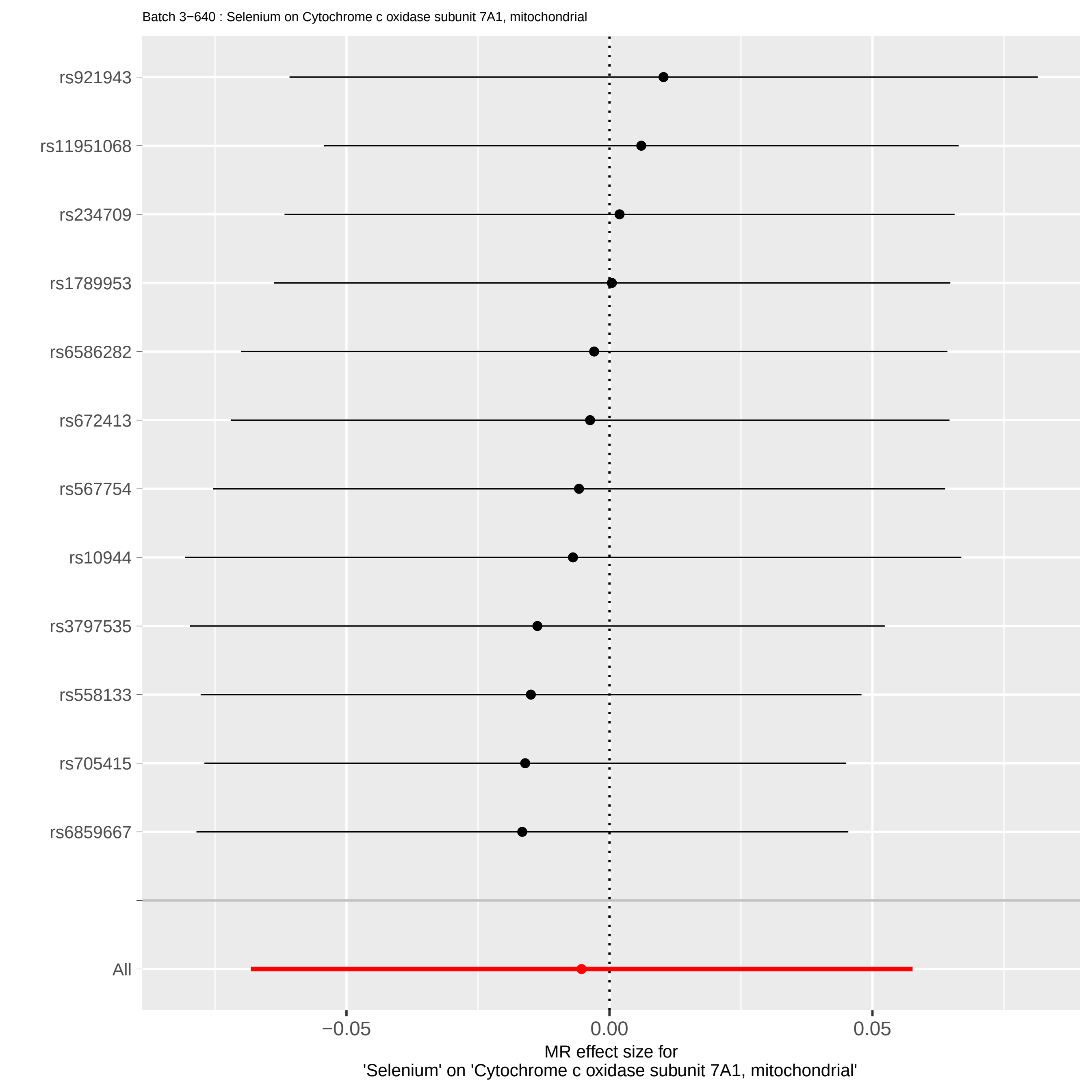

Batch 3−640 : Selenium on Cytochrome c oxidase subunit 7A1, mitochondrial
rs921943
rs11951068
rs234709
rs1789953
rs6586282
rs672413
rs567754
rs10944
rs3797535
rs558133
rs705415
rs6859667
All
−0.05
0.00
0.05
MR effect size for
'Selenium' on 'Cytochrome c oxidase subunit 7A1, mitochondrial'

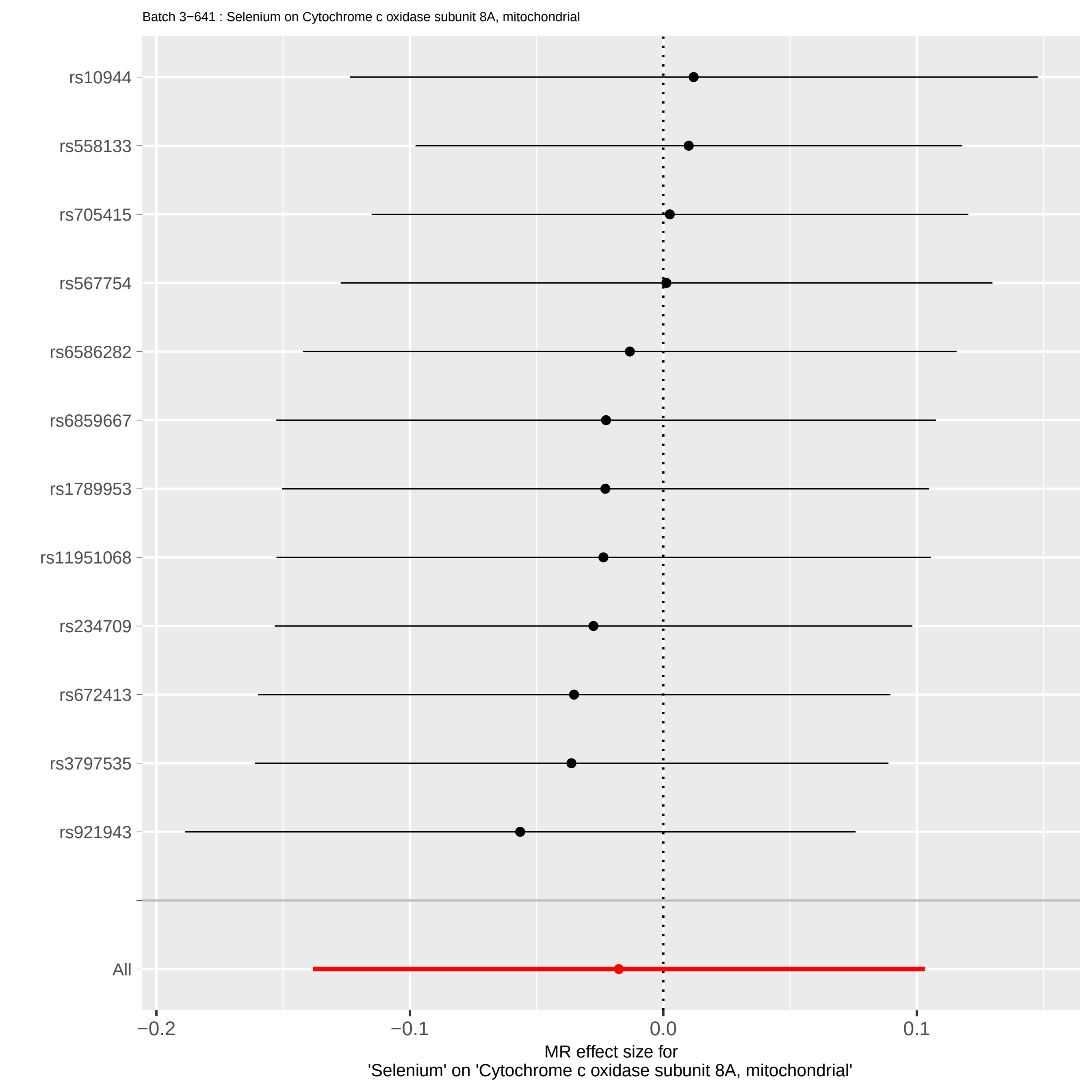

Batch 3−641 : Selenium on Cytochrome c oxidase subunit 8A, mitochondrial
rs10944
rs558133
rs705415
rs567754
rs6586282
rs6859667
rs1789953
rs11951068
rs234709
rs672413
rs3797535
rs921943
All
−0.2
−0.1
0.0
0.1
MR effect size for
'Selenium' on 'Cytochrome c oxidase subunit 8A, mitochondrial'

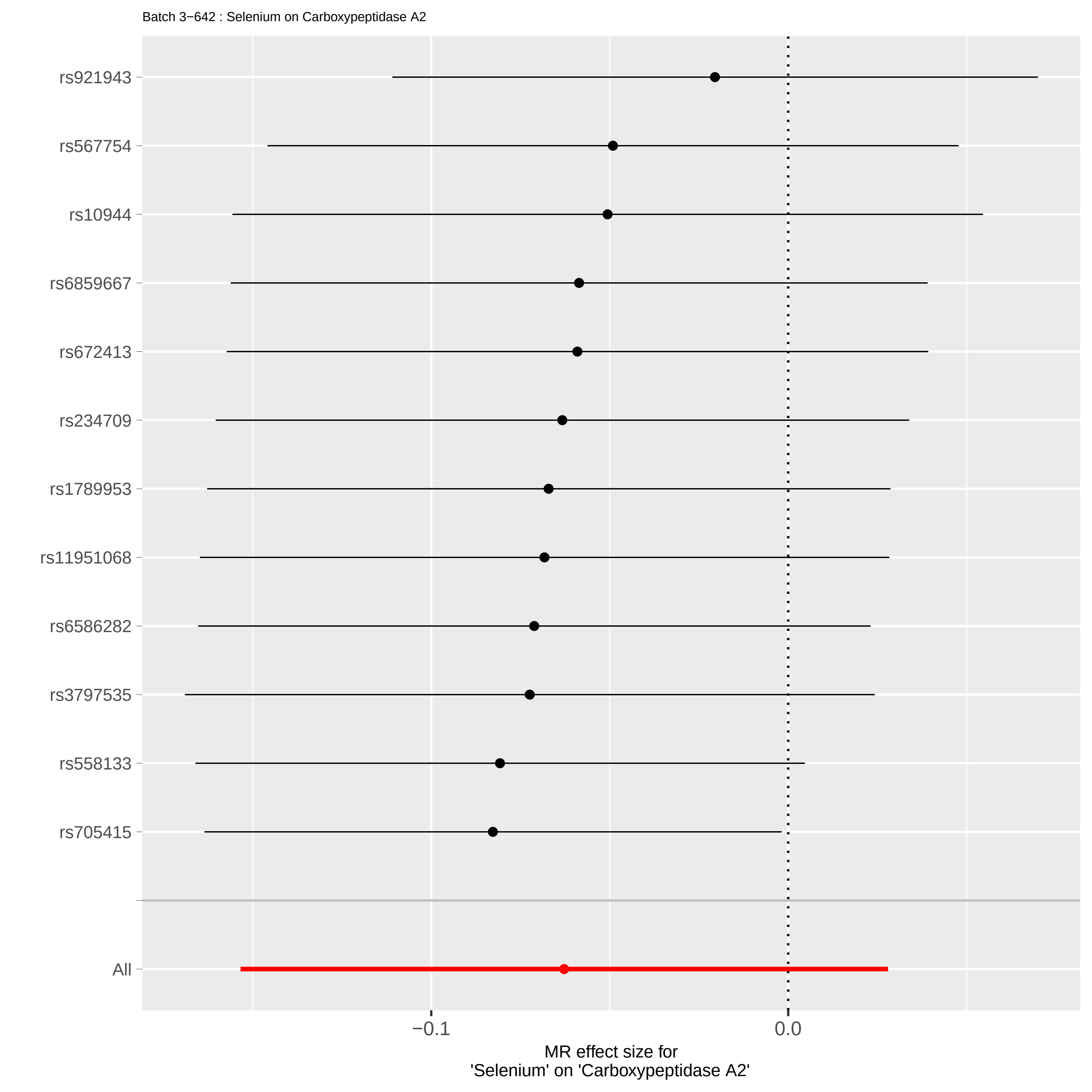

Batch 3−642 : Selenium on Carboxypeptidase A2
rs921943
rs567754
rs10944
rs6859667
rs672413
rs234709
rs1789953
rs11951068
rs6586282
rs3797535
rs558133
rs705415
All
−0.1
0.0
MR effect size for
'Selenium' on 'Carboxypeptidase A2'

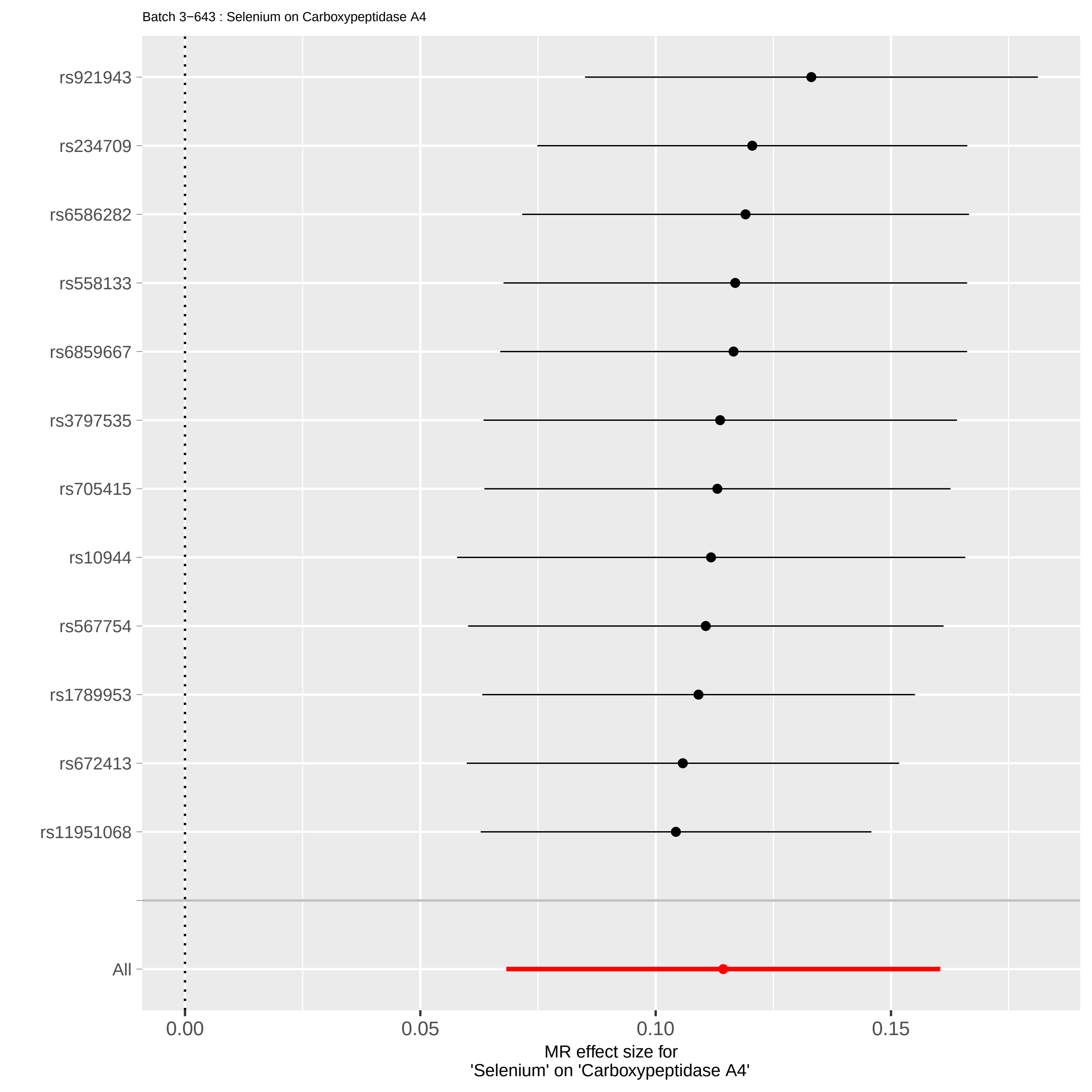

Batch 3−643 : Selenium on Carboxypeptidase A4
rs921943
rs234709
rs6586282
rs558133
rs6859667
rs3797535
rs705415
rs10944
rs567754
rs1789953
rs672413
rs11951068
All
0.00
0.05
0.10
0.15
MR effect size for
'Selenium' on 'Carboxypeptidase A4'

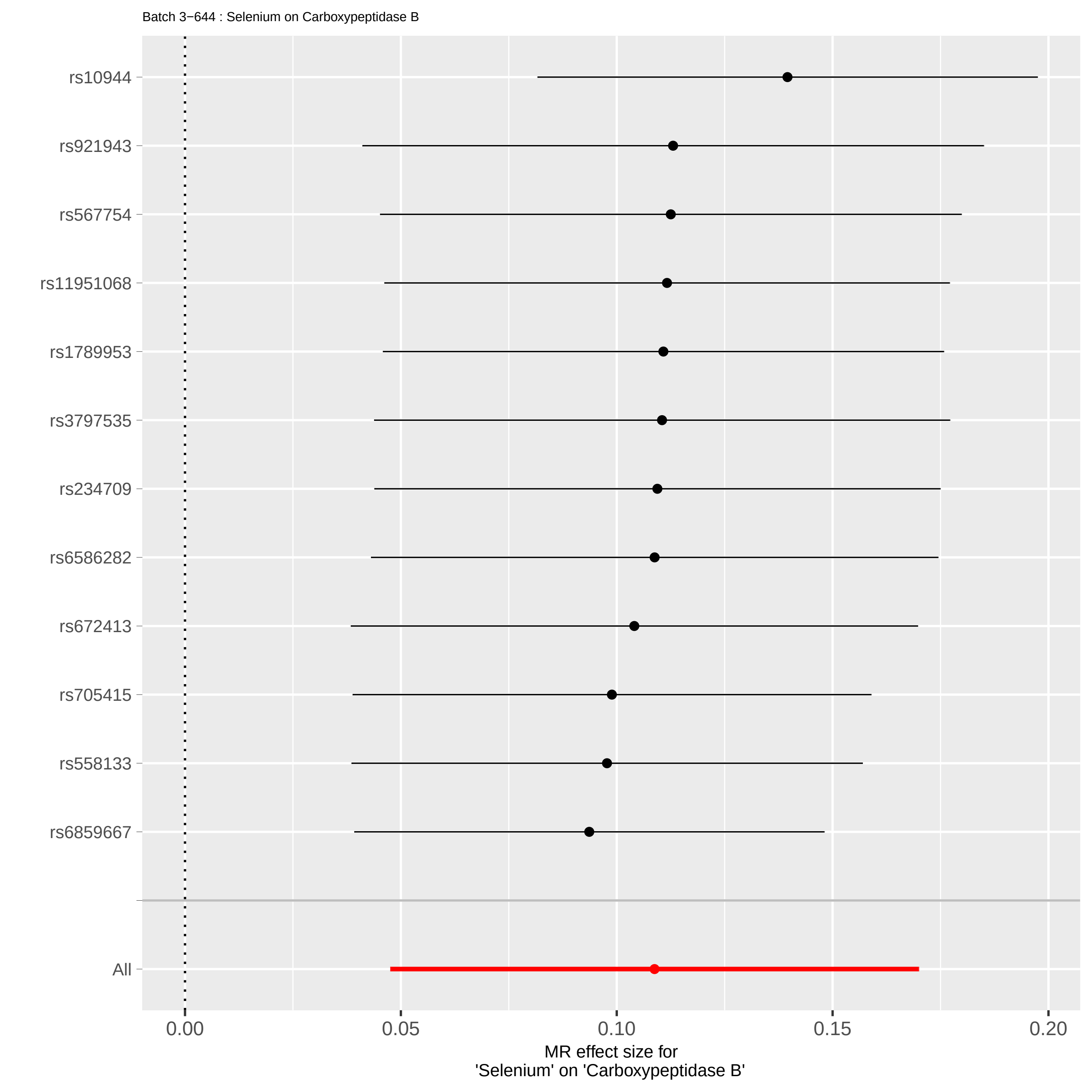

Batch 3−644 : Selenium on Carboxypeptidase B
rs10944
rs921943
rs567754
rs11951068
rs1789953
rs3797535
rs234709
rs6586282
rs672413
rs705415
rs558133
rs6859667
All
0.00
0.05
0.10
0.15
0.20
MR effect size for
'Selenium' on 'Carboxypeptidase B'

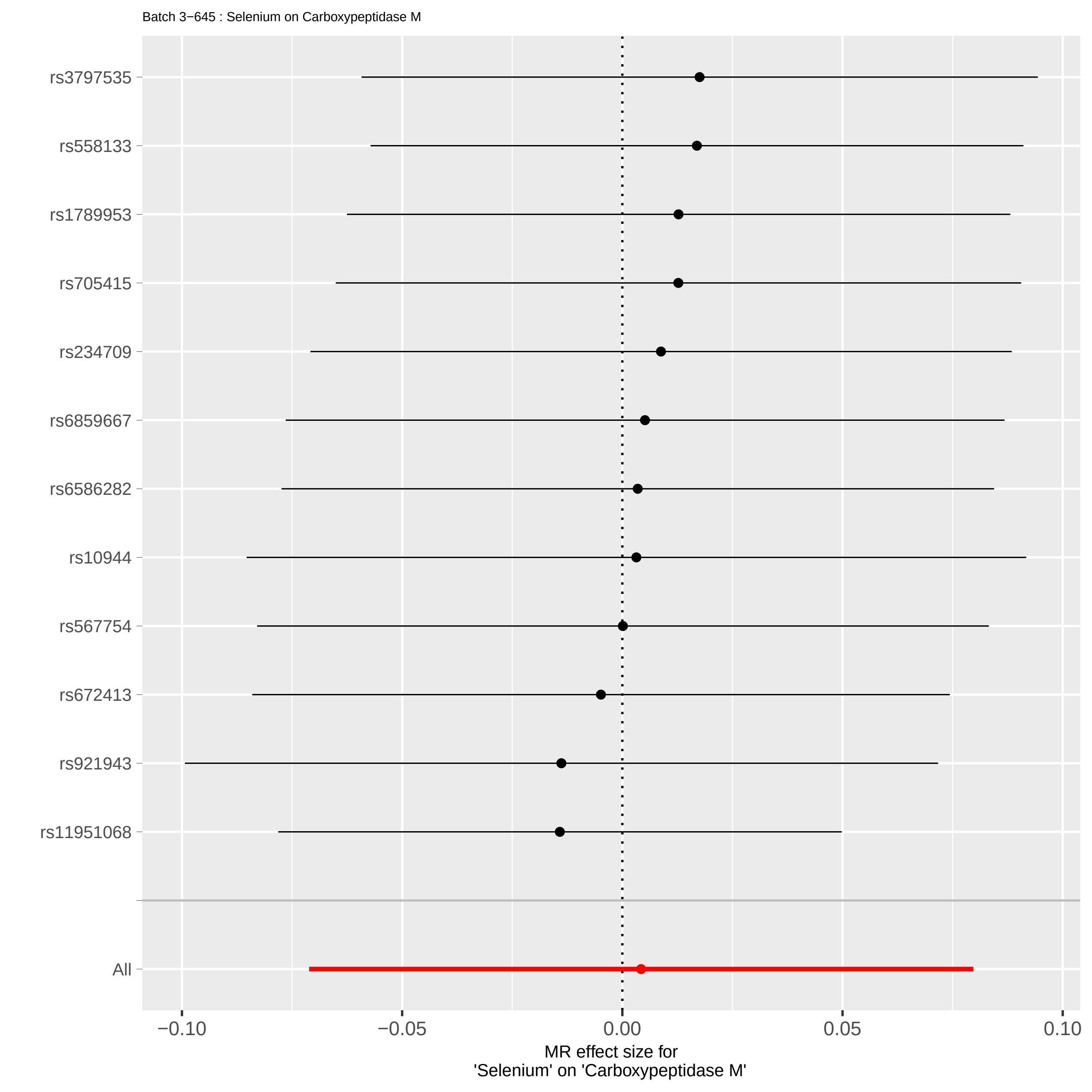

Batch 3−645 : Selenium on Carboxypeptidase M
rs3797535
rs558133
rs1789953
rs705415
rs234709
rs6859667
rs6586282
rs10944
rs567754
rs672413
rs921943
rs11951068
All
−0.10
−0.05
0.00
0.05
0.10
MR effect size for
'Selenium' on 'Carboxypeptidase M'

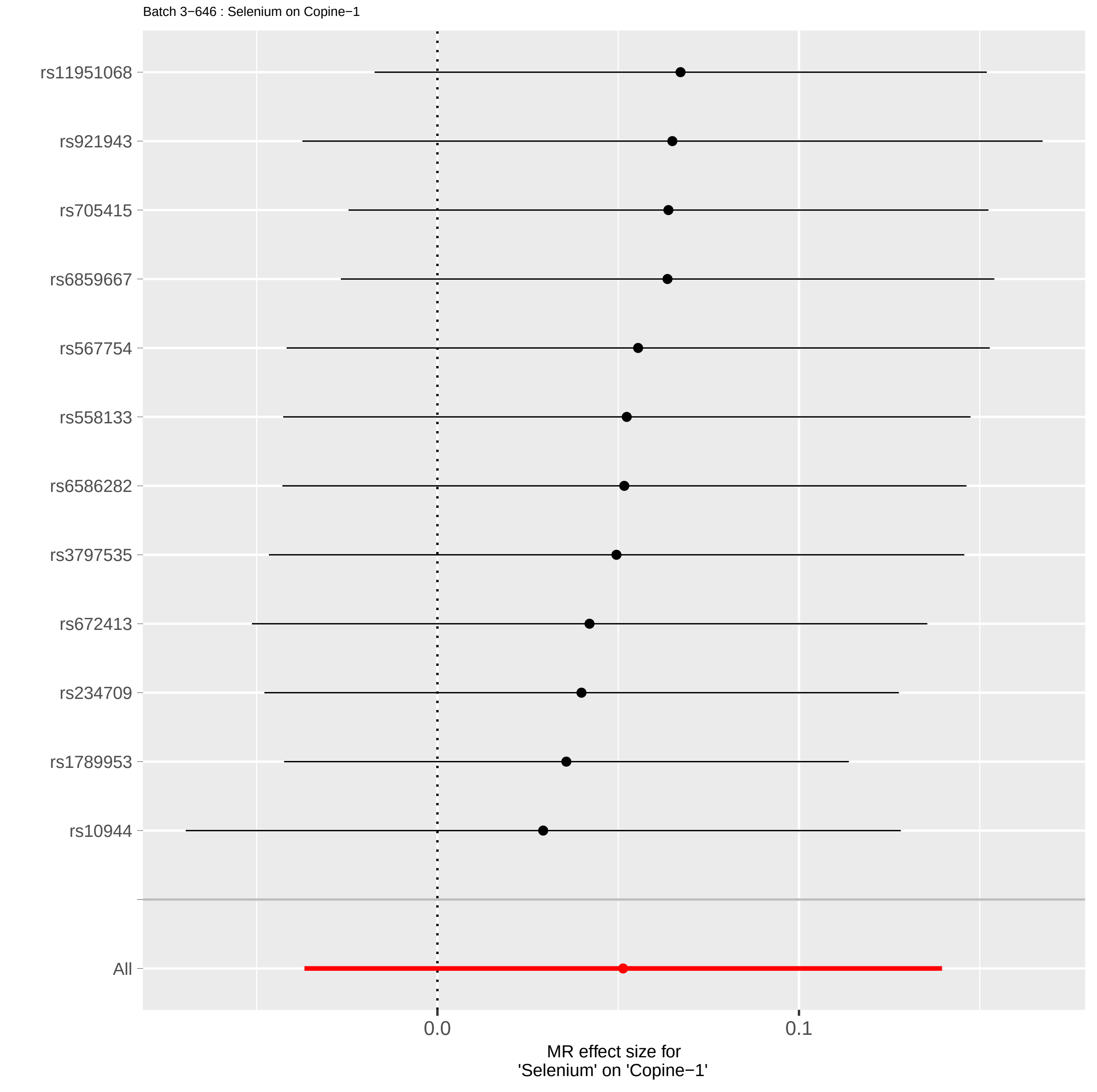
Batch 3−646 : Selenium on Copine−1
rs11951068
rs921943
rs705415
rs6859667
rs567754
rs558133
rs6586282
rs3797535
rs672413
rs234709
rs1789953
rs10944
All
0.0
0.1
MR effect size for
'Selenium' on 'Copine−1'

Batch 3−647 : Selenium on Probable serine carboxypeptidase CPVL

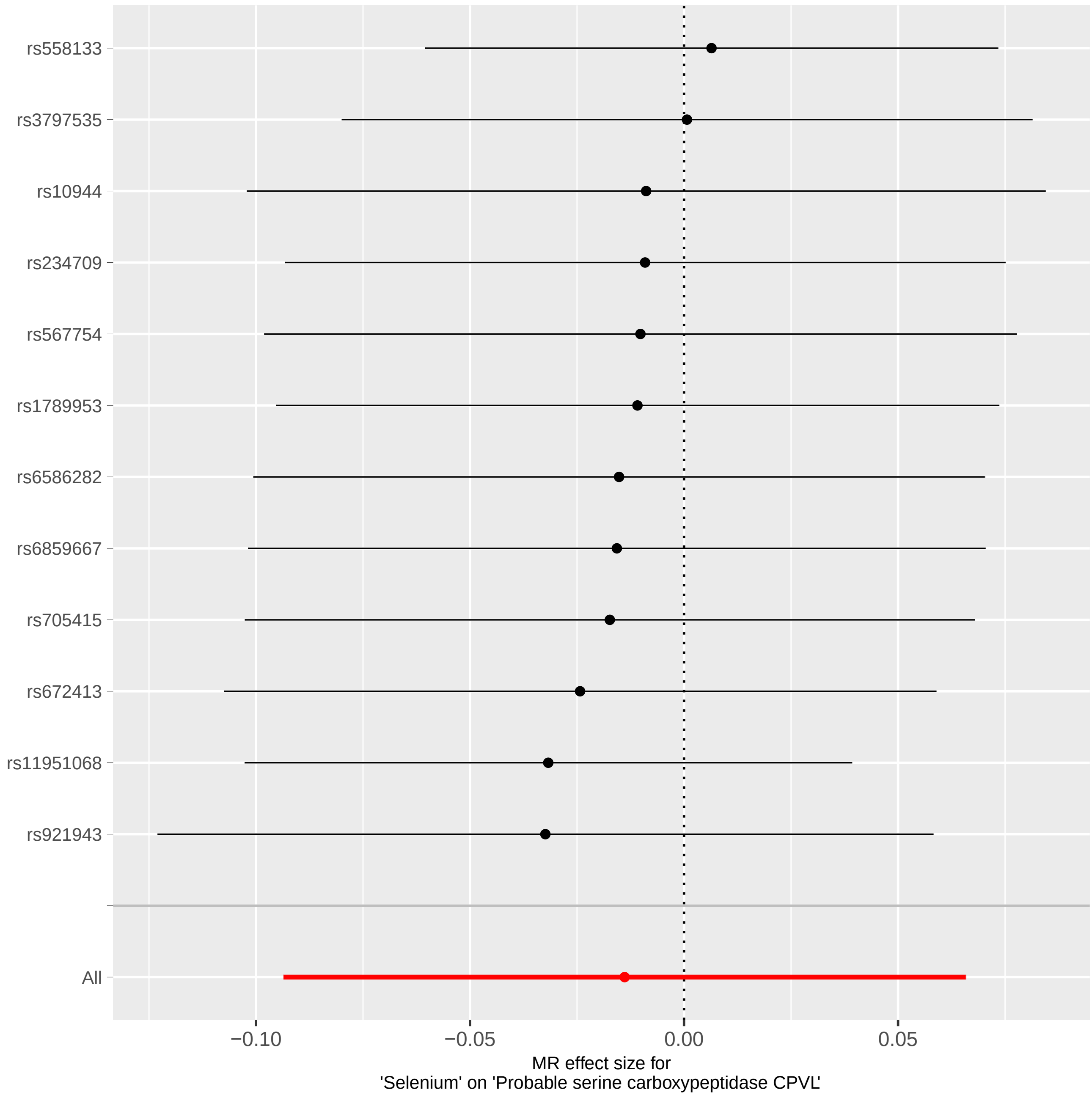

Batch 3−648 : Selenium on Probable carboxypeptidase X1

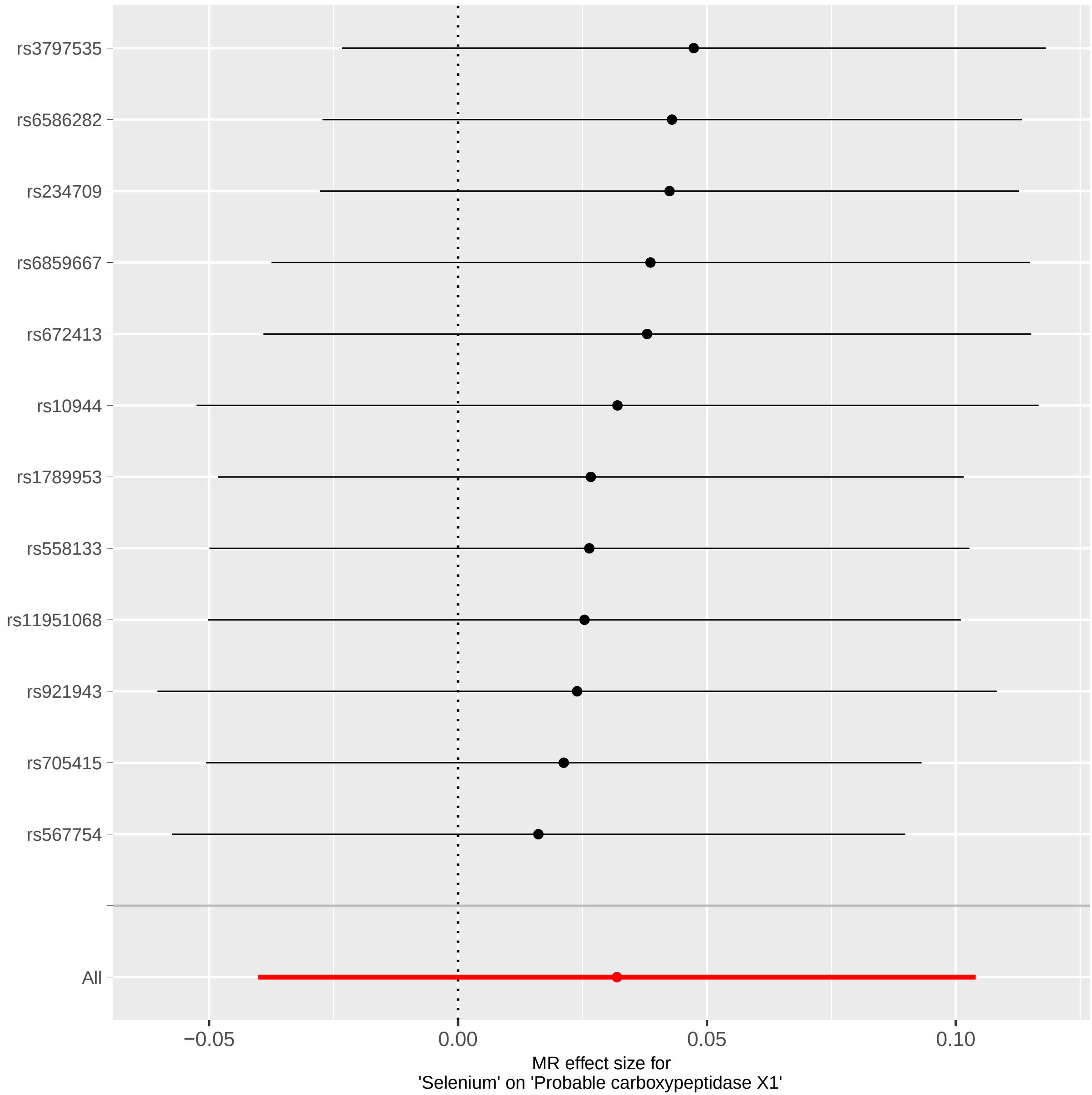

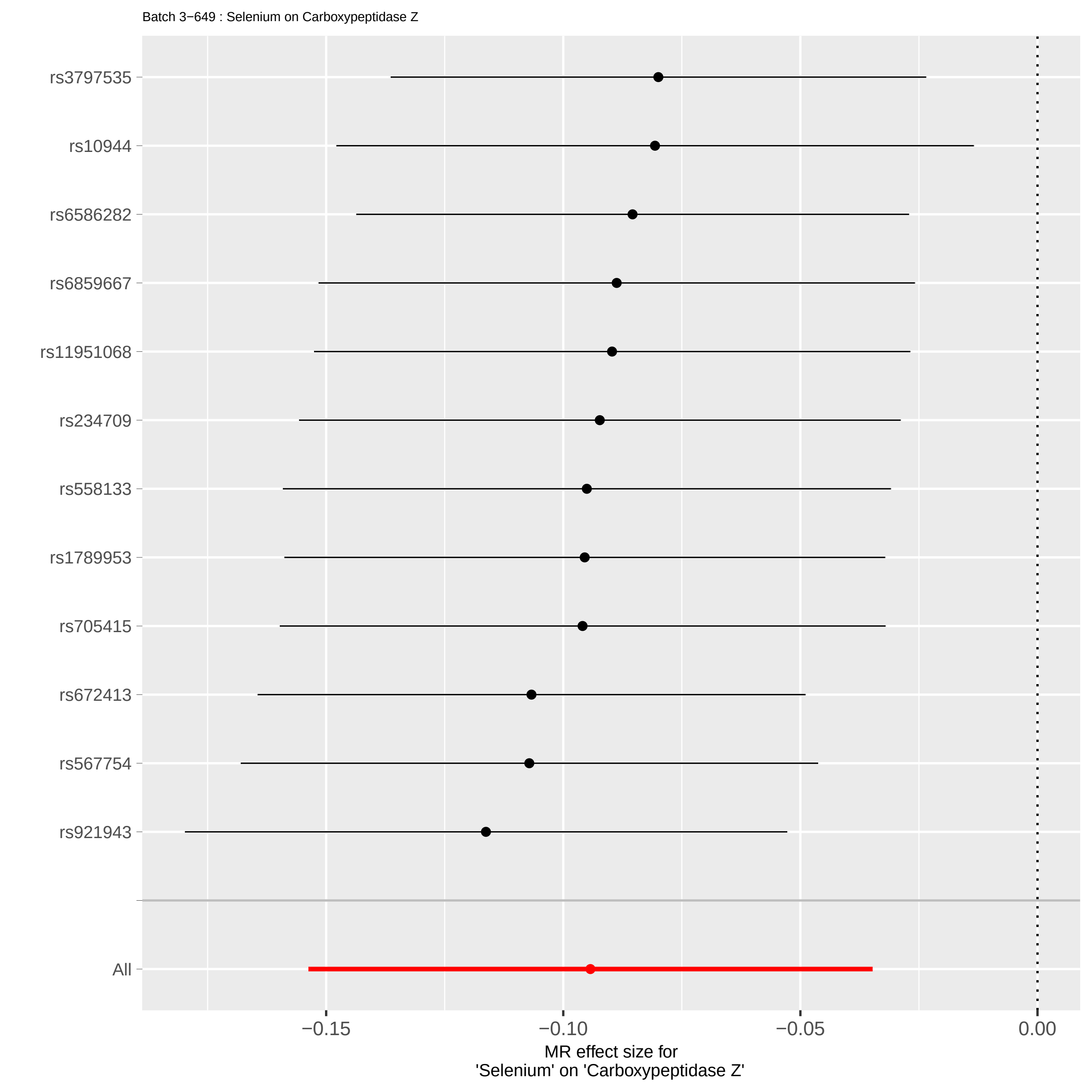

Batch 3−649 : Selenium on Carboxypeptidase Z
rs3797535
rs10944
rs6586282
rs6859667
rs11951068
rs234709
rs558133
rs1789953
rs705415
rs672413
rs567754
rs921943
All
−0.15
−0.10
−0.05
0.00
MR effect size for
'Selenium' on 'Carboxypeptidase Z'

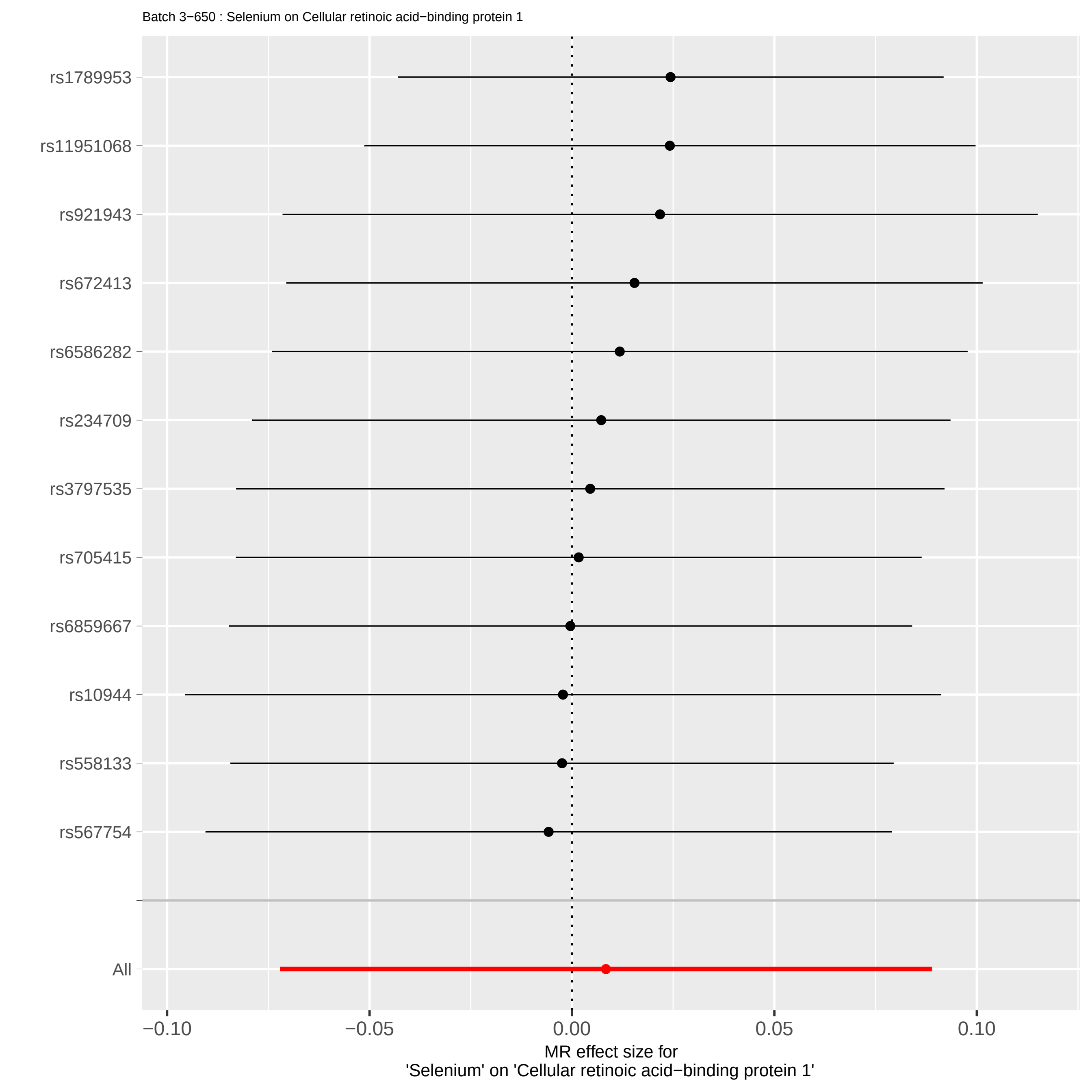
Batch 3−650 : Selenium on Cellular retinoic acid−binding protein 1
rs1789953
rs11951068
rs921943
rs672413
rs6586282
rs234709
rs3797535
rs705415
rs6859667
rs10944
rs558133
rs567754
All
−0.10
−0.05
0.00
0.05
0.10
MR effect size for
'Selenium' on 'Cellular retinoic acid−binding protein 1'

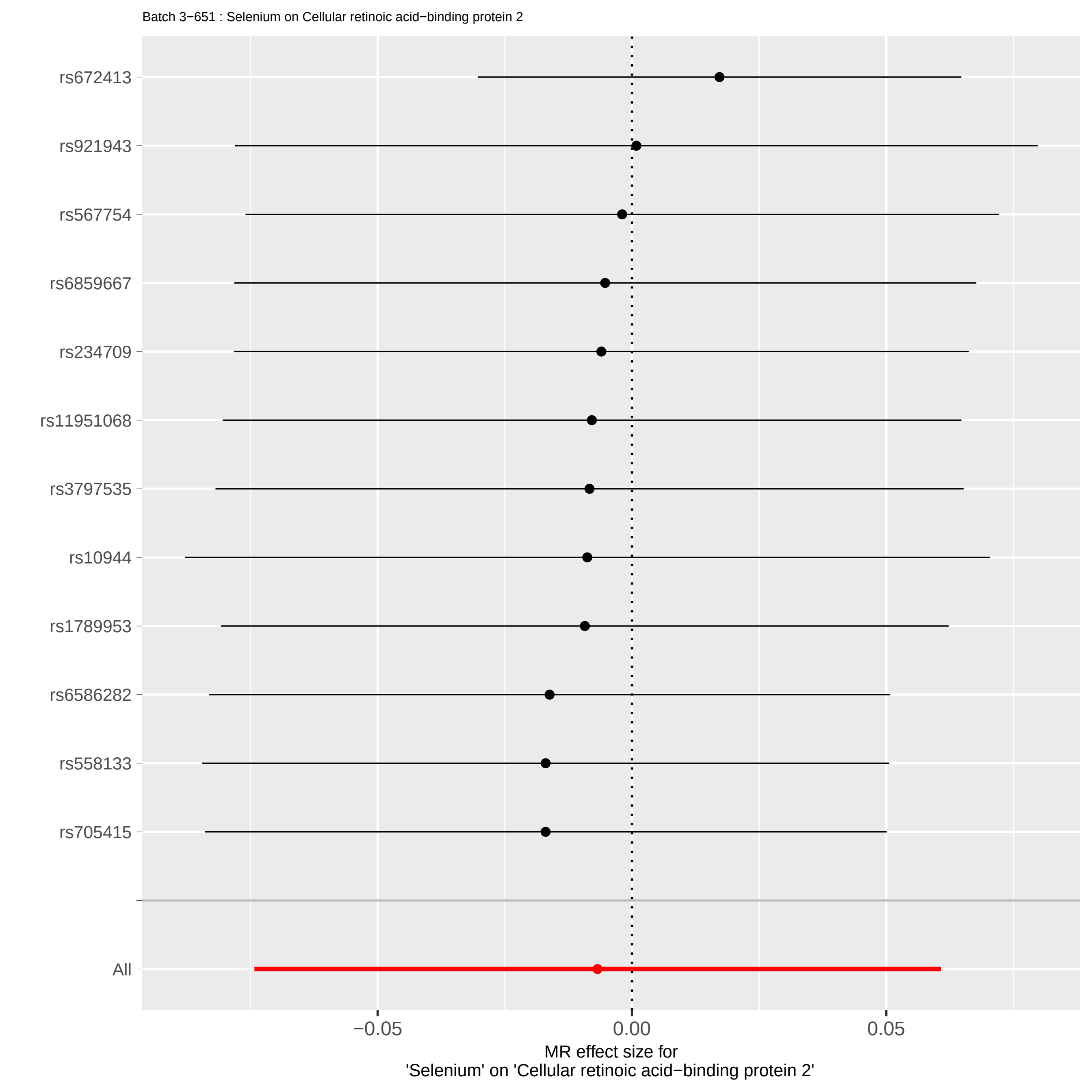

Batch 3−651 : Selenium on Cellular retinoic acid−binding protein 2
rs672413
rs921943
rs567754
rs6859667
rs234709
rs11951068
rs3797535
rs10944
rs1789953
rs6586282
rs558133
rs705415
All
−0.05
0.00
0.05
MR effect size for
'Selenium' on 'Cellular retinoic acid−binding protein 2'

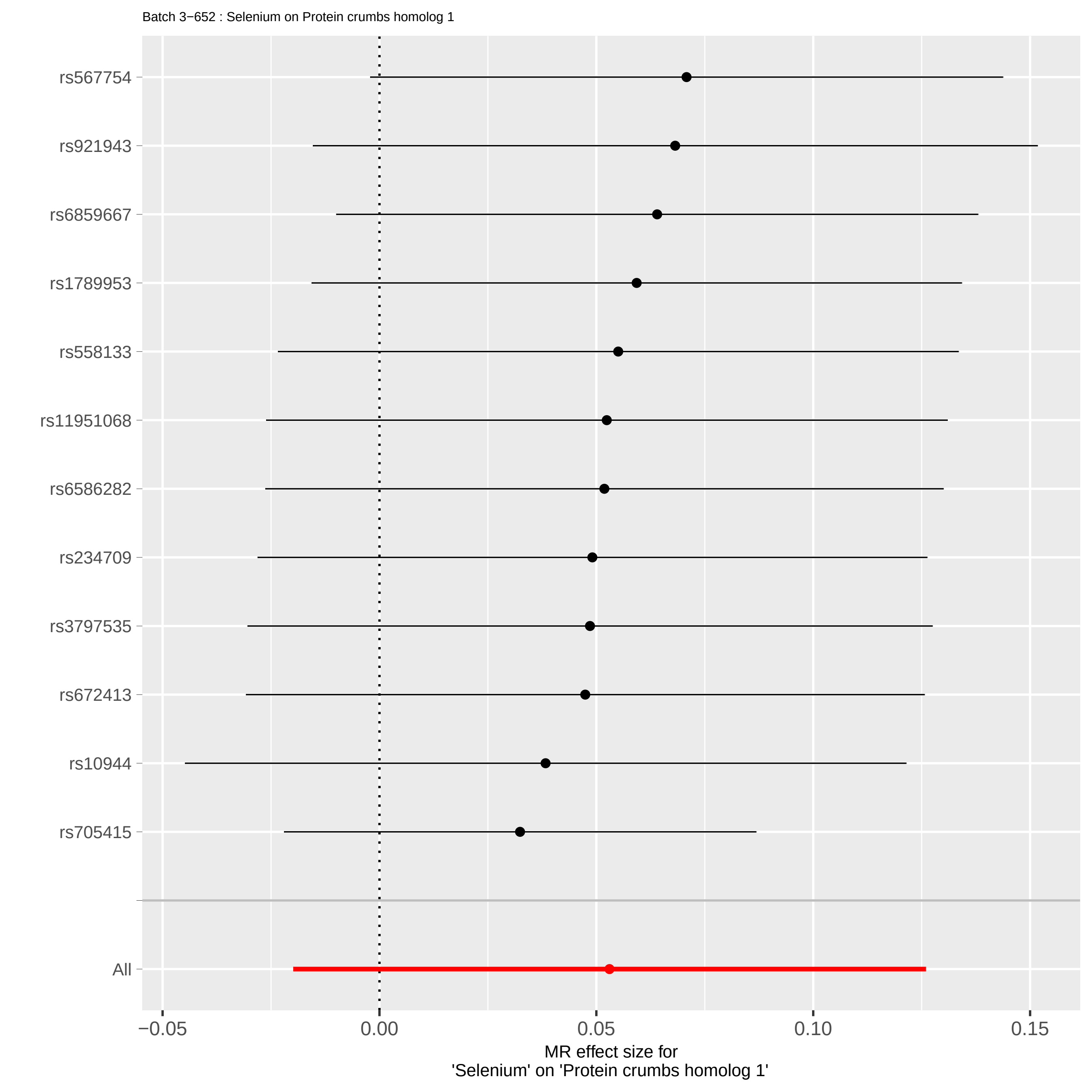

Batch 3−652 : Selenium on Protein crumbs homolog 1
rs567754
rs921943
rs6859667
rs1789953
rs558133
rs11951068
rs6586282
rs234709
rs3797535
rs672413
rs10944
rs705415
All
−0.05
0.00
0.05
0.10
0.15
MR effect size for
'Selenium' on 'Protein crumbs homolog 1'

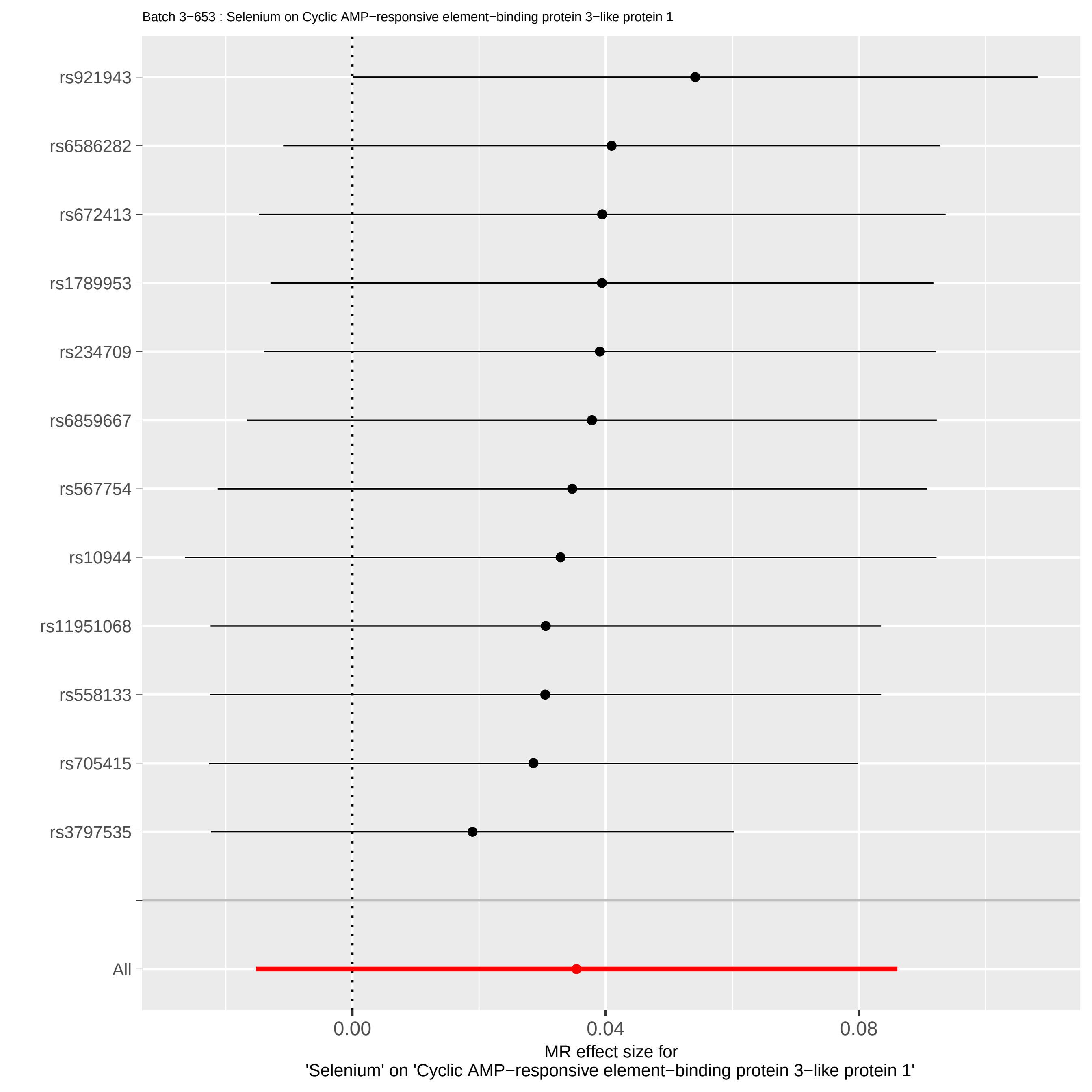

Batch 3−653 : Selenium on Cyclic AMP−responsive element−binding protein 3−like protein 1
rs921943
rs6586282
rs672413
rs1789953
rs234709
rs6859667
rs567754
rs10944
rs11951068
rs558133
rs705415
rs3797535
All
0.00
0.04
0.08
MR effect size for
'Selenium' on 'Cyclic AMP−responsive element−binding protein 3−like protein 1'

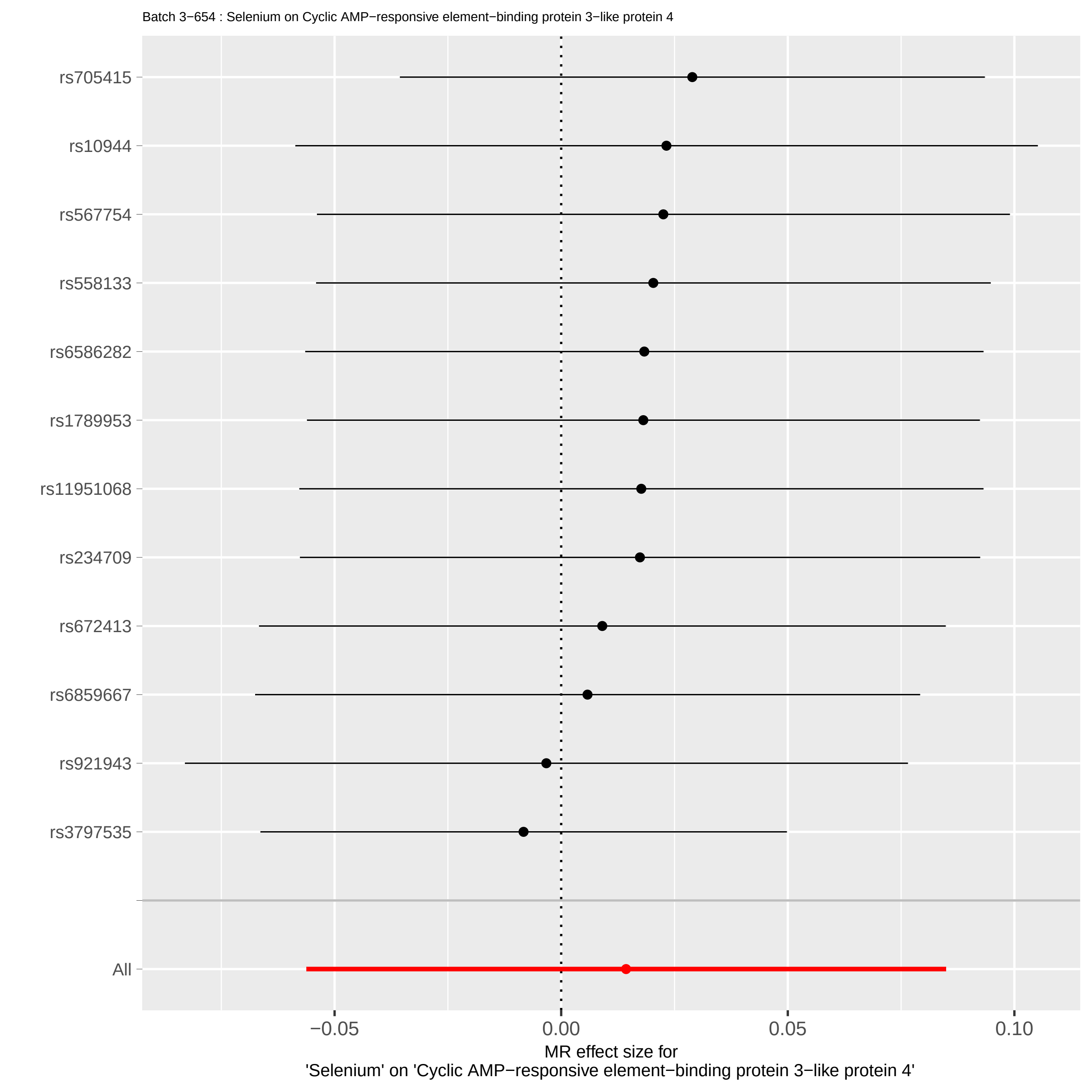

Batch 3−654 : Selenium on Cyclic AMP−responsive element−binding protein 3−like protein 4
rs705415
rs10944
rs567754
rs558133
rs6586282
rs1789953
rs11951068
rs234709
rs672413
rs6859667
rs921943
rs3797535
All
−0.05
0.00
0.05
0.10
MR effect size for
'Selenium' on 'Cyclic AMP−responsive element−binding protein 3−like protein 4'

Batch 3−655 : Selenium on CREB−binding protein

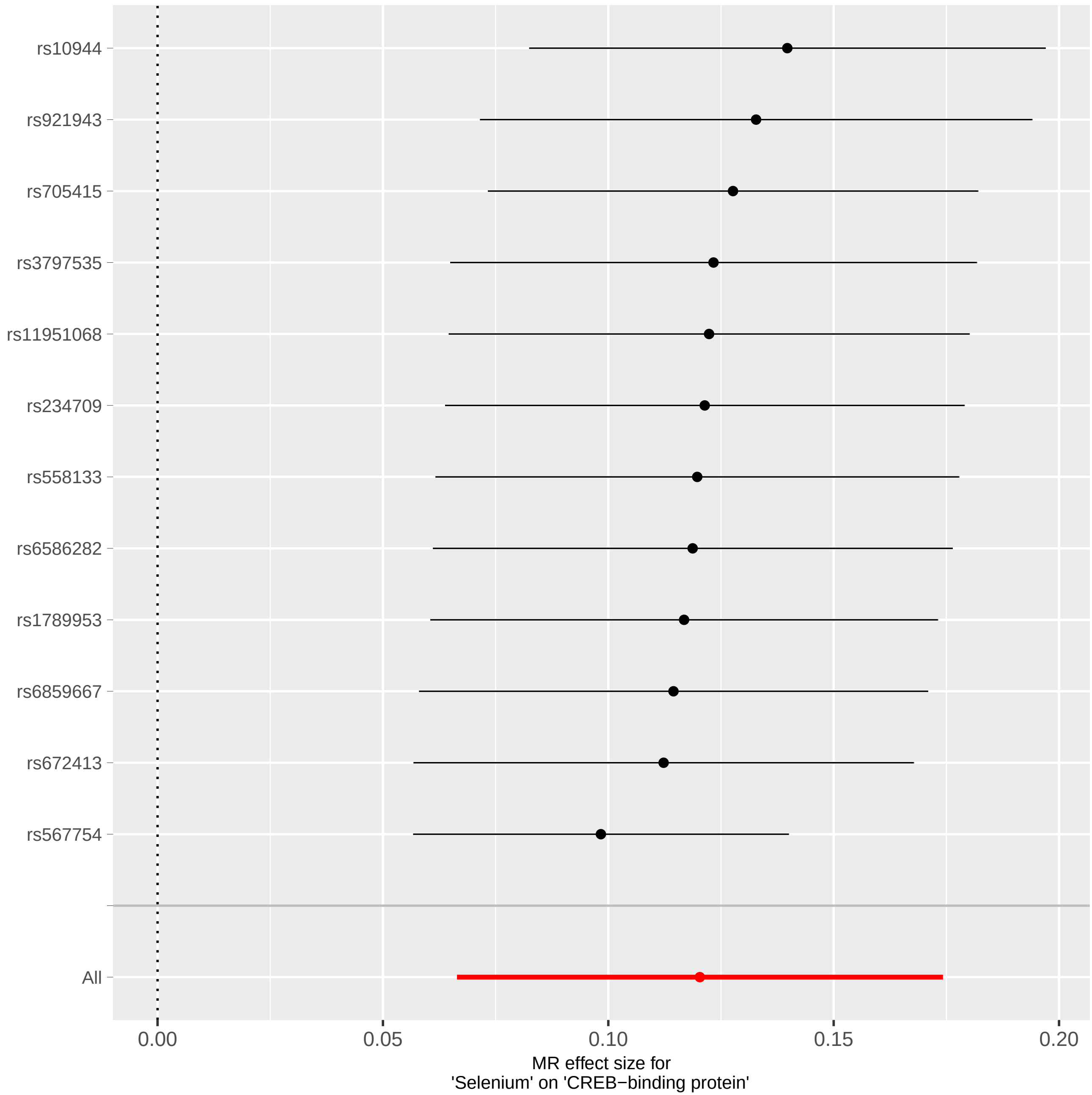

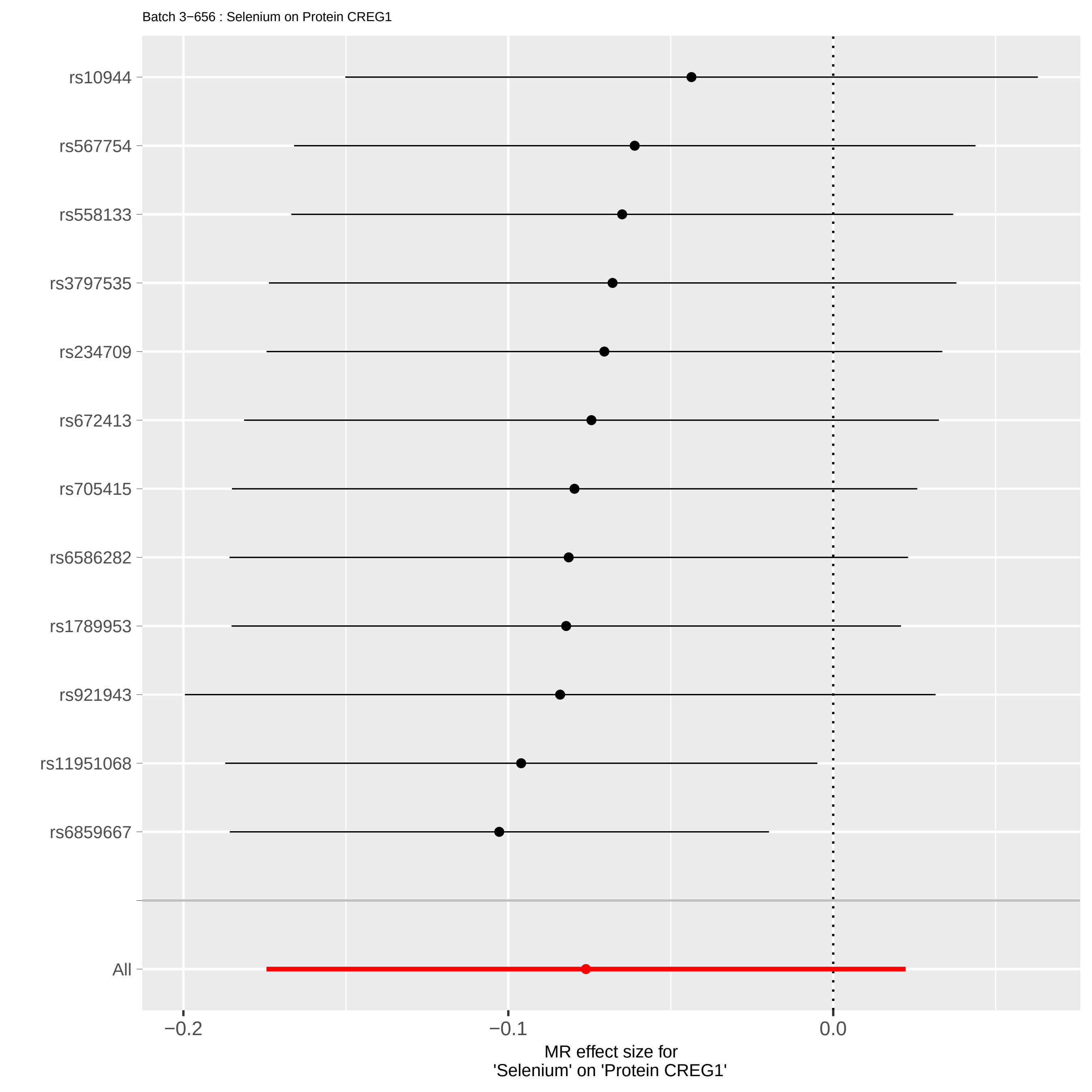

Batch 3−656 : Selenium on Protein CREG1
rs10944
rs567754
rs558133
rs3797535
rs234709
rs672413
rs705415
rs6586282
rs1789953
rs921943
rs11951068
rs6859667
All
−0.2
−0.1
0.0
MR effect size for
'Selenium' on 'Protein CREG1'

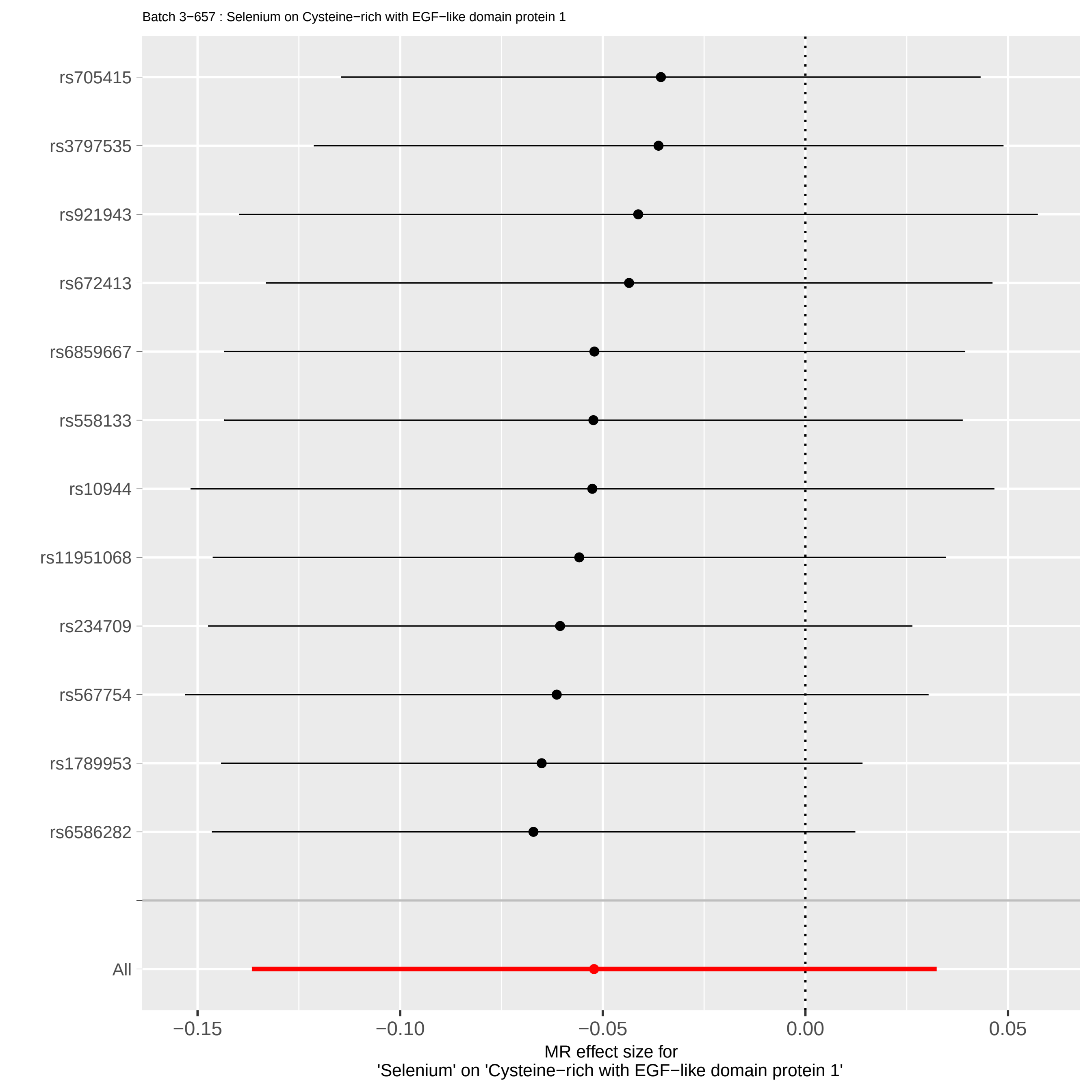

Batch 3−657 : Selenium on Cysteine−rich with EGF−like domain protein 1
rs705415
rs3797535
rs921943
rs672413
rs6859667
rs558133
rs10944
rs11951068
rs234709
rs567754
rs1789953
rs6586282
All
−0.15
−0.10
−0.05
0.00
0.05
MR effect size for
'Selenium' on 'Cysteine−rich with EGF−like domain protein 1'

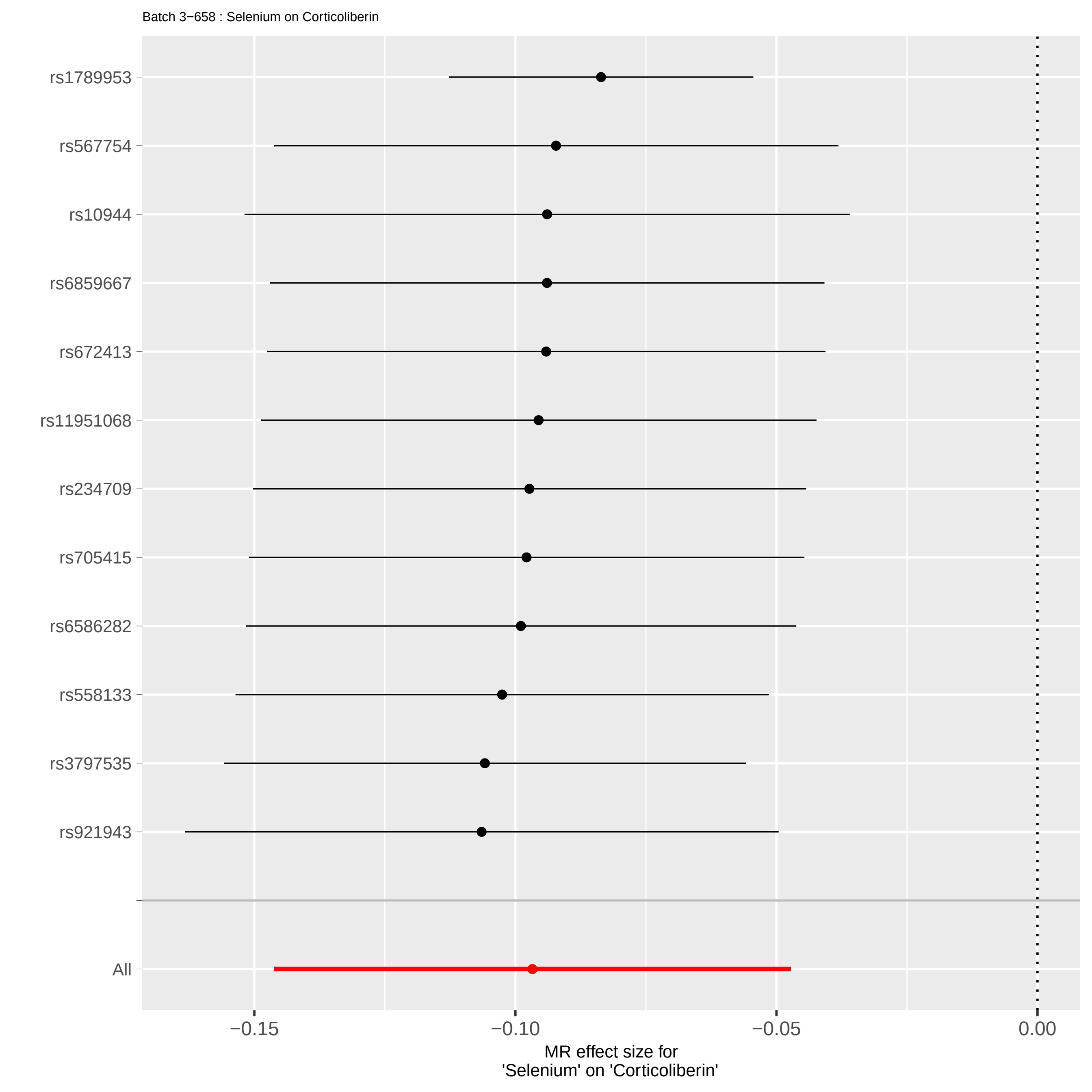
Batch 3−658 : Selenium on Corticoliberin
rs1789953
rs567754
rs10944
rs6859667
rs672413
rs11951068
rs234709
rs705415
rs6586282
rs558133
rs3797535
rs921943
All
−0.15
−0.10
−0.05
0.00
MR effect size for
'Selenium' on 'Corticoliberin'

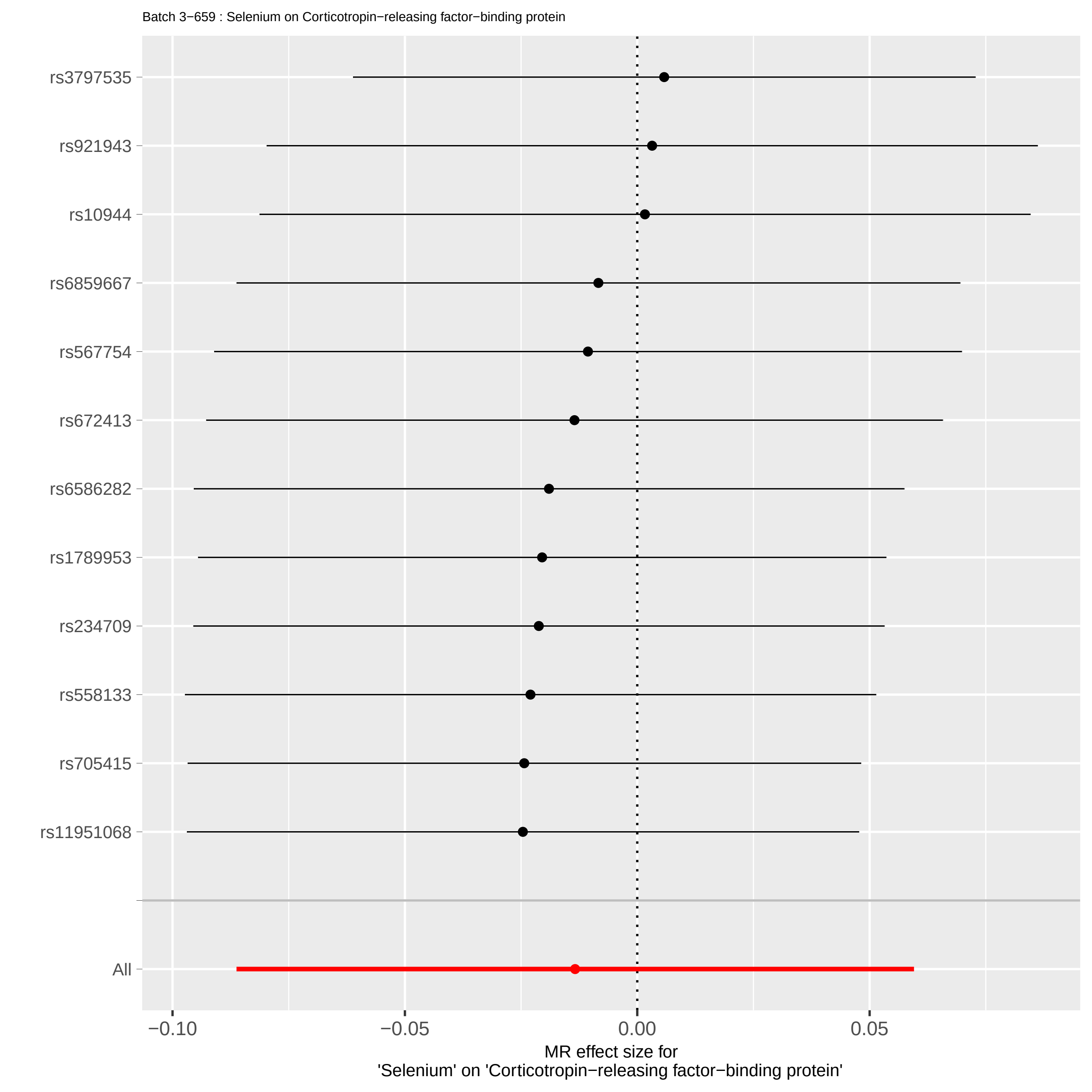

Batch 3−659 : Selenium on Corticotropin−releasing factor−binding protein
rs3797535
rs921943
rs10944
rs6859667
rs567754
rs672413
rs6586282
rs1789953
rs234709
rs558133
rs705415
rs11951068
All
−0.10
−0.05
0.00
0.05
MR effect size for
'Selenium' on 'Corticotropin−releasing factor−binding protein'

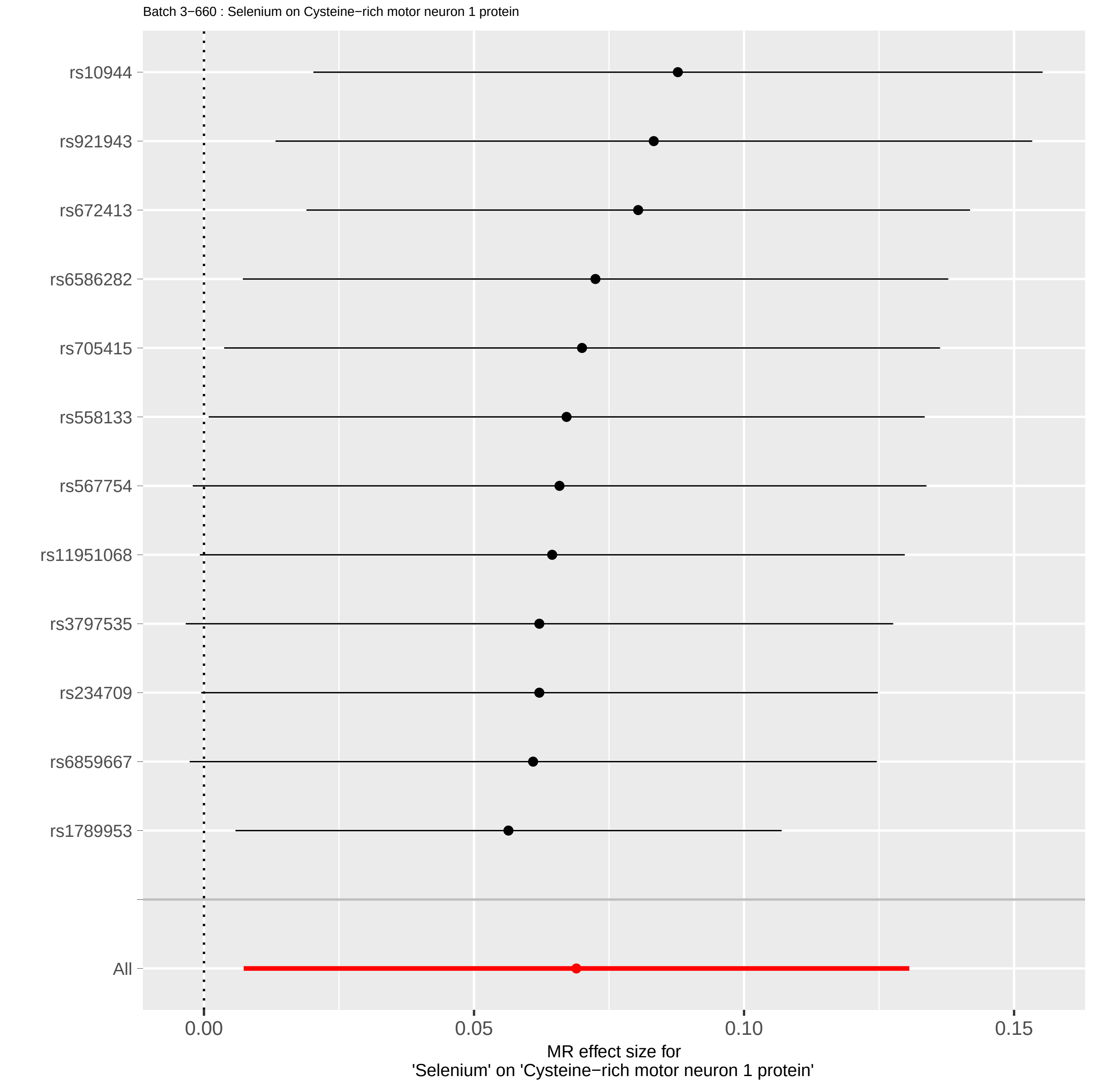

Batch 3−660 : Selenium on Cysteine−rich motor neuron 1 protein
rs10944
rs921943
rs672413
rs6586282
rs705415
rs558133
rs567754
rs11951068
rs3797535
rs234709
rs6859667
rs1789953
All
0.00
0.05
0.10
0.15
MR effect size for
'Selenium' on 'Cysteine−rich motor neuron 1 protein'

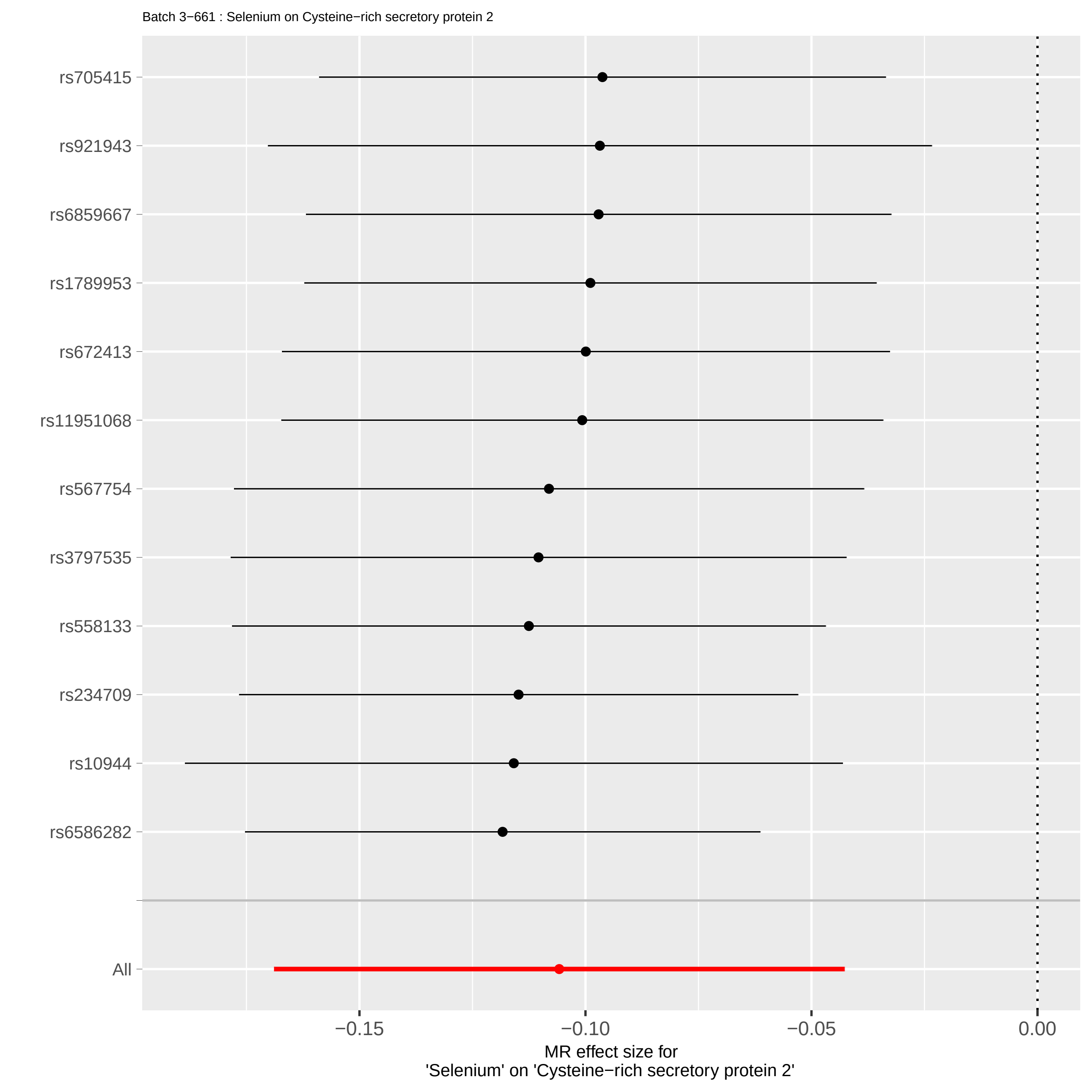

Batch 3−661 : Selenium on Cysteine−rich secretory protein 2
rs705415
rs921943
rs6859667
rs1789953
rs672413
rs11951068
rs567754
rs3797535
rs558133
rs234709
rs10944
rs6586282
All
−0.15
−0.10
−0.05
0.00
MR effect size for
'Selenium' on 'Cysteine−rich secretory protein 2'

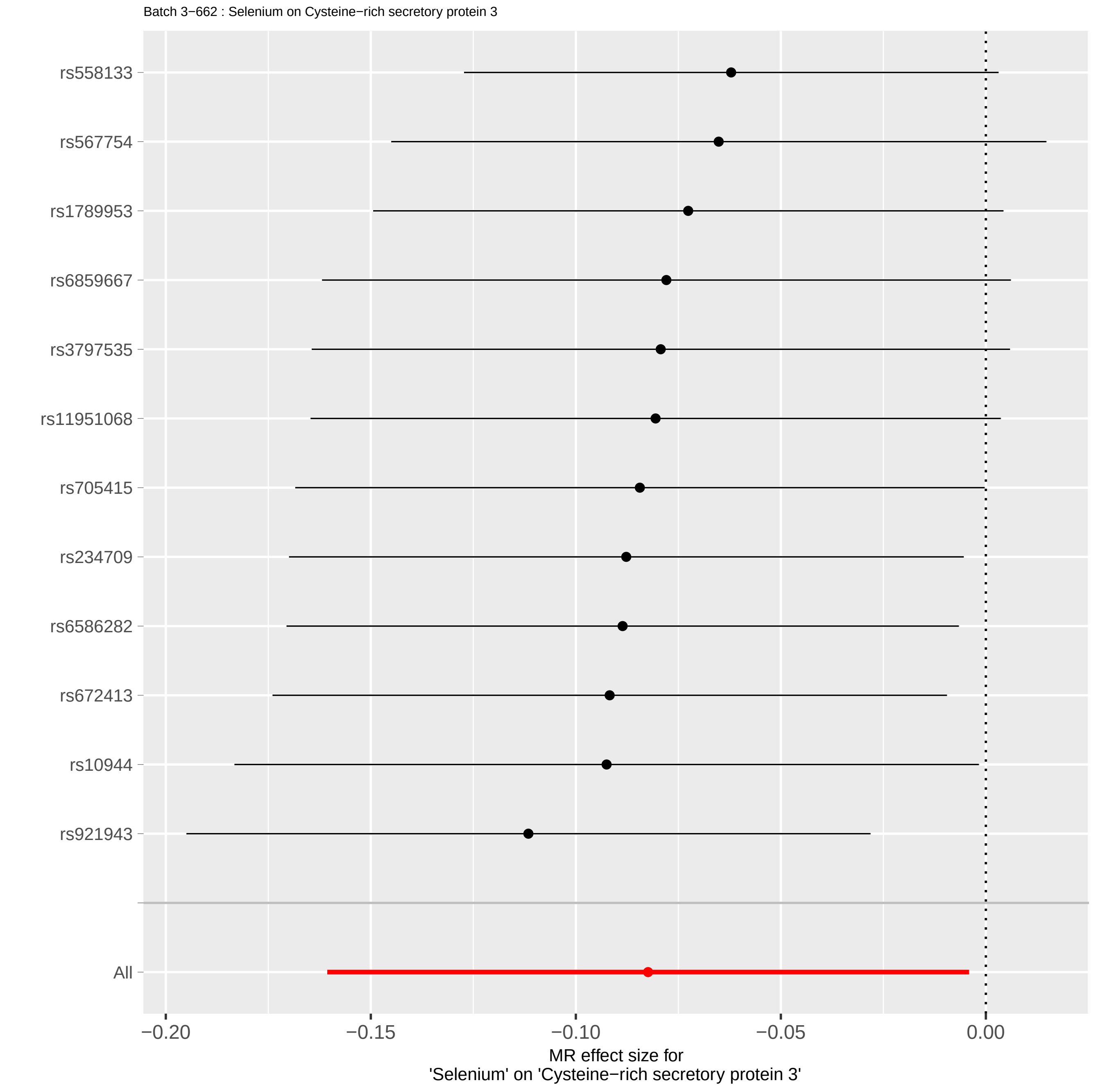

Batch 3−662 : Selenium on Cysteine−rich secretory protein 3
rs558133
rs567754
rs1789953
rs6859667
rs3797535
rs11951068
rs705415
rs234709
rs6586282
rs672413
rs10944
rs921943
All
−0.20
−0.15
−0.10
−0.05
0.00
MR effect size for
'Selenium' on 'Cysteine−rich secretory protein 3'

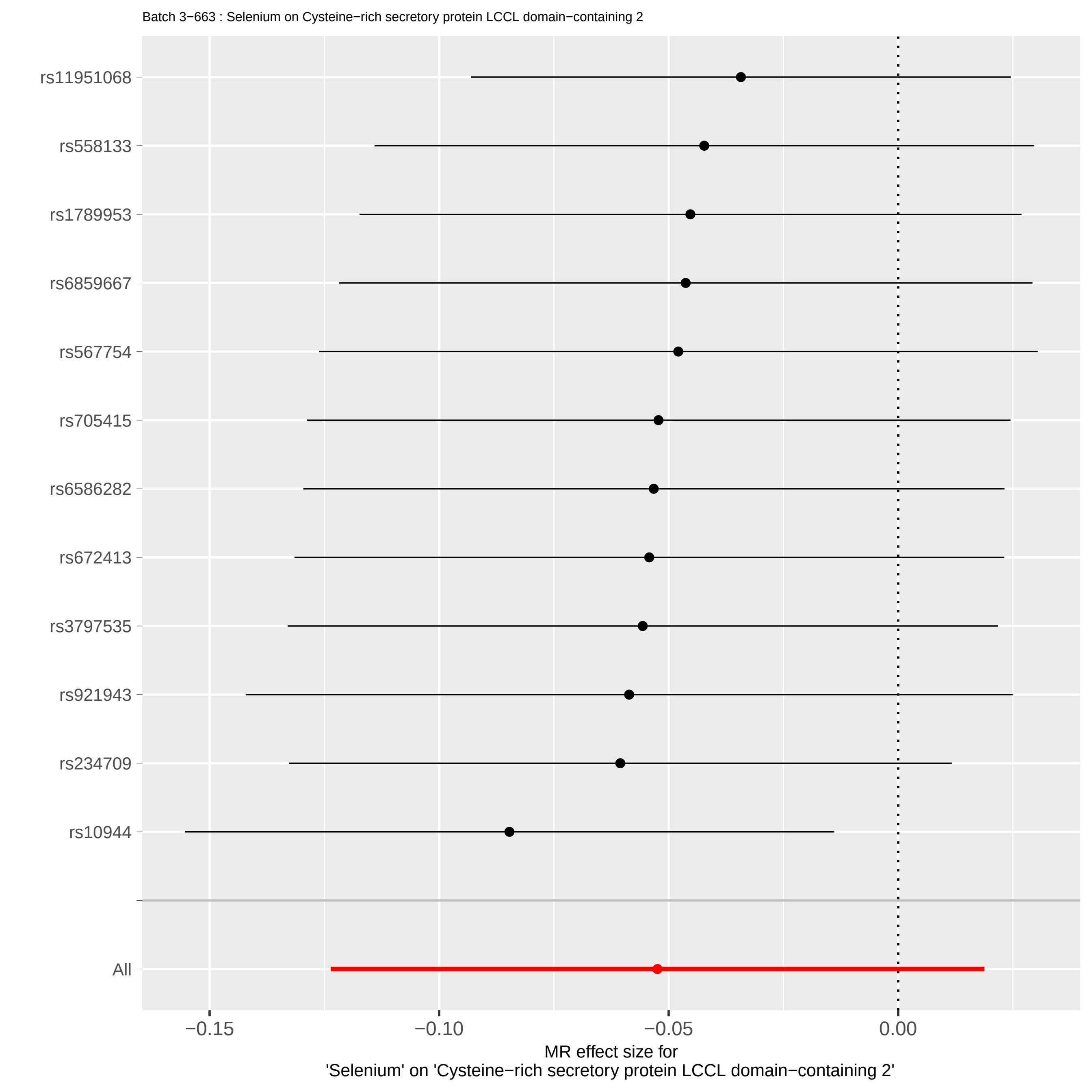
Batch 3−663 : Selenium on Cysteine−rich secretory protein LCCL domain−containing 2
rs11951068
rs558133
rs1789953
rs6859667
rs567754
rs705415
rs6586282
rs672413
rs3797535
rs921943
rs234709
rs10944
All
−0.15
−0.10
−0.05
0.00
MR effect size for
'Selenium' on 'Cysteine−rich secretory protein LCCL domain−containing 2'

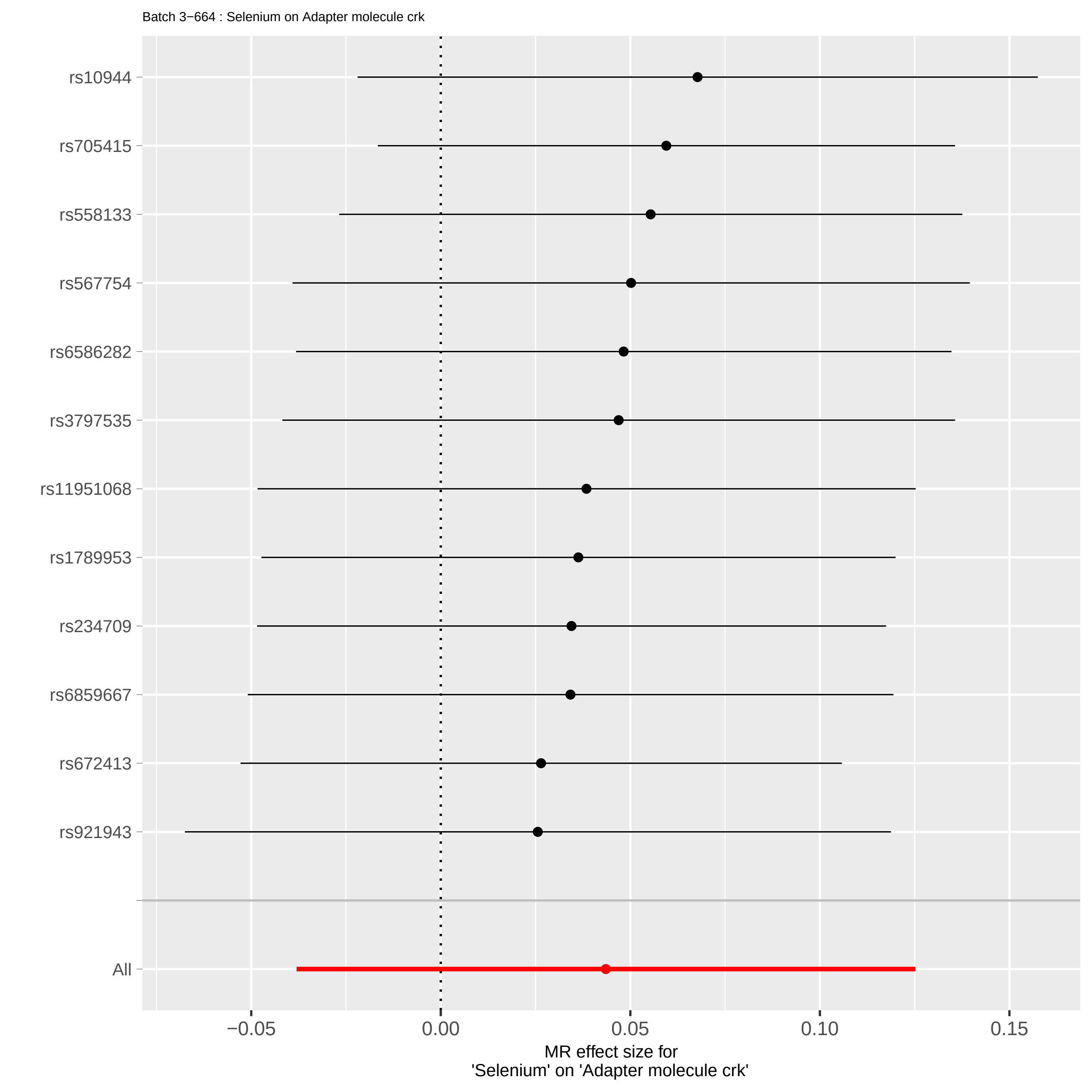

Batch 3−664 : Selenium on Adapter molecule crk
rs10944
rs705415
rs558133
rs567754
rs6586282
rs3797535
rs11951068
rs1789953
rs234709
rs6859667
rs672413
rs921943
All
−0.05
0.00
0.05
0.10
0.15
MR effect size for
'Selenium' on 'Adapter molecule crk'

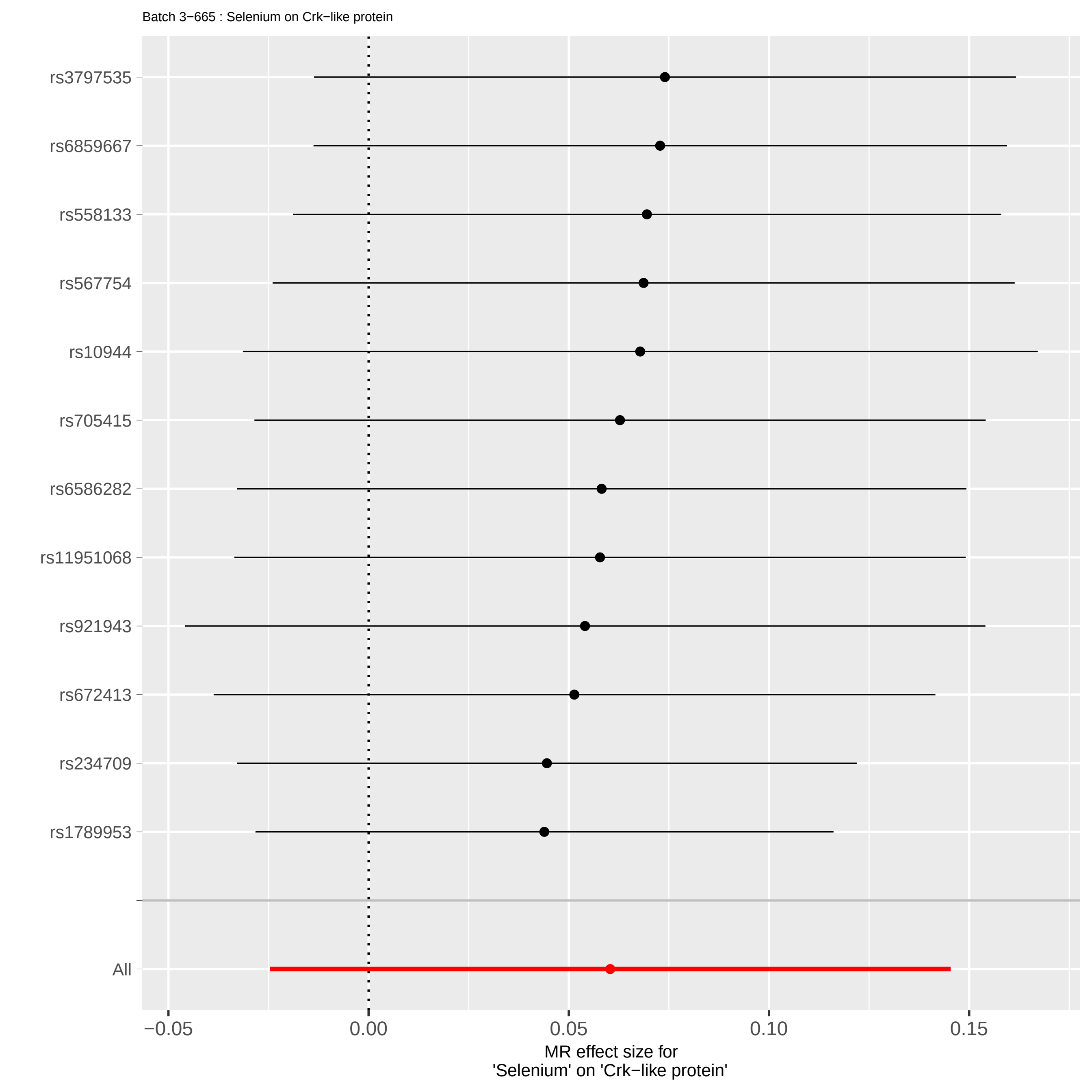

Batch 3−665 : Selenium on Crk−like protein
rs3797535
rs6859667
rs558133
rs567754
rs10944
rs705415
rs6586282
rs11951068
rs921943
rs672413
rs234709
rs1789953
All
−0.05
0.00
0.05
0.10
0.15
MR effect size for
'Selenium' on 'Crk−like protein'

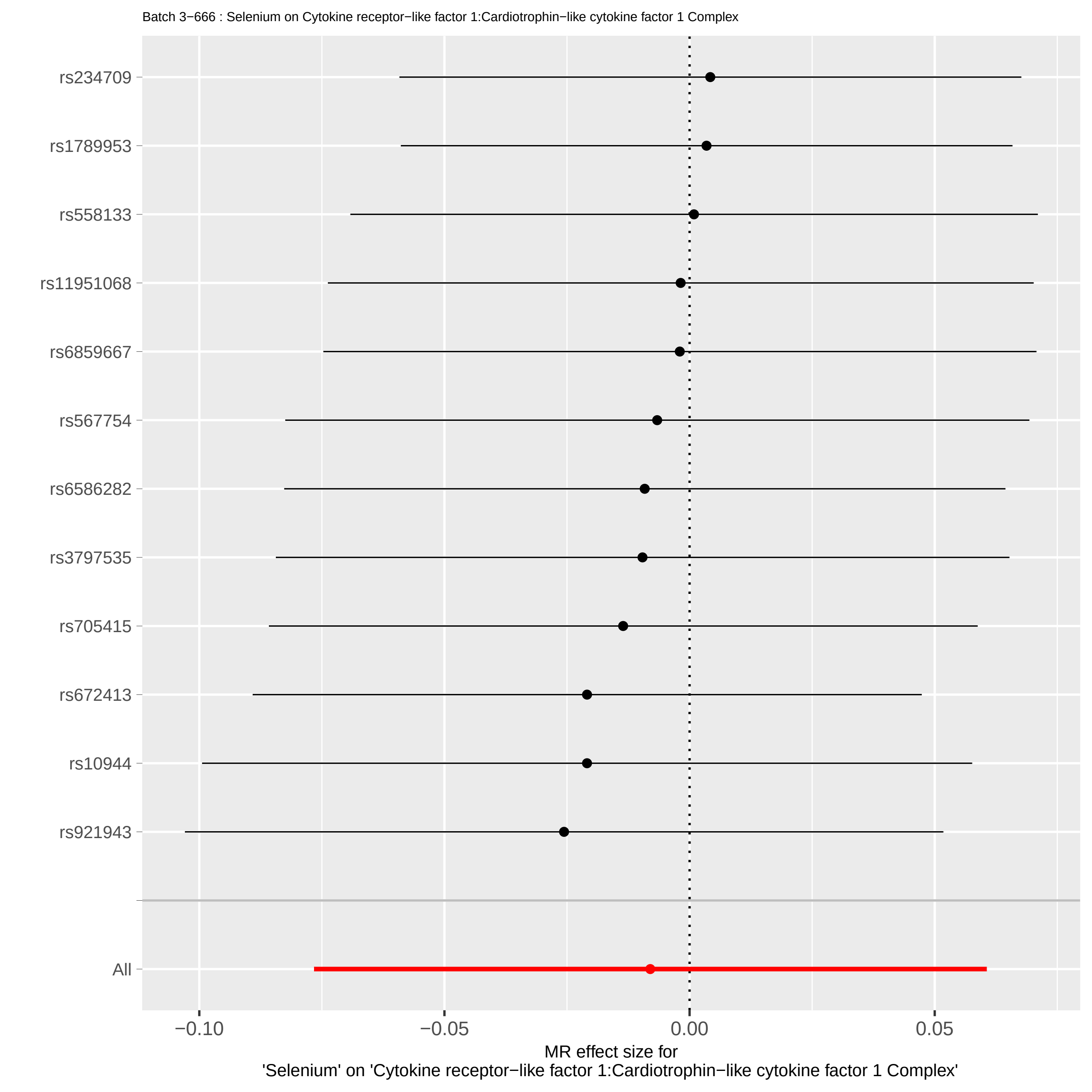
Batch 3−666 : Selenium on Cytokine receptor−like factor 1:Cardiotrophin−like cytokine factor 1 Complex
rs234709
rs1789953
rs558133
rs11951068
rs6859667
rs567754
rs6586282
rs3797535
rs705415
rs672413
rs10944
rs921943
All
−0.10
−0.05
0.00
0.05
MR effect size for
'Selenium' on 'Cytokine receptor−like factor 1:Cardiotrophin−like cytokine factor 1 Complex'

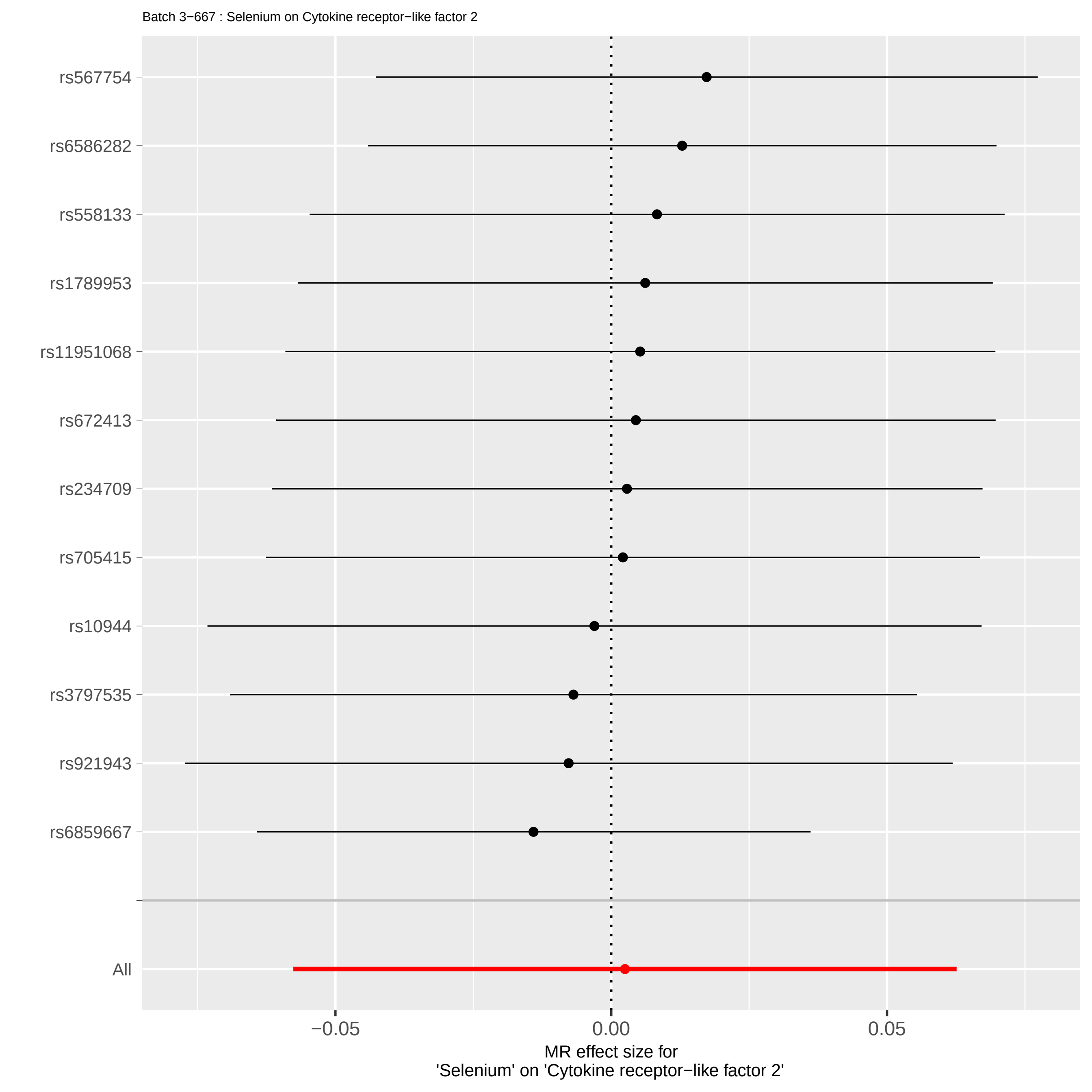

Batch 3−667 : Selenium on Cytokine receptor−like factor 2
rs567754
rs6586282
rs558133
rs1789953
rs11951068
rs672413
rs234709
rs705415
rs10944
rs3797535
rs921943
rs6859667
All
−0.05
0.00
0.05
MR effect size for
'Selenium' on 'Cytokine receptor−like factor 2'

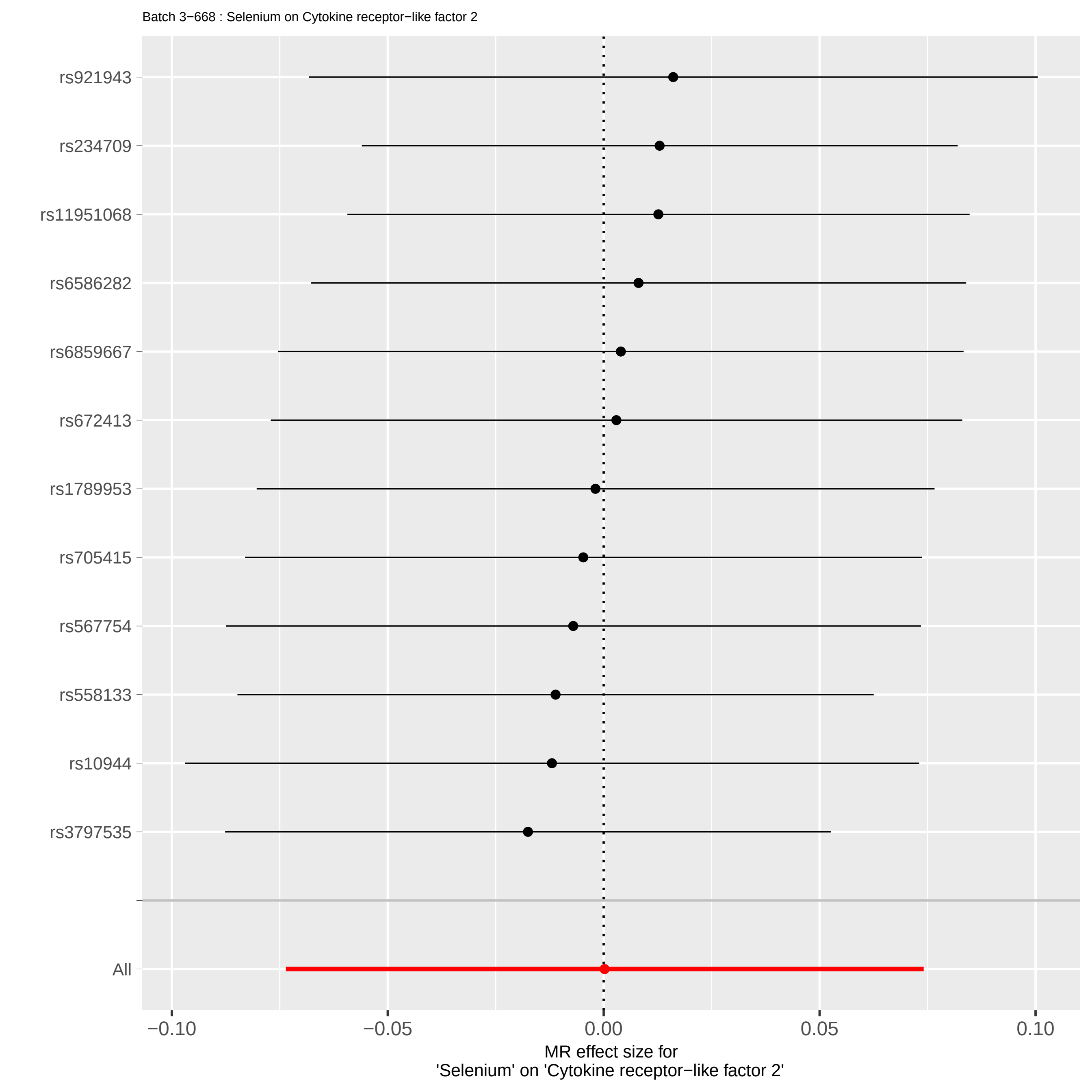

Batch 3−668 : Selenium on Cytokine receptor−like factor 2
rs921943
rs234709
rs11951068
rs6586282
rs6859667
rs672413
rs1789953
rs705415
rs567754
rs558133
rs10944
rs3797535
All
−0.10
−0.05
0.00
0.05
0.10
MR effect size for
'Selenium' on 'Cytokine receptor−like factor 2'

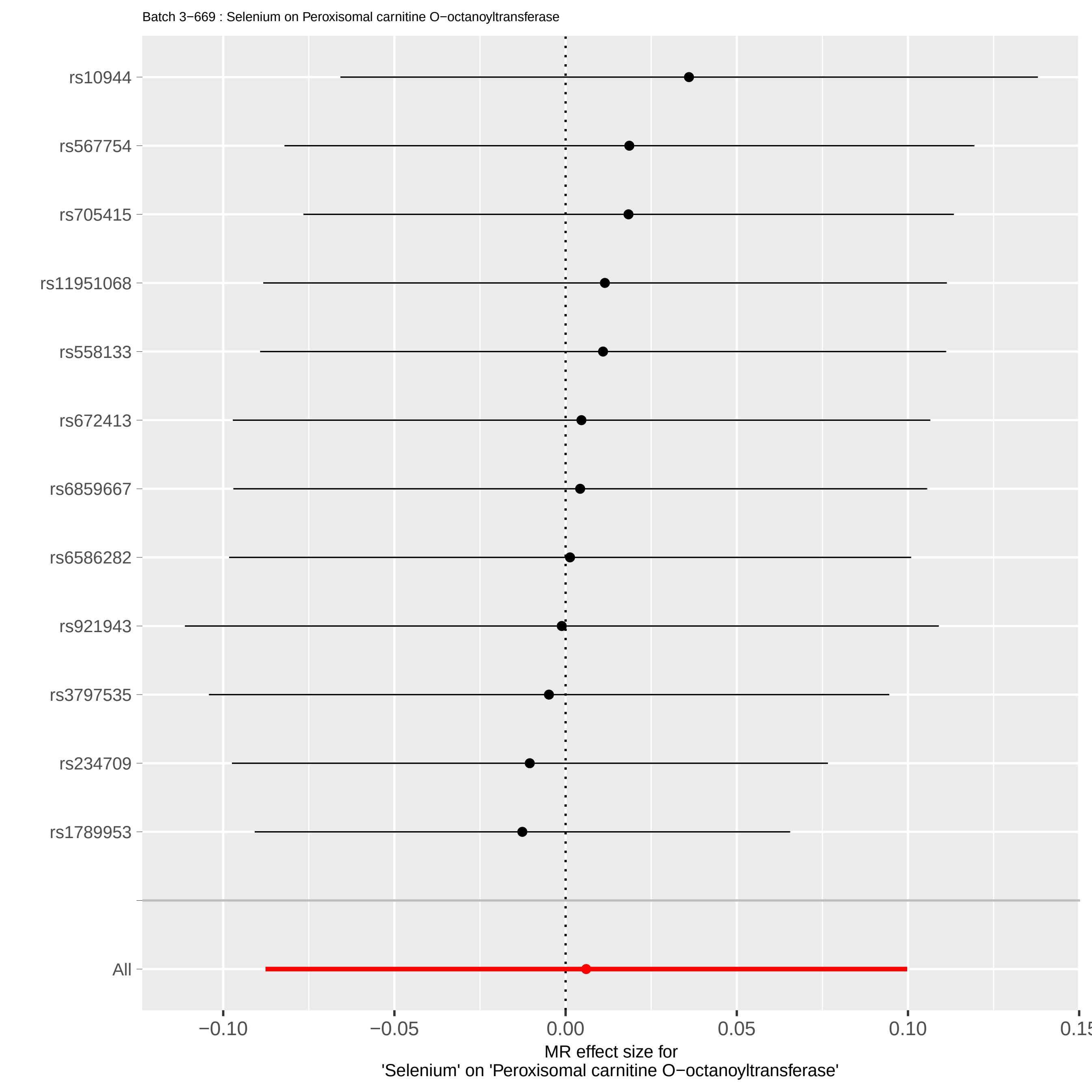

Batch 3−669 : Selenium on Peroxisomal carnitine O−octanoyltransferase
rs10944
rs567754
rs705415
rs11951068
rs558133
rs672413
rs6859667
rs6586282
rs921943
rs3797535
rs234709
rs1789953
All
−0.10
−0.05
0.00
0.05
0.10
0.15
MR effect size for
'Selenium' on 'Peroxisomal carnitine O−octanoyltransferase'

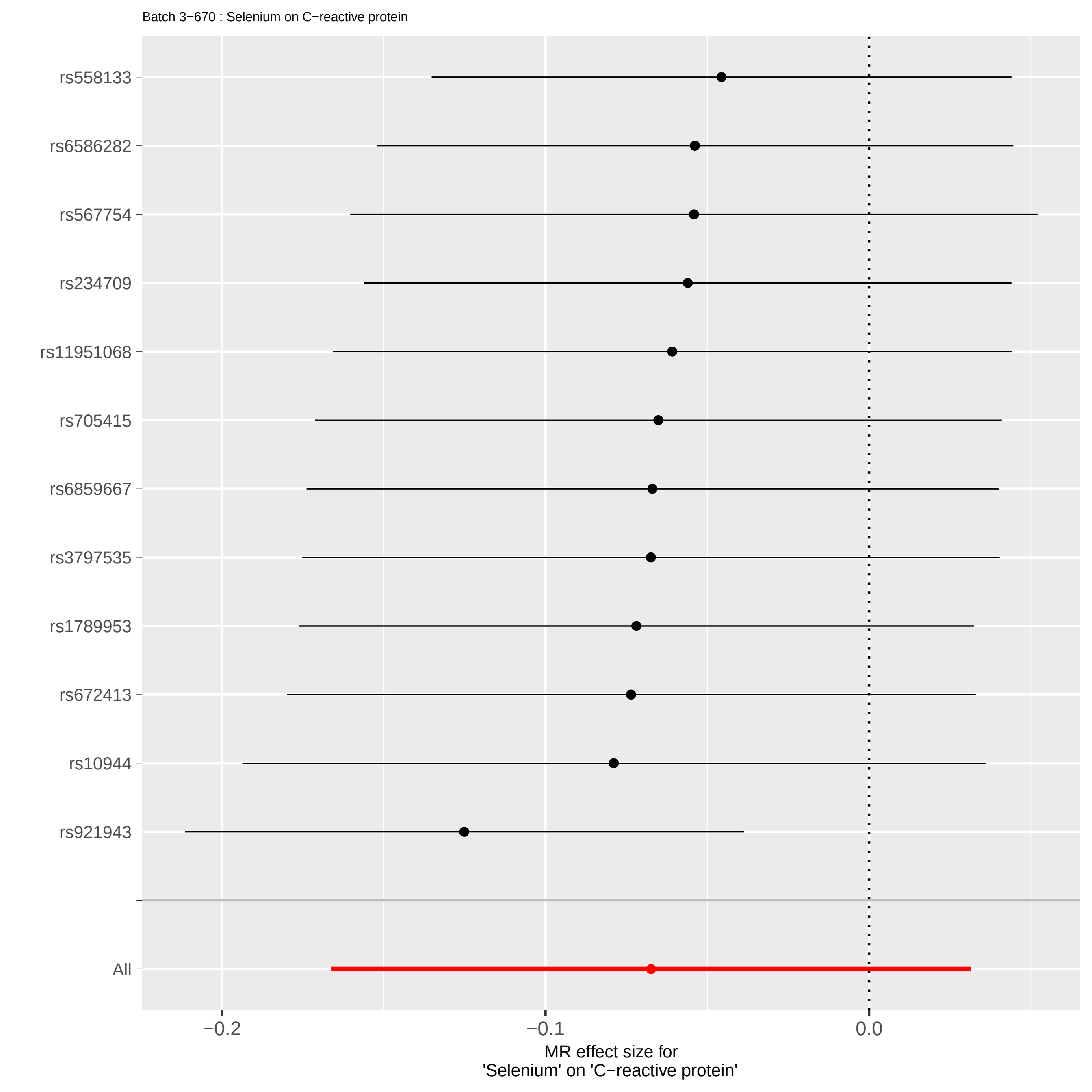

Batch 3−670 : Selenium on C−reactive protein
rs558133
rs6586282
rs567754
rs234709
rs11951068
rs705415
rs6859667
rs3797535
rs1789953
rs672413
rs10944
rs921943
All
−0.2
−0.1
0.0
MR effect size for
'Selenium' on 'C−reactive protein'

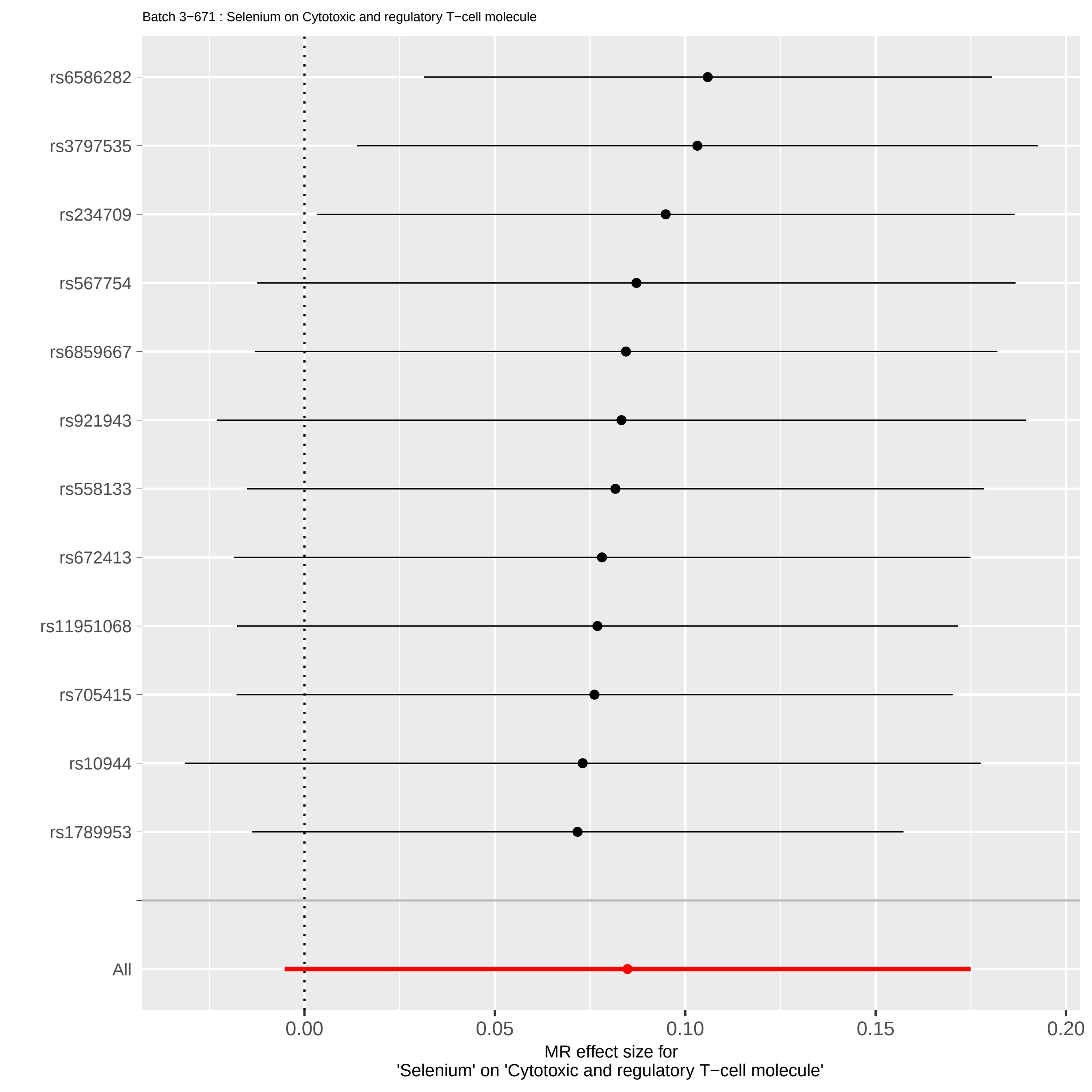

Batch 3−671 : Selenium on Cytotoxic and regulatory T−cell molecule
rs6586282
rs3797535
rs234709
rs567754
rs6859667
rs921943
rs558133
rs672413
rs11951068
rs705415
rs10944
rs1789953
All
0.00
0.05
0.10
0.15
0.20
MR effect size for
'Selenium' on 'Cytotoxic and regulatory T−cell molecule'

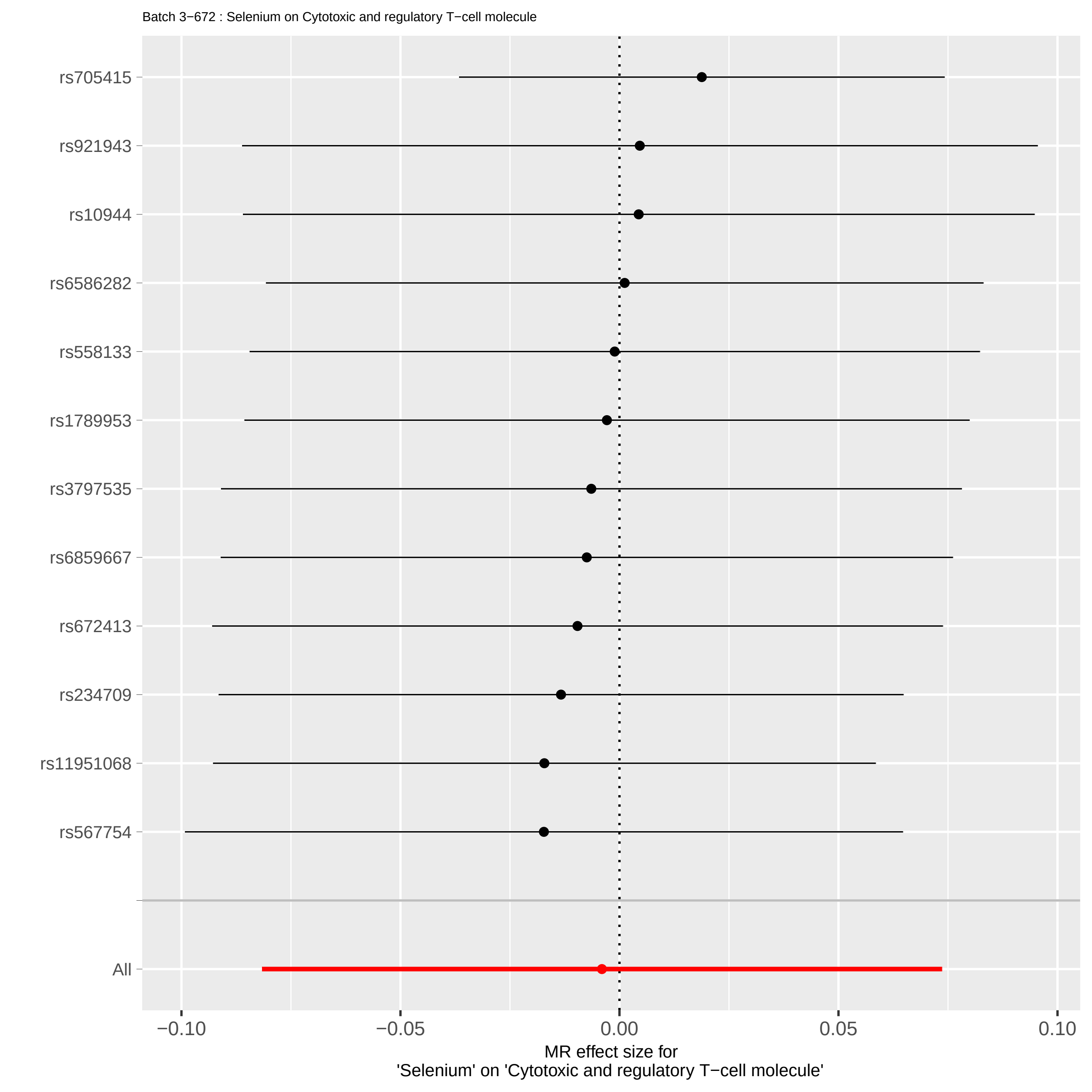
Batch 3−672 : Selenium on Cytotoxic and regulatory T−cell molecule
rs705415
rs921943
rs10944
rs6586282
rs558133
rs1789953
rs3797535
rs6859667
rs672413
rs234709
rs11951068
rs567754
All
−0.10
−0.05
0.00
0.05
0.10
MR effect size for
'Selenium' on 'Cytotoxic and regulatory T−cell molecule'

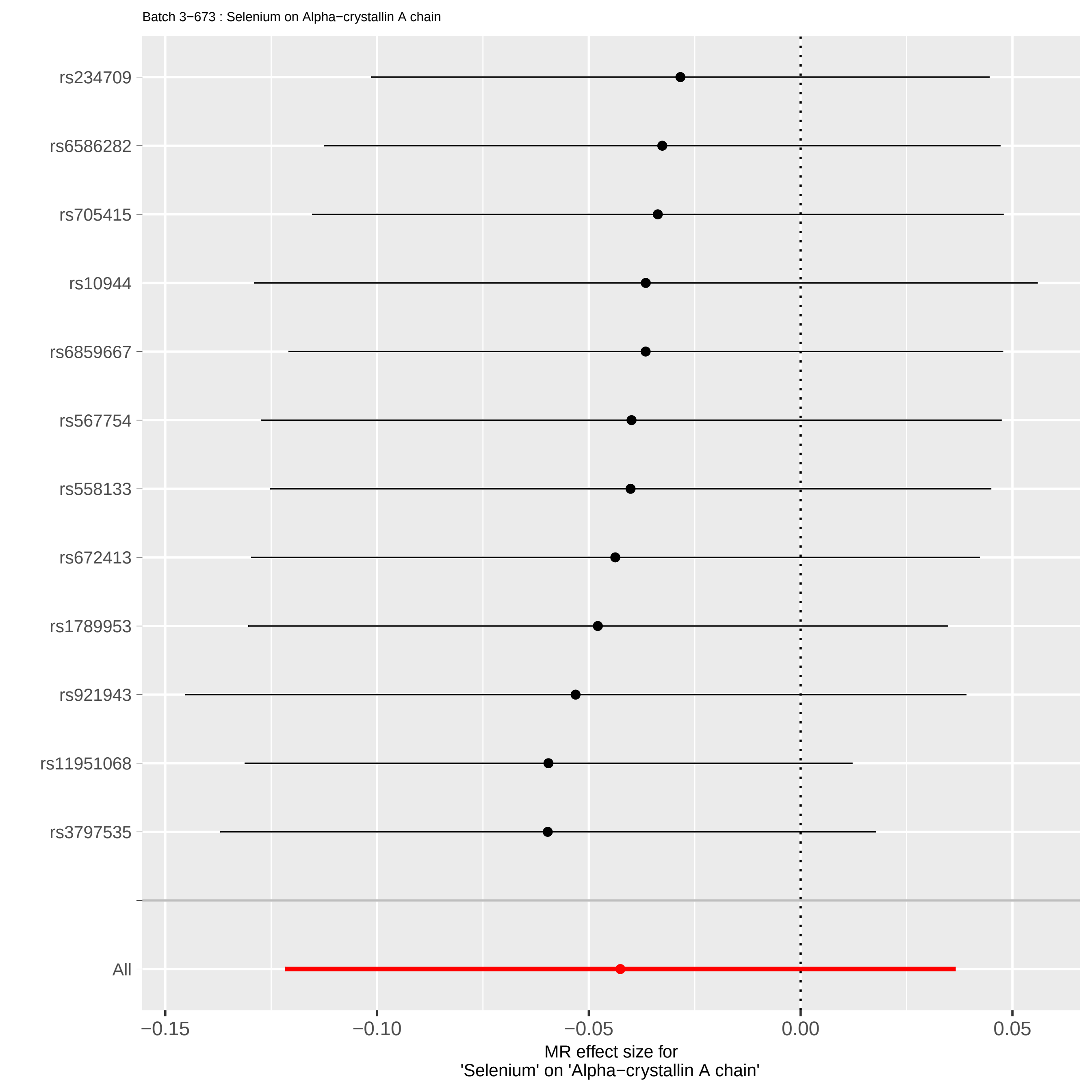

Batch 3−673 : Selenium on Alpha−crystallin A chain
rs234709
rs6586282
rs705415
rs10944
rs6859667
rs567754
rs558133
rs672413
rs1789953
rs921943
rs11951068
rs3797535
All
−0.15
−0.10
−0.05
0.00
0.05
MR effect size for
'Selenium' on 'Alpha−crystallin A chain'

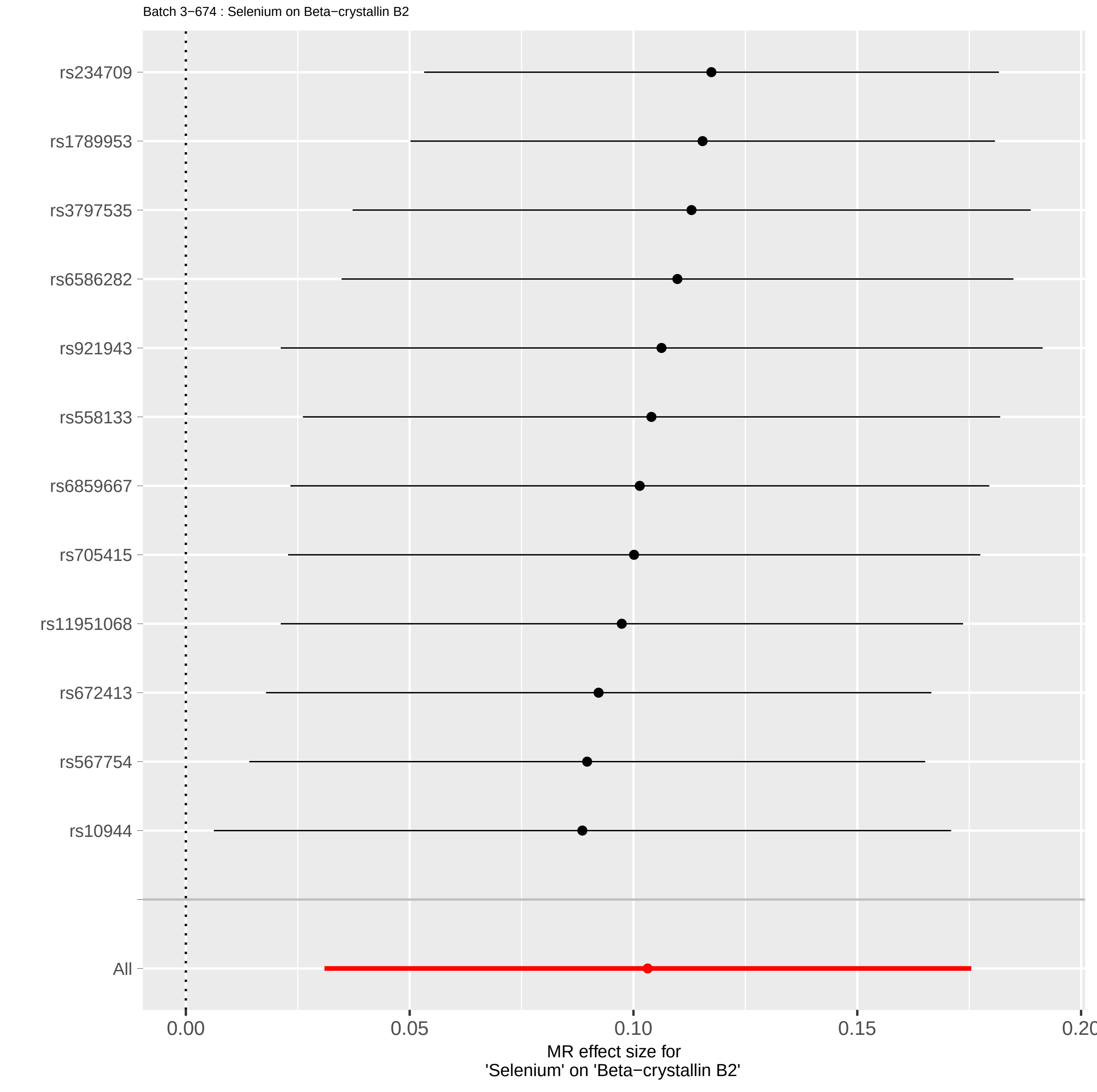

Batch 3−674 : Selenium on Beta−crystallin B2
rs234709
rs1789953
rs3797535
rs6586282
rs921943
rs558133
rs6859667
rs705415
rs11951068
rs672413
rs567754
rs10944
All
0.00
0.05
0.10
0.15
0.20
MR effect size for
'Selenium' on 'Beta−crystallin B2'

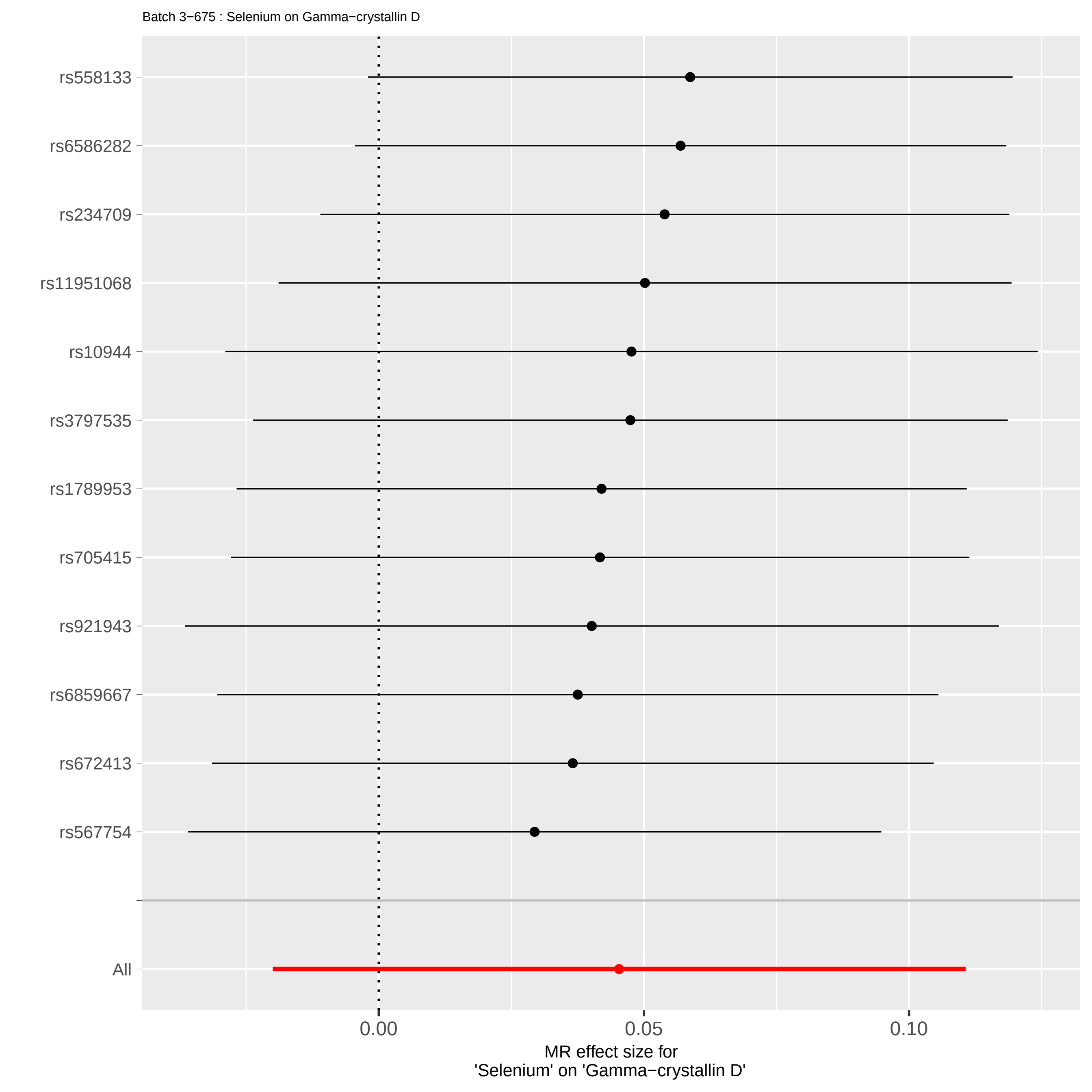
Batch 3−675 : Selenium on Gamma−crystallin D
rs558133
rs6586282
rs234709
rs11951068
rs10944
rs3797535
rs1789953
rs705415
rs921943
rs6859667
rs672413
rs567754
All
0.00
0.05
0.10
MR effect size for
'Selenium' on 'Gamma−crystallin D'

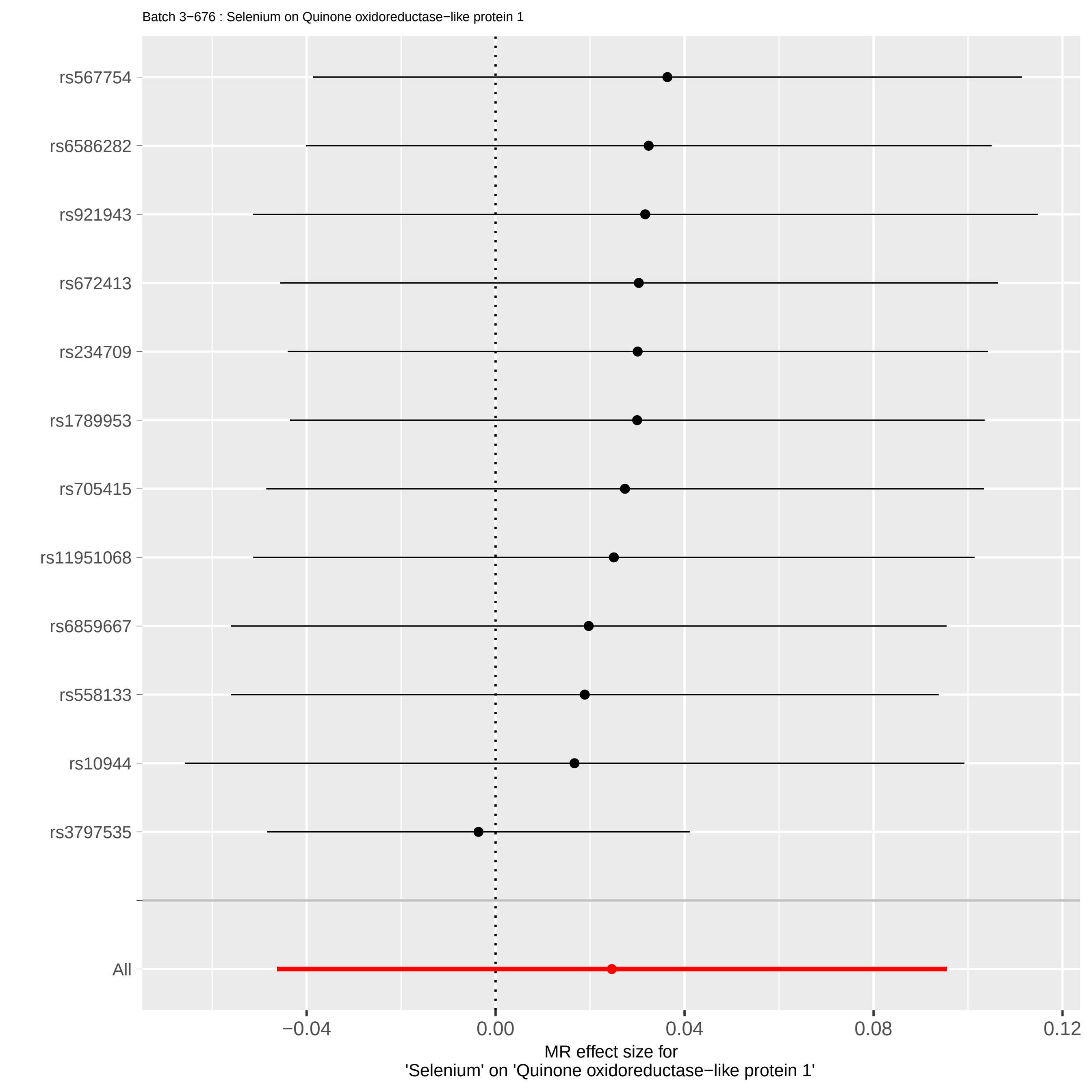

Batch 3−676 : Selenium on Quinone oxidoreductase−like protein 1
rs567754
rs6586282
rs921943
rs672413
rs234709
rs1789953
rs705415
rs11951068
rs6859667
rs558133
rs10944
rs3797535
All
−0.04
0.00
0.04
0.08
0.12
MR effect size for
'Selenium' on 'Quinone oxidoreductase−like protein 1'

Batch 3−677 : Selenium on Putative chondrosarcoma−associated gene 1 protein

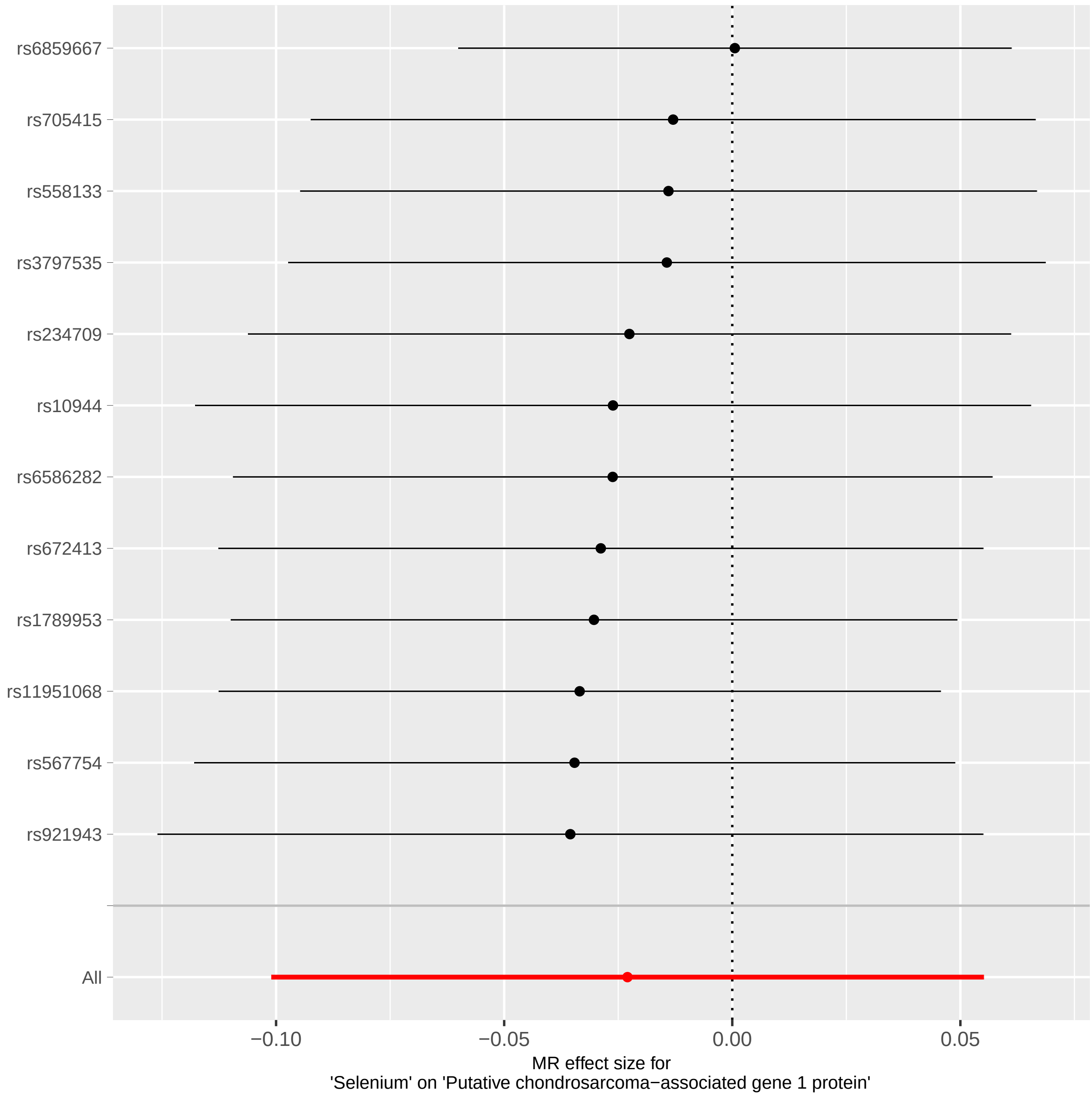

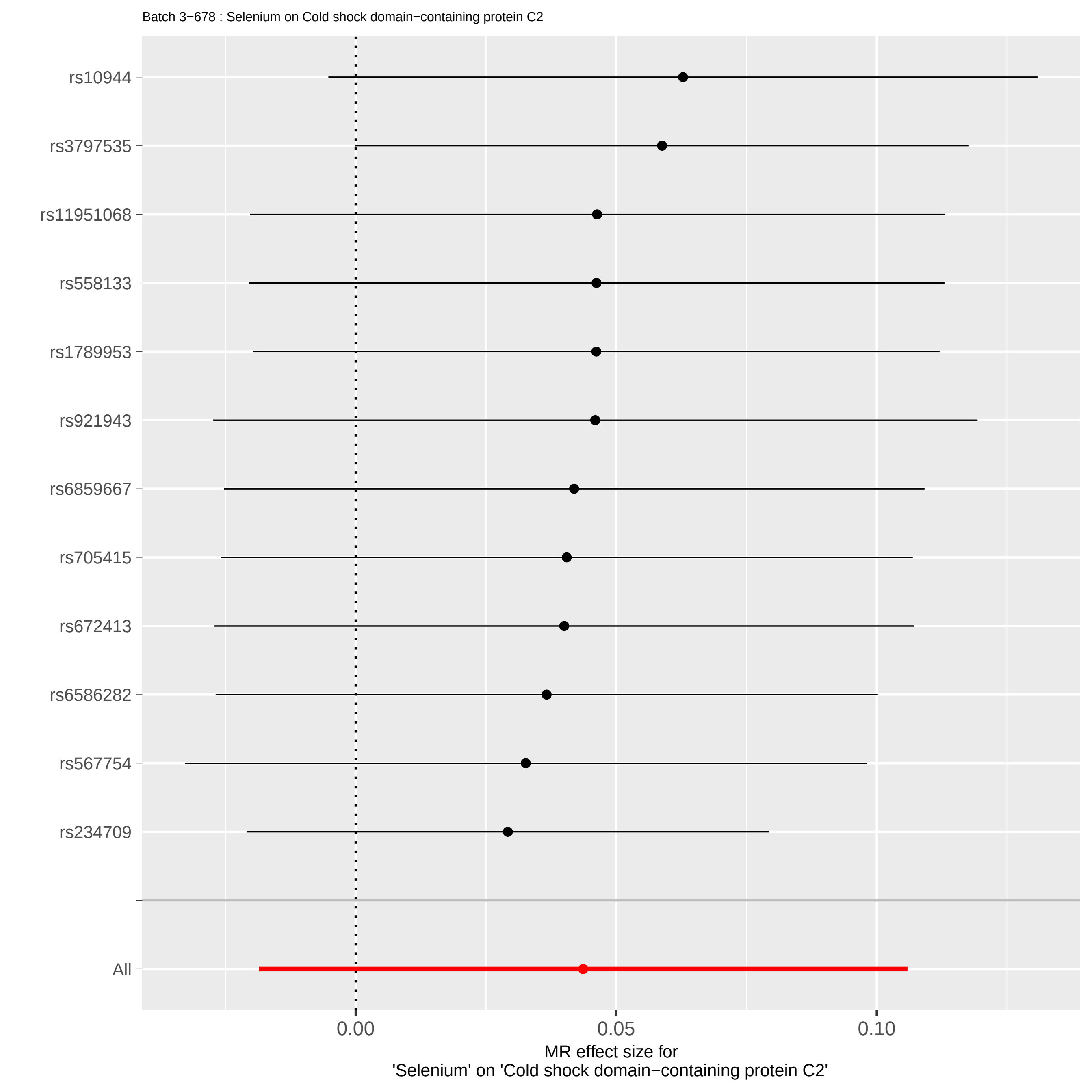

Batch 3−678 : Selenium on Cold shock domain−containing protein C2
rs10944
rs3797535
rs11951068
rs558133
rs1789953
rs921943
rs6859667
rs705415
rs672413
rs6586282
rs567754
rs234709
All
0.00
0.05
0.10
MR effect size for
'Selenium' on 'Cold shock domain−containing protein C2'

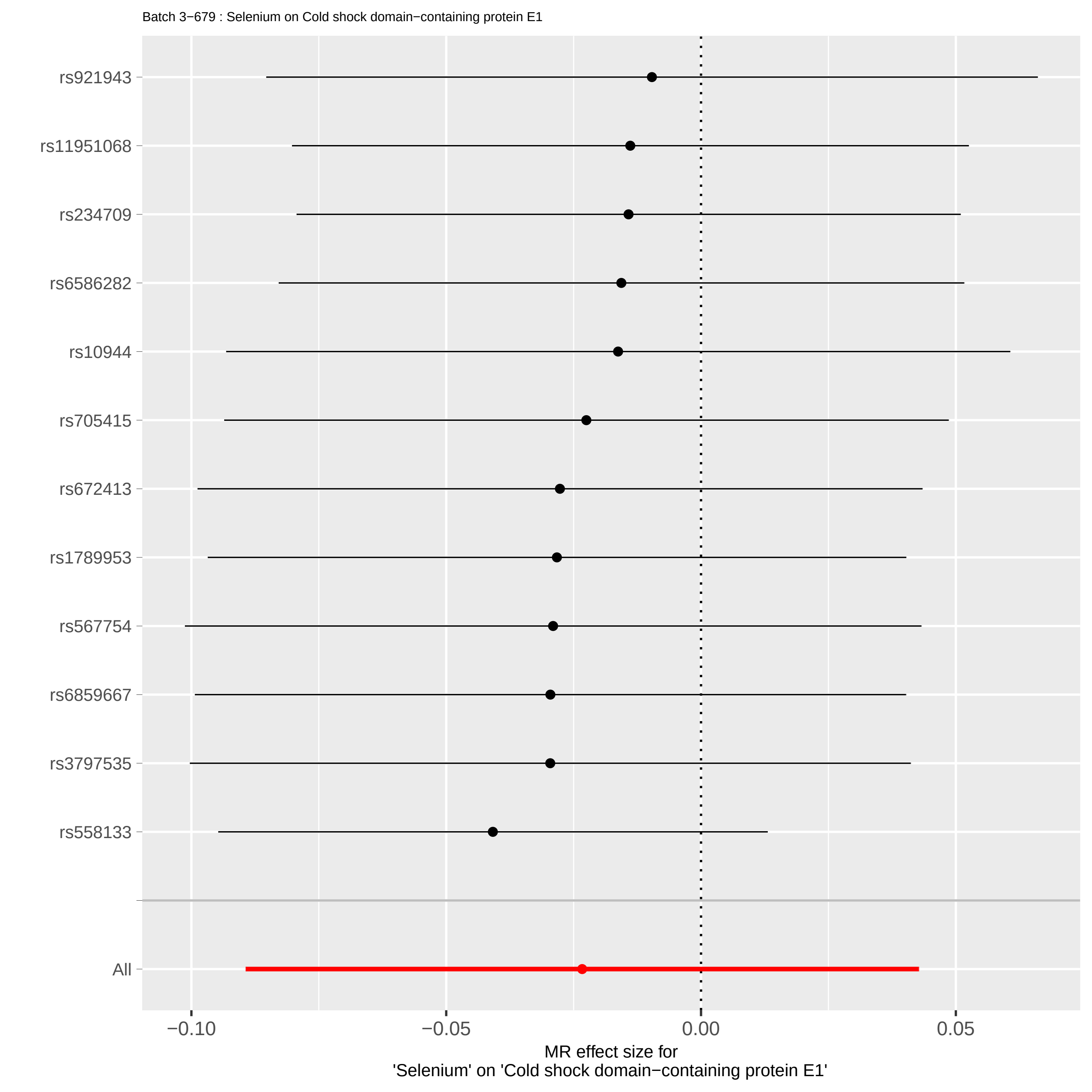
Batch 3−679 : Selenium on Cold shock domain−containing protein E1
rs921943
rs11951068
rs234709
rs6586282
rs10944
rs705415
rs672413
rs1789953
rs567754
rs6859667
rs3797535
rs558133
All
−0.10
−0.05
0.00
0.05
MR effect size for
'Selenium' on 'Cold shock domain−containing protein E1'

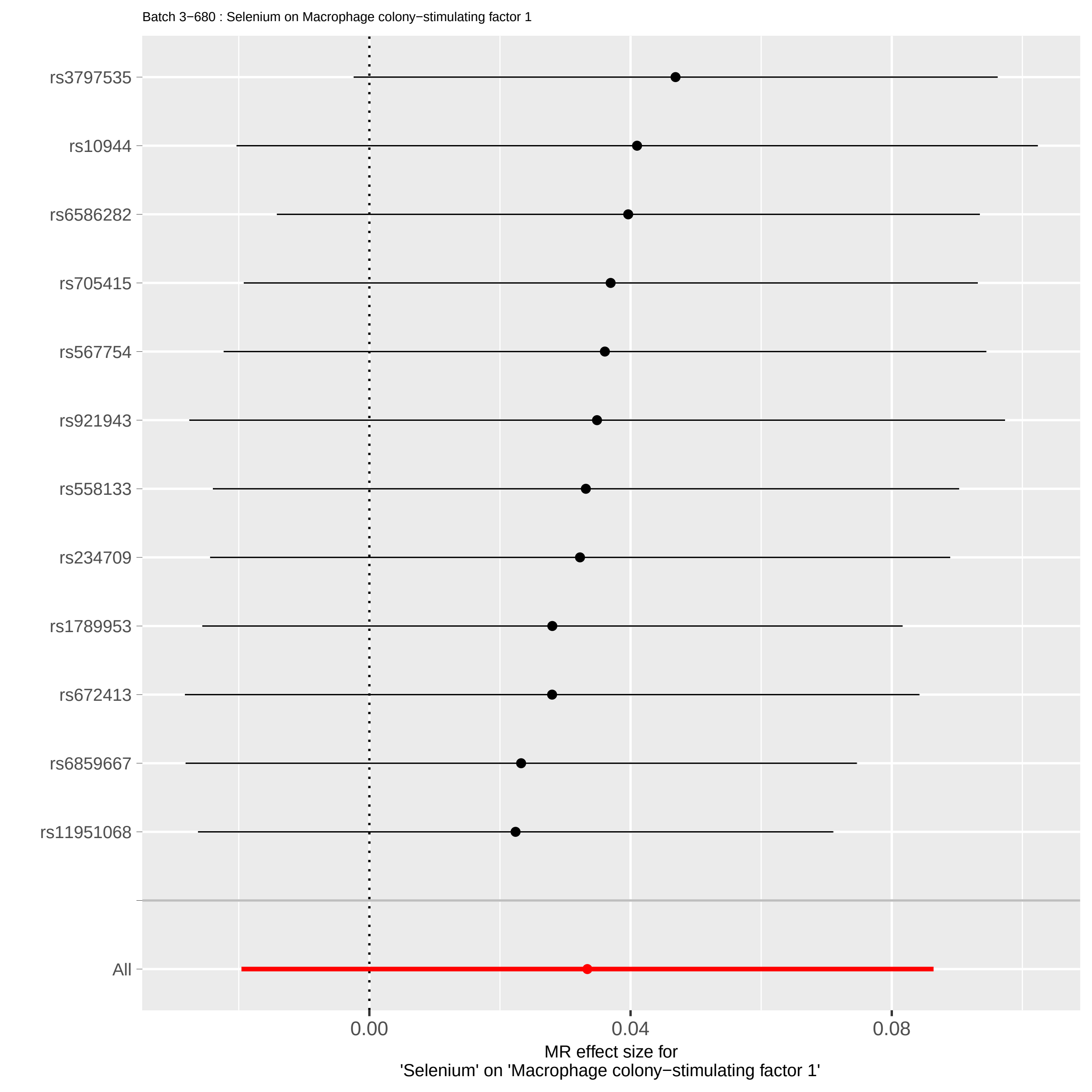

Batch 3−680 : Selenium on Macrophage colony−stimulating factor 1
rs3797535
rs10944
rs6586282
rs705415
rs567754
rs921943
rs558133
rs234709
rs1789953
rs672413
rs6859667
rs11951068
All
0.00
0.04
0.08
MR effect size for
'Selenium' on 'Macrophage colony−stimulating factor 1'

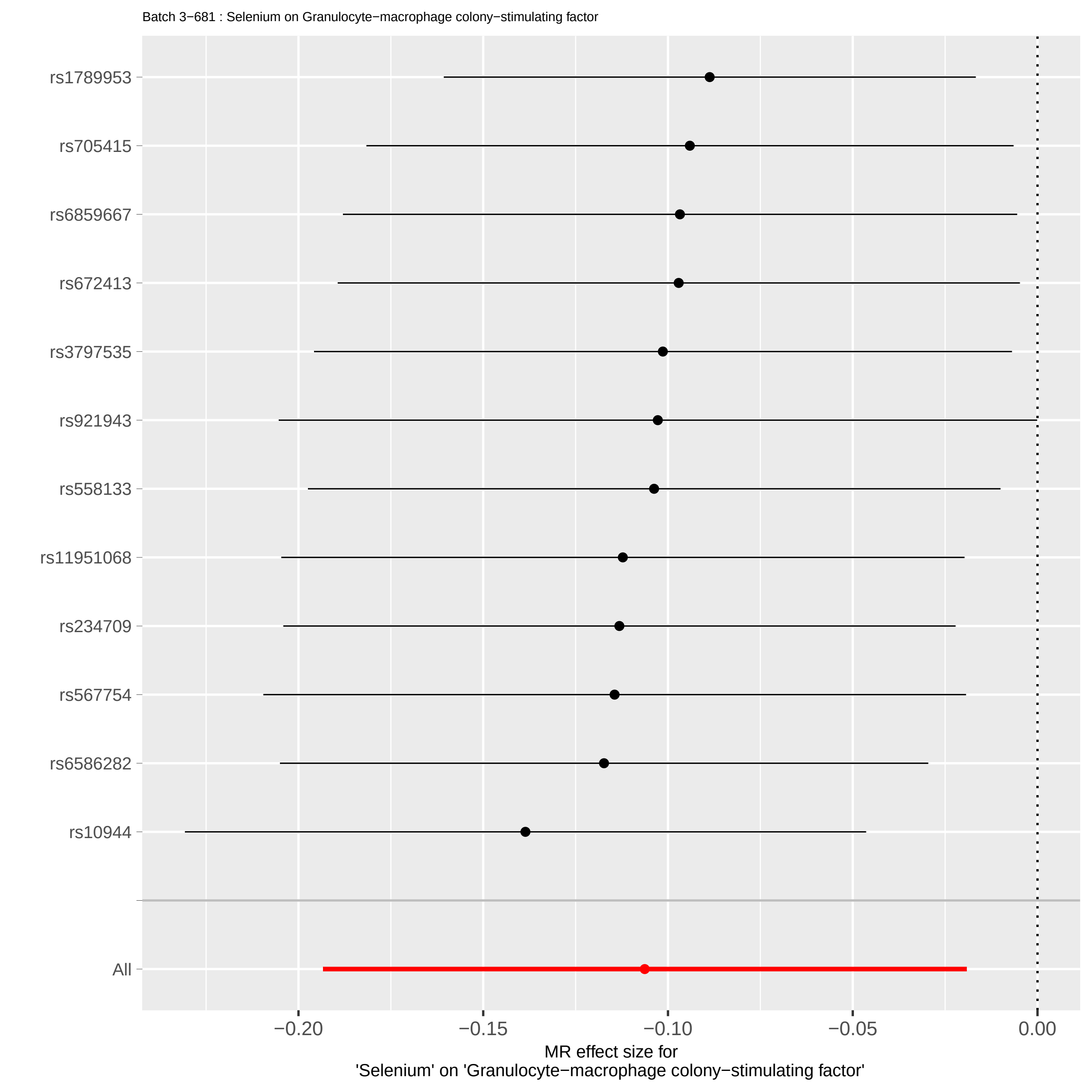

Batch 3−681 : Selenium on Granulocyte−macrophage colony−stimulating factor
rs1789953
rs705415
rs6859667
rs672413
rs3797535
rs921943
rs558133
rs11951068
rs234709
rs567754
rs6586282
rs10944
All
−0.20
−0.15
−0.10
−0.05
0.00
MR effect size for
'Selenium' on 'Granulocyte−macrophage colony−stimulating factor'

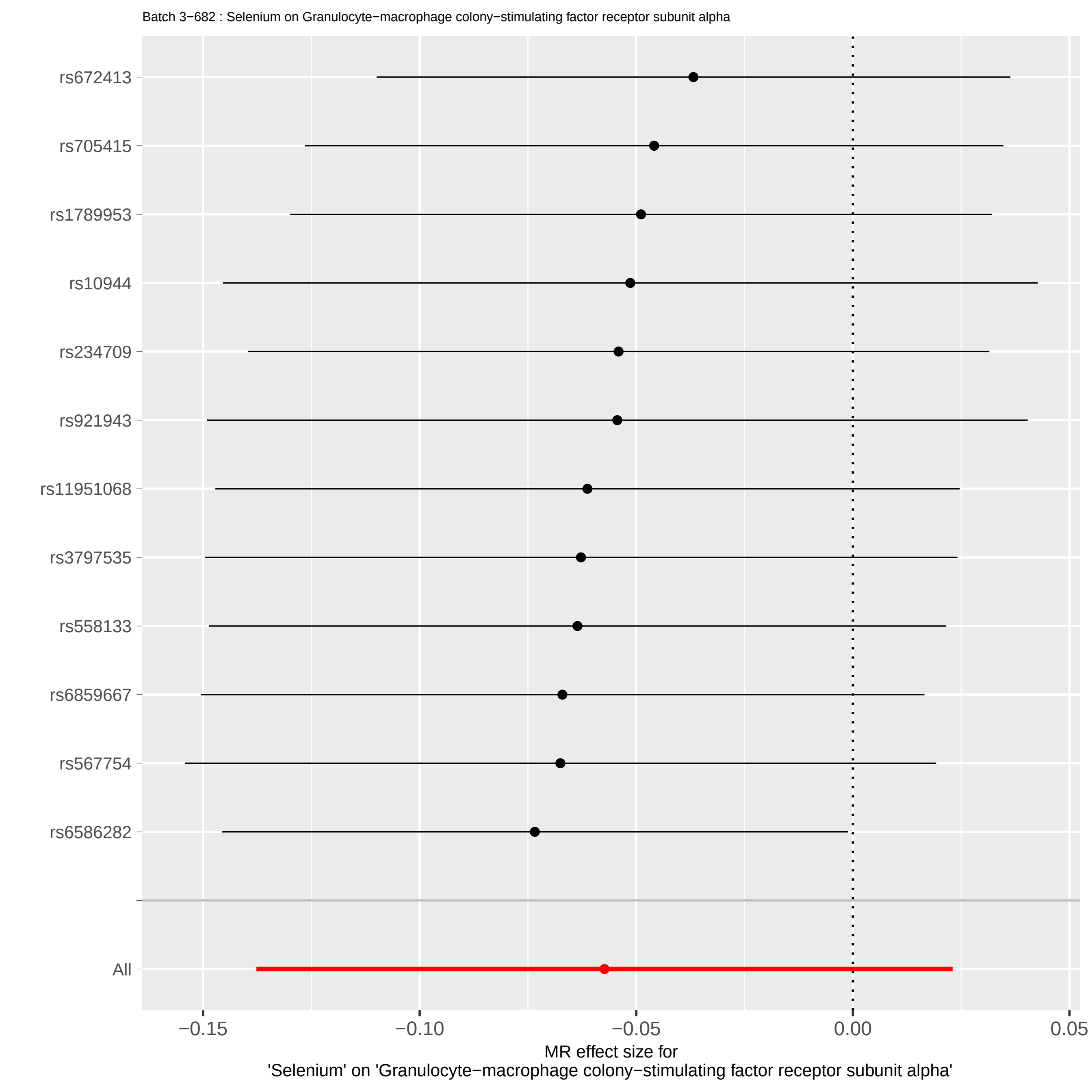

Batch 3−682 : Selenium on Granulocyte−macrophage colony−stimulating factor receptor subunit alpha
rs672413
rs705415
rs1789953
rs10944
rs234709
rs921943
rs11951068
rs3797535
rs558133
rs6859667
rs567754
rs6586282
All
−0.15
−0.10
−0.05
0.00
0.05
MR effect size for
'Selenium' on 'Granulocyte−macrophage colony−stimulating factor receptor subunit alpha'

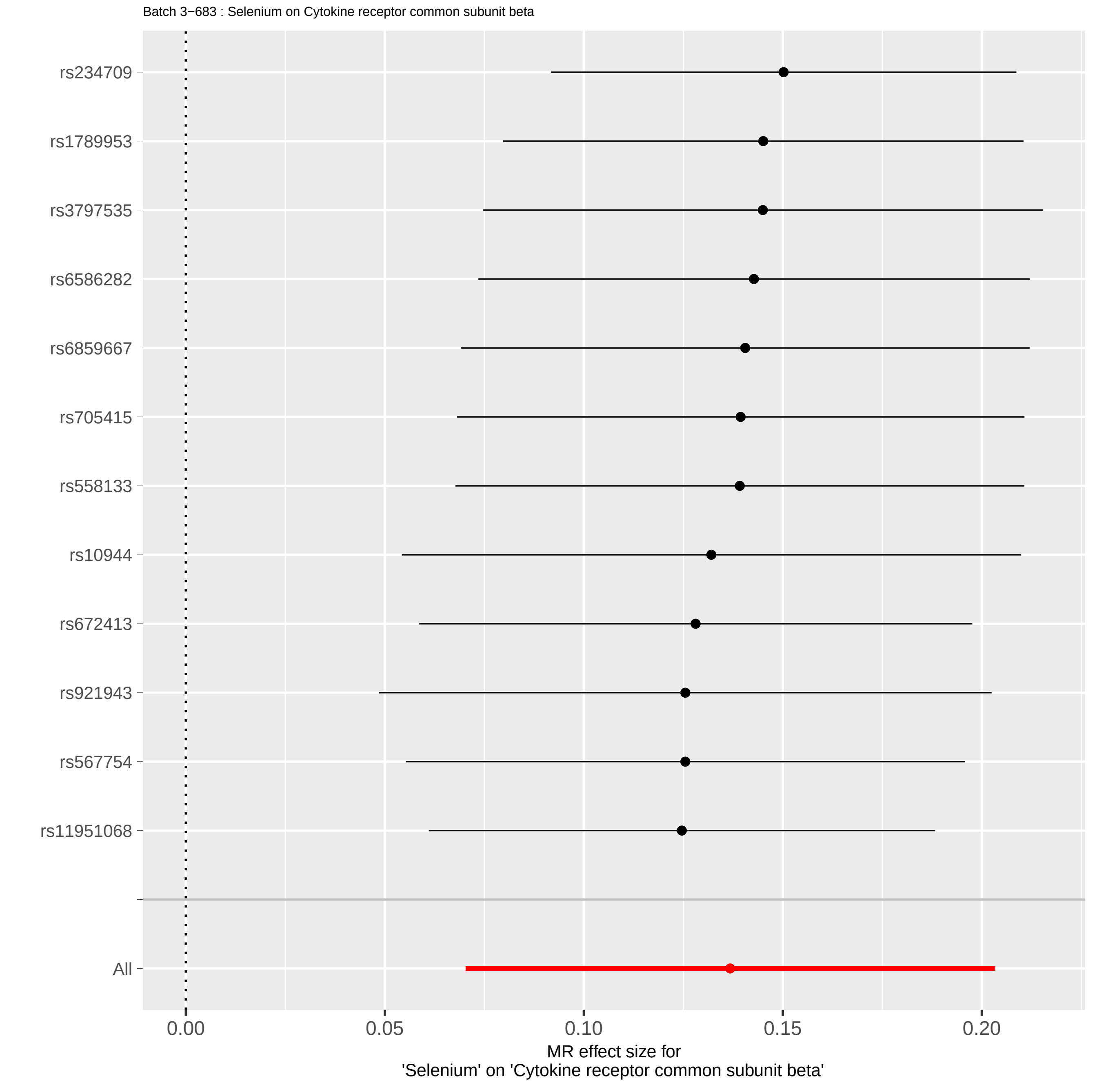
Batch 3−683 : Selenium on Cytokine receptor common subunit beta
rs234709
rs1789953
rs3797535
rs6586282
rs6859667
rs705415
rs558133
rs10944
rs672413
rs921943
rs567754
rs11951068
All
0.00
0.05
0.10
0.15
0.20
MR effect size for
'Selenium' on 'Cytokine receptor common subunit beta'

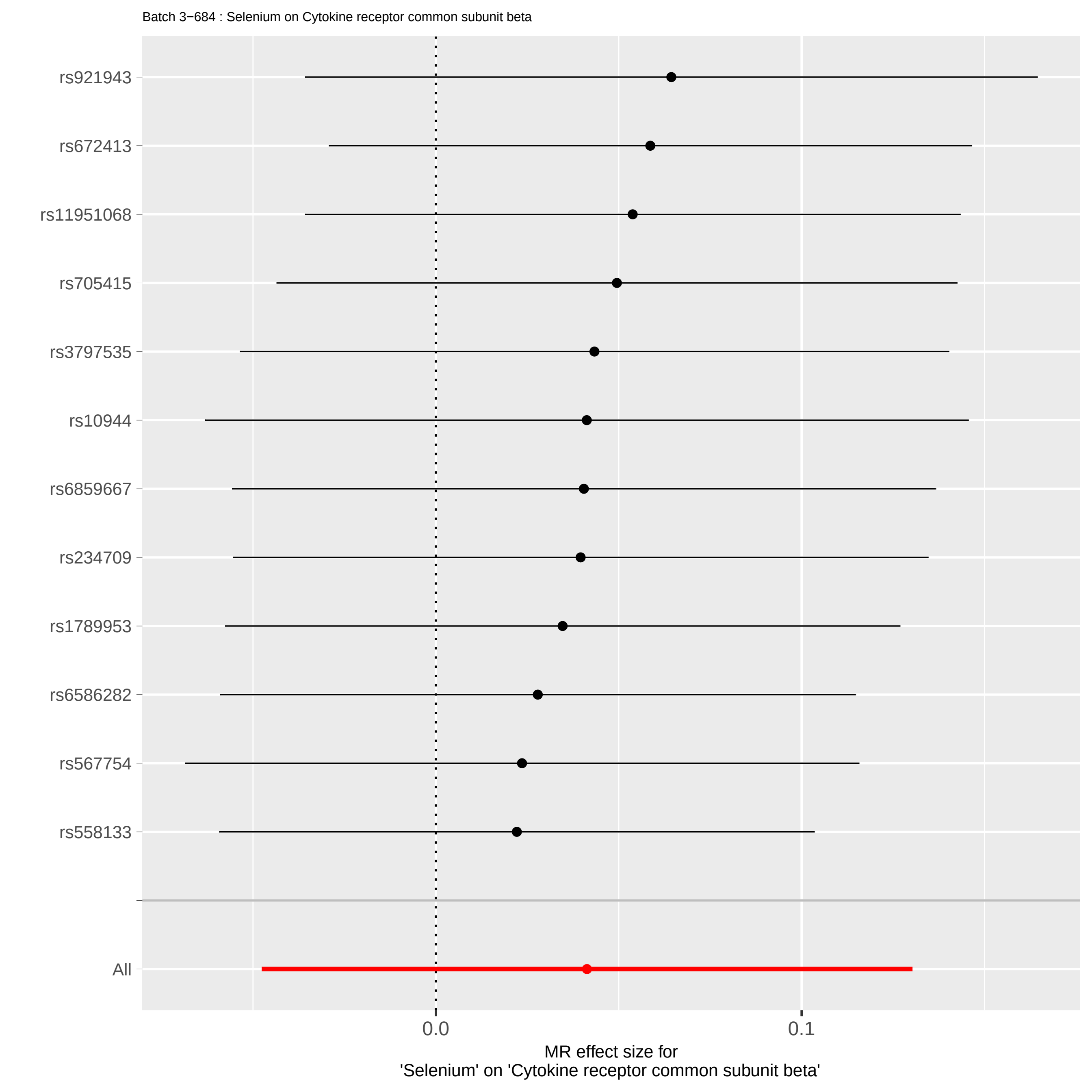

Batch 3−684 : Selenium on Cytokine receptor common subunit beta
rs921943
rs672413
rs11951068
rs705415
rs3797535
rs10944
rs6859667
rs234709
rs1789953
rs6586282
rs567754
rs558133
All
0.0
0.1
MR effect size for
'Selenium' on 'Cytokine receptor common subunit beta'

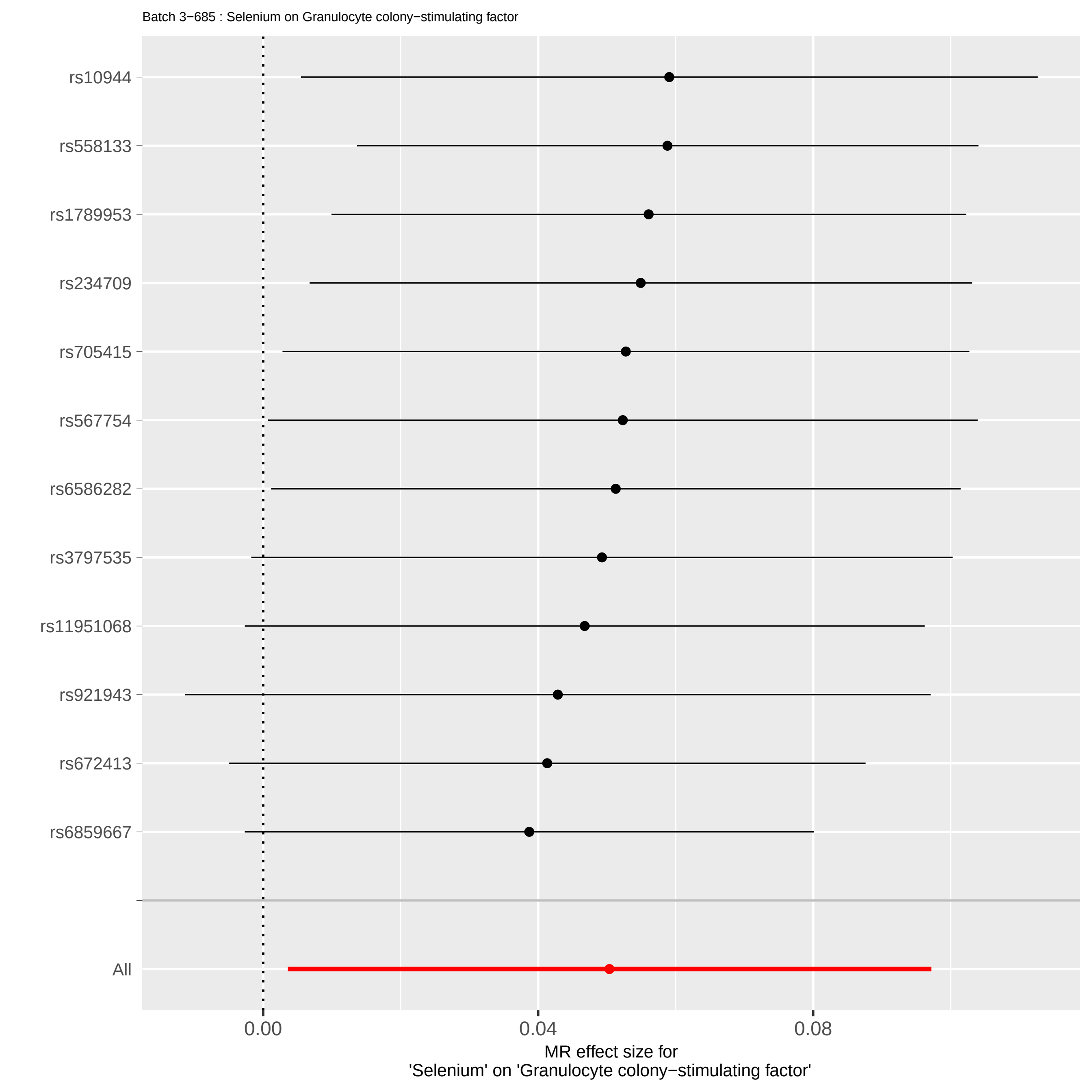
Batch 3−685 : Selenium on Granulocyte colony−stimulating factor
rs10944
rs558133
rs1789953
rs234709
rs705415
rs567754
rs6586282
rs3797535
rs11951068
rs921943
rs672413
rs6859667
All
0.00
0.04
0.08
MR effect size for
'Selenium' on 'Granulocyte colony−stimulating factor'

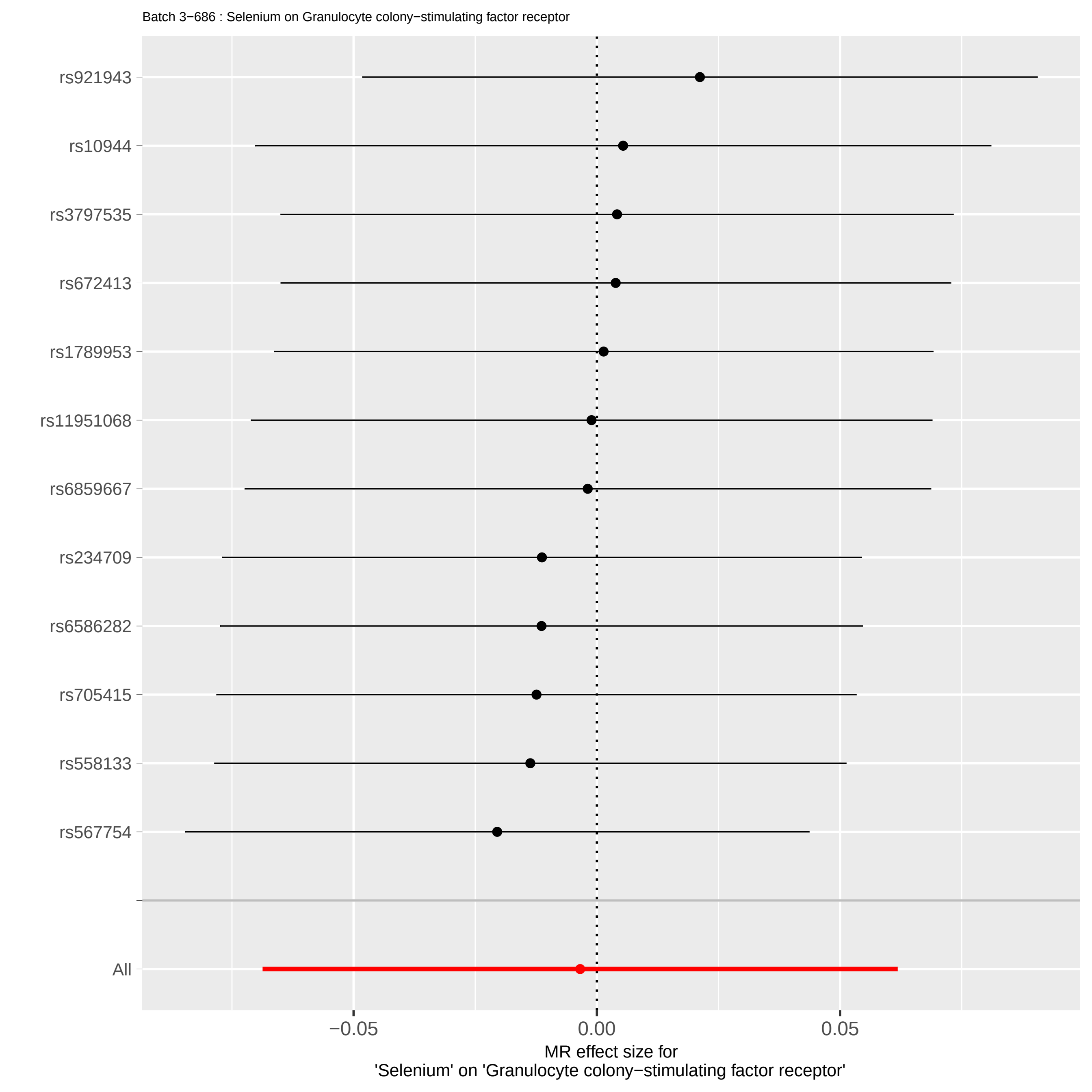

Batch 3−686 : Selenium on Granulocyte colony−stimulating factor receptor
rs921943
rs10944
rs3797535
rs672413
rs1789953
rs11951068
rs6859667
rs234709
rs6586282
rs705415
rs558133
rs567754
All
−0.05
0.00
0.05
MR effect size for
'Selenium' on 'Granulocyte colony−stimulating factor receptor'

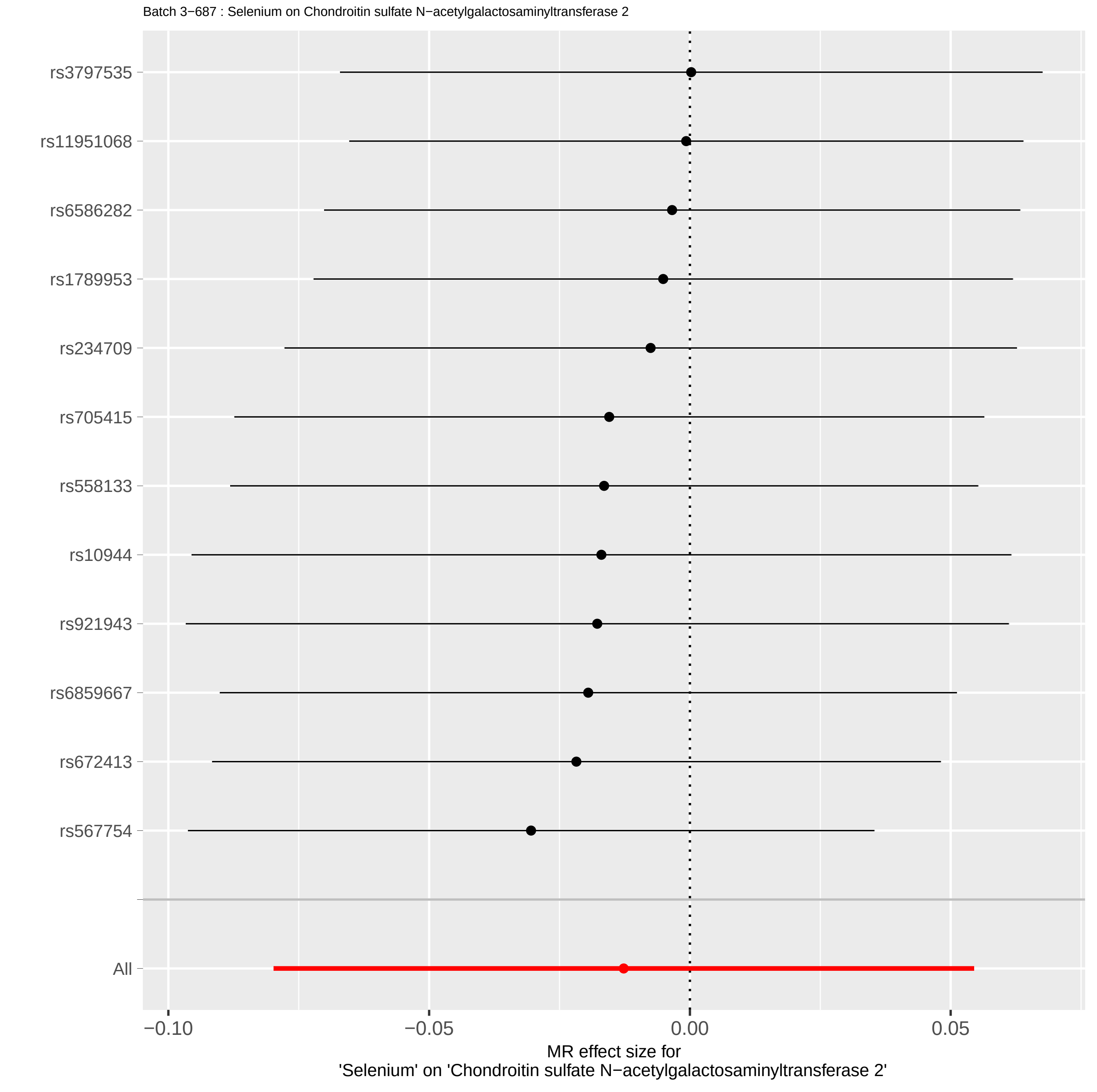

Batch 3−687 : Selenium on Chondroitin sulfate N−acetylgalactosaminyltransferase 2
rs3797535
rs11951068
rs6586282
rs1789953
rs234709
rs705415
rs558133
rs10944
rs921943
rs6859667
rs672413
rs567754
All
−0.10
−0.05
0.00
0.05
MR effect size for
'Selenium' on 'Chondroitin sulfate N−acetylgalactosaminyltransferase 2'

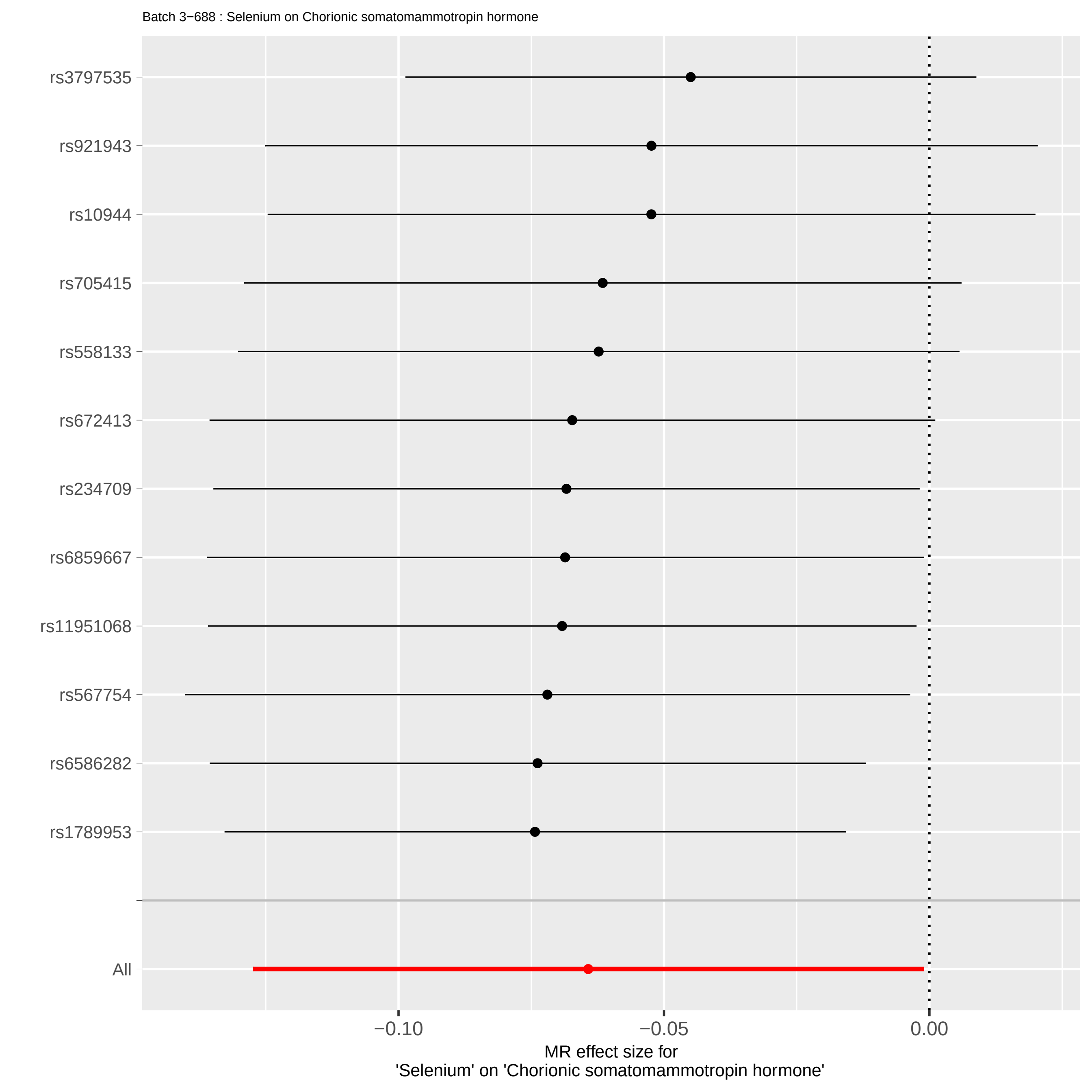

Batch 3−688 : Selenium on Chorionic somatomammotropin hormone
rs3797535
rs921943
rs10944
rs705415
rs558133
rs672413
rs234709
rs6859667
rs11951068
rs567754
rs6586282
rs1789953
All
−0.10
−0.05
0.00
MR effect size for
'Selenium' on 'Chorionic somatomammotropin hormone'

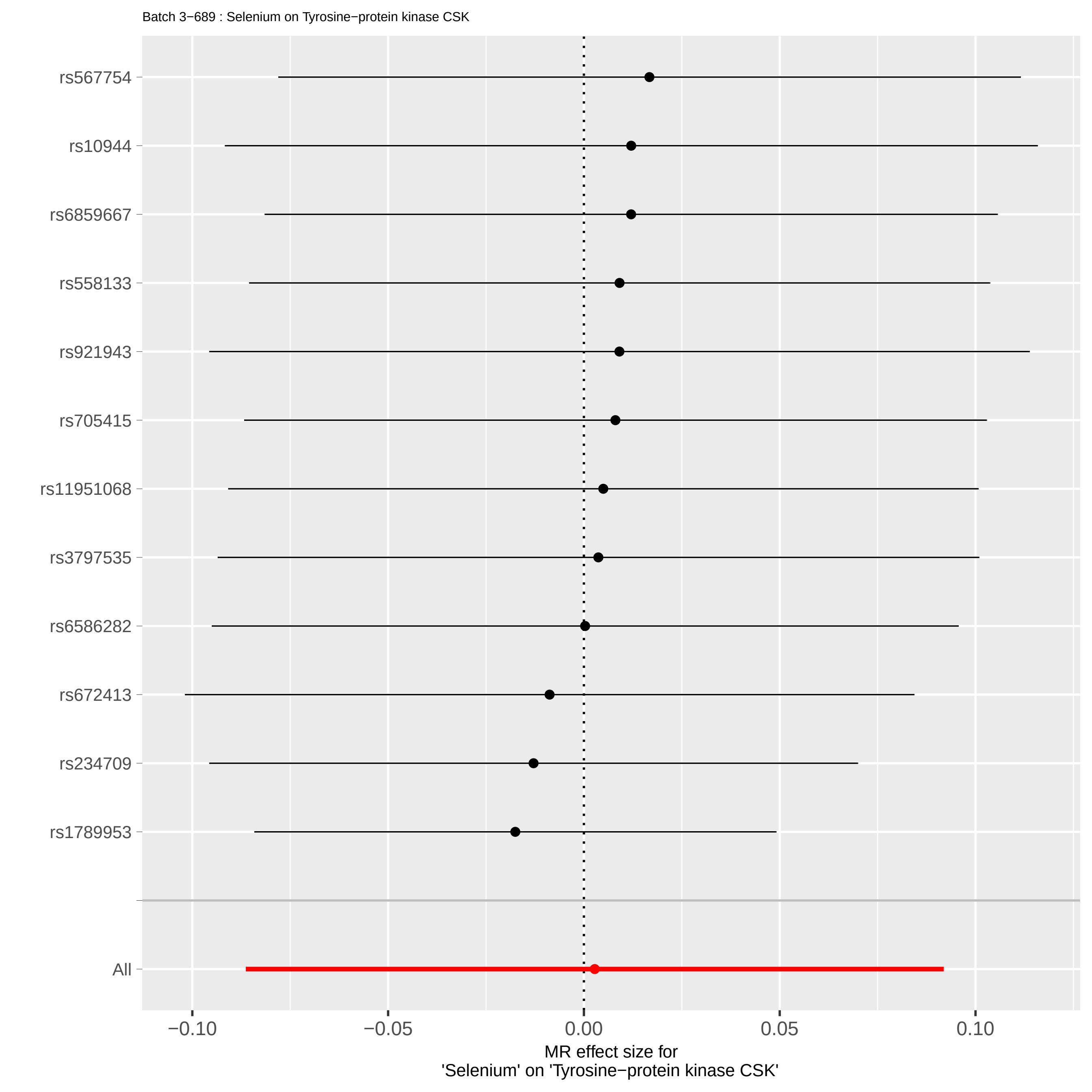

Batch 3−689 : Selenium on Tyrosine−protein kinase CSK
rs567754
rs10944
rs6859667
rs558133
rs921943
rs705415
rs11951068
rs3797535
rs6586282
rs672413
rs234709
rs1789953
All
−0.10
−0.05
0.00
0.05
0.10
MR effect size for
'Selenium' on 'Tyrosine−protein kinase CSK'

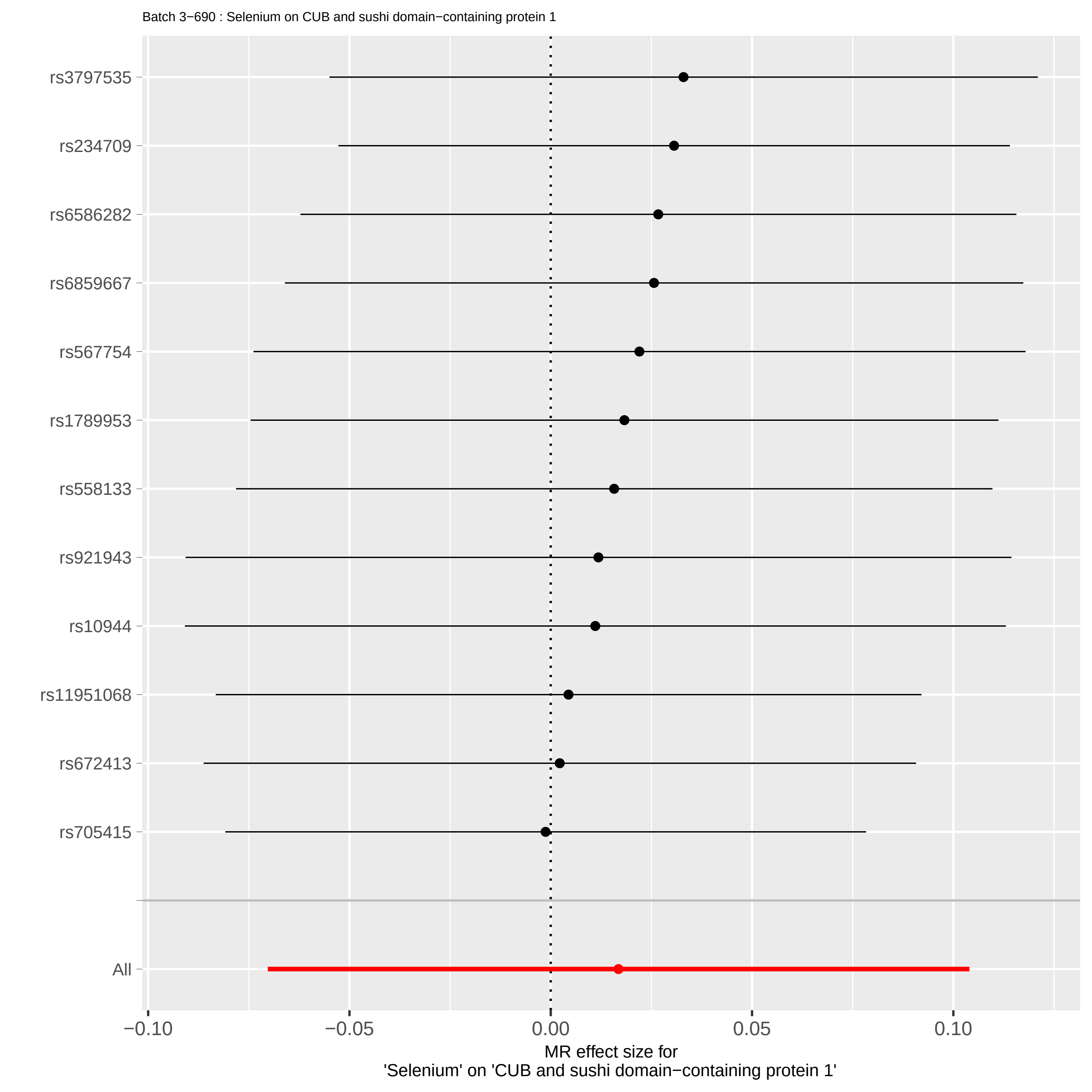

Batch 3−690 : Selenium on CUB and sushi domain−containing protein 1
rs3797535
rs234709
rs6586282
rs6859667
rs567754
rs1789953
rs558133
rs921943
rs10944
rs11951068
rs672413
rs705415
All
−0.10
−0.05
0.00
0.05
0.10
MR effect size for
'Selenium' on 'CUB and sushi domain−containing protein 1'

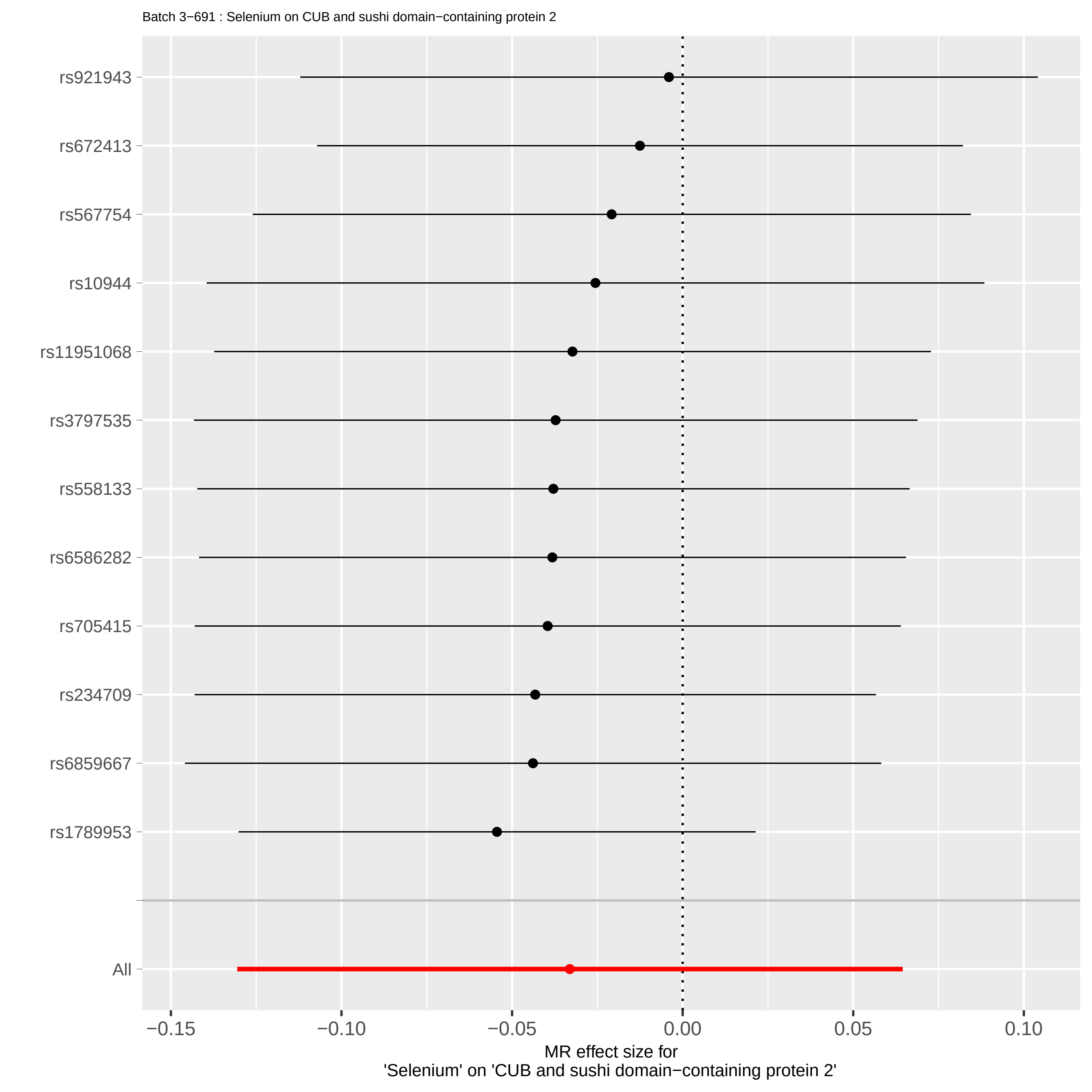

Batch 3−691 : Selenium on CUB and sushi domain−containing protein 2
rs921943
rs672413
rs567754
rs10944
rs11951068
rs3797535
rs558133
rs6586282
rs705415
rs234709
rs6859667
rs1789953
All
−0.15
−0.10
−0.05
0.00
0.05
0.10
MR effect size for
'Selenium' on 'CUB and sushi domain−containing protein 2'

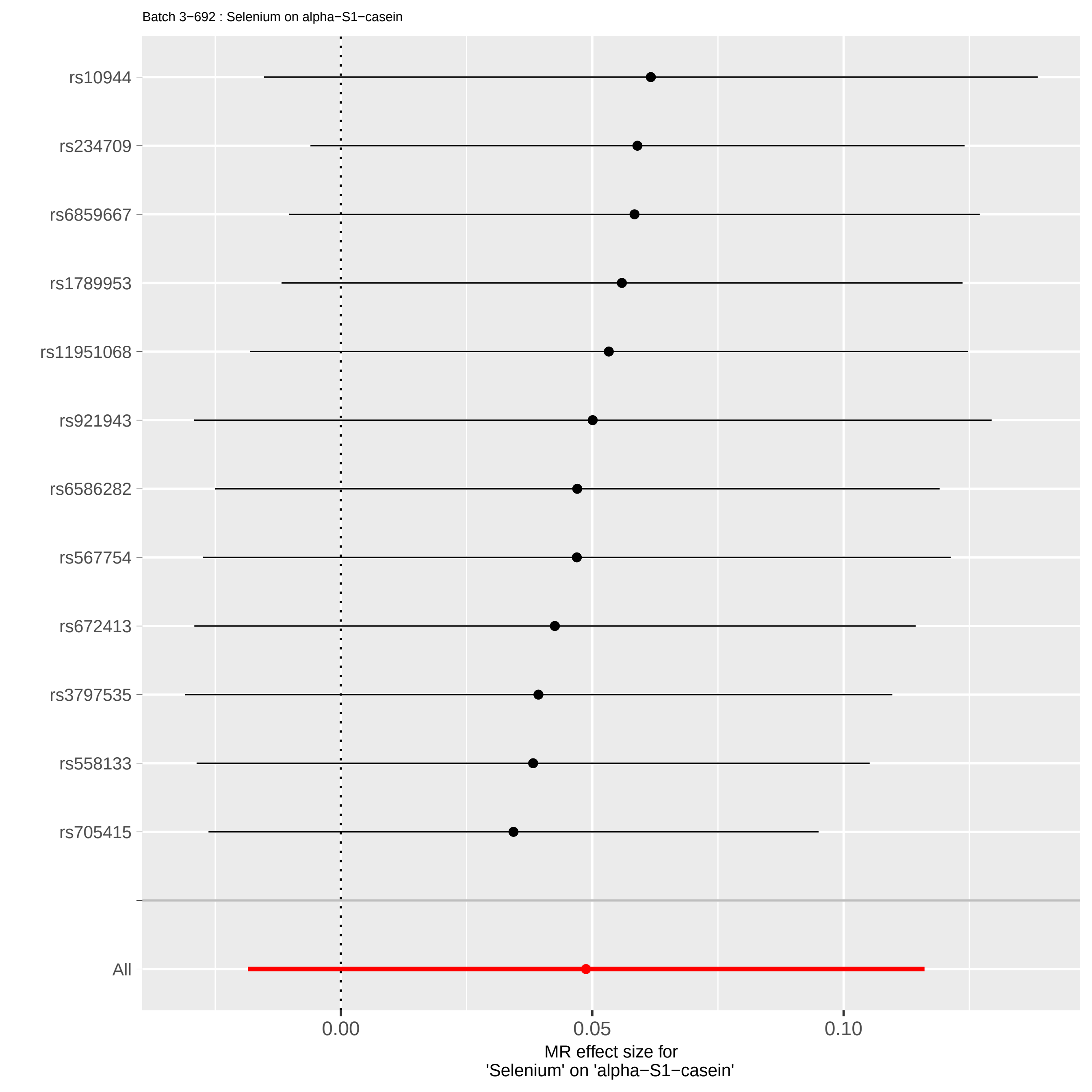

Batch 3−692 : Selenium on alpha−S1−casein
rs10944
rs234709
rs6859667
rs1789953
rs11951068
rs921943
rs6586282
rs567754
rs672413
rs3797535
rs558133
rs705415
All
0.00
0.05
0.10
MR effect size for
'Selenium' on 'alpha−S1−casein'

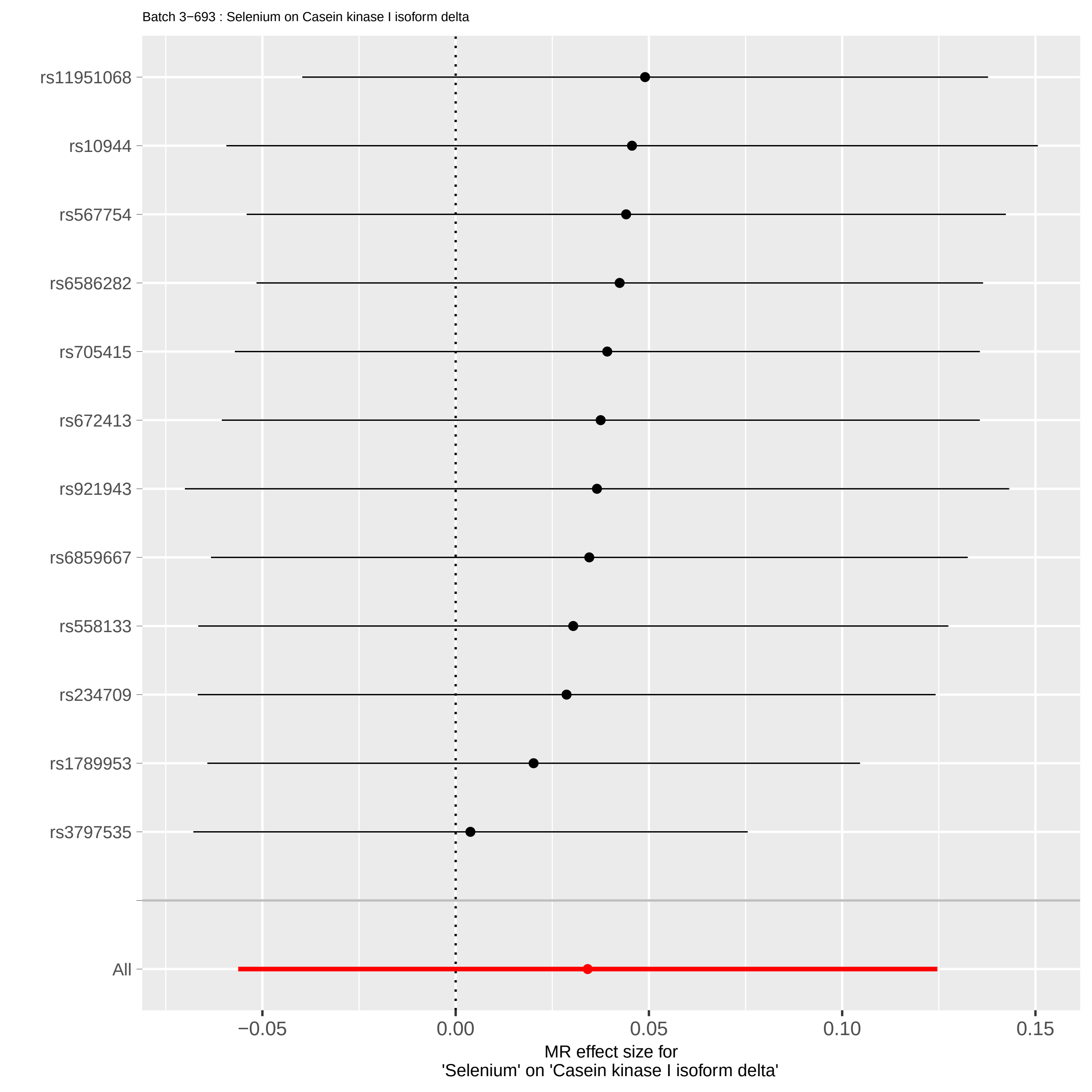

Batch 3−693 : Selenium on Casein kinase I isoform delta
rs11951068
rs10944
rs567754
rs6586282
rs705415
rs672413
rs921943
rs6859667
rs558133
rs234709
rs1789953
rs3797535
All
−0.05
0.00
0.05
0.10
0.15
MR effect size for
'Selenium' on 'Casein kinase I isoform delta'

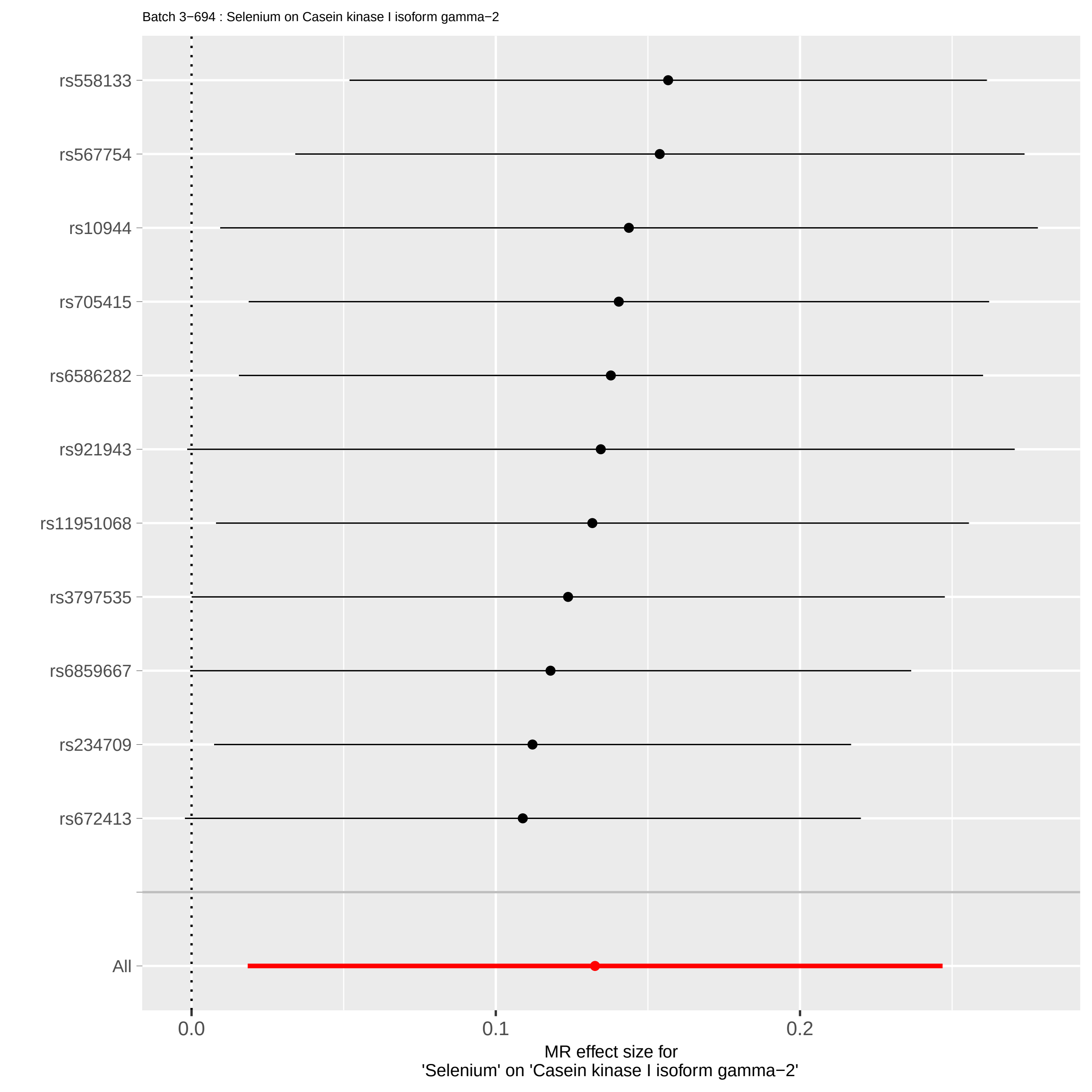

Batch 3−694 : Selenium on Casein kinase I isoform gamma−2
rs558133
rs567754
rs10944
rs705415
rs6586282
rs921943
rs11951068
rs3797535
rs6859667
rs234709
rs672413
All
0.0
0.1
0.2
MR effect size for
'Selenium' on 'Casein kinase I isoform gamma−2'

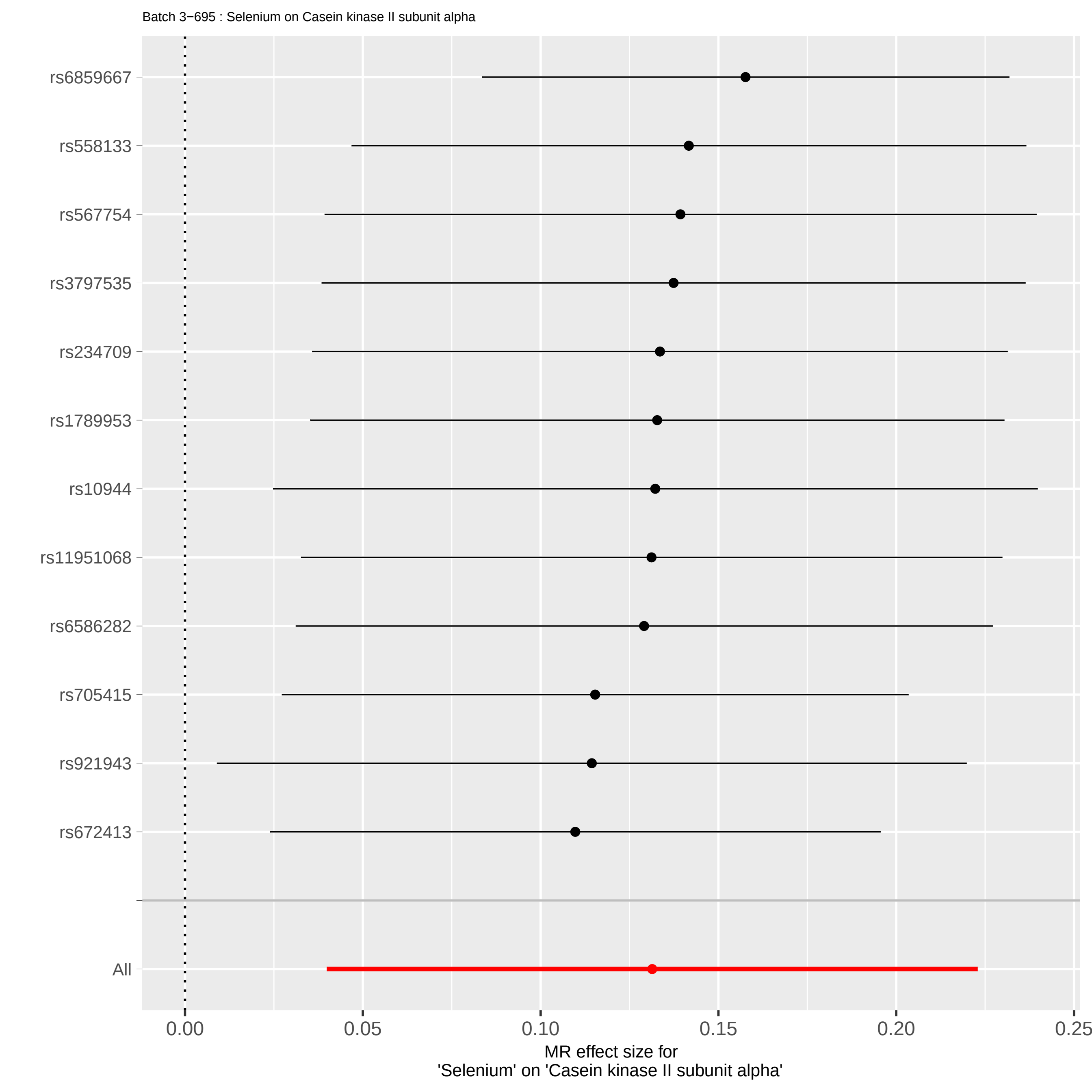
Batch 3−695 : Selenium on Casein kinase II subunit alpha
rs6859667
rs558133
rs567754
rs3797535
rs234709
rs1789953
rs10944
rs11951068
rs6586282
rs705415
rs921943
rs672413
All
0.00
0.05
0.10
0.15
0.20
0.25
MR effect size for
'Selenium' on 'Casein kinase II subunit alpha'

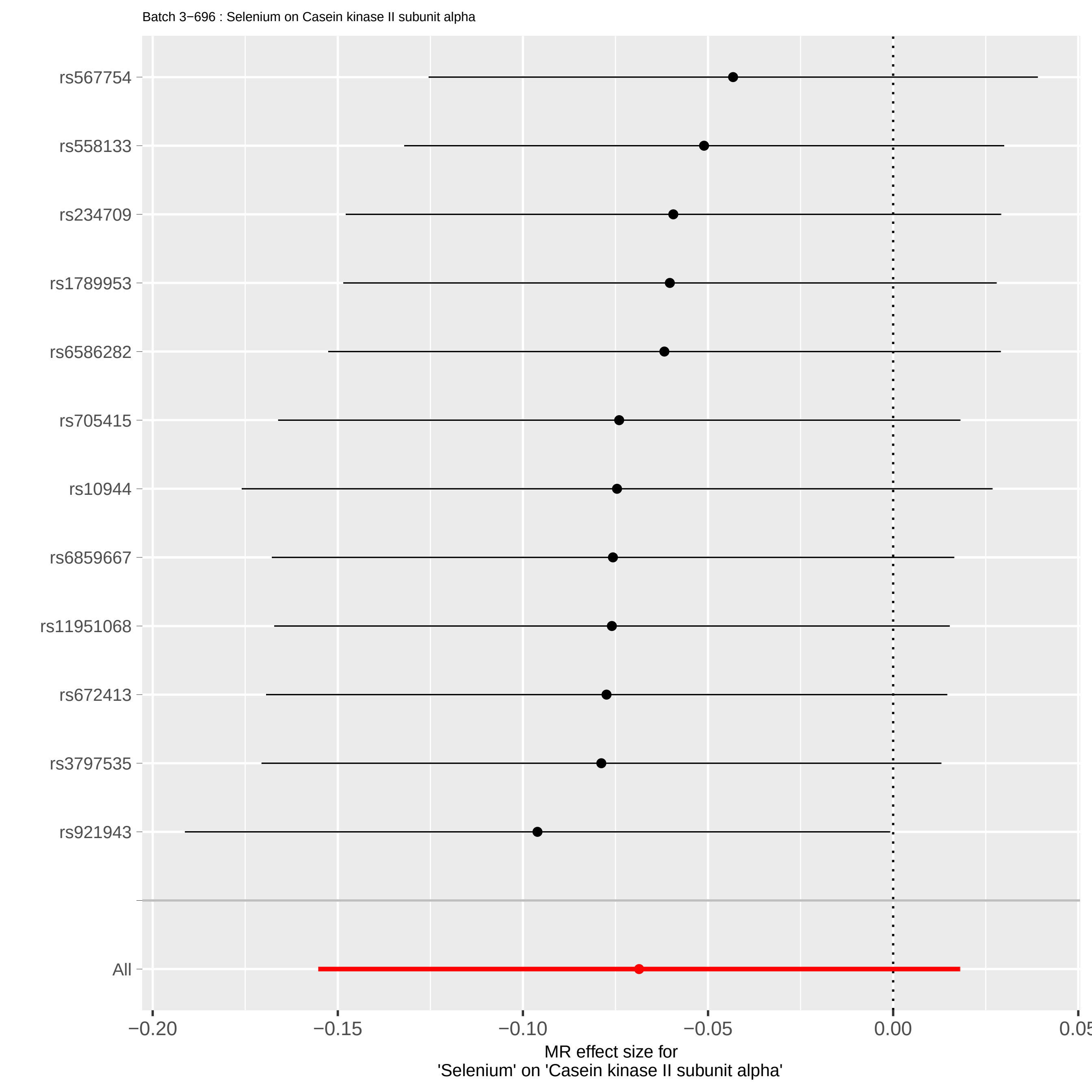
Batch 3−696 : Selenium on Casein kinase II subunit alpha
rs567754
rs558133
rs234709
rs1789953
rs6586282
rs705415
rs10944
rs6859667
rs11951068
rs672413
rs3797535
rs921943
All
−0.20
−0.15
−0.10
−0.05
0.00
0.05
MR effect size for
'Selenium' on 'Casein kinase II subunit alpha'

Batch 3−697 : Selenium on Casein kinase II 2−alpha:2−beta heterotetramer

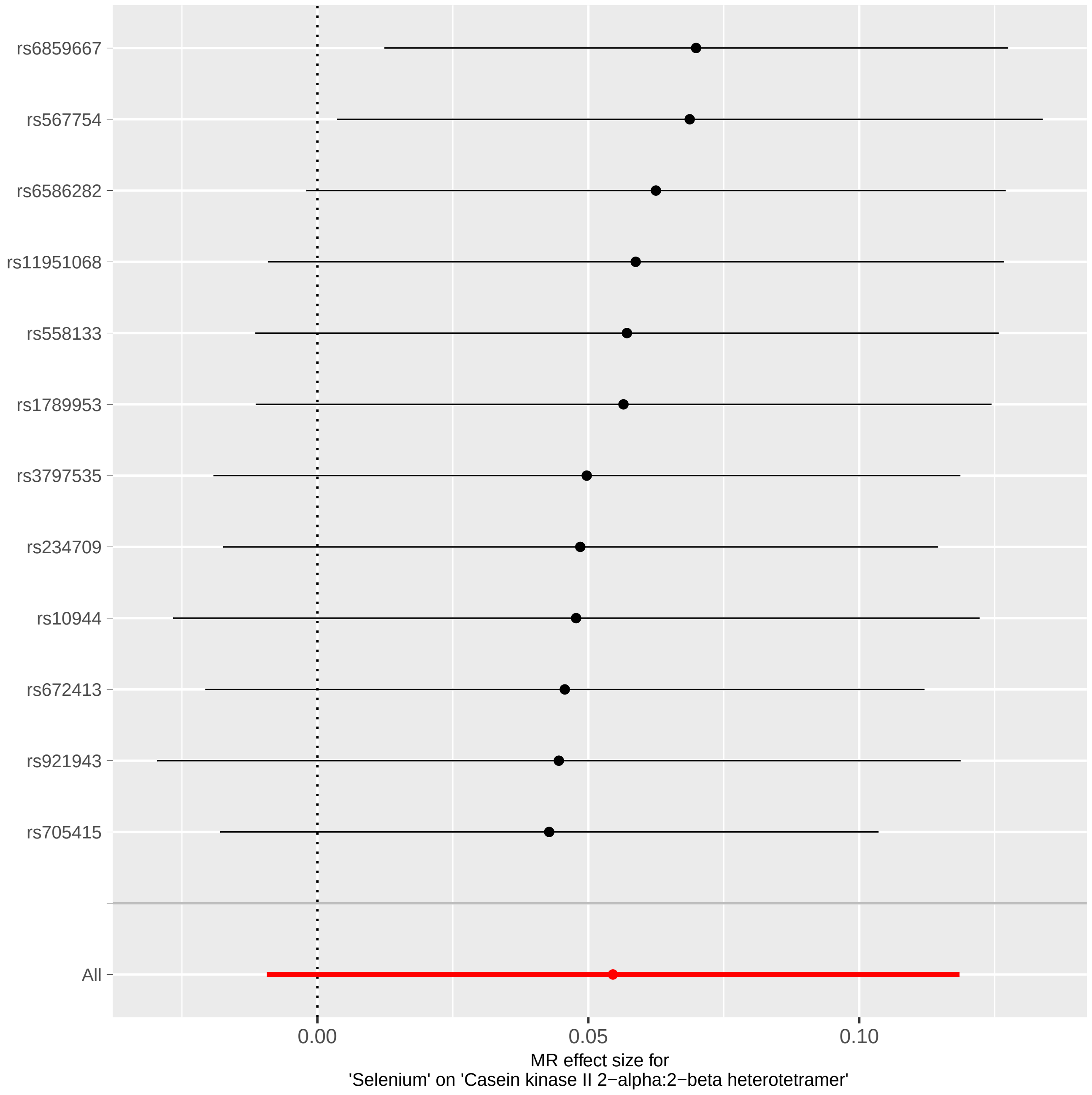

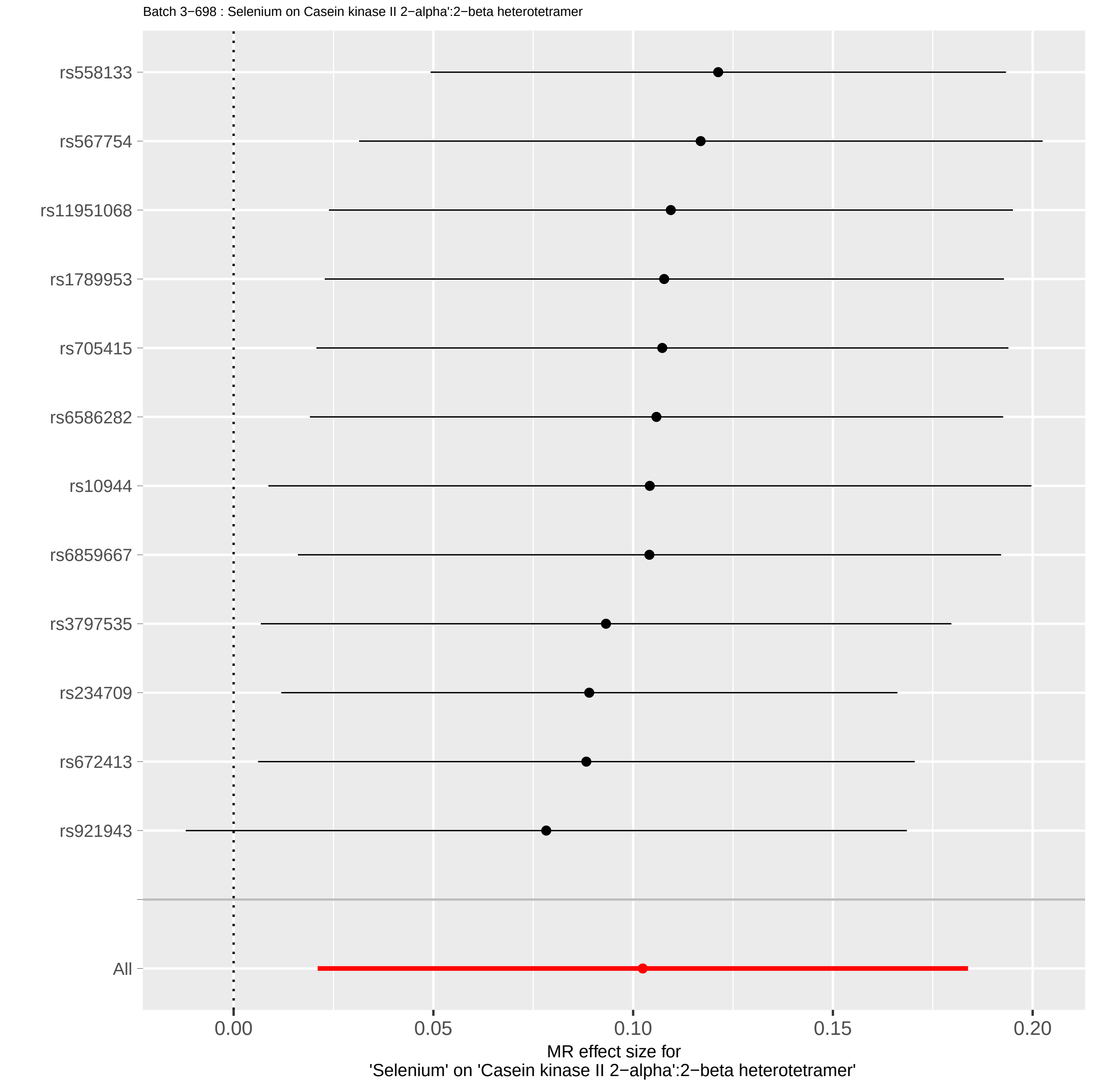

Batch 3−698 : Selenium on Casein kinase II 2−alpha':2−beta heterotetramer
rs558133
rs567754
rs11951068
rs1789953
rs705415
rs6586282
rs10944
rs6859667
rs3797535
rs234709
rs672413
rs921943
All
0.00
0.05
0.10
0.15
0.20
MR effect size for
'Selenium' on 'Casein kinase II 2−alpha':2−beta heterotetramer'

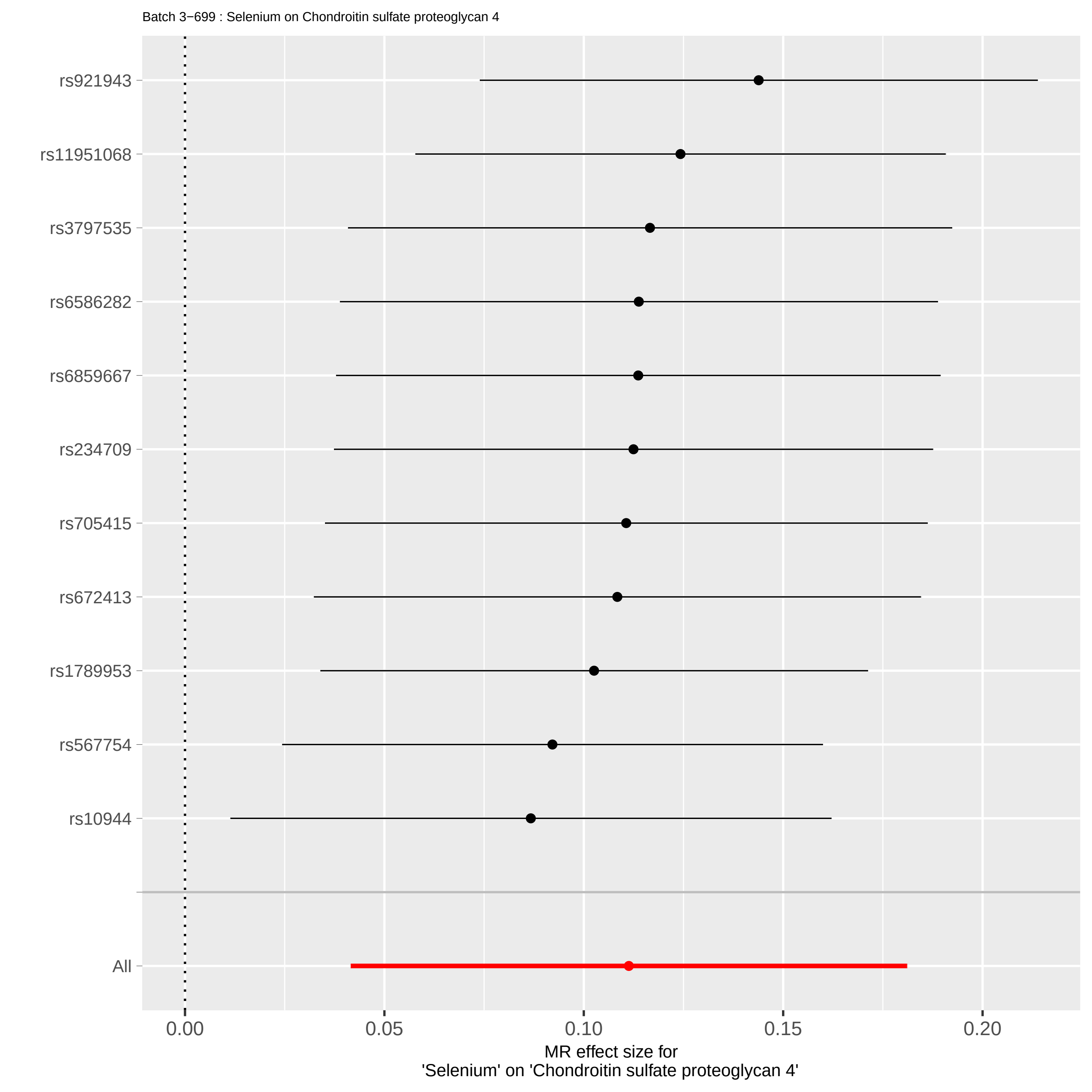

Batch 3−699 : Selenium on Chondroitin sulfate proteoglycan 4
rs921943
rs11951068
rs3797535
rs6586282
rs6859667
rs234709
rs705415
rs672413
rs1789953
rs567754
rs10944
All
0.00
0.05
0.10
0.15
0.20
MR effect size for
'Selenium' on 'Chondroitin sulfate proteoglycan 4'

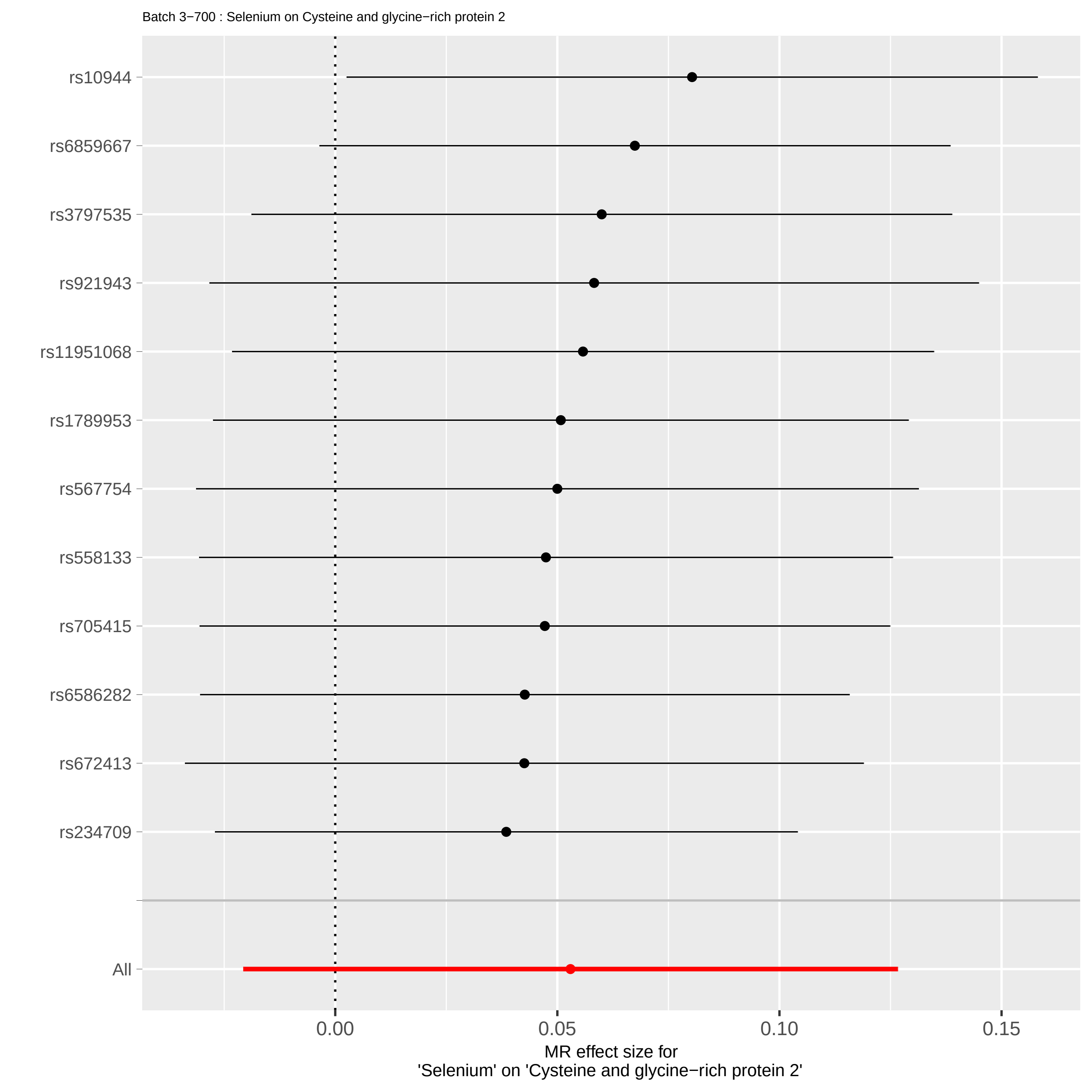

Batch 3−700 : Selenium on Cysteine and glycine−rich protein 2
rs10944
rs6859667
rs3797535
rs921943
rs11951068
rs1789953
rs567754
rs558133
rs705415
rs6586282
rs672413
rs234709
All
0.00
0.05
0.10
0.15
MR effect size for
'Selenium' on 'Cysteine and glycine−rich protein 2'

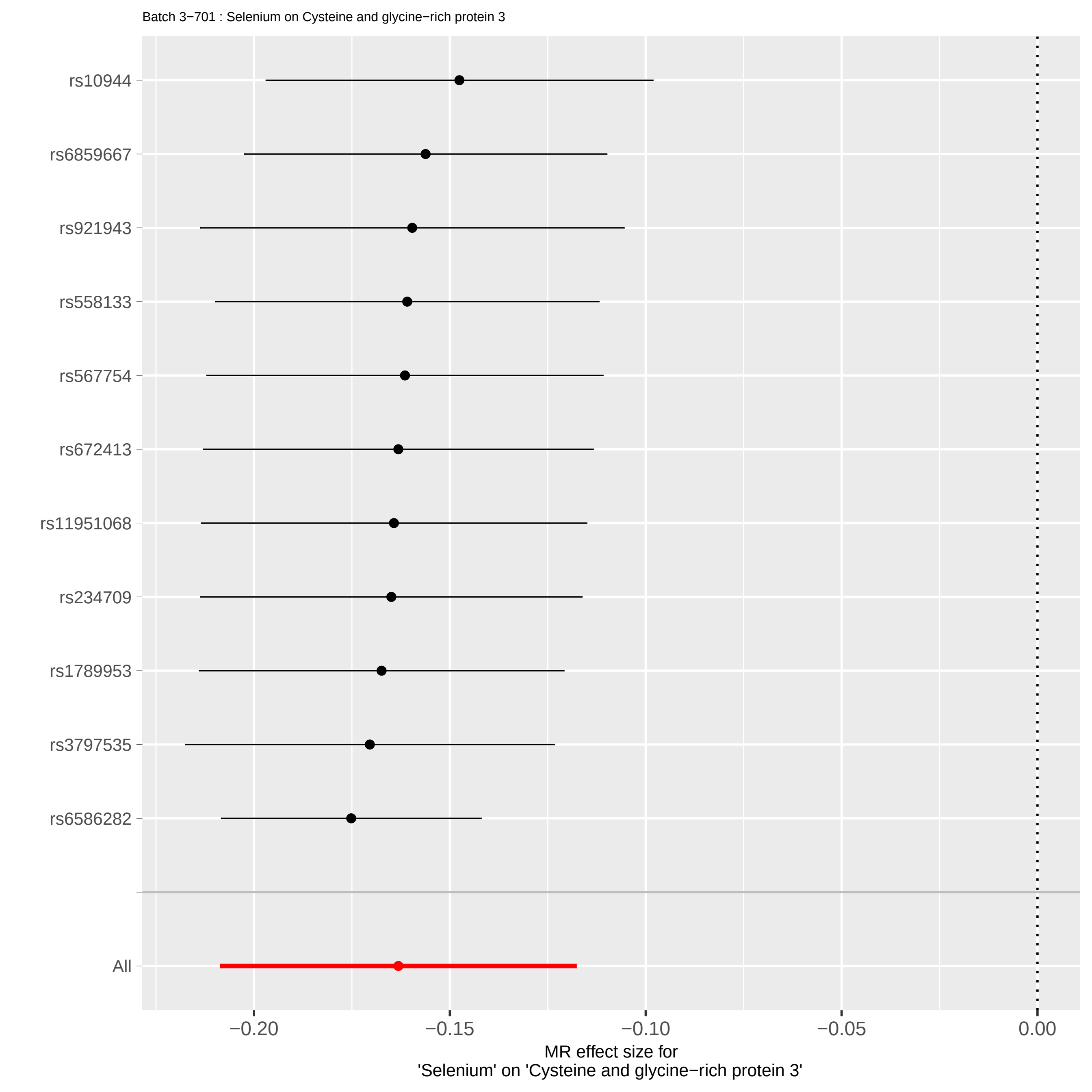

Batch 3−701 : Selenium on Cysteine and glycine−rich protein 3
rs10944
rs6859667
rs921943
rs558133
rs567754
rs672413
rs11951068
rs234709
rs1789953
rs3797535
rs6586282
All
−0.20
−0.15
−0.10
−0.05
0.00
MR effect size for
'Selenium' on 'Cysteine and glycine−rich protein 3'

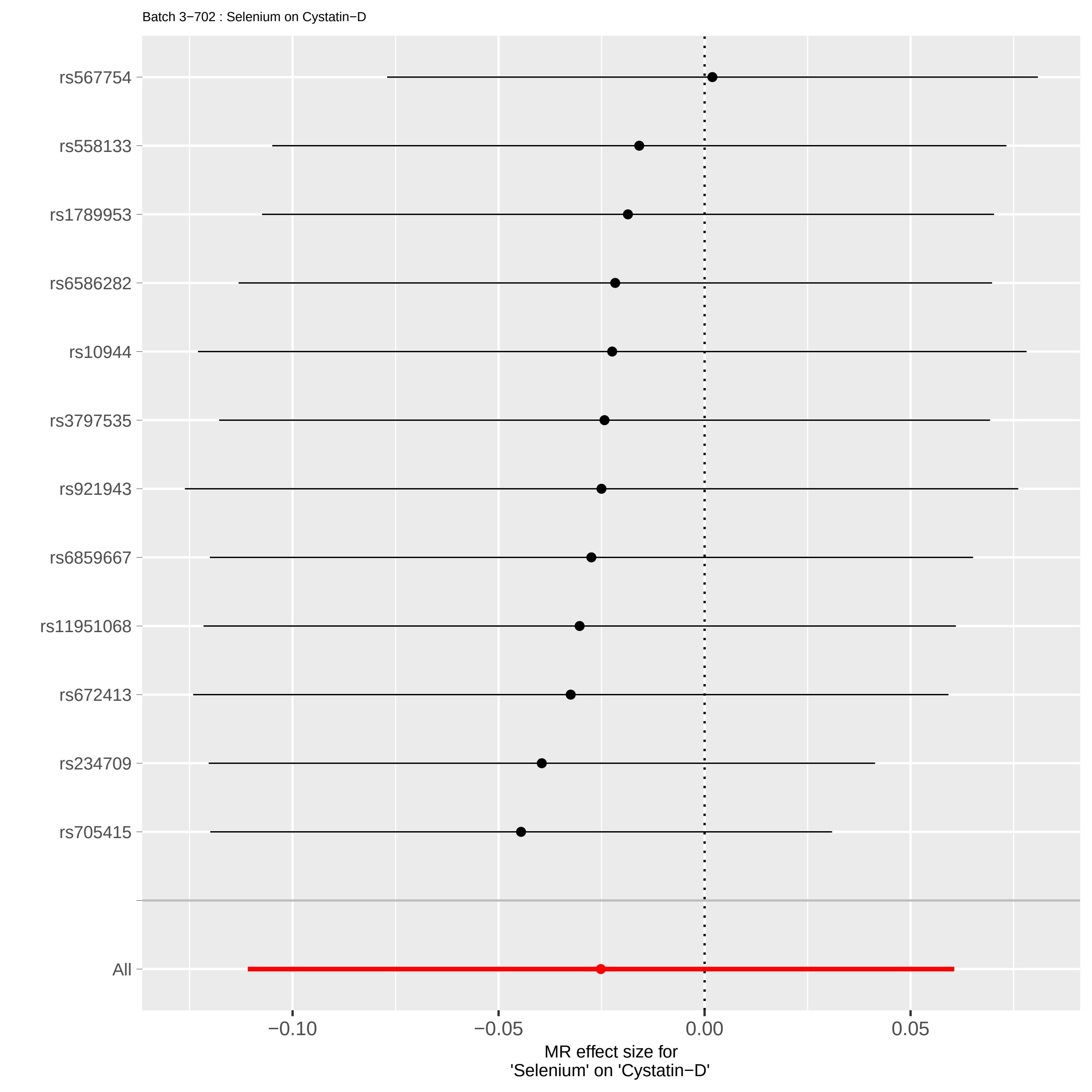

Batch 3−702 : Selenium on Cystatin−D
rs567754
rs558133
rs1789953
rs6586282
rs10944
rs3797535
rs921943
rs6859667
rs11951068
rs672413
rs234709
rs705415
All
−0.10
−0.05
0.00
0.05
MR effect size for
'Selenium' on 'Cystatin−D'

Batch 3−703 : Selenium on Cystatin−M

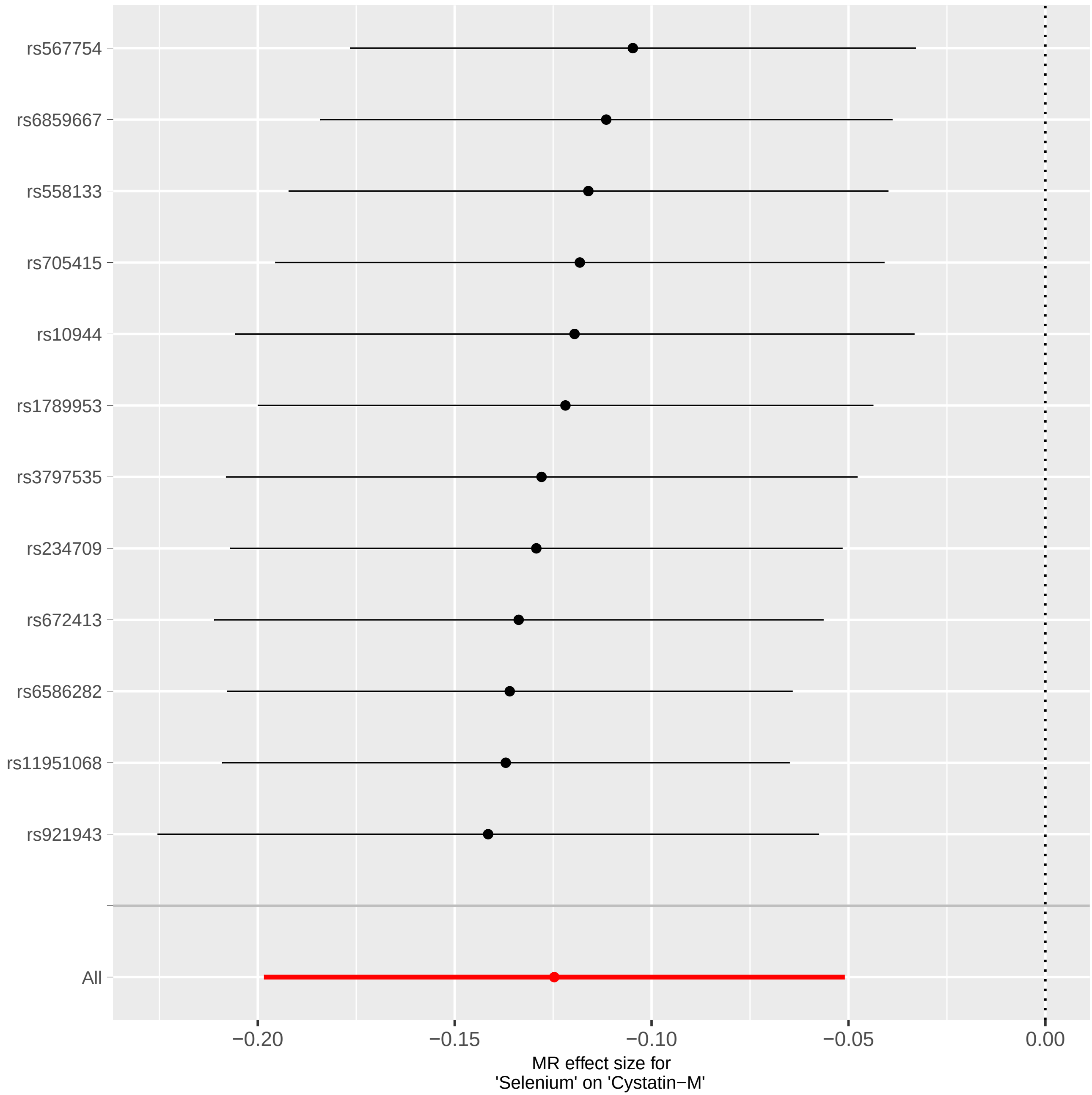

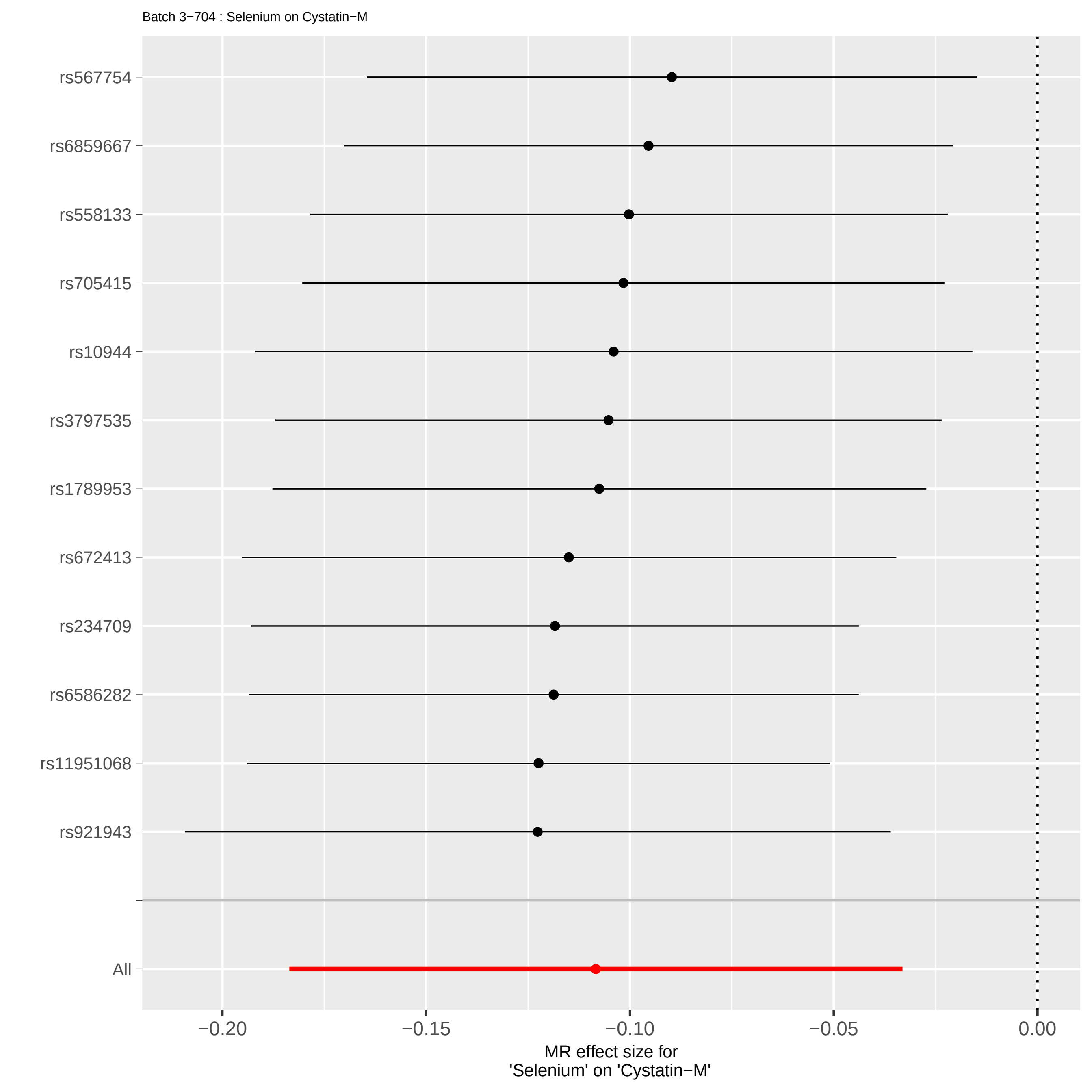

Batch 3−704 : Selenium on Cystatin−M
rs567754
rs6859667
rs558133
rs705415
rs10944
rs3797535
rs1789953
rs672413
rs234709
rs6586282
rs11951068
rs921943
All
−0.20
−0.15
−0.10
−0.05
0.00
MR effect size for
'Selenium' on 'Cystatin−M'

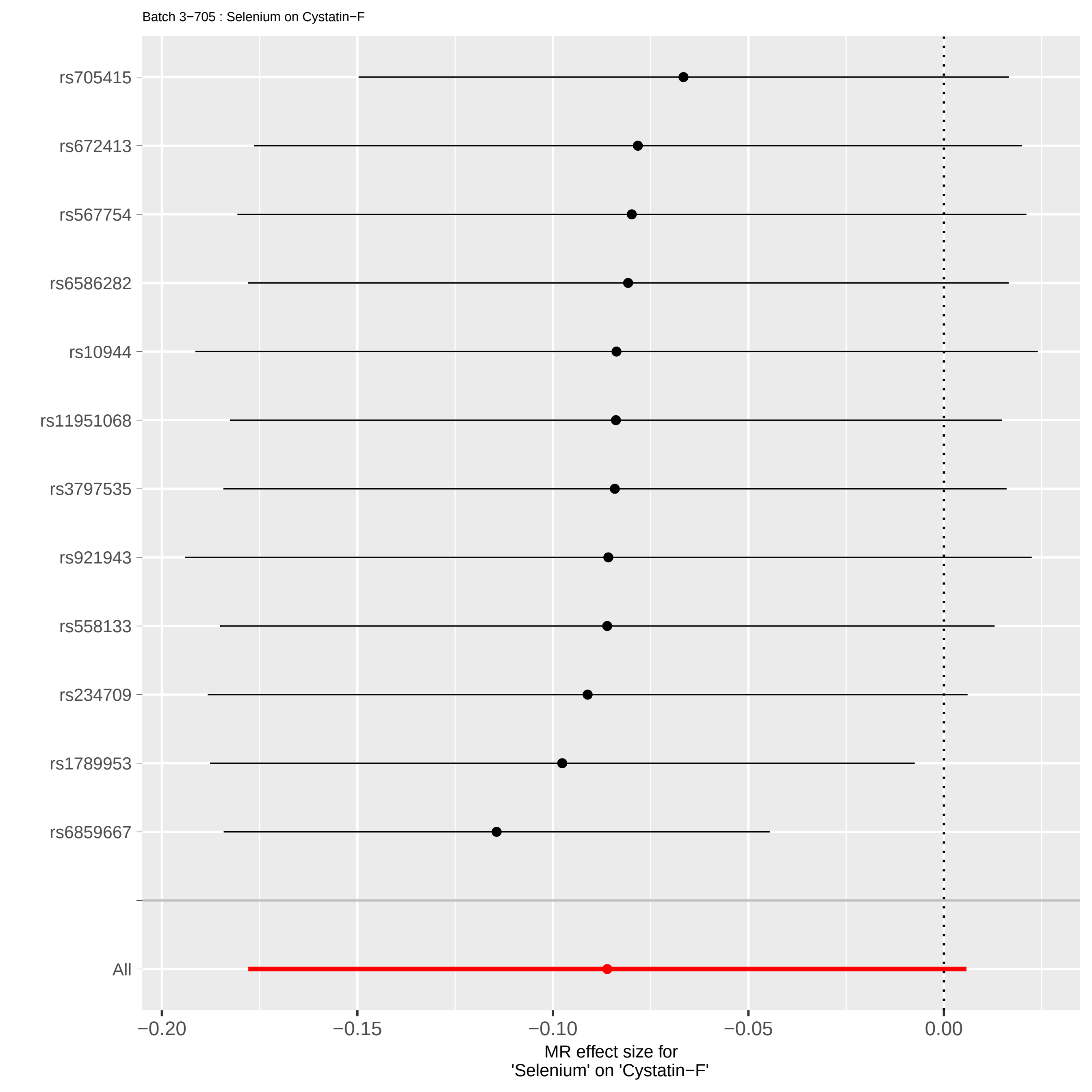

Batch 3−705 : Selenium on Cystatin−F
rs705415
rs672413
rs567754
rs6586282
rs10944
rs11951068
rs3797535
rs921943
rs558133
rs234709
rs1789953
rs6859667
All
−0.20
−0.15
−0.10
−0.05
0.00
MR effect size for
'Selenium' on 'Cystatin−F'

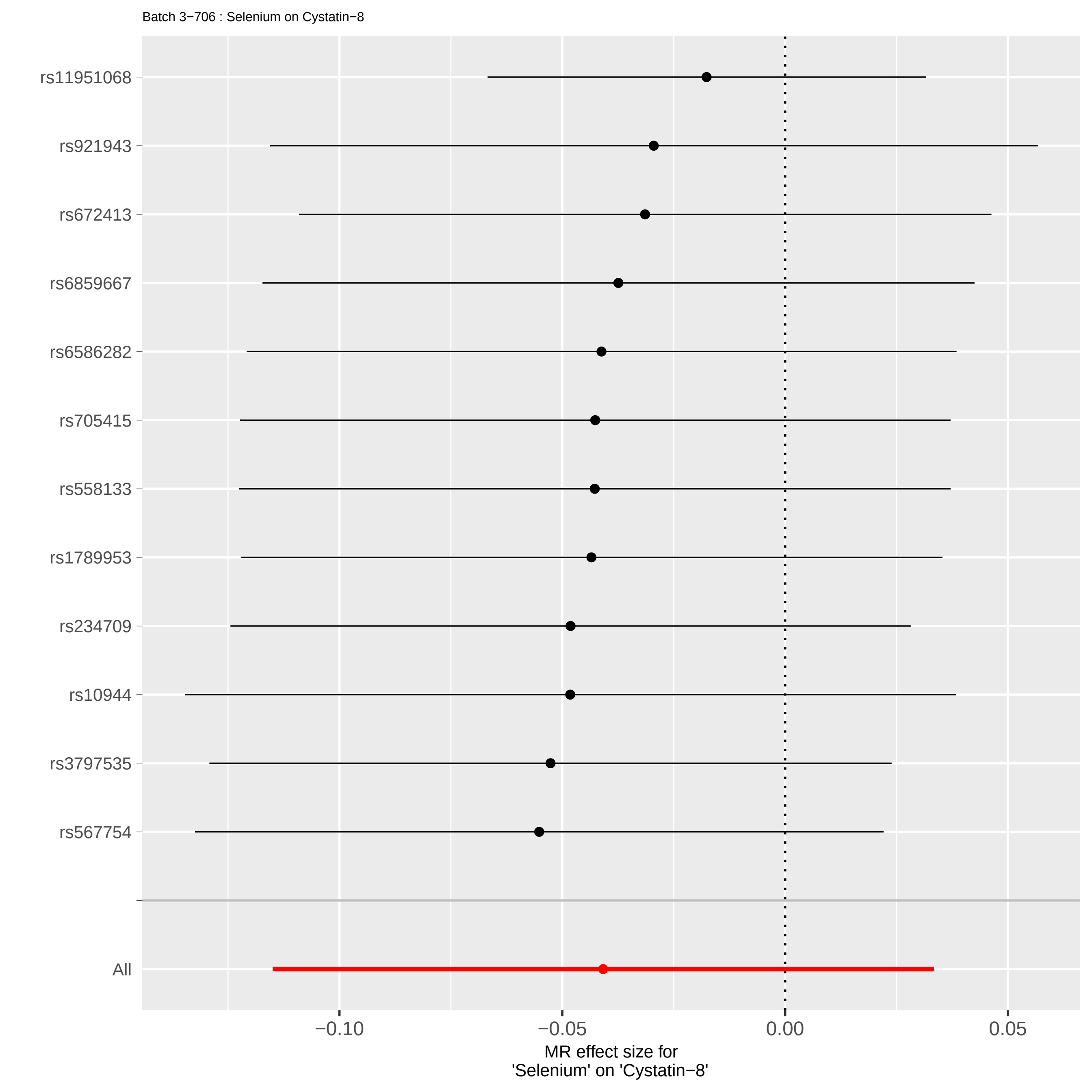

Batch 3−706 : Selenium on Cystatin−8
rs11951068
rs921943
rs672413
rs6859667
rs6586282
rs705415
rs558133
rs1789953
rs234709
rs10944
rs3797535
rs567754
All
−0.10
−0.05
0.00
0.05
MR effect size for
'Selenium' on 'Cystatin−8'

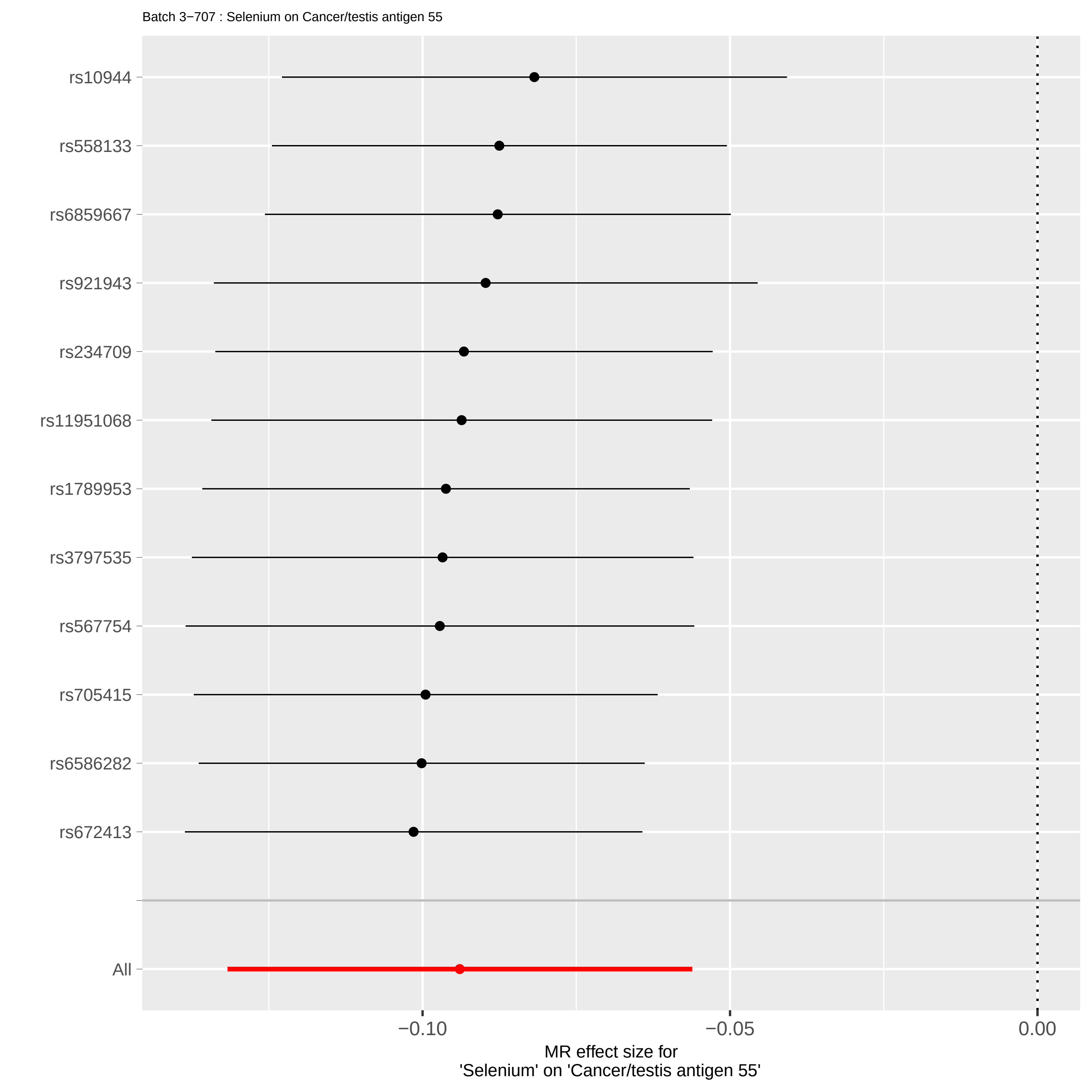

Batch 3−707 : Selenium on Cancer/testis antigen 55
rs10944
rs558133
rs6859667
rs921943
rs234709
rs11951068
rs1789953
rs3797535
rs567754
rs705415
rs6586282
rs672413
All
−0.10
−0.05
0.00
MR effect size for
'Selenium' on 'Cancer/testis antigen 55'

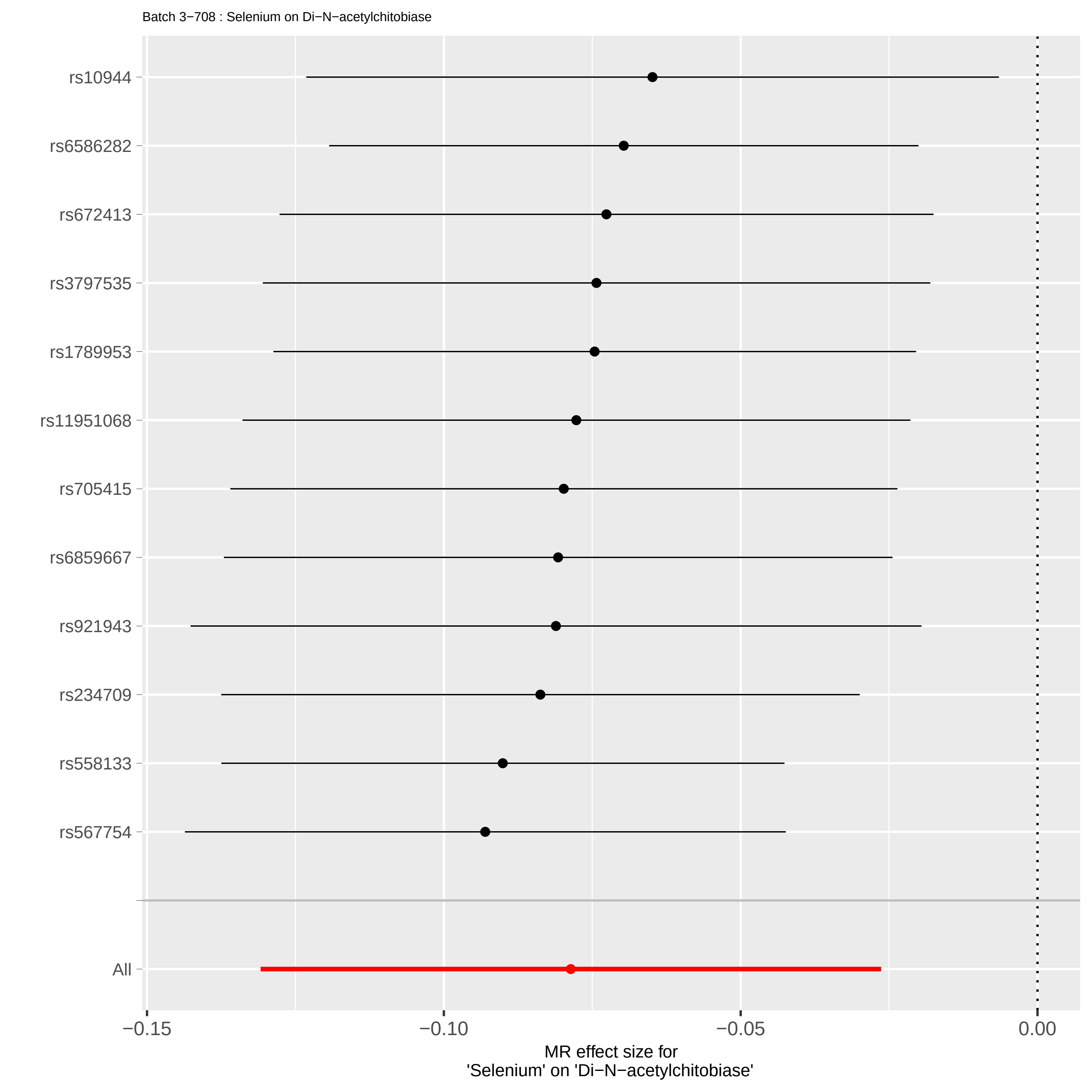

Batch 3−708 : Selenium on Di−N−acetylchitobiase
rs10944
rs6586282
rs672413
rs3797535
rs1789953
rs11951068
rs705415
rs6859667
rs921943
rs234709
rs558133
rs567754
All
−0.15
−0.10
−0.05
0.00
MR effect size for
'Selenium' on 'Di−N−acetylchitobiase'

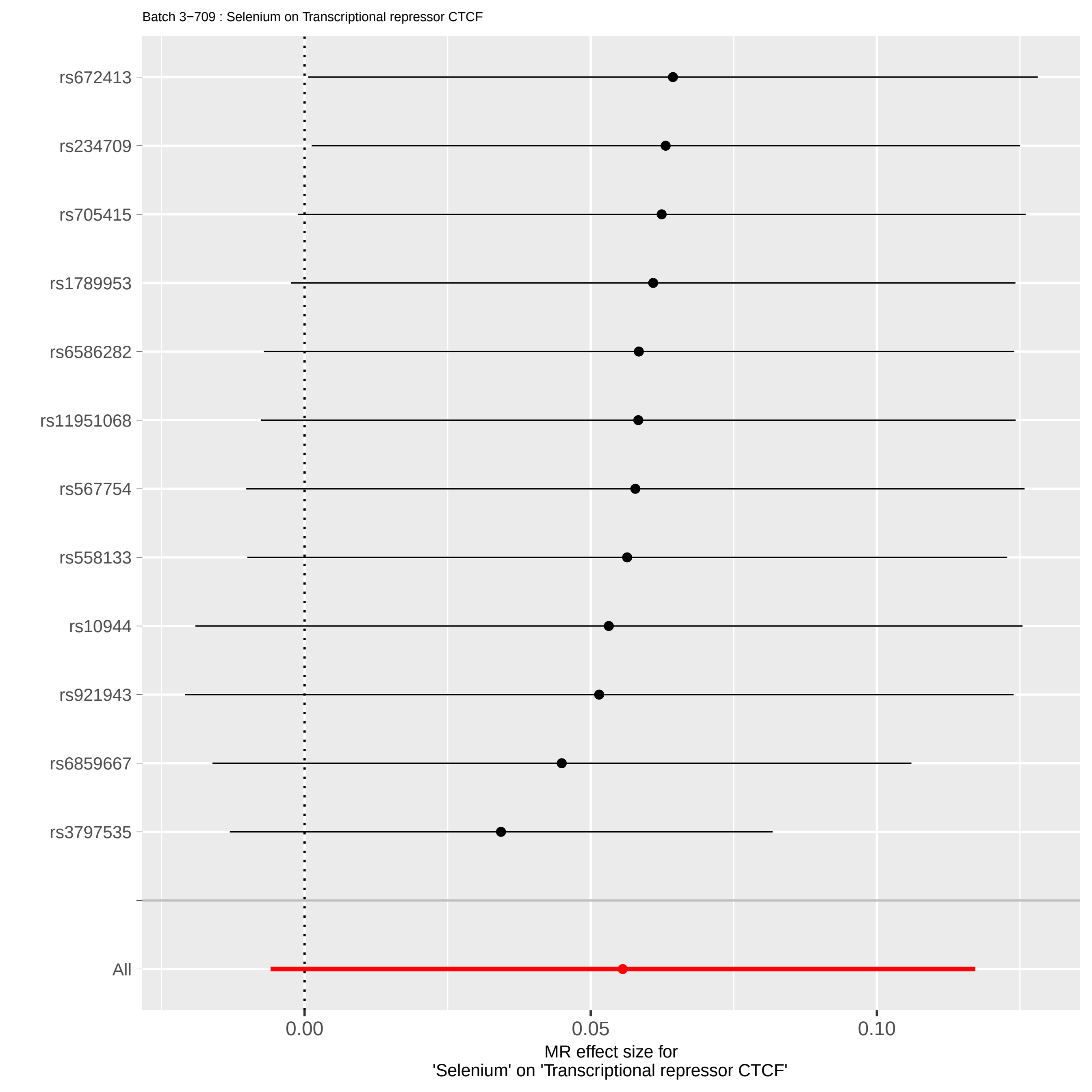

Batch 3−709 : Selenium on Transcriptional repressor CTCF
rs672413
rs234709
rs705415
rs1789953
rs6586282
rs11951068
rs567754
rs558133
rs10944
rs921943
rs6859667
rs3797535
All
0.00
0.05
0.10
MR effect size for
'Selenium' on 'Transcriptional repressor CTCF'

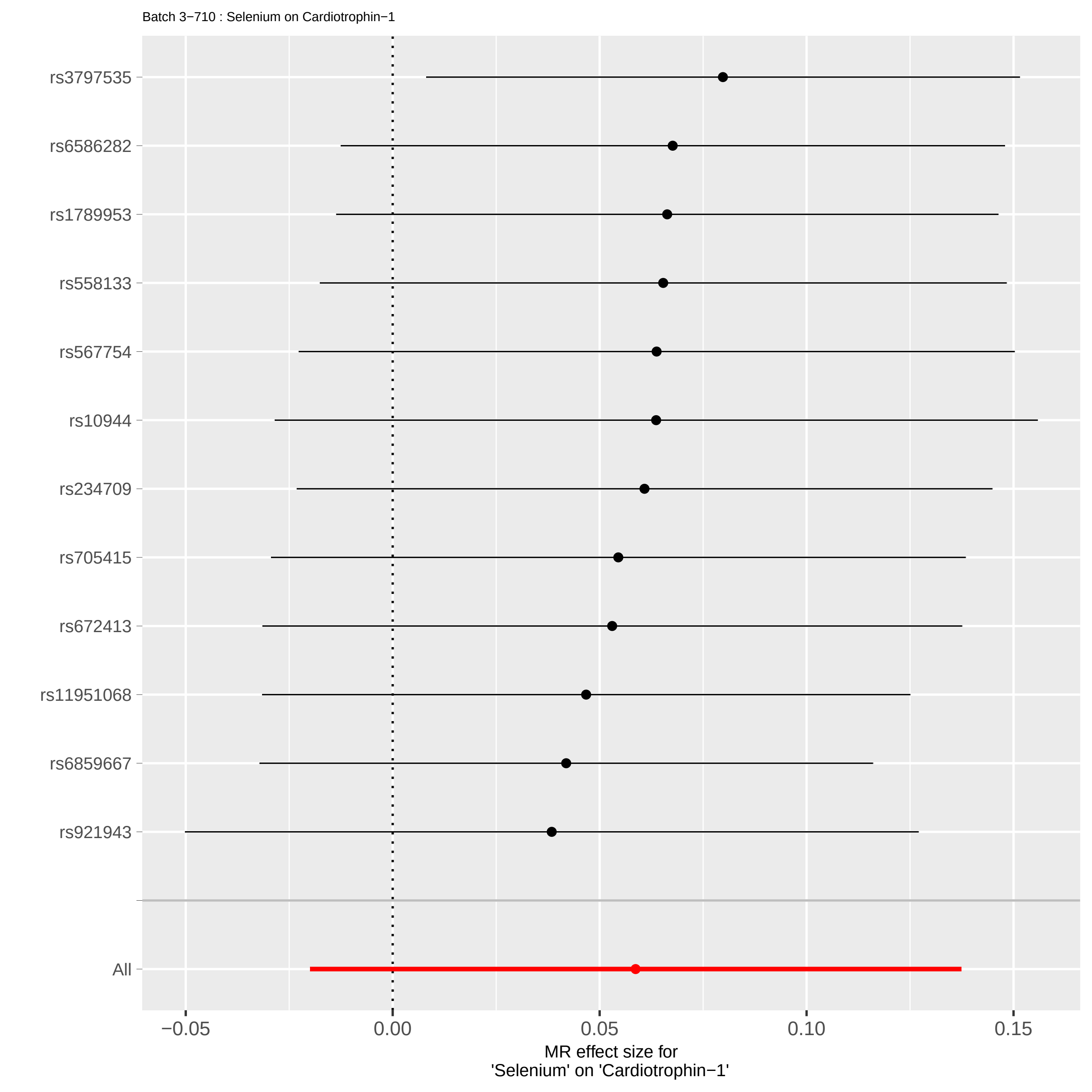

Batch 3−710 : Selenium on Cardiotrophin−1
rs3797535
rs6586282
rs1789953
rs558133
rs567754
rs10944
rs234709
rs705415
rs672413
rs11951068
rs6859667
rs921943
All
−0.05
0.00
0.05
0.10
0.15
MR effect size for
'Selenium' on 'Cardiotrophin−1'

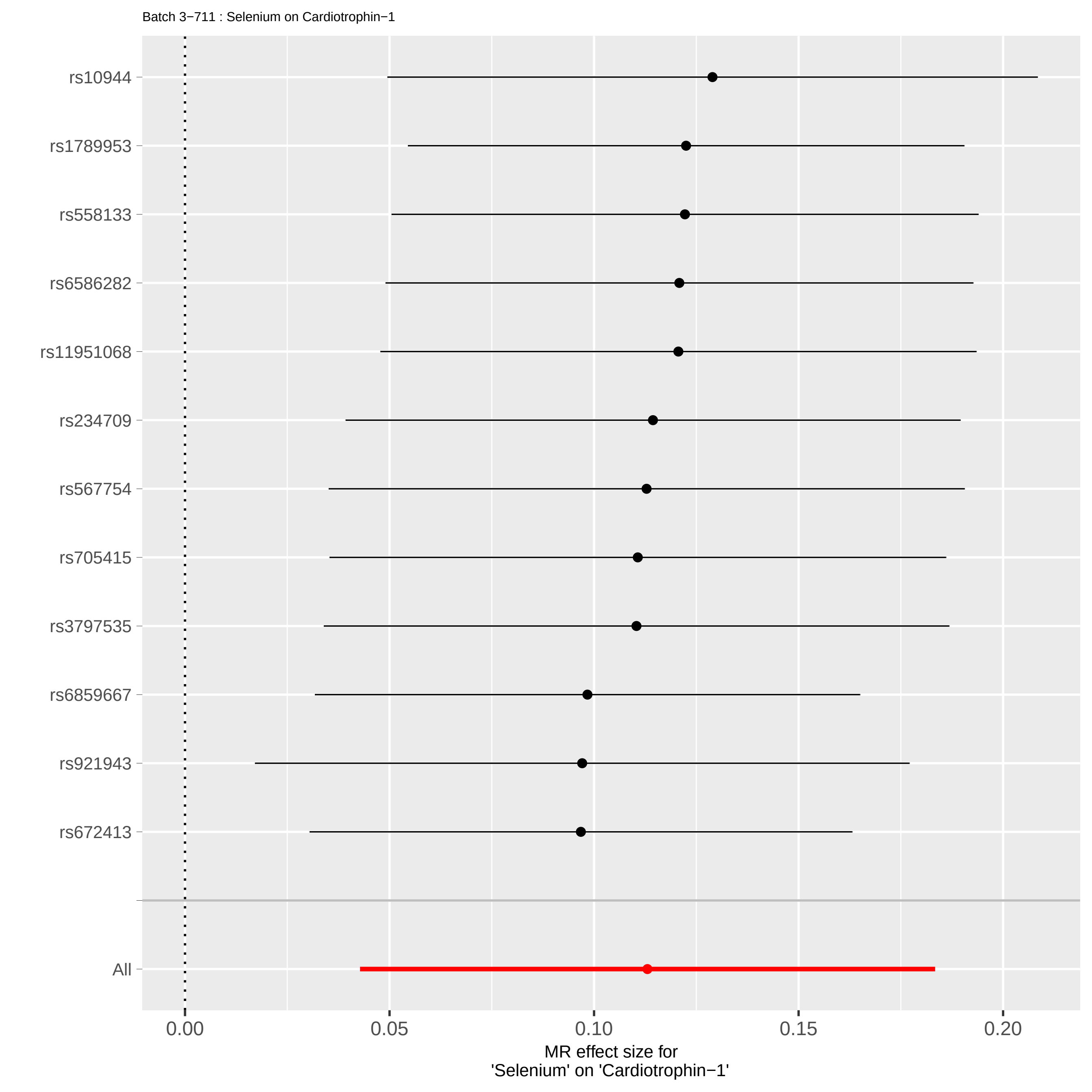

Batch 3−711 : Selenium on Cardiotrophin−1
rs10944
rs1789953
rs558133
rs6586282
rs11951068
rs234709
rs567754
rs705415
rs3797535
rs6859667
rs921943
rs672413
All
0.00
0.05
0.10
0.15
0.20
MR effect size for
'Selenium' on 'Cardiotrophin−1'

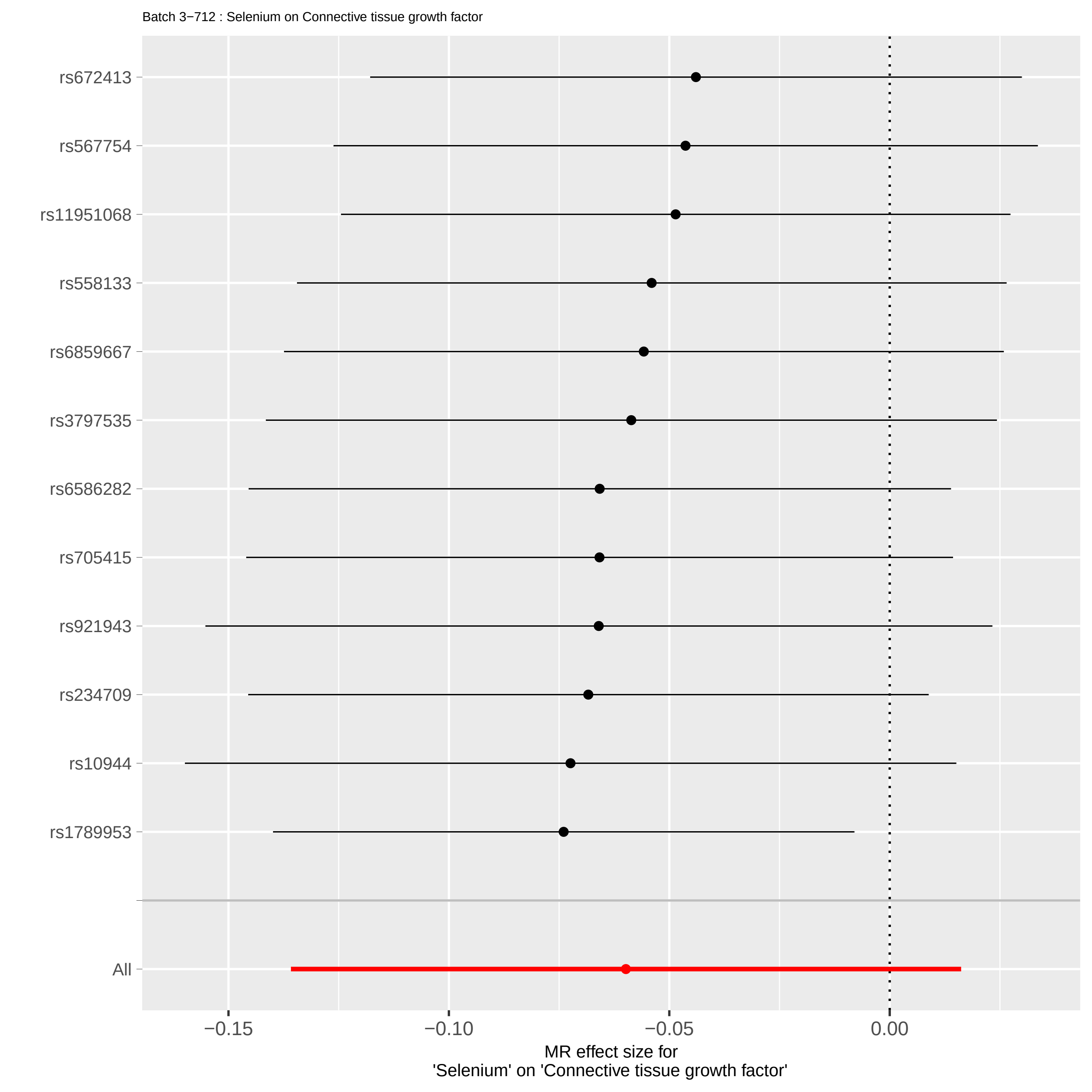
Batch 3−712 : Selenium on Connective tissue growth factor
rs672413
rs567754
rs11951068
rs558133
rs6859667
rs3797535
rs6586282
rs705415
rs921943
rs234709
rs10944
rs1789953
All
−0.15
−0.10
−0.05
0.00
MR effect size for
'Selenium' on 'Connective tissue growth factor'

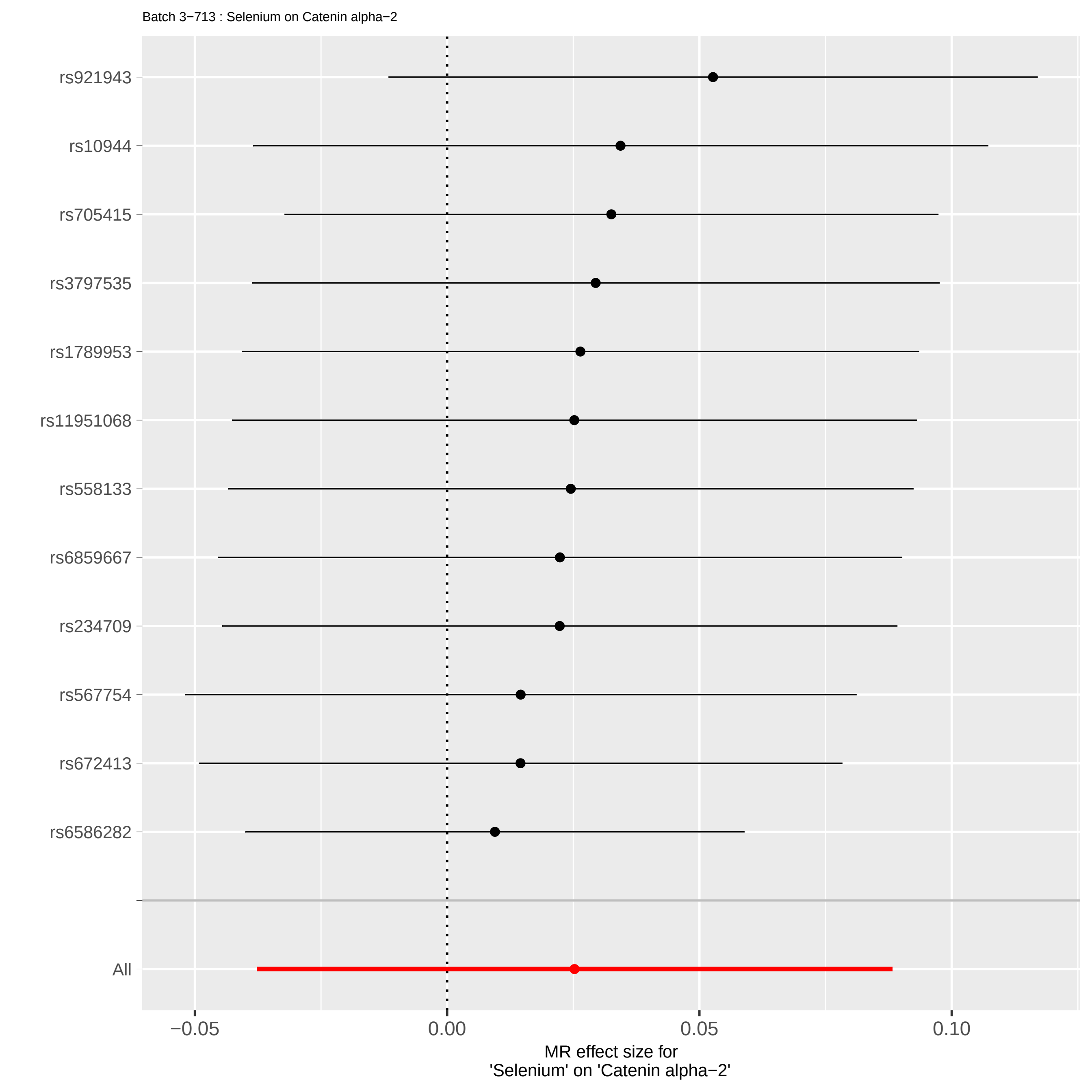

Batch 3−713 : Selenium on Catenin alpha−2
rs921943
rs10944
rs705415
rs3797535
rs1789953
rs11951068
rs558133
rs6859667
rs234709
rs567754
rs672413
rs6586282
All
−0.05
0.00
0.05
0.10
MR effect size for
'Selenium' on 'Catenin alpha−2'

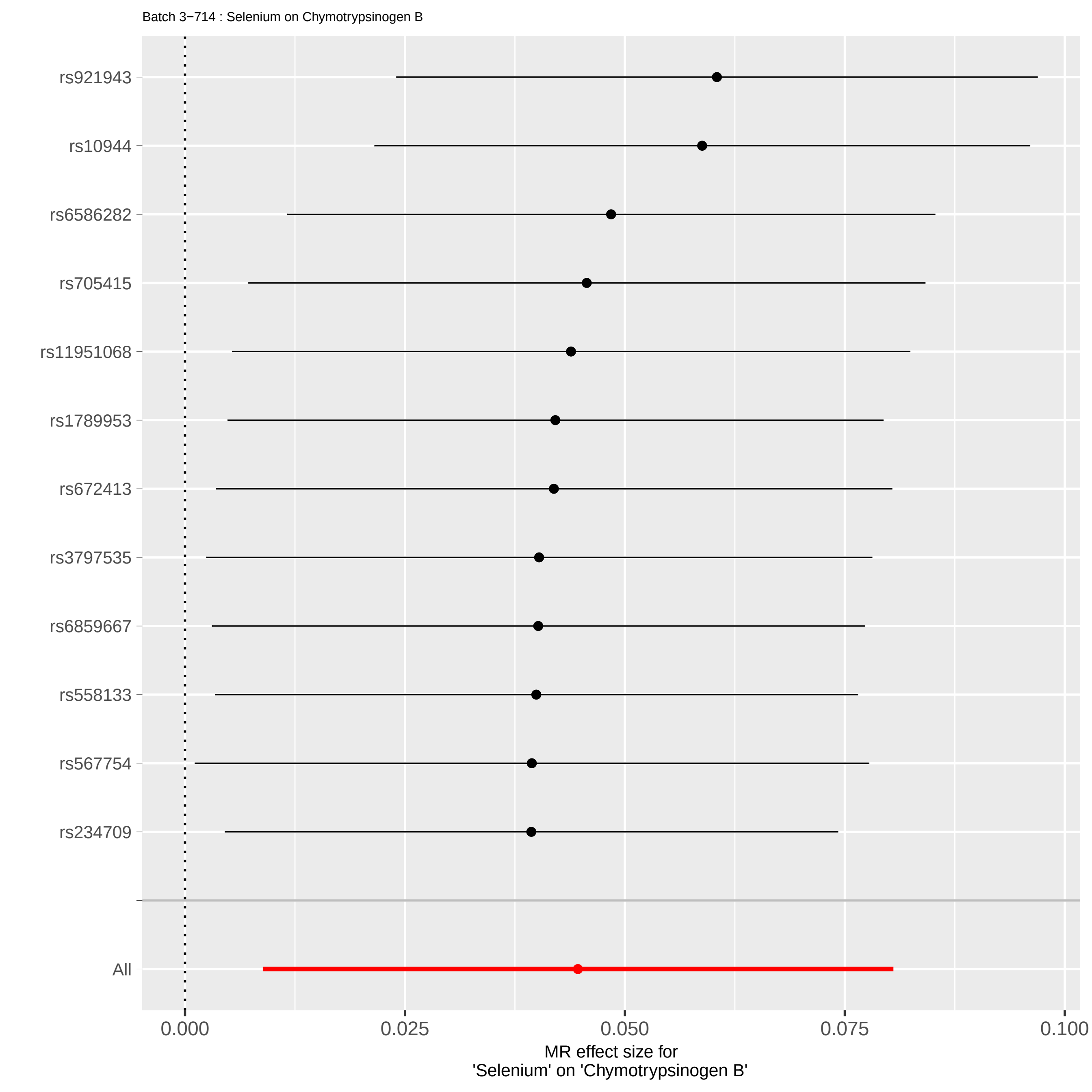

Batch 3−714 : Selenium on Chymotrypsinogen B
rs921943
rs10944
rs6586282
rs705415
rs11951068
rs1789953
rs672413
rs3797535
rs6859667
rs558133
rs567754
rs234709
All
0.000
0.025
0.050
0.075
0.100
MR effect size for
'Selenium' on 'Chymotrypsinogen B'

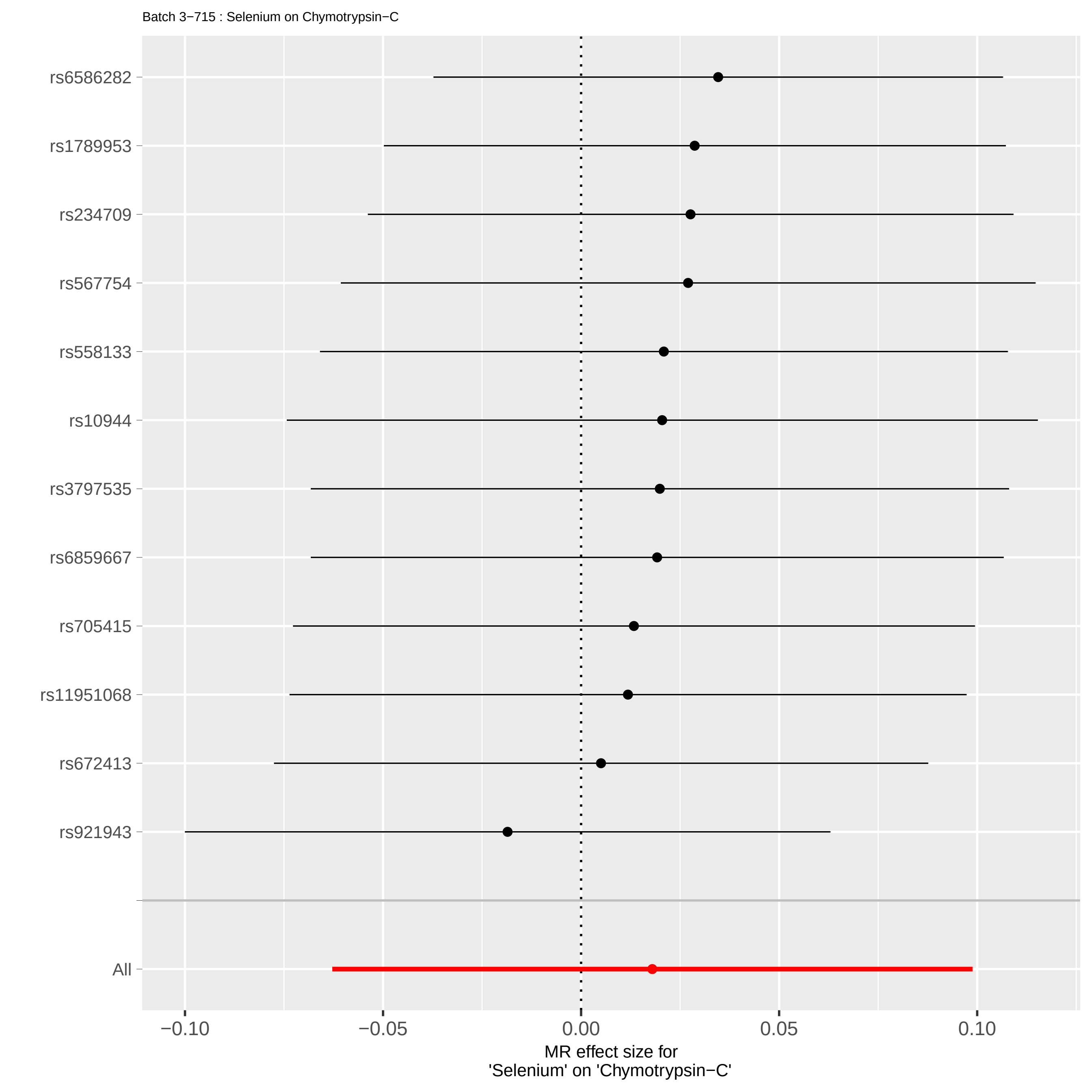

Batch 3−715 : Selenium on Chymotrypsin−C
rs6586282
rs1789953
rs234709
rs567754
rs558133
rs10944
rs3797535
rs6859667
rs705415
rs11951068
rs672413
rs921943
All
−0.10
−0.05
0.00
0.05
0.10
MR effect size for
'Selenium' on 'Chymotrypsin−C'

Batch 3−716 : Selenium on Chymotrypsin−like protease CTRL−1

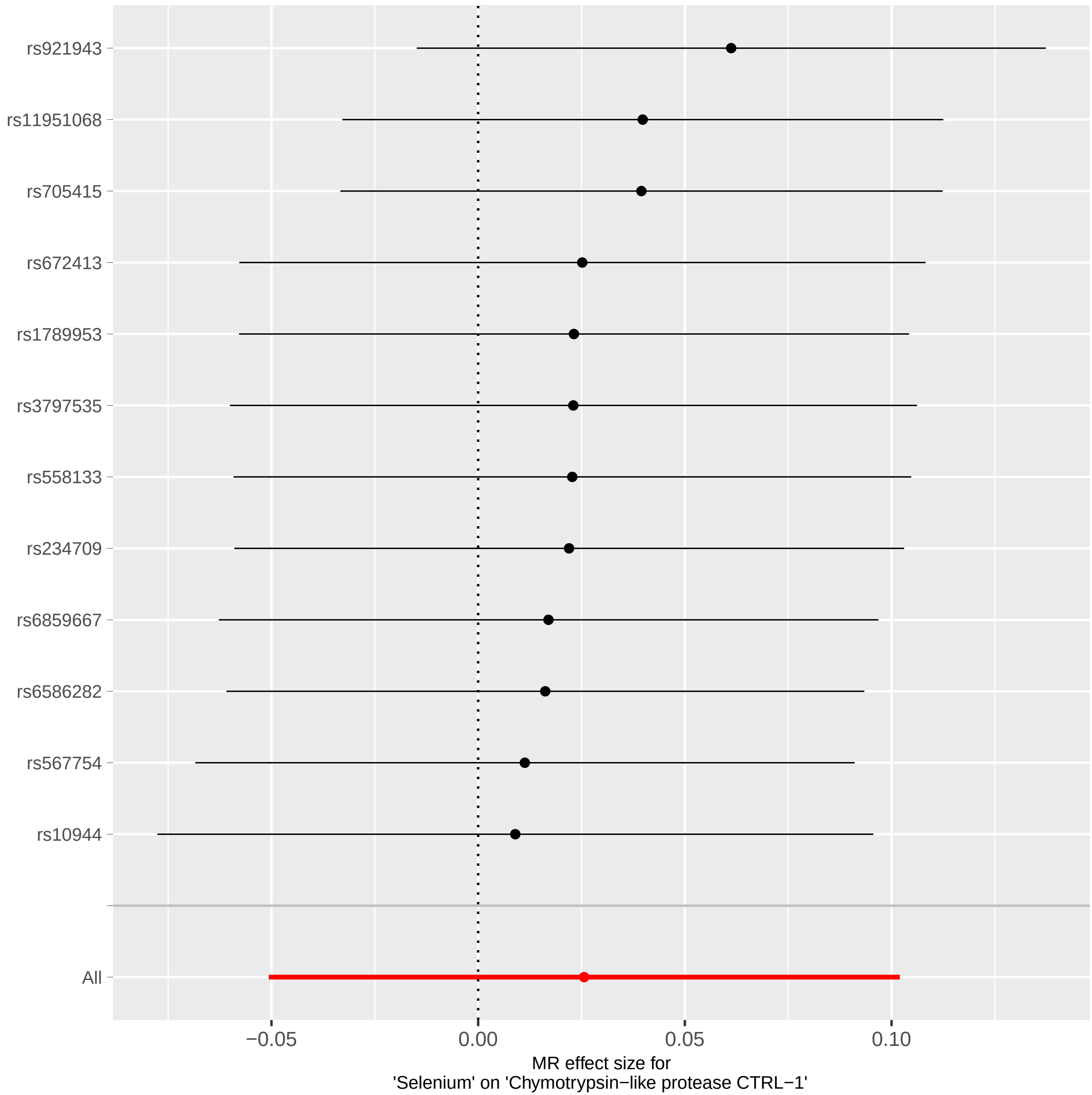

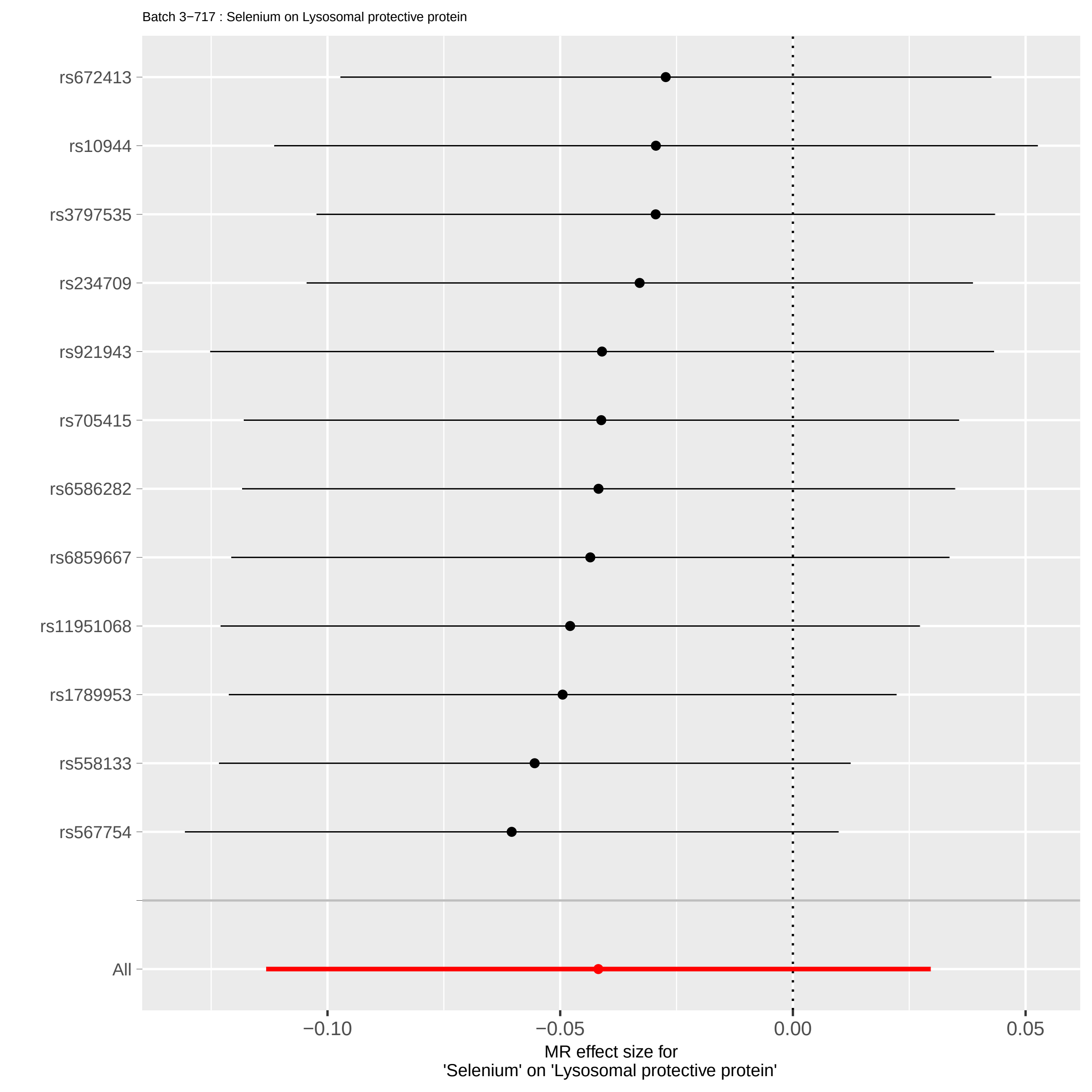

Batch 3−717 : Selenium on Lysosomal protective protein
rs672413
rs10944
rs3797535
rs234709
rs921943
rs705415
rs6586282
rs6859667
rs11951068
rs1789953
rs558133
rs567754
All
−0.10
−0.05
0.00
0.05
MR effect size for
'Selenium' on 'Lysosomal protective protein'

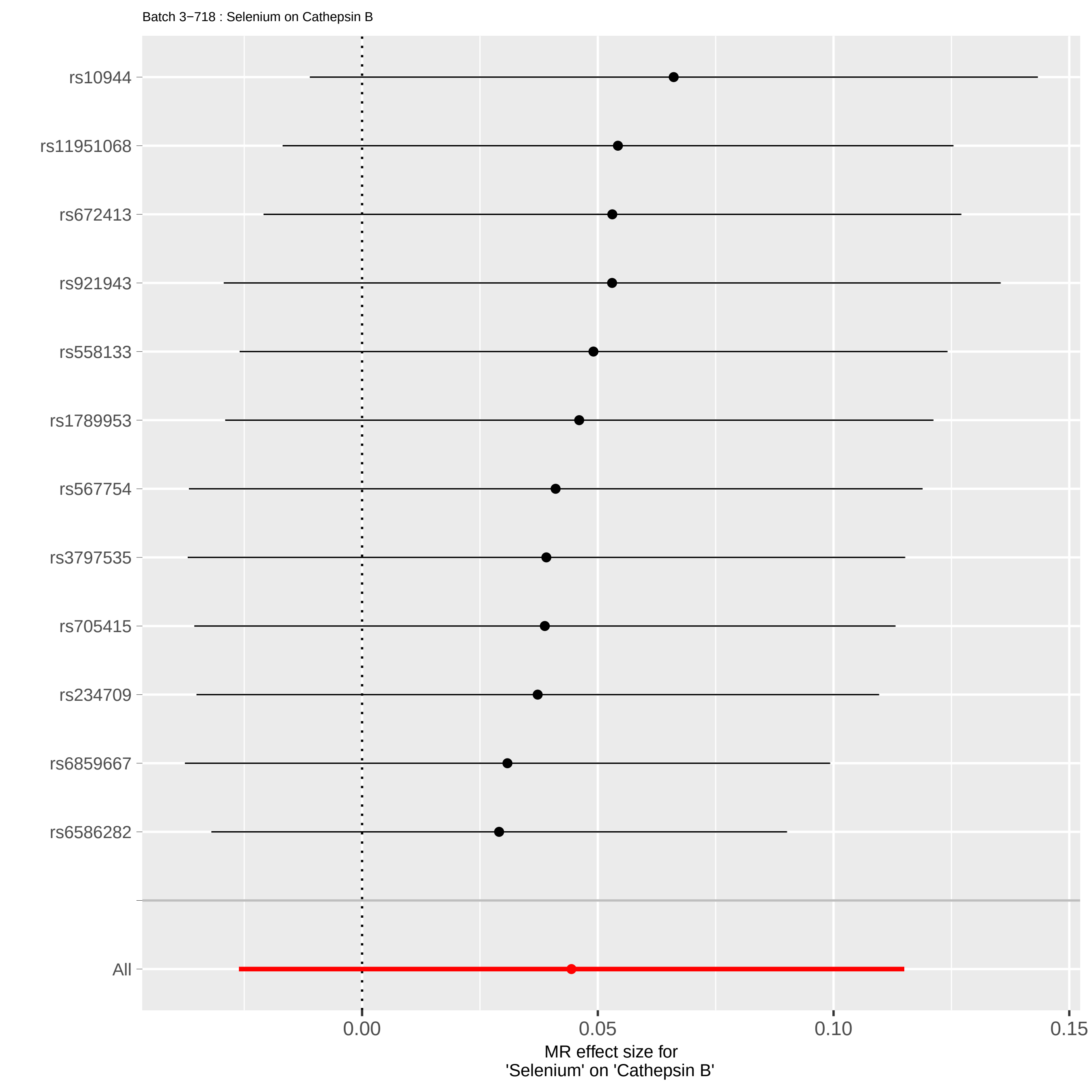
Batch 3−718 : Selenium on Cathepsin B
rs10944
rs11951068
rs672413
rs921943
rs558133
rs1789953
rs567754
rs3797535
rs705415
rs234709
rs6859667
rs6586282
All
0.00
0.05
0.10
0.15
MR effect size for
'Selenium' on 'Cathepsin B'

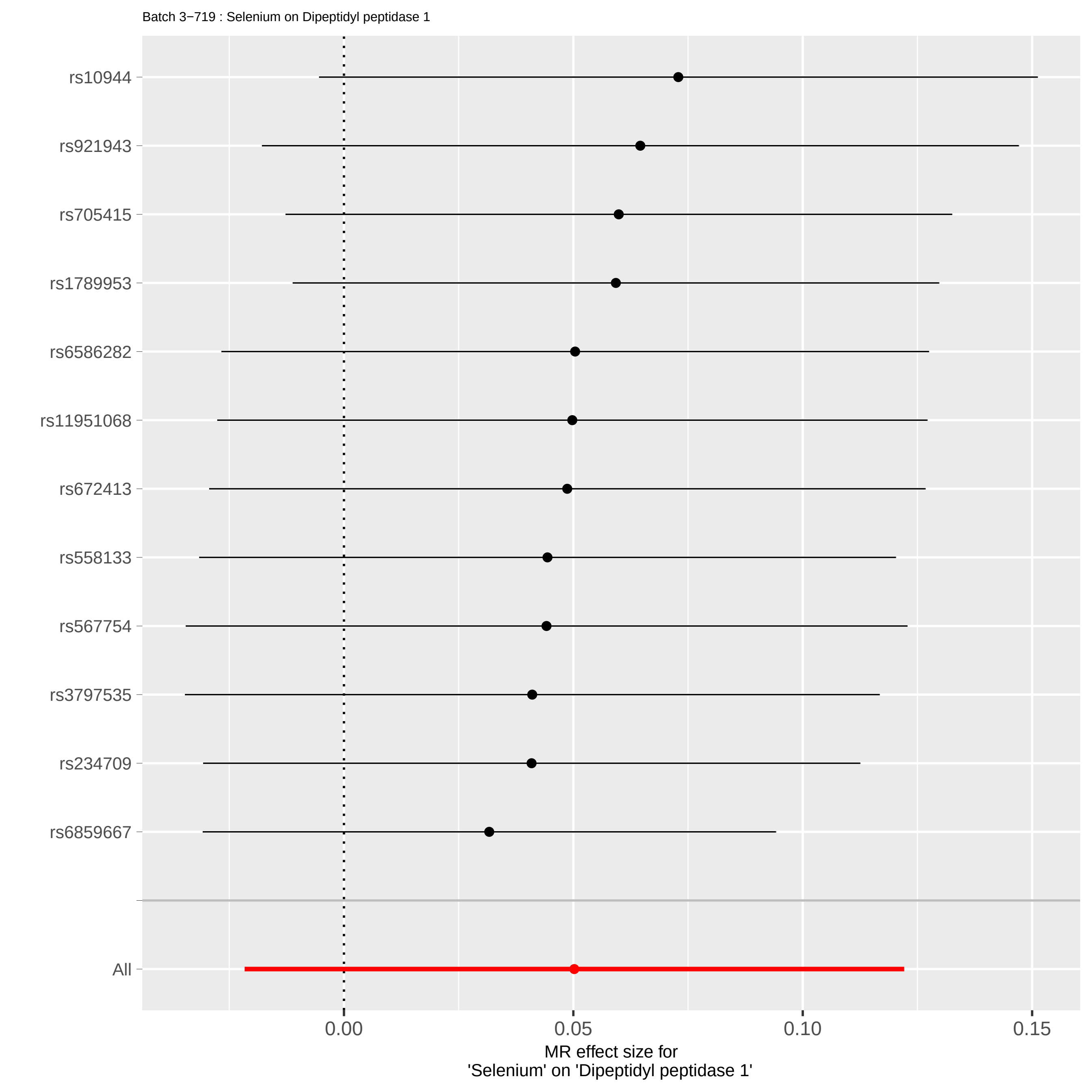

Batch 3−719 : Selenium on Dipeptidyl peptidase 1
rs10944
rs921943
rs705415
rs1789953
rs6586282
rs11951068
rs672413
rs558133
rs567754
rs3797535
rs234709
rs6859667
All
0.00
0.05
0.10
0.15
MR effect size for
'Selenium' on 'Dipeptidyl peptidase 1'

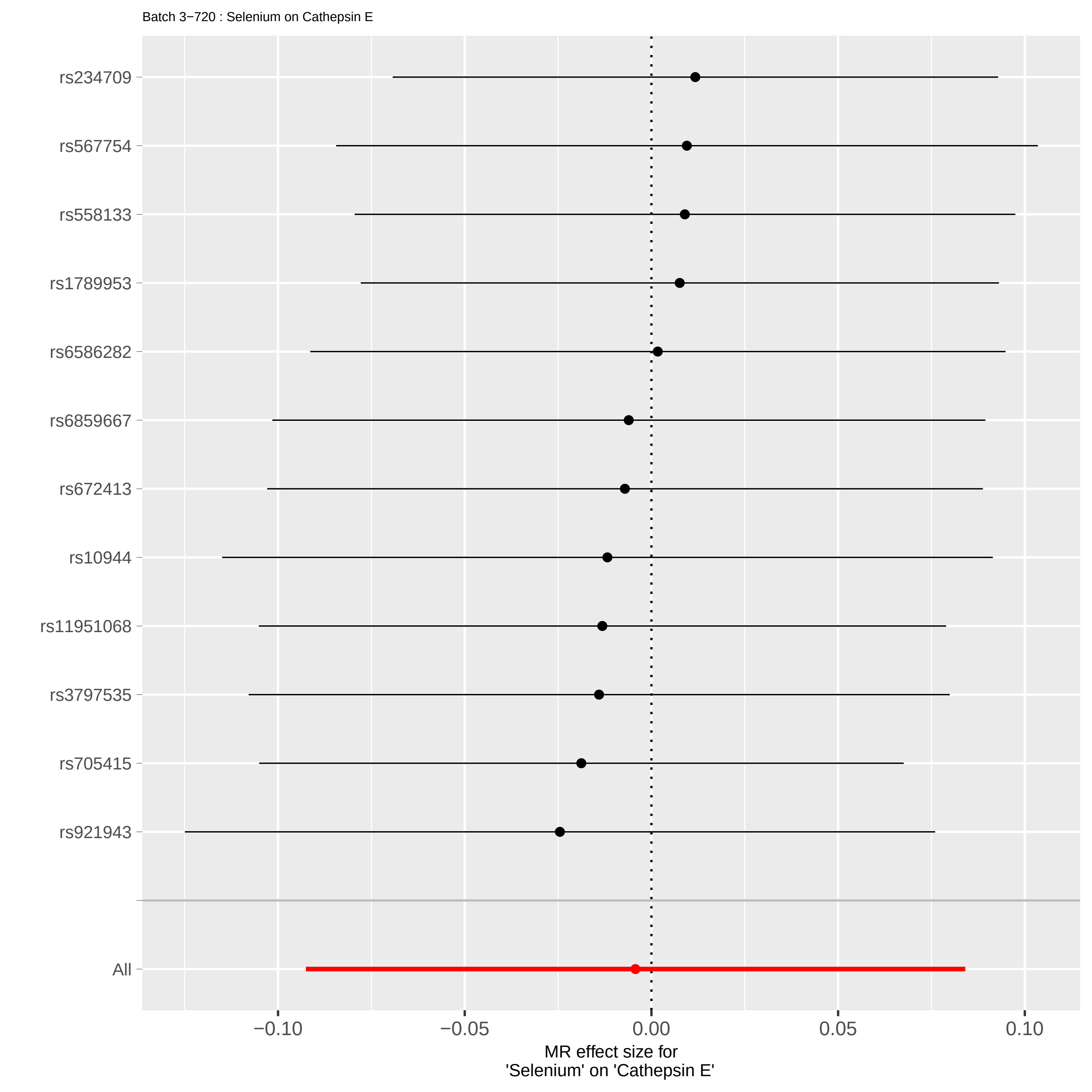
Batch 3−720 : Selenium on Cathepsin E
rs234709
rs567754
rs558133
rs1789953
rs6586282
rs6859667
rs672413
rs10944
rs11951068
rs3797535
rs705415
rs921943
All
−0.10
−0.05
0.00
0.05
0.10
MR effect size for
'Selenium' on 'Cathepsin E'

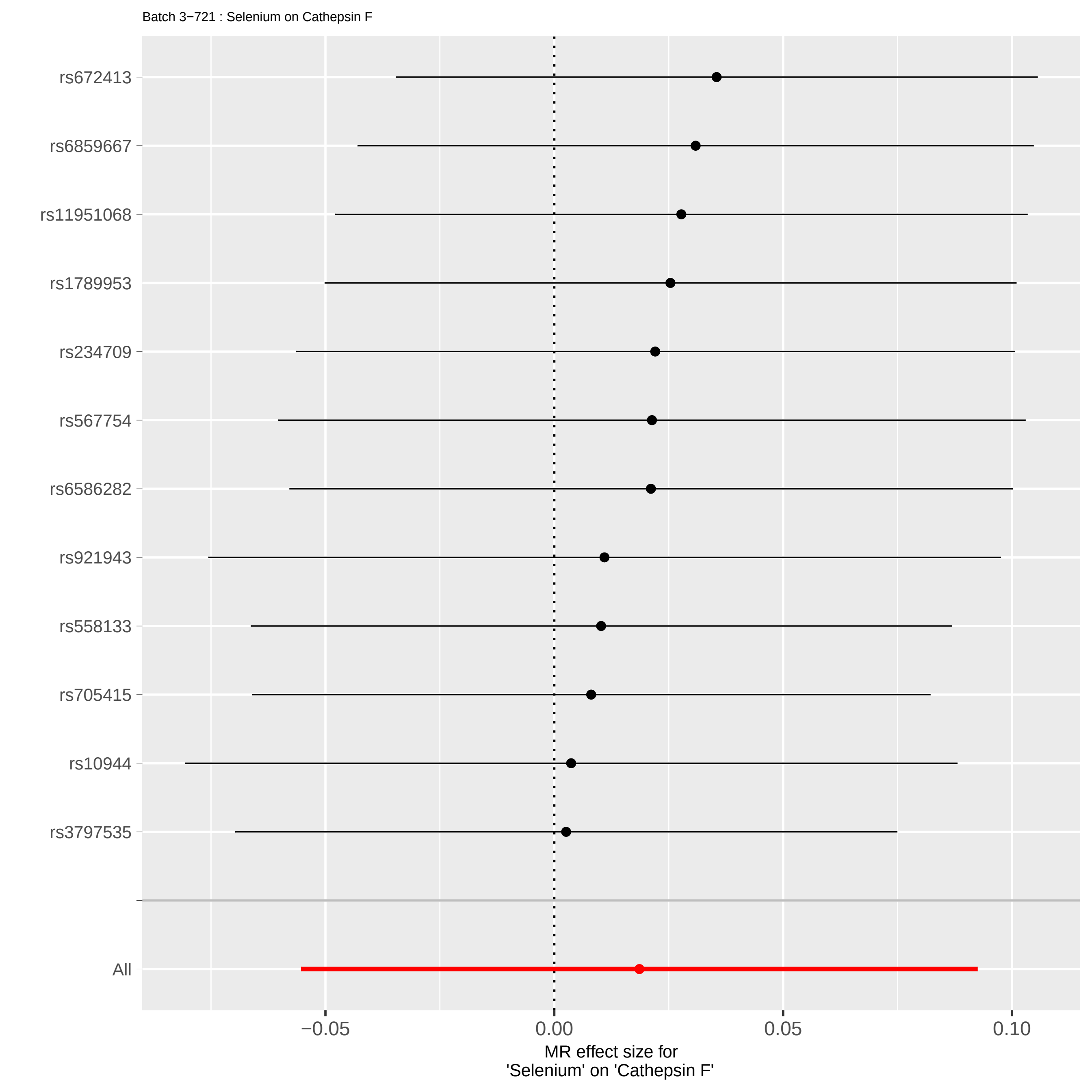
Batch 3−721 : Selenium on Cathepsin F
rs672413
rs6859667
rs11951068
rs1789953
rs234709
rs567754
rs6586282
rs921943
rs558133
rs705415
rs10944
rs3797535
All
−0.05
0.00
0.05
0.10
MR effect size for
'Selenium' on 'Cathepsin F'

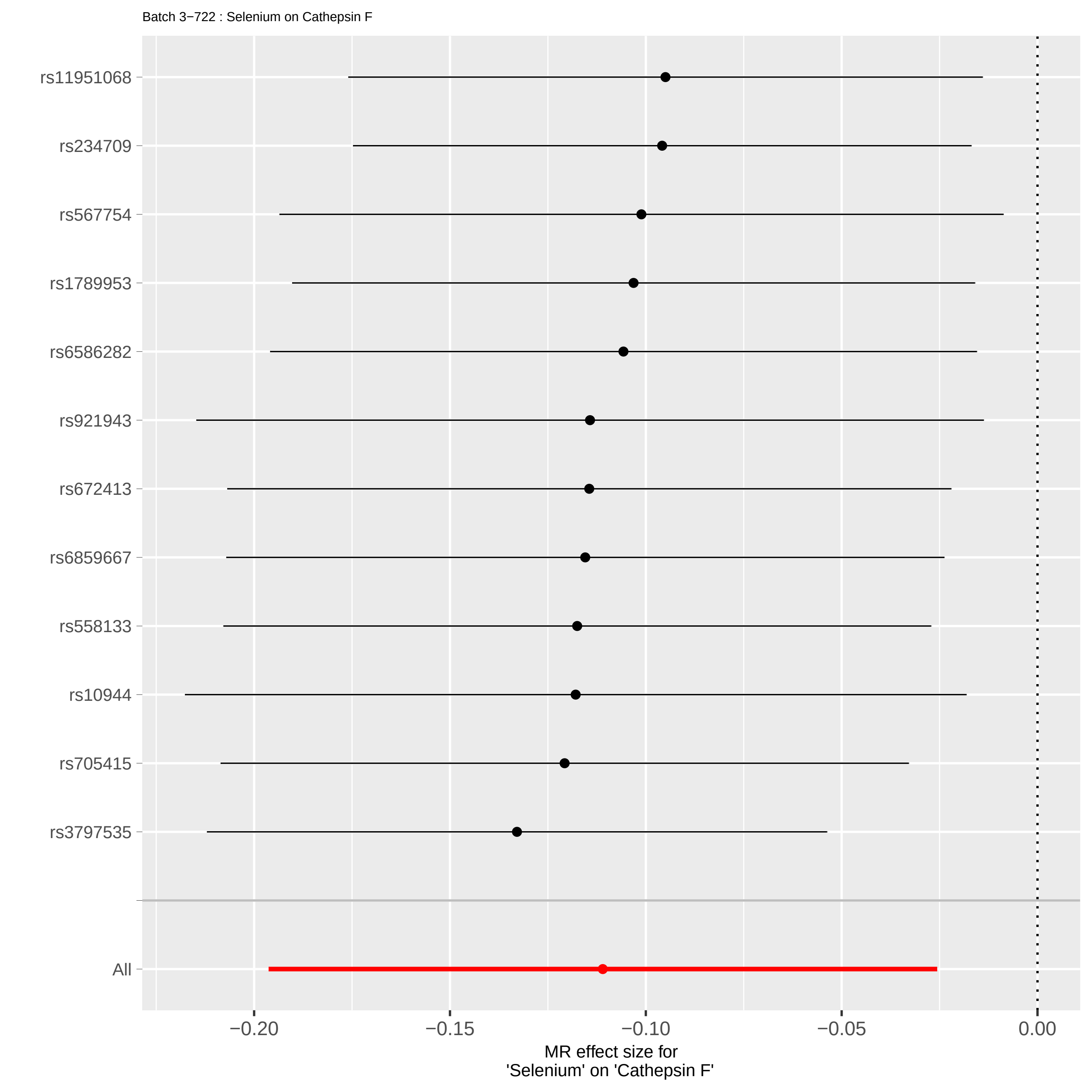

Batch 3−722 : Selenium on Cathepsin F
rs11951068
rs234709
rs567754
rs1789953
rs6586282
rs921943
rs672413
rs6859667
rs558133
rs10944
rs705415
rs3797535
All
−0.20
−0.15
−0.10
−0.05
0.00
MR effect size for
'Selenium' on 'Cathepsin F'

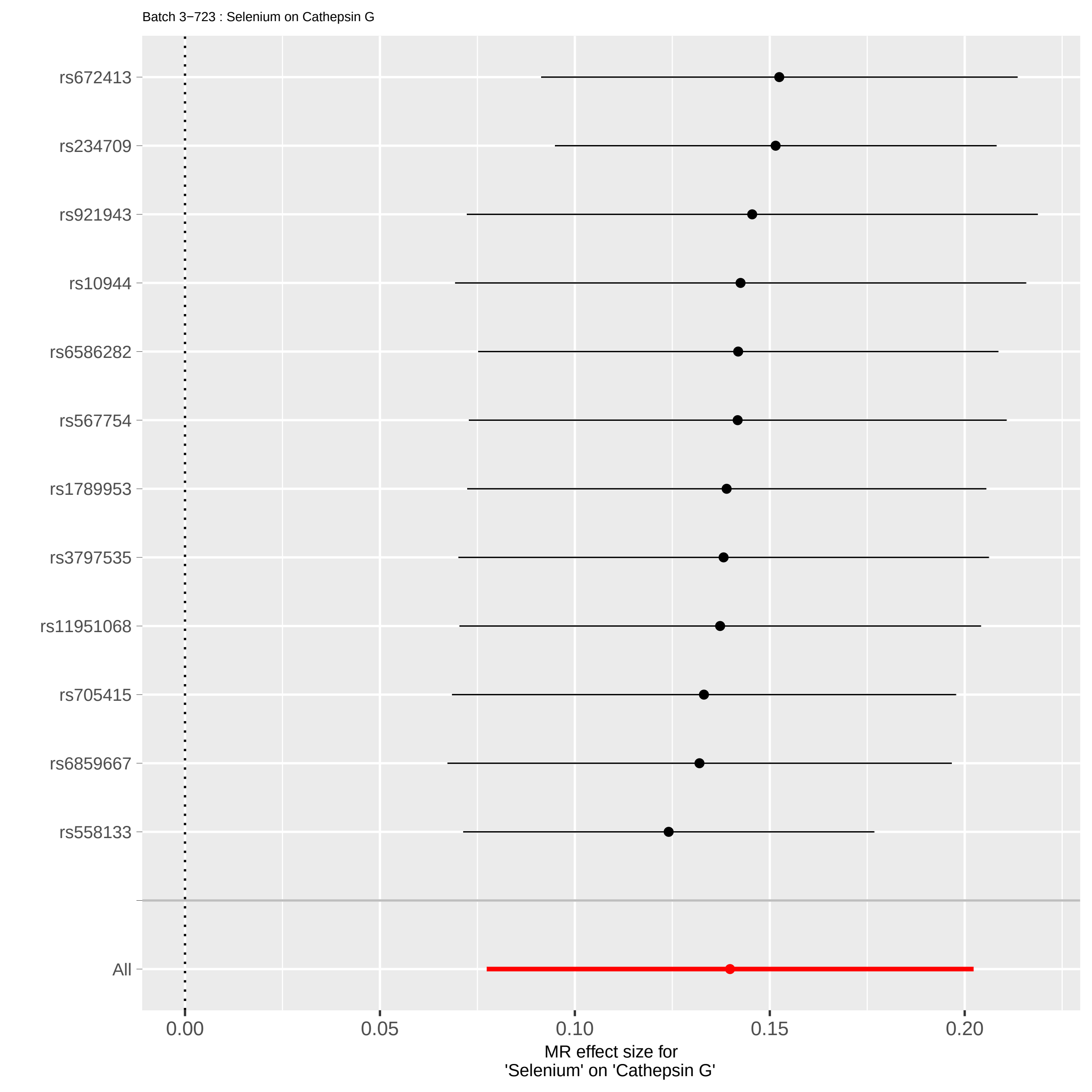

Batch 3−723 : Selenium on Cathepsin G
rs672413
rs234709
rs921943
rs10944
rs6586282
rs567754
rs1789953
rs3797535
rs11951068
rs705415
rs6859667
rs558133
All
0.00
0.05
0.10
0.15
0.20
MR effect size for
'Selenium' on 'Cathepsin G'

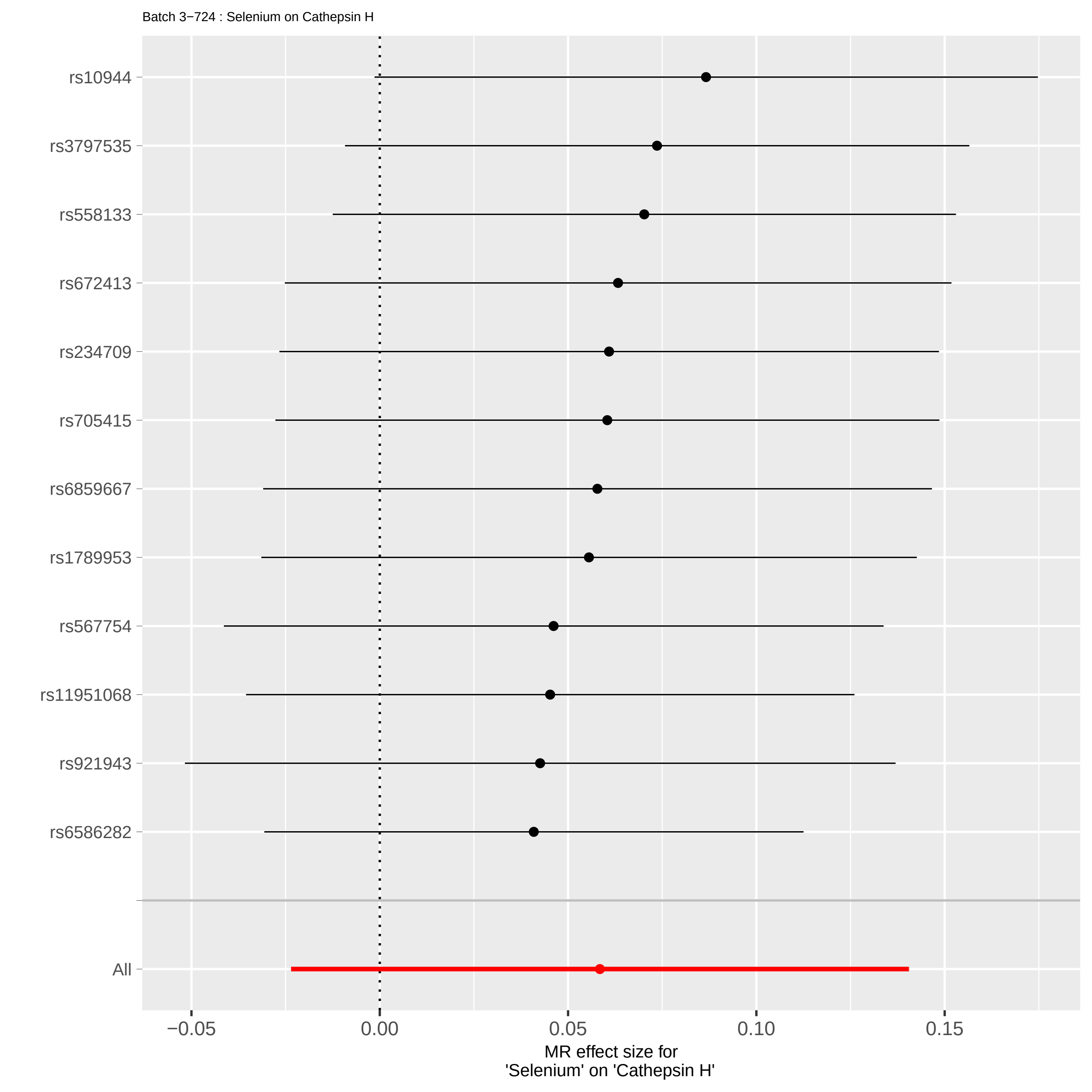

Batch 3−724 : Selenium on Cathepsin H
rs10944
rs3797535
rs558133
rs672413
rs234709
rs705415
rs6859667
rs1789953
rs567754
rs11951068
rs921943
rs6586282
All
−0.05
0.00
0.05
0.10
0.15
MR effect size for
'Selenium' on 'Cathepsin H'

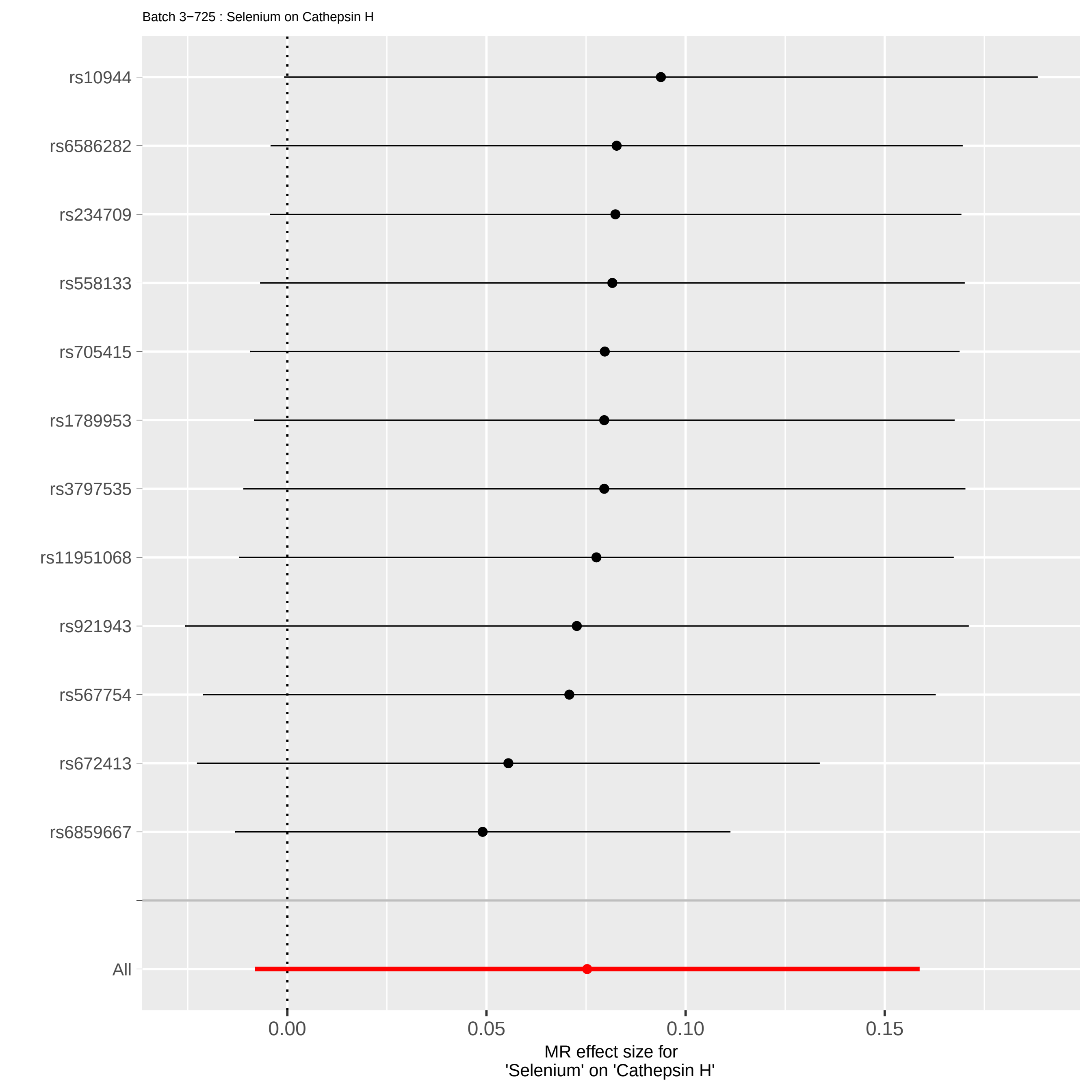

Batch 3−725 : Selenium on Cathepsin H
rs10944
rs6586282
rs234709
rs558133
rs705415
rs1789953
rs3797535
rs11951068
rs921943
rs567754
rs672413
rs6859667
All
0.00
0.05
0.10
0.15
MR effect size for
'Selenium' on 'Cathepsin H'

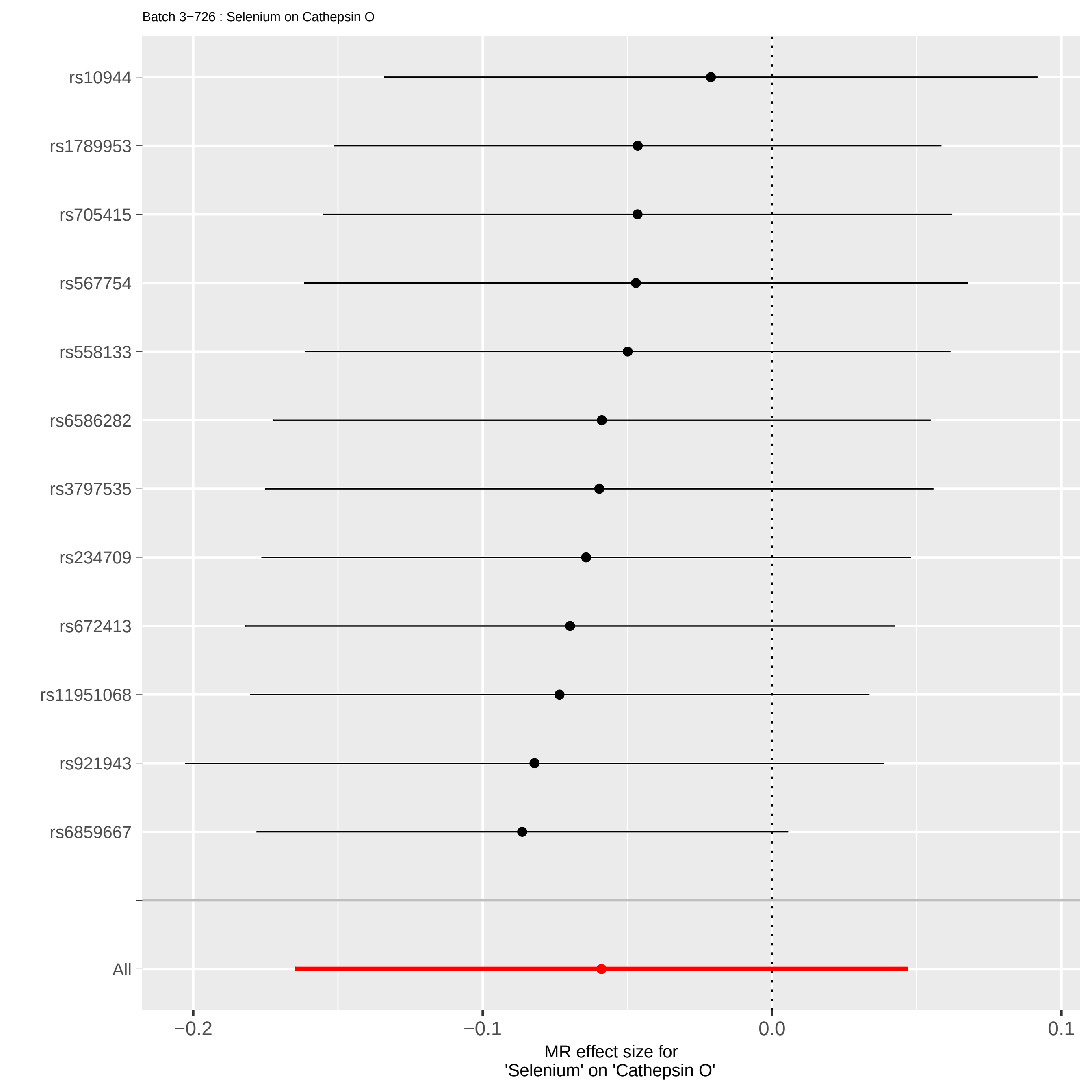

Batch 3−726 : Selenium on Cathepsin O
rs10944
rs1789953
rs705415
rs567754
rs558133
rs6586282
rs3797535
rs234709
rs672413
rs11951068
rs921943
rs6859667
All
−0.2
−0.1
0.0
0.1
MR effect size for
'Selenium' on 'Cathepsin O'

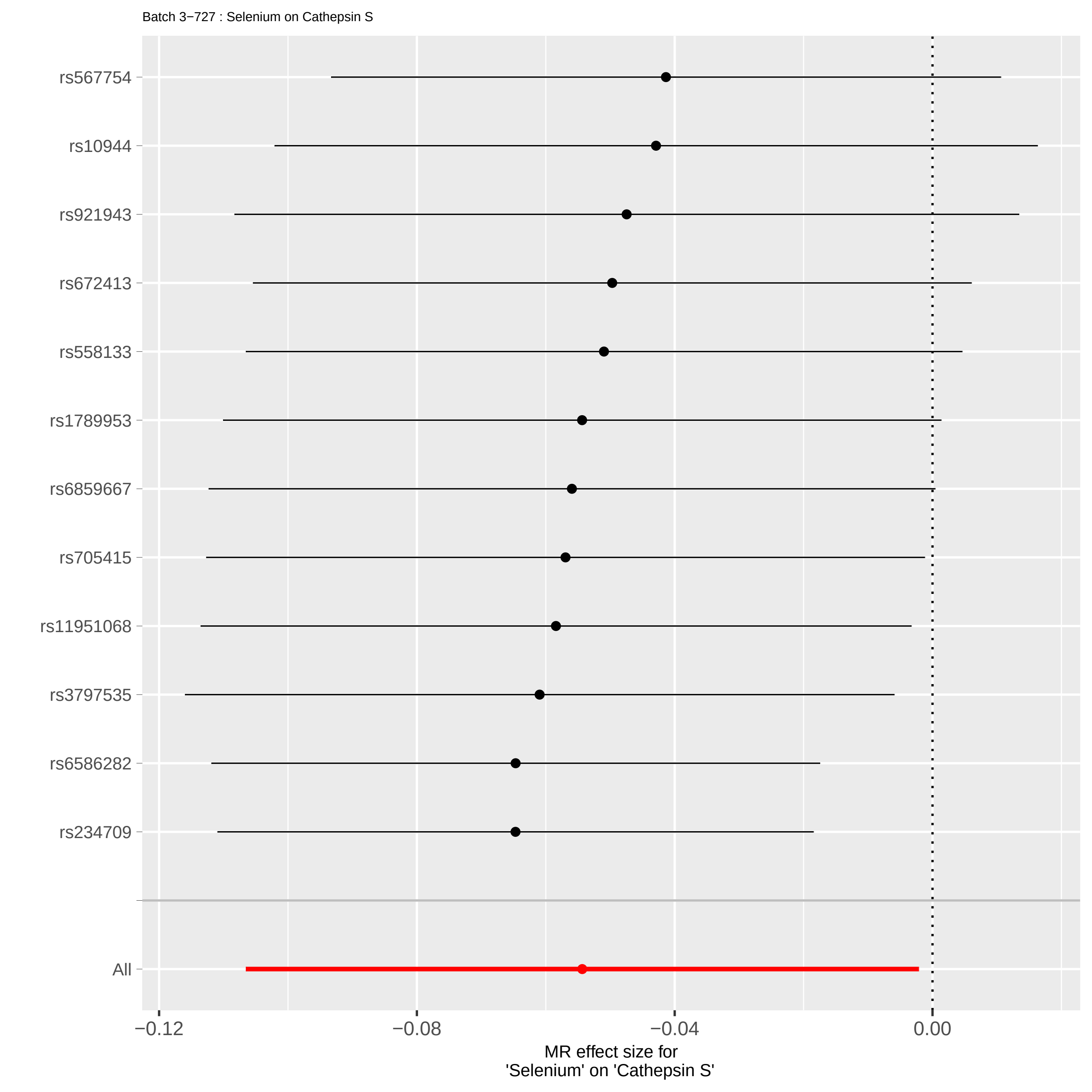

Batch 3−727 : Selenium on Cathepsin S
rs567754
rs10944
rs921943
rs672413
rs558133
rs1789953
rs6859667
rs705415
rs11951068
rs3797535
rs6586282
rs234709
All
−0.12
−0.08
−0.04
0.00
MR effect size for
'Selenium' on 'Cathepsin S'

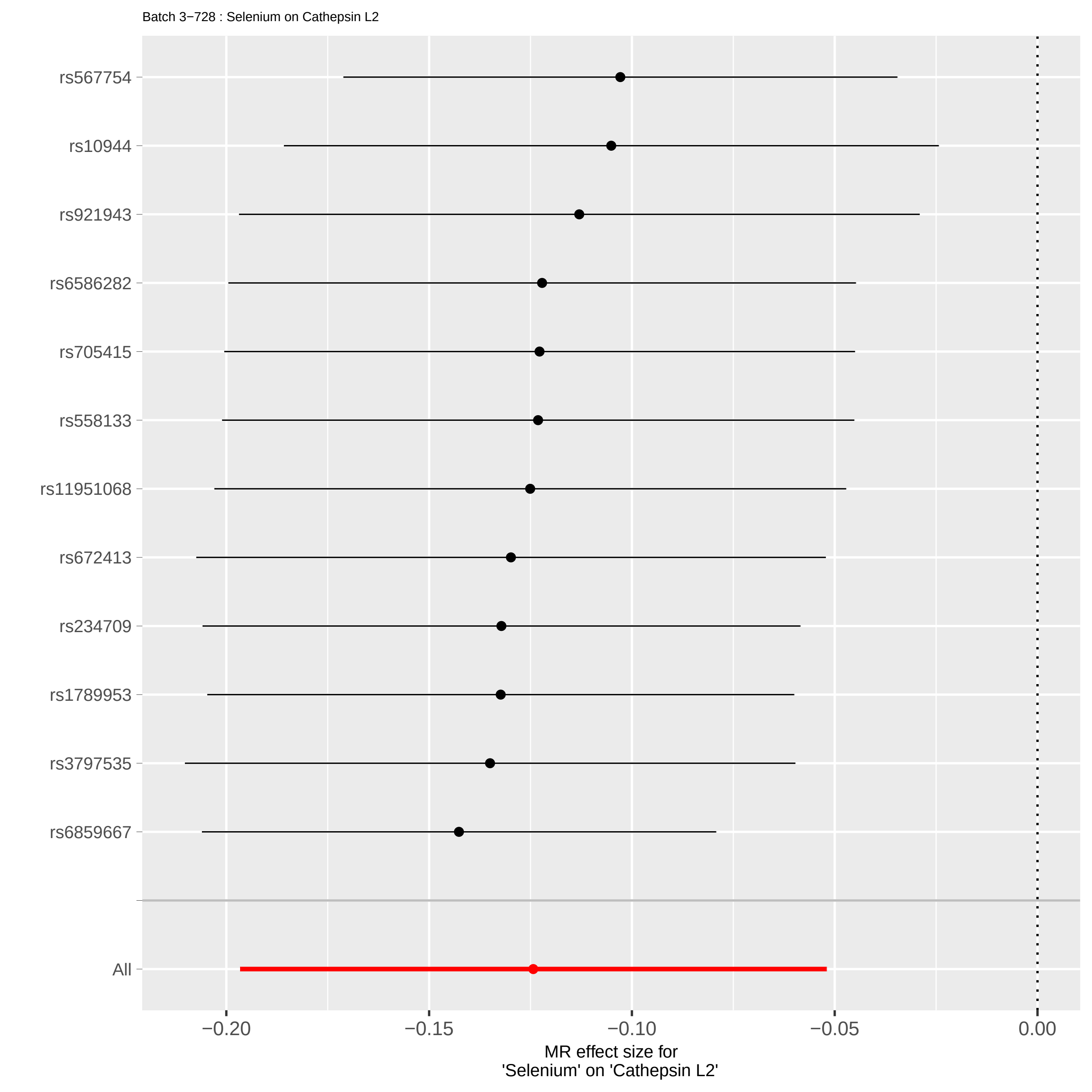

Batch 3−728 : Selenium on Cathepsin L2
rs567754
rs10944
rs921943
rs6586282
rs705415
rs558133
rs11951068
rs672413
rs234709
rs1789953
rs3797535
rs6859667
All
−0.20
−0.15
−0.10
−0.05
0.00
MR effect size for
'Selenium' on 'Cathepsin L2'

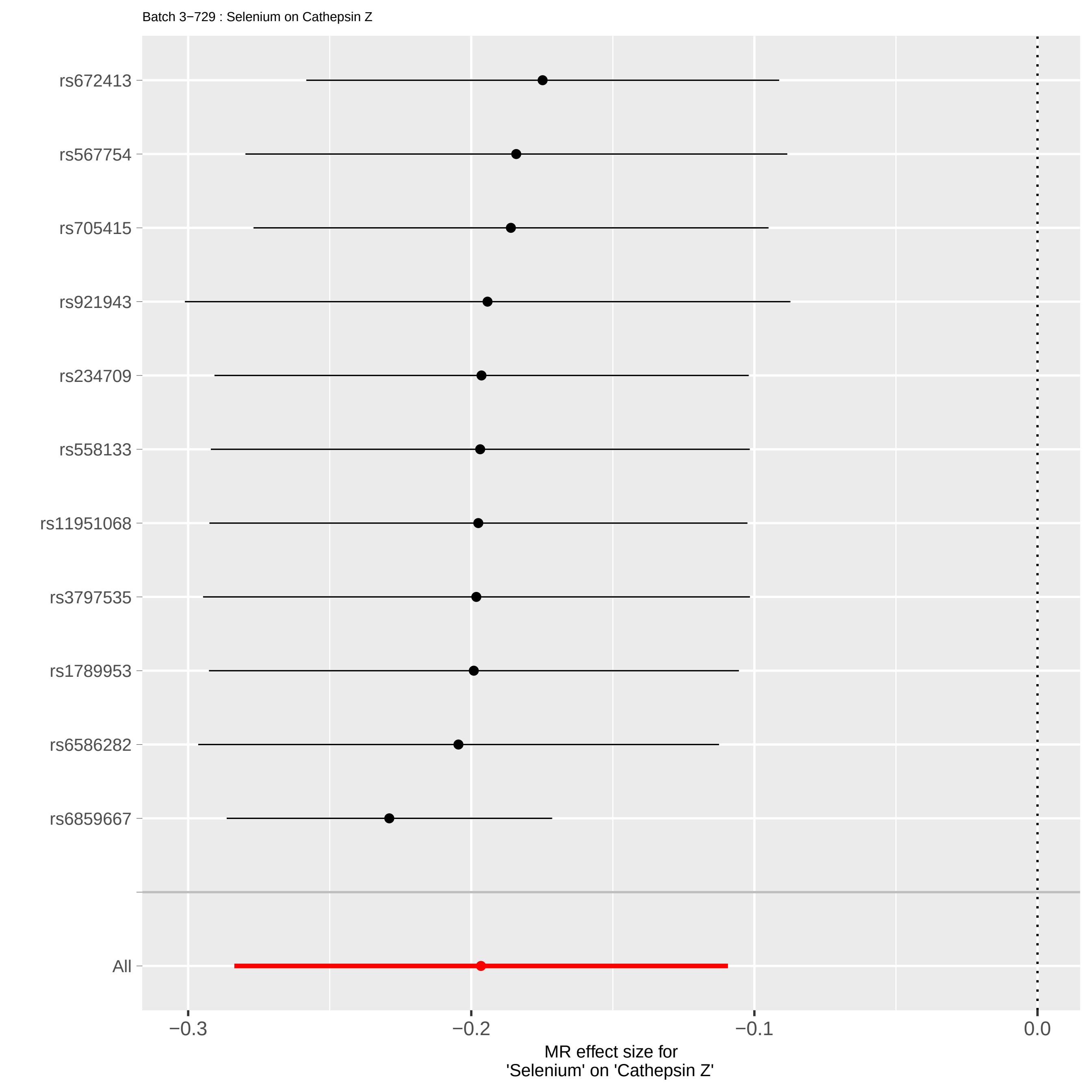

Batch 3−729 : Selenium on Cathepsin Z
rs672413
rs567754
rs705415
rs921943
rs234709
rs558133
rs11951068
rs3797535
rs1789953
rs6586282
rs6859667
All
−0.3
−0.2
−0.1
0.0
MR effect size for
'Selenium' on 'Cathepsin Z'

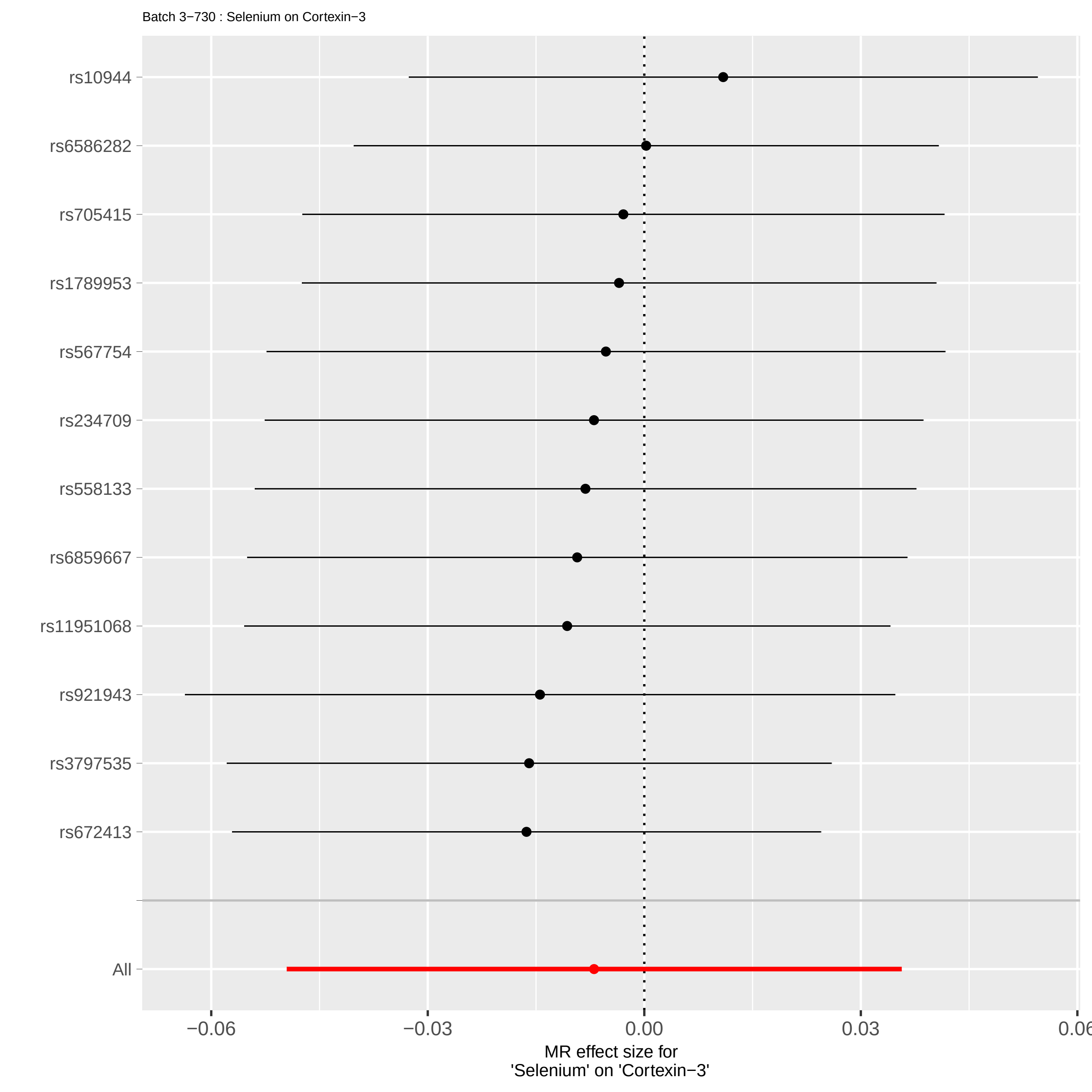

Batch 3−730 : Selenium on Cortexin−3
rs10944
rs6586282
rs705415
rs1789953
rs567754
rs234709
rs558133
rs6859667
rs11951068
rs921943
rs3797535
rs672413
All
−0.06
−0.03
0.00
0.03
0.06
MR effect size for
'Selenium' on 'Cortexin−3'

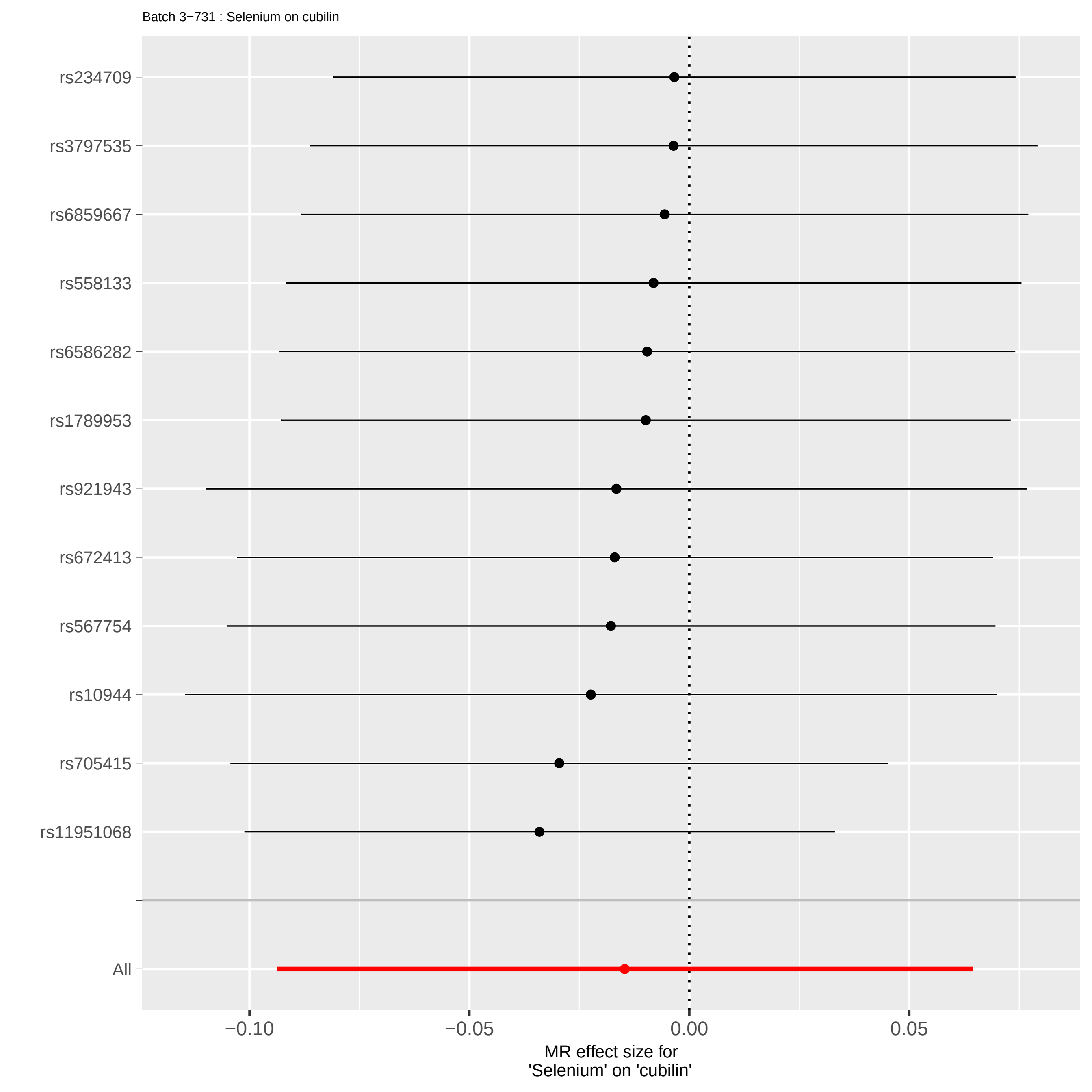
Batch 3−731 : Selenium on cubilin
rs234709
rs3797535
rs6859667
rs558133
rs6586282
rs1789953
rs921943
rs672413
rs567754
rs10944
rs705415
rs11951068
All
−0.10
−0.05
0.00
0.05
MR effect size for
'Selenium' on 'cubilin'

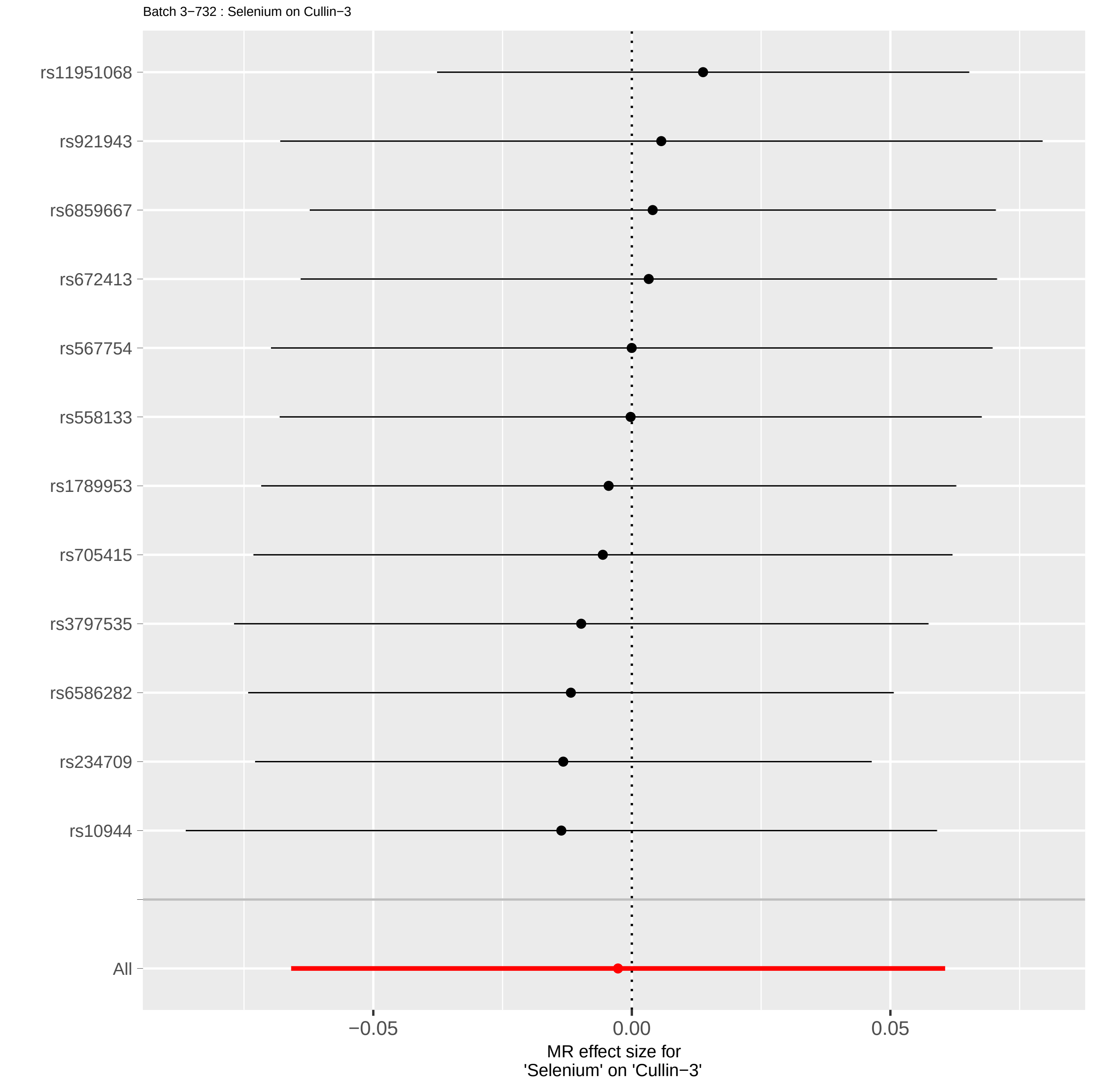
Batch 3−732 : Selenium on Cullin−3
rs11951068
rs921943
rs6859667
rs672413
rs567754
rs558133
rs1789953
rs705415
rs3797535
rs6586282
rs234709
rs10944
All
−0.05
0.00
0.05
MR effect size for
'Selenium' on 'Cullin−3'

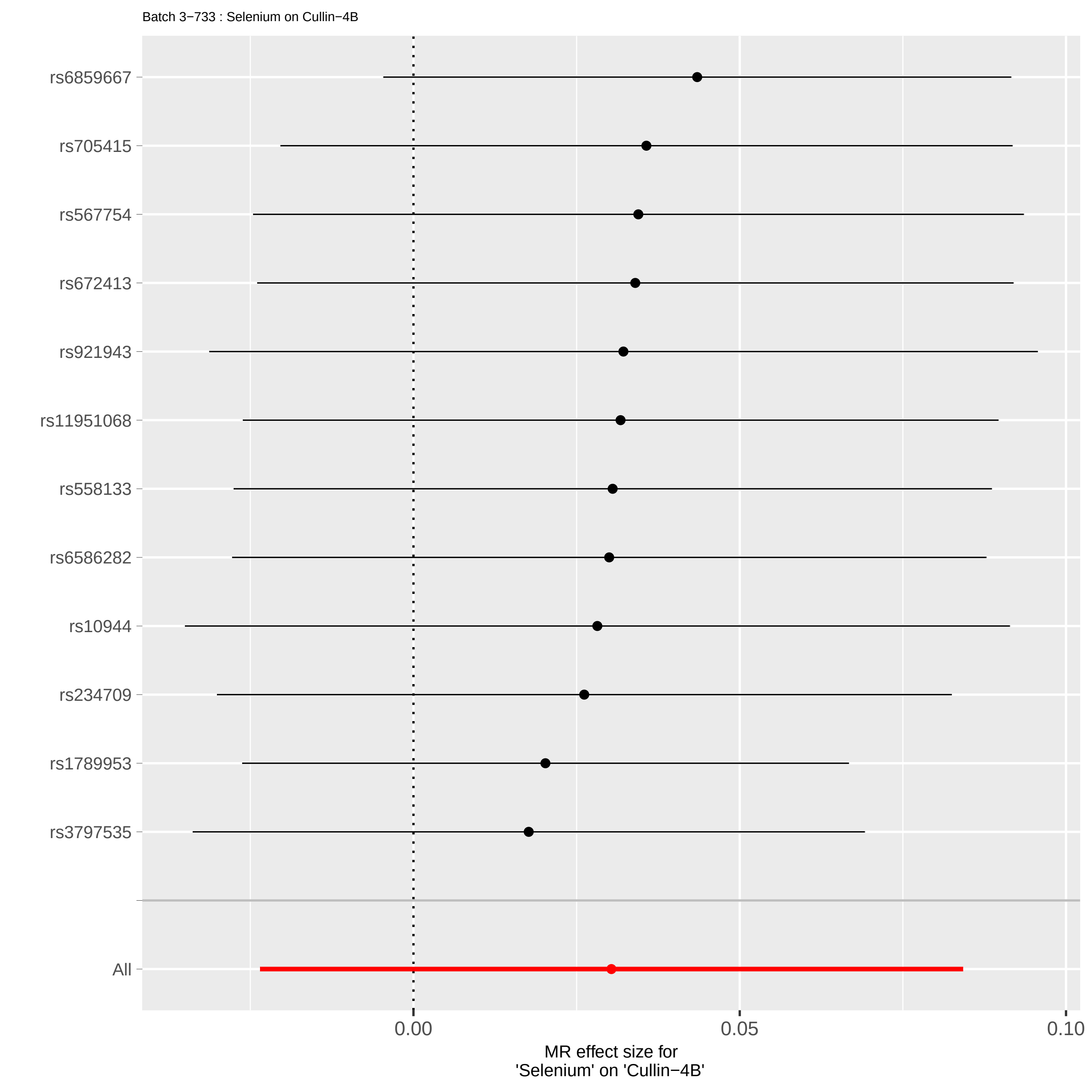
Batch 3−733 : Selenium on Cullin−4B
rs6859667
rs705415
rs567754
rs672413
rs921943
rs11951068
rs558133
rs6586282
rs10944
rs234709
rs1789953
rs3797535
All
0.00
0.05
0.10
MR effect size for
'Selenium' on 'Cullin−4B'

Batch 3−734 : Selenium on Cullin−9

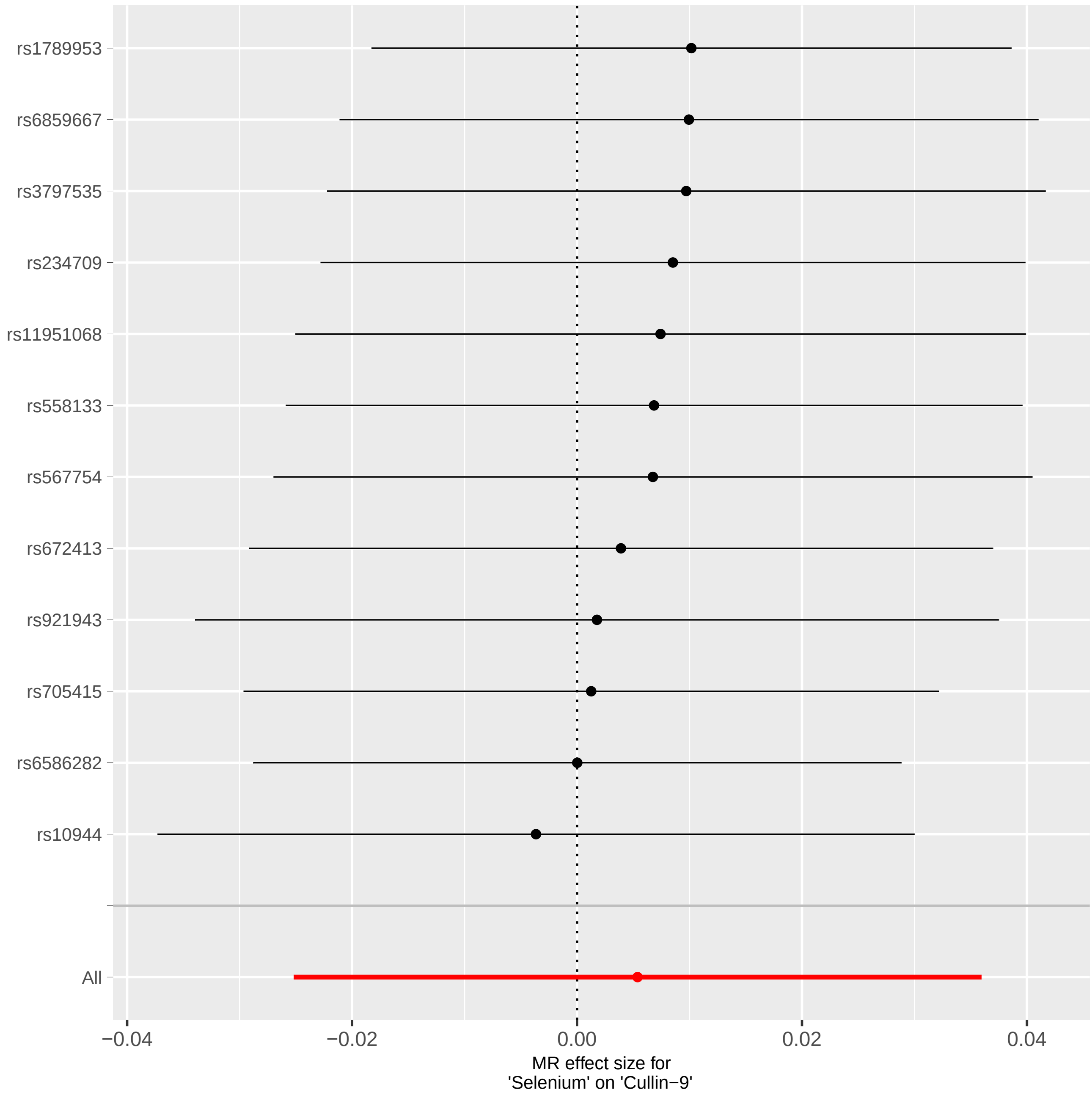

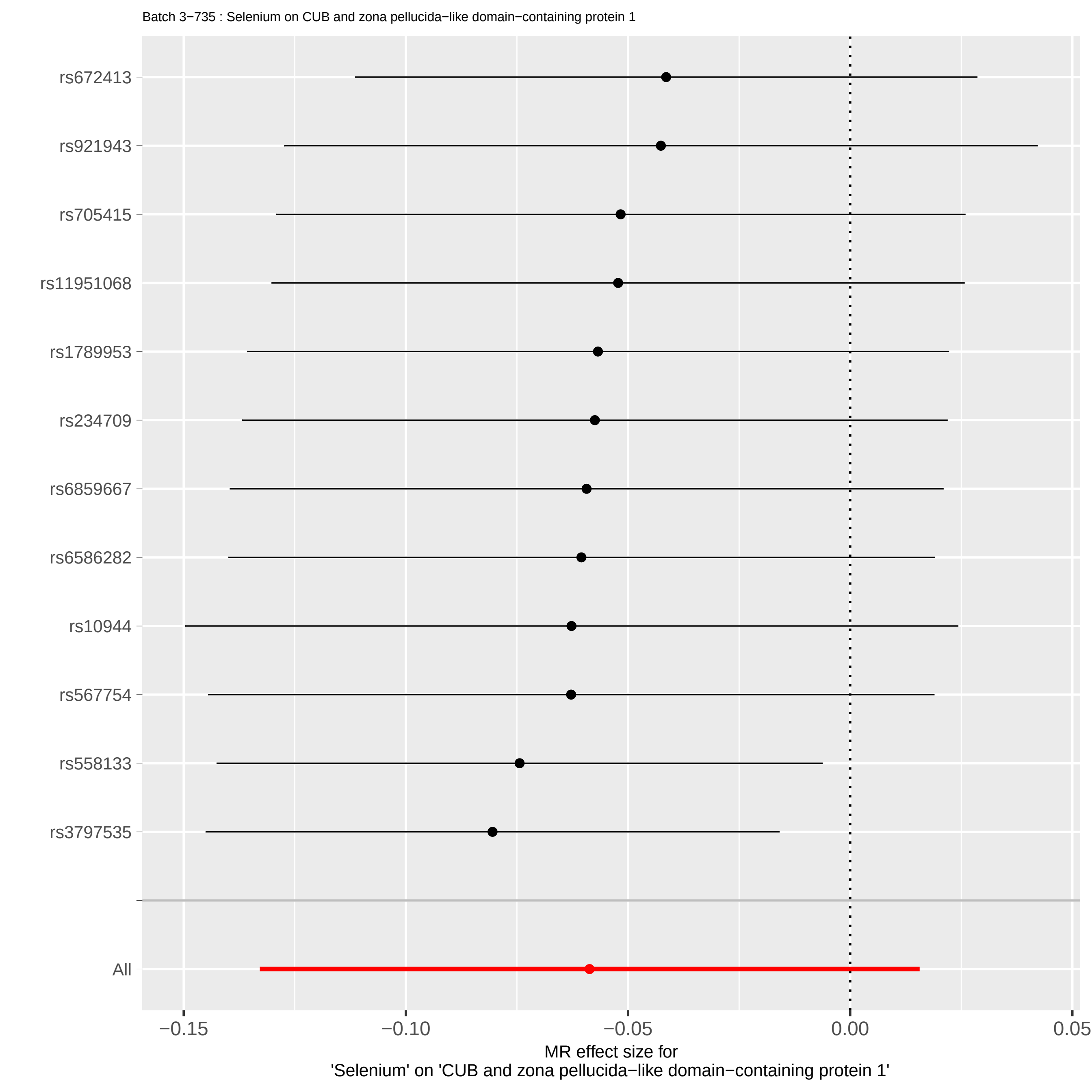

Batch 3−735 : Selenium on CUB and zona pellucida−like domain−containing protein 1
rs672413
rs921943
rs705415
rs11951068
rs1789953
rs234709
rs6859667
rs6586282
rs10944
rs567754
rs558133
rs3797535
All
−0.15
−0.10
−0.05
0.00
0.05
MR effect size for
'Selenium' on 'CUB and zona pellucida−like domain−containing protein 1'

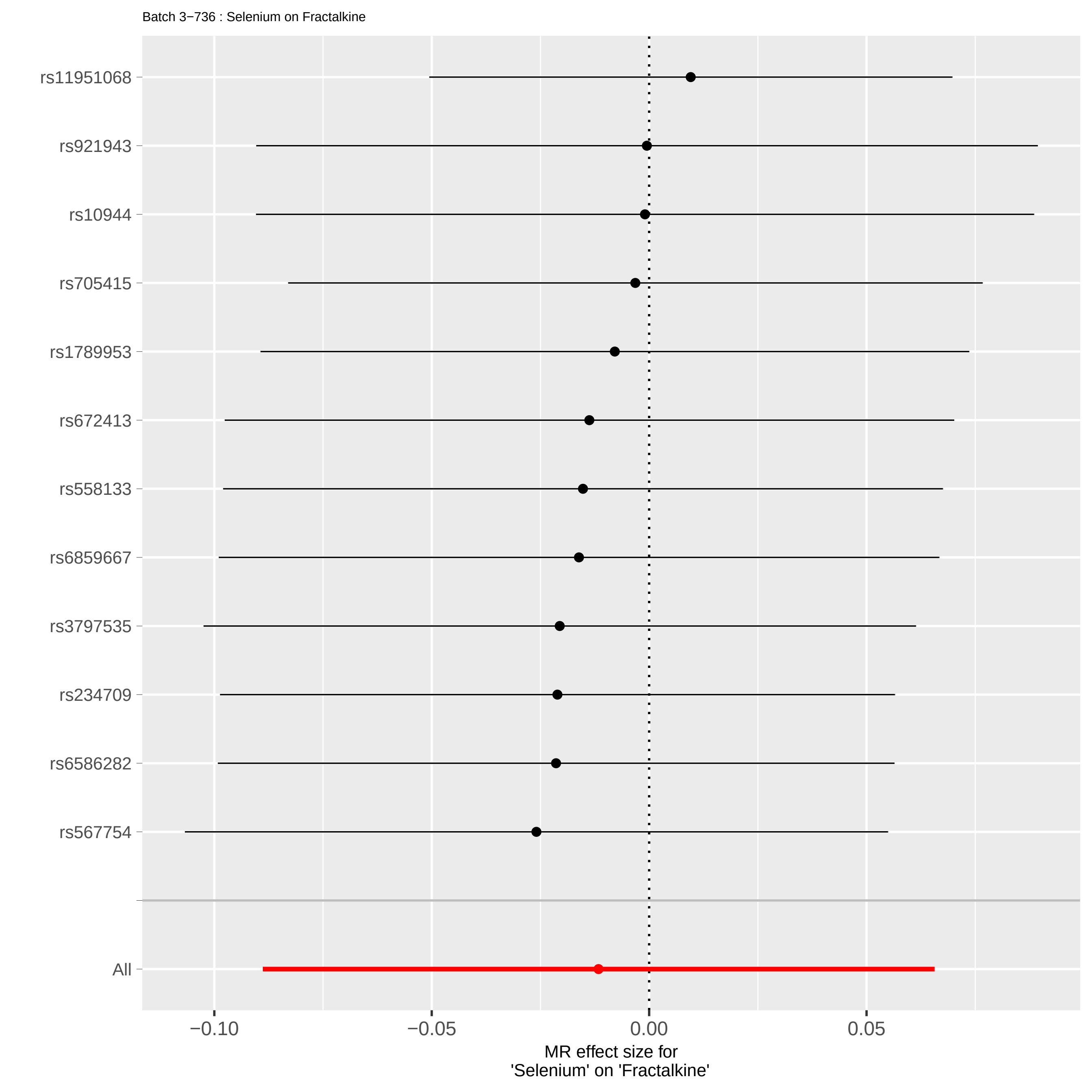
Batch 3−736 : Selenium on Fractalkine
rs11951068
rs921943
rs10944
rs705415
rs1789953
rs672413
rs558133
rs6859667
rs3797535
rs234709
rs6586282
rs567754
All
−0.10
−0.05
0.00
0.05
MR effect size for
'Selenium' on 'Fractalkine'

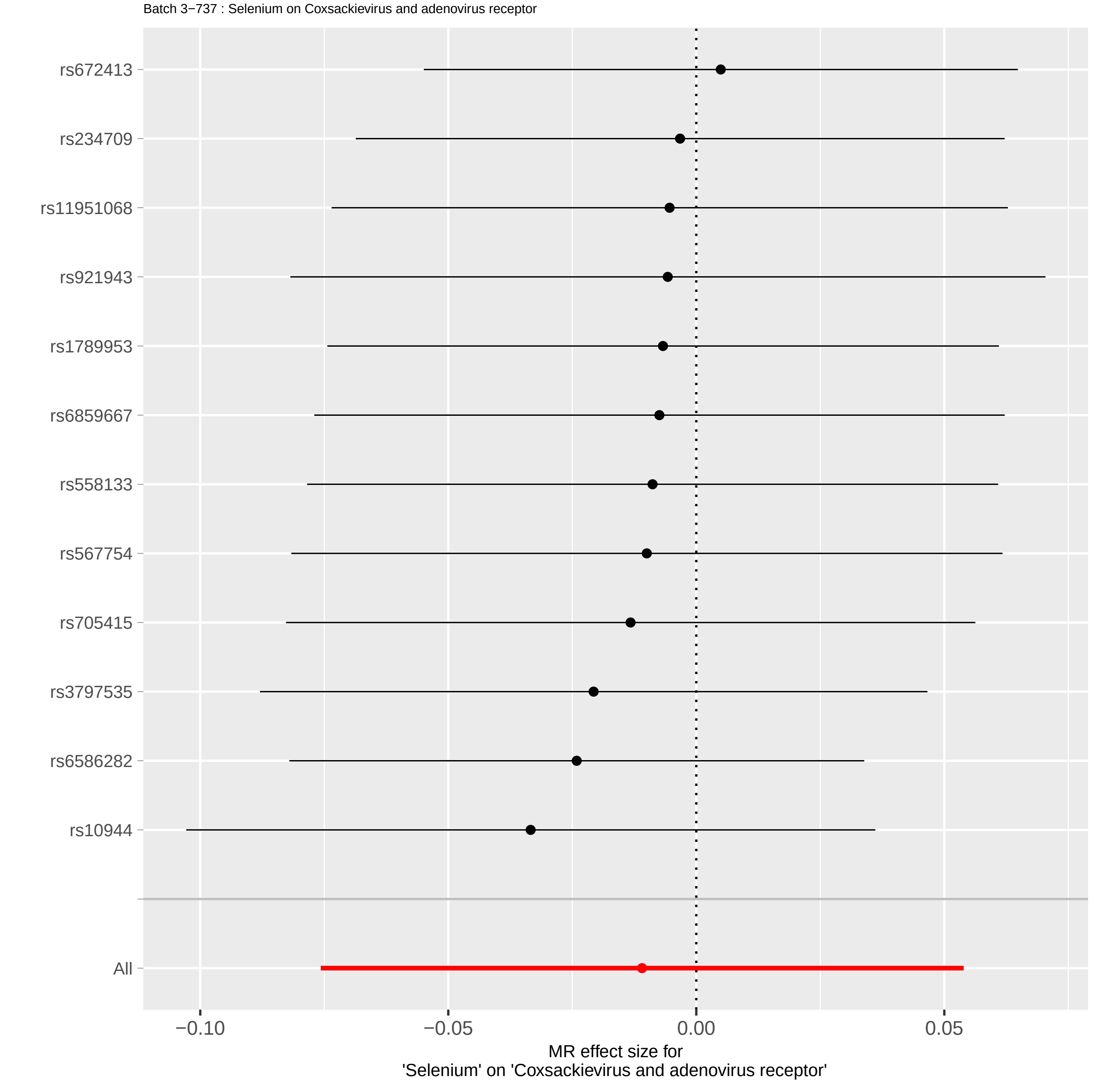

Batch 3−737 : Selenium on Coxsackievirus and adenovirus receptor
rs672413
rs234709
rs11951068
rs921943
rs1789953
rs6859667
rs558133
rs567754
rs705415
rs3797535
rs6586282
rs10944
All
−0.10
−0.05
0.00
0.05
MR effect size for
'Selenium' on 'Coxsackievirus and adenovirus receptor'

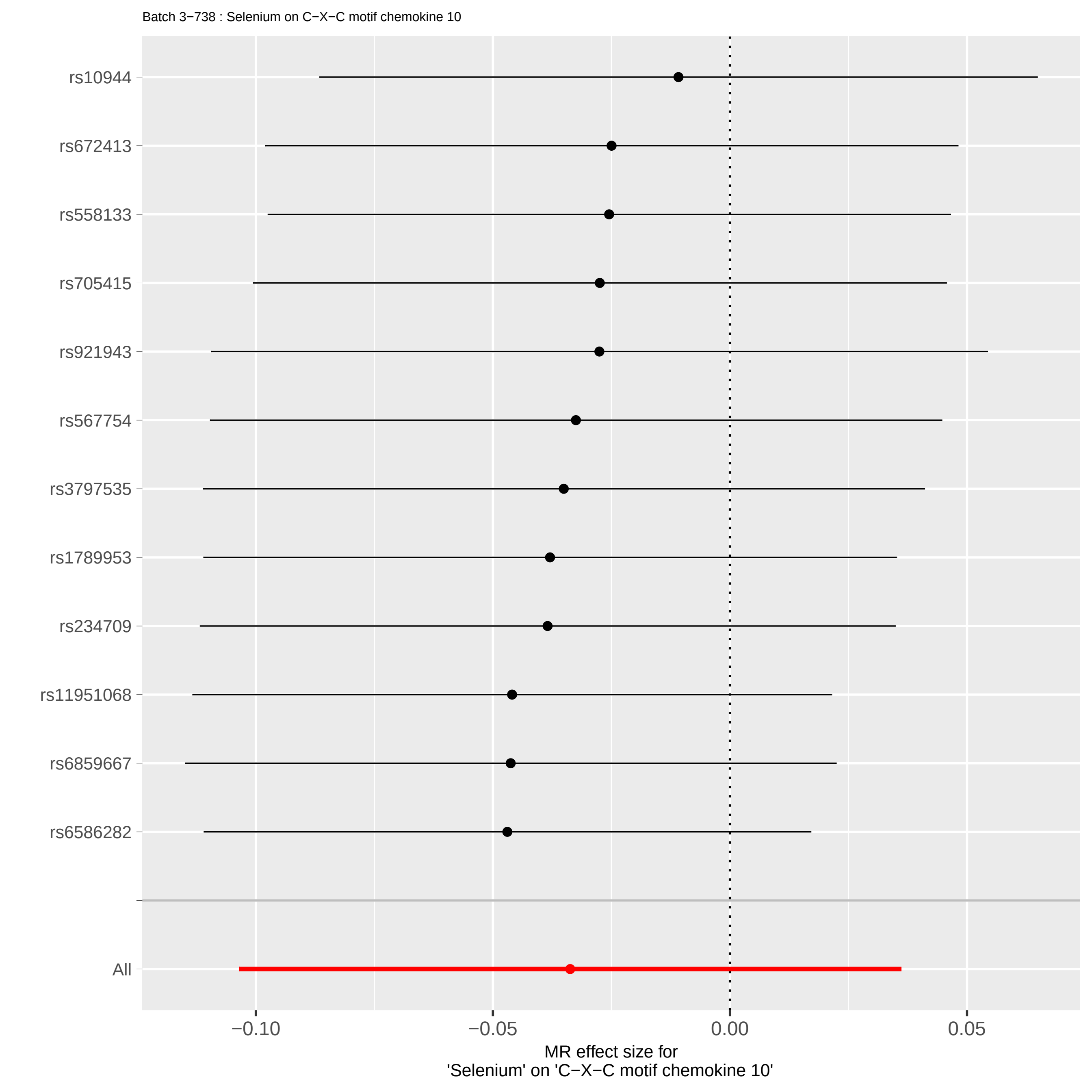

Batch 3−738 : Selenium on C−X−C motif chemokine 10
rs10944
rs672413
rs558133
rs705415
rs921943
rs567754
rs3797535
rs1789953
rs234709
rs11951068
rs6859667
rs6586282
All
−0.10
−0.05
0.00
0.05
MR effect size for
'Selenium' on 'C−X−C motif chemokine 10'

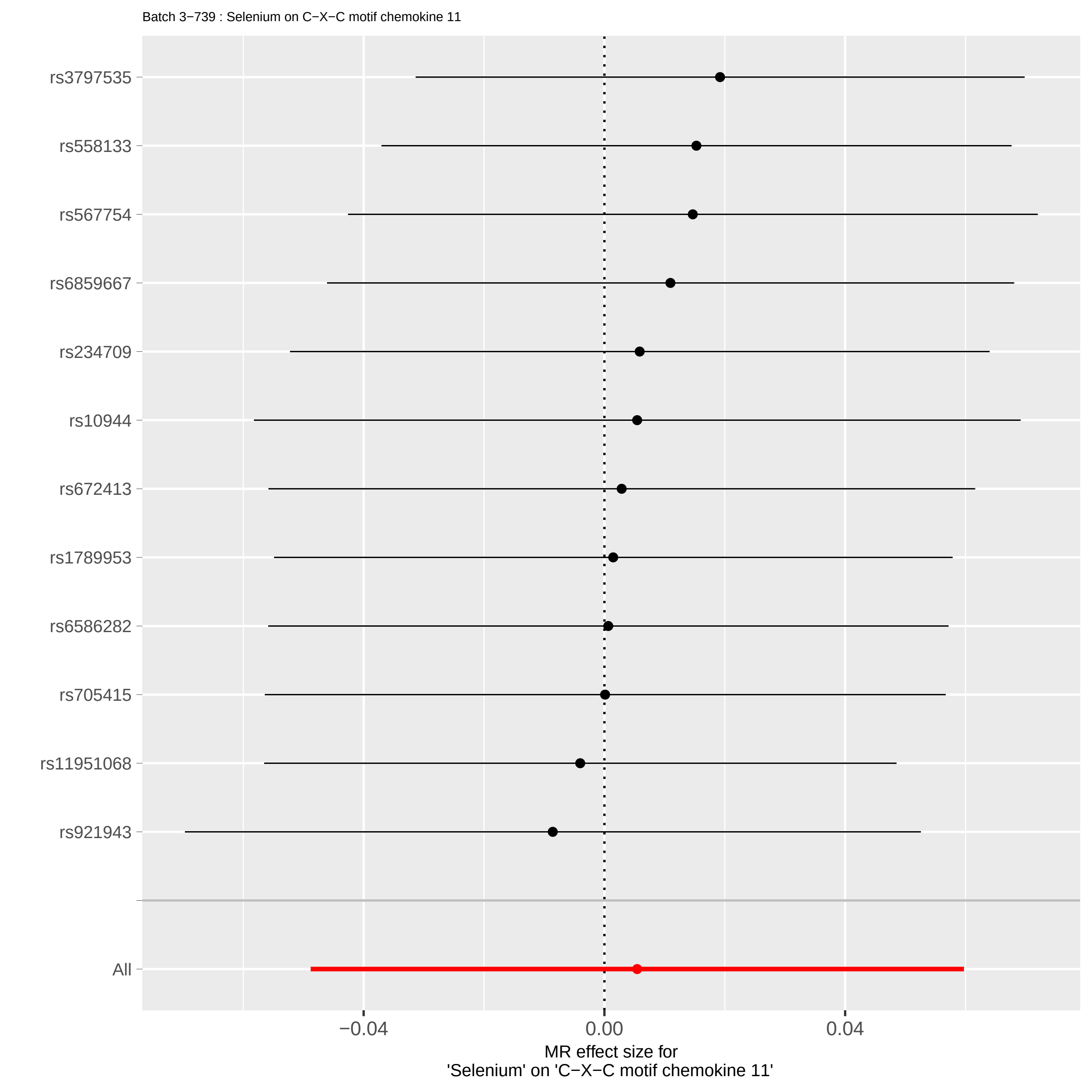

Batch 3−739 : Selenium on C−X−C motif chemokine 11
rs3797535
rs558133
rs567754
rs6859667
rs234709
rs10944
rs672413
rs1789953
rs6586282
rs705415
rs11951068
rs921943
All
−0.04
0.00
0.04
MR effect size for
'Selenium' on 'C−X−C motif chemokine 11'

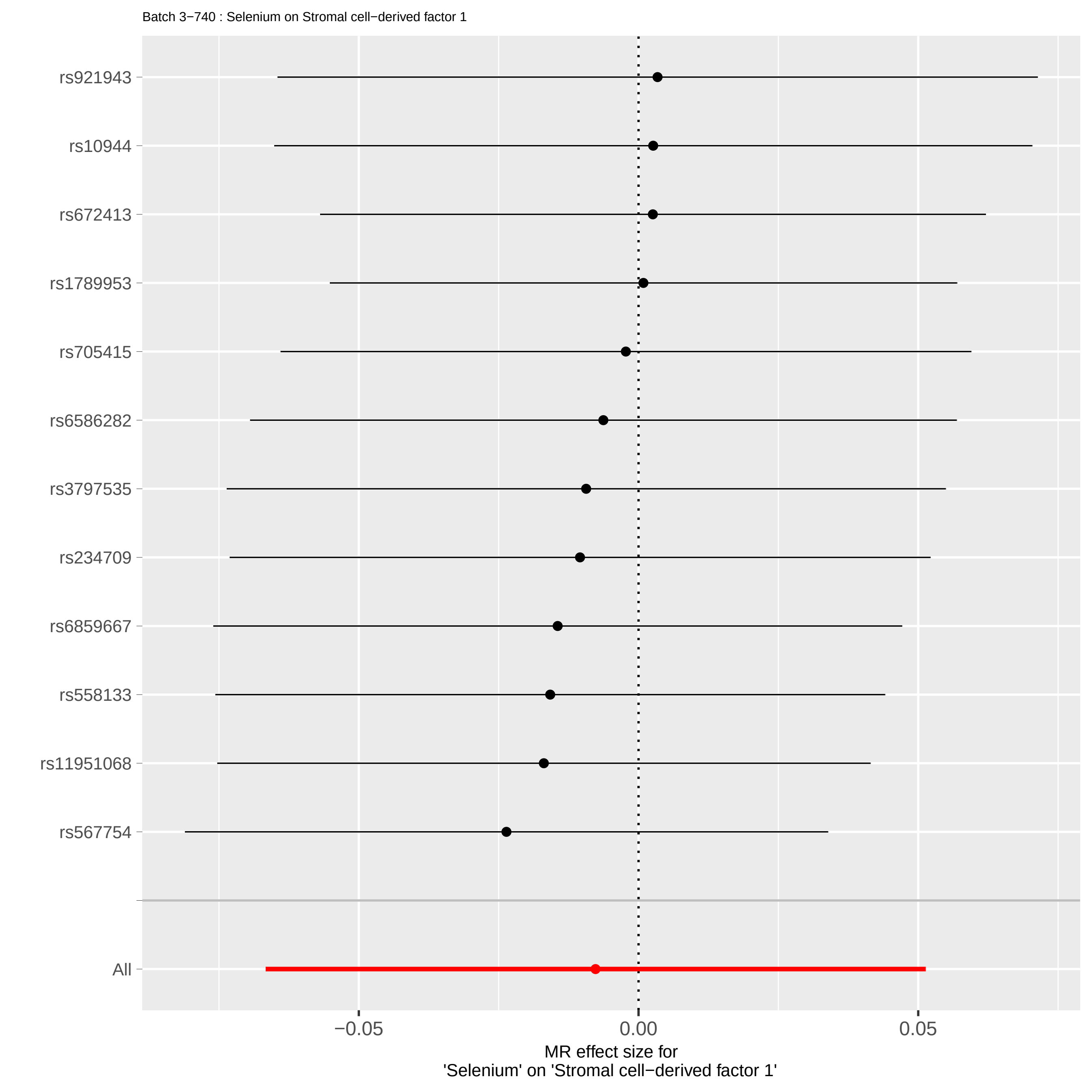

Batch 3−740 : Selenium on Stromal cell−derived factor 1
rs921943
rs10944
rs672413
rs1789953
rs705415
rs6586282
rs3797535
rs234709
rs6859667
rs558133
rs11951068
rs567754
All
−0.05
0.00
0.05
MR effect size for
'Selenium' on 'Stromal cell−derived factor 1'

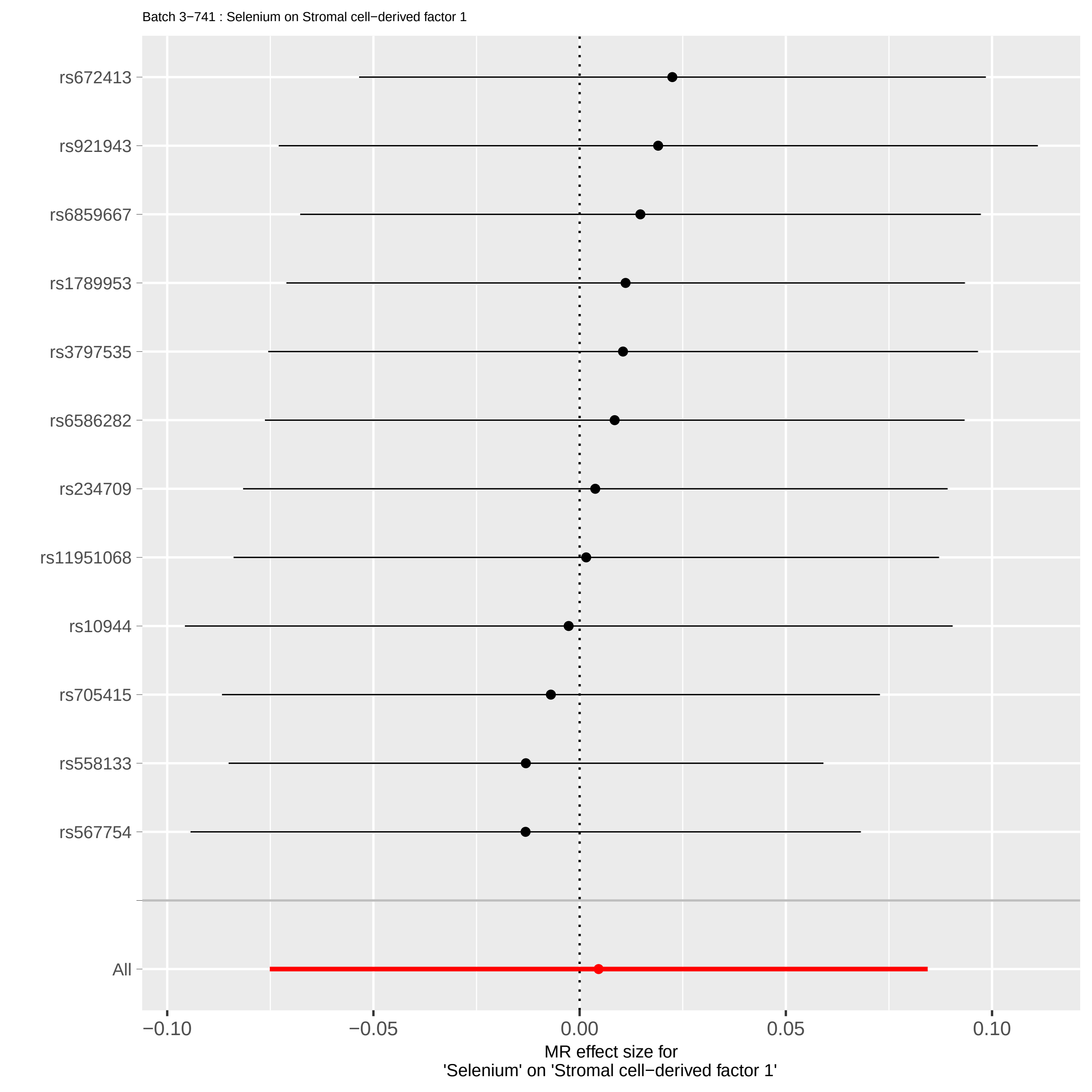

Batch 3−741 : Selenium on Stromal cell−derived factor 1
rs672413
rs921943
rs6859667
rs1789953
rs3797535
rs6586282
rs234709
rs11951068
rs10944
rs705415
rs558133
rs567754
All
−0.10
−0.05
0.00
0.05
0.10
MR effect size for
'Selenium' on 'Stromal cell−derived factor 1'

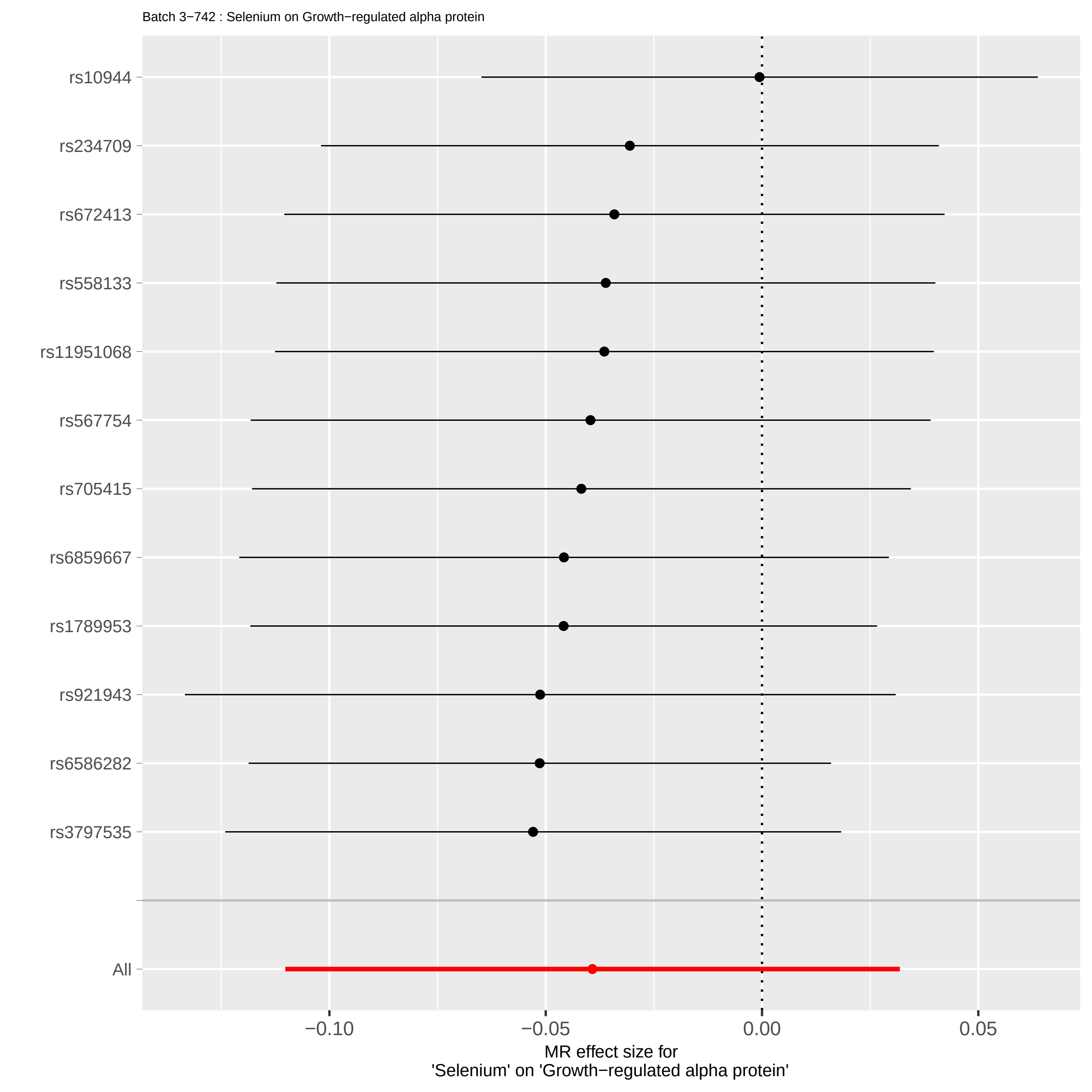

Batch 3−742 : Selenium on Growth−regulated alpha protein
rs10944
rs234709
rs672413
rs558133
rs11951068
rs567754
rs705415
rs6859667
rs1789953
rs921943
rs6586282
rs3797535
All
−0.10
−0.05
0.00
0.05
MR effect size for
'Selenium' on 'Growth−regulated alpha protein'

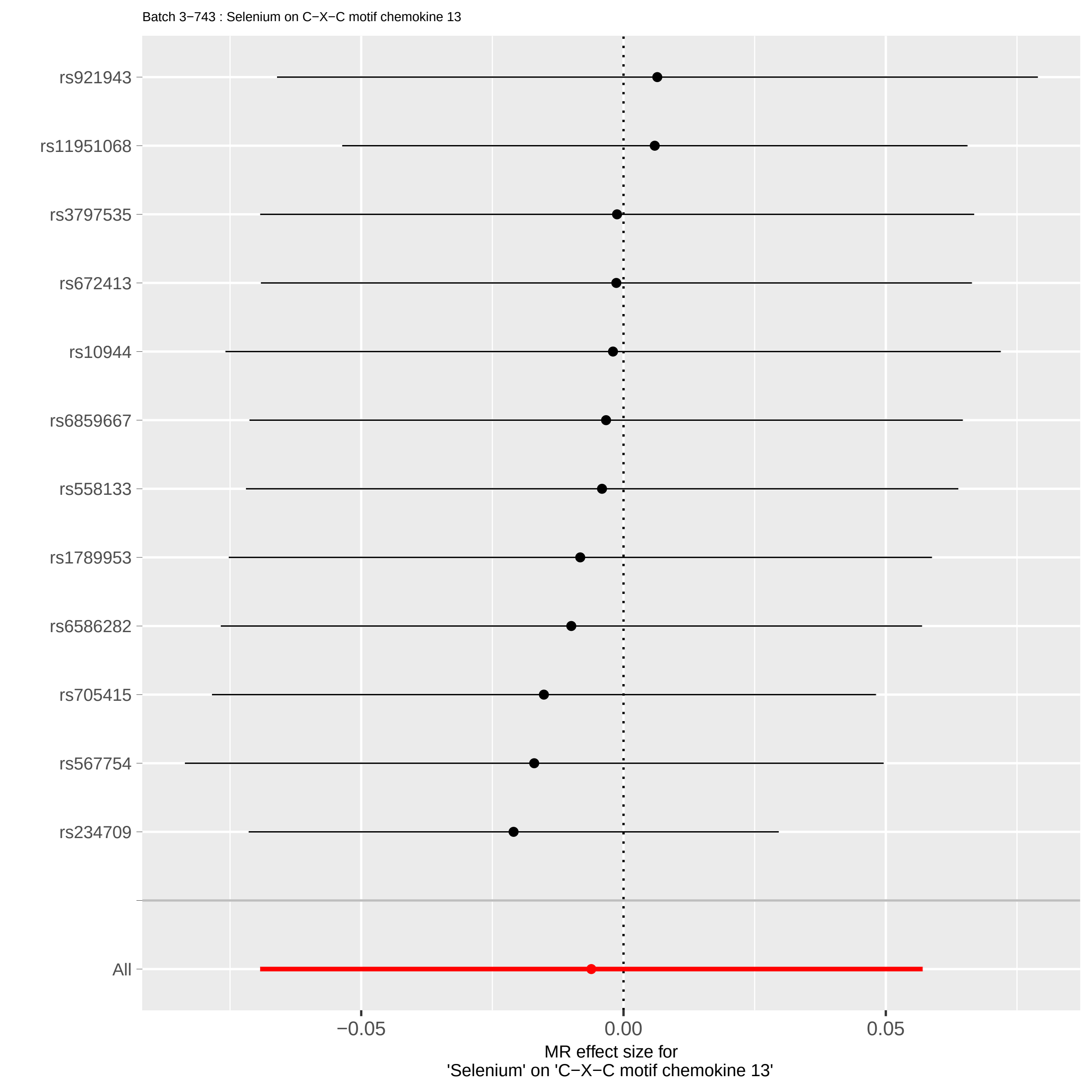

Batch 3−743 : Selenium on C−X−C motif chemokine 13
rs921943
rs11951068
rs3797535
rs672413
rs10944
rs6859667
rs558133
rs1789953
rs6586282
rs705415
rs567754
rs234709
All
−0.05
0.00
0.05
MR effect size for
'Selenium' on 'C−X−C motif chemokine 13'

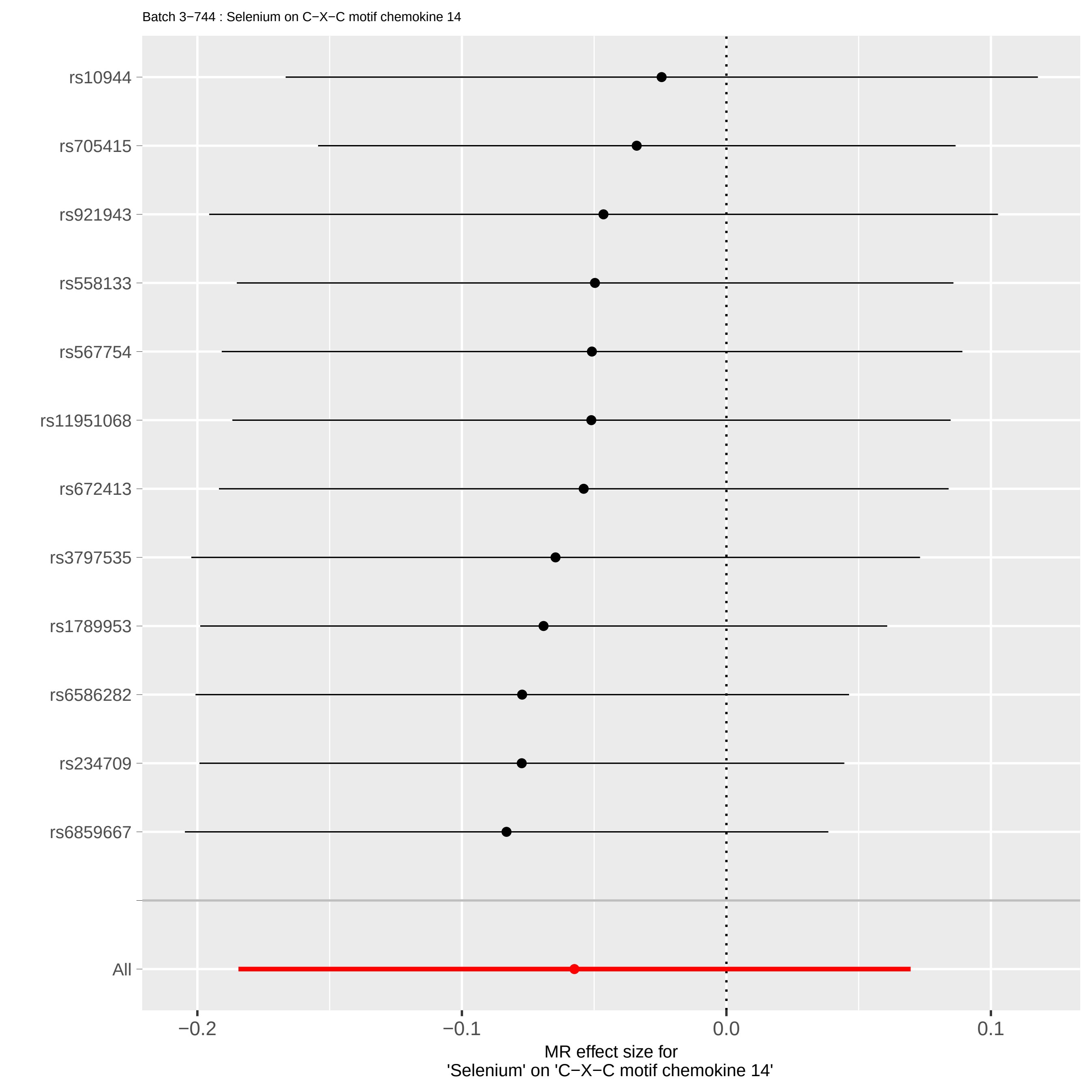

Batch 3−744 : Selenium on C−X−C motif chemokine 14
rs10944
rs705415
rs921943
rs558133
rs567754
rs11951068
rs672413
rs3797535
rs1789953
rs6586282
rs234709
rs6859667
All
−0.2
−0.1
0.0
0.1
MR effect size for
'Selenium' on 'C−X−C motif chemokine 14'

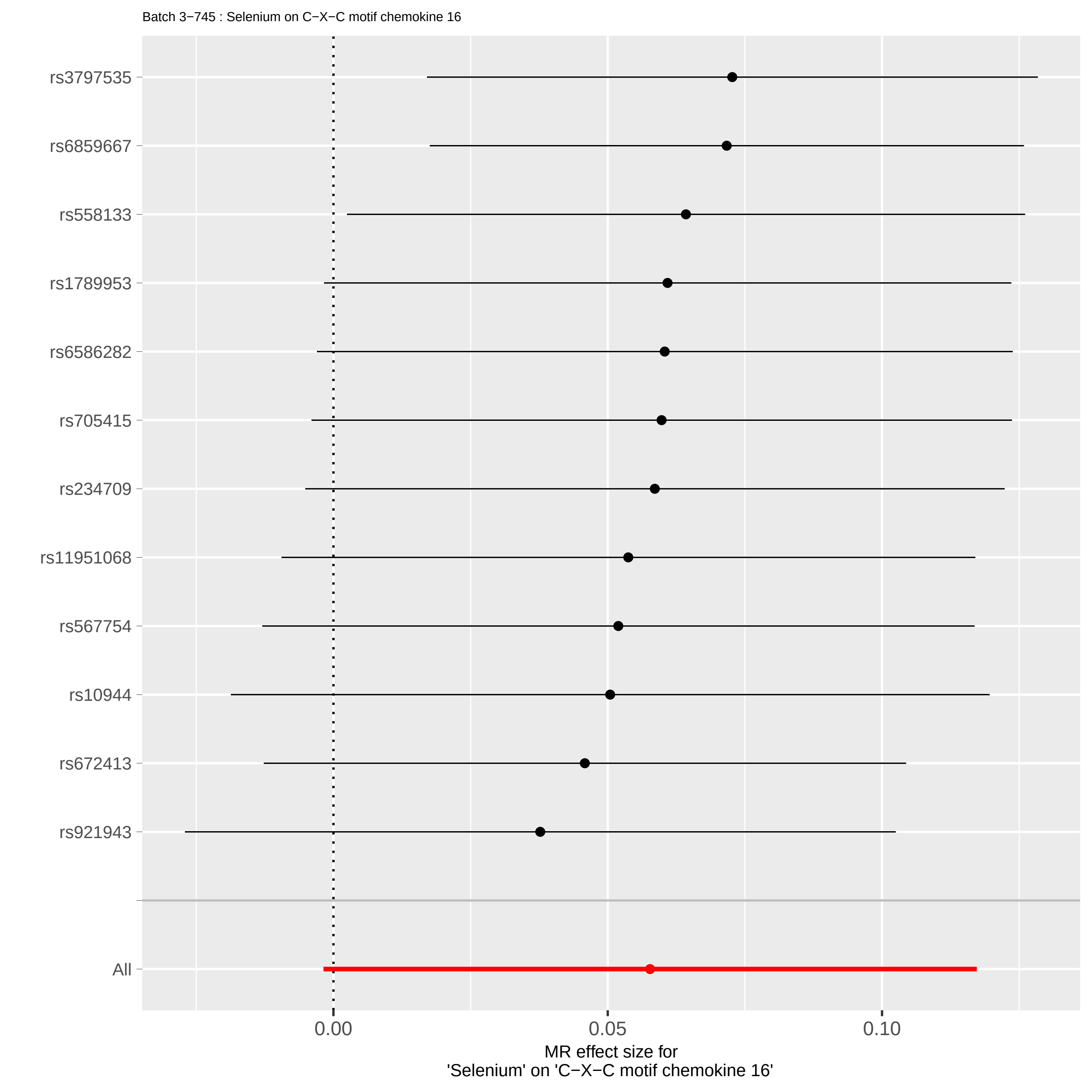

Batch 3−745 : Selenium on C−X−C motif chemokine 16
rs3797535
rs6859667
rs558133
rs1789953
rs6586282
rs705415
rs234709
rs11951068
rs567754
rs10944
rs672413
rs921943
All
0.00
0.05
0.10
MR effect size for
'Selenium' on 'C−X−C motif chemokine 16'

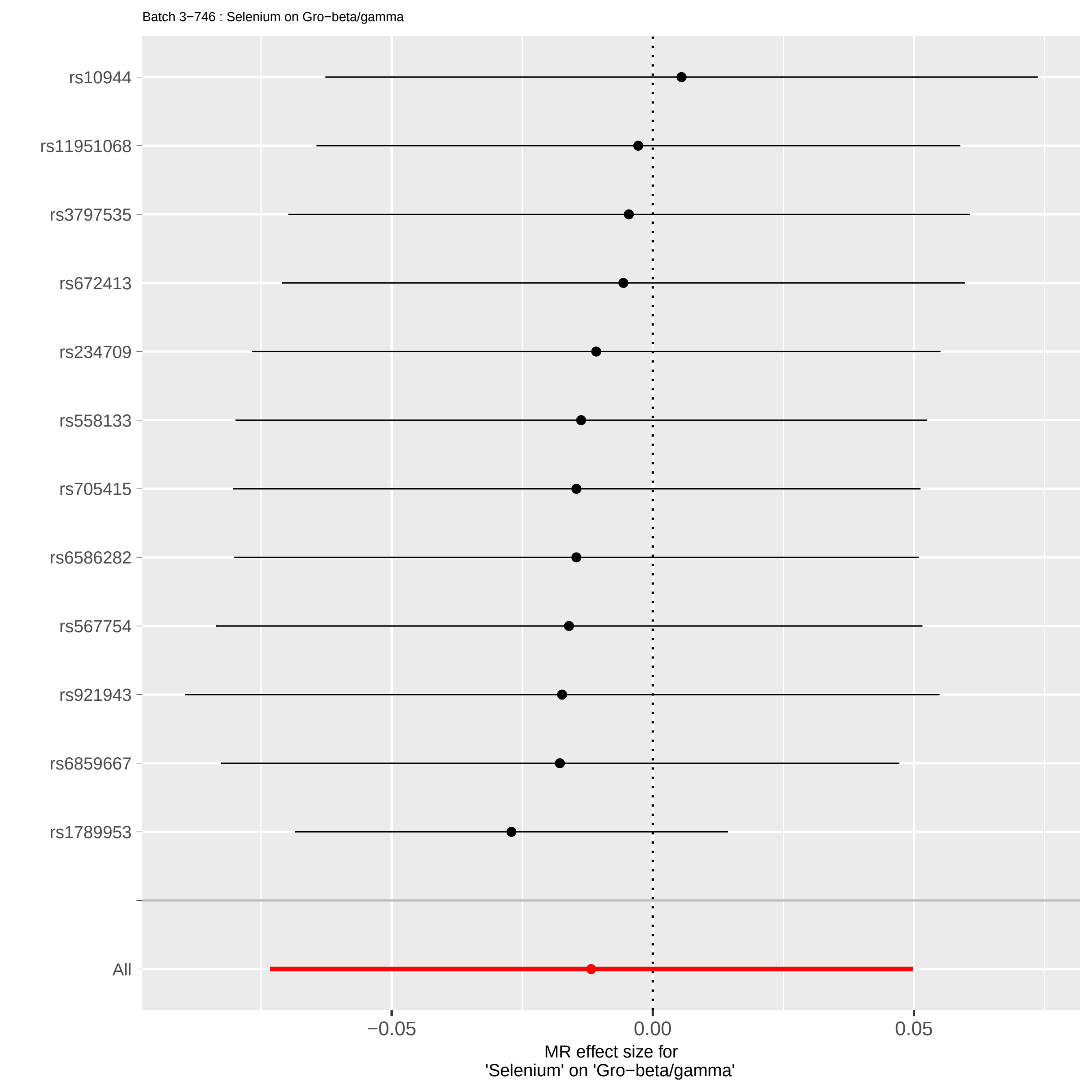

Batch 3−746 : Selenium on Gro−beta/gamma
rs10944
rs11951068
rs3797535
rs672413
rs234709
rs558133
rs705415
rs6586282
rs567754
rs921943
rs6859667
rs1789953
All
−0.05
0.00
0.05
MR effect size for
'Selenium' on 'Gro−beta/gamma'

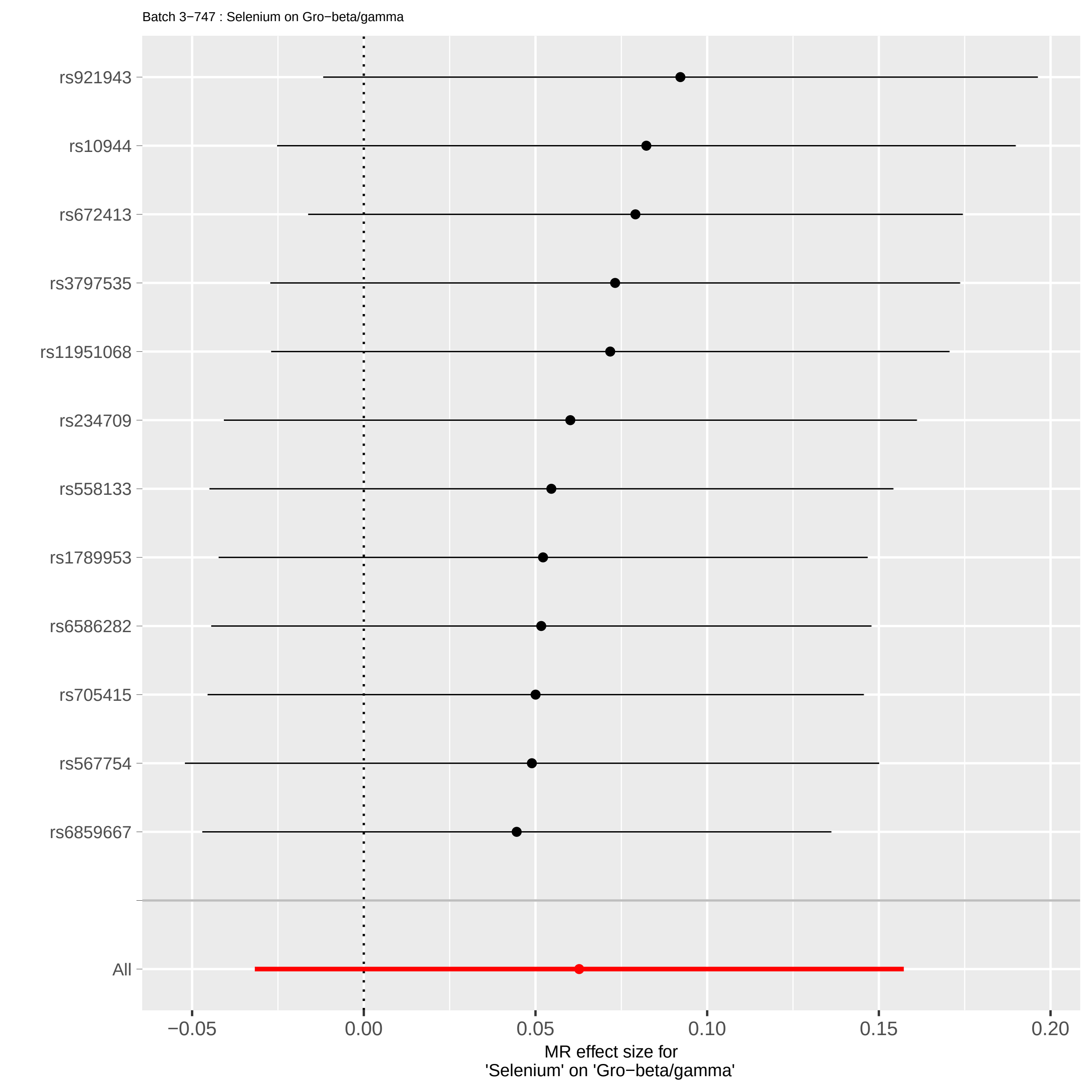

Batch 3−747 : Selenium on Gro−beta/gamma
rs921943
rs10944
rs672413
rs3797535
rs11951068
rs234709
rs558133
rs1789953
rs6586282
rs705415
rs567754
rs6859667
All
−0.05
0.00
0.05
0.10
0.15
0.20
MR effect size for
'Selenium' on 'Gro−beta/gamma'

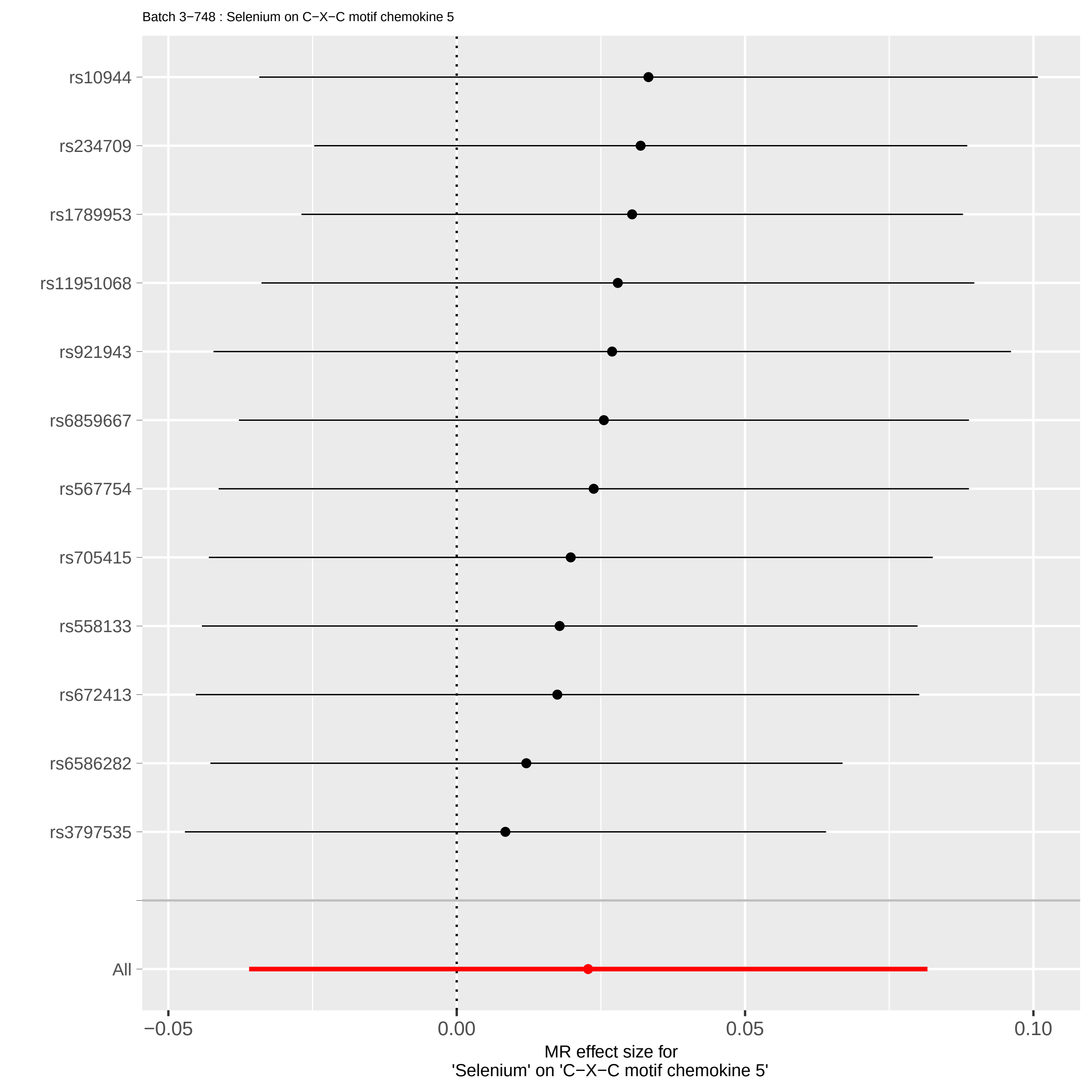
Batch 3−748 : Selenium on C−X−C motif chemokine 5
rs10944
rs234709
rs1789953
rs11951068
rs921943
rs6859667
rs567754
rs705415
rs558133
rs672413
rs6586282
rs3797535
All
−0.05
0.00
0.05
0.10
MR effect size for
'Selenium' on 'C−X−C motif chemokine 5'

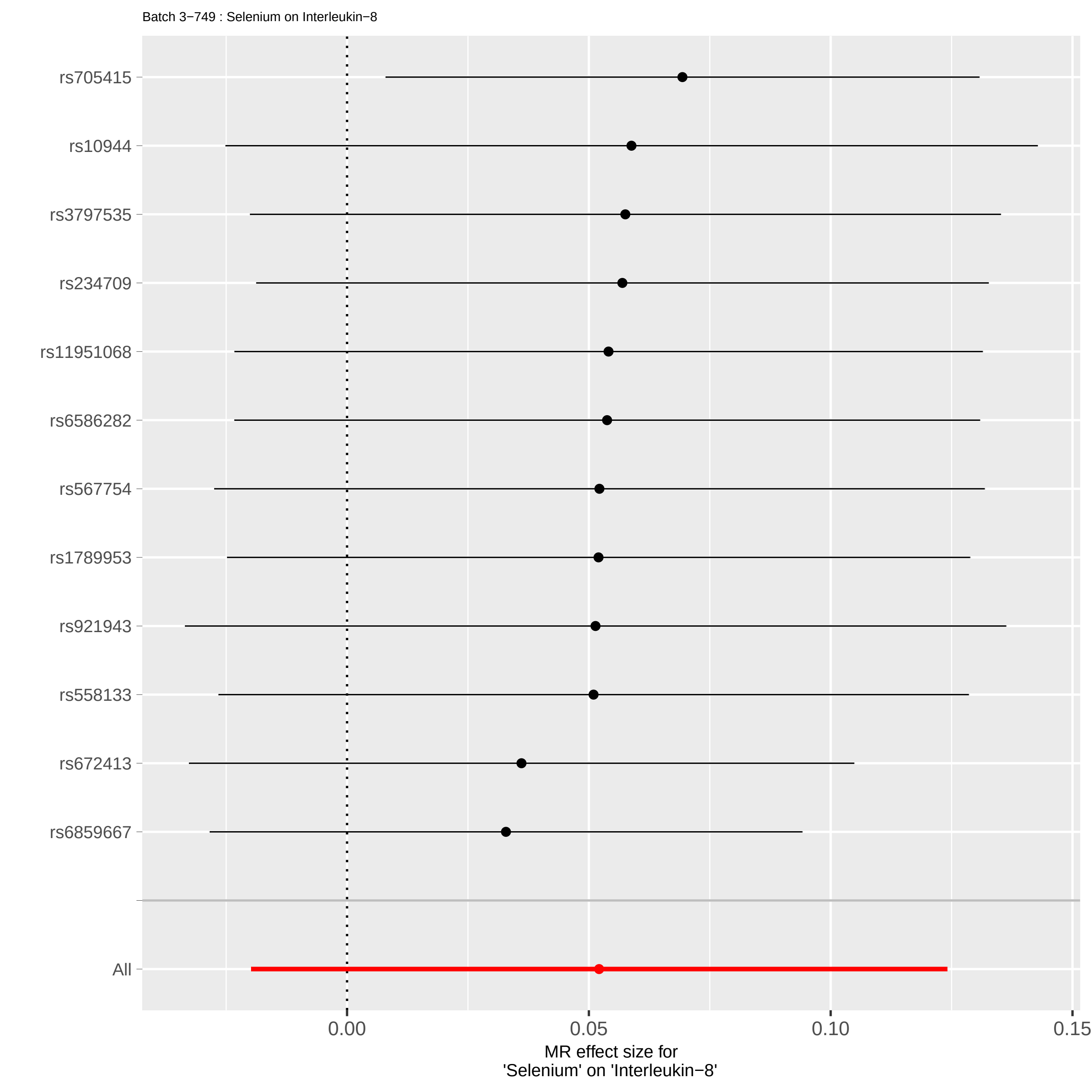

Batch 3−749 : Selenium on Interleukin−8
rs705415
rs10944
rs3797535
rs234709
rs11951068
rs6586282
rs567754
rs1789953
rs921943
rs558133
rs672413
rs6859667
All
0.00
0.05
0.10
0.15
MR effect size for
'Selenium' on 'Interleukin−8'

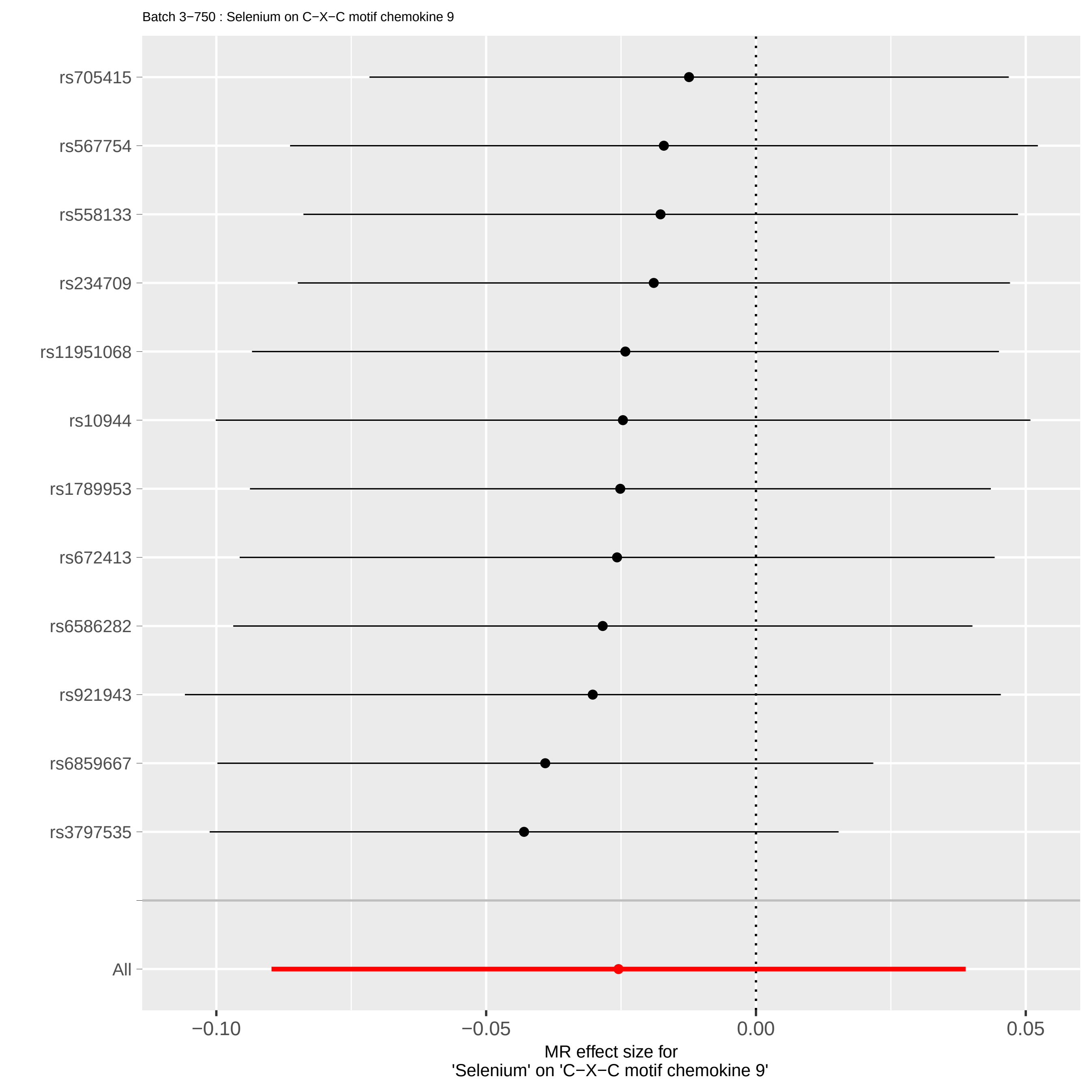

Batch 3−750 : Selenium on C−X−C motif chemokine 9
rs705415
rs567754
rs558133
rs234709
rs11951068
rs10944
rs1789953
rs672413
rs6586282
rs921943
rs6859667
rs3797535
All
−0.10
−0.05
0.00
0.05
MR effect size for
'Selenium' on 'C−X−C motif chemokine 9'

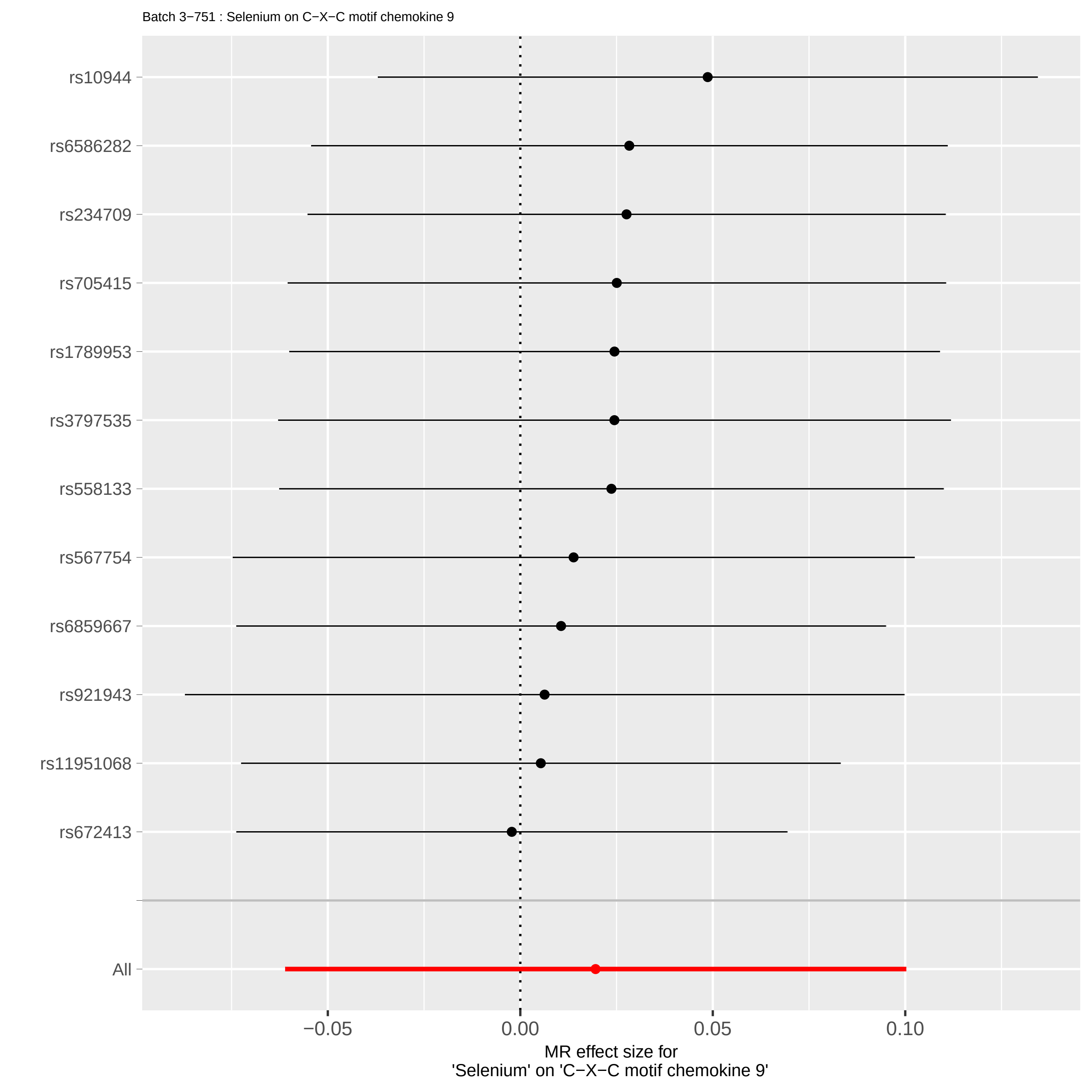

Batch 3−751 : Selenium on C−X−C motif chemokine 9
rs10944
rs6586282
rs234709
rs705415
rs1789953
rs3797535
rs558133
rs567754
rs6859667
rs921943
rs11951068
rs672413
All
−0.05
0.00
0.05
0.10
MR effect size for
'Selenium' on 'C−X−C motif chemokine 9'

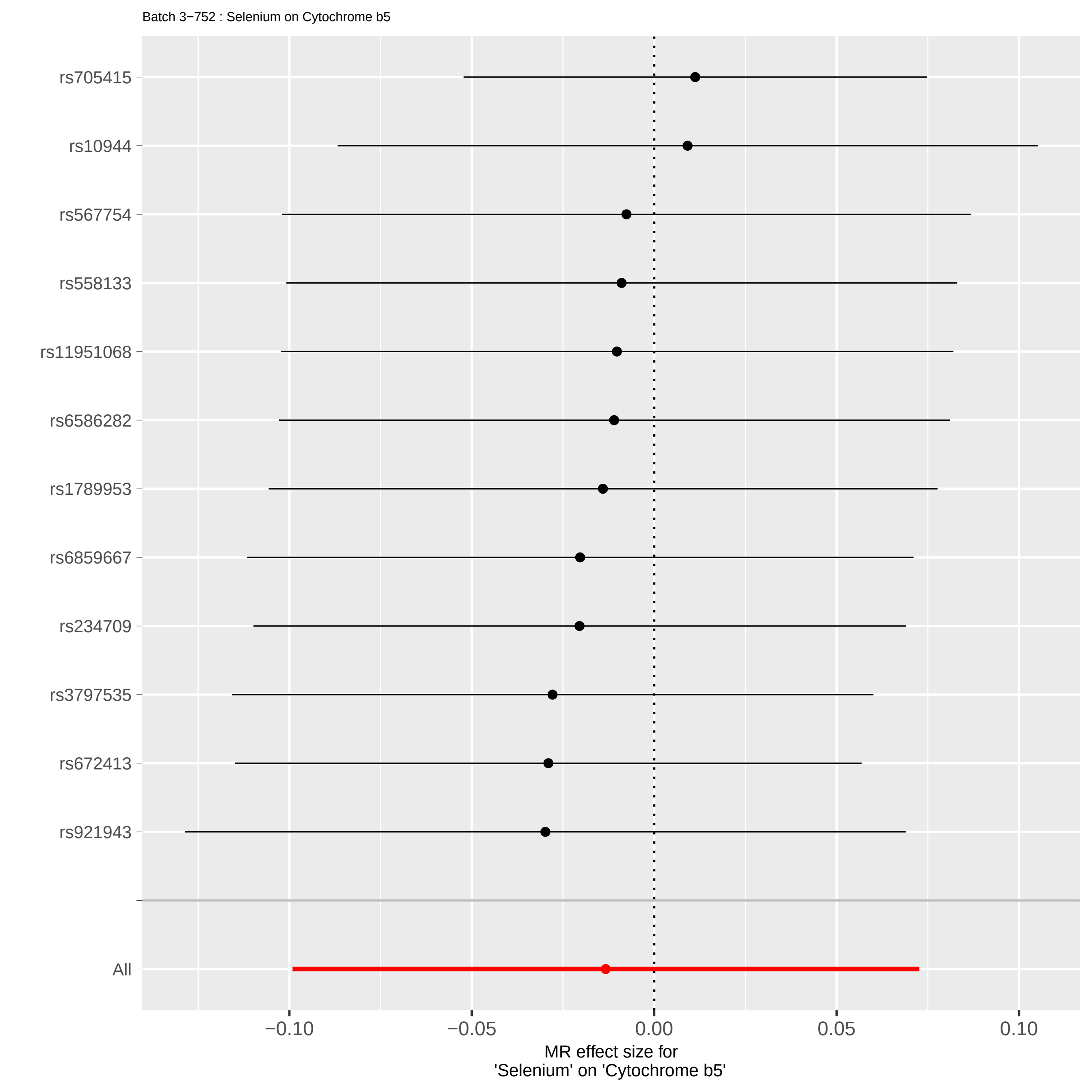
Batch 3−752 : Selenium on Cytochrome b5
rs705415
rs10944
rs567754
rs558133
rs11951068
rs6586282
rs1789953
rs6859667
rs234709
rs3797535
rs672413
rs921943
All
−0.10
−0.05
0.00
0.05
0.10
MR effect size for
'Selenium' on 'Cytochrome b5'

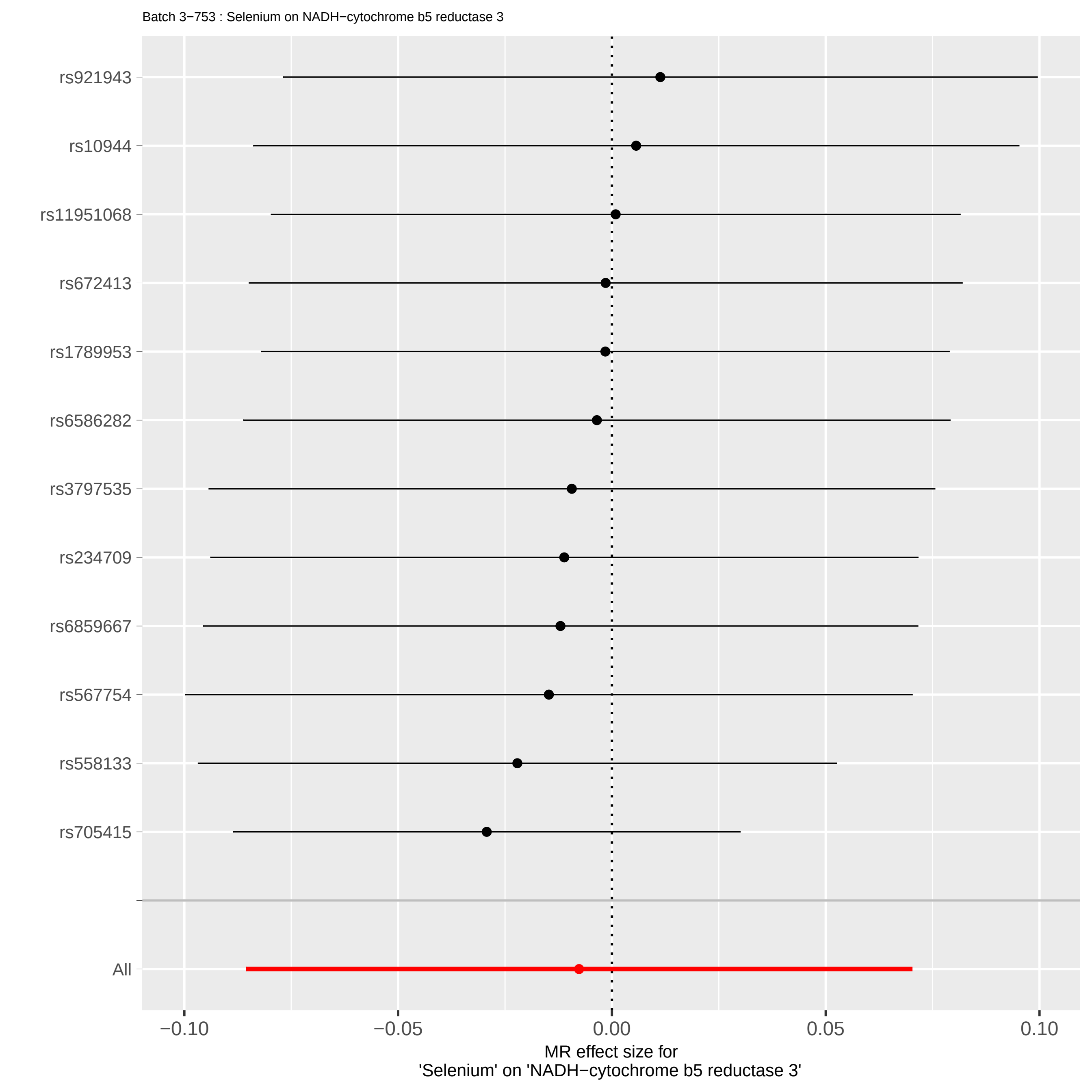

Batch 3−753 : Selenium on NADH−cytochrome b5 reductase 3
rs921943
rs10944
rs11951068
rs672413
rs1789953
rs6586282
rs3797535
rs234709
rs6859667
rs567754
rs558133
rs705415
All
−0.10
−0.05
0.00
0.05
0.10
MR effect size for
'Selenium' on 'NADH−cytochrome b5 reductase 3'

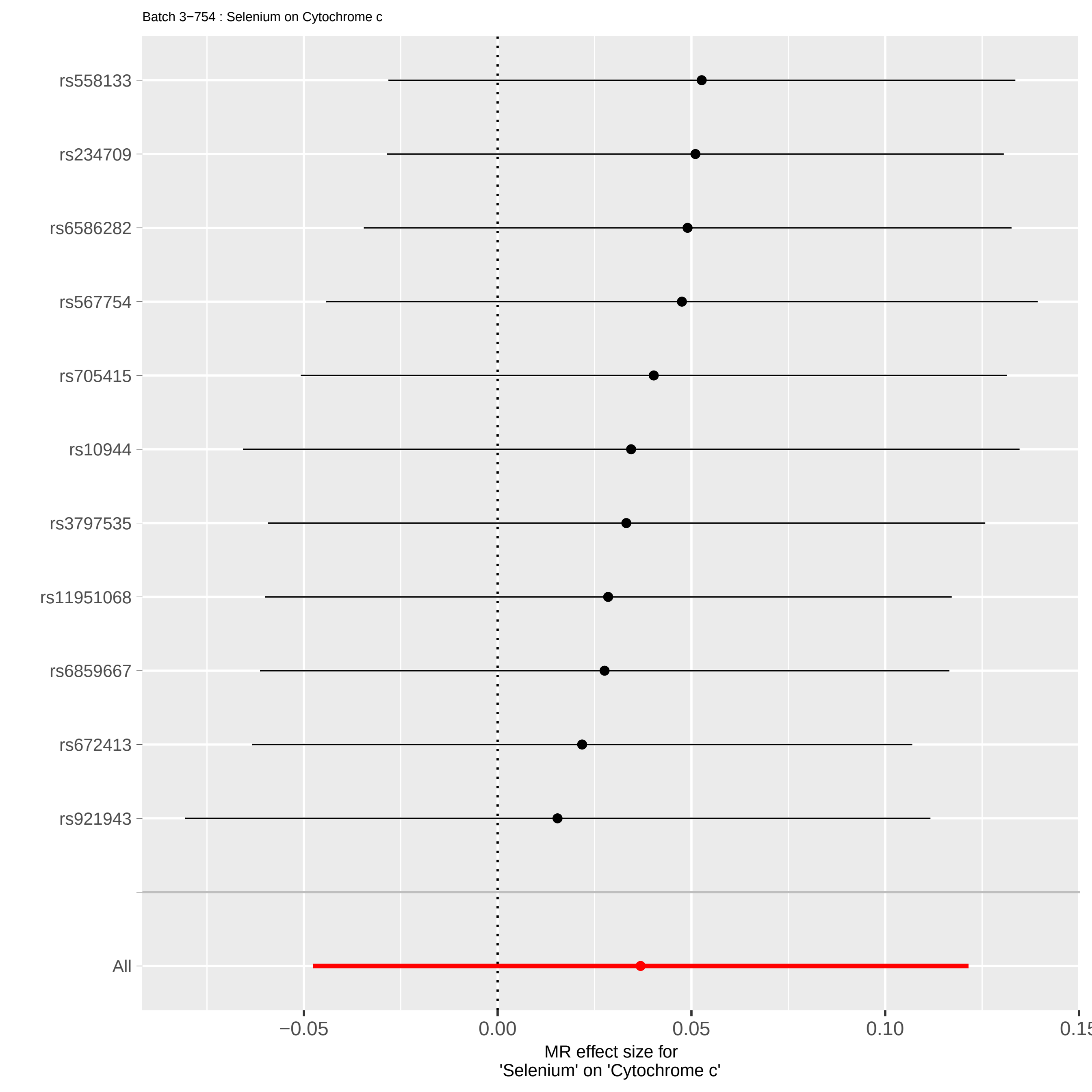

Batch 3−754 : Selenium on Cytochrome c
rs558133
rs234709
rs6586282
rs567754
rs705415
rs10944
rs3797535
rs11951068
rs6859667
rs672413
rs921943
All
−0.05
0.00
0.05
0.10
0.15
MR effect size for
'Selenium' on 'Cytochrome c'

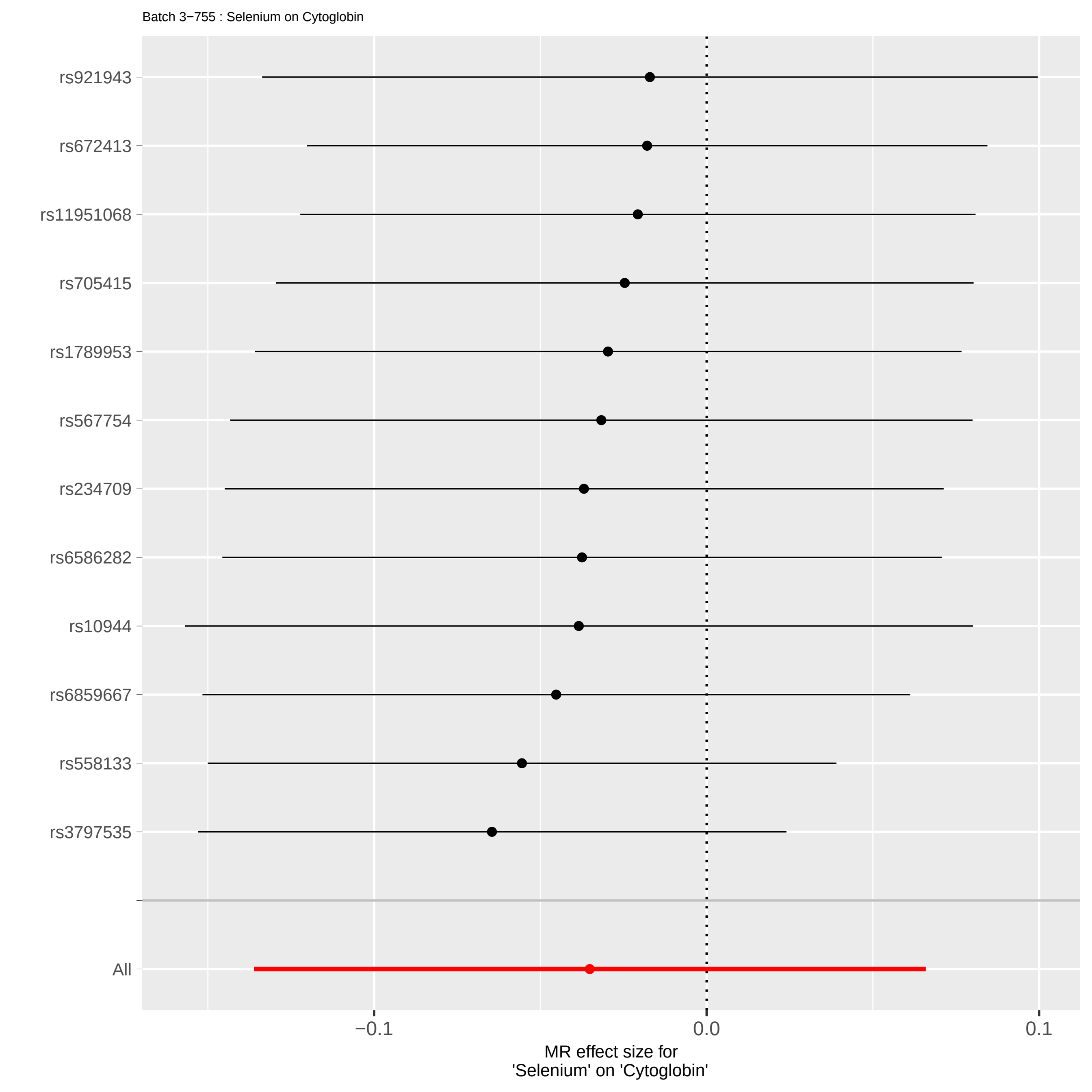
Batch 3−755 : Selenium on Cytoglobin
rs921943
rs672413
rs11951068
rs705415
rs1789953
rs567754
rs234709
rs6586282
rs10944
rs6859667
rs558133
rs3797535
All
−0.1
0.0
0.1
MR effect size for
'Selenium' on 'Cytoglobin'

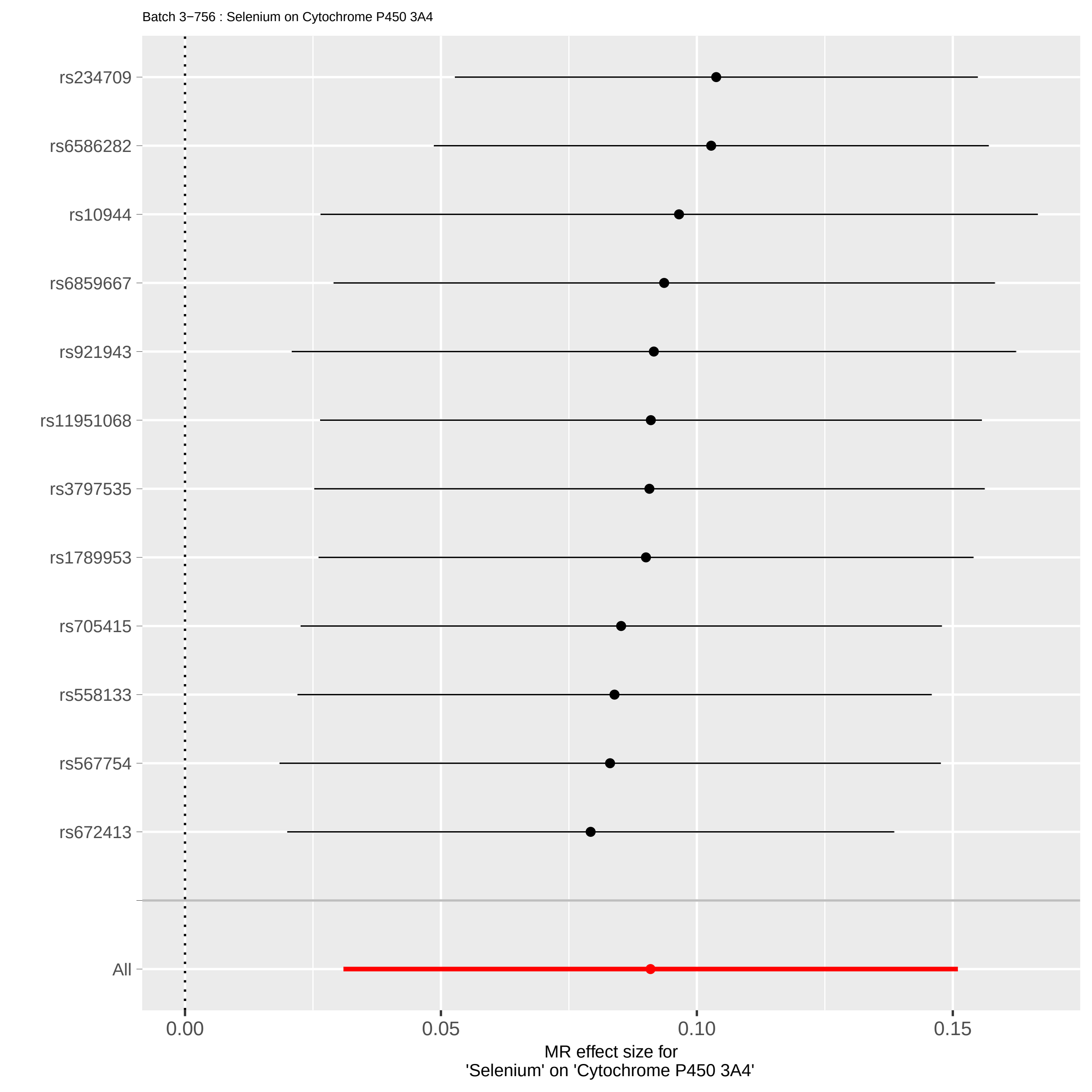

Batch 3−756 : Selenium on Cytochrome P450 3A4
rs234709
rs6586282
rs10944
rs6859667
rs921943
rs11951068
rs3797535
rs1789953
rs705415
rs558133
rs567754
rs672413
All
0.00
0.05
0.10
0.15
MR effect size for
'Selenium' on 'Cytochrome P450 3A4'

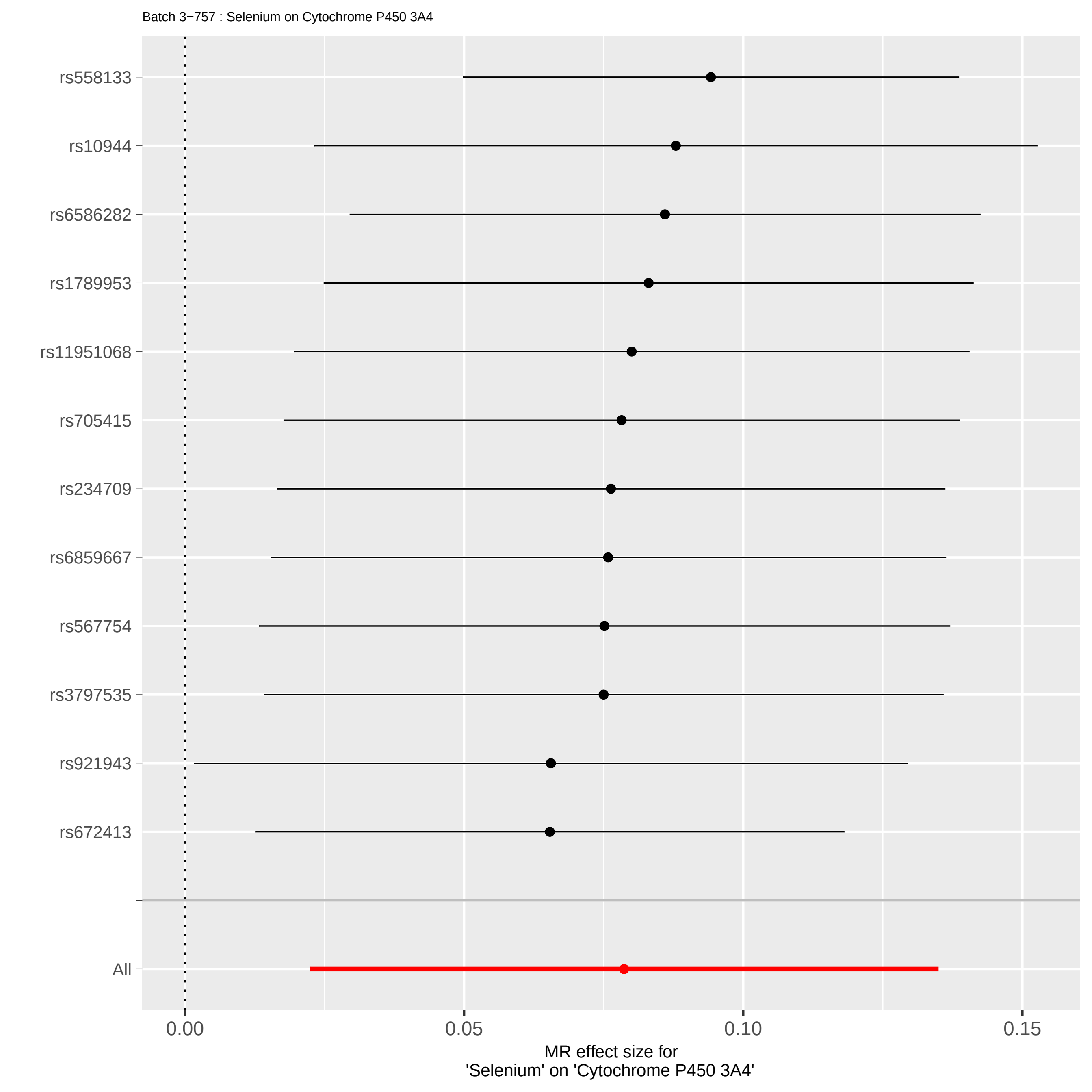

Batch 3−757 : Selenium on Cytochrome P450 3A4
rs558133
rs10944
rs6586282
rs1789953
rs11951068
rs705415
rs234709
rs6859667
rs567754
rs3797535
rs921943
rs672413
All
0.00
0.05
0.10
0.15
MR effect size for
'Selenium' on 'Cytochrome P450 3A4'

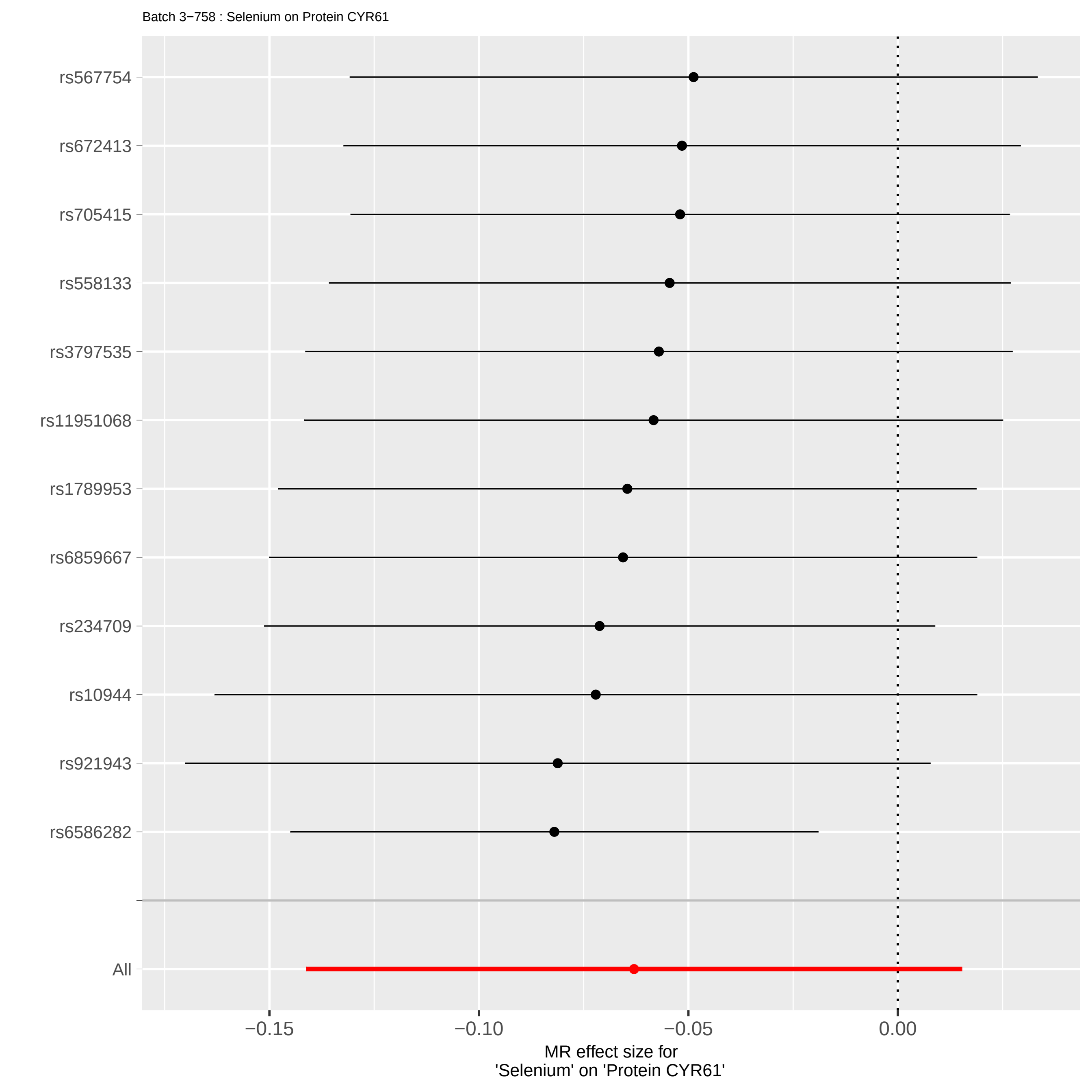

Batch 3−758 : Selenium on Protein CYR61
rs567754
rs672413
rs705415
rs558133
rs3797535
rs11951068
rs1789953
rs6859667
rs234709
rs10944
rs921943
rs6586282
All
−0.15
−0.10
−0.05
0.00
MR effect size for
'Selenium' on 'Protein CYR61'

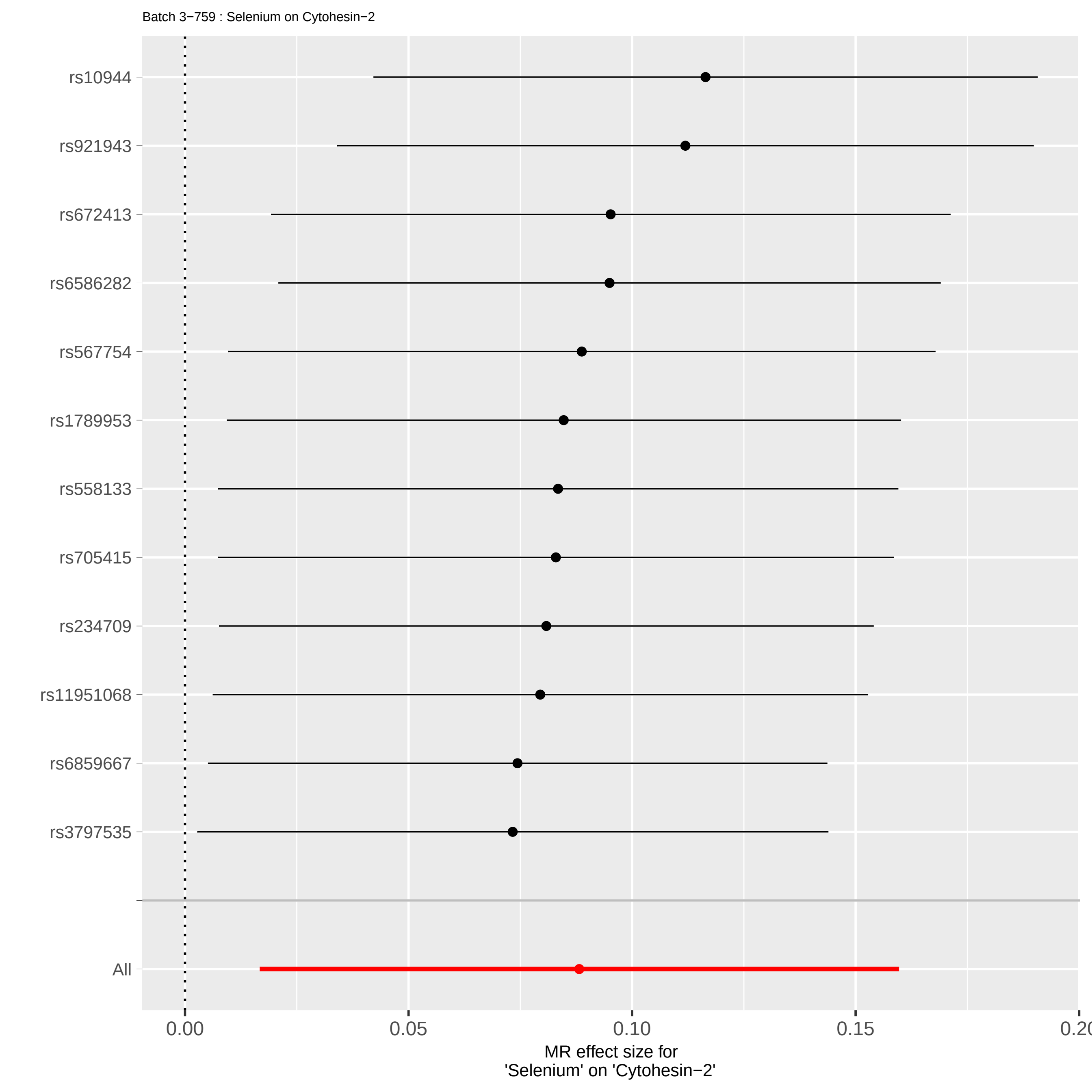

Batch 3−759 : Selenium on Cytohesin−2
rs10944
rs921943
rs672413
rs6586282
rs567754
rs1789953
rs558133
rs705415
rs234709
rs11951068
rs6859667
rs3797535
All
0.00
0.05
0.10
0.15
0.20
MR effect size for
'Selenium' on 'Cytohesin−2'

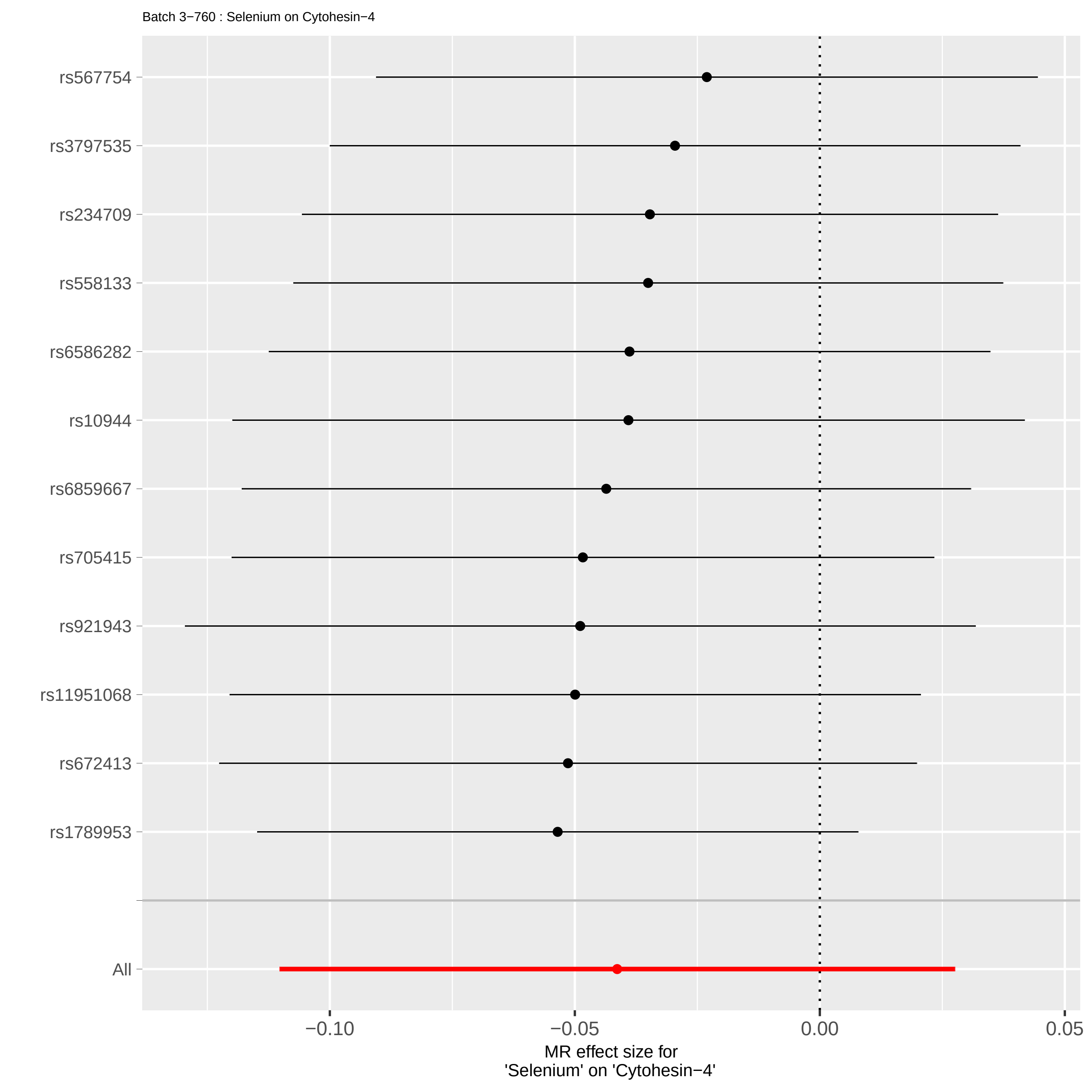
Batch 3−760 : Selenium on Cytohesin−4
rs567754
rs3797535
rs234709
rs558133
rs6586282
rs10944
rs6859667
rs705415
rs921943
rs11951068
rs672413
rs1789953
All
−0.10
−0.05
0.00
0.05
MR effect size for
'Selenium' on 'Cytohesin−4'

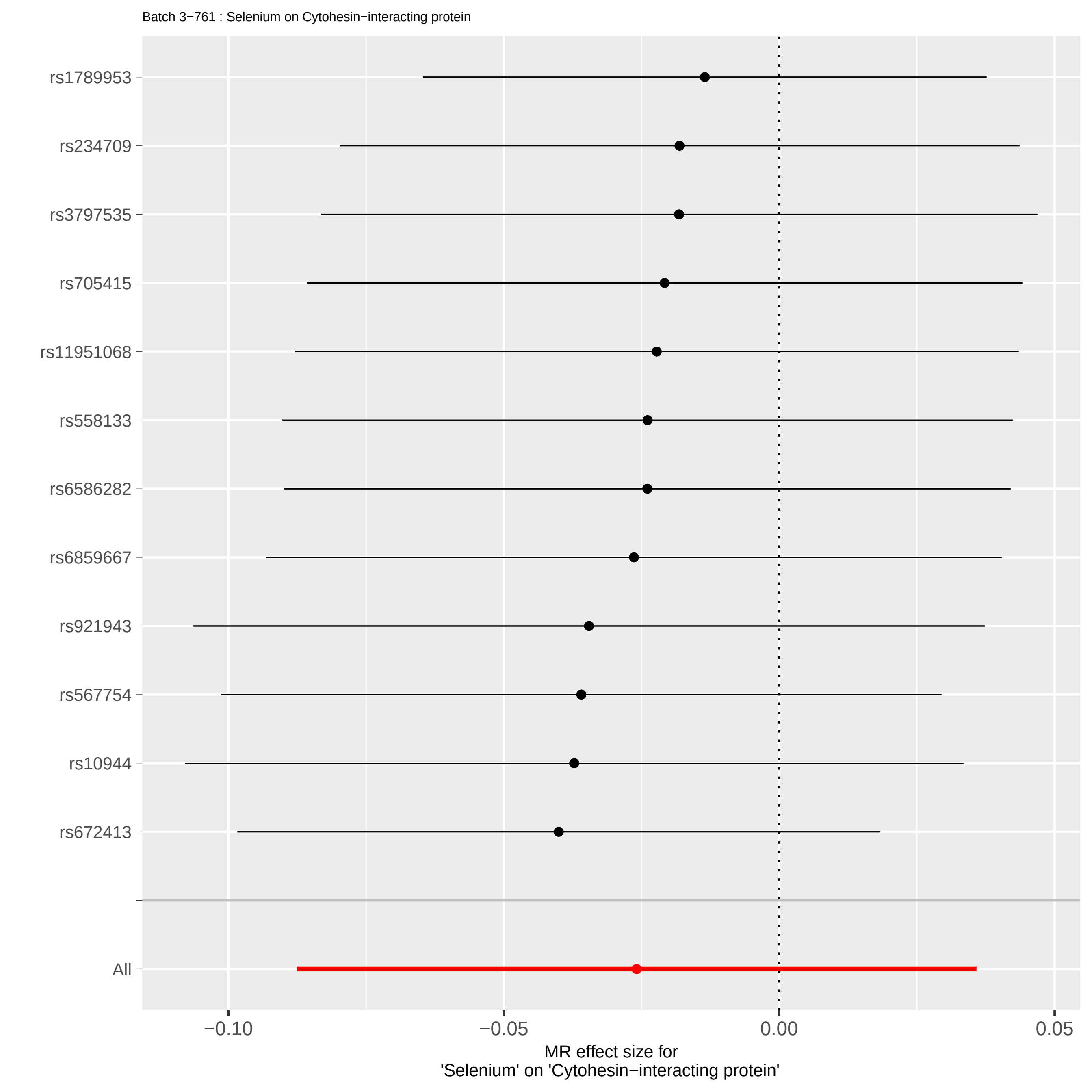

Batch 3−761 : Selenium on Cytohesin−interacting protein
rs1789953
rs234709
rs3797535
rs705415
rs11951068
rs558133
rs6586282
rs6859667
rs921943
rs567754
rs10944
rs672413
All
−0.10
−0.05
0.00
0.05
MR effect size for
'Selenium' on 'Cytohesin−interacting protein'

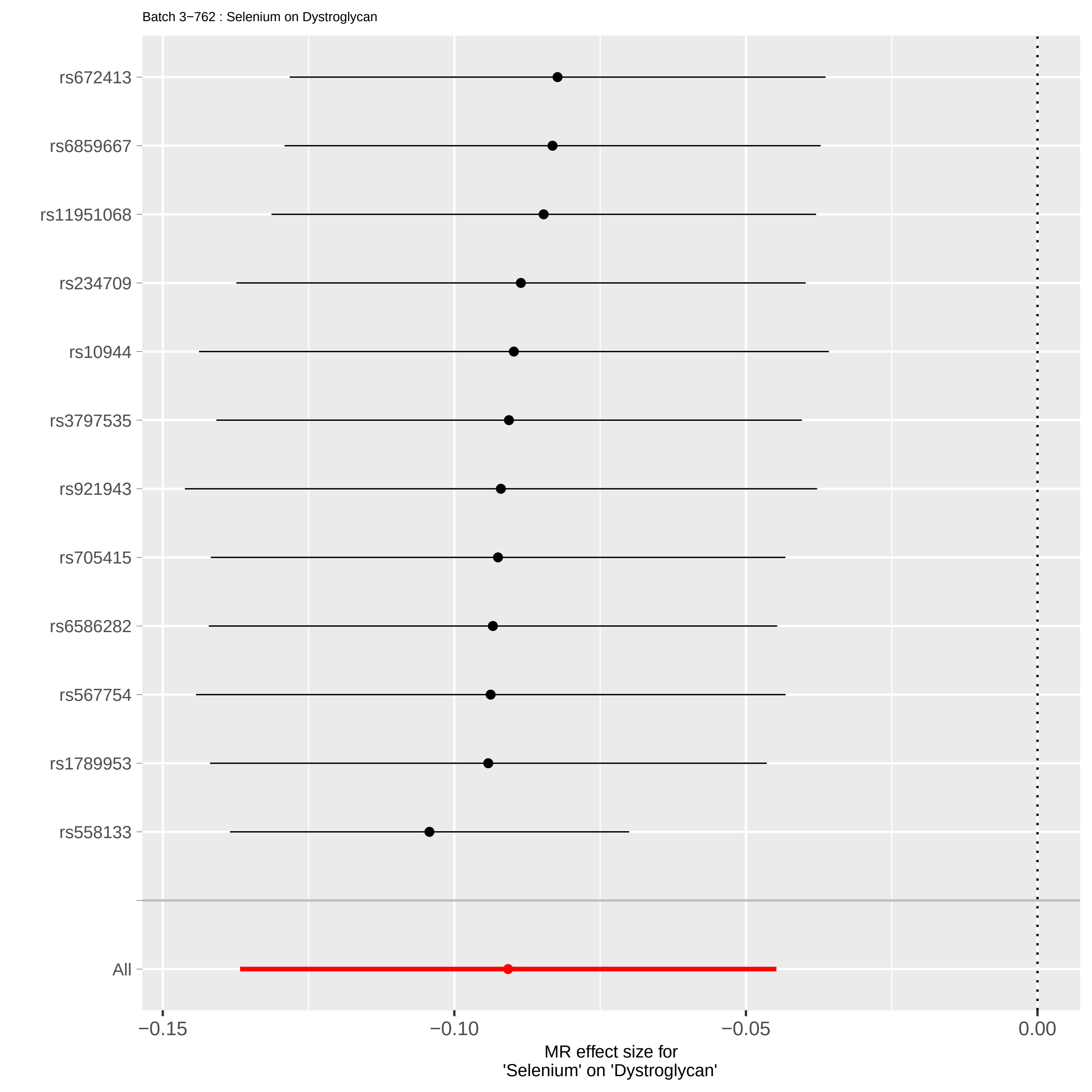

Batch 3−762 : Selenium on Dystroglycan
rs672413
rs6859667
rs11951068
rs234709
rs10944
rs3797535
rs921943
rs705415
rs6586282
rs567754
rs1789953
rs558133
All
−0.15
−0.10
−0.05
0.00
MR effect size for
'Selenium' on 'Dystroglycan'

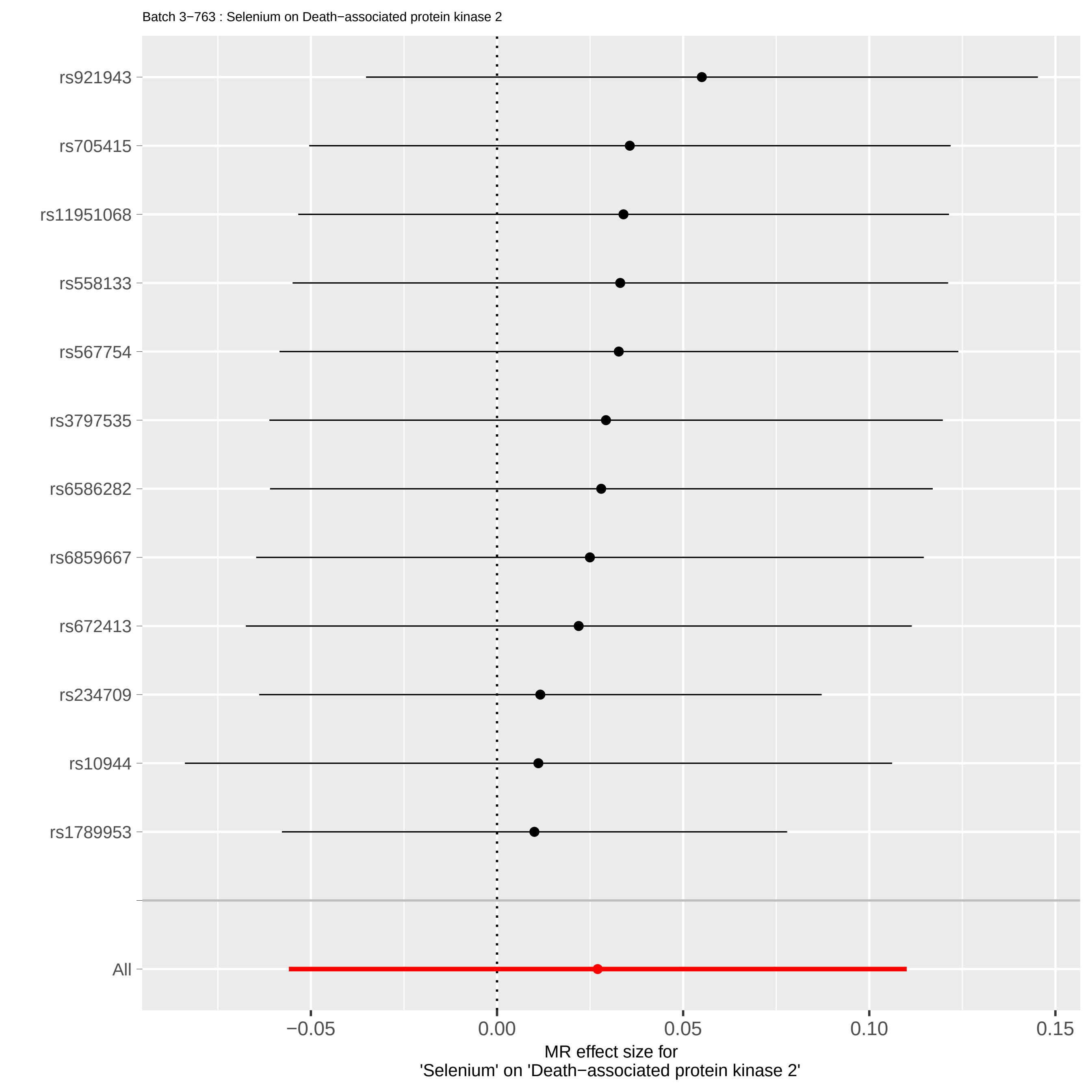

Batch 3−763 : Selenium on Death−associated protein kinase 2
rs921943
rs705415
rs11951068
rs558133
rs567754
rs3797535
rs6586282
rs6859667
rs672413
rs234709
rs10944
rs1789953
All
−0.05
0.00
0.05
0.10
0.15
MR effect size for
'Selenium' on 'Death−associated protein kinase 2'

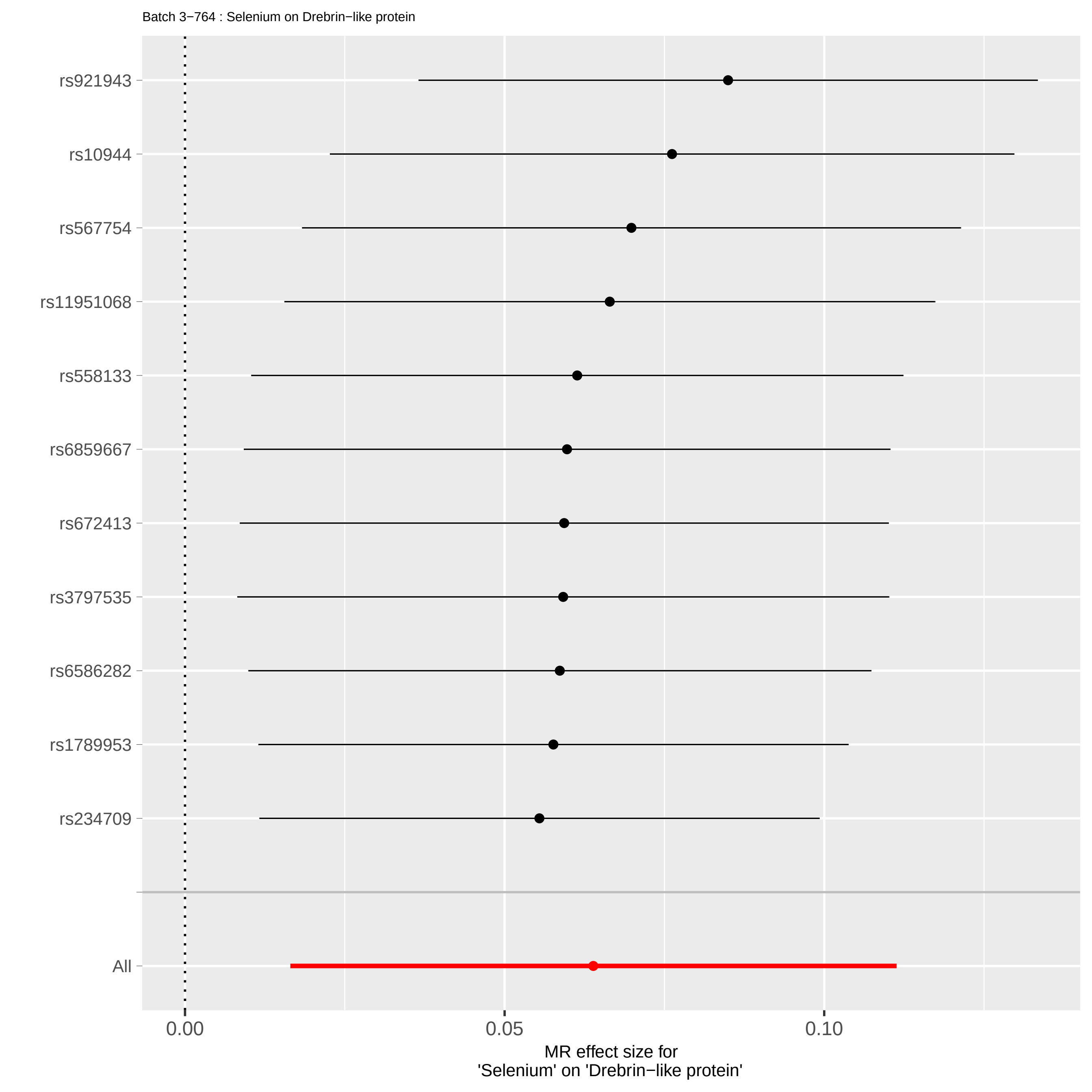
Batch 3−764 : Selenium on Drebrin−like protein
rs921943
rs10944
rs567754
rs11951068
rs558133
rs6859667
rs672413
rs3797535
rs6586282
rs1789953
rs234709
All
0.00
0.05
0.10
MR effect size for
'Selenium' on 'Drebrin−like protein'

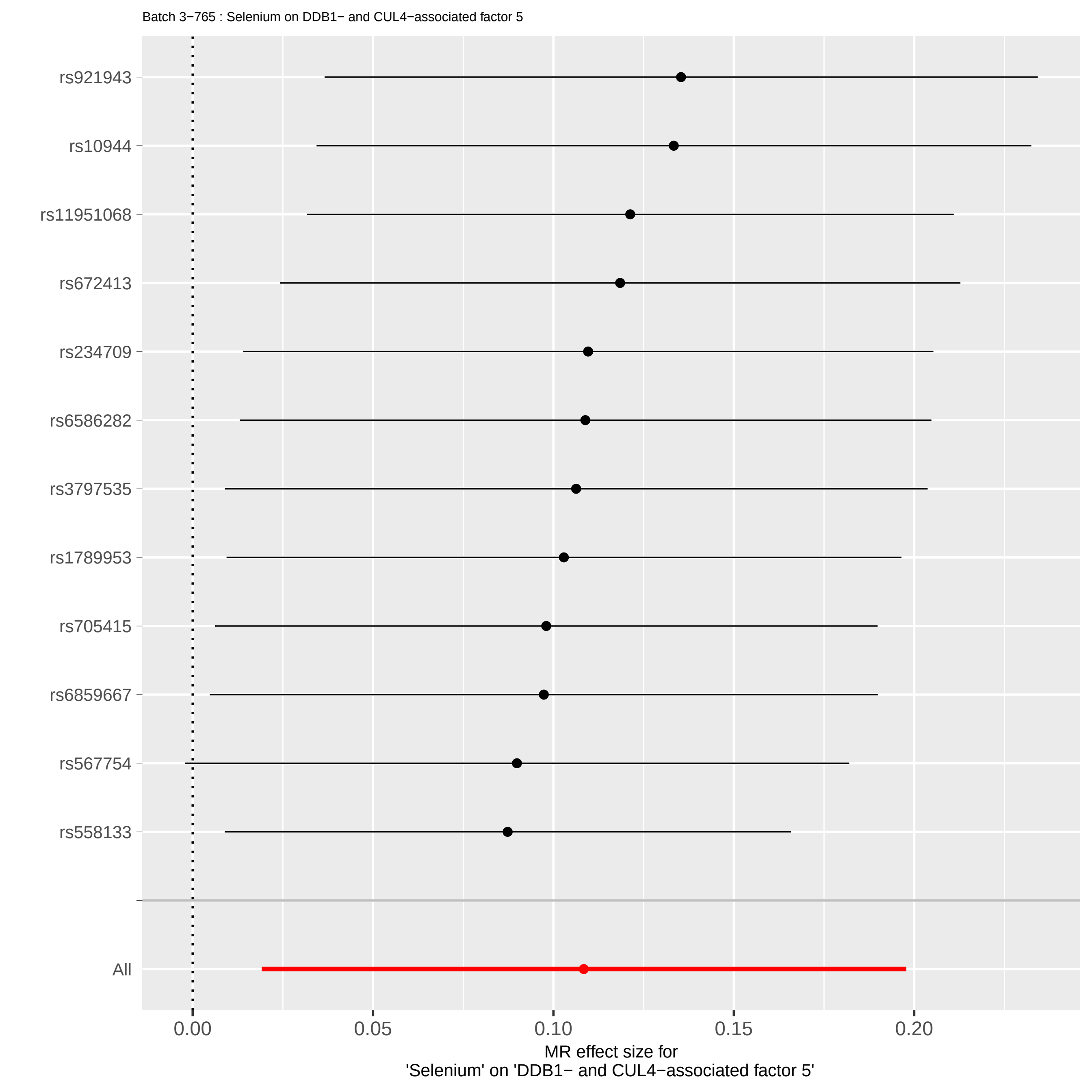
Batch 3−765 : Selenium on DDB1− and CUL4−associated factor 5
rs921943
rs10944
rs11951068
rs672413
rs234709
rs6586282
rs3797535
rs1789953
rs705415
rs6859667
rs567754
rs558133
All
0.00
0.05
0.10
0.15
0.20
MR effect size for
'Selenium' on 'DDB1− and CUL4−associated factor 5'

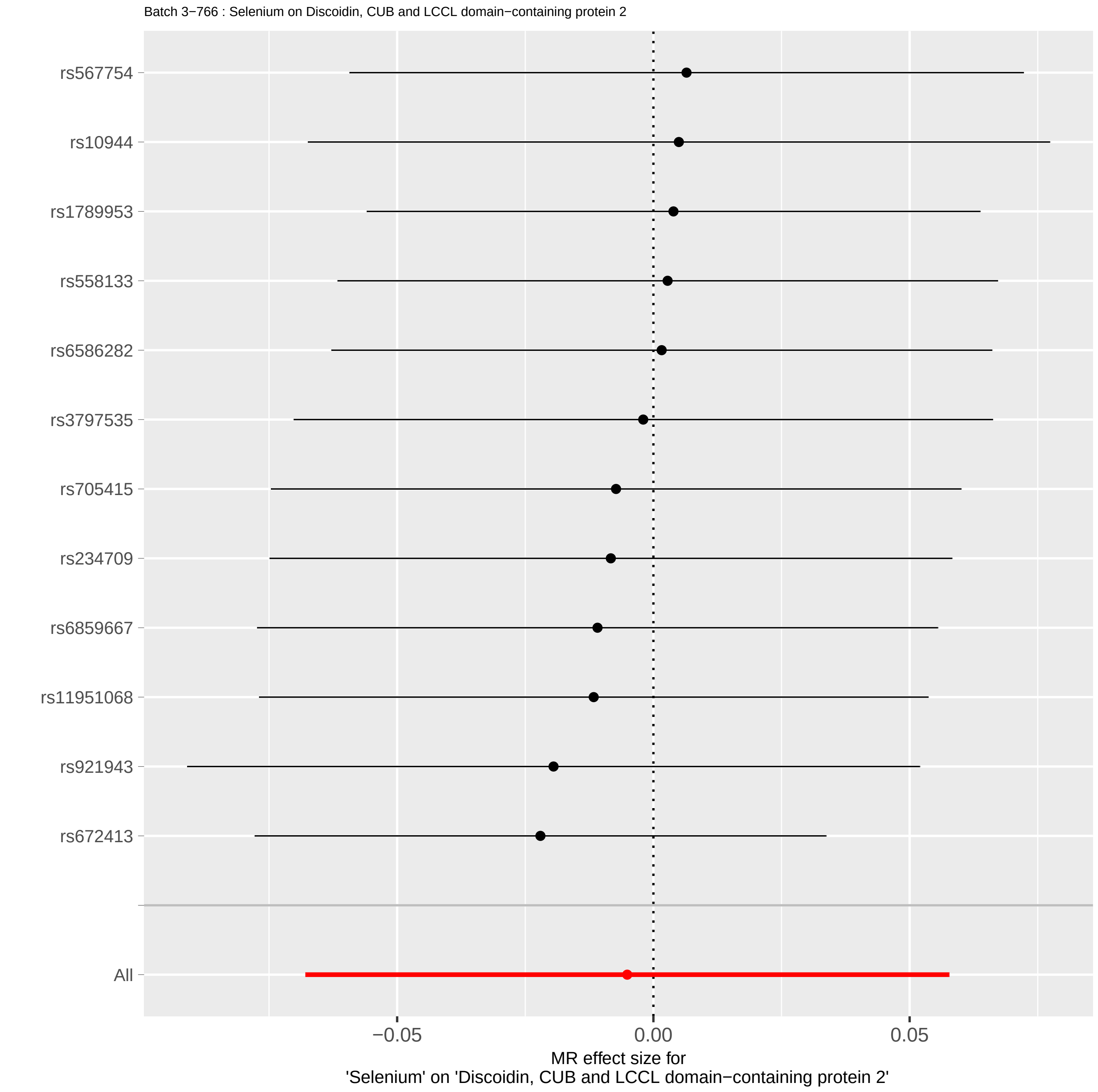
Batch 3−766 : Selenium on Discoidin, CUB and LCCL domain−containing protein 2
rs567754
rs10944
rs1789953
rs558133
rs6586282
rs3797535
rs705415
rs234709
rs6859667
rs11951068
rs921943
rs672413
All
−0.05
0.00
0.05
MR effect size for
'Selenium' on 'Discoidin, CUB and LCCL domain−containing protein 2'

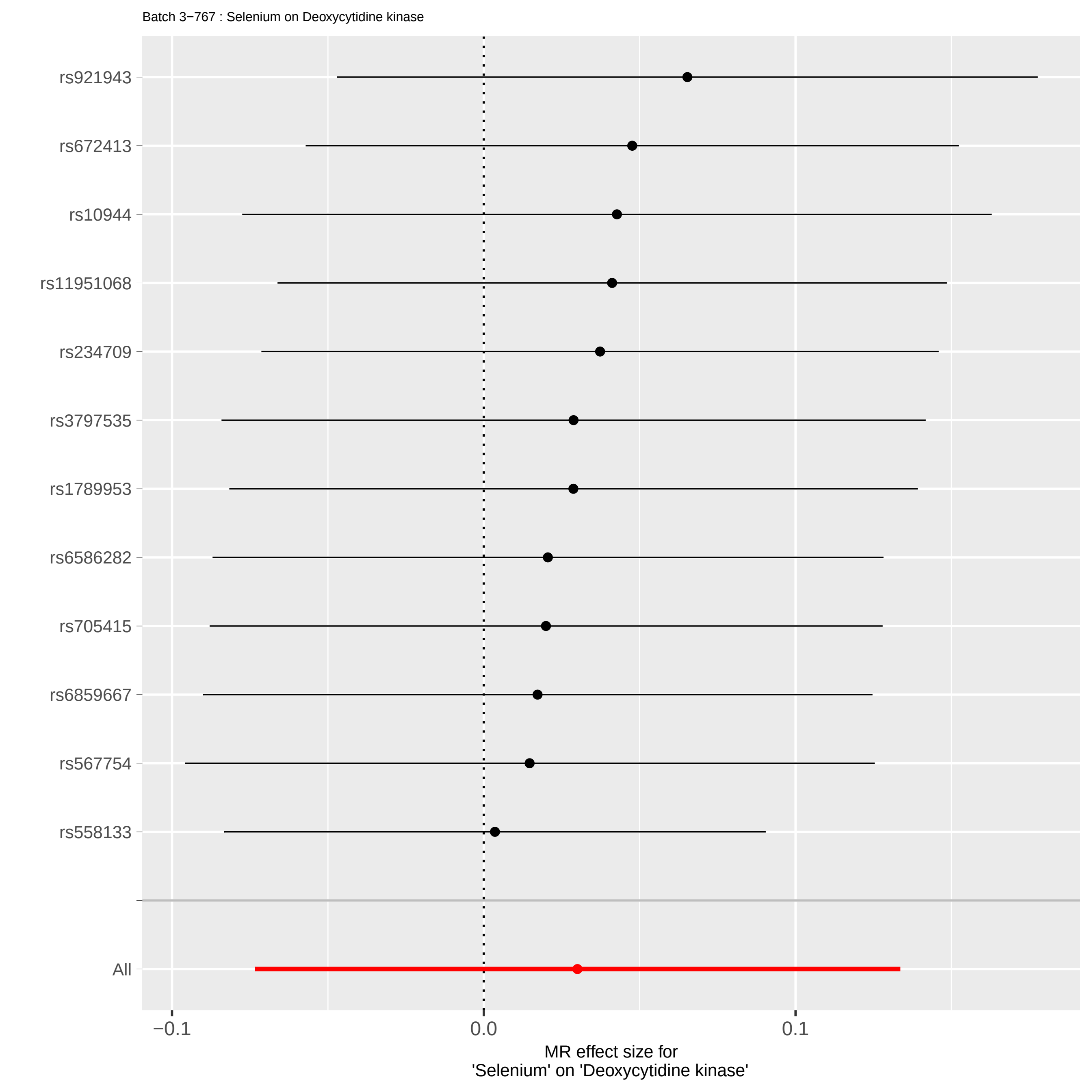

Batch 3−767 : Selenium on Deoxycytidine kinase
rs921943
rs672413
rs10944
rs11951068
rs234709
rs3797535
rs1789953
rs6586282
rs705415
rs6859667
rs567754
rs558133
All
−0.1
0.0
0.1
MR effect size for
'Selenium' on 'Deoxycytidine kinase'

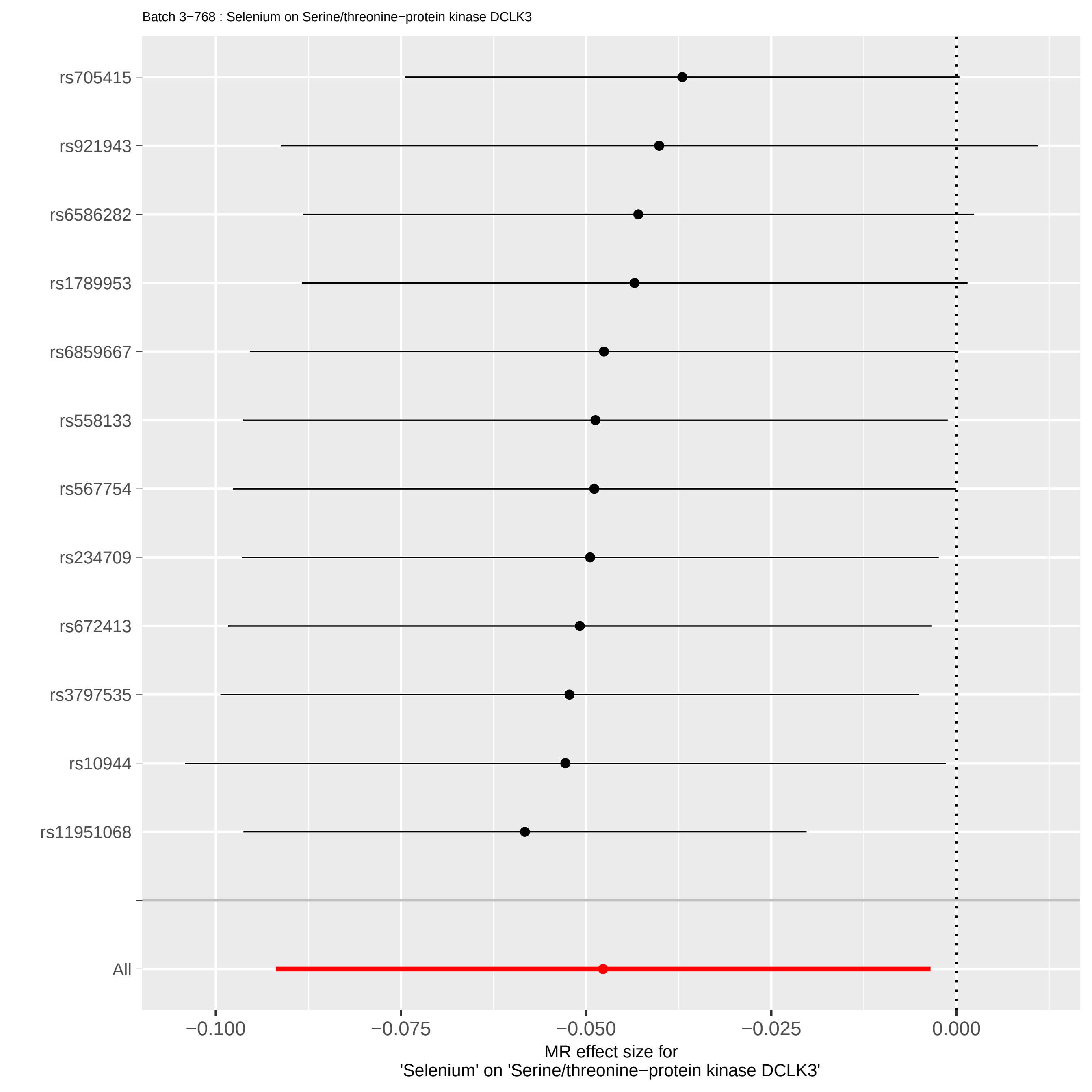

Batch 3−768 : Selenium on Serine/threonine−protein kinase DCLK3
rs705415
rs921943
rs6586282
rs1789953
rs6859667
rs558133
rs567754
rs234709
rs672413
rs3797535
rs10944
rs11951068
All
−0.100
−0.075
−0.050
−0.025
0.000
MR effect size for
'Selenium' on 'Serine/threonine−protein kinase DCLK3'

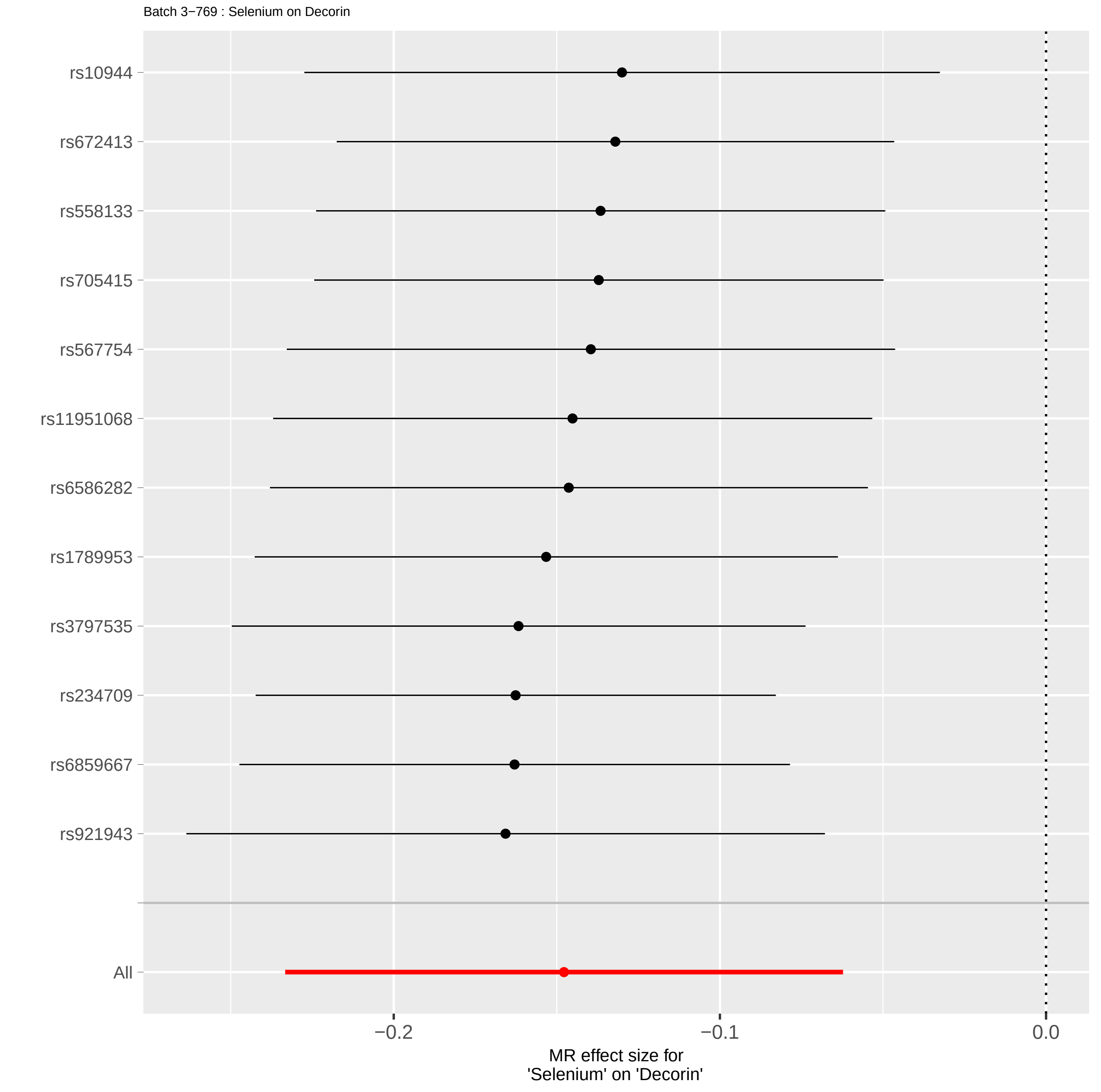

Batch 3−769 : Selenium on Decorin
rs10944
rs672413
rs558133
rs705415
rs567754
rs11951068
rs6586282
rs1789953
rs3797535
rs234709
rs6859667
rs921943
All
−0.2
−0.1
0.0
MR effect size for
'Selenium' on 'Decorin'

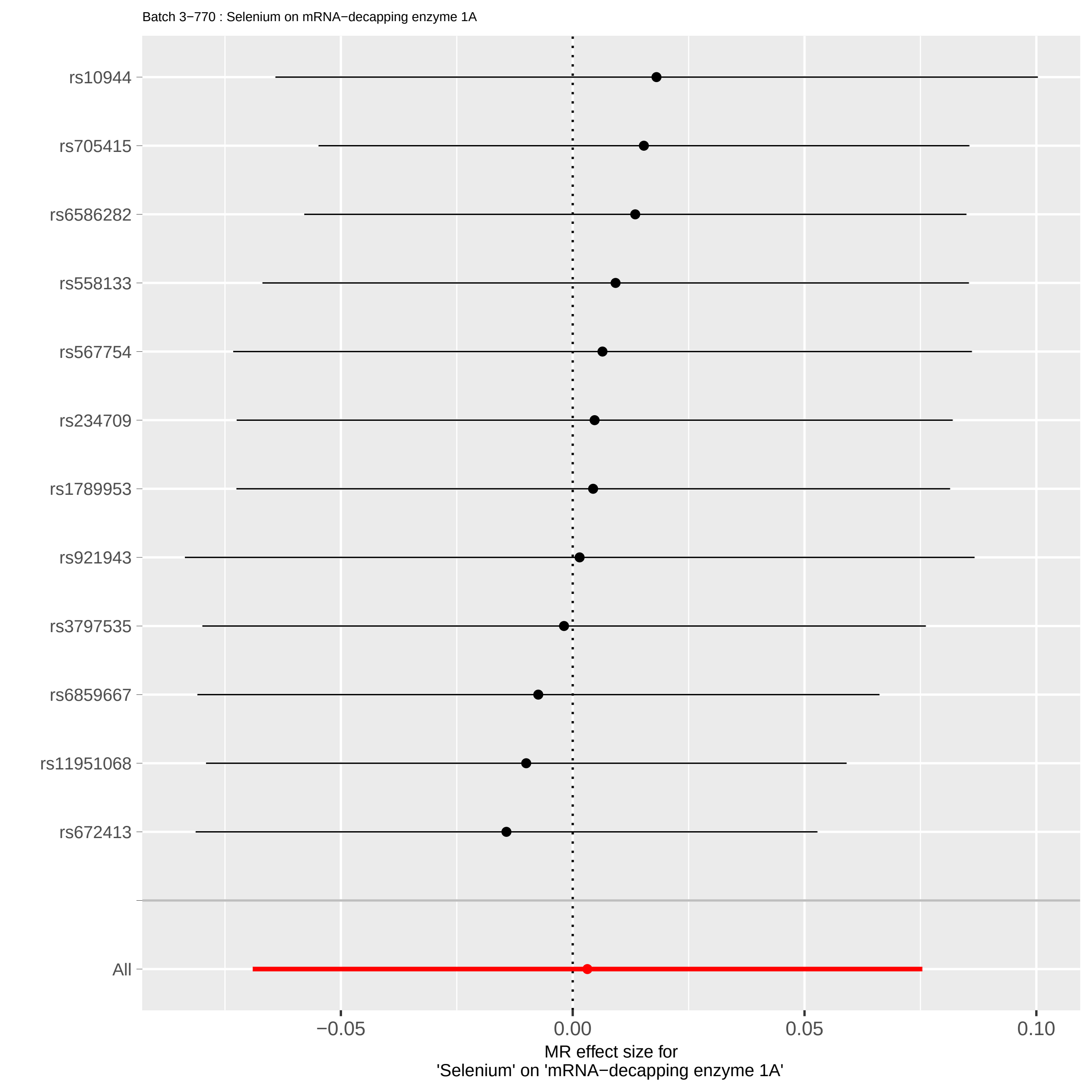

Batch 3−770 : Selenium on mRNA−decapping enzyme 1A
rs10944
rs705415
rs6586282
rs558133
rs567754
rs234709
rs1789953
rs921943
rs3797535
rs6859667
rs11951068
rs672413
All
−0.05
0.00
0.05
0.10
MR effect size for
'Selenium' on 'mRNA−decapping enzyme 1A'

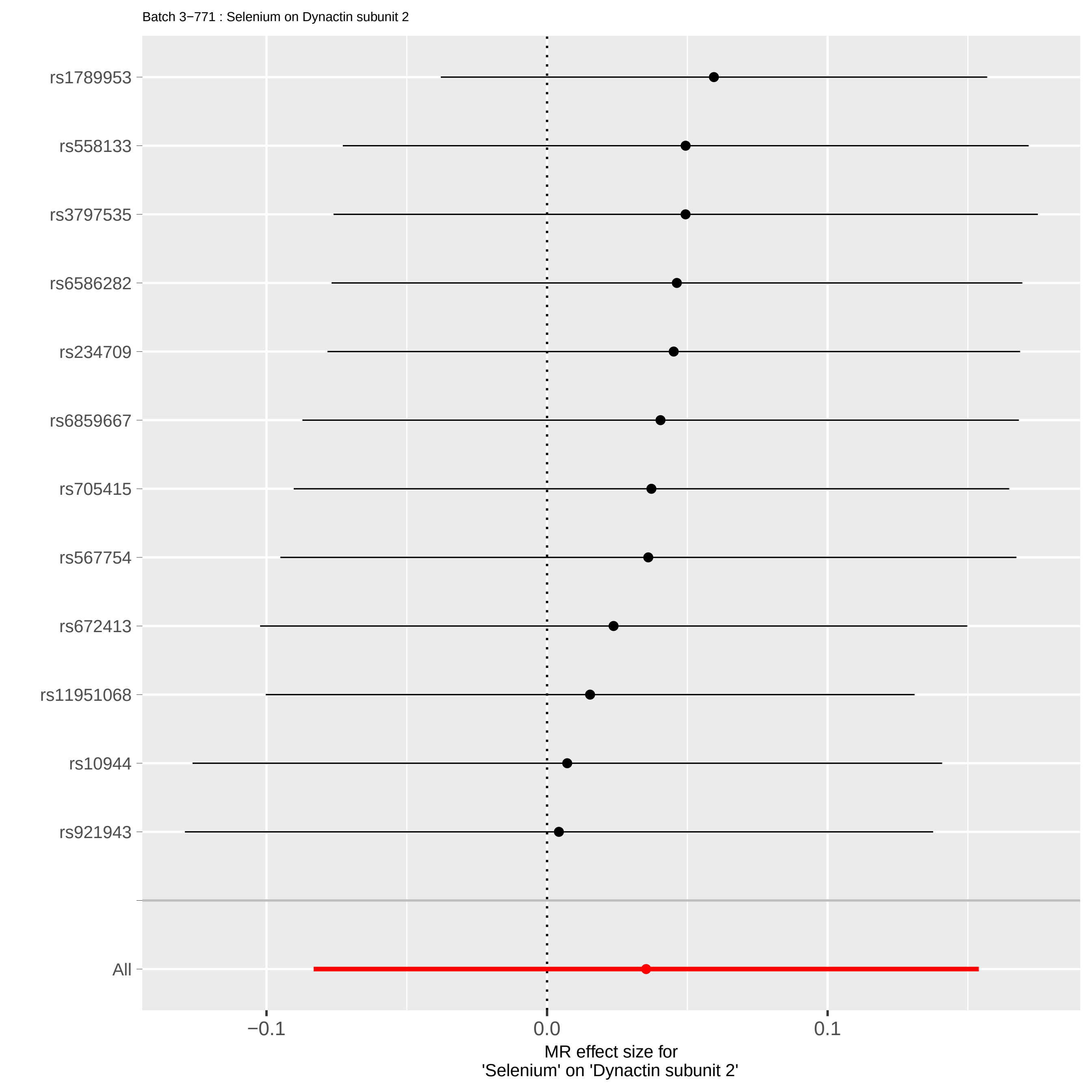

Batch 3−771 : Selenium on Dynactin subunit 2
rs1789953
rs558133
rs3797535
rs6586282
rs234709
rs6859667
rs705415
rs567754
rs672413
rs11951068
rs10944
rs921943
All
−0.1
0.0
0.1
MR effect size for
'Selenium' on 'Dynactin subunit 2'

Batch 3−772 : Selenium on dCTP pyrophosphatase 1

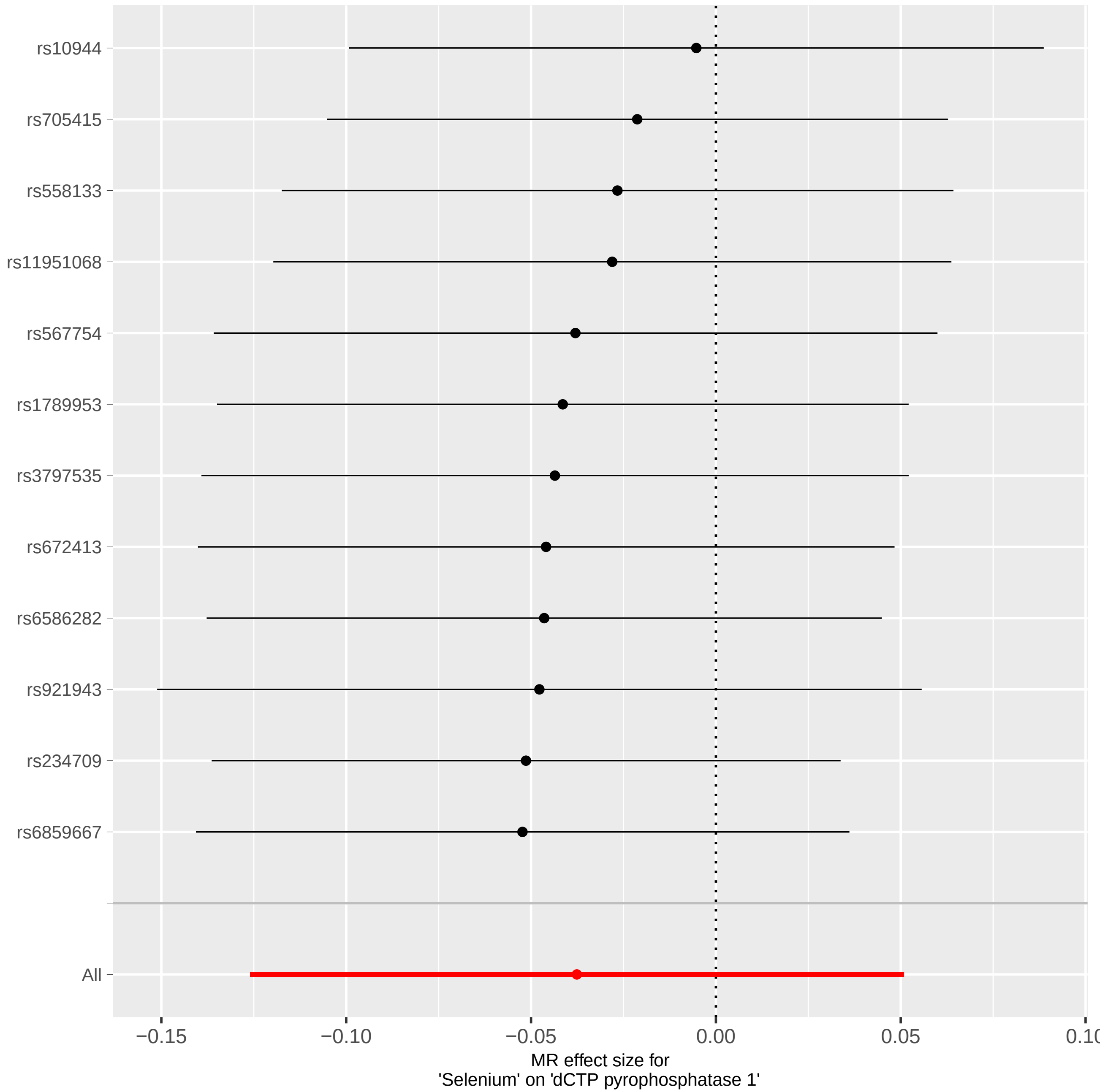

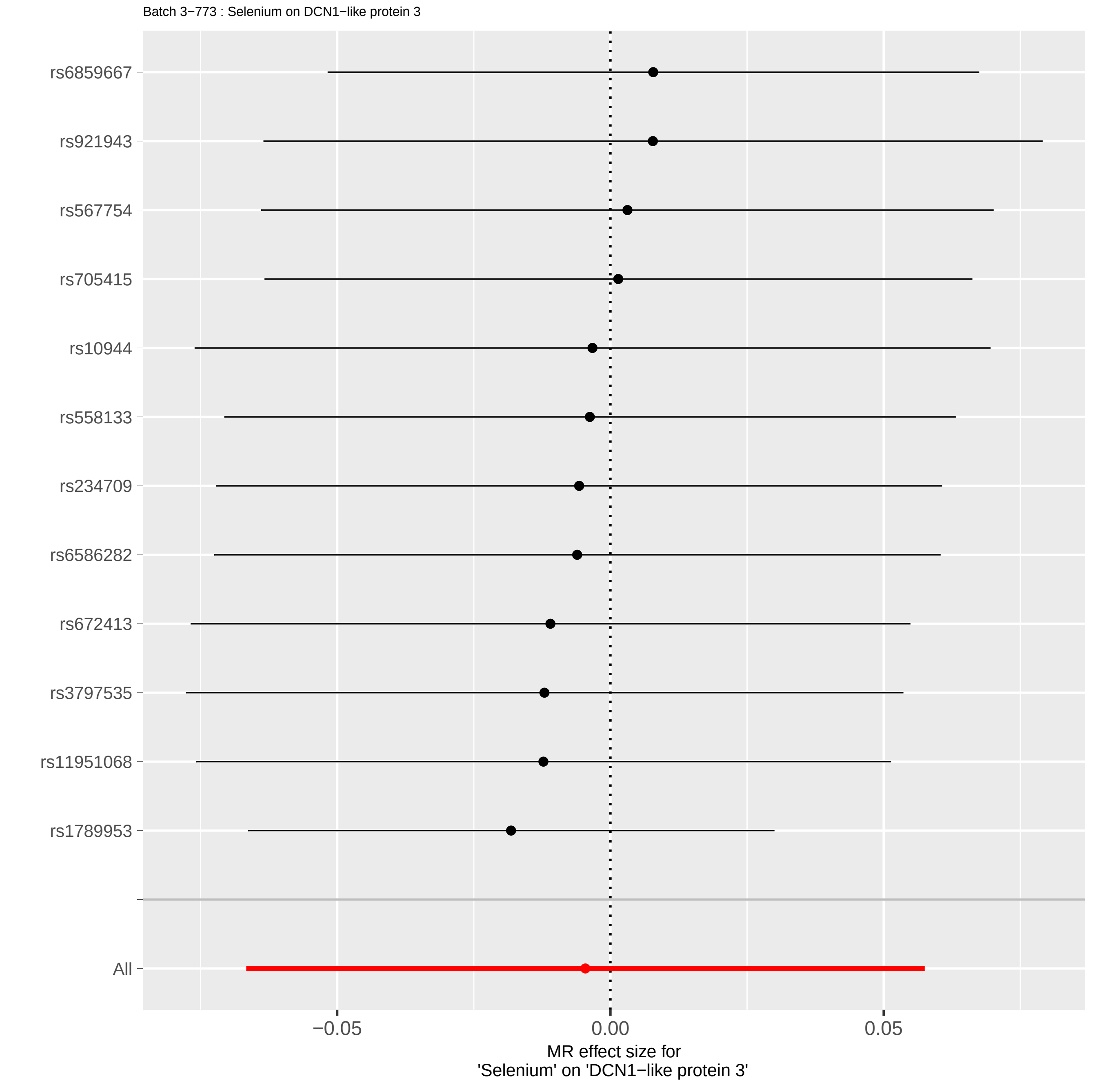
Batch 3−773 : Selenium on DCN1−like protein 3
rs6859667
rs921943
rs567754
rs705415
rs10944
rs558133
rs234709
rs6586282
rs672413
rs3797535
rs11951068
rs1789953
All
−0.05
0.00
0.05
MR effect size for
'Selenium' on 'DCN1−like protein 3'

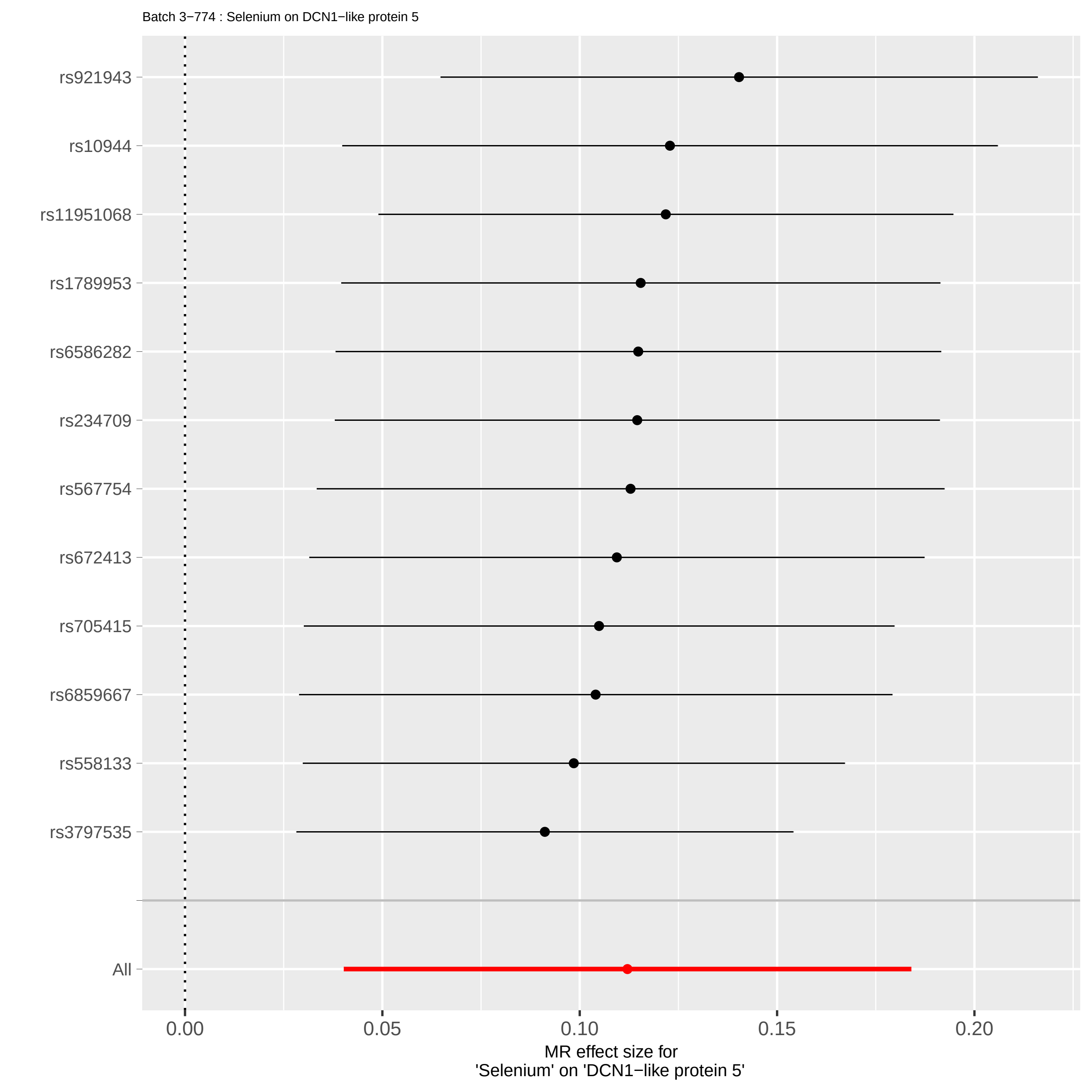
Batch 3−774 : Selenium on DCN1−like protein 5
rs921943
rs10944
rs11951068
rs1789953
rs6586282
rs234709
rs567754
rs672413
rs705415
rs6859667
rs558133
rs3797535
All
0.00
0.05
0.10
0.15
0.20
MR effect size for
'Selenium' on 'DCN1−like protein 5'

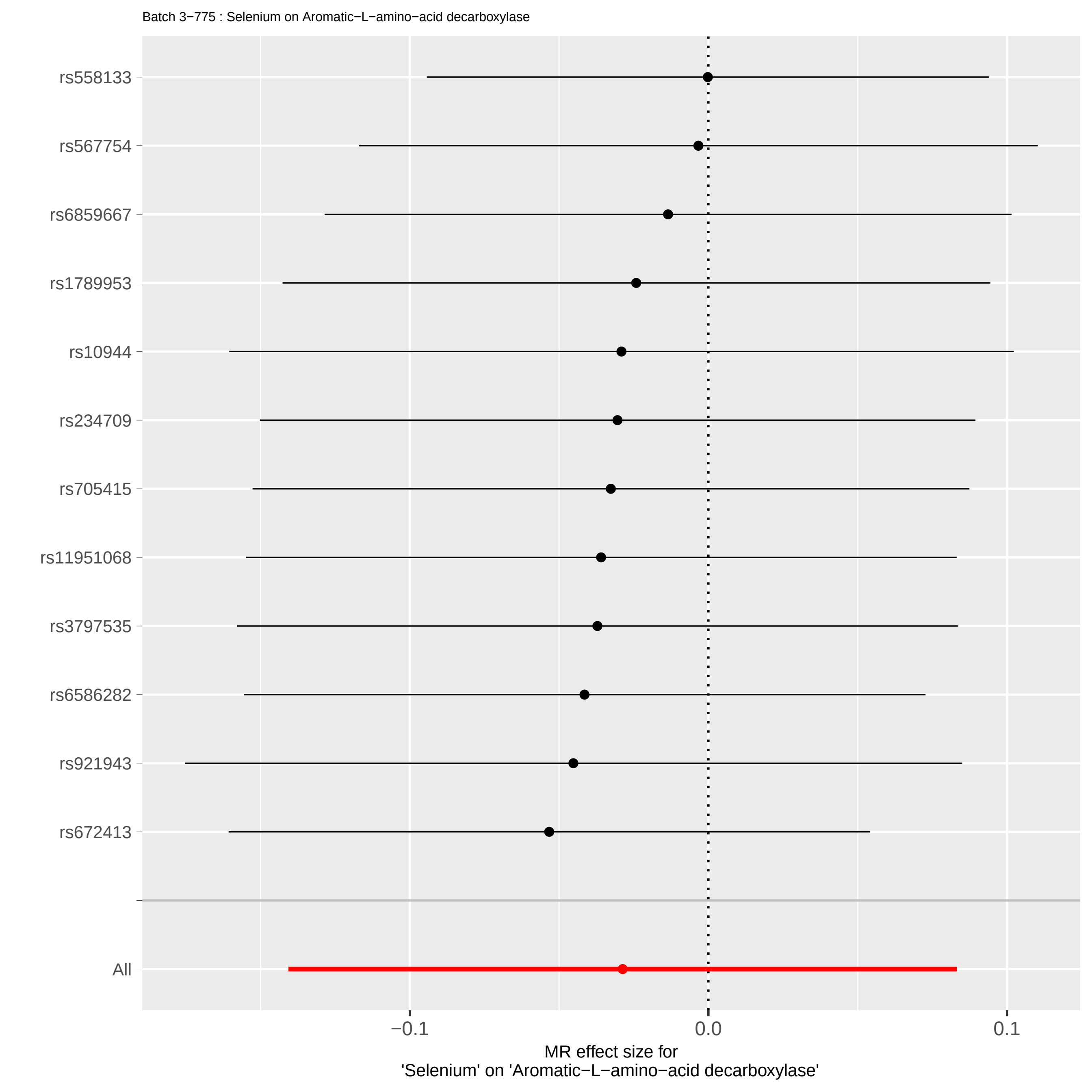

Batch 3−775 : Selenium on Aromatic−L−amino−acid decarboxylase
rs558133
rs567754
rs6859667
rs1789953
rs10944
rs234709
rs705415
rs11951068
rs3797535
rs6586282
rs921943
rs672413
All
−0.1
0.0
0.1
MR effect size for
'Selenium' on 'Aromatic−L−amino−acid decarboxylase'

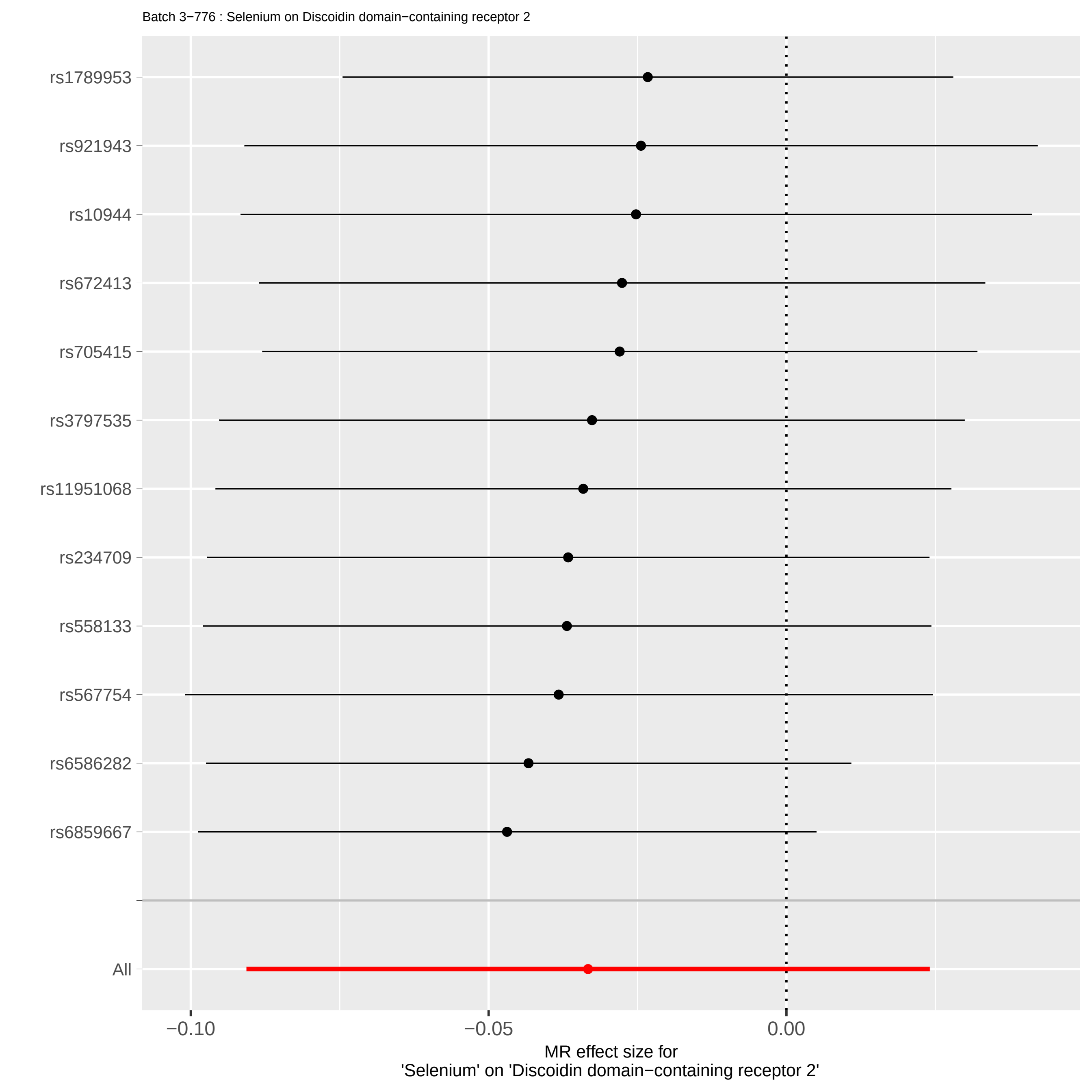

Batch 3−776 : Selenium on Discoidin domain−containing receptor 2
rs1789953
rs921943
rs10944
rs672413
rs705415
rs3797535
rs11951068
rs234709
rs558133
rs567754
rs6586282
rs6859667
All
−0.10
−0.05
0.00
MR effect size for
'Selenium' on 'Discoidin domain−containing receptor 2'

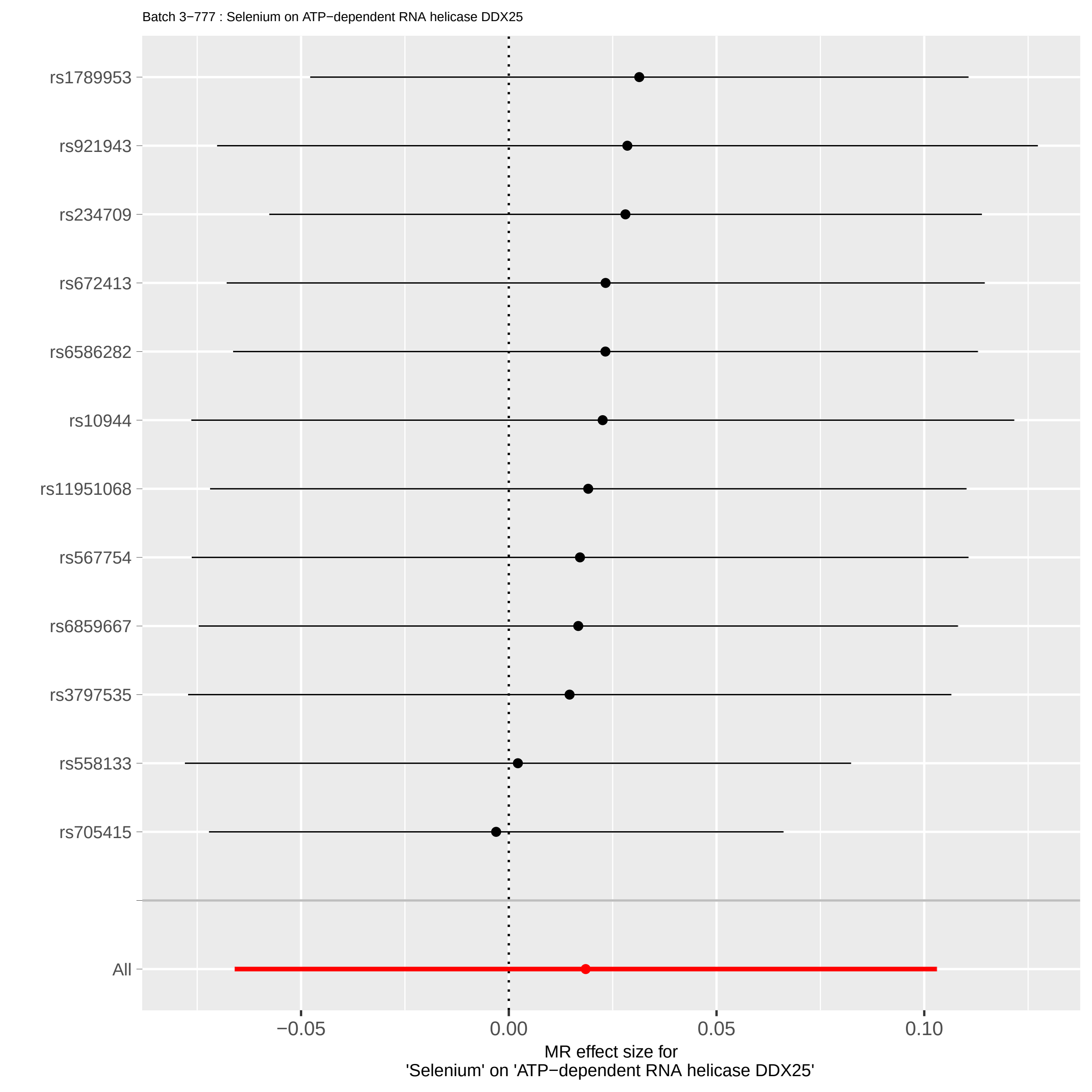

Batch 3−777 : Selenium on ATP−dependent RNA helicase DDX25
rs1789953
rs921943
rs234709
rs672413
rs6586282
rs10944
rs11951068
rs567754
rs6859667
rs3797535
rs558133
rs705415
All
−0.05
0.00
0.05
0.10
MR effect size for
'Selenium' on 'ATP−dependent RNA helicase DDX25'

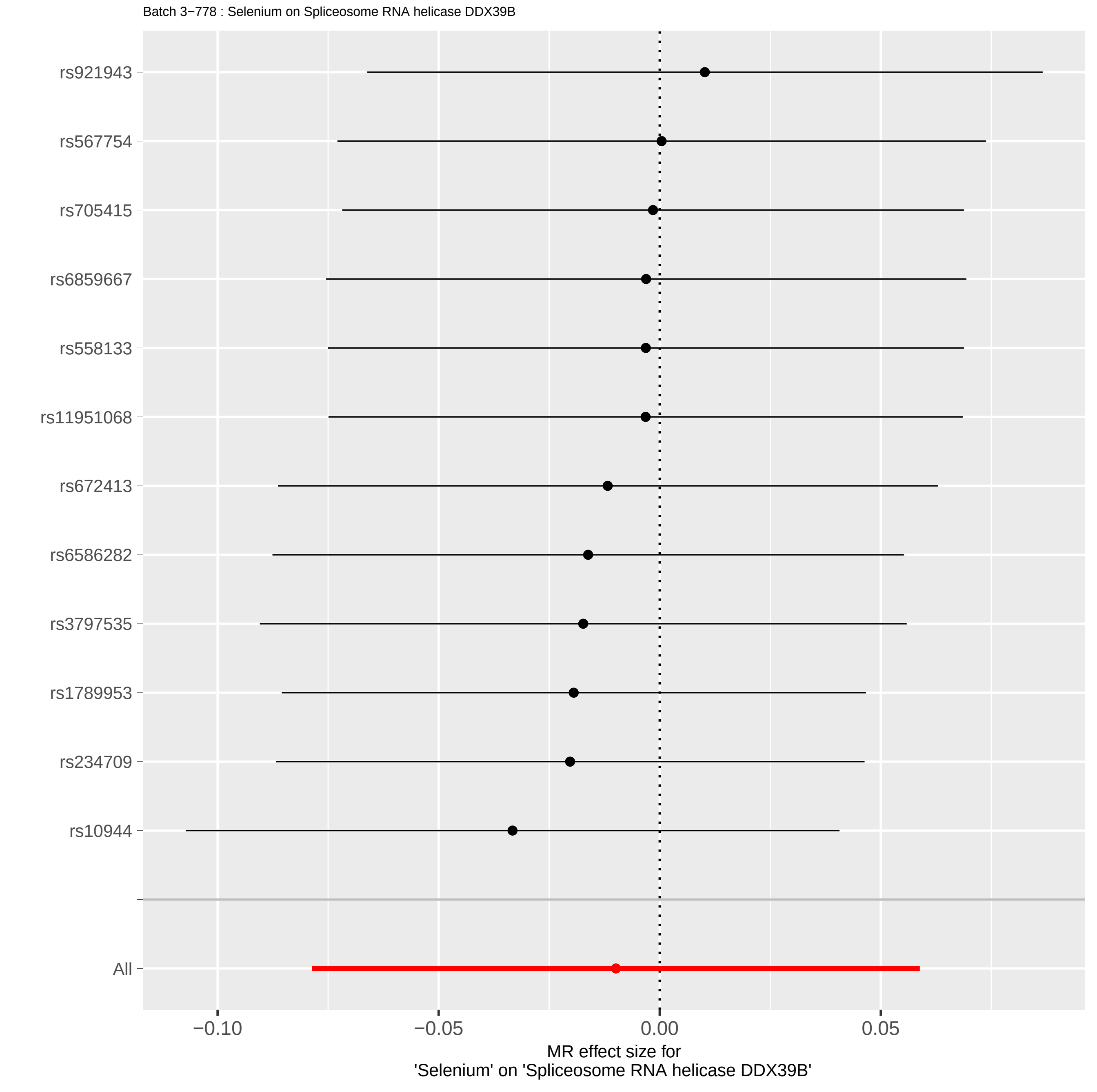

Batch 3−778 : Selenium on Spliceosome RNA helicase DDX39B
rs921943
rs567754
rs705415
rs6859667
rs558133
rs11951068
rs672413
rs6586282
rs3797535
rs1789953
rs234709
rs10944
All
−0.10
−0.05
0.00
0.05
MR effect size for
'Selenium' on 'Spliceosome RNA helicase DDX39B'

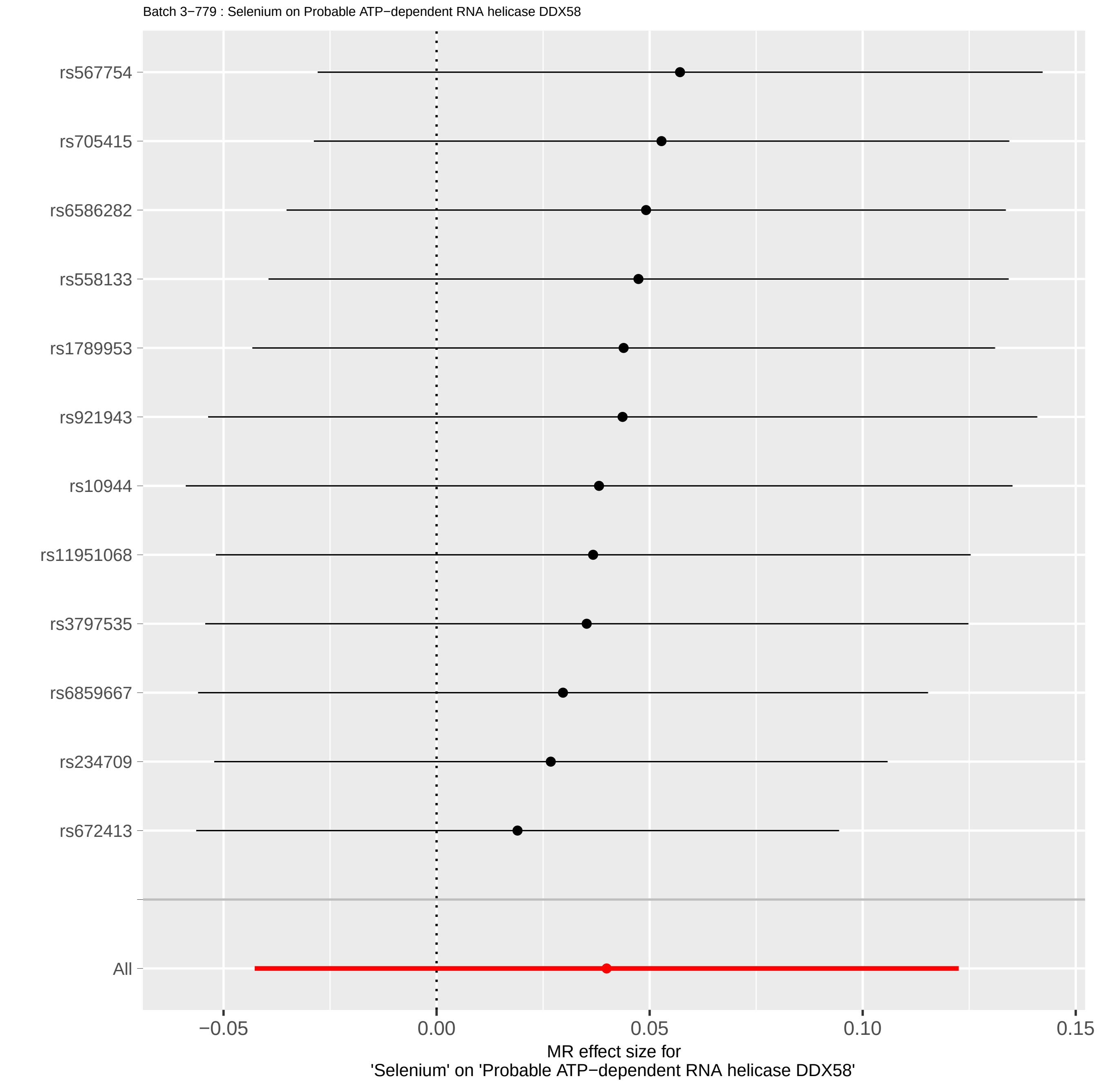

Batch 3−779 : Selenium on Probable ATP−dependent RNA helicase DDX58
rs567754
rs705415
rs6586282
rs558133
rs1789953
rs921943
rs10944
rs11951068
rs3797535
rs6859667
rs234709
rs672413
All
−0.05
0.00
0.05
0.10
0.15
MR effect size for
'Selenium' on 'Probable ATP−dependent RNA helicase DDX58'

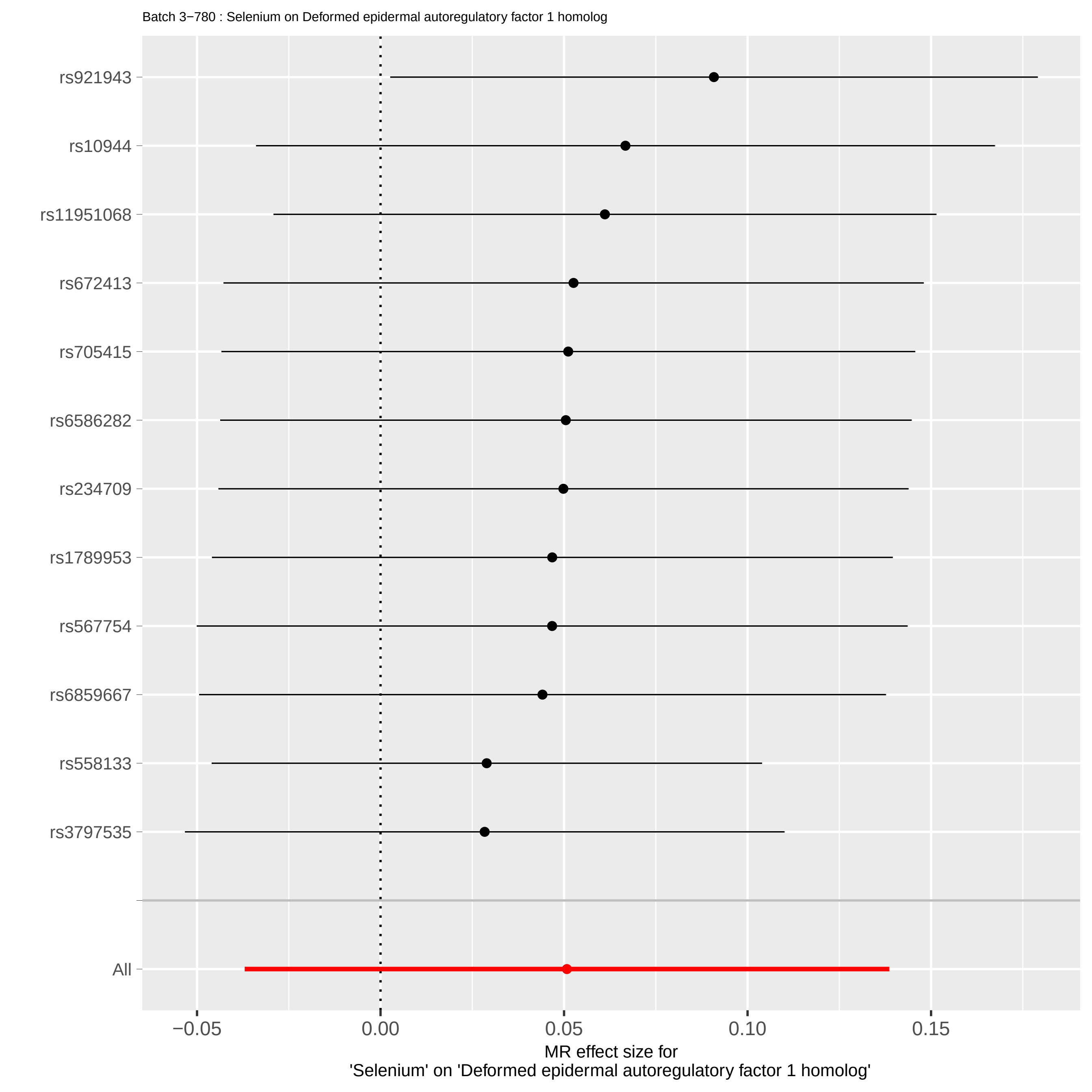

Batch 3−780 : Selenium on Deformed epidermal autoregulatory factor 1 homolog
rs921943
rs10944
rs11951068
rs672413
rs705415
rs6586282
rs234709
rs1789953
rs567754
rs6859667
rs558133
rs3797535
All
−0.05
0.00
0.05
0.10
0.15
MR effect size for
'Selenium' on 'Deformed epidermal autoregulatory factor 1 homolog'

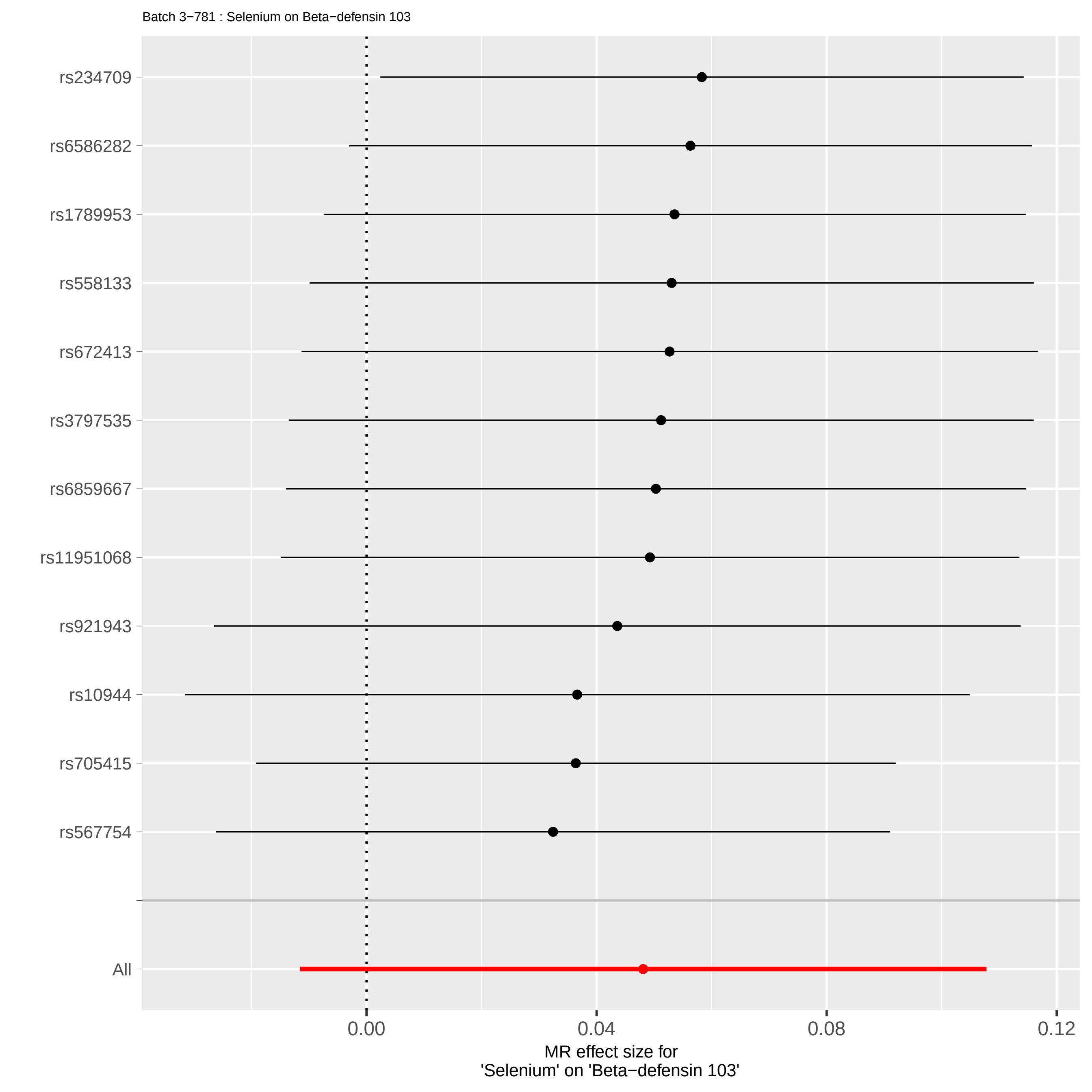

Batch 3−781 : Selenium on Beta−defensin 103
rs234709
rs6586282
rs1789953
rs558133
rs672413
rs3797535
rs6859667
rs11951068
rs921943
rs10944
rs705415
rs567754
All
0.00
0.04
0.08
0.12
MR effect size for
'Selenium' on 'Beta−defensin 103'

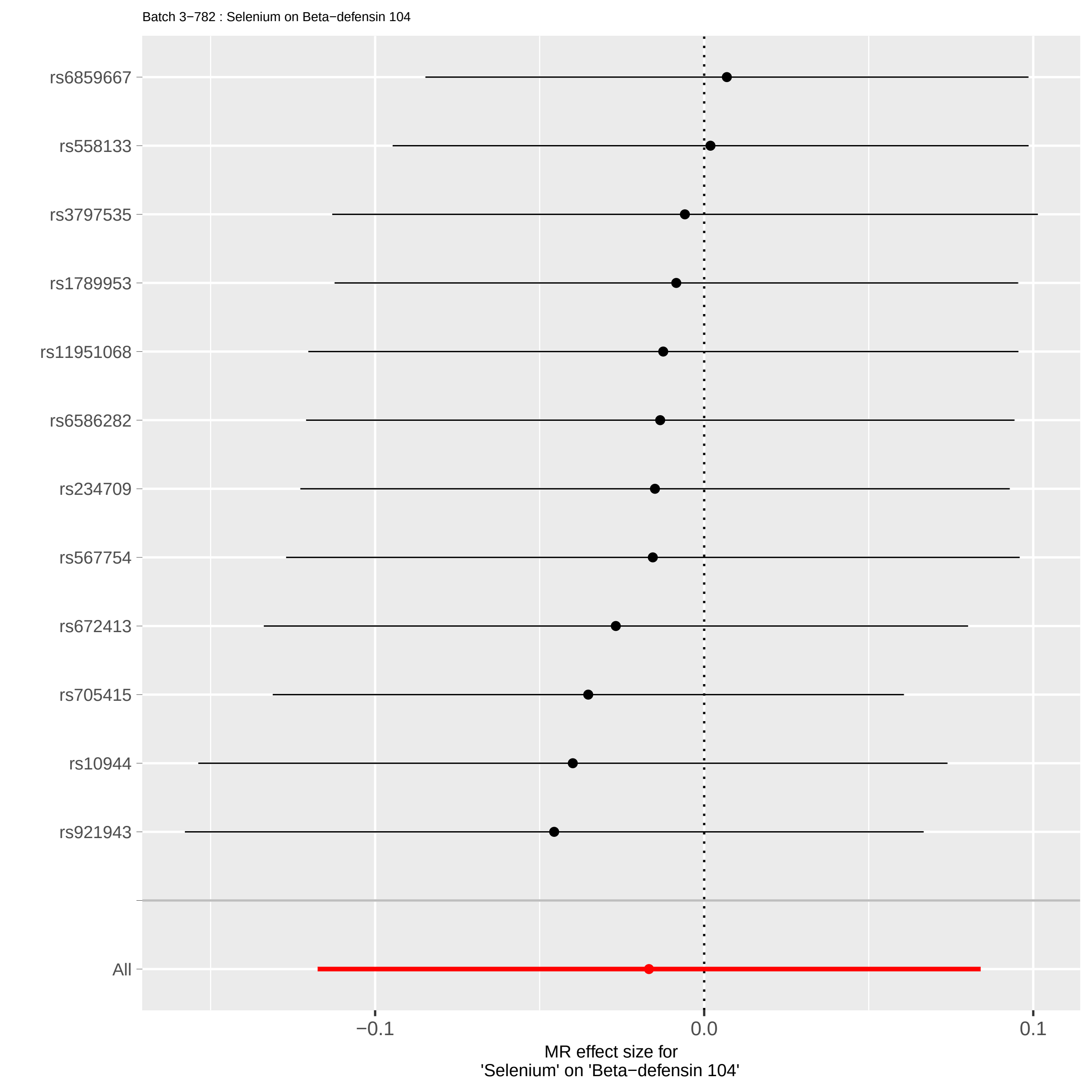
Batch 3−782 : Selenium on Beta−defensin 104
rs6859667
rs558133
rs3797535
rs1789953
rs11951068
rs6586282
rs234709
rs567754
rs672413
rs705415
rs10944
rs921943
All
−0.1
0.0
0.1
MR effect size for
'Selenium' on 'Beta−defensin 104'

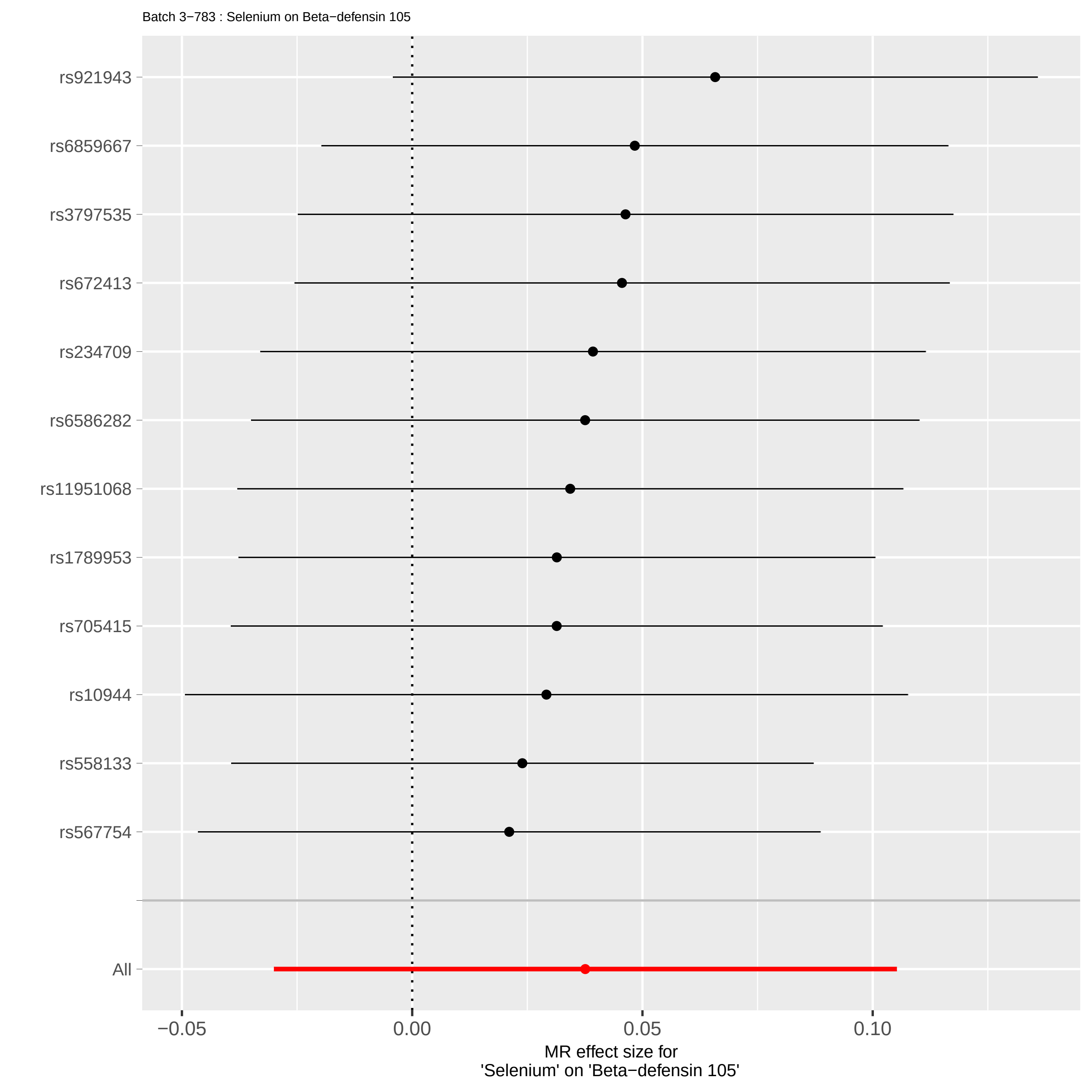

Batch 3−783 : Selenium on Beta−defensin 105
rs921943
rs6859667
rs3797535
rs672413
rs234709
rs6586282
rs11951068
rs1789953
rs705415
rs10944
rs558133
rs567754
All
−0.05
0.00
0.05
0.10
MR effect size for
'Selenium' on 'Beta−defensin 105'

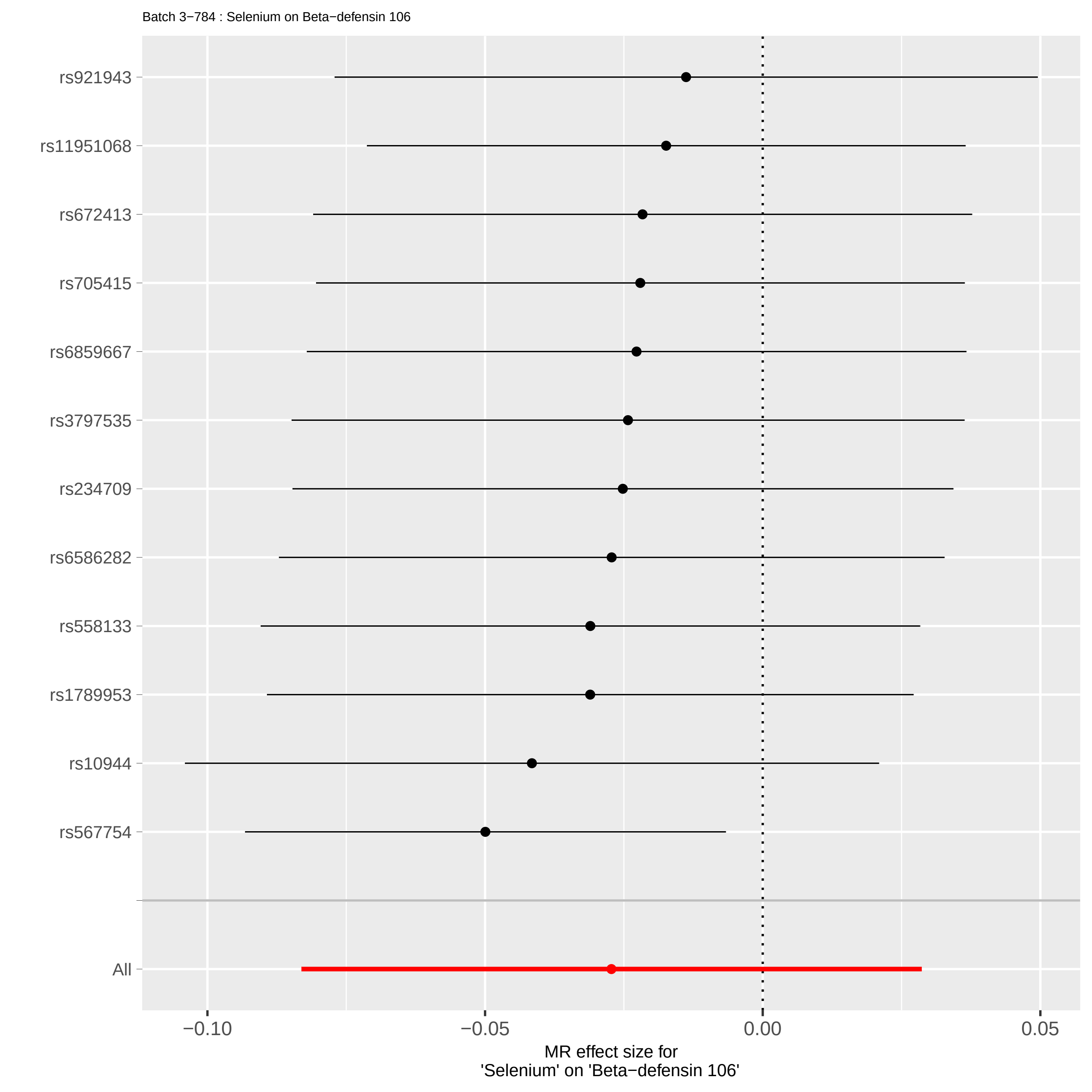

Batch 3−784 : Selenium on Beta−defensin 106
rs921943
rs11951068
rs672413
rs705415
rs6859667
rs3797535
rs234709
rs6586282
rs558133
rs1789953
rs10944
rs567754
All
−0.10
−0.05
0.00
0.05
MR effect size for
'Selenium' on 'Beta−defensin 106'

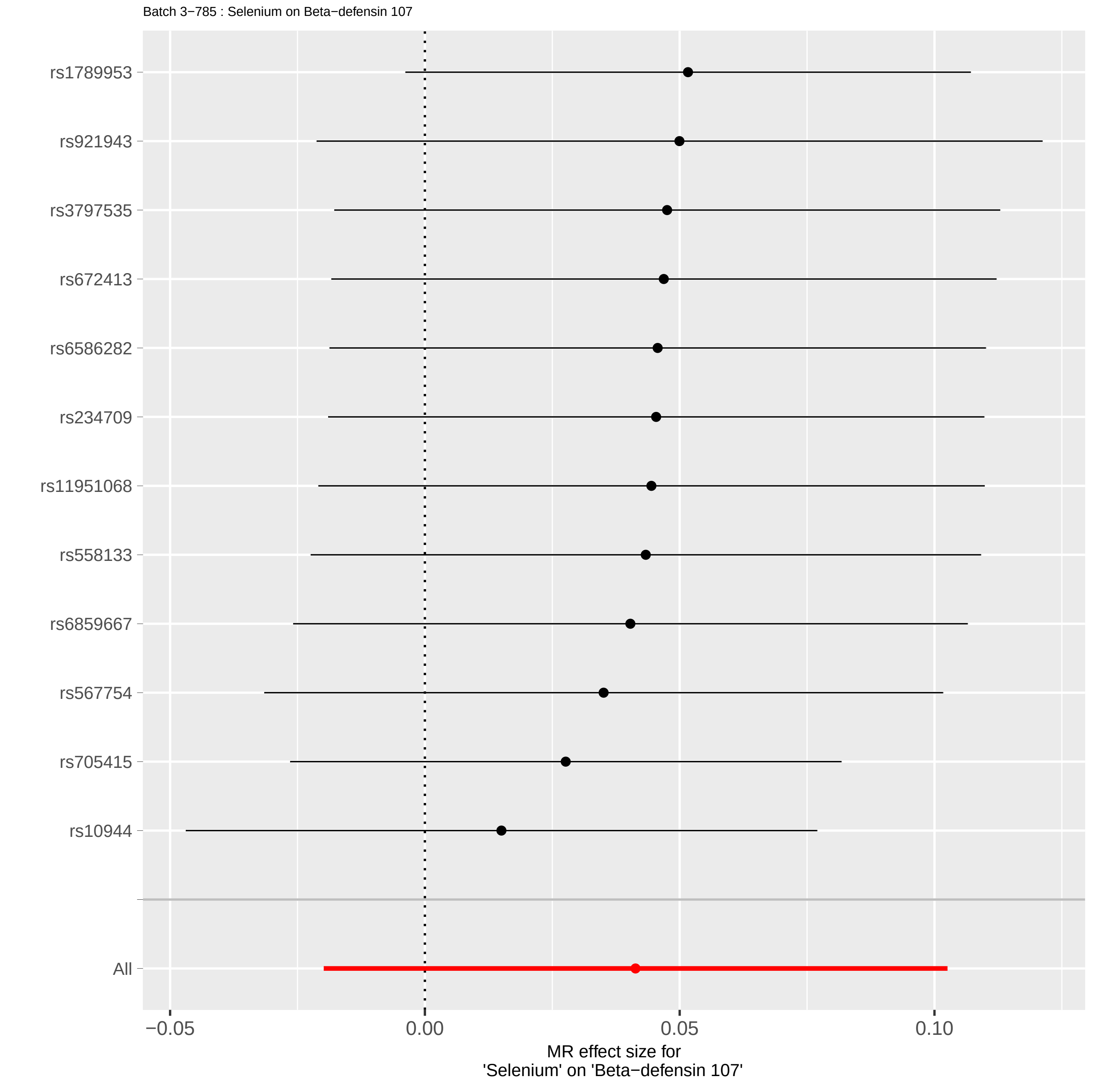

Batch 3−785 : Selenium on Beta−defensin 107
rs1789953
rs921943
rs3797535
rs672413
rs6586282
rs234709
rs11951068
rs558133
rs6859667
rs567754
rs705415
rs10944
All
−0.05
0.00
0.05
0.10
MR effect size for
'Selenium' on 'Beta−defensin 107'

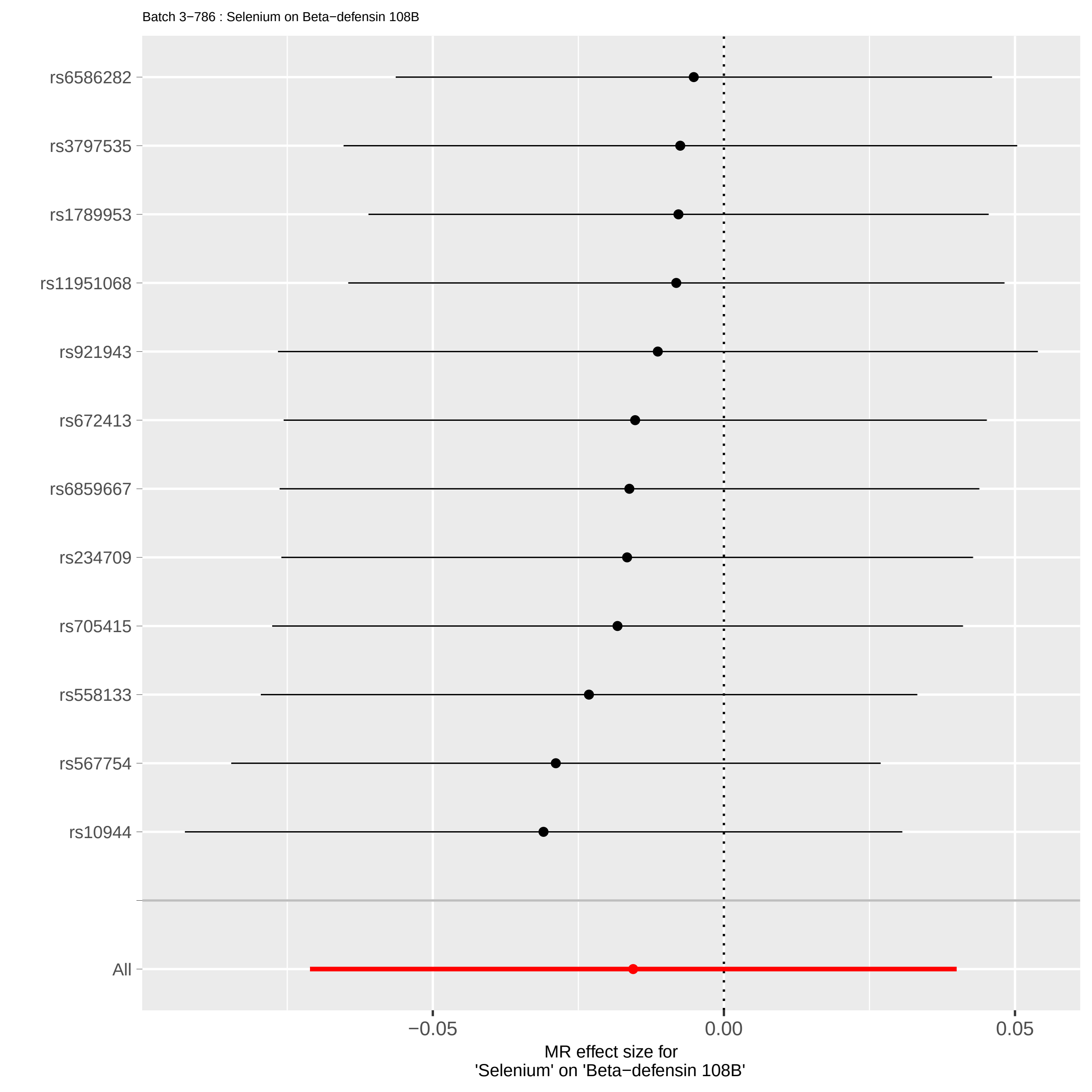

Batch 3−786 : Selenium on Beta−defensin 108B
rs6586282
rs3797535
rs1789953
rs11951068
rs921943
rs672413
rs6859667
rs234709
rs705415
rs558133
rs567754
rs10944
All
−0.05
0.00
0.05
MR effect size for
'Selenium' on 'Beta−defensin 108B'

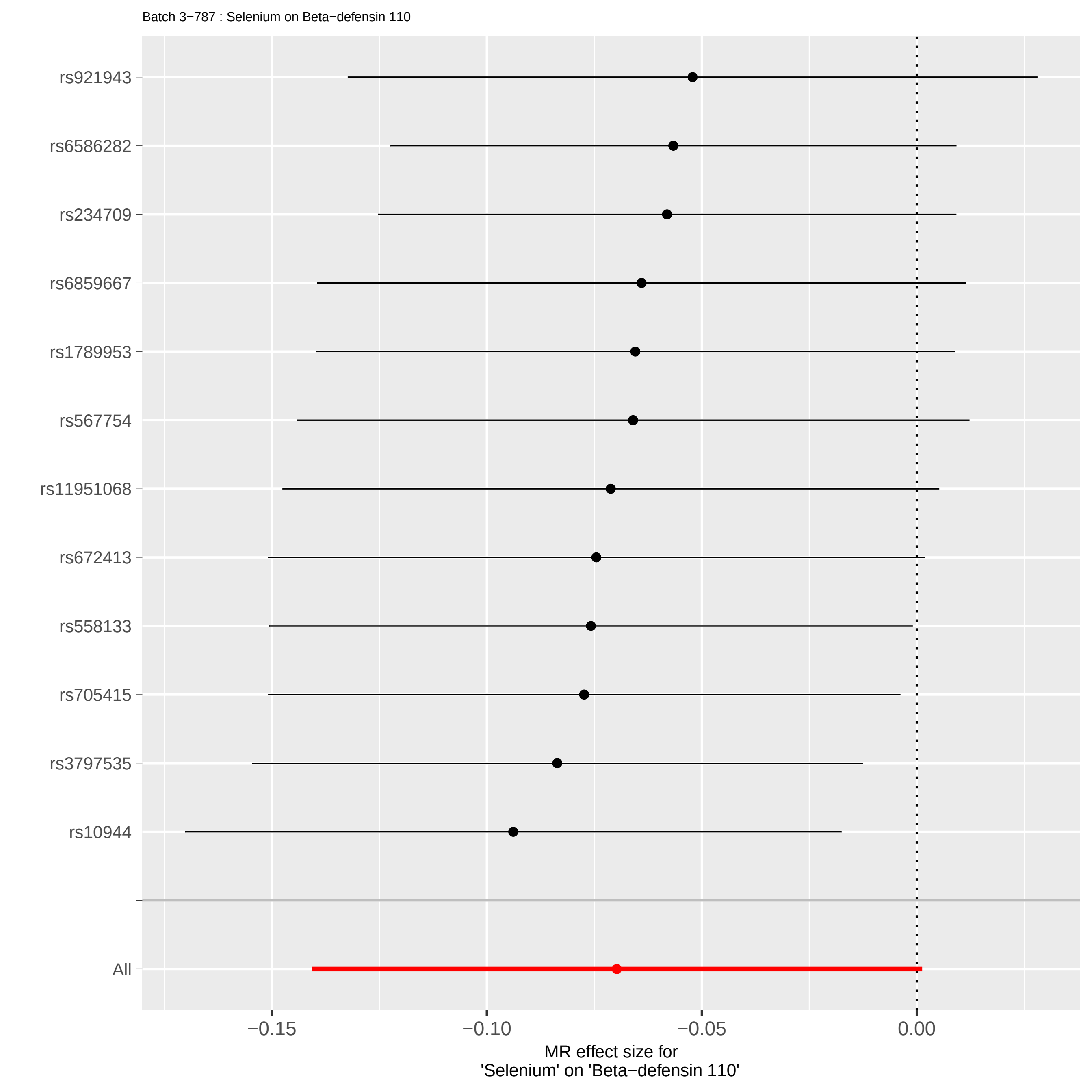

Batch 3−787 : Selenium on Beta−defensin 110
rs921943
rs6586282
rs234709
rs6859667
rs1789953
rs567754
rs11951068
rs672413
rs558133
rs705415
rs3797535
rs10944
All
−0.15
−0.10
−0.05
0.00
MR effect size for
'Selenium' on 'Beta−defensin 110'

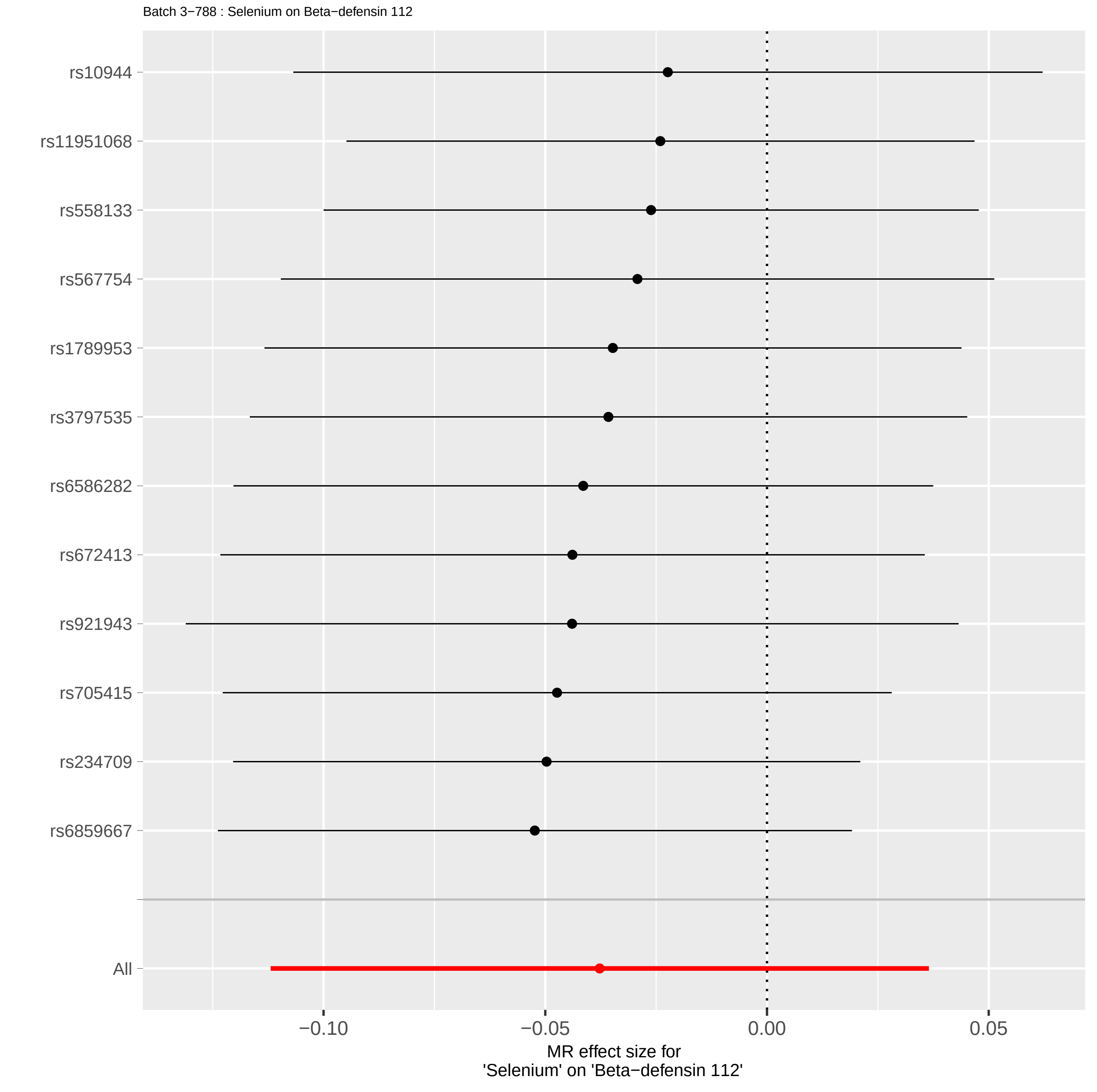

Batch 3−788 : Selenium on Beta−defensin 112
rs10944
rs11951068
rs558133
rs567754
rs1789953
rs3797535
rs6586282
rs672413
rs921943
rs705415
rs234709
rs6859667
All
−0.10
−0.05
0.00
0.05
MR effect size for
'Selenium' on 'Beta−defensin 112'

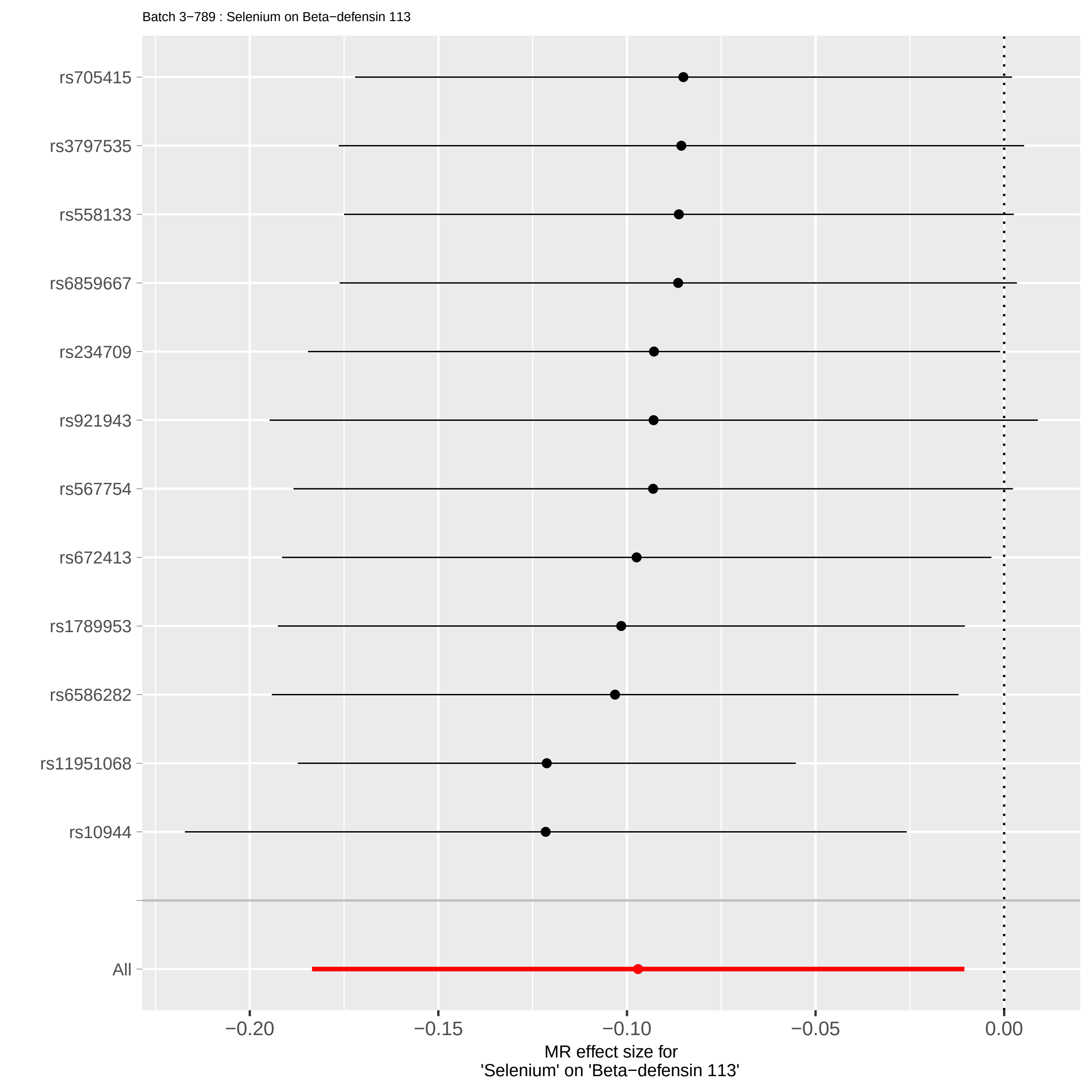
Batch 3−789 : Selenium on Beta−defensin 113
rs705415
rs3797535
rs558133
rs6859667
rs234709
rs921943
rs567754
rs672413
rs1789953
rs6586282
rs11951068
rs10944
All
−0.20
−0.15
−0.10
−0.05
0.00
MR effect size for
'Selenium' on 'Beta−defensin 113'

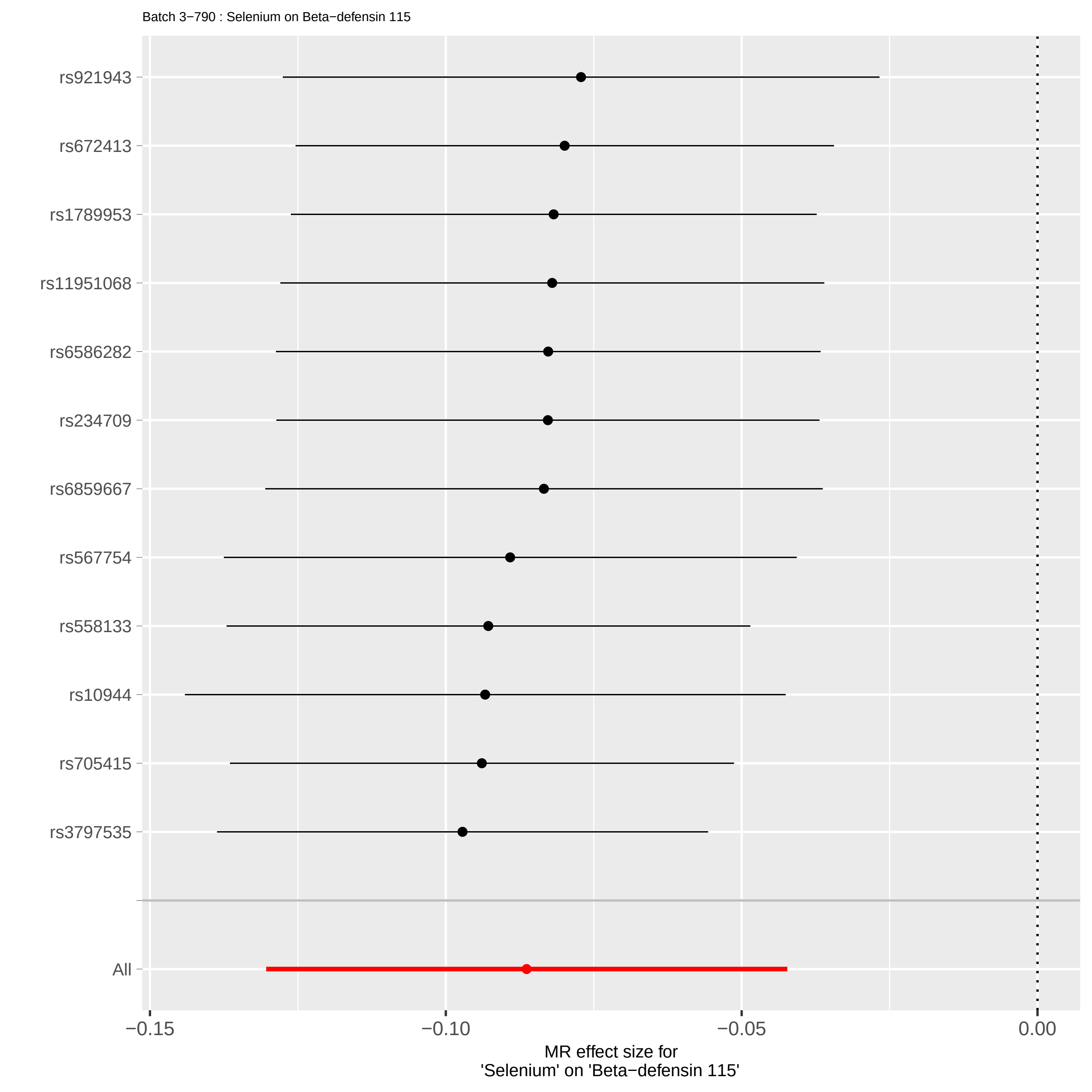
Batch 3−790 : Selenium on Beta−defensin 115
rs921943
rs672413
rs1789953
rs11951068
rs6586282
rs234709
rs6859667
rs567754
rs558133
rs10944
rs705415
rs3797535
All
−0.15
−0.10
−0.05
0.00
MR effect size for
'Selenium' on 'Beta−defensin 115'

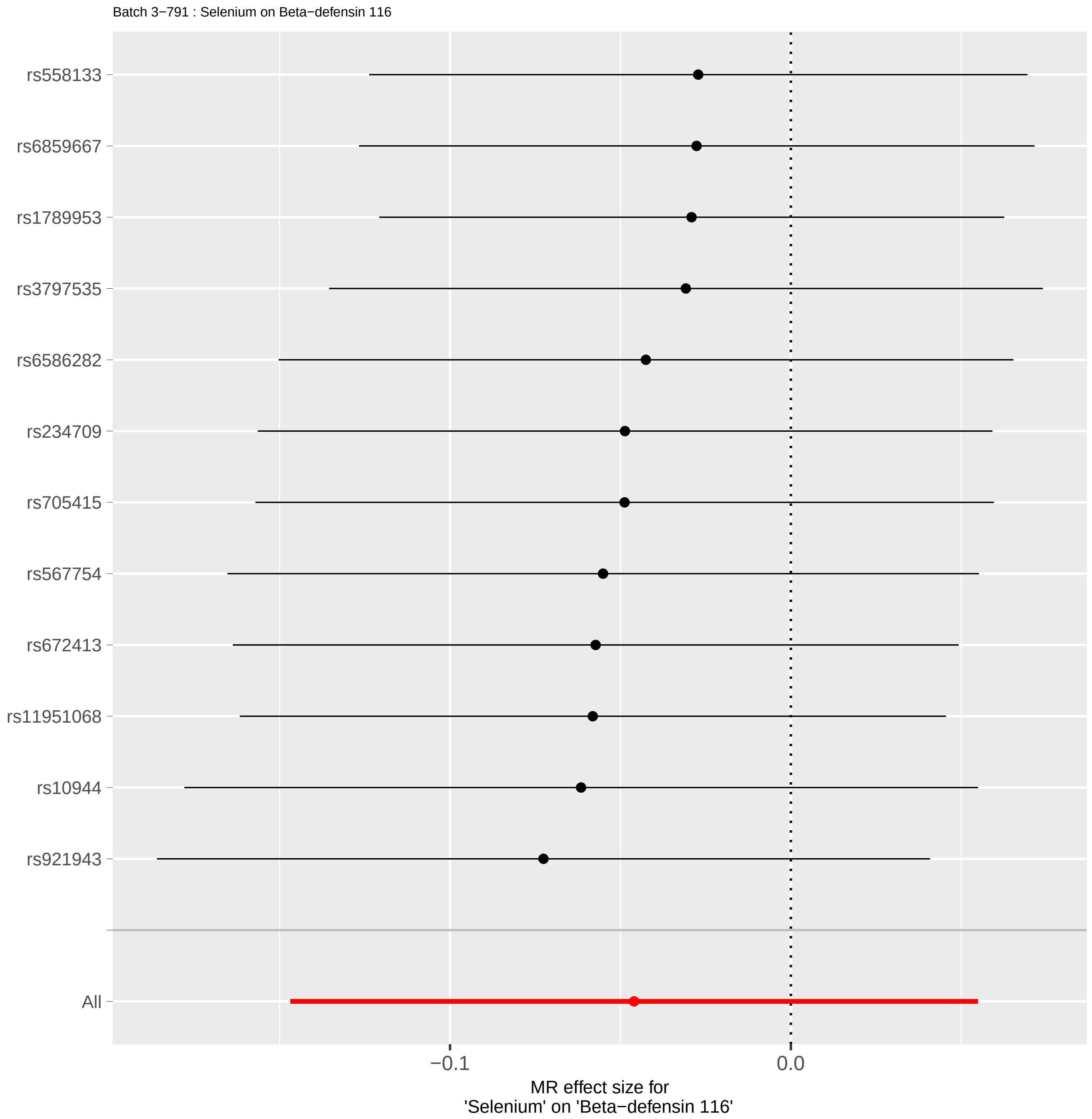
Batch 3−791 : Selenium on Beta−defensin 116
rs558133
rs6859667
rs1789953
rs3797535
rs6586282
rs234709
rs705415
rs567754
rs672413
rs11951068
rs10944
rs921943
All
−0.1
0.0
MR effect size for
'Selenium' on 'Beta−defensin 116'

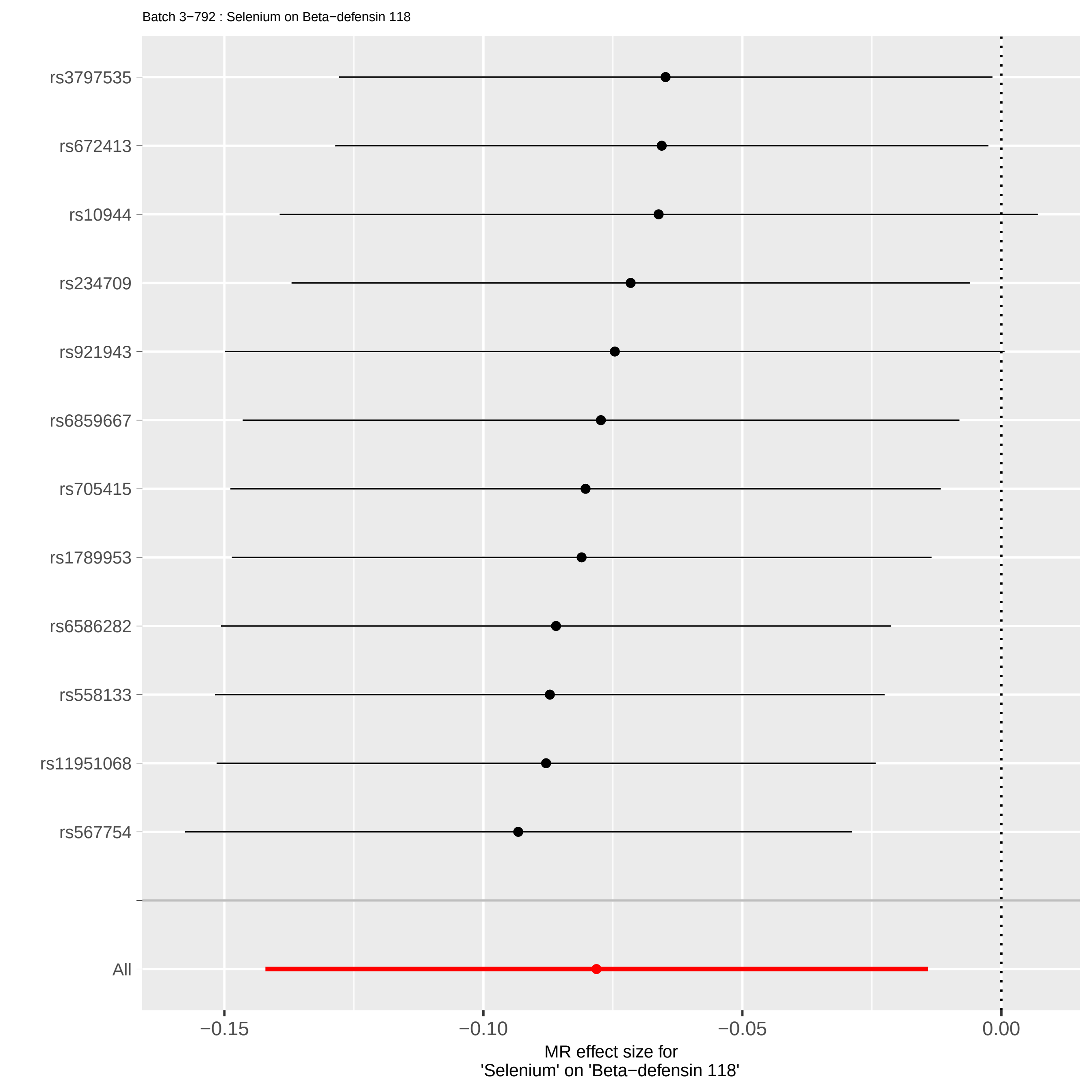

Batch 3−792 : Selenium on Beta−defensin 118
rs3797535
rs672413
rs10944
rs234709
rs921943
rs6859667
rs705415
rs1789953
rs6586282
rs558133
rs11951068
rs567754
All
−0.15
−0.10
−0.05
0.00
MR effect size for
'Selenium' on 'Beta−defensin 118'

Batch 3−793 : Selenium on Beta−defensin 119

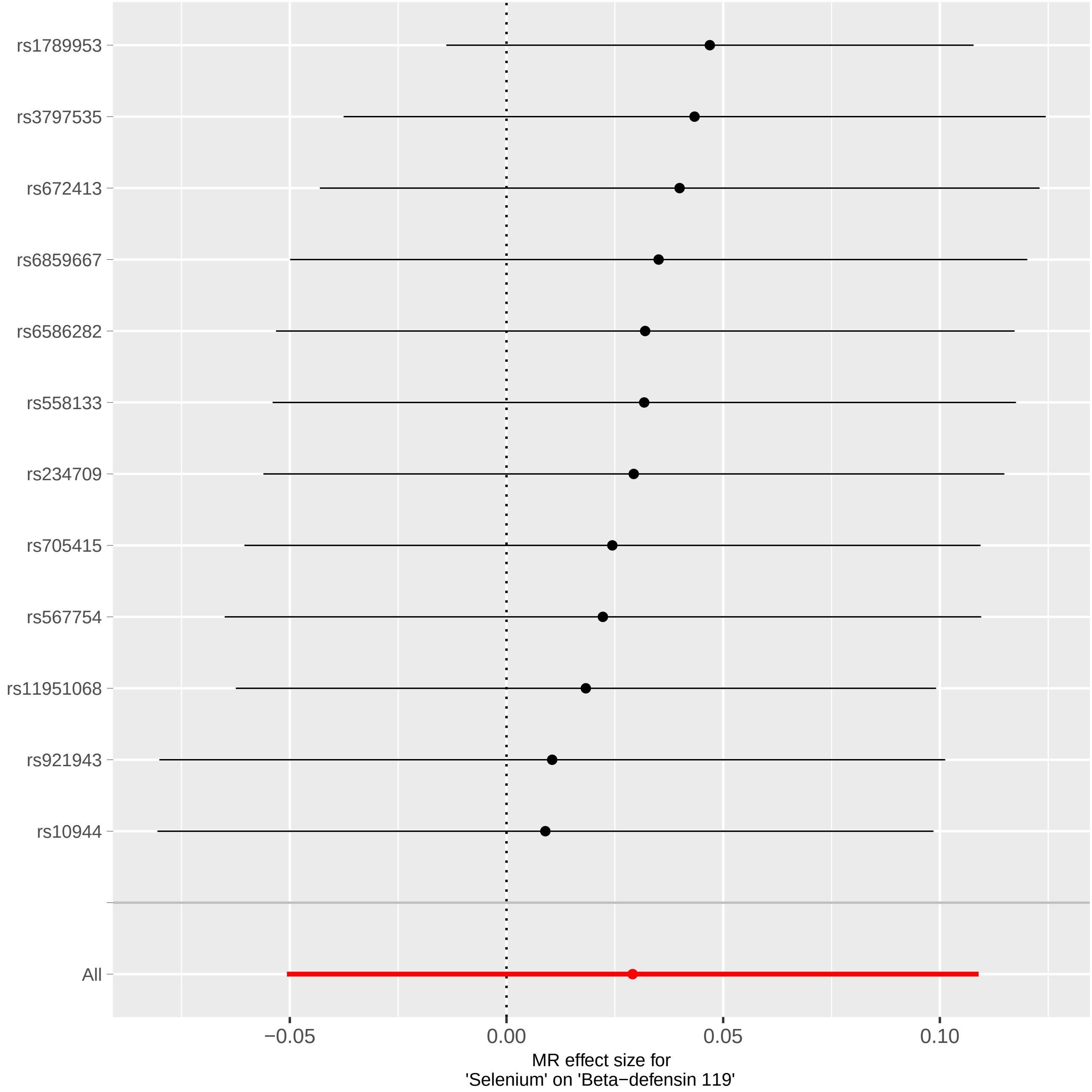

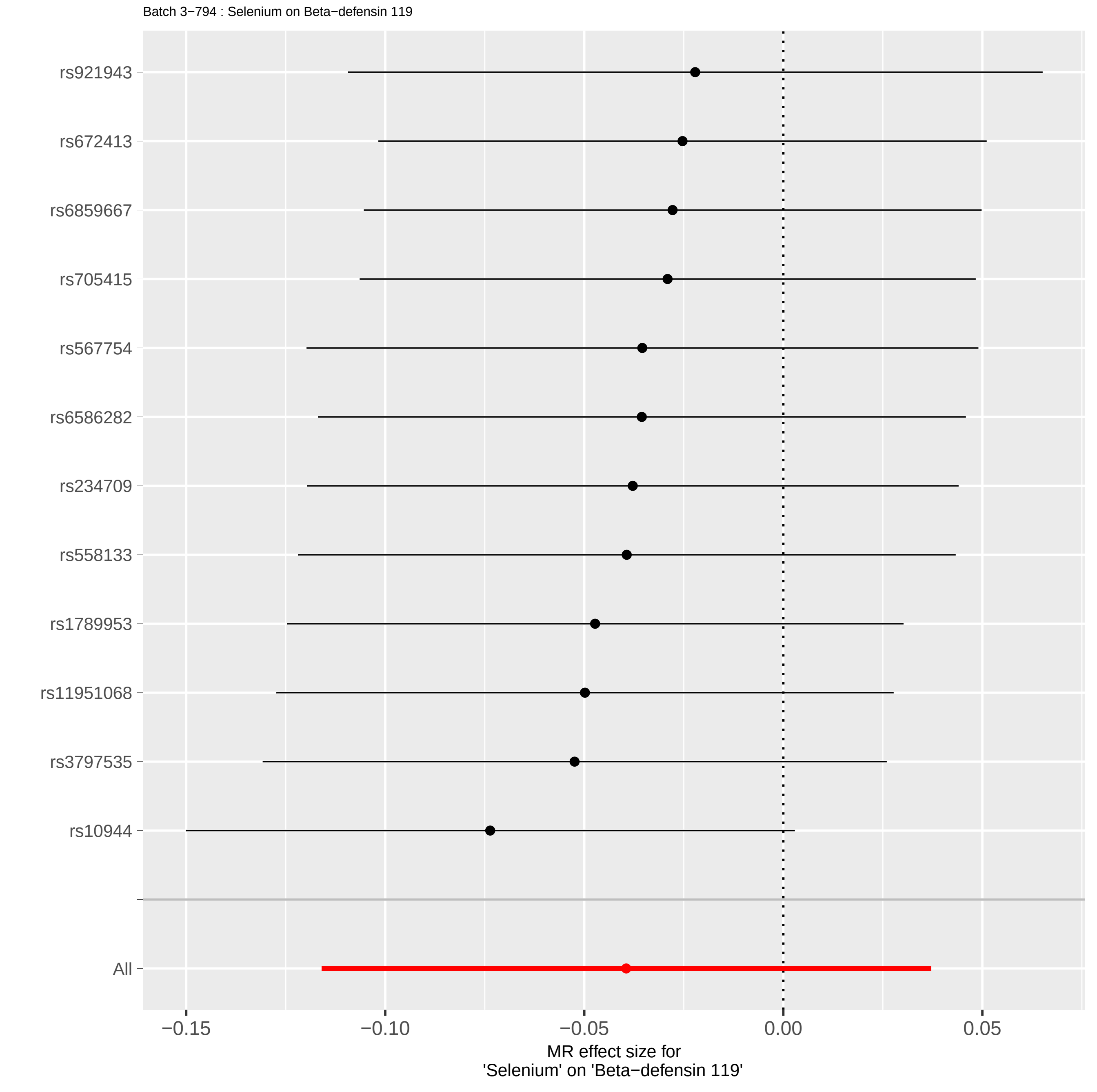

Batch 3−794 : Selenium on Beta−defensin 119
rs921943
rs672413
rs6859667
rs705415
rs567754
rs6586282
rs234709
rs558133
rs1789953
rs11951068
rs3797535
rs10944
All
−0.15
−0.10
−0.05
0.00
0.05
MR effect size for
'Selenium' on 'Beta−defensin 119'

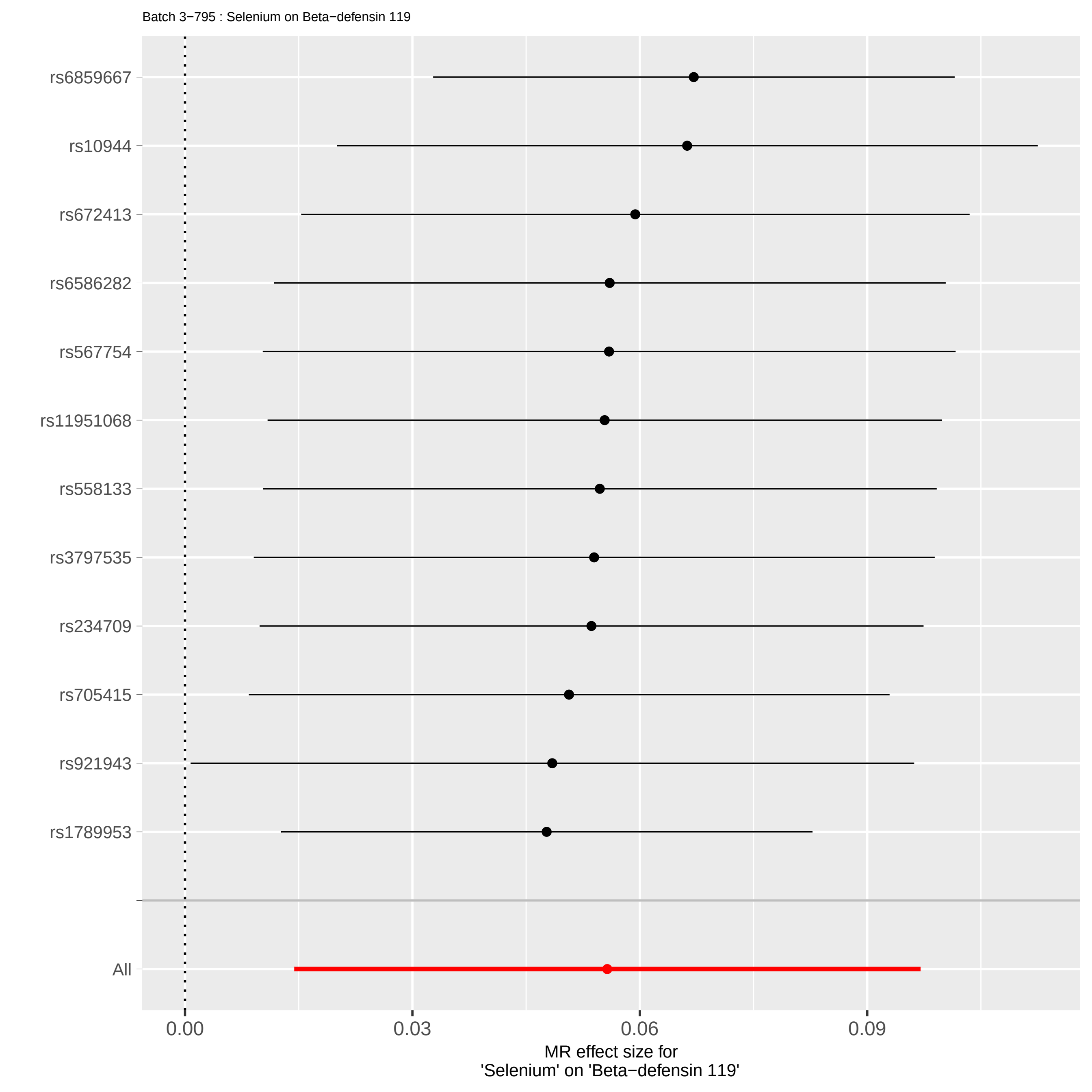

Batch 3−795 : Selenium on Beta−defensin 119
rs6859667
rs10944
rs672413
rs6586282
rs567754
rs11951068
rs558133
rs3797535
rs234709
rs705415
rs921943
rs1789953
All
0.00
0.03
0.06
0.09
MR effect size for
'Selenium' on 'Beta−defensin 119'

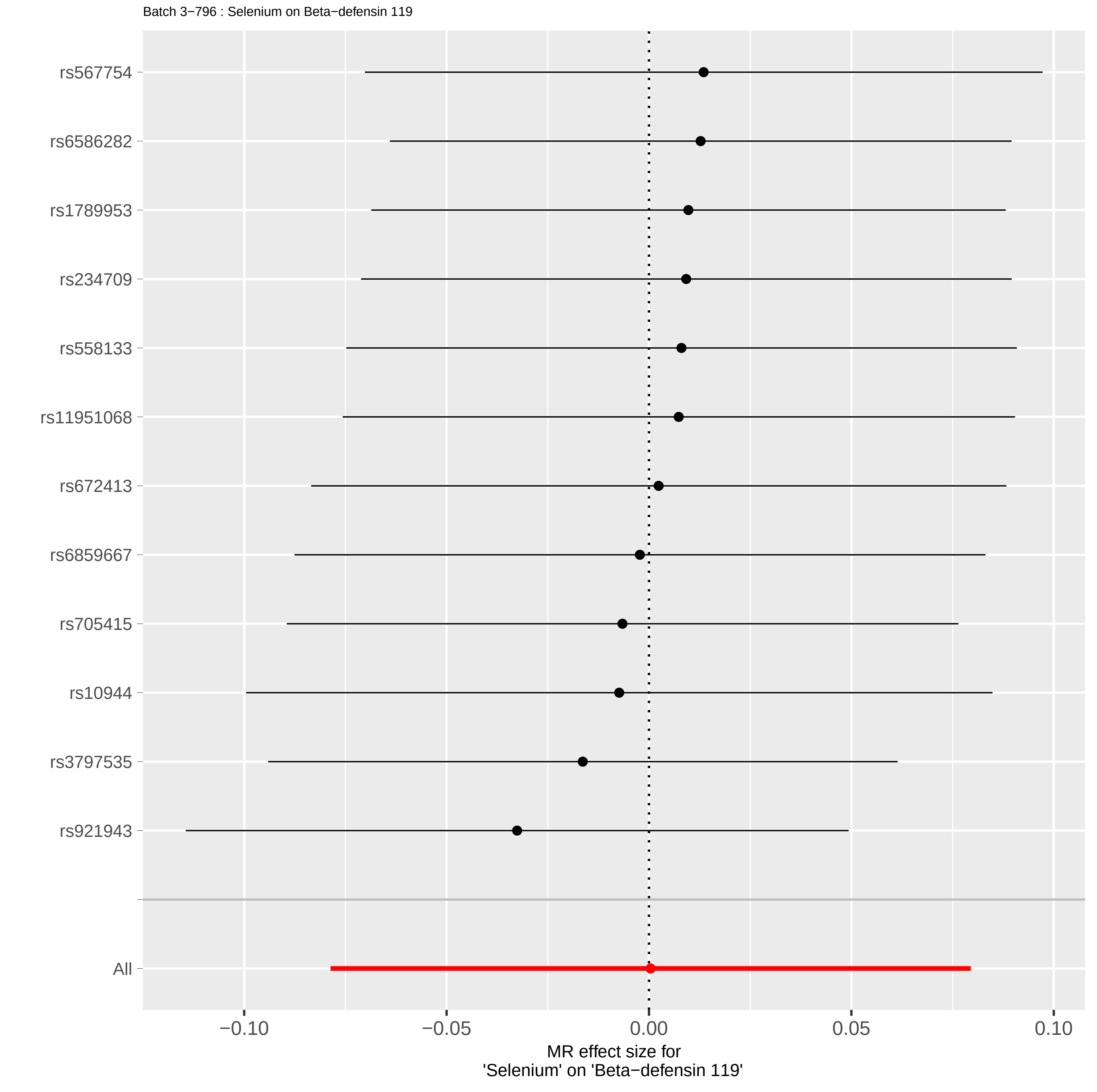

Batch 3−796 : Selenium on Beta−defensin 119
rs567754
rs6586282
rs1789953
rs234709
rs558133
rs11951068
rs672413
rs6859667
rs705415
rs10944
rs3797535
rs921943
All
−0.10
−0.05
0.00
0.05
0.10
MR effect size for
'Selenium' on 'Beta−defensin 119'

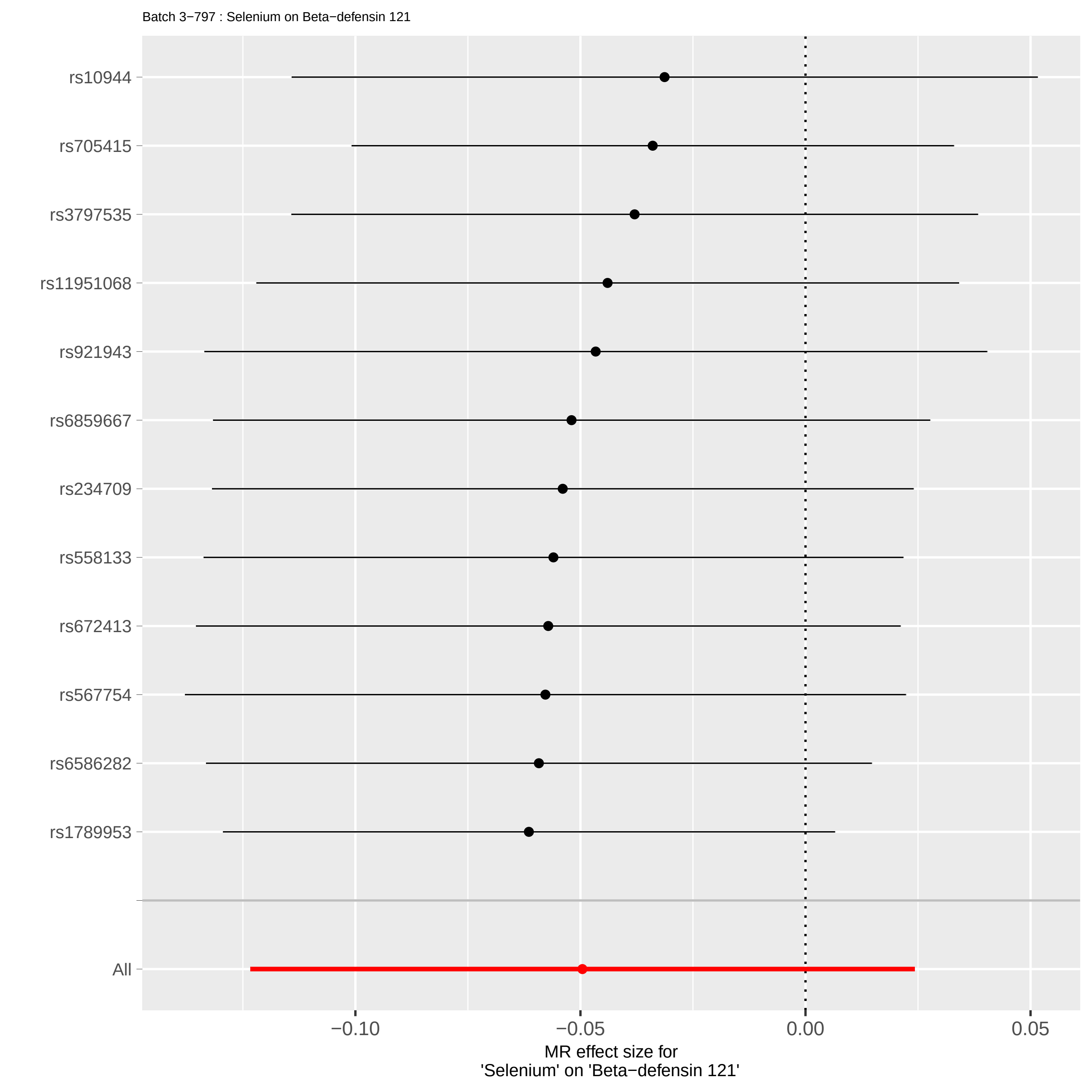

Batch 3−797 : Selenium on Beta−defensin 121
rs10944
rs705415
rs3797535
rs11951068
rs921943
rs6859667
rs234709
rs558133
rs672413
rs567754
rs6586282
rs1789953
All
−0.10
−0.05
0.00
0.05
MR effect size for
'Selenium' on 'Beta−defensin 121'

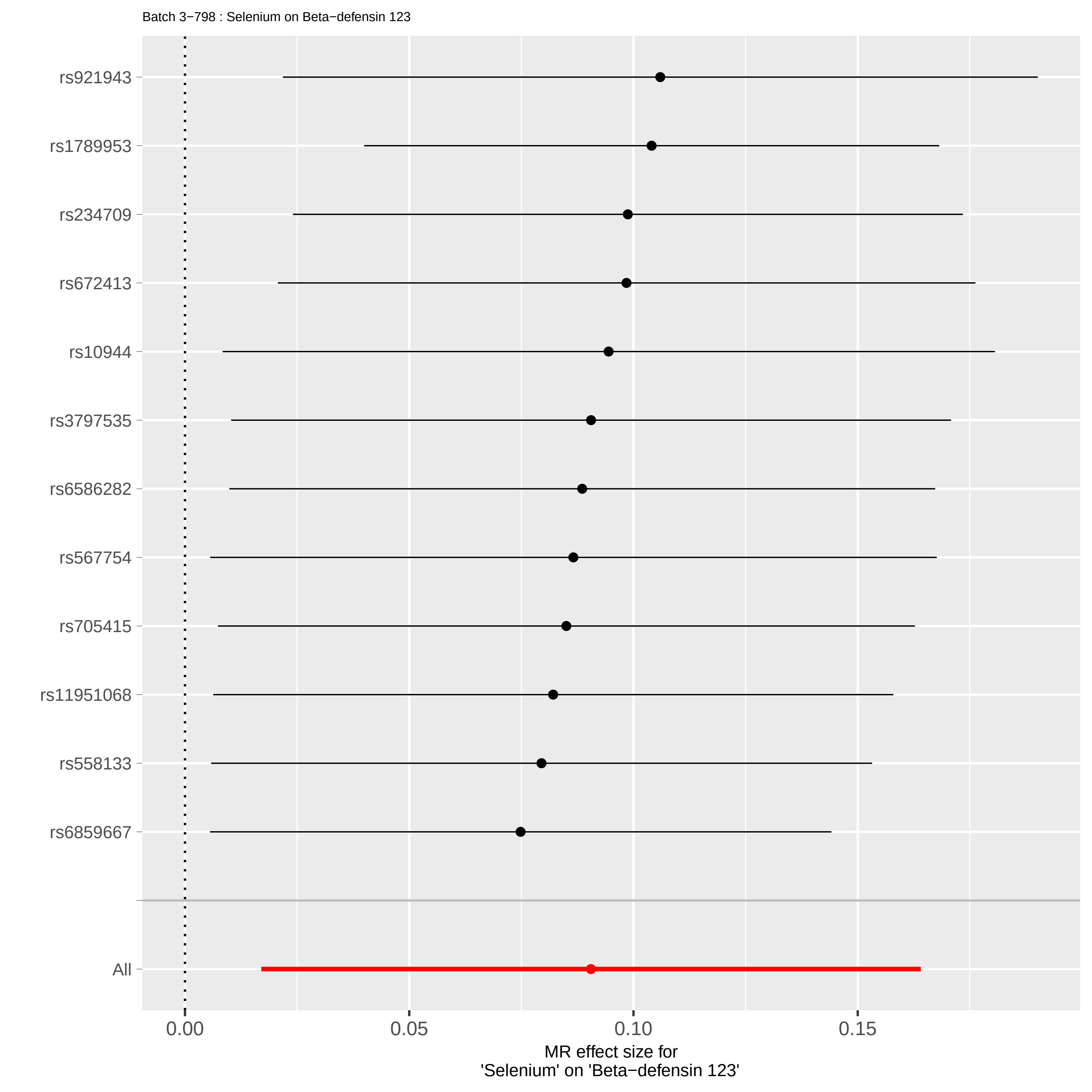

Batch 3−798 : Selenium on Beta−defensin 123
rs921943
rs1789953
rs234709
rs672413
rs10944
rs3797535
rs6586282
rs567754
rs705415
rs11951068
rs558133
rs6859667
All
0.00
0.05
0.10
0.15
MR effect size for
'Selenium' on 'Beta−defensin 123'

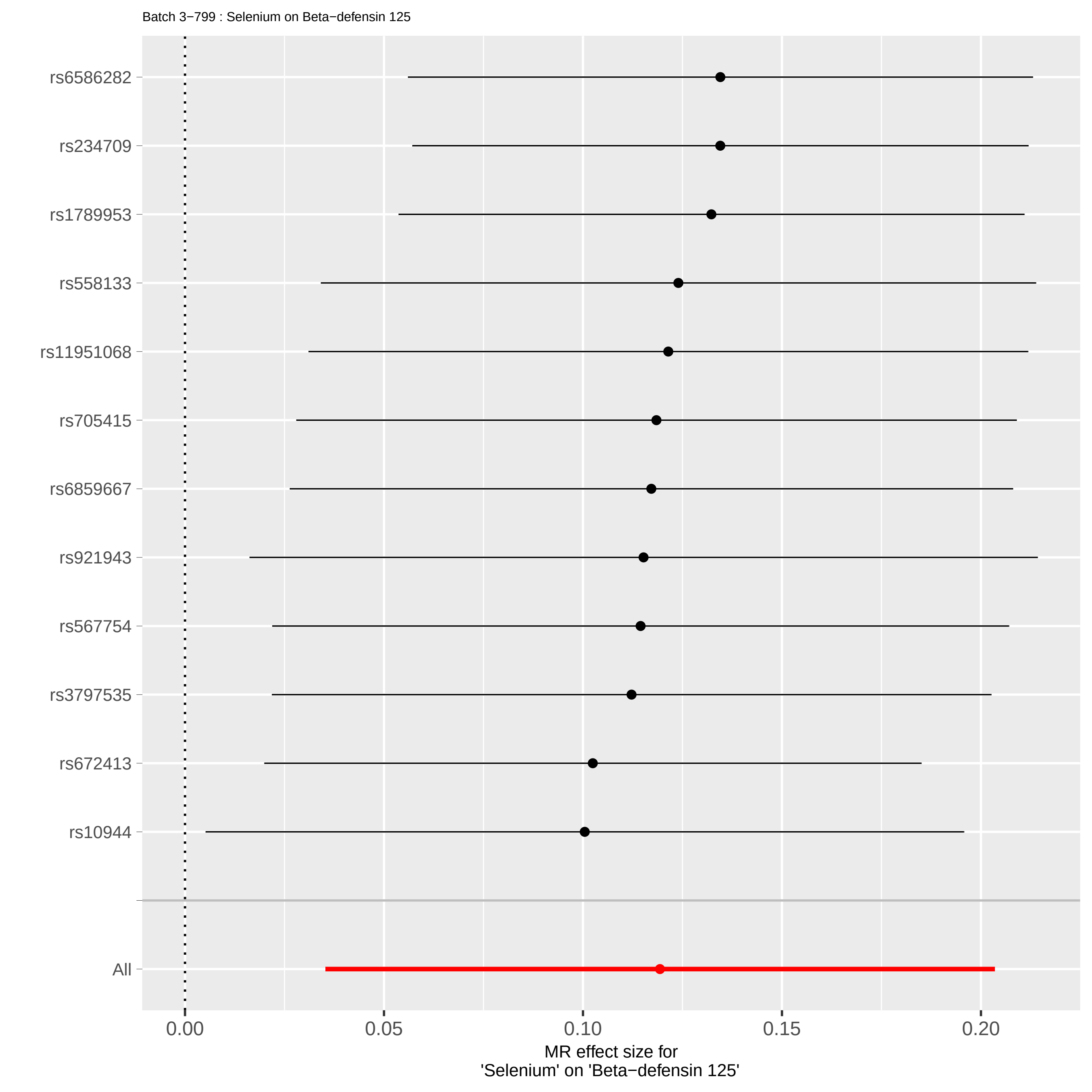

Batch 3−799 : Selenium on Beta−defensin 125
rs6586282
rs234709
rs1789953
rs558133
rs11951068
rs705415
rs6859667
rs921943
rs567754
rs3797535
rs672413
rs10944
All
0.00
0.05
0.10
0.15
0.20
MR effect size for
'Selenium' on 'Beta−defensin 125'

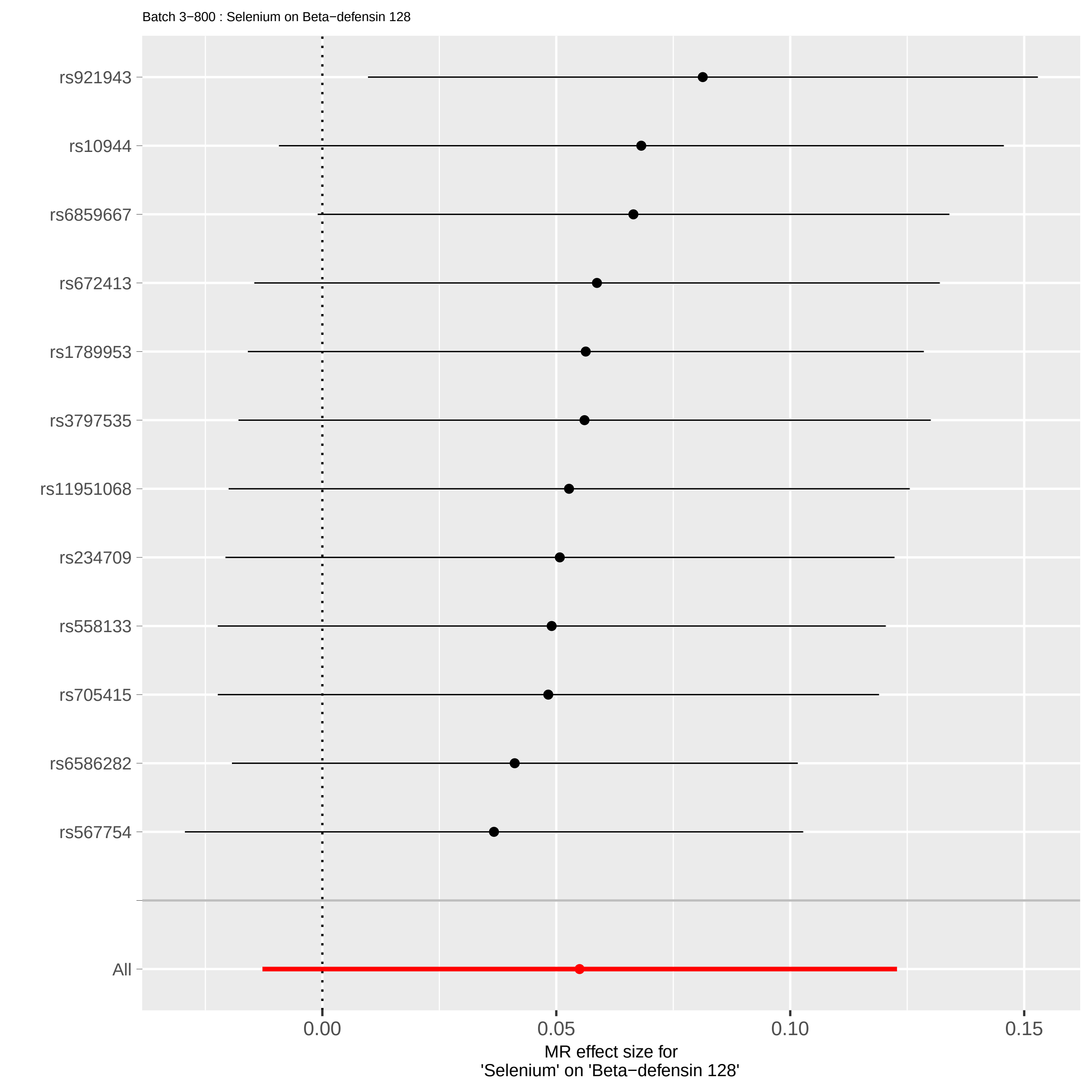
Batch 3−800 : Selenium on Beta−defensin 128
rs921943
rs10944
rs6859667
rs672413
rs1789953
rs3797535
rs11951068
rs234709
rs558133
rs705415
rs6586282
rs567754
All
0.00
0.05
0.10
0.15
MR effect size for
'Selenium' on 'Beta−defensin 128'

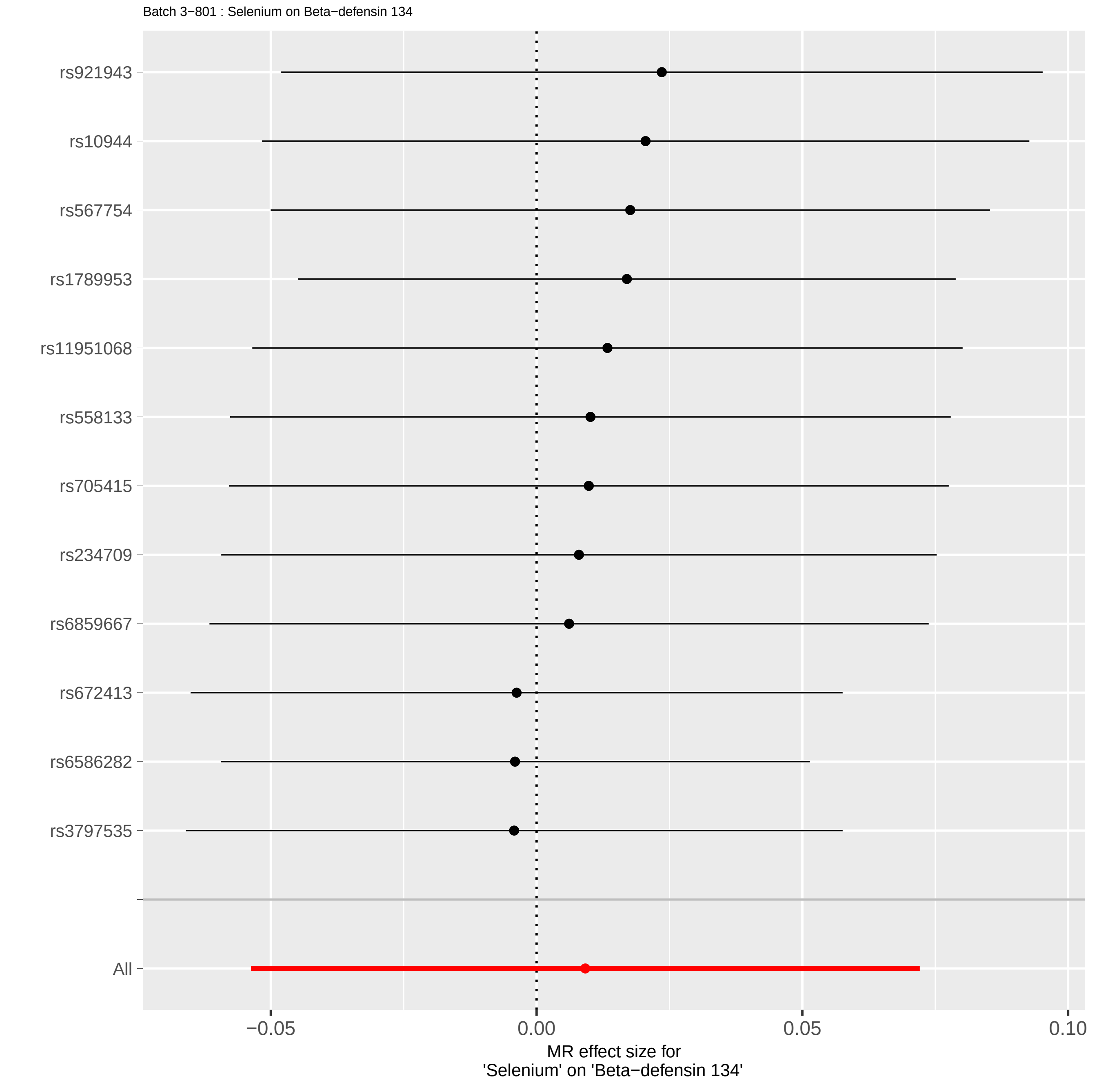

Batch 3−801 : Selenium on Beta−defensin 134
rs921943
rs10944
rs567754
rs1789953
rs11951068
rs558133
rs705415
rs234709
rs6859667
rs672413
rs6586282
rs3797535
All
−0.05
0.00
0.05
0.10
MR effect size for
'Selenium' on 'Beta−defensin 134'

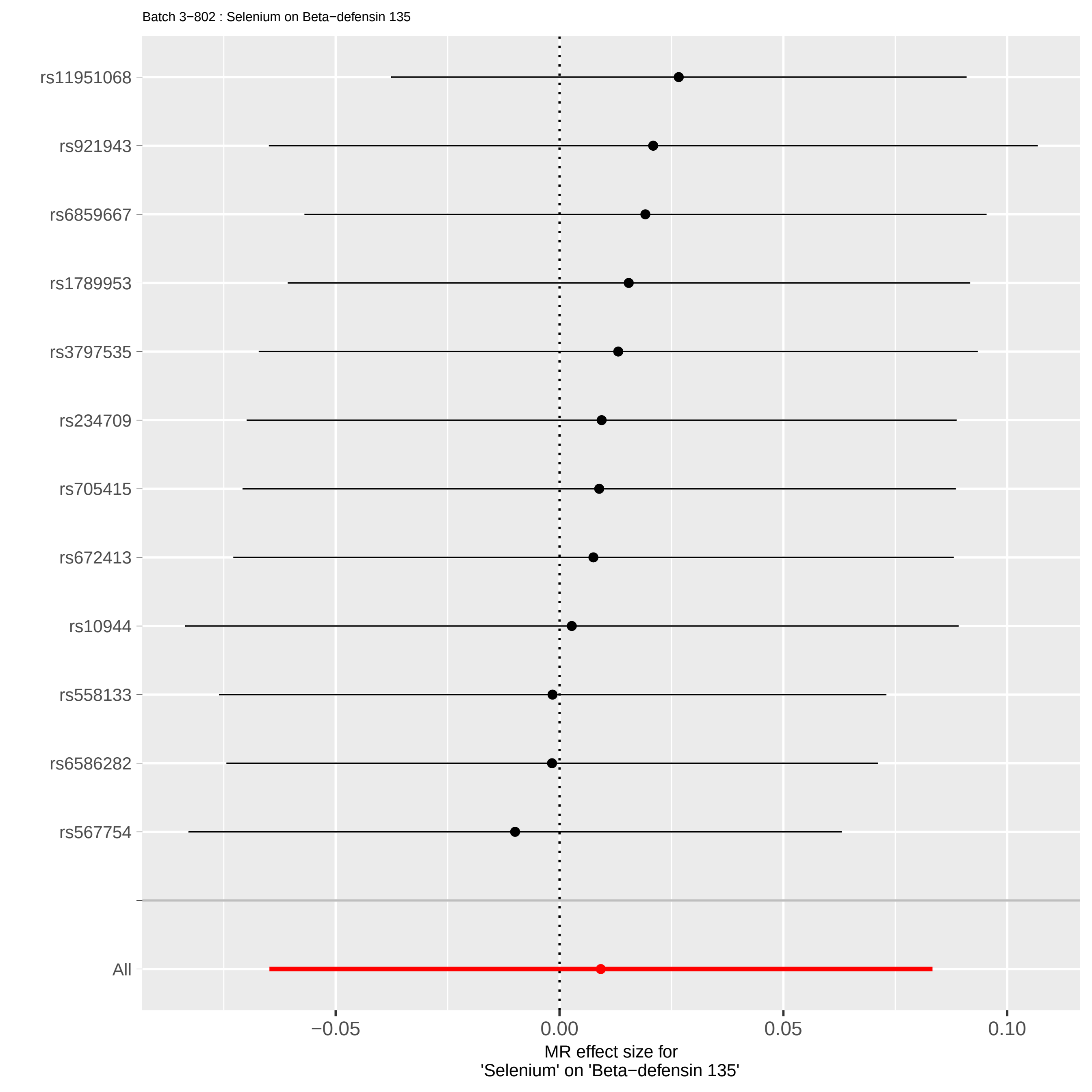

Batch 3−802 : Selenium on Beta−defensin 135
rs11951068
rs921943
rs6859667
rs1789953
rs3797535
rs234709
rs705415
rs672413
rs10944
rs558133
rs6586282
rs567754
All
−0.05
0.00
0.05
0.10
MR effect size for
'Selenium' on 'Beta−defensin 135'

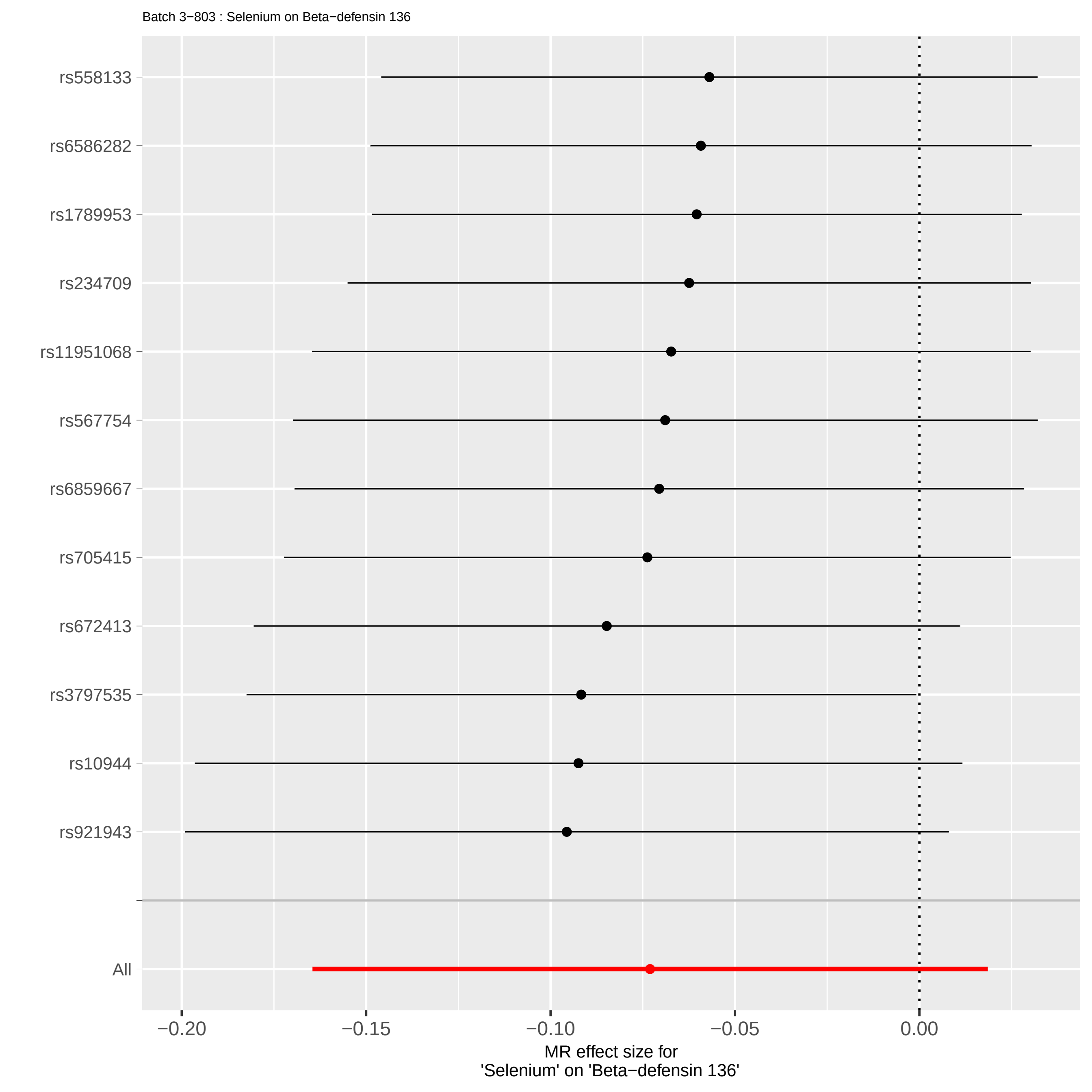

Batch 3−803 : Selenium on Beta−defensin 136
rs558133
rs6586282
rs1789953
rs234709
rs11951068
rs567754
rs6859667
rs705415
rs672413
rs3797535
rs10944
rs921943
All
−0.20
−0.15
−0.10
−0.05
0.00
MR effect size for
'Selenium' on 'Beta−defensin 136'

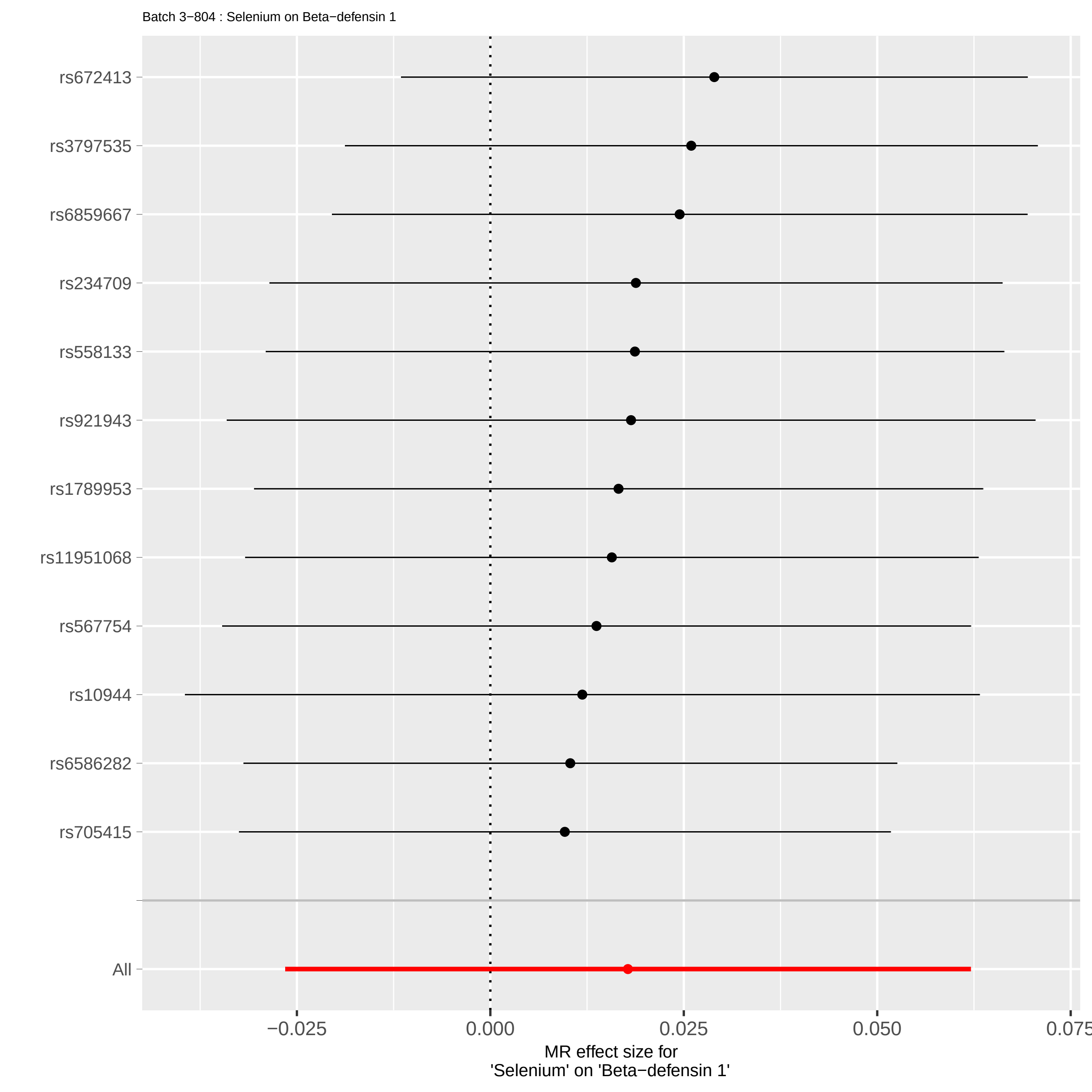

Batch 3−804 : Selenium on Beta−defensin 1
rs672413
rs3797535
rs6859667
rs234709
rs558133
rs921943
rs1789953
rs11951068
rs567754
rs10944
rs6586282
rs705415
All
−0.025
0.000
0.025
0.050
0.075
MR effect size for
'Selenium' on 'Beta−defensin 1'

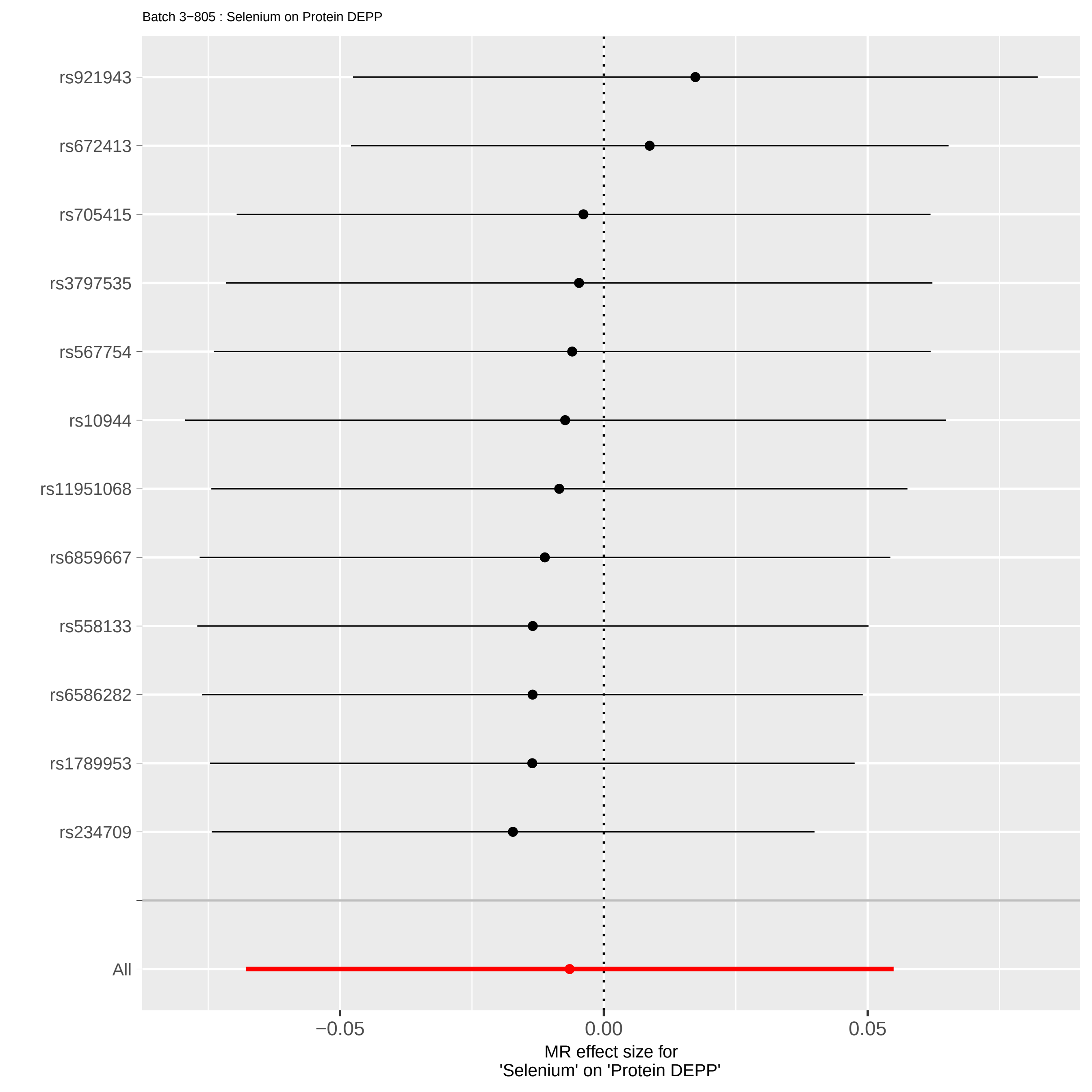

Batch 3−805 : Selenium on Protein DEPP
rs921943
rs672413
rs705415
rs3797535
rs567754
rs10944
rs11951068
rs6859667
rs558133
rs6586282
rs1789953
rs234709
All
−0.05
0.00
0.05
MR effect size for
'Selenium' on 'Protein DEPP'

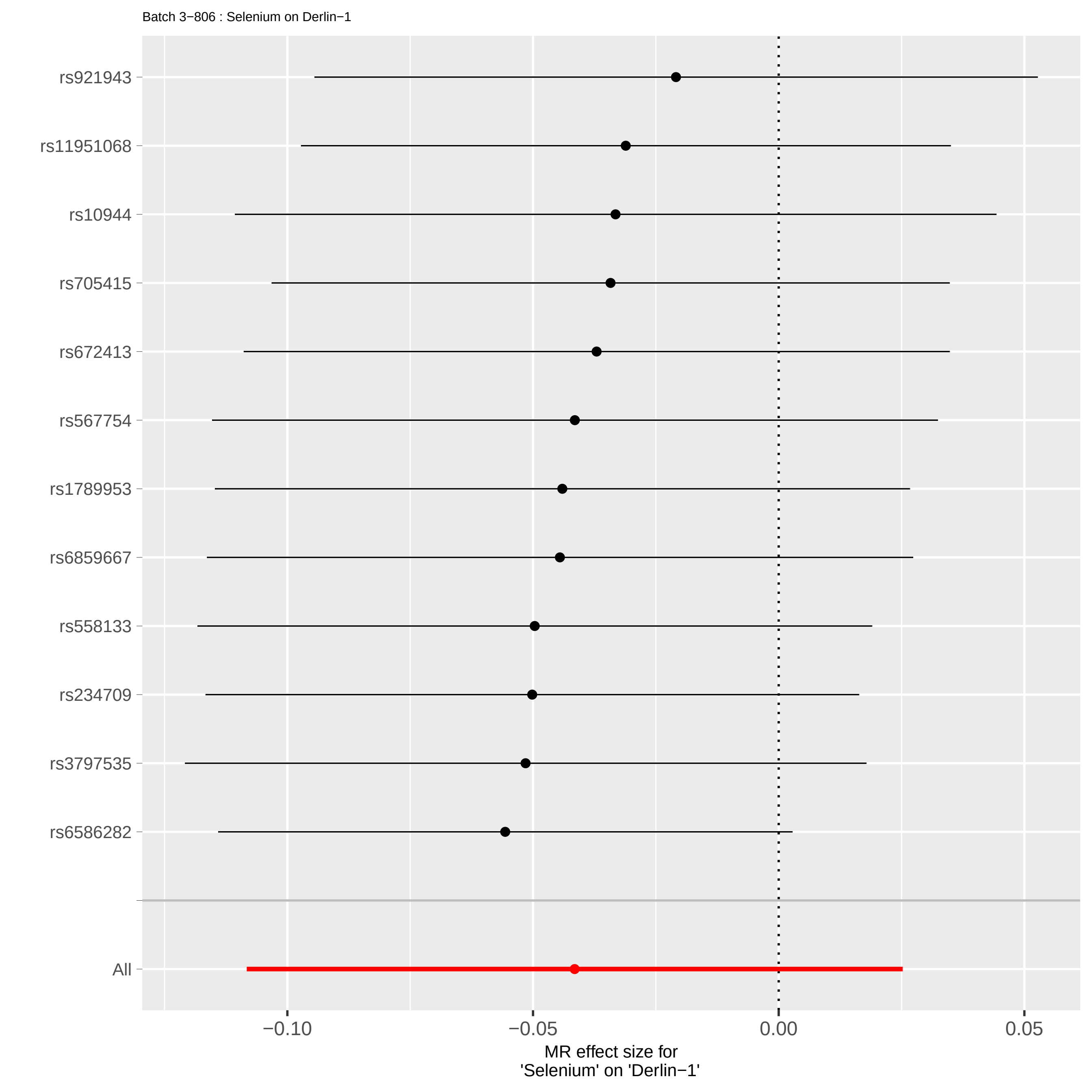
Batch 3−806 : Selenium on Derlin−1
rs921943
rs11951068
rs10944
rs705415
rs672413
rs567754
rs1789953
rs6859667
rs558133
rs234709
rs3797535
rs6586282
All
−0.10
−0.05
0.00
0.05
MR effect size for
'Selenium' on 'Derlin−1'

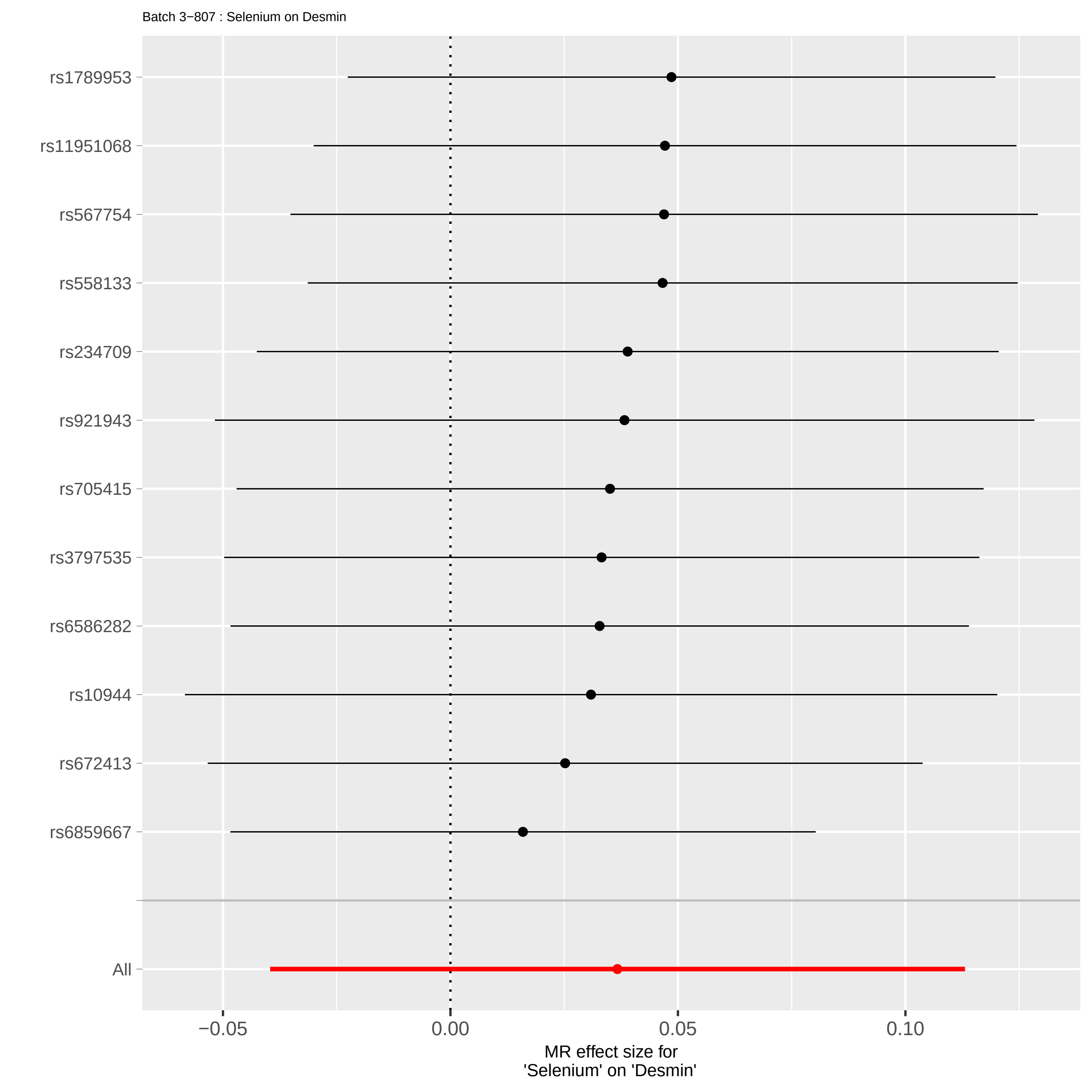
Batch 3−807 : Selenium on Desmin
rs1789953
rs11951068
rs567754
rs558133
rs234709
rs921943
rs705415
rs3797535
rs6586282
rs10944
rs672413
rs6859667
All
−0.05
0.00
0.05
0.10
MR effect size for
'Selenium' on 'Desmin'

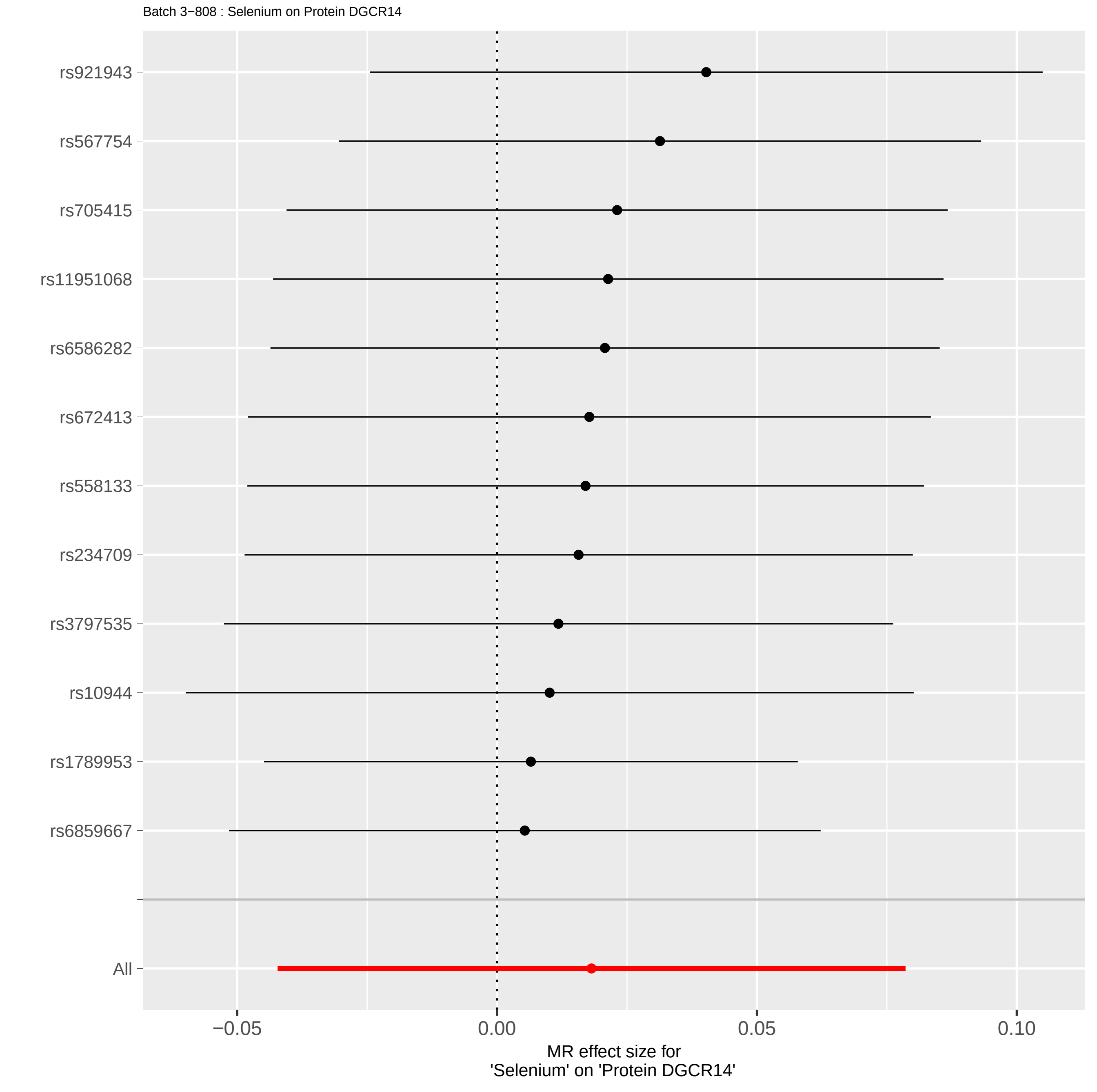
Batch 3−808 : Selenium on Protein DGCR14
rs921943
rs567754
rs705415
rs11951068
rs6586282
rs672413
rs558133
rs234709
rs3797535
rs10944
rs1789953
rs6859667
All
−0.05
0.00
0.05
0.10
MR effect size for
'Selenium' on 'Protein DGCR14'

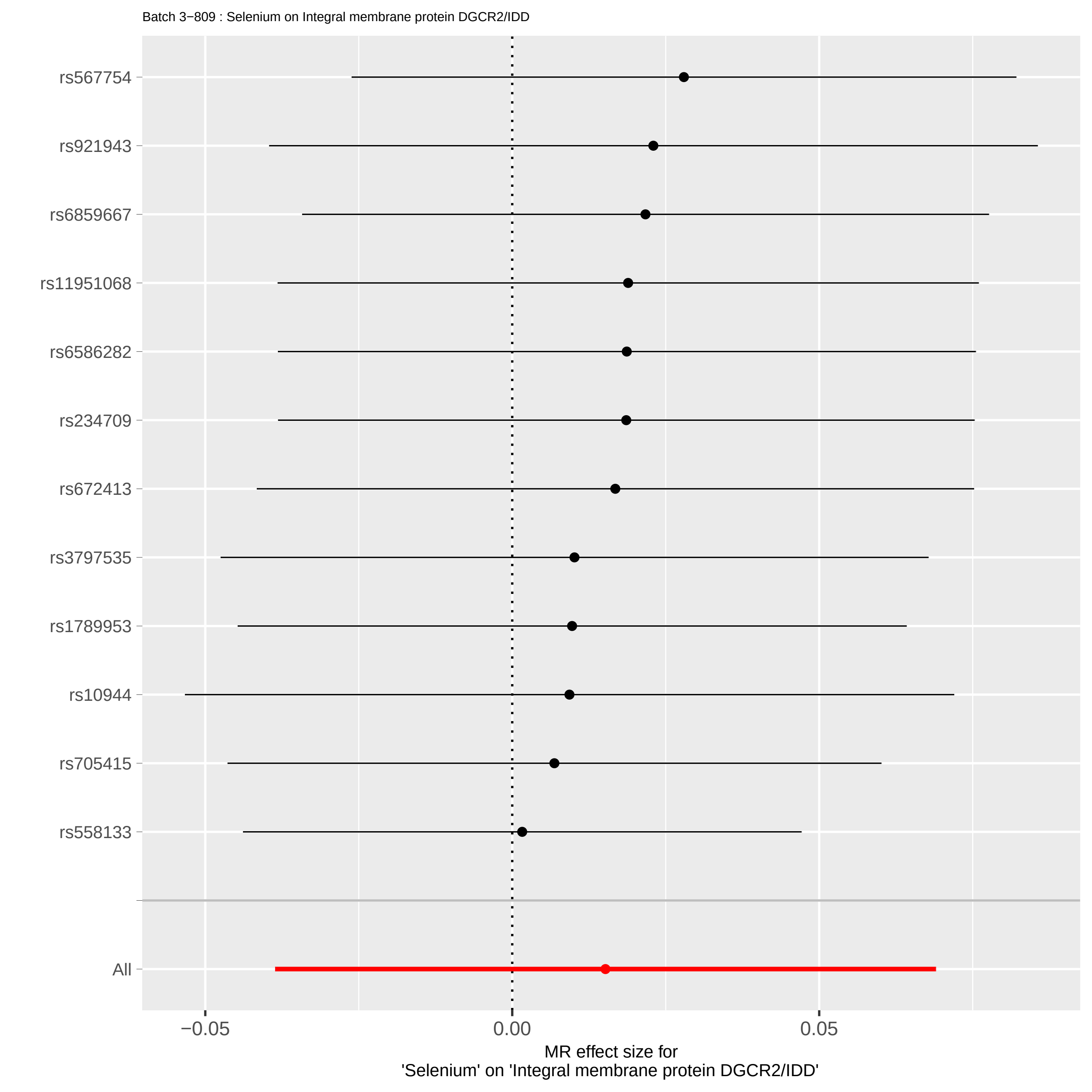

Batch 3−809 : Selenium on Integral membrane protein DGCR2/IDD
rs567754
rs921943
rs6859667
rs11951068
rs6586282
rs234709
rs672413
rs3797535
rs1789953
rs10944
rs705415
rs558133
All
−0.05
0.00
0.05
MR effect size for
'Selenium' on 'Integral membrane protein DGCR2/IDD'

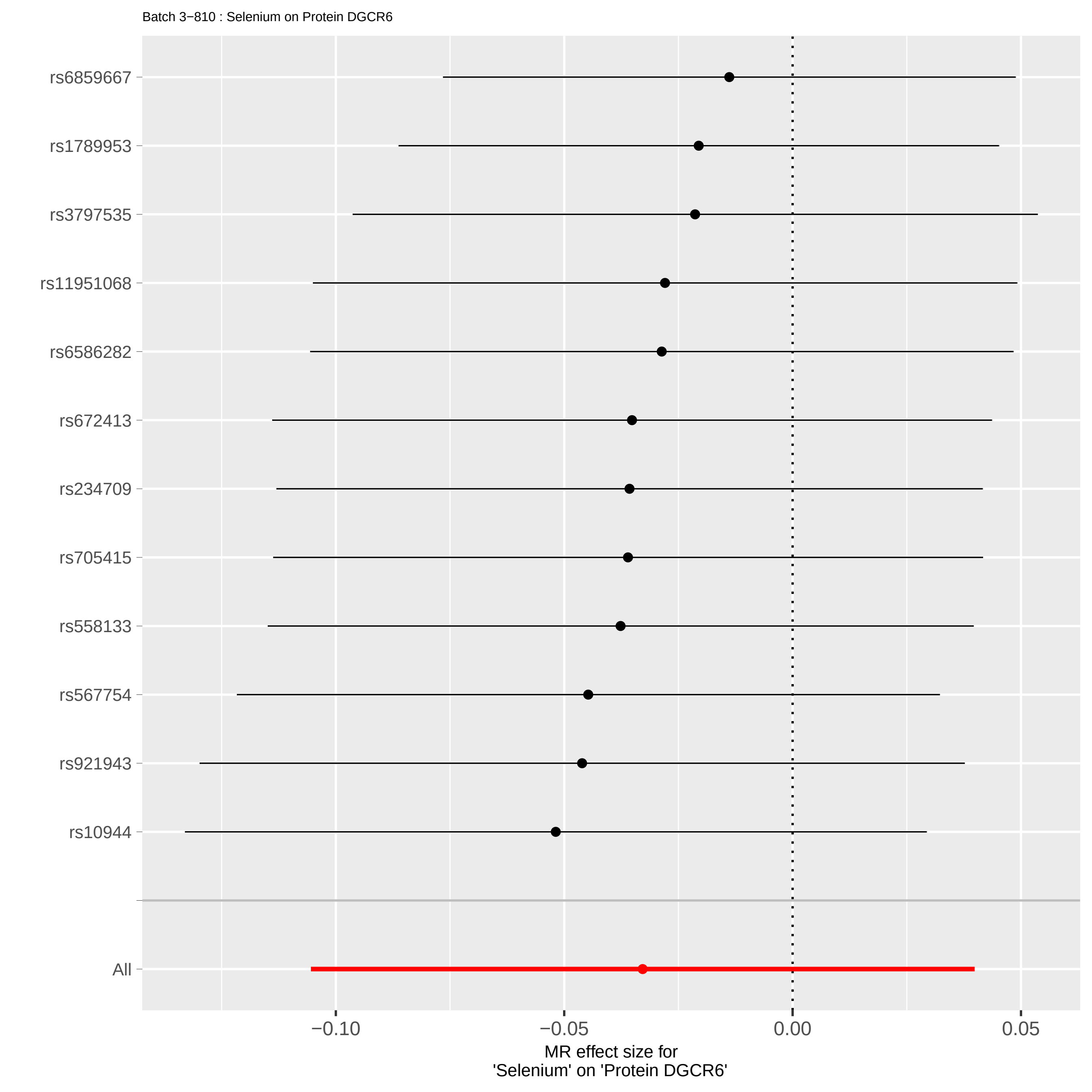

Batch 3−810 : Selenium on Protein DGCR6
rs6859667
rs1789953
rs3797535
rs11951068
rs6586282
rs672413
rs234709
rs705415
rs558133
rs567754
rs921943
rs10944
All
−0.10
−0.05
0.00
0.05
MR effect size for
'Selenium' on 'Protein DGCR6'

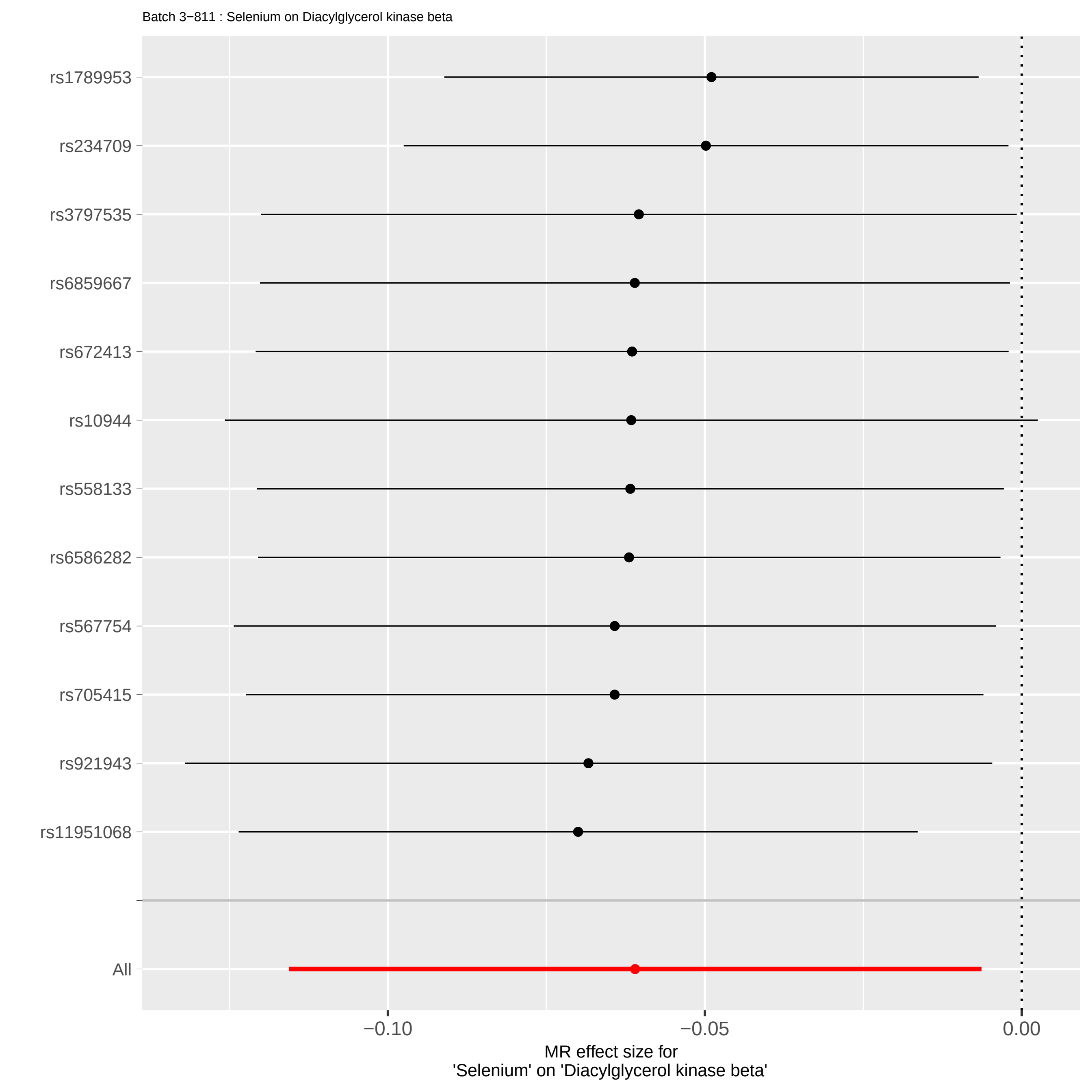
Batch 3−811 : Selenium on Diacylglycerol kinase beta
rs1789953
rs234709
rs3797535
rs6859667
rs672413
rs10944
rs558133
rs6586282
rs567754
rs705415
rs921943
rs11951068
All
−0.10
−0.05
0.00
MR effect size for
'Selenium' on 'Diacylglycerol kinase beta'

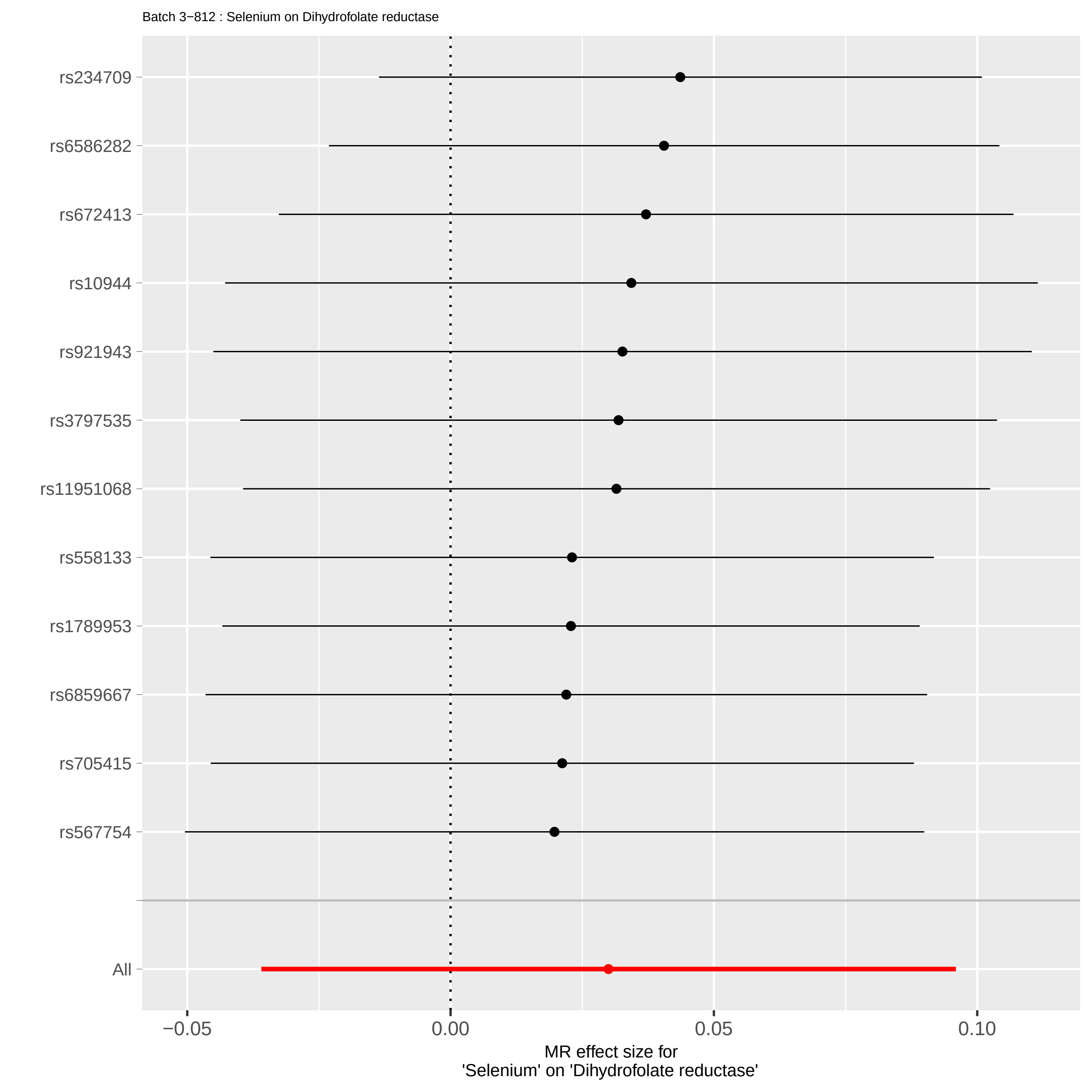

Batch 3−812 : Selenium on Dihydrofolate reductase
rs234709
rs6586282
rs672413
rs10944
rs921943
rs3797535
rs11951068
rs558133
rs1789953
rs6859667
rs705415
rs567754
All
−0.05
0.00
0.05
0.10
MR effect size for
'Selenium' on 'Dihydrofolate reductase'

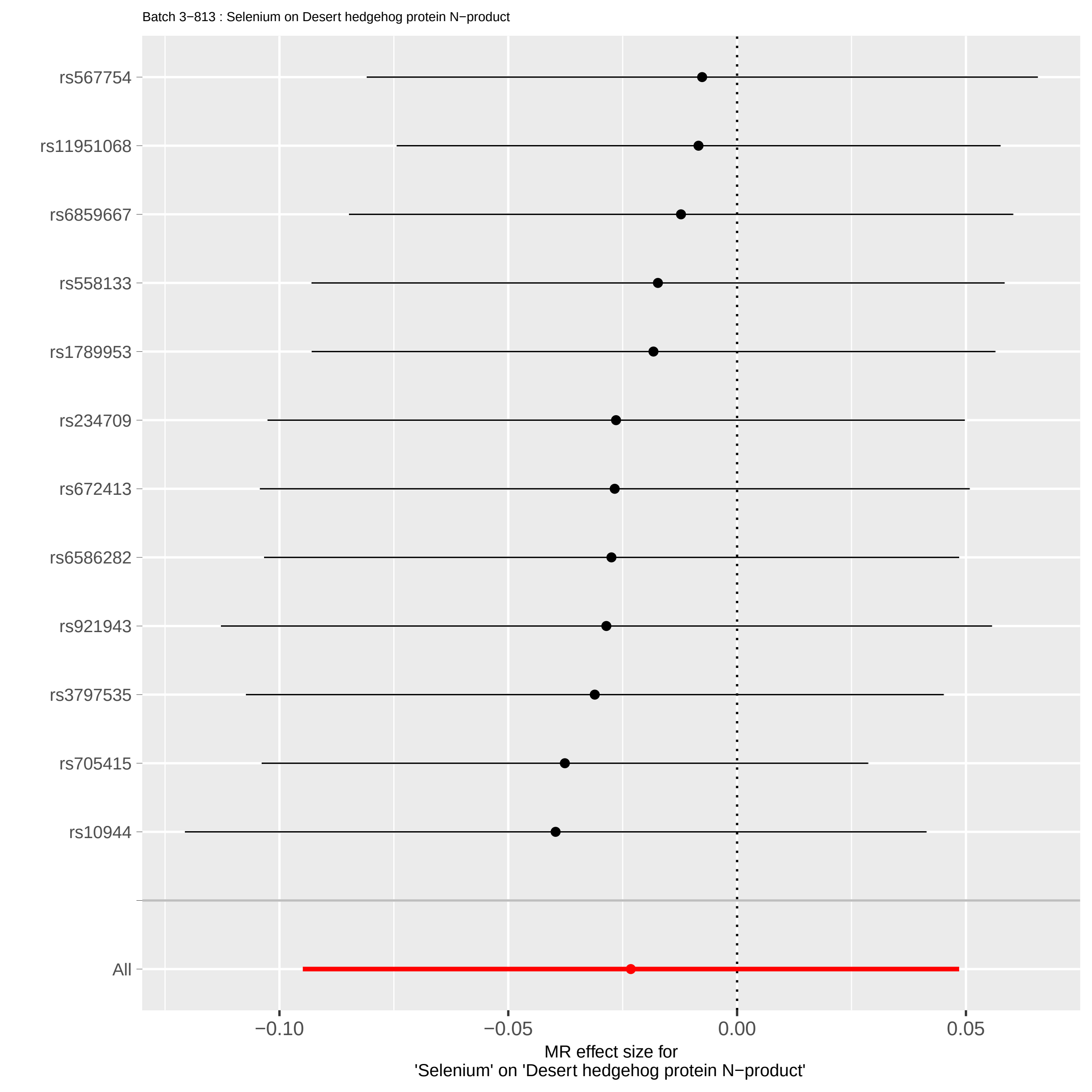

Batch 3−813 : Selenium on Desert hedgehog protein N−product
rs567754
rs11951068
rs6859667
rs558133
rs1789953
rs234709
rs672413
rs6586282
rs921943
rs3797535
rs705415
rs10944
All
−0.10
−0.05
0.00
0.05
MR effect size for
'Selenium' on 'Desert hedgehog protein N−product'

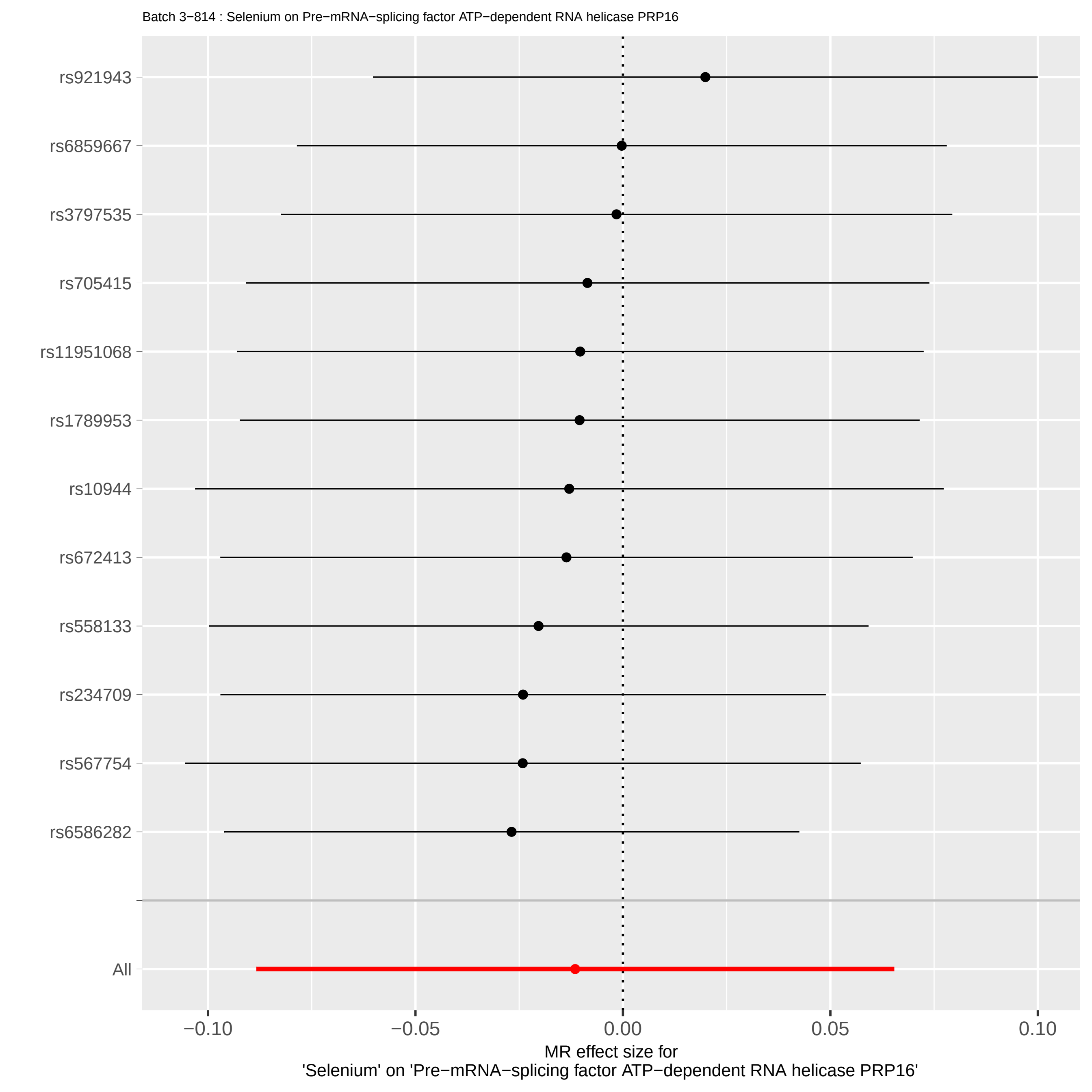

Batch 3−814 : Selenium on Pre−mRNA−splicing factor ATP−dependent RNA helicase PRP16
rs921943
rs6859667
rs3797535
rs705415
rs11951068
rs1789953
rs10944
rs672413
rs558133
rs234709
rs567754
rs6586282
All
−0.10
−0.05
0.00
0.05
0.10
MR effect size for
'Selenium' on 'Pre−mRNA−splicing factor ATP−dependent RNA helicase PRP16'

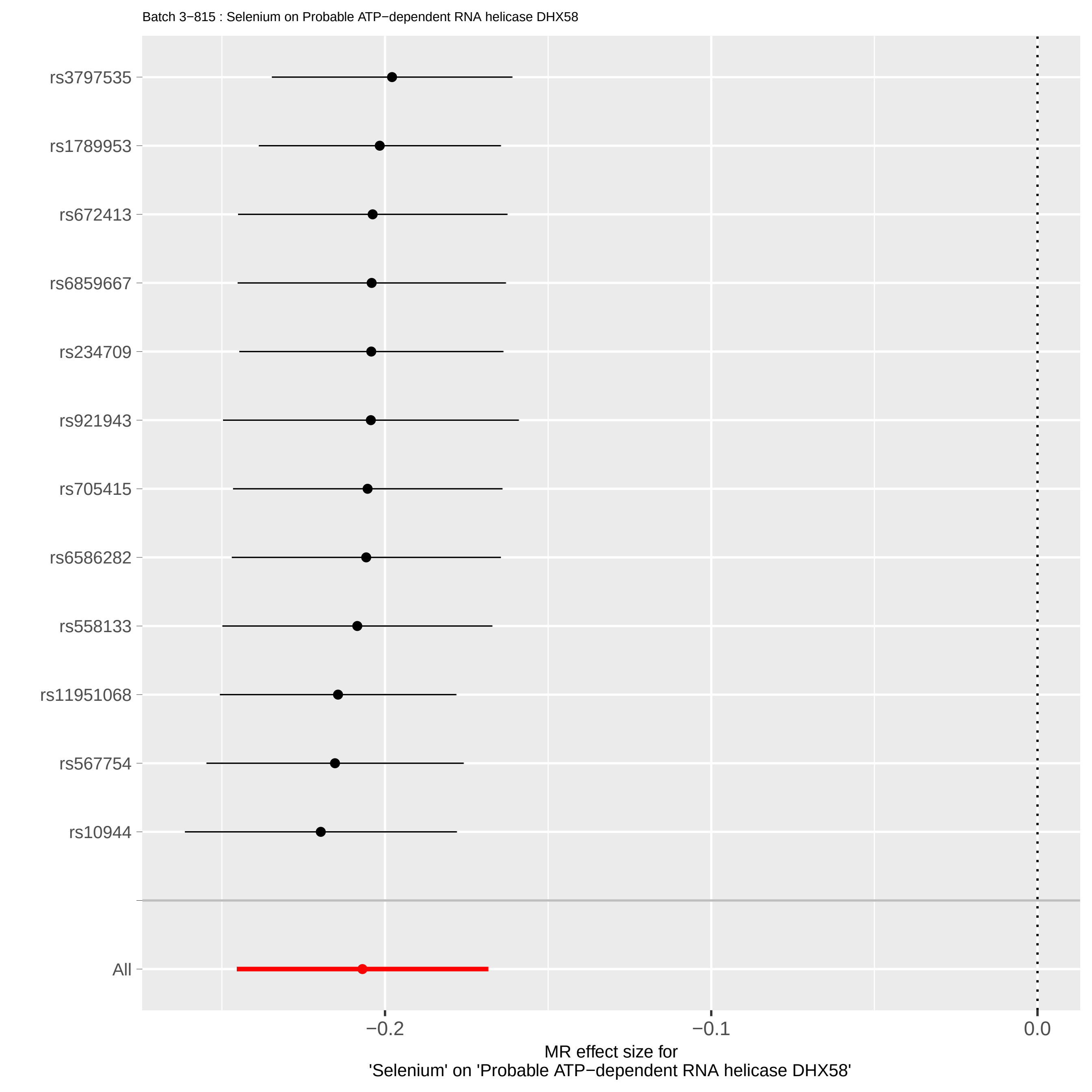
Batch 3−815 : Selenium on Probable ATP−dependent RNA helicase DHX58
rs3797535
rs1789953
rs672413
rs6859667
rs234709
rs921943
rs705415
rs6586282
rs558133
rs11951068
rs567754
rs10944
All
−0.2
−0.1
0.0
MR effect size for
'Selenium' on 'Probable ATP−dependent RNA helicase DHX58'

Batch 3−816 : Selenium on ATP−dependent RNA helicase DHX8

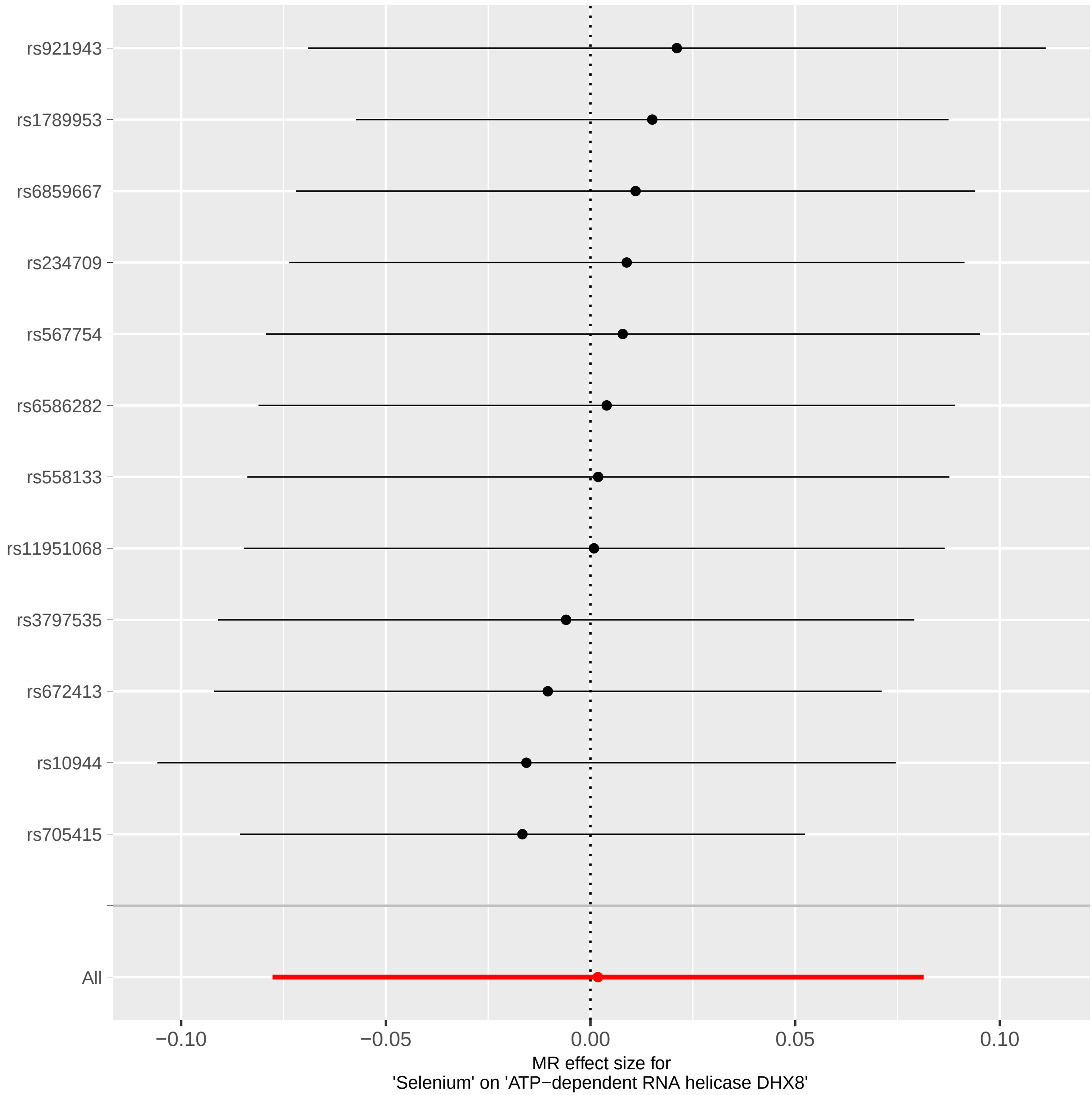

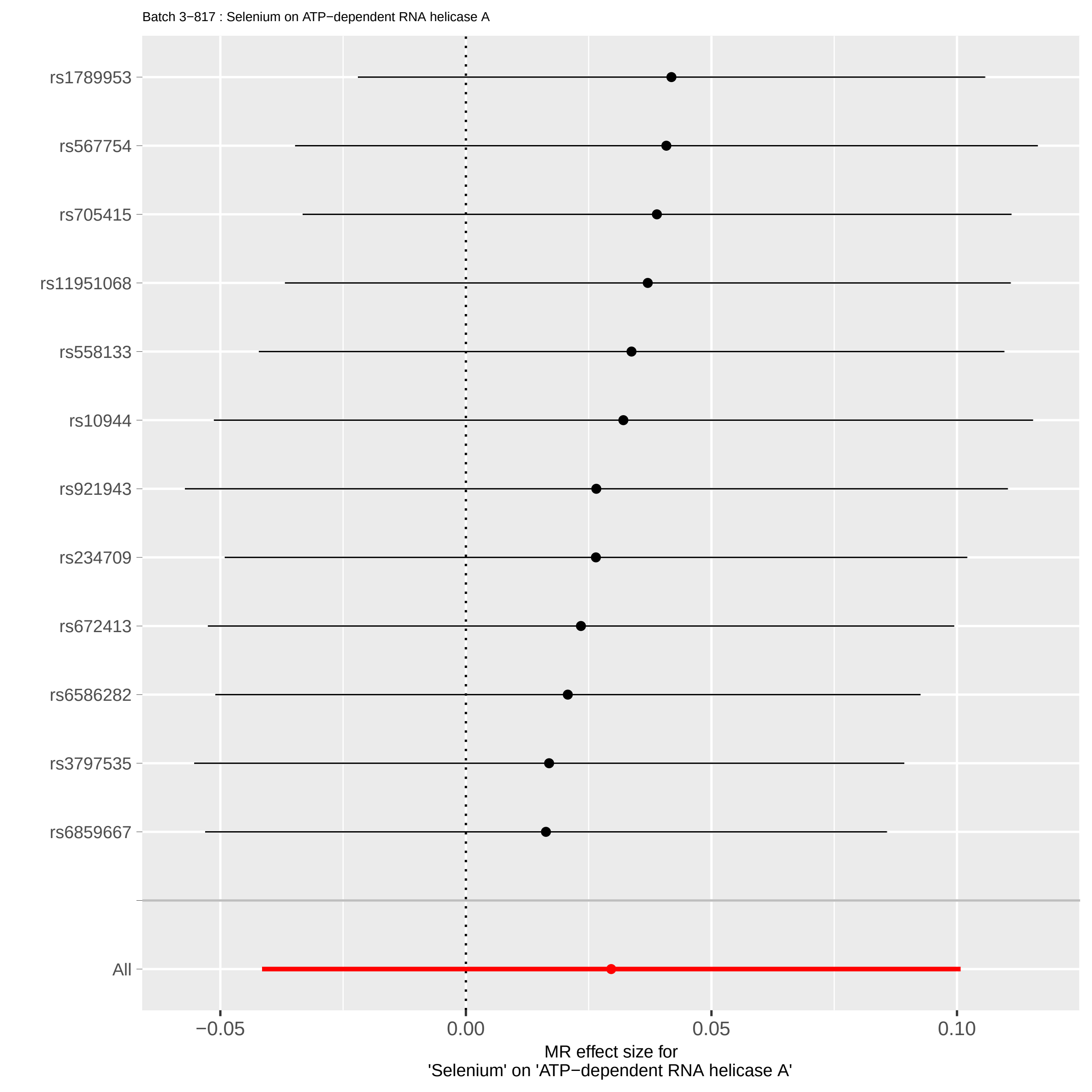
Batch 3−817 : Selenium on ATP−dependent RNA helicase A
rs1789953
rs567754
rs705415
rs11951068
rs558133
rs10944
rs921943
rs234709
rs672413
rs6586282
rs3797535
rs6859667
All
−0.05
0.00
0.05
0.10
MR effect size for
'Selenium' on 'ATP−dependent RNA helicase A'

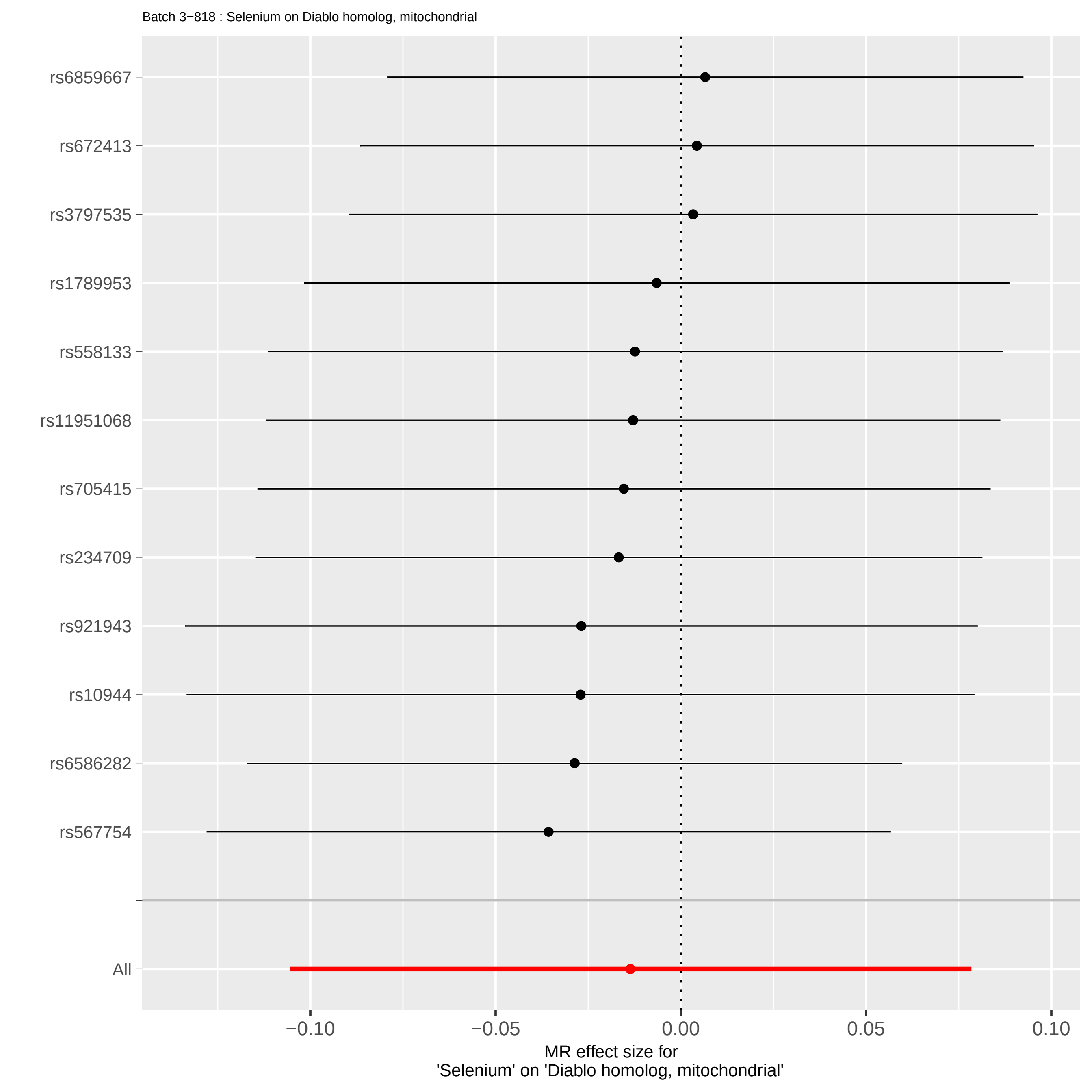

Batch 3−818 : Selenium on Diablo homolog, mitochondrial
rs6859667
rs672413
rs3797535
rs1789953
rs558133
rs11951068
rs705415
rs234709
rs921943
rs10944
rs6586282
rs567754
All
−0.10
−0.05
0.00
0.05
0.10
MR effect size for
'Selenium' on 'Diablo homolog, mitochondrial'

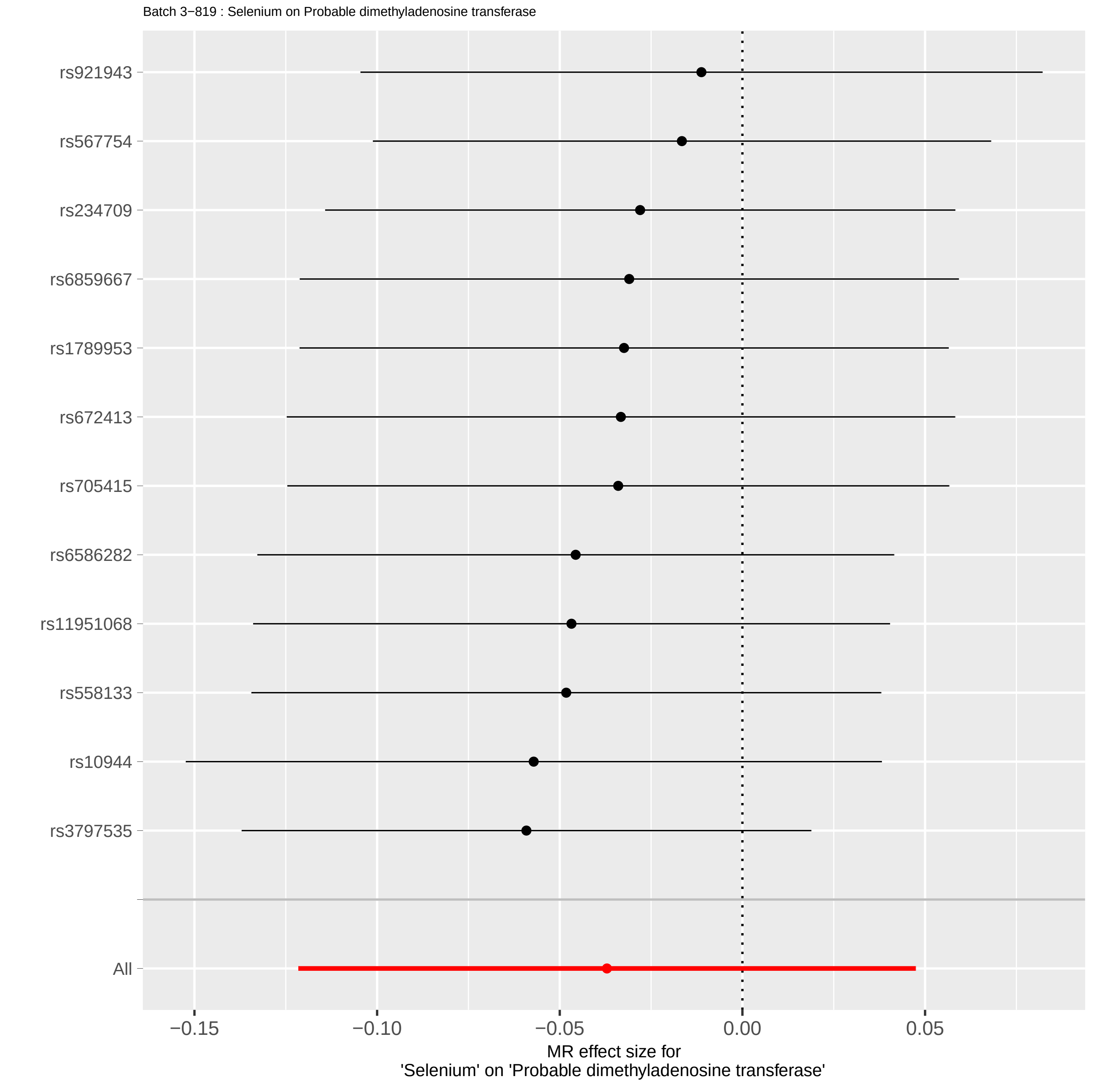

Batch 3−819 : Selenium on Probable dimethyladenosine transferase
rs921943
rs567754
rs234709
rs6859667
rs1789953
rs672413
rs705415
rs6586282
rs11951068
rs558133
rs10944
rs3797535
All
−0.15
−0.10
−0.05
0.00
0.05
MR effect size for
'Selenium' on 'Probable dimethyladenosine transferase'

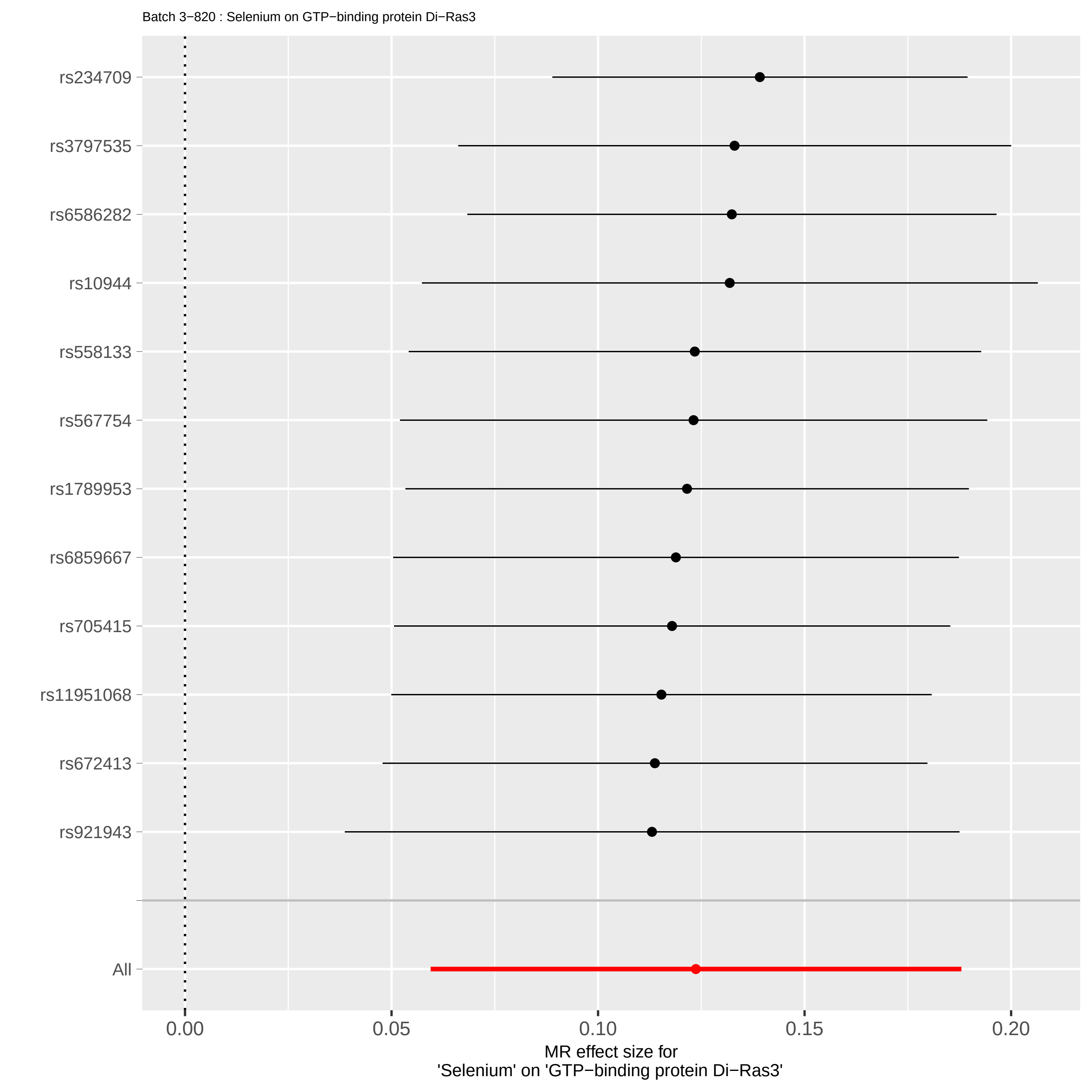

Batch 3−820 : Selenium on GTP−binding protein Di−Ras3
rs234709
rs3797535
rs6586282
rs10944
rs558133
rs567754
rs1789953
rs6859667
rs705415
rs11951068
rs672413
rs921943
All
0.00
0.05
0.10
0.15
0.20
MR effect size for
'Selenium' on 'GTP−binding protein Di−Ras3'

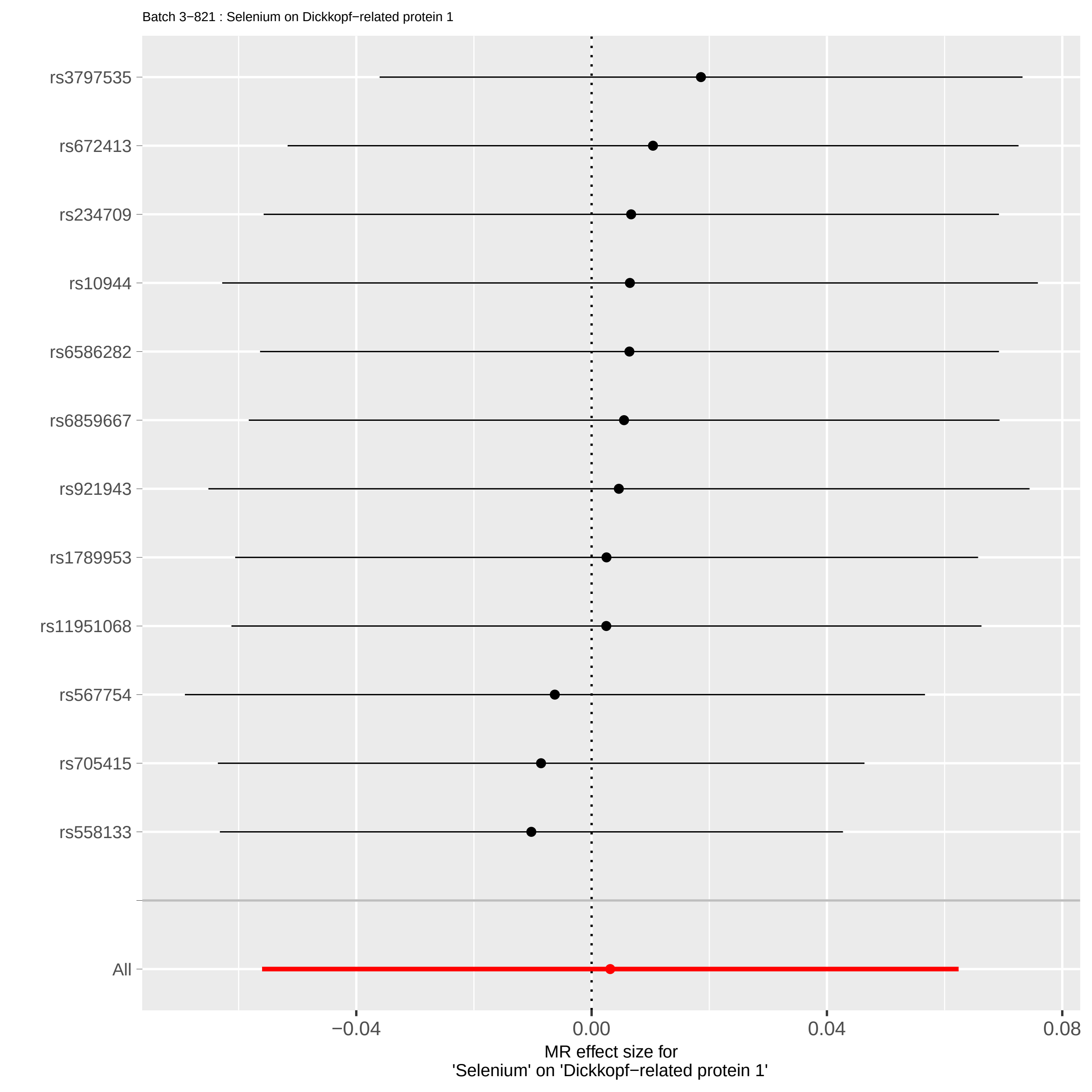
Batch 3−821 : Selenium on Dickkopf−related protein 1
rs3797535
rs672413
rs234709
rs10944
rs6586282
rs6859667
rs921943
rs1789953
rs11951068
rs567754
rs705415
rs558133
All
−0.04
0.00
0.04
0.08
MR effect size for
'Selenium' on 'Dickkopf−related protein 1'

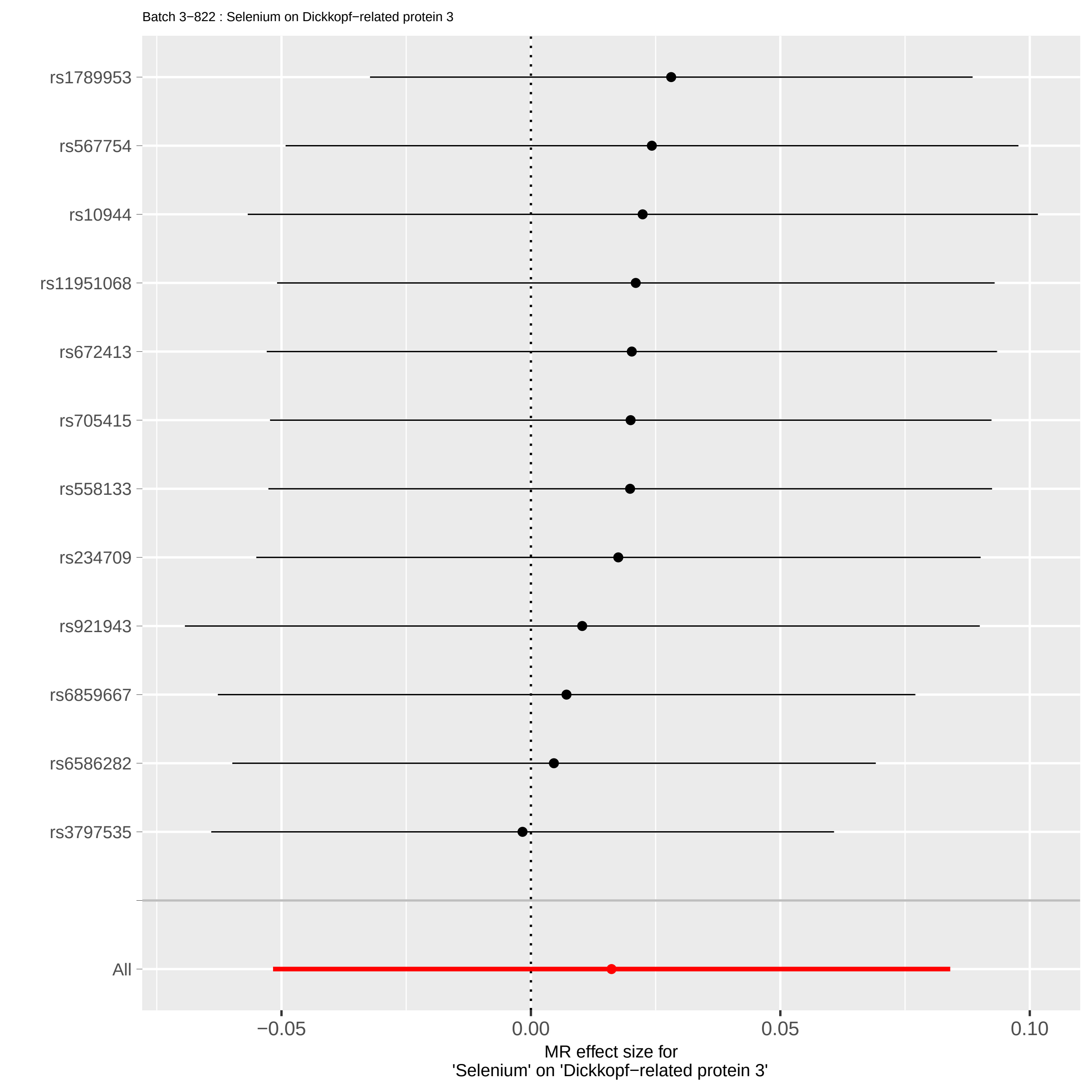

Batch 3−822 : Selenium on Dickkopf−related protein 3
rs1789953
rs567754
rs10944
rs11951068
rs672413
rs705415
rs558133
rs234709
rs921943
rs6859667
rs6586282
rs3797535
All
−0.05
0.00
0.05
0.10
MR effect size for
'Selenium' on 'Dickkopf−related protein 3'

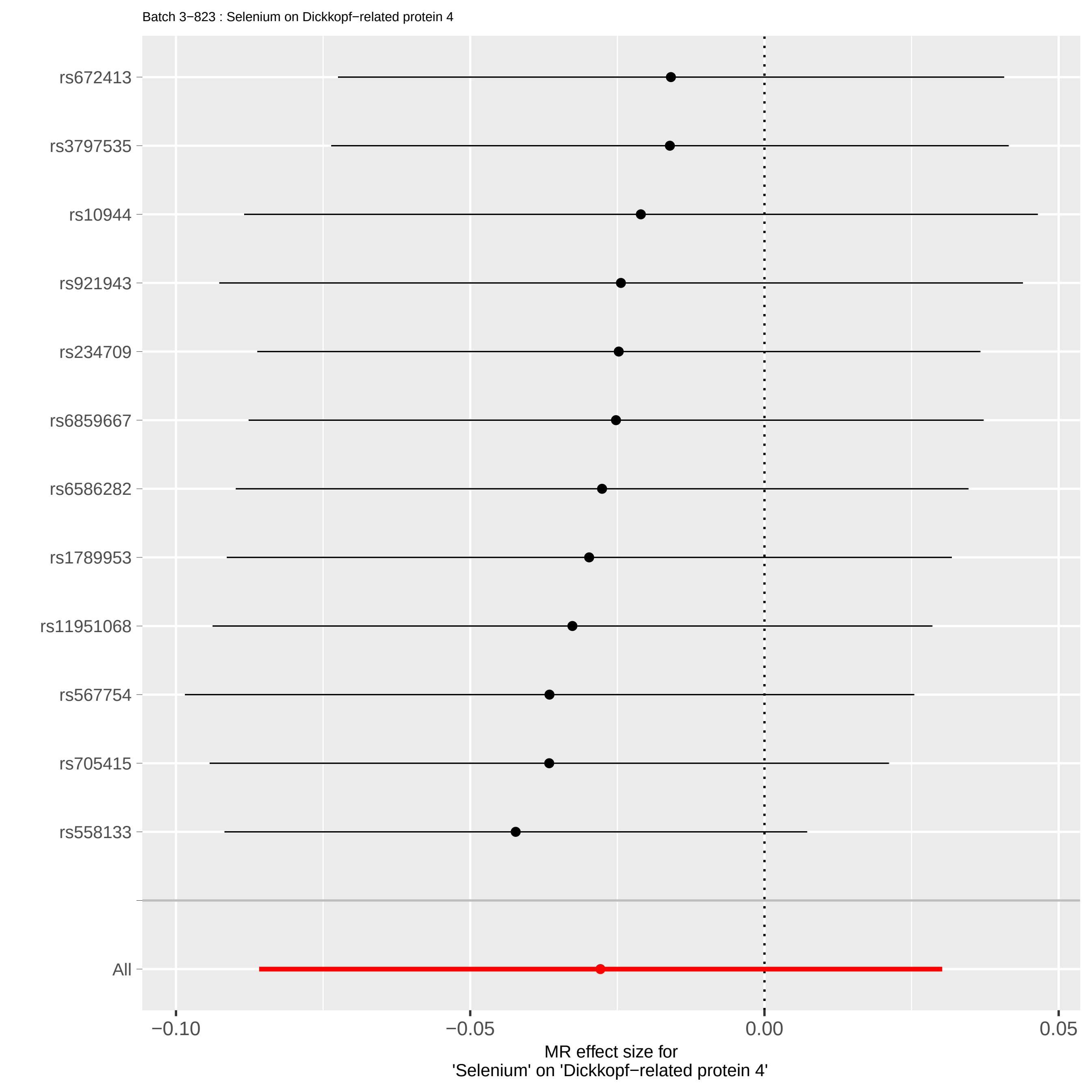

Batch 3−823 : Selenium on Dickkopf−related protein 4
rs672413
rs3797535
rs10944
rs921943
rs234709
rs6859667
rs6586282
rs1789953
rs11951068
rs567754
rs705415
rs558133
All
−0.10
−0.05
0.00
0.05
MR effect size for
'Selenium' on 'Dickkopf−related protein 4'

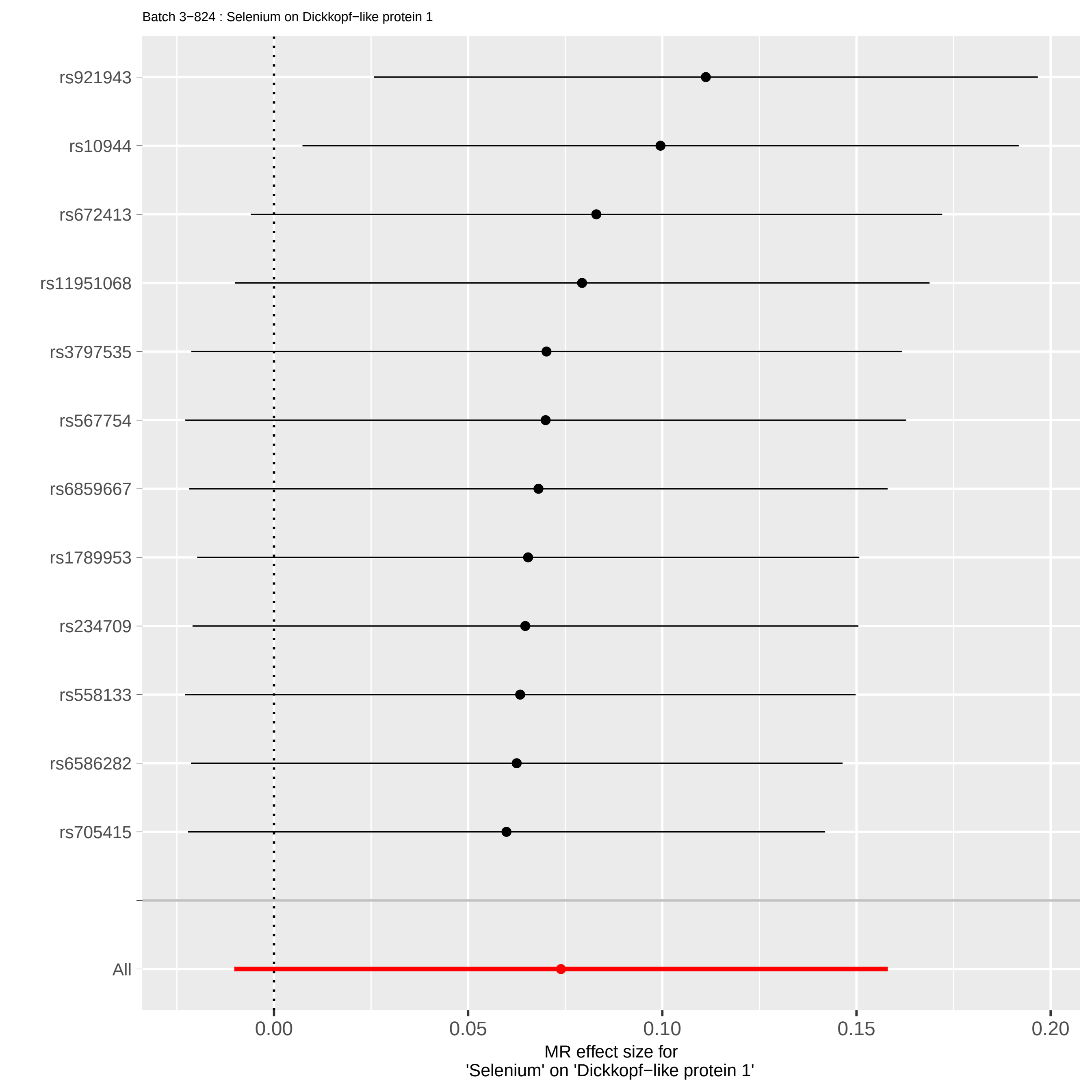

Batch 3−824 : Selenium on Dickkopf−like protein 1
rs921943
rs10944
rs672413
rs11951068
rs3797535
rs567754
rs6859667
rs1789953
rs234709
rs558133
rs6586282
rs705415
All
0.00
0.05
0.10
0.15
0.20
MR effect size for
'Selenium' on 'Dickkopf−like protein 1'

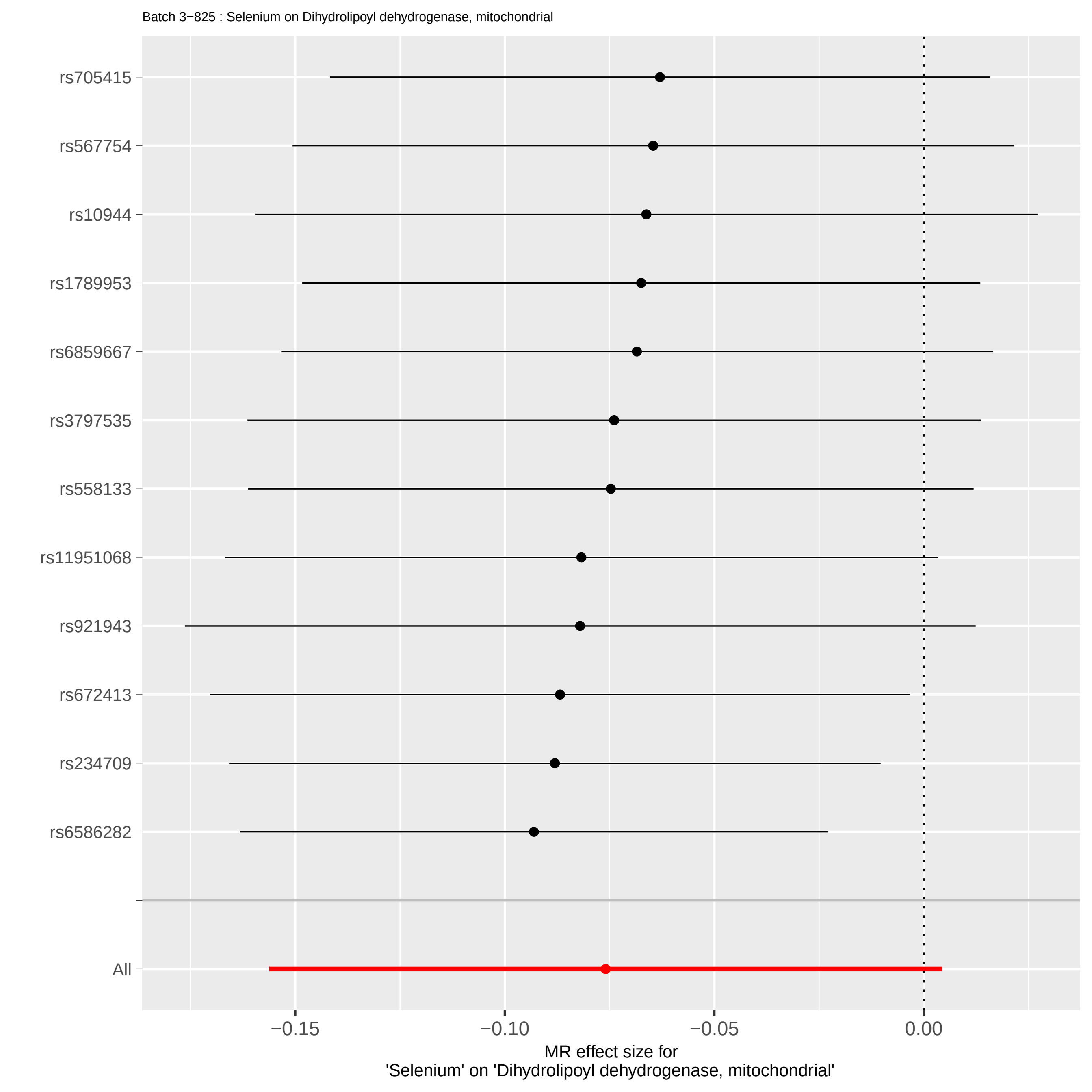

Batch 3−825 : Selenium on Dihydrolipoyl dehydrogenase, mitochondrial
rs705415
rs567754
rs10944
rs1789953
rs6859667
rs3797535
rs558133
rs11951068
rs921943
rs672413
rs234709
rs6586282
All
−0.15
−0.10
−0.05
0.00
MR effect size for
'Selenium' on 'Dihydrolipoyl dehydrogenase, mitochondrial'

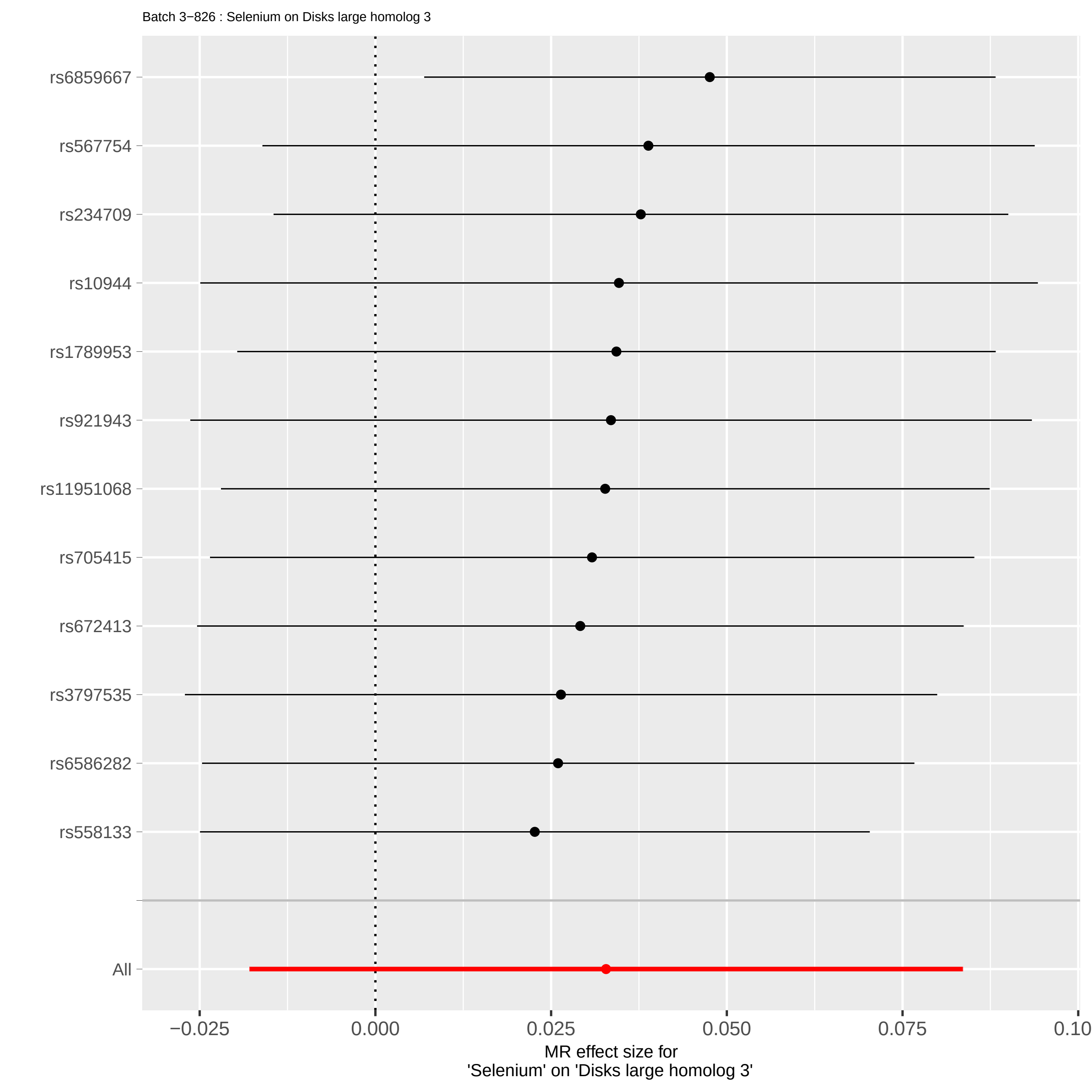
Batch 3−826 : Selenium on Disks large homolog 3
rs6859667
rs567754
rs234709
rs10944
rs1789953
rs921943
rs11951068
rs705415
rs672413
rs3797535
rs6586282
rs558133
All
−0.025
0.000
0.025
0.050
0.075
0.100
MR effect size for
'Selenium' on 'Disks large homolog 3'

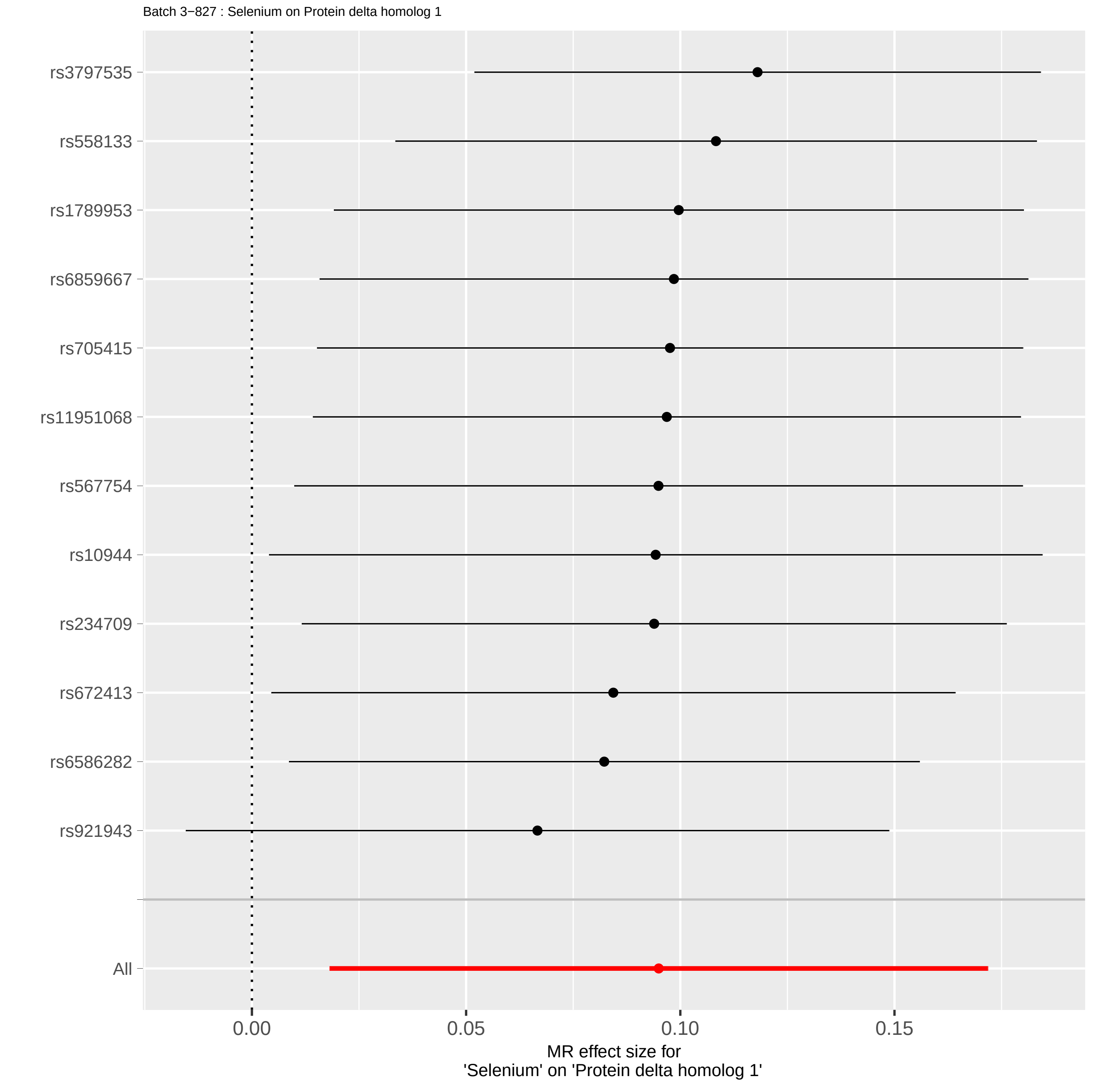

Batch 3−827 : Selenium on Protein delta homolog 1
rs3797535
rs558133
rs1789953
rs6859667
rs705415
rs11951068
rs567754
rs10944
rs234709
rs672413
rs6586282
rs921943
All
0.00
0.05
0.10
0.15
MR effect size for
'Selenium' on 'Protein delta homolog 1'

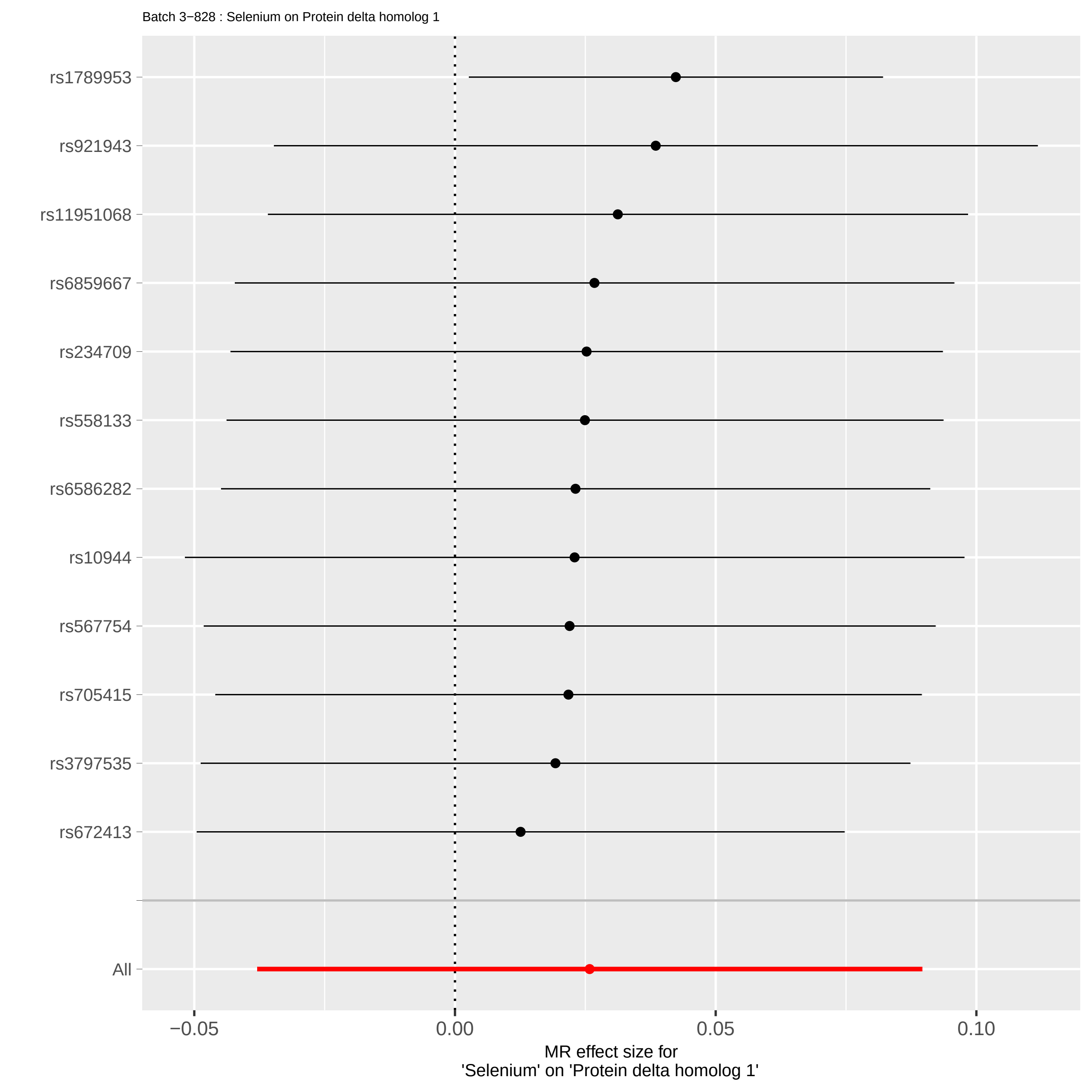

Batch 3−828 : Selenium on Protein delta homolog 1
rs1789953
rs921943
rs11951068
rs6859667
rs234709
rs558133
rs6586282
rs10944
rs567754
rs705415
rs3797535
rs672413
All
−0.05
0.00
0.05
0.10
MR effect size for
'Selenium' on 'Protein delta homolog 1'

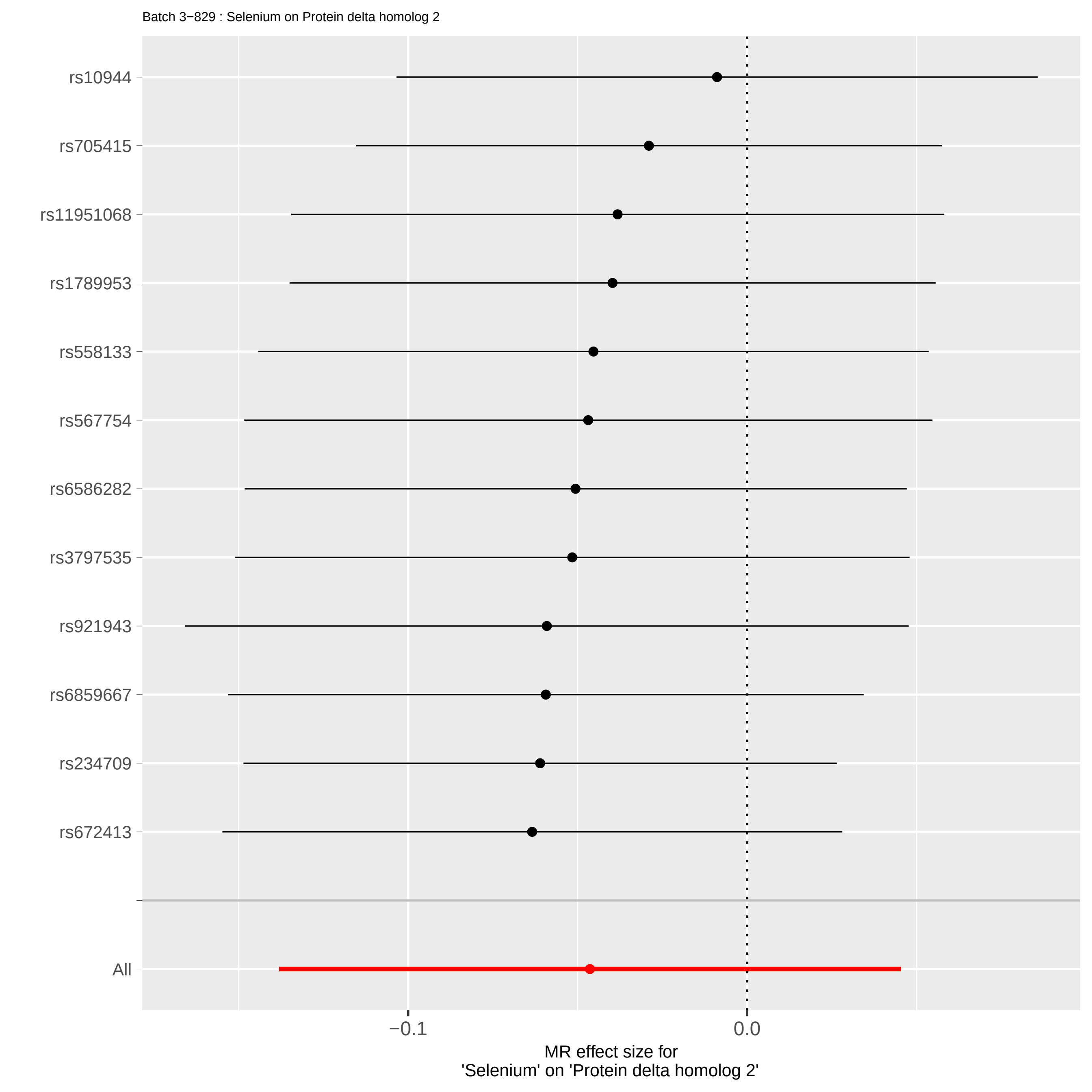
Batch 3−829 : Selenium on Protein delta homolog 2
rs10944
rs705415
rs11951068
rs1789953
rs558133
rs567754
rs6586282
rs3797535
rs921943
rs6859667
rs234709
rs672413
All
−0.1
0.0
MR effect size for
'Selenium' on 'Protein delta homolog 2'

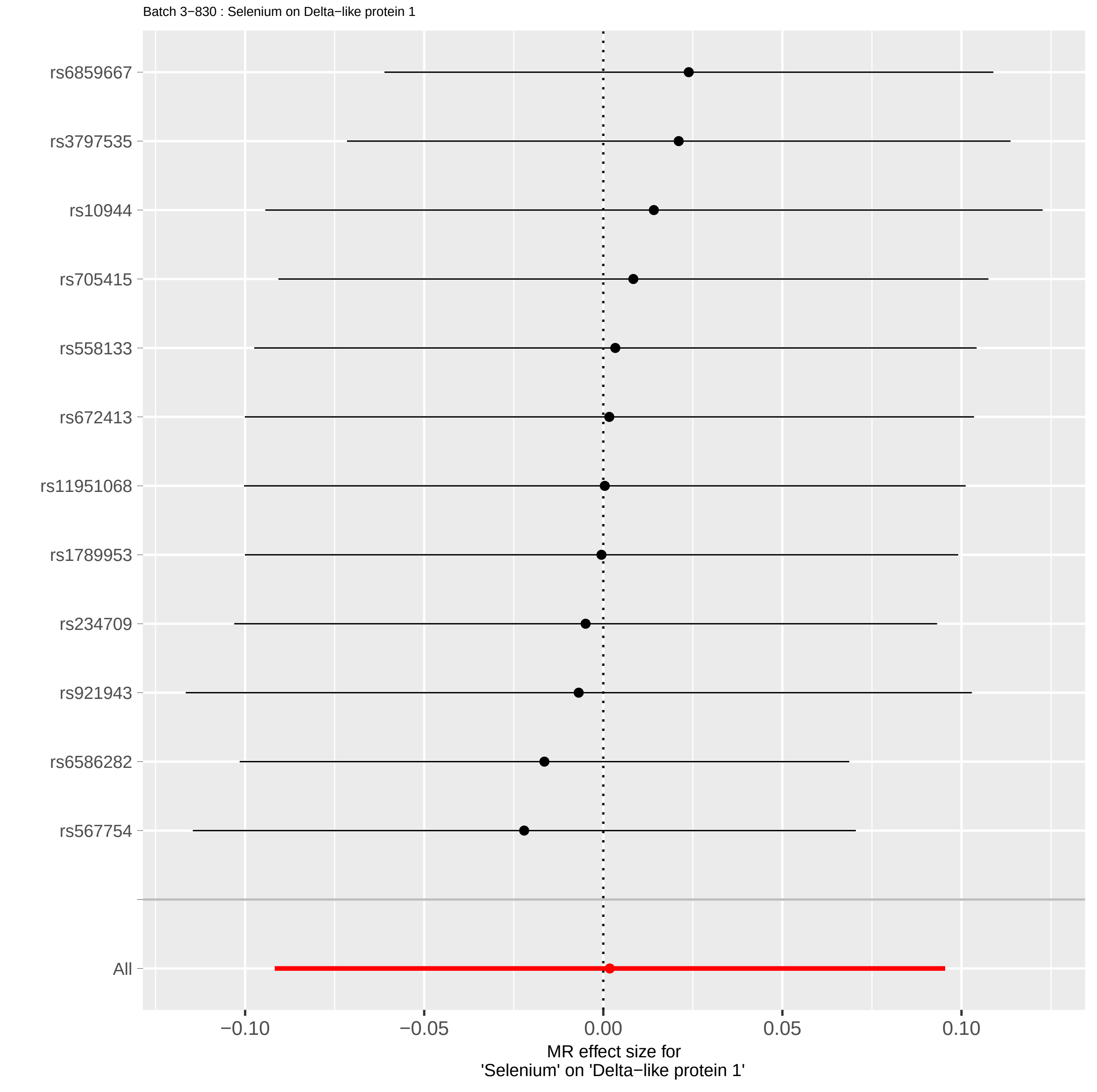

Batch 3−830 : Selenium on Delta−like protein 1
rs6859667
rs3797535
rs10944
rs705415
rs558133
rs672413
rs11951068
rs1789953
rs234709
rs921943
rs6586282
rs567754
All
−0.10
−0.05
0.00
0.05
0.10
MR effect size for
'Selenium' on 'Delta−like protein 1'

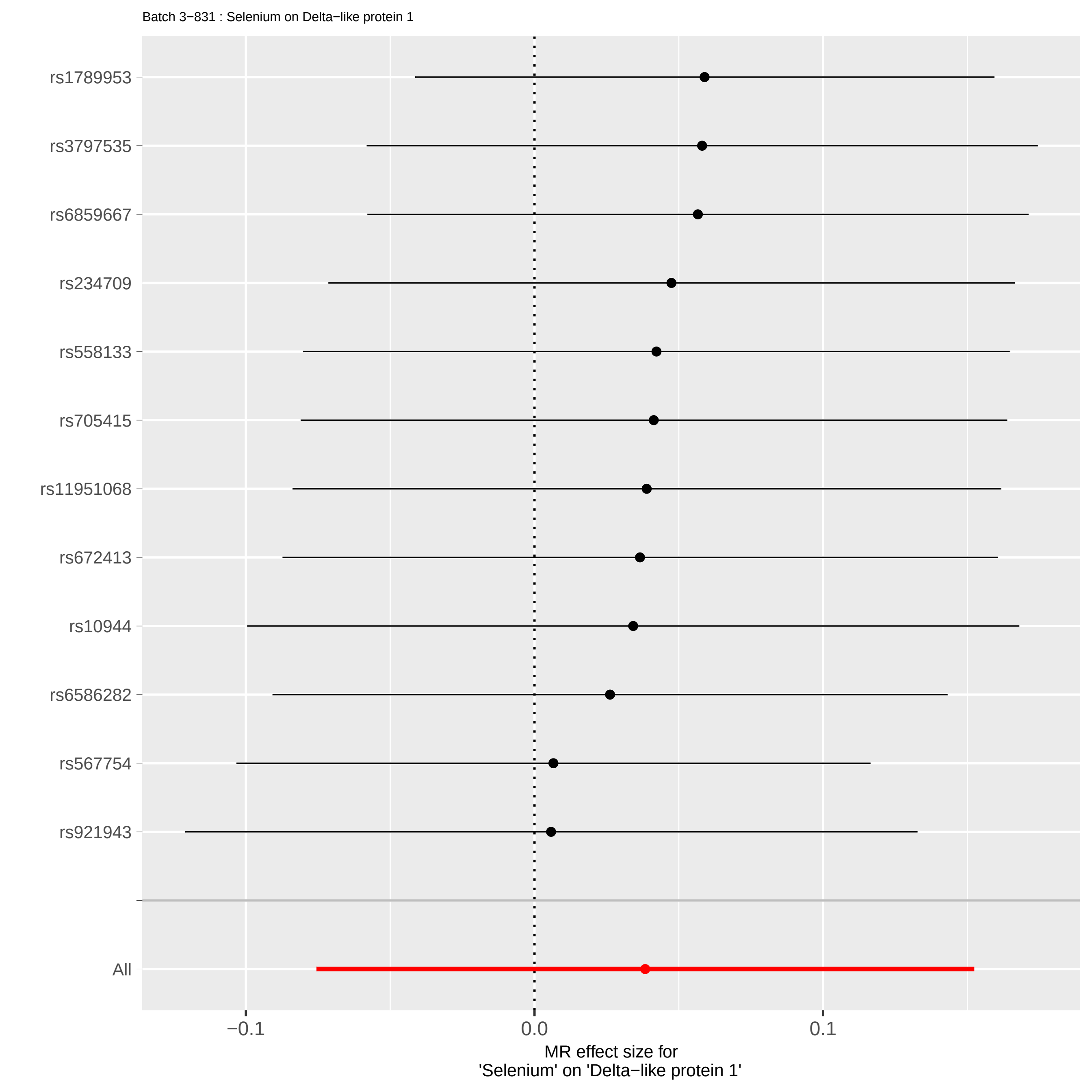

Batch 3−831 : Selenium on Delta−like protein 1
rs1789953
rs3797535
rs6859667
rs234709
rs558133
rs705415
rs11951068
rs672413
rs10944
rs6586282
rs567754
rs921943
All
−0.1
0.0
0.1
MR effect size for
'Selenium' on 'Delta−like protein 1'

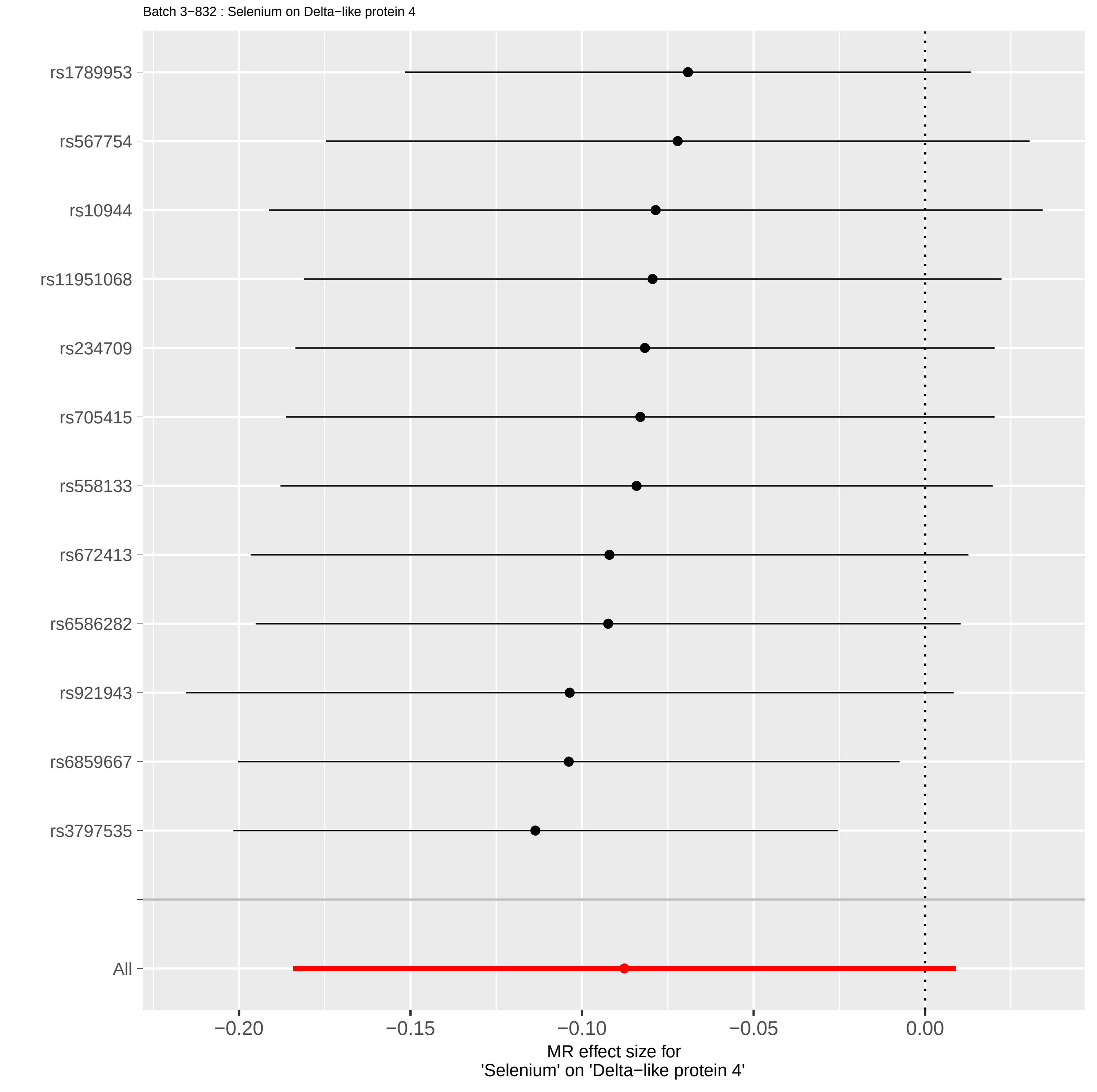

Batch 3−832 : Selenium on Delta−like protein 4
rs1789953
rs567754
rs10944
rs11951068
rs234709
rs705415
rs558133
rs672413
rs6586282
rs921943
rs6859667
rs3797535
All
−0.20
−0.15
−0.10
−0.05
0.00
MR effect size for
'Selenium' on 'Delta−like protein 4'

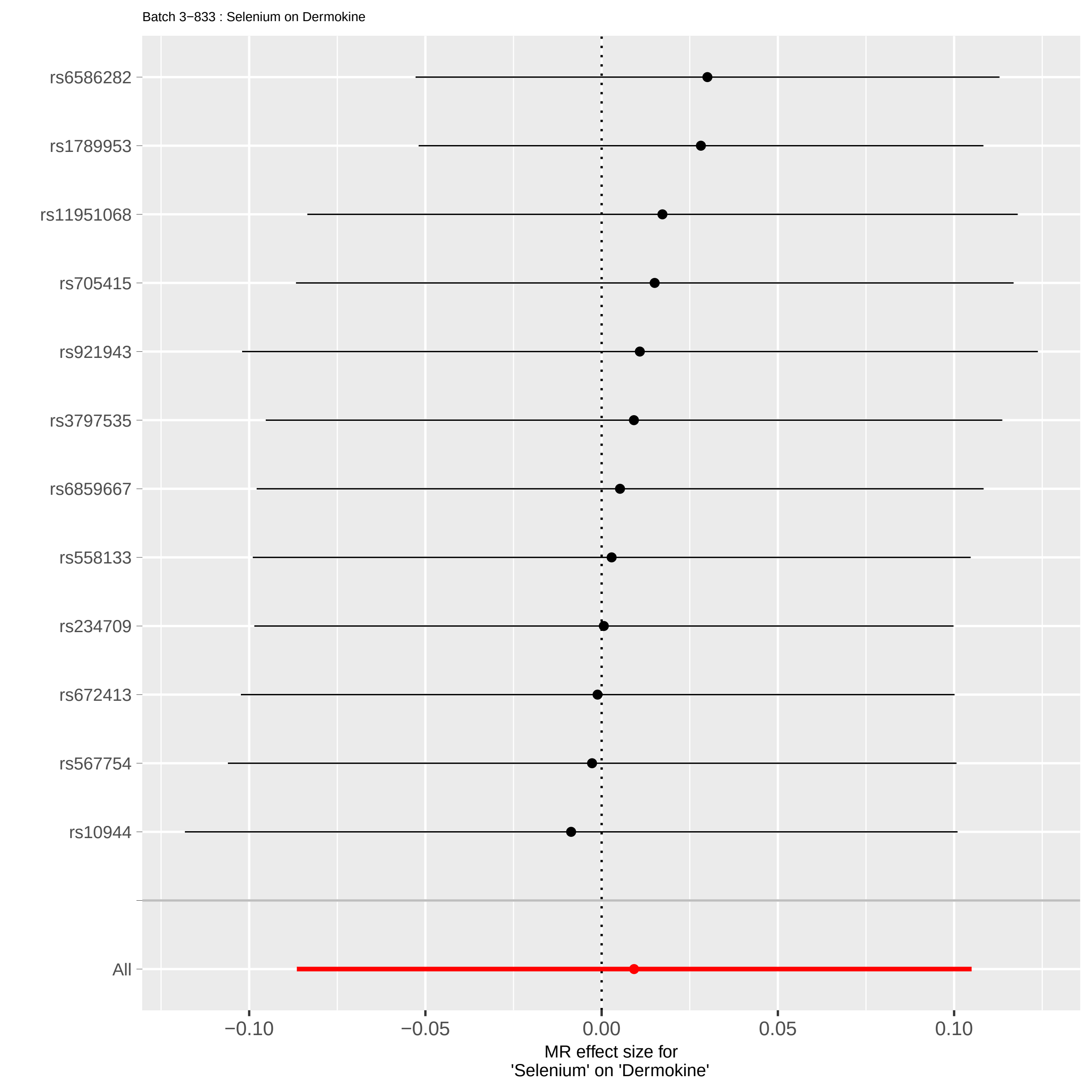

Batch 3−833 : Selenium on Dermokine
rs6586282
rs1789953
rs11951068
rs705415
rs921943
rs3797535
rs6859667
rs558133
rs234709
rs672413
rs567754
rs10944
All
−0.10
−0.05
0.00
0.05
0.10
MR effect size for
'Selenium' on 'Dermokine'

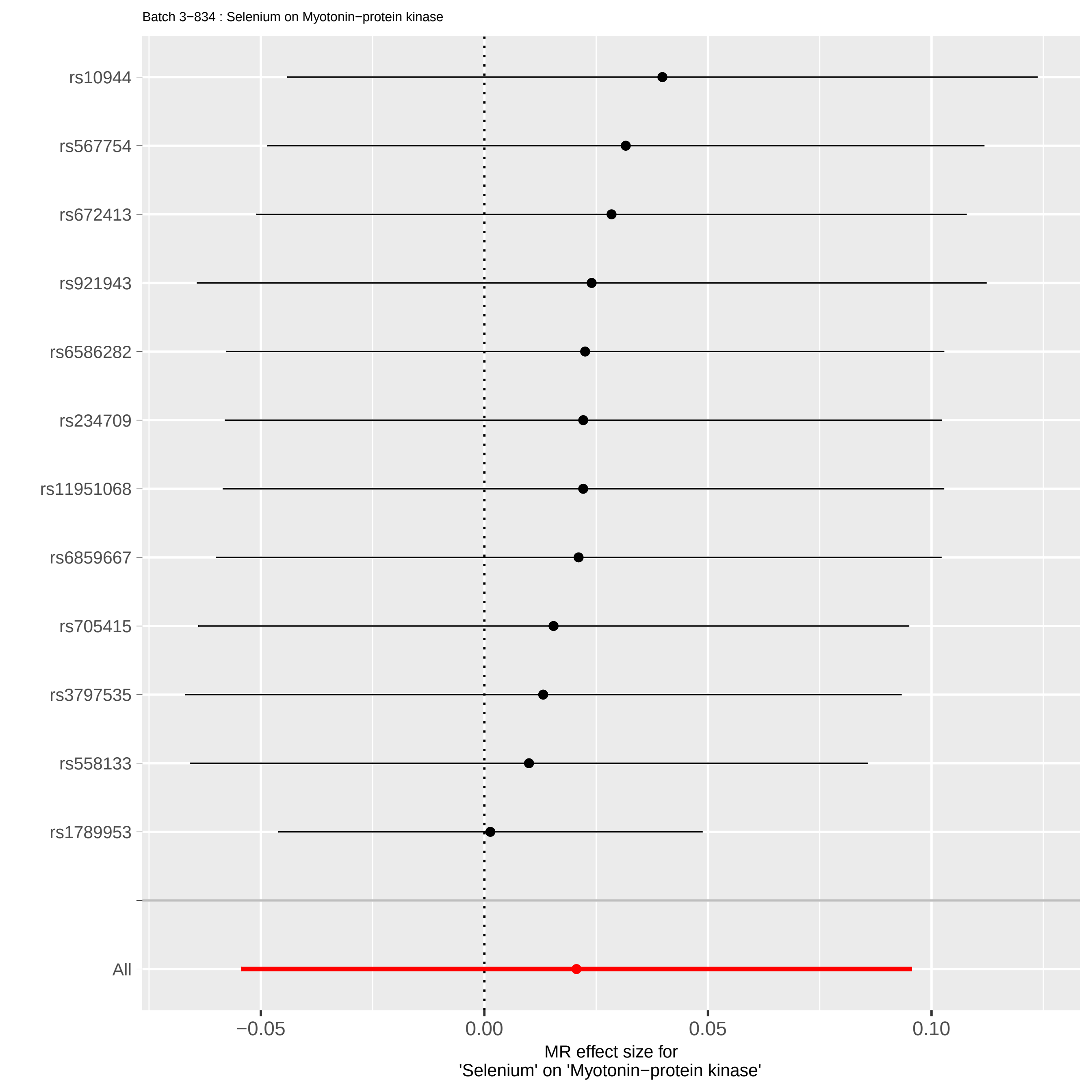

Batch 3−834 : Selenium on Myotonin−protein kinase
rs10944
rs567754
rs672413
rs921943
rs6586282
rs234709
rs11951068
rs6859667
rs705415
rs3797535
rs558133
rs1789953
All
−0.05
0.00
0.05
0.10
MR effect size for
'Selenium' on 'Myotonin−protein kinase'

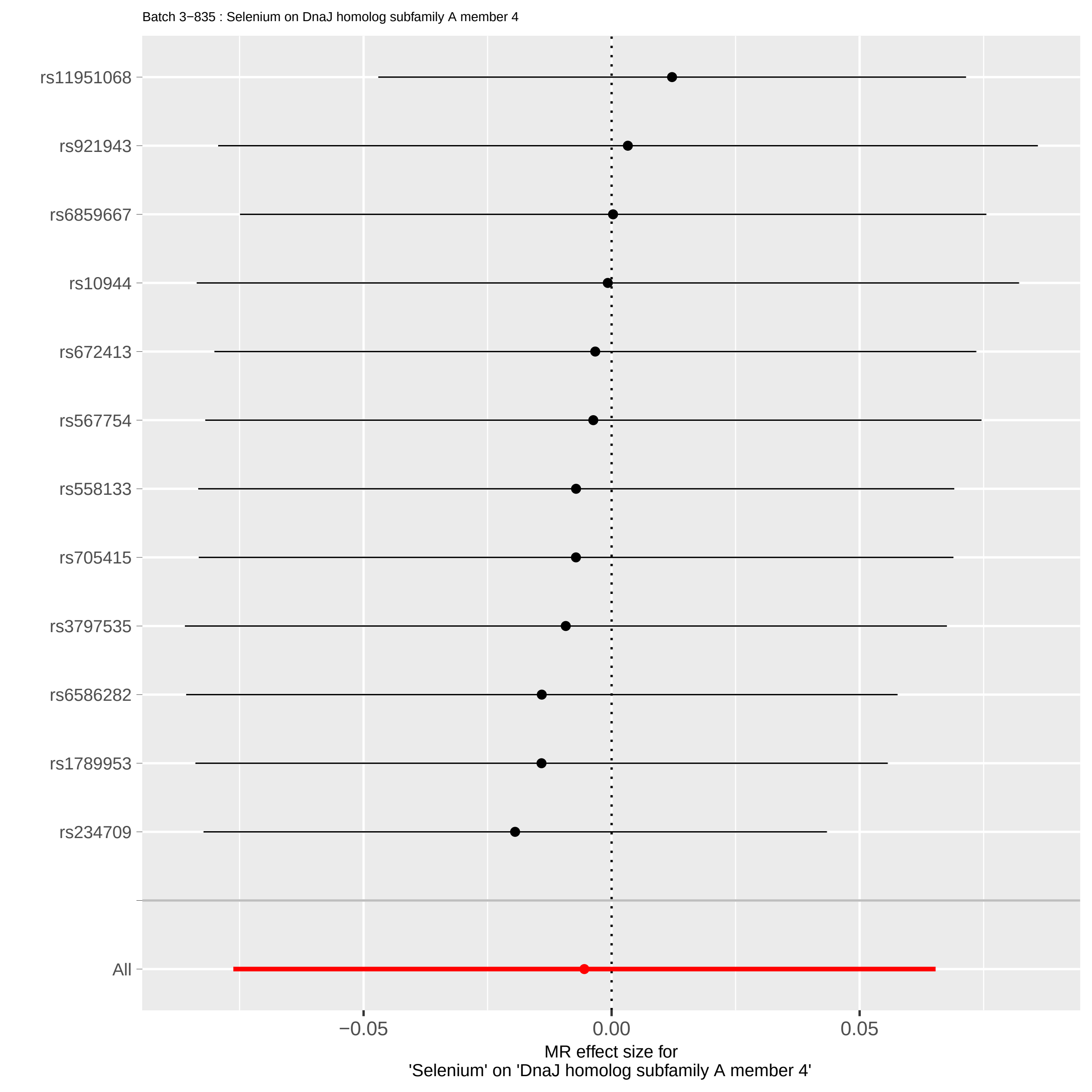

Batch 3−835 : Selenium on DnaJ homolog subfamily A member 4
rs11951068
rs921943
rs6859667
rs10944
rs672413
rs567754
rs558133
rs705415
rs3797535
rs6586282
rs1789953
rs234709
All
−0.05
0.00
0.05
MR effect size for
'Selenium' on 'DnaJ homolog subfamily A member 4'

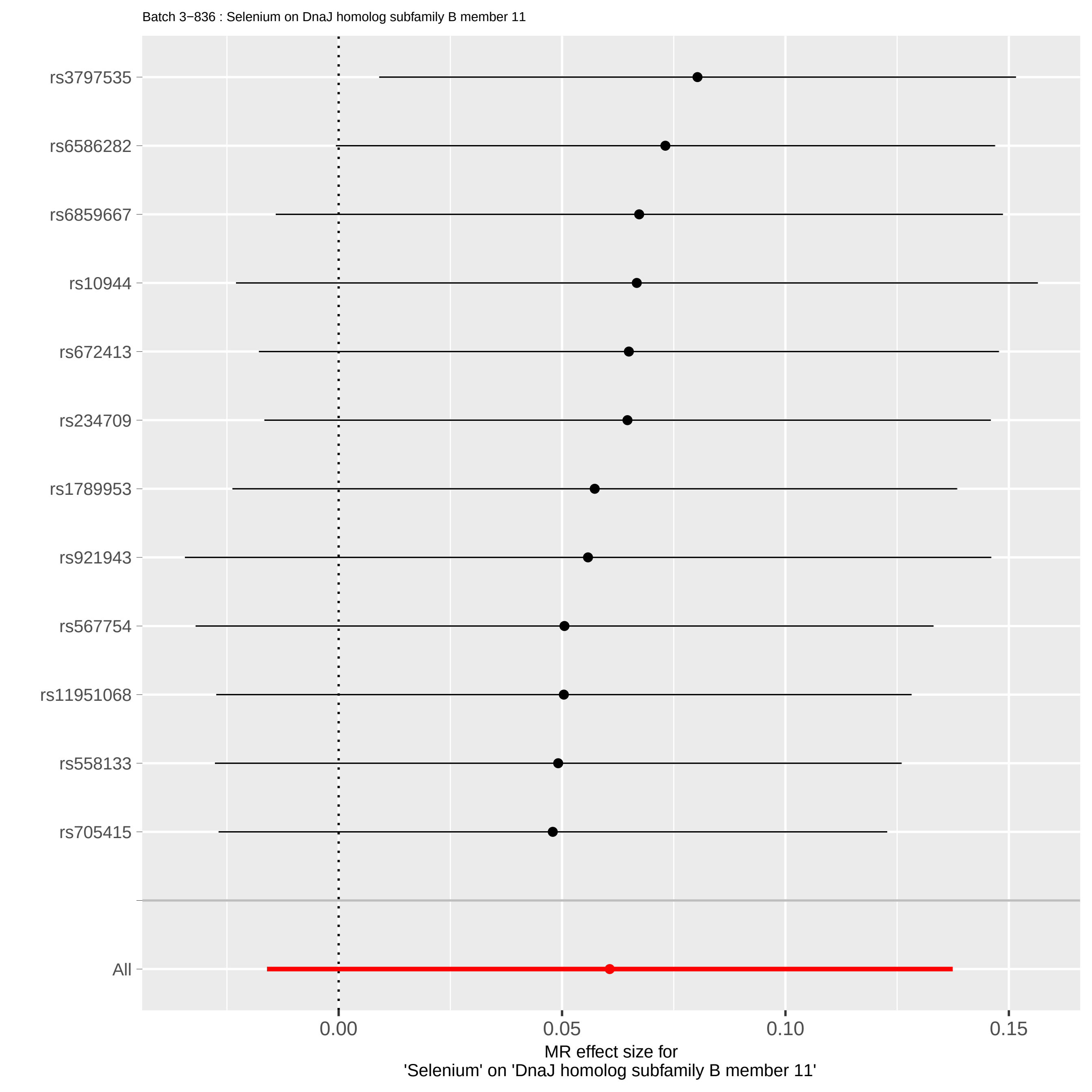
Batch 3−836 : Selenium on DnaJ homolog subfamily B member 11
rs3797535
rs6586282
rs6859667
rs10944
rs672413
rs234709
rs1789953
rs921943
rs567754
rs11951068
rs558133
rs705415
All
0.00
0.05
0.10
0.15
MR effect size for
'Selenium' on 'DnaJ homolog subfamily B member 11'

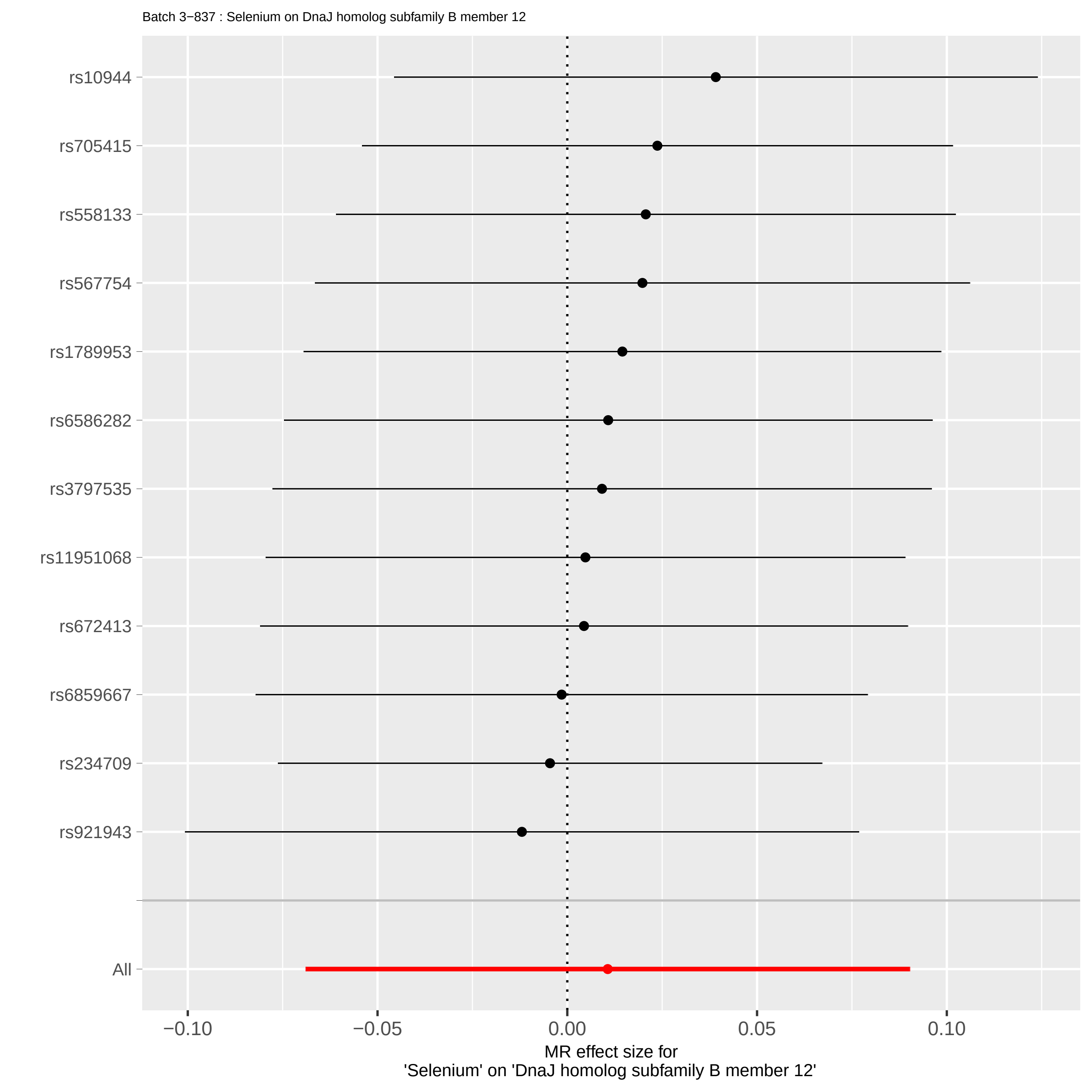
Batch 3−837 : Selenium on DnaJ homolog subfamily B member 12
rs10944
rs705415
rs558133
rs567754
rs1789953
rs6586282
rs3797535
rs11951068
rs672413
rs6859667
rs234709
rs921943
All
−0.10
−0.05
0.00
0.05
0.10
MR effect size for
'Selenium' on 'DnaJ homolog subfamily B member 12'

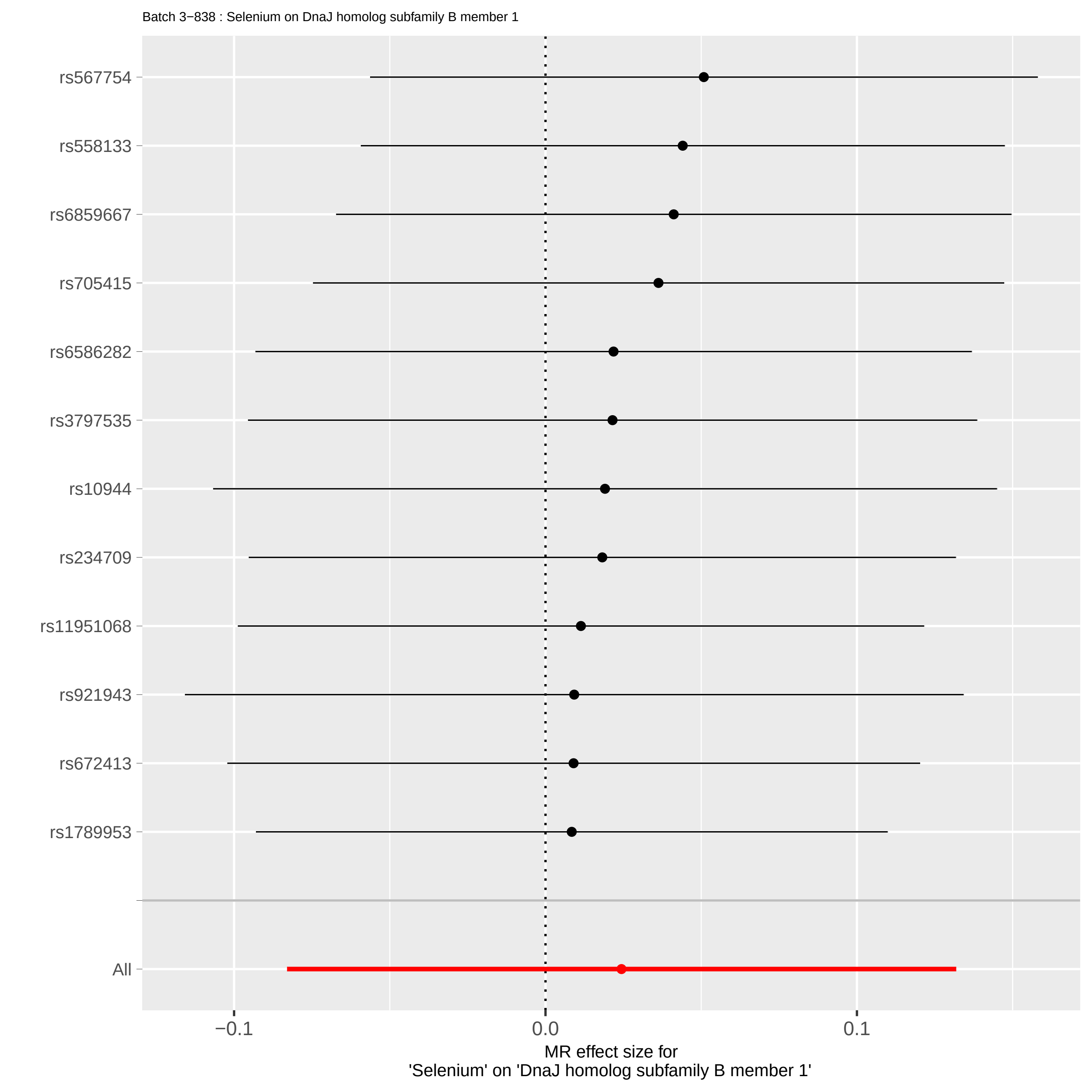

Batch 3−838 : Selenium on DnaJ homolog subfamily B member 1
rs567754
rs558133
rs6859667
rs705415
rs6586282
rs3797535
rs10944
rs234709
rs11951068
rs921943
rs672413
rs1789953
All
−0.1
0.0
0.1
MR effect size for
'Selenium' on 'DnaJ homolog subfamily B member 1'

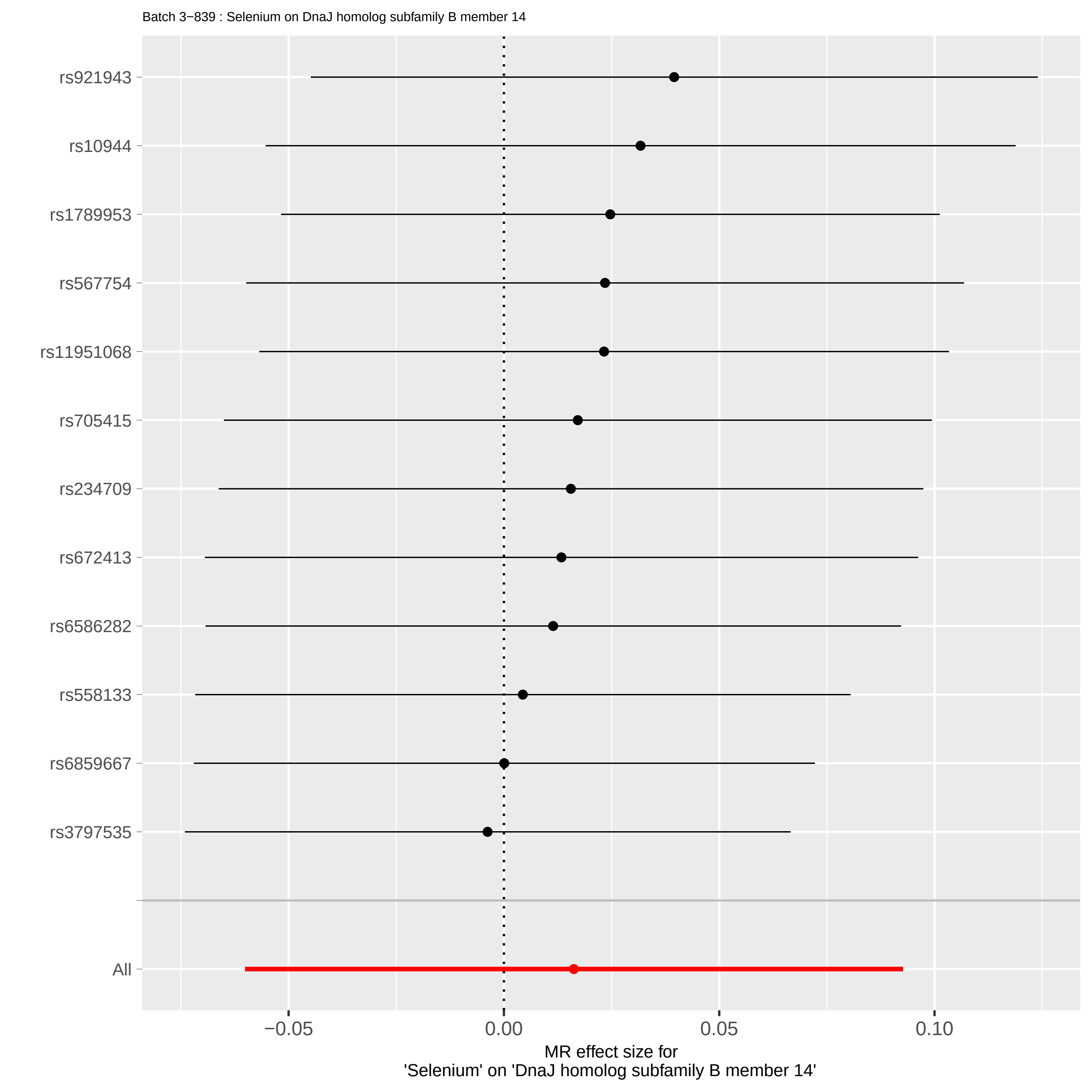

Batch 3−839 : Selenium on DnaJ homolog subfamily B member 14
rs921943
rs10944
rs1789953
rs567754
rs11951068
rs705415
rs234709
rs672413
rs6586282
rs558133
rs6859667
rs3797535
All
−0.05
0.00
0.05
0.10
MR effect size for
'Selenium' on 'DnaJ homolog subfamily B member 14'

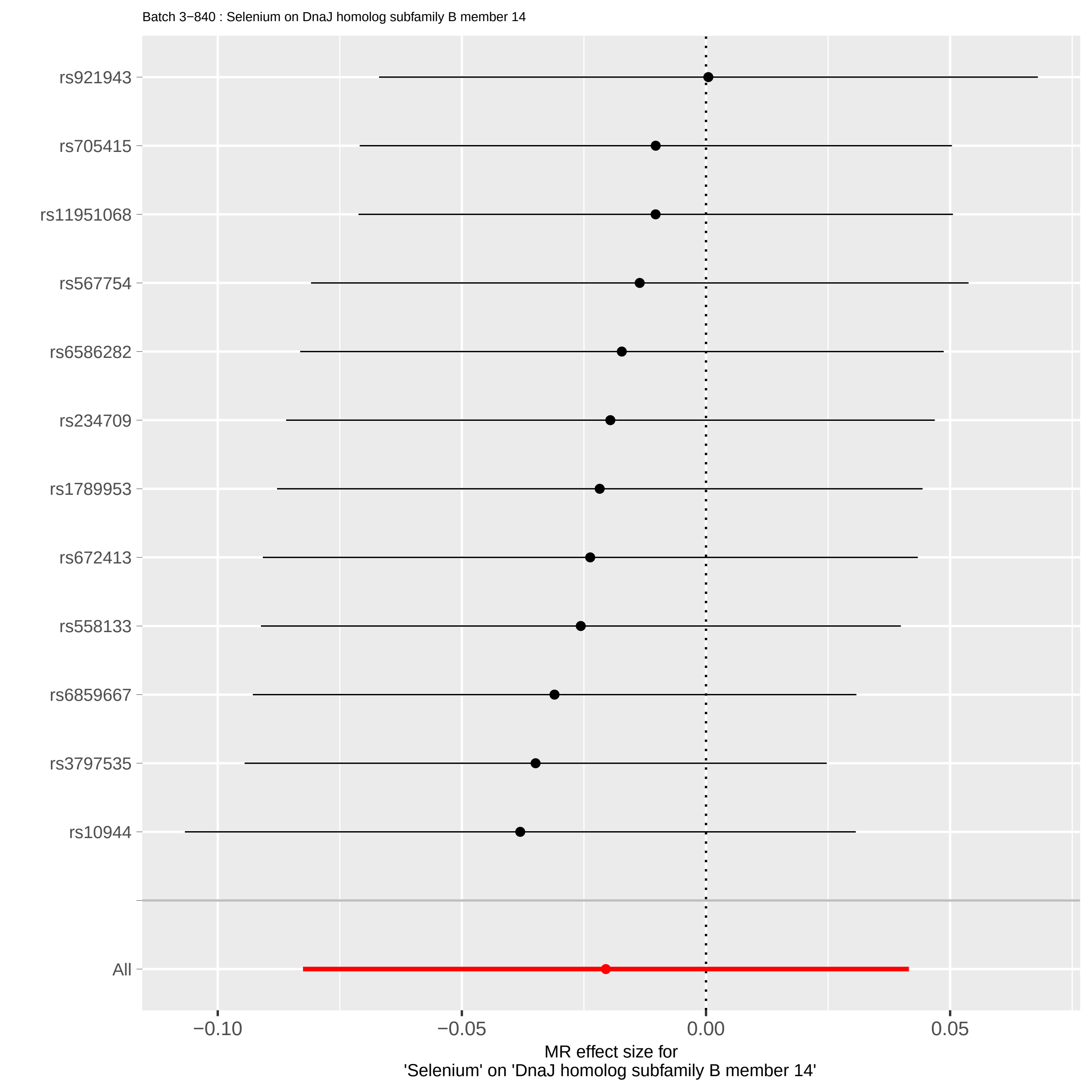

Batch 3−840 : Selenium on DnaJ homolog subfamily B member 14
rs921943
rs705415
rs11951068
rs567754
rs6586282
rs234709
rs1789953
rs672413
rs558133
rs6859667
rs3797535
rs10944
All
−0.10
−0.05
0.00
0.05
MR effect size for
'Selenium' on 'DnaJ homolog subfamily B member 14'

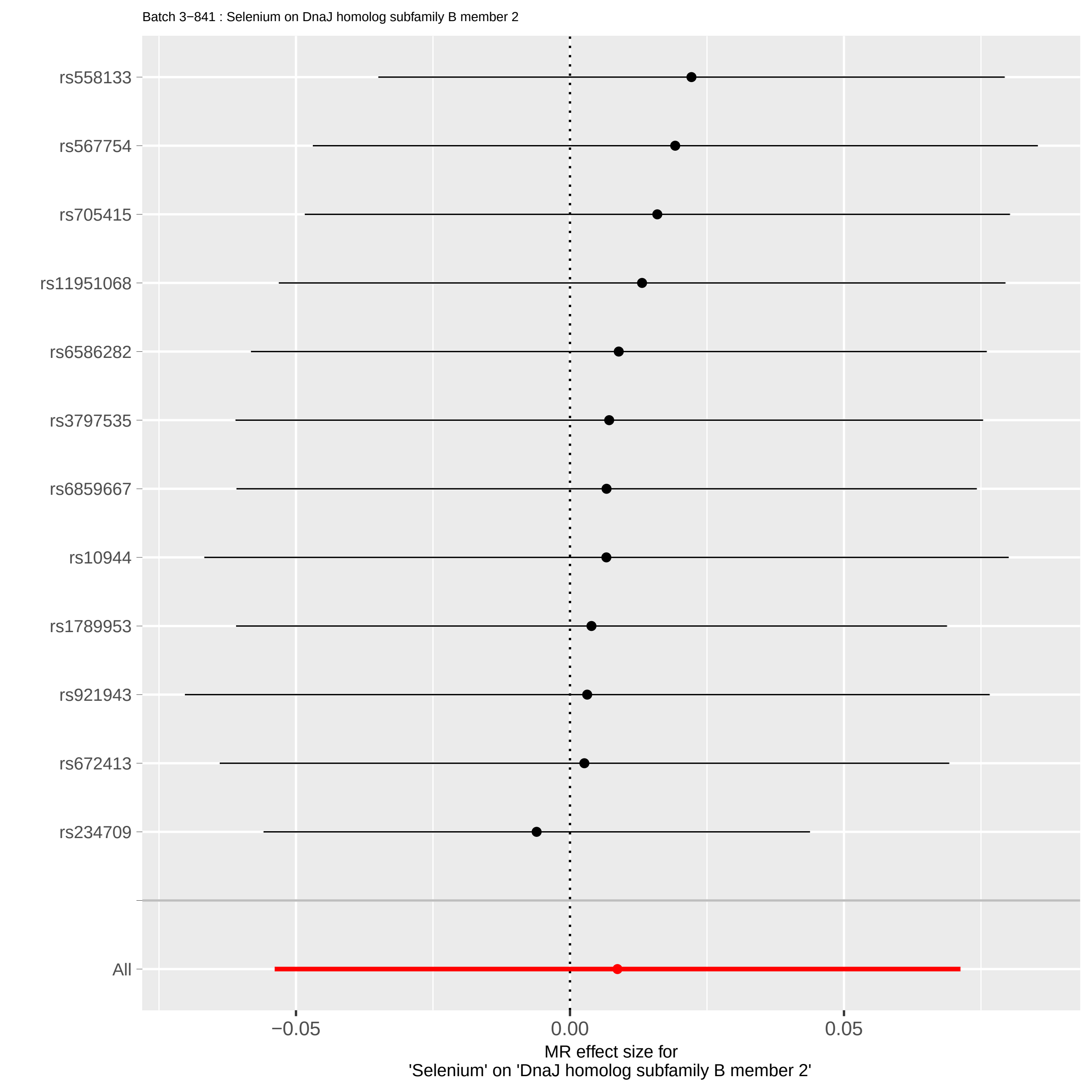

Batch 3−841 : Selenium on DnaJ homolog subfamily B member 2
rs558133
rs567754
rs705415
rs11951068
rs6586282
rs3797535
rs6859667
rs10944
rs1789953
rs921943
rs672413
rs234709
All
−0.05
0.00
0.05
MR effect size for
'Selenium' on 'DnaJ homolog subfamily B member 2'

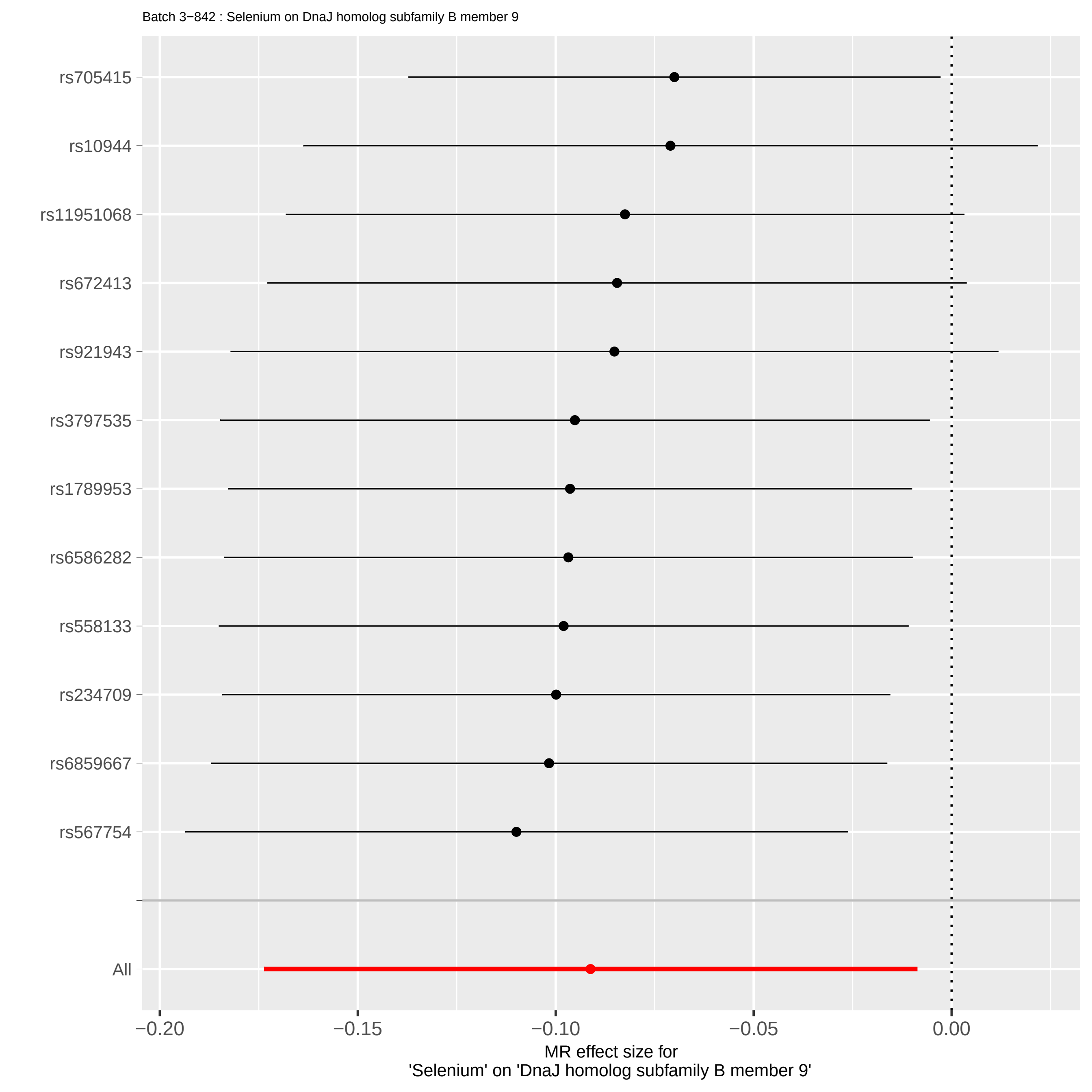
Batch 3−842 : Selenium on DnaJ homolog subfamily B member 9
rs705415
rs10944
rs11951068
rs672413
rs921943
rs3797535
rs1789953
rs6586282
rs558133
rs234709
rs6859667
rs567754
All
−0.20
−0.15
−0.10
−0.05
0.00
MR effect size for
'Selenium' on 'DnaJ homolog subfamily B member 9'

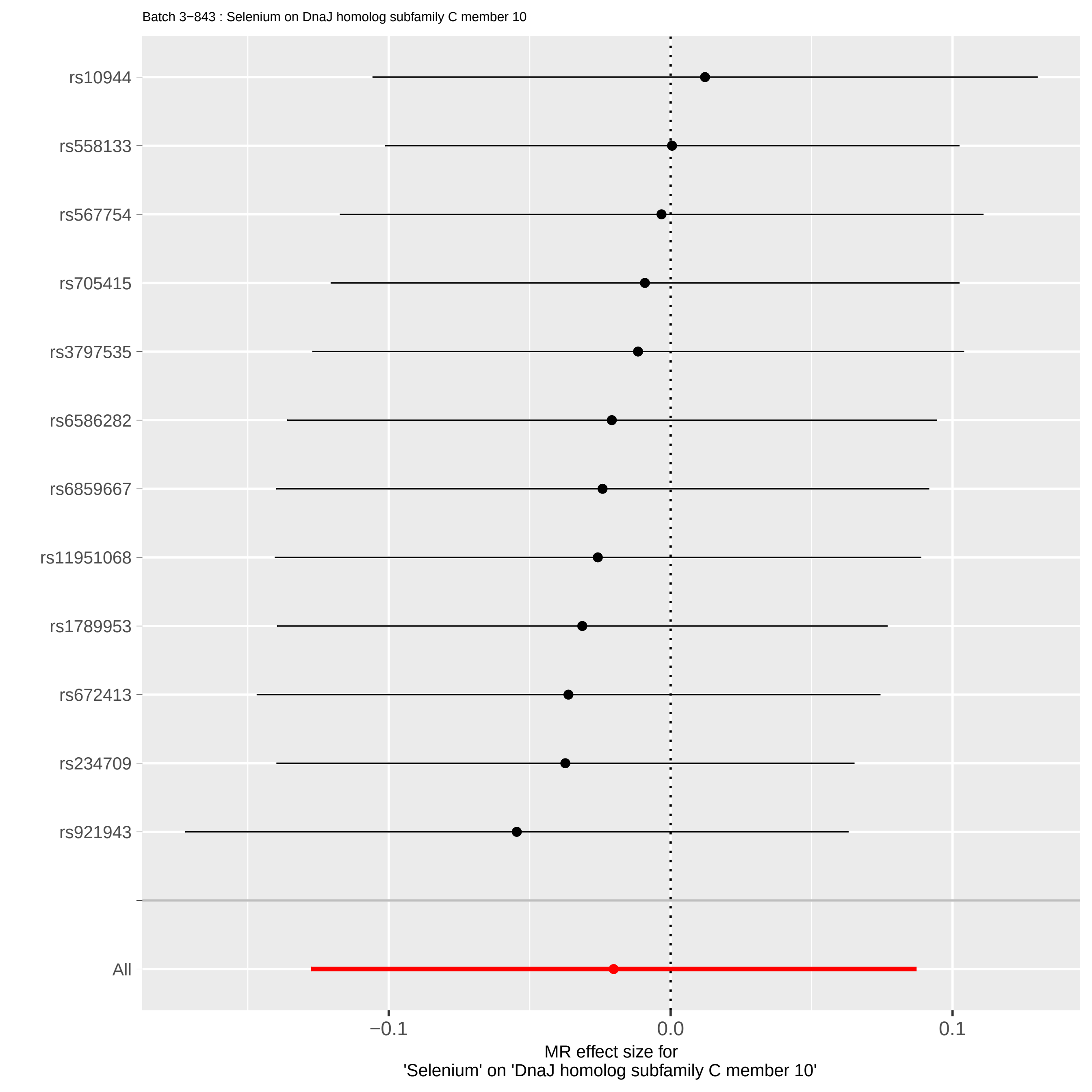

Batch 3−843 : Selenium on DnaJ homolog subfamily C member 10
rs10944
rs558133
rs567754
rs705415
rs3797535
rs6586282
rs6859667
rs11951068
rs1789953
rs672413
rs234709
rs921943
All
−0.1
0.0
0.1
MR effect size for
'Selenium' on 'DnaJ homolog subfamily C member 10'

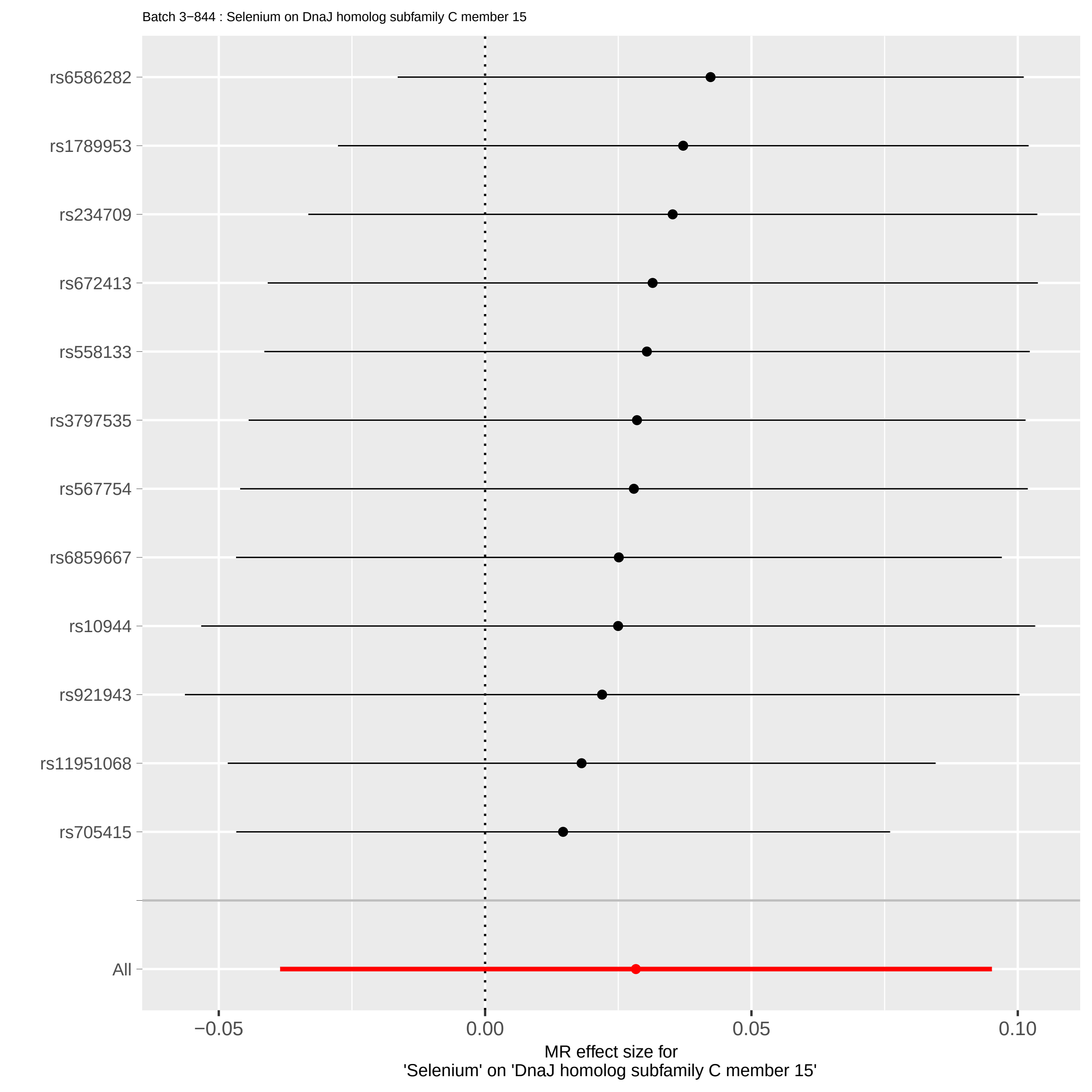

Batch 3−844 : Selenium on DnaJ homolog subfamily C member 15
rs6586282
rs1789953
rs234709
rs672413
rs558133
rs3797535
rs567754
rs6859667
rs10944
rs921943
rs11951068
rs705415
All
−0.05
0.00
0.05
0.10
MR effect size for
'Selenium' on 'DnaJ homolog subfamily C member 15'

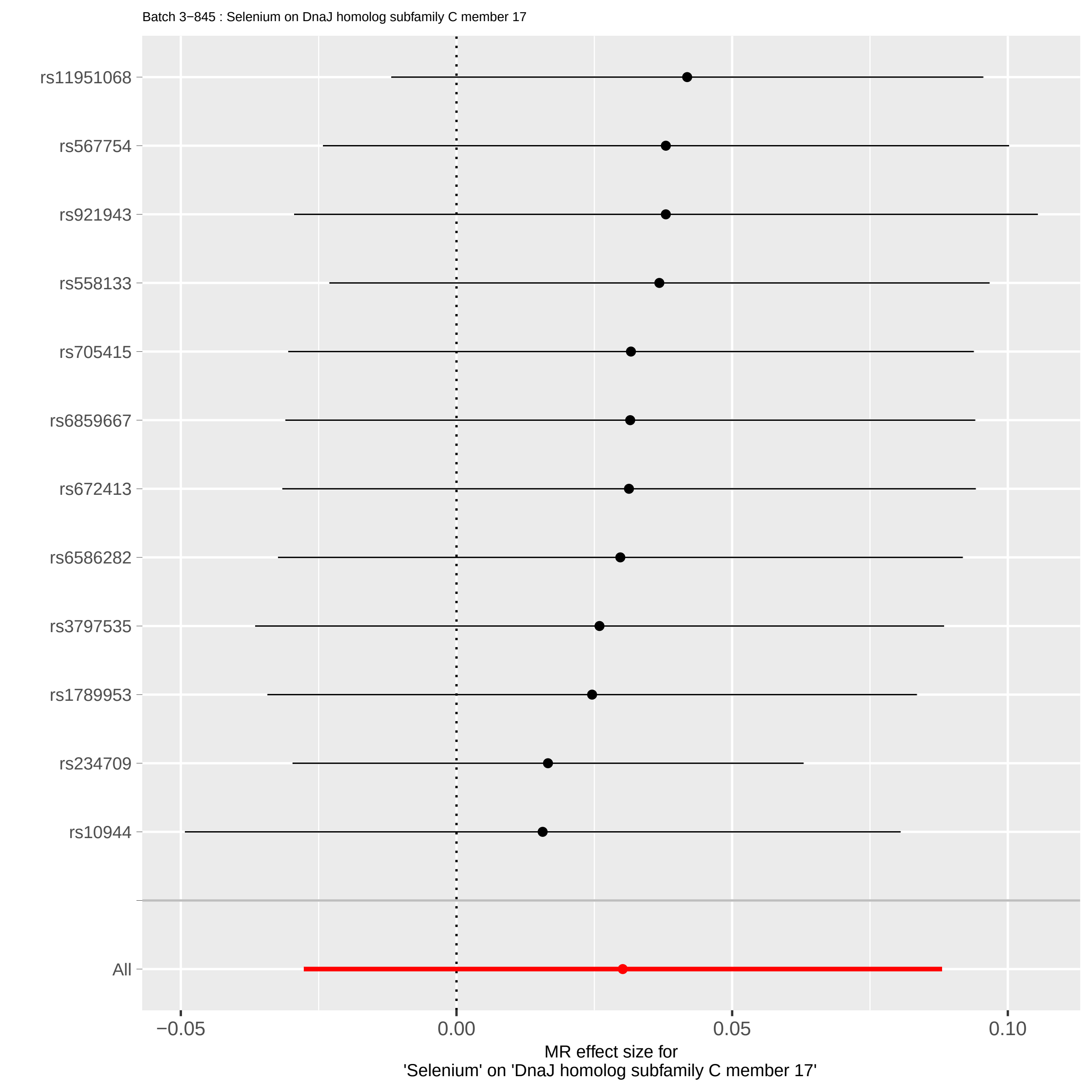

Batch 3−845 : Selenium on DnaJ homolog subfamily C member 17
rs11951068
rs567754
rs921943
rs558133
rs705415
rs6859667
rs672413
rs6586282
rs3797535
rs1789953
rs234709
rs10944
All
−0.05
0.00
0.05
0.10
MR effect size for
'Selenium' on 'DnaJ homolog subfamily C member 17'

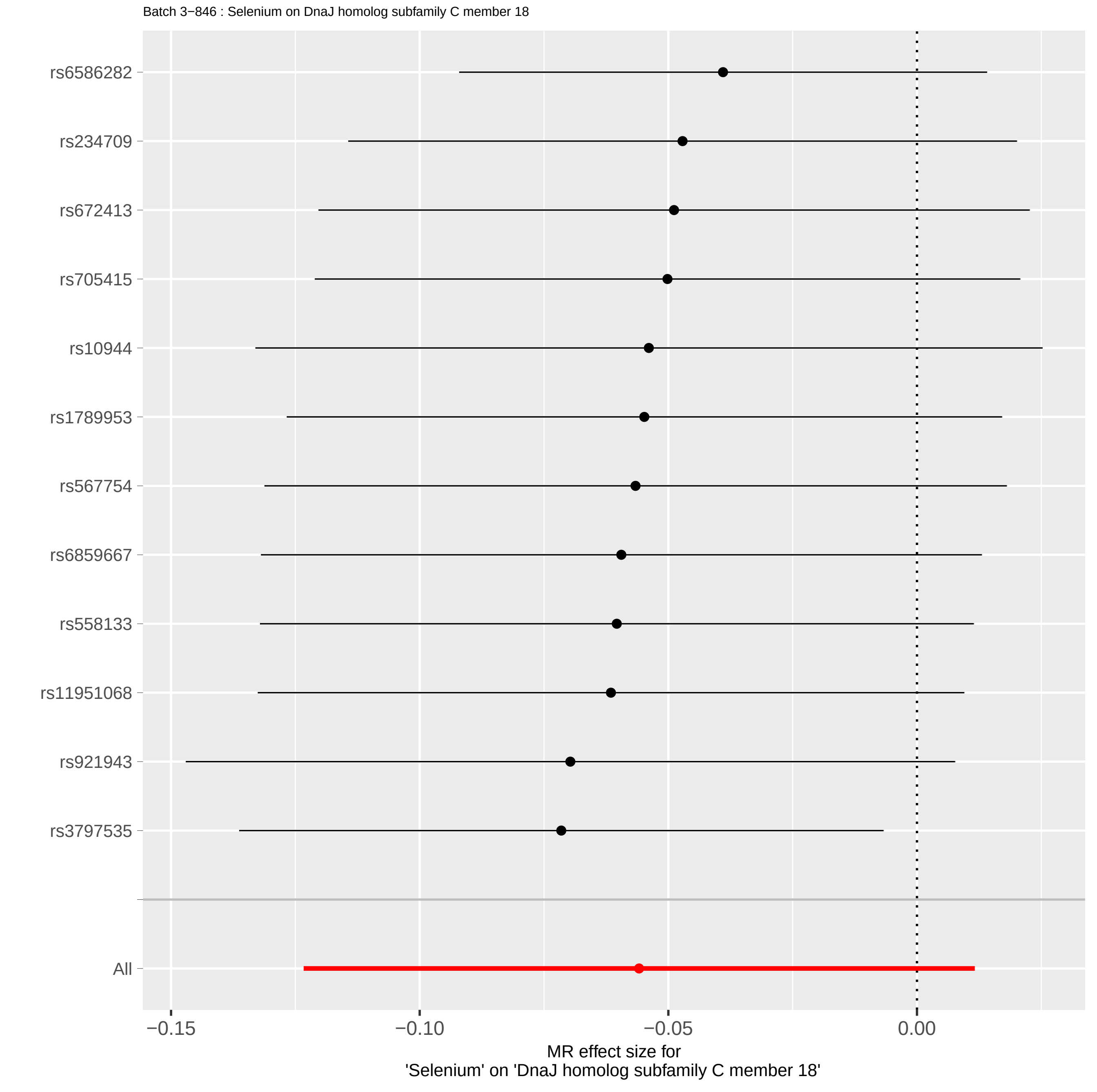

Batch 3−846 : Selenium on DnaJ homolog subfamily C member 18
rs6586282
rs234709
rs672413
rs705415
rs10944
rs1789953
rs567754
rs6859667
rs558133
rs11951068
rs921943
rs3797535
All
−0.15
−0.10
−0.05
0.00
MR effect size for
'Selenium' on 'DnaJ homolog subfamily C member 18'

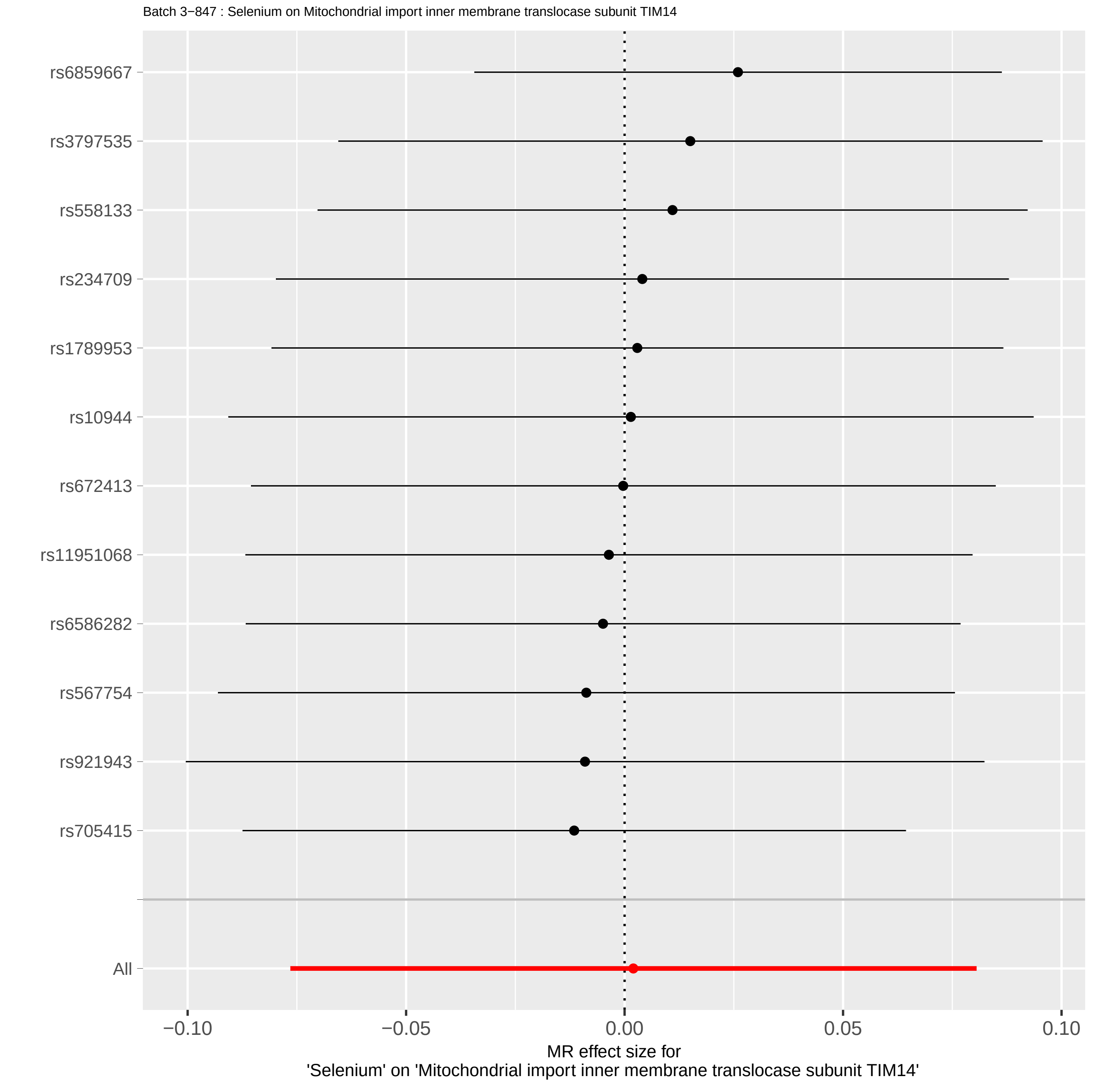
Batch 3−847 : Selenium on Mitochondrial import inner membrane translocase subunit TIM14
rs6859667
rs3797535
rs558133
rs234709
rs1789953
rs10944
rs672413
rs11951068
rs6586282
rs567754
rs921943
rs705415
All
−0.10
−0.05
0.00
0.05
0.10
MR effect size for
'Selenium' on 'Mitochondrial import inner membrane translocase subunit TIM14'

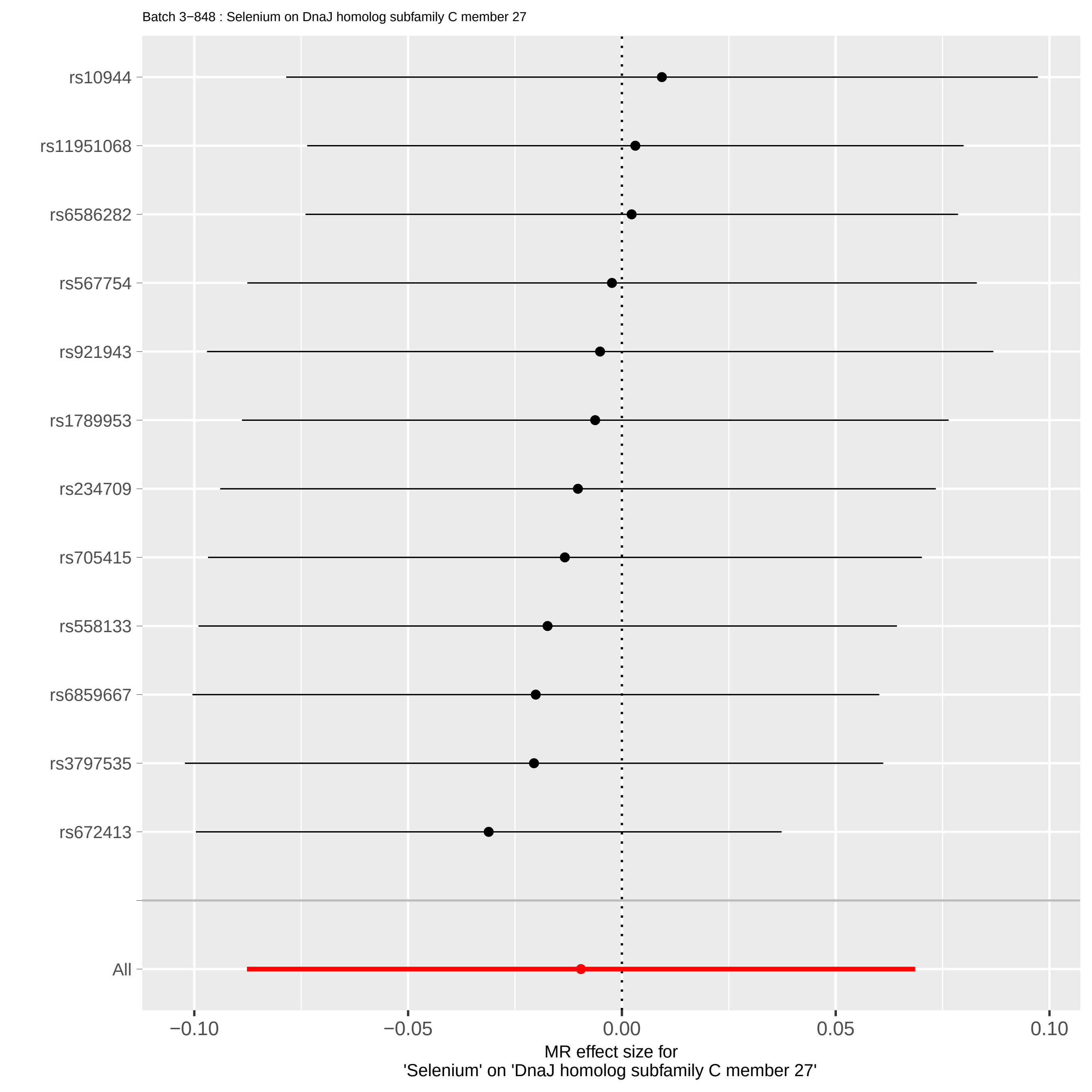

Batch 3−848 : Selenium on DnaJ homolog subfamily C member 27
rs10944
rs11951068
rs6586282
rs567754
rs921943
rs1789953
rs234709
rs705415
rs558133
rs6859667
rs3797535
rs672413
All
−0.10
−0.05
0.00
0.05
0.10
MR effect size for
'Selenium' on 'DnaJ homolog subfamily C member 27'

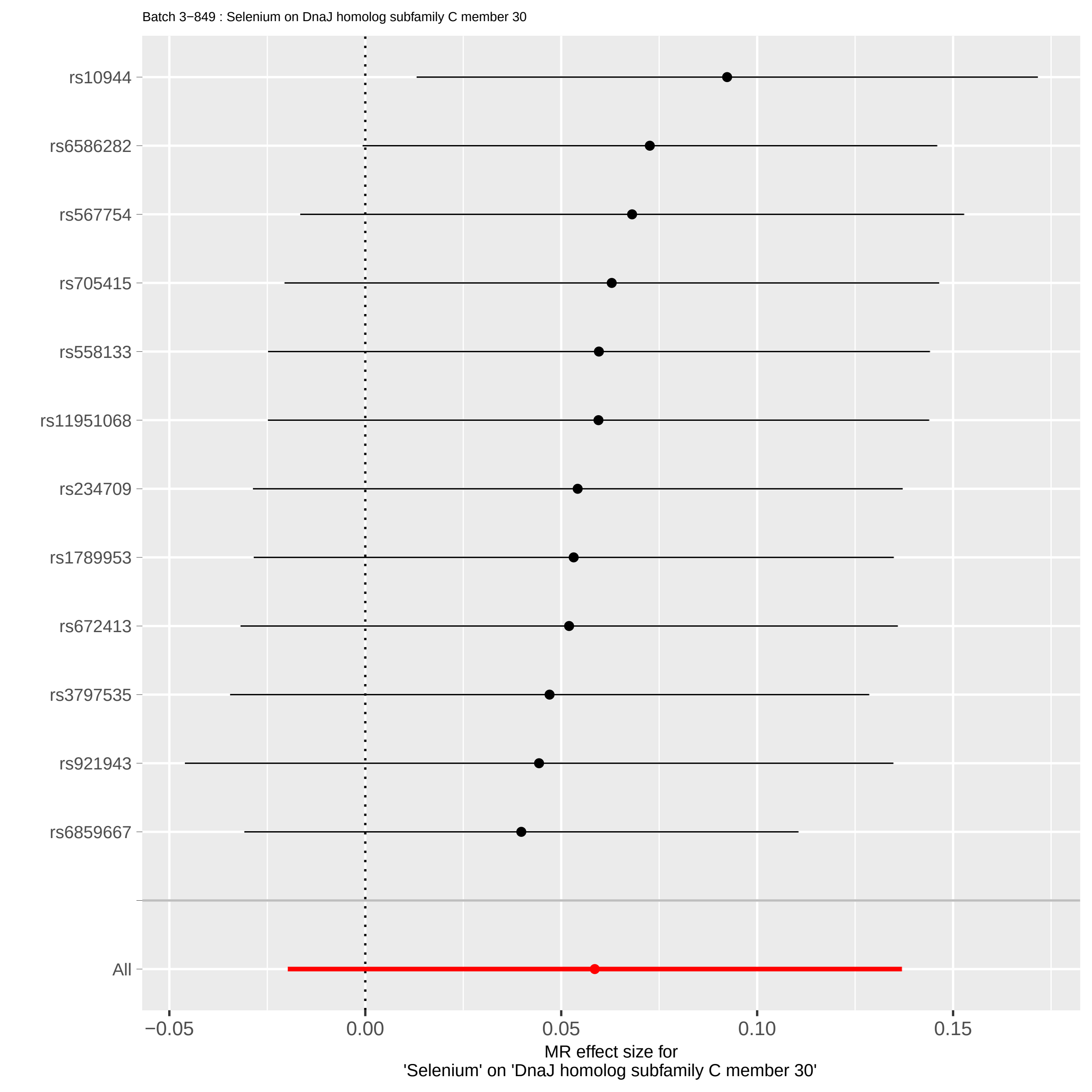

Batch 3−849 : Selenium on DnaJ homolog subfamily C member 30
rs10944
rs6586282
rs567754
rs705415
rs558133
rs11951068
rs234709
rs1789953
rs672413
rs3797535
rs921943
rs6859667
All
−0.05
0.00
0.05
0.10
0.15
MR effect size for
'Selenium' on 'DnaJ homolog subfamily C member 30'

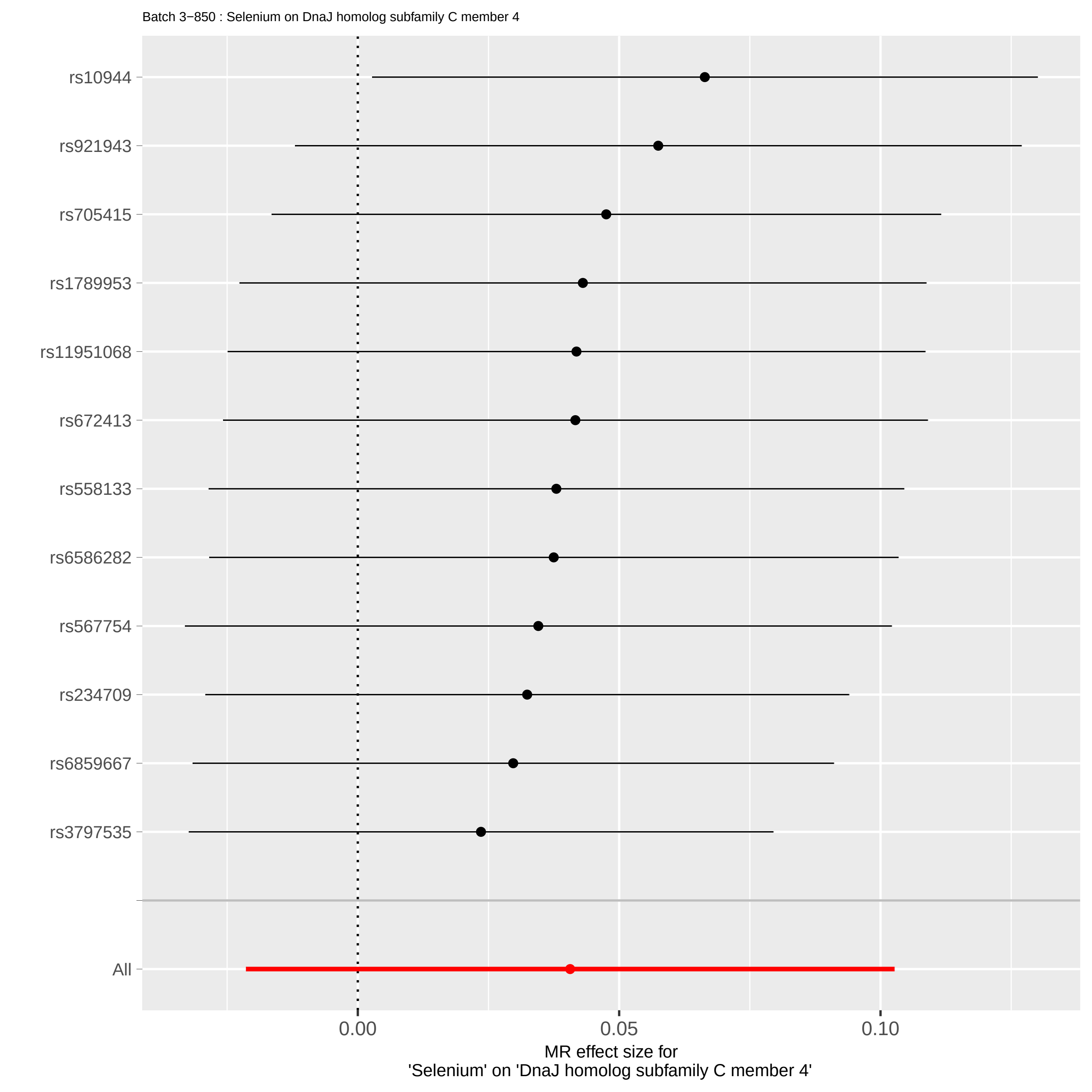
Batch 3−850 : Selenium on DnaJ homolog subfamily C member 4
rs10944
rs921943
rs705415
rs1789953
rs11951068
rs672413
rs558133
rs6586282
rs567754
rs234709
rs6859667
rs3797535
All
0.00
0.05
0.10
MR effect size for
'Selenium' on 'DnaJ homolog subfamily C member 4'

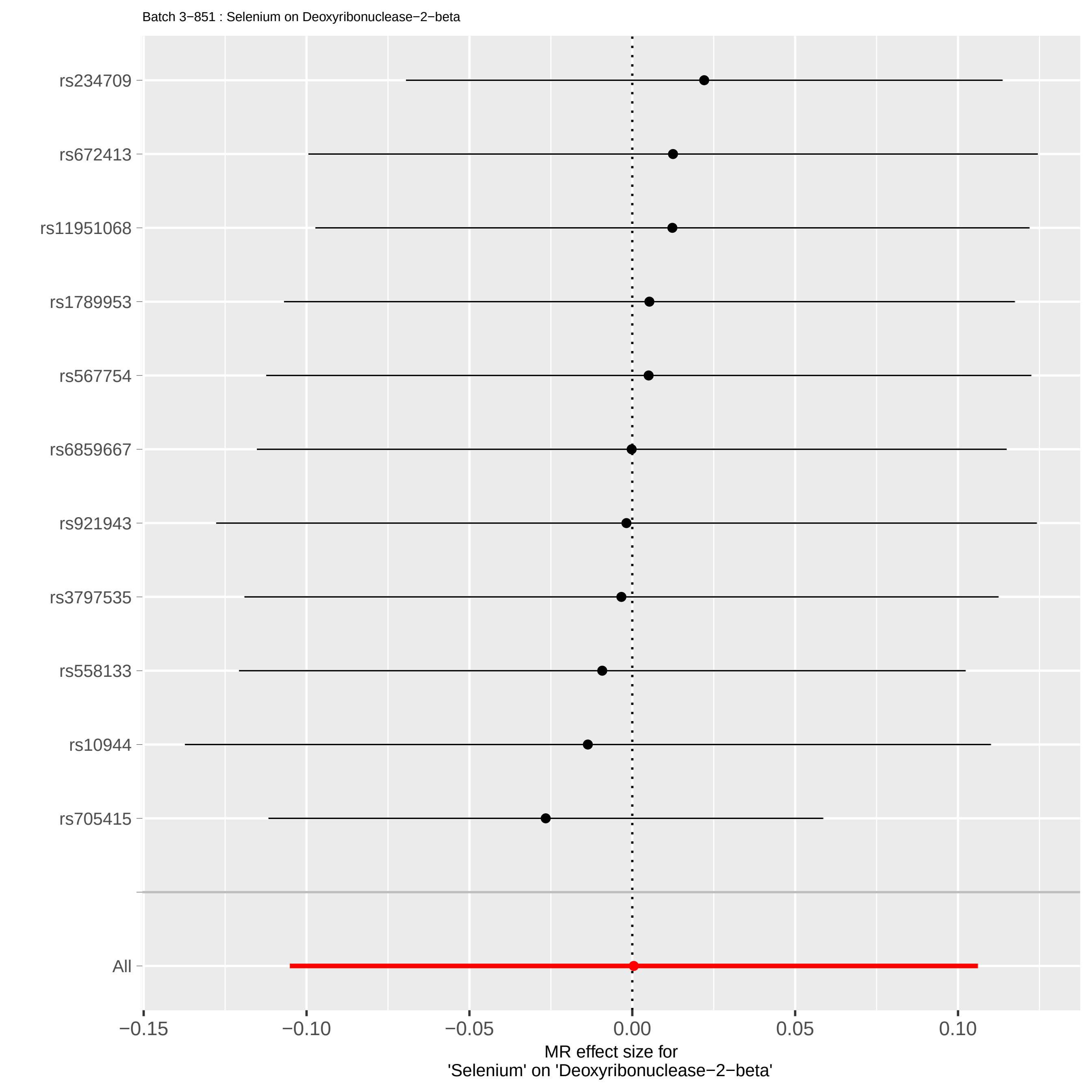

Batch 3−851 : Selenium on Deoxyribonuclease−2−beta
rs234709
rs672413
rs11951068
rs1789953
rs567754
rs6859667
rs921943
rs3797535
rs558133
rs10944
rs705415
All
−0.15
−0.10
−0.05
0.00
0.05
0.10
MR effect size for
'Selenium' on 'Deoxyribonuclease−2−beta'

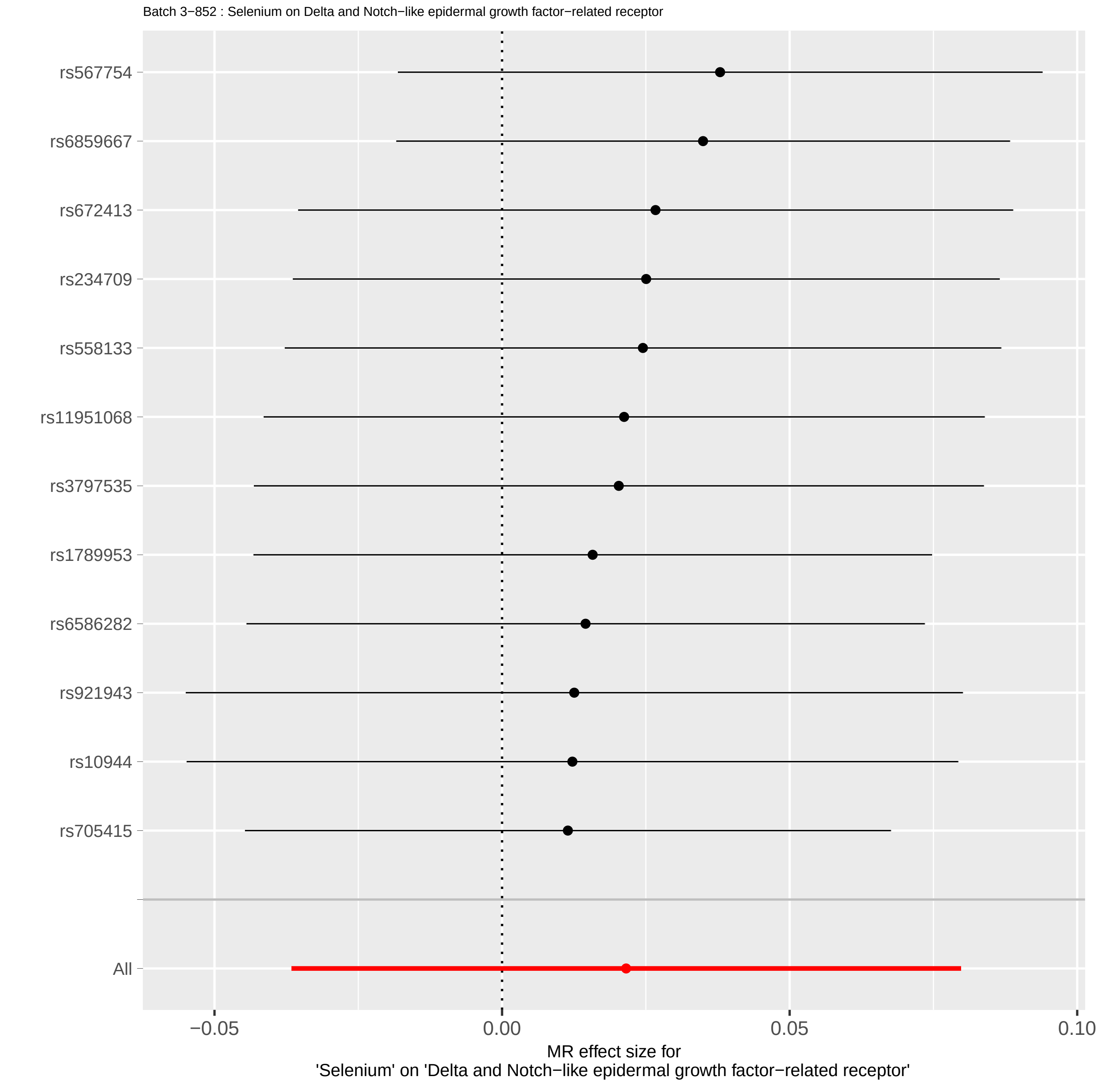

Batch 3−852 : Selenium on Delta and Notch−like epidermal growth factor−related receptor
rs567754
rs6859667
rs672413
rs234709
rs558133
rs11951068
rs3797535
rs1789953
rs6586282
rs921943
rs10944
rs705415
All
−0.05
0.00
0.05
0.10
MR effect size for
'Selenium' on 'Delta and Notch−like epidermal growth factor−related receptor'

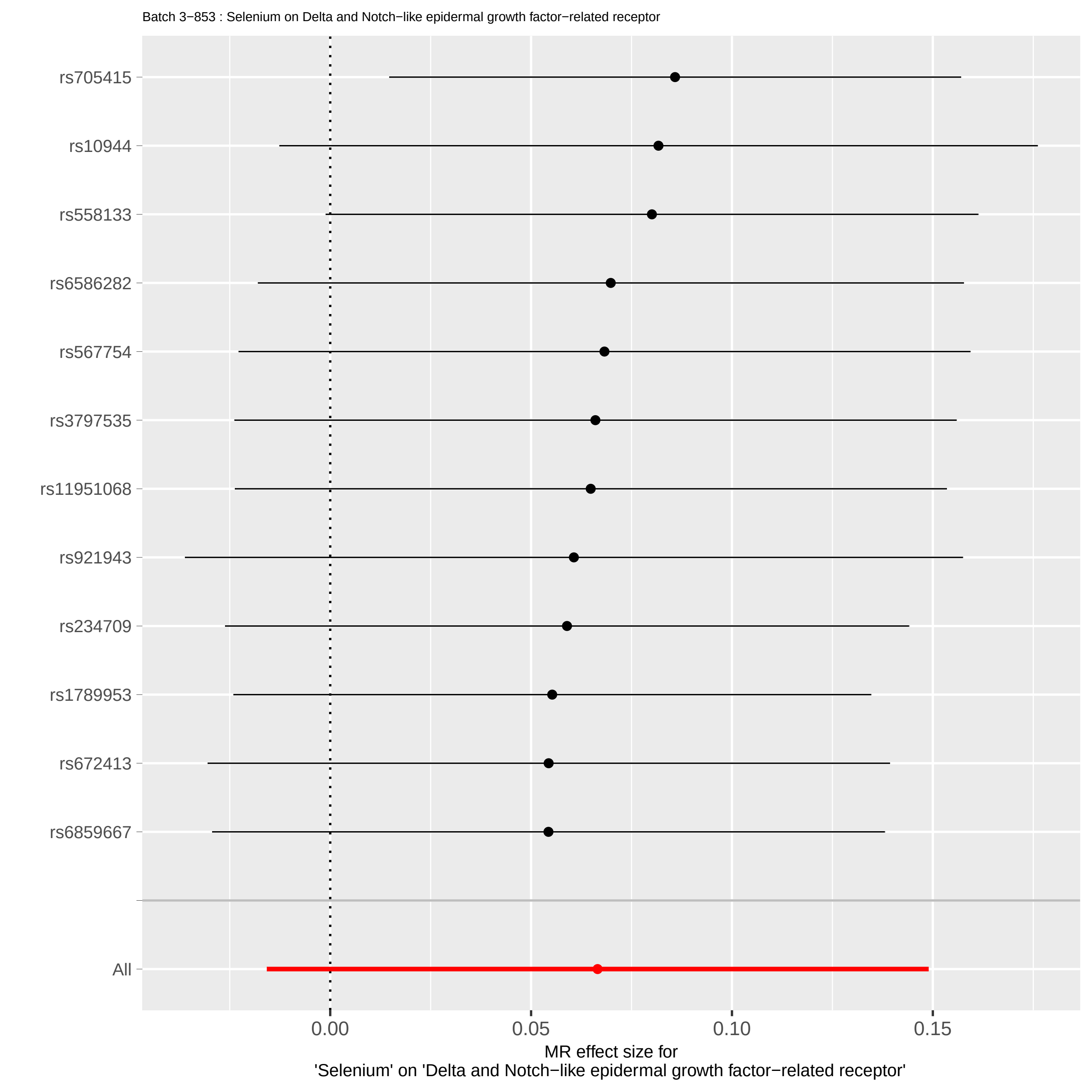

Batch 3−853 : Selenium on Delta and Notch−like epidermal growth factor−related receptor
rs705415
rs10944
rs558133
rs6586282
rs567754
rs3797535
rs11951068
rs921943
rs234709
rs1789953
rs672413
rs6859667
All
0.00
0.05
0.10
0.15
MR effect size for
'Selenium' on 'Delta and Notch−like epidermal growth factor−related receptor'

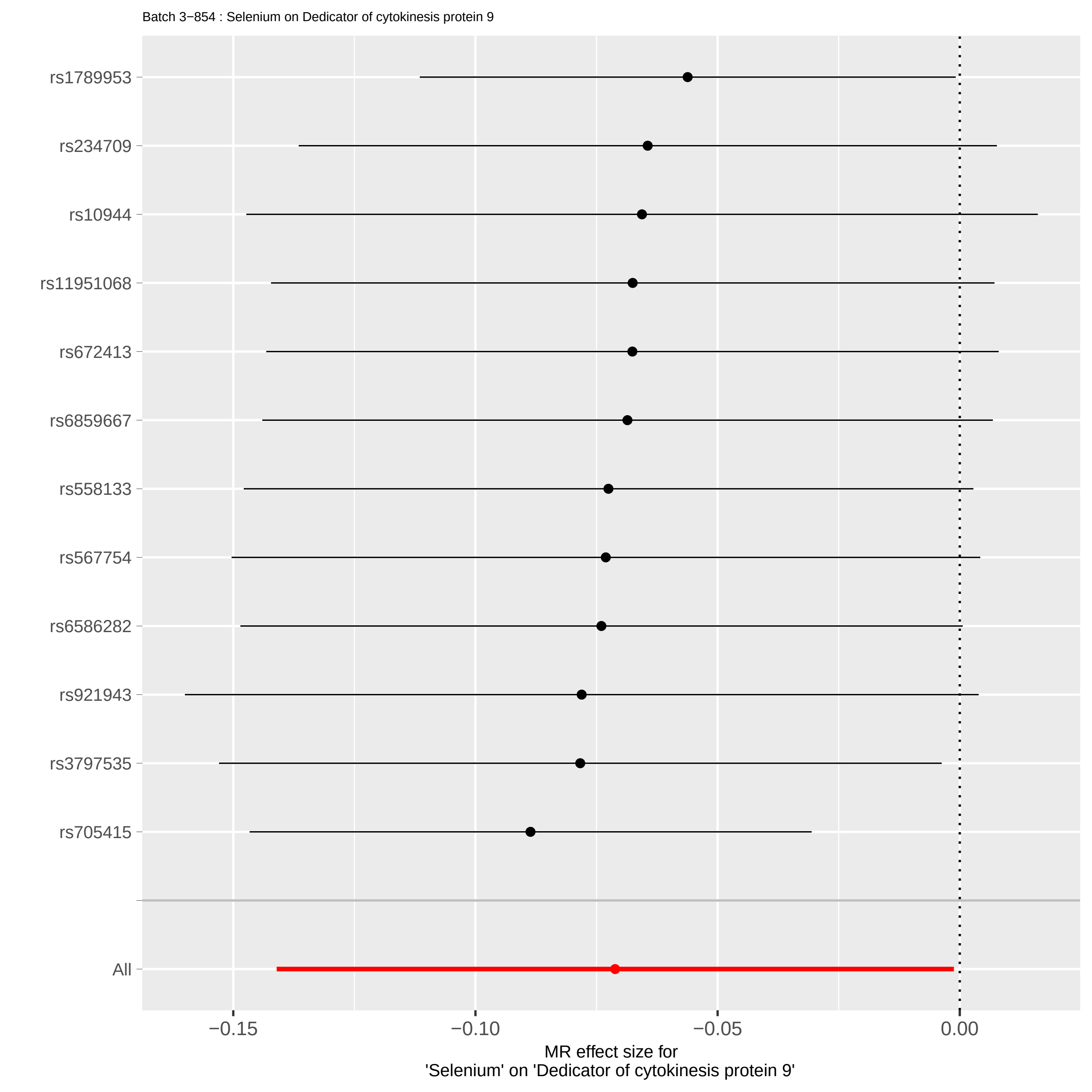

Batch 3−854 : Selenium on Dedicator of cytokinesis protein 9
rs1789953
rs234709
rs10944
rs11951068
rs672413
rs6859667
rs558133
rs567754
rs6586282
rs921943
rs3797535
rs705415
All
−0.15
−0.10
−0.05
0.00
MR effect size for
'Selenium' on 'Dedicator of cytokinesis protein 9'

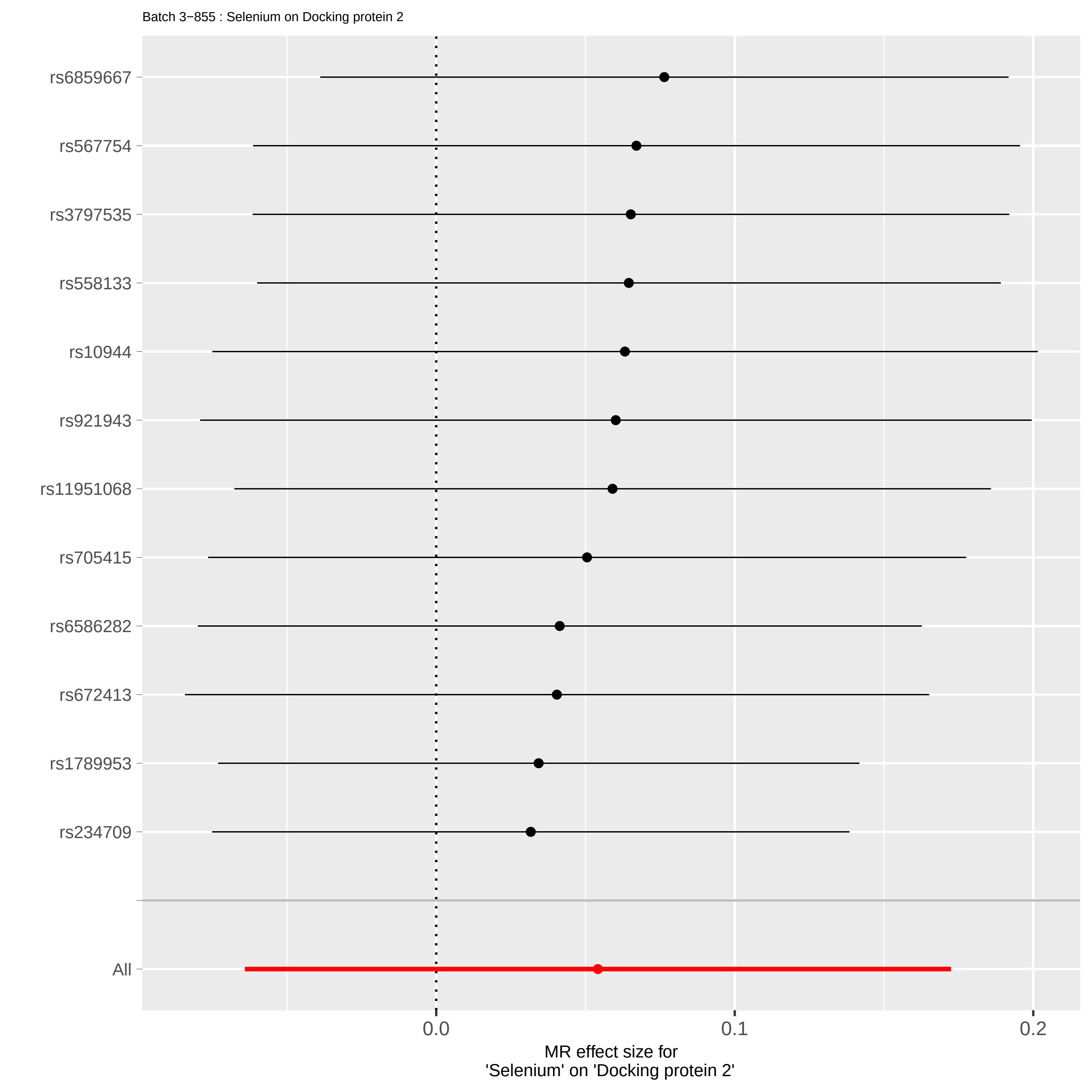
Batch 3−855 : Selenium on Docking protein 2
rs6859667
rs567754
rs3797535
rs558133
rs10944
rs921943
rs11951068
rs705415
rs6586282
rs672413
rs1789953
rs234709
All
0.0
0.1
0.2
MR effect size for
'Selenium' on 'Docking protein 2'

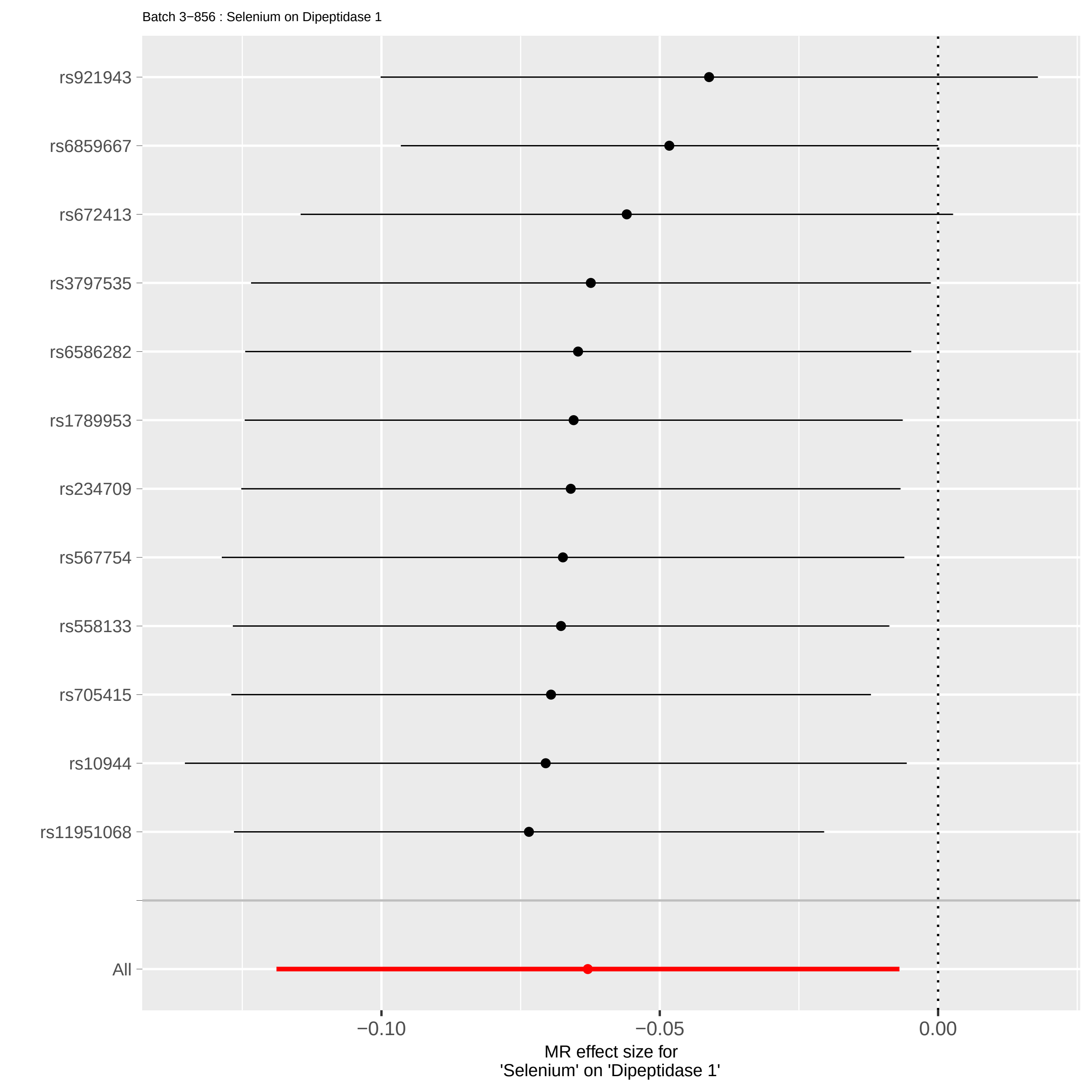

Batch 3−856 : Selenium on Dipeptidase 1
rs921943
rs6859667
rs672413
rs3797535
rs6586282
rs1789953
rs234709
rs567754
rs558133
rs705415
rs10944
rs11951068
All
−0.10
−0.05
0.00
MR effect size for
'Selenium' on 'Dipeptidase 1'

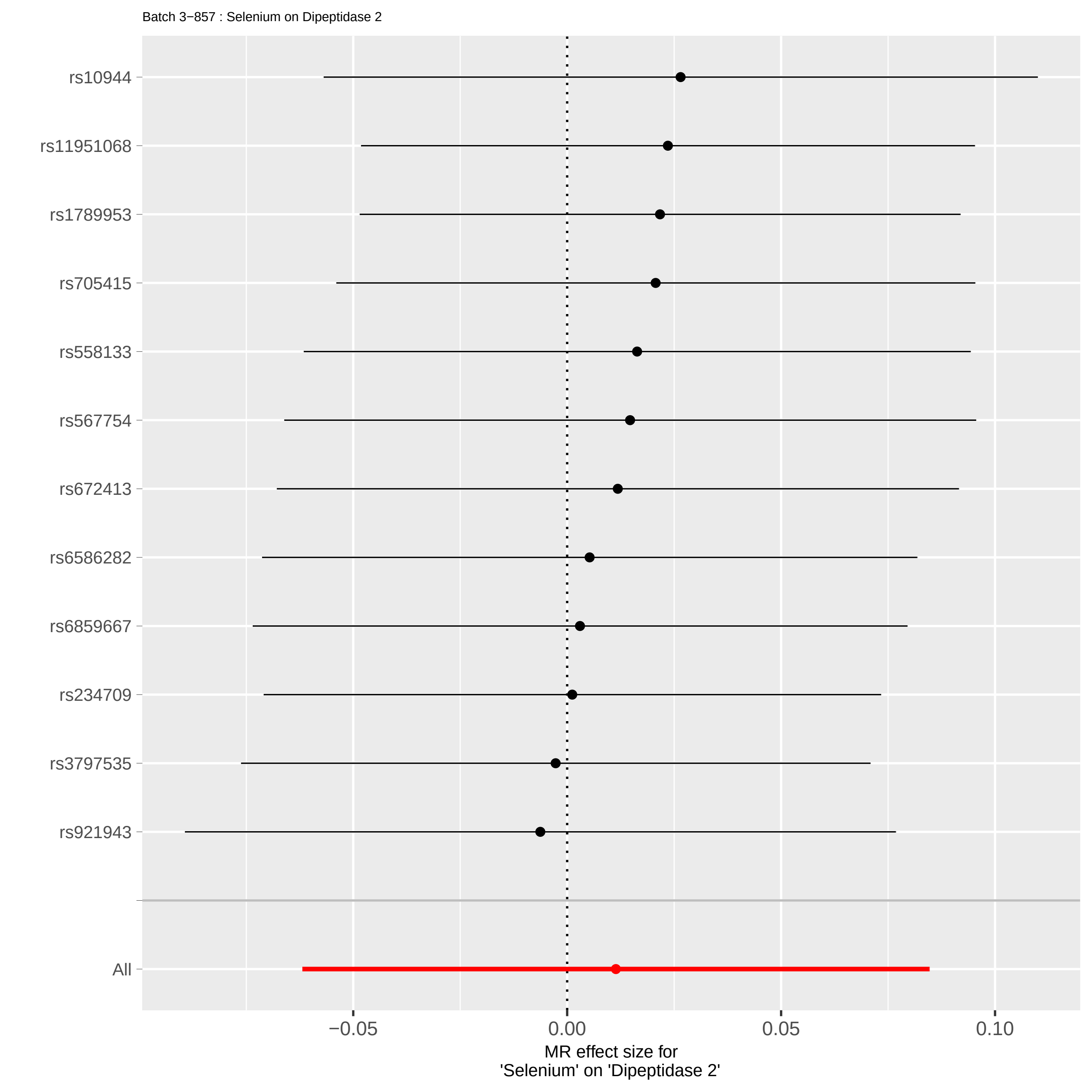

Batch 3−857 : Selenium on Dipeptidase 2
rs10944
rs11951068
rs1789953
rs705415
rs558133
rs567754
rs672413
rs6586282
rs6859667
rs234709
rs3797535
rs921943
All
−0.05
0.00
0.05
0.10
MR effect size for
'Selenium' on 'Dipeptidase 2'

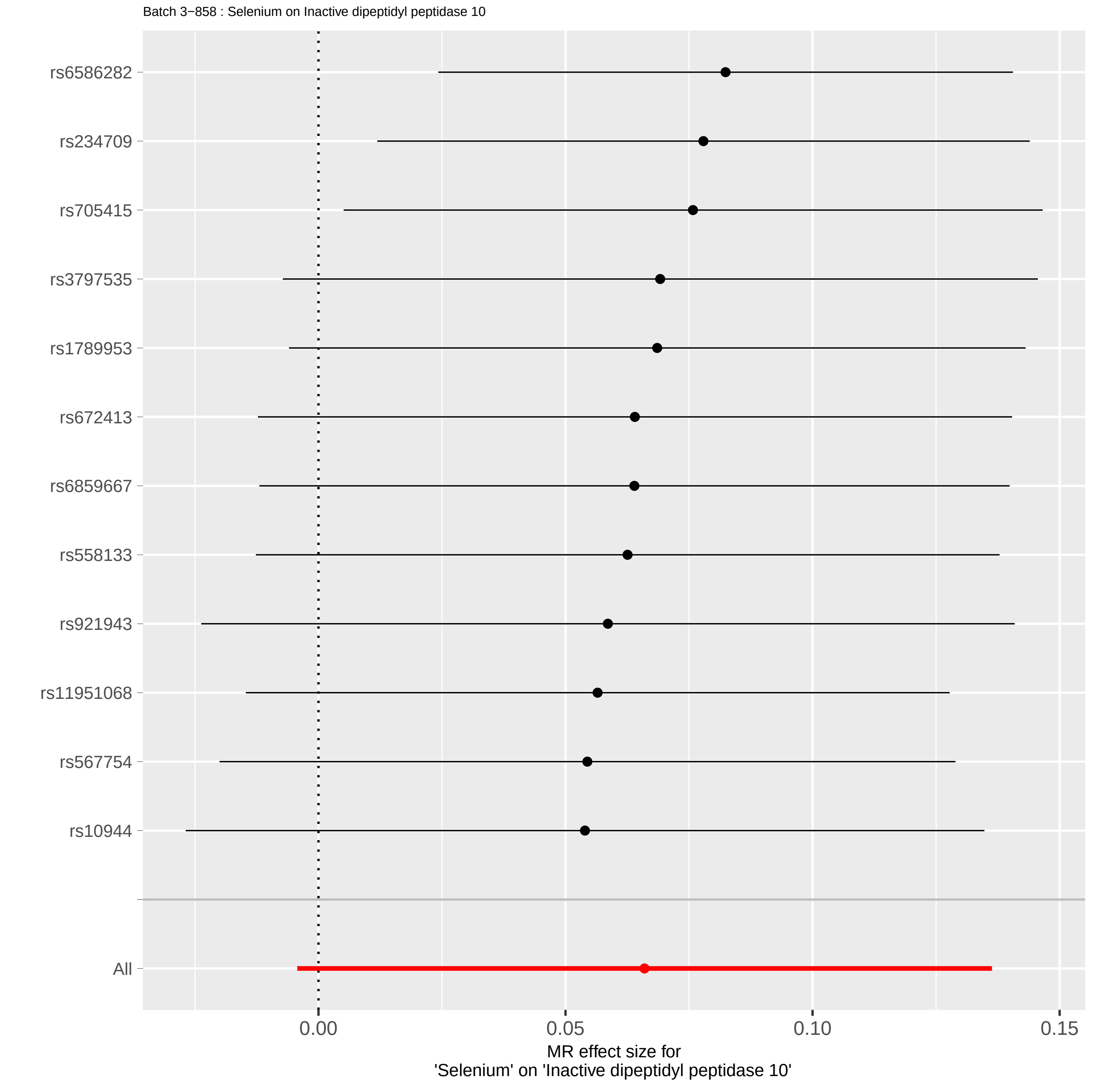

Batch 3−858 : Selenium on Inactive dipeptidyl peptidase 10
rs6586282
rs234709
rs705415
rs3797535
rs1789953
rs672413
rs6859667
rs558133
rs921943
rs11951068
rs567754
rs10944
All
0.00
0.05
0.10
0.15
MR effect size for
'Selenium' on 'Inactive dipeptidyl peptidase 10'

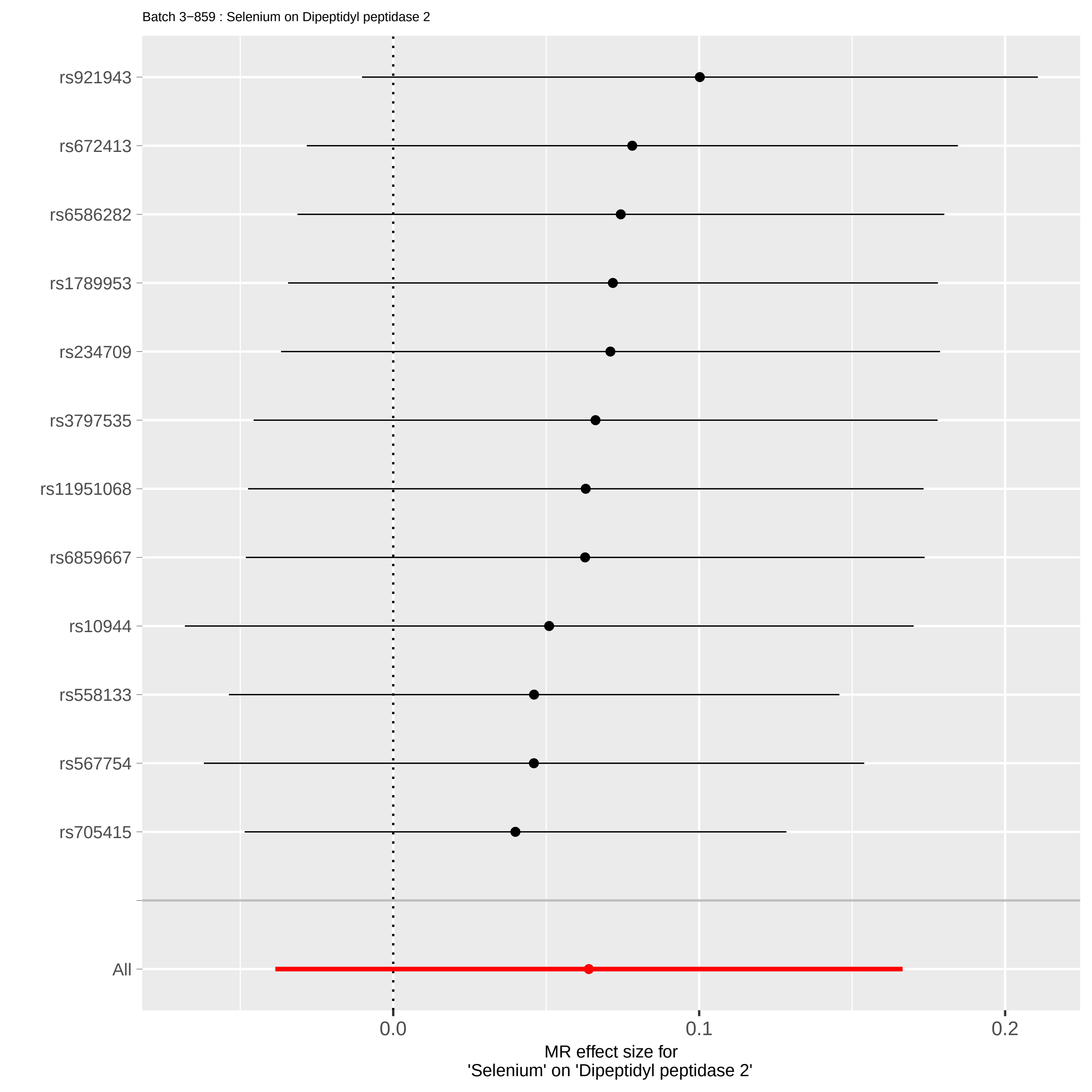
Batch 3−859 : Selenium on Dipeptidyl peptidase 2
rs921943
rs672413
rs6586282
rs1789953
rs234709
rs3797535
rs11951068
rs6859667
rs10944
rs558133
rs567754
rs705415
All
0.0
0.1
0.2
MR effect size for
'Selenium' on 'Dipeptidyl peptidase 2'

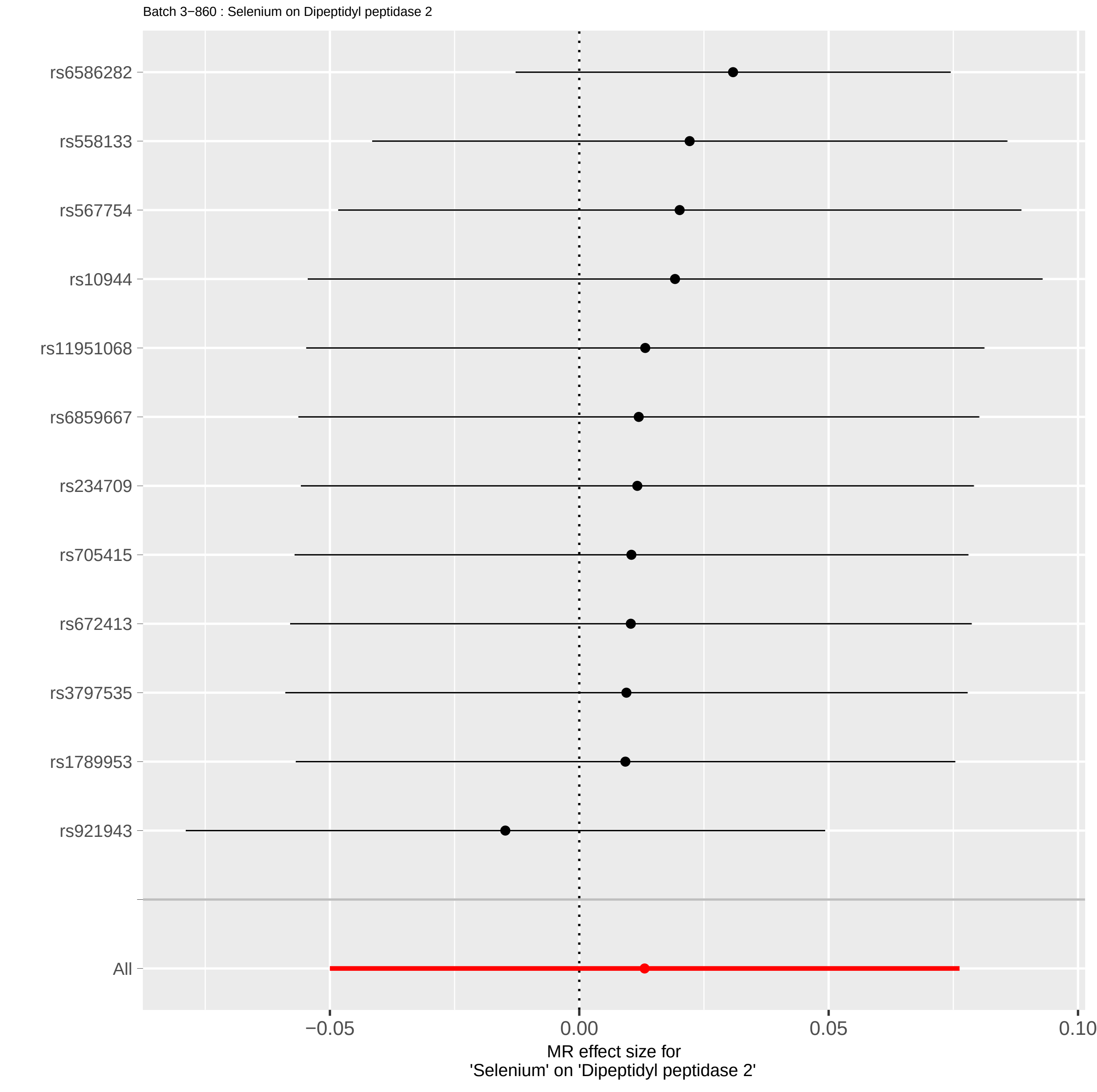

Batch 3−860 : Selenium on Dipeptidyl peptidase 2
rs6586282
rs558133
rs567754
rs10944
rs11951068
rs6859667
rs234709
rs705415
rs672413
rs3797535
rs1789953
rs921943
All
−0.05
0.00
0.05
0.10
MR effect size for
'Selenium' on 'Dipeptidyl peptidase 2'

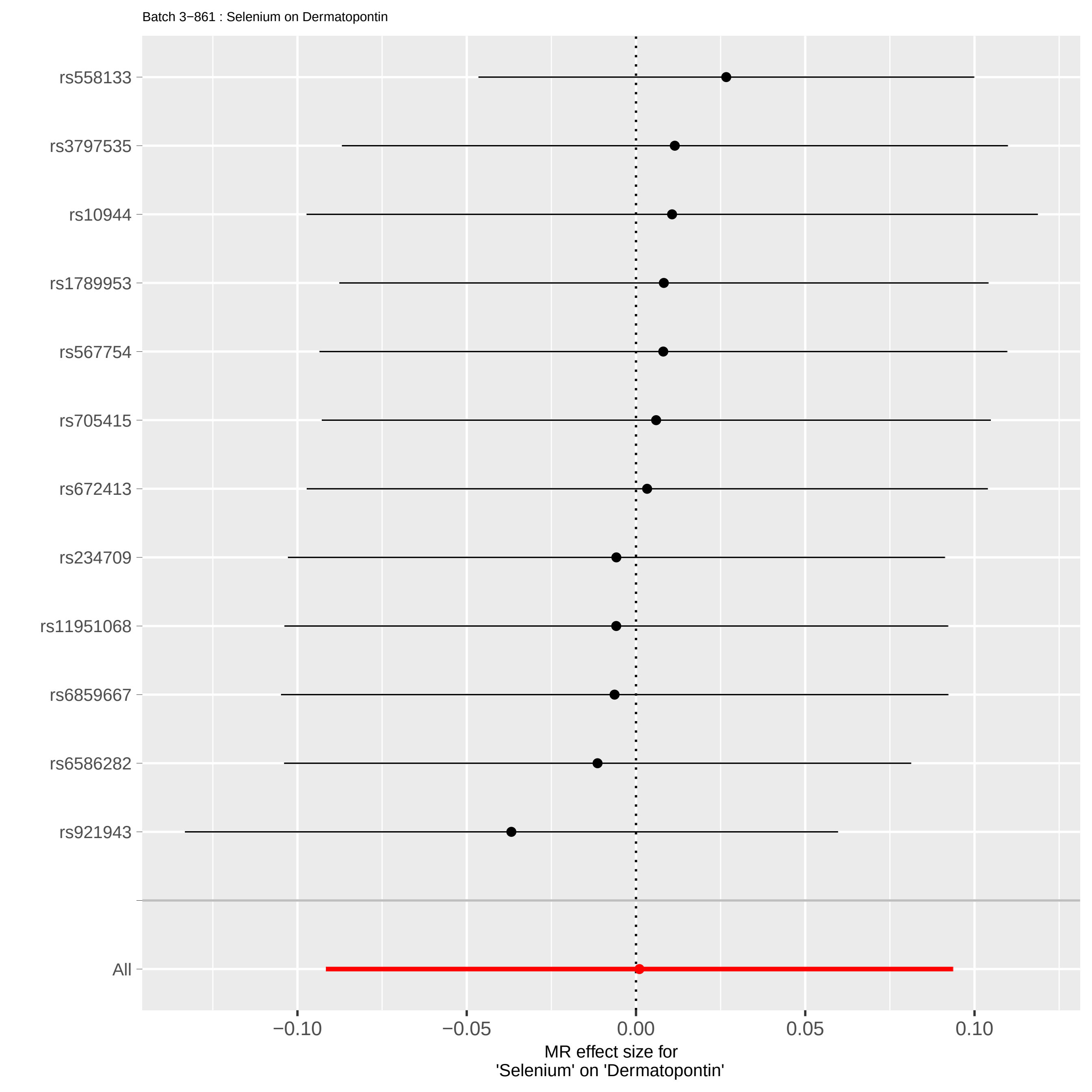

Batch 3−861 : Selenium on Dermatopontin
rs558133
rs3797535
rs10944
rs1789953
rs567754
rs705415
rs672413
rs234709
rs11951068
rs6859667
rs6586282
rs921943
All
−0.10
−0.05
0.00
0.05
0.10
MR effect size for
'Selenium' on 'Dermatopontin'

Batch 3−862 : Selenium on Protein dpy−30 homolog

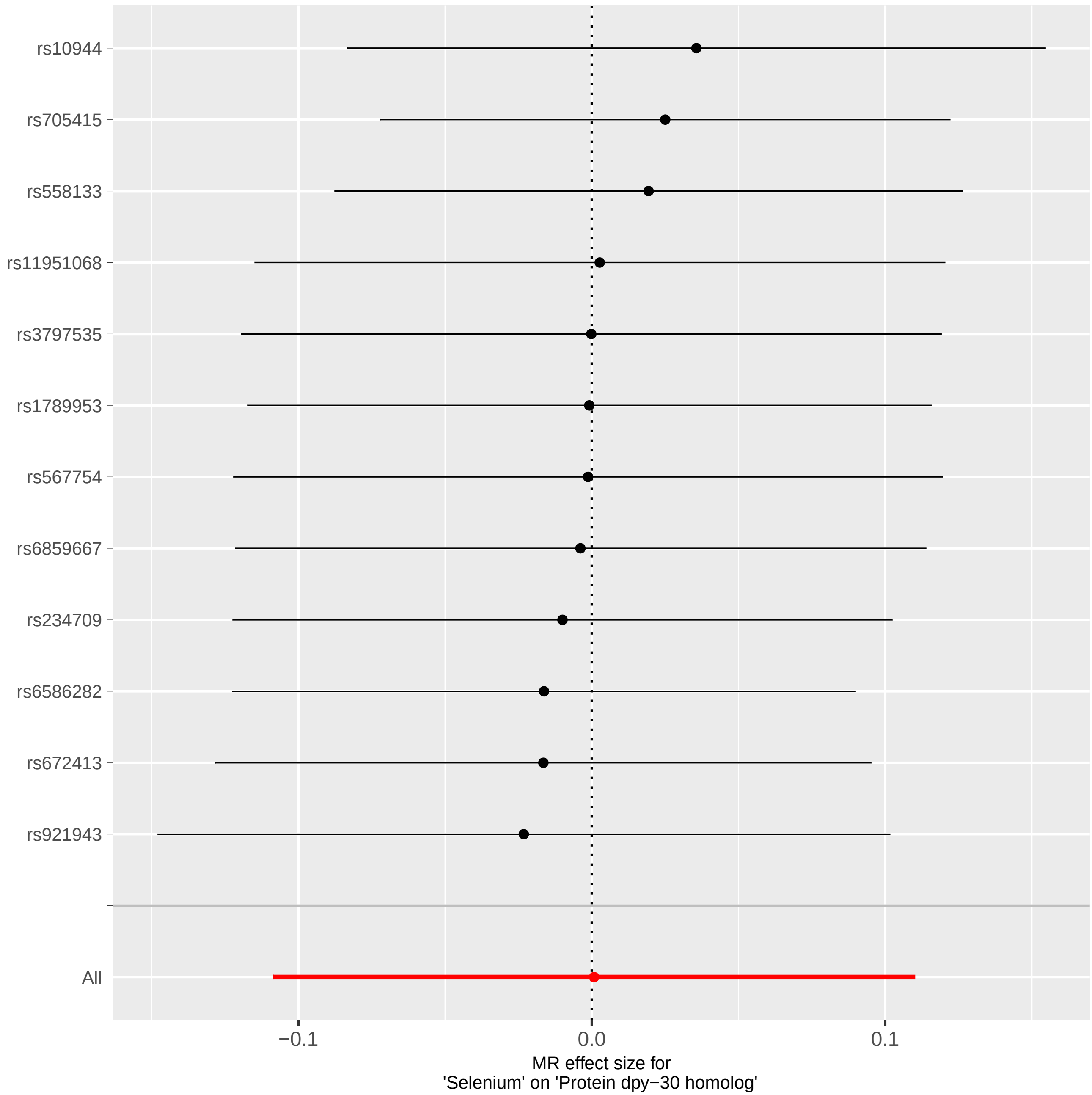

Batch 3−863 : Selenium on Dihydropyrimidinase−related protein 3

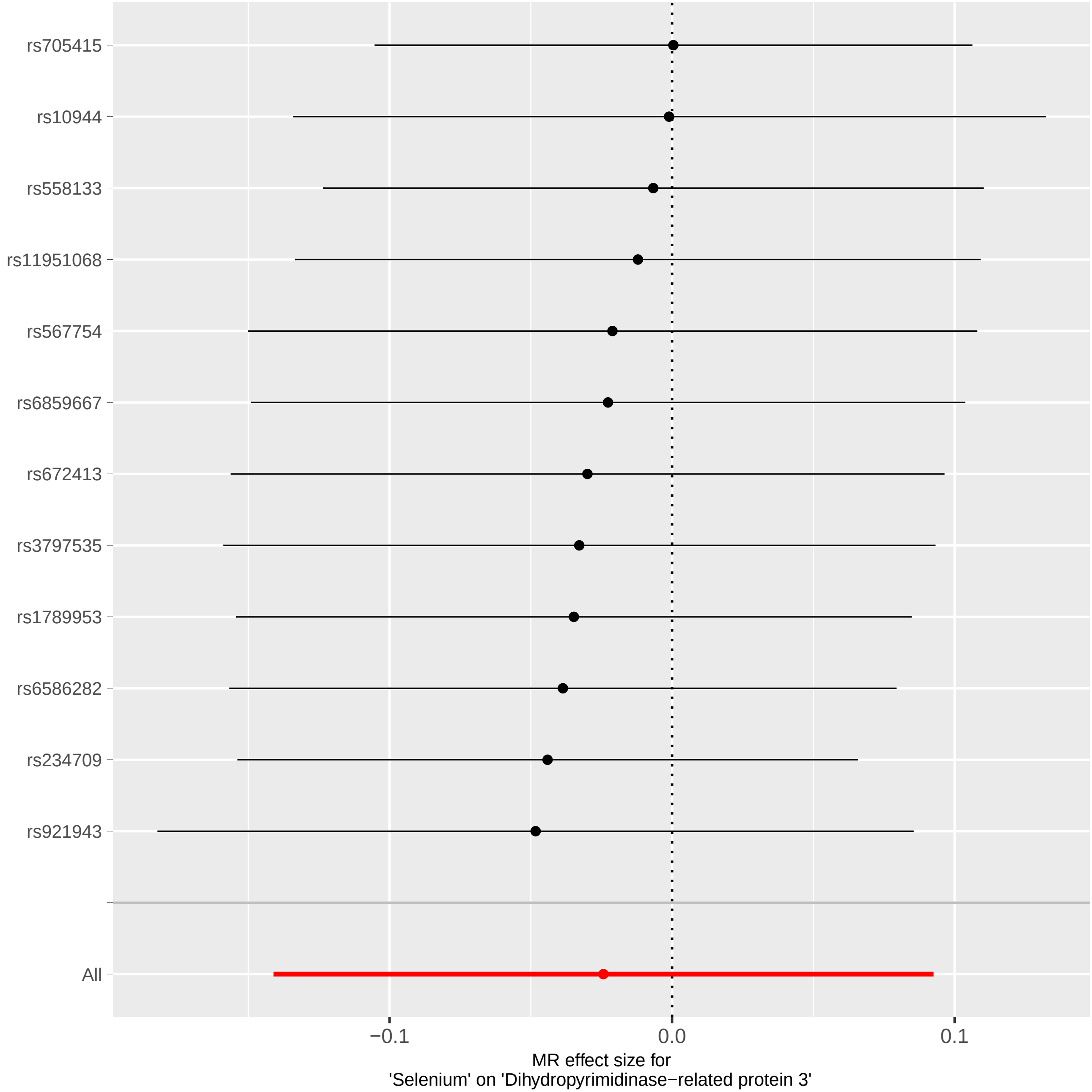

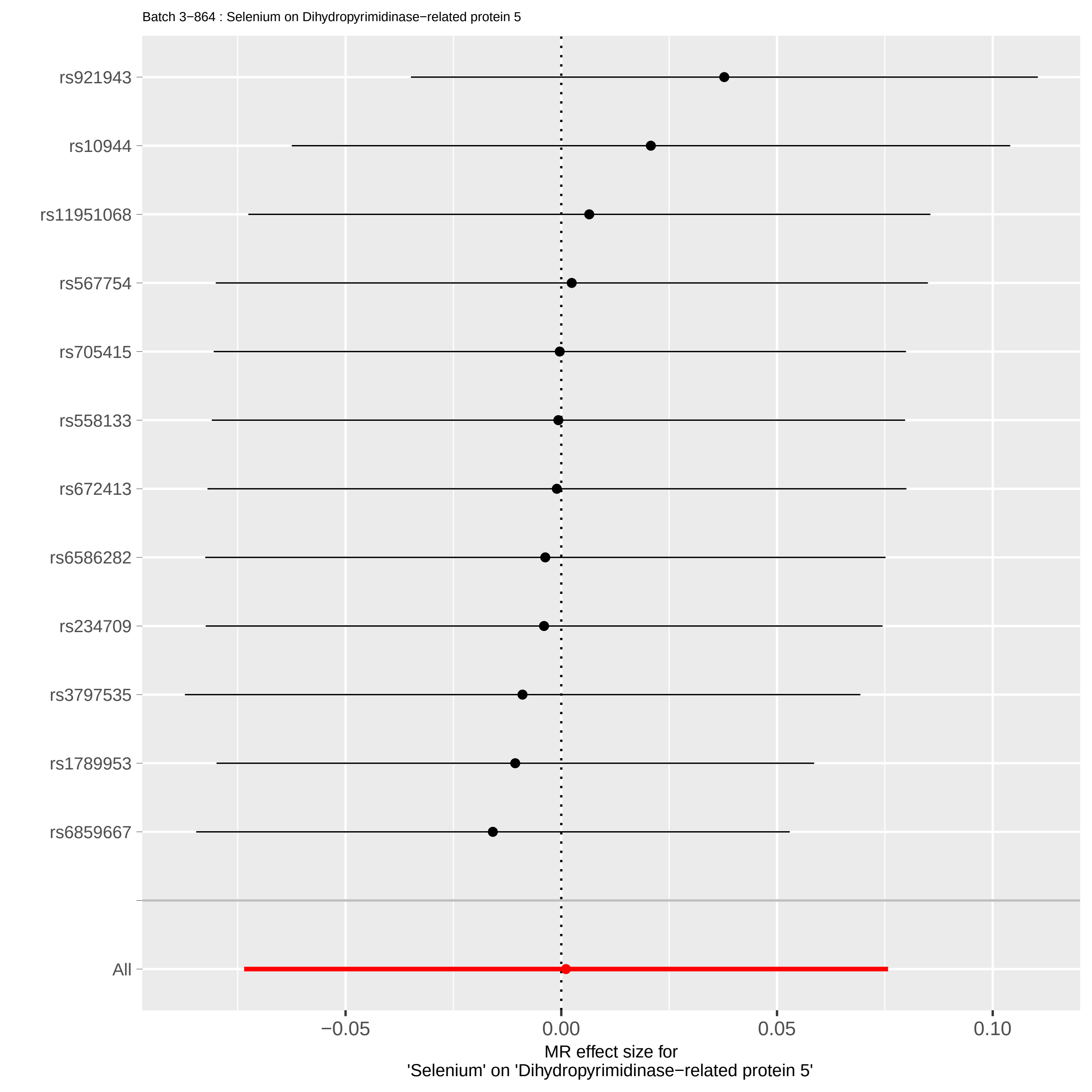
Batch 3−864 : Selenium on Dihydropyrimidinase−related protein 5
rs921943
rs10944
rs11951068
rs567754
rs705415
rs558133
rs672413
rs6586282
rs234709
rs3797535
rs1789953
rs6859667
All
−0.05
0.00
0.05
0.10
MR effect size for
'Selenium' on 'Dihydropyrimidinase−related protein 5'

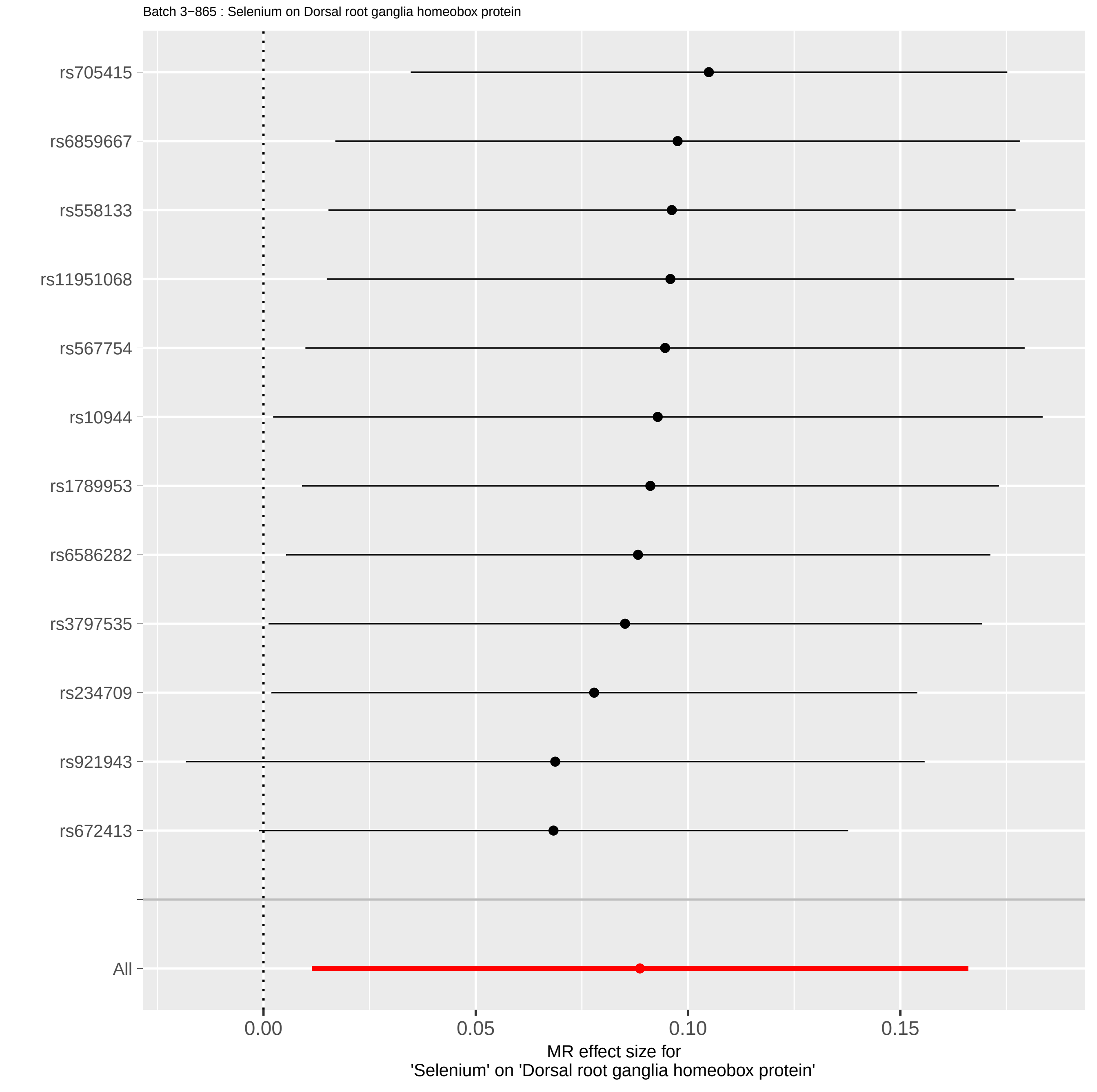

Batch 3−865 : Selenium on Dorsal root ganglia homeobox protein
rs705415
rs6859667
rs558133
rs11951068
rs567754
rs10944
rs1789953
rs6586282
rs3797535
rs234709
rs921943
rs672413
All
0.00
0.05
0.10
0.15
MR effect size for
'Selenium' on 'Dorsal root ganglia homeobox protein'

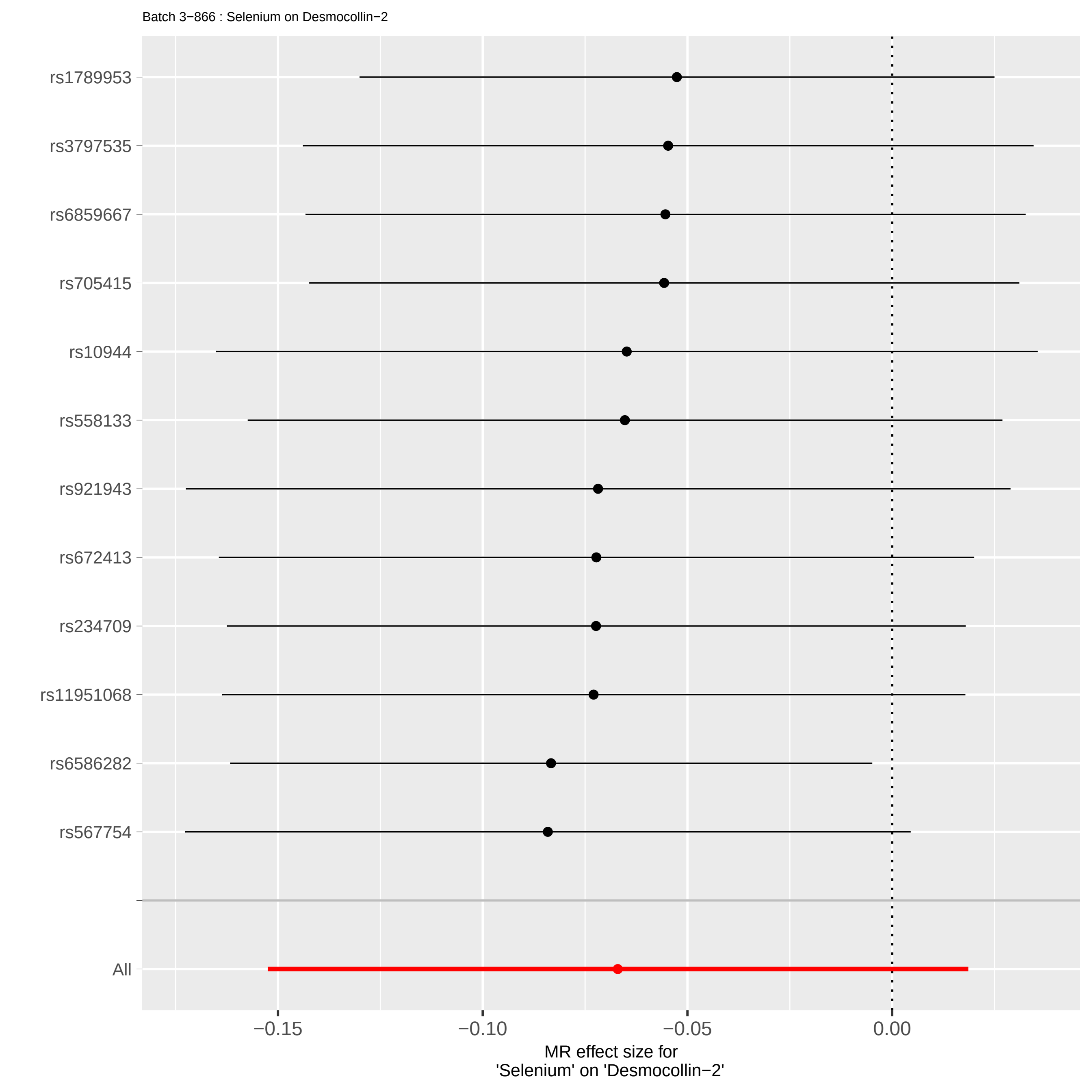

Batch 3−866 : Selenium on Desmocollin−2
rs1789953
rs3797535
rs6859667
rs705415
rs10944
rs558133
rs921943
rs672413
rs234709
rs11951068
rs6586282
rs567754
All
−0.15
−0.10
−0.05
0.00
MR effect size for
'Selenium' on 'Desmocollin−2'

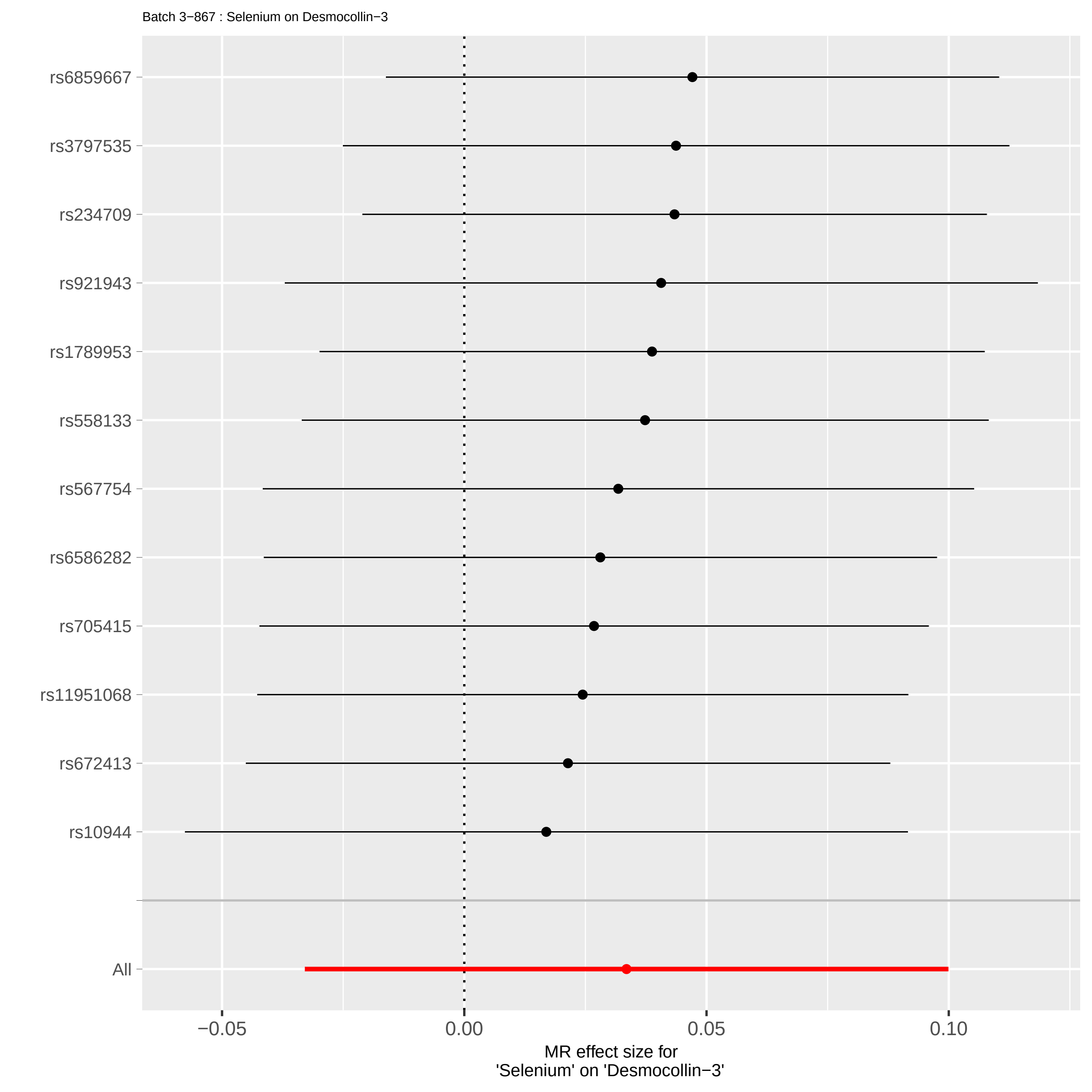

Batch 3−867 : Selenium on Desmocollin−3
rs6859667
rs3797535
rs234709
rs921943
rs1789953
rs558133
rs567754
rs6586282
rs705415
rs11951068
rs672413
rs10944
All
−0.05
0.00
0.05
0.10
MR effect size for
'Selenium' on 'Desmocollin−3'

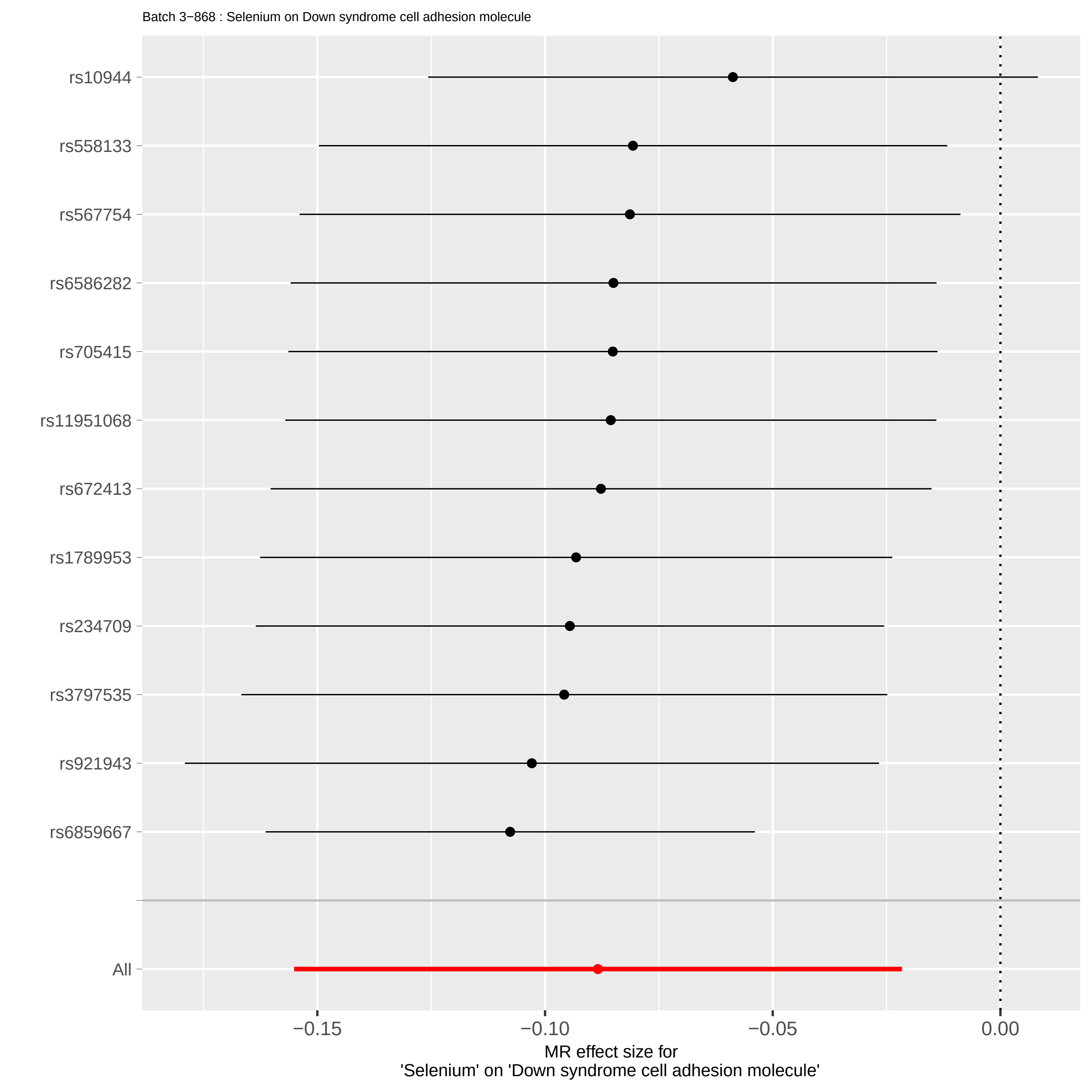

Batch 3−868 : Selenium on Down syndrome cell adhesion molecule
rs10944
rs558133
rs567754
rs6586282
rs705415
rs11951068
rs672413
rs1789953
rs234709
rs3797535
rs921943
rs6859667
All
−0.15
−0.10
−0.05
0.00
MR effect size for
'Selenium' on 'Down syndrome cell adhesion molecule'

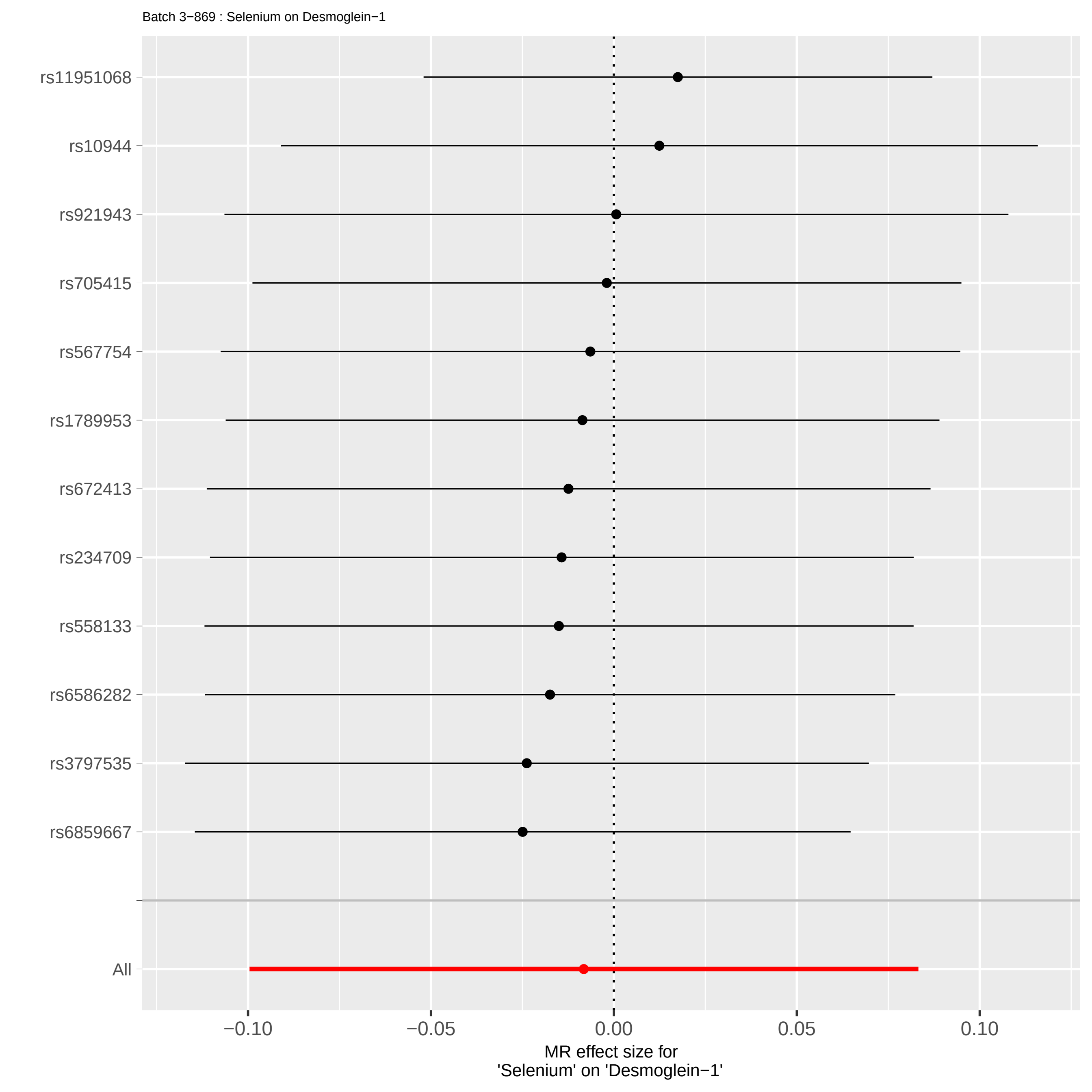

Batch 3−869 : Selenium on Desmoglein−1
rs11951068
rs10944
rs921943
rs705415
rs567754
rs1789953
rs672413
rs234709
rs558133
rs6586282
rs3797535
rs6859667
All
−0.10
−0.05
0.00
0.05
0.10
MR effect size for
'Selenium' on 'Desmoglein−1'

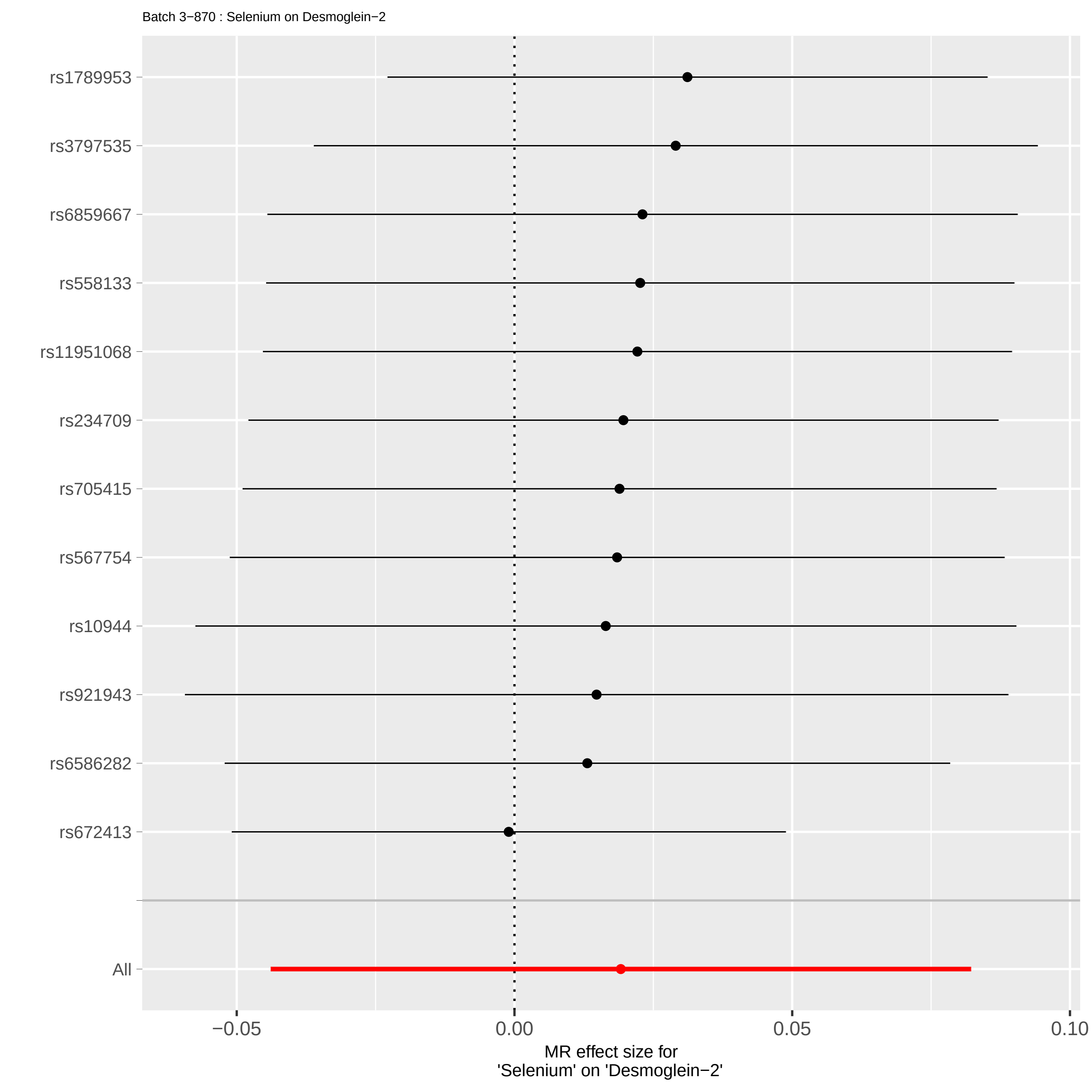
Batch 3−870 : Selenium on Desmoglein−2
rs1789953
rs3797535
rs6859667
rs558133
rs11951068
rs234709
rs705415
rs567754
rs10944
rs921943
rs6586282
rs672413
All
−0.05
0.00
0.05
0.10
MR effect size for
'Selenium' on 'Desmoglein−2'

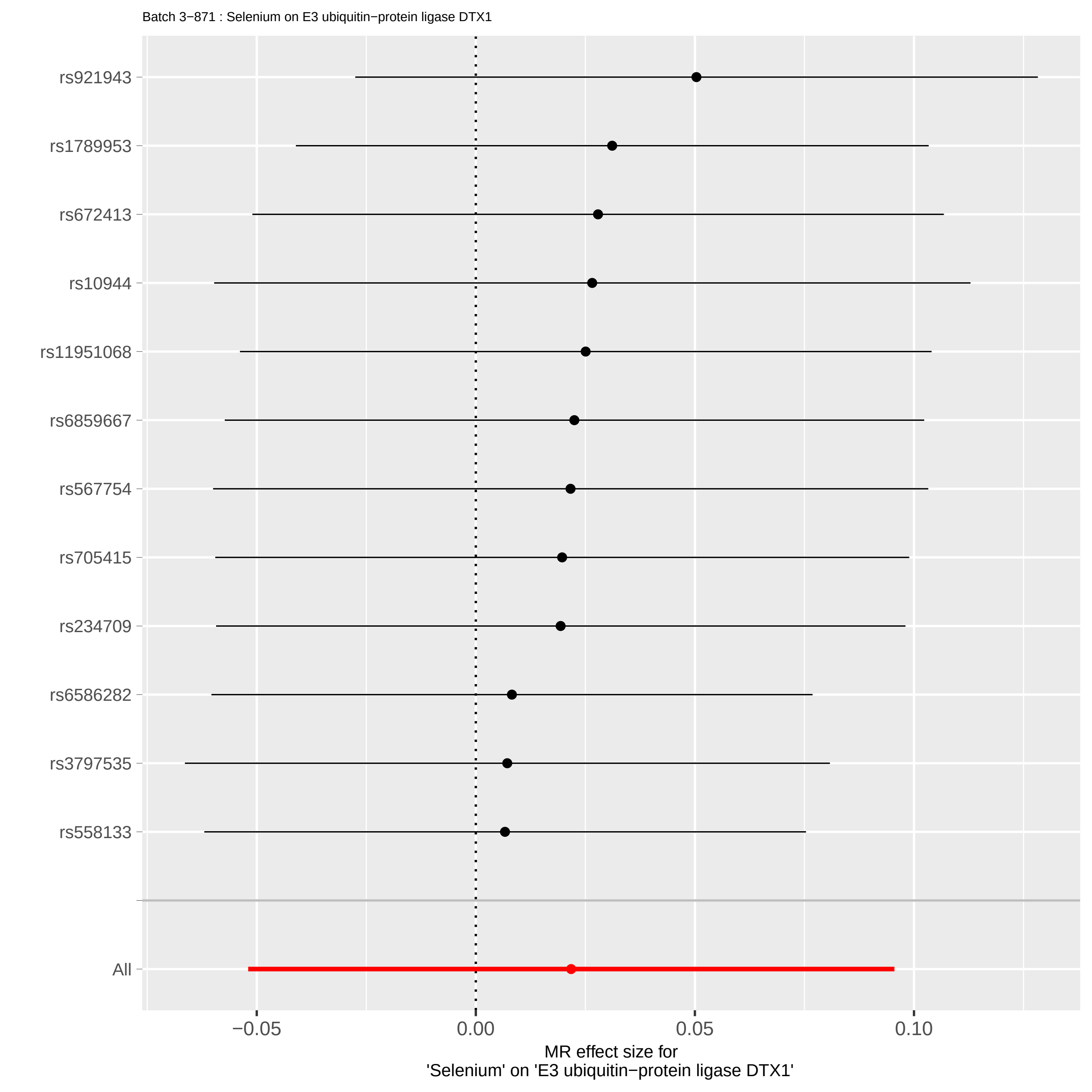
Batch 3−871 : Selenium on E3 ubiquitin−protein ligase DTX1
rs921943
rs1789953
rs672413
rs10944
rs11951068
rs6859667
rs567754
rs705415
rs234709
rs6586282
rs3797535
rs558133
All
−0.05
0.00
0.05
0.10
MR effect size for
'Selenium' on 'E3 ubiquitin−protein ligase DTX1'

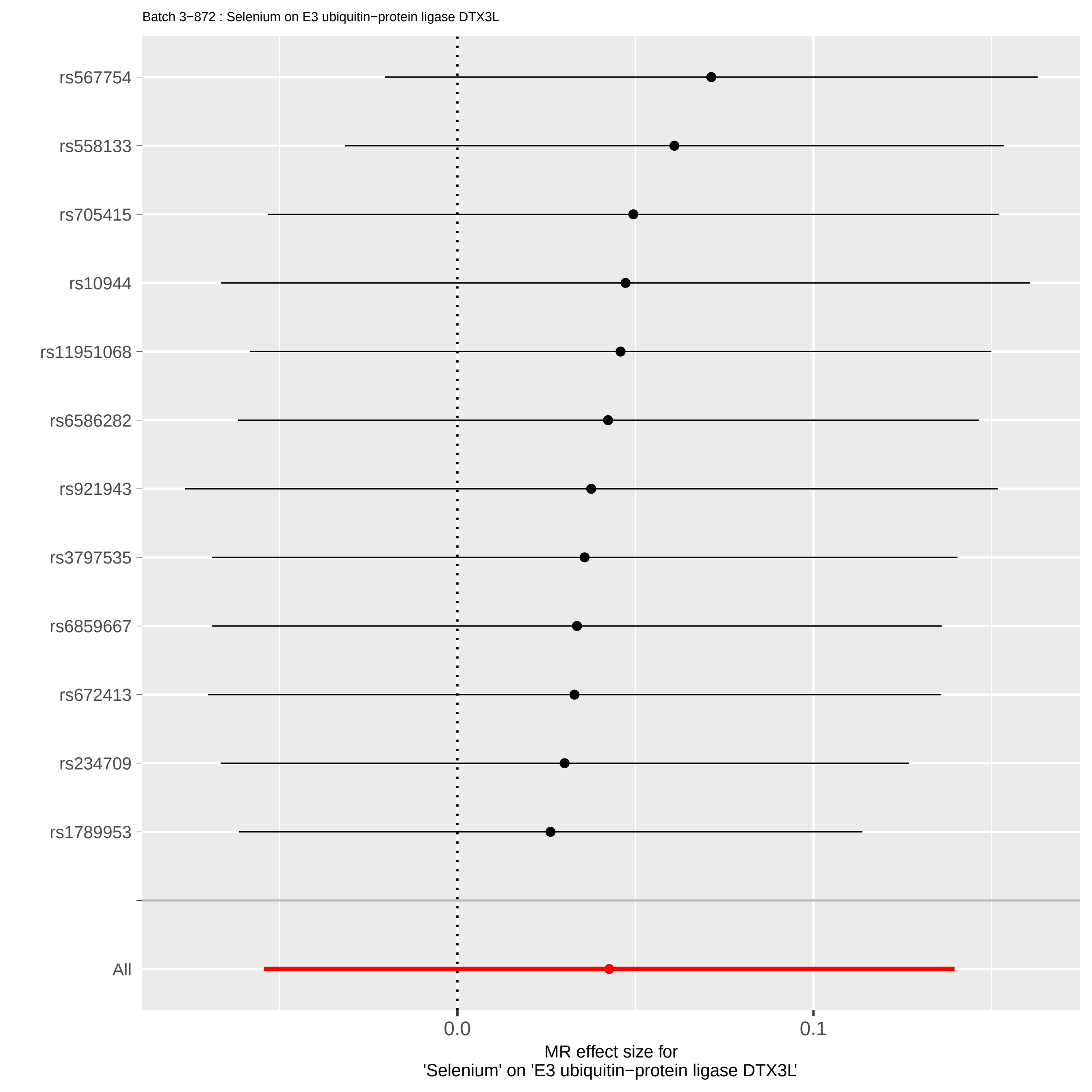
Batch 3−872 : Selenium on E3 ubiquitin−protein ligase DTX3L
rs567754
rs558133
rs705415
rs10944
rs11951068
rs6586282
rs921943
rs3797535
rs6859667
rs672413
rs234709
rs1789953
All
0.0
0.1
MR effect size for
'Selenium' on 'E3 ubiquitin−protein ligase DTX3L'

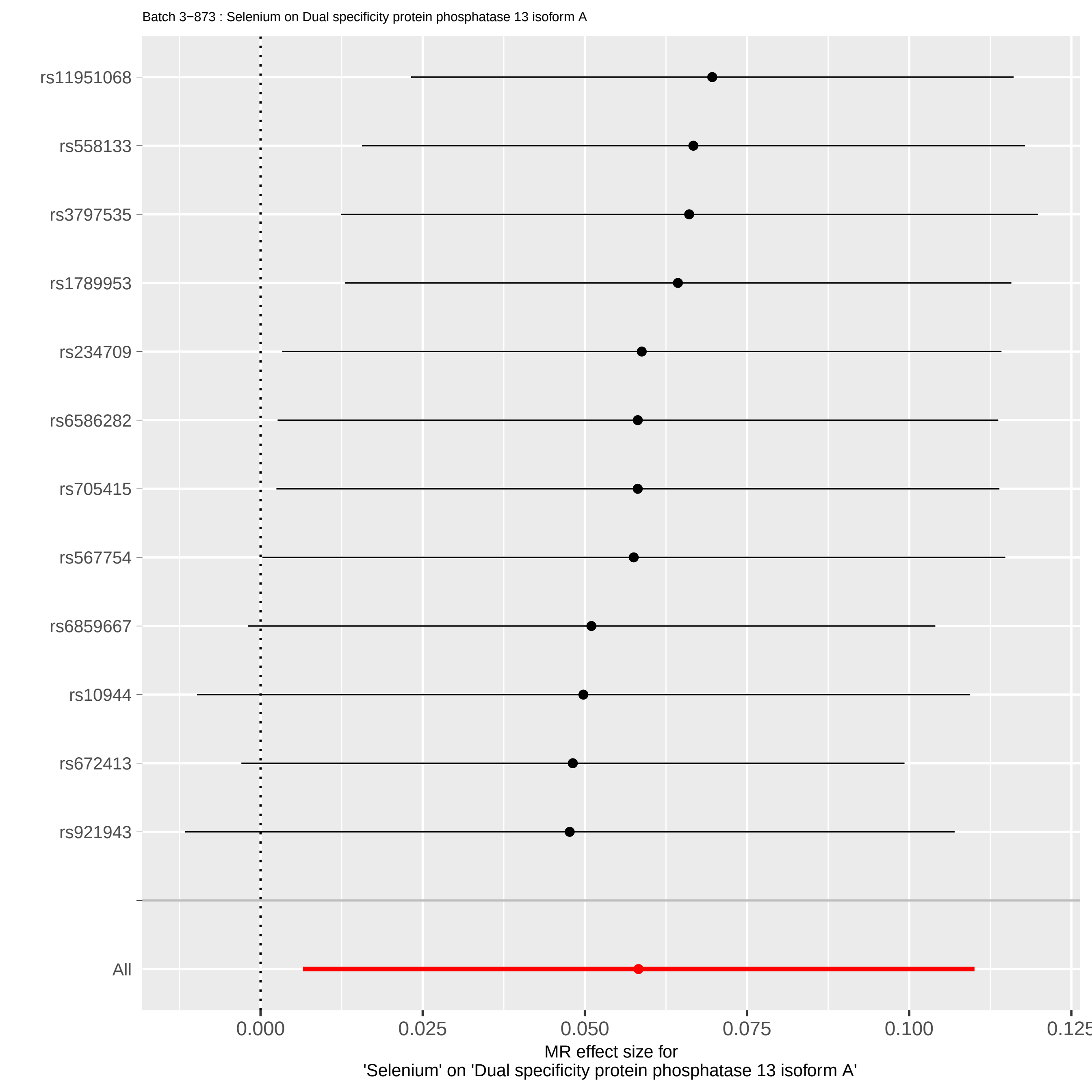
Batch 3−873 : Selenium on Dual specificity protein phosphatase 13 isoform A
rs11951068
rs558133
rs3797535
rs1789953
rs234709
rs6586282
rs705415
rs567754
rs6859667
rs10944
rs672413
rs921943
All
0.000
0.025
0.050
0.075
0.100
0.125
MR effect size for
'Selenium' on 'Dual specificity protein phosphatase 13 isoform A'

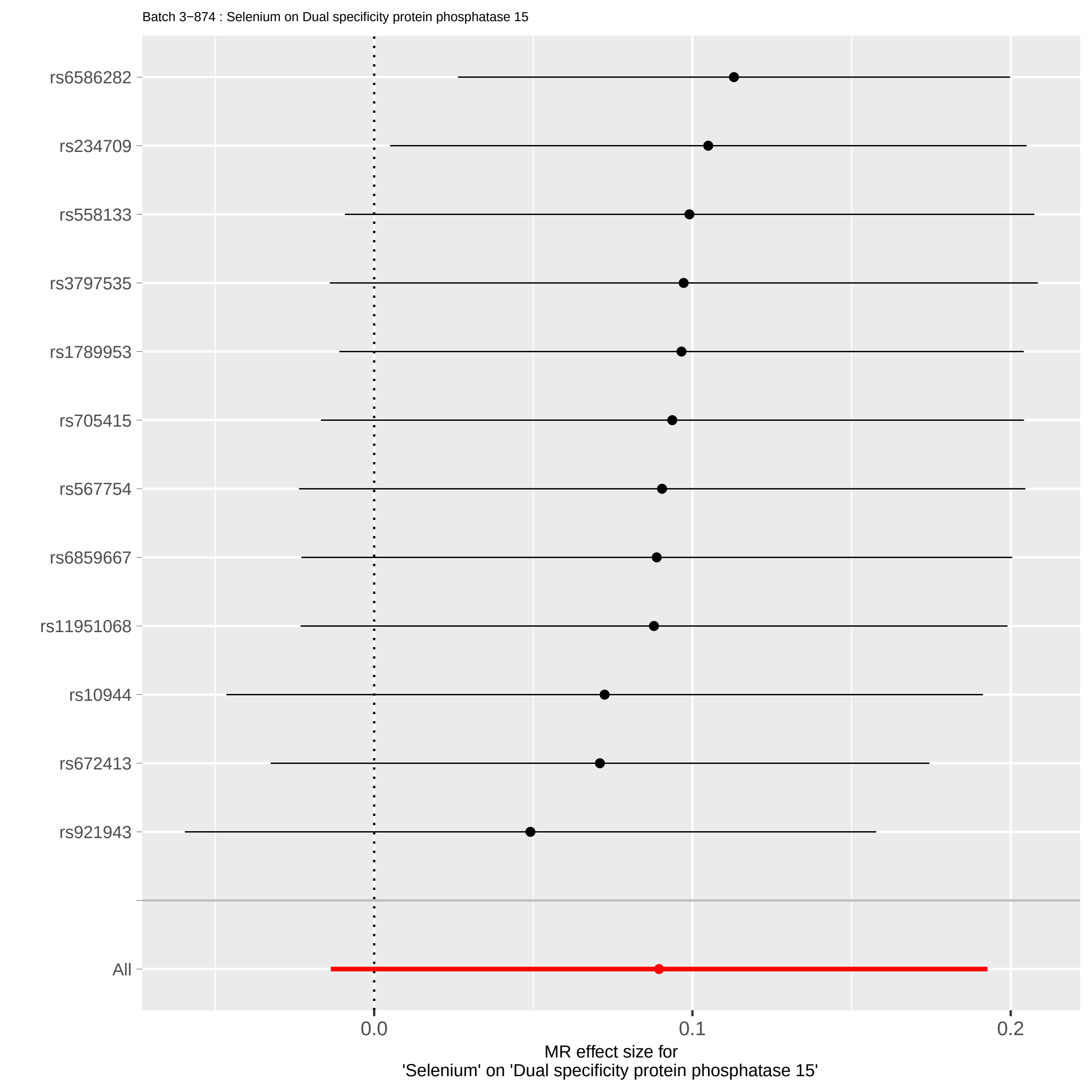
Batch 3−874 : Selenium on Dual specificity protein phosphatase 15
rs6586282
rs234709
rs558133
rs3797535
rs1789953
rs705415
rs567754
rs6859667
rs11951068
rs10944
rs672413
rs921943
All
0.0
0.1
0.2
MR effect size for
'Selenium' on 'Dual specificity protein phosphatase 15'

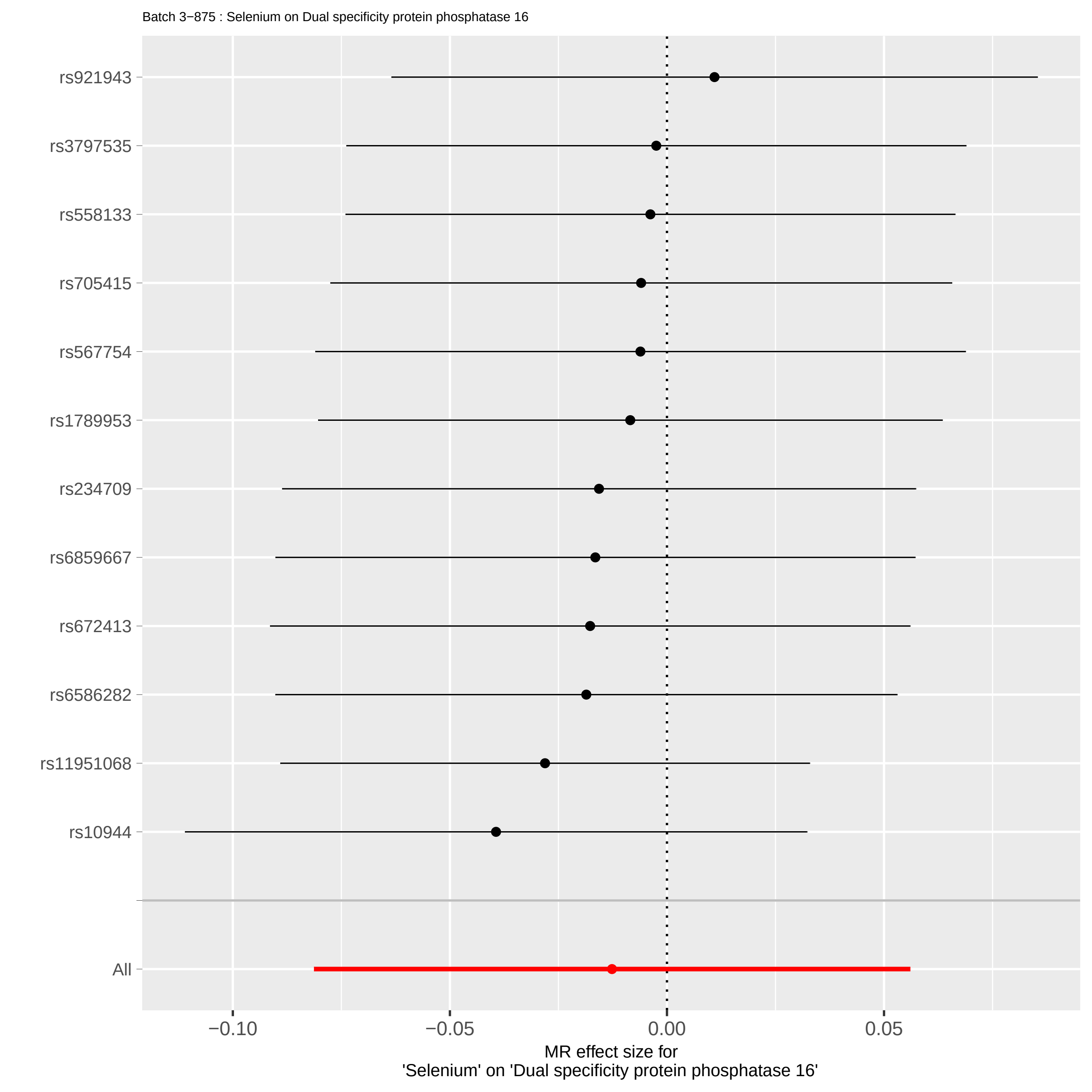

Batch 3−875 : Selenium on Dual specificity protein phosphatase 16
rs921943
rs3797535
rs558133
rs705415
rs567754
rs1789953
rs234709
rs6859667
rs672413
rs6586282
rs11951068
rs10944
All
−0.10
−0.05
0.00
0.05
MR effect size for
'Selenium' on 'Dual specificity protein phosphatase 16'

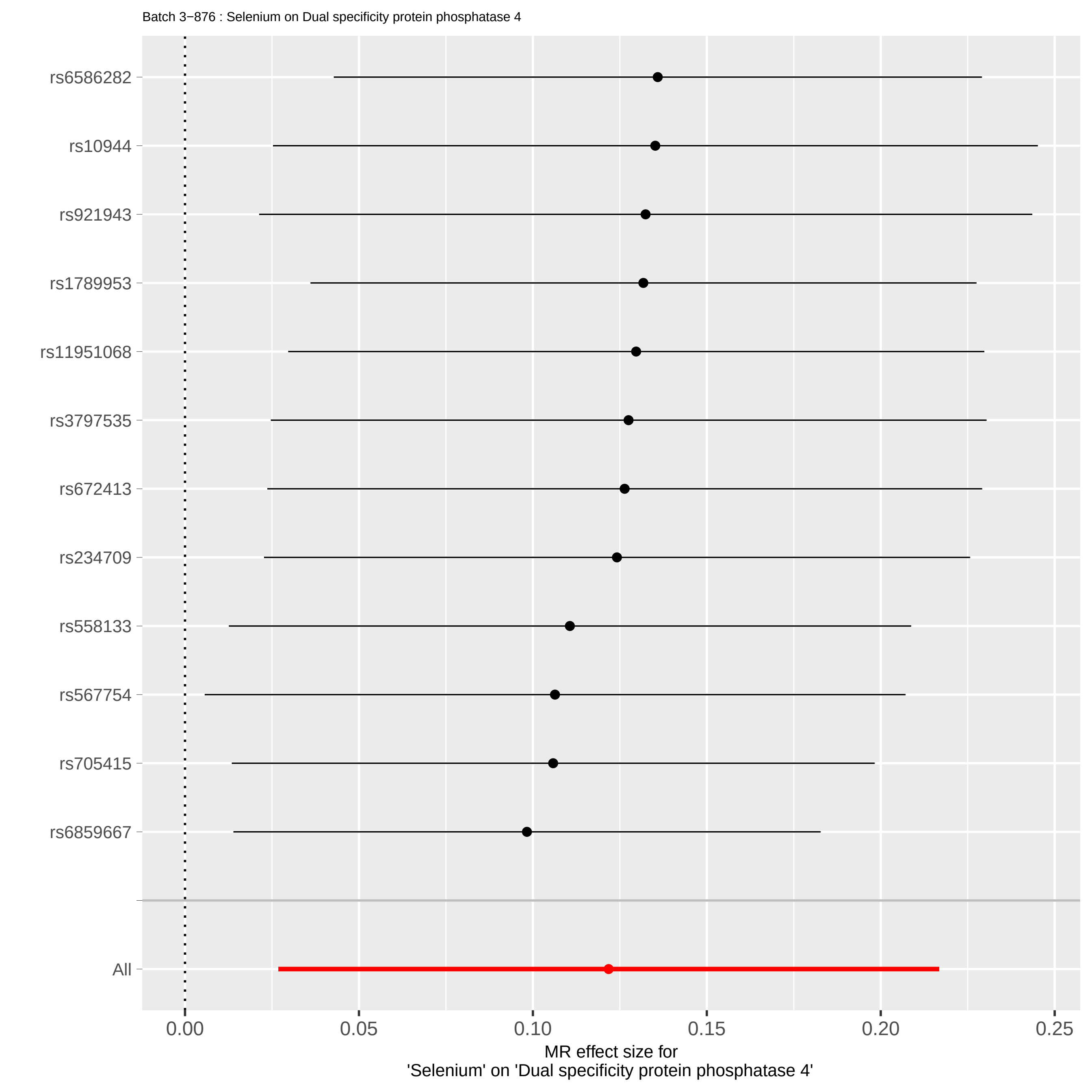

Batch 3−876 : Selenium on Dual specificity protein phosphatase 4
rs6586282
rs10944
rs921943
rs1789953
rs11951068
rs3797535
rs672413
rs234709
rs558133
rs567754
rs705415
rs6859667
All
0.00
0.05
0.10
0.15
0.20
0.25
MR effect size for
'Selenium' on 'Dual specificity protein phosphatase 4'

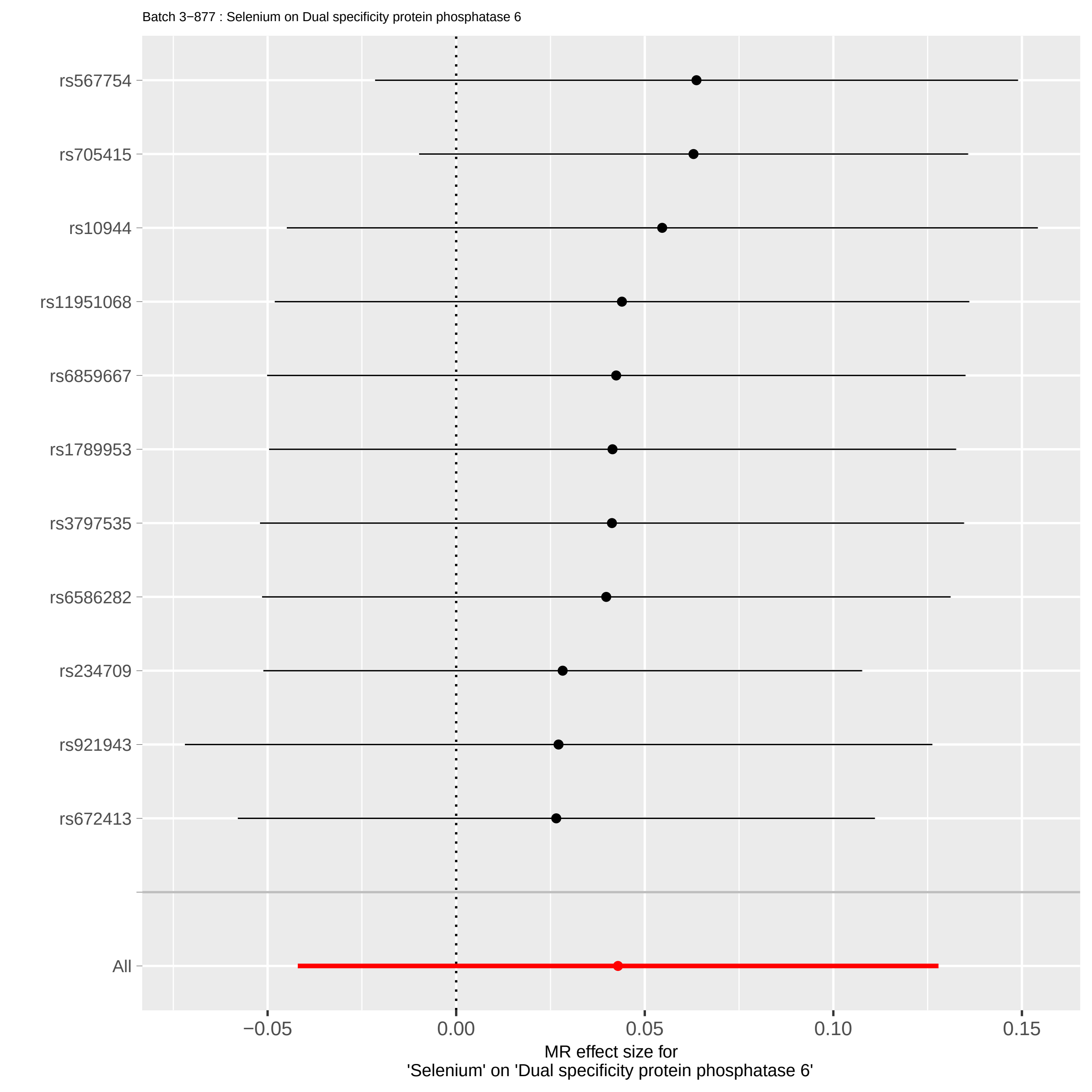

Batch 3−877 : Selenium on Dual specificity protein phosphatase 6
rs567754
rs705415
rs10944
rs11951068
rs6859667
rs1789953
rs3797535
rs6586282
rs234709
rs921943
rs672413
All
−0.05
0.00
0.05
0.10
0.15
MR effect size for
'Selenium' on 'Dual specificity protein phosphatase 6'

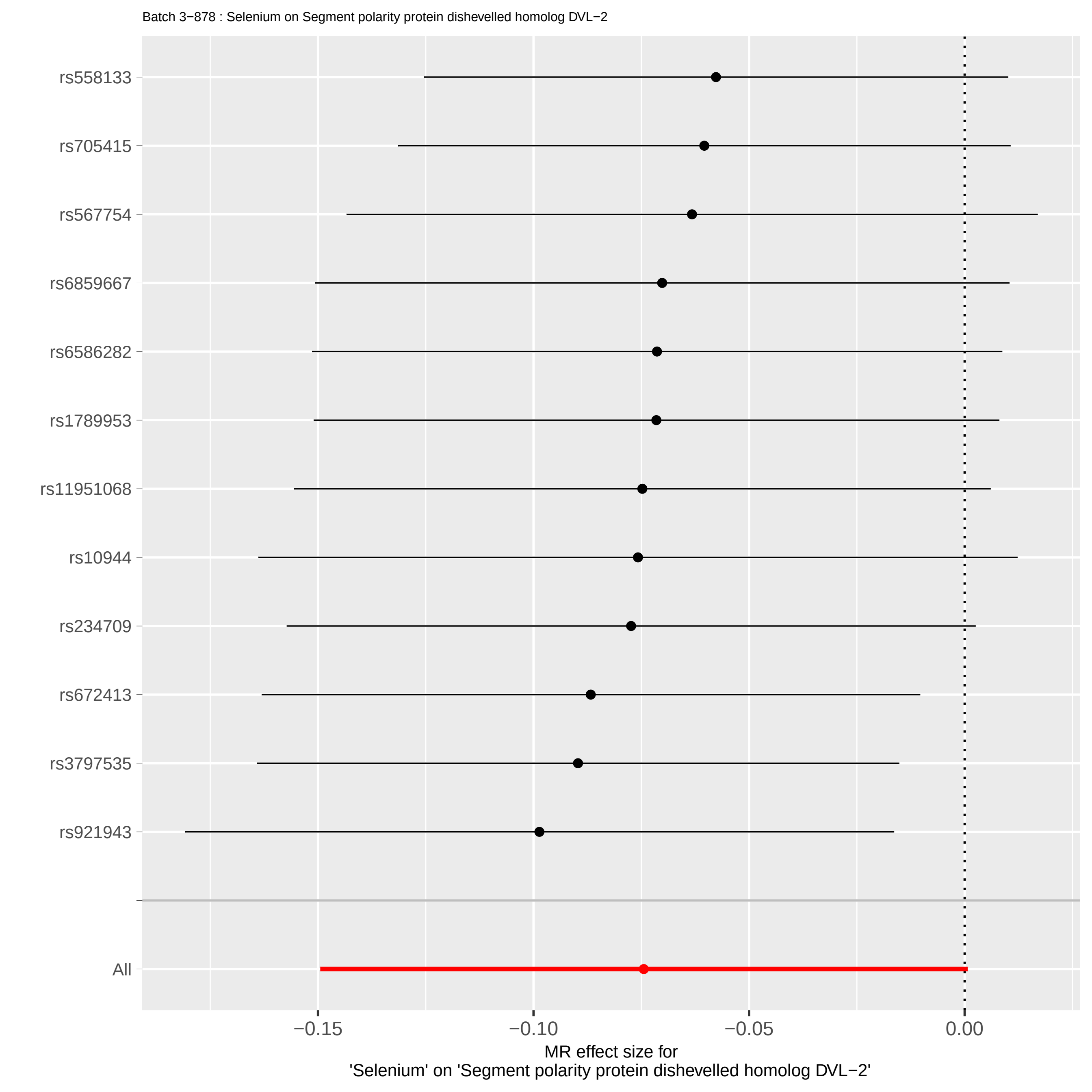
Batch 3−878 : Selenium on Segment polarity protein dishevelled homolog DVL−2
rs558133
rs705415
rs567754
rs6859667
rs6586282
rs1789953
rs11951068
rs10944
rs234709
rs672413
rs3797535
rs921943
All
−0.15
−0.10
−0.05
0.00
MR effect size for
'Selenium' on 'Segment polarity protein dishevelled homolog DVL−2'

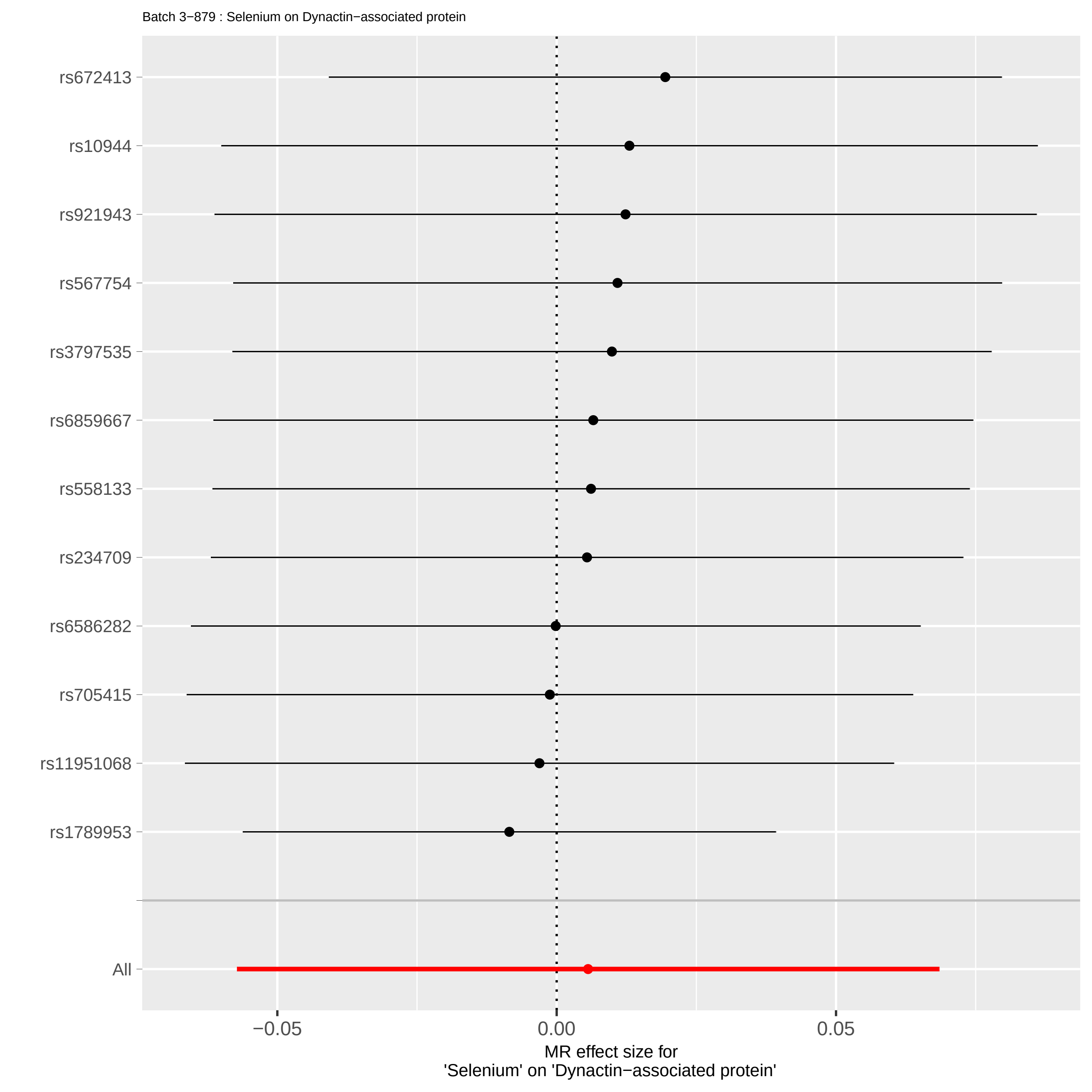
Batch 3−879 : Selenium on Dynactin−associated protein
rs672413
rs10944
rs921943
rs567754
rs3797535
rs6859667
rs558133
rs234709
rs6586282
rs705415
rs11951068
rs1789953
All
−0.05
0.00
0.05
MR effect size for
'Selenium' on 'Dynactin−associated protein'

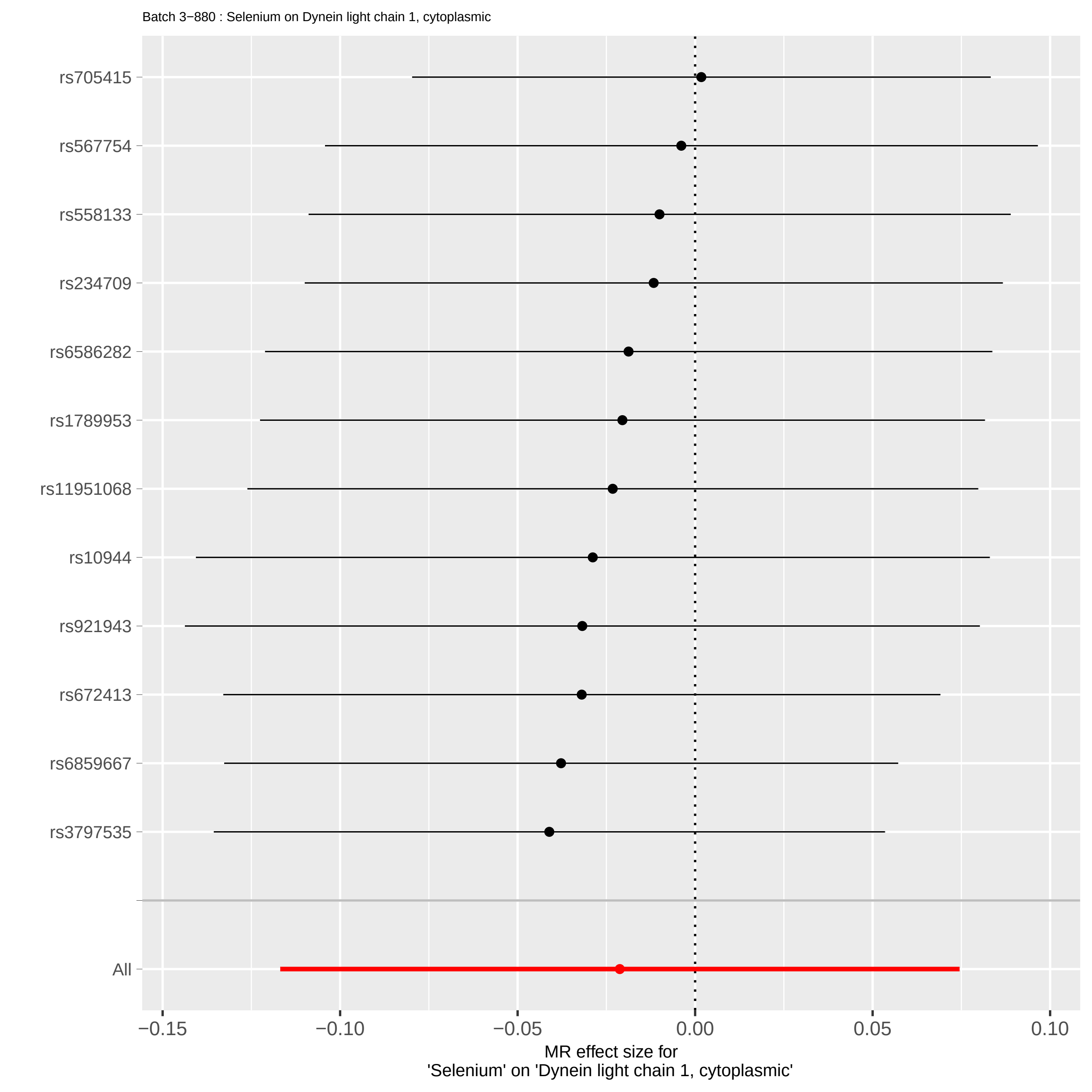
Batch 3−880 : Selenium on Dynein light chain 1, cytoplasmic
rs705415
rs567754
rs558133
rs234709
rs6586282
rs1789953
rs11951068
rs10944
rs921943
rs672413
rs6859667
rs3797535
All
−0.15
−0.10
−0.05
0.00
0.05
0.10
MR effect size for
'Selenium' on 'Dynein light chain 1, cytoplasmic'

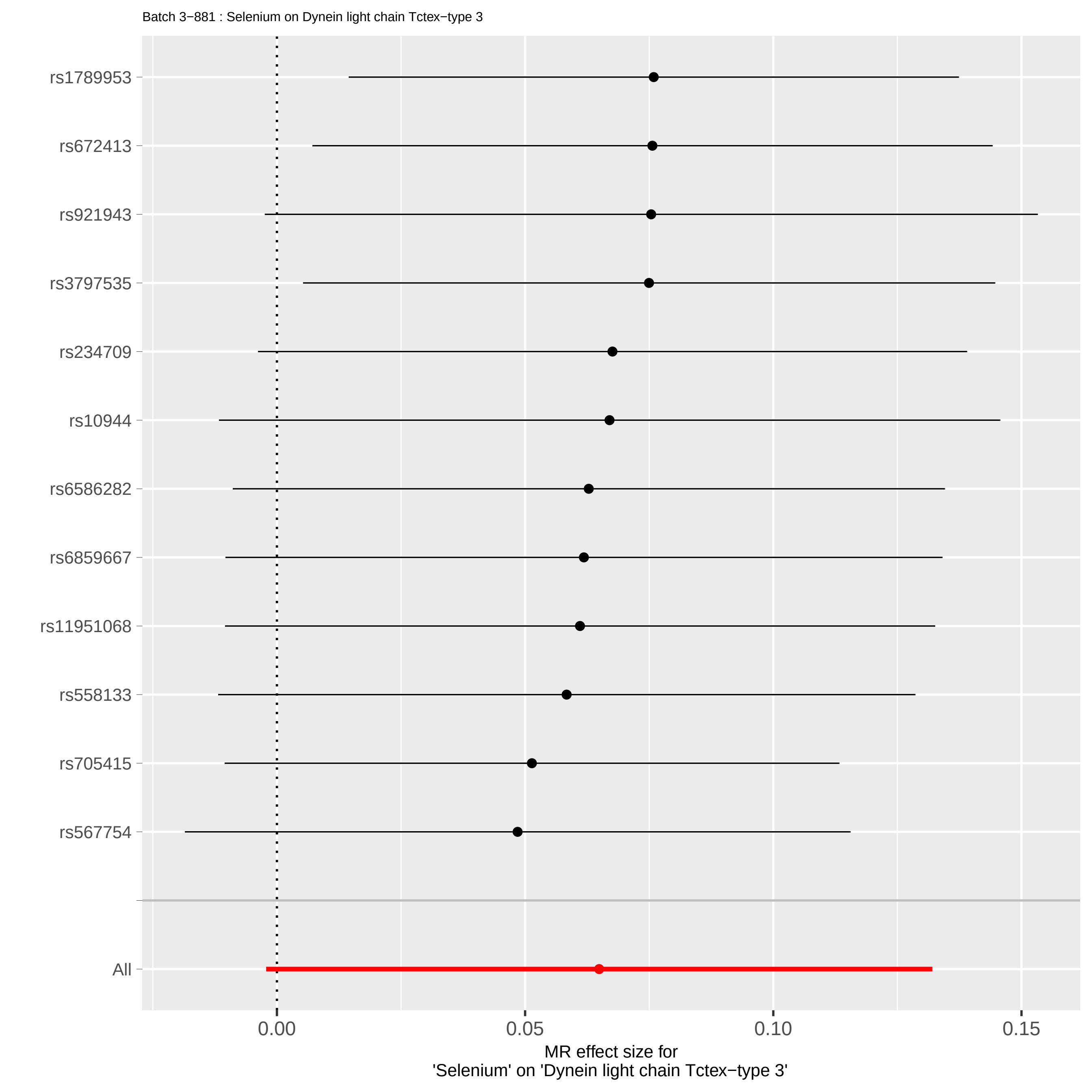
Batch 3−881 : Selenium on Dynein light chain Tctex−type 3
rs1789953
rs672413
rs921943
rs3797535
rs234709
rs10944
rs6586282
rs6859667
rs11951068
rs558133
rs705415
rs567754
All
0.00
0.05
0.10
0.15
MR effect size for
'Selenium' on 'Dynein light chain Tctex−type 3'

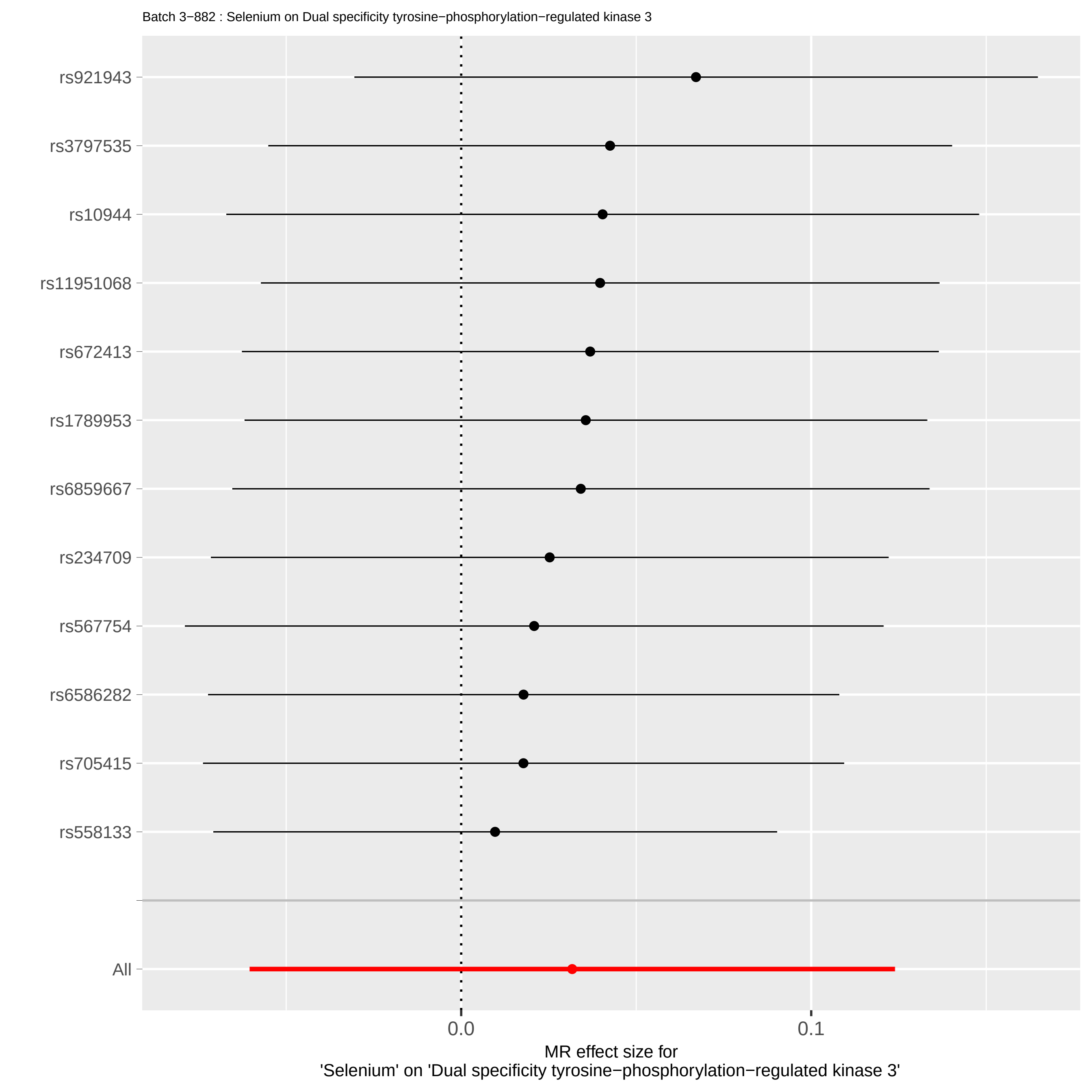
Batch 3−882 : Selenium on Dual specificity tyrosine−phosphorylation−regulated kinase 3
rs921943
rs3797535
rs10944
rs11951068
rs672413
rs1789953
rs6859667
rs234709
rs567754
rs6586282
rs705415
rs558133
All
0.0
0.1
MR effect size for
'Selenium' on 'Dual specificity tyrosine−phosphorylation−regulated kinase 3'

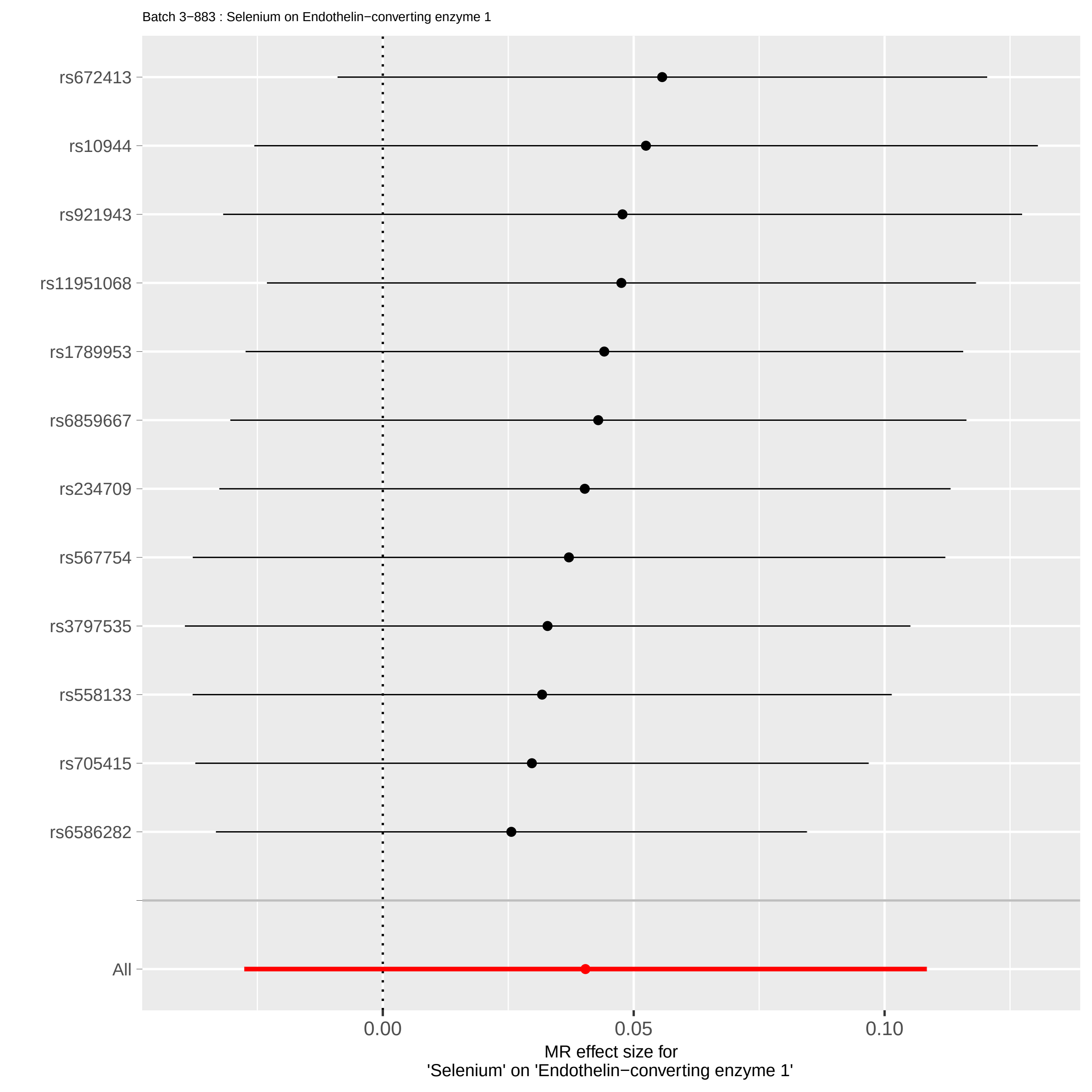
Batch 3−883 : Selenium on Endothelin−converting enzyme 1
rs672413
rs10944
rs921943
rs11951068
rs1789953
rs6859667
rs234709
rs567754
rs3797535
rs558133
rs705415
rs6586282
All
0.00
0.05
0.10
MR effect size for
'Selenium' on 'Endothelin−converting enzyme 1'

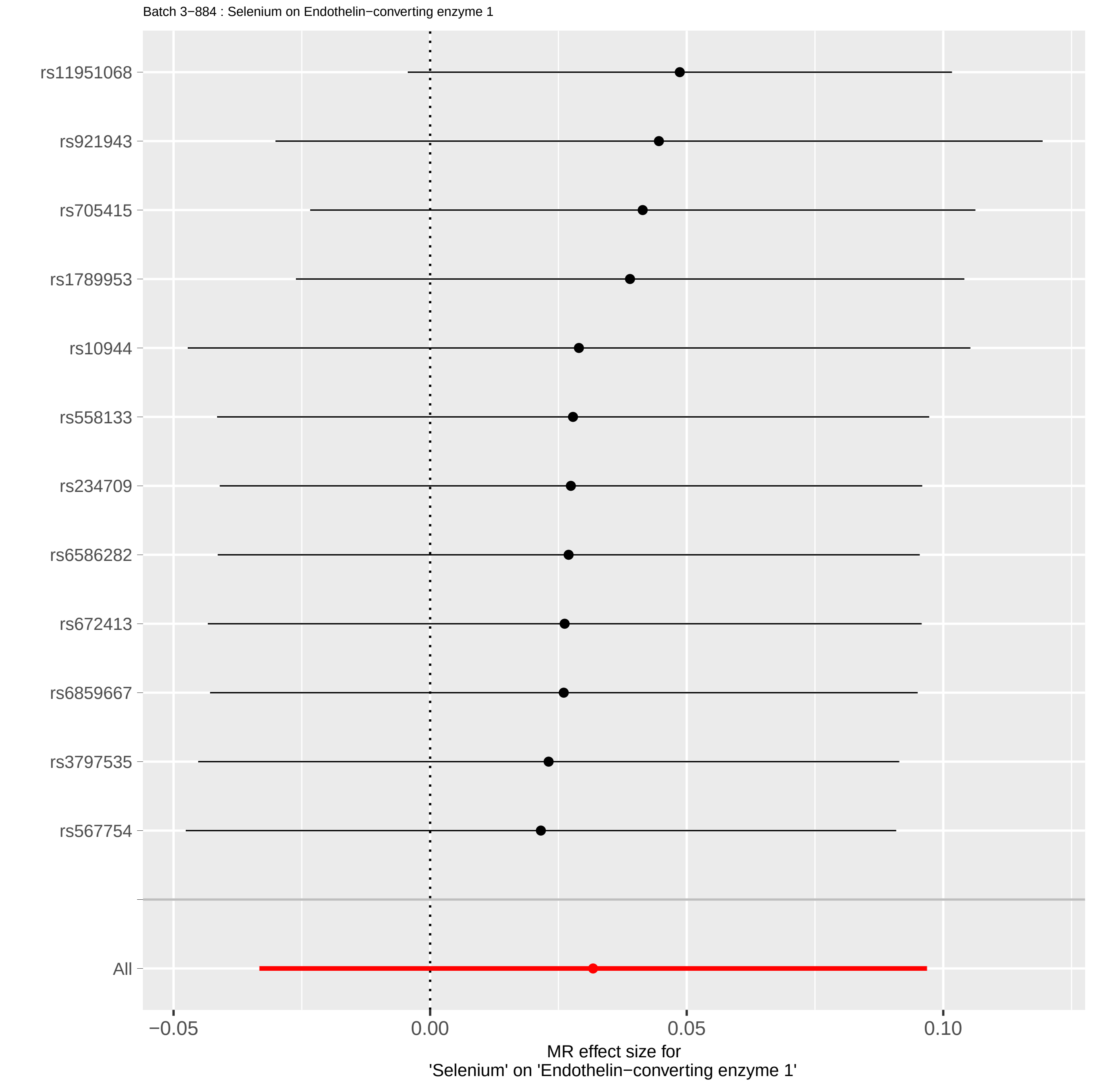
Batch 3−884 : Selenium on Endothelin−converting enzyme 1
rs11951068
rs921943
rs705415
rs1789953
rs10944
rs558133
rs234709
rs6586282
rs672413
rs6859667
rs3797535
rs567754
All
−0.05
0.00
0.05
0.10
MR effect size for
'Selenium' on 'Endothelin−converting enzyme 1'

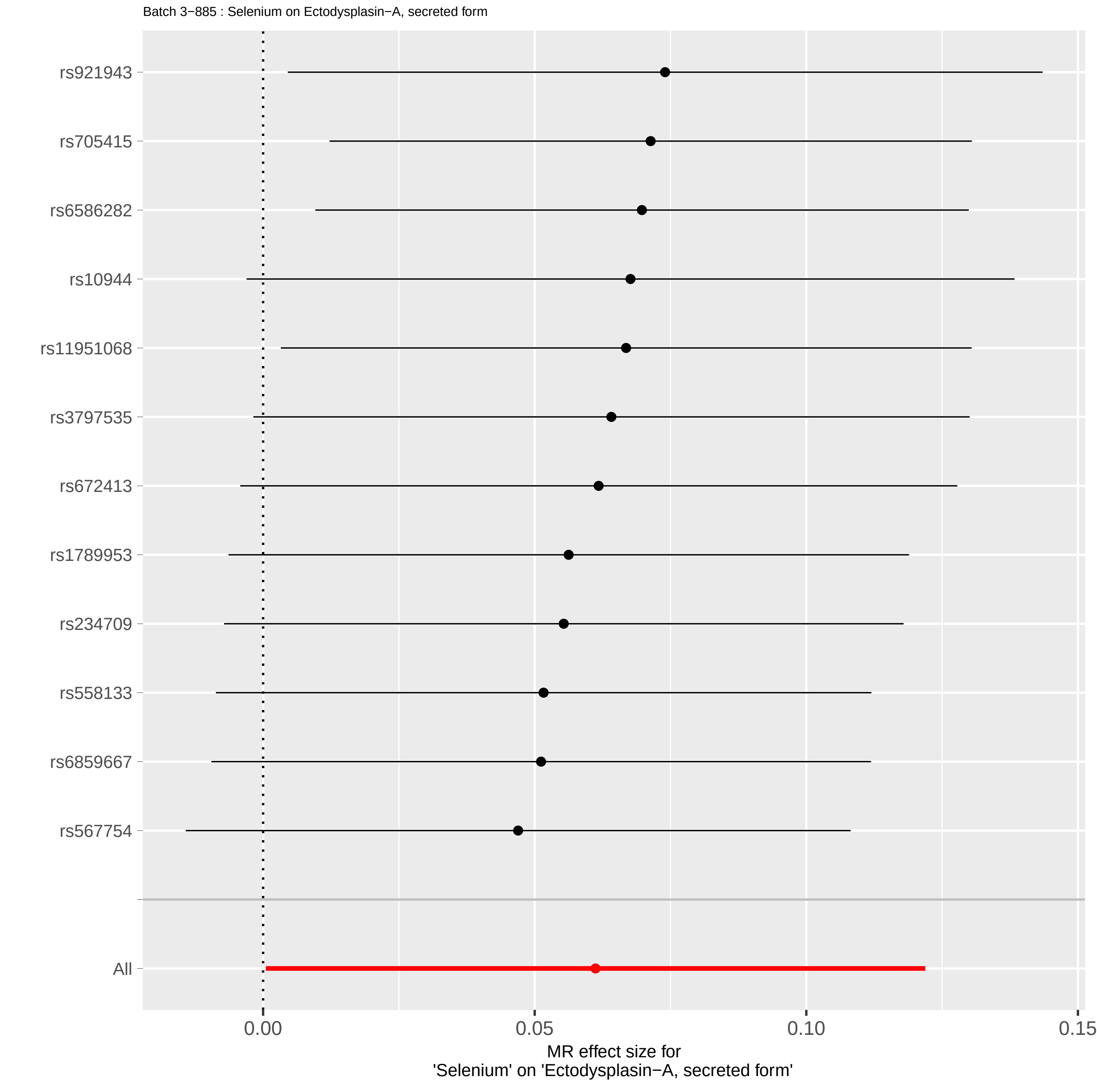

Batch 3−885 : Selenium on Ectodysplasin−A, secreted form
rs921943
rs705415
rs6586282
rs10944
rs11951068
rs3797535
rs672413
rs1789953
rs234709
rs558133
rs6859667
rs567754
All
0.00
0.05
0.10
0.15
MR effect size for
'Selenium' on 'Ectodysplasin−A, secreted form'

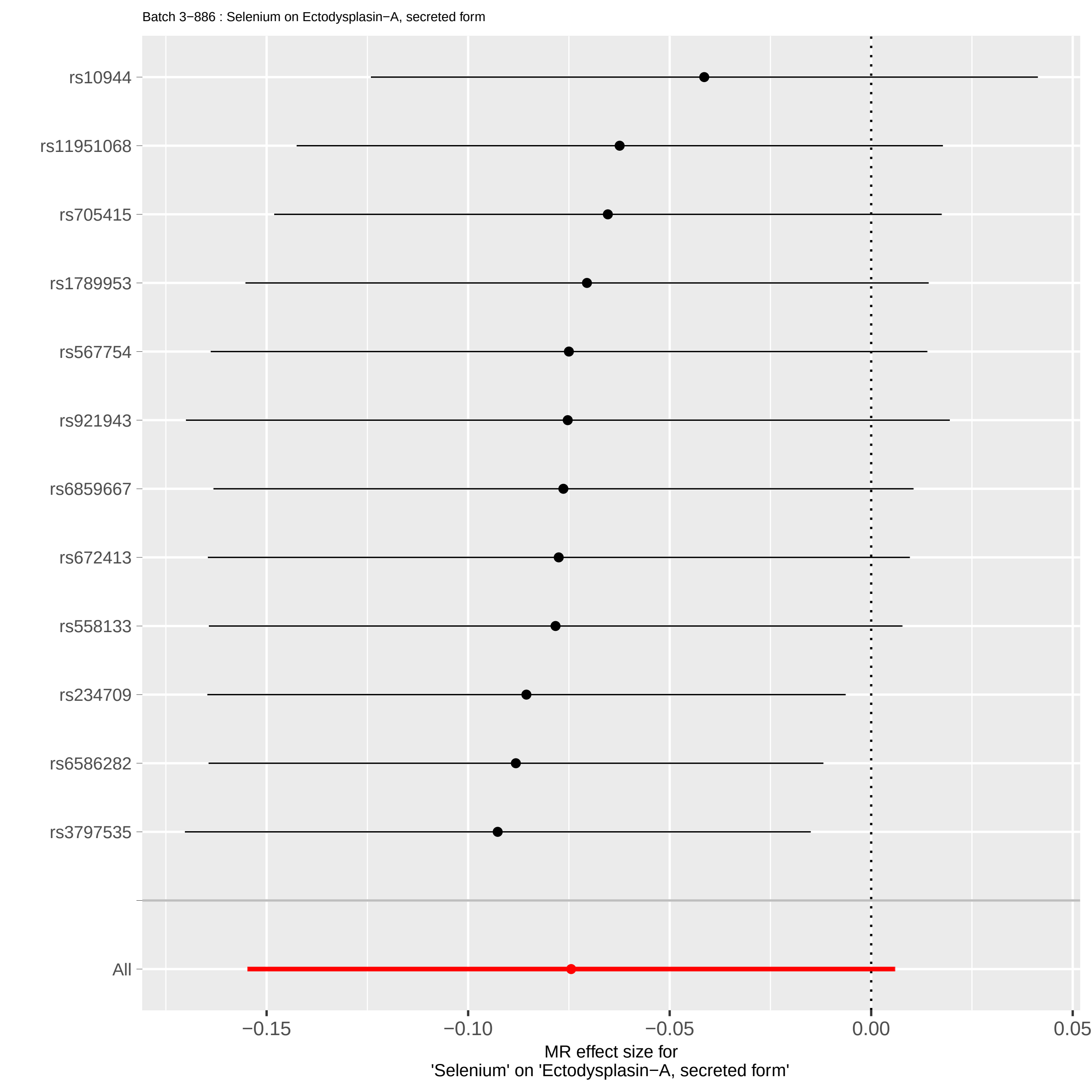

Batch 3−886 : Selenium on Ectodysplasin−A, secreted form
rs10944
rs11951068
rs705415
rs1789953
rs567754
rs921943
rs6859667
rs672413
rs558133
rs234709
rs6586282
rs3797535
All
−0.15
−0.10
−0.05
0.00
0.05
MR effect size for
'Selenium' on 'Ectodysplasin−A, secreted form'

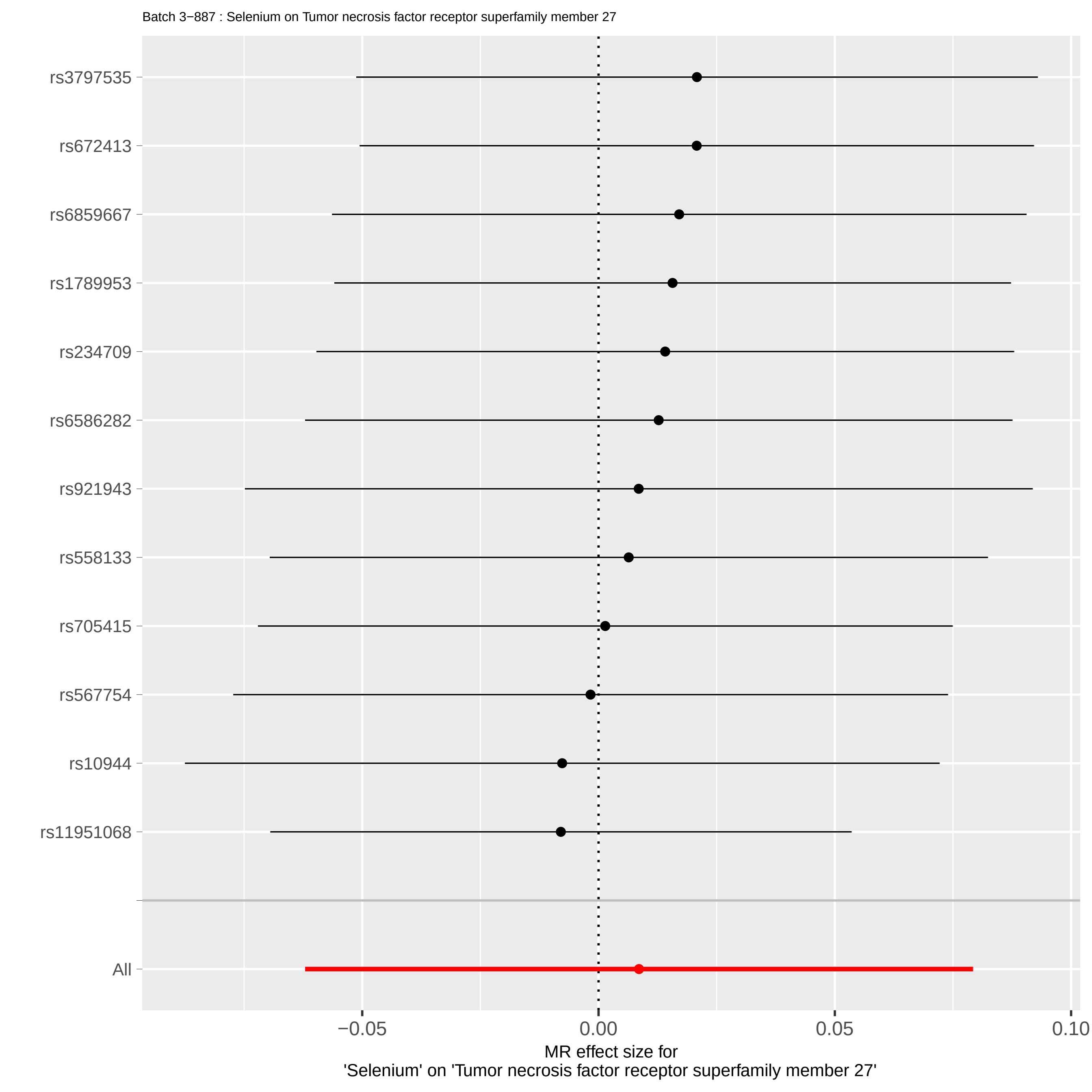

Batch 3−887 : Selenium on Tumor necrosis factor receptor superfamily member 27
rs3797535
rs672413
rs6859667
rs1789953
rs234709
rs6586282
rs921943
rs558133
rs705415
rs567754
rs10944
rs11951068
All
−0.05
0.00
0.05
0.10
MR effect size for
'Selenium' on 'Tumor necrosis factor receptor superfamily member 27'

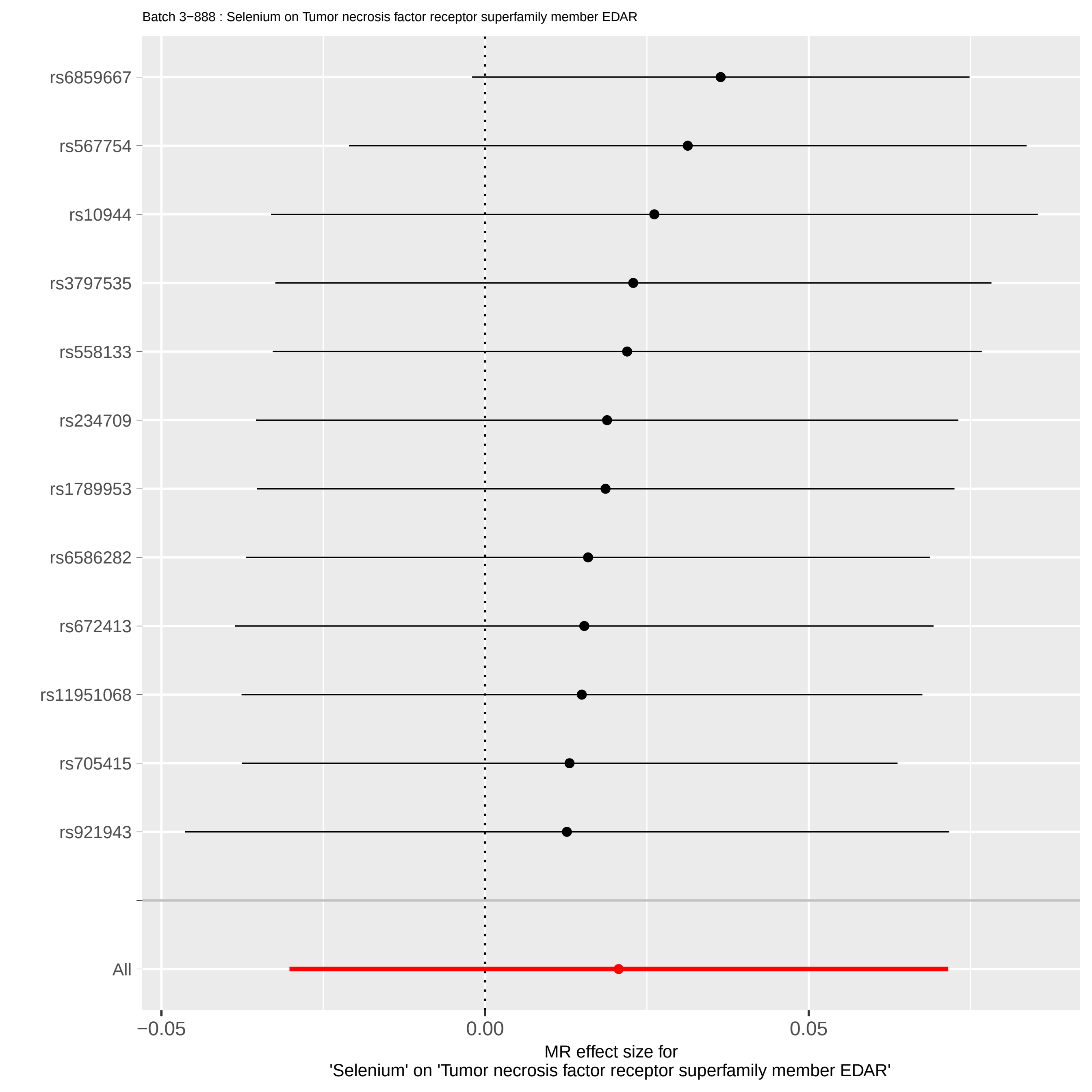

Batch 3−888 : Selenium on Tumor necrosis factor receptor superfamily member EDAR
rs6859667
rs567754
rs10944
rs3797535
rs558133
rs234709
rs1789953
rs6586282
rs672413
rs11951068
rs705415
rs921943
All
−0.05
0.00
0.05
MR effect size for
'Selenium' on 'Tumor necrosis factor receptor superfamily member EDAR'

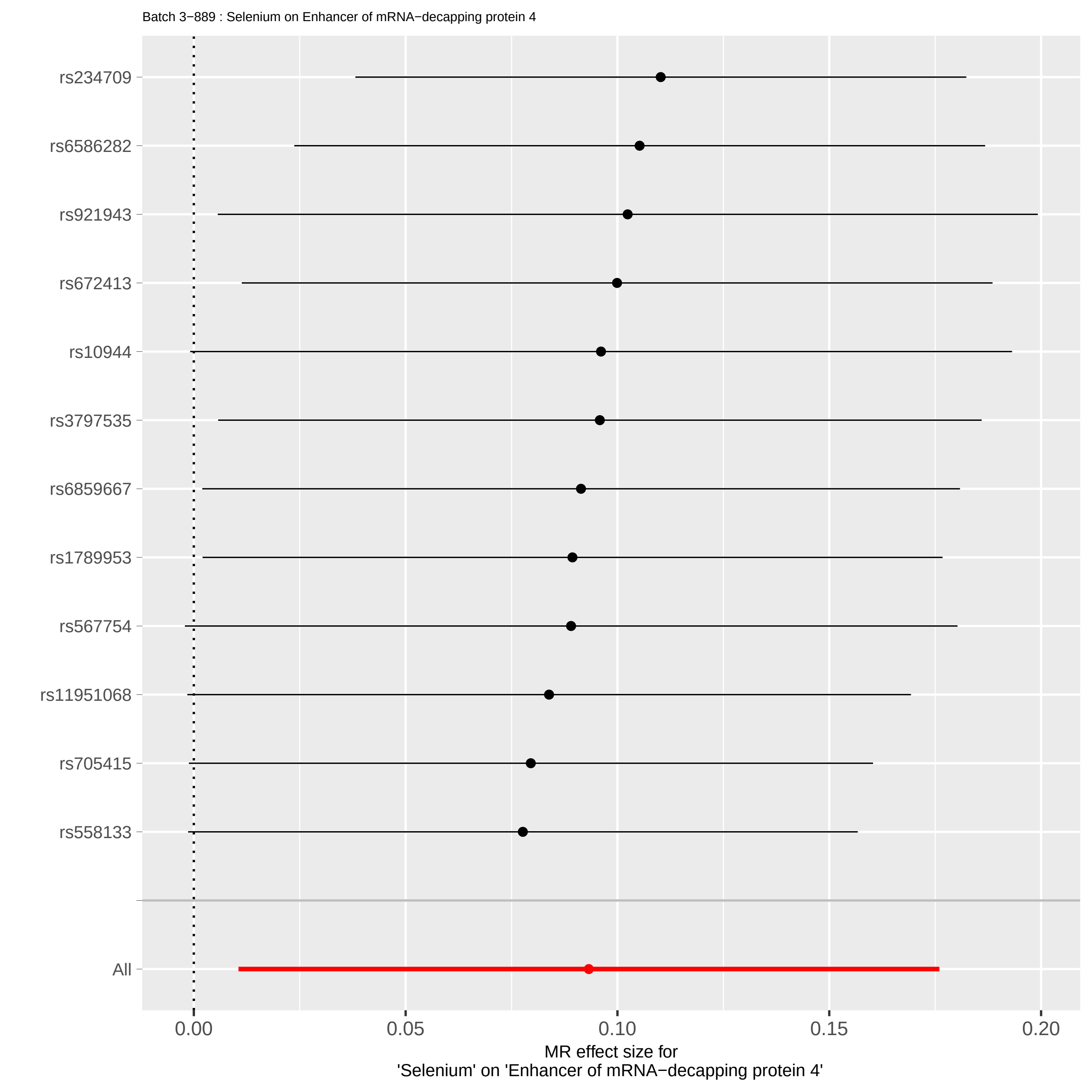
Batch 3−889 : Selenium on Enhancer of mRNA−decapping protein 4
rs234709
rs6586282
rs921943
rs672413
rs10944
rs3797535
rs6859667
rs1789953
rs567754
rs11951068
rs705415
rs558133
All
0.00
0.05
0.10
0.15
0.20
MR effect size for
'Selenium' on 'Enhancer of mRNA−decapping protein 4'

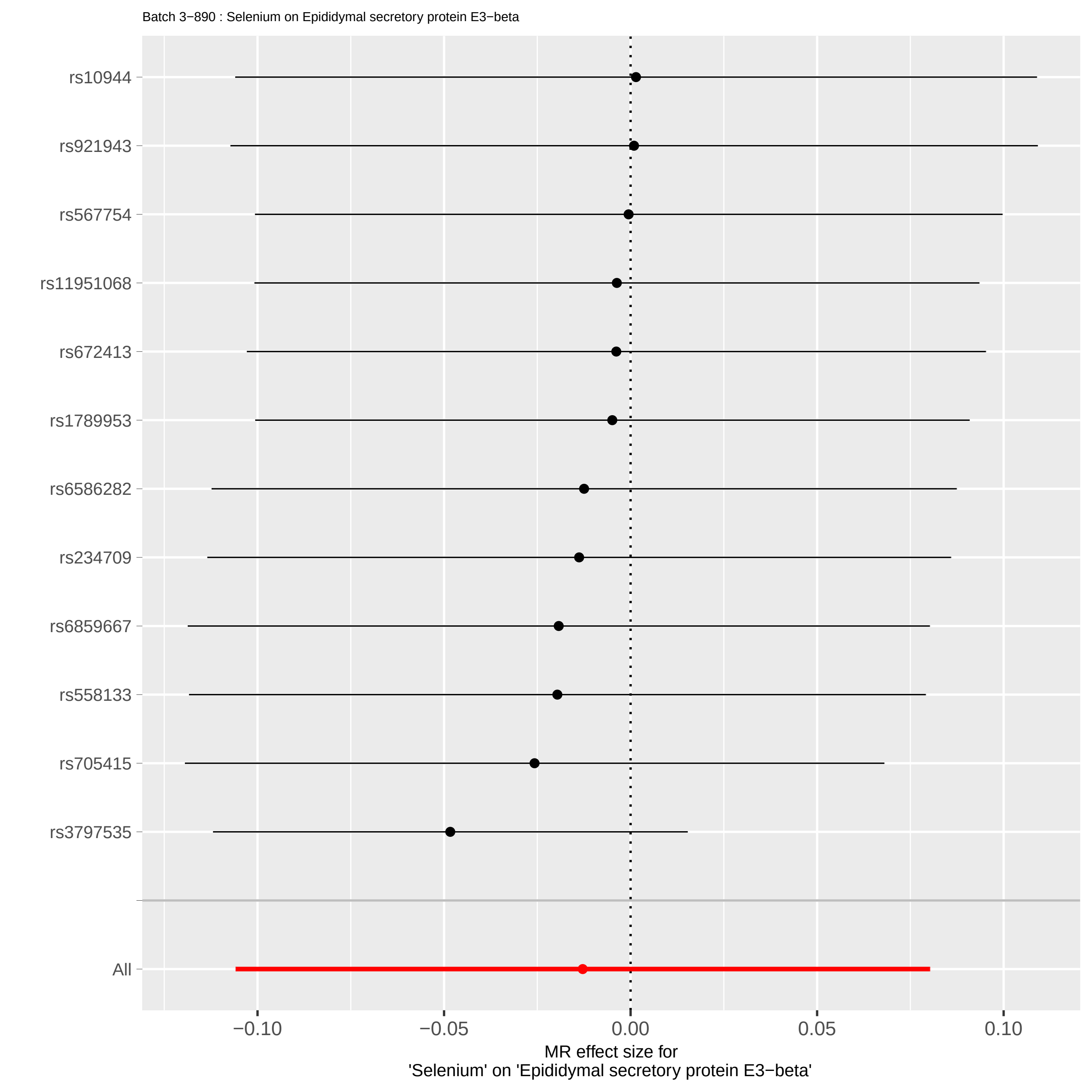

Batch 3−890 : Selenium on Epididymal secretory protein E3−beta
rs10944
rs921943
rs567754
rs11951068
rs672413
rs1789953
rs6586282
rs234709
rs6859667
rs558133
rs705415
rs3797535
All
−0.10
−0.05
0.00
0.05
0.10
MR effect size for
'Selenium' on 'Epididymal secretory protein E3−beta'

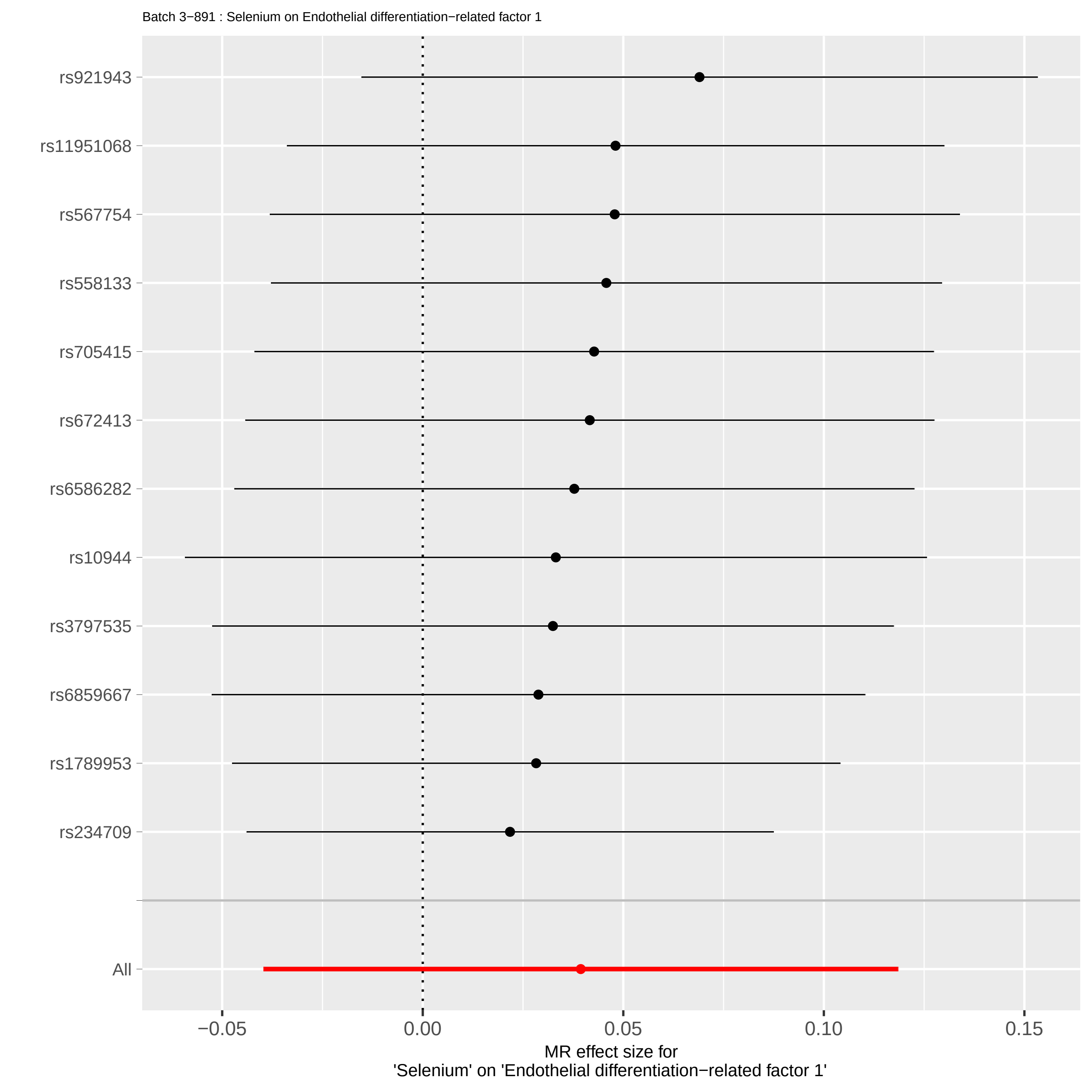

Batch 3−891 : Selenium on Endothelial differentiation−related factor 1
rs921943
rs11951068
rs567754
rs558133
rs705415
rs672413
rs6586282
rs10944
rs3797535
rs6859667
rs1789953
rs234709
All
−0.05
0.00
0.05
0.10
0.15
MR effect size for
'Selenium' on 'Endothelial differentiation−related factor 1'

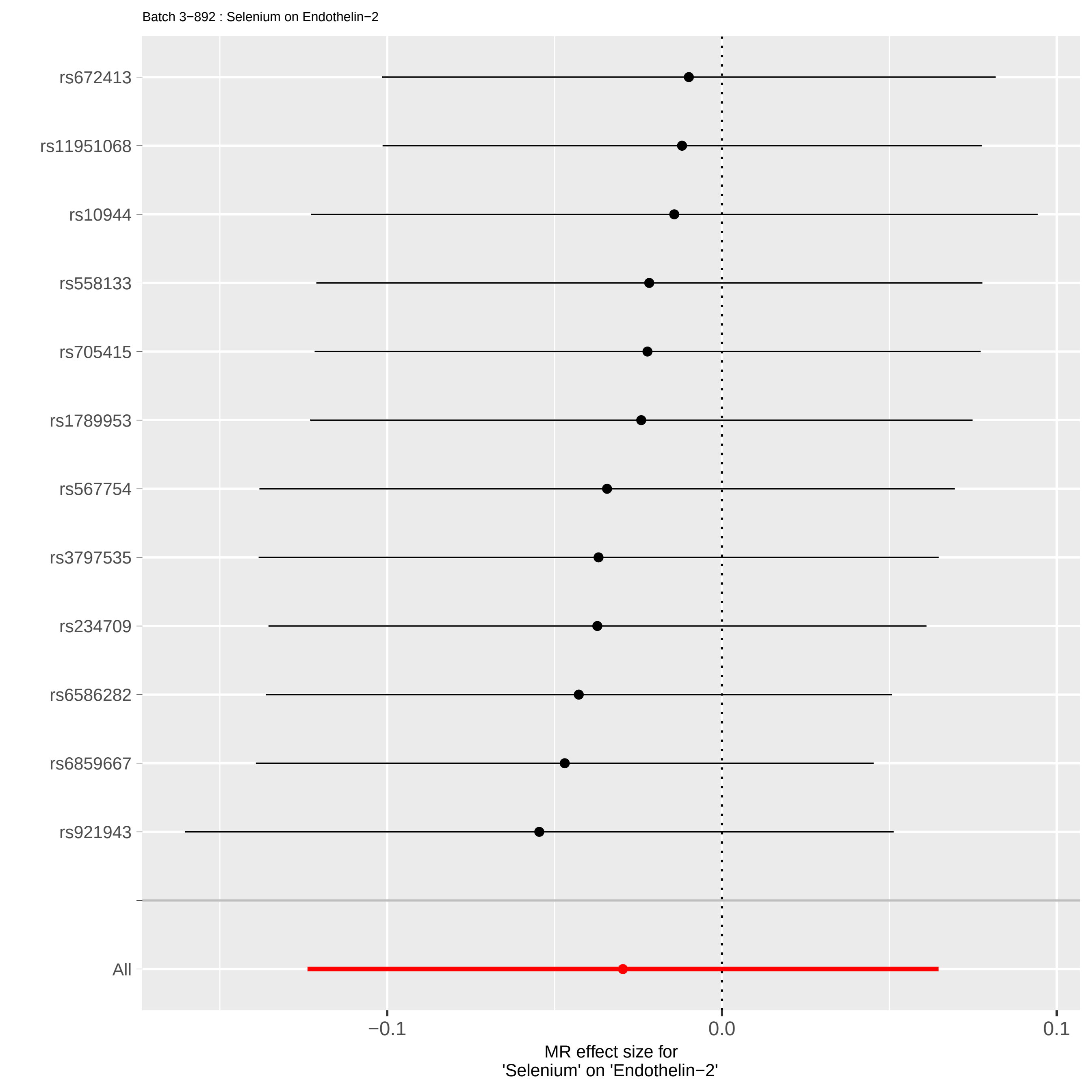

Batch 3−892 : Selenium on Endothelin−2
rs672413
rs11951068
rs10944
rs558133
rs705415
rs1789953
rs567754
rs3797535
rs234709
rs6586282
rs6859667
rs921943
All
−0.1
0.0
0.1
MR effect size for
'Selenium' on 'Endothelin−2'

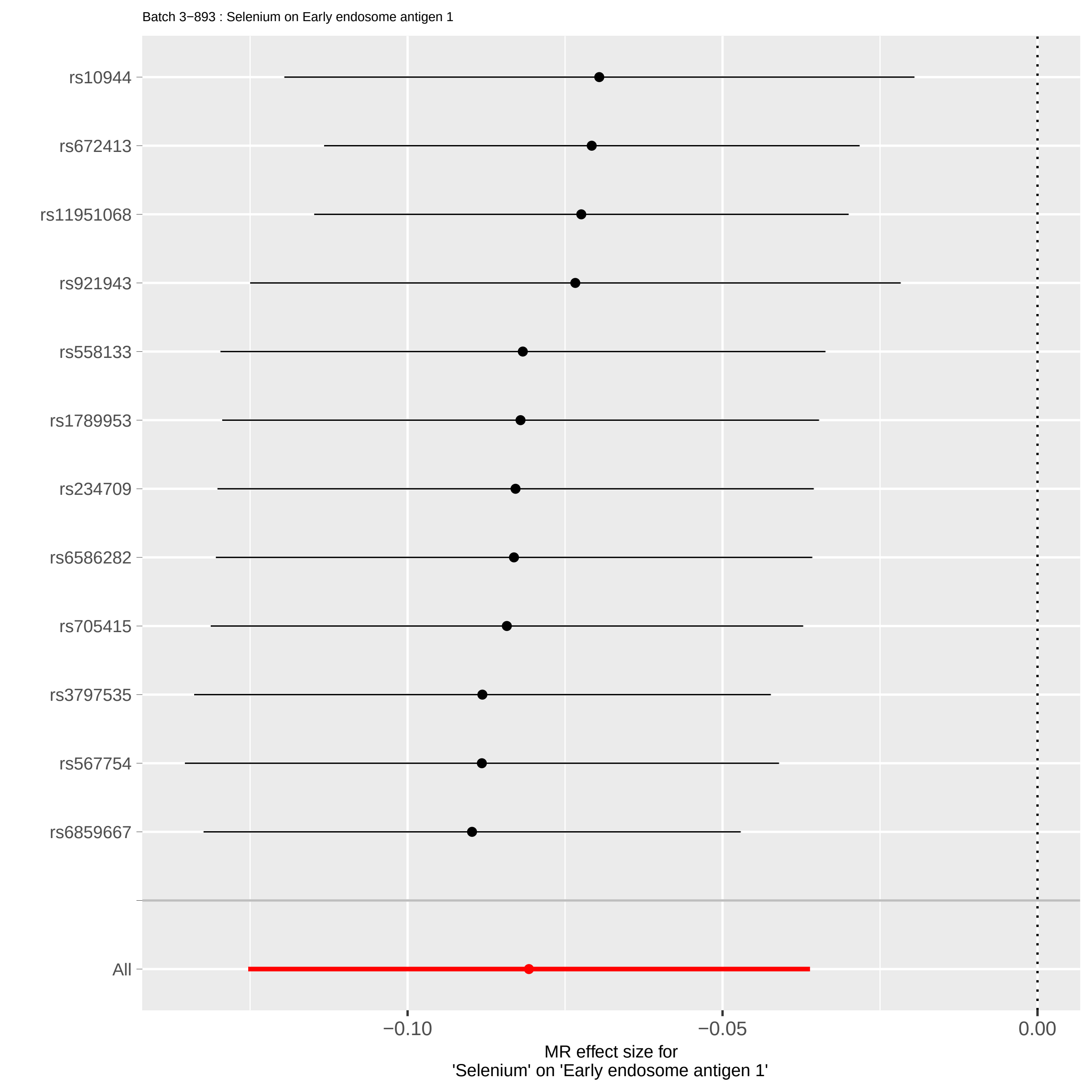
Batch 3−893 : Selenium on Early endosome antigen 1
rs10944
rs672413
rs11951068
rs921943
rs558133
rs1789953
rs234709
rs6586282
rs705415
rs3797535
rs567754
rs6859667
All
−0.10
−0.05
0.00
MR effect size for
'Selenium' on 'Early endosome antigen 1'

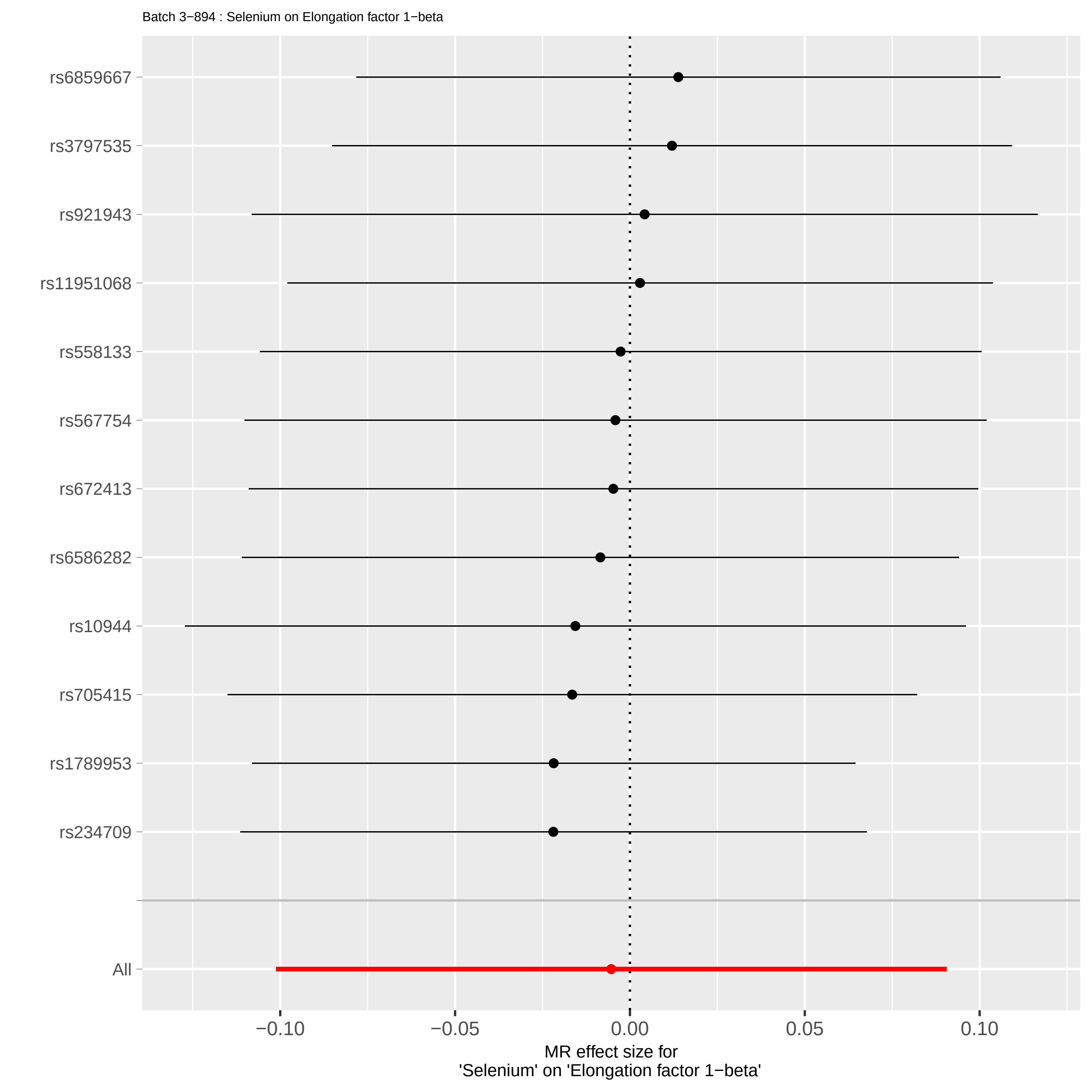

Batch 3−894 : Selenium on Elongation factor 1−beta
rs6859667
rs3797535
rs921943
rs11951068
rs558133
rs567754
rs672413
rs6586282
rs10944
rs705415
rs1789953
rs234709
All
−0.10
−0.05
0.00
0.05
0.10
MR effect size for
'Selenium' on 'Elongation factor 1−beta'

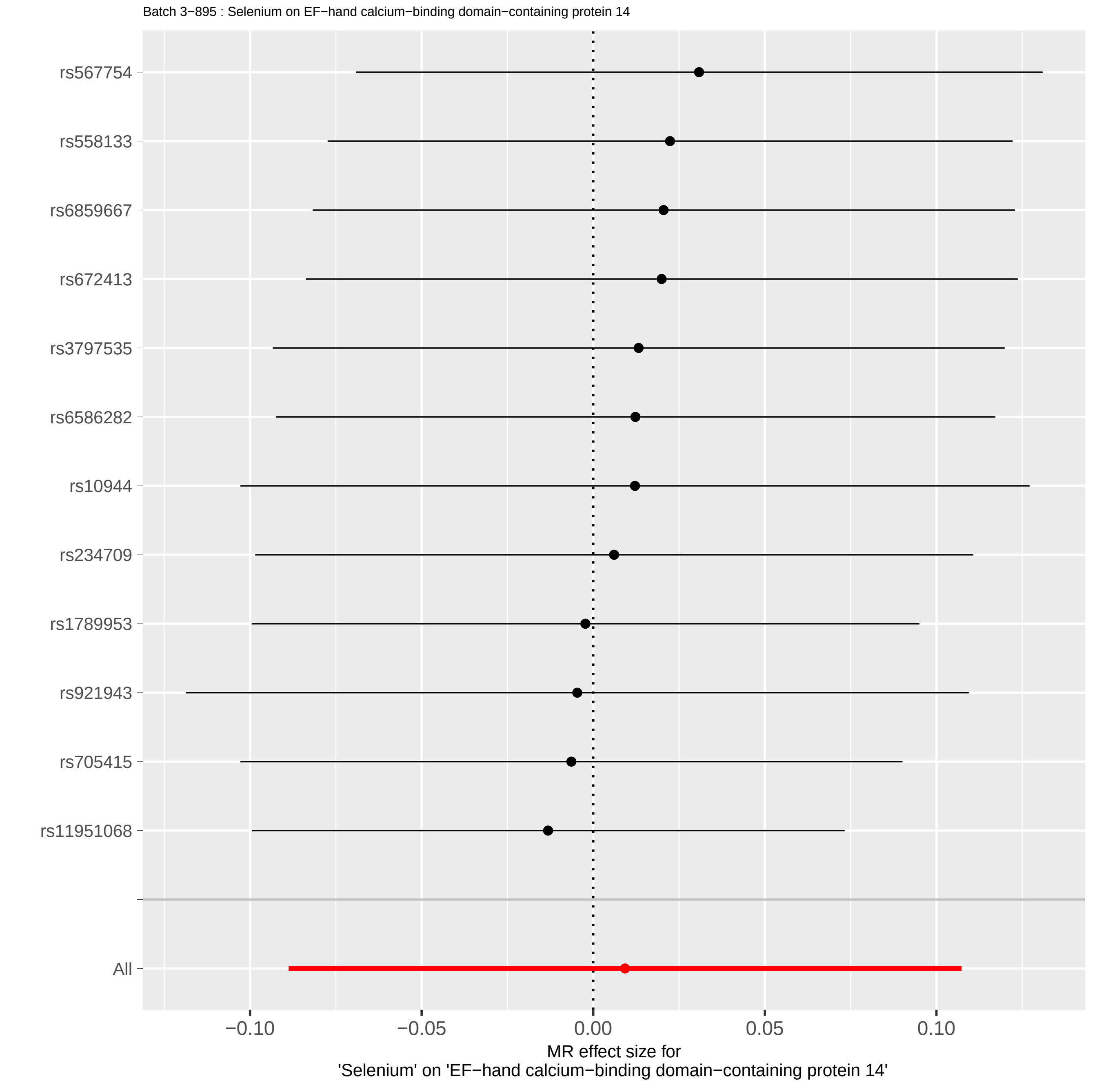

Batch 3−895 : Selenium on EF−hand calcium−binding domain−containing protein 14
rs567754
rs558133
rs6859667
rs672413
rs3797535
rs6586282
rs10944
rs234709
rs1789953
rs921943
rs705415
rs11951068
All
−0.10
−0.05
0.00
0.05
0.10
MR effect size for
'Selenium' on 'EF−hand calcium−binding domain−containing protein 14'

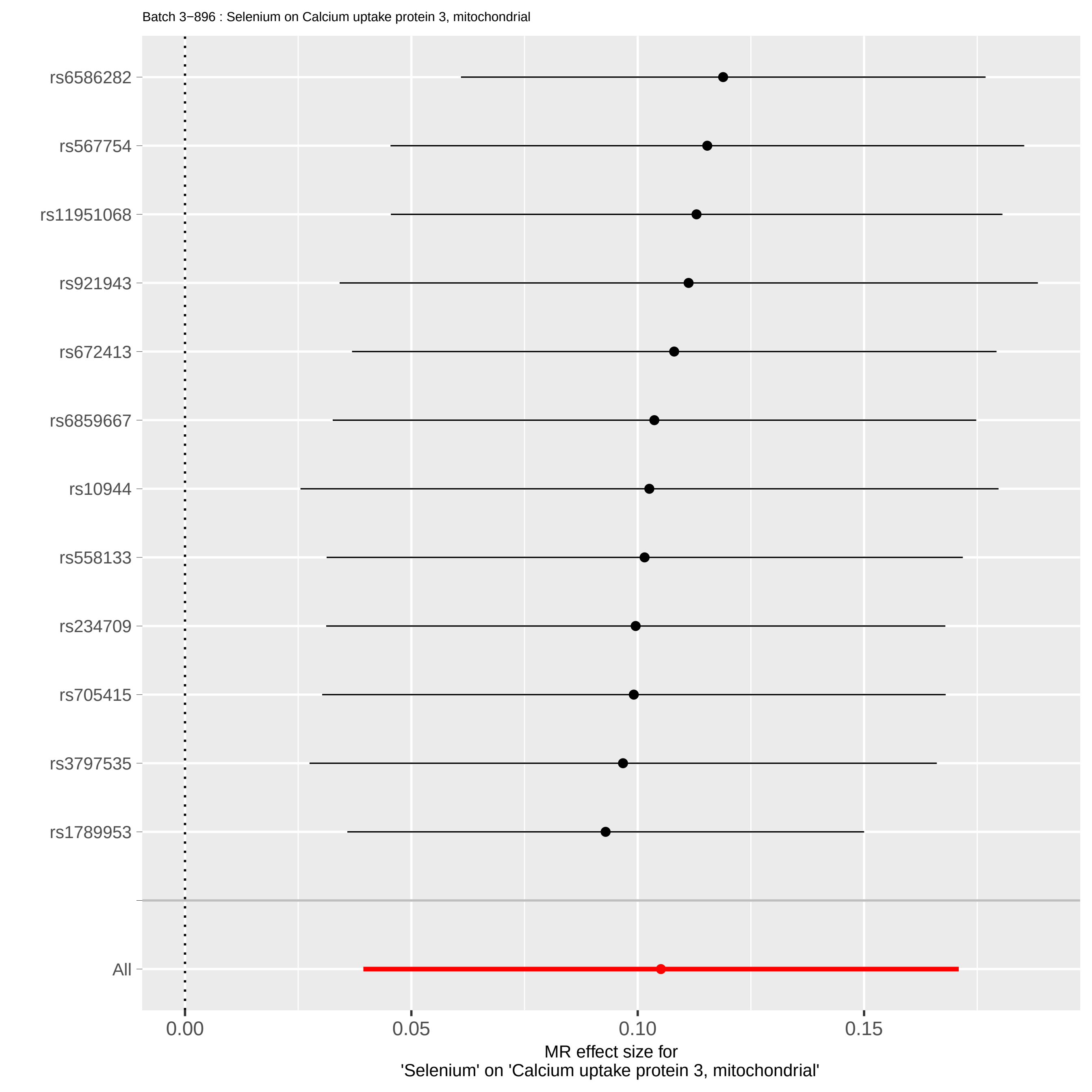

Batch 3−896 : Selenium on Calcium uptake protein 3, mitochondrial
rs6586282
rs567754
rs11951068
rs921943
rs672413
rs6859667
rs10944
rs558133
rs234709
rs705415
rs3797535
rs1789953
All
0.00
0.05
0.10
0.15
MR effect size for
'Selenium' on 'Calcium uptake protein 3, mitochondrial'

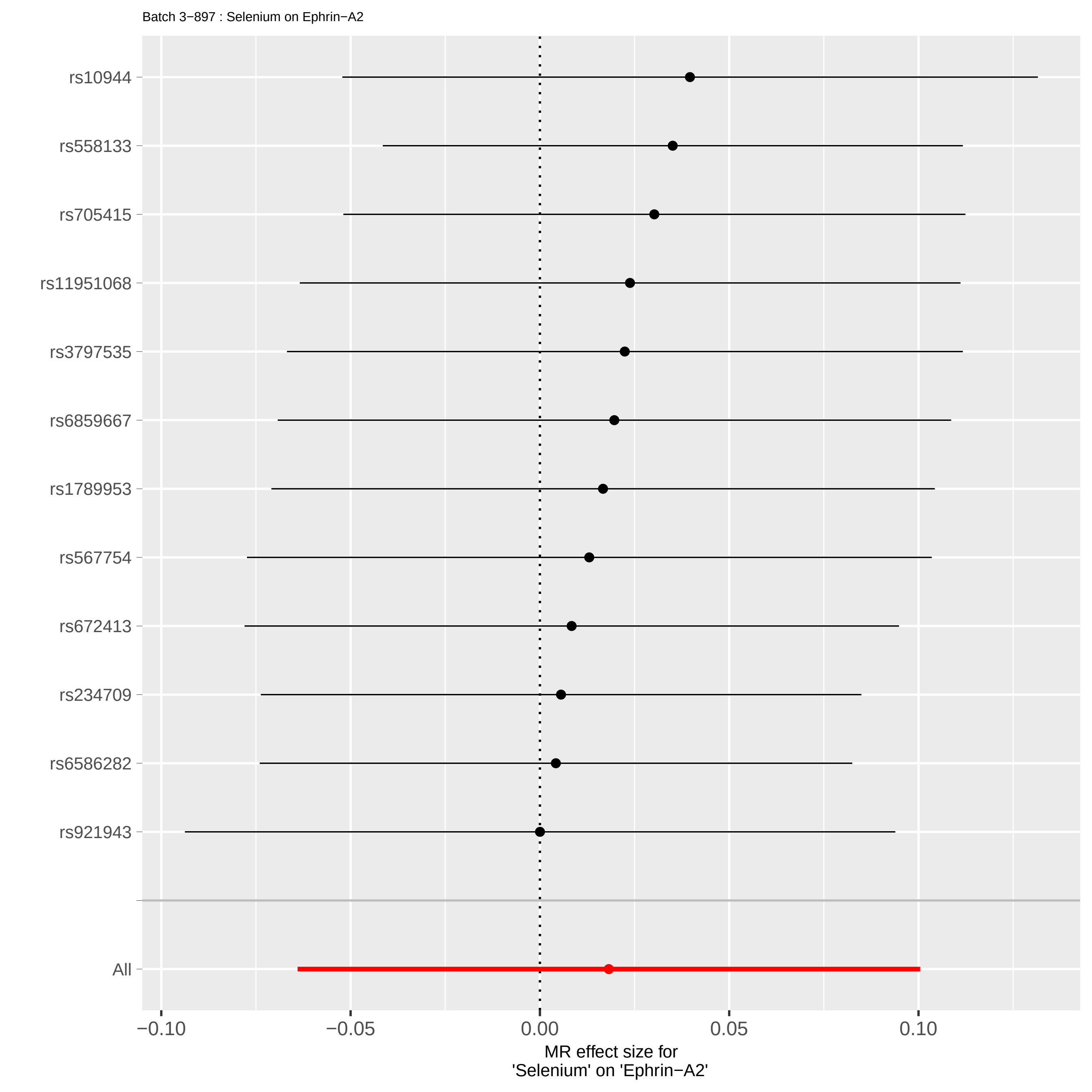

Batch 3−897 : Selenium on Ephrin−A2
rs10944
rs558133
rs705415
rs11951068
rs3797535
rs6859667
rs1789953
rs567754
rs672413
rs234709
rs6586282
rs921943
All
−0.10
−0.05
0.00
0.05
0.10
MR effect size for
'Selenium' on 'Ephrin−A2'

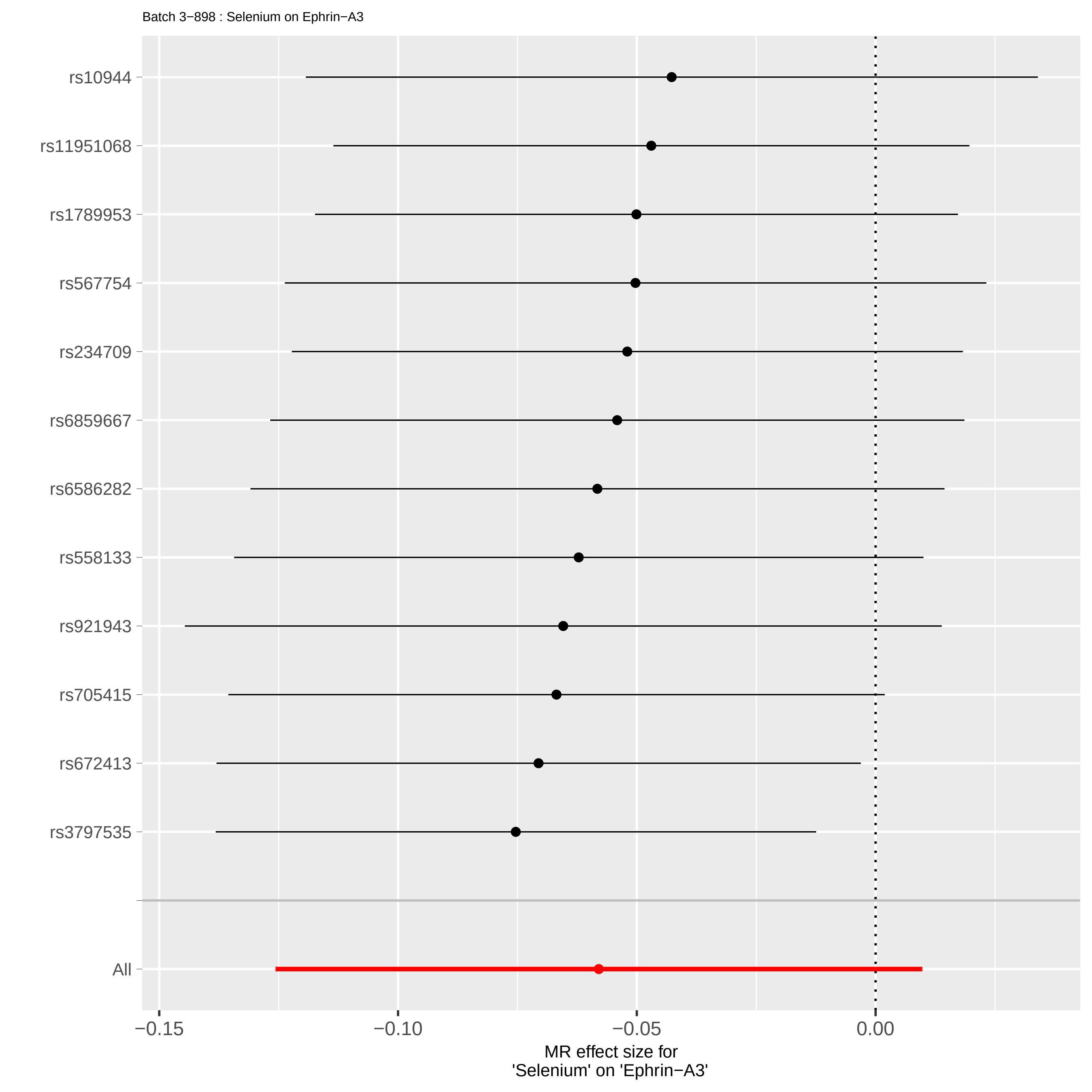

Batch 3−898 : Selenium on Ephrin−A3
rs10944
rs11951068
rs1789953
rs567754
rs234709
rs6859667
rs6586282
rs558133
rs921943
rs705415
rs672413
rs3797535
All
−0.15
−0.10
−0.05
0.00
MR effect size for
'Selenium' on 'Ephrin−A3'

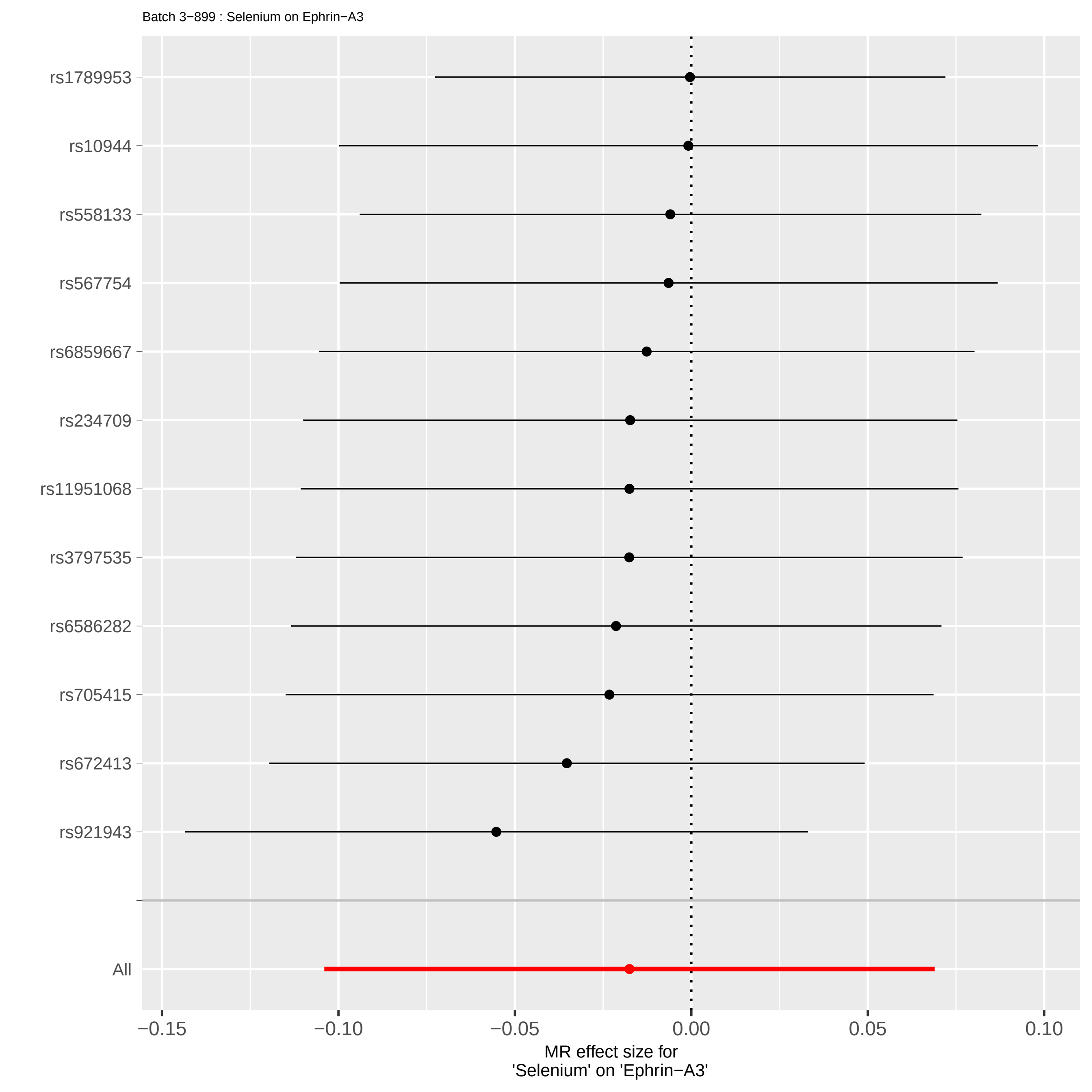

Batch 3−899 : Selenium on Ephrin−A3
rs1789953
rs10944
rs558133
rs567754
rs6859667
rs234709
rs11951068
rs3797535
rs6586282
rs705415
rs672413
rs921943
All
−0.15
−0.10
−0.05
0.00
0.05
0.10
MR effect size for
'Selenium' on 'Ephrin−A3'

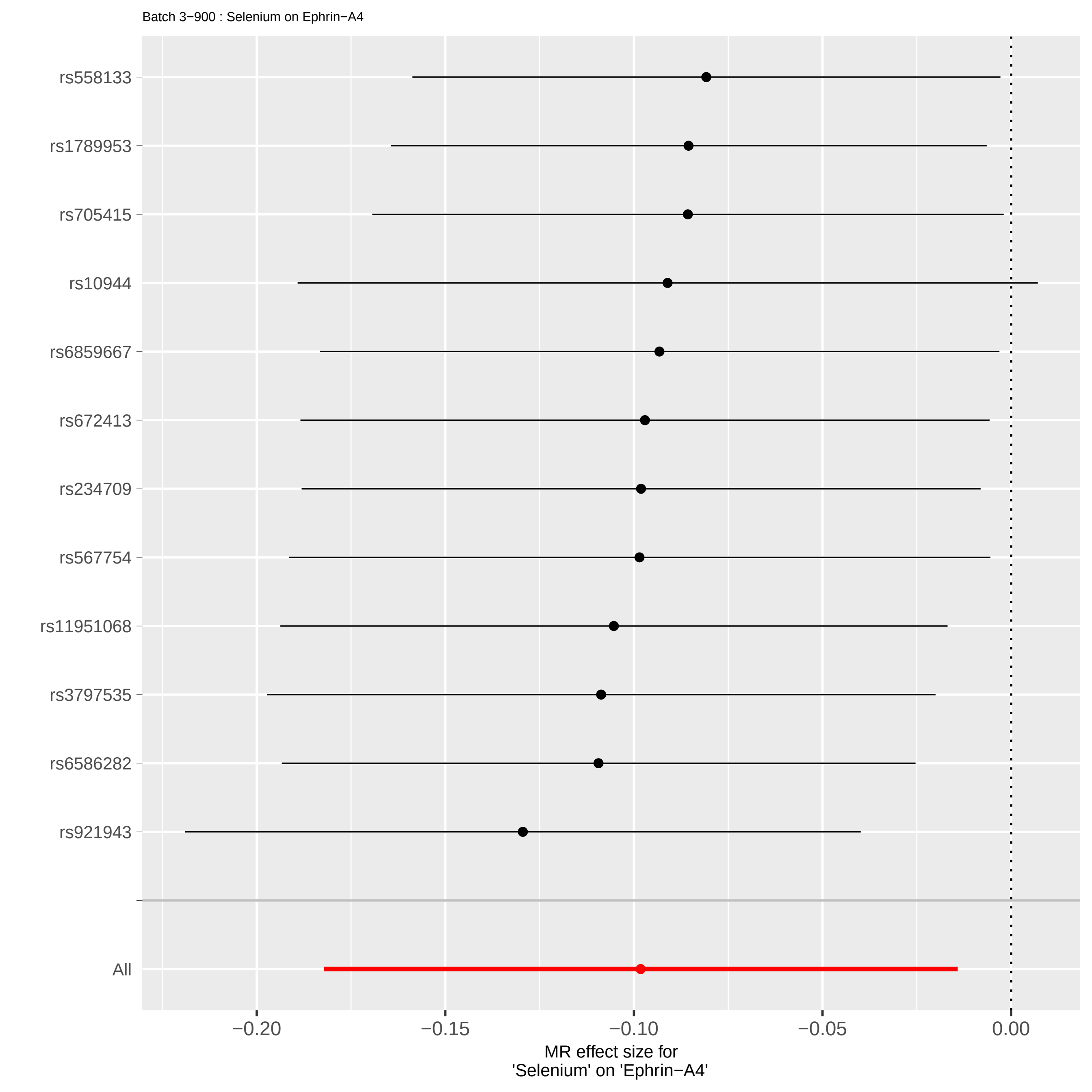

Batch 3−900 : Selenium on Ephrin−A4
rs558133
rs1789953
rs705415
rs10944
rs6859667
rs672413
rs234709
rs567754
rs11951068
rs3797535
rs6586282
rs921943
All
−0.20
−0.15
−0.10
−0.05
0.00
MR effect size for
'Selenium' on 'Ephrin−A4'

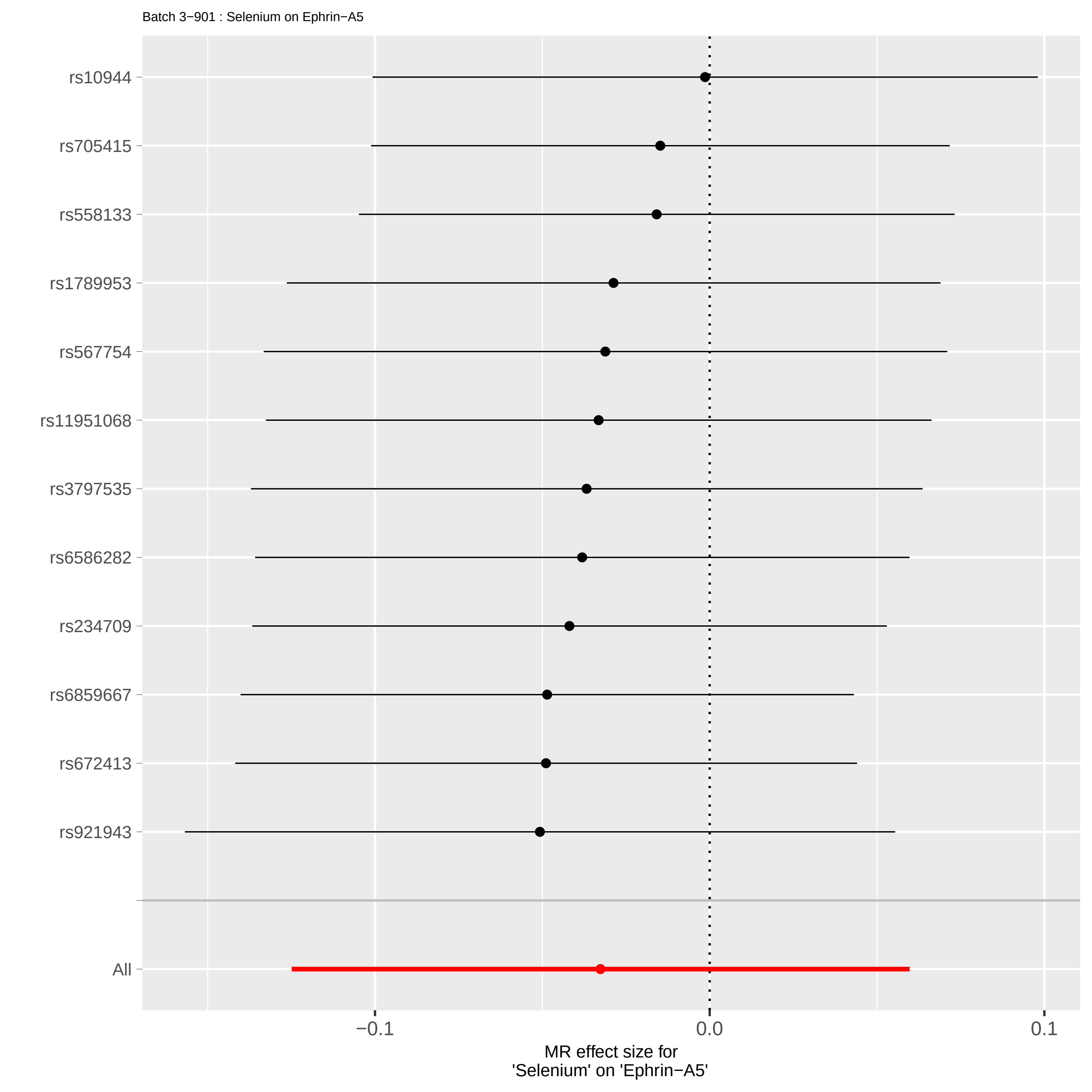

Batch 3−901 : Selenium on Ephrin−A5
rs10944
rs705415
rs558133
rs1789953
rs567754
rs11951068
rs3797535
rs6586282
rs234709
rs6859667
rs672413
rs921943
All
−0.1
0.0
0.1
MR effect size for
'Selenium' on 'Ephrin−A5'

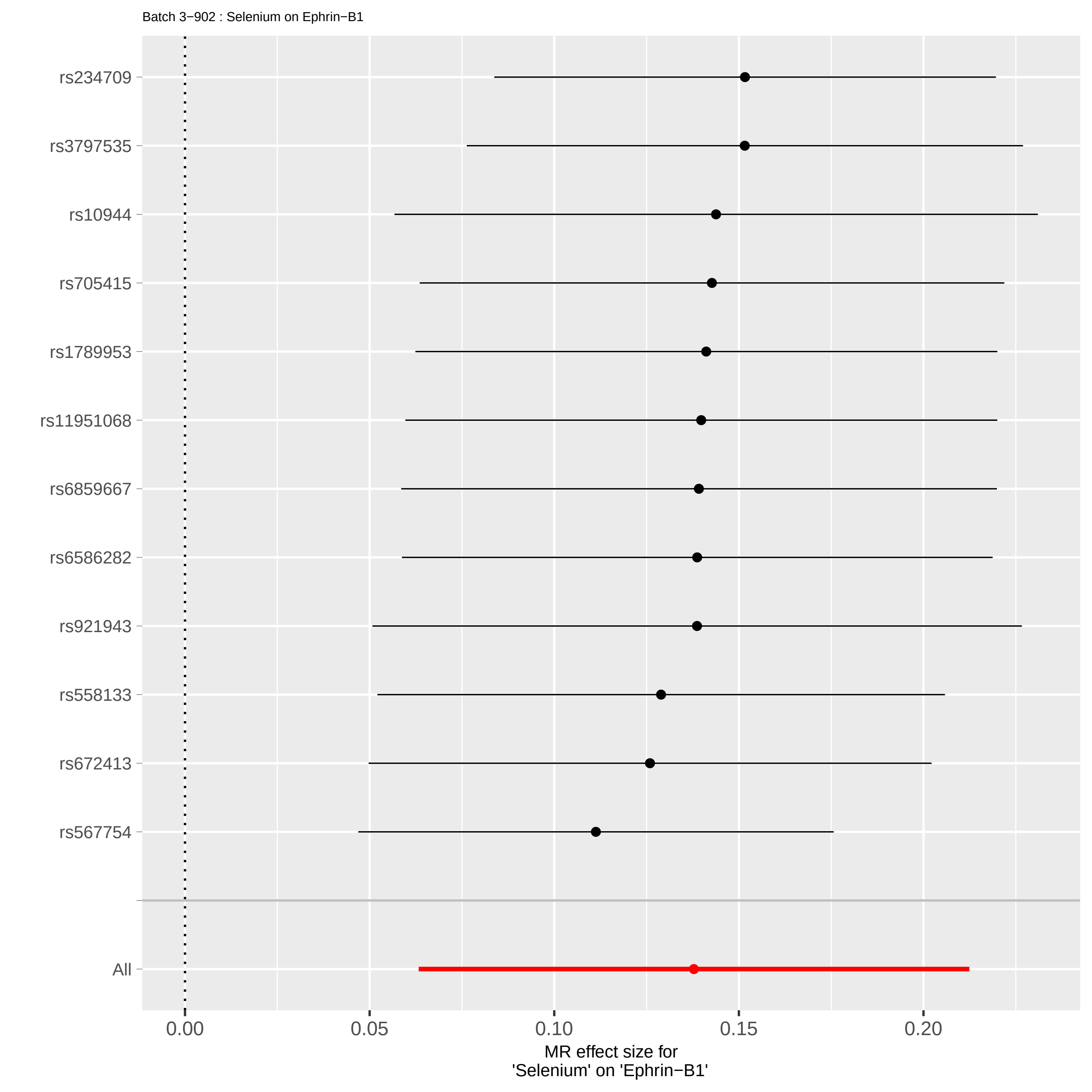
Batch 3−902 : Selenium on Ephrin−B1
rs234709
rs3797535
rs10944
rs705415
rs1789953
rs11951068
rs6859667
rs6586282
rs921943
rs558133
rs672413
rs567754
All
0.00
0.05
0.10
0.15
0.20
MR effect size for
'Selenium' on 'Ephrin−B1'

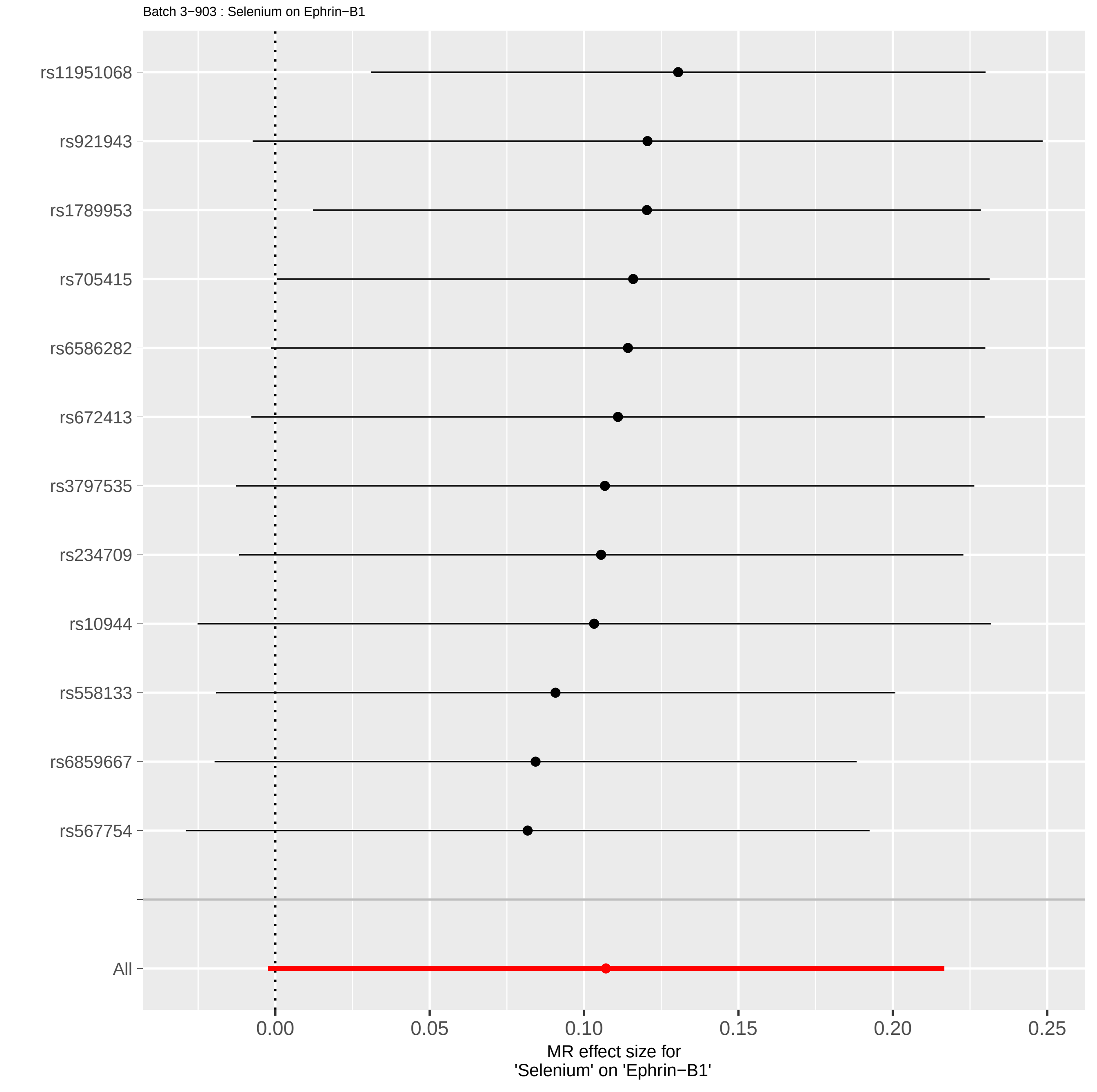

Batch 3−903 : Selenium on Ephrin−B1
rs11951068
rs921943
rs1789953
rs705415
rs6586282
rs672413
rs3797535
rs234709
rs10944
rs558133
rs6859667
rs567754
All
0.00
0.05
0.10
0.15
0.20
0.25
MR effect size for
'Selenium' on 'Ephrin−B1'

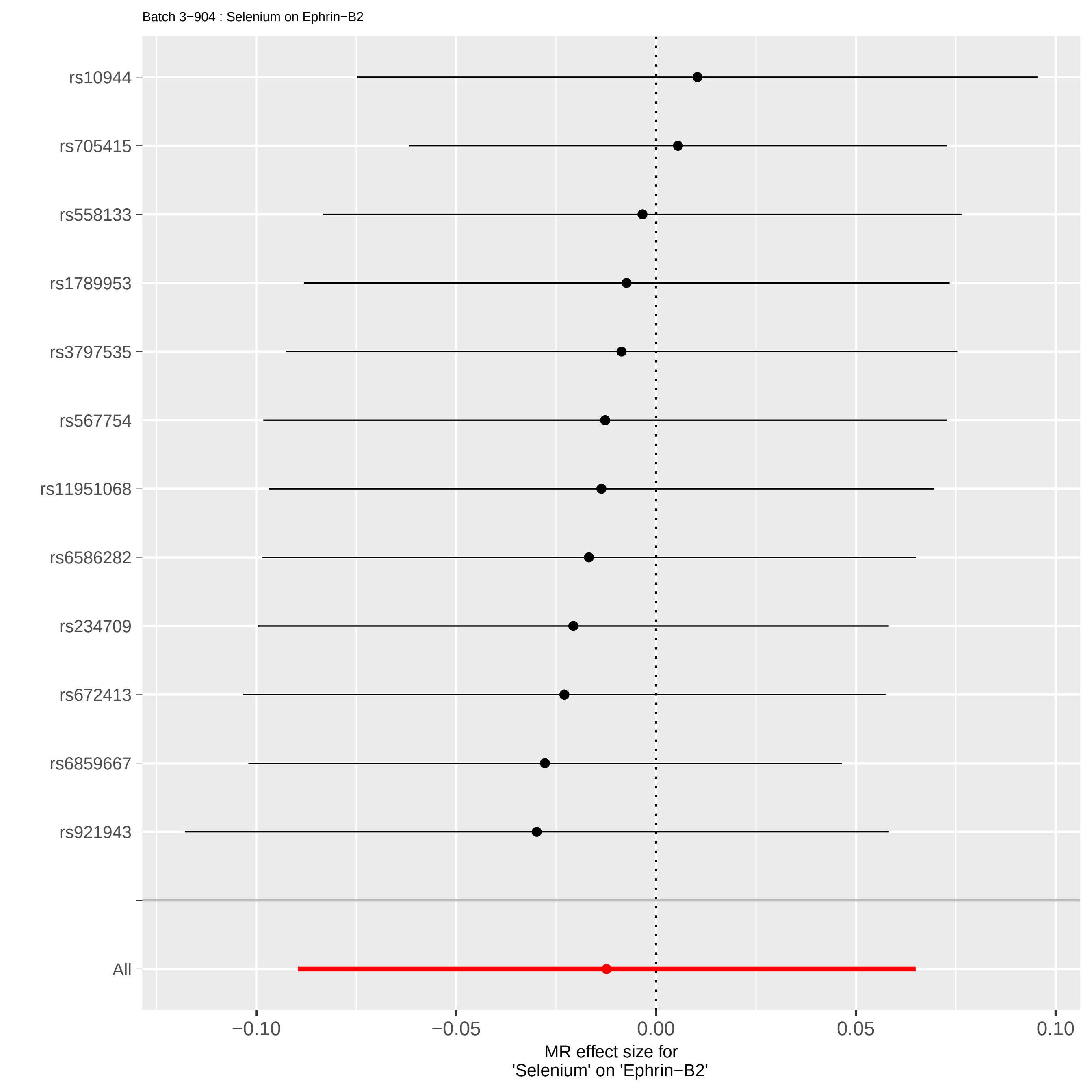

Batch 3−904 : Selenium on Ephrin−B2
rs10944
rs705415
rs558133
rs1789953
rs3797535
rs567754
rs11951068
rs6586282
rs234709
rs672413
rs6859667
rs921943
All
−0.10
−0.05
0.00
0.05
0.10
MR effect size for
'Selenium' on 'Ephrin−B2'

Batch 3−905 : Selenium on Ephrin−B2

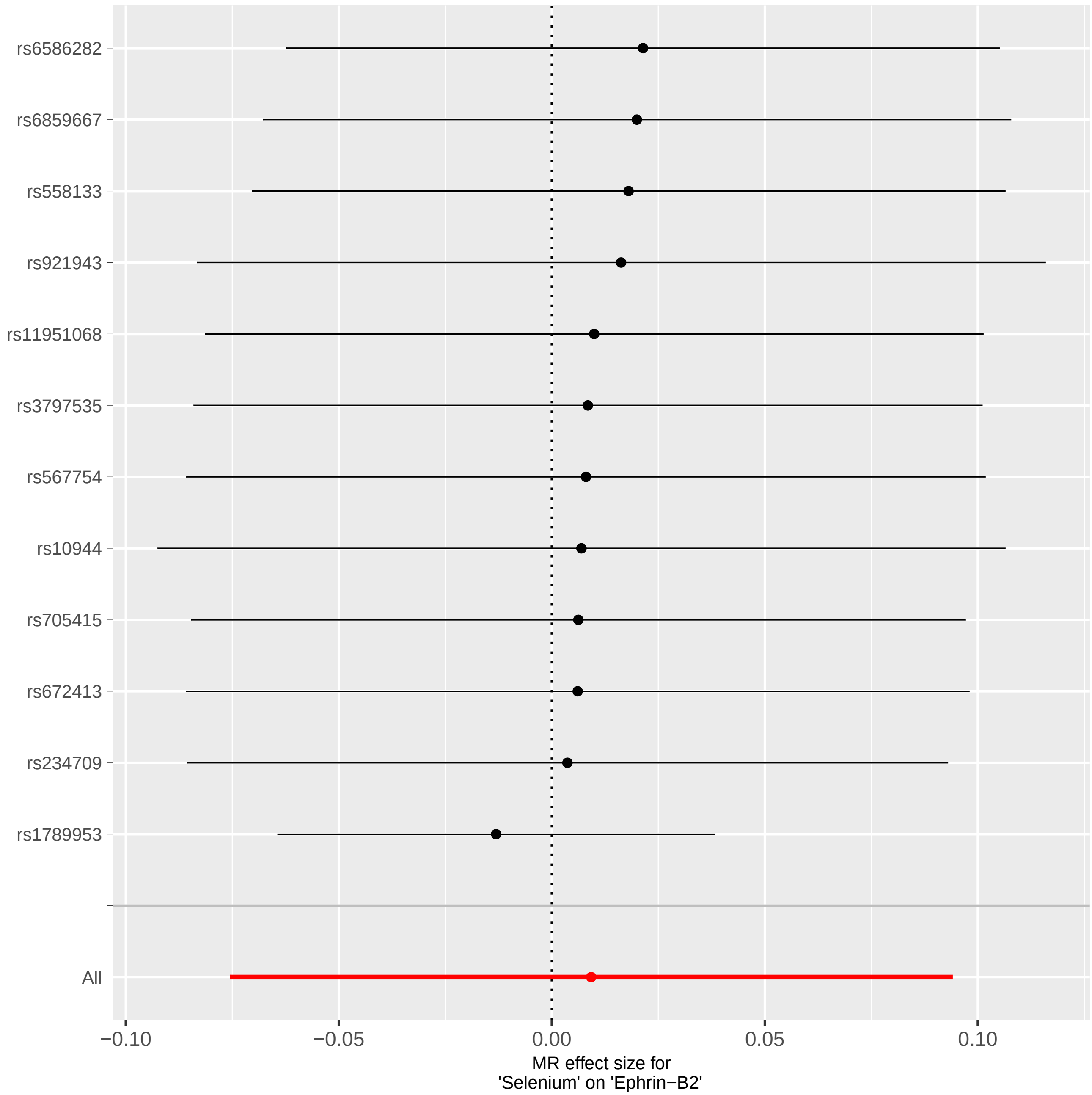

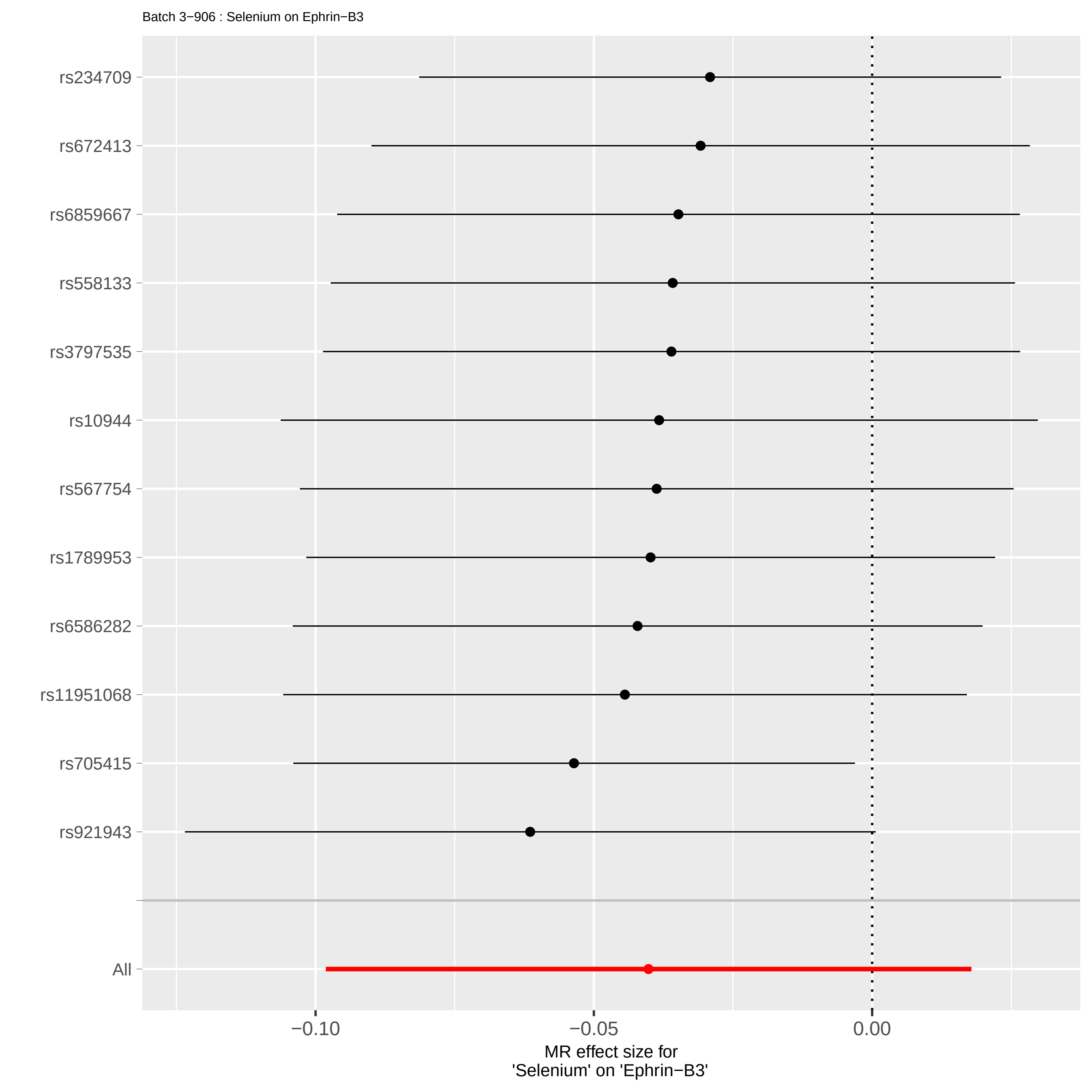

Batch 3−906 : Selenium on Ephrin−B3
rs234709
rs672413
rs6859667
rs558133
rs3797535
rs10944
rs567754
rs1789953
rs6586282
rs11951068
rs705415
rs921943
All
−0.10
−0.05
0.00
MR effect size for
'Selenium' on 'Ephrin−B3'

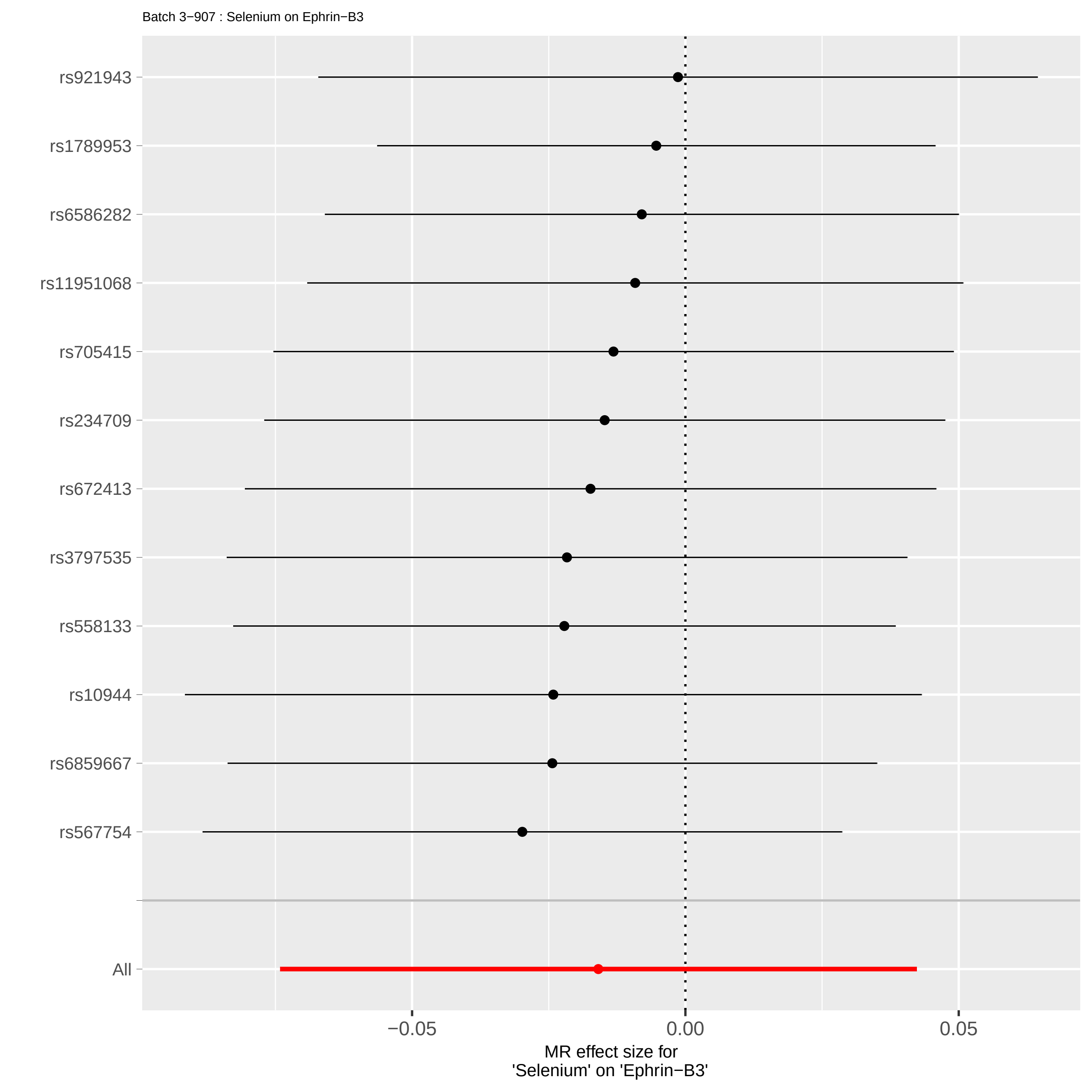
Batch 3−907 : Selenium on Ephrin−B3
rs921943
rs1789953
rs6586282
rs11951068
rs705415
rs234709
rs672413
rs3797535
rs558133
rs10944
rs6859667
rs567754
All
−0.05
0.00
0.05
MR effect size for
'Selenium' on 'Ephrin−B3'

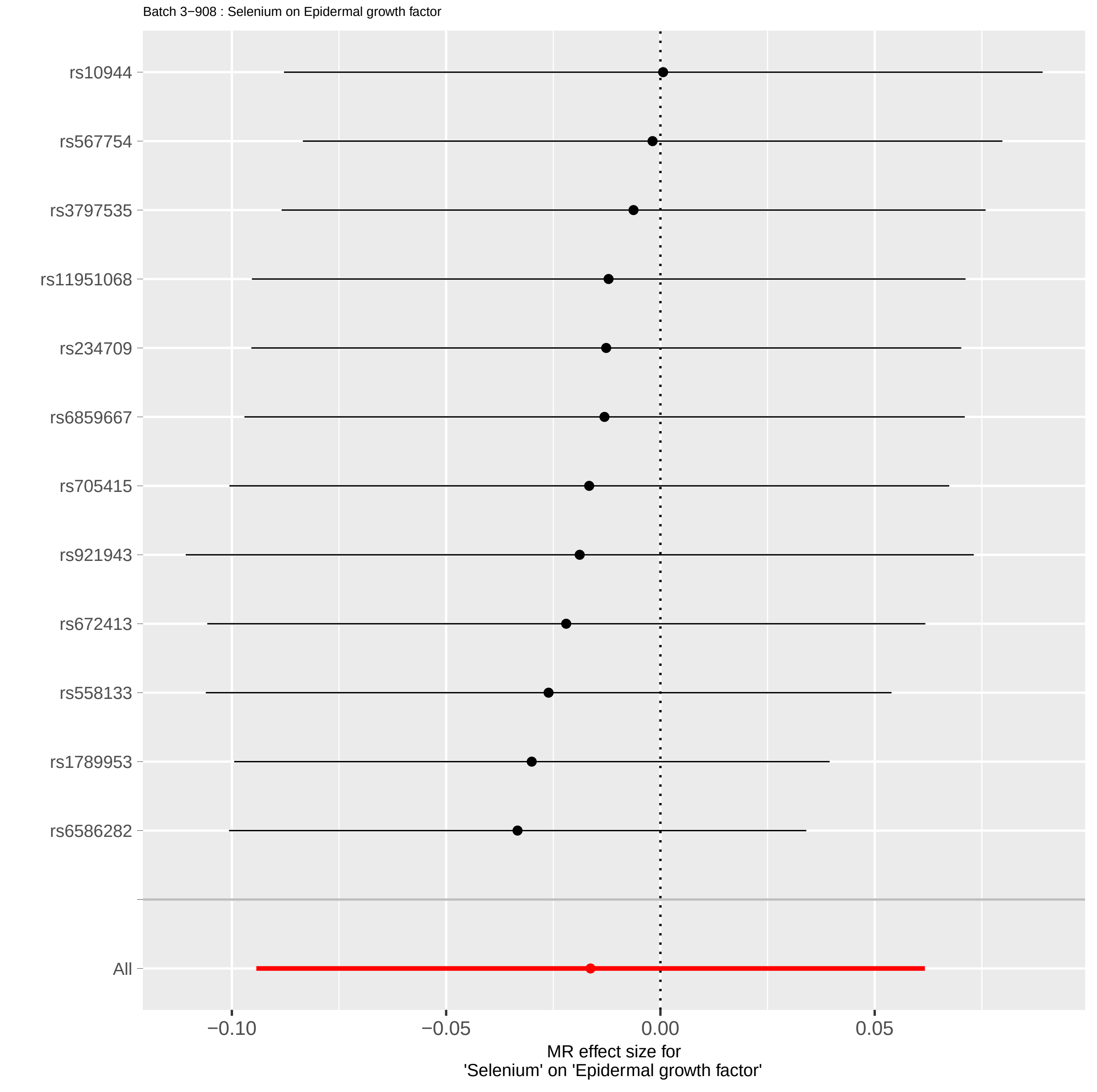

Batch 3−908 : Selenium on Epidermal growth factor
rs10944
rs567754
rs3797535
rs11951068
rs234709
rs6859667
rs705415
rs921943
rs672413
rs558133
rs1789953
rs6586282
All
−0.10
−0.05
0.00
0.05
MR effect size for
'Selenium' on 'Epidermal growth factor'

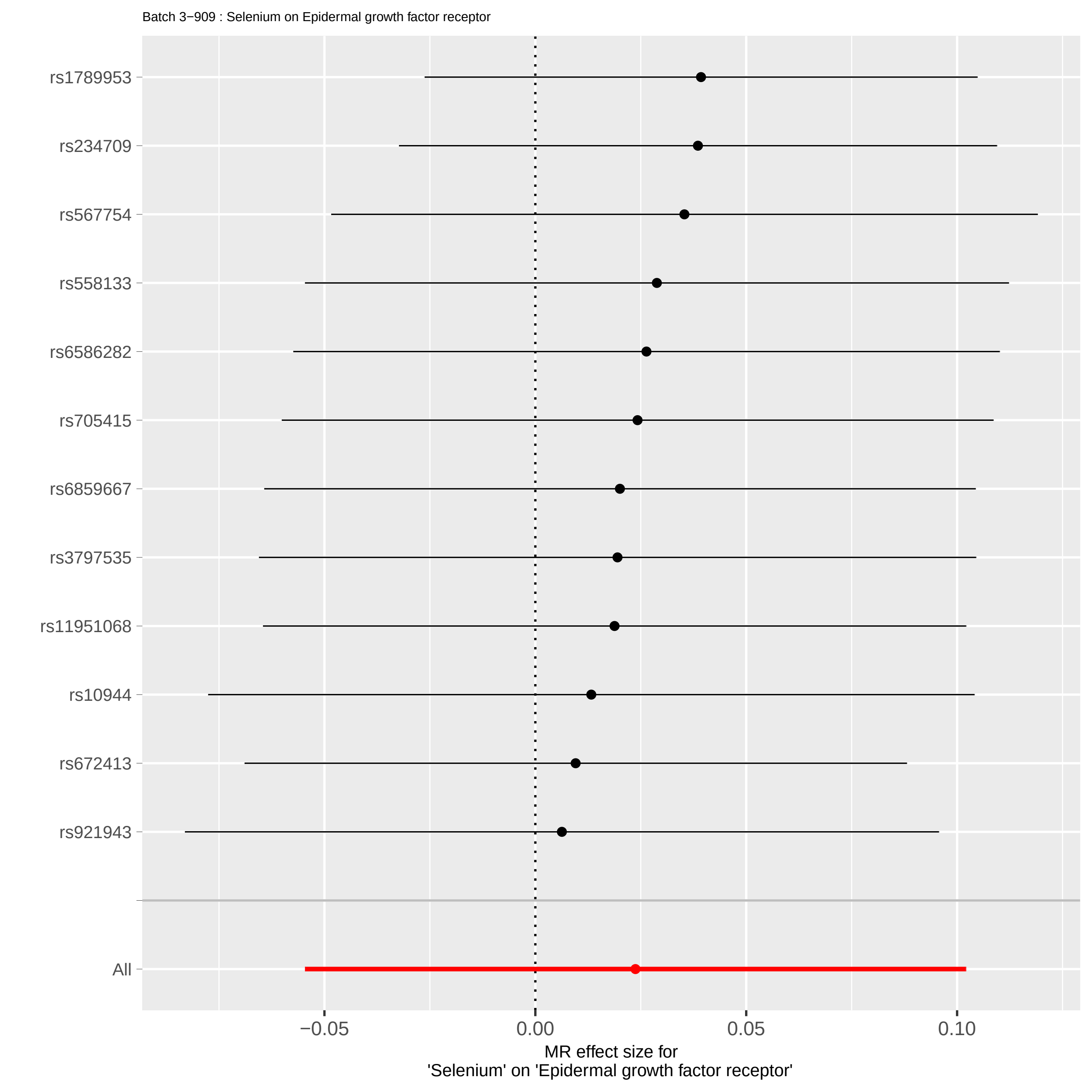

Batch 3−909 : Selenium on Epidermal growth factor receptor
rs1789953
rs234709
rs567754
rs558133
rs6586282
rs705415
rs6859667
rs3797535
rs11951068
rs10944
rs672413
rs921943
All
−0.05
0.00
0.05
0.10
MR effect size for
'Selenium' on 'Epidermal growth factor receptor'

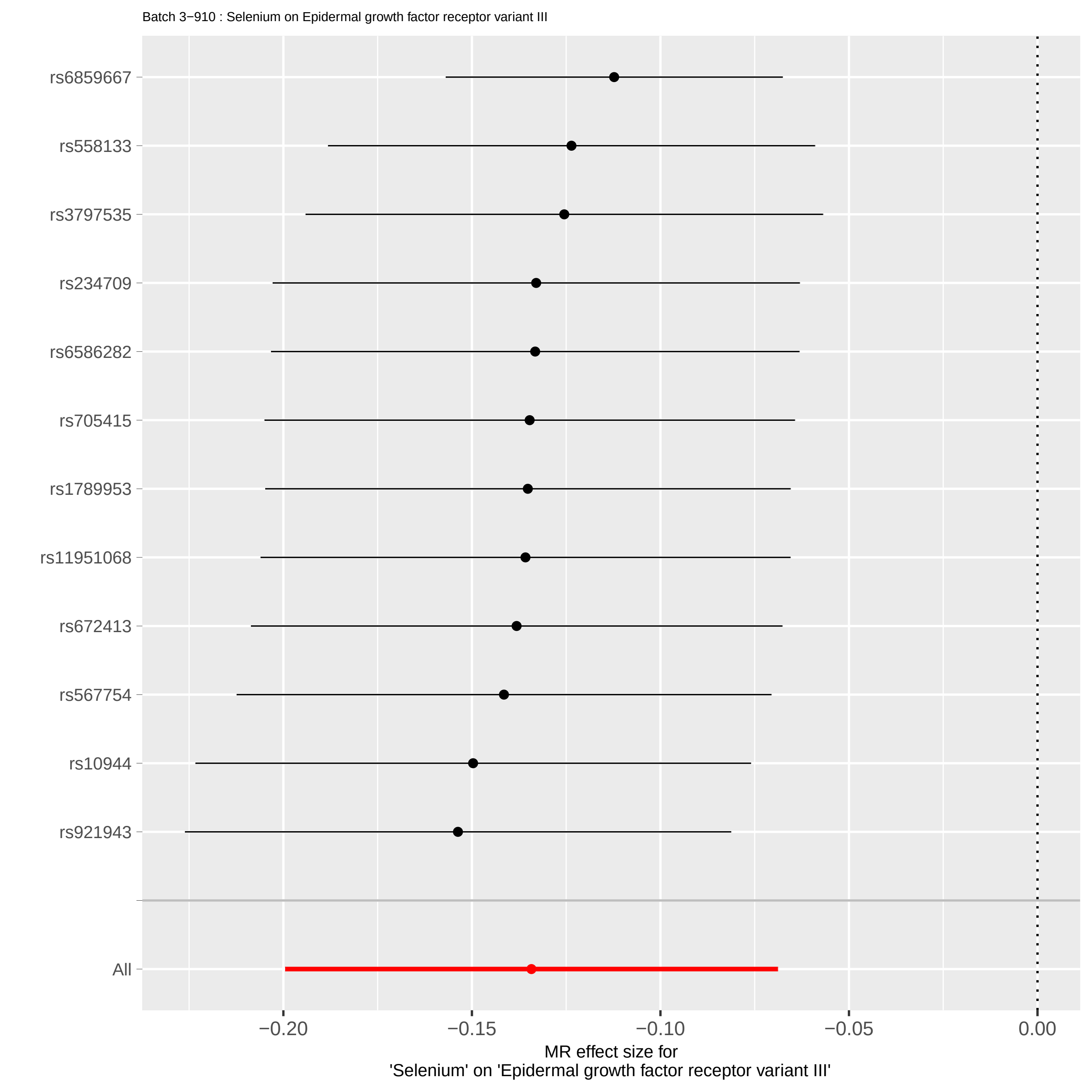

Batch 3−910 : Selenium on Epidermal growth factor receptor variant III
rs6859667
rs558133
rs3797535
rs234709
rs6586282
rs705415
rs1789953
rs11951068
rs672413
rs567754
rs10944
rs921943
All
−0.20
−0.15
−0.10
−0.05
0.00
MR effect size for
'Selenium' on 'Epidermal growth factor receptor variant III'

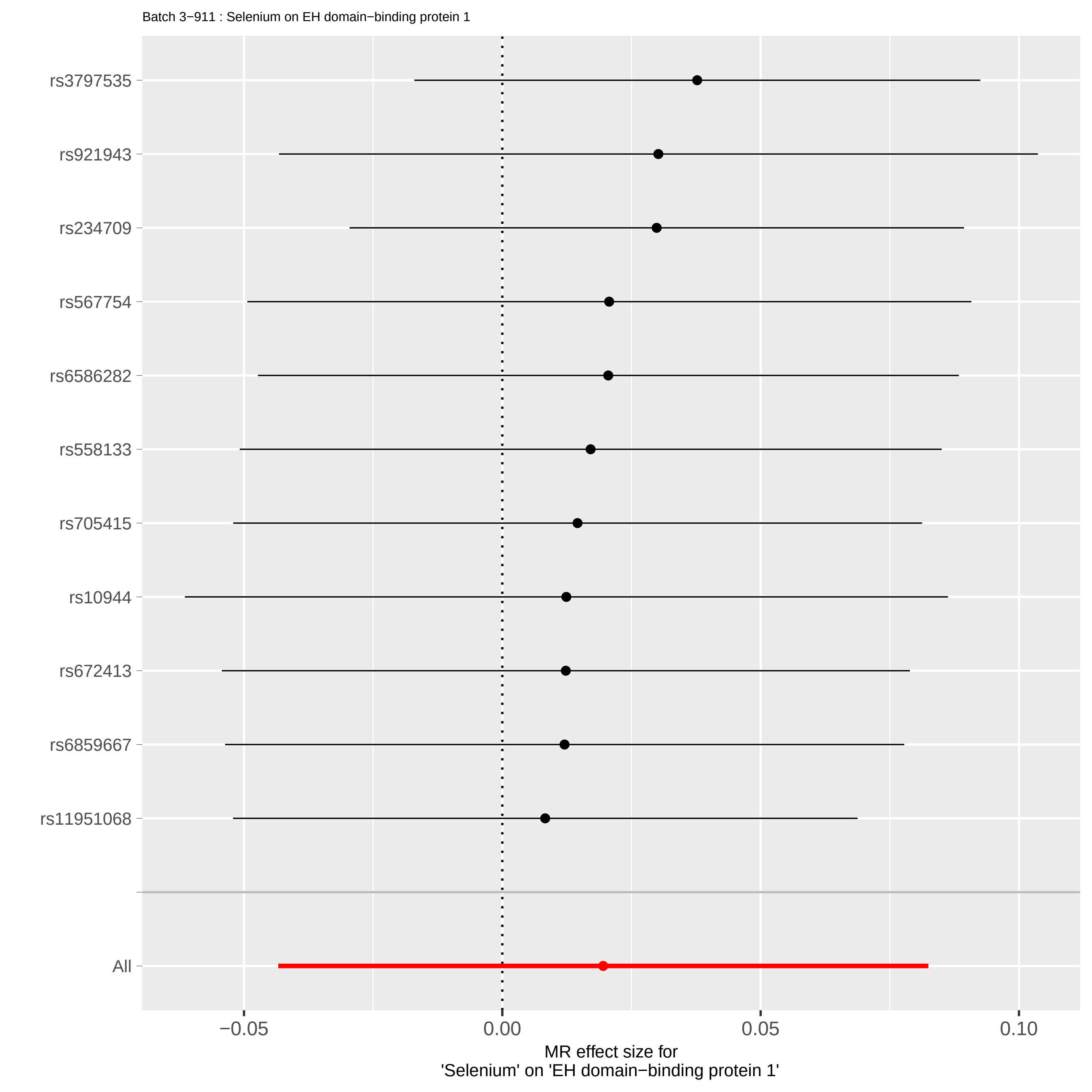
Batch 3−911 : Selenium on EH domain−binding protein 1
rs3797535
rs921943
rs234709
rs567754
rs6586282
rs558133
rs705415
rs10944
rs672413
rs6859667
rs11951068
All
−0.05
0.00
0.05
0.10
MR effect size for
'Selenium' on 'EH domain−binding protein 1'

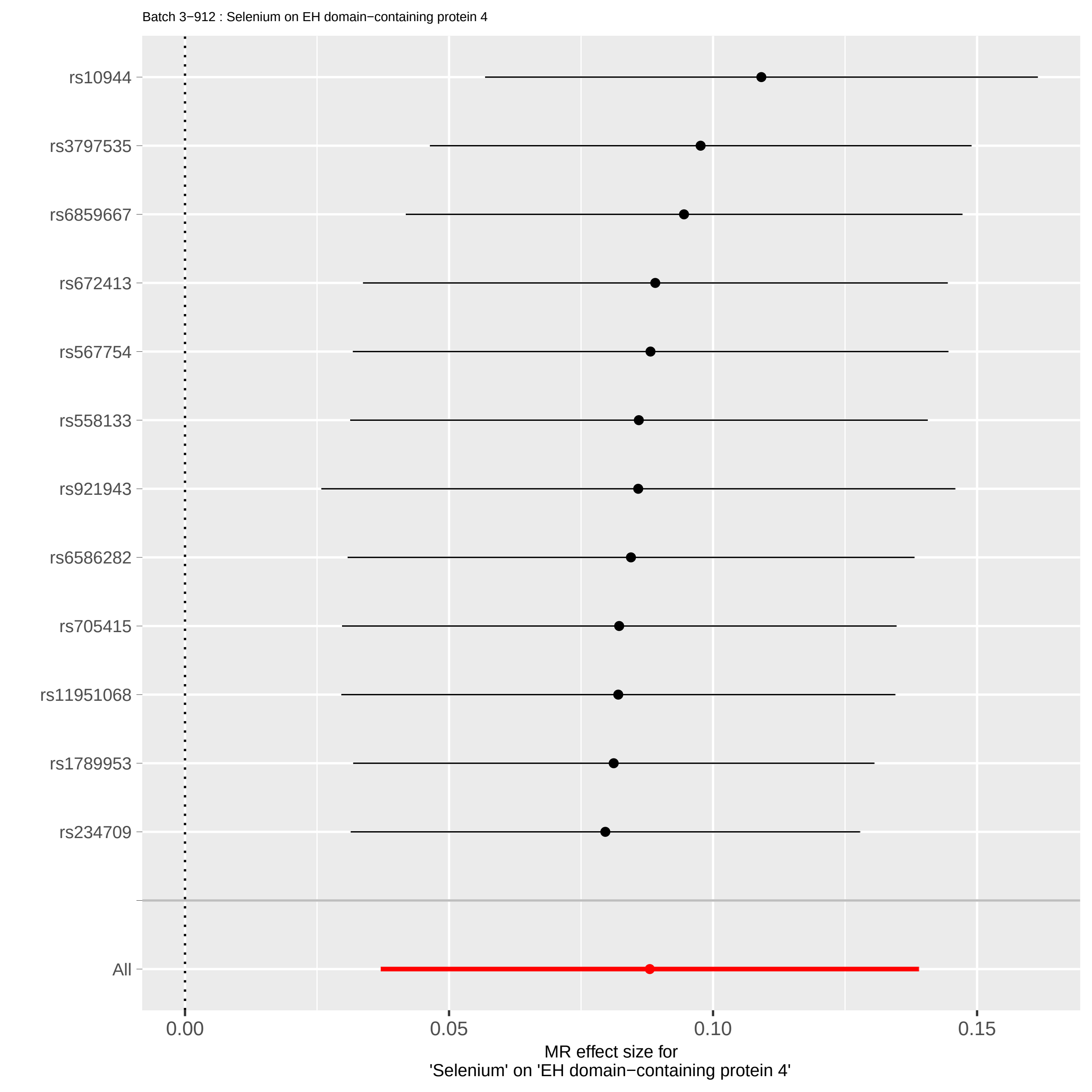
Batch 3−912 : Selenium on EH domain−containing protein 4
rs10944
rs3797535
rs6859667
rs672413
rs567754
rs558133
rs921943
rs6586282
rs705415
rs11951068
rs1789953
rs234709
All
0.00
0.05
0.10
0.15
MR effect size for
'Selenium' on 'EH domain−containing protein 4'

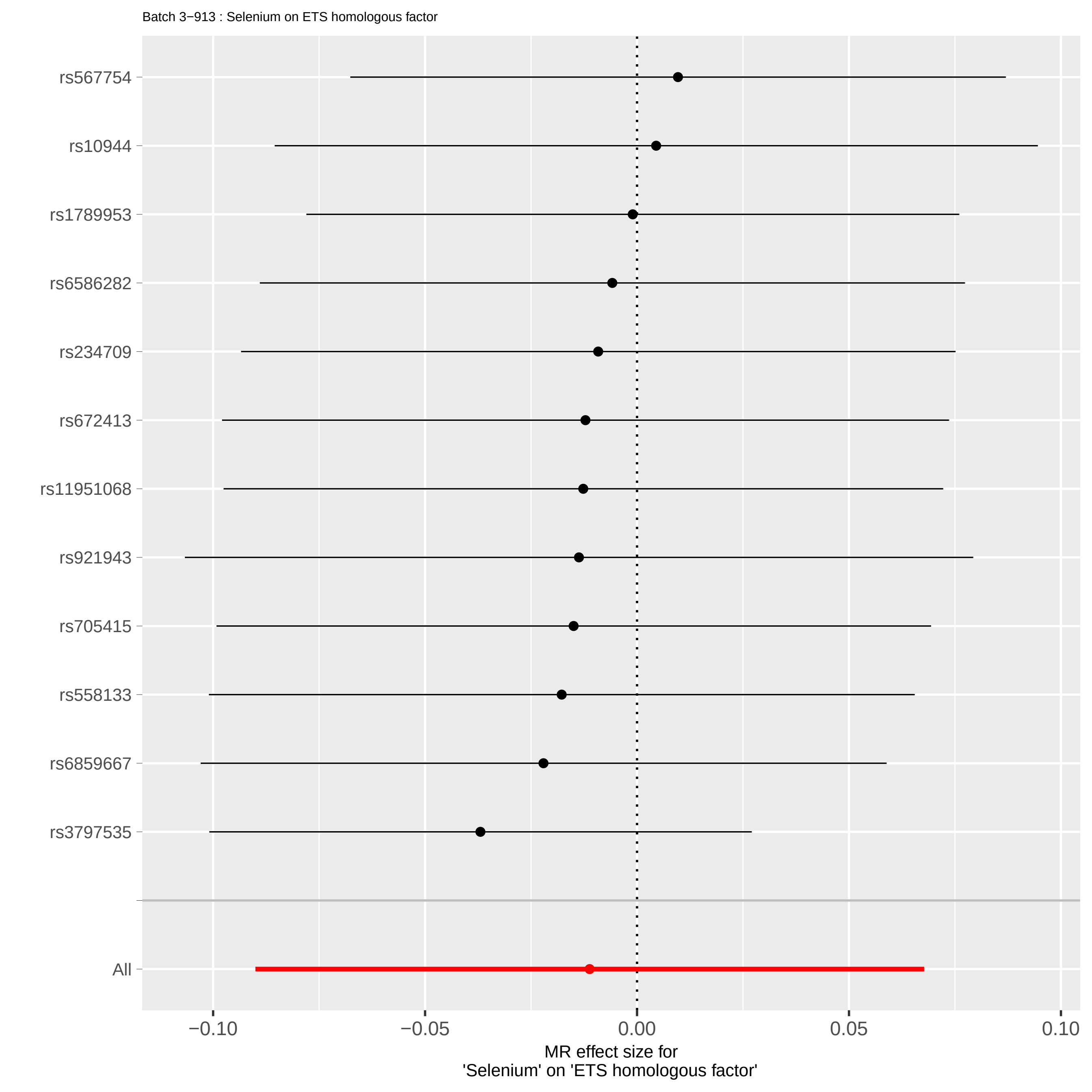

Batch 3−913 : Selenium on ETS homologous factor
rs567754
rs10944
rs1789953
rs6586282
rs234709
rs672413
rs11951068
rs921943
rs705415
rs558133
rs6859667
rs3797535
All
−0.10
−0.05
0.00
0.05
0.10
MR effect size for
'Selenium' on 'ETS homologous factor'

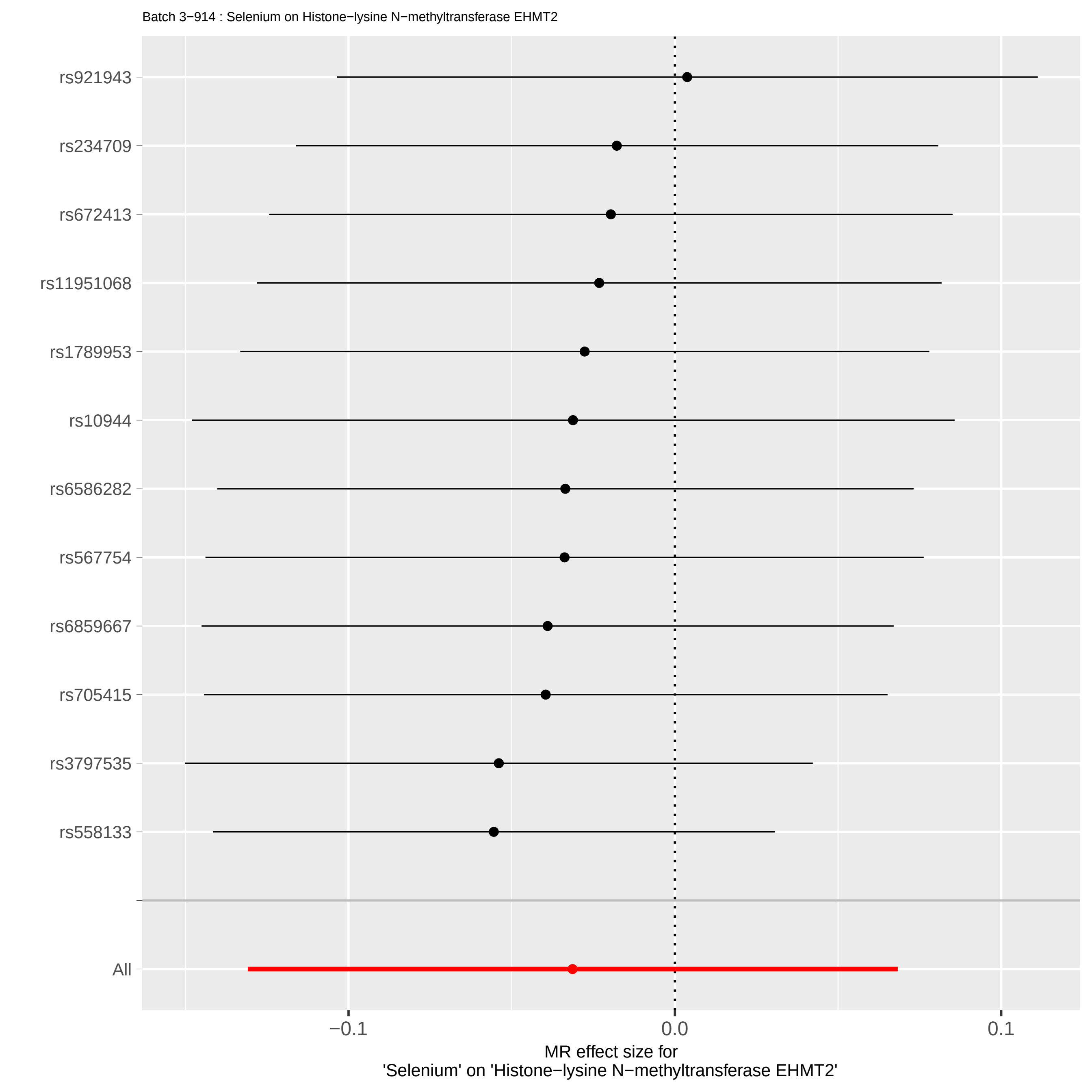
Batch 3−914 : Selenium on Histone−lysine N−methyltransferase EHMT2
rs921943
rs234709
rs672413
rs11951068
rs1789953
rs10944
rs6586282
rs567754
rs6859667
rs705415
rs3797535
rs558133
All
−0.1
0.0
0.1
MR effect size for
'Selenium' on 'Histone−lysine N−methyltransferase EHMT2'

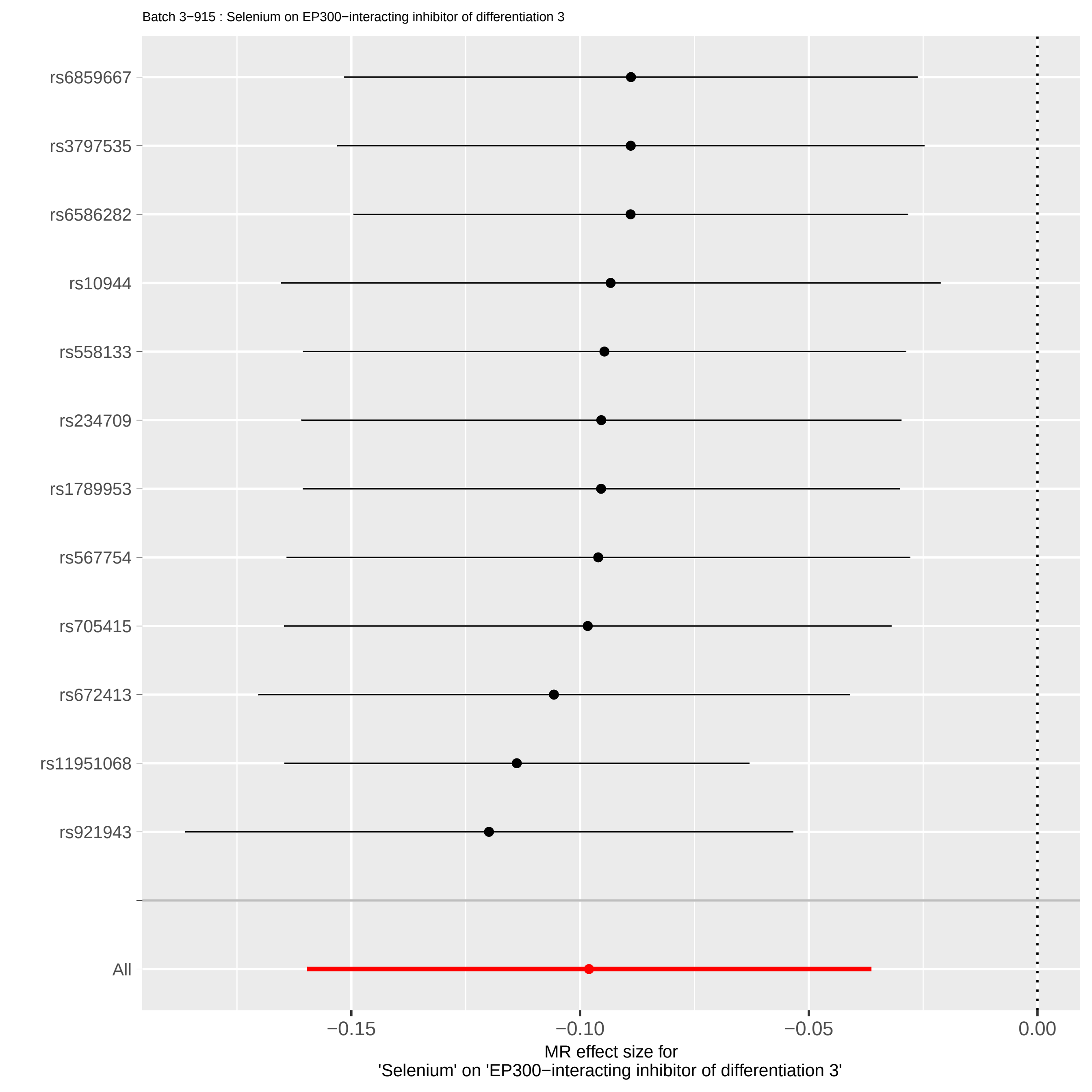

Batch 3−915 : Selenium on EP300−interacting inhibitor of differentiation 3
rs6859667
rs3797535
rs6586282
rs10944
rs558133
rs234709
rs1789953
rs567754
rs705415
rs672413
rs11951068
rs921943
All
−0.15
−0.10
−0.05
0.00
MR effect size for
'Selenium' on 'EP300−interacting inhibitor of differentiation 3'

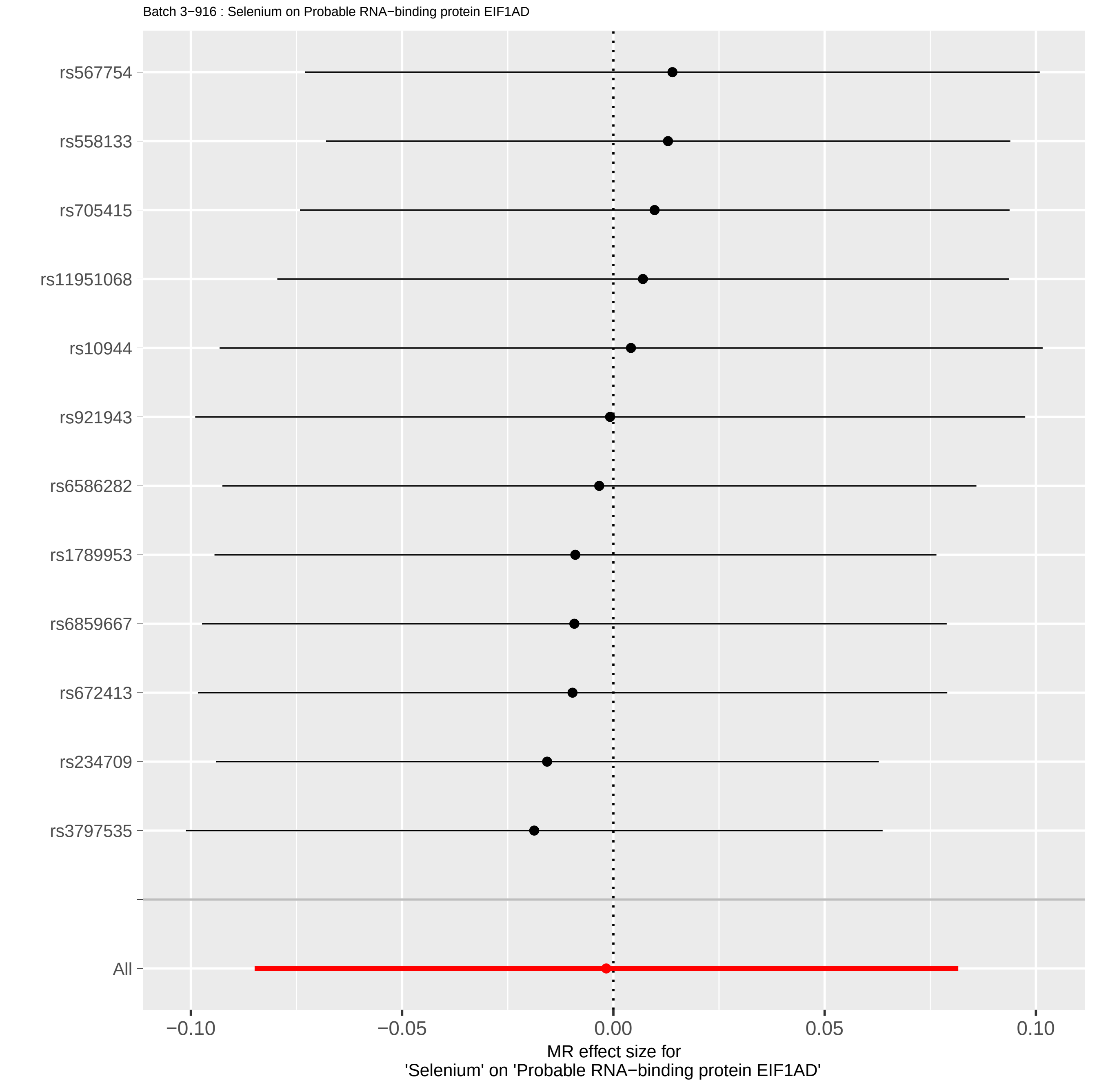
Batch 3−916 : Selenium on Probable RNA−binding protein EIF1AD
rs567754
rs558133
rs705415
rs11951068
rs10944
rs921943
rs6586282
rs1789953
rs6859667
rs672413
rs234709
rs3797535
All
−0.10
−0.05
0.00
0.05
0.10
MR effect size for
'Selenium' on 'Probable RNA−binding protein EIF1AD'

Batch 3−917 : Selenium on Eukaryotic translation initiation factor 1A, X−chromosomal

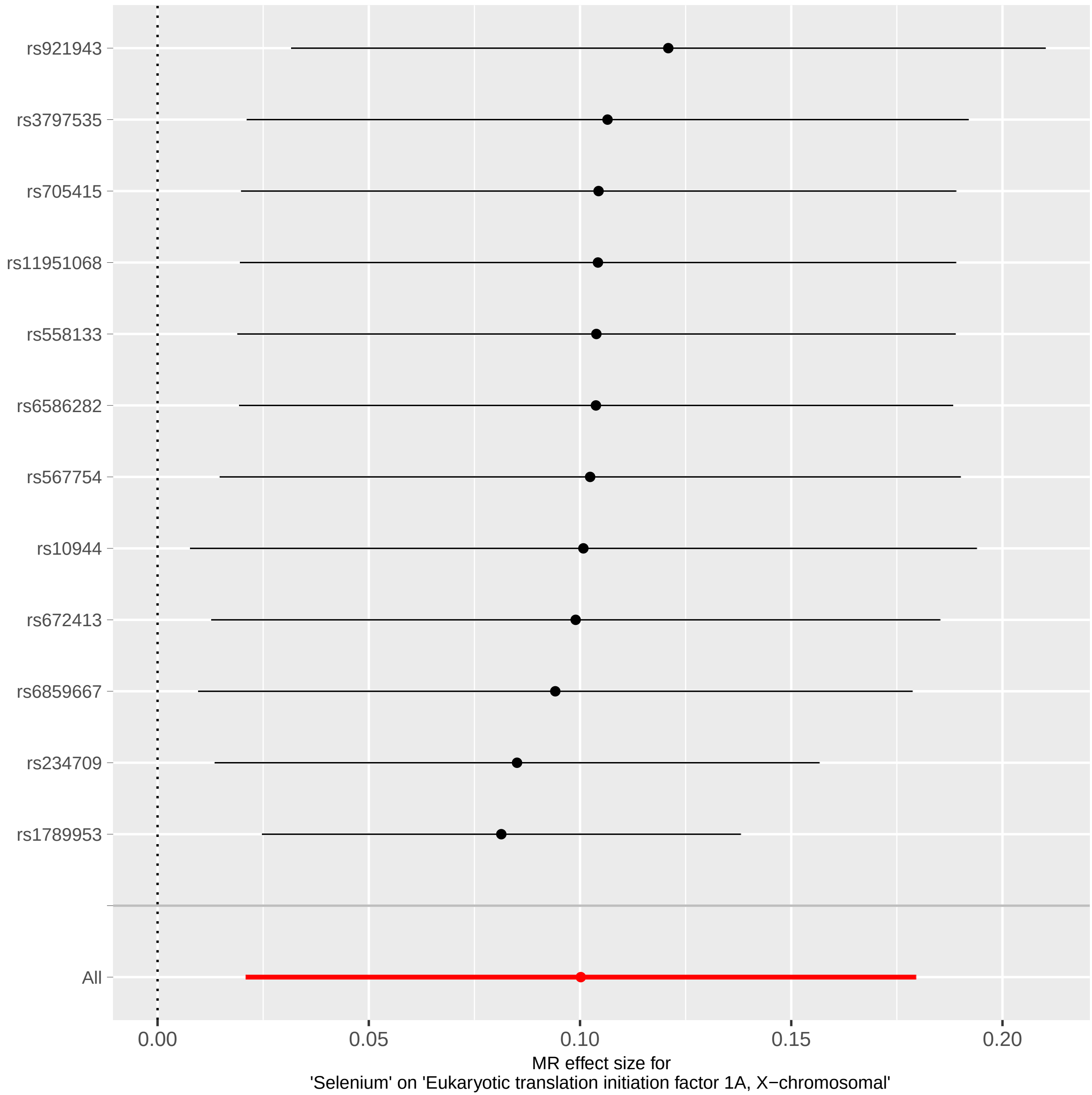

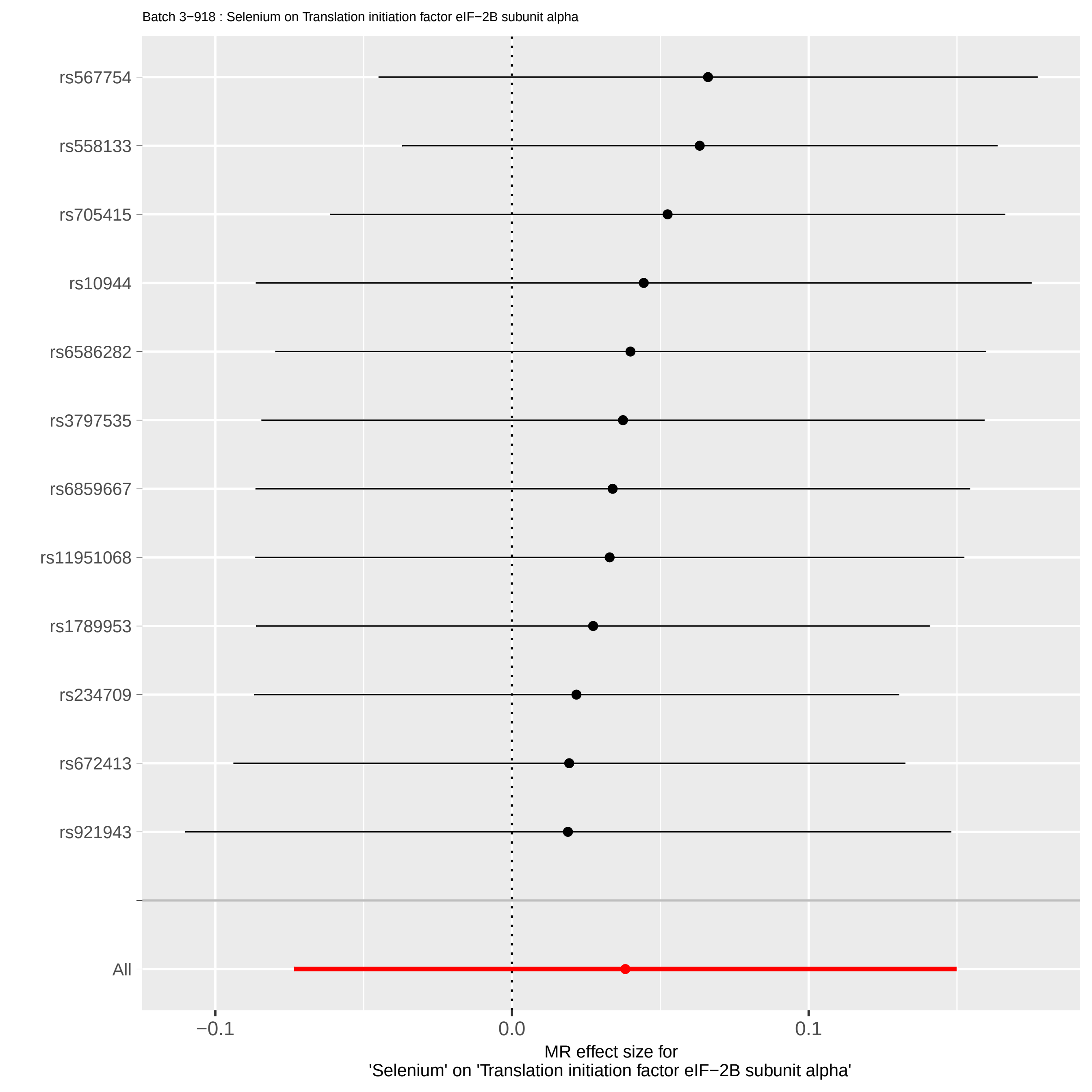
Batch 3−918 : Selenium on Translation initiation factor eIF−2B subunit alpha
rs567754
rs558133
rs705415
rs10944
rs6586282
rs3797535
rs6859667
rs11951068
rs1789953
rs234709
rs672413
rs921943
All
−0.1
0.0
0.1
MR effect size for
'Selenium' on 'Translation initiation factor eIF−2B subunit alpha'

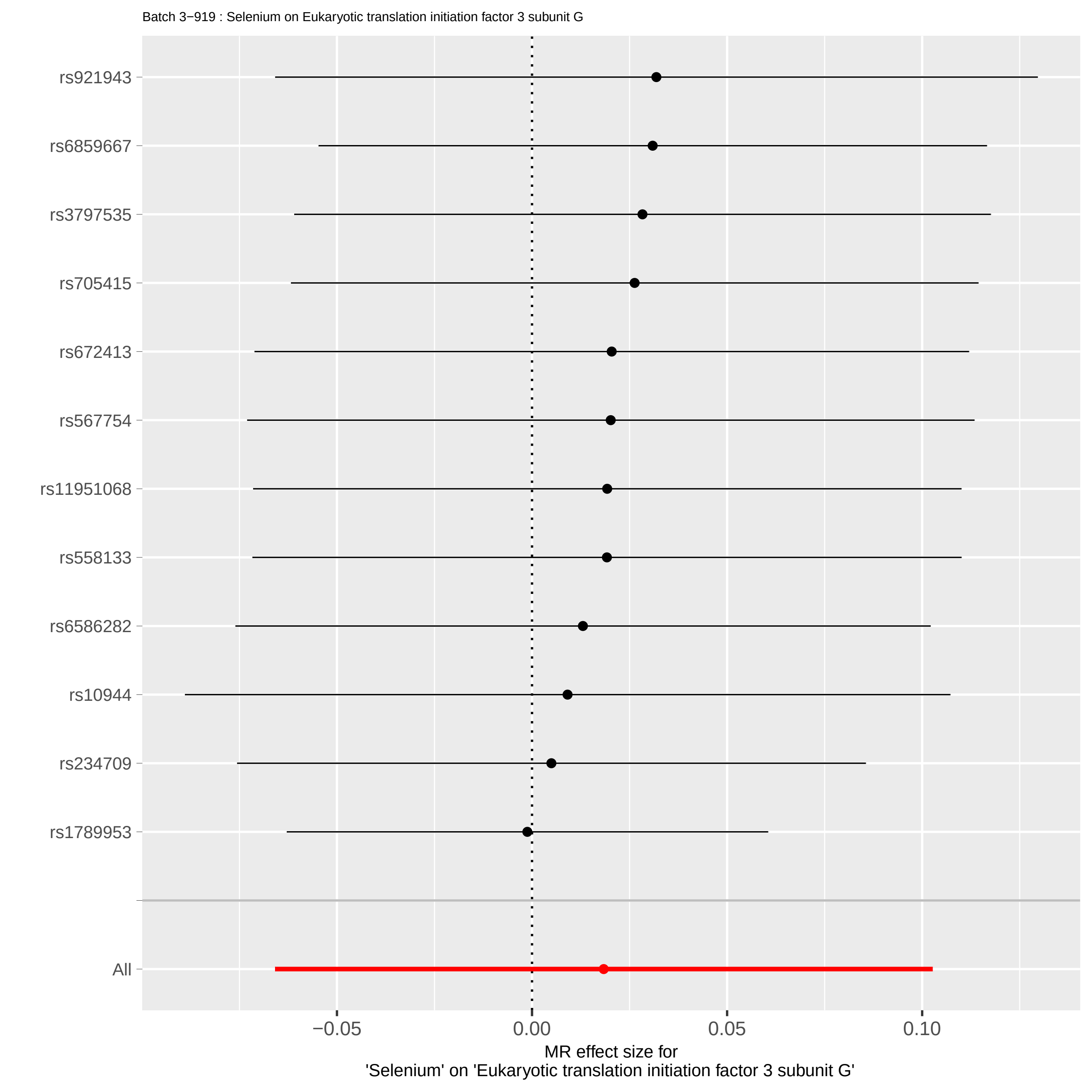

Batch 3−919 : Selenium on Eukaryotic translation initiation factor 3 subunit G
rs921943
rs6859667
rs3797535
rs705415
rs672413
rs567754
rs11951068
rs558133
rs6586282
rs10944
rs234709
rs1789953
All
−0.05
0.00
0.05
0.10
MR effect size for
'Selenium' on 'Eukaryotic translation initiation factor 3 subunit G'

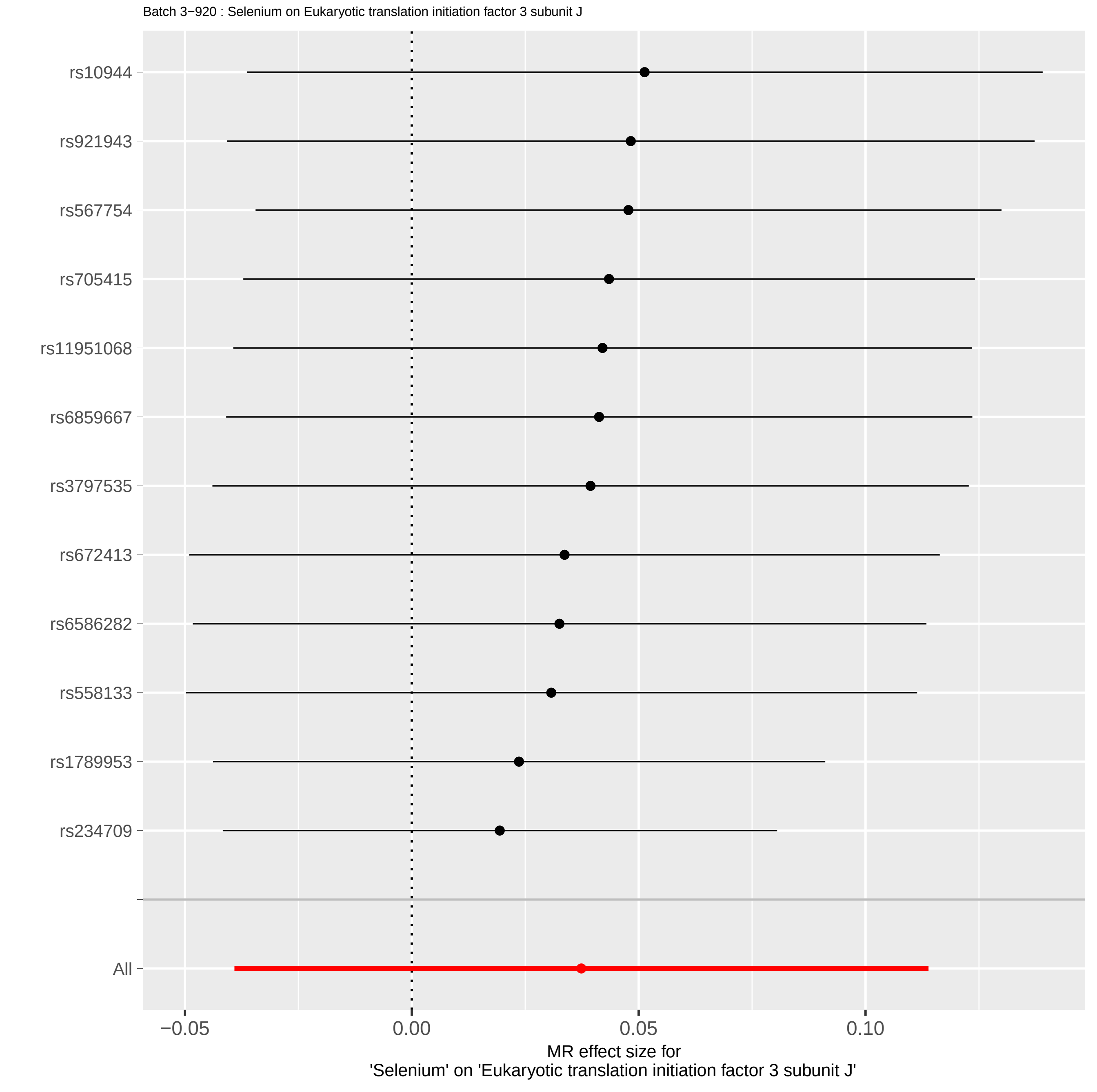

Batch 3−920 : Selenium on Eukaryotic translation initiation factor 3 subunit J
rs10944
rs921943
rs567754
rs705415
rs11951068
rs6859667
rs3797535
rs672413
rs6586282
rs558133
rs1789953
rs234709
All
−0.05
0.00
0.05
0.10
MR effect size for
'Selenium' on 'Eukaryotic translation initiation factor 3 subunit J'

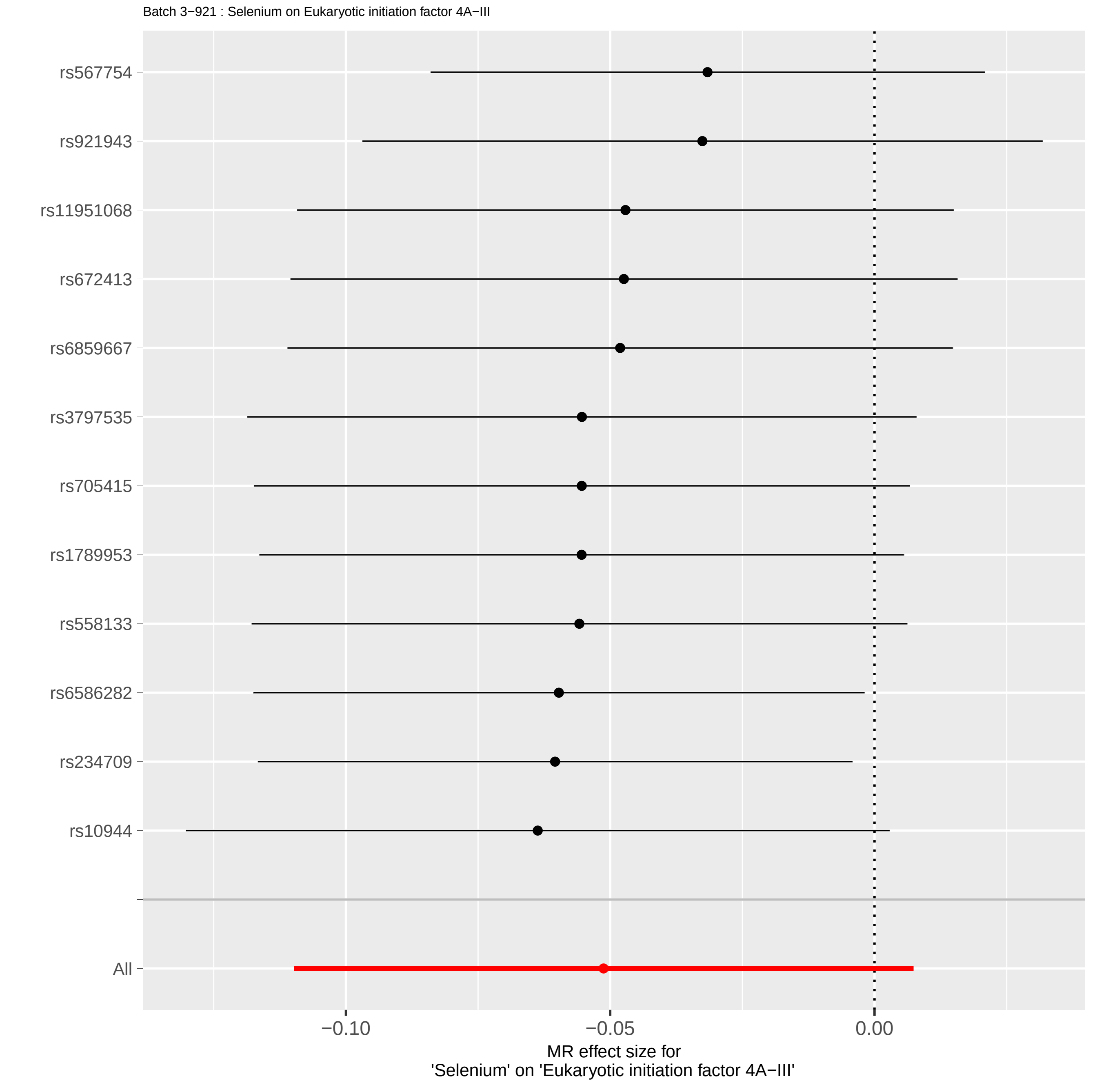
Batch 3−921 : Selenium on Eukaryotic initiation factor 4A−III
rs567754
rs921943
rs11951068
rs672413
rs6859667
rs3797535
rs705415
rs1789953
rs558133
rs6586282
rs234709
rs10944
All
−0.10
−0.05
0.00
MR effect size for
'Selenium' on 'Eukaryotic initiation factor 4A−III'

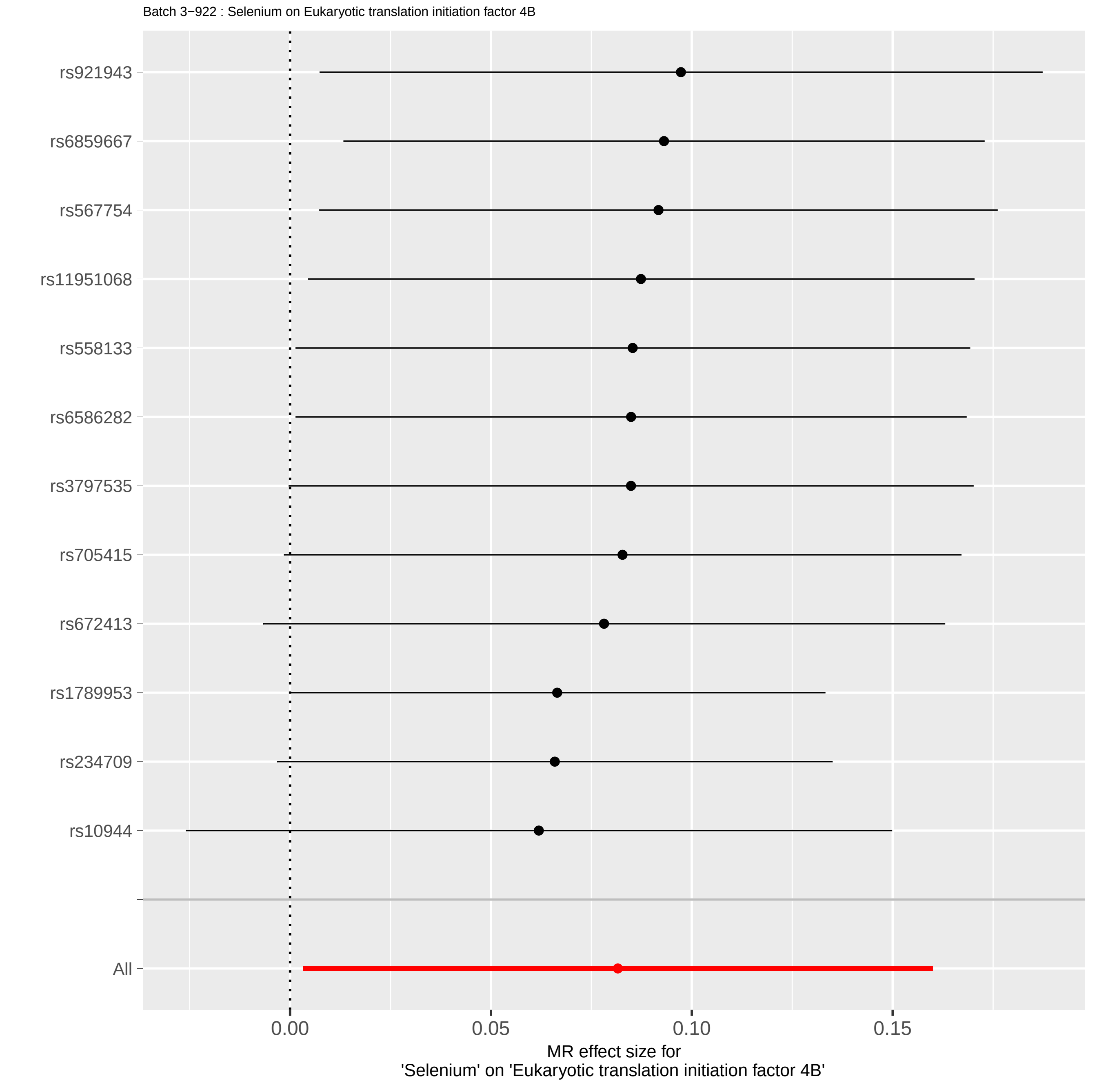
Batch 3−922 : Selenium on Eukaryotic translation initiation factor 4B
rs921943
rs6859667
rs567754
rs11951068
rs558133
rs6586282
rs3797535
rs705415
rs672413
rs1789953
rs234709
rs10944
All
0.00
0.05
0.10
0.15
MR effect size for
'Selenium' on 'Eukaryotic translation initiation factor 4B'

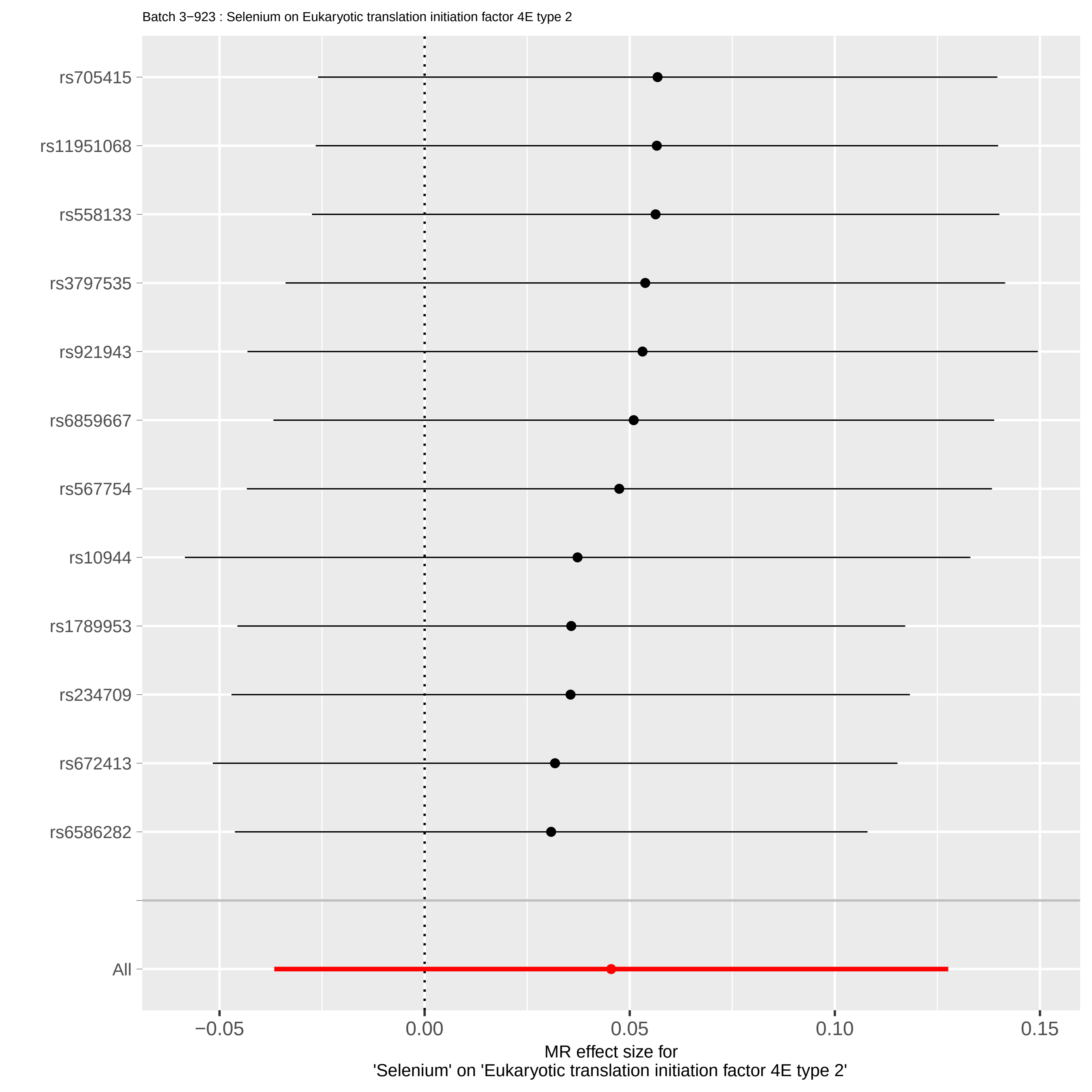

Batch 3−923 : Selenium on Eukaryotic translation initiation factor 4E type 2
rs705415
rs11951068
rs558133
rs3797535
rs921943
rs6859667
rs567754
rs10944
rs1789953
rs234709
rs672413
rs6586282
All
−0.05
0.00
0.05
0.10
0.15
MR effect size for
'Selenium' on 'Eukaryotic translation initiation factor 4E type 2'

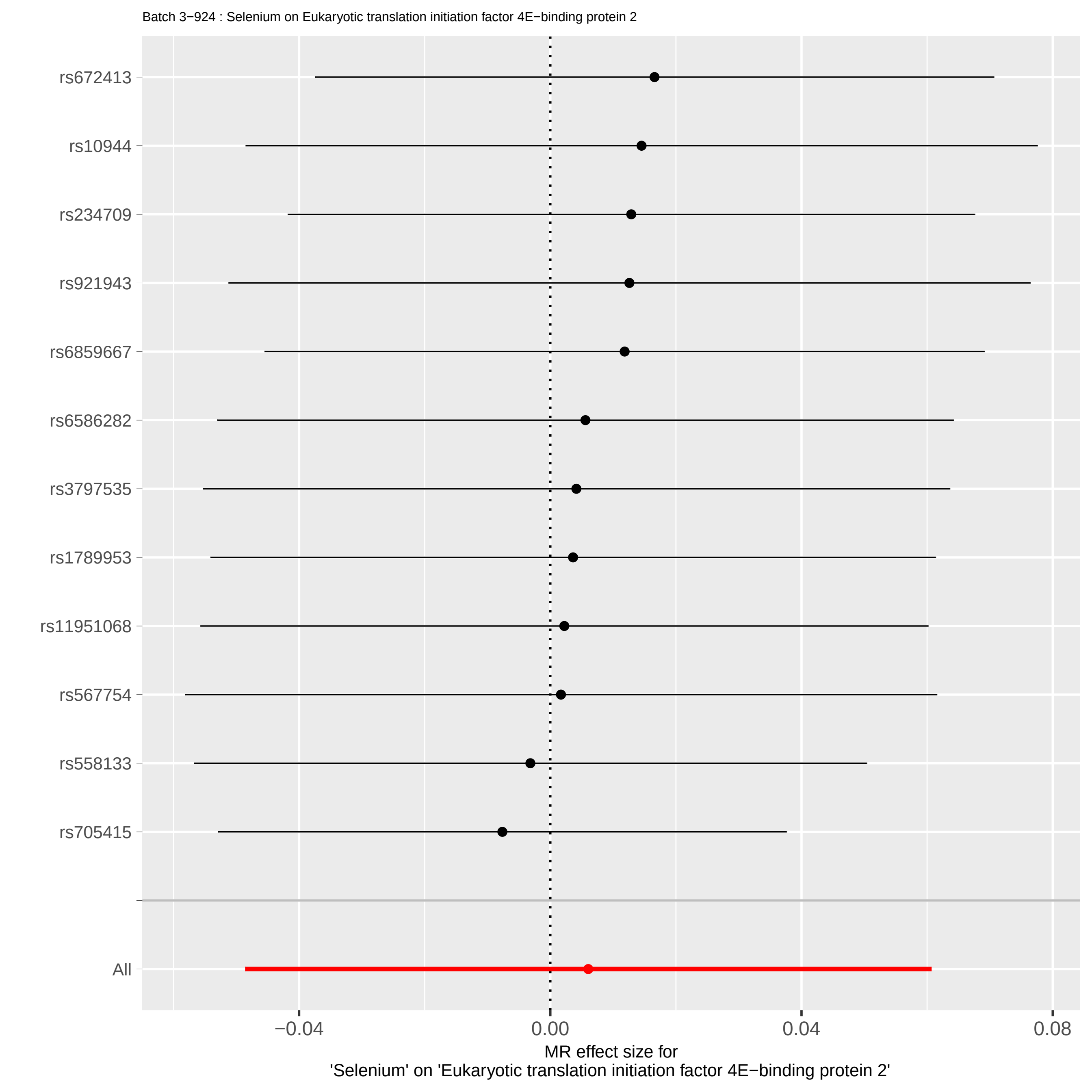

Batch 3−924 : Selenium on Eukaryotic translation initiation factor 4E−binding protein 2
rs672413
rs10944
rs234709
rs921943
rs6859667
rs6586282
rs3797535
rs1789953
rs11951068
rs567754
rs558133
rs705415
All
−0.04
0.00
0.04
0.08
MR effect size for
'Selenium' on 'Eukaryotic translation initiation factor 4E−binding protein 2'

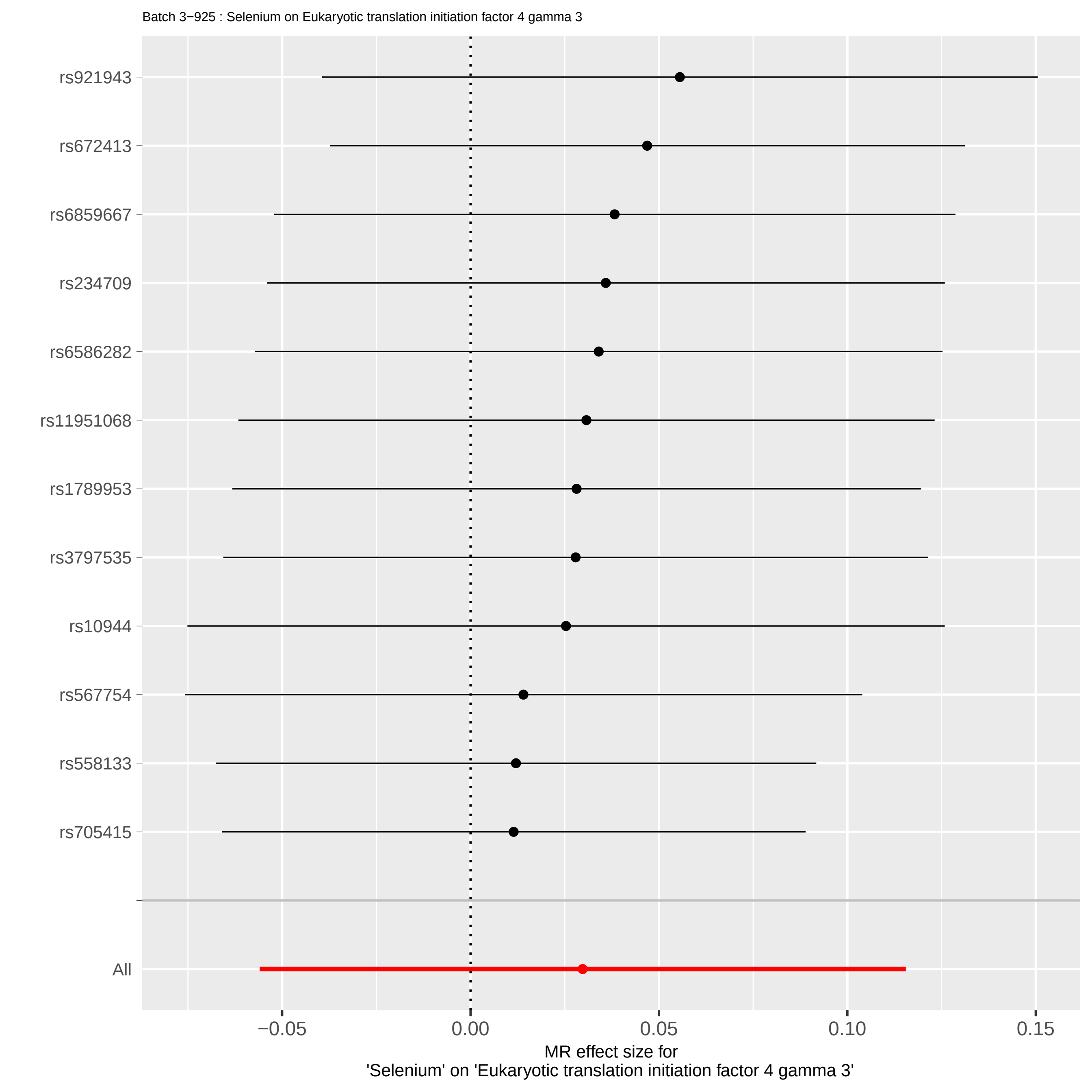

Batch 3−925 : Selenium on Eukaryotic translation initiation factor 4 gamma 3
rs921943
rs672413
rs6859667
rs234709
rs6586282
rs11951068
rs1789953
rs3797535
rs10944
rs567754
rs558133
rs705415
All
−0.05
0.00
0.05
0.10
0.15
MR effect size for
'Selenium' on 'Eukaryotic translation initiation factor 4 gamma 3'

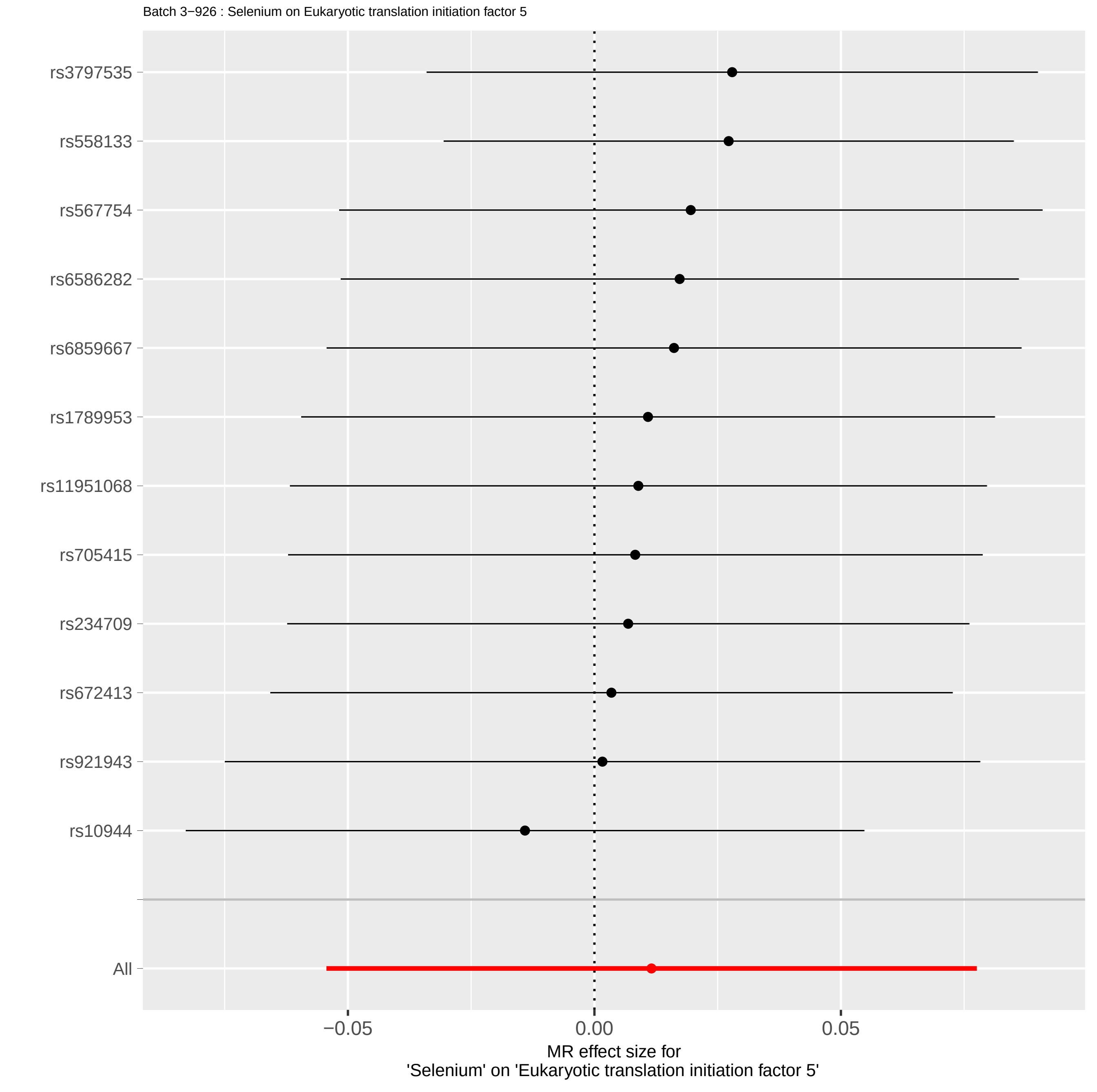

Batch 3−926 : Selenium on Eukaryotic translation initiation factor 5
rs3797535
rs558133
rs567754
rs6586282
rs6859667
rs1789953
rs11951068
rs705415
rs234709
rs672413
rs921943
rs10944
All
−0.05
0.00
0.05
MR effect size for
'Selenium' on 'Eukaryotic translation initiation factor 5'

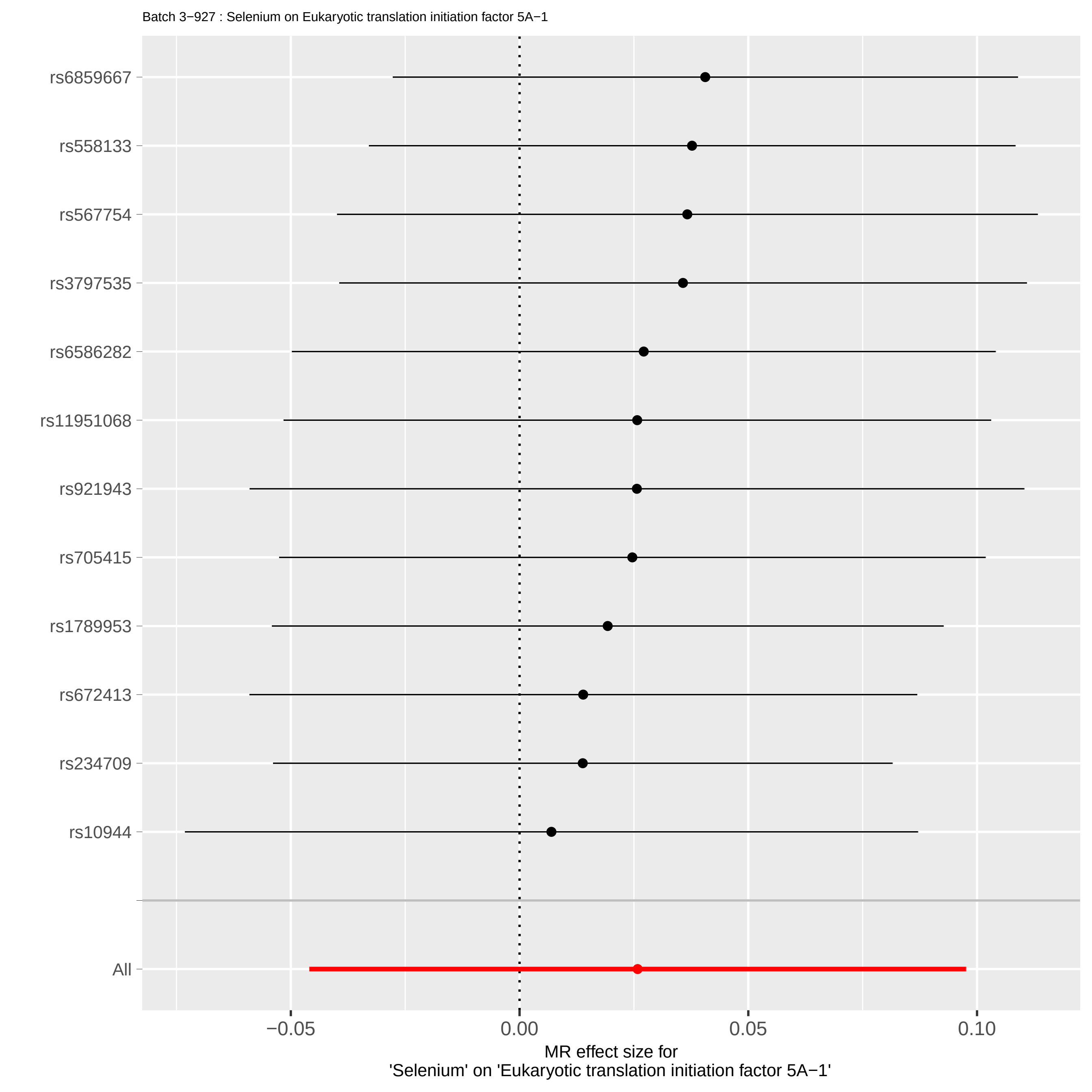

Batch 3−927 : Selenium on Eukaryotic translation initiation factor 5A−1
rs6859667
rs558133
rs567754
rs3797535
rs6586282
rs11951068
rs921943
rs705415
rs1789953
rs672413
rs234709
rs10944
All
−0.05
0.00
0.05
0.10
MR effect size for
'Selenium' on 'Eukaryotic translation initiation factor 5A−1'

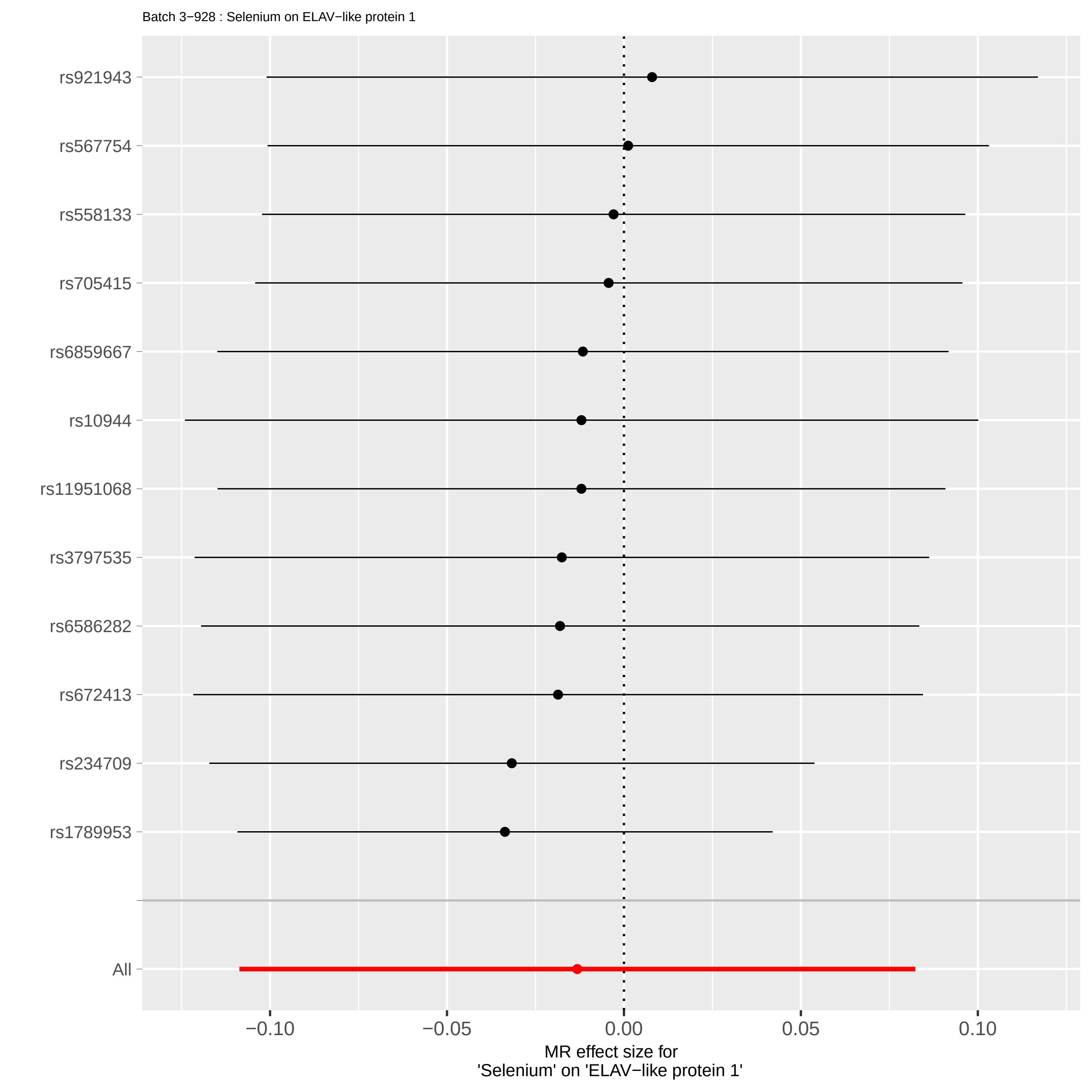

Batch 3−928 : Selenium on ELAV−like protein 1
rs921943
rs567754
rs558133
rs705415
rs6859667
rs10944
rs11951068
rs3797535
rs6586282
rs672413
rs234709
rs1789953
All
−0.10
−0.05
0.00
0.05
0.10
MR effect size for
'Selenium' on 'ELAV−like protein 1'

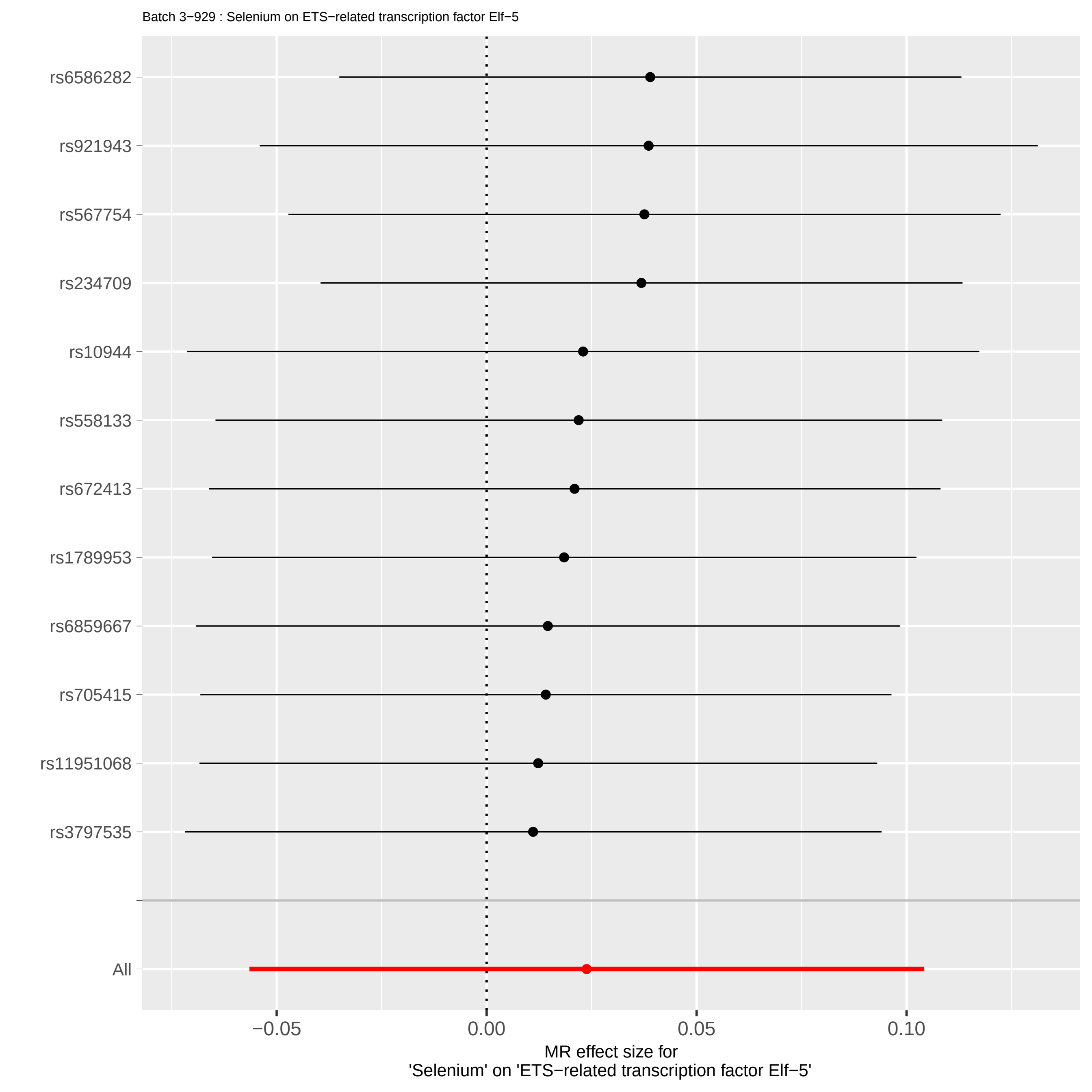

Batch 3−929 : Selenium on ETS−related transcription factor Elf−5
rs6586282
rs921943
rs567754
rs234709
rs10944
rs558133
rs672413
rs1789953
rs6859667
rs705415
rs11951068
rs3797535
All
−0.05
0.00
0.05
0.10
MR effect size for
'Selenium' on 'ETS−related transcription factor Elf−5'

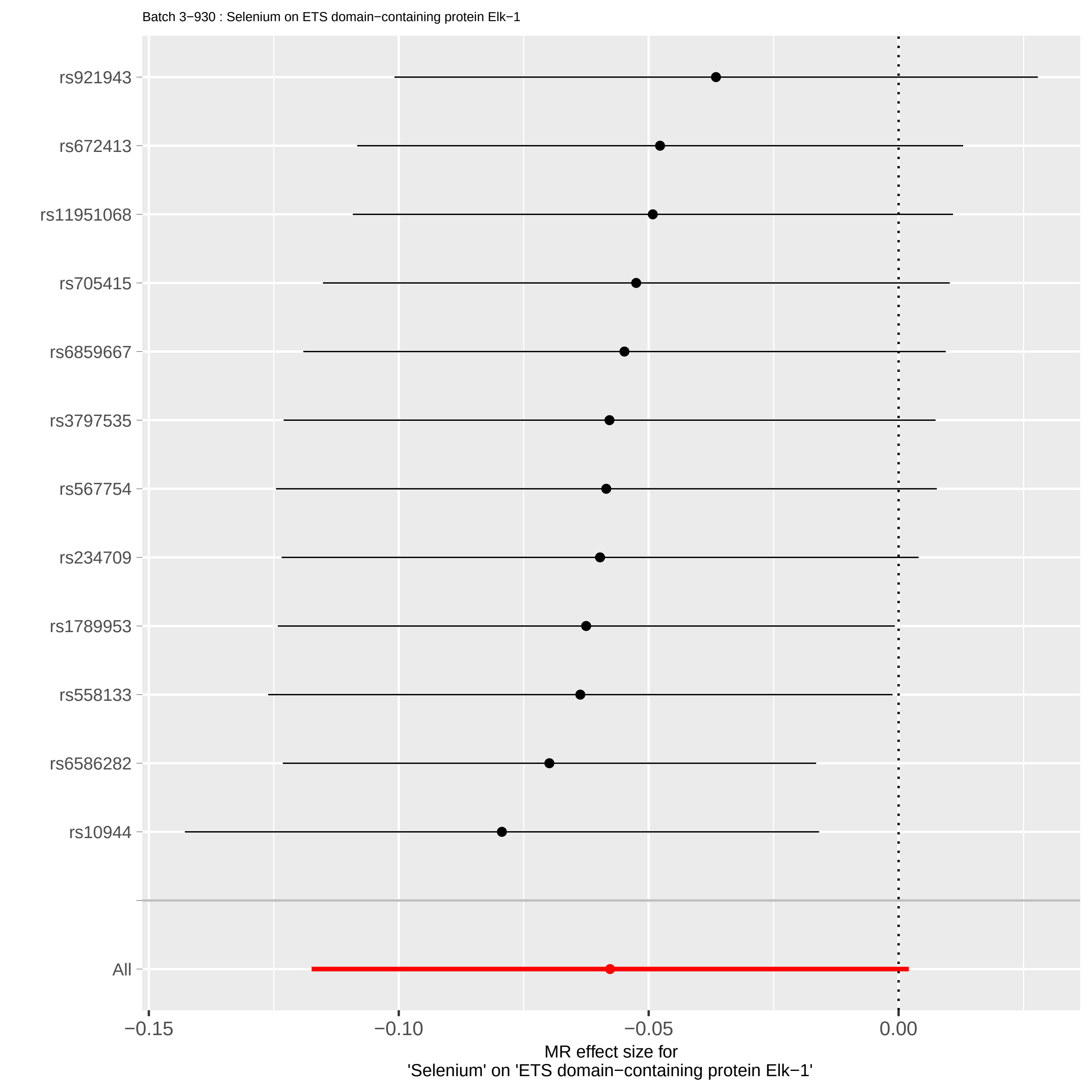

Batch 3−930 : Selenium on ETS domain−containing protein Elk−1
rs921943
rs672413
rs11951068
rs705415
rs6859667
rs3797535
rs567754
rs234709
rs1789953
rs558133
rs6586282
rs10944
All
−0.15
−0.10
−0.05
0.00
MR effect size for
'Selenium' on 'ETS domain−containing protein Elk−1'

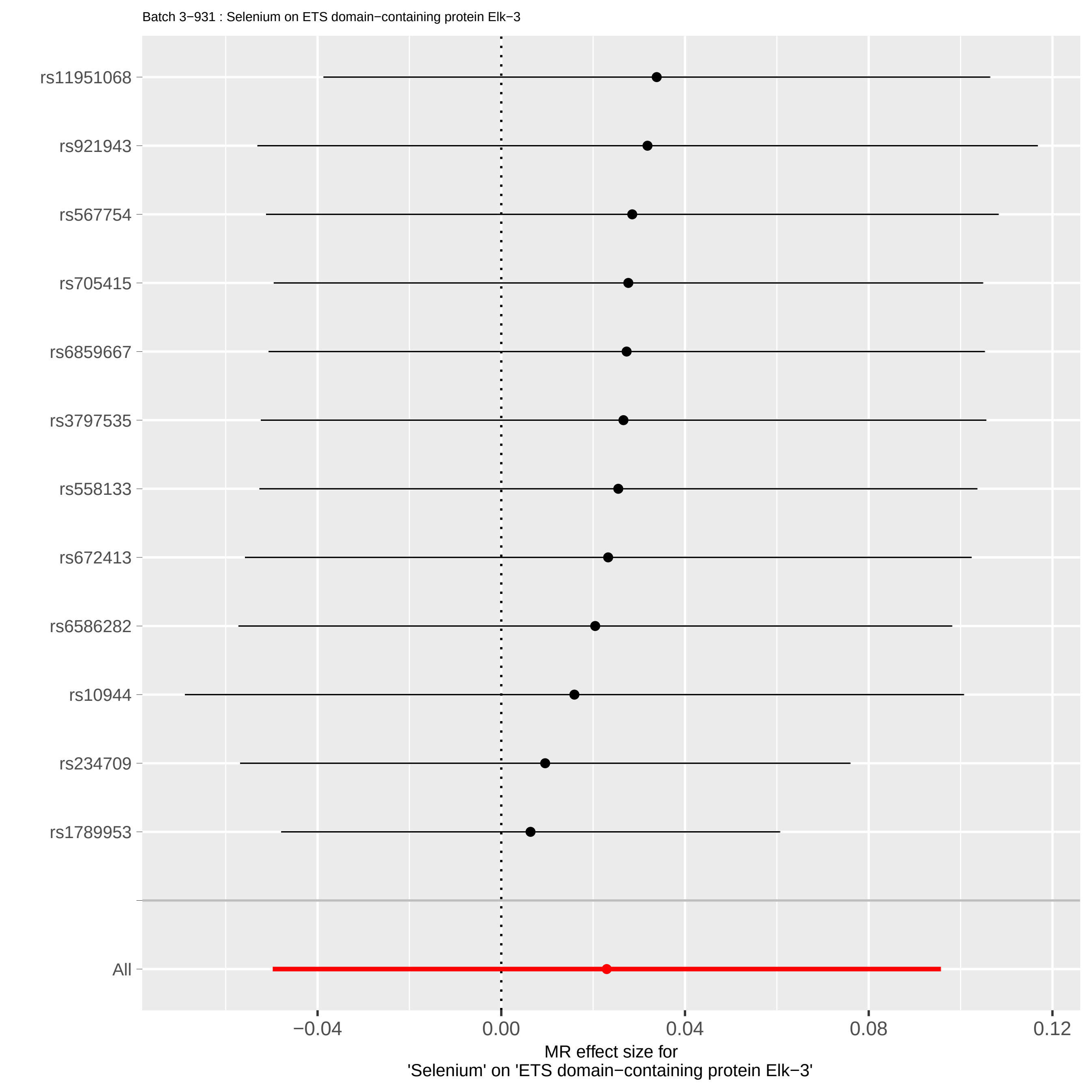

Batch 3−931 : Selenium on ETS domain−containing protein Elk−3
rs11951068
rs921943
rs567754
rs705415
rs6859667
rs3797535
rs558133
rs672413
rs6586282
rs10944
rs234709
rs1789953
All
−0.04
0.00
0.04
0.08
0.12
MR effect size for
'Selenium' on 'ETS domain−containing protein Elk−3'

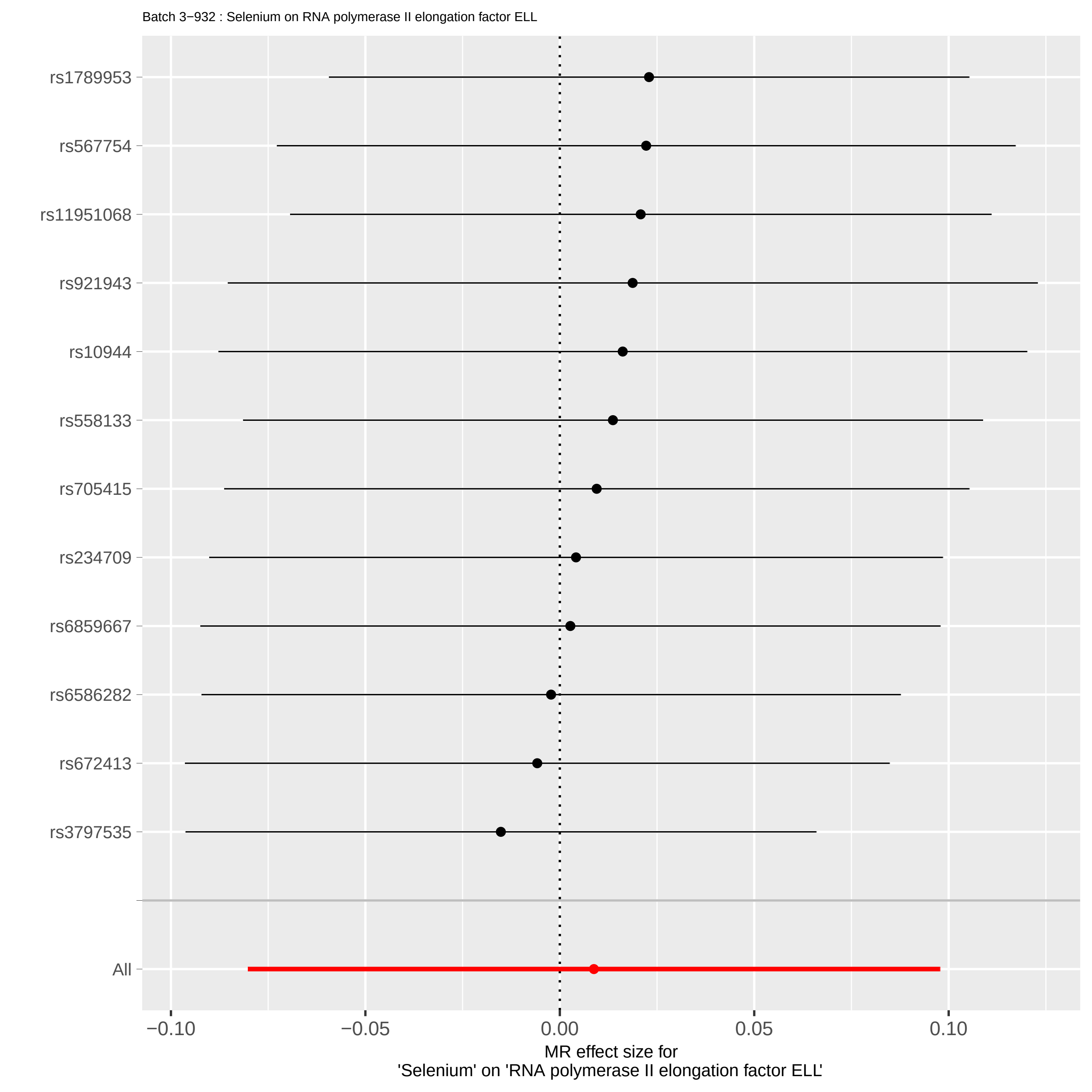
Batch 3−932 : Selenium on RNA polymerase II elongation factor ELL
rs1789953
rs567754
rs11951068
rs921943
rs10944
rs558133
rs705415
rs234709
rs6859667
rs6586282
rs672413
rs3797535
All
−0.10
−0.05
0.00
0.05
0.10
MR effect size for
'Selenium' on 'RNA polymerase II elongation factor ELL'

Batch 3−933 : Selenium on RNA polymerase II elongation factor ELL2

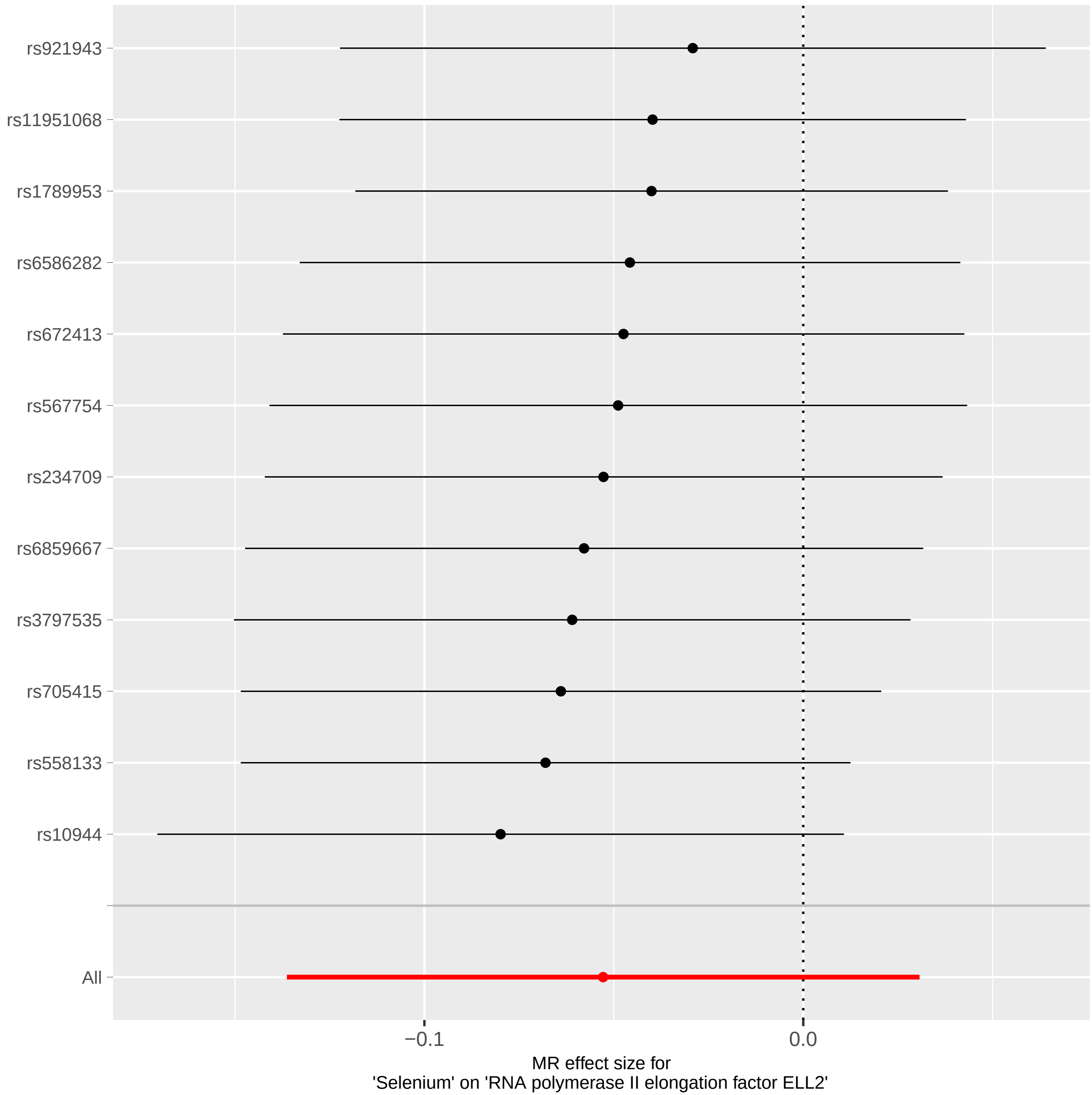

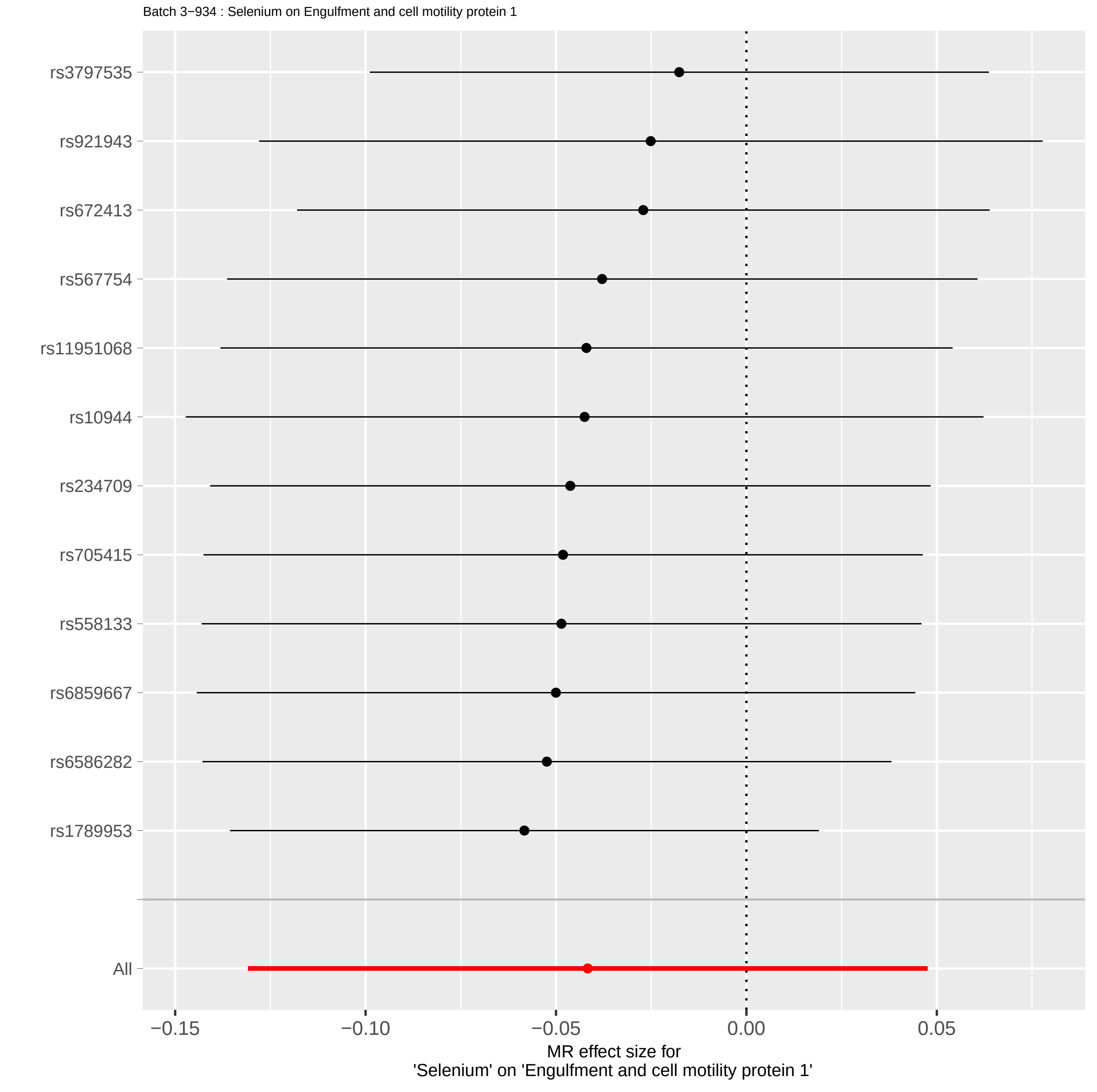
Batch 3−934 : Selenium on Engulfment and cell motility protein 1
rs3797535
rs921943
rs672413
rs567754
rs11951068
rs10944
rs234709
rs705415
rs558133
rs6859667
rs6586282
rs1789953
All
−0.15
−0.10
−0.05
0.00
0.05
MR effect size for
'Selenium' on 'Engulfment and cell motility protein 1'

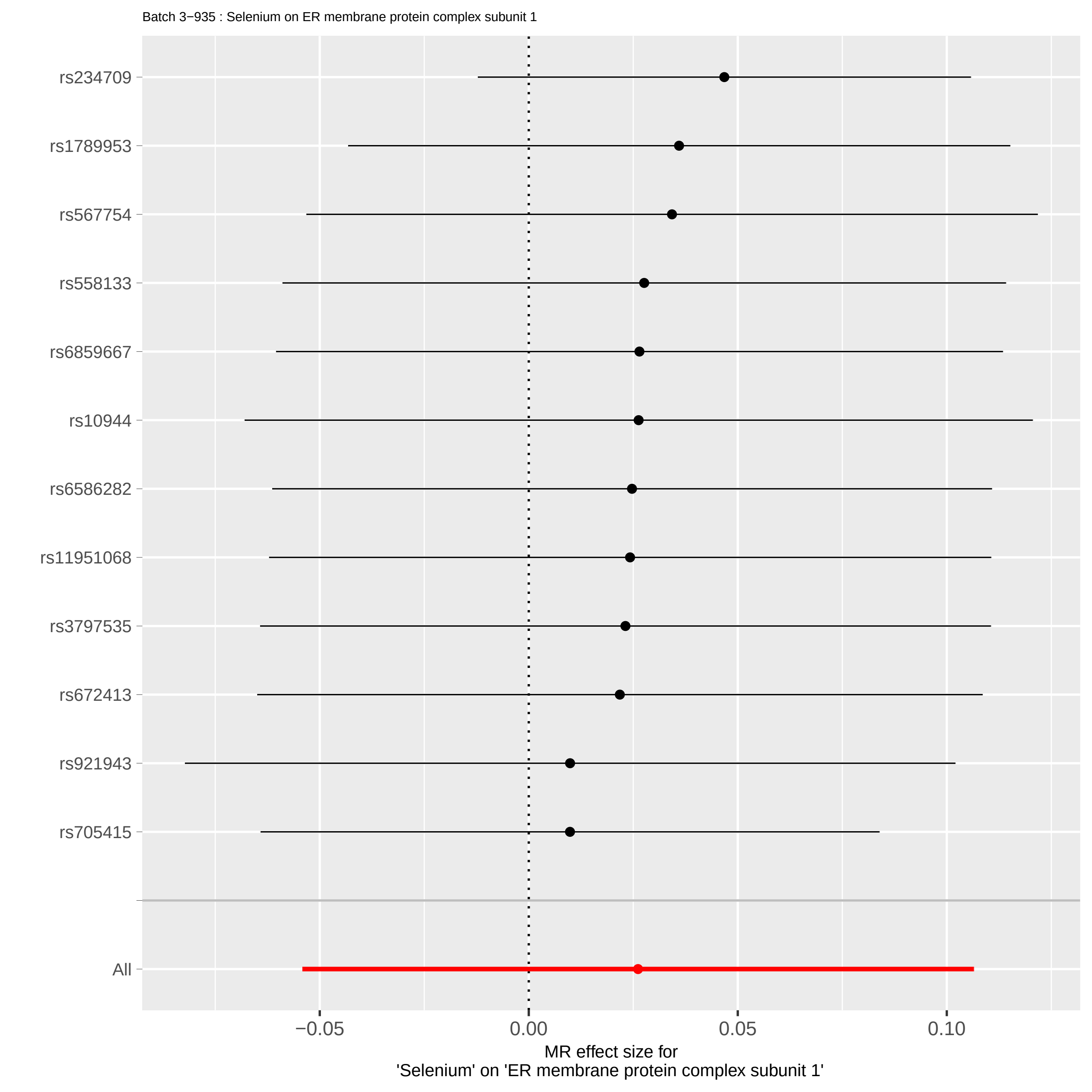

Batch 3−935 : Selenium on ER membrane protein complex subunit 1
rs234709
rs1789953
rs567754
rs558133
rs6859667
rs10944
rs6586282
rs11951068
rs3797535
rs672413
rs921943
rs705415
All
−0.05
0.00
0.05
0.10
MR effect size for
'Selenium' on 'ER membrane protein complex subunit 1'

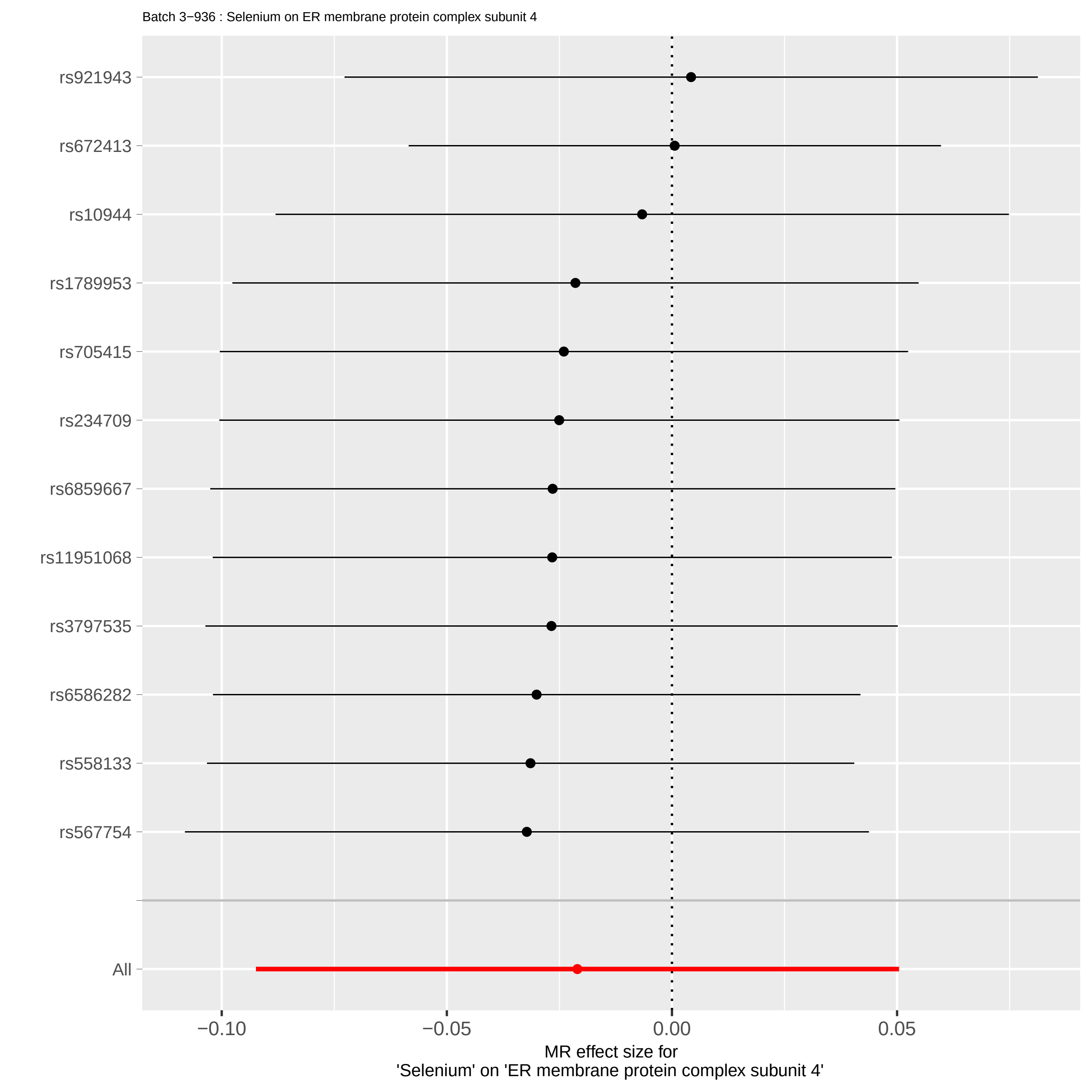

Batch 3−936 : Selenium on ER membrane protein complex subunit 4
rs921943
rs672413
rs10944
rs1789953
rs705415
rs234709
rs6859667
rs11951068
rs3797535
rs6586282
rs558133
rs567754
All
−0.10
−0.05
0.00
0.05
MR effect size for
'Selenium' on 'ER membrane protein complex subunit 4'

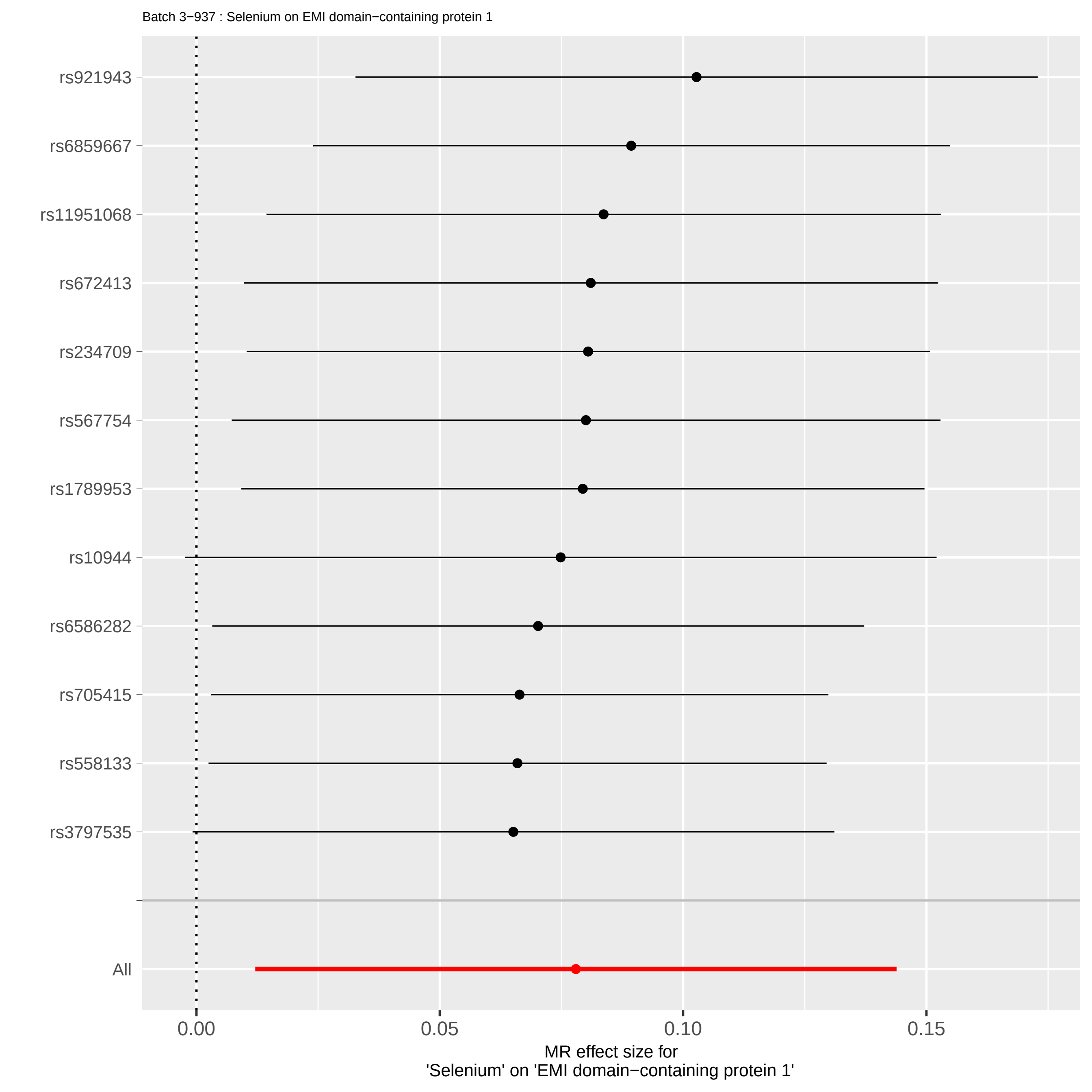
Batch 3−937 : Selenium on EMI domain−containing protein 1
rs921943
rs6859667
rs11951068
rs672413
rs234709
rs567754
rs1789953
rs10944
rs6586282
rs705415
rs558133
rs3797535
All
0.00
0.05
0.10
0.15
MR effect size for
'Selenium' on 'EMI domain−containing protein 1'

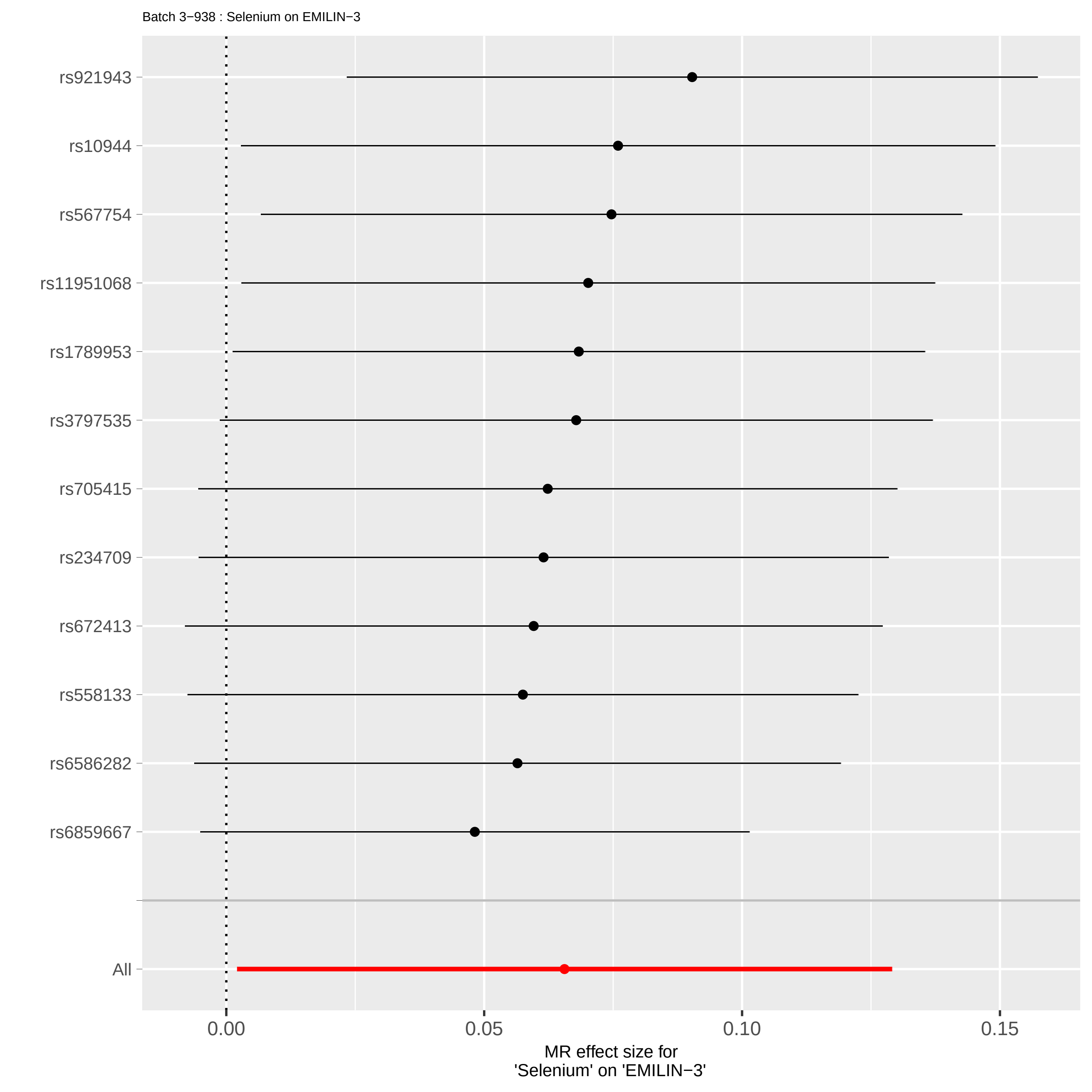

Batch 3−938 : Selenium on EMILIN−3
rs921943
rs10944
rs567754
rs11951068
rs1789953
rs3797535
rs705415
rs234709
rs672413
rs558133
rs6586282
rs6859667
All
0.00
0.05
0.10
0.15
MR effect size for
'Selenium' on 'EMILIN−3'

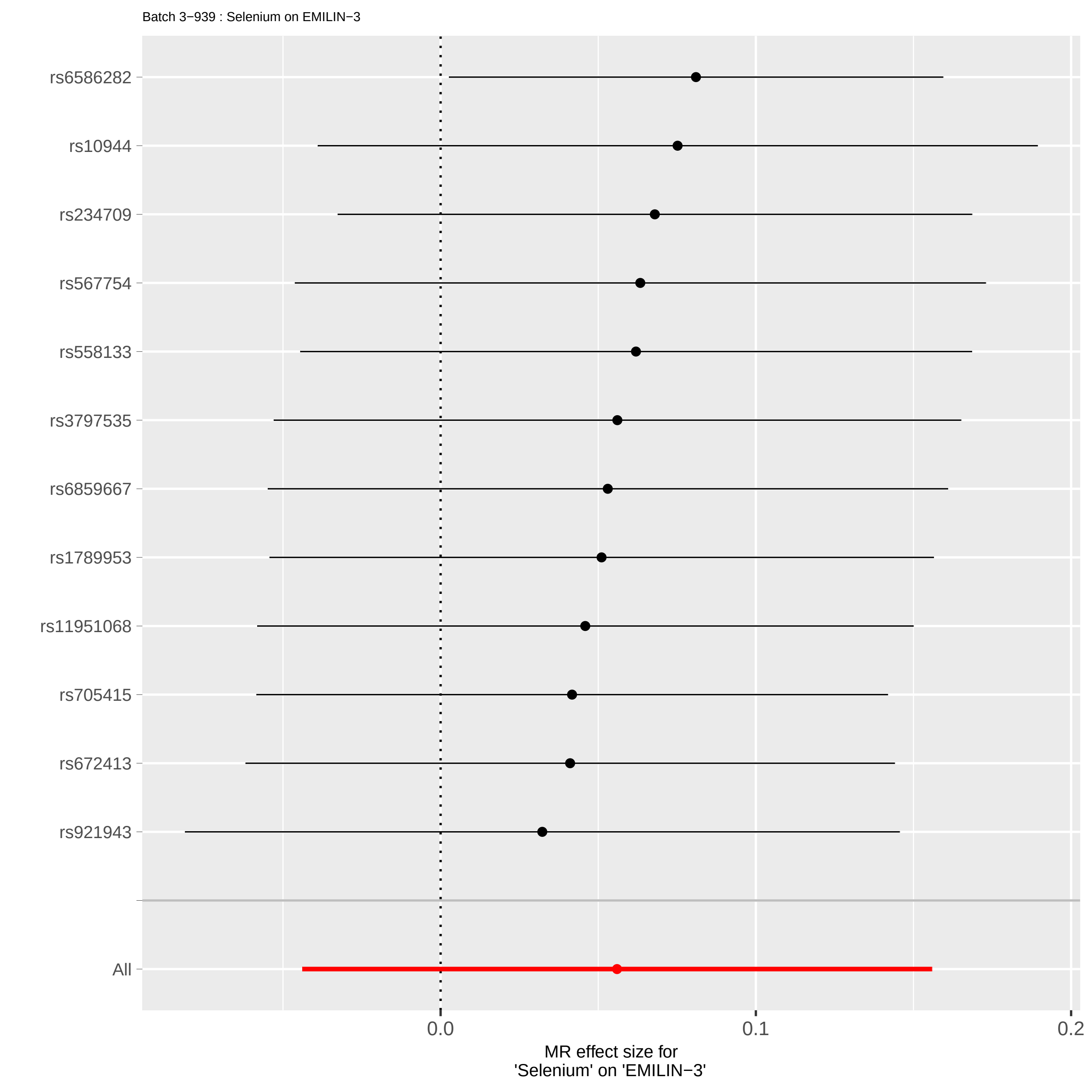

Batch 3−939 : Selenium on EMILIN−3
rs6586282
rs10944
rs234709
rs567754
rs558133
rs3797535
rs6859667
rs1789953
rs11951068
rs705415
rs672413
rs921943
All
0.0
0.1
0.2
MR effect size for
'Selenium' on 'EMILIN−3'

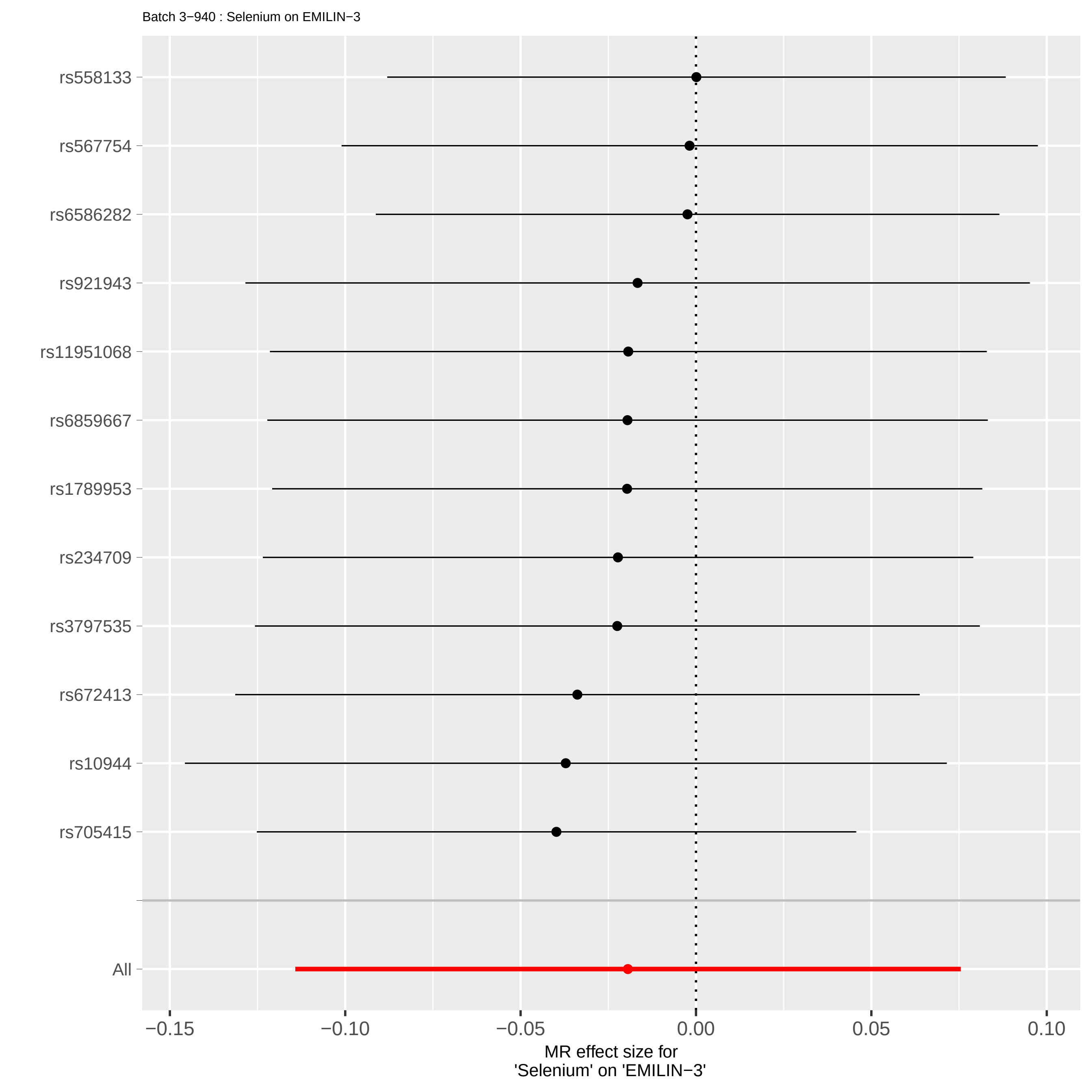
Batch 3−940 : Selenium on EMILIN−3
rs558133
rs567754
rs6586282
rs921943
rs11951068
rs6859667
rs1789953
rs234709
rs3797535
rs672413
rs10944
rs705415
All
−0.15
−0.10
−0.05
0.00
0.05
0.10
MR effect size for
'Selenium' on 'EMILIN−3'

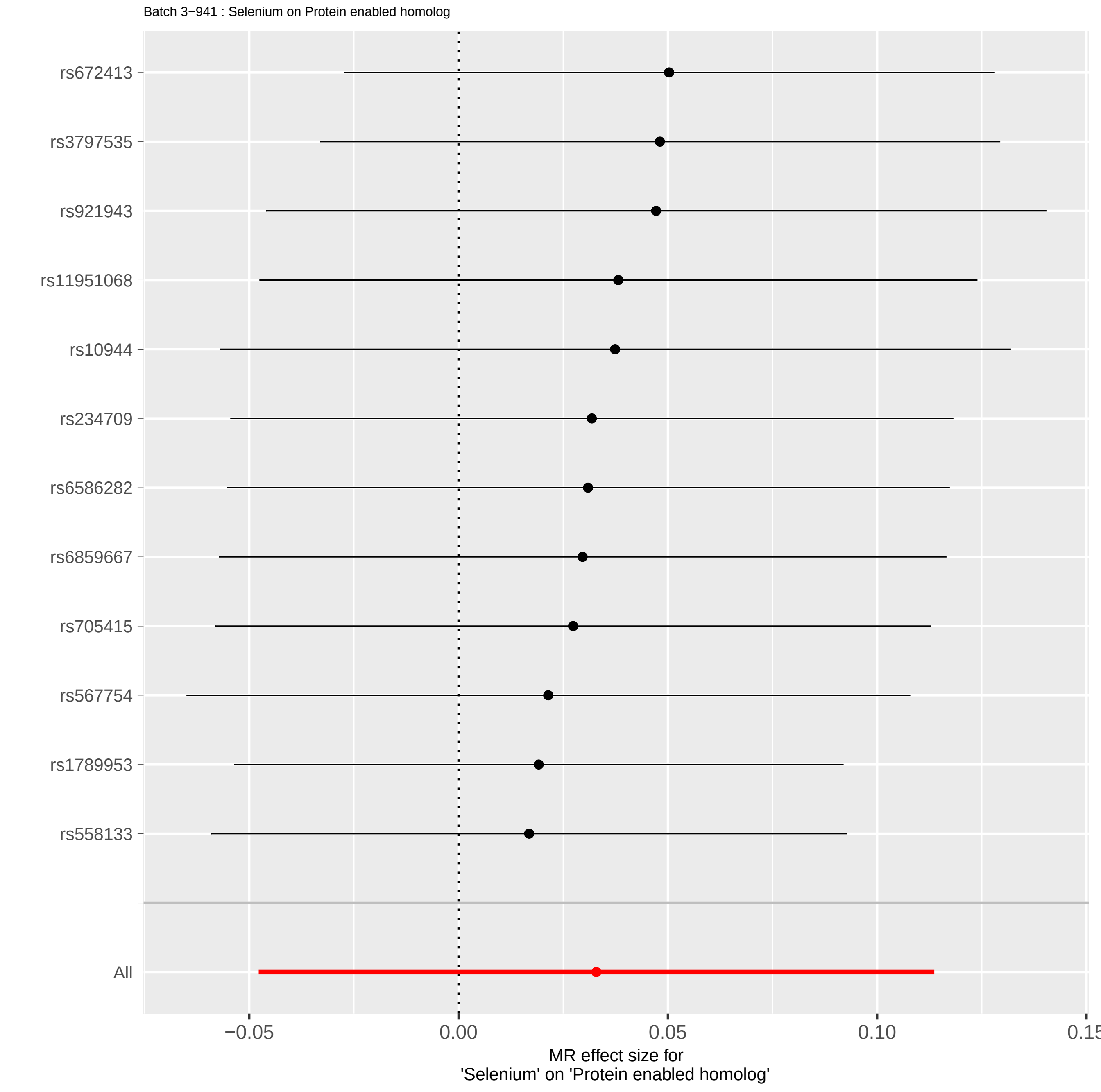

Batch 3−941 : Selenium on Protein enabled homolog
rs672413
rs3797535
rs921943
rs11951068
rs10944
rs234709
rs6586282
rs6859667
rs705415
rs567754
rs1789953
rs558133
All
−0.05
0.00
0.05
0.10
0.15
MR effect size for
'Selenium' on 'Protein enabled homolog'

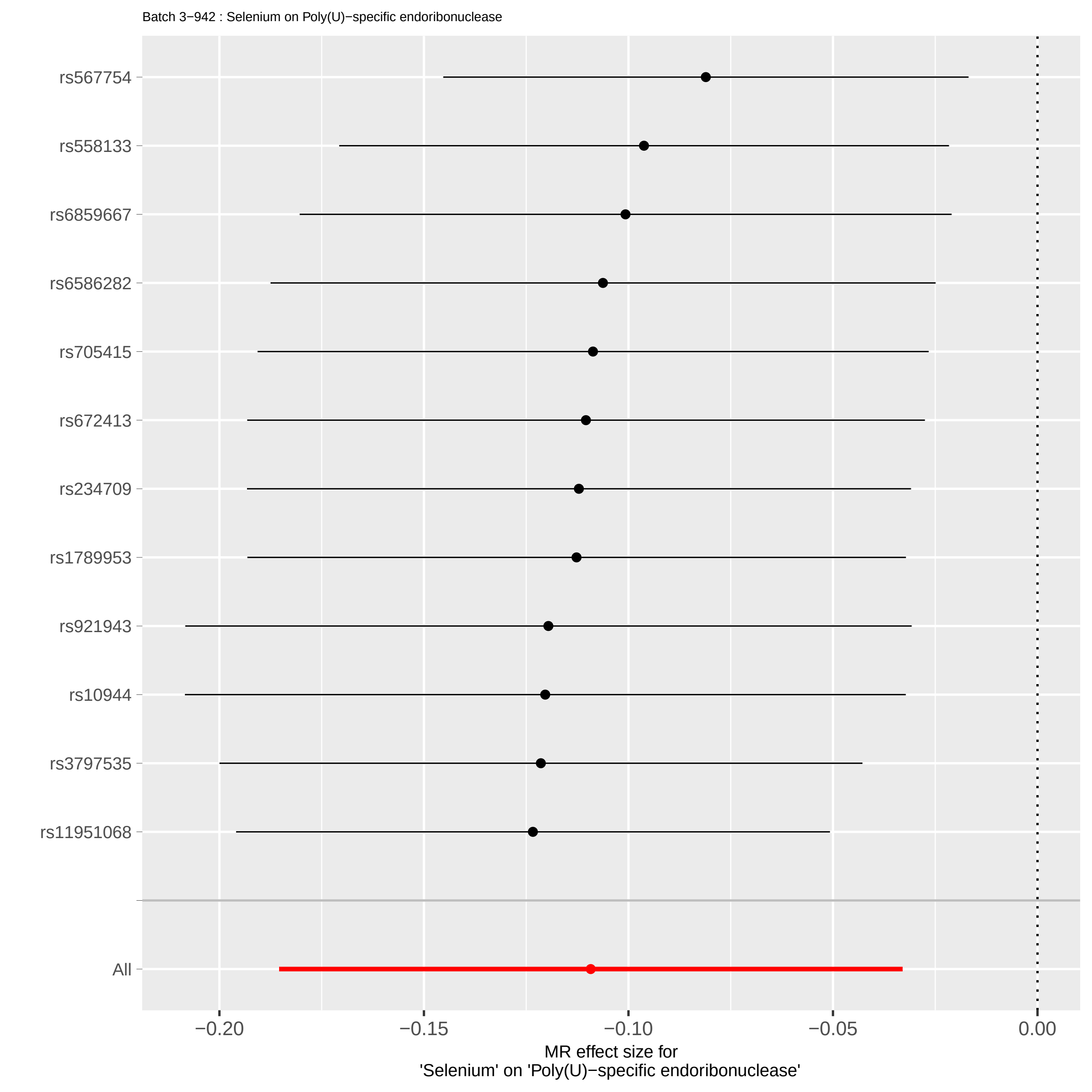

Batch 3−942 : Selenium on Poly(U)−specific endoribonuclease
rs567754
rs558133
rs6859667
rs6586282
rs705415
rs672413
rs234709
rs1789953
rs921943
rs10944
rs3797535
rs11951068
All
−0.20
−0.15
−0.10
−0.05
0.00
MR effect size for
'Selenium' on 'Poly(U)−specific endoribonuclease'

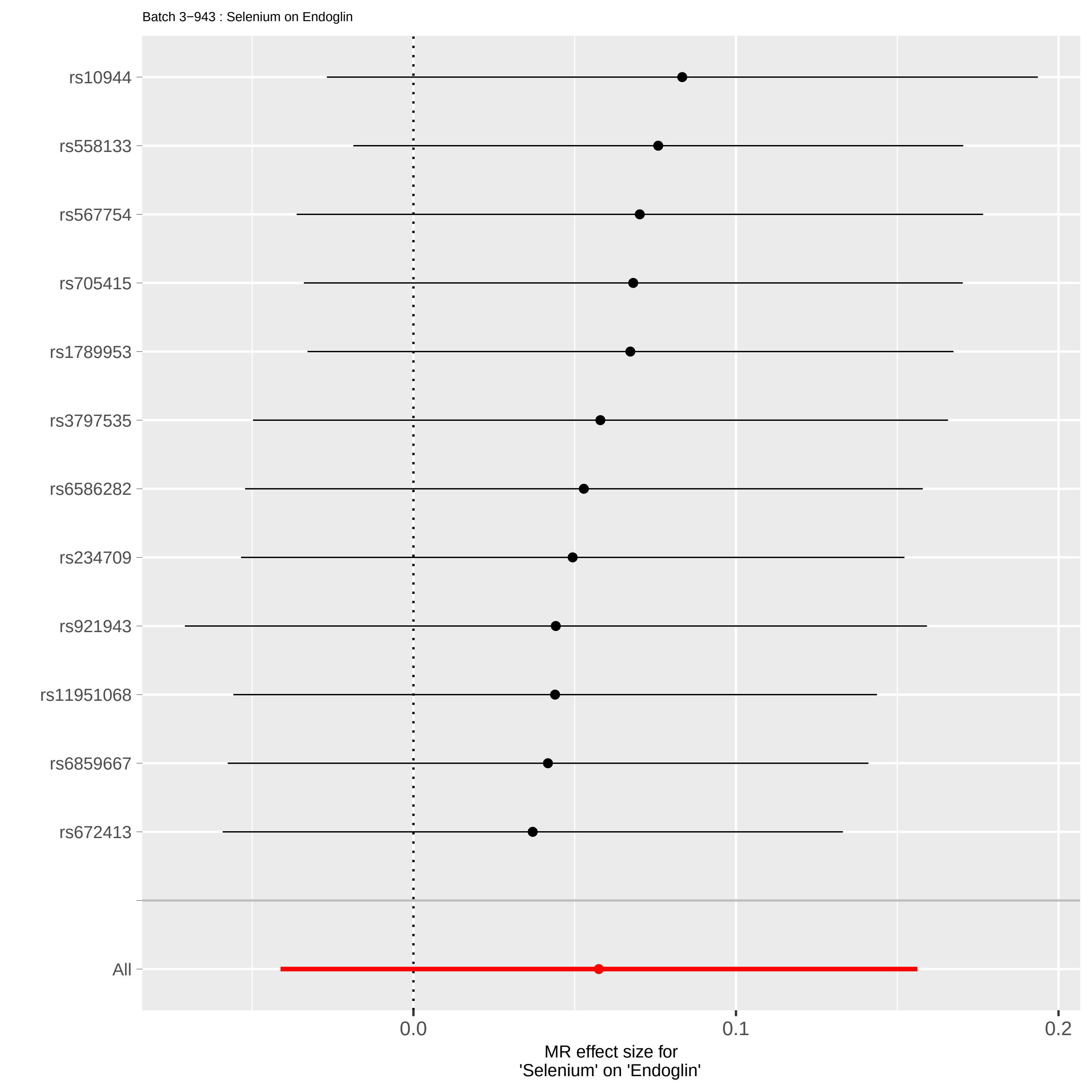

Batch 3−943 : Selenium on Endoglin
rs10944
rs558133
rs567754
rs705415
rs1789953
rs3797535
rs6586282
rs234709
rs921943
rs11951068
rs6859667
rs672413
All
0.0
0.1
0.2
MR effect size for
'Selenium' on 'Endoglin'

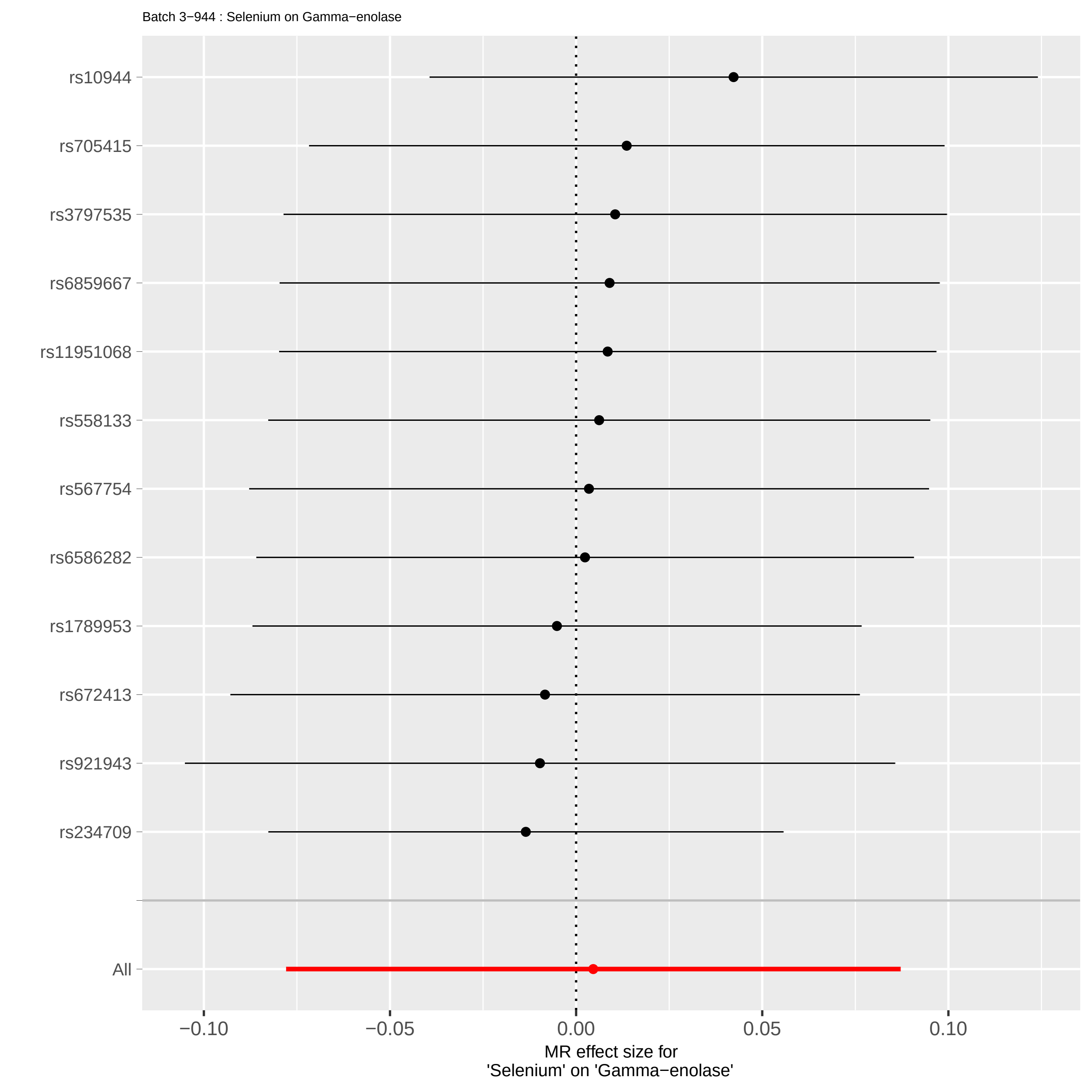

Batch 3−944 : Selenium on Gamma−enolase
rs10944
rs705415
rs3797535
rs6859667
rs11951068
rs558133
rs567754
rs6586282
rs1789953
rs672413
rs921943
rs234709
All
−0.10
−0.05
0.00
0.05
0.10
MR effect size for
'Selenium' on 'Gamma−enolase'

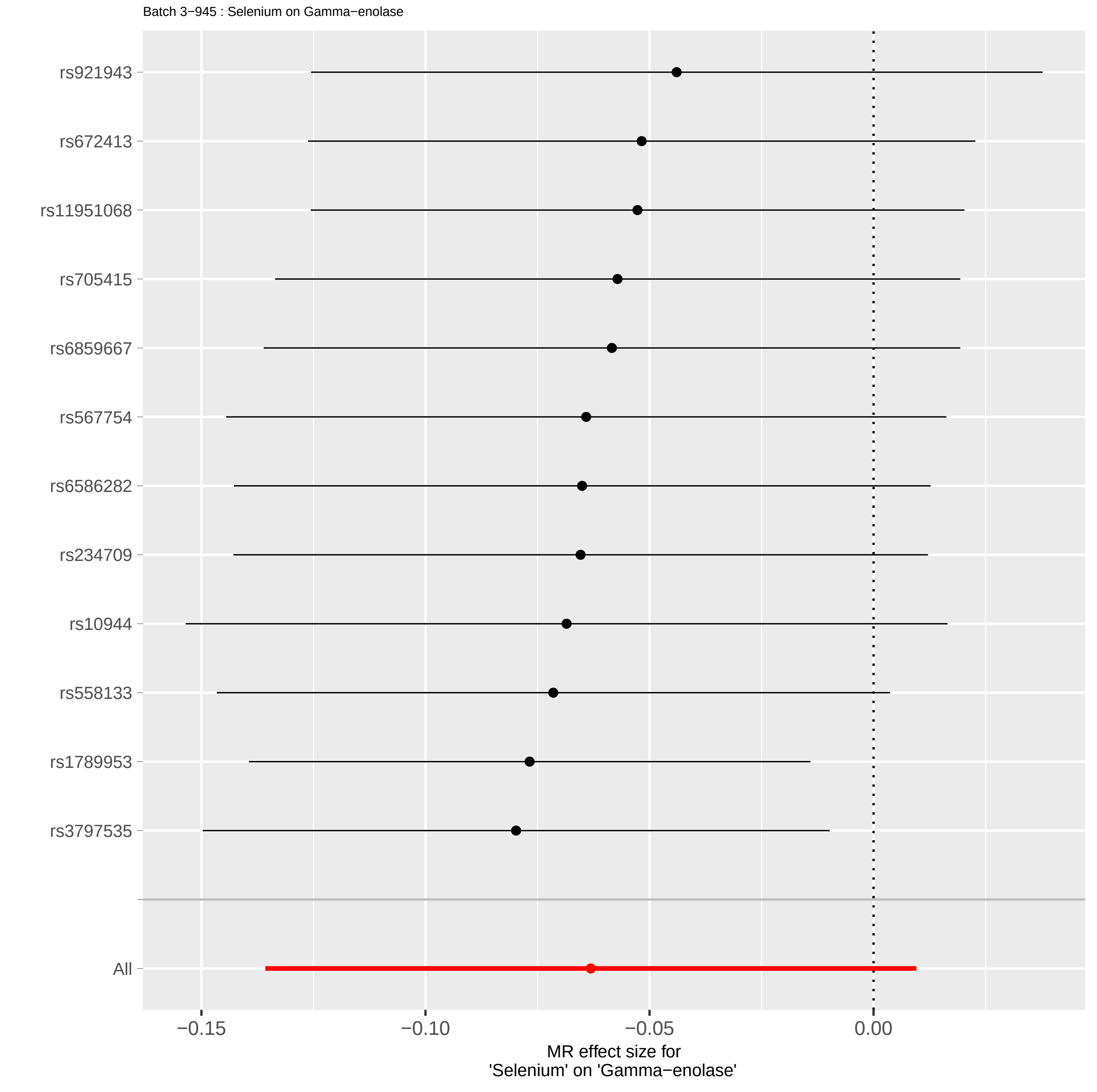

Batch 3−945 : Selenium on Gamma−enolase
rs921943
rs672413
rs11951068
rs705415
rs6859667
rs567754
rs6586282
rs234709
rs10944
rs558133
rs1789953
rs3797535
All
−0.15
−0.10
−0.05
0.00
MR effect size for
'Selenium' on 'Gamma−enolase'

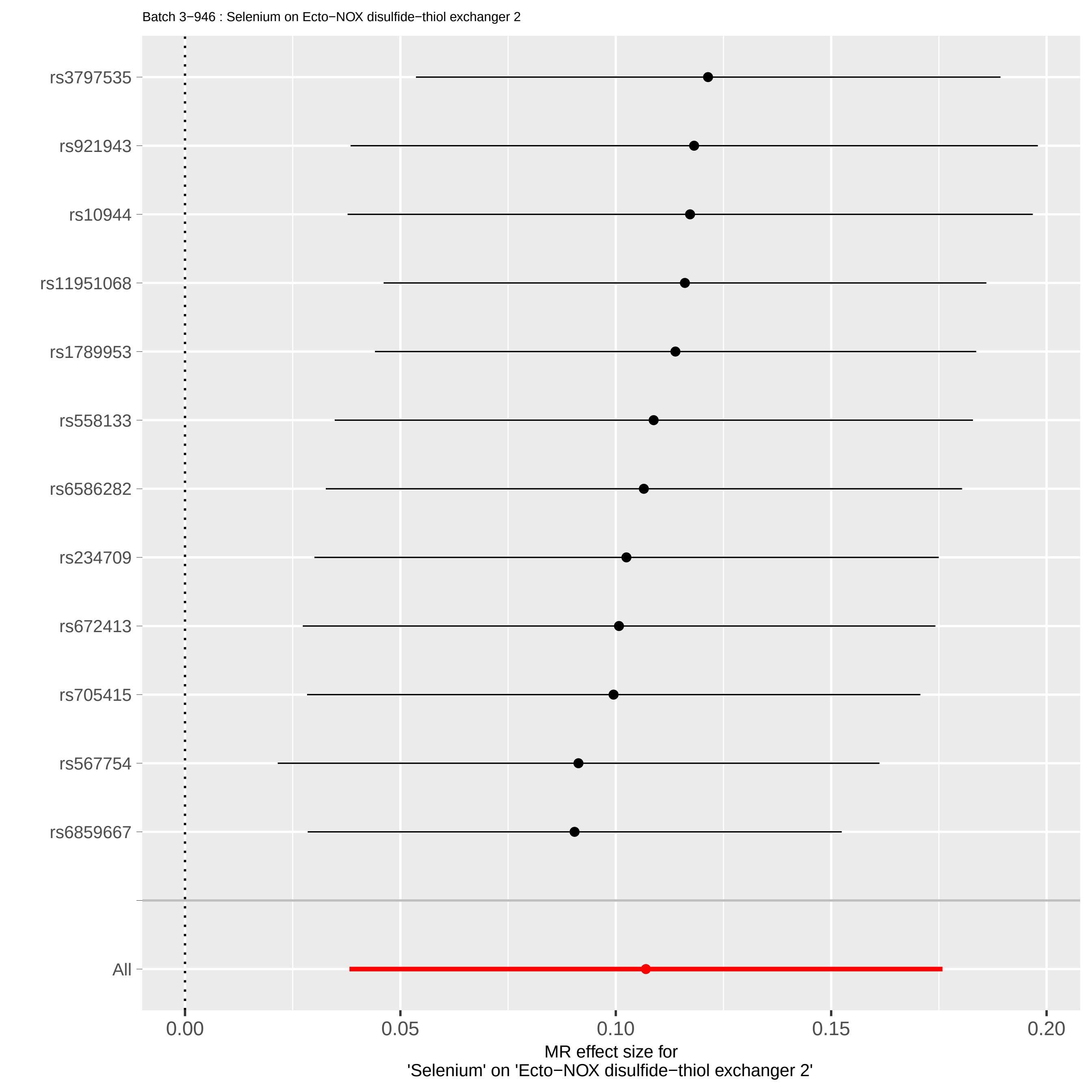

Batch 3−946 : Selenium on Ecto−NOX disulfide−thiol exchanger 2
rs3797535
rs921943
rs10944
rs11951068
rs1789953
rs558133
rs6586282
rs234709
rs672413
rs705415
rs567754
rs6859667
All
0.00
0.05
0.10
0.15
0.20
MR effect size for
'Selenium' on 'Ecto−NOX disulfide−thiol exchanger 2'

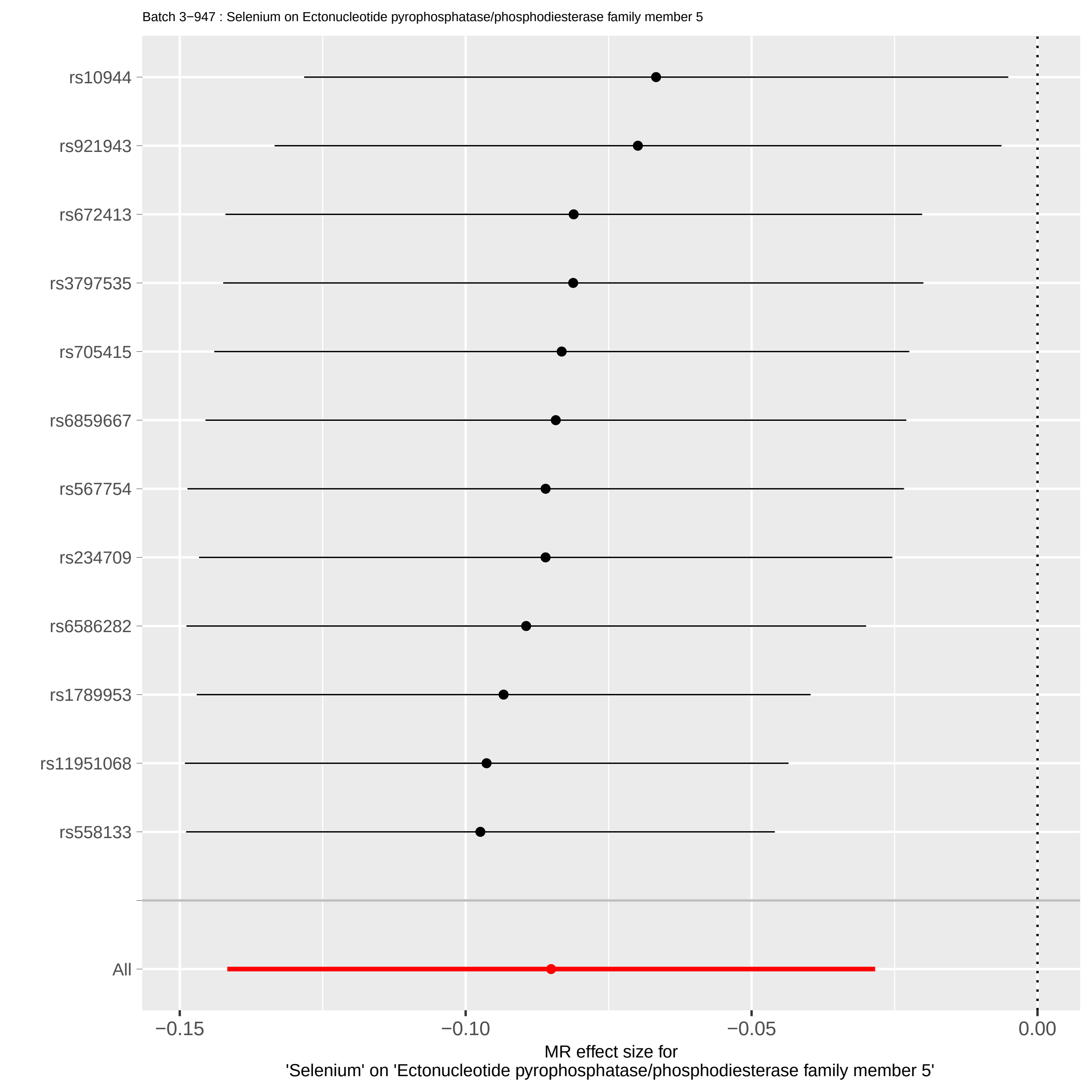

Batch 3−947 : Selenium on Ectonucleotide pyrophosphatase/phosphodiesterase family member 5
rs10944
rs921943
rs672413
rs3797535
rs705415
rs6859667
rs567754
rs234709
rs6586282
rs1789953
rs11951068
rs558133
All
−0.15
−0.10
−0.05
0.00
MR effect size for
'Selenium' on 'Ectonucleotide pyrophosphatase/phosphodiesterase family member 5'

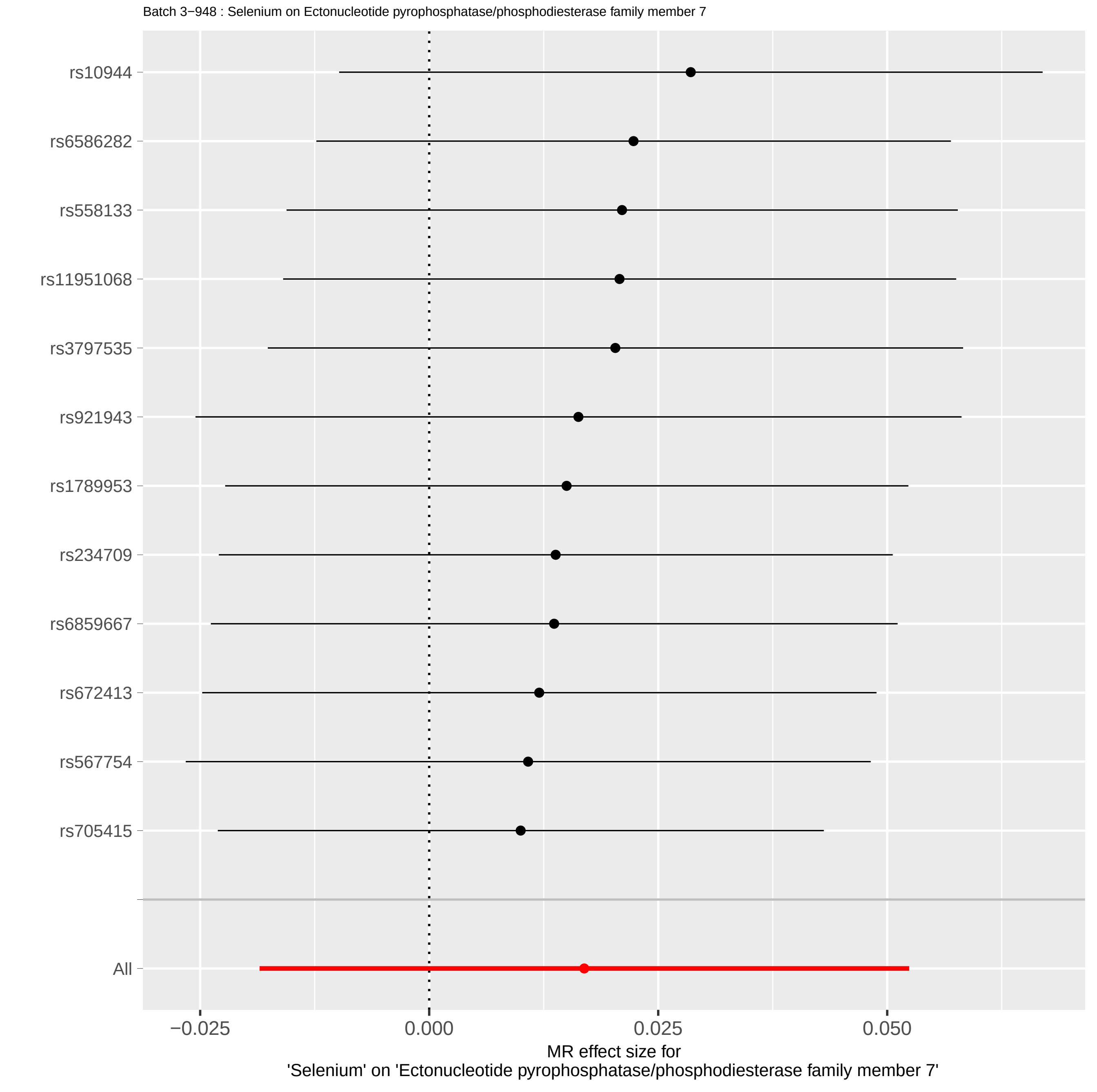

Batch 3−948 : Selenium on Ectonucleotide pyrophosphatase/phosphodiesterase family member 7
rs10944
rs6586282
rs558133
rs11951068
rs3797535
rs921943
rs1789953
rs234709
rs6859667
rs672413
rs567754
rs705415
All
−0.025
0.000
0.025
0.050
MR effect size for
'Selenium' on 'Ectonucleotide pyrophosphatase/phosphodiesterase family member 7'

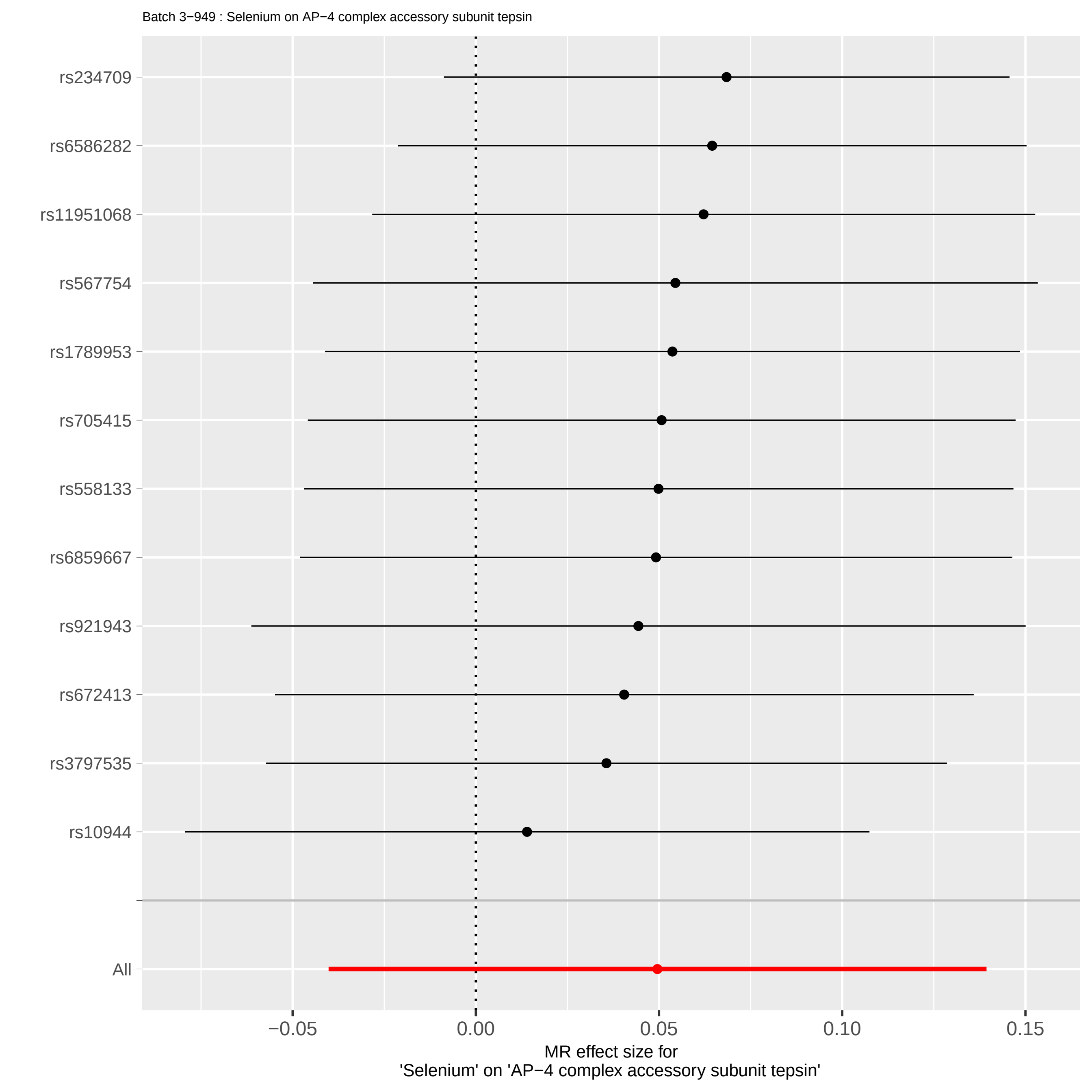

Batch 3−949 : Selenium on AP−4 complex accessory subunit tepsin
rs234709
rs6586282
rs11951068
rs567754
rs1789953
rs705415
rs558133
rs6859667
rs921943
rs672413
rs3797535
rs10944
All
−0.05
0.00
0.05
0.10
0.15
MR effect size for
'Selenium' on 'AP−4 complex accessory subunit tepsin'

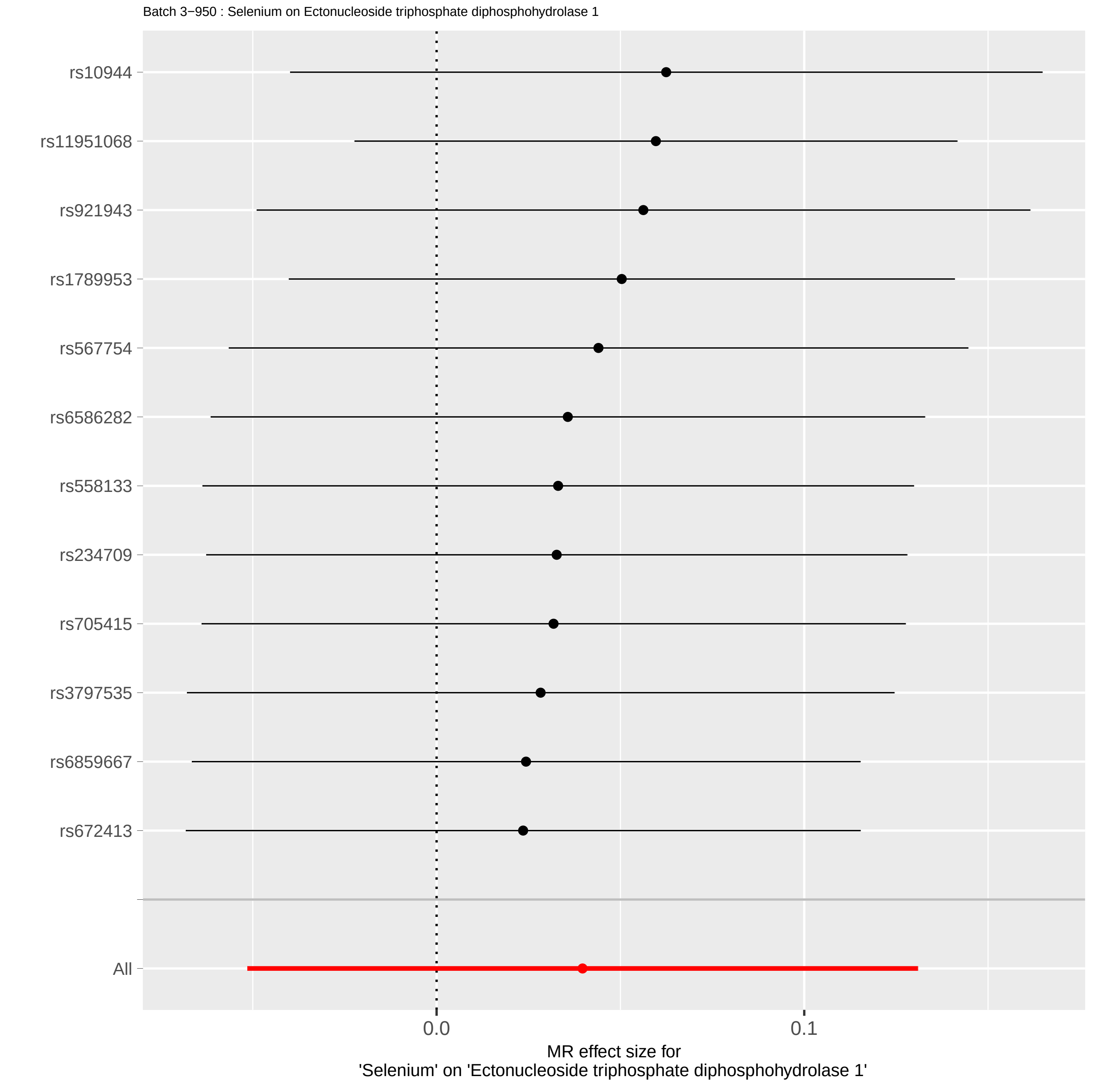
Batch 3−950 : Selenium on Ectonucleoside triphosphate diphosphohydrolase 1
rs10944
rs11951068
rs921943
rs1789953
rs567754
rs6586282
rs558133
rs234709
rs705415
rs3797535
rs6859667
rs672413
All
0.0
0.1
MR effect size for
'Selenium' on 'Ectonucleoside triphosphate diphosphohydrolase 1'

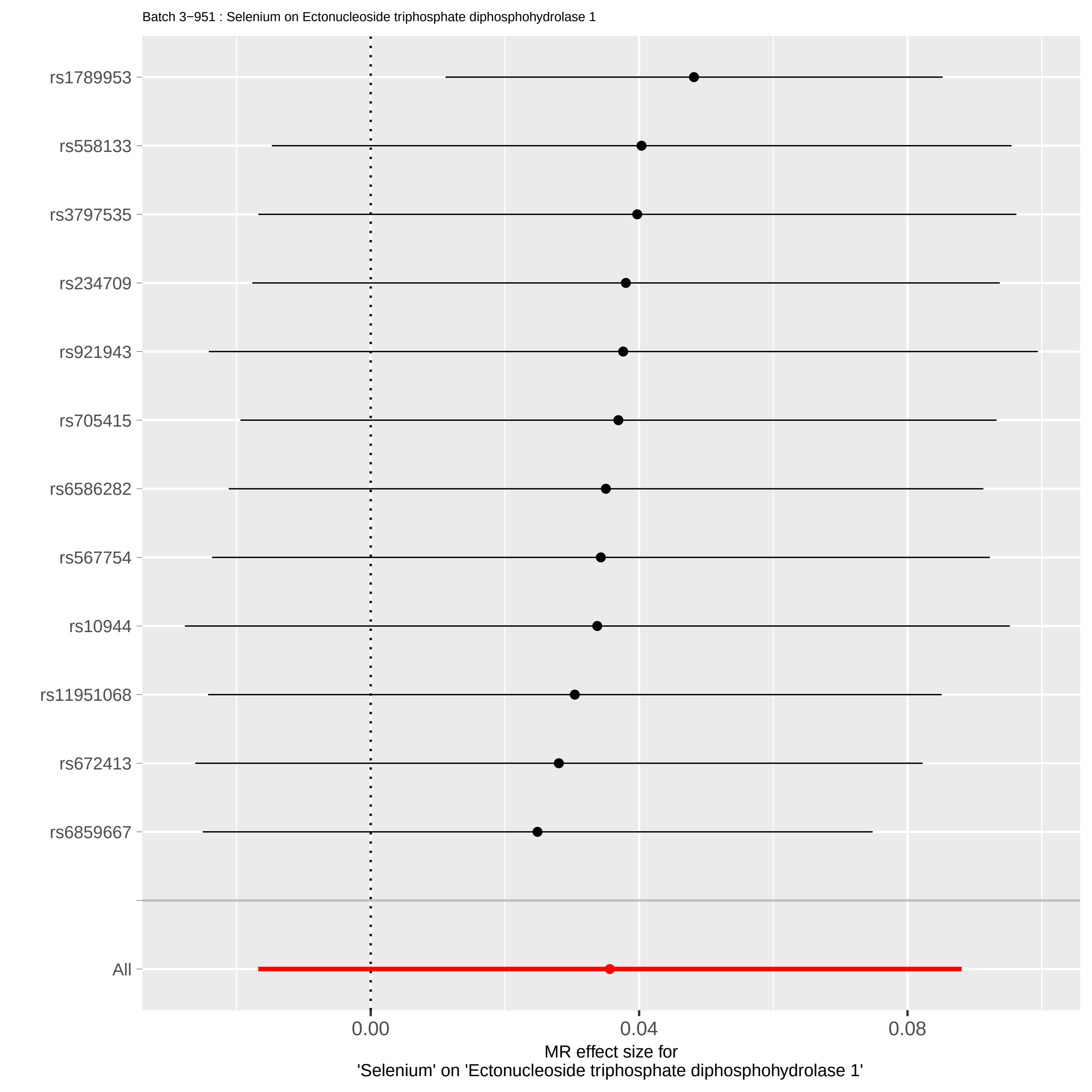
Batch 3−951 : Selenium on Ectonucleoside triphosphate diphosphohydrolase 1
rs1789953
rs558133
rs3797535
rs234709
rs921943
rs705415
rs6586282
rs567754
rs10944
rs11951068
rs672413
rs6859667
All
0.00
0.04
0.08
MR effect size for
'Selenium' on 'Ectonucleoside triphosphate diphosphohydrolase 1'

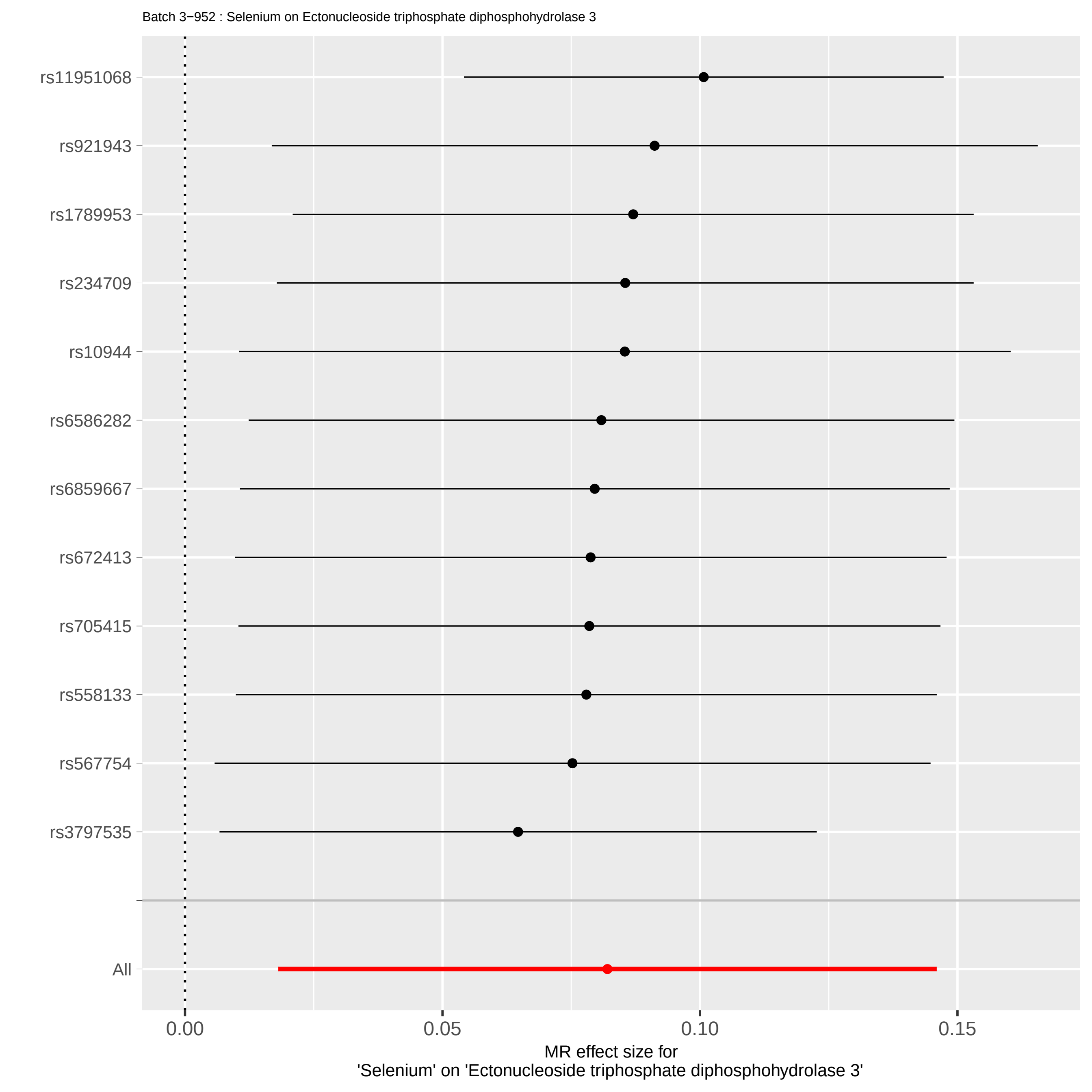

Batch 3−952 : Selenium on Ectonucleoside triphosphate diphosphohydrolase 3
rs11951068
rs921943
rs1789953
rs234709
rs10944
rs6586282
rs6859667
rs672413
rs705415
rs558133
rs567754
rs3797535
All
0.00
0.05
0.10
0.15
MR effect size for
'Selenium' on 'Ectonucleoside triphosphate diphosphohydrolase 3'

Batch 3−953 : Selenium on Ectonucleoside triphosphate diphosphohydrolase 5

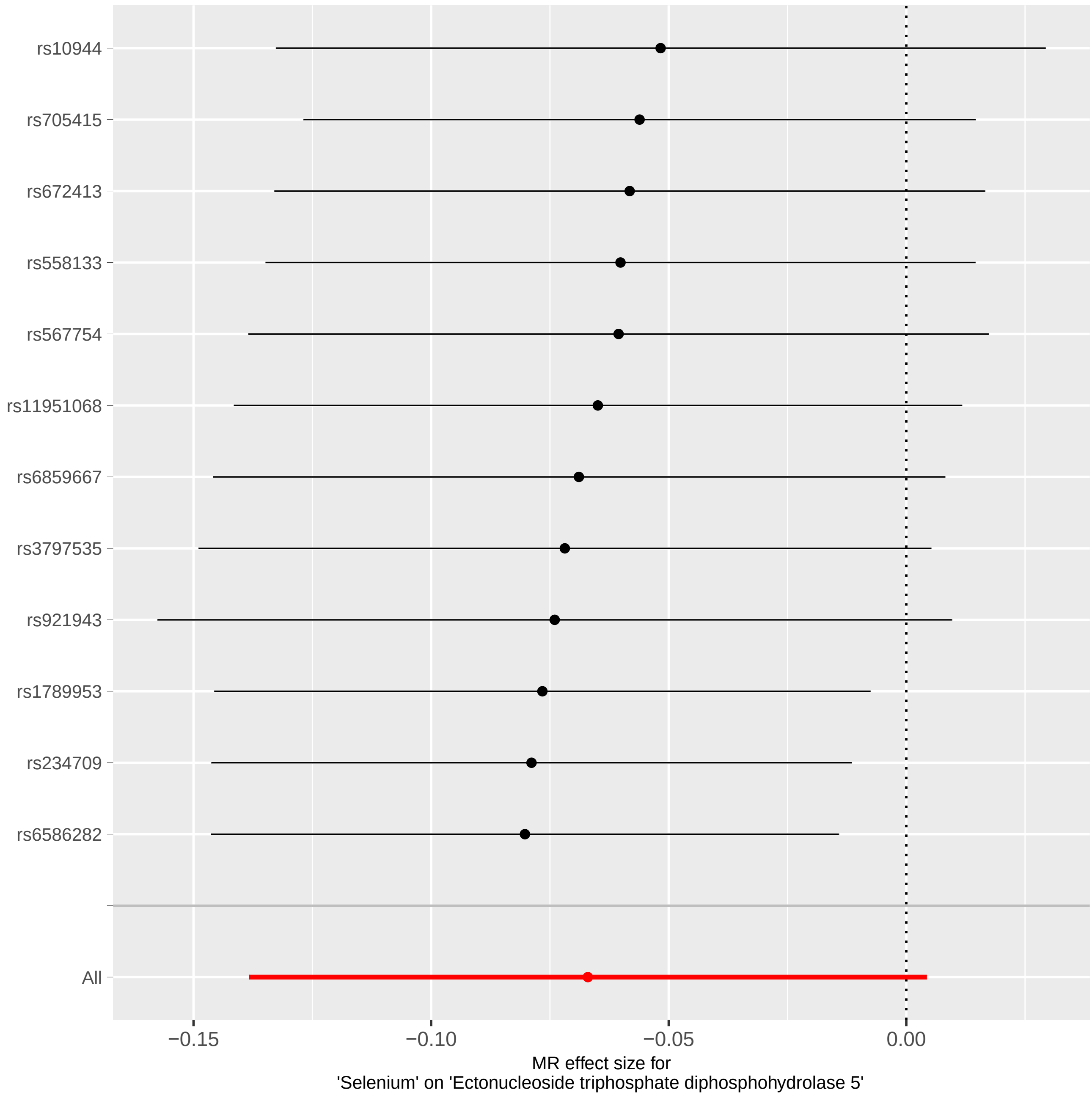

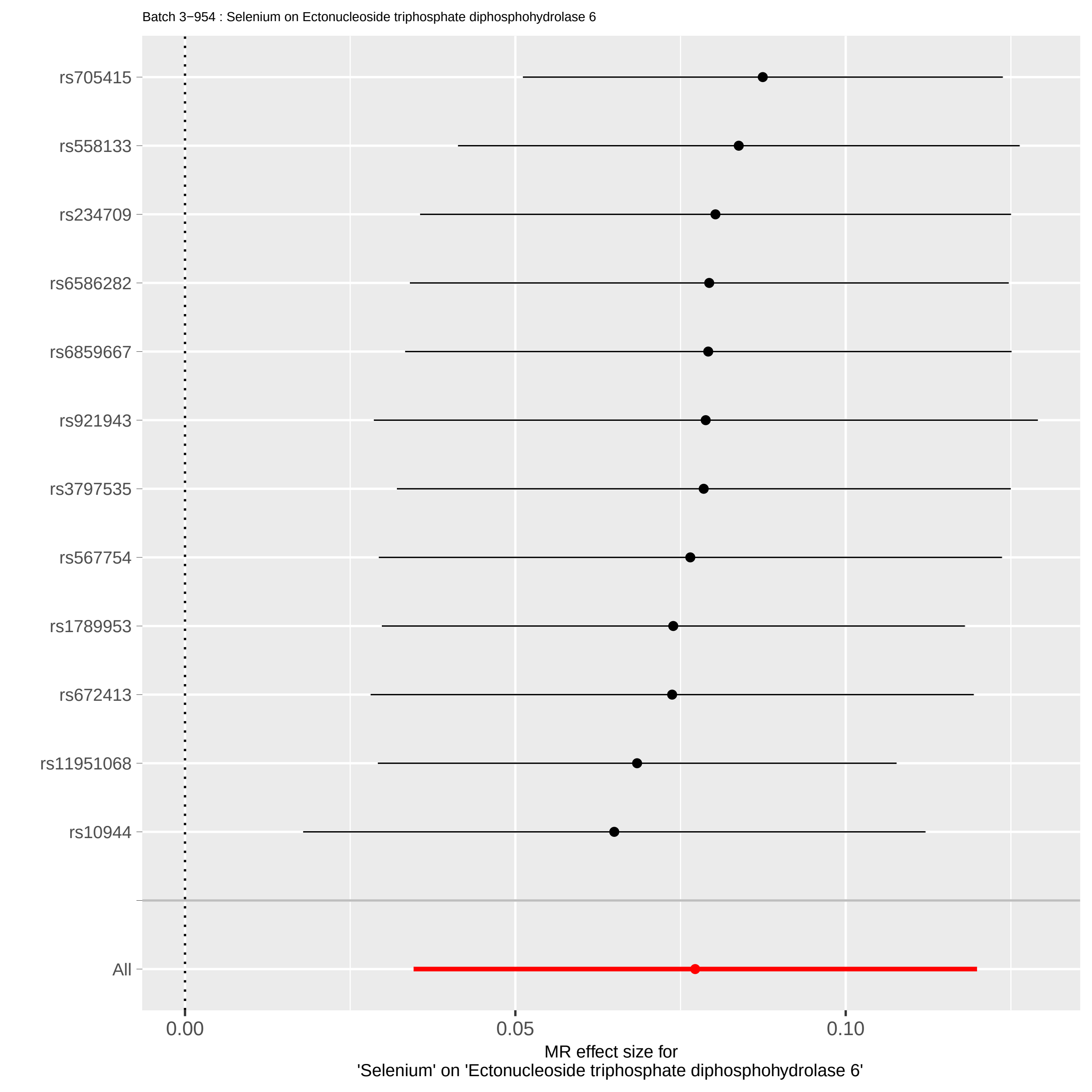

Batch 3−954 : Selenium on Ectonucleoside triphosphate diphosphohydrolase 6
rs705415
rs558133
rs234709
rs6586282
rs6859667
rs921943
rs3797535
rs567754
rs1789953
rs672413
rs11951068
rs10944
All
0.00
0.05
0.10
MR effect size for
'Selenium' on 'Ectonucleoside triphosphate diphosphohydrolase 6'

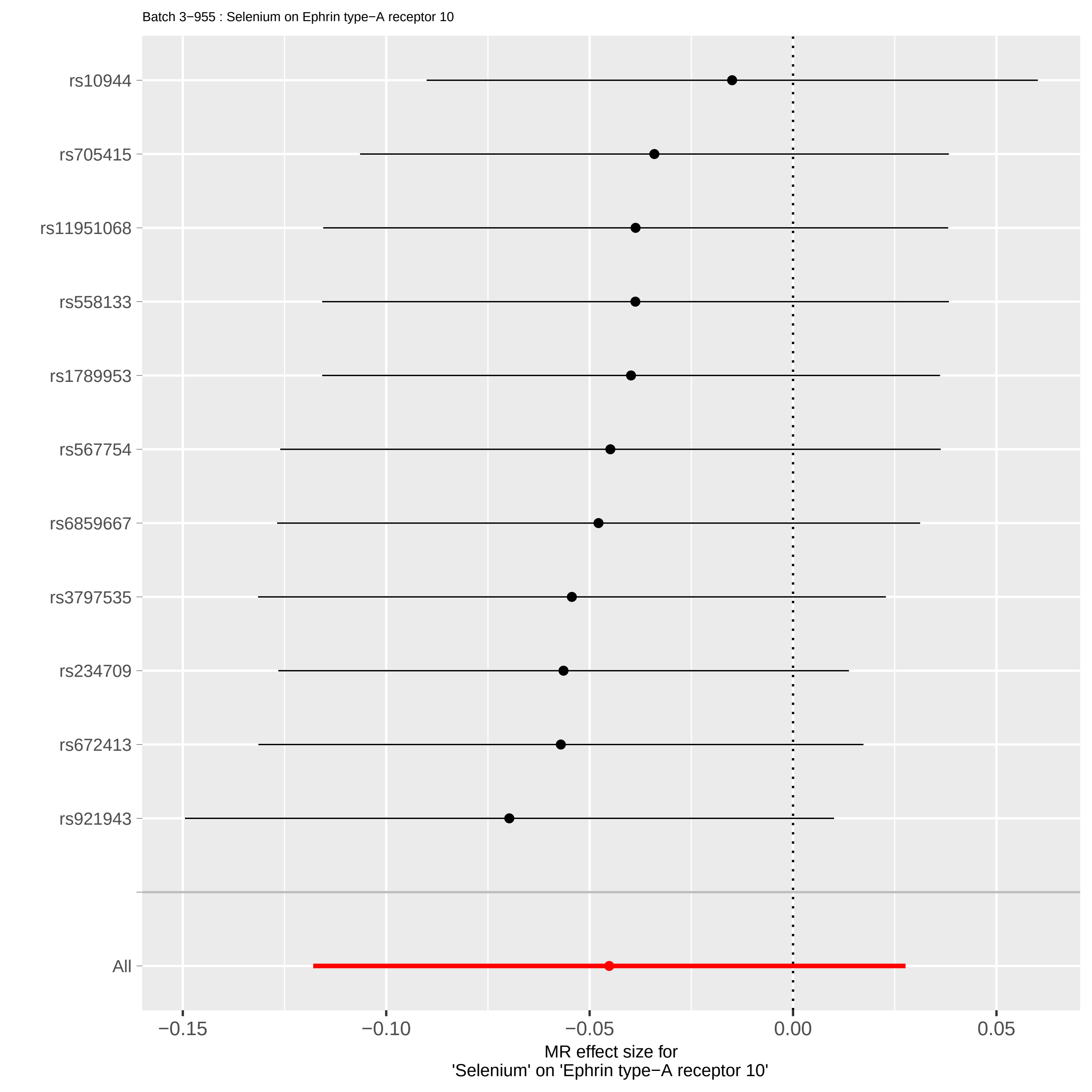

Batch 3−955 : Selenium on Ephrin type−A receptor 10
rs10944
rs705415
rs11951068
rs558133
rs1789953
rs567754
rs6859667
rs3797535
rs234709
rs672413
rs921943
All
−0.15
−0.10
−0.05
0.00
0.05
MR effect size for
'Selenium' on 'Ephrin type−A receptor 10'

Batch 3−956 : Selenium on Ephrin type−A receptor 1

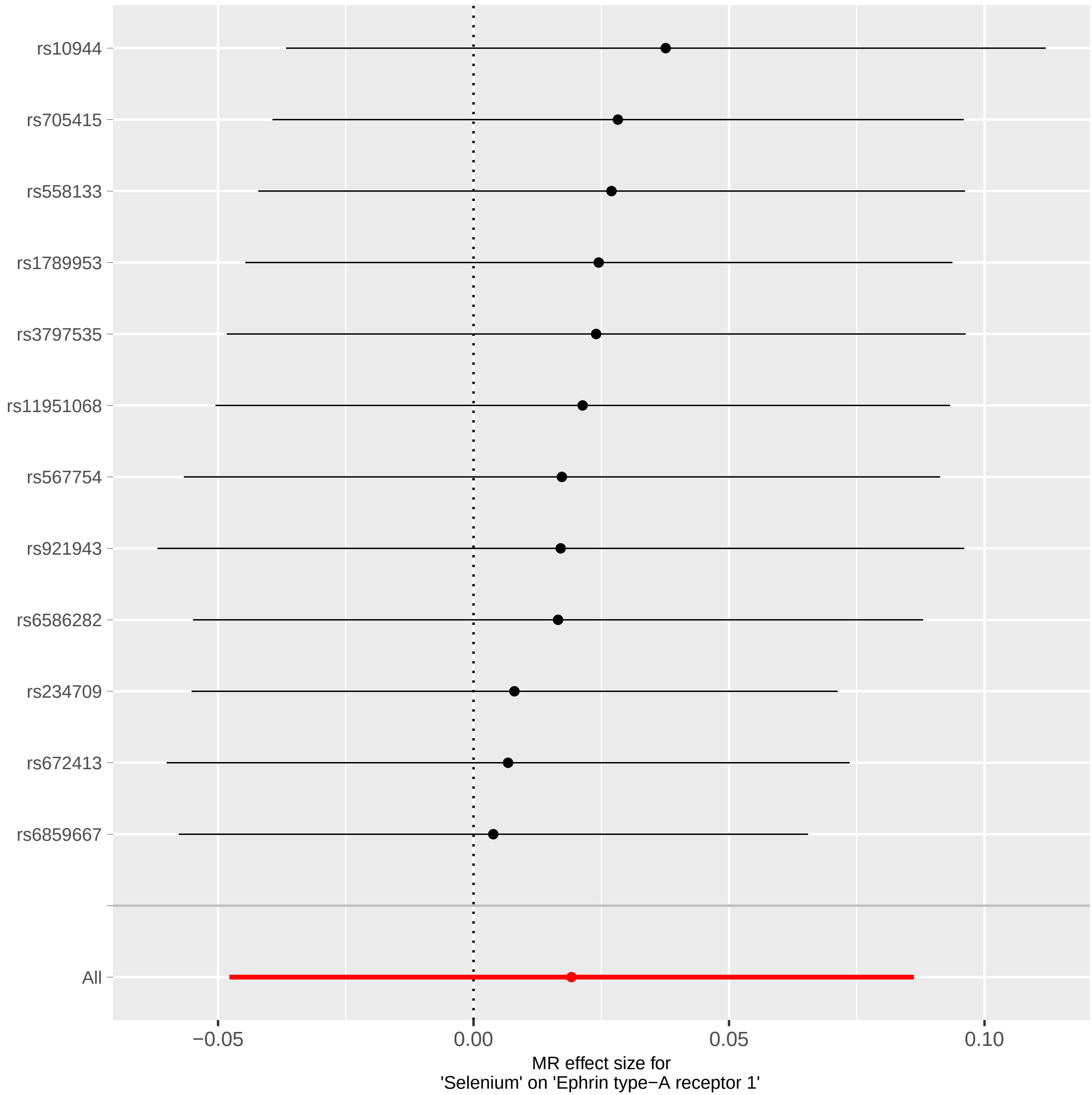

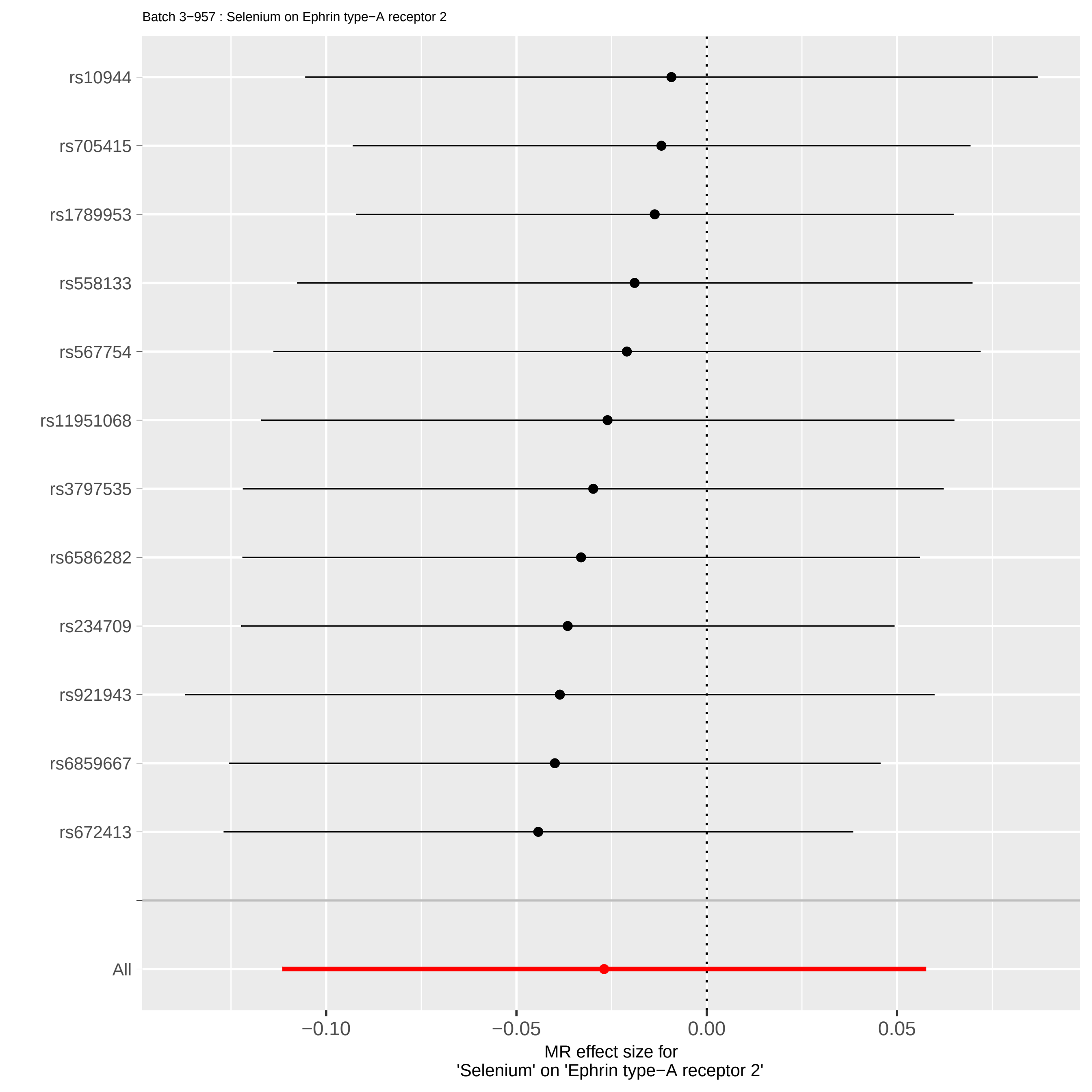

Batch 3−957 : Selenium on Ephrin type−A receptor 2
rs10944
rs705415
rs1789953
rs558133
rs567754
rs11951068
rs3797535
rs6586282
rs234709
rs921943
rs6859667
rs672413
All
−0.10
−0.05
0.00
0.05
MR effect size for
'Selenium' on 'Ephrin type−A receptor 2'

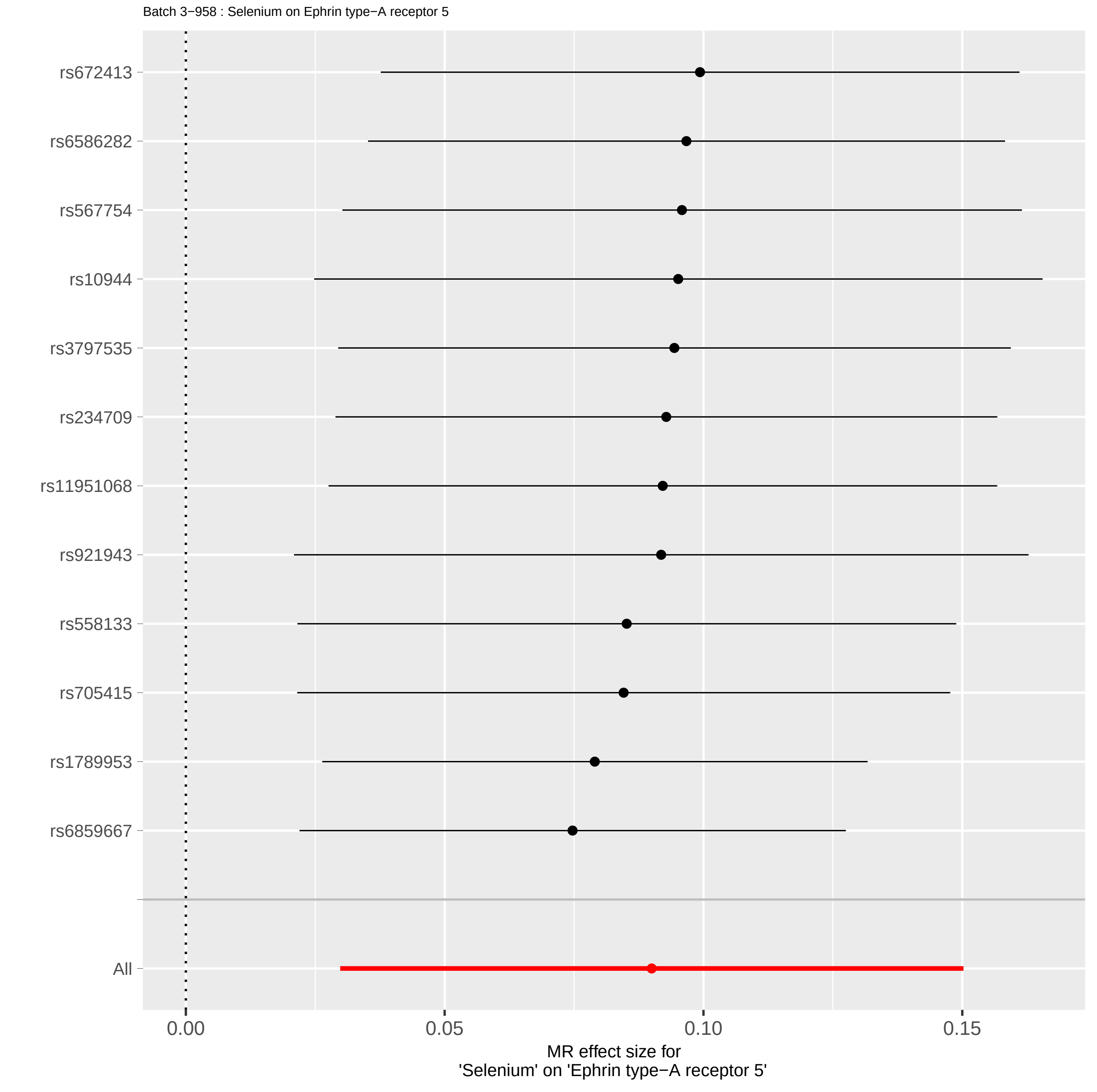

Batch 3−958 : Selenium on Ephrin type−A receptor 5
rs672413
rs6586282
rs567754
rs10944
rs3797535
rs234709
rs11951068
rs921943
rs558133
rs705415
rs1789953
rs6859667
All
0.00
0.05
0.10
0.15
MR effect size for
'Selenium' on 'Ephrin type−A receptor 5'

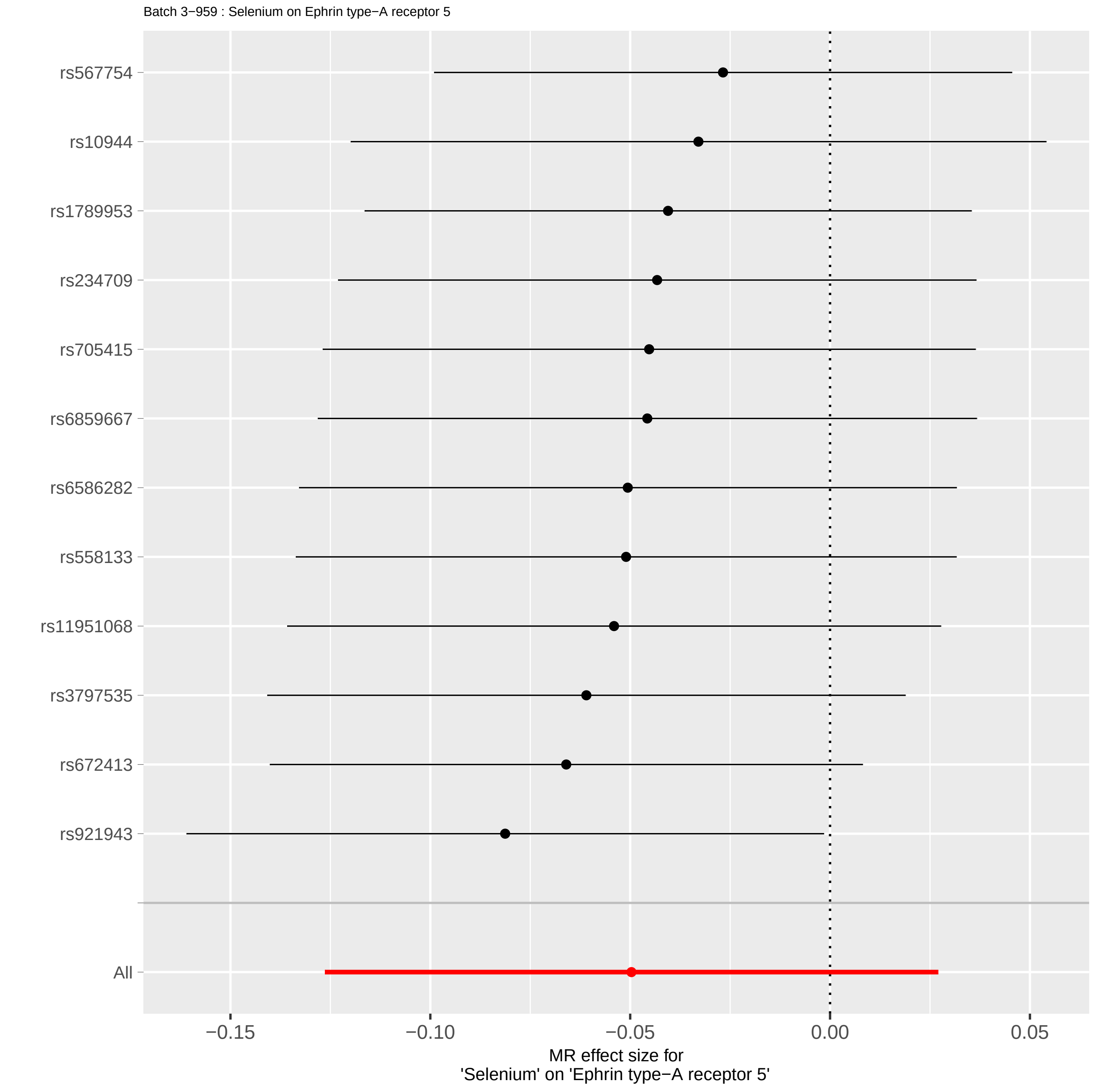
Batch 3−959 : Selenium on Ephrin type−A receptor 5
rs567754
rs10944
rs1789953
rs234709
rs705415
rs6859667
rs6586282
rs558133
rs11951068
rs3797535
rs672413
rs921943
All
−0.15
−0.10
−0.05
0.00
0.05
MR effect size for
'Selenium' on 'Ephrin type−A receptor 5'

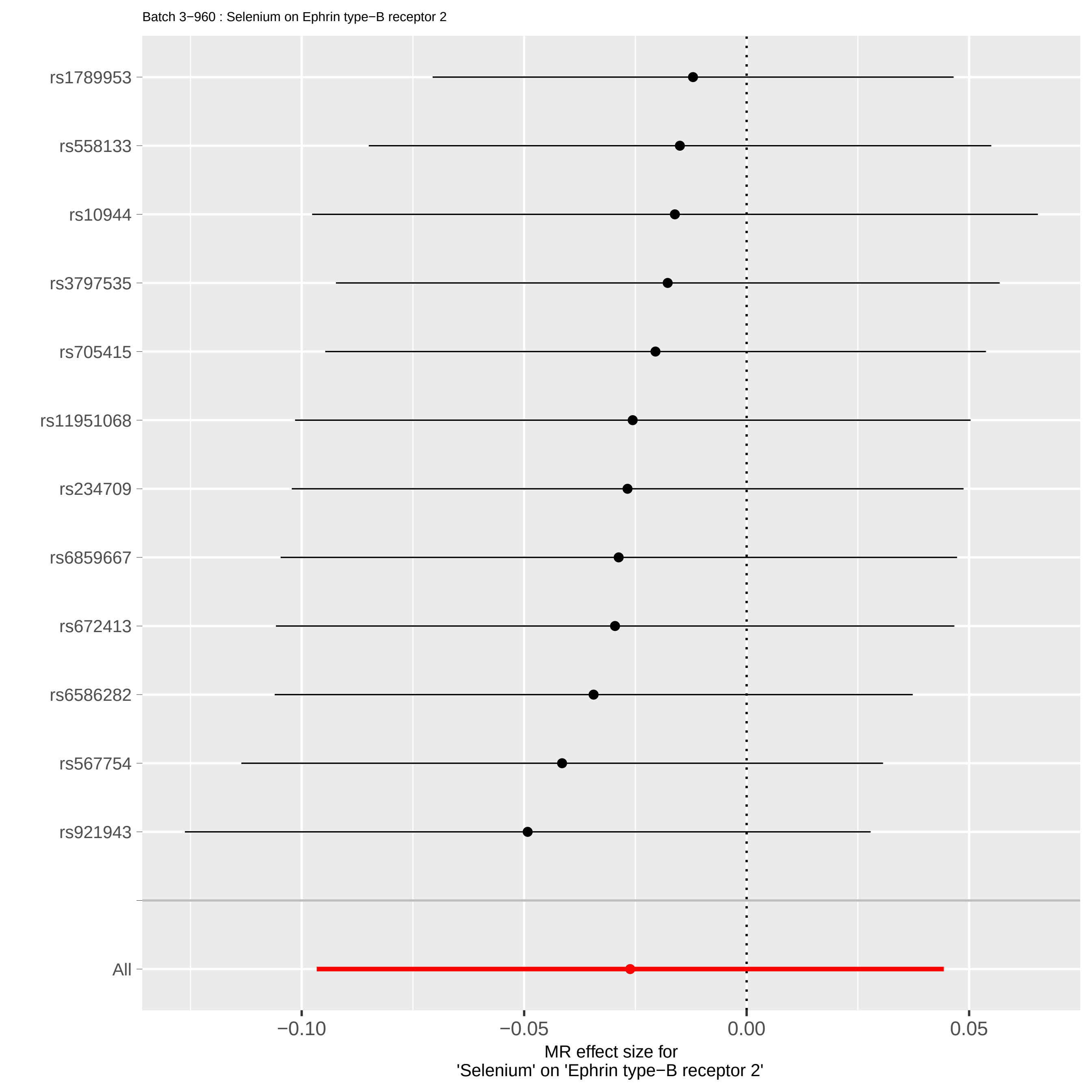

Batch 3−960 : Selenium on Ephrin type−B receptor 2
rs1789953
rs558133
rs10944
rs3797535
rs705415
rs11951068
rs234709
rs6859667
rs672413
rs6586282
rs567754
rs921943
All
−0.10
−0.05
0.00
0.05
MR effect size for
'Selenium' on 'Ephrin type−B receptor 2'

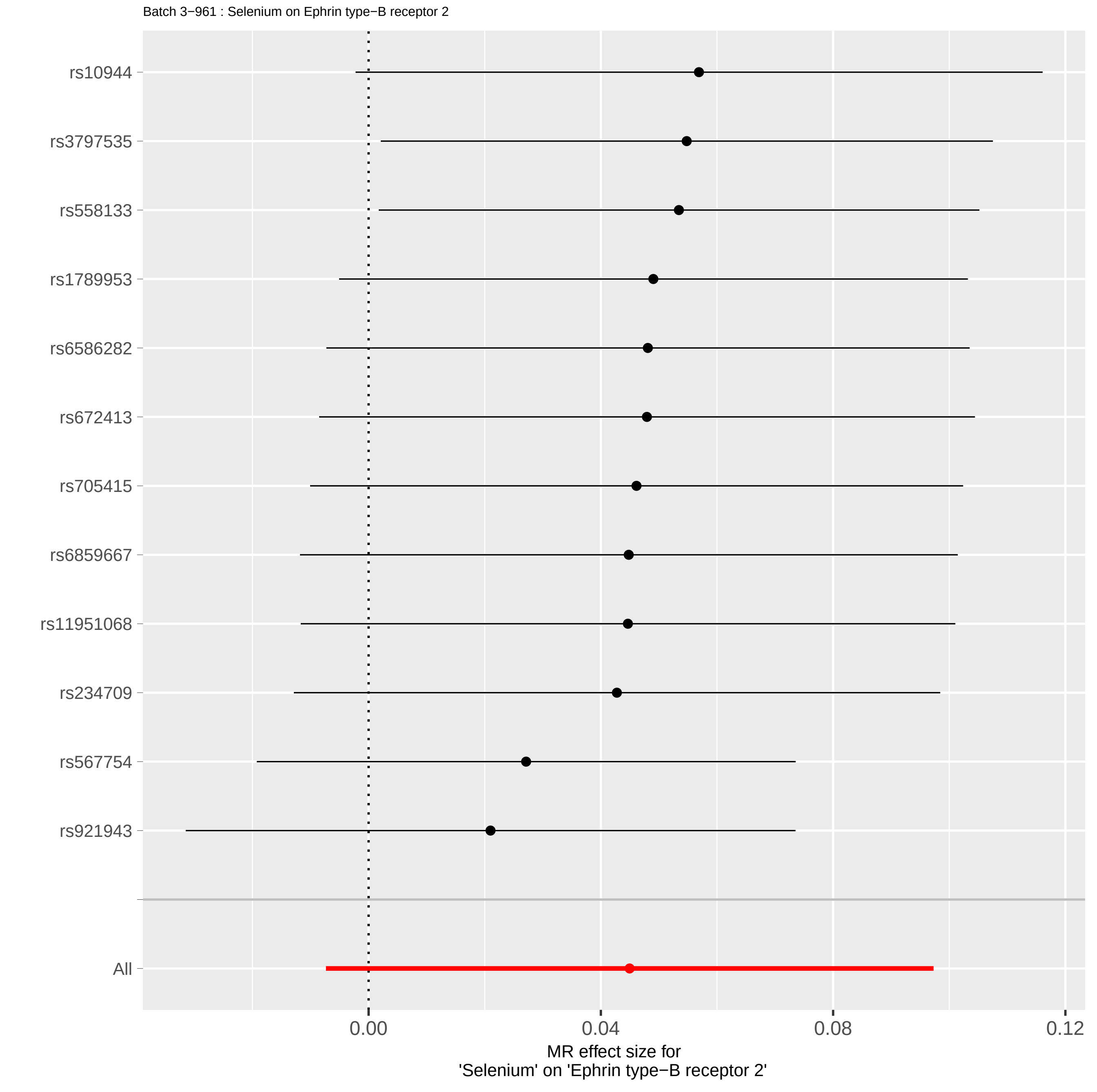

Batch 3−961 : Selenium on Ephrin type−B receptor 2
rs10944
rs3797535
rs558133
rs1789953
rs6586282
rs672413
rs705415
rs6859667
rs11951068
rs234709
rs567754
rs921943
All
0.00
0.04
0.08
0.12
MR effect size for
'Selenium' on 'Ephrin type−B receptor 2'

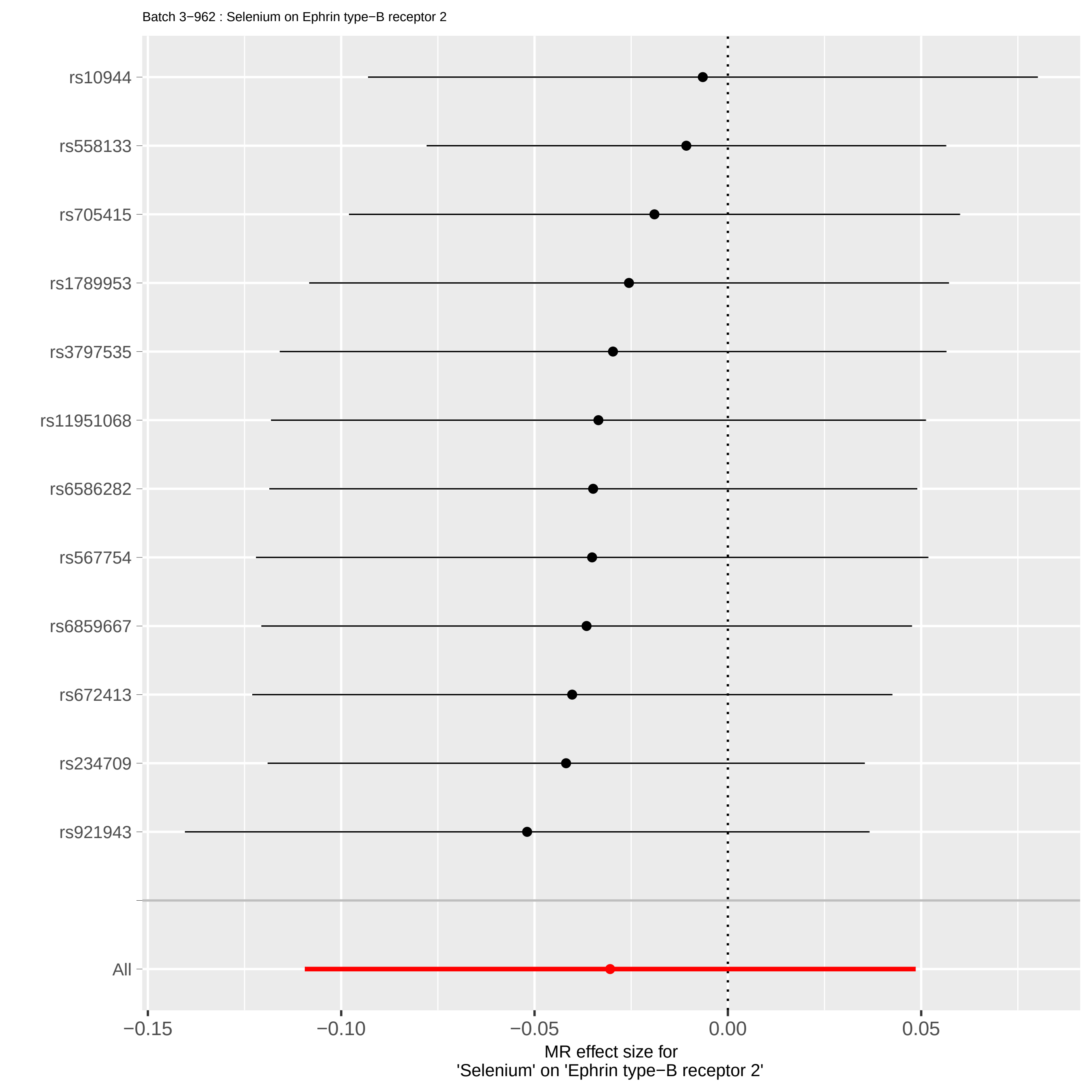
Batch 3−962 : Selenium on Ephrin type−B receptor 2
rs10944
rs558133
rs705415
rs1789953
rs3797535
rs11951068
rs6586282
rs567754
rs6859667
rs672413
rs234709
rs921943
All
−0.15
−0.10
−0.05
0.00
0.05
MR effect size for
'Selenium' on 'Ephrin type−B receptor 2'

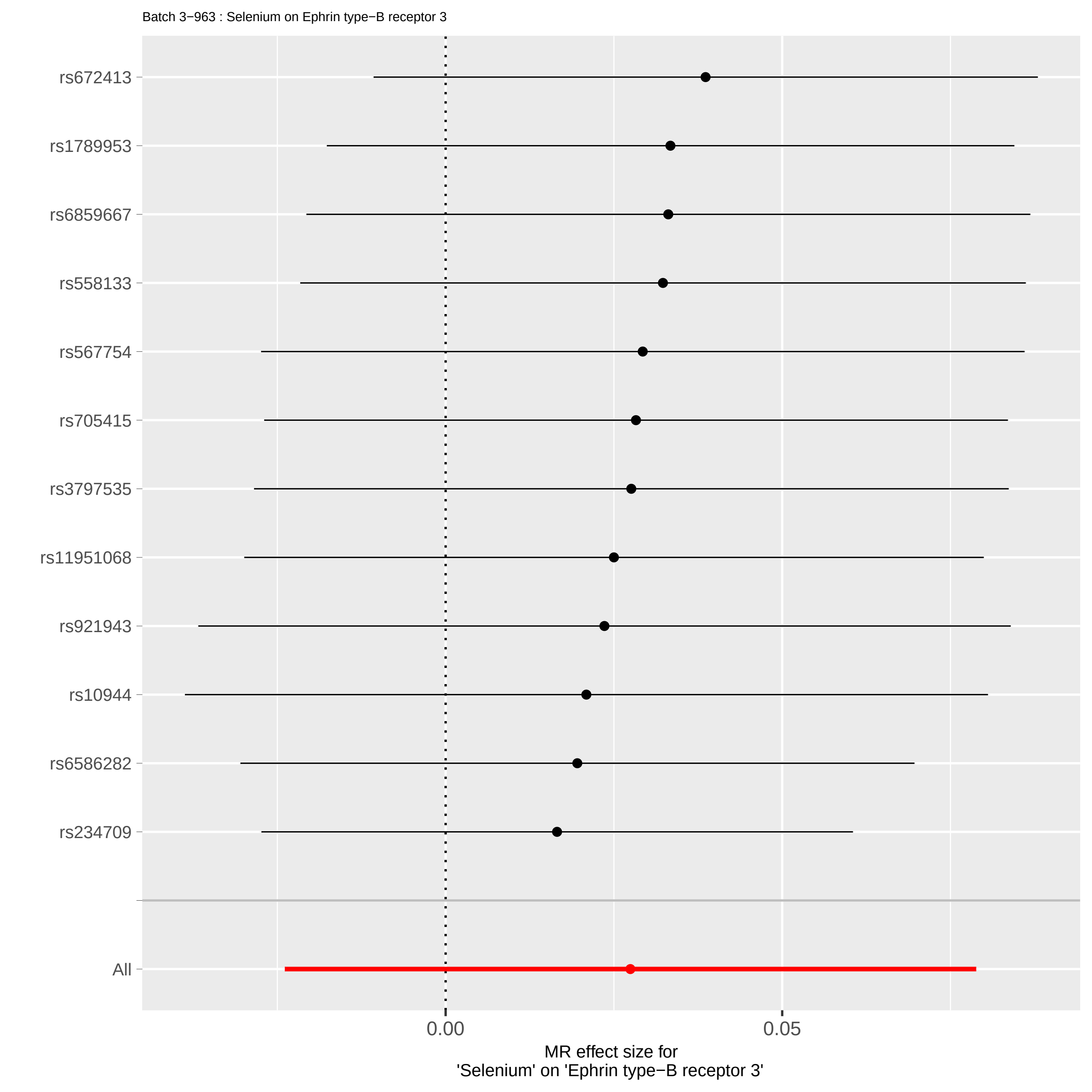

Batch 3−963 : Selenium on Ephrin type−B receptor 3
rs672413
rs1789953
rs6859667
rs558133
rs567754
rs705415
rs3797535
rs11951068
rs921943
rs10944
rs6586282
rs234709
All
0.00
0.05
MR effect size for
'Selenium' on 'Ephrin type−B receptor 3'

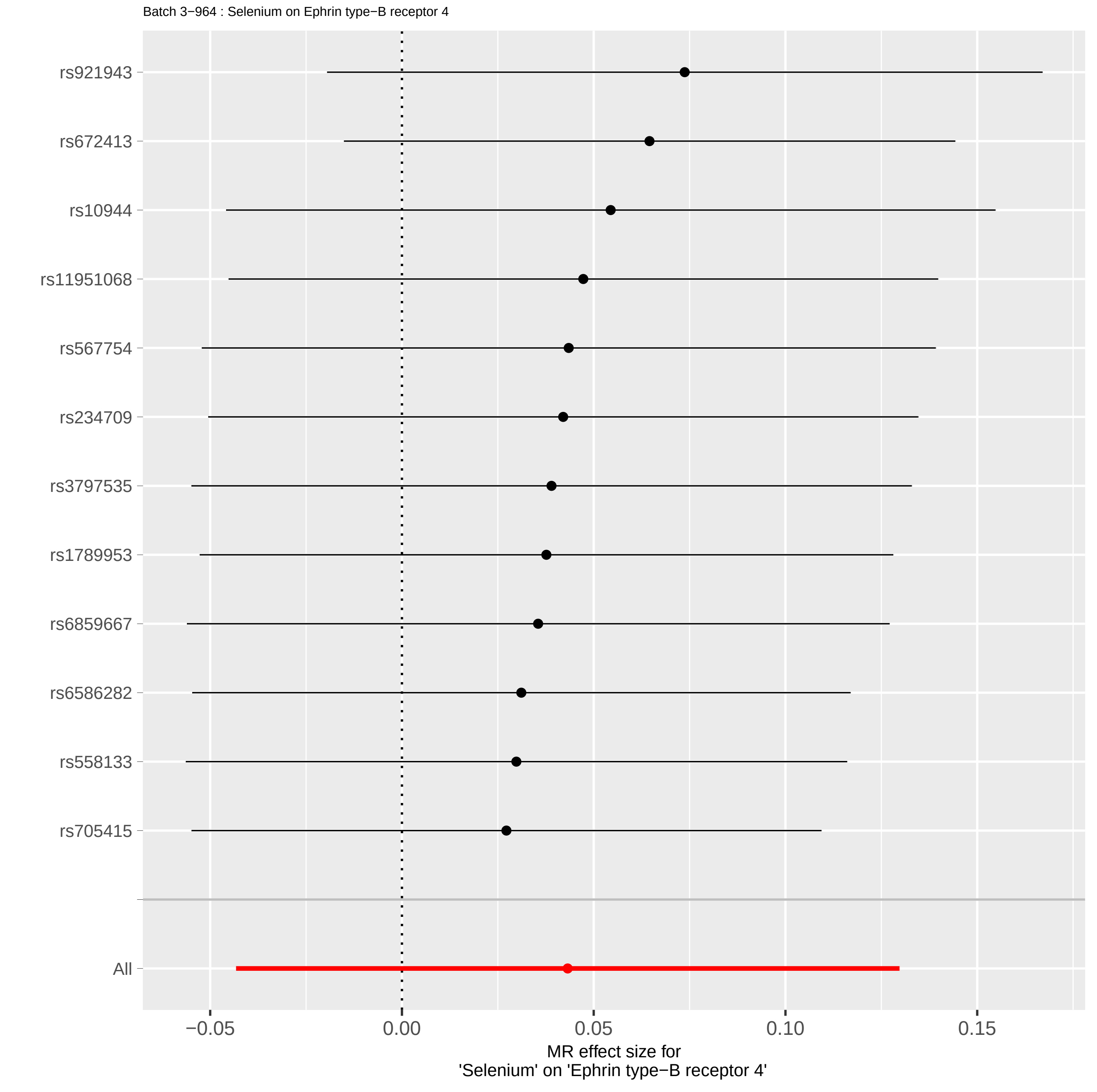

Batch 3−964 : Selenium on Ephrin type−B receptor 4
rs921943
rs672413
rs10944
rs11951068
rs567754
rs234709
rs3797535
rs1789953
rs6859667
rs6586282
rs558133
rs705415
All
−0.05
0.00
0.05
0.10
0.15
MR effect size for
'Selenium' on 'Ephrin type−B receptor 4'

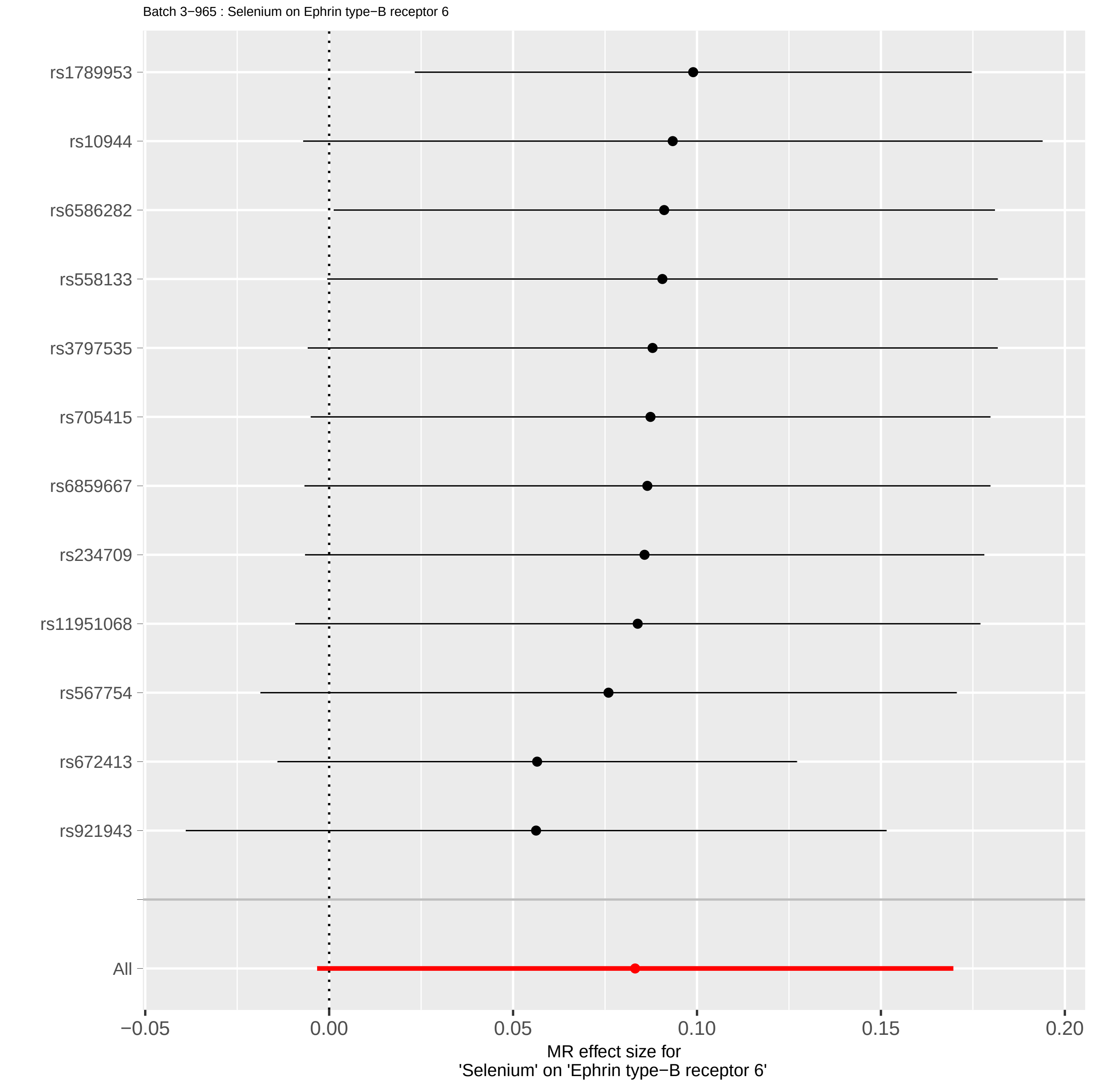
Batch 3−965 : Selenium on Ephrin type−B receptor 6
rs1789953
rs10944
rs6586282
rs558133
rs3797535
rs705415
rs6859667
rs234709
rs11951068
rs567754
rs672413
rs921943
All
−0.05
0.00
0.05
0.10
0.15
0.20
MR effect size for
'Selenium' on 'Ephrin type−B receptor 6'

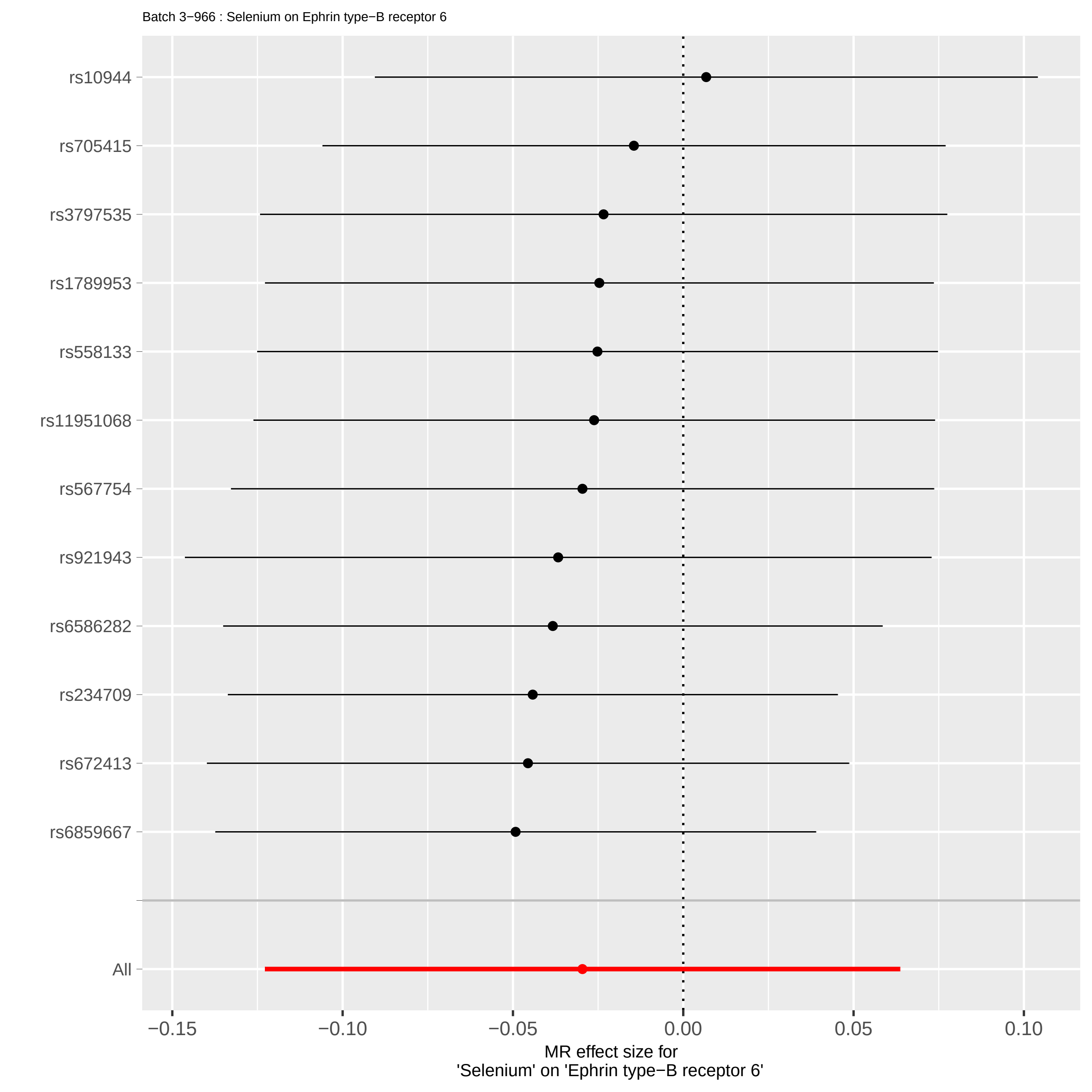
Batch 3−966 : Selenium on Ephrin type−B receptor 6
rs10944
rs705415
rs3797535
rs1789953
rs558133
rs11951068
rs567754
rs921943
rs6586282
rs234709
rs672413
rs6859667
All
−0.15
−0.10
−0.05
0.00
0.05
0.10
MR effect size for
'Selenium' on 'Ephrin type−B receptor 6'

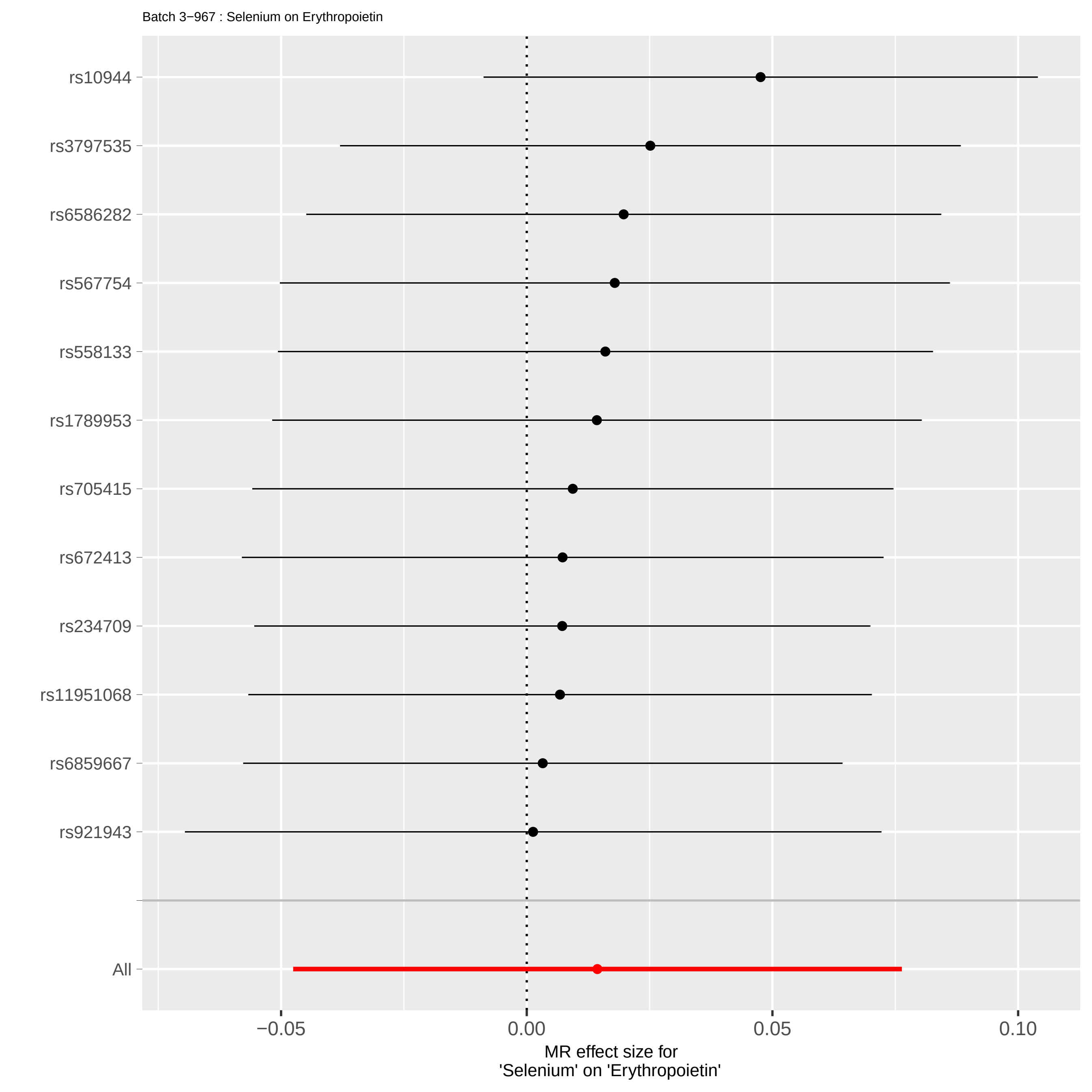

Batch 3−967 : Selenium on Erythropoietin
rs10944
rs3797535
rs6586282
rs567754
rs558133
rs1789953
rs705415
rs672413
rs234709
rs11951068
rs6859667
rs921943
All
−0.05
0.00
0.05
0.10
MR effect size for
'Selenium' on 'Erythropoietin'

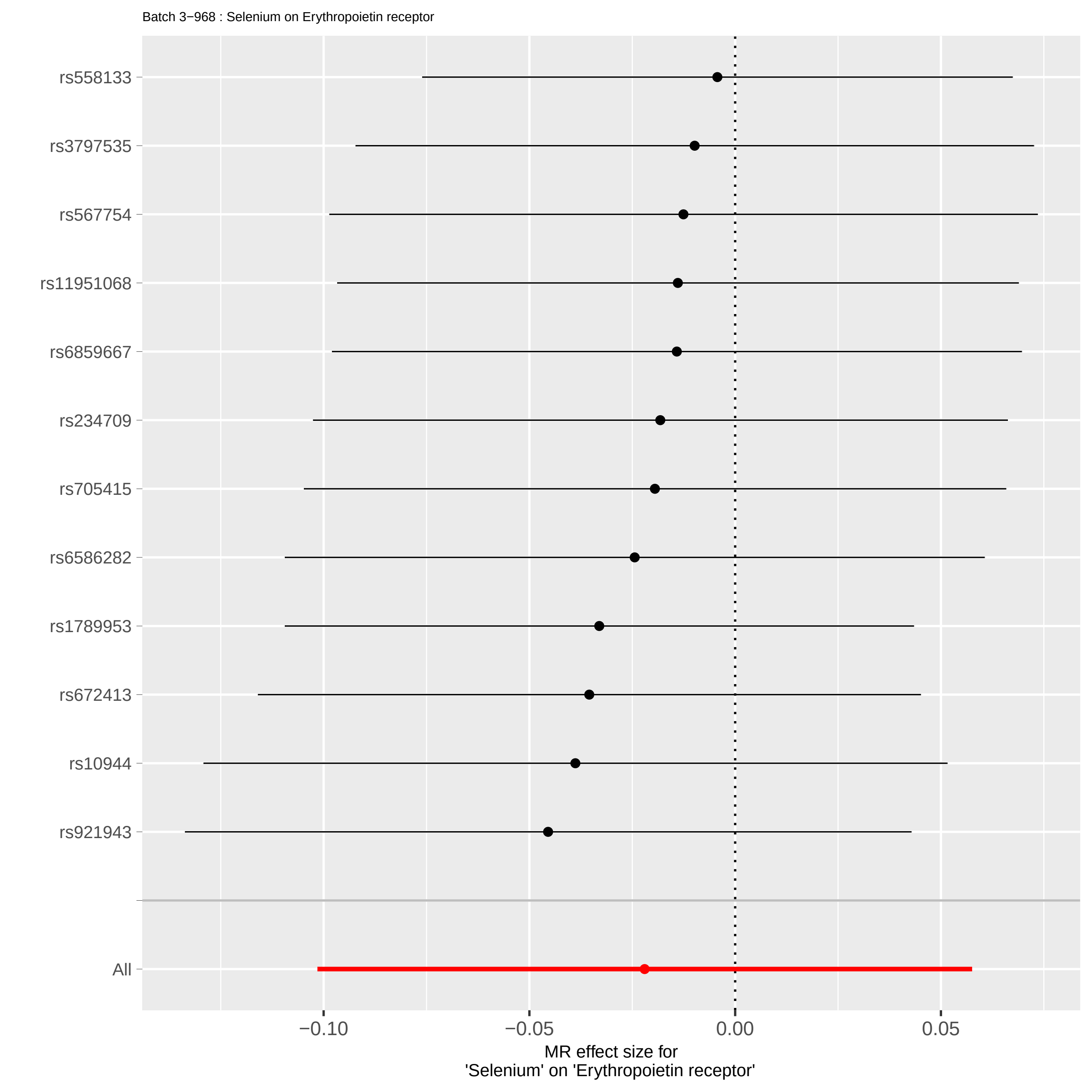

Batch 3−968 : Selenium on Erythropoietin receptor
rs558133
rs3797535
rs567754
rs11951068
rs6859667
rs234709
rs705415
rs6586282
rs1789953
rs672413
rs10944
rs921943
All
−0.10
−0.05
0.00
0.05
MR effect size for
'Selenium' on 'Erythropoietin receptor'

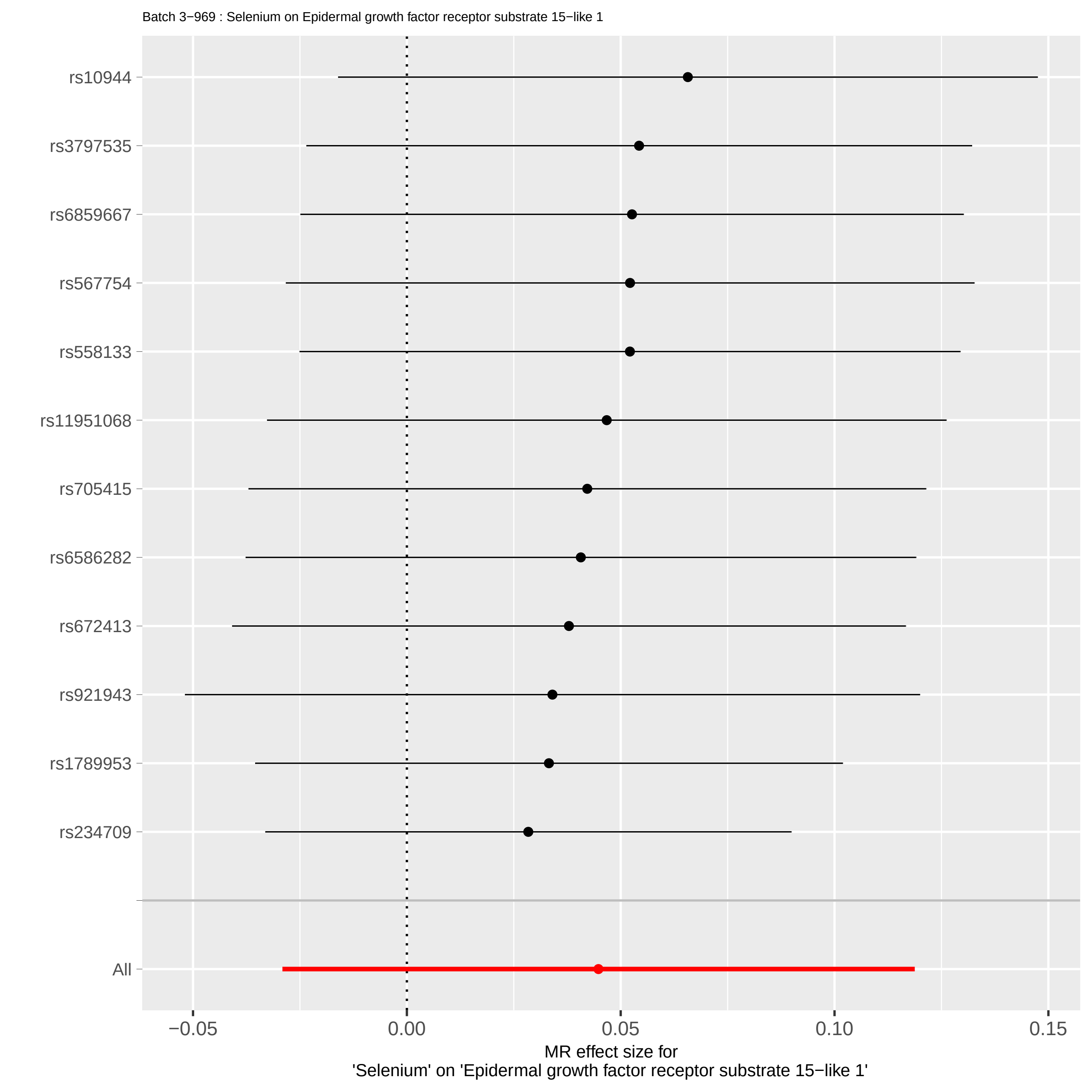
Batch 3−969 : Selenium on Epidermal growth factor receptor substrate 15−like 1
rs10944
rs3797535
rs6859667
rs567754
rs558133
rs11951068
rs705415
rs6586282
rs672413
rs921943
rs1789953
rs234709
All
−0.05
0.00
0.05
0.10
0.15
MR effect size for
'Selenium' on 'Epidermal growth factor receptor substrate 15−like 1'

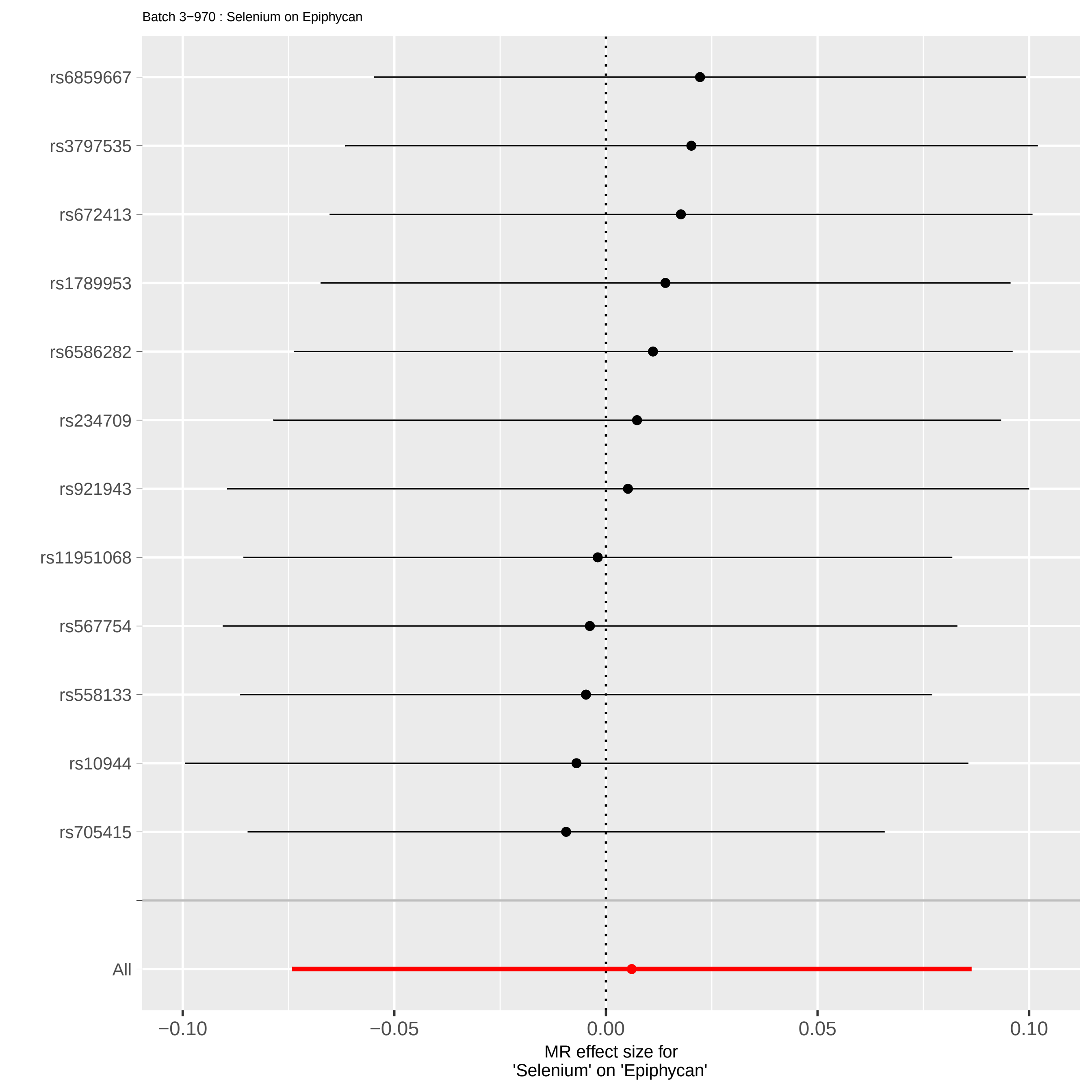
Batch 3−970 : Selenium on Epiphycan
rs6859667
rs3797535
rs672413
rs1789953
rs6586282
rs234709
rs921943
rs11951068
rs567754
rs558133
rs10944
rs705415
All
−0.10
−0.05
0.00
0.05
0.10
MR effect size for
'Selenium' on 'Epiphycan'

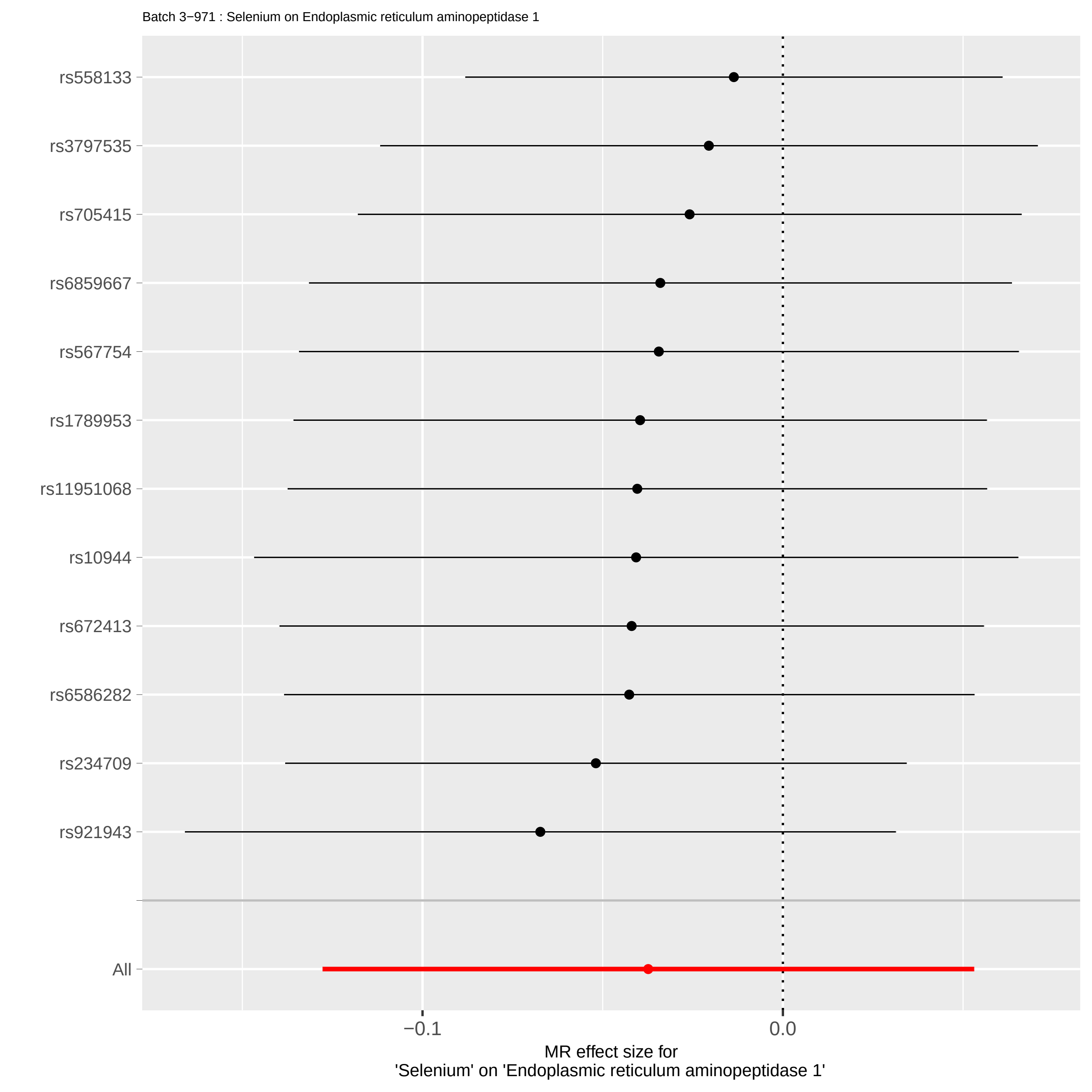

Batch 3−971 : Selenium on Endoplasmic reticulum aminopeptidase 1
rs558133
rs3797535
rs705415
rs6859667
rs567754
rs1789953
rs11951068
rs10944
rs672413
rs6586282
rs234709
rs921943
All
−0.1
0.0
MR effect size for
'Selenium' on 'Endoplasmic reticulum aminopeptidase 1'

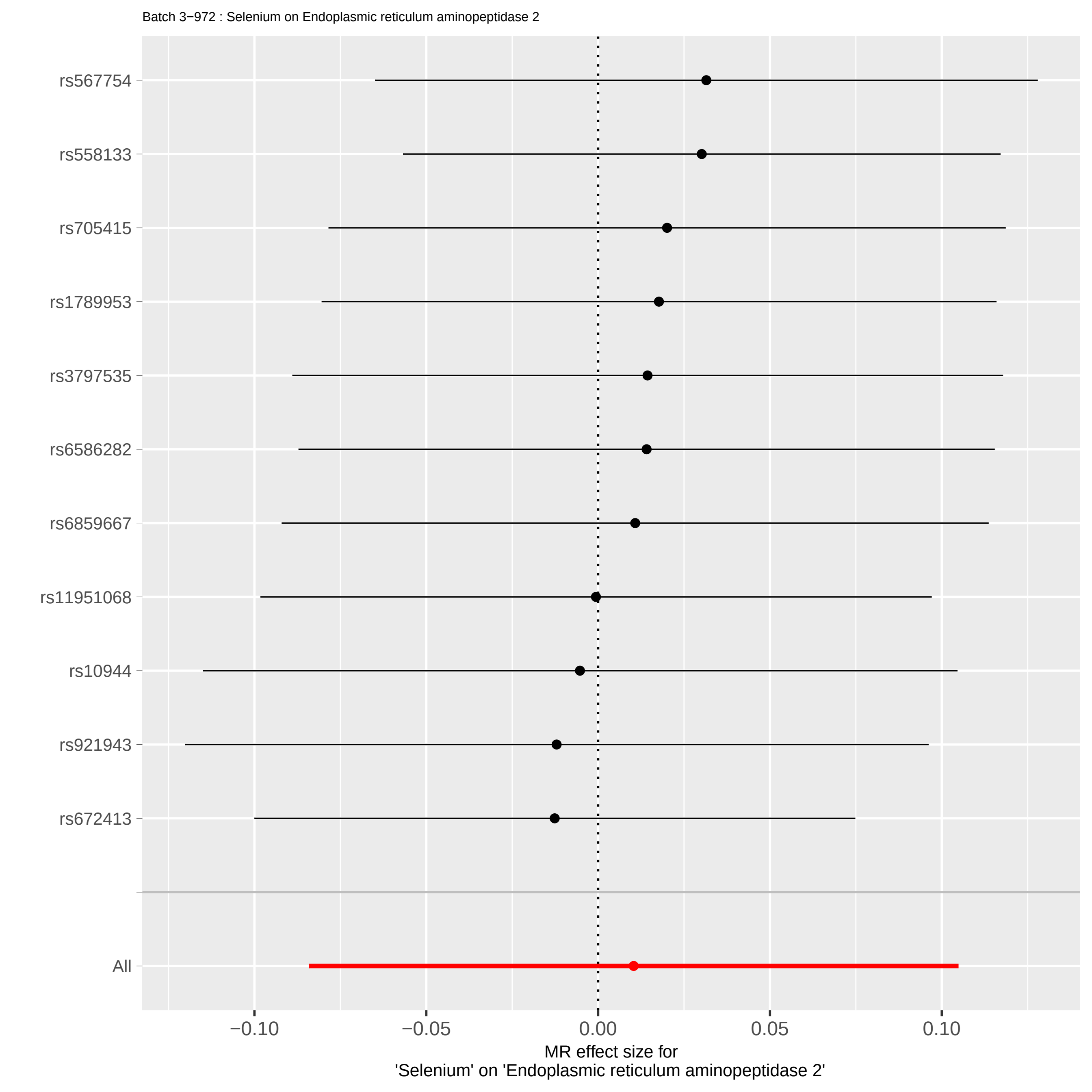
Batch 3−972 : Selenium on Endoplasmic reticulum aminopeptidase 2
rs567754
rs558133
rs705415
rs1789953
rs3797535
rs6586282
rs6859667
rs11951068
rs10944
rs921943
rs672413
All
−0.10
−0.05
0.00
0.05
0.10
MR effect size for
'Selenium' on 'Endoplasmic reticulum aminopeptidase 2'

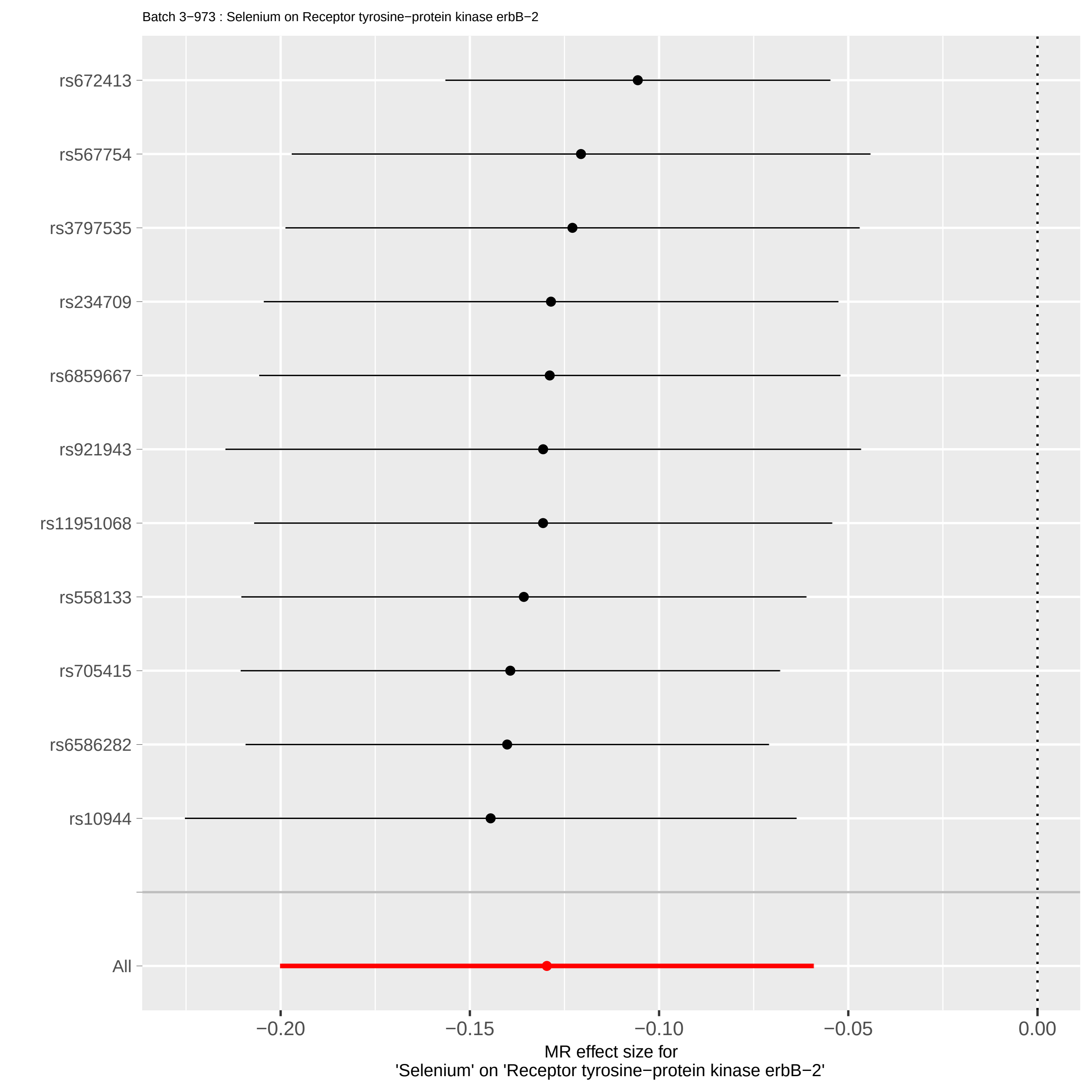

Batch 3−973 : Selenium on Receptor tyrosine−protein kinase erbB−2
rs672413
rs567754
rs3797535
rs234709
rs6859667
rs921943
rs11951068
rs558133
rs705415
rs6586282
rs10944
All
−0.20
−0.15
−0.10
−0.05
0.00
MR effect size for
'Selenium' on 'Receptor tyrosine−protein kinase erbB−2'

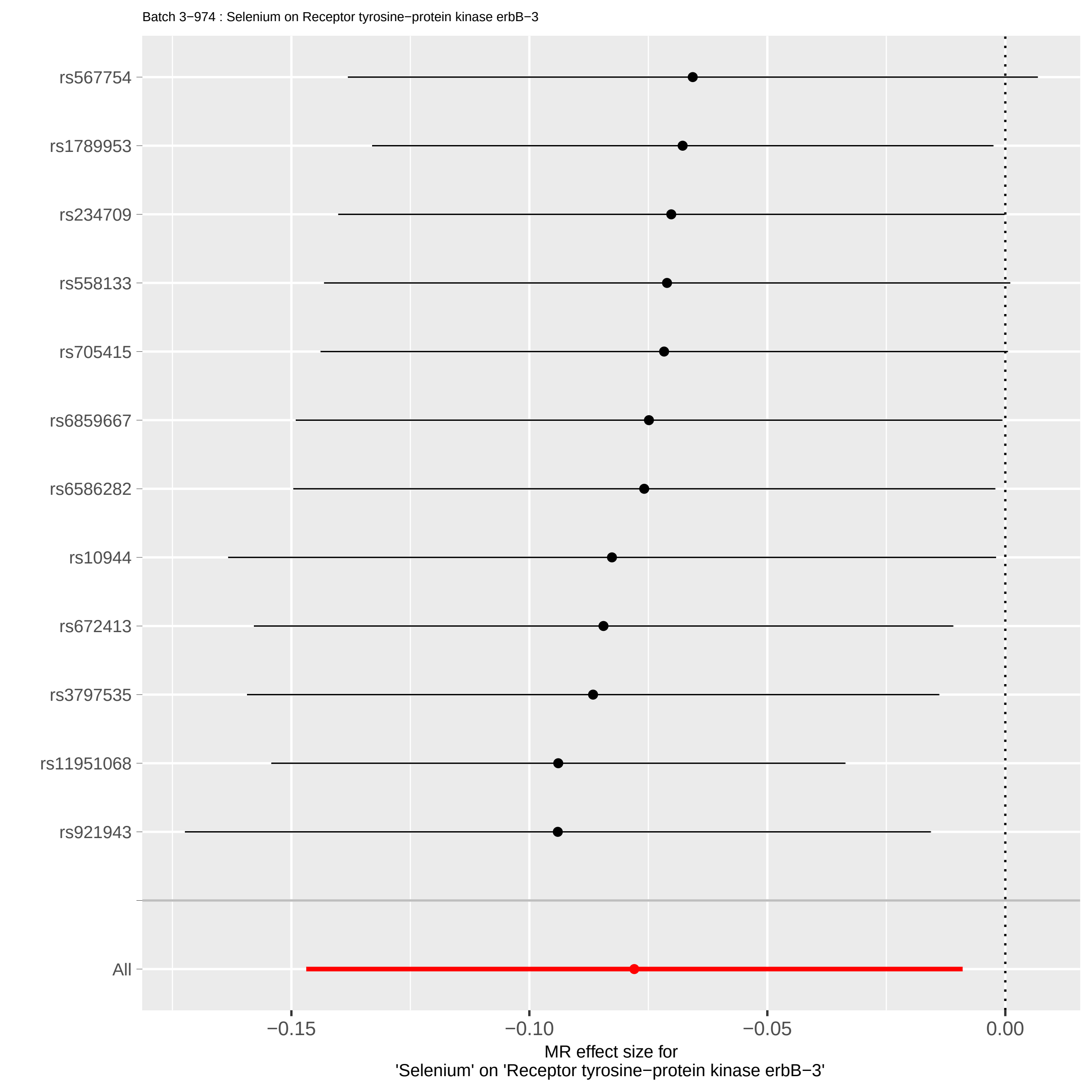

Batch 3−974 : Selenium on Receptor tyrosine−protein kinase erbB−3
rs567754
rs1789953
rs234709
rs558133
rs705415
rs6859667
rs6586282
rs10944
rs672413
rs3797535
rs11951068
rs921943
All
−0.15
−0.10
−0.05
0.00
MR effect size for
'Selenium' on 'Receptor tyrosine−protein kinase erbB−3'

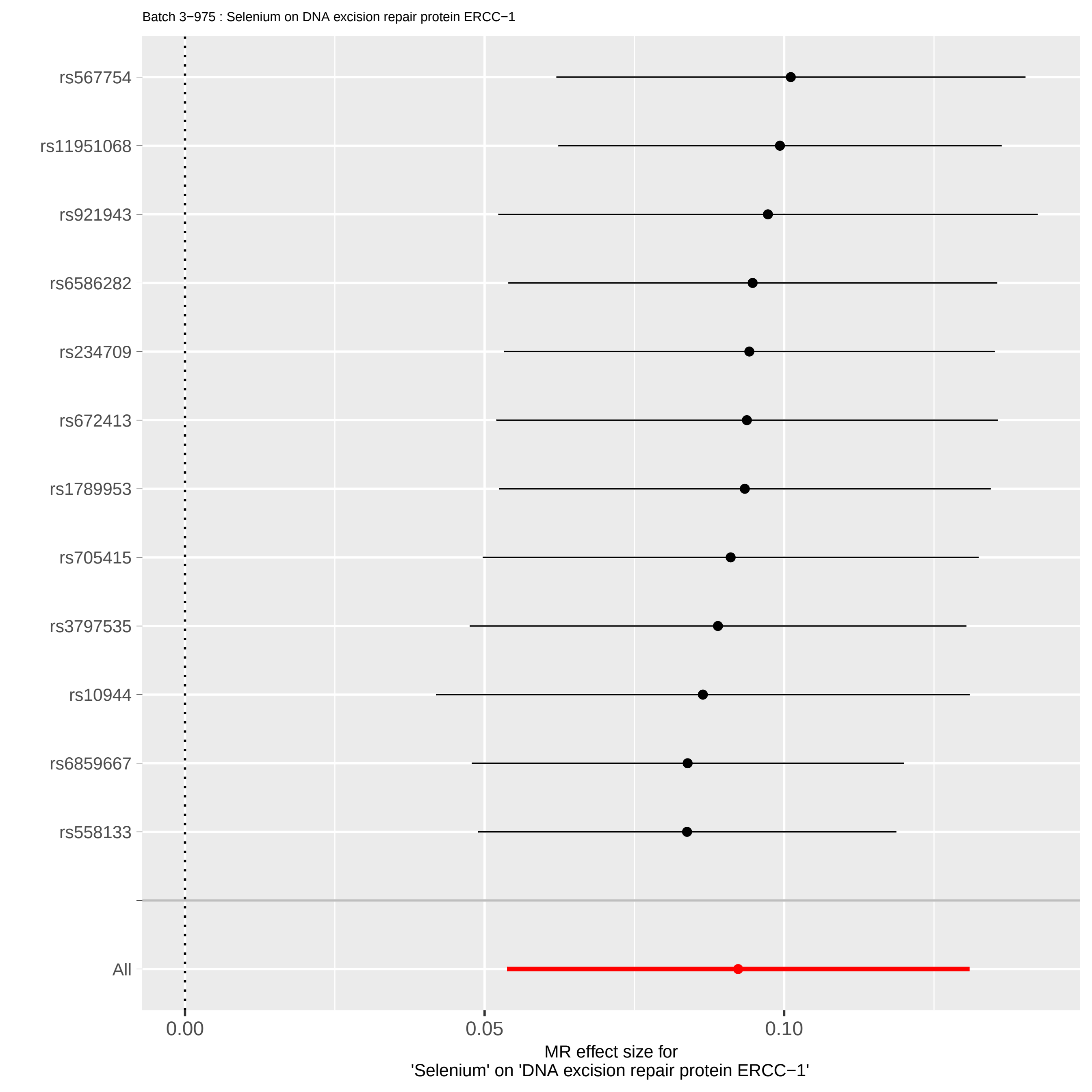

Batch 3−975 : Selenium on DNA excision repair protein ERCC−1
rs567754
rs11951068
rs921943
rs6586282
rs234709
rs672413
rs1789953
rs705415
rs3797535
rs10944
rs6859667
rs558133
All
0.00
0.05
0.10
MR effect size for
'Selenium' on 'DNA excision repair protein ERCC−1'

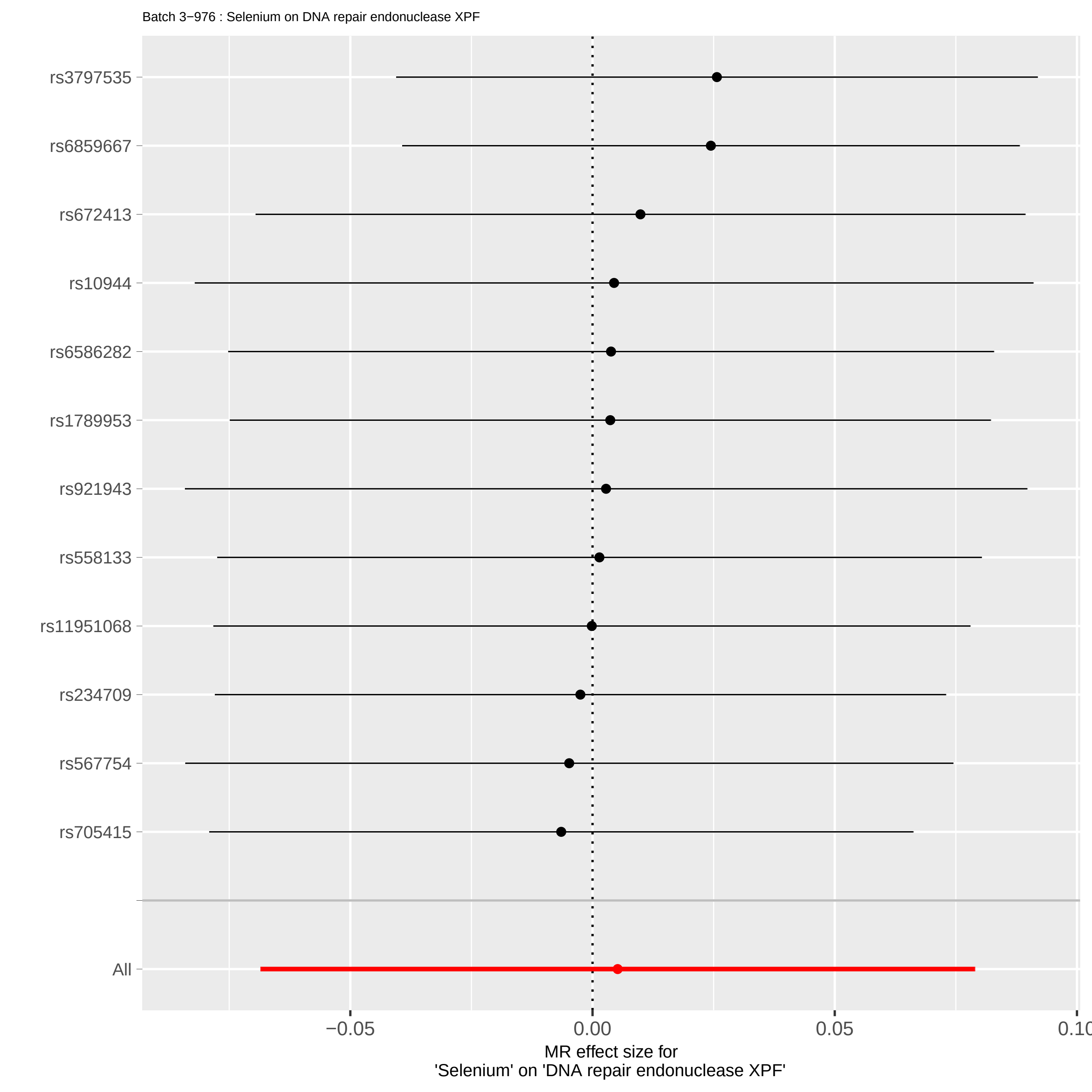

Batch 3−976 : Selenium on DNA repair endonuclease XPF
rs3797535
rs6859667
rs672413
rs10944
rs6586282
rs1789953
rs921943
rs558133
rs11951068
rs234709
rs567754
rs705415
All
−0.05
0.00
0.05
0.10
MR effect size for
'Selenium' on 'DNA repair endonuclease XPF'

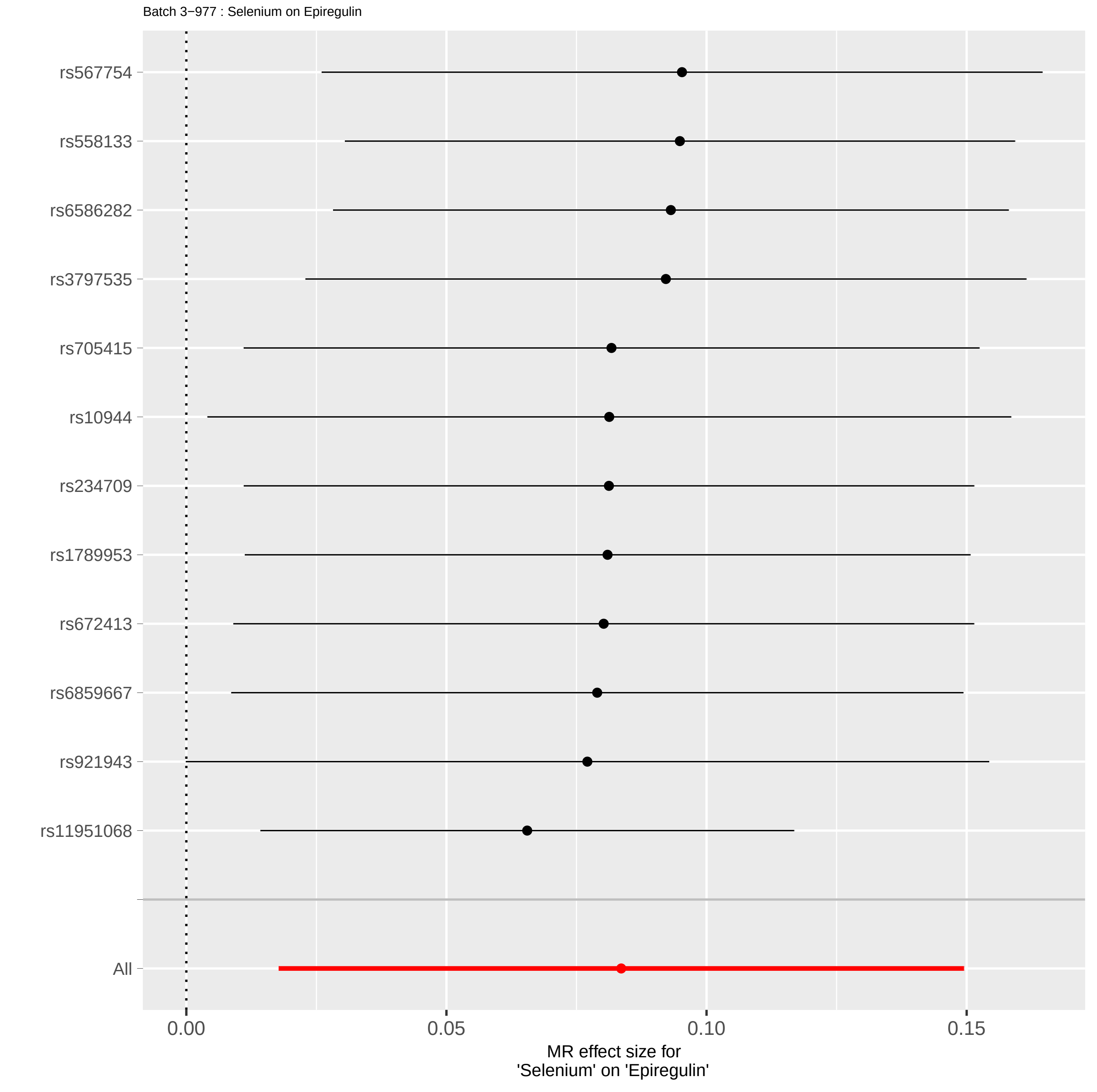
Batch 3−977 : Selenium on Epiregulin
rs567754
rs558133
rs6586282
rs3797535
rs705415
rs10944
rs234709
rs1789953
rs672413
rs6859667
rs921943
rs11951068
All
0.00
0.05
0.10
0.15
MR effect size for
'Selenium' on 'Epiregulin'

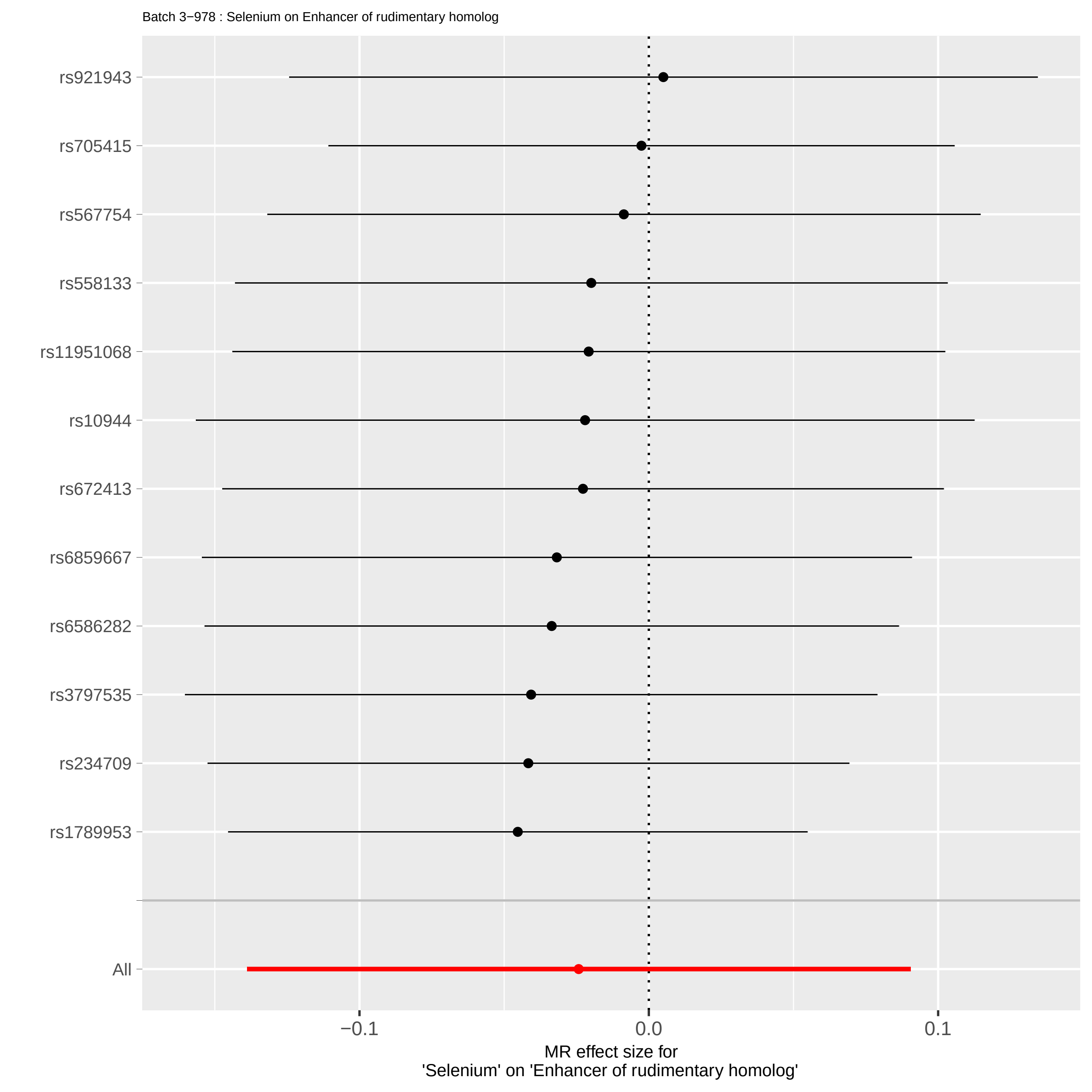
Batch 3−978 : Selenium on Enhancer of rudimentary homolog
rs921943
rs705415
rs567754
rs558133
rs11951068
rs10944
rs672413
rs6859667
rs6586282
rs3797535
rs234709
rs1789953
All
−0.1
0.0
0.1
MR effect size for
'Selenium' on 'Enhancer of rudimentary homolog'

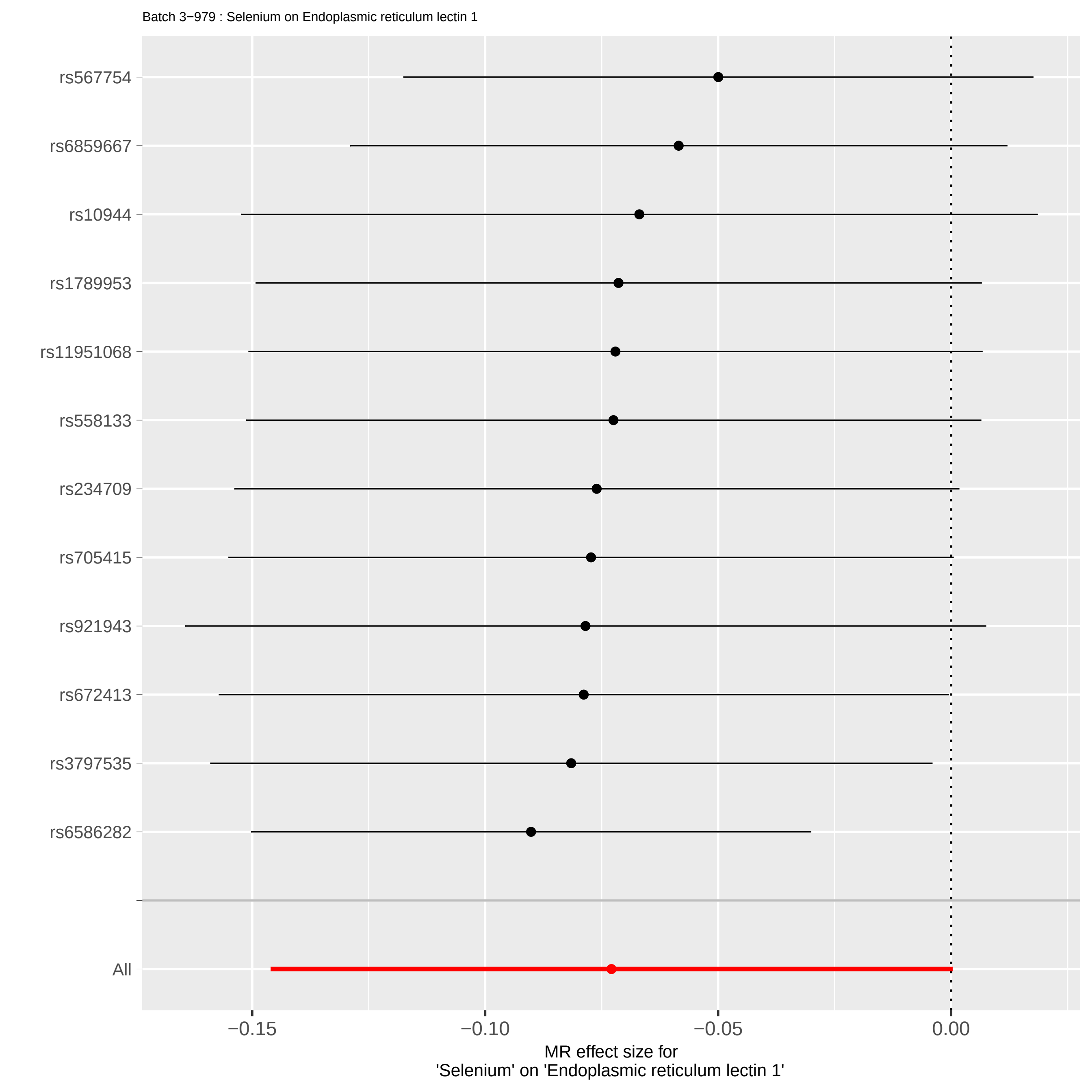

Batch 3−979 : Selenium on Endoplasmic reticulum lectin 1
rs567754
rs6859667
rs10944
rs1789953
rs11951068
rs558133
rs234709
rs705415
rs921943
rs672413
rs3797535
rs6586282
All
−0.15
−0.10
−0.05
0.00
MR effect size for
'Selenium' on 'Endoplasmic reticulum lectin 1'

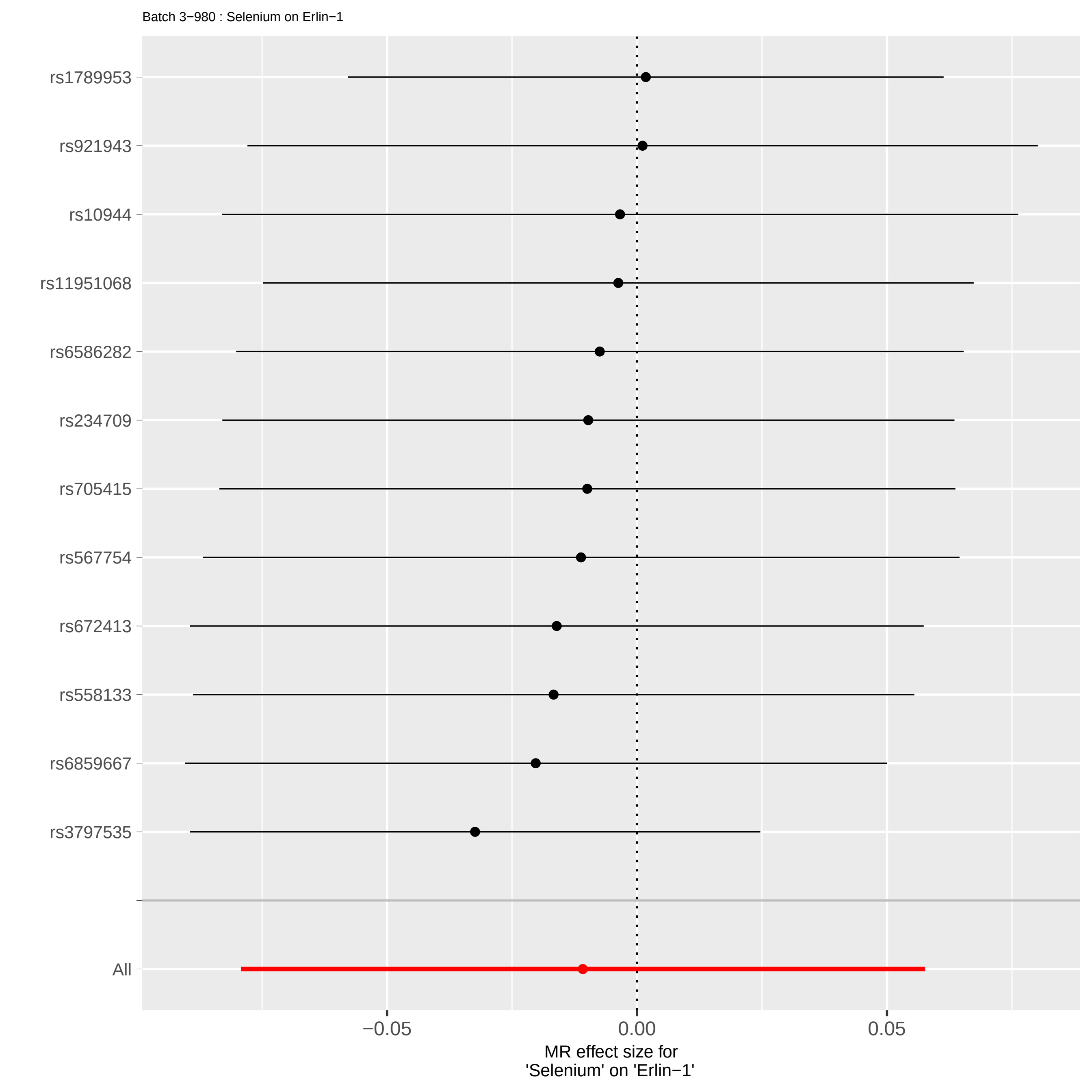
Batch 3−980 : Selenium on Erlin−1
rs1789953
rs921943
rs10944
rs11951068
rs6586282
rs234709
rs705415
rs567754
rs672413
rs558133
rs6859667
rs3797535
All
−0.05
0.00
0.05
MR effect size for
'Selenium' on 'Erlin−1'

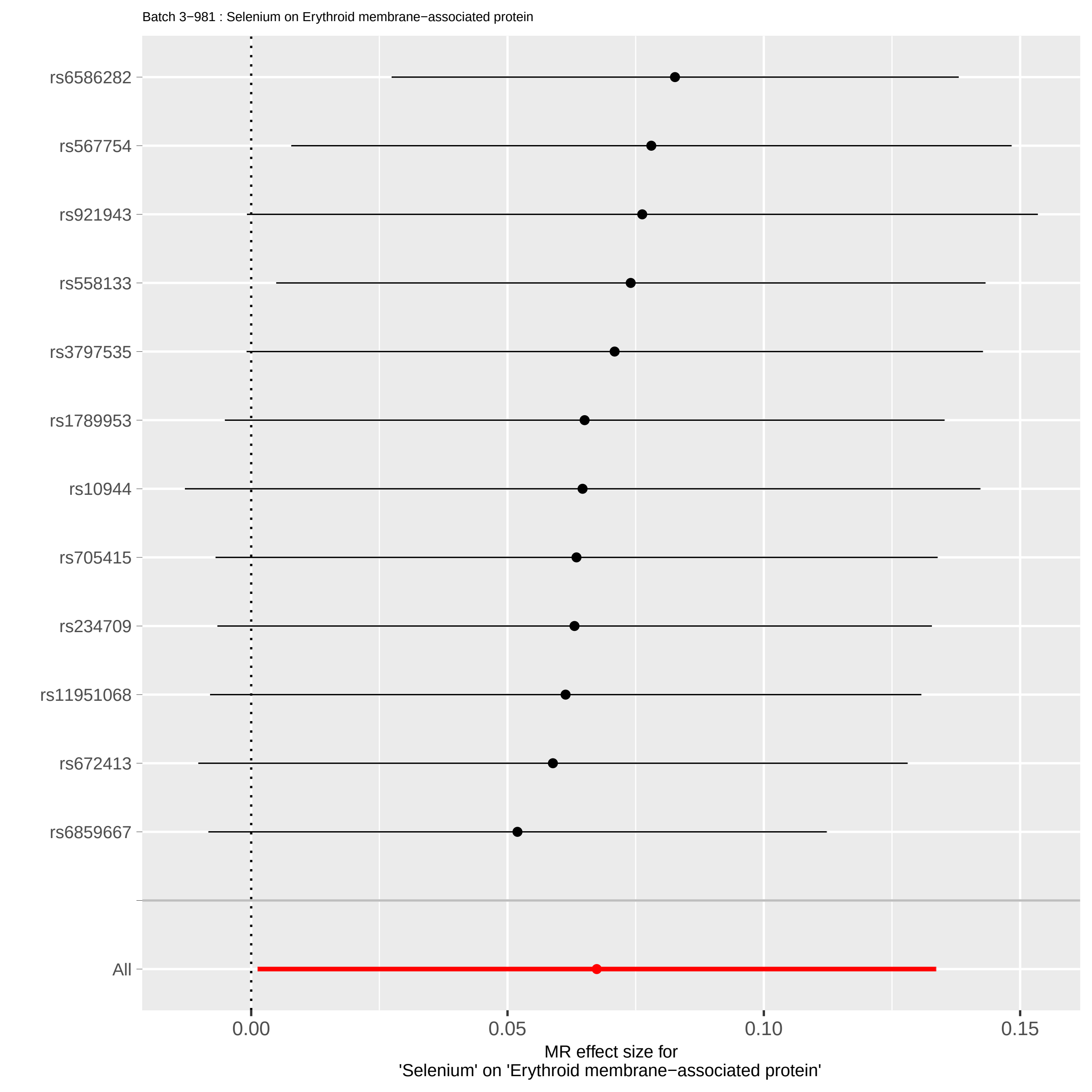

Batch 3−981 : Selenium on Erythroid membrane−associated protein
rs6586282
rs567754
rs921943
rs558133
rs3797535
rs1789953
rs10944
rs705415
rs234709
rs11951068
rs672413
rs6859667
All
0.00
0.05
0.10
0.15
MR effect size for
'Selenium' on 'Erythroid membrane−associated protein'

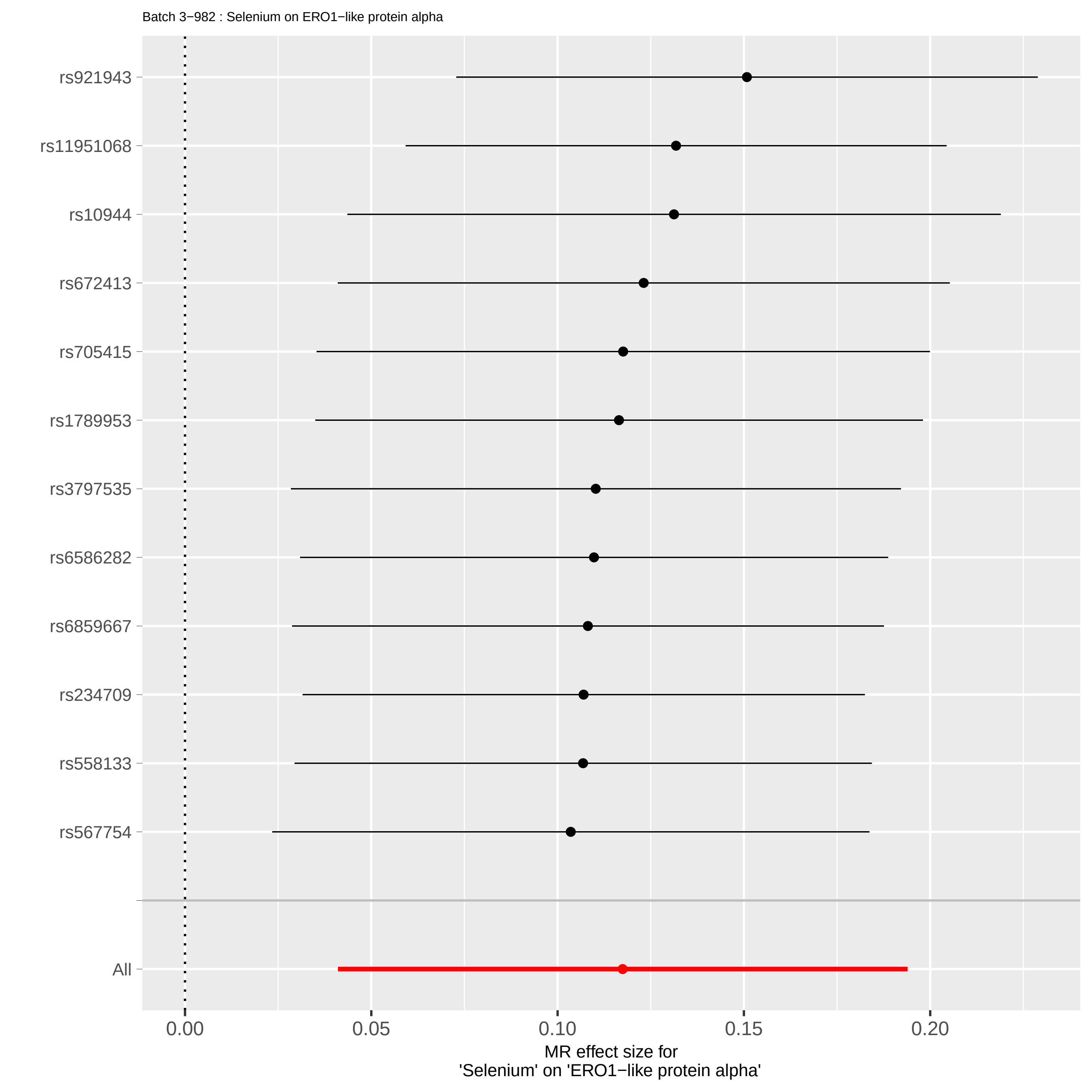
Batch 3−982 : Selenium on ERO1−like protein alpha
rs921943
rs11951068
rs10944
rs672413
rs705415
rs1789953
rs3797535
rs6586282
rs6859667
rs234709
rs558133
rs567754
All
0.00
0.05
0.10
0.15
0.20
MR effect size for
'Selenium' on 'ERO1−like protein alpha'

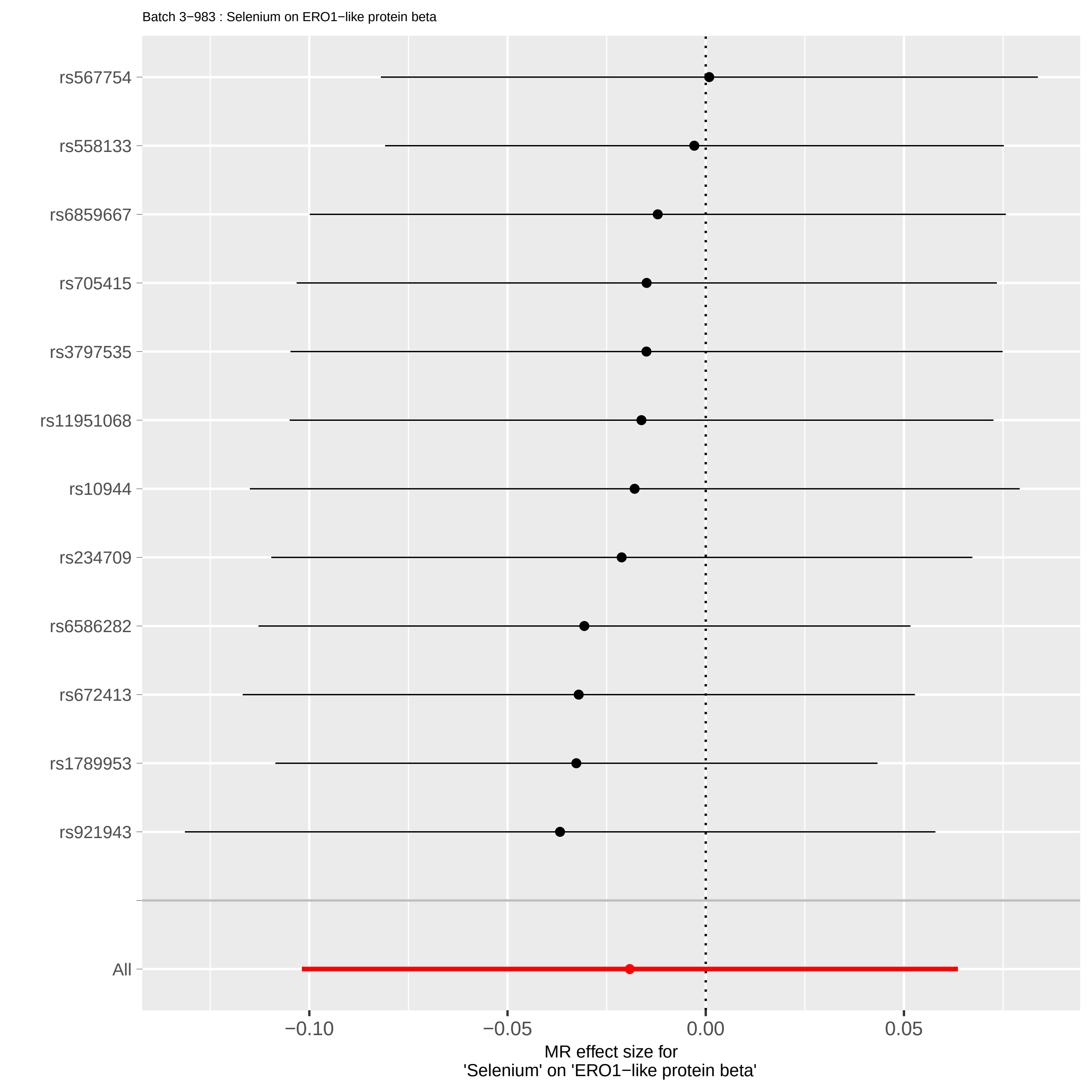
Batch 3−983 : Selenium on ERO1−like protein beta
rs567754
rs558133
rs6859667
rs705415
rs3797535
rs11951068
rs10944
rs234709
rs6586282
rs672413
rs1789953
rs921943
All
−0.10
−0.05
0.00
0.05
MR effect size for
'Selenium' on 'ERO1−like protein beta'

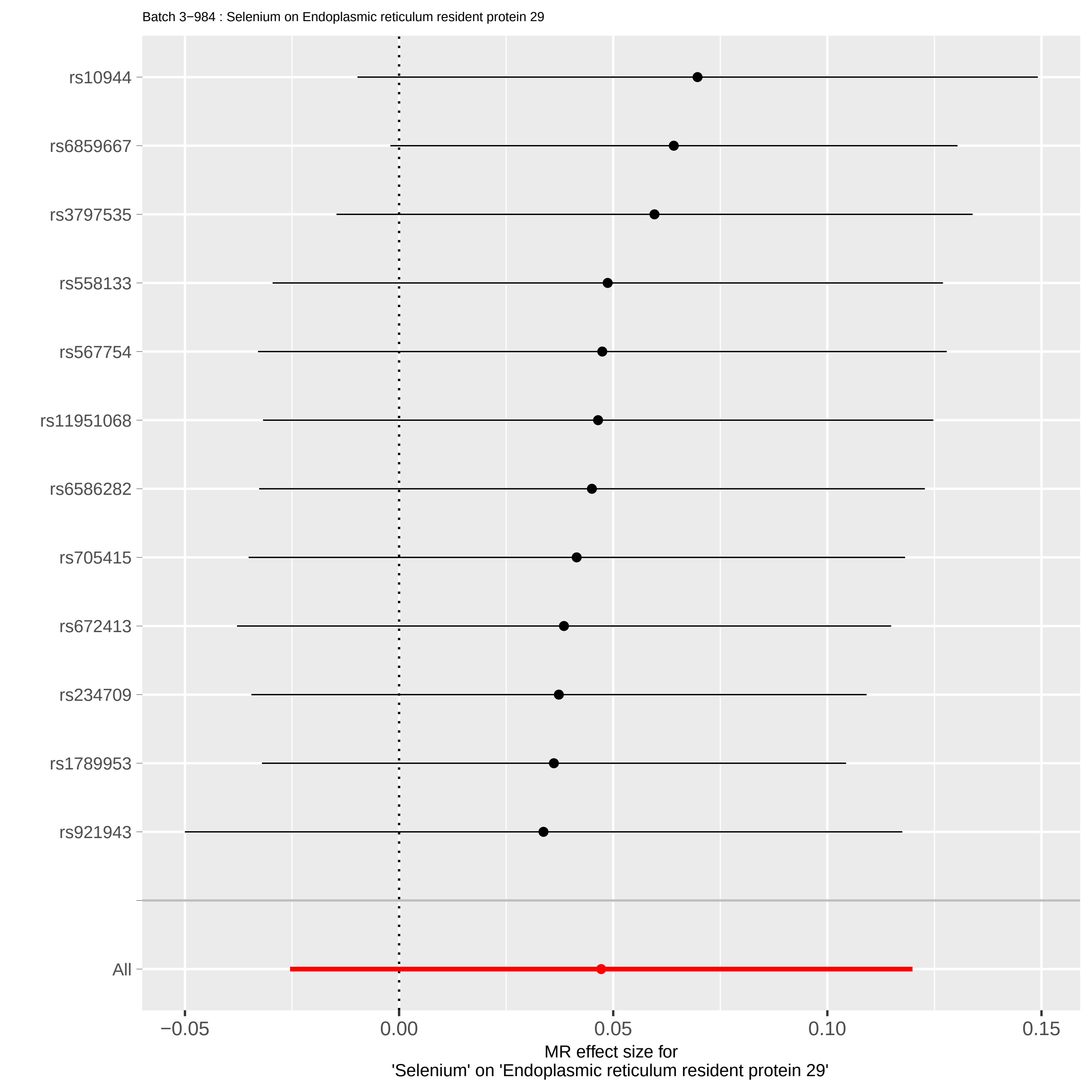

Batch 3−984 : Selenium on Endoplasmic reticulum resident protein 29
rs10944
rs6859667
rs3797535
rs558133
rs567754
rs11951068
rs6586282
rs705415
rs672413
rs234709
rs1789953
rs921943
All
−0.05
0.00
0.05
0.10
0.15
MR effect size for
'Selenium' on 'Endoplasmic reticulum resident protein 29'

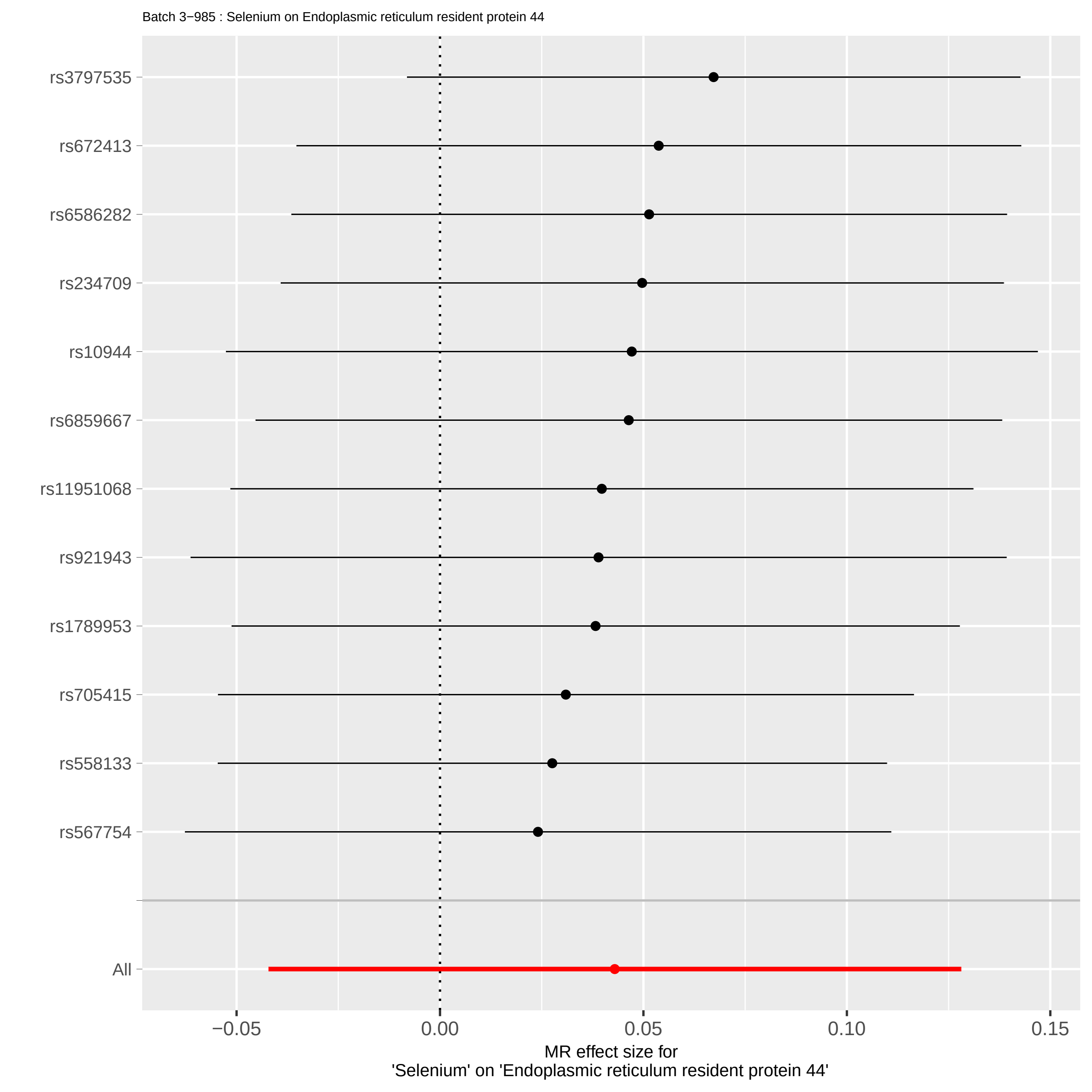

Batch 3−985 : Selenium on Endoplasmic reticulum resident protein 44
rs3797535
rs672413
rs6586282
rs234709
rs10944
rs6859667
rs11951068
rs921943
rs1789953
rs705415
rs558133
rs567754
All
−0.05
0.00
0.05
0.10
0.15
MR effect size for
'Selenium' on 'Endoplasmic reticulum resident protein 44'

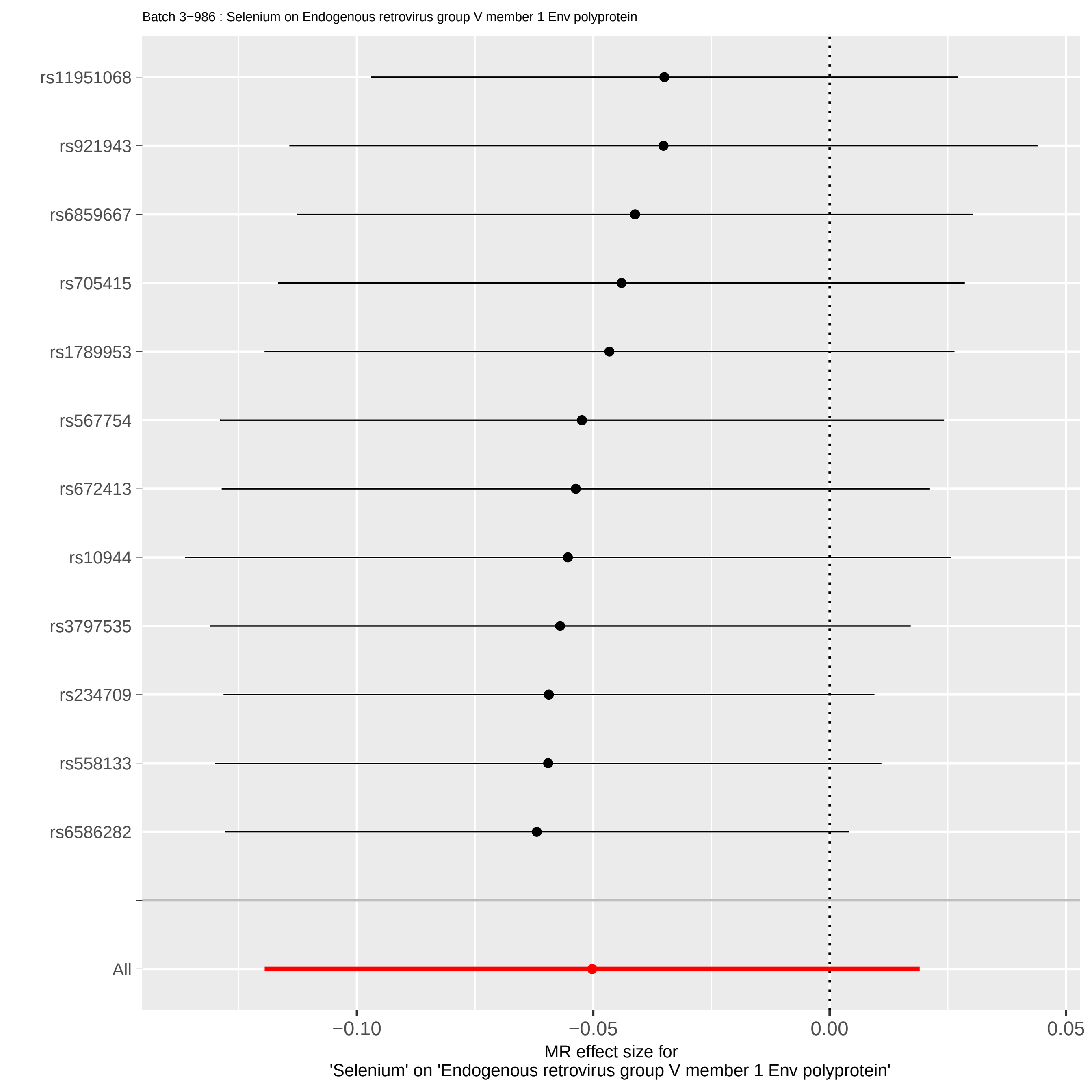

Batch 3−986 : Selenium on Endogenous retrovirus group V member 1 Env polyprotein
rs11951068
rs921943
rs6859667
rs705415
rs1789953
rs567754
rs672413
rs10944
rs3797535
rs234709
rs558133
rs6586282
All
−0.10
−0.05
0.00
0.05
MR effect size for
'Selenium' on 'Endogenous retrovirus group V member 1 Env polyprotein'

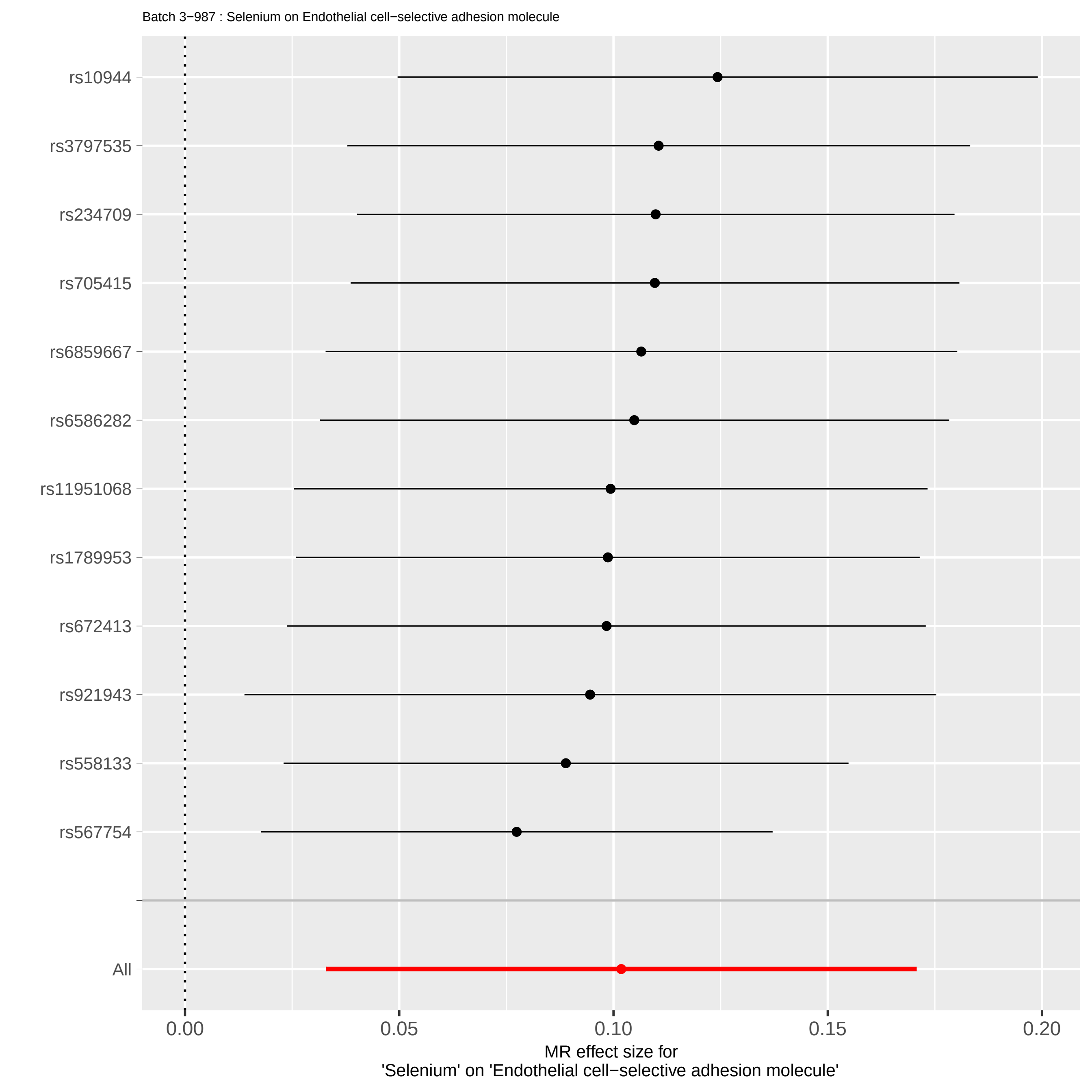

Batch 3−987 : Selenium on Endothelial cell−selective adhesion molecule
rs10944
rs3797535
rs234709
rs705415
rs6859667
rs6586282
rs11951068
rs1789953
rs672413
rs921943
rs558133
rs567754
All
0.00
0.05
0.10
0.15
0.20
MR effect size for
'Selenium' on 'Endothelial cell−selective adhesion molecule'

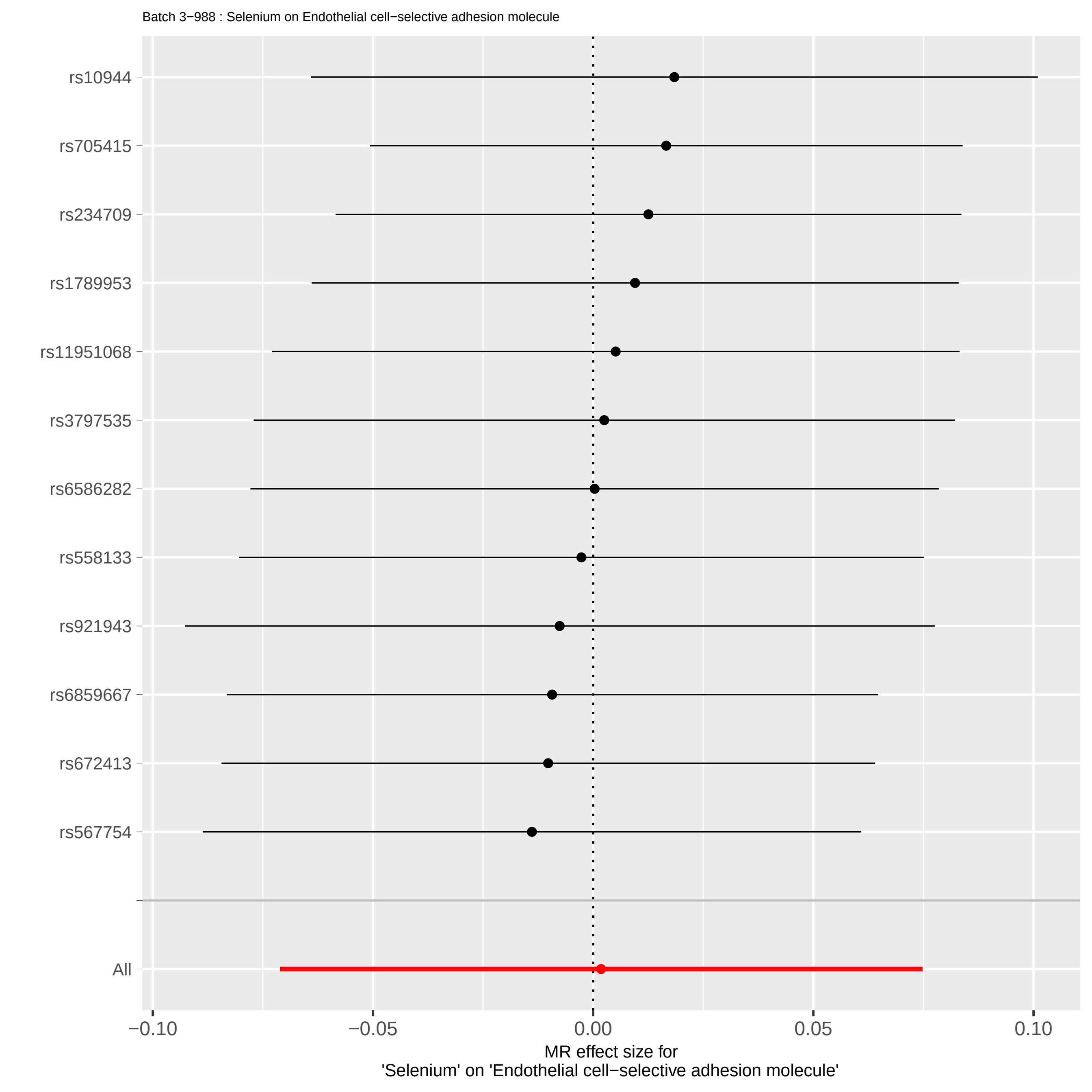
Batch 3−988 : Selenium on Endothelial cell−selective adhesion molecule
rs10944
rs705415
rs234709
rs1789953
rs11951068
rs3797535
rs6586282
rs558133
rs921943
rs6859667
rs672413
rs567754
All
−0.10
−0.05
0.00
0.05
0.10
MR effect size for
'Selenium' on 'Endothelial cell−selective adhesion molecule'

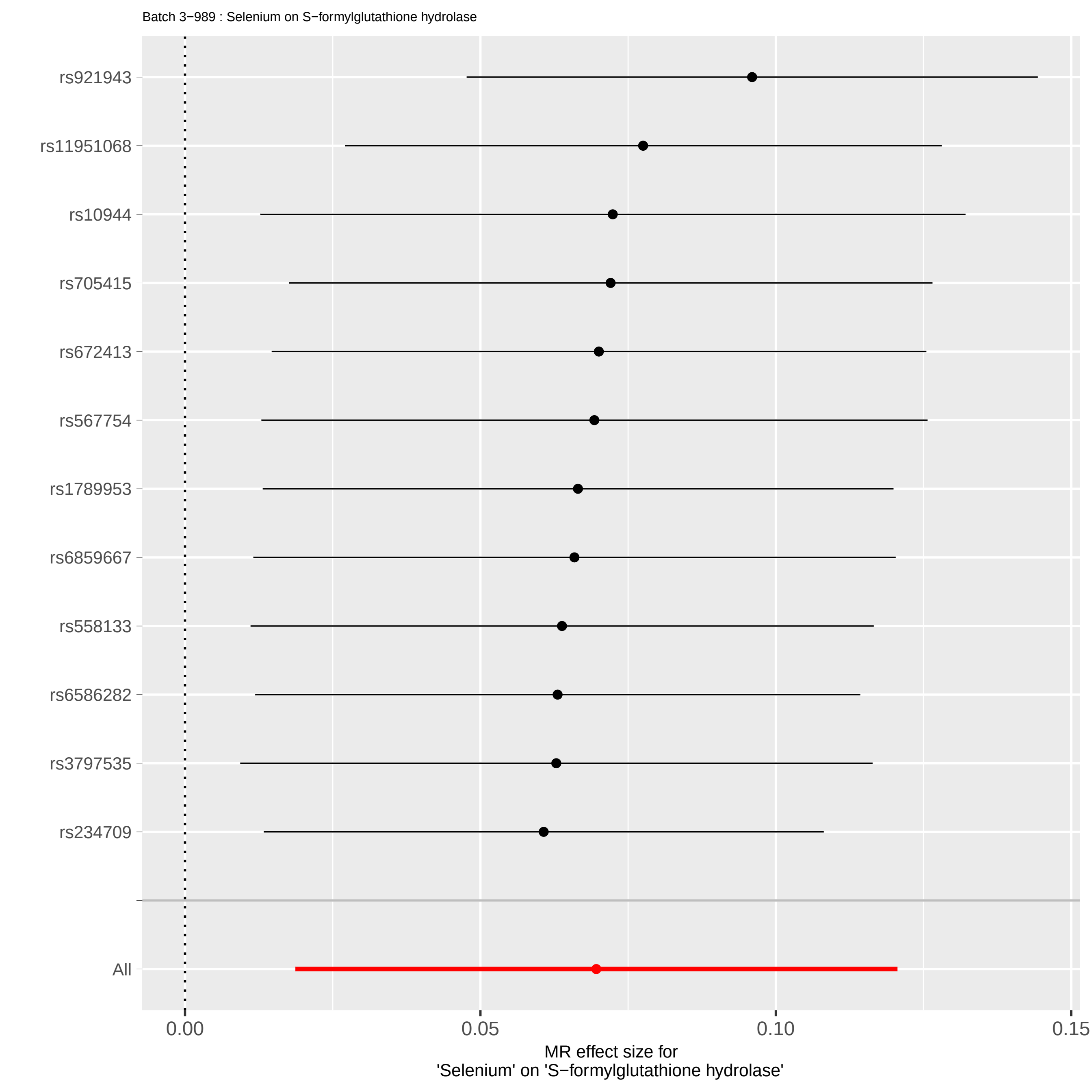

Batch 3−989 : Selenium on S−formylglutathione hydrolase
rs921943
rs11951068
rs10944
rs705415
rs672413
rs567754
rs1789953
rs6859667
rs558133
rs6586282
rs3797535
rs234709
All
0.00
0.05
0.10
0.15
MR effect size for
'Selenium' on 'S−formylglutathione hydrolase'

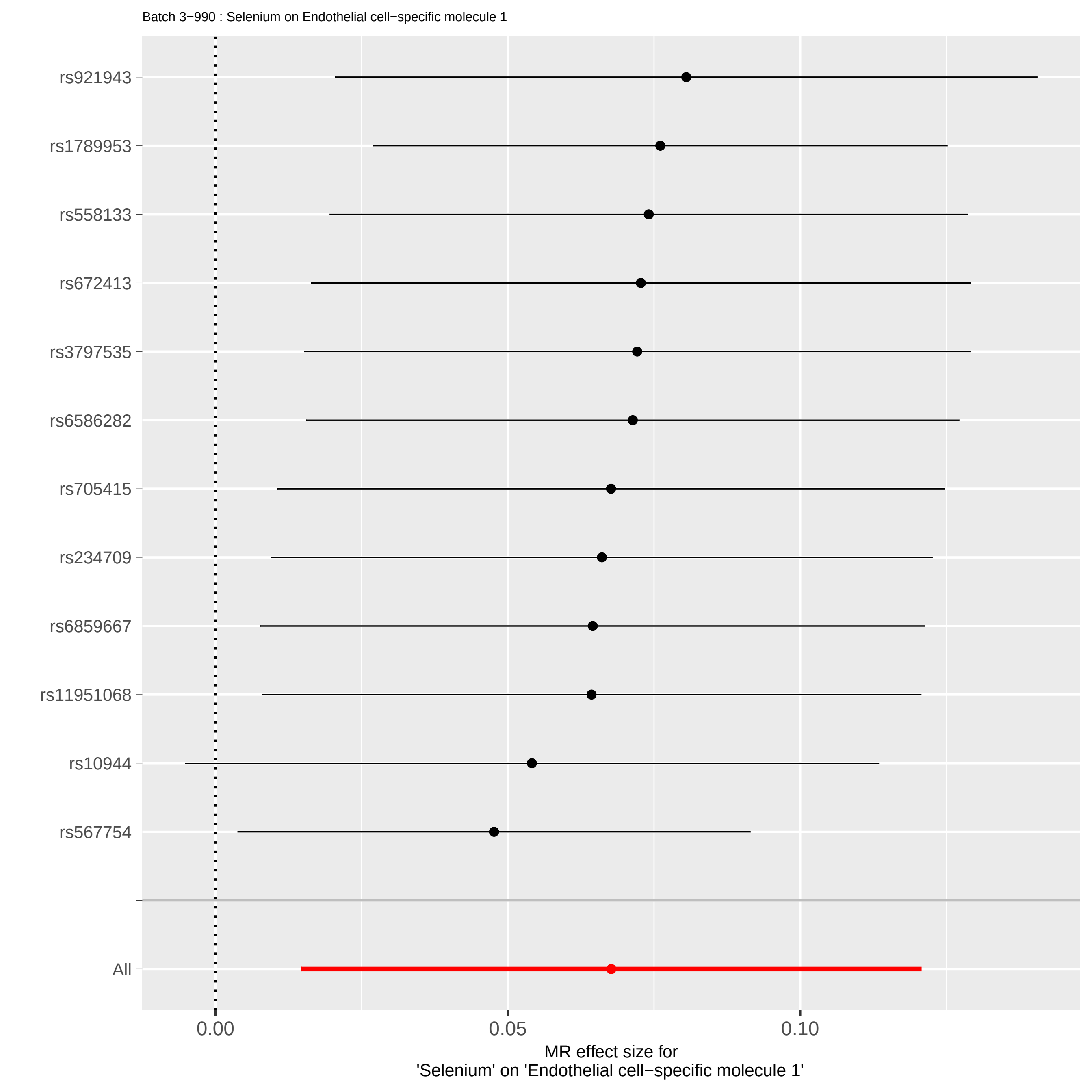
Batch 3−990 : Selenium on Endothelial cell−specific molecule 1
rs921943
rs1789953
rs558133
rs672413
rs3797535
rs6586282
rs705415
rs234709
rs6859667
rs11951068
rs10944
rs567754
All
0.00
0.05
0.10
MR effect size for
'Selenium' on 'Endothelial cell−specific molecule 1'

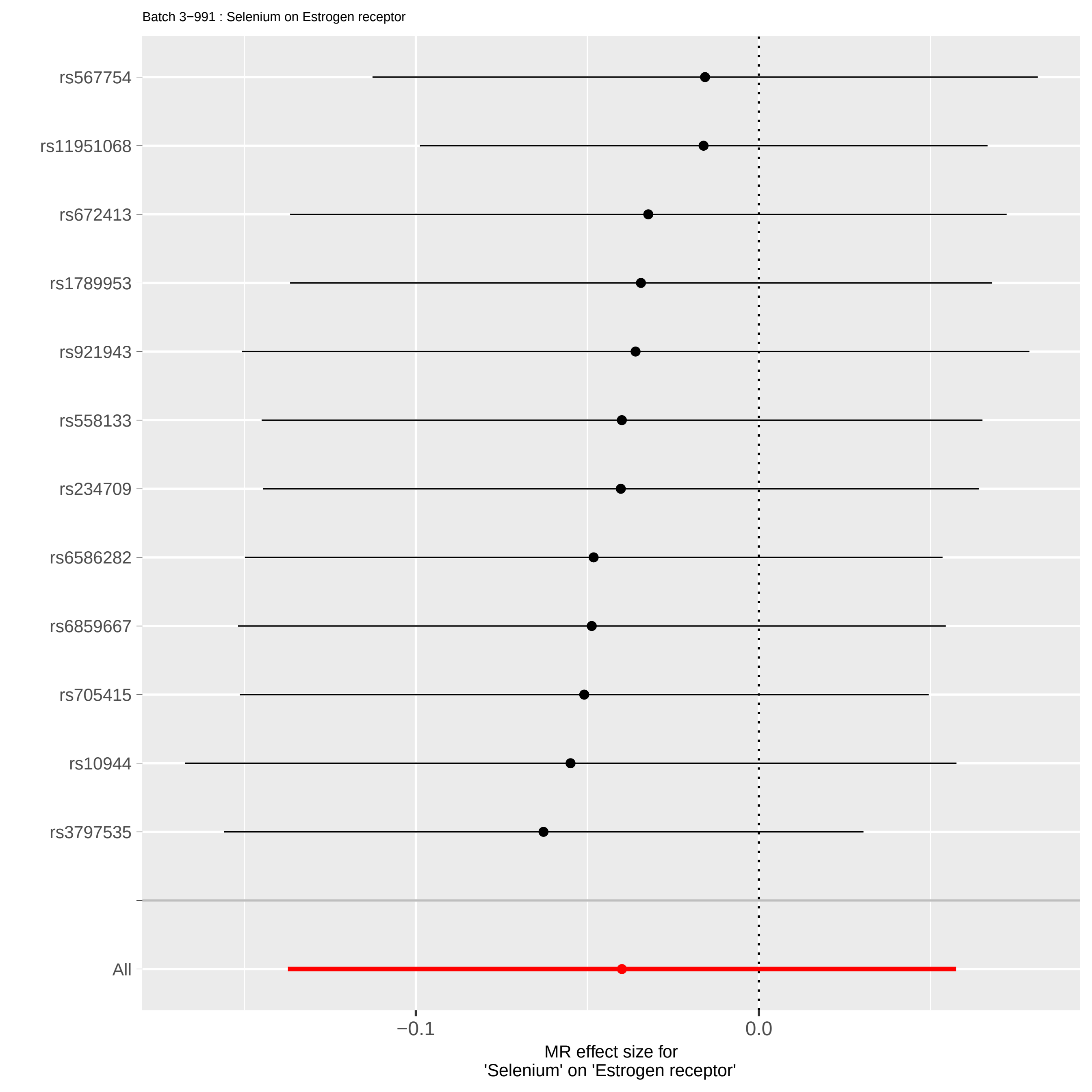

Batch 3−991 : Selenium on Estrogen receptor
rs567754
rs11951068
rs672413
rs1789953
rs921943
rs558133
rs234709
rs6586282
rs6859667
rs705415
rs10944
rs3797535
All
−0.1
0.0
MR effect size for
'Selenium' on 'Estrogen receptor'

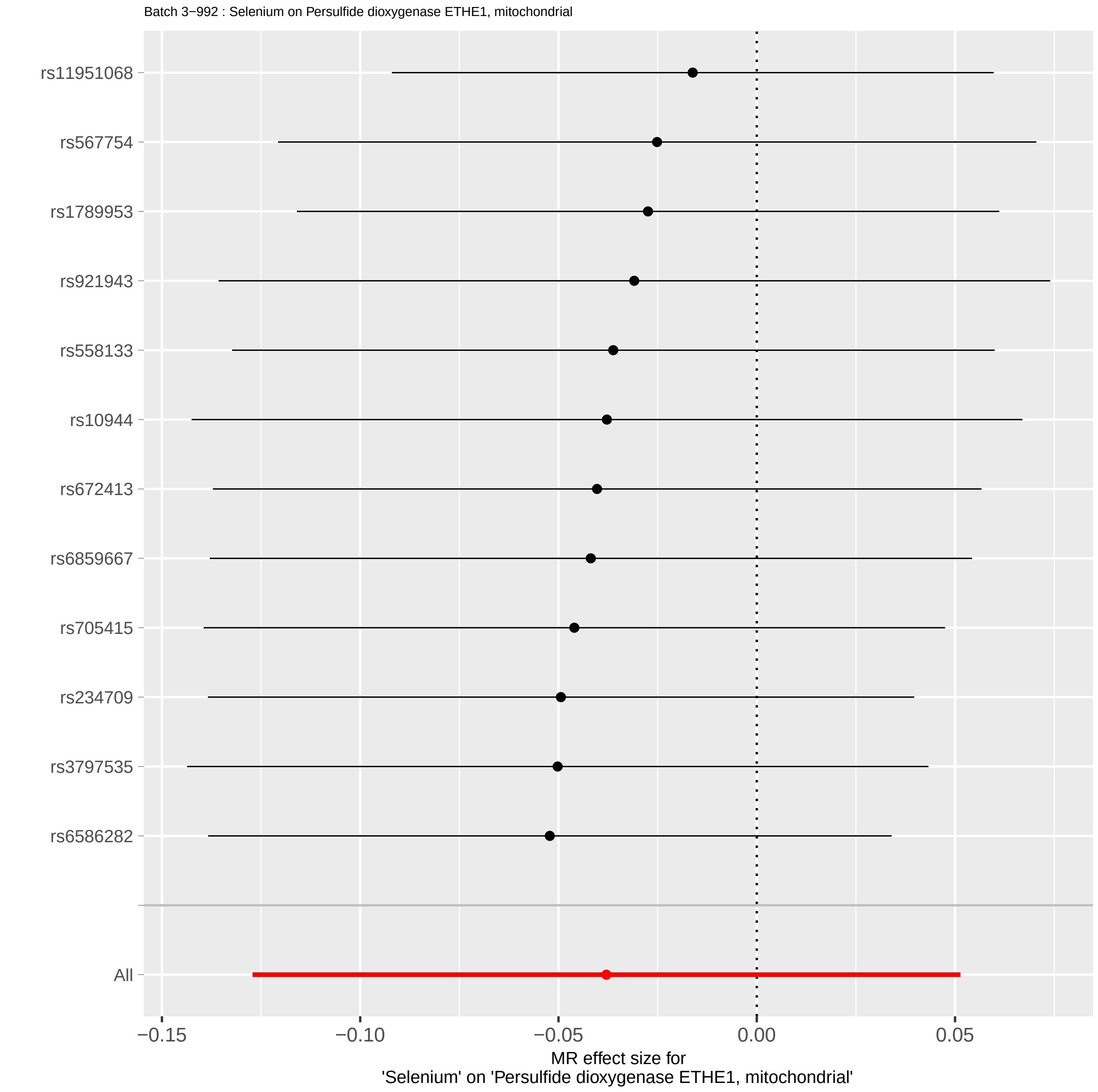

Batch 3−992 : Selenium on Persulfide dioxygenase ETHE1, mitochondrial
rs11951068
rs567754
rs1789953
rs921943
rs558133
rs10944
rs672413
rs6859667
rs705415
rs234709
rs3797535
rs6586282
All
−0.15
−0.10
−0.05
0.00
0.05
MR effect size for
'Selenium' on 'Persulfide dioxygenase ETHE1, mitochondrial'

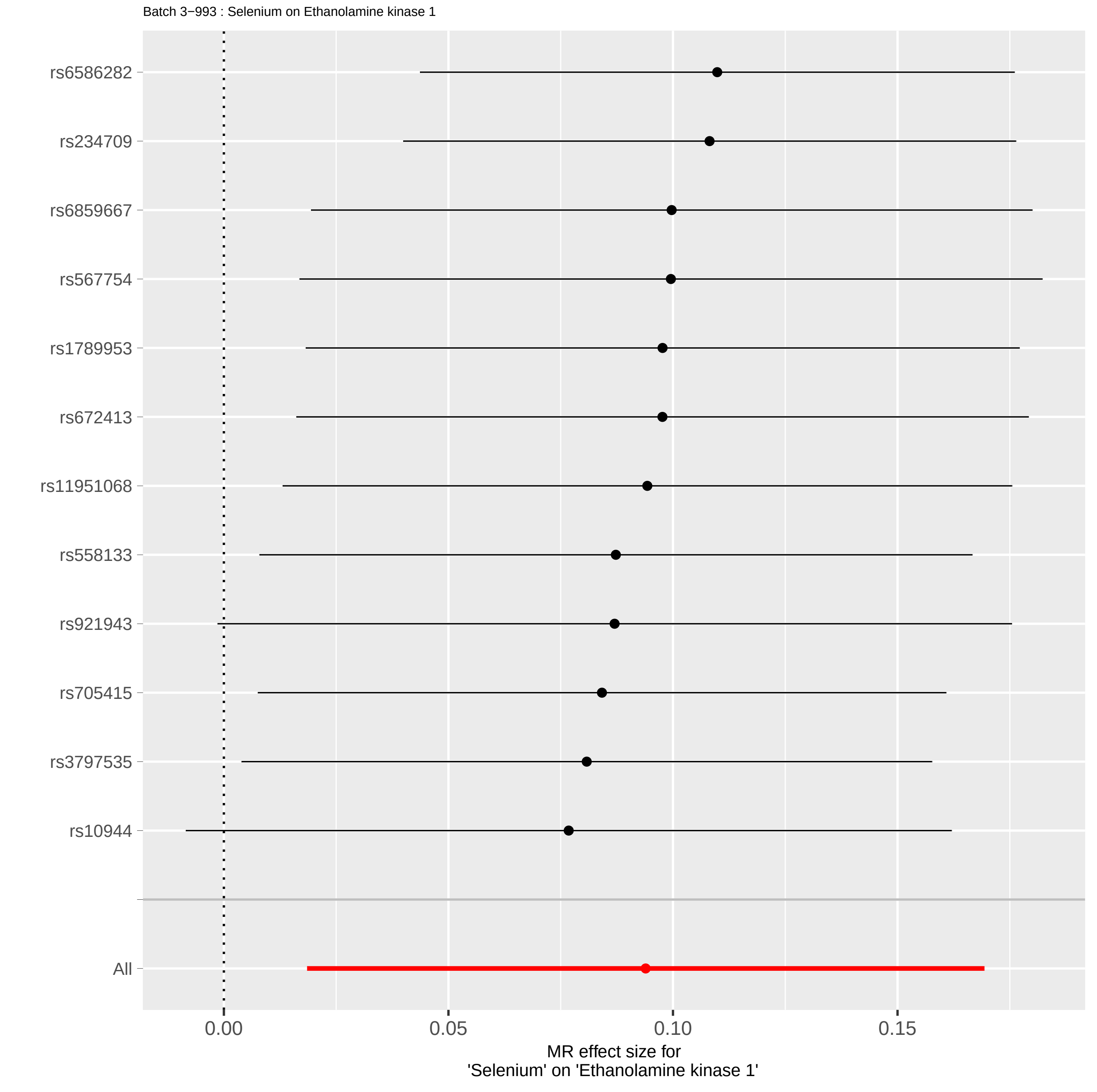
Batch 3−993 : Selenium on Ethanolamine kinase 1
rs6586282
rs234709
rs6859667
rs567754
rs1789953
rs672413
rs11951068
rs558133
rs921943
rs705415
rs3797535
rs10944
All
0.00
0.05
0.10
0.15
MR effect size for
'Selenium' on 'Ethanolamine kinase 1'

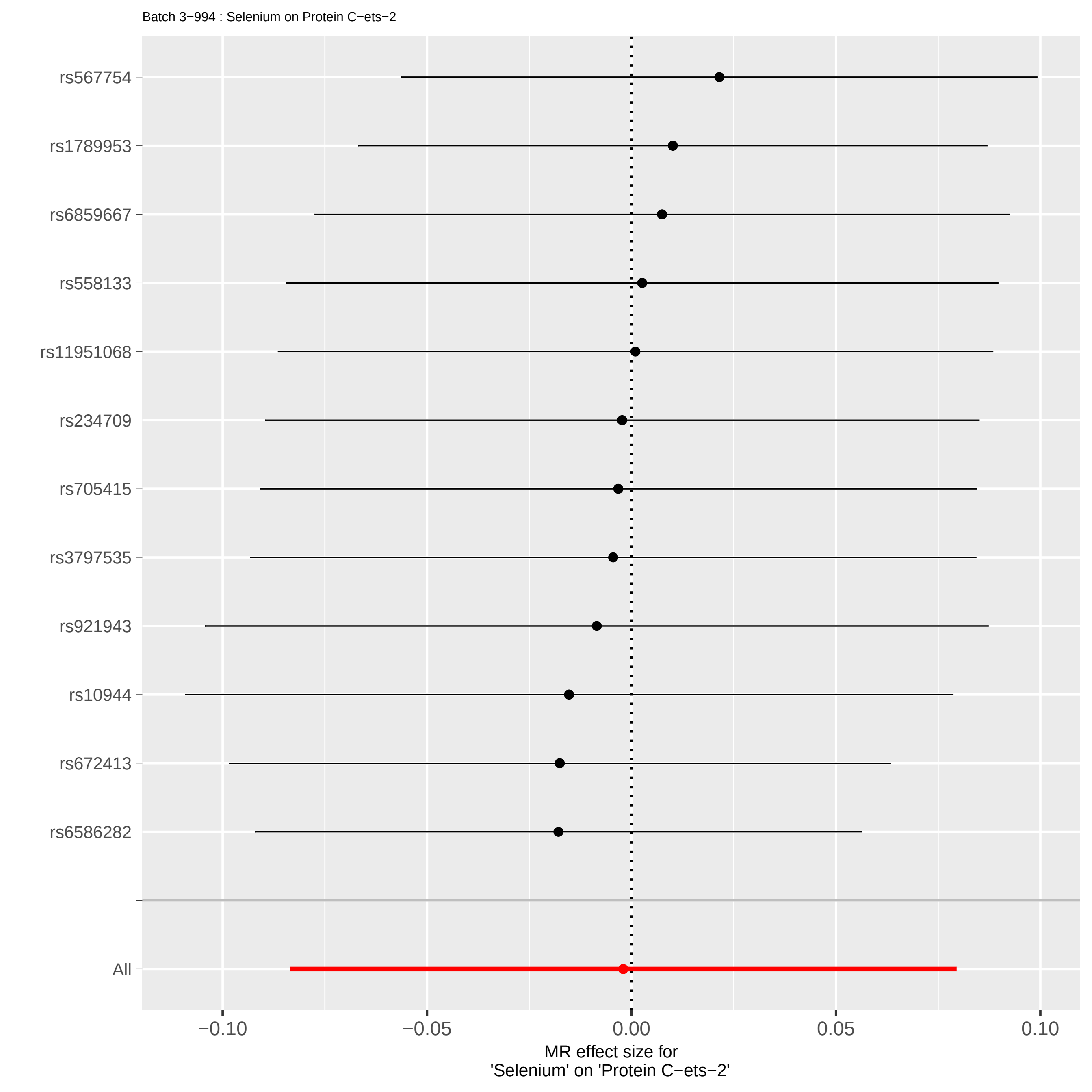

Batch 3−994 : Selenium on Protein C−ets−2
rs567754
rs1789953
rs6859667
rs558133
rs11951068
rs234709
rs705415
rs3797535
rs921943
rs10944
rs672413
rs6586282
All
−0.10
−0.05
0.00
0.05
0.10
MR effect size for
'Selenium' on 'Protein C−ets−2'

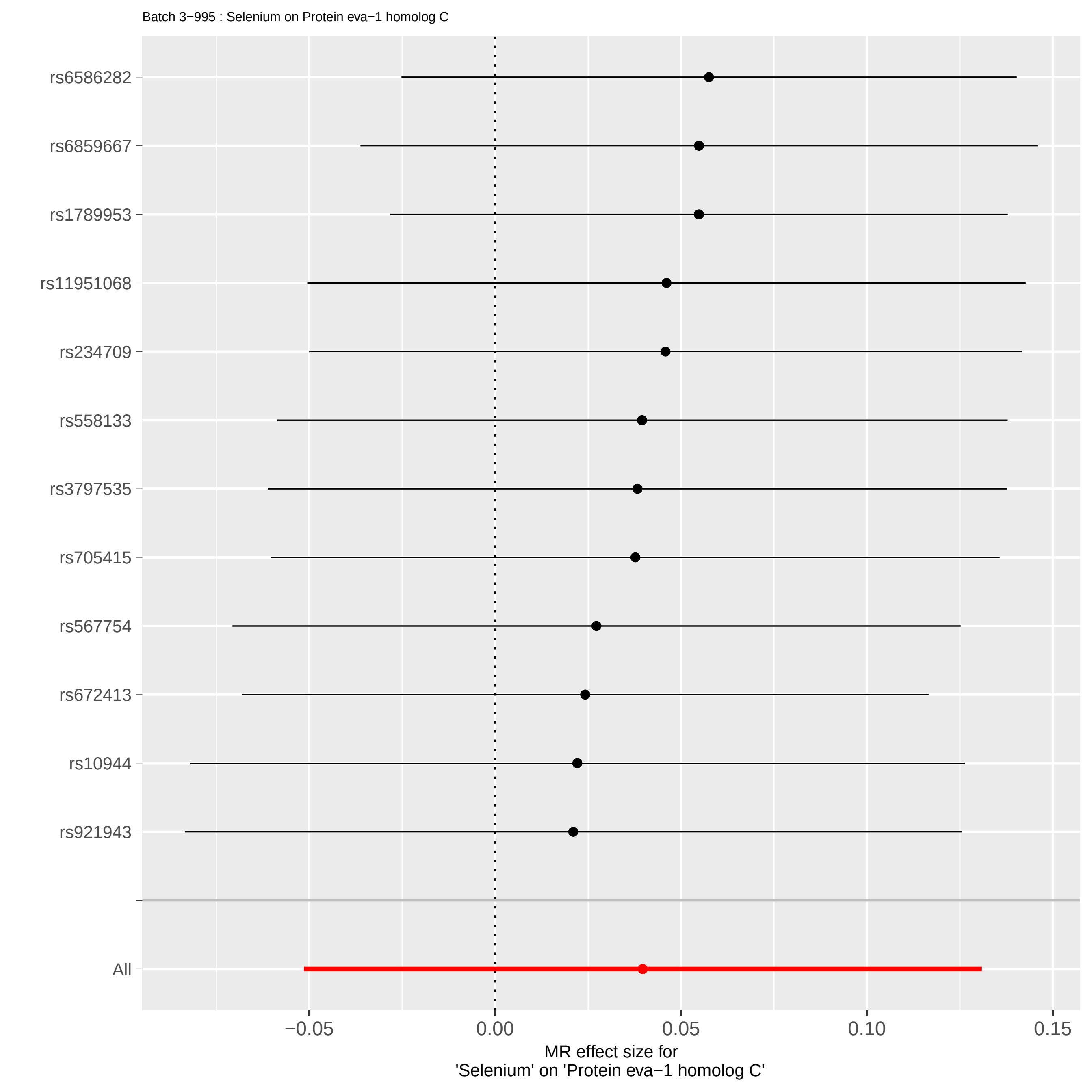

Batch 3−995 : Selenium on Protein eva−1 homolog C
rs6586282
rs6859667
rs1789953
rs11951068
rs234709
rs558133
rs3797535
rs705415
rs567754
rs672413
rs10944
rs921943
All
−0.05
0.00
0.05
0.10
0.15
MR effect size for
'Selenium' on 'Protein eva−1 homolog C'

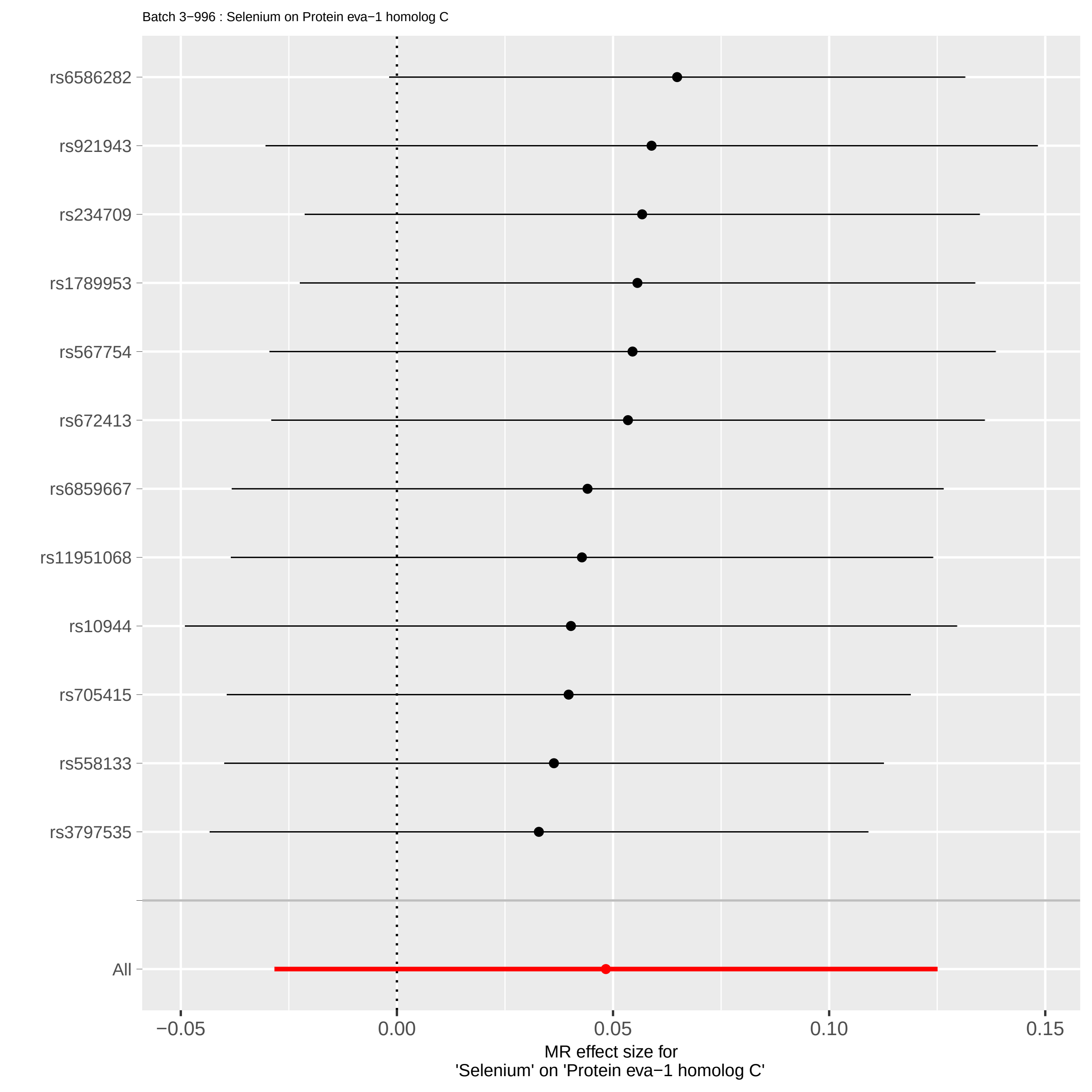

Batch 3−996 : Selenium on Protein eva−1 homolog C
rs6586282
rs921943
rs234709
rs1789953
rs567754
rs672413
rs6859667
rs11951068
rs10944
rs705415
rs558133
rs3797535
All
−0.05
0.00
0.05
0.10
0.15
MR effect size for
'Selenium' on 'Protein eva−1 homolog C'

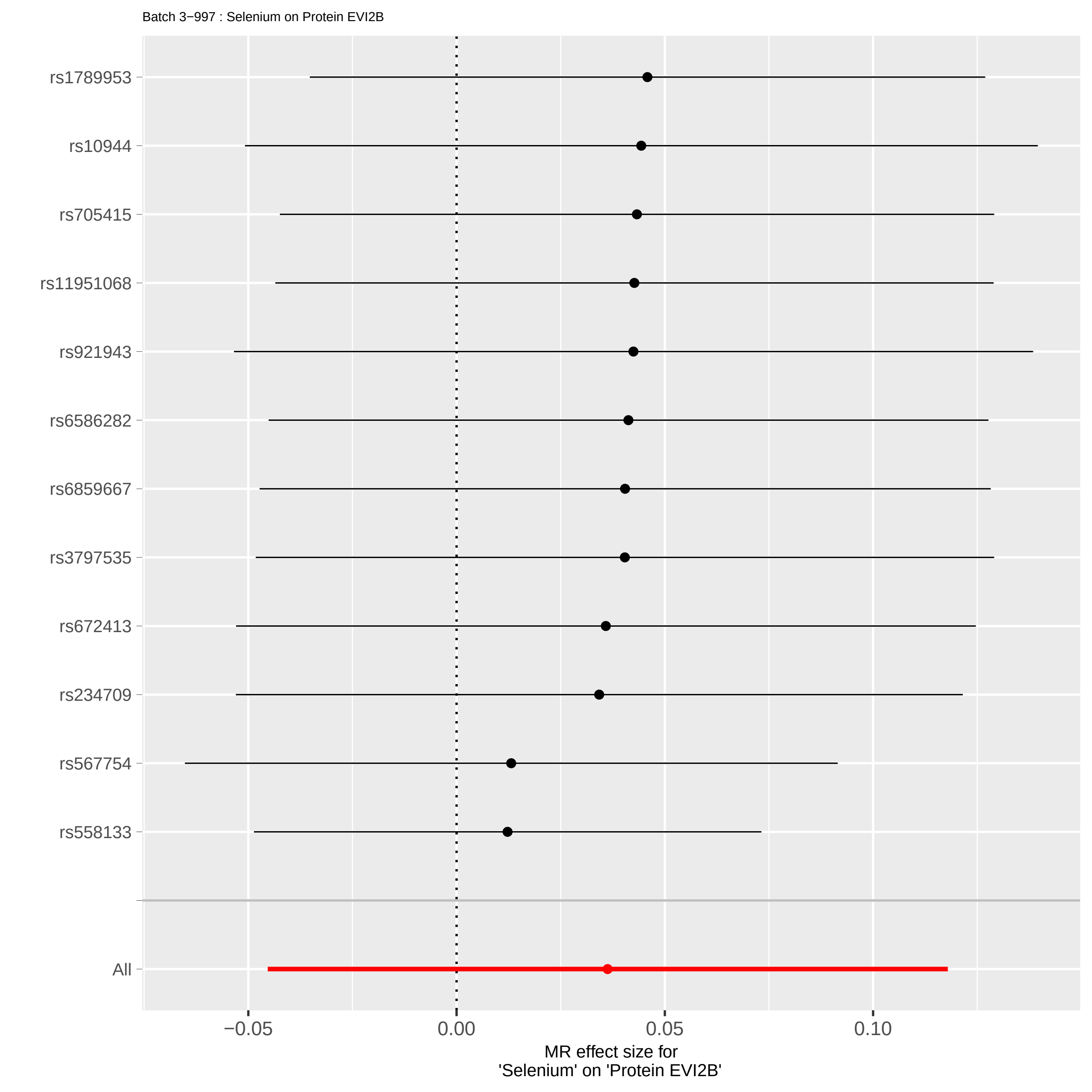

Batch 3−997 : Selenium on Protein EVI2B
rs1789953
rs10944
rs705415
rs11951068
rs921943
rs6586282
rs6859667
rs3797535
rs672413
rs234709
rs567754
rs558133
All
−0.05
0.00
0.05
0.10
MR effect size for
'Selenium' on 'Protein EVI2B'

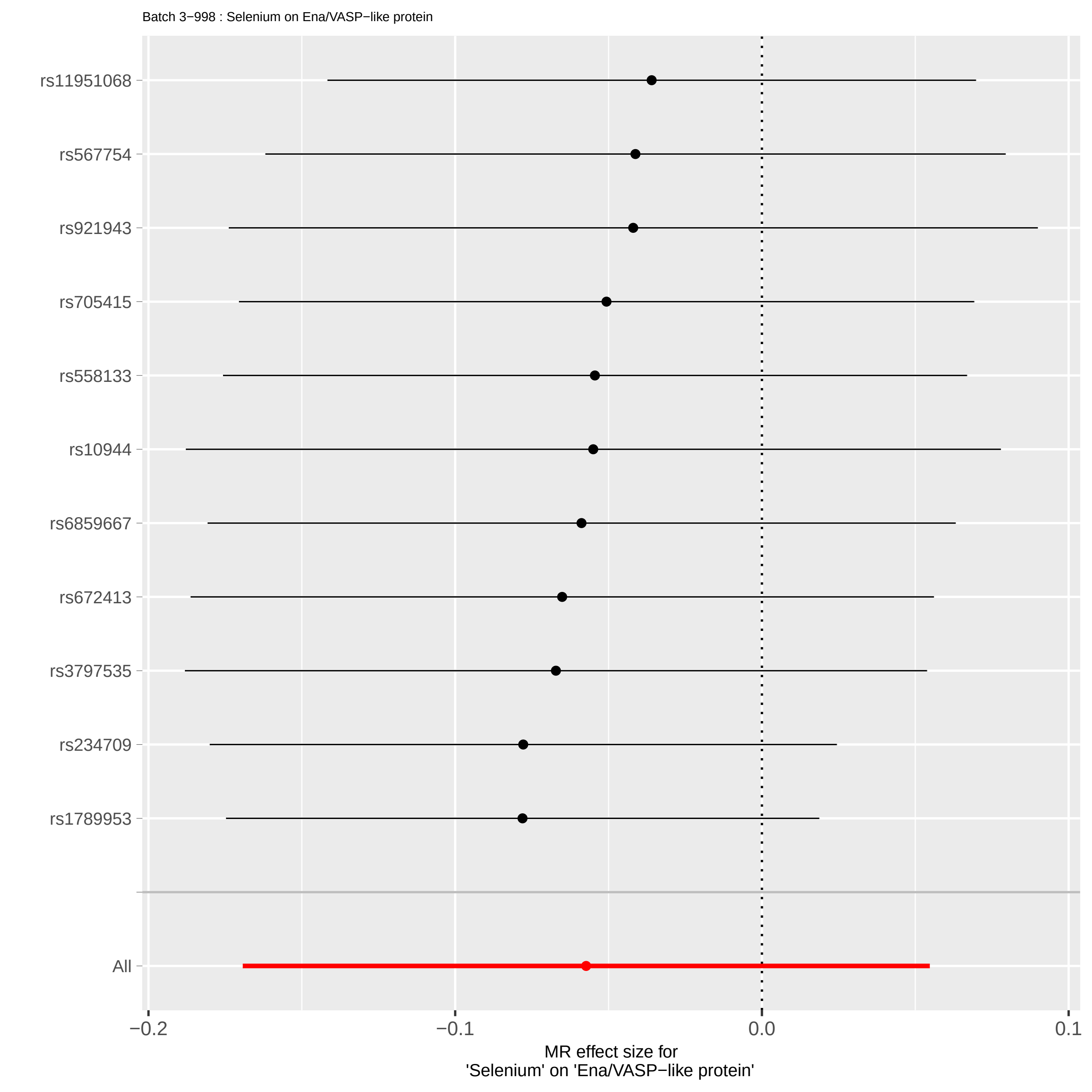

Batch 3−998 : Selenium on Ena/VASP−like protein
rs11951068
rs567754
rs921943
rs705415
rs558133
rs10944
rs6859667
rs672413
rs3797535
rs234709
rs1789953
All
−0.2
−0.1
0.0
0.1
MR effect size for
'Selenium' on 'Ena/VASP−like protein'

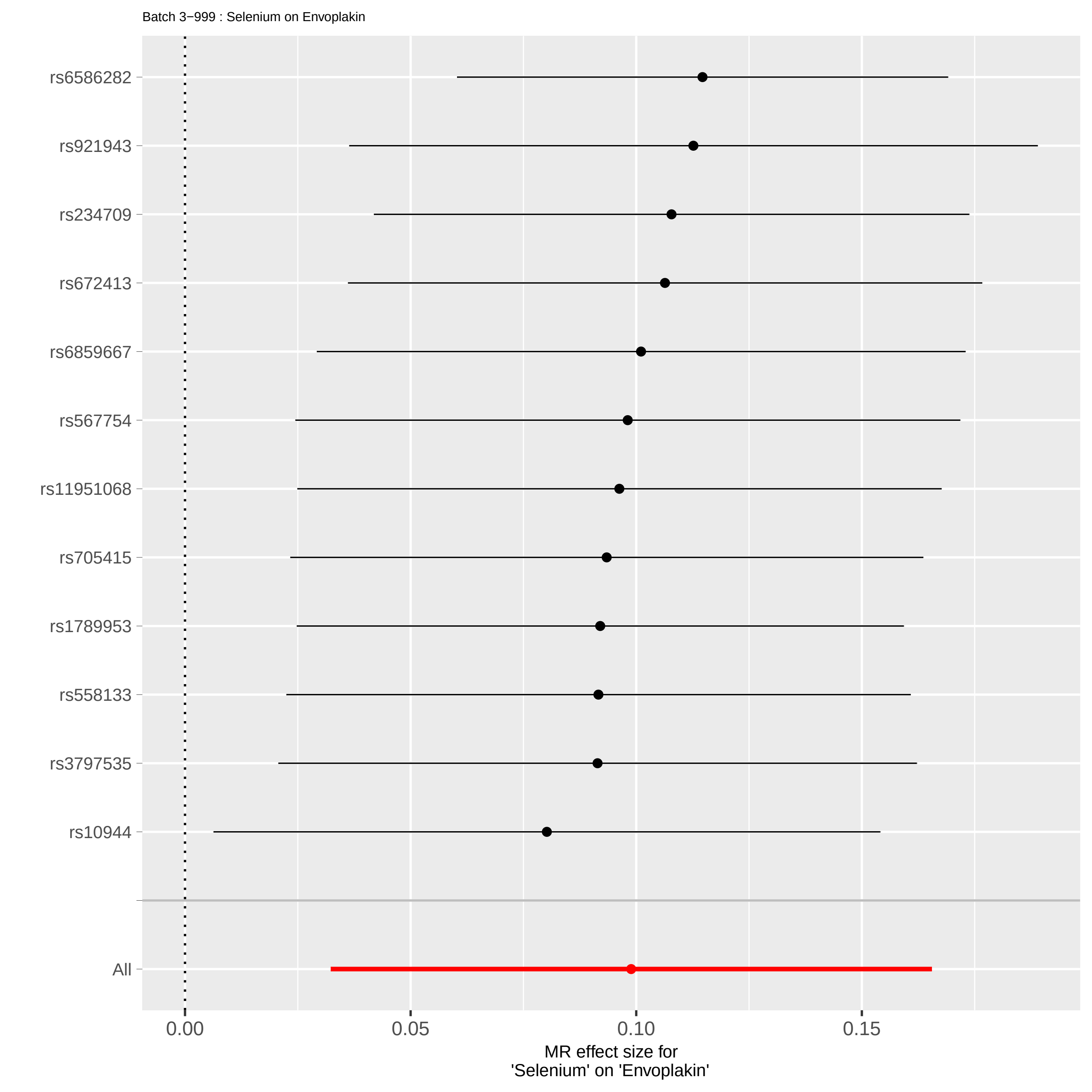

Batch 3−999 : Selenium on Envoplakin
rs6586282
rs921943
rs234709
rs672413
rs6859667
rs567754
rs11951068
rs705415
rs1789953
rs558133
rs3797535
rs10944
All
0.00
0.05
0.10
0.15
MR effect size for
'Selenium' on 'Envoplakin'

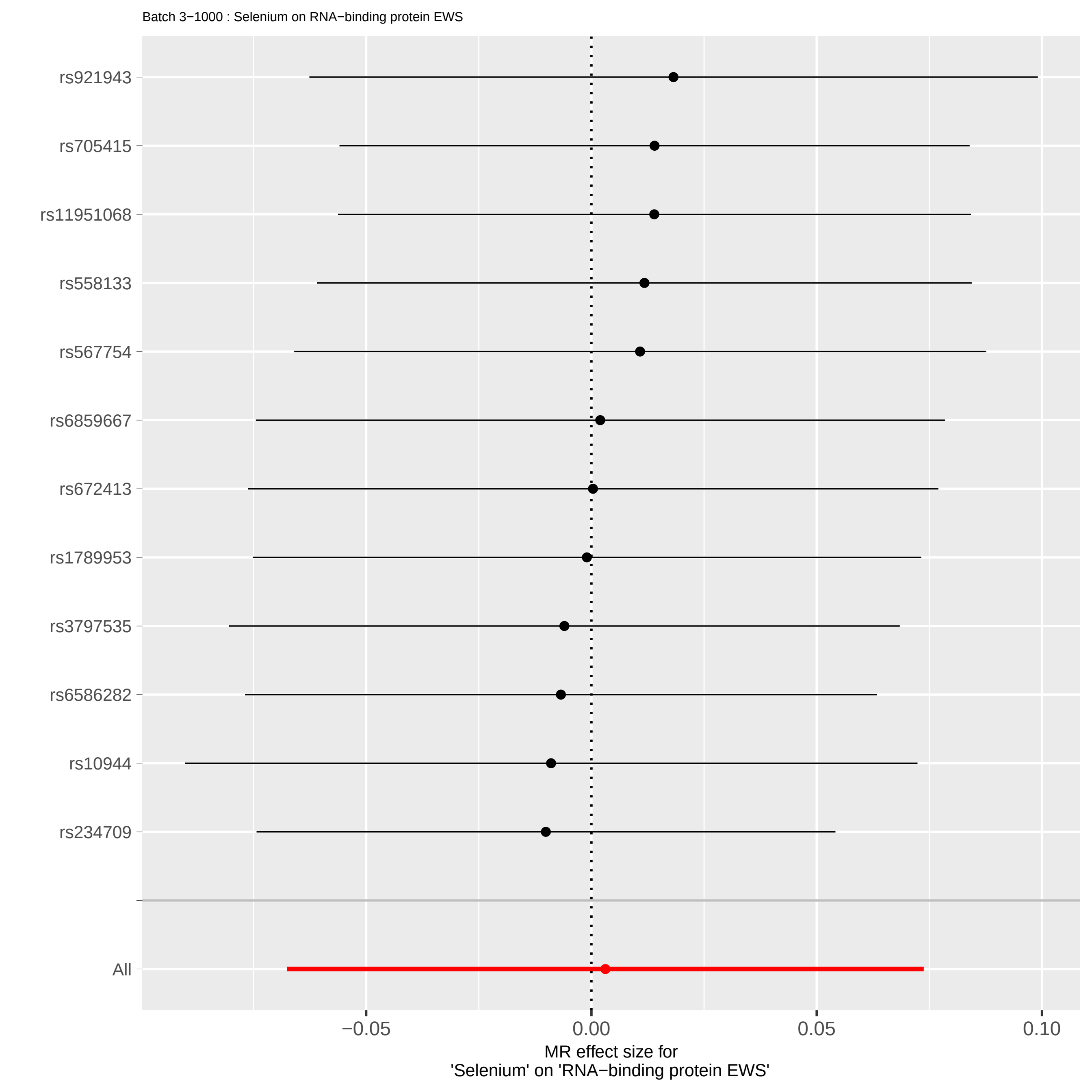
Batch 3−1000 : Selenium on RNA−binding protein EWS
rs921943
rs705415
rs11951068
rs558133
rs567754
rs6859667
rs672413
rs1789953
rs3797535
rs6586282
rs10944
rs234709
All
−0.05
0.00
0.05
0.10
MR effect size for
'Selenium' on 'RNA−binding protein EWS'

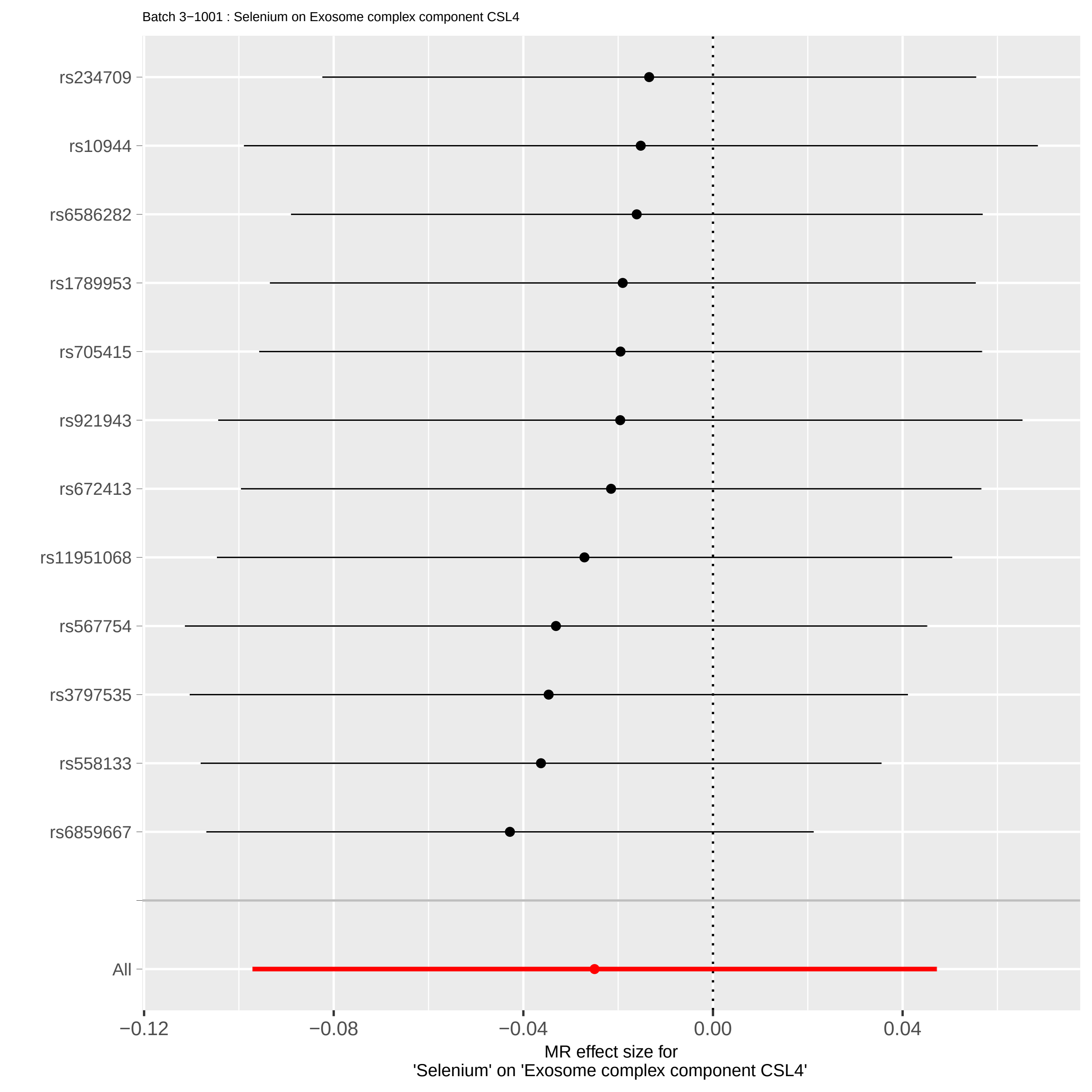

Batch 3−1001 : Selenium on Exosome complex component CSL4
rs234709
rs10944
rs6586282
rs1789953
rs705415
rs921943
rs672413
rs11951068
rs567754
rs3797535
rs558133
rs6859667
All
−0.12
−0.08
−0.04
0.00
0.04
MR effect size for
'Selenium' on 'Exosome complex component CSL4'

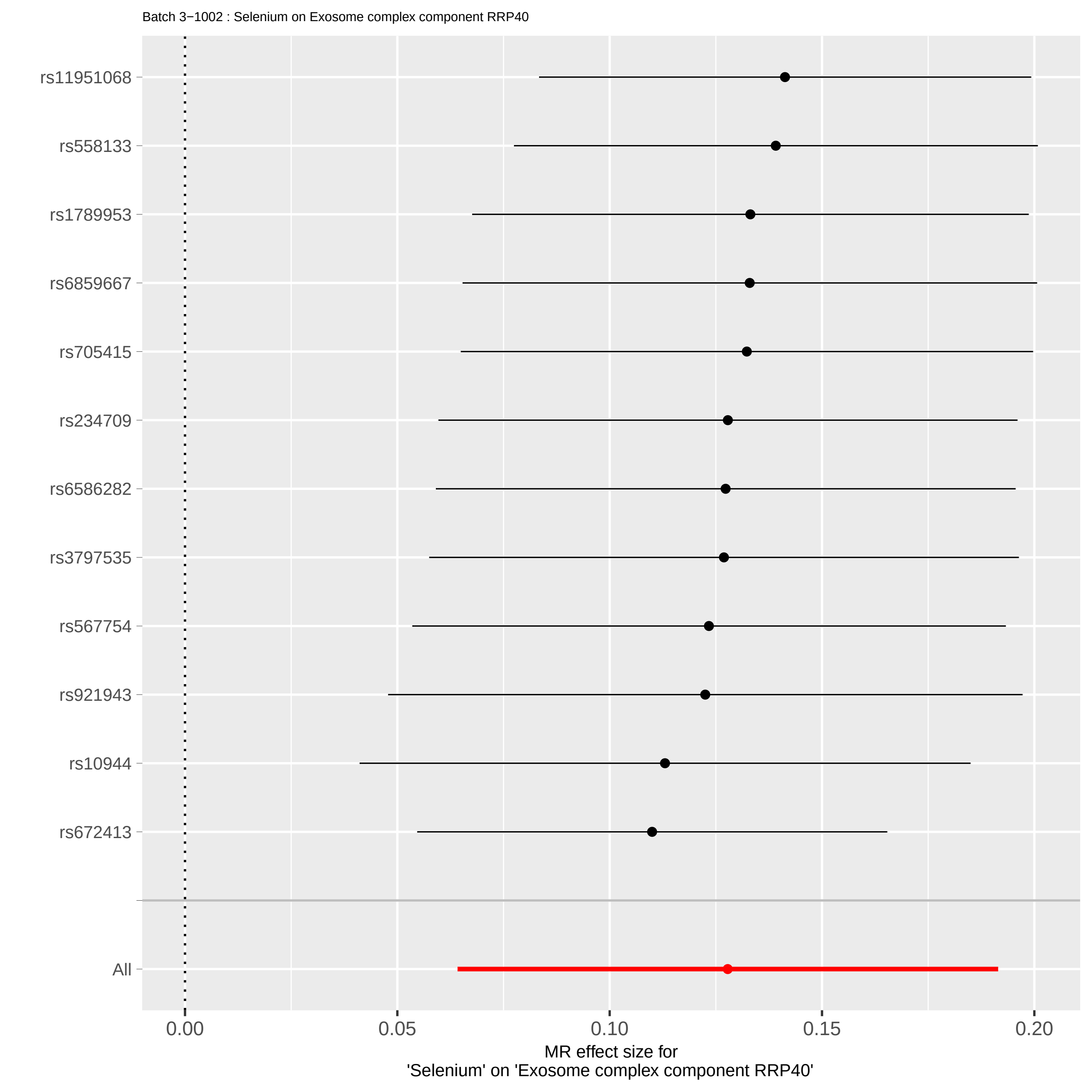

Batch 3−1002 : Selenium on Exosome complex component RRP40
rs11951068
rs558133
rs1789953
rs6859667
rs705415
rs234709
rs6586282
rs3797535
rs567754
rs921943
rs10944
rs672413
All
0.00
0.05
0.10
0.15
0.20
MR effect size for
'Selenium' on 'Exosome complex component RRP40'

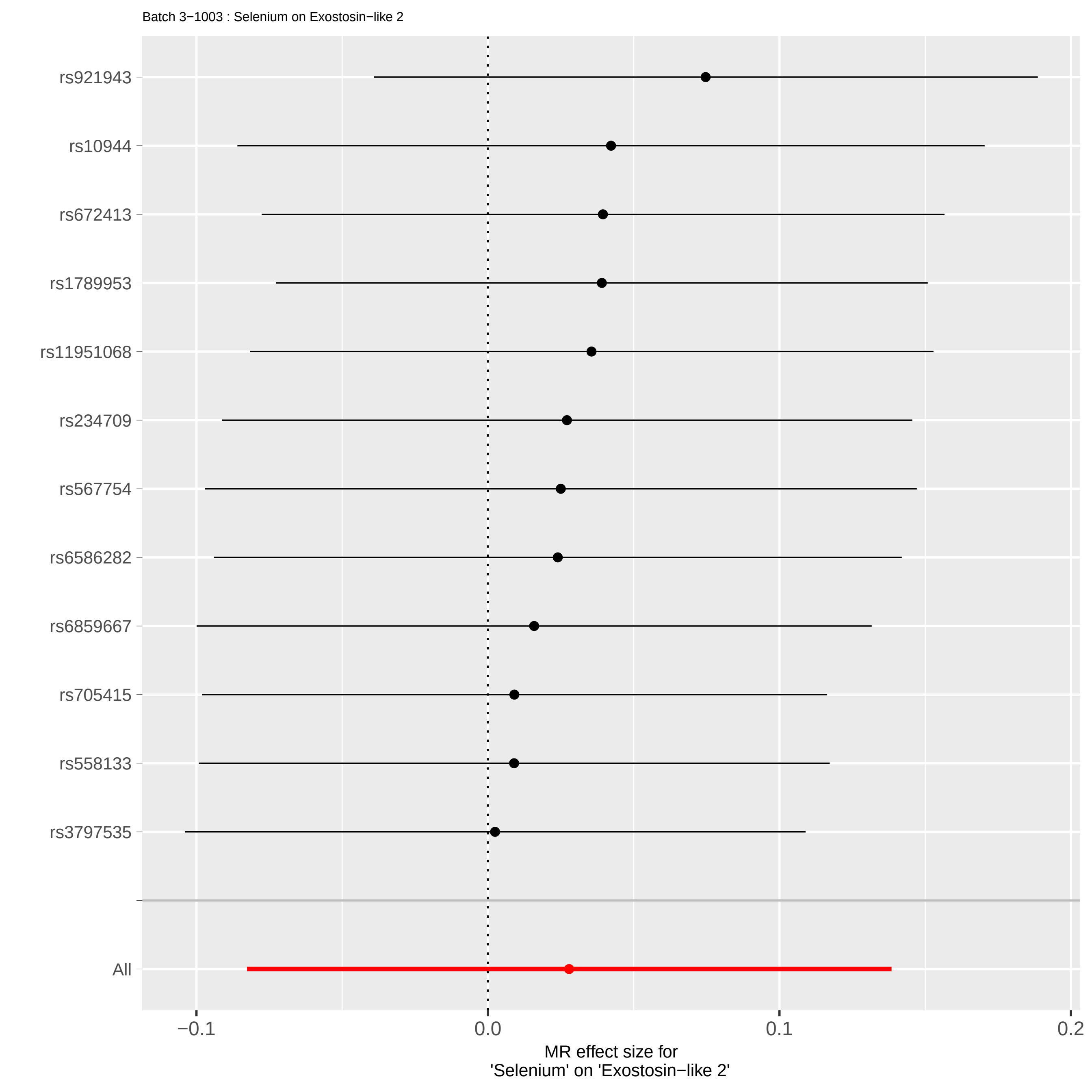

Batch 3−1003 : Selenium on Exostosin−like 2
rs921943
rs10944
rs672413
rs1789953
rs11951068
rs234709
rs567754
rs6586282
rs6859667
rs705415
rs558133
rs3797535
All
−0.1
0.0
0.1
0.2
MR effect size for
'Selenium' on 'Exostosin−like 2'

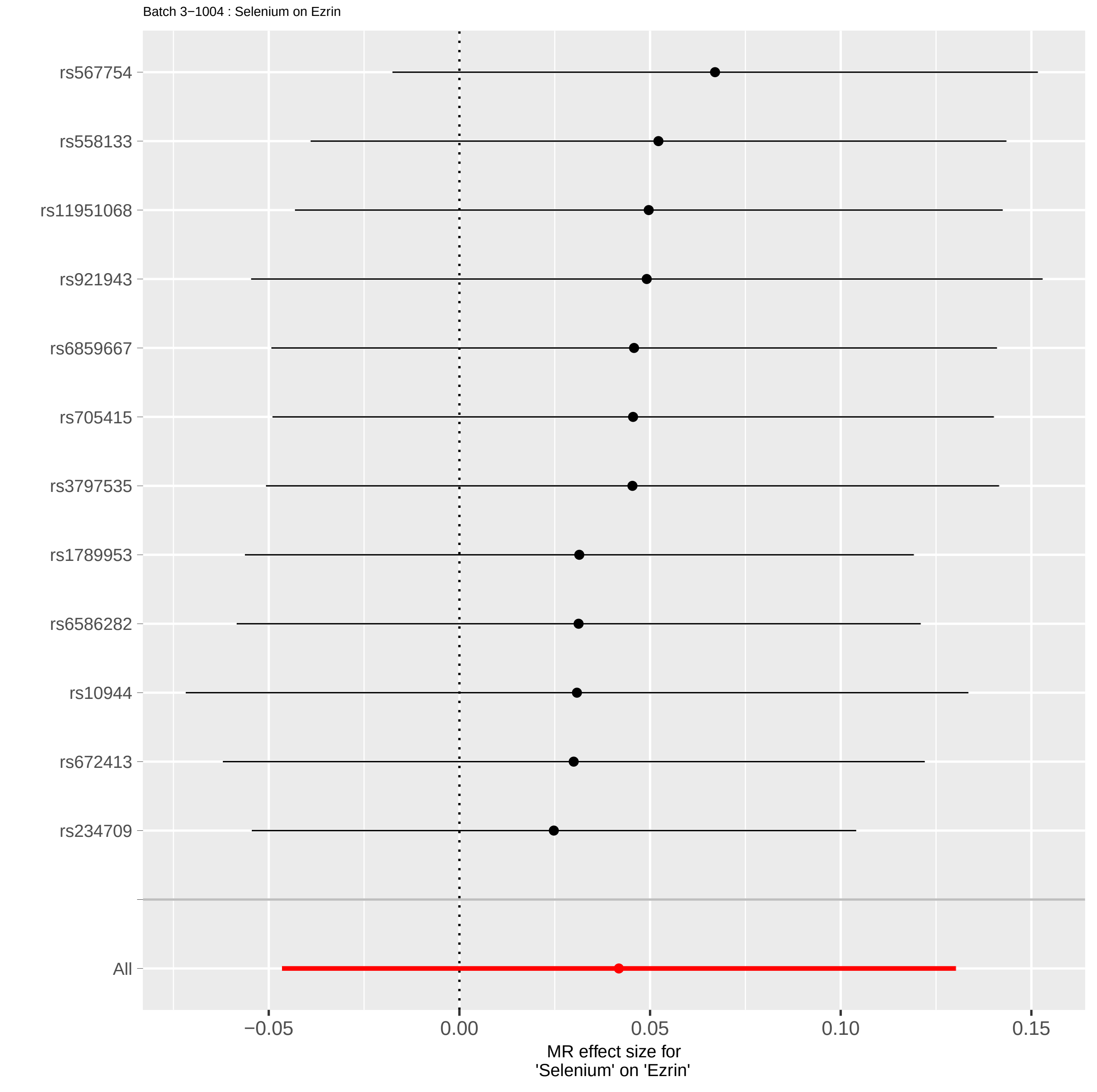

Batch 3−1004 : Selenium on Ezrin
rs567754
rs558133
rs11951068
rs921943
rs6859667
rs705415
rs3797535
rs1789953
rs6586282
rs10944
rs672413
rs234709
All
−0.05
0.00
0.05
0.10
0.15
MR effect size for
'Selenium' on 'Ezrin'

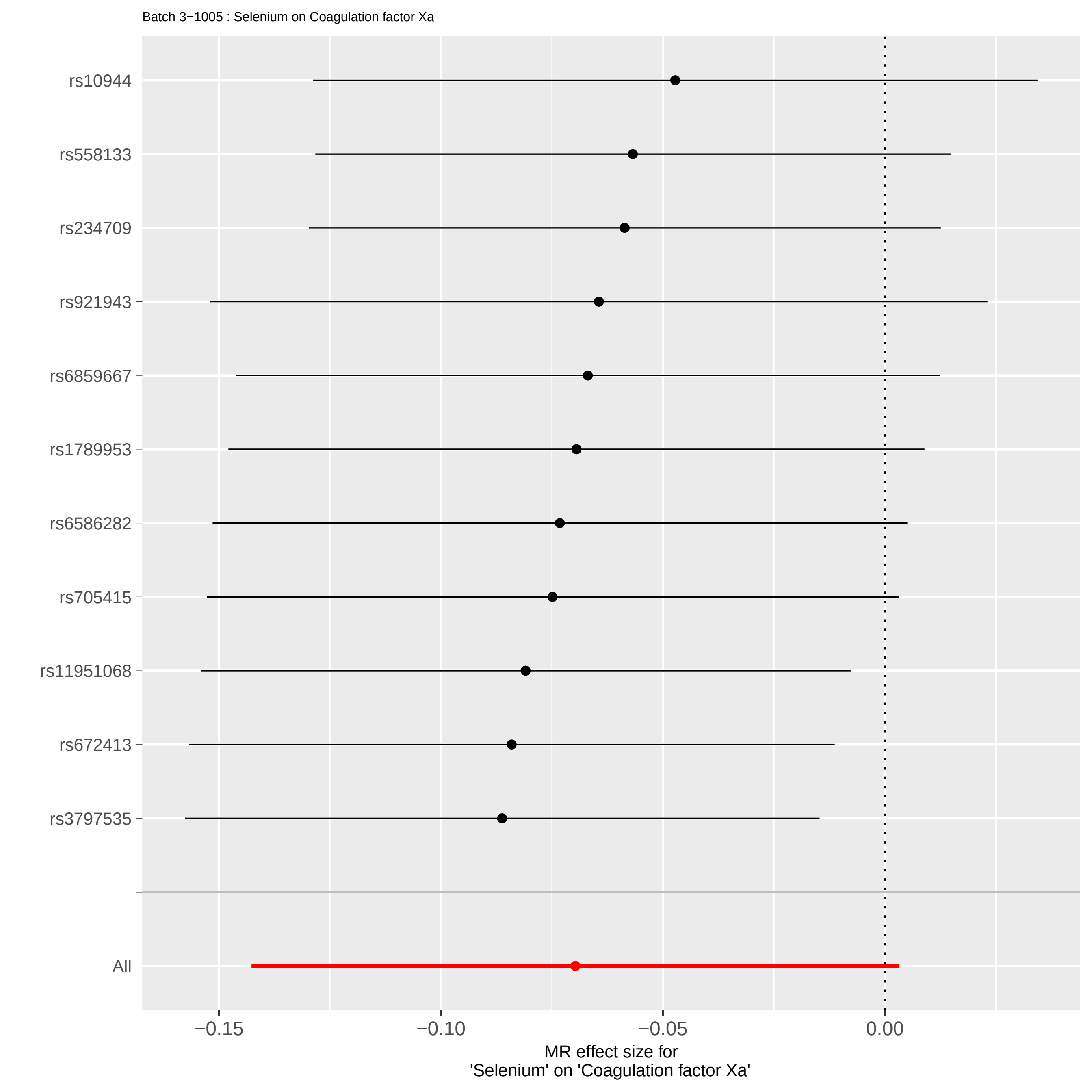

Batch 3−1005 : Selenium on Coagulation factor Xa
rs10944
rs558133
rs234709
rs921943
rs6859667
rs1789953
rs6586282
rs705415
rs11951068
rs672413
rs3797535
All
−0.15
−0.10
−0.05
0.00
MR effect size for
'Selenium' on 'Coagulation factor Xa'

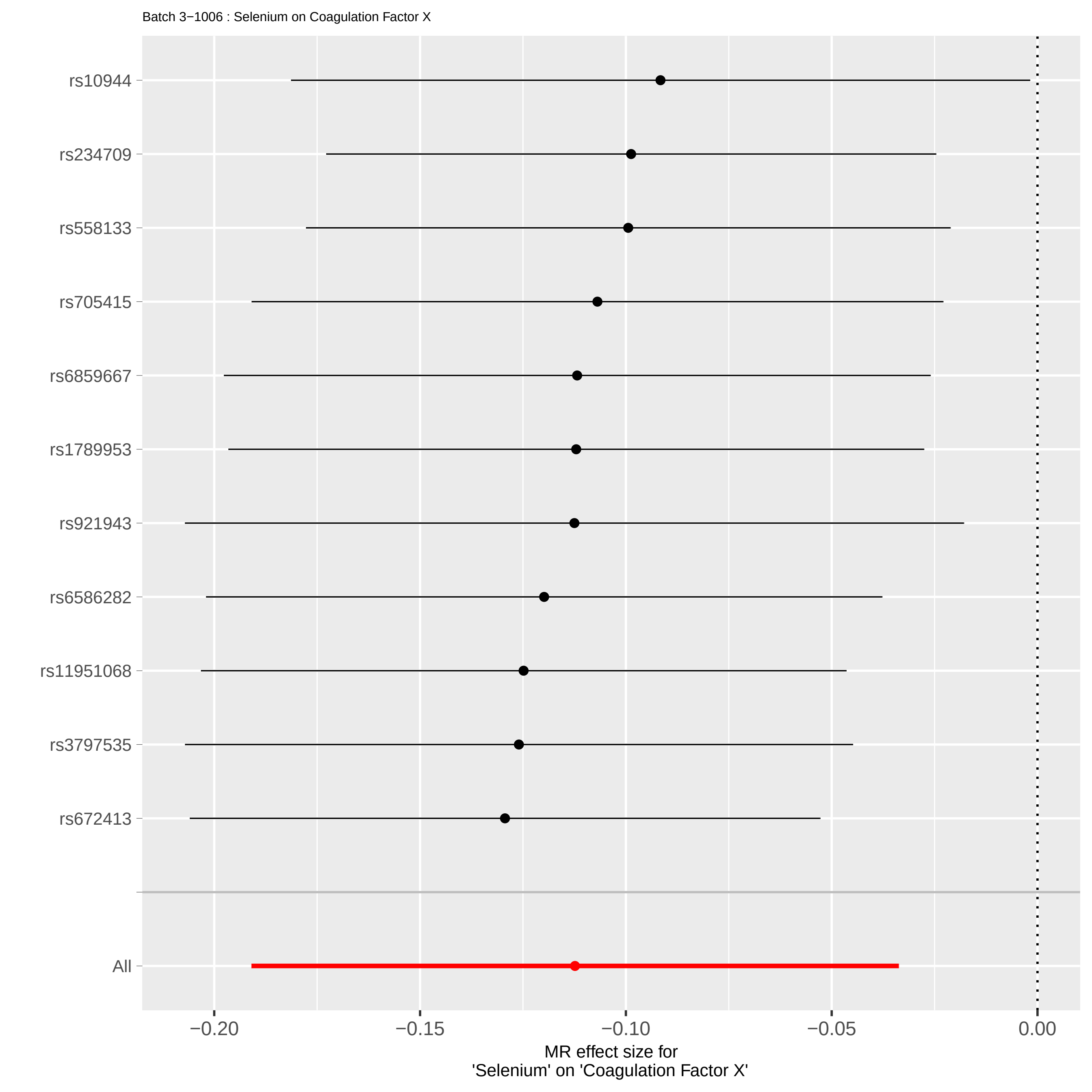

Batch 3−1006 : Selenium on Coagulation Factor X
rs10944
rs234709
rs558133
rs705415
rs6859667
rs1789953
rs921943
rs6586282
rs11951068
rs3797535
rs672413
All
−0.20
−0.15
−0.10
−0.05
0.00
MR effect size for
'Selenium' on 'Coagulation Factor X'

Batch 3−1007 : Selenium on Prothrombin

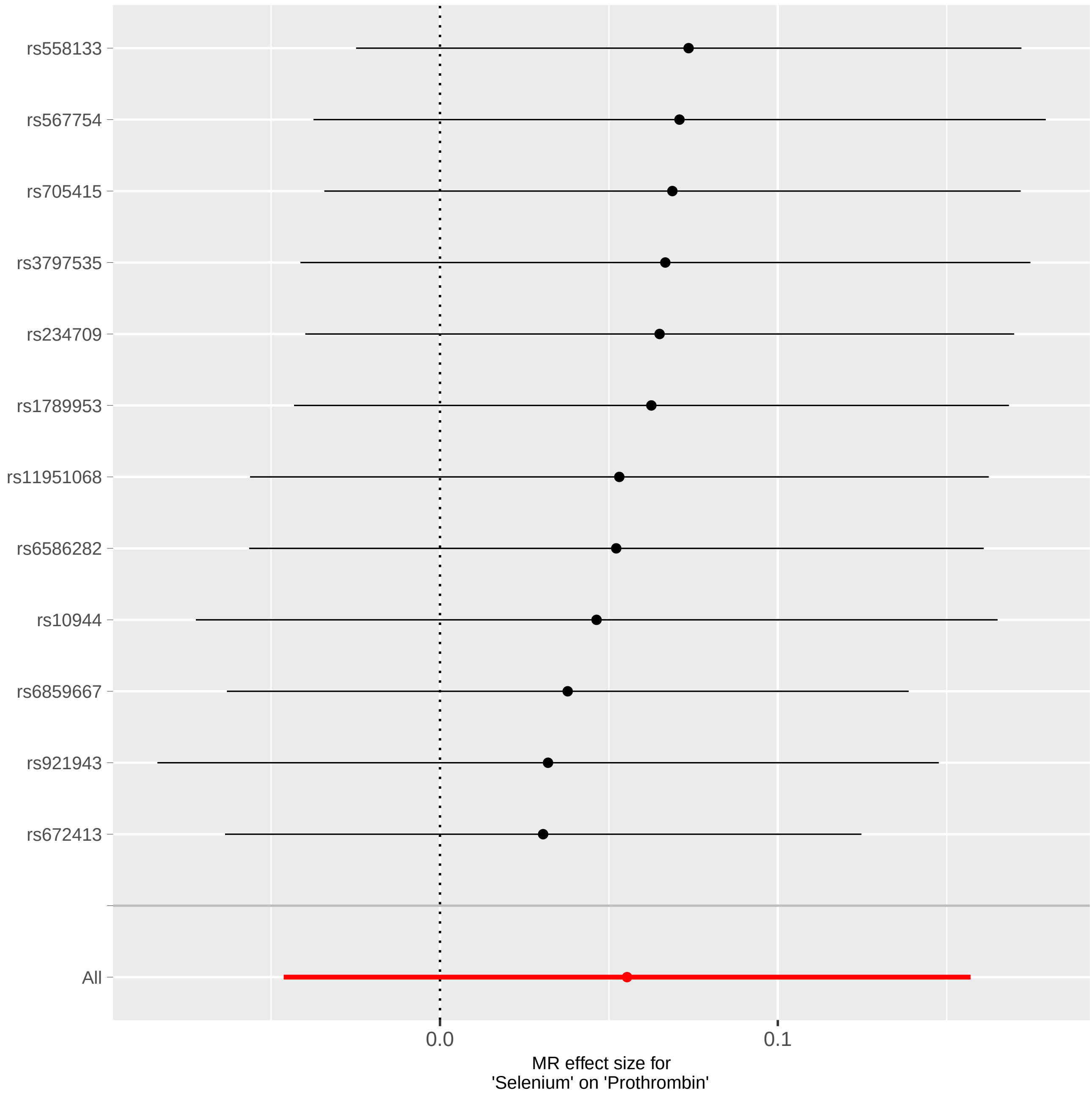

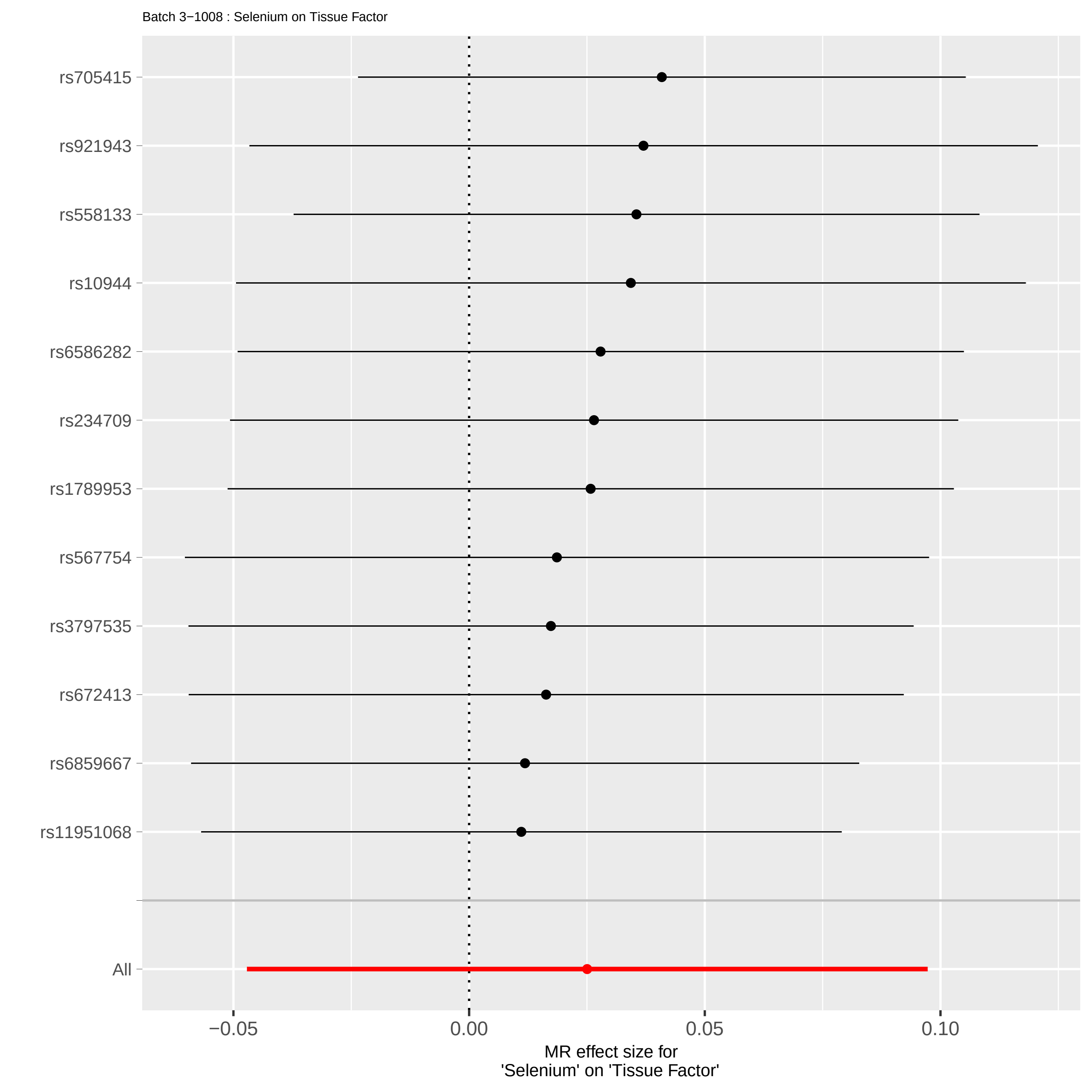
Batch 3−1008 : Selenium on Tissue Factor
rs705415
rs921943
rs558133
rs10944
rs6586282
rs234709
rs1789953
rs567754
rs3797535
rs672413
rs6859667
rs11951068
All
−0.05
0.00
0.05
0.10
MR effect size for
'Selenium' on 'Tissue Factor'

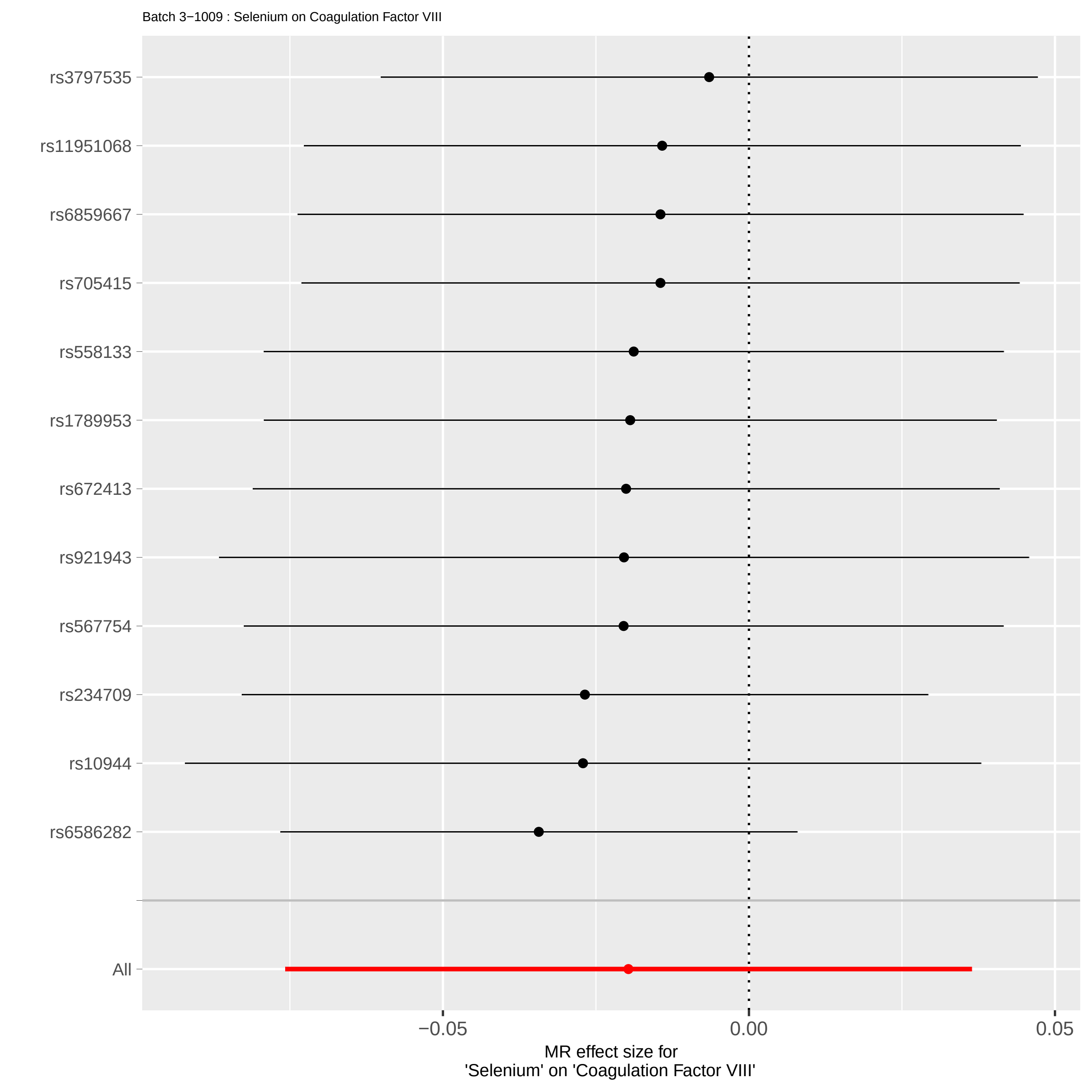
Batch 3−1009 : Selenium on Coagulation Factor VIII
rs3797535
rs11951068
rs6859667
rs705415
rs558133
rs1789953
rs672413
rs921943
rs567754
rs234709
rs10944
rs6586282
All
−0.05
0.00
0.05
MR effect size for
'Selenium' on 'Coagulation Factor VIII'

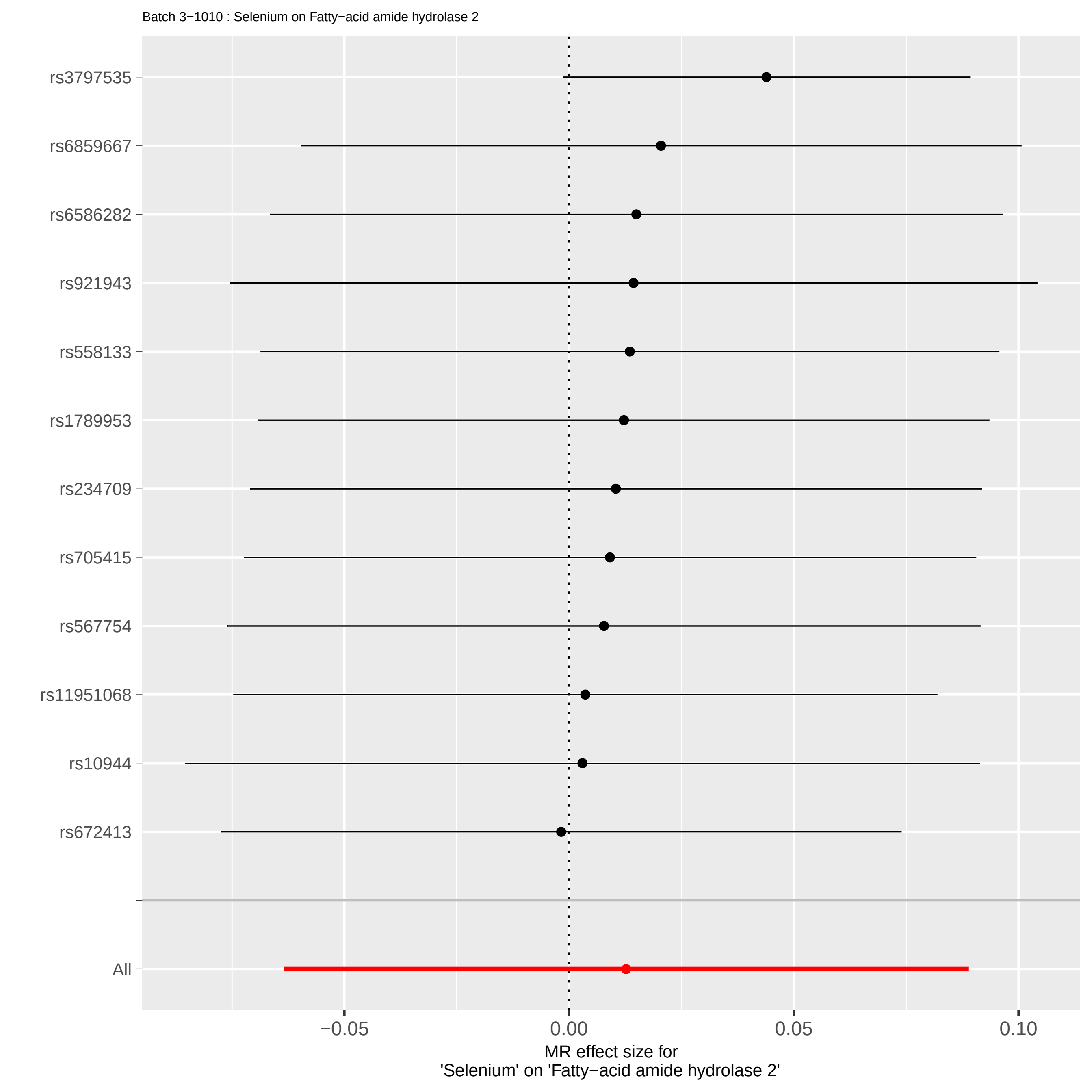
Batch 3−1010 : Selenium on Fatty−acid amide hydrolase 2
rs3797535
rs6859667
rs6586282
rs921943
rs558133
rs1789953
rs234709
rs705415
rs567754
rs11951068
rs10944
rs672413
All
−0.05
0.00
0.05
0.10
MR effect size for
'Selenium' on 'Fatty−acid amide hydrolase 2'

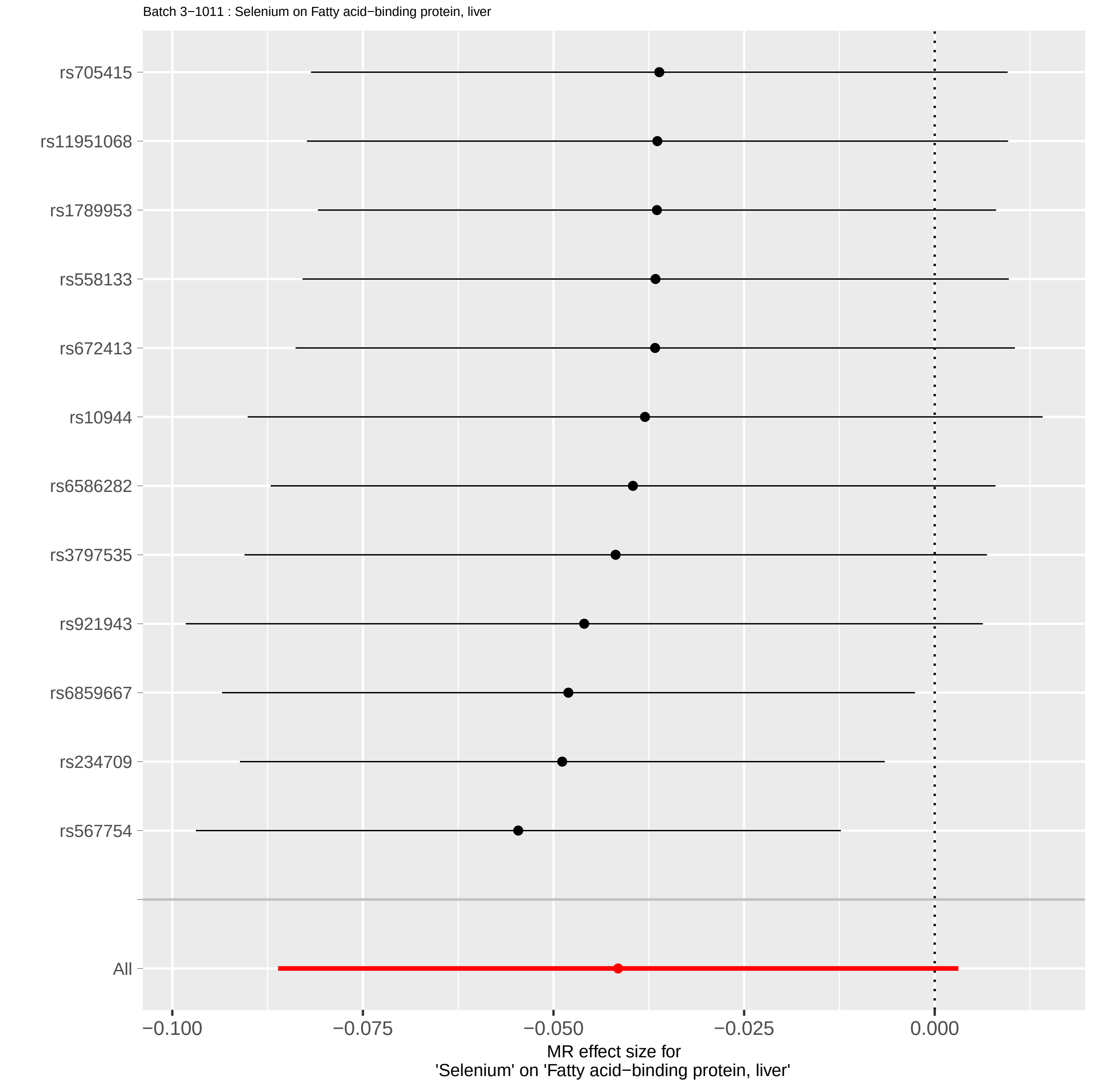

Batch 3−1011 : Selenium on Fatty acid−binding protein, liver
rs705415
rs11951068
rs1789953
rs558133
rs672413
rs10944
rs6586282
rs3797535
rs921943
rs6859667
rs234709
rs567754
All
−0.100
−0.075
−0.050
−0.025
0.000
MR effect size for
'Selenium' on 'Fatty acid−binding protein, liver'

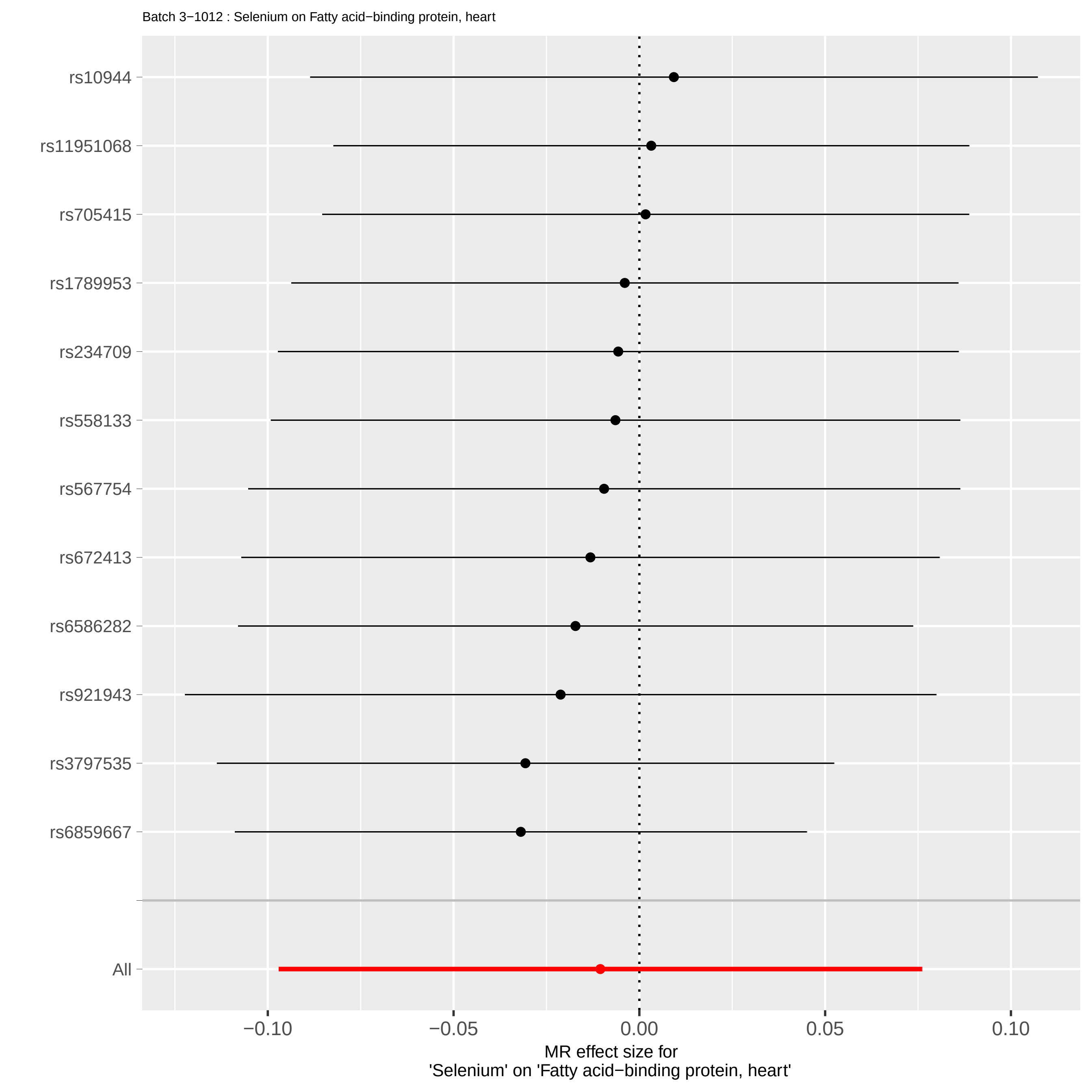

Batch 3−1012 : Selenium on Fatty acid−binding protein, heart
rs10944
rs11951068
rs705415
rs1789953
rs234709
rs558133
rs567754
rs672413
rs6586282
rs921943
rs3797535
rs6859667
All
−0.10
−0.05
0.00
0.05
0.10
MR effect size for
'Selenium' on 'Fatty acid−binding protein, heart'

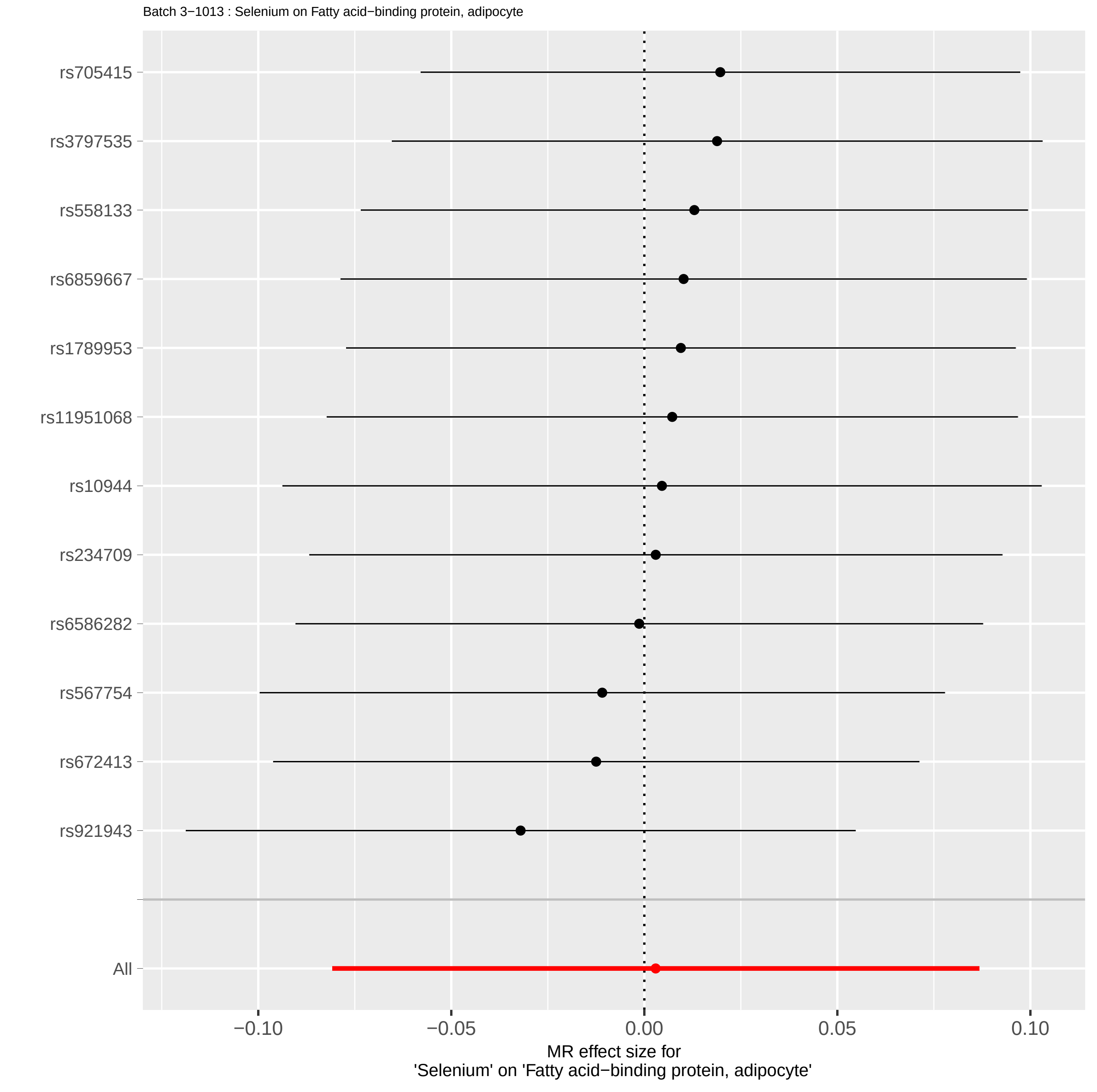

Batch 3−1013 : Selenium on Fatty acid−binding protein, adipocyte
rs705415
rs3797535
rs558133
rs6859667
rs1789953
rs11951068
rs10944
rs234709
rs6586282
rs567754
rs672413
rs921943
All
−0.10
−0.05
0.00
0.05
0.10
MR effect size for
'Selenium' on 'Fatty acid−binding protein, adipocyte'

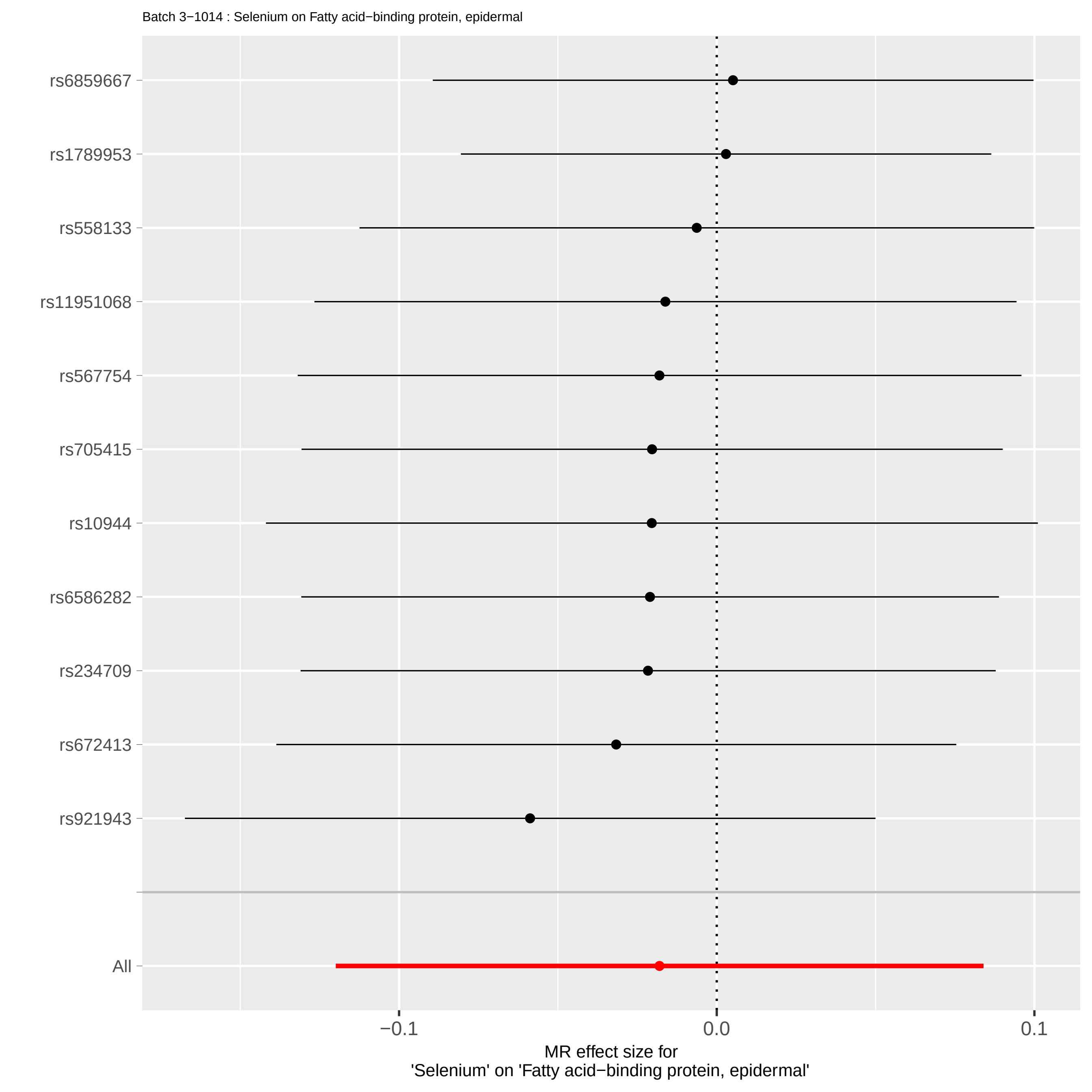

Batch 3−1014 : Selenium on Fatty acid−binding protein, epidermal
rs6859667
rs1789953
rs558133
rs11951068
rs567754
rs705415
rs10944
rs6586282
rs234709
rs672413
rs921943
All
−0.1
0.0
0.1
MR effect size for
'Selenium' on 'Fatty acid−binding protein, epidermal'

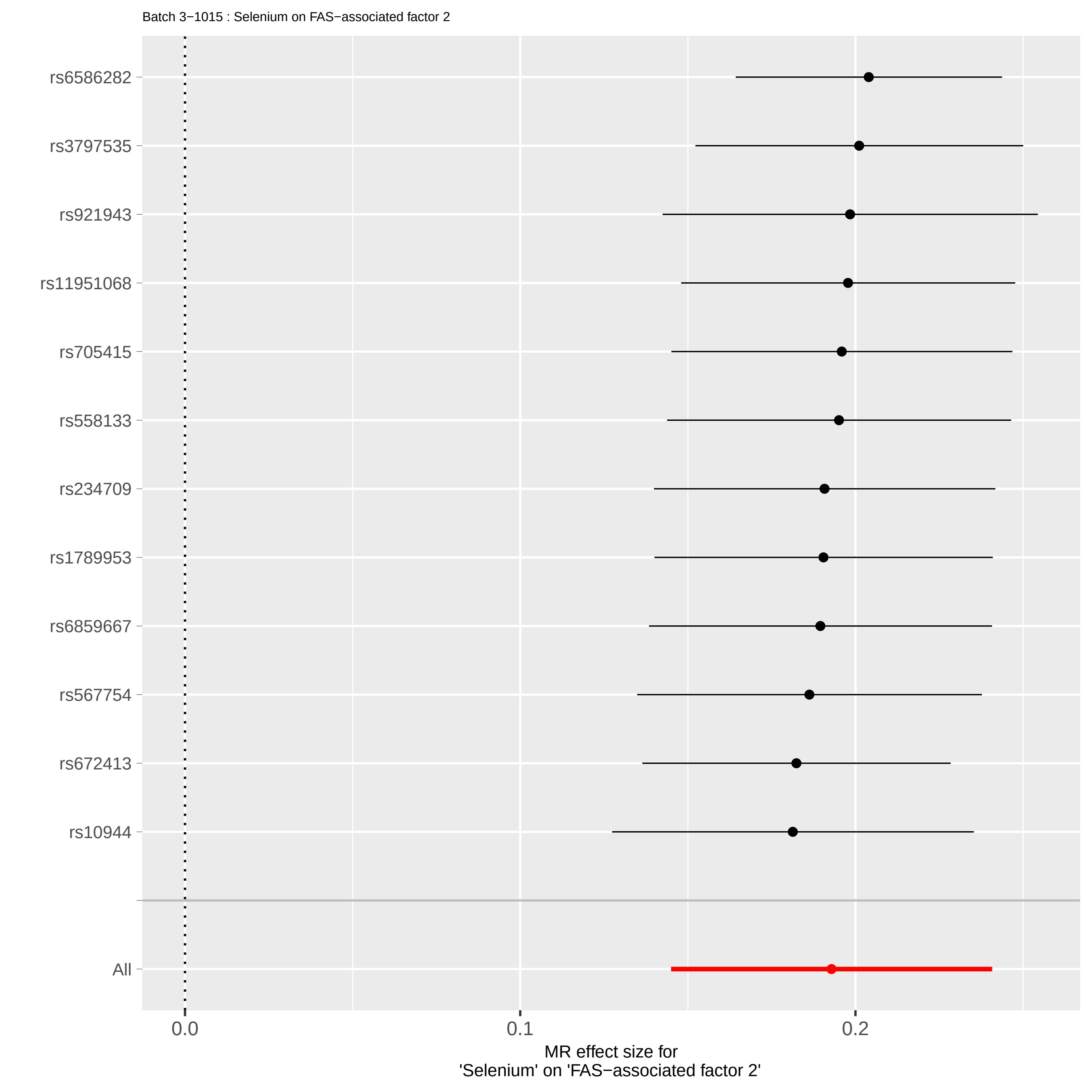
Batch 3−1015 : Selenium on FAS−associated factor 2
rs6586282
rs3797535
rs921943
rs11951068
rs705415
rs558133
rs234709
rs1789953
rs6859667
rs567754
rs672413
rs10944
All
0.0
0.1
0.2
MR effect size for
'Selenium' on 'FAS−associated factor 2'

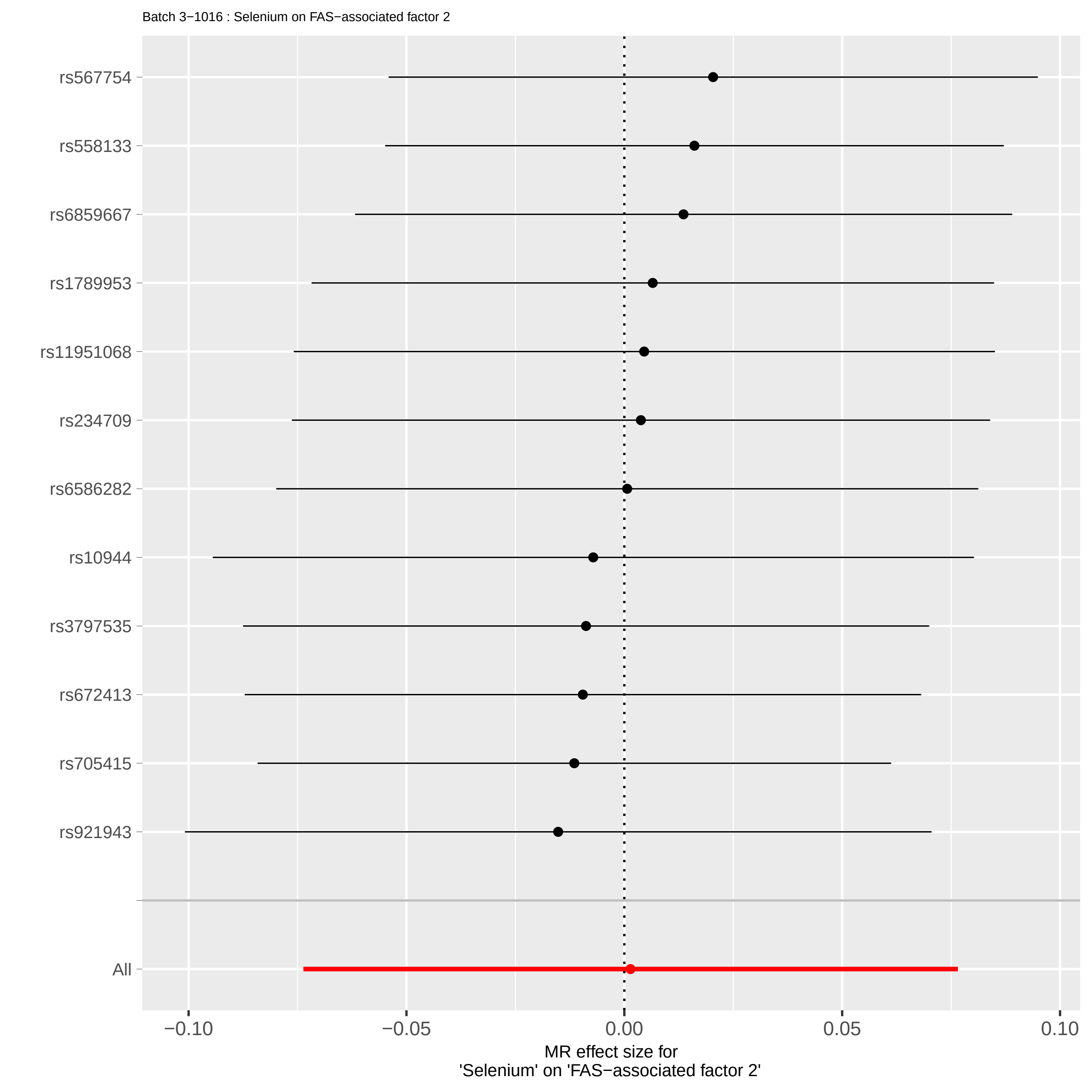
Batch 3−1016 : Selenium on FAS−associated factor 2
rs567754
rs558133
rs6859667
rs1789953
rs11951068
rs234709
rs6586282
rs10944
rs3797535
rs672413
rs705415
rs921943
All
−0.10
−0.05
0.00
0.05
0.10
MR effect size for
'Selenium' on 'FAS−associated factor 2'

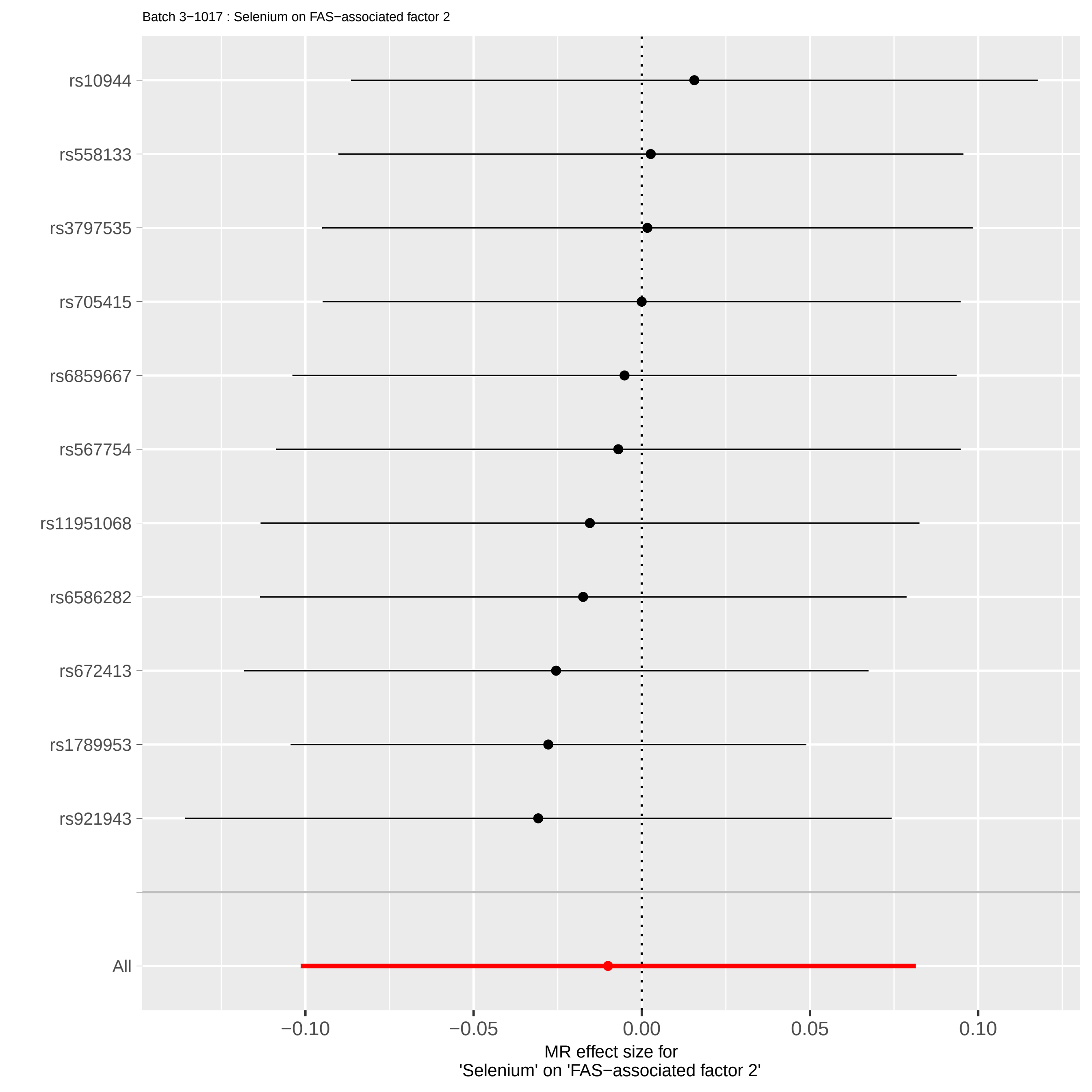
Batch 3−1017 : Selenium on FAS−associated factor 2
rs10944
rs558133
rs3797535
rs705415
rs6859667
rs567754
rs11951068
rs6586282
rs672413
rs1789953
rs921943
All
−0.10
−0.05
0.00
0.05
0.10
MR effect size for
'Selenium' on 'FAS−associated factor 2'

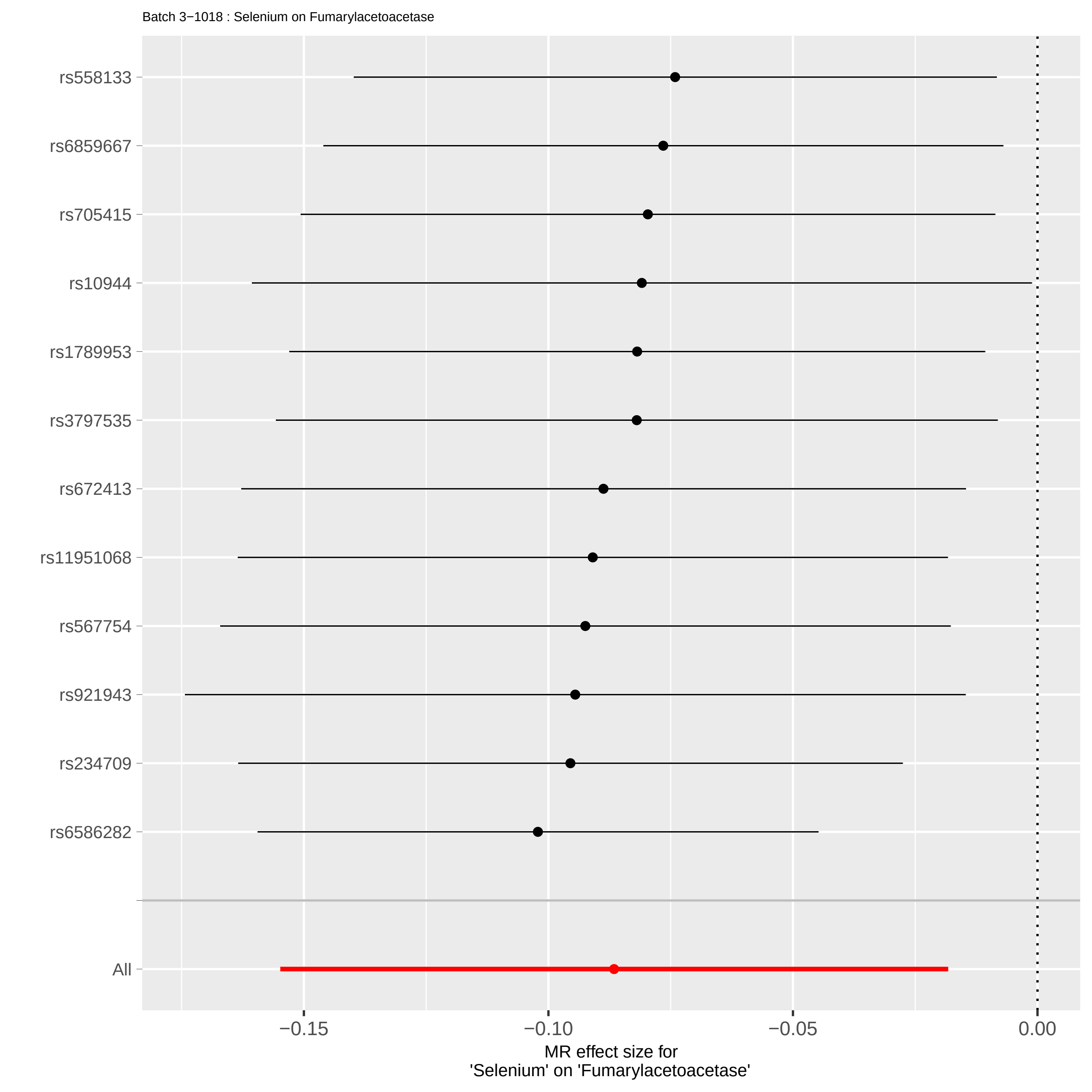

Batch 3−1018 : Selenium on Fumarylacetoacetase
rs558133
rs6859667
rs705415
rs10944
rs1789953
rs3797535
rs672413
rs11951068
rs567754
rs921943
rs234709
rs6586282
All
−0.15
−0.10
−0.05
0.00
MR effect size for
'Selenium' on 'Fumarylacetoacetase'

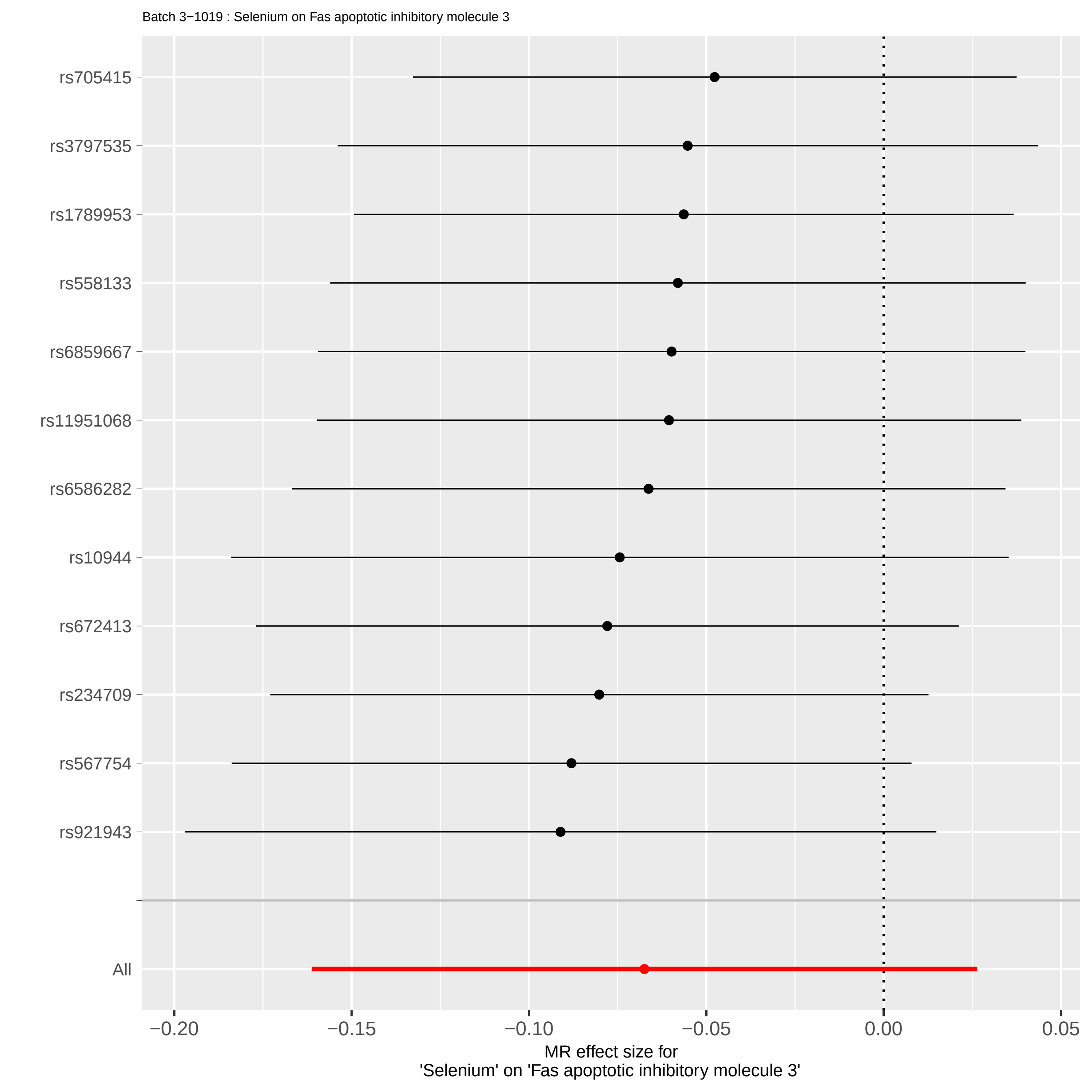

Batch 3−1019 : Selenium on Fas apoptotic inhibitory molecule 3
rs705415
rs3797535
rs1789953
rs558133
rs6859667
rs11951068
rs6586282
rs10944
rs672413
rs234709
rs567754
rs921943
All
−0.20
−0.15
−0.10
−0.05
0.00
0.05
MR effect size for
'Selenium' on 'Fas apoptotic inhibitory molecule 3'

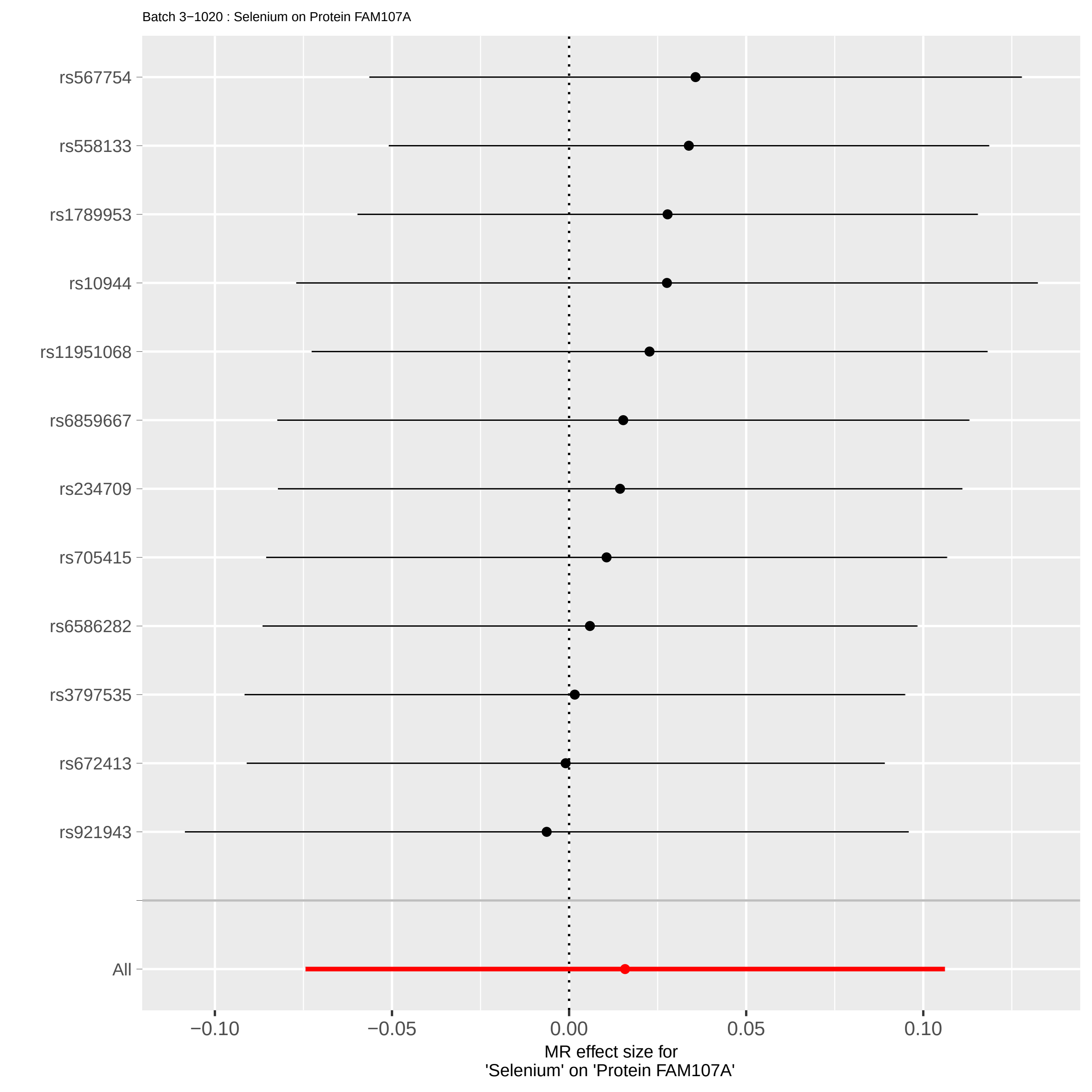

Batch 3−1020 : Selenium on Protein FAM107A
rs567754
rs558133
rs1789953
rs10944
rs11951068
rs6859667
rs234709
rs705415
rs6586282
rs3797535
rs672413
rs921943
All
−0.10
−0.05
0.00
0.05
0.10
MR effect size for
'Selenium' on 'Protein FAM107A'

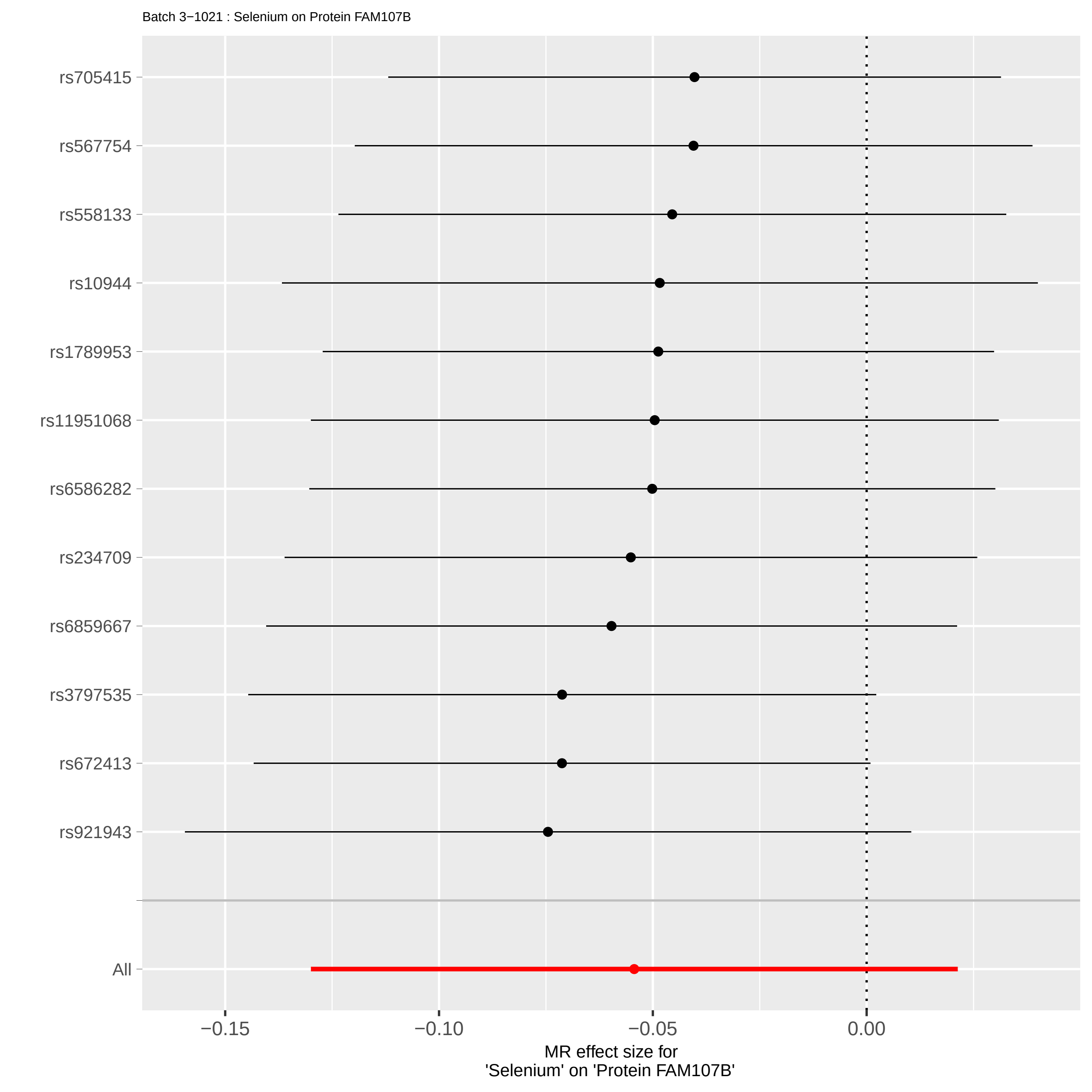

Batch 3−1021 : Selenium on Protein FAM107B
rs705415
rs567754
rs558133
rs10944
rs1789953
rs11951068
rs6586282
rs234709
rs6859667
rs3797535
rs672413
rs921943
All
−0.15
−0.10
−0.05
0.00
MR effect size for
'Selenium' on 'Protein FAM107B'

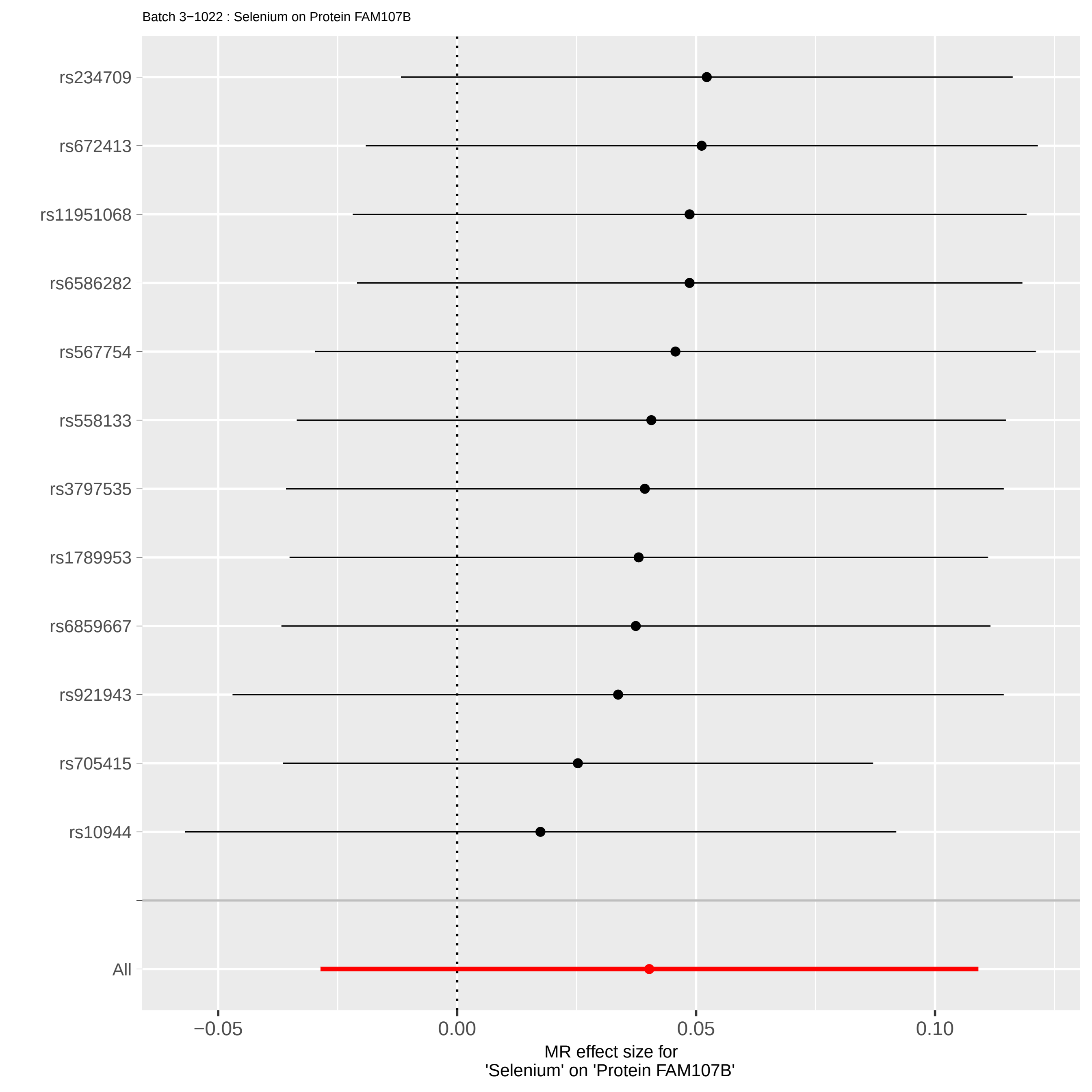

Batch 3−1022 : Selenium on Protein FAM107B
rs234709
rs672413
rs11951068
rs6586282
rs567754
rs558133
rs3797535
rs1789953
rs6859667
rs921943
rs705415
rs10944
All
−0.05
0.00
0.05
0.10
MR effect size for
'Selenium' on 'Protein FAM107B'

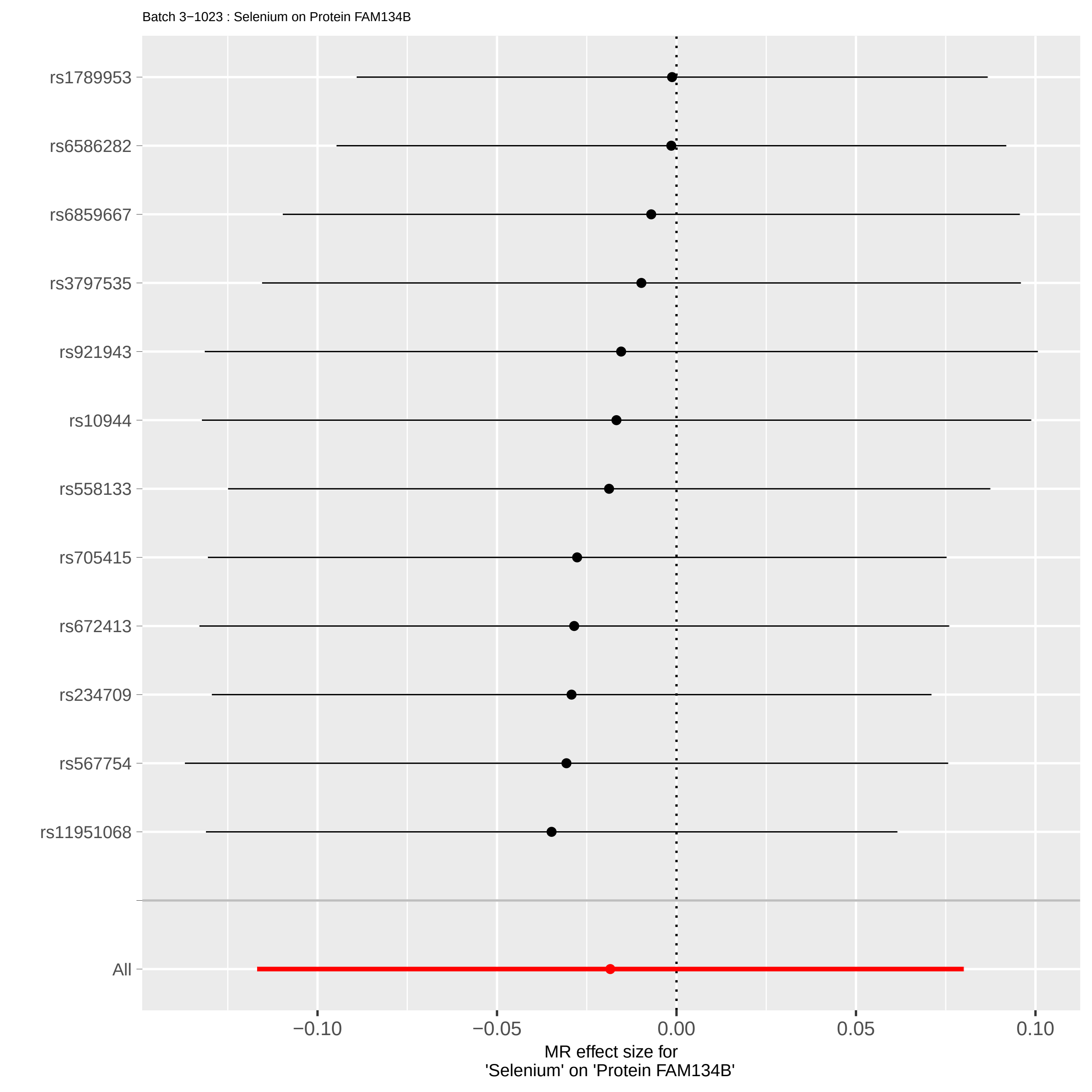

Batch 3−1023 : Selenium on Protein FAM134B
rs1789953
rs6586282
rs6859667
rs3797535
rs921943
rs10944
rs558133
rs705415
rs672413
rs234709
rs567754
rs11951068
All
−0.10
−0.05
0.00
0.05
0.10
MR effect size for
'Selenium' on 'Protein FAM134B'

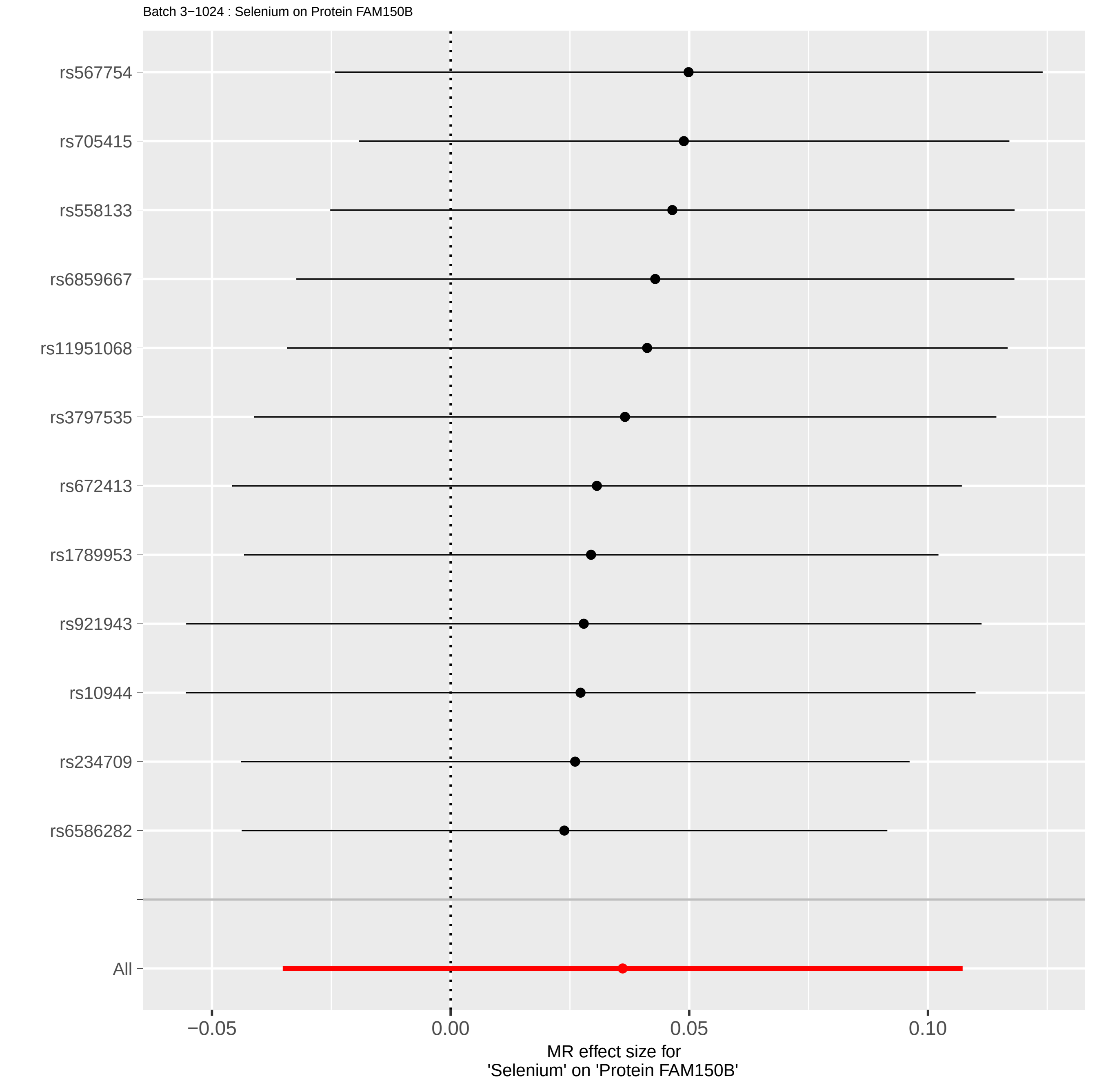

Batch 3−1024 : Selenium on Protein FAM150B
rs567754
rs705415
rs558133
rs6859667
rs11951068
rs3797535
rs672413
rs1789953
rs921943
rs10944
rs234709
rs6586282
All
−0.05
0.00
0.05
0.10
MR effect size for
'Selenium' on 'Protein FAM150B'

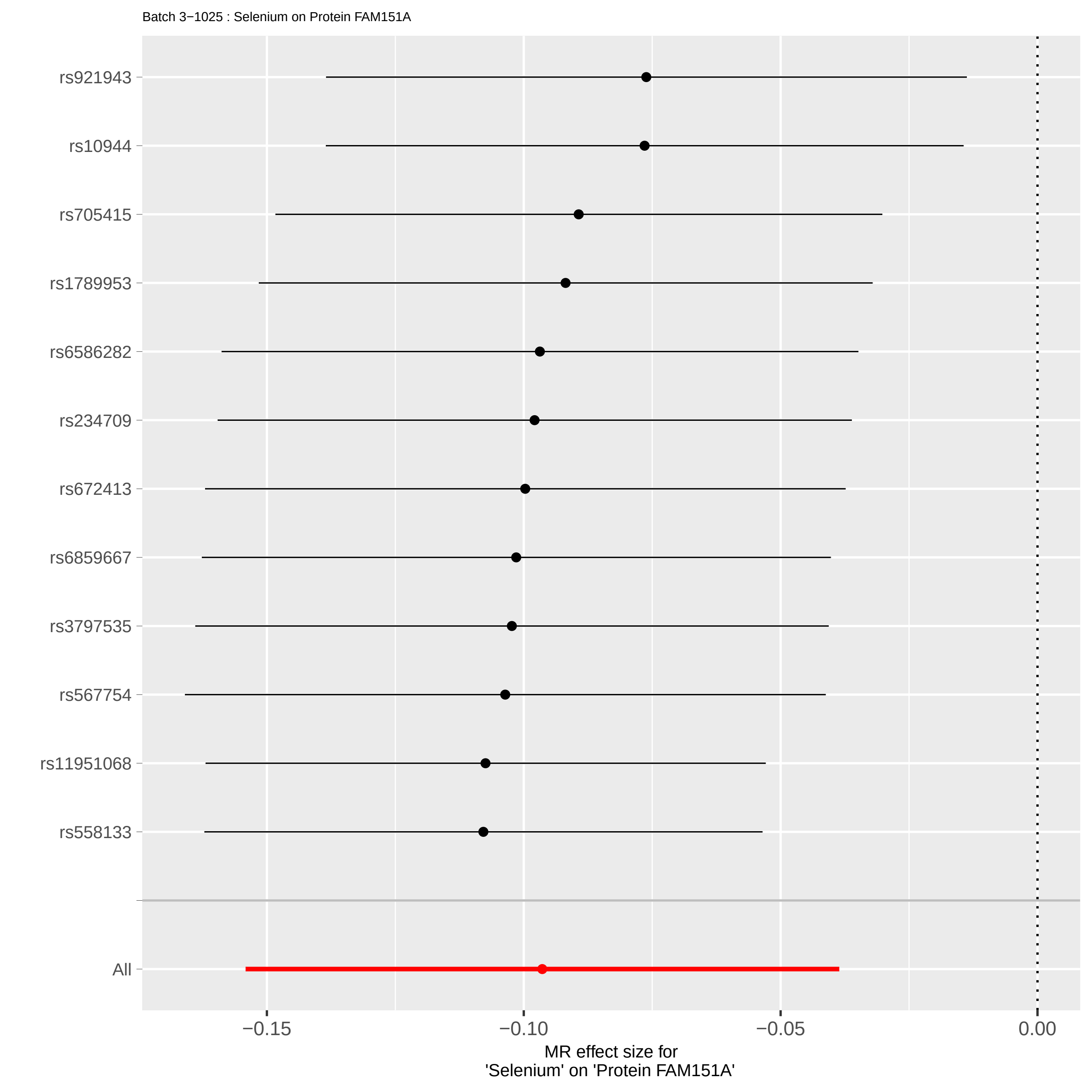

Batch 3−1025 : Selenium on Protein FAM151A
rs921943
rs10944
rs705415
rs1789953
rs6586282
rs234709
rs672413
rs6859667
rs3797535
rs567754
rs11951068
rs558133
All
−0.15
−0.10
−0.05
0.00
MR effect size for
'Selenium' on 'Protein FAM151A'

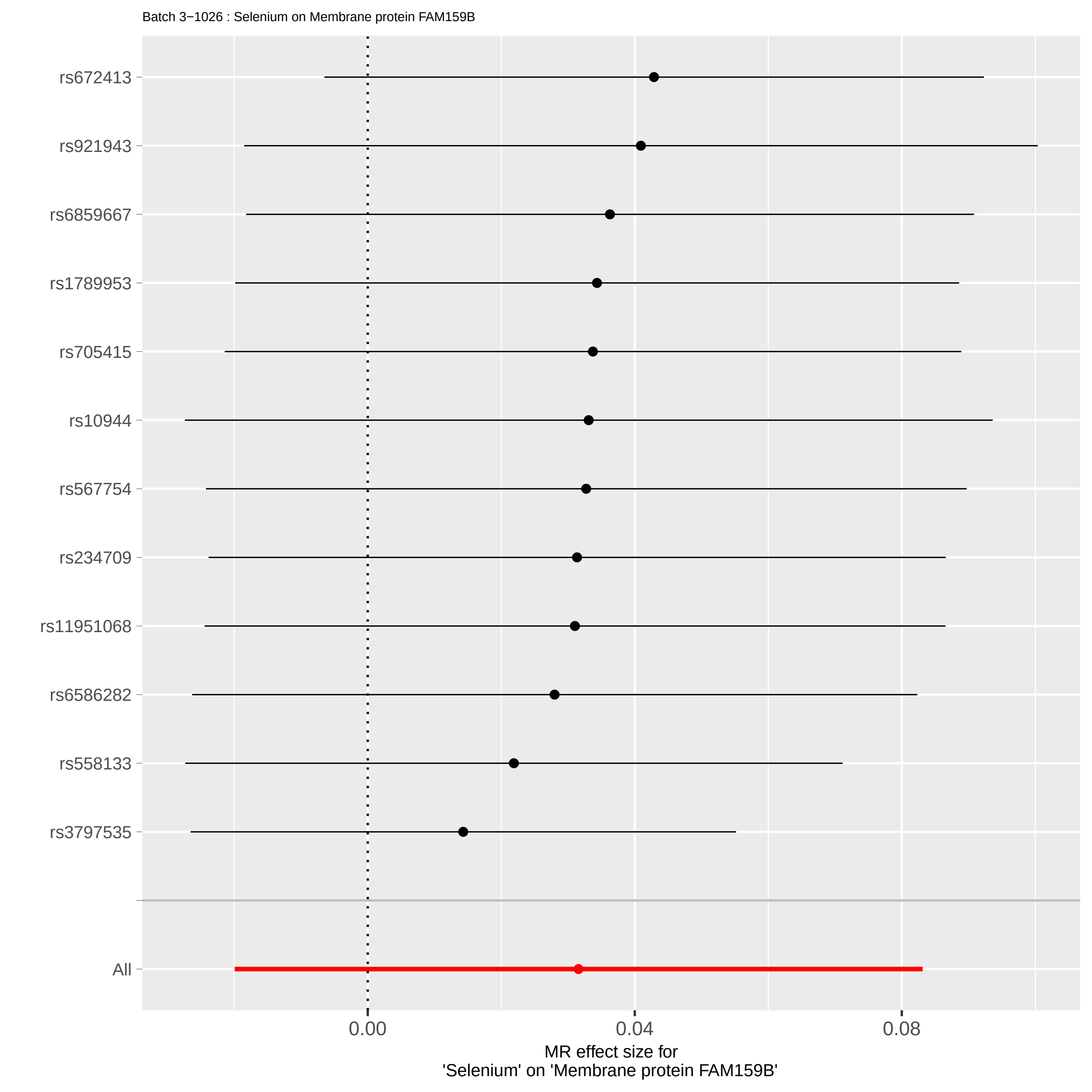
Batch 3−1026 : Selenium on Membrane protein FAM159B
rs672413
rs921943
rs6859667
rs1789953
rs705415
rs10944
rs567754
rs234709
rs11951068
rs6586282
rs558133
rs3797535
All
0.00
0.04
0.08
MR effect size for
'Selenium' on 'Membrane protein FAM159B'

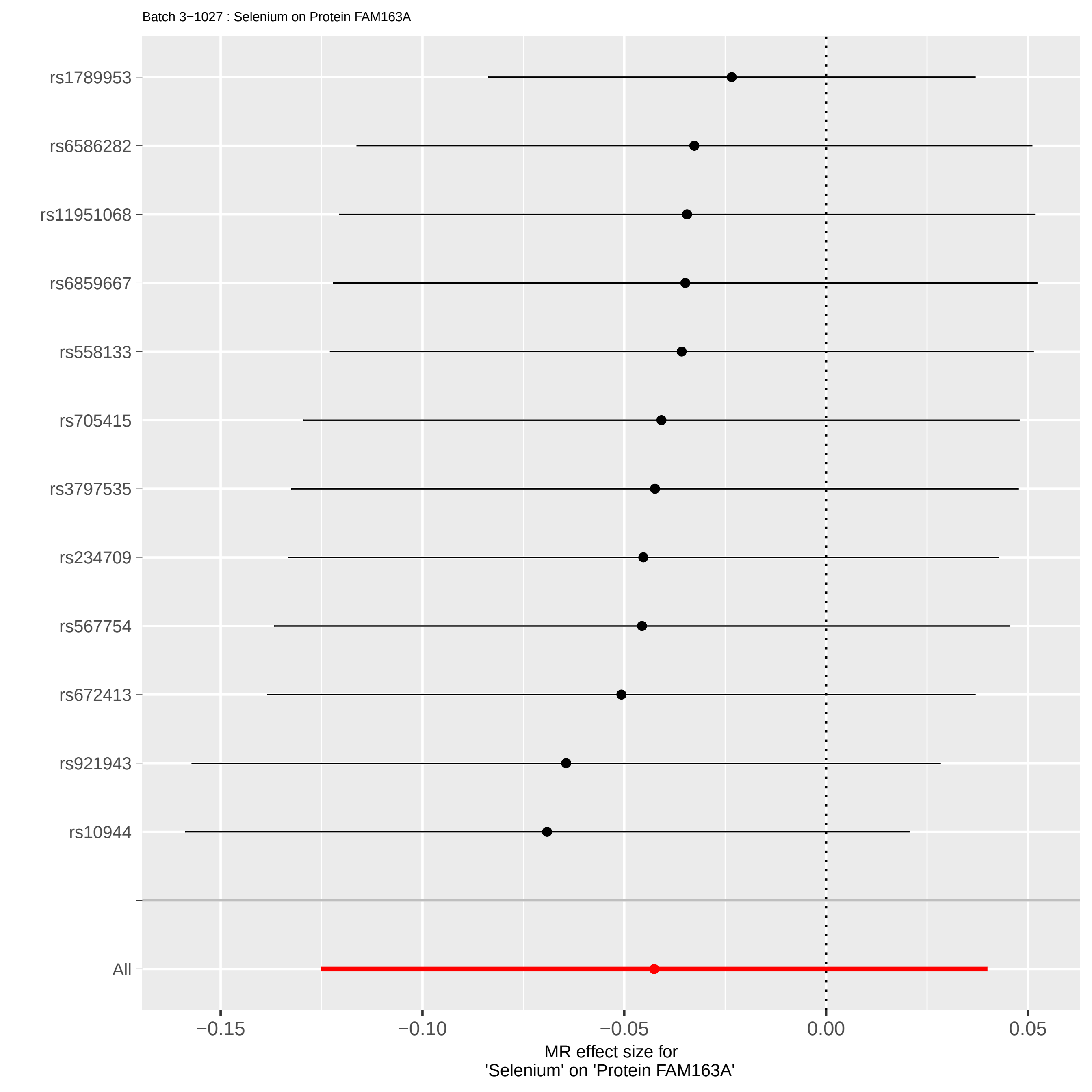

Batch 3−1027 : Selenium on Protein FAM163A
rs1789953
rs6586282
rs11951068
rs6859667
rs558133
rs705415
rs3797535
rs234709
rs567754
rs672413
rs921943
rs10944
All
−0.15
−0.10
−0.05
0.00
0.05
MR effect size for
'Selenium' on 'Protein FAM163A'

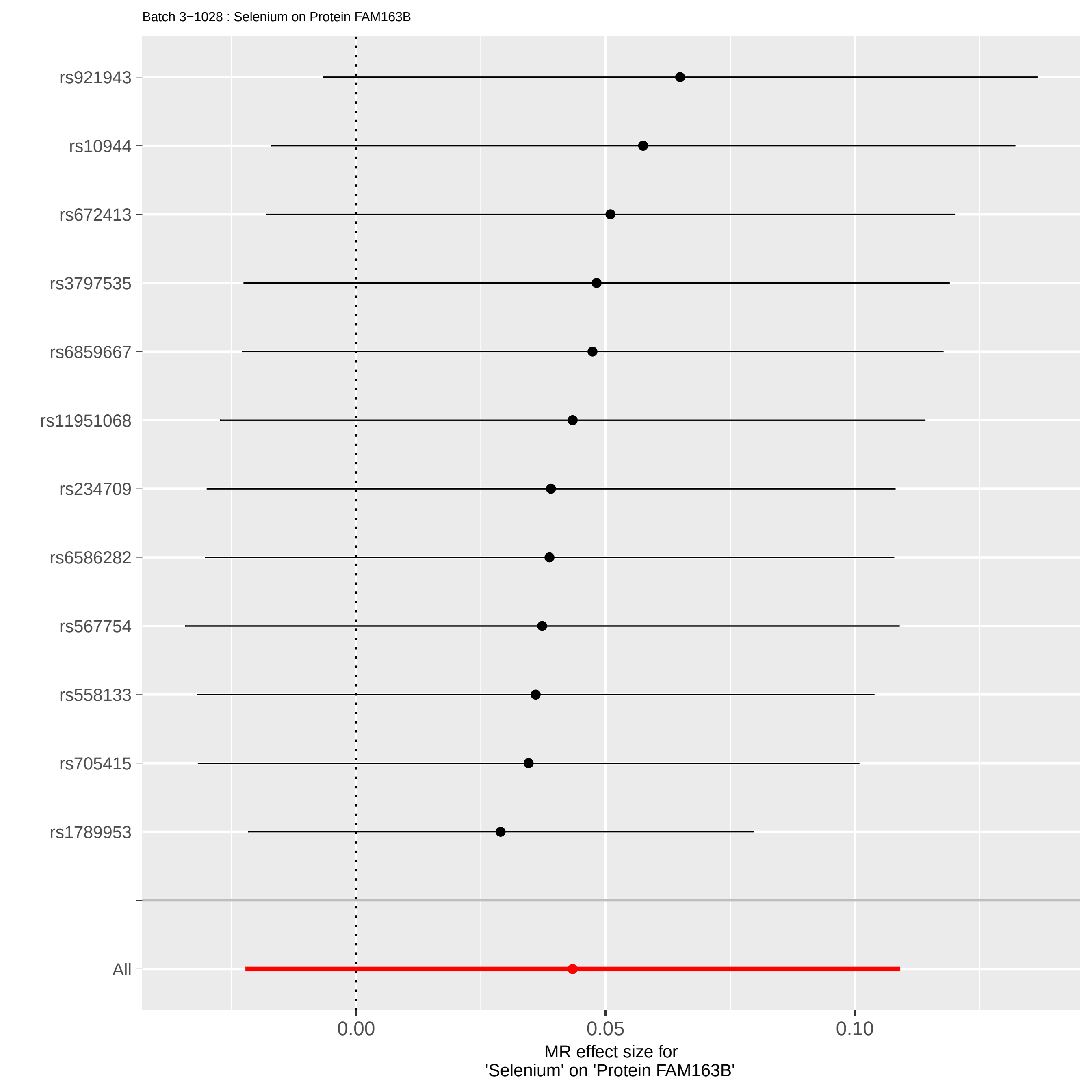

Batch 3−1028 : Selenium on Protein FAM163B
rs921943
rs10944
rs672413
rs3797535
rs6859667
rs11951068
rs234709
rs6586282
rs567754
rs558133
rs705415
rs1789953
All
0.00
0.05
0.10
MR effect size for
'Selenium' on 'Protein FAM163B'

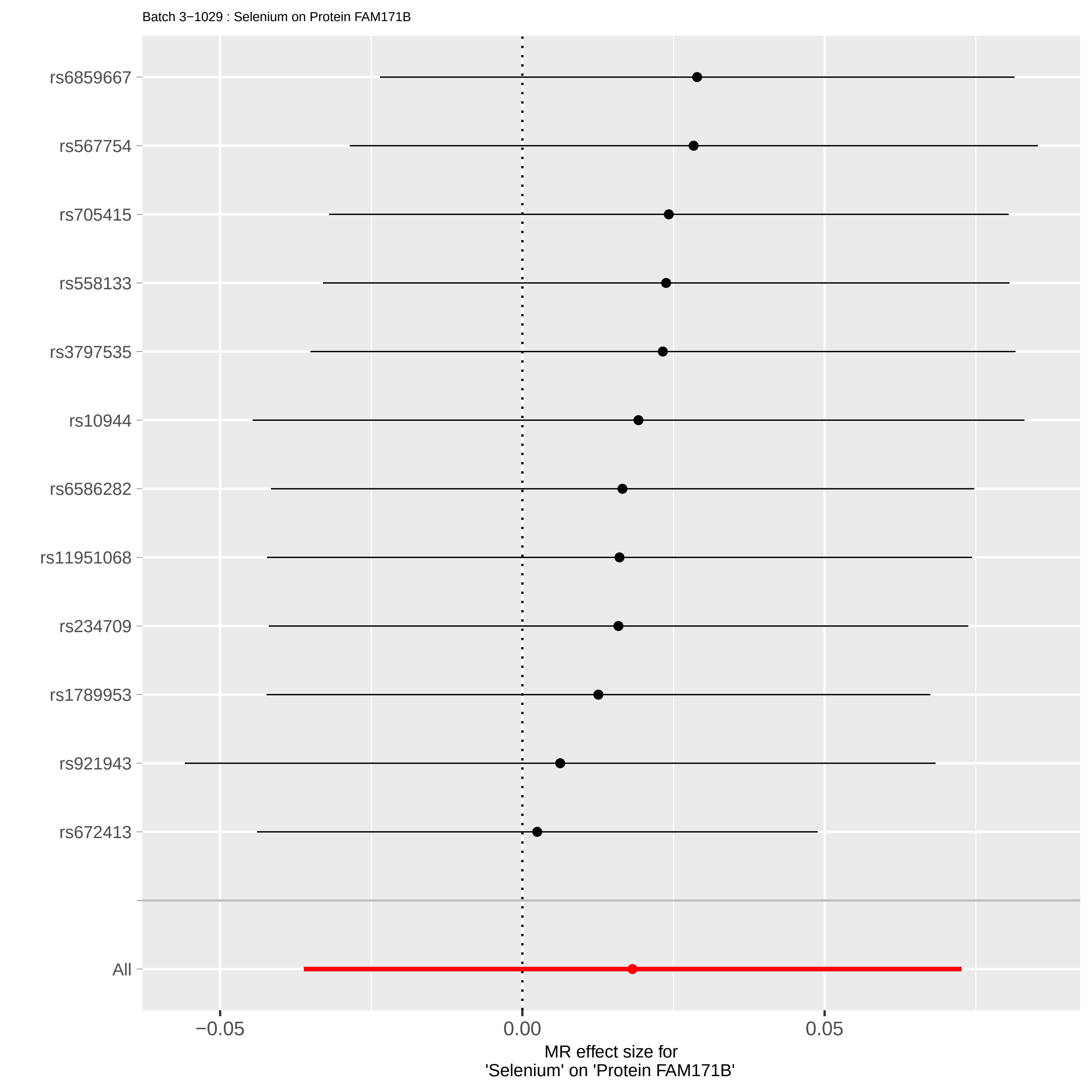
Batch 3−1029 : Selenium on Protein FAM171B
rs6859667
rs567754
rs705415
rs558133
rs3797535
rs10944
rs6586282
rs11951068
rs234709
rs1789953
rs921943
rs672413
All
−0.05
0.00
0.05
MR effect size for
'Selenium' on 'Protein FAM171B'

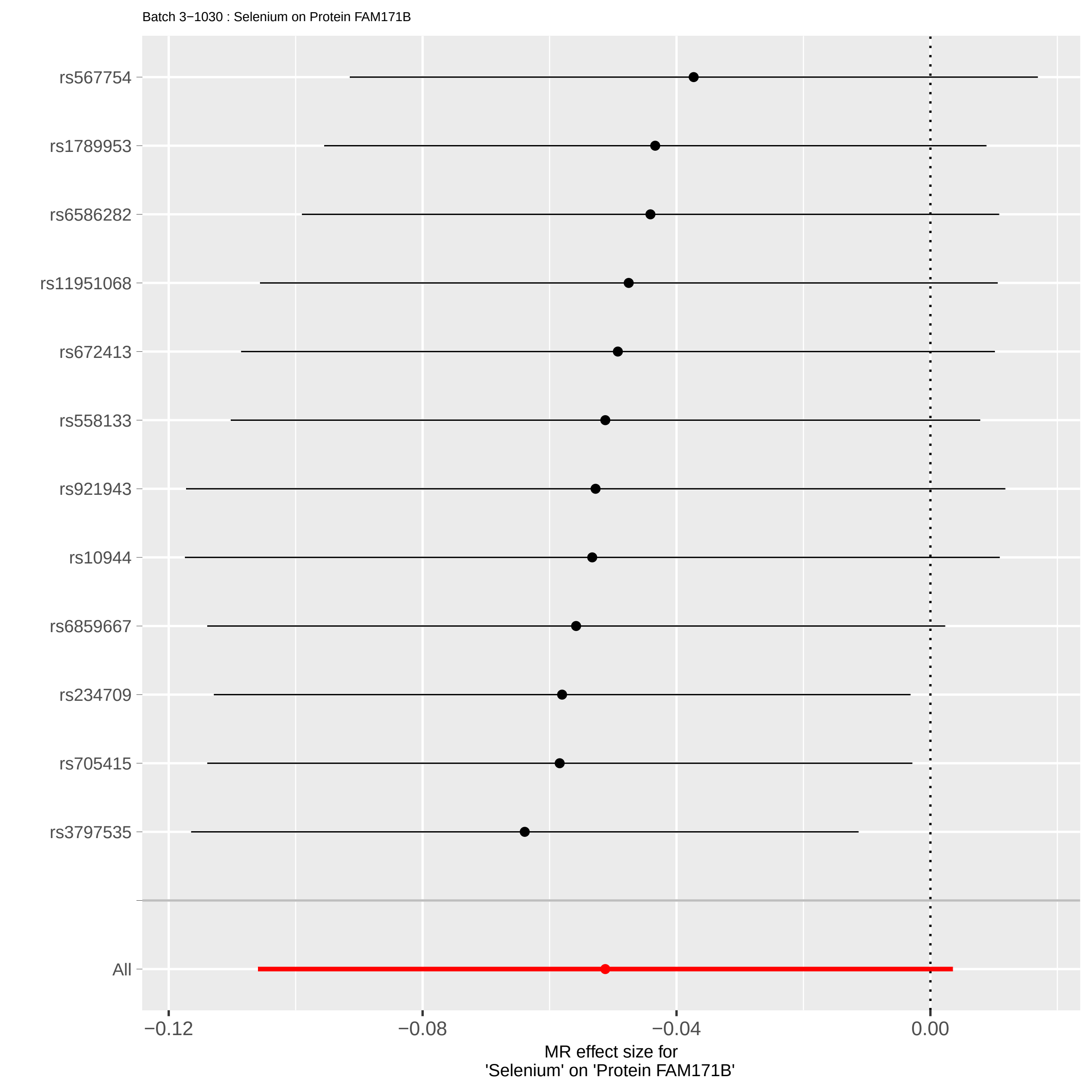

Batch 3−1030 : Selenium on Protein FAM171B
rs567754
rs1789953
rs6586282
rs11951068
rs672413
rs558133
rs921943
rs10944
rs6859667
rs234709
rs705415
rs3797535
All
−0.12
−0.08
−0.04
0.00
MR effect size for
'Selenium' on 'Protein FAM171B'

Batch 3−1031 : Selenium on Protein FAM171B

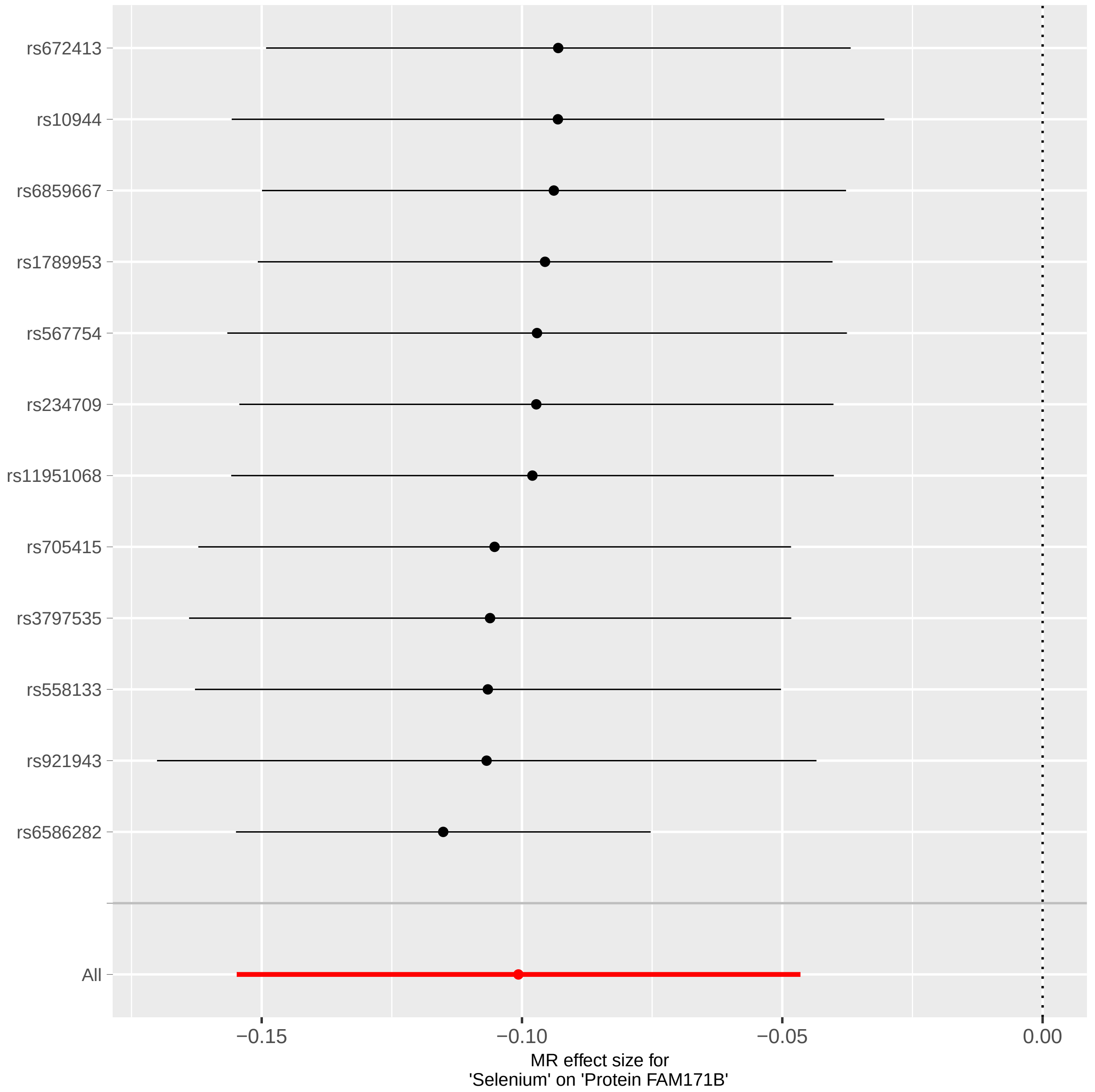

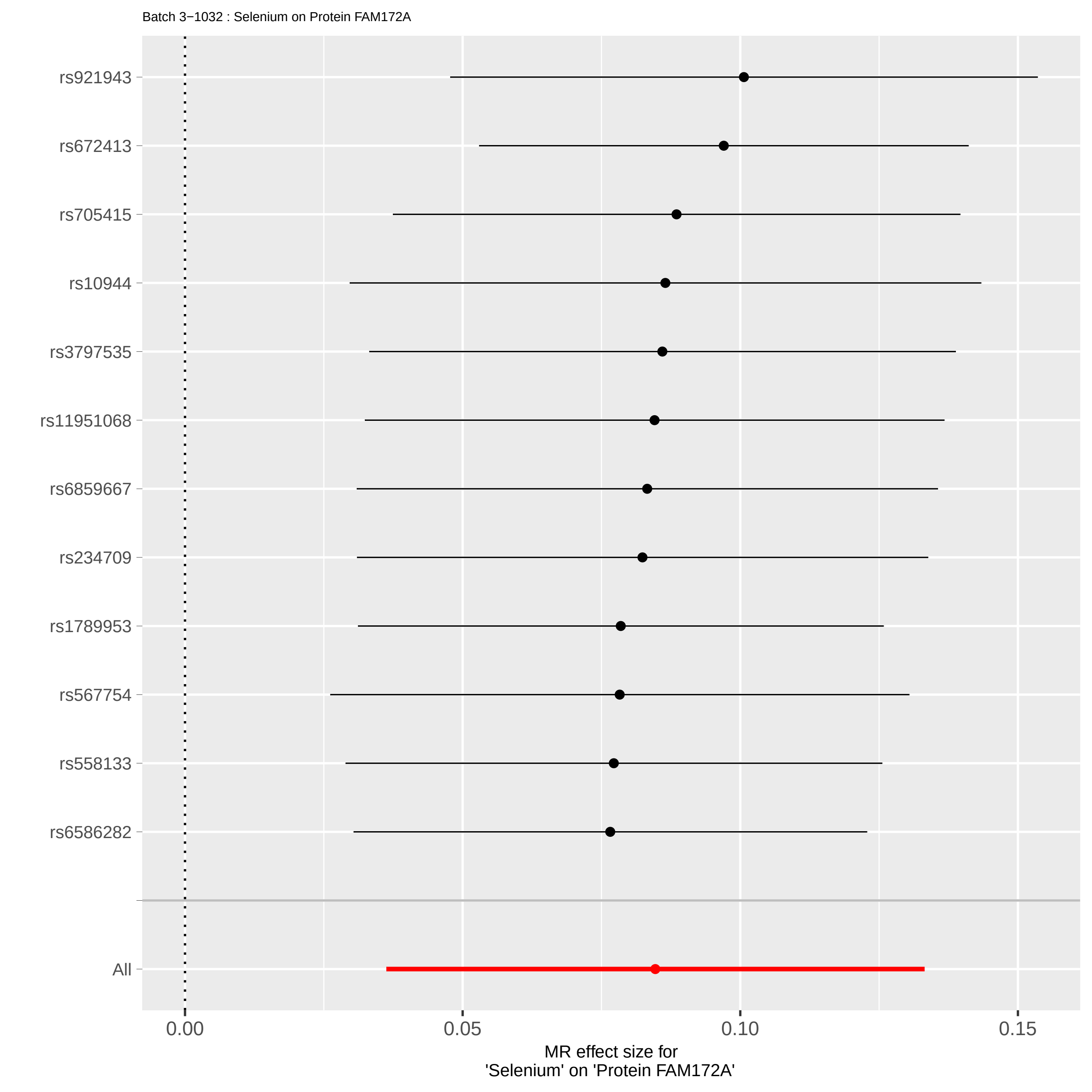

Batch 3−1032 : Selenium on Protein FAM172A
rs921943
rs672413
rs705415
rs10944
rs3797535
rs11951068
rs6859667
rs234709
rs1789953
rs567754
rs558133
rs6586282
All
0.00
0.05
0.10
0.15
MR effect size for
'Selenium' on 'Protein FAM172A'

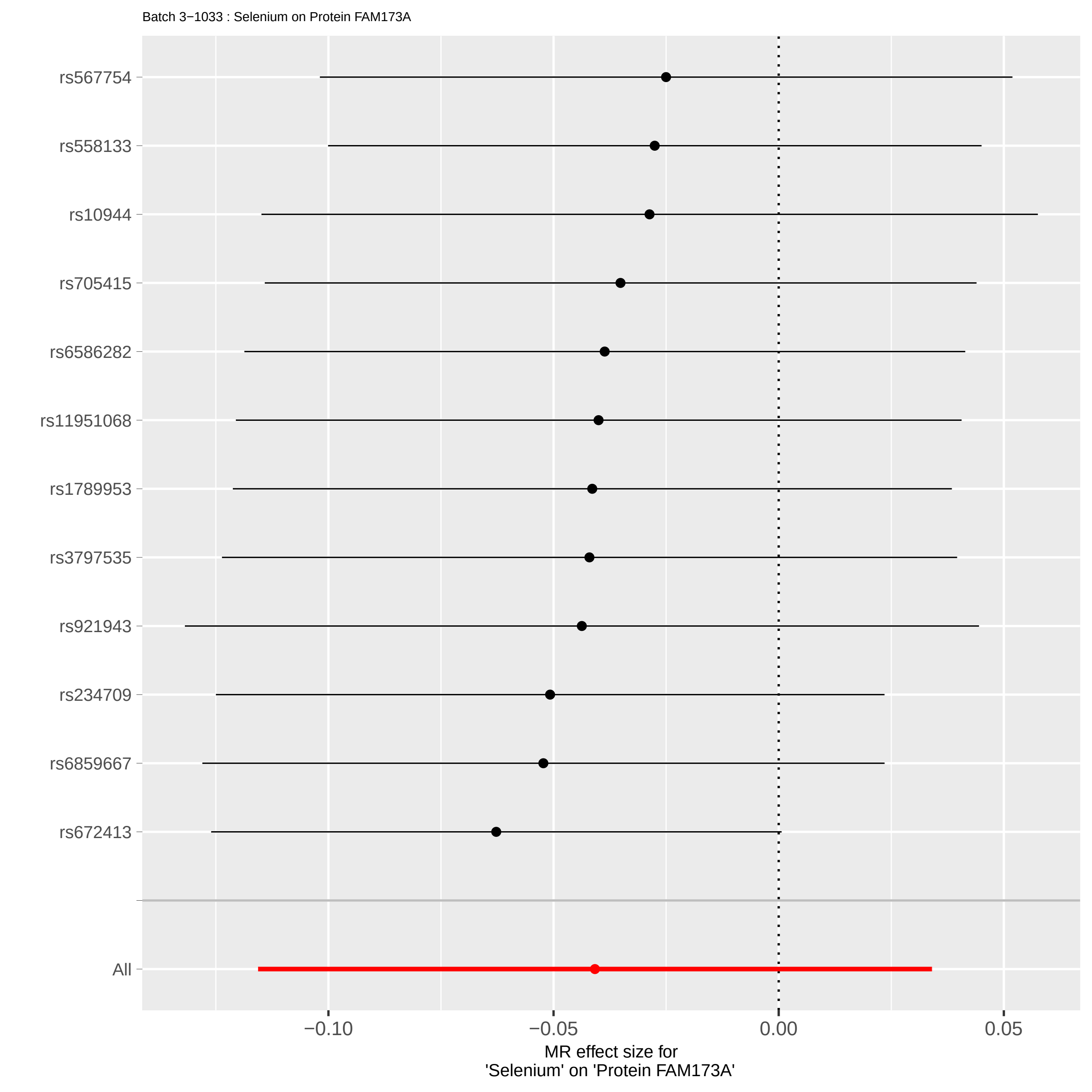

Batch 3−1033 : Selenium on Protein FAM173A
rs567754
rs558133
rs10944
rs705415
rs6586282
rs11951068
rs1789953
rs3797535
rs921943
rs234709
rs6859667
rs672413
All
−0.10
−0.05
0.00
0.05
MR effect size for
'Selenium' on 'Protein FAM173A'

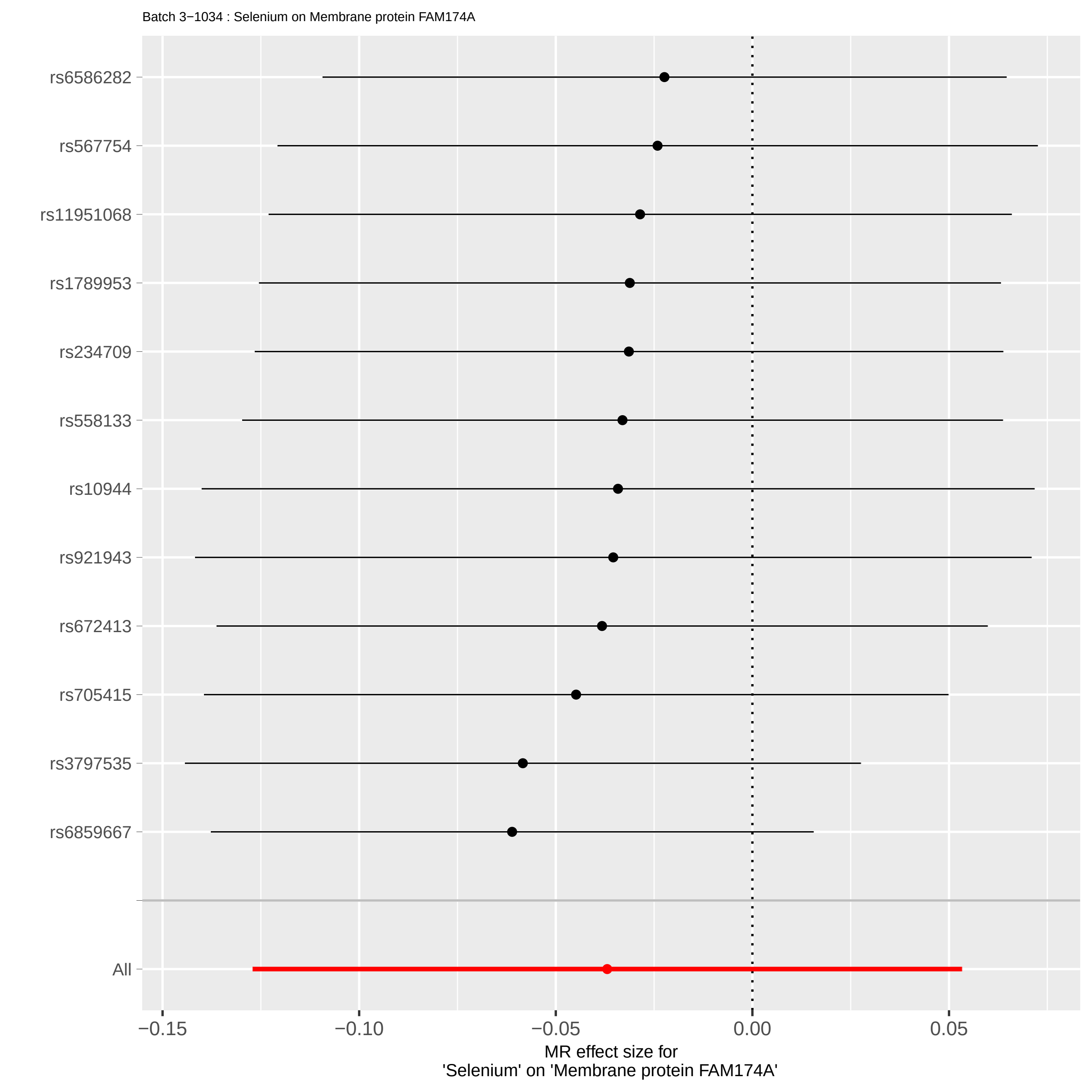

Batch 3−1034 : Selenium on Membrane protein FAM174A
rs6586282
rs567754
rs11951068
rs1789953
rs234709
rs558133
rs10944
rs921943
rs672413
rs705415
rs3797535
rs6859667
All
−0.15
−0.10
−0.05
0.00
0.05
MR effect size for
'Selenium' on 'Membrane protein FAM174A'

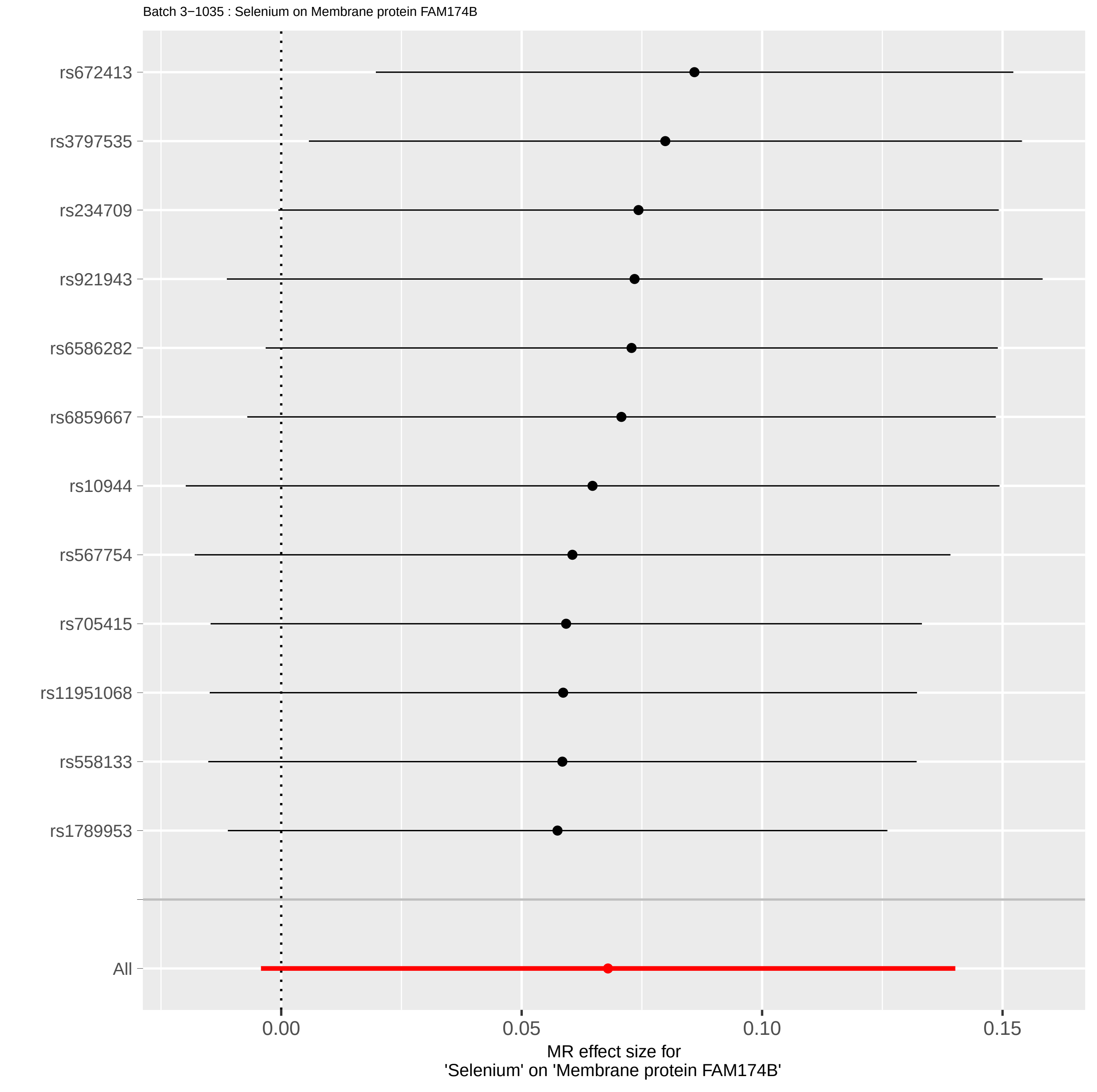
Batch 3−1035 : Selenium on Membrane protein FAM174B
rs672413
rs3797535
rs234709
rs921943
rs6586282
rs6859667
rs10944
rs567754
rs705415
rs11951068
rs558133
rs1789953
All
0.00
0.05
0.10
0.15
MR effect size for
'Selenium' on 'Membrane protein FAM174B'

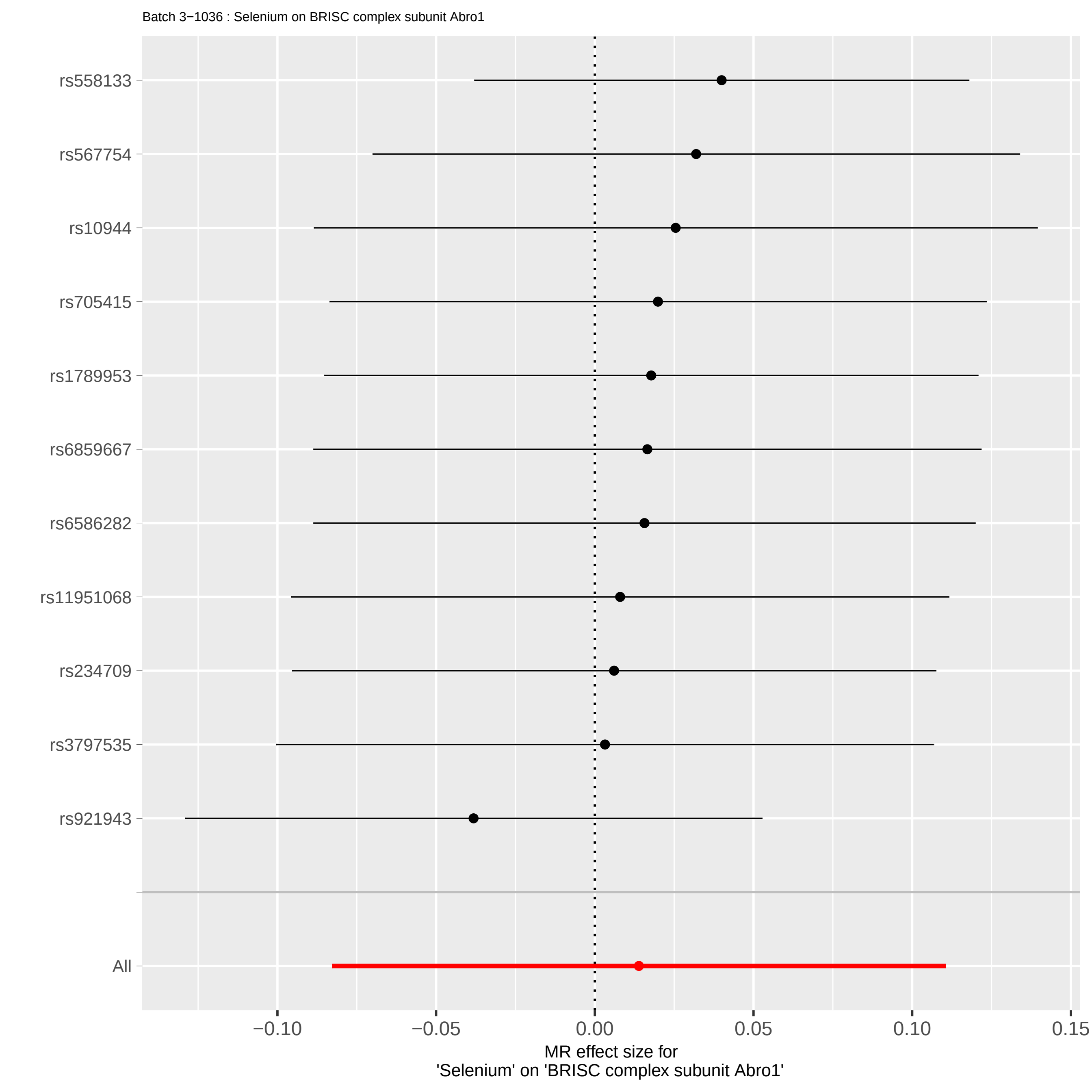
Batch 3−1036 : Selenium on BRISC complex subunit Abro1
rs558133
rs567754
rs10944
rs705415
rs1789953
rs6859667
rs6586282
rs11951068
rs234709
rs3797535
rs921943
All
−0.10
−0.05
0.00
0.05
0.10
0.15
MR effect size for
'Selenium' on 'BRISC complex subunit Abro1'

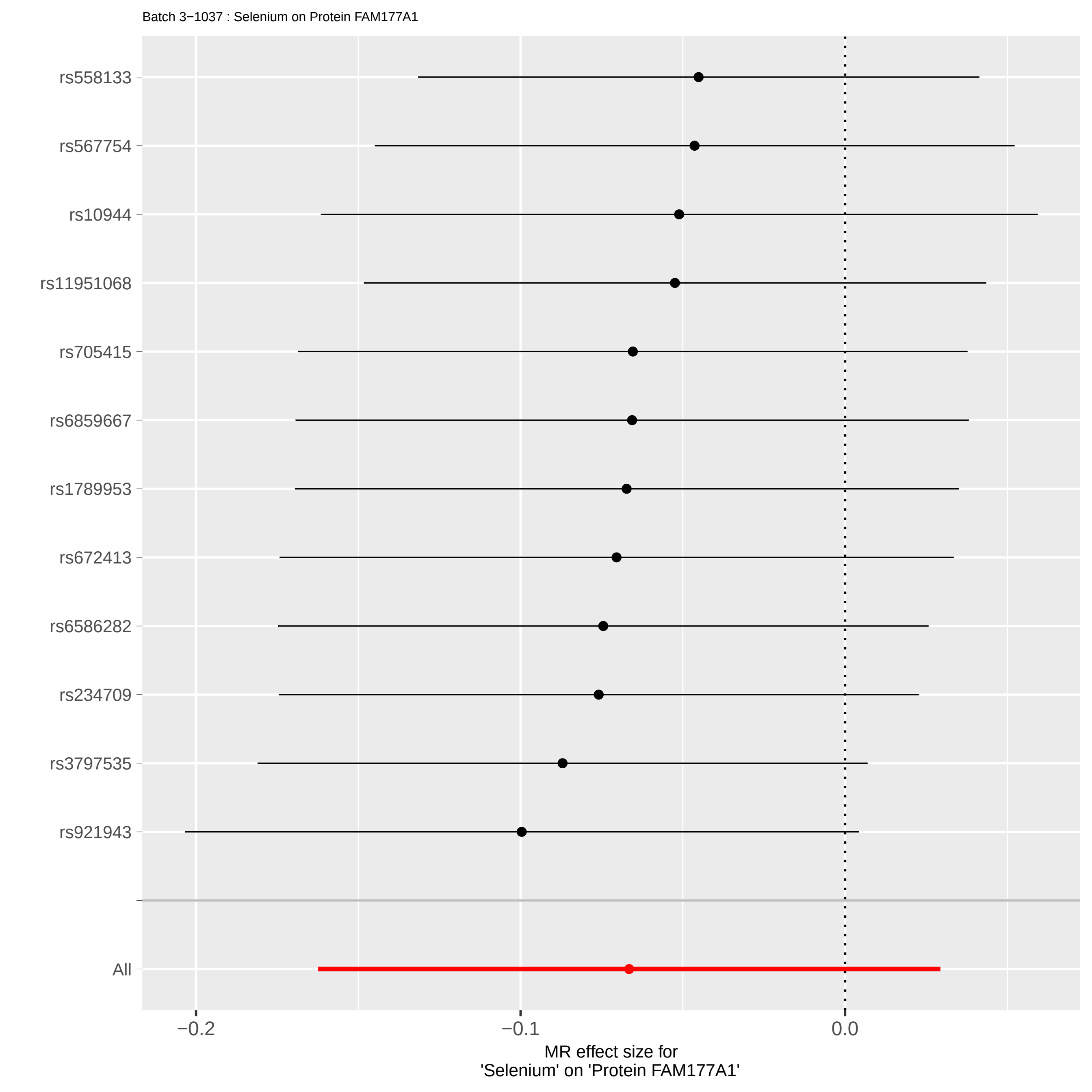

Batch 3−1037 : Selenium on Protein FAM177A1
rs558133
rs567754
rs10944
rs11951068
rs705415
rs6859667
rs1789953
rs672413
rs6586282
rs234709
rs3797535
rs921943
All
−0.2
−0.1
0.0
MR effect size for
'Selenium' on 'Protein FAM177A1'

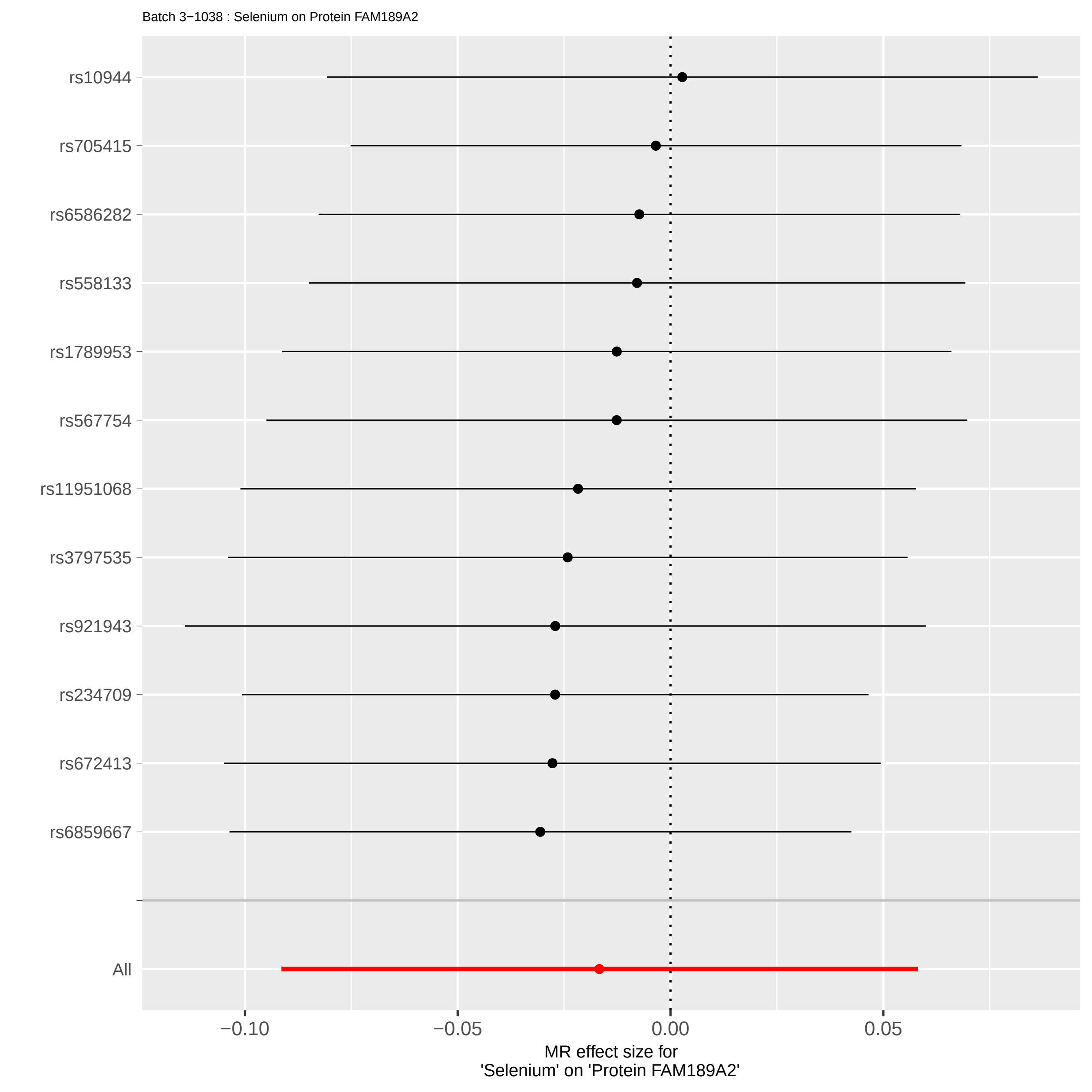
Batch 3−1038 : Selenium on Protein FAM189A2
rs10944
rs705415
rs6586282
rs558133
rs1789953
rs567754
rs11951068
rs3797535
rs921943
rs234709
rs672413
rs6859667
All
−0.10
−0.05
0.00
0.05
MR effect size for
'Selenium' on 'Protein FAM189A2'

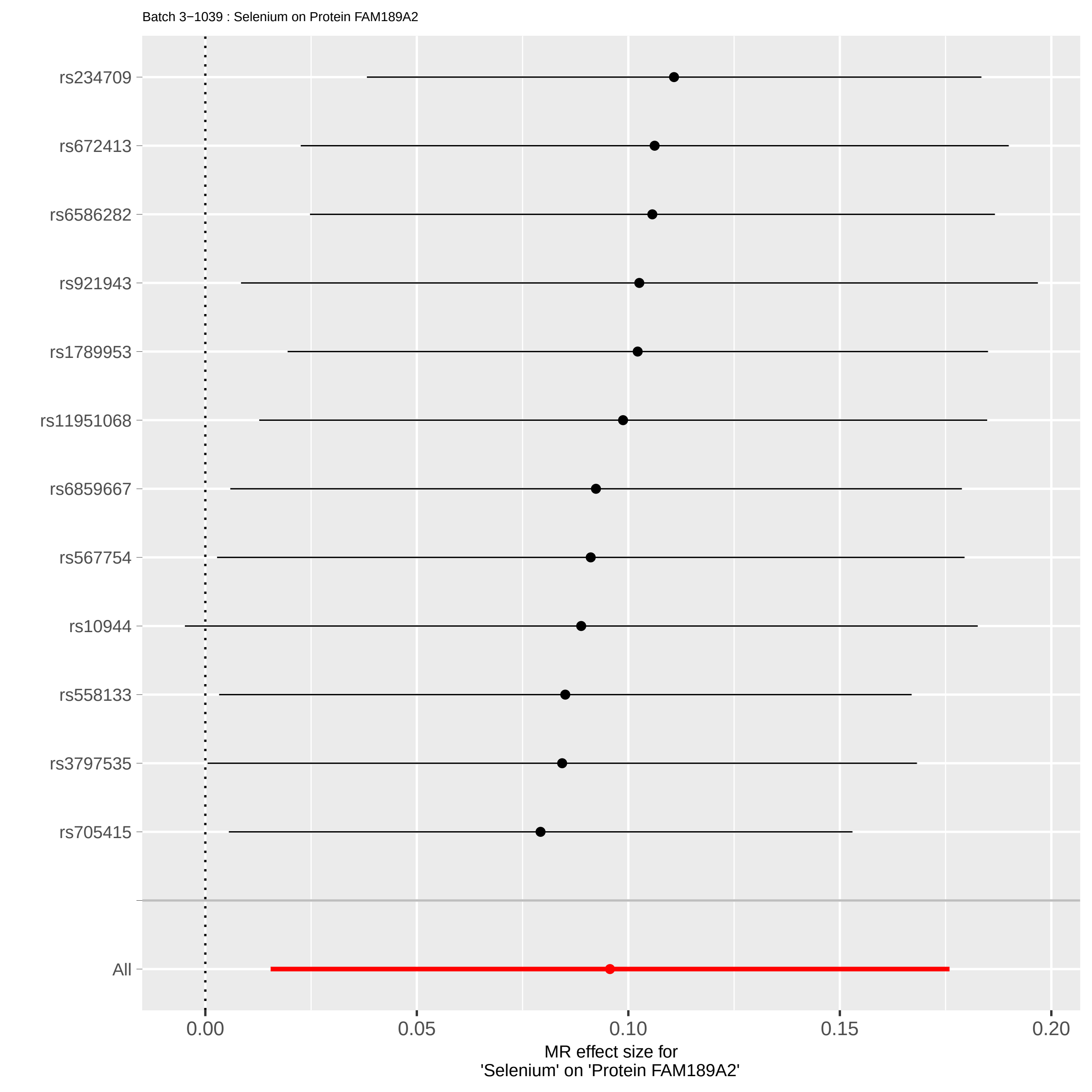
Batch 3−1039 : Selenium on Protein FAM189A2
rs234709
rs672413
rs6586282
rs921943
rs1789953
rs11951068
rs6859667
rs567754
rs10944
rs558133
rs3797535
rs705415
All
0.00
0.05
0.10
0.15
0.20
MR effect size for
'Selenium' on 'Protein FAM189A2'

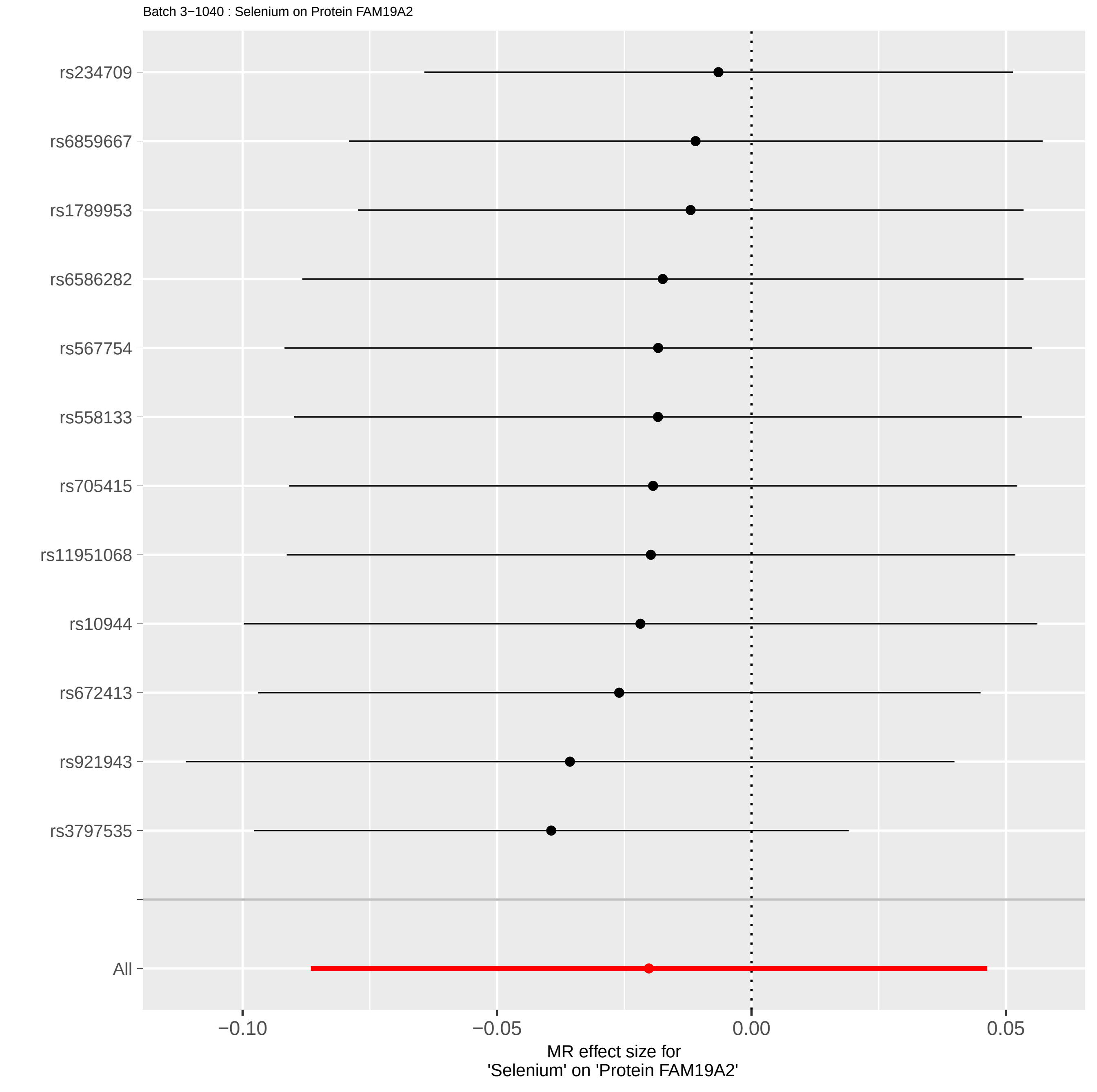

Batch 3−1040 : Selenium on Protein FAM19A2
rs234709
rs6859667
rs1789953
rs6586282
rs567754
rs558133
rs705415
rs11951068
rs10944
rs672413
rs921943
rs3797535
All
−0.10
−0.05
0.00
0.05
MR effect size for
'Selenium' on 'Protein FAM19A2'

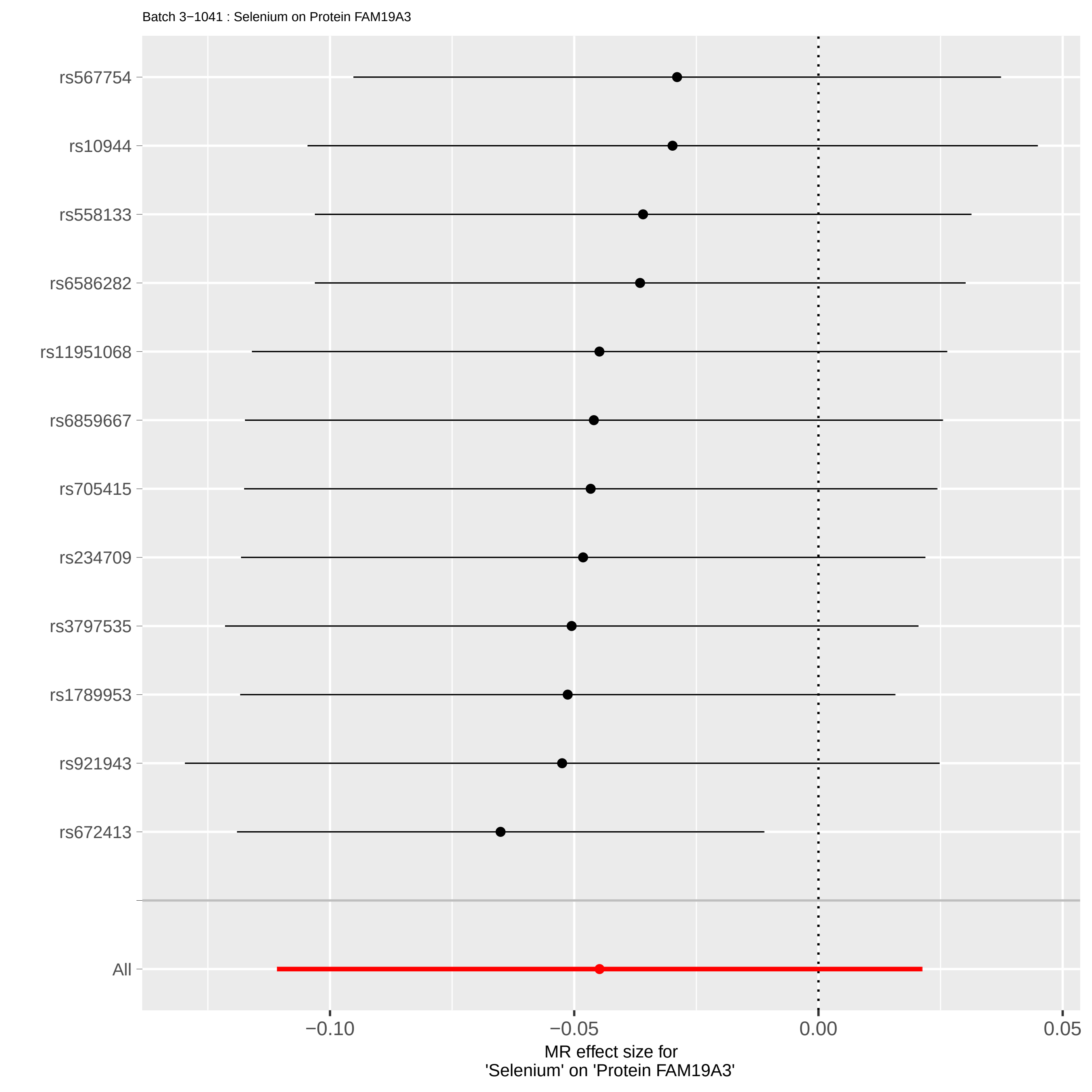
Batch 3−1041 : Selenium on Protein FAM19A3
rs567754
rs10944
rs558133
rs6586282
rs11951068
rs6859667
rs705415
rs234709
rs3797535
rs1789953
rs921943
rs672413
All
−0.10
−0.05
0.00
0.05
MR effect size for
'Selenium' on 'Protein FAM19A3'

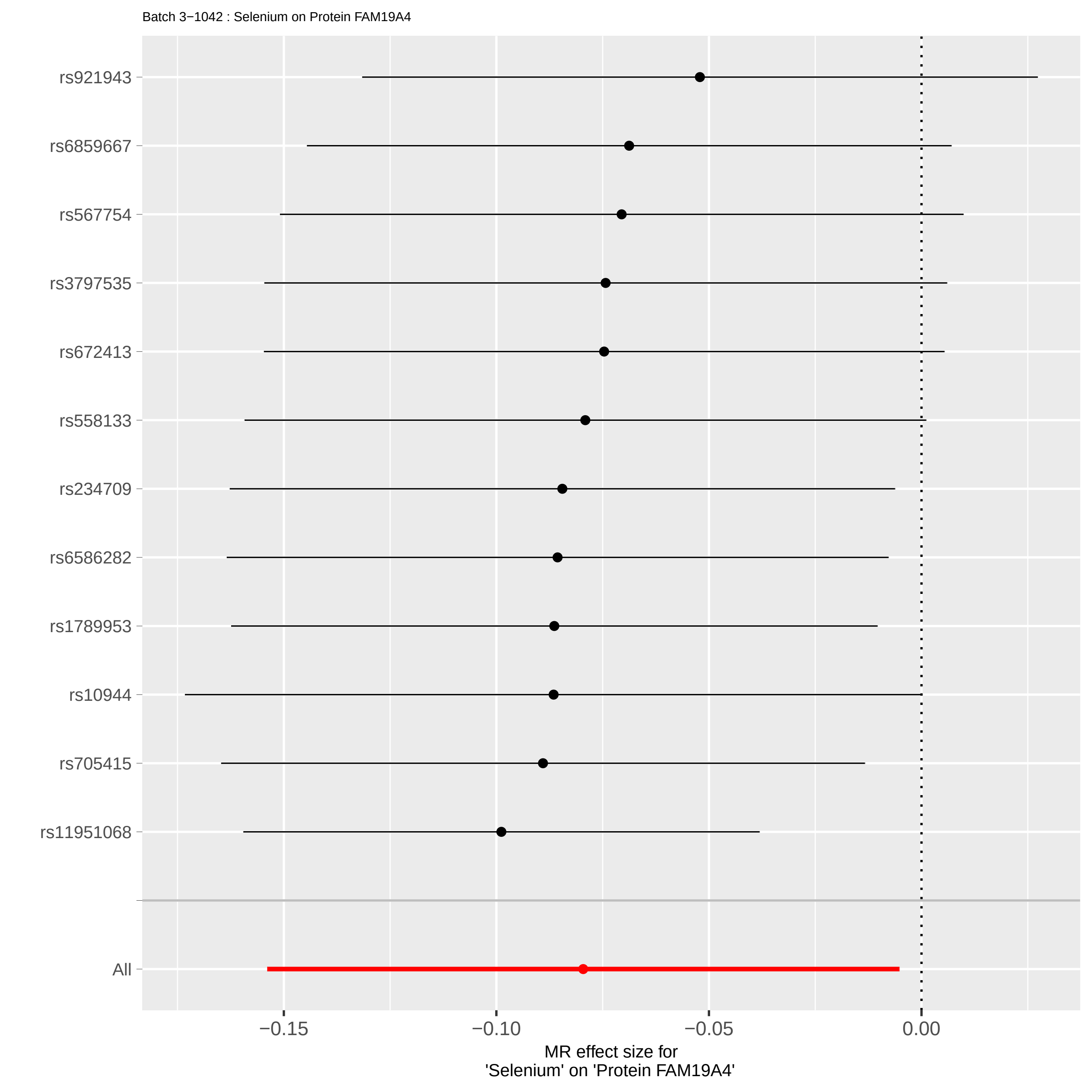

Batch 3−1042 : Selenium on Protein FAM19A4
rs921943
rs6859667
rs567754
rs3797535
rs672413
rs558133
rs234709
rs6586282
rs1789953
rs10944
rs705415
rs11951068
All
−0.15
−0.10
−0.05
0.00
MR effect size for
'Selenium' on 'Protein FAM19A4'

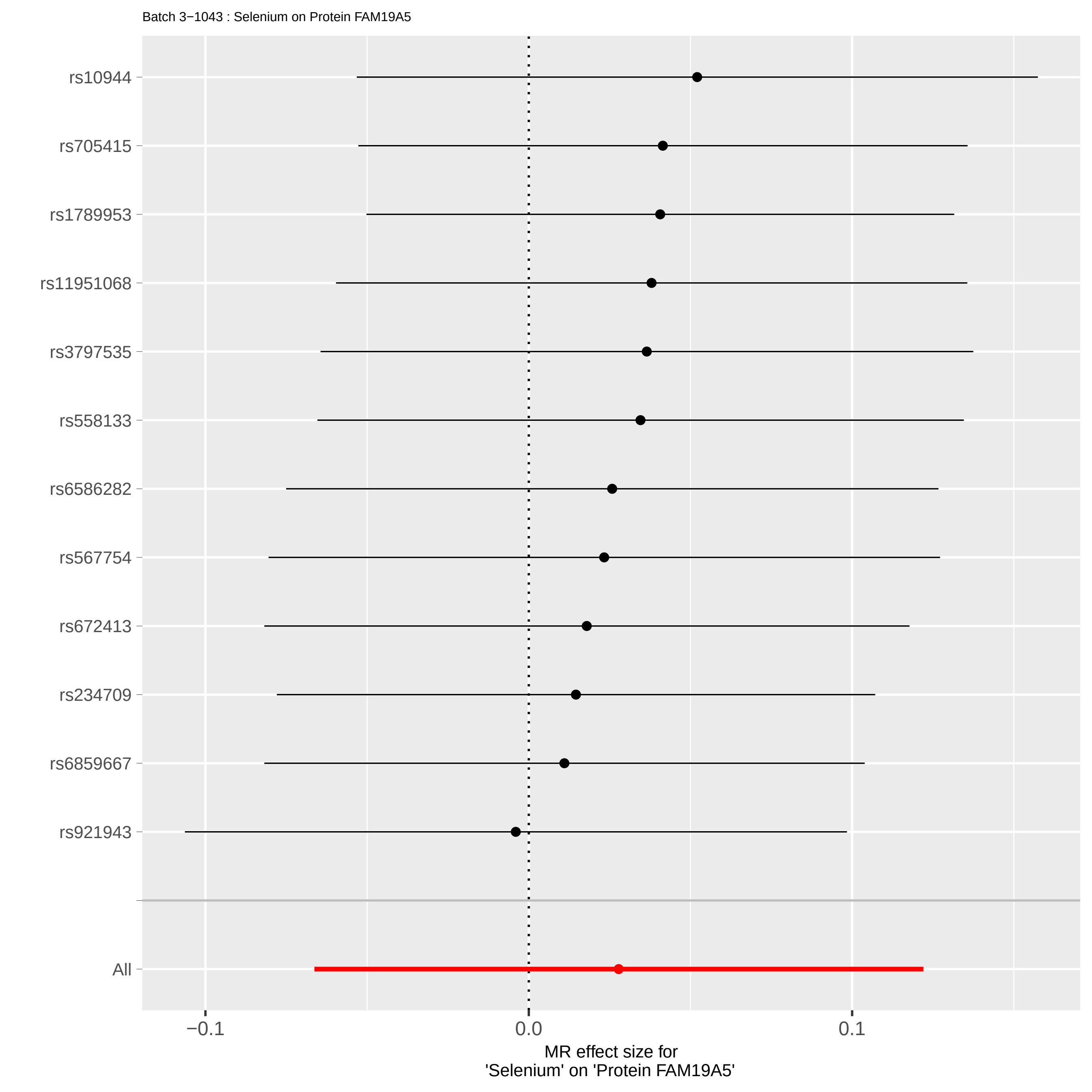

Batch 3−1043 : Selenium on Protein FAM19A5
rs10944
rs705415
rs1789953
rs11951068
rs3797535
rs558133
rs6586282
rs567754
rs672413
rs234709
rs6859667
rs921943
All
−0.1
0.0
0.1
MR effect size for
'Selenium' on 'Protein FAM19A5'

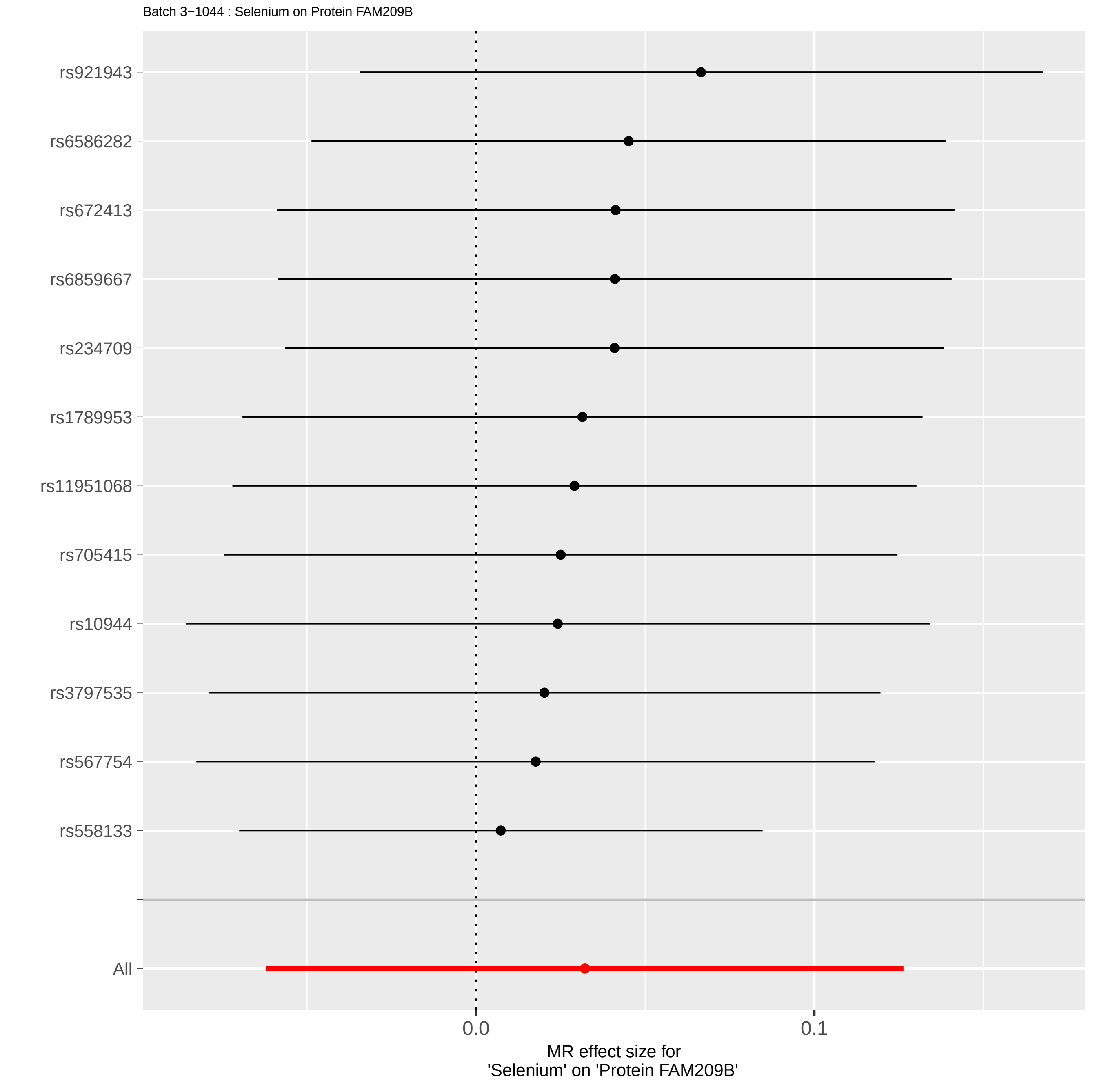
Batch 3−1044 : Selenium on Protein FAM209B
rs921943
rs6586282
rs672413
rs6859667
rs234709
rs1789953
rs11951068
rs705415
rs10944
rs3797535
rs567754
rs558133
All
0.0
0.1
MR effect size for
'Selenium' on 'Protein FAM209B'

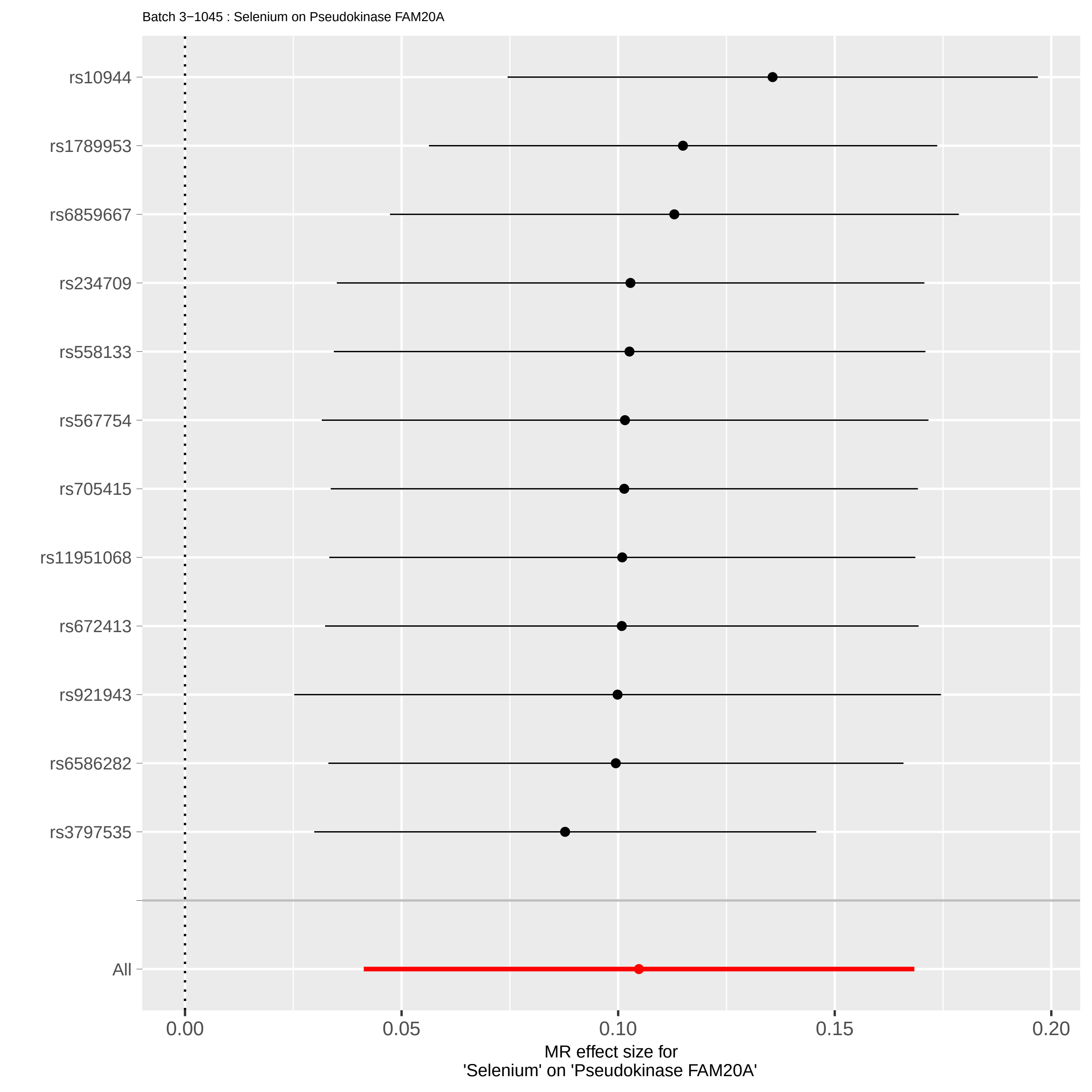

Batch 3−1045 : Selenium on Pseudokinase FAM20A
rs10944
rs1789953
rs6859667
rs234709
rs558133
rs567754
rs705415
rs11951068
rs672413
rs921943
rs6586282
rs3797535
All
0.00
0.05
0.10
0.15
0.20
MR effect size for
'Selenium' on 'Pseudokinase FAM20A'

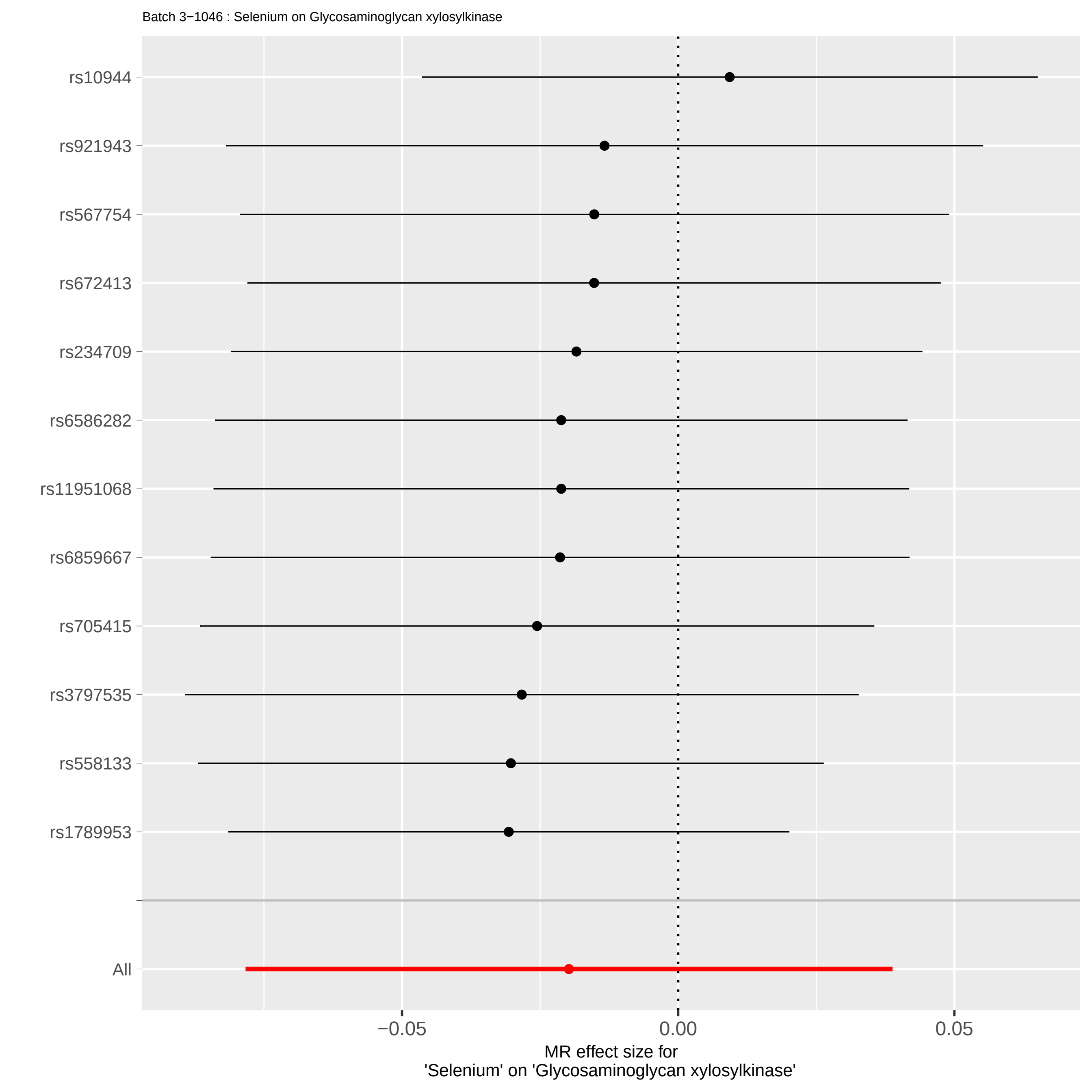

Batch 3−1046 : Selenium on Glycosaminoglycan xylosylkinase
rs10944
rs921943
rs567754
rs672413
rs234709
rs6586282
rs11951068
rs6859667
rs705415
rs3797535
rs558133
rs1789953
All
−0.05
0.00
0.05
MR effect size for
'Selenium' on 'Glycosaminoglycan xylosylkinase'

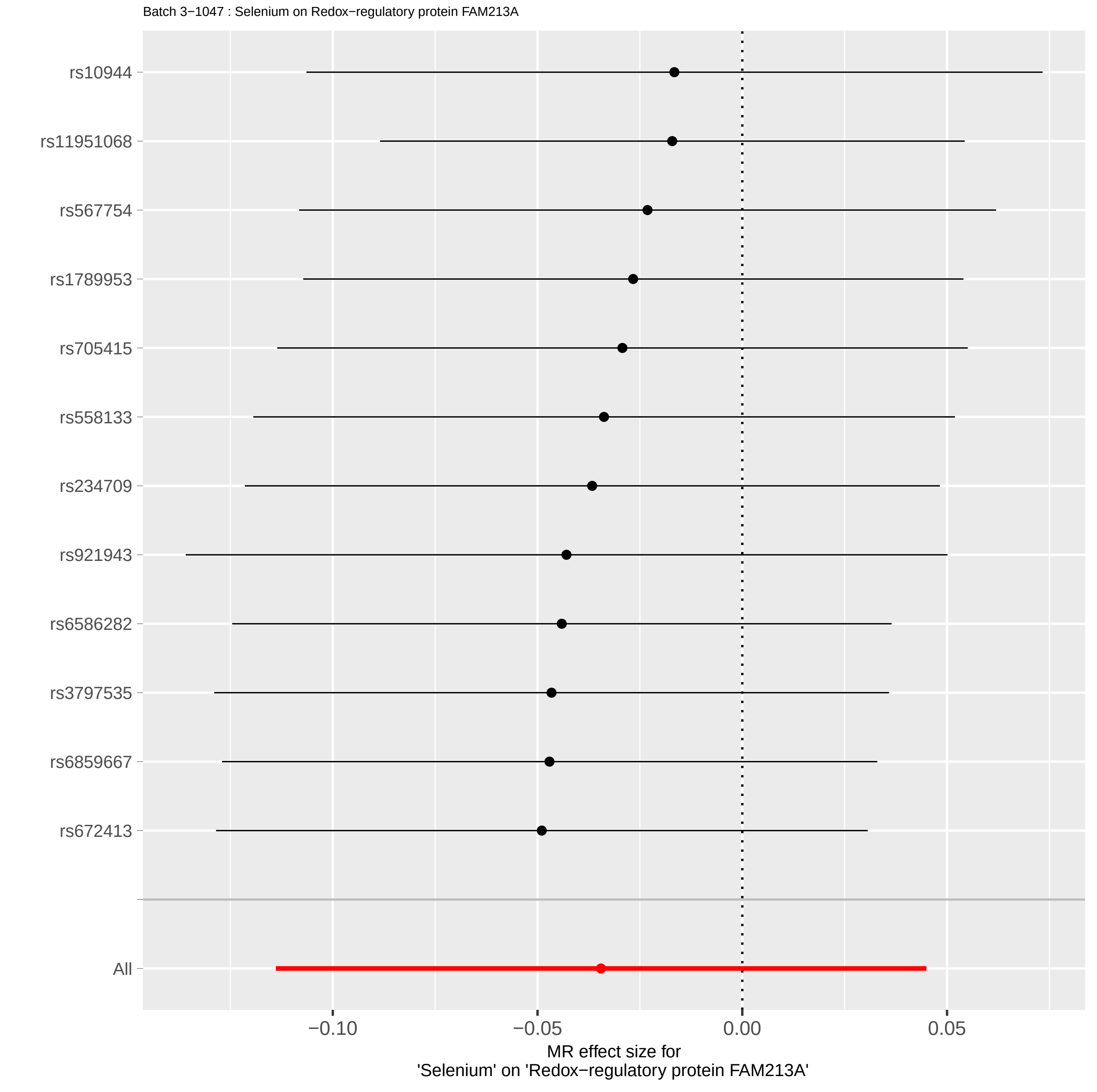
Batch 3−1047 : Selenium on Redox−regulatory protein FAM213A
rs10944
rs11951068
rs567754
rs1789953
rs705415
rs558133
rs234709
rs921943
rs6586282
rs3797535
rs6859667
rs672413
All
−0.10
−0.05
0.00
0.05
MR effect size for
'Selenium' on 'Redox−regulatory protein FAM213A'

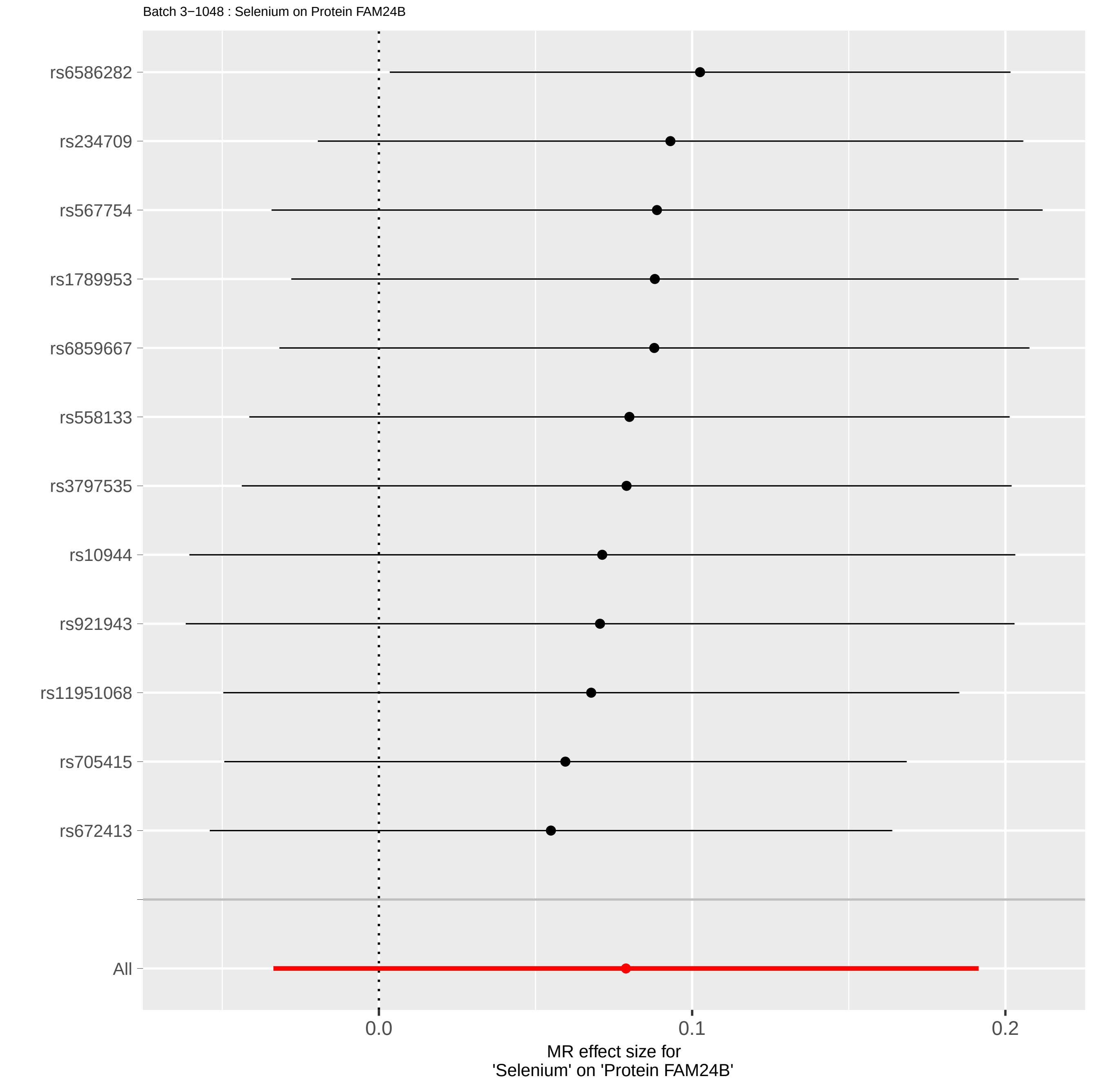

Batch 3−1048 : Selenium on Protein FAM24B
rs6586282
rs234709
rs567754
rs1789953
rs6859667
rs558133
rs3797535
rs10944
rs921943
rs11951068
rs705415
rs672413
All
0.0
0.1
0.2
MR effect size for
'Selenium' on 'Protein FAM24B'

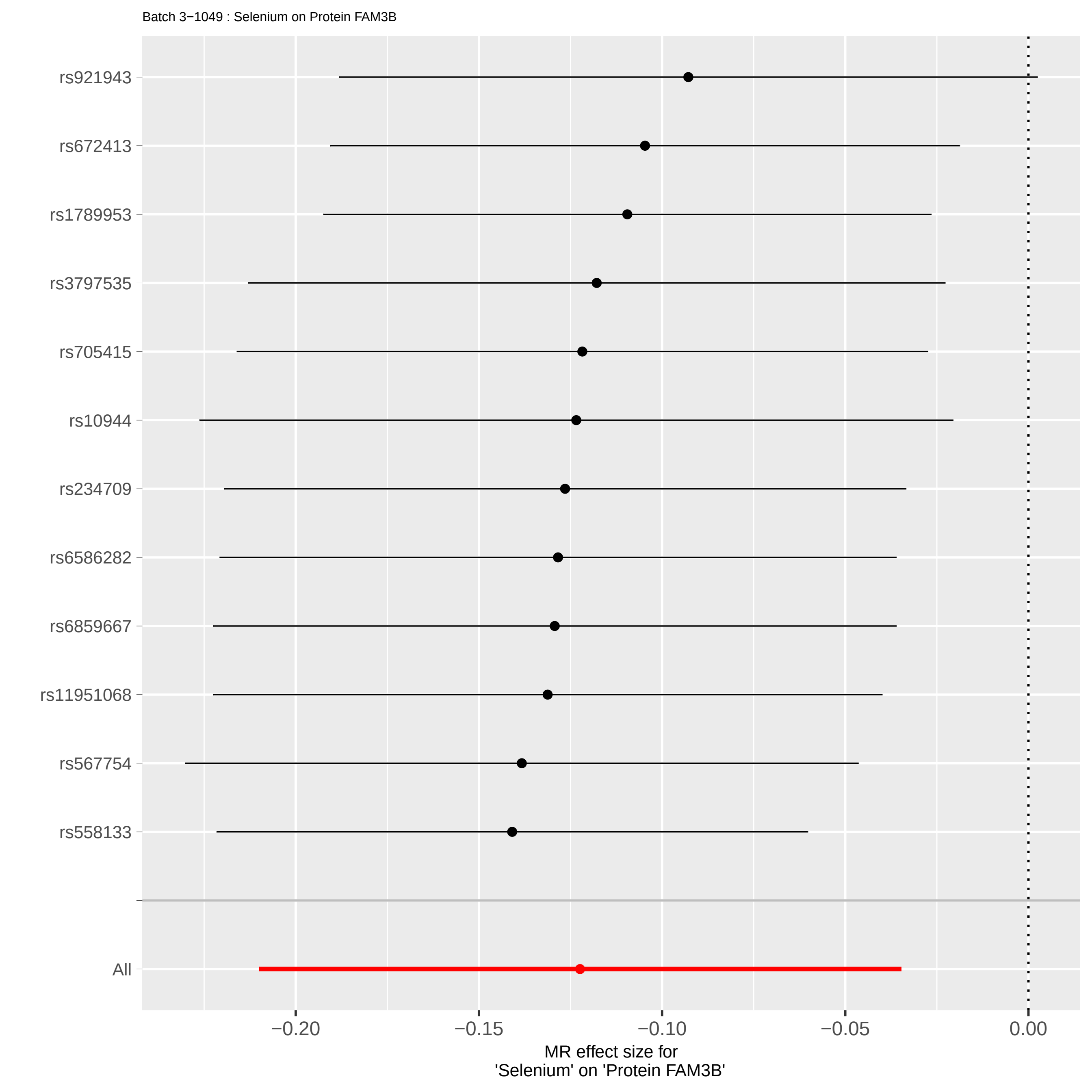

Batch 3−1049 : Selenium on Protein FAM3B
rs921943
rs672413
rs1789953
rs3797535
rs705415
rs10944
rs234709
rs6586282
rs6859667
rs11951068
rs567754
rs558133
All
−0.20
−0.15
−0.10
−0.05
0.00
MR effect size for
'Selenium' on 'Protein FAM3B'

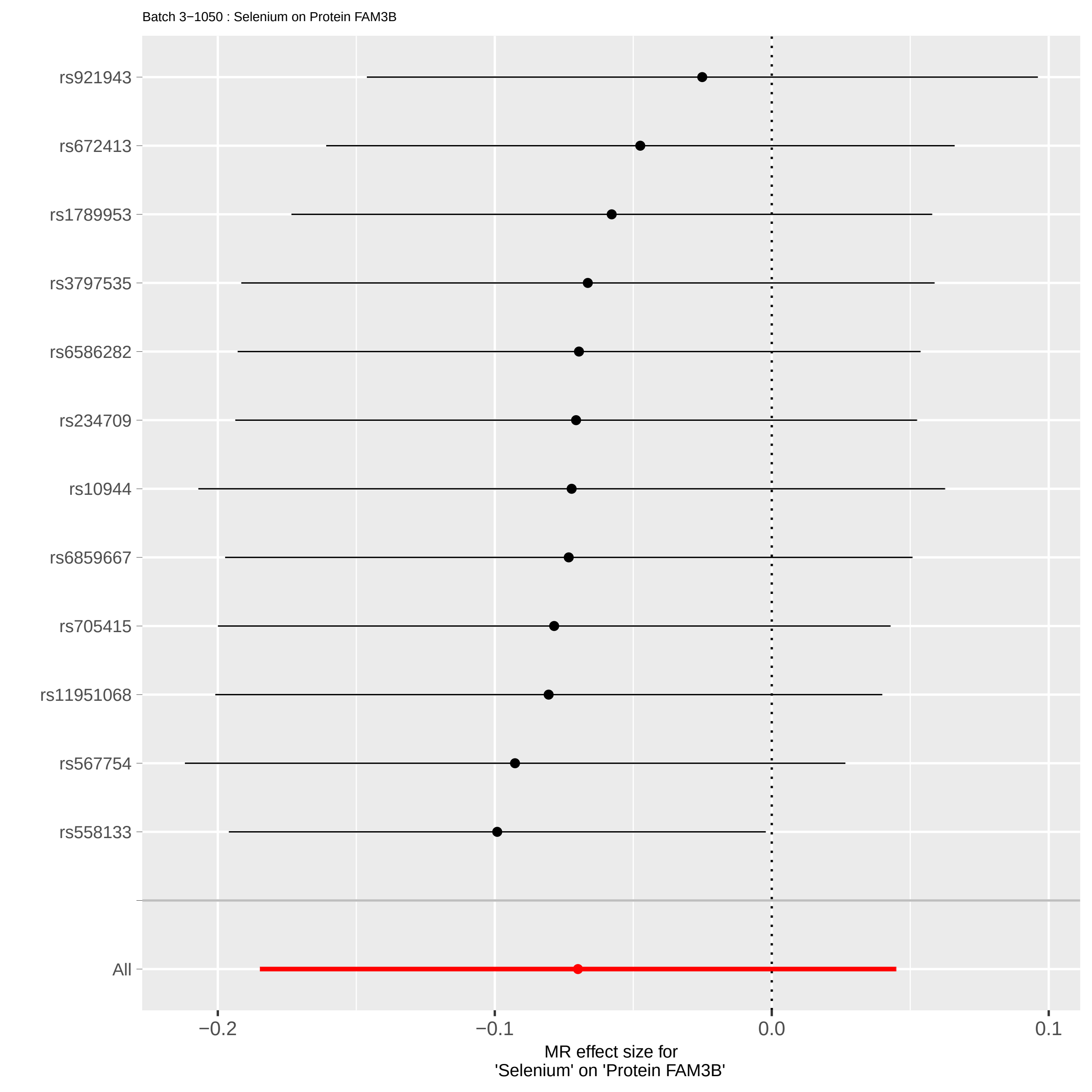

Batch 3−1050 : Selenium on Protein FAM3B
rs921943
rs672413
rs1789953
rs3797535
rs6586282
rs234709
rs10944
rs6859667
rs705415
rs11951068
rs567754
rs558133
All
−0.2
−0.1
0.0
0.1
MR effect size for
'Selenium' on 'Protein FAM3B'

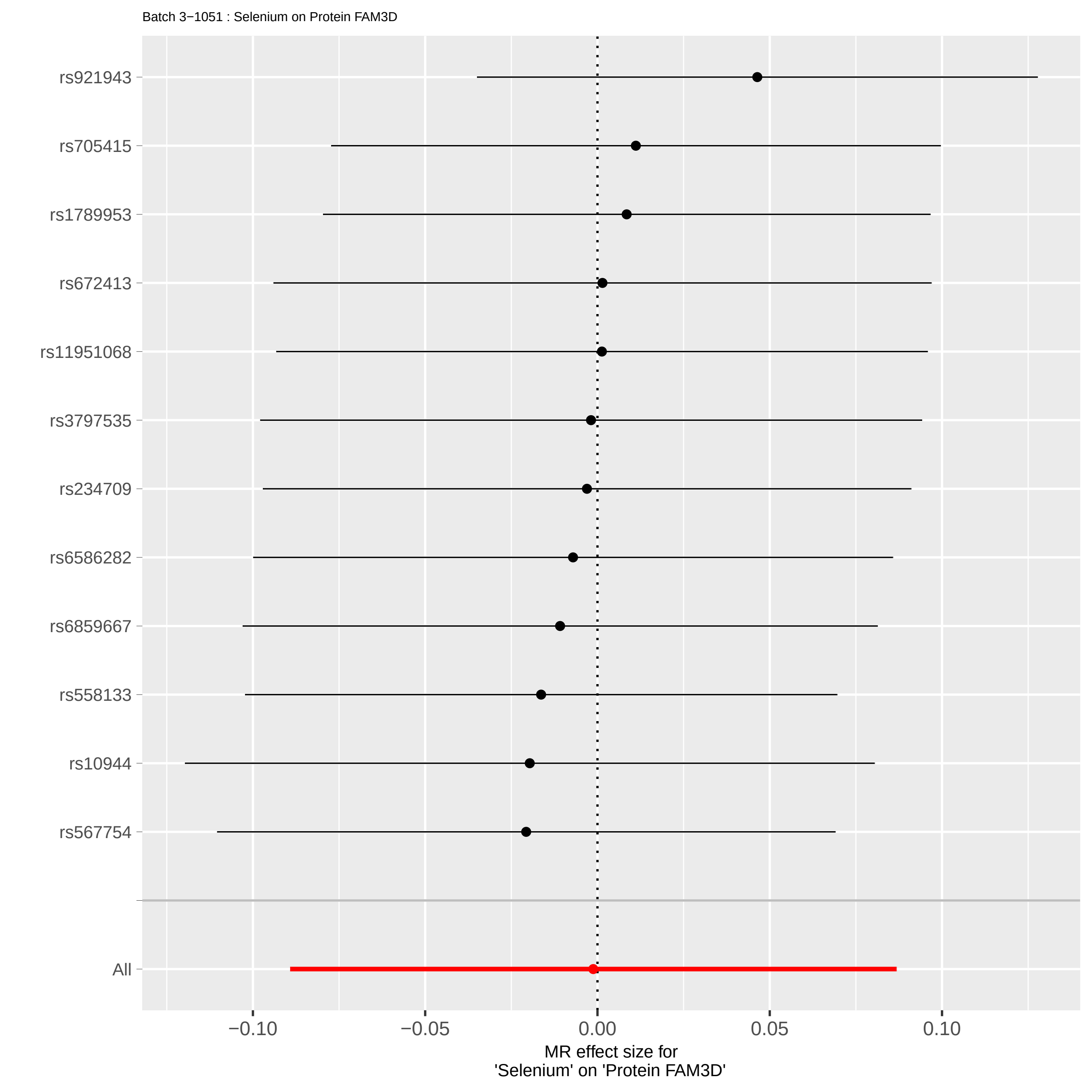

Batch 3−1051 : Selenium on Protein FAM3D
rs921943
rs705415
rs1789953
rs672413
rs11951068
rs3797535
rs234709
rs6586282
rs6859667
rs558133
rs10944
rs567754
All
−0.10
−0.05
0.00
0.05
0.10
MR effect size for
'Selenium' on 'Protein FAM3D'

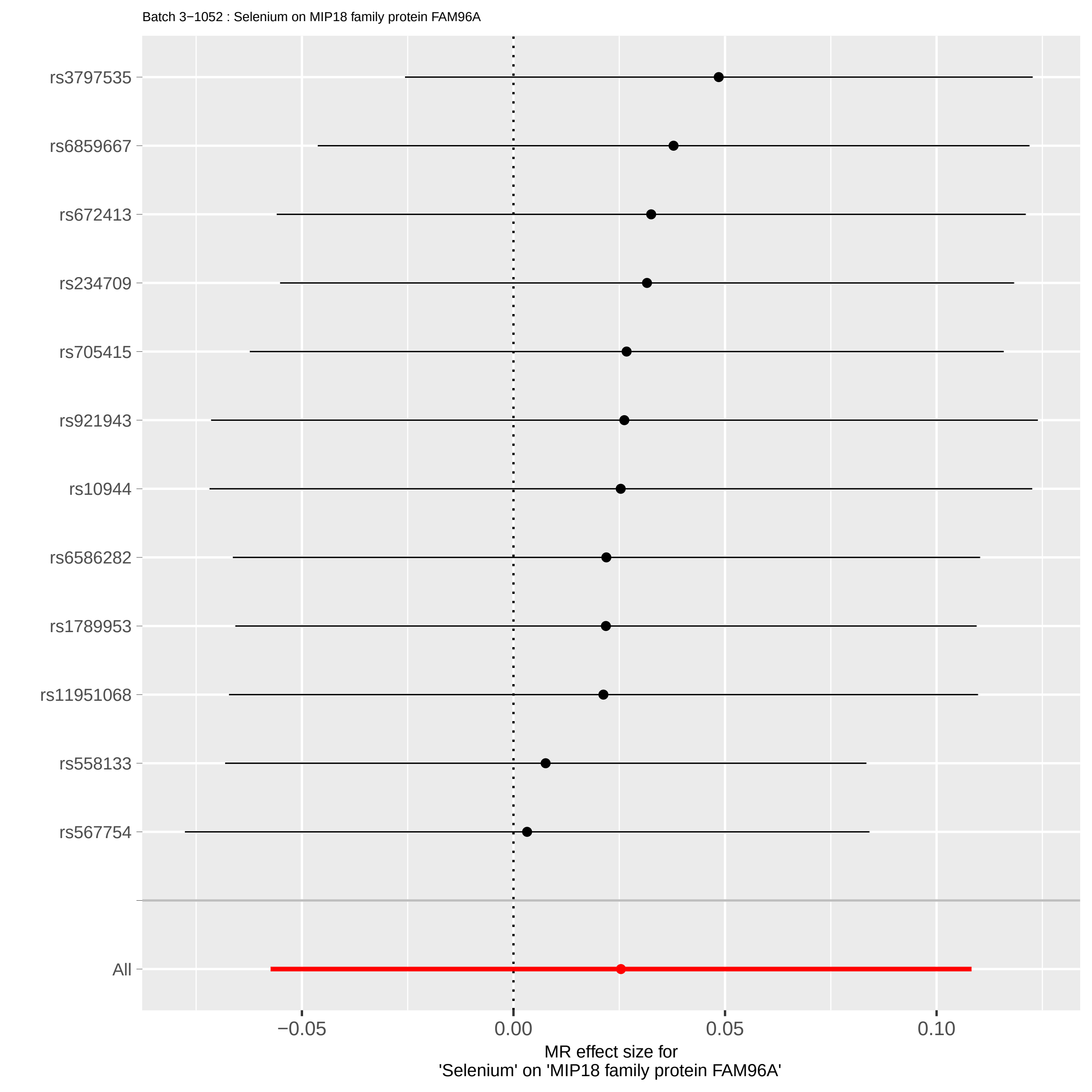

Batch 3−1052 : Selenium on MIP18 family protein FAM96A
rs3797535
rs6859667
rs672413
rs234709
rs705415
rs921943
rs10944
rs6586282
rs1789953
rs11951068
rs558133
rs567754
All
−0.05
0.00
0.05
0.10
MR effect size for
'Selenium' on 'MIP18 family protein FAM96A'

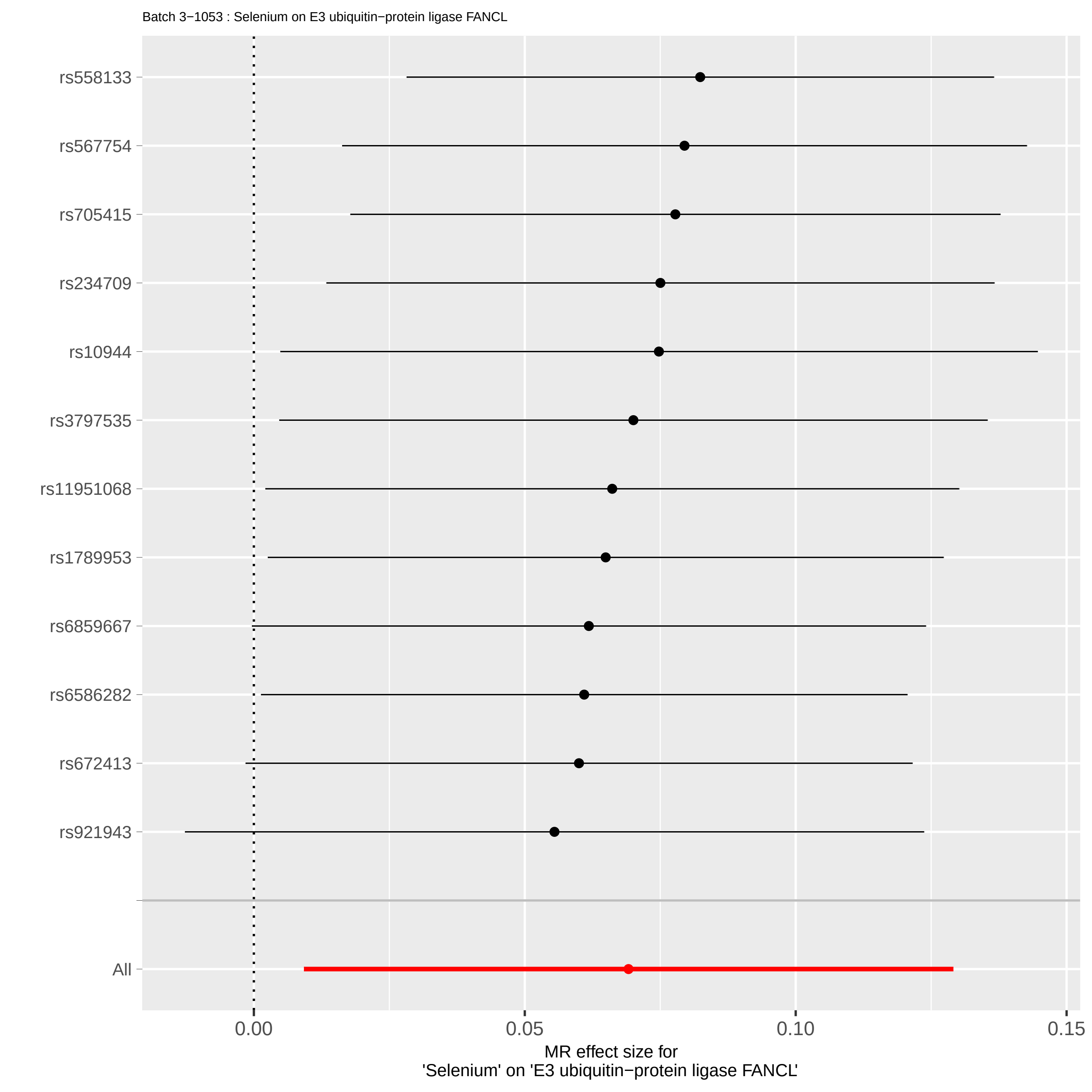

Batch 3−1053 : Selenium on E3 ubiquitin−protein ligase FANCL
rs558133
rs567754
rs705415
rs234709
rs10944
rs3797535
rs11951068
rs1789953
rs6859667
rs6586282
rs672413
rs921943
All
0.00
0.05
0.10
0.15
MR effect size for
'Selenium' on 'E3 ubiquitin−protein ligase FANCL'

Batch 3−1054 : Selenium on Prolyl endopeptidase FAP

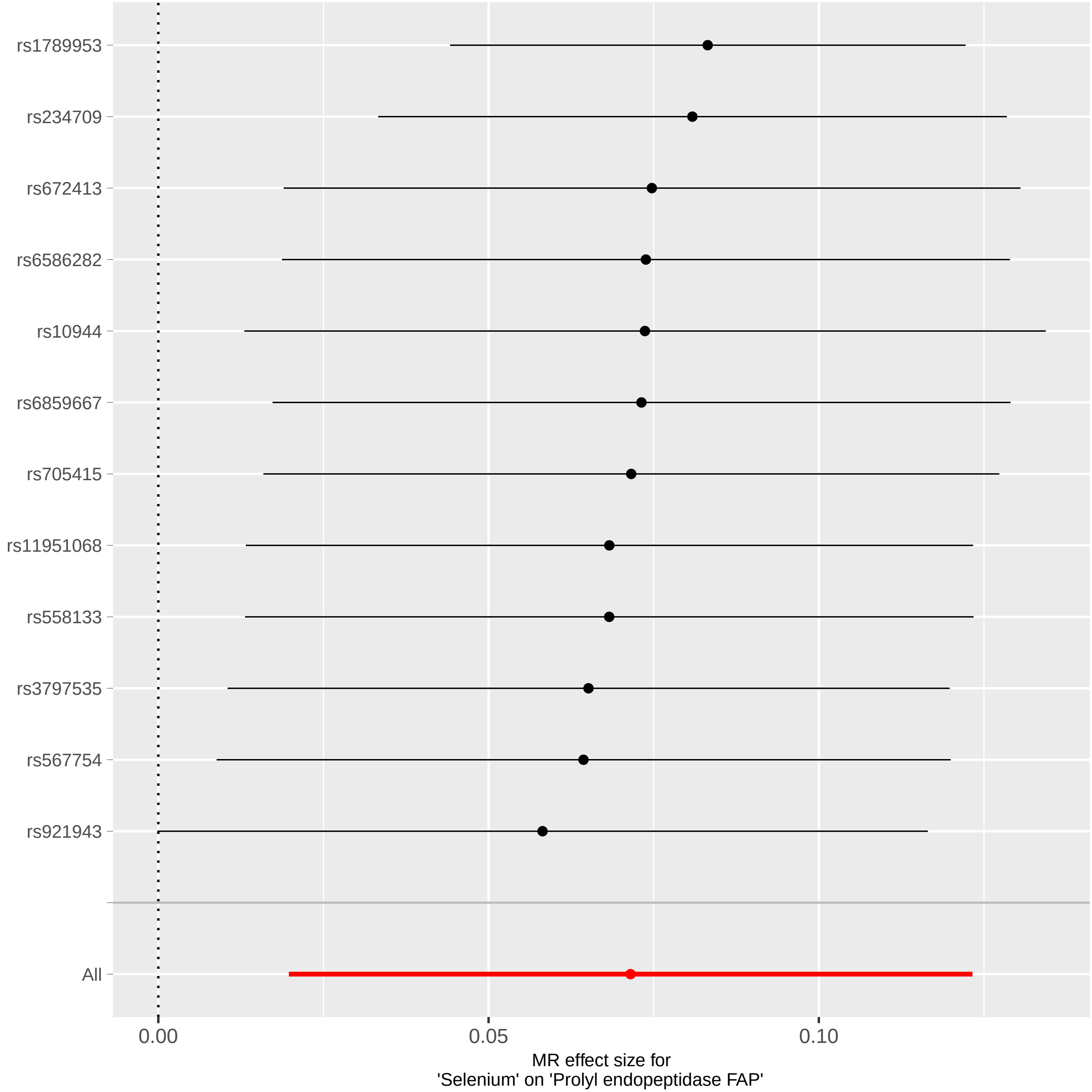

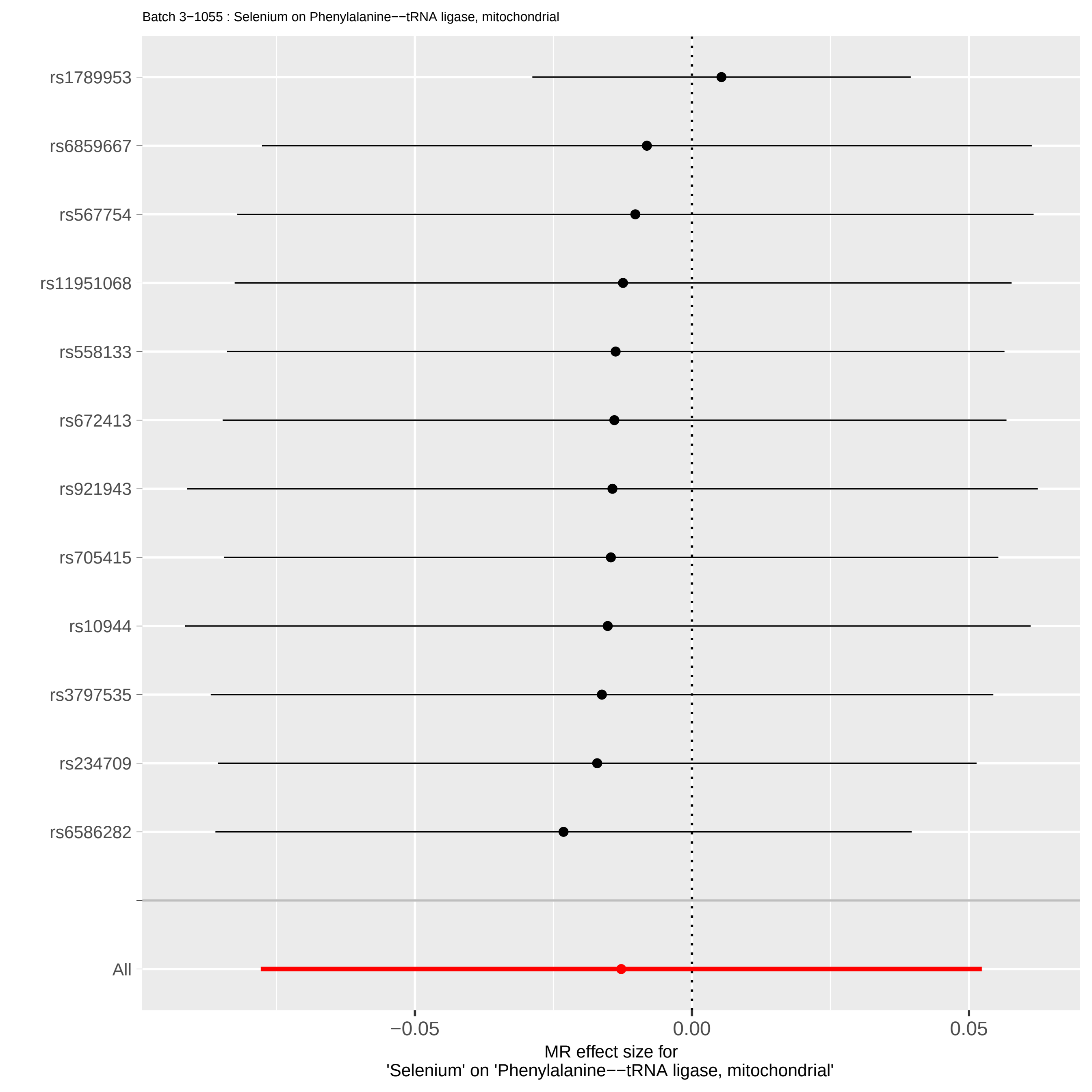

Batch 3−1055 : Selenium on Phenylalanine−−tRNA ligase, mitochondrial
rs1789953
rs6859667
rs567754
rs11951068
rs558133
rs672413
rs921943
rs705415
rs10944
rs3797535
rs234709
rs6586282
All
−0.05
0.00
0.05
MR effect size for
'Selenium' on 'Phenylalanine−−tRNA ligase, mitochondrial'

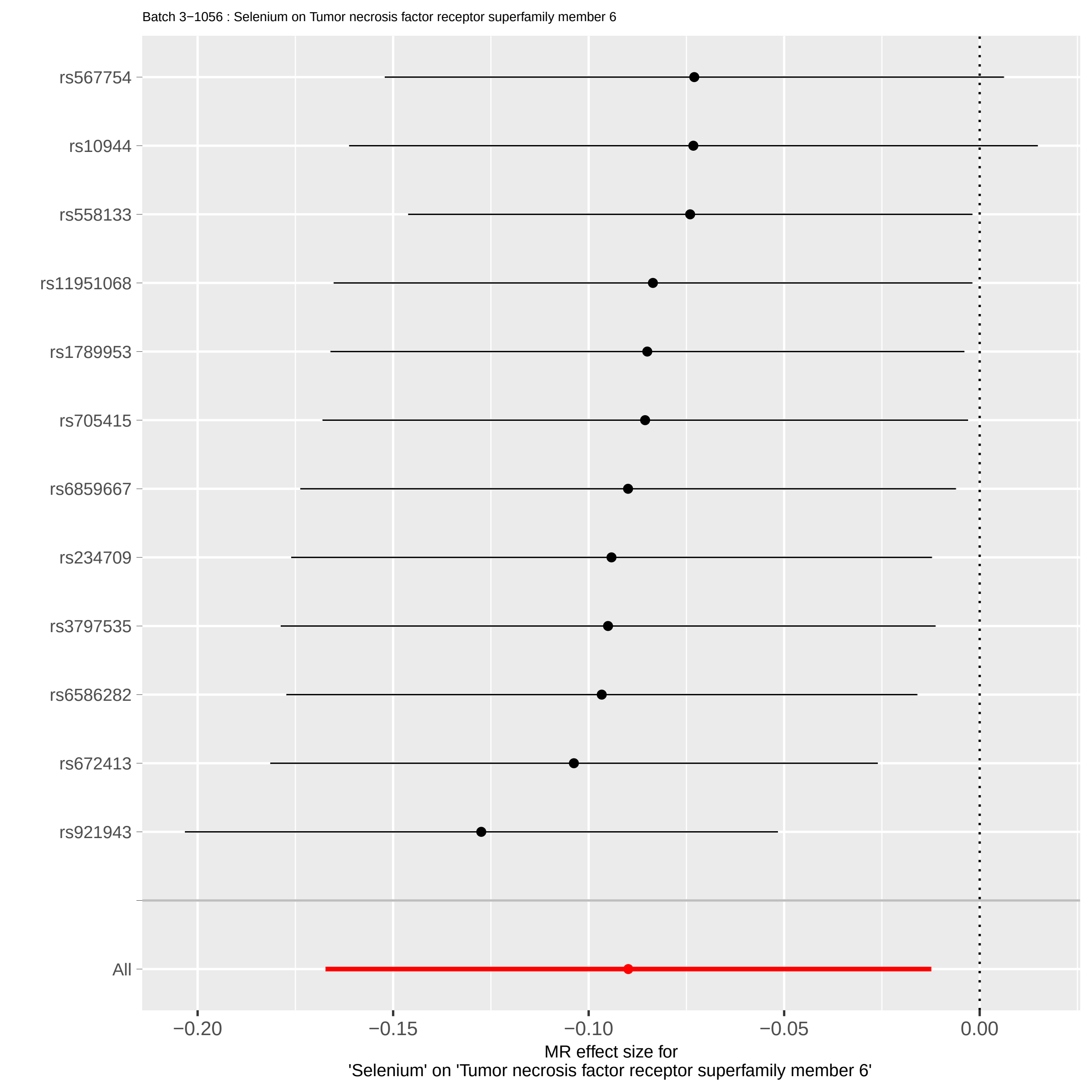

Batch 3−1056 : Selenium on Tumor necrosis factor receptor superfamily member 6
rs567754
rs10944
rs558133
rs11951068
rs1789953
rs705415
rs6859667
rs234709
rs3797535
rs6586282
rs672413
rs921943
All
−0.20
−0.15
−0.10
−0.05
0.00
MR effect size for
'Selenium' on 'Tumor necrosis factor receptor superfamily member 6'

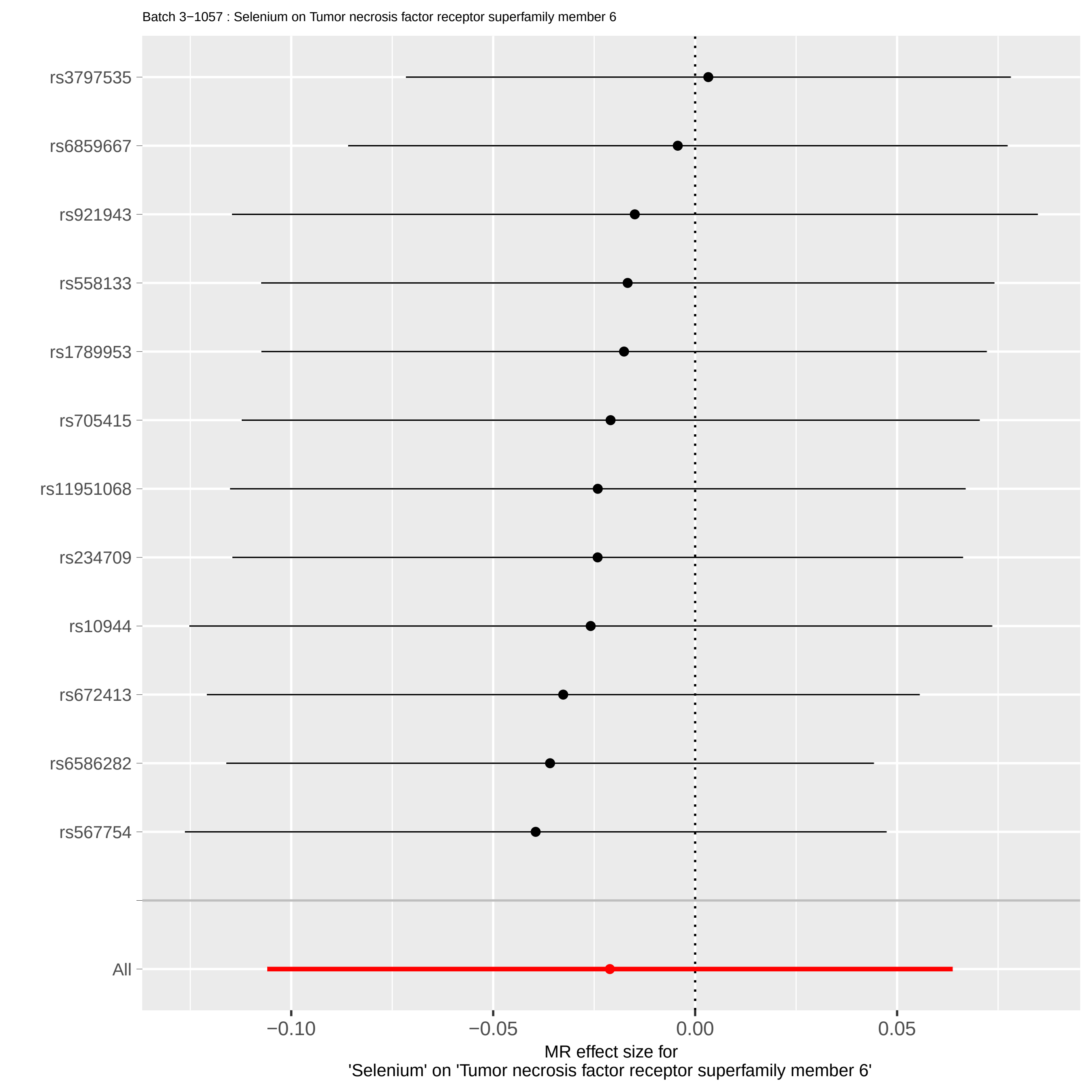

Batch 3−1057 : Selenium on Tumor necrosis factor receptor superfamily member 6
rs3797535
rs6859667
rs921943
rs558133
rs1789953
rs705415
rs11951068
rs234709
rs10944
rs672413
rs6586282
rs567754
All
−0.10
−0.05
0.00
0.05
MR effect size for
'Selenium' on 'Tumor necrosis factor receptor superfamily member 6'

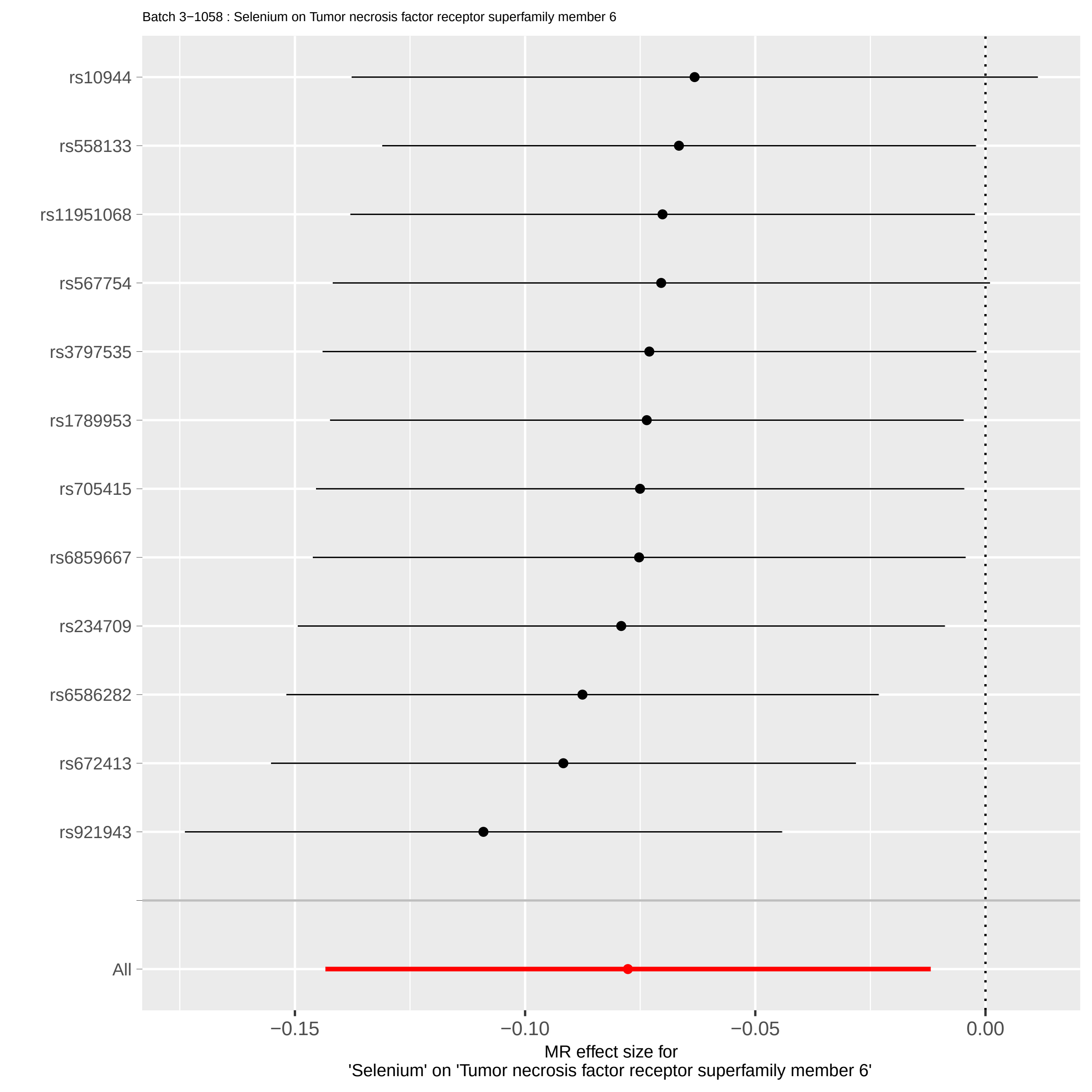
Batch 3−1058 : Selenium on Tumor necrosis factor receptor superfamily member 6
rs10944
rs558133
rs11951068
rs567754
rs3797535
rs1789953
rs705415
rs6859667
rs234709
rs6586282
rs672413
rs921943
All
−0.15
−0.10
−0.05
0.00
MR effect size for
'Selenium' on 'Tumor necrosis factor receptor superfamily member 6'

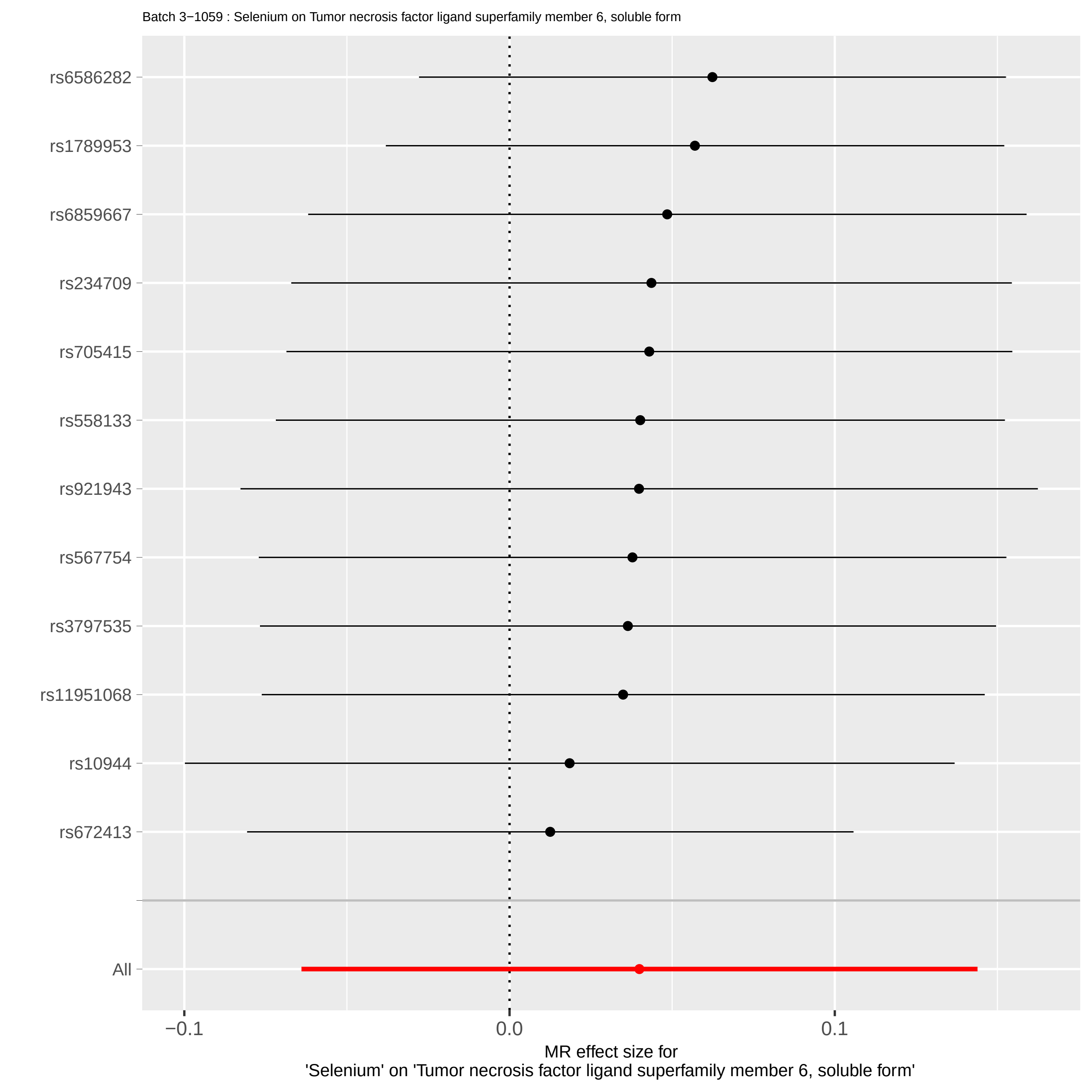

Batch 3−1059 : Selenium on Tumor necrosis factor ligand superfamily member 6, soluble form
rs6586282
rs1789953
rs6859667
rs234709
rs705415
rs558133
rs921943
rs567754
rs3797535
rs11951068
rs10944
rs672413
All
−0.1
0.0
0.1
MR effect size for
'Selenium' on 'Tumor necrosis factor ligand superfamily member 6, soluble form'

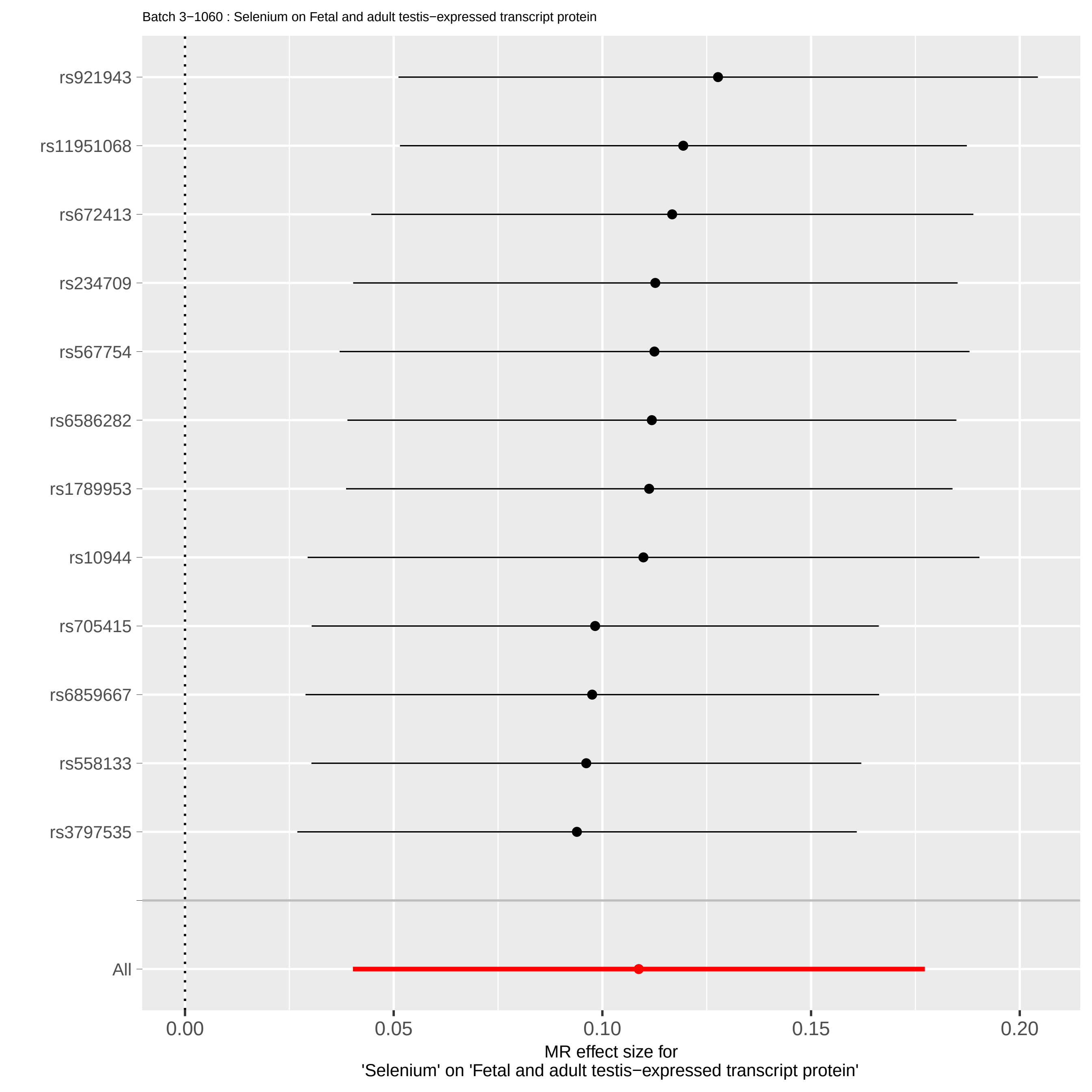

Batch 3−1060 : Selenium on Fetal and adult testis−expressed transcript protein
rs921943
rs11951068
rs672413
rs234709
rs567754
rs6586282
rs1789953
rs10944
rs705415
rs6859667
rs558133
rs3797535
All
0.00
0.05
0.10
0.15
0.20
MR effect size for
'Selenium' on 'Fetal and adult testis−expressed transcript protein'

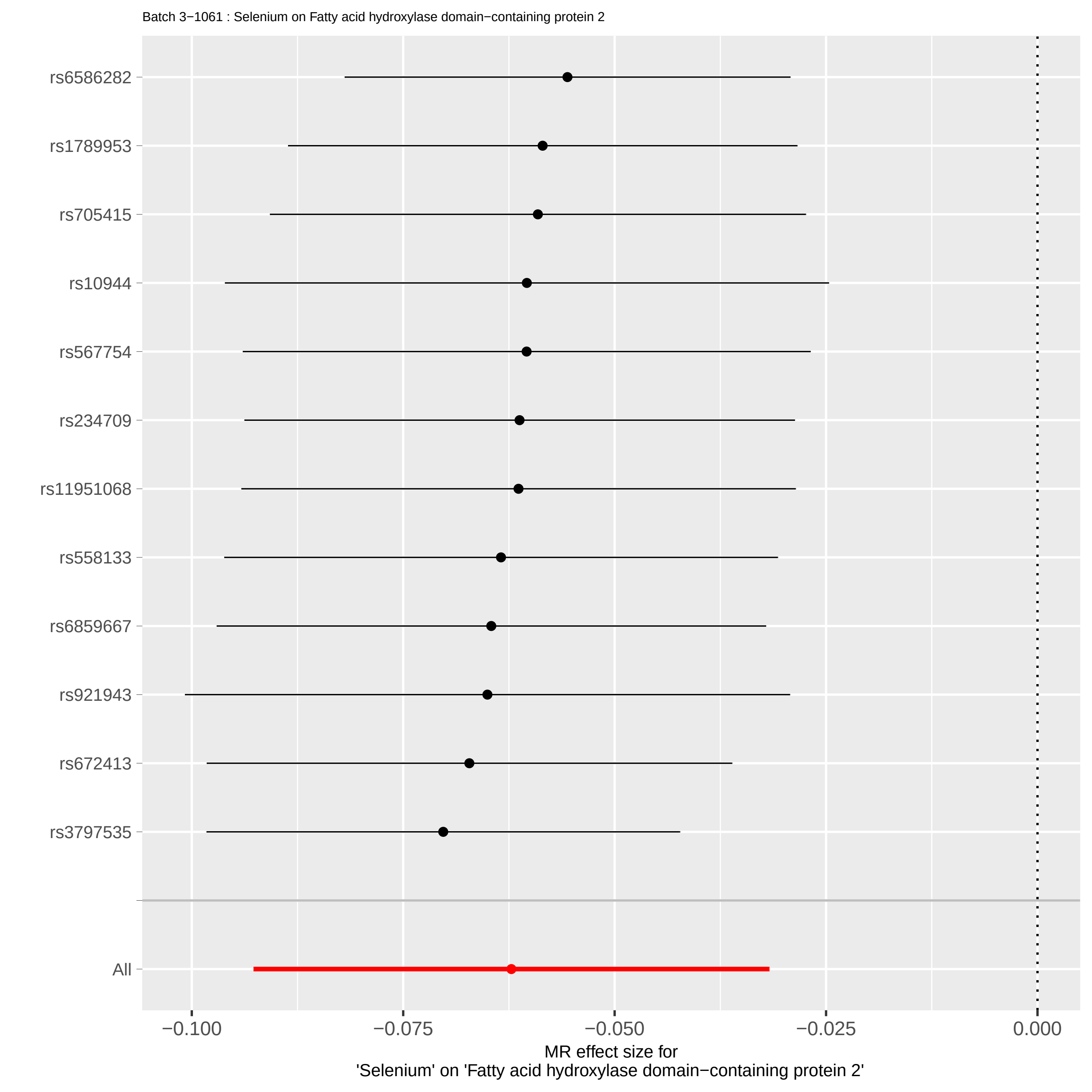

Batch 3−1061 : Selenium on Fatty acid hydroxylase domain−containing protein 2
rs6586282
rs1789953
rs705415
rs10944
rs567754
rs234709
rs11951068
rs558133
rs6859667
rs921943
rs672413
rs3797535
All
−0.100
−0.075
−0.050
−0.025
0.000
MR effect size for
'Selenium' on 'Fatty acid hydroxylase domain−containing protein 2'

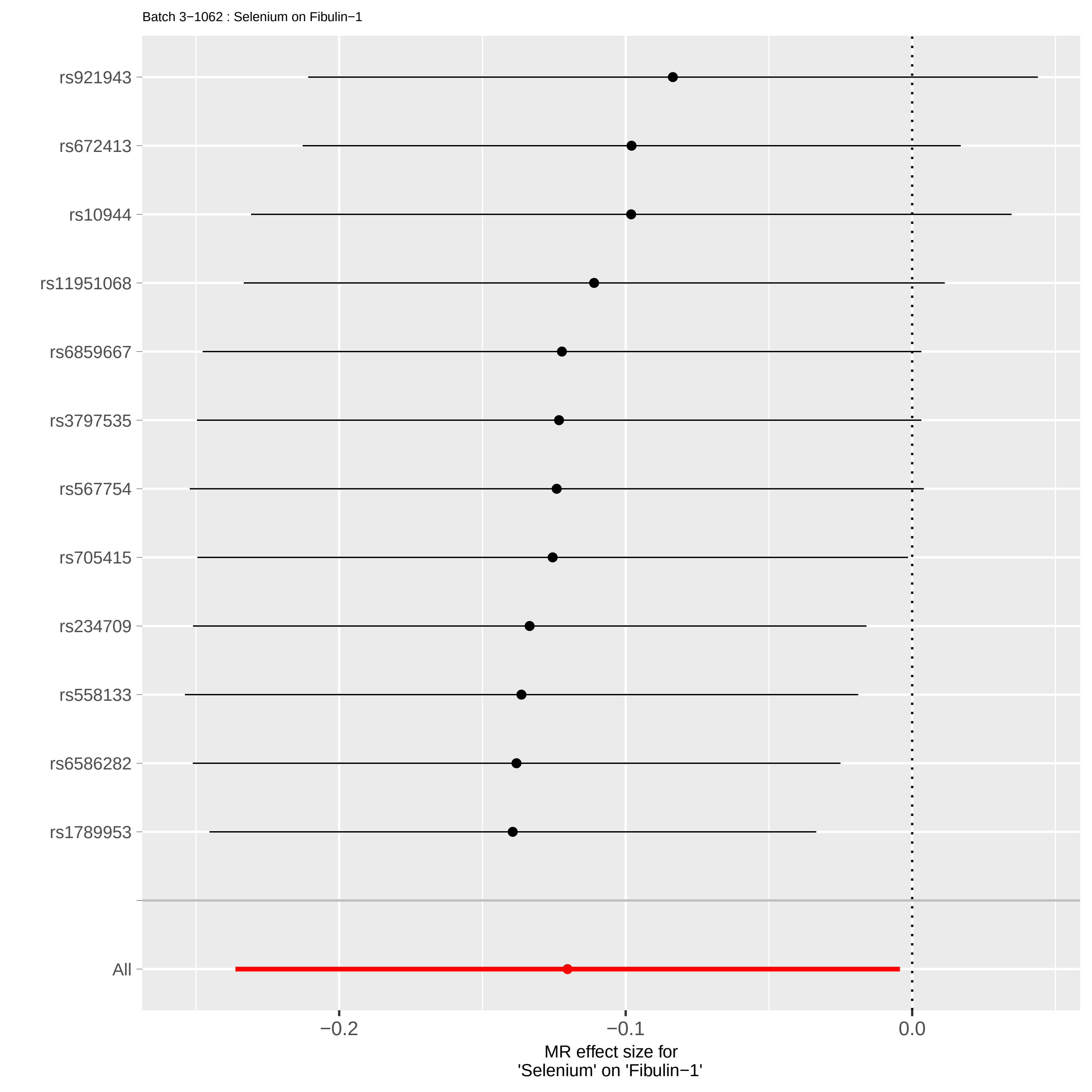

Batch 3−1062 : Selenium on Fibulin−1
rs921943
rs672413
rs10944
rs11951068
rs6859667
rs3797535
rs567754
rs705415
rs234709
rs558133
rs6586282
rs1789953
All
−0.2
−0.1
0.0
MR effect size for
'Selenium' on 'Fibulin−1'

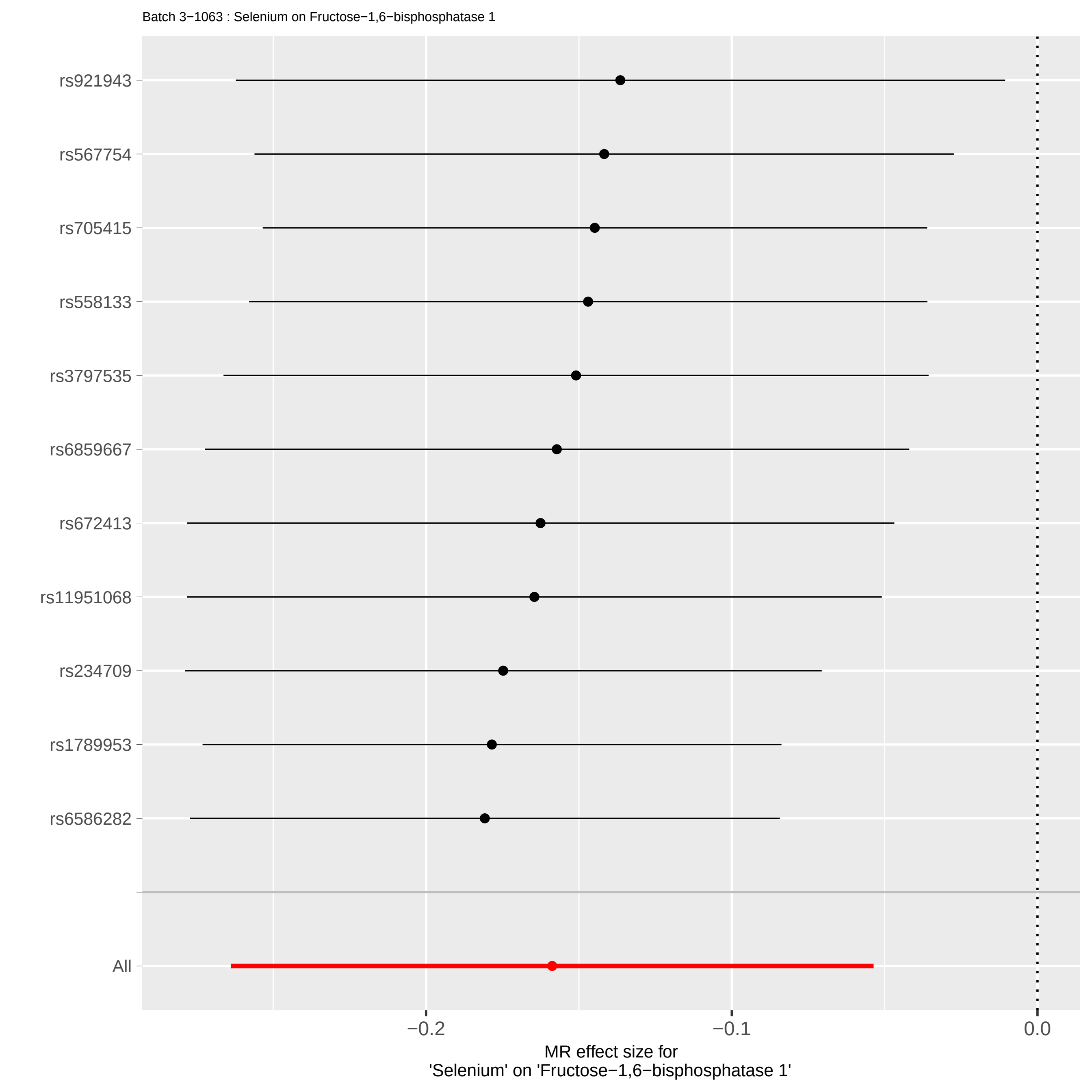

Batch 3−1063 : Selenium on Fructose−1,6−bisphosphatase 1
rs921943
rs567754
rs705415
rs558133
rs3797535
rs6859667
rs672413
rs11951068
rs234709
rs1789953
rs6586282
All
−0.2
−0.1
0.0
MR effect size for
'Selenium' on 'Fructose−1,6−bisphosphatase 1'

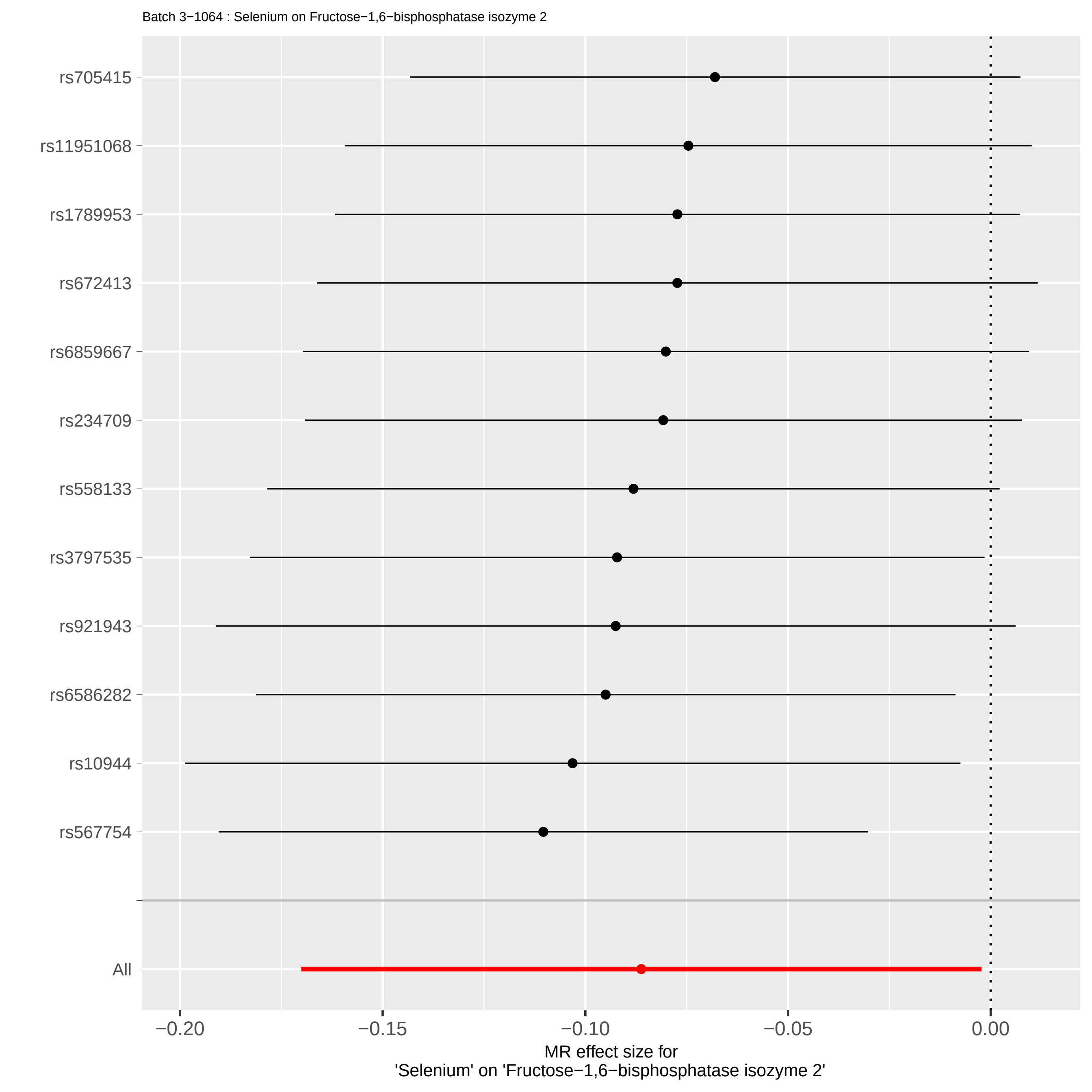

Batch 3−1064 : Selenium on Fructose−1,6−bisphosphatase isozyme 2
rs705415
rs11951068
rs1789953
rs672413
rs6859667
rs234709
rs558133
rs3797535
rs921943
rs6586282
rs10944
rs567754
All
−0.20
−0.15
−0.10
−0.05
0.00
MR effect size for
'Selenium' on 'Fructose−1,6−bisphosphatase isozyme 2'

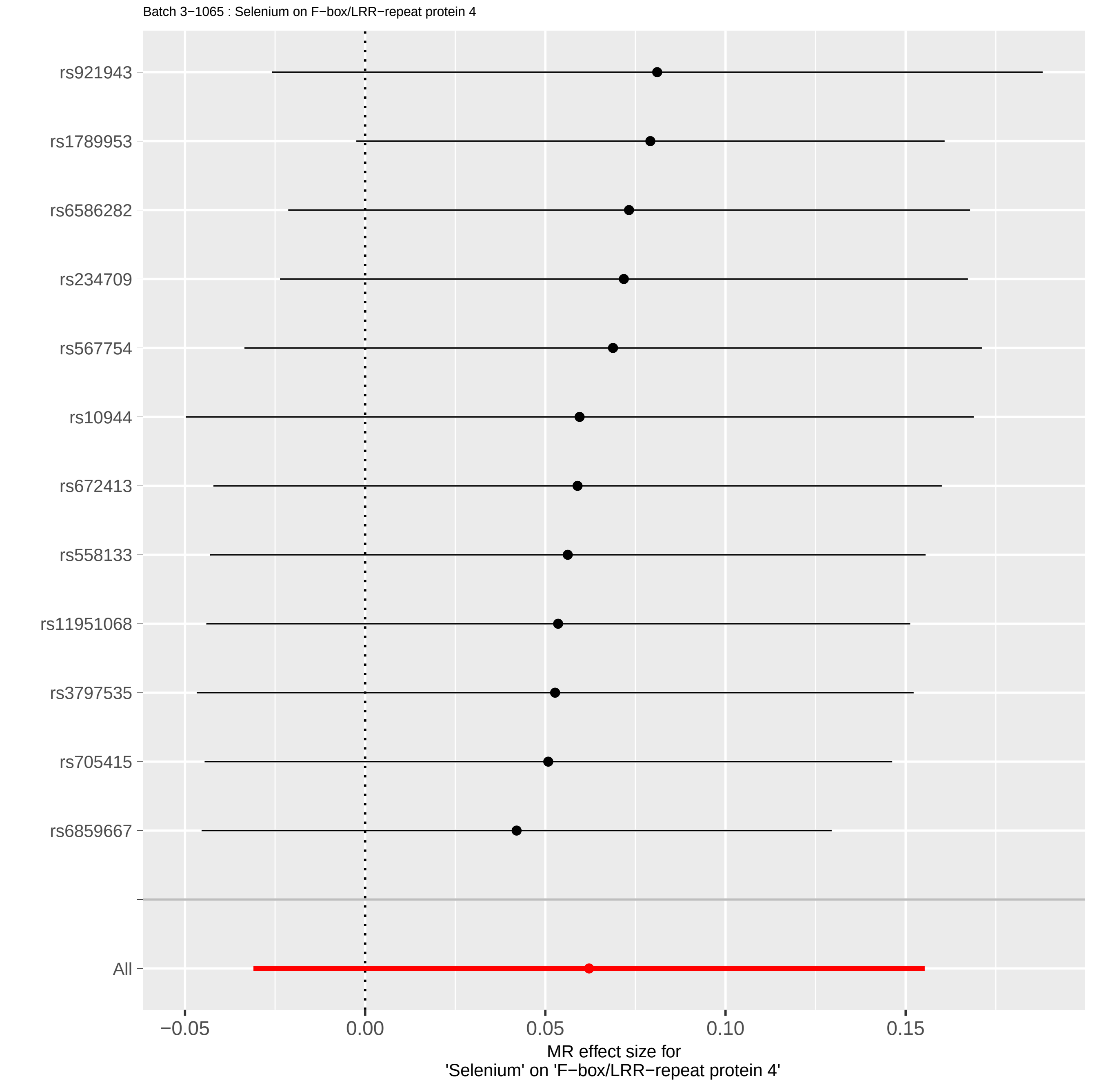

Batch 3−1065 : Selenium on F−box/LRR−repeat protein 4
rs921943
rs1789953
rs6586282
rs234709
rs567754
rs10944
rs672413
rs558133
rs11951068
rs3797535
rs705415
rs6859667
All
−0.05
0.00
0.05
0.10
0.15
MR effect size for
'Selenium' on 'F−box/LRR−repeat protein 4'

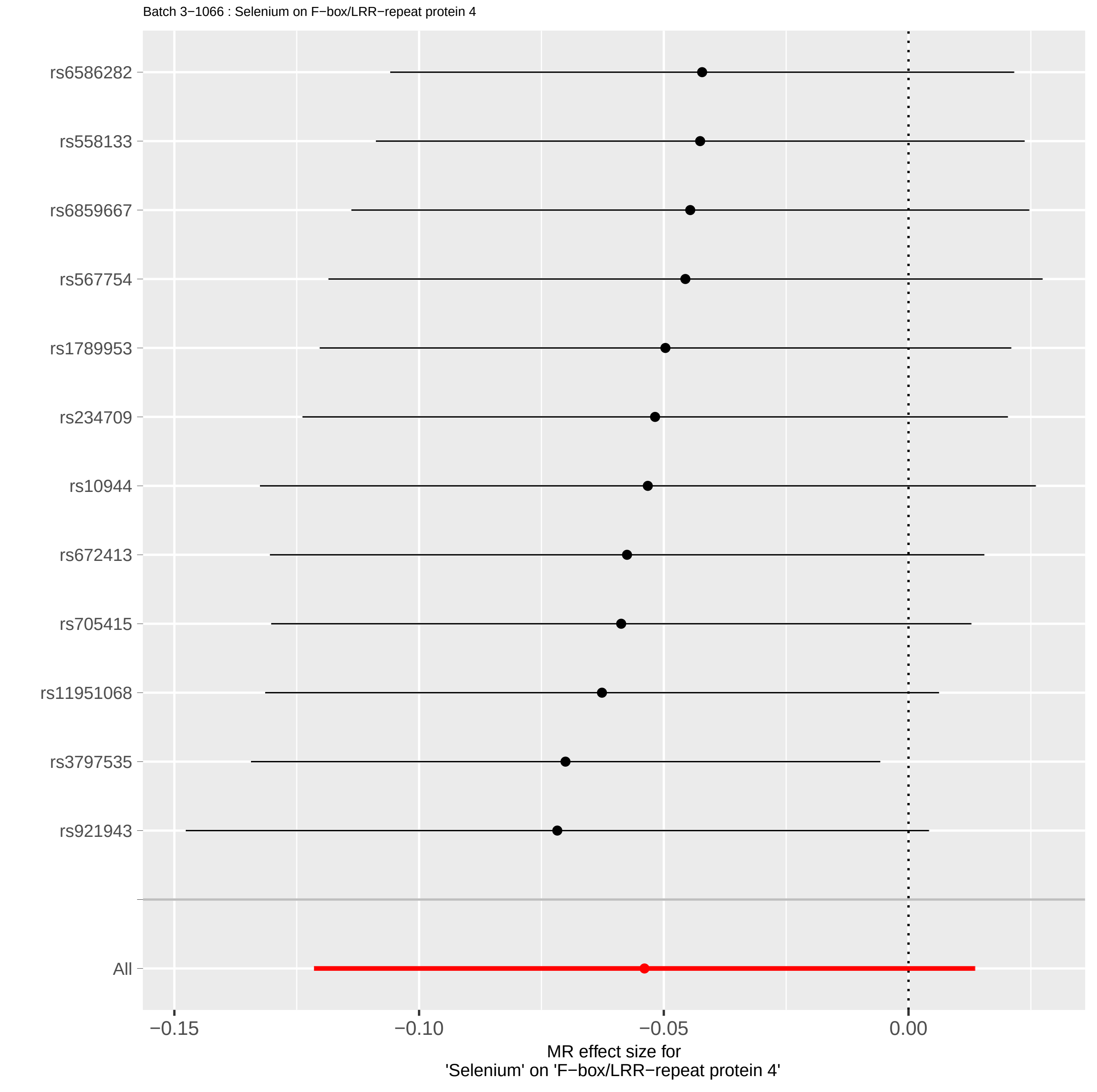

Batch 3−1066 : Selenium on F−box/LRR−repeat protein 4
rs6586282
rs558133
rs6859667
rs567754
rs1789953
rs234709
rs10944
rs672413
rs705415
rs11951068
rs3797535
rs921943
All
−0.15
−0.10
−0.05
0.00
MR effect size for
'Selenium' on 'F−box/LRR−repeat protein 4'

Batch 3−1067 : Selenium on F−box/LRR−repeat protein 4

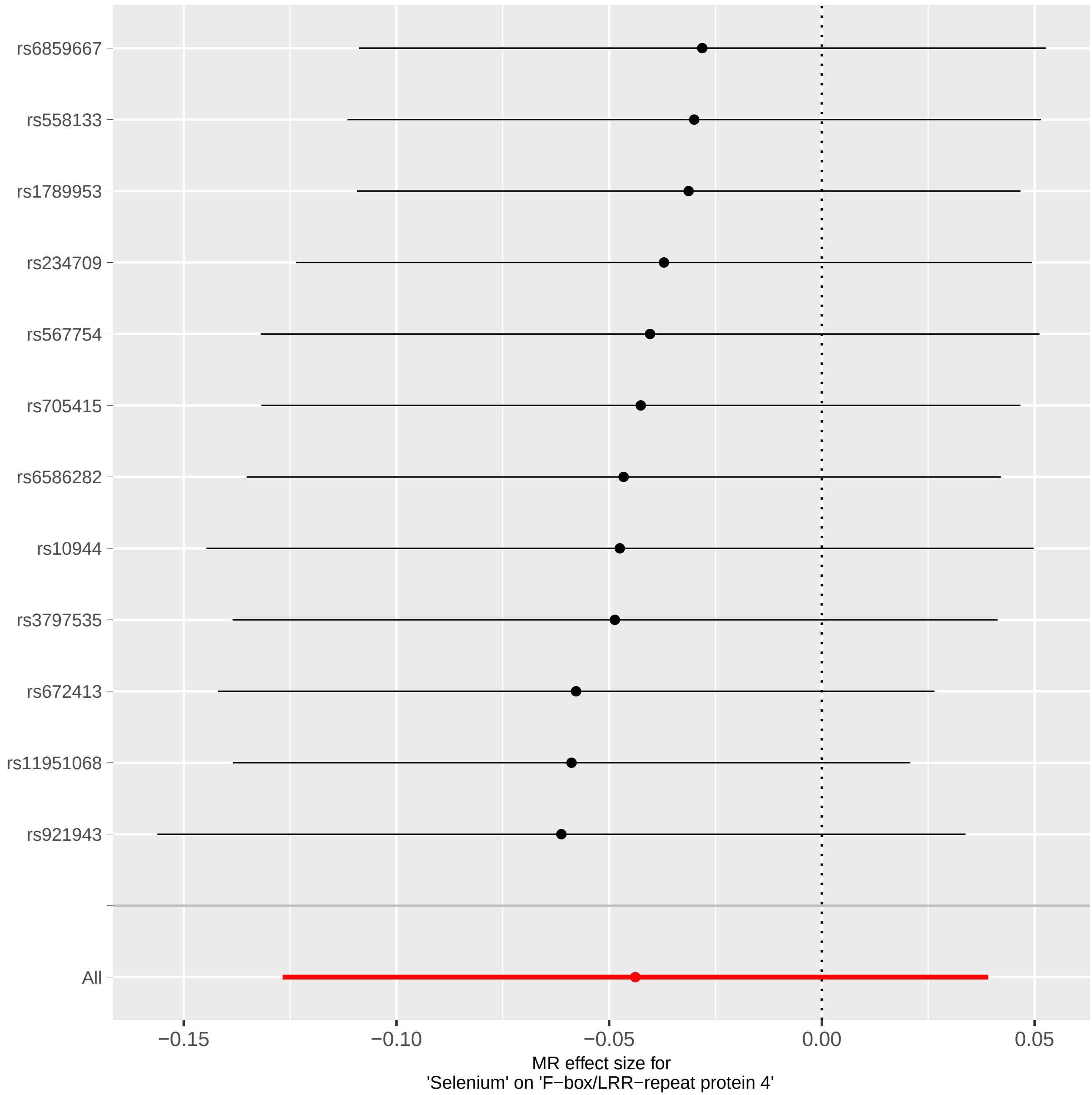

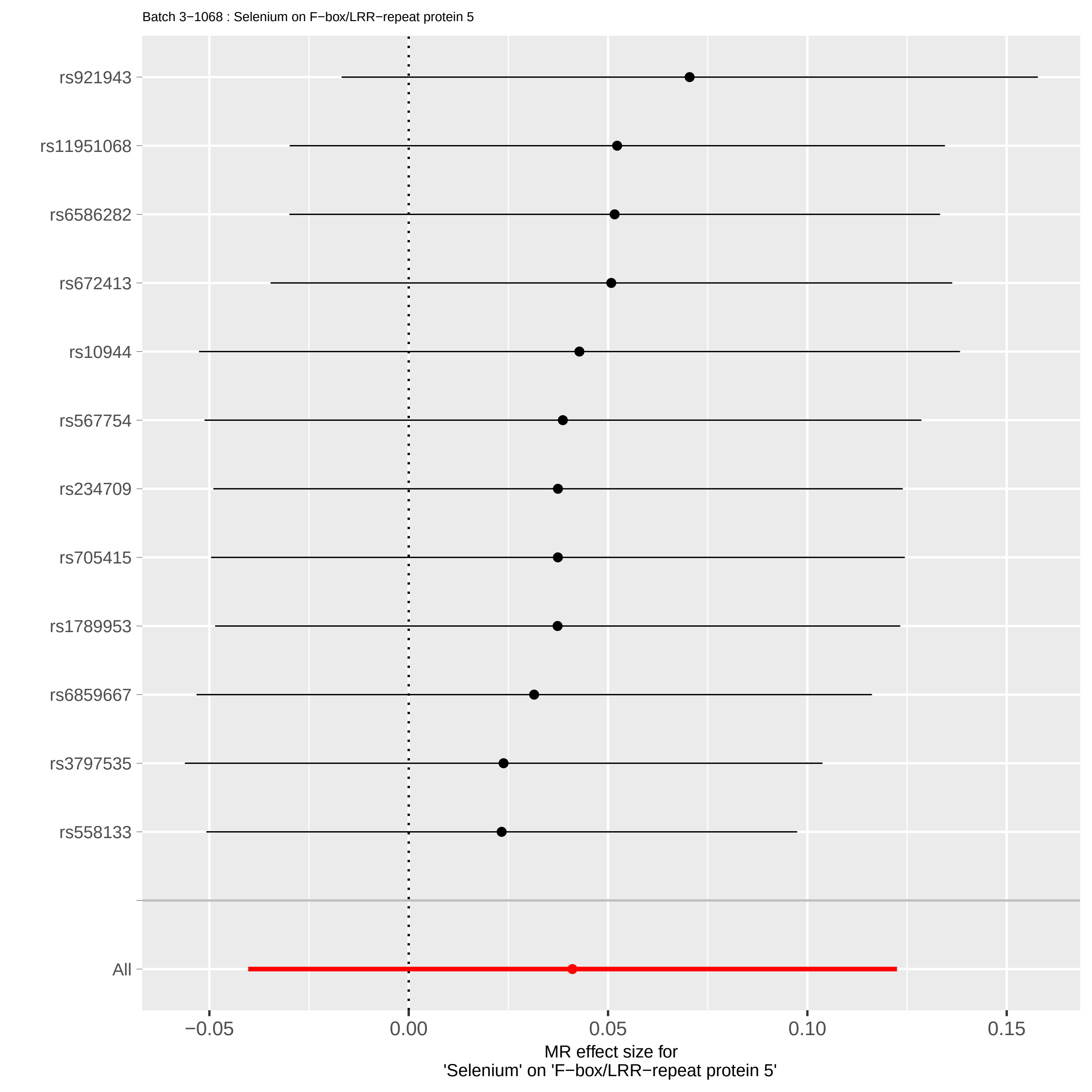

Batch 3−1068 : Selenium on F−box/LRR−repeat protein 5
rs921943
rs11951068
rs6586282
rs672413
rs10944
rs567754
rs234709
rs705415
rs1789953
rs6859667
rs3797535
rs558133
All
−0.05
0.00
0.05
0.10
0.15
MR effect size for
'Selenium' on 'F−box/LRR−repeat protein 5'

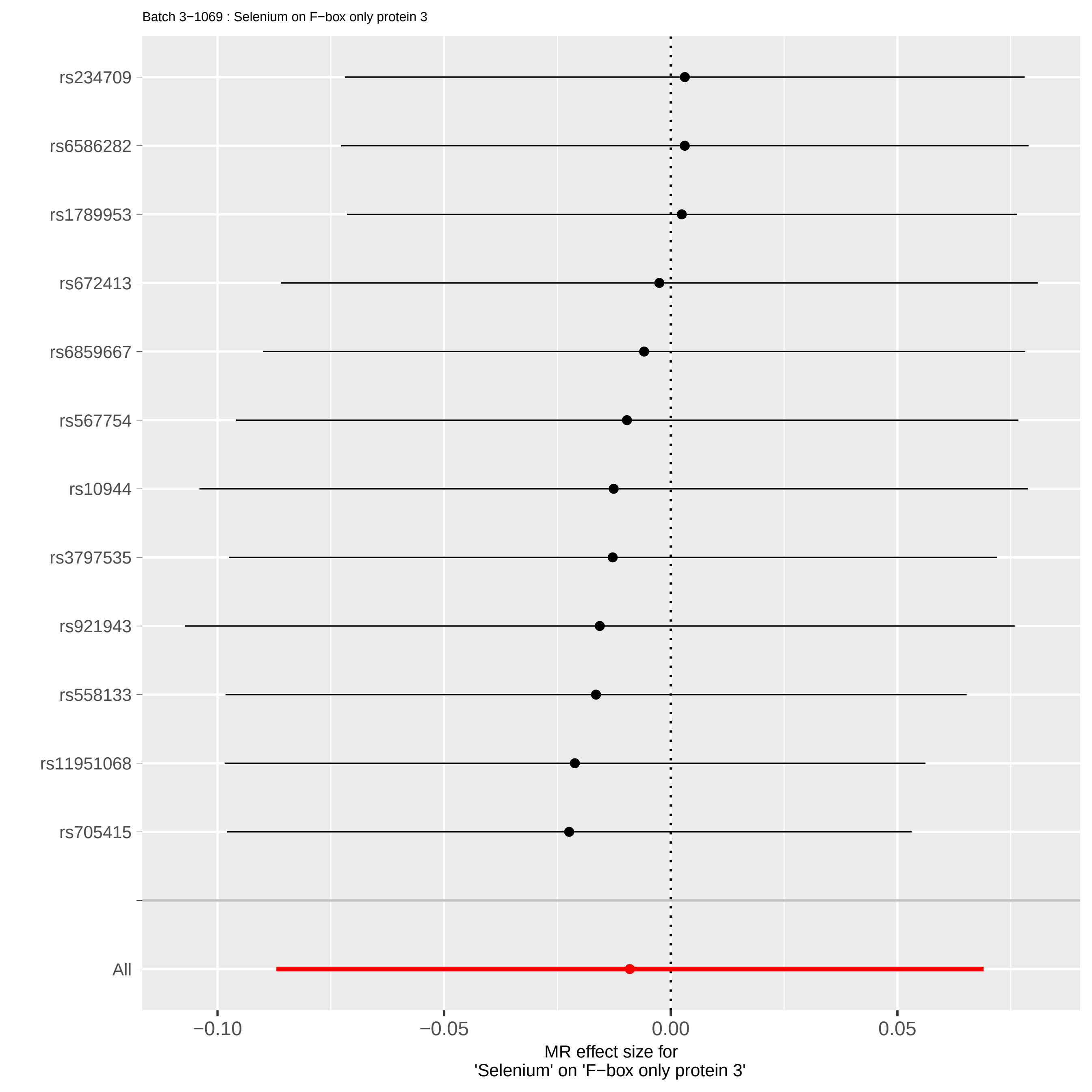

Batch 3−1069 : Selenium on F−box only protein 3
rs234709
rs6586282
rs1789953
rs672413
rs6859667
rs567754
rs10944
rs3797535
rs921943
rs558133
rs11951068
rs705415
All
−0.10
−0.05
0.00
0.05
MR effect size for
'Selenium' on 'F−box only protein 3'

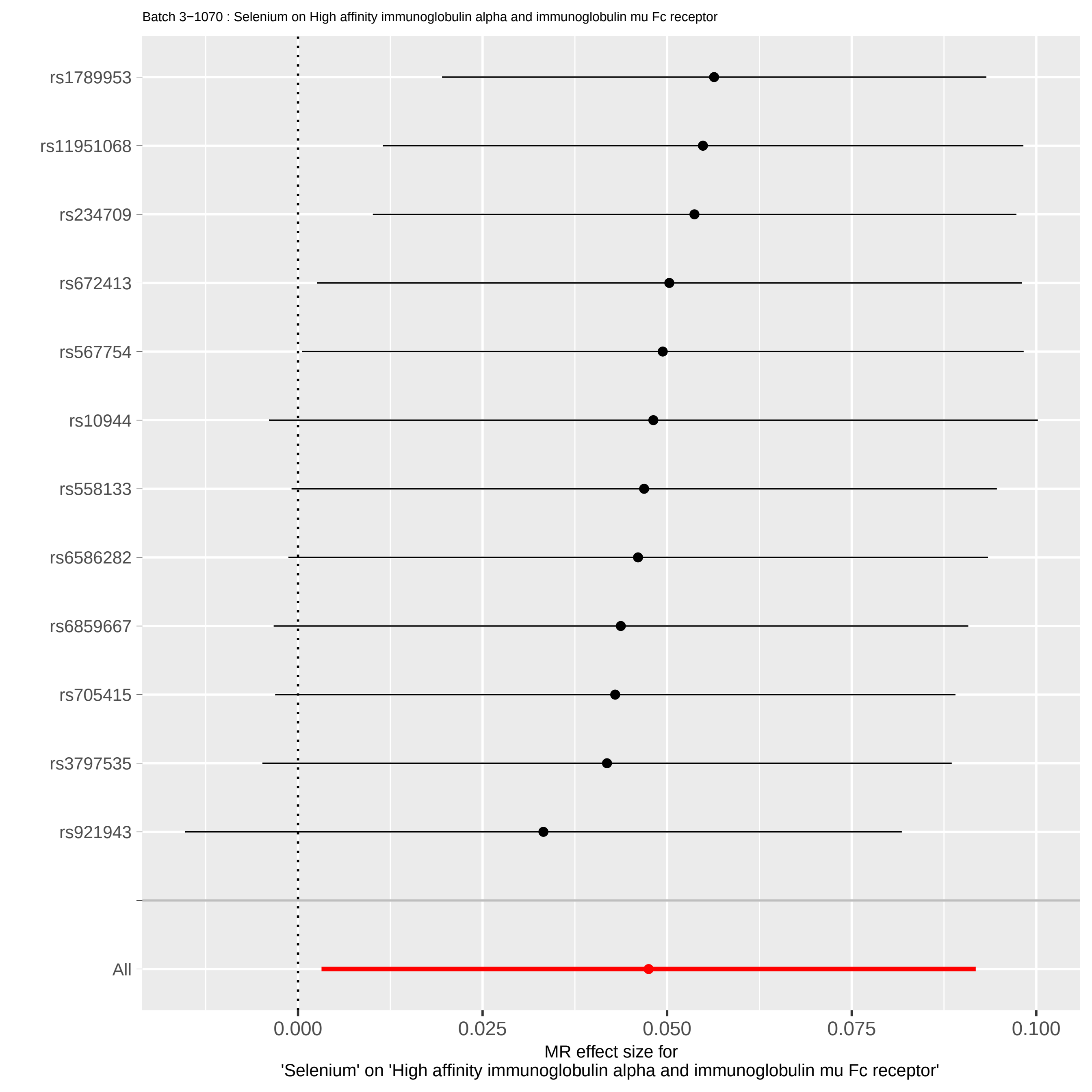

Batch 3−1070 : Selenium on High affinity immunoglobulin alpha and immunoglobulin mu Fc receptor
rs1789953
rs11951068
rs234709
rs672413
rs567754
rs10944
rs558133
rs6586282
rs6859667
rs705415
rs3797535
rs921943
All
0.000
0.025
0.050
0.075
0.100
MR effect size for
'Selenium' on 'High affinity immunoglobulin alpha and immunoglobulin mu Fc receptor'

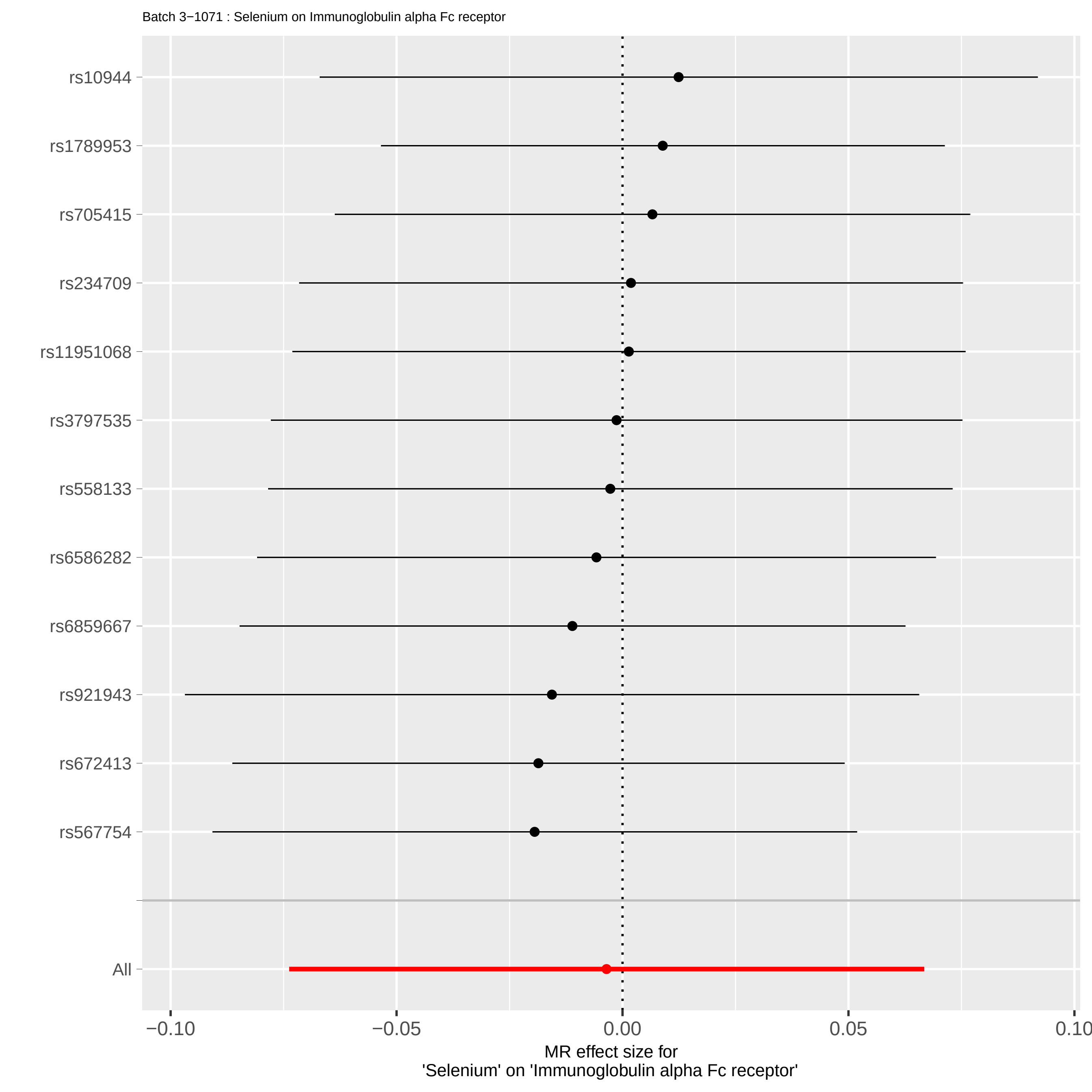

Batch 3−1071 : Selenium on Immunoglobulin alpha Fc receptor
rs10944
rs1789953
rs705415
rs234709
rs11951068
rs3797535
rs558133
rs6586282
rs6859667
rs921943
rs672413
rs567754
All
−0.10
−0.05
0.00
0.05
0.10
MR effect size for
'Selenium' on 'Immunoglobulin alpha Fc receptor'

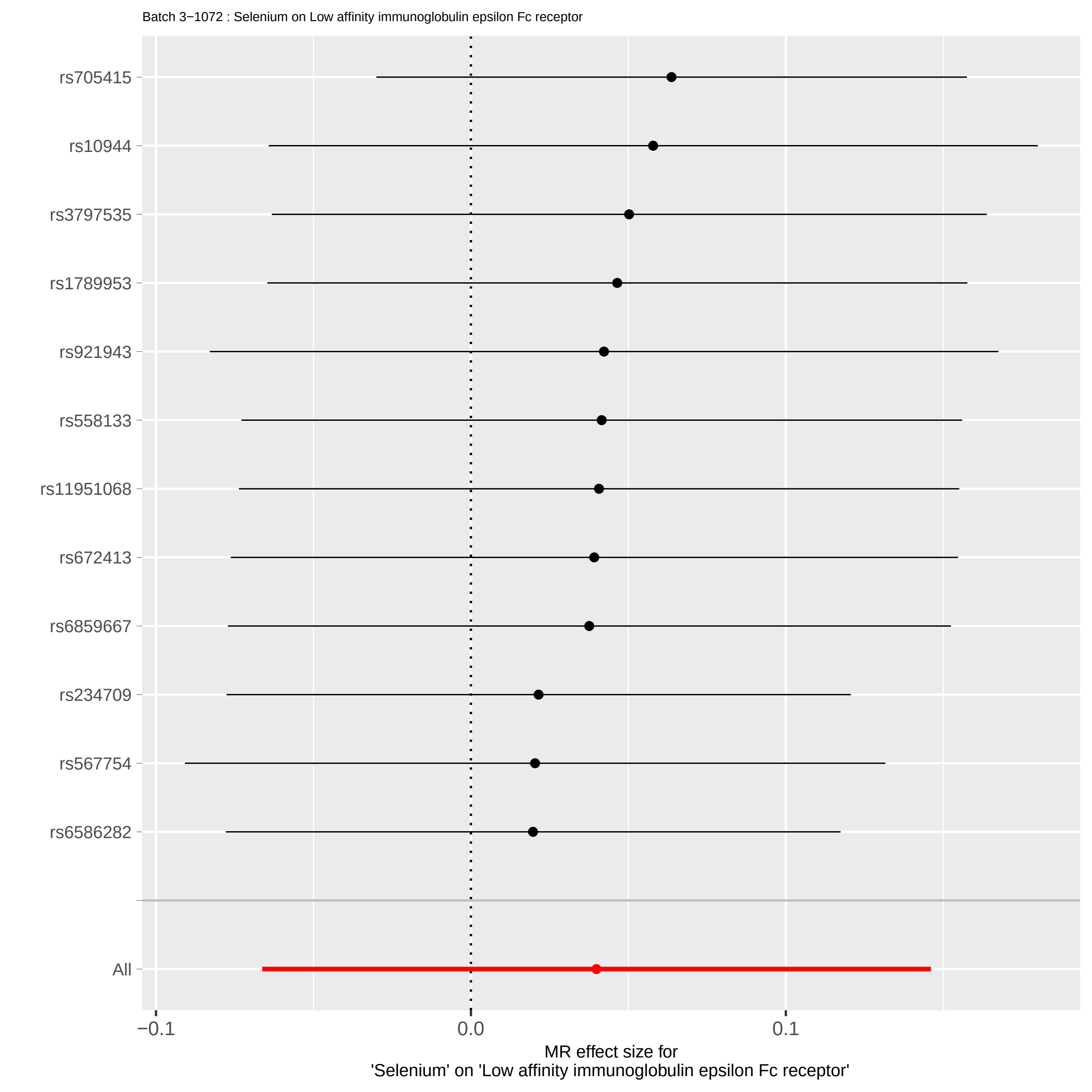

Batch 3−1072 : Selenium on Low affinity immunoglobulin epsilon Fc receptor
rs705415
rs10944
rs3797535
rs1789953
rs921943
rs558133
rs11951068
rs672413
rs6859667
rs234709
rs567754
rs6586282
All
−0.1
0.0
0.1
MR effect size for
'Selenium' on 'Low affinity immunoglobulin epsilon Fc receptor'

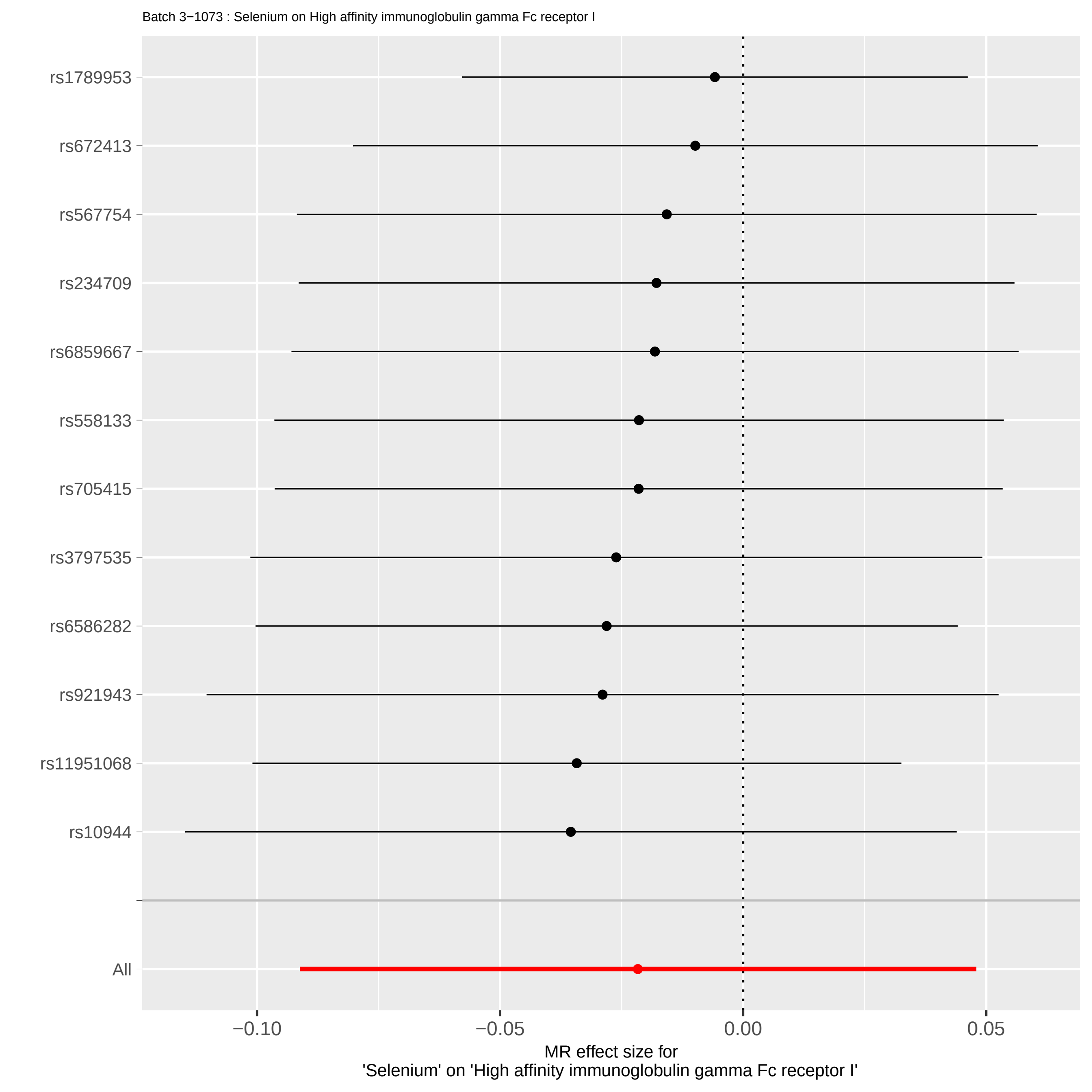

Batch 3−1073 : Selenium on High affinity immunoglobulin gamma Fc receptor I
rs1789953
rs672413
rs567754
rs234709
rs6859667
rs558133
rs705415
rs3797535
rs6586282
rs921943
rs11951068
rs10944
All
−0.10
−0.05
0.00
0.05
MR effect size for
'Selenium' on 'High affinity immunoglobulin gamma Fc receptor I'

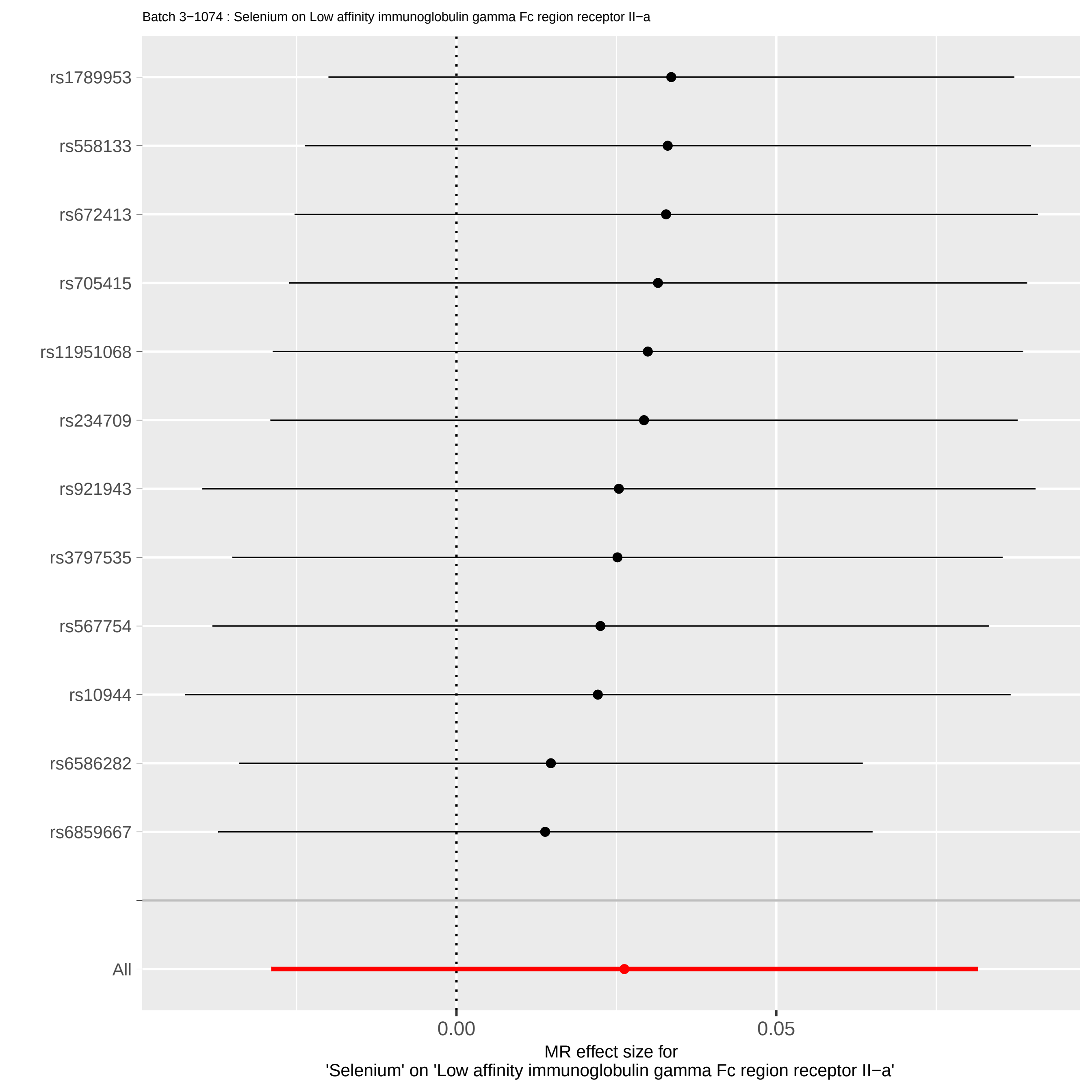
Batch 3−1074 : Selenium on Low affinity immunoglobulin gamma Fc region receptor II−a
rs1789953
rs558133
rs672413
rs705415
rs11951068
rs234709
rs921943
rs3797535
rs567754
rs10944
rs6586282
rs6859667
All
0.00
0.05
MR effect size for
'Selenium' on 'Low affinity immunoglobulin gamma Fc region receptor II−a'

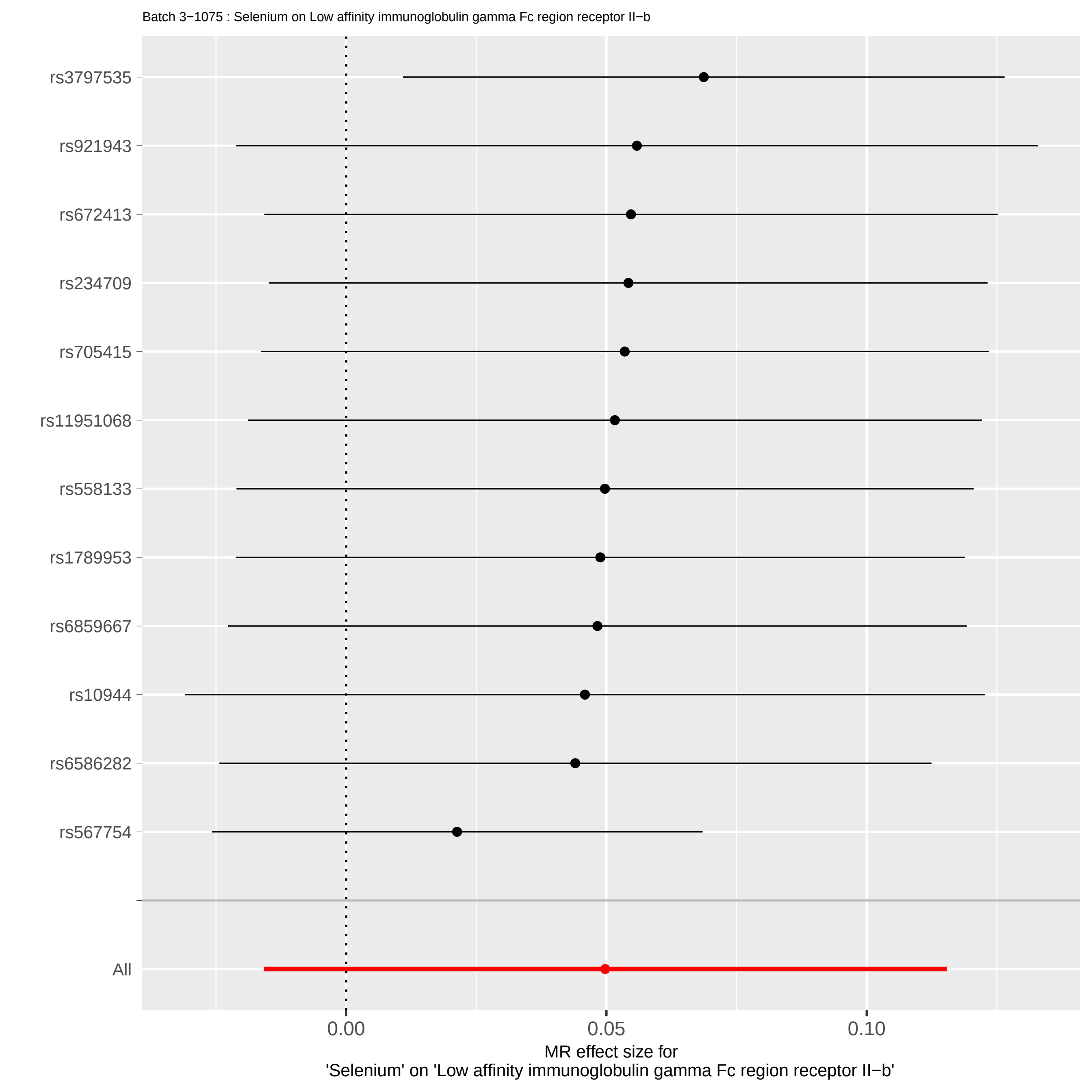

Batch 3−1075 : Selenium on Low affinity immunoglobulin gamma Fc region receptor II−b
rs3797535
rs921943
rs672413
rs234709
rs705415
rs11951068
rs558133
rs1789953
rs6859667
rs10944
rs6586282
rs567754
All
0.00
0.05
0.10
MR effect size for
'Selenium' on 'Low affinity immunoglobulin gamma Fc region receptor II−b'

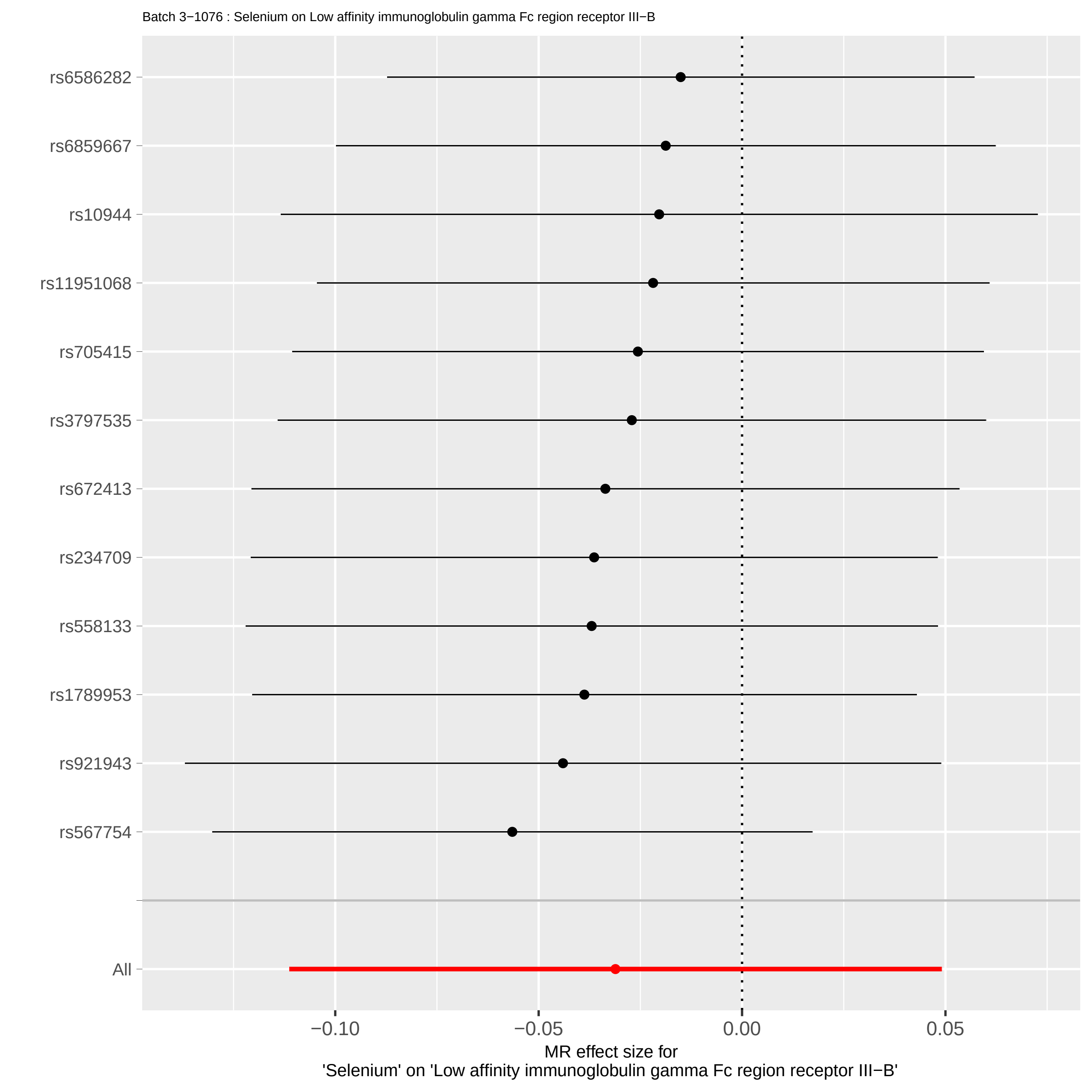

Batch 3−1076 : Selenium on Low affinity immunoglobulin gamma Fc region receptor III−B
rs6586282
rs6859667
rs10944
rs11951068
rs705415
rs3797535
rs672413
rs234709
rs558133
rs1789953
rs921943
rs567754
All
−0.10
−0.05
0.00
0.05
MR effect size for
'Selenium' on 'Low affinity immunoglobulin gamma Fc region receptor III−B'

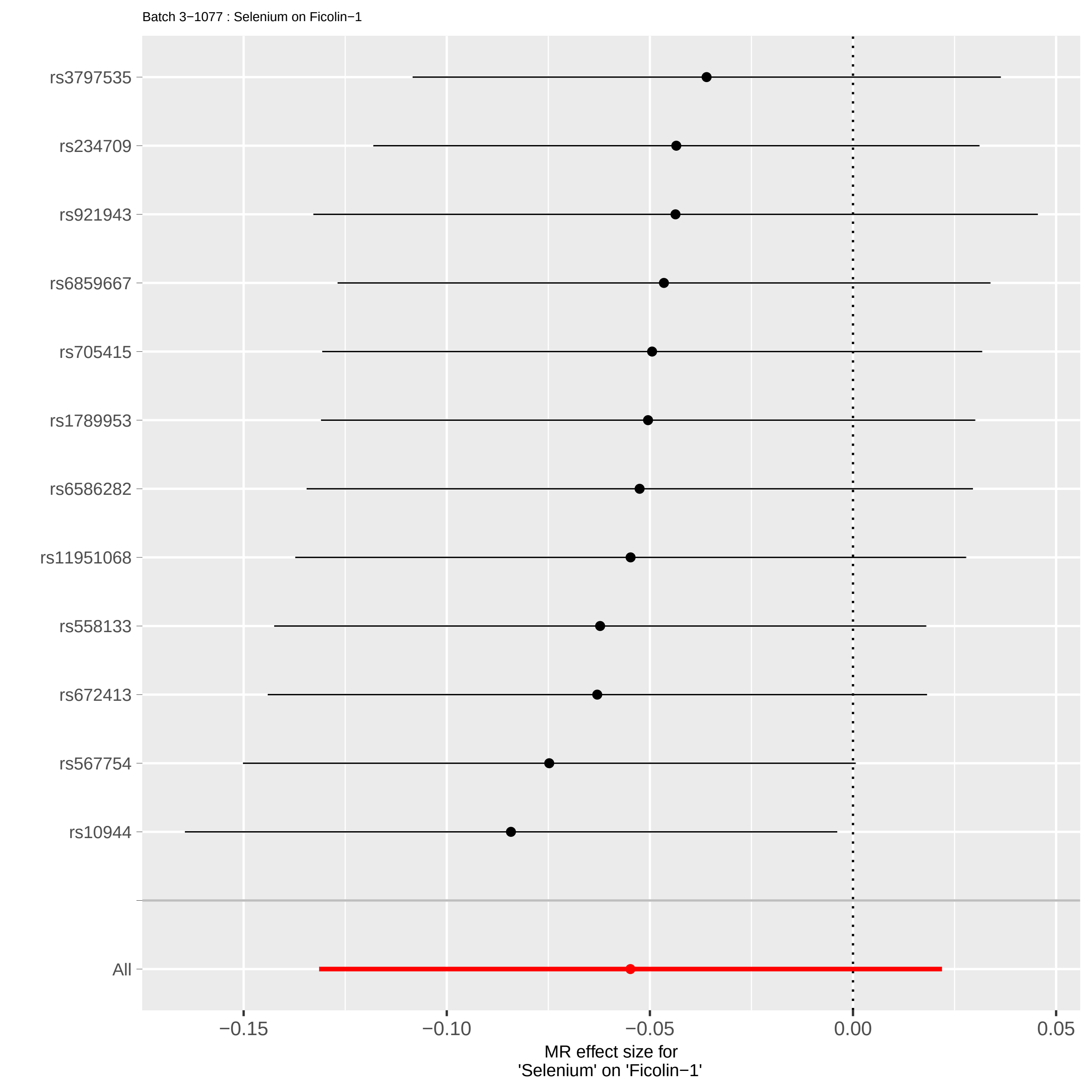

Batch 3−1077 : Selenium on Ficolin−1
rs3797535
rs234709
rs921943
rs6859667
rs705415
rs1789953
rs6586282
rs11951068
rs558133
rs672413
rs567754
rs10944
All
−0.15
−0.10
−0.05
0.00
0.05
MR effect size for
'Selenium' on 'Ficolin−1'

Batch 3−1078 : Selenium on Ficolin−2

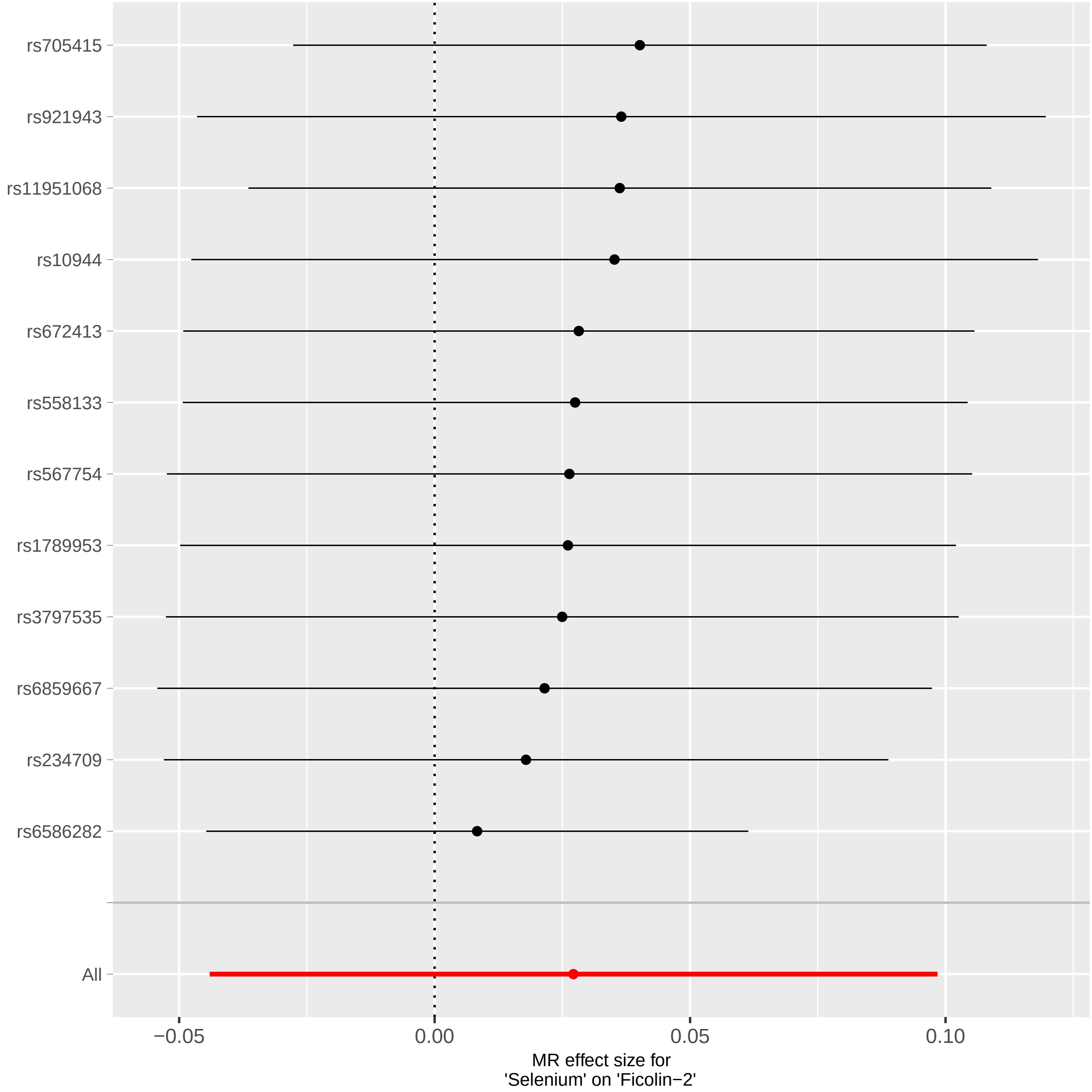

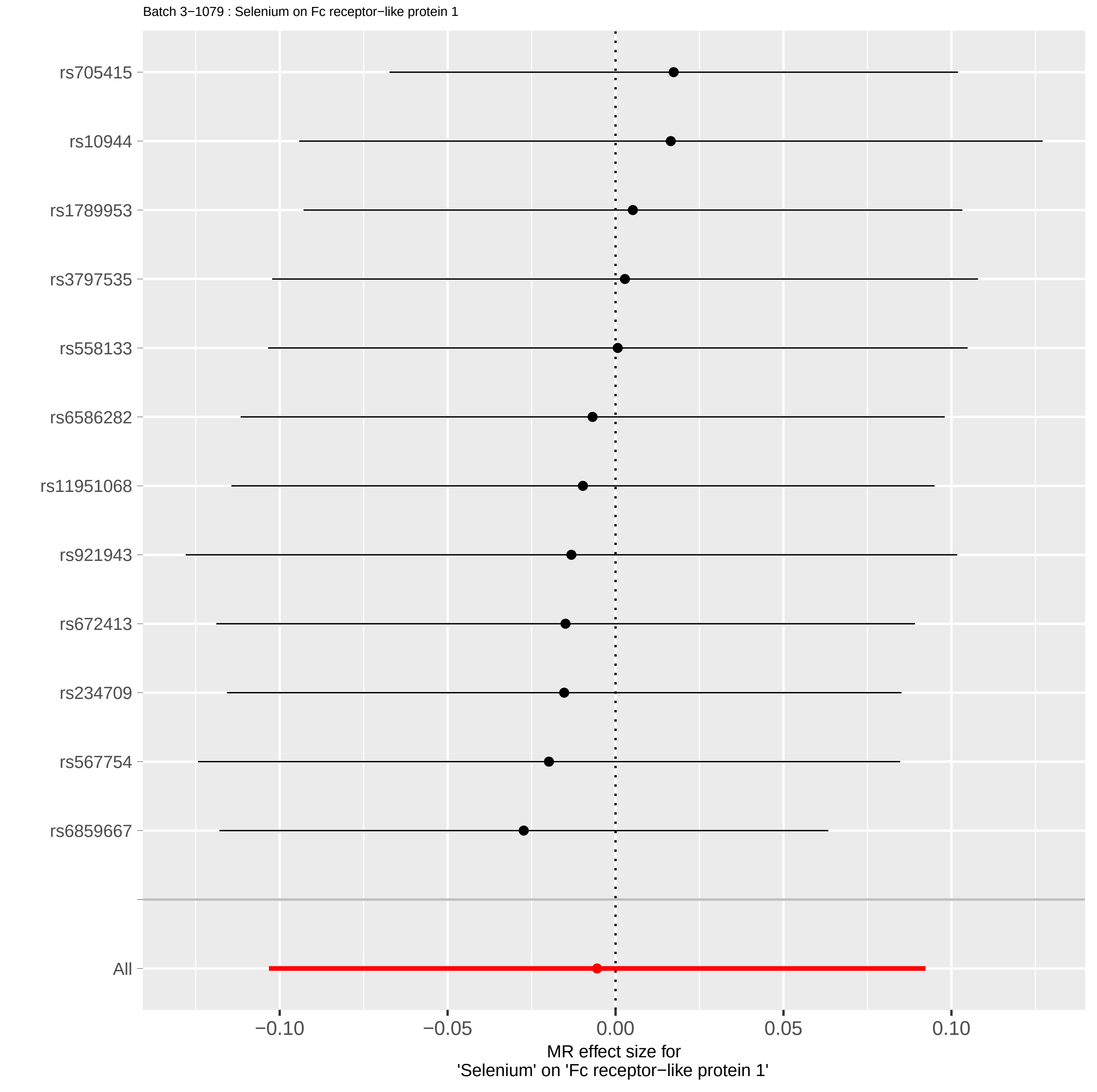

Batch 3−1079 : Selenium on Fc receptor−like protein 1
rs705415
rs10944
rs1789953
rs3797535
rs558133
rs6586282
rs11951068
rs921943
rs672413
rs234709
rs567754
rs6859667
All
−0.10
−0.05
0.00
0.05
0.10
MR effect size for
'Selenium' on 'Fc receptor−like protein 1'

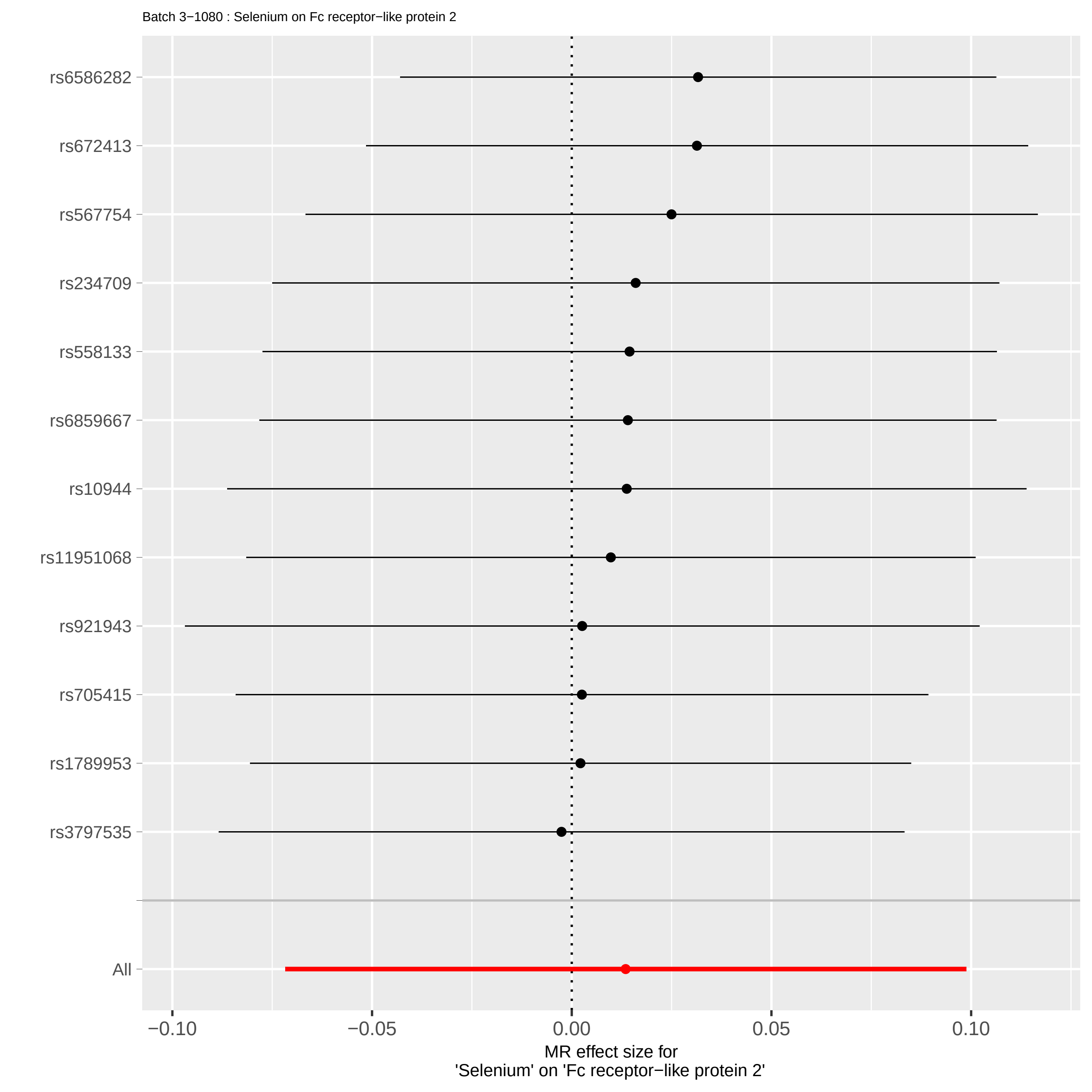

Batch 3−1080 : Selenium on Fc receptor−like protein 2
rs6586282
rs672413
rs567754
rs234709
rs558133
rs6859667
rs10944
rs11951068
rs921943
rs705415
rs1789953
rs3797535
All
−0.10
−0.05
0.00
0.05
0.10
MR effect size for
'Selenium' on 'Fc receptor−like protein 2'

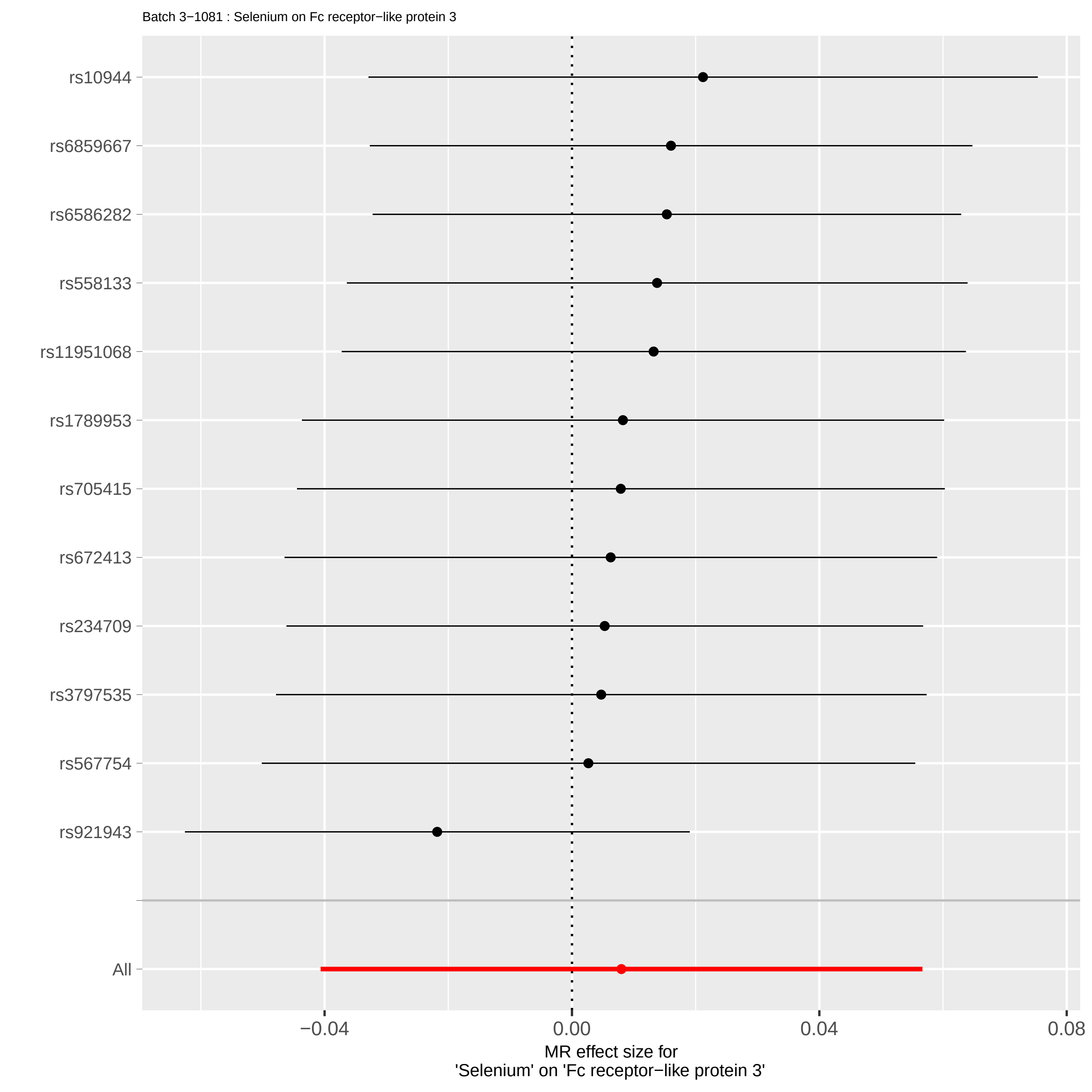

Batch 3−1081 : Selenium on Fc receptor−like protein 3
rs10944
rs6859667
rs6586282
rs558133
rs11951068
rs1789953
rs705415
rs672413
rs234709
rs3797535
rs567754
rs921943
All
−0.04
0.00
0.04
0.08
MR effect size for
'Selenium' on 'Fc receptor−like protein 3'

Batch 3−1082 : Selenium on Fc receptor−like protein 4

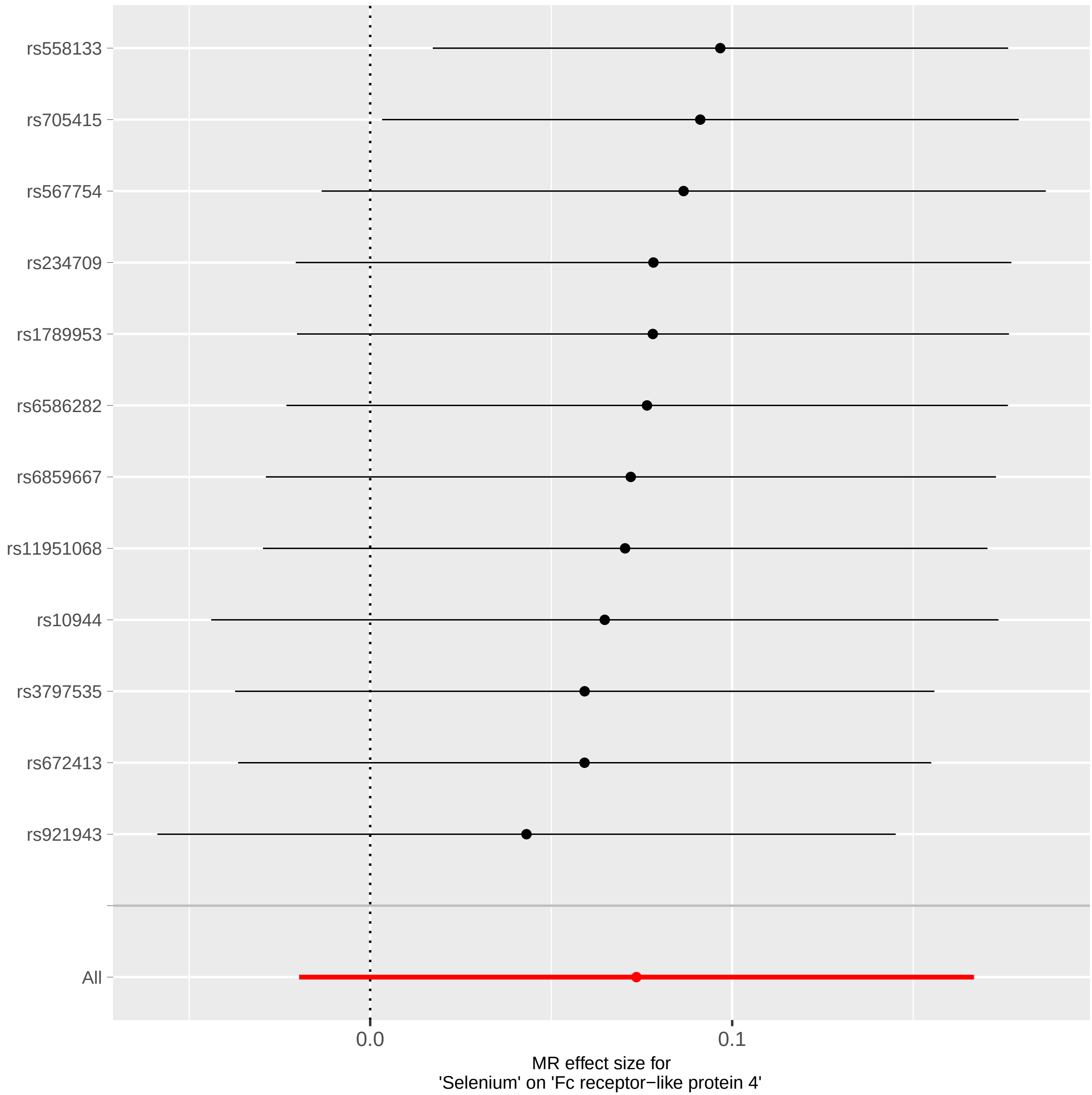

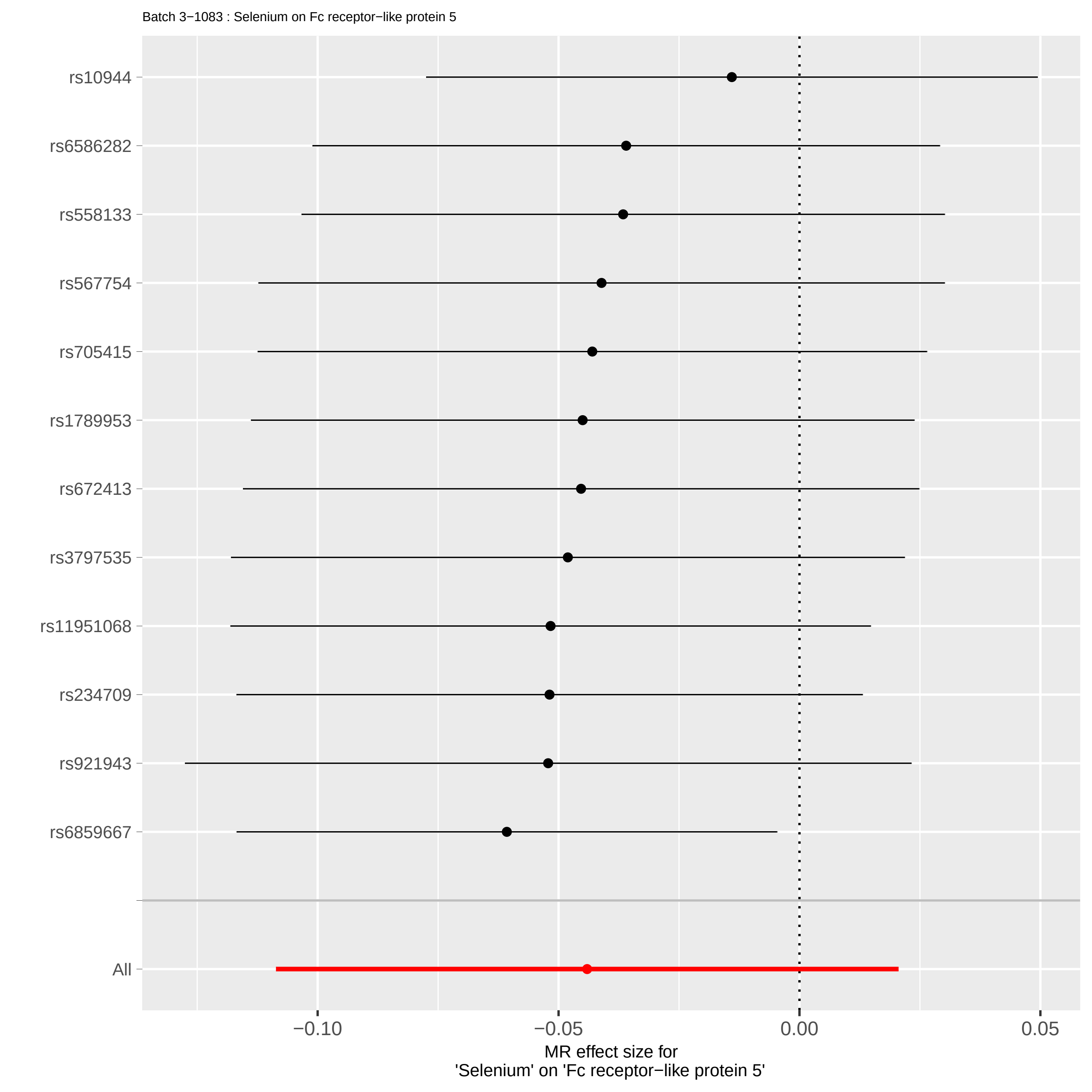

Batch 3−1083 : Selenium on Fc receptor−like protein 5
rs10944
rs6586282
rs558133
rs567754
rs705415
rs1789953
rs672413
rs3797535
rs11951068
rs234709
rs921943
rs6859667
All
−0.10
−0.05
0.00
0.05
MR effect size for
'Selenium' on 'Fc receptor−like protein 5'

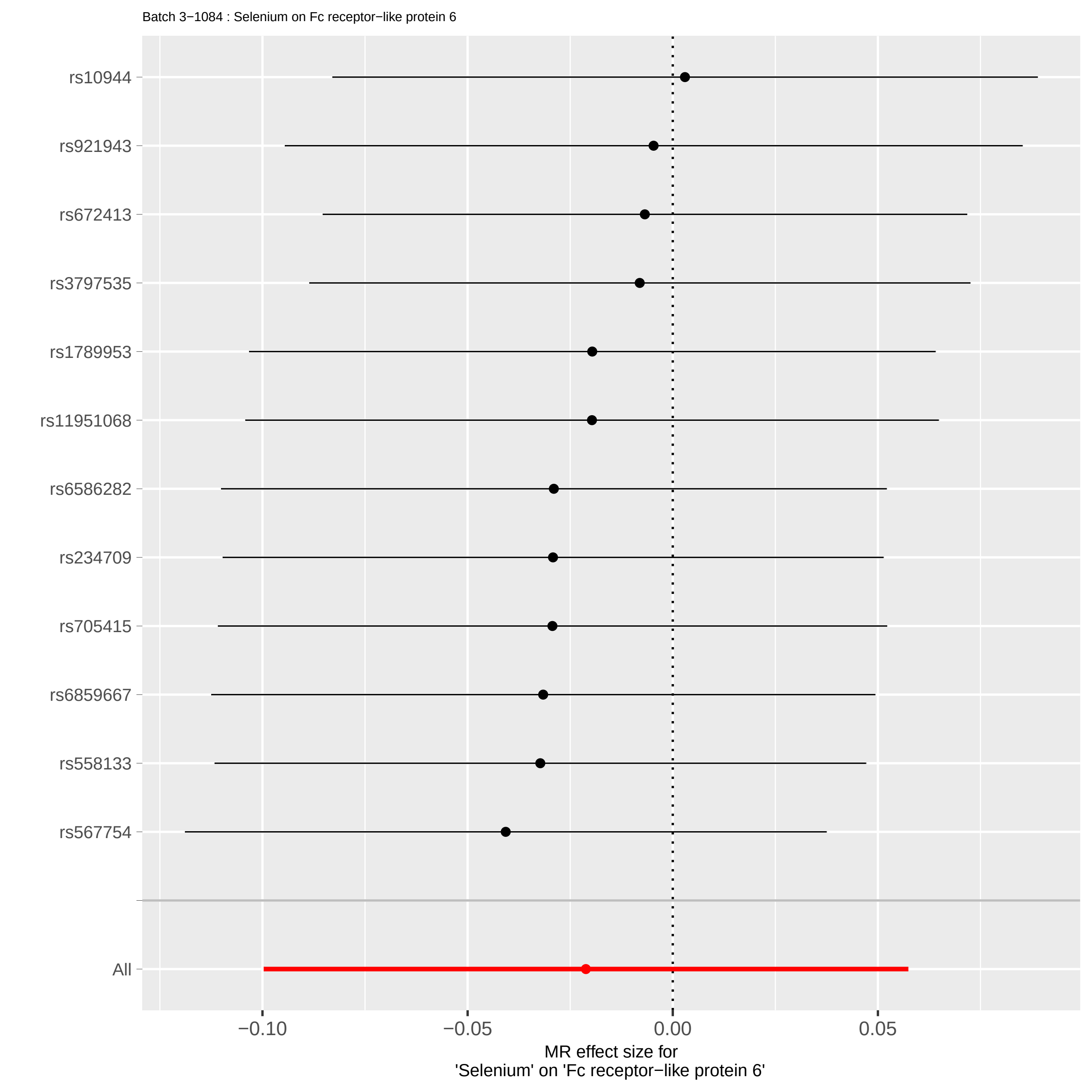

Batch 3−1084 : Selenium on Fc receptor−like protein 6
rs10944
rs921943
rs672413
rs3797535
rs1789953
rs11951068
rs6586282
rs234709
rs705415
rs6859667
rs558133
rs567754
All
−0.10
−0.05
0.00
0.05
MR effect size for
'Selenium' on 'Fc receptor−like protein 6'

Batch 3−1085 : Selenium on Flap endonuclease 1

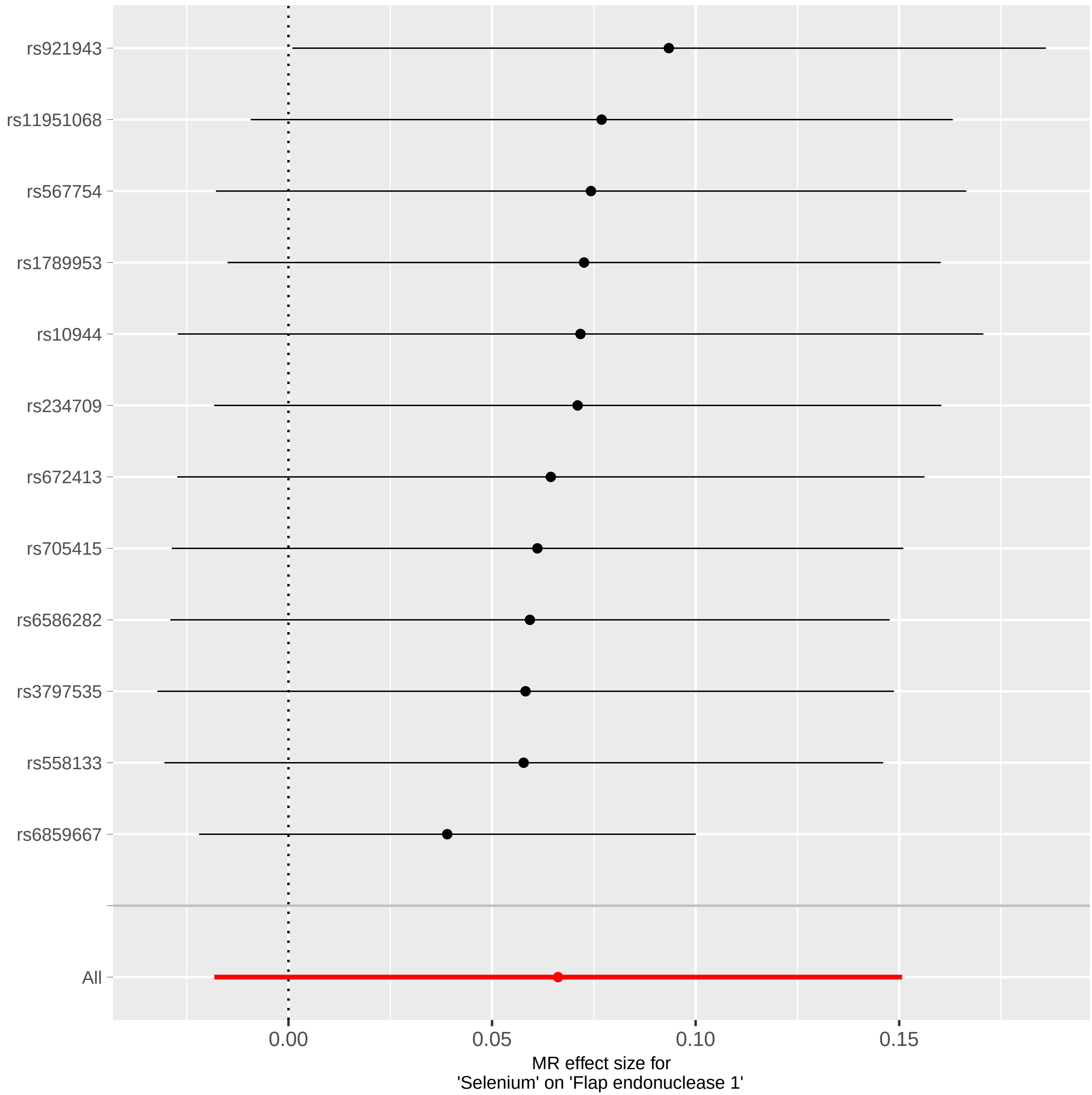

Batch 3−1086 : Selenium on D−dimer

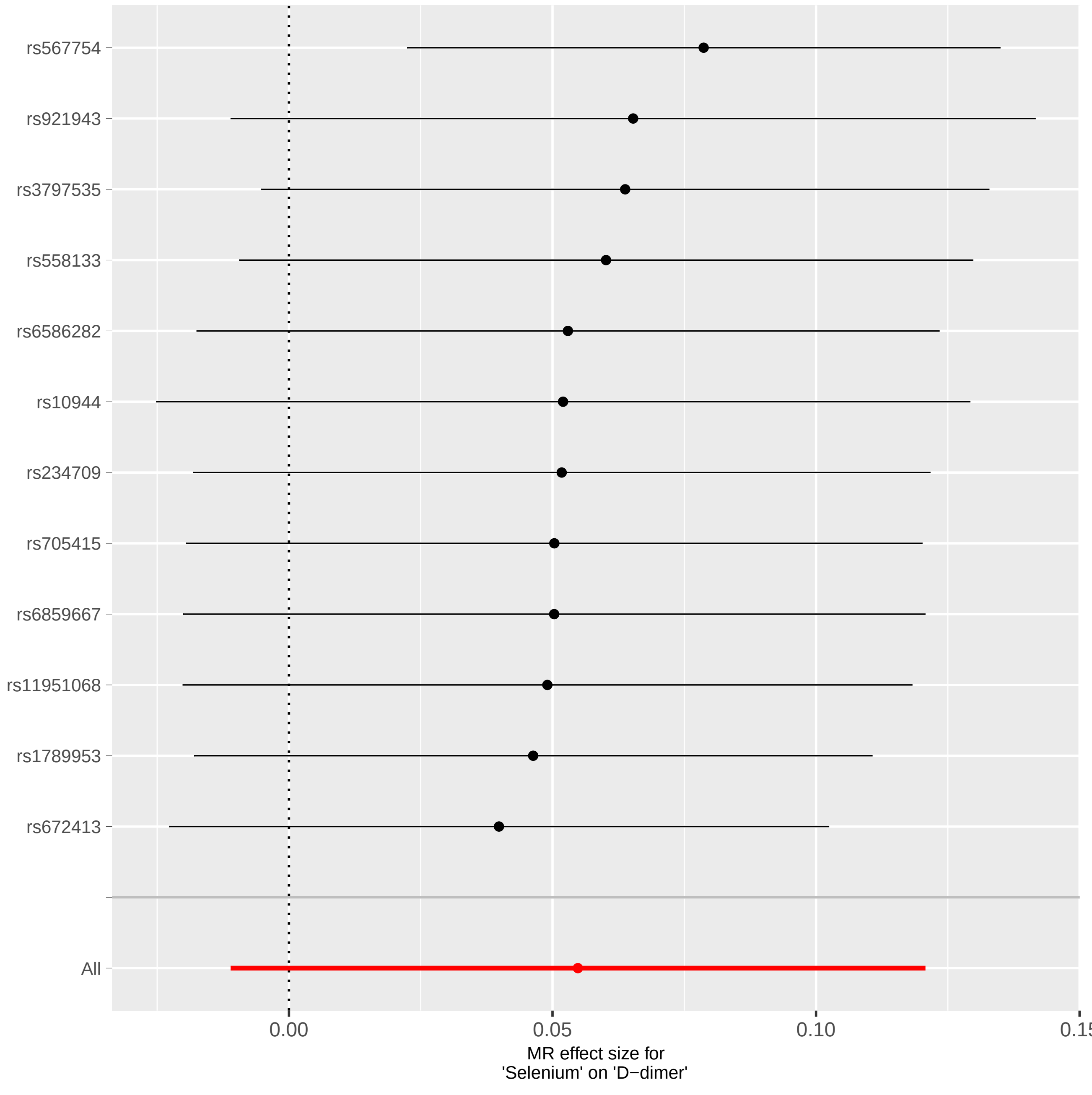

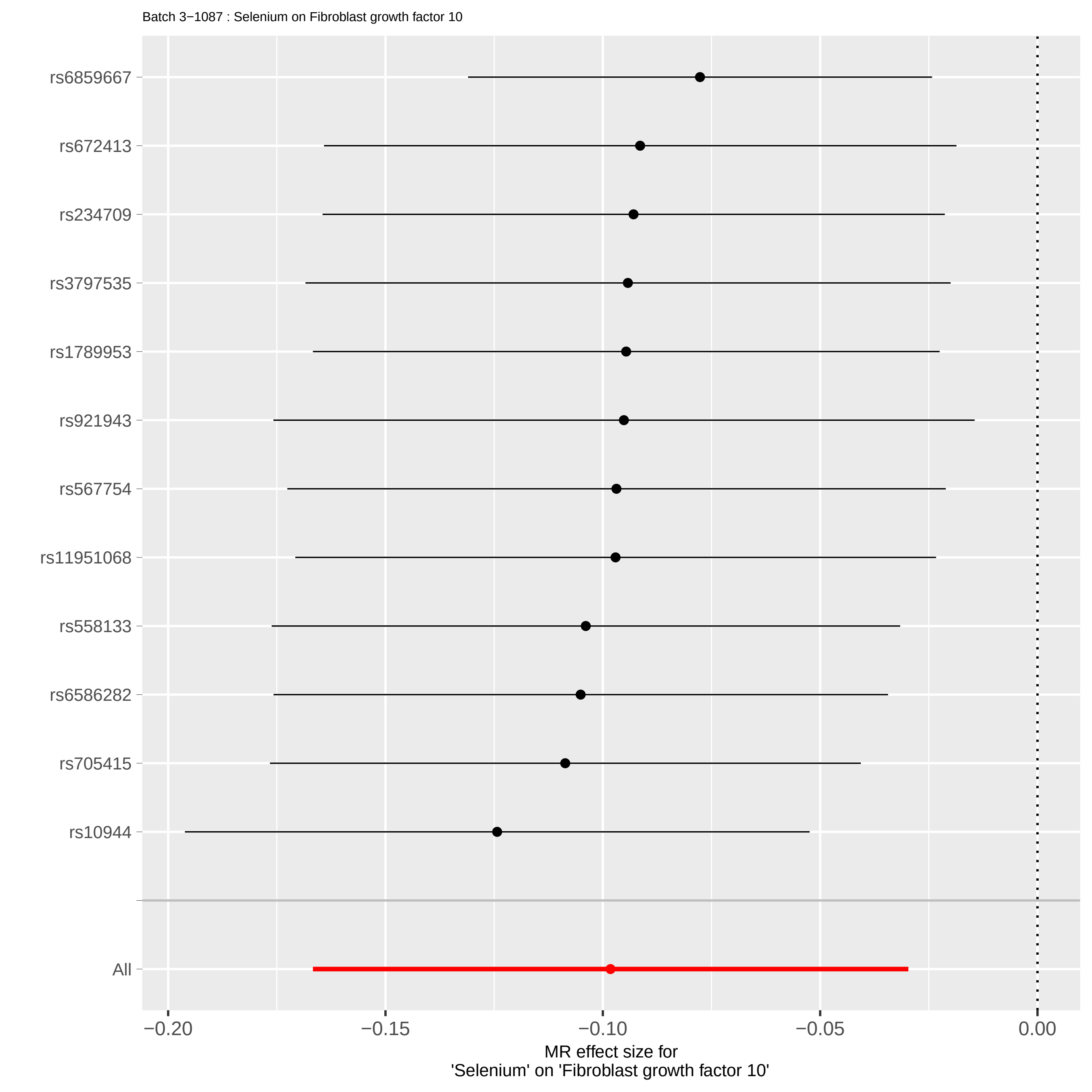

Batch 3−1087 : Selenium on Fibroblast growth factor 10
rs6859667
rs672413
rs234709
rs3797535
rs1789953
rs921943
rs567754
rs11951068
rs558133
rs6586282
rs705415
rs10944
All
−0.20
−0.15
−0.10
−0.05
0.00
MR effect size for
'Selenium' on 'Fibroblast growth factor 10'

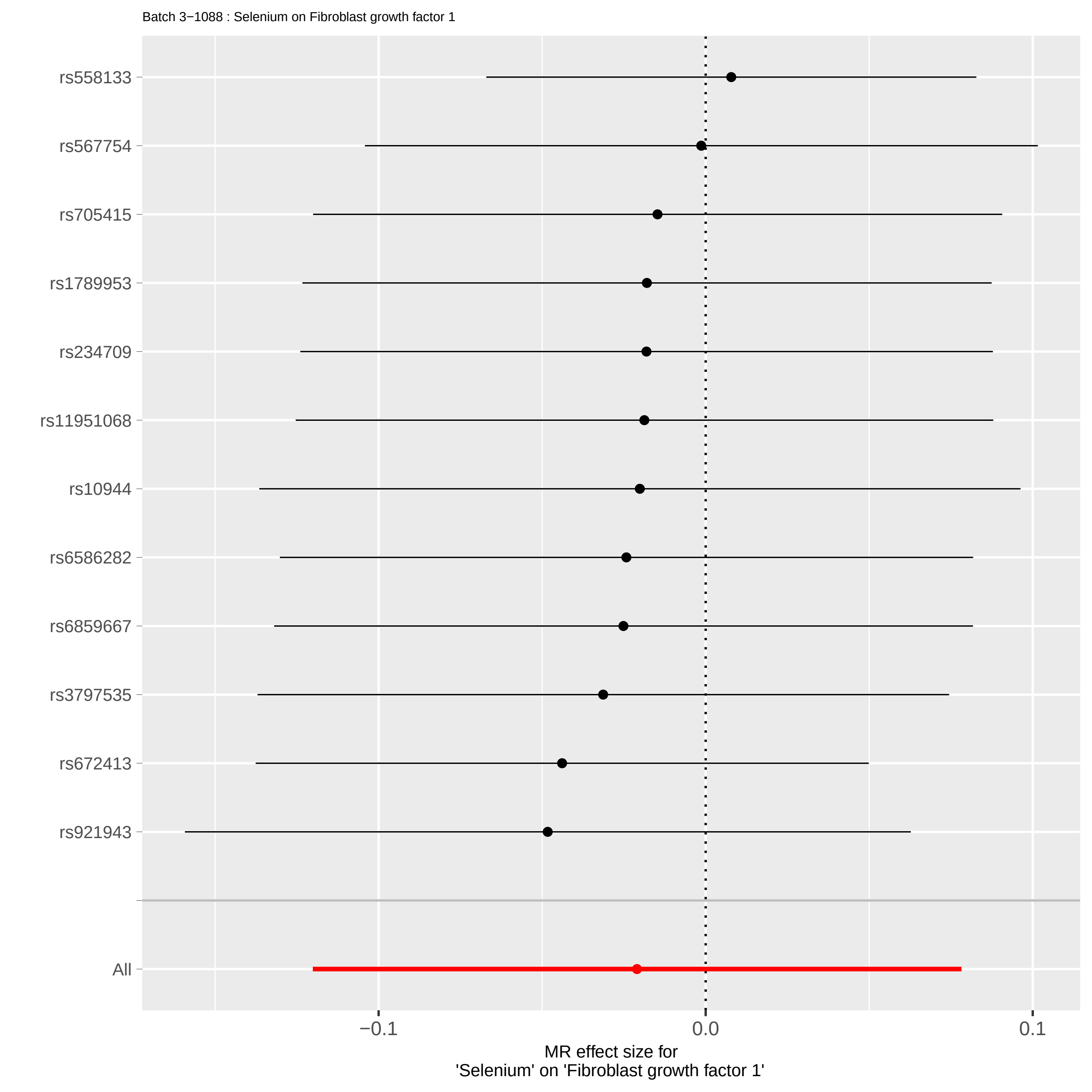

Batch 3−1088 : Selenium on Fibroblast growth factor 1
rs558133
rs567754
rs705415
rs1789953
rs234709
rs11951068
rs10944
rs6586282
rs6859667
rs3797535
rs672413
rs921943
All
−0.1
0.0
0.1
MR effect size for
'Selenium' on 'Fibroblast growth factor 1'

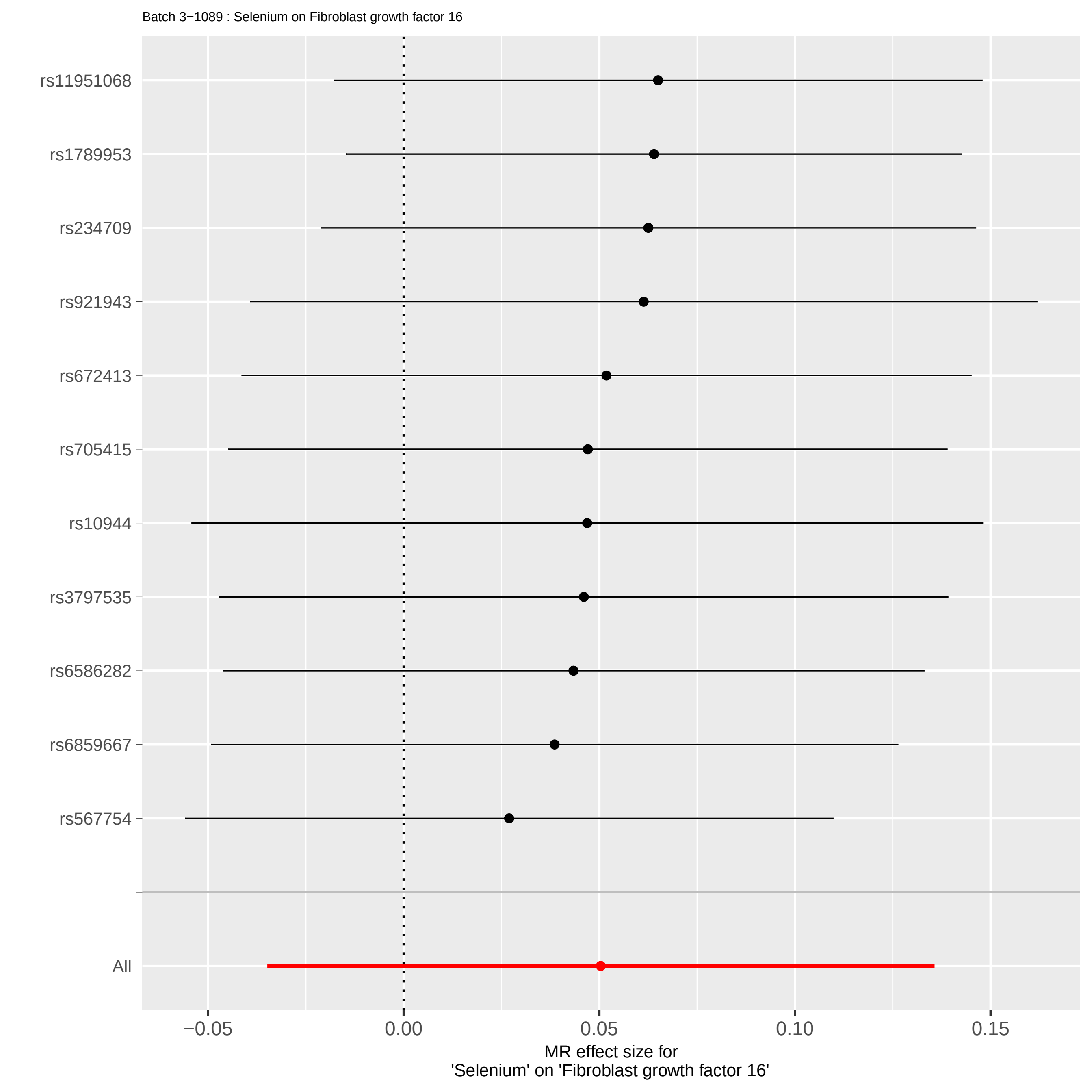

Batch 3−1089 : Selenium on Fibroblast growth factor 16
rs11951068
rs1789953
rs234709
rs921943
rs672413
rs705415
rs10944
rs3797535
rs6586282
rs6859667
rs567754
All
−0.05
0.00
0.05
0.10
0.15
MR effect size for
'Selenium' on 'Fibroblast growth factor 16'

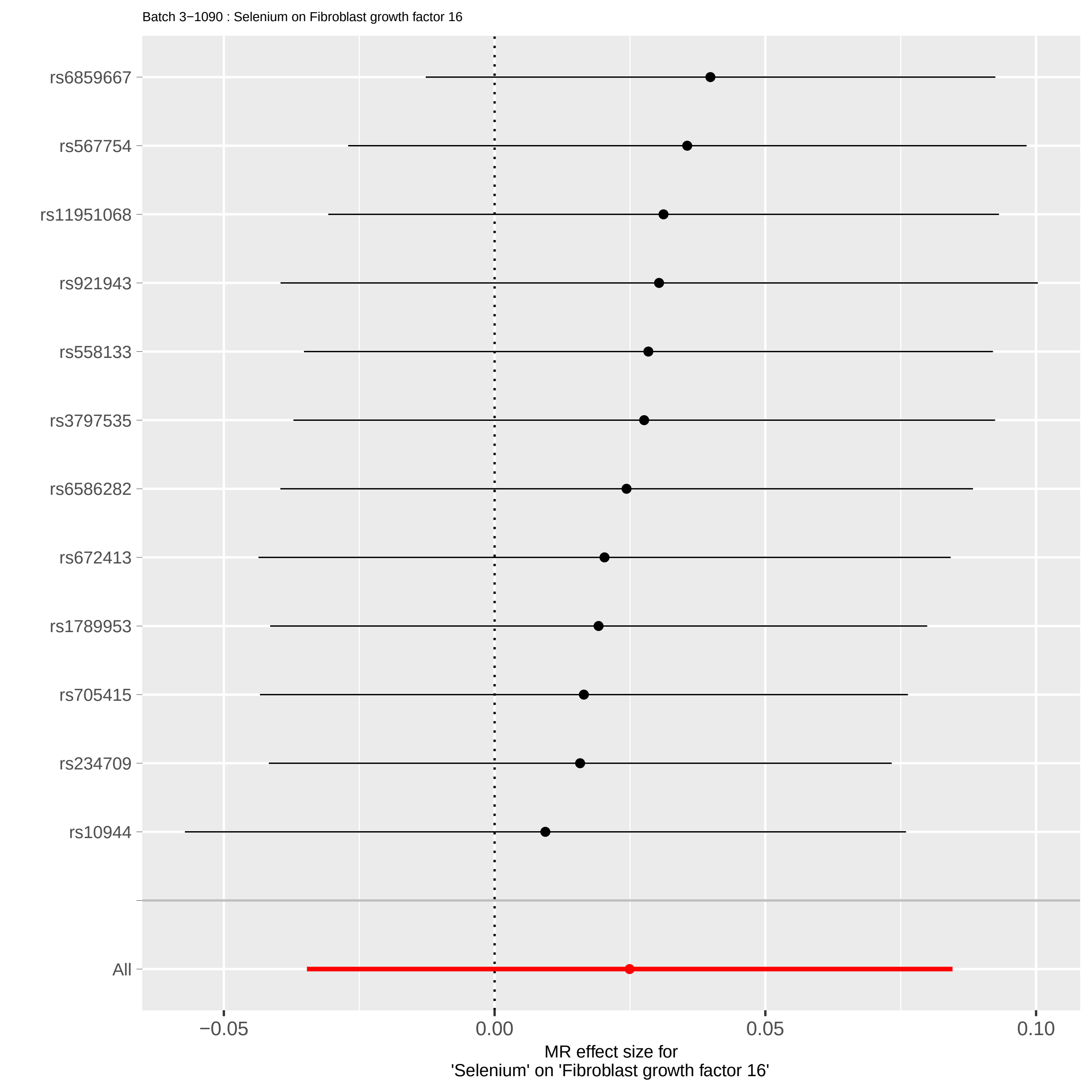

Batch 3−1090 : Selenium on Fibroblast growth factor 16
rs6859667
rs567754
rs11951068
rs921943
rs558133
rs3797535
rs6586282
rs672413
rs1789953
rs705415
rs234709
rs10944
All
−0.05
0.00
0.05
0.10
MR effect size for
'Selenium' on 'Fibroblast growth factor 16'

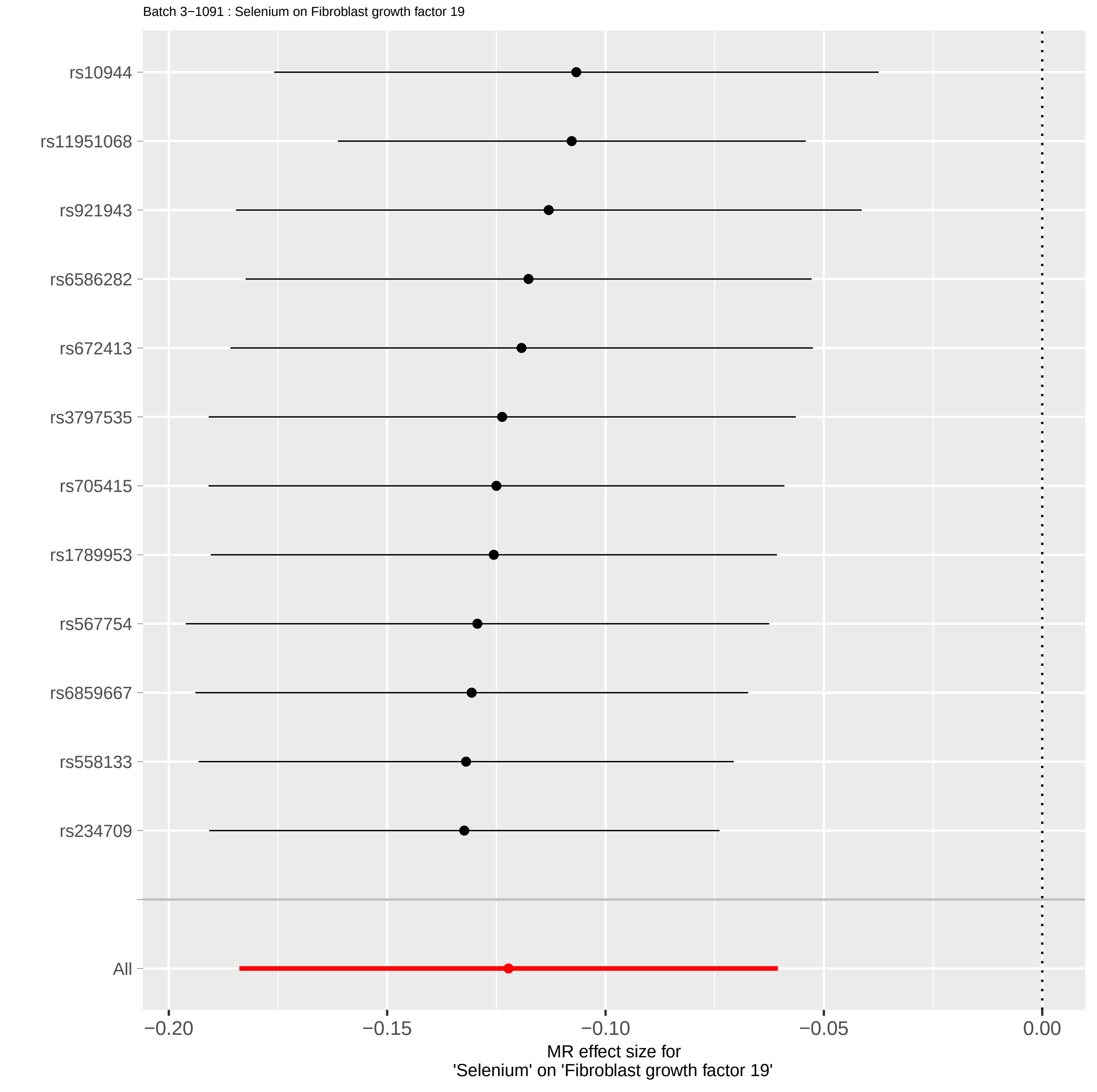

Batch 3−1091 : Selenium on Fibroblast growth factor 19
rs10944
rs11951068
rs921943
rs6586282
rs672413
rs3797535
rs705415
rs1789953
rs567754
rs6859667
rs558133
rs234709
All
−0.20
−0.15
−0.10
−0.05
0.00
MR effect size for
'Selenium' on 'Fibroblast growth factor 19'

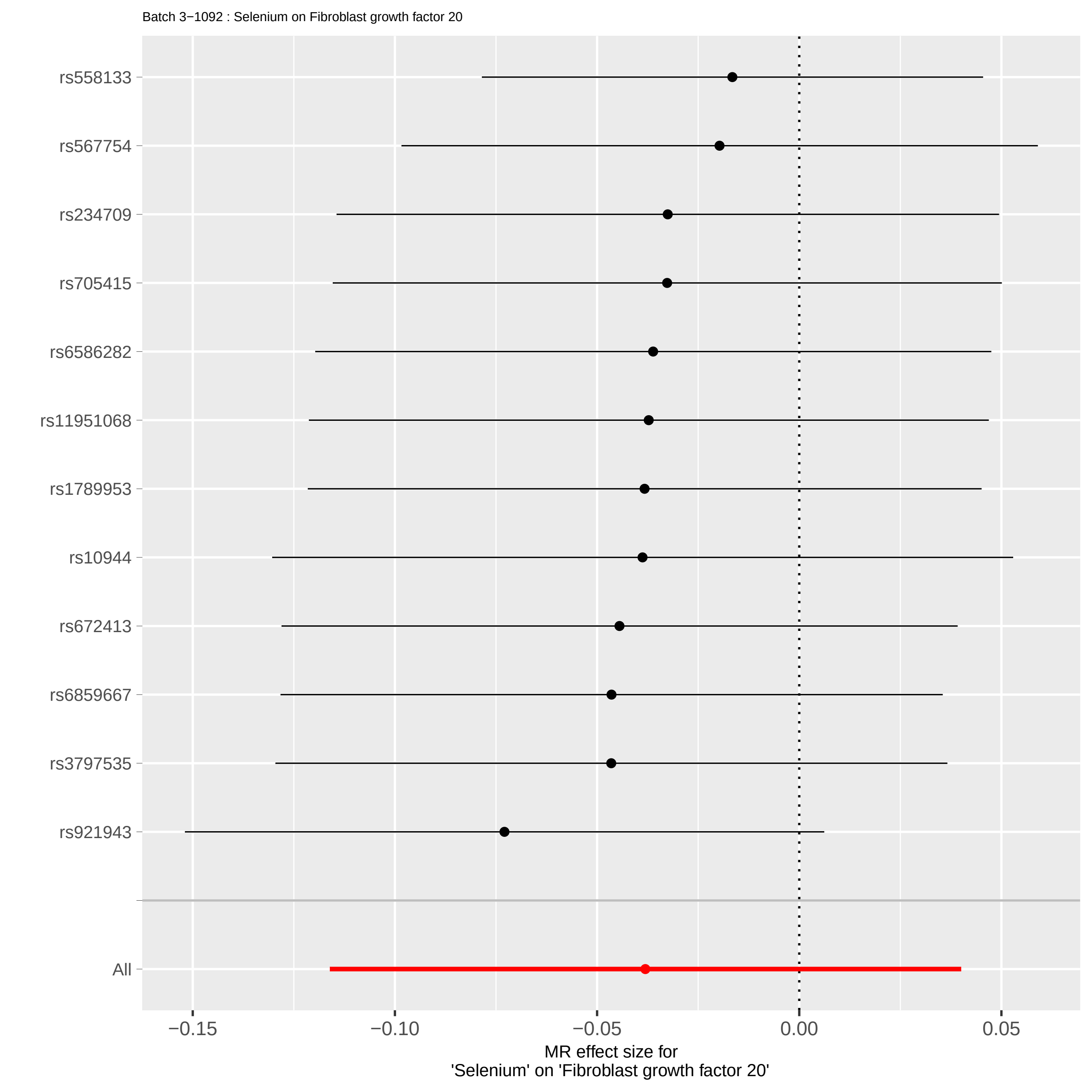
Batch 3−1092 : Selenium on Fibroblast growth factor 20
rs558133
rs567754
rs234709
rs705415
rs6586282
rs11951068
rs1789953
rs10944
rs672413
rs6859667
rs3797535
rs921943
All
−0.15
−0.10
−0.05
0.00
0.05
MR effect size for
'Selenium' on 'Fibroblast growth factor 20'

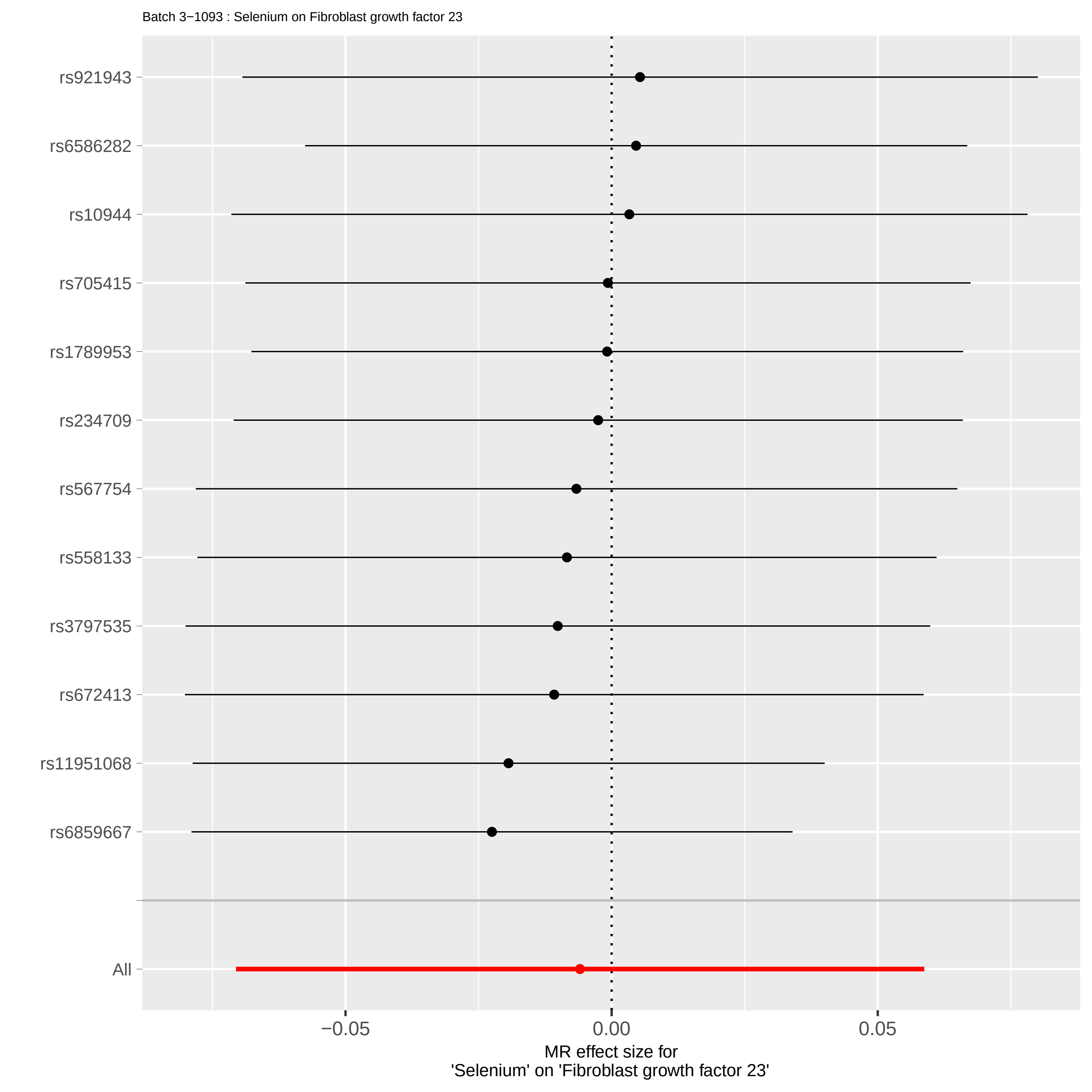
Batch 3−1093 : Selenium on Fibroblast growth factor 23
rs921943
rs6586282
rs10944
rs705415
rs1789953
rs234709
rs567754
rs558133
rs3797535
rs672413
rs11951068
rs6859667
All
−0.05
0.00
0.05
MR effect size for
'Selenium' on 'Fibroblast growth factor 23'

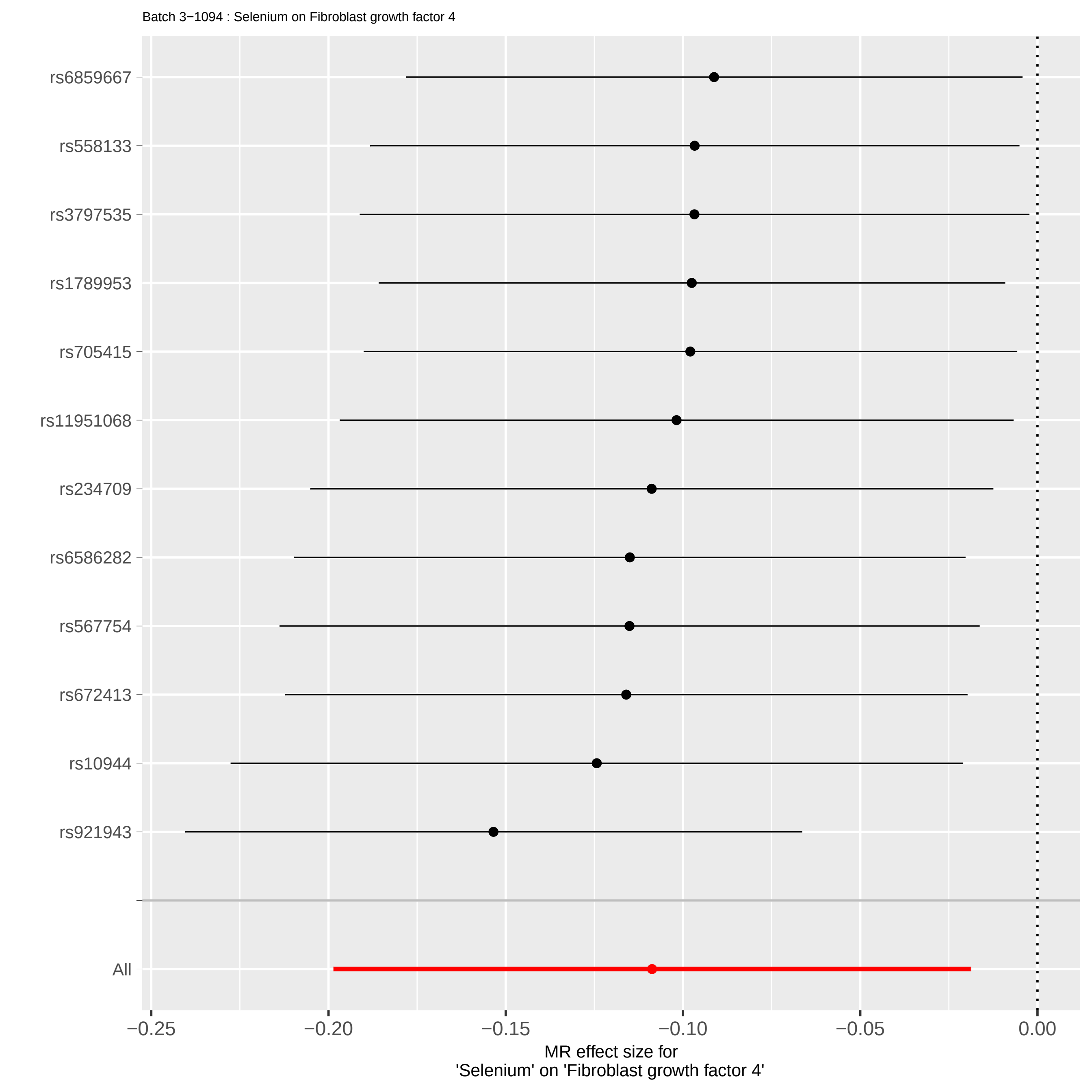

Batch 3−1094 : Selenium on Fibroblast growth factor 4
rs6859667
rs558133
rs3797535
rs1789953
rs705415
rs11951068
rs234709
rs6586282
rs567754
rs672413
rs10944
rs921943
All
−0.25
−0.20
−0.15
−0.10
−0.05
0.00
MR effect size for
'Selenium' on 'Fibroblast growth factor 4'

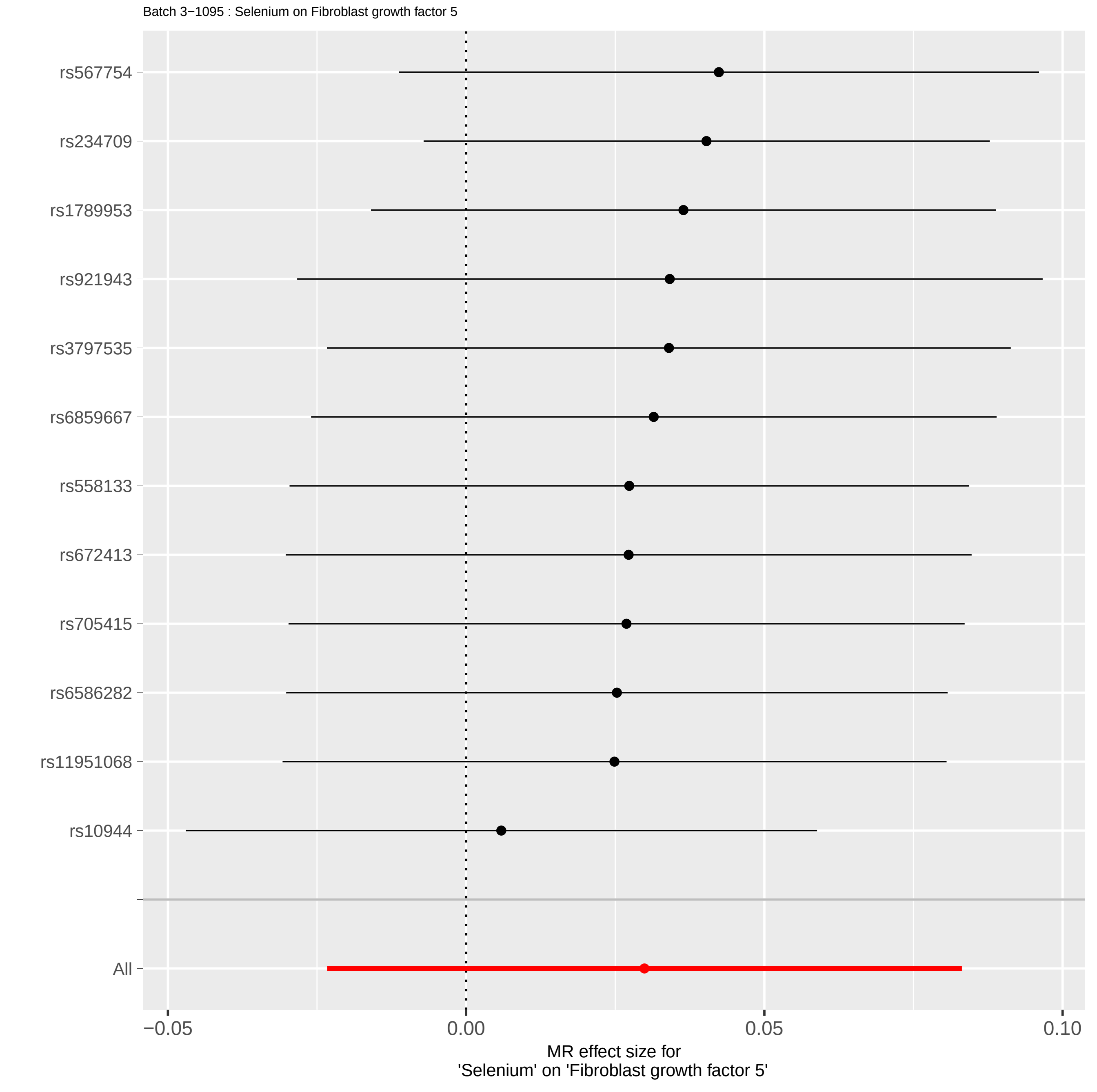
Batch 3−1095 : Selenium on Fibroblast growth factor 5
rs567754
rs234709
rs1789953
rs921943
rs3797535
rs6859667
rs558133
rs672413
rs705415
rs6586282
rs11951068
rs10944
All
−0.05
0.00
0.05
0.10
MR effect size for
'Selenium' on 'Fibroblast growth factor 5'

Batch 3−1096 : Selenium on Fibroblast growth factor 6

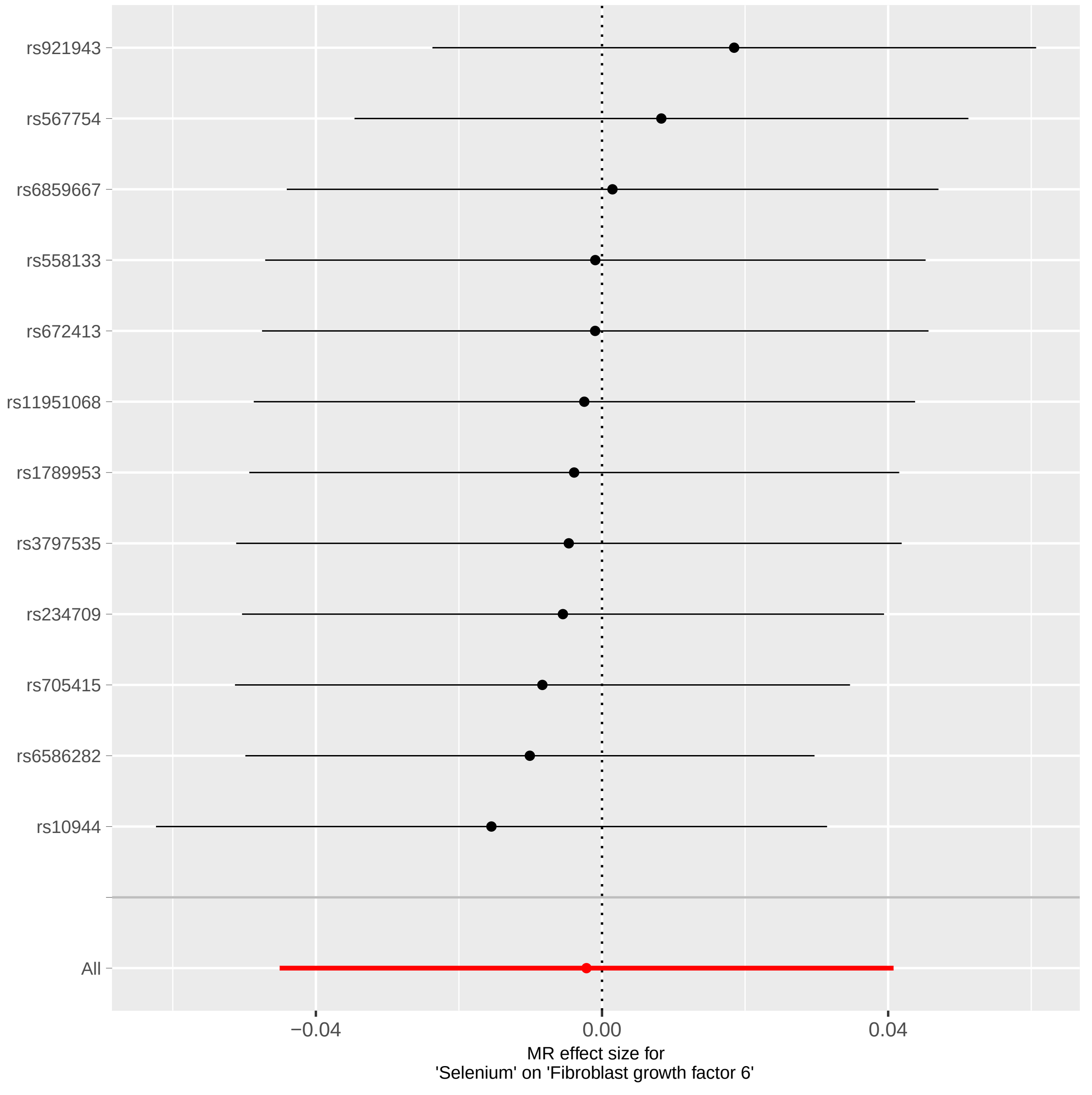

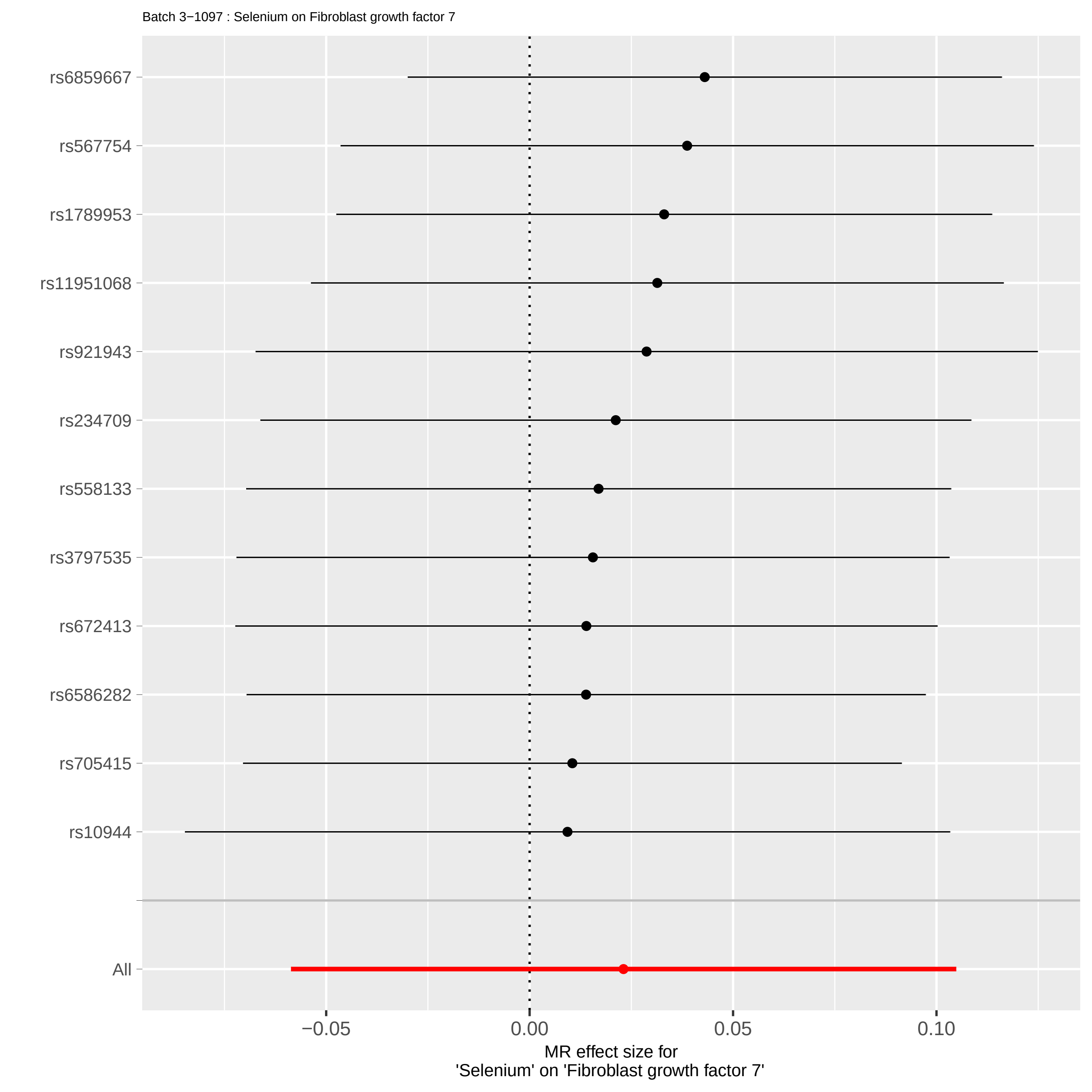

Batch 3−1097 : Selenium on Fibroblast growth factor 7
rs6859667
rs567754
rs1789953
rs11951068
rs921943
rs234709
rs558133
rs3797535
rs672413
rs6586282
rs705415
rs10944
All
−0.05
0.00
0.05
0.10
MR effect size for
'Selenium' on 'Fibroblast growth factor 7'

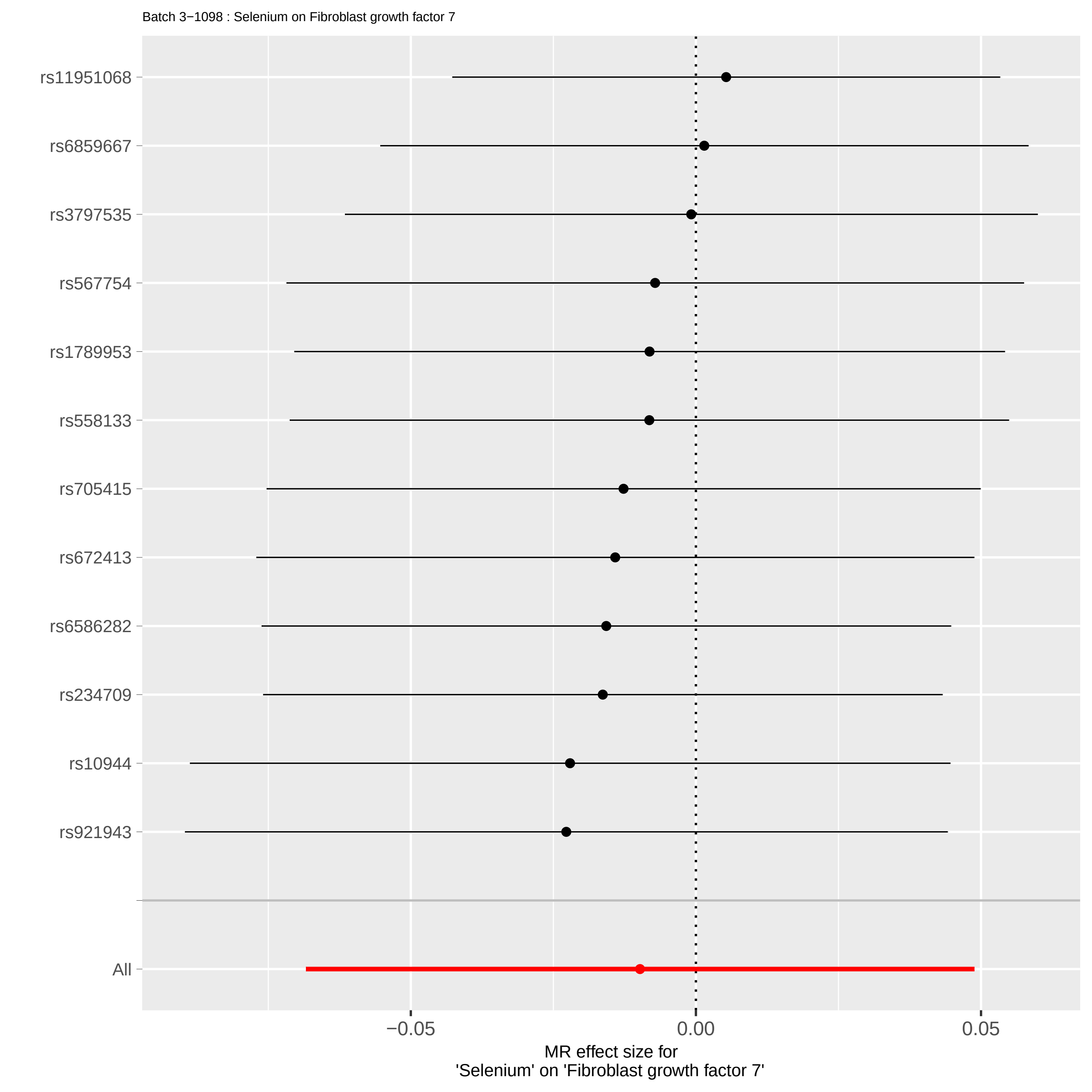

Batch 3−1098 : Selenium on Fibroblast growth factor 7
rs11951068
rs6859667
rs3797535
rs567754
rs1789953
rs558133
rs705415
rs672413
rs6586282
rs234709
rs10944
rs921943
All
−0.05
0.00
0.05
MR effect size for
'Selenium' on 'Fibroblast growth factor 7'

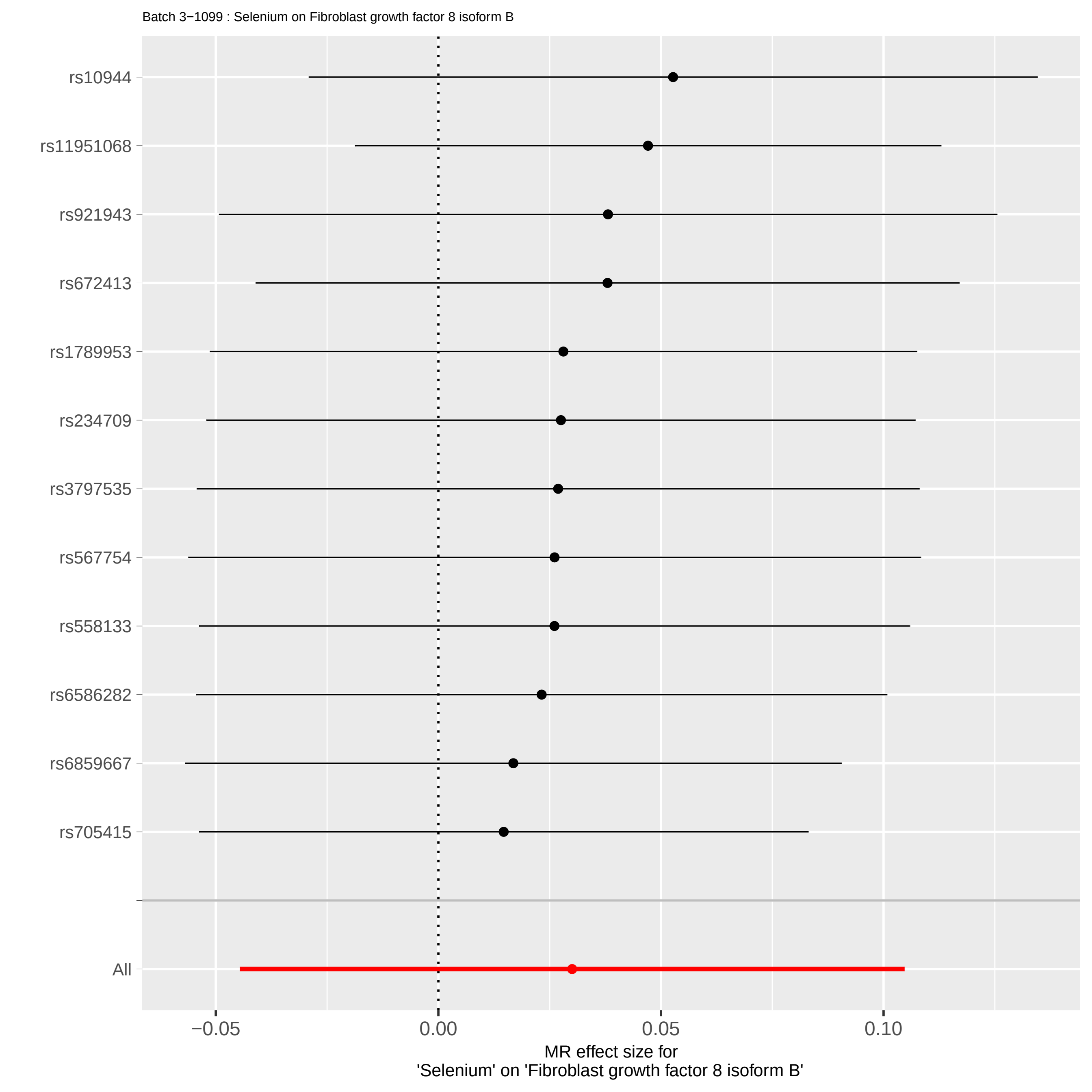

Batch 3−1099 : Selenium on Fibroblast growth factor 8 isoform B
rs10944
rs11951068
rs921943
rs672413
rs1789953
rs234709
rs3797535
rs567754
rs558133
rs6586282
rs6859667
rs705415
All
−0.05
0.00
0.05
0.10
MR effect size for
'Selenium' on 'Fibroblast growth factor 8 isoform B'

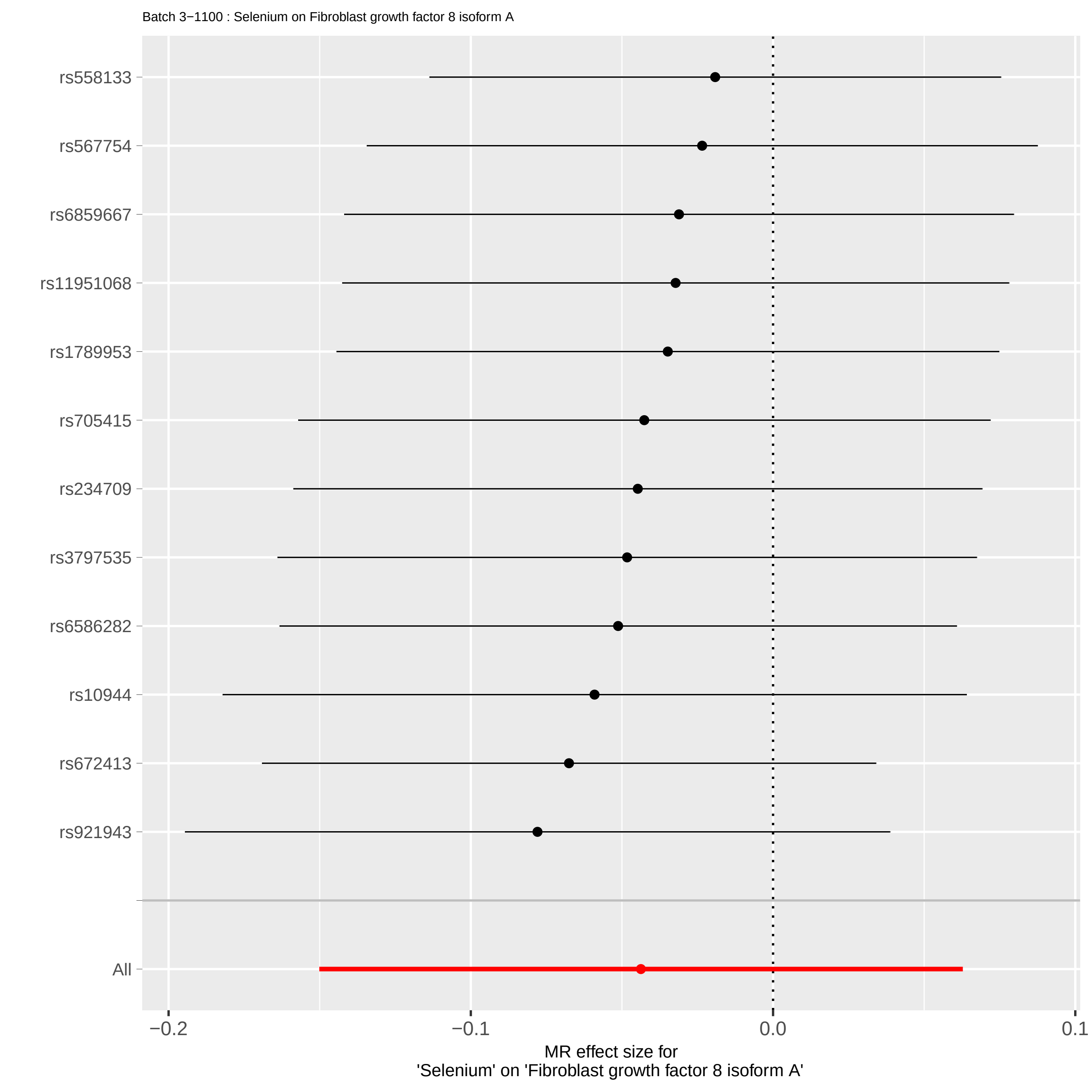

Batch 3−1100 : Selenium on Fibroblast growth factor 8 isoform A
rs558133
rs567754
rs6859667
rs11951068
rs1789953
rs705415
rs234709
rs3797535
rs6586282
rs10944
rs672413
rs921943
All
−0.2
−0.1
0.0
0.1
MR effect size for
'Selenium' on 'Fibroblast growth factor 8 isoform A'

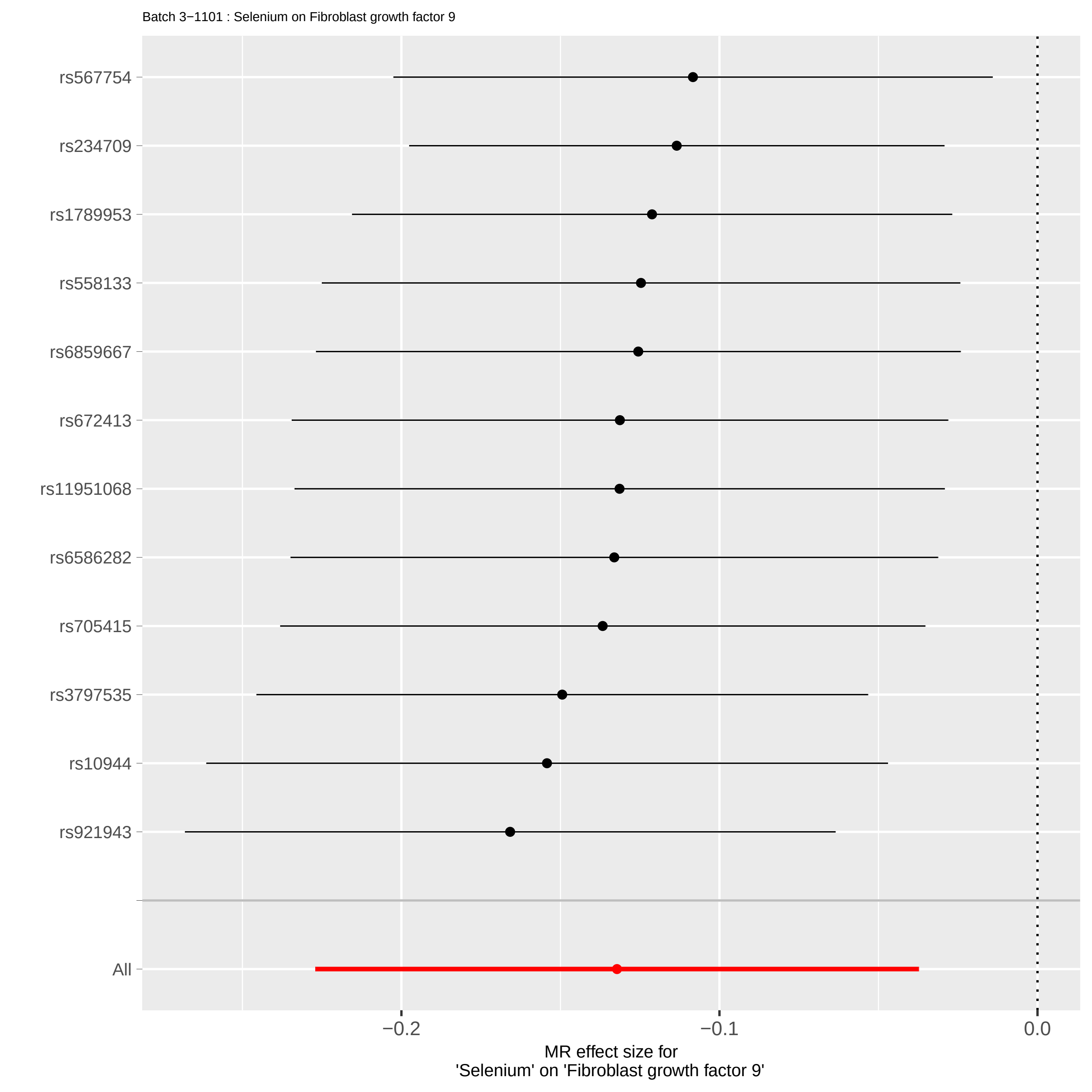

Batch 3−1101 : Selenium on Fibroblast growth factor 9
rs567754
rs234709
rs1789953
rs558133
rs6859667
rs672413
rs11951068
rs6586282
rs705415
rs3797535
rs10944
rs921943
All
−0.2
−0.1
0.0
MR effect size for
'Selenium' on 'Fibroblast growth factor 9'

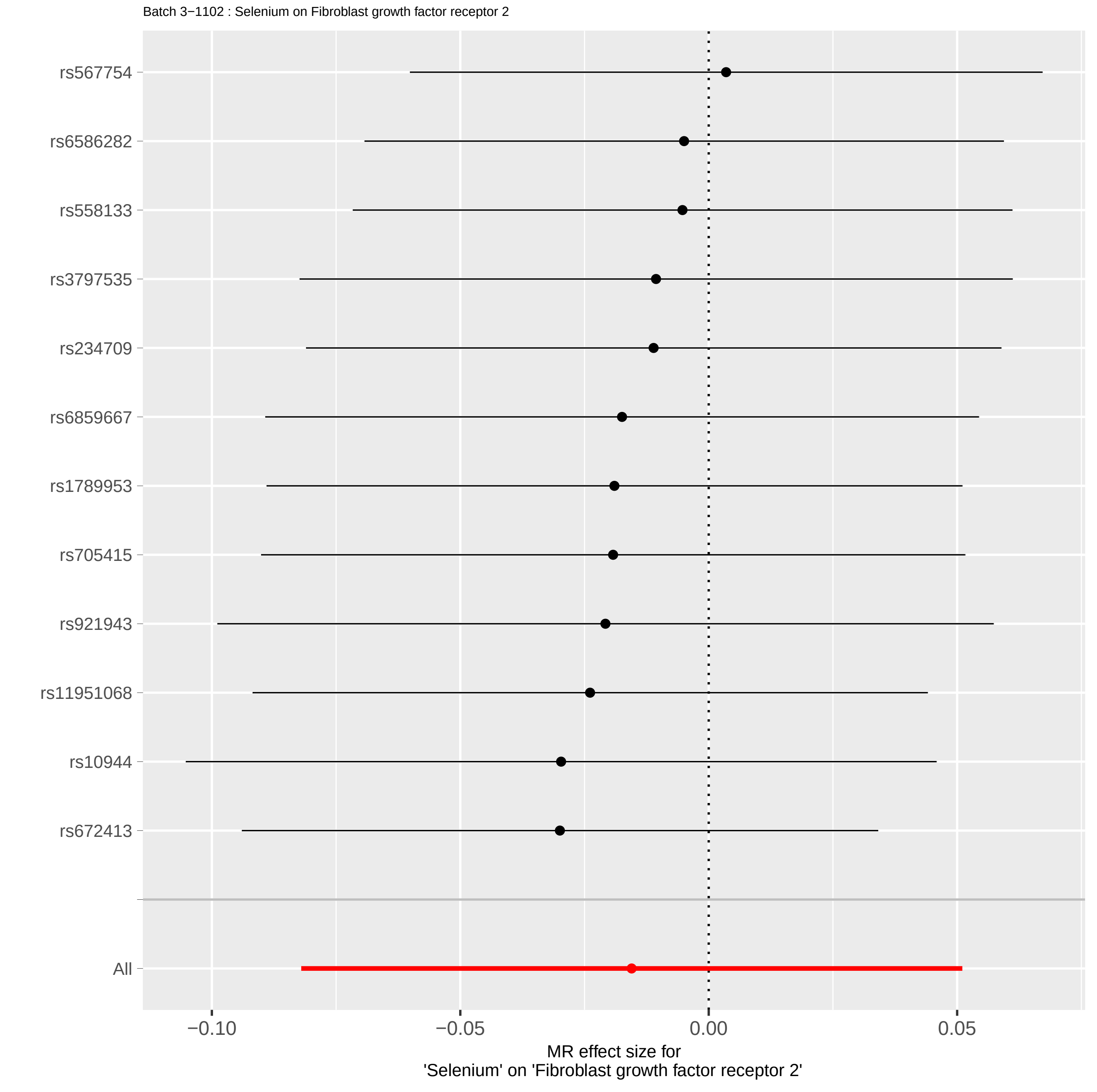
Batch 3−1102 : Selenium on Fibroblast growth factor receptor 2
rs567754
rs6586282
rs558133
rs3797535
rs234709
rs6859667
rs1789953
rs705415
rs921943
rs11951068
rs10944
rs672413
All
−0.10
−0.05
0.00
0.05
MR effect size for
'Selenium' on 'Fibroblast growth factor receptor 2'

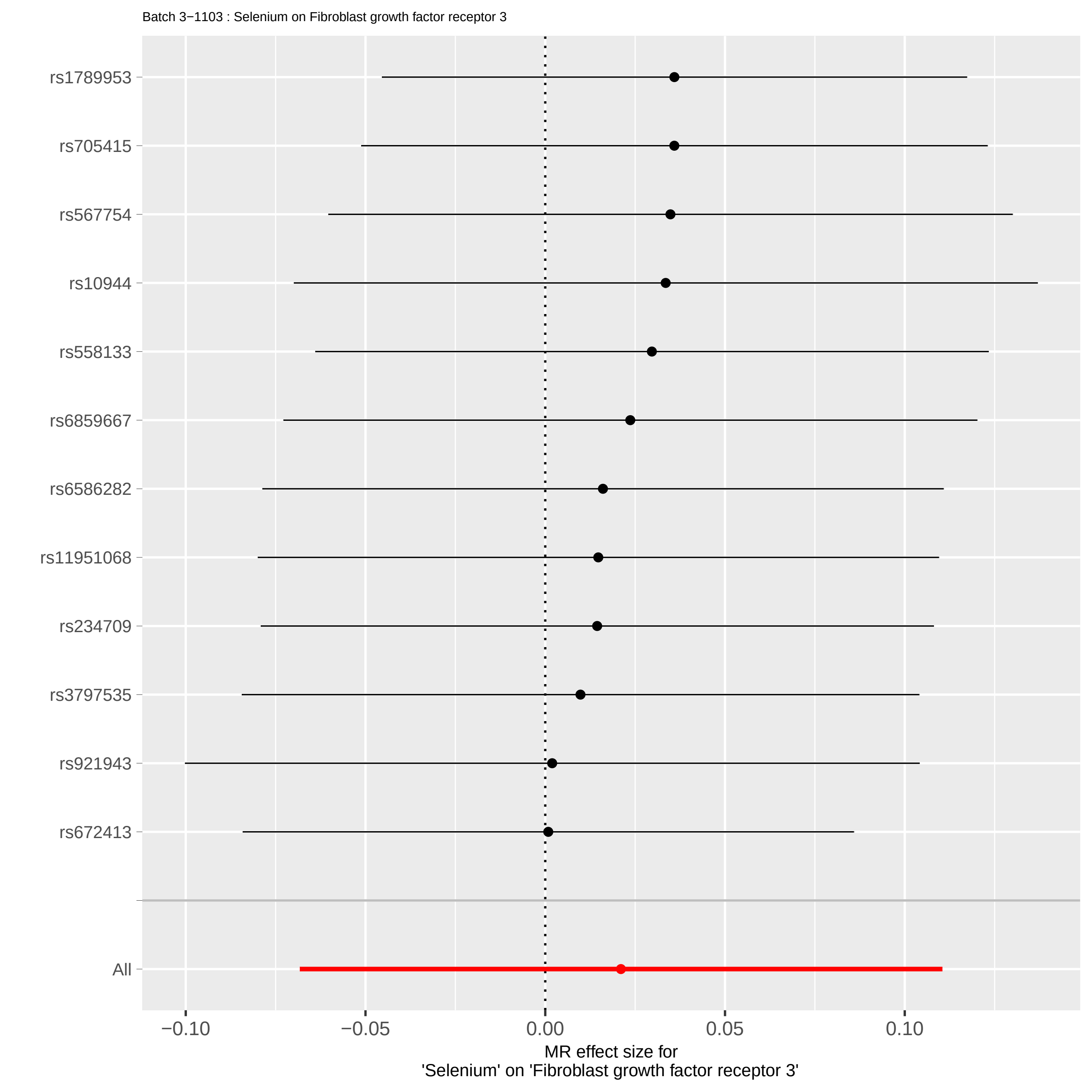

Batch 3−1103 : Selenium on Fibroblast growth factor receptor 3
rs1789953
rs705415
rs567754
rs10944
rs558133
rs6859667
rs6586282
rs11951068
rs234709
rs3797535
rs921943
rs672413
All
−0.10
−0.05
0.00
0.05
0.10
MR effect size for
'Selenium' on 'Fibroblast growth factor receptor 3'

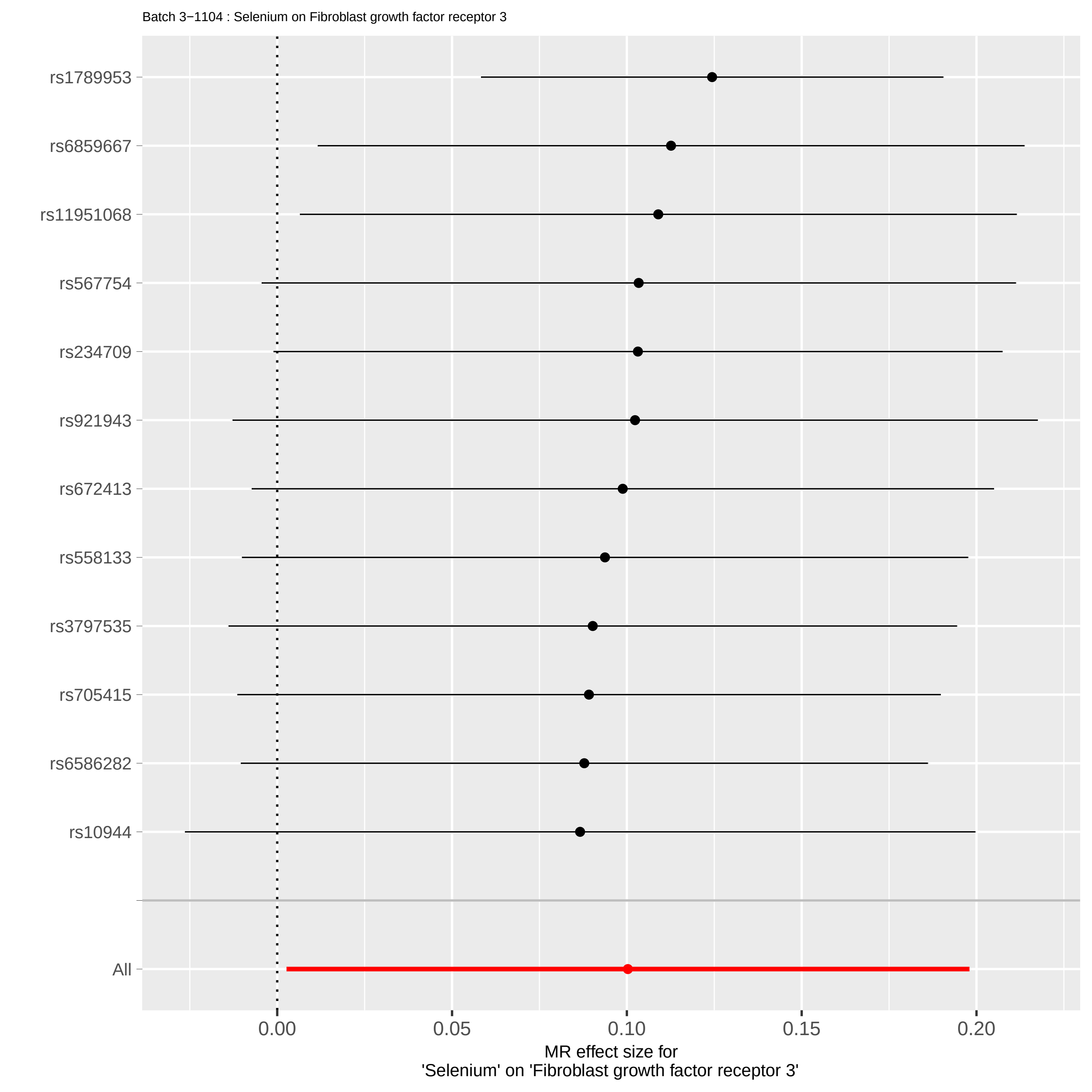

Batch 3−1104 : Selenium on Fibroblast growth factor receptor 3
rs1789953
rs6859667
rs11951068
rs567754
rs234709
rs921943
rs672413
rs558133
rs3797535
rs705415
rs6586282
rs10944
All
0.00
0.05
0.10
0.15
0.20
MR effect size for
'Selenium' on 'Fibroblast growth factor receptor 3'

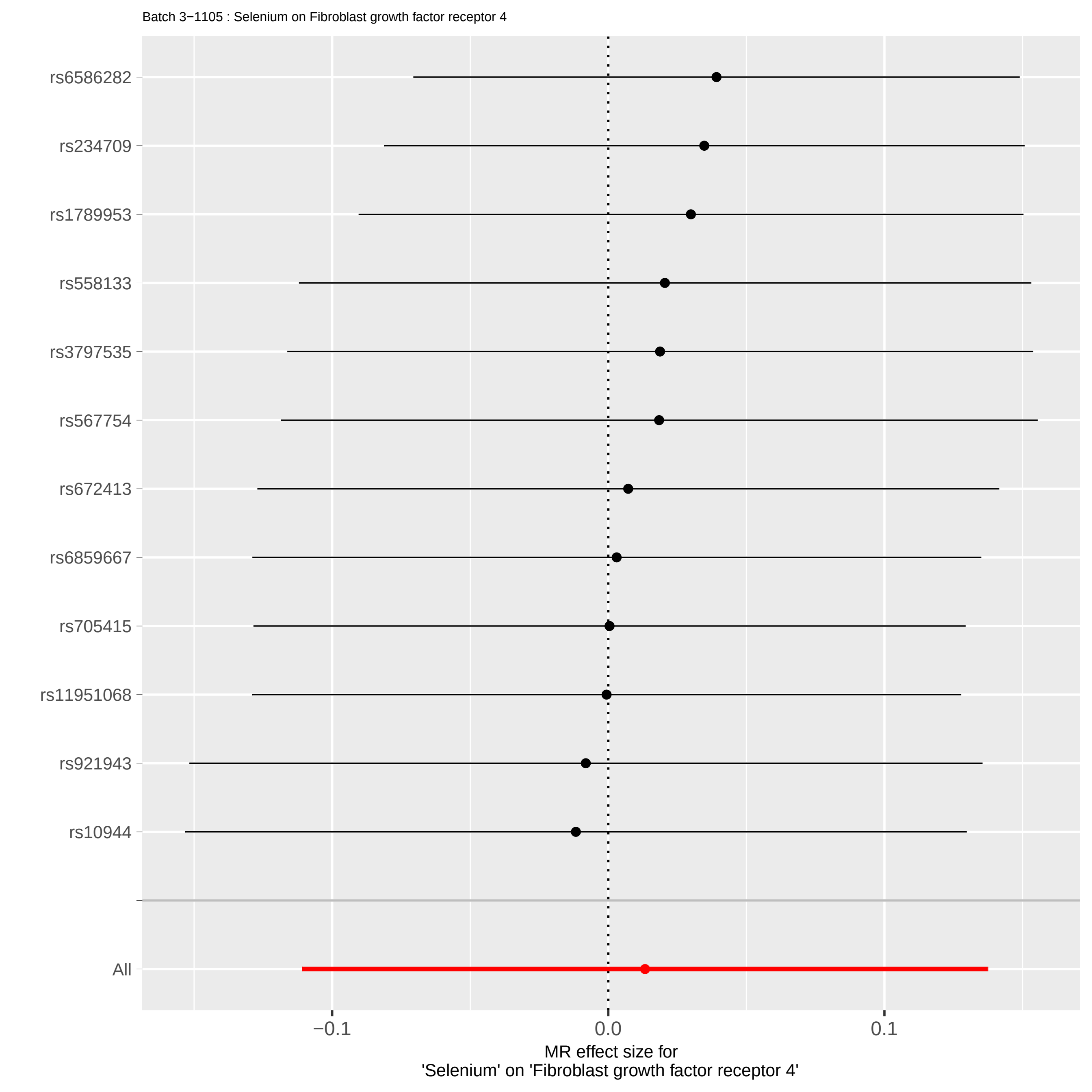
Batch 3−1105 : Selenium on Fibroblast growth factor receptor 4
rs6586282
rs234709
rs1789953
rs558133
rs3797535
rs567754
rs672413
rs6859667
rs705415
rs11951068
rs921943
rs10944
All
−0.1
0.0
0.1
MR effect size for
'Selenium' on 'Fibroblast growth factor receptor 4'

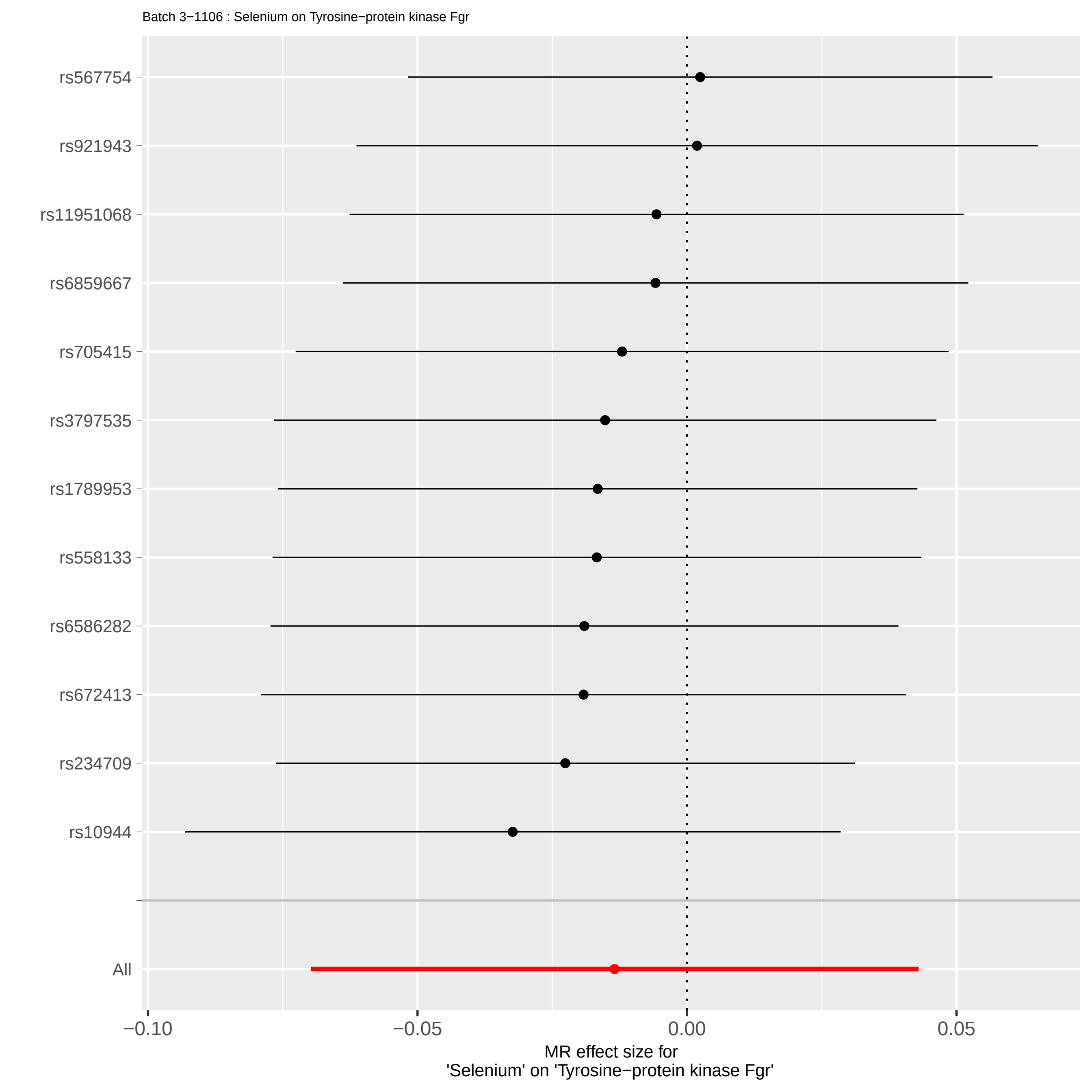

Batch 3−1106 : Selenium on Tyrosine−protein kinase Fgr
rs567754
rs921943
rs11951068
rs6859667
rs705415
rs3797535
rs1789953
rs558133
rs6586282
rs672413
rs234709
rs10944
All
−0.10
−0.05
0.00
0.05
MR effect size for
'Selenium' on 'Tyrosine−protein kinase Fgr'

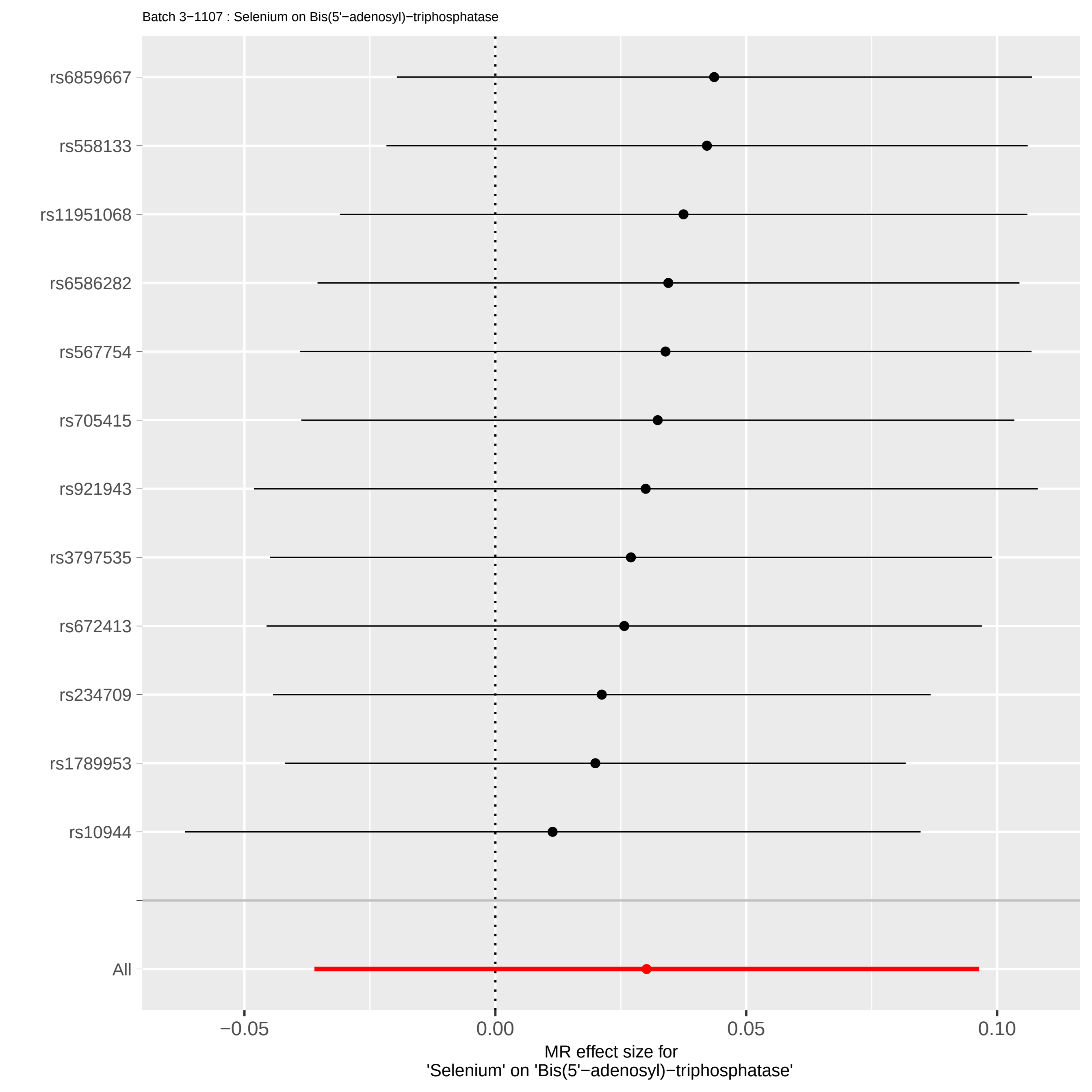

Batch 3−1107 : Selenium on Bis(5'−adenosyl)−triphosphatase
rs6859667
rs558133
rs11951068
rs6586282
rs567754
rs705415
rs921943
rs3797535
rs672413
rs234709
rs1789953
rs10944
All
−0.05
0.00
0.05
0.10
MR effect size for
'Selenium' on 'Bis(5'−adenosyl)−triphosphatase'

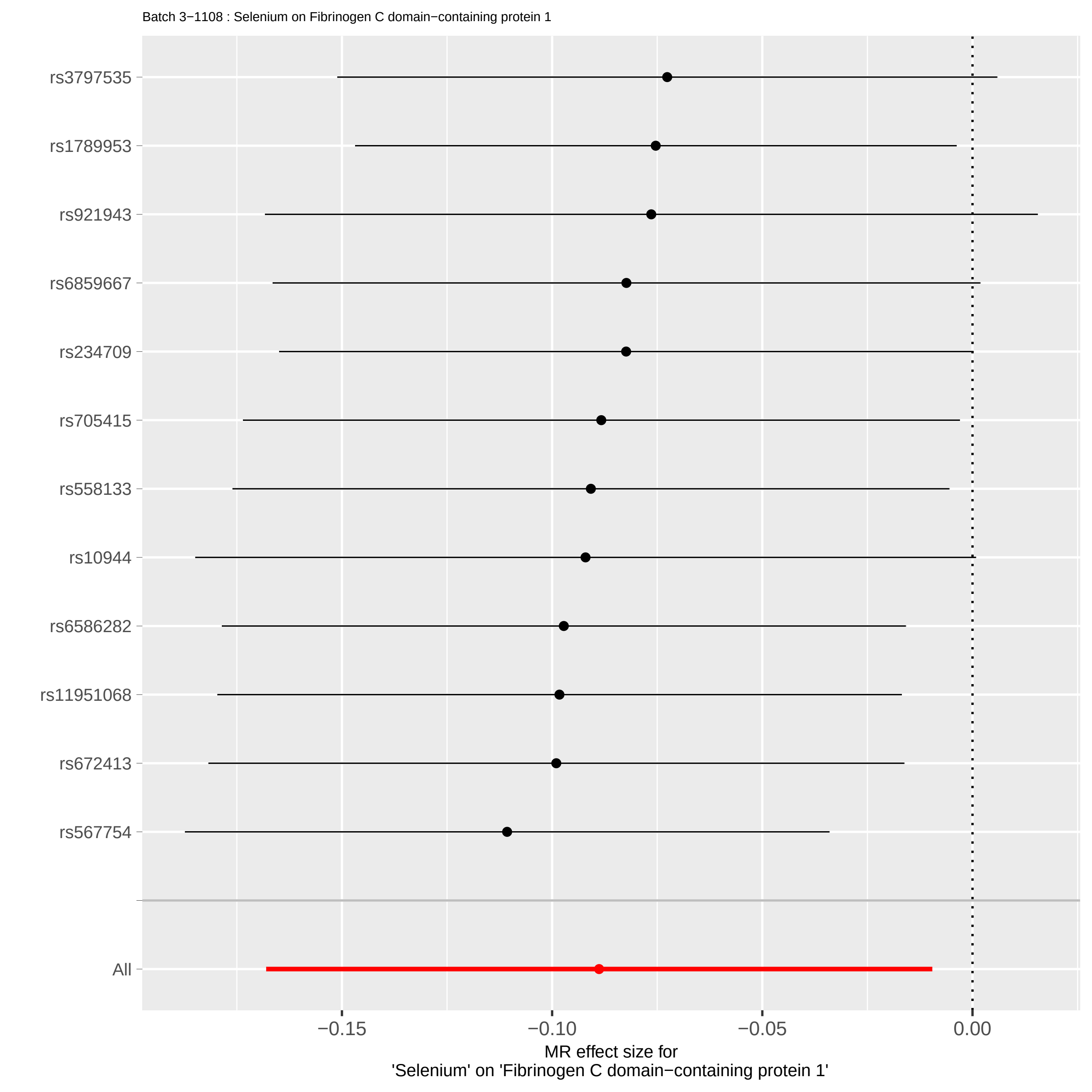

Batch 3−1108 : Selenium on Fibrinogen C domain−containing protein 1
rs3797535
rs1789953
rs921943
rs6859667
rs234709
rs705415
rs558133
rs10944
rs6586282
rs11951068
rs672413
rs567754
All
−0.15
−0.10
−0.05
0.00
MR effect size for
'Selenium' on 'Fibrinogen C domain−containing protein 1'

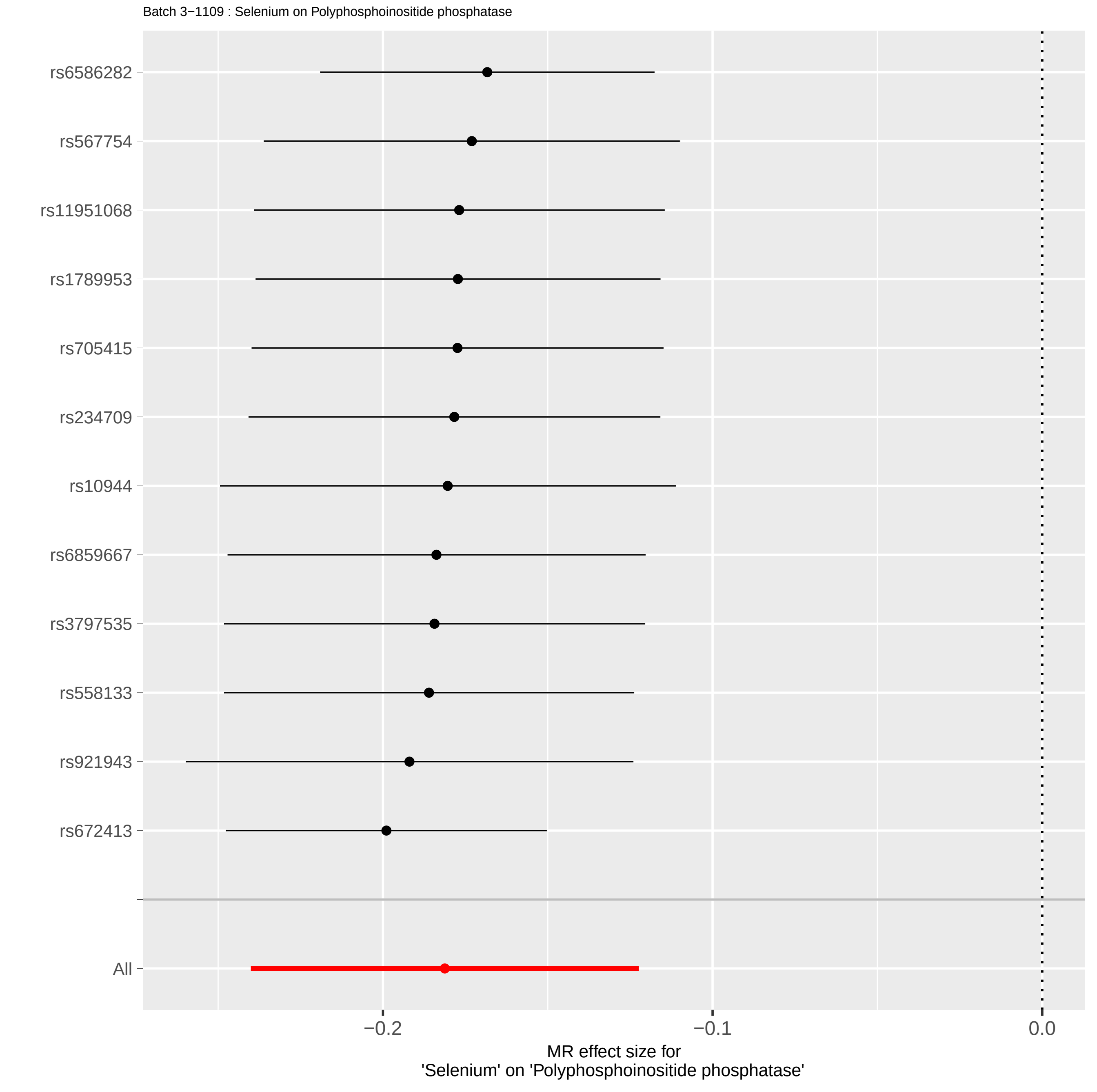

Batch 3−1109 : Selenium on Polyphosphoinositide phosphatase
rs6586282
rs567754
rs11951068
rs1789953
rs705415
rs234709
rs10944
rs6859667
rs3797535
rs558133
rs921943
rs672413
All
−0.2
−0.1
0.0
MR effect size for
'Selenium' on 'Polyphosphoinositide phosphatase'

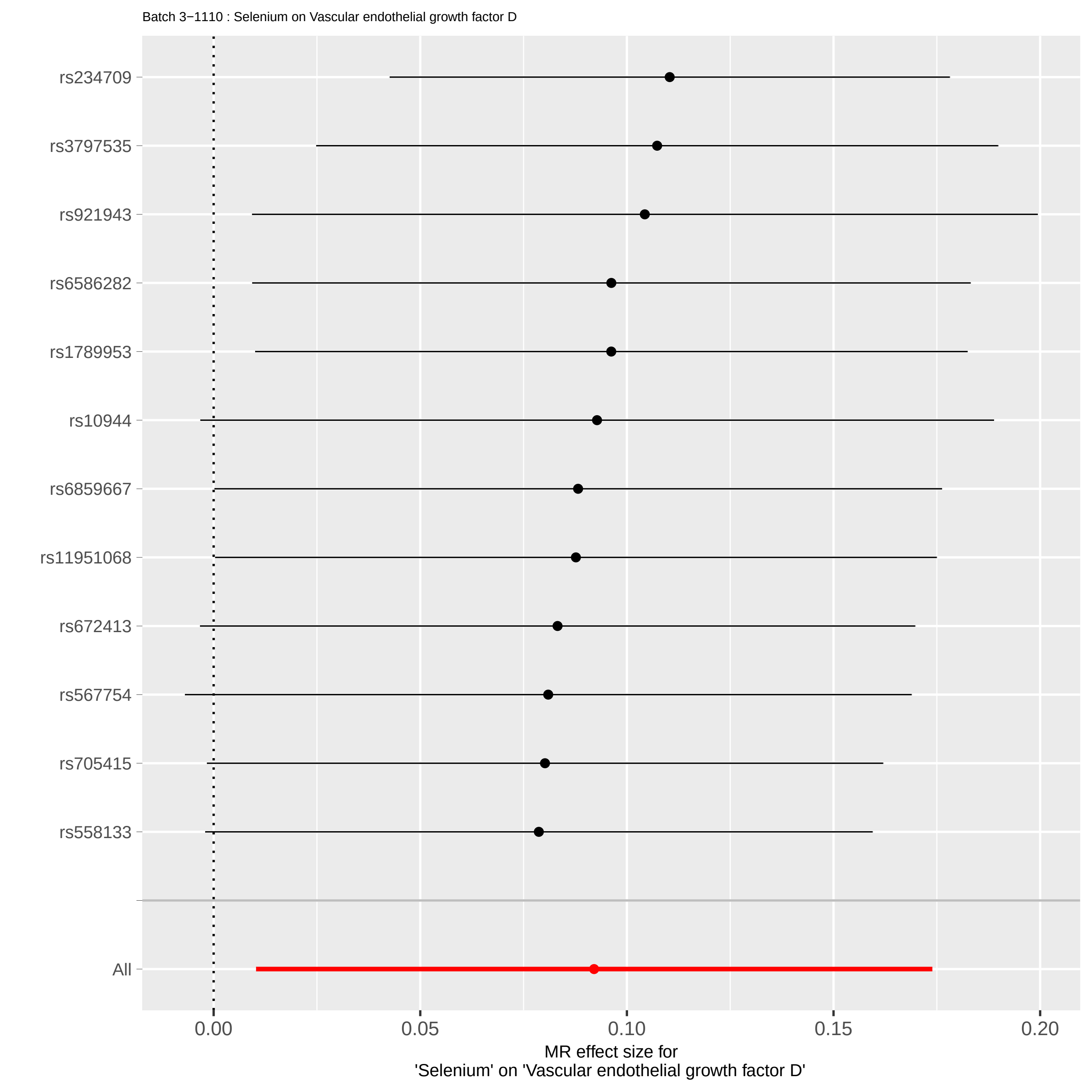
Batch 3−1110 : Selenium on Vascular endothelial growth factor D
rs234709
rs3797535
rs921943
rs6586282
rs1789953
rs10944
rs6859667
rs11951068
rs672413
rs567754
rs705415
rs558133
All
0.00
0.05
0.10
0.15
0.20
MR effect size for
'Selenium' on 'Vascular endothelial growth factor D'

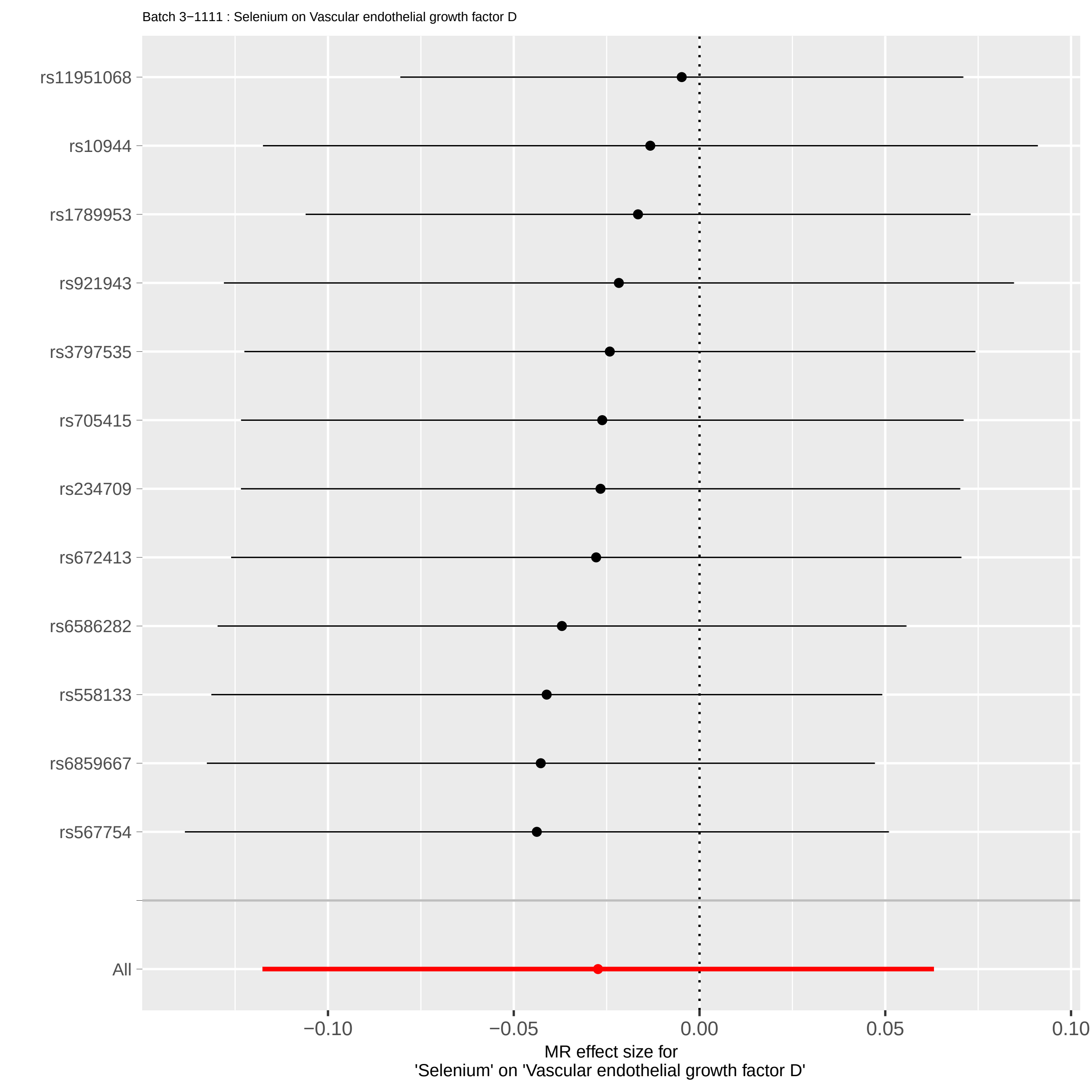
Batch 3−1111 : Selenium on Vascular endothelial growth factor D
rs11951068
rs10944
rs1789953
rs921943
rs3797535
rs705415
rs234709
rs672413
rs6586282
rs558133
rs6859667
rs567754
All
−0.10
−0.05
0.00
0.05
0.10
MR effect size for
'Selenium' on 'Vascular endothelial growth factor D'

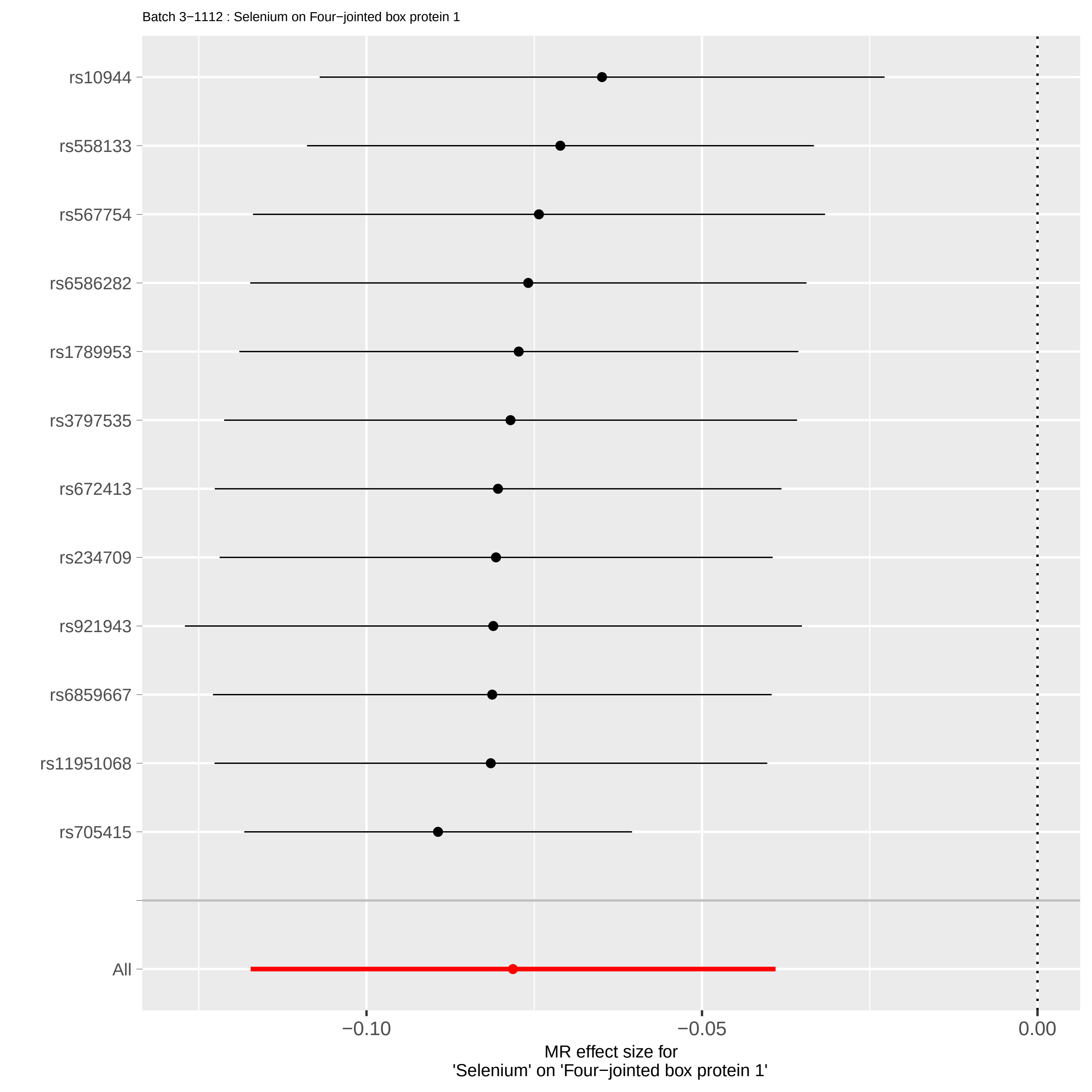
Batch 3−1112 : Selenium on Four−jointed box protein 1
rs10944
rs558133
rs567754
rs6586282
rs1789953
rs3797535
rs672413
rs234709
rs921943
rs6859667
rs11951068
rs705415
All
−0.10
−0.05
0.00
MR effect size for
'Selenium' on 'Four−jointed box protein 1'

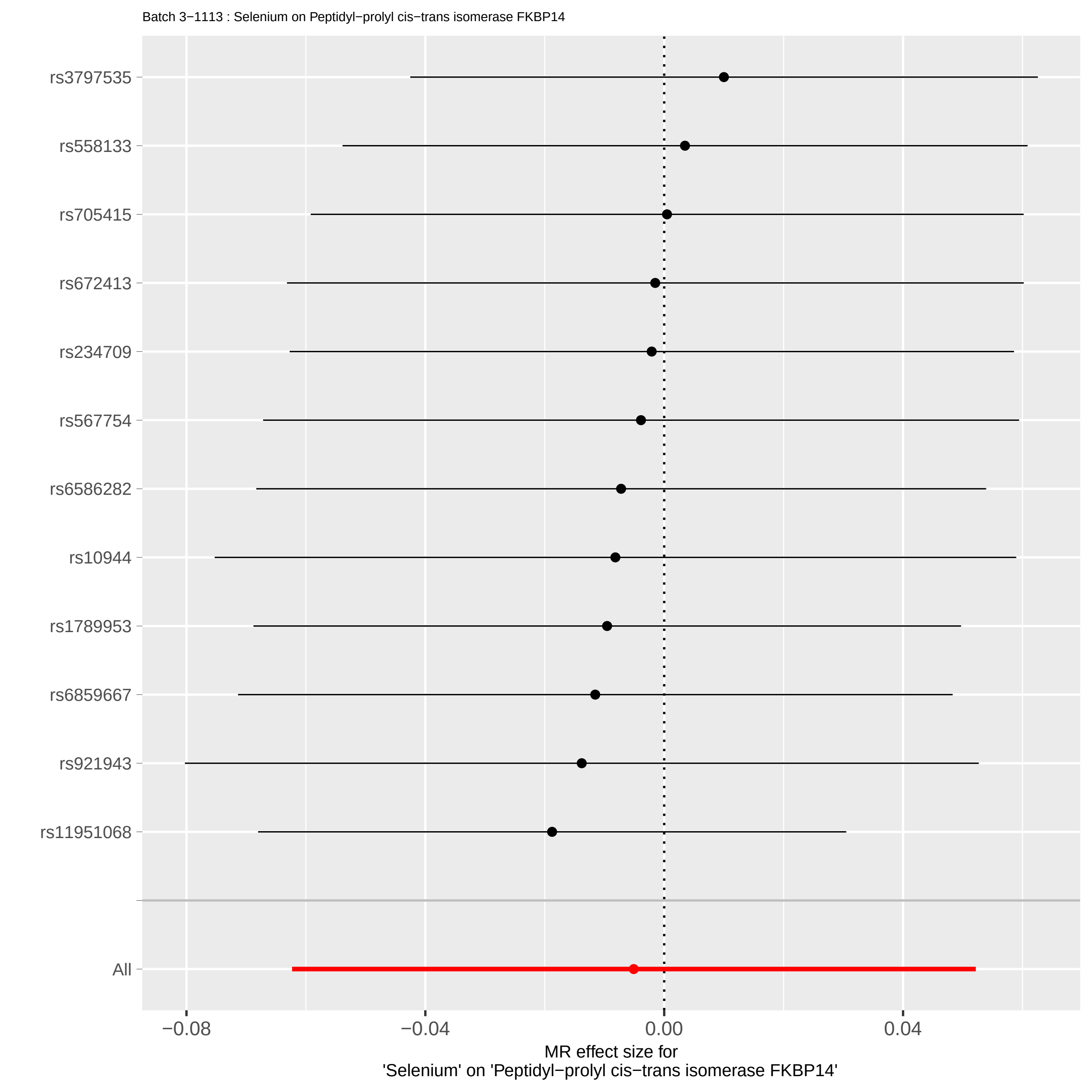

Batch 3−1113 : Selenium on Peptidyl−prolyl cis−trans isomerase FKBP14
rs3797535
rs558133
rs705415
rs672413
rs234709
rs567754
rs6586282
rs10944
rs1789953
rs6859667
rs921943
rs11951068
All
−0.08
−0.04
0.00
0.04
MR effect size for
'Selenium' on 'Peptidyl−prolyl cis−trans isomerase FKBP14'

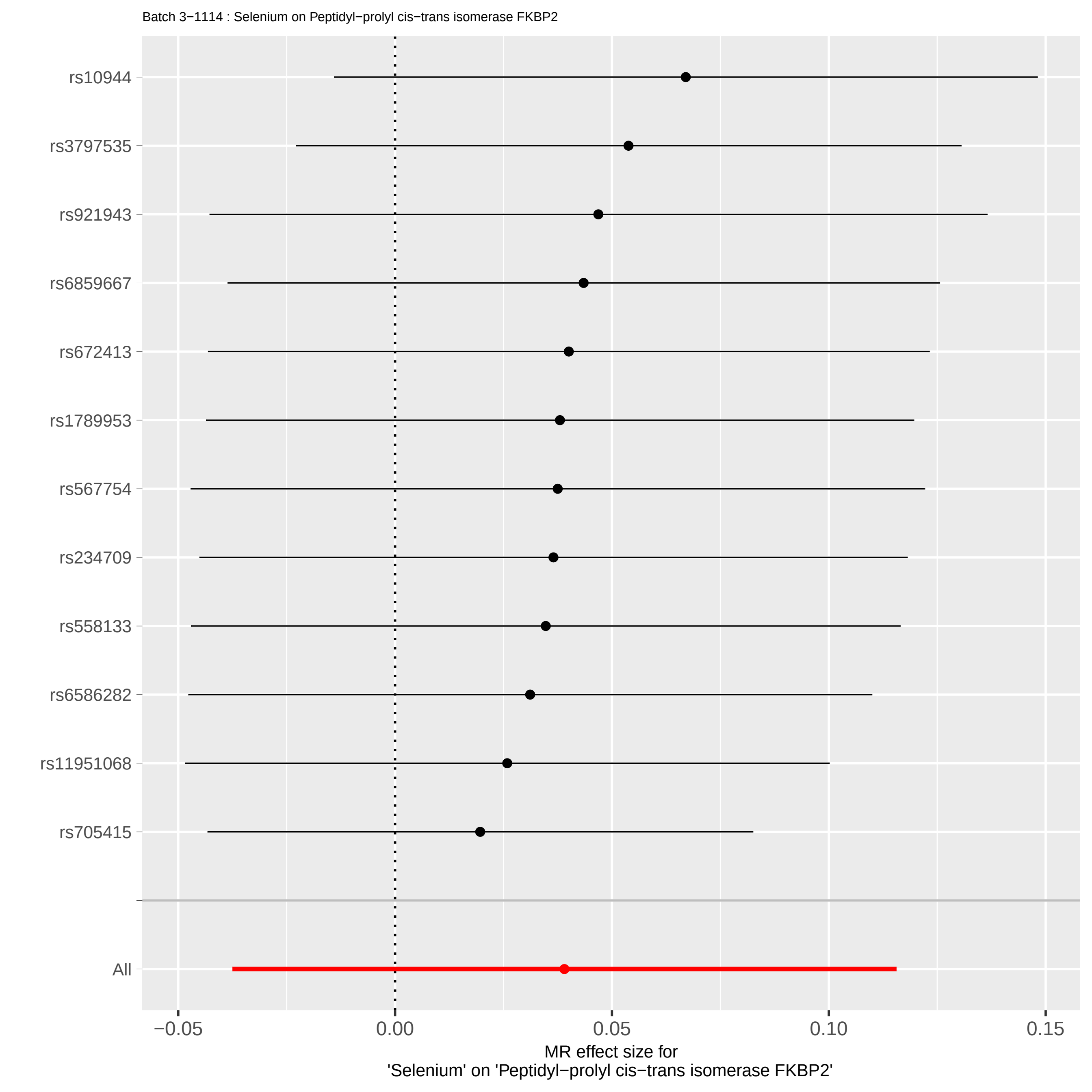

Batch 3−1114 : Selenium on Peptidyl−prolyl cis−trans isomerase FKBP2
rs10944
rs3797535
rs921943
rs6859667
rs672413
rs1789953
rs567754
rs234709
rs558133
rs6586282
rs11951068
rs705415
All
−0.05
0.00
0.05
0.10
0.15
MR effect size for
'Selenium' on 'Peptidyl−prolyl cis−trans isomerase FKBP2'

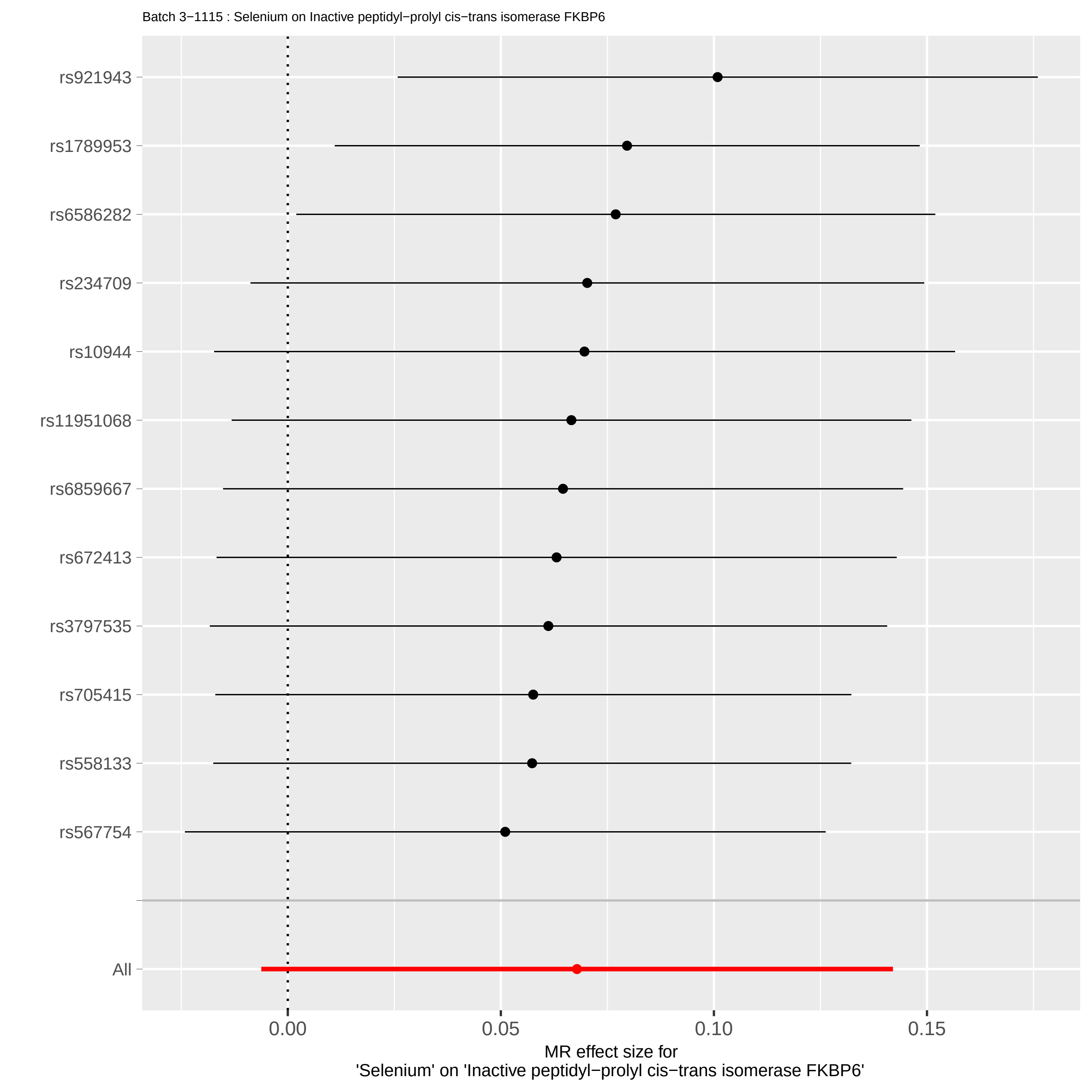

Batch 3−1115 : Selenium on Inactive peptidyl−prolyl cis−trans isomerase FKBP6
rs921943
rs1789953
rs6586282
rs234709
rs10944
rs11951068
rs6859667
rs672413
rs3797535
rs705415
rs558133
rs567754
All
0.00
0.05
0.10
0.15
MR effect size for
'Selenium' on 'Inactive peptidyl−prolyl cis−trans isomerase FKBP6'

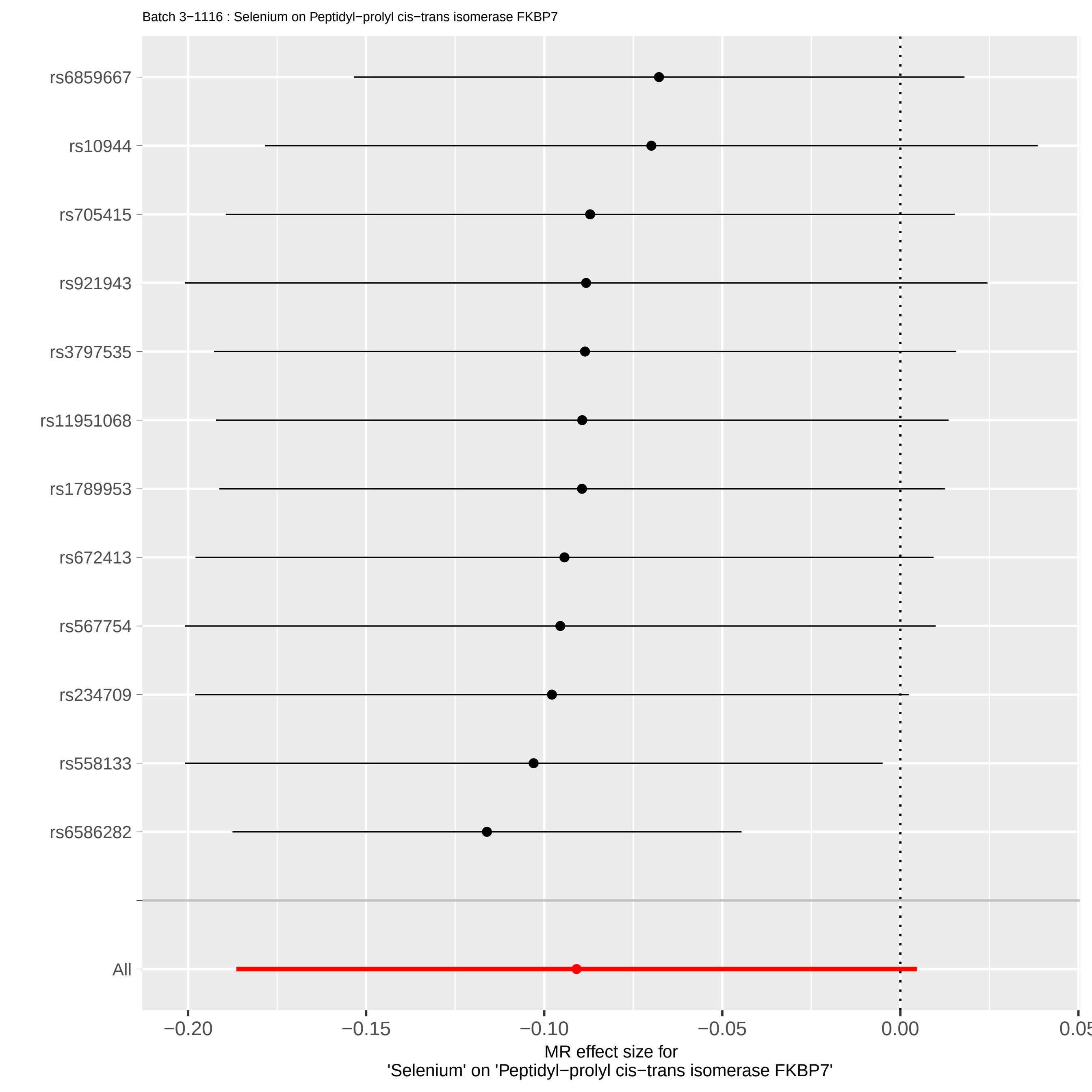

Batch 3−1116 : Selenium on Peptidyl−prolyl cis−trans isomerase FKBP7
rs6859667
rs10944
rs705415
rs921943
rs3797535
rs11951068
rs1789953
rs672413
rs567754
rs234709
rs558133
rs6586282
All
−0.20
−0.15
−0.10
−0.05
0.00
0.05
MR effect size for
'Selenium' on 'Peptidyl−prolyl cis−trans isomerase FKBP7'

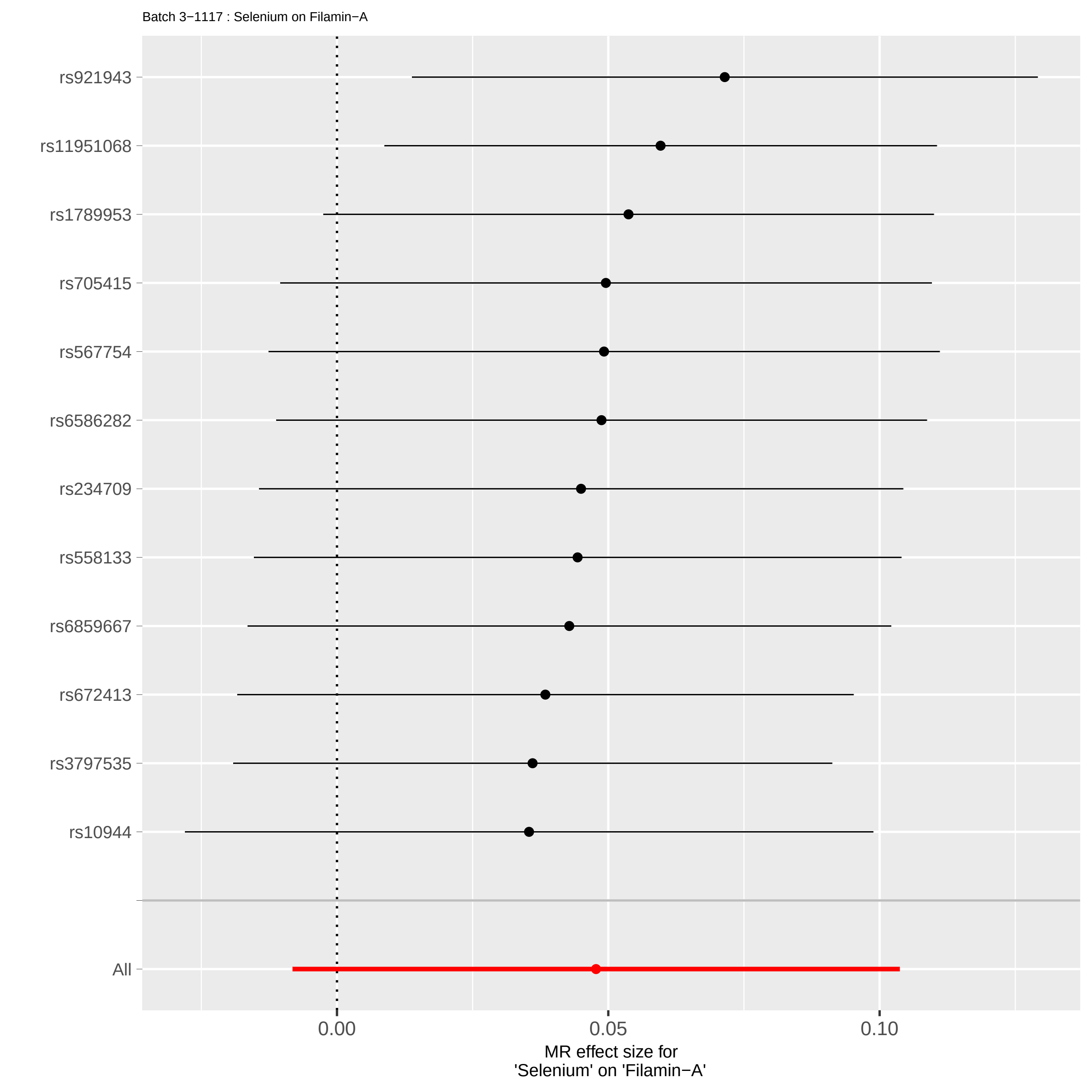
Batch 3−1117 : Selenium on Filamin−A
rs921943
rs11951068
rs1789953
rs705415
rs567754
rs6586282
rs234709
rs558133
rs6859667
rs672413
rs3797535
rs10944
All
0.00
0.05
0.10
MR effect size for
'Selenium' on 'Filamin−A'

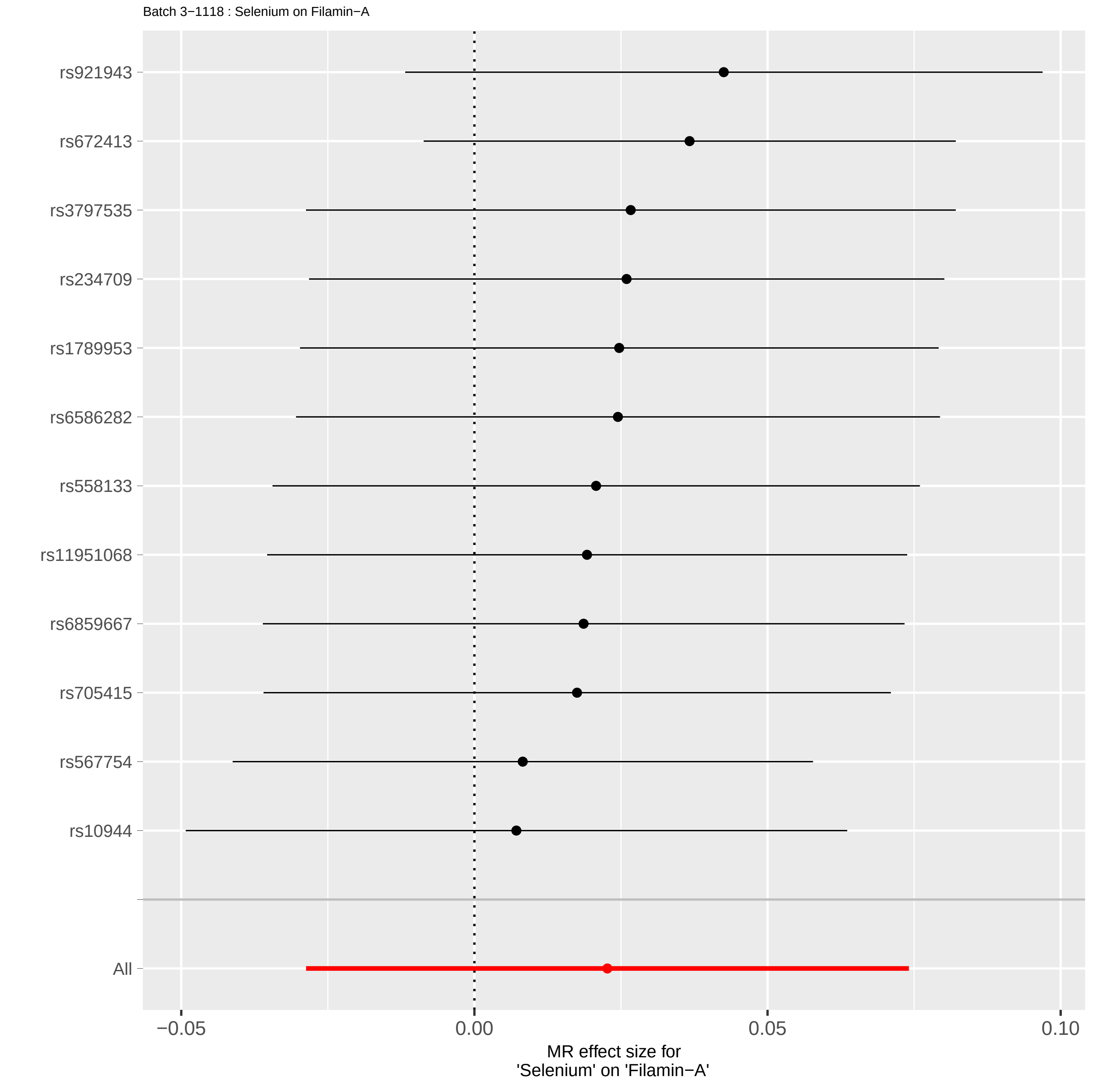
Batch 3−1118 : Selenium on Filamin−A
rs921943
rs672413
rs3797535
rs234709
rs1789953
rs6586282
rs558133
rs11951068
rs6859667
rs705415
rs567754
rs10944
All
−0.05
0.00
0.05
0.10
MR effect size for
'Selenium' on 'Filamin−A'

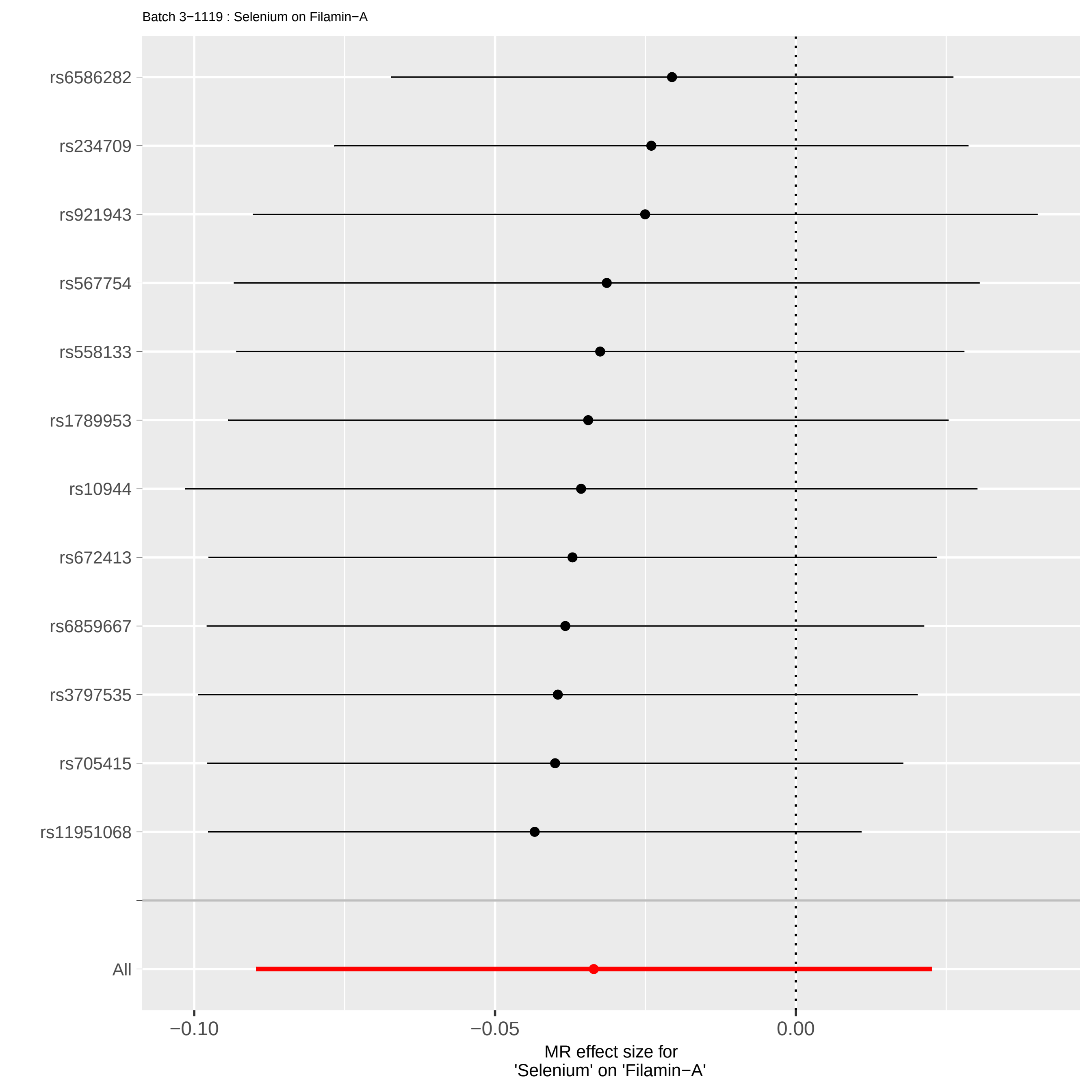
Batch 3−1119 : Selenium on Filamin−A
rs6586282
rs234709
rs921943
rs567754
rs558133
rs1789953
rs10944
rs672413
rs6859667
rs3797535
rs705415
rs11951068
All
−0.10
−0.05
0.00
MR effect size for
'Selenium' on 'Filamin−A'

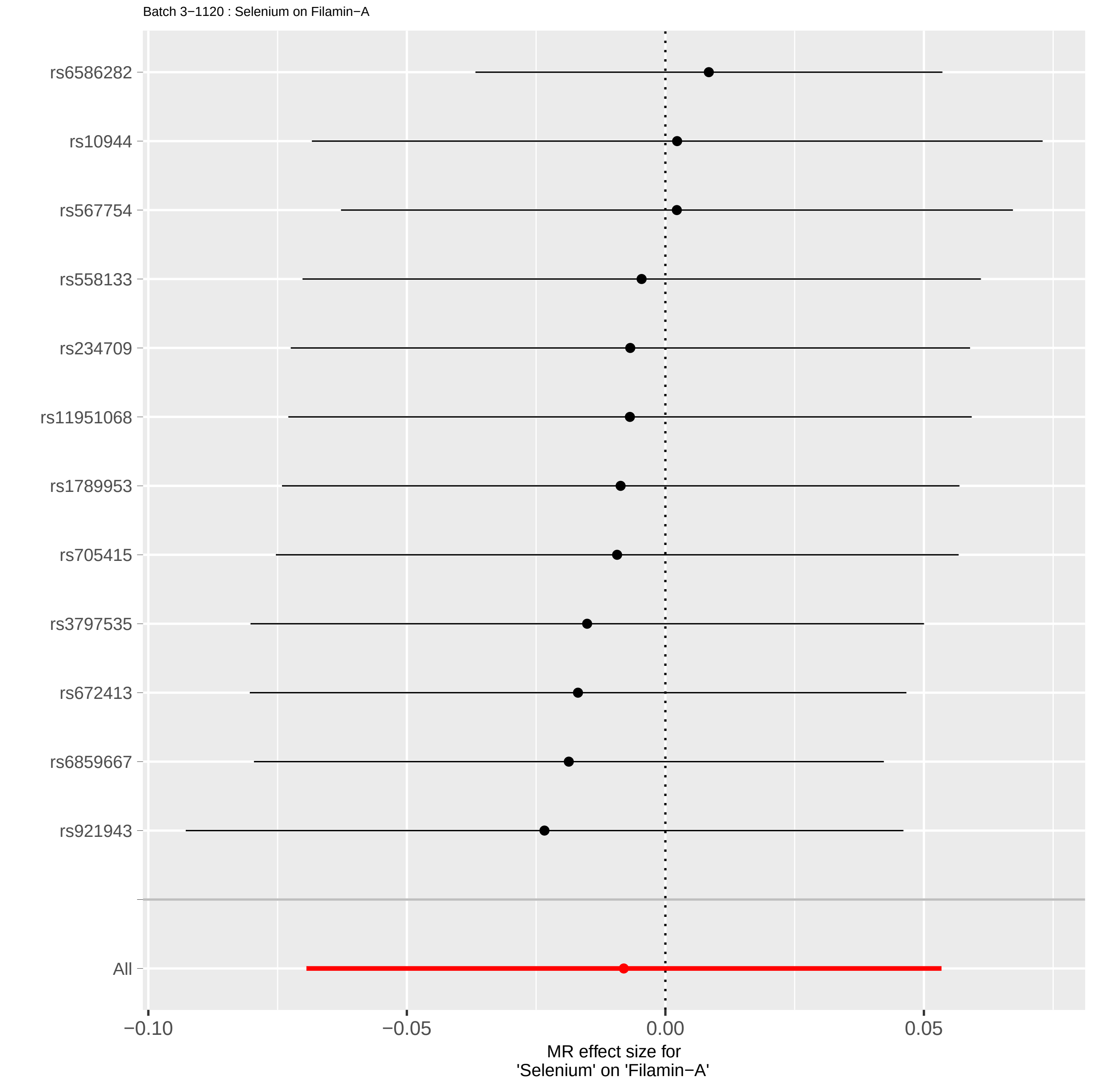
Batch 3−1120 : Selenium on Filamin−A
rs6586282
rs10944
rs567754
rs558133
rs234709
rs11951068
rs1789953
rs705415
rs3797535
rs672413
rs6859667
rs921943
All
−0.10
−0.05
0.00
0.05
MR effect size for
'Selenium' on 'Filamin−A'

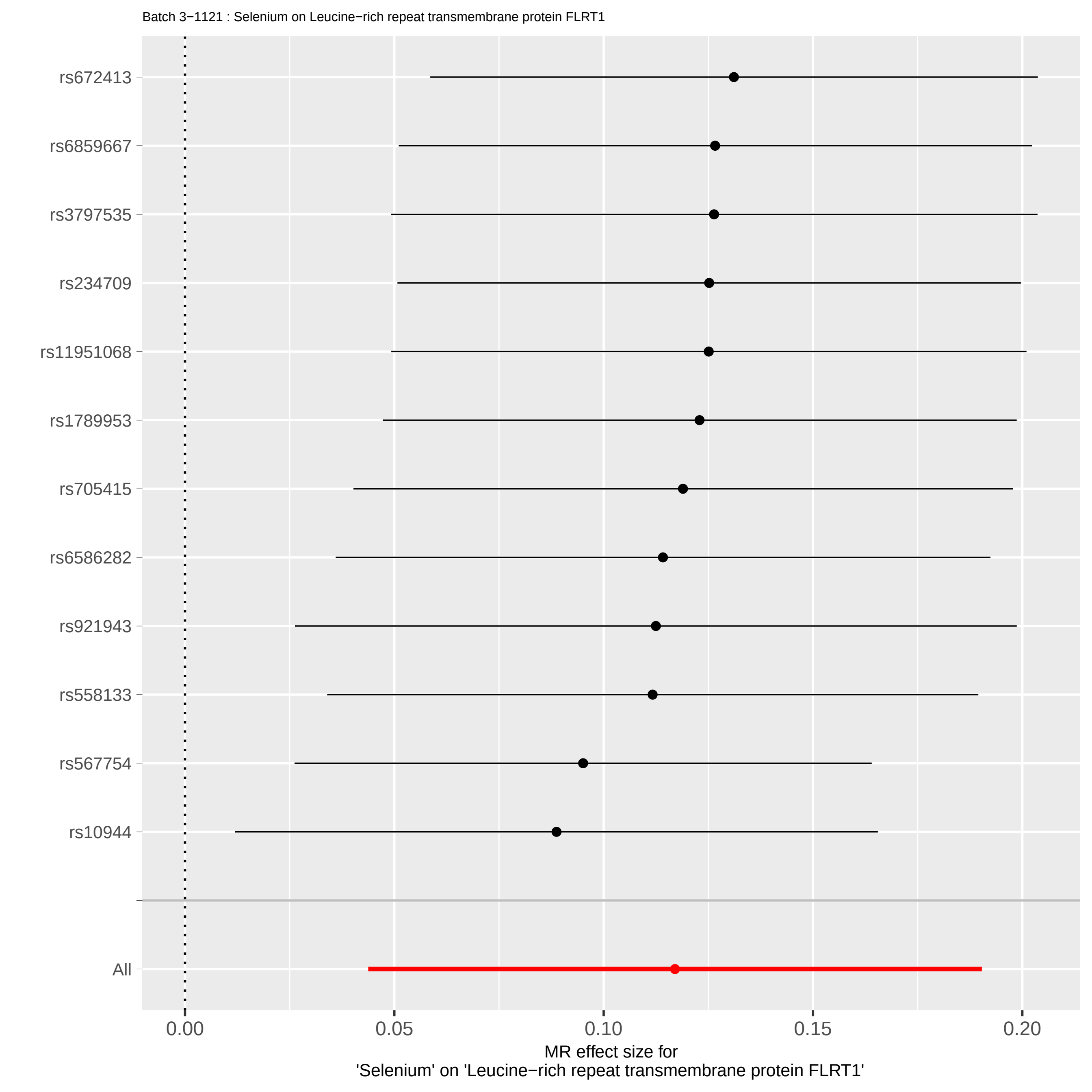
Batch 3−1121 : Selenium on Leucine−rich repeat transmembrane protein FLRT1
rs672413
rs6859667
rs3797535
rs234709
rs11951068
rs1789953
rs705415
rs6586282
rs921943
rs558133
rs567754
rs10944
All
0.00
0.05
0.10
0.15
0.20
MR effect size for
'Selenium' on 'Leucine−rich repeat transmembrane protein FLRT1'

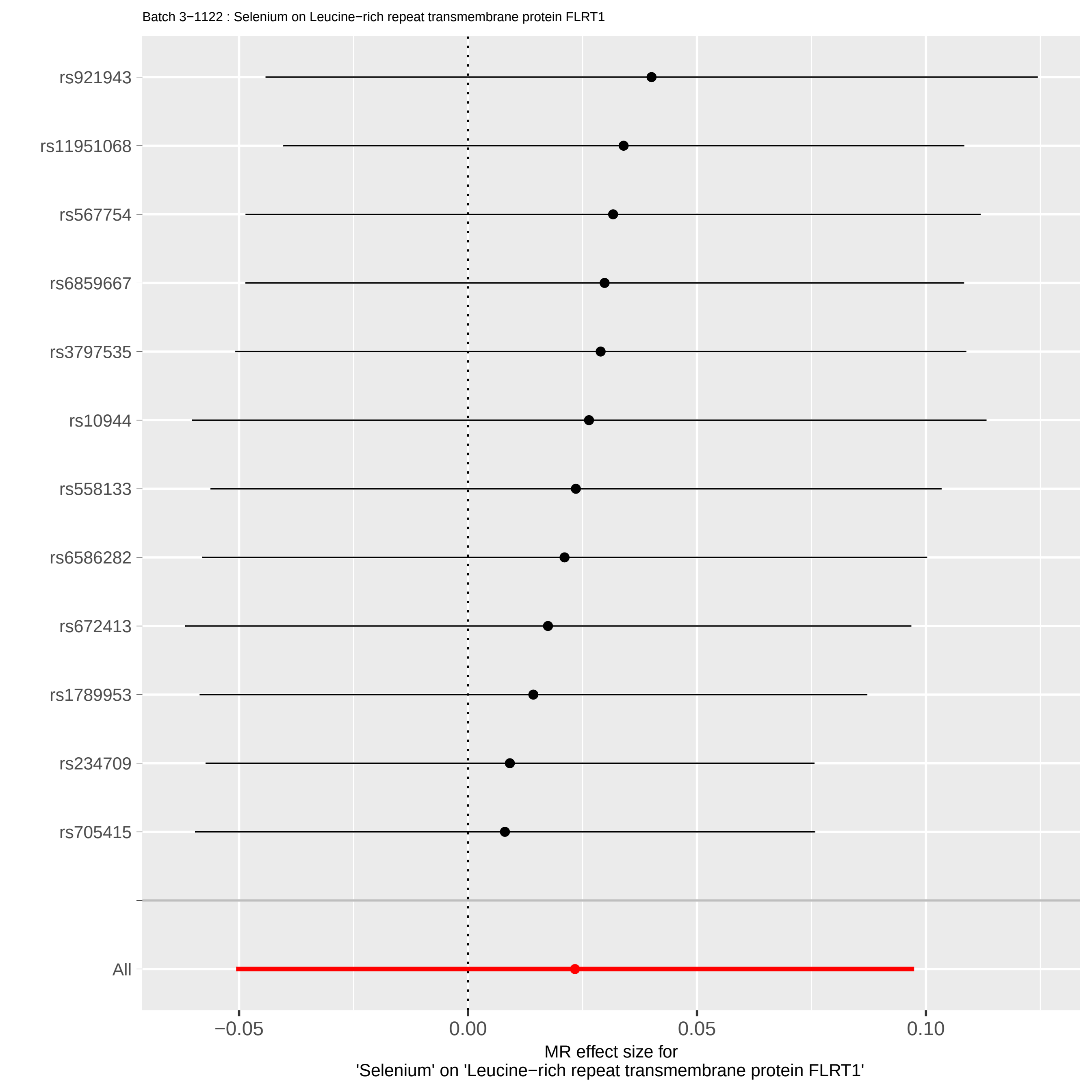
Batch 3−1122 : Selenium on Leucine−rich repeat transmembrane protein FLRT1
rs921943
rs11951068
rs567754
rs6859667
rs3797535
rs10944
rs558133
rs6586282
rs672413
rs1789953
rs234709
rs705415
All
−0.05
0.00
0.05
0.10
MR effect size for
'Selenium' on 'Leucine−rich repeat transmembrane protein FLRT1'

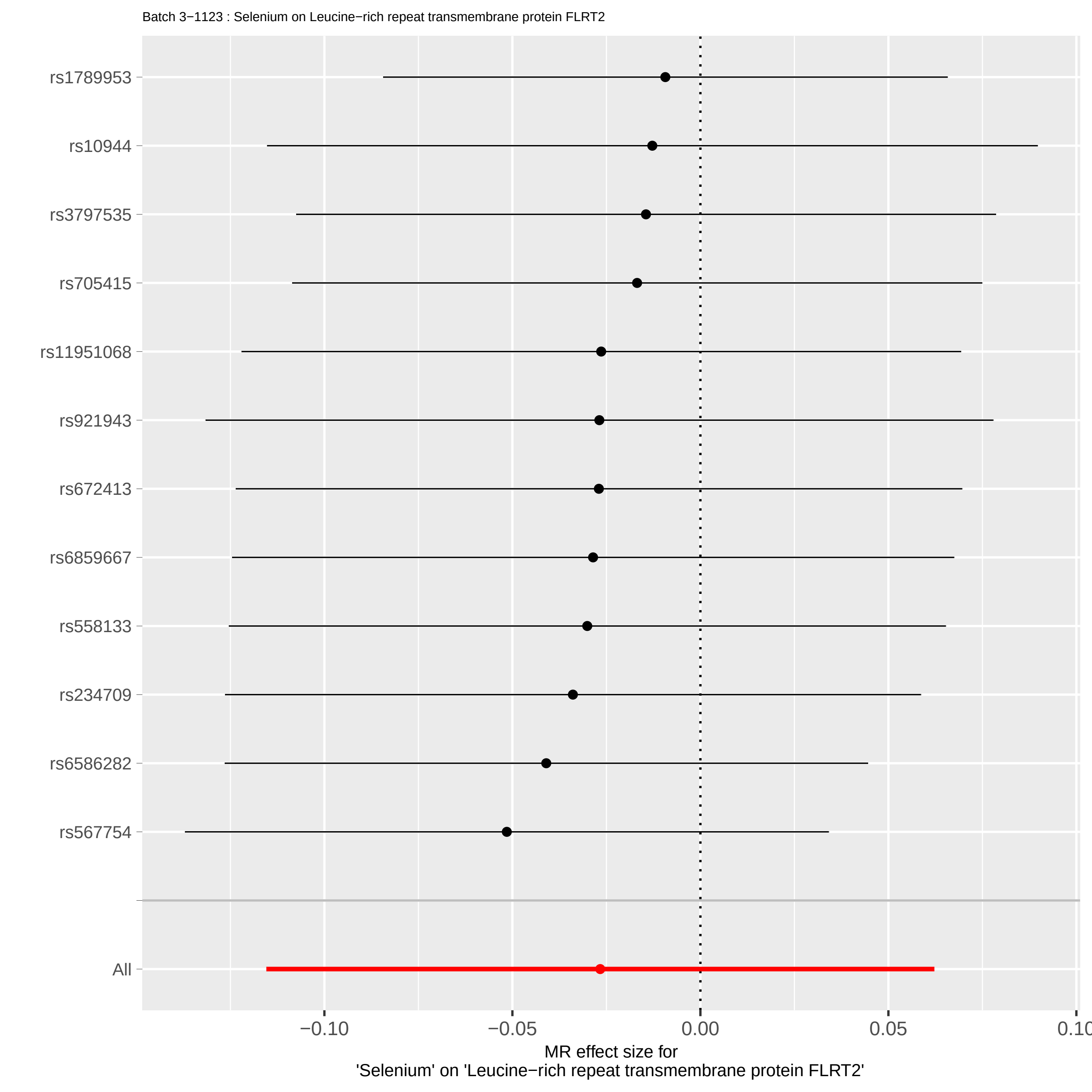
Batch 3−1123 : Selenium on Leucine−rich repeat transmembrane protein FLRT2
rs1789953
rs10944
rs3797535
rs705415
rs11951068
rs921943
rs672413
rs6859667
rs558133
rs234709
rs6586282
rs567754
All
−0.10
−0.05
0.00
0.05
0.10
MR effect size for
'Selenium' on 'Leucine−rich repeat transmembrane protein FLRT2'

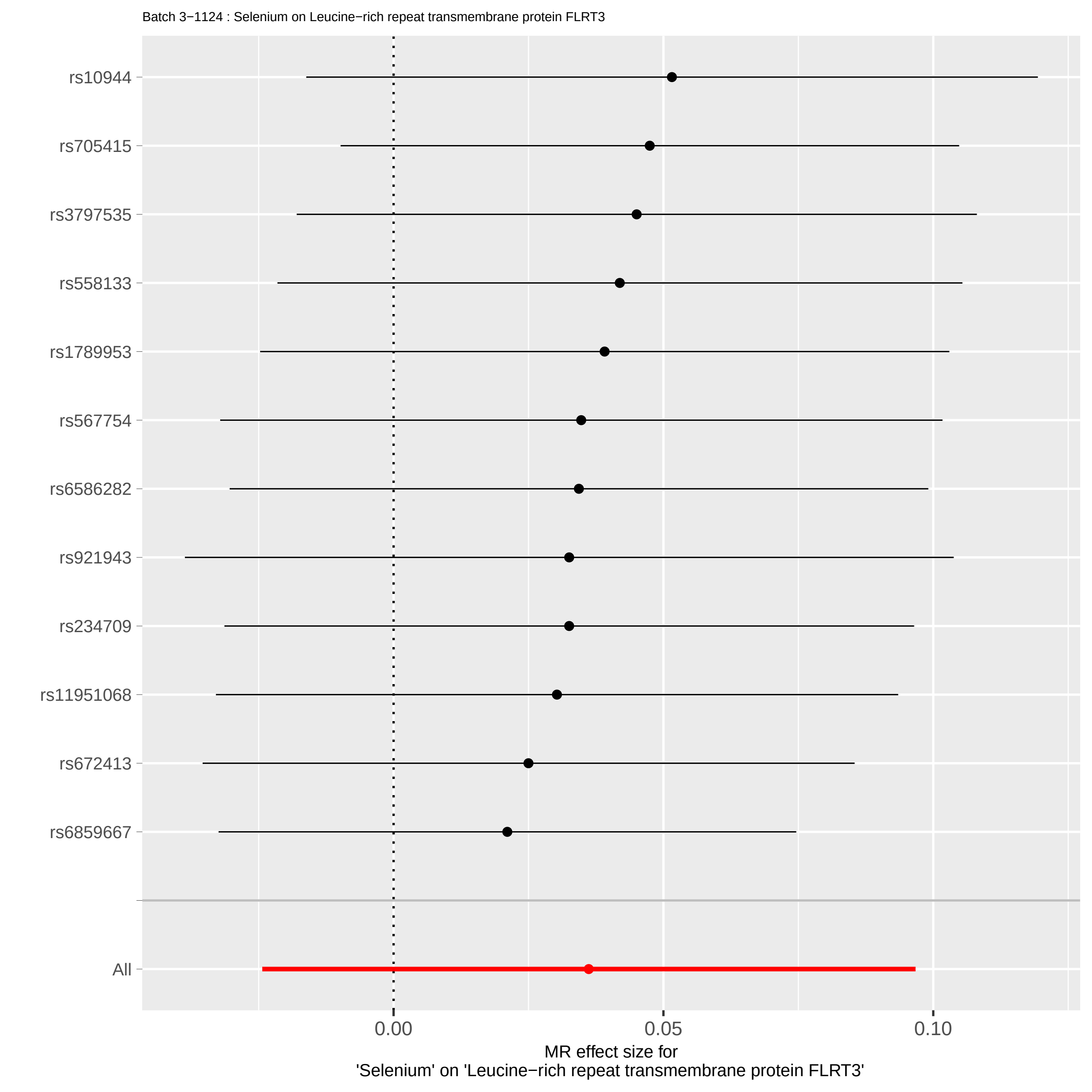

Batch 3−1124 : Selenium on Leucine−rich repeat transmembrane protein FLRT3
rs10944
rs705415
rs3797535
rs558133
rs1789953
rs567754
rs6586282
rs921943
rs234709
rs11951068
rs672413
rs6859667
All
0.00
0.05
0.10
MR effect size for
'Selenium' on 'Leucine−rich repeat transmembrane protein FLRT3'

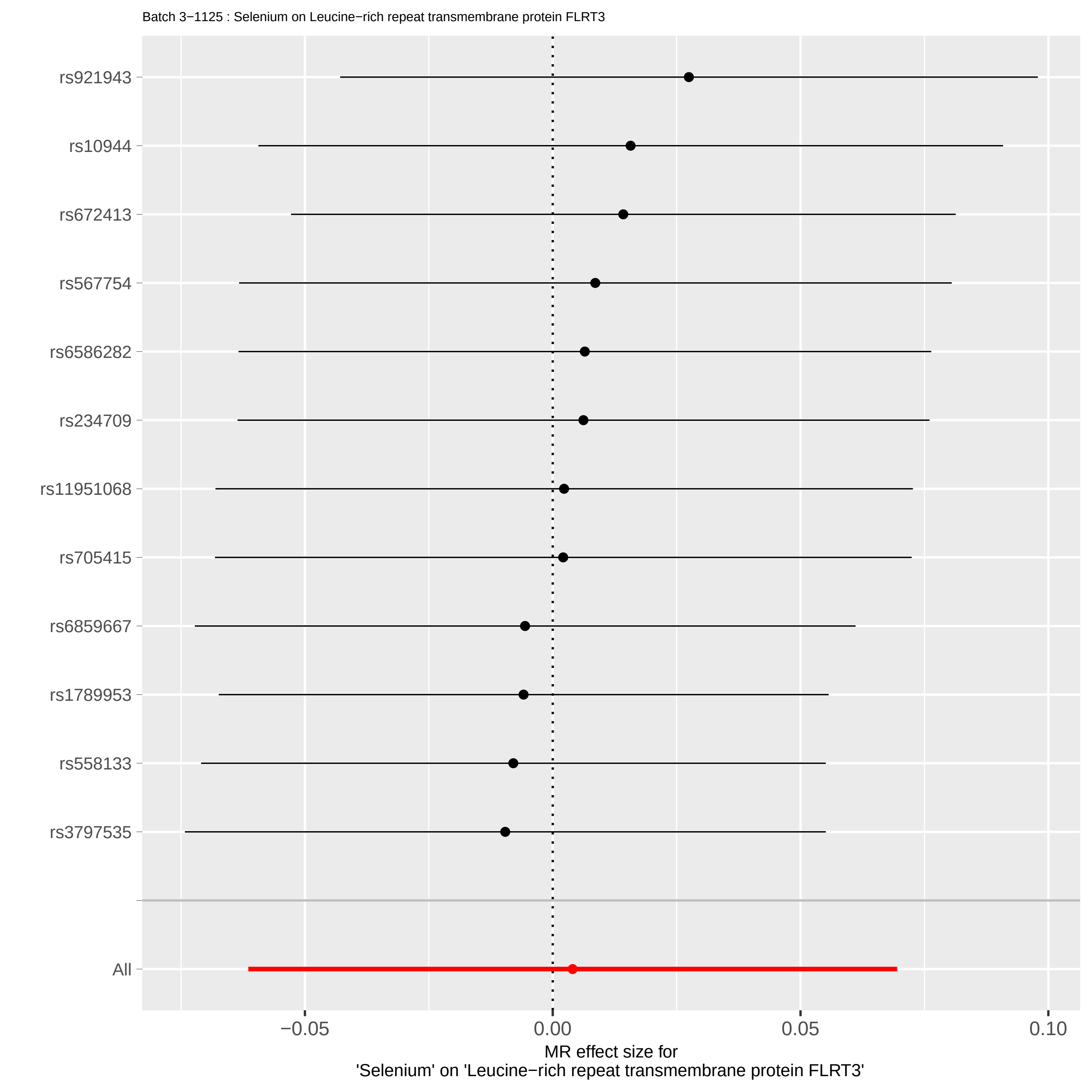
Batch 3−1125 : Selenium on Leucine−rich repeat transmembrane protein FLRT3
rs921943
rs10944
rs672413
rs567754
rs6586282
rs234709
rs11951068
rs705415
rs6859667
rs1789953
rs558133
rs3797535
All
−0.05
0.00
0.05
0.10
MR effect size for
'Selenium' on 'Leucine−rich repeat transmembrane protein FLRT3'

Batch 3−1126 : Selenium on Receptor−type tyrosine−protein kinase FLT3

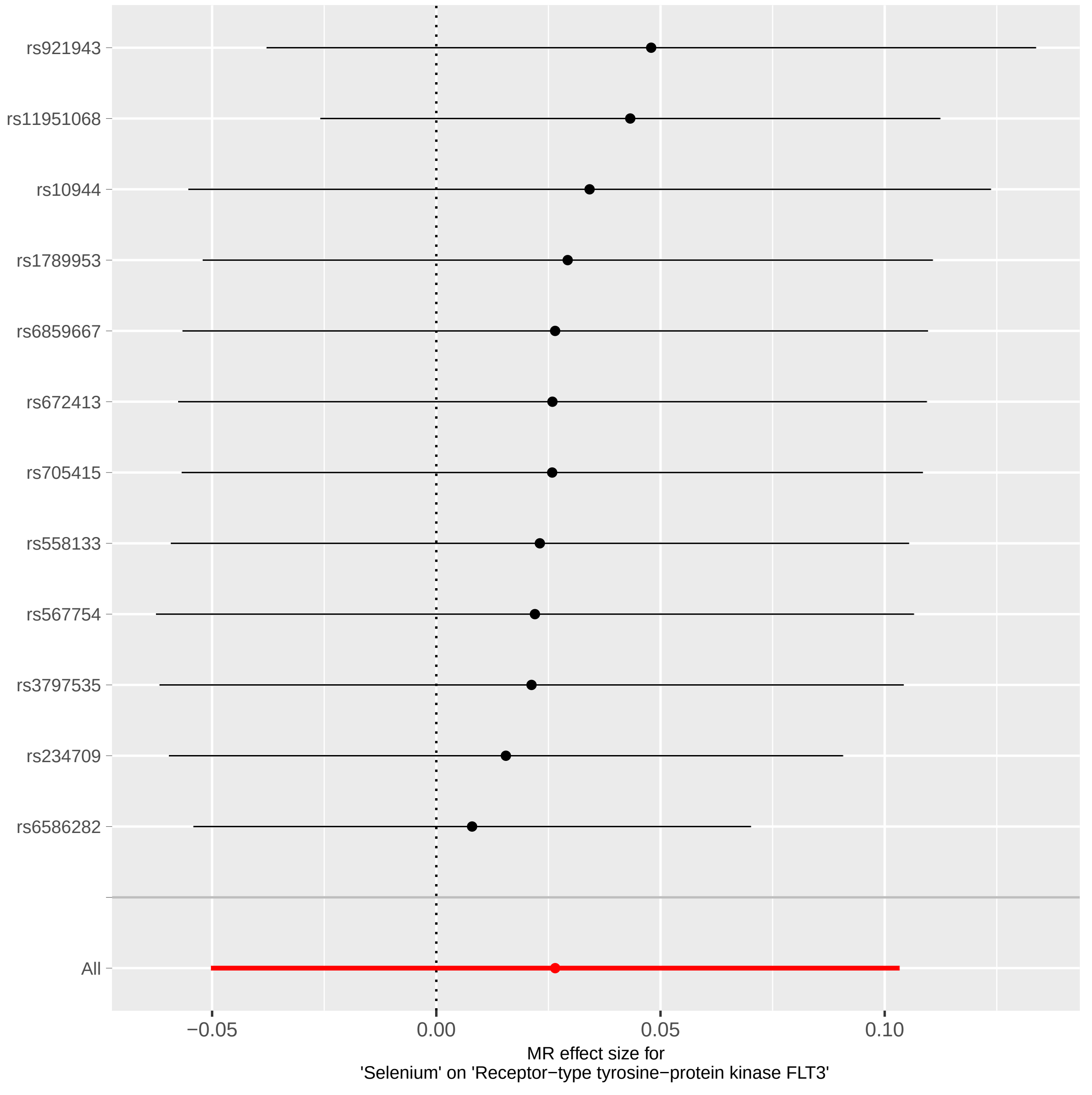

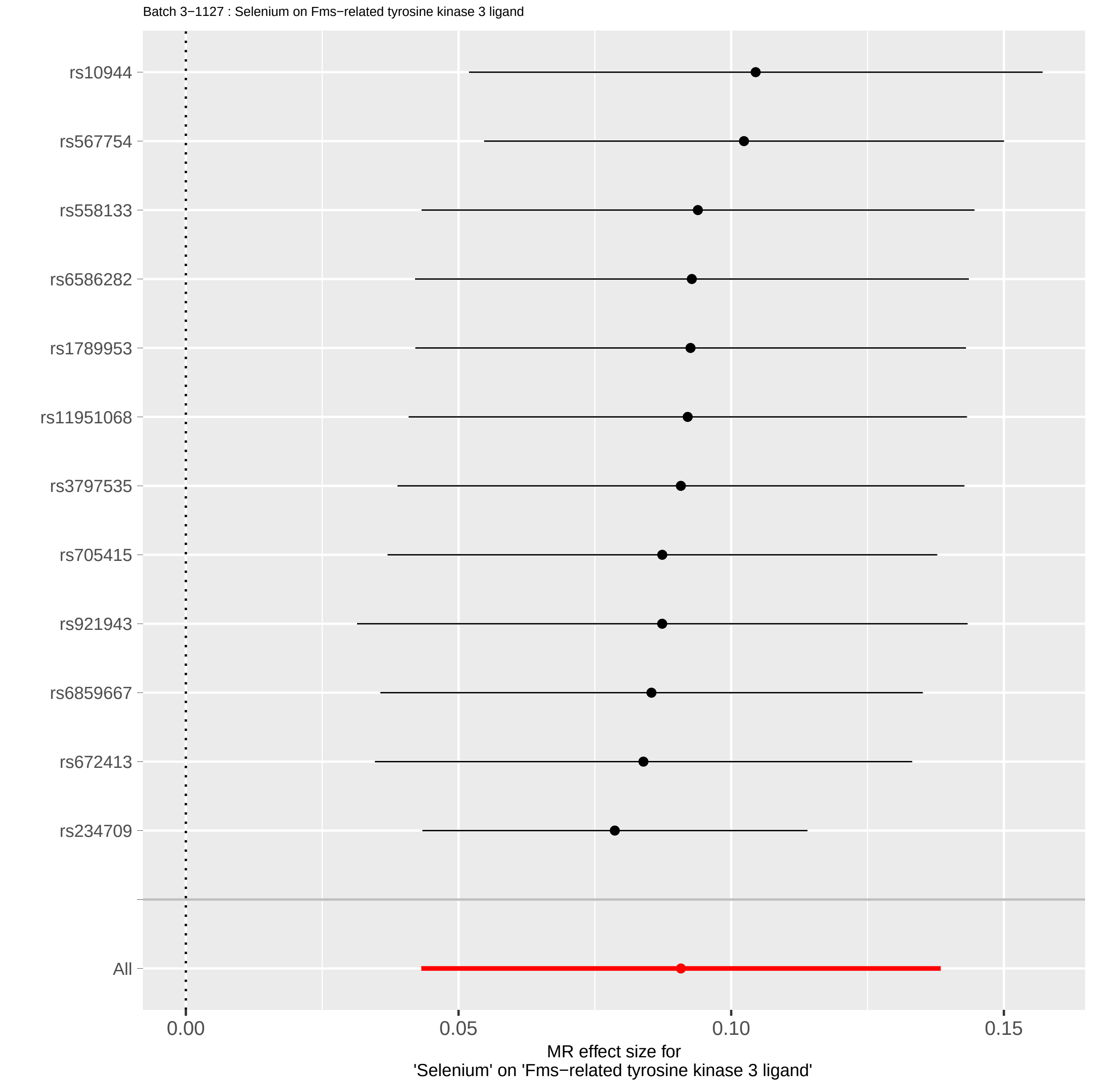

Batch 3−1127 : Selenium on Fms−related tyrosine kinase 3 ligand
rs10944
rs567754
rs558133
rs6586282
rs1789953
rs11951068
rs3797535
rs705415
rs921943
rs6859667
rs672413
rs234709
All
0.00
0.05
0.10
0.15
MR effect size for
'Selenium' on 'Fms−related tyrosine kinase 3 ligand'

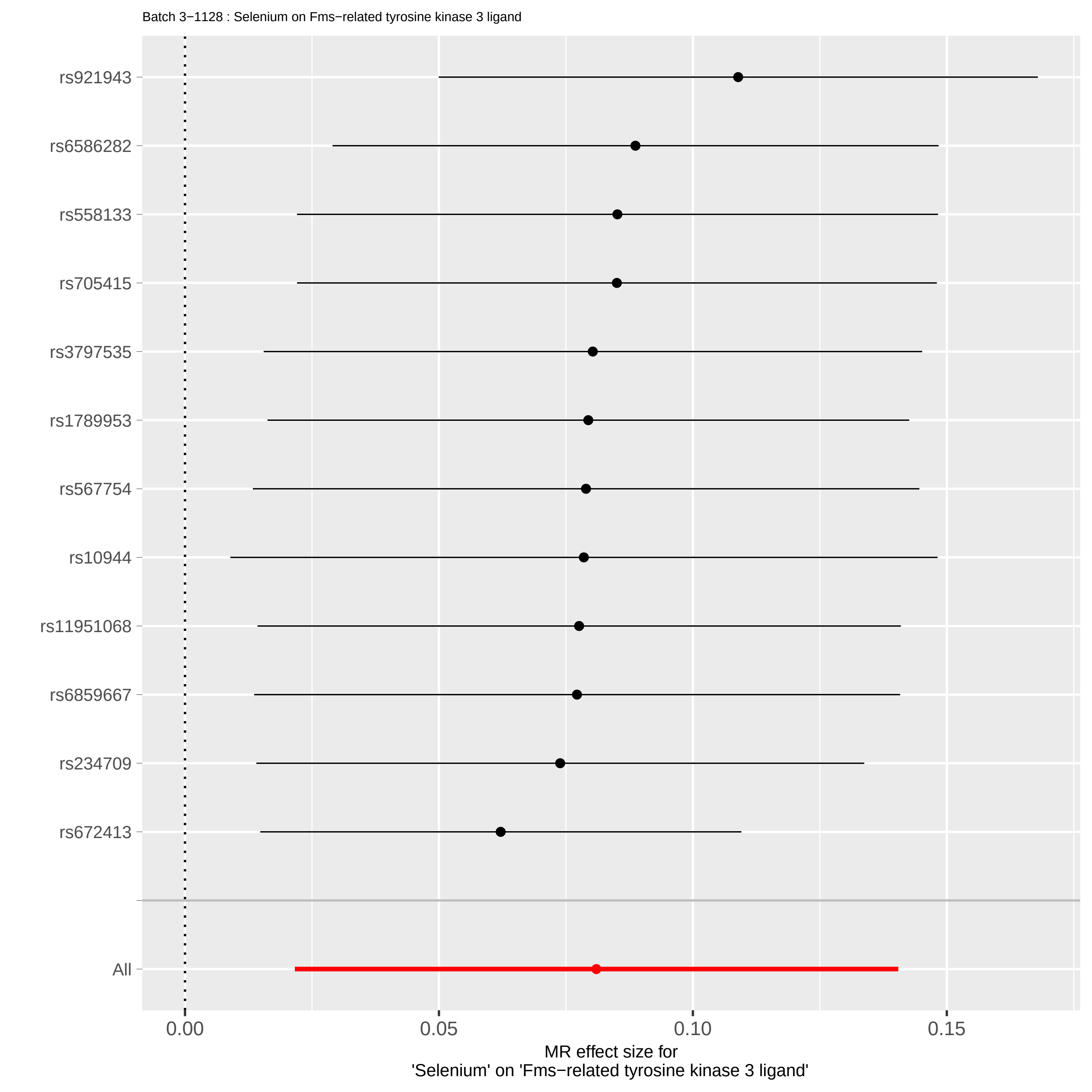

Batch 3−1128 : Selenium on Fms−related tyrosine kinase 3 ligand
rs921943
rs6586282
rs558133
rs705415
rs3797535
rs1789953
rs567754
rs10944
rs11951068
rs6859667
rs234709
rs672413
All
0.00
0.05
0.10
0.15
MR effect size for
'Selenium' on 'Fms−related tyrosine kinase 3 ligand'

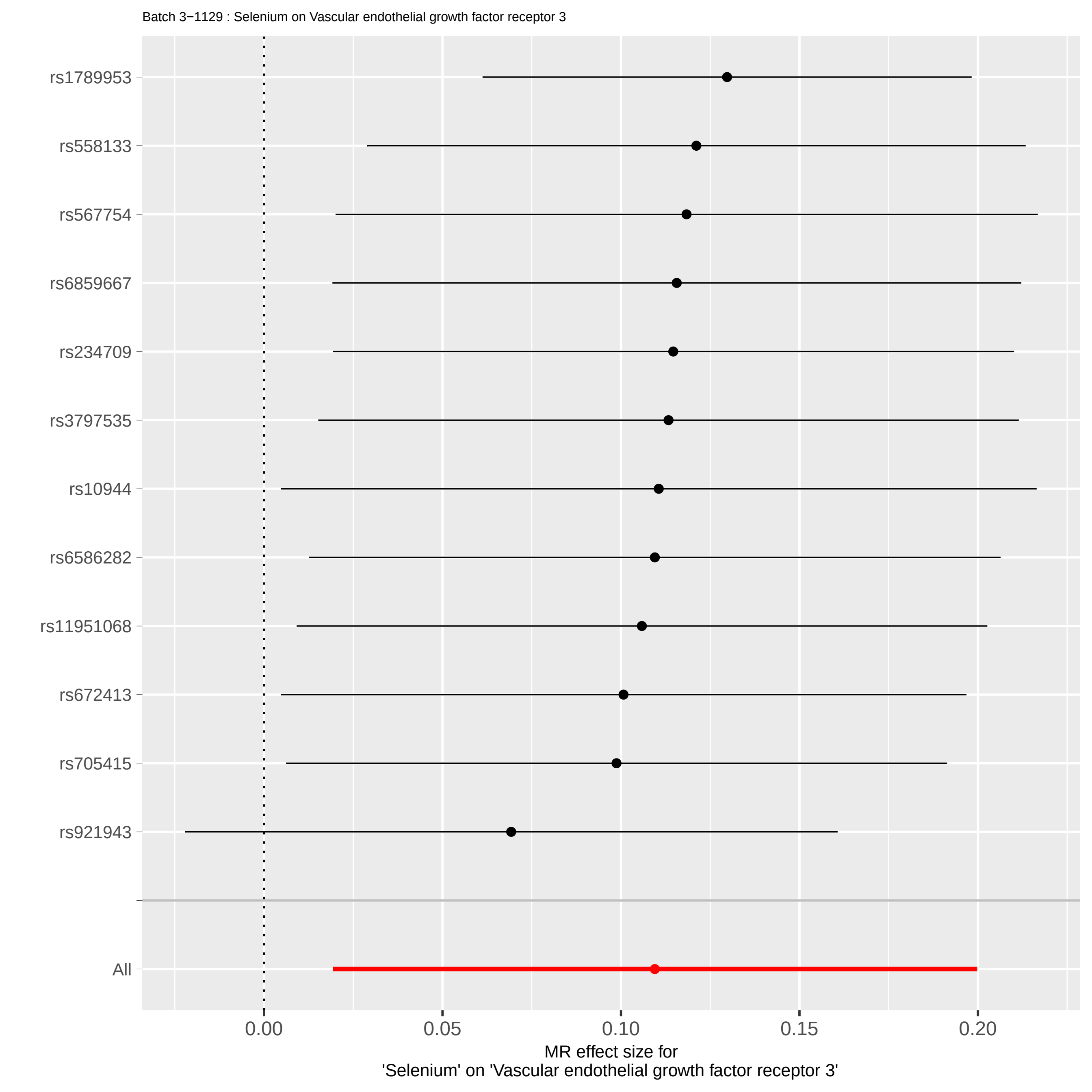

Batch 3−1129 : Selenium on Vascular endothelial growth factor receptor 3
rs1789953
rs558133
rs567754
rs6859667
rs234709
rs3797535
rs10944
rs6586282
rs11951068
rs672413
rs705415
rs921943
All
0.00
0.05
0.10
0.15
0.20
MR effect size for
'Selenium' on 'Vascular endothelial growth factor receptor 3'

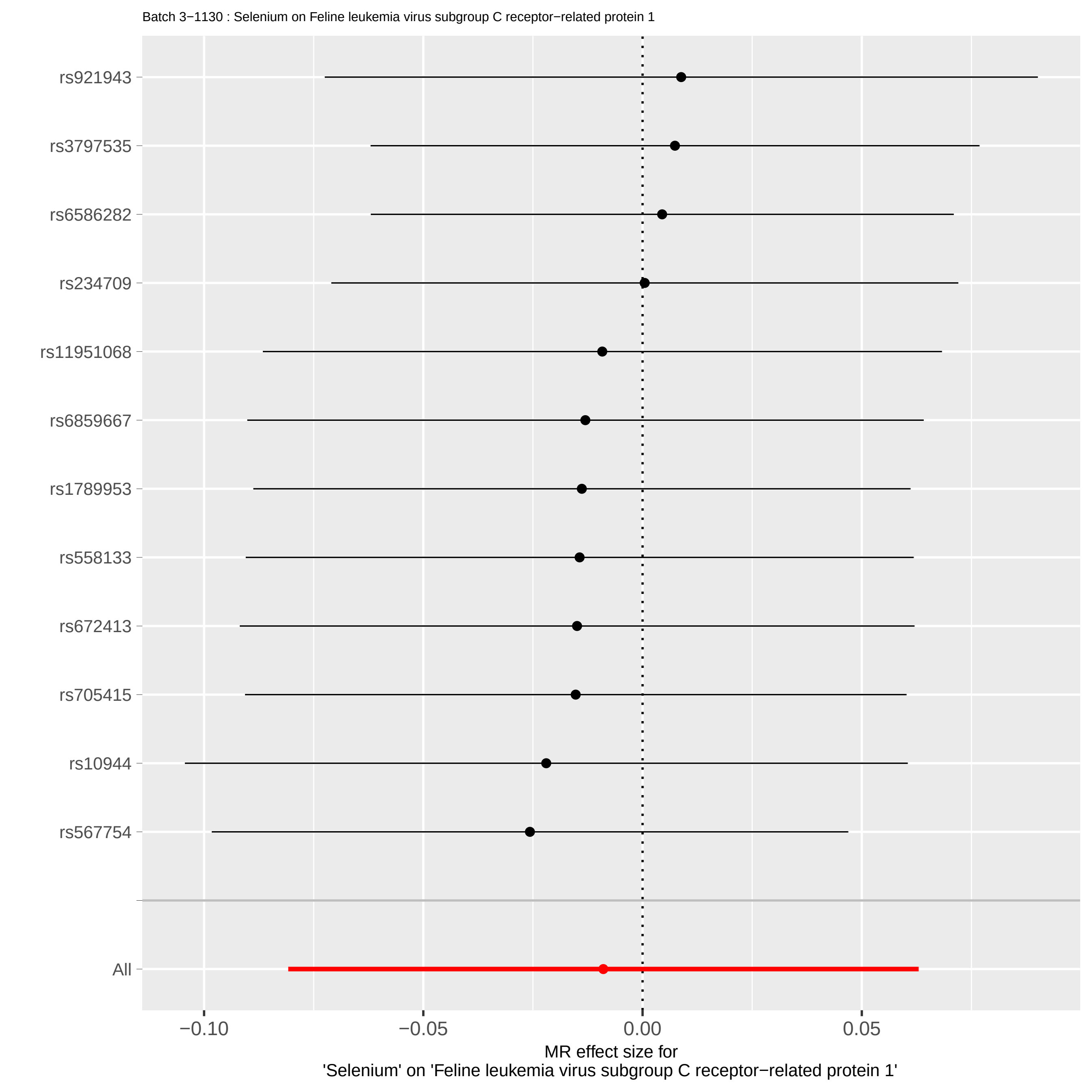

Batch 3−1130 : Selenium on Feline leukemia virus subgroup C receptor−related protein 1
rs921943
rs3797535
rs6586282
rs234709
rs11951068
rs6859667
rs1789953
rs558133
rs672413
rs705415
rs10944
rs567754
All
−0.10
−0.05
0.00
0.05
MR effect size for
'Selenium' on 'Feline leukemia virus subgroup C receptor−related protein 1'

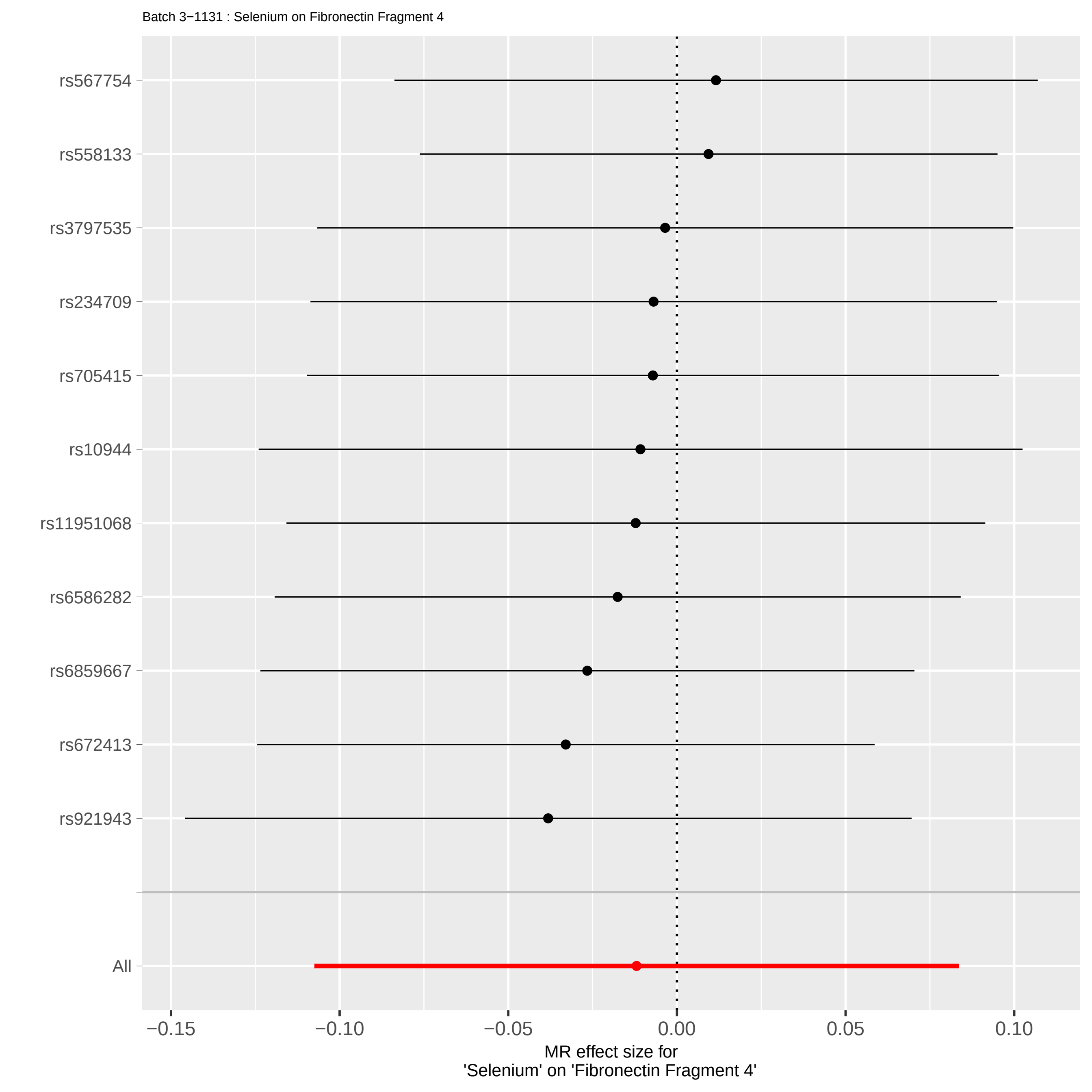

Batch 3−1131 : Selenium on Fibronectin Fragment 4
rs567754
rs558133
rs3797535
rs234709
rs705415
rs10944
rs11951068
rs6586282
rs6859667
rs672413
rs921943
All
−0.15
−0.10
−0.05
0.00
0.05
0.10
MR effect size for
'Selenium' on 'Fibronectin Fragment 4'

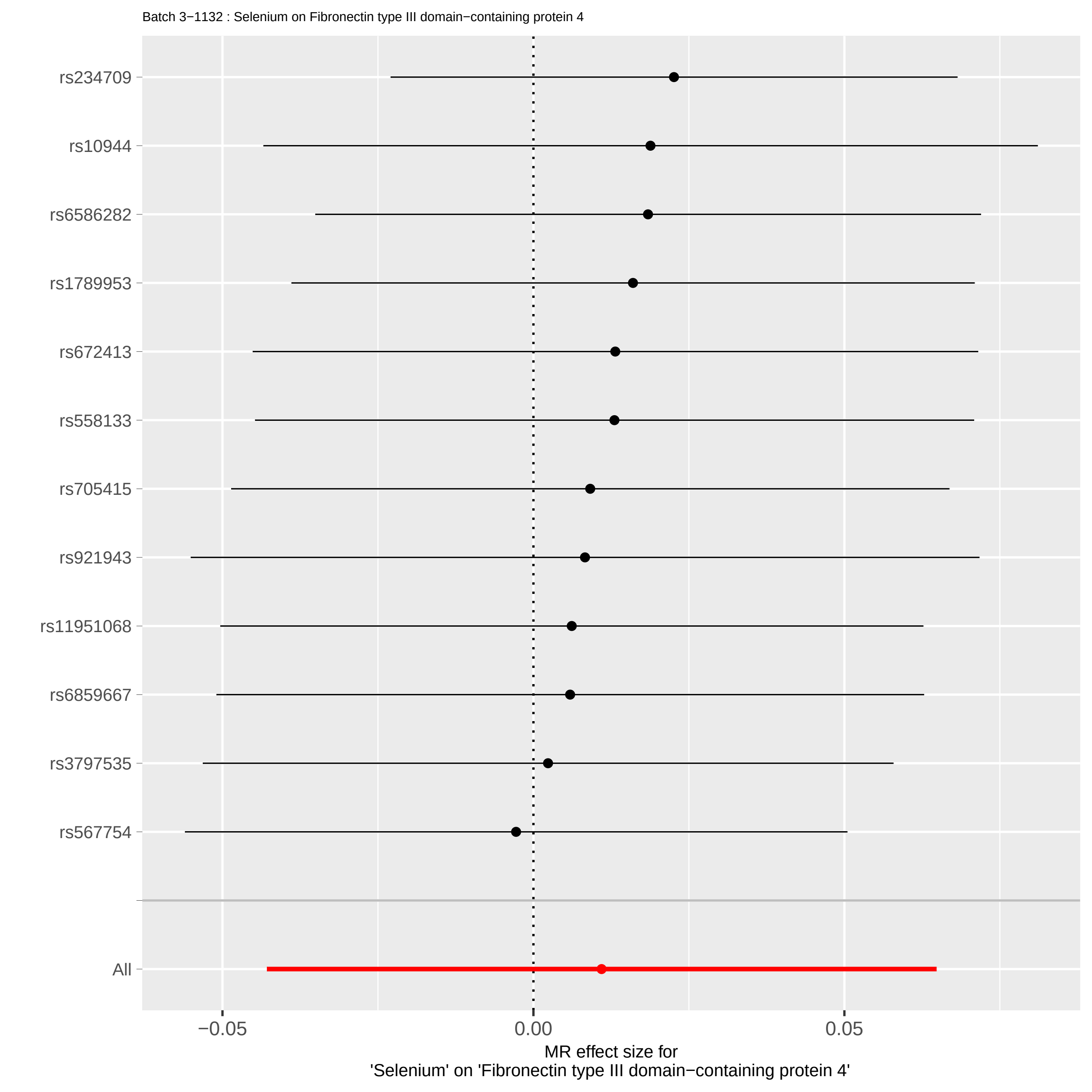

Batch 3−1132 : Selenium on Fibronectin type III domain−containing protein 4
rs234709
rs10944
rs6586282
rs1789953
rs672413
rs558133
rs705415
rs921943
rs11951068
rs6859667
rs3797535
rs567754
All
−0.05
0.00
0.05
MR effect size for
'Selenium' on 'Fibronectin type III domain−containing protein 4'

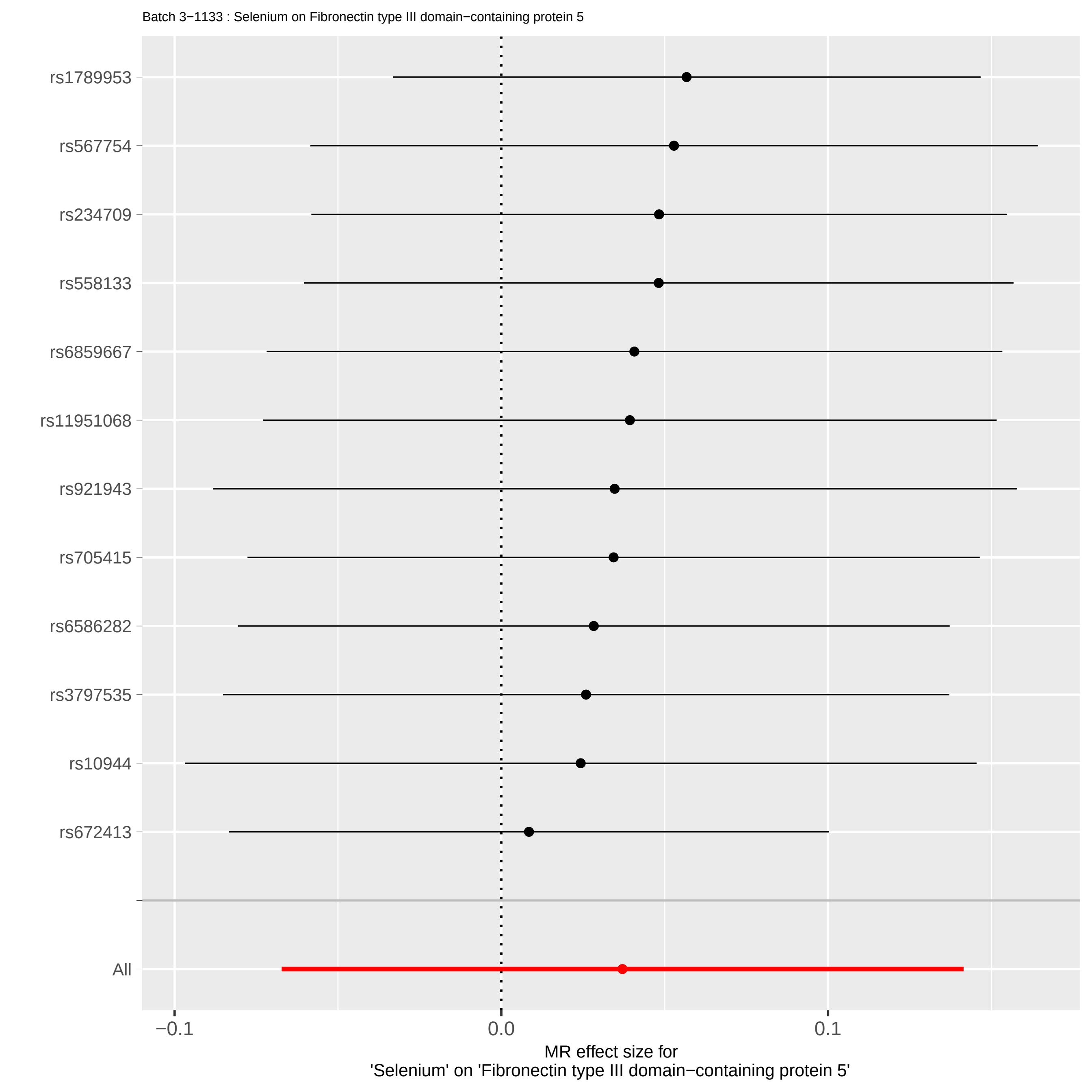

Batch 3−1133 : Selenium on Fibronectin type III domain−containing protein 5
rs1789953
rs567754
rs234709
rs558133
rs6859667
rs11951068
rs921943
rs705415
rs6586282
rs3797535
rs10944
rs672413
All
−0.1
0.0
0.1
MR effect size for
'Selenium' on 'Fibronectin type III domain−containing protein 5'

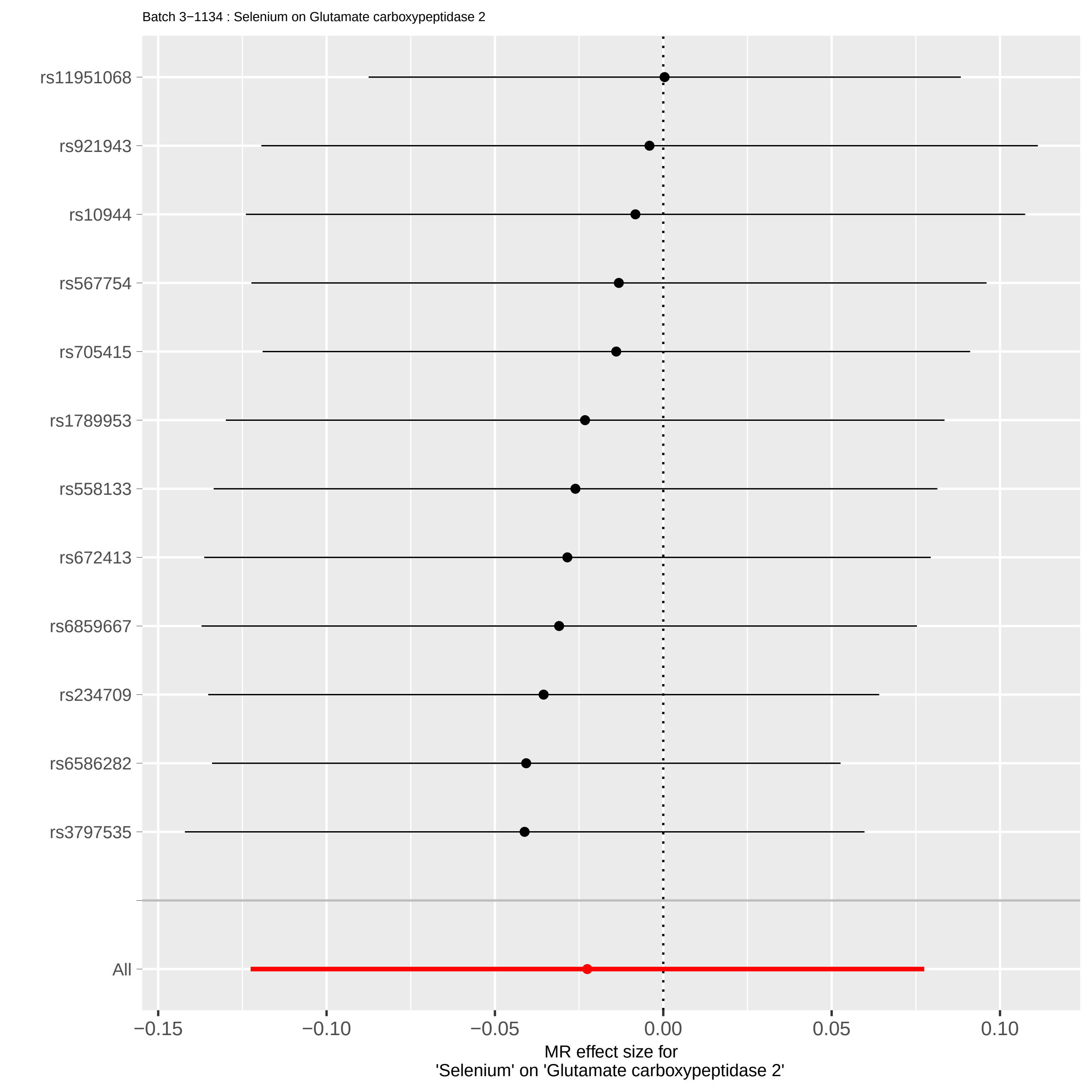
Batch 3−1134 : Selenium on Glutamate carboxypeptidase 2
rs11951068
rs921943
rs10944
rs567754
rs705415
rs1789953
rs558133
rs672413
rs6859667
rs234709
rs6586282
rs3797535
All
−0.15
−0.10
−0.05
0.00
0.05
0.10
MR effect size for
'Selenium' on 'Glutamate carboxypeptidase 2'

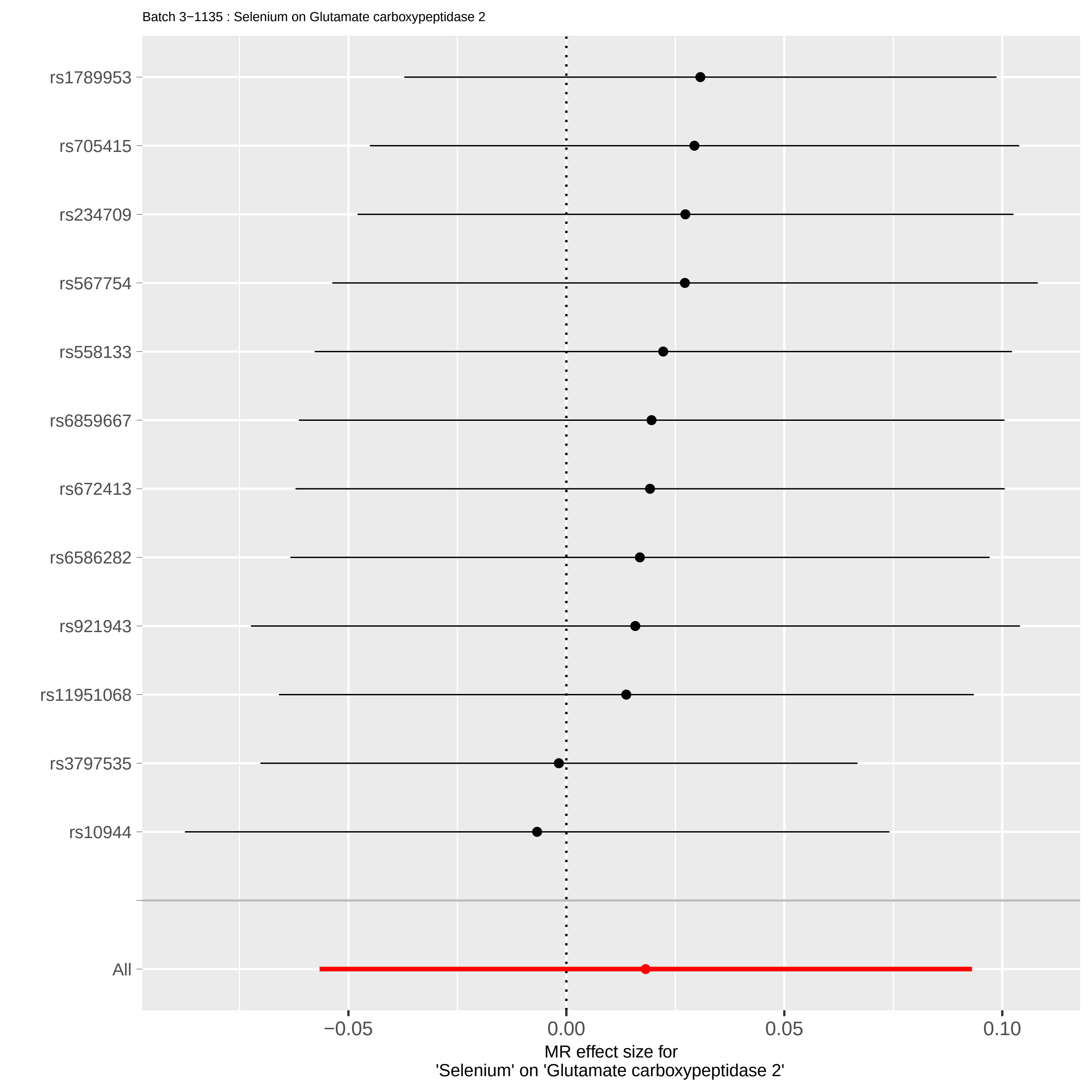

Batch 3−1135 : Selenium on Glutamate carboxypeptidase 2
rs1789953
rs705415
rs234709
rs567754
rs558133
rs6859667
rs672413
rs6586282
rs921943
rs11951068
rs3797535
rs10944
All
−0.05
0.00
0.05
0.10
MR effect size for
'Selenium' on 'Glutamate carboxypeptidase 2'

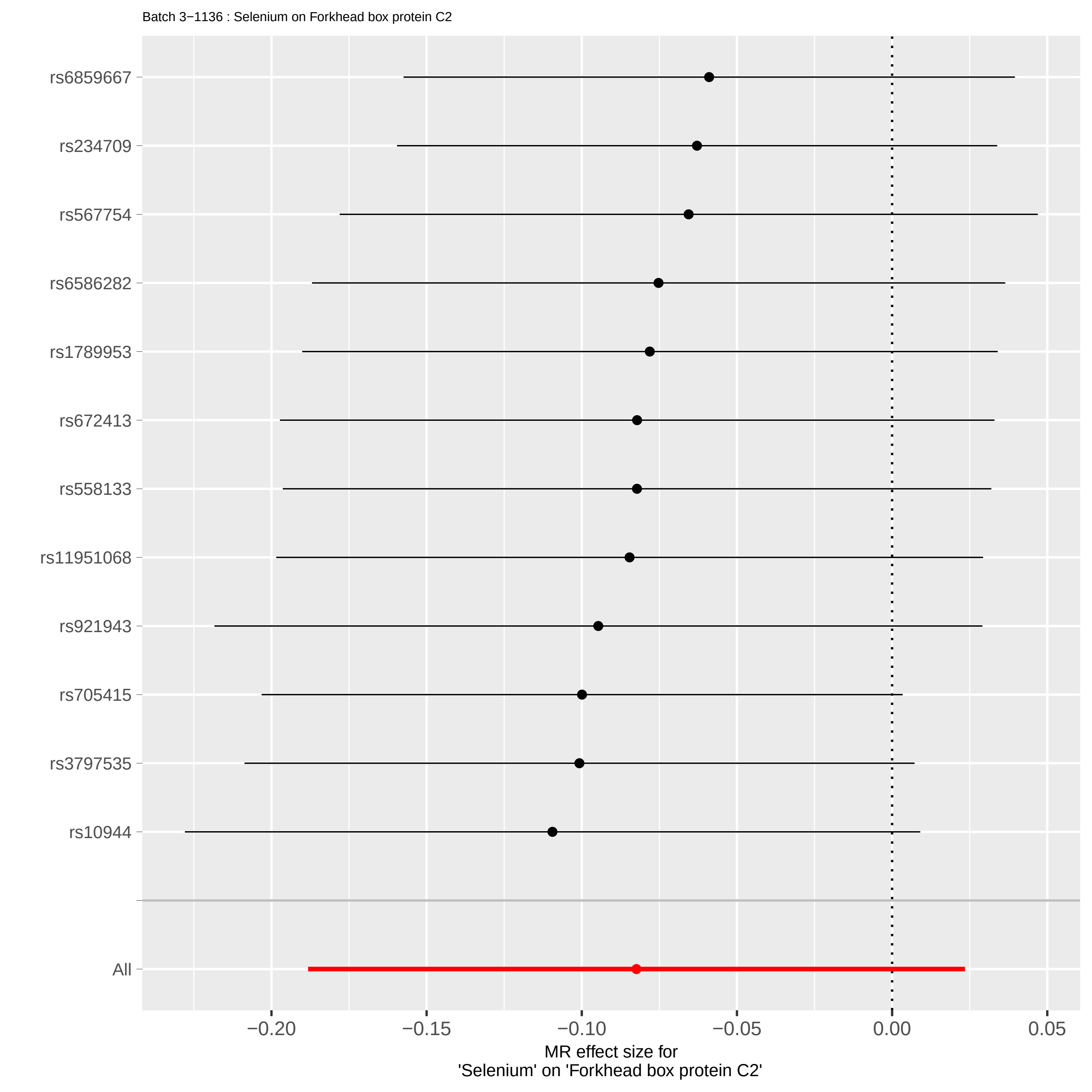

Batch 3−1136 : Selenium on Forkhead box protein C2
rs6859667
rs234709
rs567754
rs6586282
rs1789953
rs672413
rs558133
rs11951068
rs921943
rs705415
rs3797535
rs10944
All
−0.20
−0.15
−0.10
−0.05
0.00
0.05
MR effect size for
'Selenium' on 'Forkhead box protein C2'

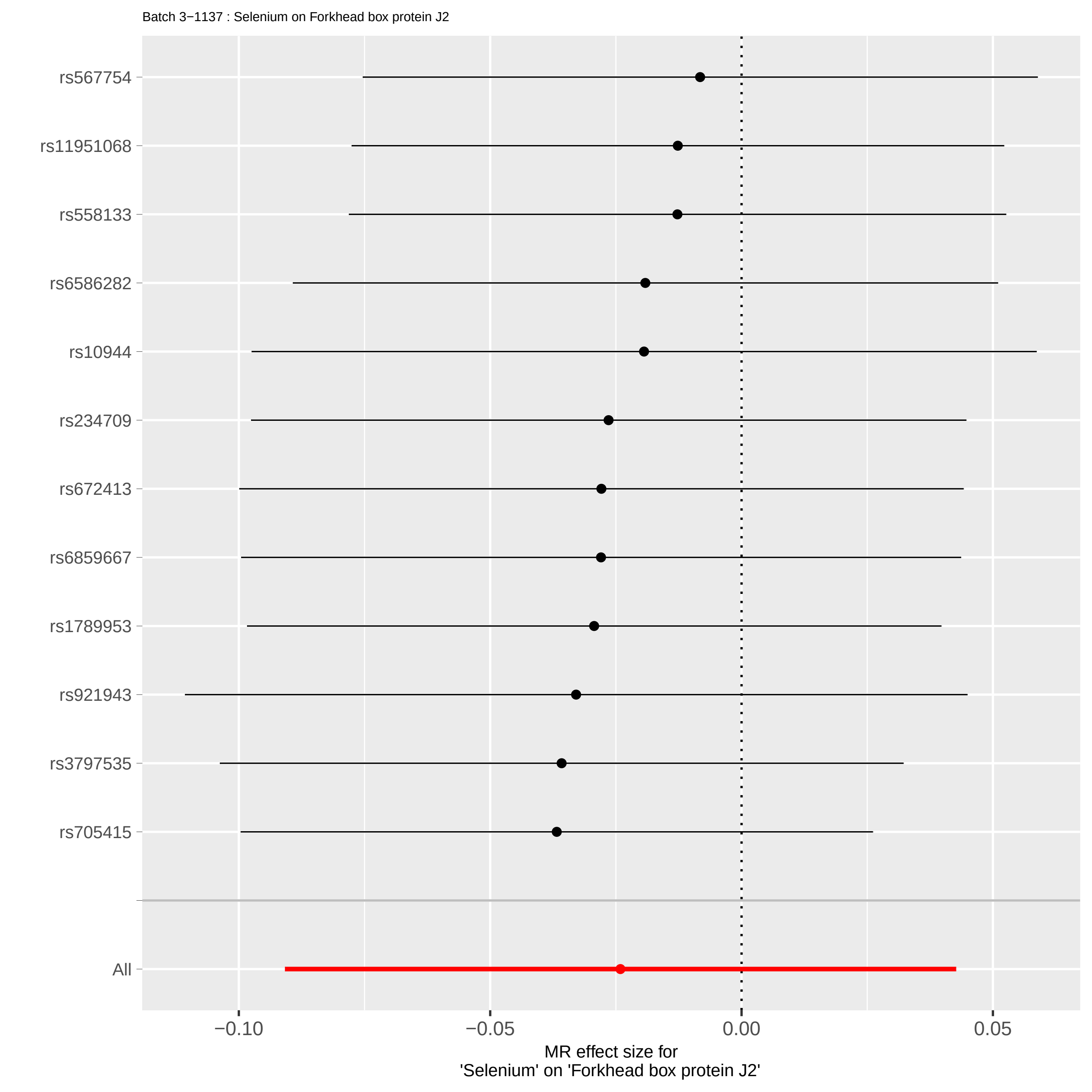
Batch 3−1137 : Selenium on Forkhead box protein J2
rs567754
rs11951068
rs558133
rs6586282
rs10944
rs234709
rs672413
rs6859667
rs1789953
rs921943
rs3797535
rs705415
All
−0.10
−0.05
0.00
0.05
MR effect size for
'Selenium' on 'Forkhead box protein J2'

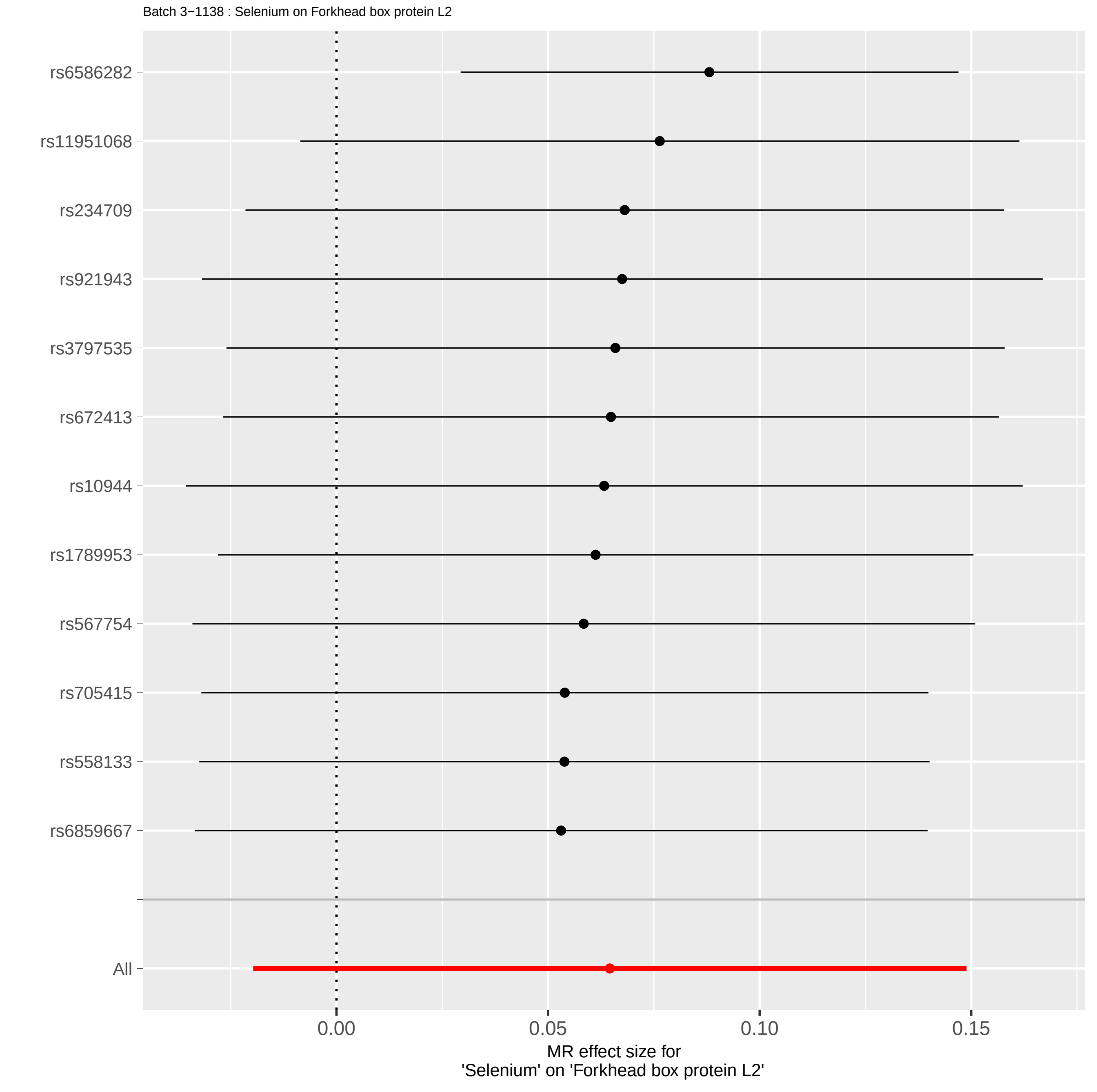

Batch 3−1138 : Selenium on Forkhead box protein L2
rs6586282
rs11951068
rs234709
rs921943
rs3797535
rs672413
rs10944
rs1789953
rs567754
rs705415
rs558133
rs6859667
All
0.00
0.05
0.10
0.15
MR effect size for
'Selenium' on 'Forkhead box protein L2'

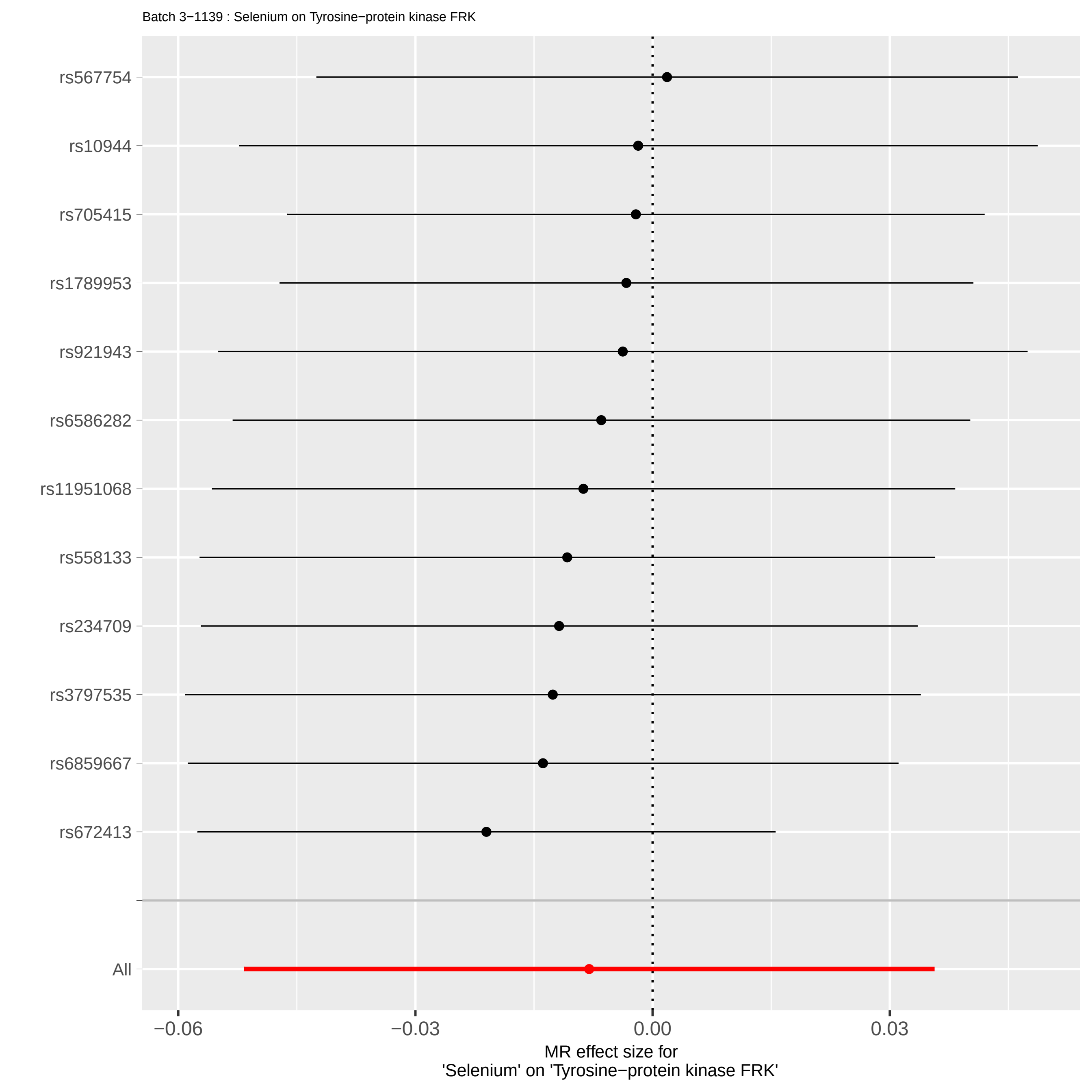
Batch 3−1139 : Selenium on Tyrosine−protein kinase FRK
rs567754
rs10944
rs705415
rs1789953
rs921943
rs6586282
rs11951068
rs558133
rs234709
rs3797535
rs6859667
rs672413
All
−0.06
−0.03
0.00
0.03
MR effect size for
'Selenium' on 'Tyrosine−protein kinase FRK'

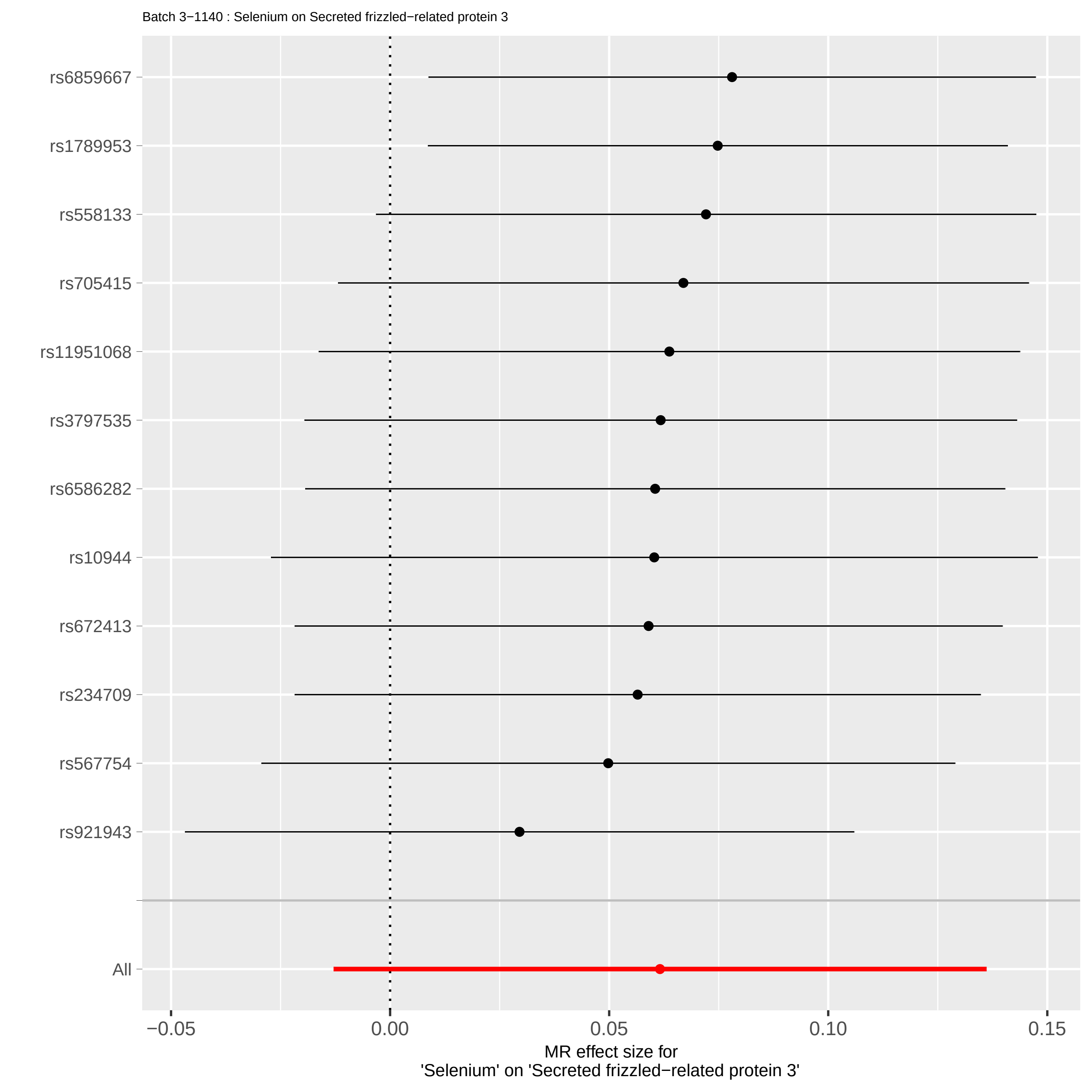

Batch 3−1140 : Selenium on Secreted frizzled−related protein 3
rs6859667
rs1789953
rs558133
rs705415
rs11951068
rs3797535
rs6586282
rs10944
rs672413
rs234709
rs567754
rs921943
All
−0.05
0.00
0.05
0.10
0.15
MR effect size for
'Selenium' on 'Secreted frizzled−related protein 3'

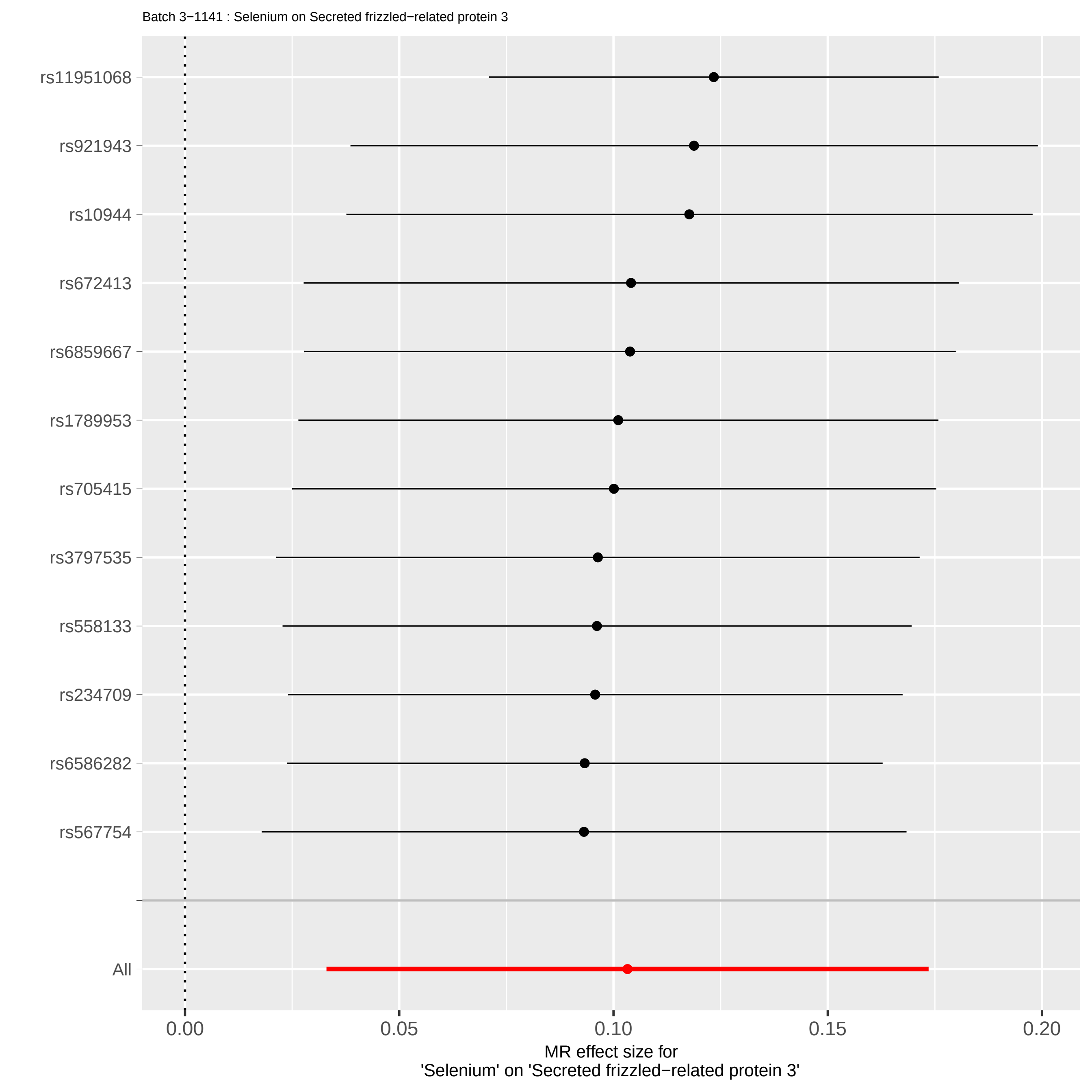

Batch 3−1141 : Selenium on Secreted frizzled−related protein 3
rs11951068
rs921943
rs10944
rs672413
rs6859667
rs1789953
rs705415
rs3797535
rs558133
rs234709
rs6586282
rs567754
All
0.00
0.05
0.10
0.15
0.20
MR effect size for
'Selenium' on 'Secreted frizzled−related protein 3'

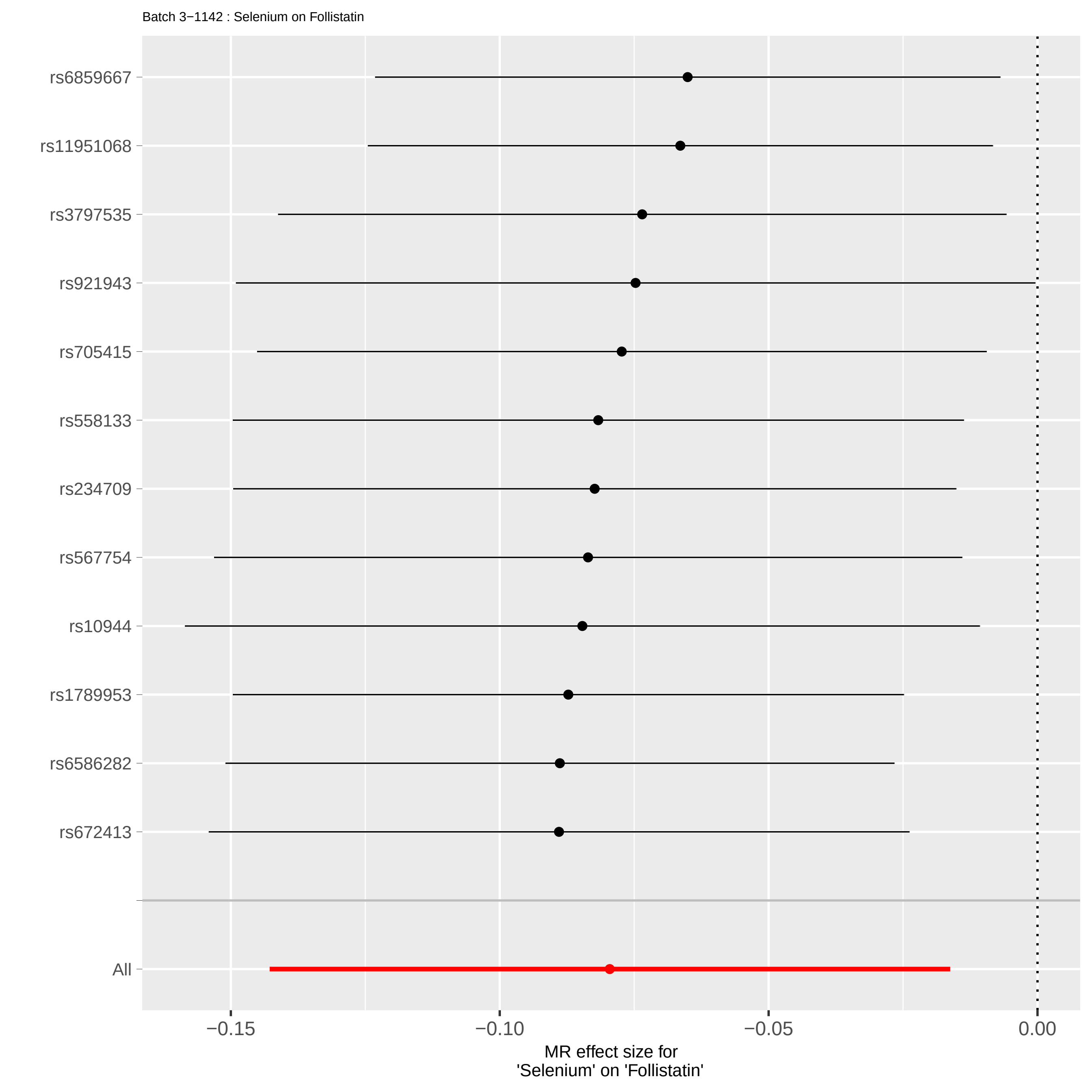
Batch 3−1142 : Selenium on Follistatin
rs6859667
rs11951068
rs3797535
rs921943
rs705415
rs558133
rs234709
rs567754
rs10944
rs1789953
rs6586282
rs672413
All
−0.15
−0.10
−0.05
0.00
MR effect size for
'Selenium' on 'Follistatin'

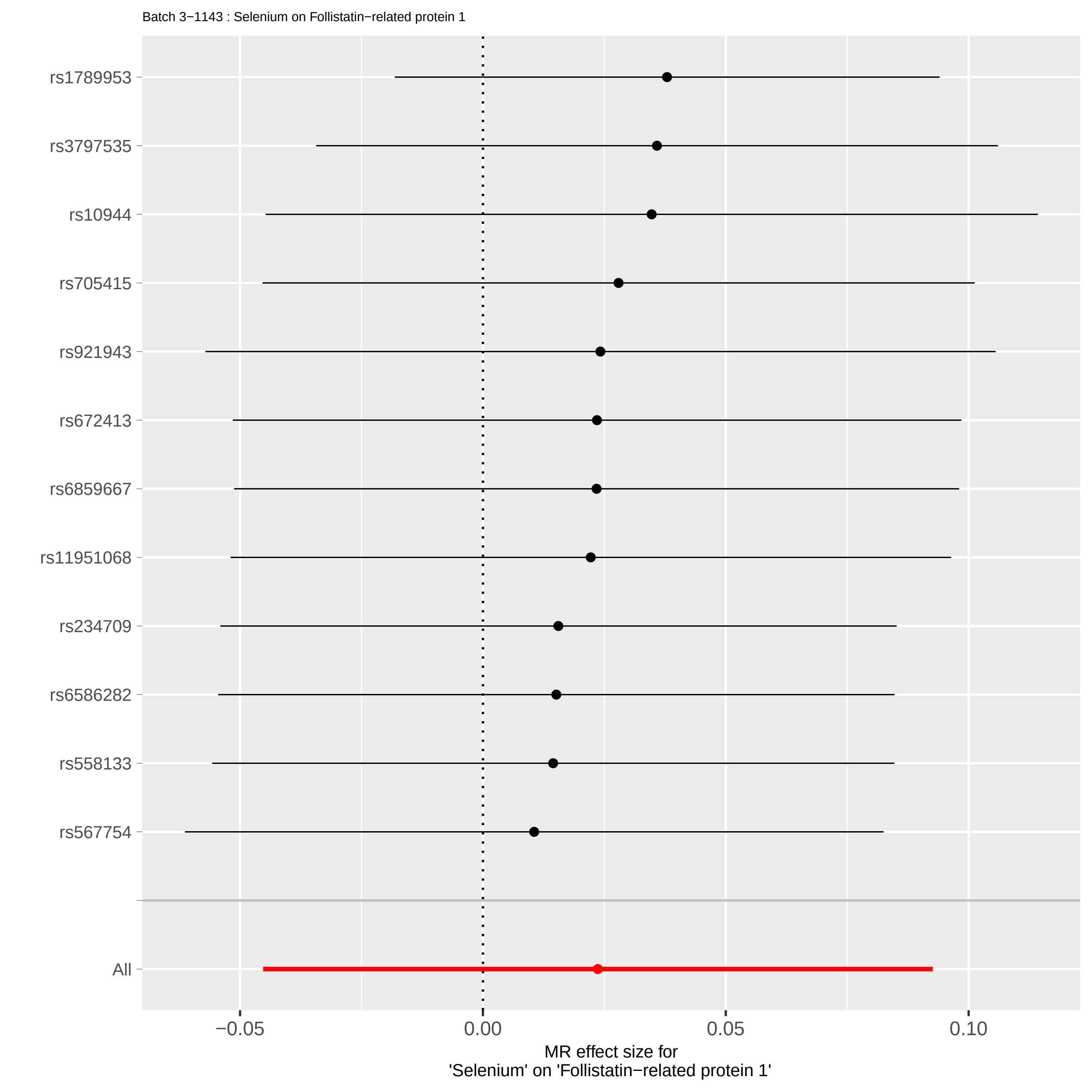
Batch 3−1143 : Selenium on Follistatin−related protein 1
rs1789953
rs3797535
rs10944
rs705415
rs921943
rs672413
rs6859667
rs11951068
rs234709
rs6586282
rs558133
rs567754
All
−0.05
0.00
0.05
0.10
MR effect size for
'Selenium' on 'Follistatin−related protein 1'

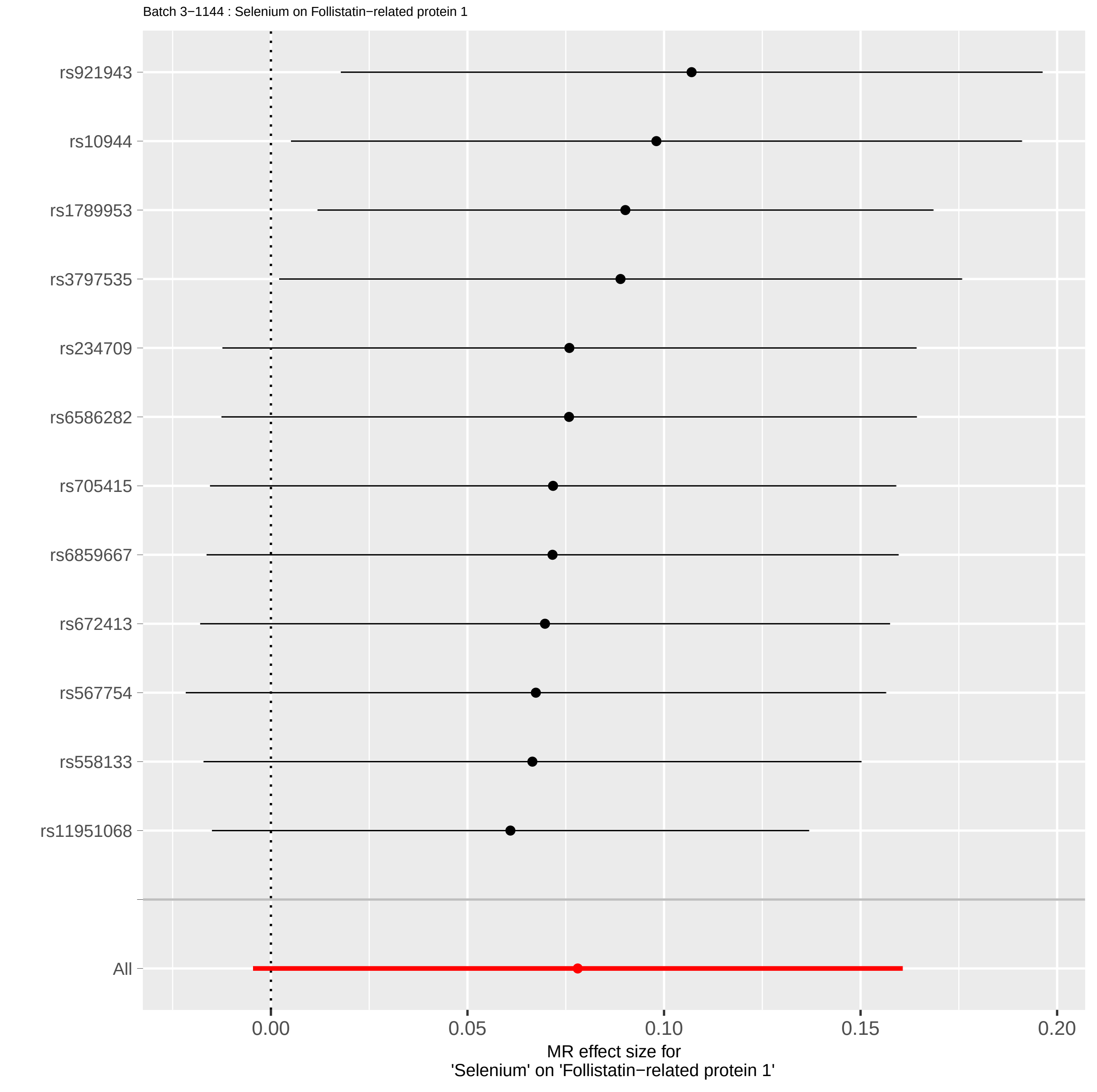

Batch 3−1144 : Selenium on Follistatin−related protein 1
rs921943
rs10944
rs1789953
rs3797535
rs234709
rs6586282
rs705415
rs6859667
rs672413
rs567754
rs558133
rs11951068
All
0.00
0.05
0.10
0.15
0.20
MR effect size for
'Selenium' on 'Follistatin−related protein 1'

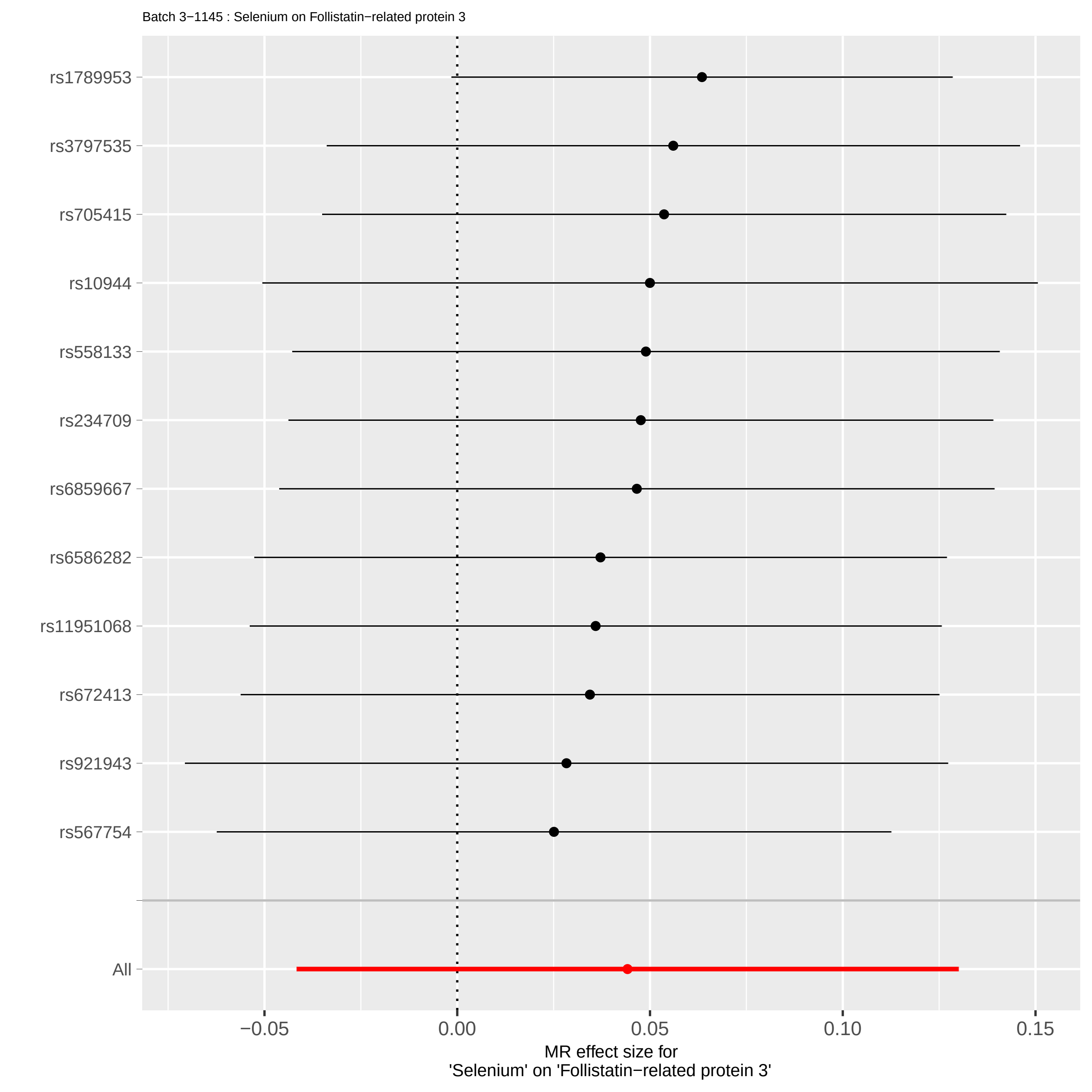

Batch 3−1145 : Selenium on Follistatin−related protein 3
rs1789953
rs3797535
rs705415
rs10944
rs558133
rs234709
rs6859667
rs6586282
rs11951068
rs672413
rs921943
rs567754
All
−0.05
0.00
0.05
0.10
0.15
MR effect size for
'Selenium' on 'Follistatin−related protein 3'

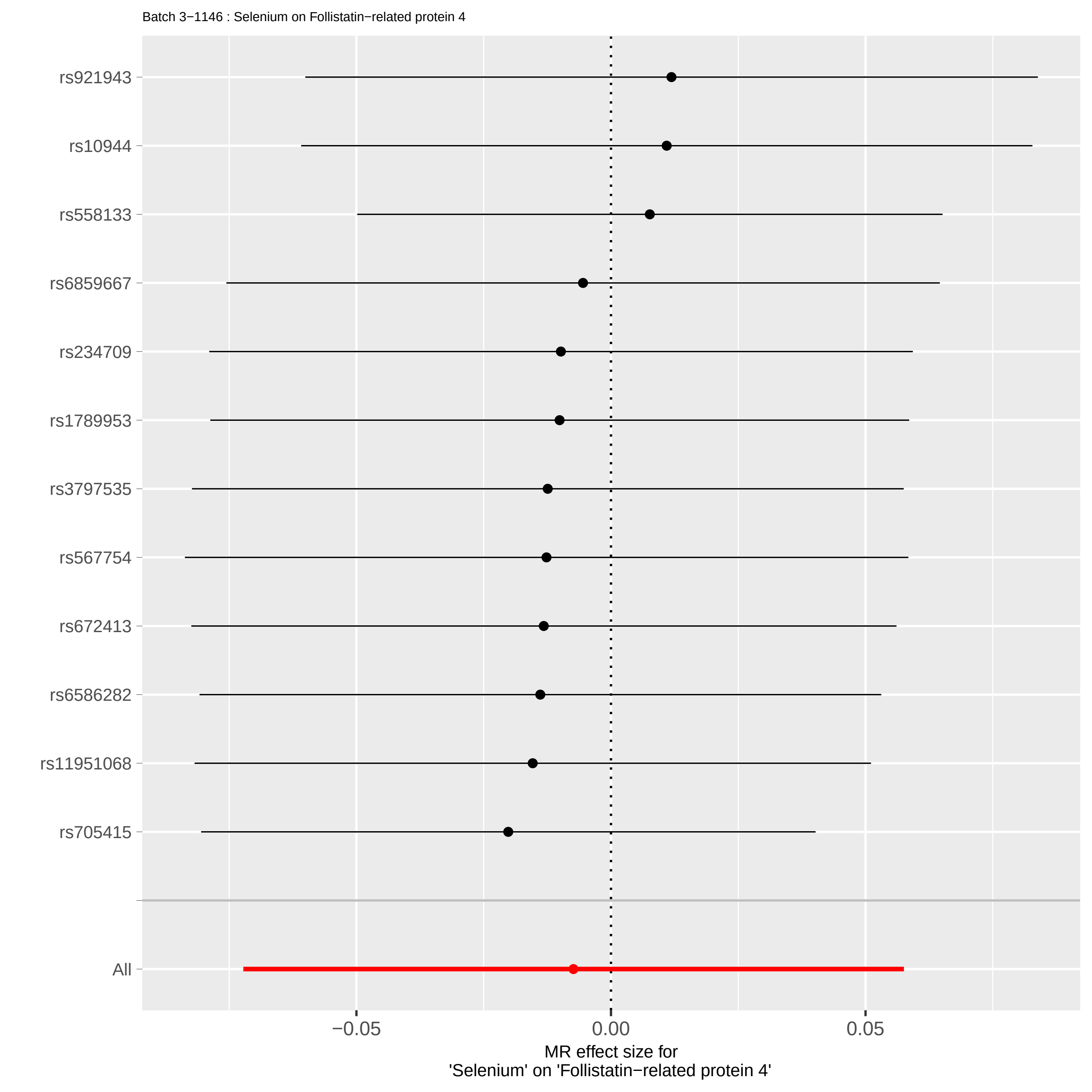

Batch 3−1146 : Selenium on Follistatin−related protein 4
rs921943
rs10944
rs558133
rs6859667
rs234709
rs1789953
rs3797535
rs567754
rs672413
rs6586282
rs11951068
rs705415
All
−0.05
0.00
0.05
MR effect size for
'Selenium' on 'Follistatin−related protein 4'

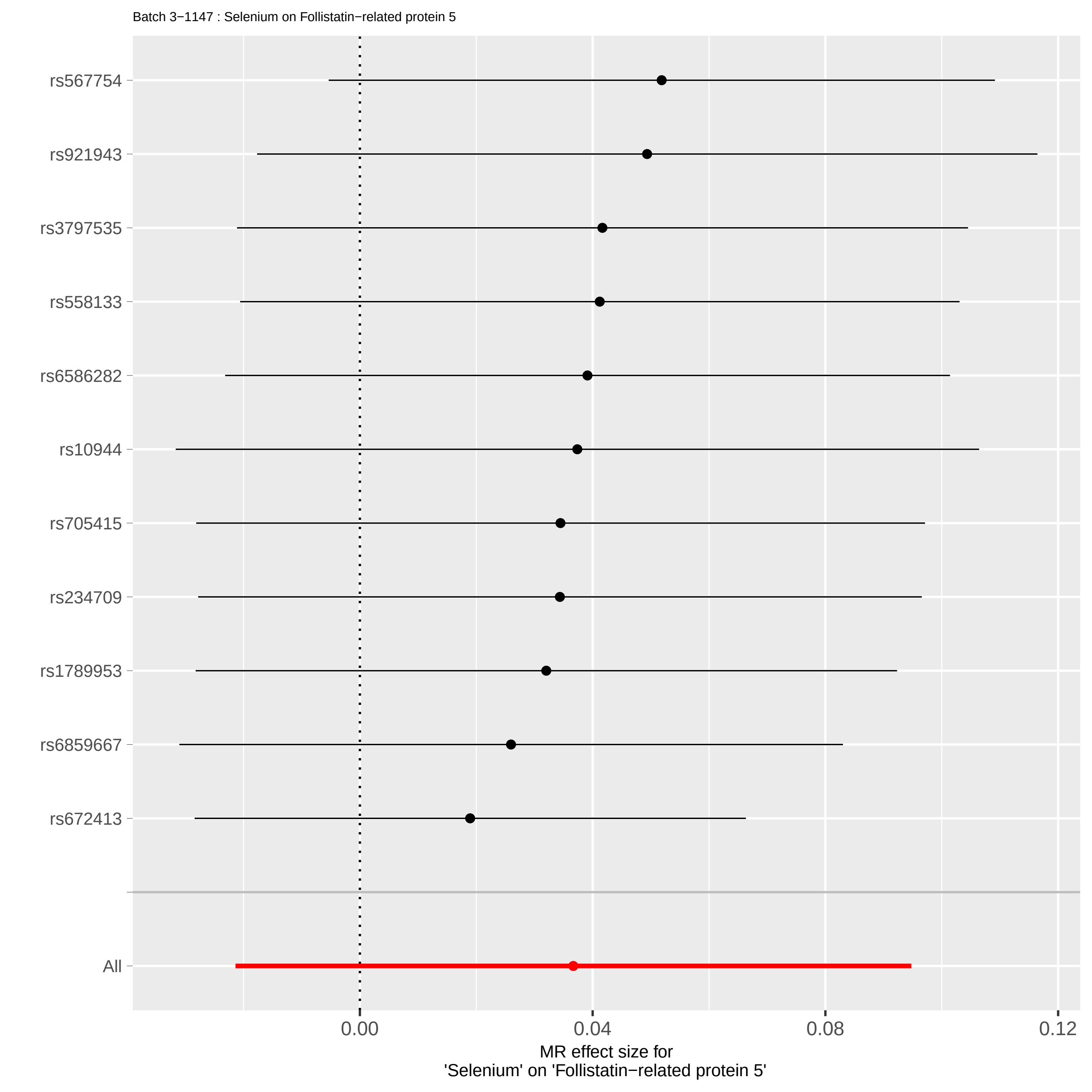

Batch 3−1147 : Selenium on Follistatin−related protein 5
rs567754
rs921943
rs3797535
rs558133
rs6586282
rs10944
rs705415
rs234709
rs1789953
rs6859667
rs672413
All
0.00
0.04
0.08
0.12
MR effect size for
'Selenium' on 'Follistatin−related protein 5'

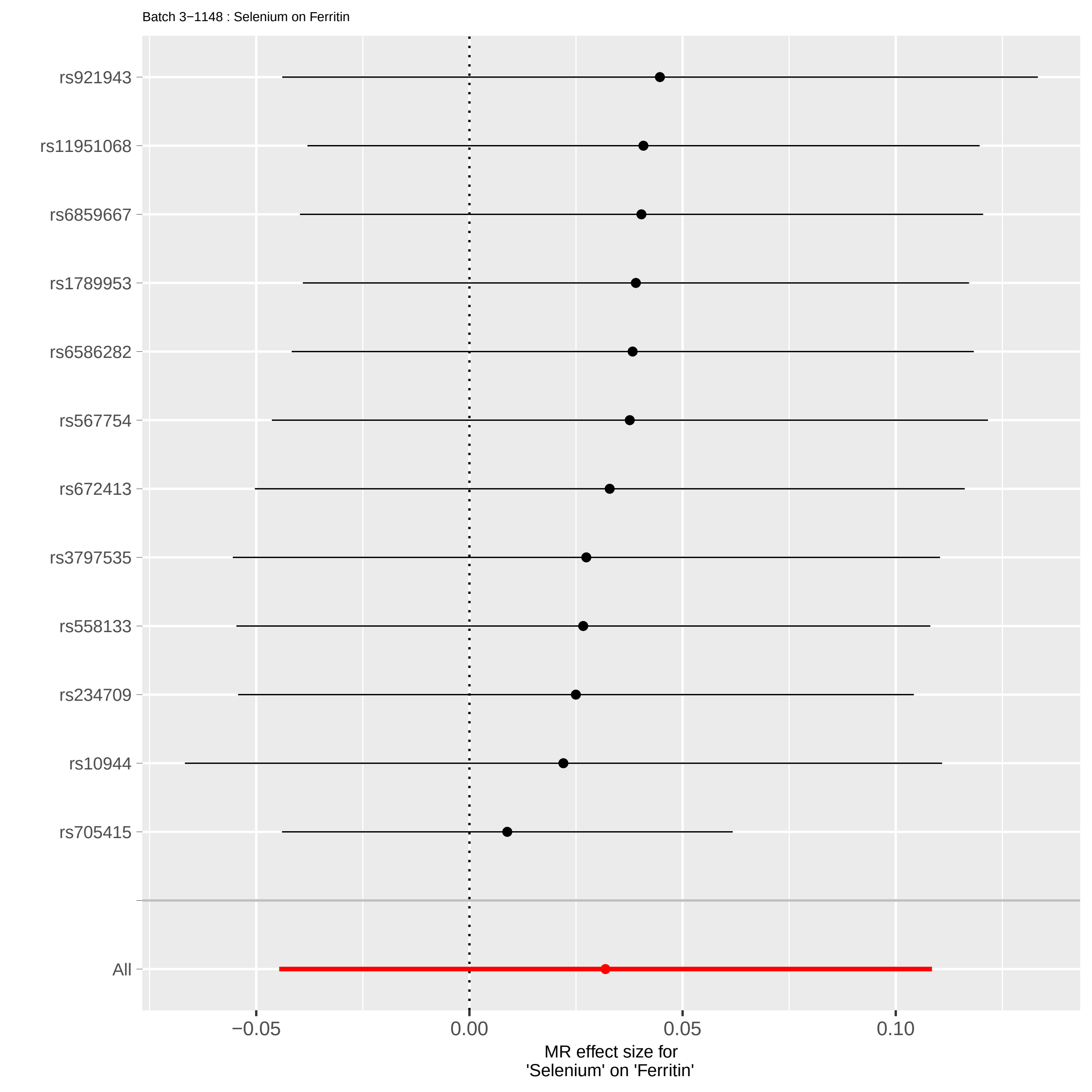

Batch 3−1148 : Selenium on Ferritin
rs921943
rs11951068
rs6859667
rs1789953
rs6586282
rs567754
rs672413
rs3797535
rs558133
rs234709
rs10944
rs705415
All
−0.05
0.00
0.05
0.10
MR effect size for
'Selenium' on 'Ferritin'

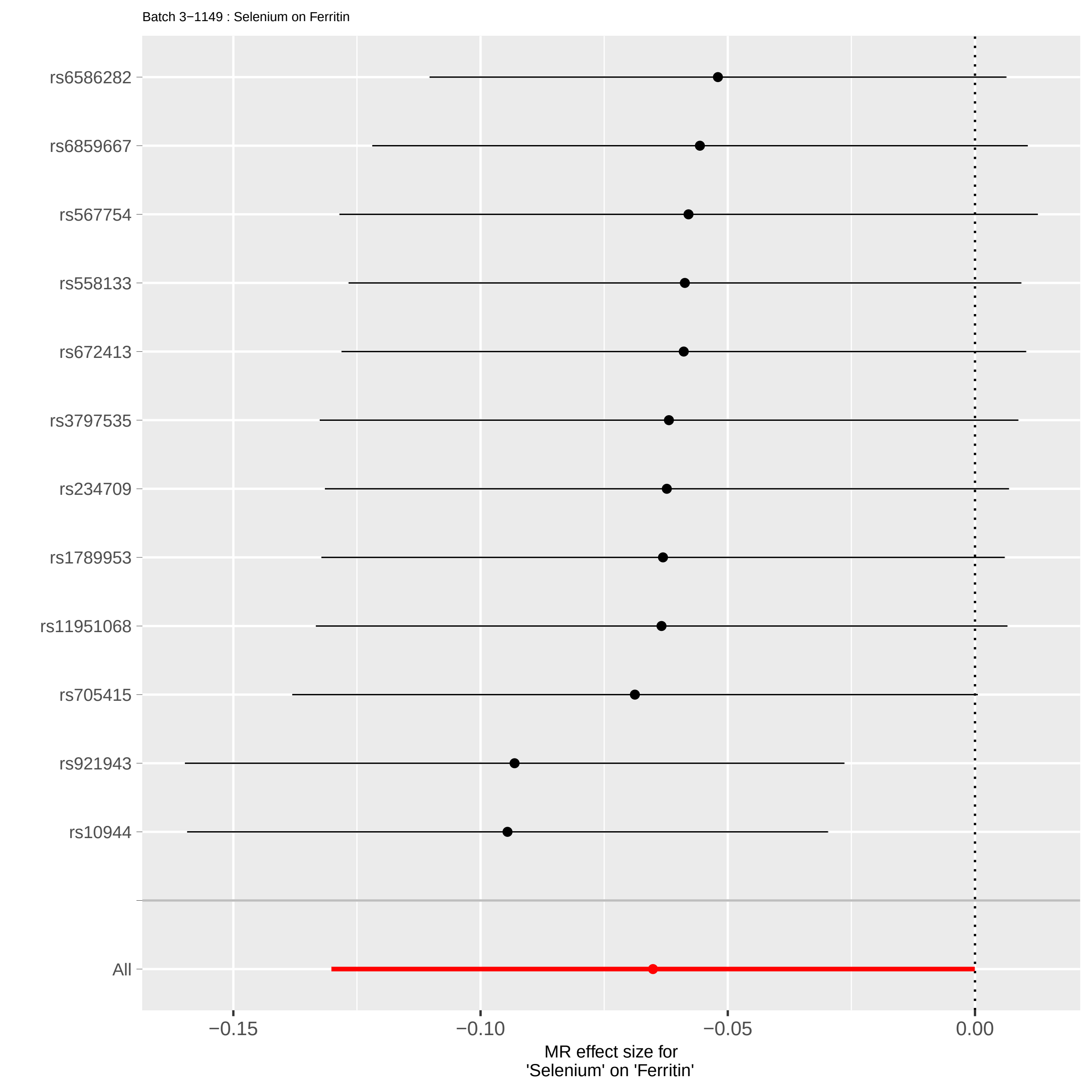

Batch 3−1149 : Selenium on Ferritin
rs6586282
rs6859667
rs567754
rs558133
rs672413
rs3797535
rs234709
rs1789953
rs11951068
rs705415
rs921943
rs10944
All
−0.15
−0.10
−0.05
0.00
MR effect size for
'Selenium' on 'Ferritin'

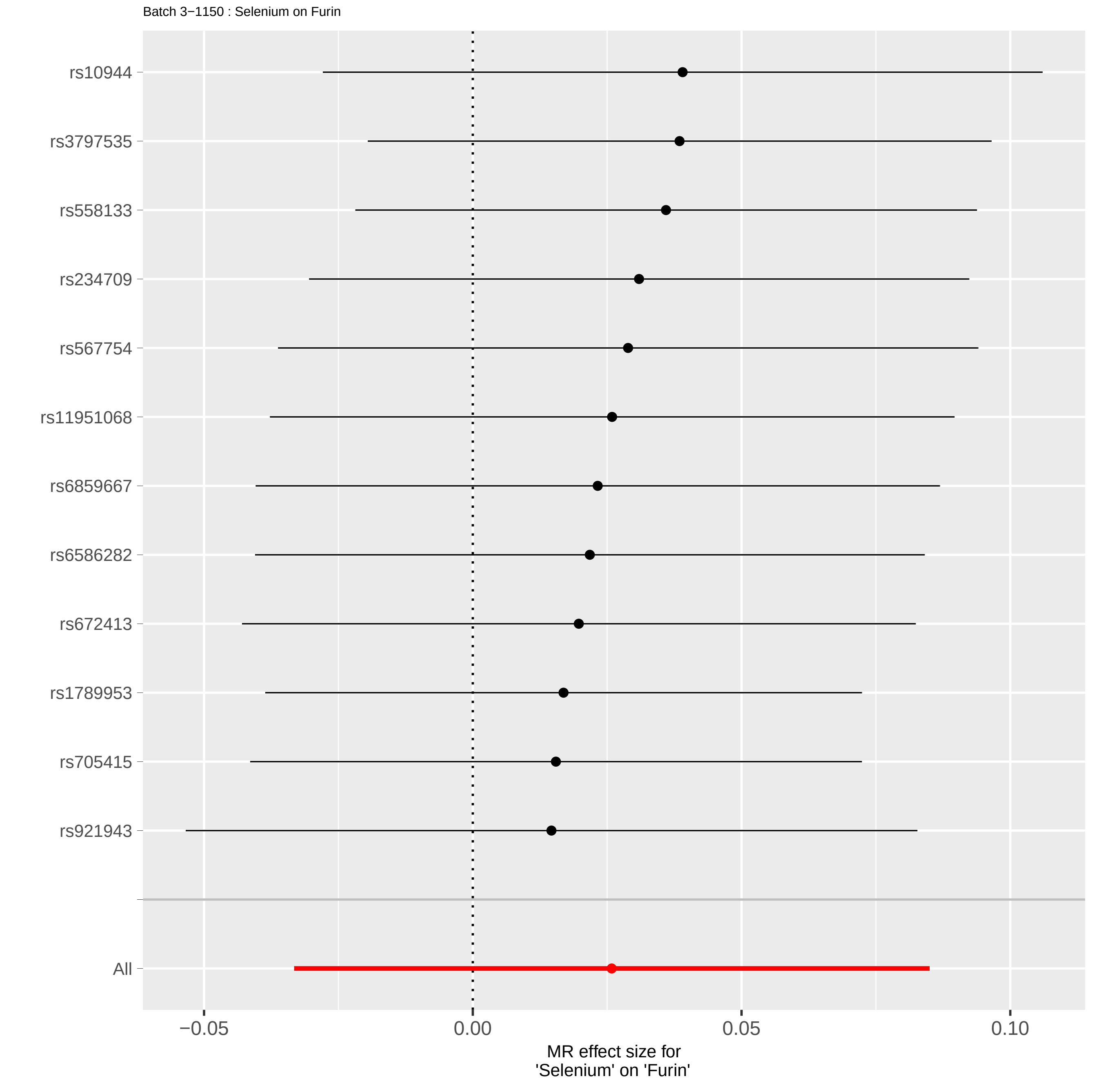

Batch 3−1150 : Selenium on Furin
rs10944
rs3797535
rs558133
rs234709
rs567754
rs11951068
rs6859667
rs6586282
rs672413
rs1789953
rs705415
rs921943
All
−0.05
0.00
0.05
0.10
MR effect size for
'Selenium' on 'Furin'

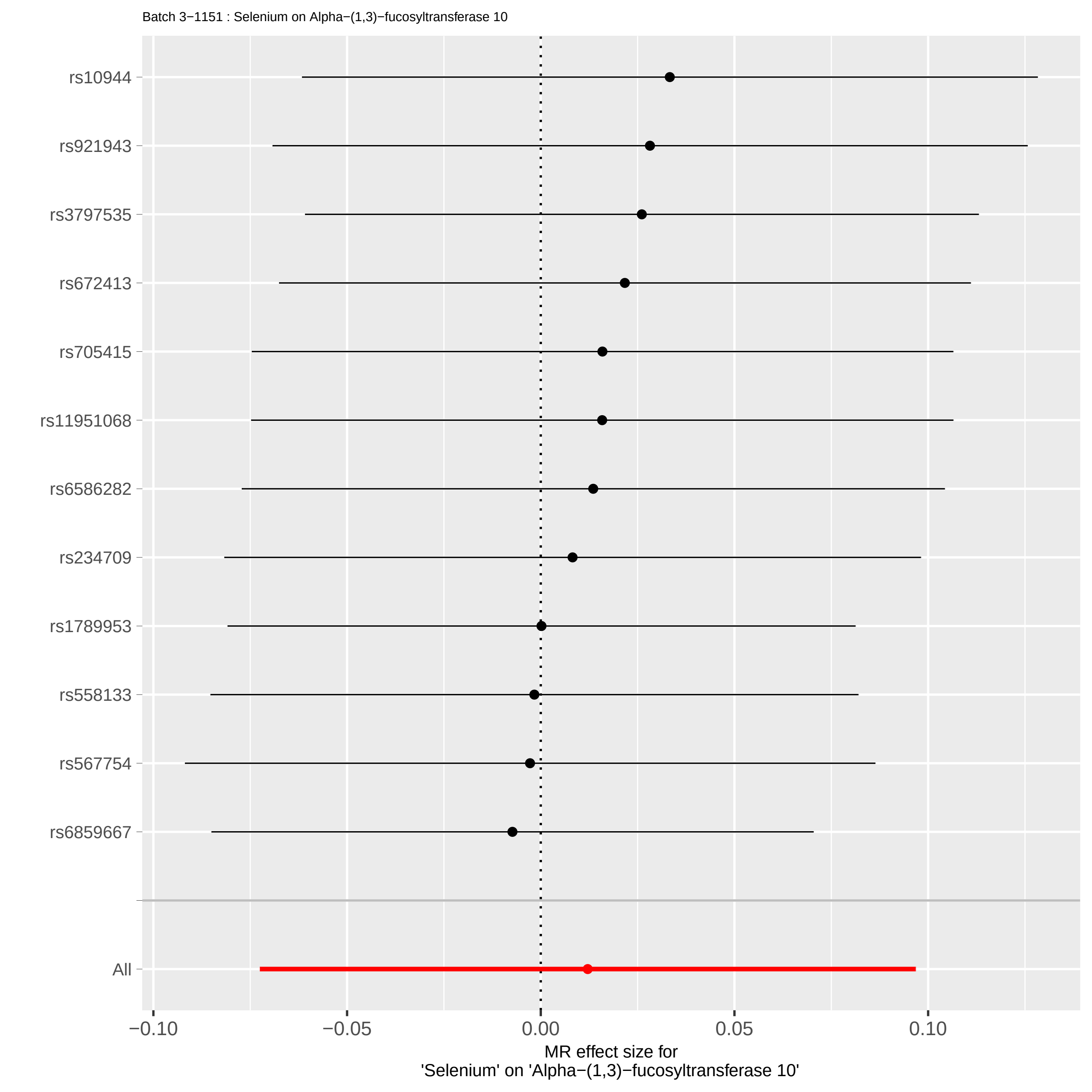

Batch 3−1151 : Selenium on Alpha−(1,3)−fucosyltransferase 10
rs10944
rs921943
rs3797535
rs672413
rs705415
rs11951068
rs6586282
rs234709
rs1789953
rs558133
rs567754
rs6859667
All
−0.10
−0.05
0.00
0.05
0.10
MR effect size for
'Selenium' on 'Alpha−(1,3)−fucosyltransferase 10'

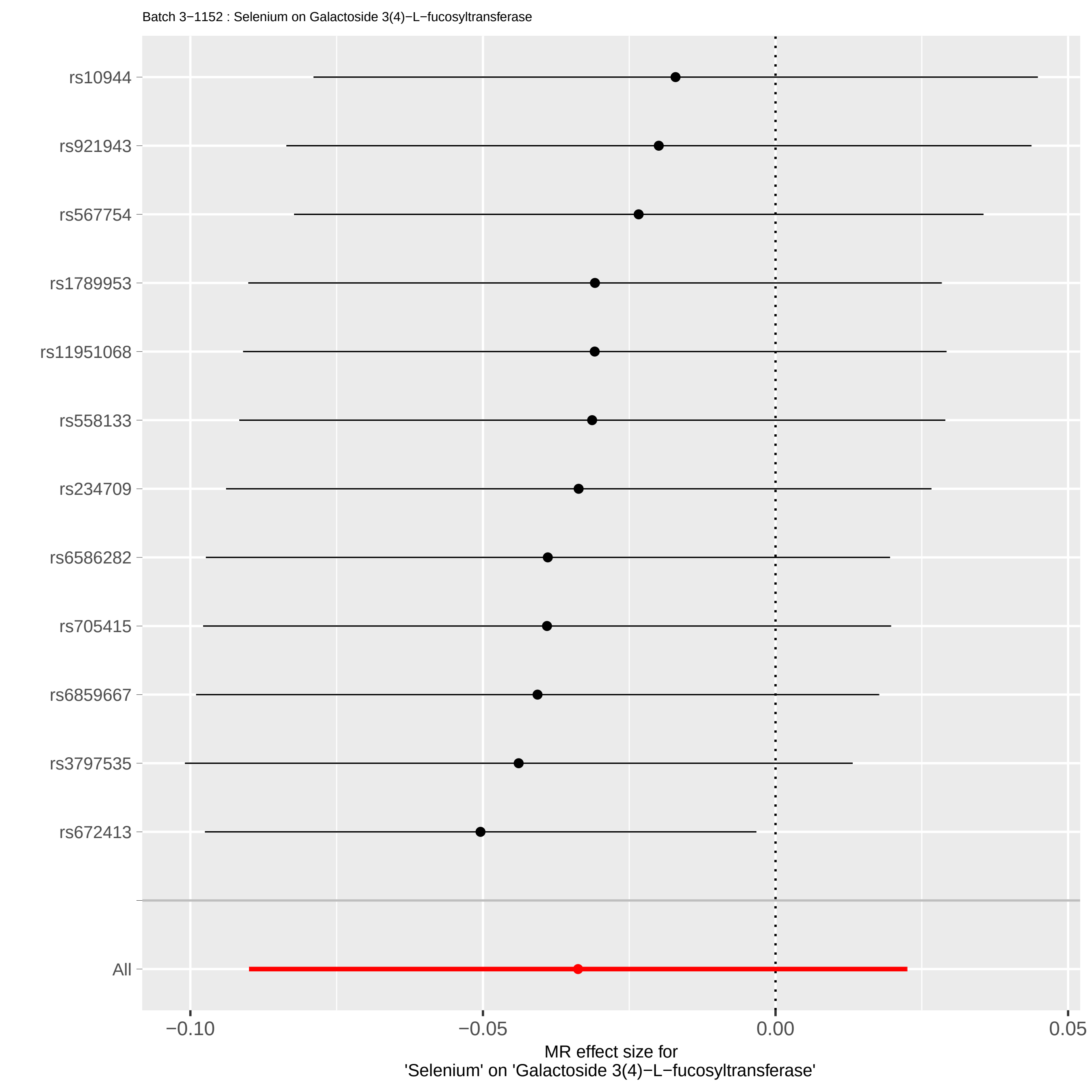

Batch 3−1152 : Selenium on Galactoside 3(4)−L−fucosyltransferase
rs10944
rs921943
rs567754
rs1789953
rs11951068
rs558133
rs234709
rs6586282
rs705415
rs6859667
rs3797535
rs672413
All
−0.10
−0.05
0.00
0.05
MR effect size for
'Selenium' on 'Galactoside 3(4)−L−fucosyltransferase'

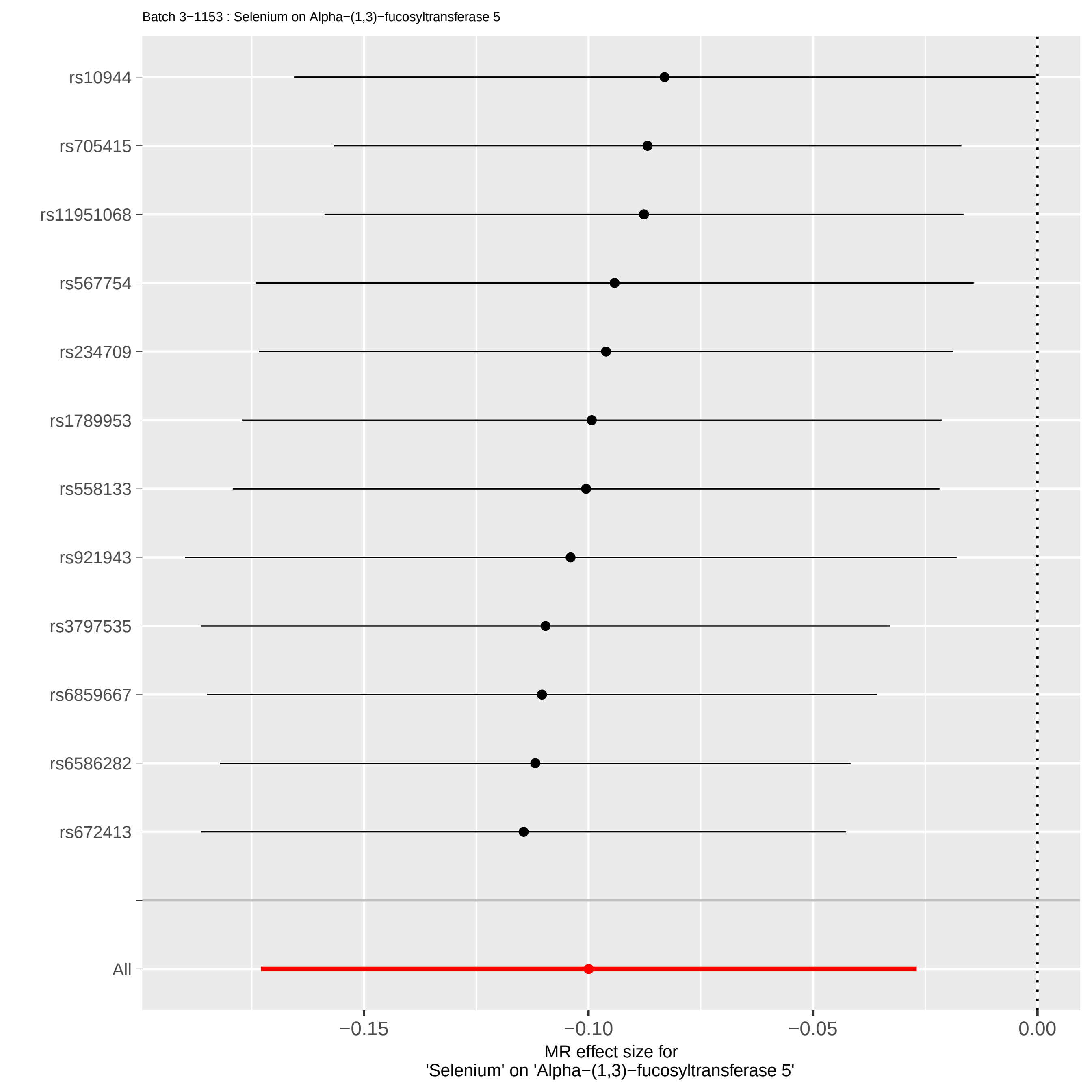
Batch 3−1153 : Selenium on Alpha−(1,3)−fucosyltransferase 5
rs10944
rs705415
rs11951068
rs567754
rs234709
rs1789953
rs558133
rs921943
rs3797535
rs6859667
rs6586282
rs672413
All
−0.15
−0.10
−0.05
0.00
MR effect size for
'Selenium' on 'Alpha−(1,3)−fucosyltransferase 5'

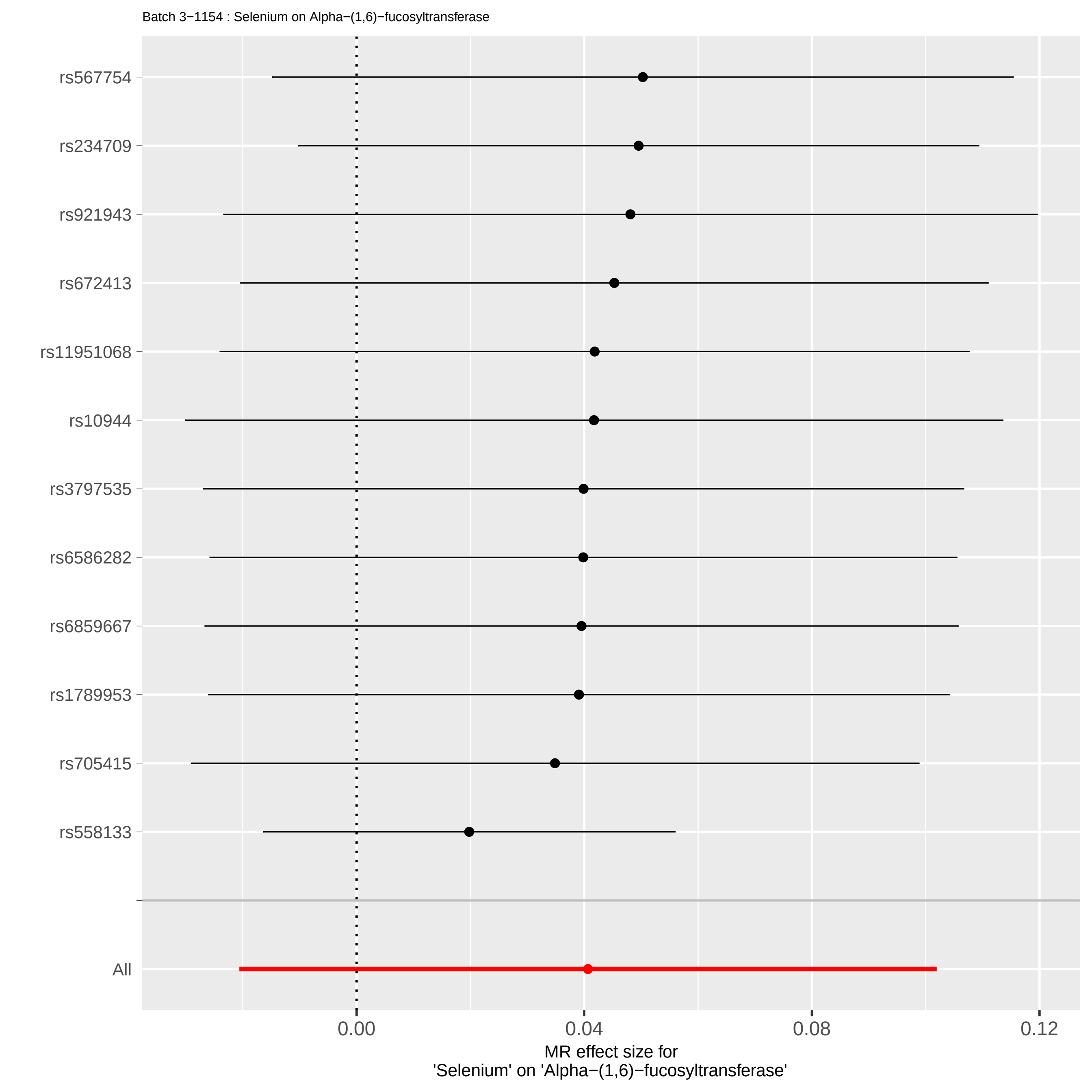

Batch 3−1154 : Selenium on Alpha−(1,6)−fucosyltransferase
rs567754
rs234709
rs921943
rs672413
rs11951068
rs10944
rs3797535
rs6586282
rs6859667
rs1789953
rs705415
rs558133
All
0.00
0.04
0.08
0.12
MR effect size for
'Selenium' on 'Alpha−(1,6)−fucosyltransferase'

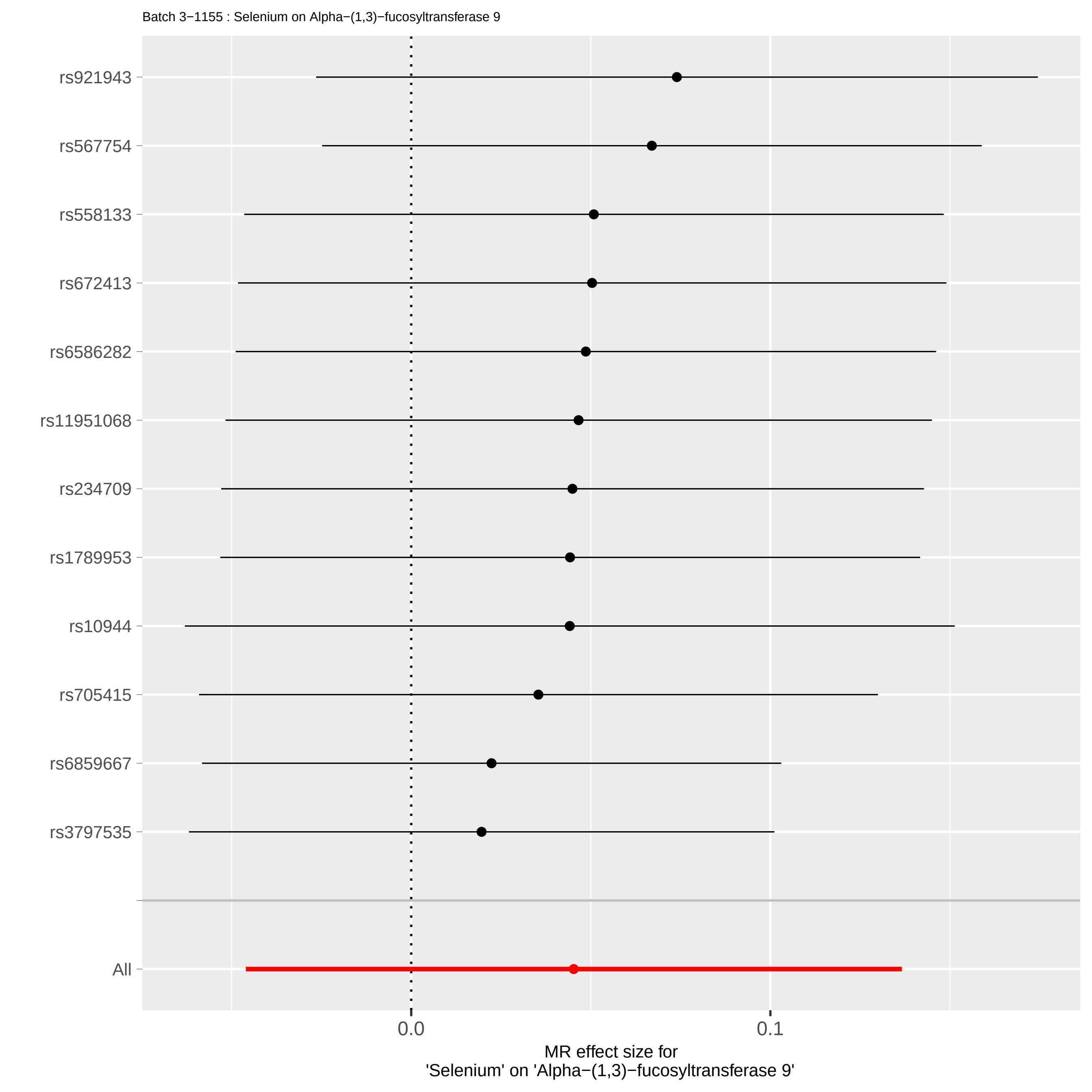

Batch 3−1155 : Selenium on Alpha−(1,3)−fucosyltransferase 9
rs921943
rs567754
rs558133
rs672413
rs6586282
rs11951068
rs234709
rs1789953
rs10944
rs705415
rs6859667
rs3797535
All
0.0
0.1
MR effect size for
'Selenium' on 'Alpha−(1,3)−fucosyltransferase 9'

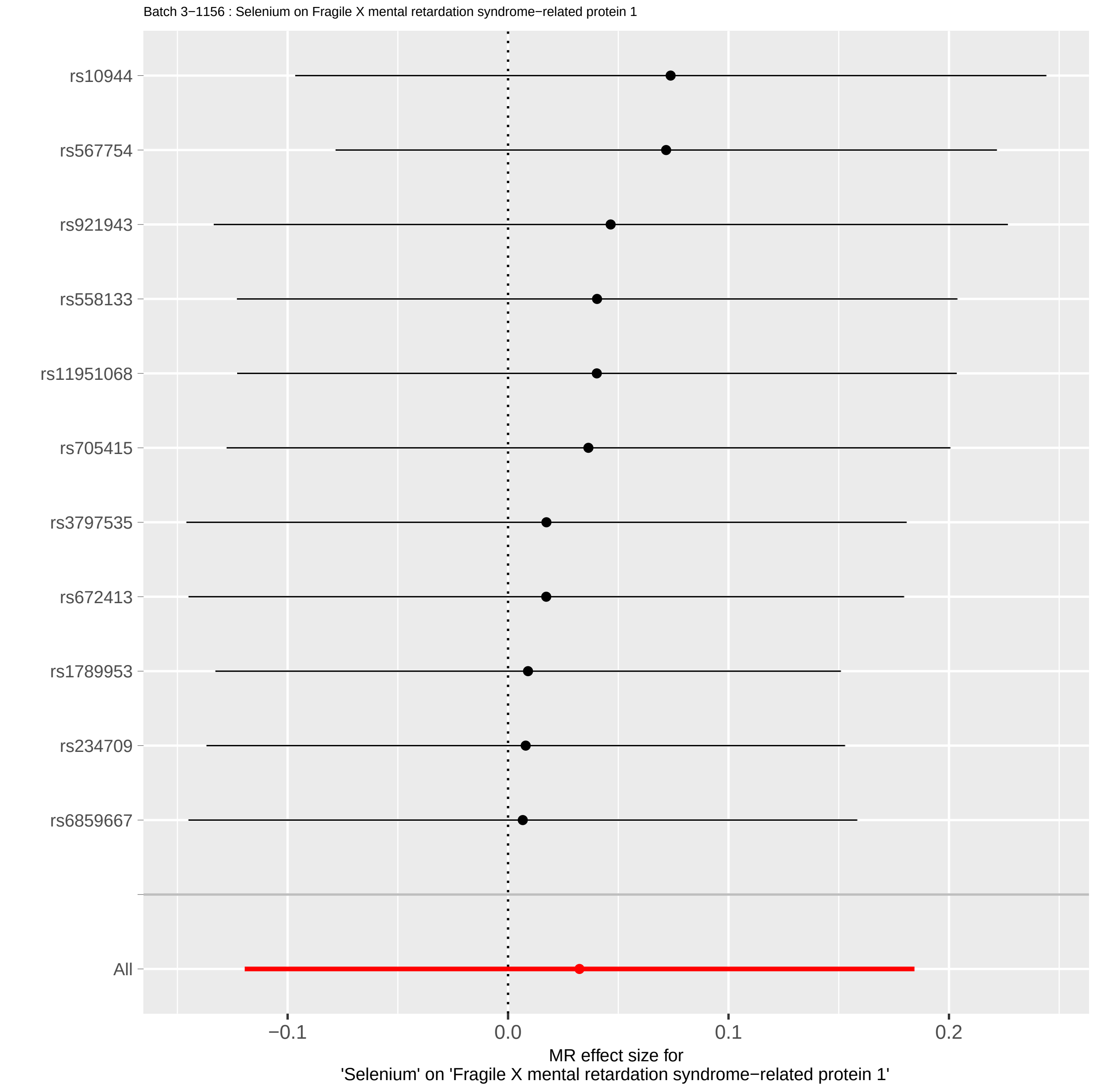

Batch 3−1156 : Selenium on Fragile X mental retardation syndrome−related protein 1
rs10944
rs567754
rs921943
rs558133
rs11951068
rs705415
rs3797535
rs672413
rs1789953
rs234709
rs6859667
All
−0.1
0.0
0.1
0.2
MR effect size for
'Selenium' on 'Fragile X mental retardation syndrome−related protein 1'

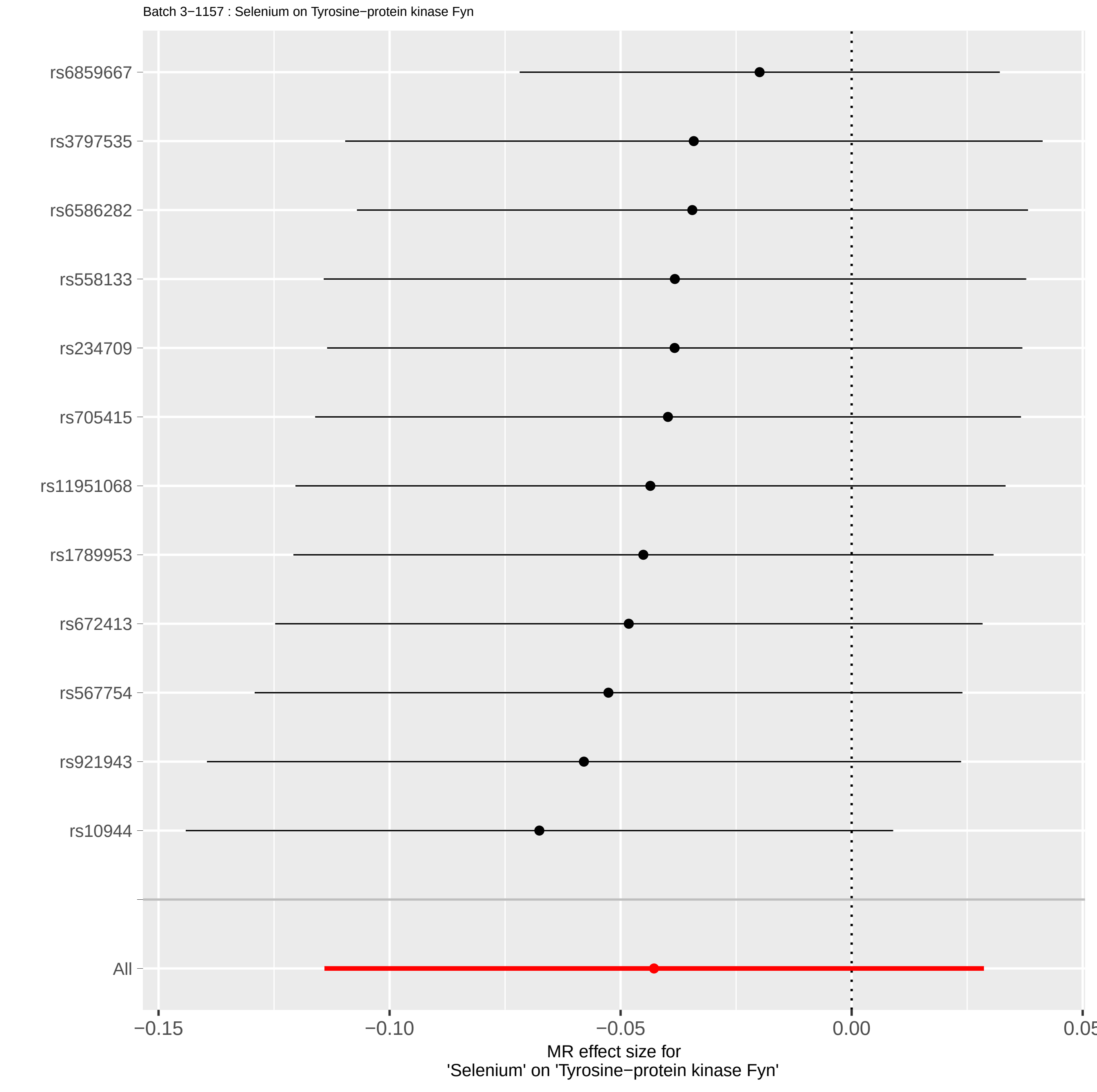

Batch 3−1157 : Selenium on Tyrosine−protein kinase Fyn
rs6859667
rs3797535
rs6586282
rs558133
rs234709
rs705415
rs11951068
rs1789953
rs672413
rs567754
rs921943
rs10944
All
−0.15
−0.10
−0.05
0.00
0.05
MR effect size for
'Selenium' on 'Tyrosine−protein kinase Fyn'

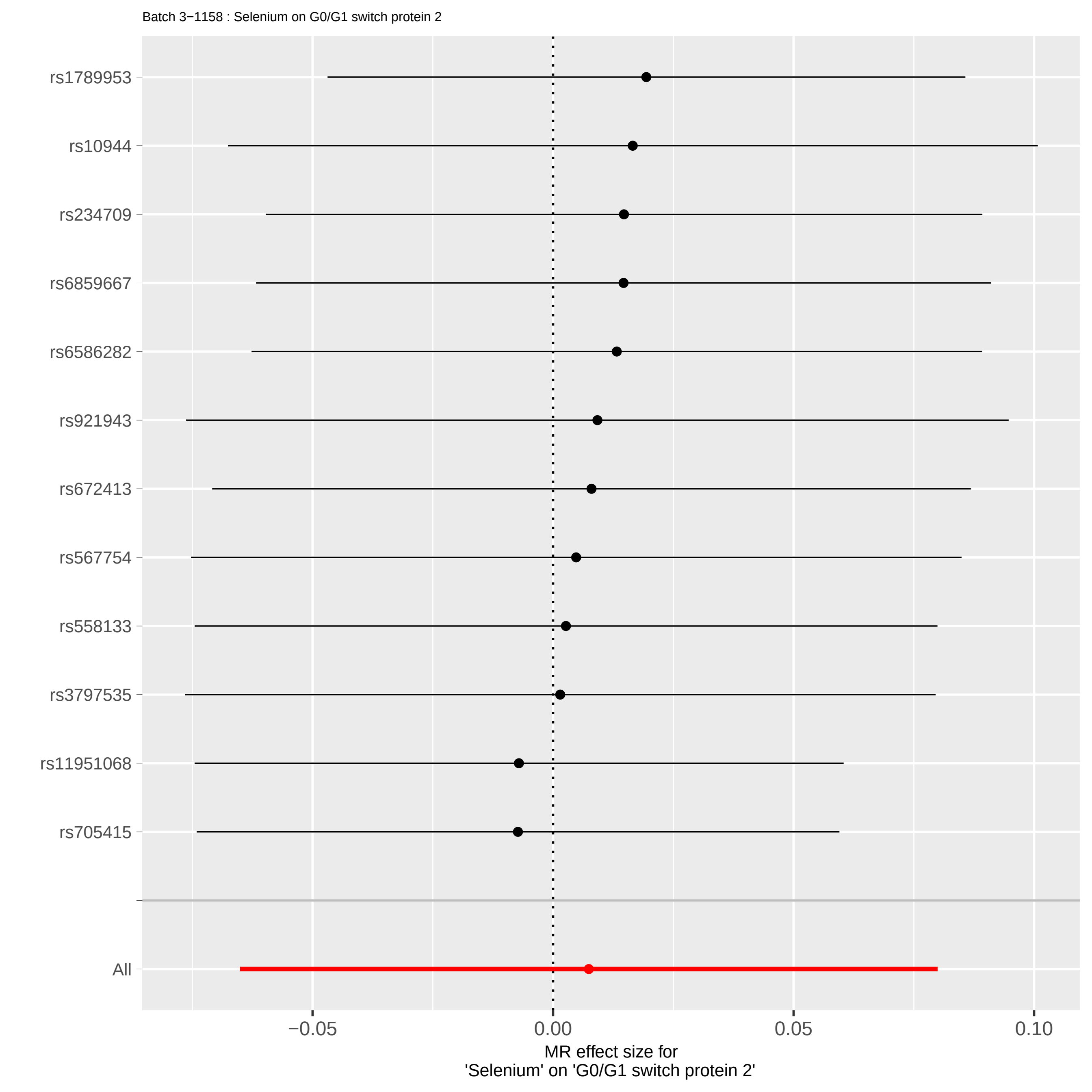
Batch 3−1158 : Selenium on G0/G1 switch protein 2
rs1789953
rs10944
rs234709
rs6859667
rs6586282
rs921943
rs672413
rs567754
rs558133
rs3797535
rs11951068
rs705415
All
−0.05
0.00
0.05
0.10
MR effect size for
'Selenium' on 'G0/G1 switch protein 2'

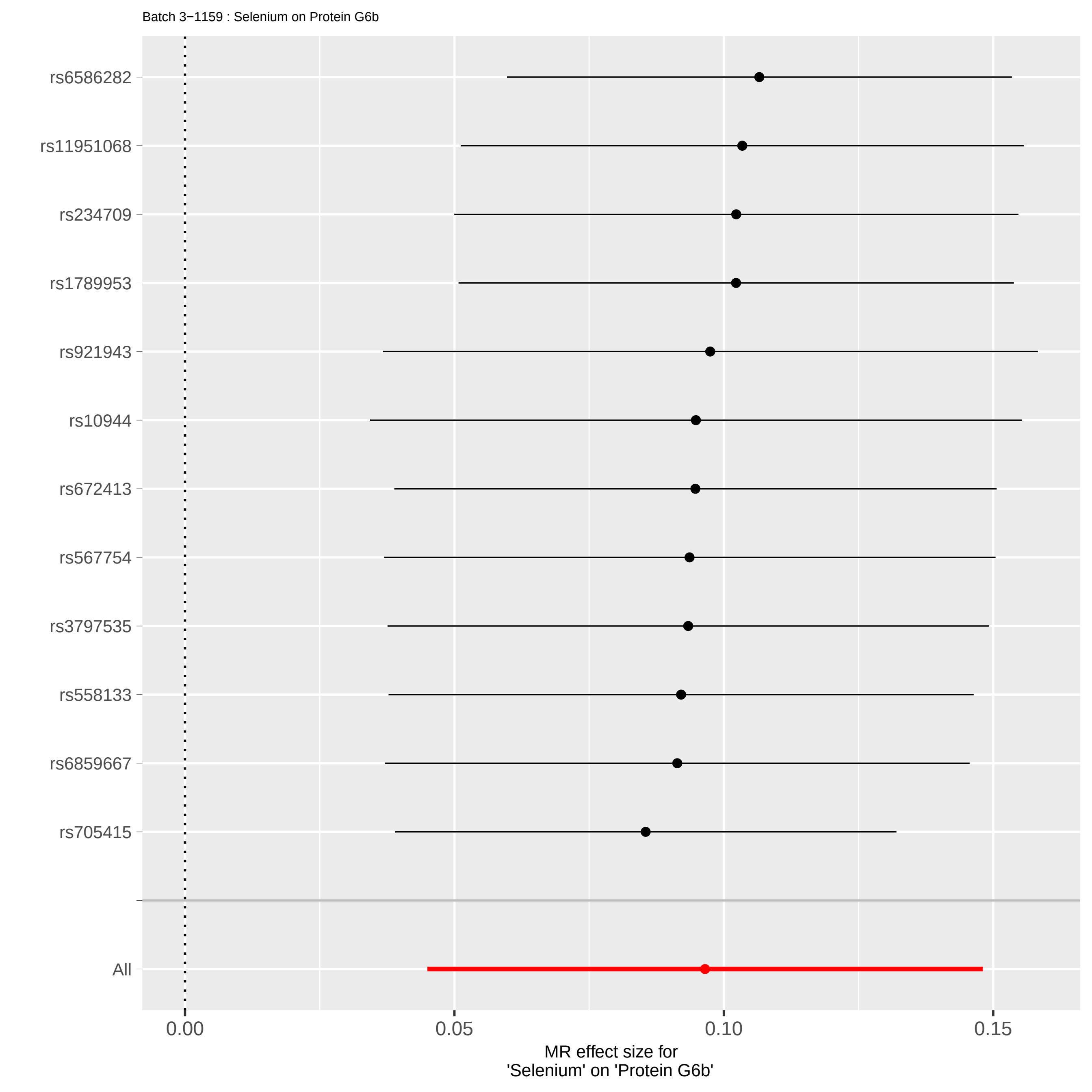

Batch 3−1159 : Selenium on Protein G6b
rs6586282
rs11951068
rs234709
rs1789953
rs921943
rs10944
rs672413
rs567754
rs3797535
rs558133
rs6859667
rs705415
All
0.00
0.05
0.10
0.15
MR effect size for
'Selenium' on 'Protein G6b'

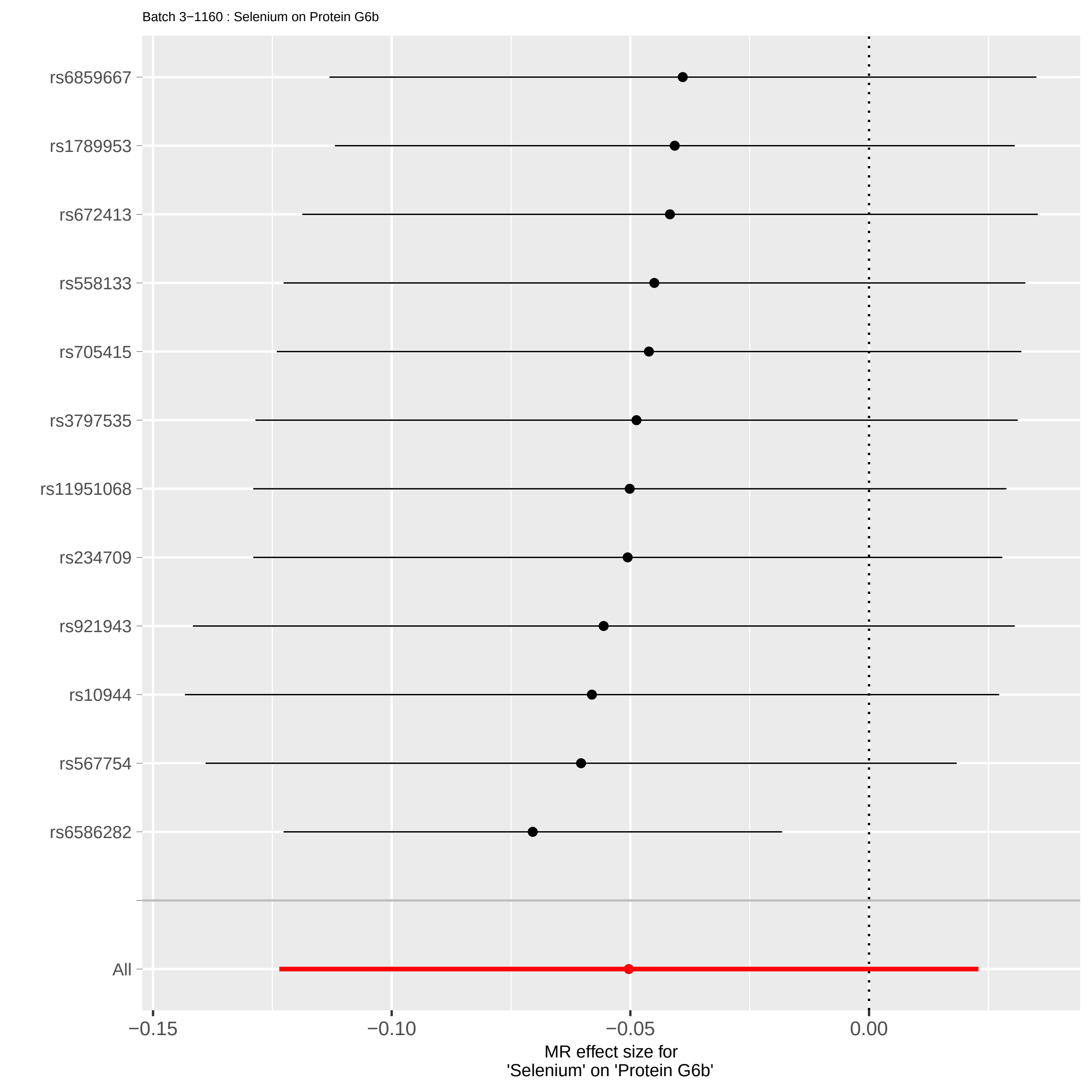

Batch 3−1160 : Selenium on Protein G6b
rs6859667
rs1789953
rs672413
rs558133
rs705415
rs3797535
rs11951068
rs234709
rs921943
rs10944
rs567754
rs6586282
All
−0.15
−0.10
−0.05
0.00
MR effect size for
'Selenium' on 'Protein G6b'

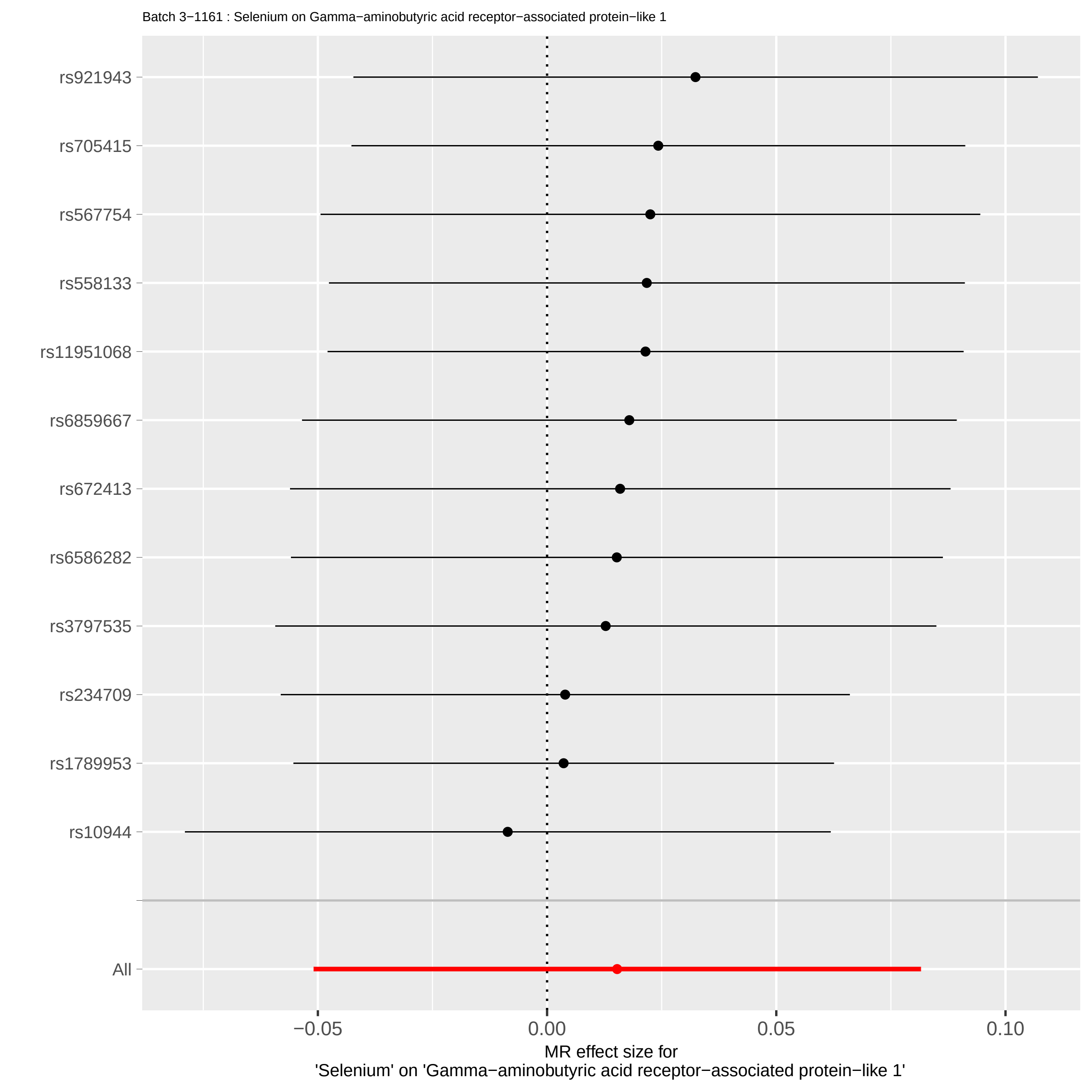

Batch 3−1161 : Selenium on Gamma−aminobutyric acid receptor−associated protein−like 1
rs921943
rs705415
rs567754
rs558133
rs11951068
rs6859667
rs672413
rs6586282
rs3797535
rs234709
rs1789953
rs10944
All
−0.05
0.00
0.05
0.10
MR effect size for
'Selenium' on 'Gamma−aminobutyric acid receptor−associated protein−like 1'

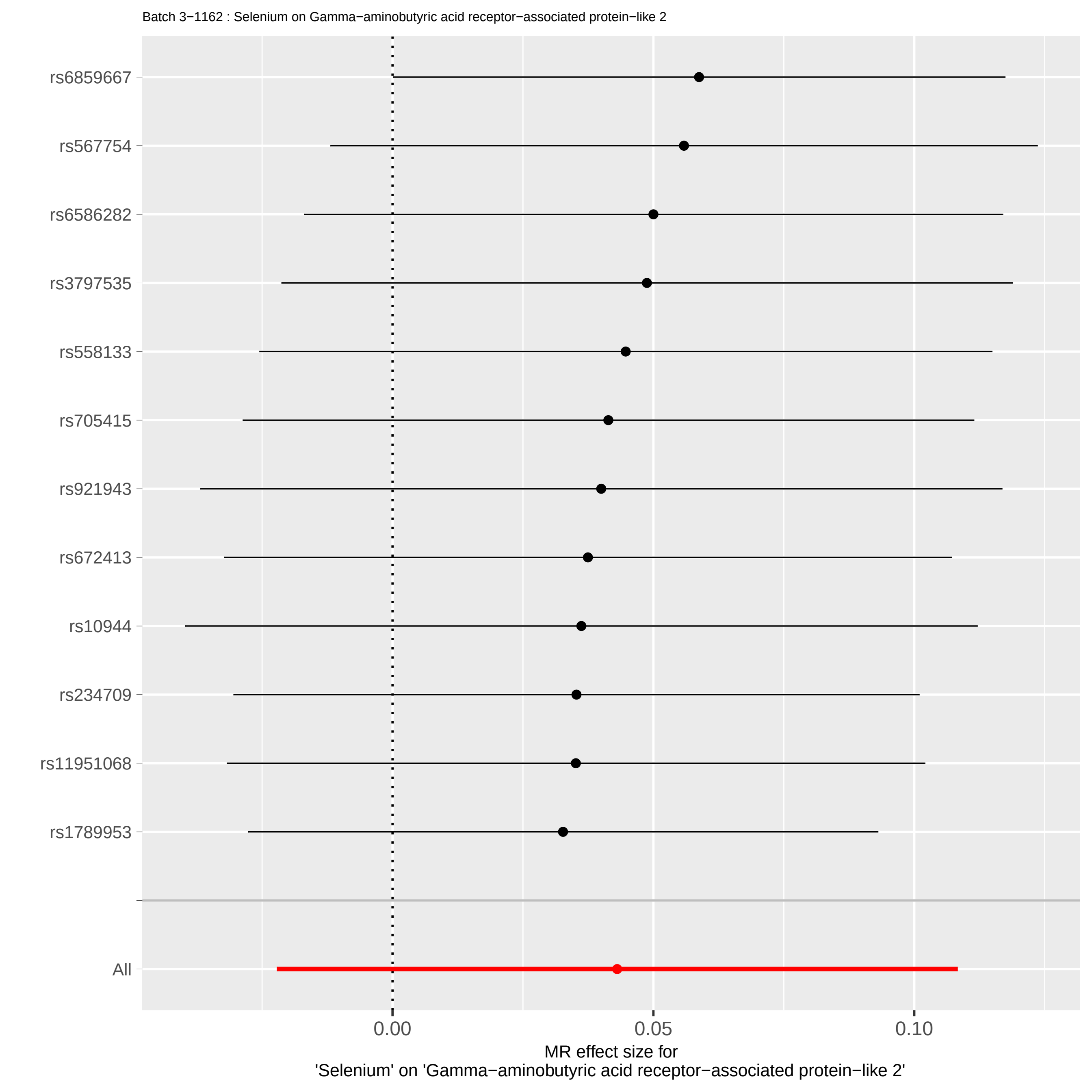
Batch 3−1162 : Selenium on Gamma−aminobutyric acid receptor−associated protein−like 2
rs6859667
rs567754
rs6586282
rs3797535
rs558133
rs705415
rs921943
rs672413
rs10944
rs234709
rs11951068
rs1789953
All
0.00
0.05
0.10
MR effect size for
'Selenium' on 'Gamma−aminobutyric acid receptor−associated protein−like 2'

Batch 3−1163 : Selenium on Gamma−aminobutyric acid type B receptor subunit 2

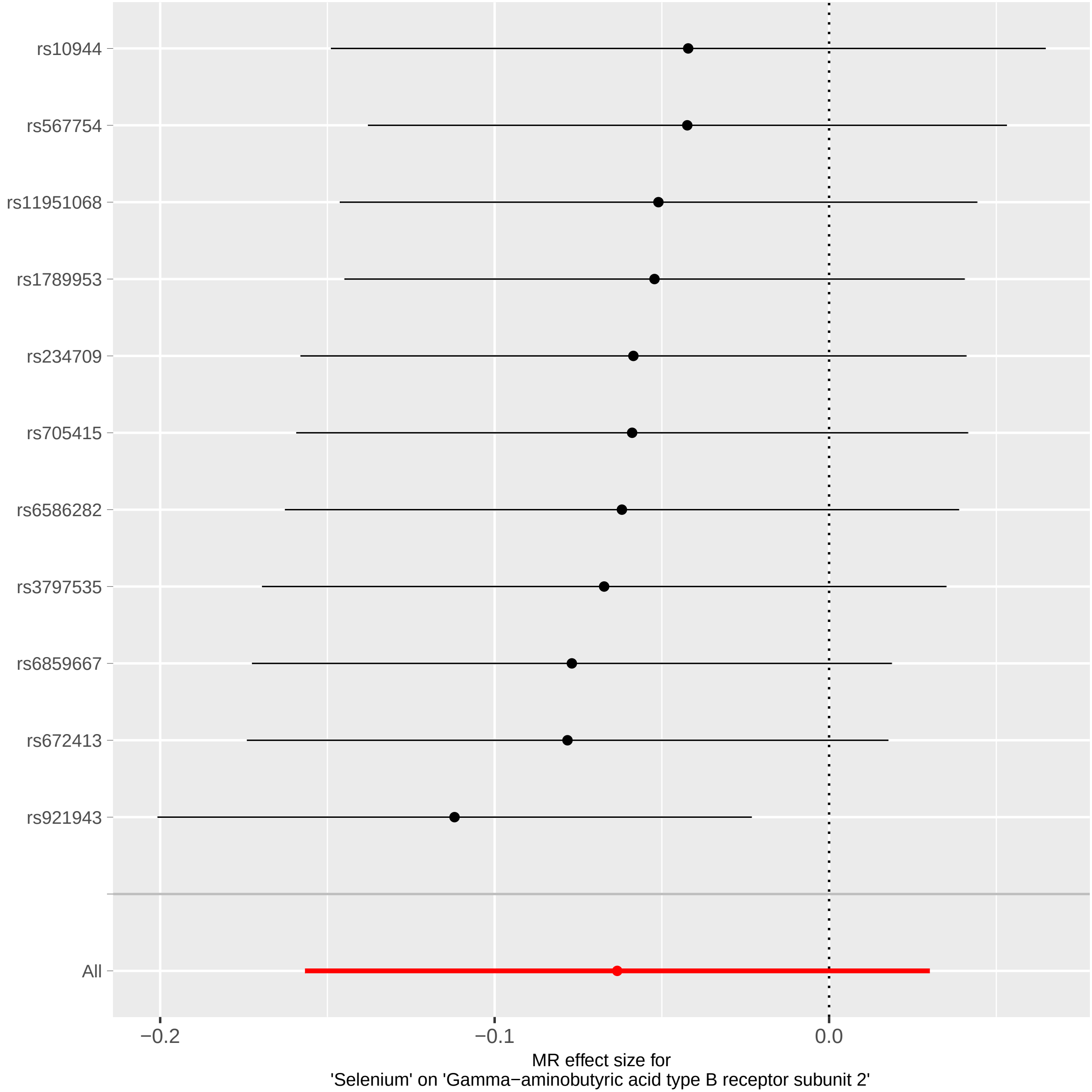

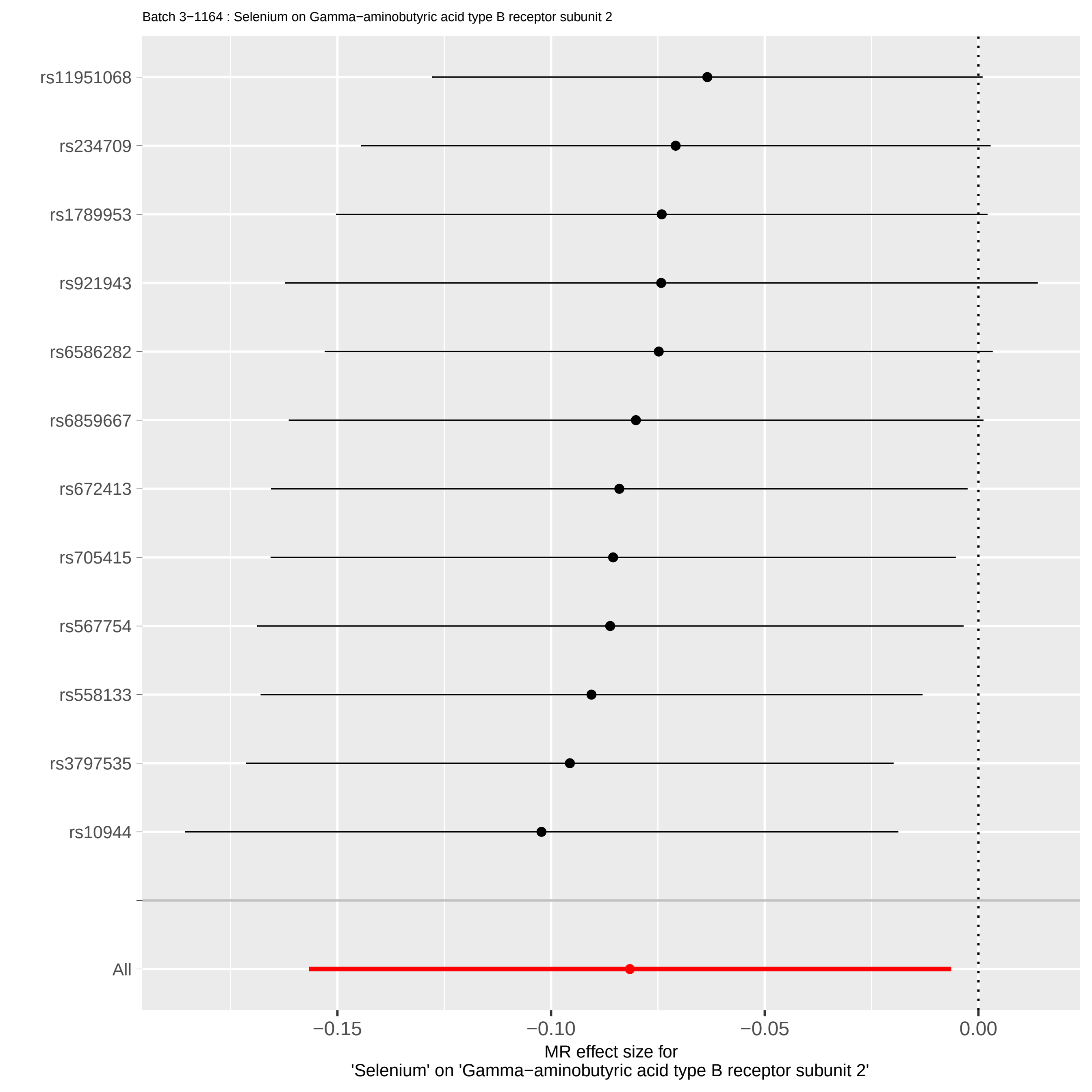

Batch 3−1164 : Selenium on Gamma−aminobutyric acid type B receptor subunit 2
rs11951068
rs234709
rs1789953
rs921943
rs6586282
rs6859667
rs672413
rs705415
rs567754
rs558133
rs3797535
rs10944
All
−0.15
−0.10
−0.05
0.00
MR effect size for
'Selenium' on 'Gamma−aminobutyric acid type B receptor subunit 2'

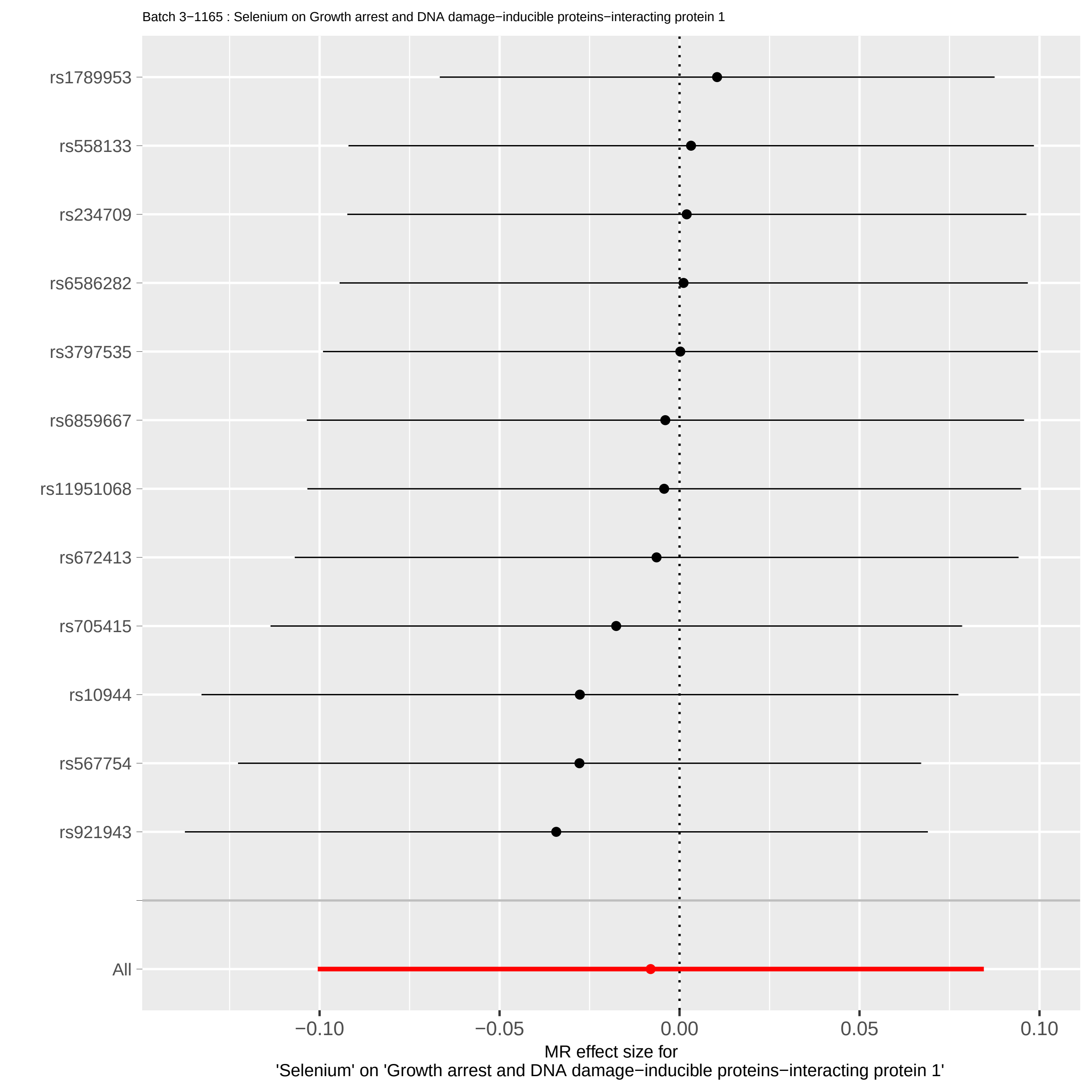
Batch 3−1165 : Selenium on Growth arrest and DNA damage−inducible proteins−interacting protein 1
rs1789953
rs558133
rs234709
rs6586282
rs3797535
rs6859667
rs11951068
rs672413
rs705415
rs10944
rs567754
rs921943
All
−0.10
−0.05
0.00
0.05
0.10
MR effect size for
'Selenium' on 'Growth arrest and DNA damage−inducible proteins−interacting protein 1'

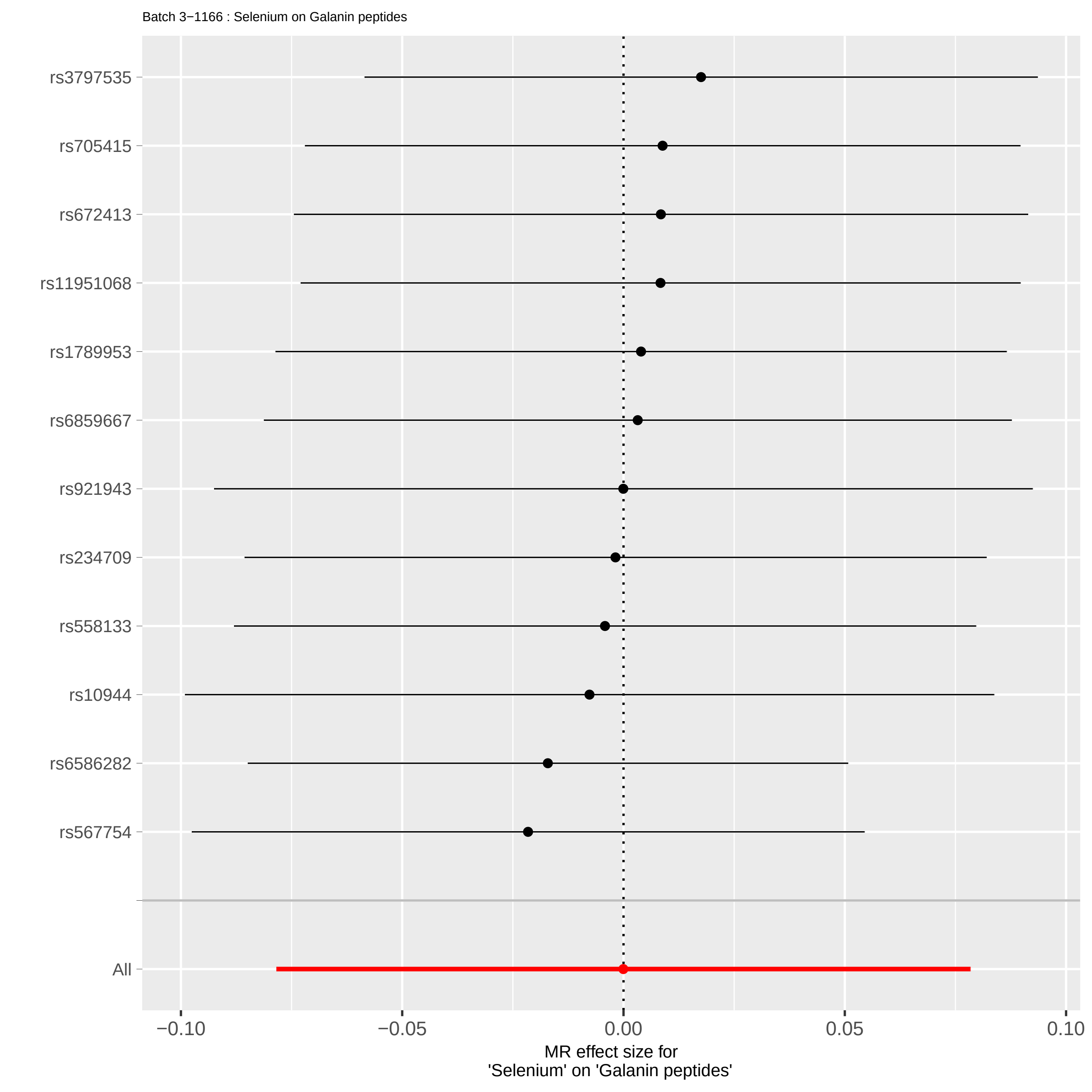
Batch 3−1166 : Selenium on Galanin peptides
rs3797535
rs705415
rs672413
rs11951068
rs1789953
rs6859667
rs921943
rs234709
rs558133
rs10944
rs6586282
rs567754
All
−0.10
−0.05
0.00
0.05
0.10
MR effect size for
'Selenium' on 'Galanin peptides'

Batch 3−1167 : Selenium on Polypeptide N−acetylgalactosaminyltransferase 10

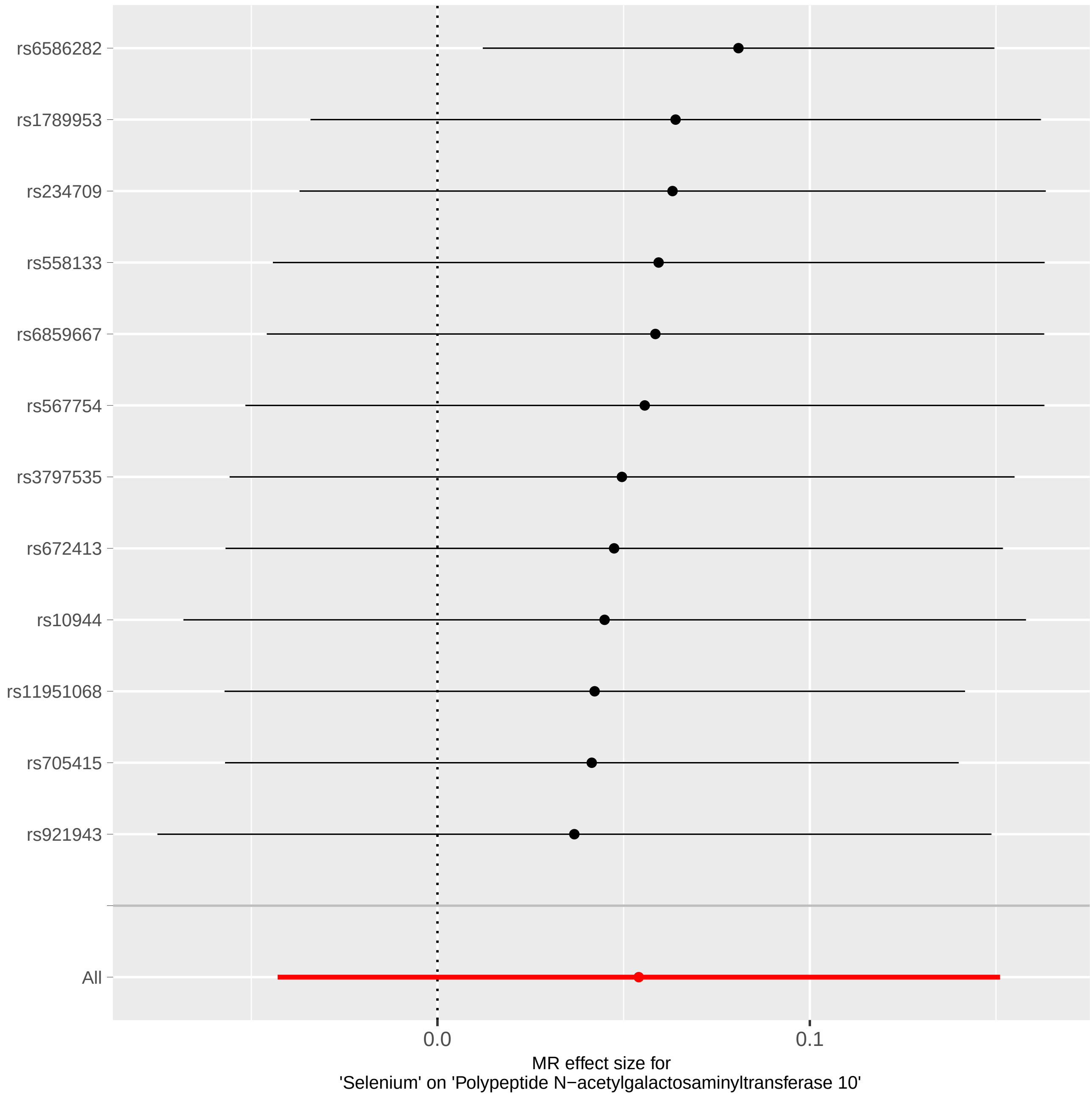

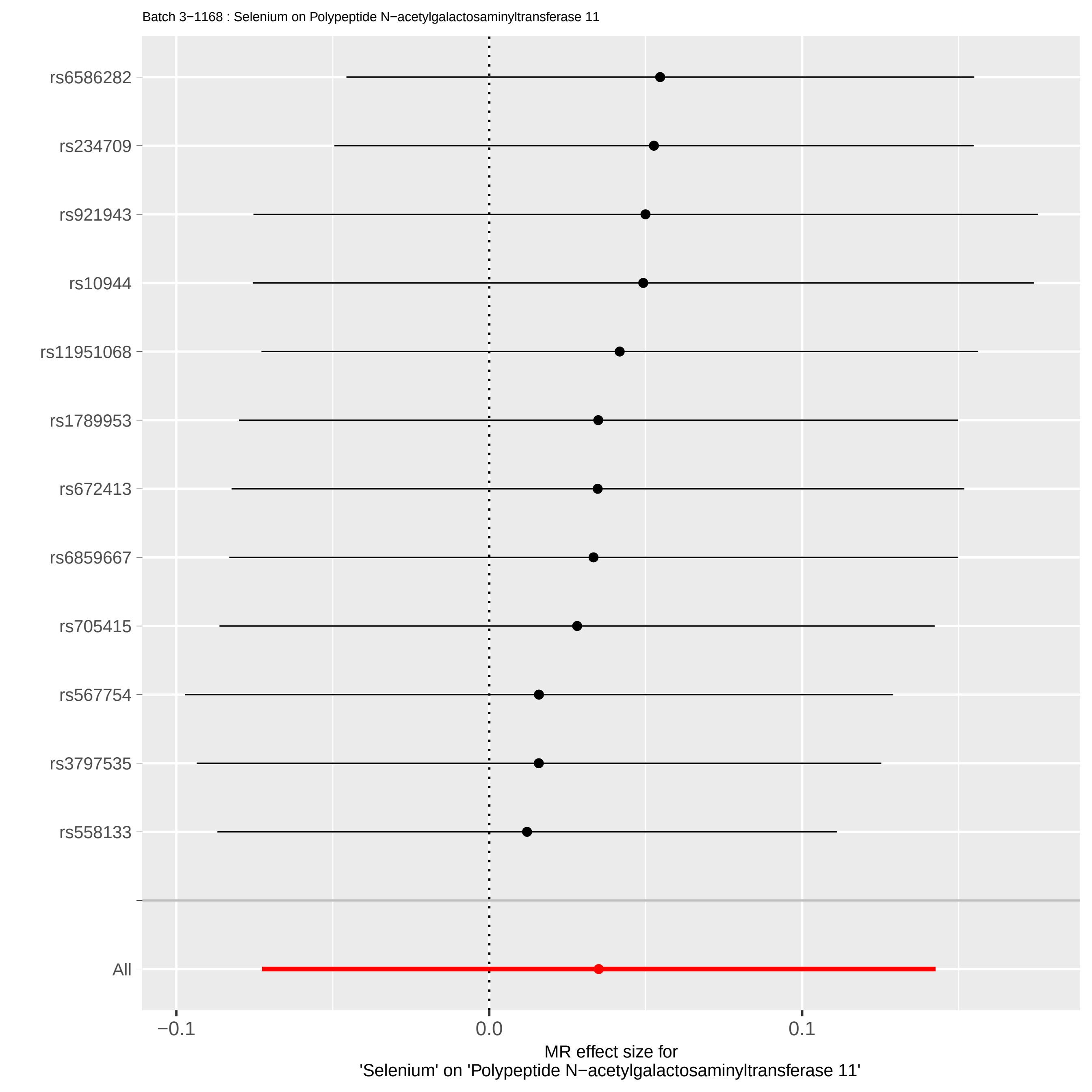

Batch 3−1168 : Selenium on Polypeptide N−acetylgalactosaminyltransferase 11
rs6586282
rs234709
rs921943
rs10944
rs11951068
rs1789953
rs672413
rs6859667
rs705415
rs567754
rs3797535
rs558133
All
−0.1
0.0
0.1
MR effect size for
'Selenium' on 'Polypeptide N−acetylgalactosaminyltransferase 11'

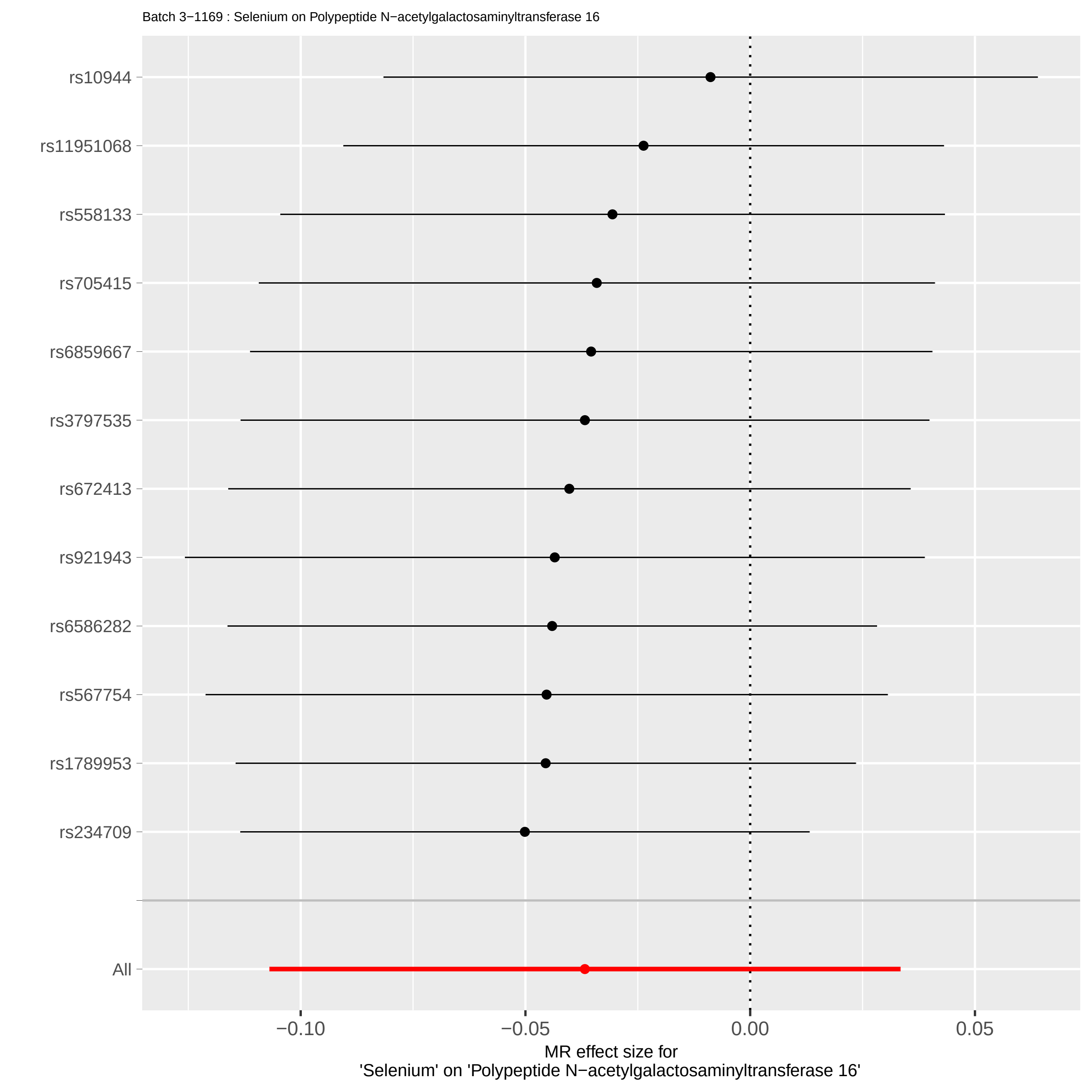

Batch 3−1169 : Selenium on Polypeptide N−acetylgalactosaminyltransferase 16
rs10944
rs11951068
rs558133
rs705415
rs6859667
rs3797535
rs672413
rs921943
rs6586282
rs567754
rs1789953
rs234709
All
−0.10
−0.05
0.00
0.05
MR effect size for
'Selenium' on 'Polypeptide N−acetylgalactosaminyltransferase 16'

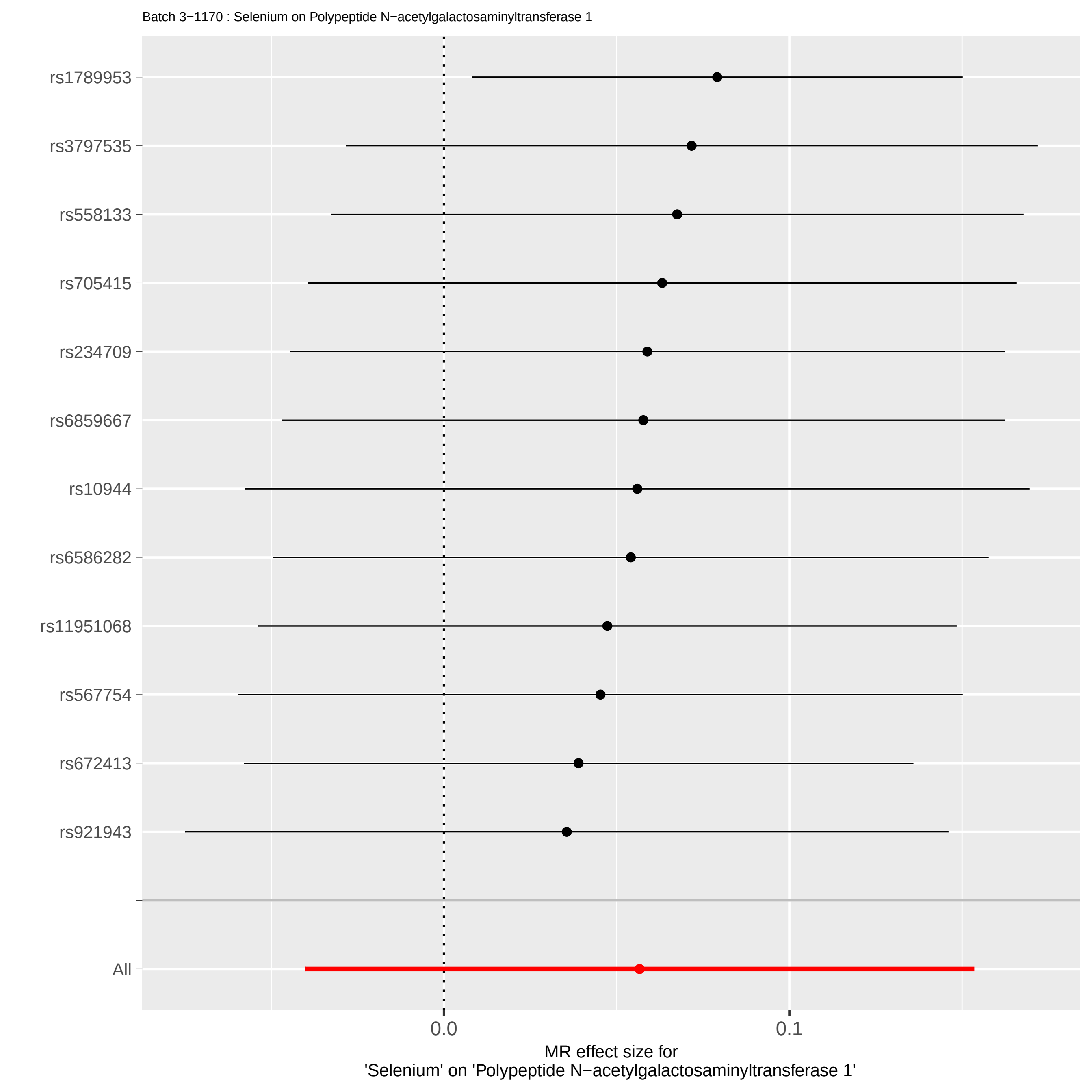
Batch 3−1170 : Selenium on Polypeptide N−acetylgalactosaminyltransferase 1
rs1789953
rs3797535
rs558133
rs705415
rs234709
rs6859667
rs10944
rs6586282
rs11951068
rs567754
rs672413
rs921943
All
0.0
0.1
MR effect size for
'Selenium' on 'Polypeptide N−acetylgalactosaminyltransferase 1'

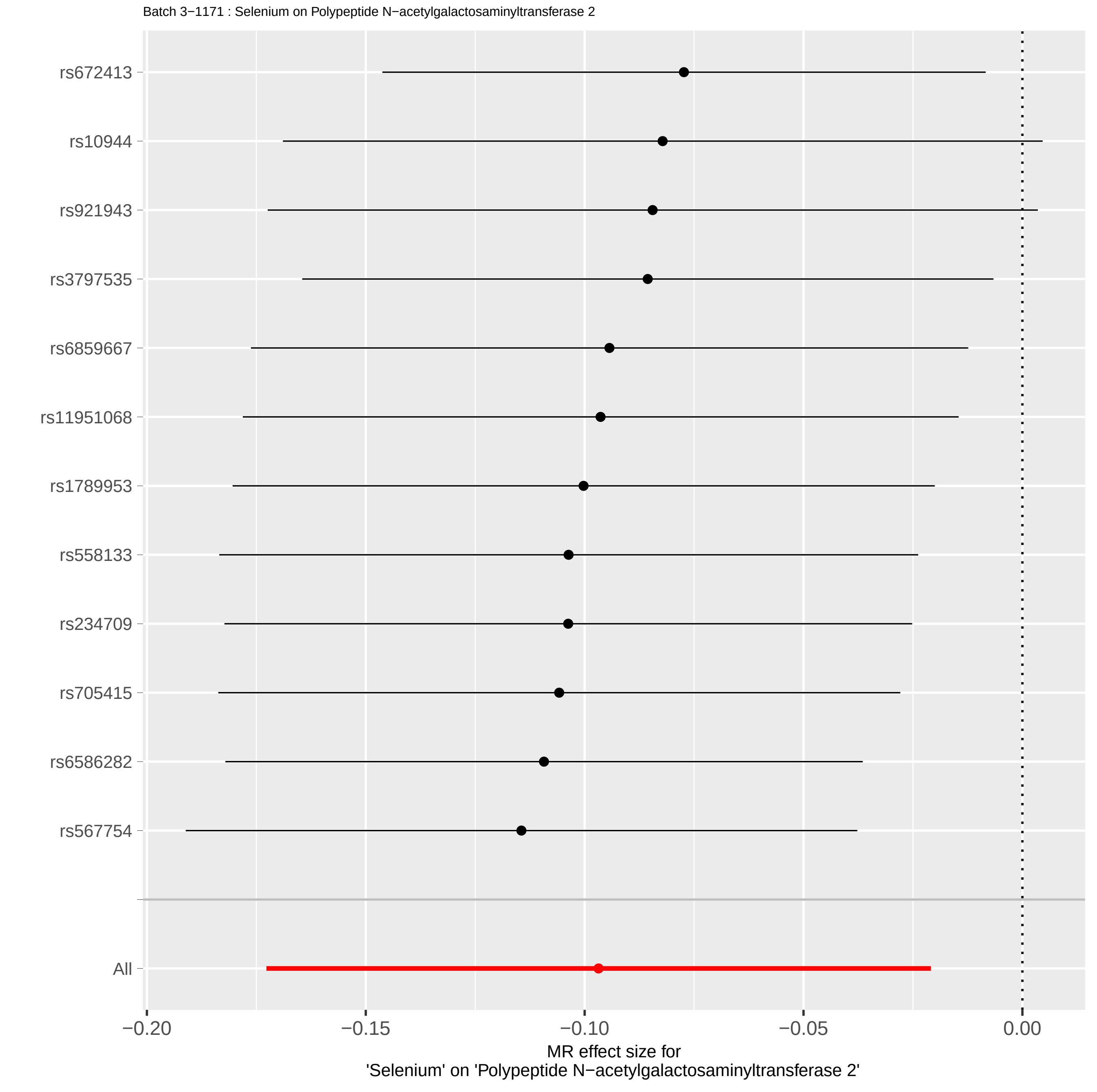

Batch 3−1171 : Selenium on Polypeptide N−acetylgalactosaminyltransferase 2
rs672413
rs10944
rs921943
rs3797535
rs6859667
rs11951068
rs1789953
rs558133
rs234709
rs705415
rs6586282
rs567754
All
−0.20
−0.15
−0.10
−0.05
0.00
MR effect size for
'Selenium' on 'Polypeptide N−acetylgalactosaminyltransferase 2'

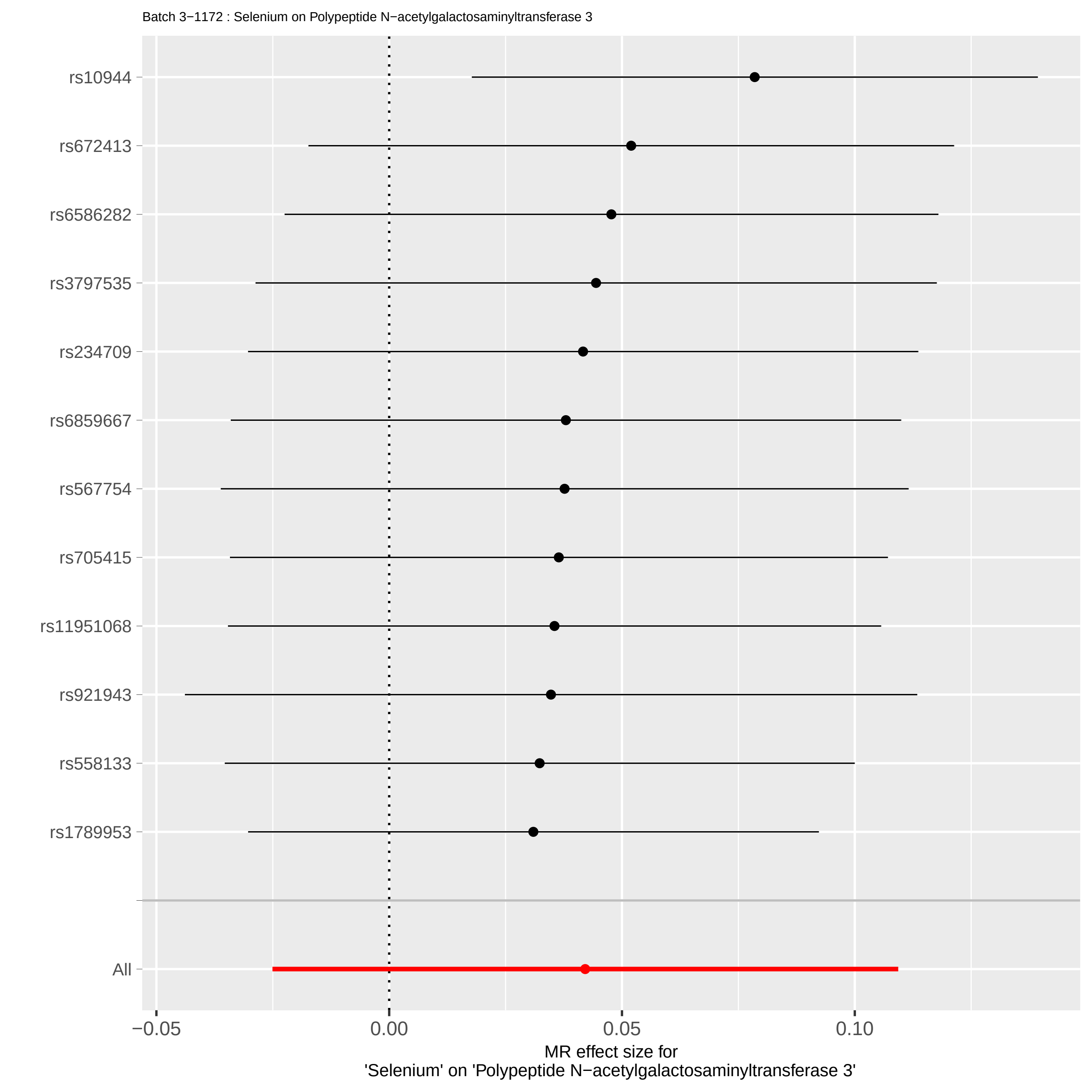

Batch 3−1172 : Selenium on Polypeptide N−acetylgalactosaminyltransferase 3
rs10944
rs672413
rs6586282
rs3797535
rs234709
rs6859667
rs567754
rs705415
rs11951068
rs921943
rs558133
rs1789953
All
−0.05
0.00
0.05
0.10
MR effect size for
'Selenium' on 'Polypeptide N−acetylgalactosaminyltransferase 3'

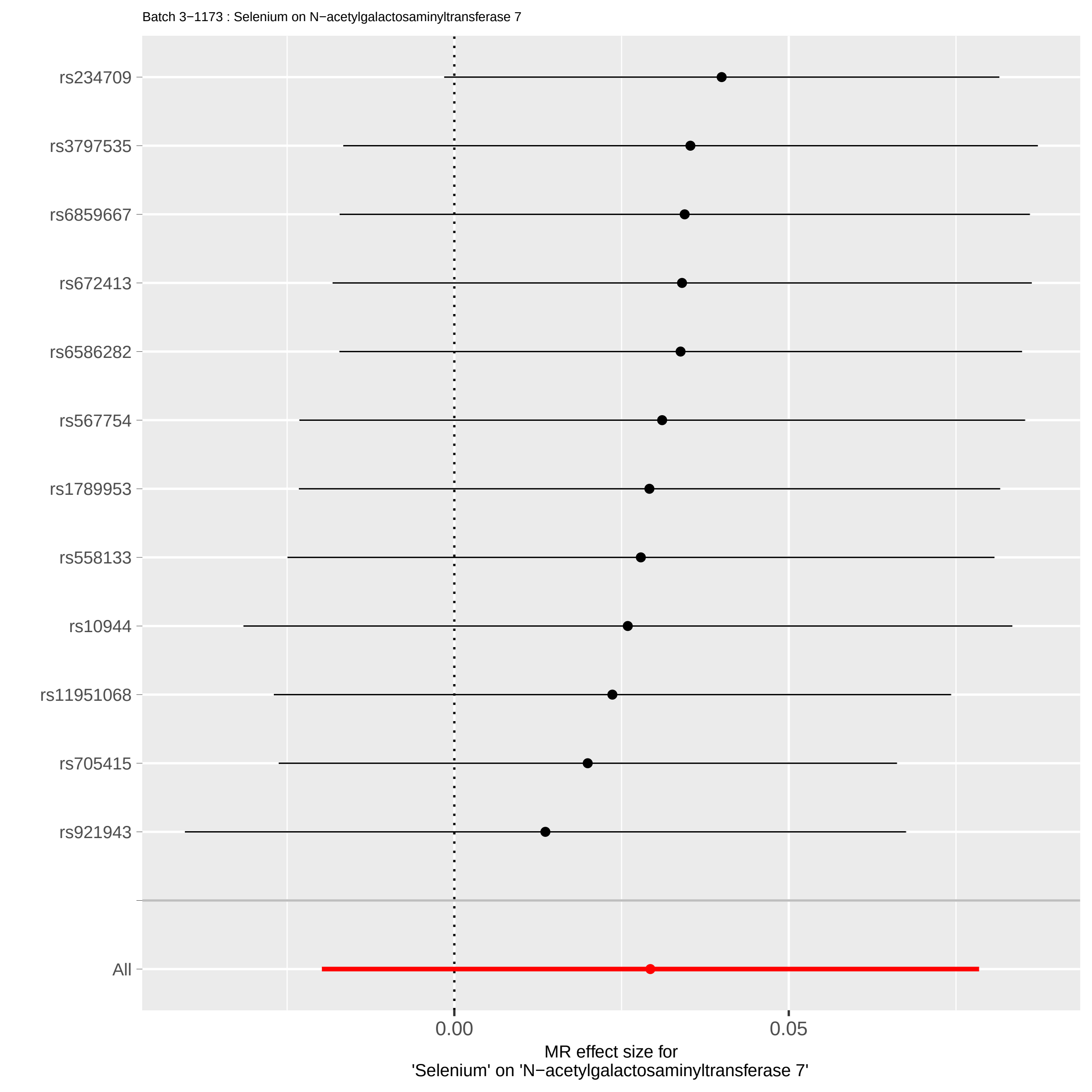

Batch 3−1173 : Selenium on N−acetylgalactosaminyltransferase 7
rs234709
rs3797535
rs6859667
rs672413
rs6586282
rs567754
rs1789953
rs558133
rs10944
rs11951068
rs705415
rs921943
All
0.00
0.05
MR effect size for
'Selenium' on 'N−acetylgalactosaminyltransferase 7'

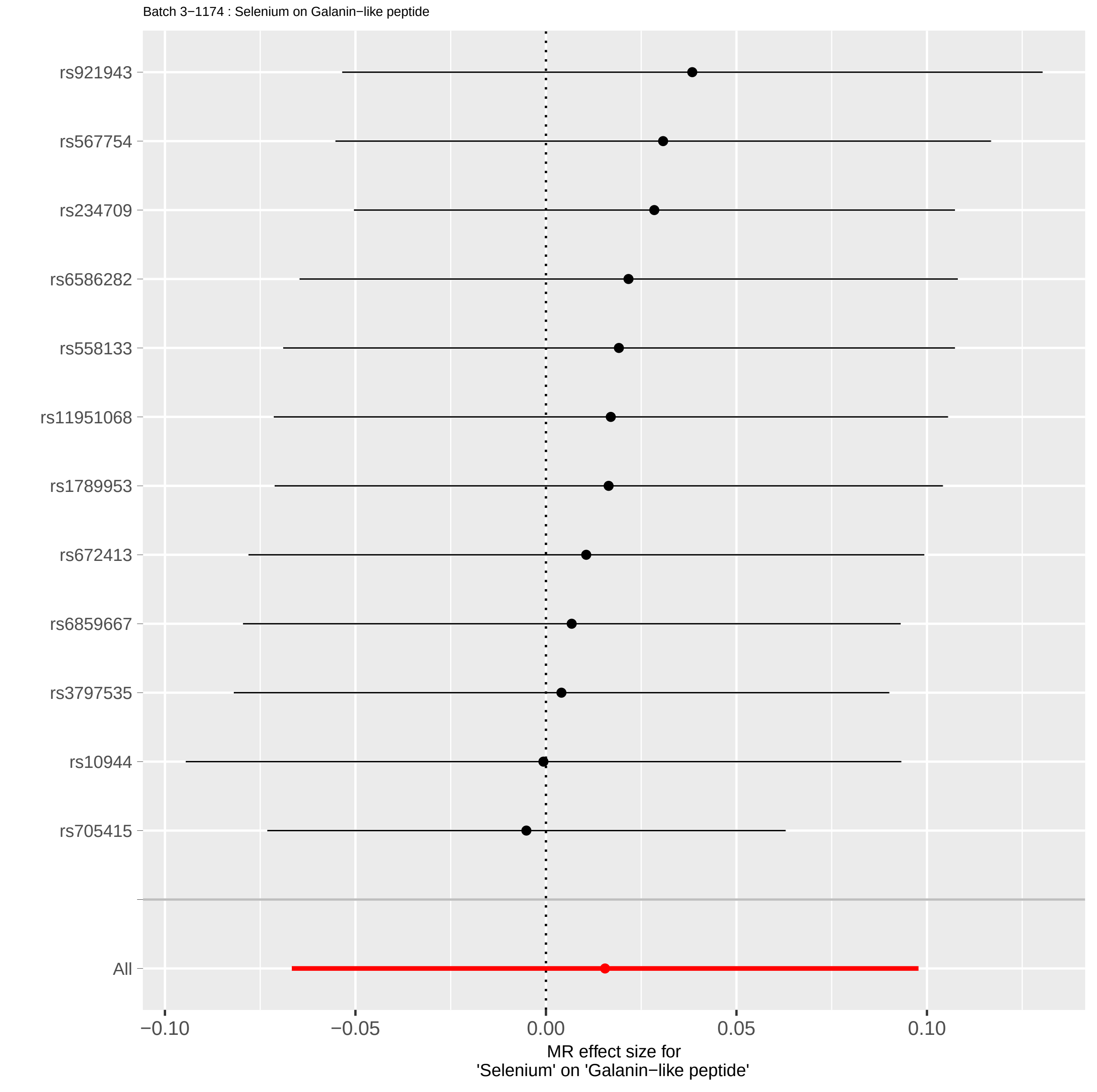

Batch 3−1174 : Selenium on Galanin−like peptide
rs921943
rs567754
rs234709
rs6586282
rs558133
rs11951068
rs1789953
rs672413
rs6859667
rs3797535
rs10944
rs705415
All
−0.10
−0.05
0.00
0.05
0.10
MR effect size for
'Selenium' on 'Galanin−like peptide'

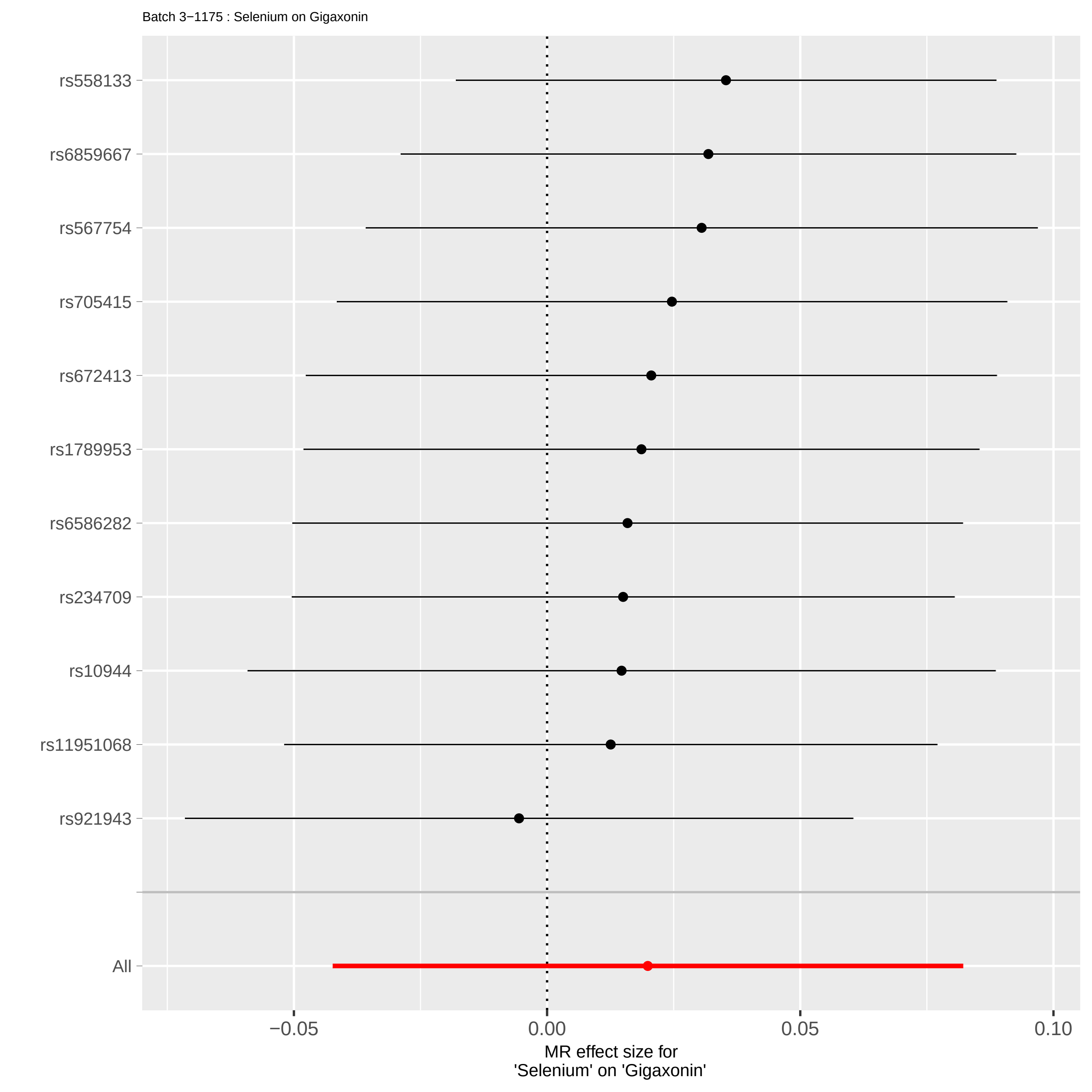

Batch 3−1175 : Selenium on Gigaxonin
rs558133
rs6859667
rs567754
rs705415
rs672413
rs1789953
rs6586282
rs234709
rs10944
rs11951068
rs921943
All
−0.05
0.00
0.05
0.10
MR effect size for
'Selenium' on 'Gigaxonin'

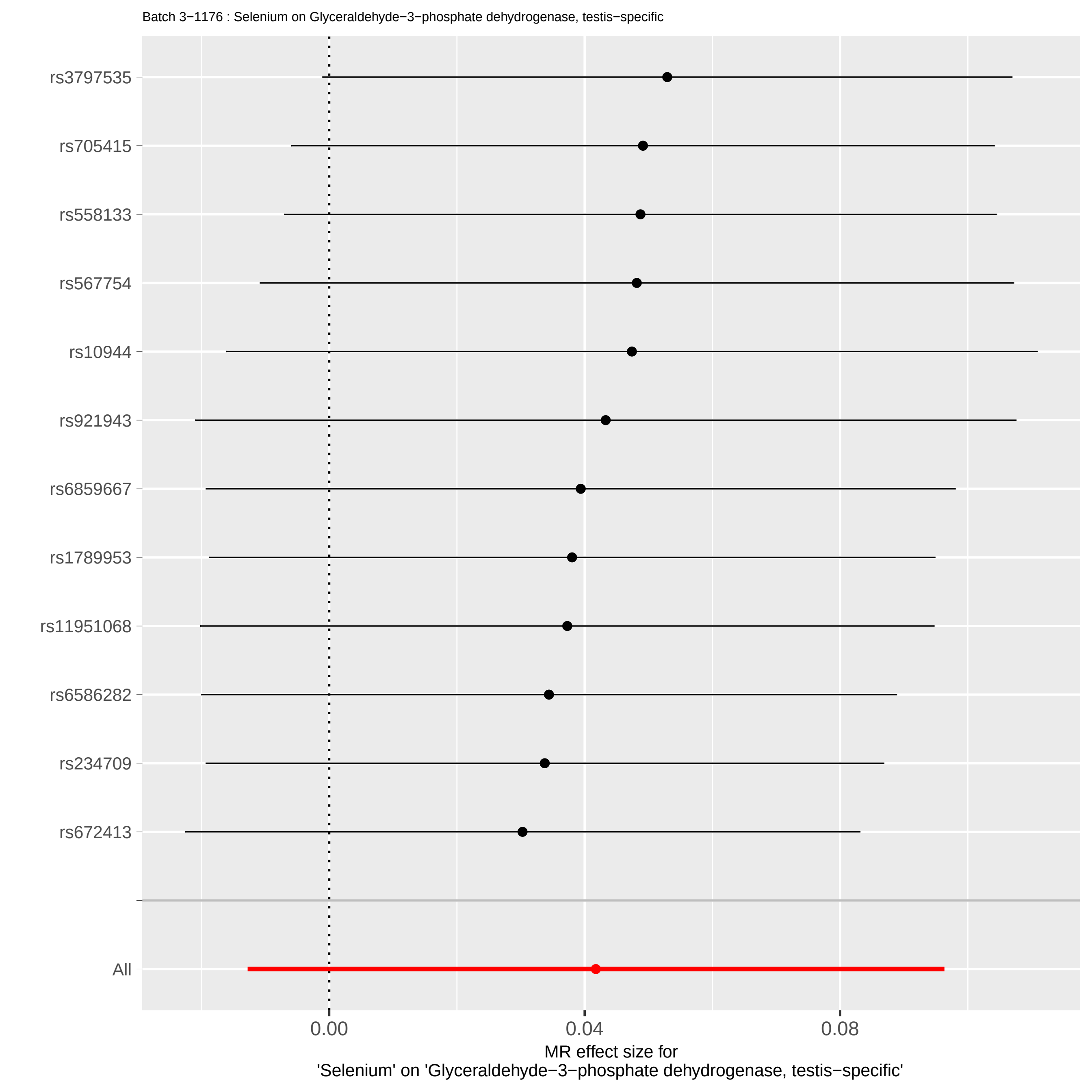
Batch 3−1176 : Selenium on Glyceraldehyde−3−phosphate dehydrogenase, testis−specific
rs3797535
rs705415
rs558133
rs567754
rs10944
rs921943
rs6859667
rs1789953
rs11951068
rs6586282
rs234709
rs672413
All
0.00
0.04
0.08
MR effect size for
'Selenium' on 'Glyceraldehyde−3−phosphate dehydrogenase, testis−specific'

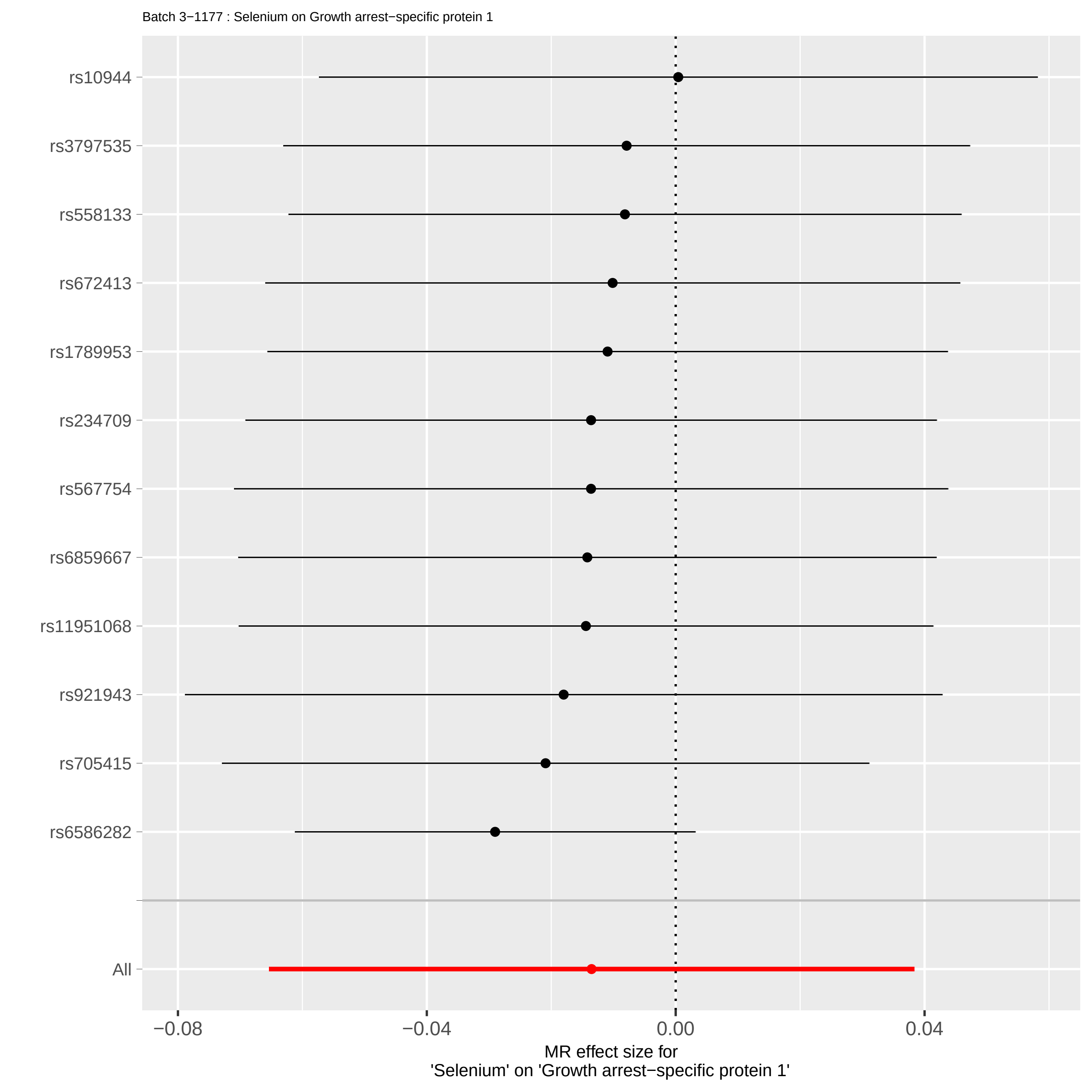

Batch 3−1177 : Selenium on Growth arrest−specific protein 1
rs10944
rs3797535
rs558133
rs672413
rs1789953
rs234709
rs567754
rs6859667
rs11951068
rs921943
rs705415
rs6586282
All
−0.08
−0.04
0.00
0.04
MR effect size for
'Selenium' on 'Growth arrest−specific protein 1'

Batch 3−1178 : Selenium on Guanylate−binding protein 6

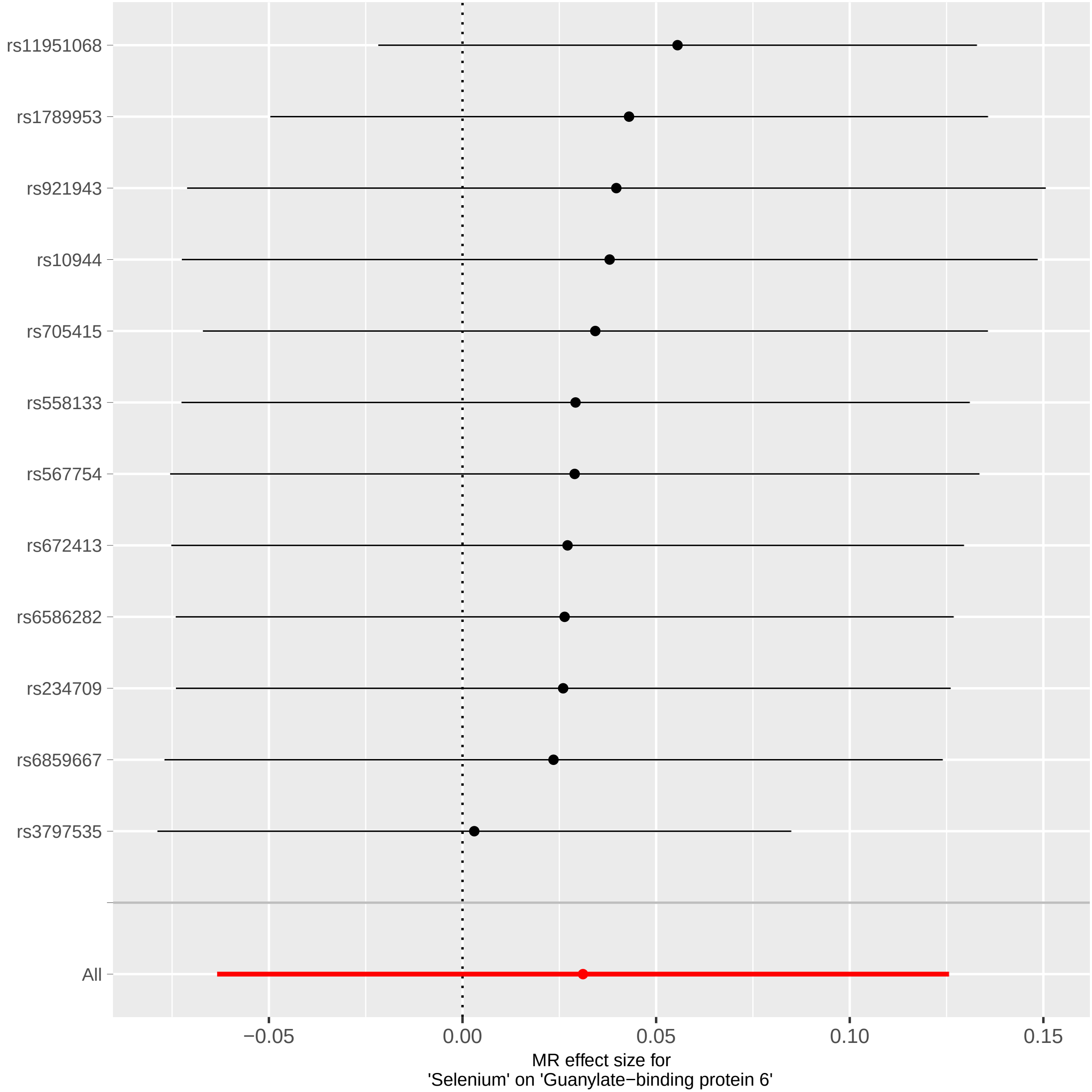

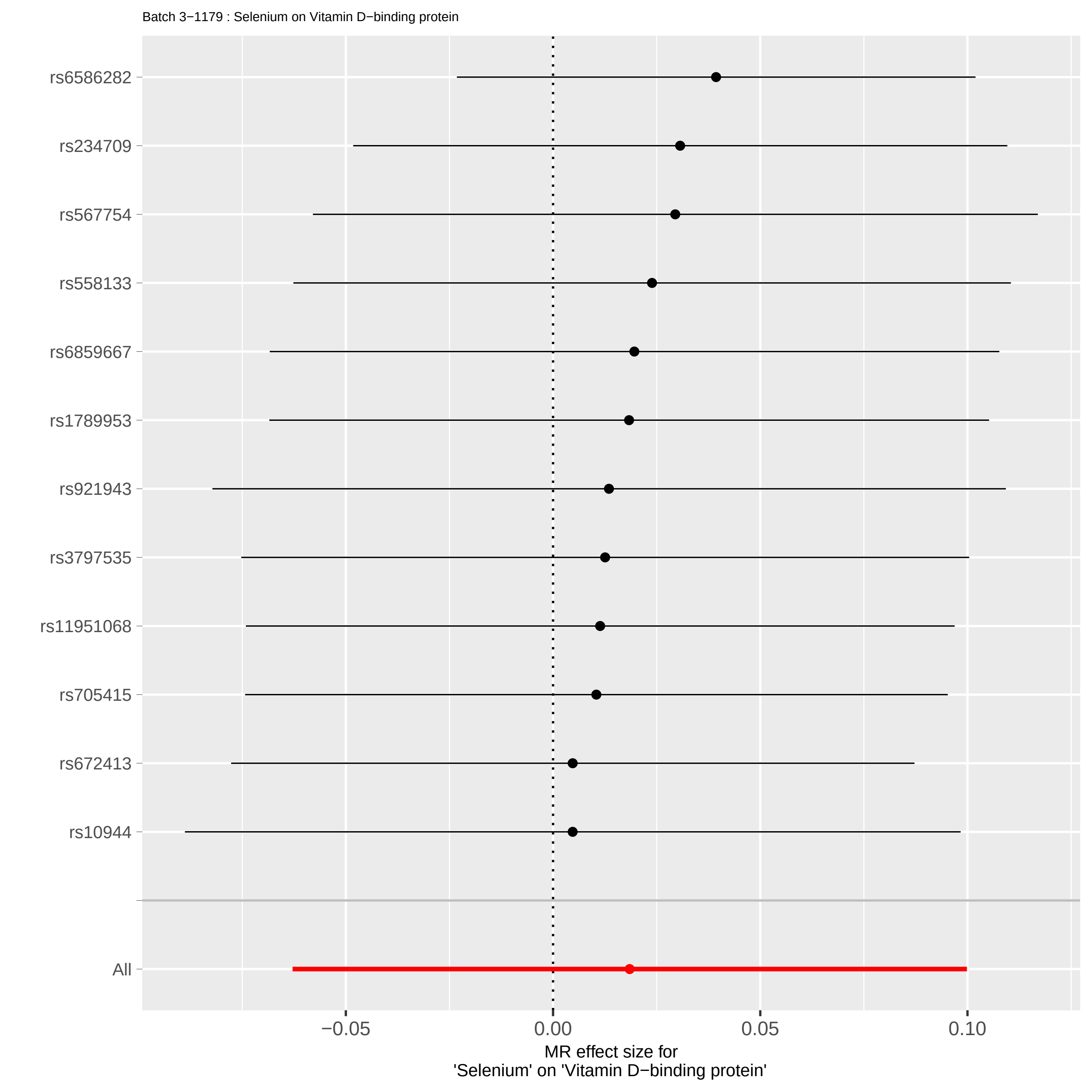

Batch 3−1179 : Selenium on Vitamin D−binding protein
rs6586282
rs234709
rs567754
rs558133
rs6859667
rs1789953
rs921943
rs3797535
rs11951068
rs705415
rs672413
rs10944
All
−0.05
0.00
0.05
0.10
MR effect size for
'Selenium' on 'Vitamin D−binding protein'

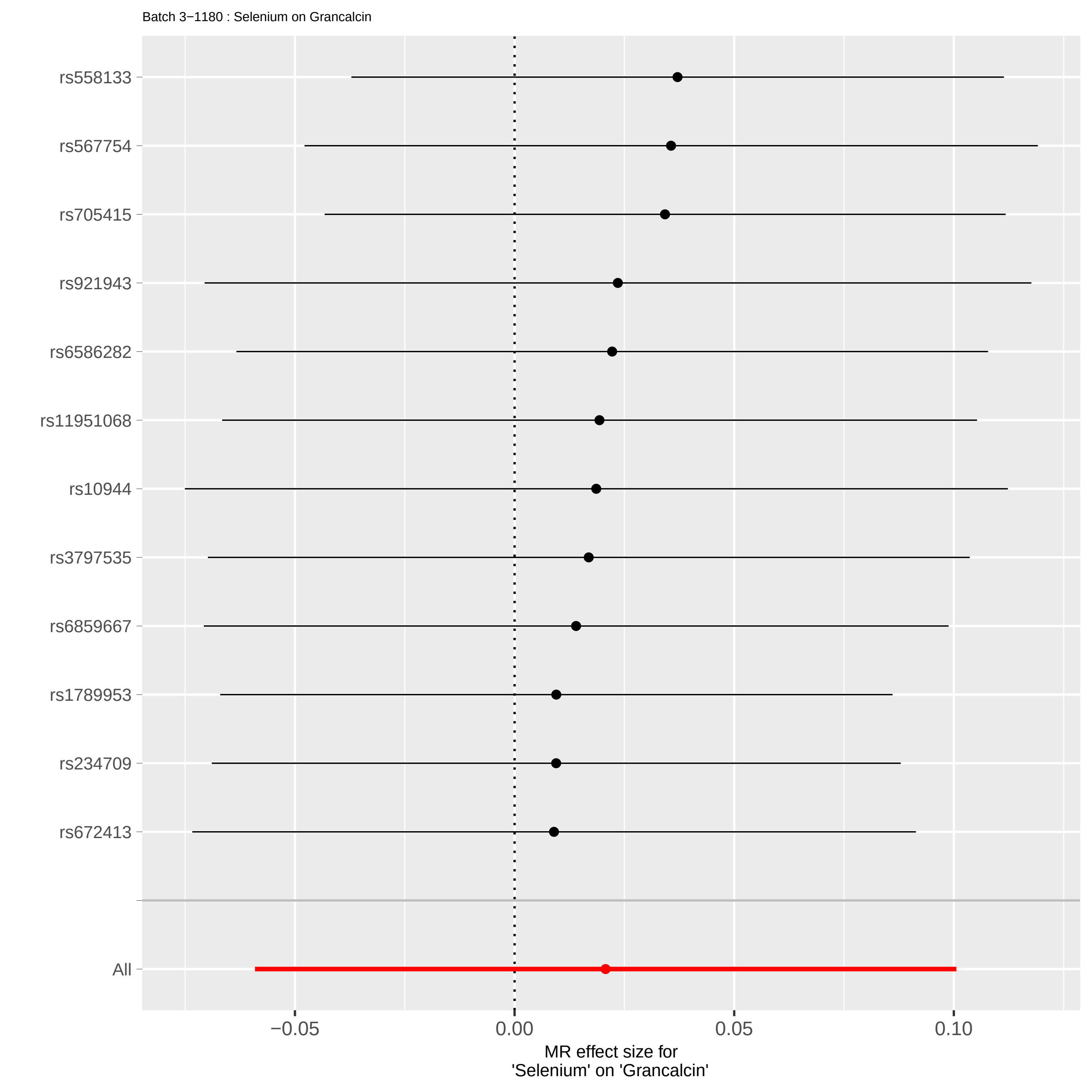

Batch 3−1180 : Selenium on Grancalcin
rs558133
rs567754
rs705415
rs921943
rs6586282
rs11951068
rs10944
rs3797535
rs6859667
rs1789953
rs234709
rs672413
All
−0.05
0.00
0.05
0.10
MR effect size for
'Selenium' on 'Grancalcin'

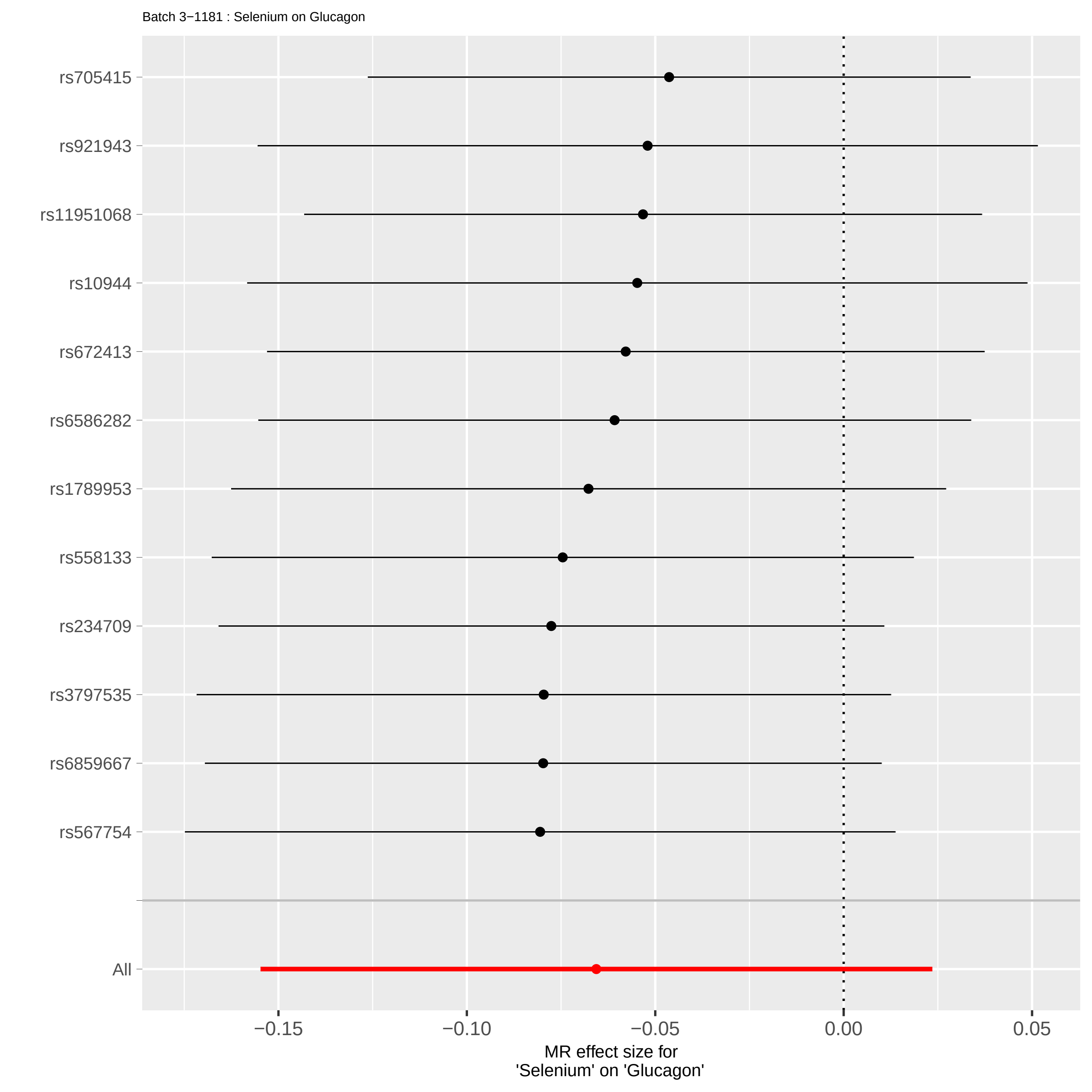

Batch 3−1181 : Selenium on Glucagon
rs705415
rs921943
rs11951068
rs10944
rs672413
rs6586282
rs1789953
rs558133
rs234709
rs3797535
rs6859667
rs567754
All
−0.15
−0.10
−0.05
0.00
0.05
MR effect size for
'Selenium' on 'Glucagon'

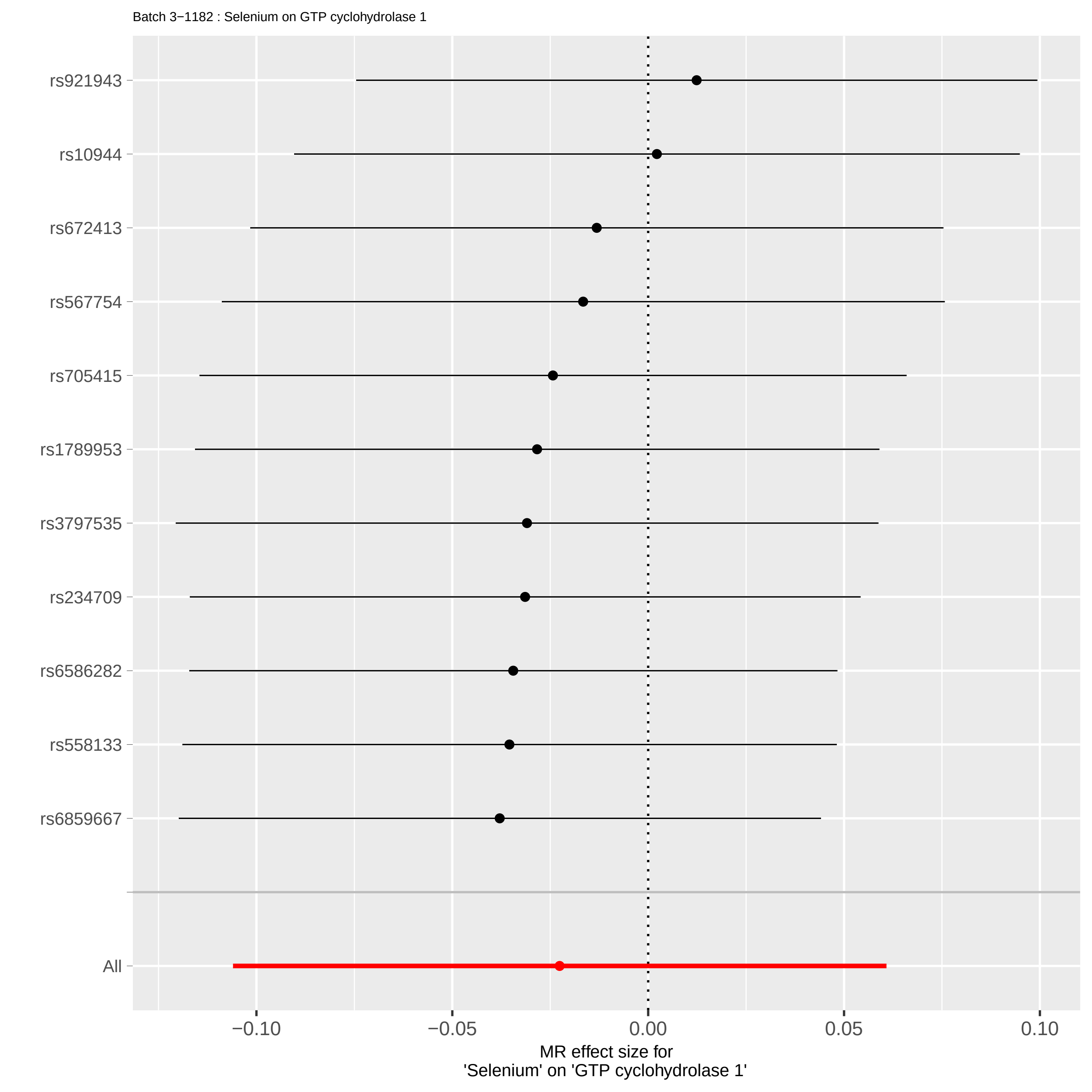
Batch 3−1182 : Selenium on GTP cyclohydrolase 1
rs921943
rs10944
rs672413
rs567754
rs705415
rs1789953
rs3797535
rs234709
rs6586282
rs558133
rs6859667
All
−0.10
−0.05
0.00
0.05
0.10
MR effect size for
'Selenium' on 'GTP cyclohydrolase 1'

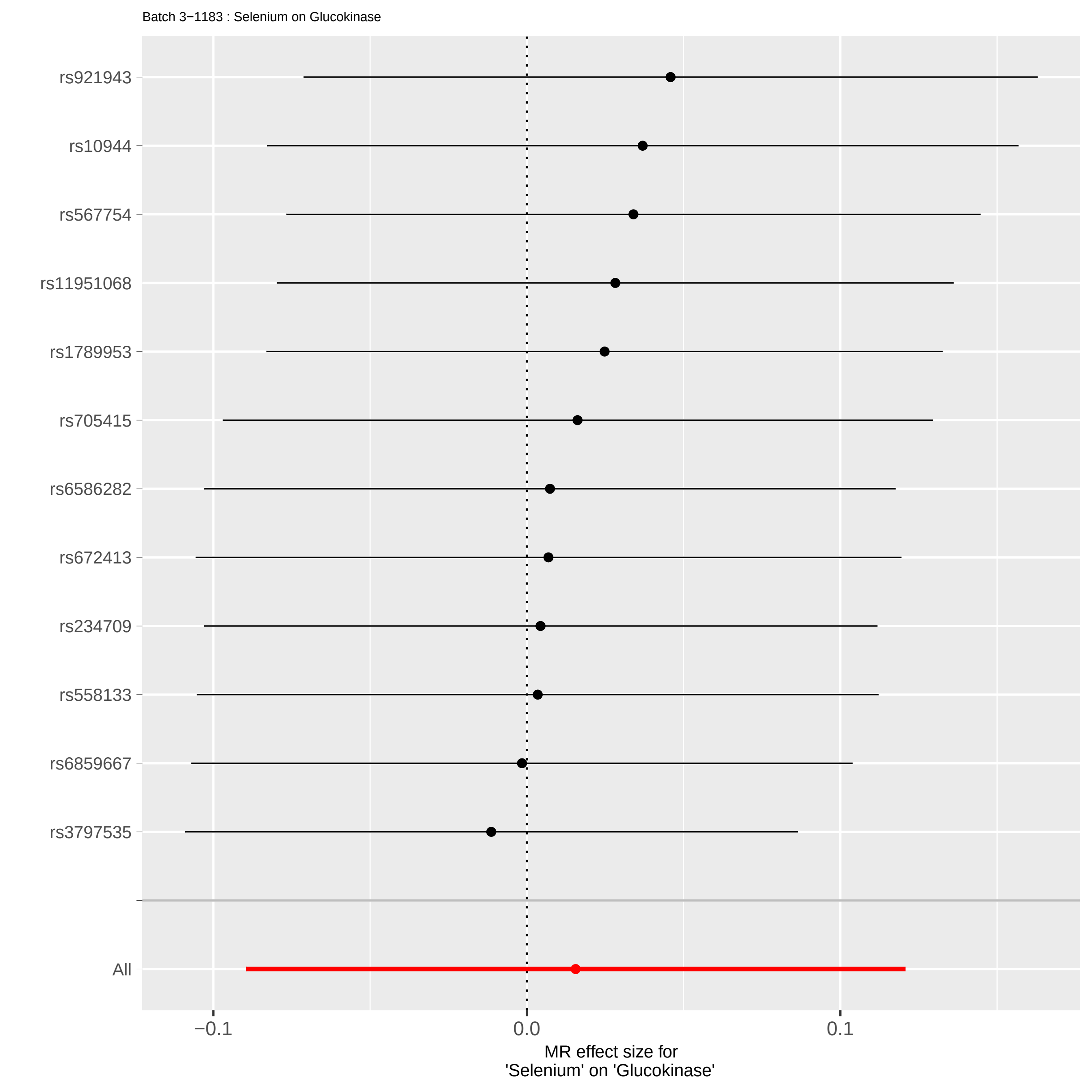

Batch 3−1183 : Selenium on Glucokinase
rs921943
rs10944
rs567754
rs11951068
rs1789953
rs705415
rs6586282
rs672413
rs234709
rs558133
rs6859667
rs3797535
All
−0.1
0.0
0.1
MR effect size for
'Selenium' on 'Glucokinase'

Batch 3−1184 : Selenium on Glucokinase regulatory protein

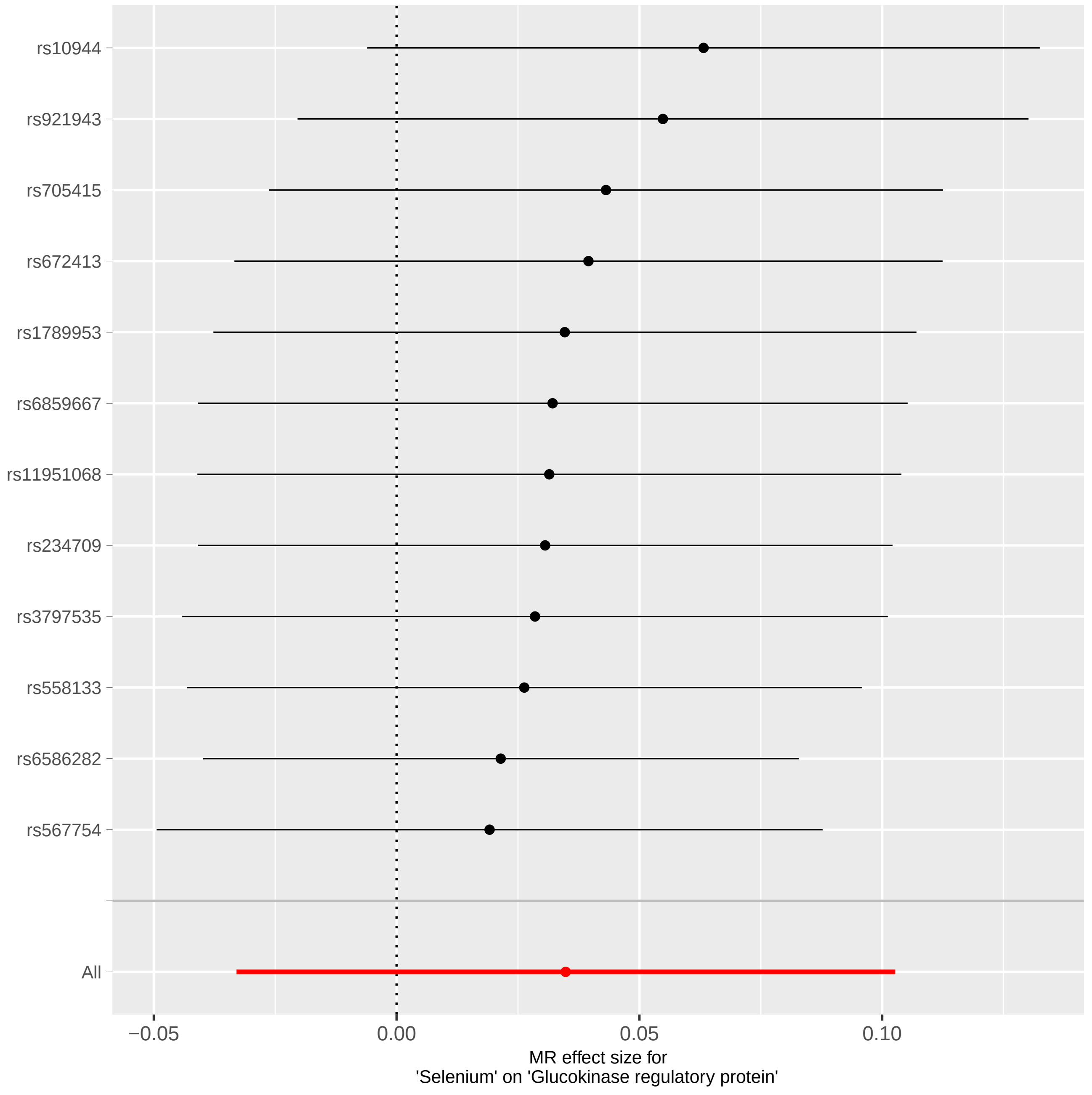

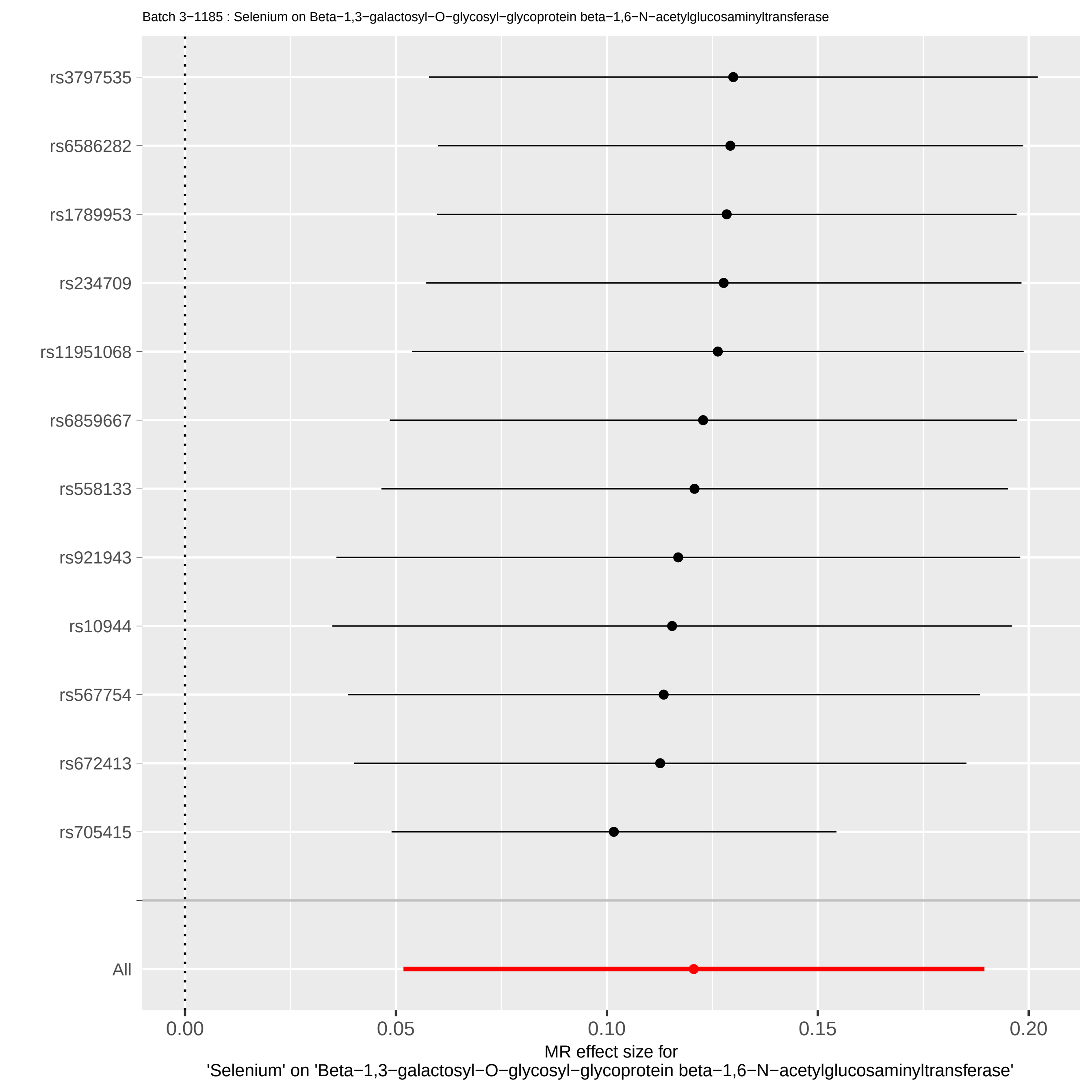

Batch 3−1185 : Selenium on Beta−1,3−galactosyl−O−glycosyl−glycoprotein beta−1,6−N−acetylglucosaminyltransferase
rs3797535
rs6586282
rs1789953
rs234709
rs11951068
rs6859667
rs558133
rs921943
rs10944
rs567754
rs672413
rs705415
All
0.00
0.05
0.10
0.15
0.20
MR effect size for
'Selenium' on 'Beta−1,3−galactosyl−O−glycosyl−glycoprotein beta−1,6−N−acetylglucosaminyltransferase'

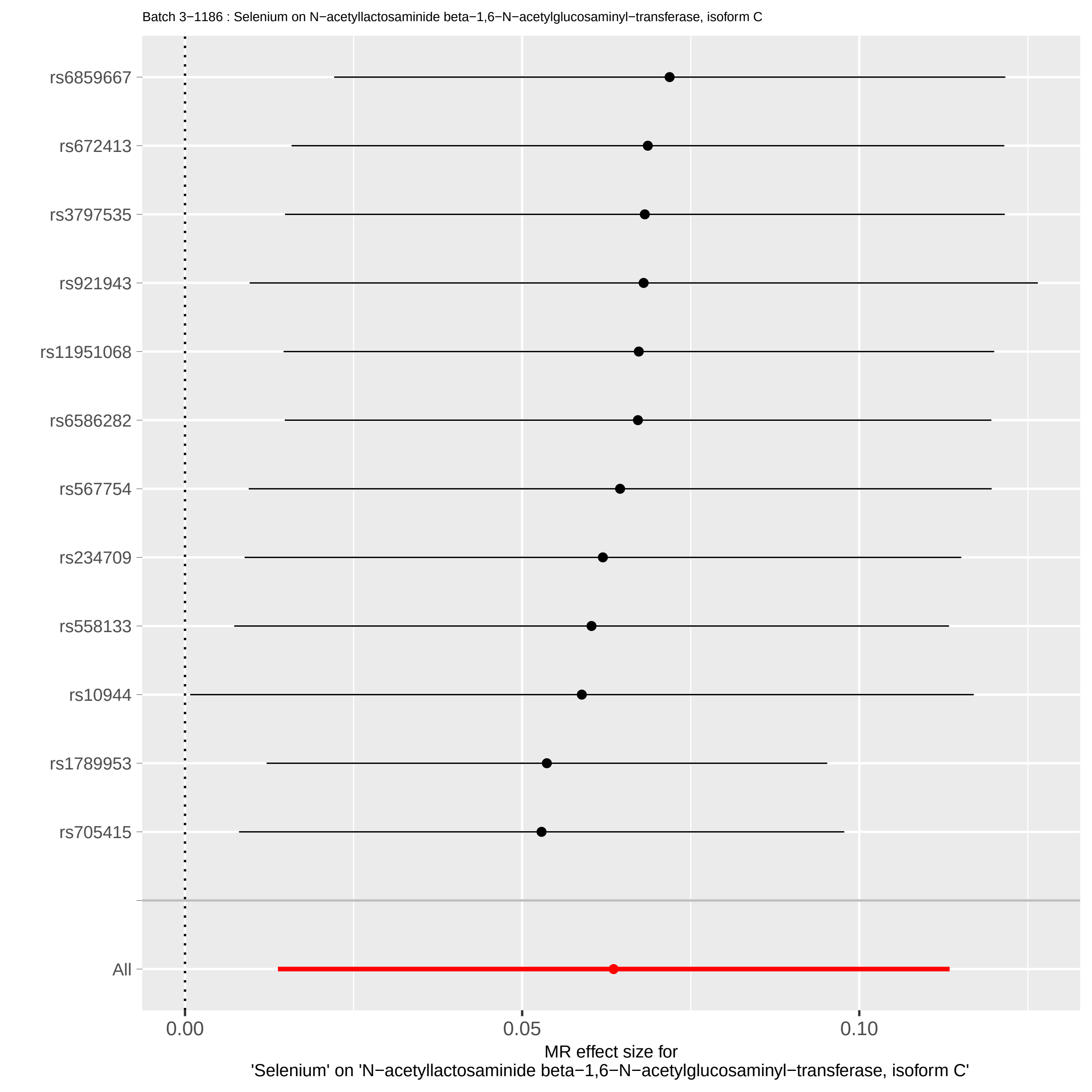
Batch 3−1186 : Selenium on N−acetyllactosaminide beta−1,6−N−acetylglucosaminyl−transferase, isoform C
rs6859667
rs672413
rs3797535
rs921943
rs11951068
rs6586282
rs567754
rs234709
rs558133
rs10944
rs1789953
rs705415
All
0.00
0.05
0.10
MR effect size for
'Selenium' on 'N−acetyllactosaminide beta−1,6−N−acetylglucosaminyl−transferase, isoform C'

Batch 3−1187 : Selenium on Beta−1,3−galactosyl−O−glycosyl−glycoprotein beta−1,6−N−acetylglucosaminyltransferase 4

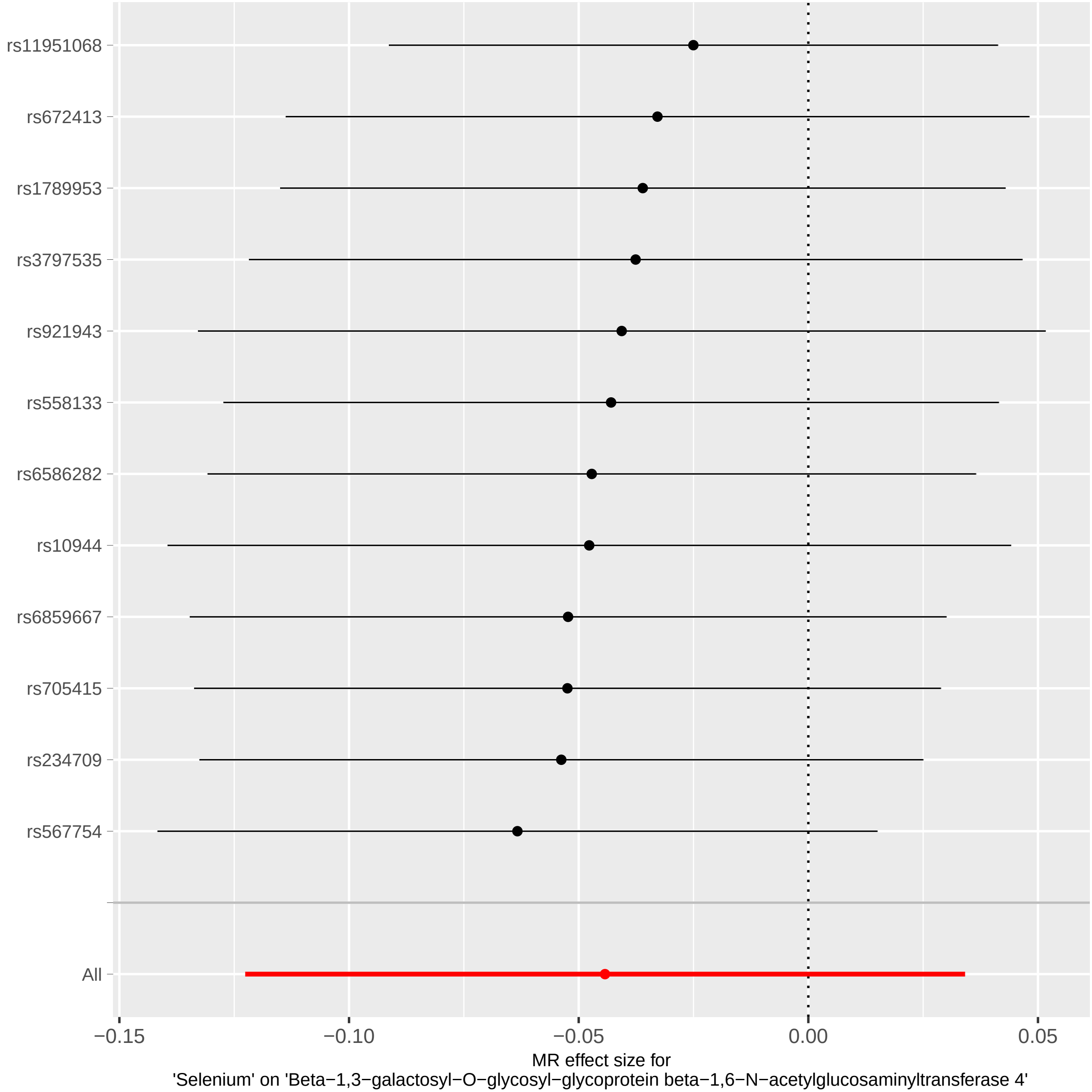

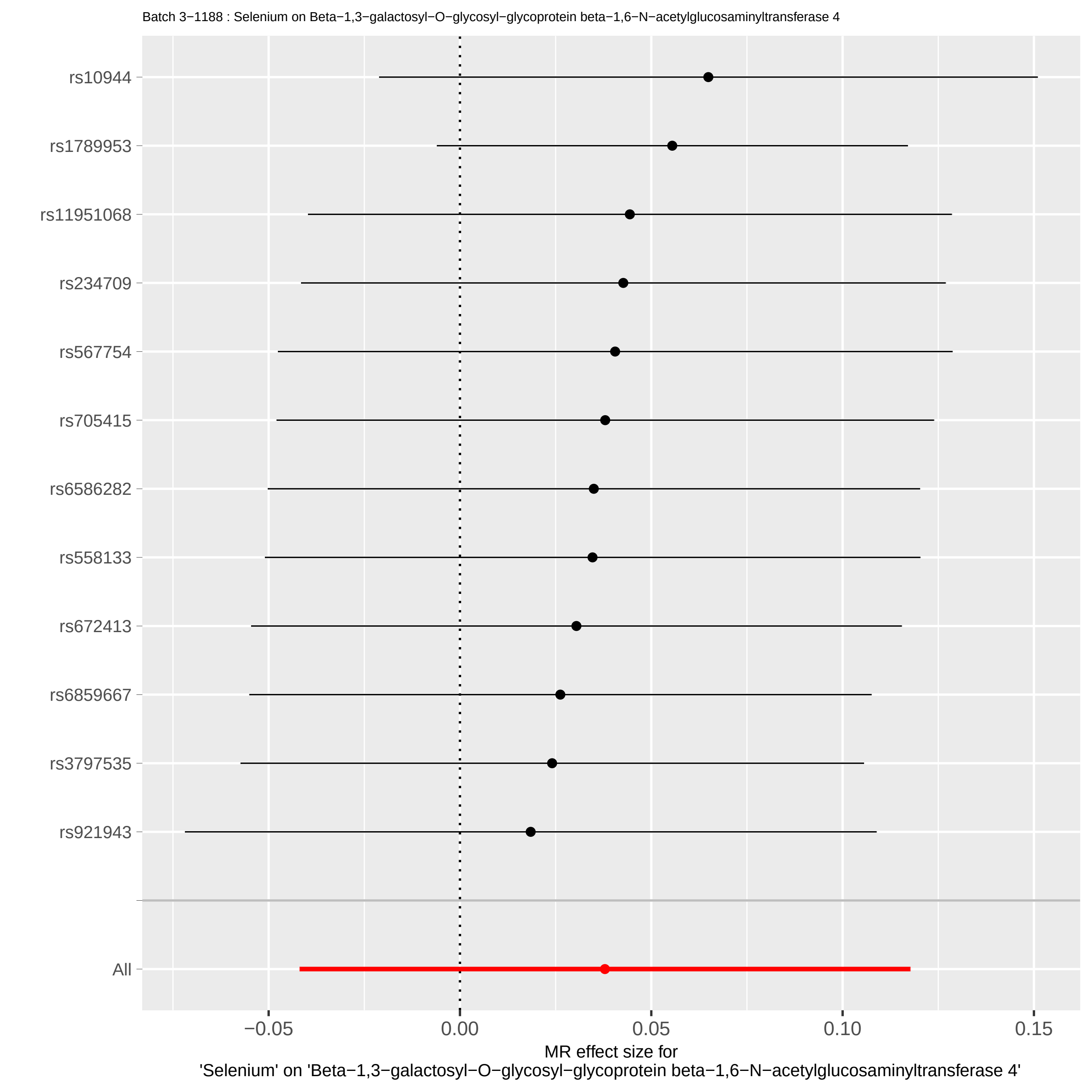
Batch 3−1188 : Selenium on Beta−1,3−galactosyl−O−glycosyl−glycoprotein beta−1,6−N−acetylglucosaminyltransferase 4
rs10944
rs1789953
rs11951068
rs234709
rs567754
rs705415
rs6586282
rs558133
rs672413
rs6859667
rs3797535
rs921943
All
−0.05
0.00
0.05
0.10
0.15
MR effect size for
'Selenium' on 'Beta−1,3−galactosyl−O−glycosyl−glycoprotein beta−1,6−N−acetylglucosaminyltransferase 4'

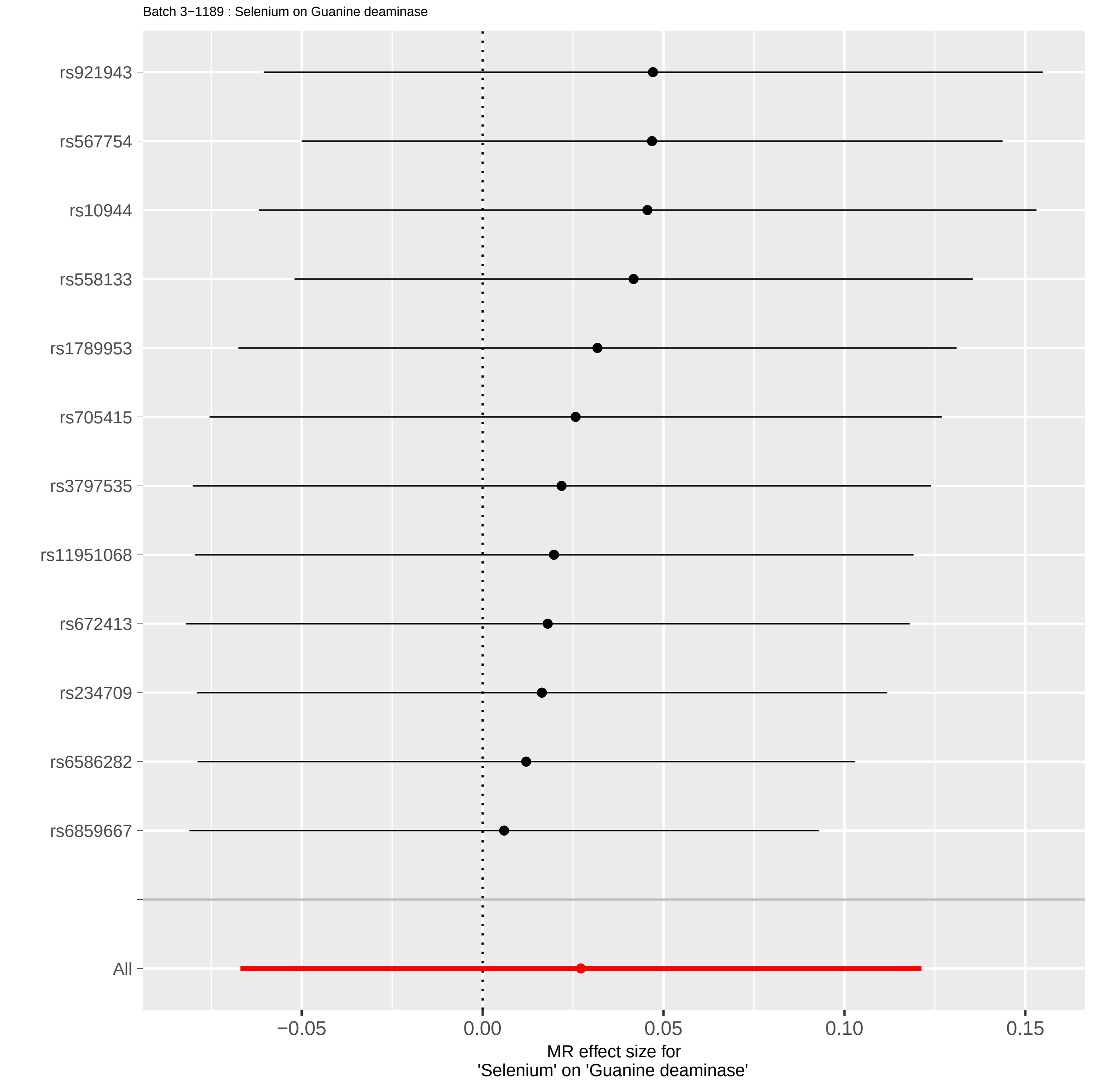

Batch 3−1189 : Selenium on Guanine deaminase
rs921943
rs567754
rs10944
rs558133
rs1789953
rs705415
rs3797535
rs11951068
rs672413
rs234709
rs6586282
rs6859667
All
−0.05
0.00
0.05
0.10
0.15
MR effect size for
'Selenium' on 'Guanine deaminase'

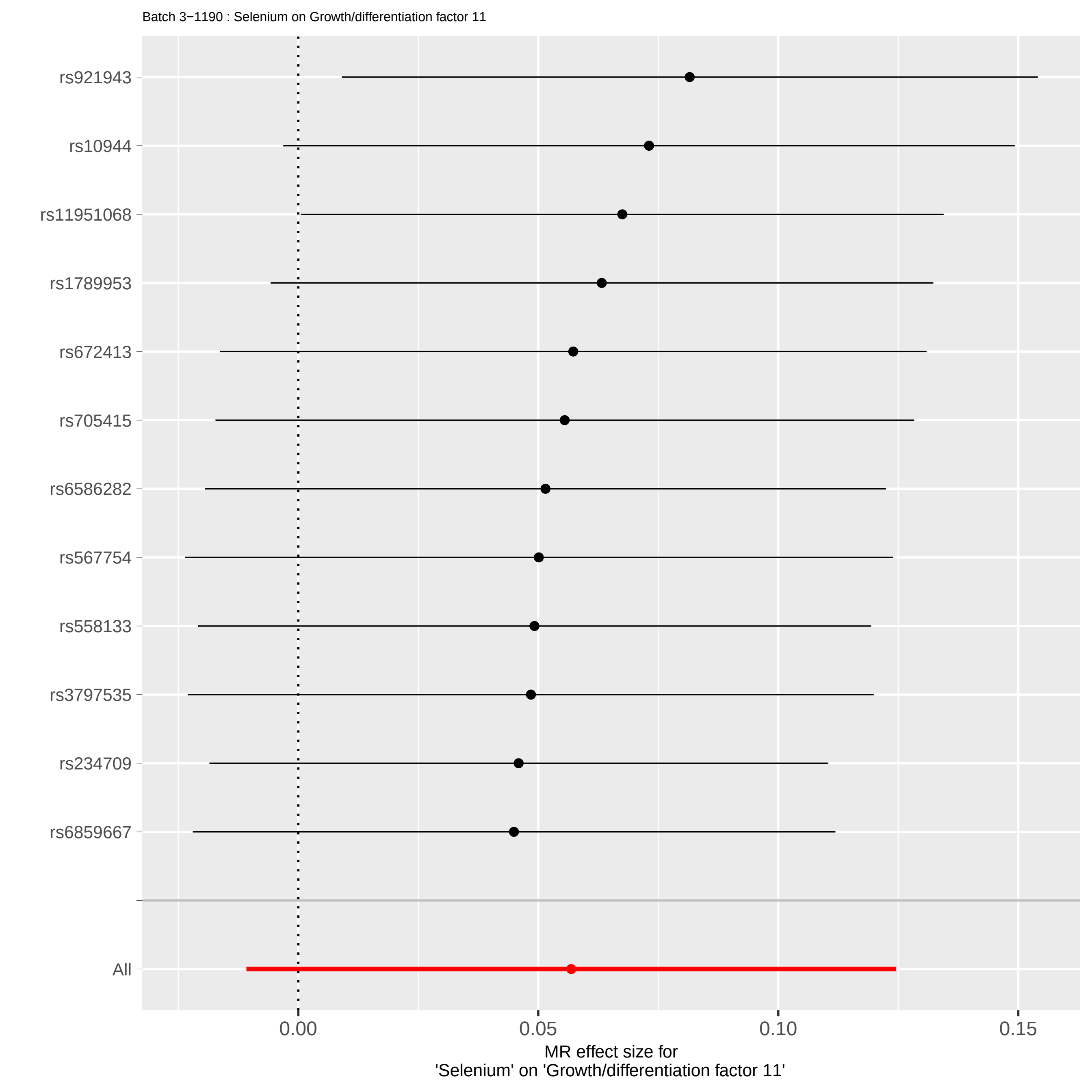
Batch 3−1190 : Selenium on Growth/differentiation factor 11
rs921943
rs10944
rs11951068
rs1789953
rs672413
rs705415
rs6586282
rs567754
rs558133
rs3797535
rs234709
rs6859667
All
0.00
0.05
0.10
0.15
MR effect size for
'Selenium' on 'Growth/differentiation factor 11'

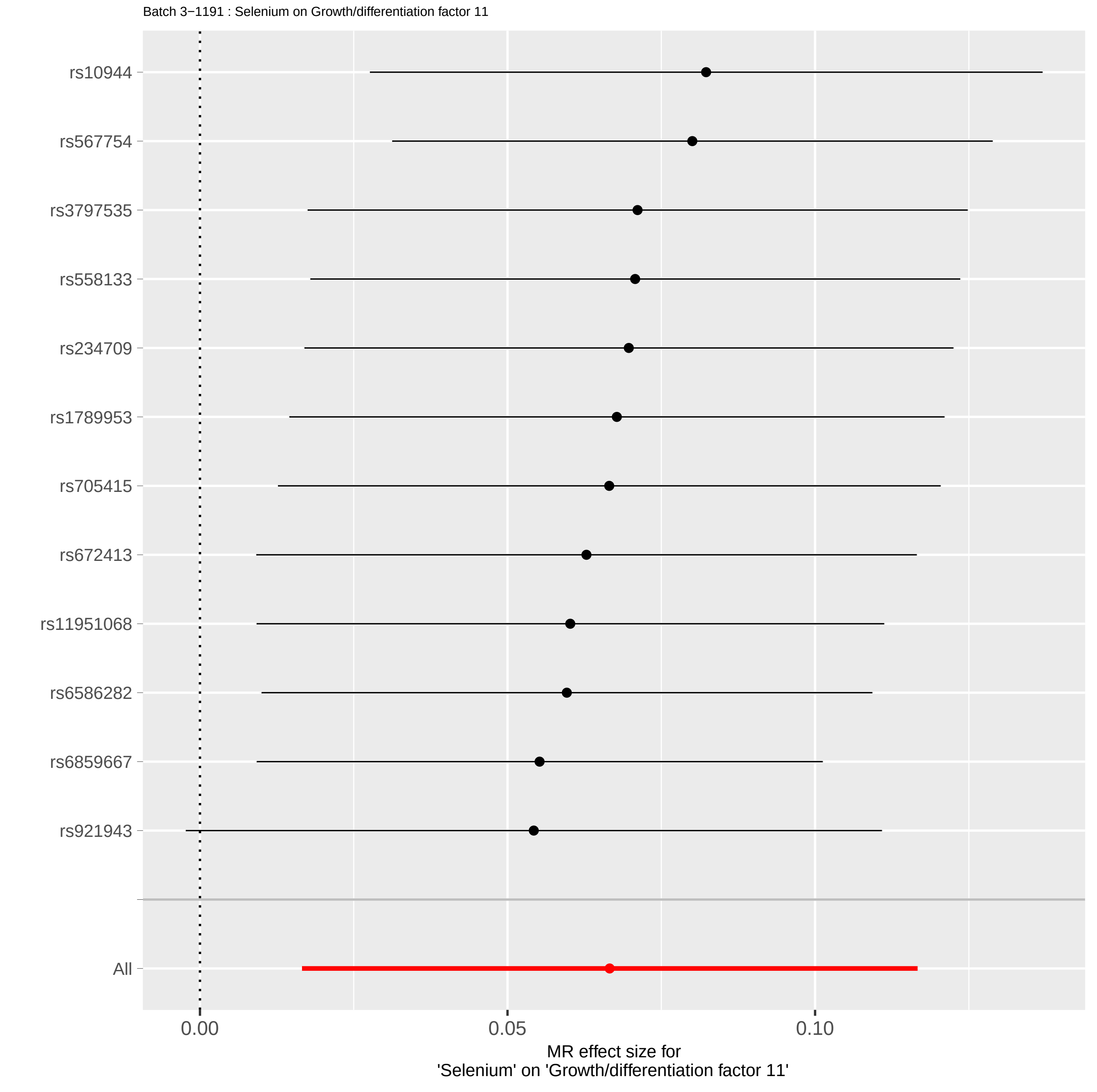

Batch 3−1191 : Selenium on Growth/differentiation factor 11
rs10944
rs567754
rs3797535
rs558133
rs234709
rs1789953
rs705415
rs672413
rs11951068
rs6586282
rs6859667
rs921943
All
0.00
0.05
0.10
MR effect size for
'Selenium' on 'Growth/differentiation factor 11'

Batch 3−1192 : Selenium on Growth/differentiation factor 11

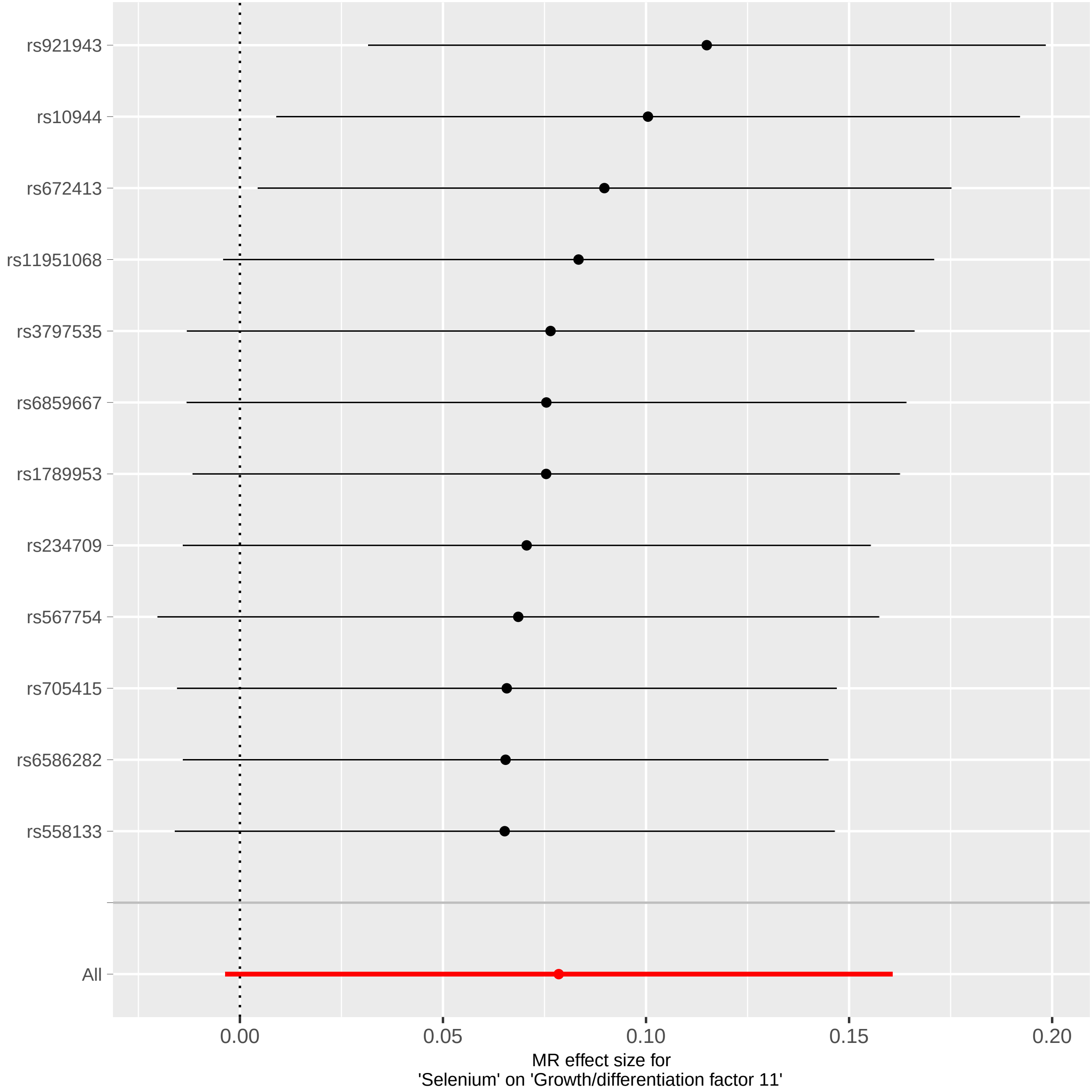

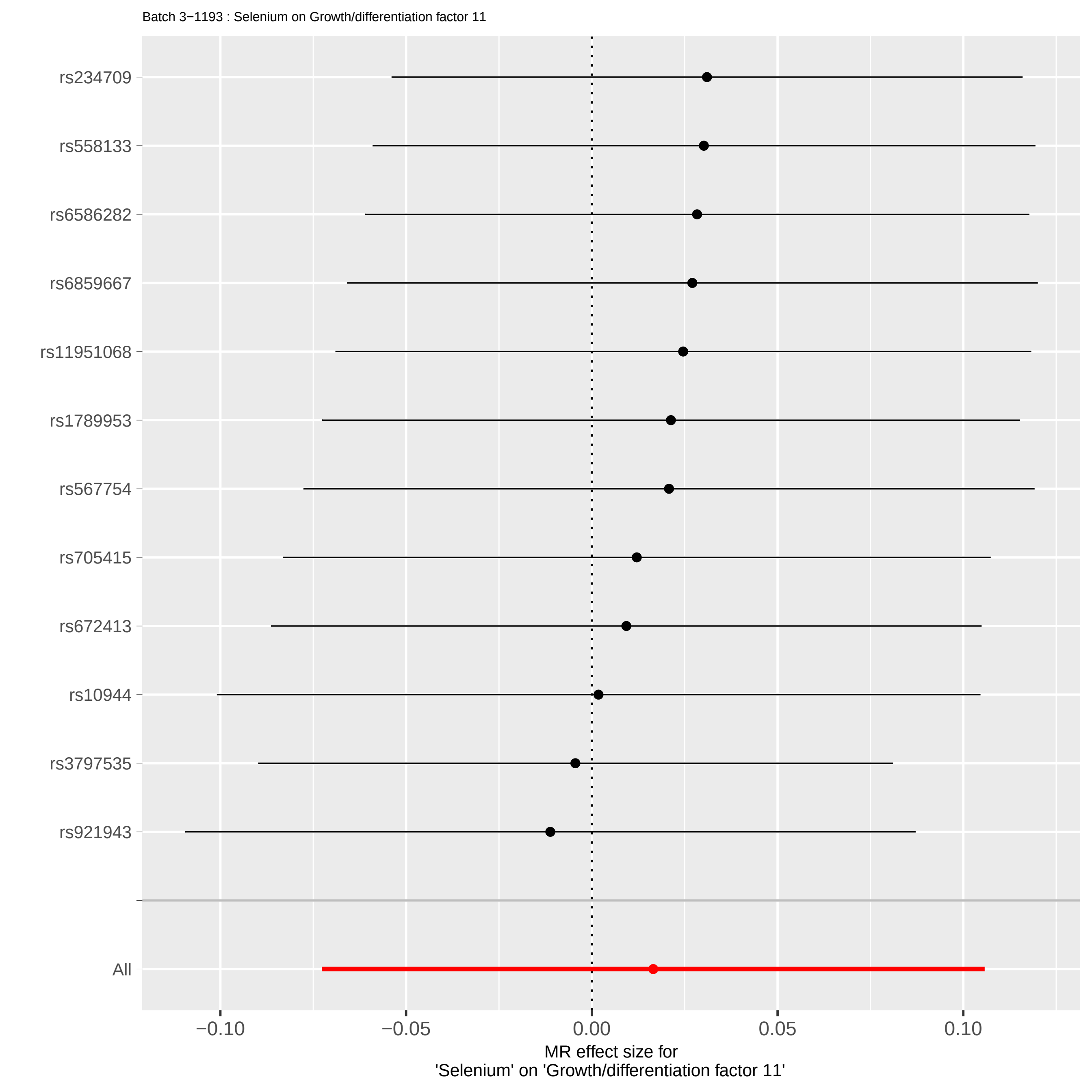

Batch 3−1193 : Selenium on Growth/differentiation factor 11
rs234709
rs558133
rs6586282
rs6859667
rs11951068
rs1789953
rs567754
rs705415
rs672413
rs10944
rs3797535
rs921943
All
−0.10
−0.05
0.00
0.05
0.10
MR effect size for
'Selenium' on 'Growth/differentiation factor 11'

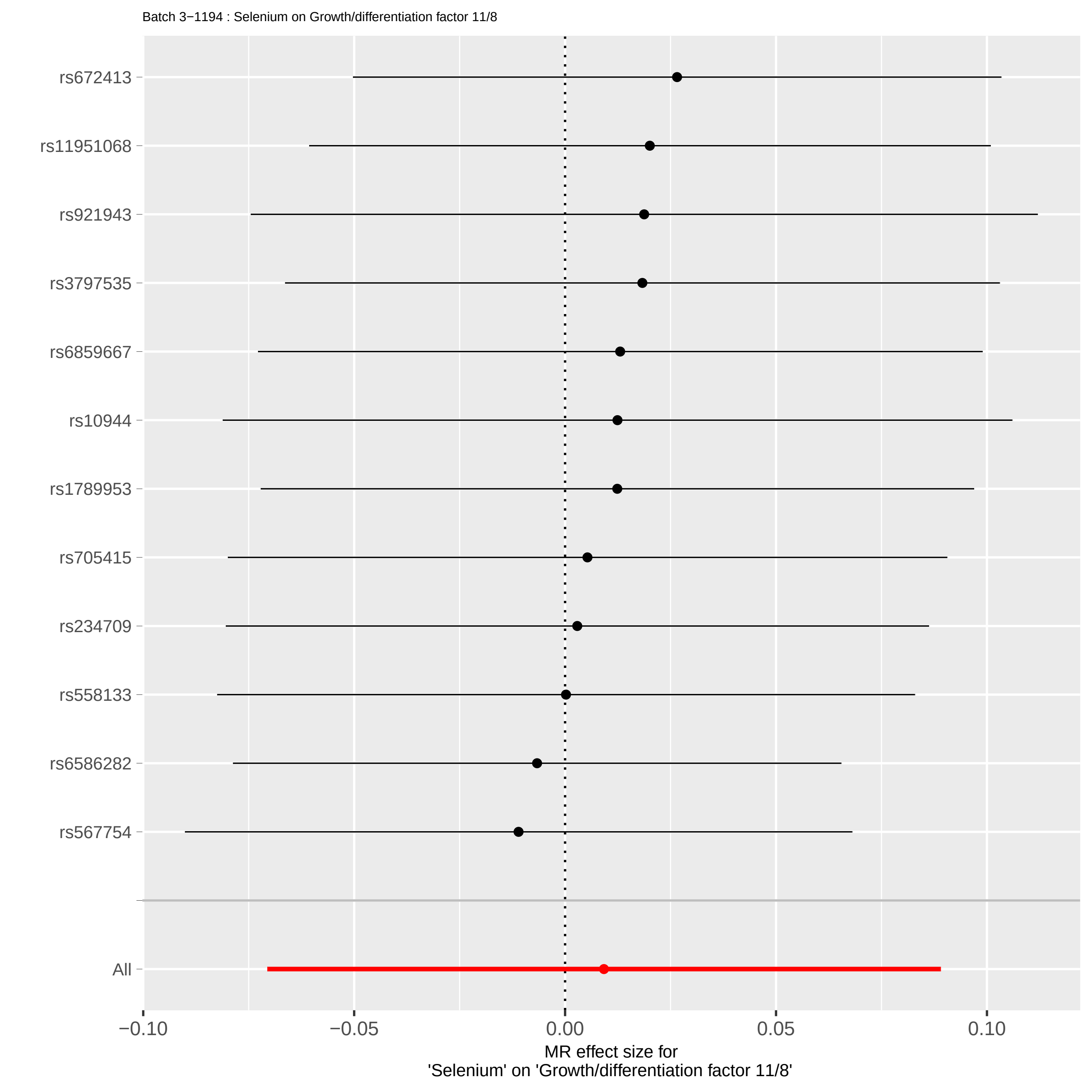
Batch 3−1194 : Selenium on Growth/differentiation factor 11/8
rs672413
rs11951068
rs921943
rs3797535
rs6859667
rs10944
rs1789953
rs705415
rs234709
rs558133
rs6586282
rs567754
All
−0.10
−0.05
0.00
0.05
0.10
MR effect size for
'Selenium' on 'Growth/differentiation factor 11/8'

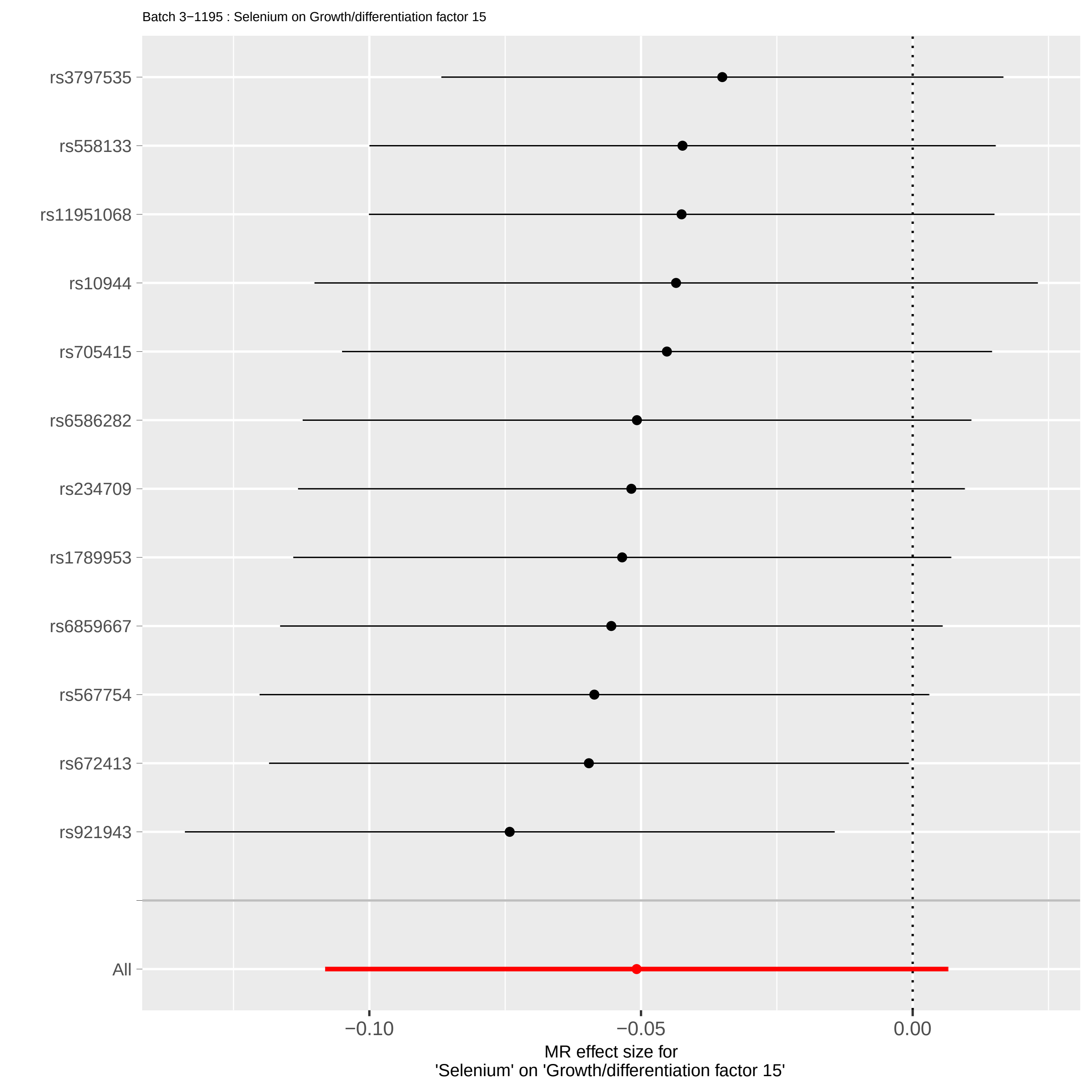

Batch 3−1195 : Selenium on Growth/differentiation factor 15
rs3797535
rs558133
rs11951068
rs10944
rs705415
rs6586282
rs234709
rs1789953
rs6859667
rs567754
rs672413
rs921943
All
−0.10
−0.05
0.00
MR effect size for
'Selenium' on 'Growth/differentiation factor 15'

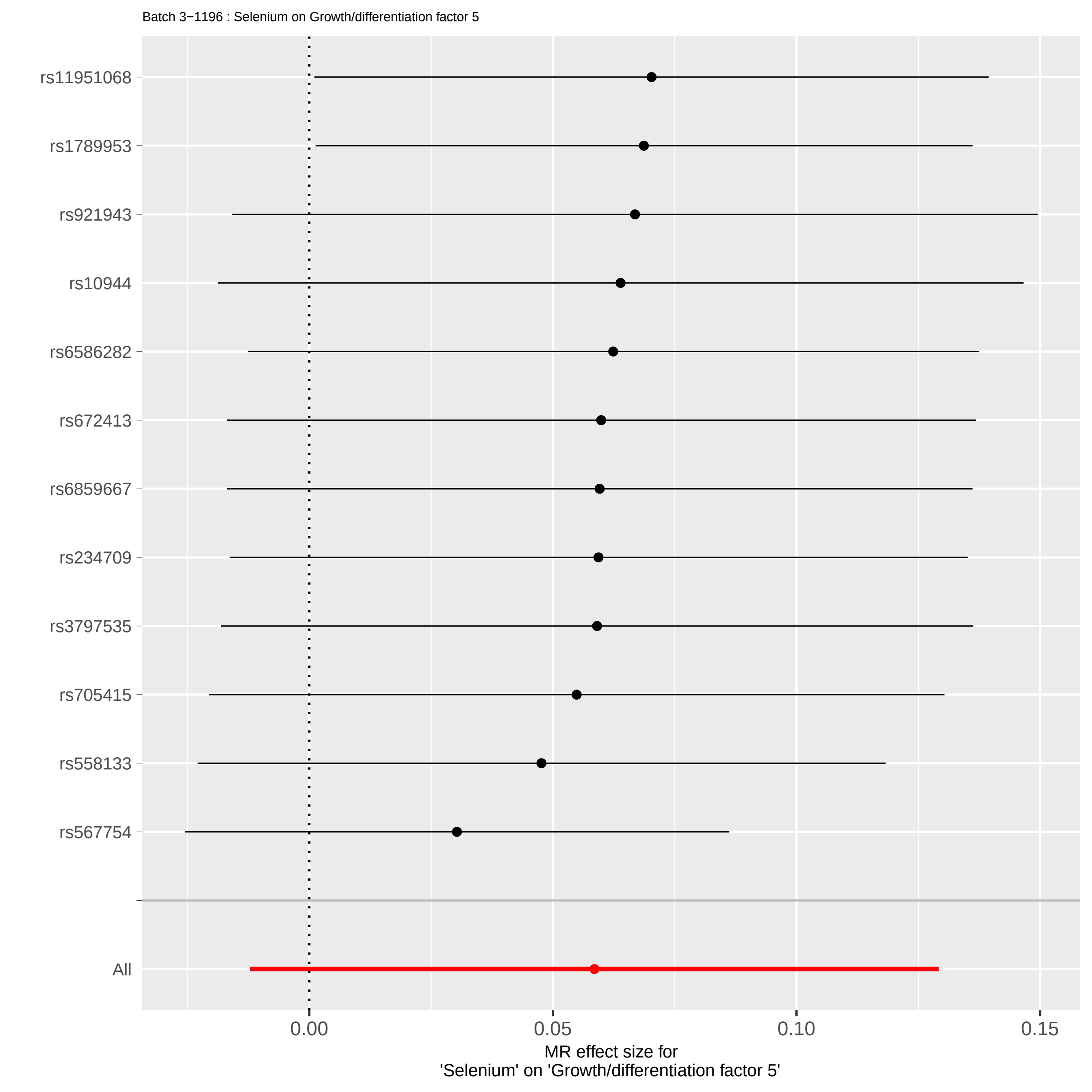
Batch 3−1196 : Selenium on Growth/differentiation factor 5
rs11951068
rs1789953
rs921943
rs10944
rs6586282
rs672413
rs6859667
rs234709
rs3797535
rs705415
rs558133
rs567754
All
0.00
0.05
0.10
0.15
MR effect size for
'Selenium' on 'Growth/differentiation factor 5'

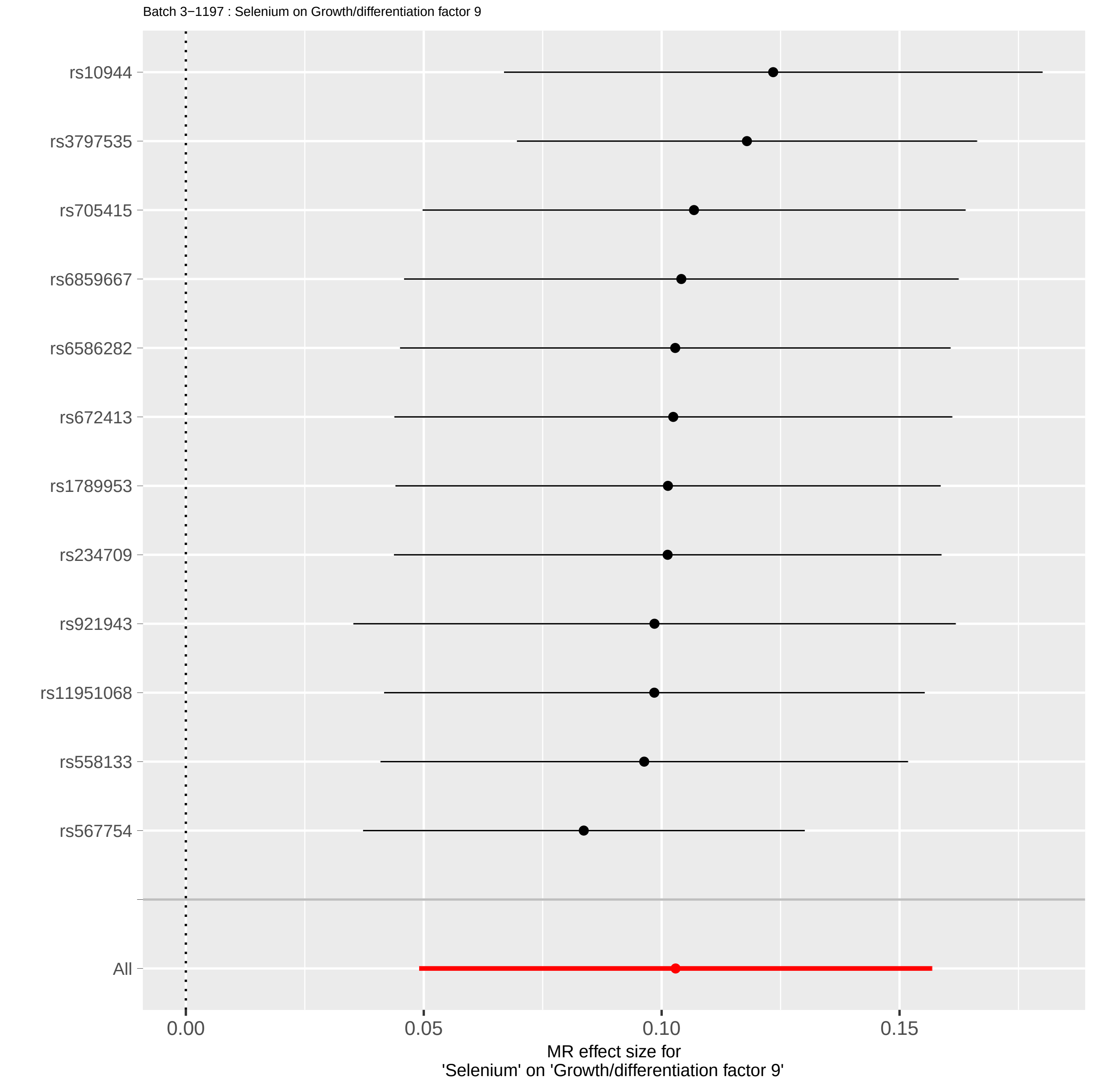

Batch 3−1197 : Selenium on Growth/differentiation factor 9
rs10944
rs3797535
rs705415
rs6859667
rs6586282
rs672413
rs1789953
rs234709
rs921943
rs11951068
rs558133
rs567754
All
0.00
0.05
0.10
0.15
MR effect size for
'Selenium' on 'Growth/differentiation factor 9'

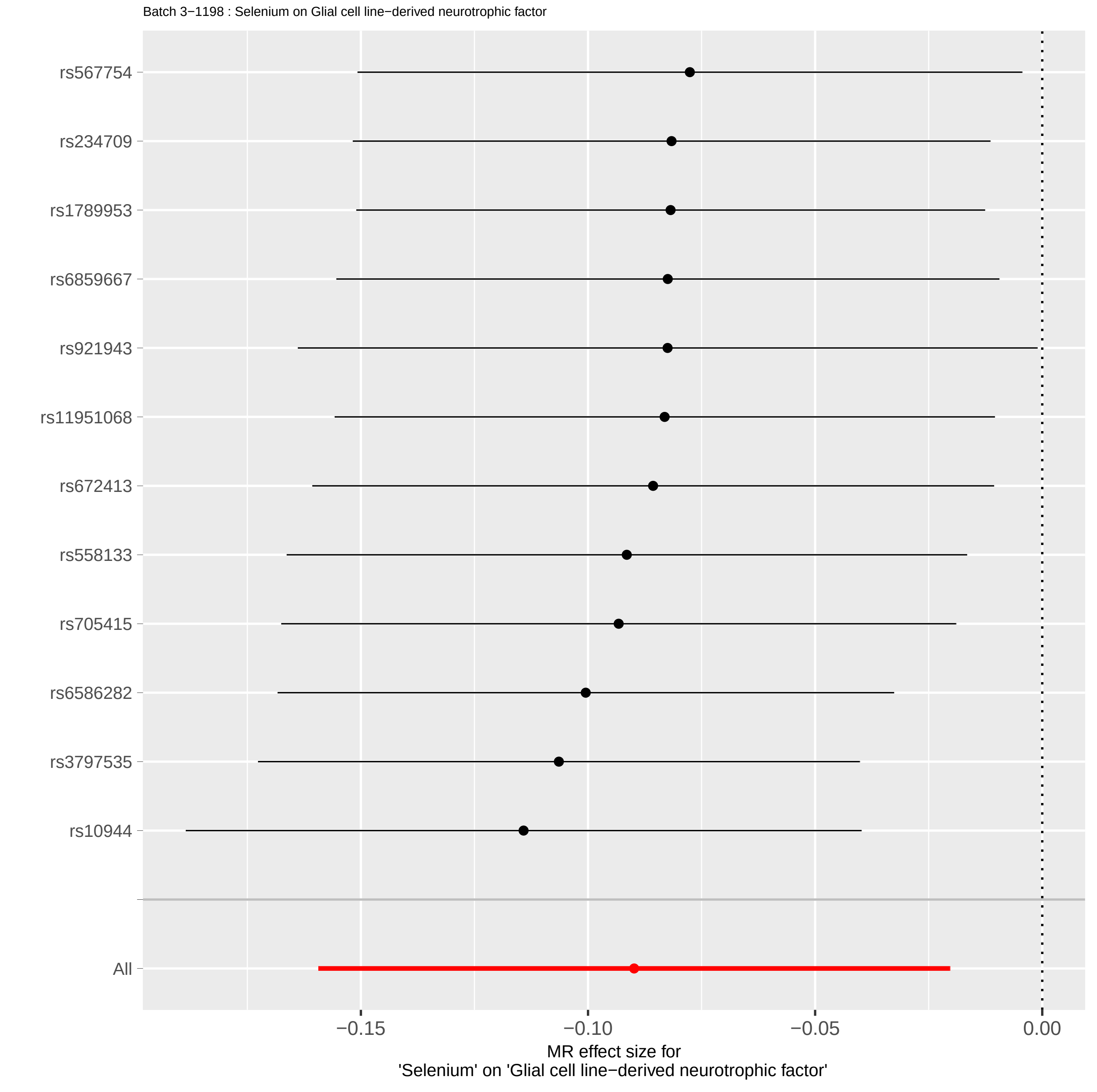

Batch 3−1198 : Selenium on Glial cell line−derived neurotrophic factor
rs567754
rs234709
rs1789953
rs6859667
rs921943
rs11951068
rs672413
rs558133
rs705415
rs6586282
rs3797535
rs10944
All
−0.15
−0.10
−0.05
0.00
MR effect size for
'Selenium' on 'Glial cell line−derived neurotrophic factor'

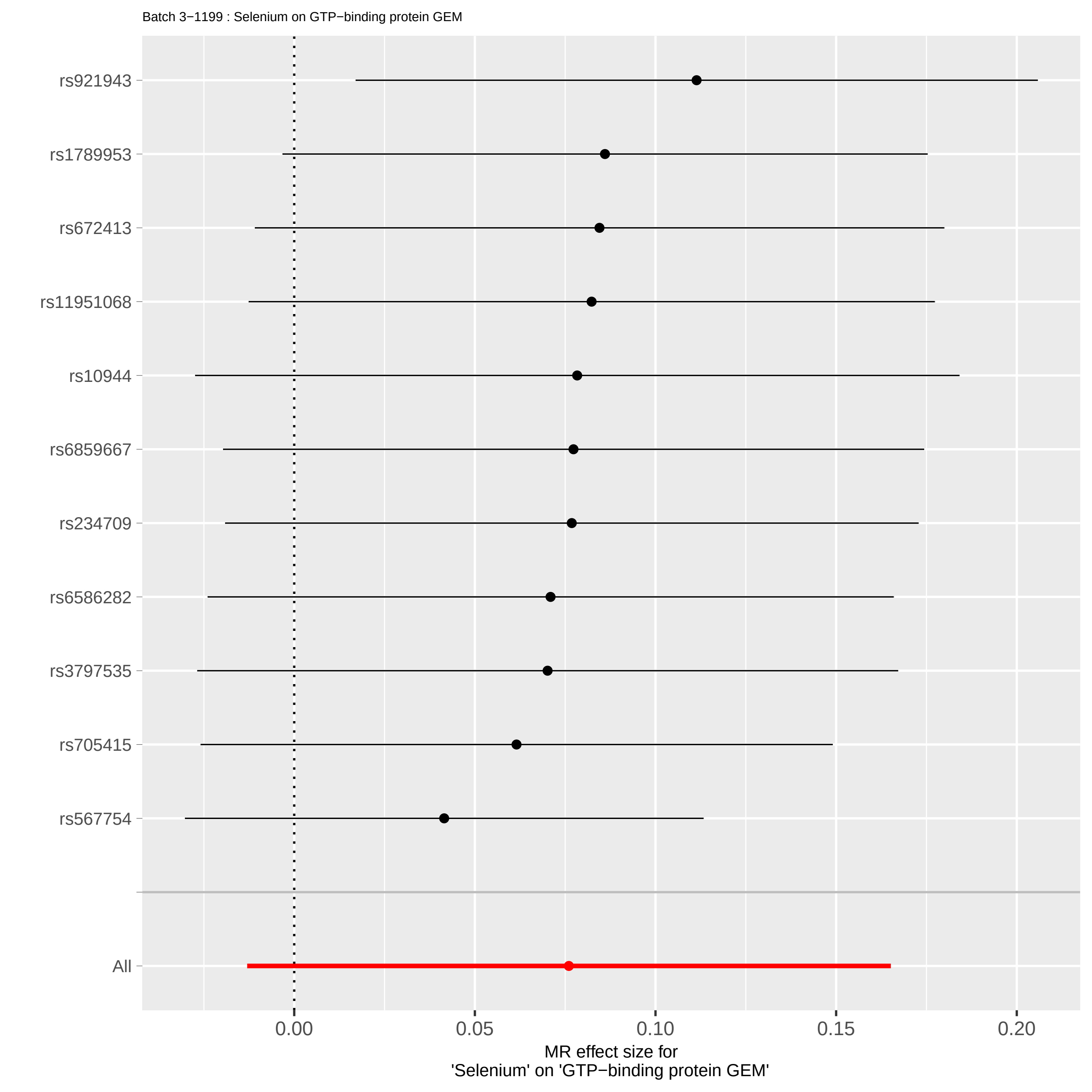

Batch 3−1199 : Selenium on GTP−binding protein GEM
rs921943
rs1789953
rs672413
rs11951068
rs10944
rs6859667
rs234709
rs6586282
rs3797535
rs705415
rs567754
All
0.00
0.05
0.10
0.15
0.20
MR effect size for
'Selenium' on 'GTP−binding protein GEM'

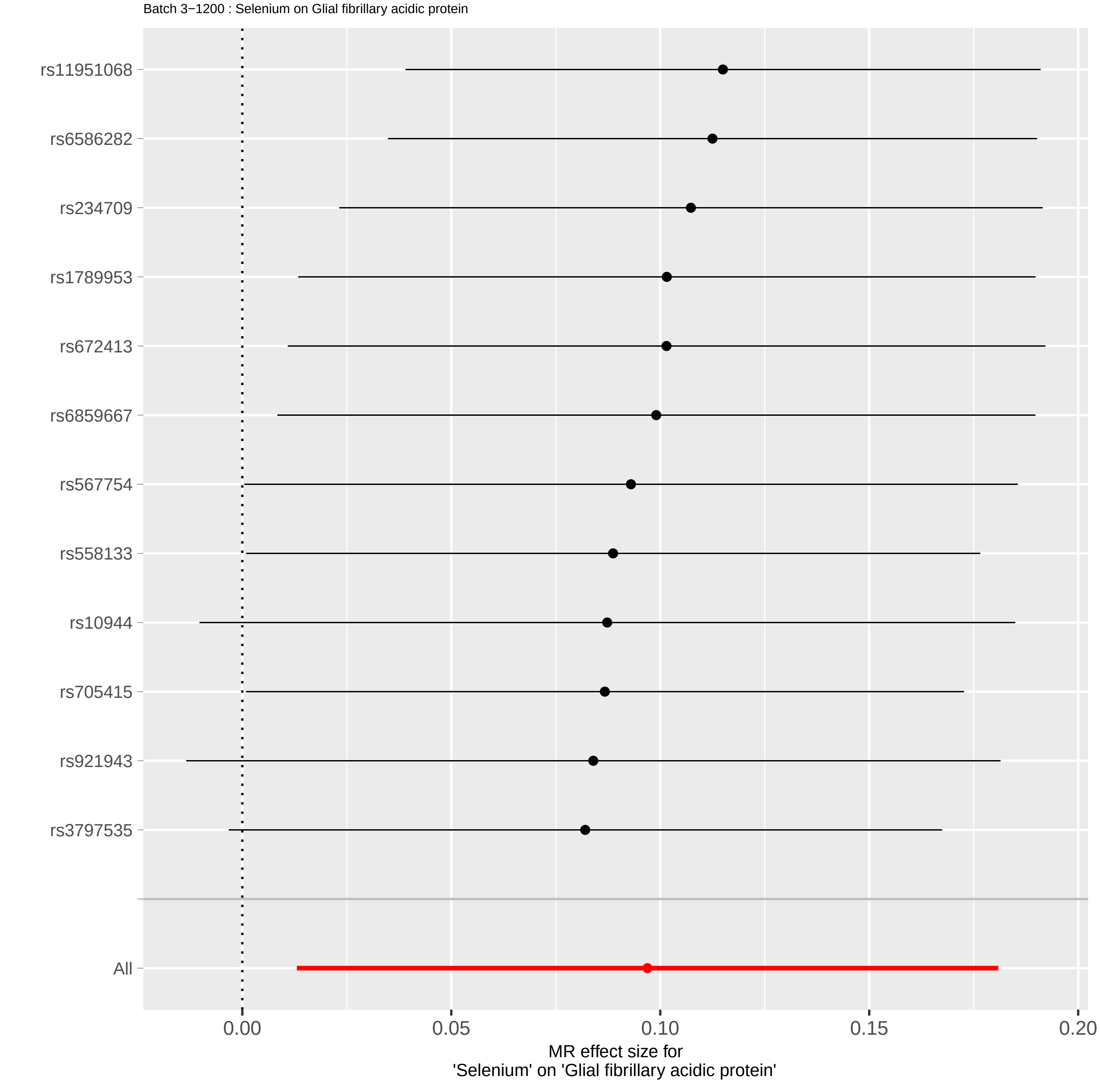
Batch 3−1200 : Selenium on Glial fibrillary acidic protein
rs11951068
rs6586282
rs234709
rs1789953
rs672413
rs6859667
rs567754
rs558133
rs10944
rs705415
rs921943
rs3797535
All
0.00
0.05
0.10
0.15
0.20
MR effect size for
'Selenium' on 'Glial fibrillary acidic protein'

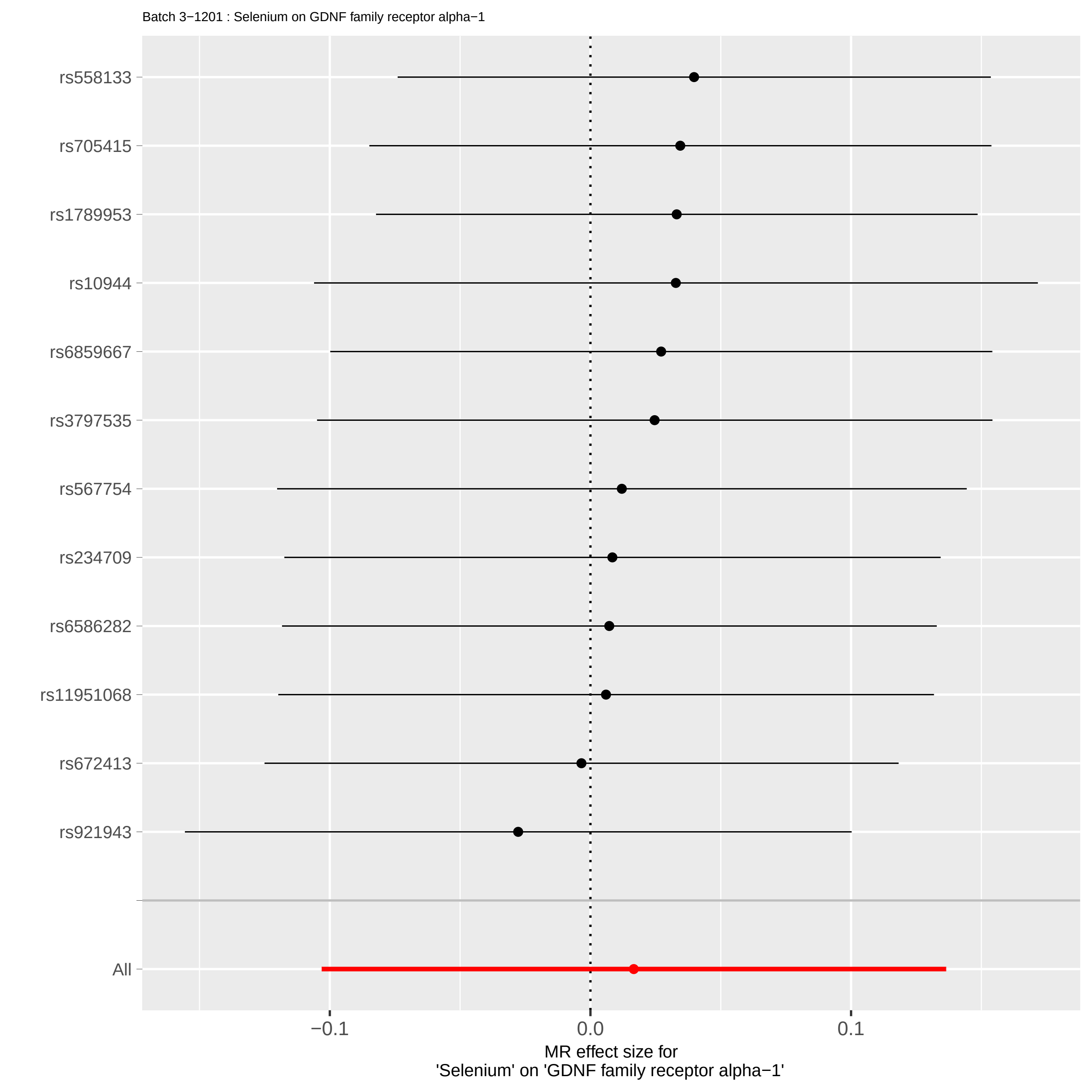

Batch 3−1201 : Selenium on GDNF family receptor alpha−1
rs558133
rs705415
rs1789953
rs10944
rs6859667
rs3797535
rs567754
rs234709
rs6586282
rs11951068
rs672413
rs921943
All
−0.1
0.0
0.1
MR effect size for
'Selenium' on 'GDNF family receptor alpha−1'

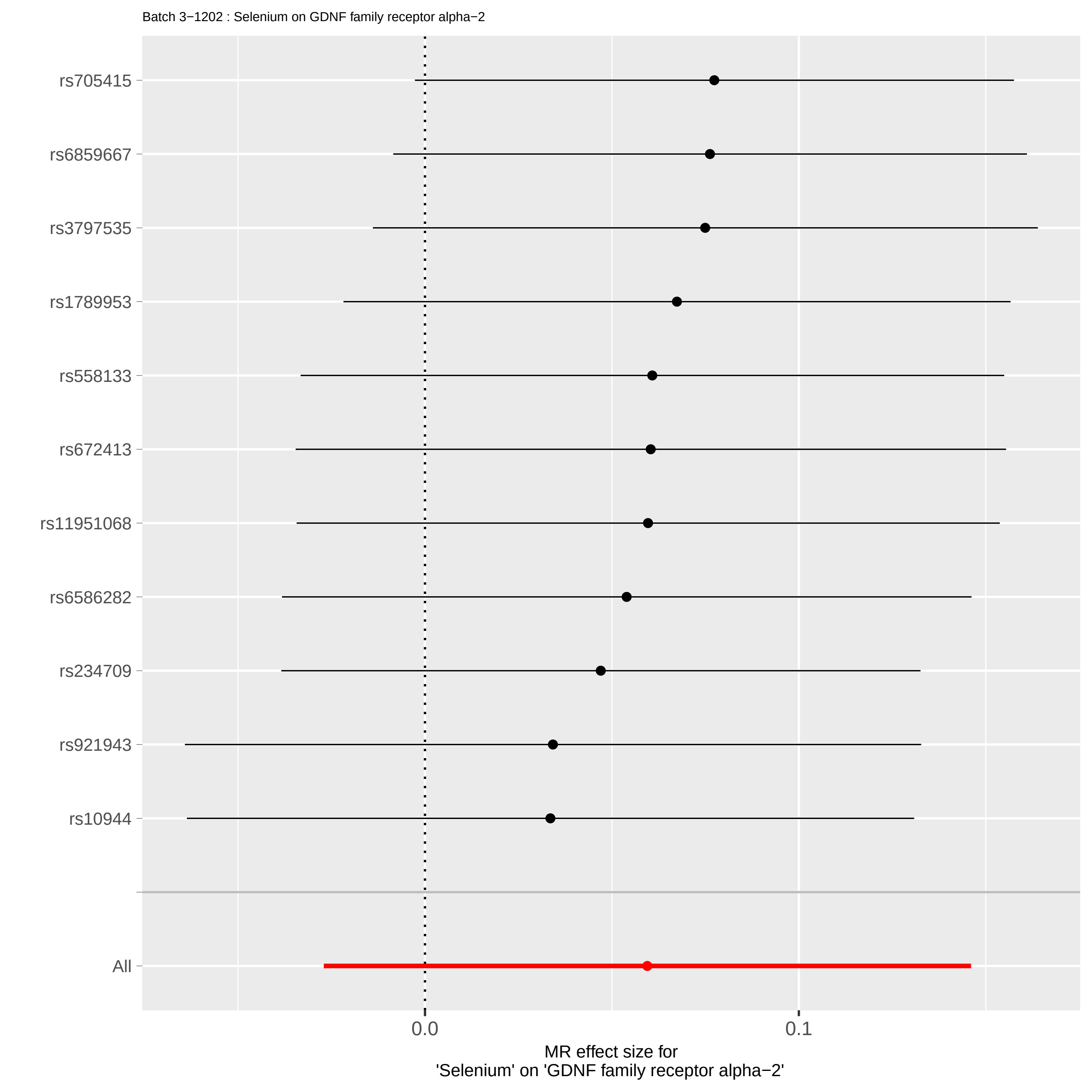
Batch 3−1202 : Selenium on GDNF family receptor alpha−2
rs705415
rs6859667
rs3797535
rs1789953
rs558133
rs672413
rs11951068
rs6586282
rs234709
rs921943
rs10944
All
0.0
0.1
MR effect size for
'Selenium' on 'GDNF family receptor alpha−2'

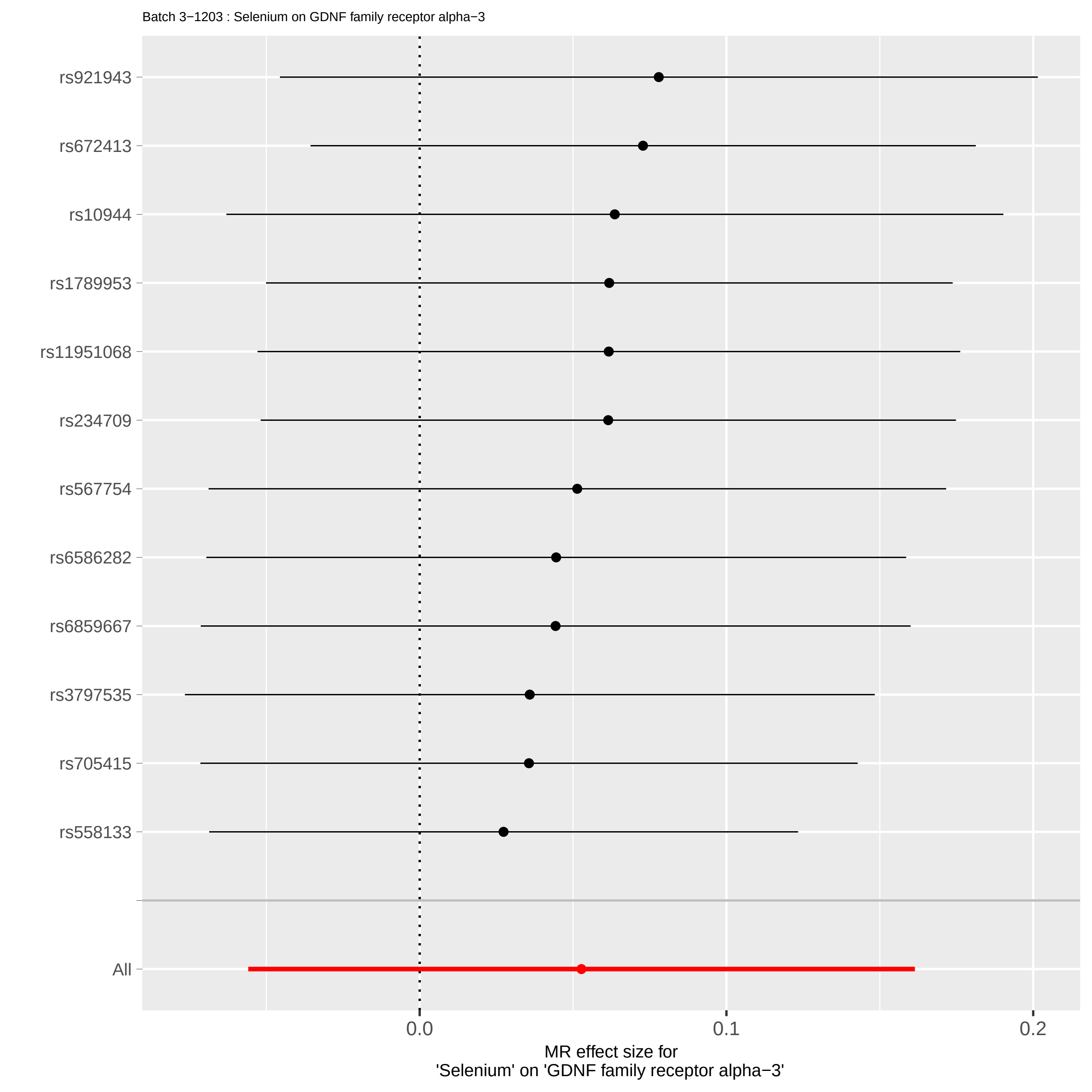
Batch 3−1203 : Selenium on GDNF family receptor alpha−3
rs921943
rs672413
rs10944
rs1789953
rs11951068
rs234709
rs567754
rs6586282
rs6859667
rs3797535
rs705415
rs558133
All
0.0
0.1
0.2
MR effect size for
'Selenium' on 'GDNF family receptor alpha−3'

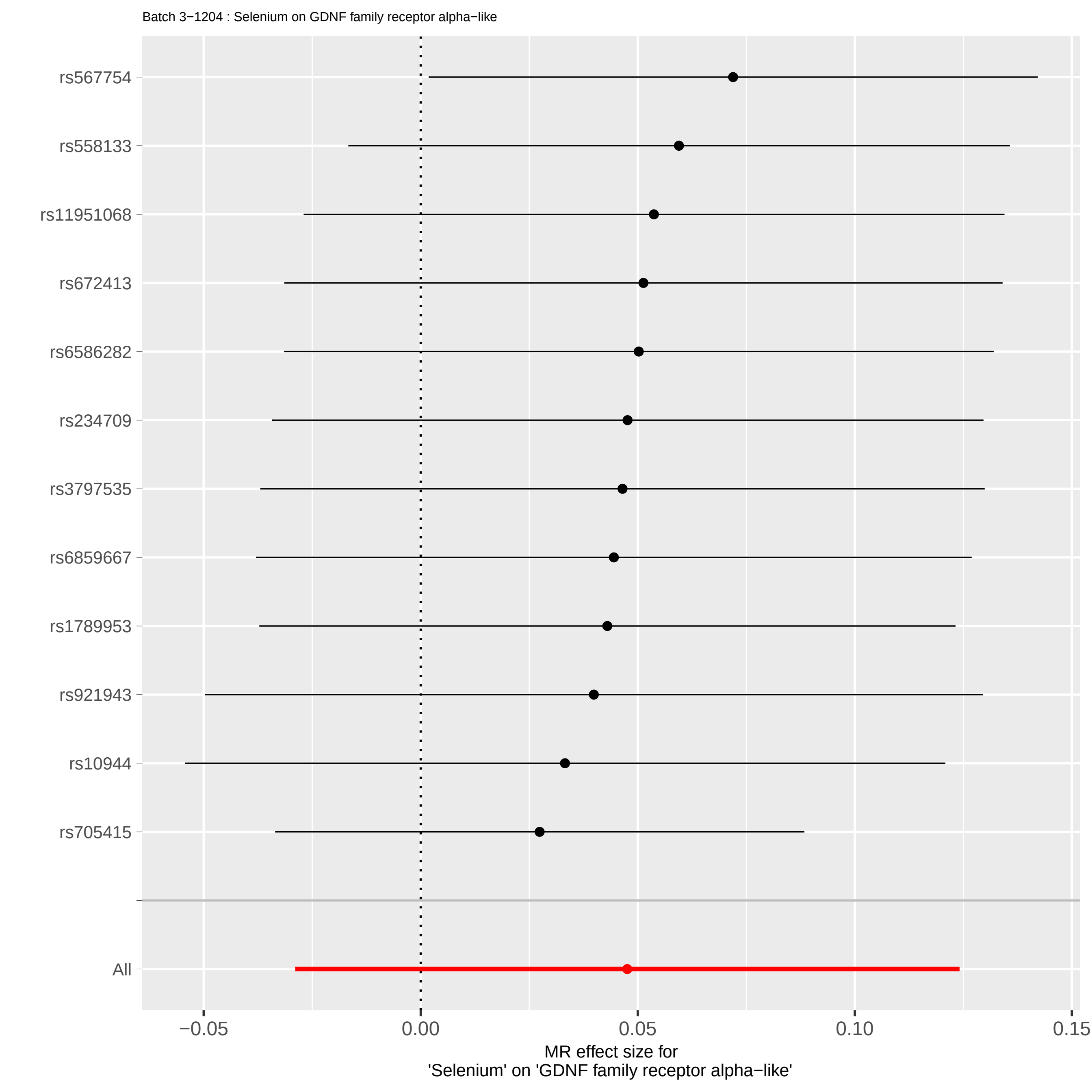

Batch 3−1204 : Selenium on GDNF family receptor alpha−like
rs567754
rs558133
rs11951068
rs672413
rs6586282
rs234709
rs3797535
rs6859667
rs1789953
rs921943
rs10944
rs705415
All
−0.05
0.00
0.05
0.10
0.15
MR effect size for
'Selenium' on 'GDNF family receptor alpha−like'

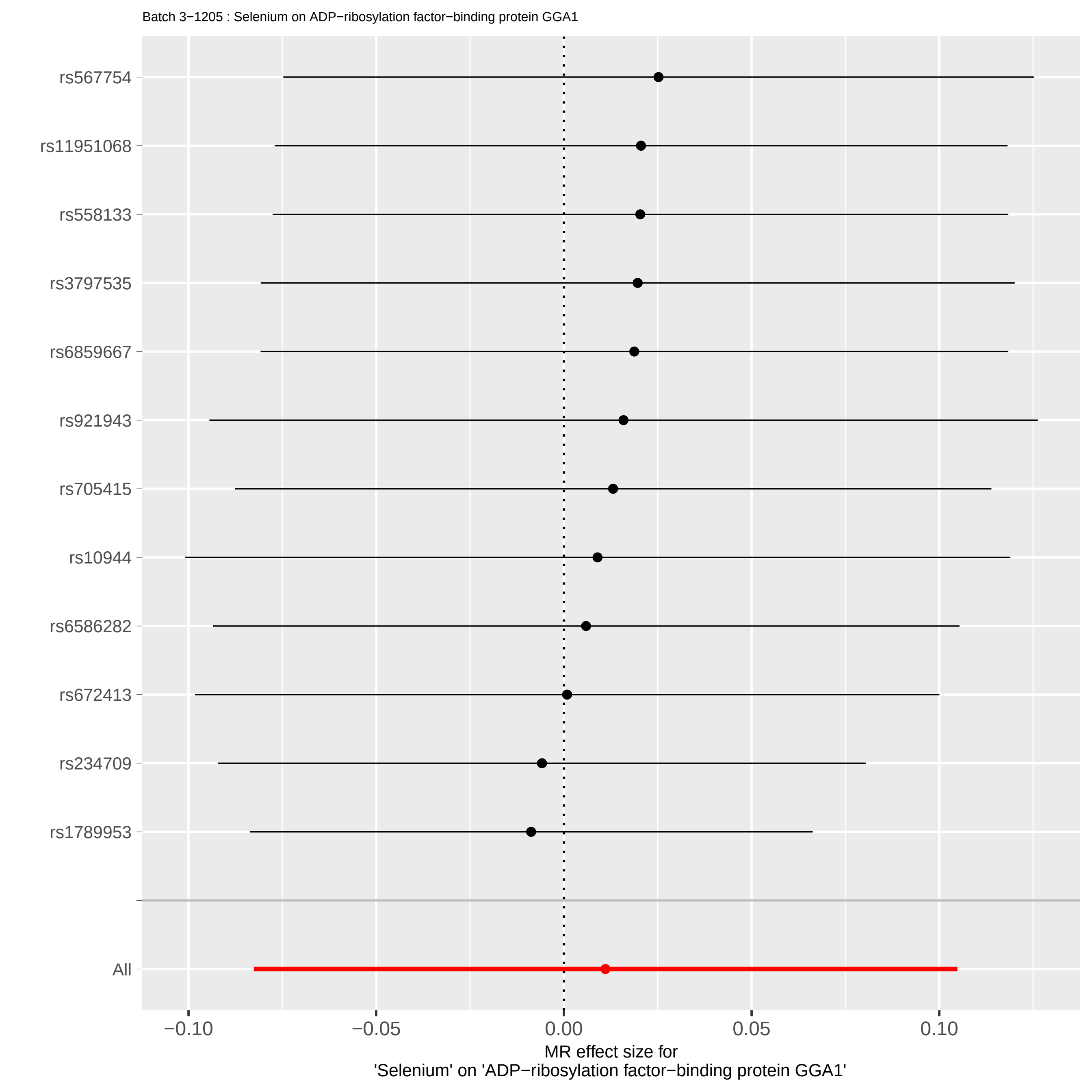
Batch 3−1205 : Selenium on ADP−ribosylation factor−binding protein GGA1
rs567754
rs11951068
rs558133
rs3797535
rs6859667
rs921943
rs705415
rs10944
rs6586282
rs672413
rs234709
rs1789953
All
−0.10
−0.05
0.00
0.05
0.10
MR effect size for
'Selenium' on 'ADP−ribosylation factor−binding protein GGA1'

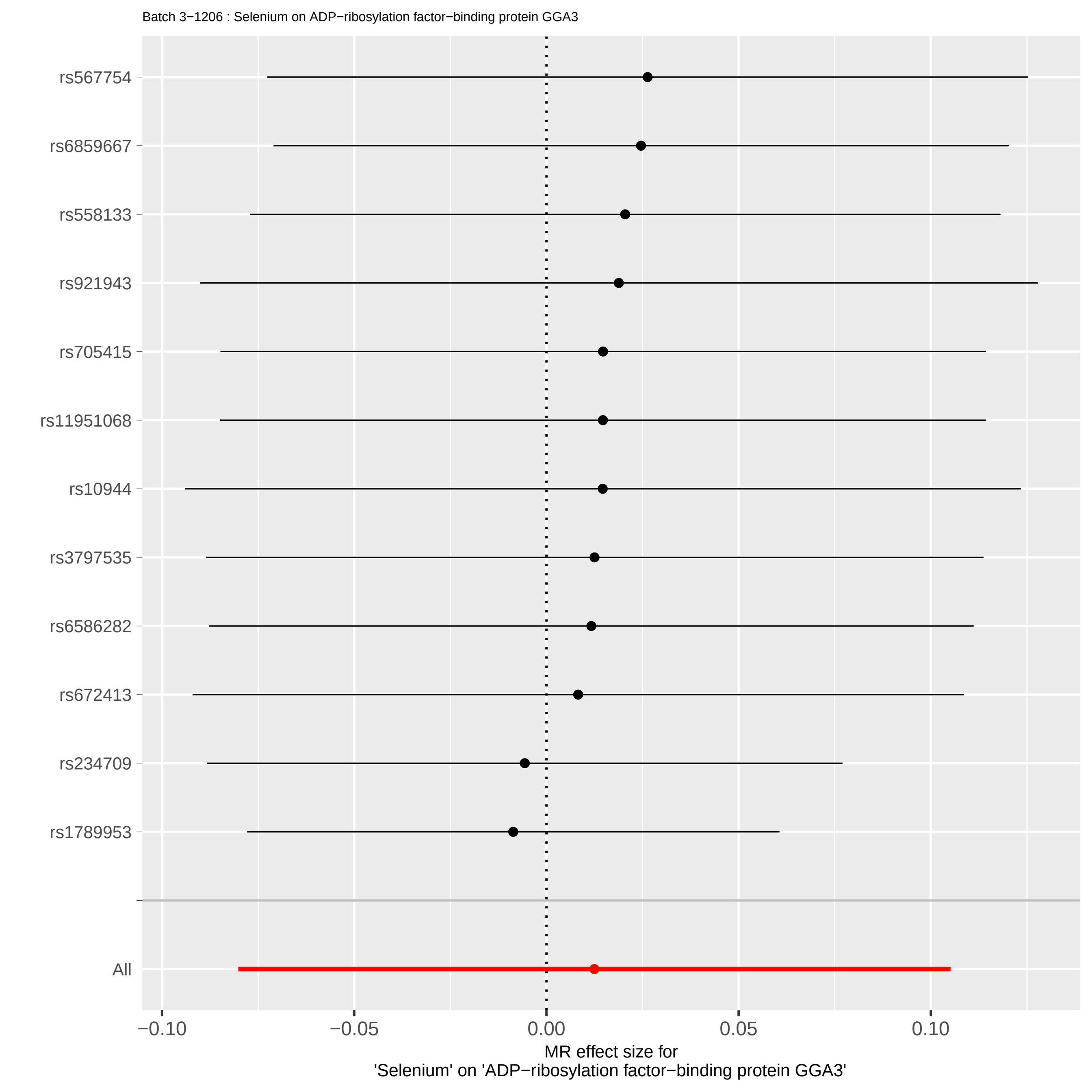

Batch 3−1206 : Selenium on ADP−ribosylation factor−binding protein GGA3
rs567754
rs6859667
rs558133
rs921943
rs705415
rs11951068
rs10944
rs3797535
rs6586282
rs672413
rs234709
rs1789953
All
−0.10
−0.05
0.00
0.05
0.10
MR effect size for
'Selenium' on 'ADP−ribosylation factor−binding protein GGA3'

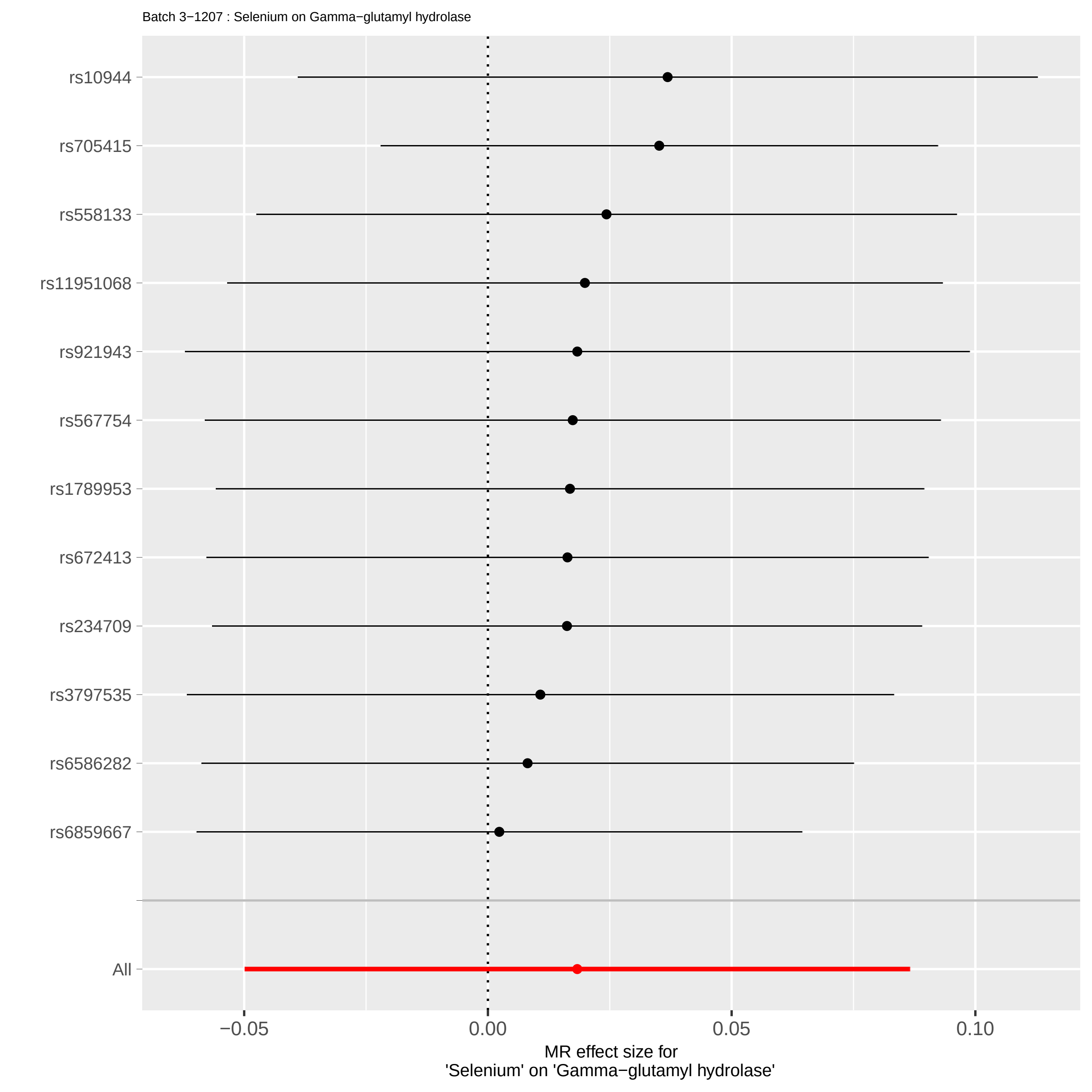
Batch 3−1207 : Selenium on Gamma−glutamyl hydrolase
rs10944
rs705415
rs558133
rs11951068
rs921943
rs567754
rs1789953
rs672413
rs234709
rs3797535
rs6586282
rs6859667
All
−0.05
0.00
0.05
0.10
MR effect size for
'Selenium' on 'Gamma−glutamyl hydrolase'

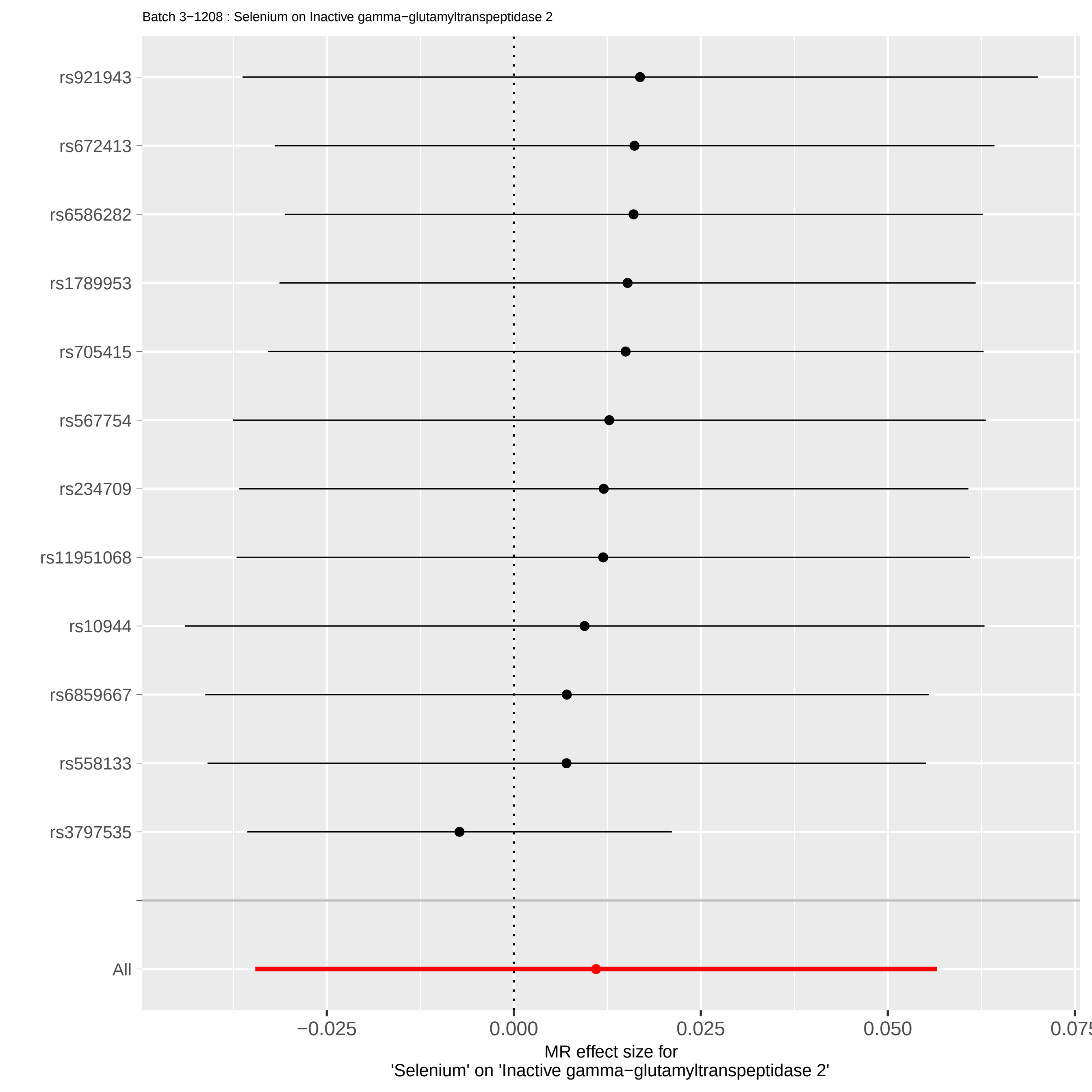

Batch 3−1208 : Selenium on Inactive gamma−glutamyltranspeptidase 2
rs921943
rs672413
rs6586282
rs1789953
rs705415
rs567754
rs234709
rs11951068
rs10944
rs6859667
rs558133
rs3797535
All
−0.025
0.000
0.025
0.050
0.075
MR effect size for
'Selenium' on 'Inactive gamma−glutamyltranspeptidase 2'

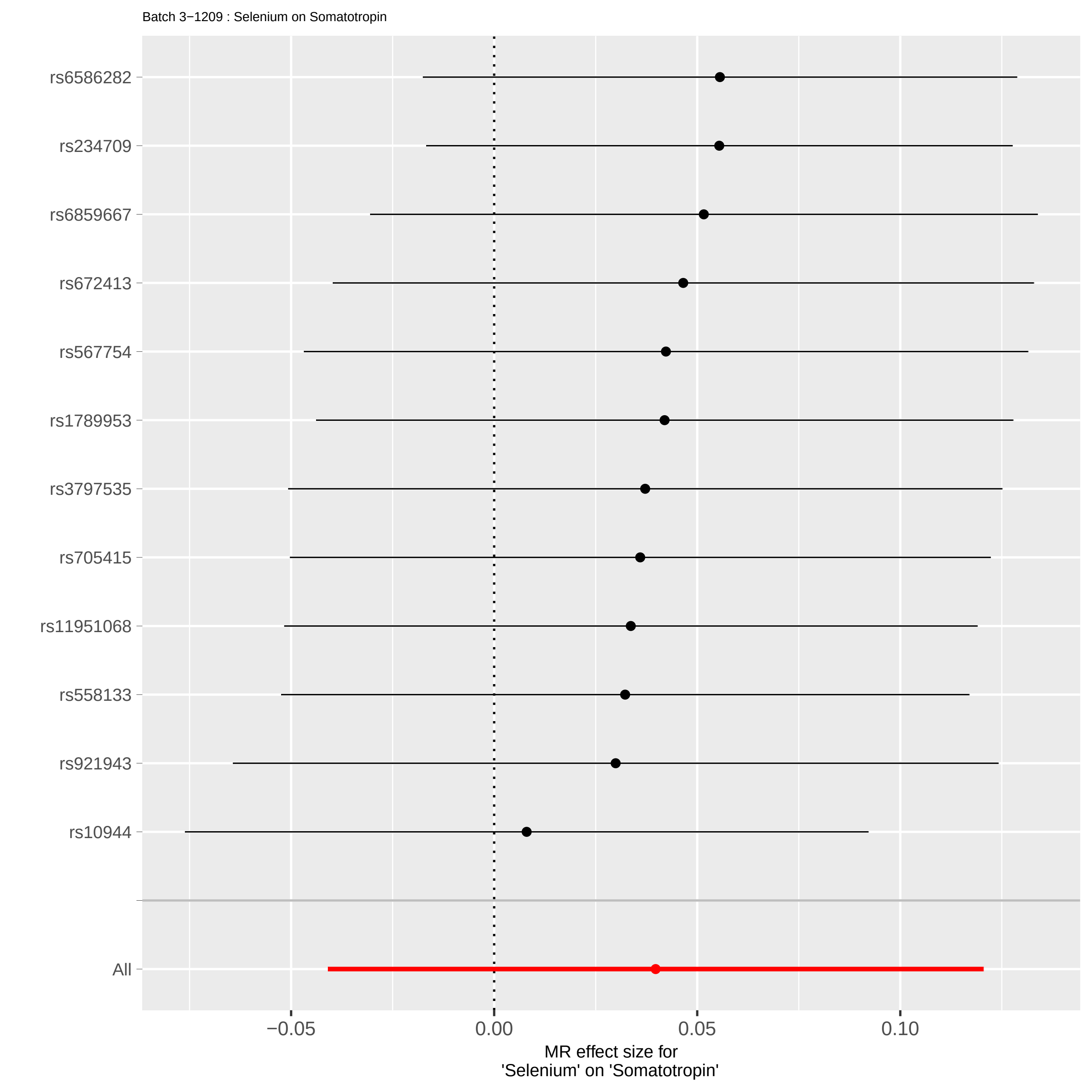

Batch 3−1209 : Selenium on Somatotropin
rs6586282
rs234709
rs6859667
rs672413
rs567754
rs1789953
rs3797535
rs705415
rs11951068
rs558133
rs921943
rs10944
All
−0.05
0.00
0.05
0.10
MR effect size for
'Selenium' on 'Somatotropin'

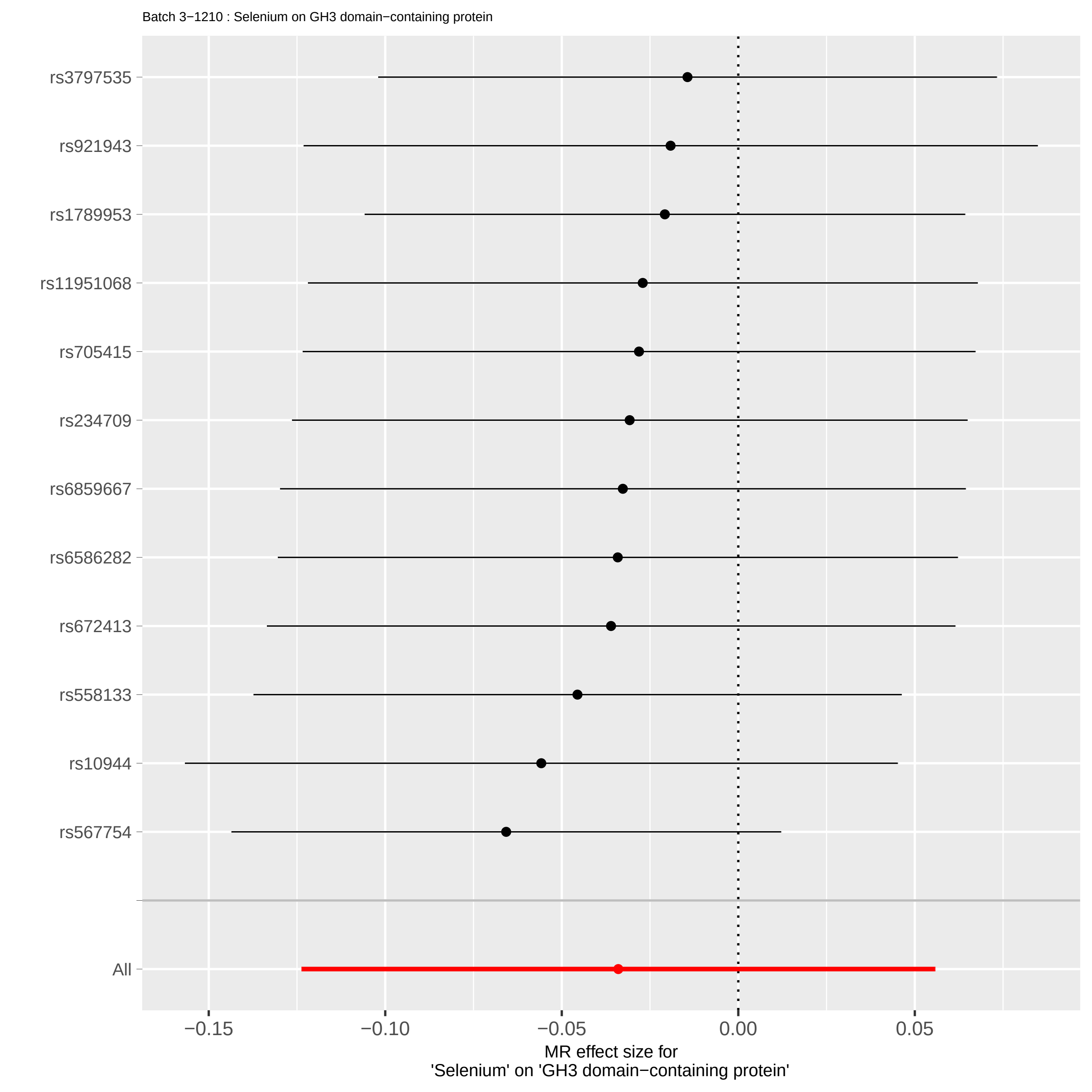
Batch 3−1210 : Selenium on GH3 domain−containing protein
rs3797535
rs921943
rs1789953
rs11951068
rs705415
rs234709
rs6859667
rs6586282
rs672413
rs558133
rs10944
rs567754
All
−0.15
−0.10
−0.05
0.00
0.05
MR effect size for
'Selenium' on 'GH3 domain−containing protein'

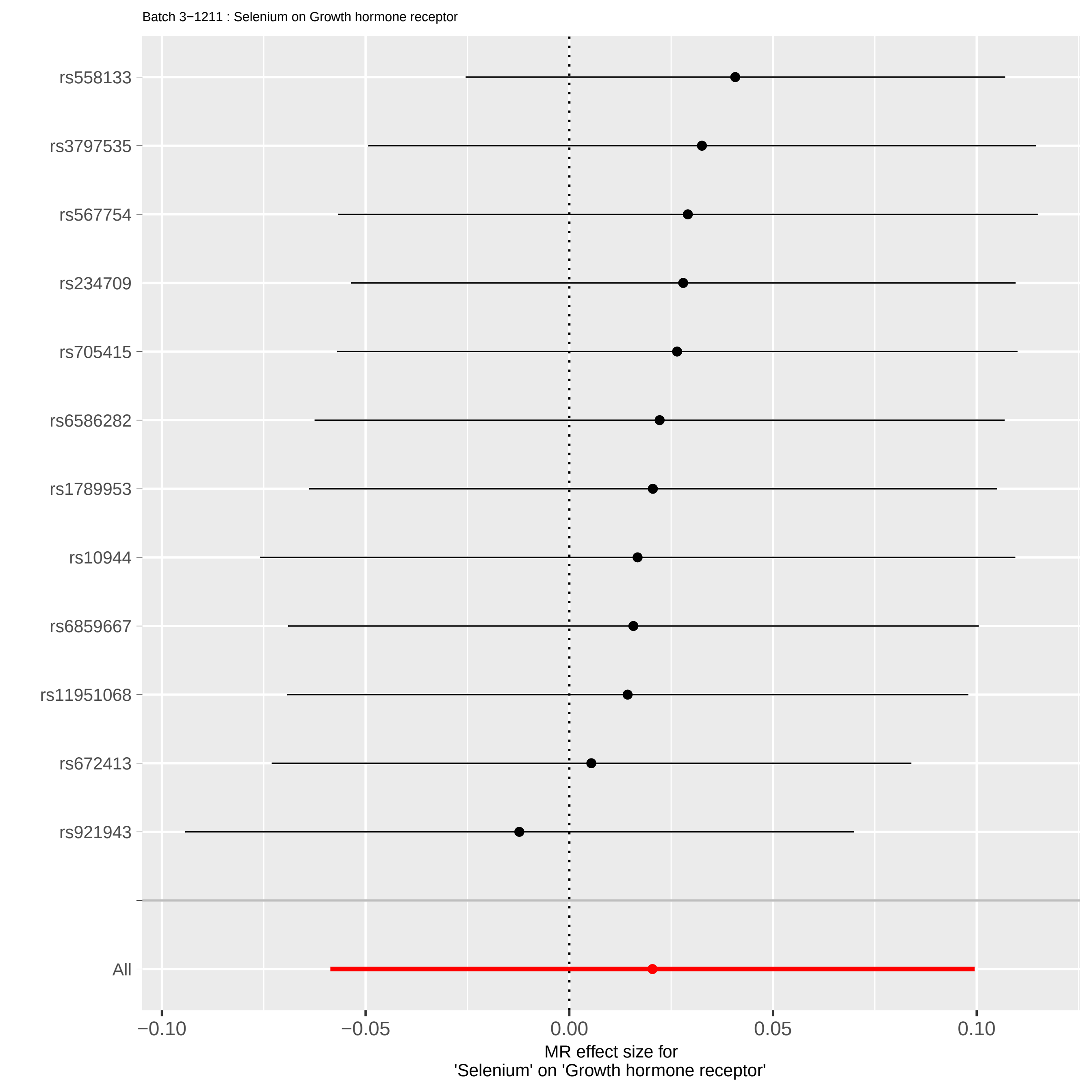

Batch 3−1211 : Selenium on Growth hormone receptor
rs558133
rs3797535
rs567754
rs234709
rs705415
rs6586282
rs1789953
rs10944
rs6859667
rs11951068
rs672413
rs921943
All
−0.10
−0.05
0.00
0.05
0.10
MR effect size for
'Selenium' on 'Growth hormone receptor'

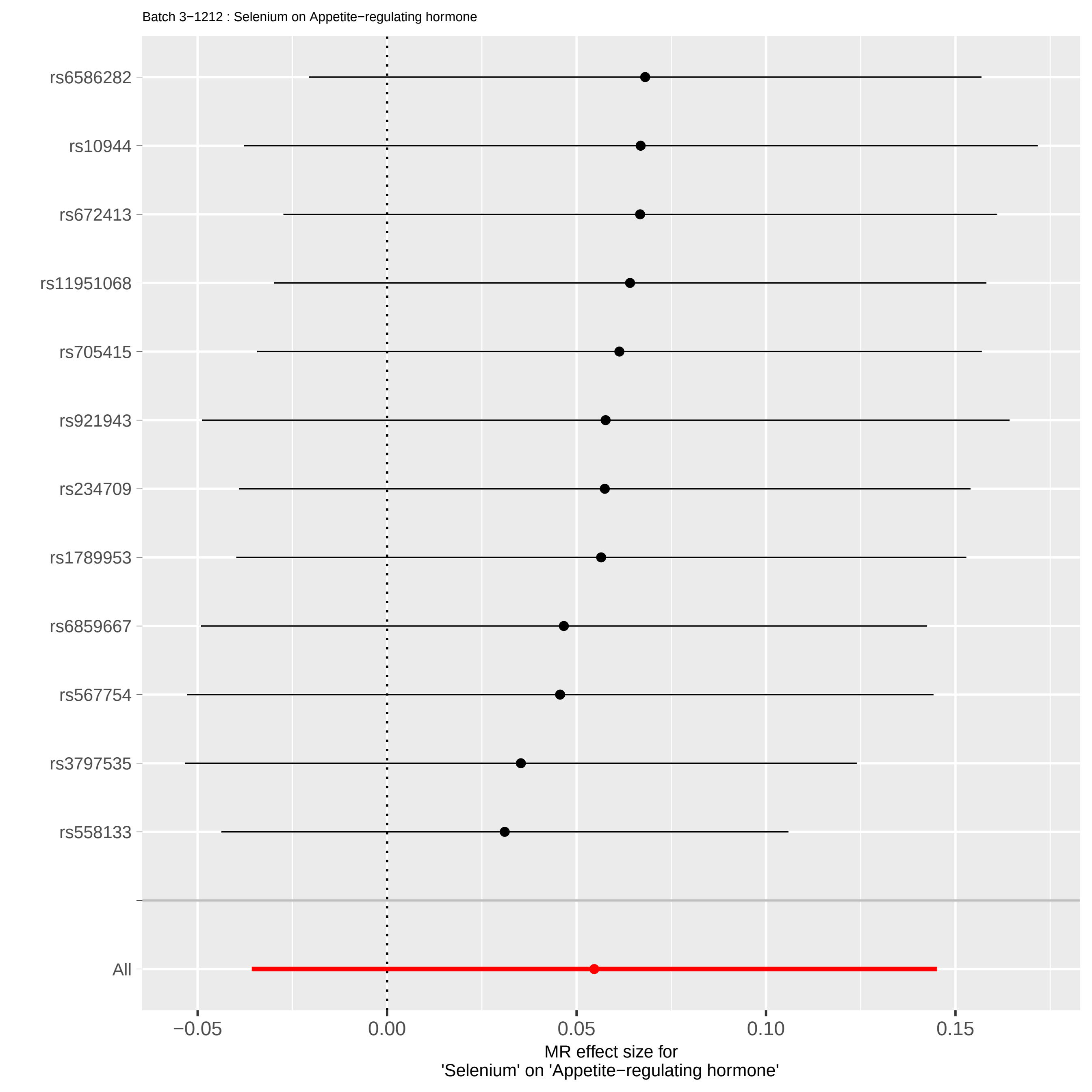

Batch 3−1212 : Selenium on Appetite−regulating hormone
rs6586282
rs10944
rs672413
rs11951068
rs705415
rs921943
rs234709
rs1789953
rs6859667
rs567754
rs3797535
rs558133
All
−0.05
0.00
0.05
0.10
0.15
MR effect size for
'Selenium' on 'Appetite−regulating hormone'

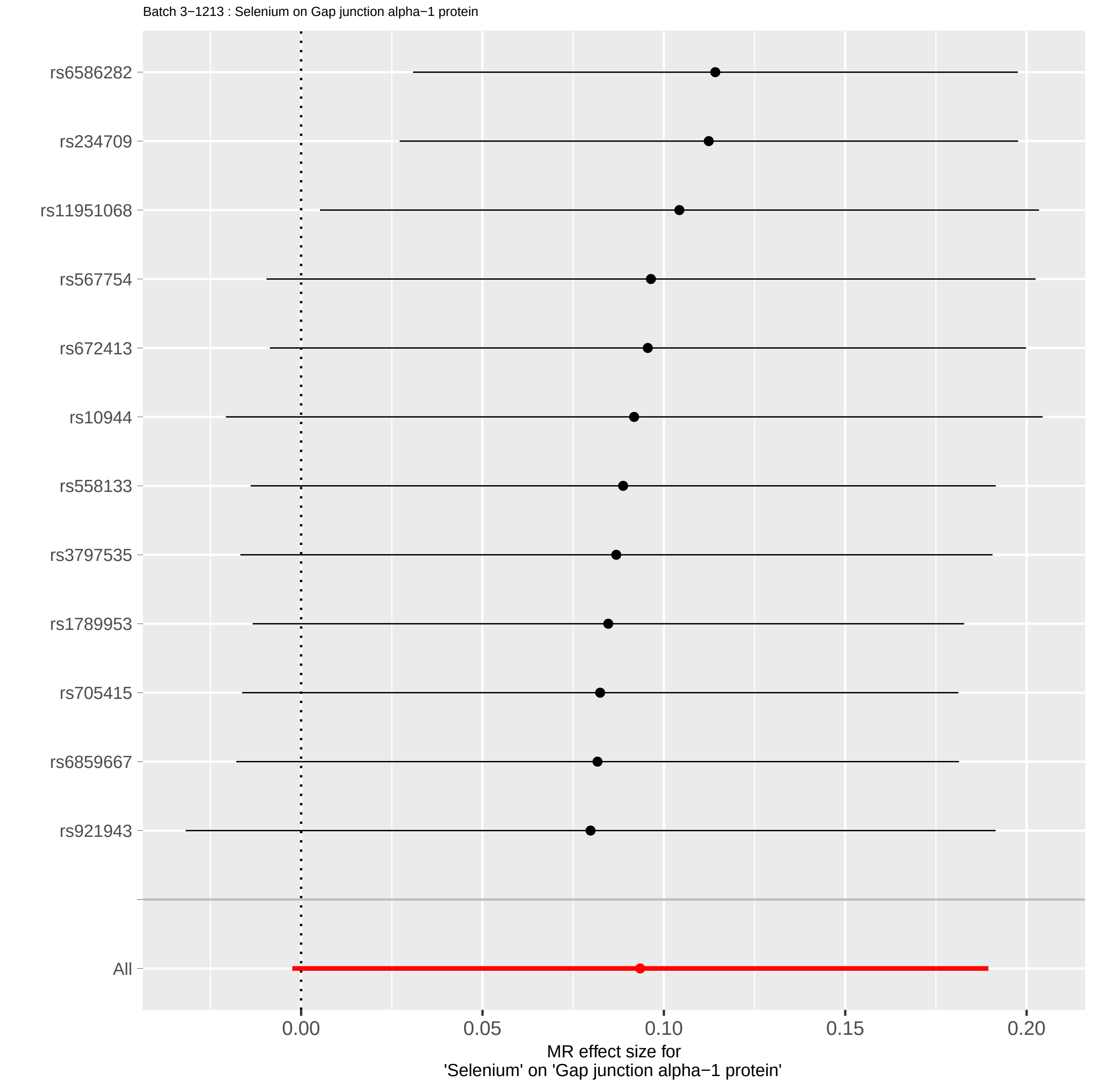

Batch 3−1213 : Selenium on Gap junction alpha−1 protein
rs6586282
rs234709
rs11951068
rs567754
rs672413
rs10944
rs558133
rs3797535
rs1789953
rs705415
rs6859667
rs921943
All
0.00
0.05
0.10
0.15
0.20
MR effect size for
'Selenium' on 'Gap junction alpha−1 protein'

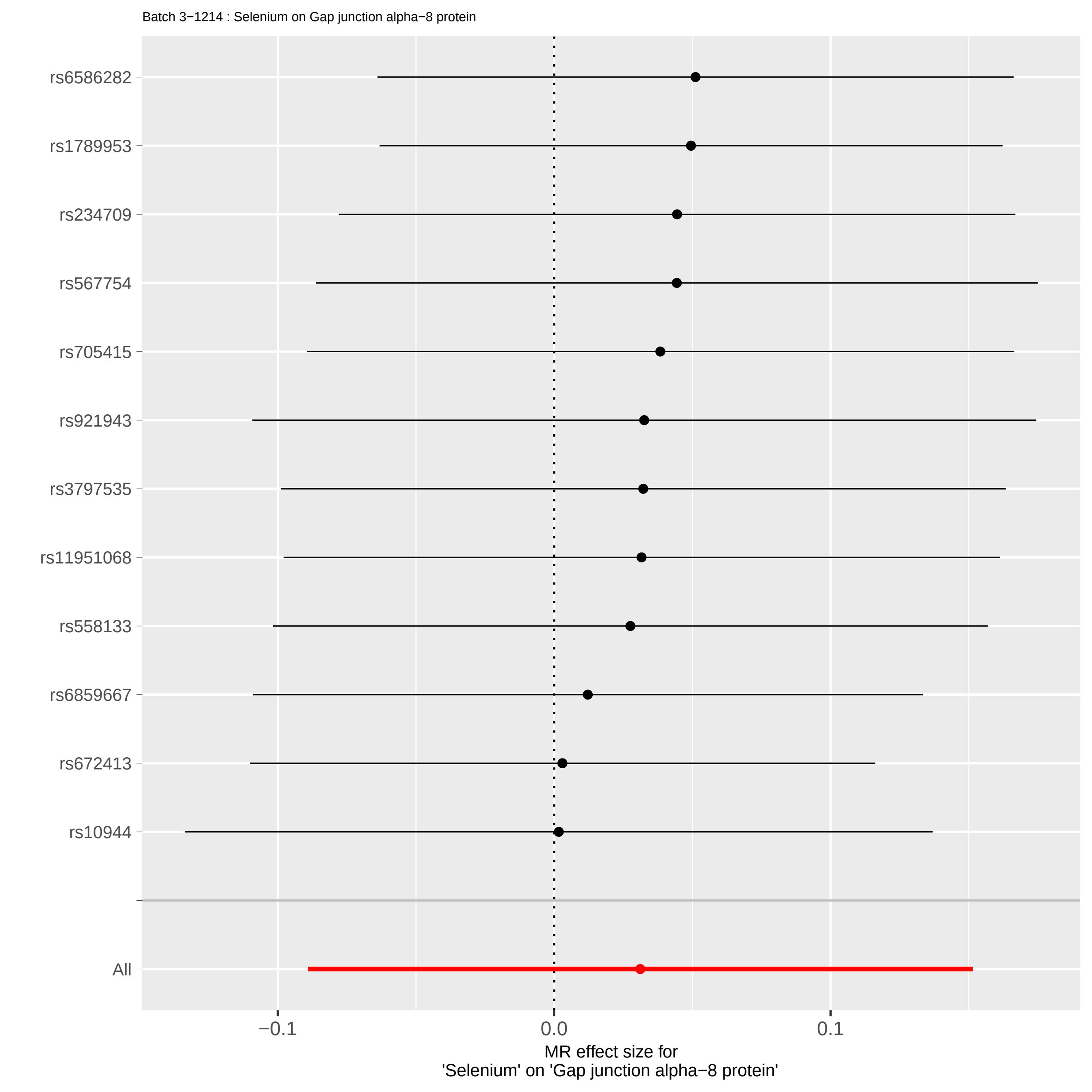
Batch 3−1214 : Selenium on Gap junction alpha−8 protein
rs6586282
rs1789953
rs234709
rs567754
rs705415
rs921943
rs3797535
rs11951068
rs558133
rs6859667
rs672413
rs10944
All
−0.1
0.0
0.1
MR effect size for
'Selenium' on 'Gap junction alpha−8 protein'

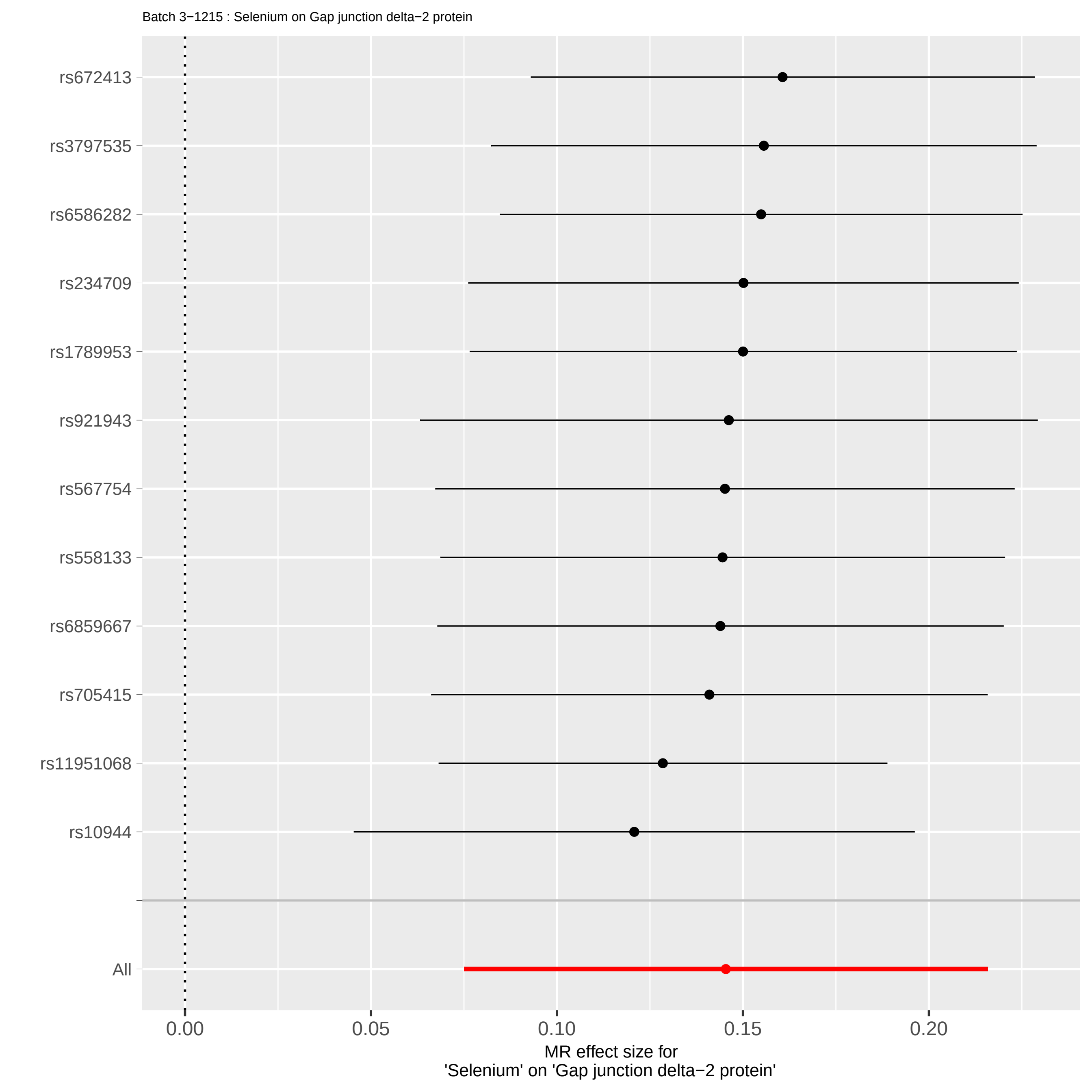

Batch 3−1215 : Selenium on Gap junction delta−2 protein
rs672413
rs3797535
rs6586282
rs234709
rs1789953
rs921943
rs567754
rs558133
rs6859667
rs705415
rs11951068
rs10944
All
0.00
0.05
0.10
0.15
0.20
MR effect size for
'Selenium' on 'Gap junction delta−2 protein'

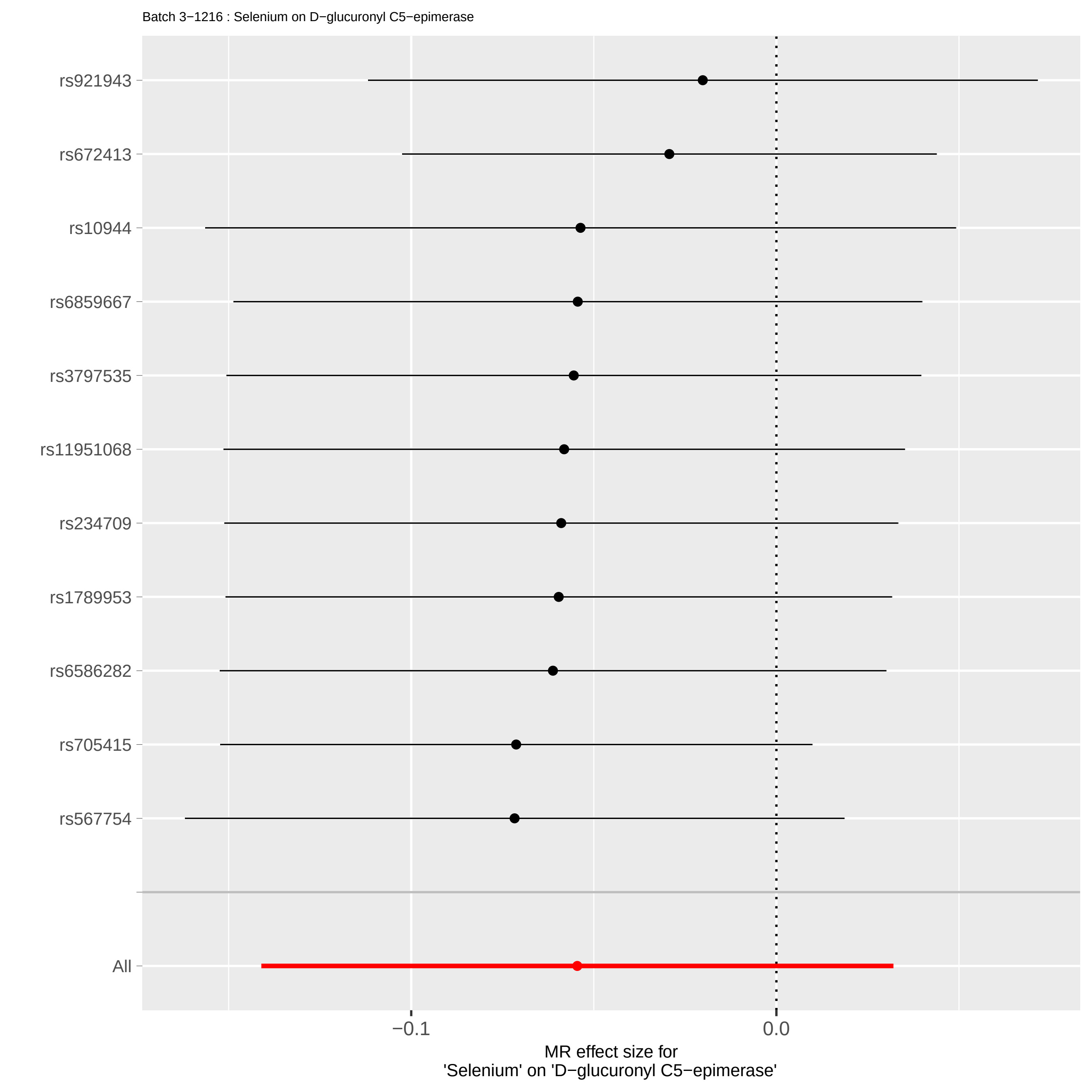

Batch 3−1216 : Selenium on D−glucuronyl C5−epimerase
rs921943
rs672413
rs10944
rs6859667
rs3797535
rs11951068
rs234709
rs1789953
rs6586282
rs705415
rs567754
All
−0.1
0.0
MR effect size for
'Selenium' on 'D−glucuronyl C5−epimerase'

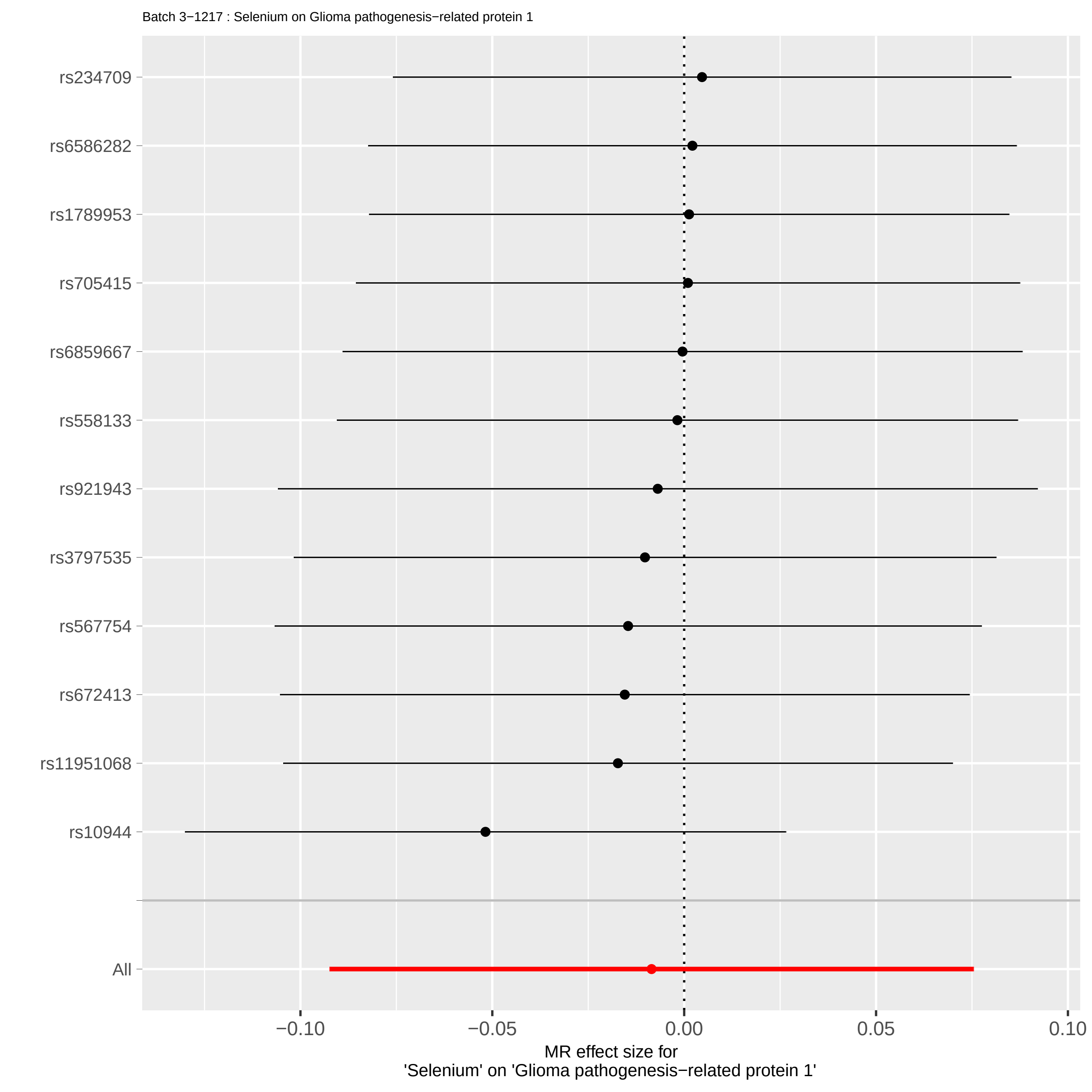

Batch 3−1217 : Selenium on Glioma pathogenesis−related protein 1
rs234709
rs6586282
rs1789953
rs705415
rs6859667
rs558133
rs921943
rs3797535
rs567754
rs672413
rs11951068
rs10944
All
−0.10
−0.05
0.00
0.05
0.10
MR effect size for
'Selenium' on 'Glioma pathogenesis−related protein 1'

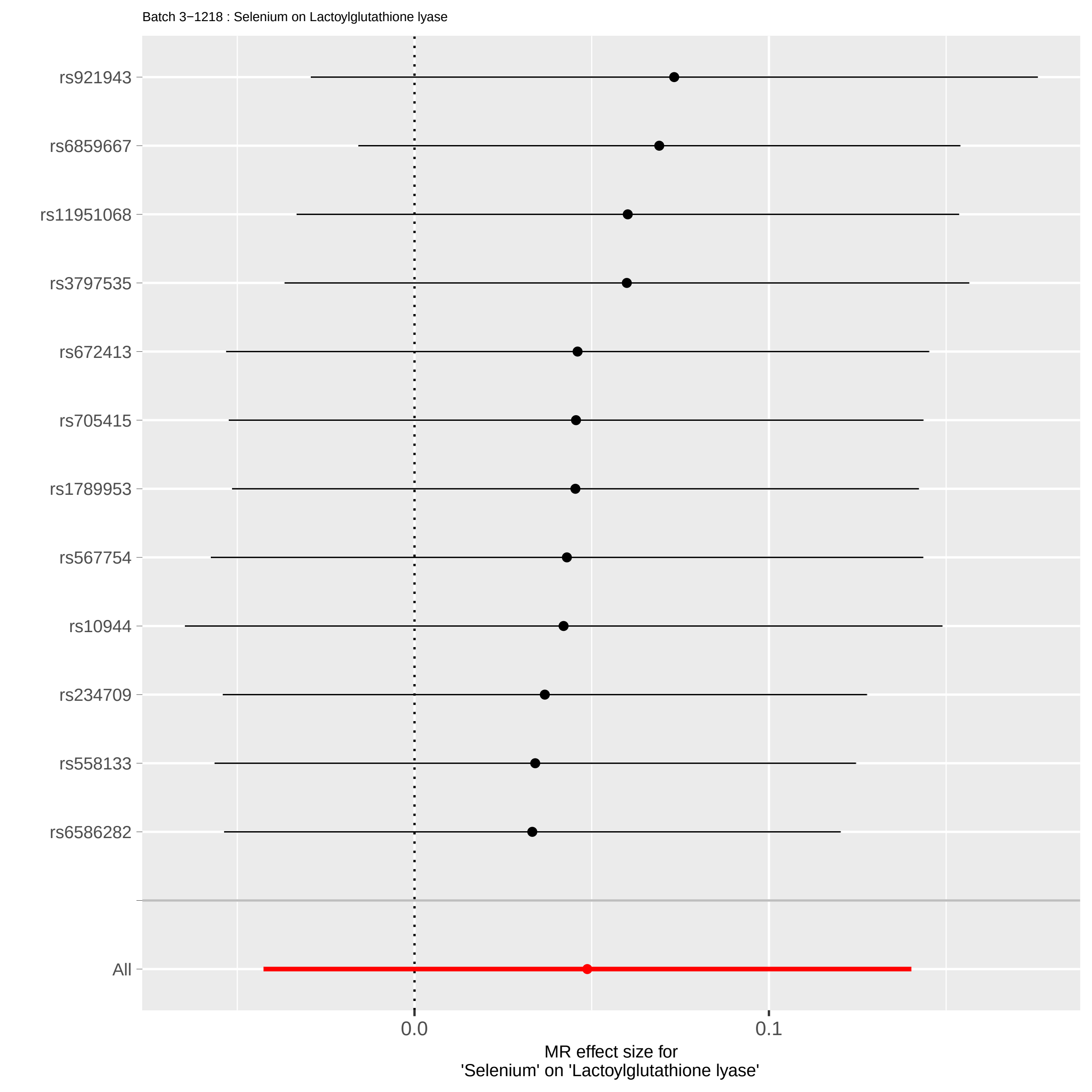

Batch 3−1218 : Selenium on Lactoylglutathione lyase
rs921943
rs6859667
rs11951068
rs3797535
rs672413
rs705415
rs1789953
rs567754
rs10944
rs234709
rs558133
rs6586282
All
0.0
0.1
MR effect size for
'Selenium' on 'Lactoylglutathione lyase'

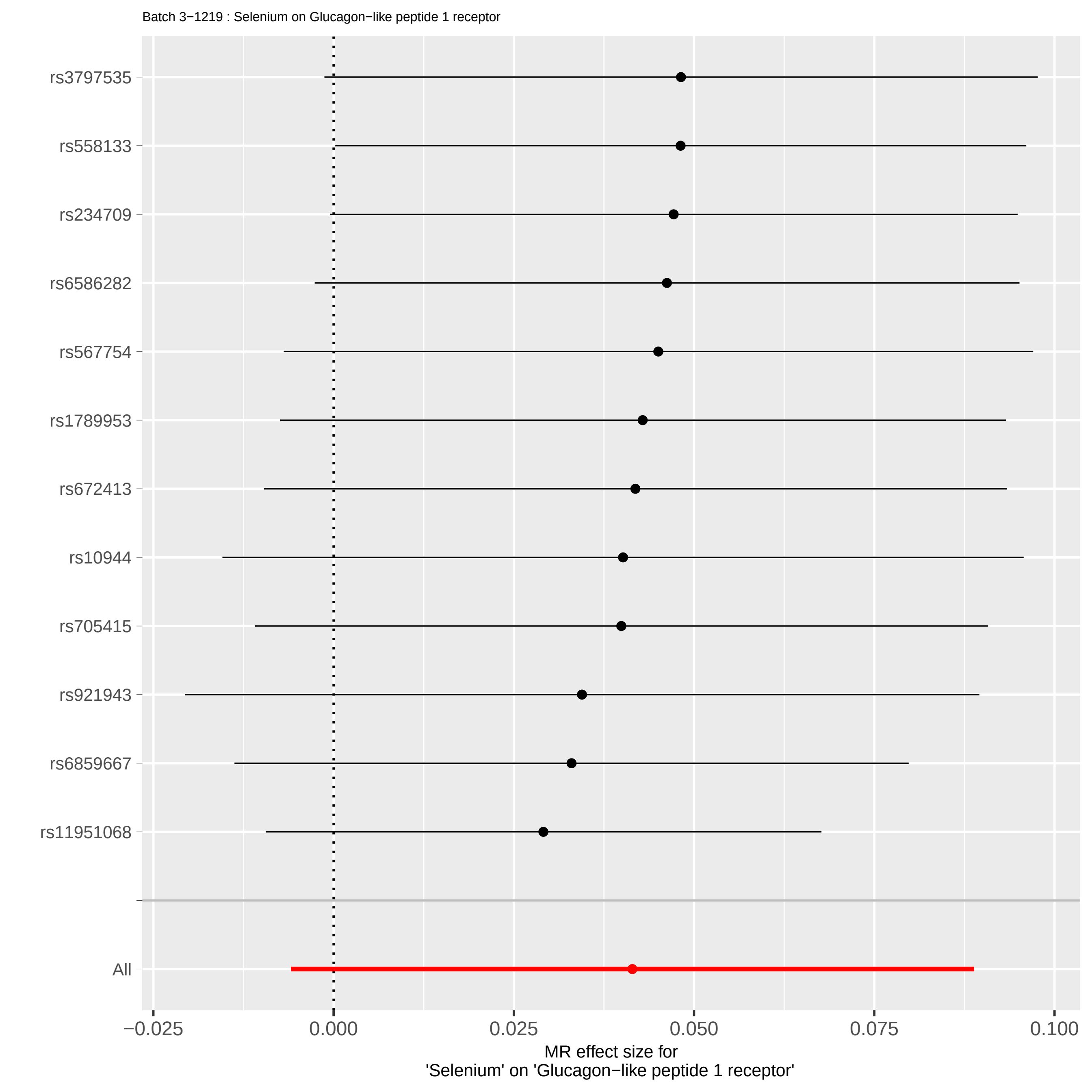
Batch 3−1219 : Selenium on Glucagon−like peptide 1 receptor
rs3797535
rs558133
rs234709
rs6586282
rs567754
rs1789953
rs672413
rs10944
rs705415
rs921943
rs6859667
rs11951068
All
−0.025
0.000
0.025
0.050
0.075
0.100
MR effect size for
'Selenium' on 'Glucagon−like peptide 1 receptor'

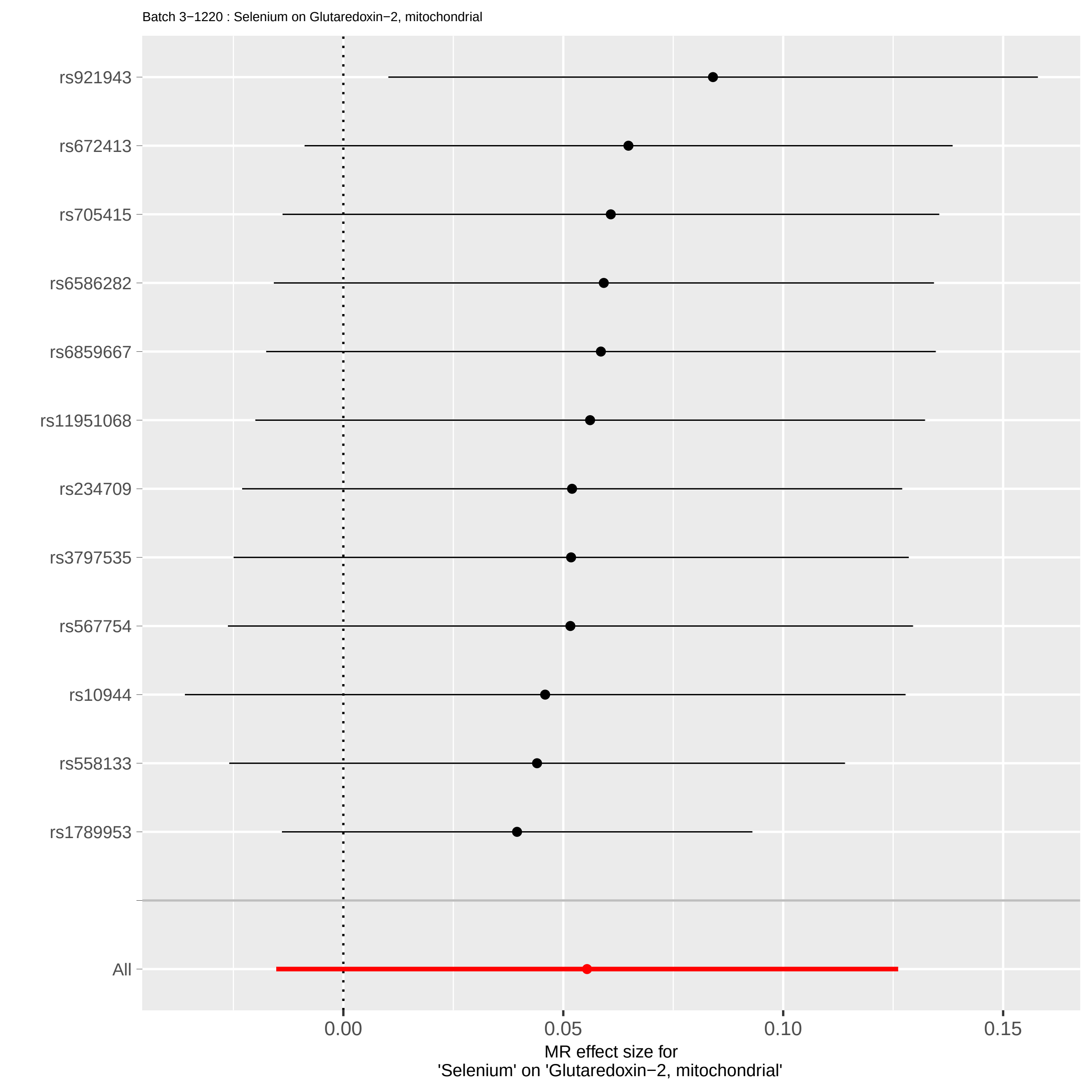
Batch 3−1220 : Selenium on Glutaredoxin−2, mitochondrial
rs921943
rs672413
rs705415
rs6586282
rs6859667
rs11951068
rs234709
rs3797535
rs567754
rs10944
rs558133
rs1789953
All
0.00
0.05
0.10
0.15
MR effect size for
'Selenium' on 'Glutaredoxin−2, mitochondrial'

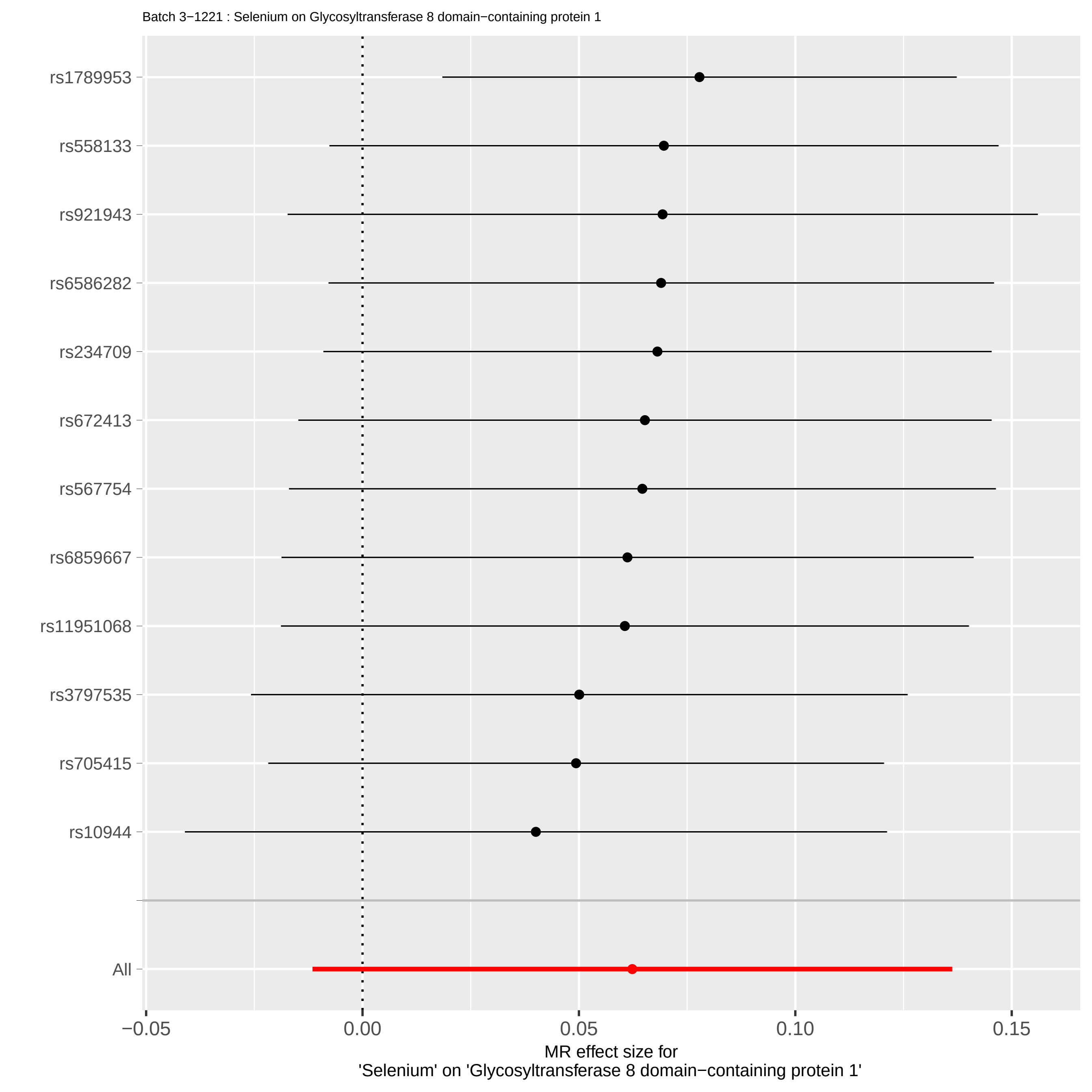

Batch 3−1221 : Selenium on Glycosyltransferase 8 domain−containing protein 1
rs1789953
rs558133
rs921943
rs6586282
rs234709
rs672413
rs567754
rs6859667
rs11951068
rs3797535
rs705415
rs10944
All
−0.05
0.00
0.05
0.10
0.15
MR effect size for
'Selenium' on 'Glycosyltransferase 8 domain−containing protein 1'

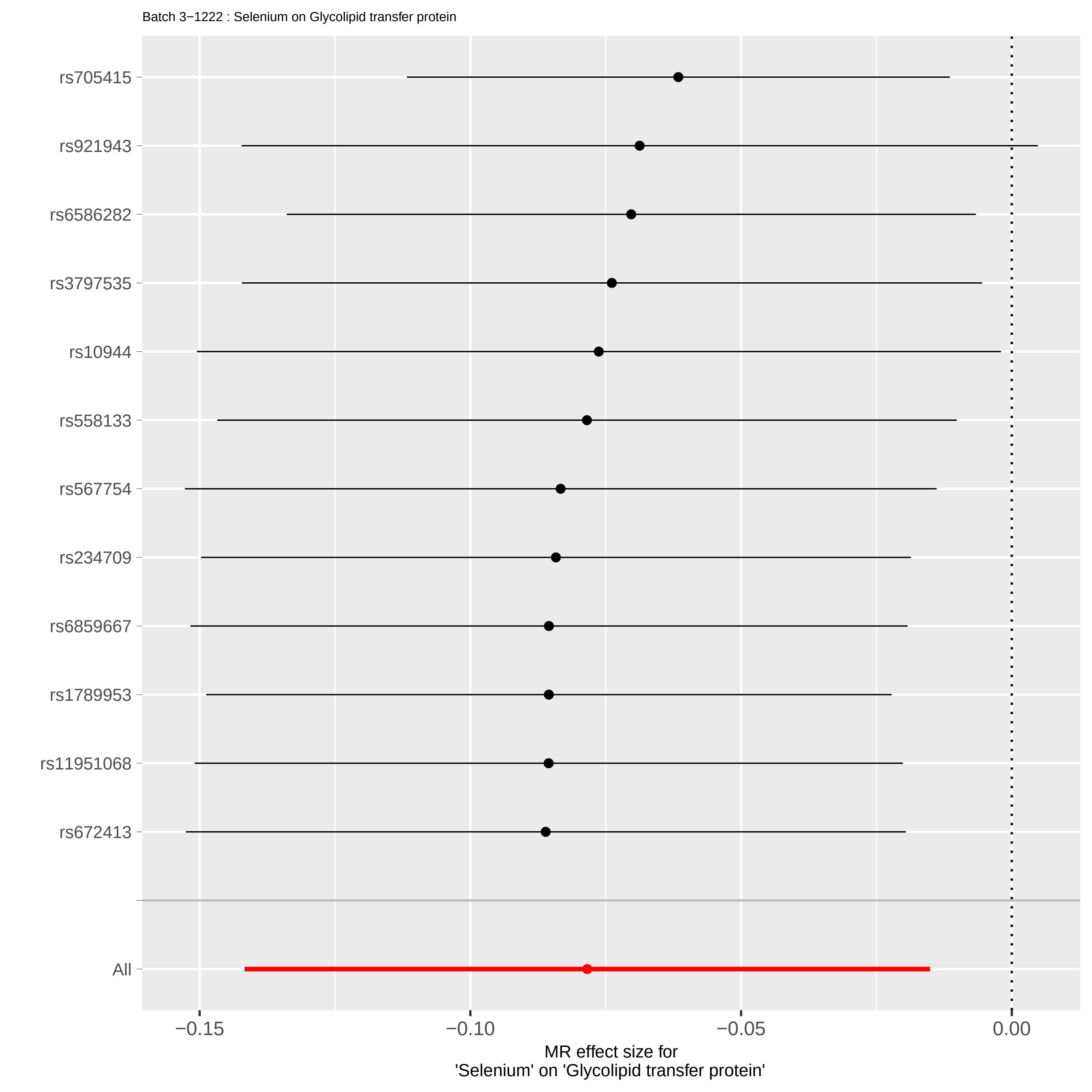

Batch 3−1222 : Selenium on Glycolipid transfer protein
rs705415
rs921943
rs6586282
rs3797535
rs10944
rs558133
rs567754
rs234709
rs6859667
rs1789953
rs11951068
rs672413
All
−0.15
−0.10
−0.05
0.00
MR effect size for
'Selenium' on 'Glycolipid transfer protein'

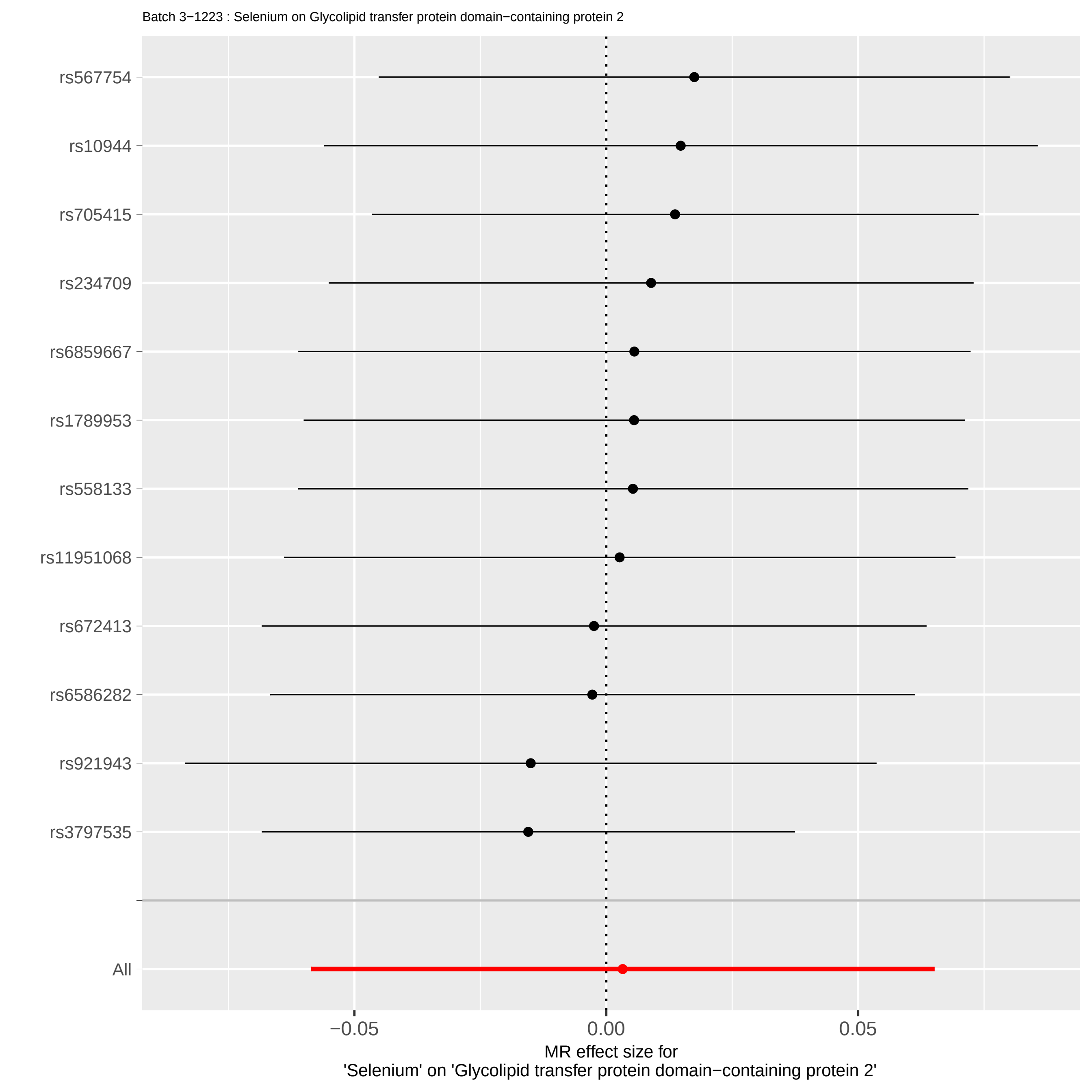

Batch 3−1223 : Selenium on Glycolipid transfer protein domain−containing protein 2
rs567754
rs10944
rs705415
rs234709
rs6859667
rs1789953
rs558133
rs11951068
rs672413
rs6586282
rs921943
rs3797535
All
−0.05
0.00
0.05
MR effect size for
'Selenium' on 'Glycolipid transfer protein domain−containing protein 2'

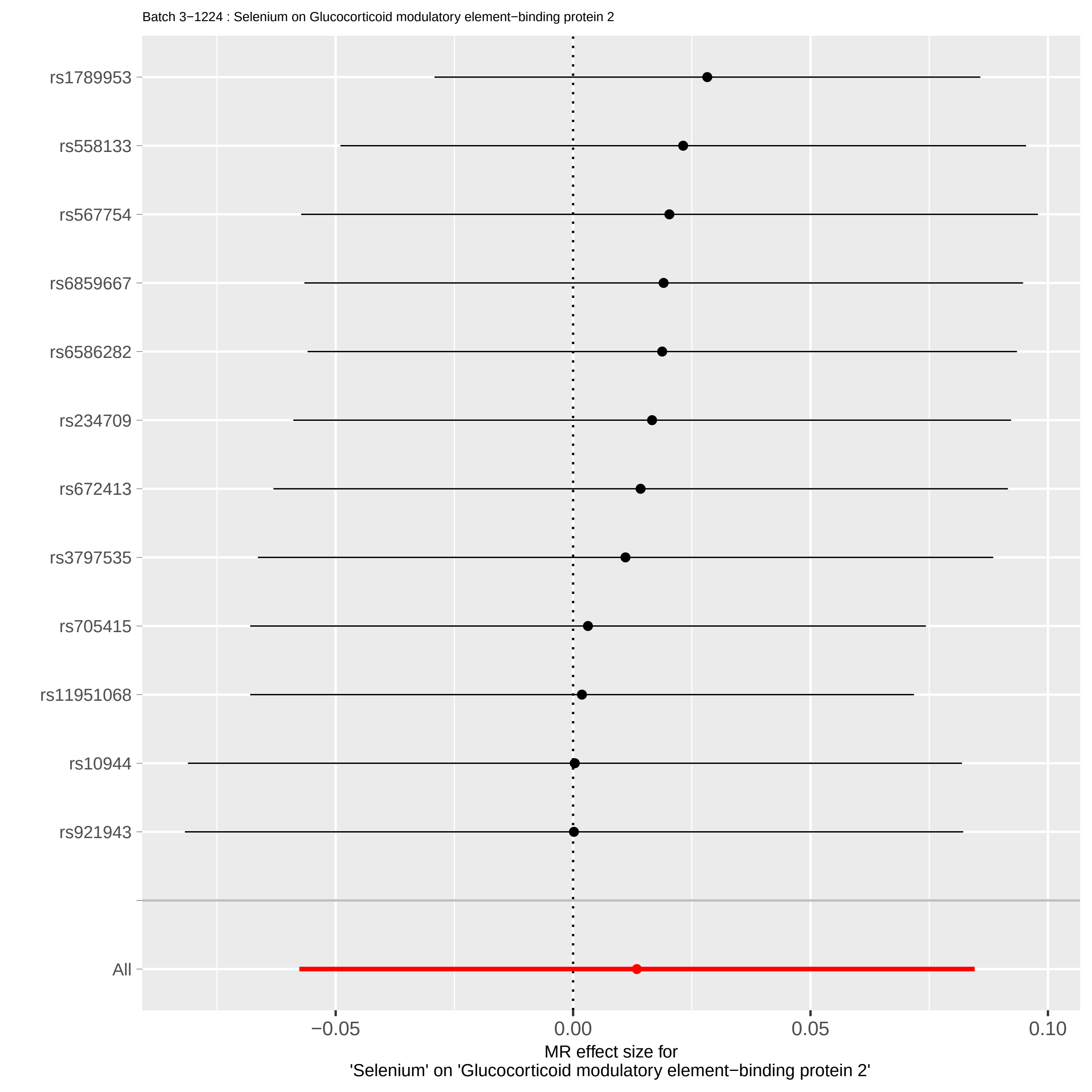
Batch 3−1224 : Selenium on Glucocorticoid modulatory element−binding protein 2
rs1789953
rs558133
rs567754
rs6859667
rs6586282
rs234709
rs672413
rs3797535
rs705415
rs11951068
rs10944
rs921943
All
−0.05
0.00
0.05
0.10
MR effect size for
'Selenium' on 'Glucocorticoid modulatory element−binding protein 2'

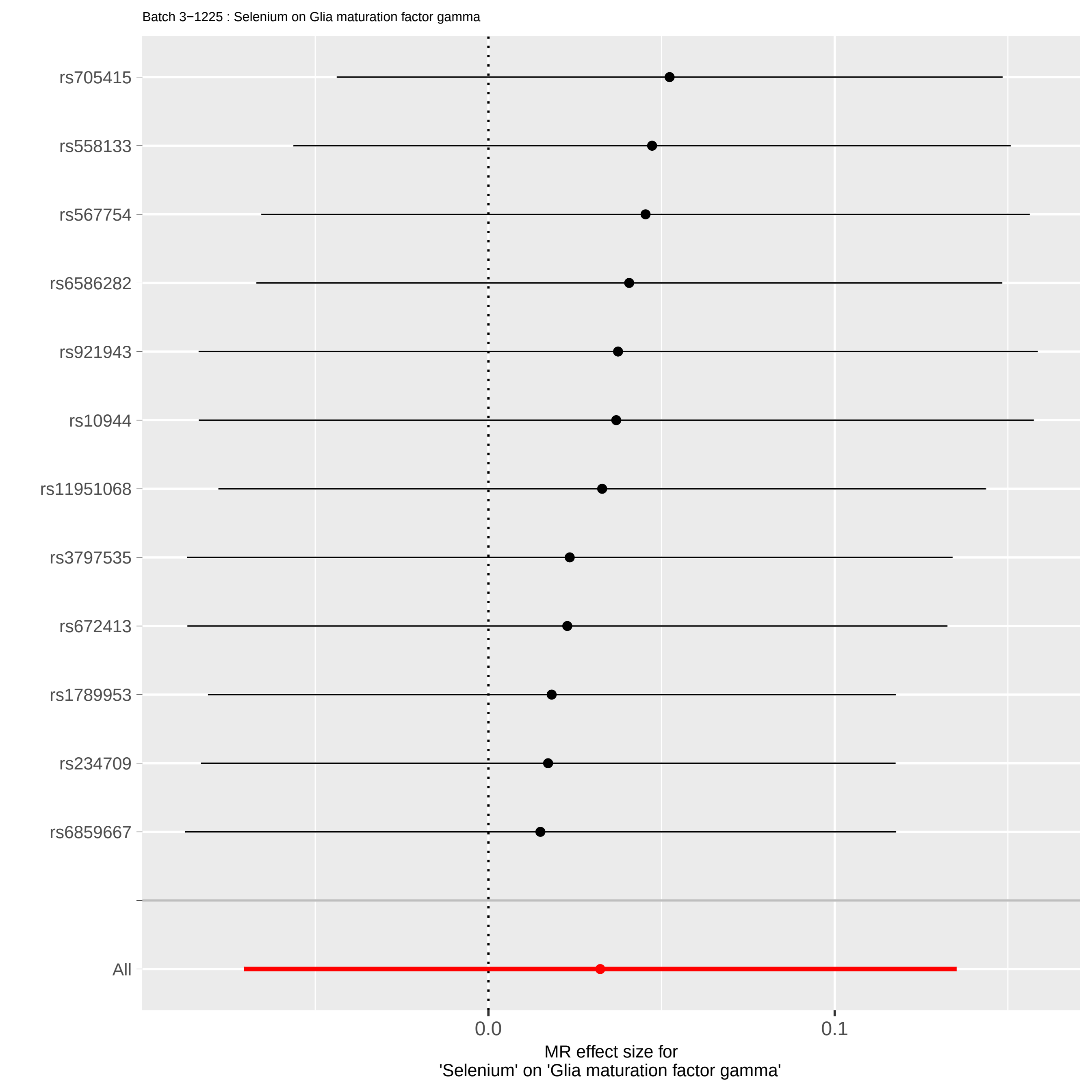
Batch 3−1225 : Selenium on Glia maturation factor gamma
rs705415
rs558133
rs567754
rs6586282
rs921943
rs10944
rs11951068
rs3797535
rs672413
rs1789953
rs234709
rs6859667
All
0.0
0.1
MR effect size for
'Selenium' on 'Glia maturation factor gamma'

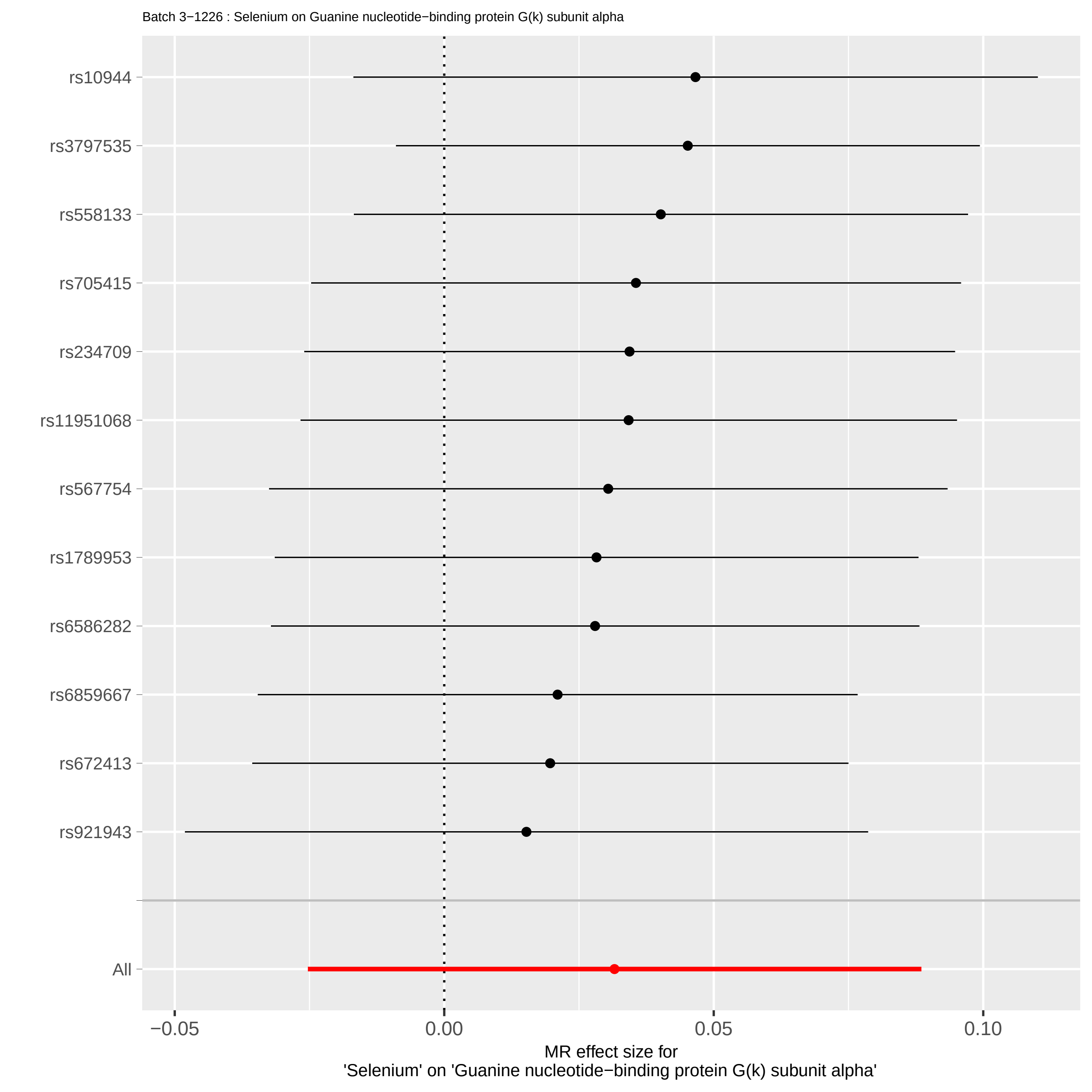

Batch 3−1226 : Selenium on Guanine nucleotide−binding protein G(k) subunit alpha
rs10944
rs3797535
rs558133
rs705415
rs234709
rs11951068
rs567754
rs1789953
rs6586282
rs6859667
rs672413
rs921943
All
−0.05
0.00
0.05
0.10
MR effect size for
'Selenium' on 'Guanine nucleotide−binding protein G(k) subunit alpha'

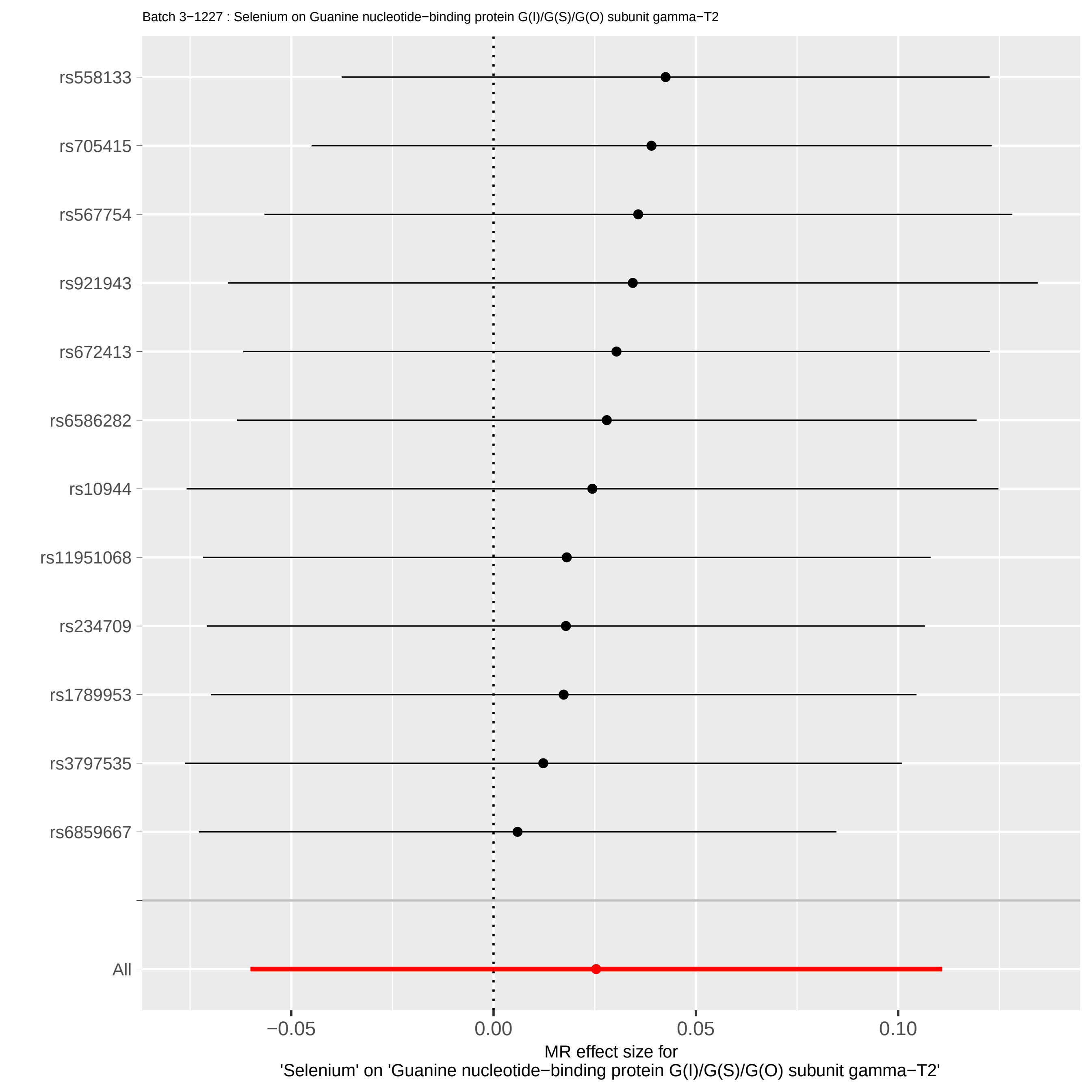
Batch 3−1227 : Selenium on Guanine nucleotide−binding protein G(I)/G(S)/G(O) subunit gamma−T2
rs558133
rs705415
rs567754
rs921943
rs672413
rs6586282
rs10944
rs11951068
rs234709
rs1789953
rs3797535
rs6859667
All
−0.05
0.00
0.05
0.10
MR effect size for
'Selenium' on 'Guanine nucleotide−binding protein G(I)/G(S)/G(O) subunit gamma−T2'

Batch 3−1228 : Selenium on Granulysin

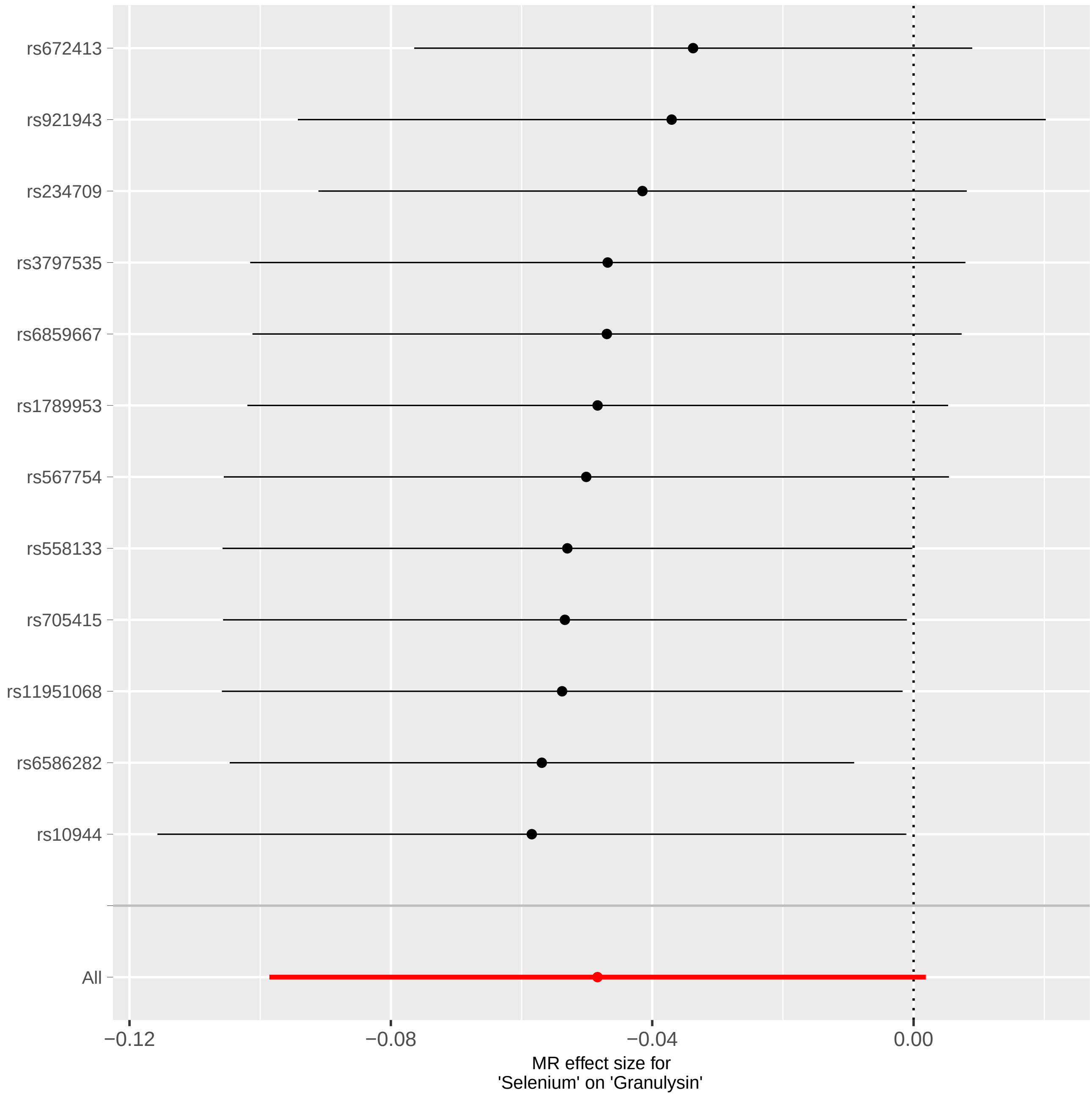

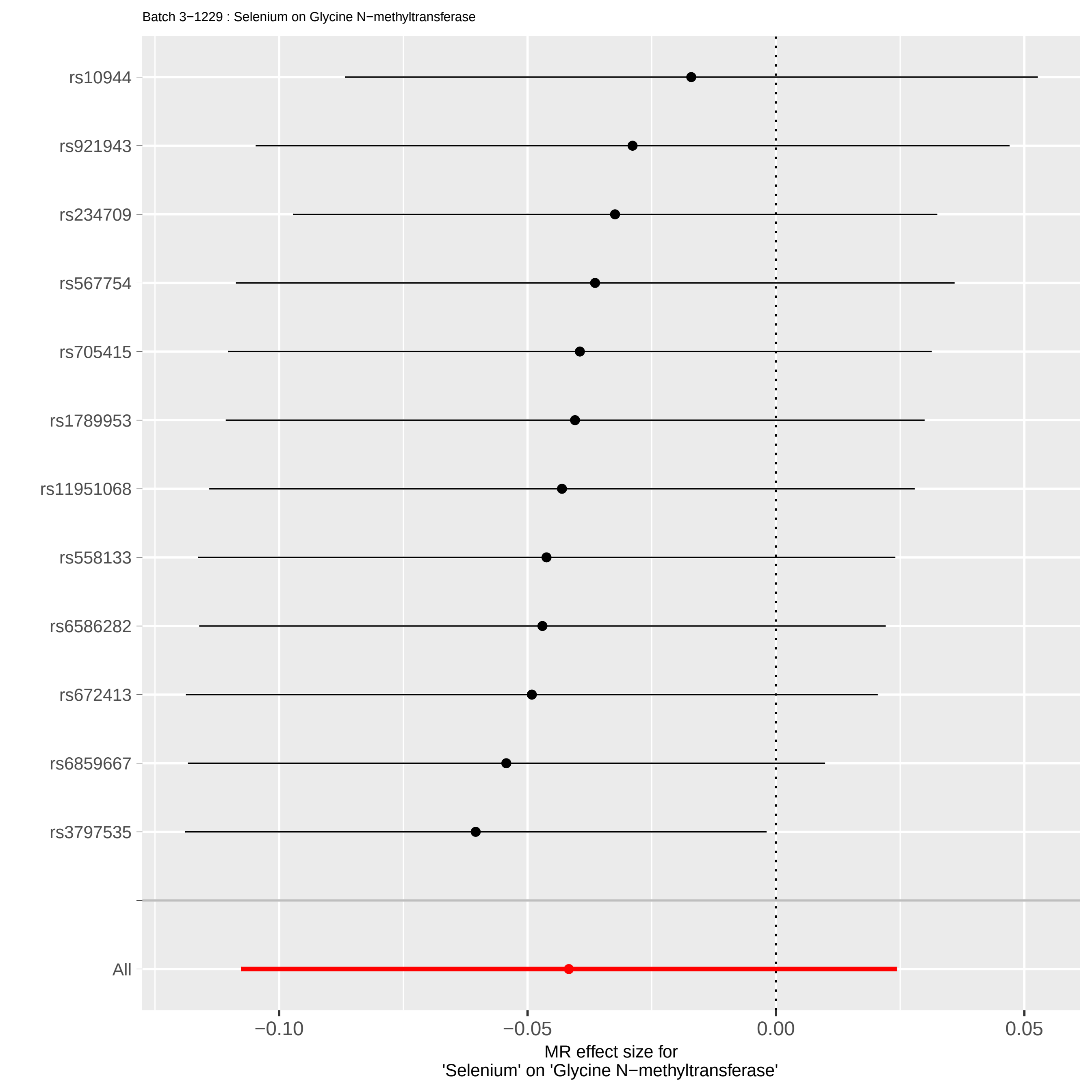

Batch 3−1229 : Selenium on Glycine N−methyltransferase
rs10944
rs921943
rs234709
rs567754
rs705415
rs1789953
rs11951068
rs558133
rs6586282
rs672413
rs6859667
rs3797535
All
−0.10
−0.05
0.00
0.05
MR effect size for
'Selenium' on 'Glycine N−methyltransferase'

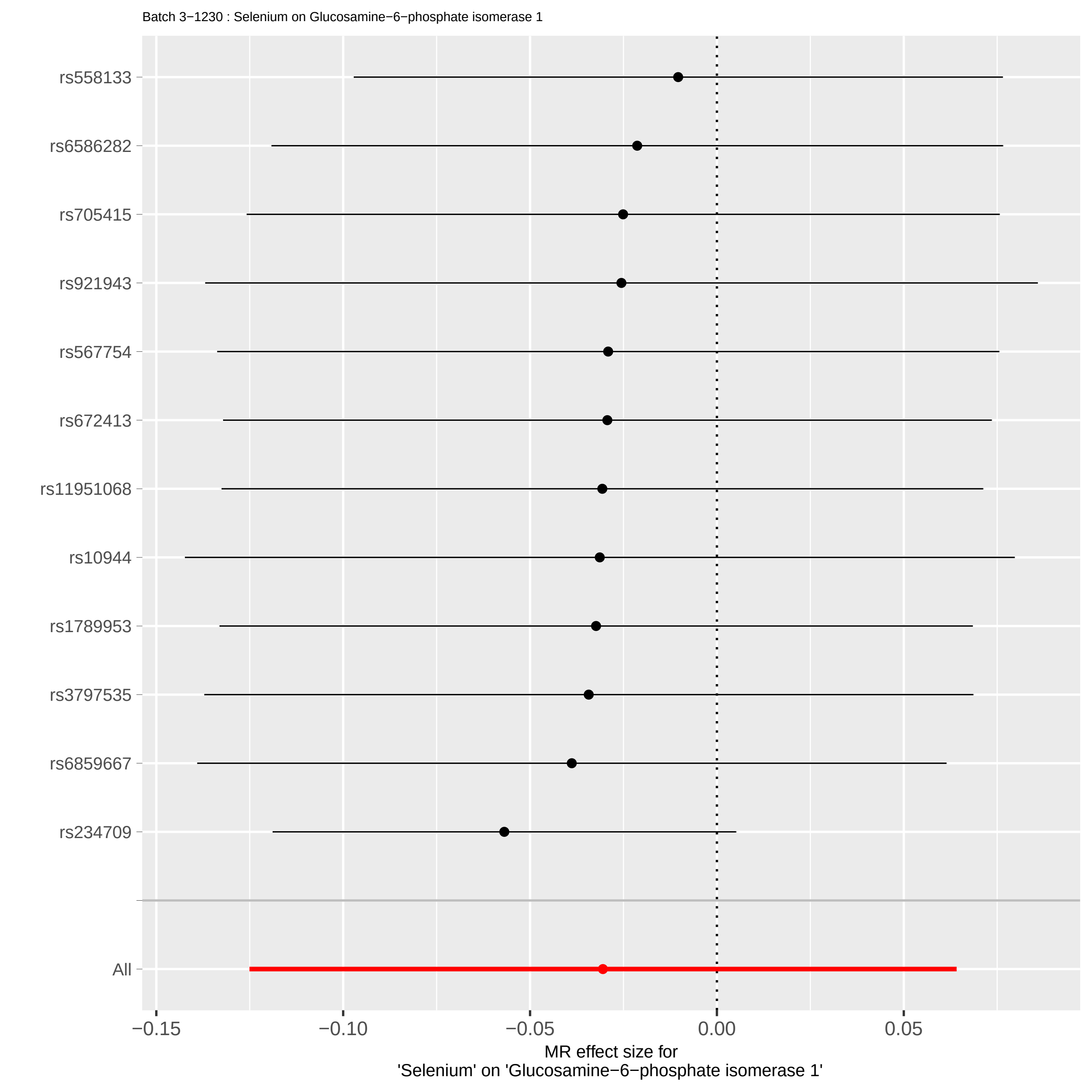

Batch 3−1230 : Selenium on Glucosamine−6−phosphate isomerase 1
rs558133
rs6586282
rs705415
rs921943
rs567754
rs672413
rs11951068
rs10944
rs1789953
rs3797535
rs6859667
rs234709
All
−0.15
−0.10
−0.05
0.00
0.05
MR effect size for
'Selenium' on 'Glucosamine−6−phosphate isomerase 1'

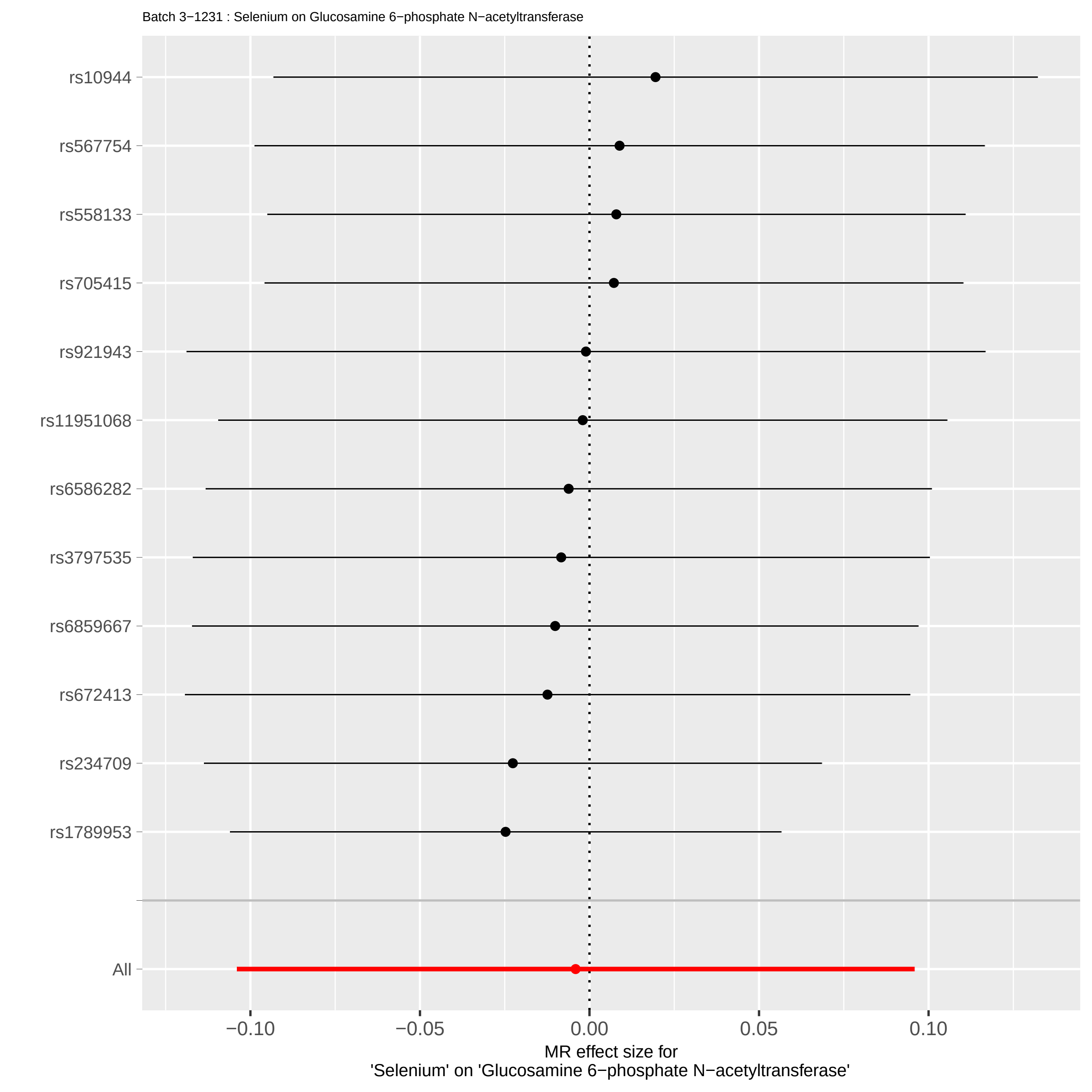

Batch 3−1231 : Selenium on Glucosamine 6−phosphate N−acetyltransferase
rs10944
rs567754
rs558133
rs705415
rs921943
rs11951068
rs6586282
rs3797535
rs6859667
rs672413
rs234709
rs1789953
All
−0.10
−0.05
0.00
0.05
0.10
MR effect size for
'Selenium' on 'Glucosamine 6−phosphate N−acetyltransferase'

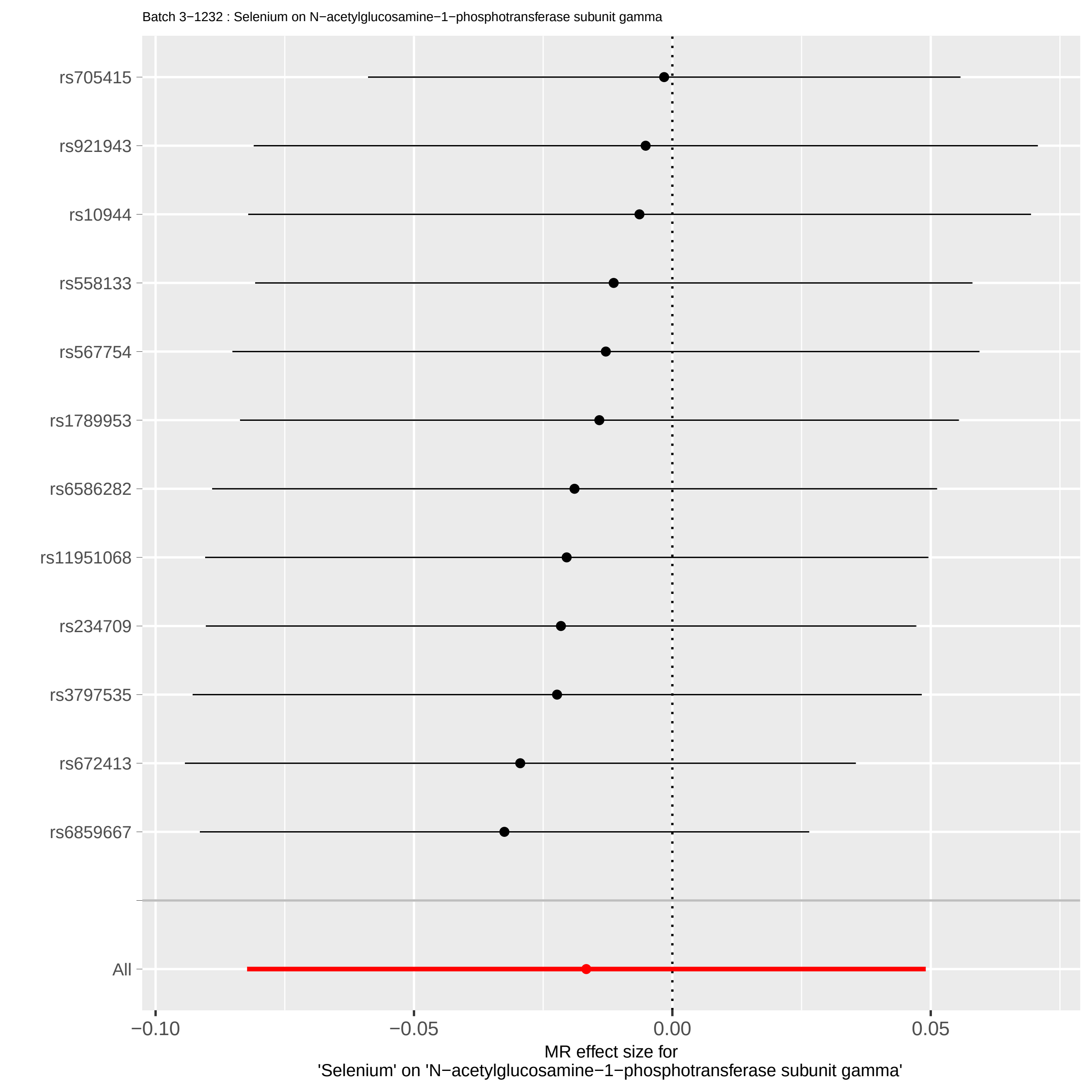

Batch 3−1232 : Selenium on N−acetylglucosamine−1−phosphotransferase subunit gamma
rs705415
rs921943
rs10944
rs558133
rs567754
rs1789953
rs6586282
rs11951068
rs234709
rs3797535
rs672413
rs6859667
All
−0.10
−0.05
0.00
0.05
MR effect size for
'Selenium' on 'N−acetylglucosamine−1−phosphotransferase subunit gamma'

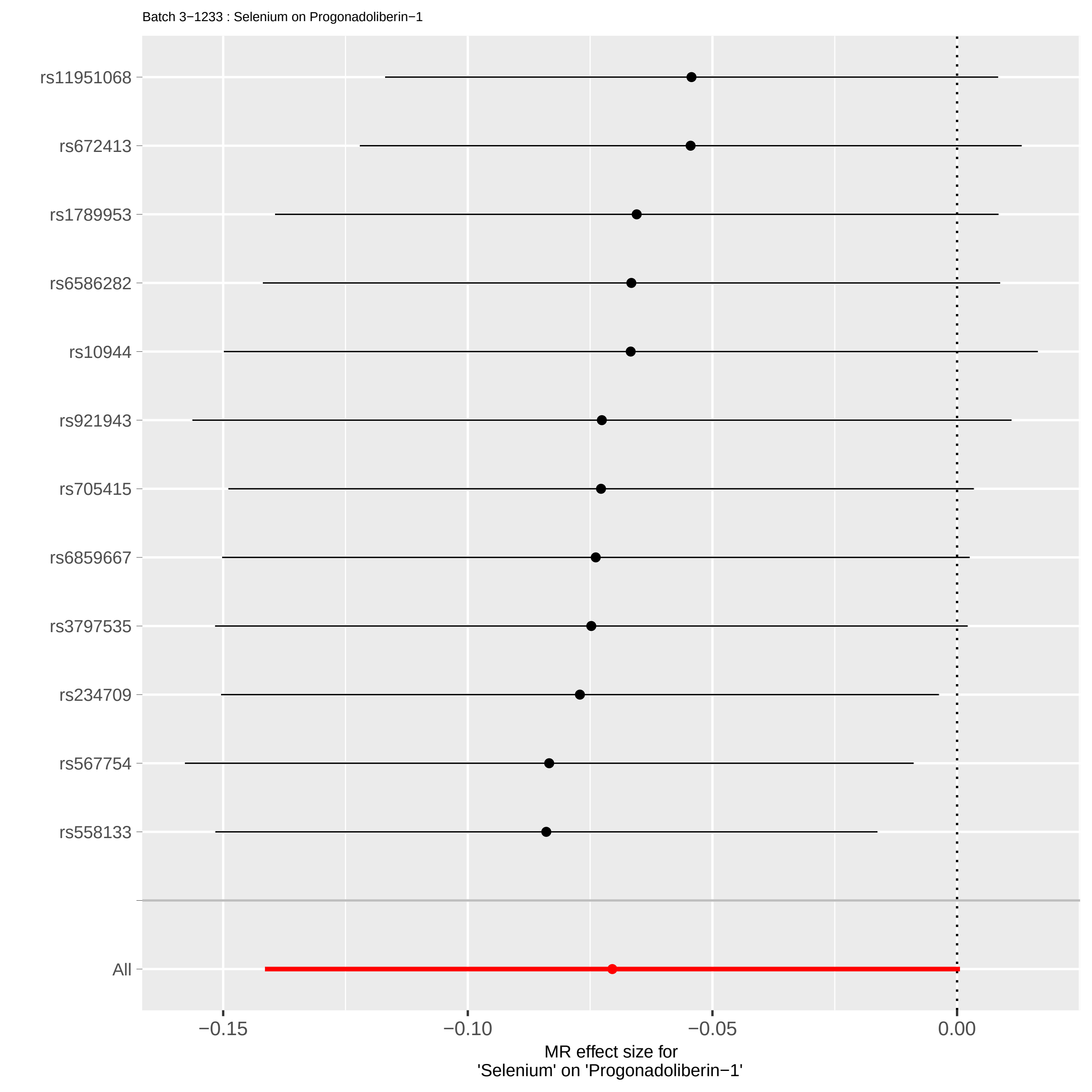

Batch 3−1233 : Selenium on Progonadoliberin−1
rs11951068
rs672413
rs1789953
rs6586282
rs10944
rs921943
rs705415
rs6859667
rs3797535
rs234709
rs567754
rs558133
All
−0.15
−0.10
−0.05
0.00
MR effect size for
'Selenium' on 'Progonadoliberin−1'

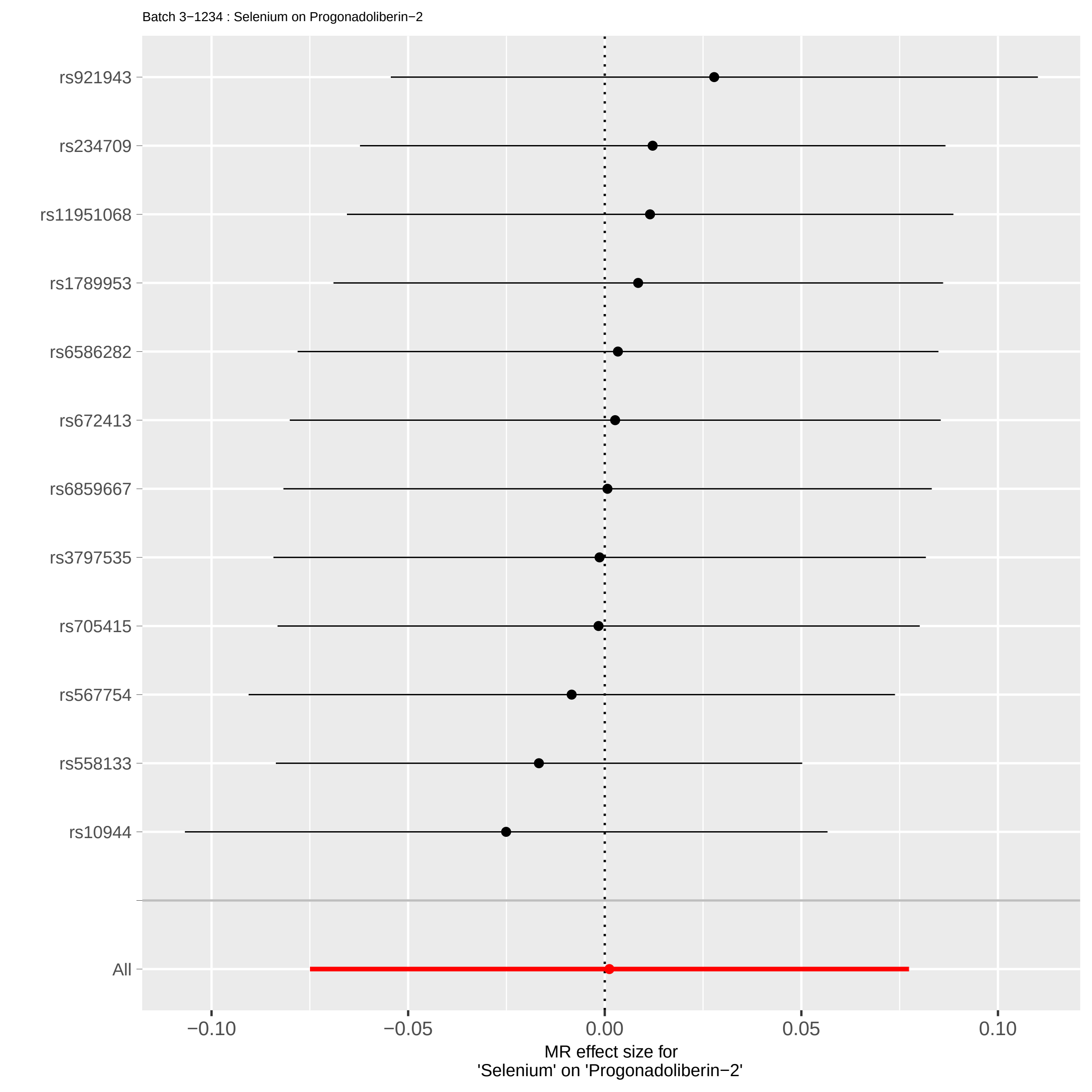

Batch 3−1234 : Selenium on Progonadoliberin−2
rs921943
rs234709
rs11951068
rs1789953
rs6586282
rs672413
rs6859667
rs3797535
rs705415
rs567754
rs558133
rs10944
All
−0.10
−0.05
0.00
0.05
0.10
MR effect size for
'Selenium' on 'Progonadoliberin−2'

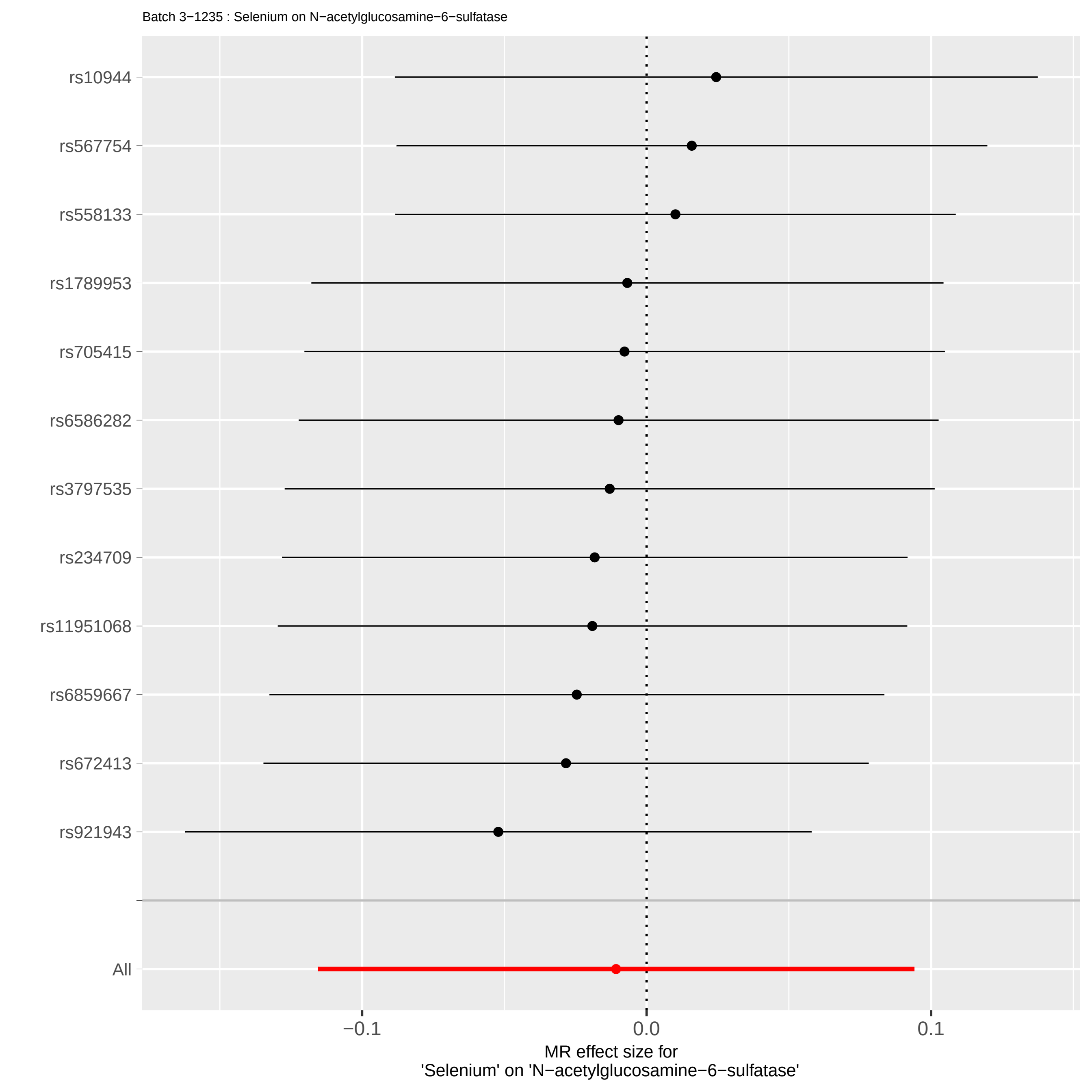

Batch 3−1235 : Selenium on N−acetylglucosamine−6−sulfatase
rs10944
rs567754
rs558133
rs1789953
rs705415
rs6586282
rs3797535
rs234709
rs11951068
rs6859667
rs672413
rs921943
All
−0.1
0.0
0.1
MR effect size for
'Selenium' on 'N−acetylglucosamine−6−sulfatase'

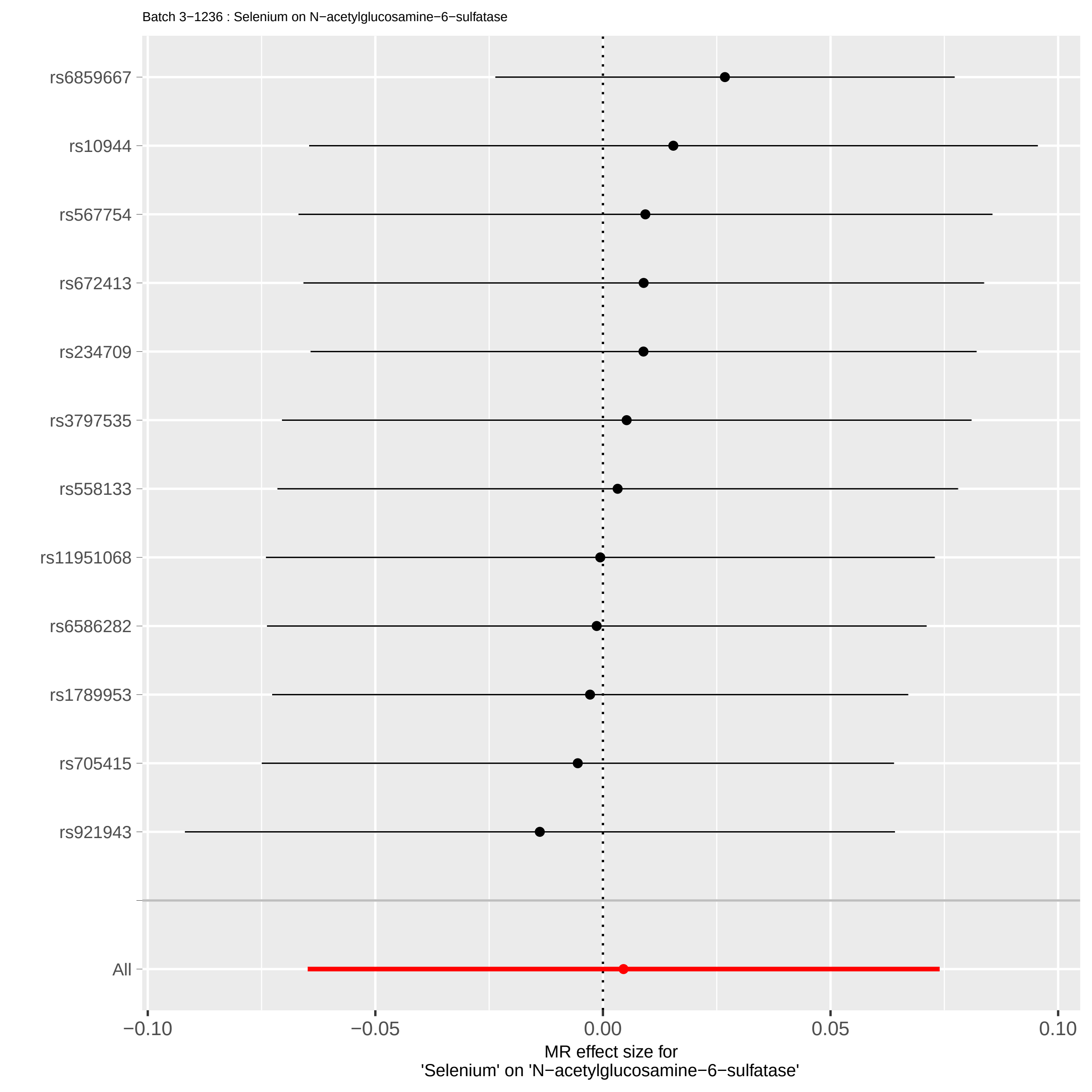
Batch 3−1236 : Selenium on N−acetylglucosamine−6−sulfatase
rs6859667
rs10944
rs567754
rs672413
rs234709
rs3797535
rs558133
rs11951068
rs6586282
rs1789953
rs705415
rs921943
All
−0.10
−0.05
0.00
0.05
0.10
MR effect size for
'Selenium' on 'N−acetylglucosamine−6−sulfatase'

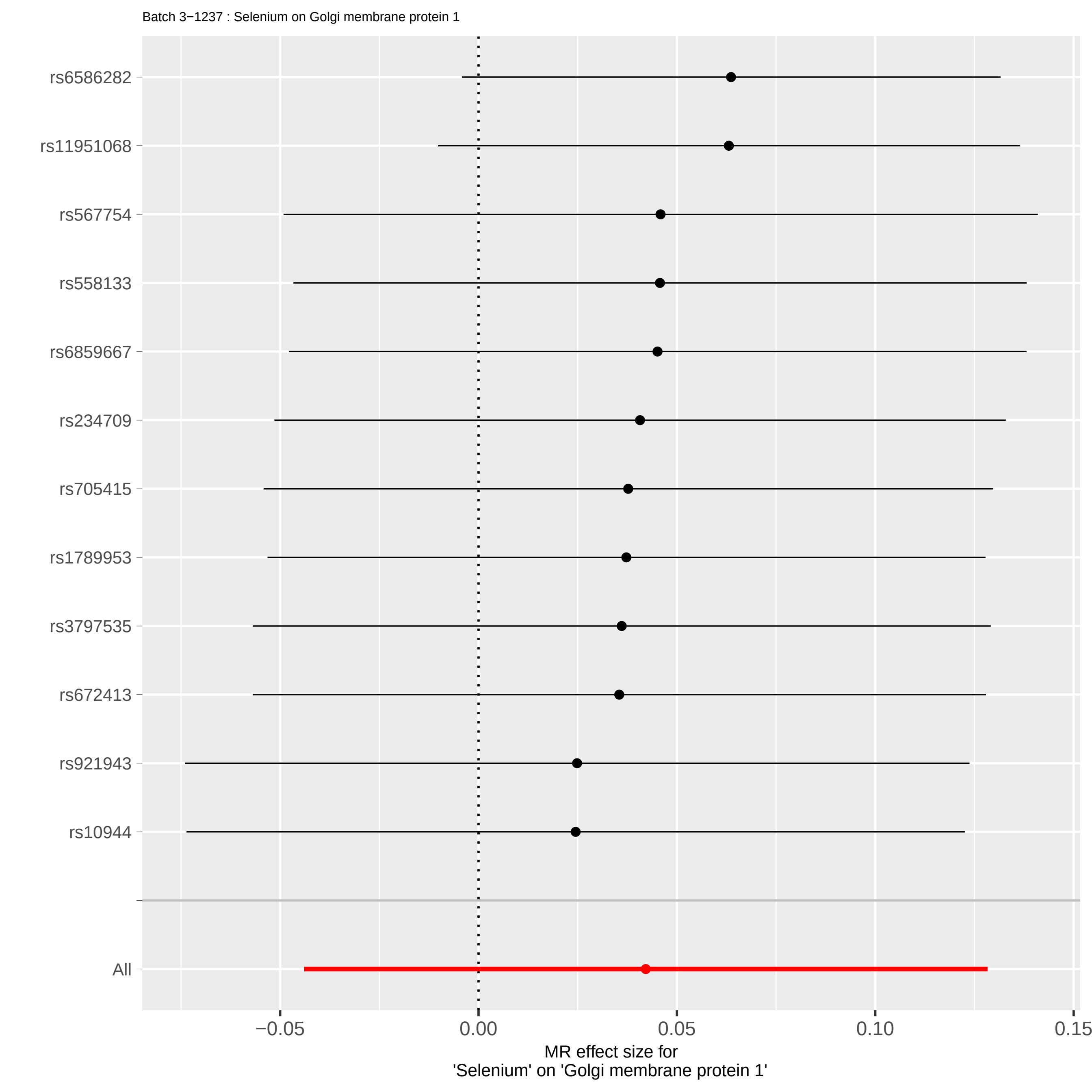
Batch 3−1237 : Selenium on Golgi membrane protein 1
rs6586282
rs11951068
rs567754
rs558133
rs6859667
rs234709
rs705415
rs1789953
rs3797535
rs672413
rs921943
rs10944
All
−0.05
0.00
0.05
0.10
0.15
MR effect size for
'Selenium' on 'Golgi membrane protein 1'

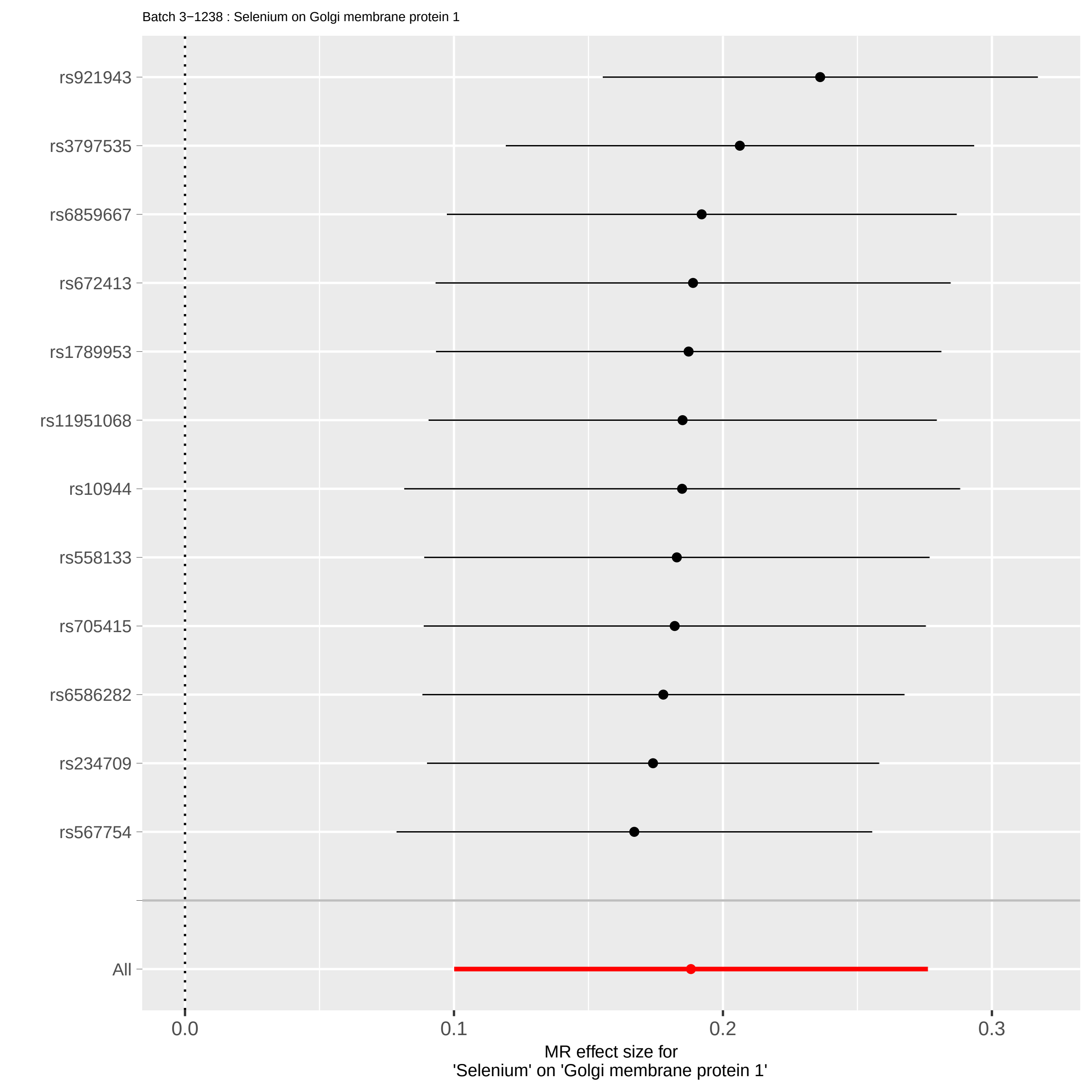

Batch 3−1238 : Selenium on Golgi membrane protein 1
rs921943
rs3797535
rs6859667
rs672413
rs1789953
rs11951068
rs10944
rs558133
rs705415
rs6586282
rs234709
rs567754
All
0.0
0.1
0.2
0.3
MR effect size for
'Selenium' on 'Golgi membrane protein 1'

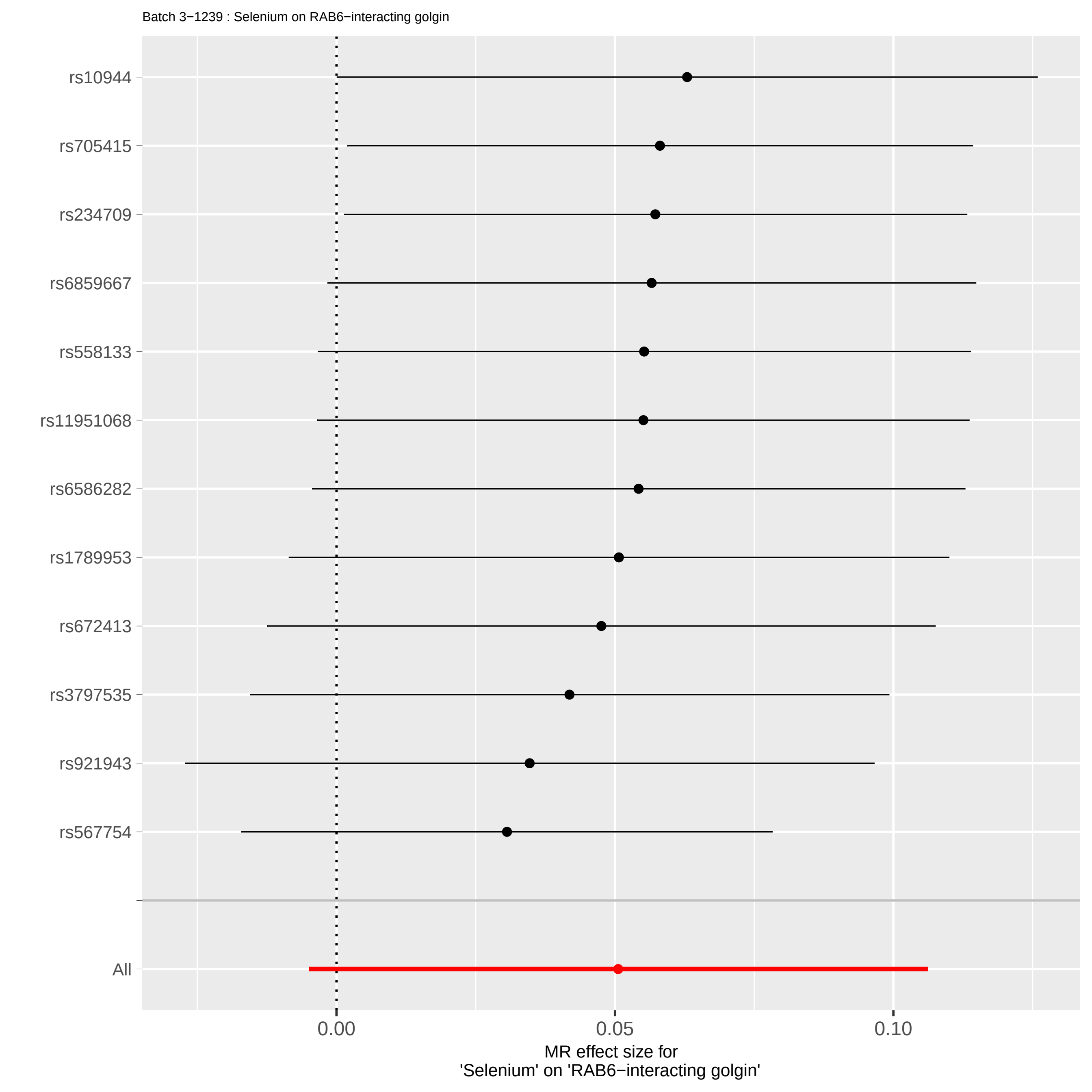
Batch 3−1239 : Selenium on RAB6−interacting golgin
rs10944
rs705415
rs234709
rs6859667
rs558133
rs11951068
rs6586282
rs1789953
rs672413
rs3797535
rs921943
rs567754
All
0.00
0.05
0.10
MR effect size for
'Selenium' on 'RAB6−interacting golgin'

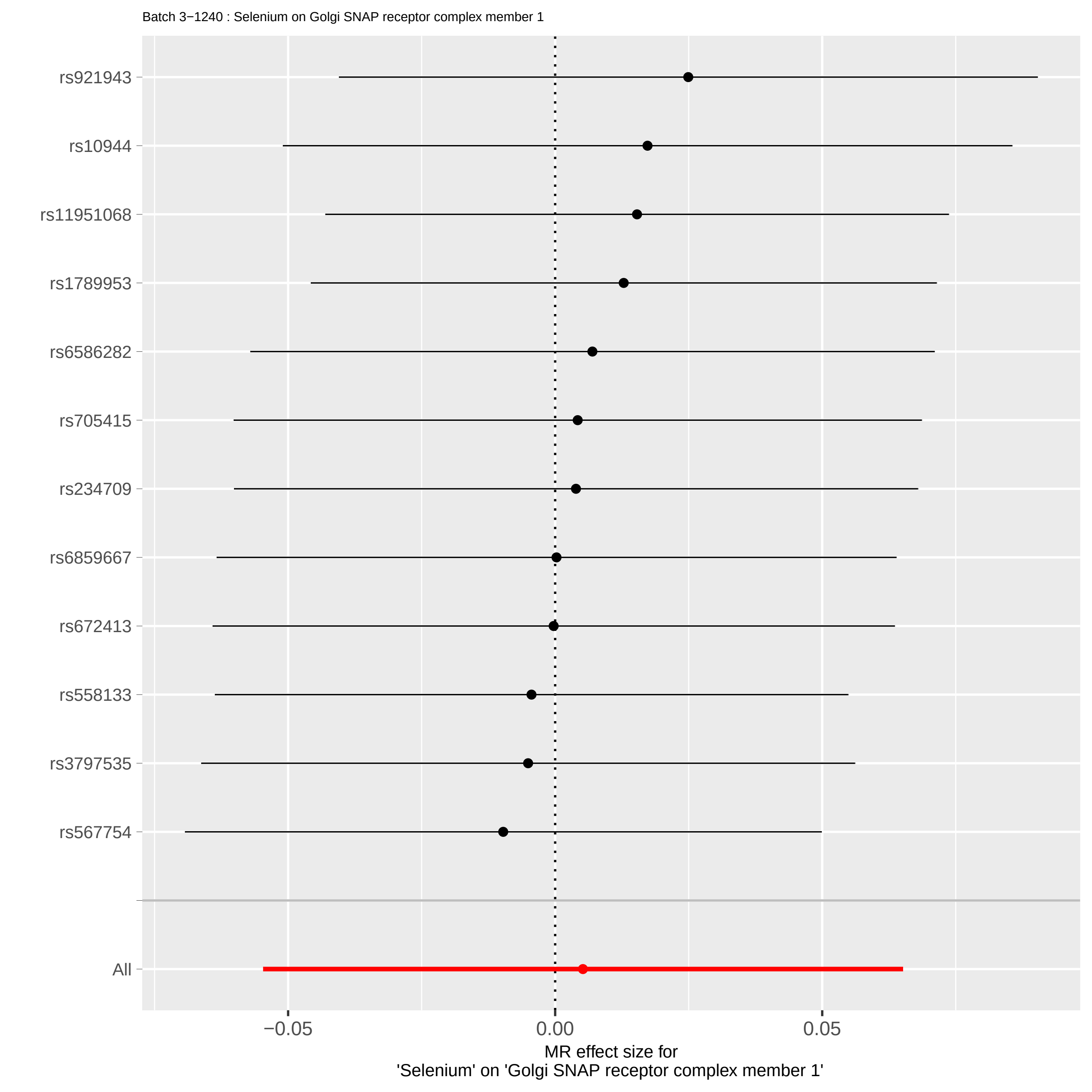

Batch 3−1240 : Selenium on Golgi SNAP receptor complex member 1
rs921943
rs10944
rs11951068
rs1789953
rs6586282
rs705415
rs234709
rs6859667
rs672413
rs558133
rs3797535
rs567754
All
−0.05
0.00
0.05
MR effect size for
'Selenium' on 'Golgi SNAP receptor complex member 1'

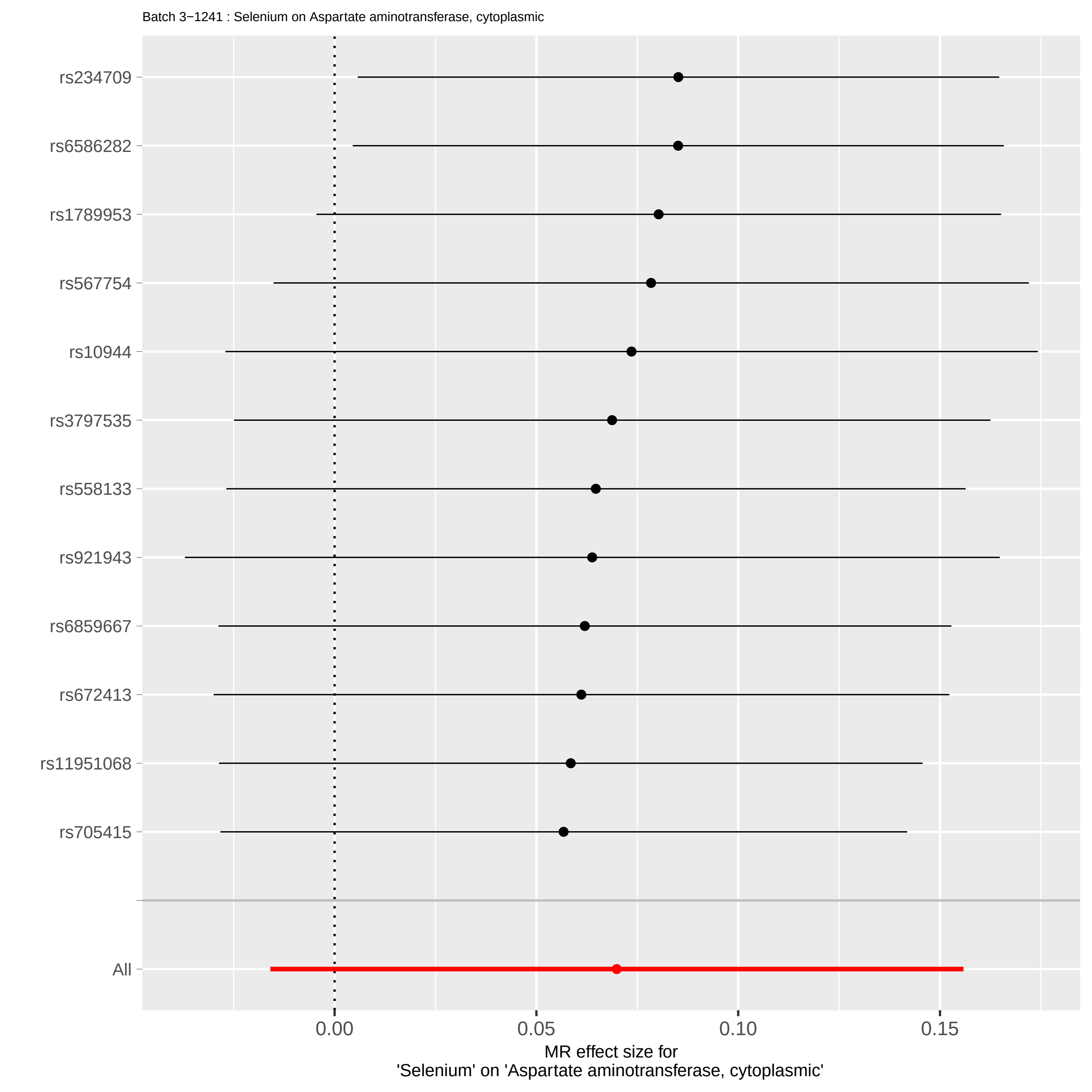

Batch 3−1241 : Selenium on Aspartate aminotransferase, cytoplasmic
rs234709
rs6586282
rs1789953
rs567754
rs10944
rs3797535
rs558133
rs921943
rs6859667
rs672413
rs11951068
rs705415
All
0.00
0.05
0.10
0.15
MR effect size for
'Selenium' on 'Aspartate aminotransferase, cytoplasmic'

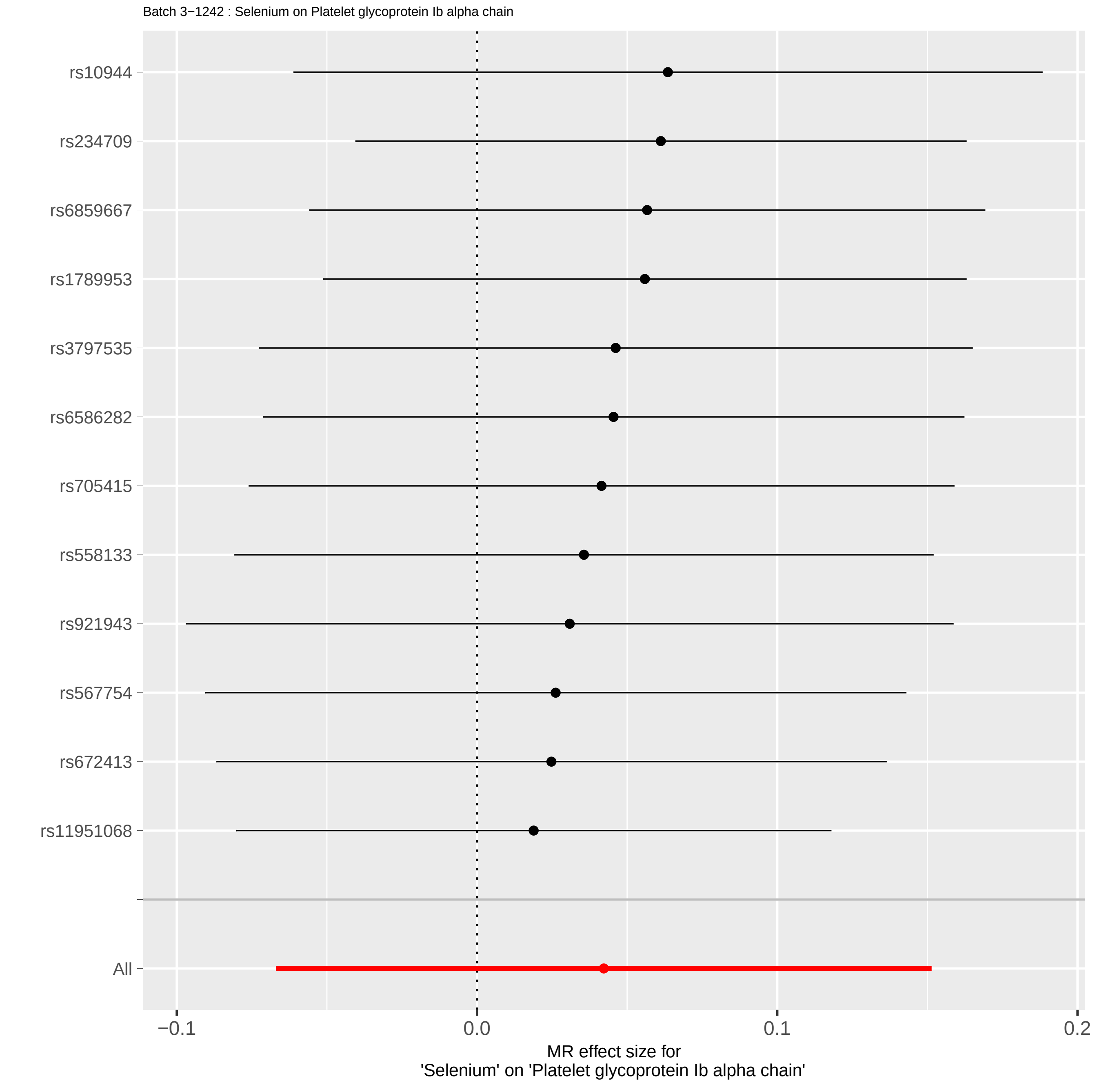
Batch 3−1242 : Selenium on Platelet glycoprotein Ib alpha chain
rs10944
rs234709
rs6859667
rs1789953
rs3797535
rs6586282
rs705415
rs558133
rs921943
rs567754
rs672413
rs11951068
All
−0.1
0.0
0.1
0.2
MR effect size for
'Selenium' on 'Platelet glycoprotein Ib alpha chain'

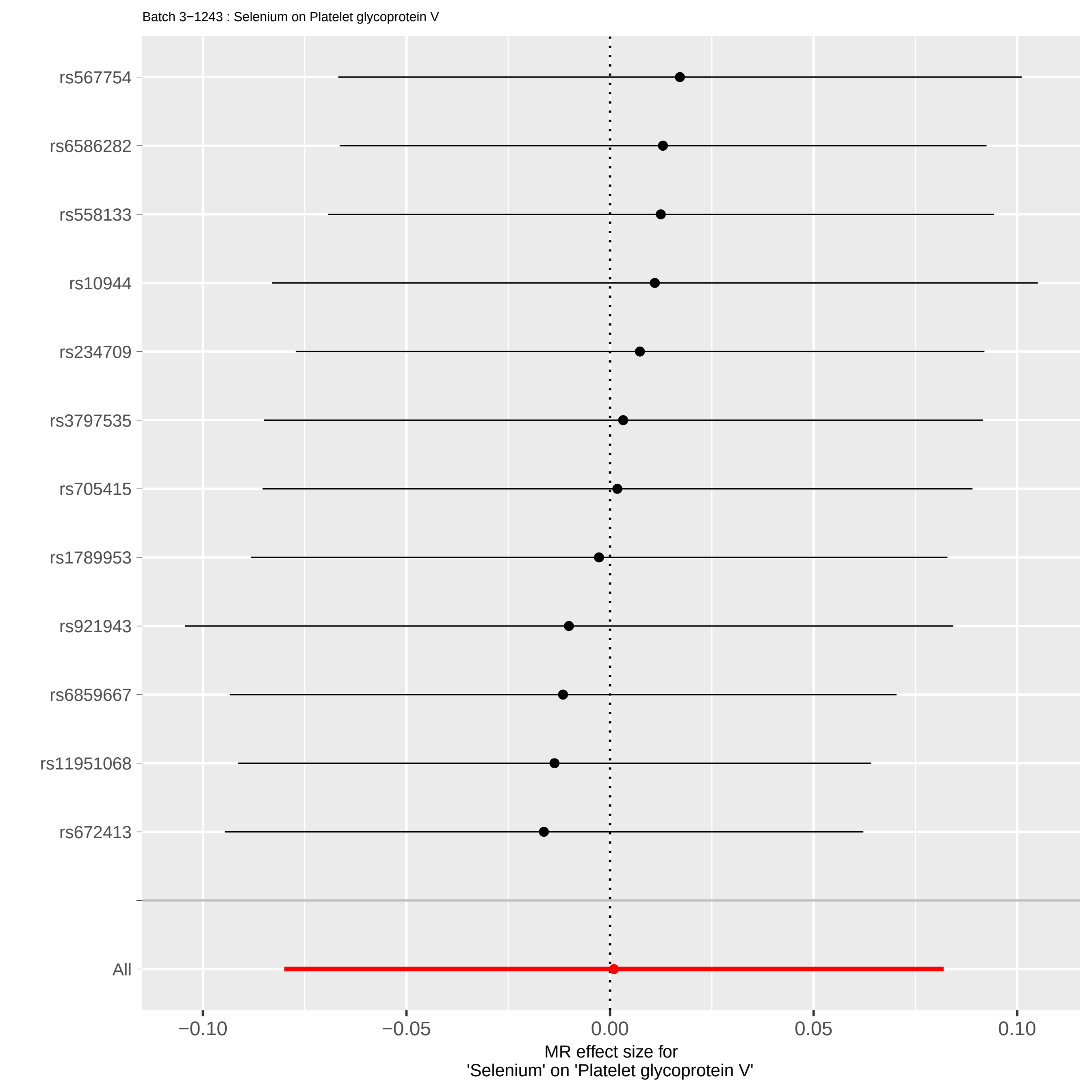

Batch 3−1243 : Selenium on Platelet glycoprotein V
rs567754
rs6586282
rs558133
rs10944
rs234709
rs3797535
rs705415
rs1789953
rs921943
rs6859667
rs11951068
rs672413
All
−0.10
−0.05
0.00
0.05
0.10
MR effect size for
'Selenium' on 'Platelet glycoprotein V'

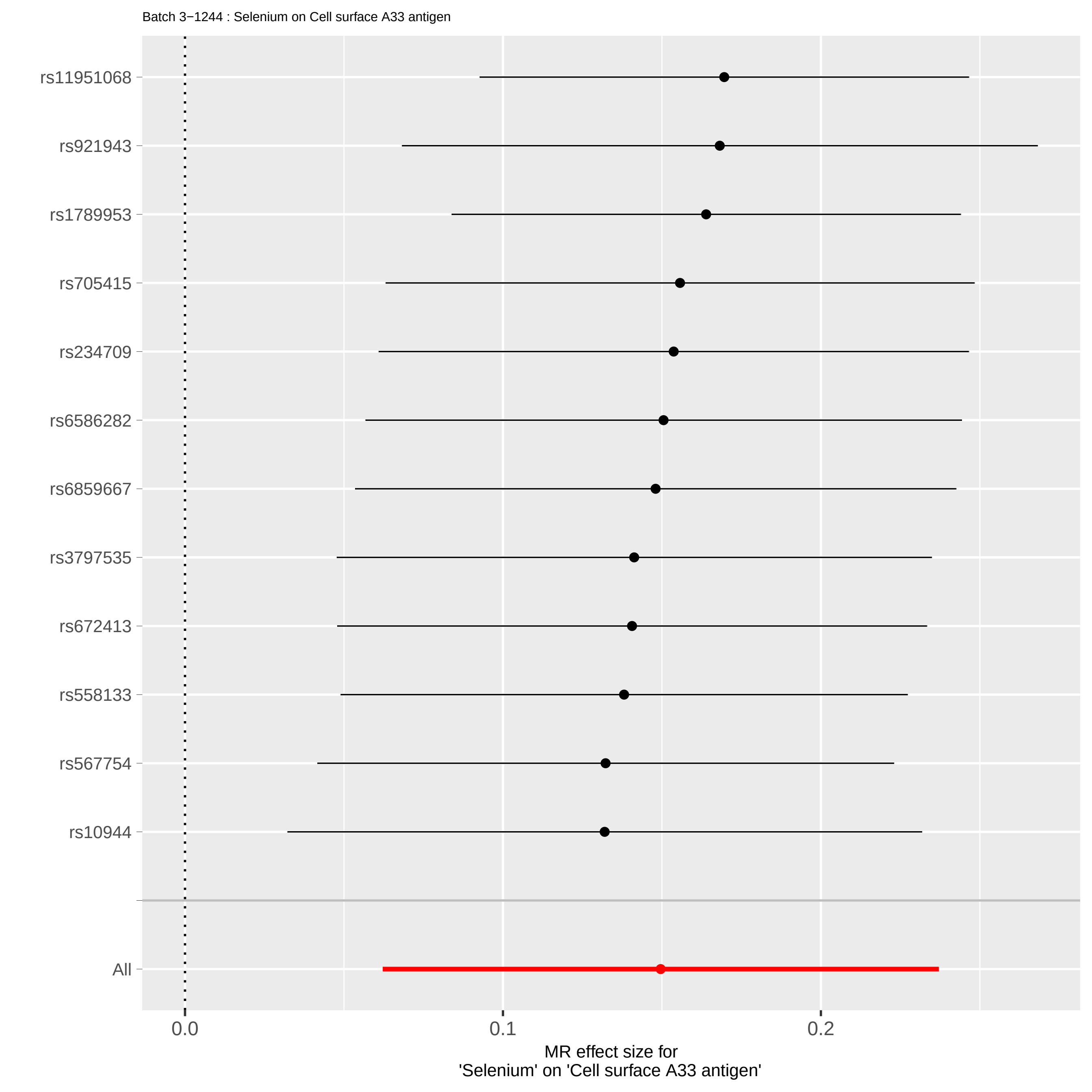

Batch 3−1244 : Selenium on Cell surface A33 antigen
rs11951068
rs921943
rs1789953
rs705415
rs234709
rs6586282
rs6859667
rs3797535
rs672413
rs558133
rs567754
rs10944
All
0.0
0.1
0.2
MR effect size for
'Selenium' on 'Cell surface A33 antigen'

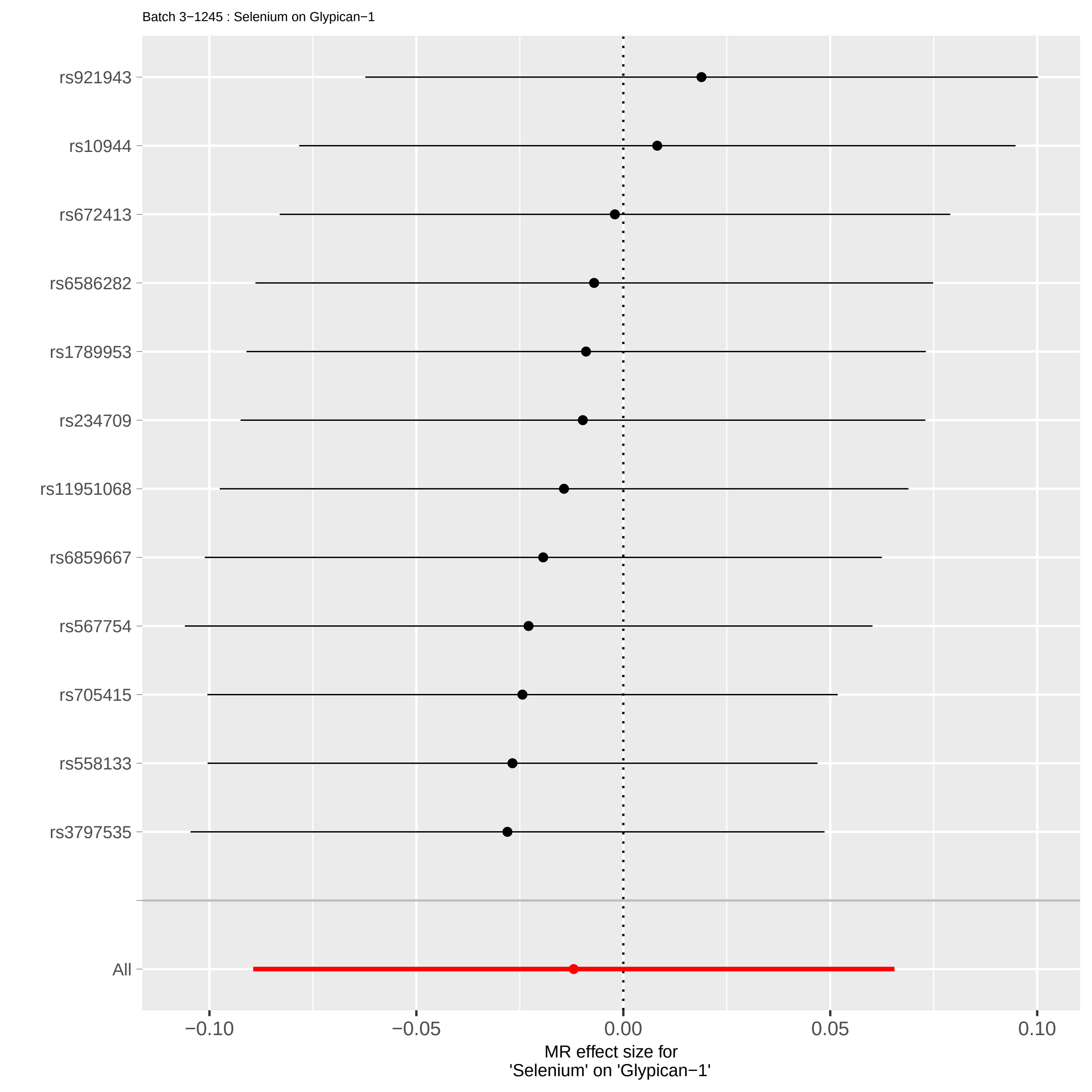

Batch 3−1245 : Selenium on Glypican−1
rs921943
rs10944
rs672413
rs6586282
rs1789953
rs234709
rs11951068
rs6859667
rs567754
rs705415
rs558133
rs3797535
All
−0.10
−0.05
0.00
0.05
0.10
MR effect size for
'Selenium' on 'Glypican−1'

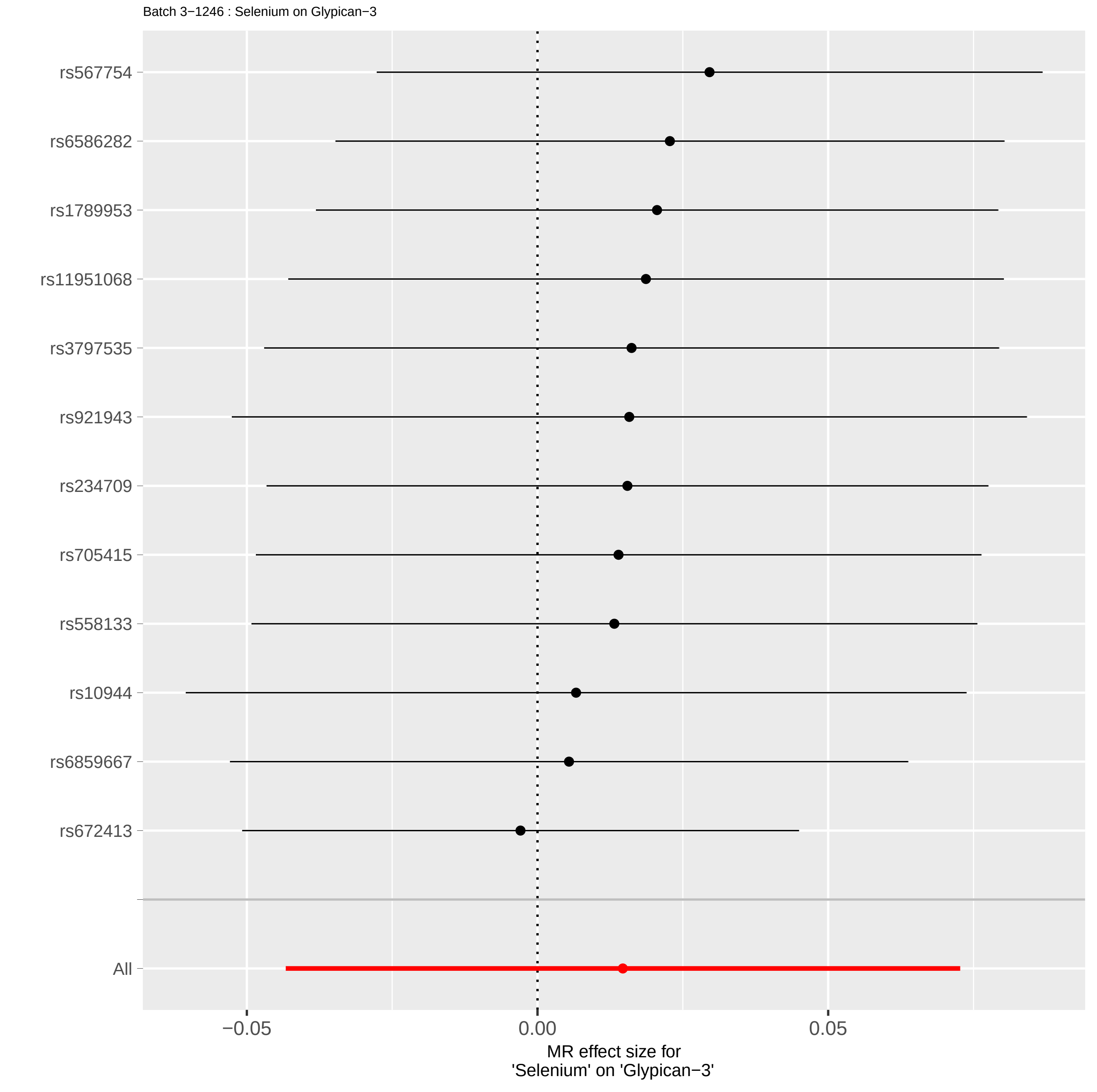

Batch 3−1246 : Selenium on Glypican−3
rs567754
rs6586282
rs1789953
rs11951068
rs3797535
rs921943
rs234709
rs705415
rs558133
rs10944
rs6859667
rs672413
All
−0.05
0.00
0.05
MR effect size for
'Selenium' on 'Glypican−3'

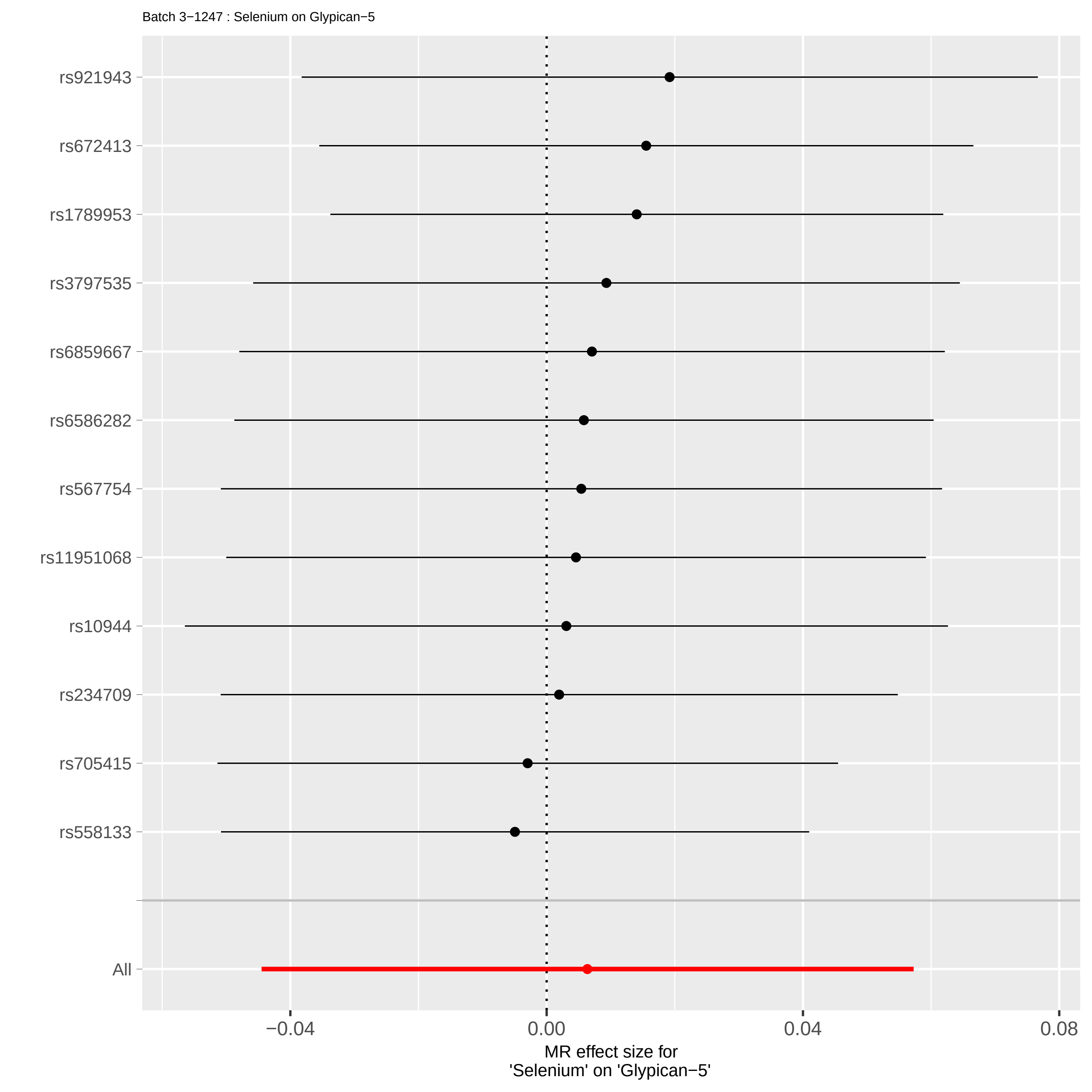
Batch 3−1247 : Selenium on Glypican−5
rs921943
rs672413
rs1789953
rs3797535
rs6859667
rs6586282
rs567754
rs11951068
rs10944
rs234709
rs705415
rs558133
All
−0.04
0.00
0.04
0.08
MR effect size for
'Selenium' on 'Glypican−5'

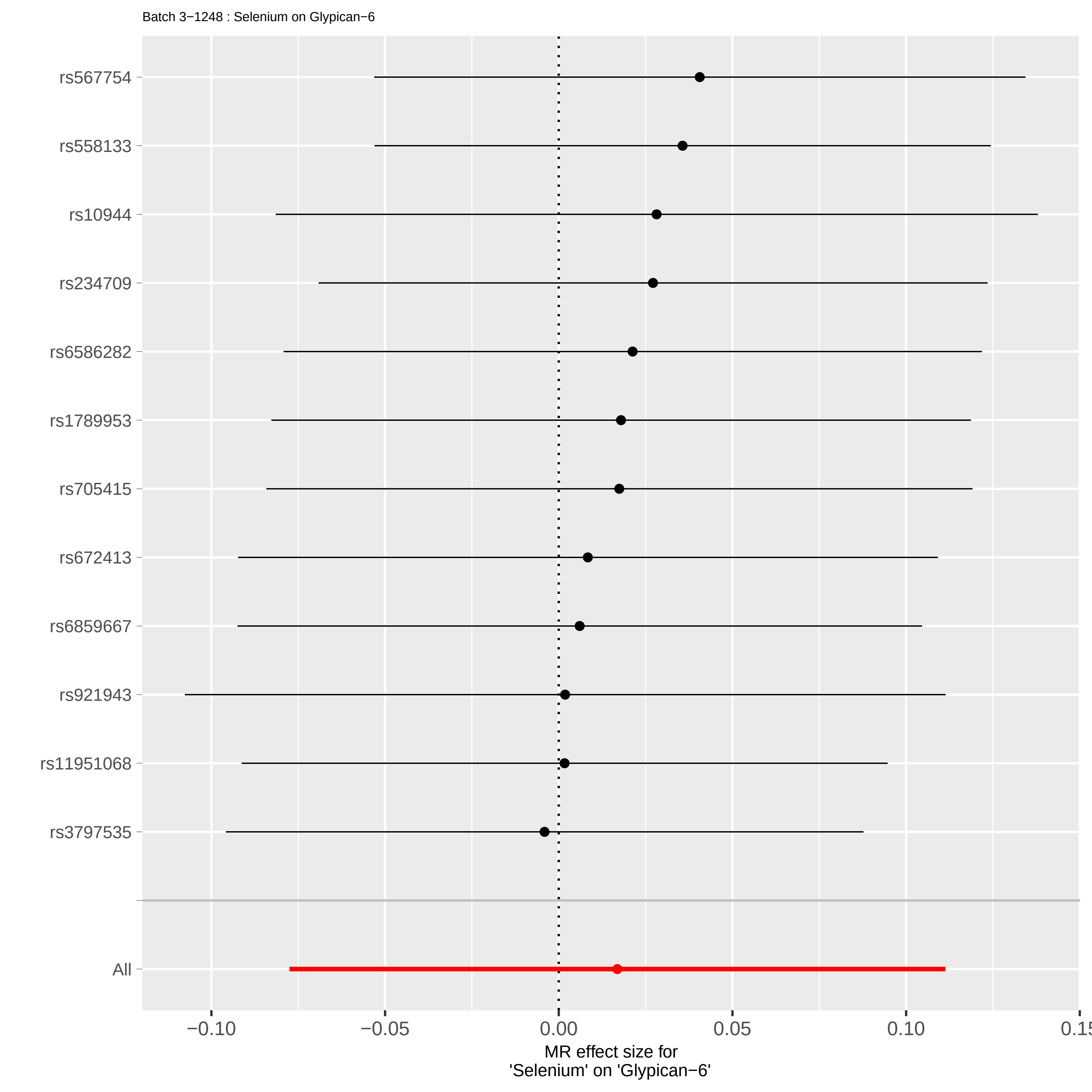
Batch 3−1248 : Selenium on Glypican−6
rs567754
rs558133
rs10944
rs234709
rs6586282
rs1789953
rs705415
rs672413
rs6859667
rs921943
rs11951068
rs3797535
All
−0.10
−0.05
0.00
0.05
0.10
0.15
MR effect size for
'Selenium' on 'Glypican−6'

Batch 3−1249 : Selenium on Glycerophosphocholine phosphodiesterase GPCPD1

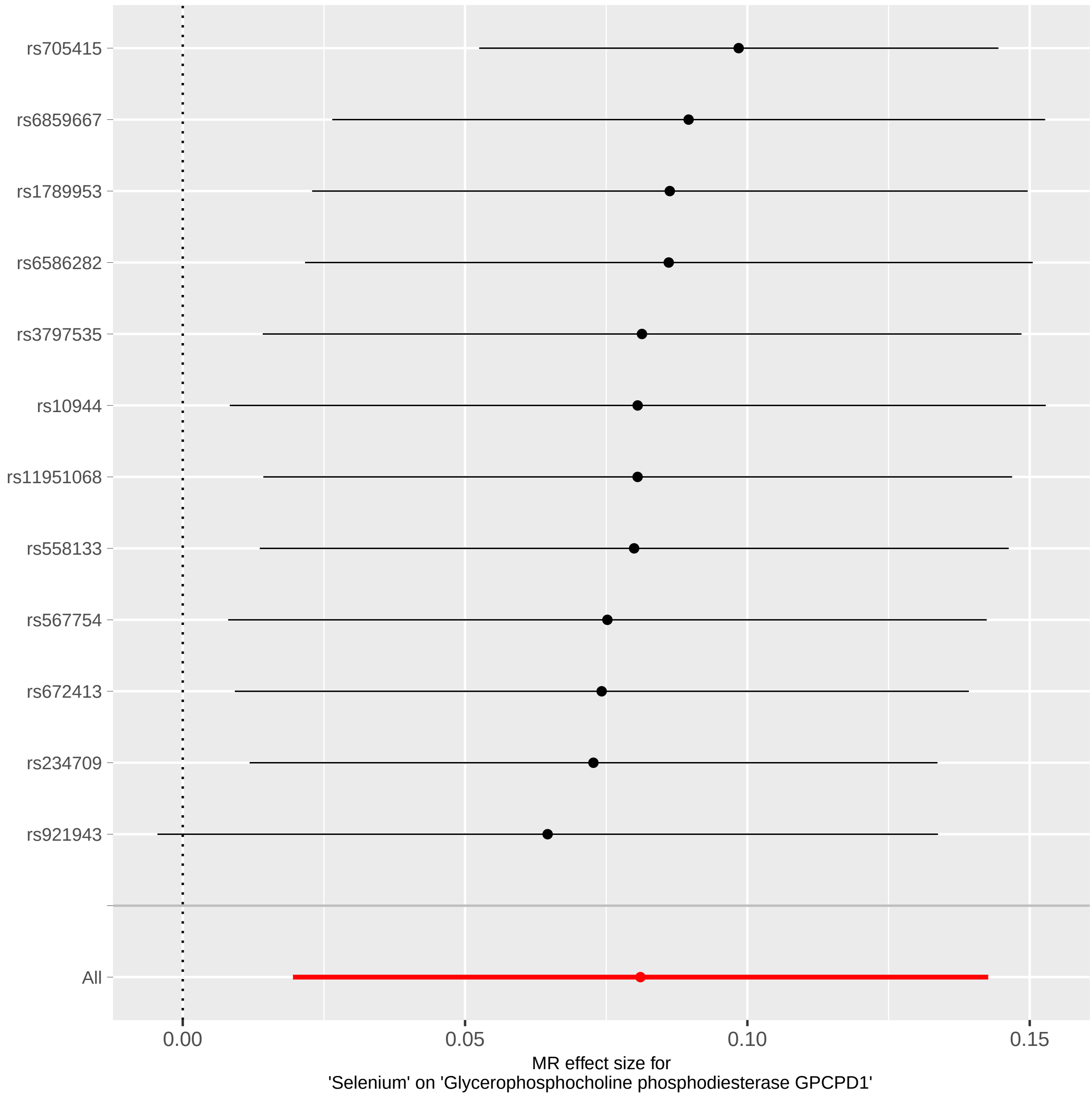

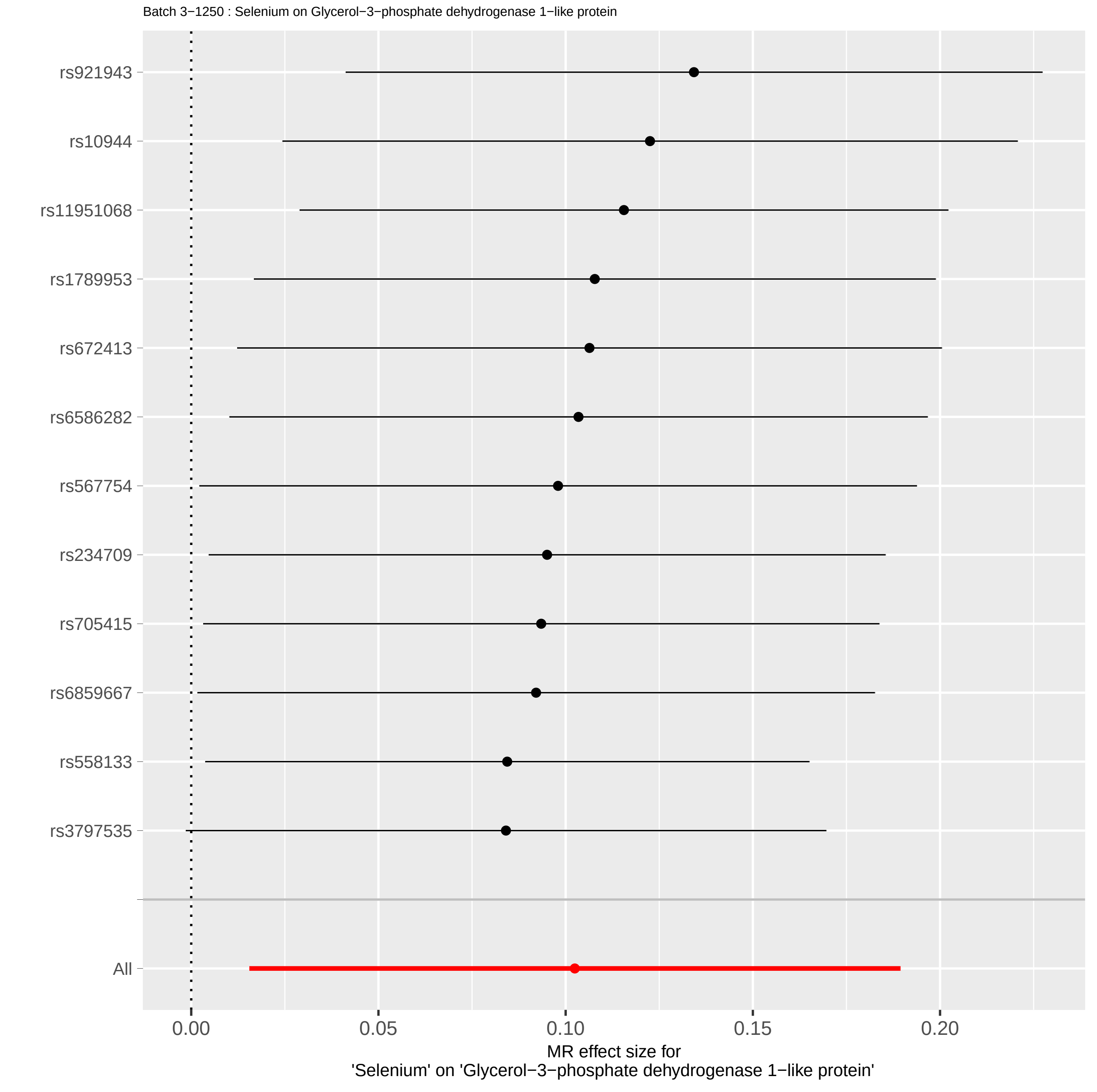

Batch 3−1250 : Selenium on Glycerol−3−phosphate dehydrogenase 1−like protein
rs921943
rs10944
rs11951068
rs1789953
rs672413
rs6586282
rs567754
rs234709
rs705415
rs6859667
rs558133
rs3797535
All
0.00
0.05
0.10
0.15
0.20
MR effect size for
'Selenium' on 'Glycerol−3−phosphate dehydrogenase 1−like protein'

Batch 3−1251 : Selenium on Glycoprotein hormone alpha−2

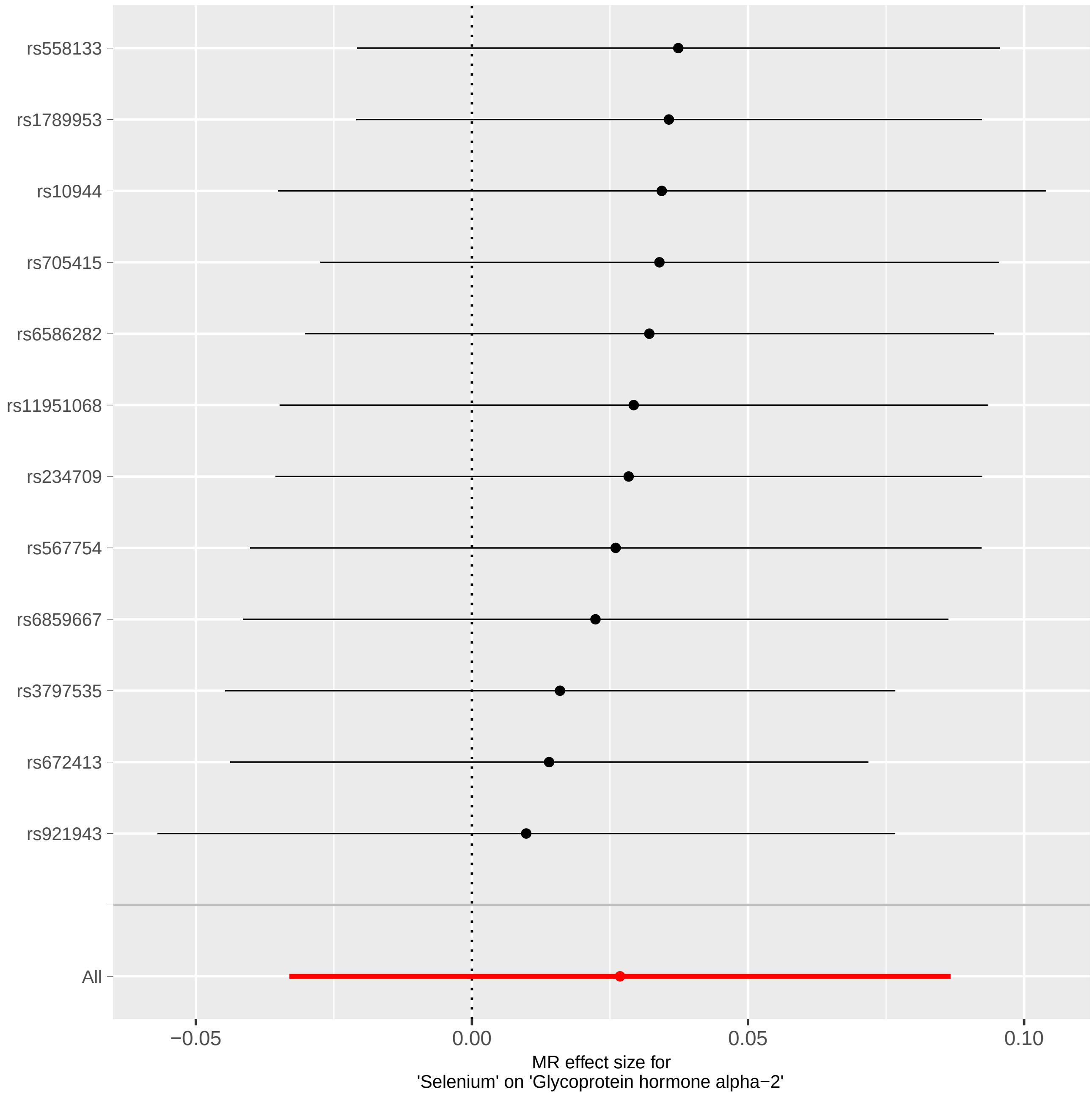

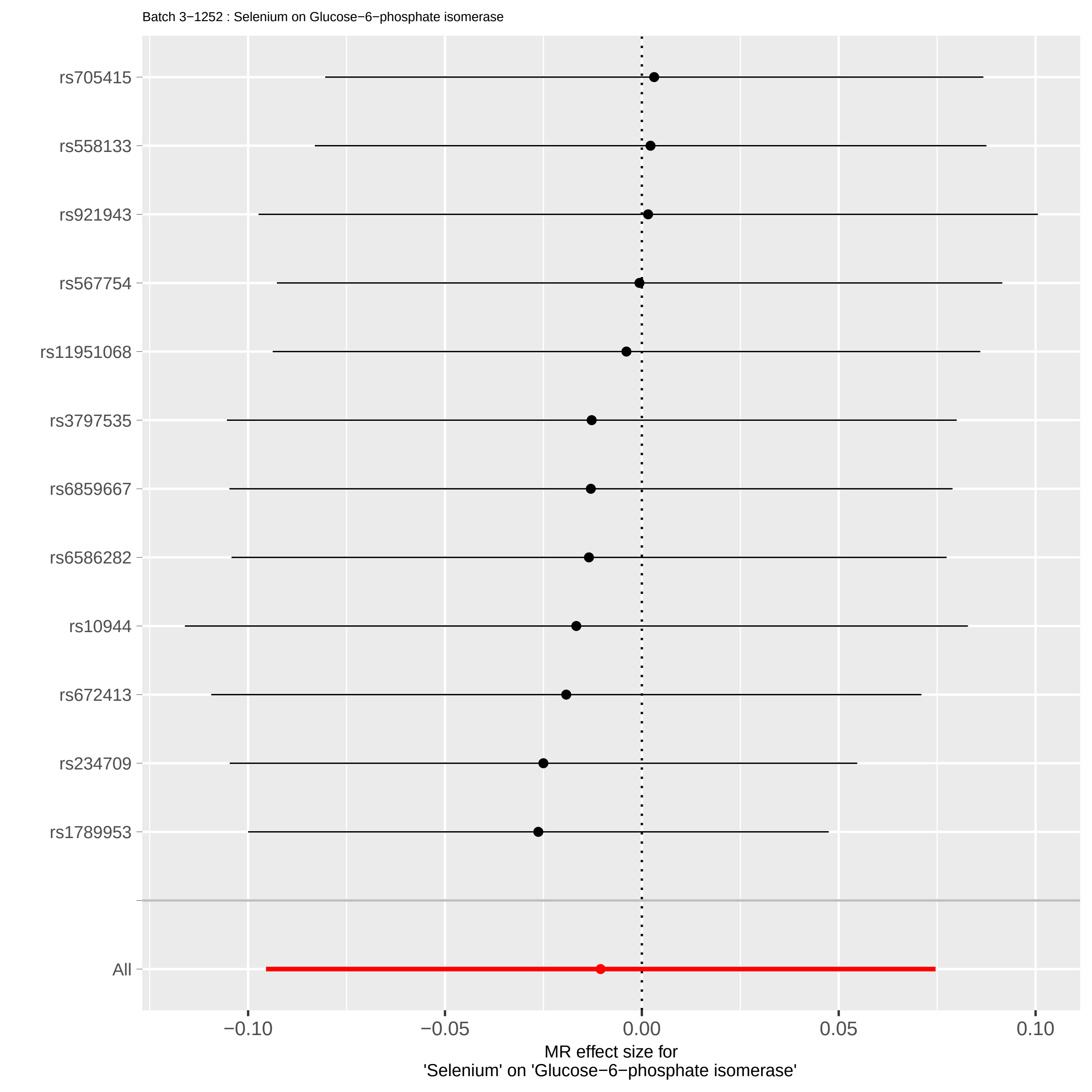

Batch 3−1252 : Selenium on Glucose−6−phosphate isomerase
rs705415
rs558133
rs921943
rs567754
rs11951068
rs3797535
rs6859667
rs6586282
rs10944
rs672413
rs234709
rs1789953
All
−0.10
−0.05
0.00
0.05
0.10
MR effect size for
'Selenium' on 'Glucose−6−phosphate isomerase'

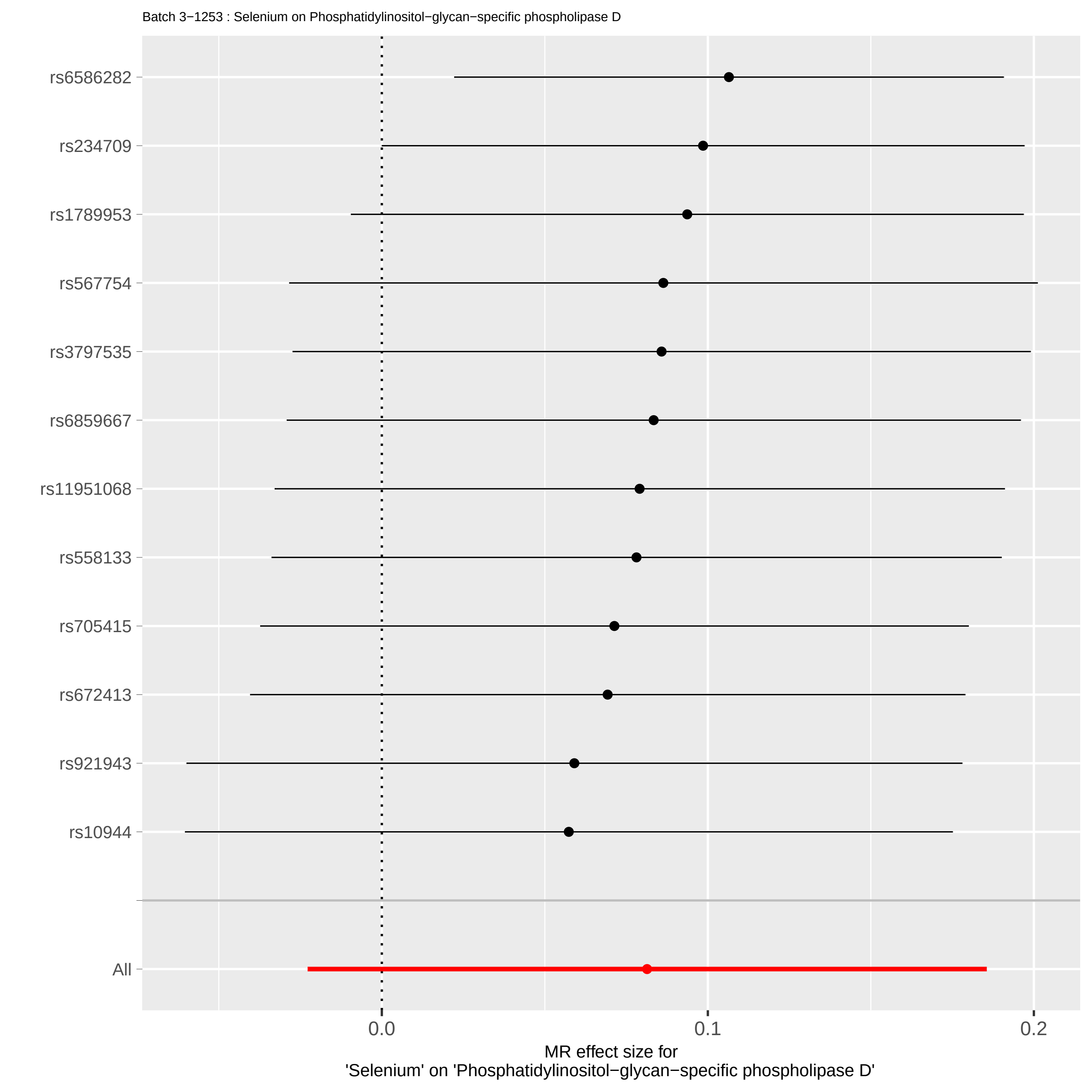
Batch 3−1253 : Selenium on Phosphatidylinositol−glycan−specific phospholipase D
rs6586282
rs234709
rs1789953
rs567754
rs3797535
rs6859667
rs11951068
rs558133
rs705415
rs672413
rs921943
rs10944
All
0.0
0.1
0.2
MR effect size for
'Selenium' on 'Phosphatidylinositol−glycan−specific phospholipase D'

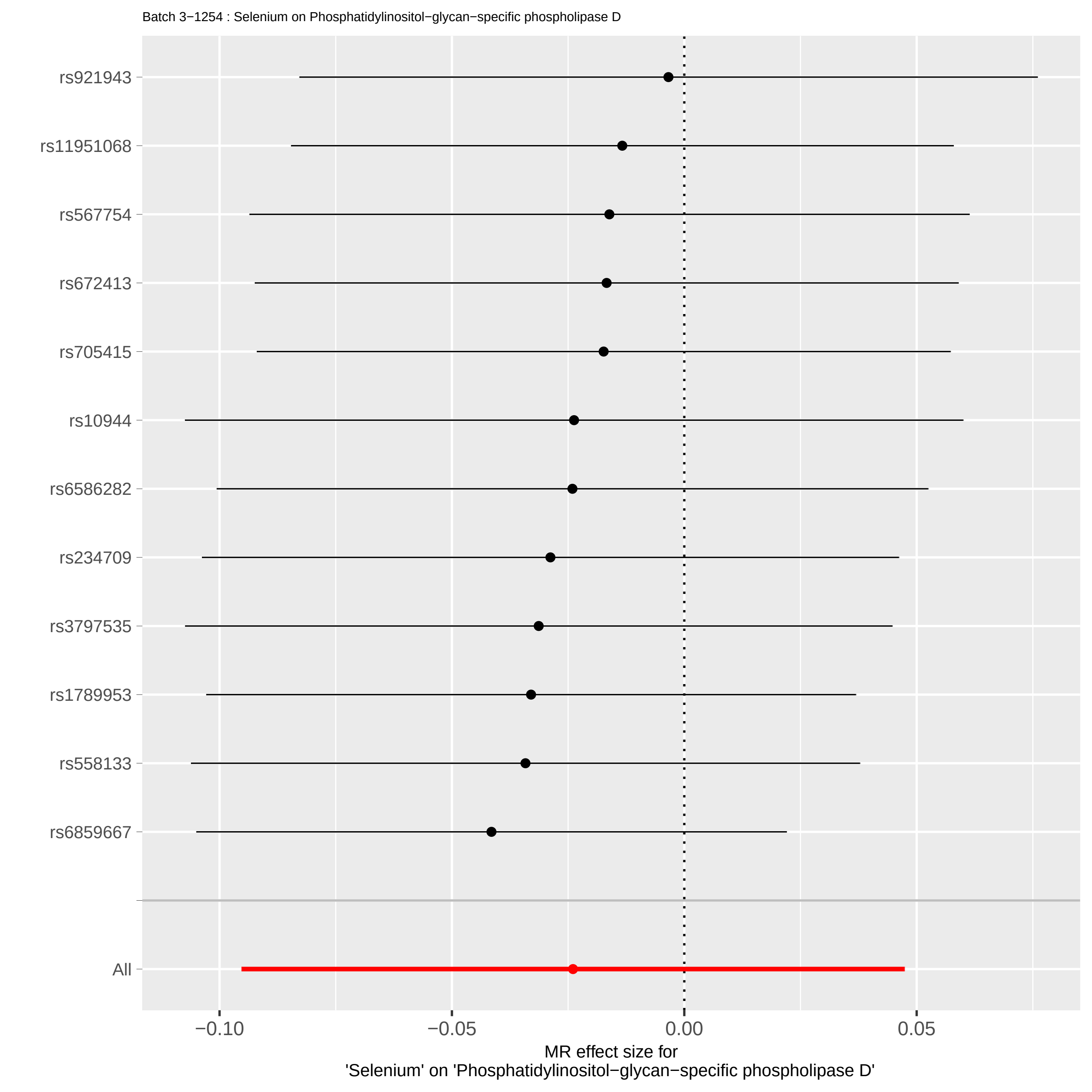
Batch 3−1254 : Selenium on Phosphatidylinositol−glycan−specific phospholipase D
rs921943
rs11951068
rs567754
rs672413
rs705415
rs10944
rs6586282
rs234709
rs3797535
rs1789953
rs558133
rs6859667
All
−0.10
−0.05
0.00
0.05
MR effect size for
'Selenium' on 'Phosphatidylinositol−glycan−specific phospholipase D'

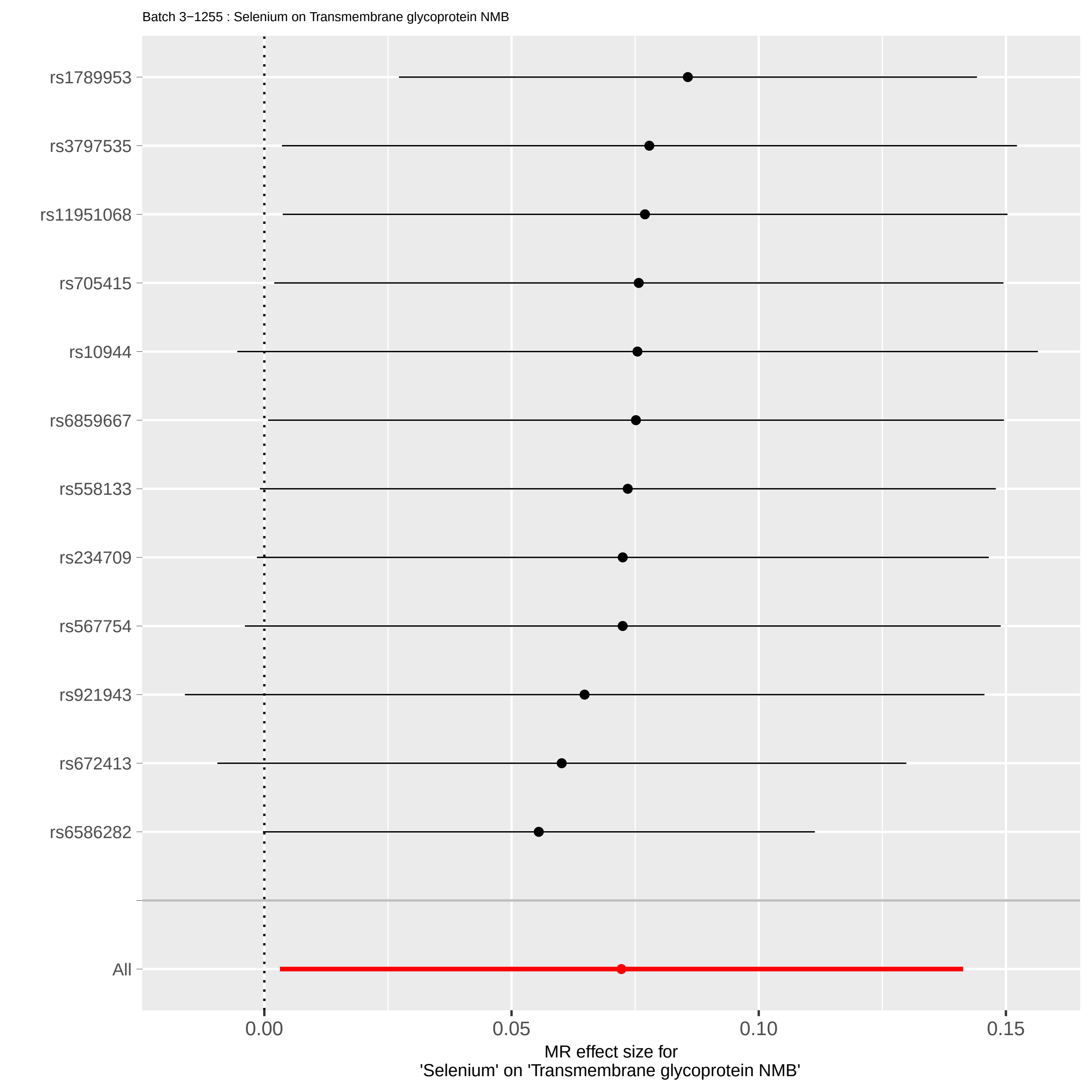
Batch 3−1255 : Selenium on Transmembrane glycoprotein NMB
rs1789953
rs3797535
rs11951068
rs705415
rs10944
rs6859667
rs558133
rs234709
rs567754
rs921943
rs672413
rs6586282
All
0.00
0.05
0.10
0.15
MR effect size for
'Selenium' on 'Transmembrane glycoprotein NMB'

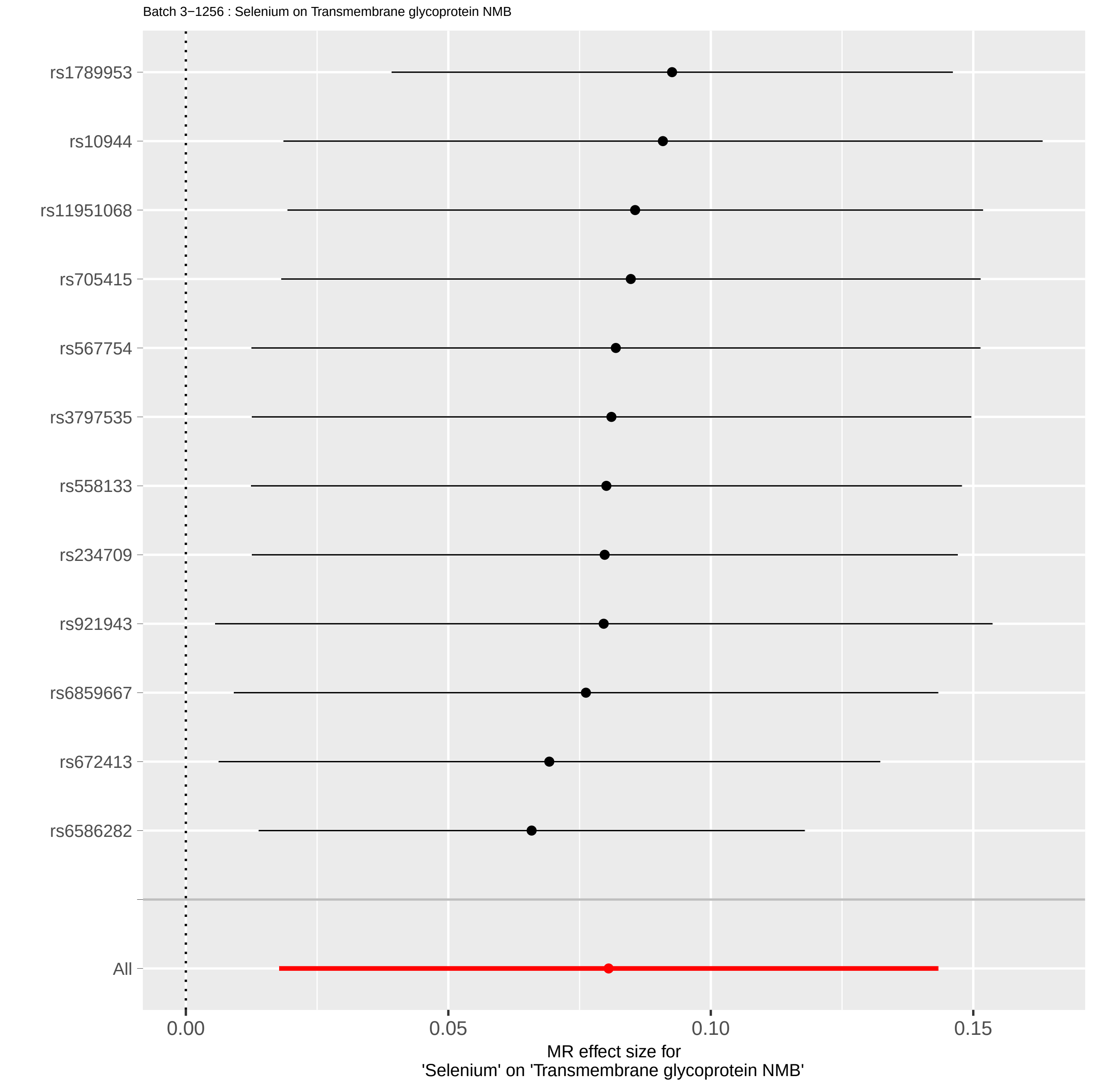

Batch 3−1256 : Selenium on Transmembrane glycoprotein NMB
rs1789953
rs10944
rs11951068
rs705415
rs567754
rs3797535
rs558133
rs234709
rs921943
rs6859667
rs672413
rs6586282
All
0.00
0.05
0.10
0.15
MR effect size for
'Selenium' on 'Transmembrane glycoprotein NMB'

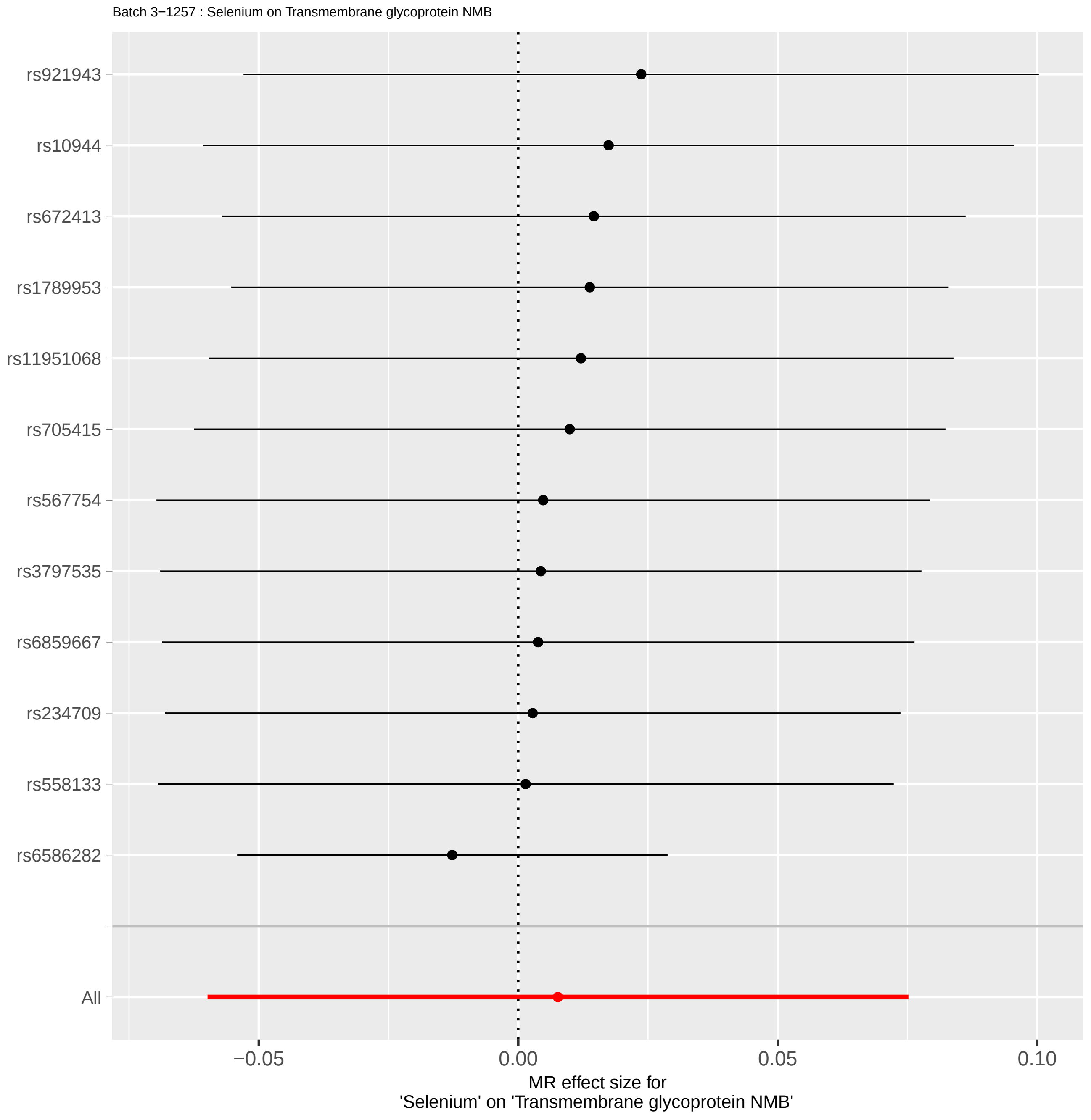

Batch 3−1257 : Selenium on Transmembrane glycoprotein NMB
rs921943
rs10944
rs672413
rs1789953
rs11951068
rs705415
rs567754
rs3797535
rs6859667
rs234709
rs558133
rs6586282
All
−0.05
0.00
0.05
0.10
MR effect size for
'Selenium' on 'Transmembrane glycoprotein NMB'

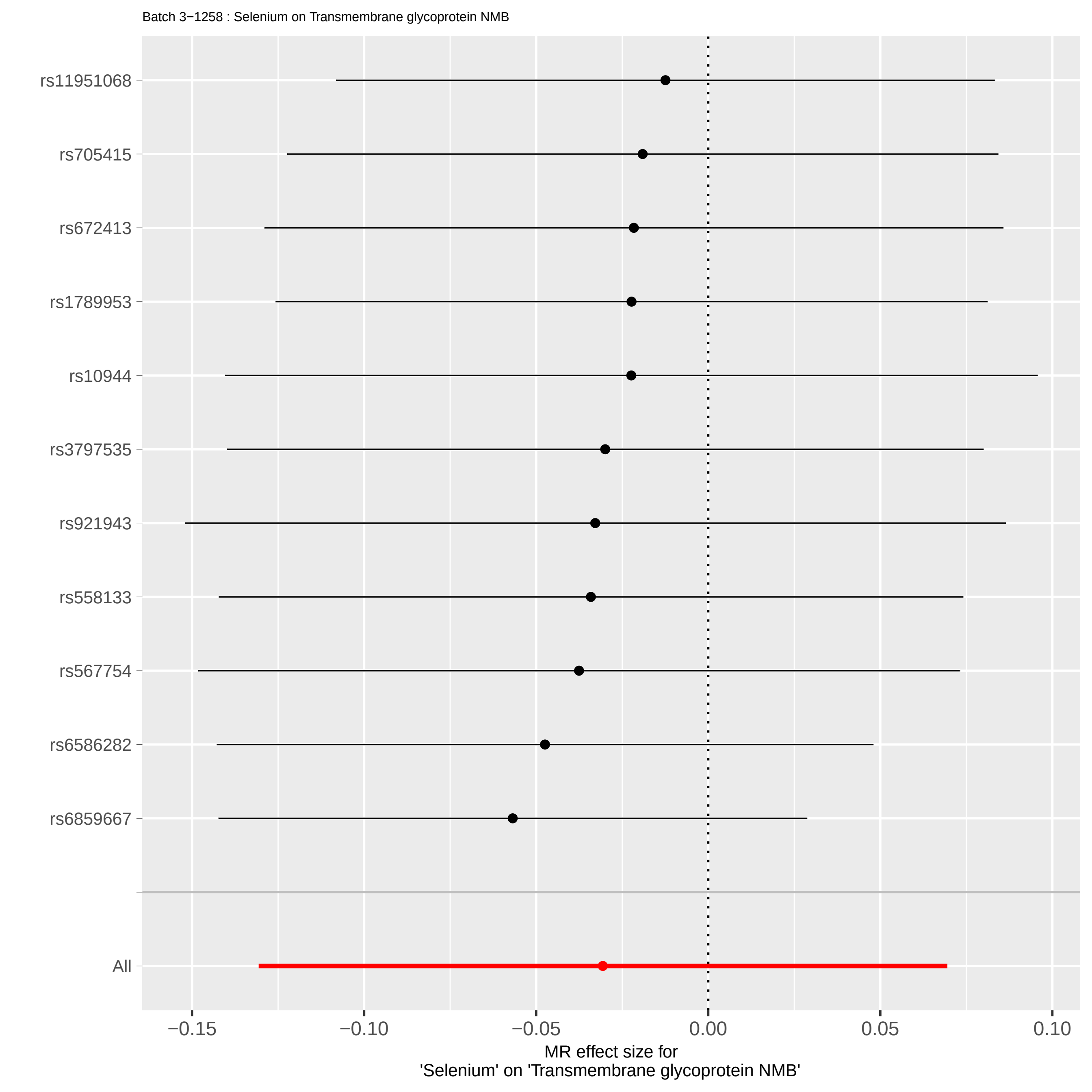
Batch 3−1258 : Selenium on Transmembrane glycoprotein NMB
rs11951068
rs705415
rs672413
rs1789953
rs10944
rs3797535
rs921943
rs558133
rs567754
rs6586282
rs6859667
All
−0.15
−0.10
−0.05
0.00
0.05
0.10
MR effect size for
'Selenium' on 'Transmembrane glycoprotein NMB'

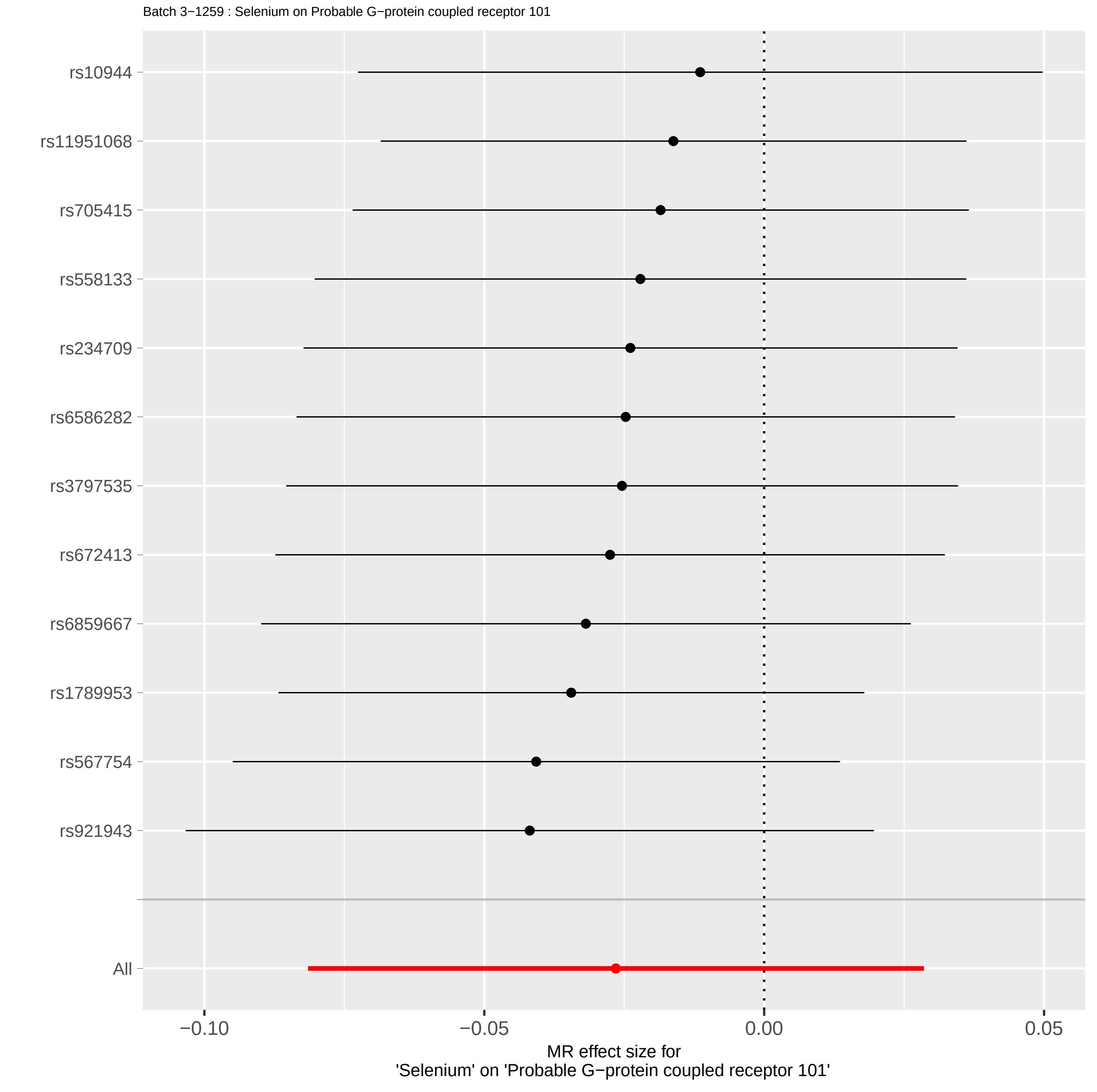

Batch 3−1259 : Selenium on Probable G−protein coupled receptor 101
rs10944
rs11951068
rs705415
rs558133
rs234709
rs6586282
rs3797535
rs672413
rs6859667
rs1789953
rs567754
rs921943
All
−0.10
−0.05
0.00
0.05
MR effect size for
'Selenium' on 'Probable G−protein coupled receptor 101'

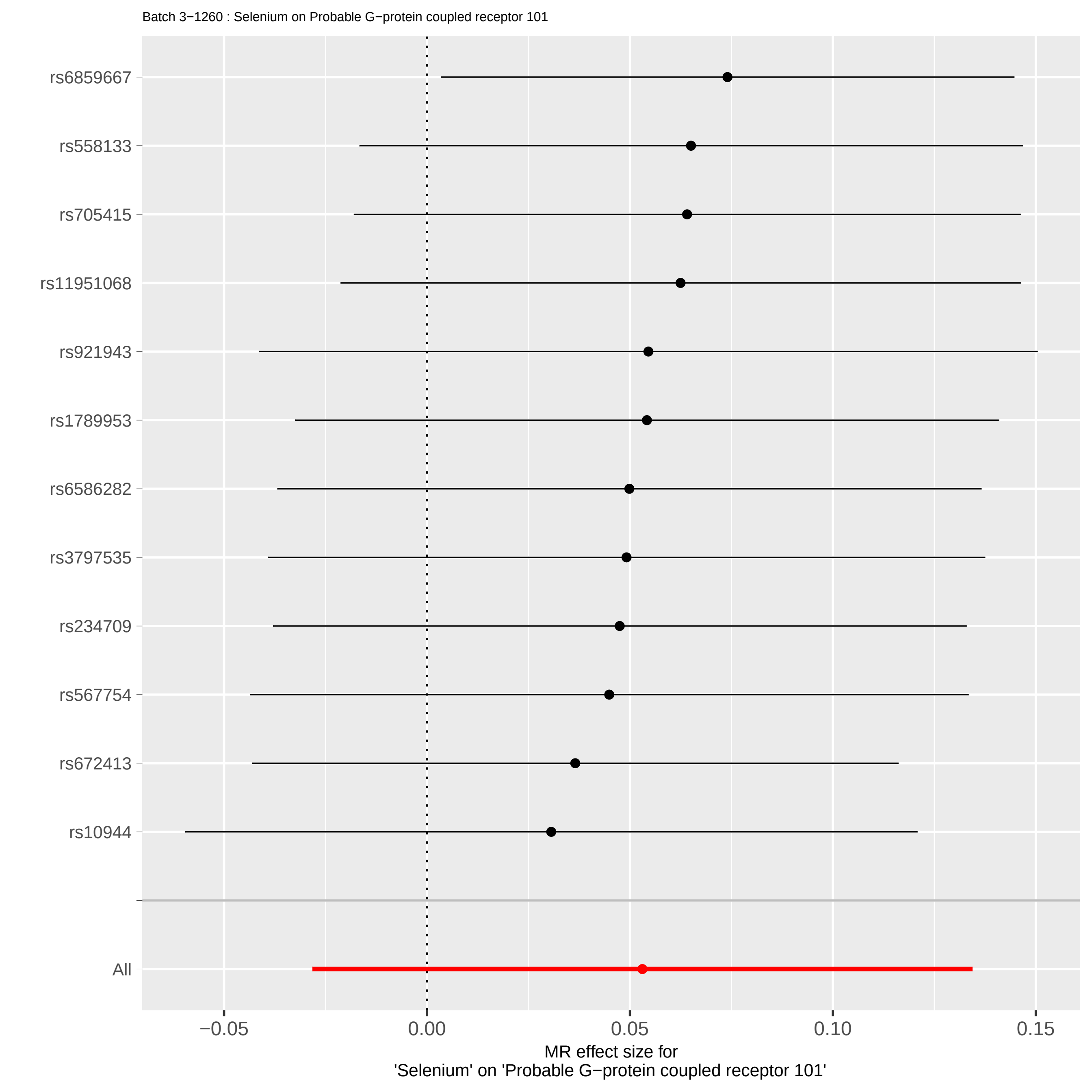

Batch 3−1260 : Selenium on Probable G−protein coupled receptor 101
rs6859667
rs558133
rs705415
rs11951068
rs921943
rs1789953
rs6586282
rs3797535
rs234709
rs567754
rs672413
rs10944
All
−0.05
0.00
0.05
0.10
0.15
MR effect size for
'Selenium' on 'Probable G−protein coupled receptor 101'

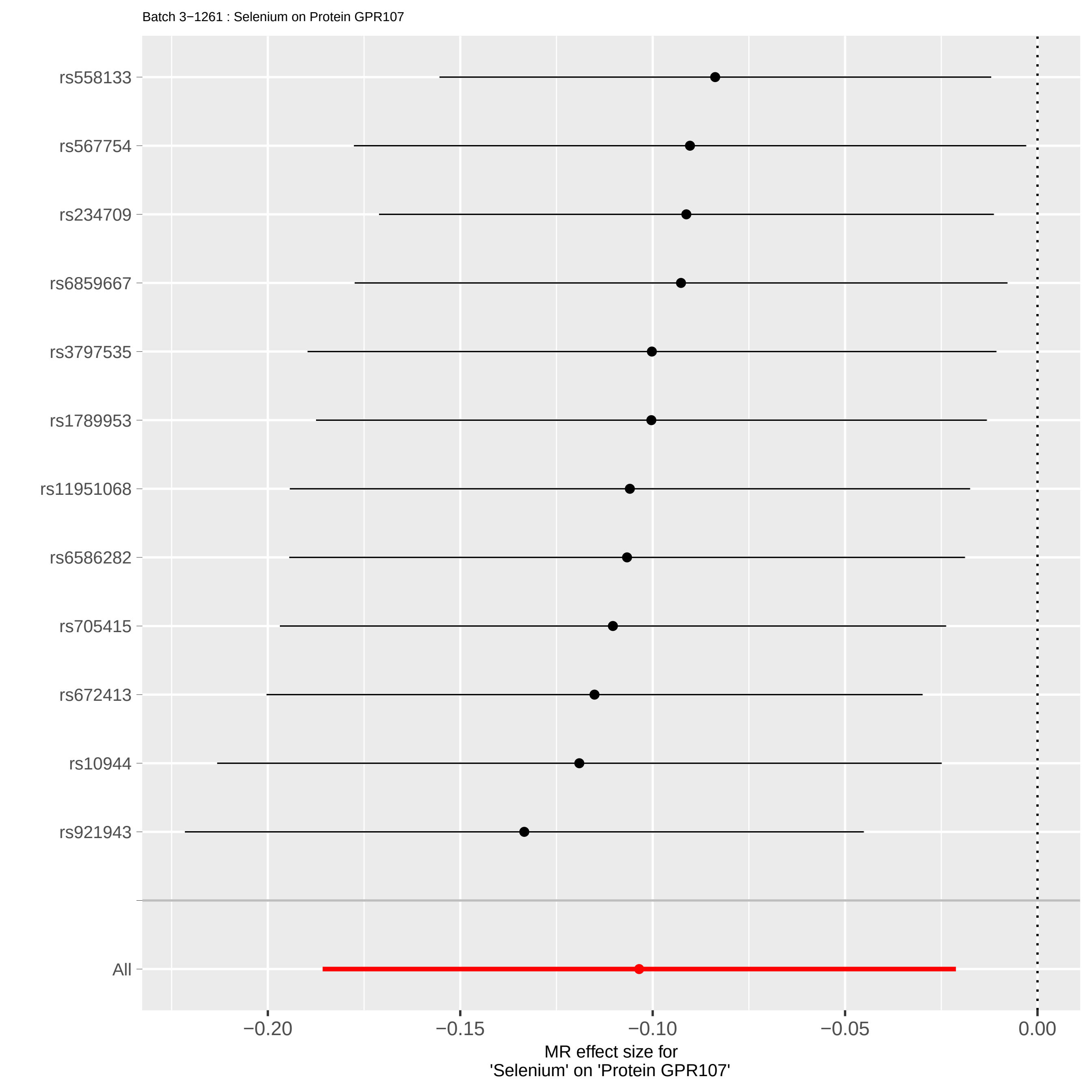
Batch 3−1261 : Selenium on Protein GPR107
rs558133
rs567754
rs234709
rs6859667
rs3797535
rs1789953
rs11951068
rs6586282
rs705415
rs672413
rs10944
rs921943
All
−0.20
−0.15
−0.10
−0.05
0.00
MR effect size for
'Selenium' on 'Protein GPR107'

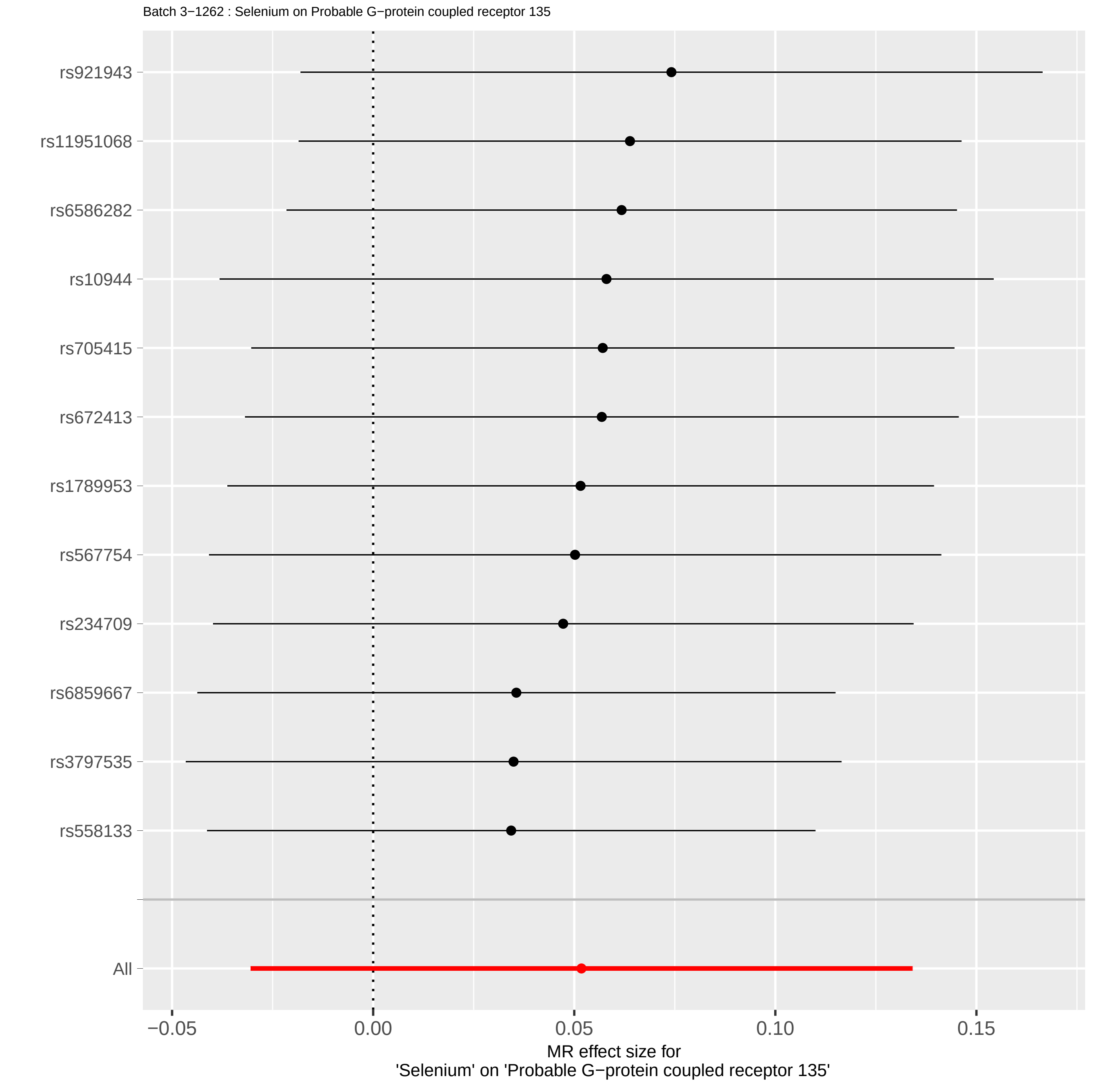
Batch 3−1262 : Selenium on Probable G−protein coupled receptor 135
rs921943
rs11951068
rs6586282
rs10944
rs705415
rs672413
rs1789953
rs567754
rs234709
rs6859667
rs3797535
rs558133
All
−0.05
0.00
0.05
0.10
0.15
MR effect size for
'Selenium' on 'Probable G−protein coupled receptor 135'

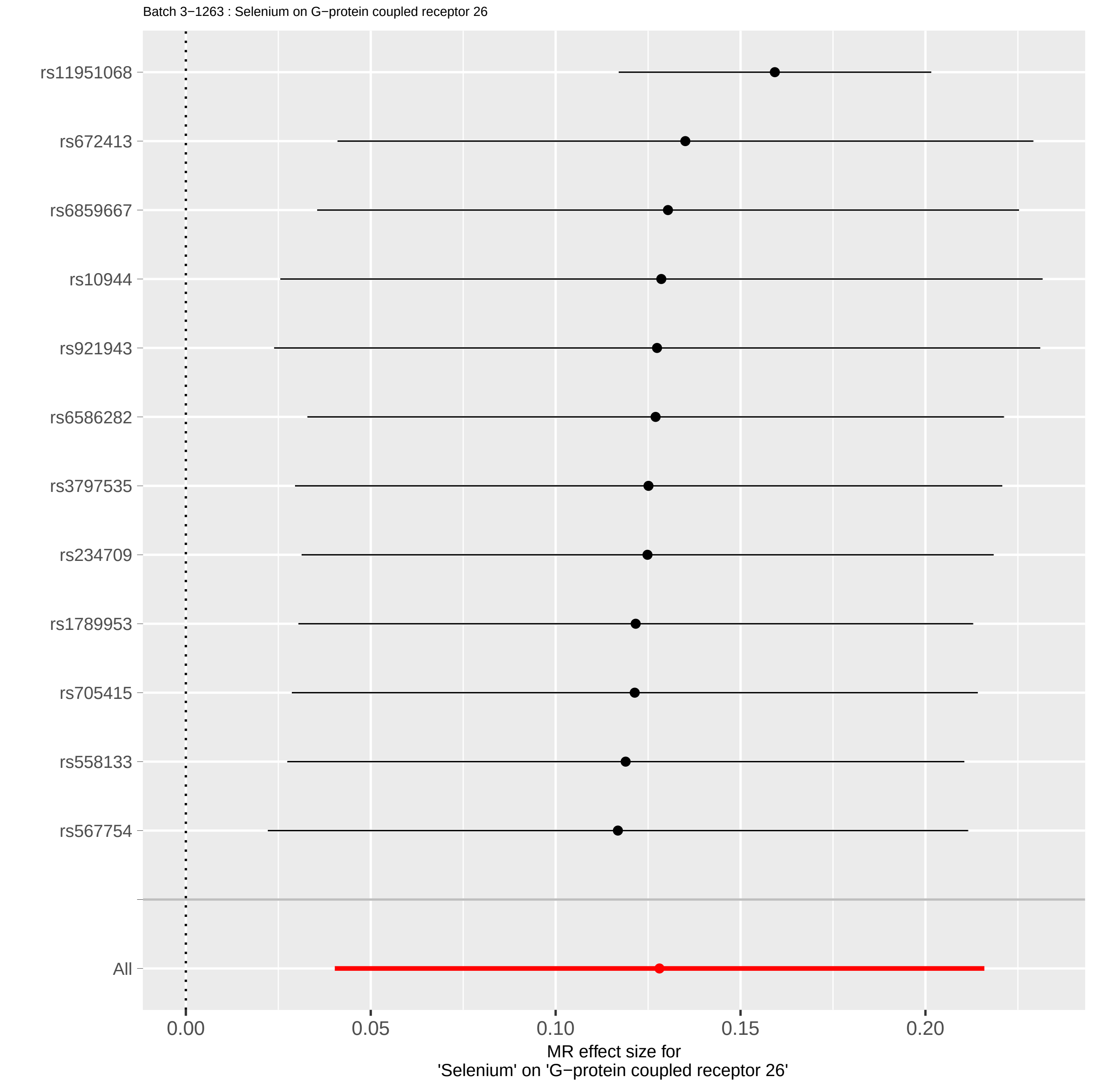

Batch 3−1263 : Selenium on G−protein coupled receptor 26
rs11951068
rs672413
rs6859667
rs10944
rs921943
rs6586282
rs3797535
rs234709
rs1789953
rs705415
rs558133
rs567754
All
0.00
0.05
0.10
0.15
0.20
MR effect size for
'Selenium' on 'G−protein coupled receptor 26'

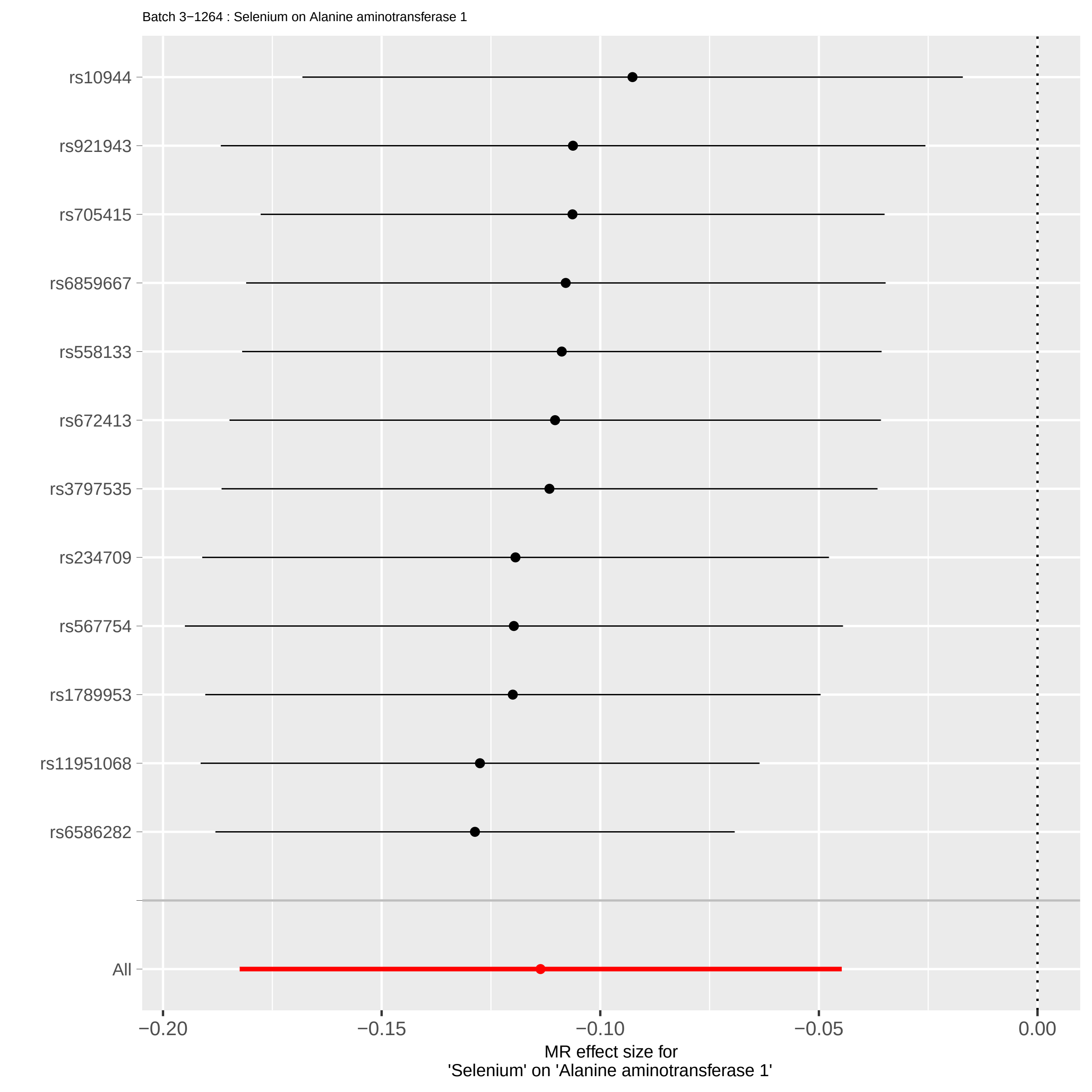

Batch 3−1264 : Selenium on Alanine aminotransferase 1
rs10944
rs921943
rs705415
rs6859667
rs558133
rs672413
rs3797535
rs234709
rs567754
rs1789953
rs11951068
rs6586282
All
−0.20
−0.15
−0.10
−0.05
0.00
MR effect size for
'Selenium' on 'Alanine aminotransferase 1'

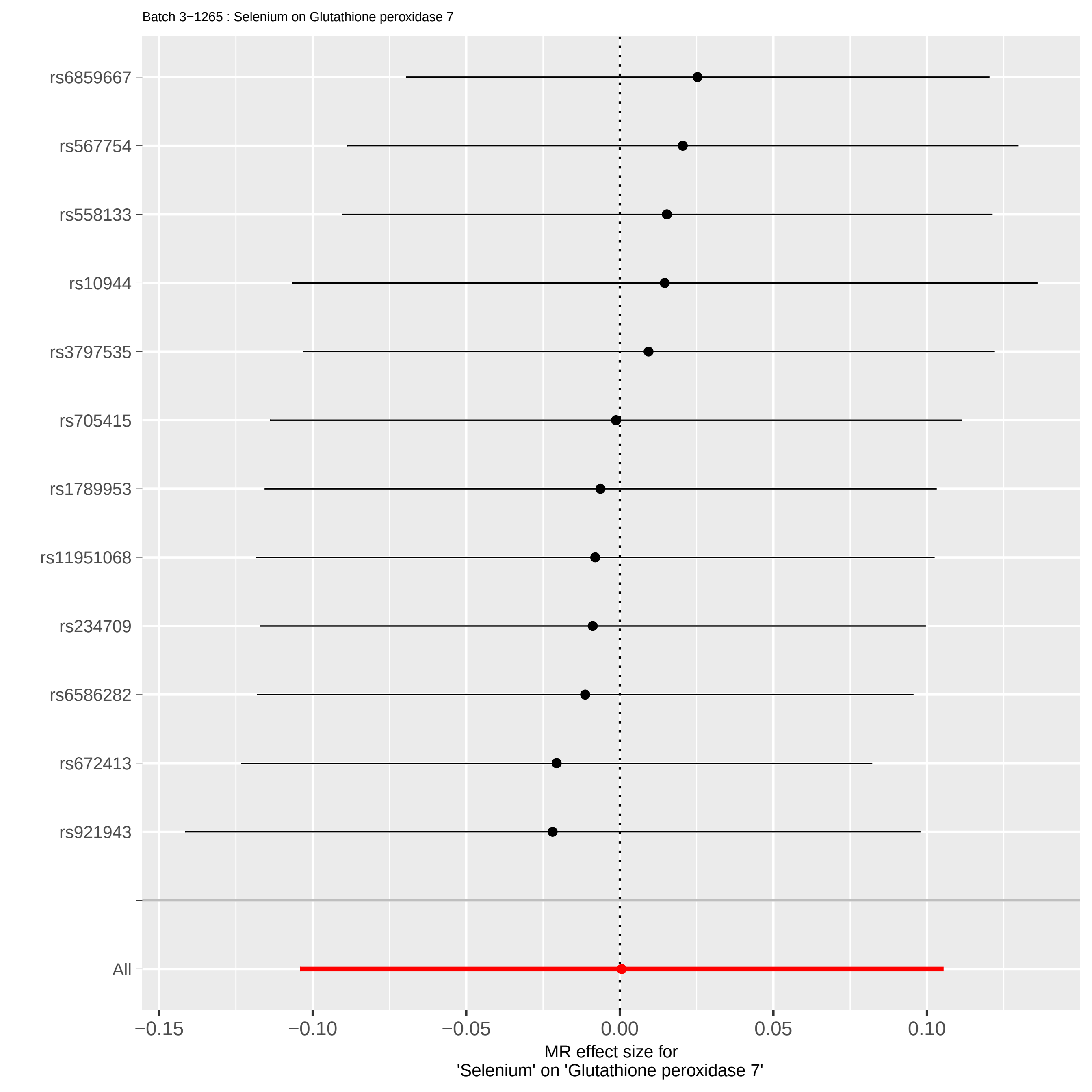

Batch 3−1265 : Selenium on Glutathione peroxidase 7
rs6859667
rs567754
rs558133
rs10944
rs3797535
rs705415
rs1789953
rs11951068
rs234709
rs6586282
rs672413
rs921943
All
−0.15
−0.10
−0.05
0.00
0.05
0.10
MR effect size for
'Selenium' on 'Glutathione peroxidase 7'

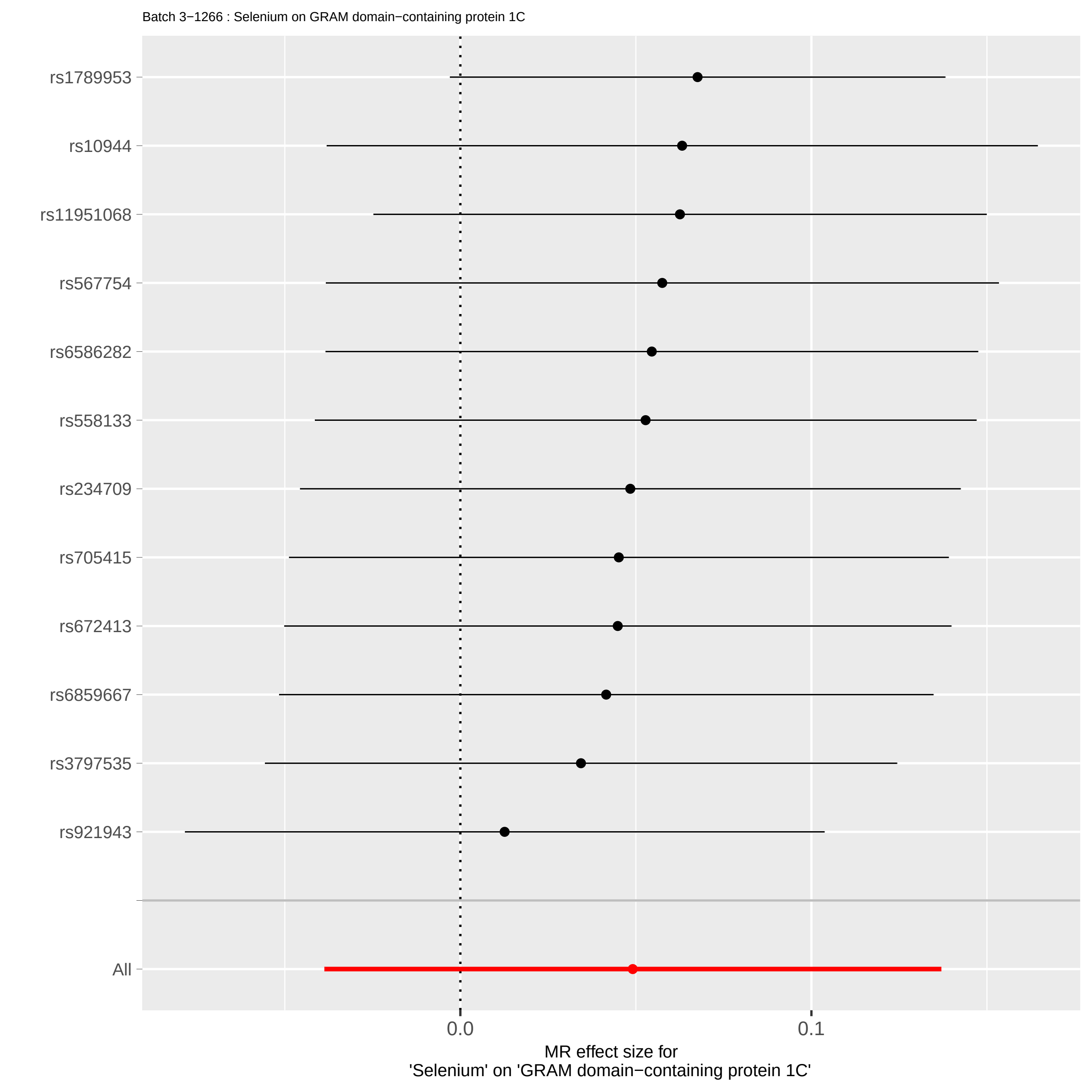

Batch 3−1266 : Selenium on GRAM domain−containing protein 1C
rs1789953
rs10944
rs11951068
rs567754
rs6586282
rs558133
rs234709
rs705415
rs672413
rs6859667
rs3797535
rs921943
All
0.0
0.1
MR effect size for
'Selenium' on 'GRAM domain−containing protein 1C'

Batch 3−1267 : Selenium on GRAM domain−containing protein 1C

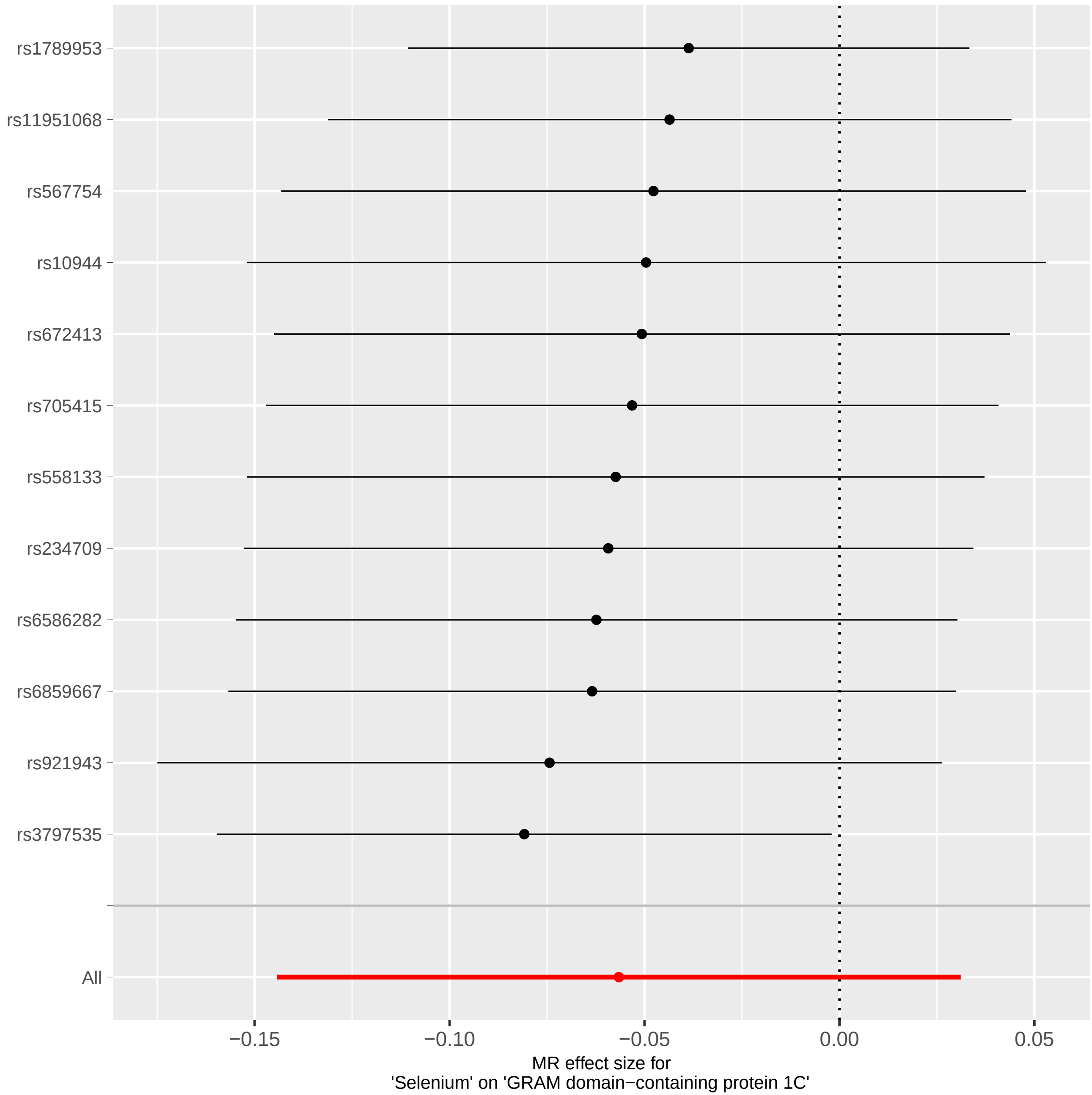

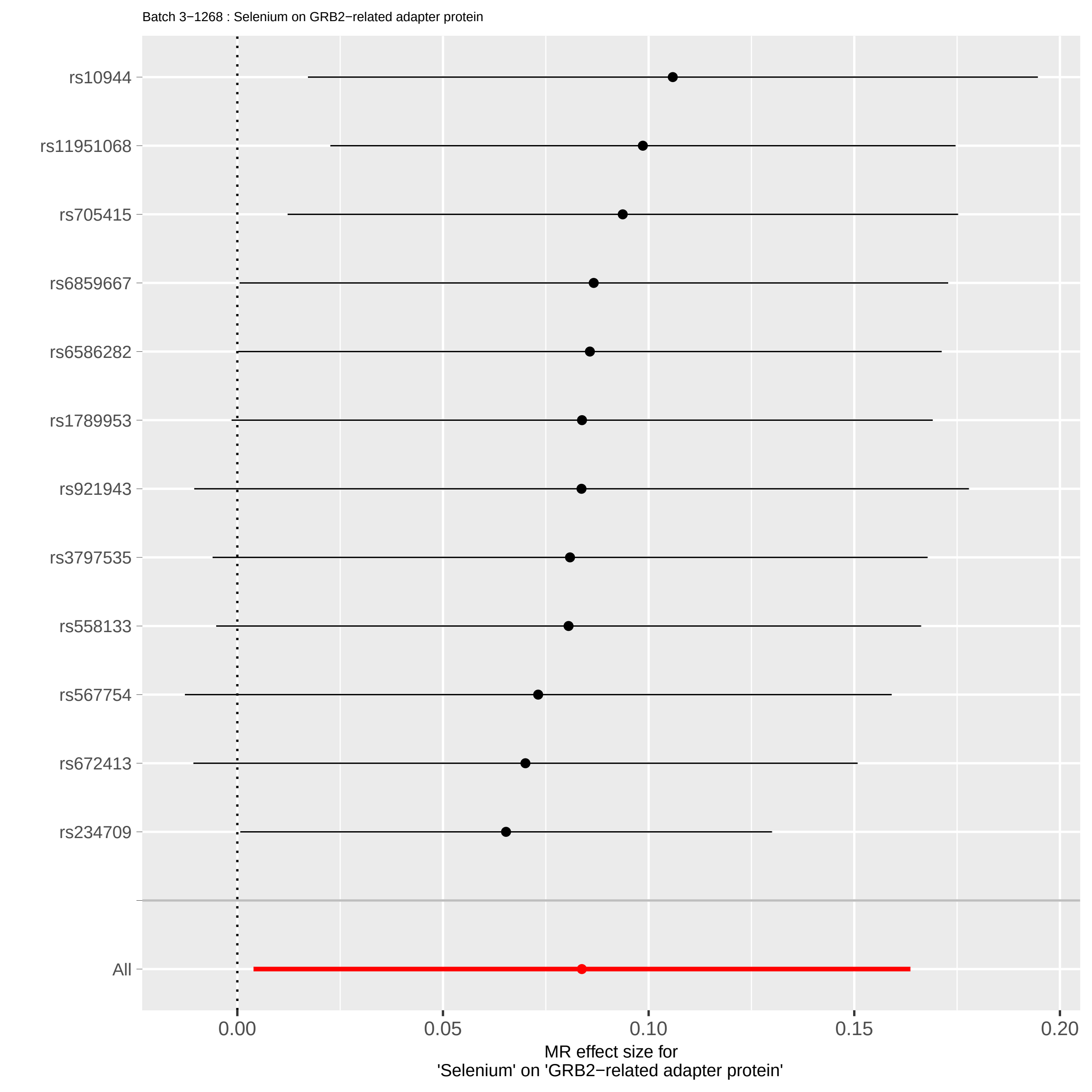

Batch 3−1268 : Selenium on GRB2−related adapter protein
rs10944
rs11951068
rs705415
rs6859667
rs6586282
rs1789953
rs921943
rs3797535
rs558133
rs567754
rs672413
rs234709
All
0.00
0.05
0.10
0.15
0.20
MR effect size for
'Selenium' on 'GRB2−related adapter protein'

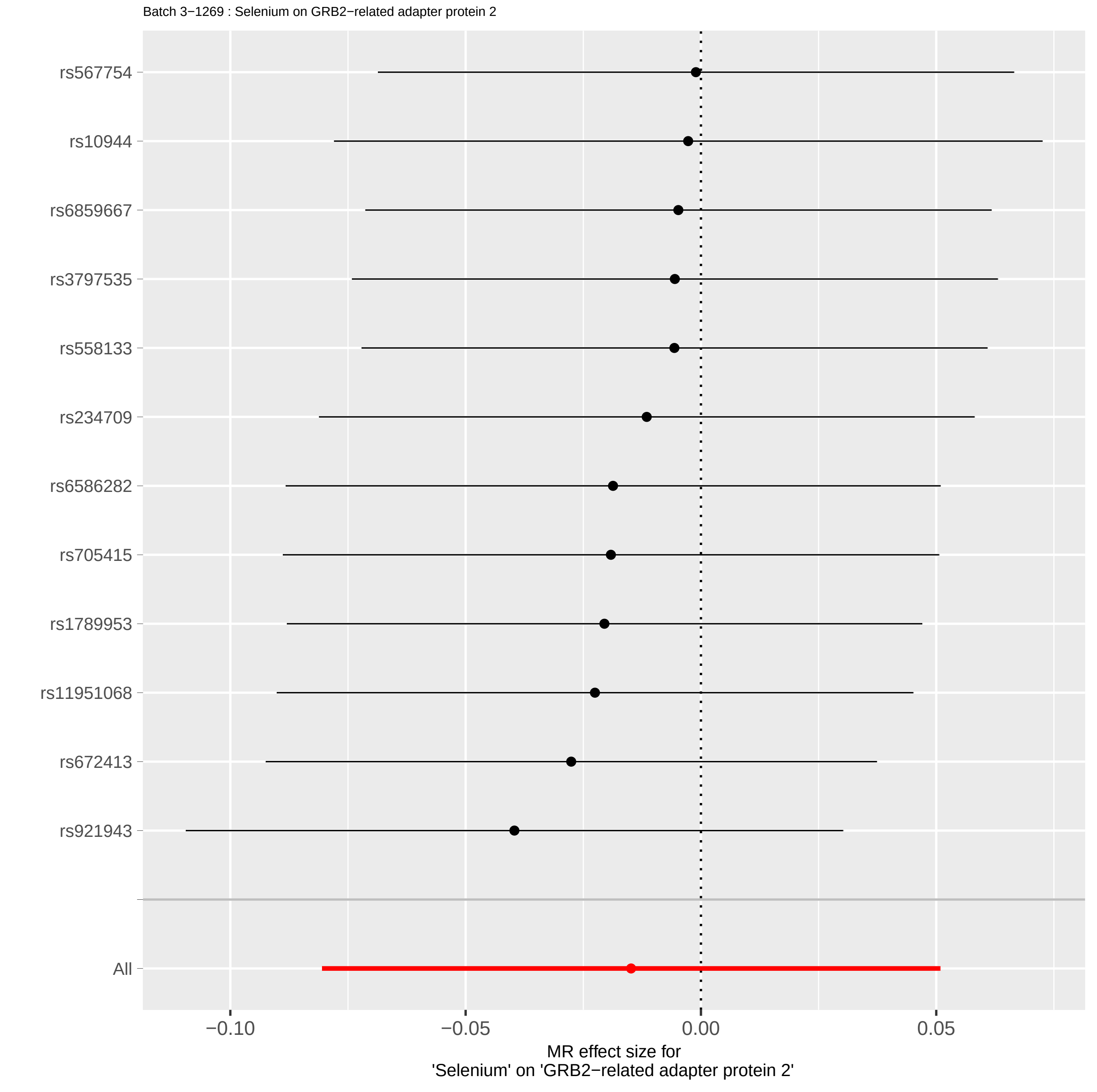

Batch 3−1269 : Selenium on GRB2−related adapter protein 2
rs567754
rs10944
rs6859667
rs3797535
rs558133
rs234709
rs6586282
rs705415
rs1789953
rs11951068
rs672413
rs921943
All
−0.10
−0.05
0.00
0.05
MR effect size for
'Selenium' on 'GRB2−related adapter protein 2'

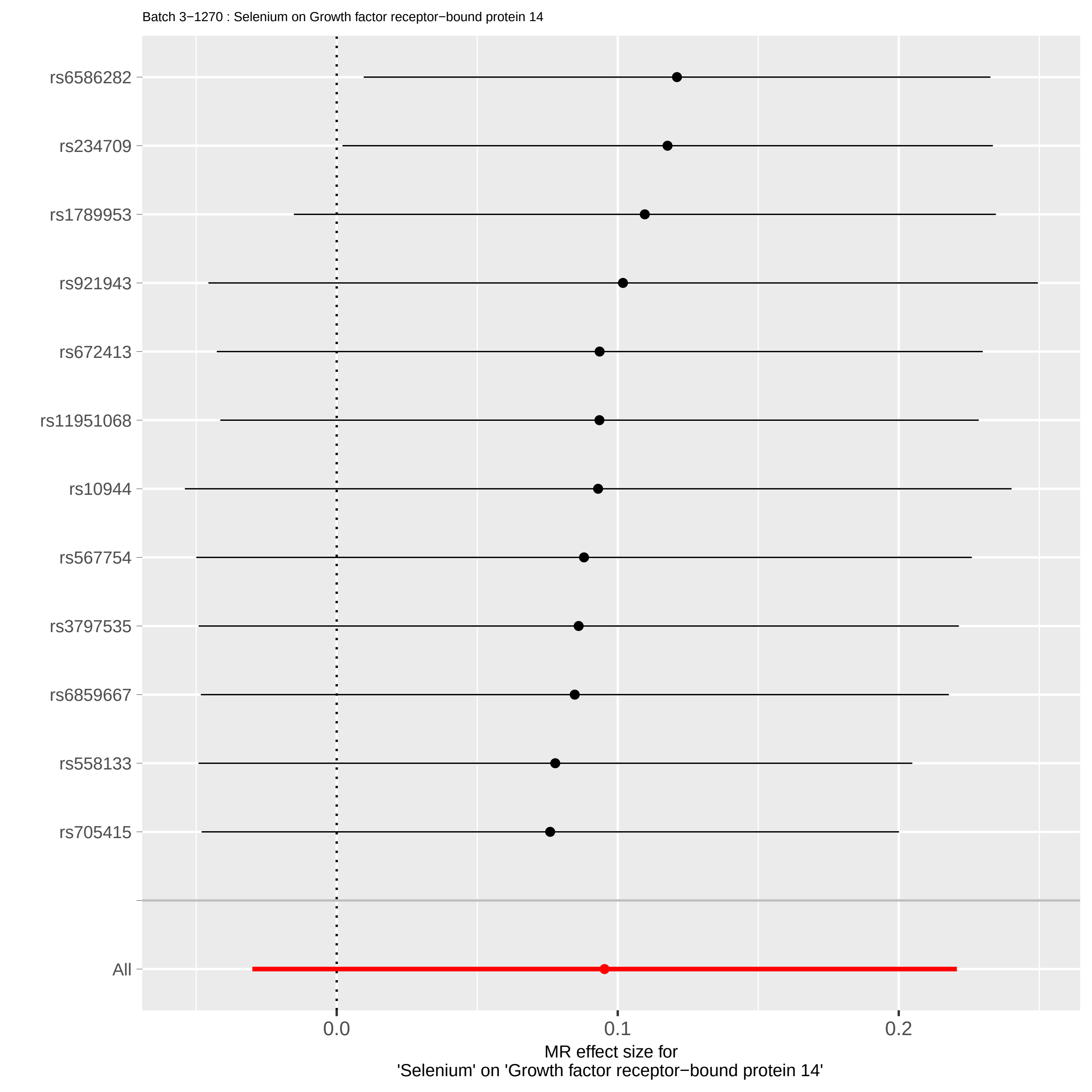
Batch 3−1270 : Selenium on Growth factor receptor−bound protein 14
rs6586282
rs234709
rs1789953
rs921943
rs672413
rs11951068
rs10944
rs567754
rs3797535
rs6859667
rs558133
rs705415
All
0.0
0.1
0.2
MR effect size for
'Selenium' on 'Growth factor receptor−bound protein 14'

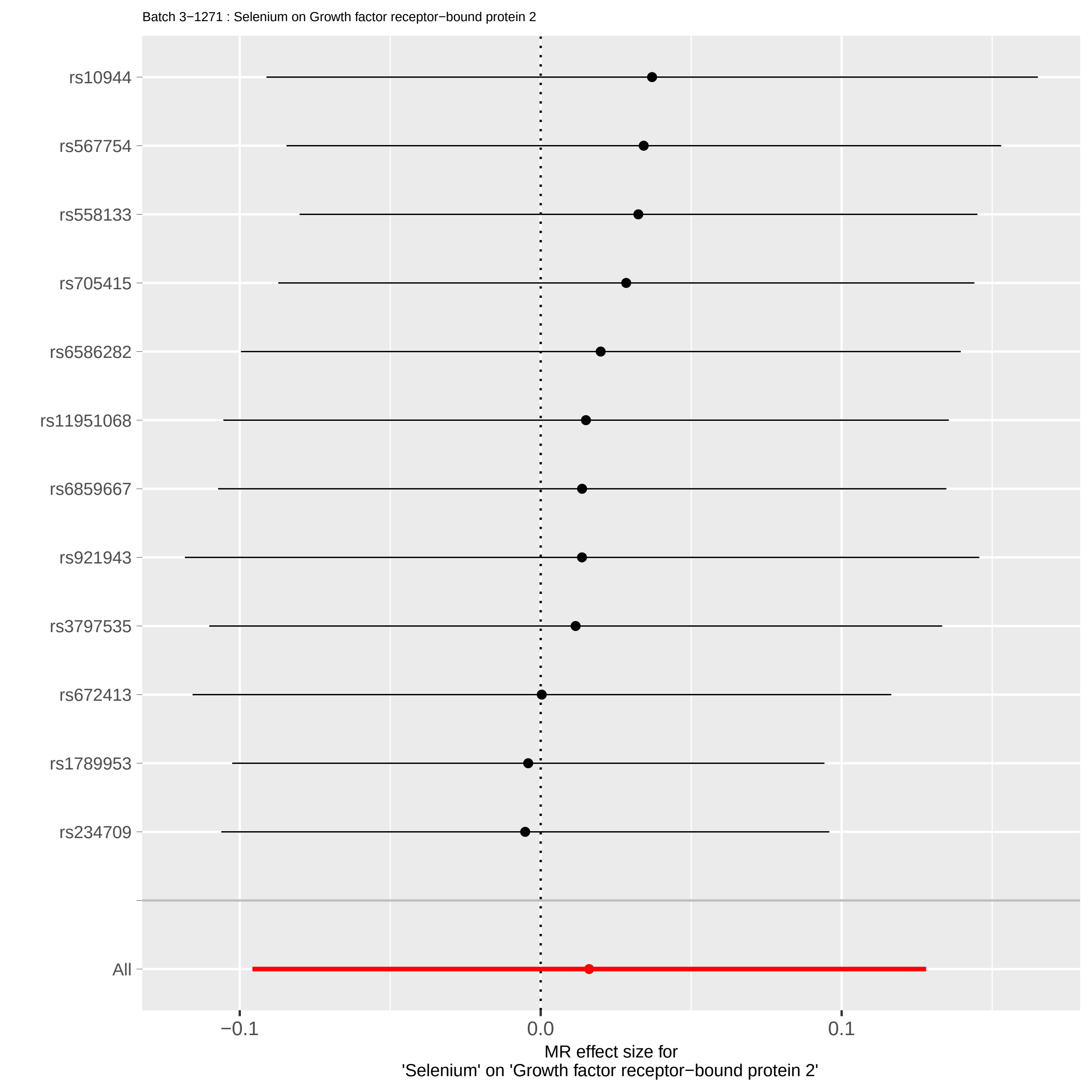
Batch 3−1271 : Selenium on Growth factor receptor−bound protein 2
rs10944
rs567754
rs558133
rs705415
rs6586282
rs11951068
rs6859667
rs921943
rs3797535
rs672413
rs1789953
rs234709
All
−0.1
0.0
0.1
MR effect size for
'Selenium' on 'Growth factor receptor−bound protein 2'

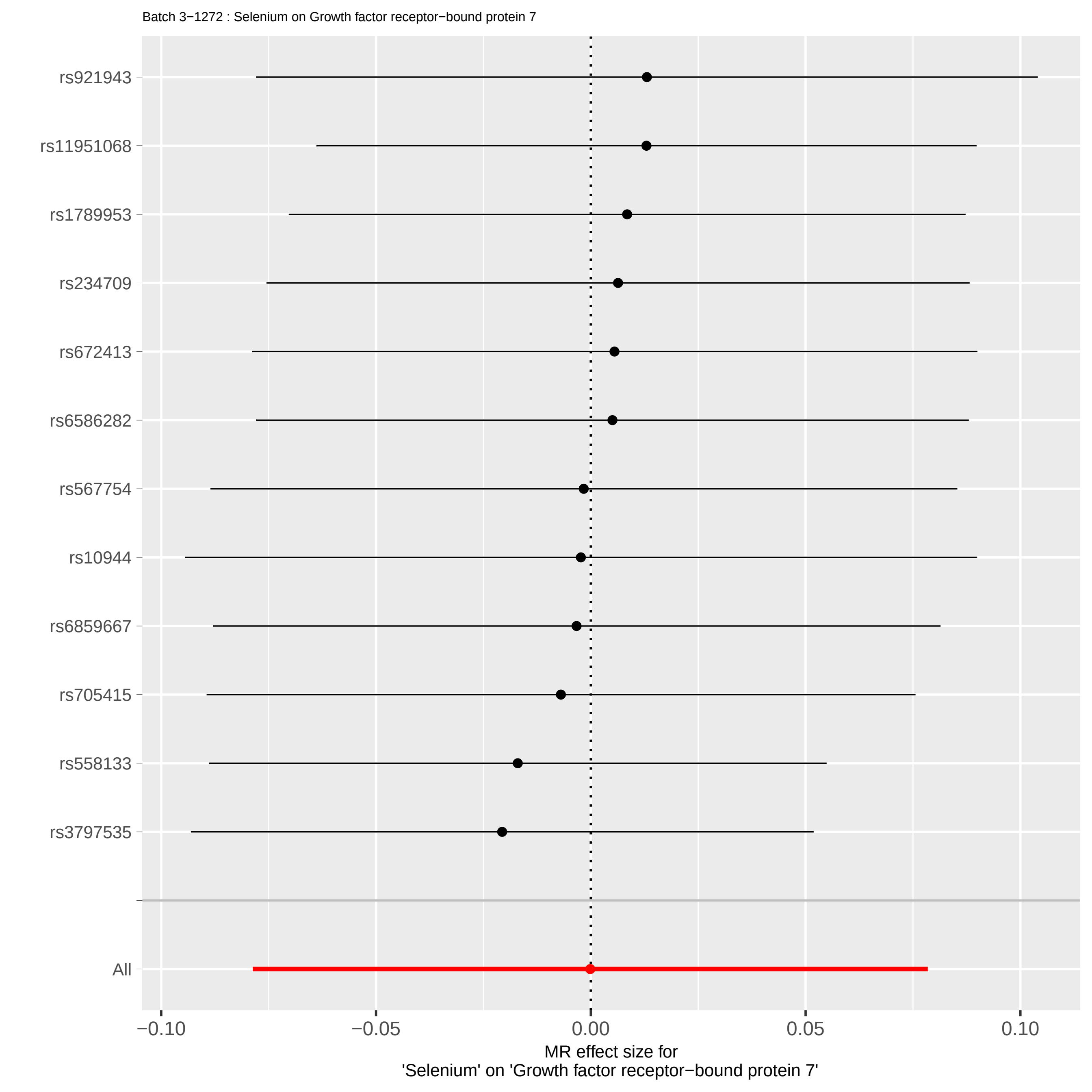

Batch 3−1272 : Selenium on Growth factor receptor−bound protein 7
rs921943
rs11951068
rs1789953
rs234709
rs672413
rs6586282
rs567754
rs10944
rs6859667
rs705415
rs558133
rs3797535
All
−0.10
−0.05
0.00
0.05
0.10
MR effect size for
'Selenium' on 'Growth factor receptor−bound protein 7'

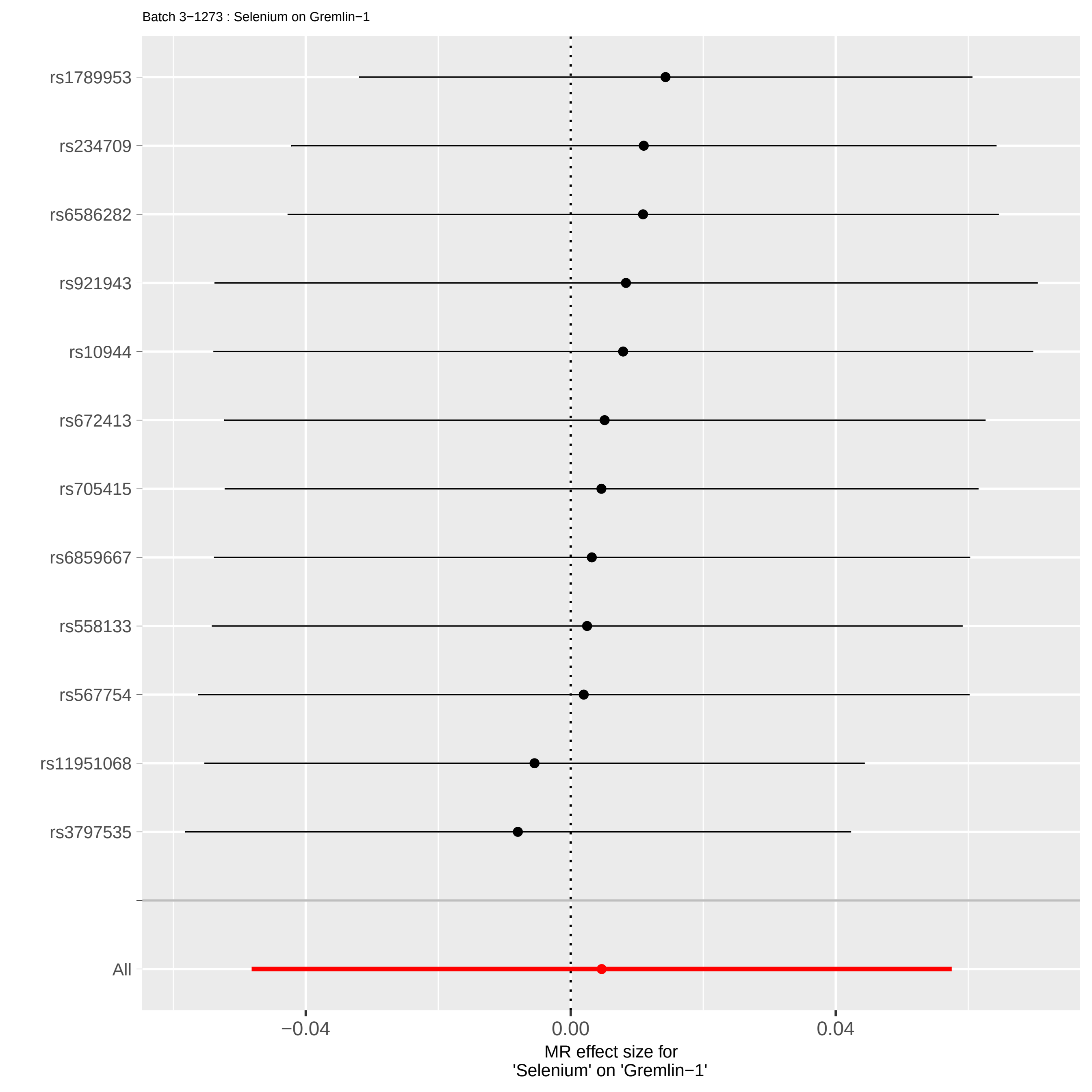

Batch 3−1273 : Selenium on Gremlin−1
rs1789953
rs234709
rs6586282
rs921943
rs10944
rs672413
rs705415
rs6859667
rs558133
rs567754
rs11951068
rs3797535
All
−0.04
0.00
0.04
MR effect size for
'Selenium' on 'Gremlin−1'

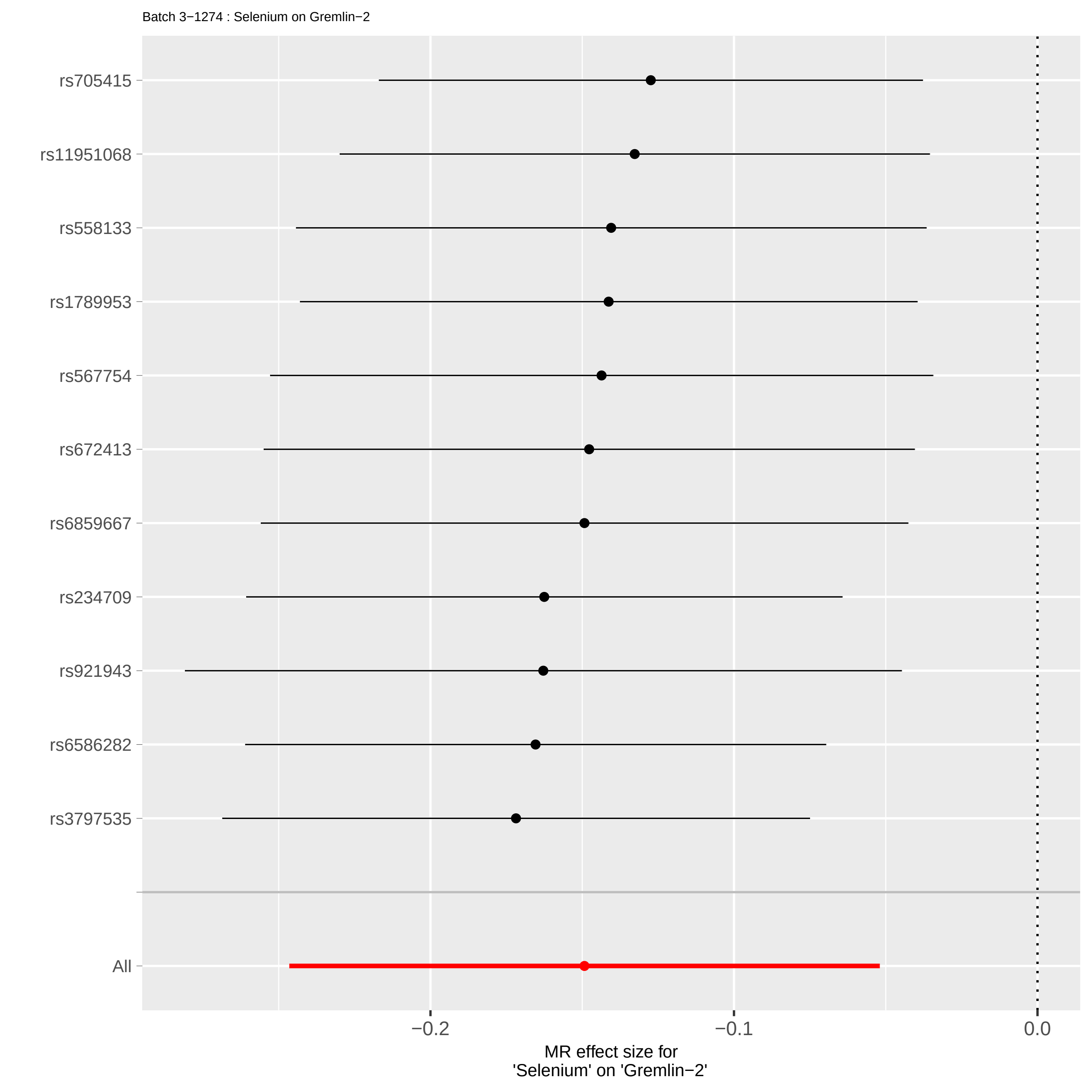
Batch 3−1274 : Selenium on Gremlin−2
rs705415
rs11951068
rs558133
rs1789953
rs567754
rs672413
rs6859667
rs234709
rs921943
rs6586282
rs3797535
All
−0.2
−0.1
0.0
MR effect size for
'Selenium' on 'Gremlin−2'

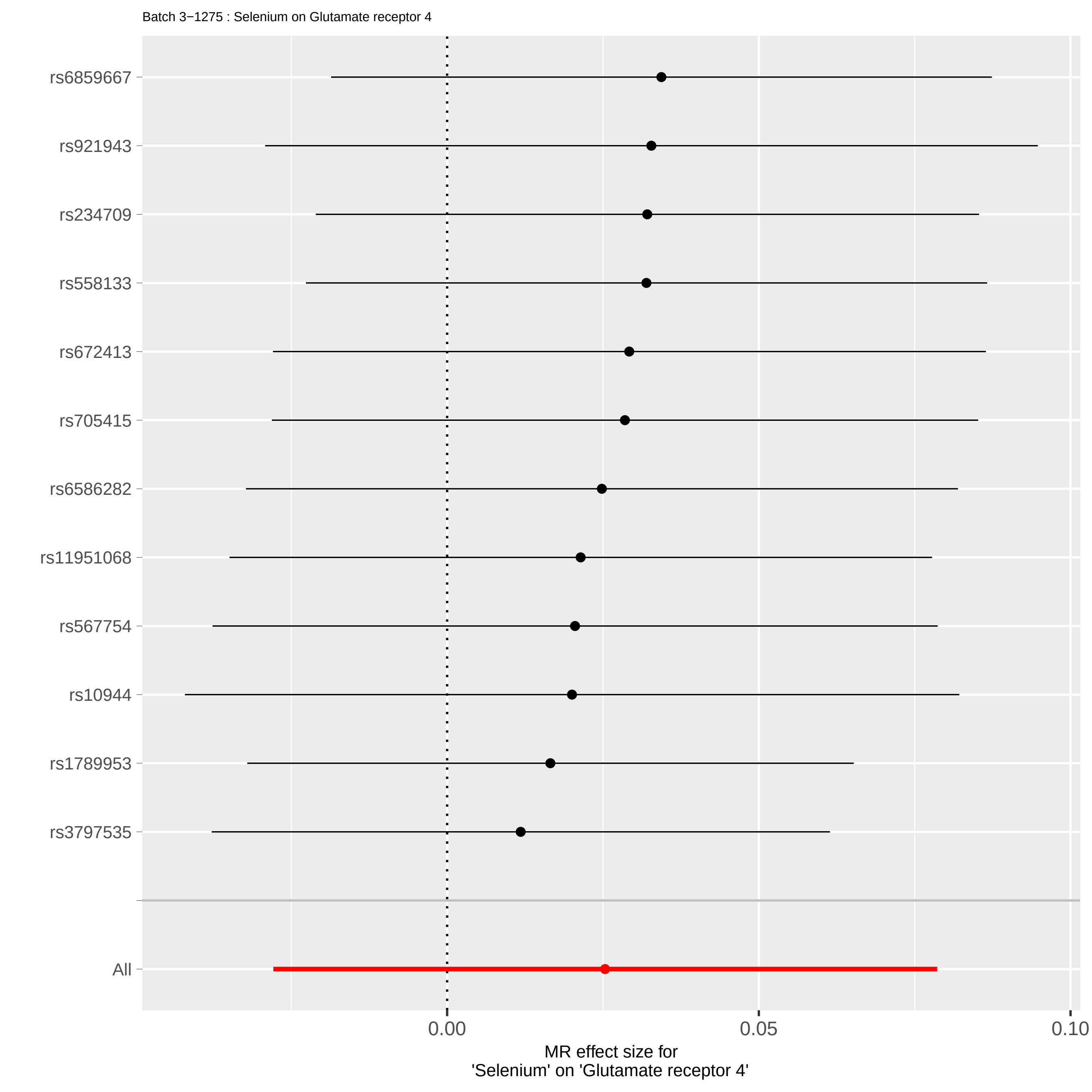

Batch 3−1275 : Selenium on Glutamate receptor 4
rs6859667
rs921943
rs234709
rs558133
rs672413
rs705415
rs6586282
rs11951068
rs567754
rs10944
rs1789953
rs3797535
All
0.00
0.05
0.10
MR effect size for
'Selenium' on 'Glutamate receptor 4'

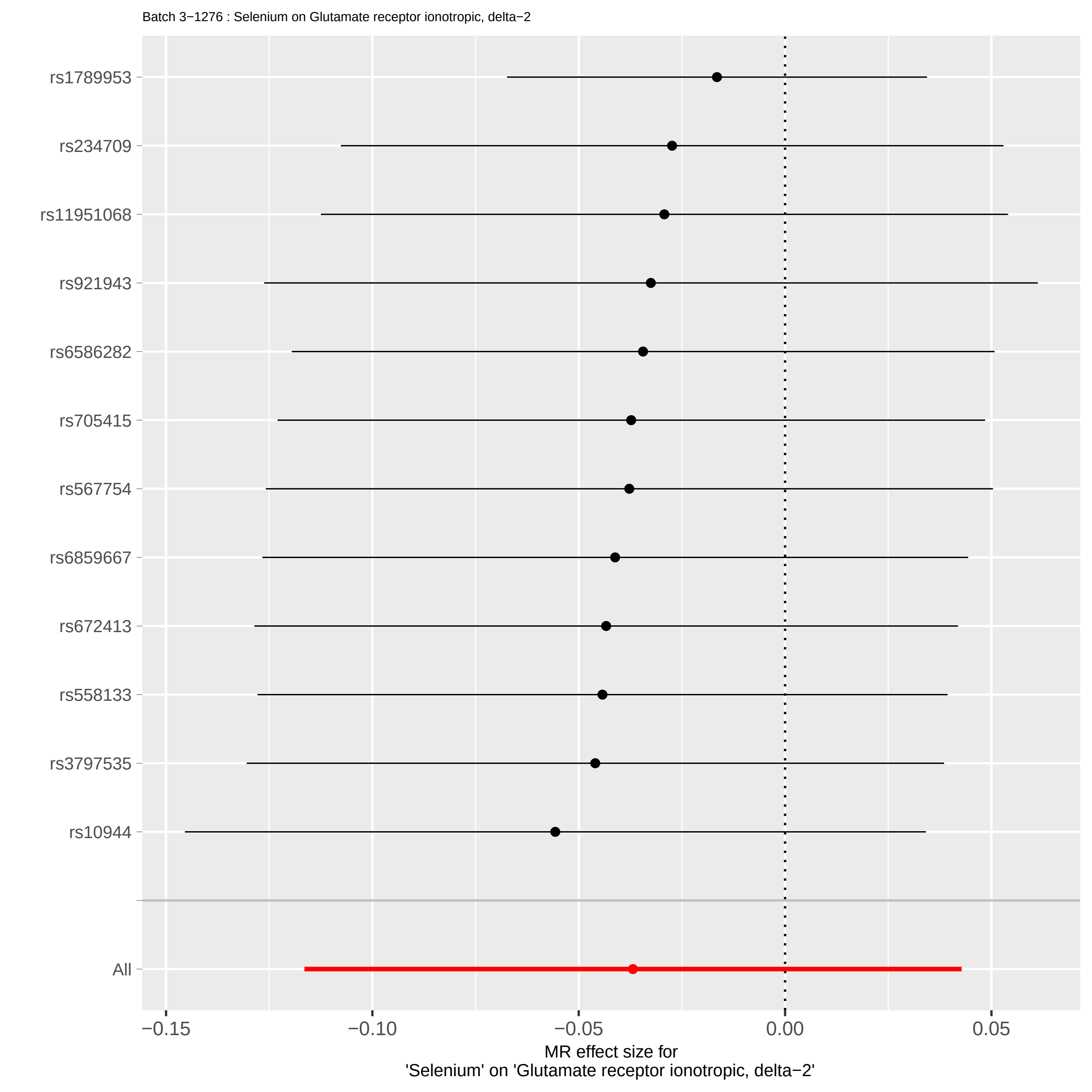

Batch 3−1276 : Selenium on Glutamate receptor ionotropic, delta−2
rs1789953
rs234709
rs11951068
rs921943
rs6586282
rs705415
rs567754
rs6859667
rs672413
rs558133
rs3797535
rs10944
All
−0.15
−0.10
−0.05
0.00
0.05
MR effect size for
'Selenium' on 'Glutamate receptor ionotropic, delta−2'

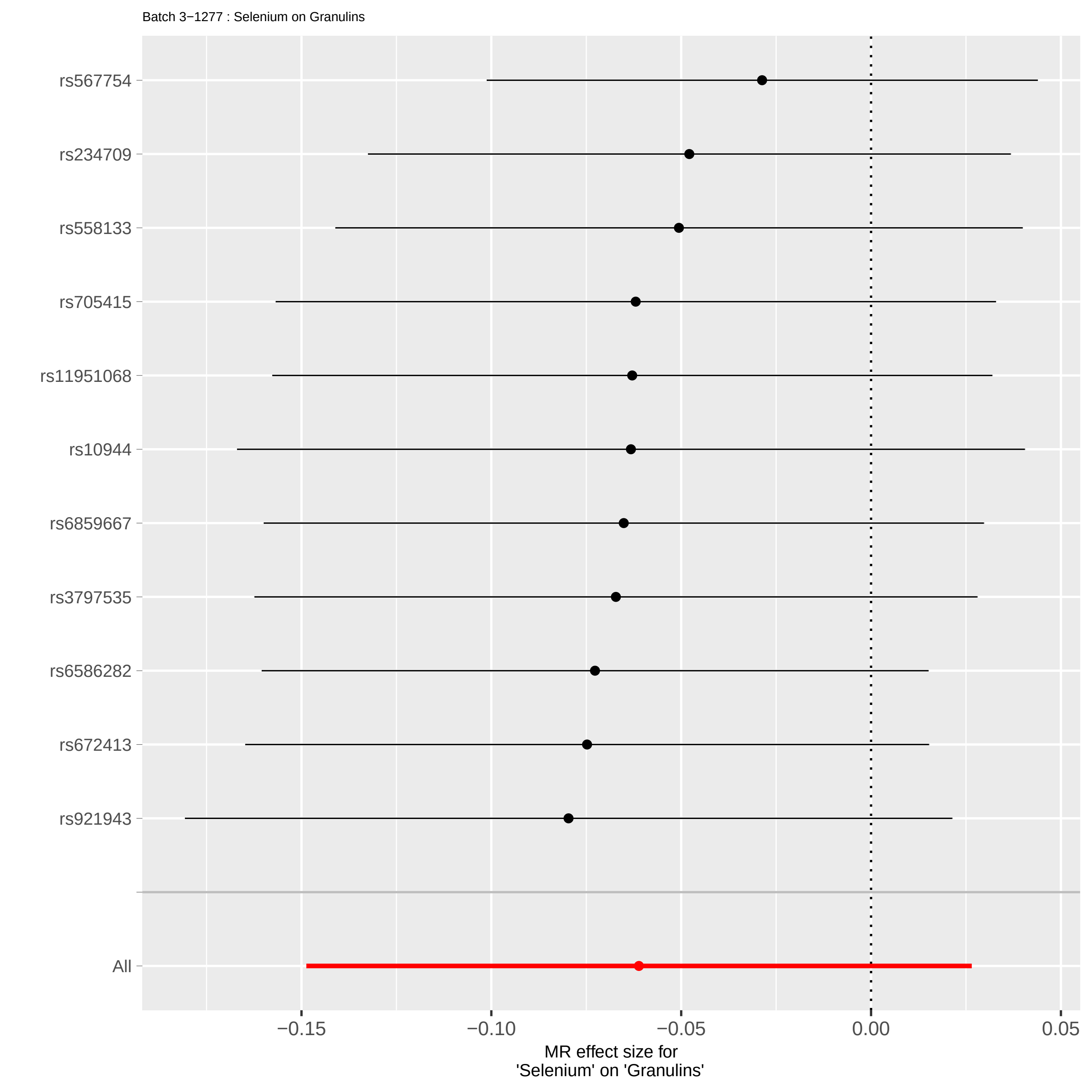

Batch 3−1277 : Selenium on Granulins
rs567754
rs234709
rs558133
rs705415
rs11951068
rs10944
rs6859667
rs3797535
rs6586282
rs672413
rs921943
All
−0.15
−0.10
−0.05
0.00
0.05
MR effect size for
'Selenium' on 'Granulins'

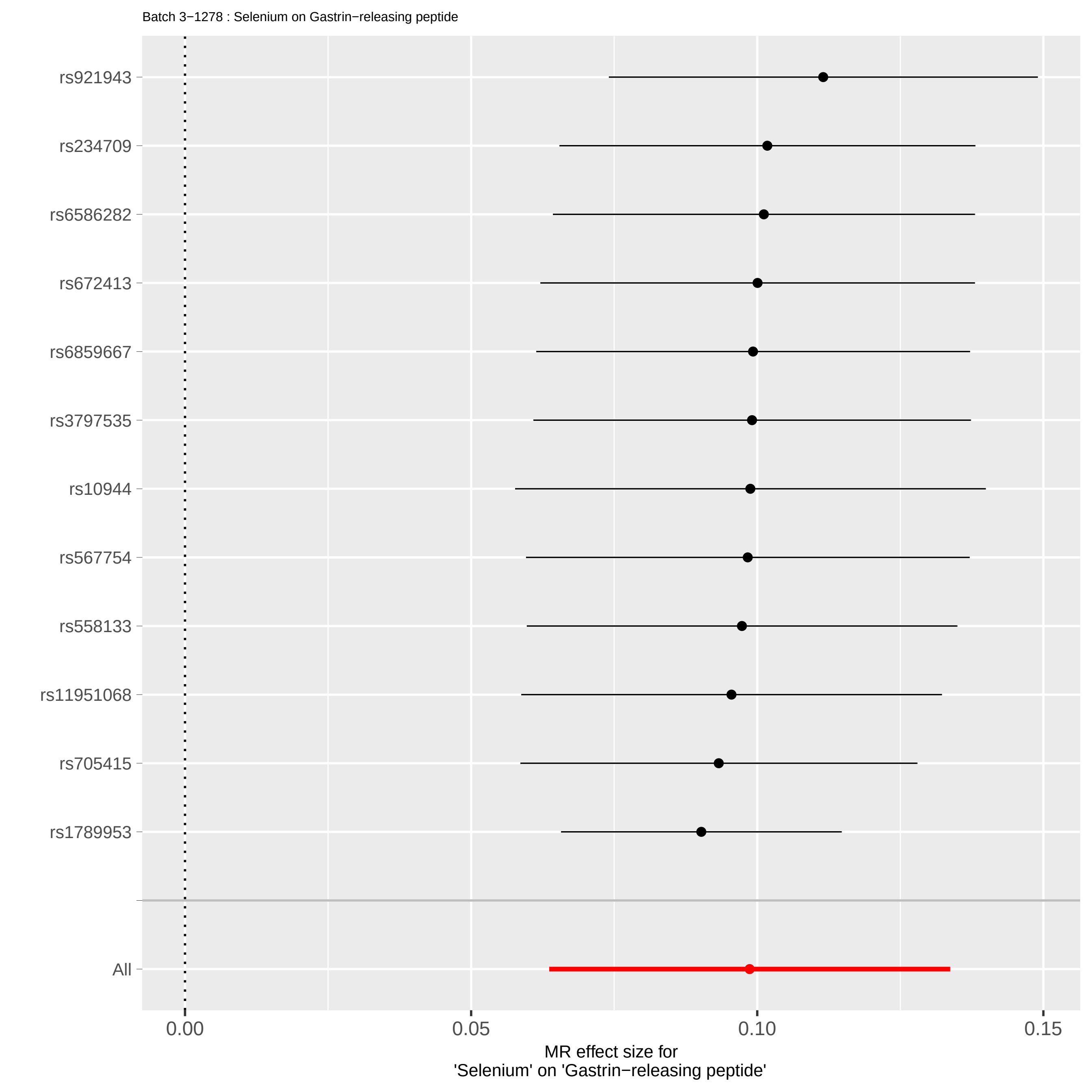

Batch 3−1278 : Selenium on Gastrin−releasing peptide
rs921943
rs234709
rs6586282
rs672413
rs6859667
rs3797535
rs10944
rs567754
rs558133
rs11951068
rs705415
rs1789953
All
0.00
0.05
0.10
0.15
MR effect size for
'Selenium' on 'Gastrin−releasing peptide'

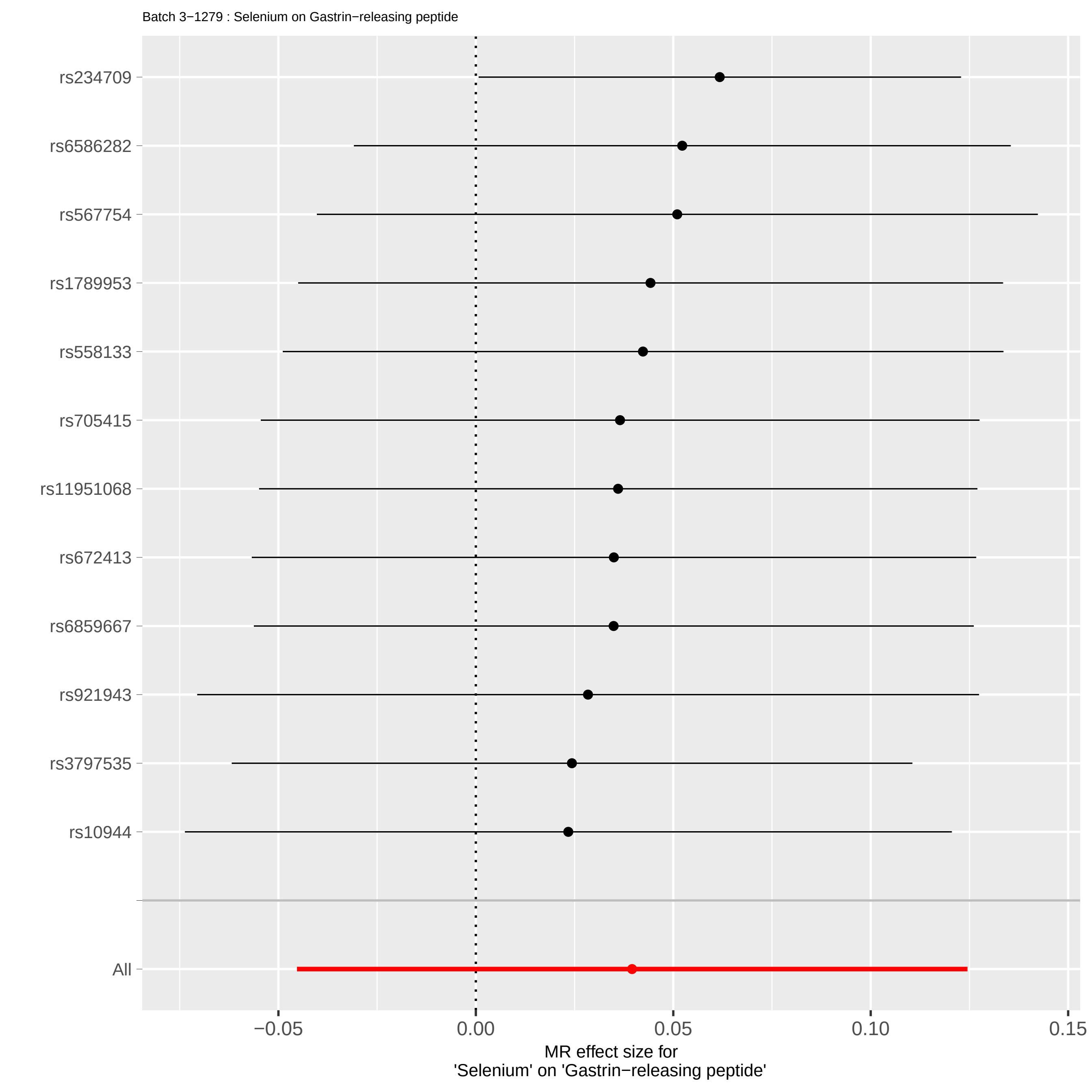

Batch 3−1279 : Selenium on Gastrin−releasing peptide
rs234709
rs6586282
rs567754
rs1789953
rs558133
rs705415
rs11951068
rs672413
rs6859667
rs921943
rs3797535
rs10944
All
−0.05
0.00
0.05
0.10
0.15
MR effect size for
'Selenium' on 'Gastrin−releasing peptide'

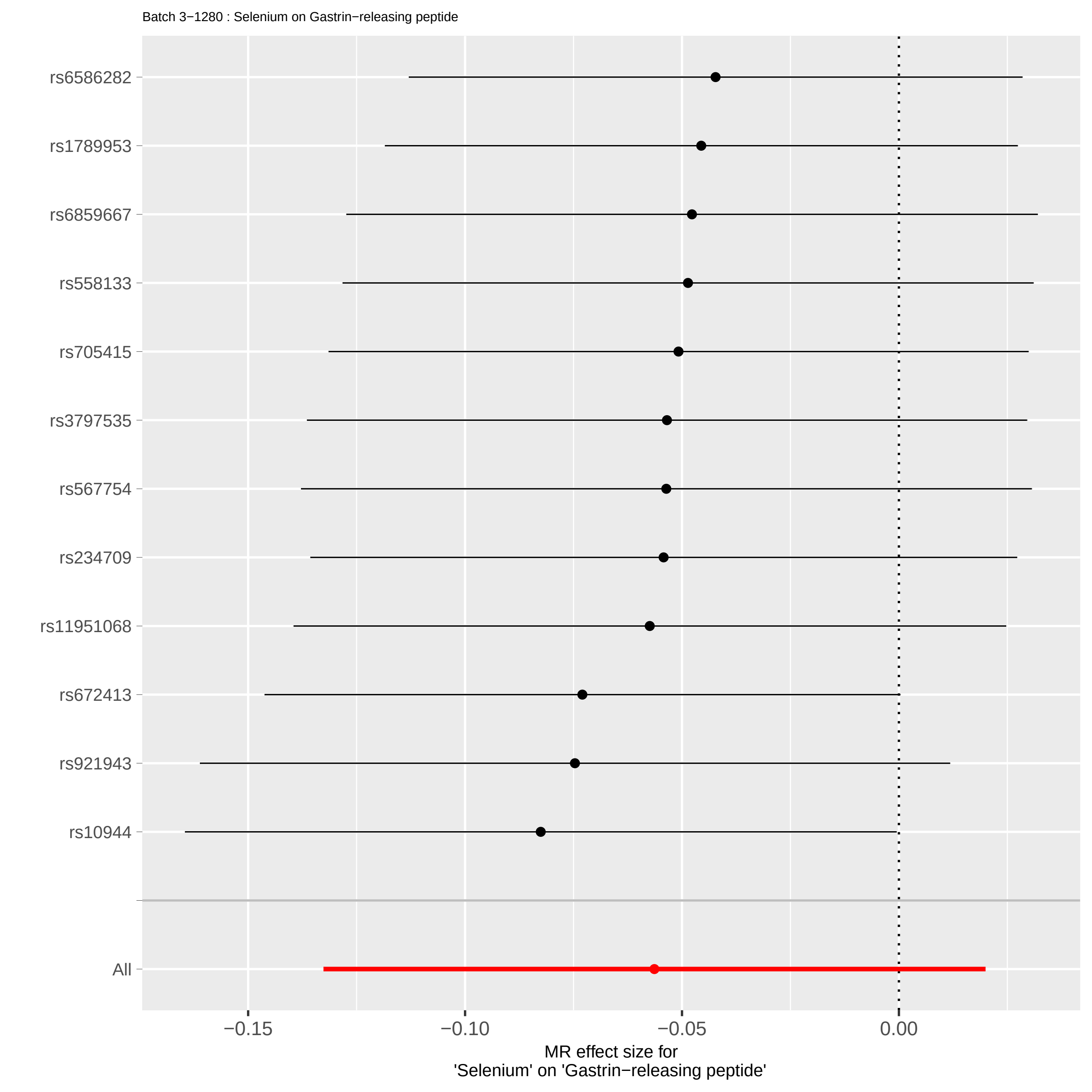

Batch 3−1280 : Selenium on Gastrin−releasing peptide
rs6586282
rs1789953
rs6859667
rs558133
rs705415
rs3797535
rs567754
rs234709
rs11951068
rs672413
rs921943
rs10944
All
−0.15
−0.10
−0.05
0.00
MR effect size for
'Selenium' on 'Gastrin−releasing peptide'

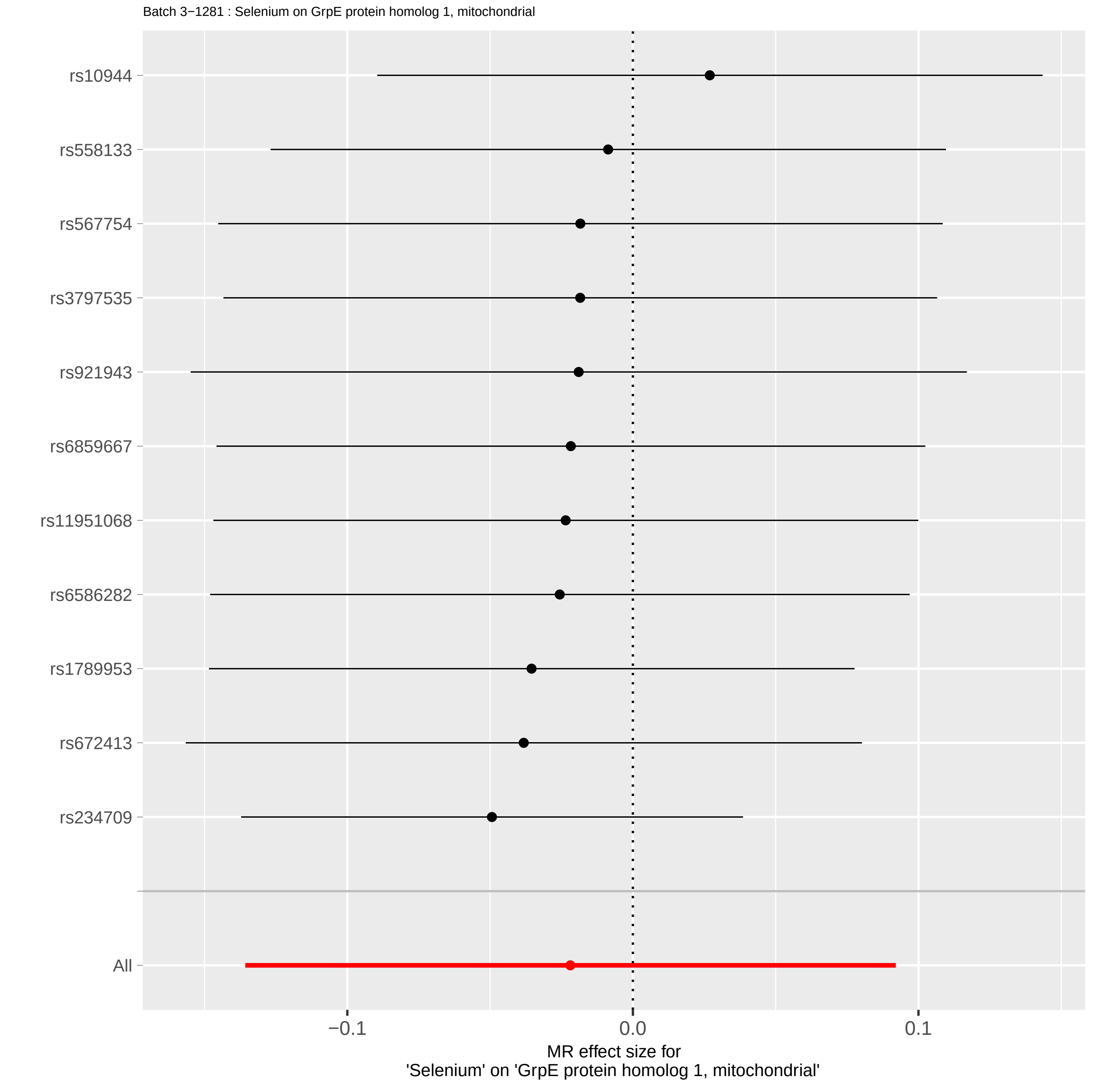

Batch 3−1281 : Selenium on GrpE protein homolog 1, mitochondrial
rs10944
rs558133
rs567754
rs3797535
rs921943
rs6859667
rs11951068
rs6586282
rs1789953
rs672413
rs234709
All
−0.1
0.0
0.1
MR effect size for
'Selenium' on 'GrpE protein homolog 1, mitochondrial'

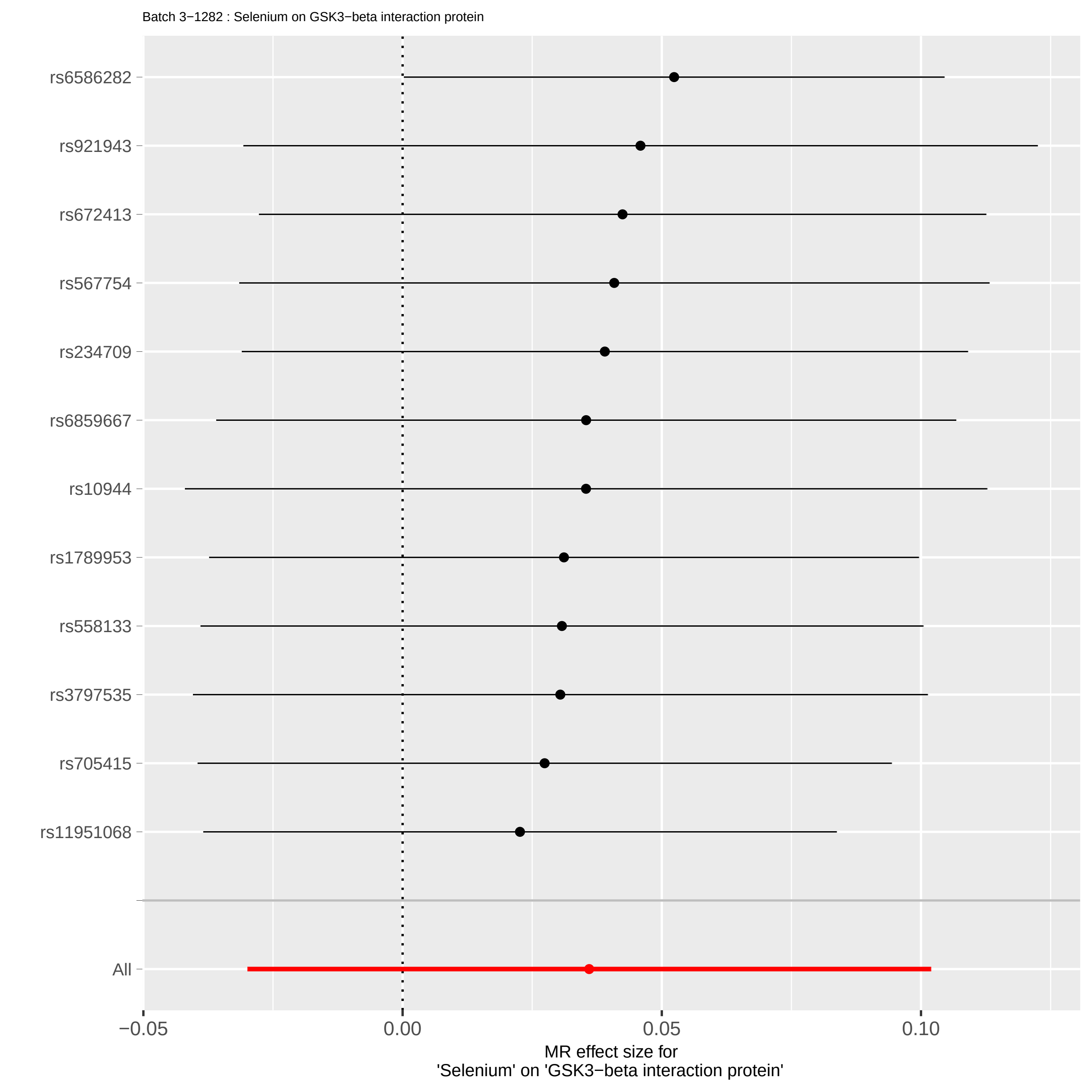

Batch 3−1282 : Selenium on GSK3−beta interaction protein
rs6586282
rs921943
rs672413
rs567754
rs234709
rs6859667
rs10944
rs1789953
rs558133
rs3797535
rs705415
rs11951068
All
−0.05
0.00
0.05
0.10
MR effect size for
'Selenium' on 'GSK3−beta interaction protein'

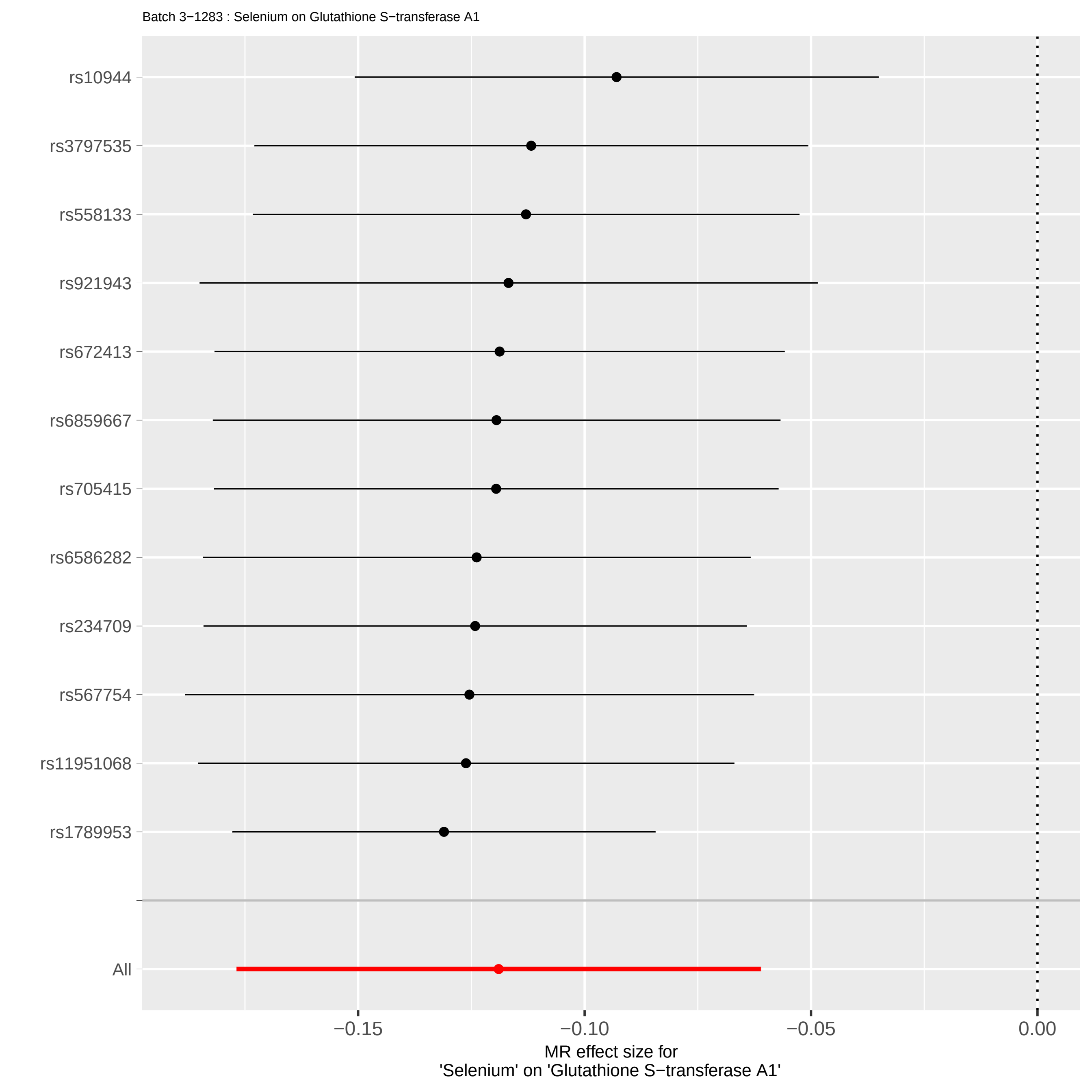
Batch 3−1283 : Selenium on Glutathione S−transferase A1
rs10944
rs3797535
rs558133
rs921943
rs672413
rs6859667
rs705415
rs6586282
rs234709
rs567754
rs11951068
rs1789953
All
−0.15
−0.10
−0.05
0.00
MR effect size for
'Selenium' on 'Glutathione S−transferase A1'

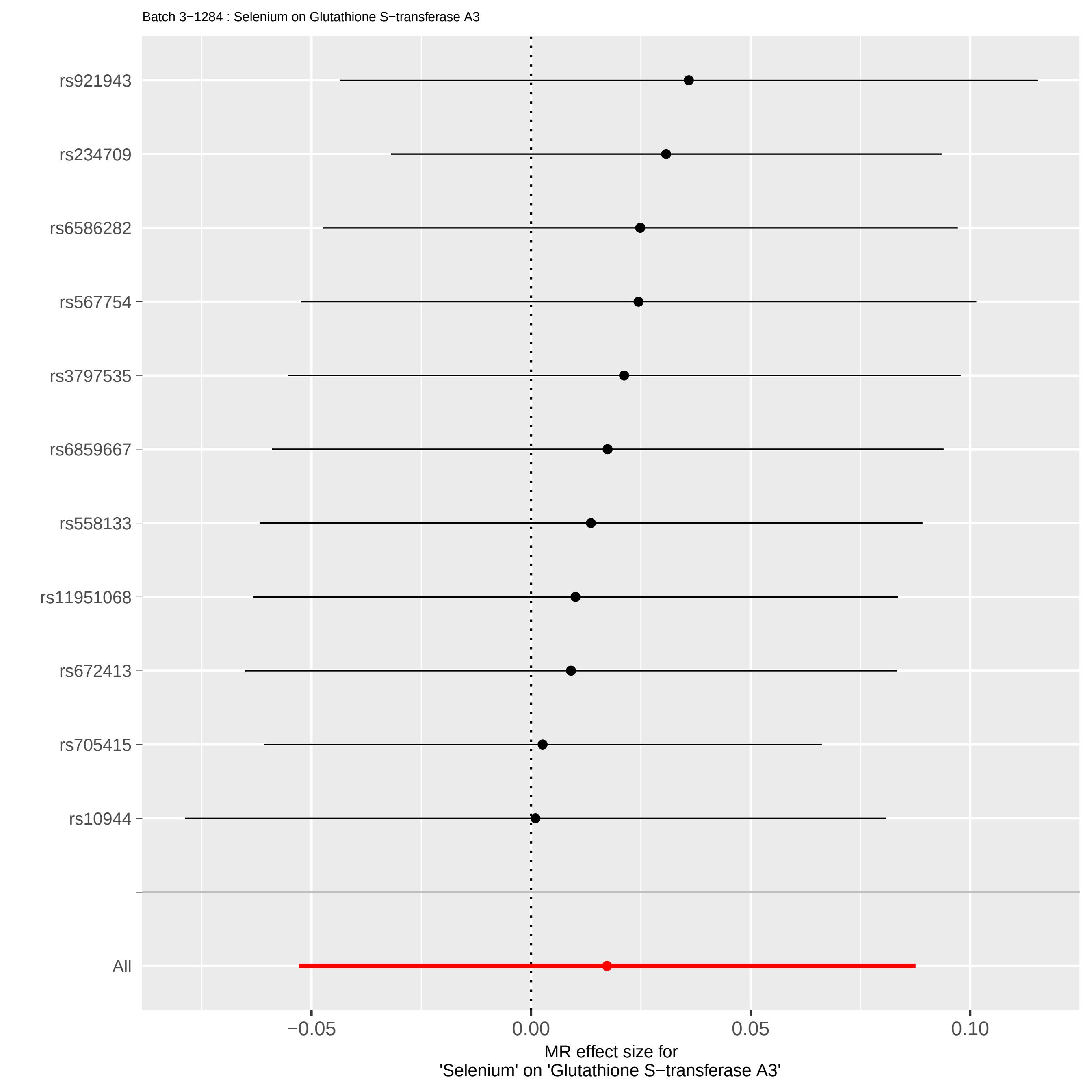

Batch 3−1284 : Selenium on Glutathione S−transferase A3
rs921943
rs234709
rs6586282
rs567754
rs3797535
rs6859667
rs558133
rs11951068
rs672413
rs705415
rs10944
All
−0.05
0.00
0.05
0.10
MR effect size for
'Selenium' on 'Glutathione S−transferase A3'

Batch 3−1285 : Selenium on Glutathione S−transferase A4

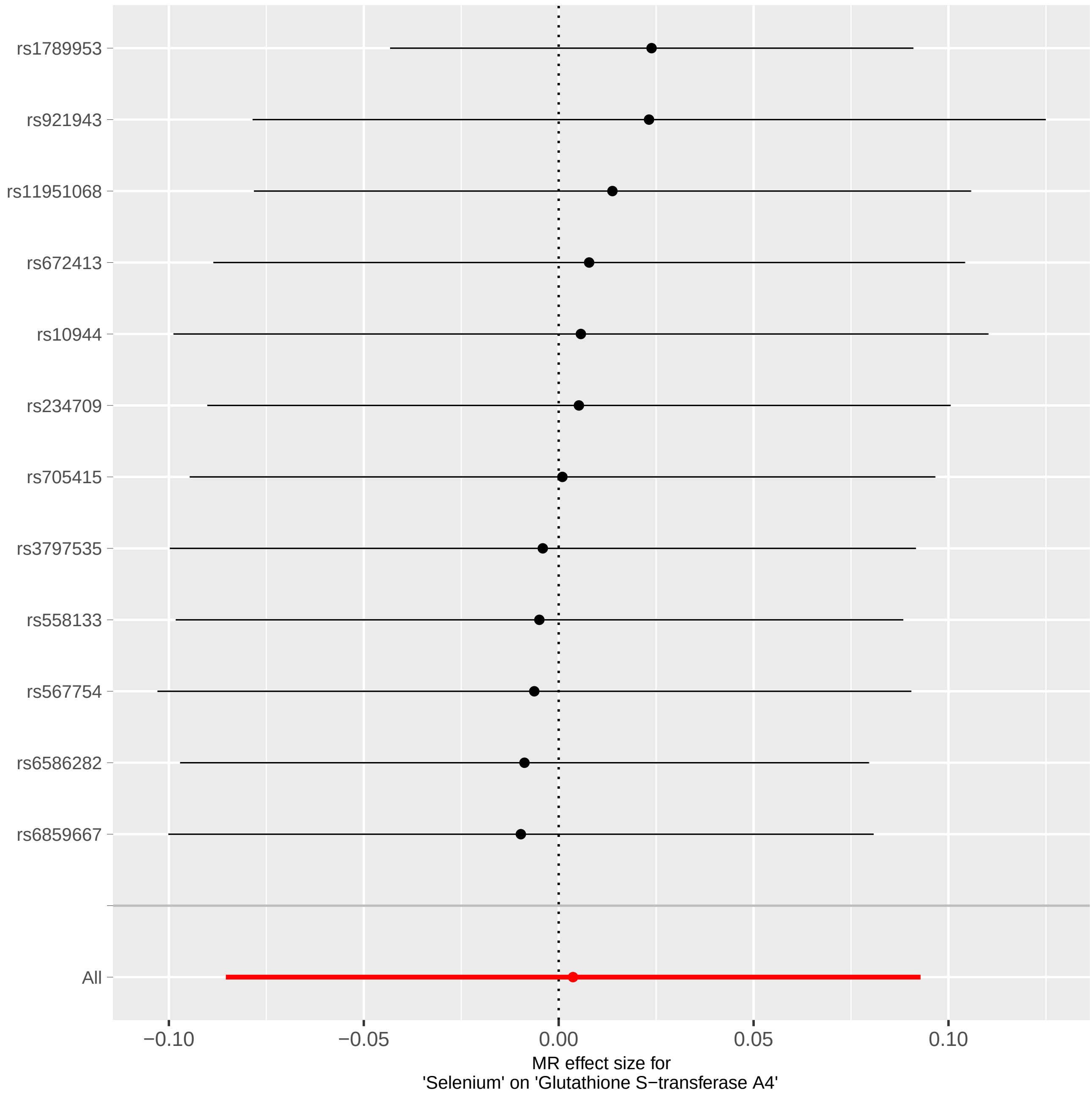

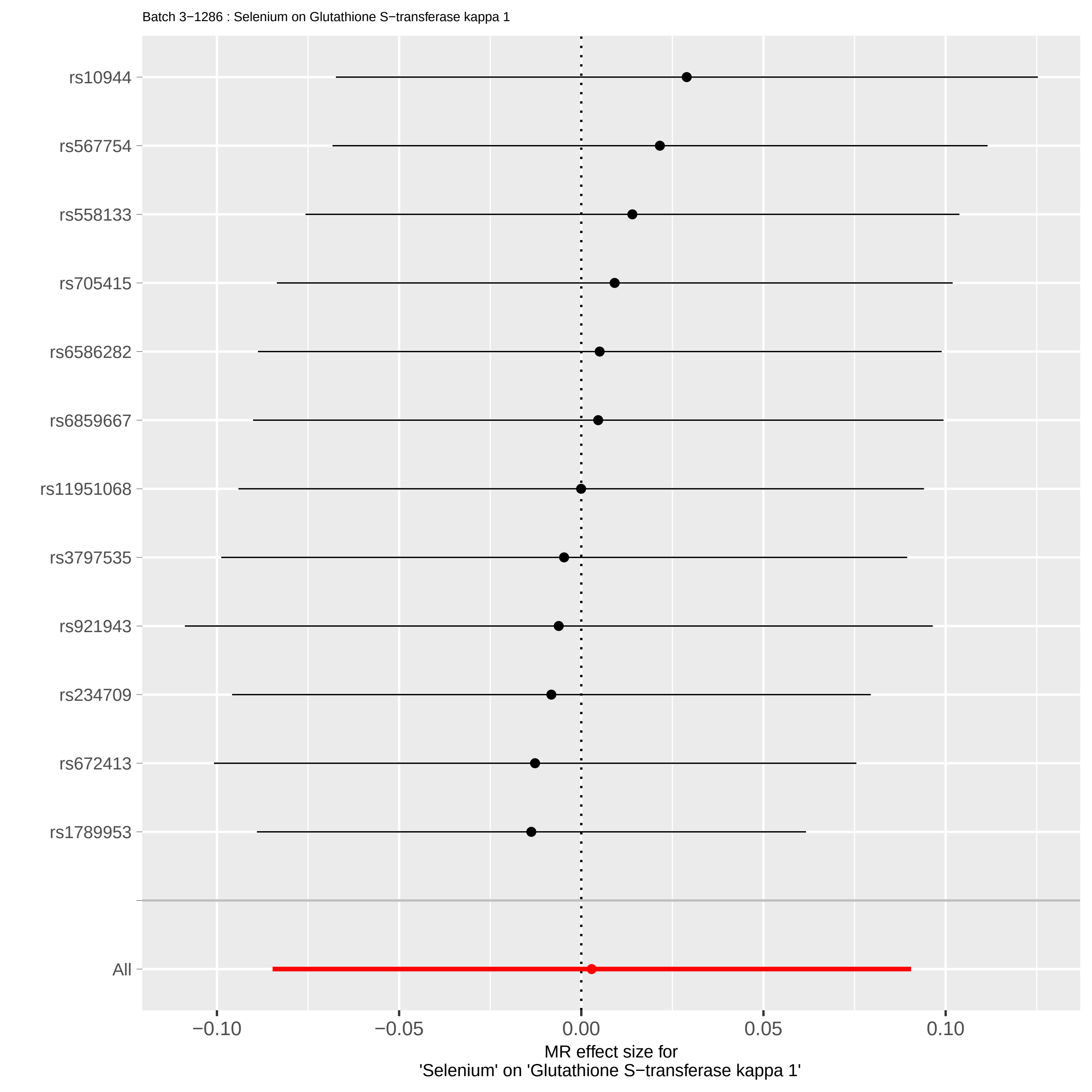
Batch 3−1286 : Selenium on Glutathione S−transferase kappa 1
rs10944
rs567754
rs558133
rs705415
rs6586282
rs6859667
rs11951068
rs3797535
rs921943
rs234709
rs672413
rs1789953
All
−0.10
−0.05
0.00
0.05
0.10
MR effect size for
'Selenium' on 'Glutathione S−transferase kappa 1'

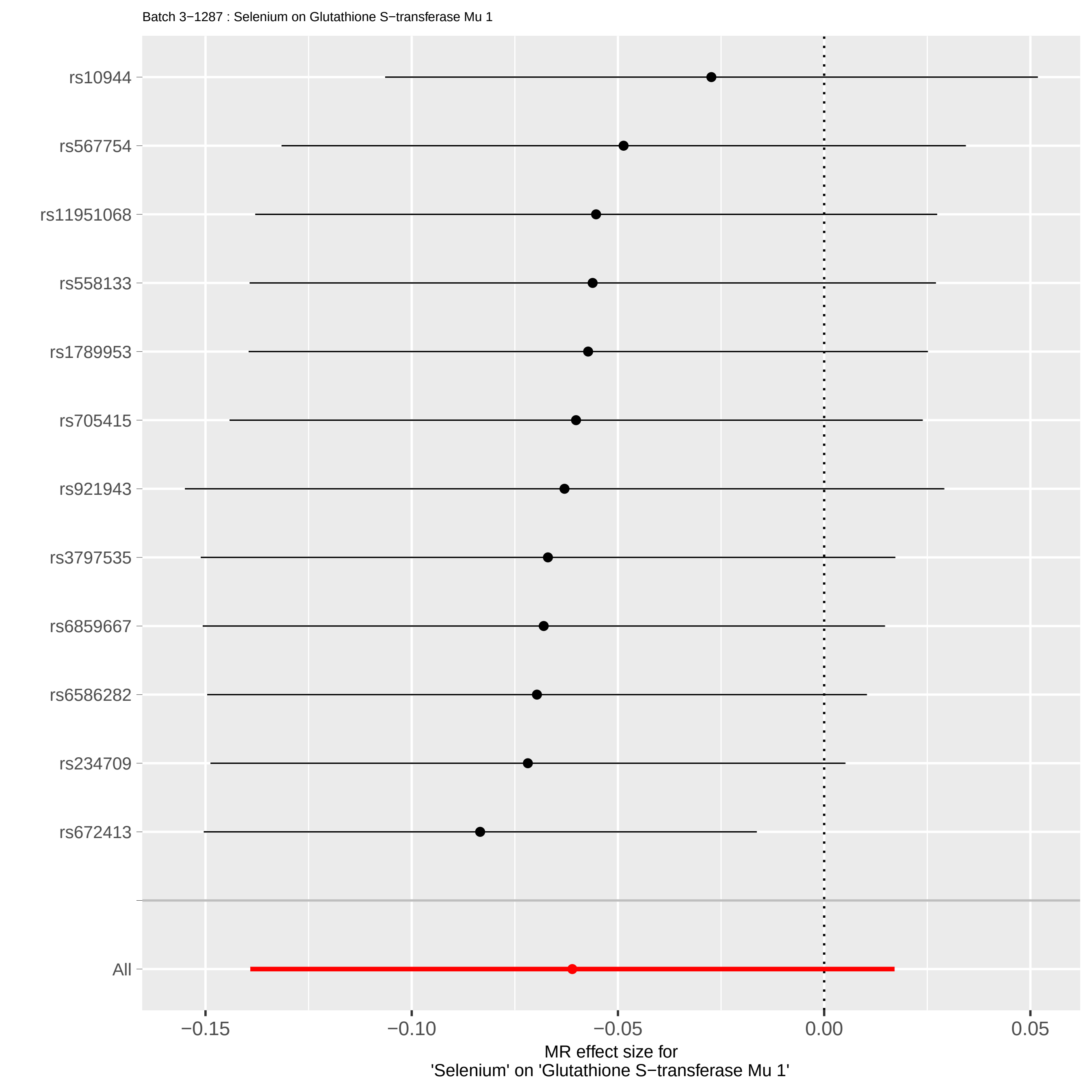
Batch 3−1287 : Selenium on Glutathione S−transferase Mu 1
rs10944
rs567754
rs11951068
rs558133
rs1789953
rs705415
rs921943
rs3797535
rs6859667
rs6586282
rs234709
rs672413
All
−0.15
−0.10
−0.05
0.00
0.05
MR effect size for
'Selenium' on 'Glutathione S−transferase Mu 1'

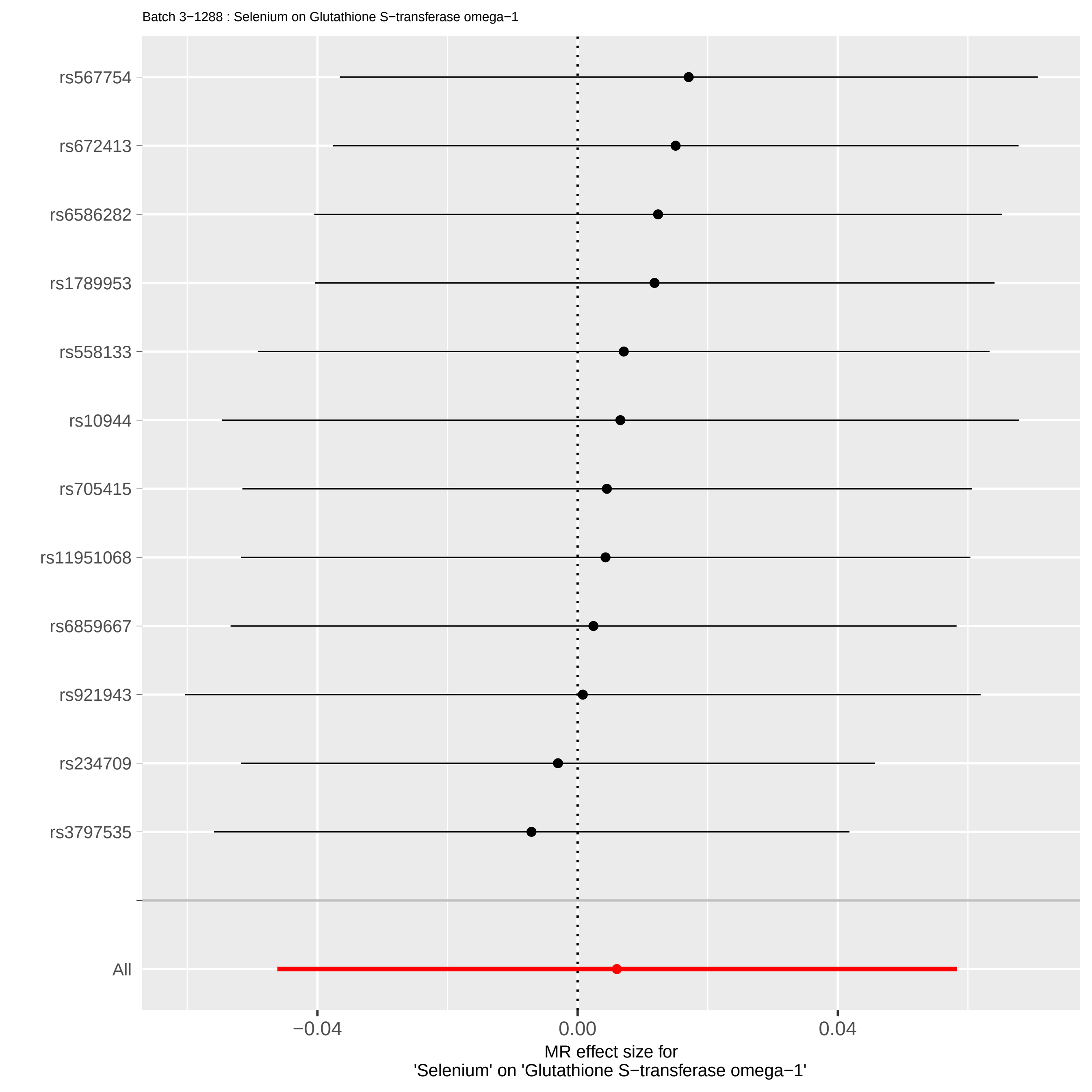

Batch 3−1288 : Selenium on Glutathione S−transferase omega−1
rs567754
rs672413
rs6586282
rs1789953
rs558133
rs10944
rs705415
rs11951068
rs6859667
rs921943
rs234709
rs3797535
All
−0.04
0.00
0.04
MR effect size for
'Selenium' on 'Glutathione S−transferase omega−1'

Batch 3−1289 : Selenium on Glutathione S−transferase P

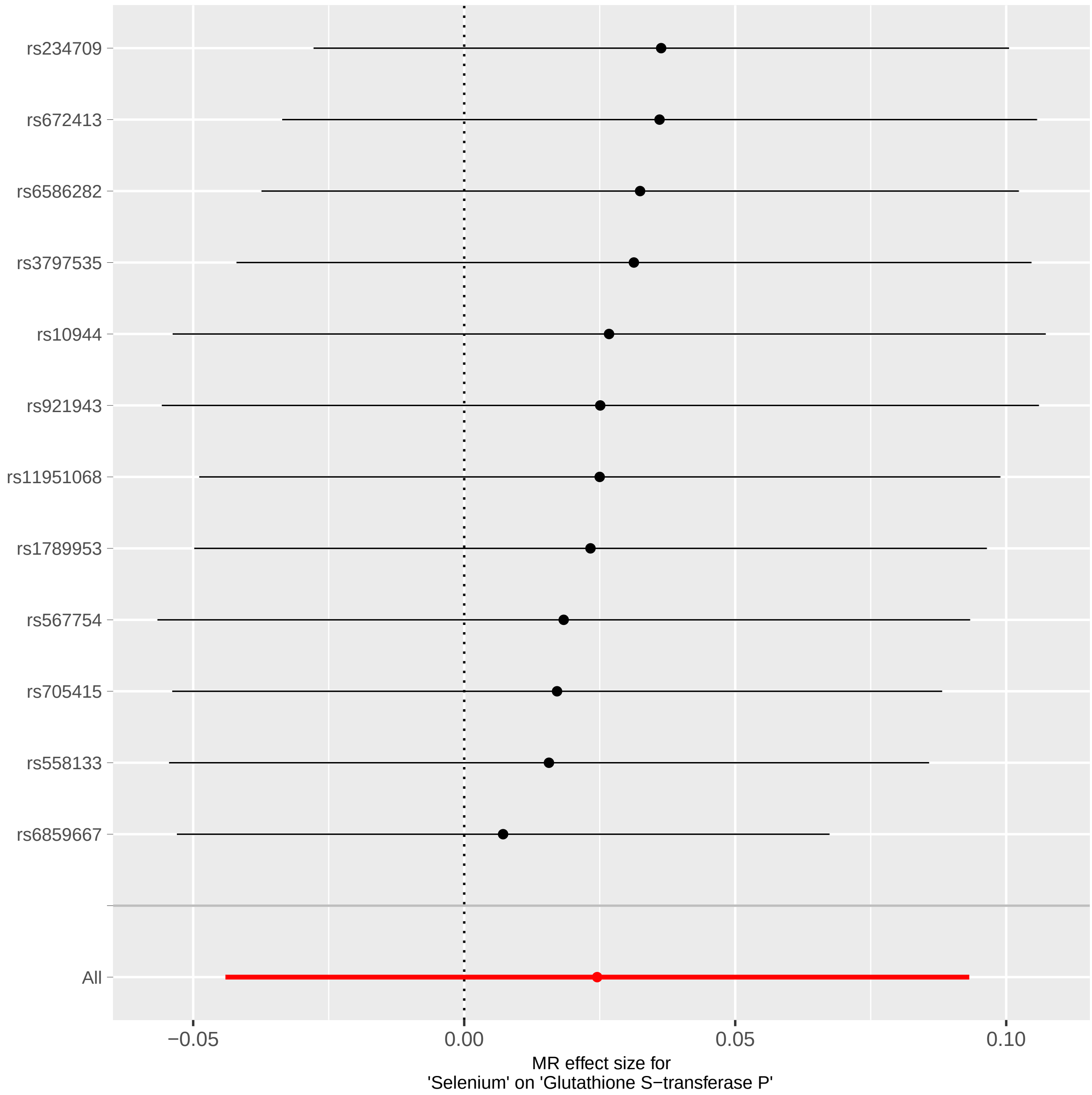

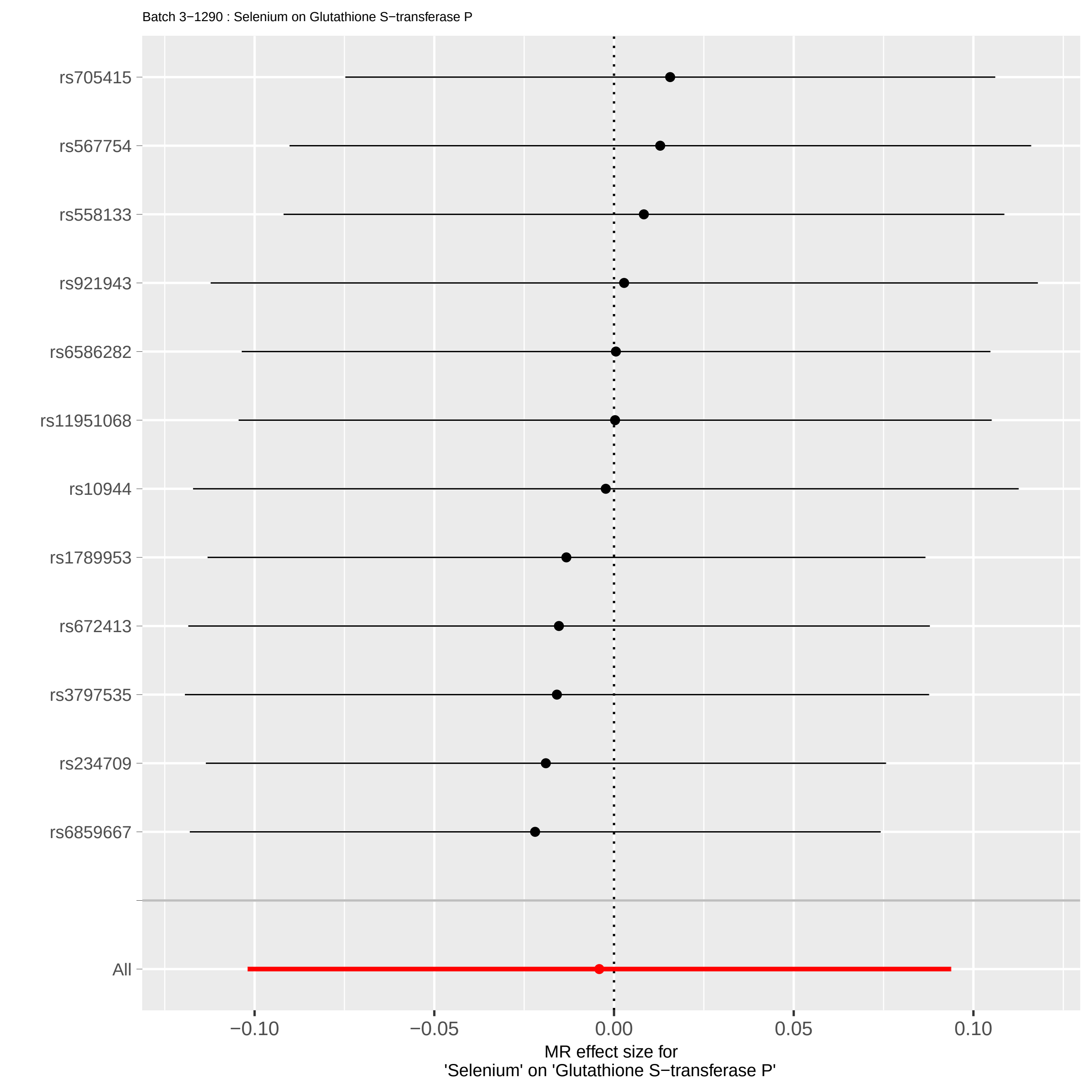

Batch 3−1290 : Selenium on Glutathione S−transferase P
rs705415
rs567754
rs558133
rs921943
rs6586282
rs11951068
rs10944
rs1789953
rs672413
rs3797535
rs234709
rs6859667
All
−0.10
−0.05
0.00
0.05
0.10
MR effect size for
'Selenium' on 'Glutathione S−transferase P'

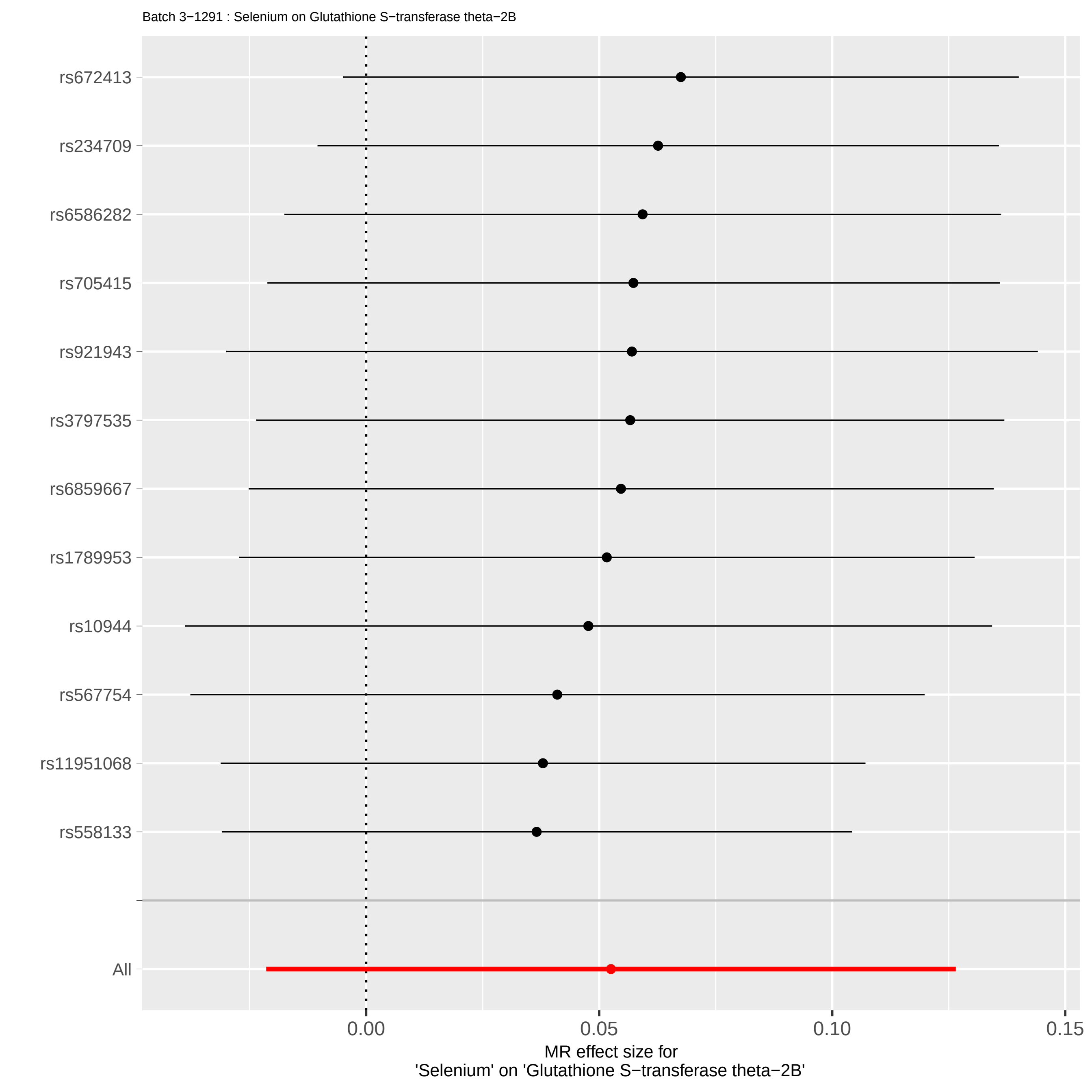
Batch 3−1291 : Selenium on Glutathione S−transferase theta−2B
rs672413
rs234709
rs6586282
rs705415
rs921943
rs3797535
rs6859667
rs1789953
rs10944
rs567754
rs11951068
rs558133
All
0.00
0.05
0.10
0.15
MR effect size for
'Selenium' on 'Glutathione S−transferase theta−2B'

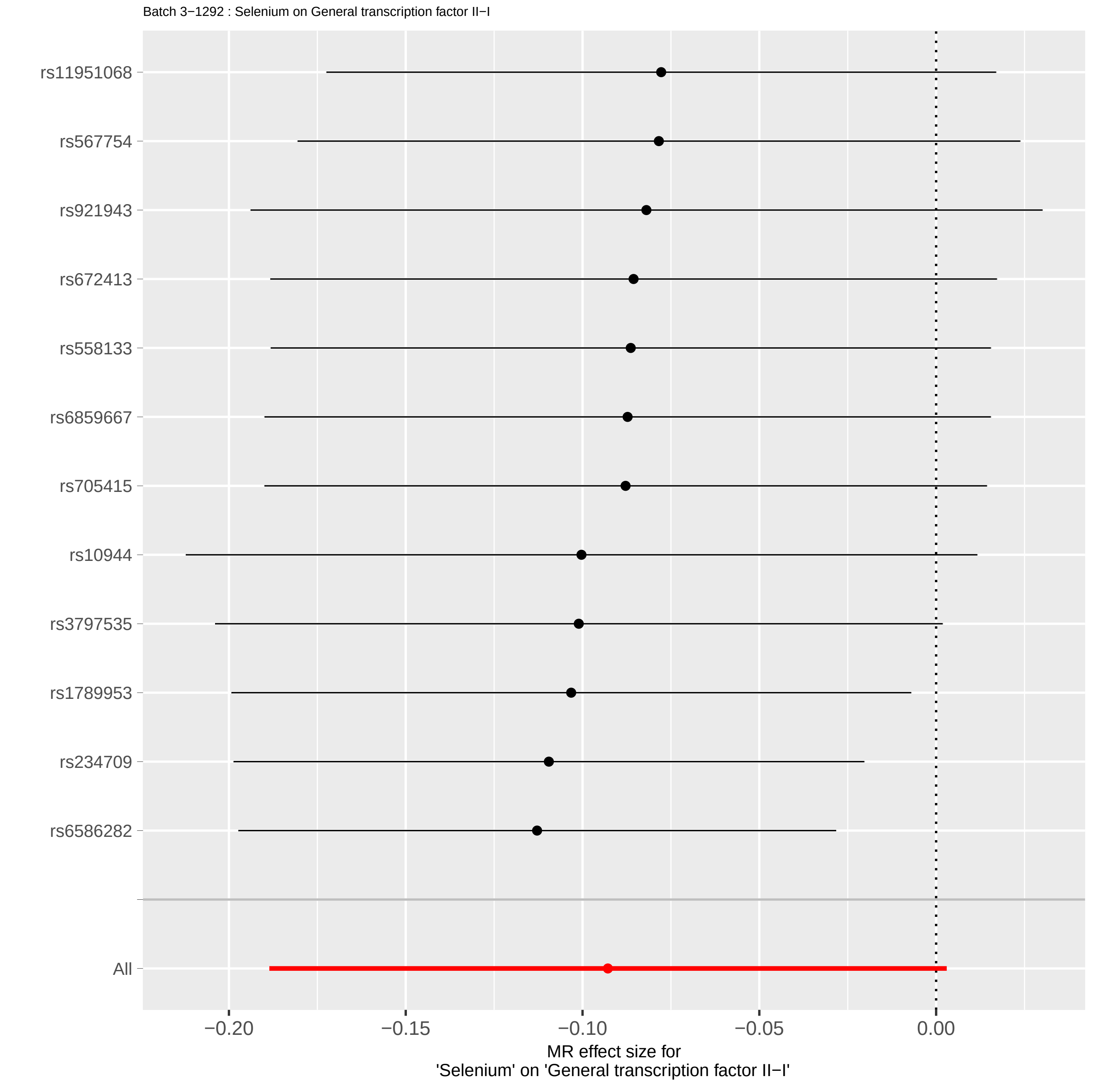
Batch 3−1292 : Selenium on General transcription factor II−I
rs11951068
rs567754
rs921943
rs672413
rs558133
rs6859667
rs705415
rs10944
rs3797535
rs1789953
rs234709
rs6586282
All
−0.20
−0.15
−0.10
−0.05
0.00
MR effect size for
'Selenium' on 'General transcription factor II−I'

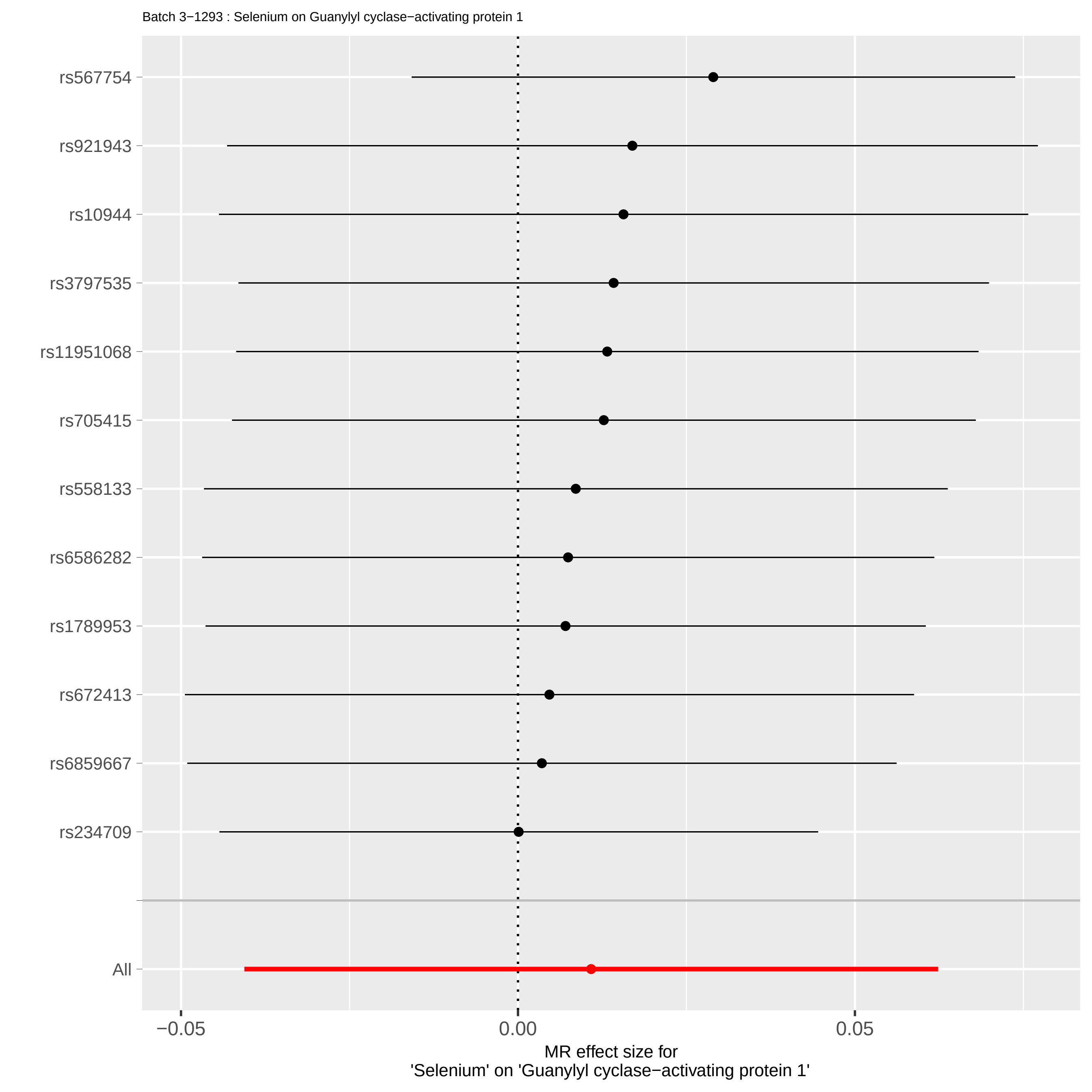

Batch 3−1293 : Selenium on Guanylyl cyclase−activating protein 1
rs567754
rs921943
rs10944
rs3797535
rs11951068
rs705415
rs558133
rs6586282
rs1789953
rs672413
rs6859667
rs234709
All
−0.05
0.00
0.05
MR effect size for
'Selenium' on 'Guanylyl cyclase−activating protein 1'

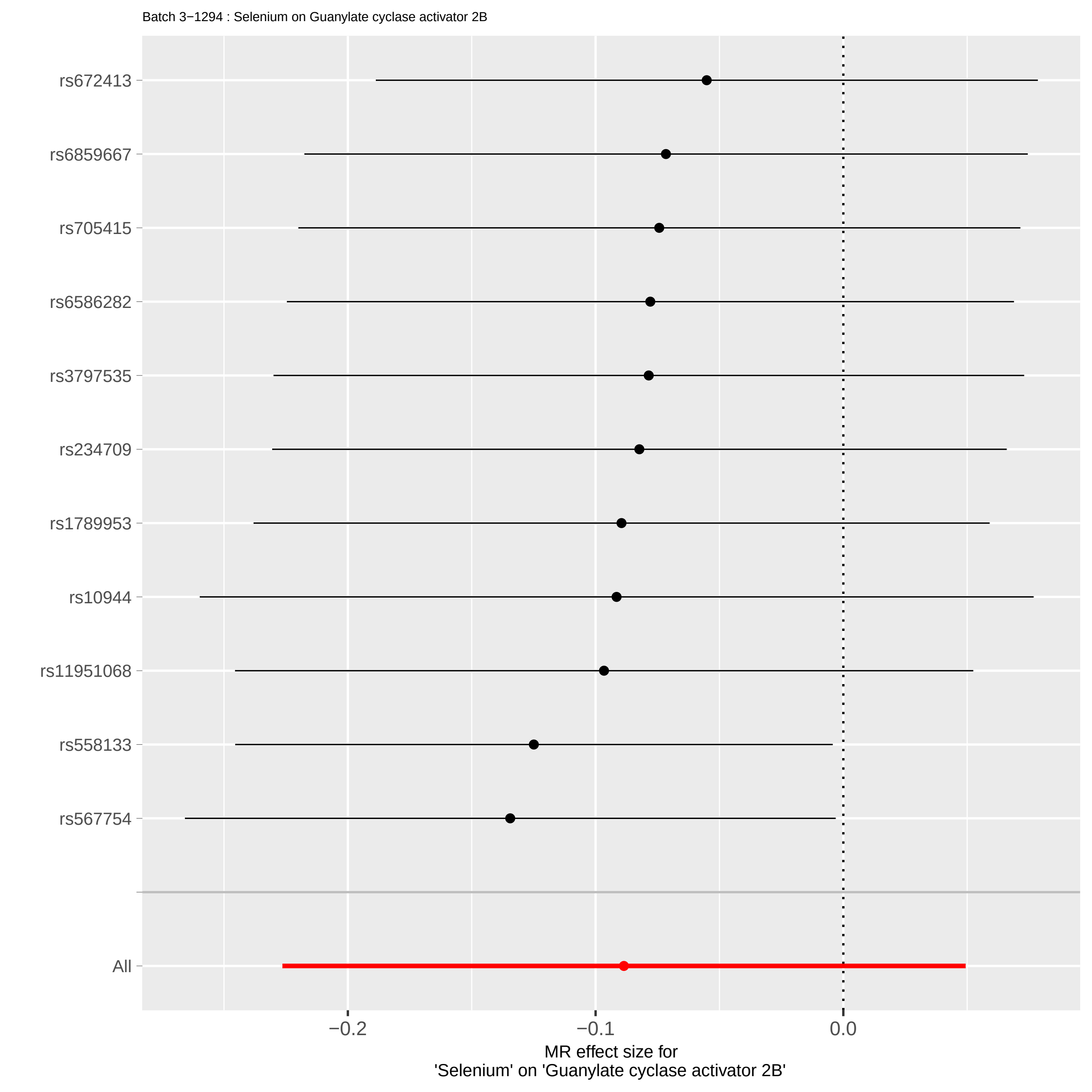

Batch 3−1294 : Selenium on Guanylate cyclase activator 2B
rs672413
rs6859667
rs705415
rs6586282
rs3797535
rs234709
rs1789953
rs10944
rs11951068
rs558133
rs567754
All
−0.2
−0.1
0.0
MR effect size for
'Selenium' on 'Guanylate cyclase activator 2B'

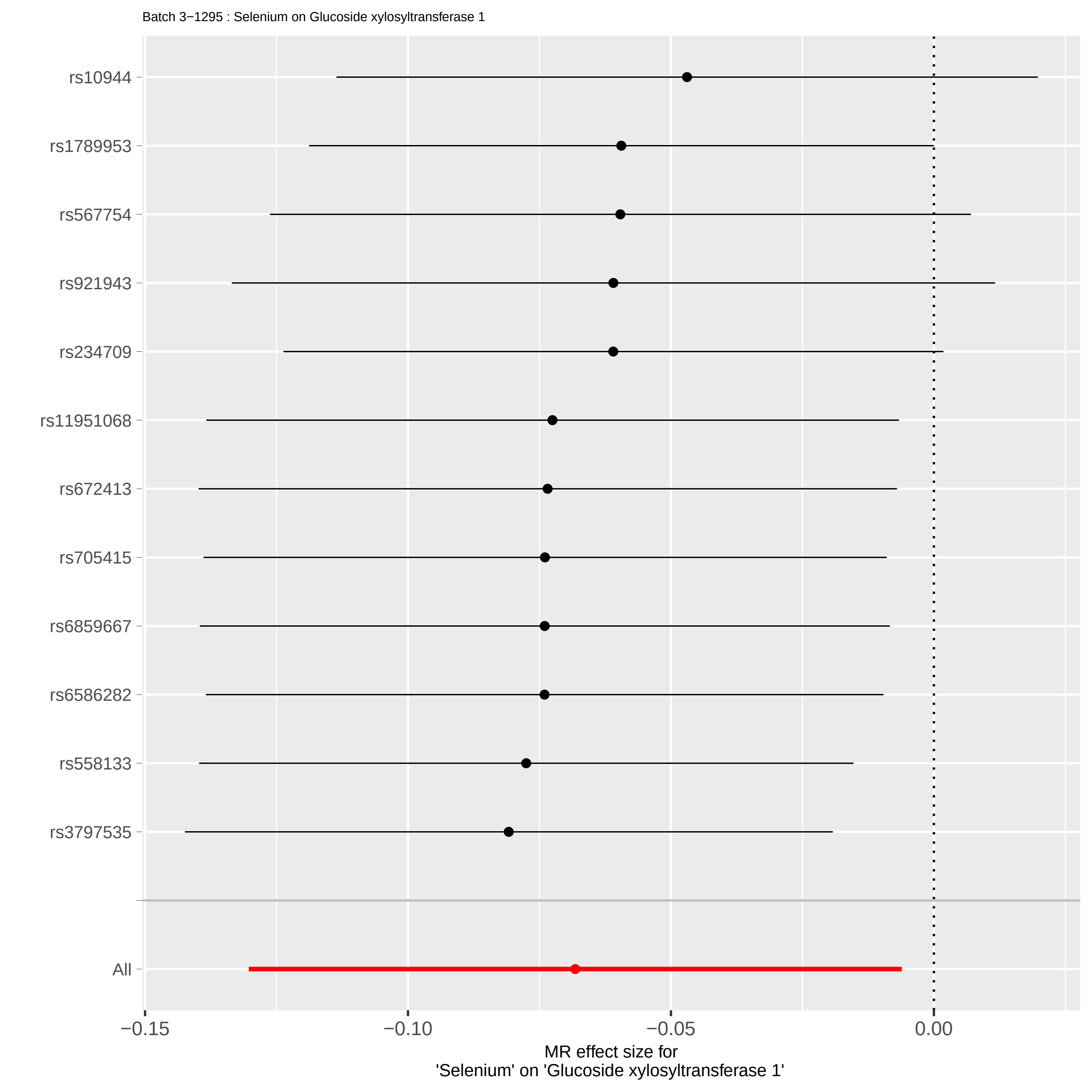

Batch 3−1295 : Selenium on Glucoside xylosyltransferase 1
rs10944
rs1789953
rs567754
rs921943
rs234709
rs11951068
rs672413
rs705415
rs6859667
rs6586282
rs558133
rs3797535
All
−0.15
−0.10
−0.05
0.00
MR effect size for
'Selenium' on 'Glucoside xylosyltransferase 1'

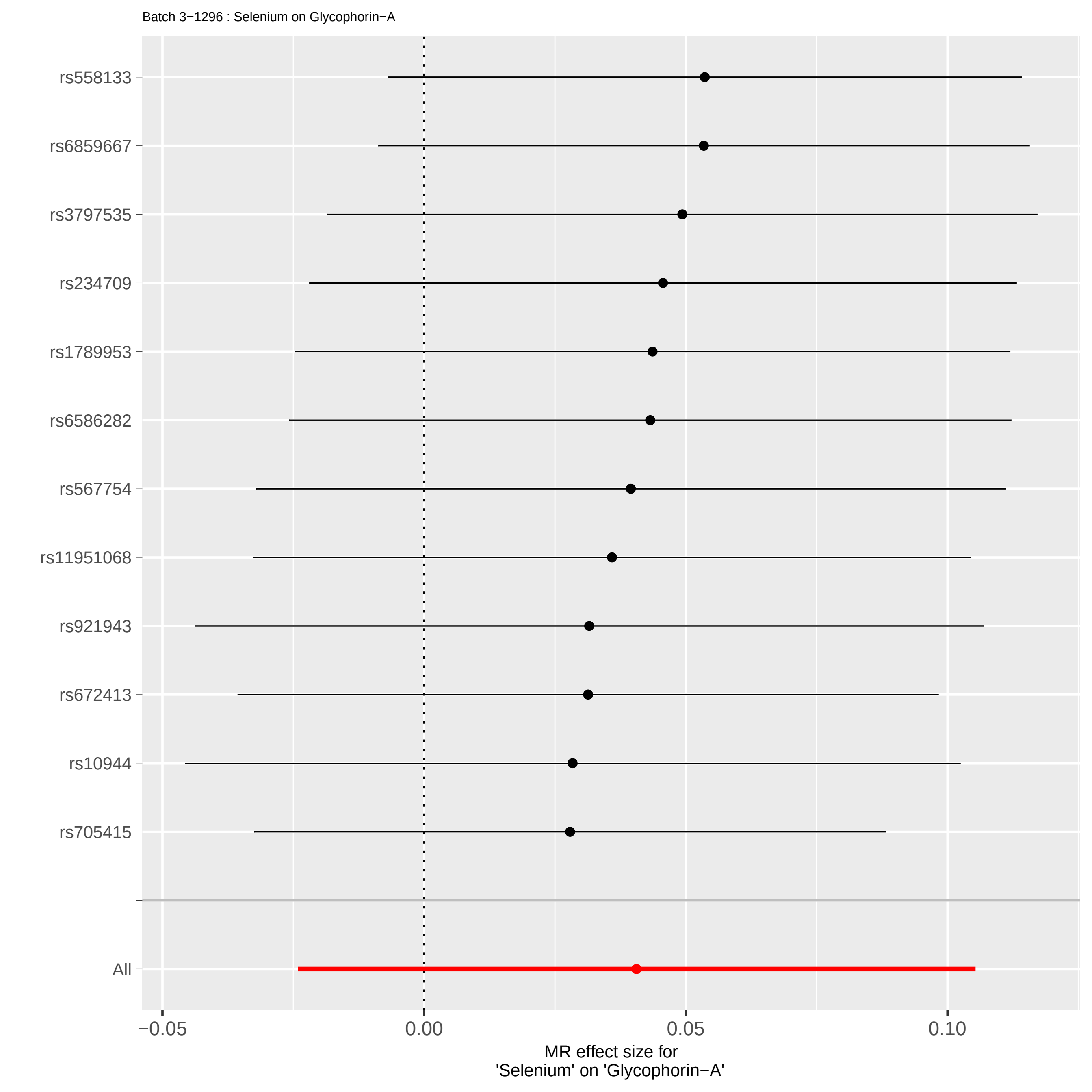

Batch 3−1296 : Selenium on Glycophorin−A
rs558133
rs6859667
rs3797535
rs234709
rs1789953
rs6586282
rs567754
rs11951068
rs921943
rs672413
rs10944
rs705415
All
−0.05
0.00
0.05
0.10
MR effect size for
'Selenium' on 'Glycophorin−A'

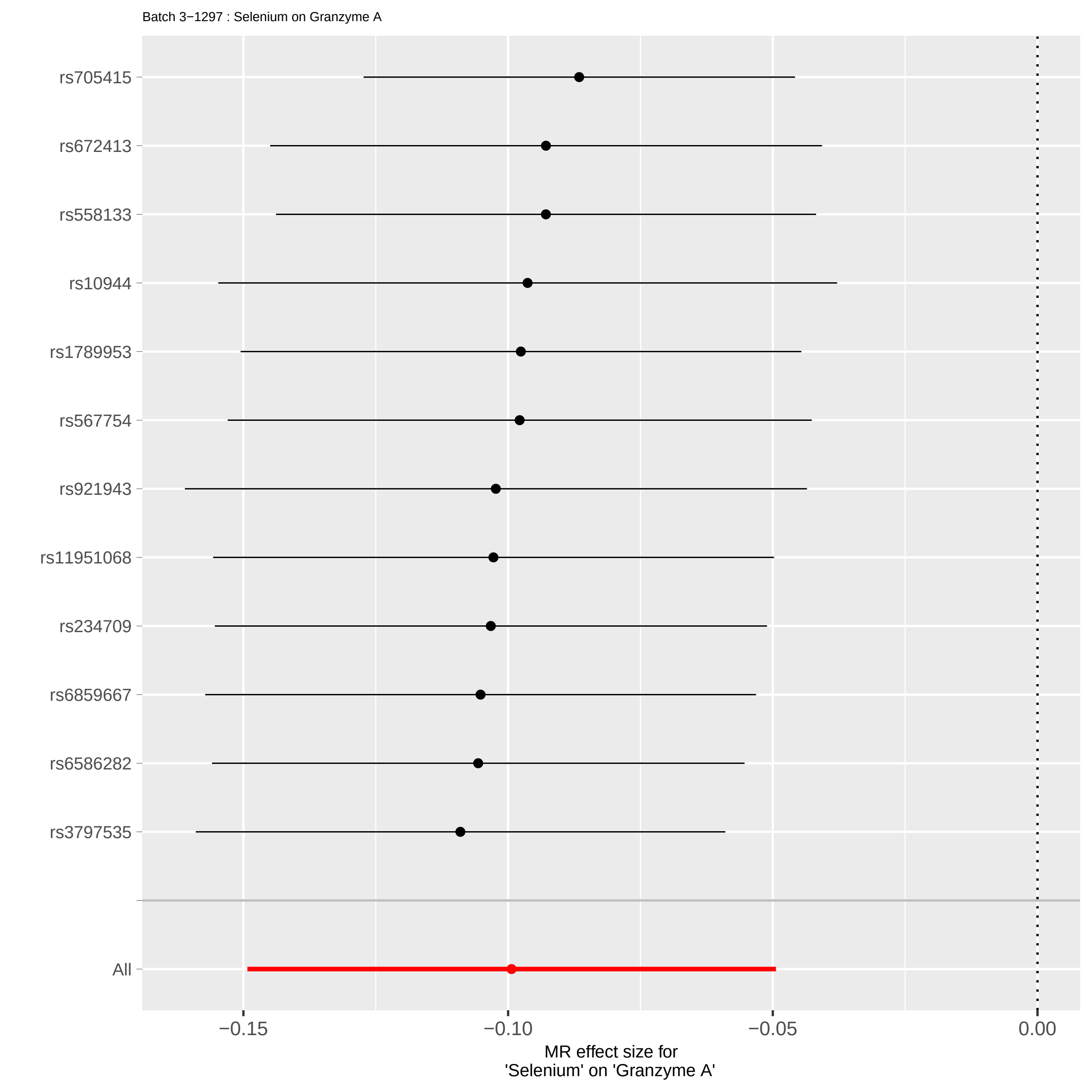

Batch 3−1297 : Selenium on Granzyme A
rs705415
rs672413
rs558133
rs10944
rs1789953
rs567754
rs921943
rs11951068
rs234709
rs6859667
rs6586282
rs3797535
All
−0.15
−0.10
−0.05
0.00
MR effect size for
'Selenium' on 'Granzyme A'

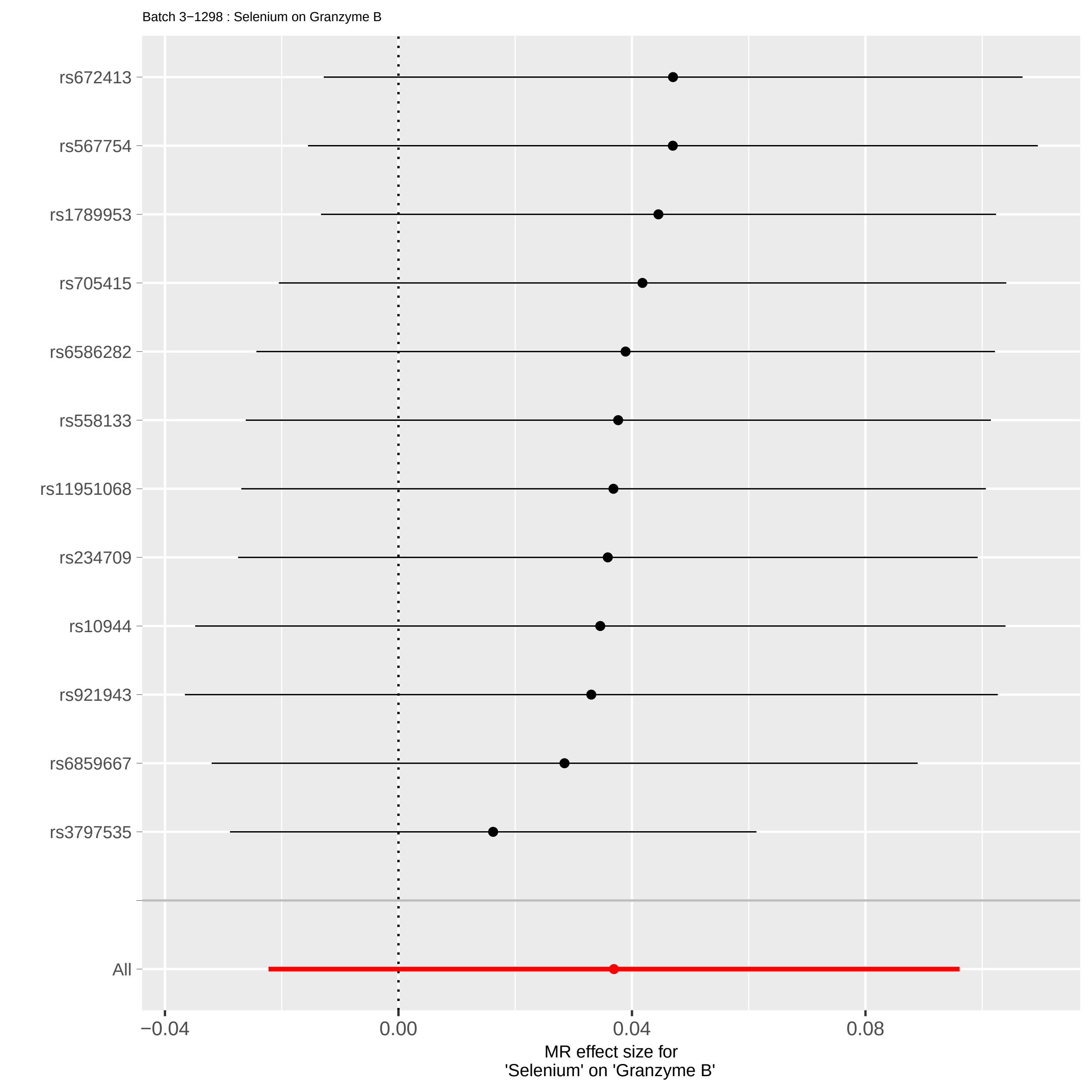

Batch 3−1298 : Selenium on Granzyme B
rs672413
rs567754
rs1789953
rs705415
rs6586282
rs558133
rs11951068
rs234709
rs10944
rs921943
rs6859667
rs3797535
All
−0.04
0.00
0.04
0.08
MR effect size for
'Selenium' on 'Granzyme B'

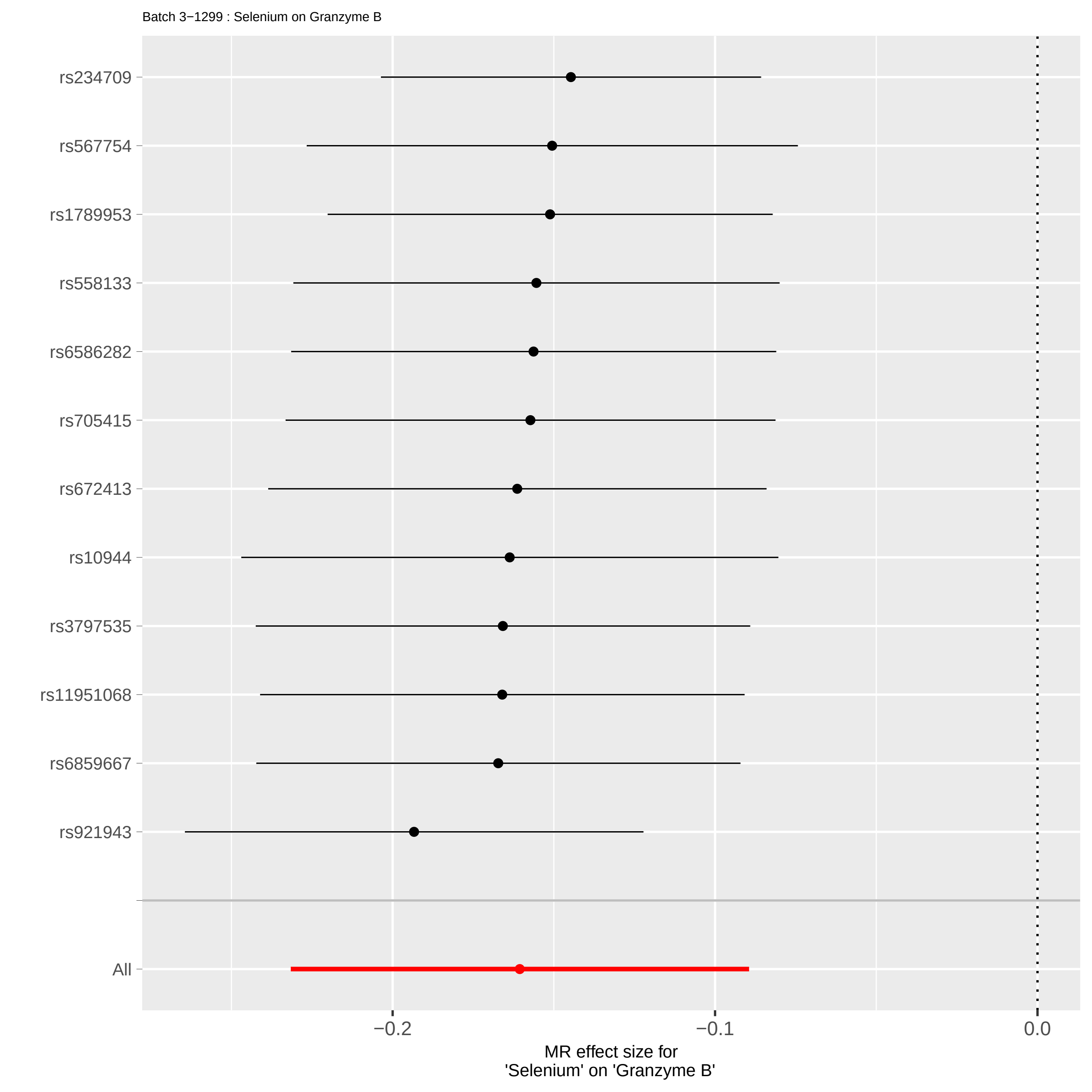

Batch 3−1299 : Selenium on Granzyme B
rs234709
rs567754
rs1789953
rs558133
rs6586282
rs705415
rs672413
rs10944
rs3797535
rs11951068
rs6859667
rs921943
All
−0.2
−0.1
0.0
MR effect size for
'Selenium' on 'Granzyme B'

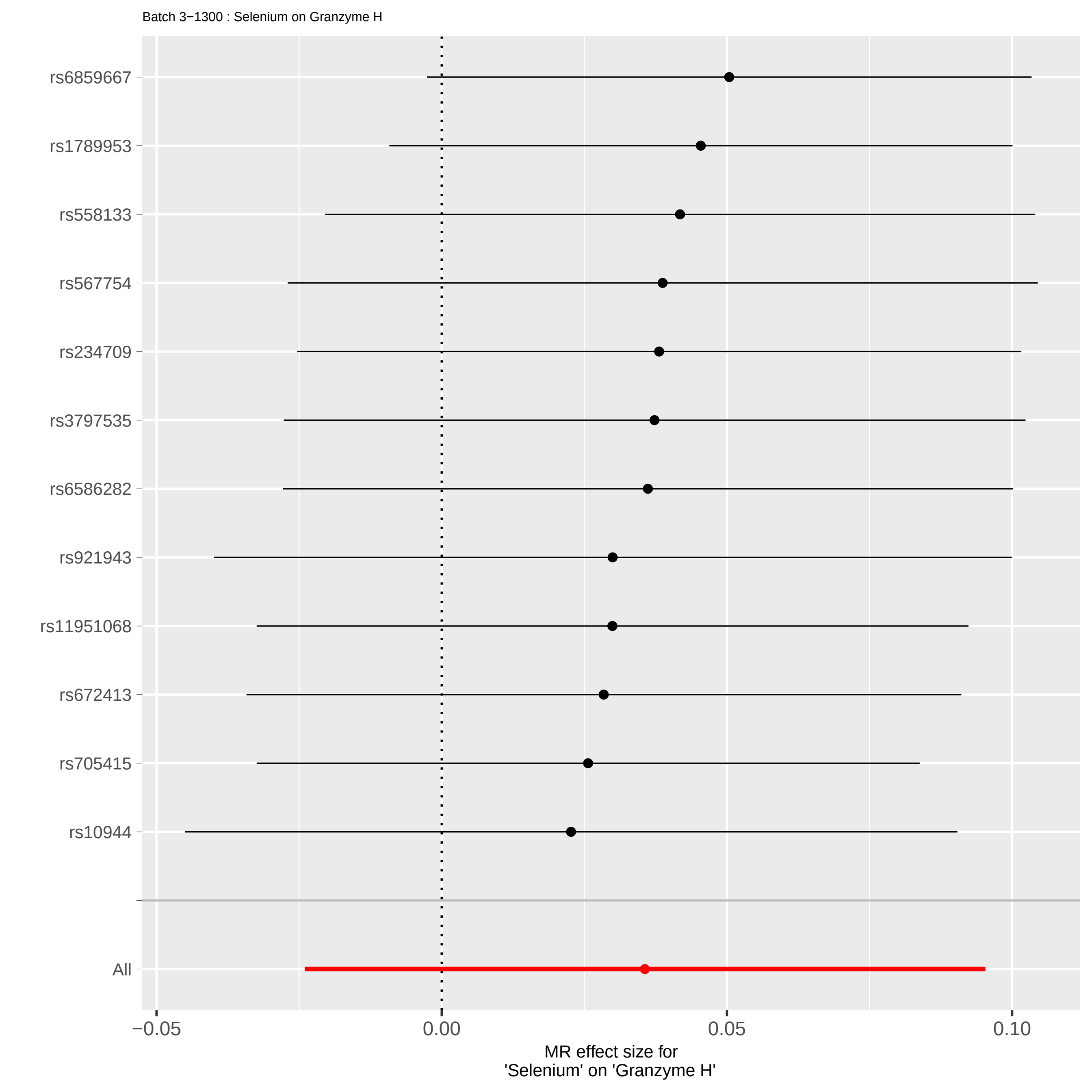

Batch 3−1300 : Selenium on Granzyme H
rs6859667
rs1789953
rs558133
rs567754
rs234709
rs3797535
rs6586282
rs921943
rs11951068
rs672413
rs705415
rs10944
All
−0.05
0.00
0.05
0.10
MR effect size for
'Selenium' on 'Granzyme H'

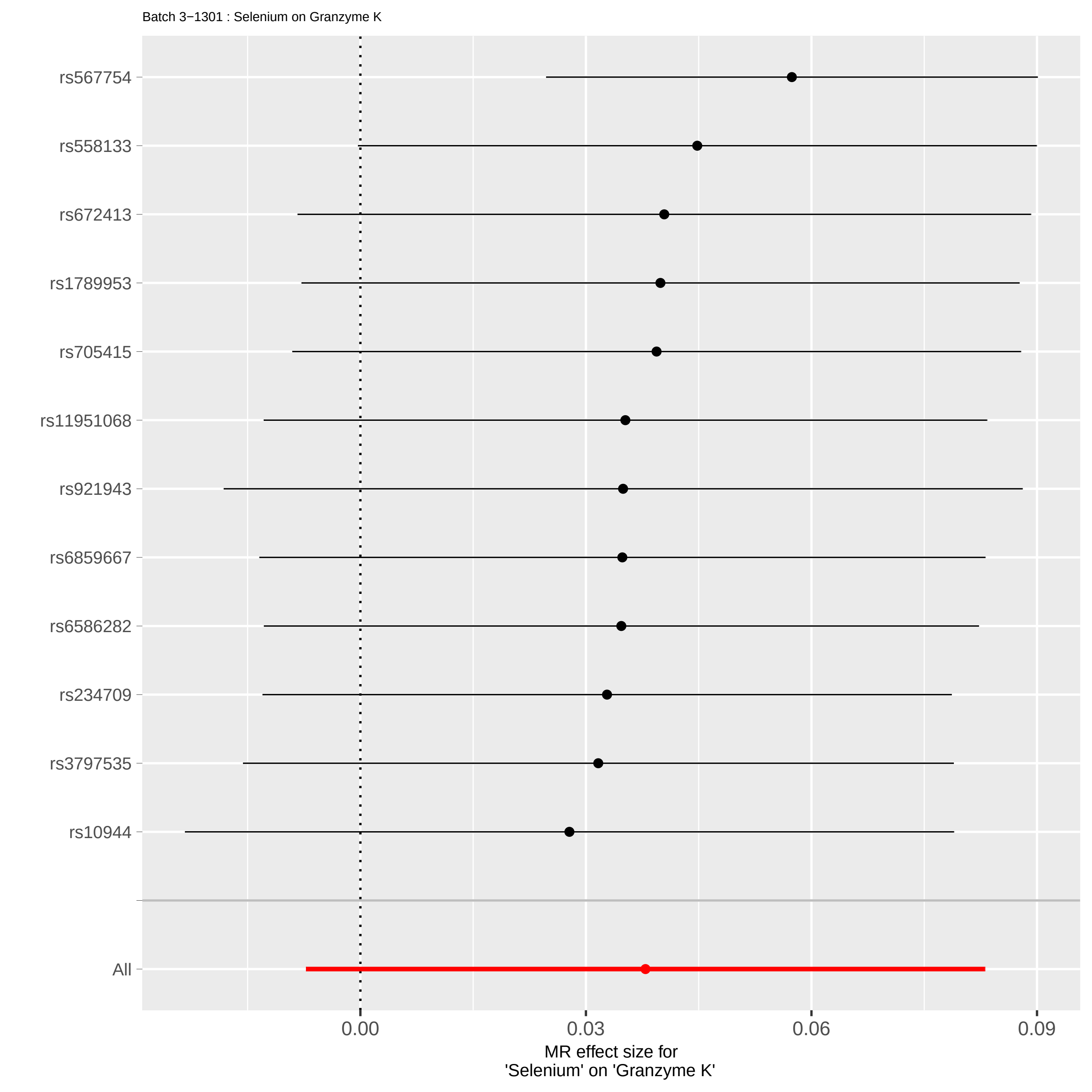
Batch 3−1301 : Selenium on Granzyme K
rs567754
rs558133
rs672413
rs1789953
rs705415
rs11951068
rs921943
rs6859667
rs6586282
rs234709
rs3797535
rs10944
All
0.00
0.03
0.06
0.09
MR effect size for
'Selenium' on 'Granzyme K'

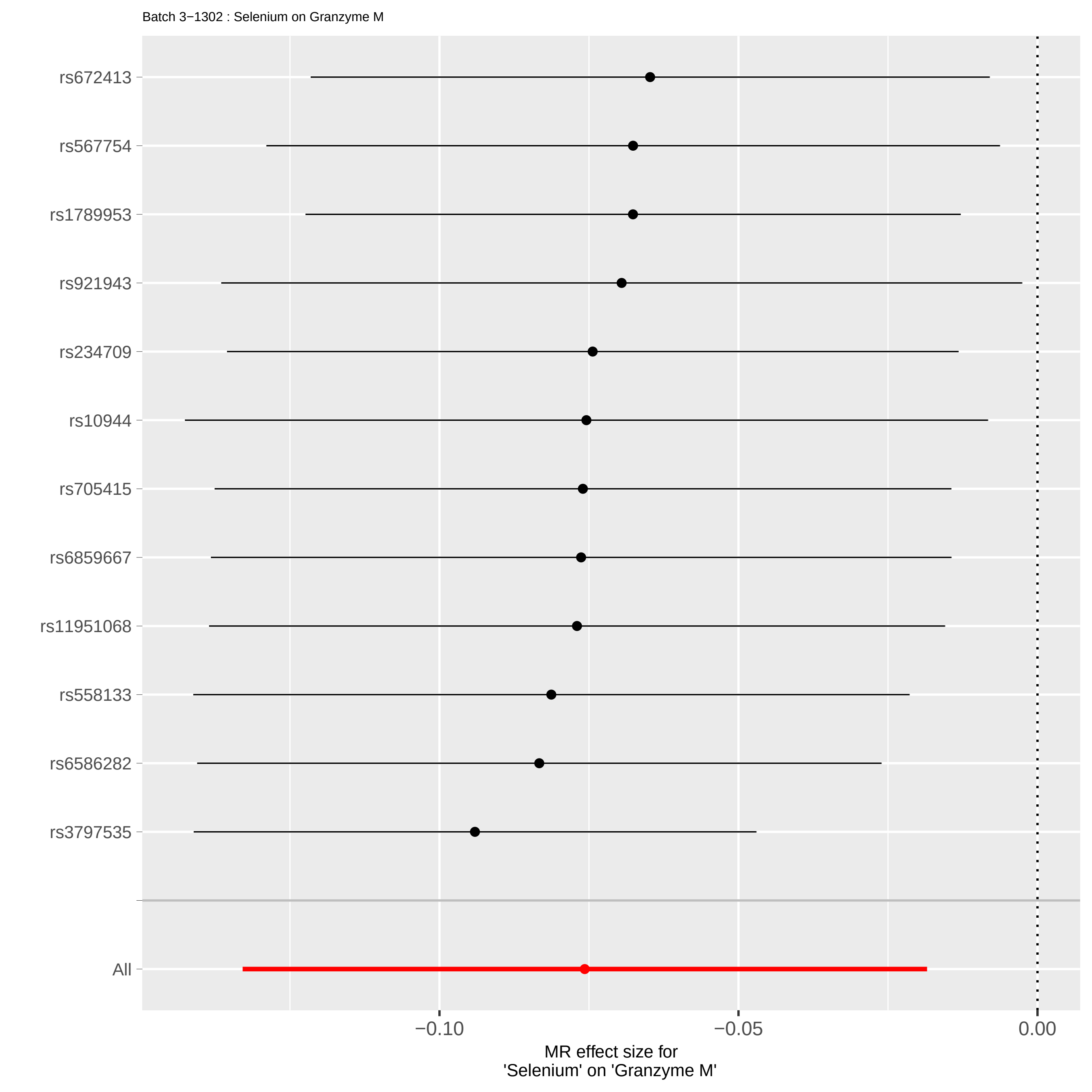

Batch 3−1302 : Selenium on Granzyme M
rs672413
rs567754
rs1789953
rs921943
rs234709
rs10944
rs705415
rs6859667
rs11951068
rs558133
rs6586282
rs3797535
All
−0.10
−0.05
0.00
MR effect size for
'Selenium' on 'Granzyme M'

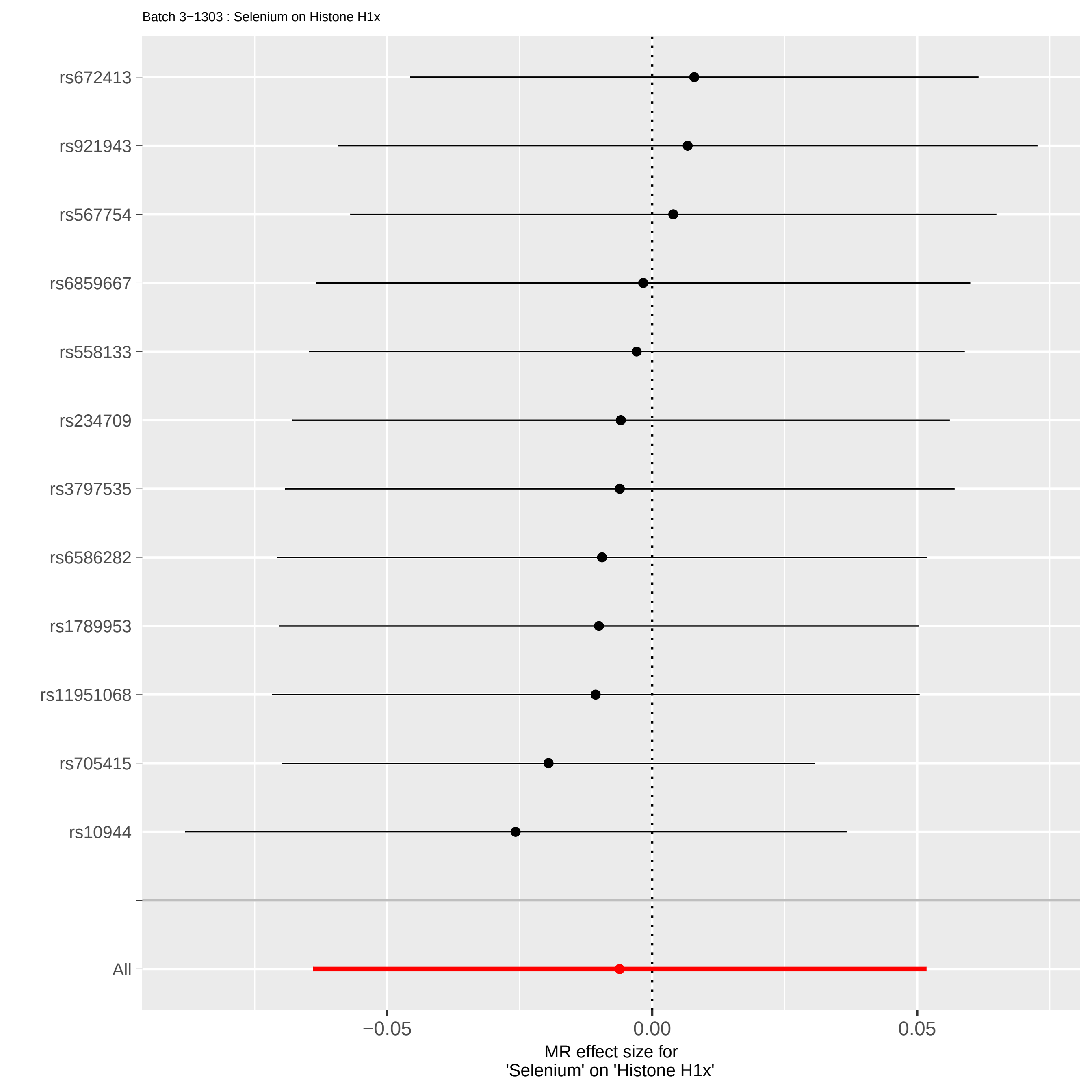

Batch 3−1303 : Selenium on Histone H1x
rs672413
rs921943
rs567754
rs6859667
rs558133
rs234709
rs3797535
rs6586282
rs1789953
rs11951068
rs705415
rs10944
All
−0.05
0.00
0.05
MR effect size for
'Selenium' on 'Histone H1x'

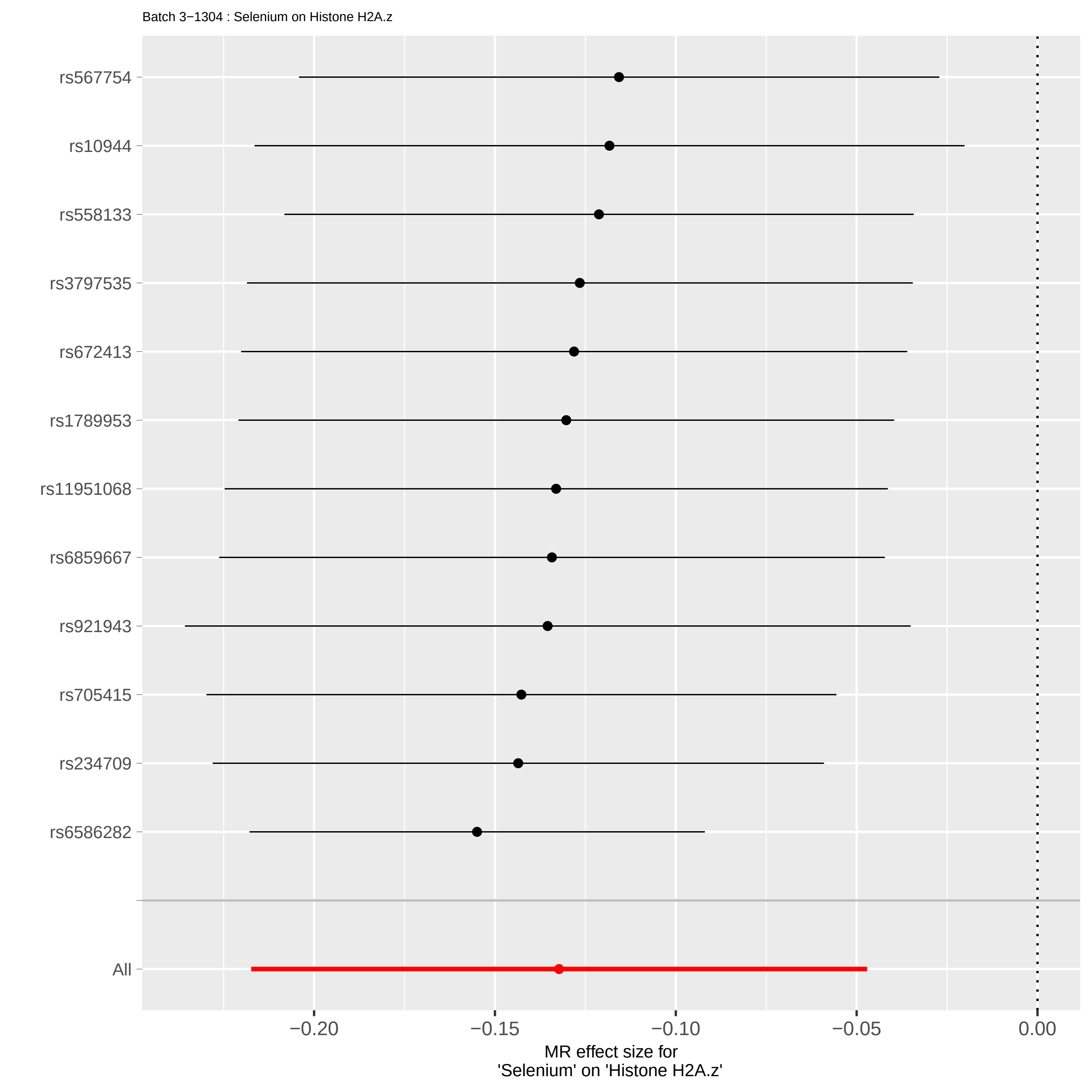

Batch 3−1304 : Selenium on Histone H2A.z
rs567754
rs10944
rs558133
rs3797535
rs672413
rs1789953
rs11951068
rs6859667
rs921943
rs705415
rs234709
rs6586282
All
−0.20
−0.15
−0.10
−0.05
0.00
MR effect size for
'Selenium' on 'Histone H2A.z'

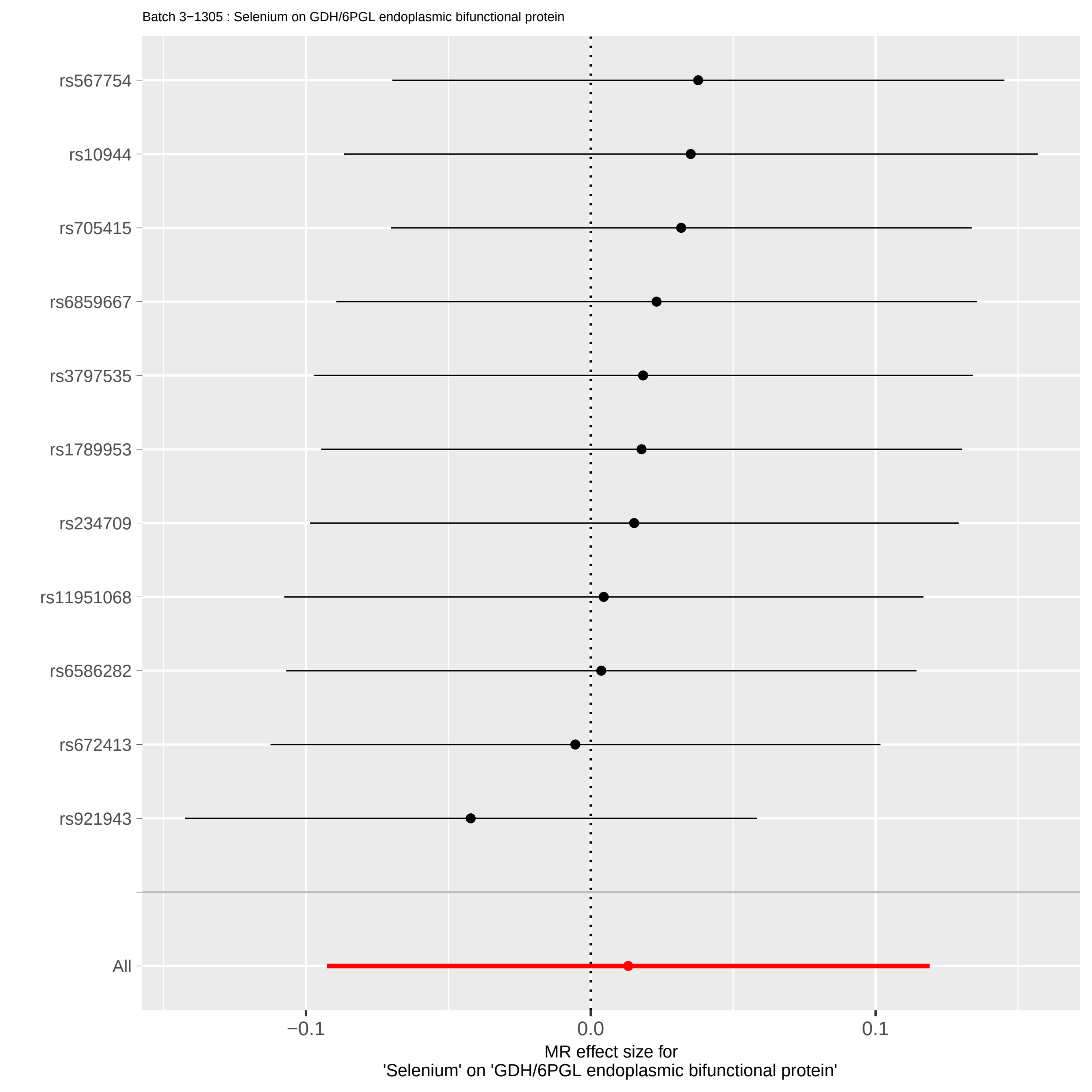

Batch 3−1305 : Selenium on GDH/6PGL endoplasmic bifunctional protein
rs567754
rs10944
rs705415
rs6859667
rs3797535
rs1789953
rs234709
rs11951068
rs6586282
rs672413
rs921943
All
−0.1
0.0
0.1
MR effect size for
'Selenium' on 'GDH/6PGL endoplasmic bifunctional protein'

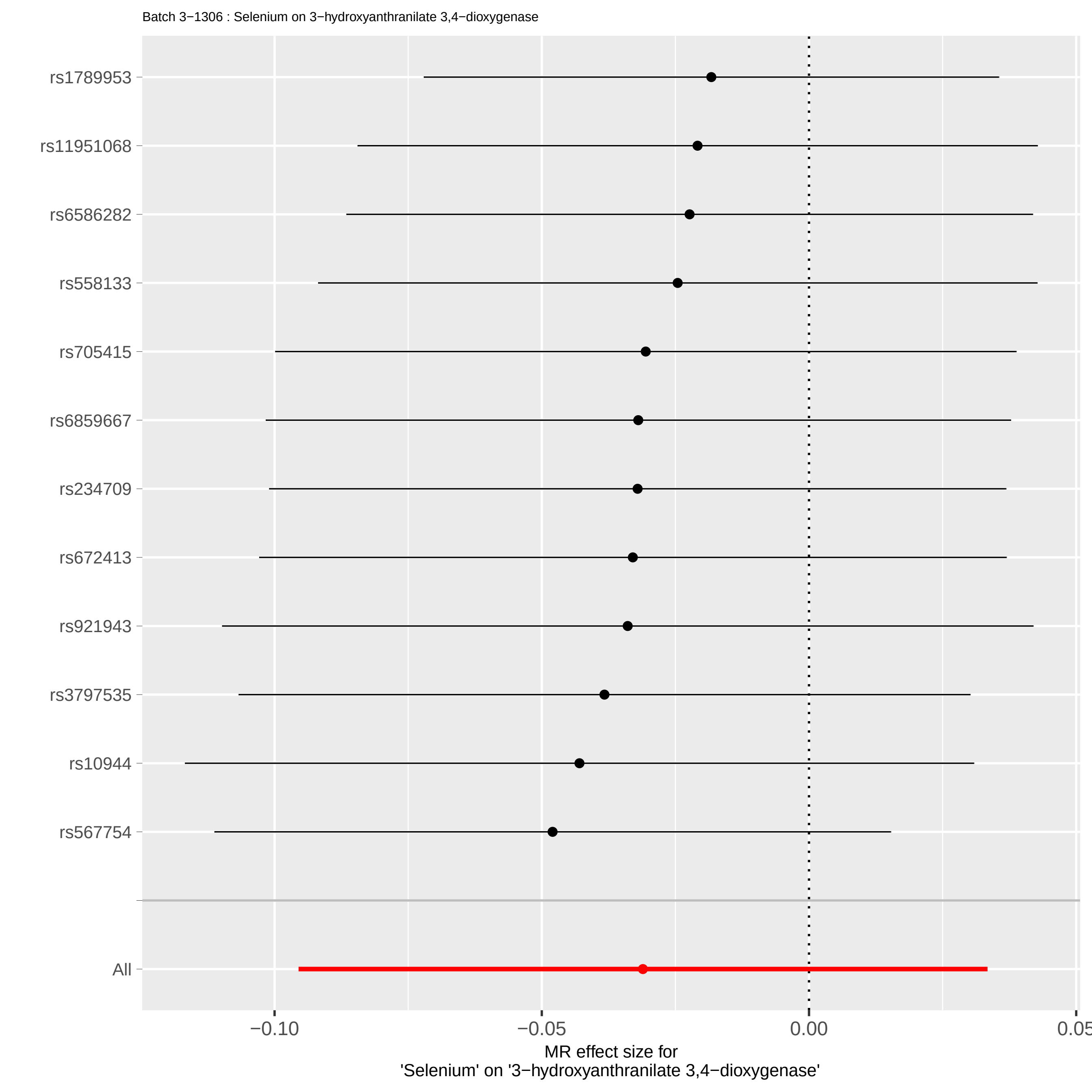
Batch 3−1306 : Selenium on 3−hydroxyanthranilate 3,4−dioxygenase
rs1789953
rs11951068
rs6586282
rs558133
rs705415
rs6859667
rs234709
rs672413
rs921943
rs3797535
rs10944
rs567754
All
−0.10
−0.05
0.00
0.05
MR effect size for
'Selenium' on '3−hydroxyanthranilate 3,4−dioxygenase'

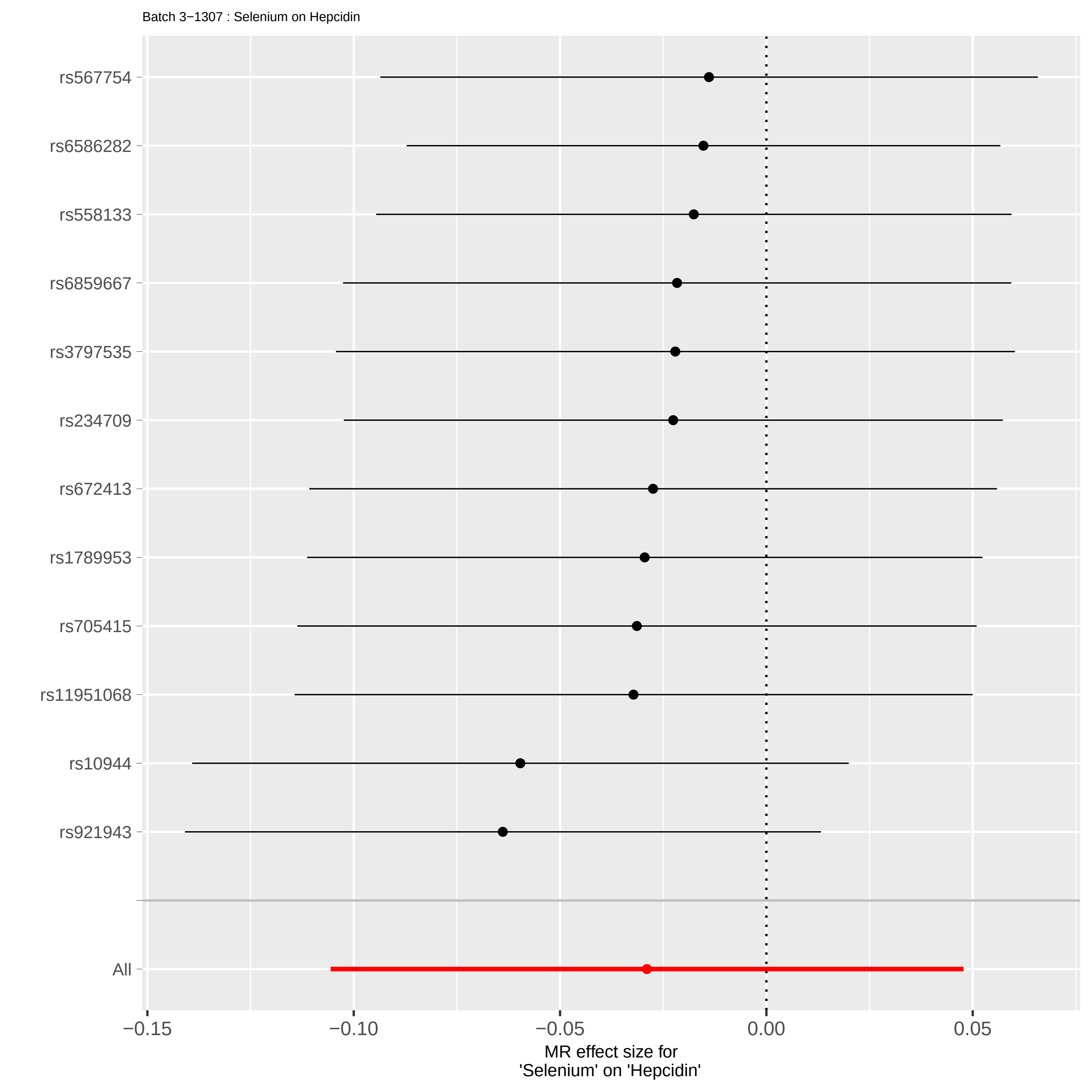

Batch 3−1307 : Selenium on Hepcidin
rs567754
rs6586282
rs558133
rs6859667
rs3797535
rs234709
rs672413
rs1789953
rs705415
rs11951068
rs10944
rs921943
All
−0.15
−0.10
−0.05
0.00
0.05
MR effect size for
'Selenium' on 'Hepcidin'

Batch 3−1308 : Selenium on Hyaluronan and proteoglycan link protein 1

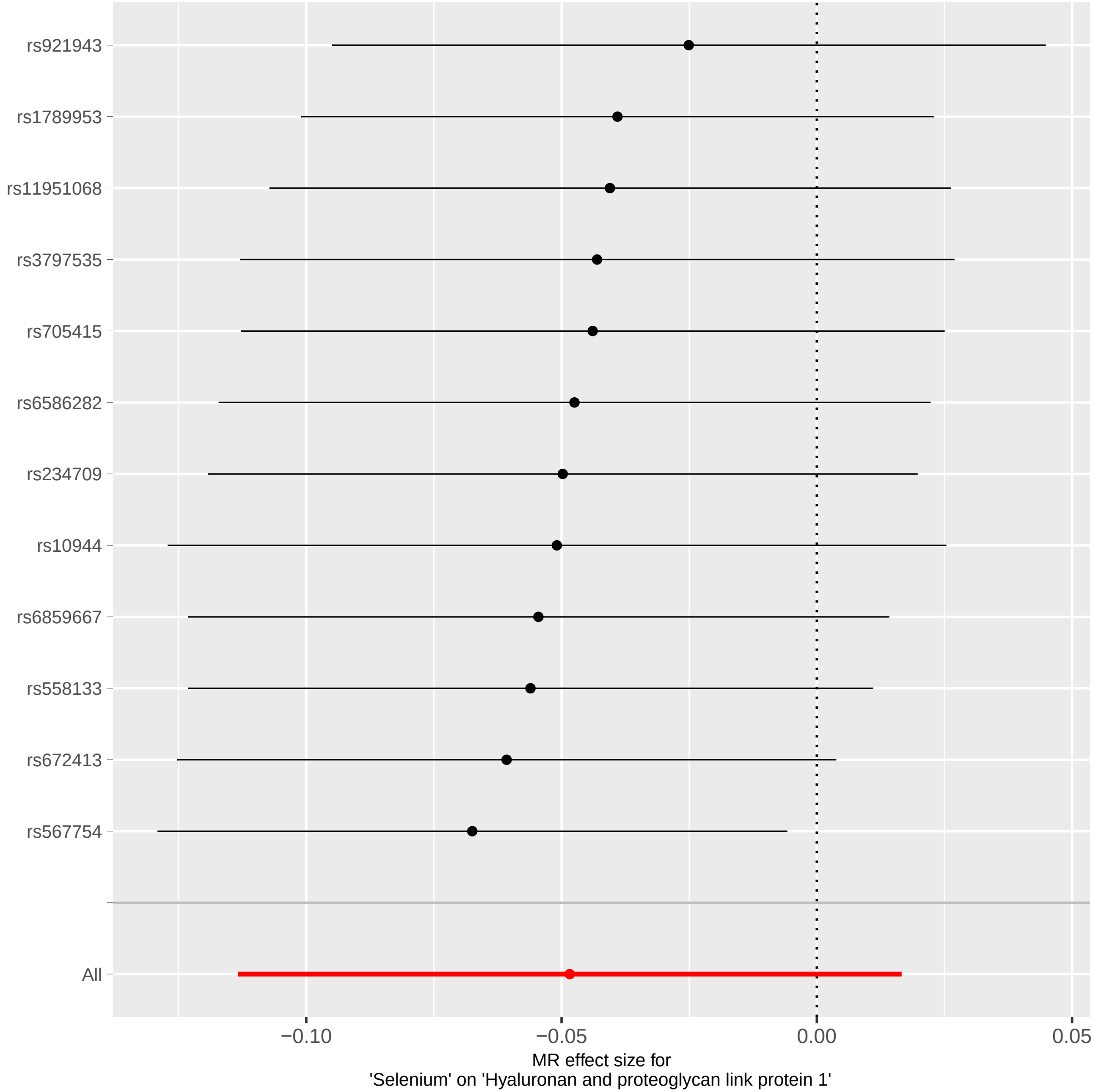

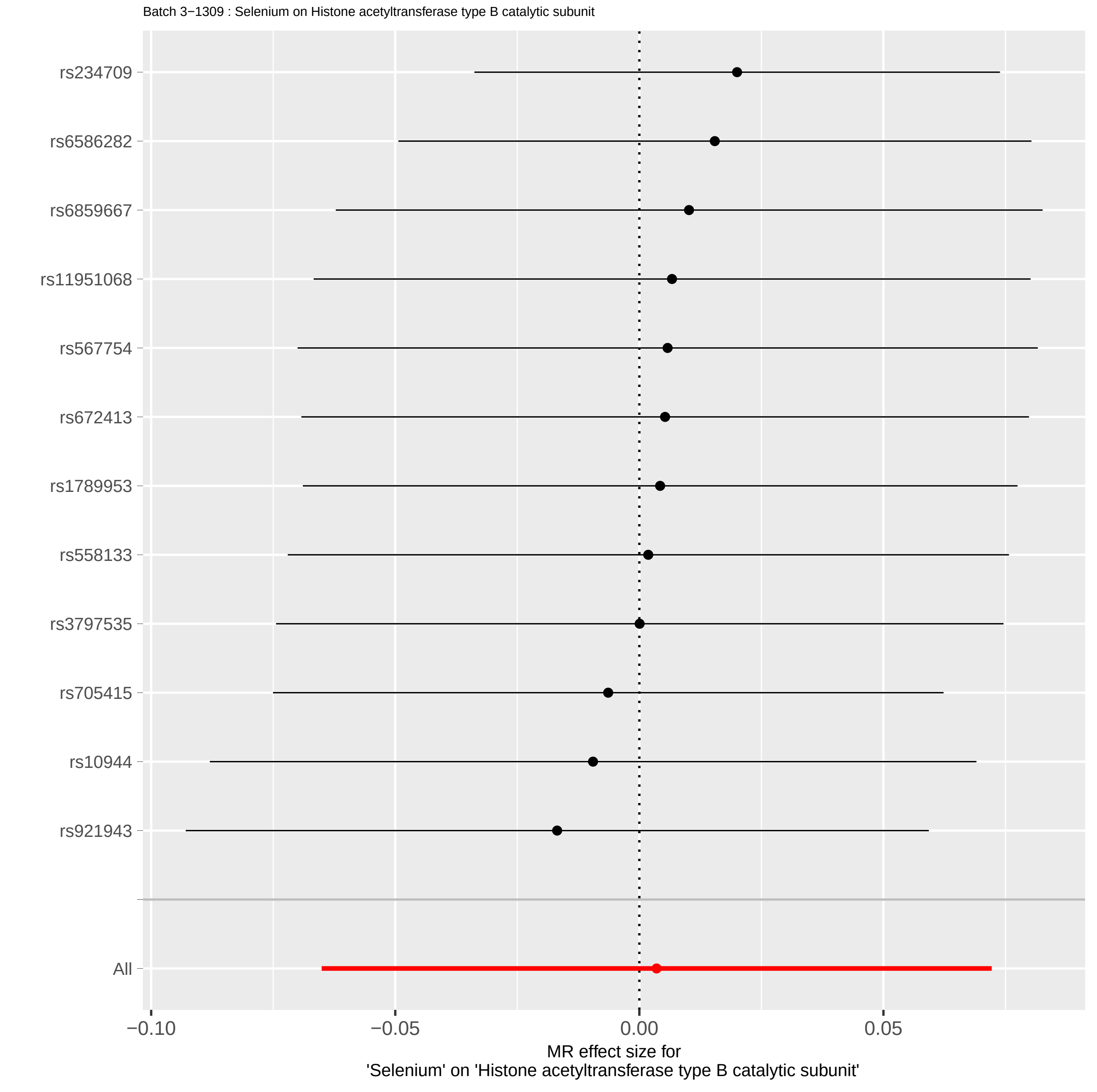
Batch 3−1309 : Selenium on Histone acetyltransferase type B catalytic subunit
rs234709
rs6586282
rs6859667
rs11951068
rs567754
rs672413
rs1789953
rs558133
rs3797535
rs705415
rs10944
rs921943
All
−0.10
−0.05
0.00
0.05
MR effect size for
'Selenium' on 'Histone acetyltransferase type B catalytic subunit'

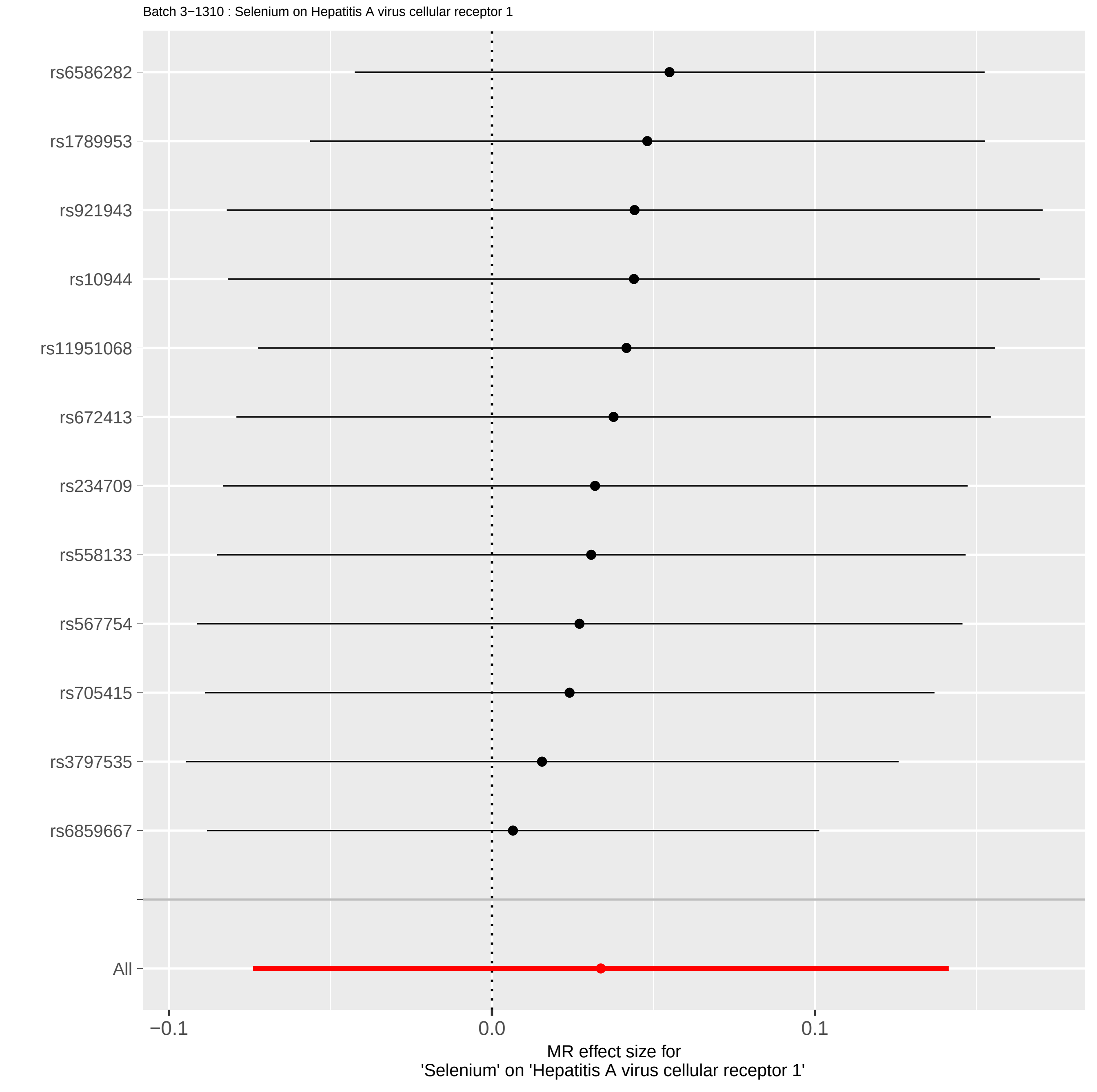

Batch 3−1310 : Selenium on Hepatitis A virus cellular receptor 1
rs6586282
rs1789953
rs921943
rs10944
rs11951068
rs672413
rs234709
rs558133
rs567754
rs705415
rs3797535
rs6859667
All
−0.1
0.0
0.1
MR effect size for
'Selenium' on 'Hepatitis A virus cellular receptor 1'

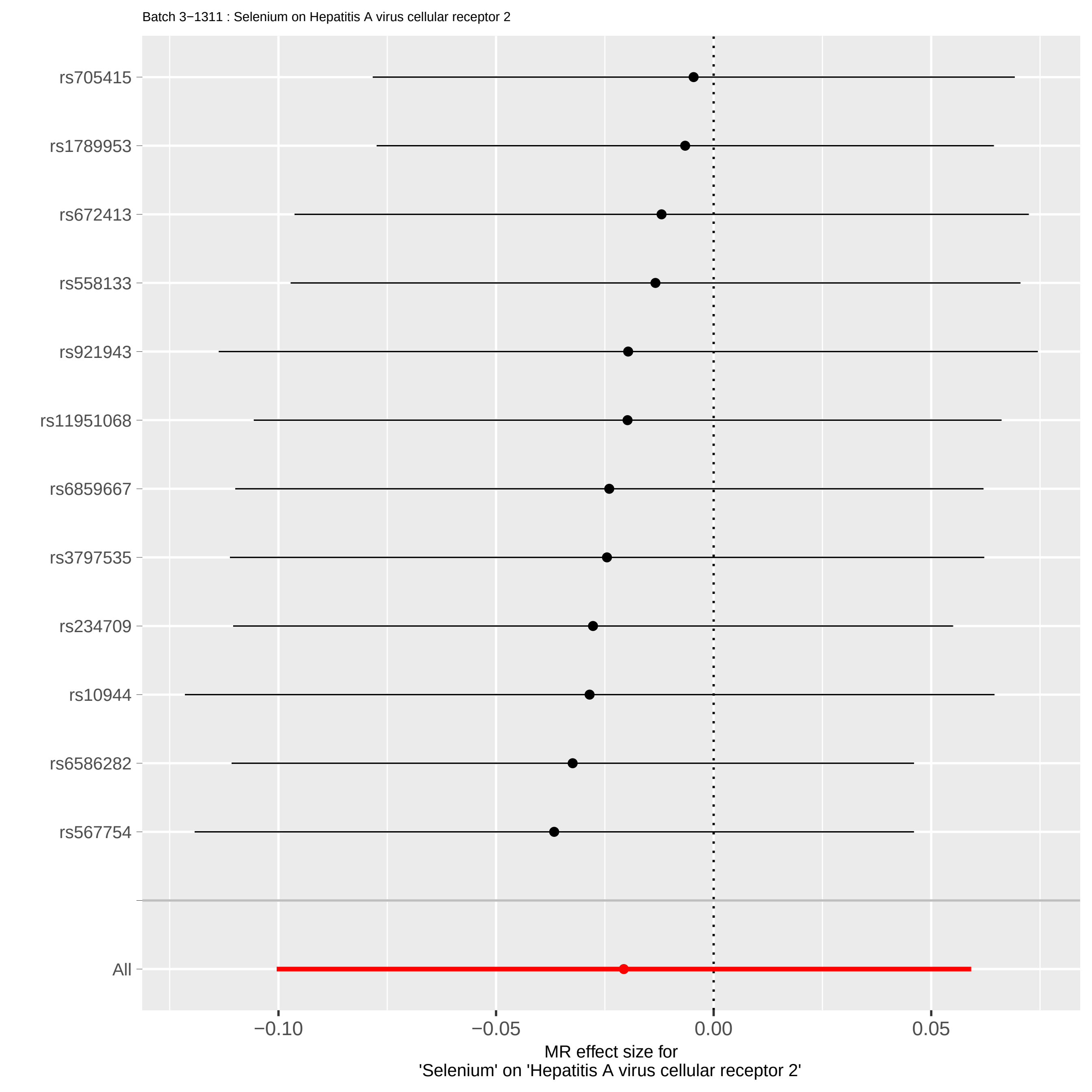

Batch 3−1311 : Selenium on Hepatitis A virus cellular receptor 2
rs705415
rs1789953
rs672413
rs558133
rs921943
rs11951068
rs6859667
rs3797535
rs234709
rs10944
rs6586282
rs567754
All
−0.10
−0.05
0.00
0.05
MR effect size for
'Selenium' on 'Hepatitis A virus cellular receptor 2'

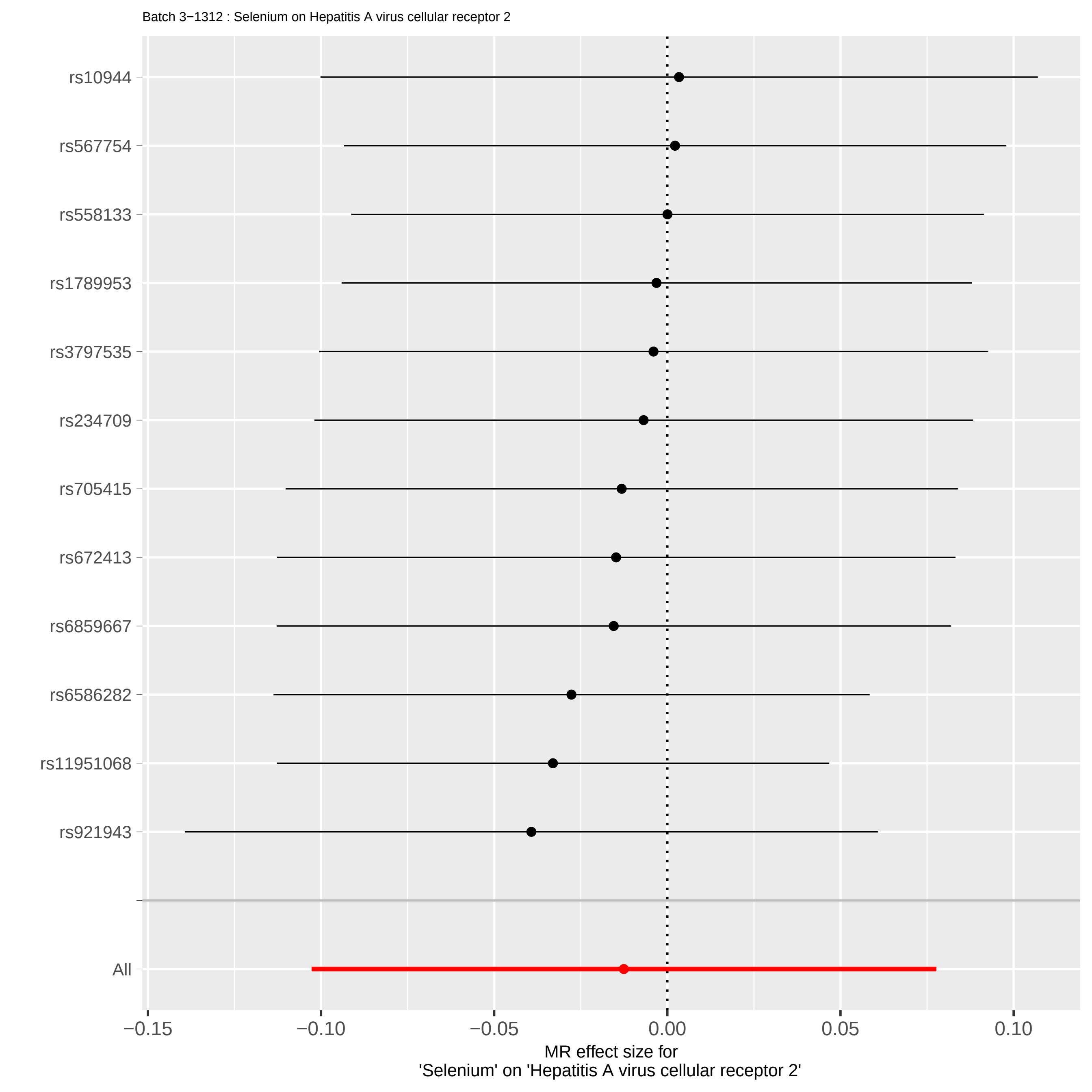
Batch 3−1312 : Selenium on Hepatitis A virus cellular receptor 2
rs10944
rs567754
rs558133
rs1789953
rs3797535
rs234709
rs705415
rs672413
rs6859667
rs6586282
rs11951068
rs921943
All
−0.15
−0.10
−0.05
0.00
0.05
0.10
MR effect size for
'Selenium' on 'Hepatitis A virus cellular receptor 2'

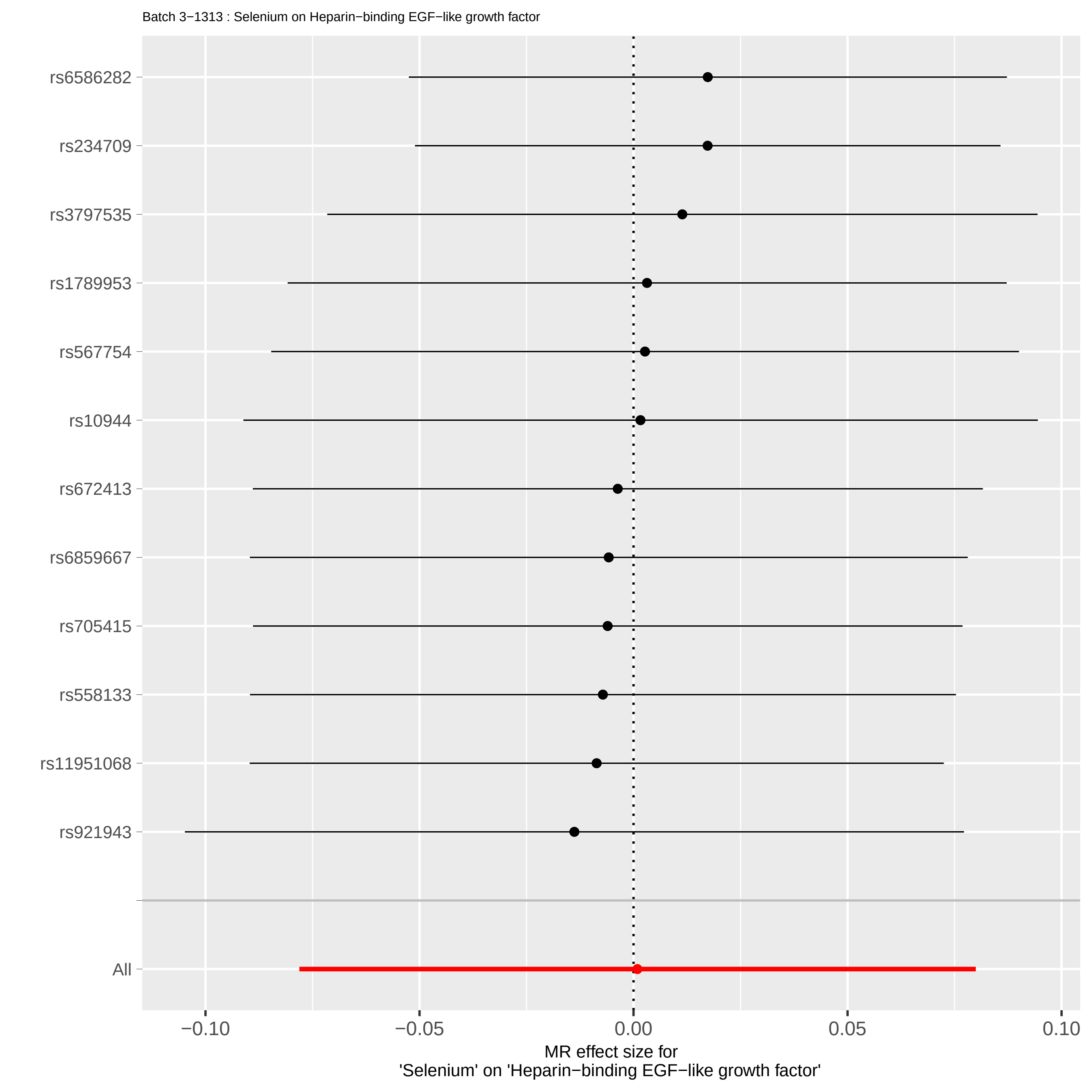

Batch 3−1313 : Selenium on Heparin−binding EGF−like growth factor
rs6586282
rs234709
rs3797535
rs1789953
rs567754
rs10944
rs672413
rs6859667
rs705415
rs558133
rs11951068
rs921943
All
−0.10
−0.05
0.00
0.05
0.10
MR effect size for
'Selenium' on 'Heparin−binding EGF−like growth factor'

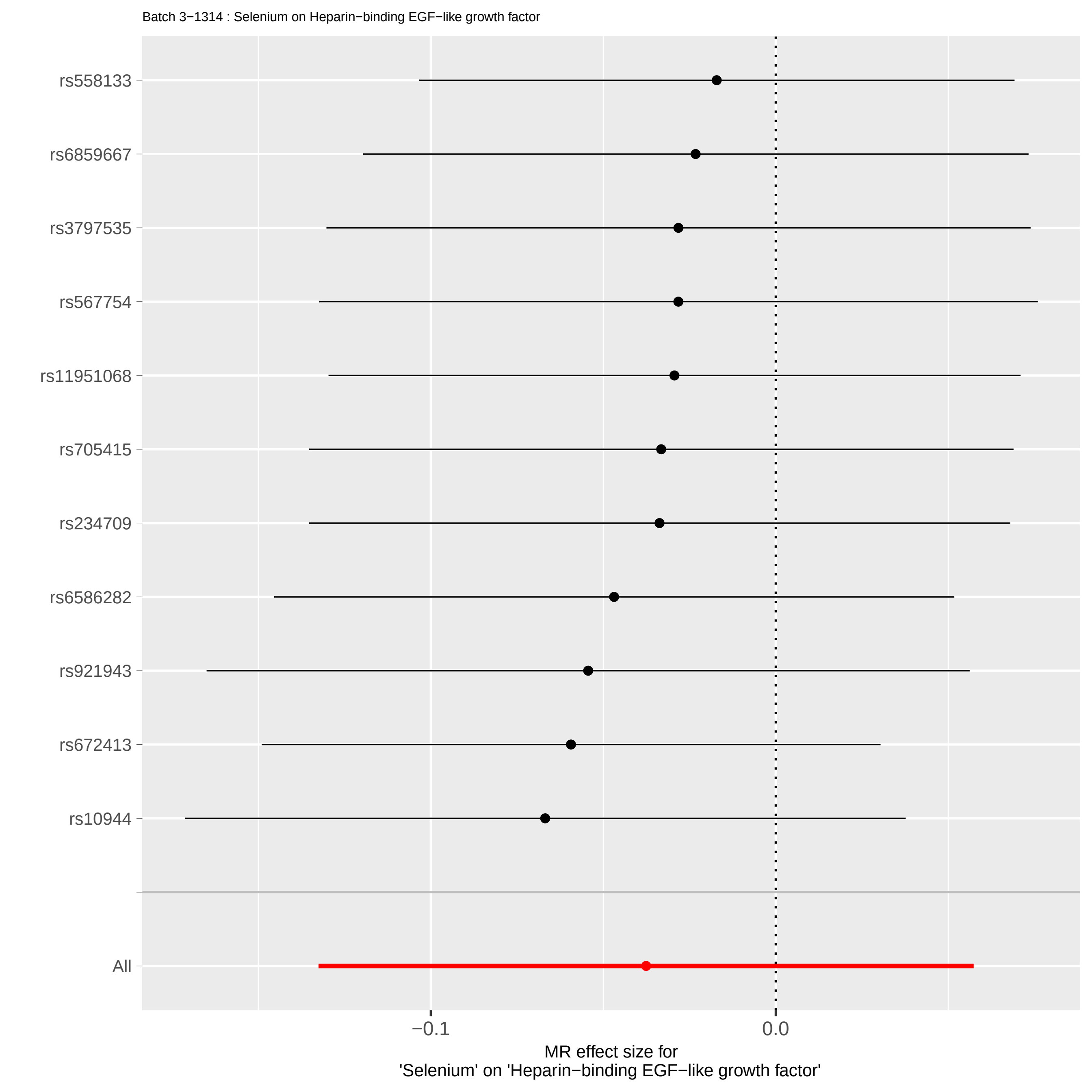

Batch 3−1314 : Selenium on Heparin−binding EGF−like growth factor
rs558133
rs6859667
rs3797535
rs567754
rs11951068
rs705415
rs234709
rs6586282
rs921943
rs672413
rs10944
All
−0.1
0.0
MR effect size for
'Selenium' on 'Heparin−binding EGF−like growth factor'

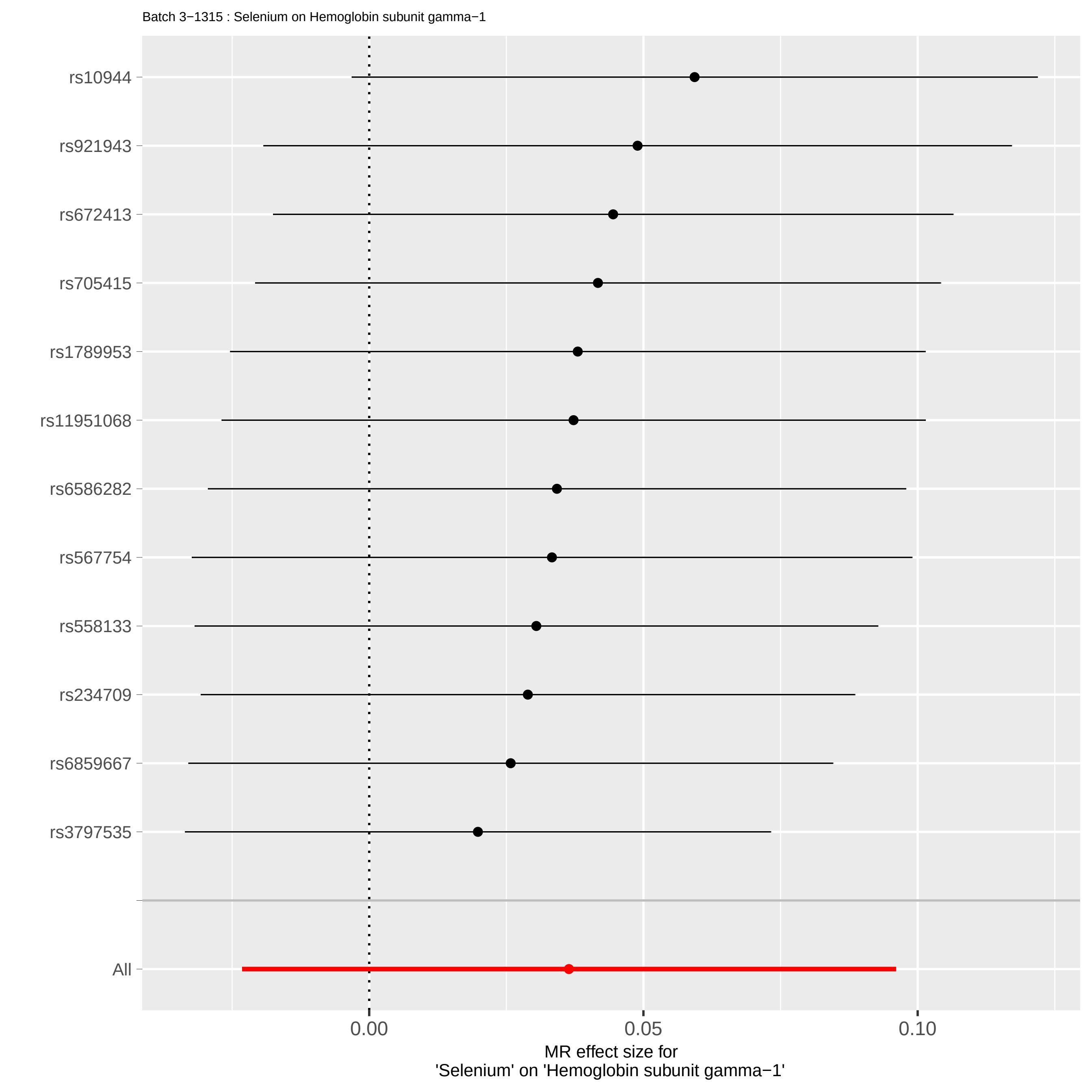

Batch 3−1315 : Selenium on Hemoglobin subunit gamma−1
rs10944
rs921943
rs672413
rs705415
rs1789953
rs11951068
rs6586282
rs567754
rs558133
rs234709
rs6859667
rs3797535
All
0.00
0.05
0.10
MR effect size for
'Selenium' on 'Hemoglobin subunit gamma−1'

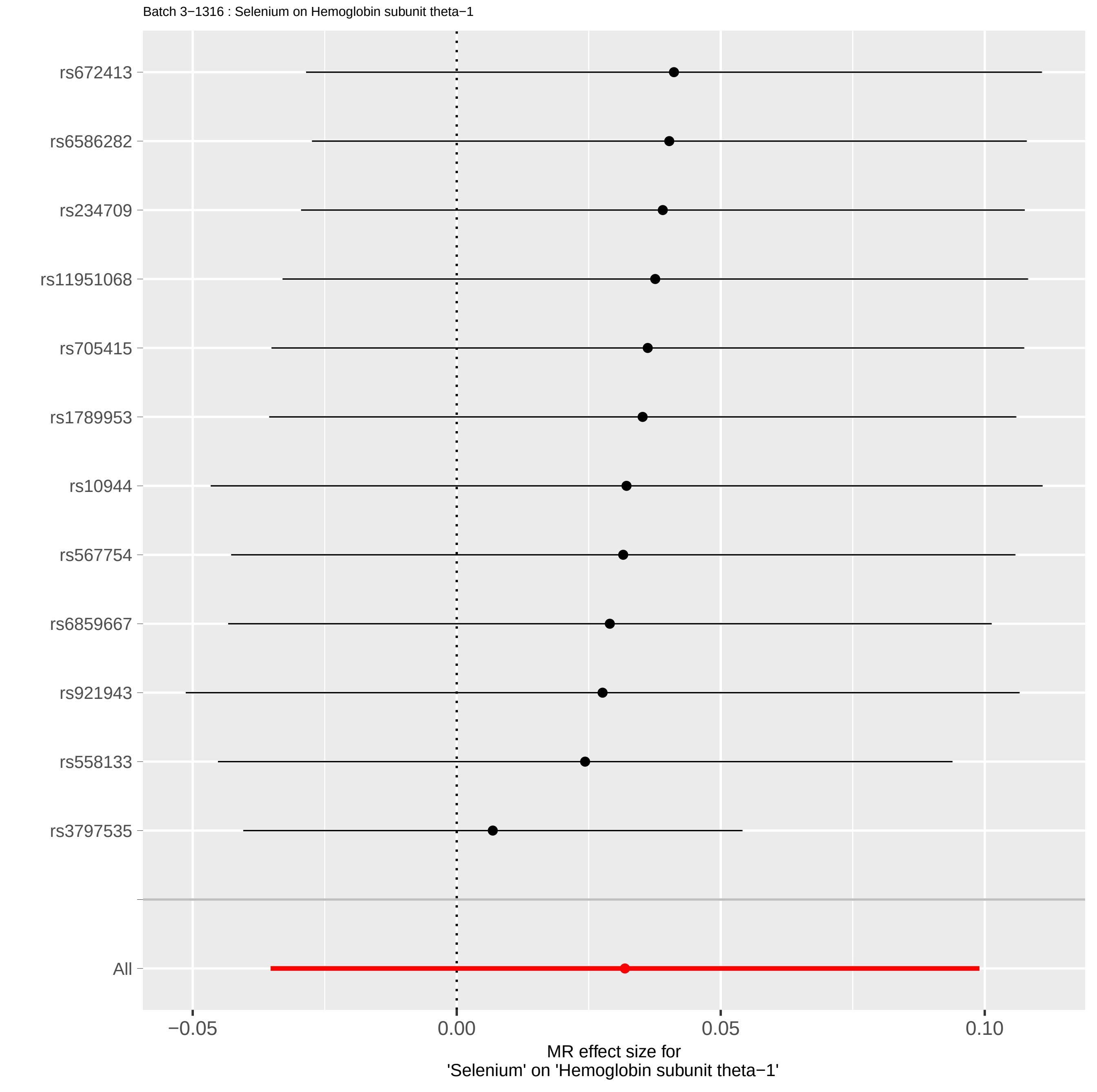

Batch 3−1316 : Selenium on Hemoglobin subunit theta−1
rs672413
rs6586282
rs234709
rs11951068
rs705415
rs1789953
rs10944
rs567754
rs6859667
rs921943
rs558133
rs3797535
All
−0.05
0.00
0.05
0.10
MR effect size for
'Selenium' on 'Hemoglobin subunit theta−1'

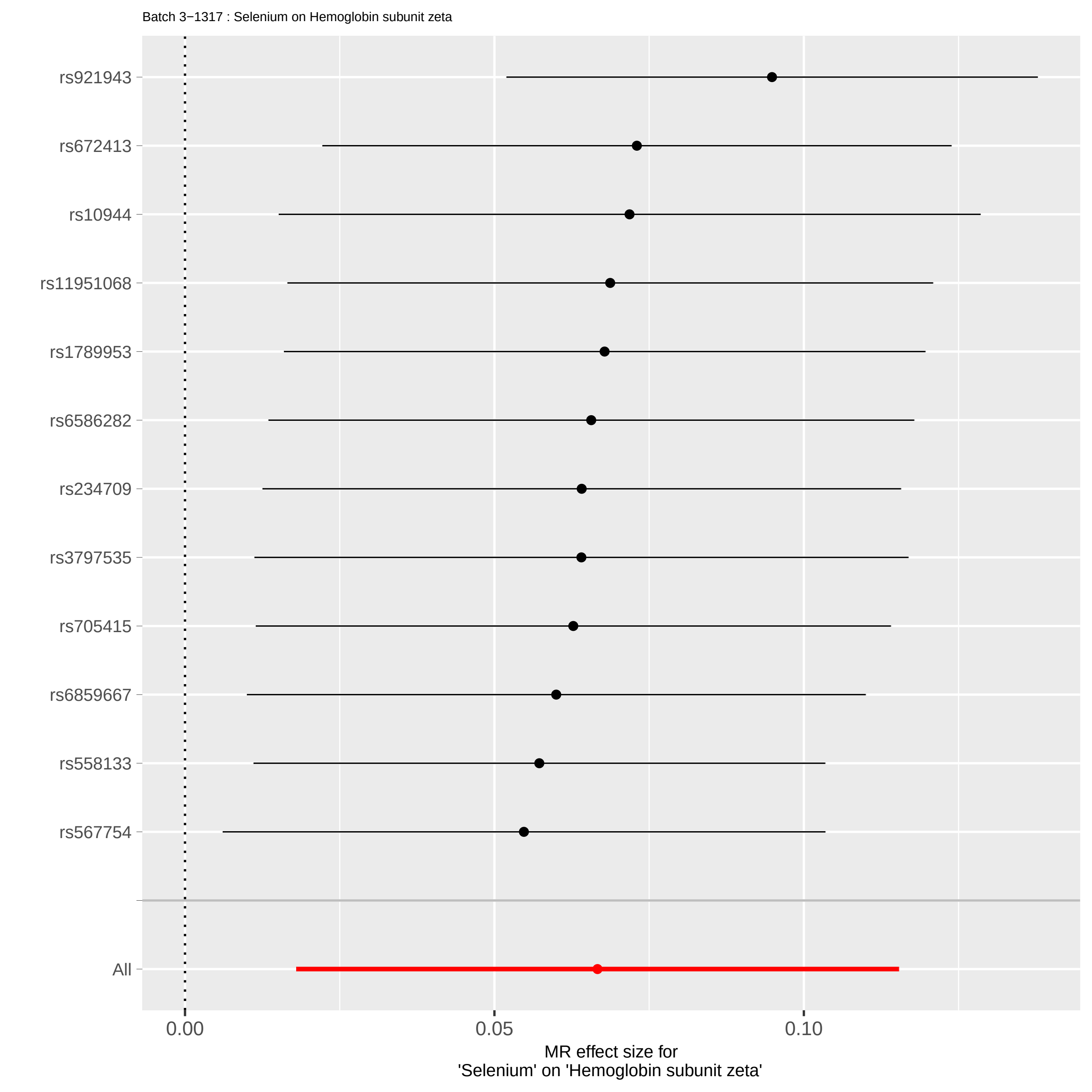

Batch 3−1317 : Selenium on Hemoglobin subunit zeta
rs921943
rs672413
rs10944
rs11951068
rs1789953
rs6586282
rs234709
rs3797535
rs705415
rs6859667
rs558133
rs567754
All
0.00
0.05
0.10
MR effect size for
'Selenium' on 'Hemoglobin subunit zeta'

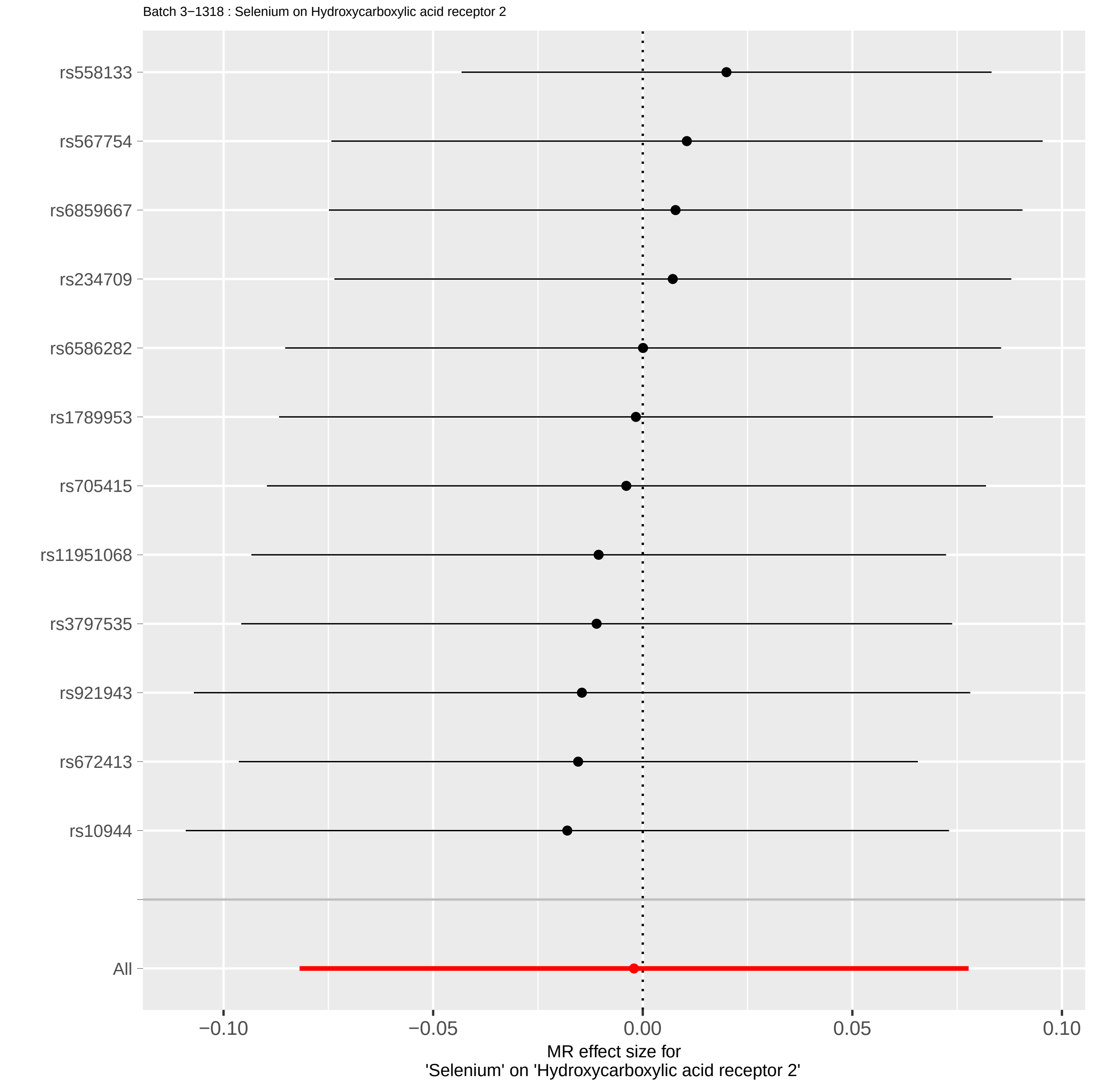
Batch 3−1318 : Selenium on Hydroxycarboxylic acid receptor 2
rs558133
rs567754
rs6859667
rs234709
rs6586282
rs1789953
rs705415
rs11951068
rs3797535
rs921943
rs672413
rs10944
All
−0.10
−0.05
0.00
0.05
0.10
MR effect size for
'Selenium' on 'Hydroxycarboxylic acid receptor 2'

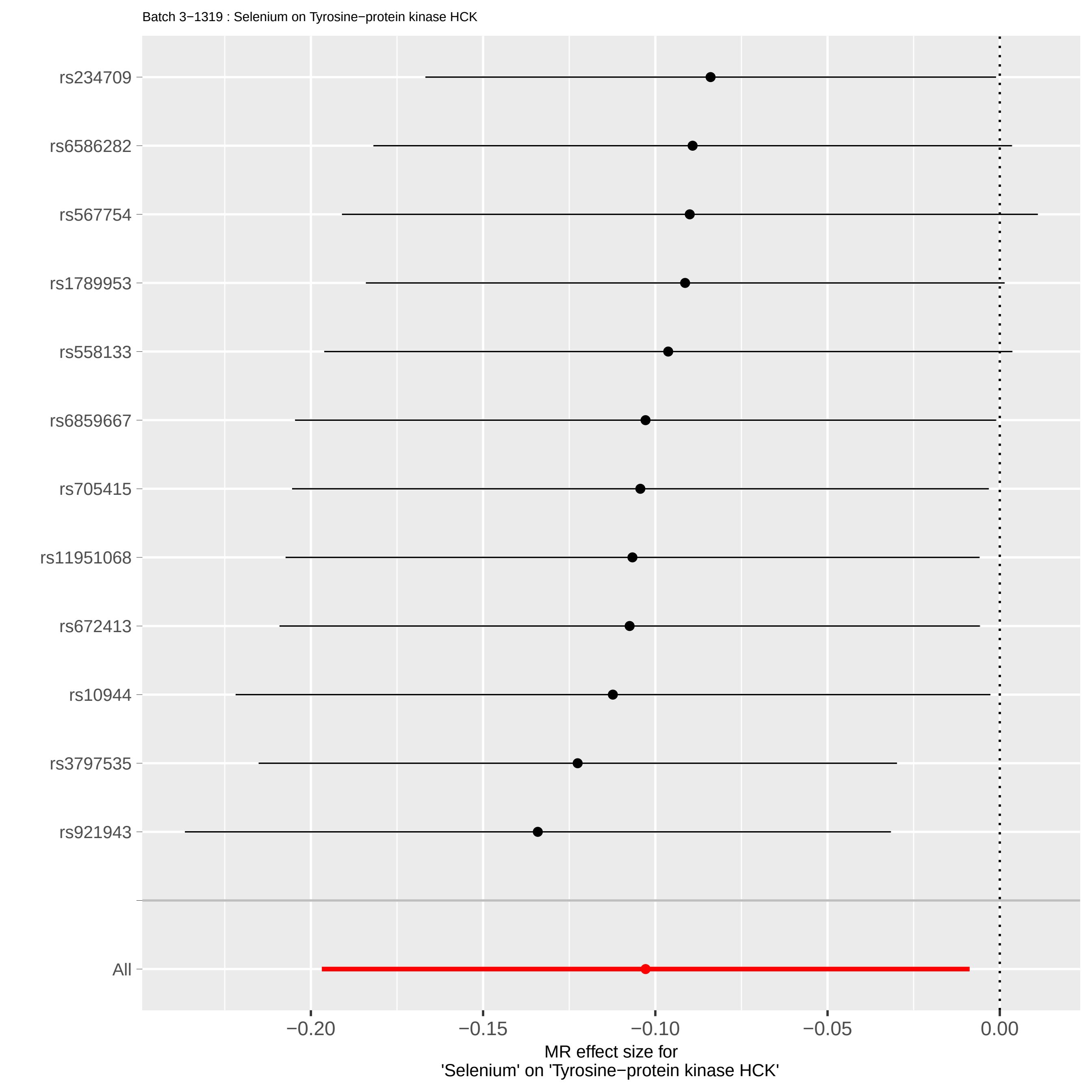

Batch 3−1319 : Selenium on Tyrosine−protein kinase HCK
rs234709
rs6586282
rs567754
rs1789953
rs558133
rs6859667
rs705415
rs11951068
rs672413
rs10944
rs3797535
rs921943
All
−0.20
−0.15
−0.10
−0.05
0.00
MR effect size for
'Selenium' on 'Tyrosine−protein kinase HCK'

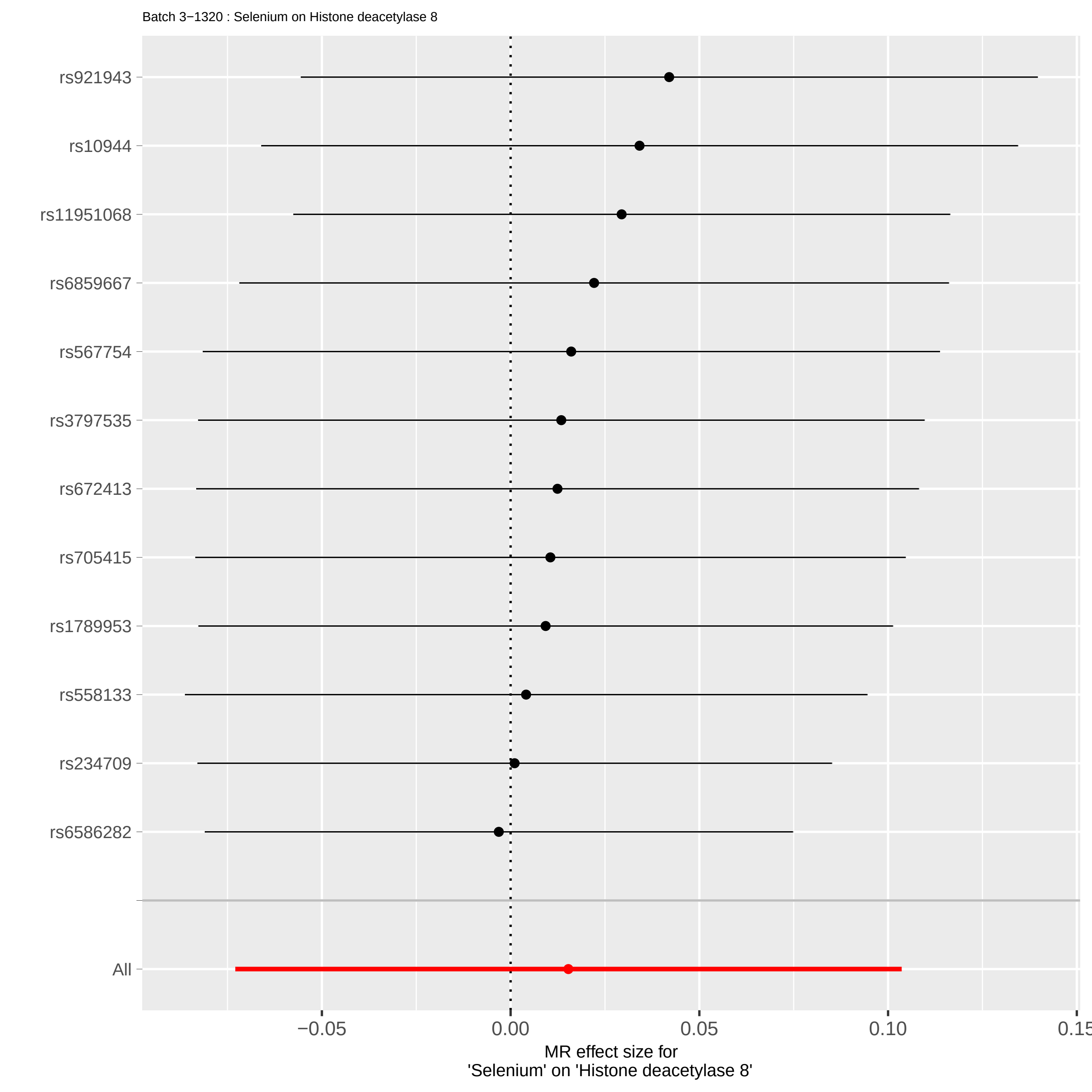

Batch 3−1320 : Selenium on Histone deacetylase 8
rs921943
rs10944
rs11951068
rs6859667
rs567754
rs3797535
rs672413
rs705415
rs1789953
rs558133
rs234709
rs6586282
All
−0.05
0.00
0.05
0.10
0.15
MR effect size for
'Selenium' on 'Histone deacetylase 8'

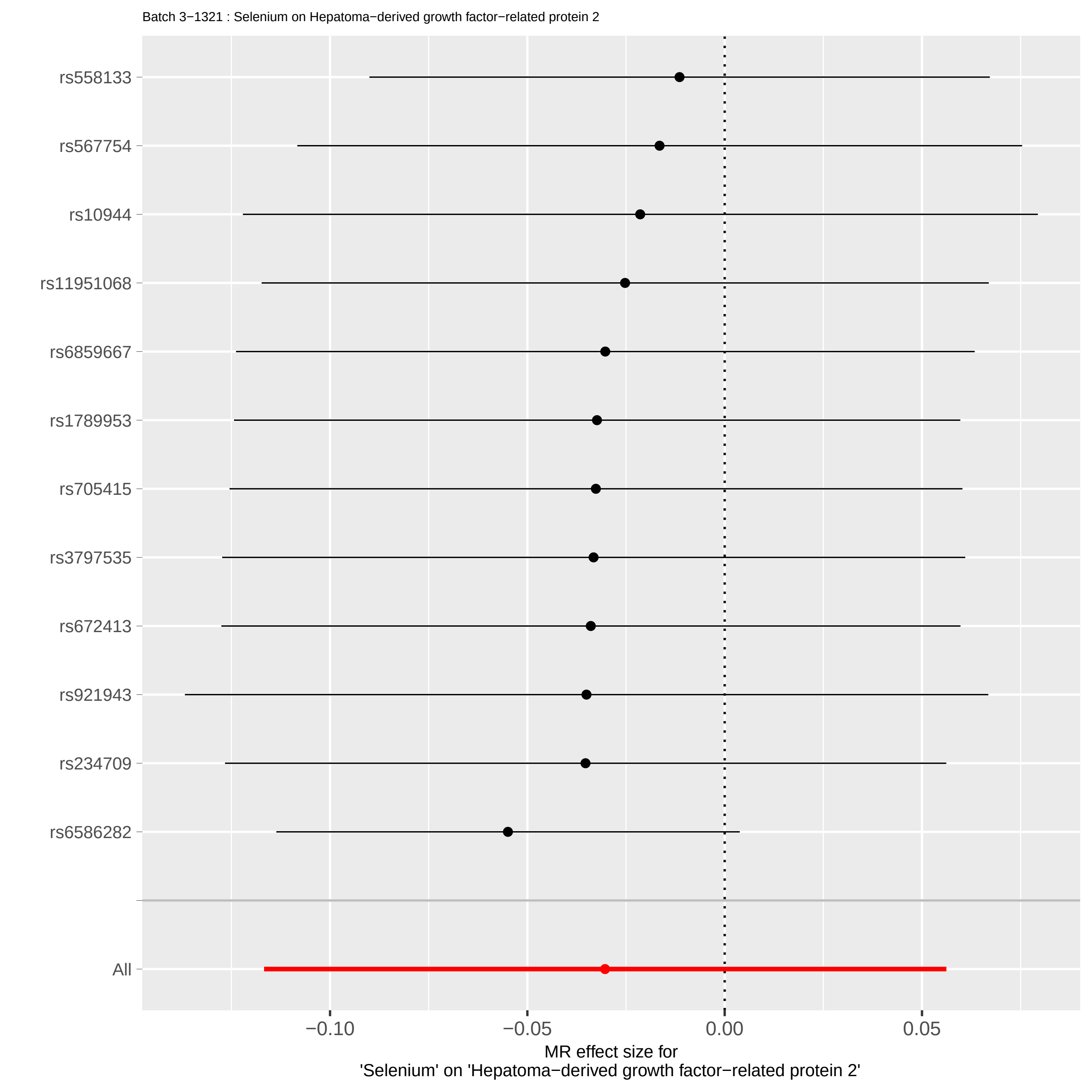

Batch 3−1321 : Selenium on Hepatoma−derived growth factor−related protein 2
rs558133
rs567754
rs10944
rs11951068
rs6859667
rs1789953
rs705415
rs3797535
rs672413
rs921943
rs234709
rs6586282
All
−0.10
−0.05
0.00
0.05
MR effect size for
'Selenium' on 'Hepatoma−derived growth factor−related protein 2'

Batch 3−1322 : Selenium on Haloacid dehalogenase−like hydrolase domain−containing protein 2

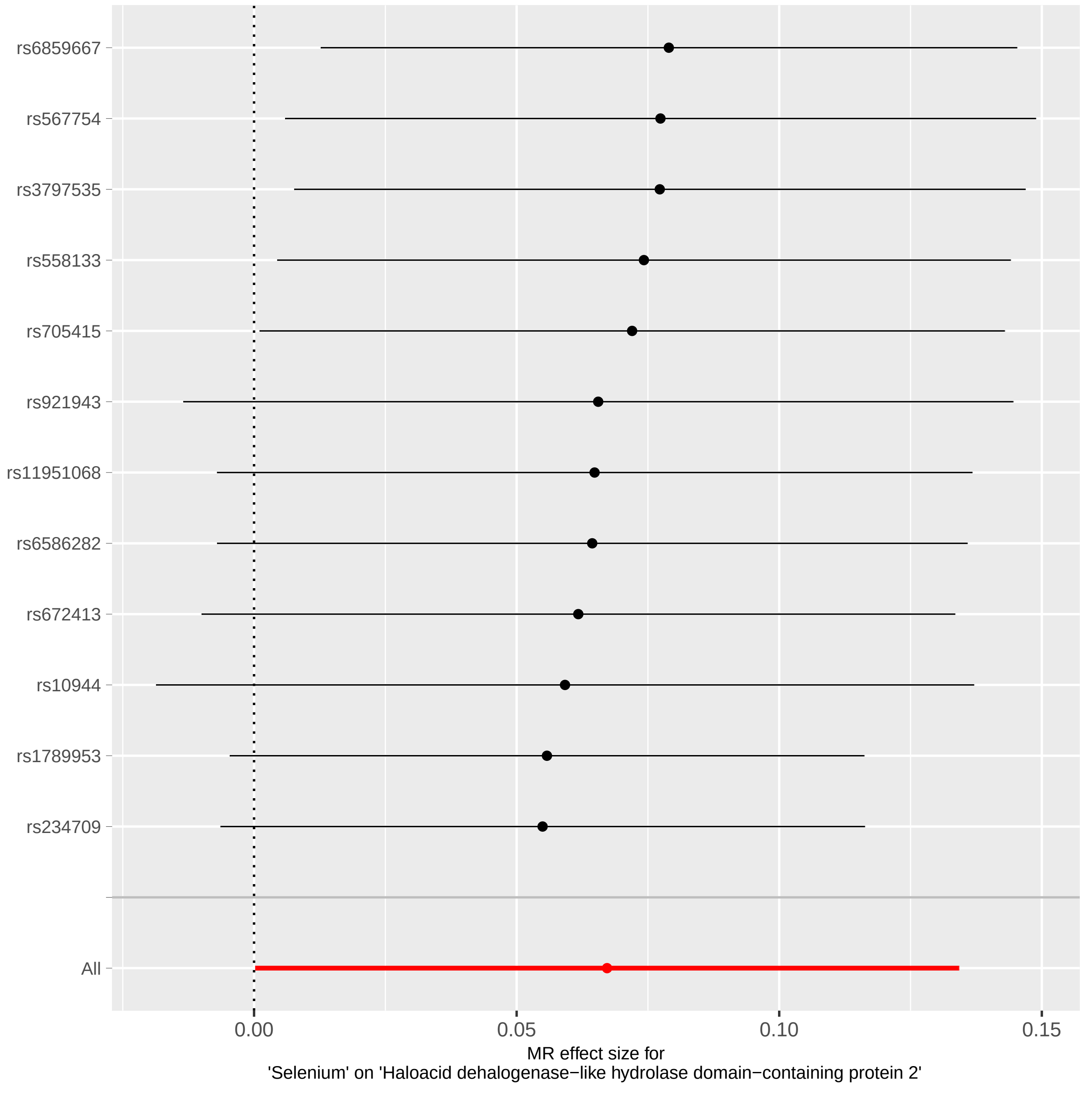

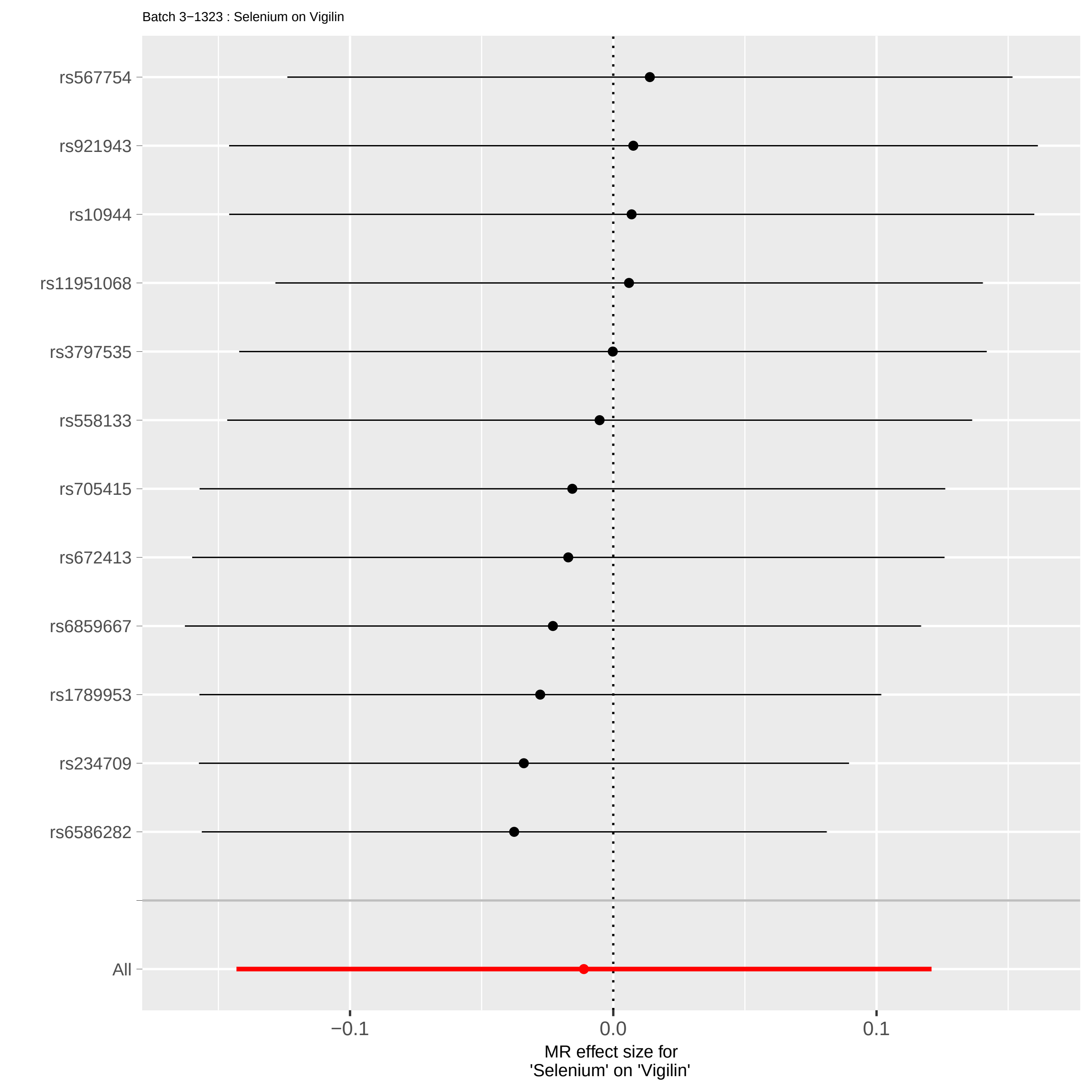

Batch 3−1323 : Selenium on Vigilin
rs567754
rs921943
rs10944
rs11951068
rs3797535
rs558133
rs705415
rs672413
rs6859667
rs1789953
rs234709
rs6586282
All
−0.1
0.0
0.1
MR effect size for
'Selenium' on 'Vigilin'

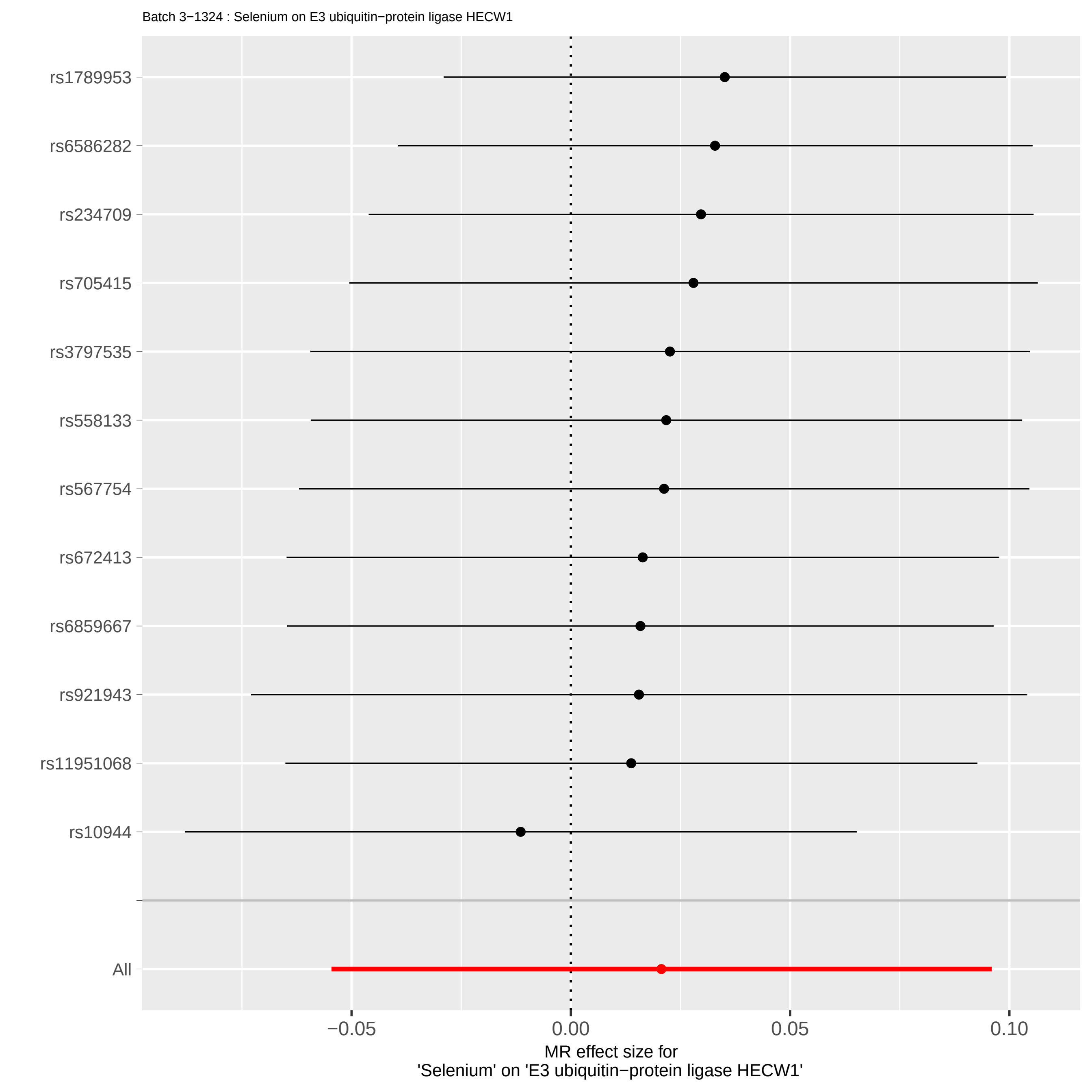

Batch 3−1324 : Selenium on E3 ubiquitin−protein ligase HECW1
rs1789953
rs6586282
rs234709
rs705415
rs3797535
rs558133
rs567754
rs672413
rs6859667
rs921943
rs11951068
rs10944
All
−0.05
0.00
0.05
0.10
MR effect size for
'Selenium' on 'E3 ubiquitin−protein ligase HECW1'

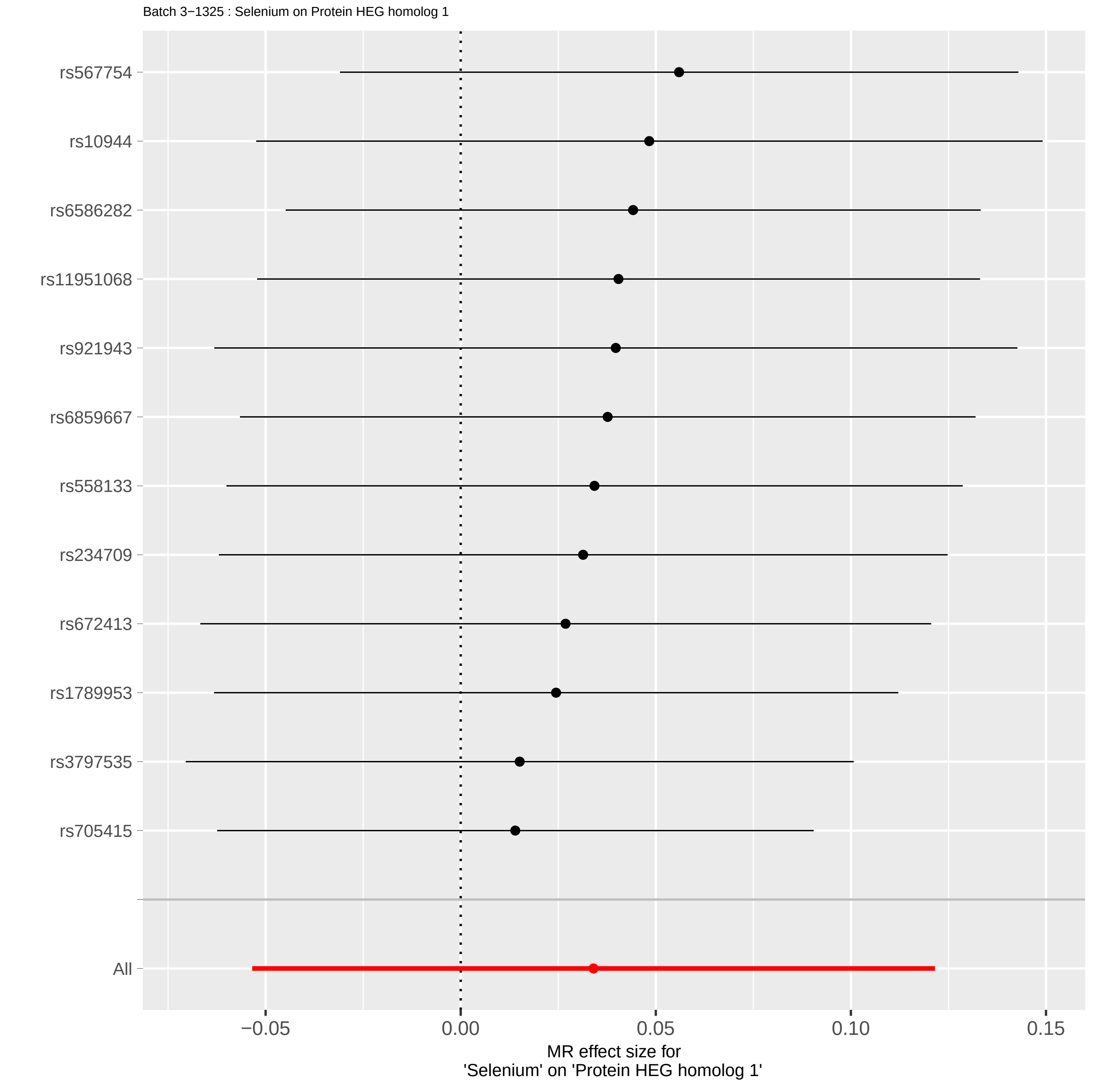

Batch 3−1325 : Selenium on Protein HEG homolog 1
rs567754
rs10944
rs6586282
rs11951068
rs921943
rs6859667
rs558133
rs234709
rs672413
rs1789953
rs3797535
rs705415
All
−0.05
0.00
0.05
0.10
0.15
MR effect size for
'Selenium' on 'Protein HEG homolog 1'

Batch 3−1326 : Selenium on HEPACAM family member 2

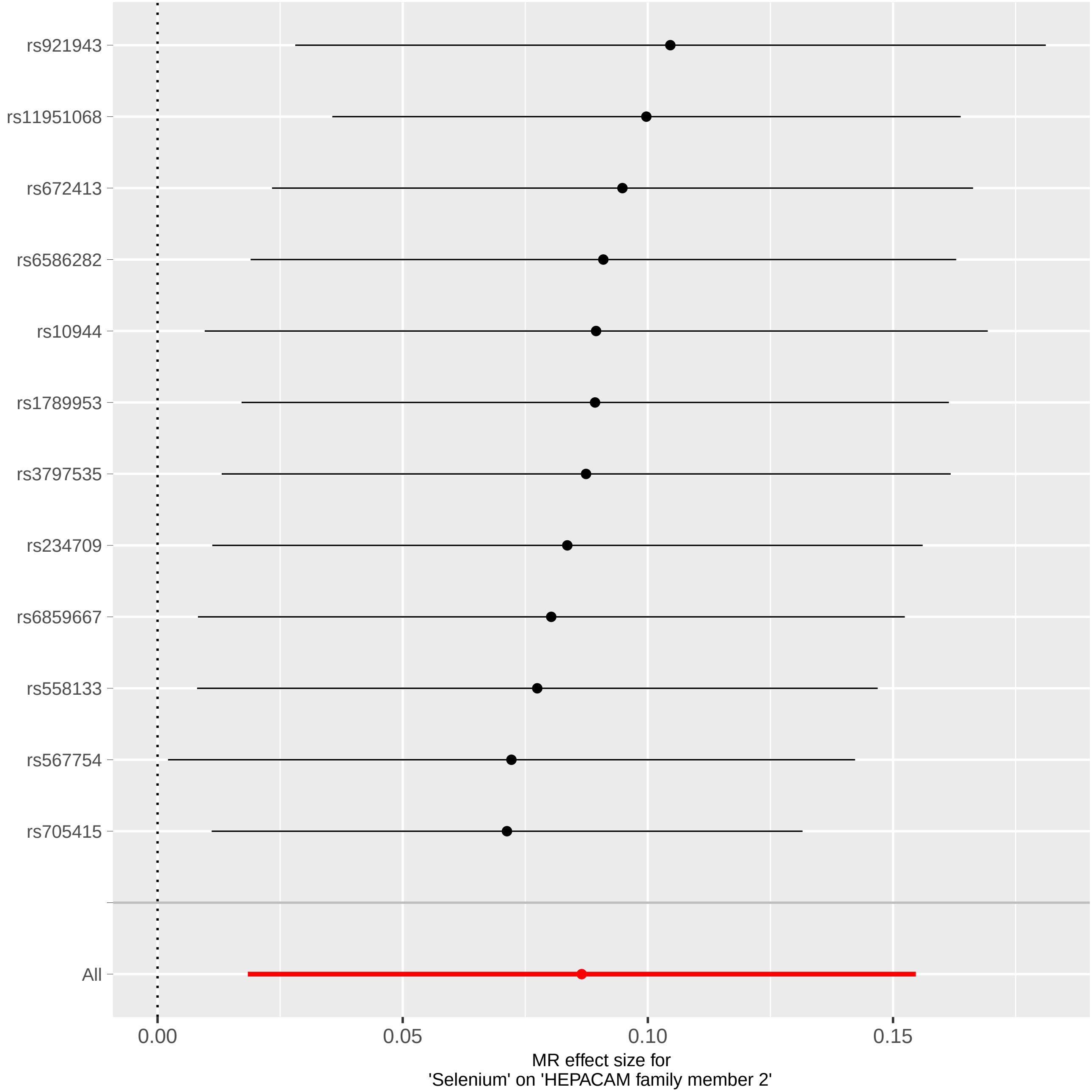

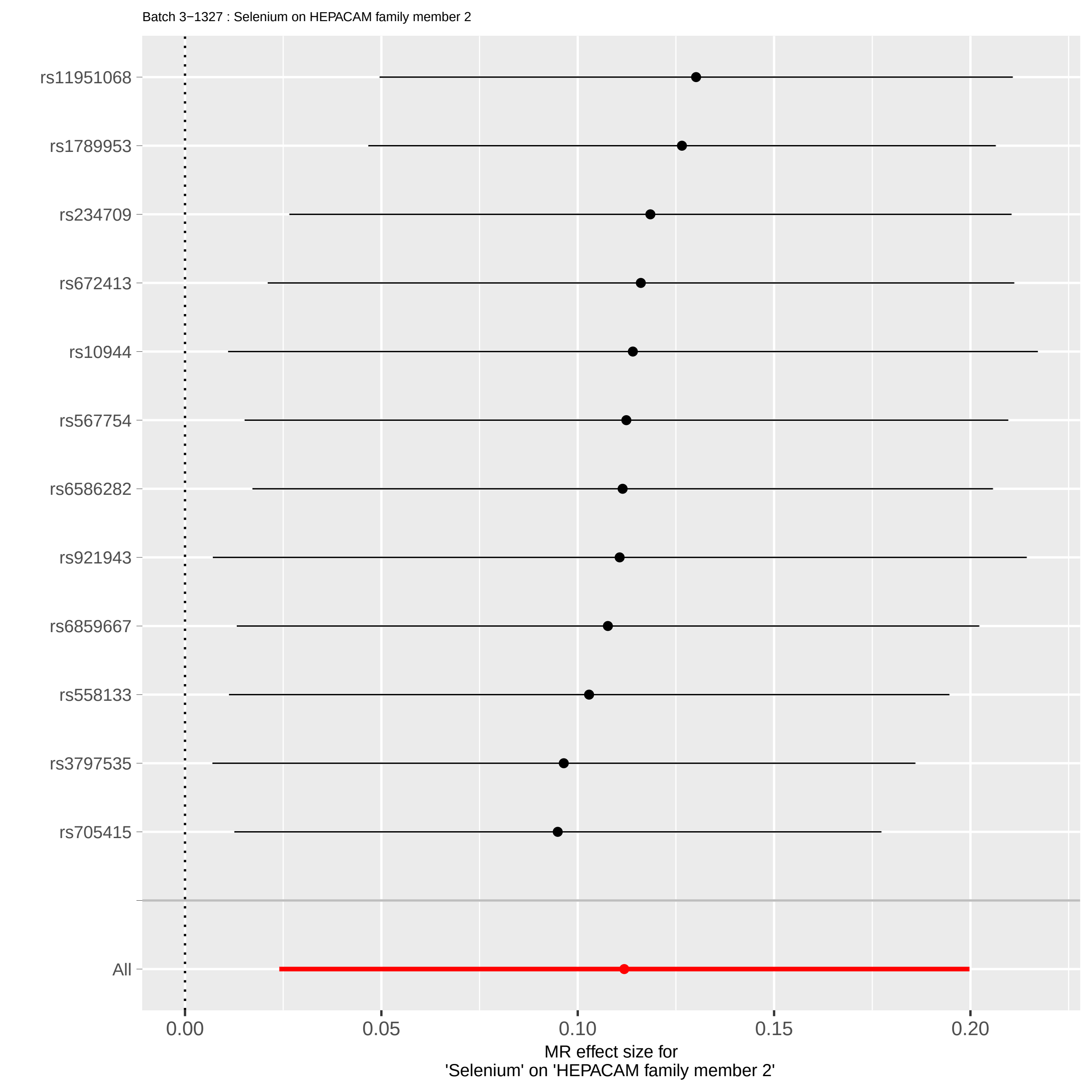

Batch 3−1327 : Selenium on HEPACAM family member 2
rs11951068
rs1789953
rs234709
rs672413
rs10944
rs567754
rs6586282
rs921943
rs6859667
rs558133
rs3797535
rs705415
All
0.00
0.05
0.10
0.15
0.20
MR effect size for
'Selenium' on 'HEPACAM family member 2'

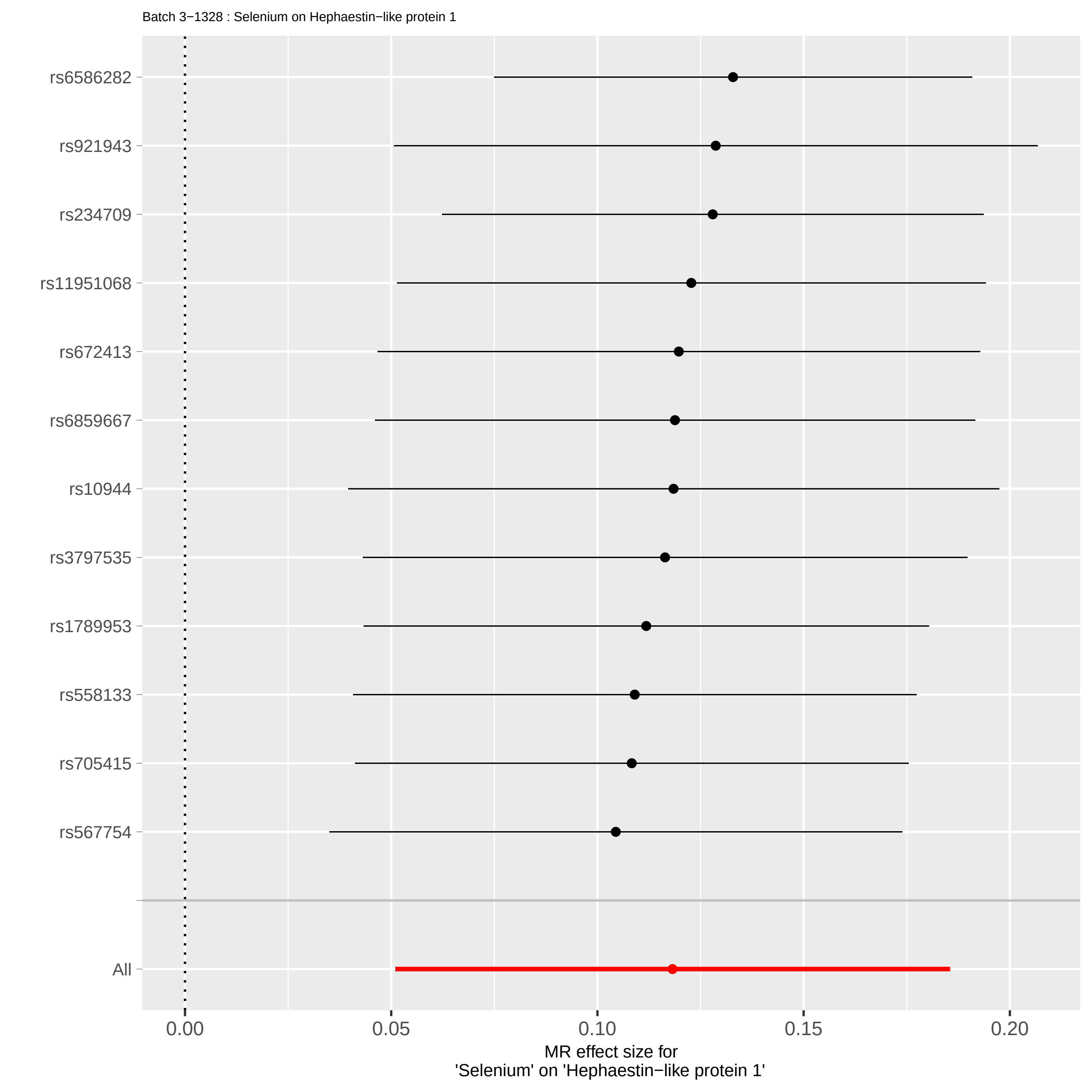
Batch 3−1328 : Selenium on Hephaestin−like protein 1
rs6586282
rs921943
rs234709
rs11951068
rs672413
rs6859667
rs10944
rs3797535
rs1789953
rs558133
rs705415
rs567754
All
0.00
0.05
0.10
0.15
0.20
MR effect size for
'Selenium' on 'Hephaestin−like protein 1'

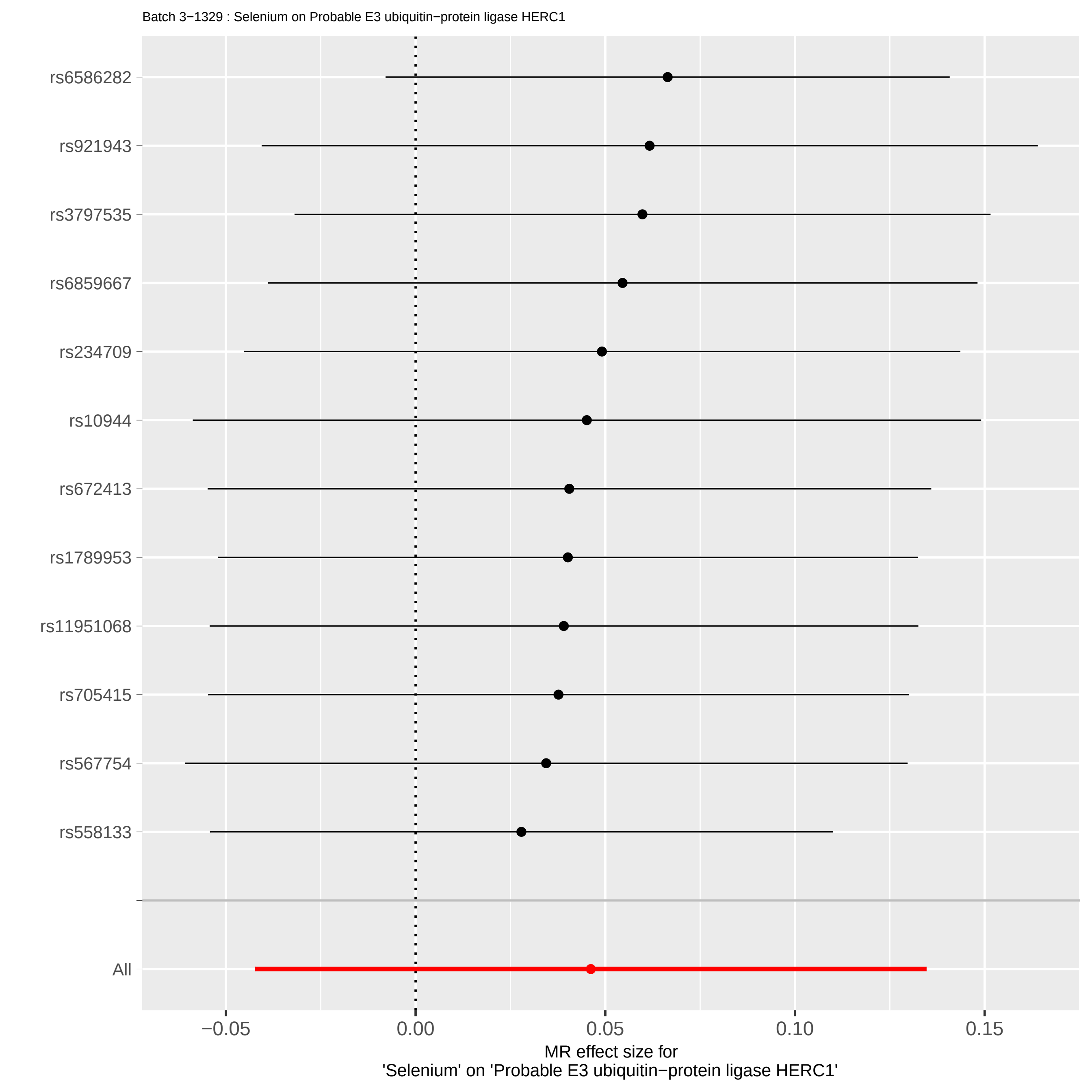

Batch 3−1329 : Selenium on Probable E3 ubiquitin−protein ligase HERC1
rs6586282
rs921943
rs3797535
rs6859667
rs234709
rs10944
rs672413
rs1789953
rs11951068
rs705415
rs567754
rs558133
All
−0.05
0.00
0.05
0.10
0.15
MR effect size for
'Selenium' on 'Probable E3 ubiquitin−protein ligase HERC1'

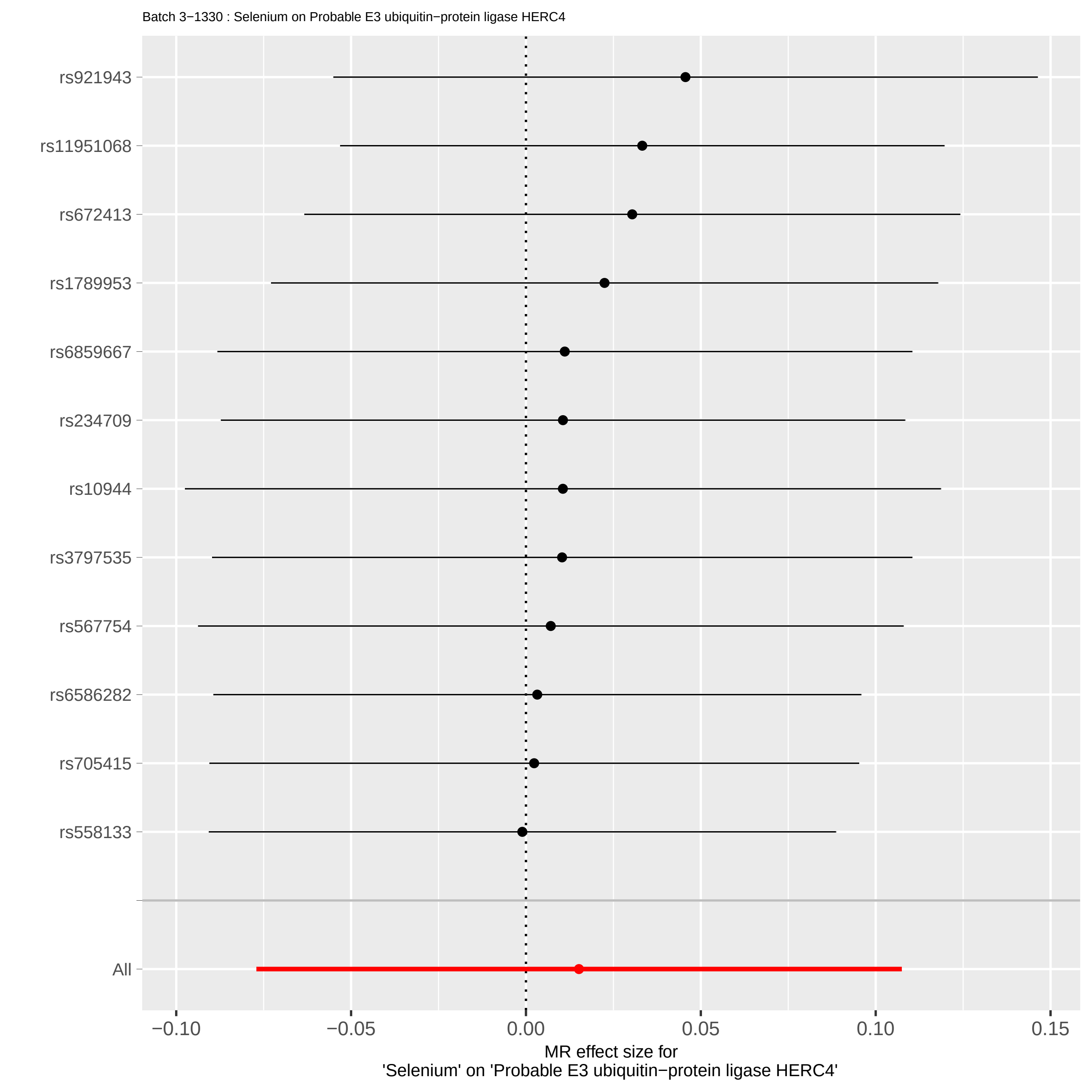

Batch 3−1330 : Selenium on Probable E3 ubiquitin−protein ligase HERC4
rs921943
rs11951068
rs672413
rs1789953
rs6859667
rs234709
rs10944
rs3797535
rs567754
rs6586282
rs705415
rs558133
All
−0.10
−0.05
0.00
0.05
0.10
0.15
MR effect size for
'Selenium' on 'Probable E3 ubiquitin−protein ligase HERC4'

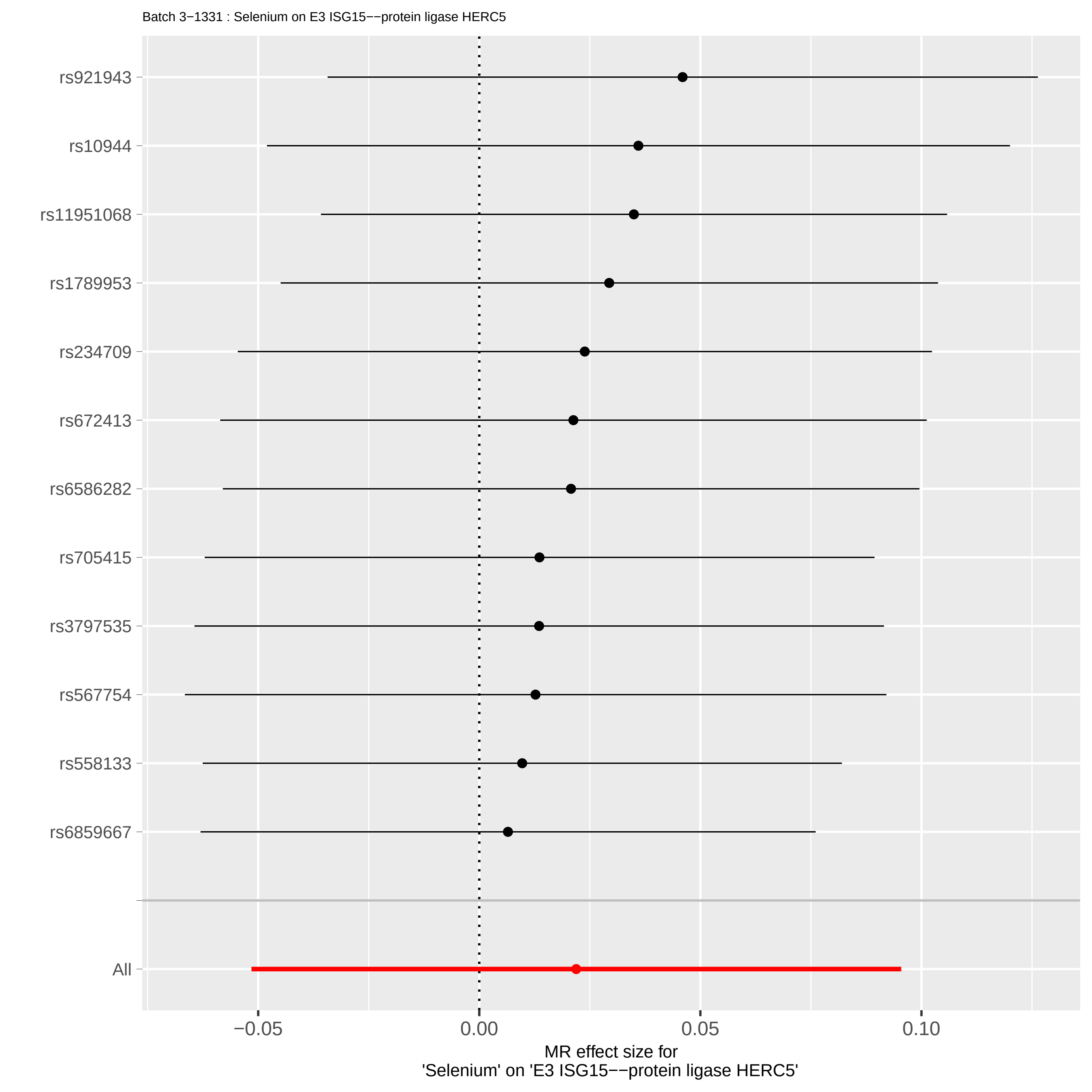

Batch 3−1331 : Selenium on E3 ISG15−−protein ligase HERC5
rs921943
rs10944
rs11951068
rs1789953
rs234709
rs672413
rs6586282
rs705415
rs3797535
rs567754
rs558133
rs6859667
All
−0.05
0.00
0.05
0.10
MR effect size for
'Selenium' on 'E3 ISG15−−protein ligase HERC5'

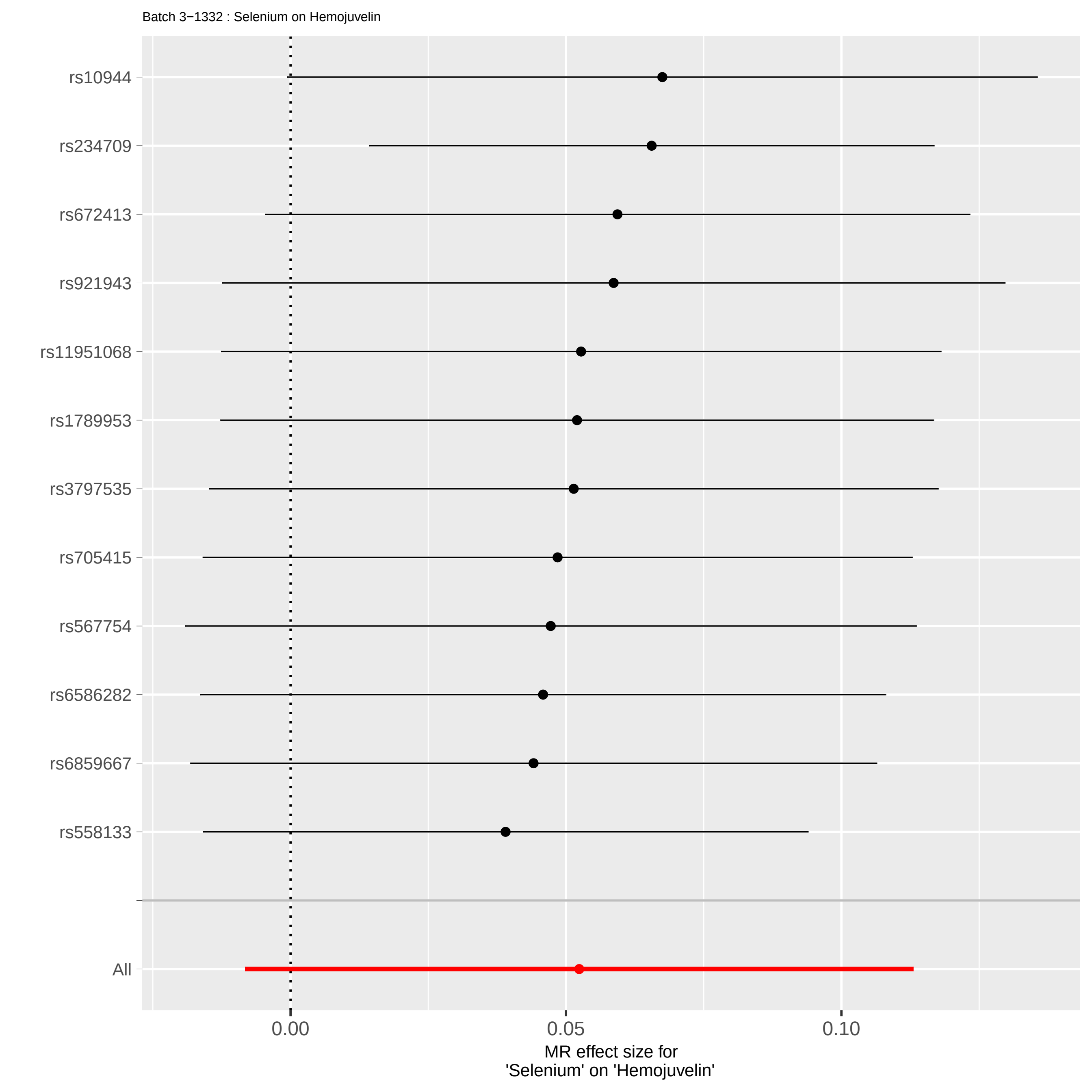

Batch 3−1332 : Selenium on Hemojuvelin
rs10944
rs234709
rs672413
rs921943
rs11951068
rs1789953
rs3797535
rs705415
rs567754
rs6586282
rs6859667
rs558133
All
0.00
0.05
0.10
MR effect size for
'Selenium' on 'Hemojuvelin'

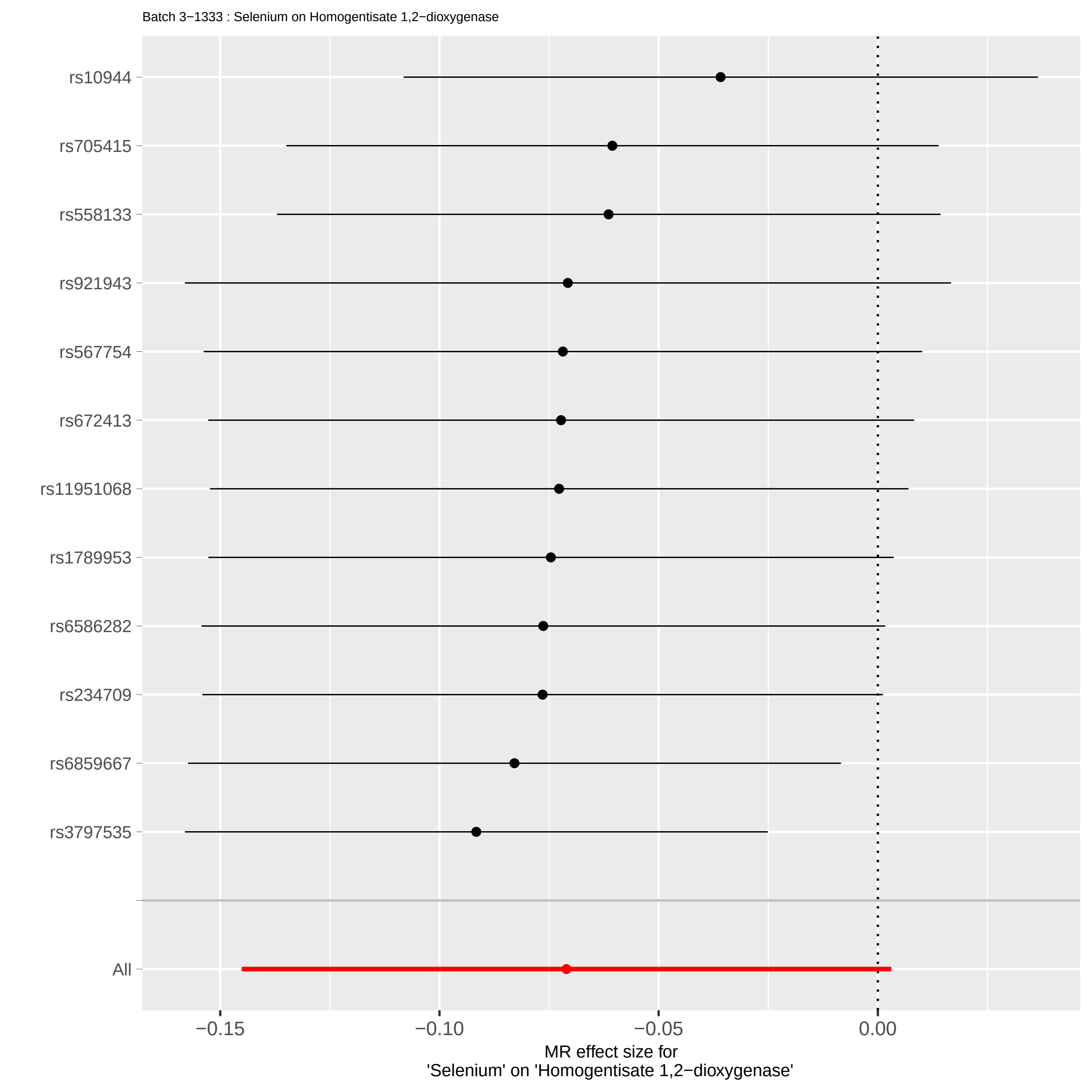

Batch 3−1333 : Selenium on Homogentisate 1,2−dioxygenase
rs10944
rs705415
rs558133
rs921943
rs567754
rs672413
rs11951068
rs1789953
rs6586282
rs234709
rs6859667
rs3797535
All
−0.15
−0.10
−0.05
0.00
MR effect size for
'Selenium' on 'Homogentisate 1,2−dioxygenase'

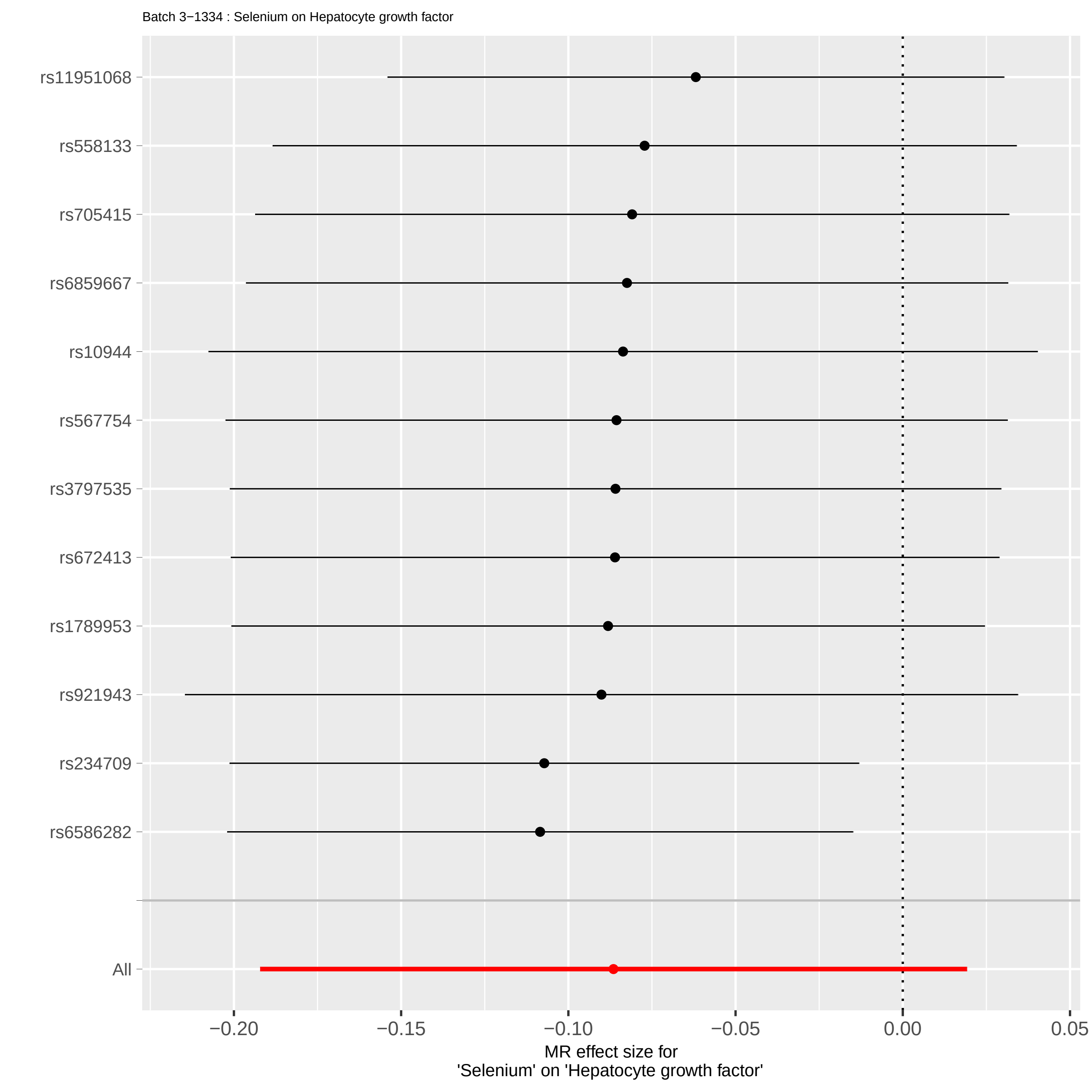

Batch 3−1334 : Selenium on Hepatocyte growth factor
rs11951068
rs558133
rs705415
rs6859667
rs10944
rs567754
rs3797535
rs672413
rs1789953
rs921943
rs234709
rs6586282
All
−0.20
−0.15
−0.10
−0.05
0.00
0.05
MR effect size for
'Selenium' on 'Hepatocyte growth factor'

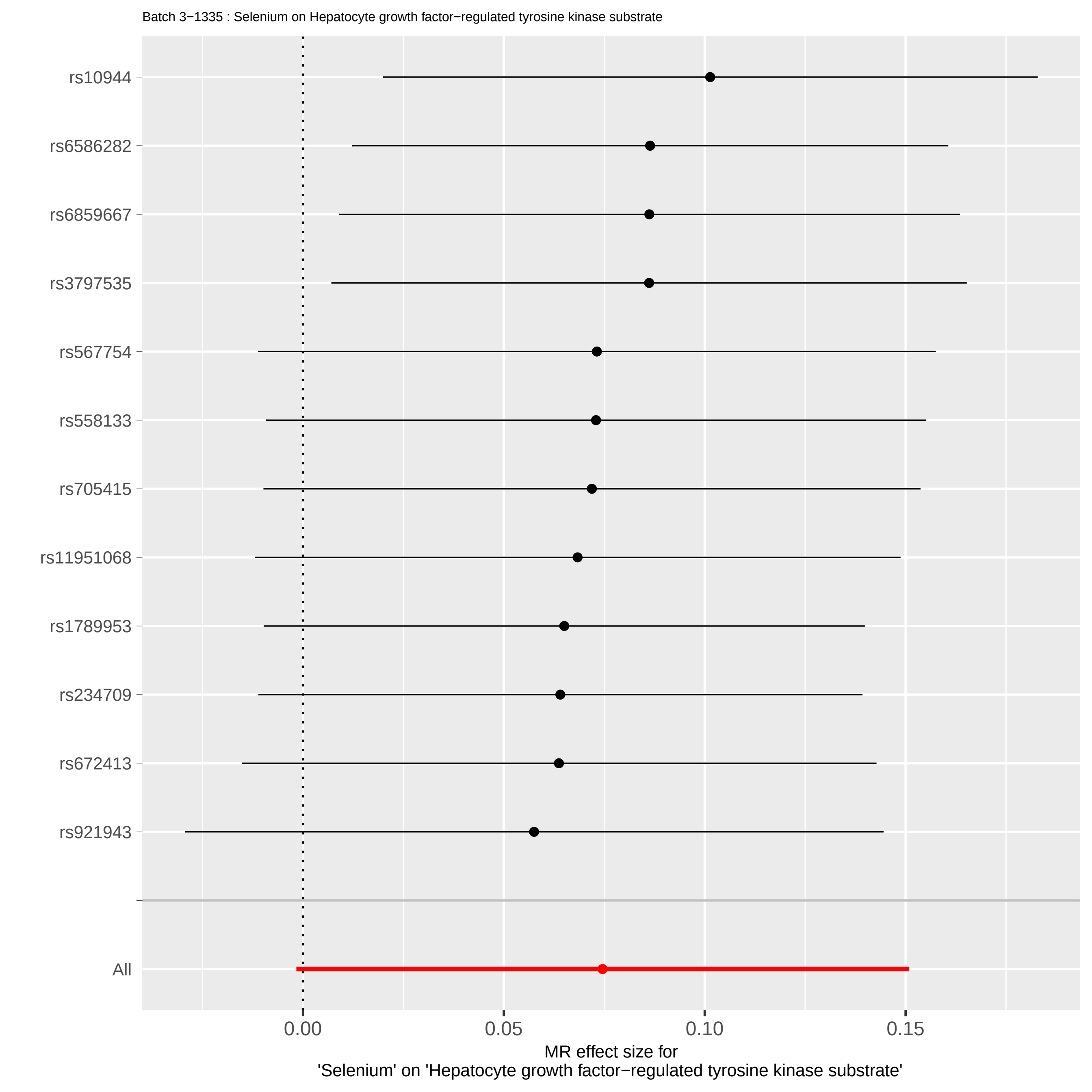

Batch 3−1335 : Selenium on Hepatocyte growth factor−regulated tyrosine kinase substrate
rs10944
rs6586282
rs6859667
rs3797535
rs567754
rs558133
rs705415
rs11951068
rs1789953
rs234709
rs672413
rs921943
All
0.00
0.05
0.10
0.15
MR effect size for
'Selenium' on 'Hepatocyte growth factor−regulated tyrosine kinase substrate'

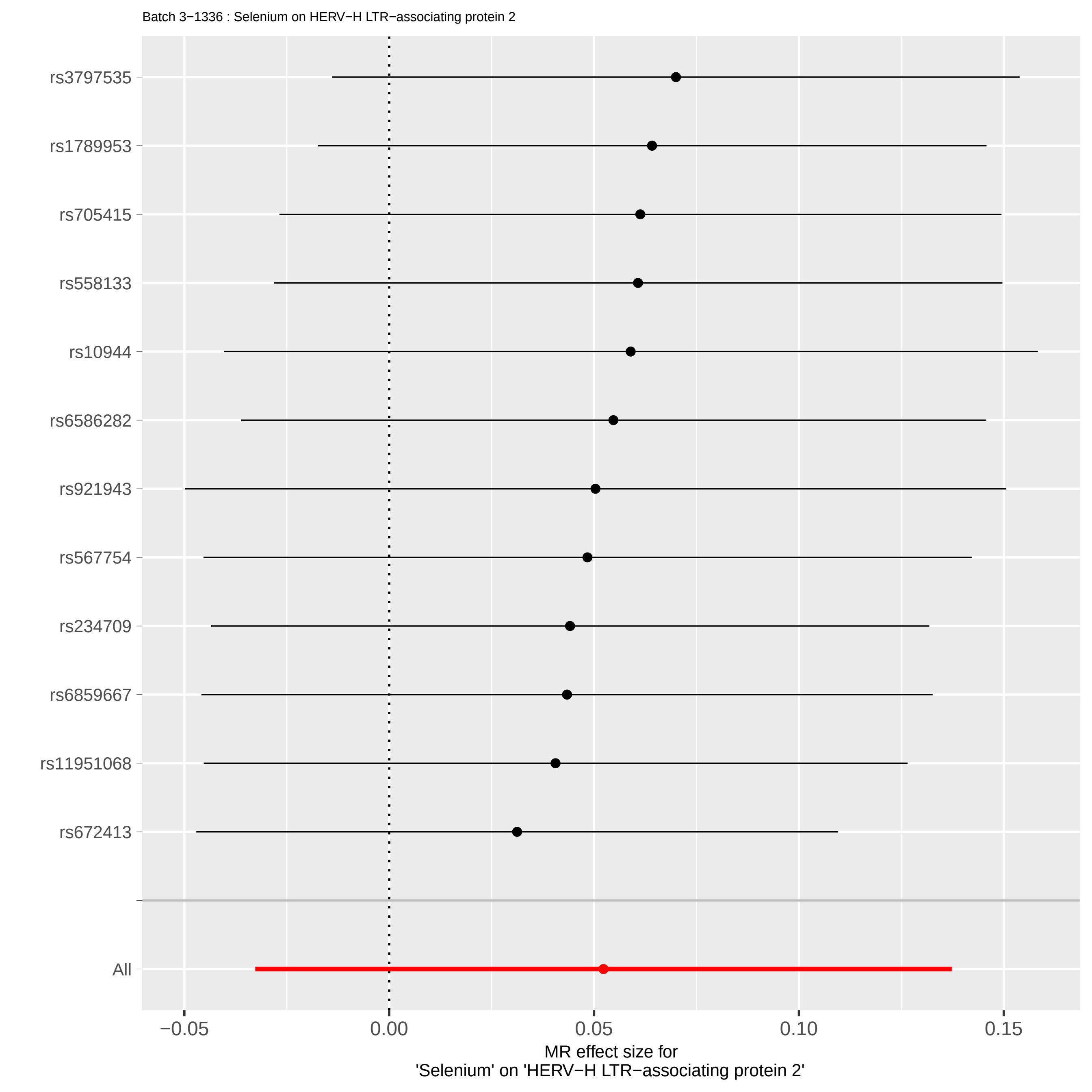
Batch 3−1336 : Selenium on HERV−H LTR−associating protein 2
rs3797535
rs1789953
rs705415
rs558133
rs10944
rs6586282
rs921943
rs567754
rs234709
rs6859667
rs11951068
rs672413
All
−0.05
0.00
0.05
0.10
0.15
MR effect size for
'Selenium' on 'HERV−H LTR−associating protein 2'

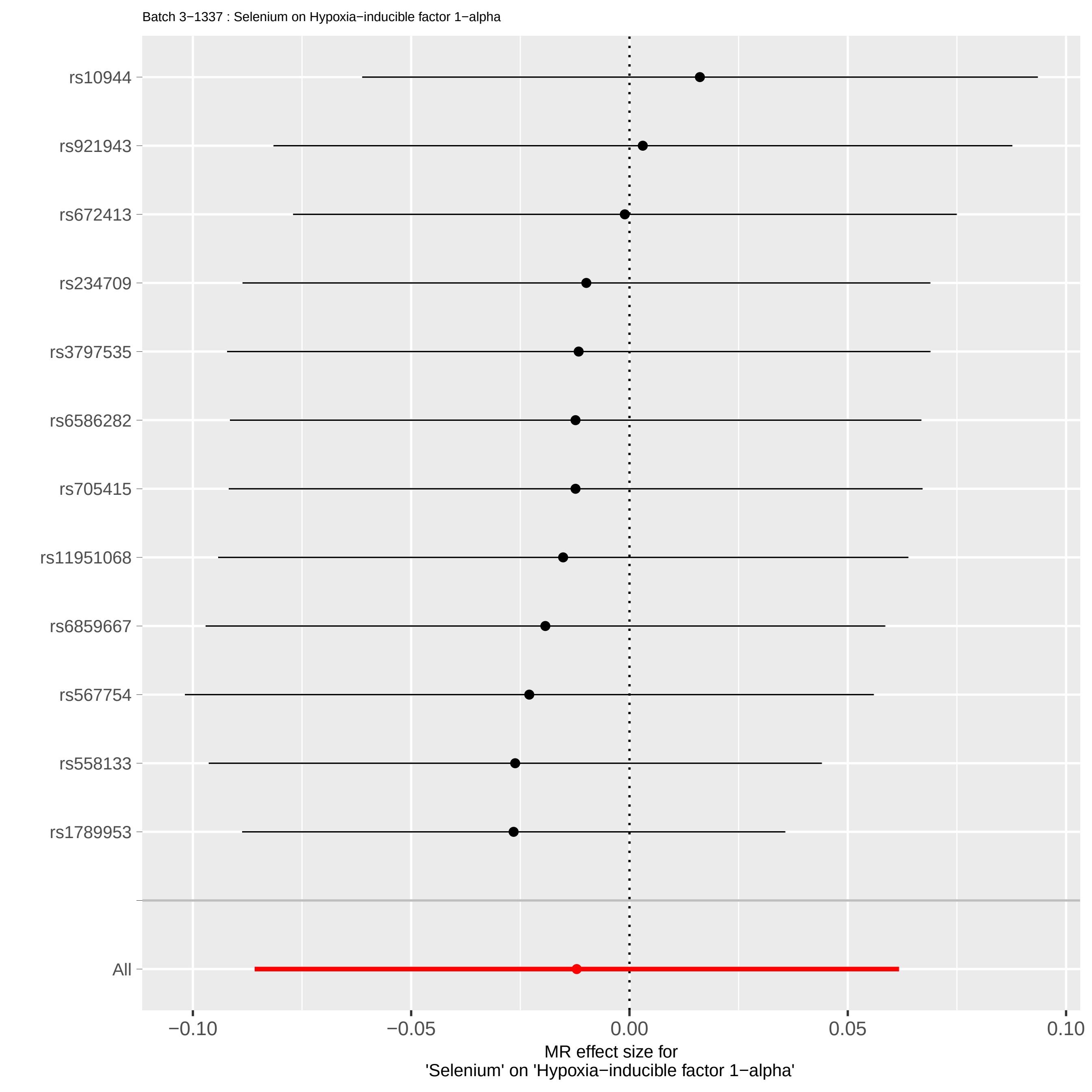

Batch 3−1337 : Selenium on Hypoxia−inducible factor 1−alpha
rs10944
rs921943
rs672413
rs234709
rs3797535
rs6586282
rs705415
rs11951068
rs6859667
rs567754
rs558133
rs1789953
All
−0.10
−0.05
0.00
0.05
0.10
MR effect size for
'Selenium' on 'Hypoxia−inducible factor 1−alpha'

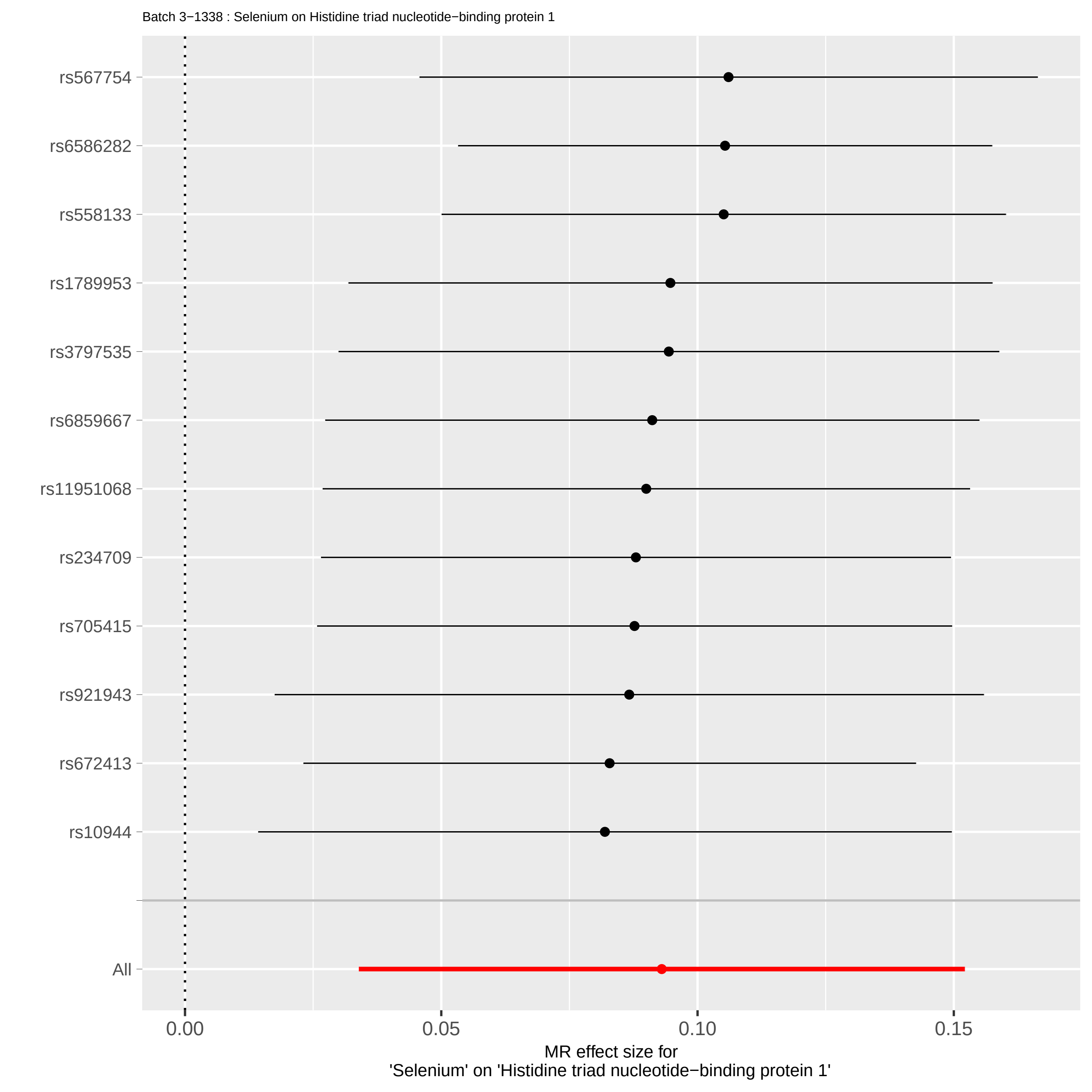

Batch 3−1338 : Selenium on Histidine triad nucleotide−binding protein 1
rs567754
rs6586282
rs558133
rs1789953
rs3797535
rs6859667
rs11951068
rs234709
rs705415
rs921943
rs672413
rs10944
All
0.00
0.05
0.10
0.15
MR effect size for
'Selenium' on 'Histidine triad nucleotide−binding protein 1'

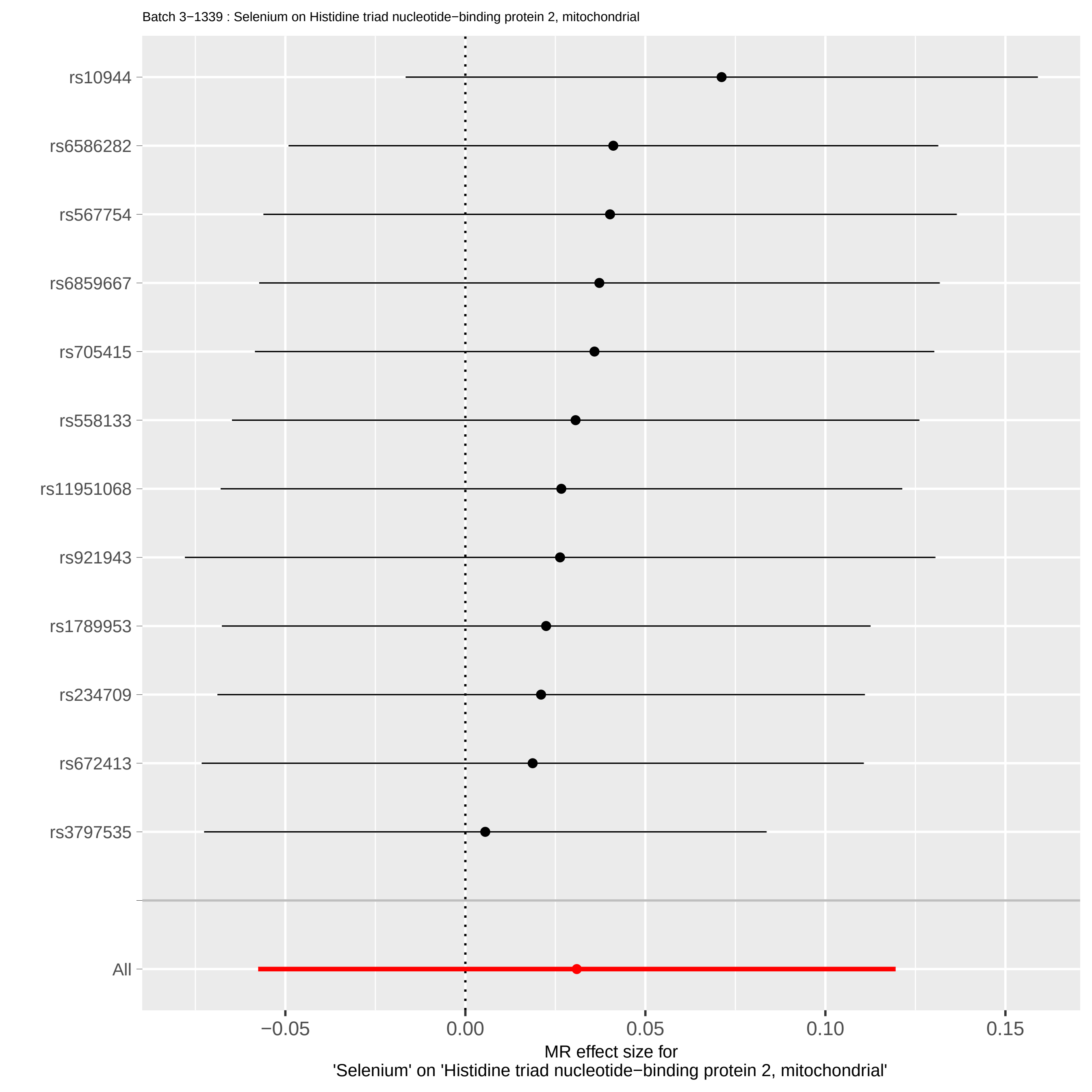
Batch 3−1339 : Selenium on Histidine triad nucleotide−binding protein 2, mitochondrial
rs10944
rs6586282
rs567754
rs6859667
rs705415
rs558133
rs11951068
rs921943
rs1789953
rs234709
rs672413
rs3797535
All
−0.05
0.00
0.05
0.10
0.15
MR effect size for
'Selenium' on 'Histidine triad nucleotide−binding protein 2, mitochondrial'

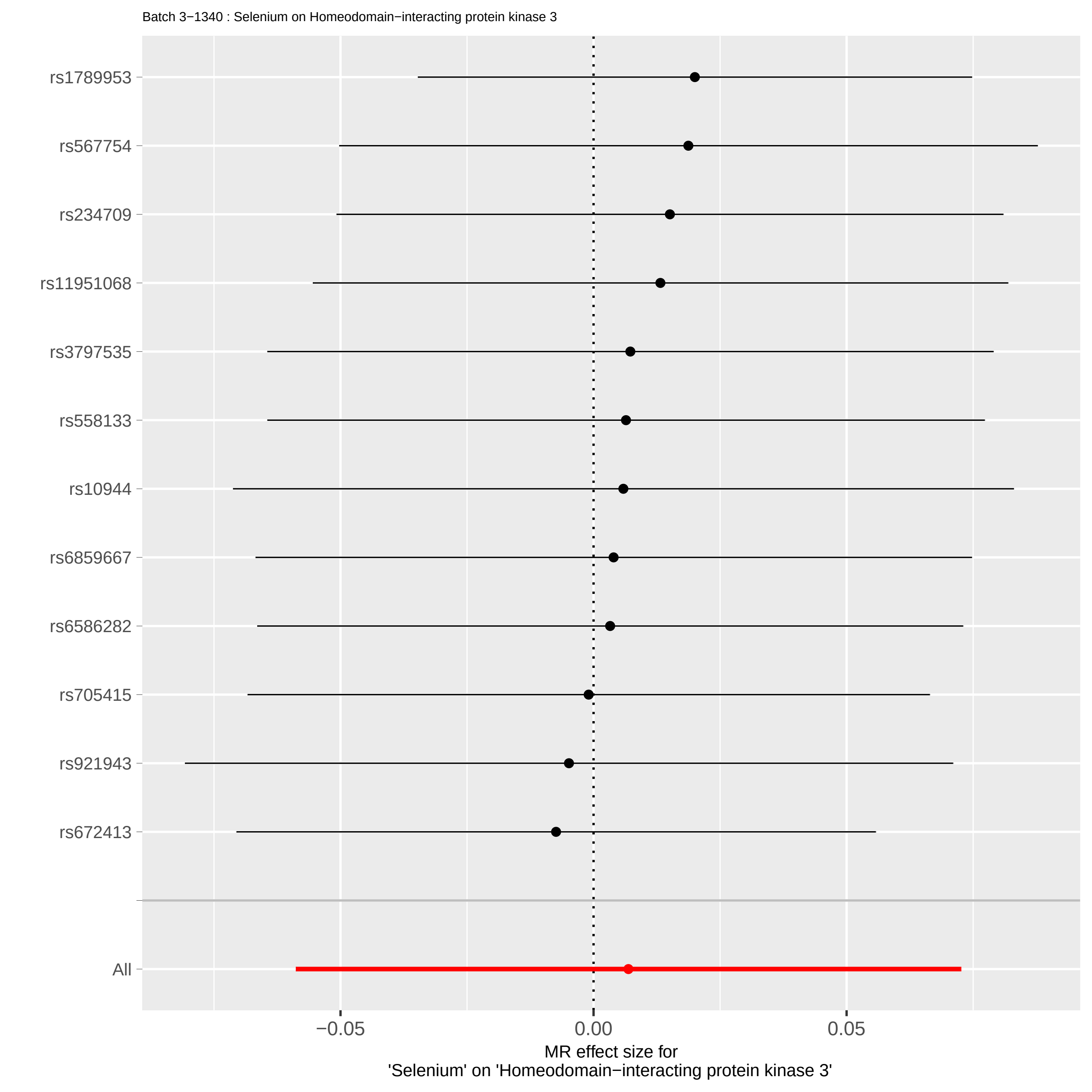
Batch 3−1340 : Selenium on Homeodomain−interacting protein kinase 3
rs1789953
rs567754
rs234709
rs11951068
rs3797535
rs558133
rs10944
rs6859667
rs6586282
rs705415
rs921943
rs672413
All
−0.05
0.00
0.05
MR effect size for
'Selenium' on 'Homeodomain−interacting protein kinase 3'

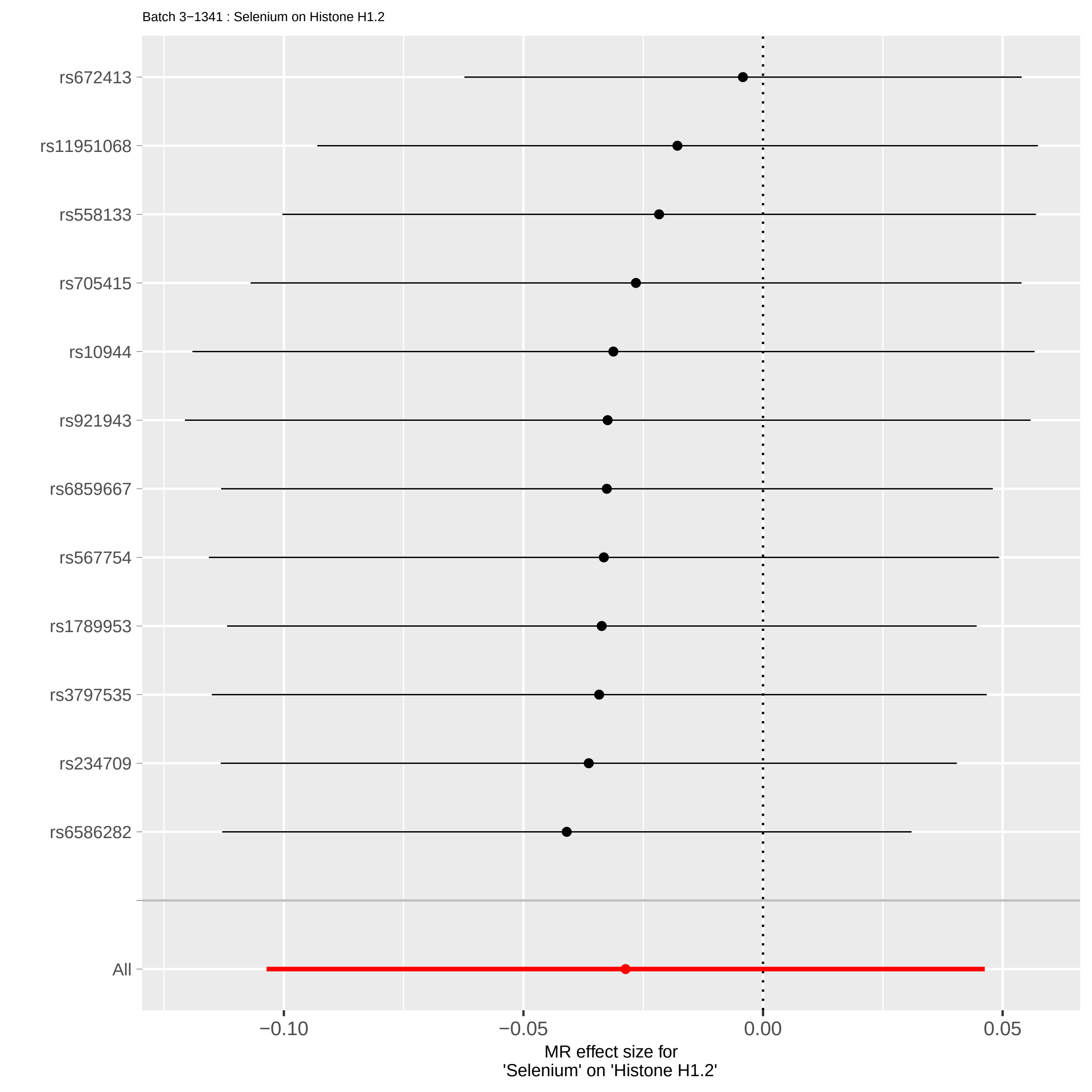

Batch 3−1341 : Selenium on Histone H1.2
rs672413
rs11951068
rs558133
rs705415
rs10944
rs921943
rs6859667
rs567754
rs1789953
rs3797535
rs234709
rs6586282
All
−0.10
−0.05
0.00
0.05
MR effect size for
'Selenium' on 'Histone H1.2'

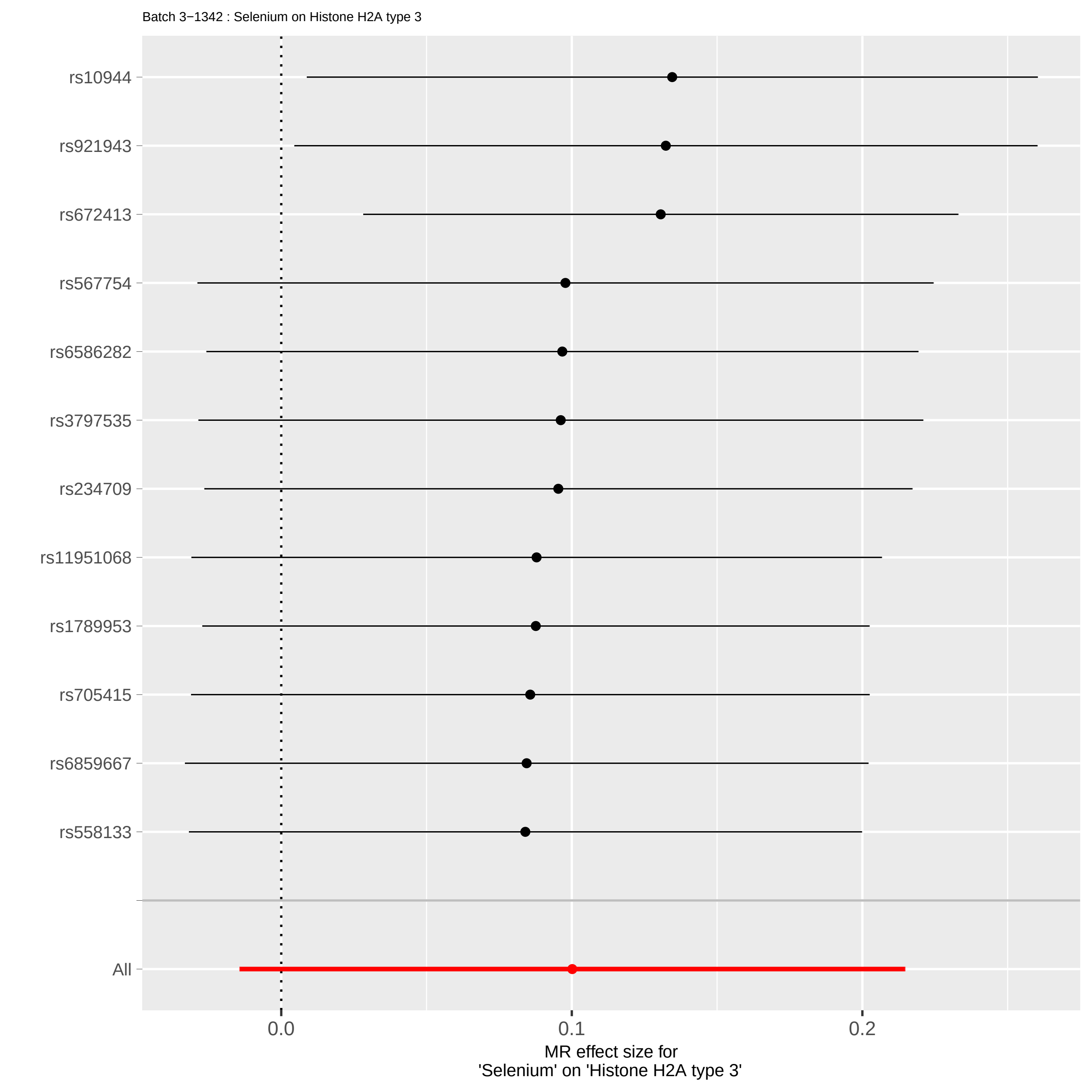
Batch 3−1342 : Selenium on Histone H2A type 3
rs10944
rs921943
rs672413
rs567754
rs6586282
rs3797535
rs234709
rs11951068
rs1789953
rs705415
rs6859667
rs558133
All
0.0
0.1
0.2
MR effect size for
'Selenium' on 'Histone H2A type 3'

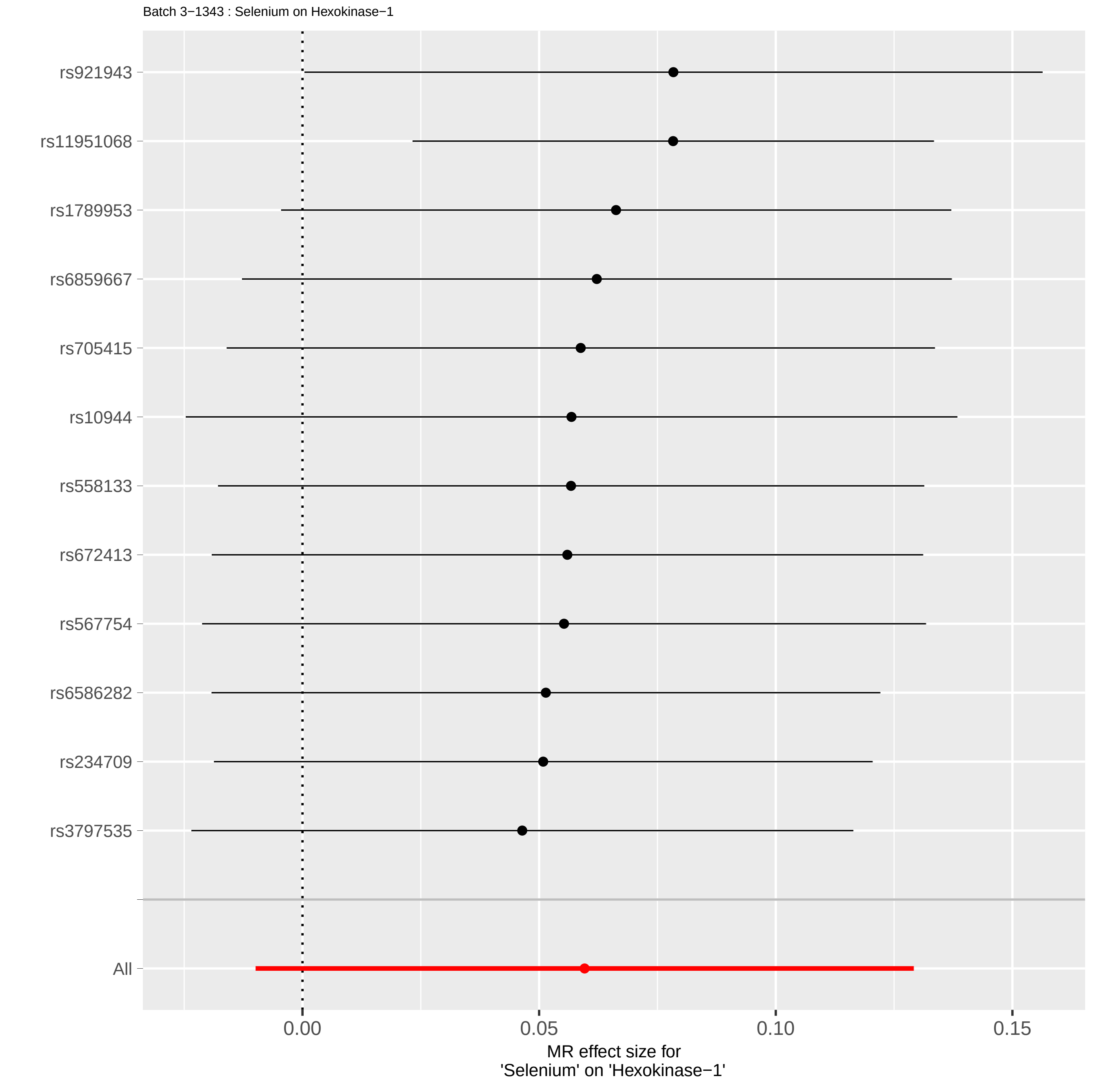

Batch 3−1343 : Selenium on Hexokinase−1
rs921943
rs11951068
rs1789953
rs6859667
rs705415
rs10944
rs558133
rs672413
rs567754
rs6586282
rs234709
rs3797535
All
0.00
0.05
0.10
0.15
MR effect size for
'Selenium' on 'Hexokinase−1'

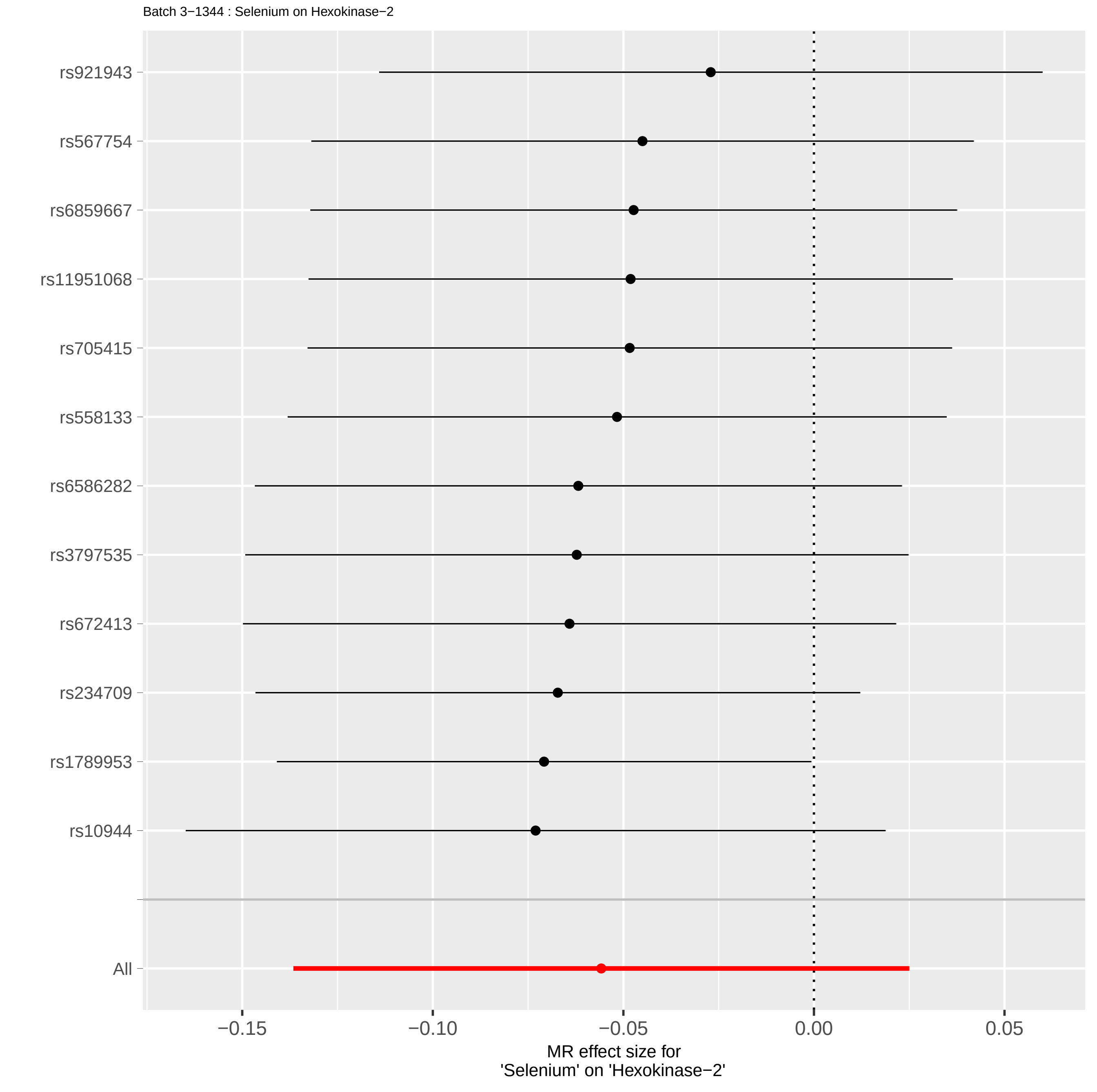

Batch 3−1344 : Selenium on Hexokinase−2
rs921943
rs567754
rs6859667
rs11951068
rs705415
rs558133
rs6586282
rs3797535
rs672413
rs234709
rs1789953
rs10944
All
−0.15
−0.10
−0.05
0.00
0.05
MR effect size for
'Selenium' on 'Hexokinase−2'

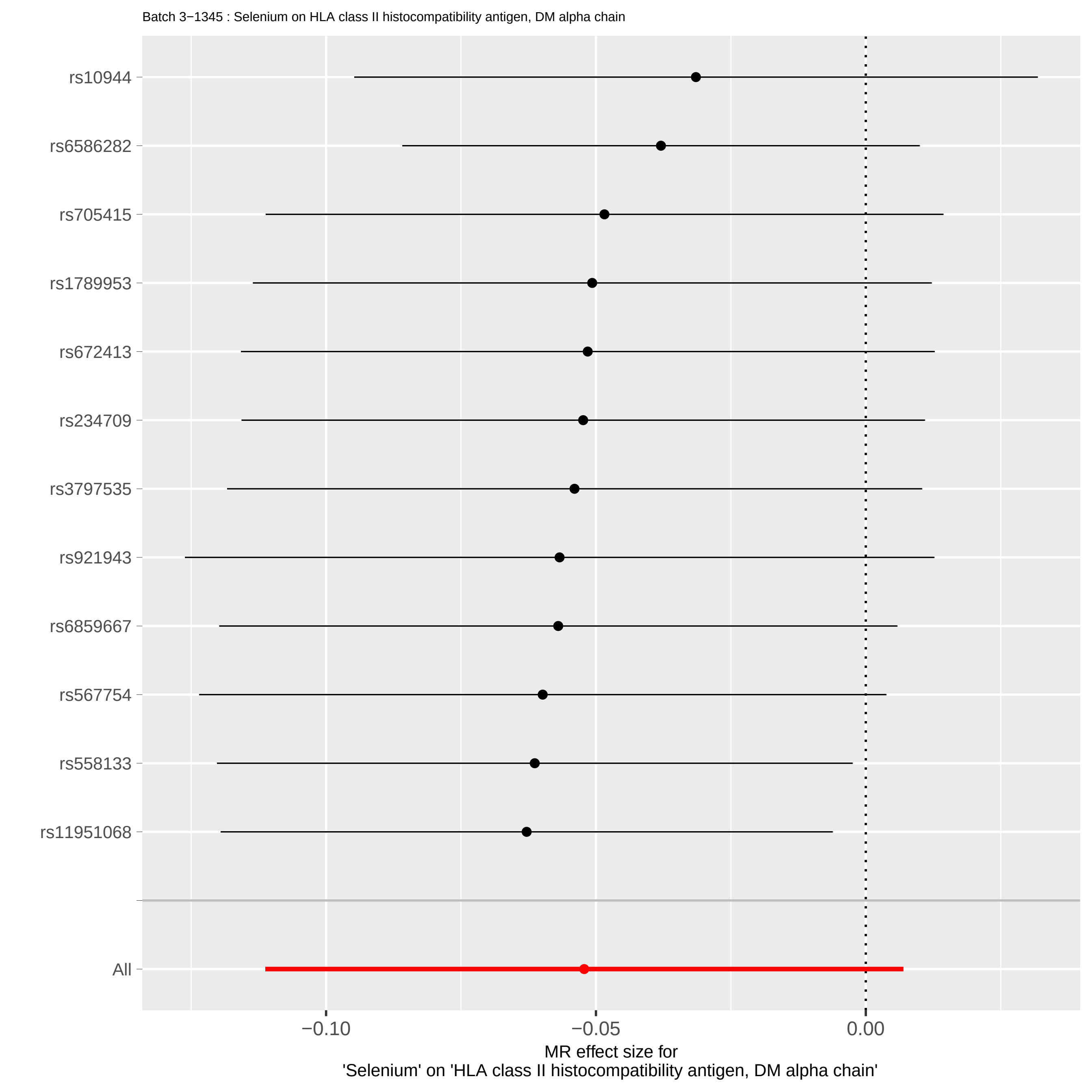

Batch 3−1345 : Selenium on HLA class II histocompatibility antigen, DM alpha chain
rs10944
rs6586282
rs705415
rs1789953
rs672413
rs234709
rs3797535
rs921943
rs6859667
rs567754
rs558133
rs11951068
All
−0.10
−0.05
0.00
MR effect size for
'Selenium' on 'HLA class II histocompatibility antigen, DM alpha chain'

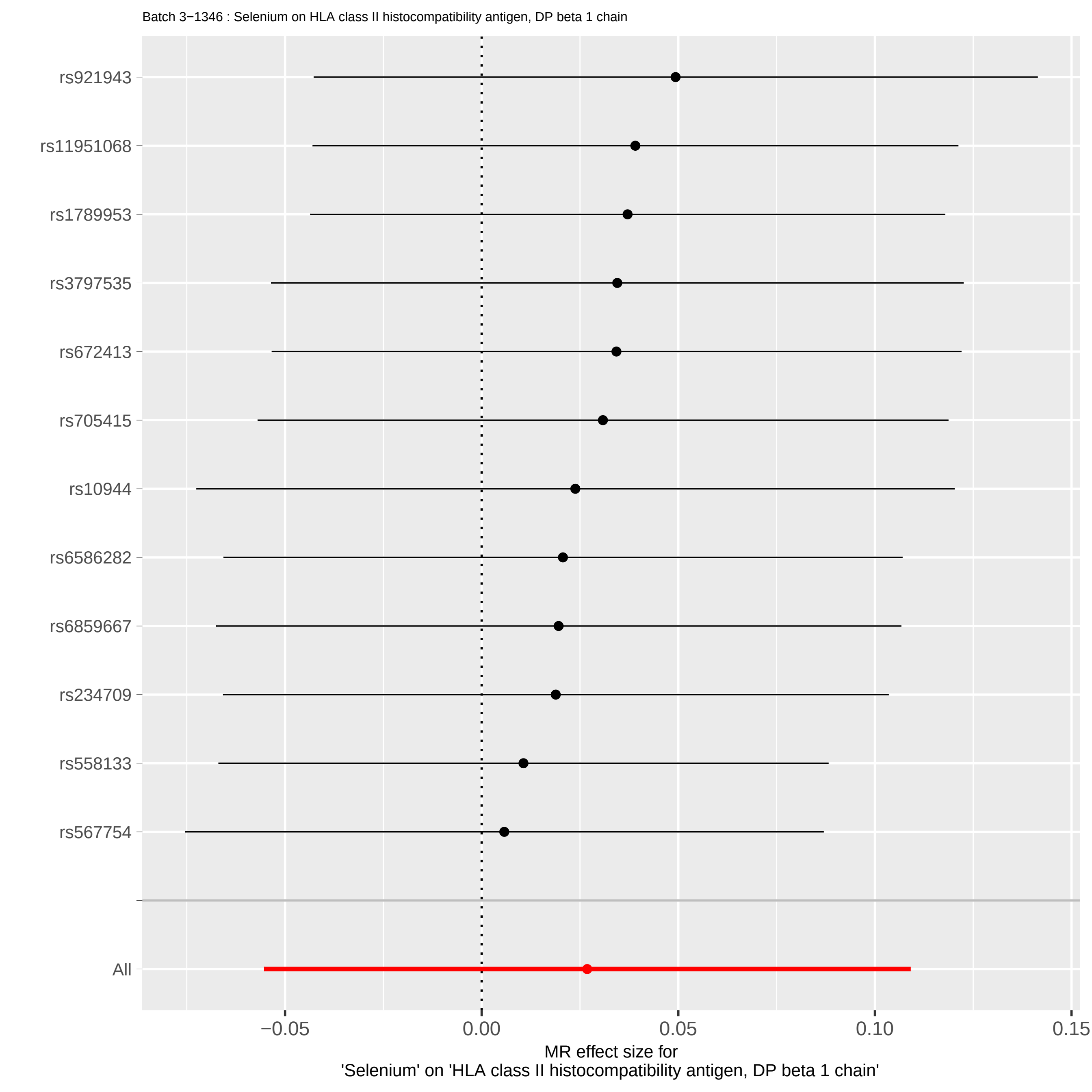

Batch 3−1346 : Selenium on HLA class II histocompatibility antigen, DP beta 1 chain
rs921943
rs11951068
rs1789953
rs3797535
rs672413
rs705415
rs10944
rs6586282
rs6859667
rs234709
rs558133
rs567754
All
−0.05
0.00
0.05
0.10
0.15
MR effect size for
'Selenium' on 'HLA class II histocompatibility antigen, DP beta 1 chain'

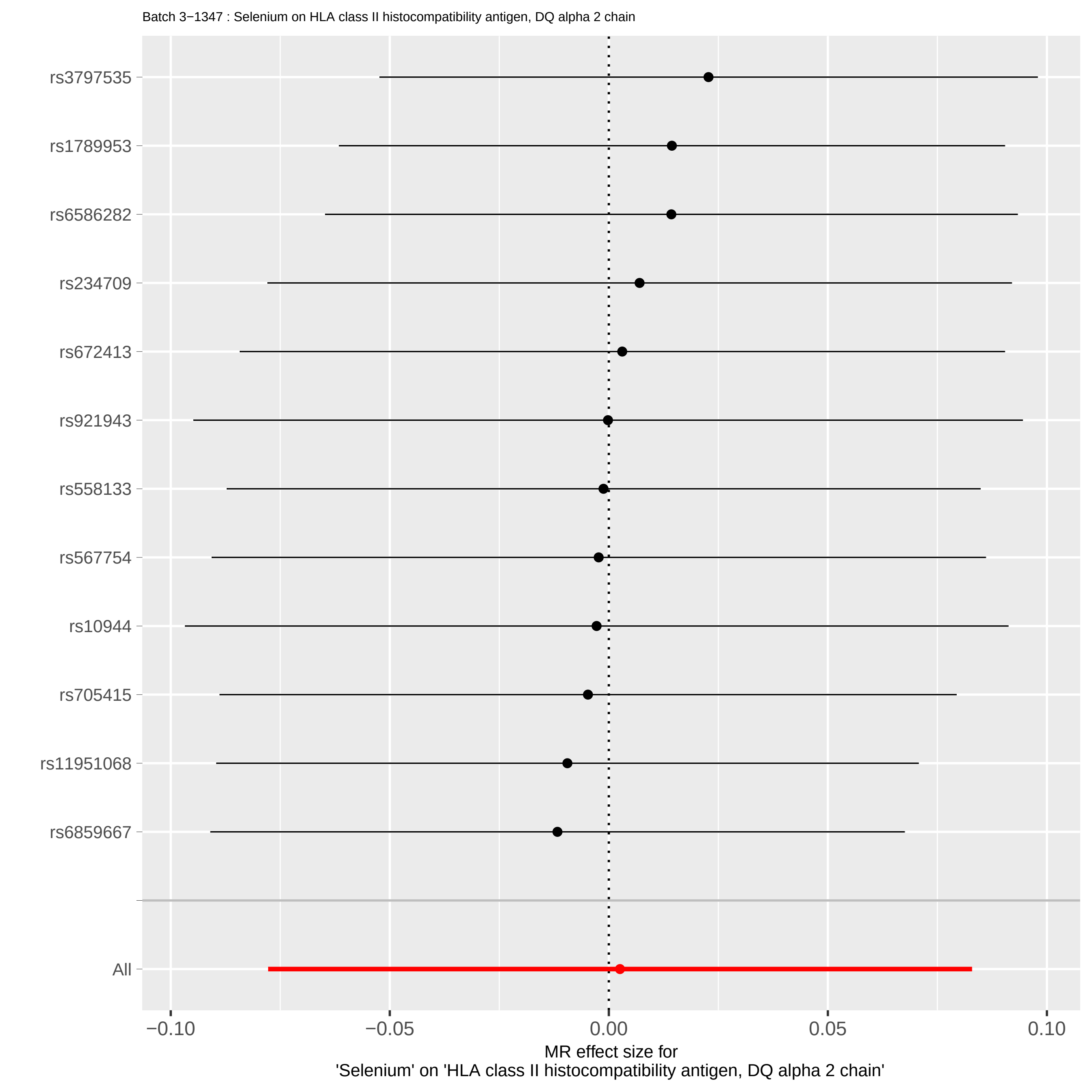

Batch 3−1347 : Selenium on HLA class II histocompatibility antigen, DQ alpha 2 chain
rs3797535
rs1789953
rs6586282
rs234709
rs672413
rs921943
rs558133
rs567754
rs10944
rs705415
rs11951068
rs6859667
All
−0.10
−0.05
0.00
0.05
0.10
MR effect size for
'Selenium' on 'HLA class II histocompatibility antigen, DQ alpha 2 chain'

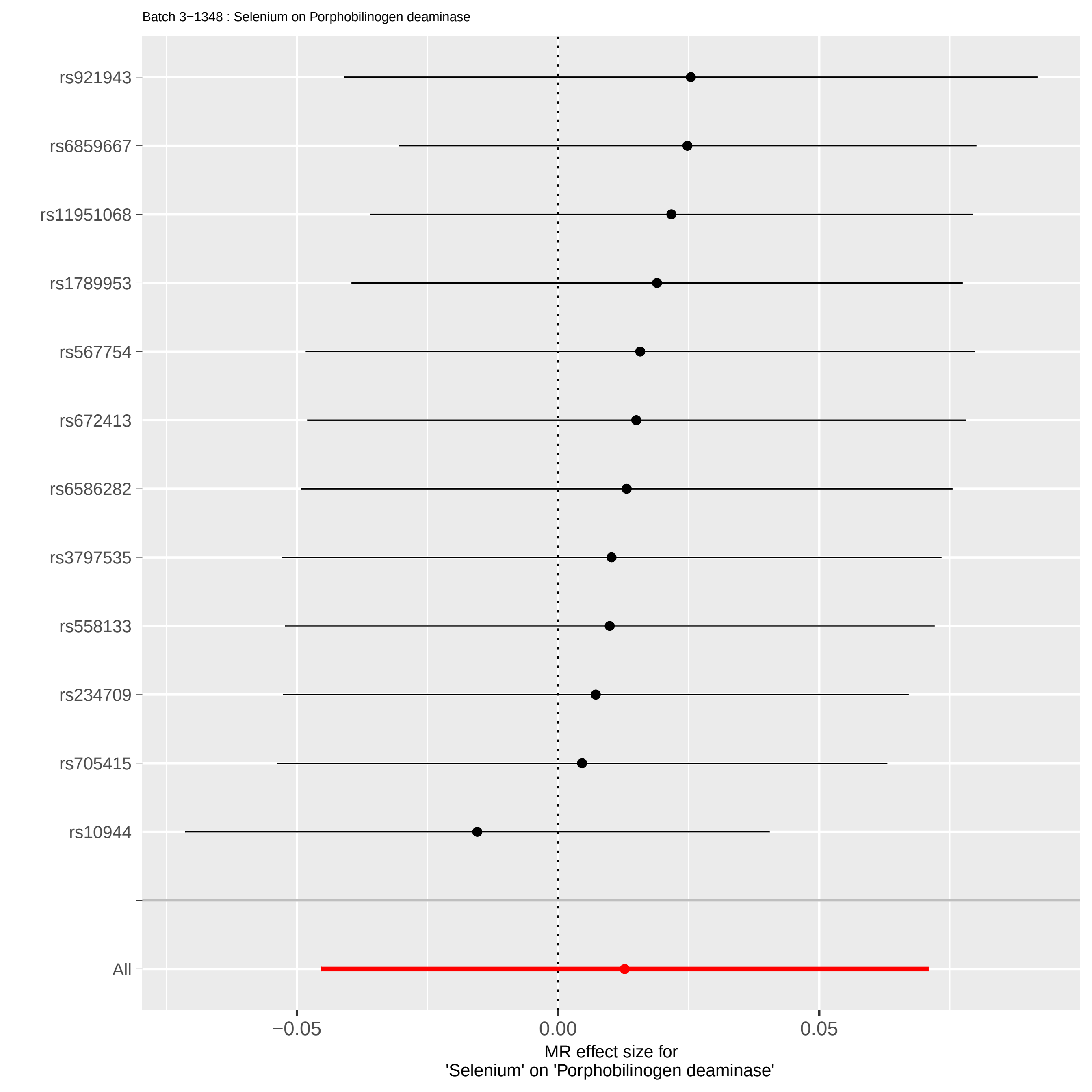

Batch 3−1348 : Selenium on Porphobilinogen deaminase
rs921943
rs6859667
rs11951068
rs1789953
rs567754
rs672413
rs6586282
rs3797535
rs558133
rs234709
rs705415
rs10944
All
−0.05
0.00
0.05
MR effect size for
'Selenium' on 'Porphobilinogen deaminase'

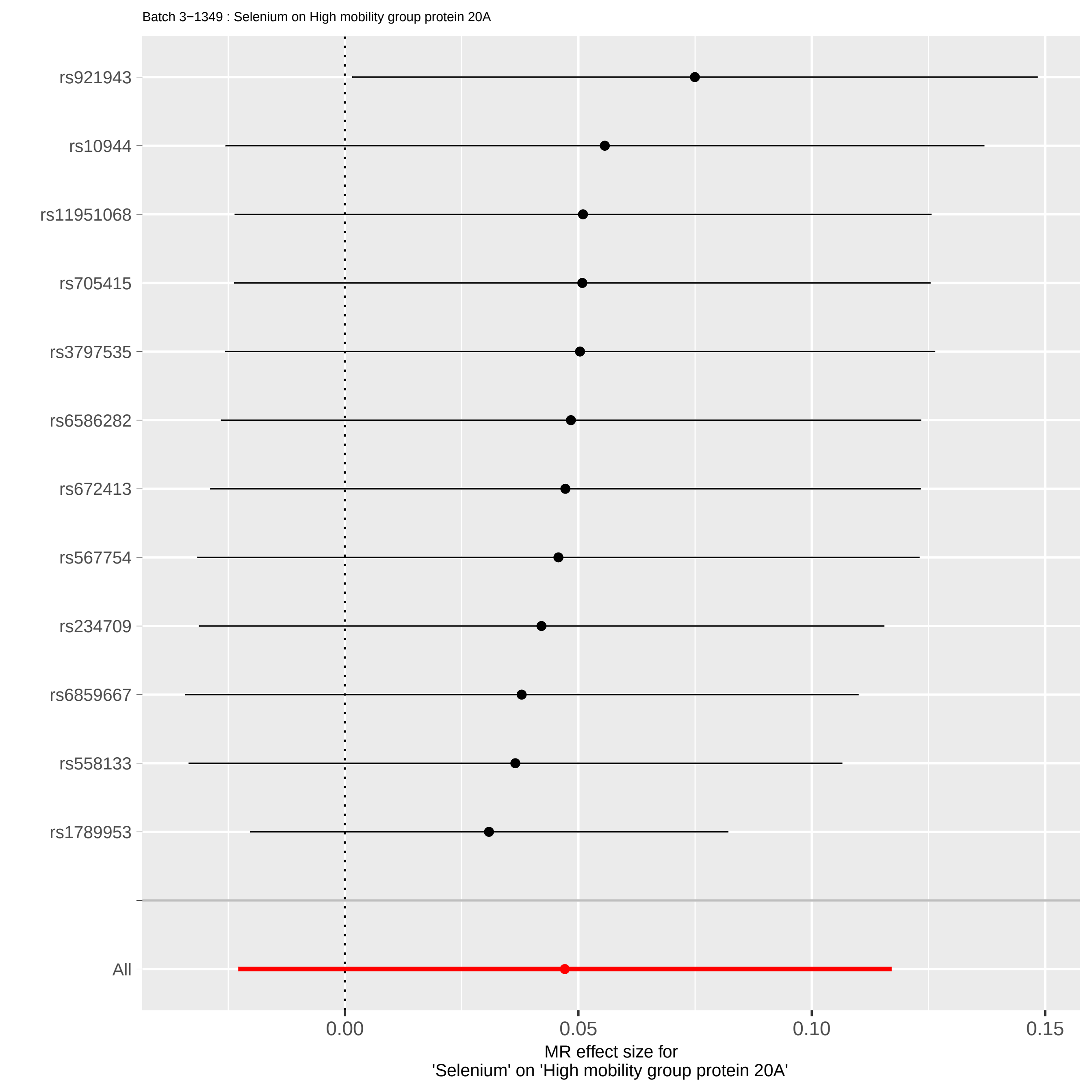

Batch 3−1349 : Selenium on High mobility group protein 20A
rs921943
rs10944
rs11951068
rs705415
rs3797535
rs6586282
rs672413
rs567754
rs234709
rs6859667
rs558133
rs1789953
All
0.00
0.05
0.10
0.15
MR effect size for
'Selenium' on 'High mobility group protein 20A'

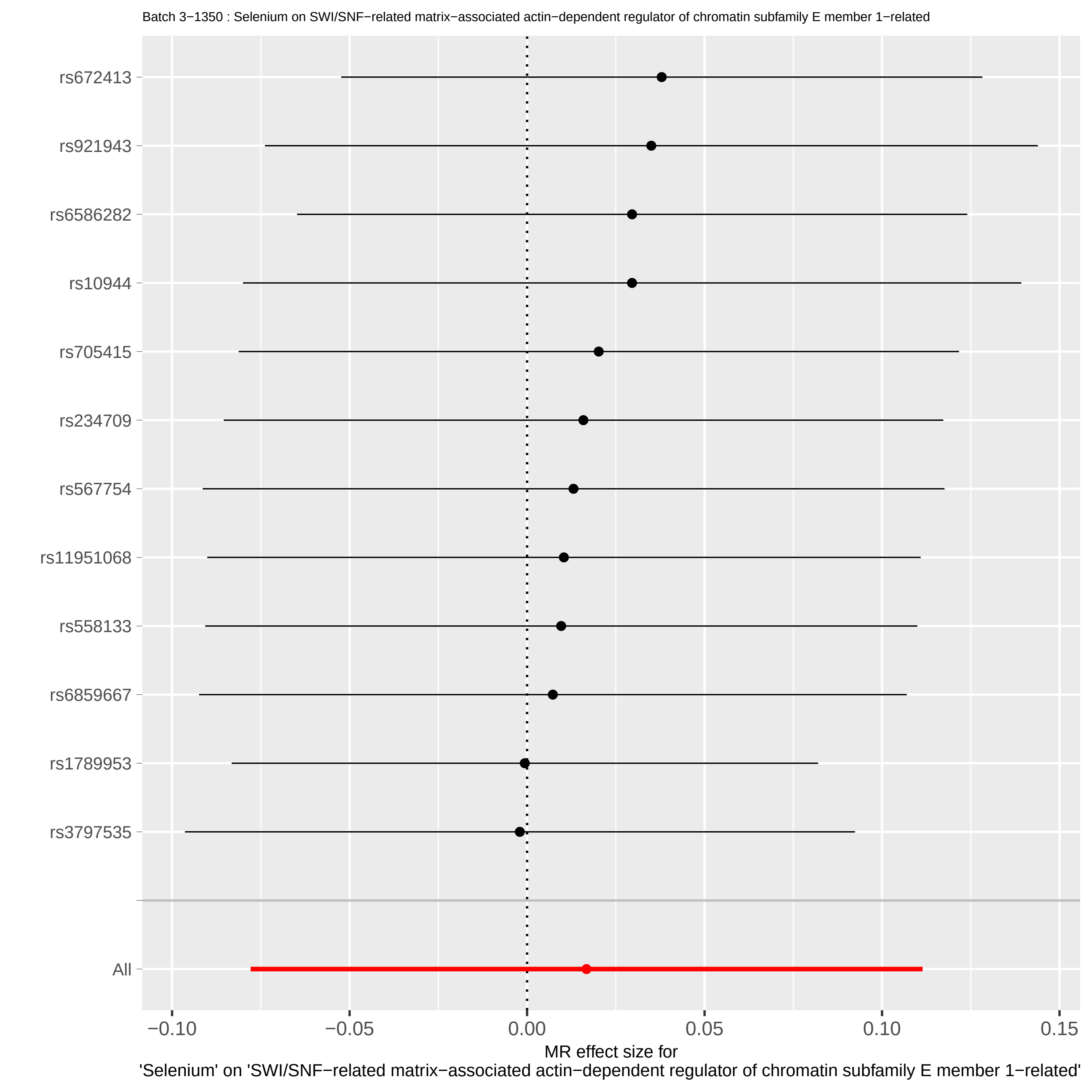
Batch 3−1350 : Selenium on SWI/SNF−related matrix−associated actin−dependent regulator of chromatin subfamily E member 1−related
rs672413
rs921943
rs6586282
rs10944
rs705415
rs234709
rs567754
rs11951068
rs558133
rs6859667
rs1789953
rs3797535
All
−0.10
−0.05
0.00
0.05
0.10
0.15
MR effect size for
'Selenium' on 'SWI/SNF−related matrix−associated actin−dependent regulator of chromatin subfamily E member 1−related'

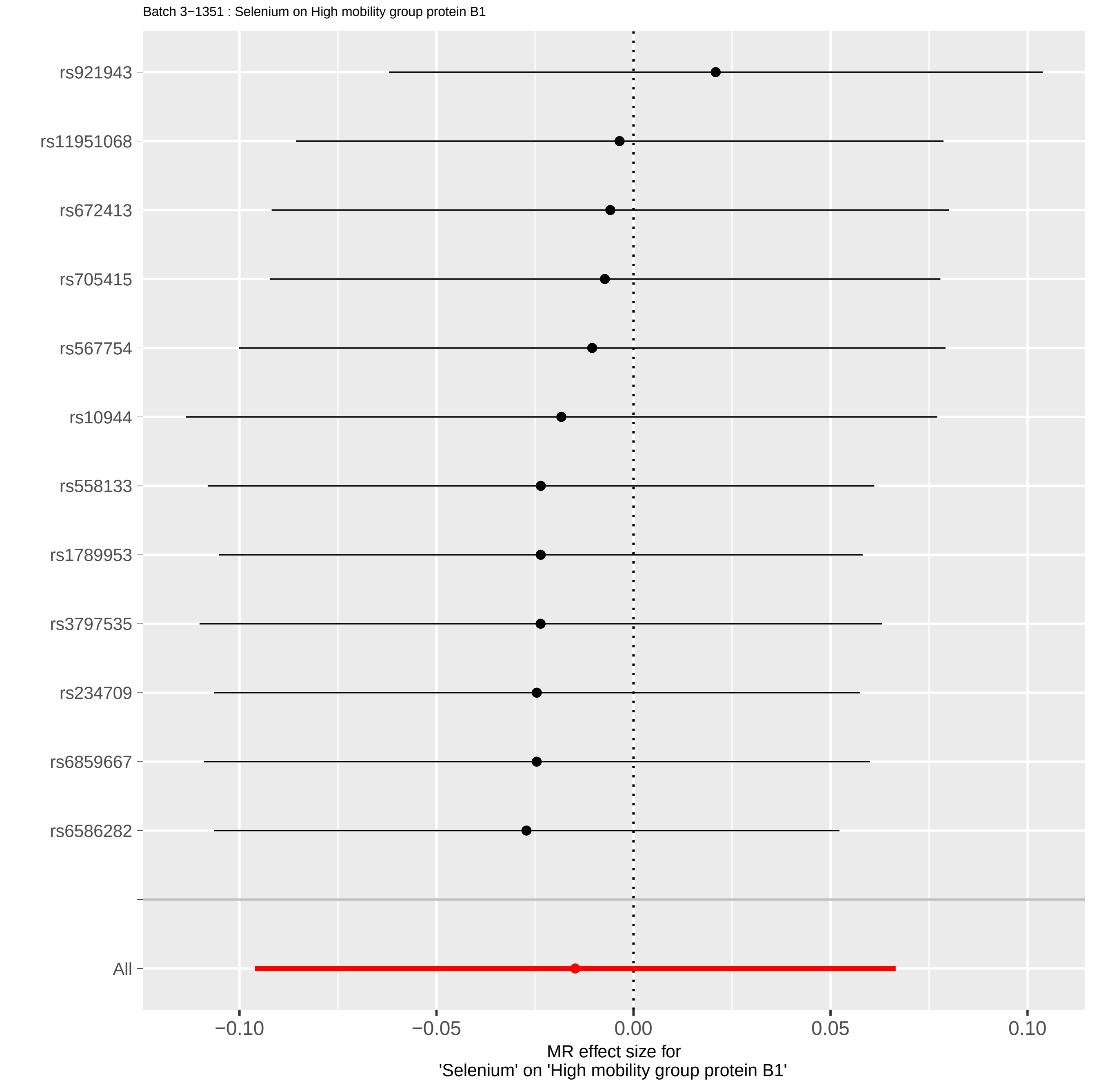

Batch 3−1351 : Selenium on High mobility group protein B1
rs921943
rs11951068
rs672413
rs705415
rs567754
rs10944
rs558133
rs1789953
rs3797535
rs234709
rs6859667
rs6586282
All
−0.10
−0.05
0.00
0.05
0.10
MR effect size for
'Selenium' on 'High mobility group protein B1'

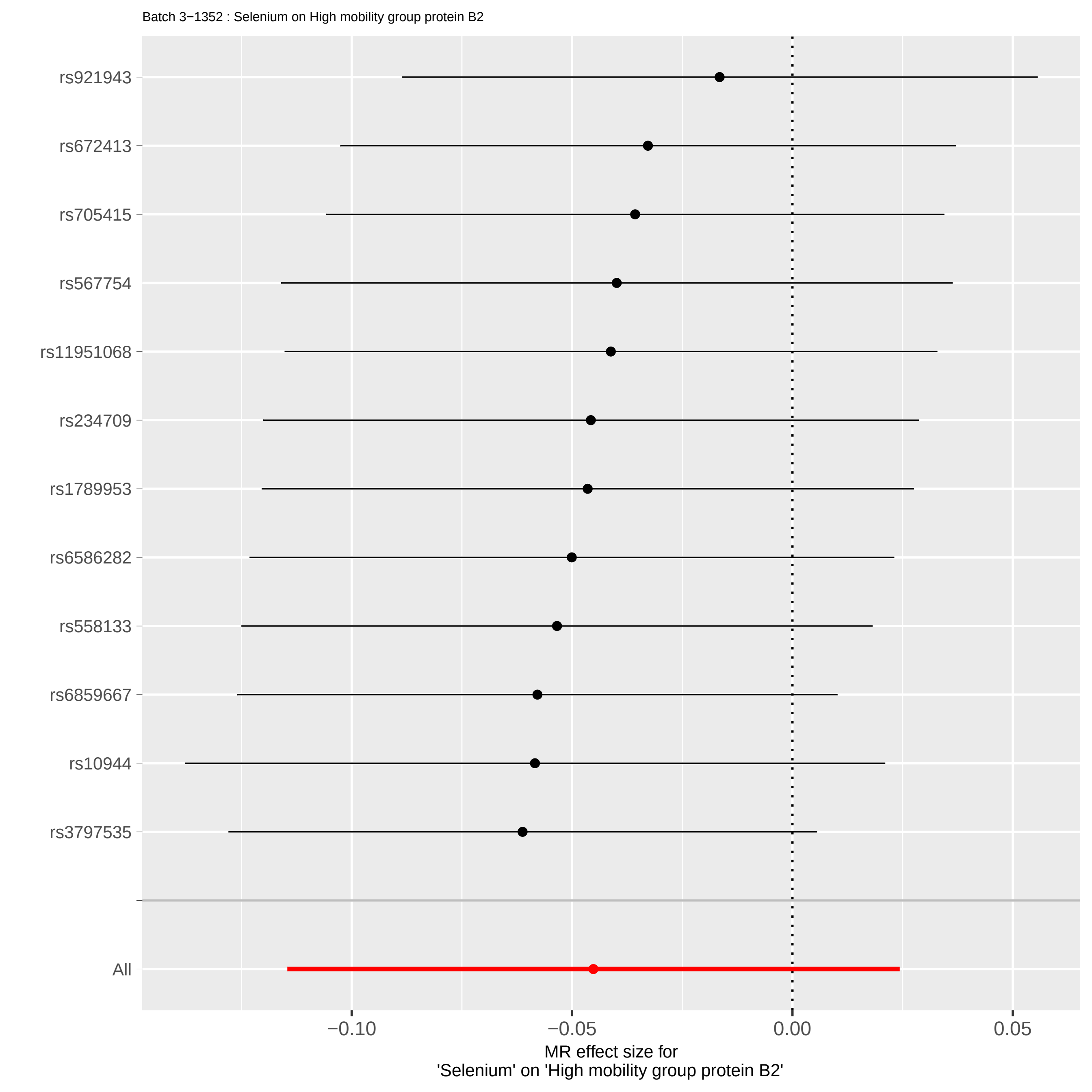

Batch 3−1352 : Selenium on High mobility group protein B2
rs921943
rs672413
rs705415
rs567754
rs11951068
rs234709
rs1789953
rs6586282
rs558133
rs6859667
rs10944
rs3797535
All
−0.10
−0.05
0.00
0.05
MR effect size for
'Selenium' on 'High mobility group protein B2'

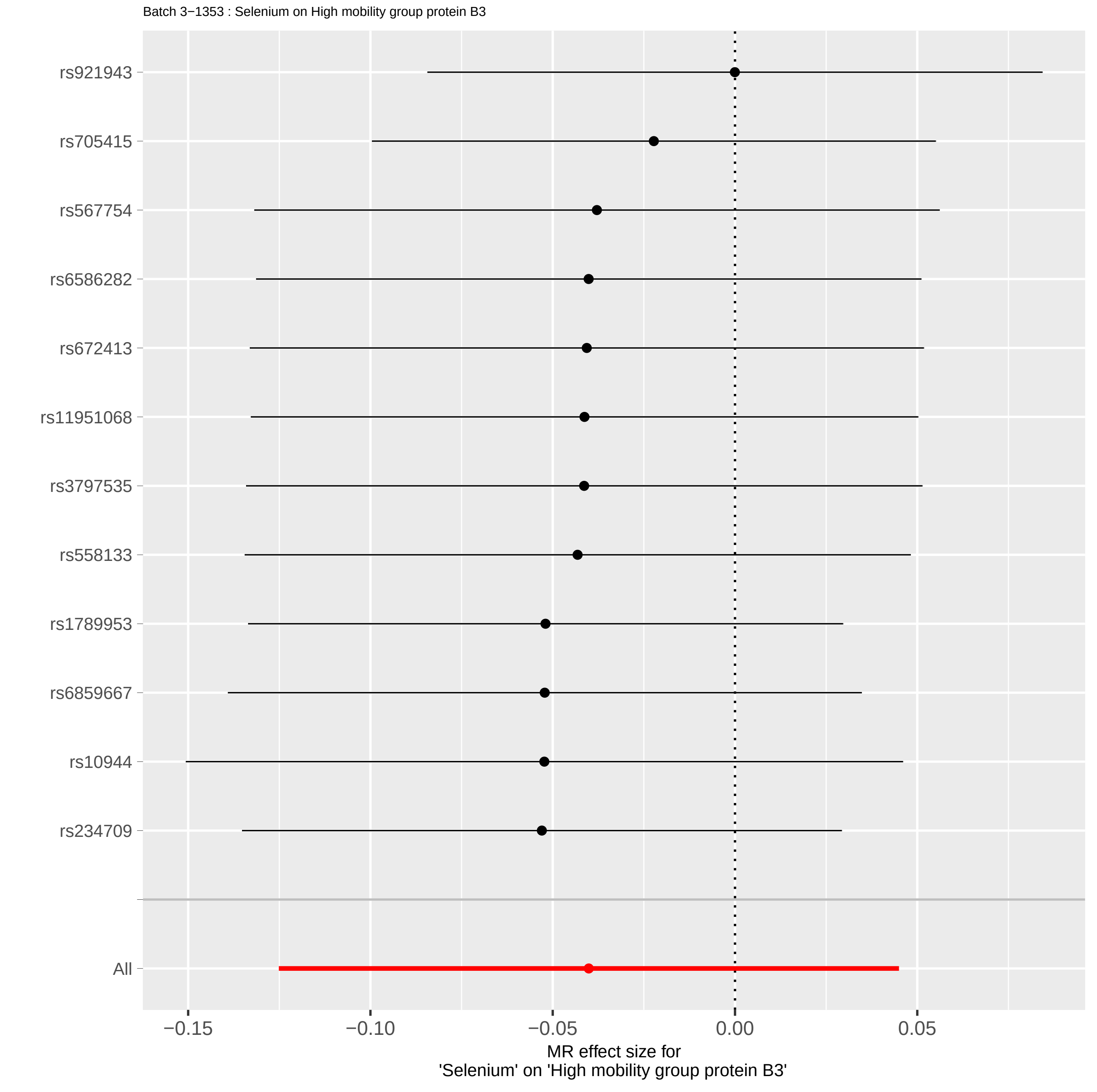

Batch 3−1353 : Selenium on High mobility group protein B3
rs921943
rs705415
rs567754
rs6586282
rs672413
rs11951068
rs3797535
rs558133
rs1789953
rs6859667
rs10944
rs234709
All
−0.15
−0.10
−0.05
0.00
0.05
MR effect size for
'Selenium' on 'High mobility group protein B3'

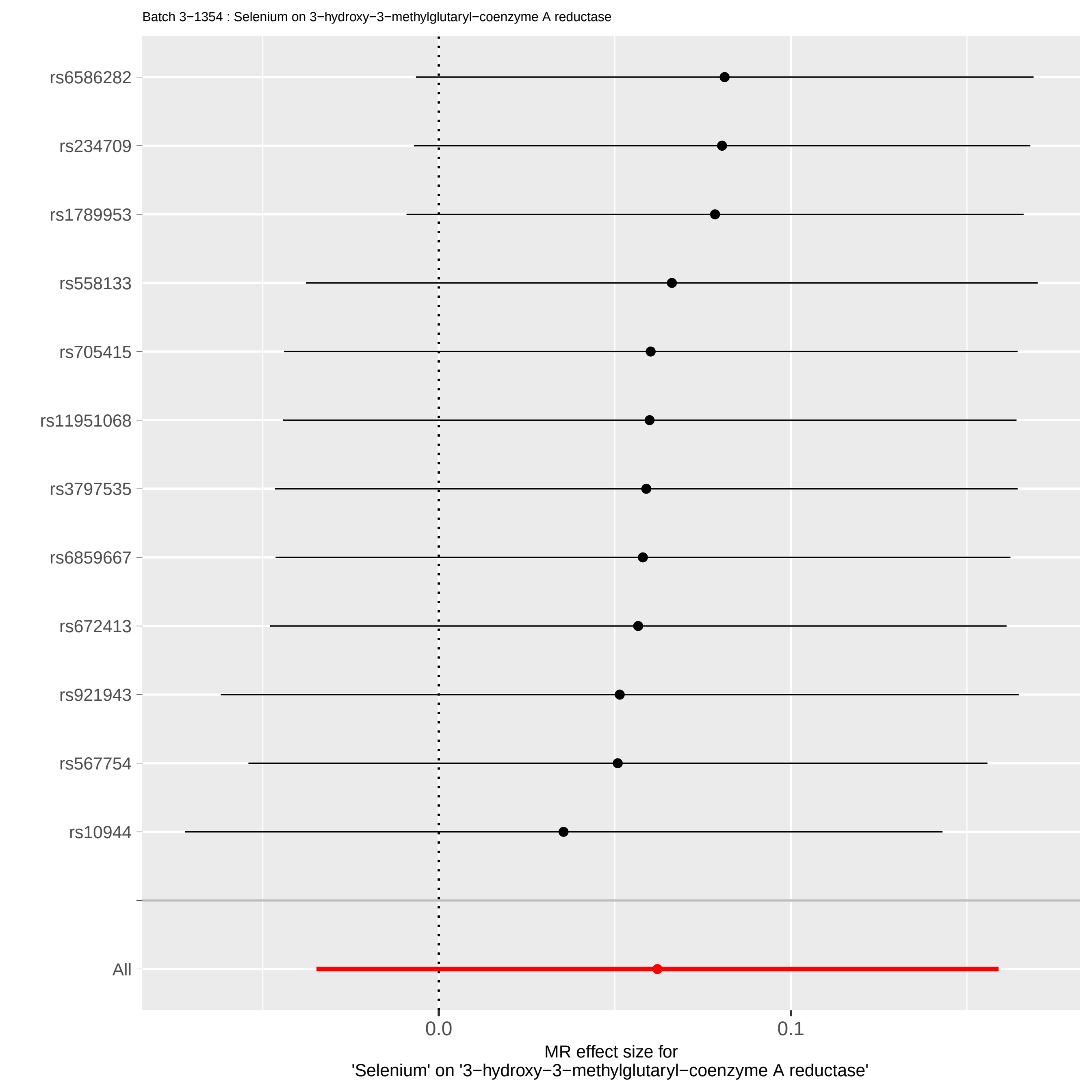
Batch 3−1354 : Selenium on 3−hydroxy−3−methylglutaryl−coenzyme A reductase
rs6586282
rs234709
rs1789953
rs558133
rs705415
rs11951068
rs3797535
rs6859667
rs672413
rs921943
rs567754
rs10944
All
0.0
0.1
MR effect size for
'Selenium' on '3−hydroxy−3−methylglutaryl−coenzyme A reductase'

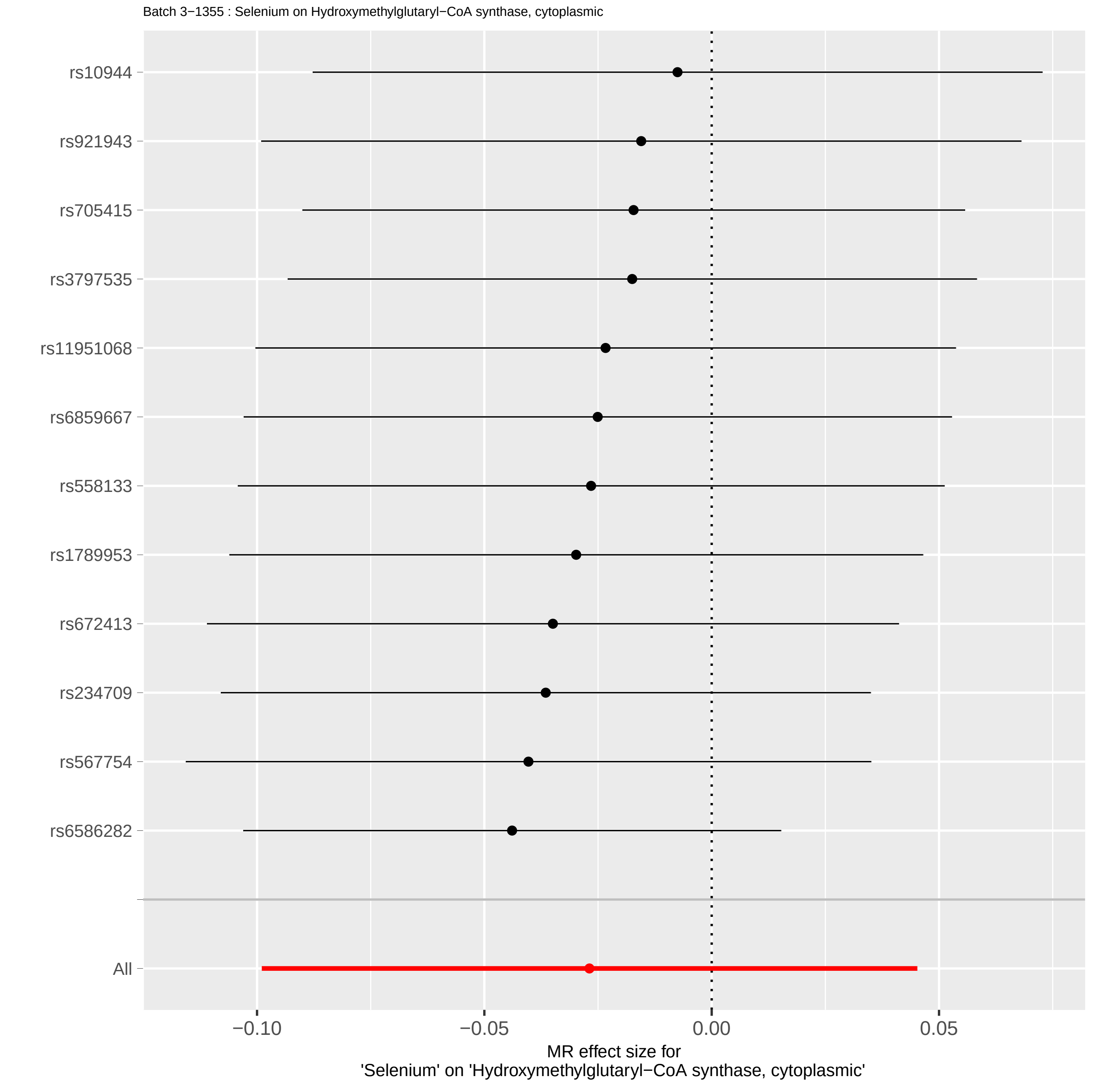

Batch 3−1355 : Selenium on Hydroxymethylglutaryl−CoA synthase, cytoplasmic
rs10944
rs921943
rs705415
rs3797535
rs11951068
rs6859667
rs558133
rs1789953
rs672413
rs234709
rs567754
rs6586282
All
−0.10
−0.05
0.00
0.05
MR effect size for
'Selenium' on 'Hydroxymethylglutaryl−CoA synthase, cytoplasmic'

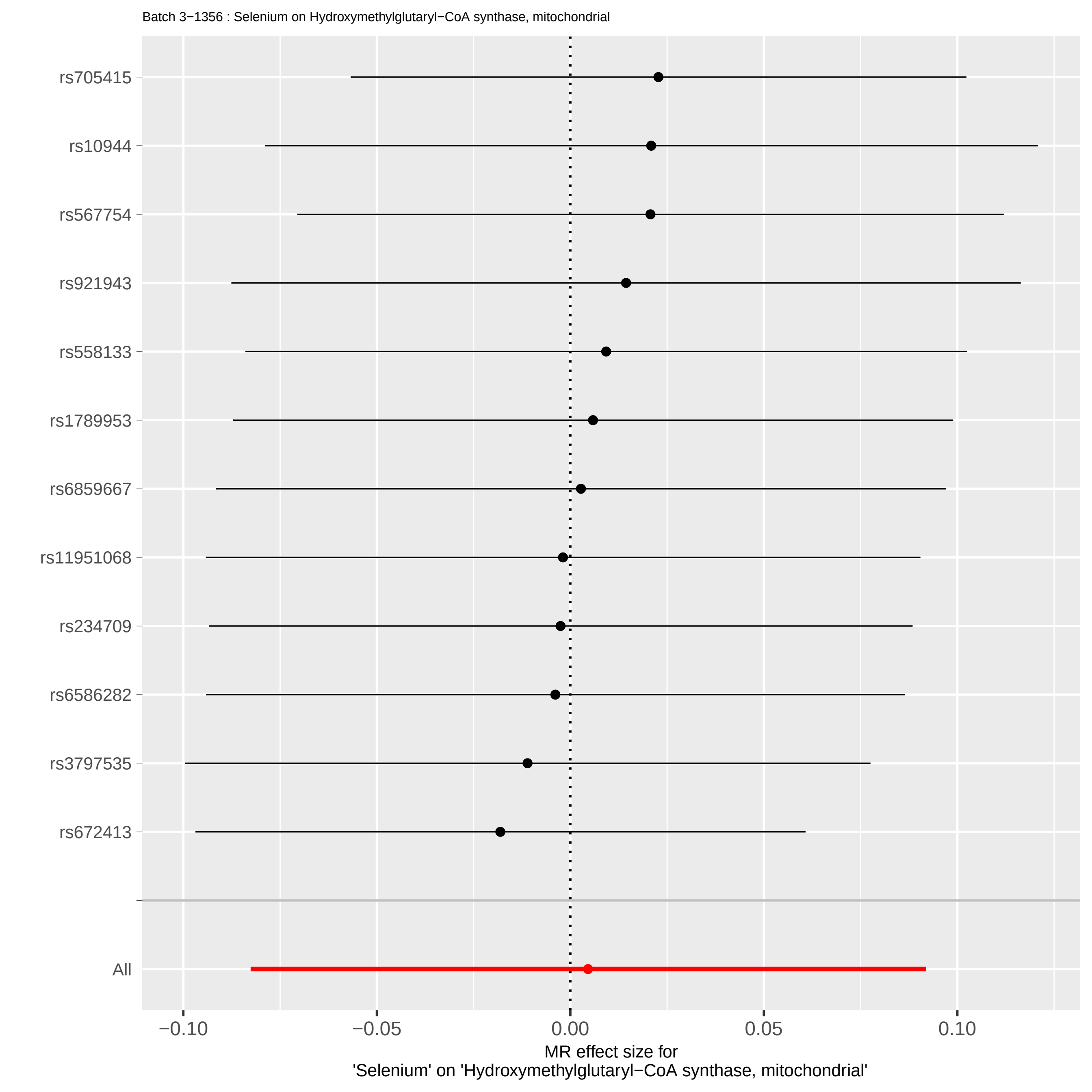

Batch 3−1356 : Selenium on Hydroxymethylglutaryl−CoA synthase, mitochondrial
rs705415
rs10944
rs567754
rs921943
rs558133
rs1789953
rs6859667
rs11951068
rs234709
rs6586282
rs3797535
rs672413
All
−0.10
−0.05
0.00
0.05
0.10
MR effect size for
'Selenium' on 'Hydroxymethylglutaryl−CoA synthase, mitochondrial'

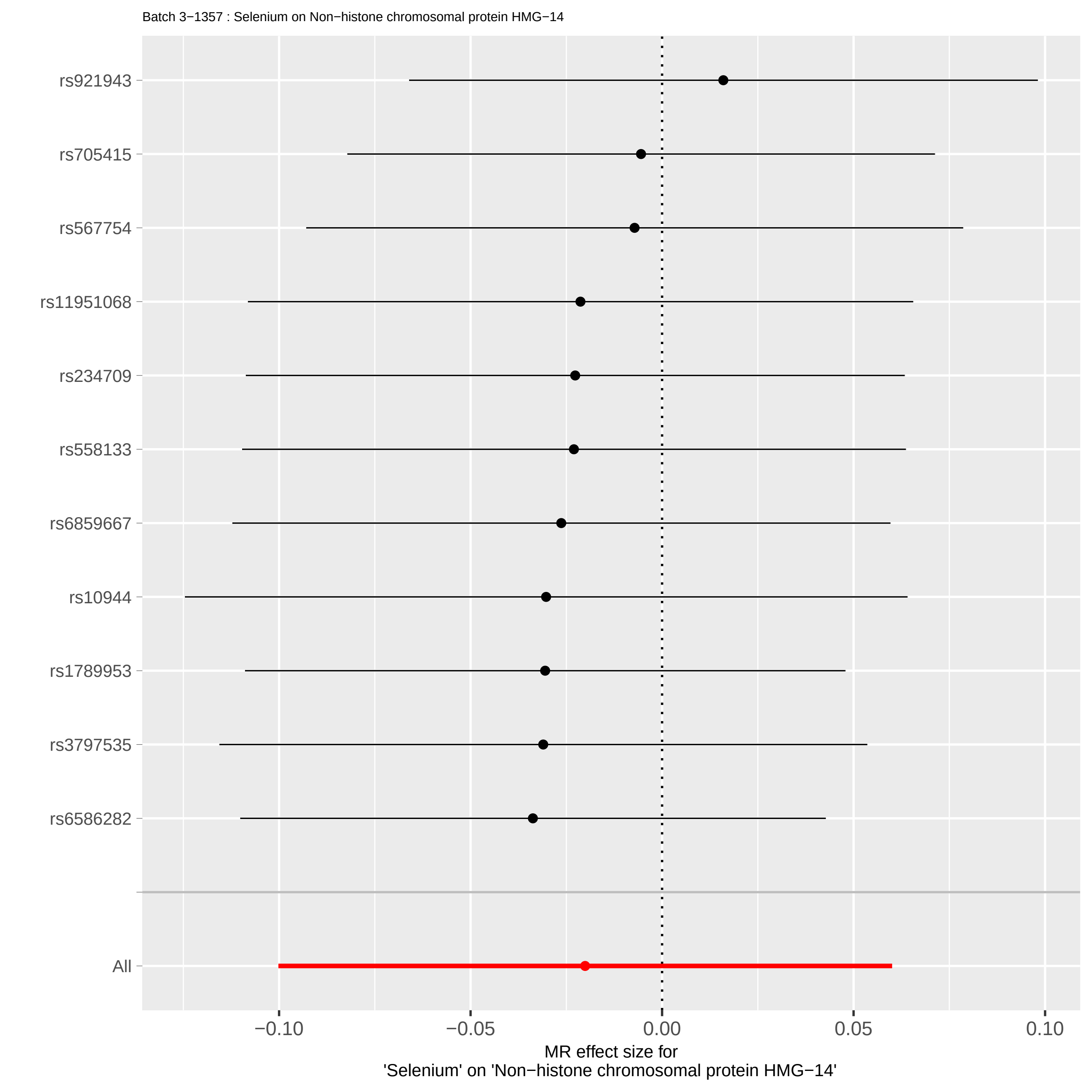

Batch 3−1357 : Selenium on Non−histone chromosomal protein HMG−14
rs921943
rs705415
rs567754
rs11951068
rs234709
rs558133
rs6859667
rs10944
rs1789953
rs3797535
rs6586282
All
−0.10
−0.05
0.00
0.05
0.10
MR effect size for
'Selenium' on 'Non−histone chromosomal protein HMG−14'

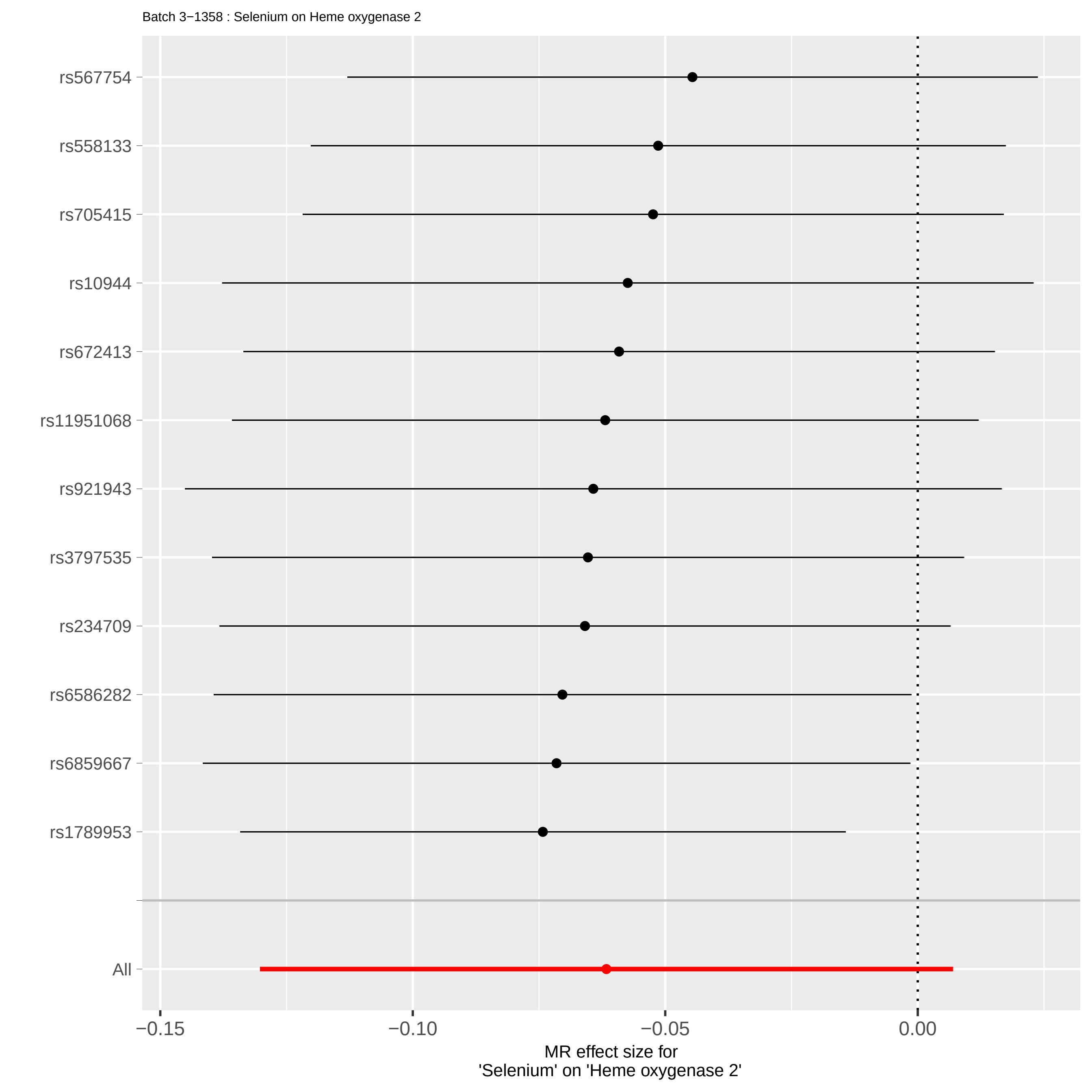

Batch 3−1358 : Selenium on Heme oxygenase 2
rs567754
rs558133
rs705415
rs10944
rs672413
rs11951068
rs921943
rs3797535
rs234709
rs6586282
rs6859667
rs1789953
All
−0.15
−0.10
−0.05
0.00
MR effect size for
'Selenium' on 'Heme oxygenase 2'

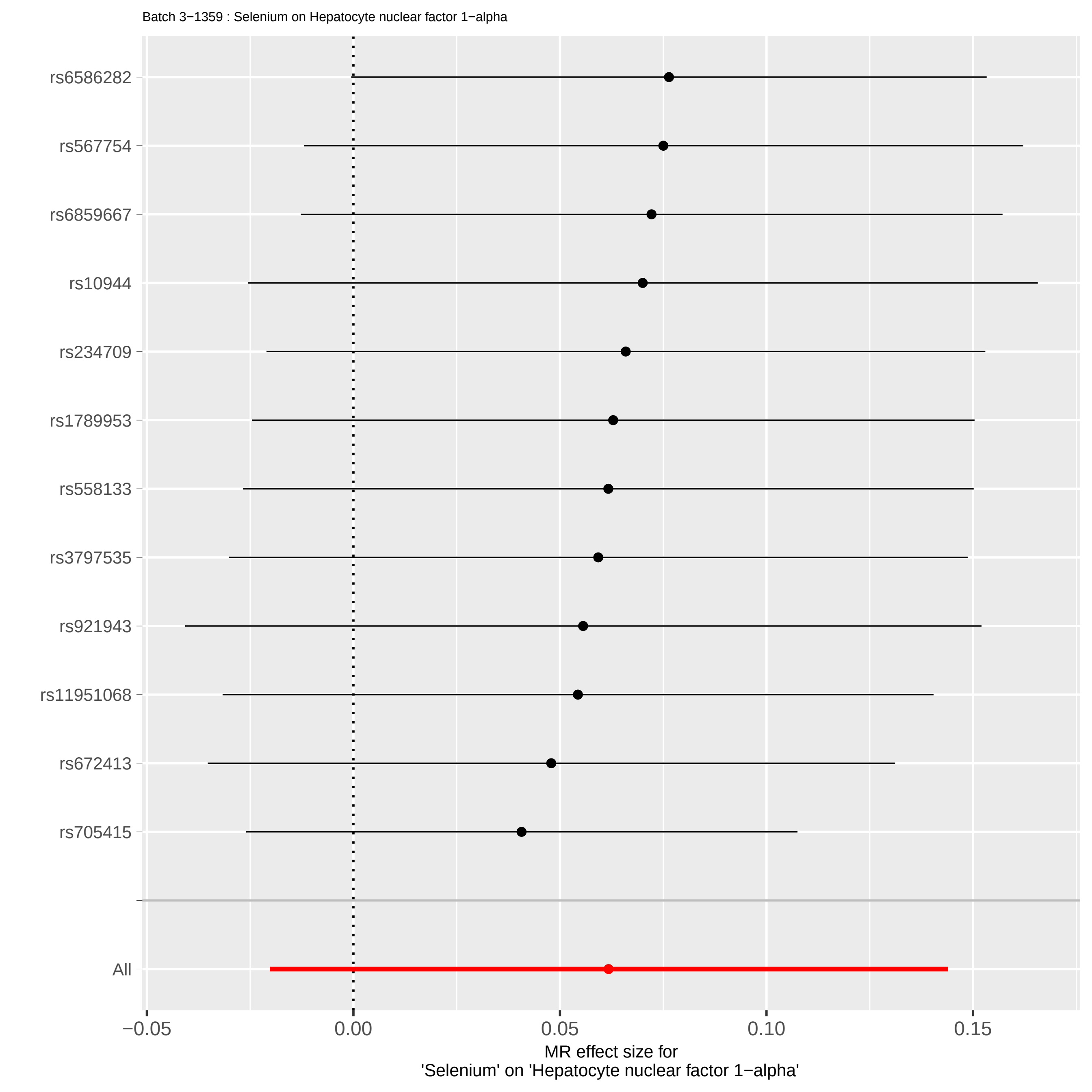
Batch 3−1359 : Selenium on Hepatocyte nuclear factor 1−alpha
rs6586282
rs567754
rs6859667
rs10944
rs234709
rs1789953
rs558133
rs3797535
rs921943
rs11951068
rs672413
rs705415
All
−0.05
0.00
0.05
0.10
0.15
MR effect size for
'Selenium' on 'Hepatocyte nuclear factor 1−alpha'

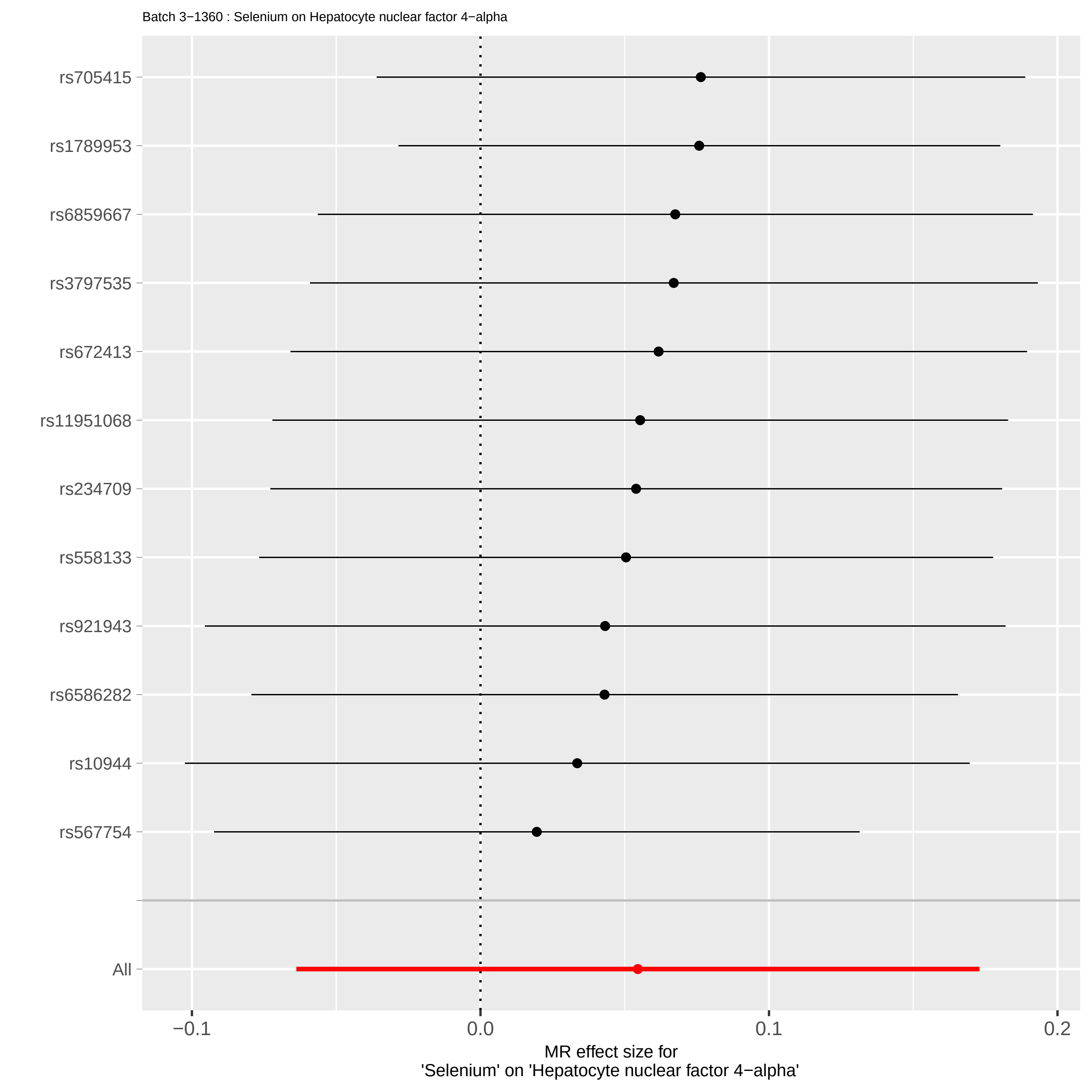

Batch 3−1360 : Selenium on Hepatocyte nuclear factor 4−alpha
rs705415
rs1789953
rs6859667
rs3797535
rs672413
rs11951068
rs234709
rs558133
rs921943
rs6586282
rs10944
rs567754
All
−0.1
0.0
0.1
0.2
MR effect size for
'Selenium' on 'Hepatocyte nuclear factor 4−alpha'

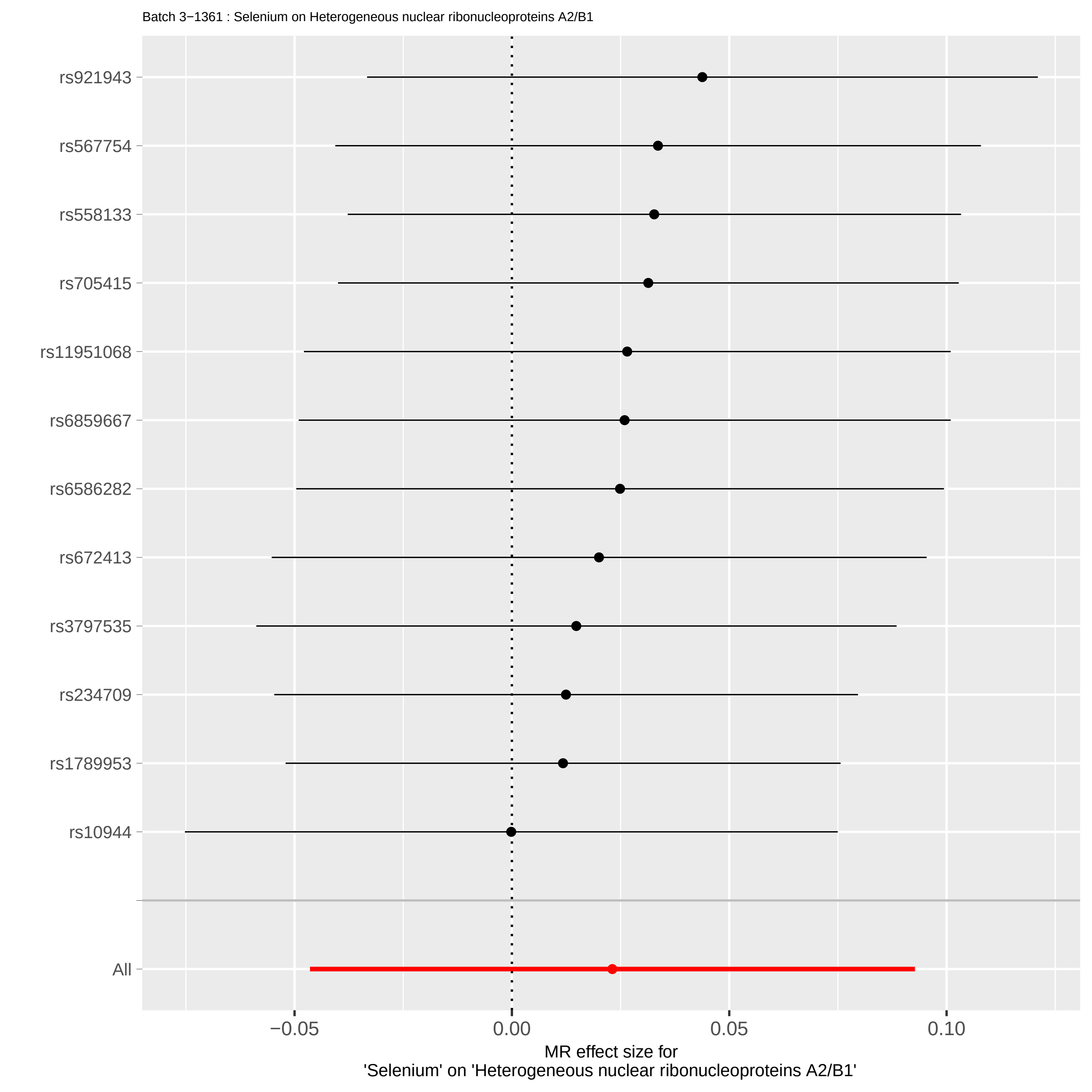

Batch 3−1361 : Selenium on Heterogeneous nuclear ribonucleoproteins A2/B1
rs921943
rs567754
rs558133
rs705415
rs11951068
rs6859667
rs6586282
rs672413
rs3797535
rs234709
rs1789953
rs10944
All
−0.05
0.00
0.05
0.10
MR effect size for
'Selenium' on 'Heterogeneous nuclear ribonucleoproteins A2/B1'

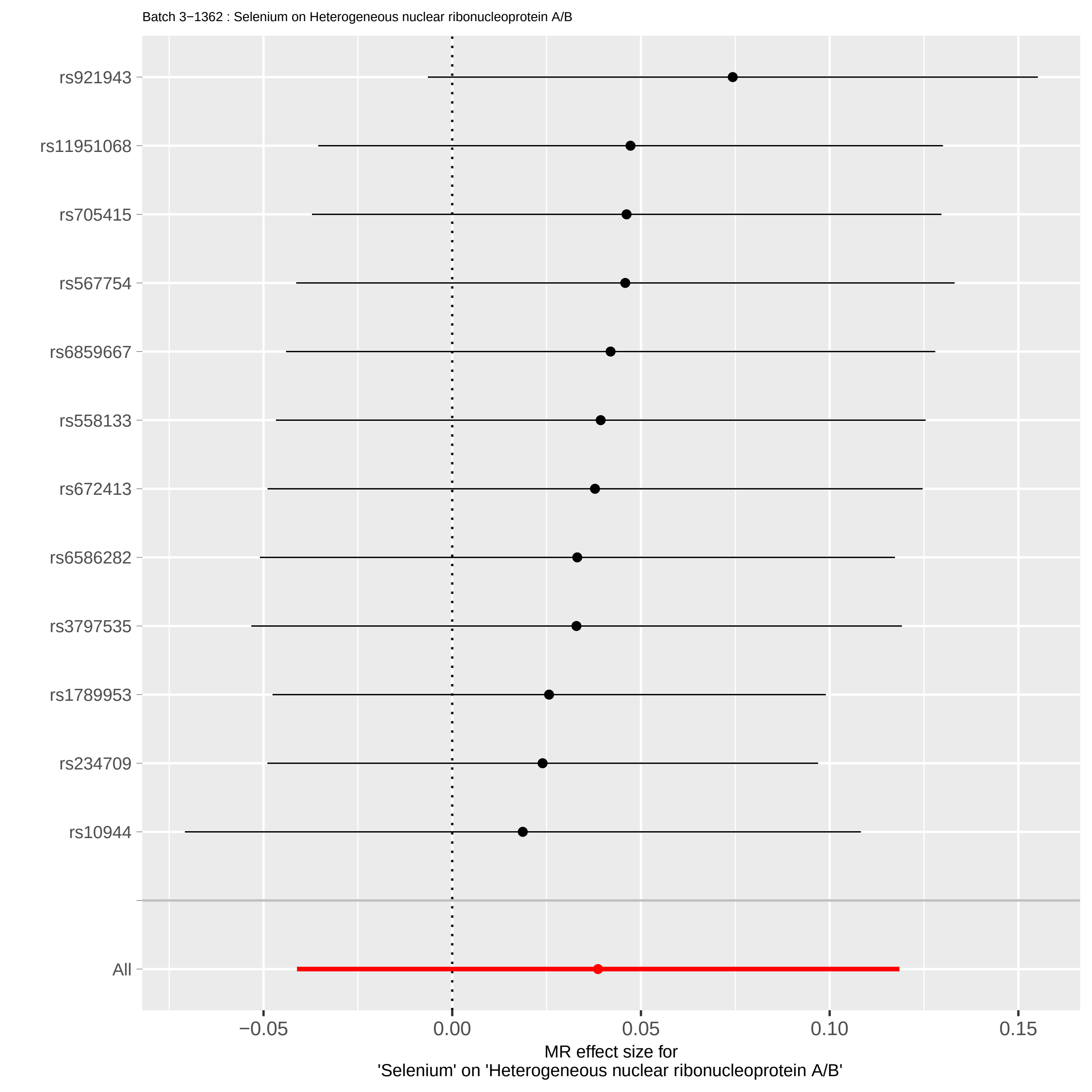

Batch 3−1362 : Selenium on Heterogeneous nuclear ribonucleoprotein A/B
rs921943
rs11951068
rs705415
rs567754
rs6859667
rs558133
rs672413
rs6586282
rs3797535
rs1789953
rs234709
rs10944
All
−0.05
0.00
0.05
0.10
0.15
MR effect size for
'Selenium' on 'Heterogeneous nuclear ribonucleoprotein A/B'

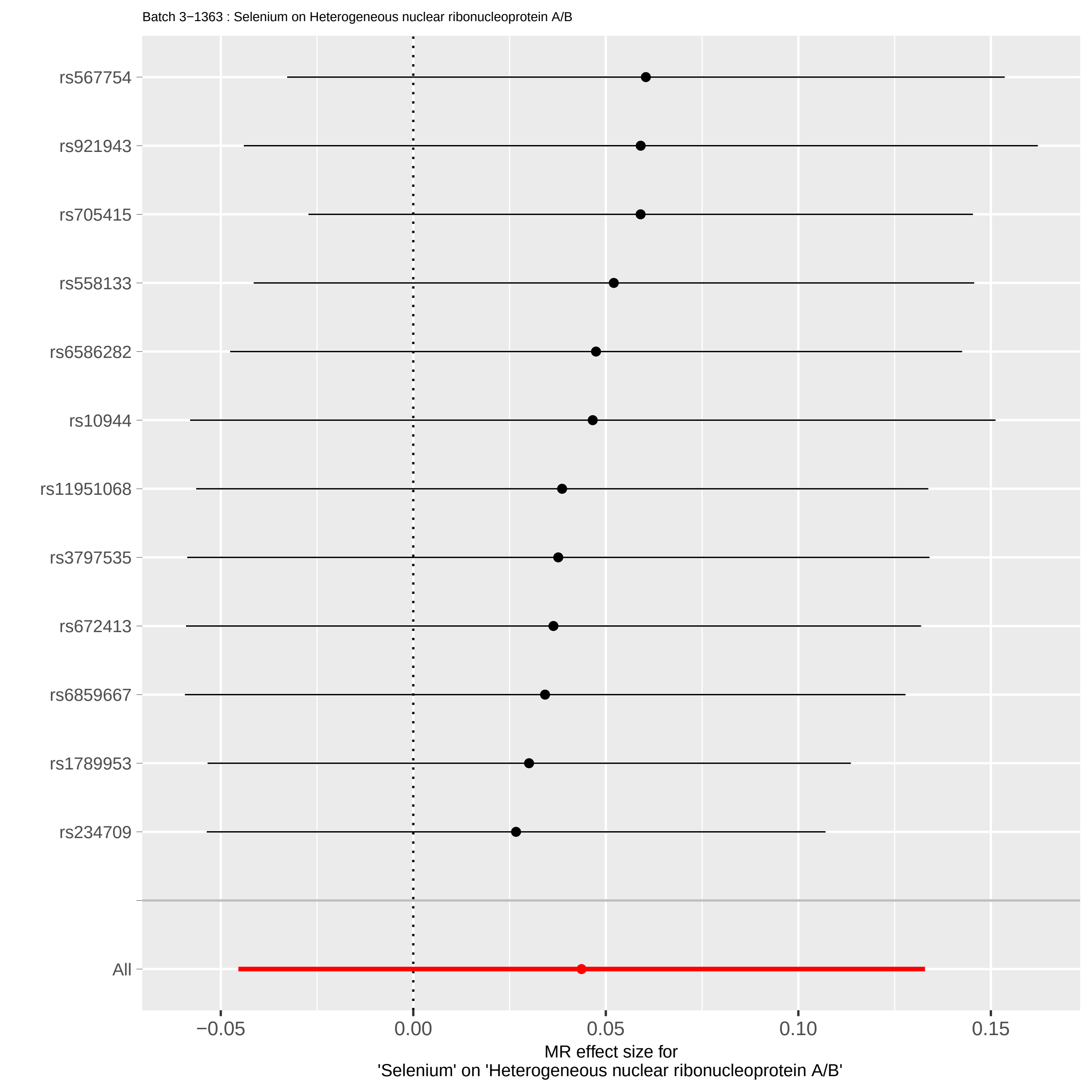
Batch 3−1363 : Selenium on Heterogeneous nuclear ribonucleoprotein A/B
rs567754
rs921943
rs705415
rs558133
rs6586282
rs10944
rs11951068
rs3797535
rs672413
rs6859667
rs1789953
rs234709
All
−0.05
0.00
0.05
0.10
0.15
MR effect size for
'Selenium' on 'Heterogeneous nuclear ribonucleoprotein A/B'

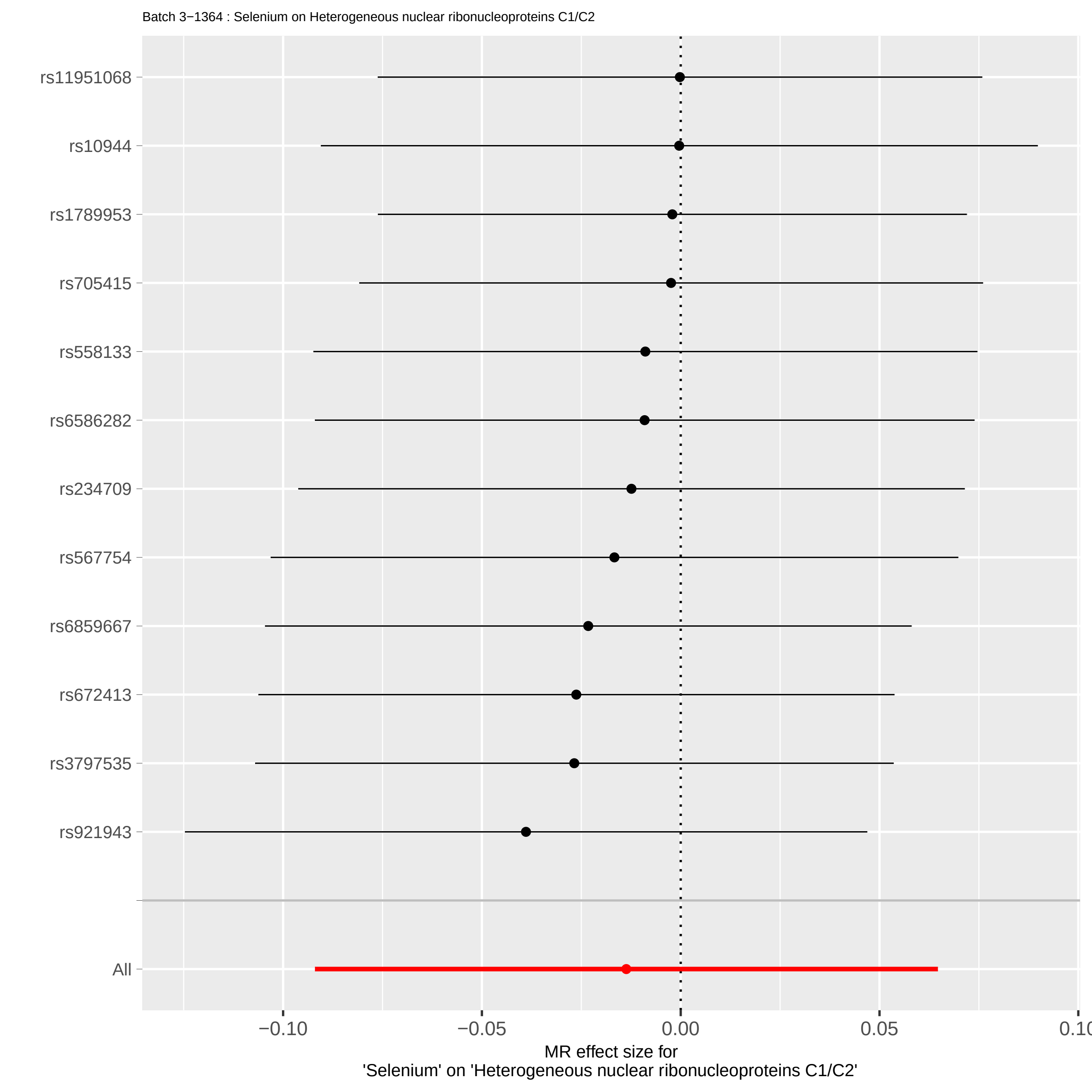

Batch 3−1364 : Selenium on Heterogeneous nuclear ribonucleoproteins C1/C2
rs11951068
rs10944
rs1789953
rs705415
rs558133
rs6586282
rs234709
rs567754
rs6859667
rs672413
rs3797535
rs921943
All
−0.10
−0.05
0.00
0.05
0.10
MR effect size for
'Selenium' on 'Heterogeneous nuclear ribonucleoproteins C1/C2'

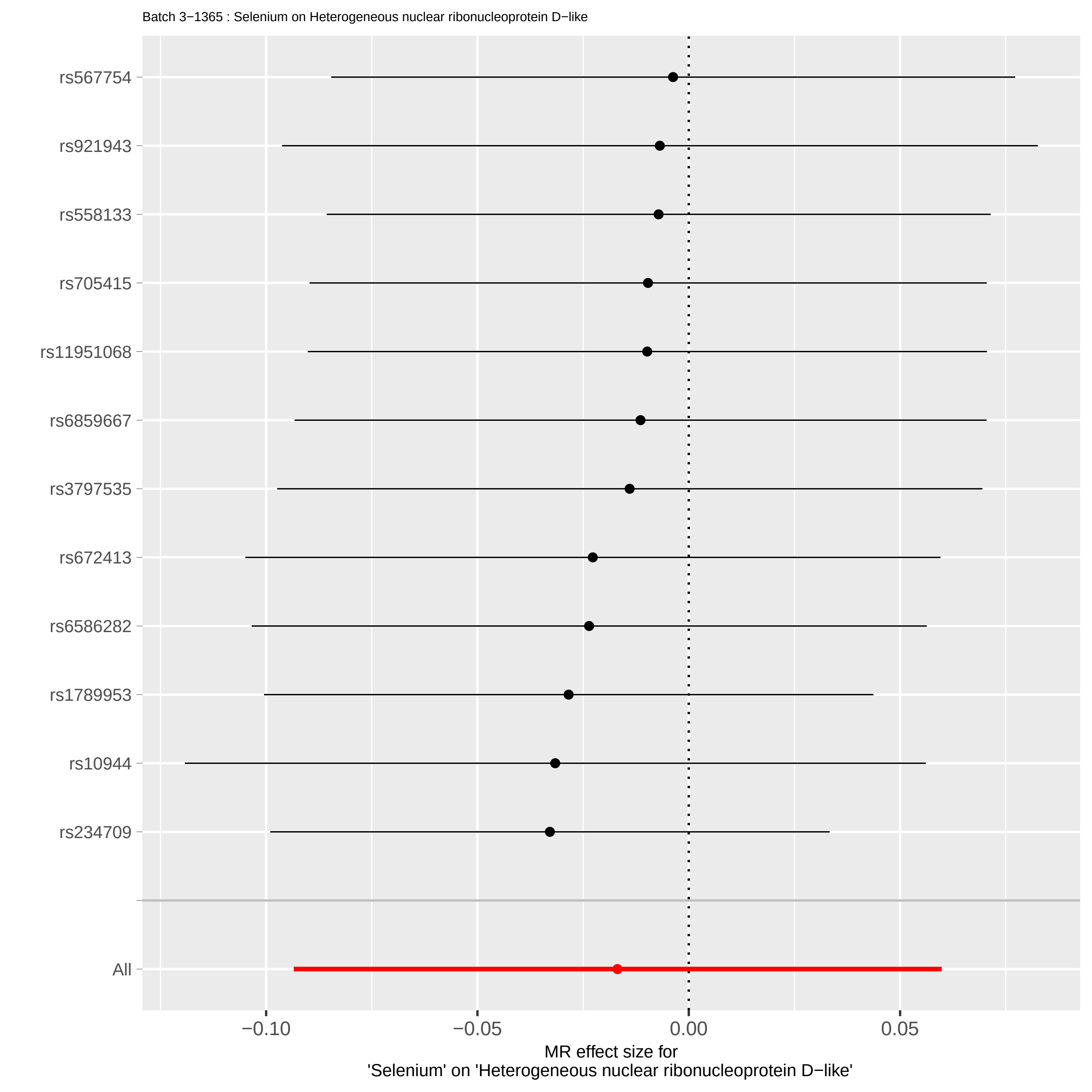
Batch 3−1365 : Selenium on Heterogeneous nuclear ribonucleoprotein D−like
rs567754
rs921943
rs558133
rs705415
rs11951068
rs6859667
rs3797535
rs672413
rs6586282
rs1789953
rs10944
rs234709
All
−0.10
−0.05
0.00
0.05
MR effect size for
'Selenium' on 'Heterogeneous nuclear ribonucleoprotein D−like'

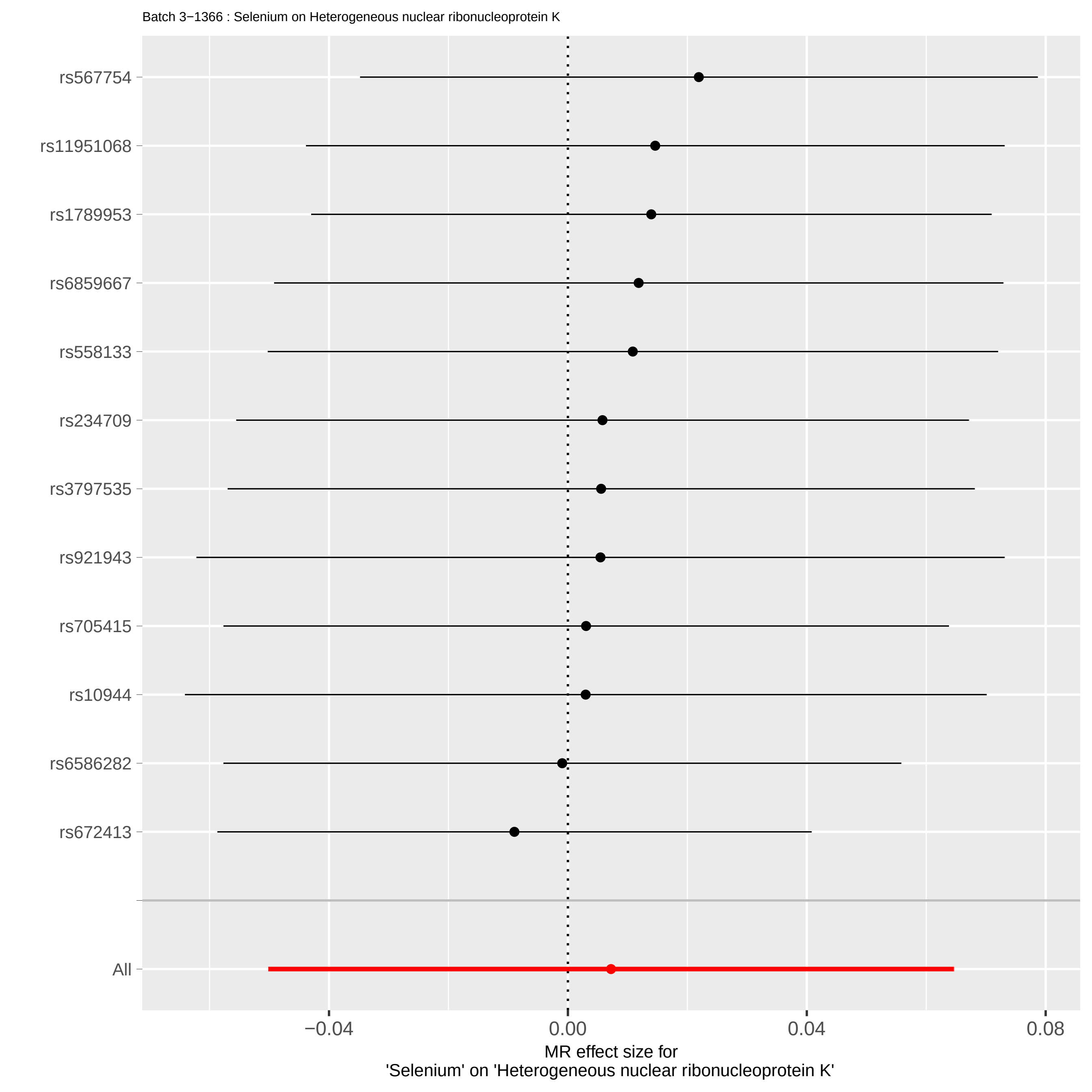
Batch 3−1366 : Selenium on Heterogeneous nuclear ribonucleoprotein K
rs567754
rs11951068
rs1789953
rs6859667
rs558133
rs234709
rs3797535
rs921943
rs705415
rs10944
rs6586282
rs672413
All
−0.04
0.00
0.04
0.08
MR effect size for
'Selenium' on 'Heterogeneous nuclear ribonucleoprotein K'

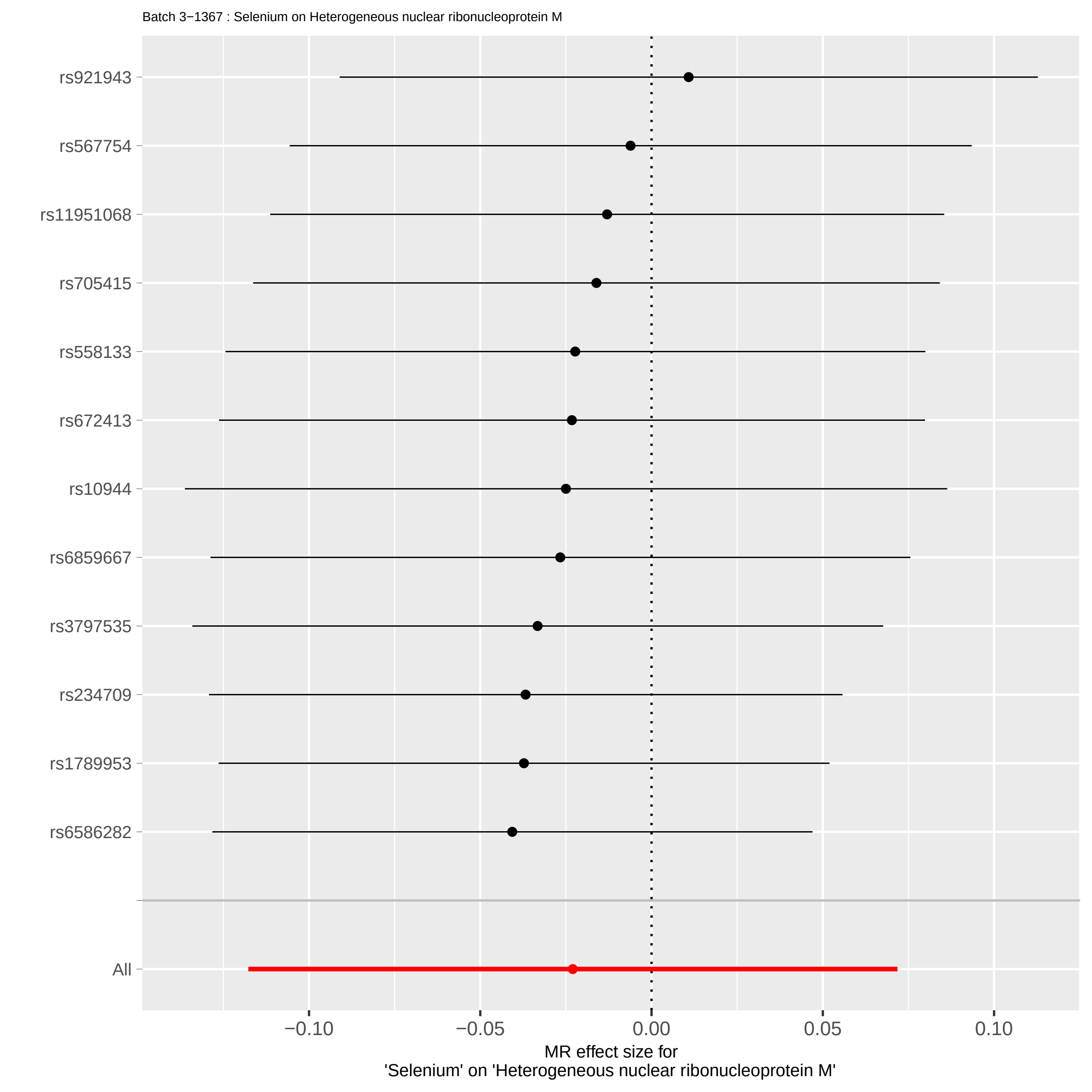
Batch 3−1367 : Selenium on Heterogeneous nuclear ribonucleoprotein M
rs921943
rs567754
rs11951068
rs705415
rs558133
rs672413
rs10944
rs6859667
rs3797535
rs234709
rs1789953
rs6586282
All
−0.10
−0.05
0.00
0.05
0.10
MR effect size for
'Selenium' on 'Heterogeneous nuclear ribonucleoprotein M'

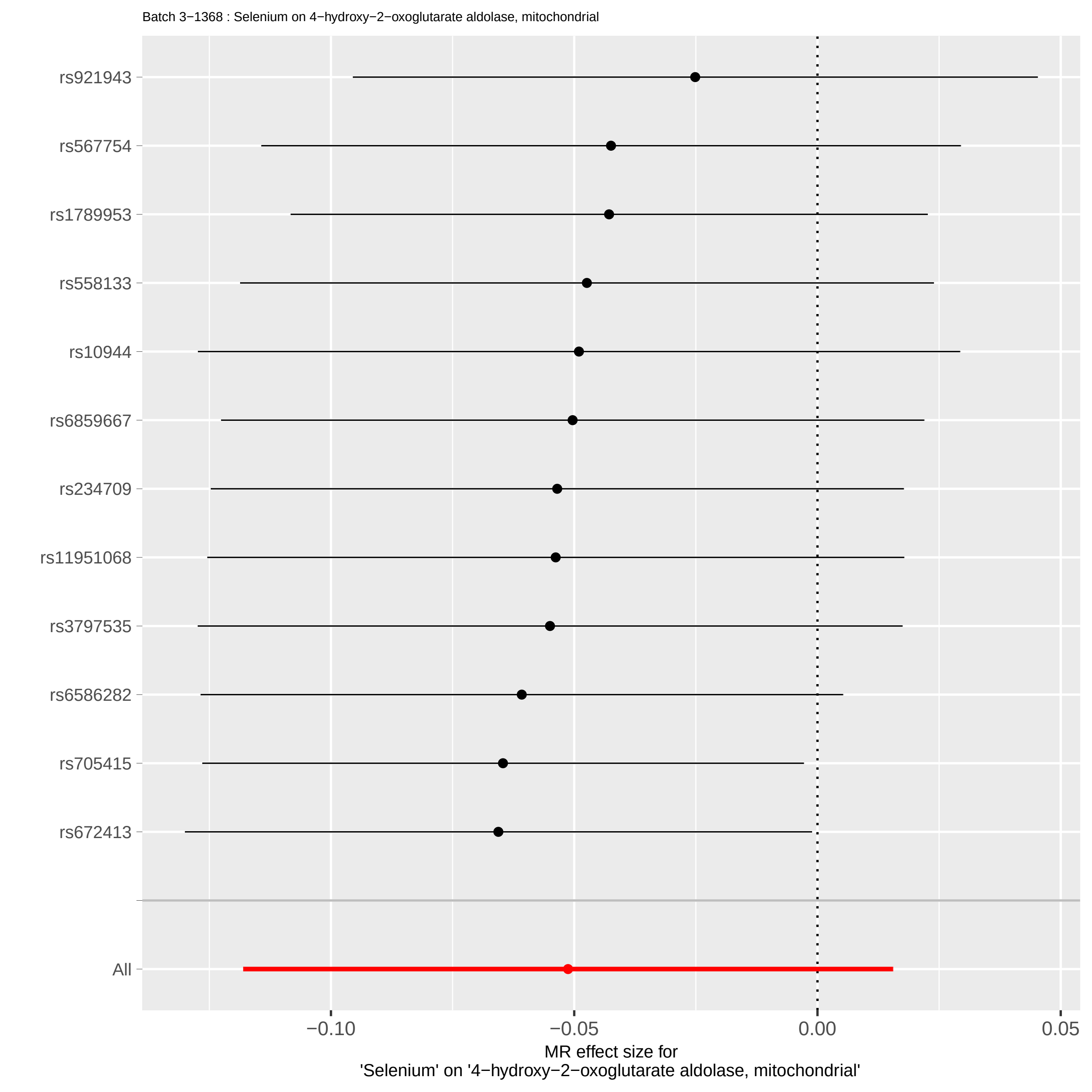
Batch 3−1368 : Selenium on 4−hydroxy−2−oxoglutarate aldolase, mitochondrial
rs921943
rs567754
rs1789953
rs558133
rs10944
rs6859667
rs234709
rs11951068
rs3797535
rs6586282
rs705415
rs672413
All
−0.10
−0.05
0.00
0.05
MR effect size for
'Selenium' on '4−hydroxy−2−oxoglutarate aldolase, mitochondrial'

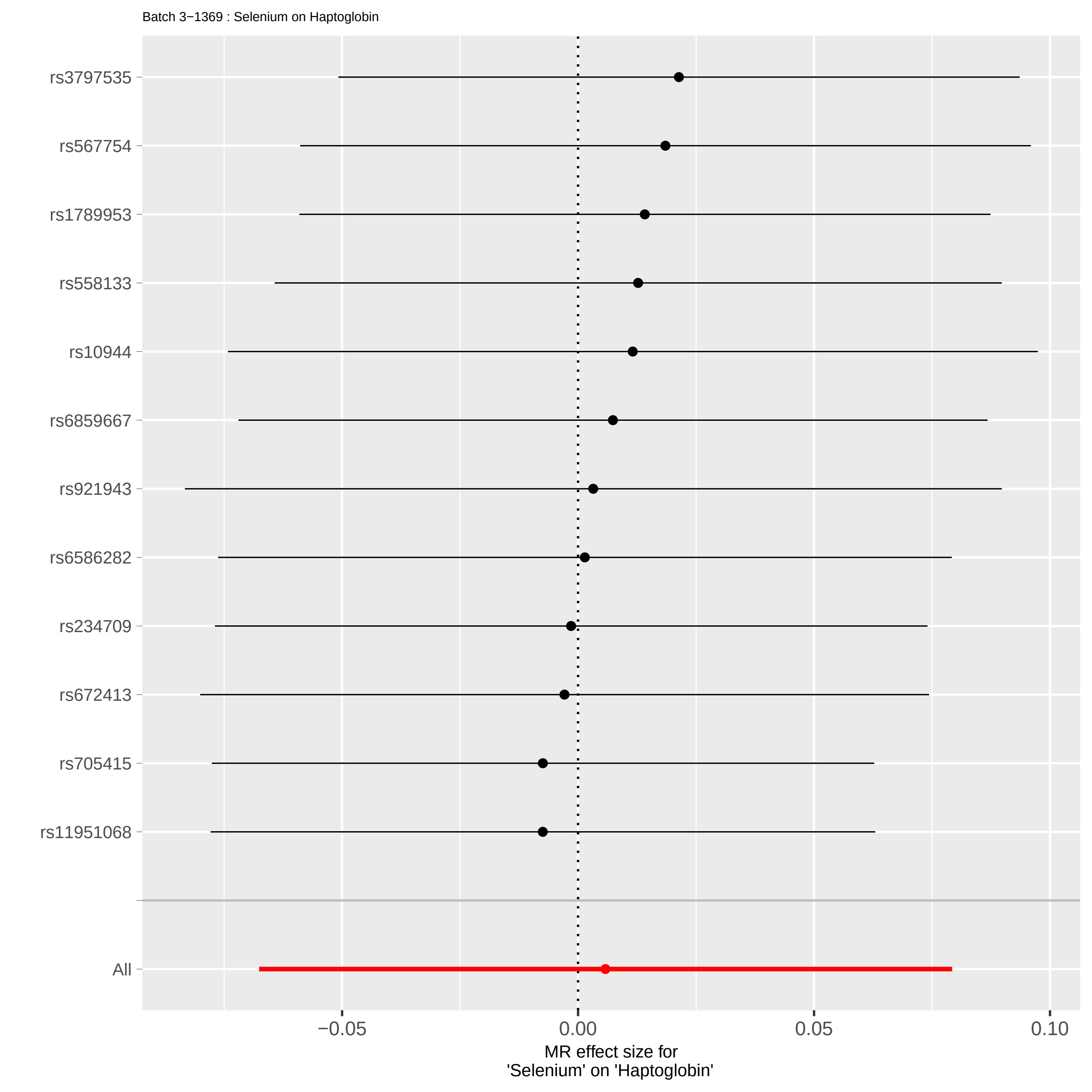

Batch 3−1369 : Selenium on Haptoglobin
rs3797535
rs567754
rs1789953
rs558133
rs10944
rs6859667
rs921943
rs6586282
rs234709
rs672413
rs705415
rs11951068
All
−0.05
0.00
0.05
0.10
MR effect size for
'Selenium' on 'Haptoglobin'

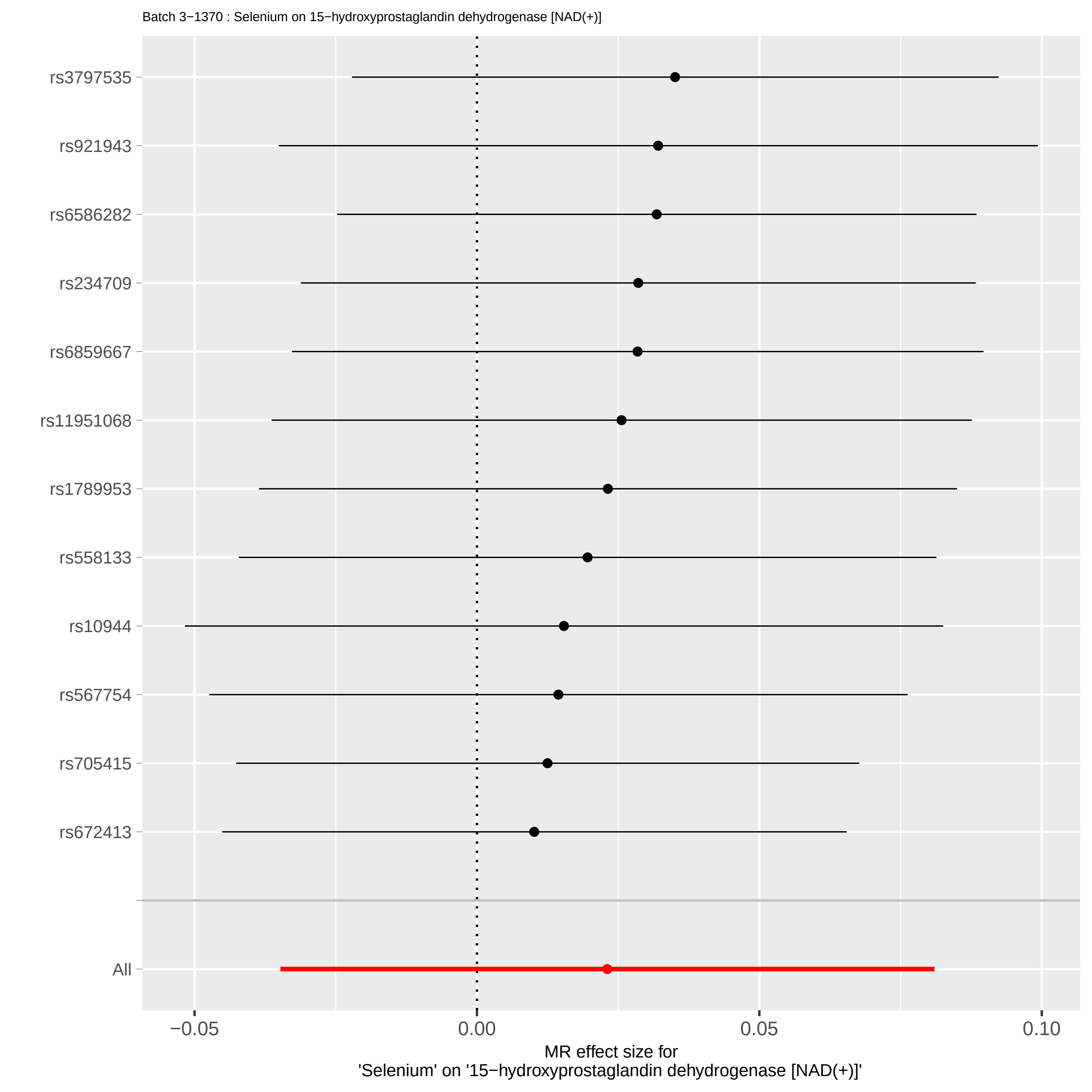
Batch 3−1370 : Selenium on 15−hydroxyprostaglandin dehydrogenase [NAD(+)]
rs3797535
rs921943
rs6586282
rs234709
rs6859667
rs11951068
rs1789953
rs558133
rs10944
rs567754
rs705415
rs672413
All
−0.05
0.00
0.05
0.10
MR effect size for
'Selenium' on '15−hydroxyprostaglandin dehydrogenase [NAD(+)]'

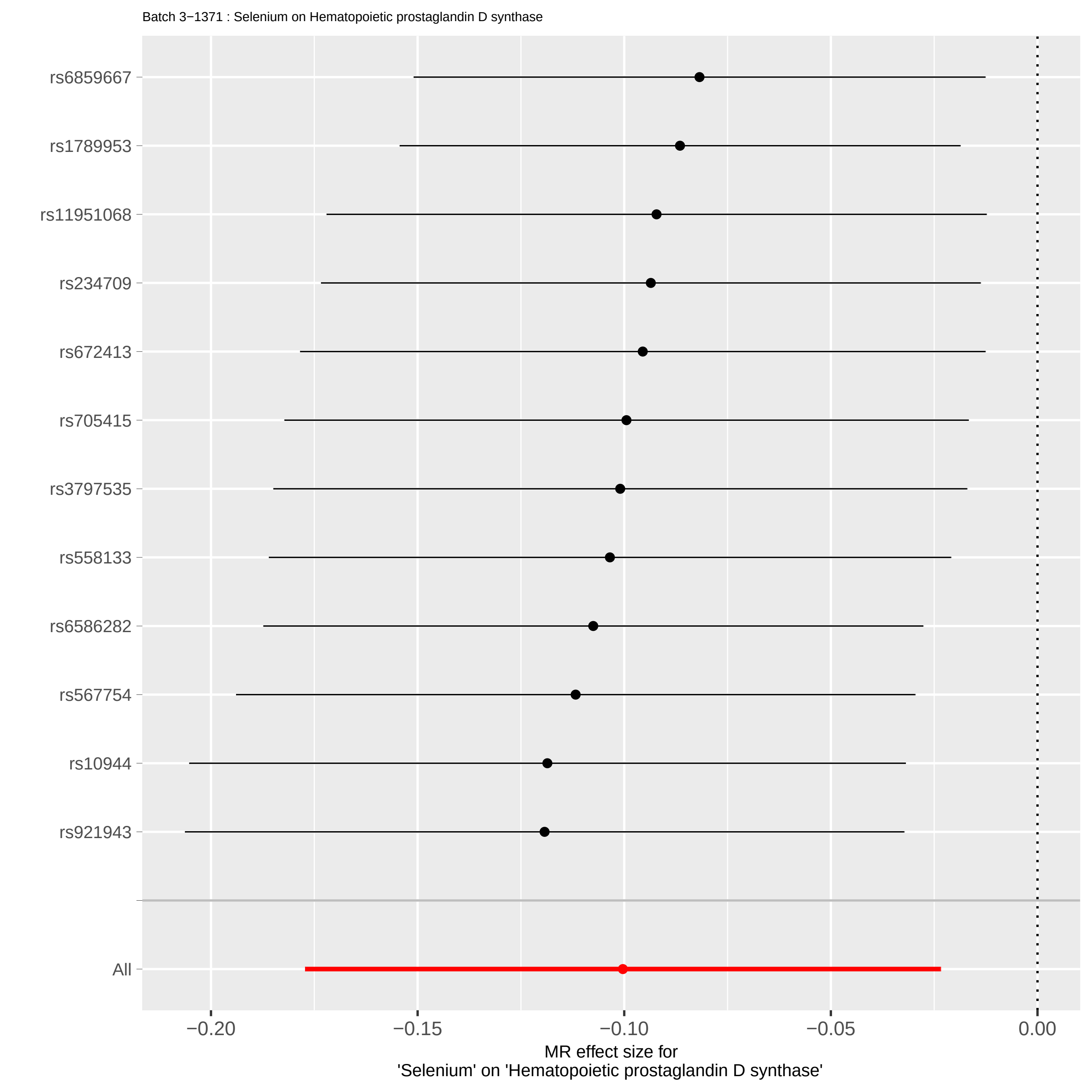
Batch 3−1371 : Selenium on Hematopoietic prostaglandin D synthase
rs6859667
rs1789953
rs11951068
rs234709
rs672413
rs705415
rs3797535
rs558133
rs6586282
rs567754
rs10944
rs921943
All
−0.20
−0.15
−0.10
−0.05
0.00
MR effect size for
'Selenium' on 'Hematopoietic prostaglandin D synthase'

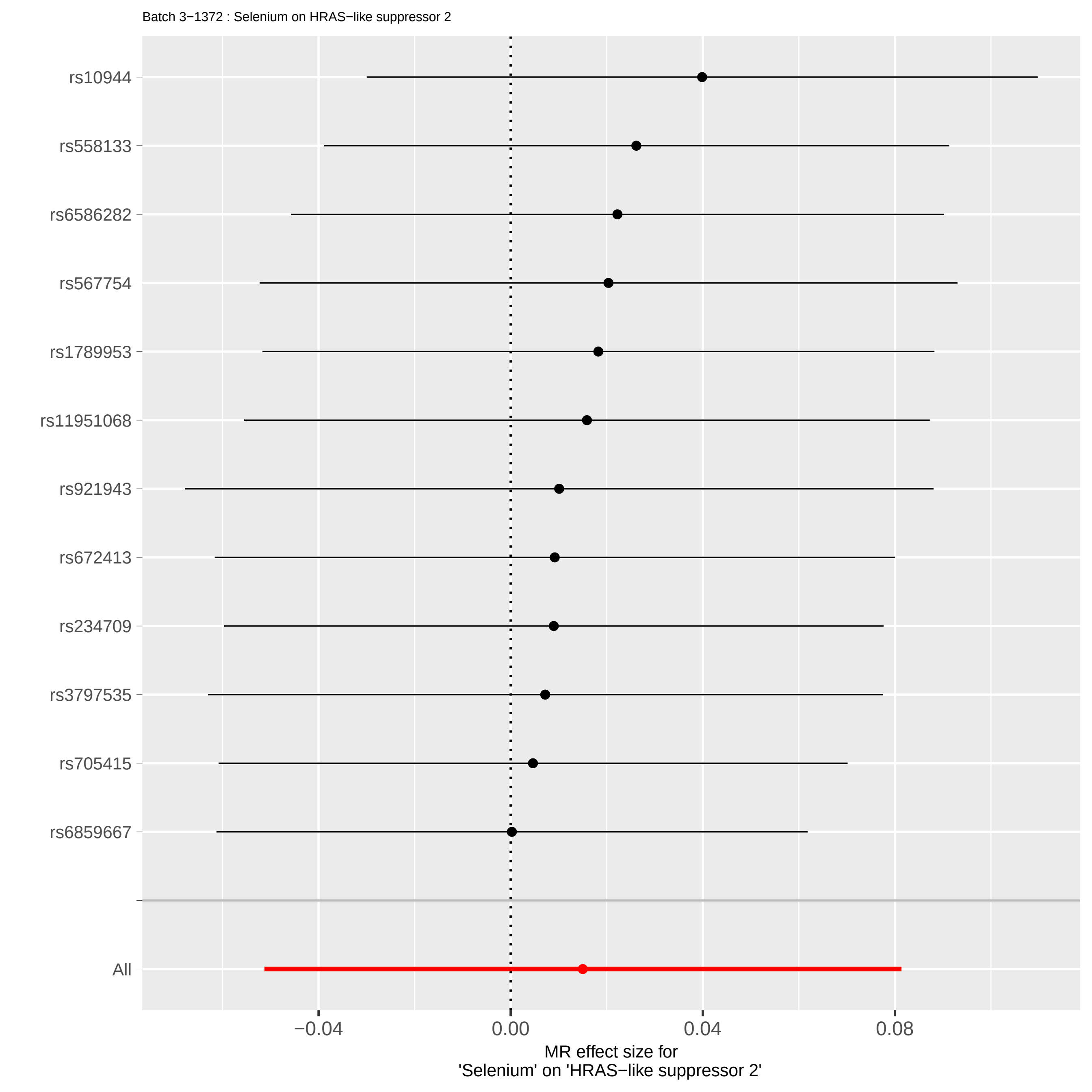

Batch 3−1372 : Selenium on HRAS−like suppressor 2
rs10944
rs558133
rs6586282
rs567754
rs1789953
rs11951068
rs921943
rs672413
rs234709
rs3797535
rs705415
rs6859667
All
−0.04
0.00
0.04
0.08
MR effect size for
'Selenium' on 'HRAS−like suppressor 2'

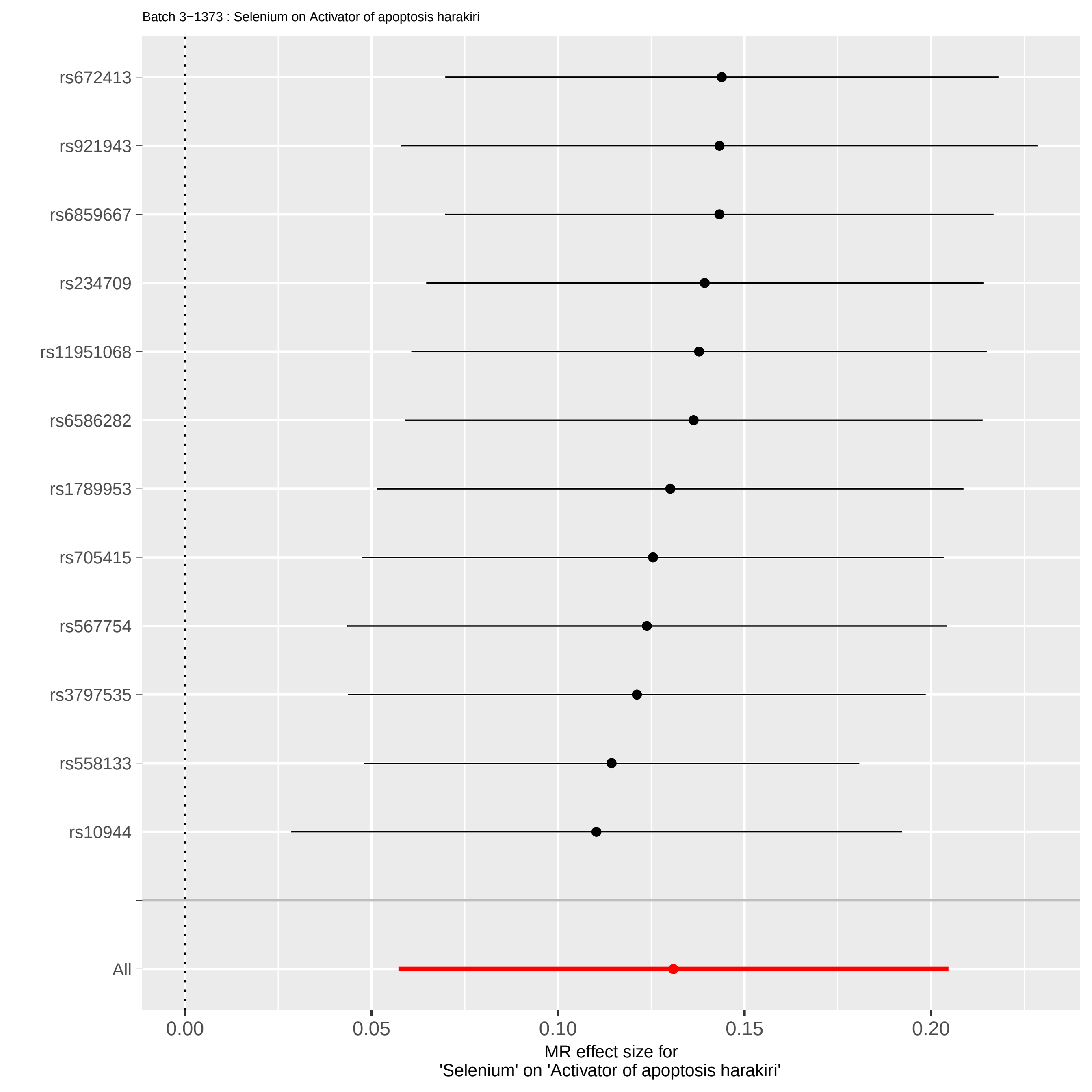

Batch 3−1373 : Selenium on Activator of apoptosis harakiri
rs672413
rs921943
rs6859667
rs234709
rs11951068
rs6586282
rs1789953
rs705415
rs567754
rs3797535
rs558133
rs10944
All
0.00
0.05
0.10
0.15
0.20
MR effect size for
'Selenium' on 'Activator of apoptosis harakiri'

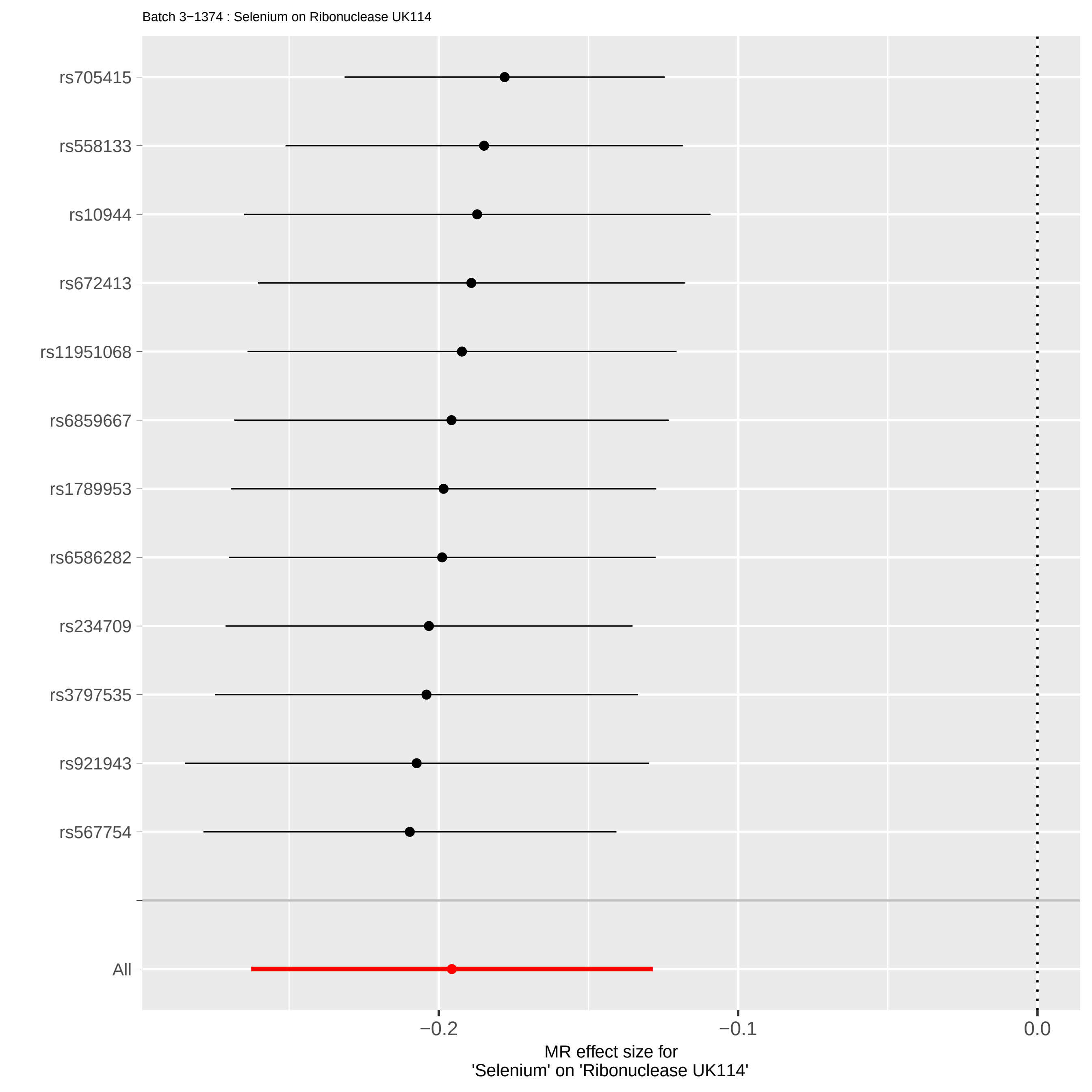

Batch 3−1374 : Selenium on Ribonuclease UK114
rs705415
rs558133
rs10944
rs672413
rs11951068
rs6859667
rs1789953
rs6586282
rs234709
rs3797535
rs921943
rs567754
All
−0.2
−0.1
0.0
MR effect size for
'Selenium' on 'Ribonuclease UK114'

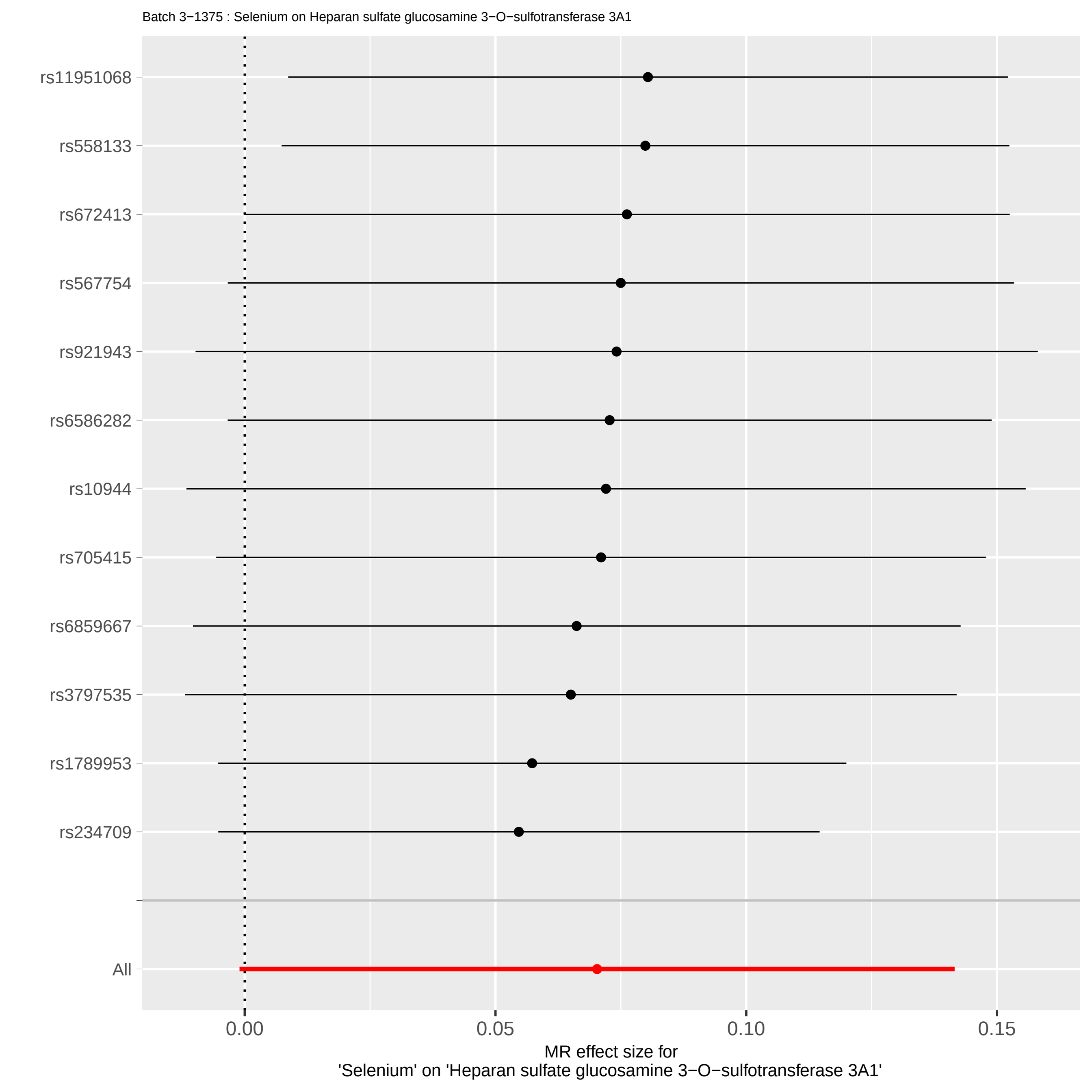
Batch 3−1375 : Selenium on Heparan sulfate glucosamine 3−O−sulfotransferase 3A1
rs11951068
rs558133
rs672413
rs567754
rs921943
rs6586282
rs10944
rs705415
rs6859667
rs3797535
rs1789953
rs234709
All
0.00
0.05
0.10
0.15
MR effect size for
'Selenium' on 'Heparan sulfate glucosamine 3−O−sulfotransferase 3A1'

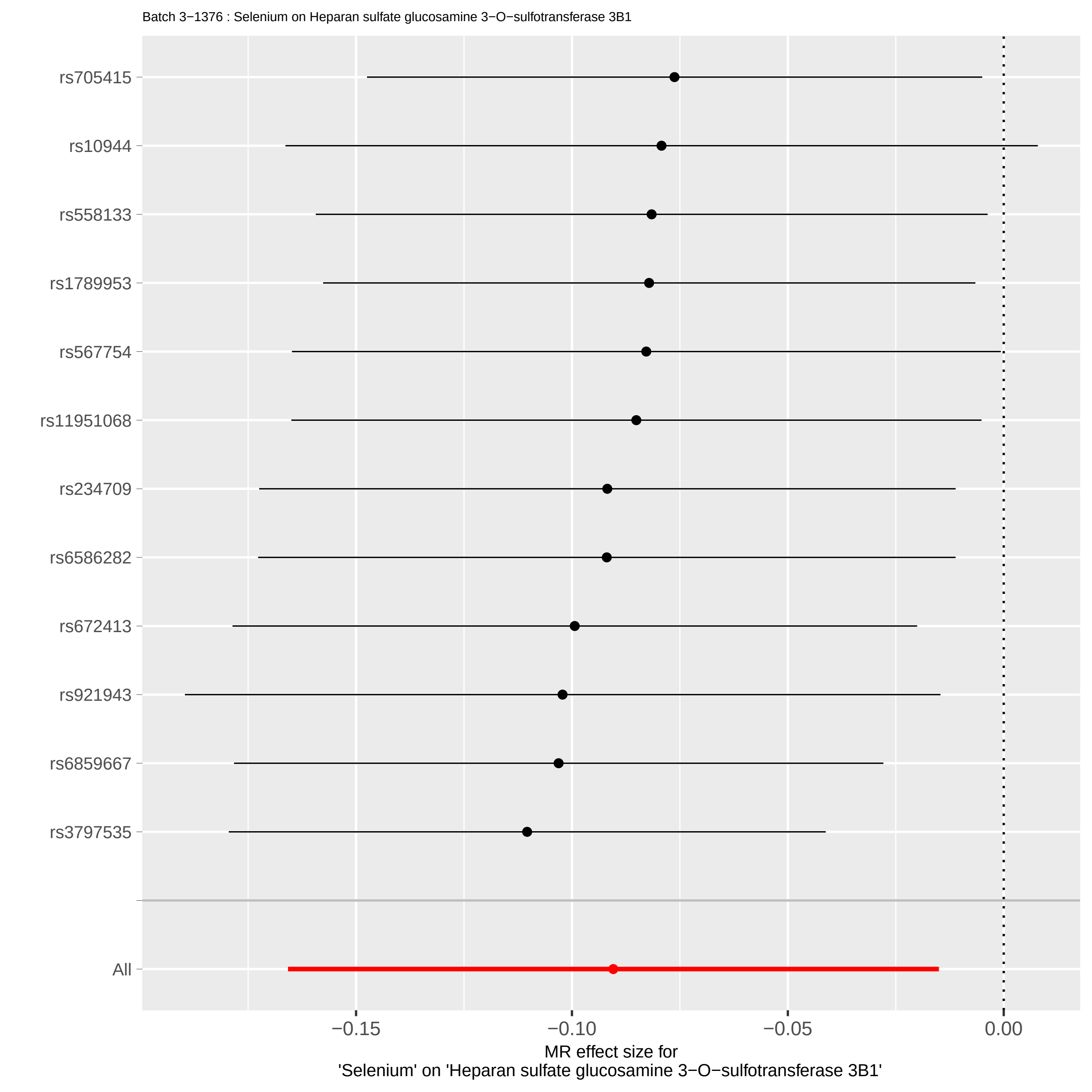
Batch 3−1376 : Selenium on Heparan sulfate glucosamine 3−O−sulfotransferase 3B1
rs705415
rs10944
rs558133
rs1789953
rs567754
rs11951068
rs234709
rs6586282
rs672413
rs921943
rs6859667
rs3797535
All
−0.15
−0.10
−0.05
0.00
MR effect size for
'Selenium' on 'Heparan sulfate glucosamine 3−O−sulfotransferase 3B1'

Batch 3−1377 : Selenium on Heparan sulfate glucosamine 3−O−sulfotransferase 4

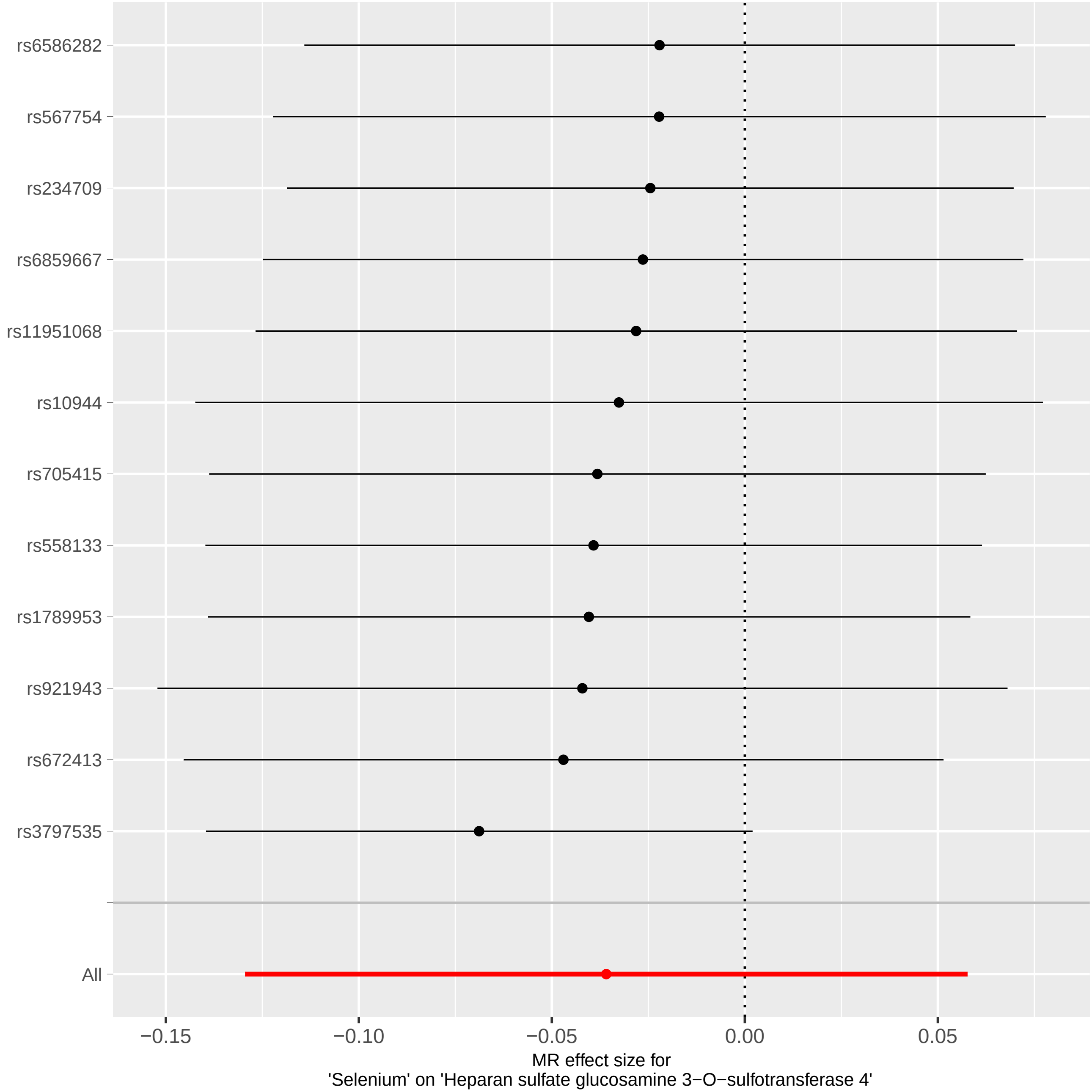

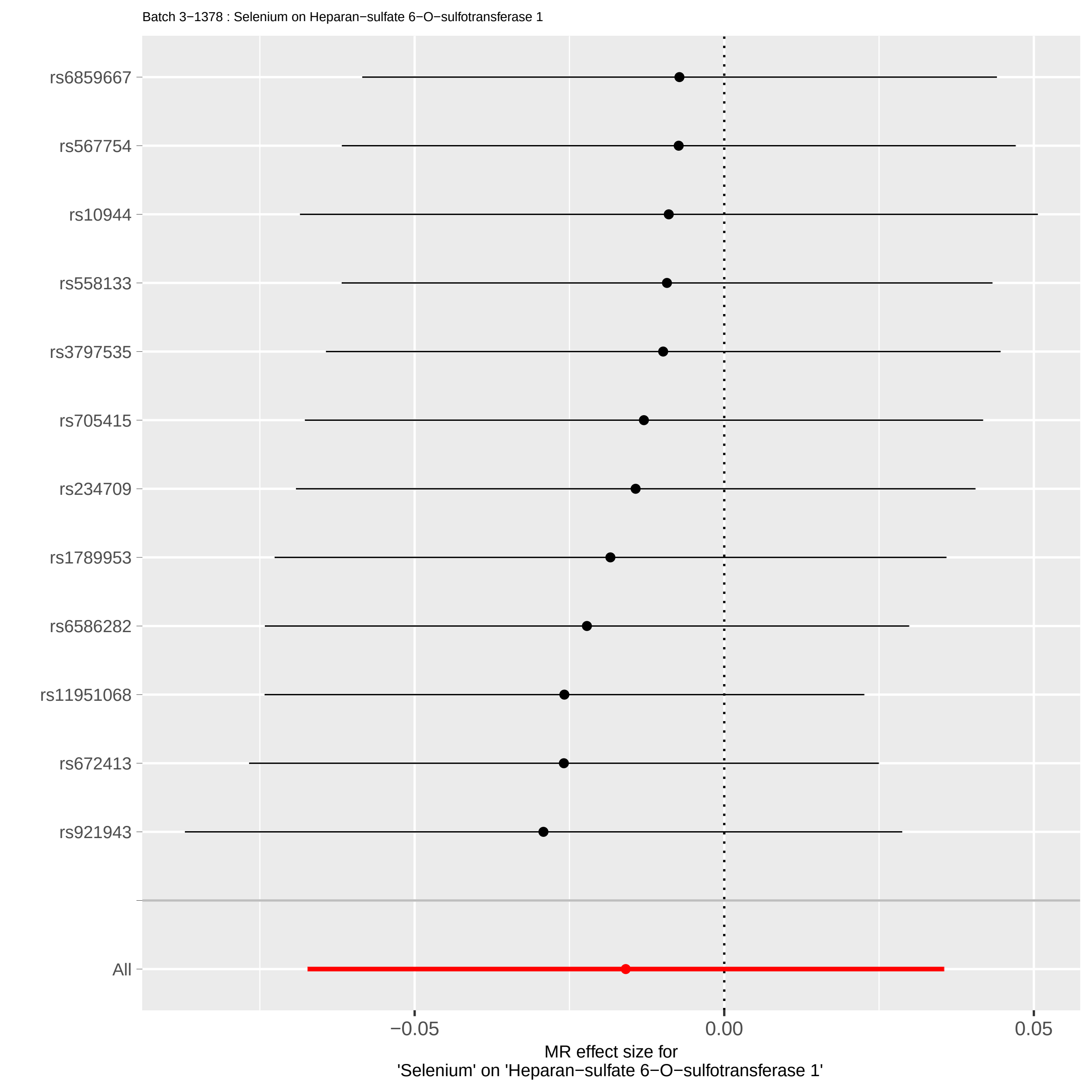

Batch 3−1378 : Selenium on Heparan−sulfate 6−O−sulfotransferase 1
rs6859667
rs567754
rs10944
rs558133
rs3797535
rs705415
rs234709
rs1789953
rs6586282
rs11951068
rs672413
rs921943
All
−0.05
0.00
0.05
MR effect size for
'Selenium' on 'Heparan−sulfate 6−O−sulfotransferase 1'

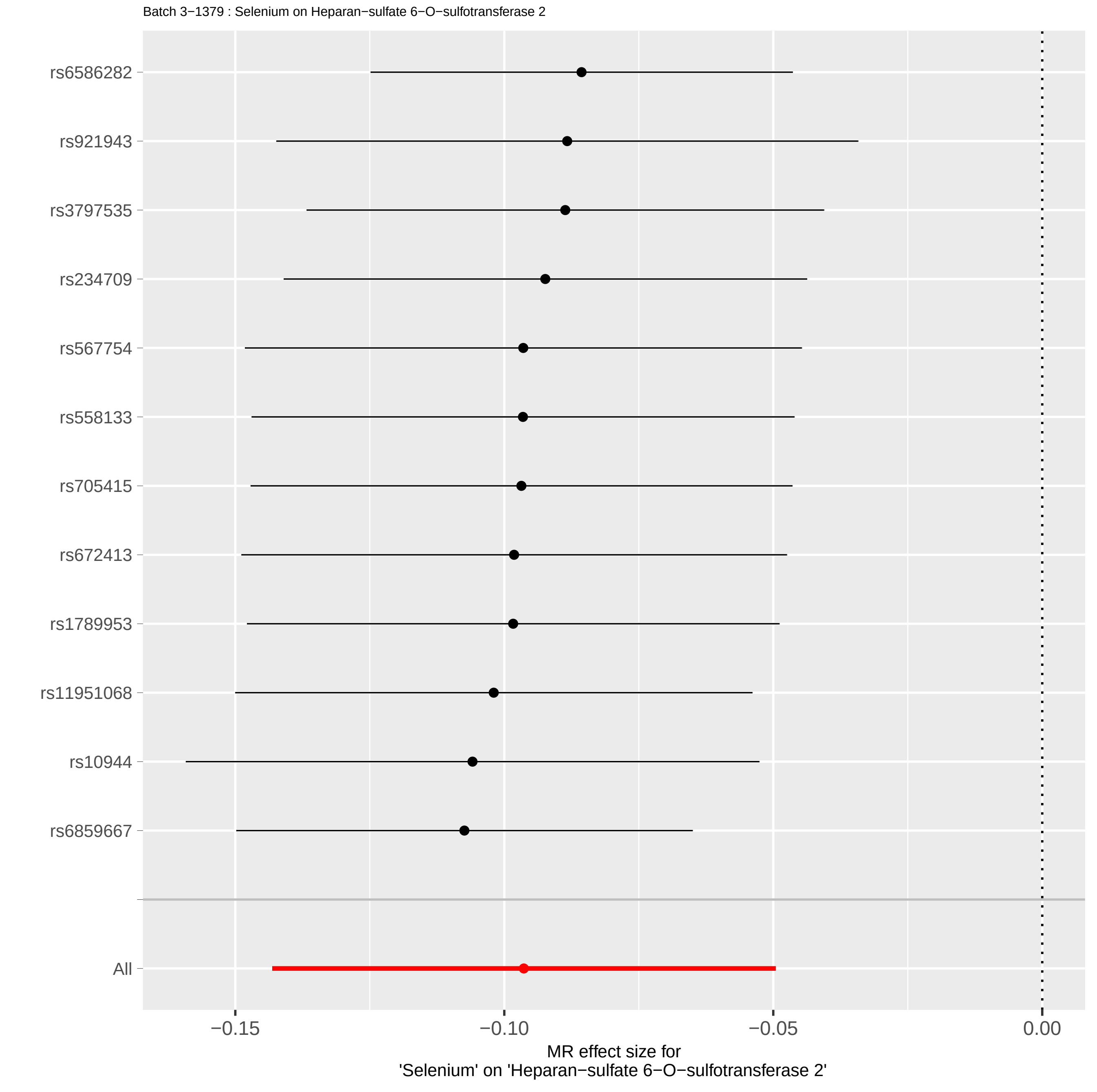

Batch 3−1379 : Selenium on Heparan−sulfate 6−O−sulfotransferase 2
rs6586282
rs921943
rs3797535
rs234709
rs567754
rs558133
rs705415
rs672413
rs1789953
rs11951068
rs10944
rs6859667
All
−0.15
−0.10
−0.05
0.00
MR effect size for
'Selenium' on 'Heparan−sulfate 6−O−sulfotransferase 2'

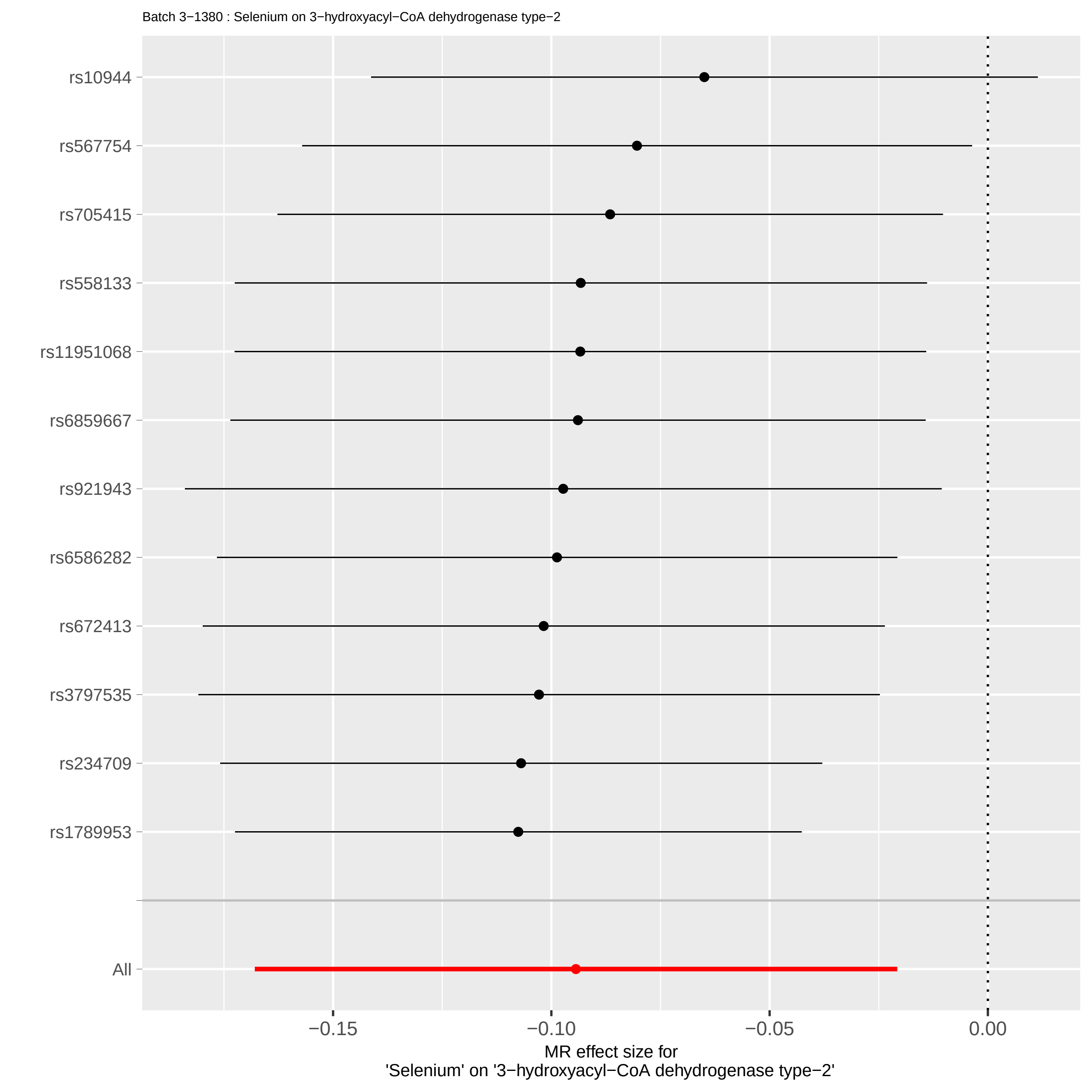
Batch 3−1380 : Selenium on 3−hydroxyacyl−CoA dehydrogenase type−2
rs10944
rs567754
rs705415
rs558133
rs11951068
rs6859667
rs921943
rs6586282
rs672413
rs3797535
rs234709
rs1789953
All
−0.15
−0.10
−0.05
0.00
MR effect size for
'Selenium' on '3−hydroxyacyl−CoA dehydrogenase type−2'

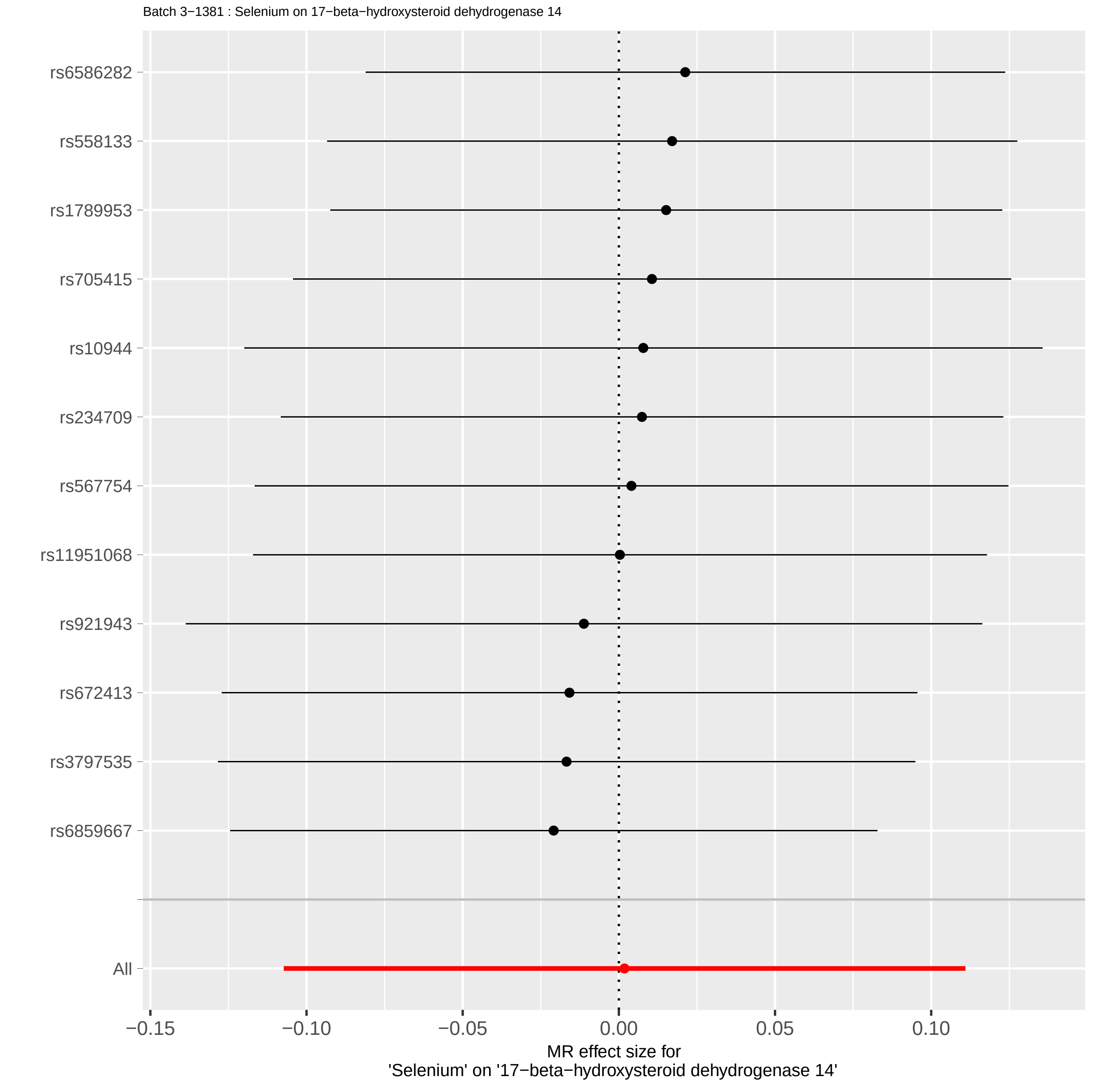

Batch 3−1381 : Selenium on 17−beta−hydroxysteroid dehydrogenase 14
rs6586282
rs558133
rs1789953
rs705415
rs10944
rs234709
rs567754
rs11951068
rs921943
rs672413
rs3797535
rs6859667
All
−0.15
−0.10
−0.05
0.00
0.05
0.10
MR effect size for
'Selenium' on '17−beta−hydroxysteroid dehydrogenase 14'

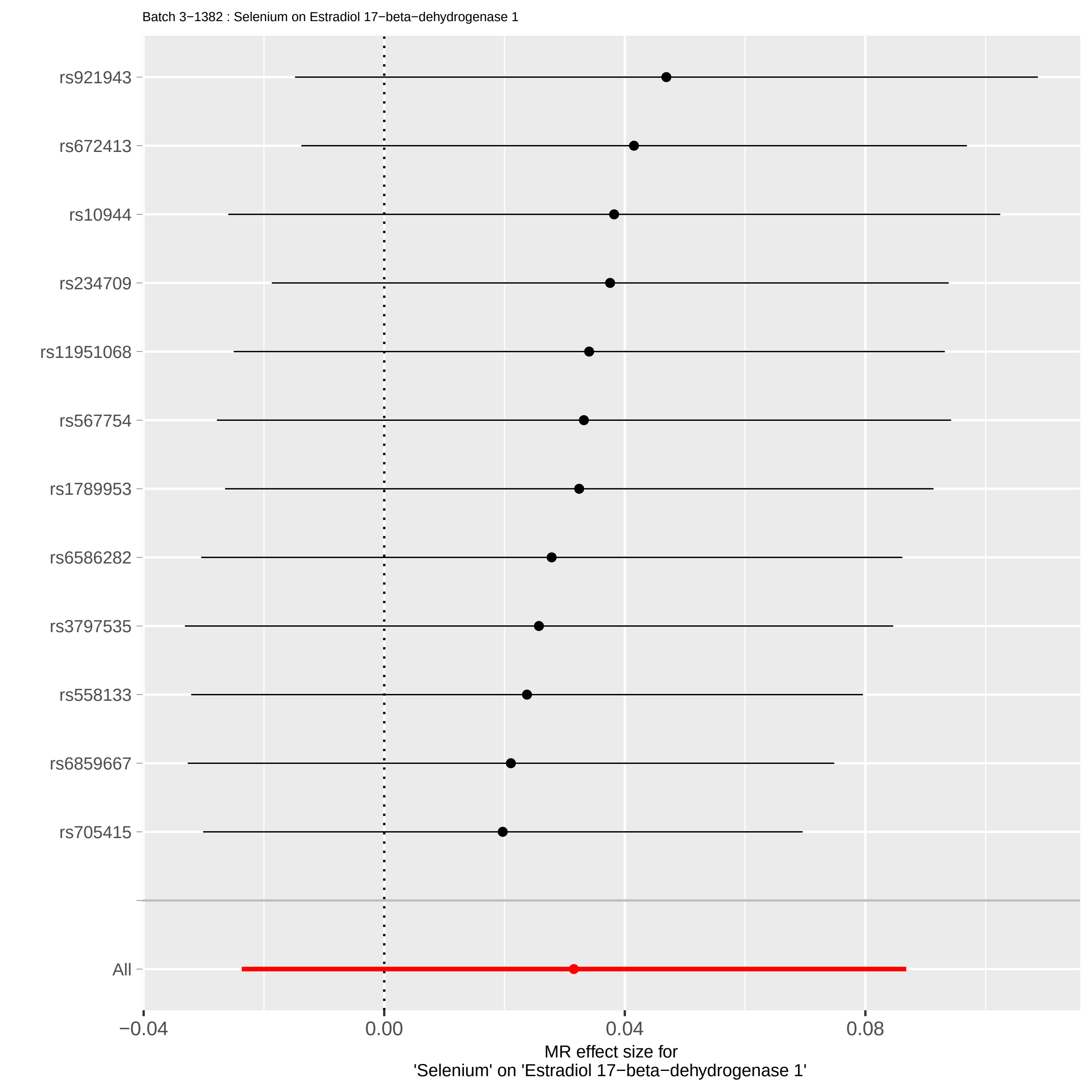

Batch 3−1382 : Selenium on Estradiol 17−beta−dehydrogenase 1
rs921943
rs672413
rs10944
rs234709
rs11951068
rs567754
rs1789953
rs6586282
rs3797535
rs558133
rs6859667
rs705415
All
−0.04
0.00
0.04
0.08
MR effect size for
'Selenium' on 'Estradiol 17−beta−dehydrogenase 1'

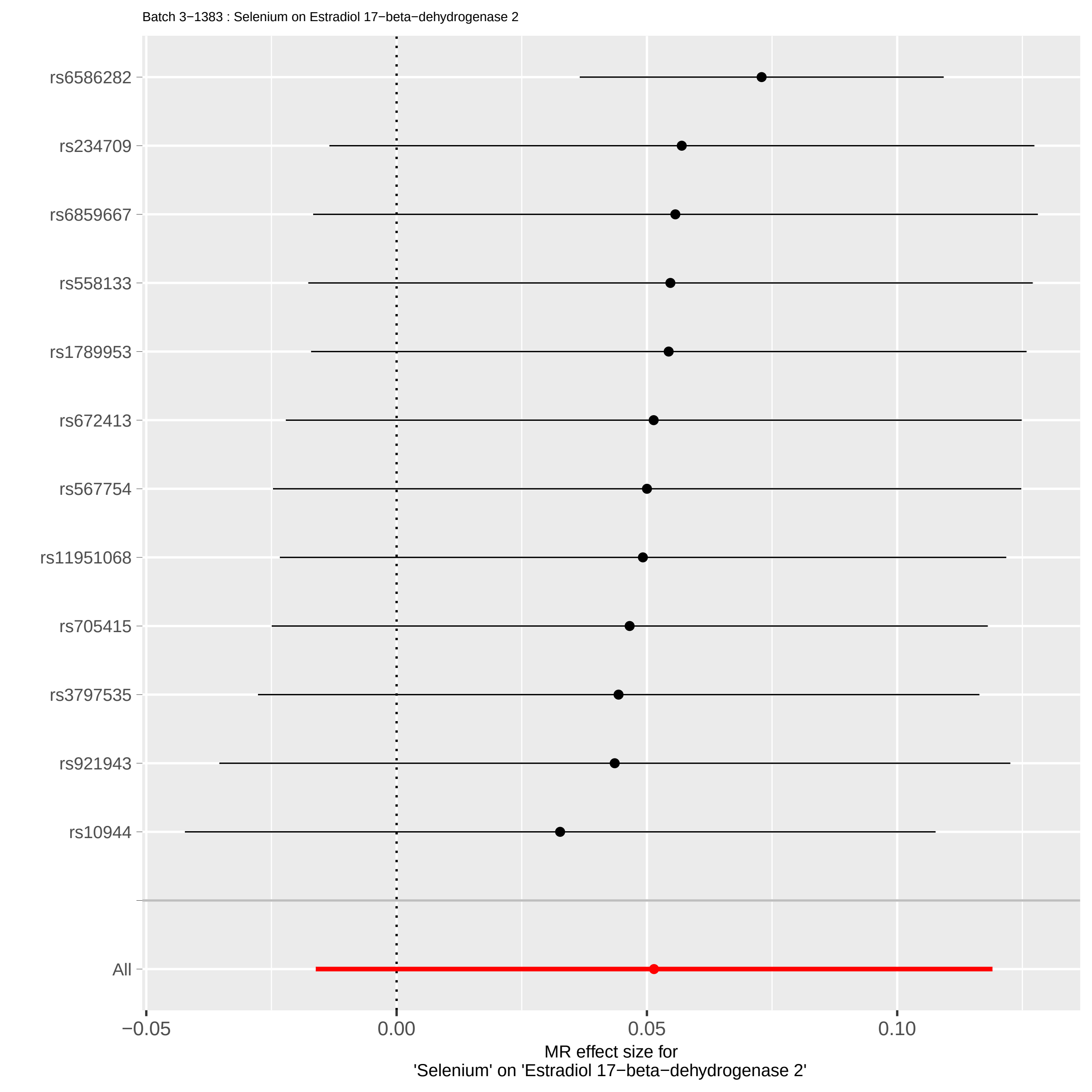
Batch 3−1383 : Selenium on Estradiol 17−beta−dehydrogenase 2
rs6586282
rs234709
rs6859667
rs558133
rs1789953
rs672413
rs567754
rs11951068
rs705415
rs3797535
rs921943
rs10944
All
−0.05
0.00
0.05
0.10
MR effect size for
'Selenium' on 'Estradiol 17−beta−dehydrogenase 2'

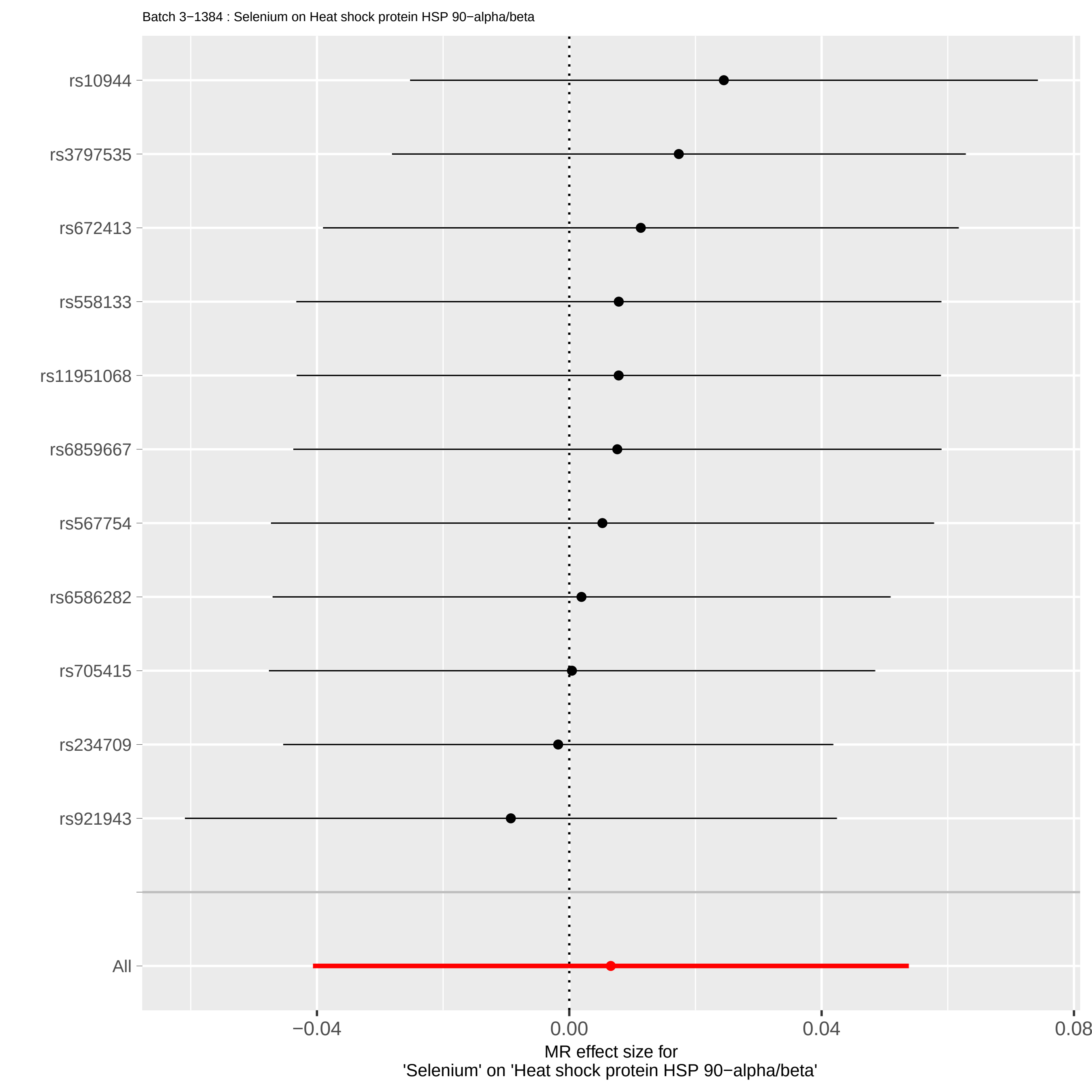
Batch 3−1384 : Selenium on Heat shock protein HSP 90−alpha/beta
rs10944
rs3797535
rs672413
rs558133
rs11951068
rs6859667
rs567754
rs6586282
rs705415
rs234709
rs921943
All
−0.04
0.00
0.04
0.08
MR effect size for
'Selenium' on 'Heat shock protein HSP 90−alpha/beta'

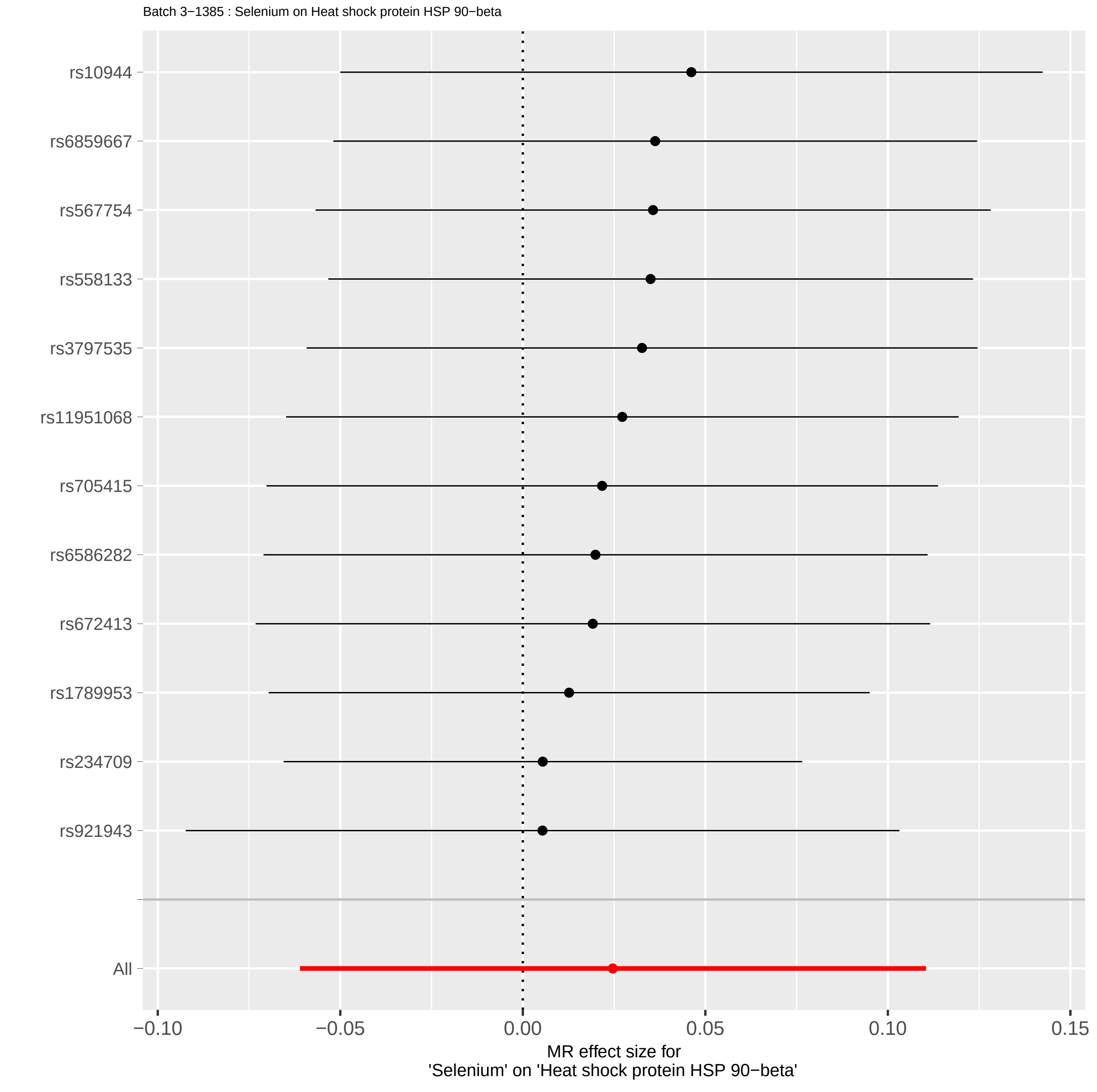

Batch 3−1385 : Selenium on Heat shock protein HSP 90−beta
rs10944
rs6859667
rs567754
rs558133
rs3797535
rs11951068
rs705415
rs6586282
rs672413
rs1789953
rs234709
rs921943
All
−0.10
−0.05
0.00
0.05
0.10
0.15
MR effect size for
'Selenium' on 'Heat shock protein HSP 90−beta'

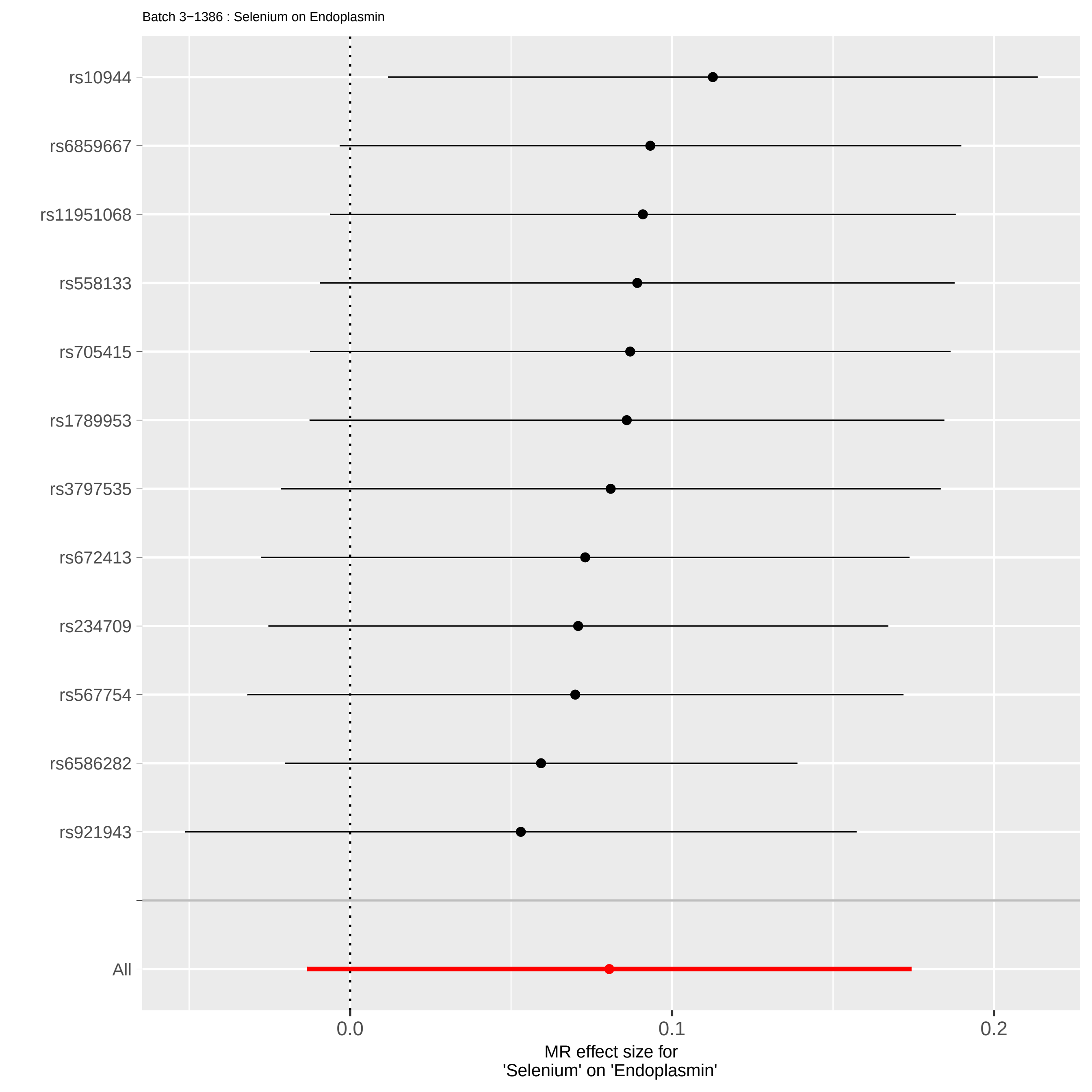

Batch 3−1386 : Selenium on Endoplasmin
rs10944
rs6859667
rs11951068
rs558133
rs705415
rs1789953
rs3797535
rs672413
rs234709
rs567754
rs6586282
rs921943
All
0.0
0.1
0.2
MR effect size for
'Selenium' on 'Endoplasmin'

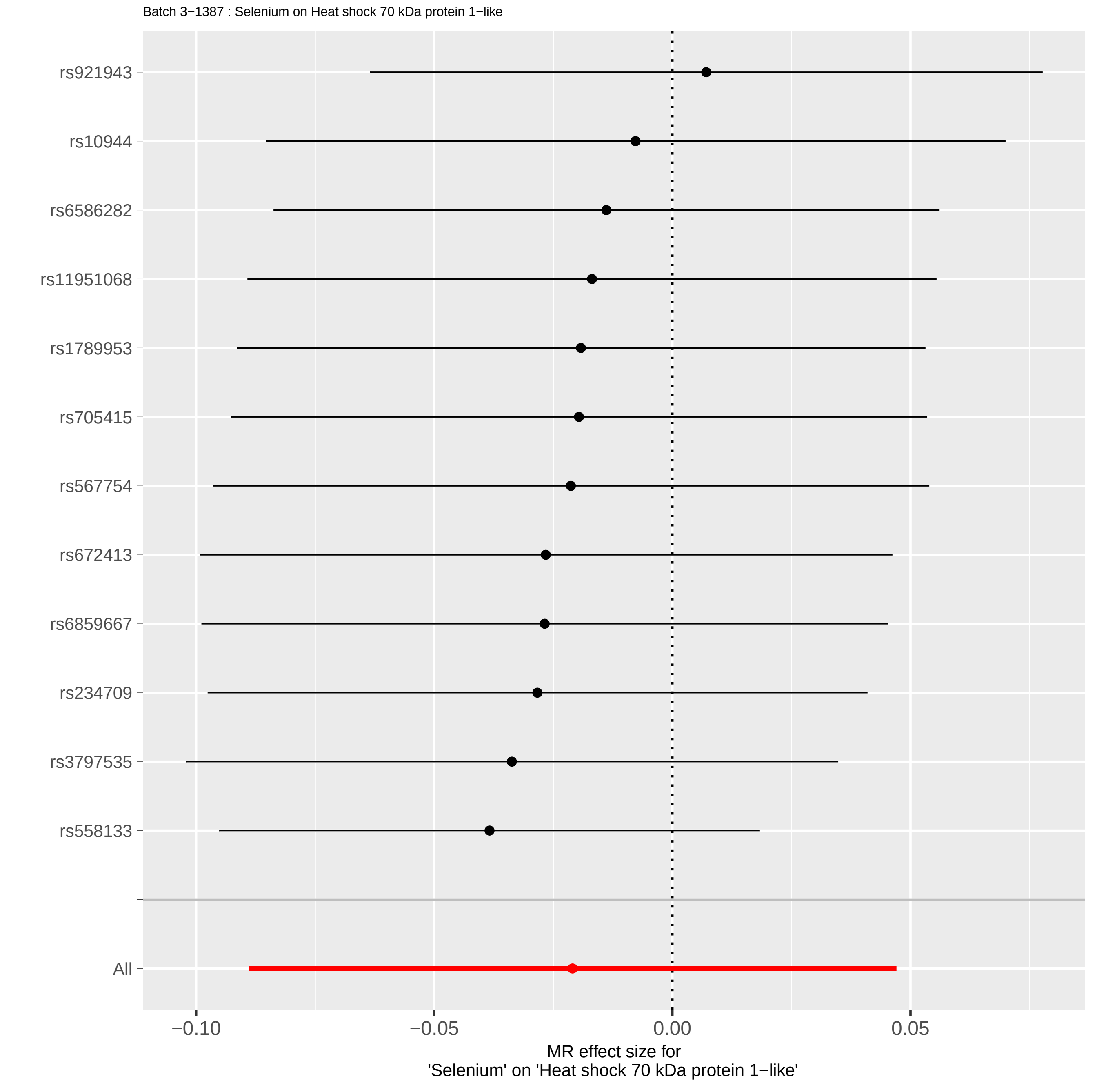

Batch 3−1387 : Selenium on Heat shock 70 kDa protein 1−like
rs921943
rs10944
rs6586282
rs11951068
rs1789953
rs705415
rs567754
rs672413
rs6859667
rs234709
rs3797535
rs558133
All
−0.10
−0.05
0.00
0.05
MR effect size for
'Selenium' on 'Heat shock 70 kDa protein 1−like'

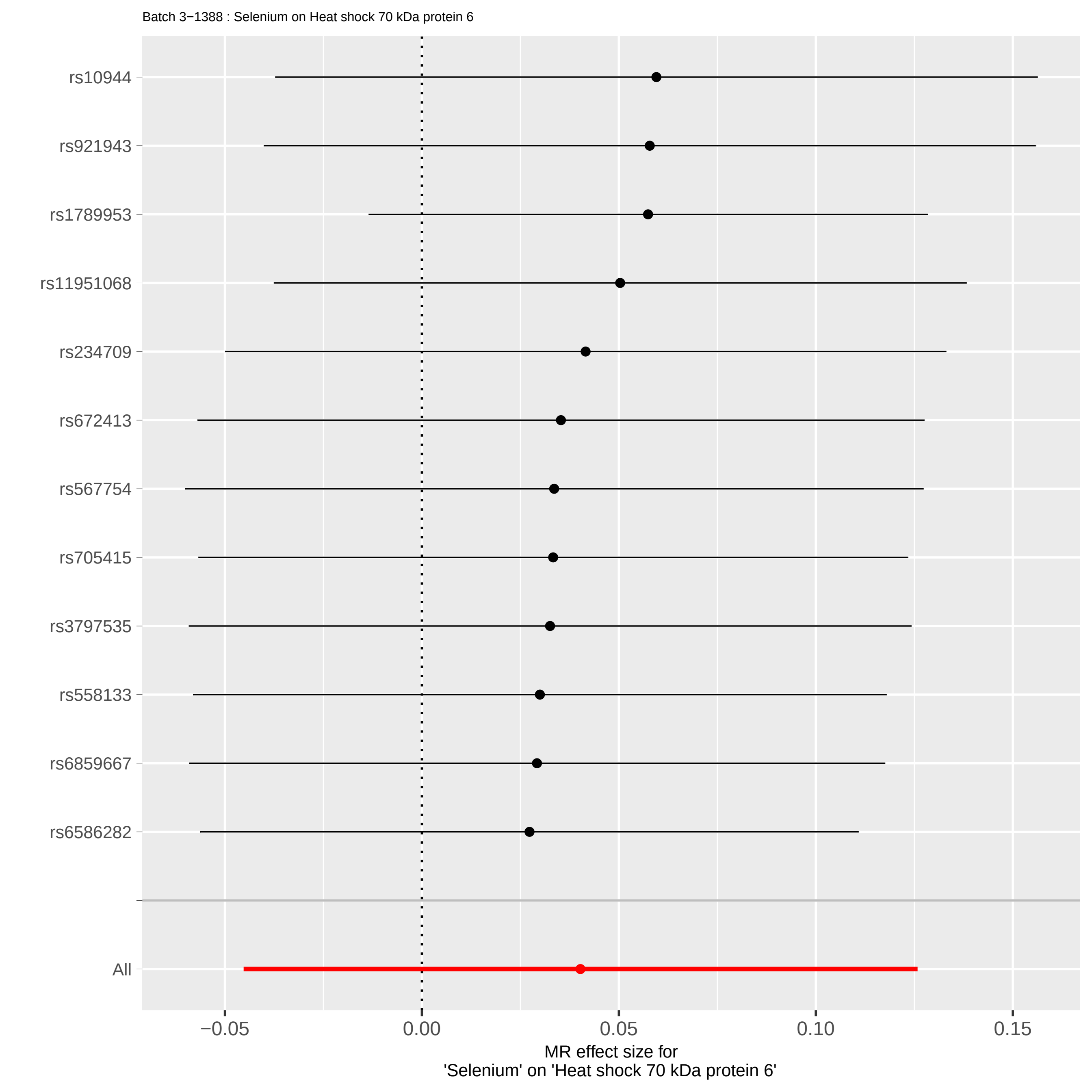

Batch 3−1388 : Selenium on Heat shock 70 kDa protein 6
rs10944
rs921943
rs1789953
rs11951068
rs234709
rs672413
rs567754
rs705415
rs3797535
rs558133
rs6859667
rs6586282
All
−0.05
0.00
0.05
0.10
0.15
MR effect size for
'Selenium' on 'Heat shock 70 kDa protein 6'

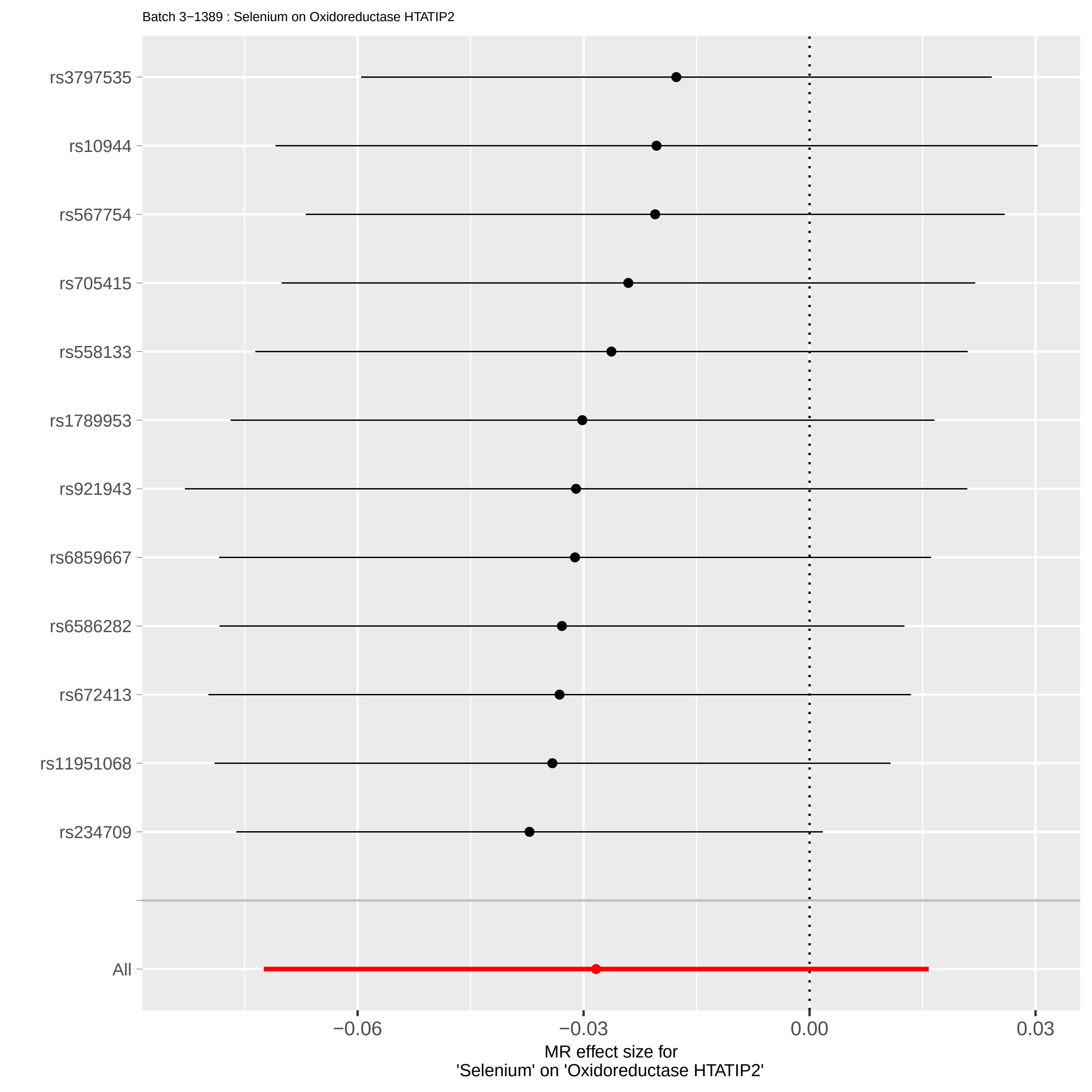

Batch 3−1389 : Selenium on Oxidoreductase HTATIP2
rs3797535
rs10944
rs567754
rs705415
rs558133
rs1789953
rs921943
rs6859667
rs6586282
rs672413
rs11951068
rs234709
All
−0.06
−0.03
0.00
0.03
MR effect size for
'Selenium' on 'Oxidoreductase HTATIP2'

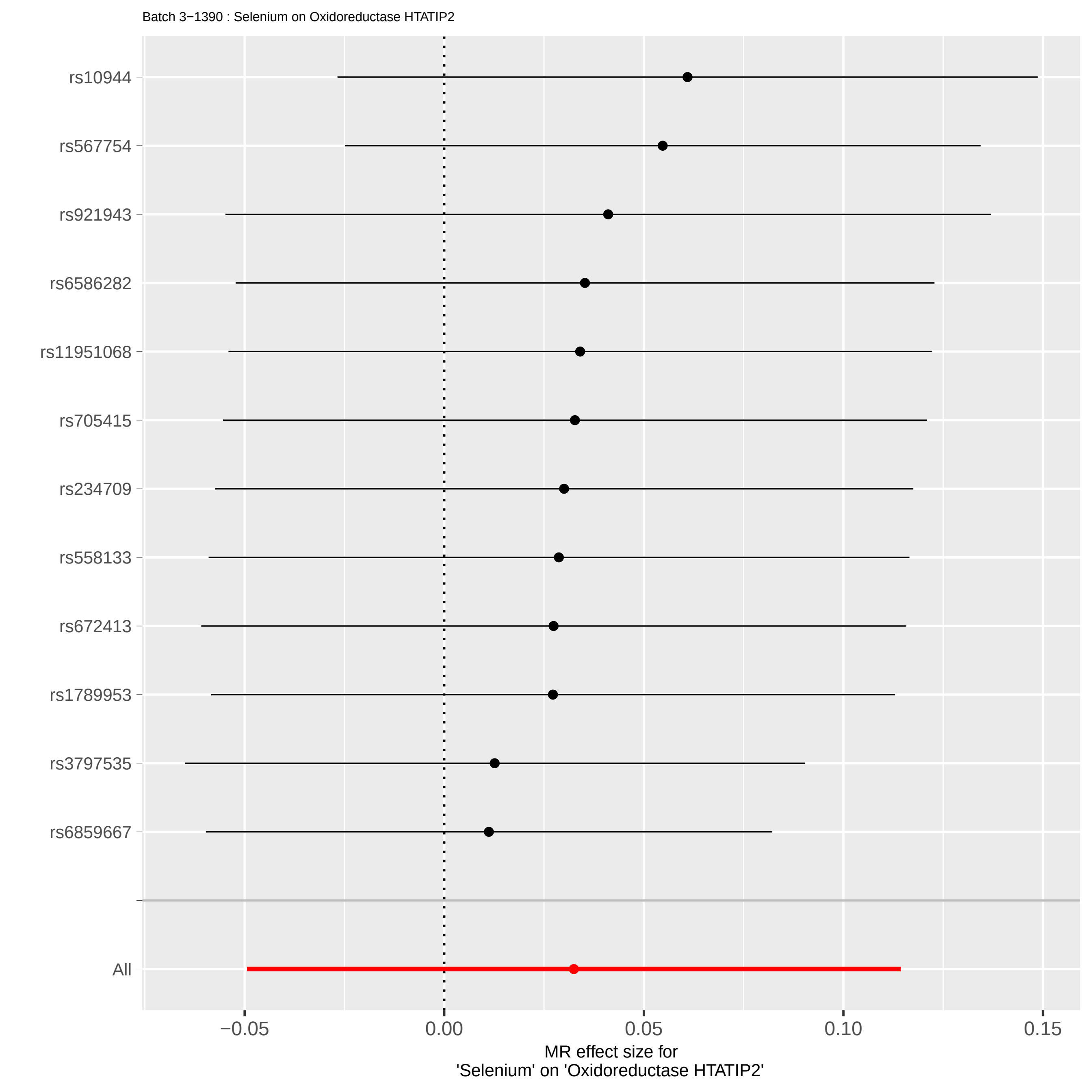

Batch 3−1390 : Selenium on Oxidoreductase HTATIP2
rs10944
rs567754
rs921943
rs6586282
rs11951068
rs705415
rs234709
rs558133
rs672413
rs1789953
rs3797535
rs6859667
All
−0.05
0.00
0.05
0.10
0.15
MR effect size for
'Selenium' on 'Oxidoreductase HTATIP2'

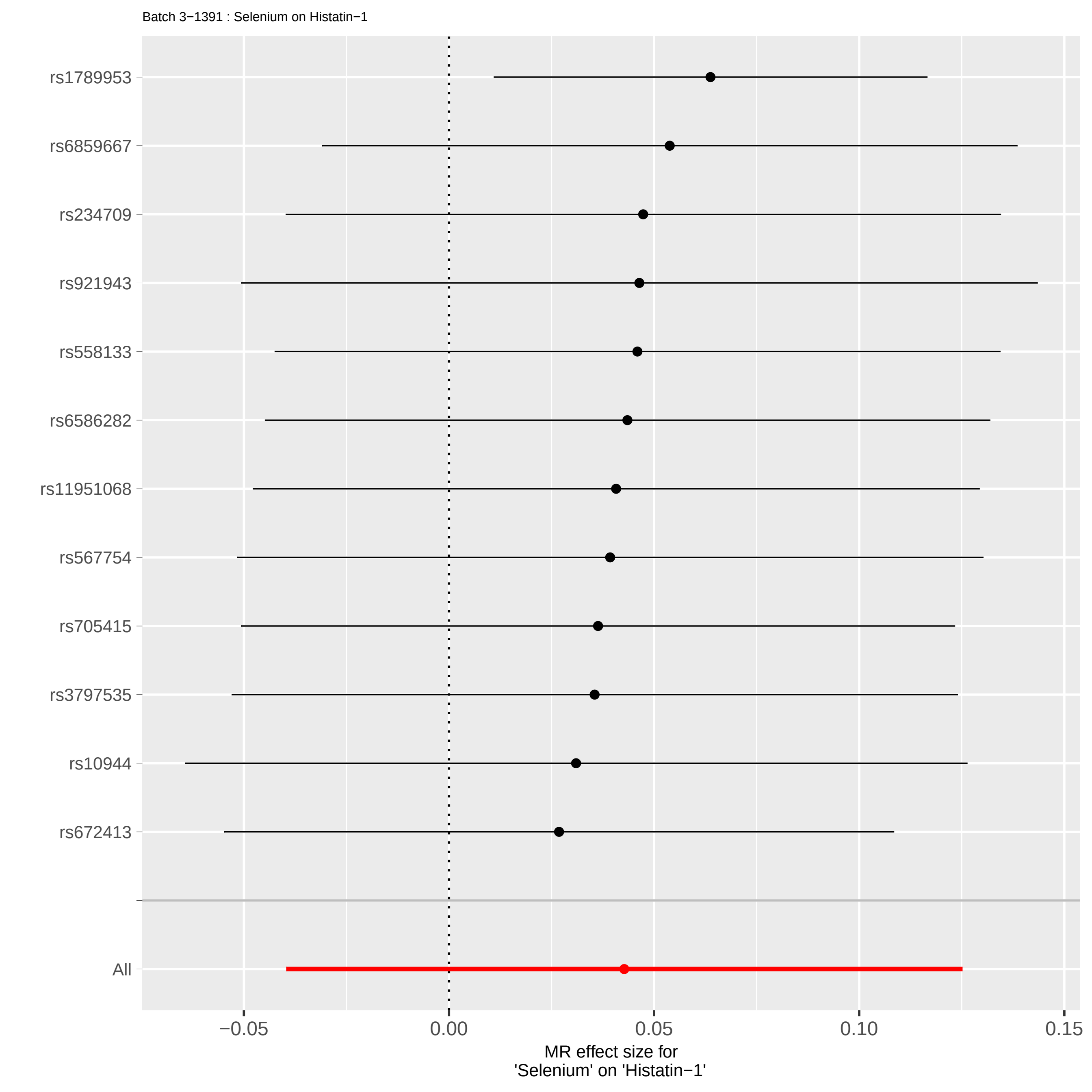
Batch 3−1391 : Selenium on Histatin−1
rs1789953
rs6859667
rs234709
rs921943
rs558133
rs6586282
rs11951068
rs567754
rs705415
rs3797535
rs10944
rs672413
All
−0.05
0.00
0.05
0.10
0.15
MR effect size for
'Selenium' on 'Histatin−1'

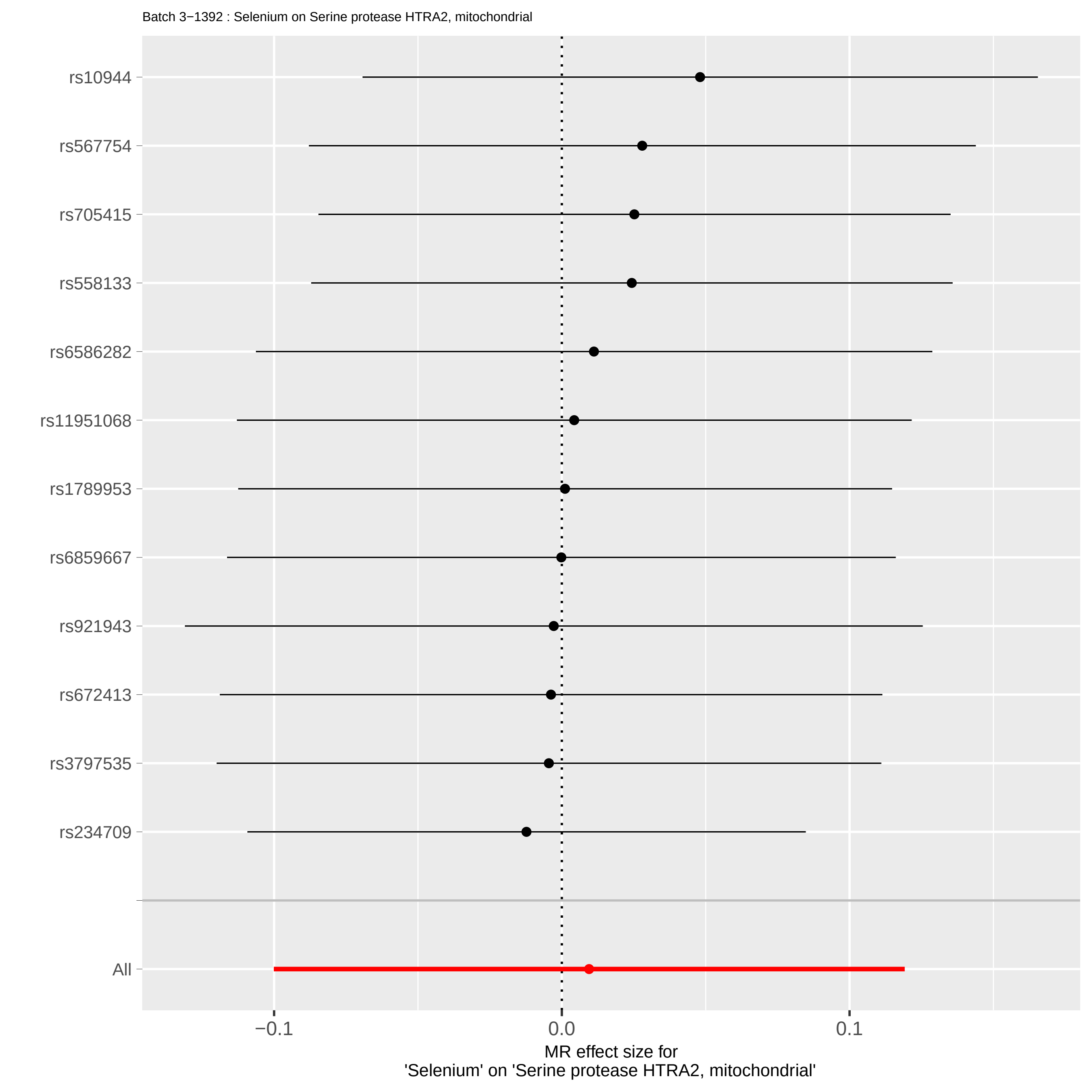
Batch 3−1392 : Selenium on Serine protease HTRA2, mitochondrial
rs10944
rs567754
rs705415
rs558133
rs6586282
rs11951068
rs1789953
rs6859667
rs921943
rs672413
rs3797535
rs234709
All
−0.1
0.0
0.1
MR effect size for
'Selenium' on 'Serine protease HTRA2, mitochondrial'

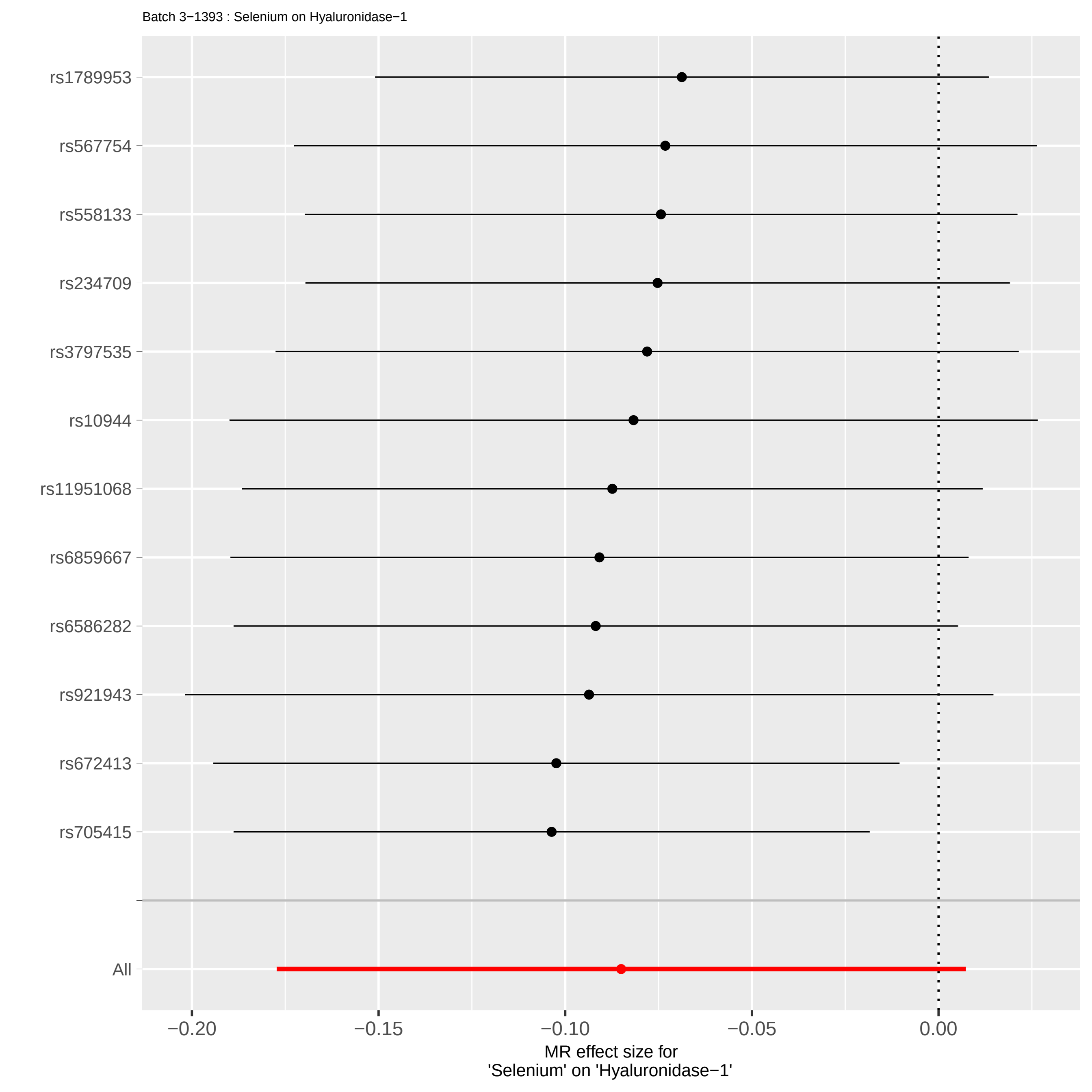

Batch 3−1393 : Selenium on Hyaluronidase−1
rs1789953
rs567754
rs558133
rs234709
rs3797535
rs10944
rs11951068
rs6859667
rs6586282
rs921943
rs672413
rs705415
All
−0.20
−0.15
−0.10
−0.05
0.00
MR effect size for
'Selenium' on 'Hyaluronidase−1'

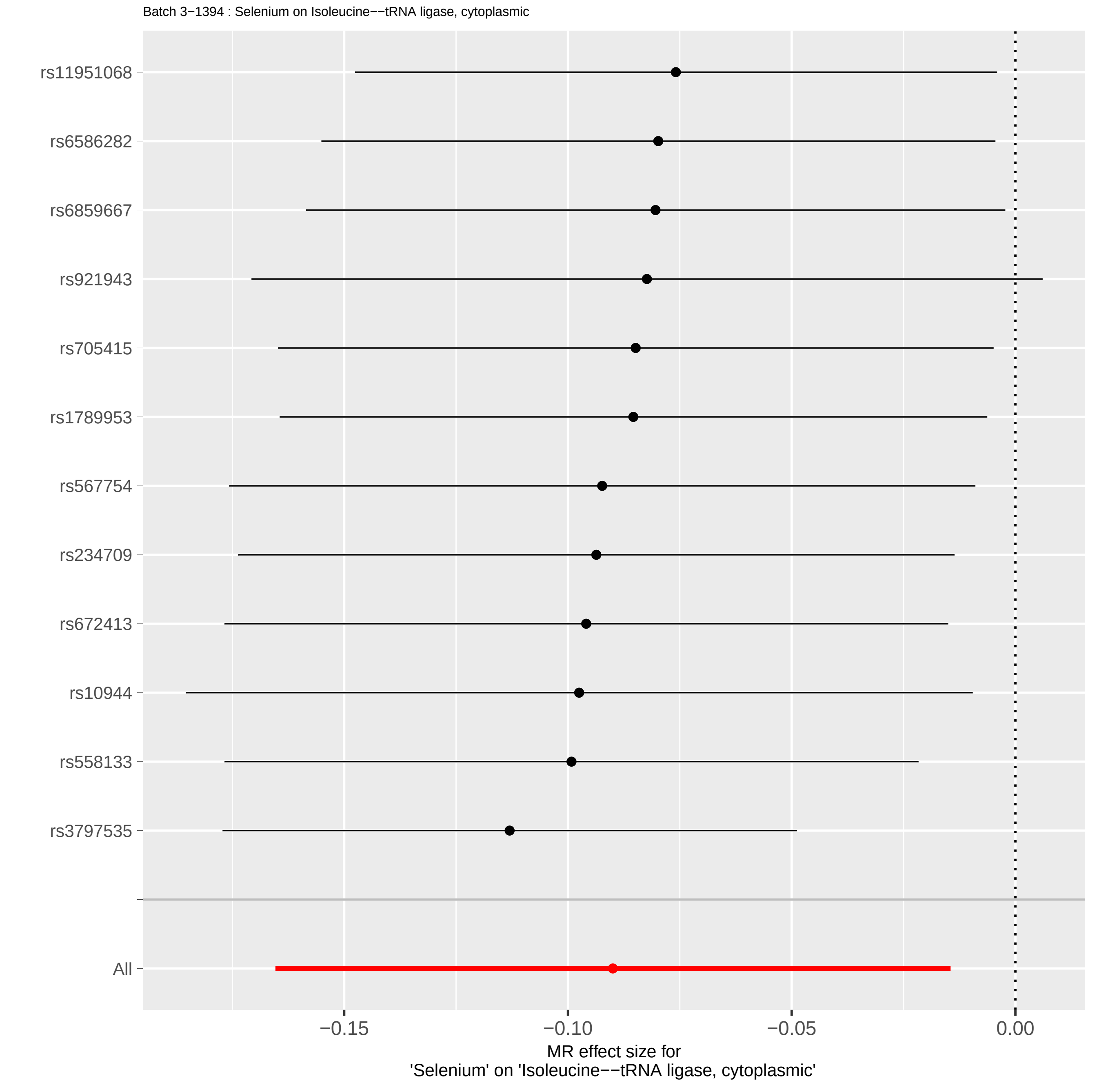
Batch 3−1394 : Selenium on Isoleucine−−tRNA ligase, cytoplasmic
rs11951068
rs6586282
rs6859667
rs921943
rs705415
rs1789953
rs567754
rs234709
rs672413
rs10944
rs558133
rs3797535
All
−0.15
−0.10
−0.05
0.00
MR effect size for
'Selenium' on 'Isoleucine−−tRNA ligase, cytoplasmic'

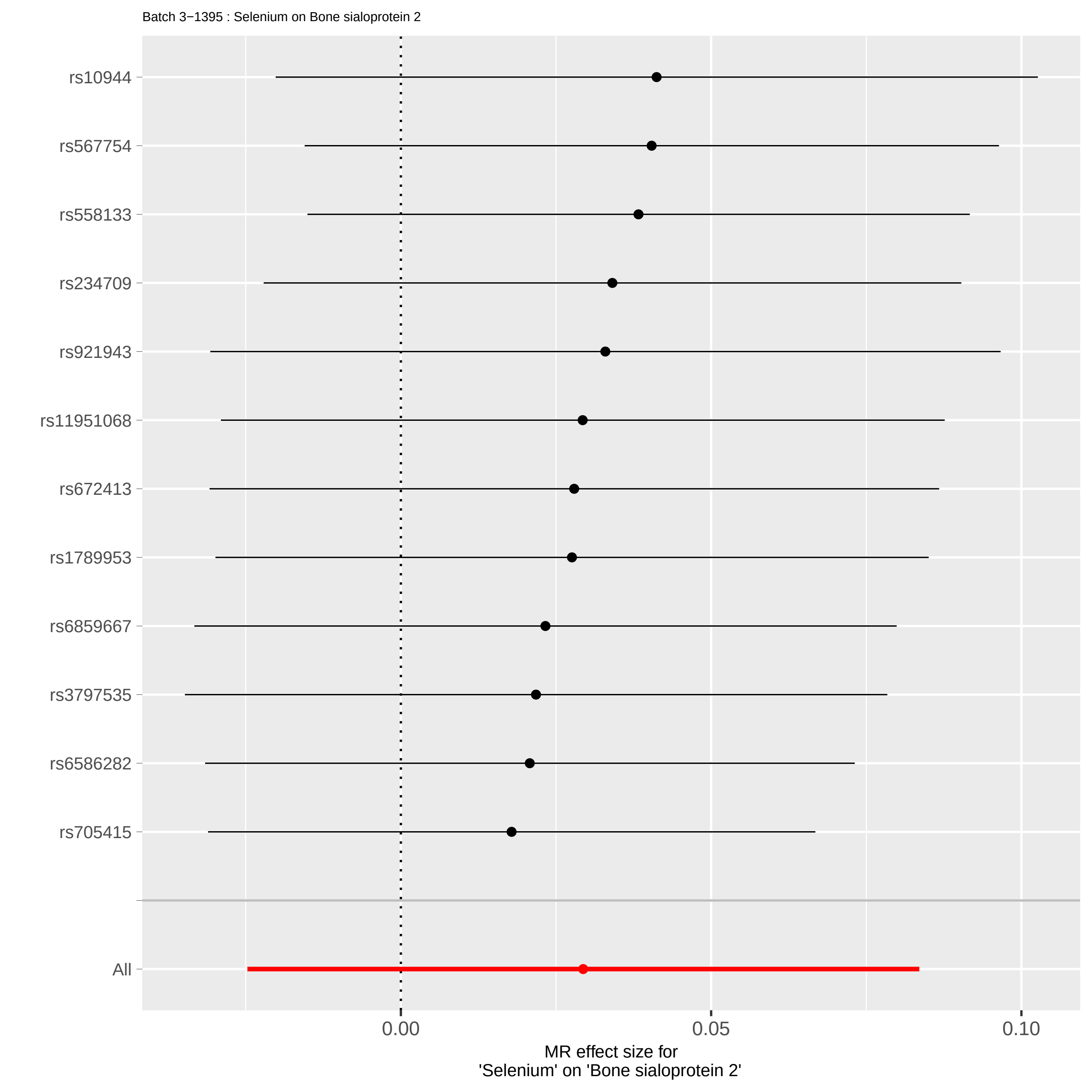

Batch 3−1395 : Selenium on Bone sialoprotein 2
rs10944
rs567754
rs558133
rs234709
rs921943
rs11951068
rs672413
rs1789953
rs6859667
rs3797535
rs6586282
rs705415
All
0.00
0.05
0.10
MR effect size for
'Selenium' on 'Bone sialoprotein 2'

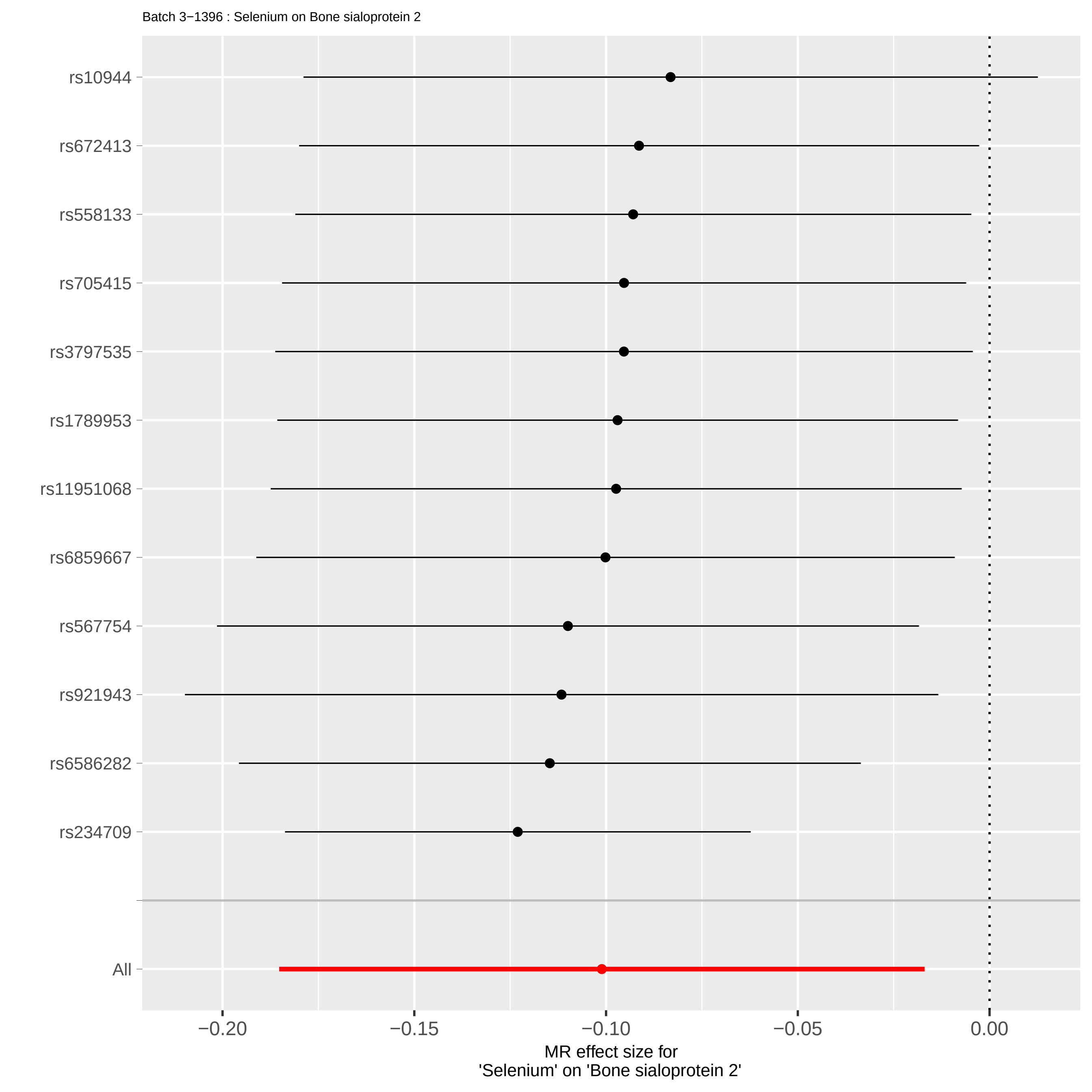

Batch 3−1396 : Selenium on Bone sialoprotein 2
rs10944
rs672413
rs558133
rs705415
rs3797535
rs1789953
rs11951068
rs6859667
rs567754
rs921943
rs6586282
rs234709
All
−0.20
−0.15
−0.10
−0.05
0.00
MR effect size for
'Selenium' on 'Bone sialoprotein 2'

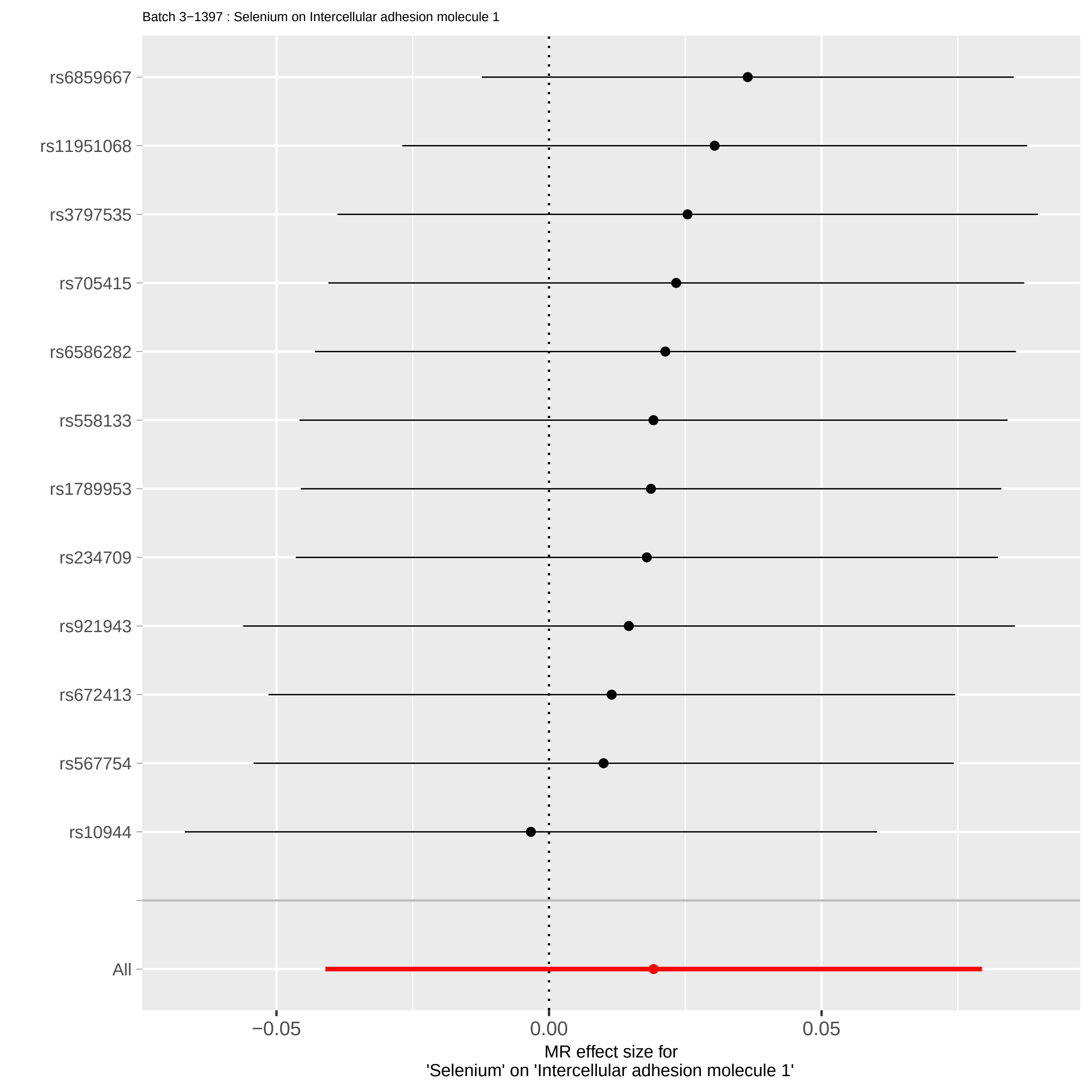
Batch 3−1397 : Selenium on Intercellular adhesion molecule 1
rs6859667
rs11951068
rs3797535
rs705415
rs6586282
rs558133
rs1789953
rs234709
rs921943
rs672413
rs567754
rs10944
All
−0.05
0.00
0.05
MR effect size for
'Selenium' on 'Intercellular adhesion molecule 1'

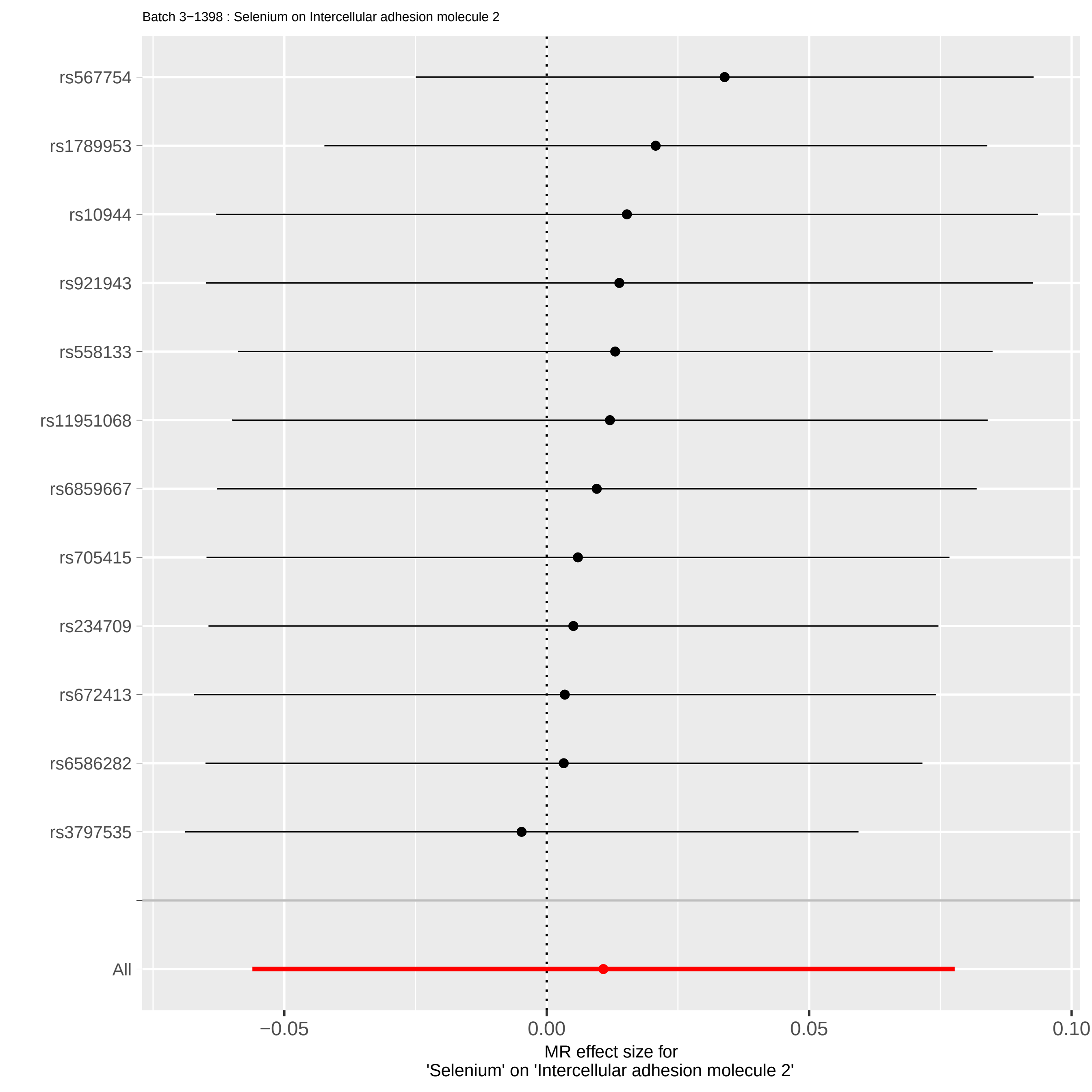
Batch 3−1398 : Selenium on Intercellular adhesion molecule 2
rs567754
rs1789953
rs10944
rs921943
rs558133
rs11951068
rs6859667
rs705415
rs234709
rs672413
rs6586282
rs3797535
All
−0.05
0.00
0.05
0.10
MR effect size for
'Selenium' on 'Intercellular adhesion molecule 2'

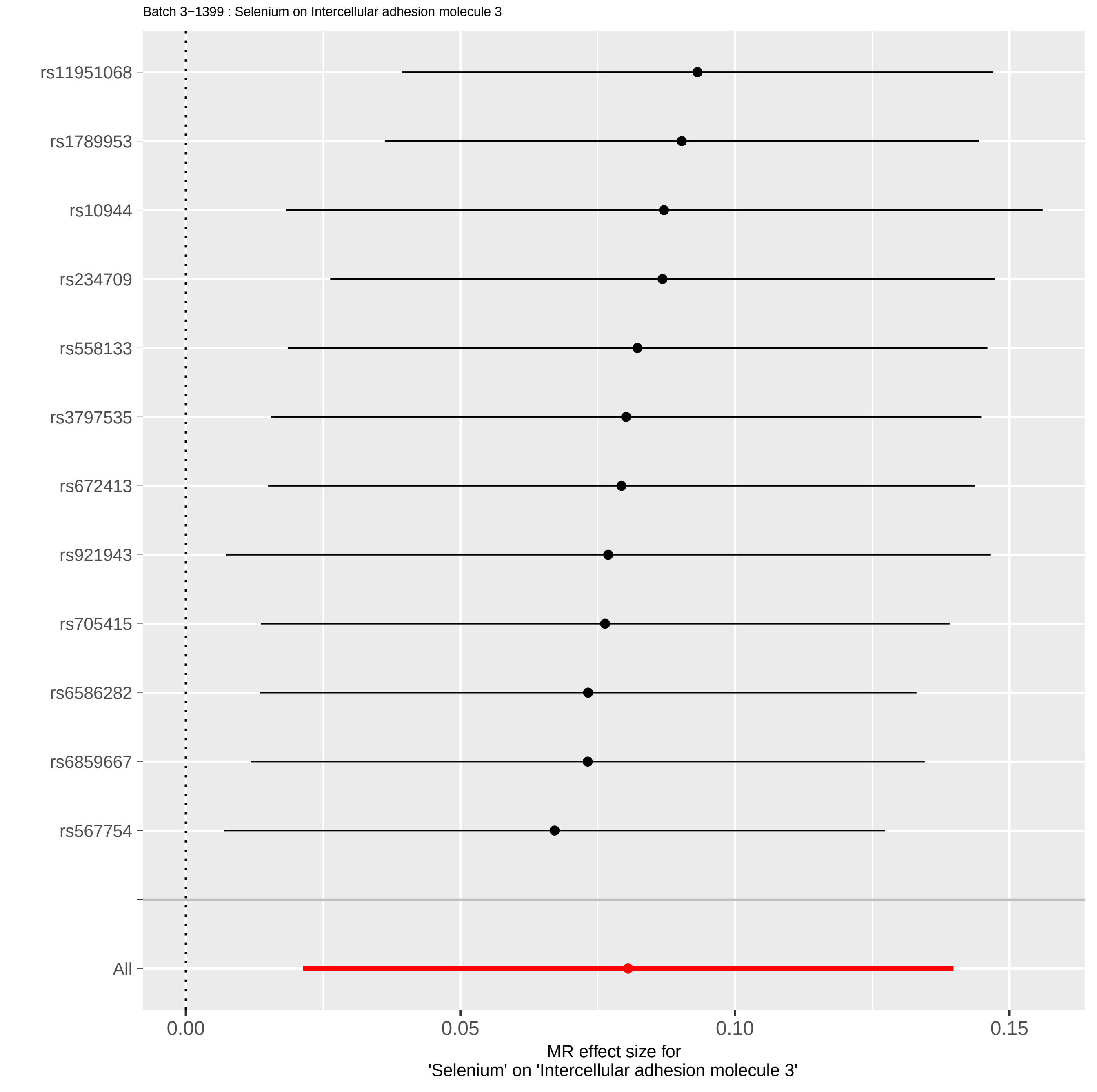
Batch 3−1399 : Selenium on Intercellular adhesion molecule 3
rs11951068
rs1789953
rs10944
rs234709
rs558133
rs3797535
rs672413
rs921943
rs705415
rs6586282
rs6859667
rs567754
All
0.00
0.05
0.10
0.15
MR effect size for
'Selenium' on 'Intercellular adhesion molecule 3'

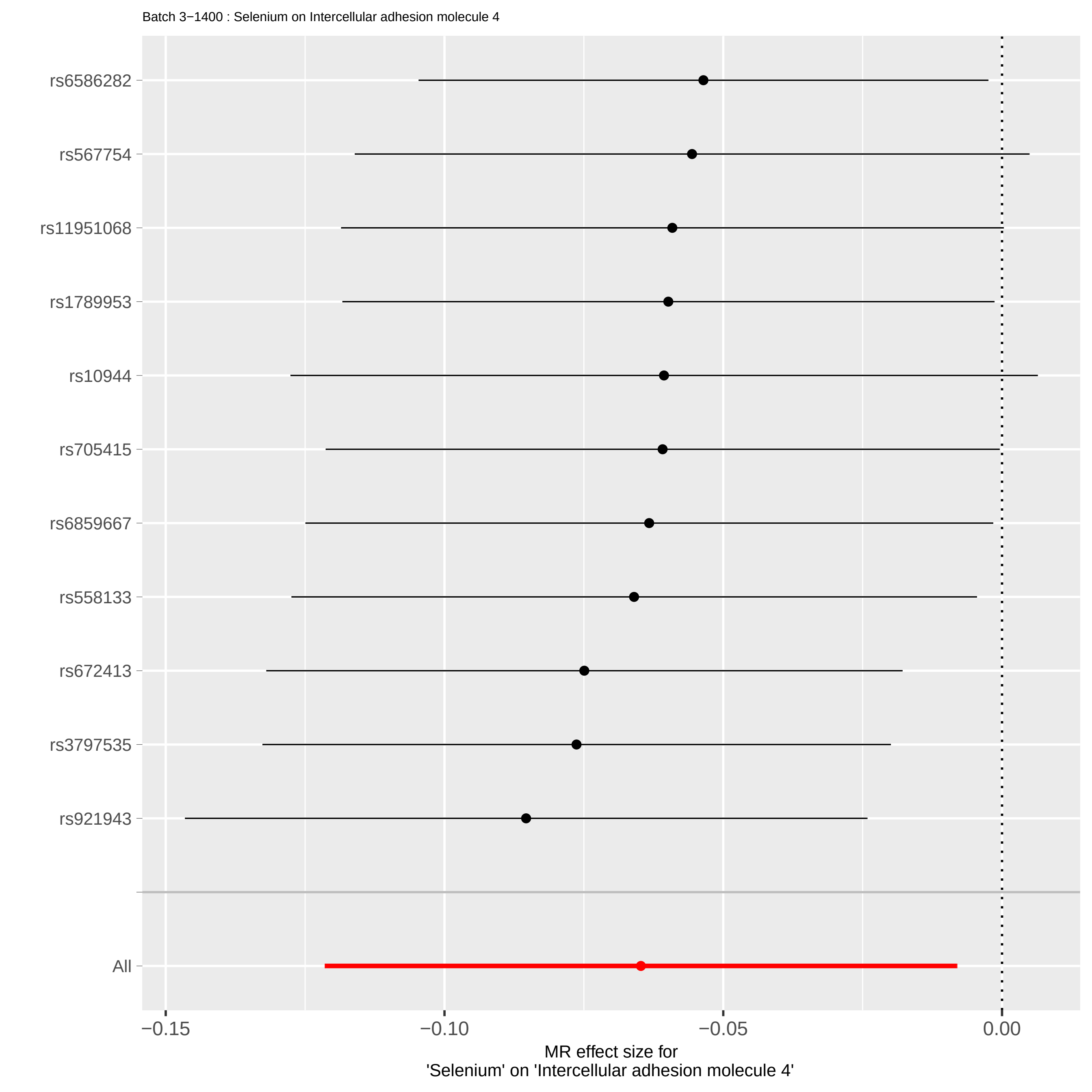

Batch 3−1400 : Selenium on Intercellular adhesion molecule 4
rs6586282
rs567754
rs11951068
rs1789953
rs10944
rs705415
rs6859667
rs558133
rs672413
rs3797535
rs921943
All
−0.15
−0.10
−0.05
0.00
MR effect size for
'Selenium' on 'Intercellular adhesion molecule 4'

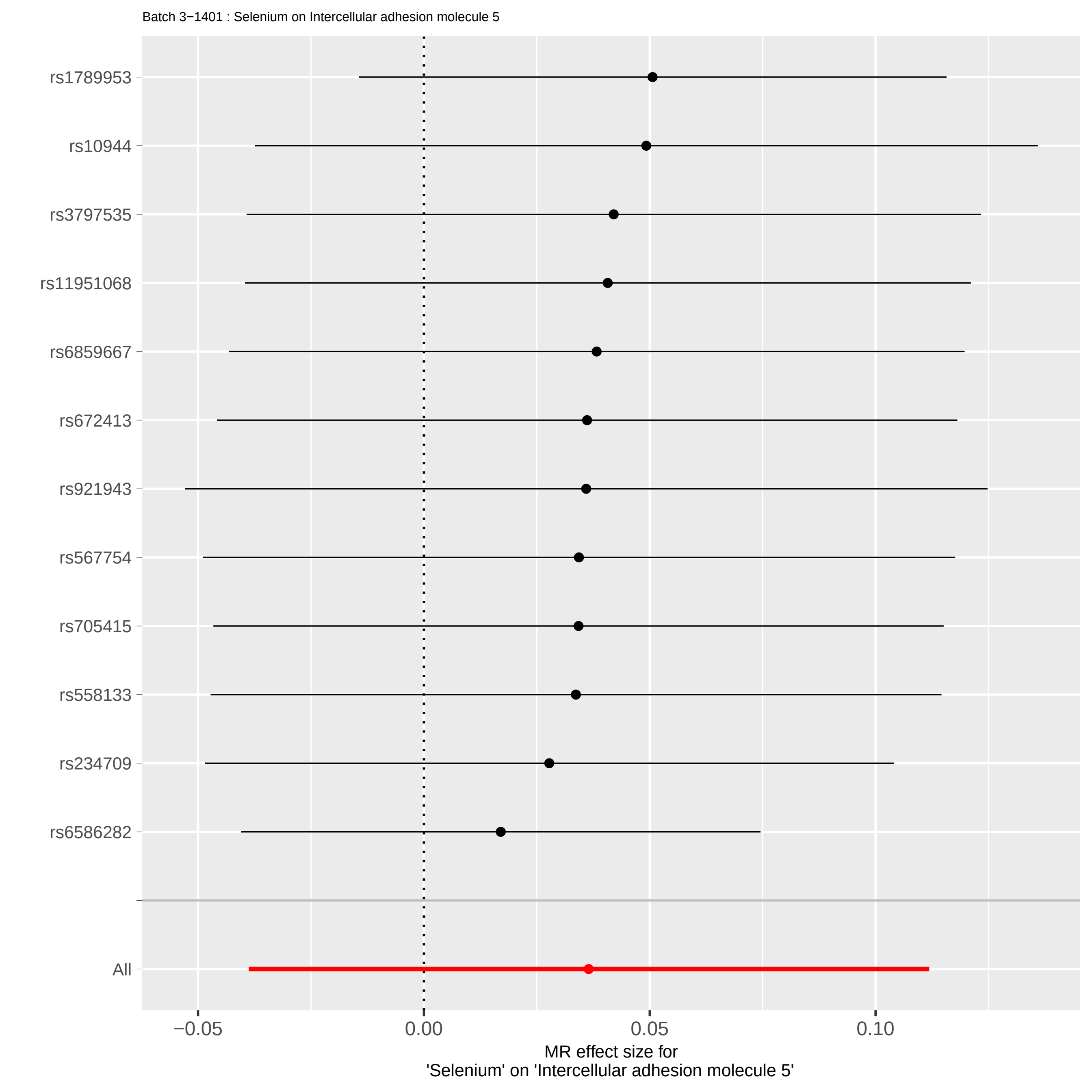

Batch 3−1401 : Selenium on Intercellular adhesion molecule 5
rs1789953
rs10944
rs3797535
rs11951068
rs6859667
rs672413
rs921943
rs567754
rs705415
rs558133
rs234709
rs6586282
All
−0.05
0.00
0.05
0.10
MR effect size for
'Selenium' on 'Intercellular adhesion molecule 5'

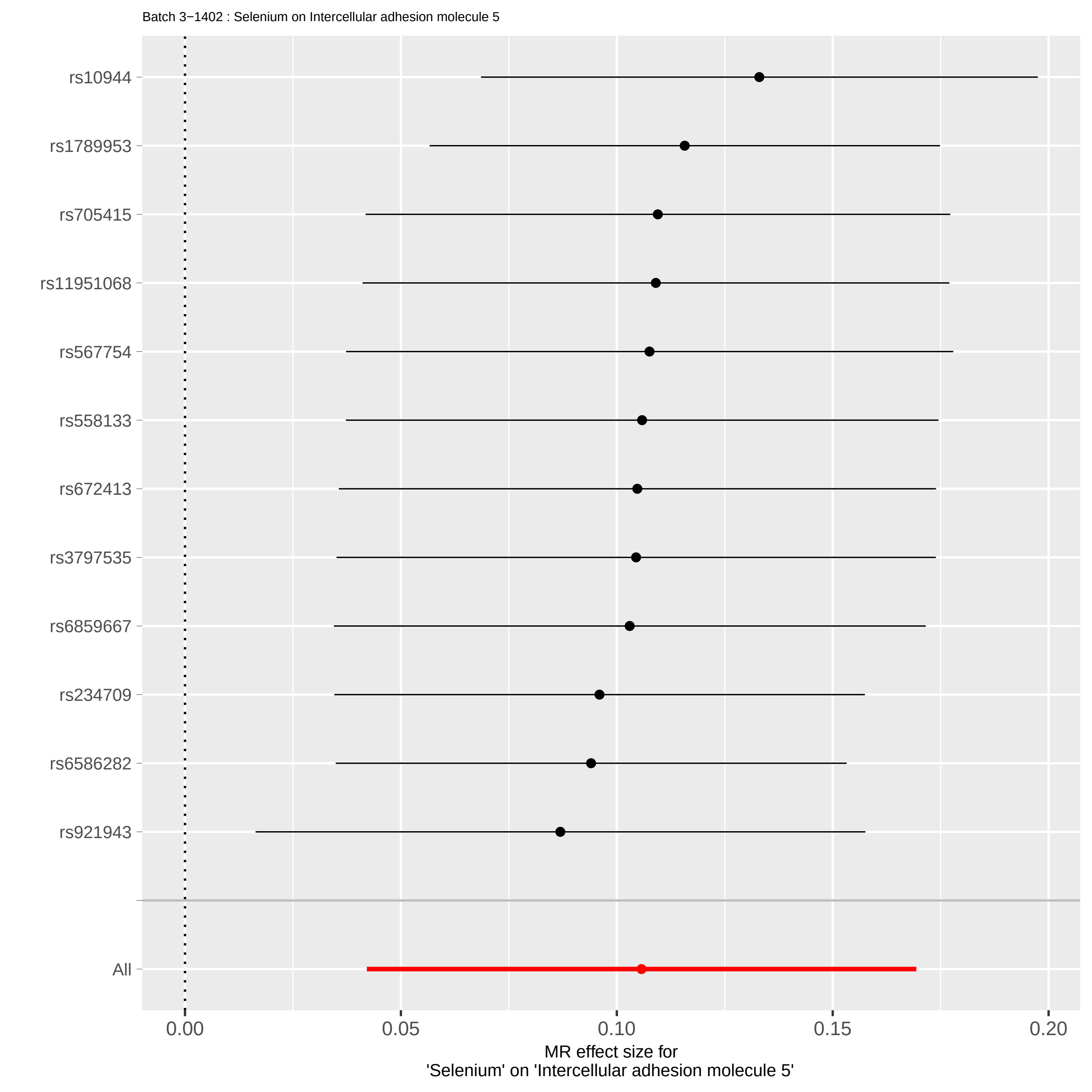
Batch 3−1402 : Selenium on Intercellular adhesion molecule 5
rs10944
rs1789953
rs705415
rs11951068
rs567754
rs558133
rs672413
rs3797535
rs6859667
rs234709
rs6586282
rs921943
All
0.00
0.05
0.10
0.15
0.20
MR effect size for
'Selenium' on 'Intercellular adhesion molecule 5'

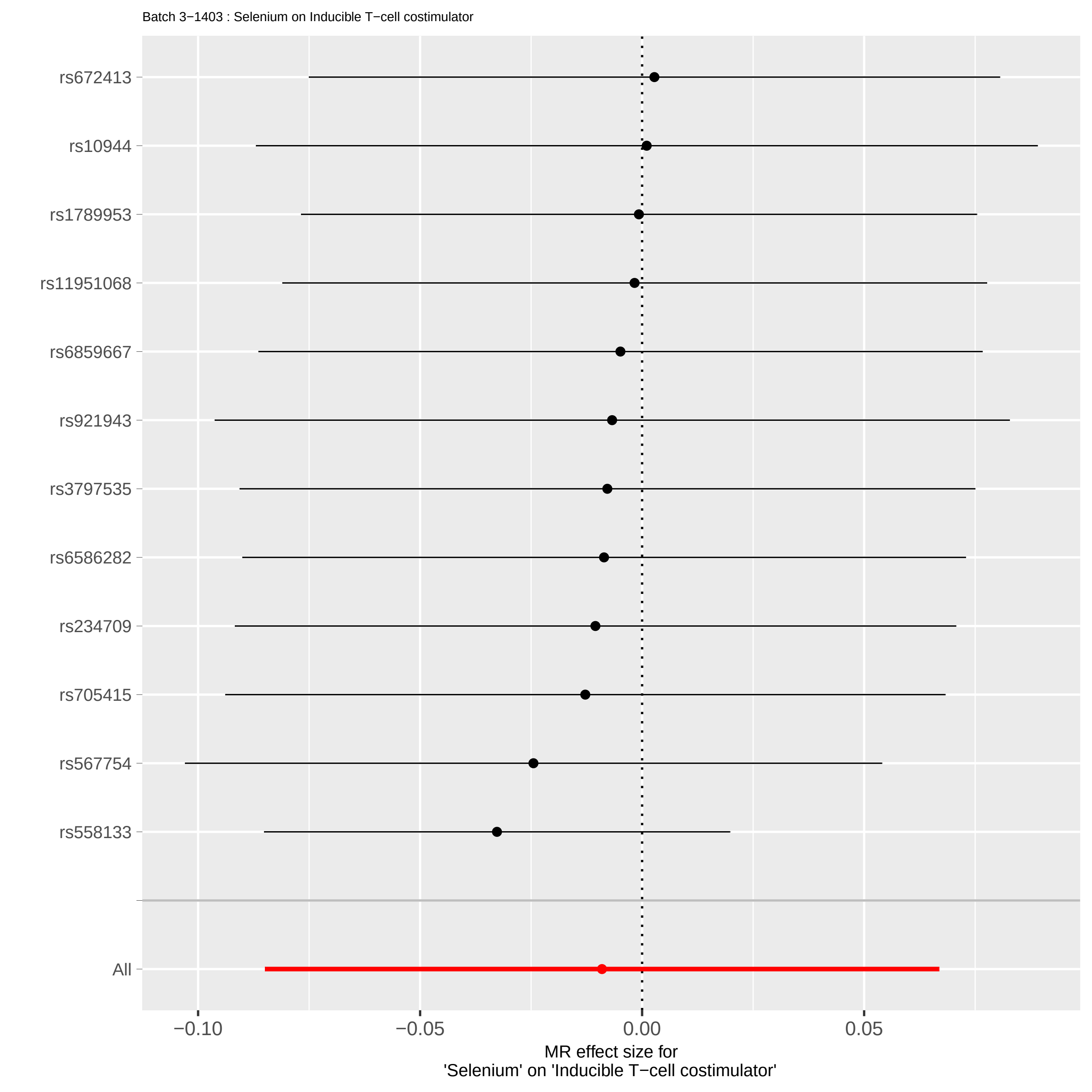

Batch 3−1403 : Selenium on Inducible T−cell costimulator
rs672413
rs10944
rs1789953
rs11951068
rs6859667
rs921943
rs3797535
rs6586282
rs234709
rs705415
rs567754
rs558133
All
−0.10
−0.05
0.00
0.05
MR effect size for
'Selenium' on 'Inducible T−cell costimulator'

Batch 3−1404 : Selenium on Inducible T−cell costimulator

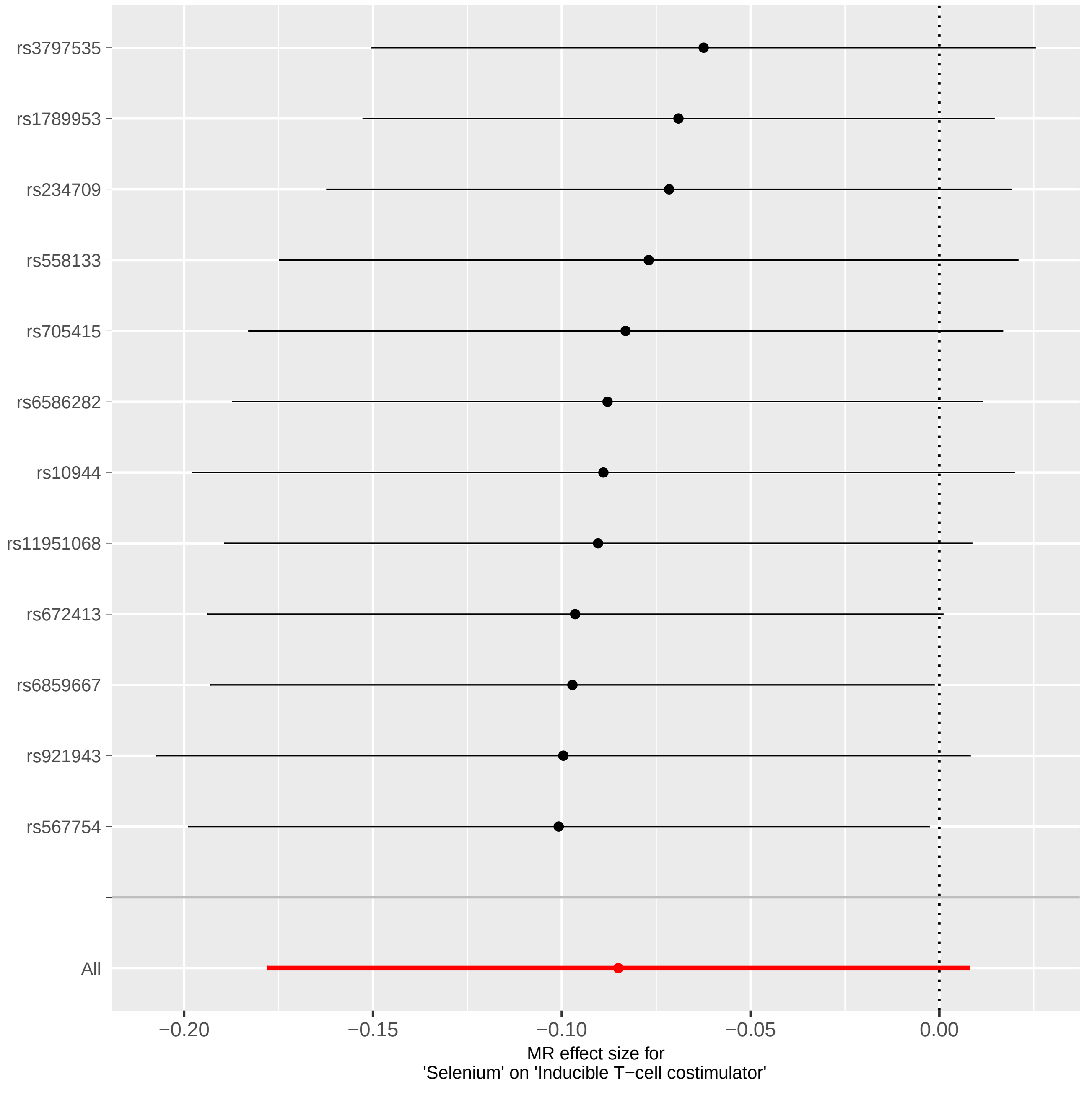

Batch 3−1405 : Selenium on ICOS ligand

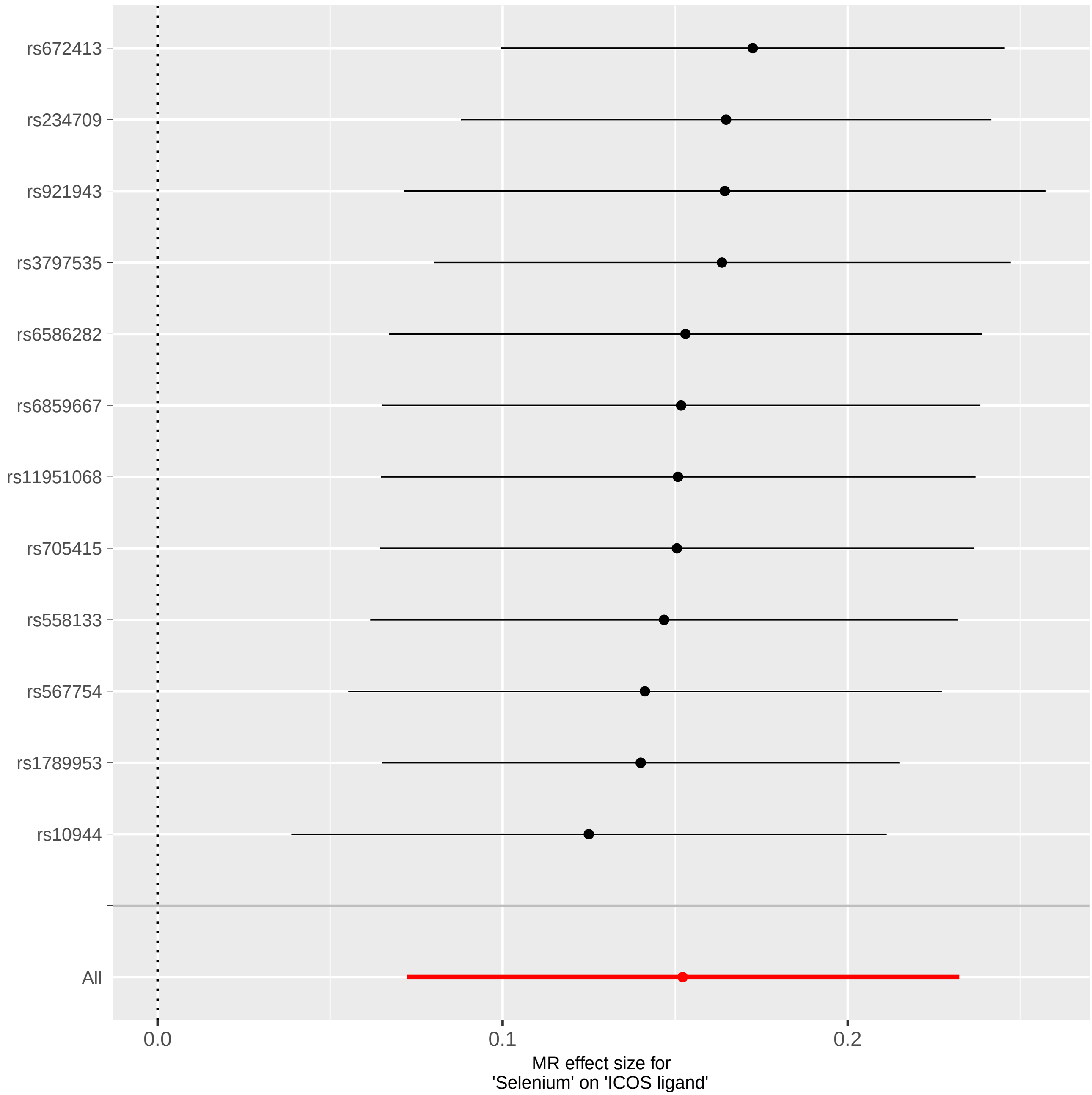

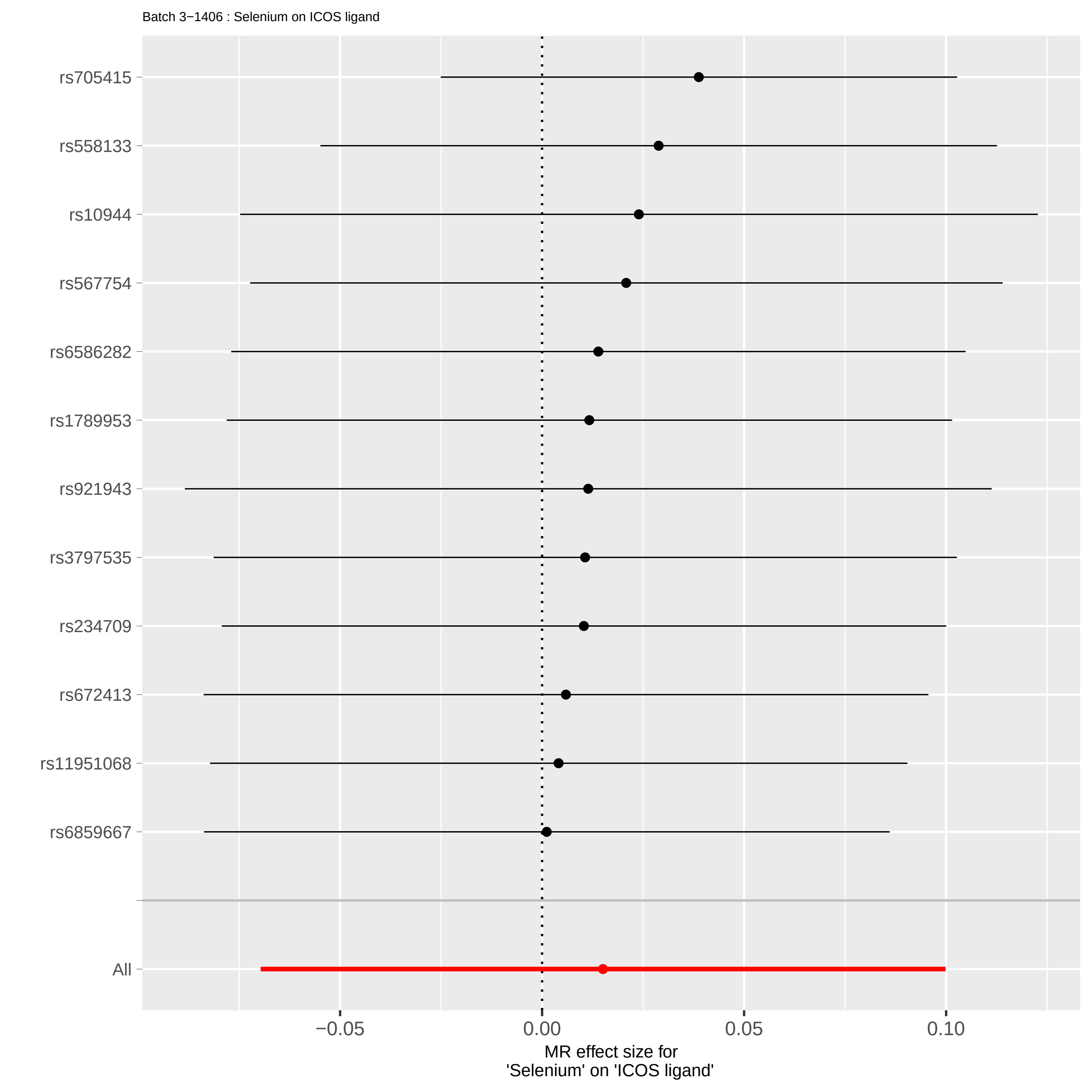

Batch 3−1406 : Selenium on ICOS ligand
rs705415
rs558133
rs10944
rs567754
rs6586282
rs1789953
rs921943
rs3797535
rs234709
rs672413
rs11951068
rs6859667
All
−0.05
0.00
0.05
0.10
MR effect size for
'Selenium' on 'ICOS ligand'

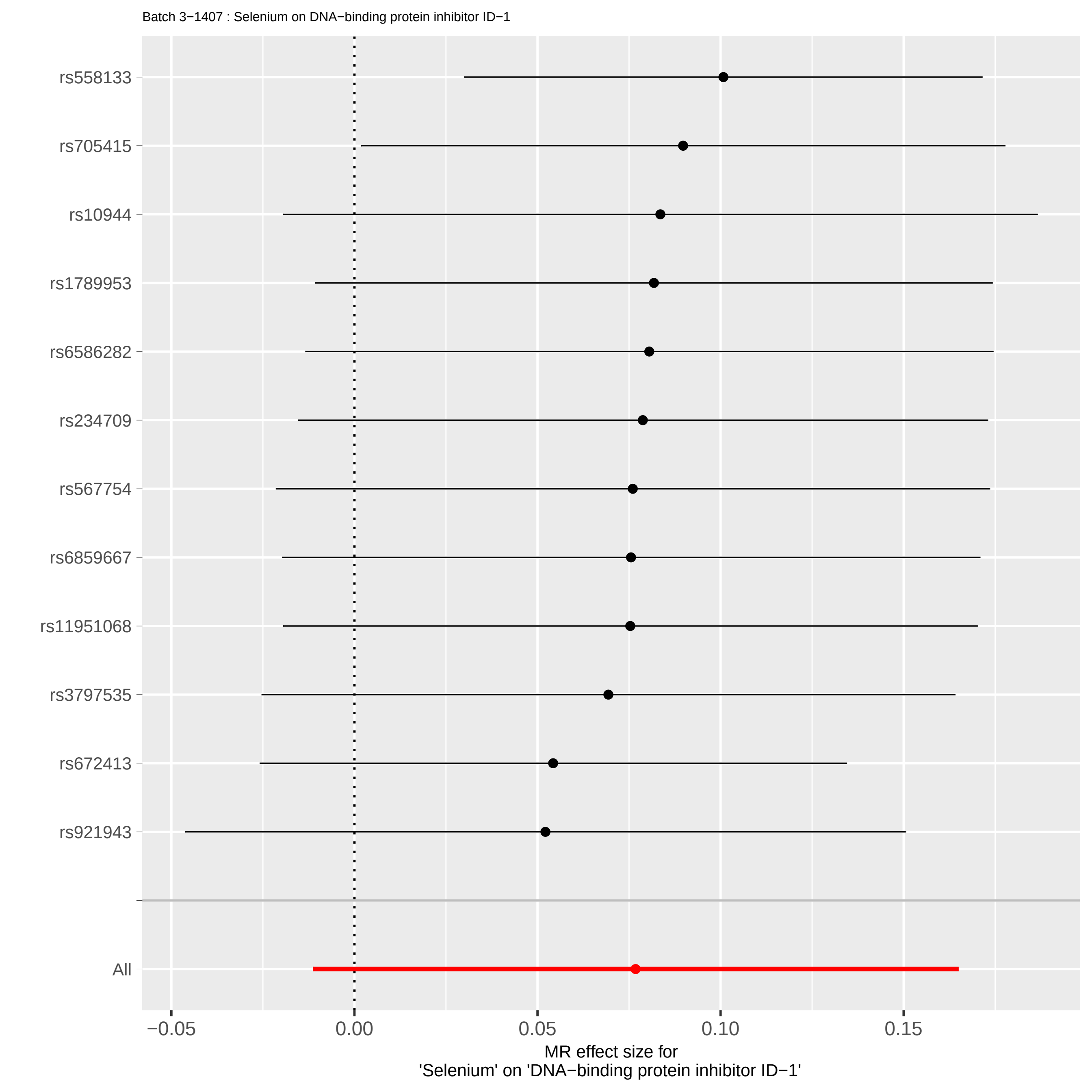

Batch 3−1407 : Selenium on DNA−binding protein inhibitor ID−1
rs558133
rs705415
rs10944
rs1789953
rs6586282
rs234709
rs567754
rs6859667
rs11951068
rs3797535
rs672413
rs921943
All
−0.05
0.00
0.05
0.10
0.15
MR effect size for
'Selenium' on 'DNA−binding protein inhibitor ID−1'

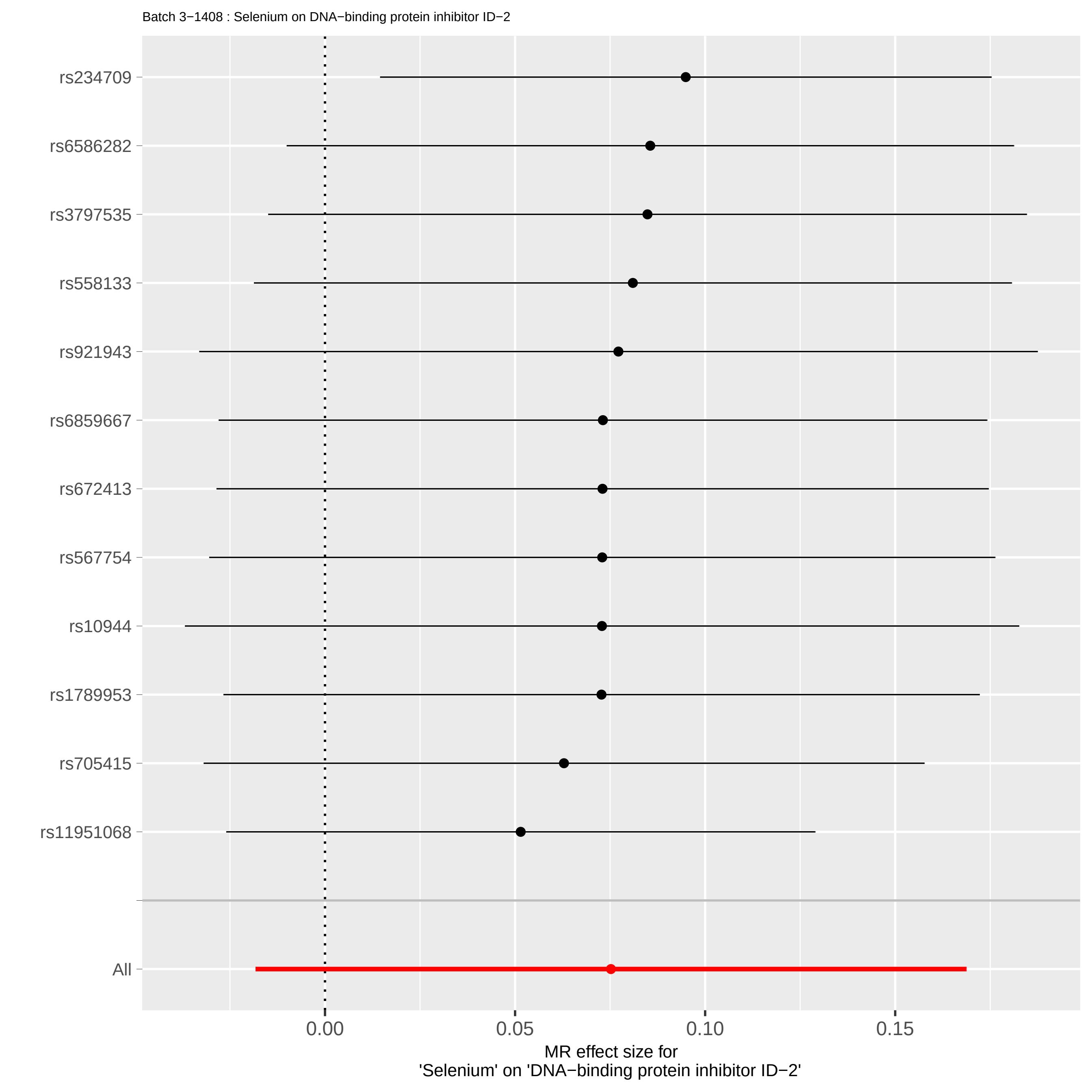

Batch 3−1408 : Selenium on DNA−binding protein inhibitor ID−2
rs234709
rs6586282
rs3797535
rs558133
rs921943
rs6859667
rs672413
rs567754
rs10944
rs1789953
rs705415
rs11951068
All
0.00
0.05
0.10
0.15
MR effect size for
'Selenium' on 'DNA−binding protein inhibitor ID−2'

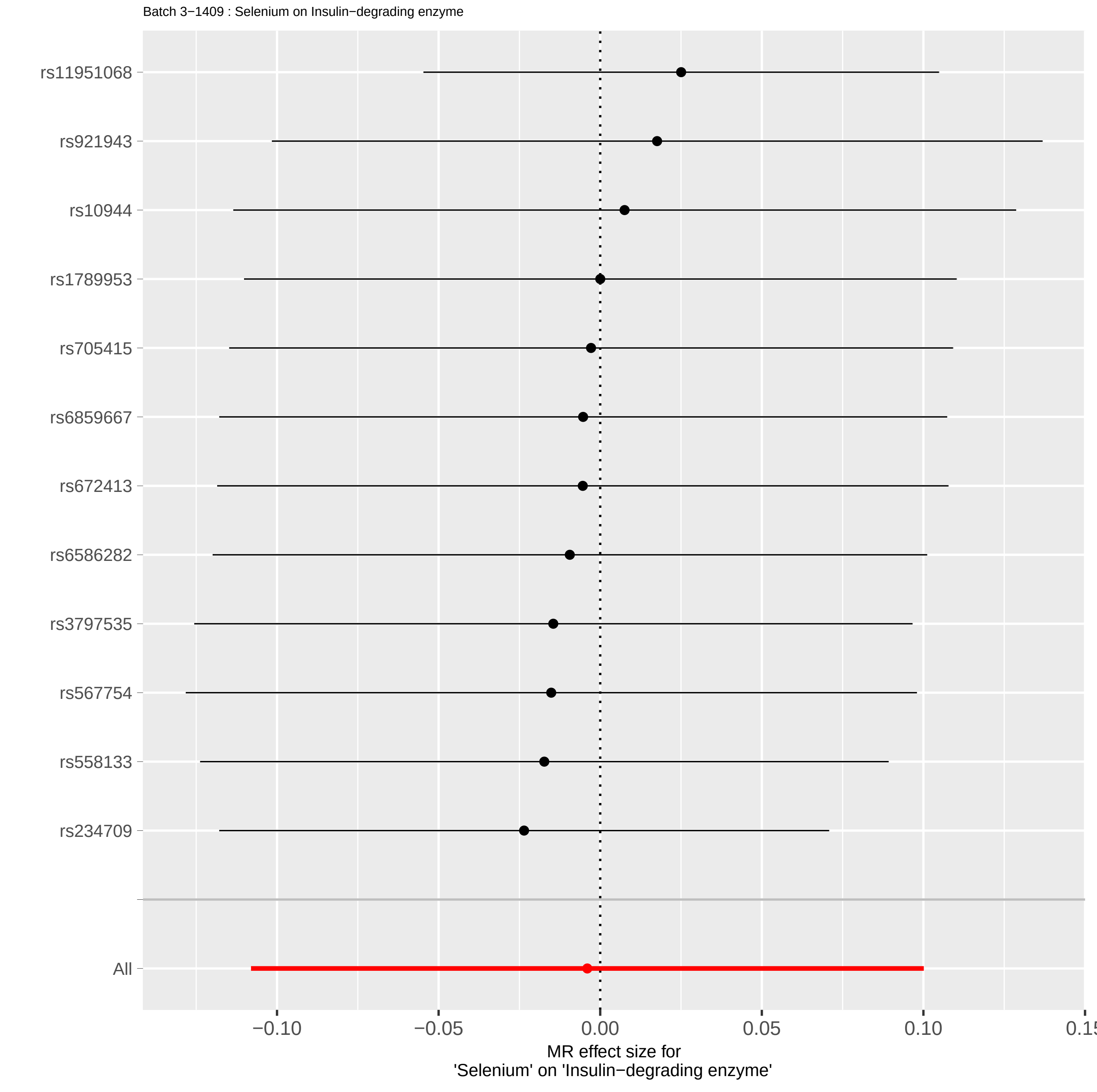

Batch 3−1409 : Selenium on Insulin−degrading enzyme
rs11951068
rs921943
rs10944
rs1789953
rs705415
rs6859667
rs672413
rs6586282
rs3797535
rs567754
rs558133
rs234709
All
−0.10
−0.05
0.00
0.05
0.10
MR effect size for
'Selenium' on 'Insulin−degrading enzyme'

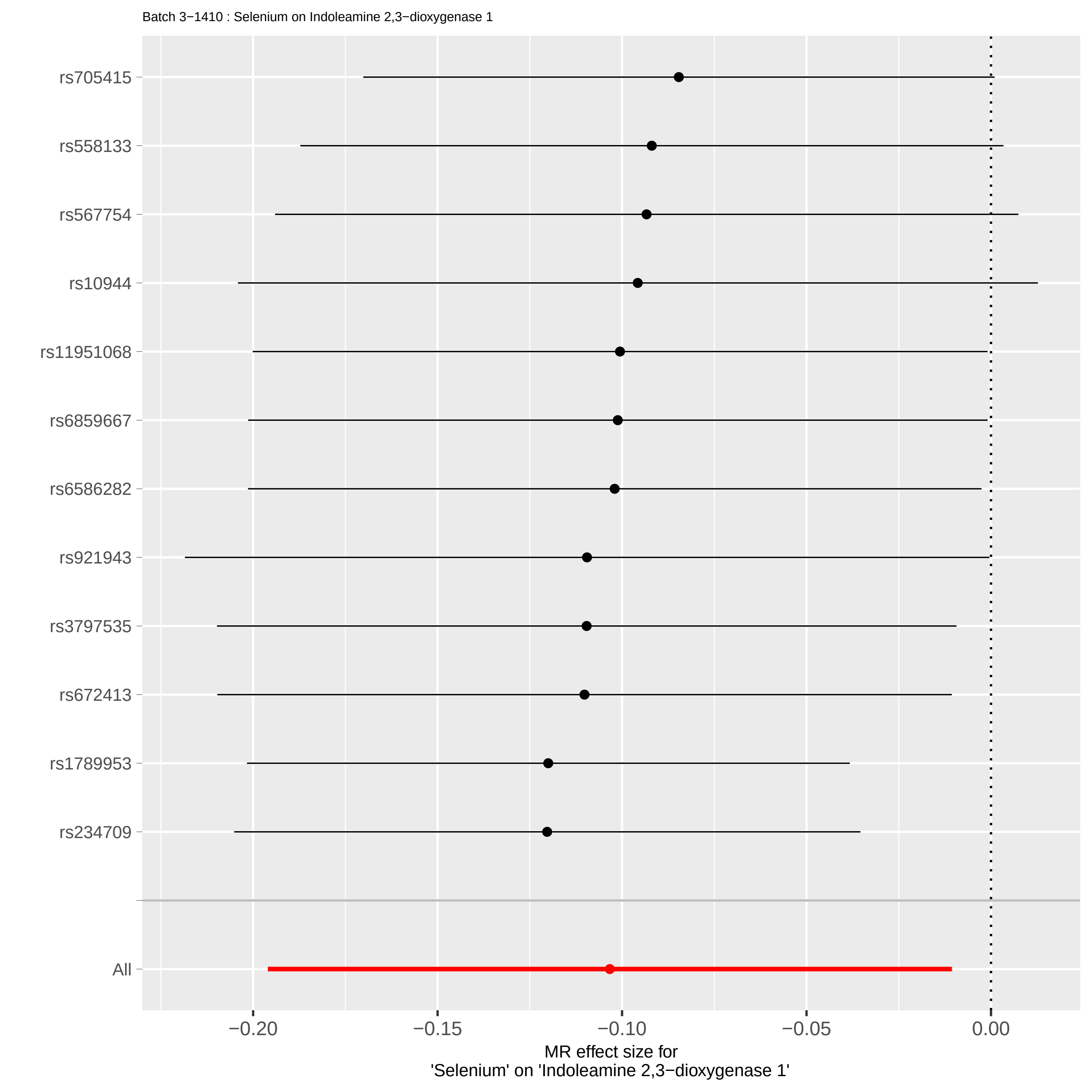
Batch 3−1410 : Selenium on Indoleamine 2,3−dioxygenase 1
rs705415
rs558133
rs567754
rs10944
rs11951068
rs6859667
rs6586282
rs921943
rs3797535
rs672413
rs1789953
rs234709
All
−0.20
−0.15
−0.10
−0.05
0.00
MR effect size for
'Selenium' on 'Indoleamine 2,3−dioxygenase 1'

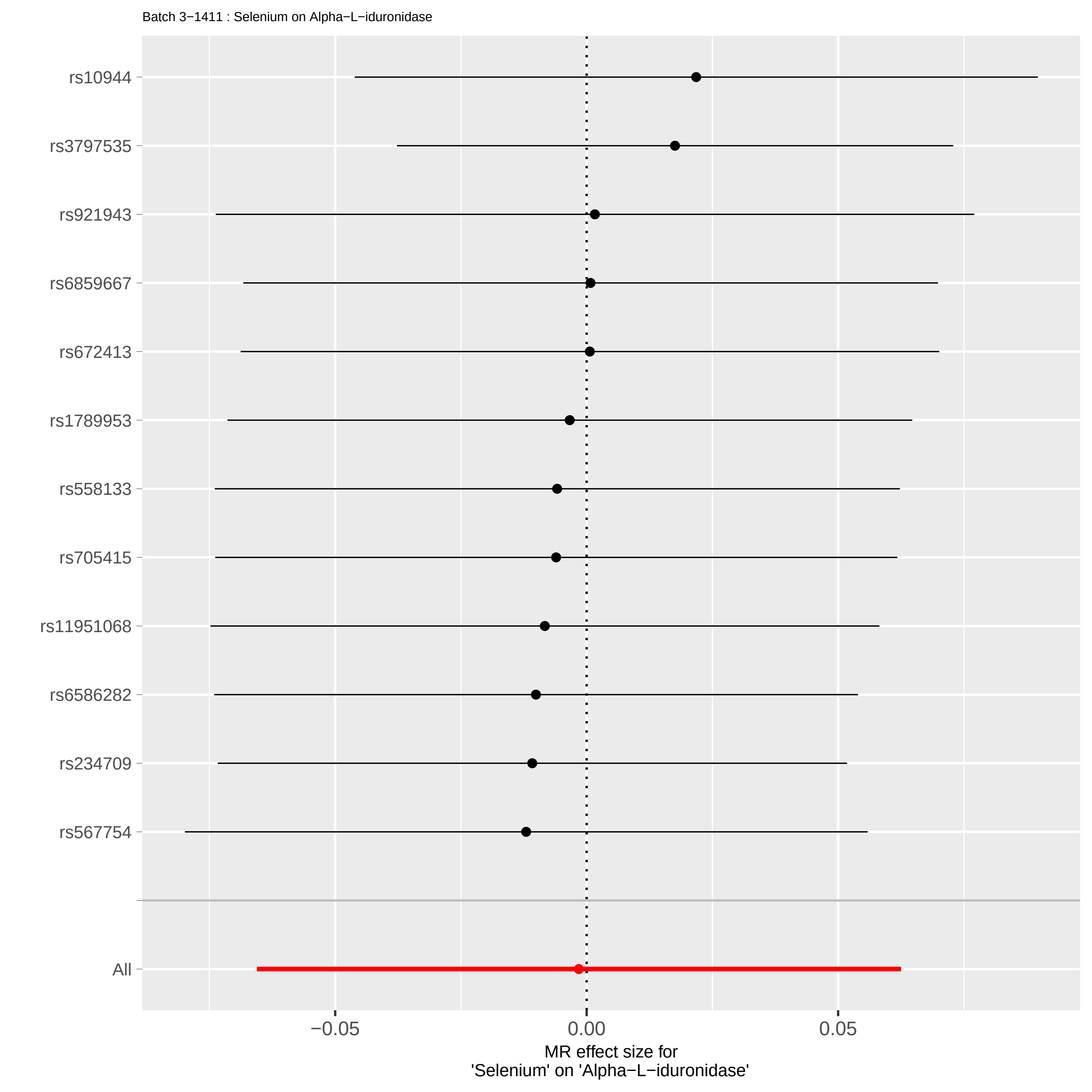
Batch 3−1411 : Selenium on Alpha−L−iduronidase
rs10944
rs3797535
rs921943
rs6859667
rs672413
rs1789953
rs558133
rs705415
rs11951068
rs6586282
rs234709
rs567754
All
−0.05
0.00
0.05
MR effect size for
'Selenium' on 'Alpha−L−iduronidase'

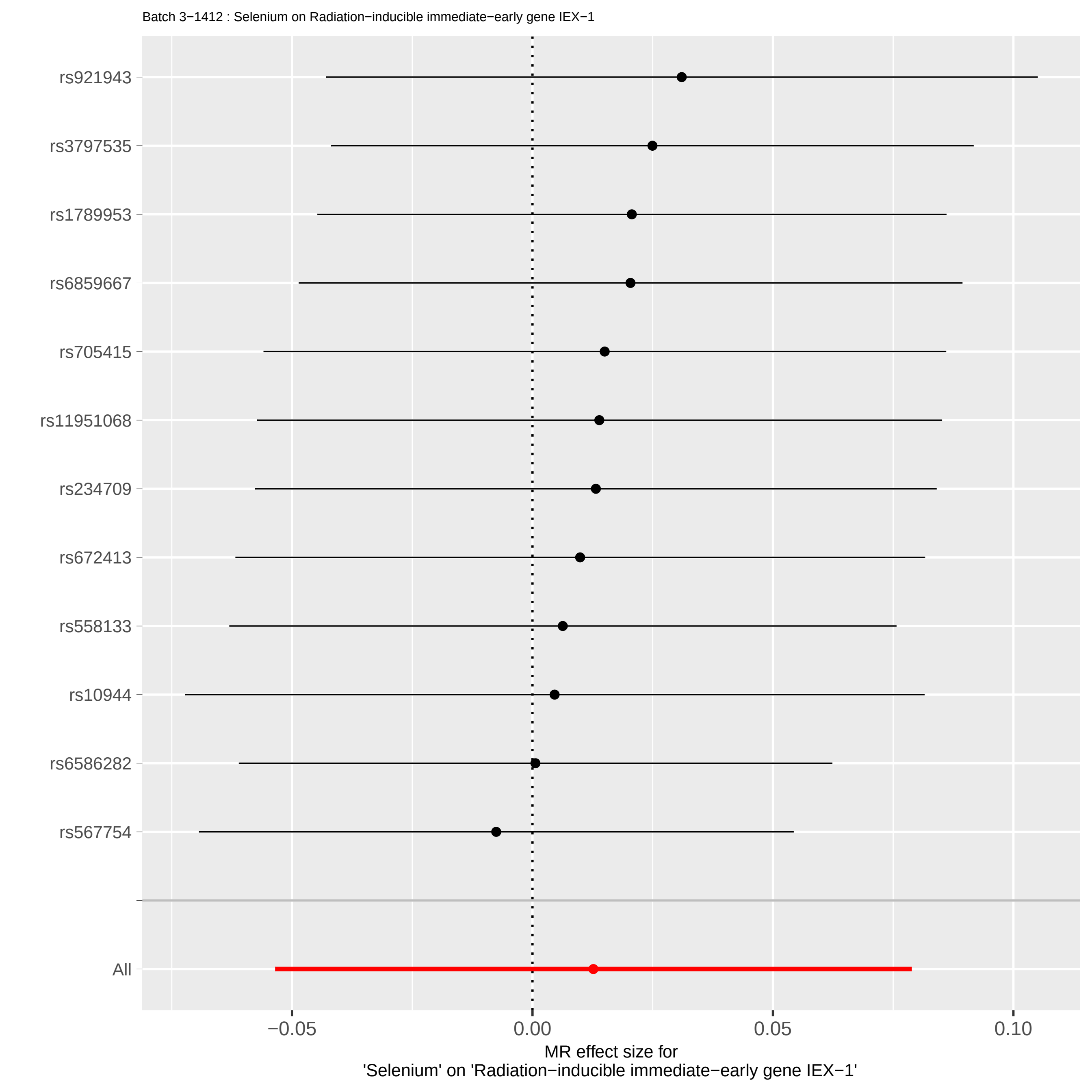

Batch 3−1412 : Selenium on Radiation−inducible immediate−early gene IEX−1
rs921943
rs3797535
rs1789953
rs6859667
rs705415
rs11951068
rs234709
rs672413
rs558133
rs10944
rs6586282
rs567754
All
−0.05
0.00
0.05
0.10
MR effect size for
'Selenium' on 'Radiation−inducible immediate−early gene IEX−1'

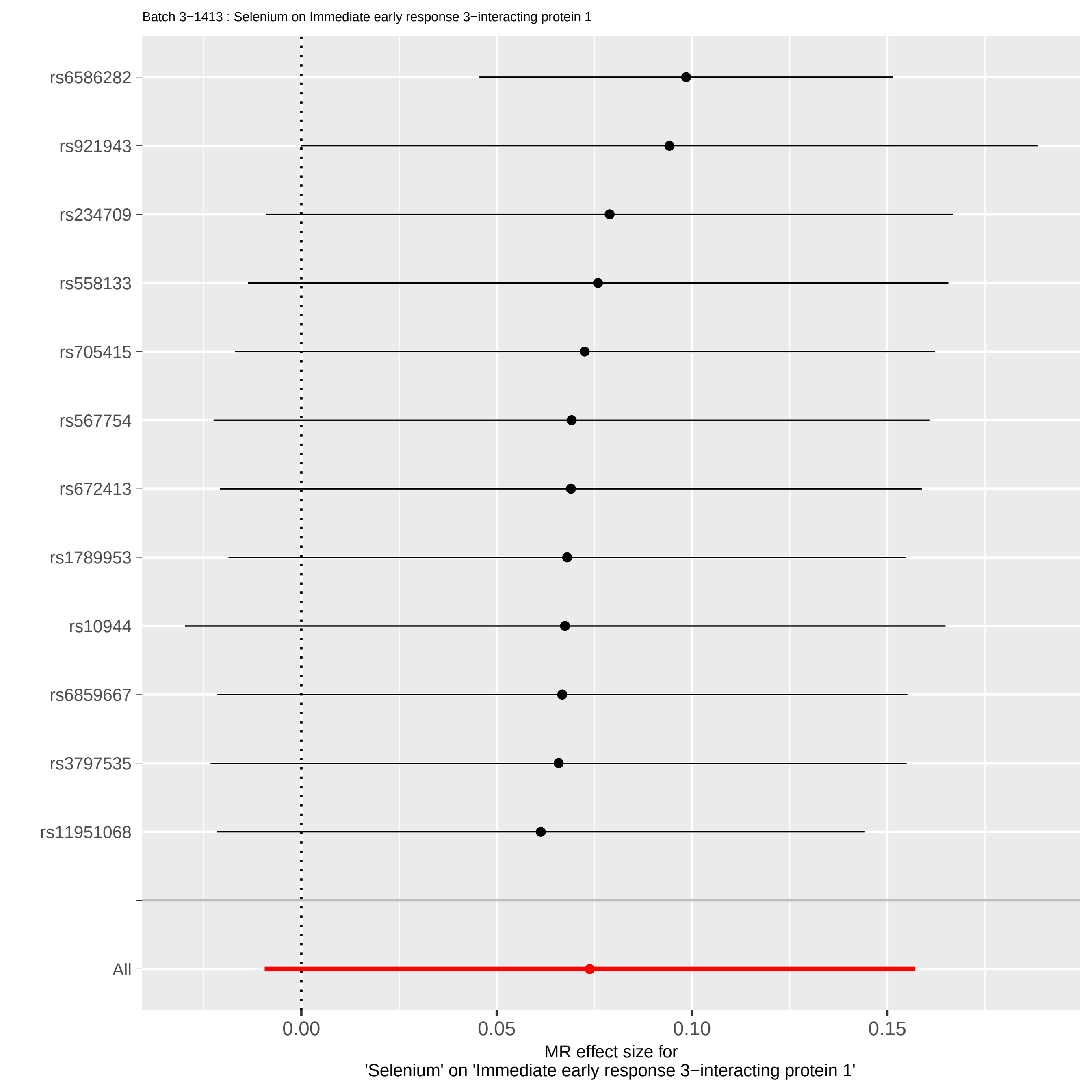

Batch 3−1413 : Selenium on Immediate early response 3−interacting protein 1
rs6586282
rs921943
rs234709
rs558133
rs705415
rs567754
rs672413
rs1789953
rs10944
rs6859667
rs3797535
rs11951068
All
0.00
0.05
0.10
0.15
MR effect size for
'Selenium' on 'Immediate early response 3−interacting protein 1'

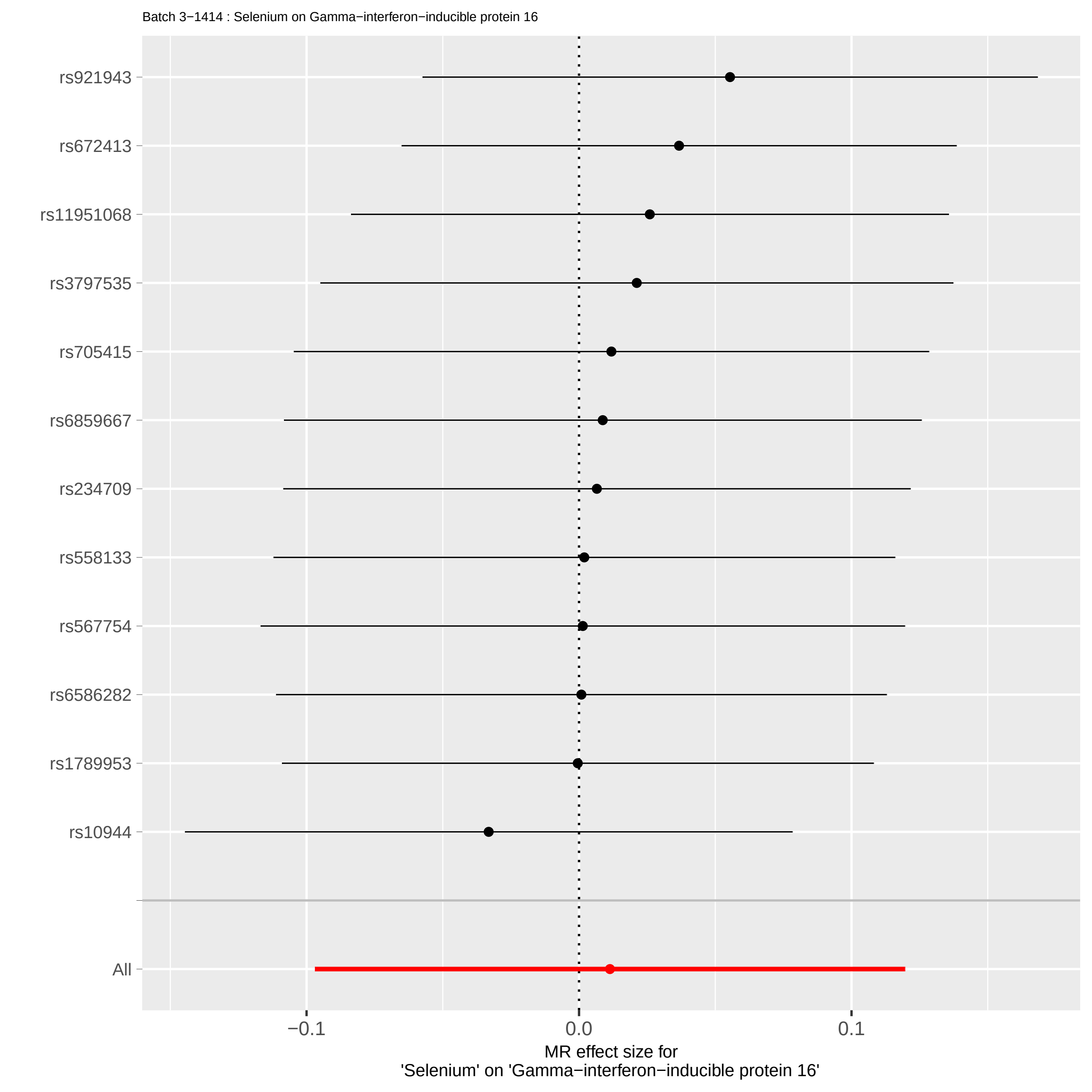

Batch 3−1414 : Selenium on Gamma−interferon−inducible protein 16
rs921943
rs672413
rs11951068
rs3797535
rs705415
rs6859667
rs234709
rs558133
rs567754
rs6586282
rs1789953
rs10944
All
−0.1
0.0
0.1
MR effect size for
'Selenium' on 'Gamma−interferon−inducible protein 16'

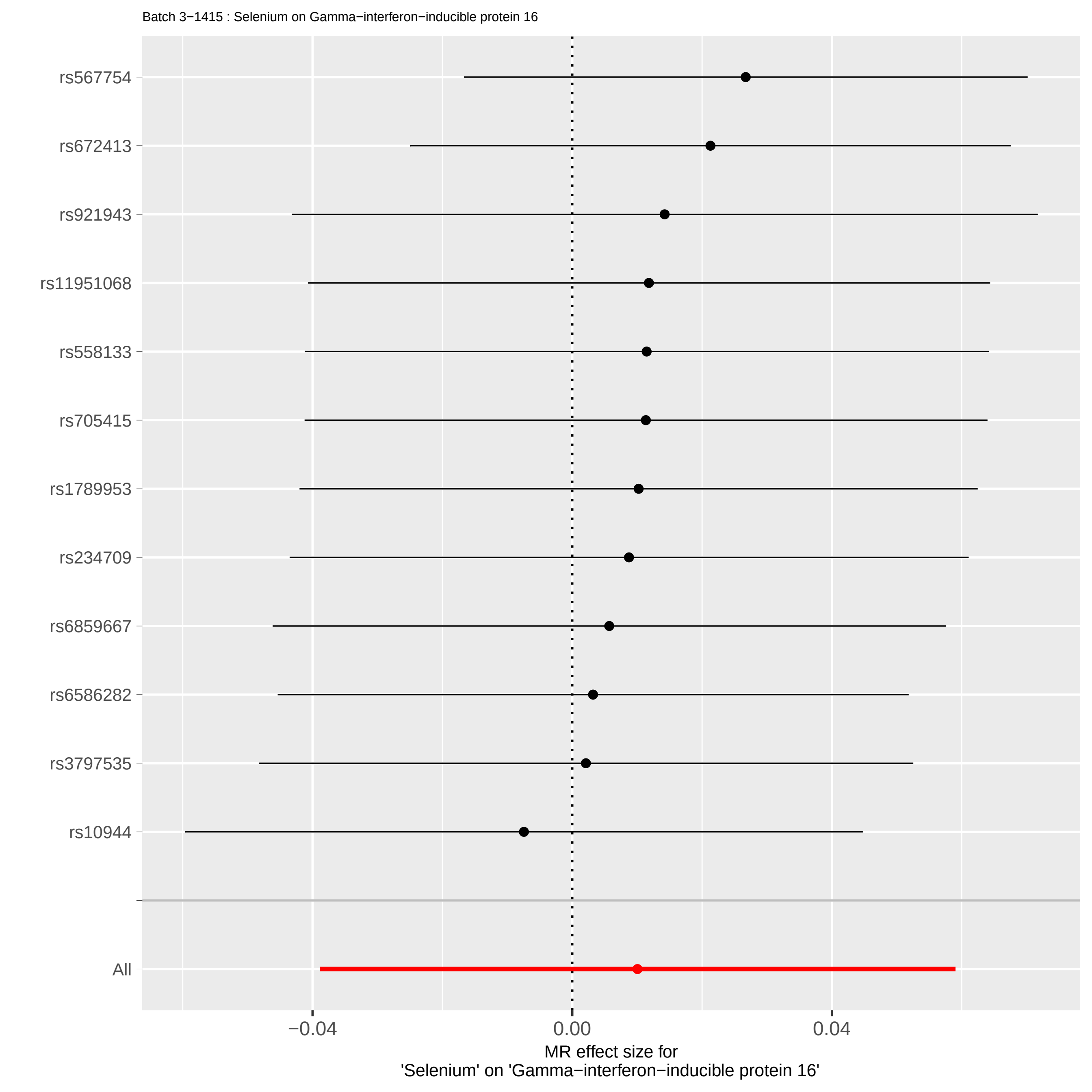

Batch 3−1415 : Selenium on Gamma−interferon−inducible protein 16
rs567754
rs672413
rs921943
rs11951068
rs558133
rs705415
rs1789953
rs234709
rs6859667
rs6586282
rs3797535
rs10944
All
−0.04
0.00
0.04
MR effect size for
'Selenium' on 'Gamma−interferon−inducible protein 16'

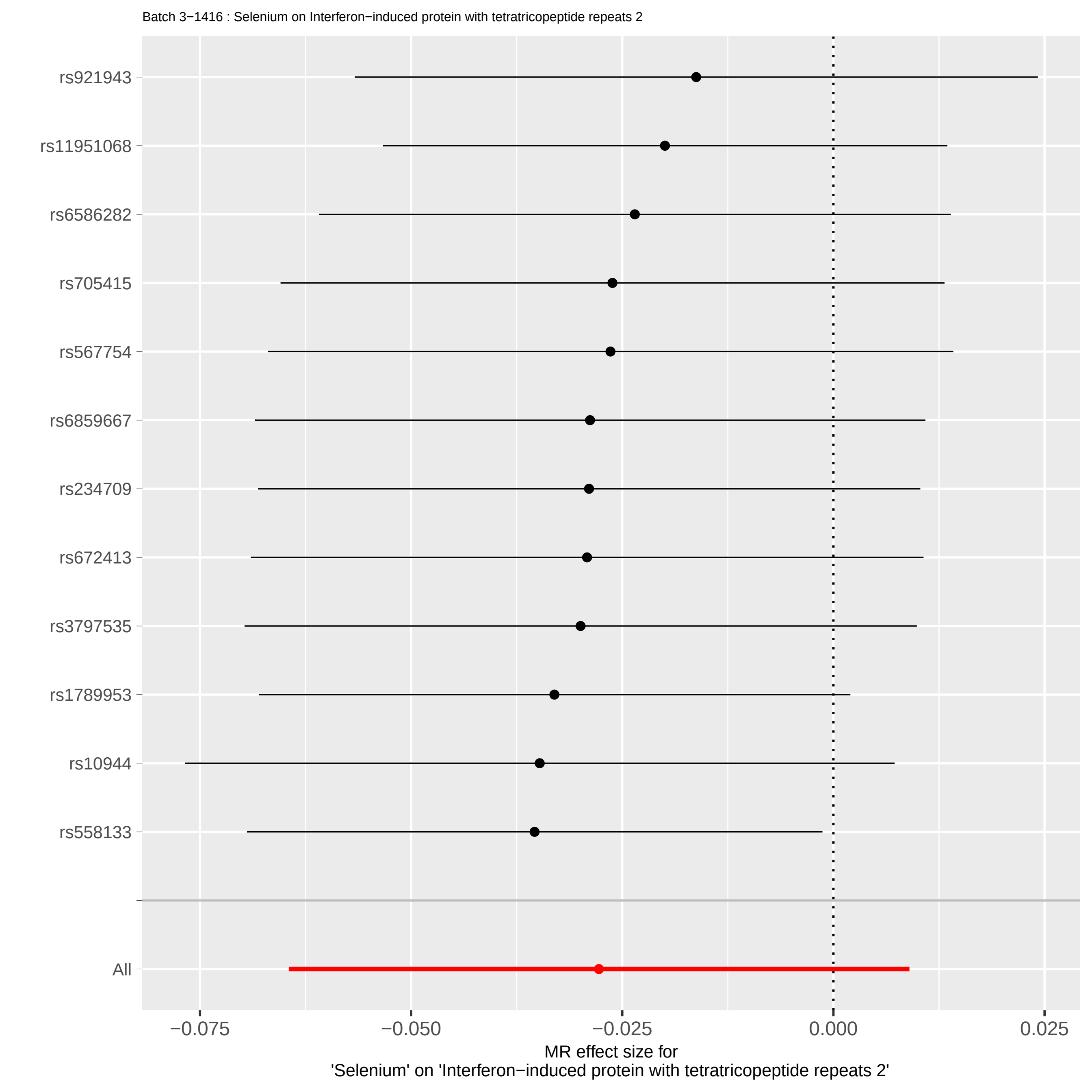
Batch 3−1416 : Selenium on Interferon−induced protein with tetratricopeptide repeats 2
rs921943
rs11951068
rs6586282
rs705415
rs567754
rs6859667
rs234709
rs672413
rs3797535
rs1789953
rs10944
rs558133
All
−0.075
−0.050
−0.025
0.000
0.025
MR effect size for
'Selenium' on 'Interferon−induced protein with tetratricopeptide repeats 2'

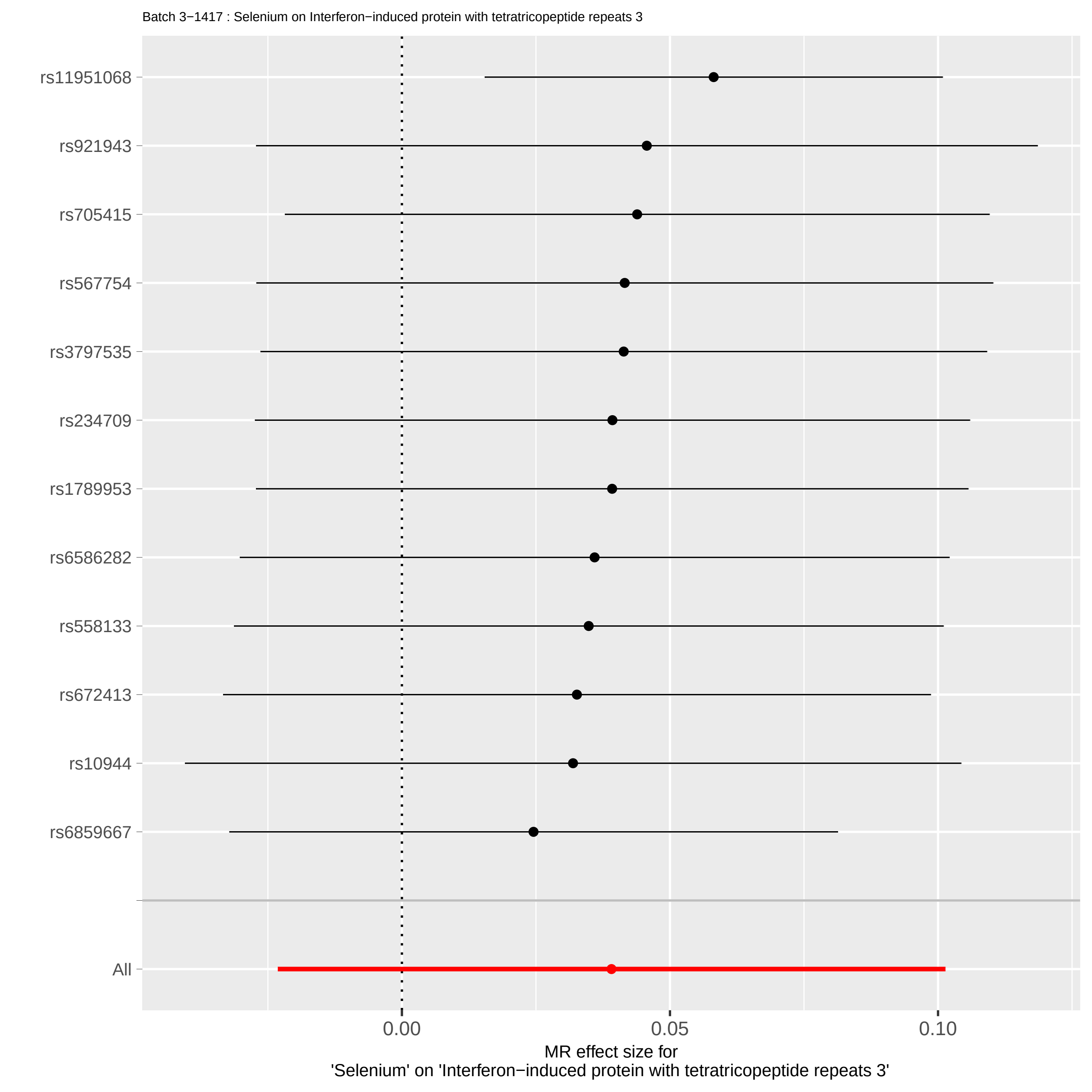
Batch 3−1417 : Selenium on Interferon−induced protein with tetratricopeptide repeats 3
rs11951068
rs921943
rs705415
rs567754
rs3797535
rs234709
rs1789953
rs6586282
rs558133
rs672413
rs10944
rs6859667
All
0.00
0.05
0.10
MR effect size for
'Selenium' on 'Interferon−induced protein with tetratricopeptide repeats 3'

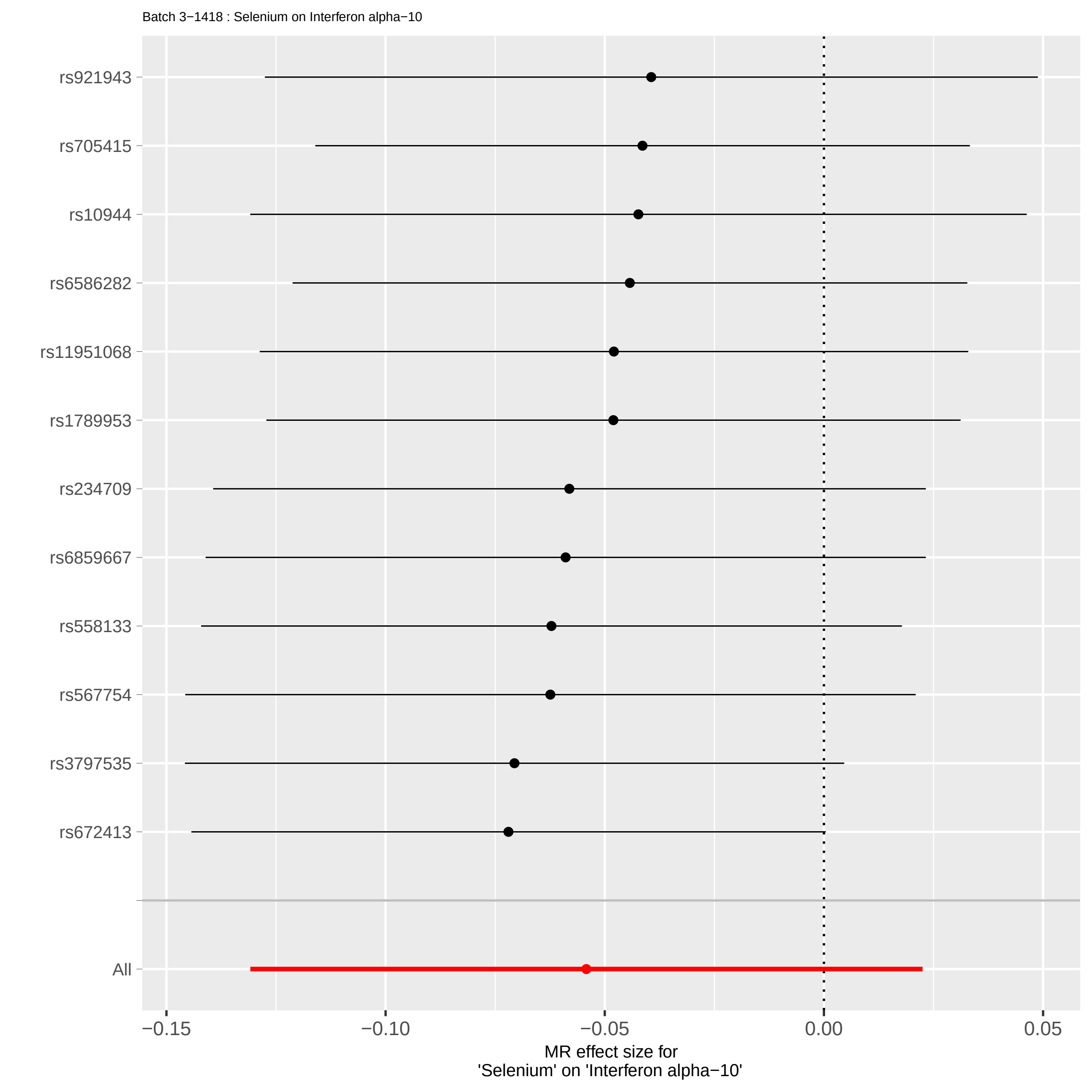

Batch 3−1418 : Selenium on Interferon alpha−10
rs921943
rs705415
rs10944
rs6586282
rs11951068
rs1789953
rs234709
rs6859667
rs558133
rs567754
rs3797535
rs672413
All
−0.15
−0.10
−0.05
0.00
0.05
MR effect size for
'Selenium' on 'Interferon alpha−10'

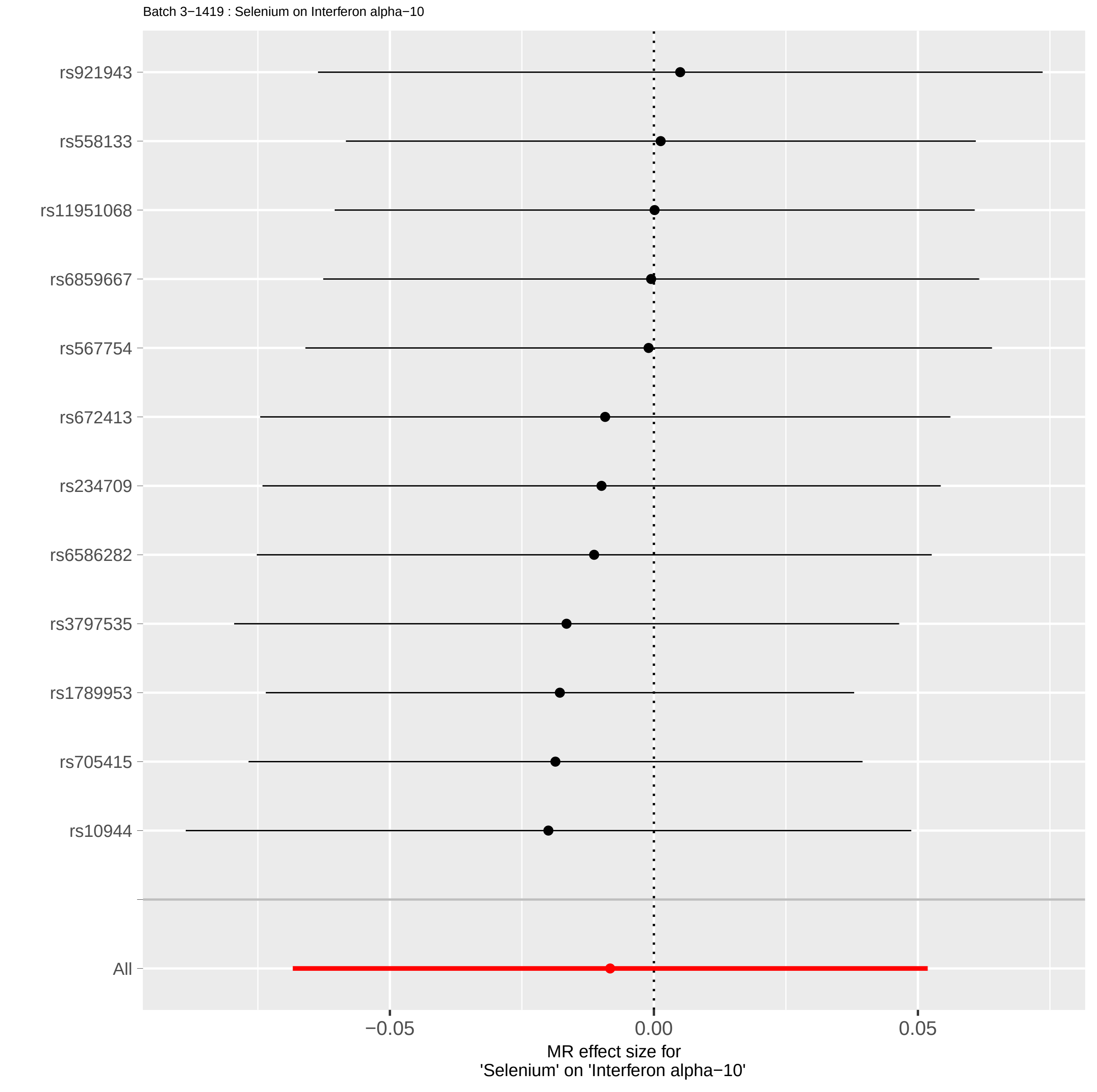

Batch 3−1419 : Selenium on Interferon alpha−10
rs921943
rs558133
rs11951068
rs6859667
rs567754
rs672413
rs234709
rs6586282
rs3797535
rs1789953
rs705415
rs10944
All
−0.05
0.00
0.05
MR effect size for
'Selenium' on 'Interferon alpha−10'

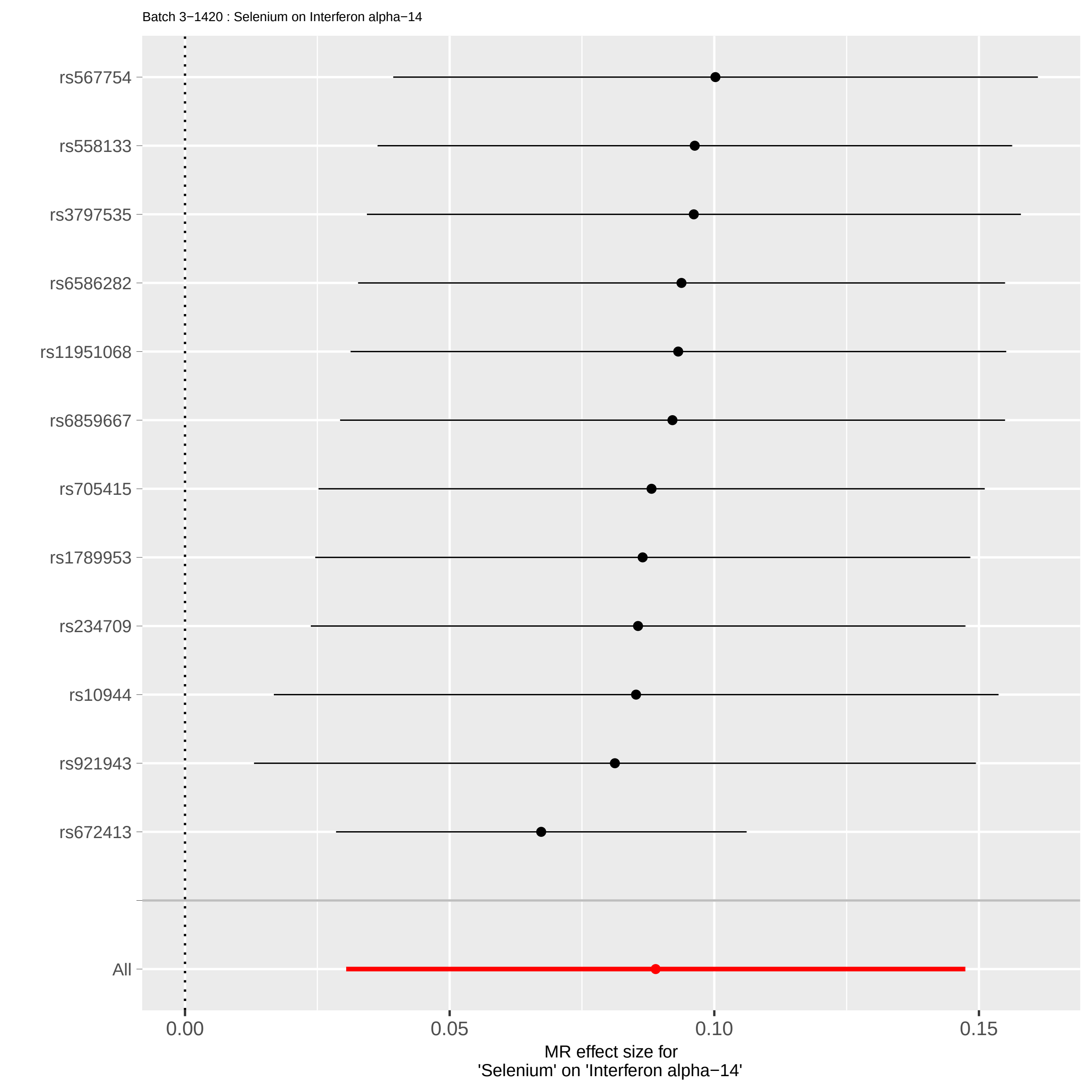
Batch 3−1420 : Selenium on Interferon alpha−14
rs567754
rs558133
rs3797535
rs6586282
rs11951068
rs6859667
rs705415
rs1789953
rs234709
rs10944
rs921943
rs672413
All
0.00
0.05
0.10
0.15
MR effect size for
'Selenium' on 'Interferon alpha−14'

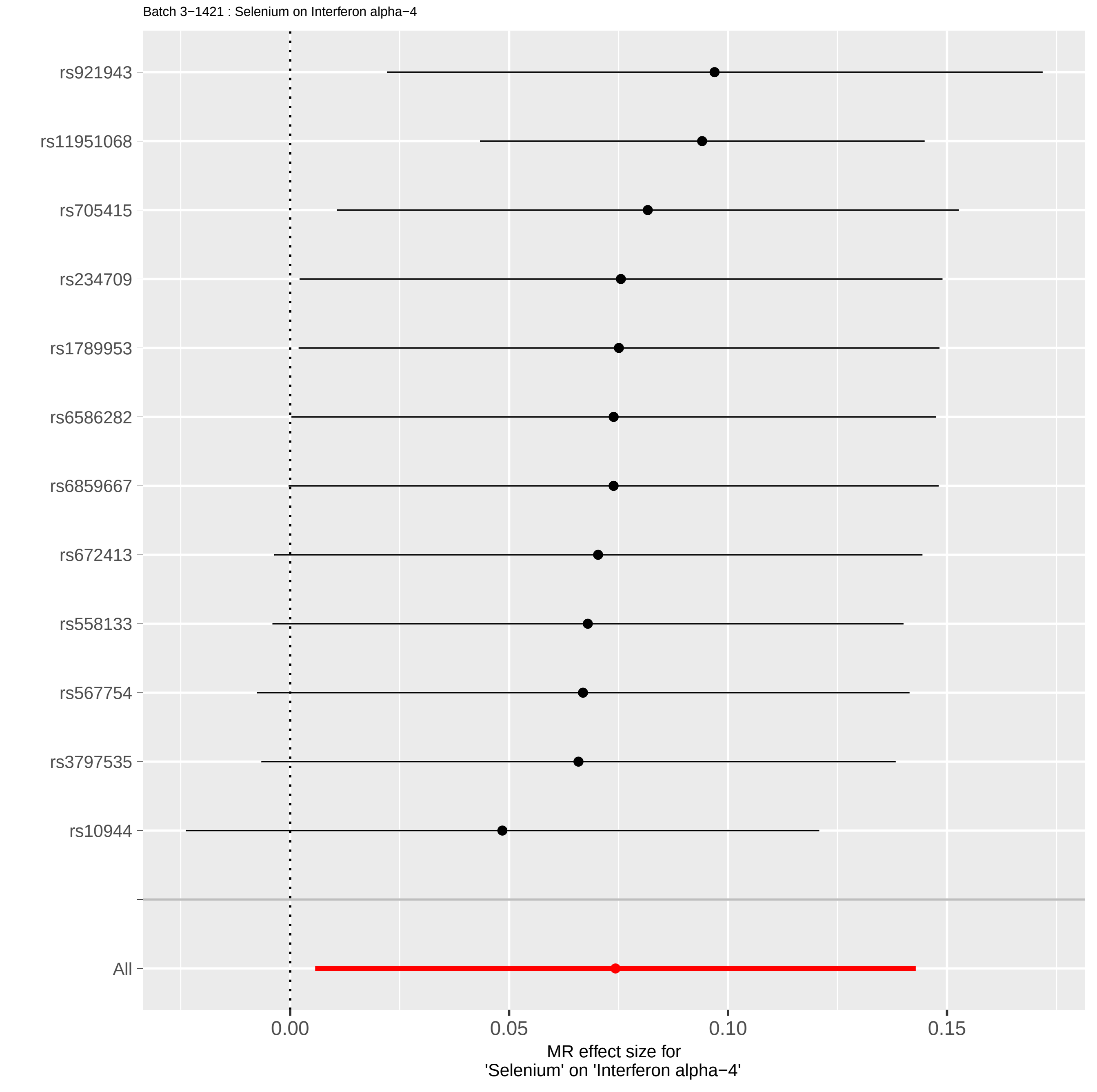

Batch 3−1421 : Selenium on Interferon alpha−4
rs921943
rs11951068
rs705415
rs234709
rs1789953
rs6586282
rs6859667
rs672413
rs558133
rs567754
rs3797535
rs10944
All
0.00
0.05
0.10
0.15
MR effect size for
'Selenium' on 'Interferon alpha−4'

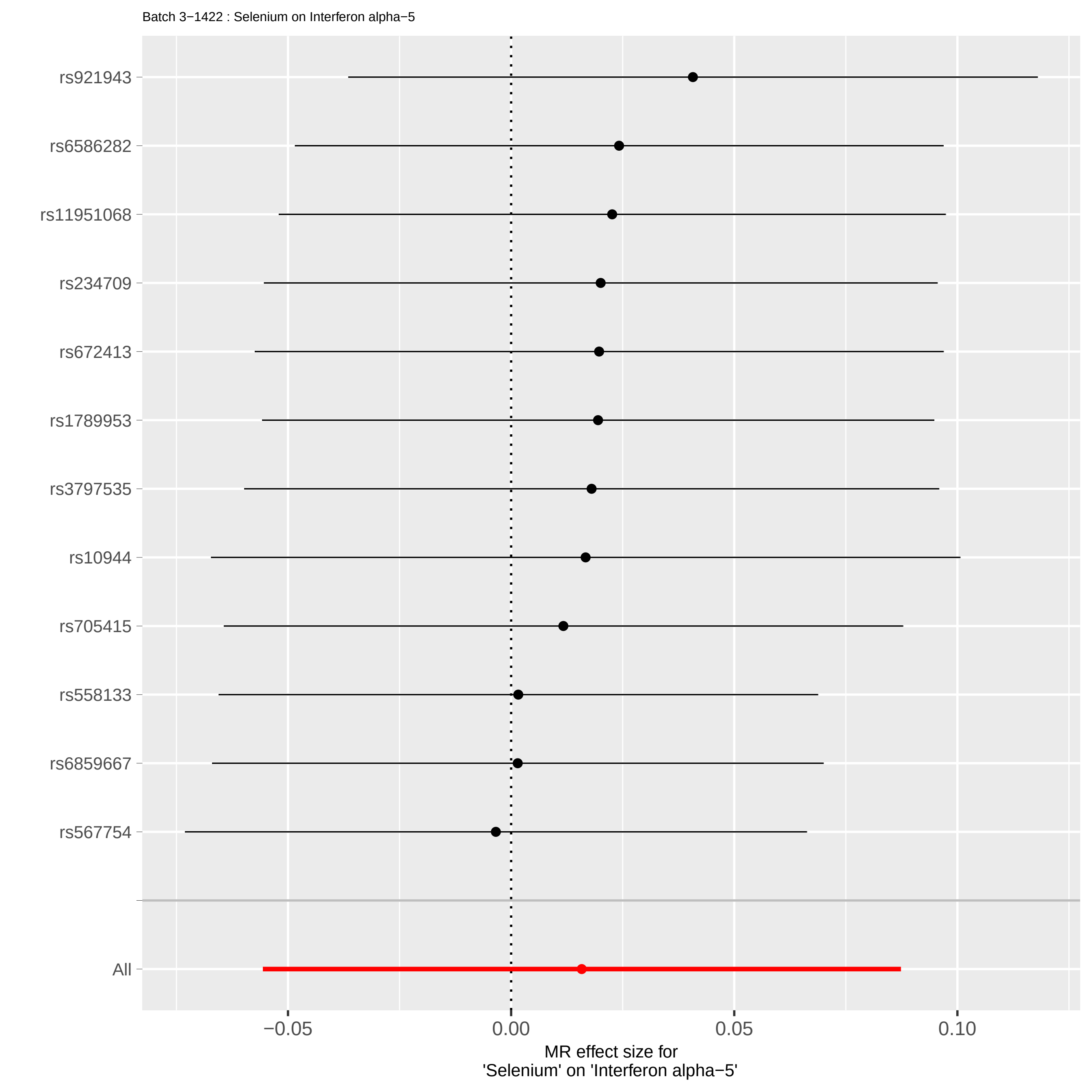

Batch 3−1422 : Selenium on Interferon alpha−5
rs921943
rs6586282
rs11951068
rs234709
rs672413
rs1789953
rs3797535
rs10944
rs705415
rs558133
rs6859667
rs567754
All
−0.05
0.00
0.05
0.10
MR effect size for
'Selenium' on 'Interferon alpha−5'

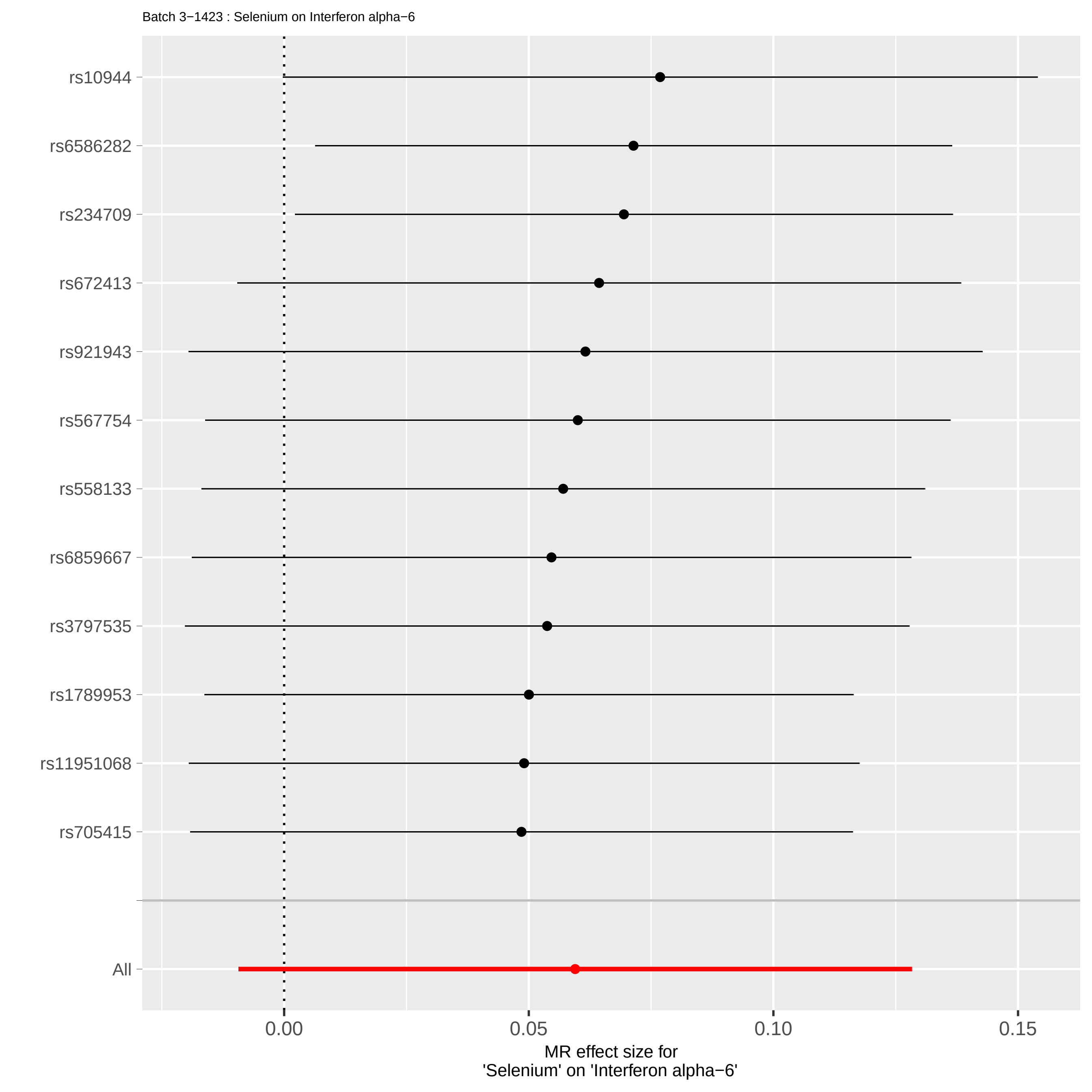

Batch 3−1423 : Selenium on Interferon alpha−6
rs10944
rs6586282
rs234709
rs672413
rs921943
rs567754
rs558133
rs6859667
rs3797535
rs1789953
rs11951068
rs705415
All
0.00
0.05
0.10
0.15
MR effect size for
'Selenium' on 'Interferon alpha−6'

Batch 3−1424 : Selenium on Interferon alpha−7

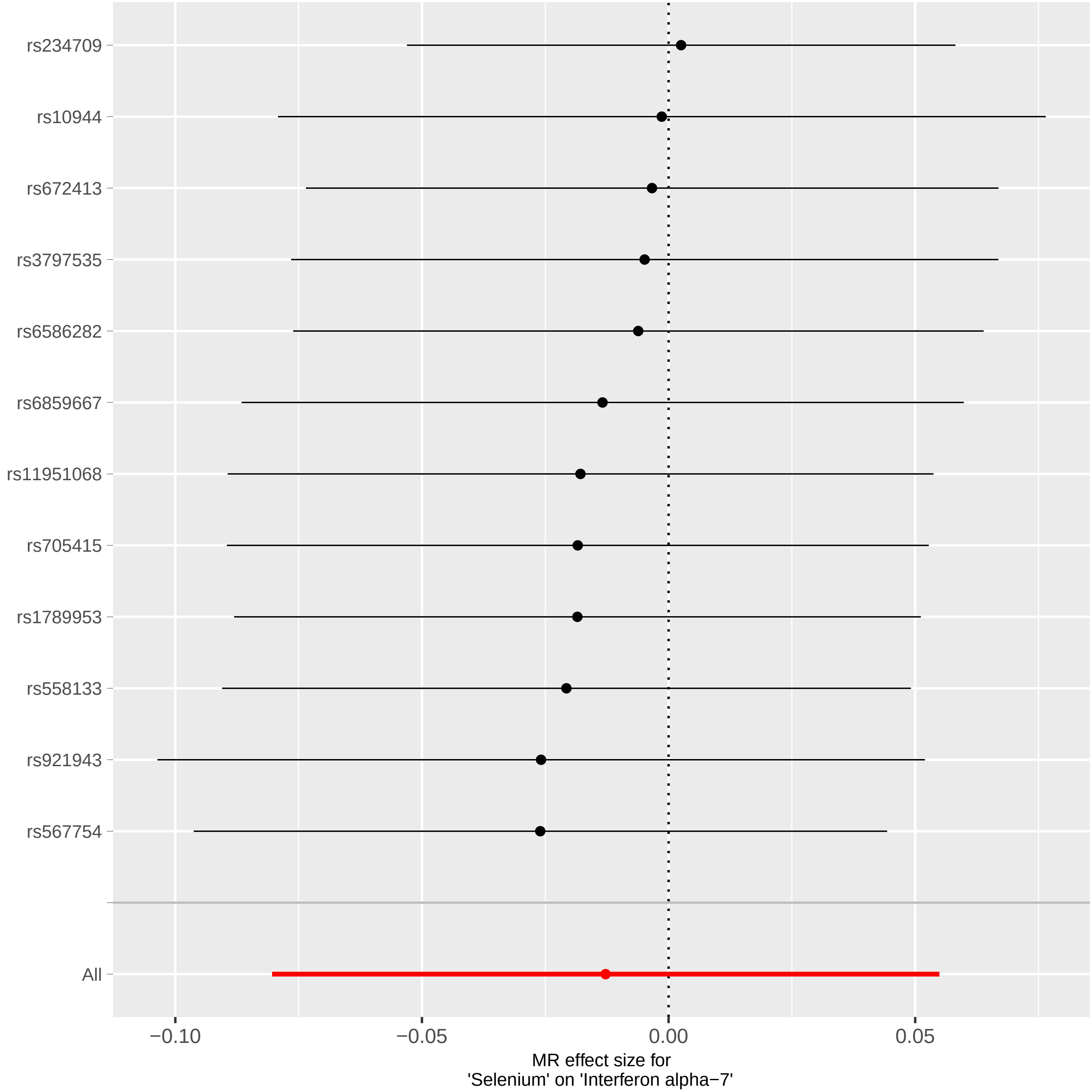

Batch 3−1425 : Selenium on Interferon alpha−8

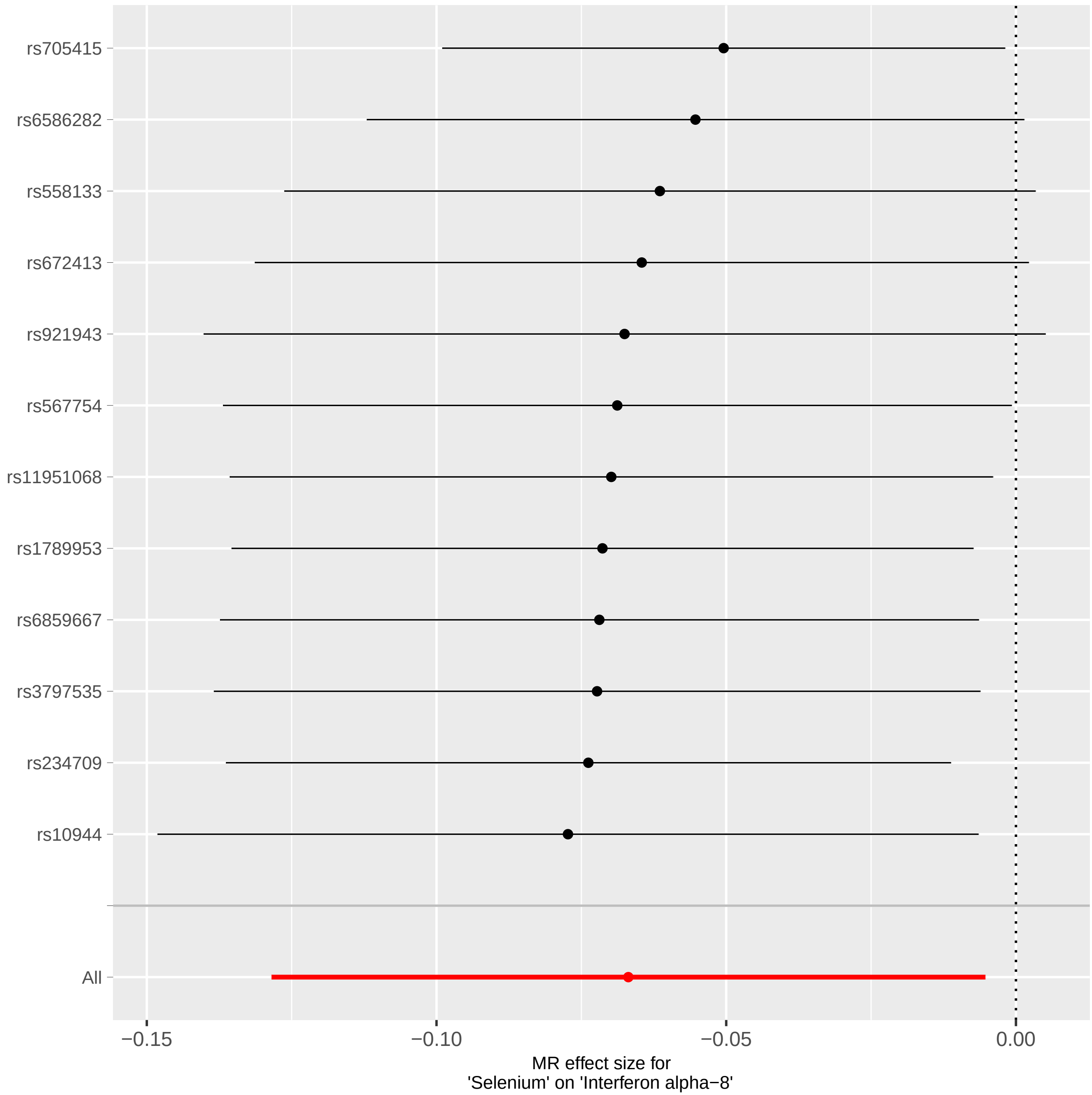

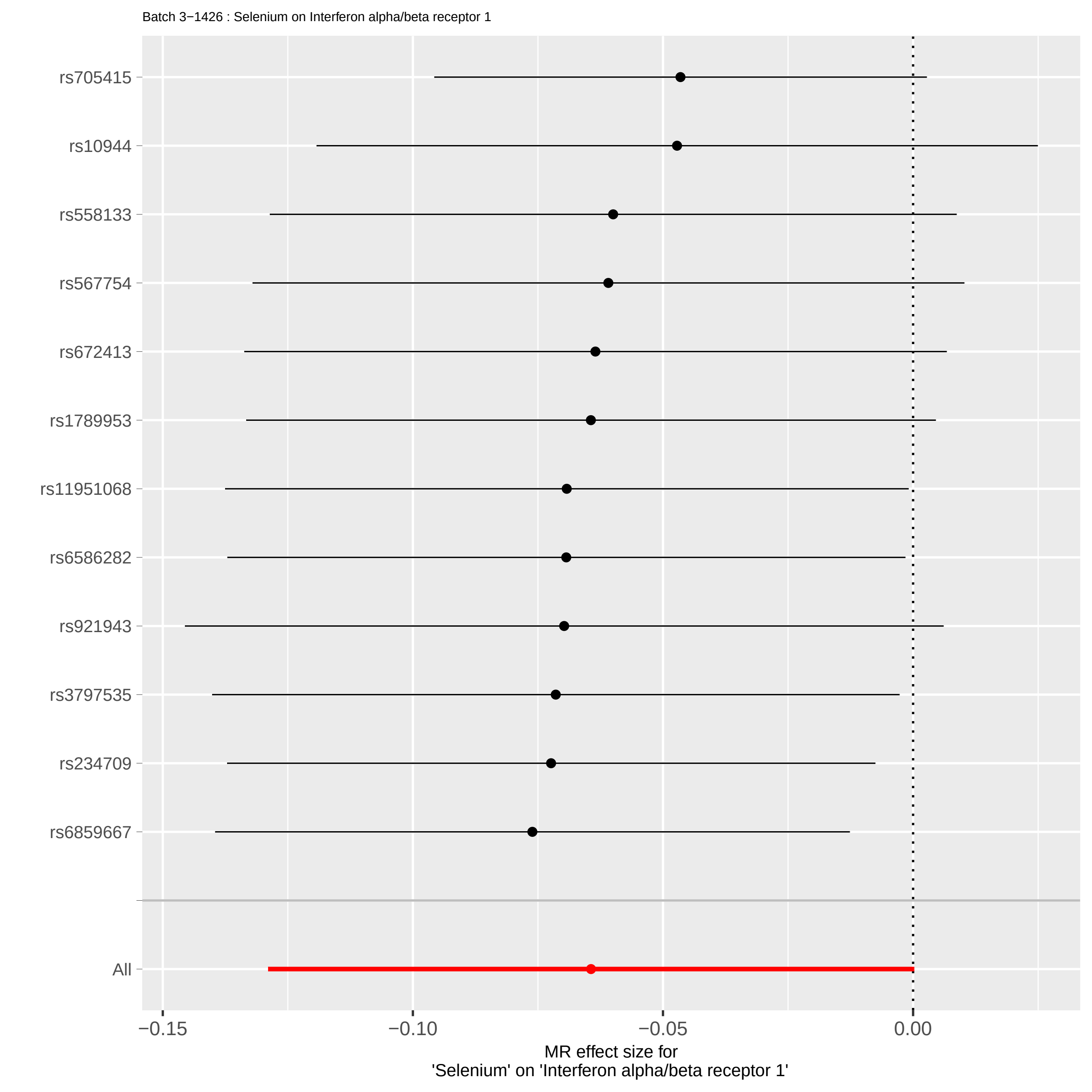
Batch 3−1426 : Selenium on Interferon alpha/beta receptor 1
rs705415
rs10944
rs558133
rs567754
rs672413
rs1789953
rs11951068
rs6586282
rs921943
rs3797535
rs234709
rs6859667
All
−0.15
−0.10
−0.05
0.00
MR effect size for
'Selenium' on 'Interferon alpha/beta receptor 1'

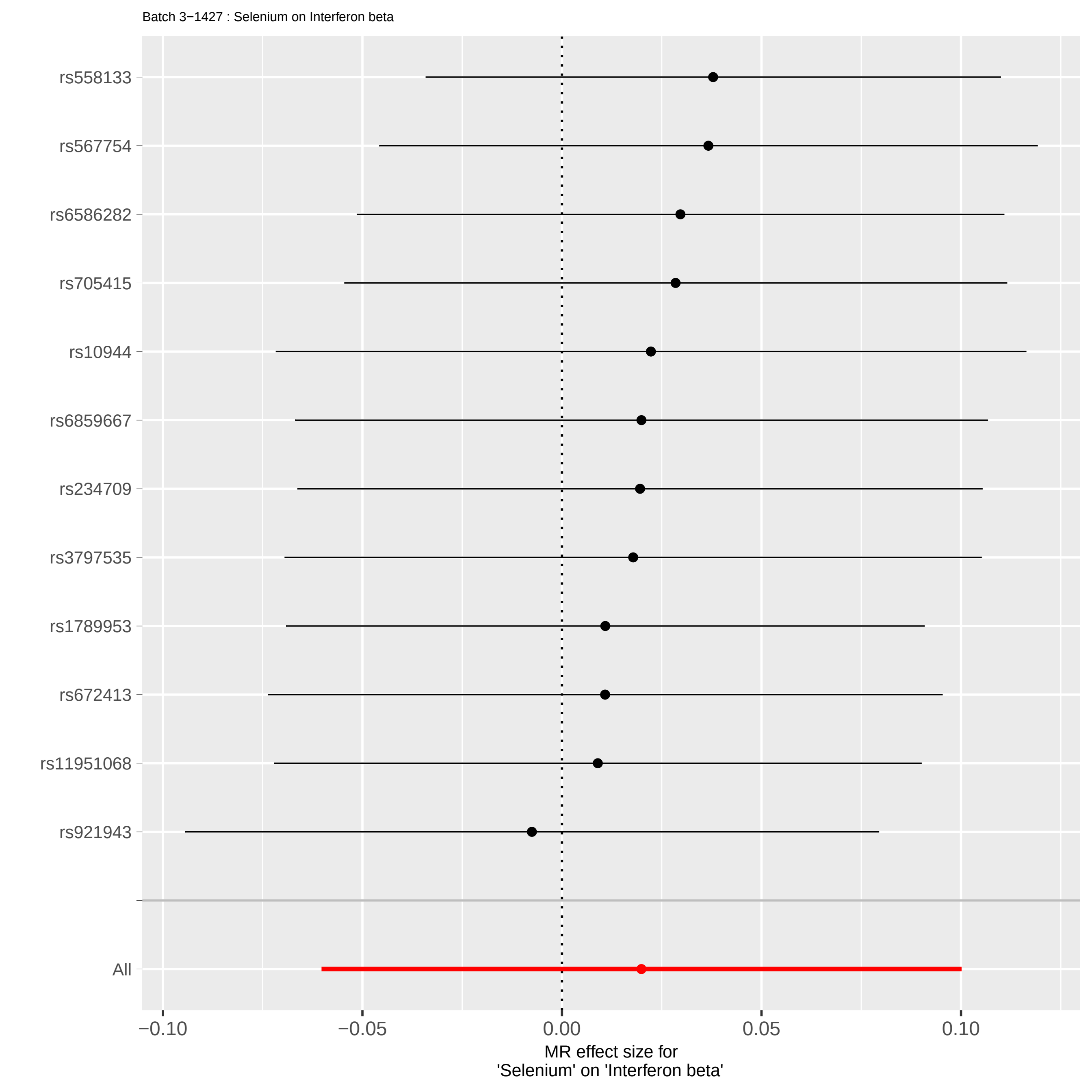

Batch 3−1427 : Selenium on Interferon beta
rs558133
rs567754
rs6586282
rs705415
rs10944
rs6859667
rs234709
rs3797535
rs1789953
rs672413
rs11951068
rs921943
All
−0.10
−0.05
0.00
0.05
0.10
MR effect size for
'Selenium' on 'Interferon beta'

Batch 3−1428 : Selenium on Interferon gamma

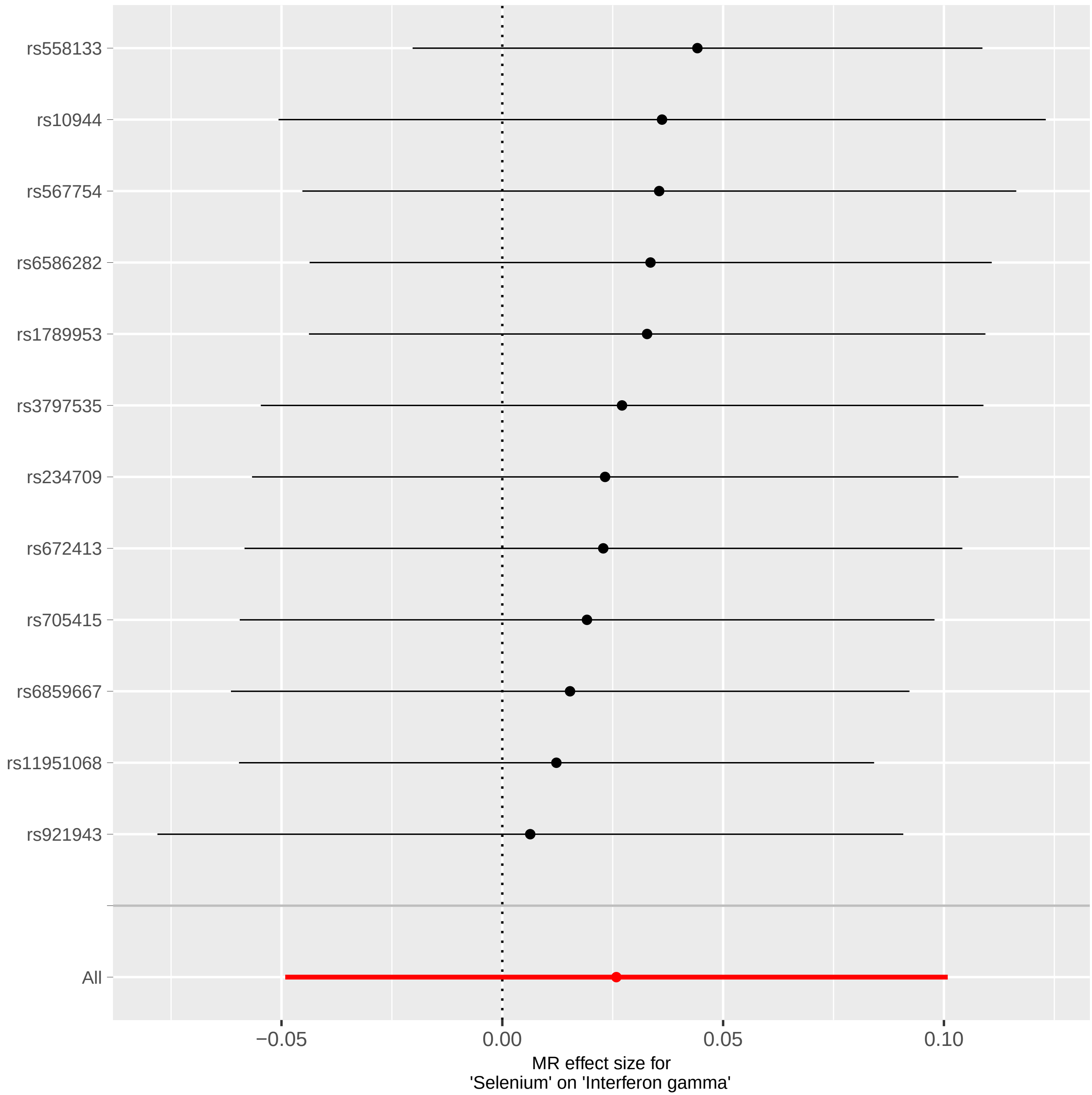

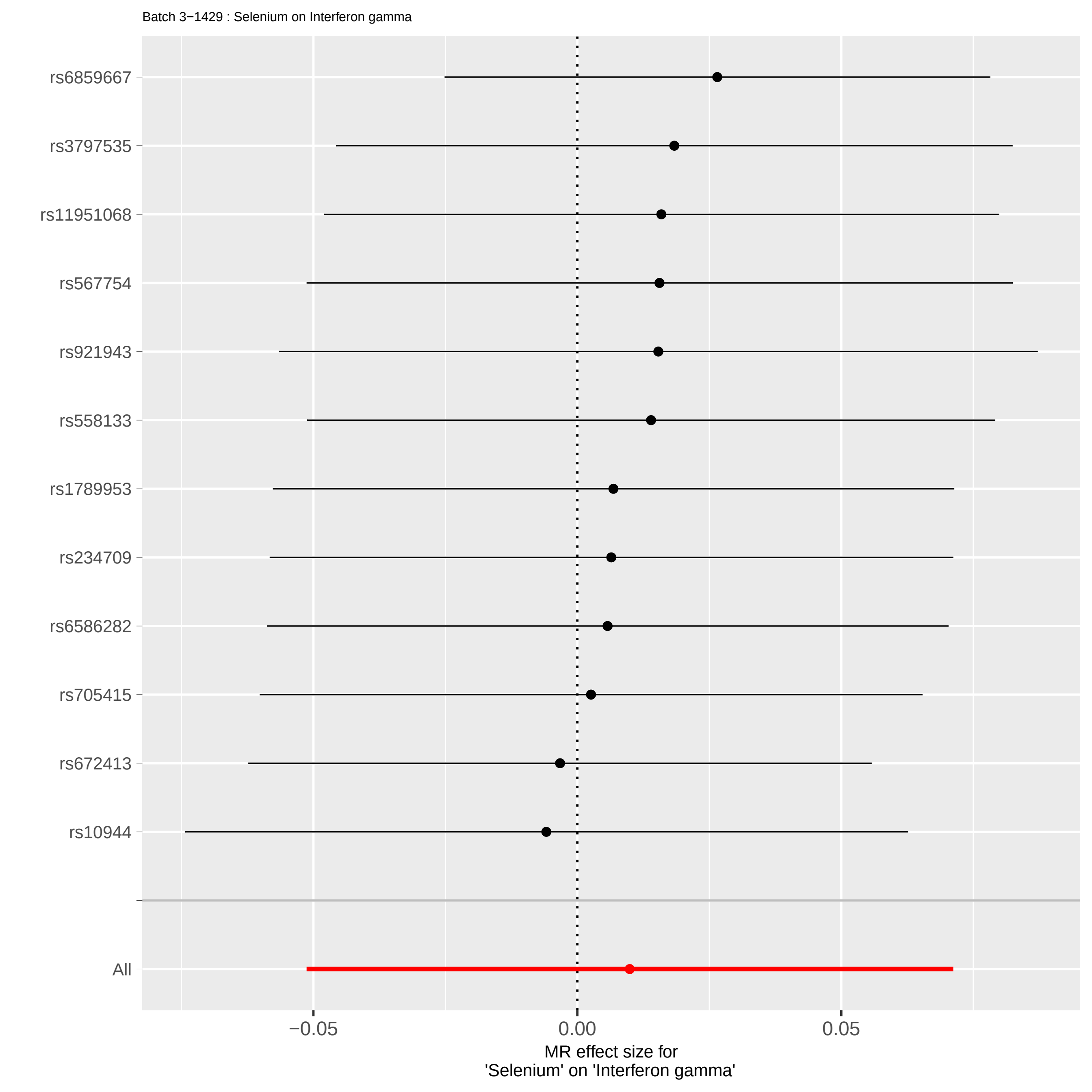

Batch 3−1429 : Selenium on Interferon gamma
rs6859667
rs3797535
rs11951068
rs567754
rs921943
rs558133
rs1789953
rs234709
rs6586282
rs705415
rs672413
rs10944
All
−0.05
0.00
0.05
MR effect size for
'Selenium' on 'Interferon gamma'

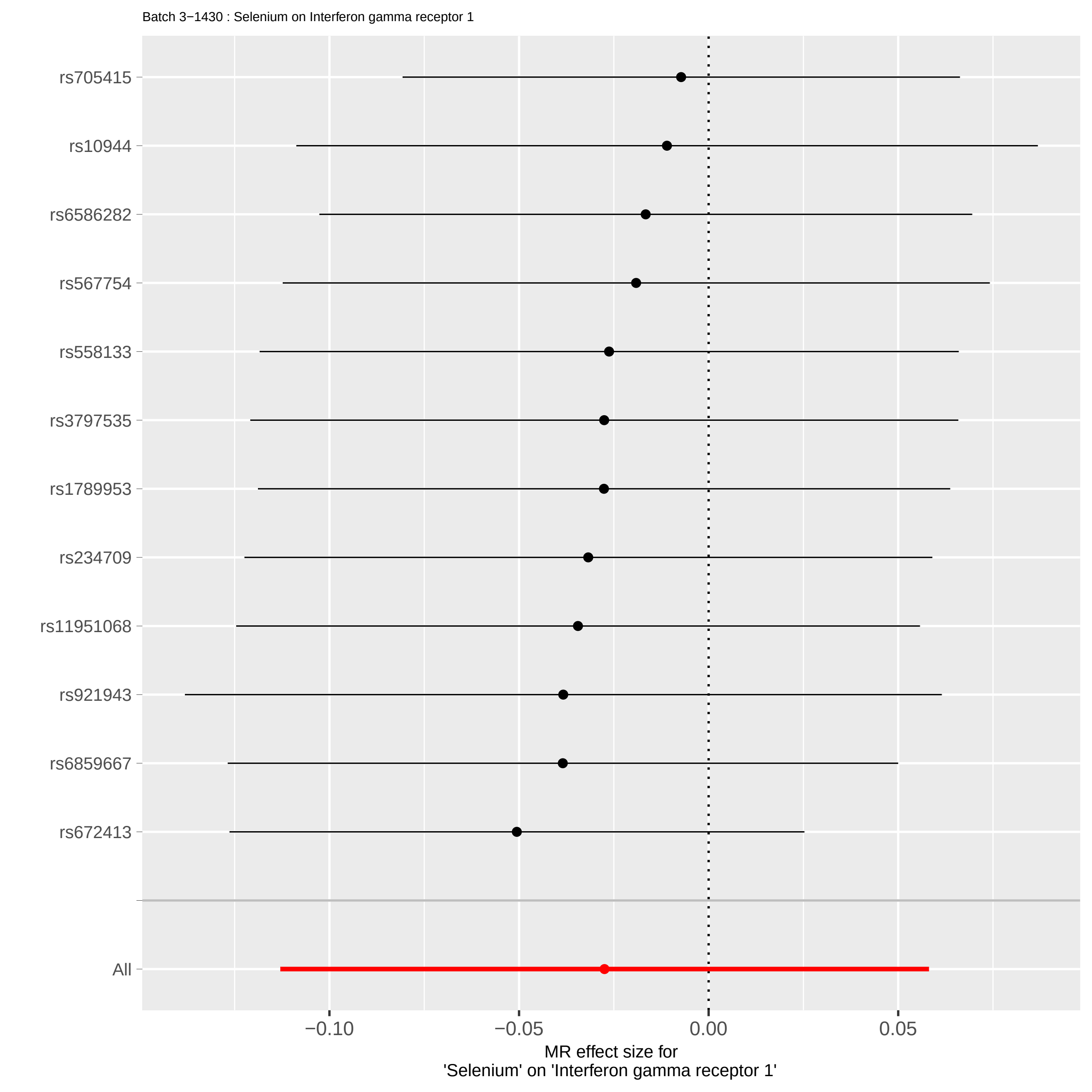

Batch 3−1430 : Selenium on Interferon gamma receptor 1
rs705415
rs10944
rs6586282
rs567754
rs558133
rs3797535
rs1789953
rs234709
rs11951068
rs921943
rs6859667
rs672413
All
−0.10
−0.05
0.00
0.05
MR effect size for
'Selenium' on 'Interferon gamma receptor 1'

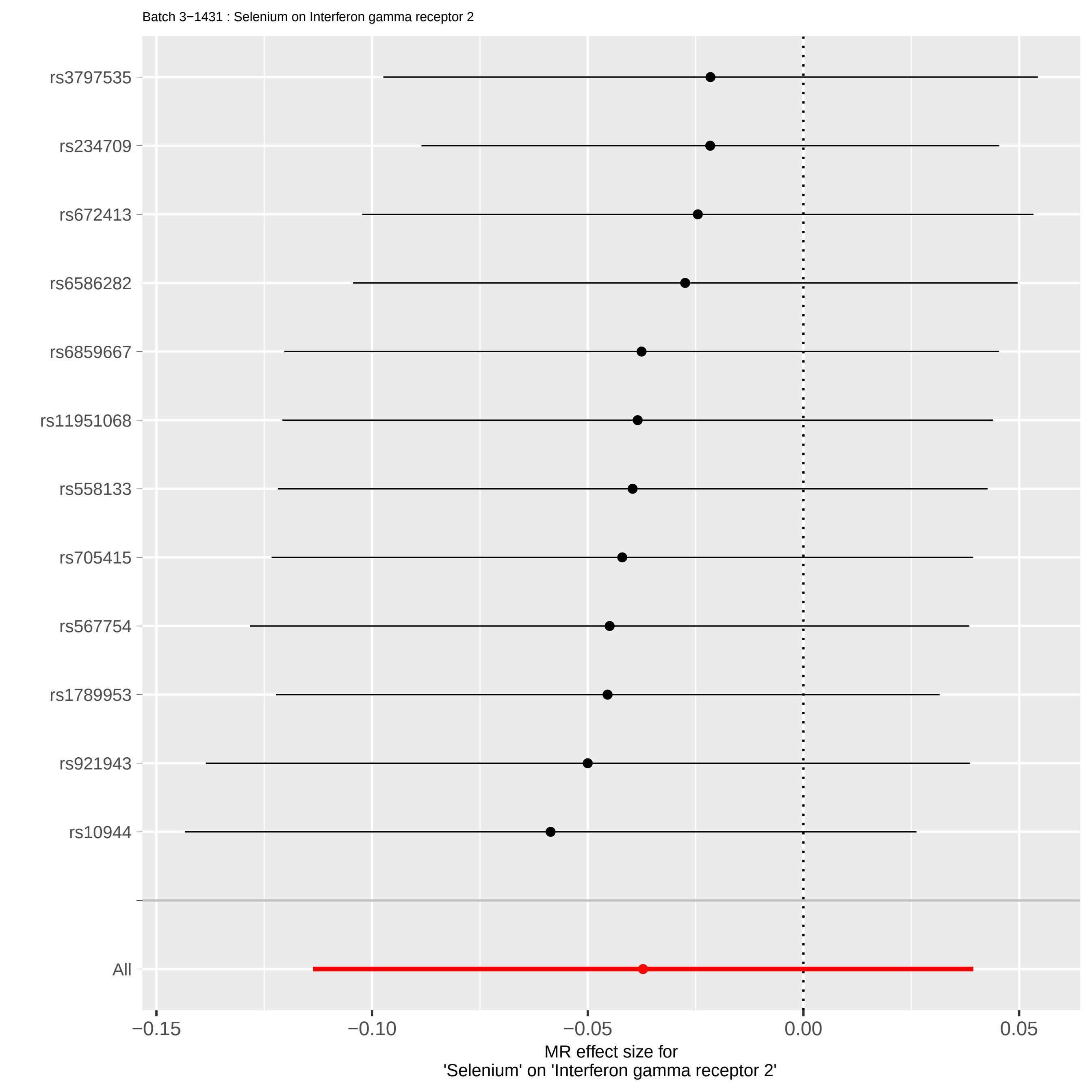

Batch 3−1431 : Selenium on Interferon gamma receptor 2
rs3797535
rs234709
rs672413
rs6586282
rs6859667
rs11951068
rs558133
rs705415
rs567754
rs1789953
rs921943
rs10944
All
−0.15
−0.10
−0.05
0.00
0.05
MR effect size for
'Selenium' on 'Interferon gamma receptor 2'

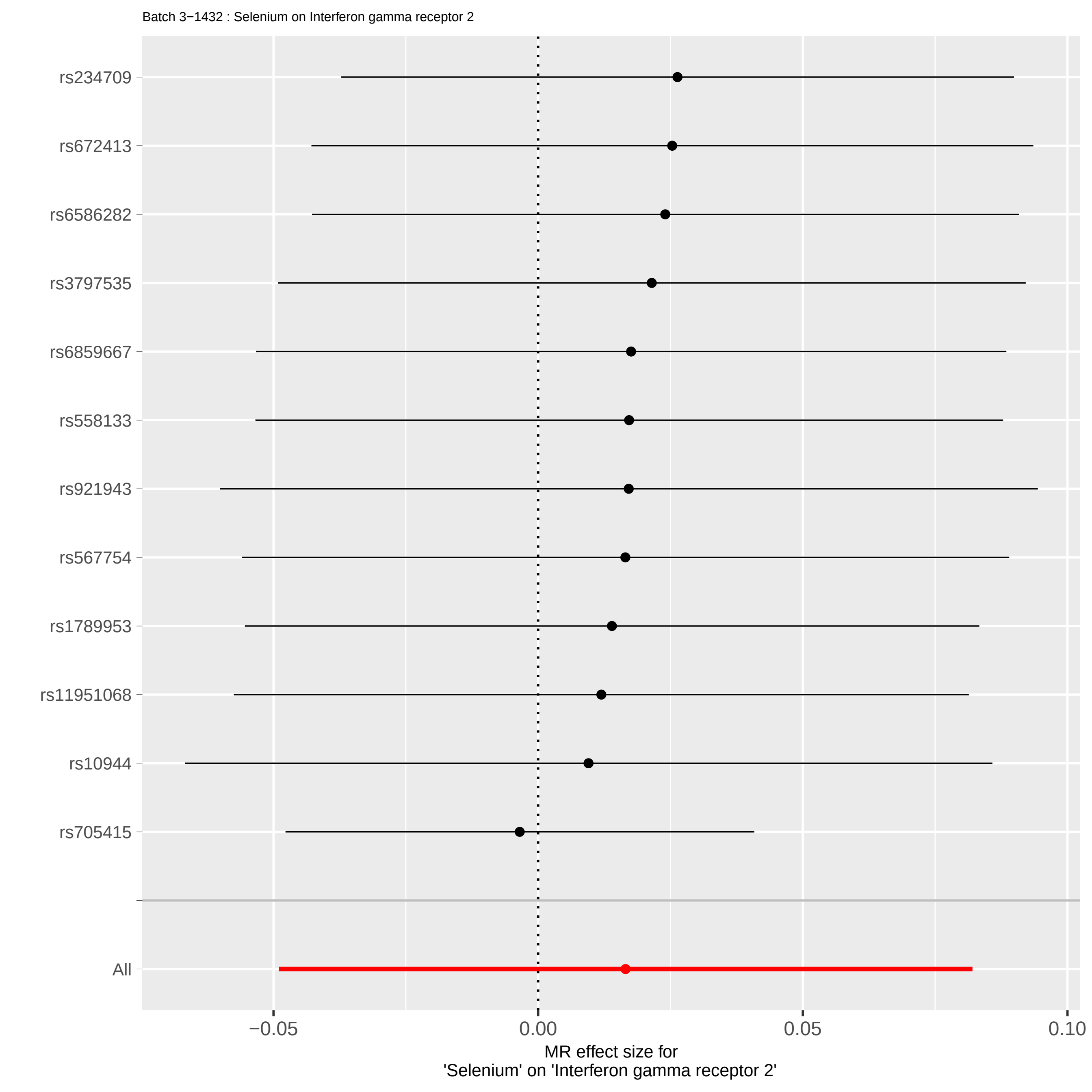

Batch 3−1432 : Selenium on Interferon gamma receptor 2
rs234709
rs672413
rs6586282
rs3797535
rs6859667
rs558133
rs921943
rs567754
rs1789953
rs11951068
rs10944
rs705415
All
−0.05
0.00
0.05
0.10
MR effect size for
'Selenium' on 'Interferon gamma receptor 2'

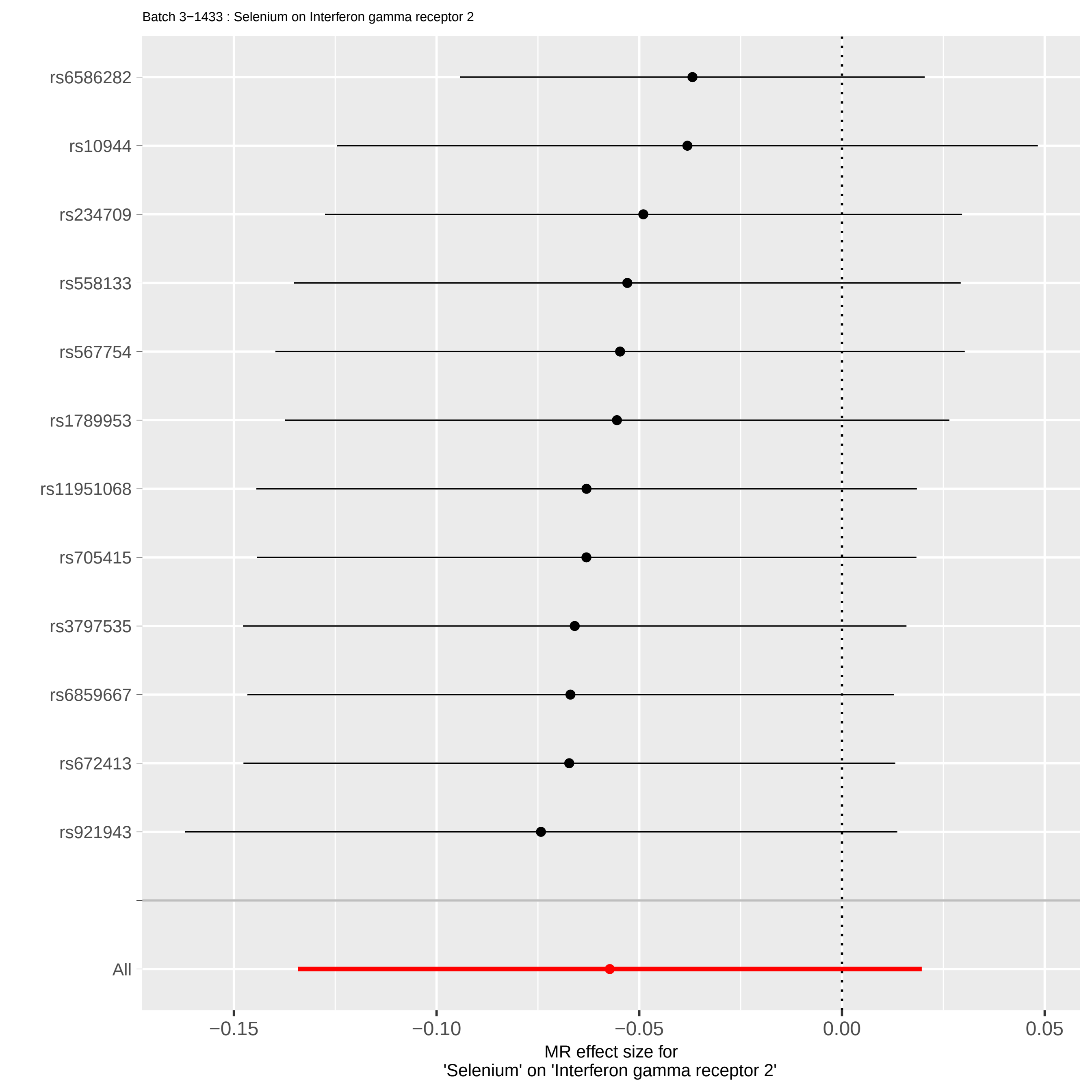
Batch 3−1433 : Selenium on Interferon gamma receptor 2
rs6586282
rs10944
rs234709
rs558133
rs567754
rs1789953
rs11951068
rs705415
rs3797535
rs6859667
rs672413
rs921943
All
−0.15
−0.10
−0.05
0.00
0.05
MR effect size for
'Selenium' on 'Interferon gamma receptor 2'

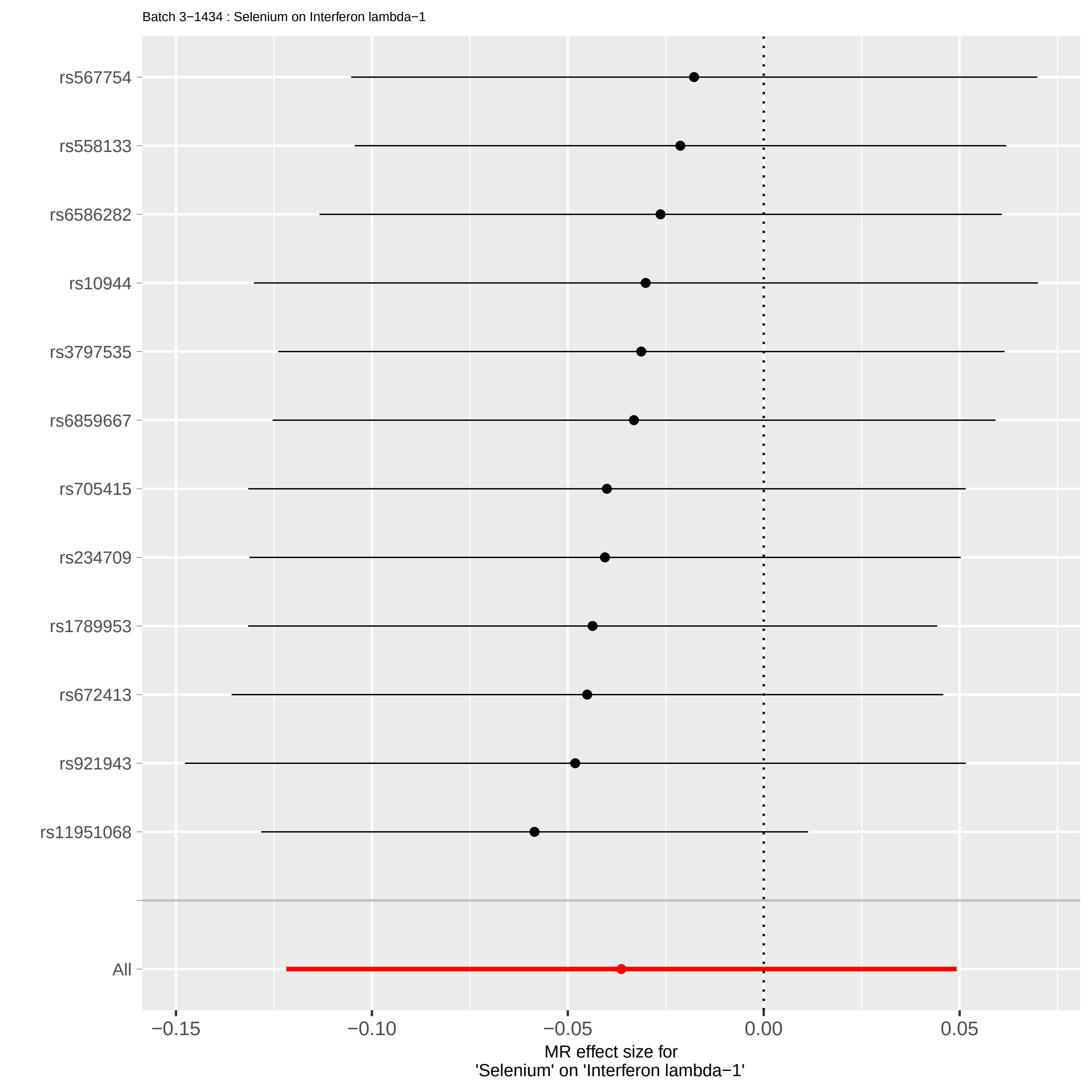
Batch 3−1434 : Selenium on Interferon lambda−1
rs567754
rs558133
rs6586282
rs10944
rs3797535
rs6859667
rs705415
rs234709
rs1789953
rs672413
rs921943
rs11951068
All
−0.15
−0.10
−0.05
0.00
0.05
MR effect size for
'Selenium' on 'Interferon lambda−1'

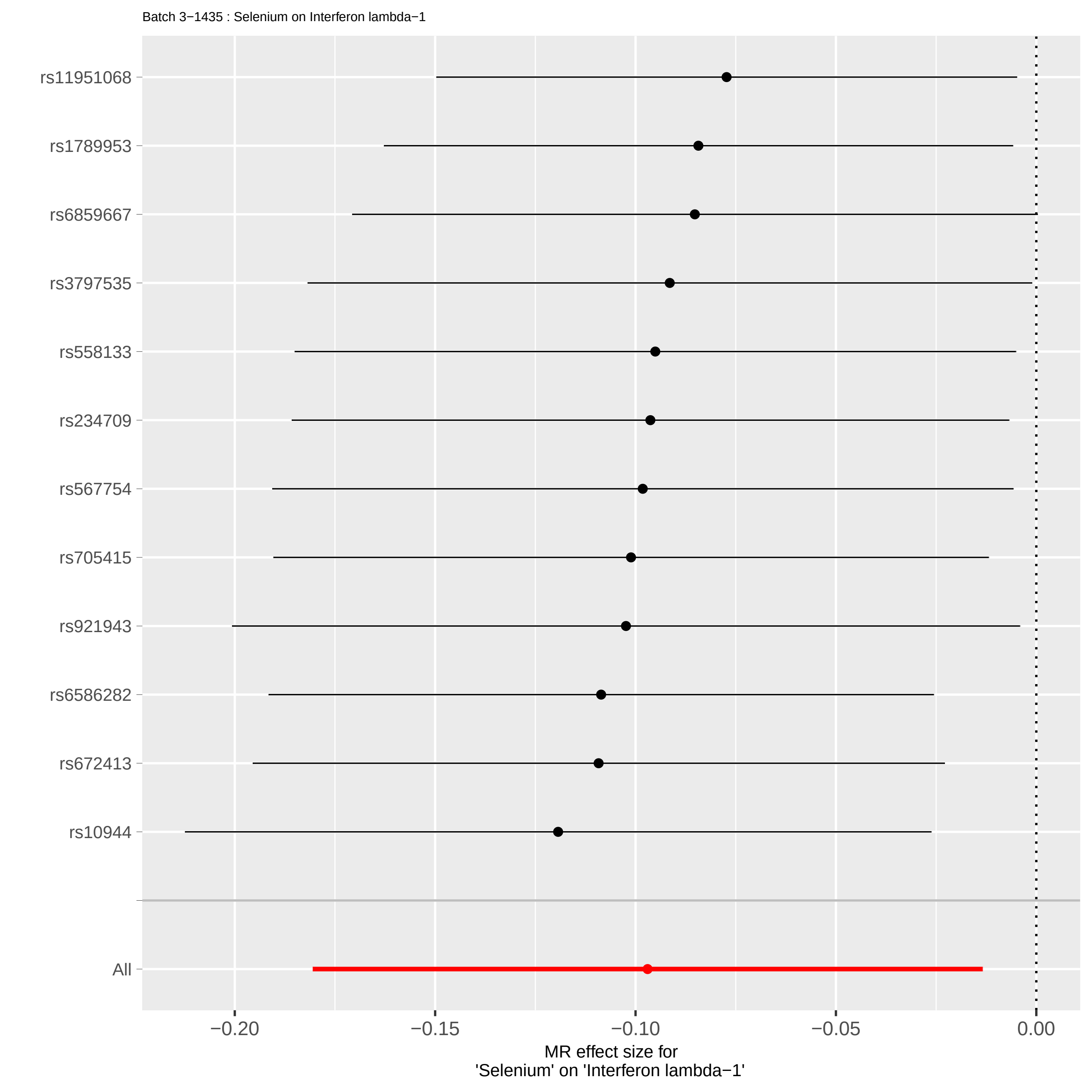

Batch 3−1435 : Selenium on Interferon lambda−1
rs11951068
rs1789953
rs6859667
rs3797535
rs558133
rs234709
rs567754
rs705415
rs921943
rs6586282
rs672413
rs10944
All
−0.20
−0.15
−0.10
−0.05
0.00
MR effect size for
'Selenium' on 'Interferon lambda−1'

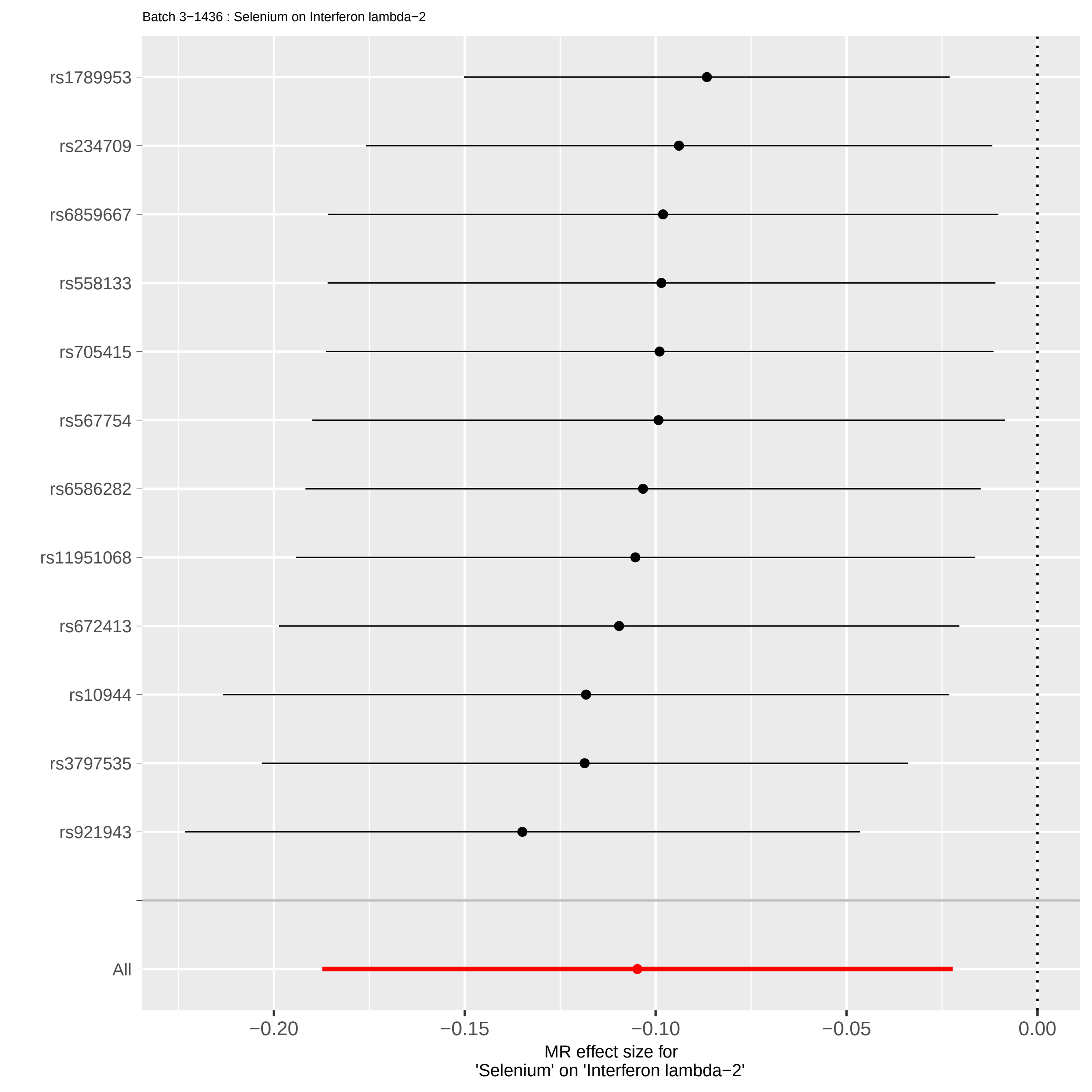
Batch 3−1436 : Selenium on Interferon lambda−2
rs1789953
rs234709
rs6859667
rs558133
rs705415
rs567754
rs6586282
rs11951068
rs672413
rs10944
rs3797535
rs921943
All
−0.20
−0.15
−0.10
−0.05
0.00
MR effect size for
'Selenium' on 'Interferon lambda−2'

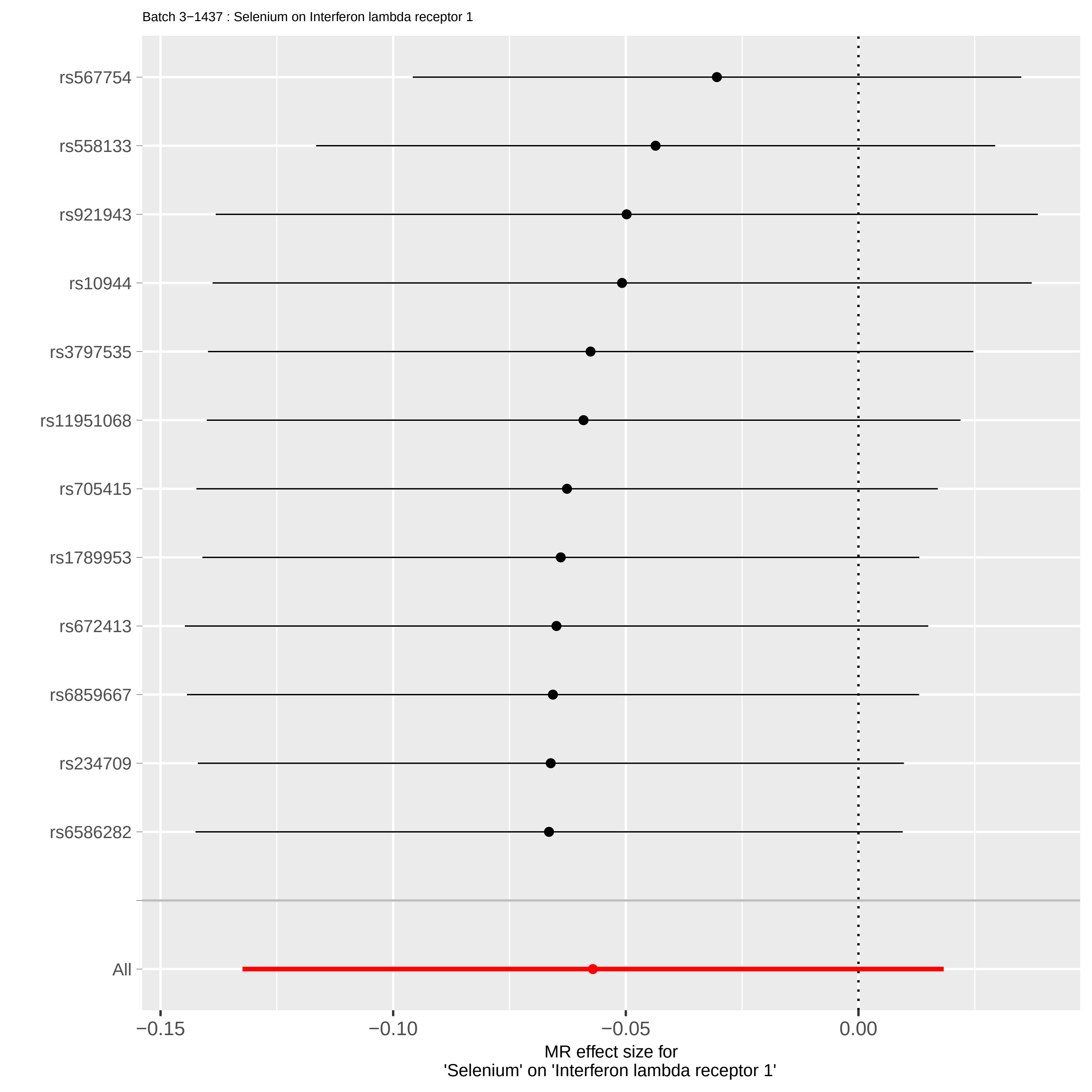

Batch 3−1437 : Selenium on Interferon lambda receptor 1
rs567754
rs558133
rs921943
rs10944
rs3797535
rs11951068
rs705415
rs1789953
rs672413
rs6859667
rs234709
rs6586282
All
−0.15
−0.10
−0.05
0.00
MR effect size for
'Selenium' on 'Interferon lambda receptor 1'

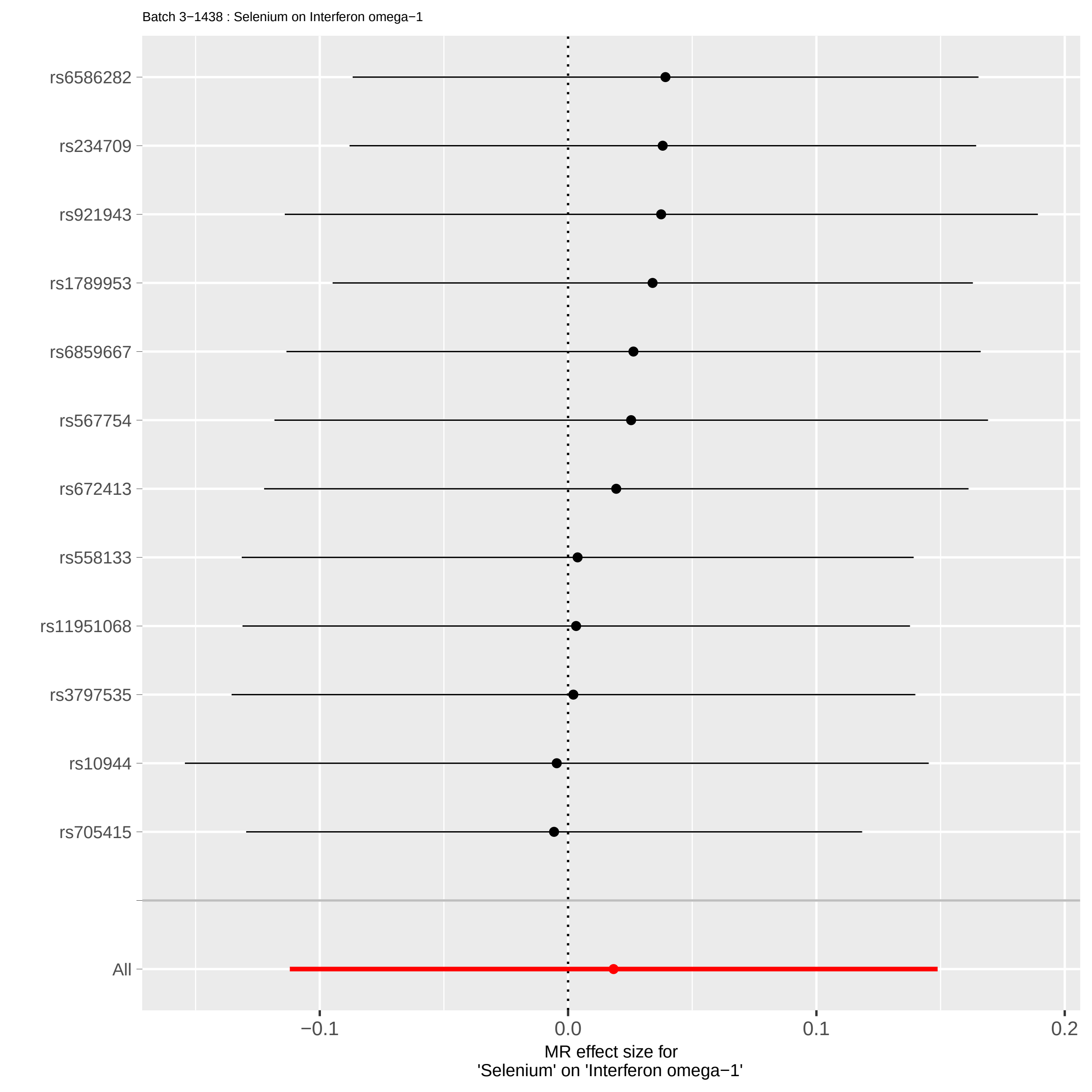

Batch 3−1438 : Selenium on Interferon omega−1
rs6586282
rs234709
rs921943
rs1789953
rs6859667
rs567754
rs672413
rs558133
rs11951068
rs3797535
rs10944
rs705415
All
−0.1
0.0
0.1
0.2
MR effect size for
'Selenium' on 'Interferon omega−1'

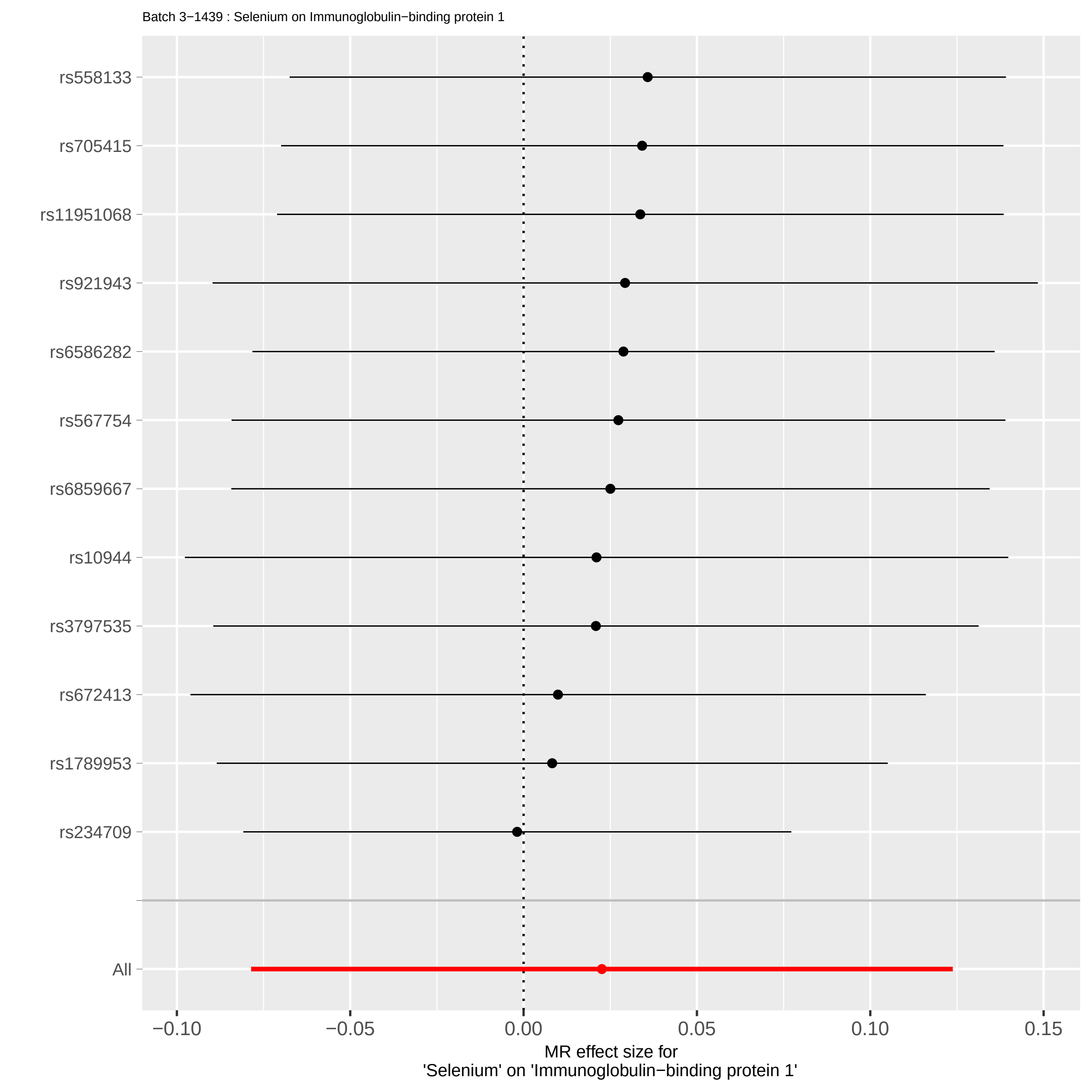

Batch 3−1439 : Selenium on Immunoglobulin−binding protein 1
rs558133
rs705415
rs11951068
rs921943
rs6586282
rs567754
rs6859667
rs10944
rs3797535
rs672413
rs1789953
rs234709
All
−0.10
−0.05
0.00
0.05
0.10
0.15
MR effect size for
'Selenium' on 'Immunoglobulin−binding protein 1'

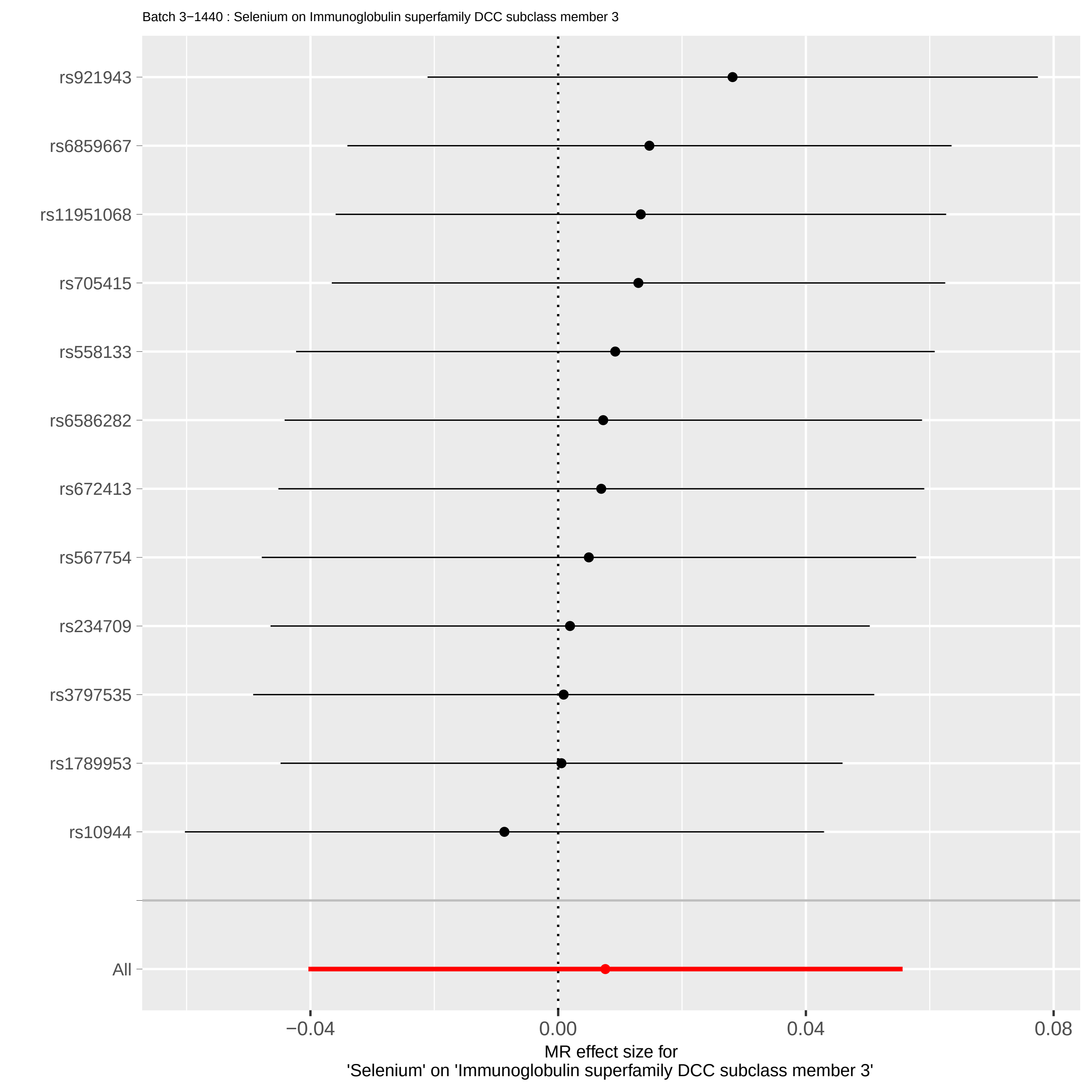

Batch 3−1440 : Selenium on Immunoglobulin superfamily DCC subclass member 3
rs921943
rs6859667
rs11951068
rs705415
rs558133
rs6586282
rs672413
rs567754
rs234709
rs3797535
rs1789953
rs10944
All
−0.04
0.00
0.04
0.08
MR effect size for
'Selenium' on 'Immunoglobulin superfamily DCC subclass member 3'

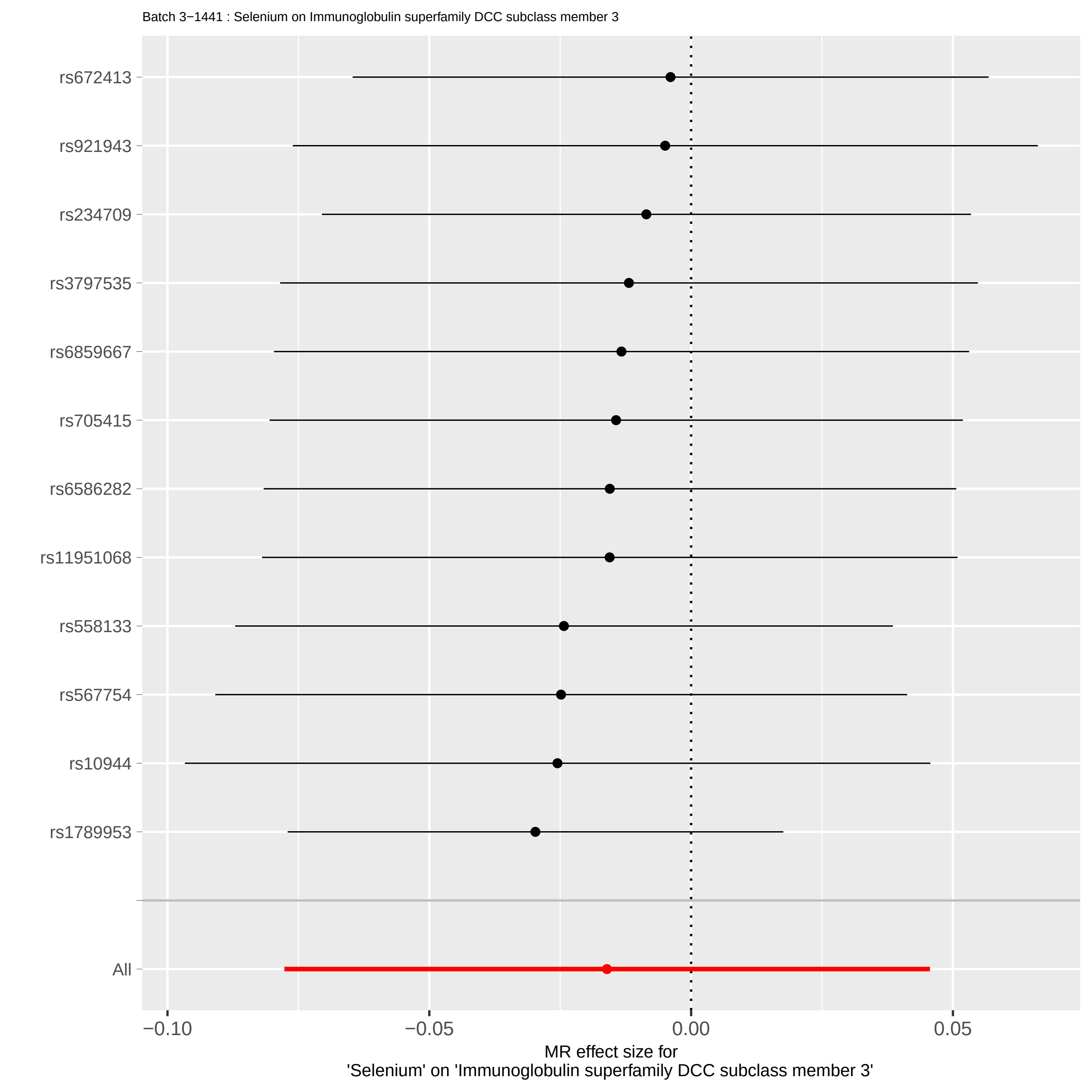

Batch 3−1441 : Selenium on Immunoglobulin superfamily DCC subclass member 3
rs672413
rs921943
rs234709
rs3797535
rs6859667
rs705415
rs6586282
rs11951068
rs558133
rs567754
rs10944
rs1789953
All
−0.10
−0.05
0.00
0.05
MR effect size for
'Selenium' on 'Immunoglobulin superfamily DCC subclass member 3'

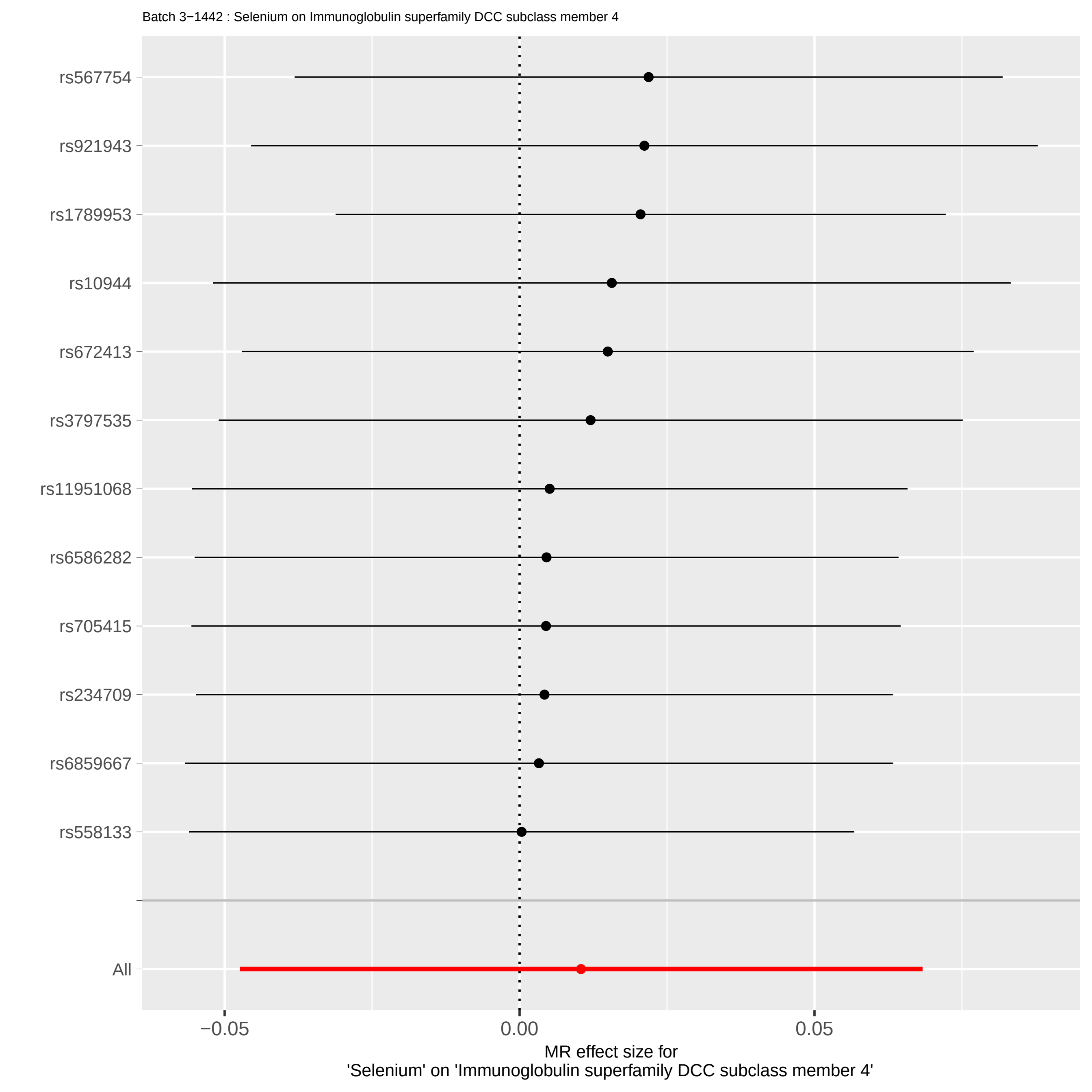

Batch 3−1442 : Selenium on Immunoglobulin superfamily DCC subclass member 4
rs567754
rs921943
rs1789953
rs10944
rs672413
rs3797535
rs11951068
rs6586282
rs705415
rs234709
rs6859667
rs558133
All
−0.05
0.00
0.05
MR effect size for
'Selenium' on 'Immunoglobulin superfamily DCC subclass member 4'

Batch 3−1443 : Selenium on Insulin−like growth factor I

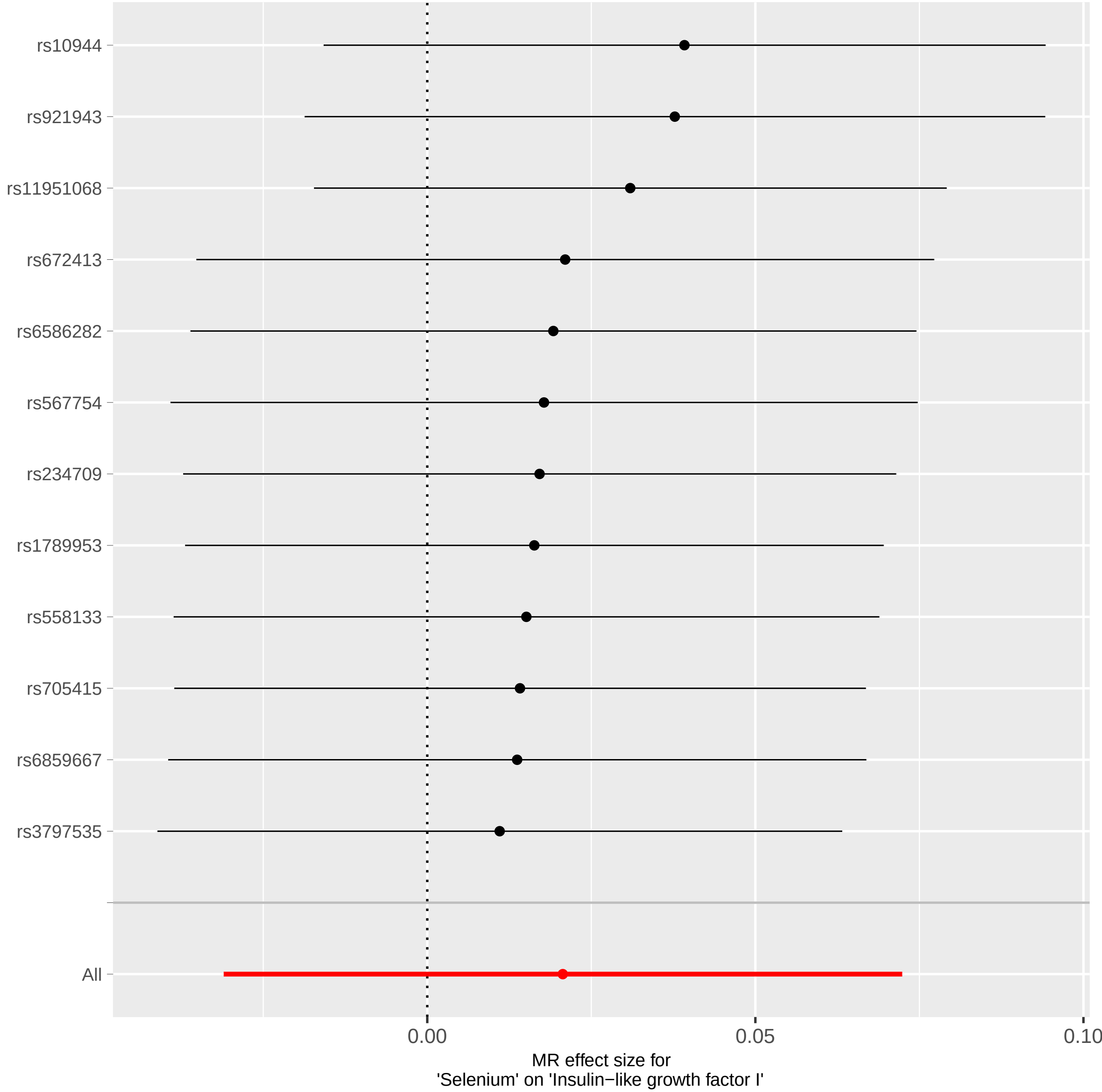

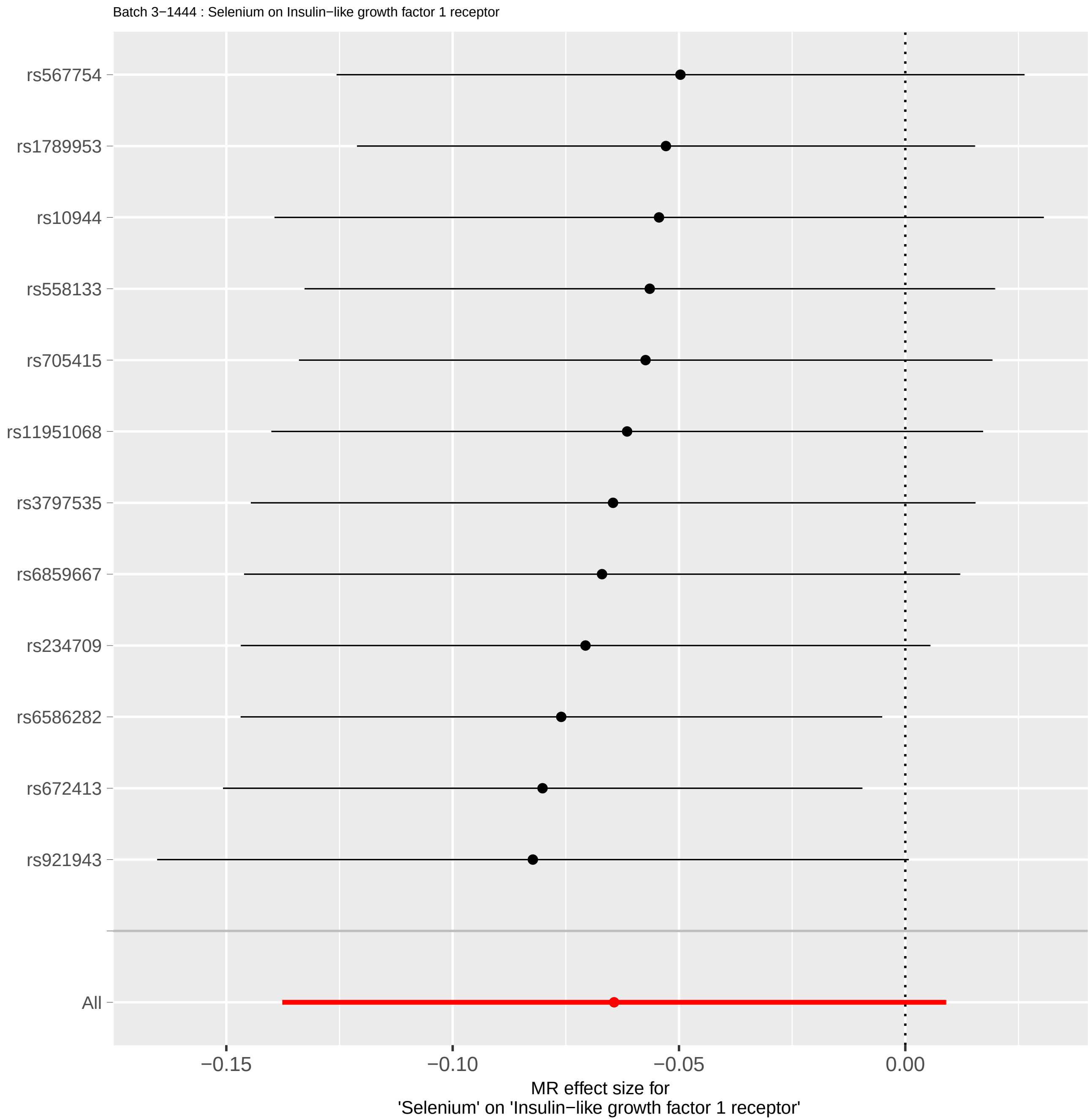

Batch 3−1444 : Selenium on Insulin−like growth factor 1 receptor
rs567754
rs1789953
rs10944
rs558133
rs705415
rs11951068
rs3797535
rs6859667
rs234709
rs6586282
rs672413
rs921943
All
−0.15
−0.10
−0.05
0.00
MR effect size for
'Selenium' on 'Insulin−like growth factor 1 receptor'

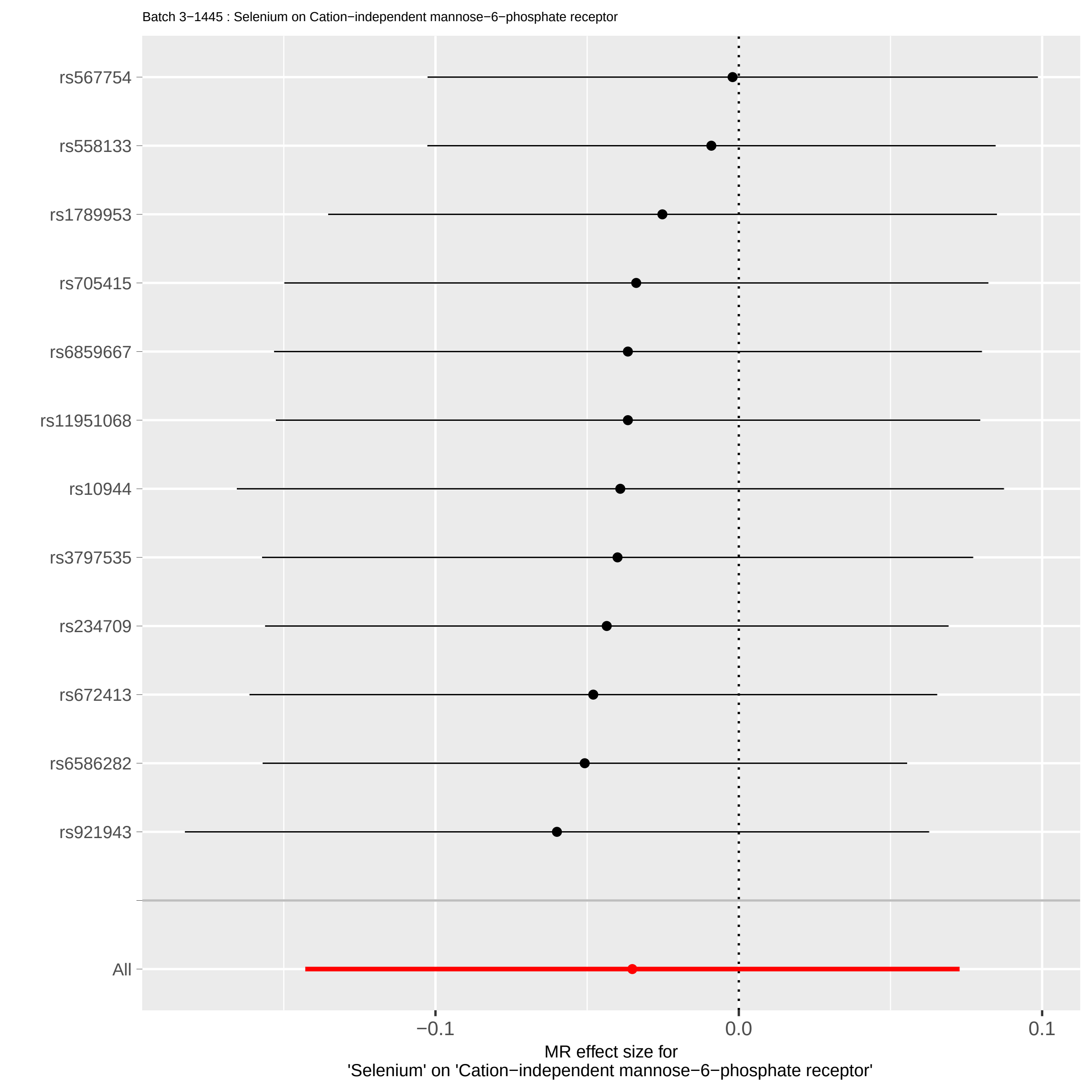

Batch 3−1445 : Selenium on Cation−independent mannose−6−phosphate receptor
rs567754
rs558133
rs1789953
rs705415
rs6859667
rs11951068
rs10944
rs3797535
rs234709
rs672413
rs6586282
rs921943
All
−0.1
0.0
0.1
MR effect size for
'Selenium' on 'Cation−independent mannose−6−phosphate receptor'

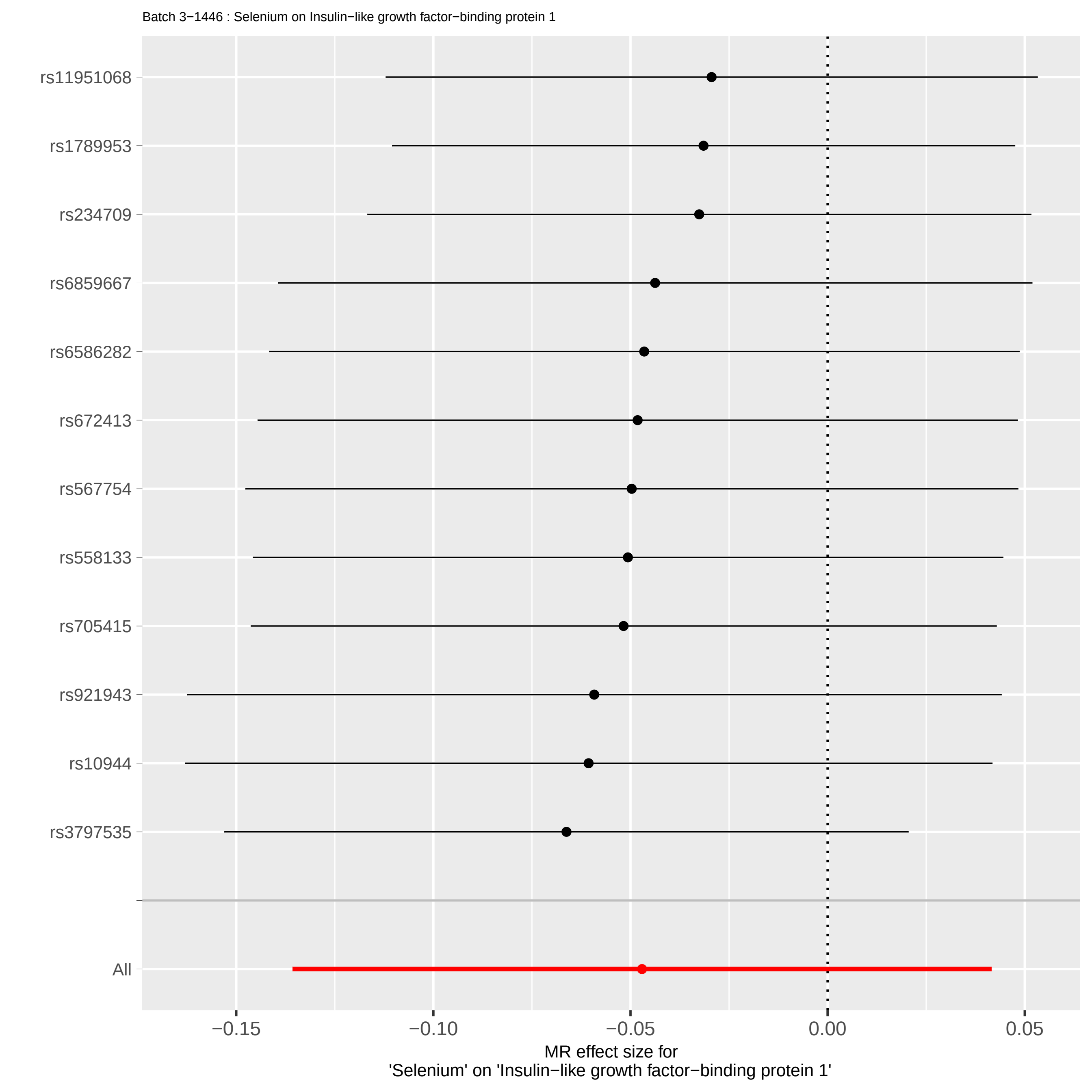
Batch 3−1446 : Selenium on Insulin−like growth factor−binding protein 1
rs11951068
rs1789953
rs234709
rs6859667
rs6586282
rs672413
rs567754
rs558133
rs705415
rs921943
rs10944
rs3797535
All
−0.15
−0.10
−0.05
0.00
0.05
MR effect size for
'Selenium' on 'Insulin−like growth factor−binding protein 1'

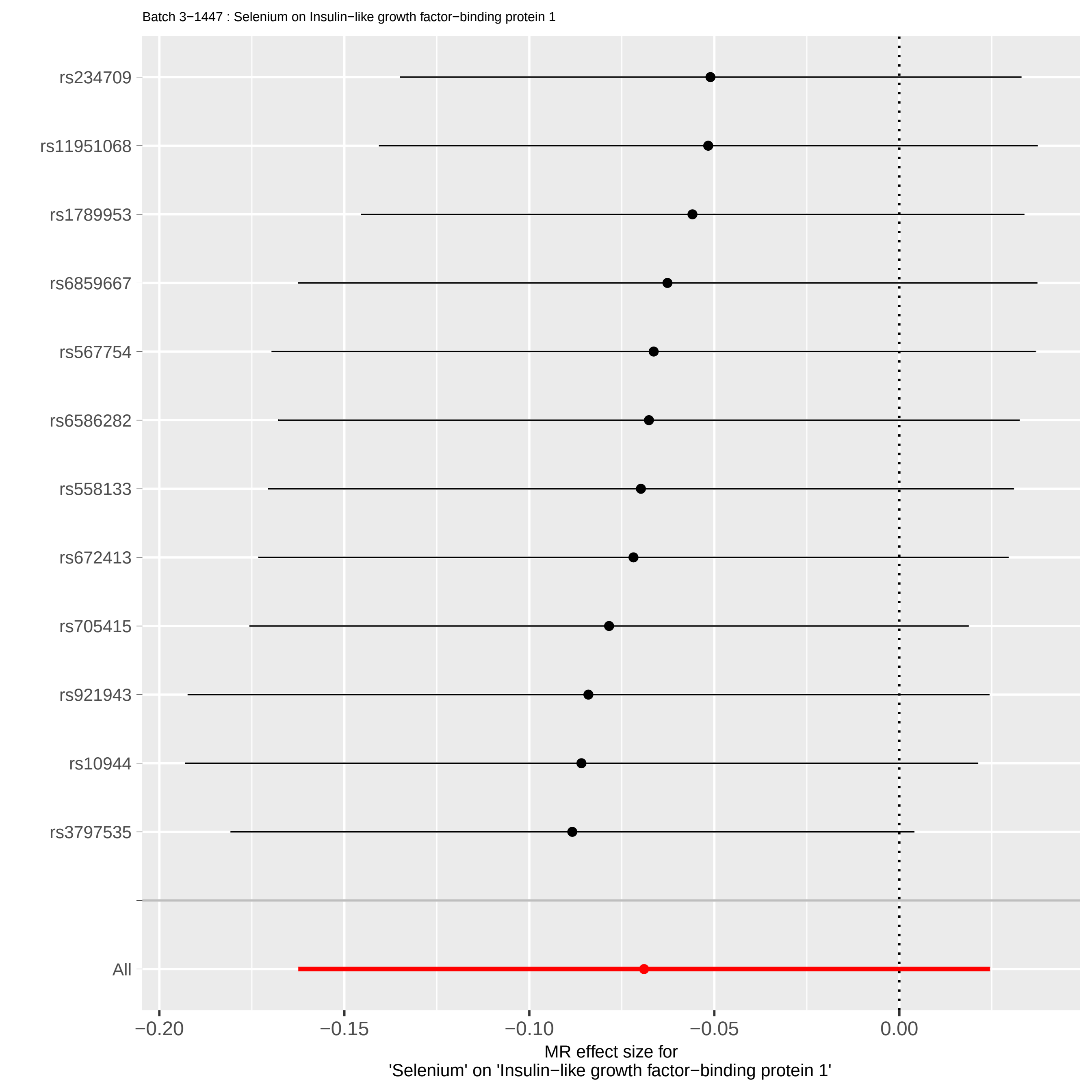

Batch 3−1447 : Selenium on Insulin−like growth factor−binding protein 1
rs234709
rs11951068
rs1789953
rs6859667
rs567754
rs6586282
rs558133
rs672413
rs705415
rs921943
rs10944
rs3797535
All
−0.20
−0.15
−0.10
−0.05
0.00
MR effect size for
'Selenium' on 'Insulin−like growth factor−binding protein 1'

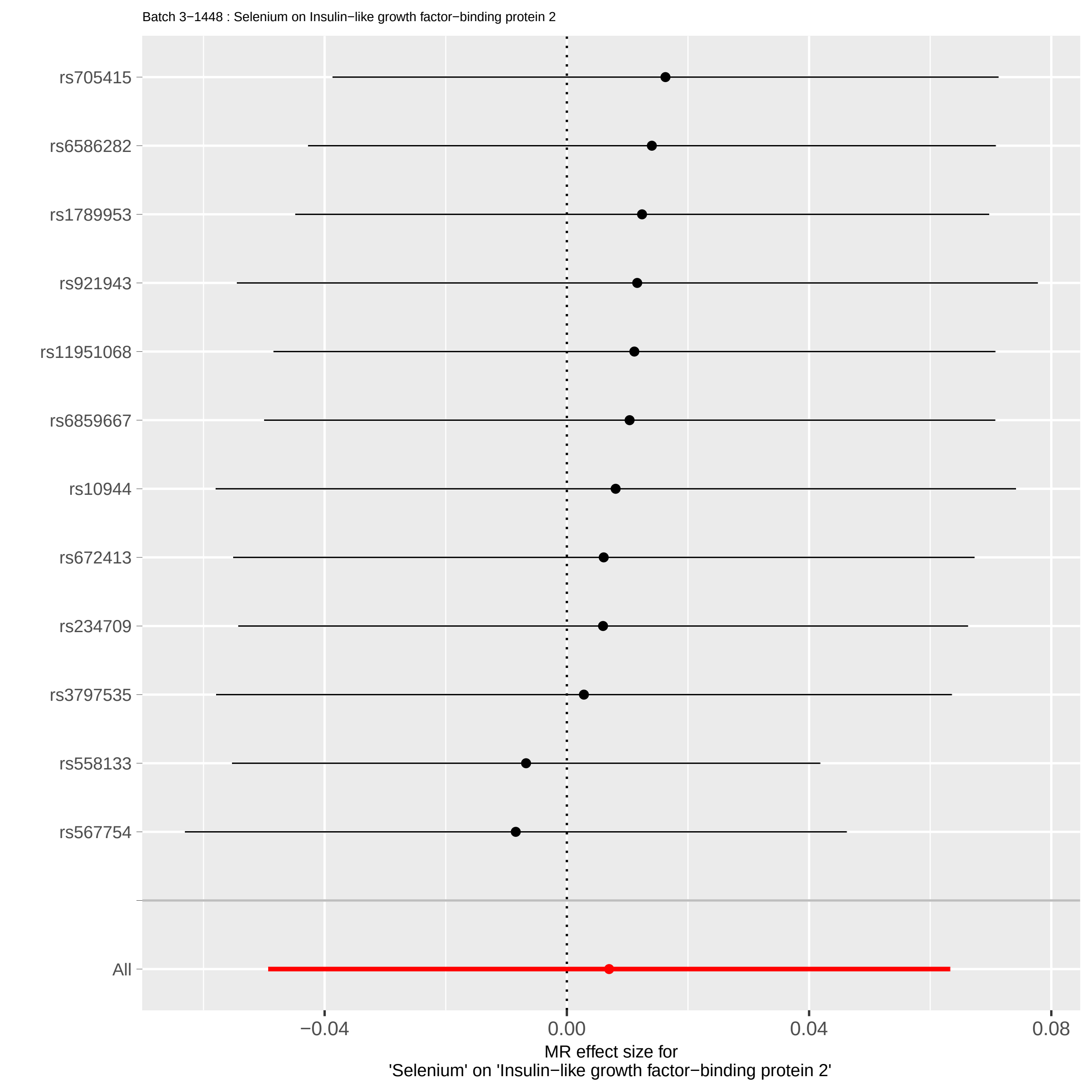

Batch 3−1448 : Selenium on Insulin−like growth factor−binding protein 2
rs705415
rs6586282
rs1789953
rs921943
rs11951068
rs6859667
rs10944
rs672413
rs234709
rs3797535
rs558133
rs567754
All
−0.04
0.00
0.04
0.08
MR effect size for
'Selenium' on 'Insulin−like growth factor−binding protein 2'

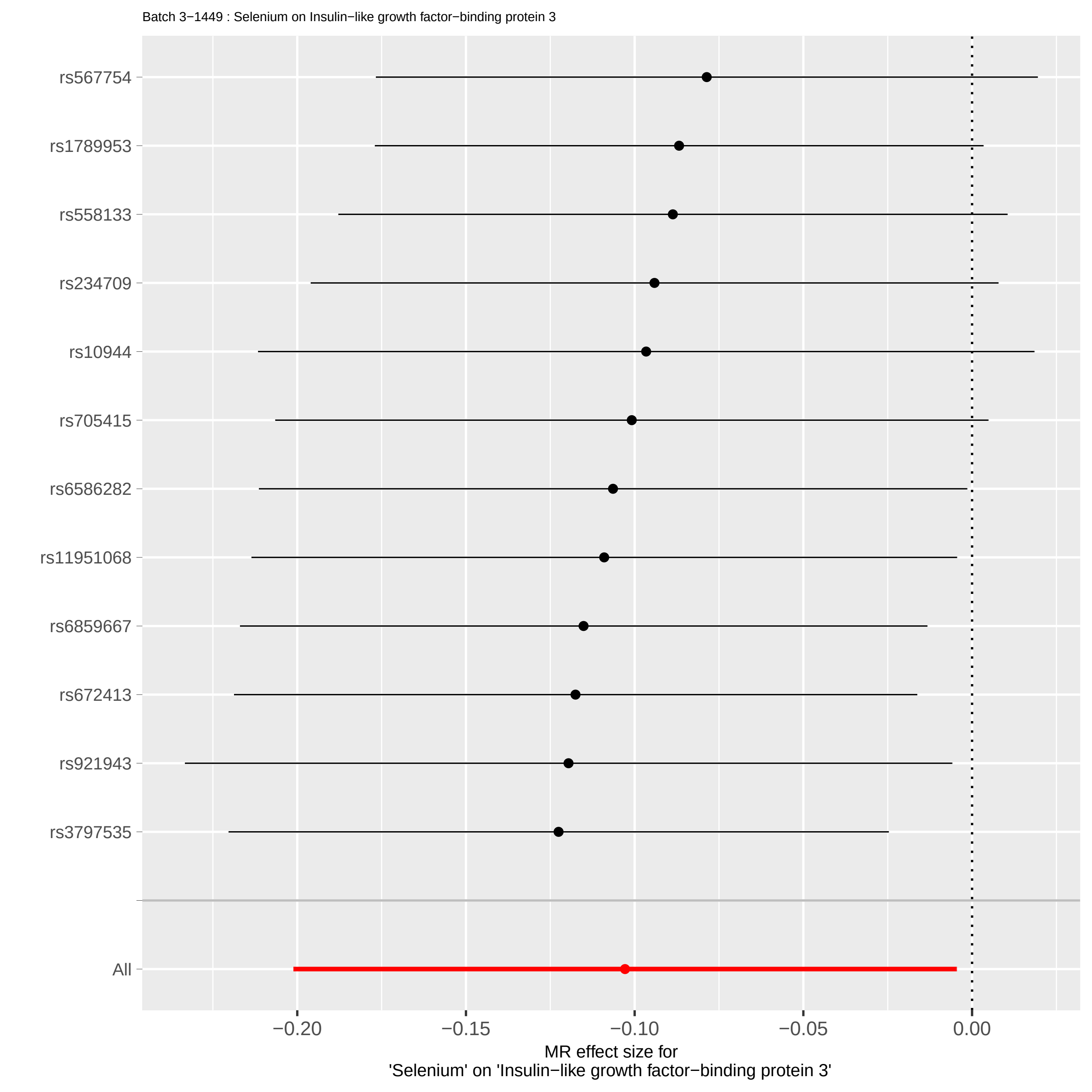
Batch 3−1449 : Selenium on Insulin−like growth factor−binding protein 3
rs567754
rs1789953
rs558133
rs234709
rs10944
rs705415
rs6586282
rs11951068
rs6859667
rs672413
rs921943
rs3797535
All
−0.20
−0.15
−0.10
−0.05
0.00
MR effect size for
'Selenium' on 'Insulin−like growth factor−binding protein 3'

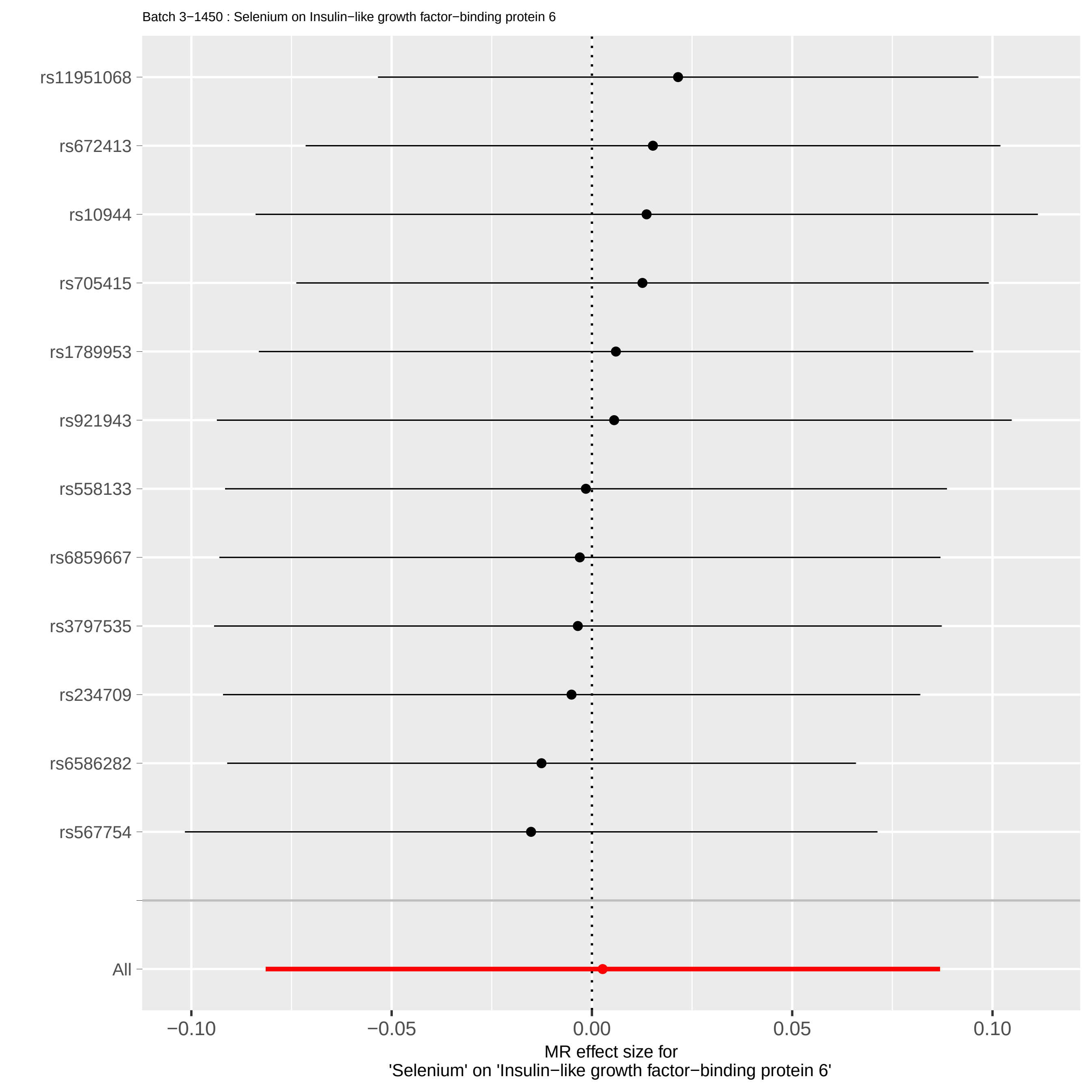
Batch 3−1450 : Selenium on Insulin−like growth factor−binding protein 6
rs11951068
rs672413
rs10944
rs705415
rs1789953
rs921943
rs558133
rs6859667
rs3797535
rs234709
rs6586282
rs567754
All
−0.10
−0.05
0.00
0.05
0.10
MR effect size for
'Selenium' on 'Insulin−like growth factor−binding protein 6'

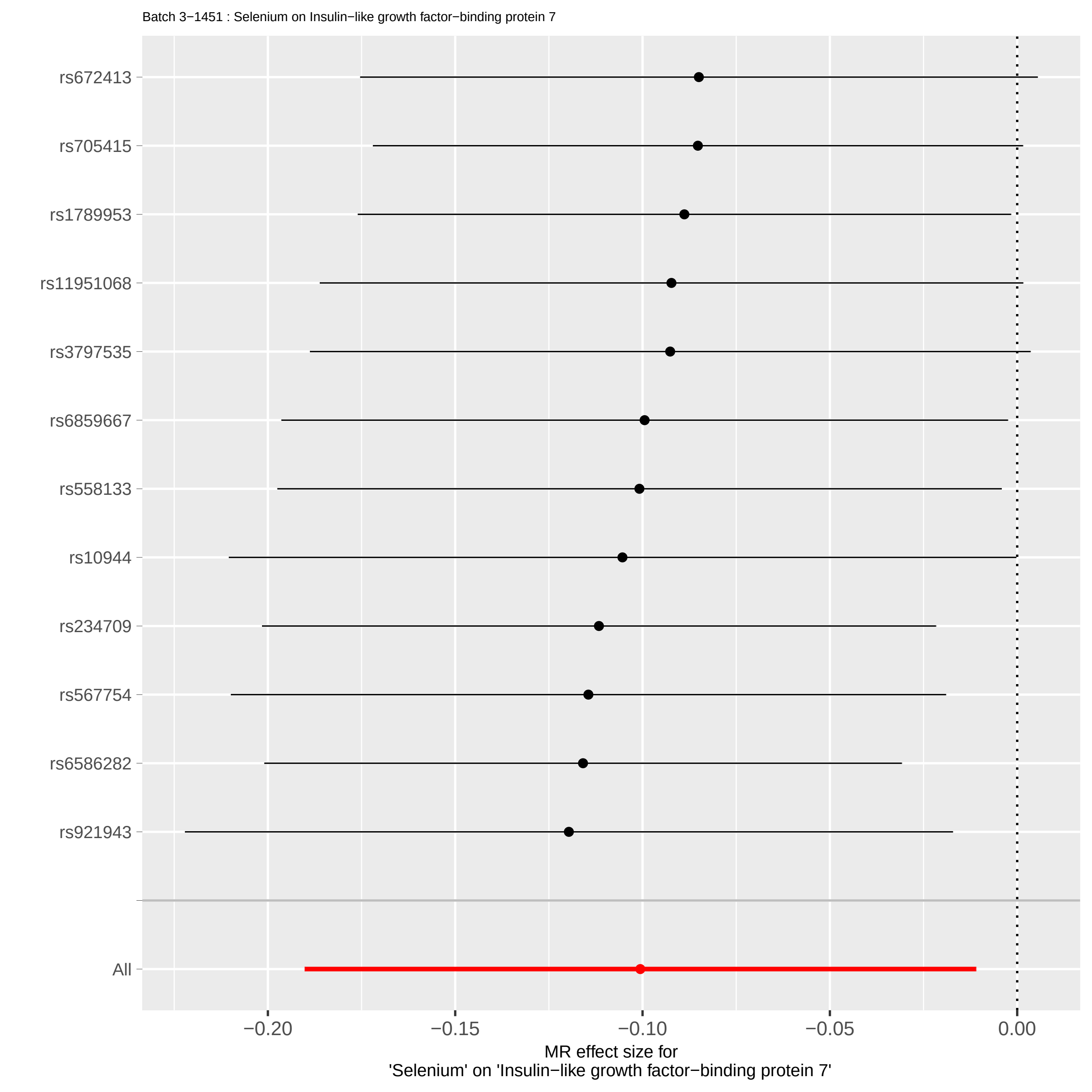

Batch 3−1451 : Selenium on Insulin−like growth factor−binding protein 7
rs672413
rs705415
rs1789953
rs11951068
rs3797535
rs6859667
rs558133
rs10944
rs234709
rs567754
rs6586282
rs921943
All
−0.20
−0.15
−0.10
−0.05
0.00
MR effect size for
'Selenium' on 'Insulin−like growth factor−binding protein 7'

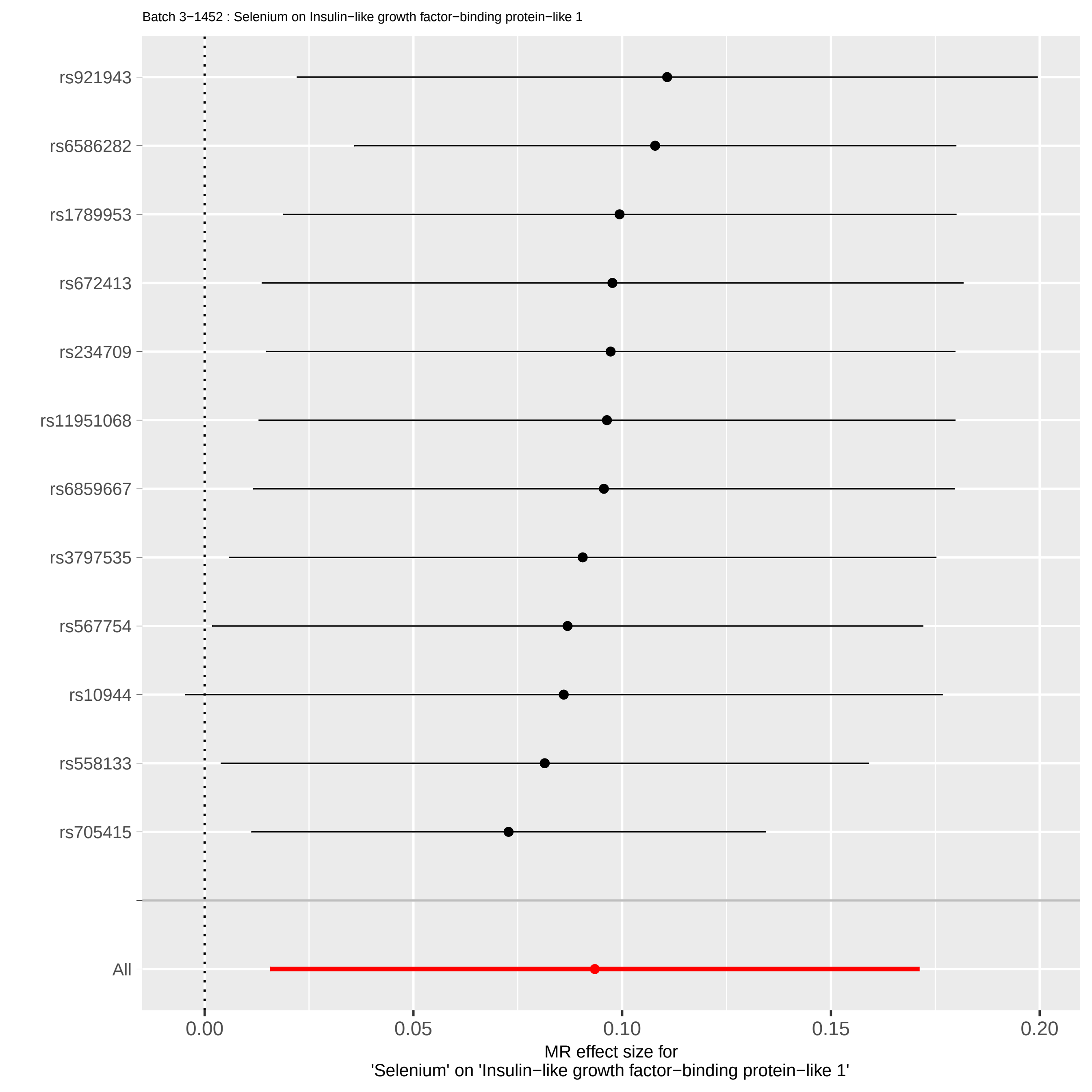

Batch 3−1452 : Selenium on Insulin−like growth factor−binding protein−like 1
rs921943
rs6586282
rs1789953
rs672413
rs234709
rs11951068
rs6859667
rs3797535
rs567754
rs10944
rs558133
rs705415
All
0.00
0.05
0.10
0.15
0.20
MR effect size for
'Selenium' on 'Insulin−like growth factor−binding protein−like 1'

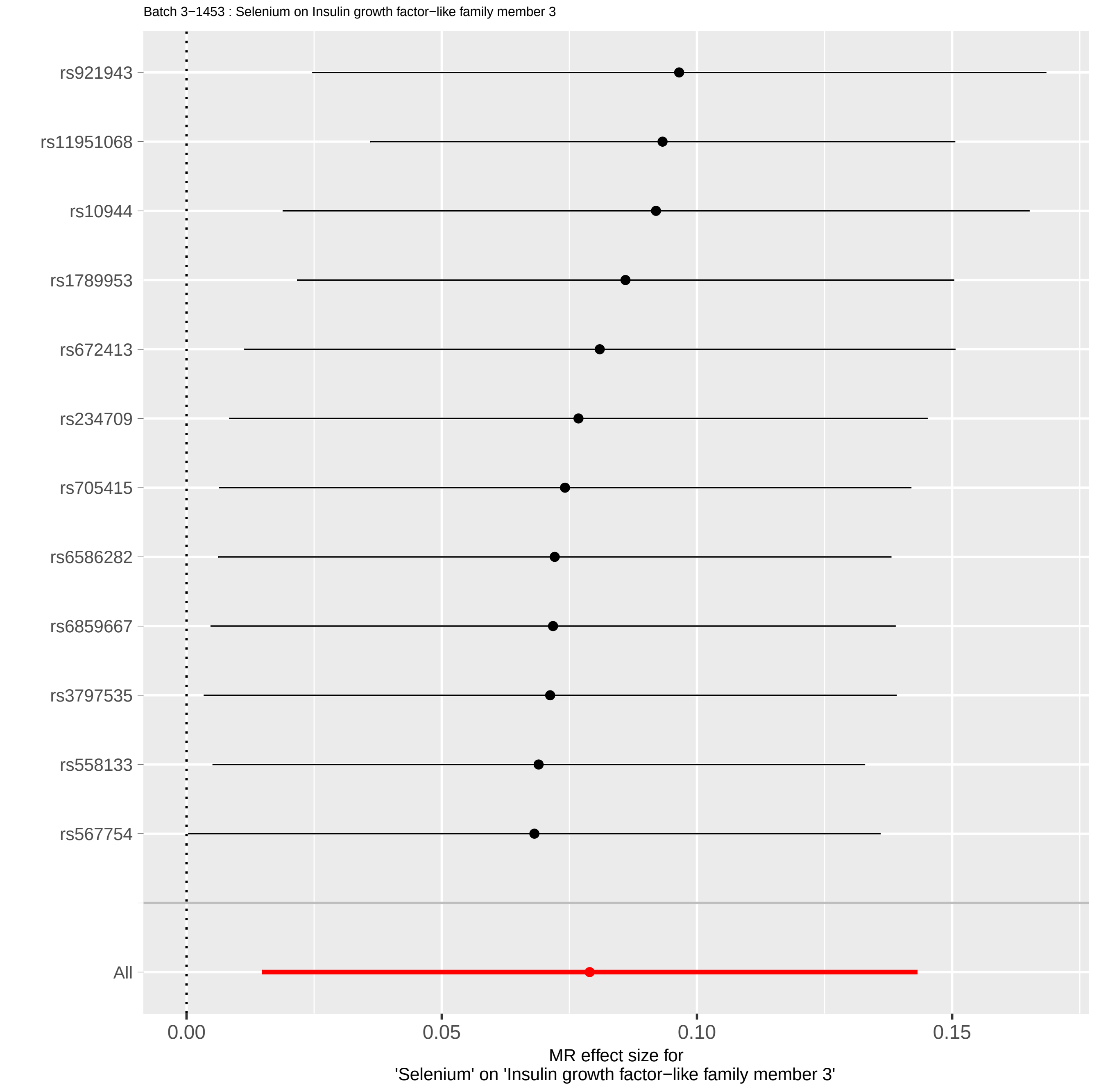

Batch 3−1453 : Selenium on Insulin growth factor−like family member 3
rs921943
rs11951068
rs10944
rs1789953
rs672413
rs234709
rs705415
rs6586282
rs6859667
rs3797535
rs558133
rs567754
All
0.00
0.05
0.10
0.15
MR effect size for
'Selenium' on 'Insulin growth factor−like family member 3'

Batch 3−1454 : Selenium on Insulin growth factor−like family member 4

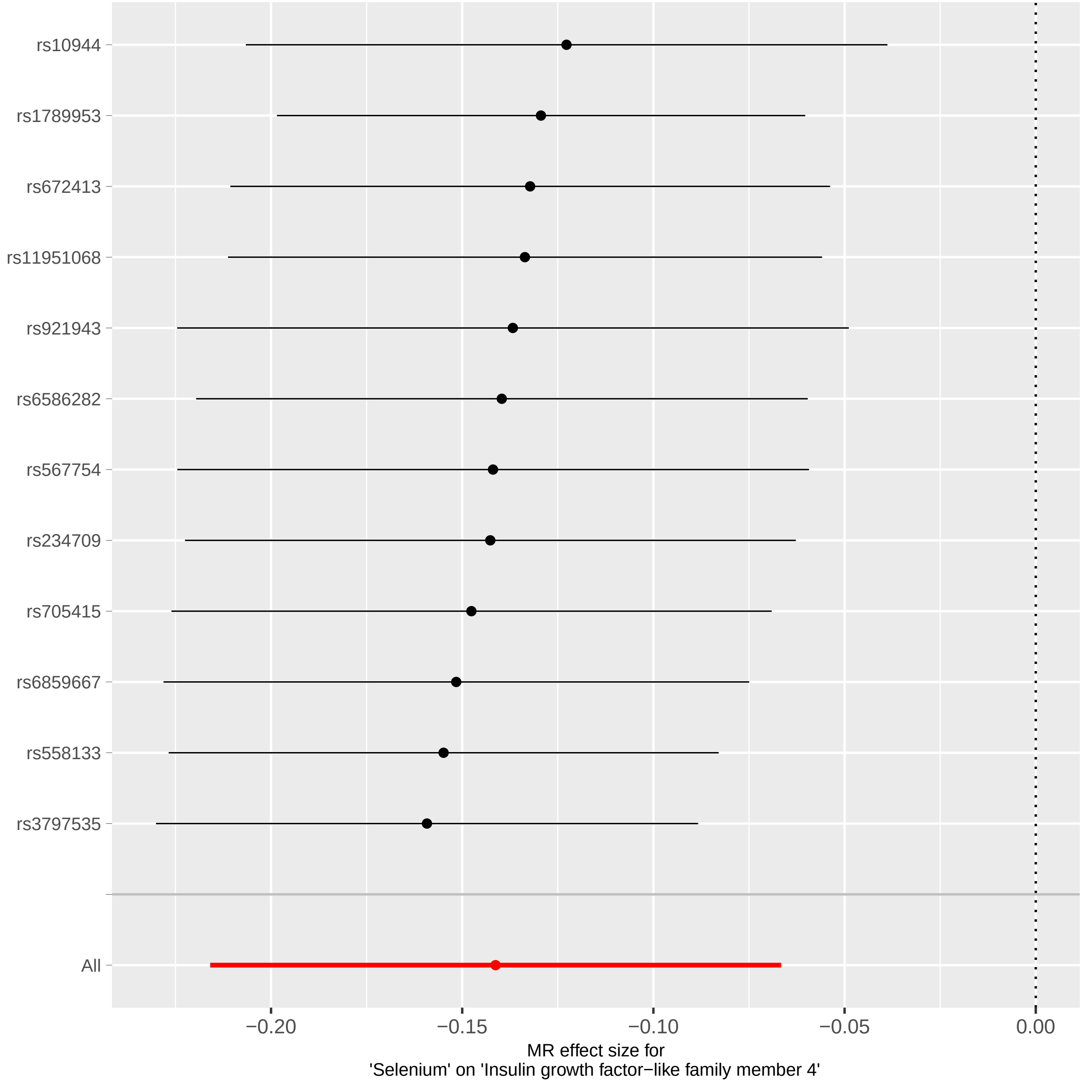

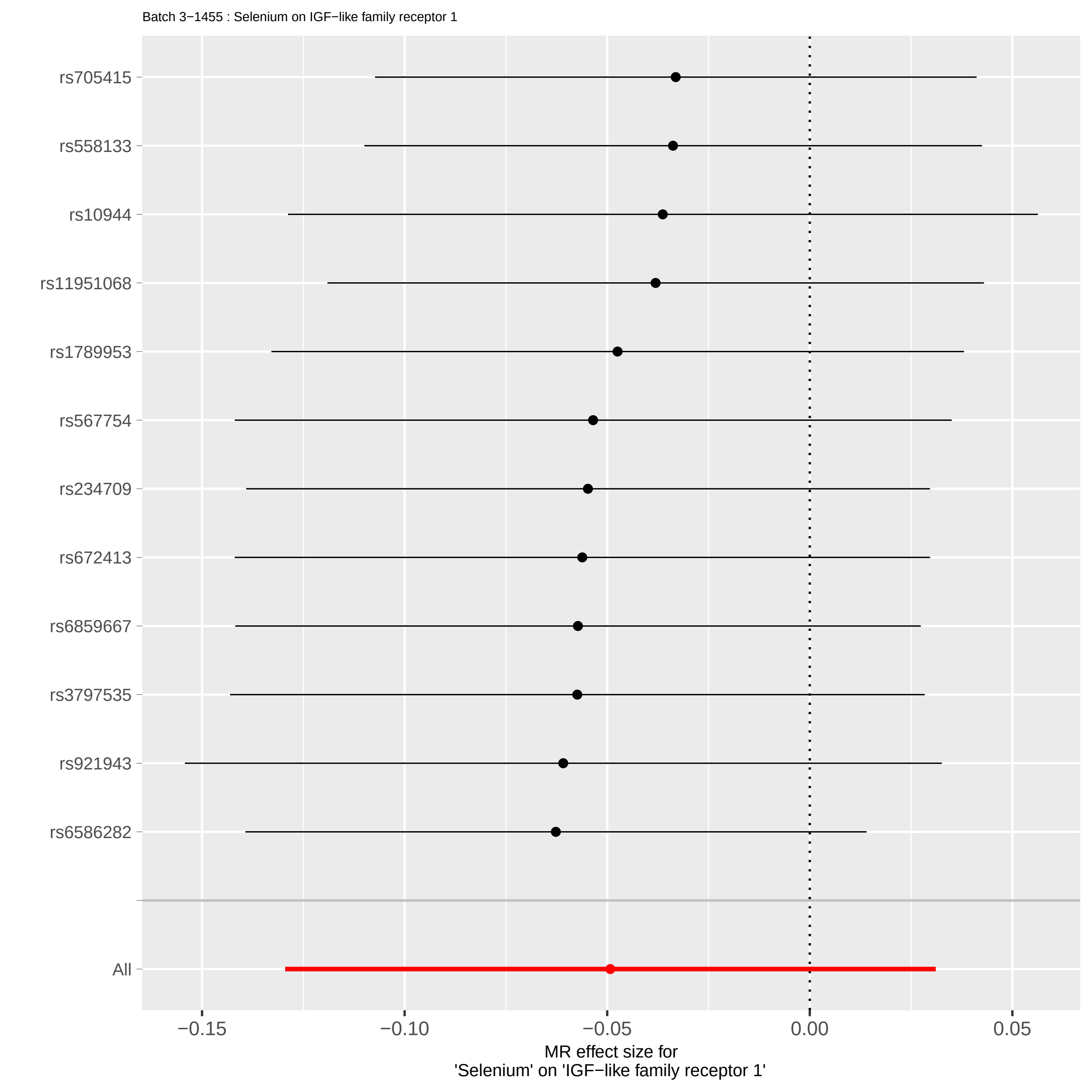

Batch 3−1455 : Selenium on IGF−like family receptor 1
rs705415
rs558133
rs10944
rs11951068
rs1789953
rs567754
rs234709
rs672413
rs6859667
rs3797535
rs921943
rs6586282
All
−0.15
−0.10
−0.05
0.00
0.05
MR effect size for
'Selenium' on 'IGF−like family receptor 1'

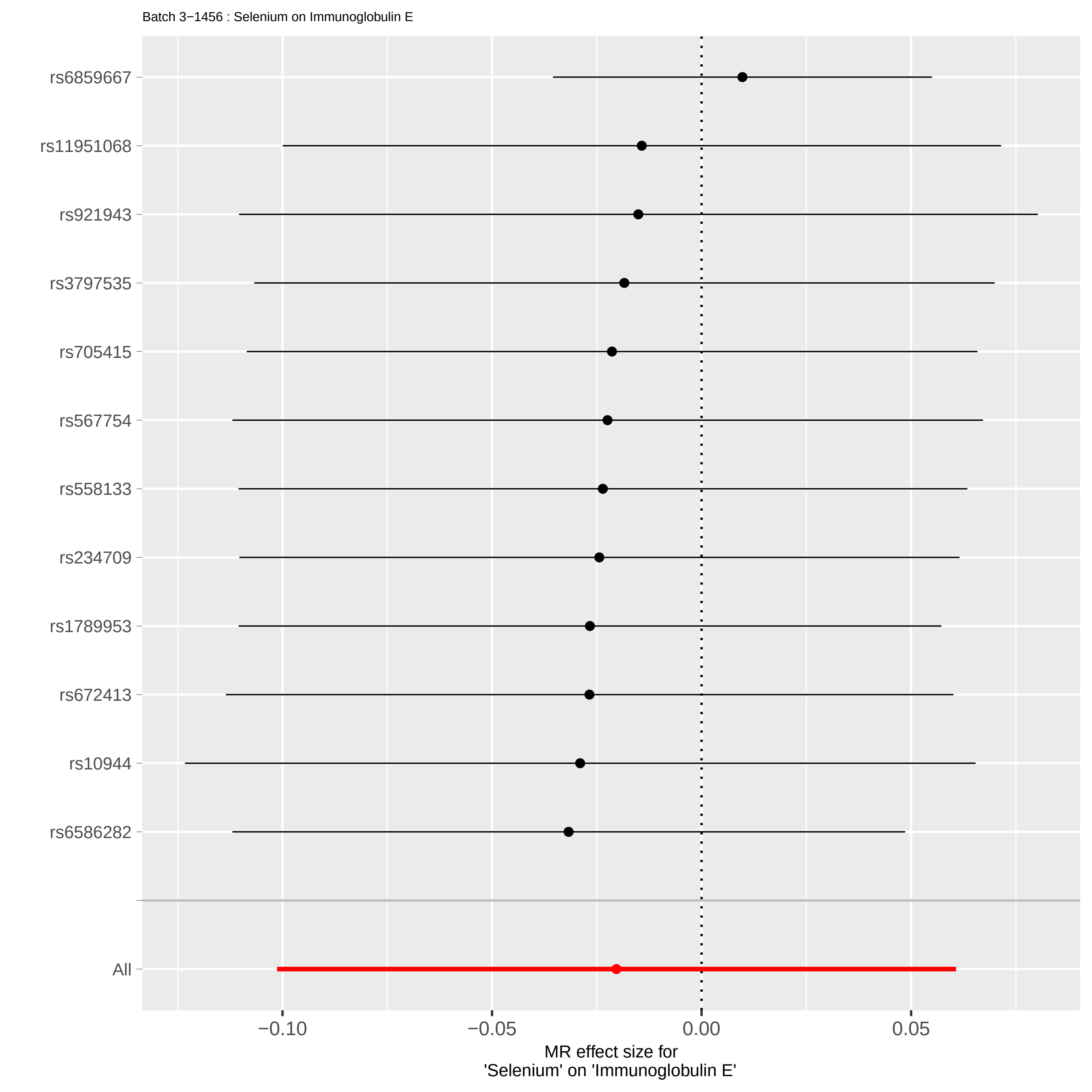
Batch 3−1456 : Selenium on Immunoglobulin E
rs6859667
rs11951068
rs921943
rs3797535
rs705415
rs567754
rs558133
rs234709
rs1789953
rs672413
rs10944
rs6586282
All
−0.10
−0.05
0.00
0.05
MR effect size for
'Selenium' on 'Immunoglobulin E'

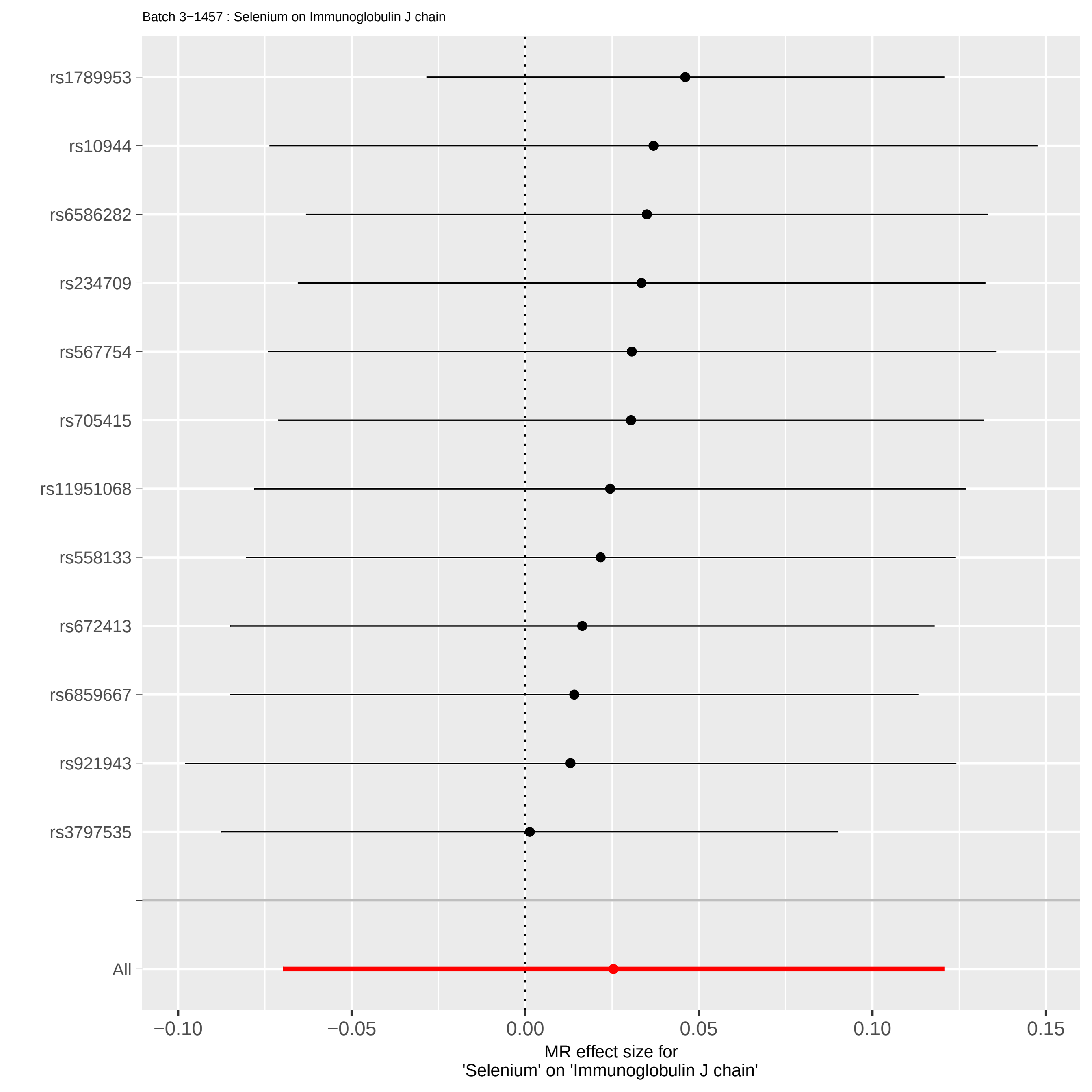

Batch 3−1457 : Selenium on Immunoglobulin J chain
rs1789953
rs10944
rs6586282
rs234709
rs567754
rs705415
rs11951068
rs558133
rs672413
rs6859667
rs921943
rs3797535
All
−0.10
−0.05
0.00
0.05
0.10
0.15
MR effect size for
'Selenium' on 'Immunoglobulin J chain'

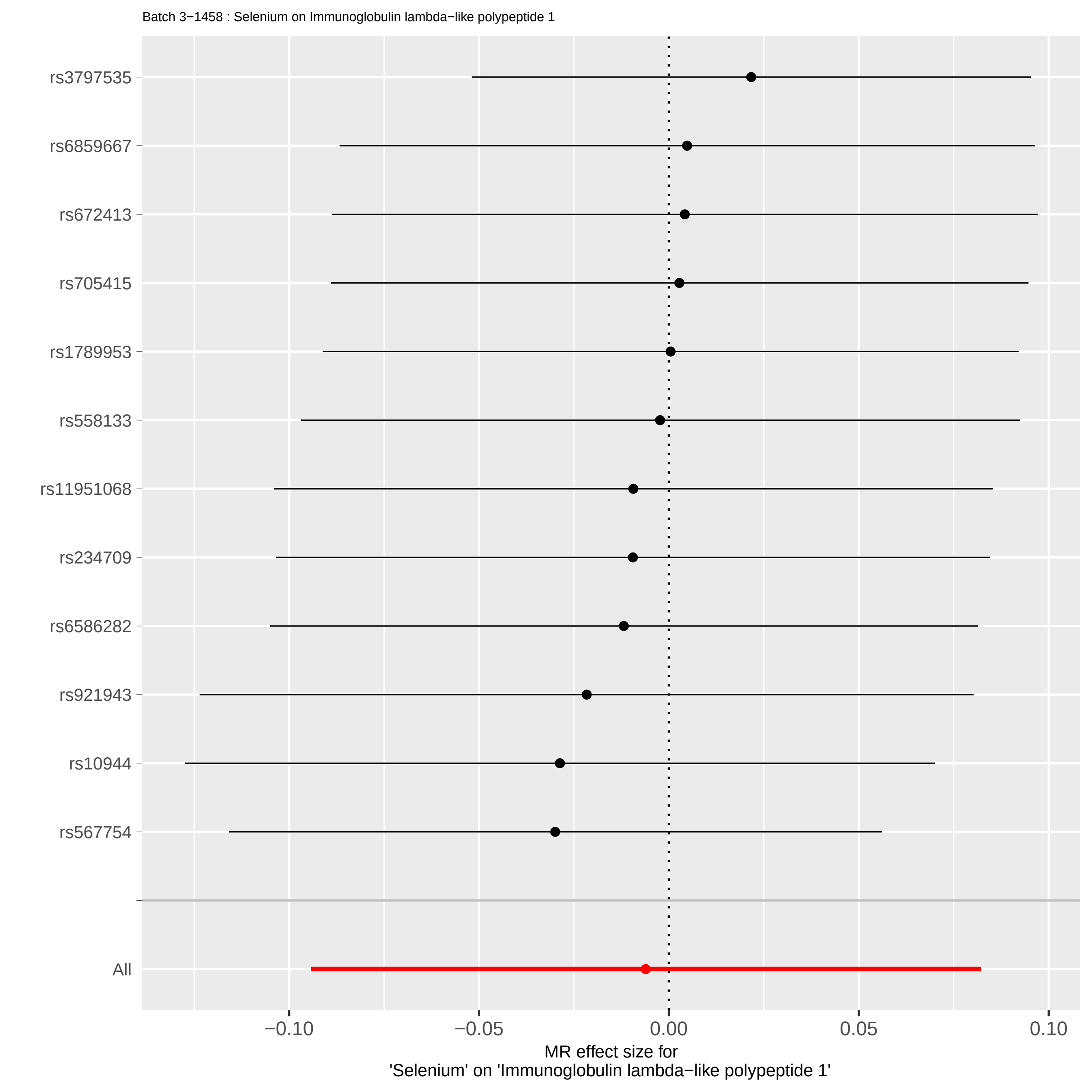
Batch 3−1458 : Selenium on Immunoglobulin lambda−like polypeptide 1
rs3797535
rs6859667
rs672413
rs705415
rs1789953
rs558133
rs11951068
rs234709
rs6586282
rs921943
rs10944
rs567754
All
−0.10
−0.05
0.00
0.05
0.10
MR effect size for
'Selenium' on 'Immunoglobulin lambda−like polypeptide 1'

Batch 3−1459 : Selenium on IgLON family member 5

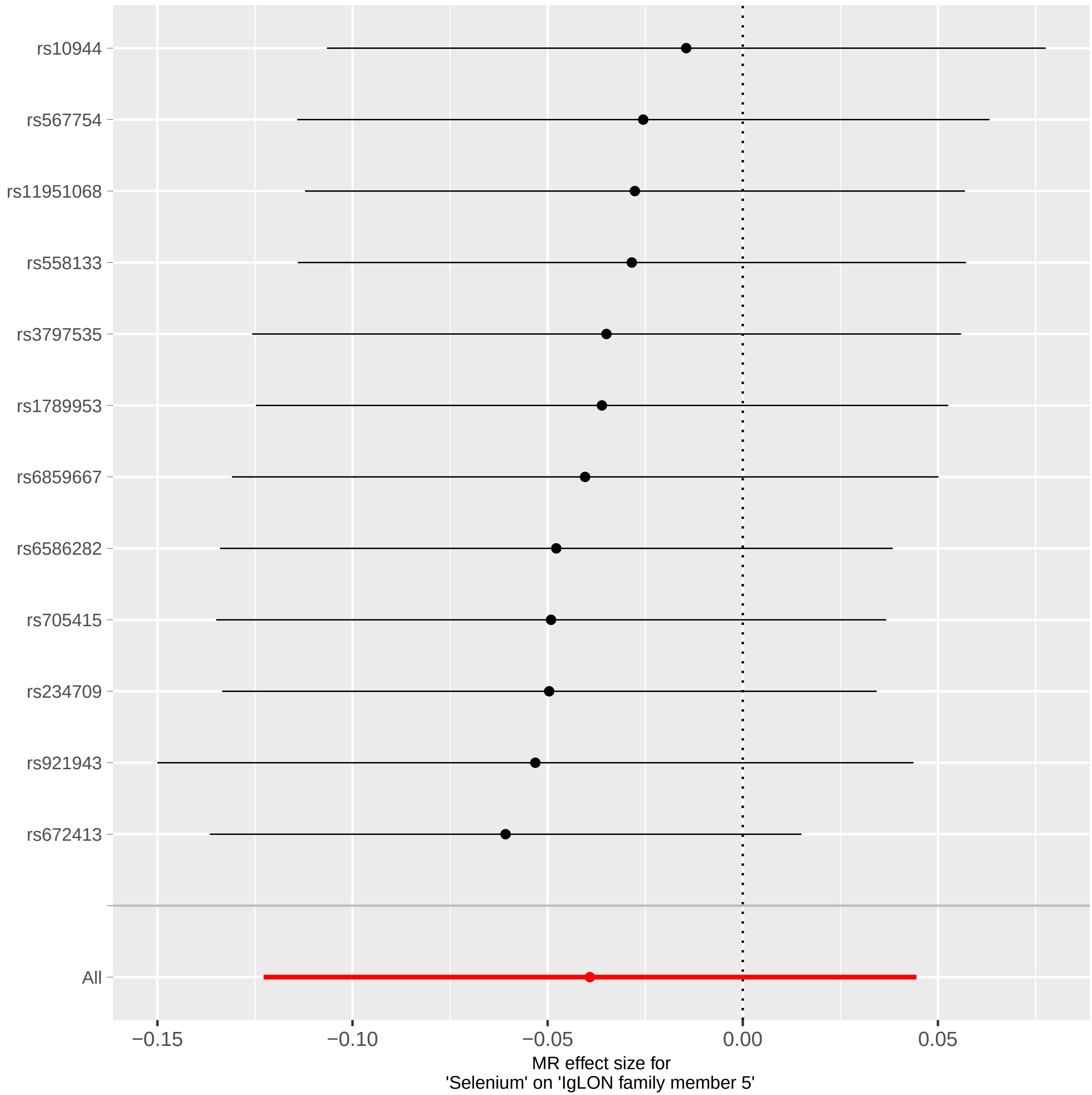

Batch 3−1460 : Selenium on Immunoglobulin superfamily member 11

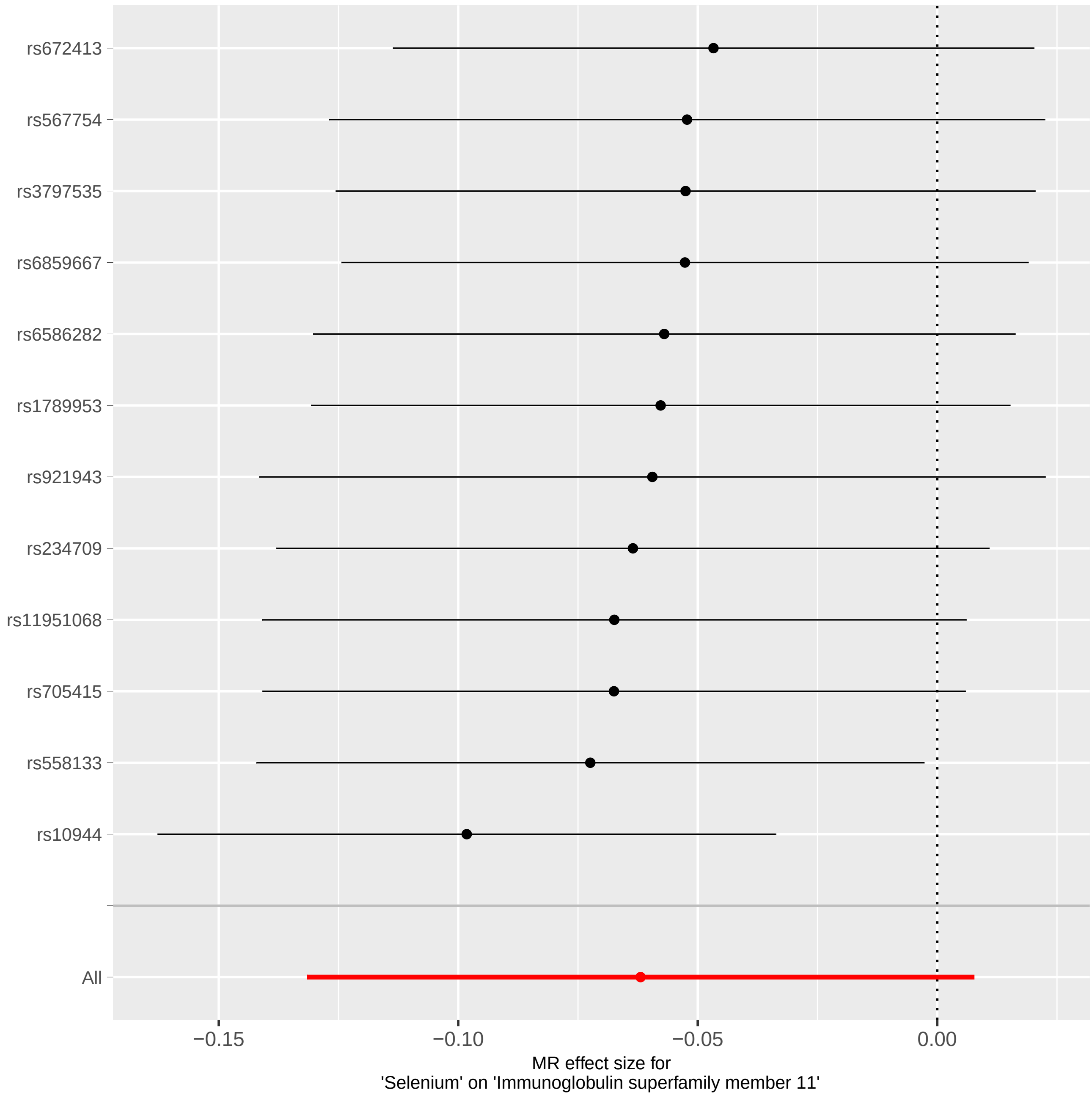

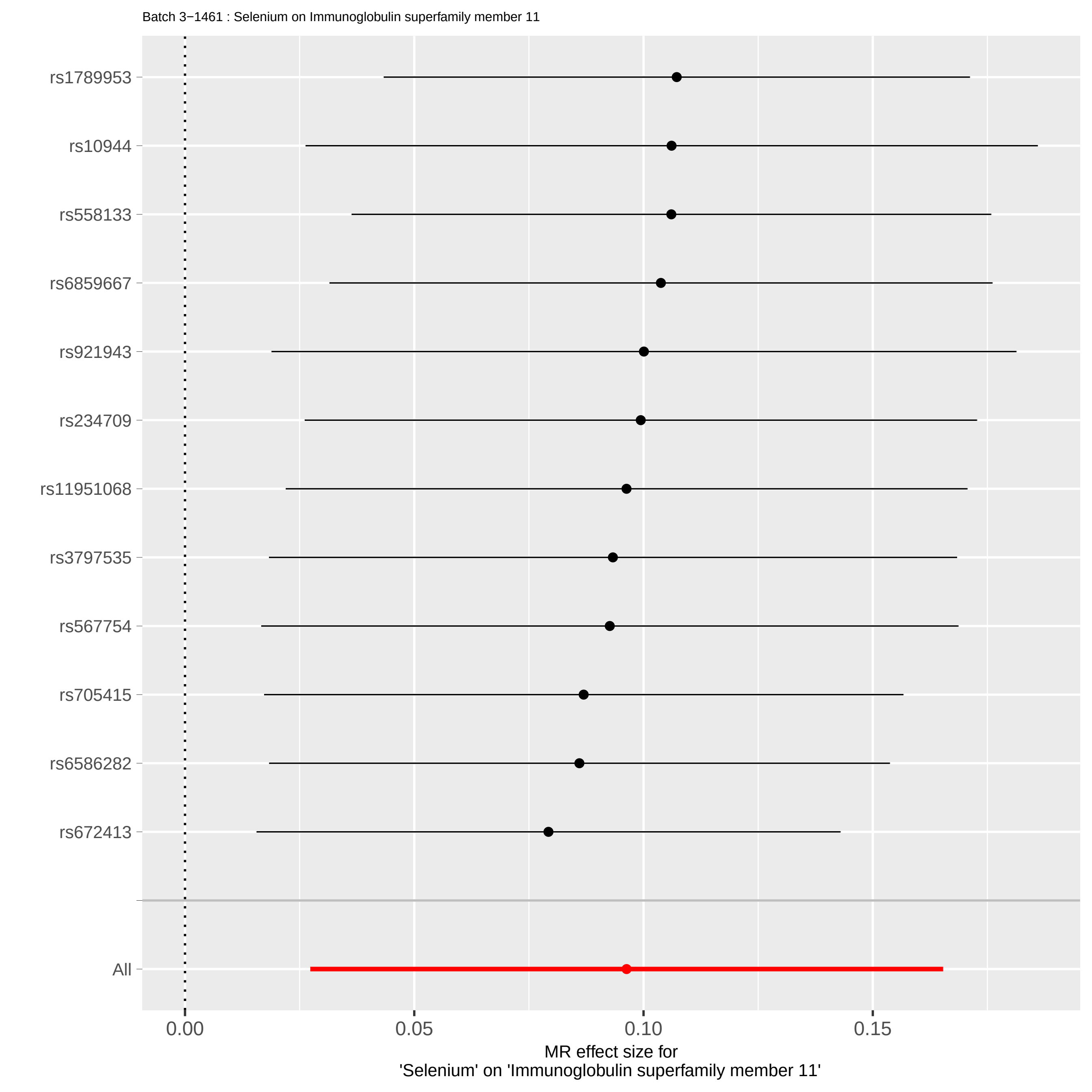

Batch 3−1461 : Selenium on Immunoglobulin superfamily member 11
rs1789953
rs10944
rs558133
rs6859667
rs921943
rs234709
rs11951068
rs3797535
rs567754
rs705415
rs6586282
rs672413
All
0.00
0.05
0.10
0.15
MR effect size for
'Selenium' on 'Immunoglobulin superfamily member 11'

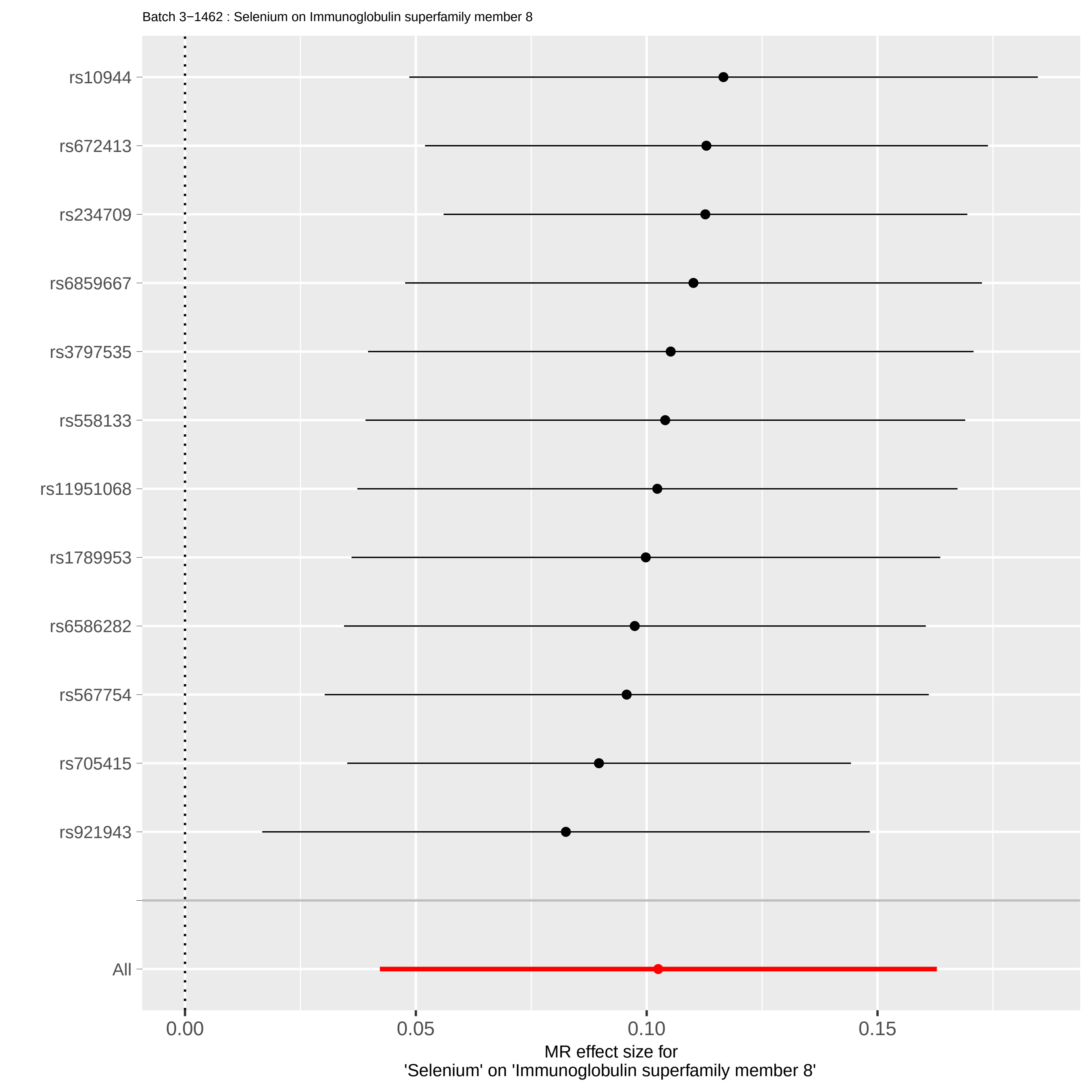
Batch 3−1462 : Selenium on Immunoglobulin superfamily member 8
rs10944
rs672413
rs234709
rs6859667
rs3797535
rs558133
rs11951068
rs1789953
rs6586282
rs567754
rs705415
rs921943
All
0.00
0.05
0.10
0.15
MR effect size for
'Selenium' on 'Immunoglobulin superfamily member 8'

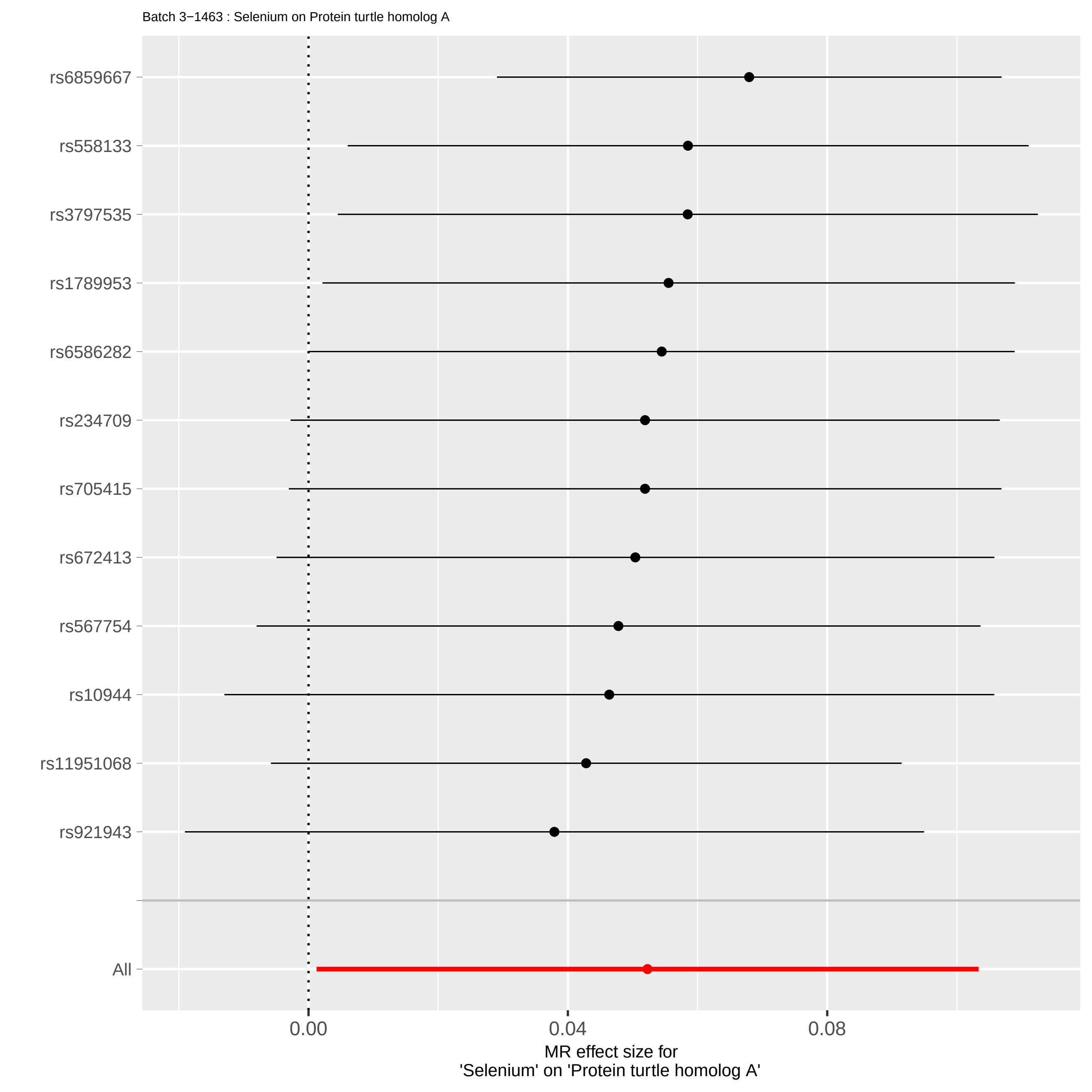
Batch 3−1463 : Selenium on Protein turtle homolog A
rs6859667
rs558133
rs3797535
rs1789953
rs6586282
rs234709
rs705415
rs672413
rs567754
rs10944
rs11951068
rs921943
All
0.00
0.04
0.08
MR effect size for
'Selenium' on 'Protein turtle homolog A'

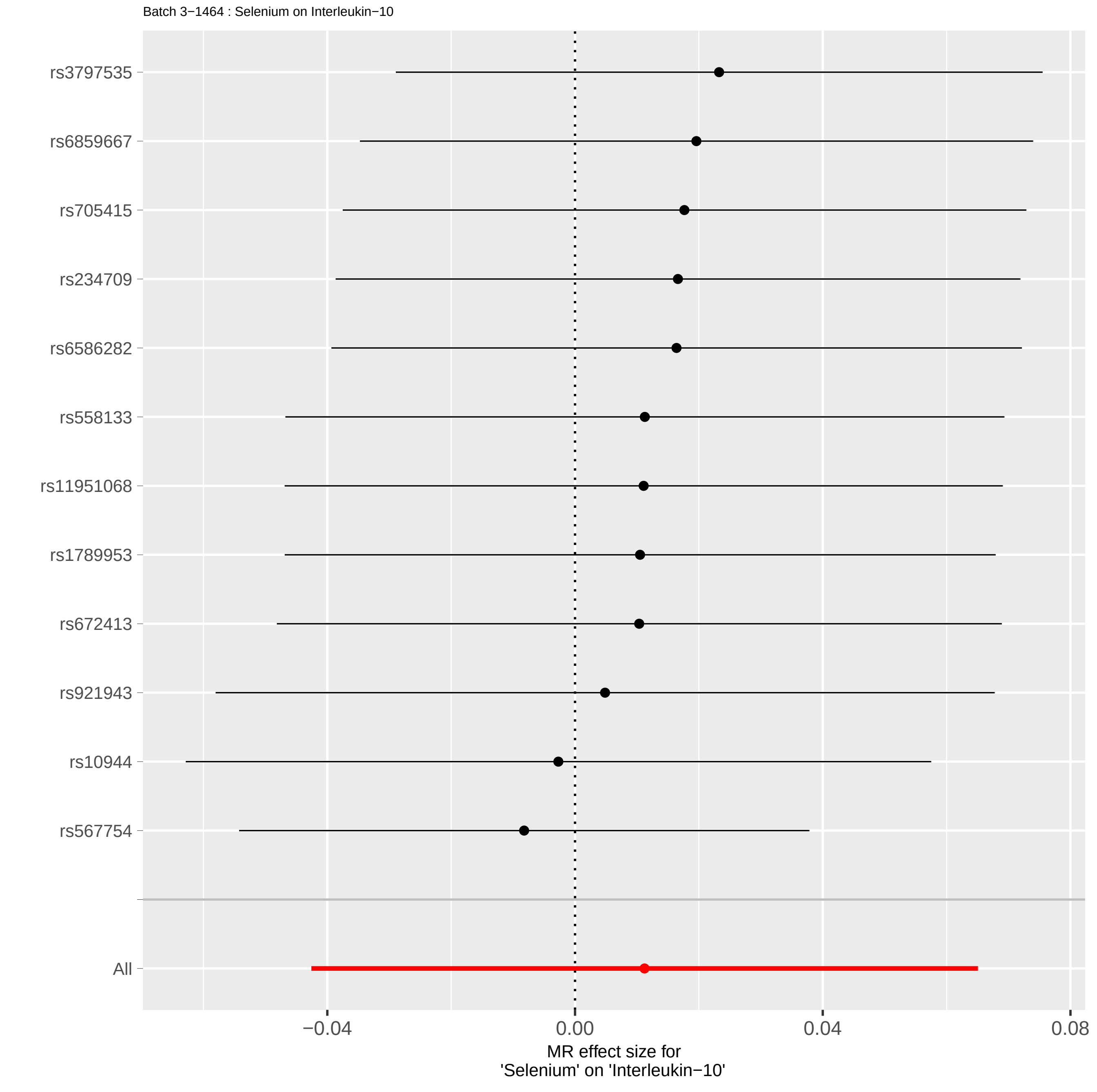

Batch 3−1464 : Selenium on Interleukin−10
rs3797535
rs6859667
rs705415
rs234709
rs6586282
rs558133
rs11951068
rs1789953
rs672413
rs921943
rs10944
rs567754
All
−0.04
0.00
0.04
0.08
MR effect size for
'Selenium' on 'Interleukin−10'

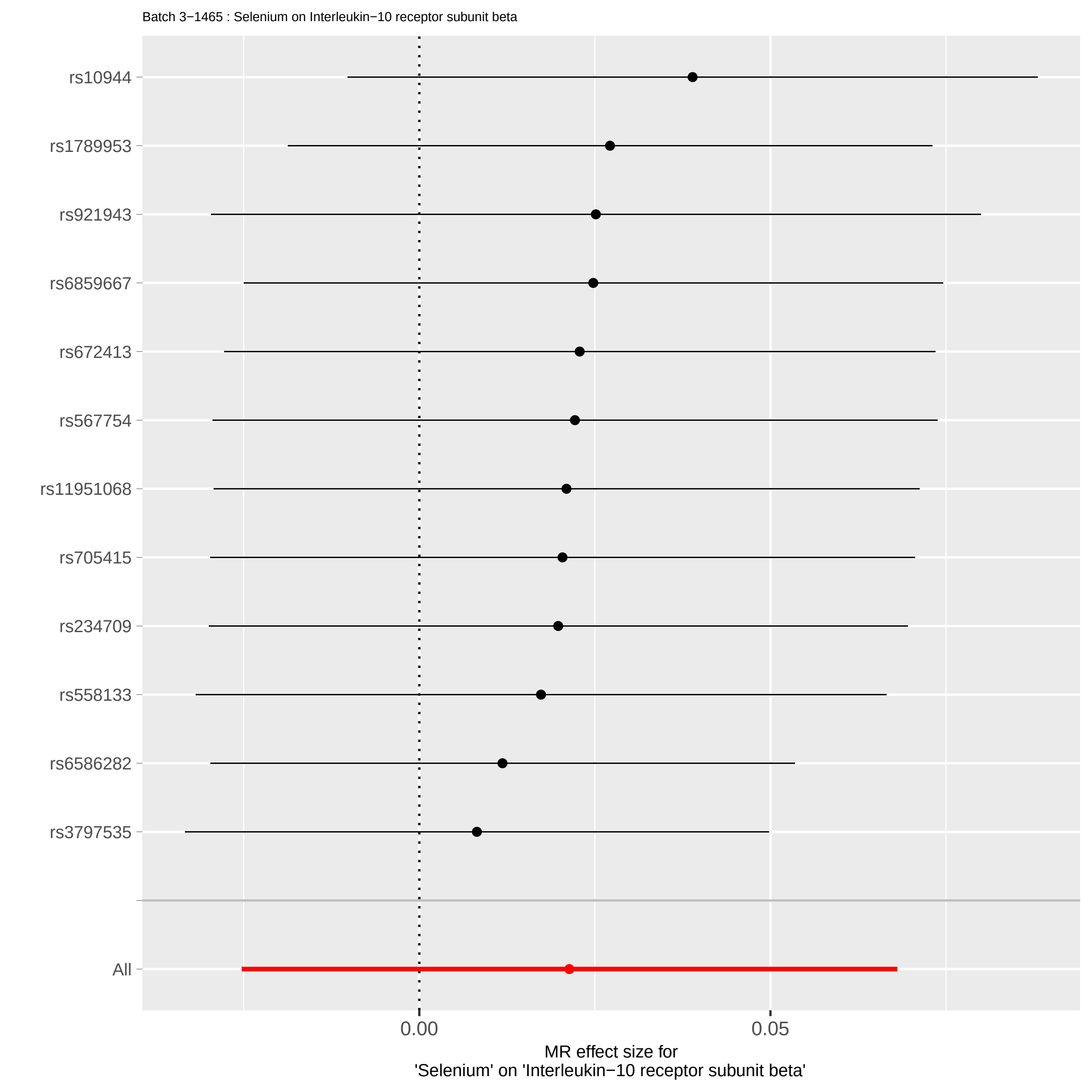

Batch 3−1465 : Selenium on Interleukin−10 receptor subunit beta
rs10944
rs1789953
rs921943
rs6859667
rs672413
rs567754
rs11951068
rs705415
rs234709
rs558133
rs6586282
rs3797535
All
0.00
0.05
MR effect size for
'Selenium' on 'Interleukin−10 receptor subunit beta'

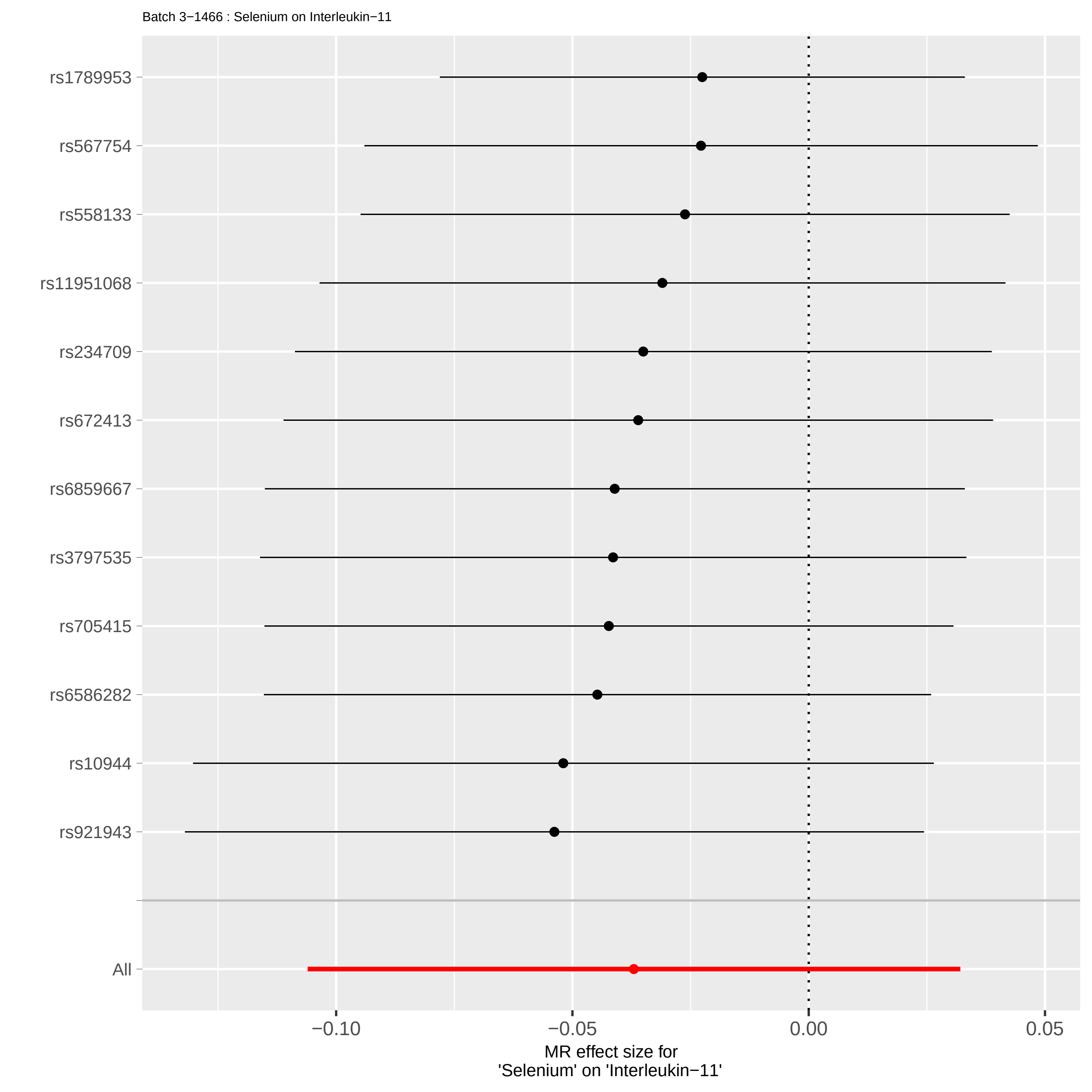
Batch 3−1466 : Selenium on Interleukin−11
rs1789953
rs567754
rs558133
rs11951068
rs234709
rs672413
rs6859667
rs3797535
rs705415
rs6586282
rs10944
rs921943
All
−0.10
−0.05
0.00
0.05
MR effect size for
'Selenium' on 'Interleukin−11'

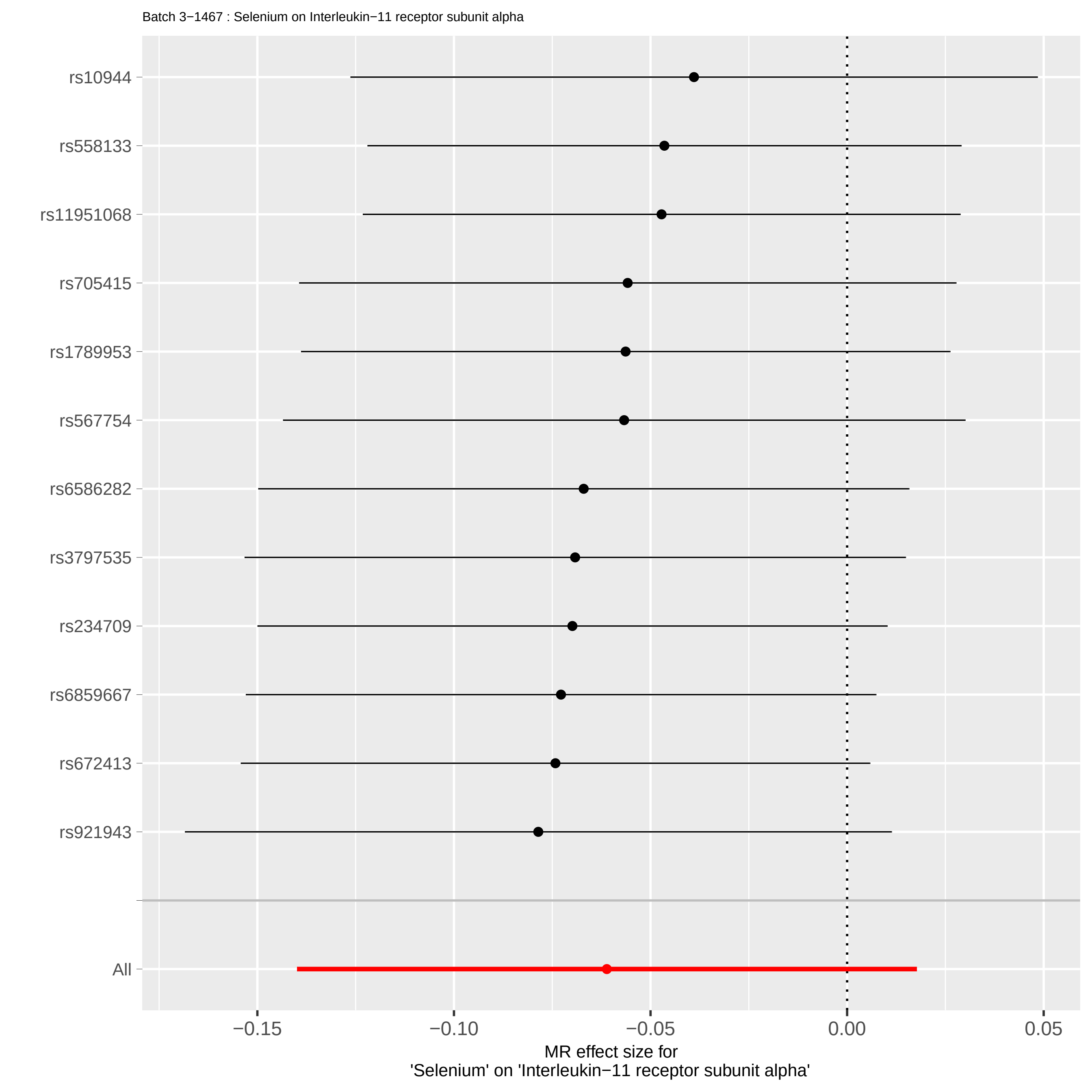

Batch 3−1467 : Selenium on Interleukin−11 receptor subunit alpha
rs10944
rs558133
rs11951068
rs705415
rs1789953
rs567754
rs6586282
rs3797535
rs234709
rs6859667
rs672413
rs921943
All
−0.15
−0.10
−0.05
0.00
0.05
MR effect size for
'Selenium' on 'Interleukin−11 receptor subunit alpha'

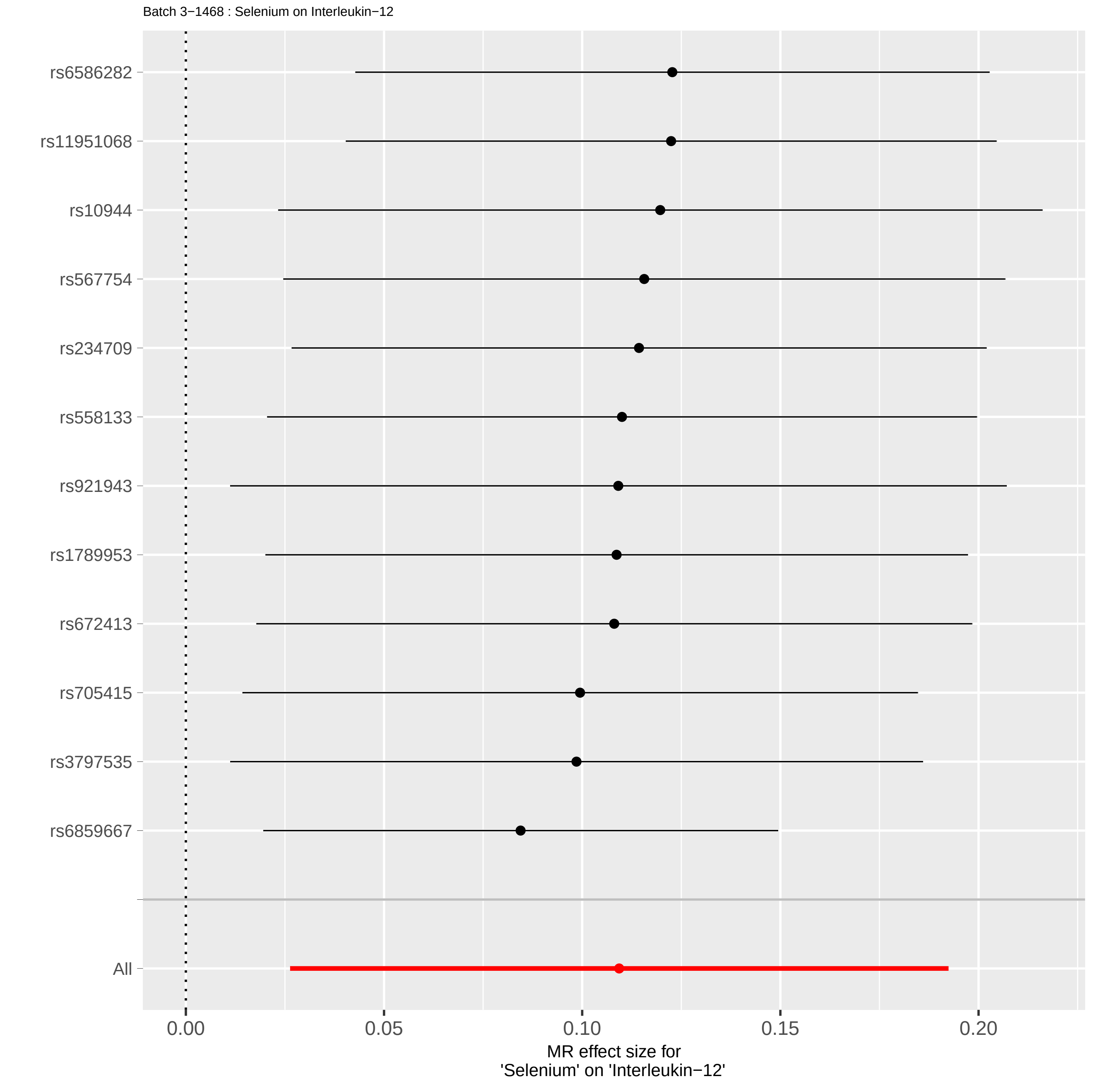

Batch 3−1468 : Selenium on Interleukin−12
rs6586282
rs11951068
rs10944
rs567754
rs234709
rs558133
rs921943
rs1789953
rs672413
rs705415
rs3797535
rs6859667
All
0.00
0.05
0.10
0.15
0.20
MR effect size for
'Selenium' on 'Interleukin−12'

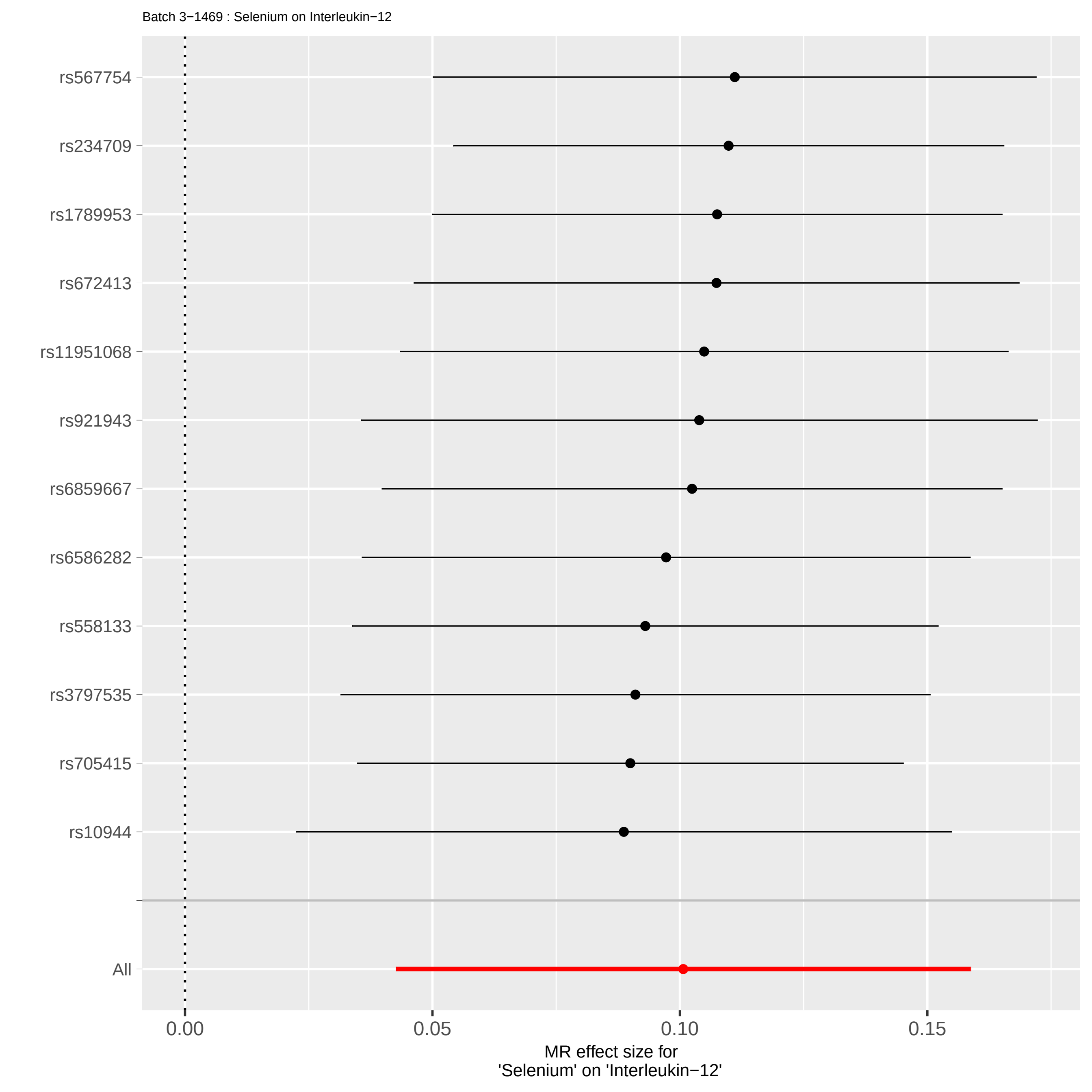

Batch 3−1469 : Selenium on Interleukin−12
rs567754
rs234709
rs1789953
rs672413
rs11951068
rs921943
rs6859667
rs6586282
rs558133
rs3797535
rs705415
rs10944
All
0.00
0.05
0.10
0.15
MR effect size for
'Selenium' on 'Interleukin−12'

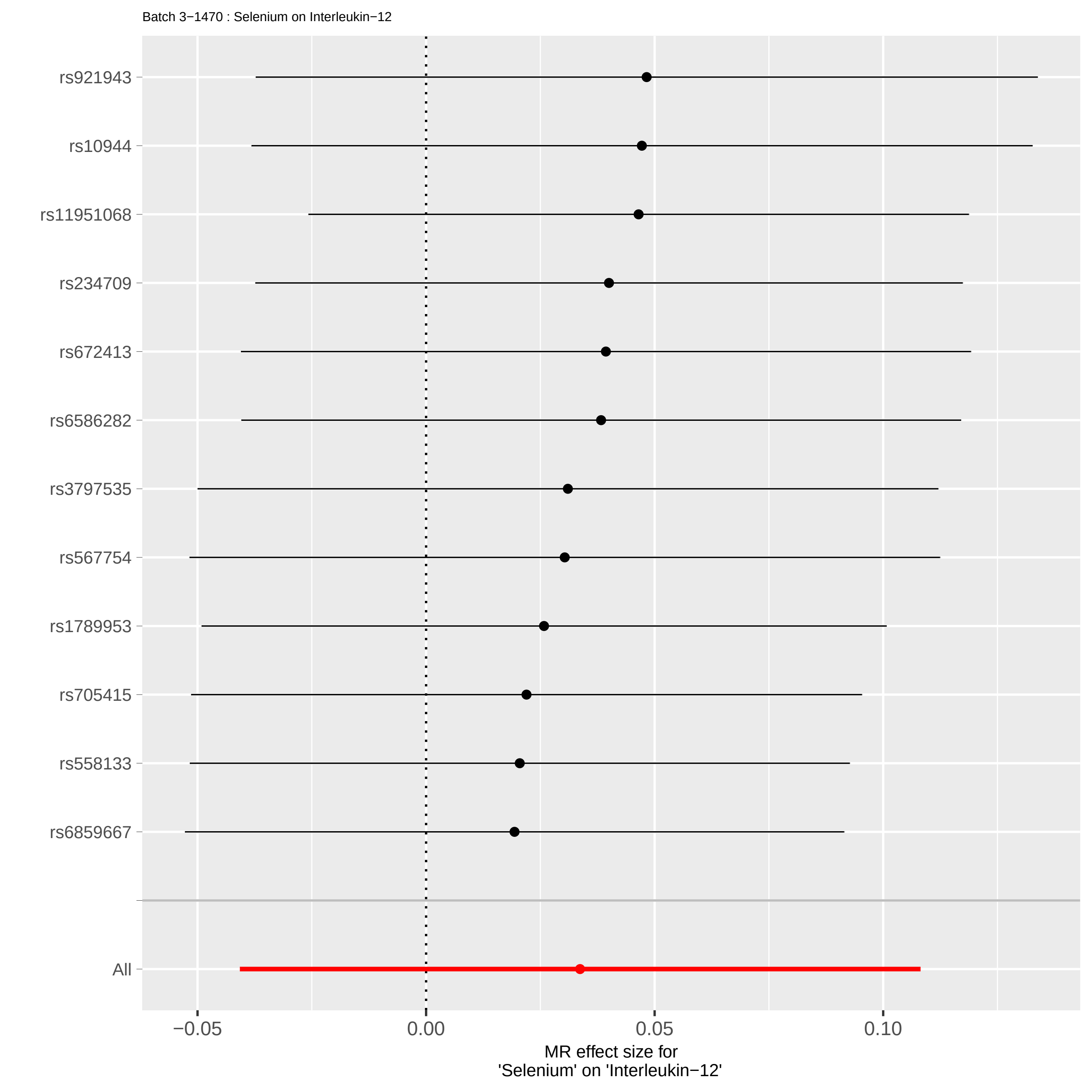

Batch 3−1470 : Selenium on Interleukin−12
rs921943
rs10944
rs11951068
rs234709
rs672413
rs6586282
rs3797535
rs567754
rs1789953
rs705415
rs558133
rs6859667
All
−0.05
0.00
0.05
0.10
MR effect size for
'Selenium' on 'Interleukin−12'

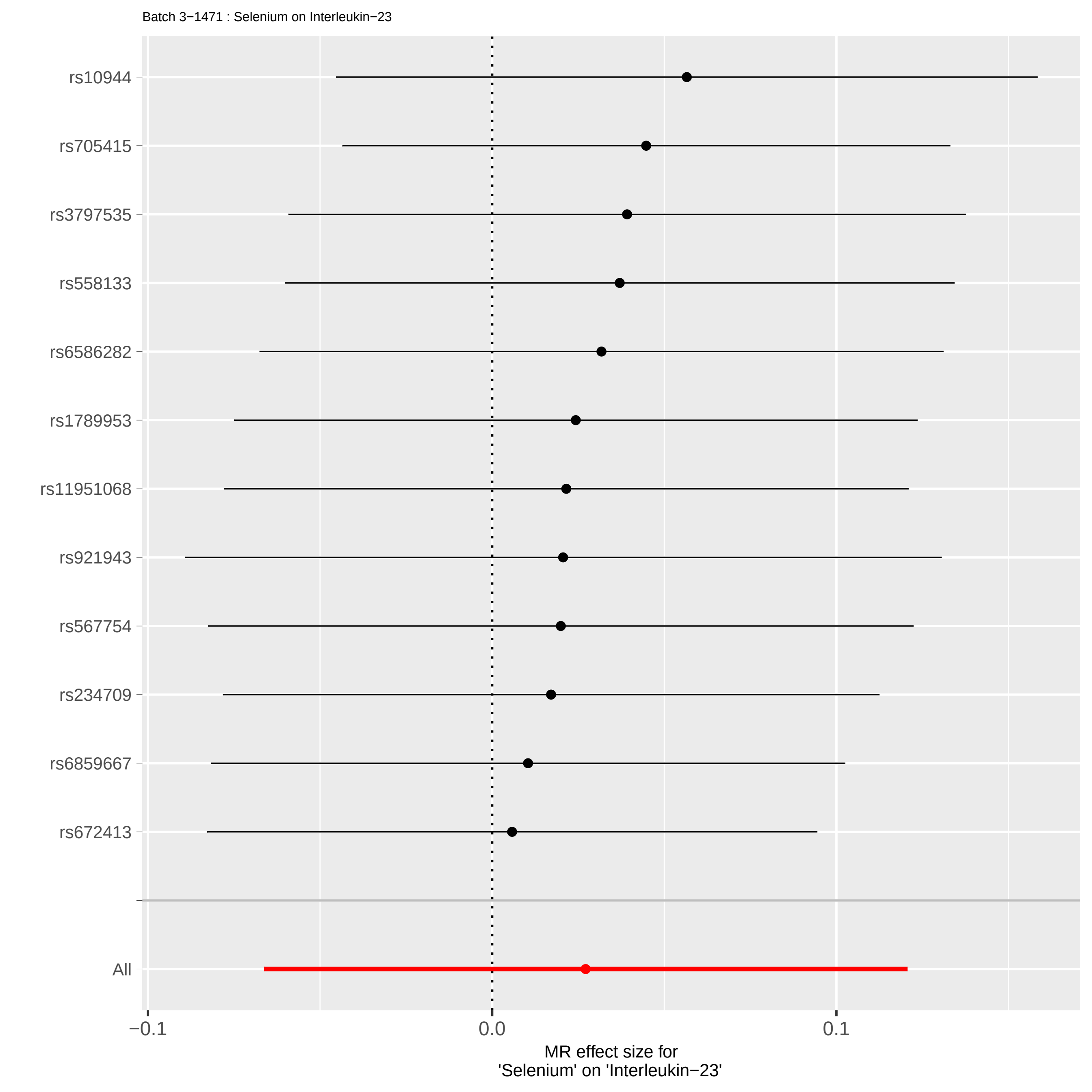

Batch 3−1471 : Selenium on Interleukin−23
rs10944
rs705415
rs3797535
rs558133
rs6586282
rs1789953
rs11951068
rs921943
rs567754
rs234709
rs6859667
rs672413
All
−0.1
0.0
0.1
MR effect size for
'Selenium' on 'Interleukin−23'

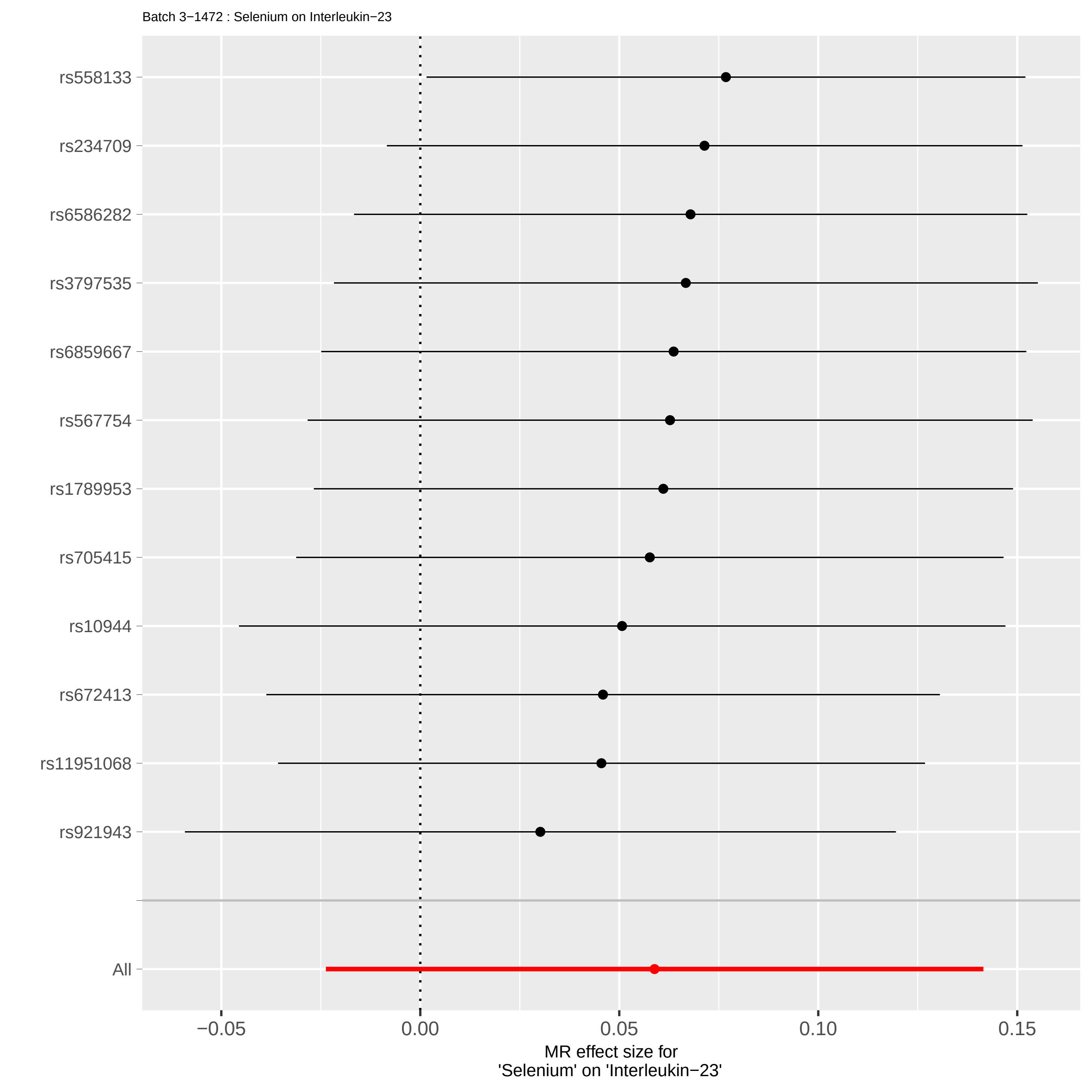

Batch 3−1472 : Selenium on Interleukin−23
rs558133
rs234709
rs6586282
rs3797535
rs6859667
rs567754
rs1789953
rs705415
rs10944
rs672413
rs11951068
rs921943
All
−0.05
0.00
0.05
0.10
0.15
MR effect size for
'Selenium' on 'Interleukin−23'

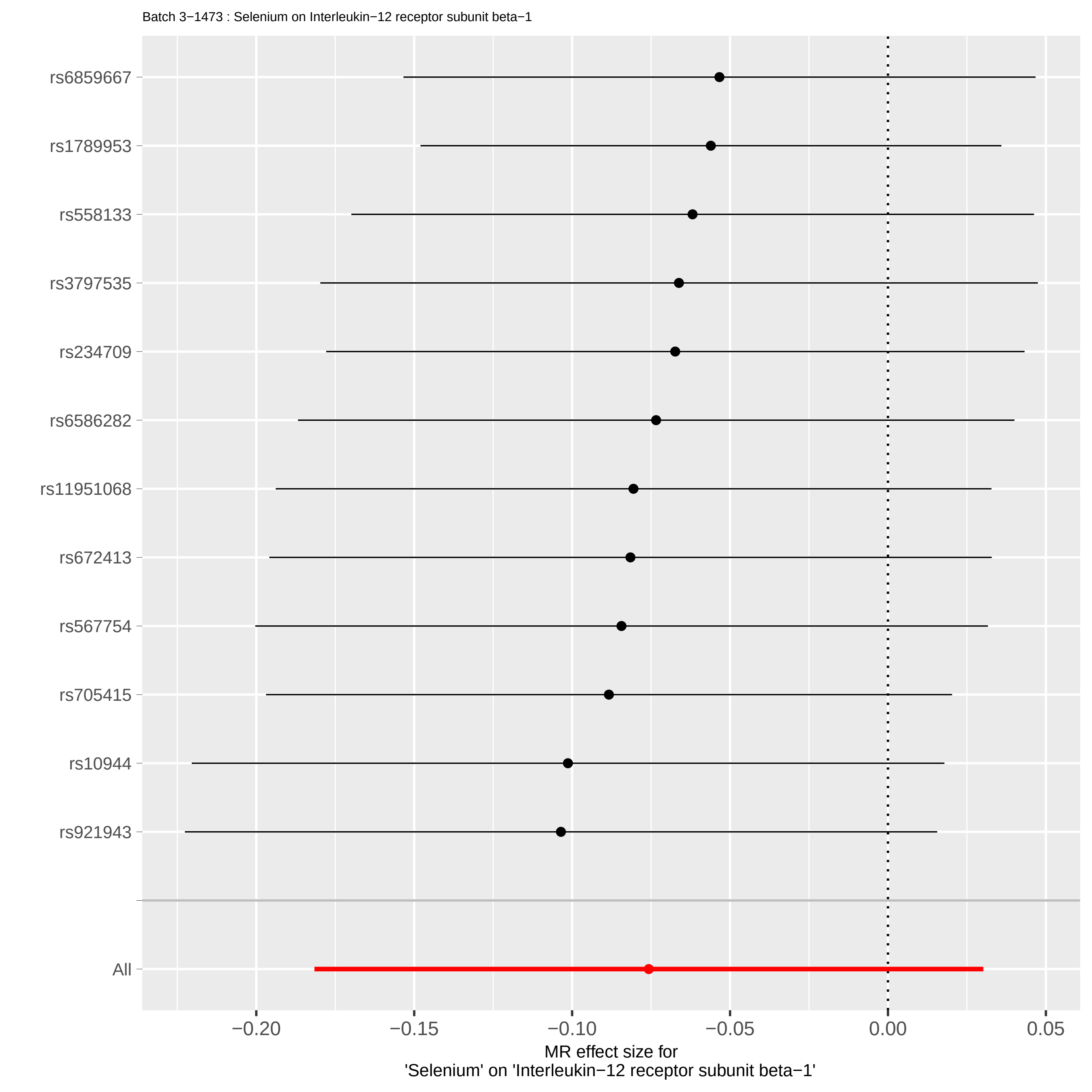

Batch 3−1473 : Selenium on Interleukin−12 receptor subunit beta−1
rs6859667
rs1789953
rs558133
rs3797535
rs234709
rs6586282
rs11951068
rs672413
rs567754
rs705415
rs10944
rs921943
All
−0.20
−0.15
−0.10
−0.05
0.00
0.05
MR effect size for
'Selenium' on 'Interleukin−12 receptor subunit beta−1'

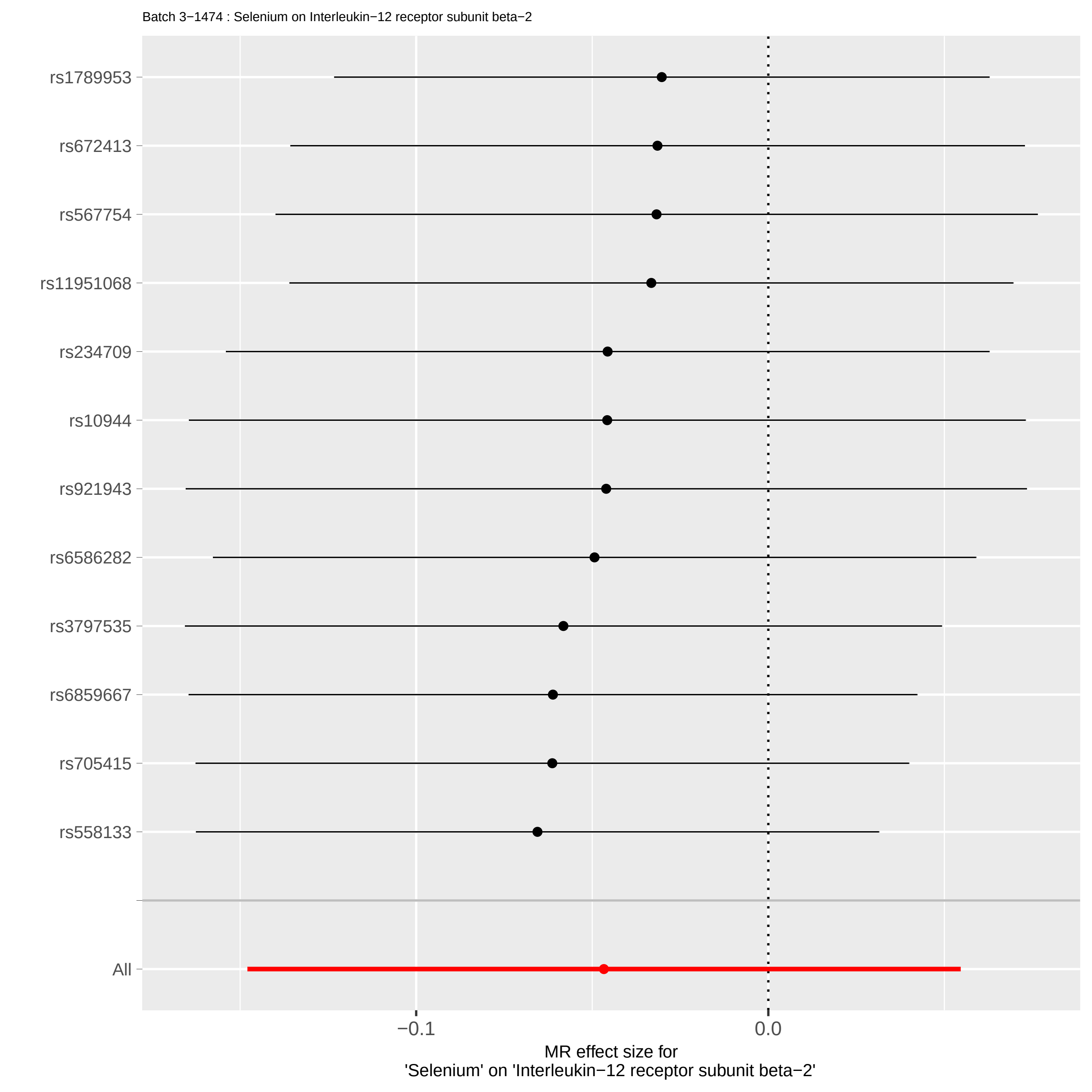

Batch 3−1474 : Selenium on Interleukin−12 receptor subunit beta−2
rs1789953
rs672413
rs567754
rs11951068
rs234709
rs10944
rs921943
rs6586282
rs3797535
rs6859667
rs705415
rs558133
All
−0.1
0.0
MR effect size for
'Selenium' on 'Interleukin−12 receptor subunit beta−2'

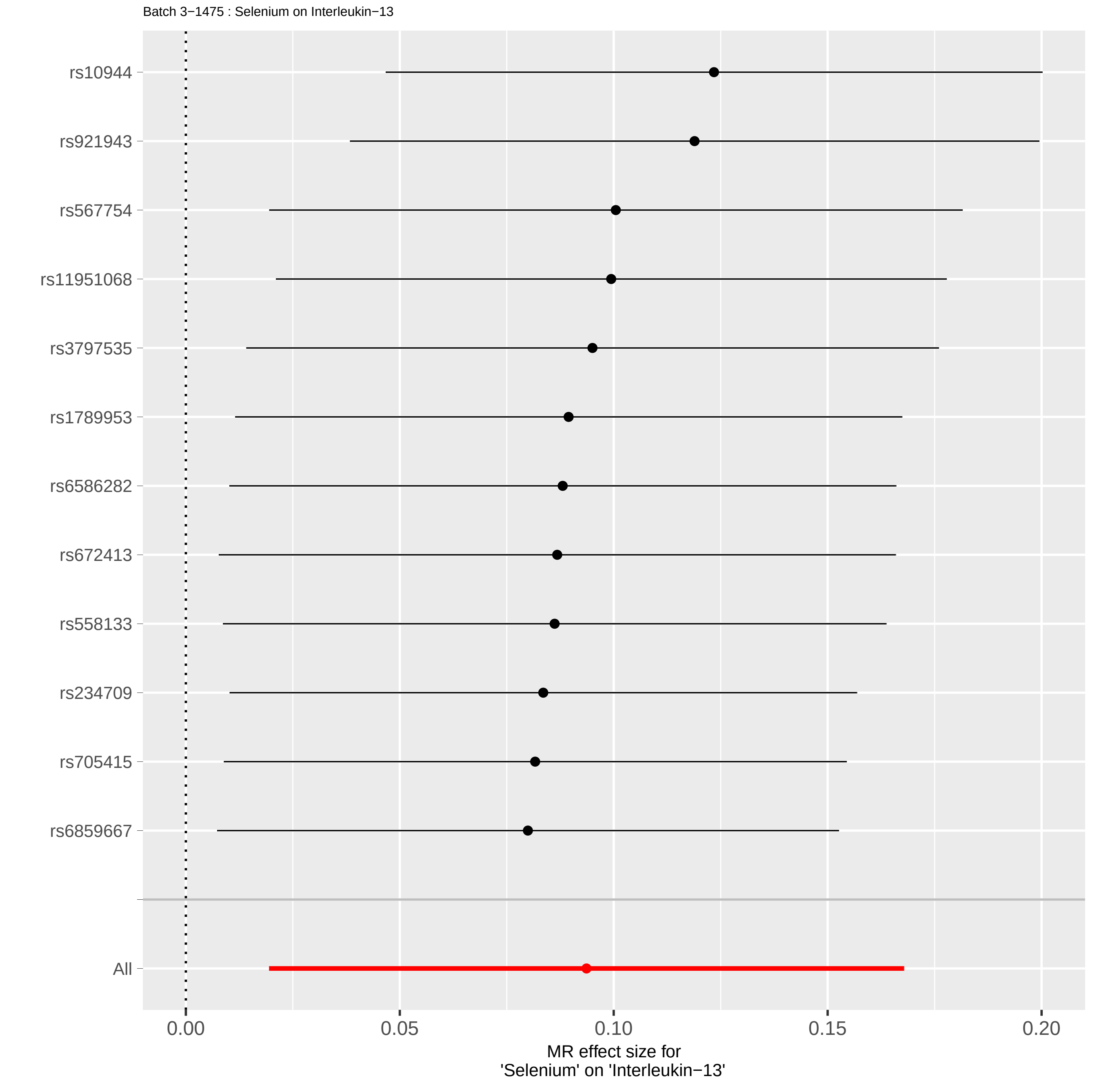

Batch 3−1475 : Selenium on Interleukin−13
rs10944
rs921943
rs567754
rs11951068
rs3797535
rs1789953
rs6586282
rs672413
rs558133
rs234709
rs705415
rs6859667
All
0.00
0.05
0.10
0.15
0.20
MR effect size for
'Selenium' on 'Interleukin−13'

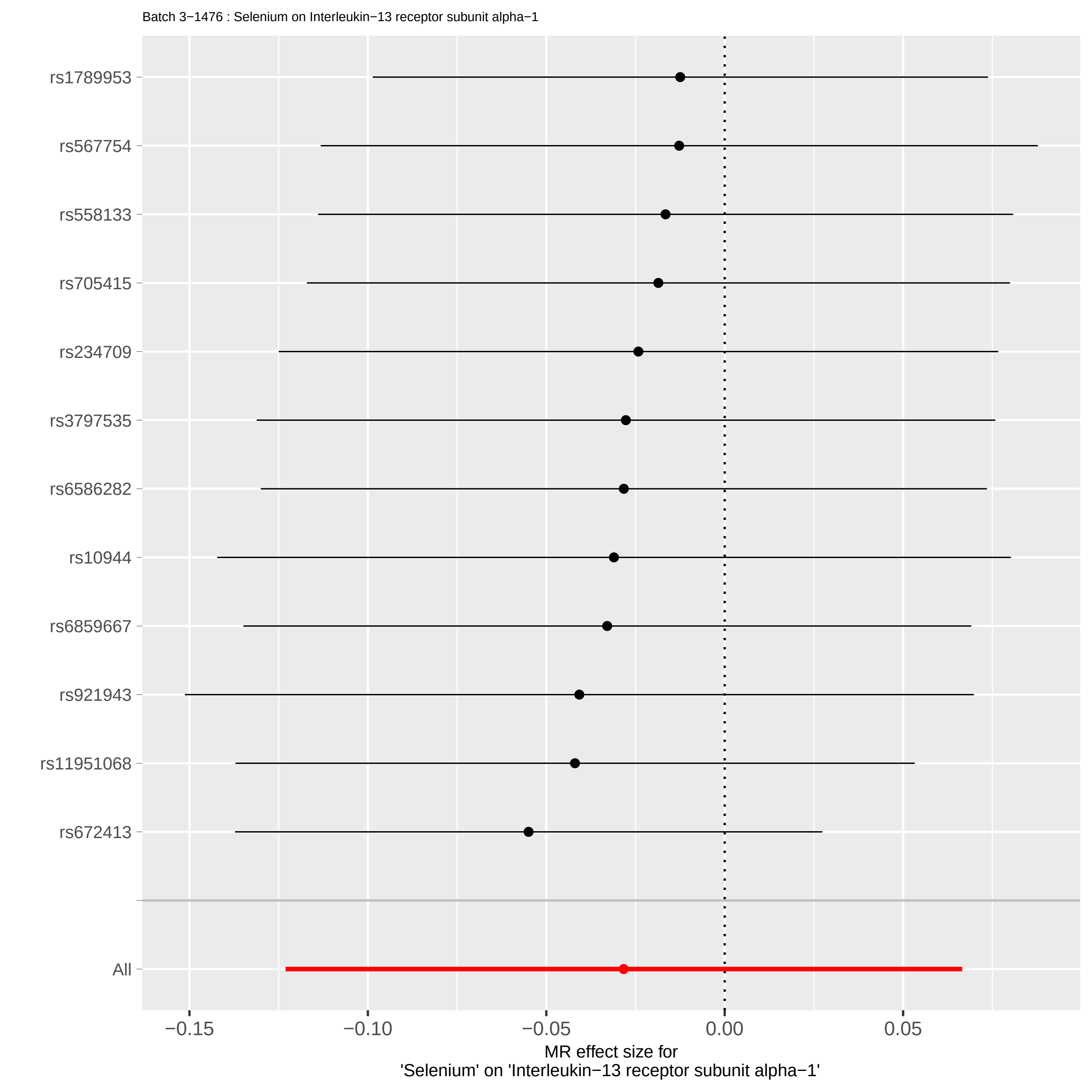

Batch 3−1476 : Selenium on Interleukin−13 receptor subunit alpha−1
rs1789953
rs567754
rs558133
rs705415
rs234709
rs3797535
rs6586282
rs10944
rs6859667
rs921943
rs11951068
rs672413
All
−0.15
−0.10
−0.05
0.00
0.05
MR effect size for
'Selenium' on 'Interleukin−13 receptor subunit alpha−1'

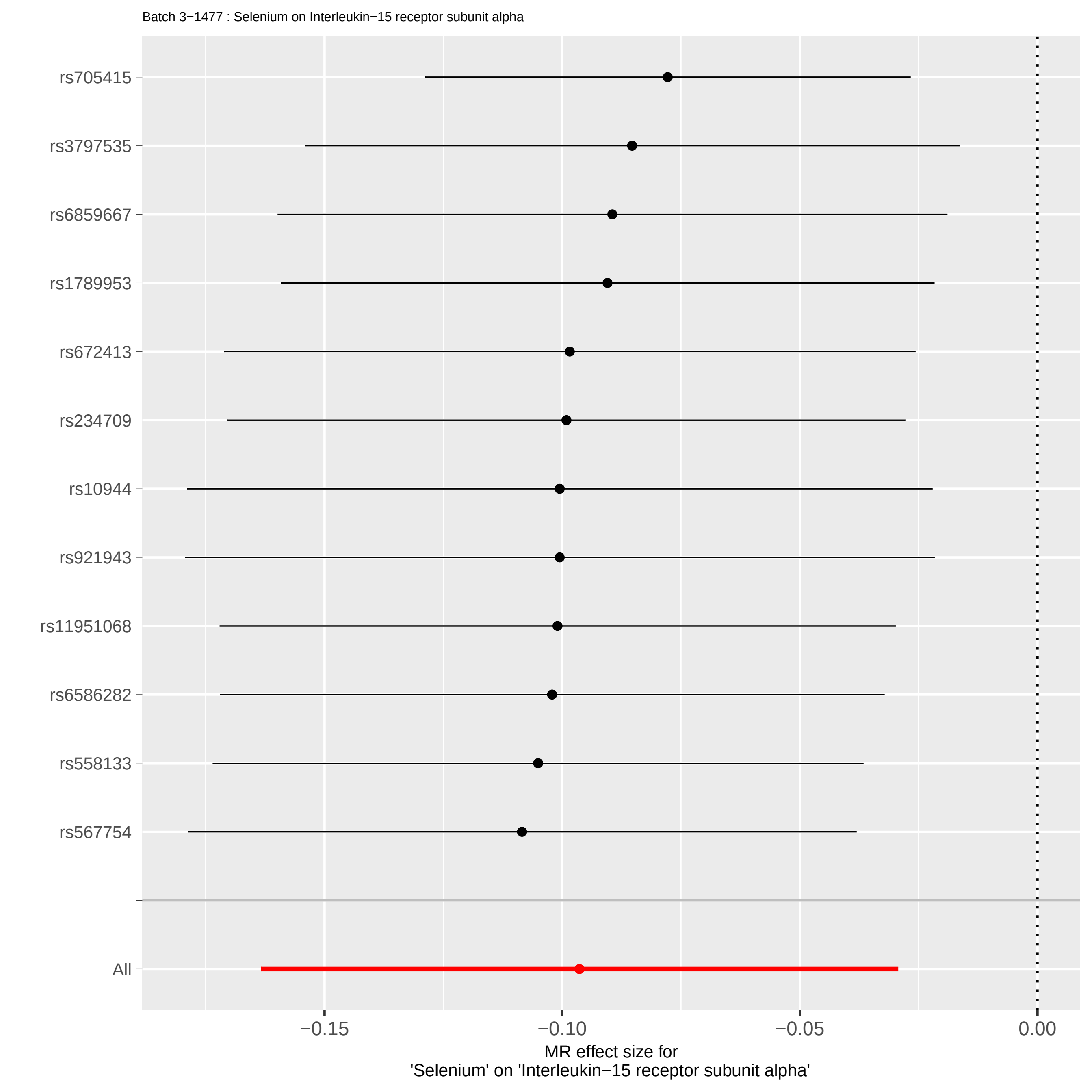

Batch 3−1477 : Selenium on Interleukin−15 receptor subunit alpha
rs705415
rs3797535
rs6859667
rs1789953
rs672413
rs234709
rs10944
rs921943
rs11951068
rs6586282
rs558133
rs567754
All
−0.15
−0.10
−0.05
0.00
MR effect size for
'Selenium' on 'Interleukin−15 receptor subunit alpha'

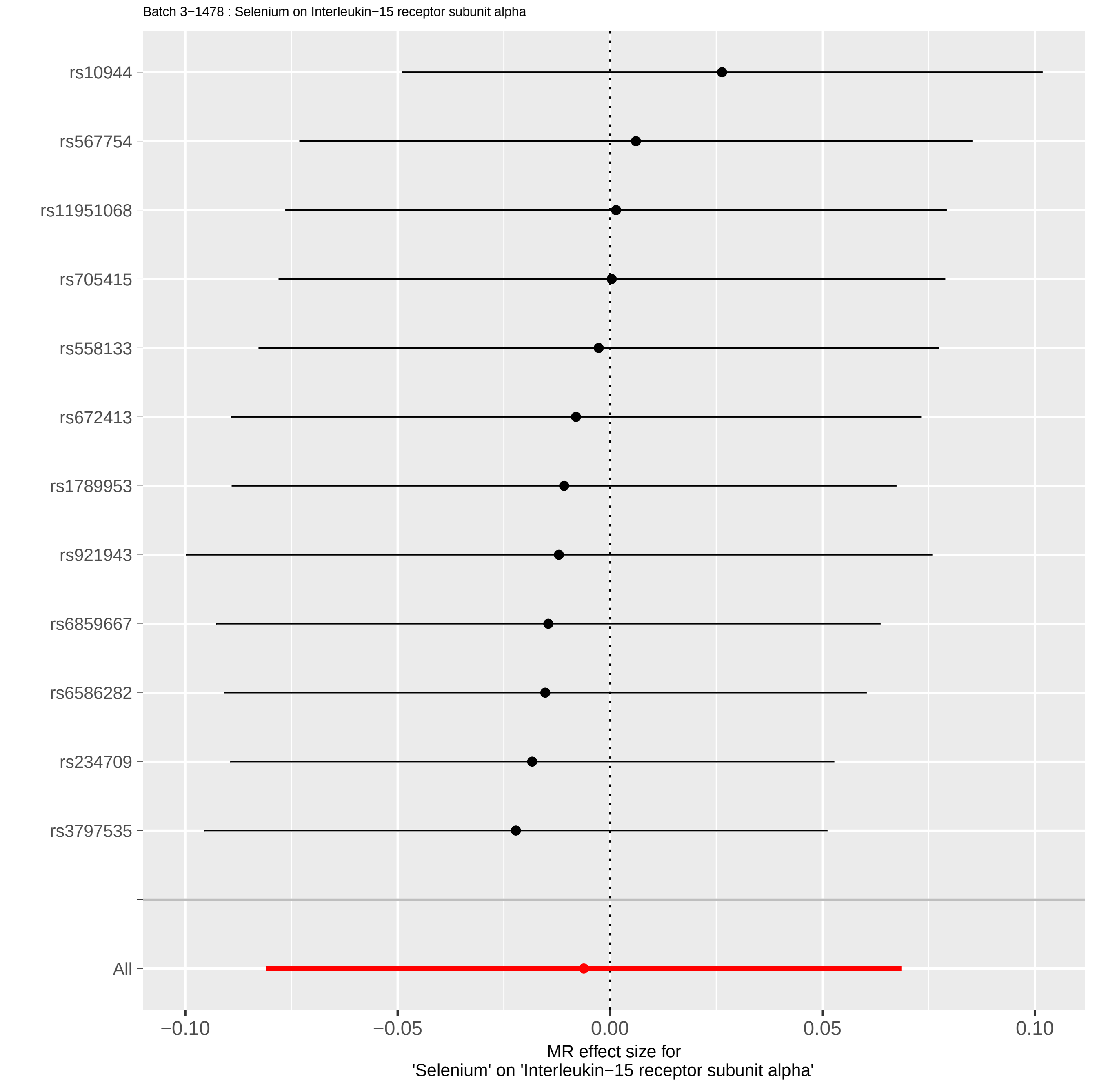

Batch 3−1478 : Selenium on Interleukin−15 receptor subunit alpha
rs10944
rs567754
rs11951068
rs705415
rs558133
rs672413
rs1789953
rs921943
rs6859667
rs6586282
rs234709
rs3797535
All
−0.10
−0.05
0.00
0.05
0.10
MR effect size for
'Selenium' on 'Interleukin−15 receptor subunit alpha'

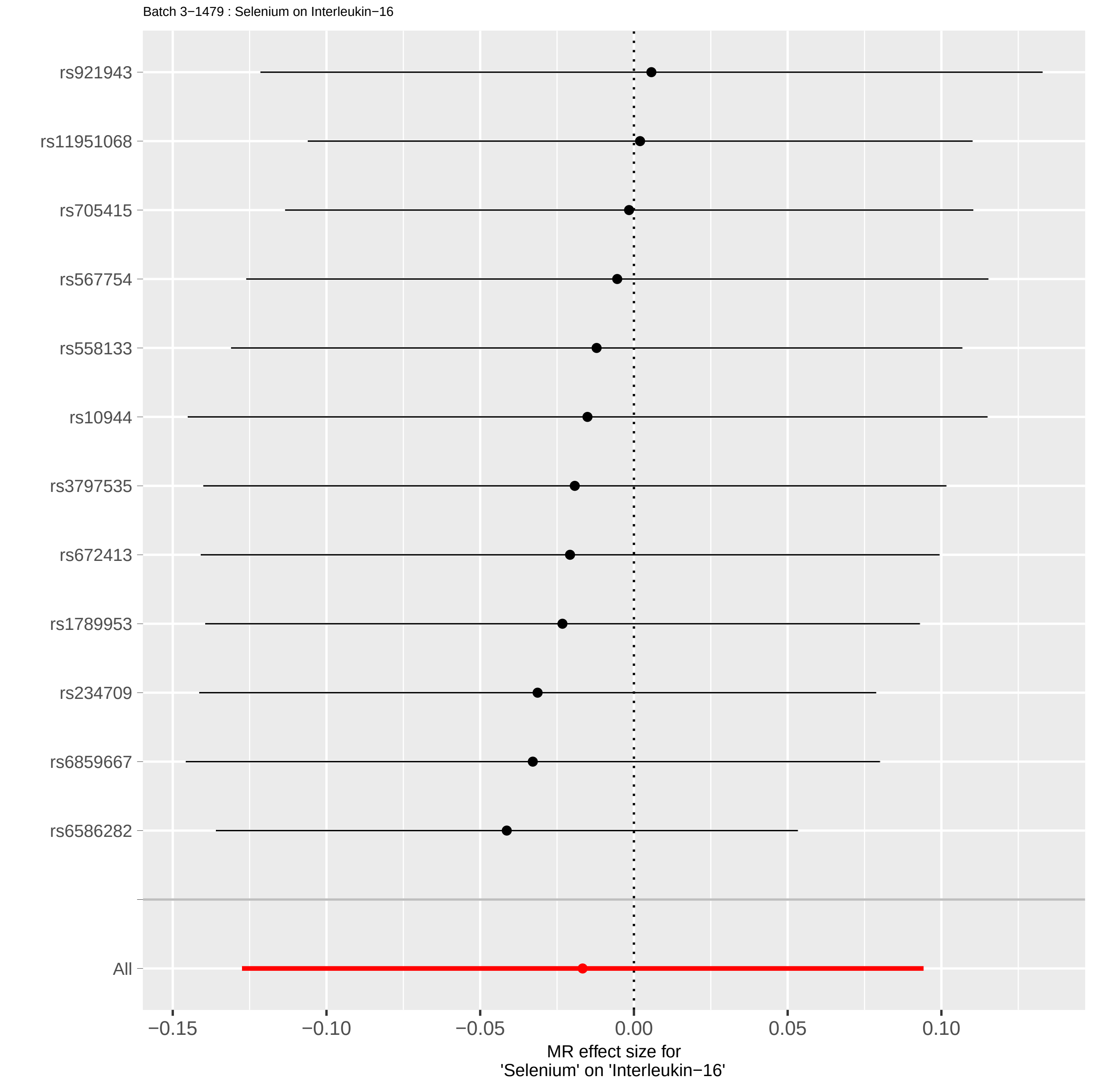

Batch 3−1479 : Selenium on Interleukin−16
rs921943
rs11951068
rs705415
rs567754
rs558133
rs10944
rs3797535
rs672413
rs1789953
rs234709
rs6859667
rs6586282
All
−0.15
−0.10
−0.05
0.00
0.05
0.10
MR effect size for
'Selenium' on 'Interleukin−16'

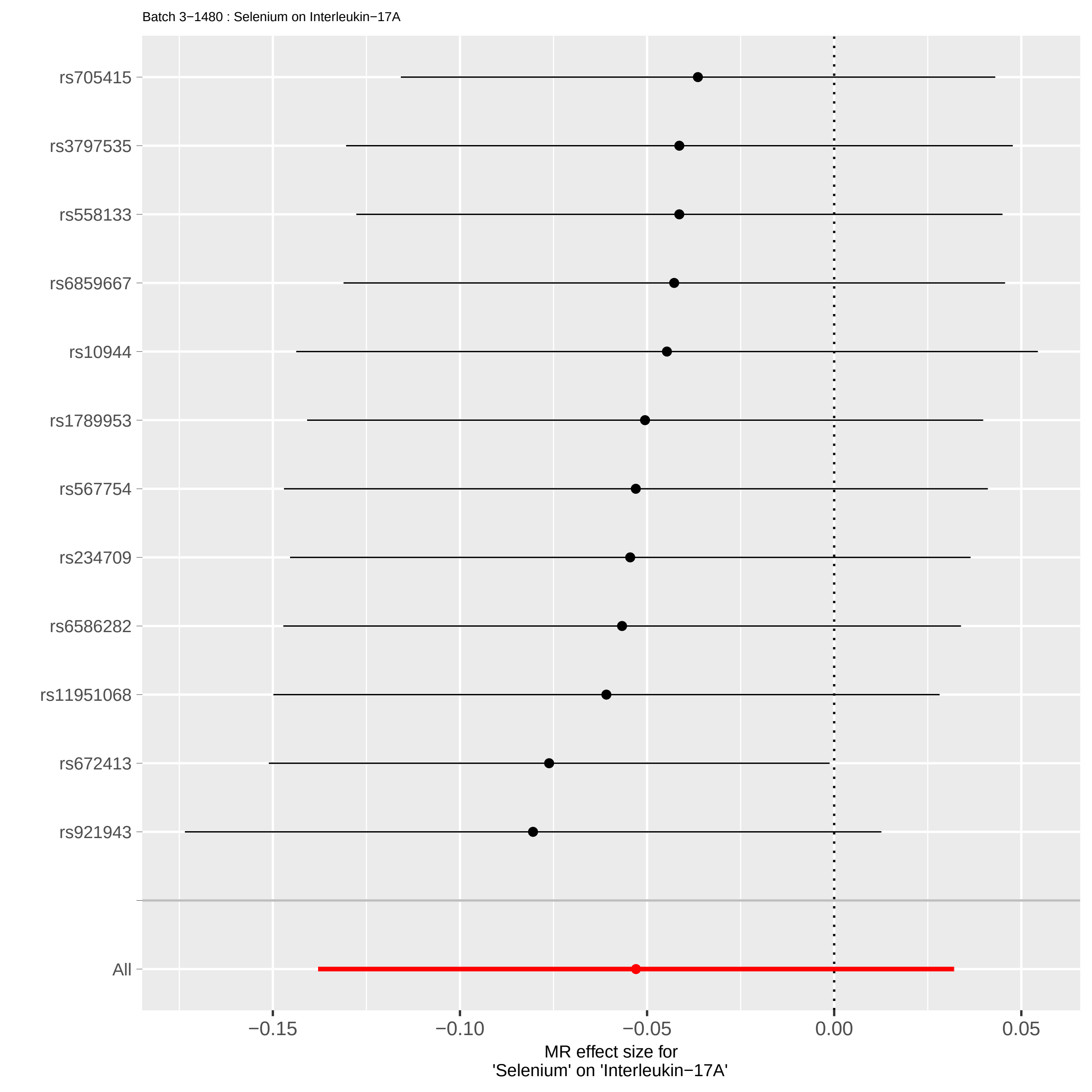
Batch 3−1480 : Selenium on Interleukin−17A
rs705415
rs3797535
rs558133
rs6859667
rs10944
rs1789953
rs567754
rs234709
rs6586282
rs11951068
rs672413
rs921943
All
−0.15
−0.10
−0.05
0.00
0.05
MR effect size for
'Selenium' on 'Interleukin−17A'

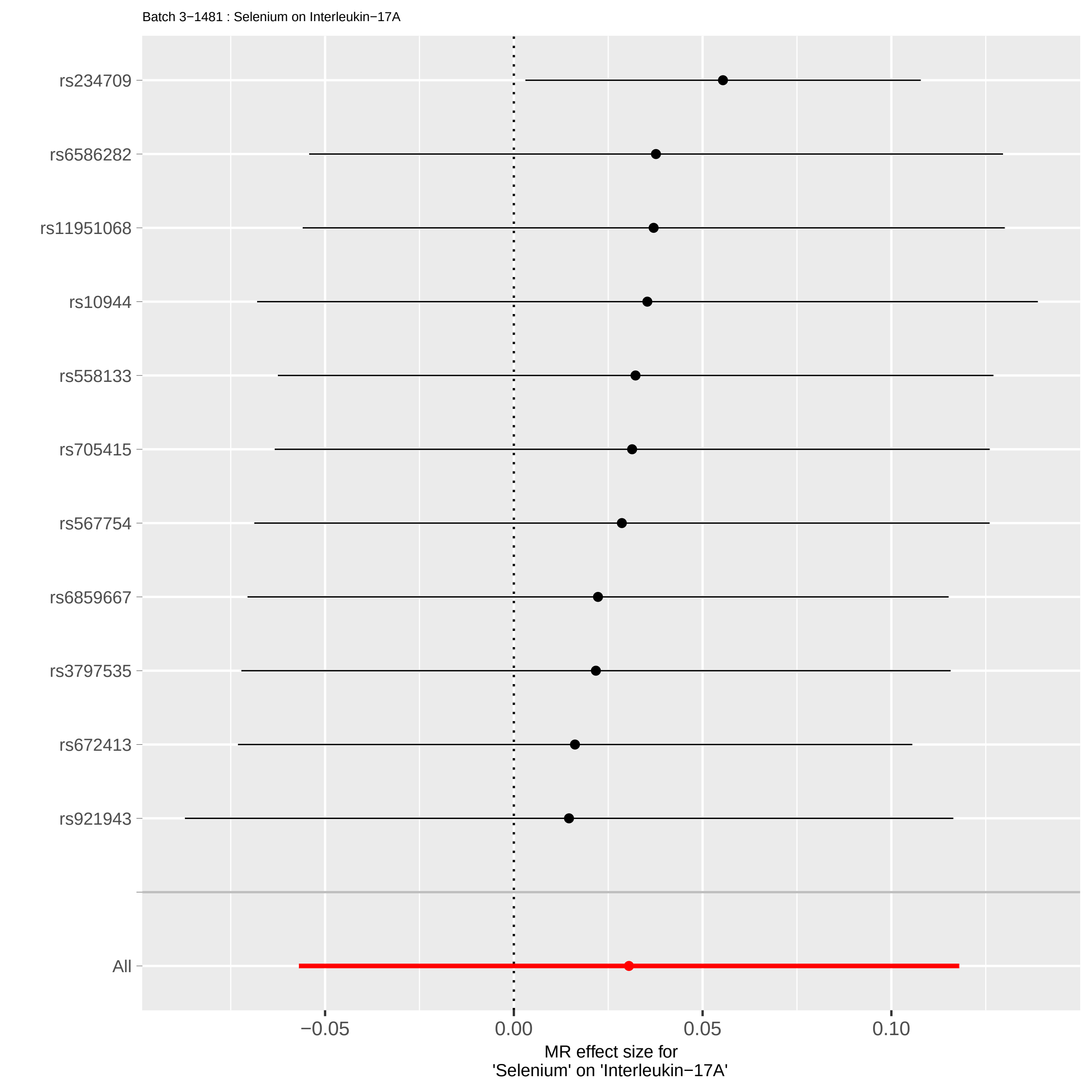

Batch 3−1481 : Selenium on Interleukin−17A
rs234709
rs6586282
rs11951068
rs10944
rs558133
rs705415
rs567754
rs6859667
rs3797535
rs672413
rs921943
All
−0.05
0.00
0.05
0.10
MR effect size for
'Selenium' on 'Interleukin−17A'

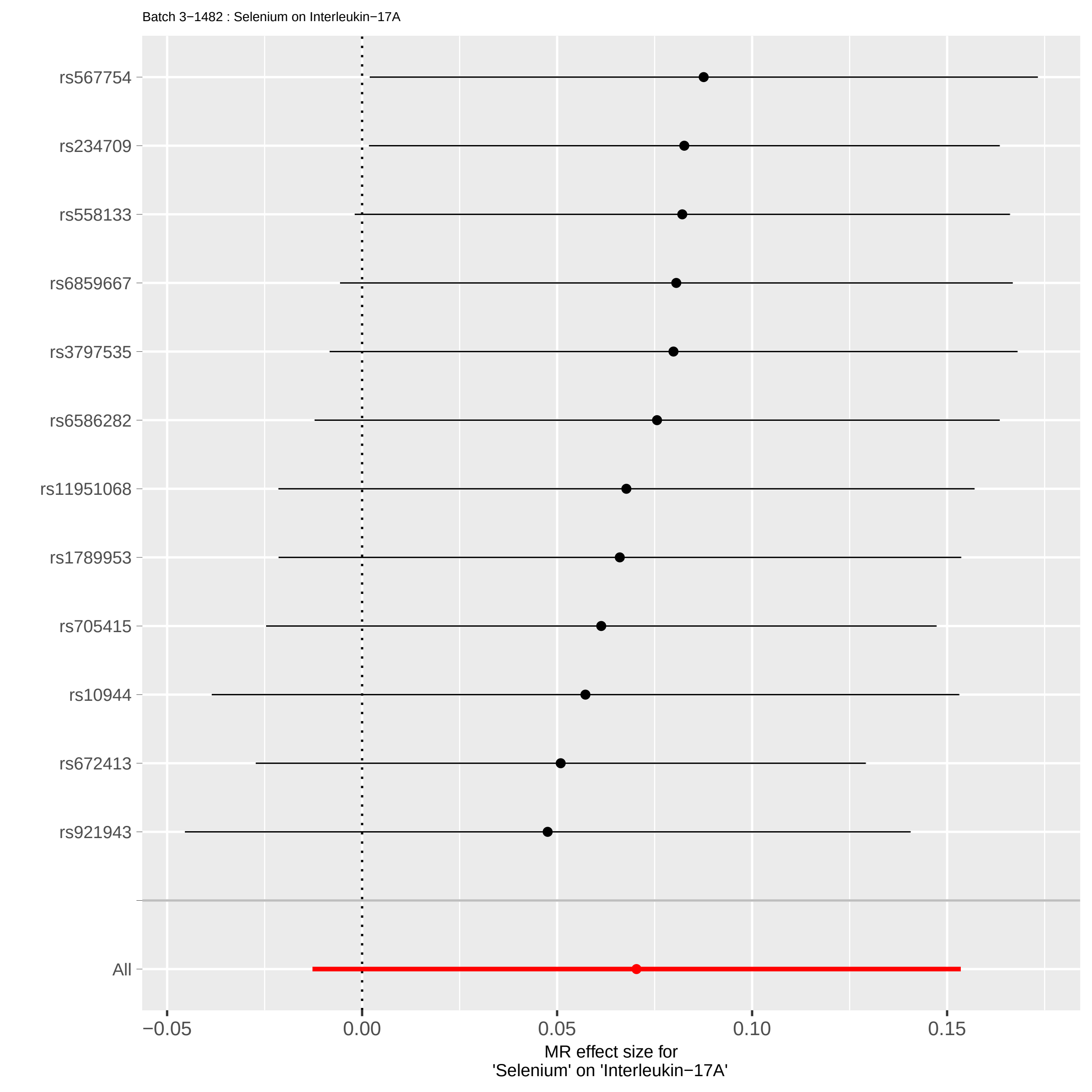

Batch 3−1482 : Selenium on Interleukin−17A
rs567754
rs234709
rs558133
rs6859667
rs3797535
rs6586282
rs11951068
rs1789953
rs705415
rs10944
rs672413
rs921943
All
−0.05
0.00
0.05
0.10
0.15
MR effect size for
'Selenium' on 'Interleukin−17A'

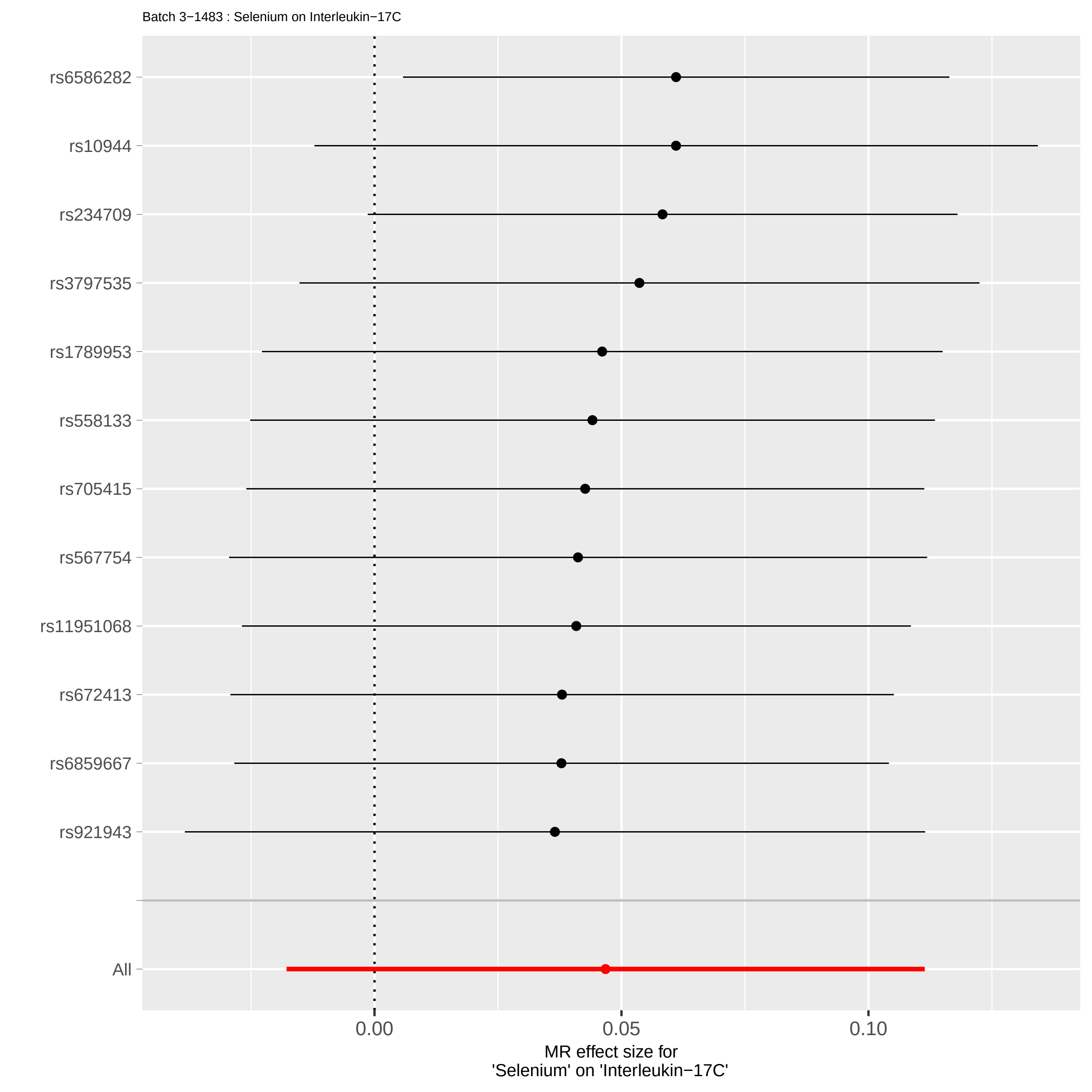

Batch 3−1483 : Selenium on Interleukin−17C
rs6586282
rs10944
rs234709
rs3797535
rs1789953
rs558133
rs705415
rs567754
rs11951068
rs672413
rs6859667
rs921943
All
0.00
0.05
0.10
MR effect size for
'Selenium' on 'Interleukin−17C'

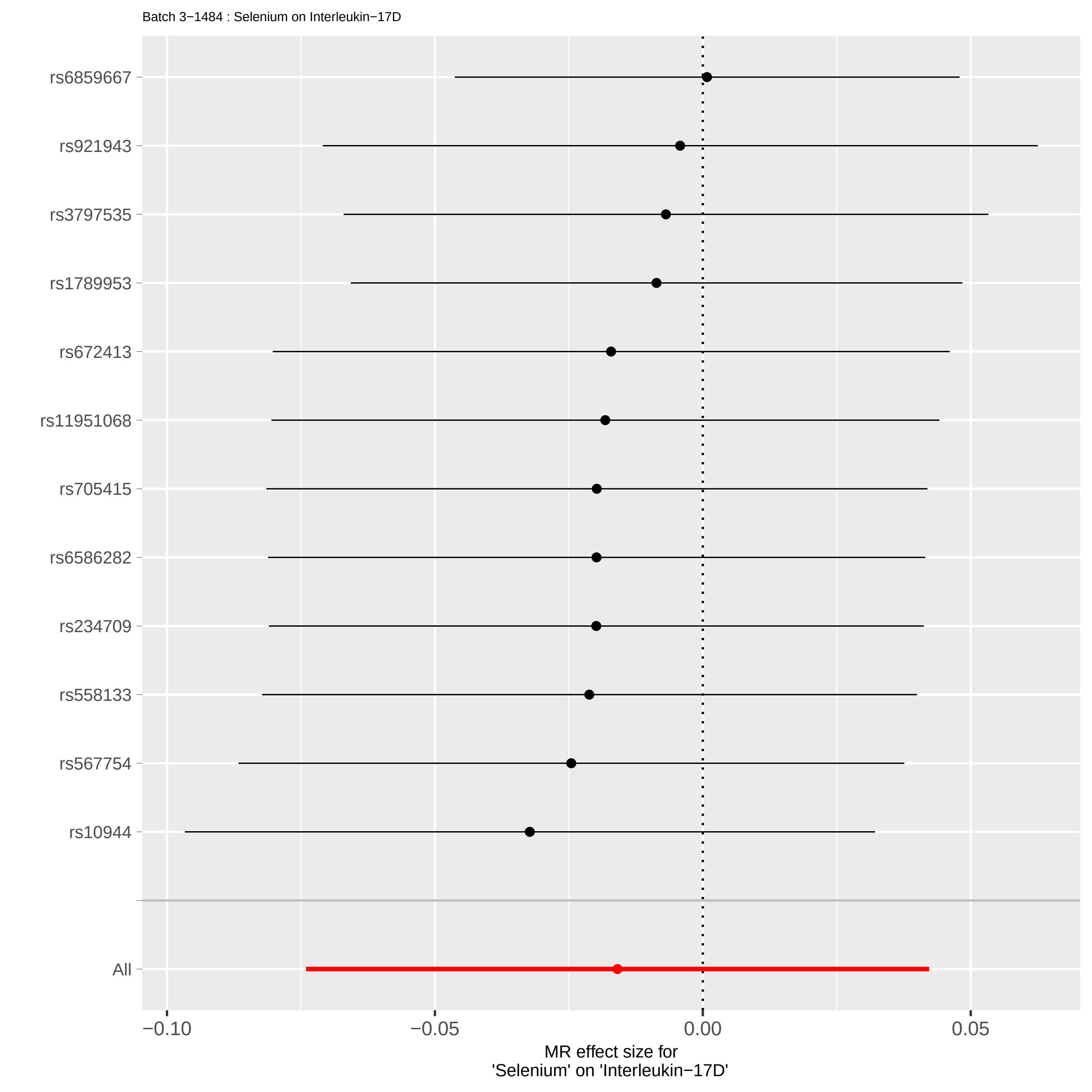
Batch 3−1484 : Selenium on Interleukin−17D
rs6859667
rs921943
rs3797535
rs1789953
rs672413
rs11951068
rs705415
rs6586282
rs234709
rs558133
rs567754
rs10944
All
−0.10
−0.05
0.00
0.05
MR effect size for
'Selenium' on 'Interleukin−17D'

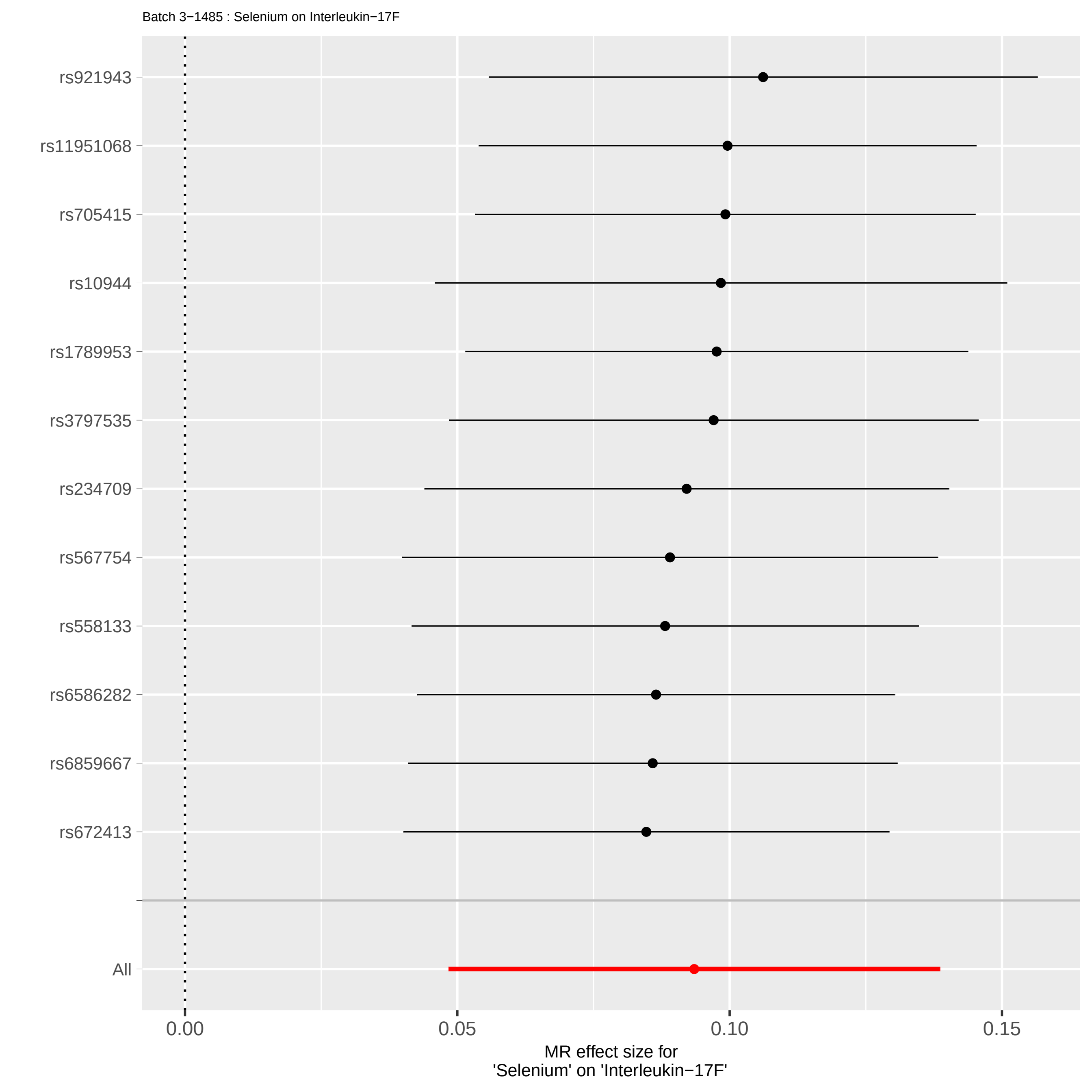

Batch 3−1485 : Selenium on Interleukin−17F
rs921943
rs11951068
rs705415
rs10944
rs1789953
rs3797535
rs234709
rs567754
rs558133
rs6586282
rs6859667
rs672413
All
0.00
0.05
0.10
0.15
MR effect size for
'Selenium' on 'Interleukin−17F'

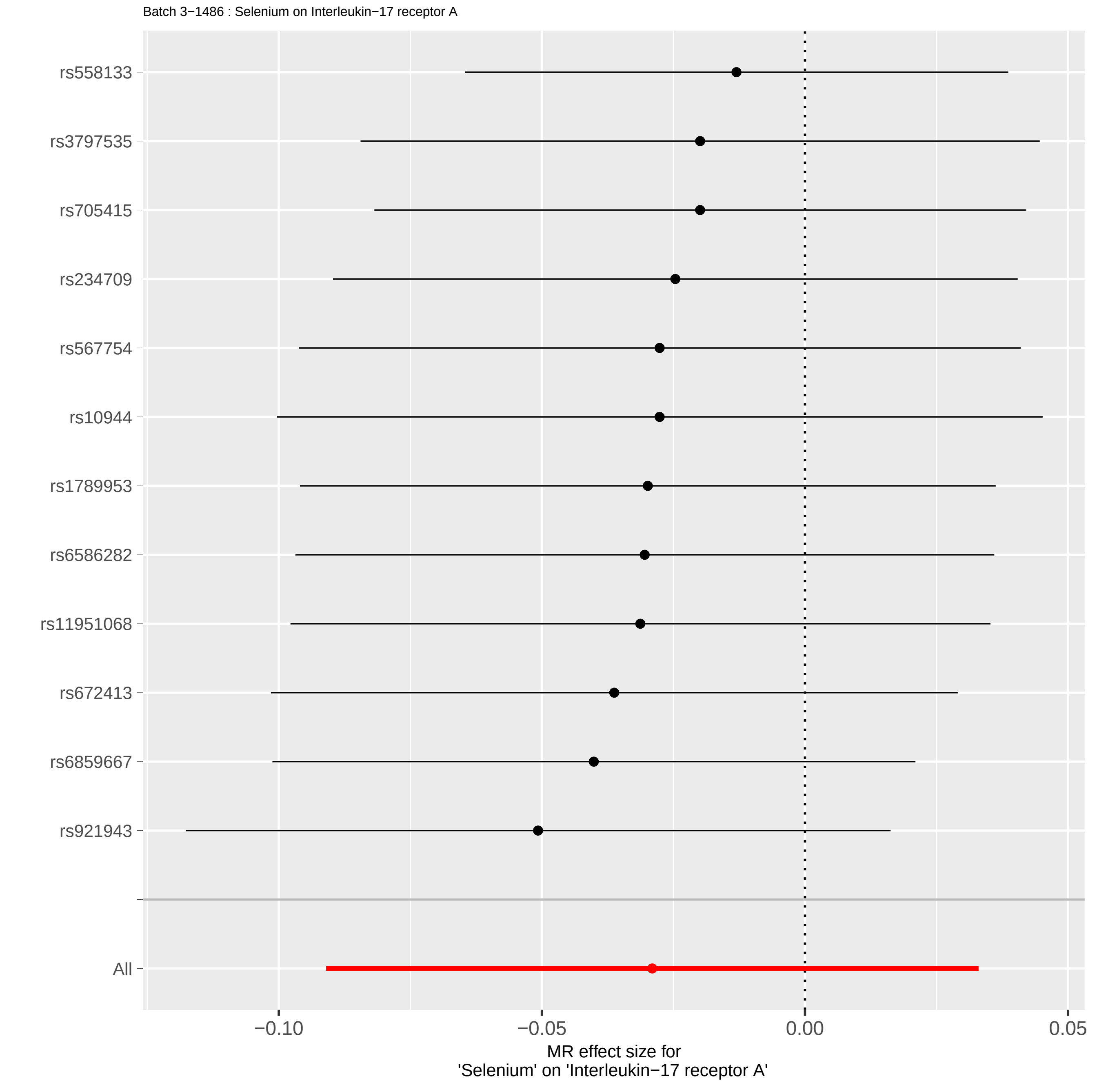

Batch 3−1486 : Selenium on Interleukin−17 receptor A
rs558133
rs3797535
rs705415
rs234709
rs567754
rs10944
rs1789953
rs6586282
rs11951068
rs672413
rs6859667
rs921943
All
−0.10
−0.05
0.00
0.05
MR effect size for
'Selenium' on 'Interleukin−17 receptor A'

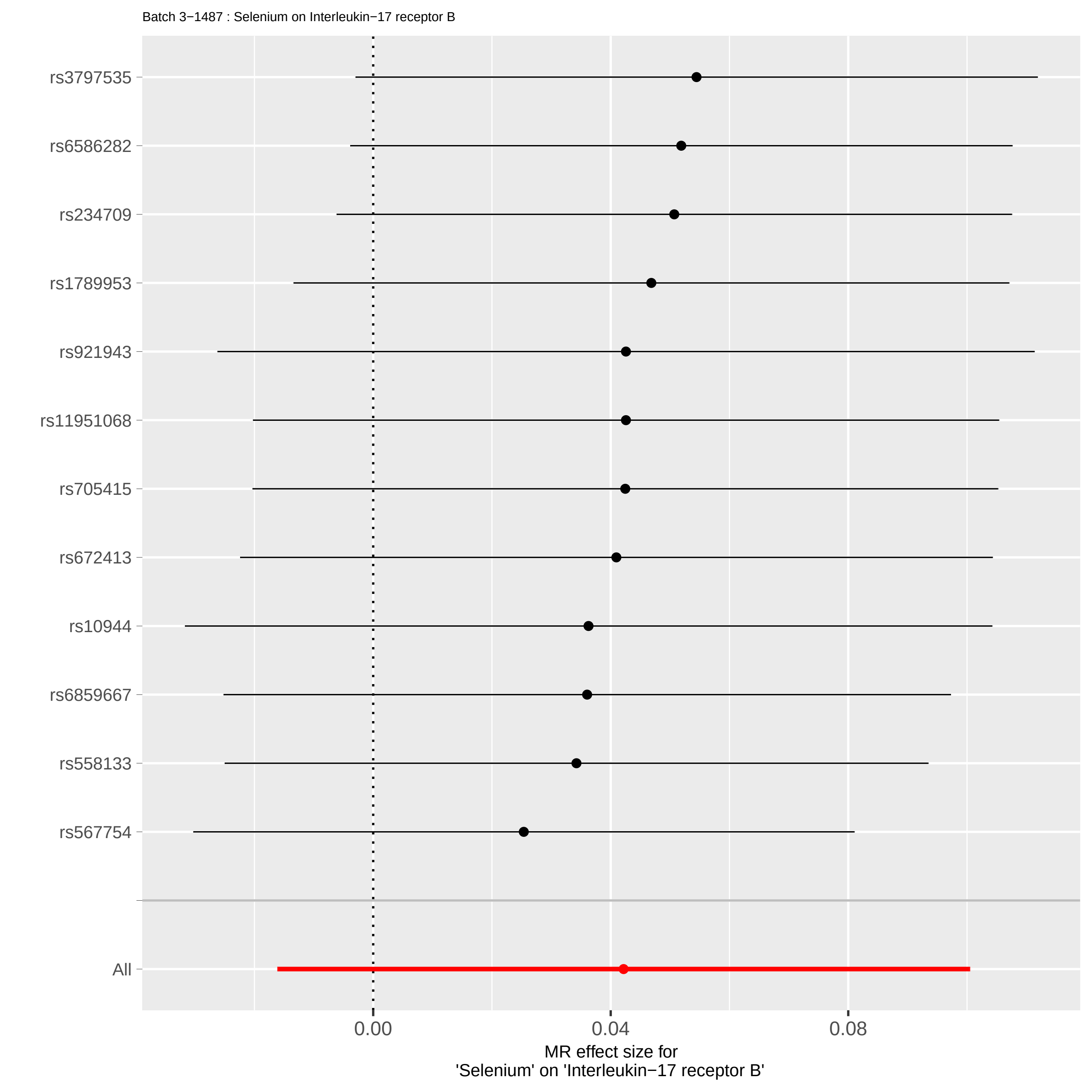

Batch 3−1487 : Selenium on Interleukin−17 receptor B
rs3797535
rs6586282
rs234709
rs1789953
rs921943
rs11951068
rs705415
rs672413
rs10944
rs6859667
rs558133
rs567754
All
0.00
0.04
0.08
MR effect size for
'Selenium' on 'Interleukin−17 receptor B'

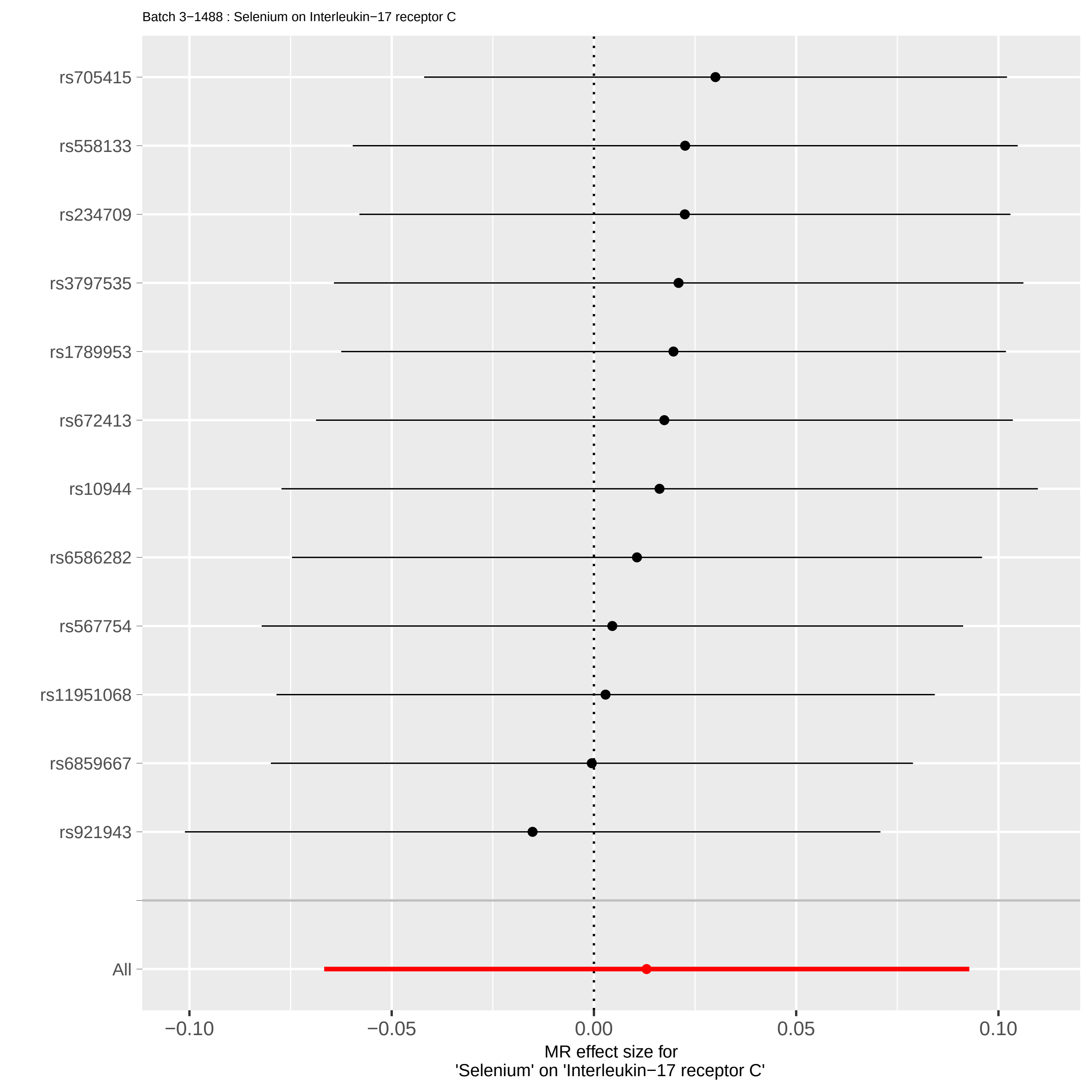

Batch 3−1488 : Selenium on Interleukin−17 receptor C
rs705415
rs558133
rs234709
rs3797535
rs1789953
rs672413
rs10944
rs6586282
rs567754
rs11951068
rs6859667
rs921943
All
−0.10
−0.05
0.00
0.05
0.10
MR effect size for
'Selenium' on 'Interleukin−17 receptor C'

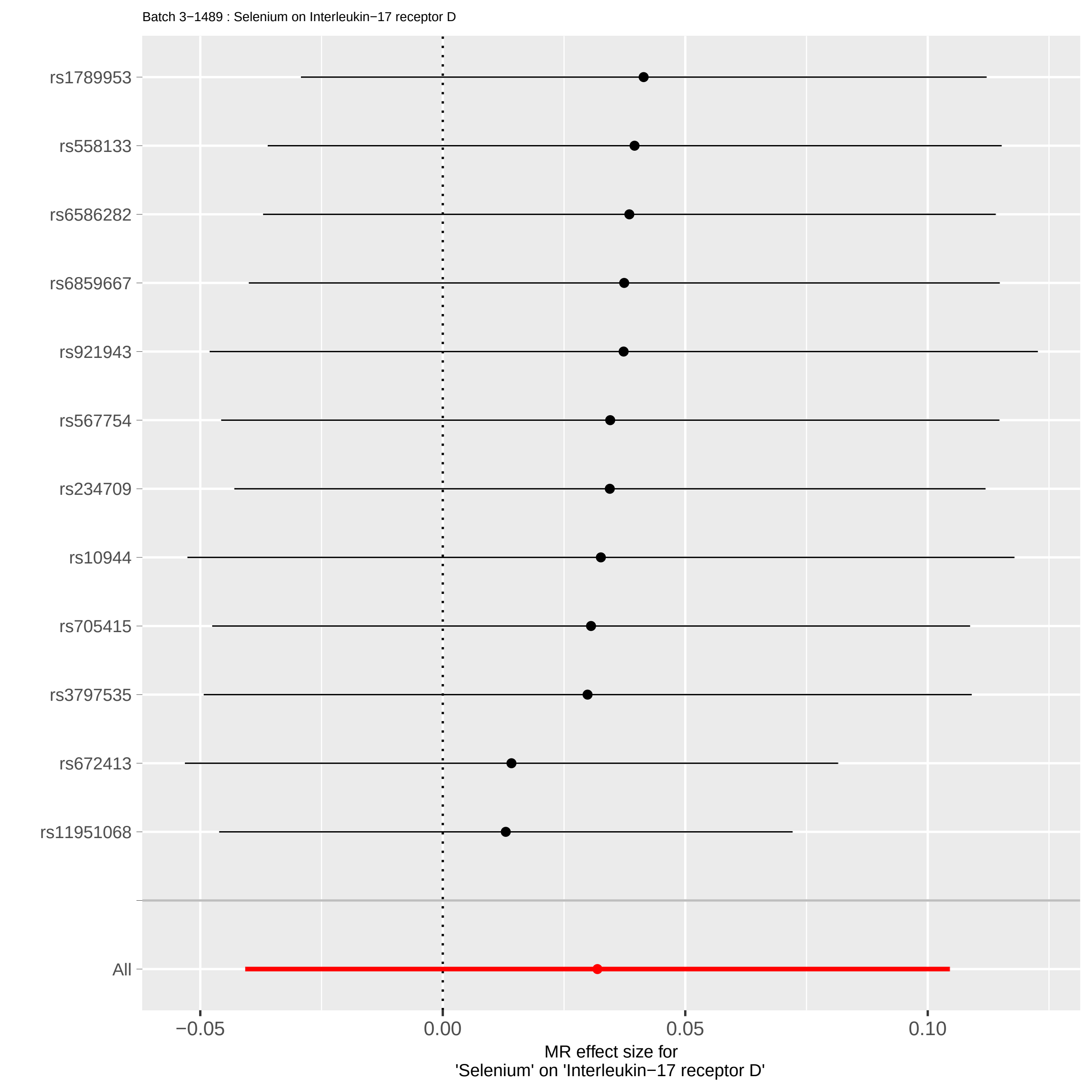

Batch 3−1489 : Selenium on Interleukin−17 receptor D
rs1789953
rs558133
rs6586282
rs6859667
rs921943
rs567754
rs234709
rs10944
rs705415
rs3797535
rs672413
rs11951068
All
−0.05
0.00
0.05
0.10
MR effect size for
'Selenium' on 'Interleukin−17 receptor D'

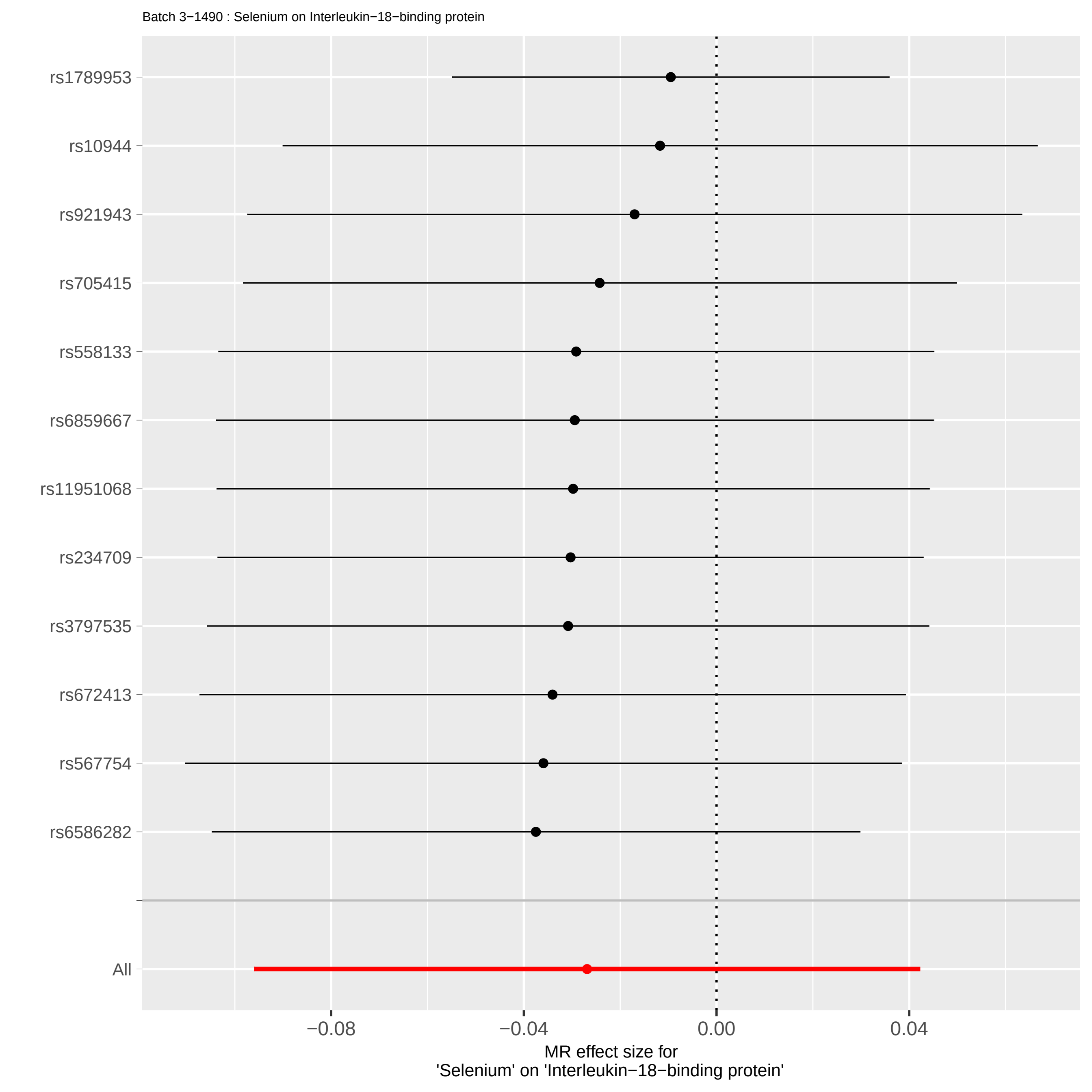

Batch 3−1490 : Selenium on Interleukin−18−binding protein
rs1789953
rs10944
rs921943
rs705415
rs558133
rs6859667
rs11951068
rs234709
rs3797535
rs672413
rs567754
rs6586282
All
−0.08
−0.04
0.00
0.04
MR effect size for
'Selenium' on 'Interleukin−18−binding protein'

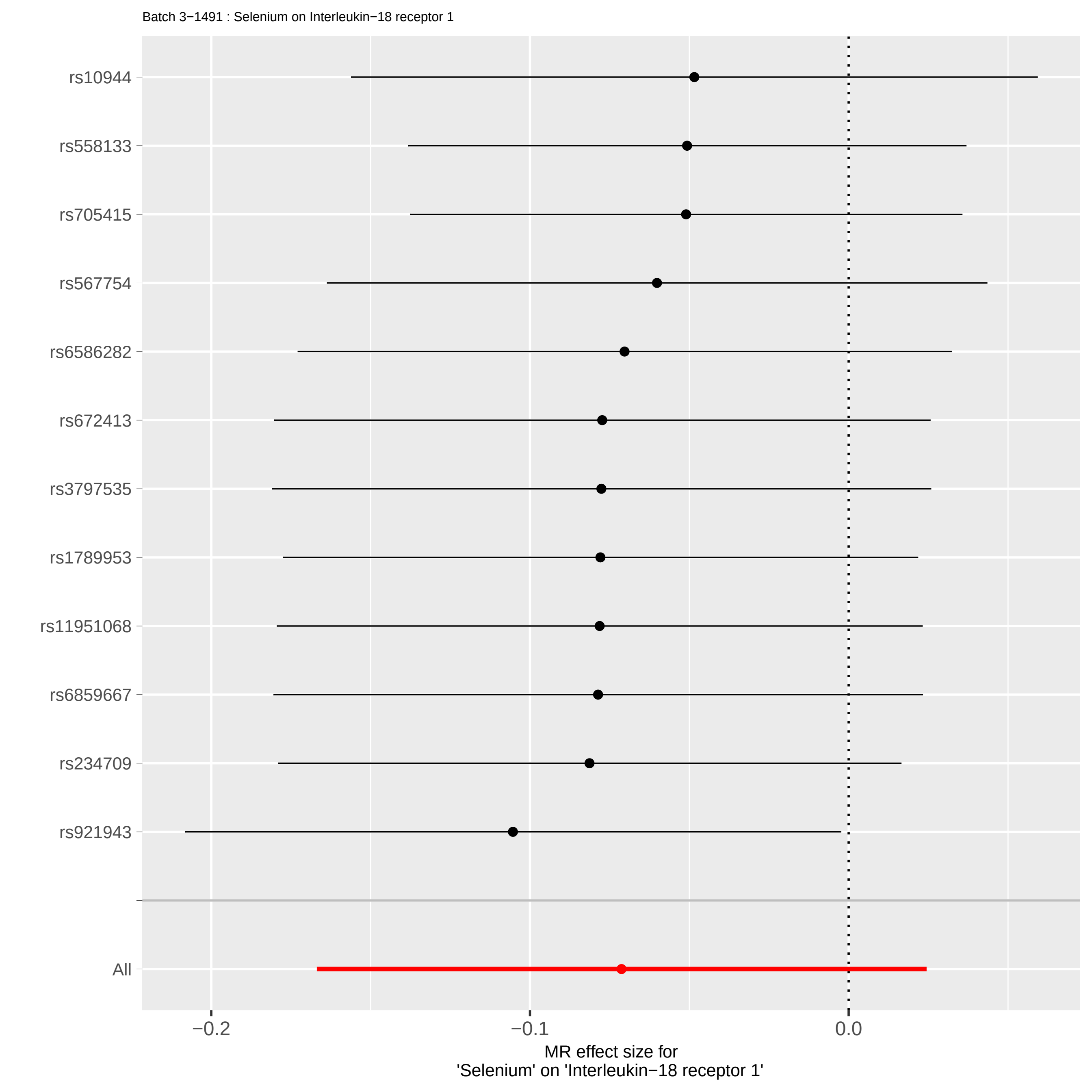

Batch 3−1491 : Selenium on Interleukin−18 receptor 1
rs10944
rs558133
rs705415
rs567754
rs6586282
rs672413
rs3797535
rs1789953
rs11951068
rs6859667
rs234709
rs921943
All
−0.2
−0.1
0.0
MR effect size for
'Selenium' on 'Interleukin−18 receptor 1'

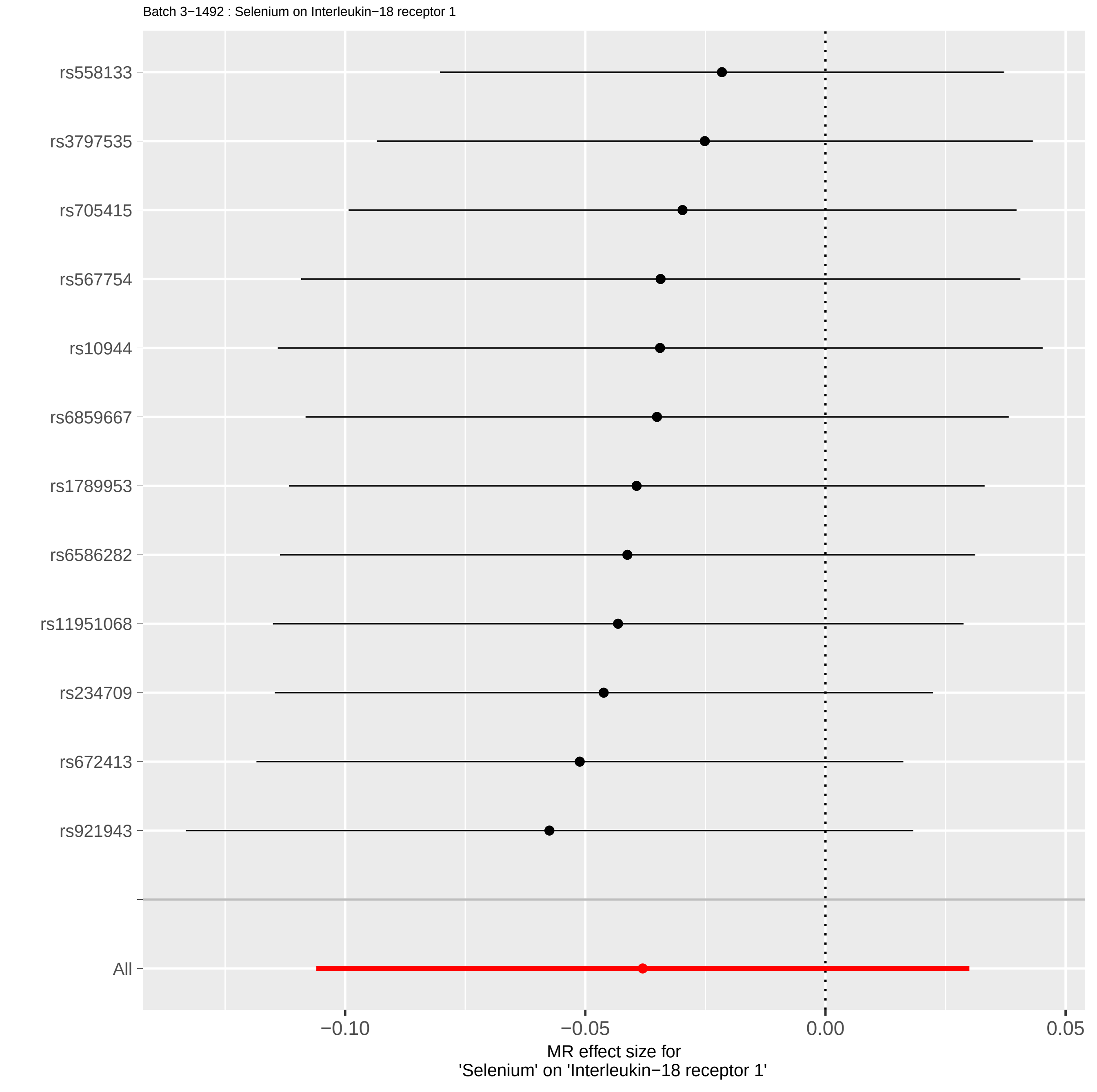

Batch 3−1492 : Selenium on Interleukin−18 receptor 1
rs558133
rs3797535
rs705415
rs567754
rs10944
rs6859667
rs1789953
rs6586282
rs11951068
rs234709
rs672413
rs921943
All
−0.10
−0.05
0.00
0.05
MR effect size for
'Selenium' on 'Interleukin−18 receptor 1'

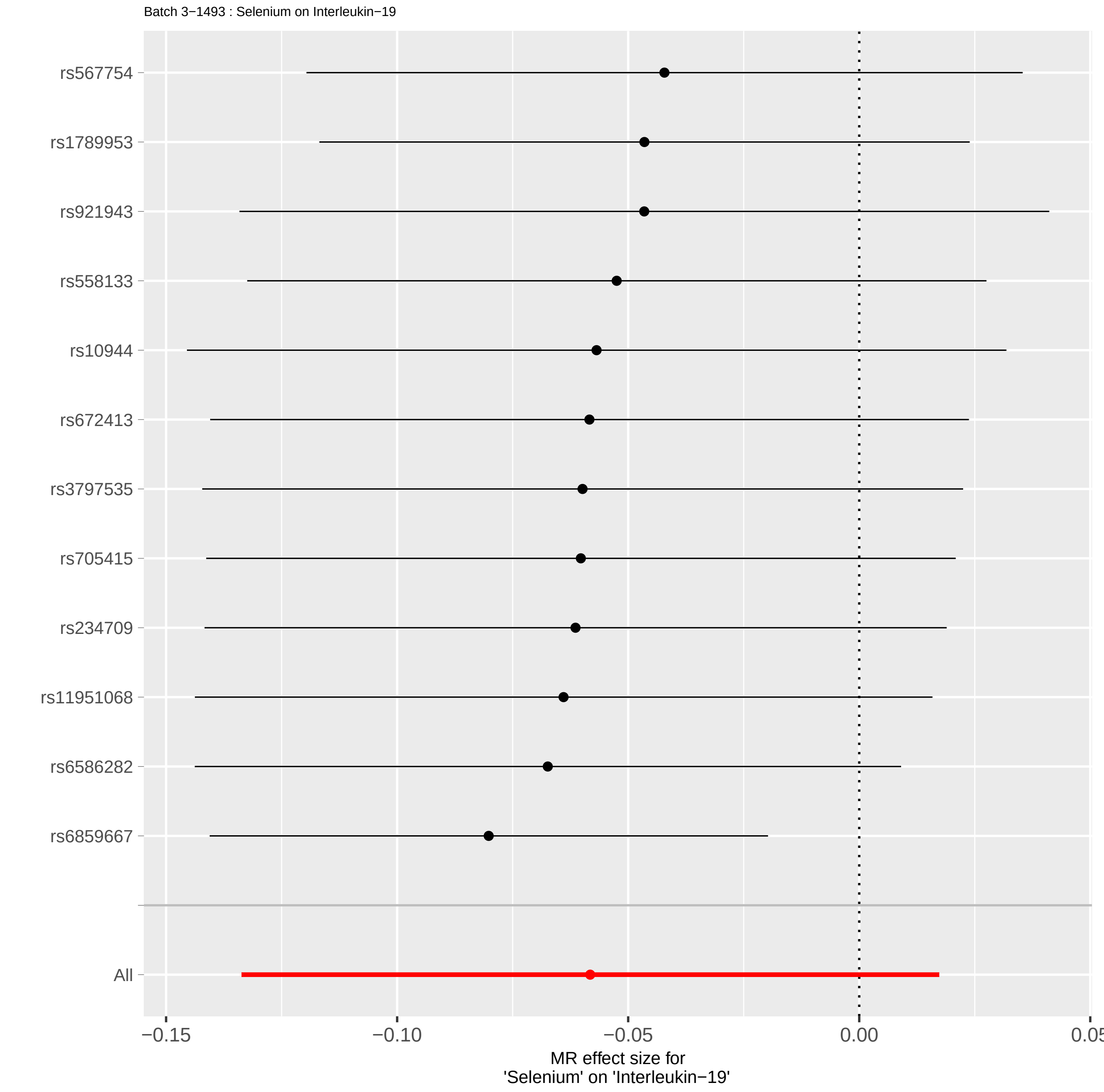

Batch 3−1493 : Selenium on Interleukin−19
rs567754
rs1789953
rs921943
rs558133
rs10944
rs672413
rs3797535
rs705415
rs234709
rs11951068
rs6586282
rs6859667
All
−0.15
−0.10
−0.05
0.00
0.05
MR effect size for
'Selenium' on 'Interleukin−19'

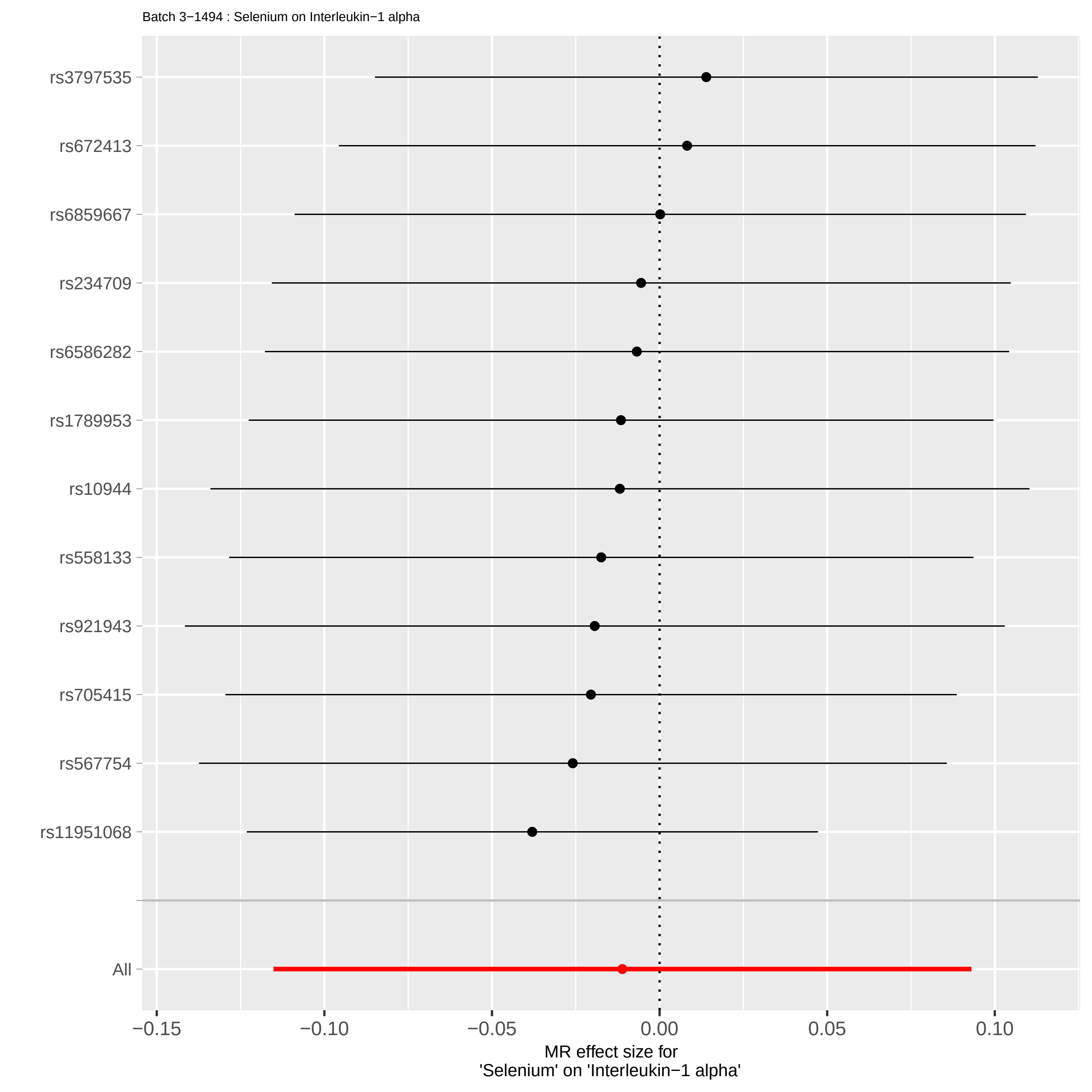

Batch 3−1494 : Selenium on Interleukin−1 alpha
rs3797535
rs672413
rs6859667
rs234709
rs6586282
rs1789953
rs10944
rs558133
rs921943
rs705415
rs567754
rs11951068
All
−0.15
−0.10
−0.05
0.00
0.05
0.10
MR effect size for
'Selenium' on 'Interleukin−1 alpha'

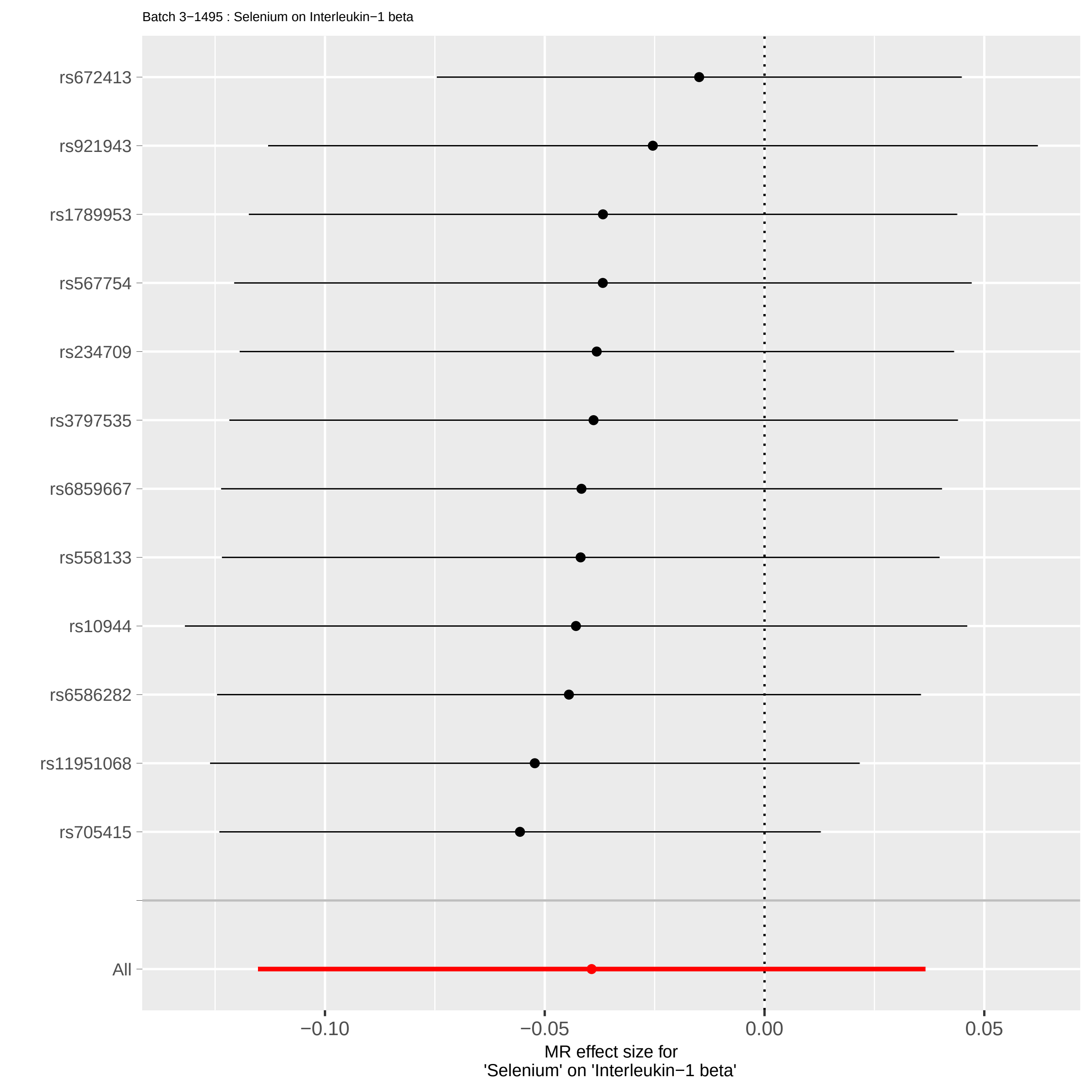

Batch 3−1495 : Selenium on Interleukin−1 beta
rs672413
rs921943
rs1789953
rs567754
rs234709
rs3797535
rs6859667
rs558133
rs10944
rs6586282
rs11951068
rs705415
All
−0.10
−0.05
0.00
0.05
MR effect size for
'Selenium' on 'Interleukin−1 beta'

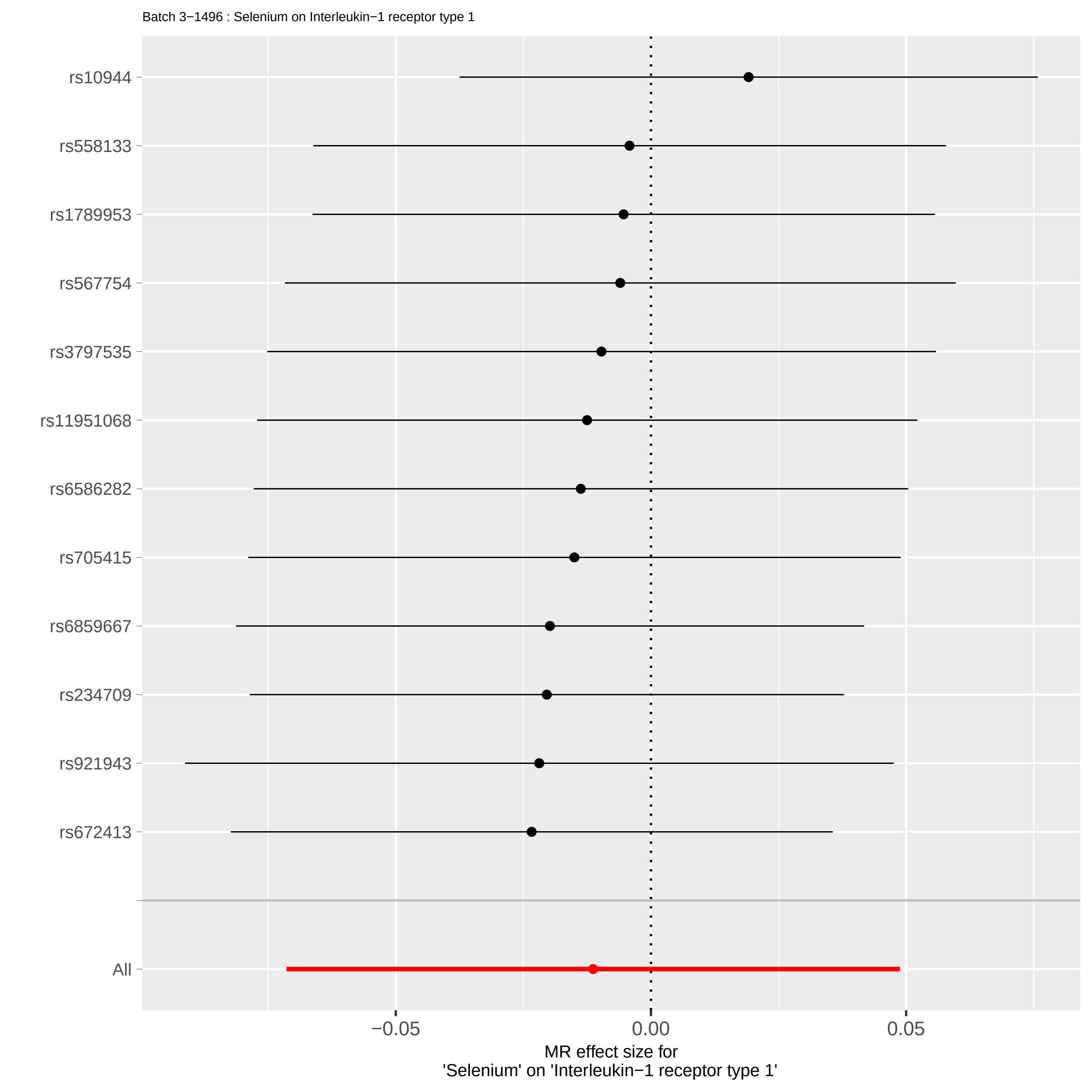

Batch 3−1496 : Selenium on Interleukin−1 receptor type 1
rs10944
rs558133
rs1789953
rs567754
rs3797535
rs11951068
rs6586282
rs705415
rs6859667
rs234709
rs921943
rs672413
All
−0.05
0.00
0.05
MR effect size for
'Selenium' on 'Interleukin−1 receptor type 1'

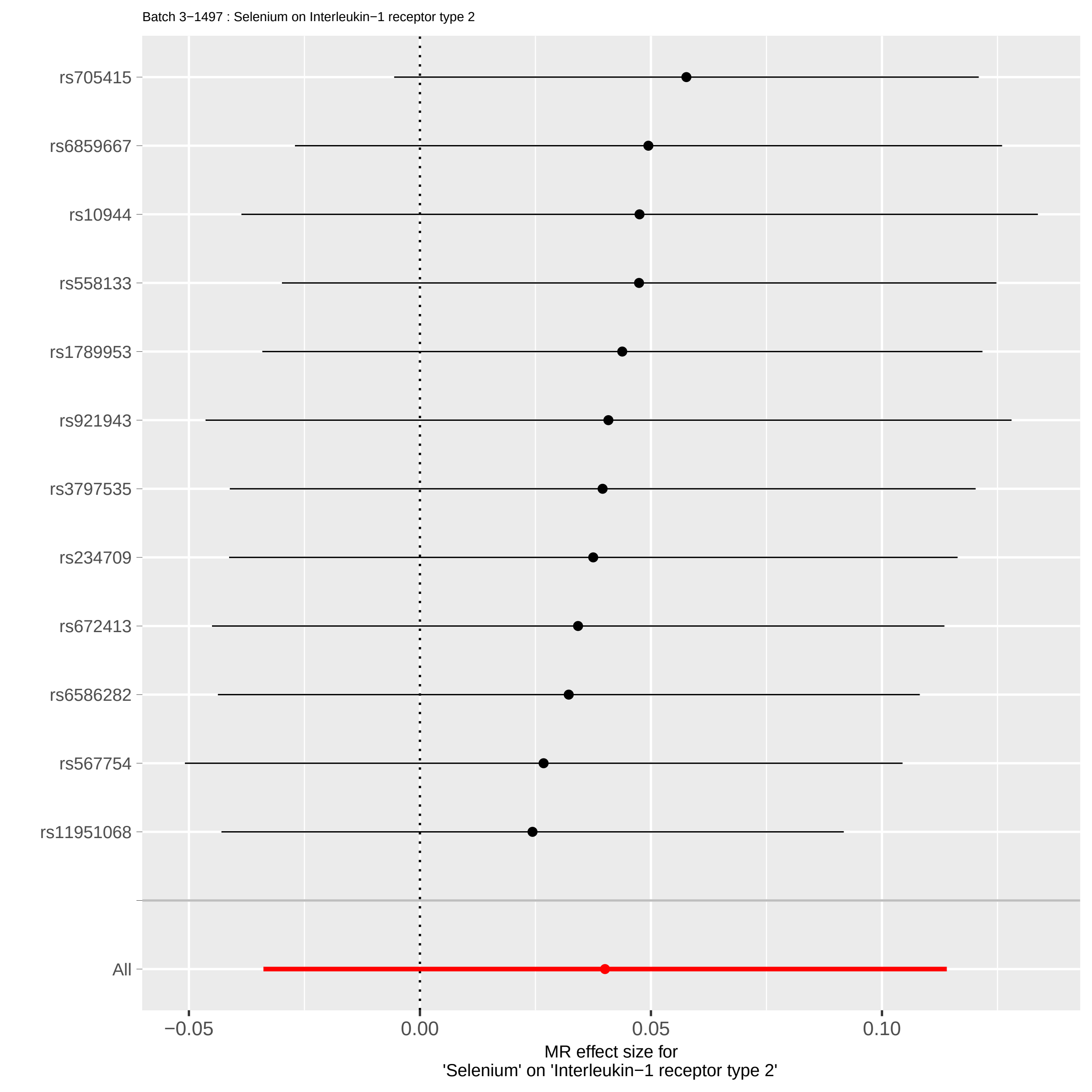
Batch 3−1497 : Selenium on Interleukin−1 receptor type 2
rs705415
rs6859667
rs10944
rs558133
rs1789953
rs921943
rs3797535
rs234709
rs672413
rs6586282
rs567754
rs11951068
All
−0.05
0.00
0.05
0.10
MR effect size for
'Selenium' on 'Interleukin−1 receptor type 2'

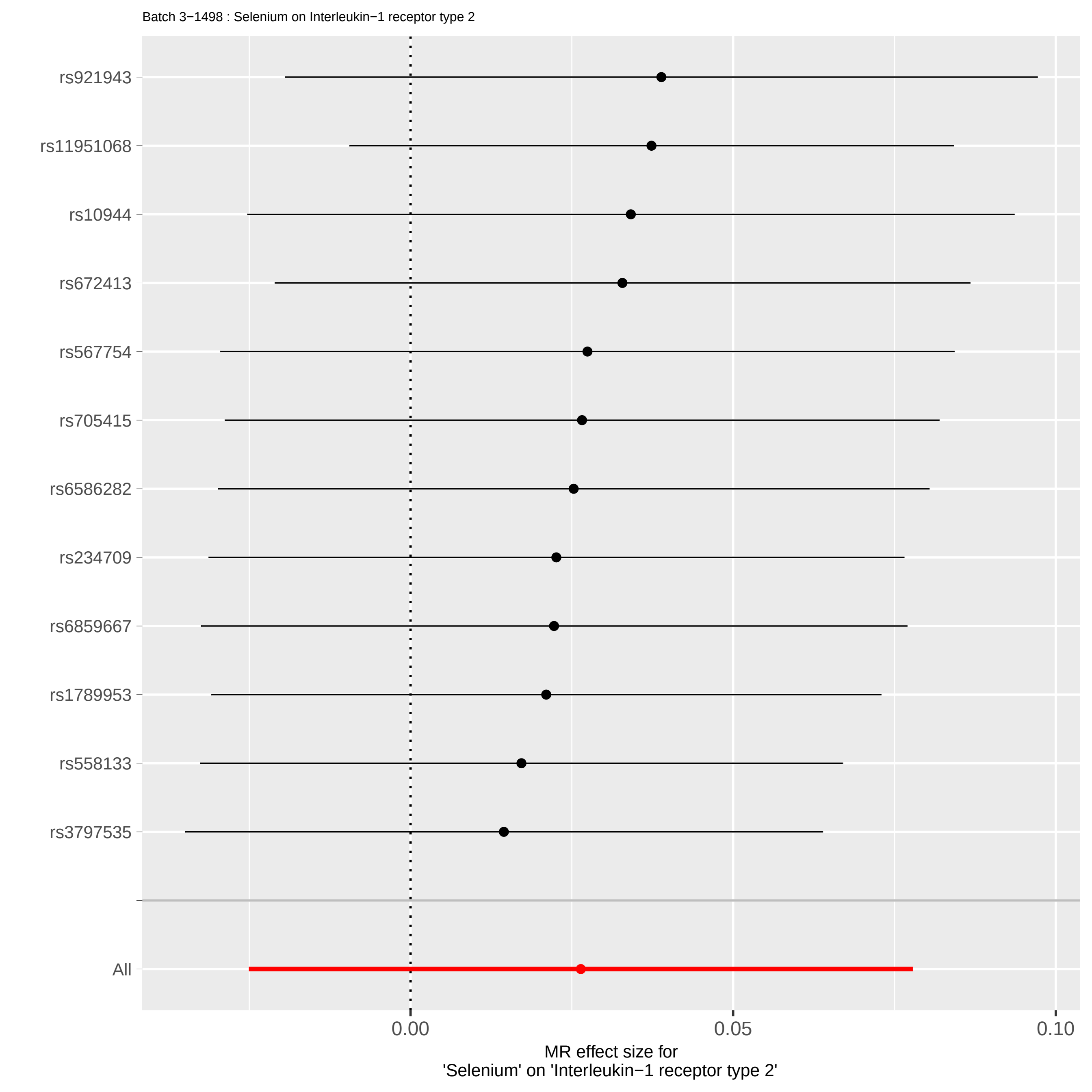

Batch 3−1498 : Selenium on Interleukin−1 receptor type 2
rs921943
rs11951068
rs10944
rs672413
rs567754
rs705415
rs6586282
rs234709
rs6859667
rs1789953
rs558133
rs3797535
All
0.00
0.05
0.10
MR effect size for
'Selenium' on 'Interleukin−1 receptor type 2'

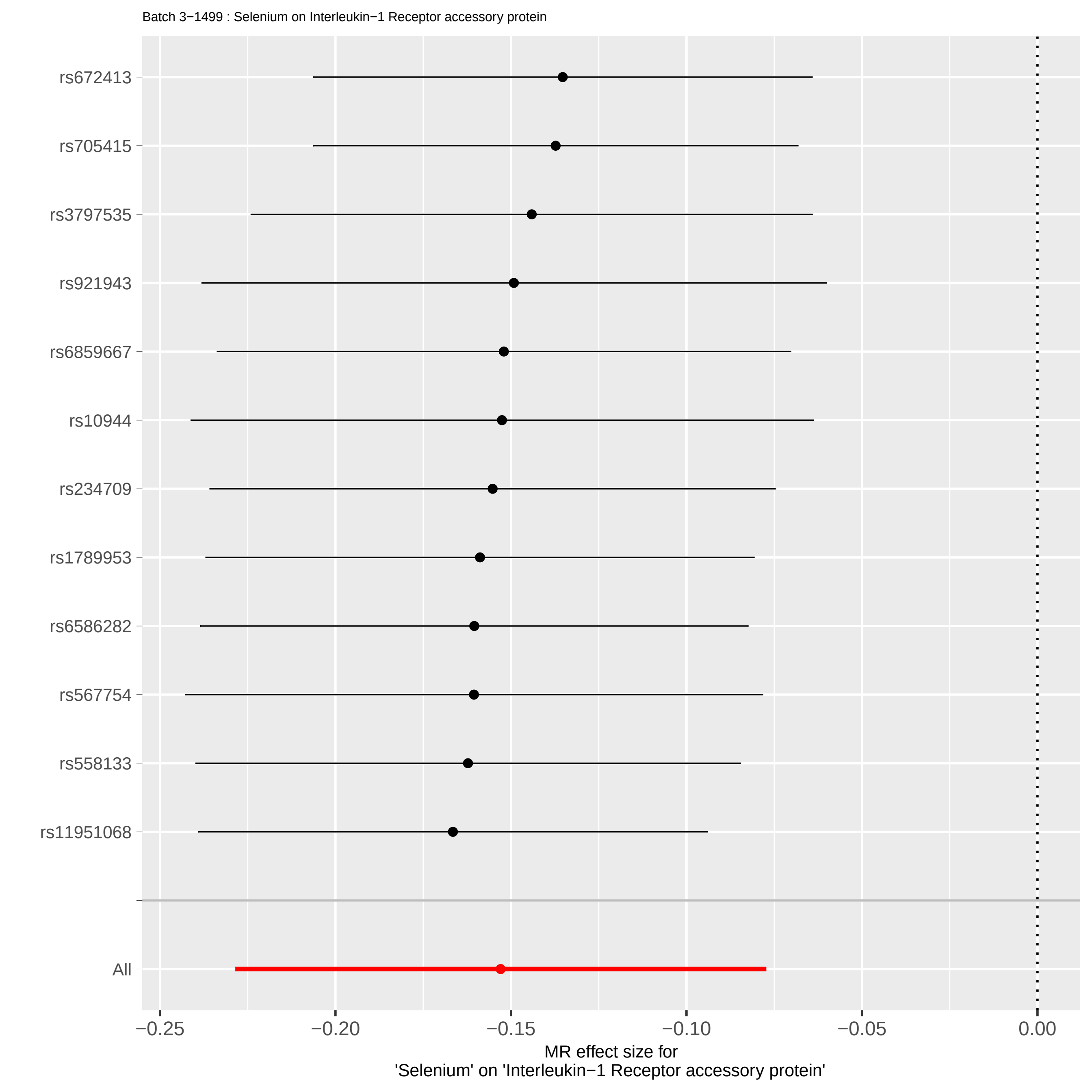

Batch 3−1499 : Selenium on Interleukin−1 Receptor accessory protein
rs672413
rs705415
rs3797535
rs921943
rs6859667
rs10944
rs234709
rs1789953
rs6586282
rs567754
rs558133
rs11951068
All
−0.25
−0.20
−0.15
−0.10
−0.05
0.00
MR effect size for
'Selenium' on 'Interleukin−1 Receptor accessory protein'

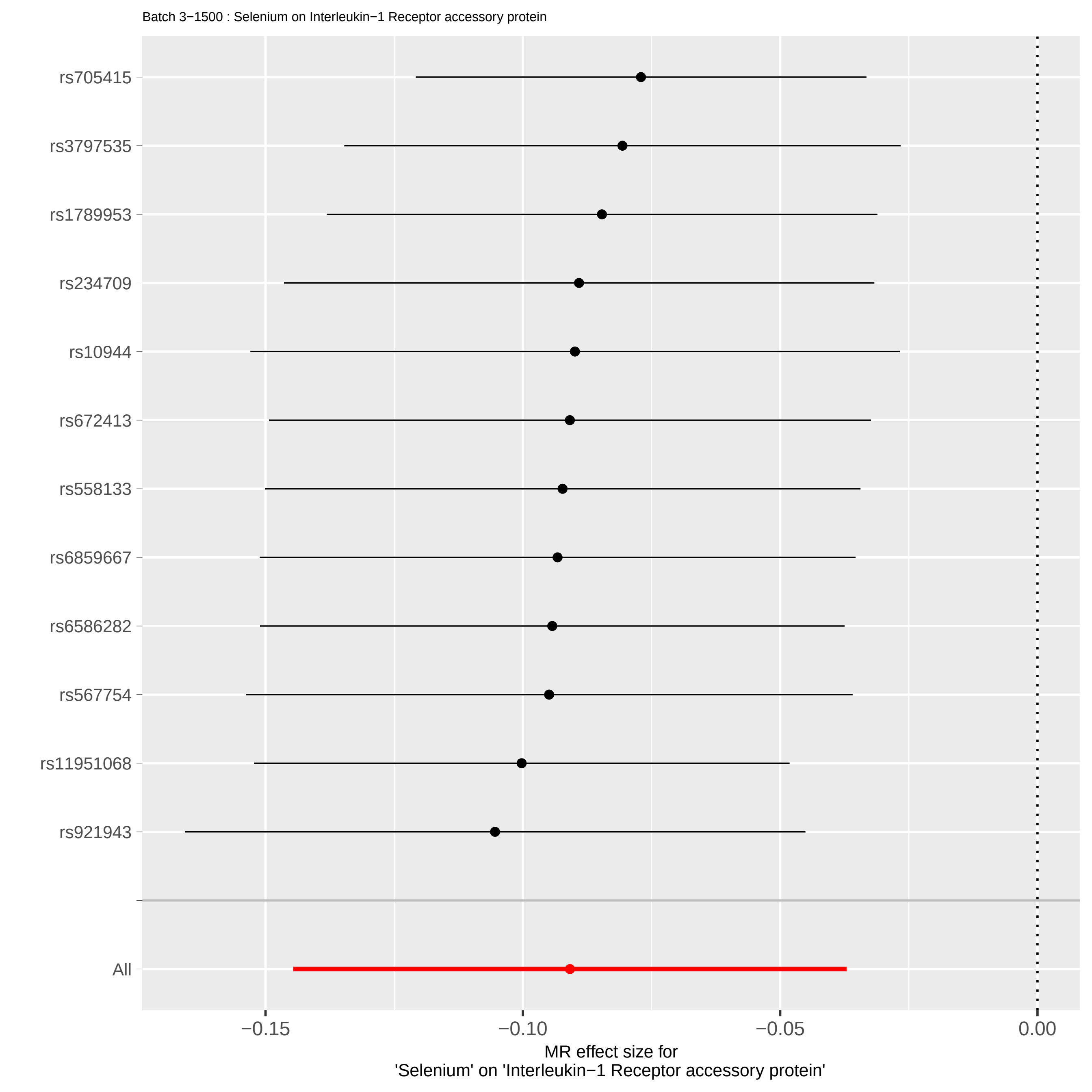

Batch 3−1500 : Selenium on Interleukin−1 Receptor accessory protein
rs705415
rs3797535
rs1789953
rs234709
rs10944
rs672413
rs558133
rs6859667
rs6586282
rs567754
rs11951068
rs921943
All
−0.15
−0.10
−0.05
0.00
MR effect size for
'Selenium' on 'Interleukin−1 Receptor accessory protein'

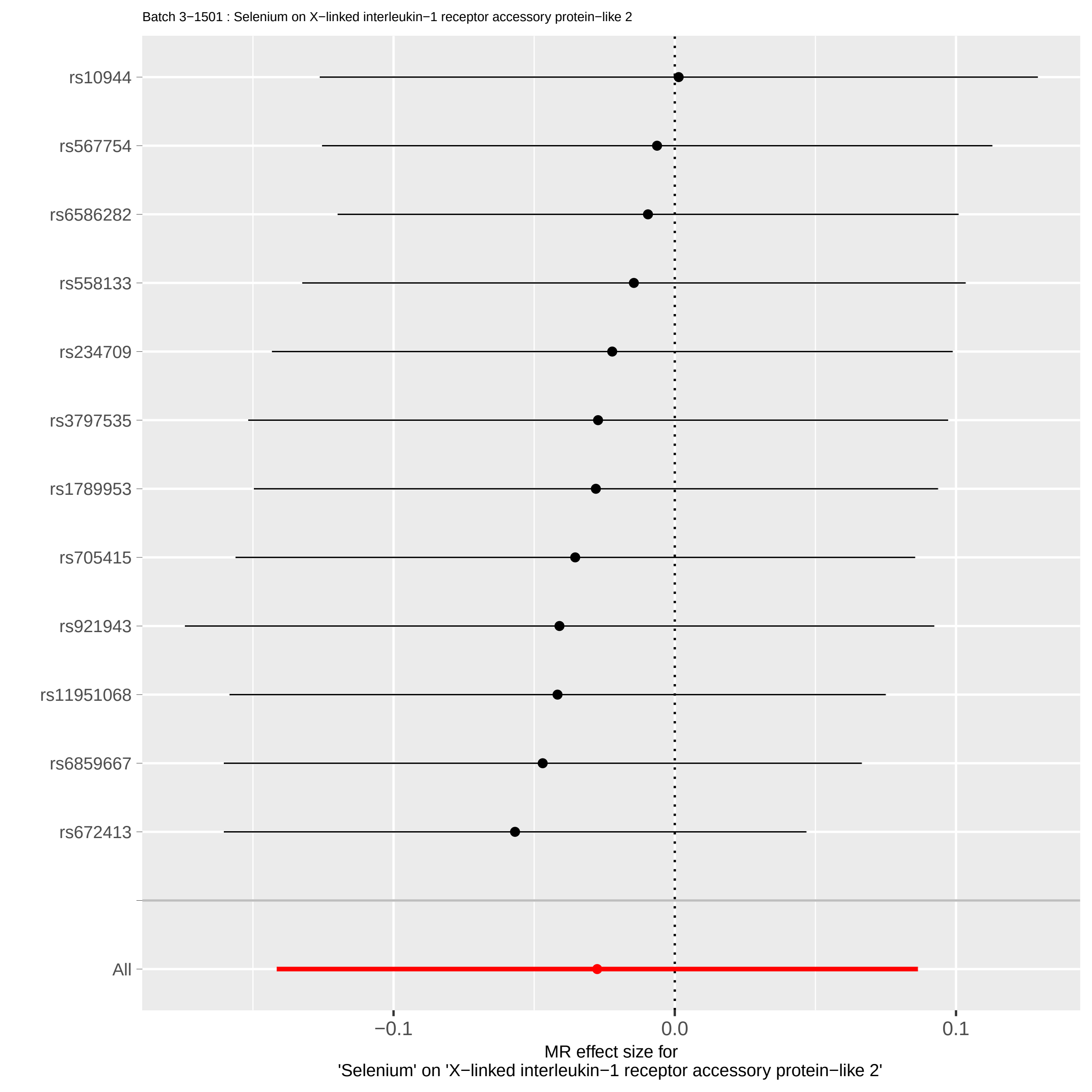
Batch 3−1501 : Selenium on X−linked interleukin−1 receptor accessory protein−like 2
rs10944
rs567754
rs6586282
rs558133
rs234709
rs3797535
rs1789953
rs705415
rs921943
rs11951068
rs6859667
rs672413
All
−0.1
0.0
0.1
MR effect size for
'Selenium' on 'X−linked interleukin−1 receptor accessory protein−like 2'

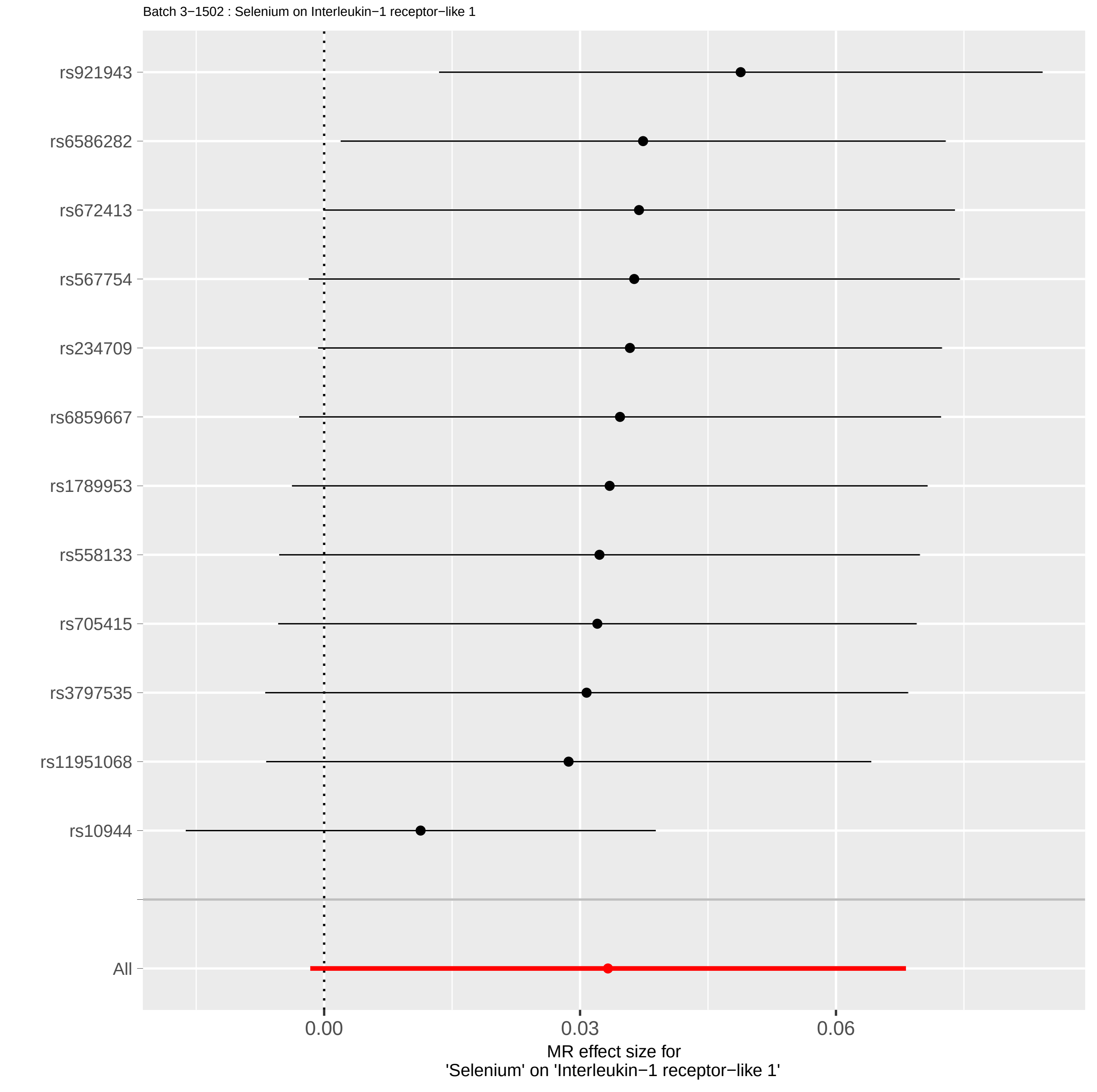

Batch 3−1502 : Selenium on Interleukin−1 receptor−like 1
rs921943
rs6586282
rs672413
rs567754
rs234709
rs6859667
rs1789953
rs558133
rs705415
rs3797535
rs11951068
rs10944
All
0.00
0.03
0.06
MR effect size for
'Selenium' on 'Interleukin−1 receptor−like 1'

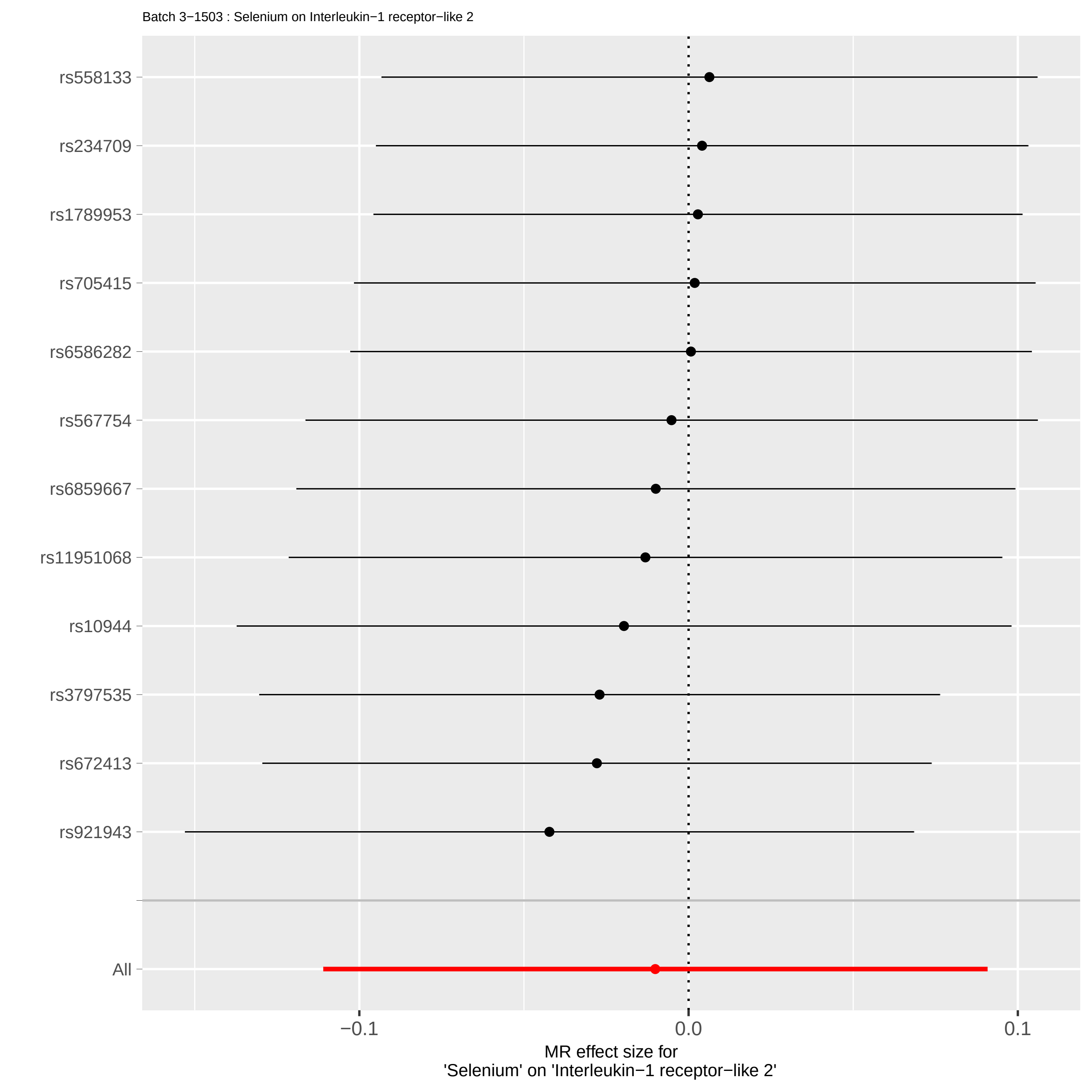

Batch 3−1503 : Selenium on Interleukin−1 receptor−like 2
rs558133
rs234709
rs1789953
rs705415
rs6586282
rs567754
rs6859667
rs11951068
rs10944
rs3797535
rs672413
rs921943
All
−0.1
0.0
0.1
MR effect size for
'Selenium' on 'Interleukin−1 receptor−like 2'

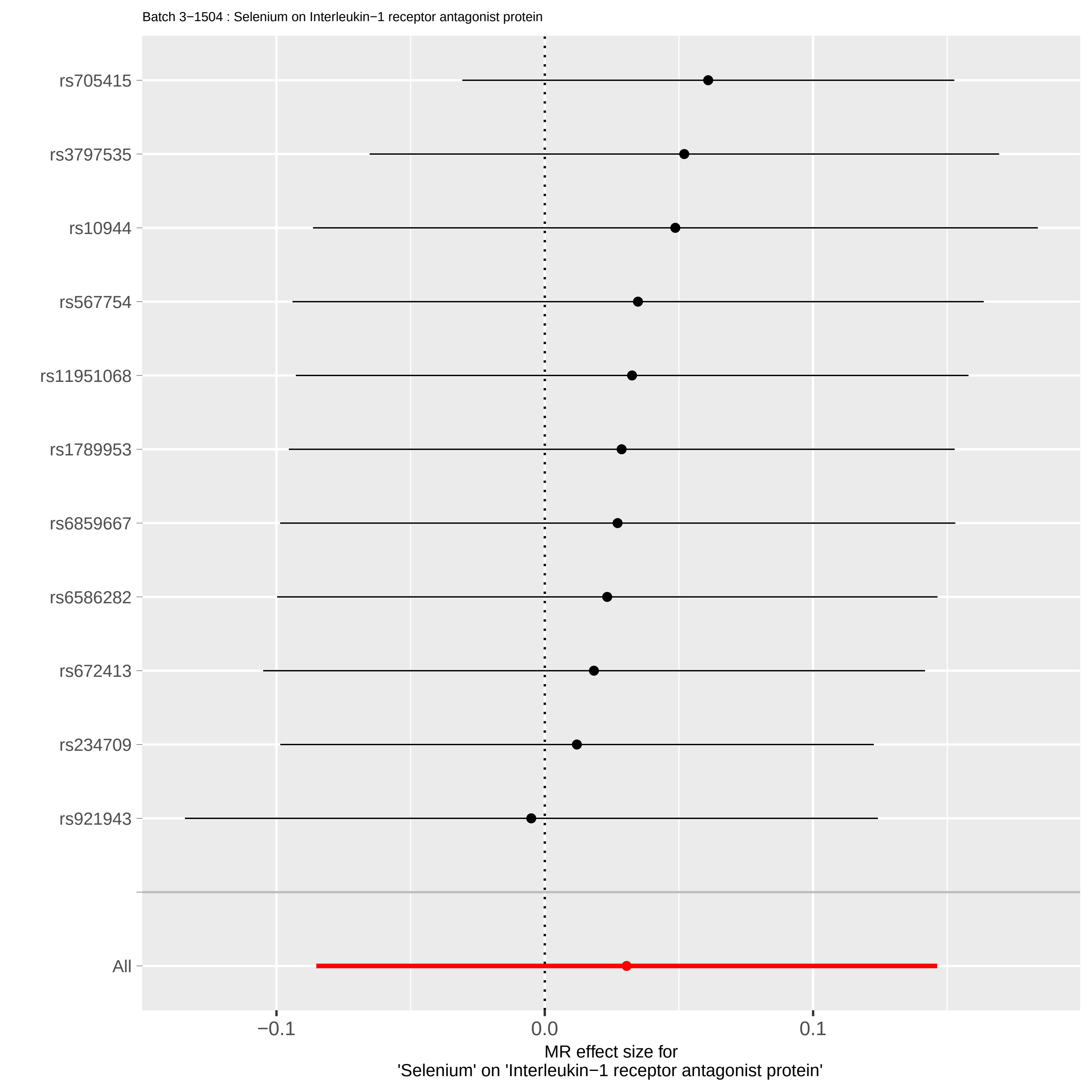

Batch 3−1504 : Selenium on Interleukin−1 receptor antagonist protein
rs705415
rs3797535
rs10944
rs567754
rs11951068
rs1789953
rs6859667
rs6586282
rs672413
rs234709
rs921943
All
−0.1
0.0
0.1
MR effect size for
'Selenium' on 'Interleukin−1 receptor antagonist protein'

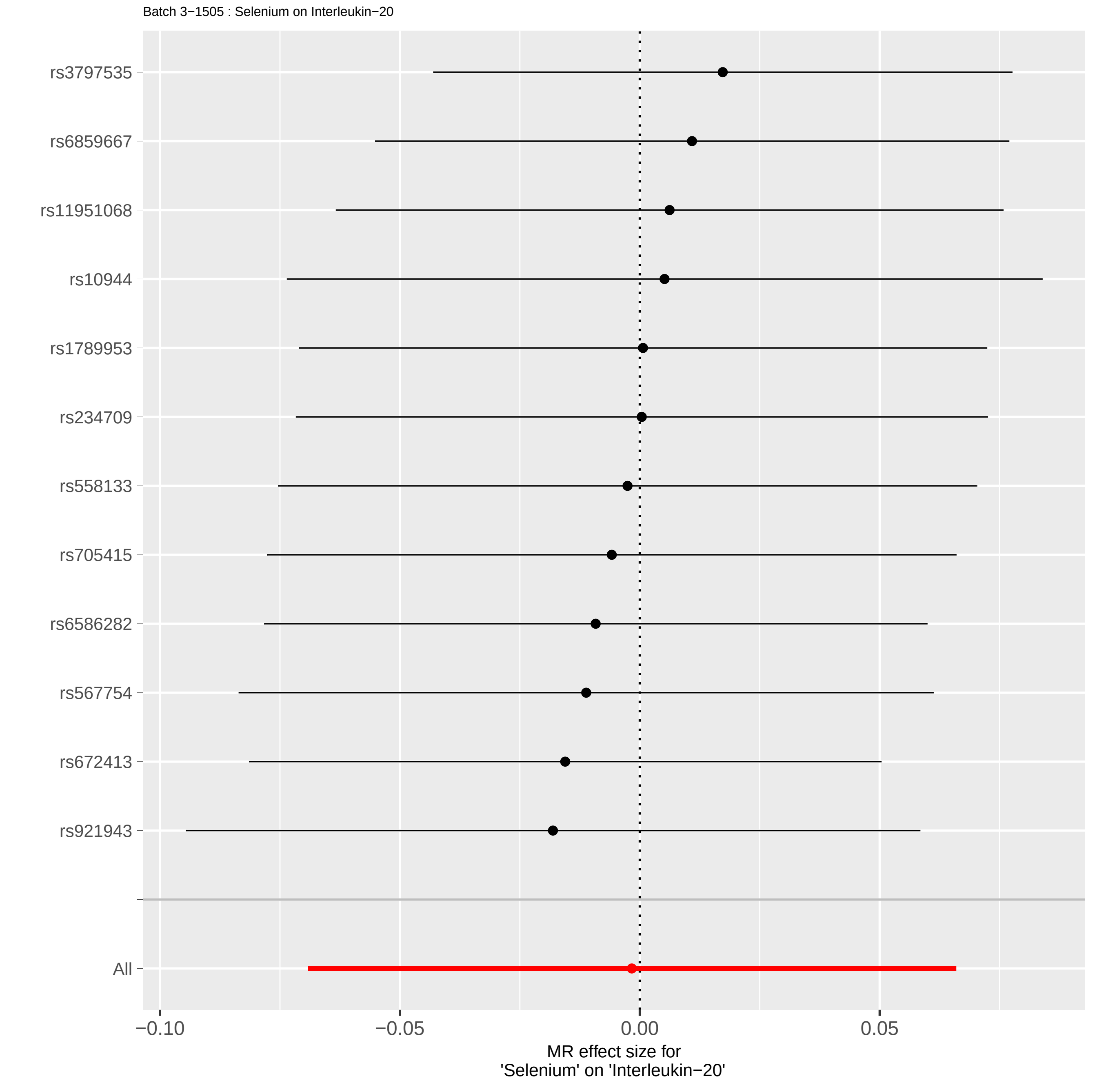

Batch 3−1505 : Selenium on Interleukin−20
rs3797535
rs6859667
rs11951068
rs10944
rs1789953
rs234709
rs558133
rs705415
rs6586282
rs567754
rs672413
rs921943
All
−0.10
−0.05
0.00
0.05
MR effect size for
'Selenium' on 'Interleukin−20'

Batch 3−1506 : Selenium on Interleukin−21

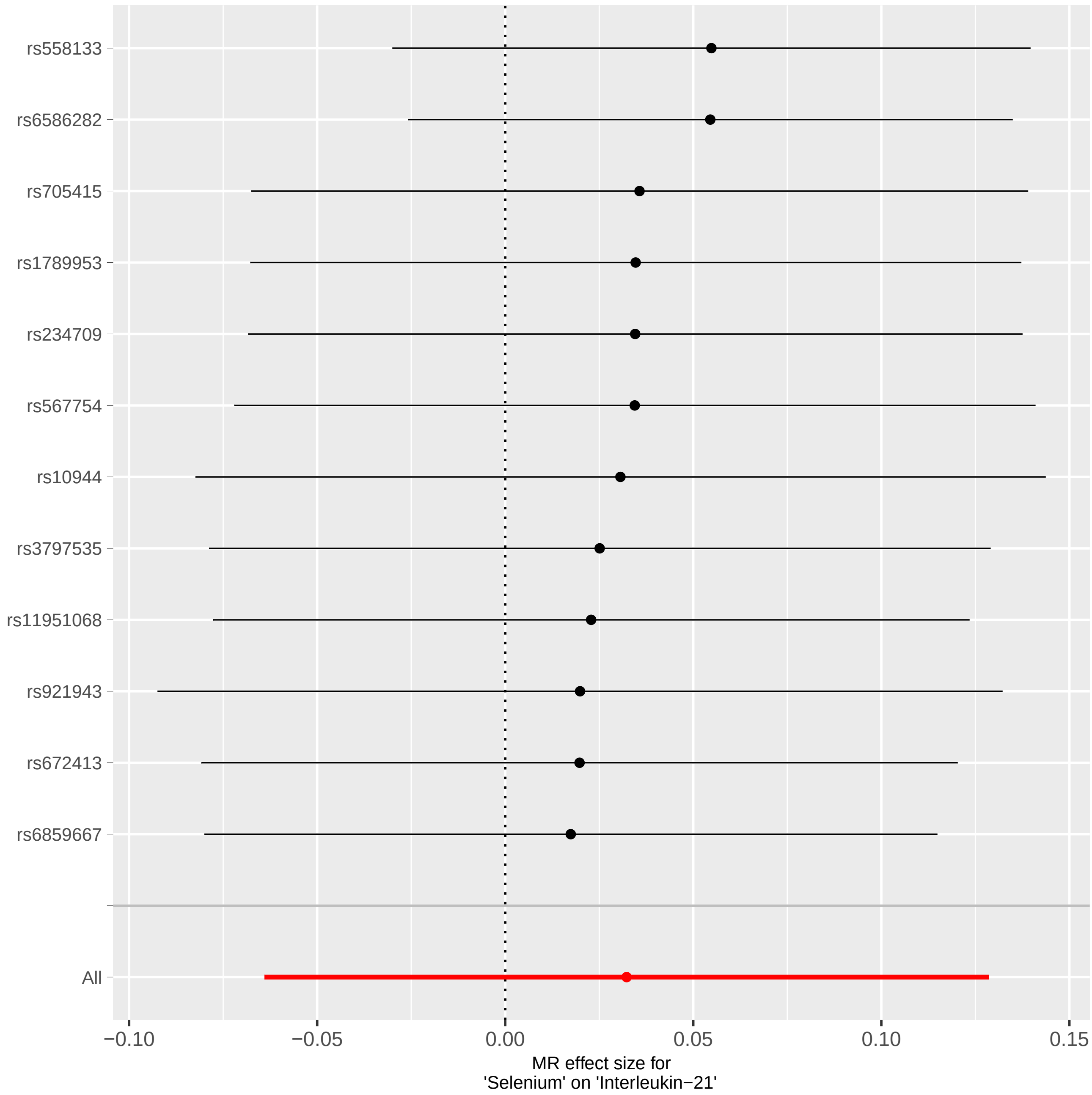

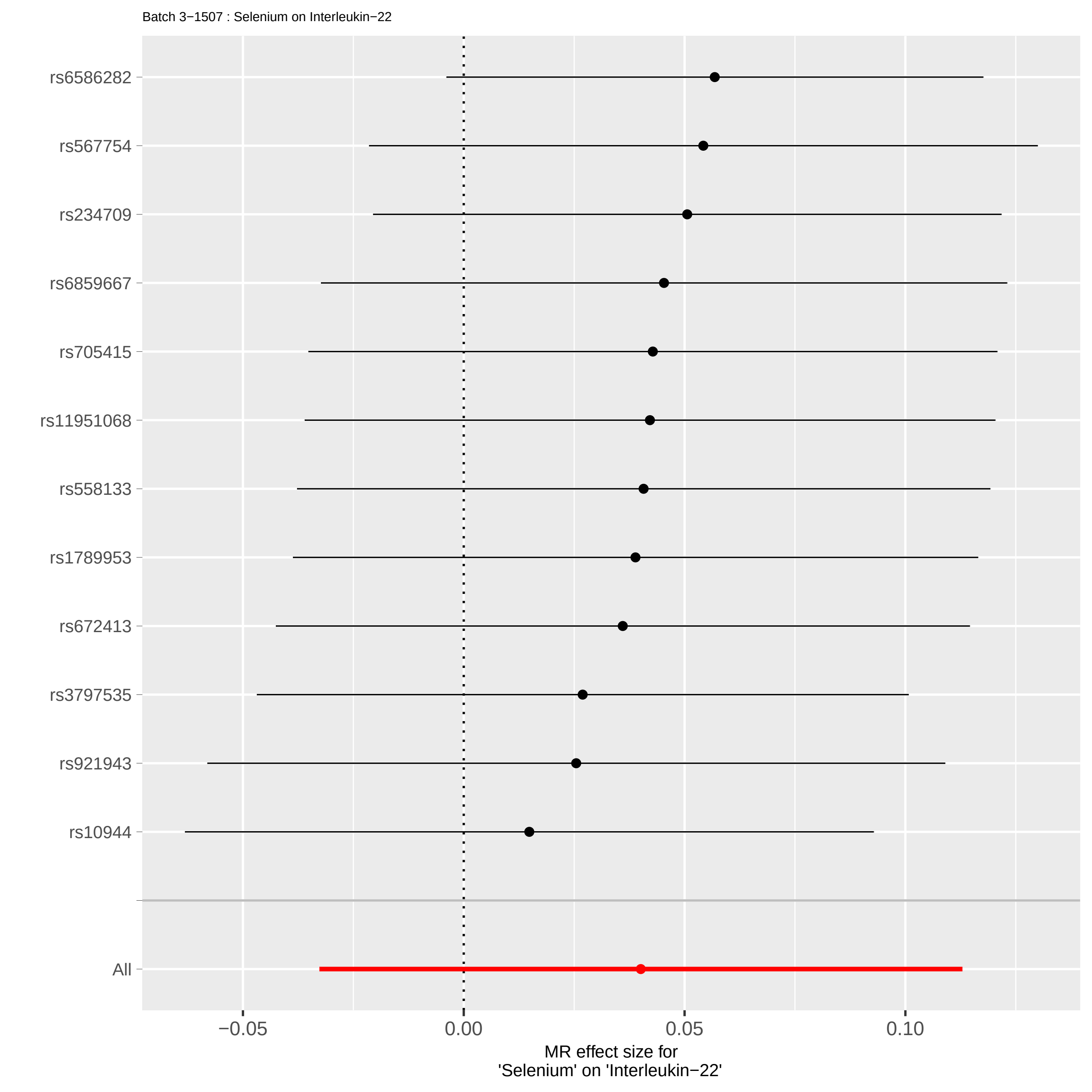

Batch 3−1507 : Selenium on Interleukin−22
rs6586282
rs567754
rs234709
rs6859667
rs705415
rs11951068
rs558133
rs1789953
rs672413
rs3797535
rs921943
rs10944
All
−0.05
0.00
0.05
0.10
MR effect size for
'Selenium' on 'Interleukin−22'

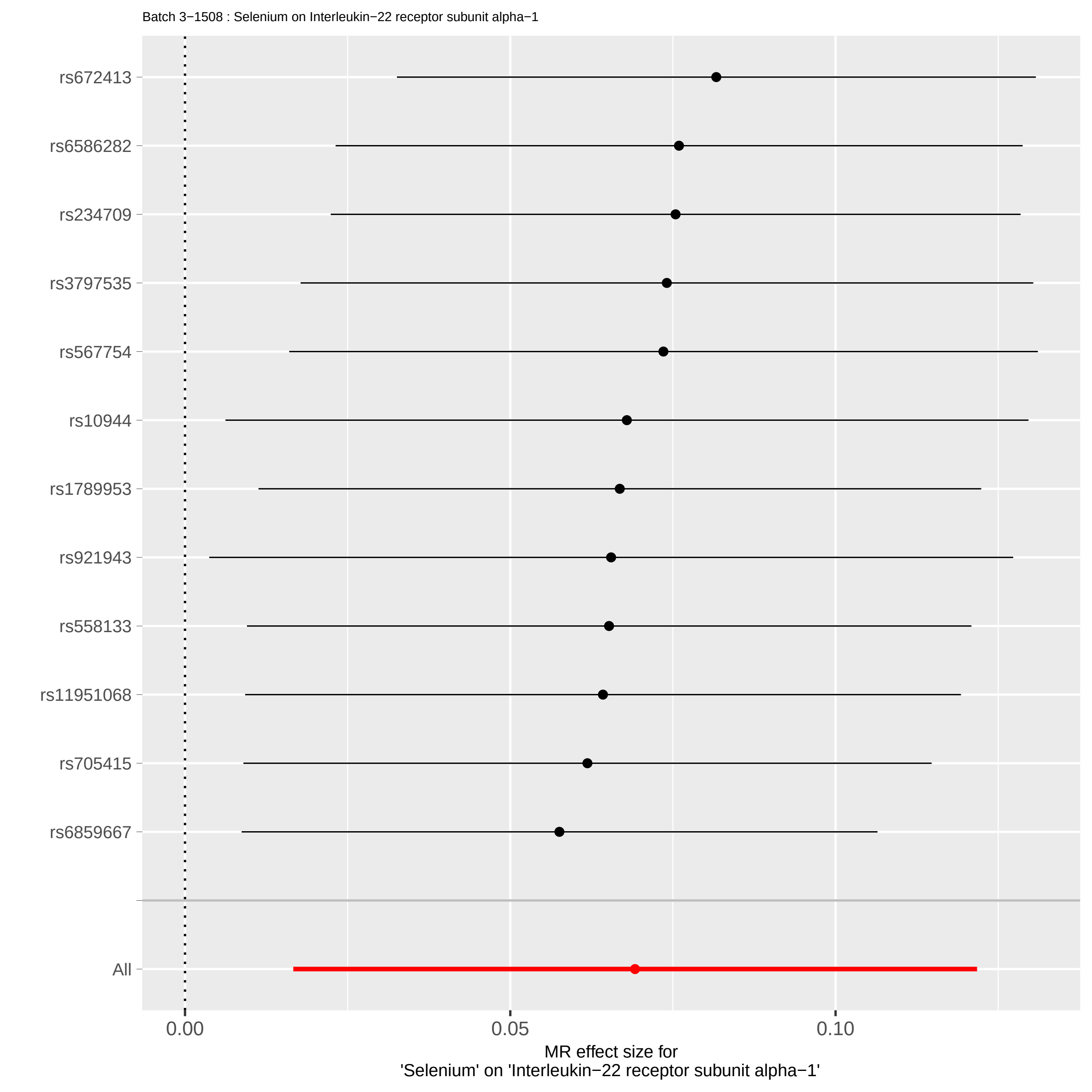
Batch 3−1508 : Selenium on Interleukin−22 receptor subunit alpha−1
rs672413
rs6586282
rs234709
rs3797535
rs567754
rs10944
rs1789953
rs921943
rs558133
rs11951068
rs705415
rs6859667
All
0.00
0.05
0.10
MR effect size for
'Selenium' on 'Interleukin−22 receptor subunit alpha−1'

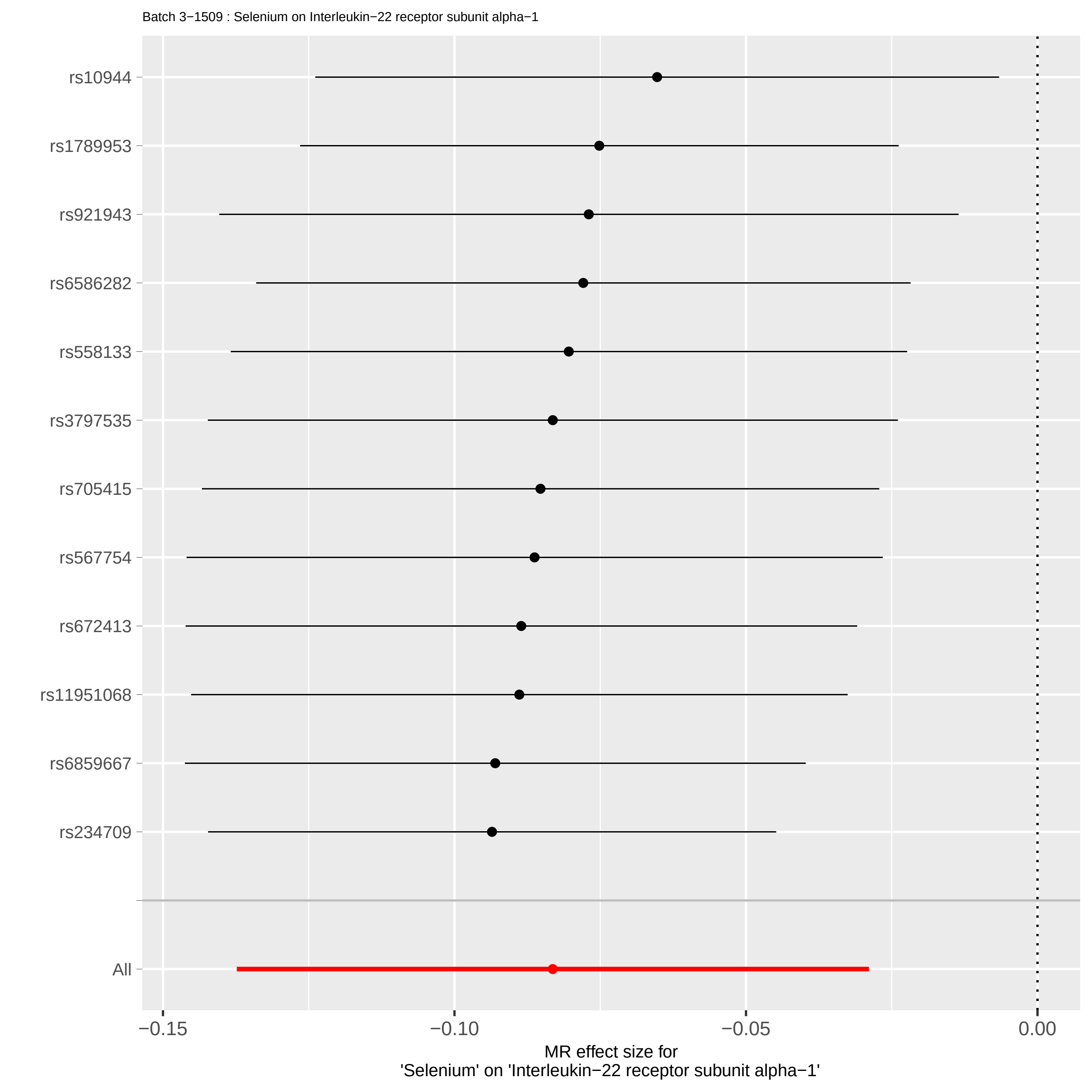
Batch 3−1509 : Selenium on Interleukin−22 receptor subunit alpha−1
rs10944
rs1789953
rs921943
rs6586282
rs558133
rs3797535
rs705415
rs567754
rs672413
rs11951068
rs6859667
rs234709
All
−0.15
−0.10
−0.05
0.00
MR effect size for
'Selenium' on 'Interleukin−22 receptor subunit alpha−1'

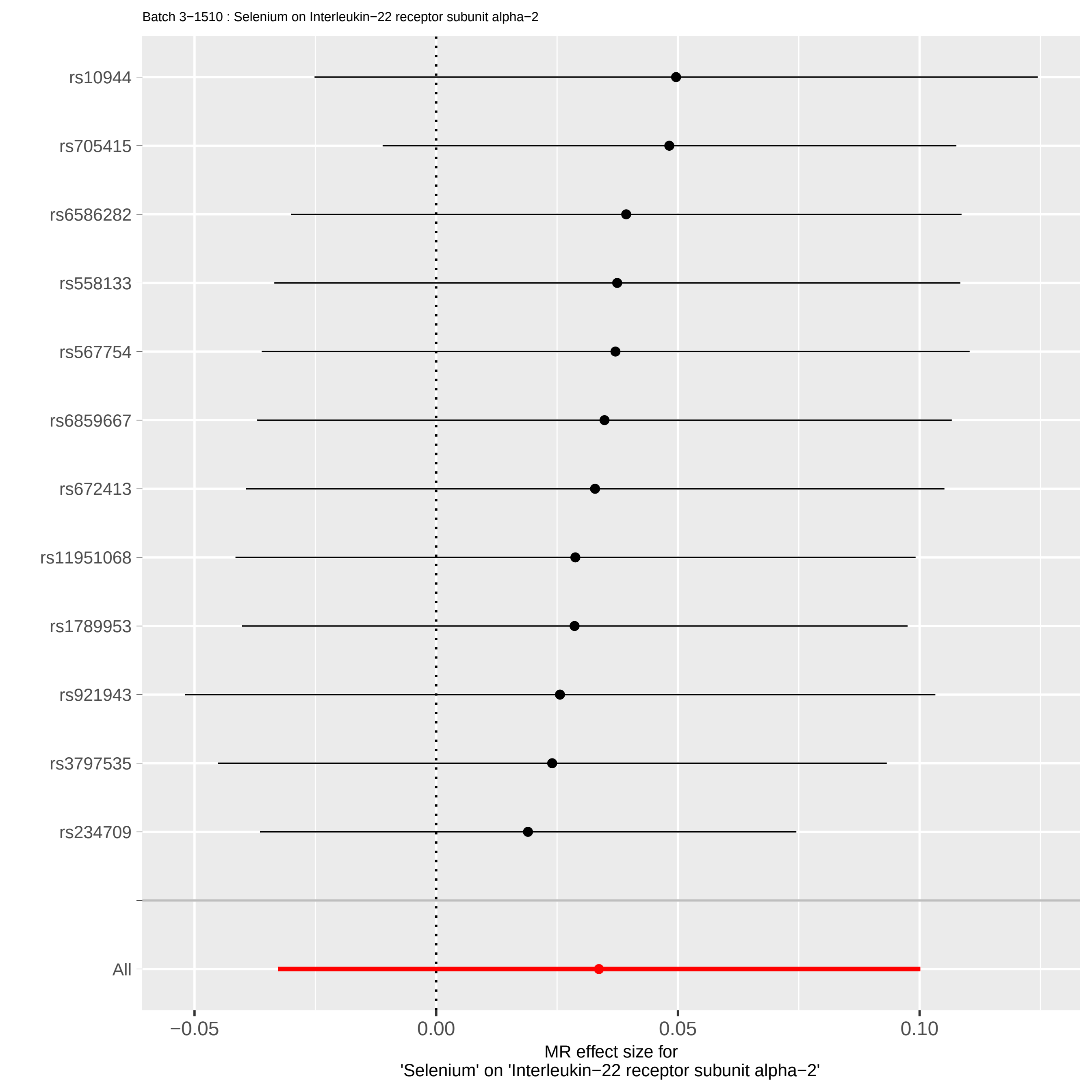

Batch 3−1510 : Selenium on Interleukin−22 receptor subunit alpha−2
rs10944
rs705415
rs6586282
rs558133
rs567754
rs6859667
rs672413
rs11951068
rs1789953
rs921943
rs3797535
rs234709
All
−0.05
0.00
0.05
0.10
MR effect size for
'Selenium' on 'Interleukin−22 receptor subunit alpha−2'

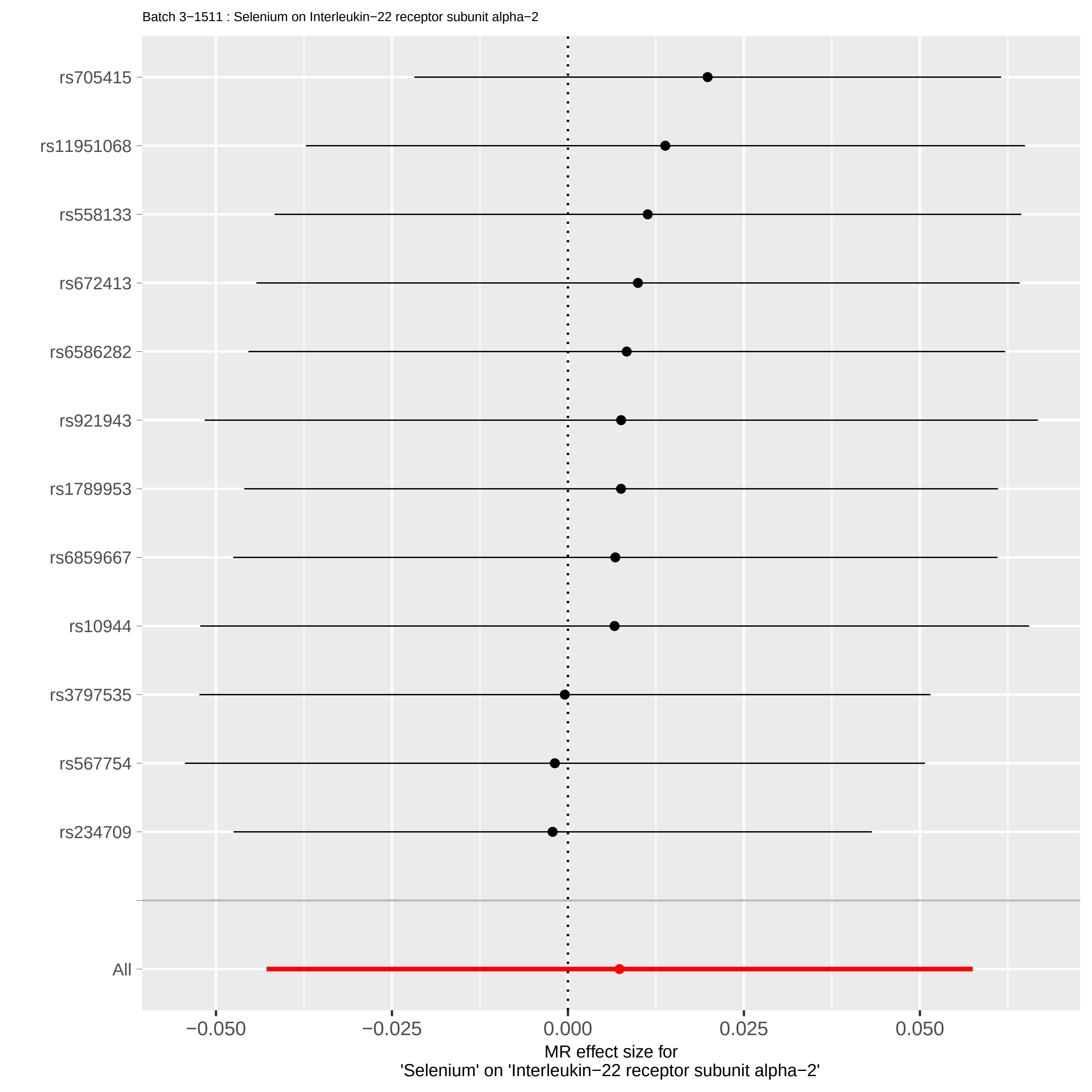

Batch 3−1511 : Selenium on Interleukin−22 receptor subunit alpha−2
rs705415
rs11951068
rs558133
rs672413
rs6586282
rs921943
rs1789953
rs6859667
rs10944
rs3797535
rs567754
rs234709
All
−0.050
−0.025
0.000
0.025
0.050
MR effect size for
'Selenium' on 'Interleukin−22 receptor subunit alpha−2'

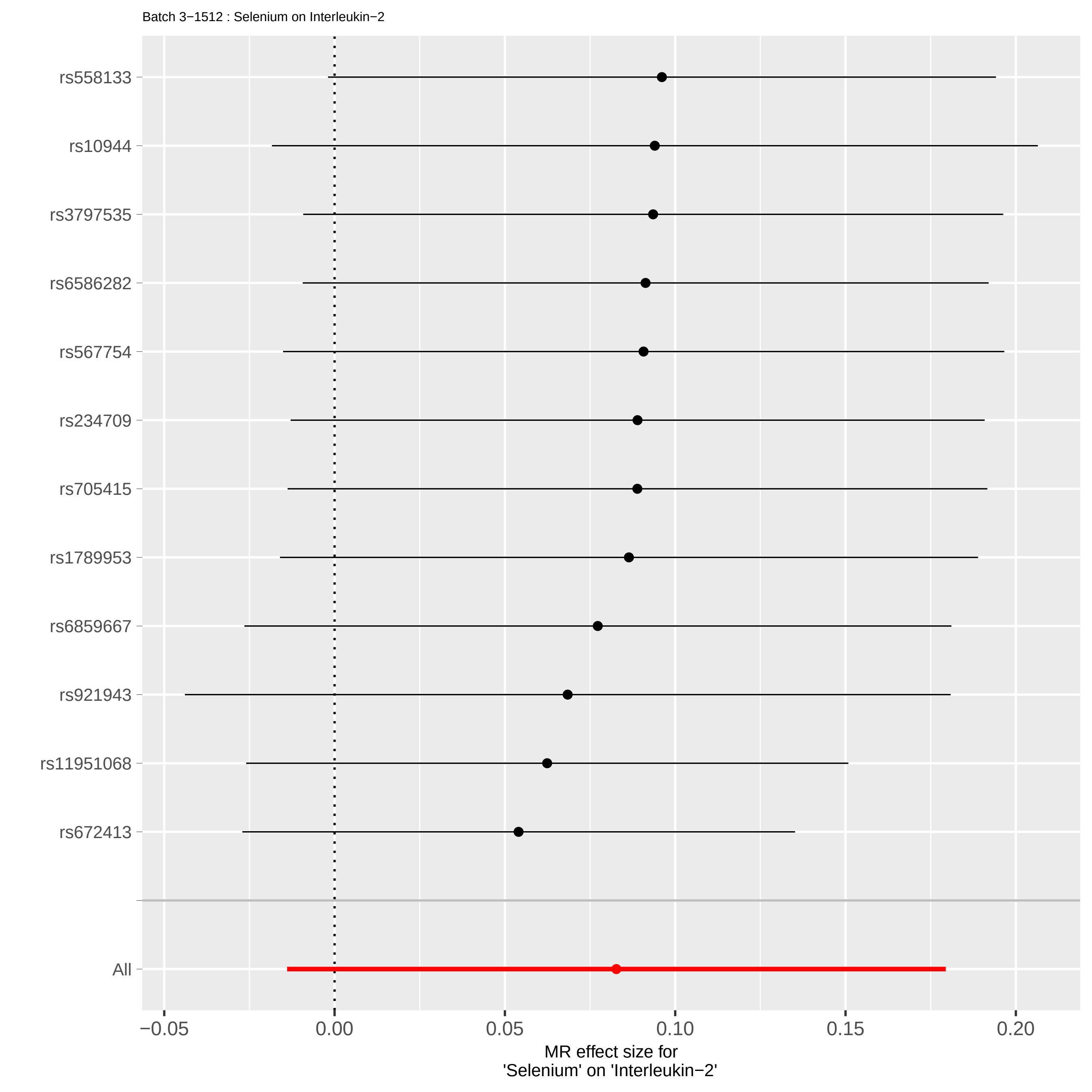

Batch 3−1512 : Selenium on Interleukin−2
rs558133
rs10944
rs3797535
rs6586282
rs567754
rs234709
rs705415
rs1789953
rs6859667
rs921943
rs11951068
rs672413
All
−0.05
0.00
0.05
0.10
0.15
0.20
MR effect size for
'Selenium' on 'Interleukin−2'

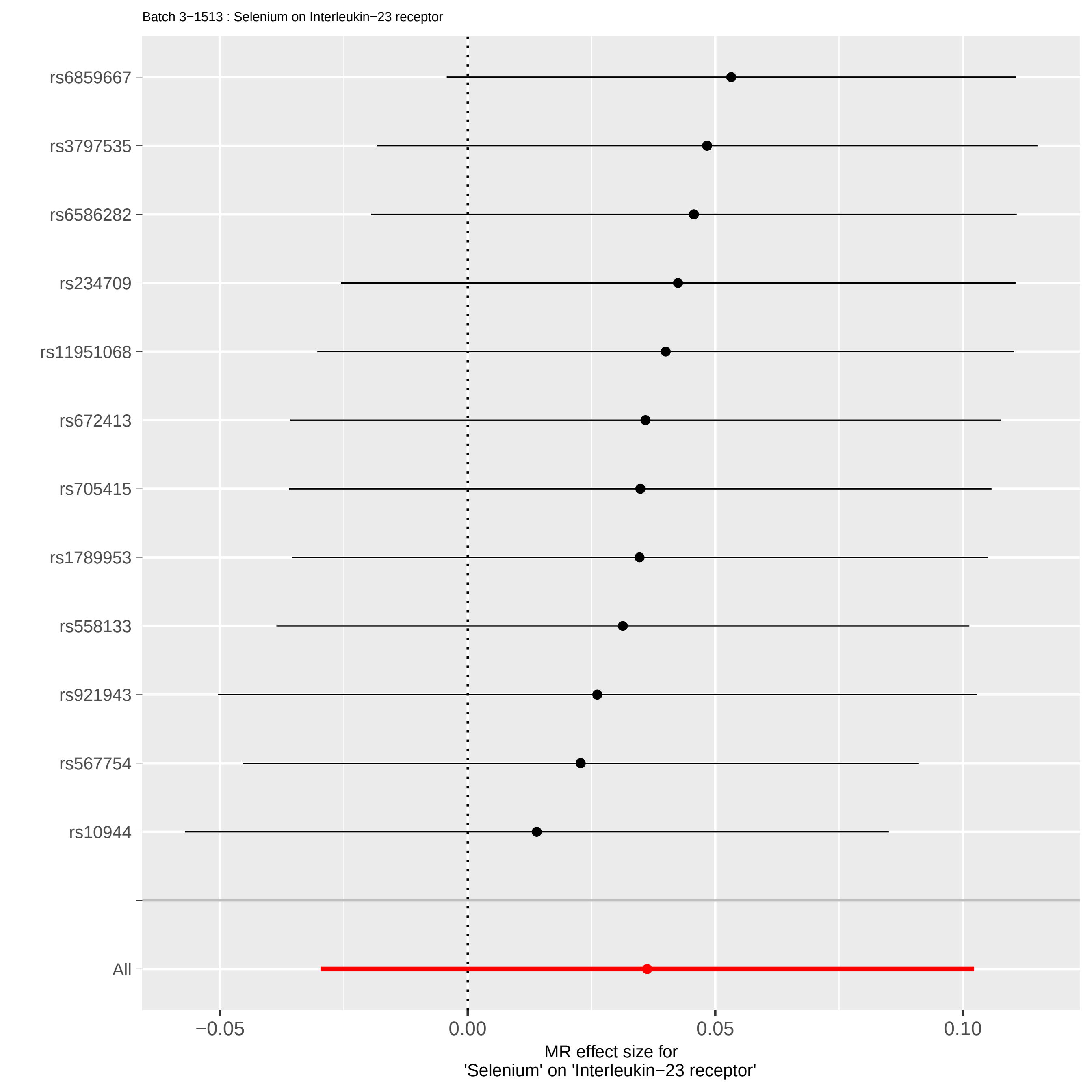
Batch 3−1513 : Selenium on Interleukin−23 receptor
rs6859667
rs3797535
rs6586282
rs234709
rs11951068
rs672413
rs705415
rs1789953
rs558133
rs921943
rs567754
rs10944
All
−0.05
0.00
0.05
0.10
MR effect size for
'Selenium' on 'Interleukin−23 receptor'

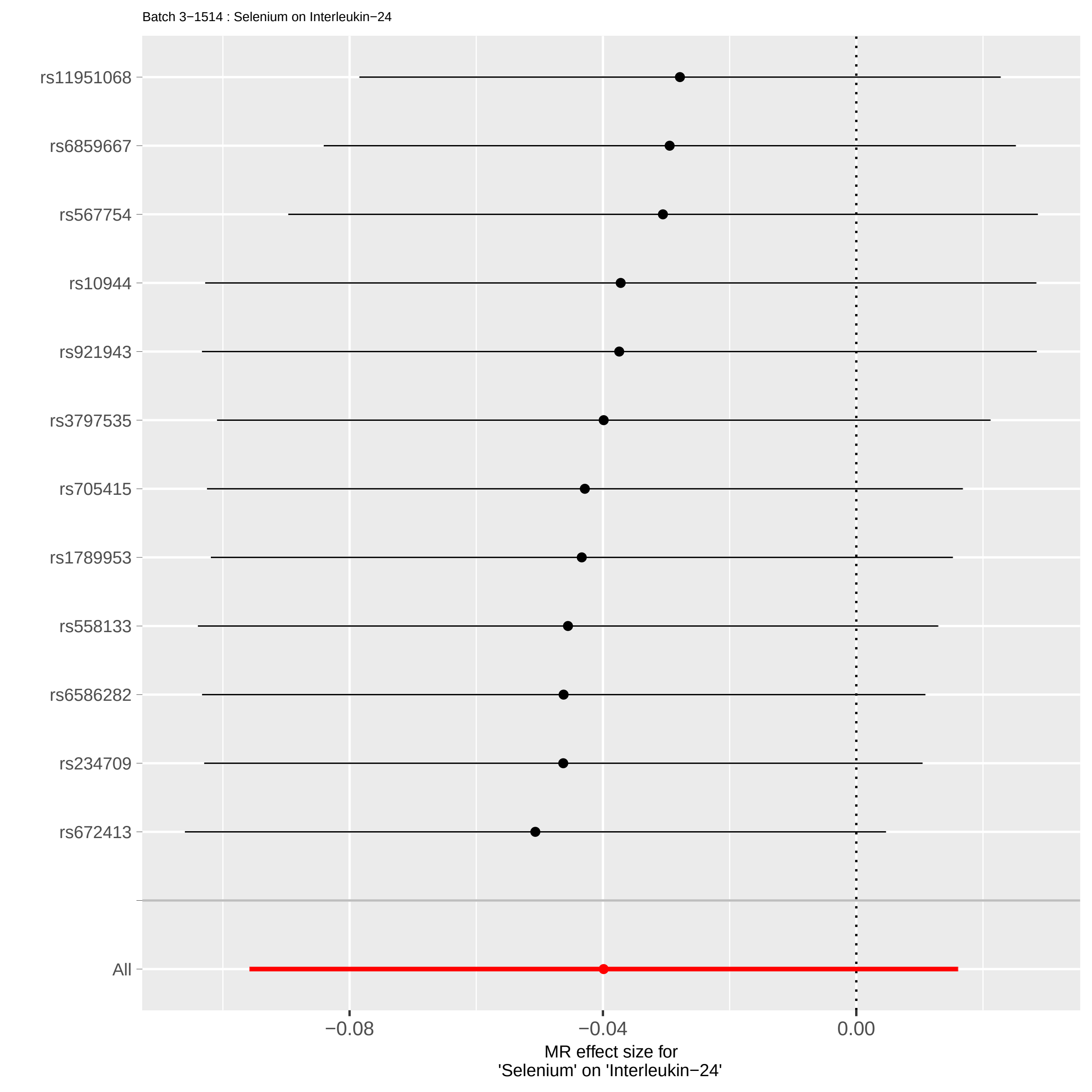

Batch 3−1514 : Selenium on Interleukin−24
rs11951068
rs6859667
rs567754
rs10944
rs921943
rs3797535
rs705415
rs1789953
rs558133
rs6586282
rs234709
rs672413
All
−0.08
−0.04
0.00
MR effect size for
'Selenium' on 'Interleukin−24'

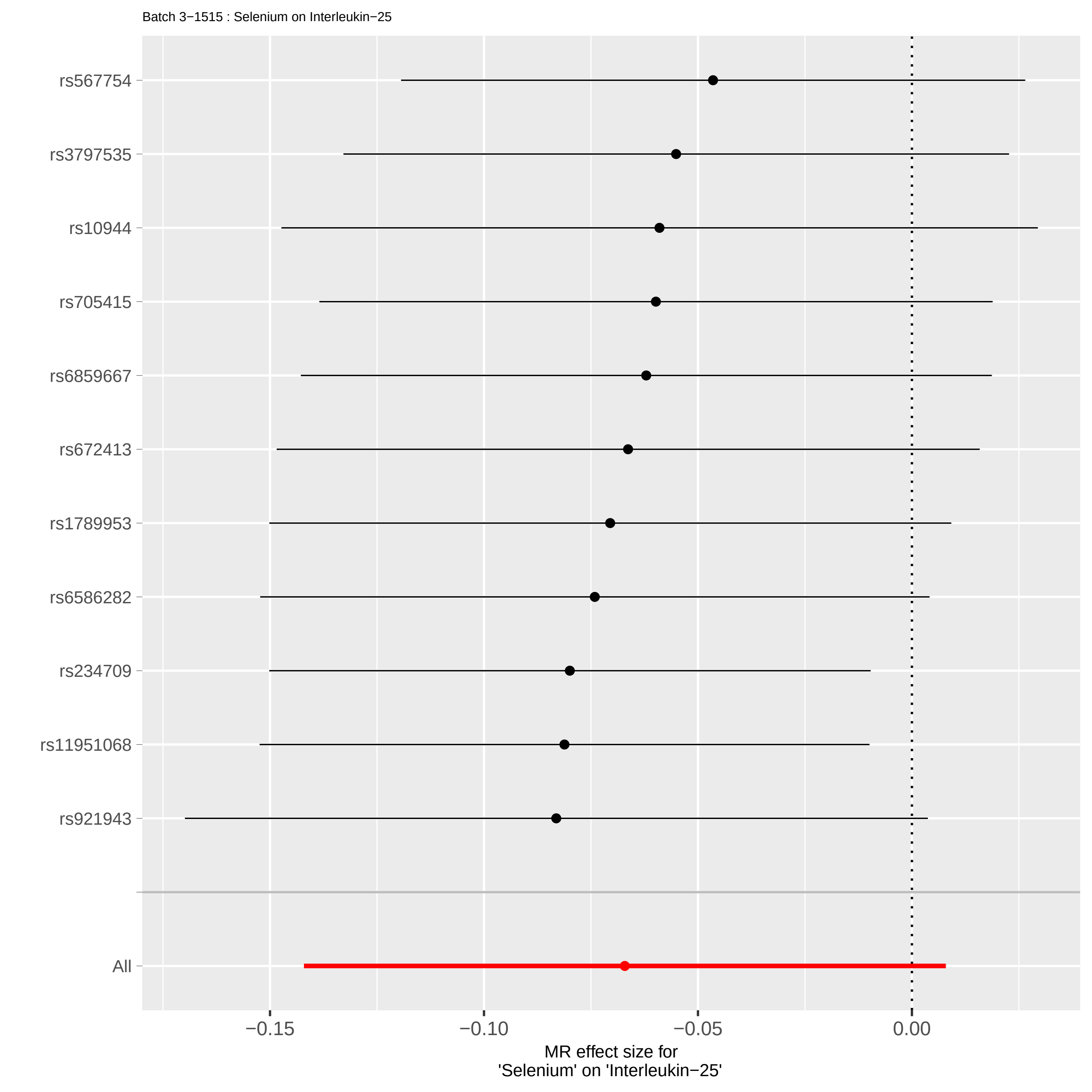

Batch 3−1515 : Selenium on Interleukin−25
rs567754
rs3797535
rs10944
rs705415
rs6859667
rs672413
rs1789953
rs6586282
rs234709
rs11951068
rs921943
All
−0.15
−0.10
−0.05
0.00
MR effect size for
'Selenium' on 'Interleukin−25'

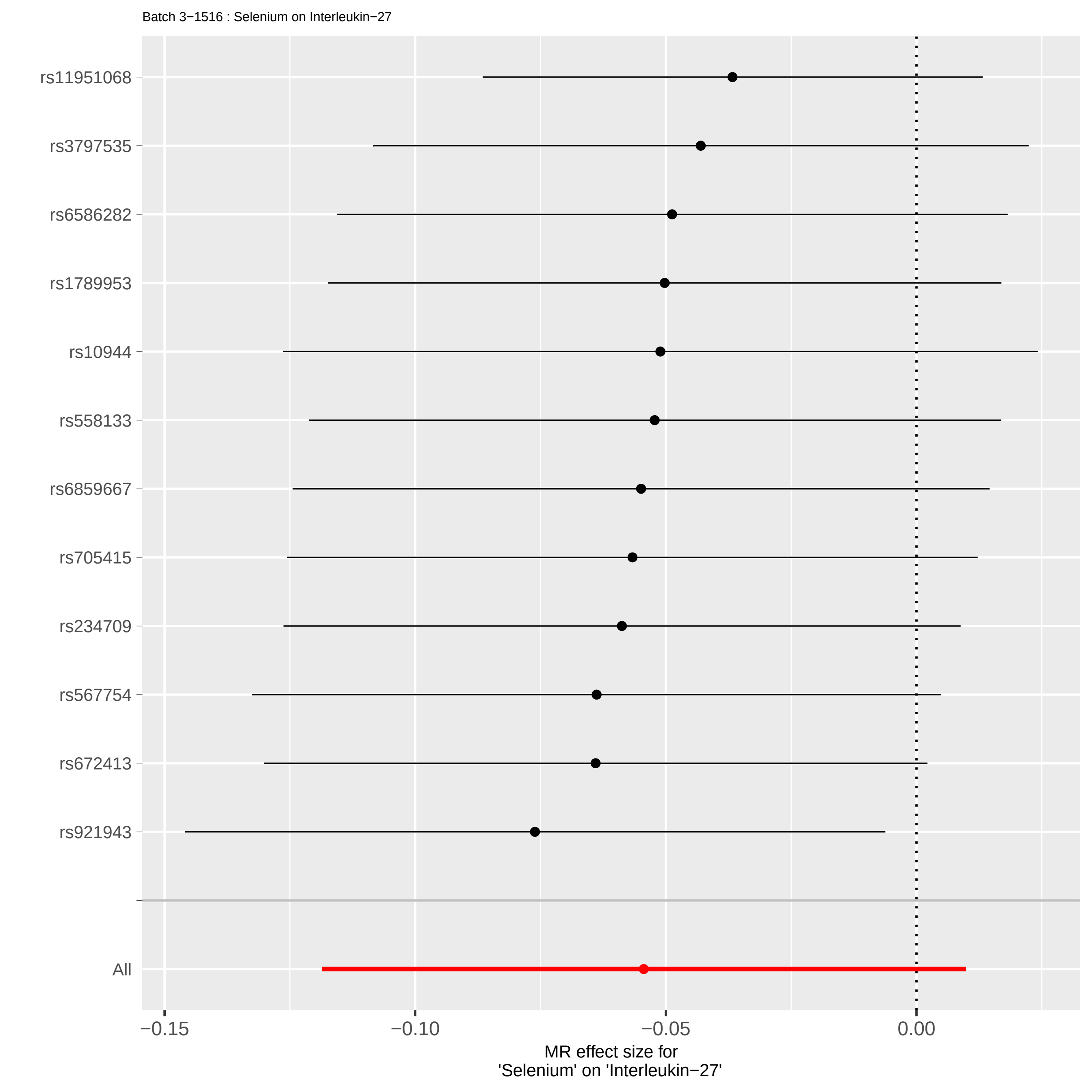

Batch 3−1516 : Selenium on Interleukin−27
rs11951068
rs3797535
rs6586282
rs1789953
rs10944
rs558133
rs6859667
rs705415
rs234709
rs567754
rs672413
rs921943
All
−0.15
−0.10
−0.05
0.00
MR effect size for
'Selenium' on 'Interleukin−27'

Batch 3−1517 : Selenium on Interleukin−27 receptor subunit alpha

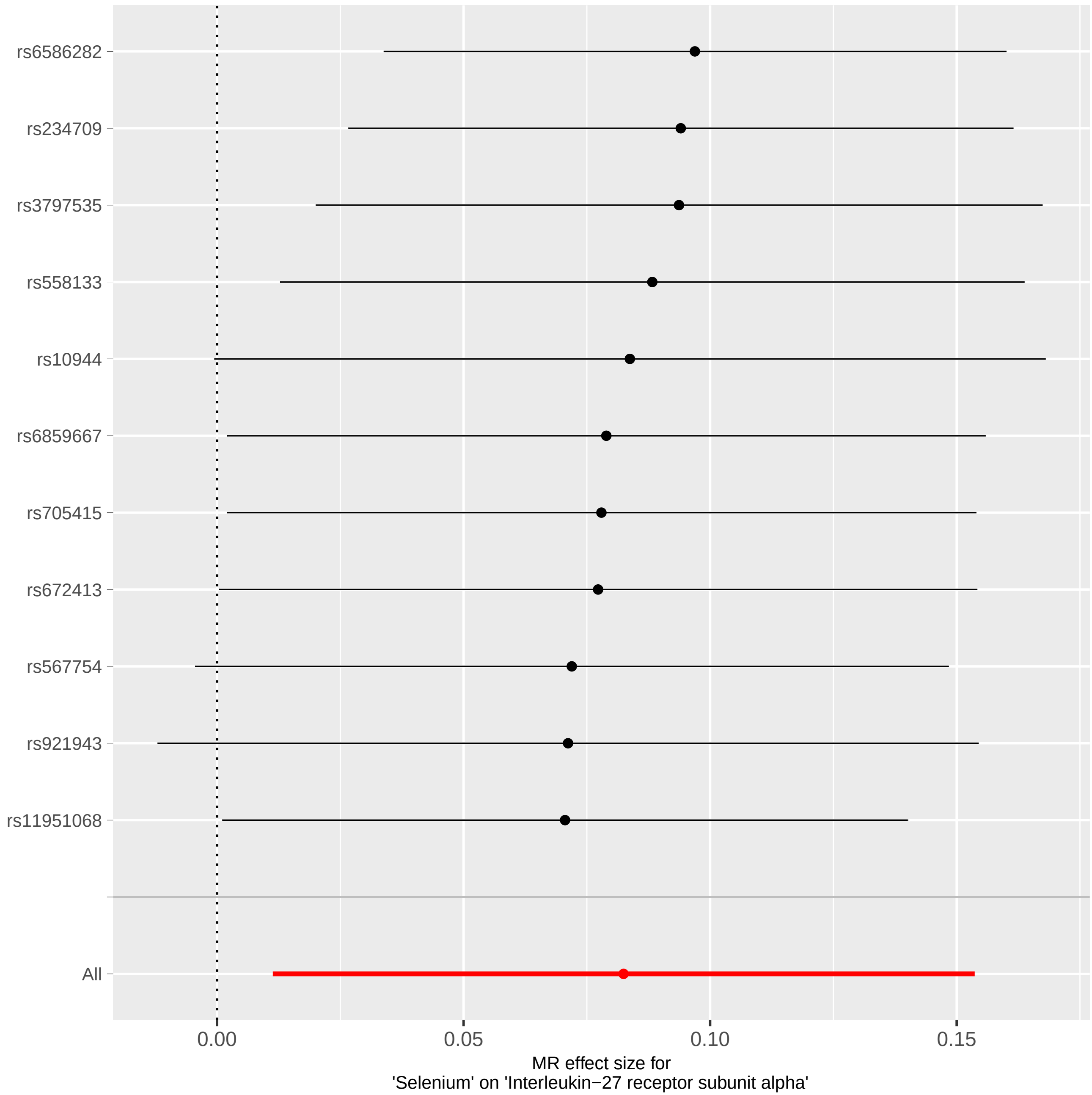

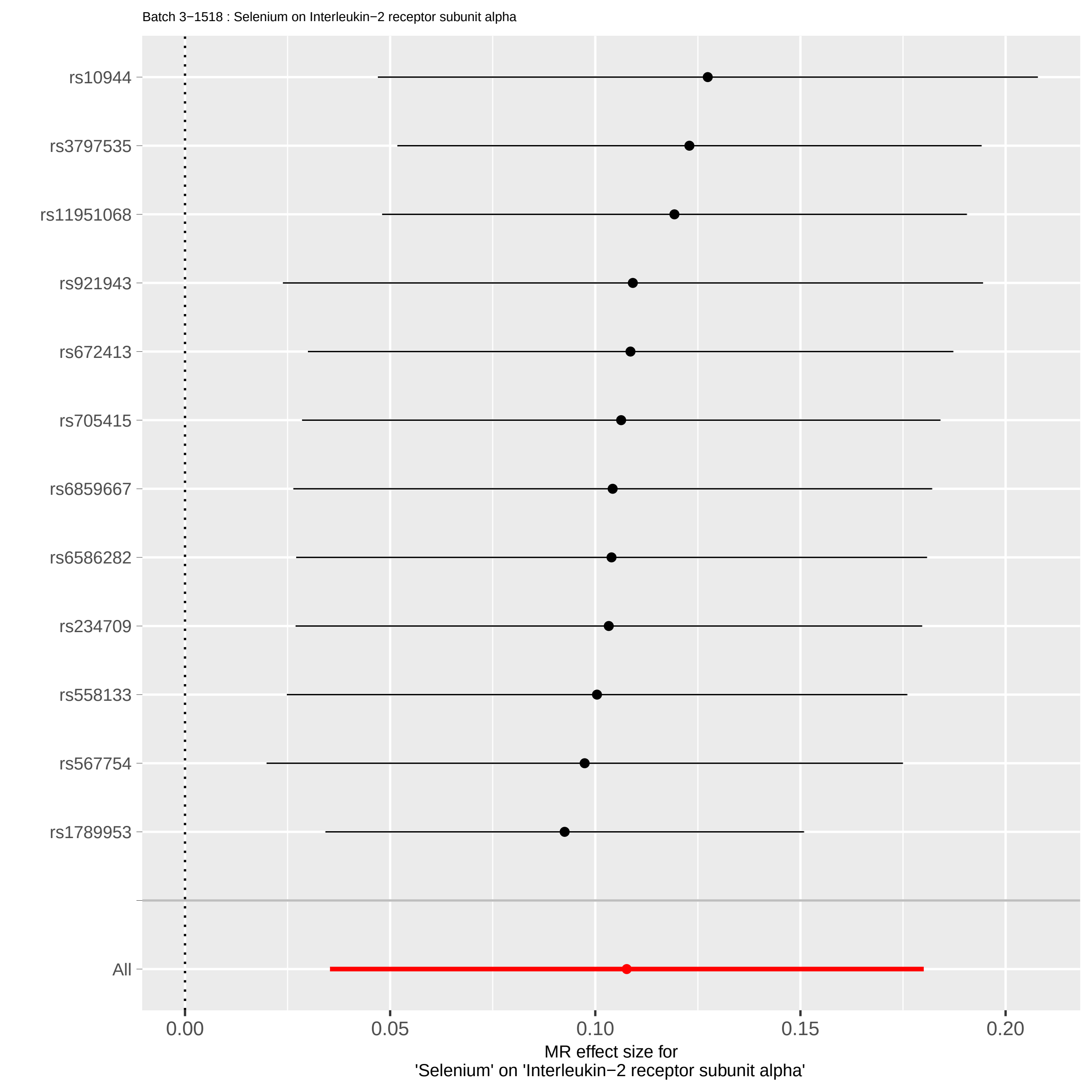

Batch 3−1518 : Selenium on Interleukin−2 receptor subunit alpha
rs10944
rs3797535
rs11951068
rs921943
rs672413
rs705415
rs6859667
rs6586282
rs234709
rs558133
rs567754
rs1789953
All
0.00
0.05
0.10
0.15
0.20
MR effect size for
'Selenium' on 'Interleukin−2 receptor subunit alpha'

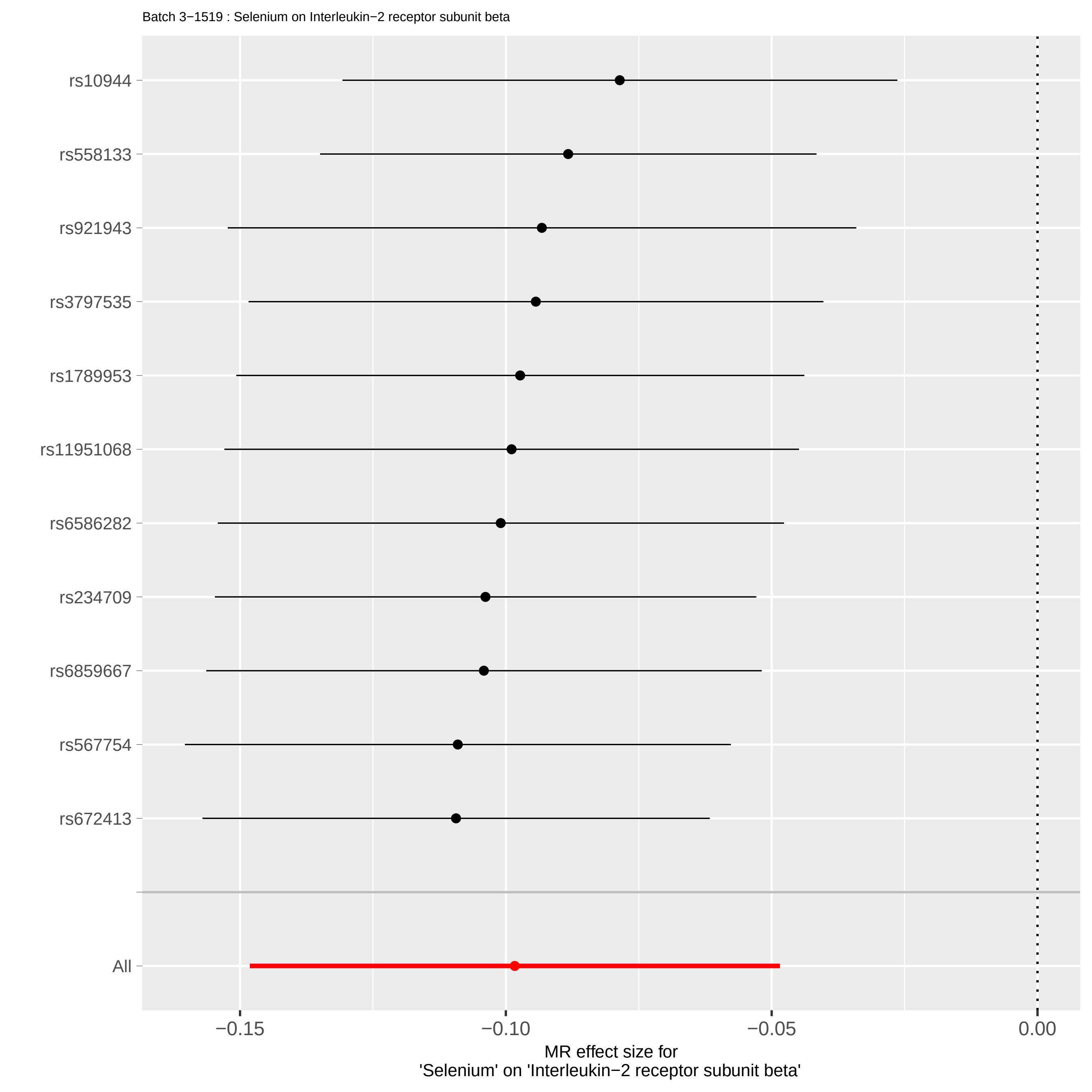

Batch 3−1519 : Selenium on Interleukin−2 receptor subunit beta
rs10944
rs558133
rs921943
rs3797535
rs1789953
rs11951068
rs6586282
rs234709
rs6859667
rs567754
rs672413
All
−0.15
−0.10
−0.05
0.00
MR effect size for
'Selenium' on 'Interleukin−2 receptor subunit beta'

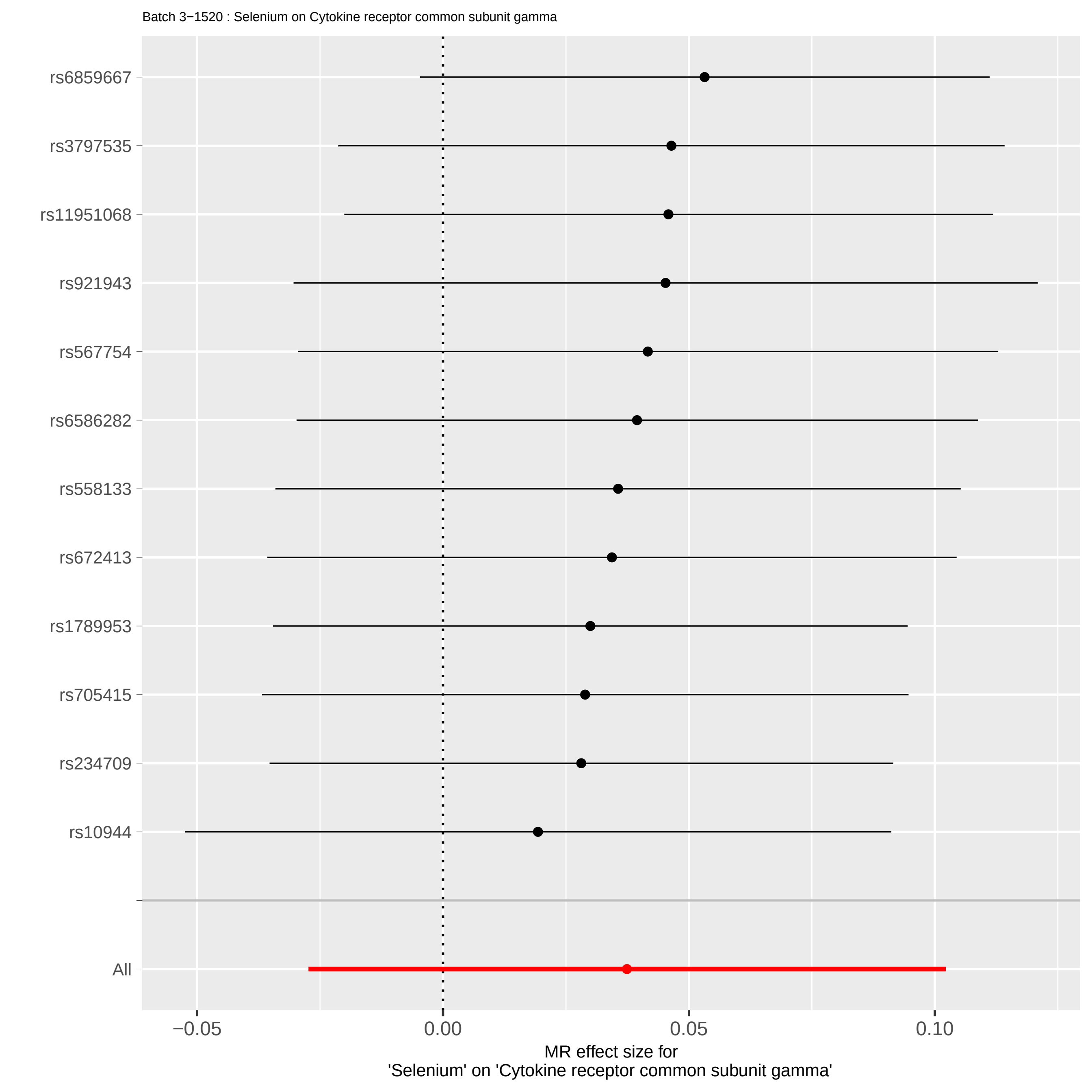

Batch 3−1520 : Selenium on Cytokine receptor common subunit gamma
rs6859667
rs3797535
rs11951068
rs921943
rs567754
rs6586282
rs558133
rs672413
rs1789953
rs705415
rs234709
rs10944
All
−0.05
0.00
0.05
0.10
MR effect size for
'Selenium' on 'Cytokine receptor common subunit gamma'

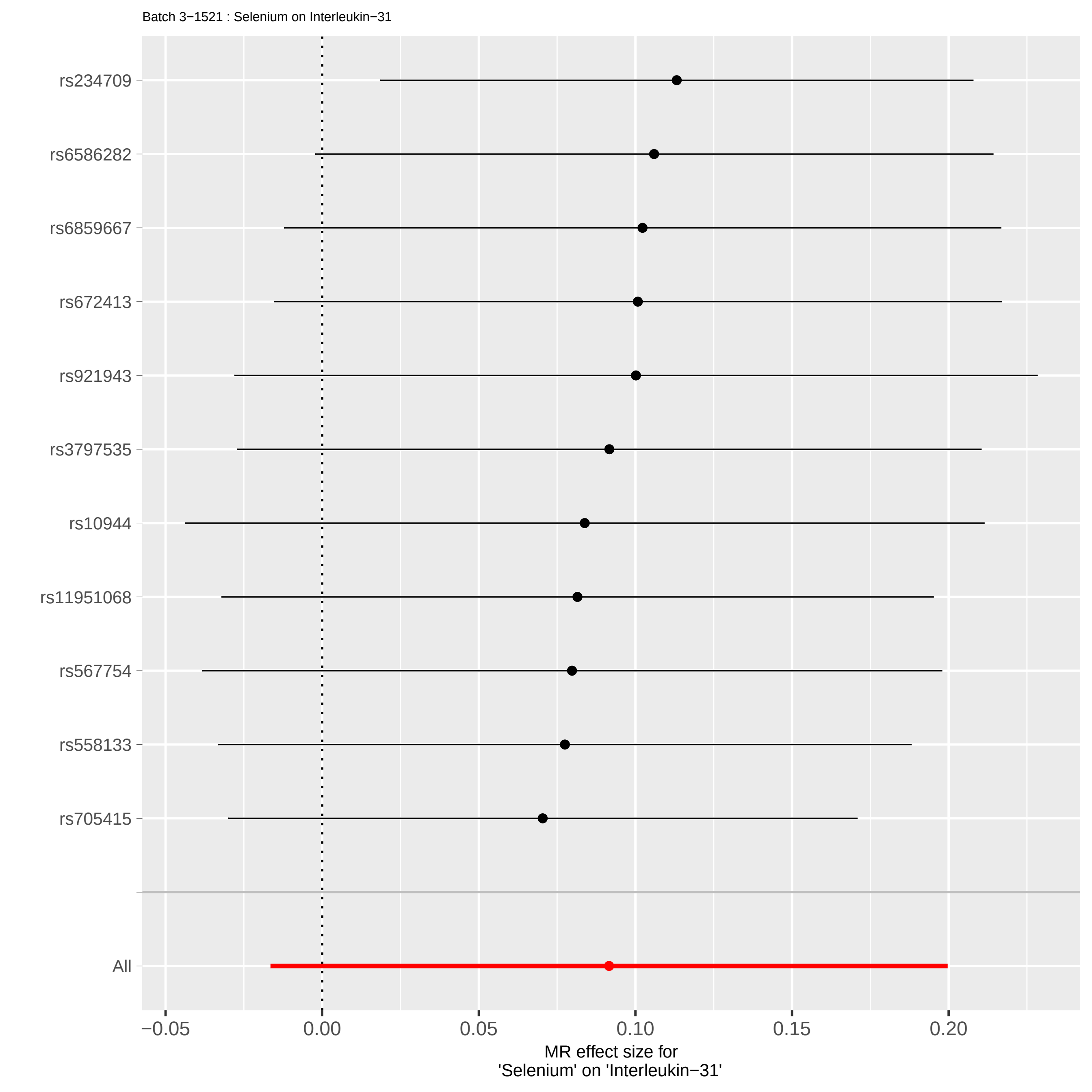
Batch 3−1521 : Selenium on Interleukin−31
rs234709
rs6586282
rs6859667
rs672413
rs921943
rs3797535
rs10944
rs11951068
rs567754
rs558133
rs705415
All
−0.05
0.00
0.05
0.10
0.15
0.20
MR effect size for
'Selenium' on 'Interleukin−31'

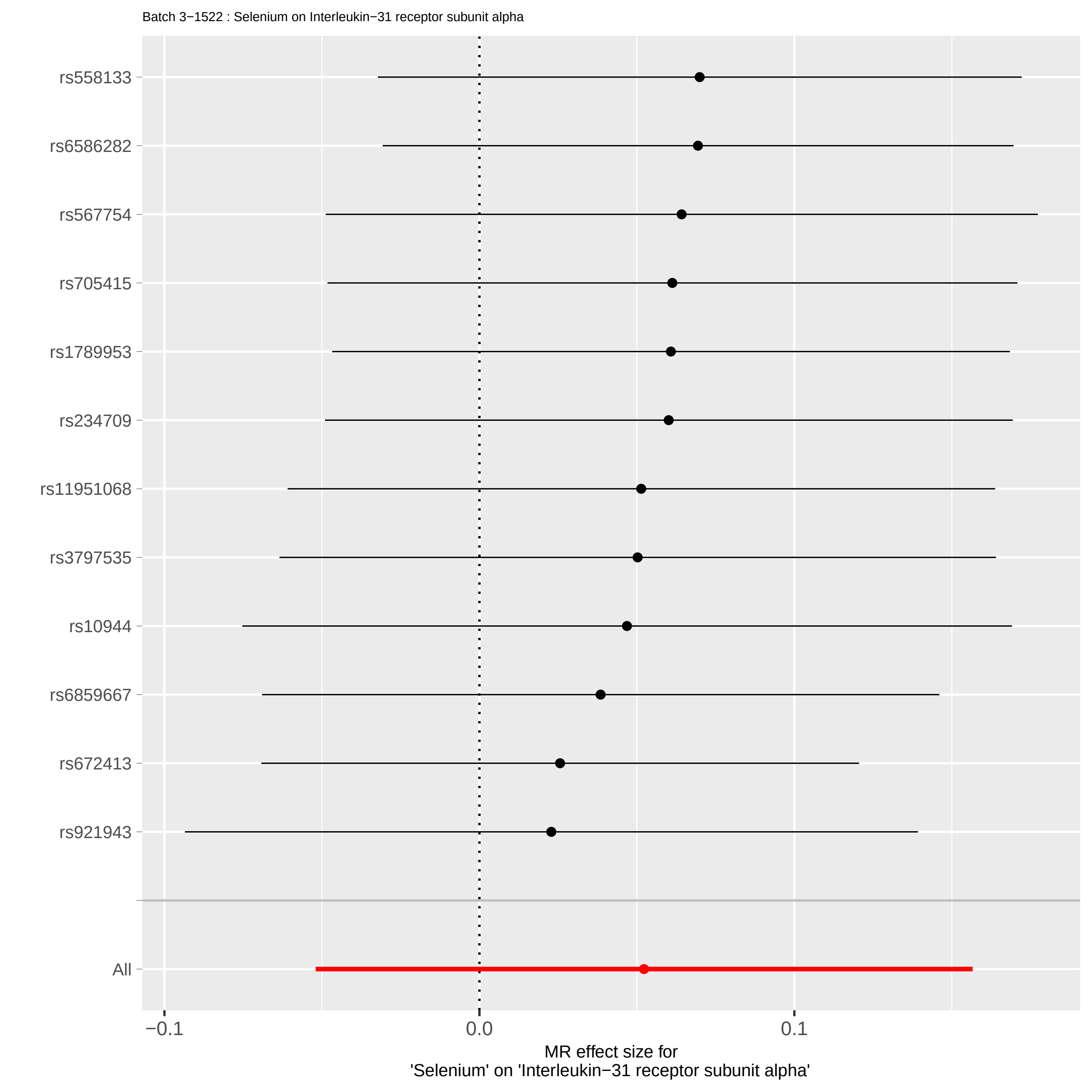

Batch 3−1522 : Selenium on Interleukin−31 receptor subunit alpha
rs558133
rs6586282
rs567754
rs705415
rs1789953
rs234709
rs11951068
rs3797535
rs10944
rs6859667
rs672413
rs921943
All
−0.1
0.0
0.1
MR effect size for
'Selenium' on 'Interleukin−31 receptor subunit alpha'

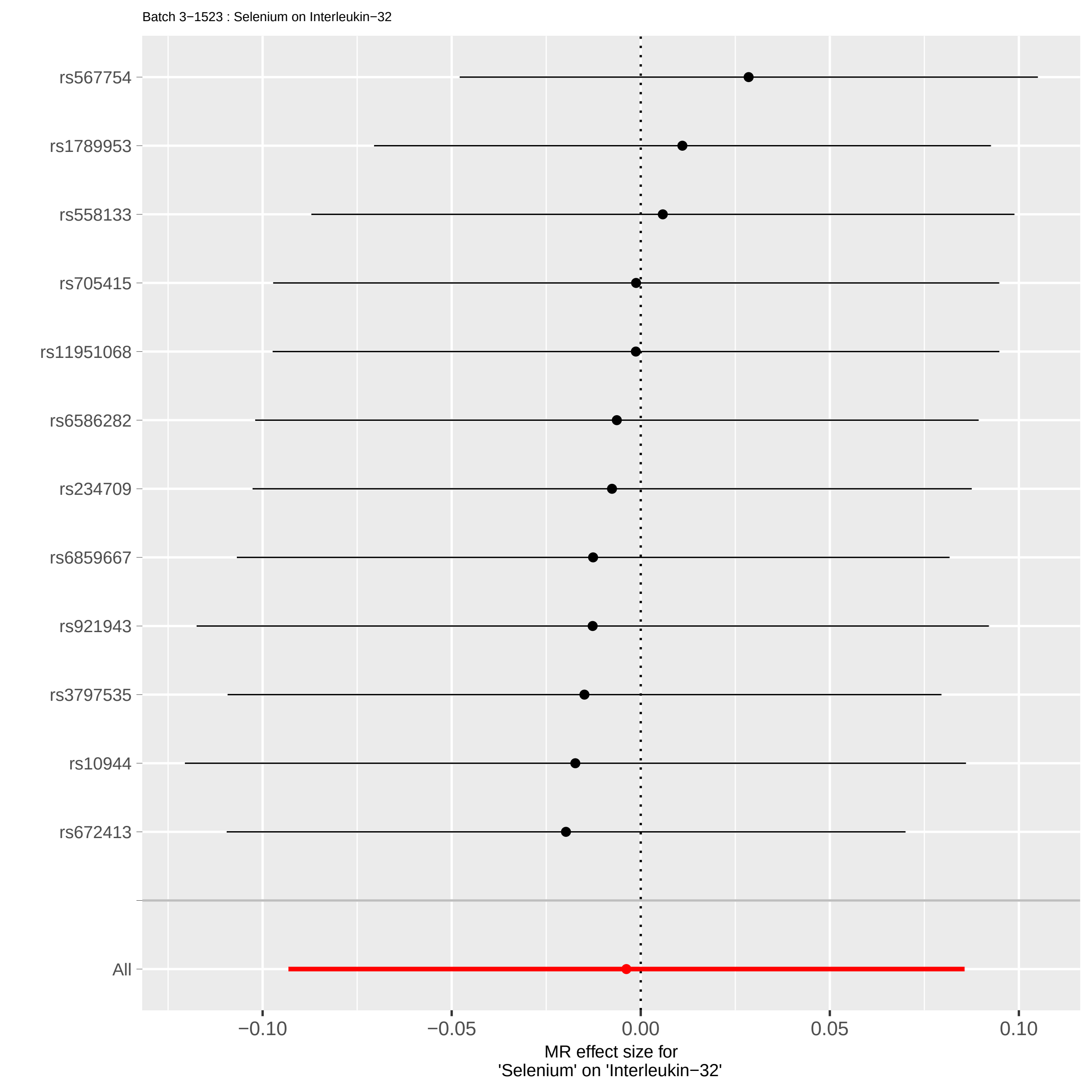
Batch 3−1523 : Selenium on Interleukin−32
rs567754
rs1789953
rs558133
rs705415
rs11951068
rs6586282
rs234709
rs6859667
rs921943
rs3797535
rs10944
rs672413
All
−0.10
−0.05
0.00
0.05
0.10
MR effect size for
'Selenium' on 'Interleukin−32'

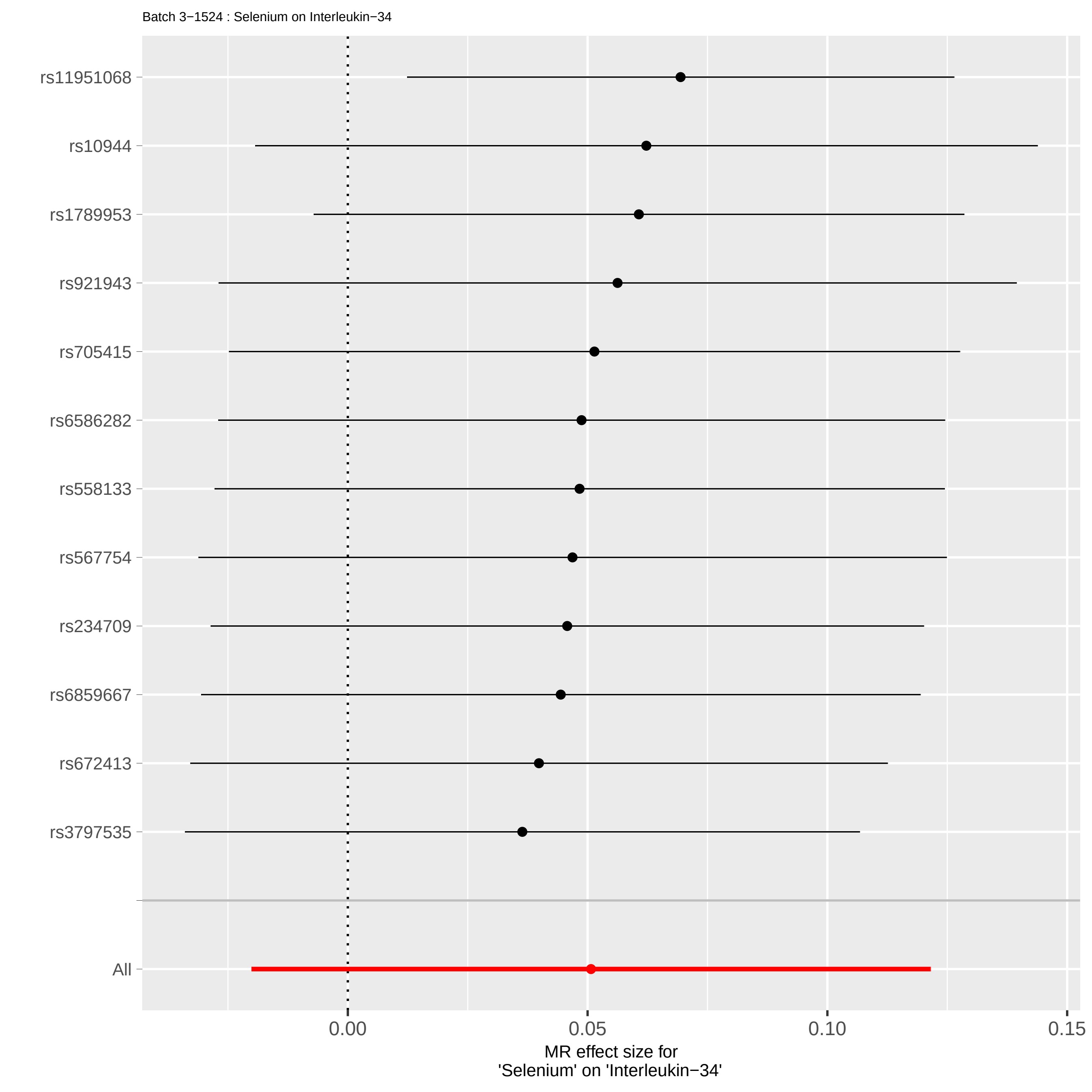

Batch 3−1524 : Selenium on Interleukin−34
rs11951068
rs10944
rs1789953
rs921943
rs705415
rs6586282
rs558133
rs567754
rs234709
rs6859667
rs672413
rs3797535
All
0.00
0.05
0.10
0.15
MR effect size for
'Selenium' on 'Interleukin−34'

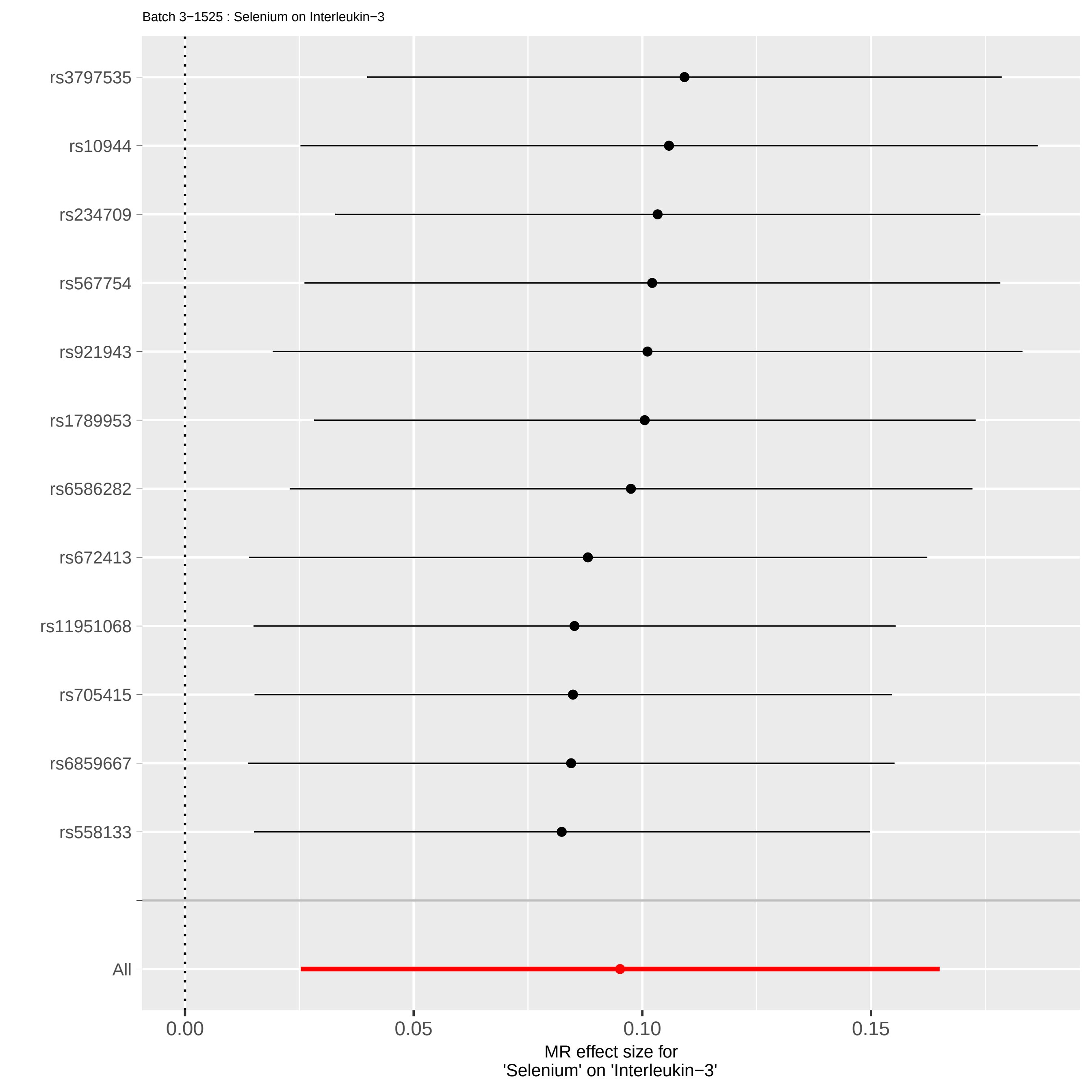

Batch 3−1525 : Selenium on Interleukin−3
rs3797535
rs10944
rs234709
rs567754
rs921943
rs1789953
rs6586282
rs672413
rs11951068
rs705415
rs6859667
rs558133
All
0.00
0.05
0.10
0.15
MR effect size for
'Selenium' on 'Interleukin−3'

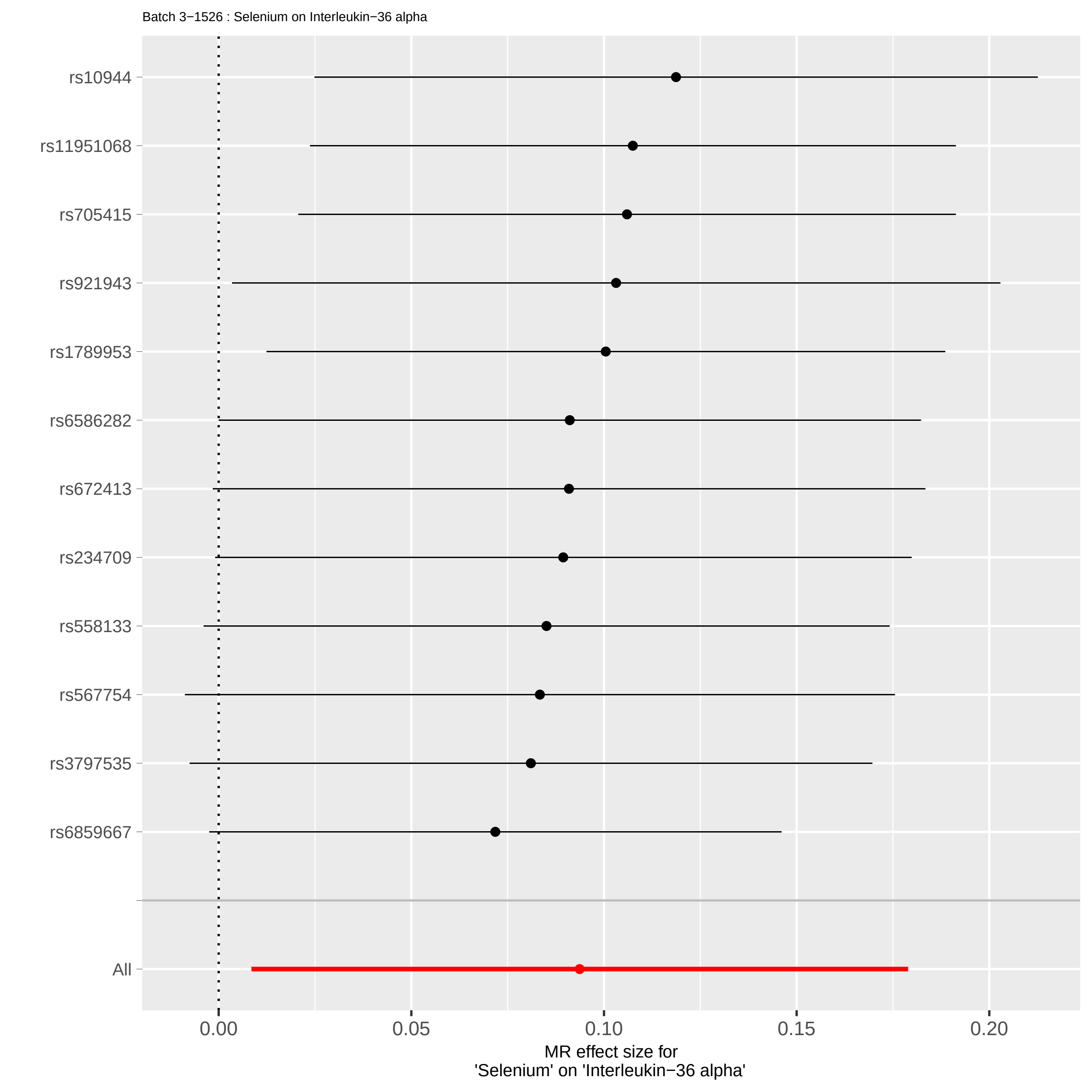

Batch 3−1526 : Selenium on Interleukin−36 alpha
rs10944
rs11951068
rs705415
rs921943
rs1789953
rs6586282
rs672413
rs234709
rs558133
rs567754
rs3797535
rs6859667
All
0.00
0.05
0.10
0.15
0.20
MR effect size for
'Selenium' on 'Interleukin−36 alpha'

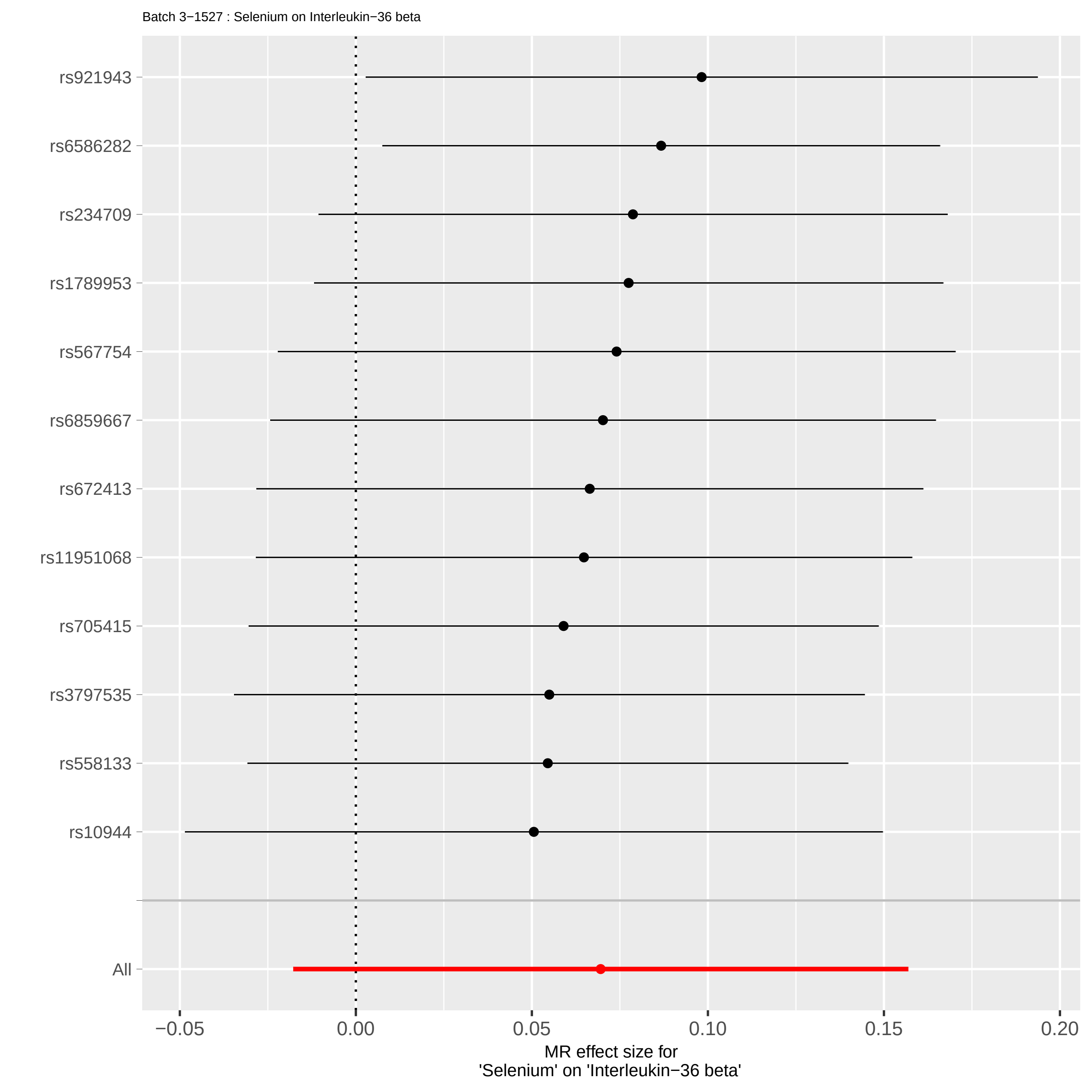

Batch 3−1527 : Selenium on Interleukin−36 beta
rs921943
rs6586282
rs234709
rs1789953
rs567754
rs6859667
rs672413
rs11951068
rs705415
rs3797535
rs558133
rs10944
All
−0.05
0.00
0.05
0.10
0.15
0.20
MR effect size for
'Selenium' on 'Interleukin−36 beta'

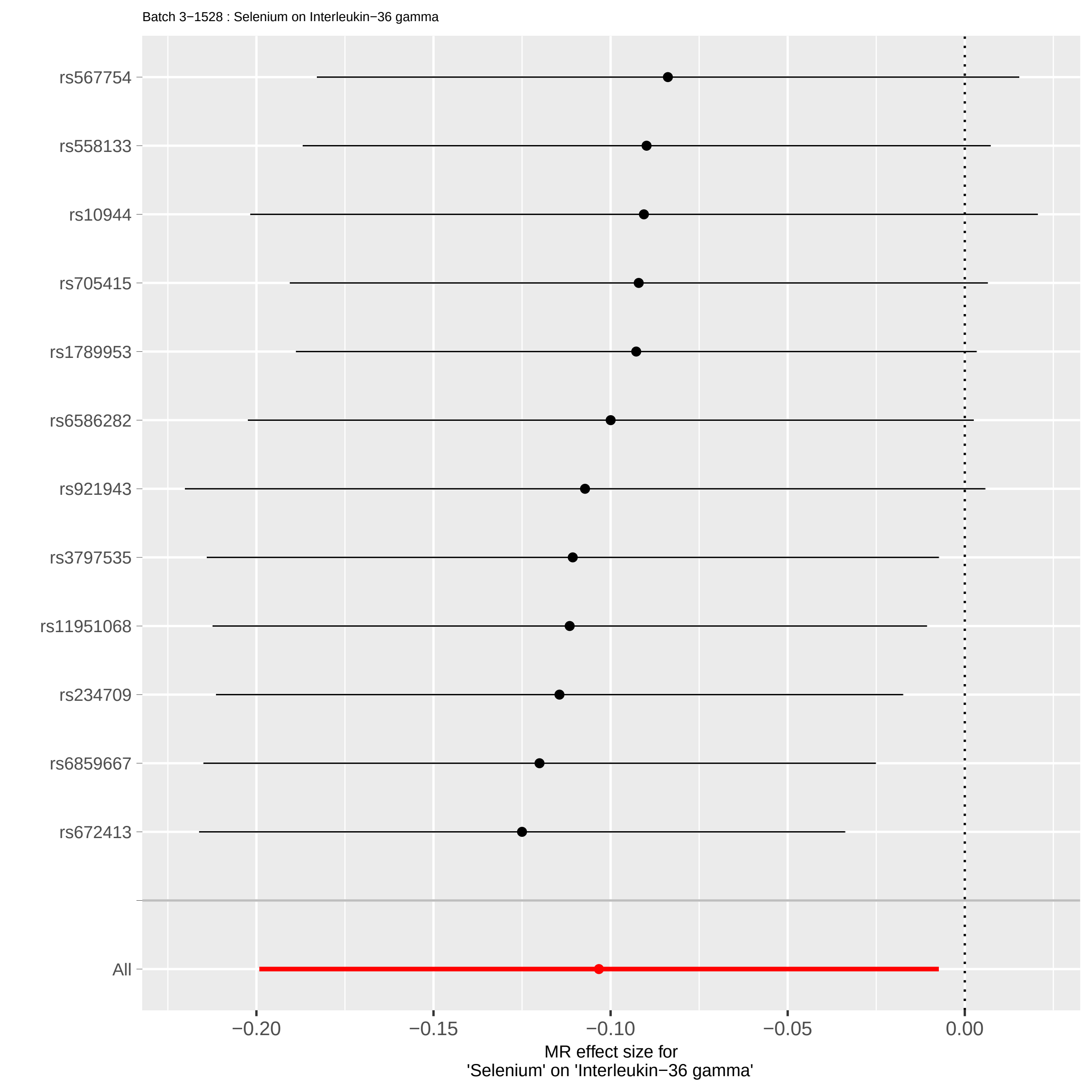

Batch 3−1528 : Selenium on Interleukin−36 gamma
rs567754
rs558133
rs10944
rs705415
rs1789953
rs6586282
rs921943
rs3797535
rs11951068
rs234709
rs6859667
rs672413
All
−0.20
−0.15
−0.10
−0.05
0.00
MR effect size for
'Selenium' on 'Interleukin−36 gamma'

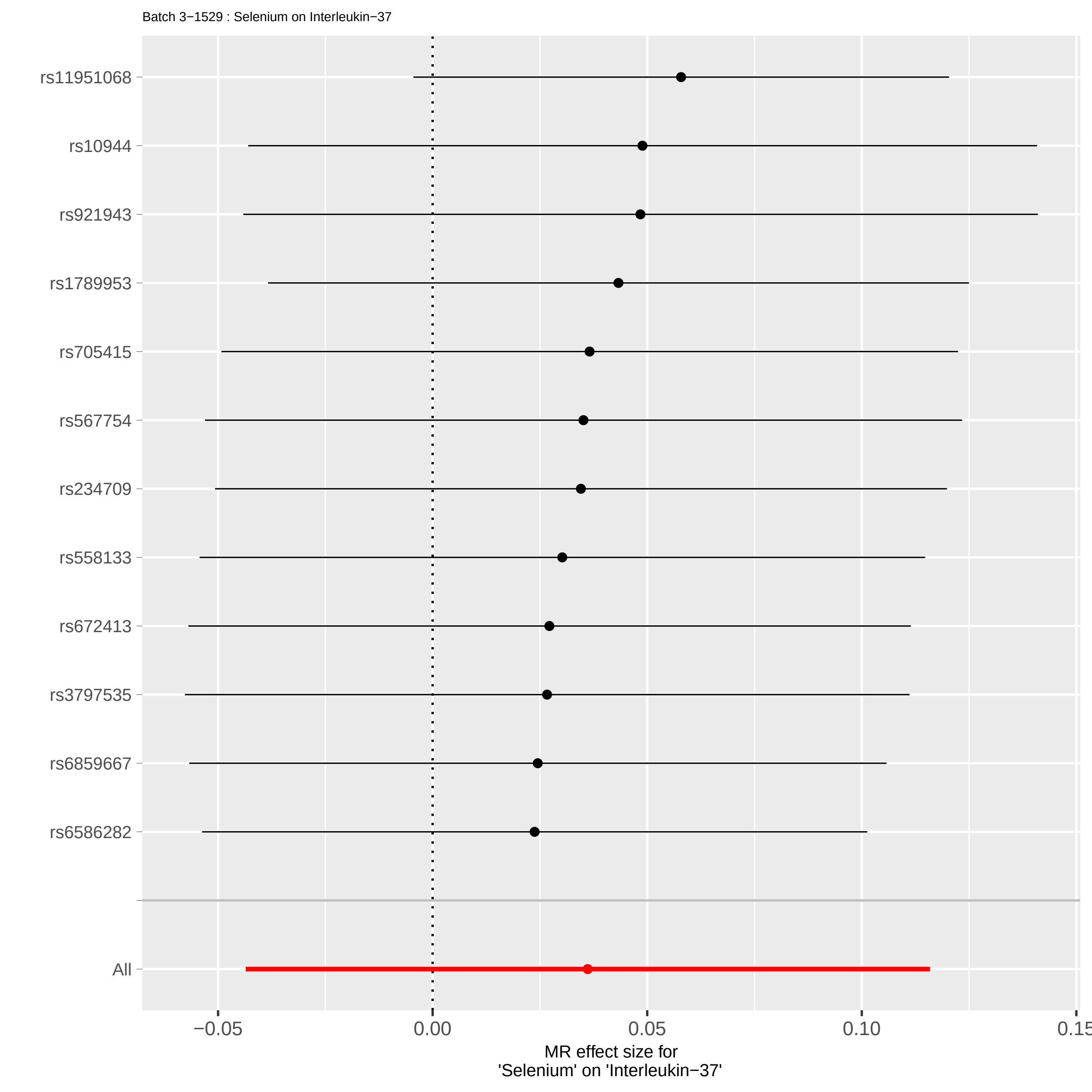

Batch 3−1529 : Selenium on Interleukin−37
rs11951068
rs10944
rs921943
rs1789953
rs705415
rs567754
rs234709
rs558133
rs672413
rs3797535
rs6859667
rs6586282
All
−0.05
0.00
0.05
0.10
0.15
MR effect size for
'Selenium' on 'Interleukin−37'

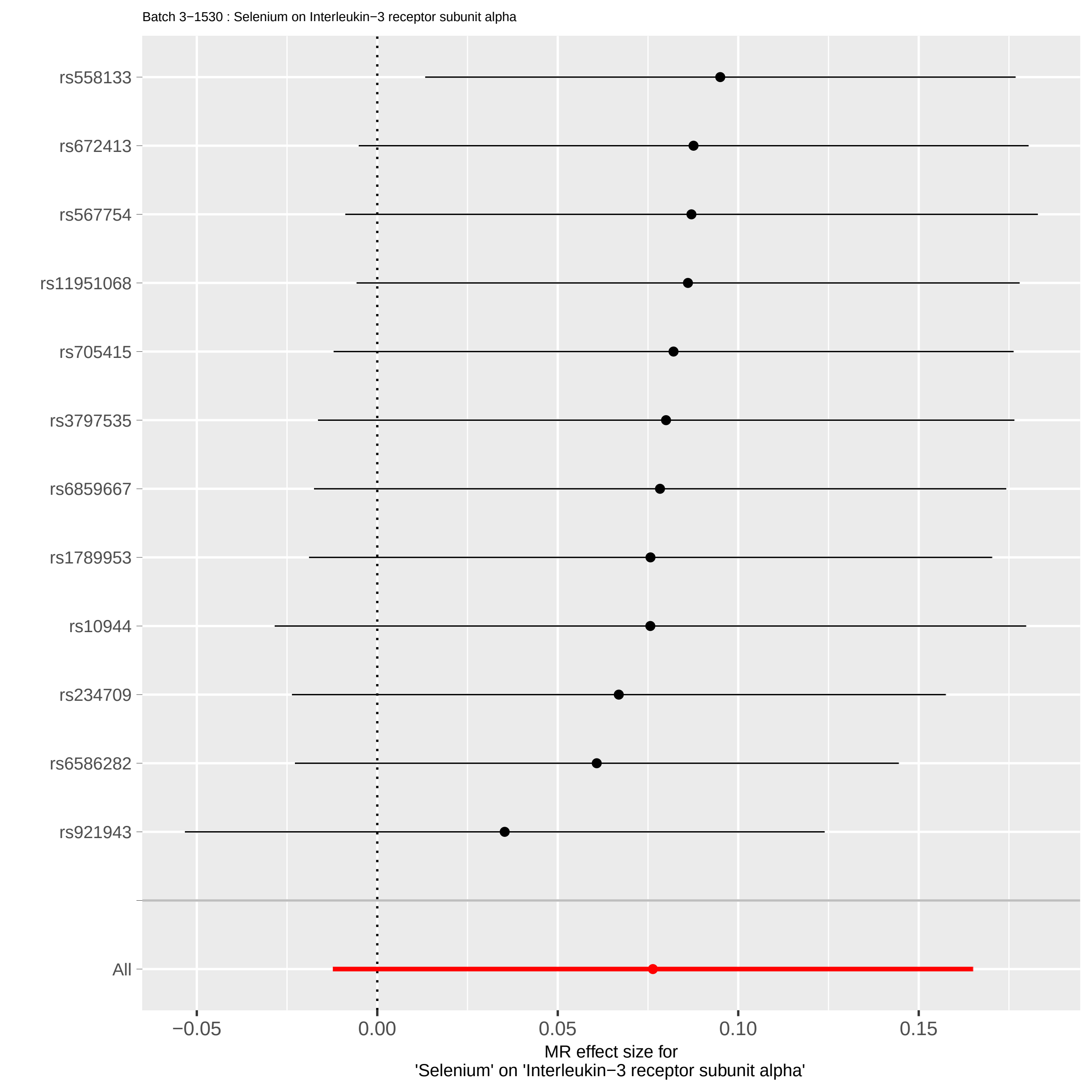

Batch 3−1530 : Selenium on Interleukin−3 receptor subunit alpha
rs558133
rs672413
rs567754
rs11951068
rs705415
rs3797535
rs6859667
rs1789953
rs10944
rs234709
rs6586282
rs921943
All
−0.05
0.00
0.05
0.10
0.15
MR effect size for
'Selenium' on 'Interleukin−3 receptor subunit alpha'

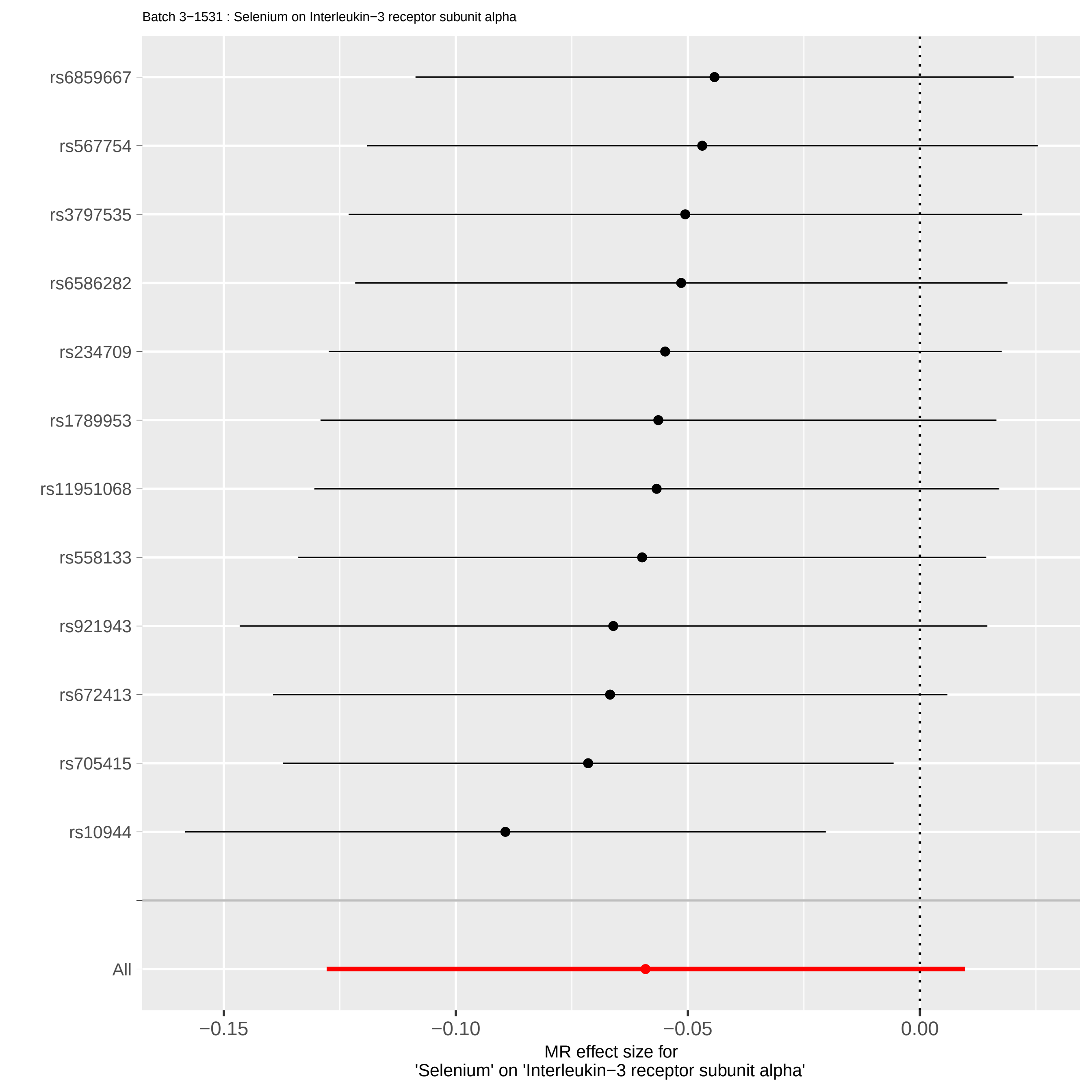
Batch 3−1531 : Selenium on Interleukin−3 receptor subunit alpha
rs6859667
rs567754
rs3797535
rs6586282
rs234709
rs1789953
rs11951068
rs558133
rs921943
rs672413
rs705415
rs10944
All
−0.15
−0.10
−0.05
0.00
MR effect size for
'Selenium' on 'Interleukin−3 receptor subunit alpha'

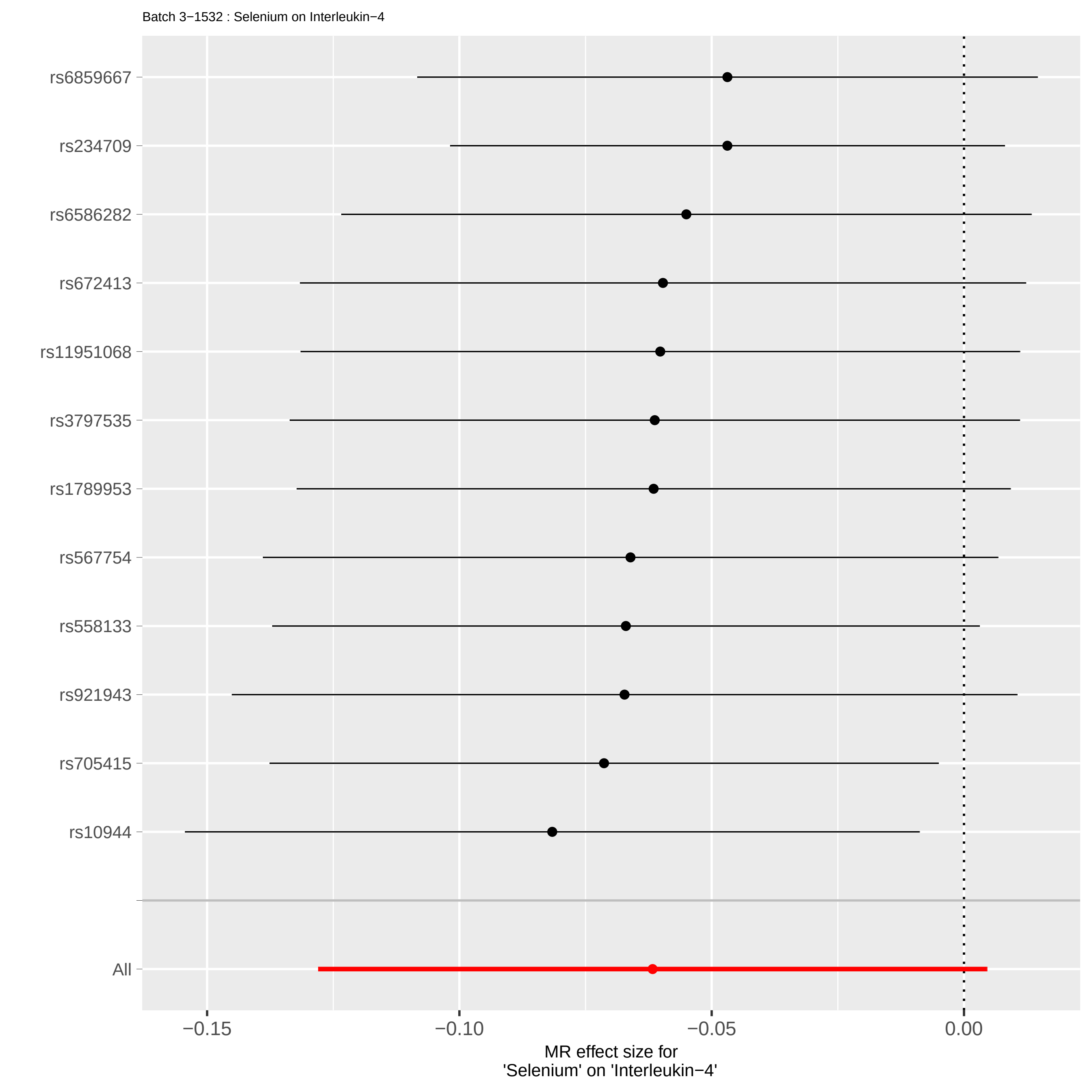
Batch 3−1532 : Selenium on Interleukin−4
rs6859667
rs234709
rs6586282
rs672413
rs11951068
rs3797535
rs1789953
rs567754
rs558133
rs921943
rs705415
rs10944
All
−0.15
−0.10
−0.05
0.00
MR effect size for
'Selenium' on 'Interleukin−4'

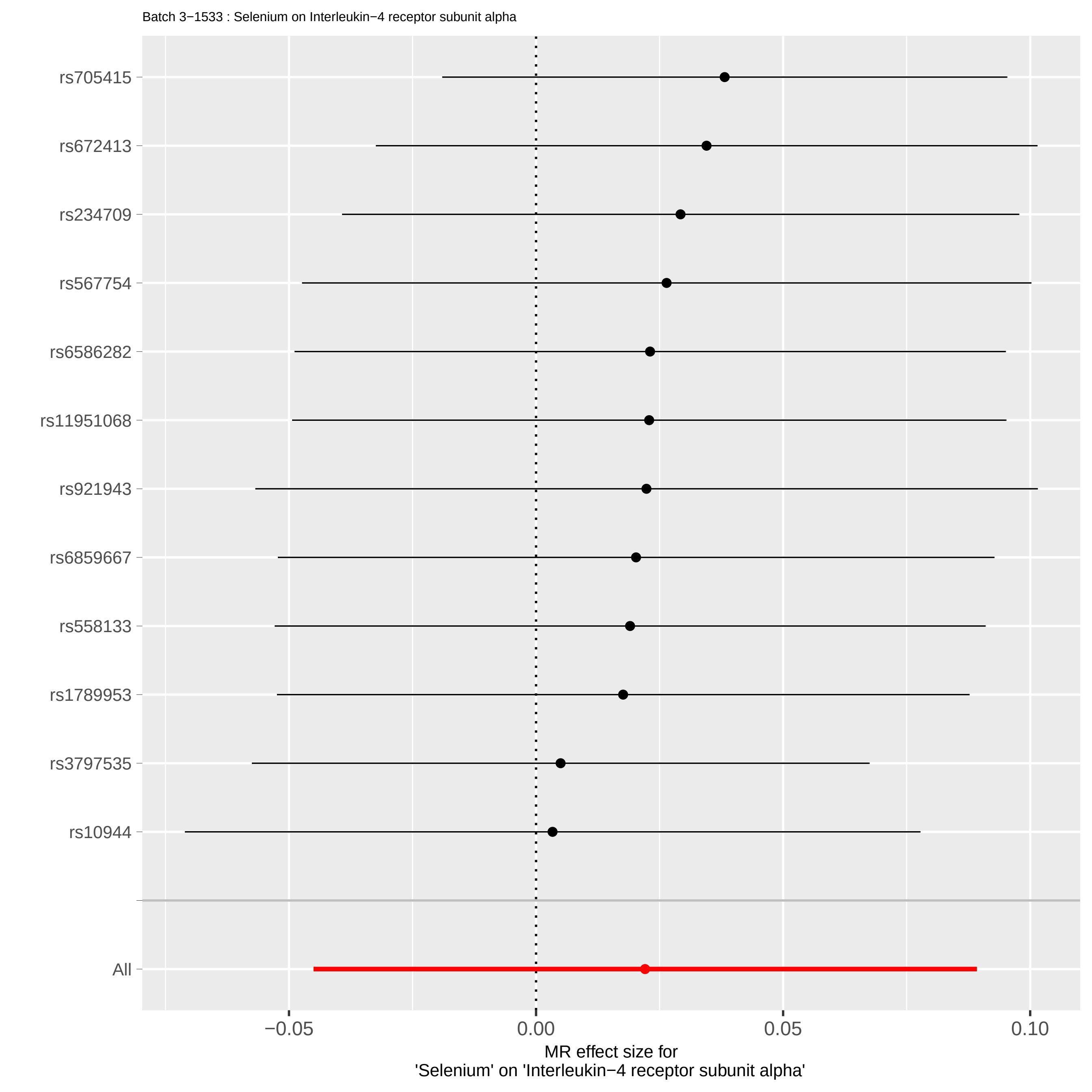

Batch 3−1533 : Selenium on Interleukin−4 receptor subunit alpha
rs705415
rs672413
rs234709
rs567754
rs6586282
rs11951068
rs921943
rs6859667
rs558133
rs1789953
rs3797535
rs10944
All
−0.05
0.00
0.05
0.10
MR effect size for
'Selenium' on 'Interleukin−4 receptor subunit alpha'

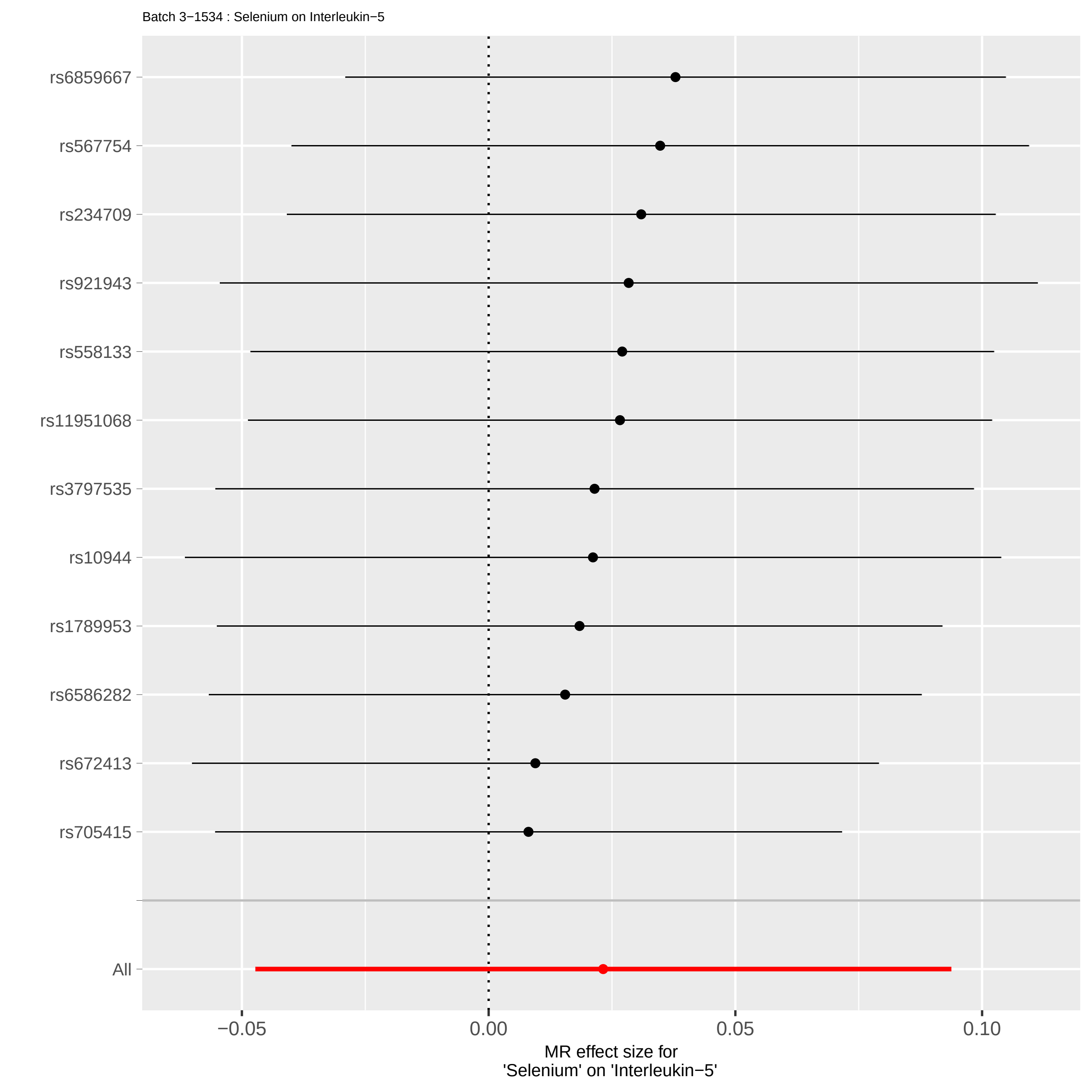

Batch 3−1534 : Selenium on Interleukin−5
rs6859667
rs567754
rs234709
rs921943
rs558133
rs11951068
rs3797535
rs10944
rs1789953
rs6586282
rs672413
rs705415
All
−0.05
0.00
0.05
0.10
MR effect size for
'Selenium' on 'Interleukin−5'

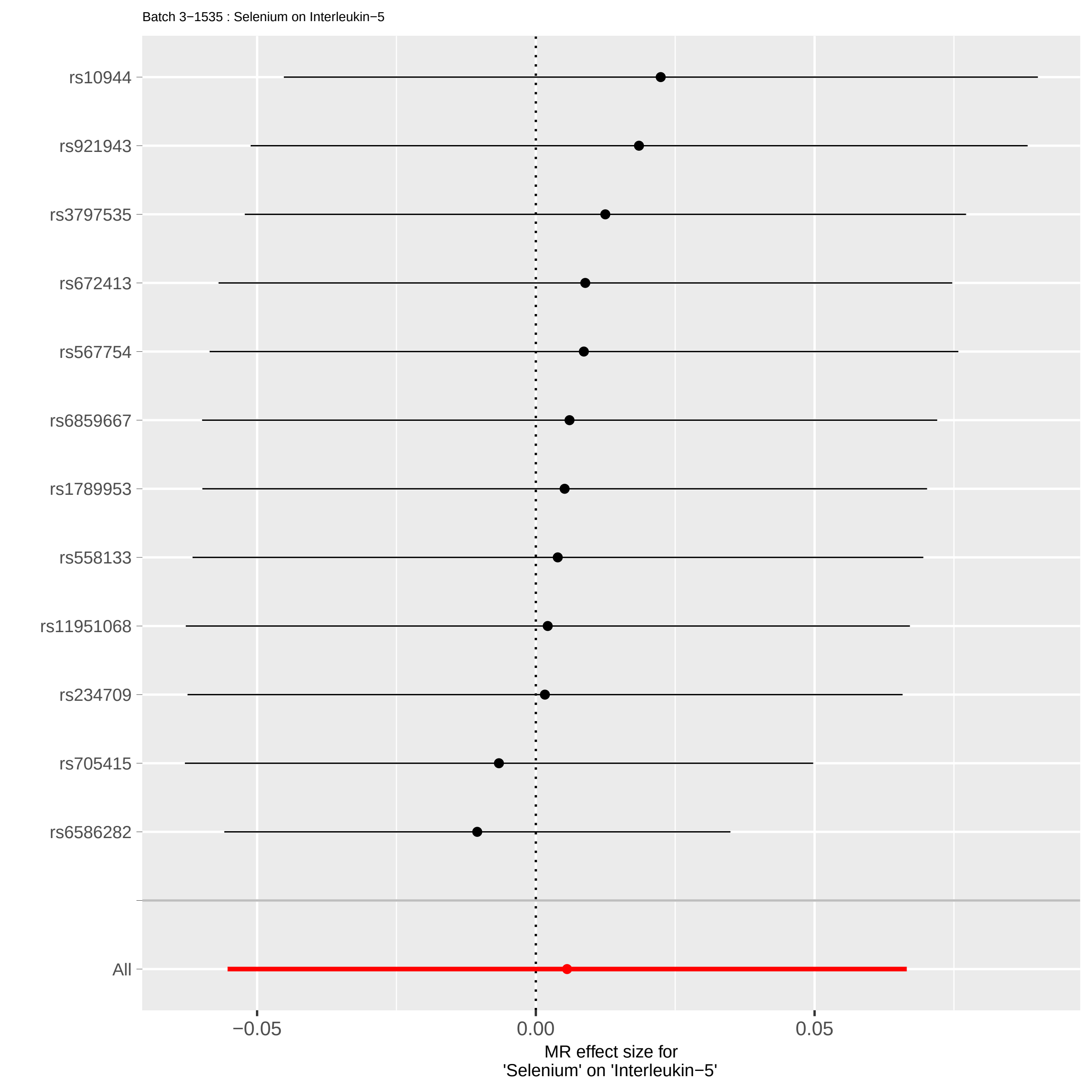

Batch 3−1535 : Selenium on Interleukin−5
rs10944
rs921943
rs3797535
rs672413
rs567754
rs6859667
rs1789953
rs558133
rs11951068
rs234709
rs705415
rs6586282
All
−0.05
0.00
0.05
MR effect size for
'Selenium' on 'Interleukin−5'

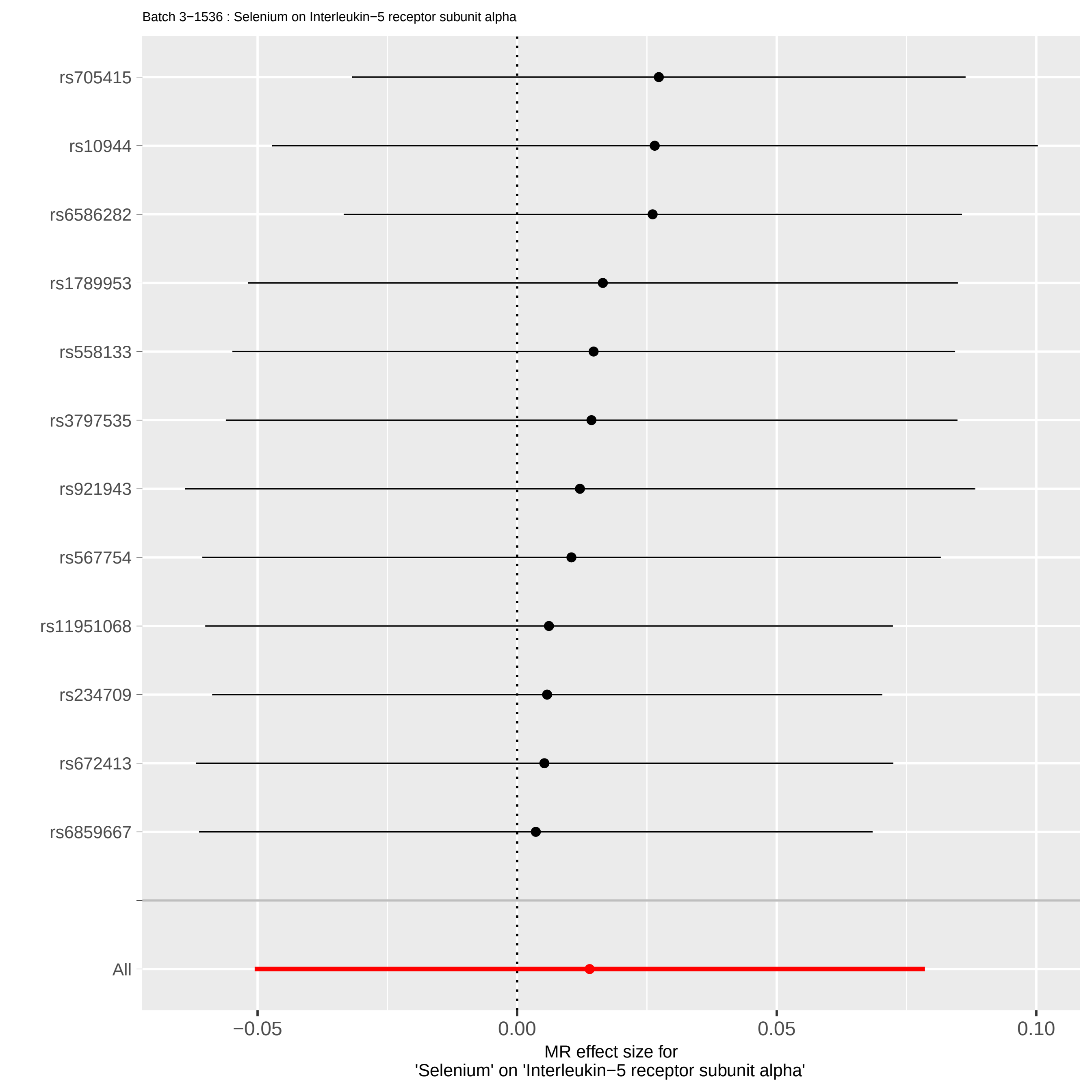

Batch 3−1536 : Selenium on Interleukin−5 receptor subunit alpha
rs705415
rs10944
rs6586282
rs1789953
rs558133
rs3797535
rs921943
rs567754
rs11951068
rs234709
rs672413
rs6859667
All
−0.05
0.00
0.05
0.10
MR effect size for
'Selenium' on 'Interleukin−5 receptor subunit alpha'

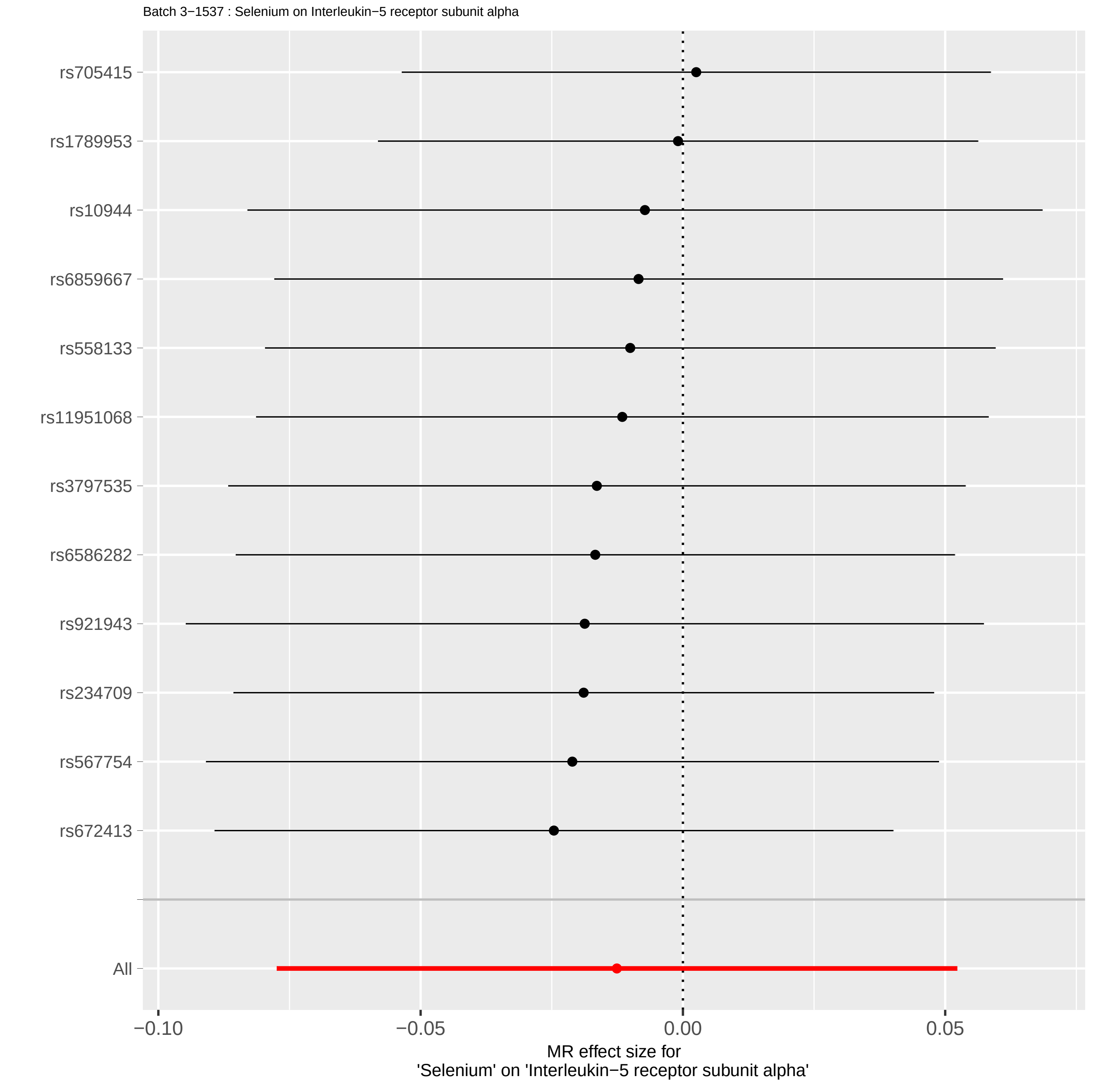

Batch 3−1537 : Selenium on Interleukin−5 receptor subunit alpha
rs705415
rs1789953
rs10944
rs6859667
rs558133
rs11951068
rs3797535
rs6586282
rs921943
rs234709
rs567754
rs672413
All
−0.10
−0.05
0.00
0.05
MR effect size for
'Selenium' on 'Interleukin−5 receptor subunit alpha'

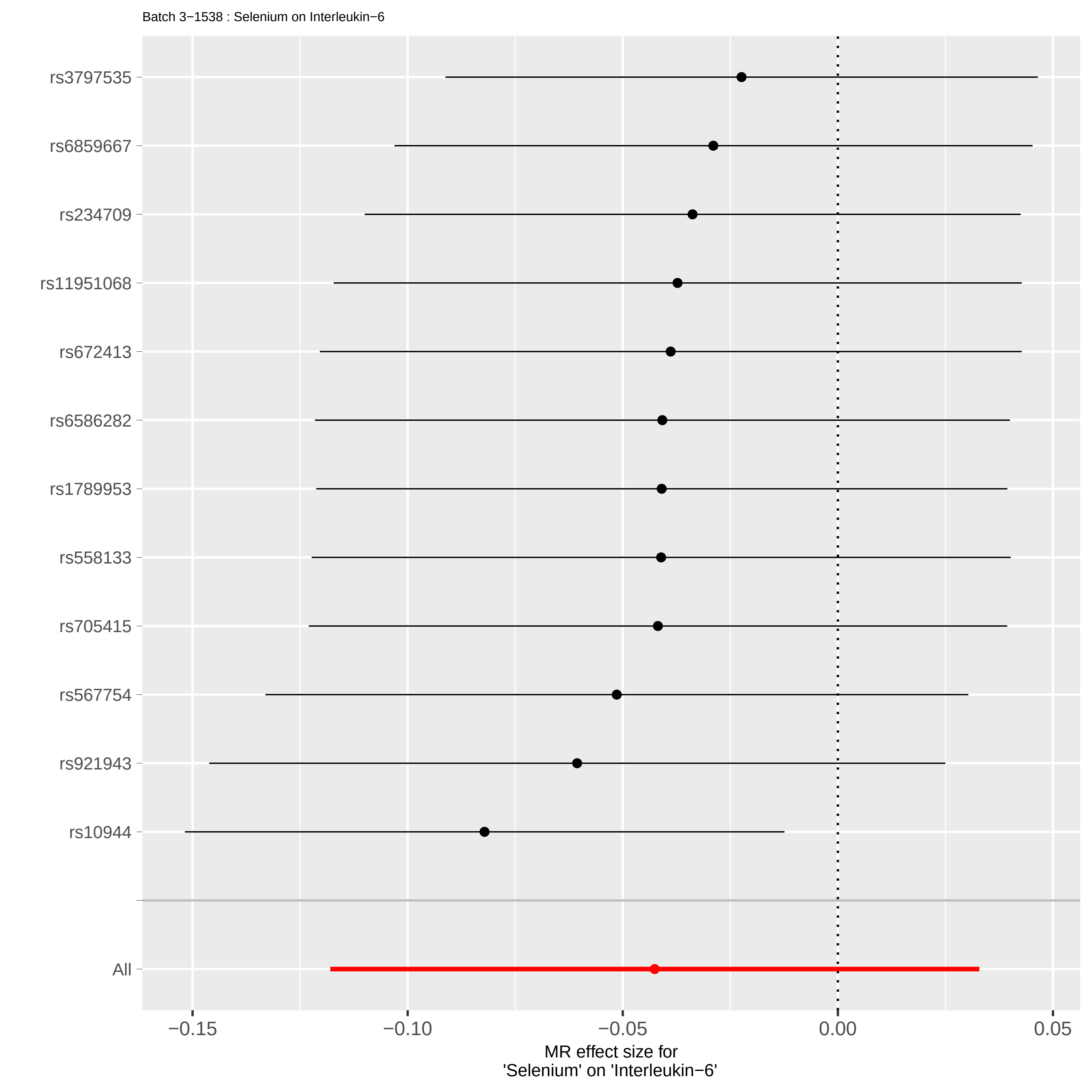

Batch 3−1538 : Selenium on Interleukin−6
rs3797535
rs6859667
rs234709
rs11951068
rs672413
rs6586282
rs1789953
rs558133
rs705415
rs567754
rs921943
rs10944
All
−0.15
−0.10
−0.05
0.00
0.05
MR effect size for
'Selenium' on 'Interleukin−6'

Batch 3−1539 : Selenium on Interleukin−6

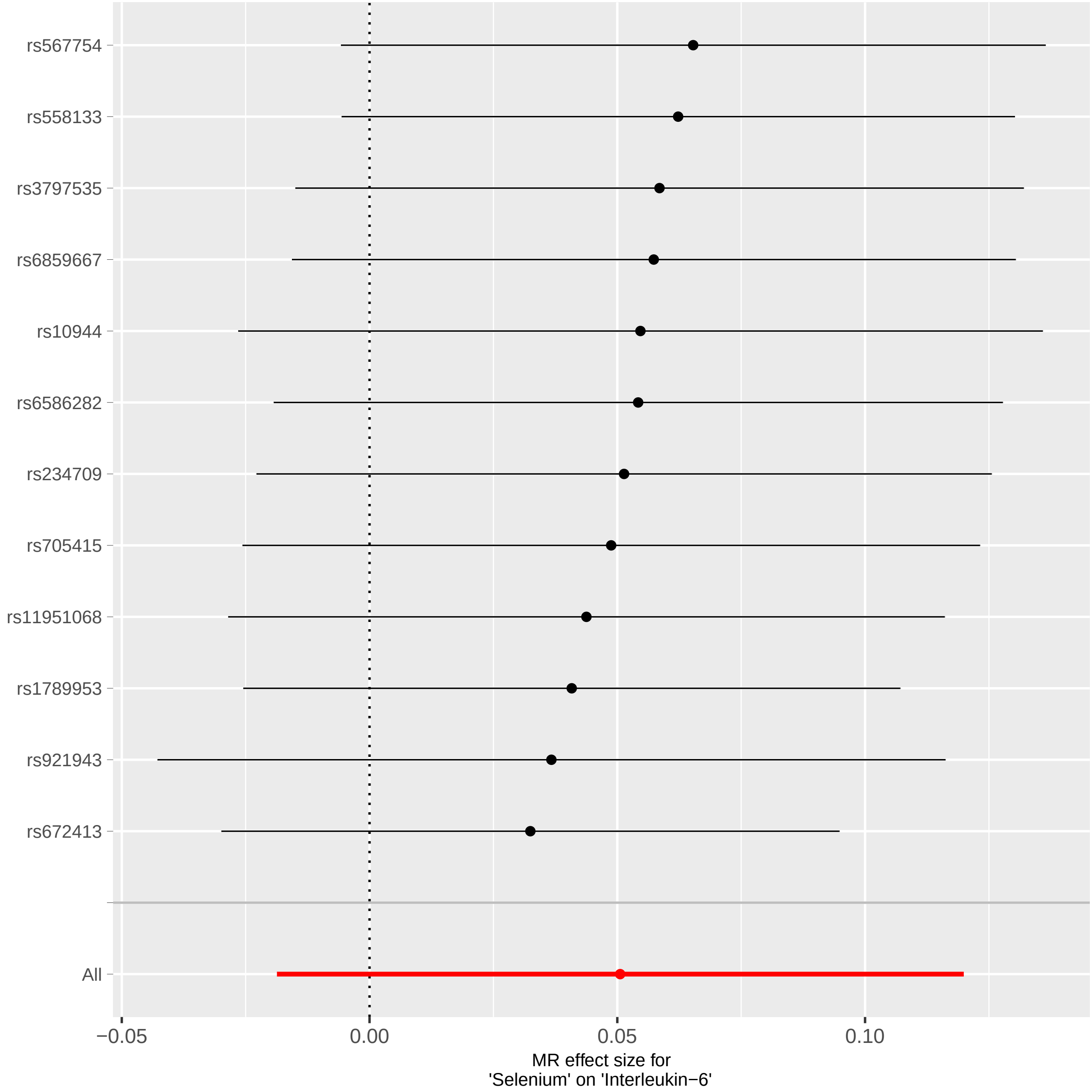

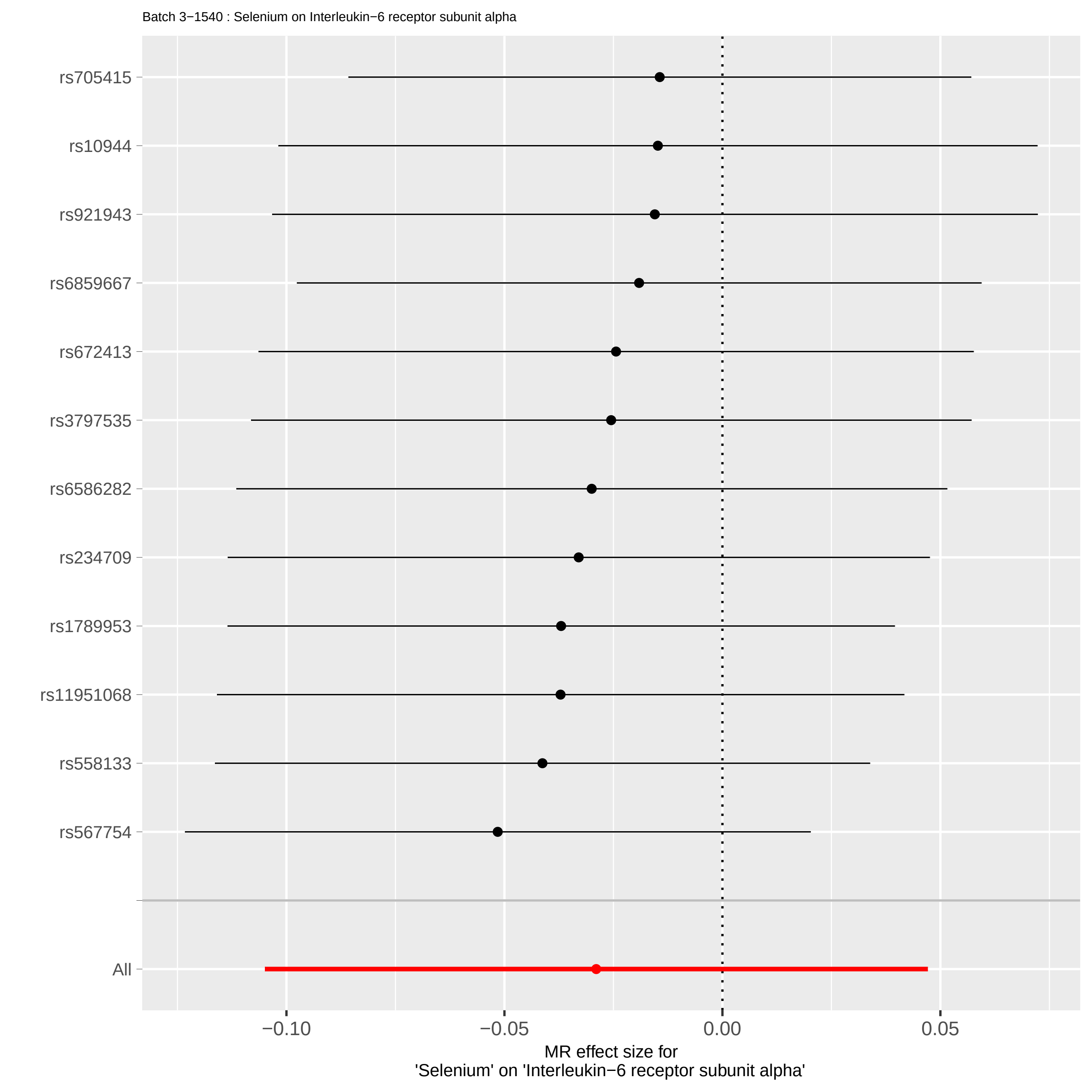

Batch 3−1540 : Selenium on Interleukin−6 receptor subunit alpha
rs705415
rs10944
rs921943
rs6859667
rs672413
rs3797535
rs6586282
rs234709
rs1789953
rs11951068
rs558133
rs567754
All
−0.10
−0.05
0.00
0.05
MR effect size for
'Selenium' on 'Interleukin−6 receptor subunit alpha'

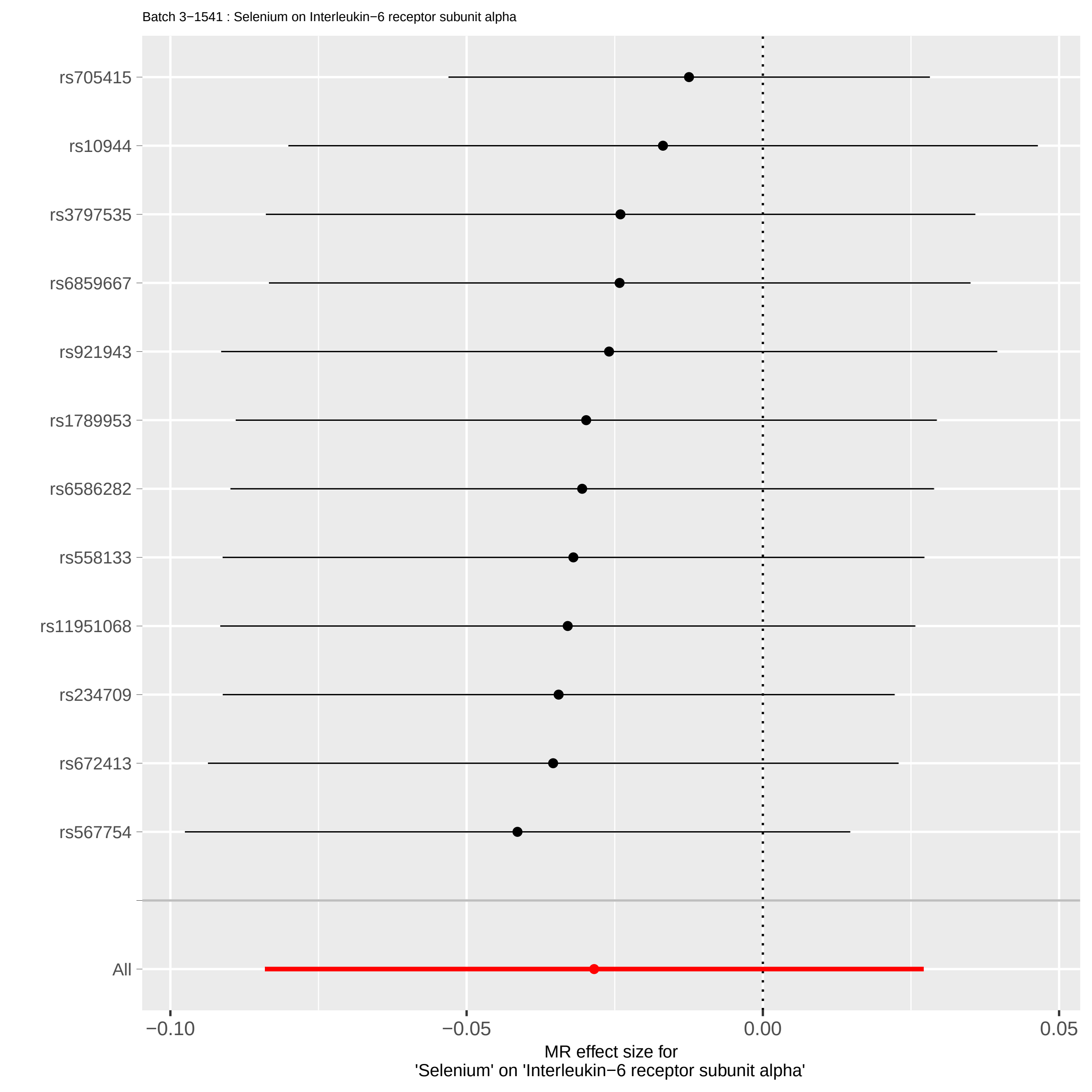

Batch 3−1541 : Selenium on Interleukin−6 receptor subunit alpha
rs705415
rs10944
rs3797535
rs6859667
rs921943
rs1789953
rs6586282
rs558133
rs11951068
rs234709
rs672413
rs567754
All
−0.10
−0.05
0.00
0.05
MR effect size for
'Selenium' on 'Interleukin−6 receptor subunit alpha'

Batch 3−1542 : Selenium on Interleukin−6 receptor subunit beta

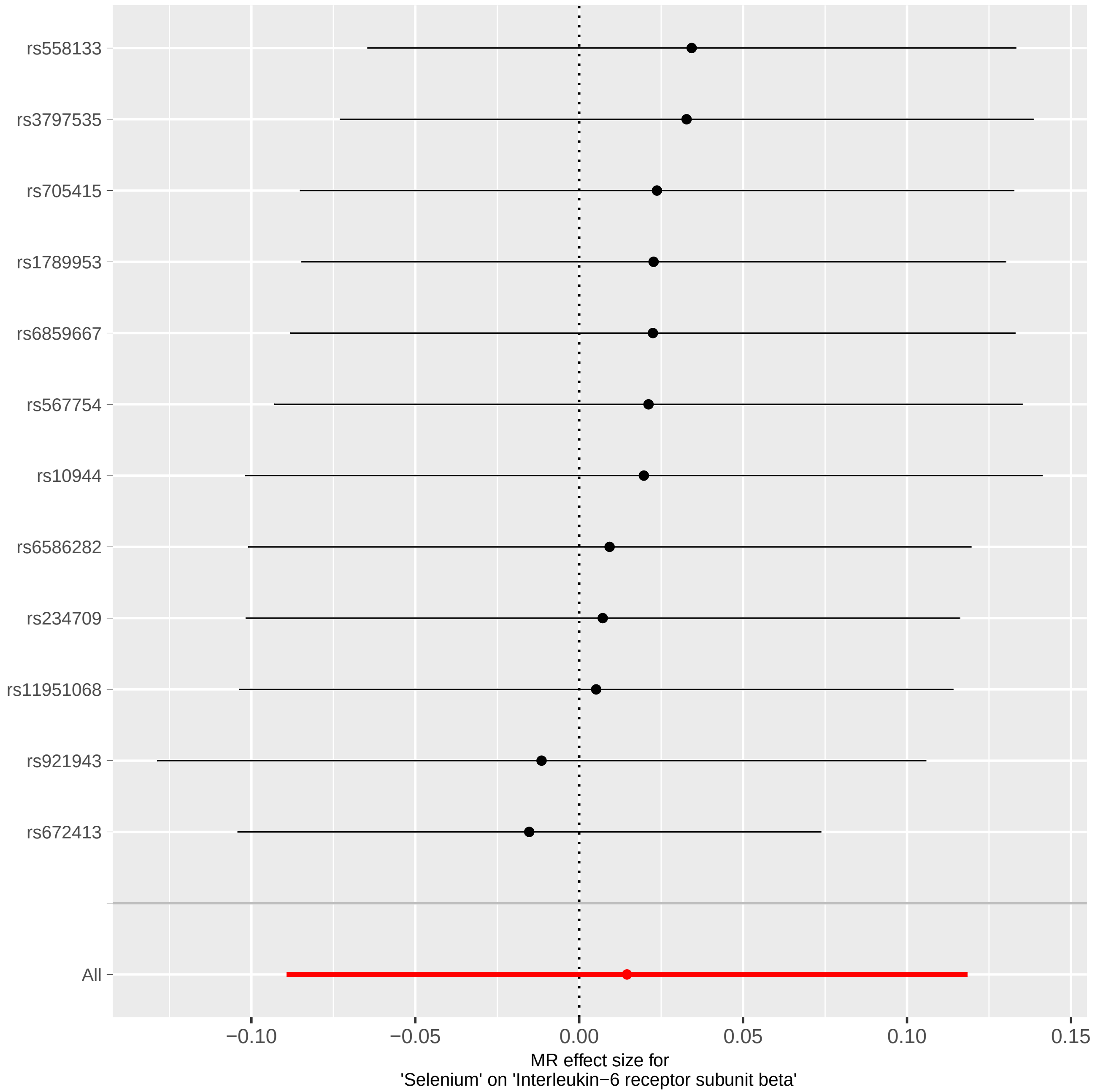

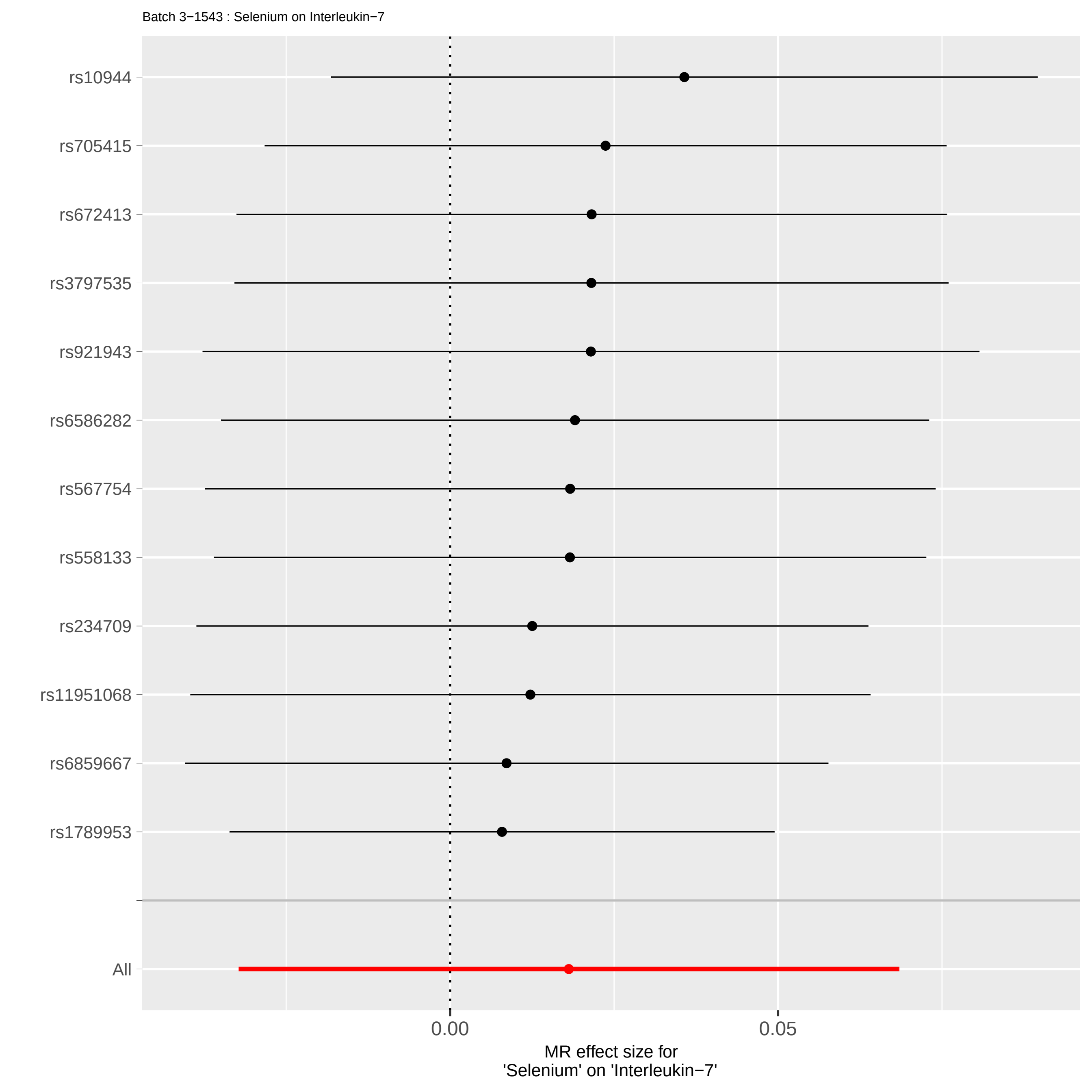

Batch 3−1543 : Selenium on Interleukin−7
rs10944
rs705415
rs672413
rs3797535
rs921943
rs6586282
rs567754
rs558133
rs234709
rs11951068
rs6859667
rs1789953
All
0.00
0.05
MR effect size for
'Selenium' on 'Interleukin−7'

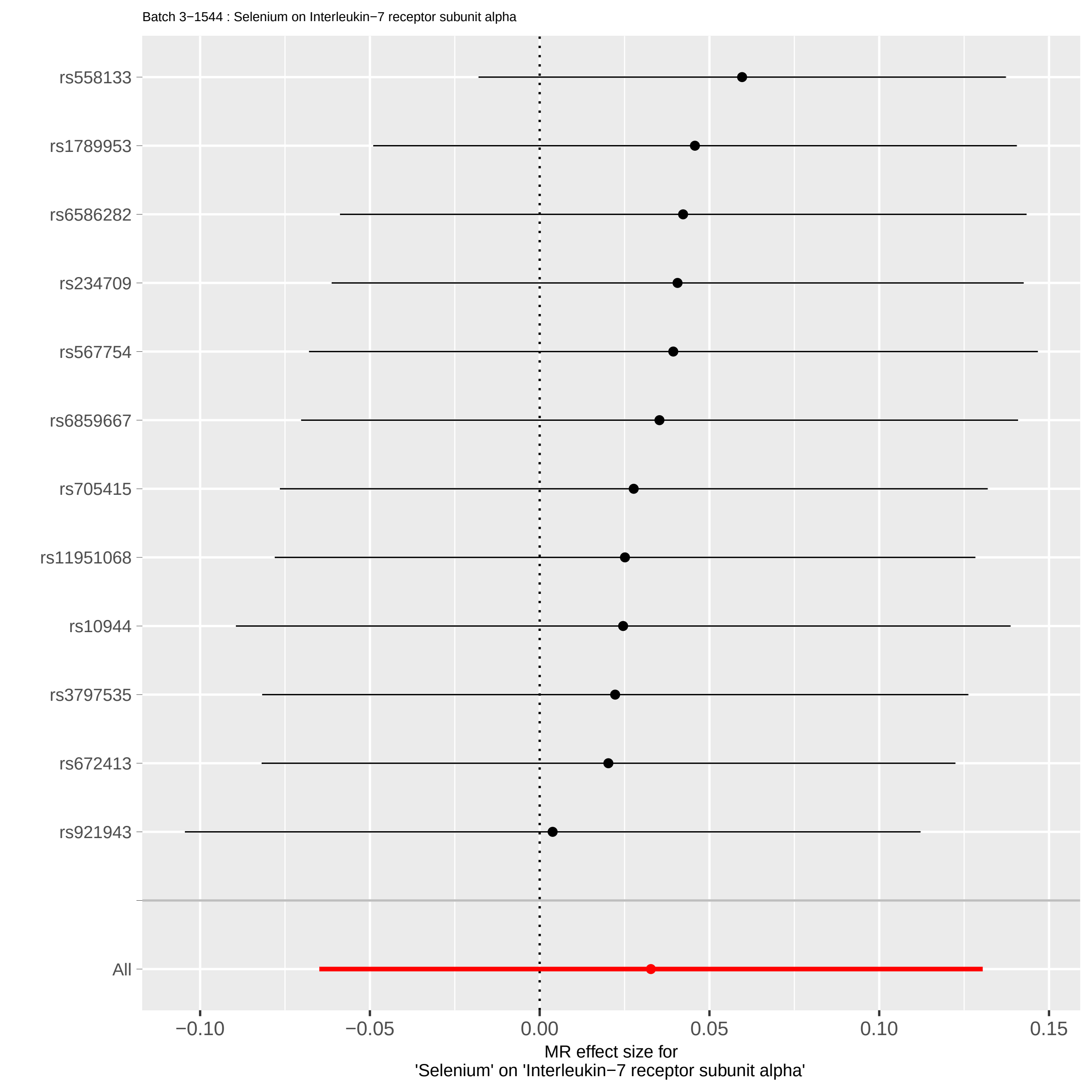

Batch 3−1544 : Selenium on Interleukin−7 receptor subunit alpha
rs558133
rs1789953
rs6586282
rs234709
rs567754
rs6859667
rs705415
rs11951068
rs10944
rs3797535
rs672413
rs921943
All
−0.10
−0.05
0.00
0.05
0.10
0.15
MR effect size for
'Selenium' on 'Interleukin−7 receptor subunit alpha'

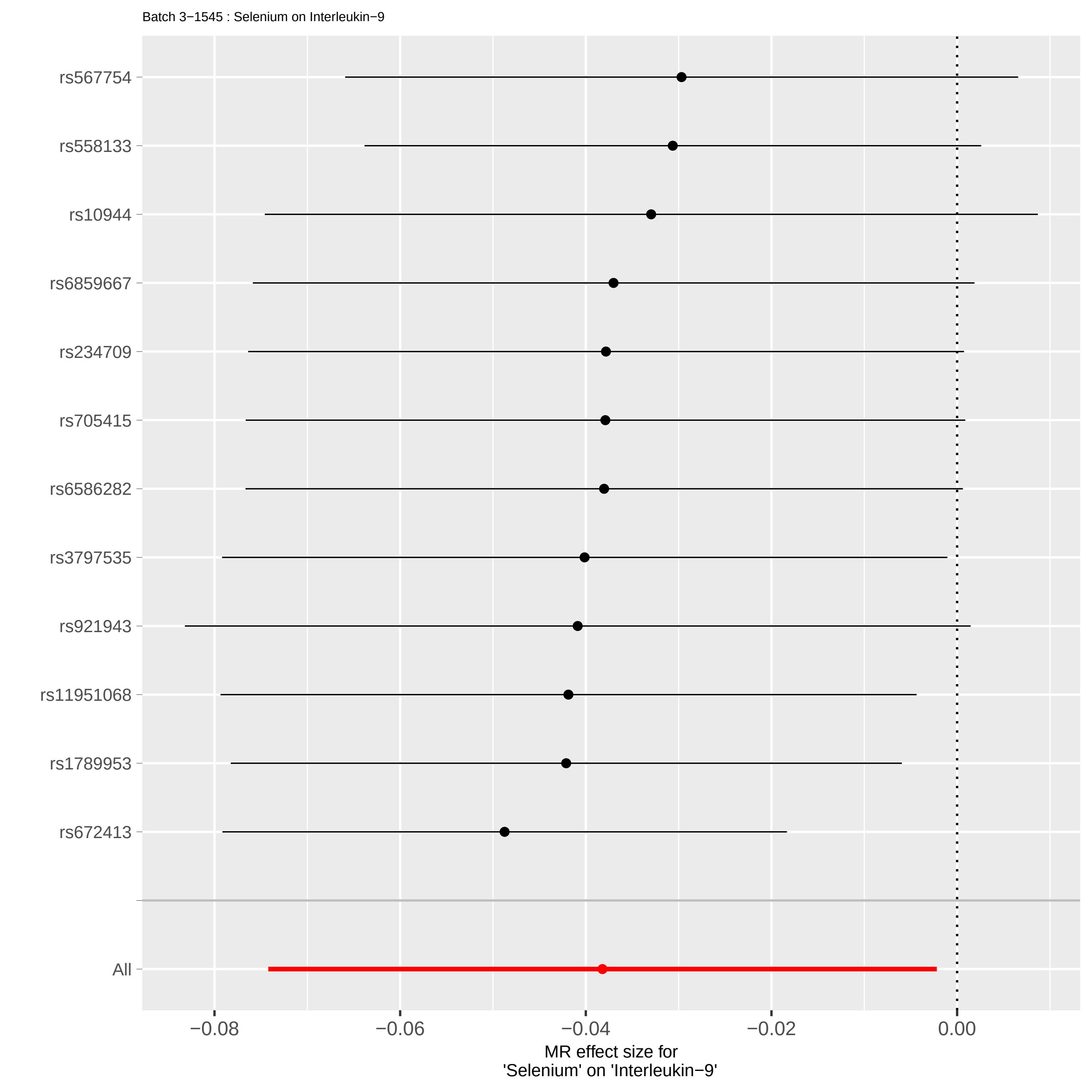

Batch 3−1545 : Selenium on Interleukin−9
rs567754
rs558133
rs10944
rs6859667
rs234709
rs705415
rs6586282
rs3797535
rs921943
rs11951068
rs1789953
rs672413
All
−0.08
−0.06
−0.04
−0.02
0.00
MR effect size for
'Selenium' on 'Interleukin−9'

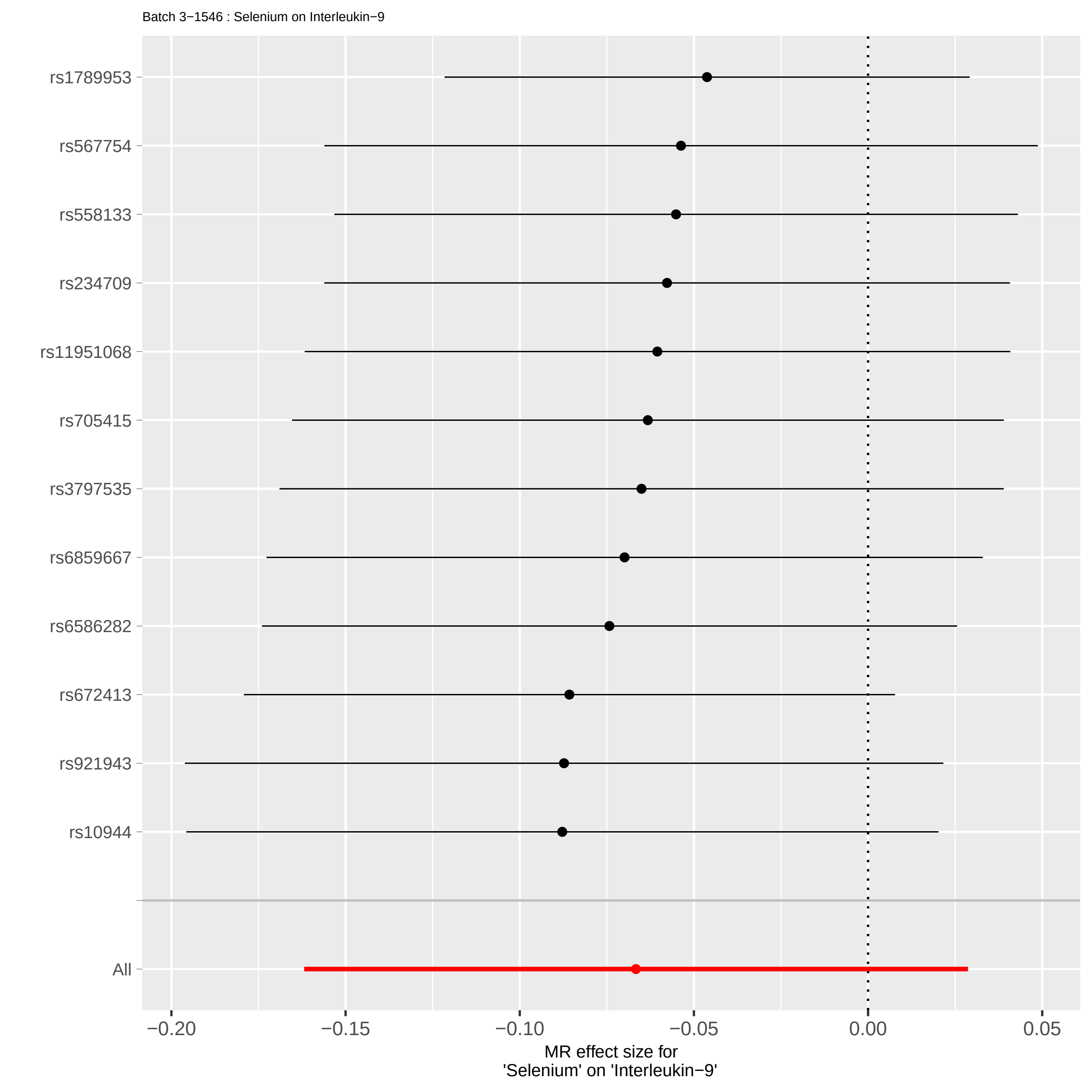

Batch 3−1546 : Selenium on Interleukin−9
rs1789953
rs567754
rs558133
rs234709
rs11951068
rs705415
rs3797535
rs6859667
rs6586282
rs672413
rs921943
rs10944
All
−0.20
−0.15
−0.10
−0.05
0.00
0.05
MR effect size for
'Selenium' on 'Interleukin−9'

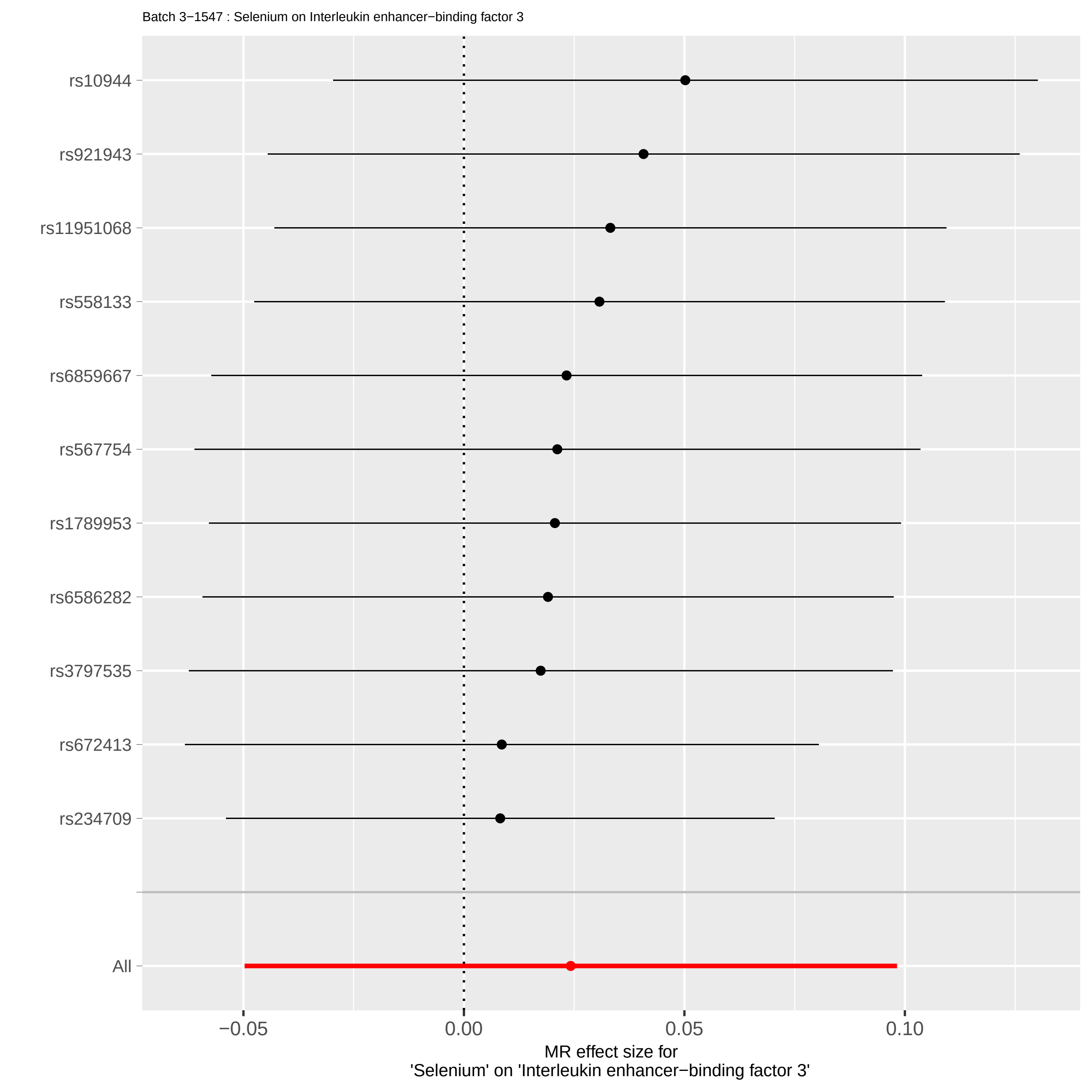
Batch 3−1547 : Selenium on Interleukin enhancer−binding factor 3
rs10944
rs921943
rs11951068
rs558133
rs6859667
rs567754
rs1789953
rs6586282
rs3797535
rs672413
rs234709
All
−0.05
0.00
0.05
0.10
MR effect size for
'Selenium' on 'Interleukin enhancer−binding factor 3'

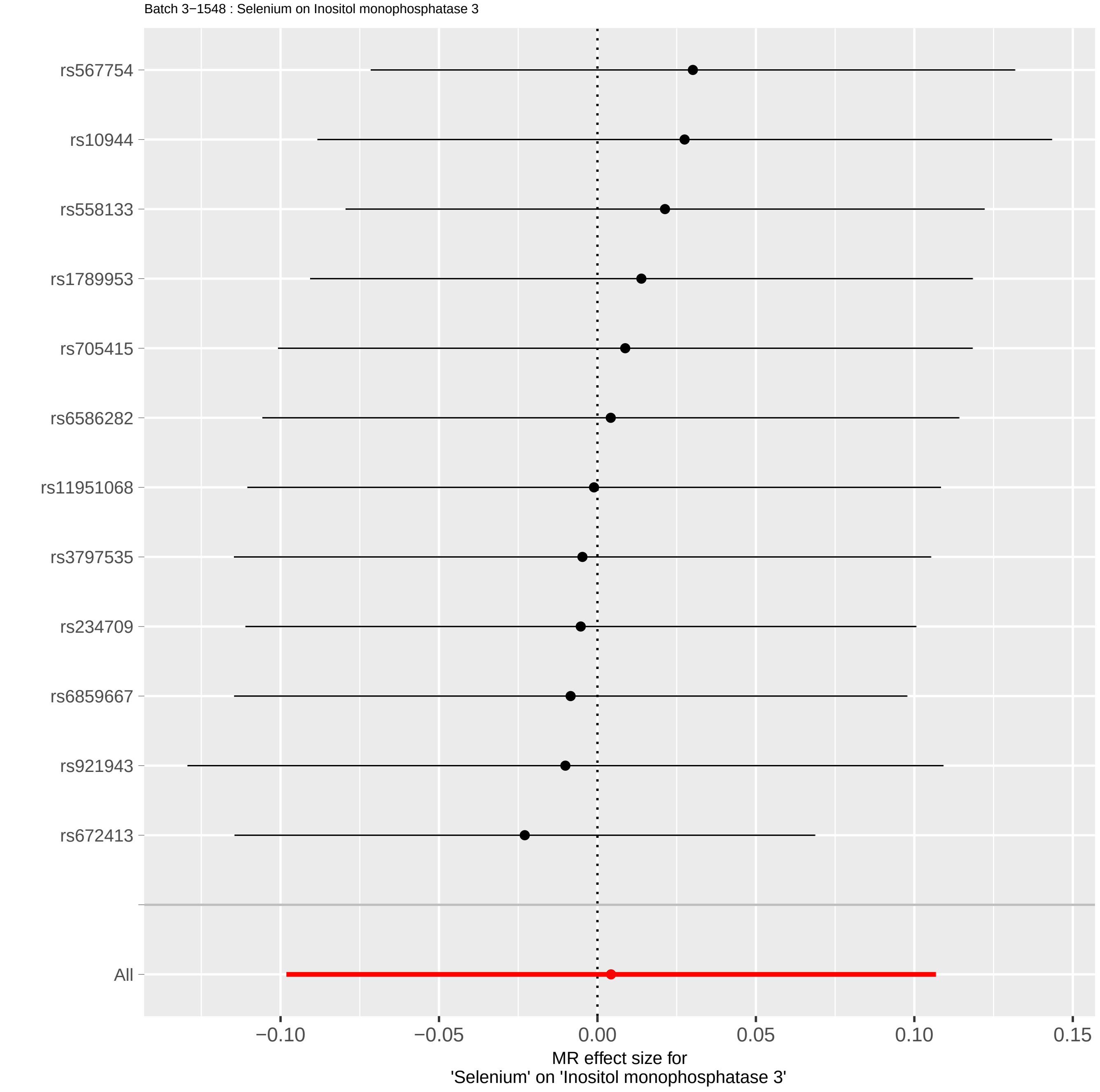

Batch 3−1548 : Selenium on Inositol monophosphatase 3
rs567754
rs10944
rs558133
rs1789953
rs705415
rs6586282
rs11951068
rs3797535
rs234709
rs6859667
rs921943
rs672413
All
−0.10
−0.05
0.00
0.05
0.10
0.15
MR effect size for
'Selenium' on 'Inositol monophosphatase 3'

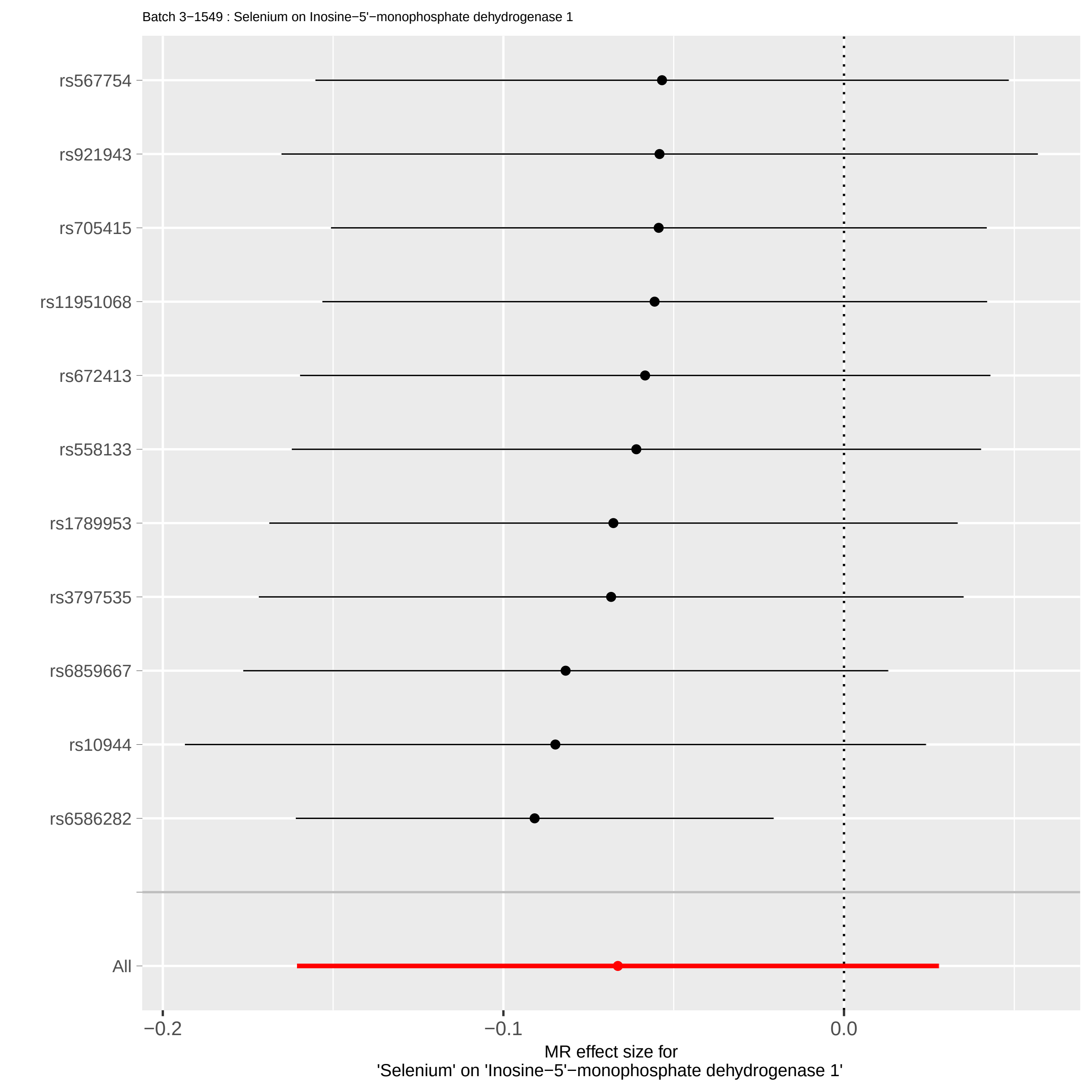
Batch 3−1549 : Selenium on Inosine−5'−monophosphate dehydrogenase 1
rs567754
rs921943
rs705415
rs11951068
rs672413
rs558133
rs1789953
rs3797535
rs6859667
rs10944
rs6586282
All
−0.2
−0.1
0.0
MR effect size for
'Selenium' on 'Inosine−5'−monophosphate dehydrogenase 1'

Batch 3−1550 : Selenium on Inosine−5'−monophosphate dehydrogenase 2

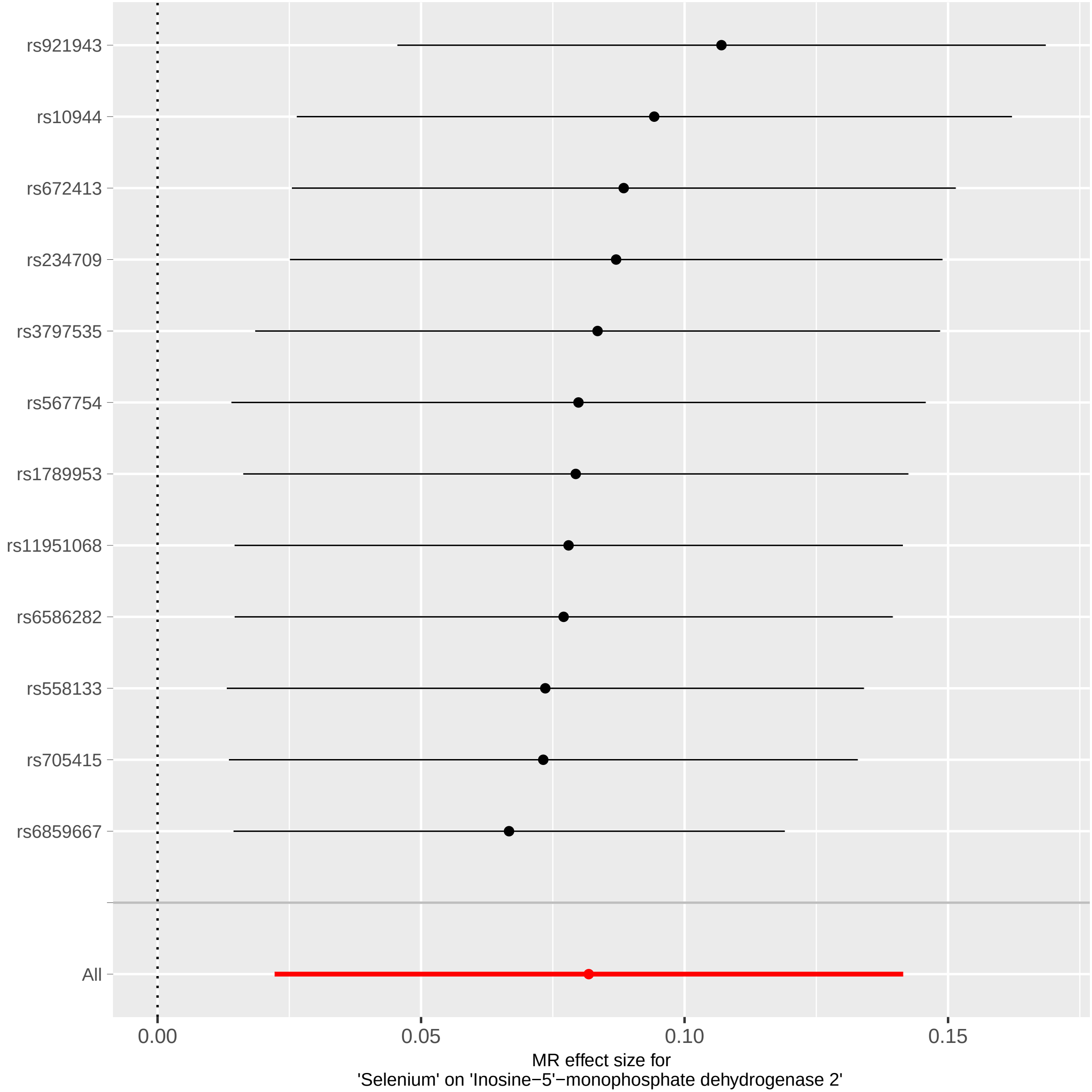

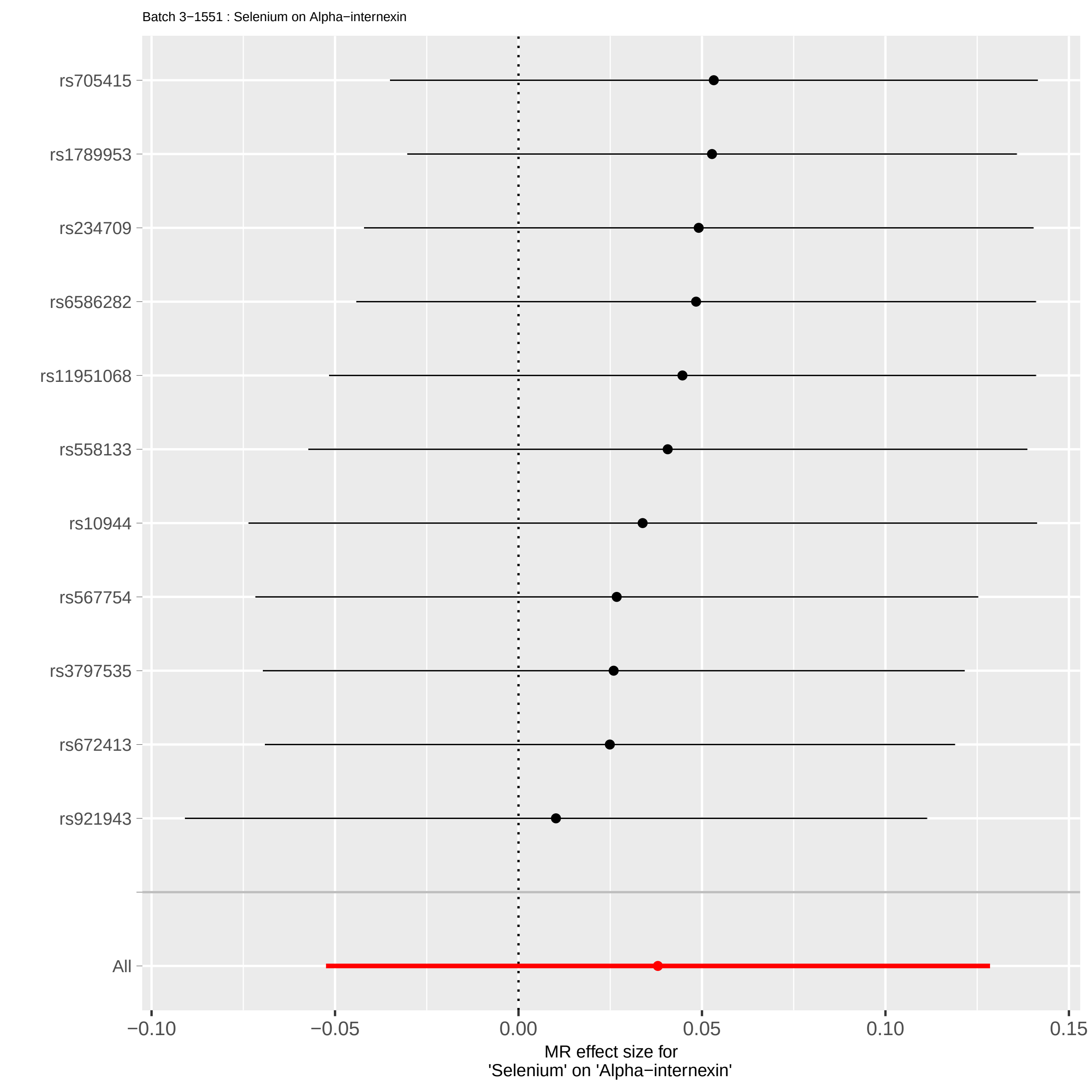

Batch 3−1551 : Selenium on Alpha−internexin
rs705415
rs1789953
rs234709
rs6586282
rs11951068
rs558133
rs10944
rs567754
rs3797535
rs672413
rs921943
All
−0.10
−0.05
0.00
0.05
0.10
0.15
MR effect size for
'Selenium' on 'Alpha−internexin'

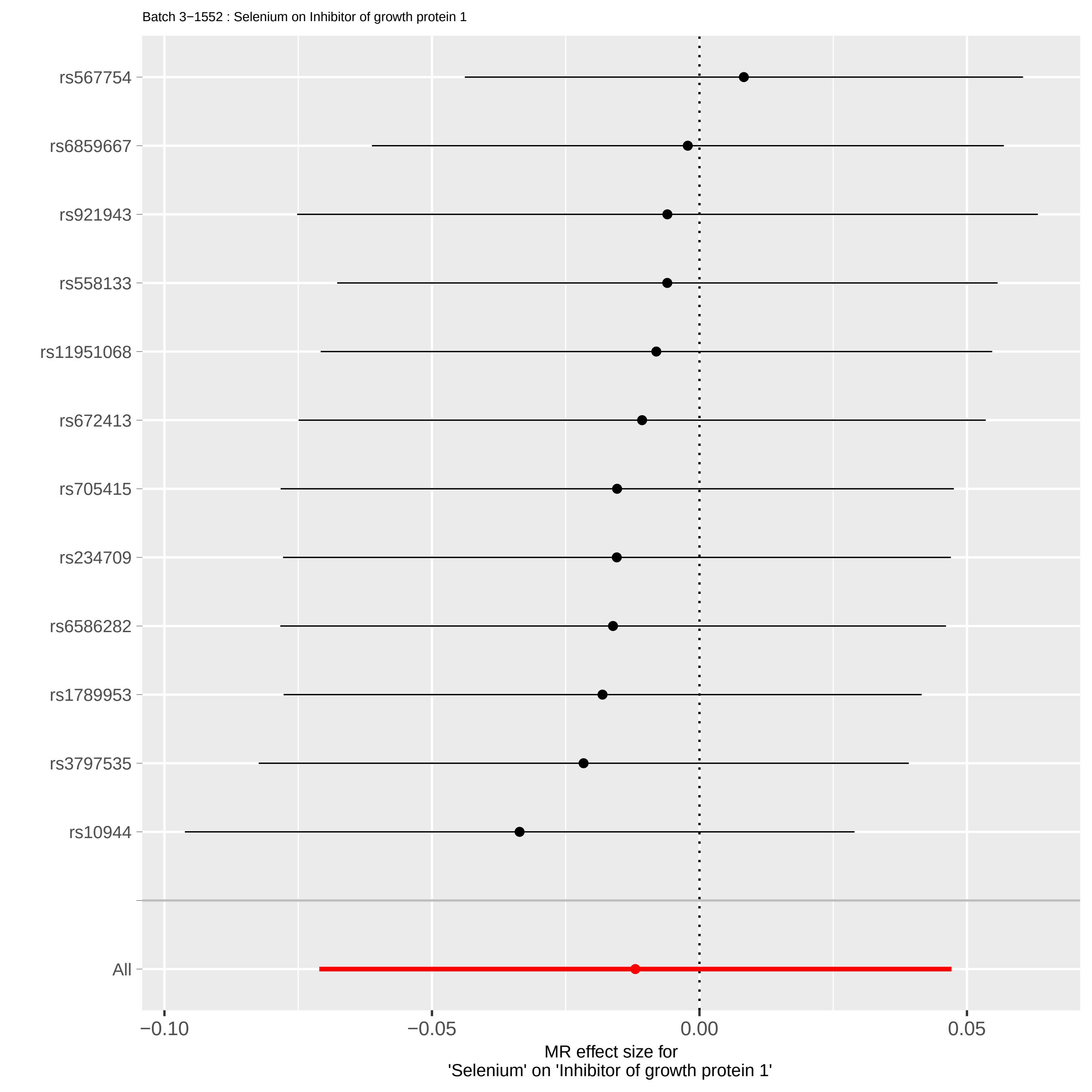

Batch 3−1552 : Selenium on Inhibitor of growth protein 1
rs567754
rs6859667
rs921943
rs558133
rs11951068
rs672413
rs705415
rs234709
rs6586282
rs1789953
rs3797535
rs10944
All
−0.10
−0.05
0.00
0.05
MR effect size for
'Selenium' on 'Inhibitor of growth protein 1'

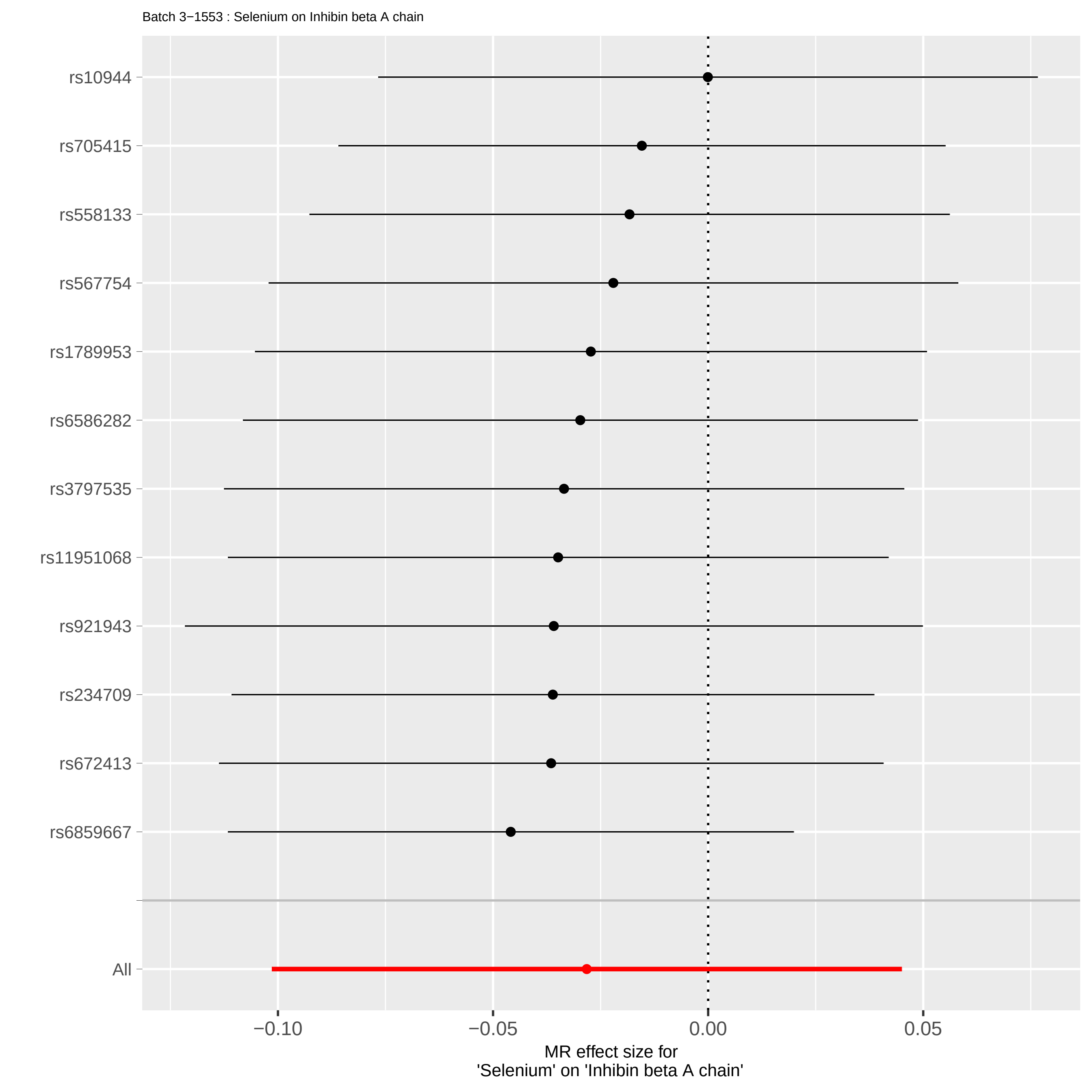
Batch 3−1553 : Selenium on Inhibin beta A chain
rs10944
rs705415
rs558133
rs567754
rs1789953
rs6586282
rs3797535
rs11951068
rs921943
rs234709
rs672413
rs6859667
All
−0.10
−0.05
0.00
0.05
MR effect size for
'Selenium' on 'Inhibin beta A chain'

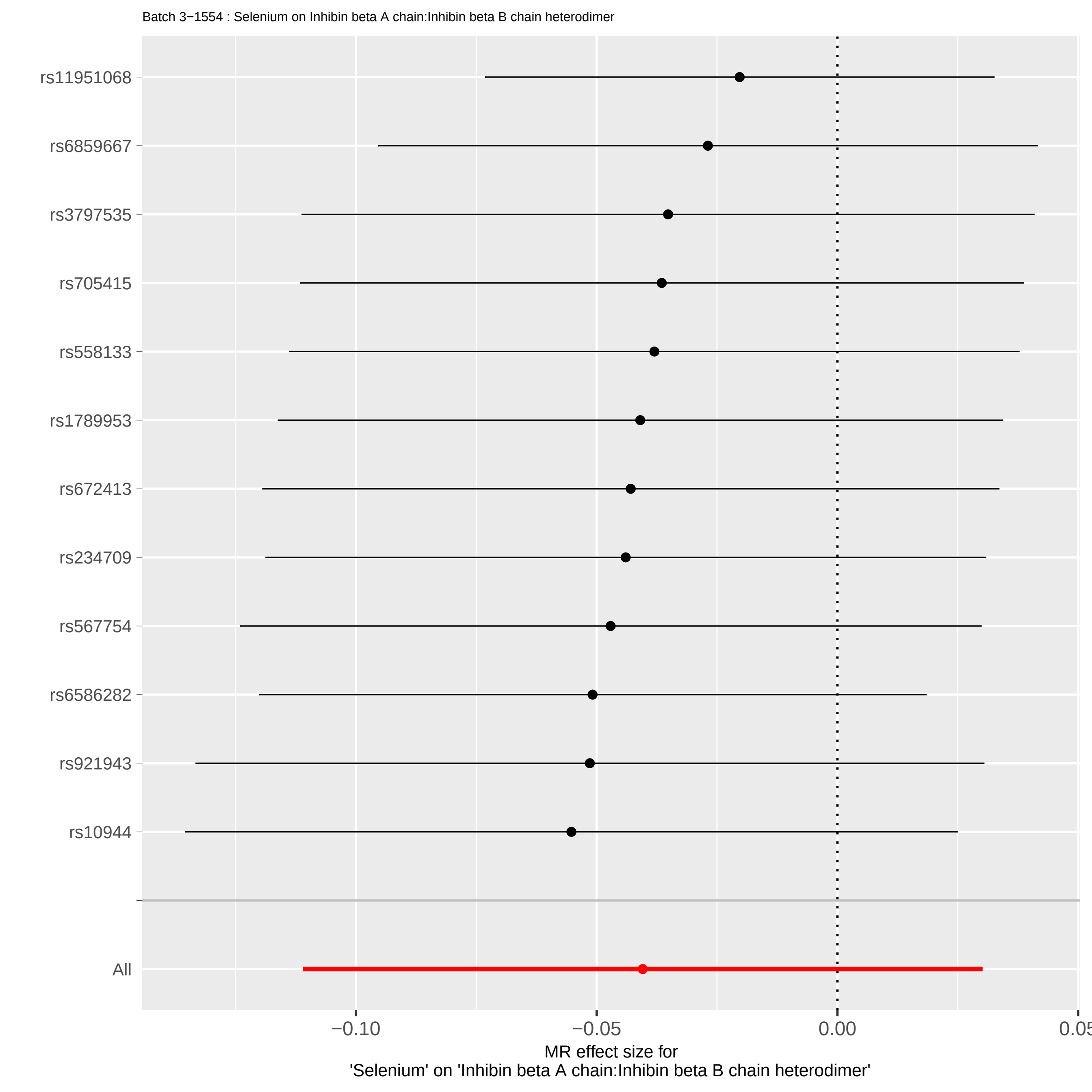
Batch 3−1554 : Selenium on Inhibin beta A chain:Inhibin beta B chain heterodimer
rs11951068
rs6859667
rs3797535
rs705415
rs558133
rs1789953
rs672413
rs234709
rs567754
rs6586282
rs921943
rs10944
All
−0.10
−0.05
0.00
0.05
MR effect size for
'Selenium' on 'Inhibin beta A chain:Inhibin beta B chain heterodimer'

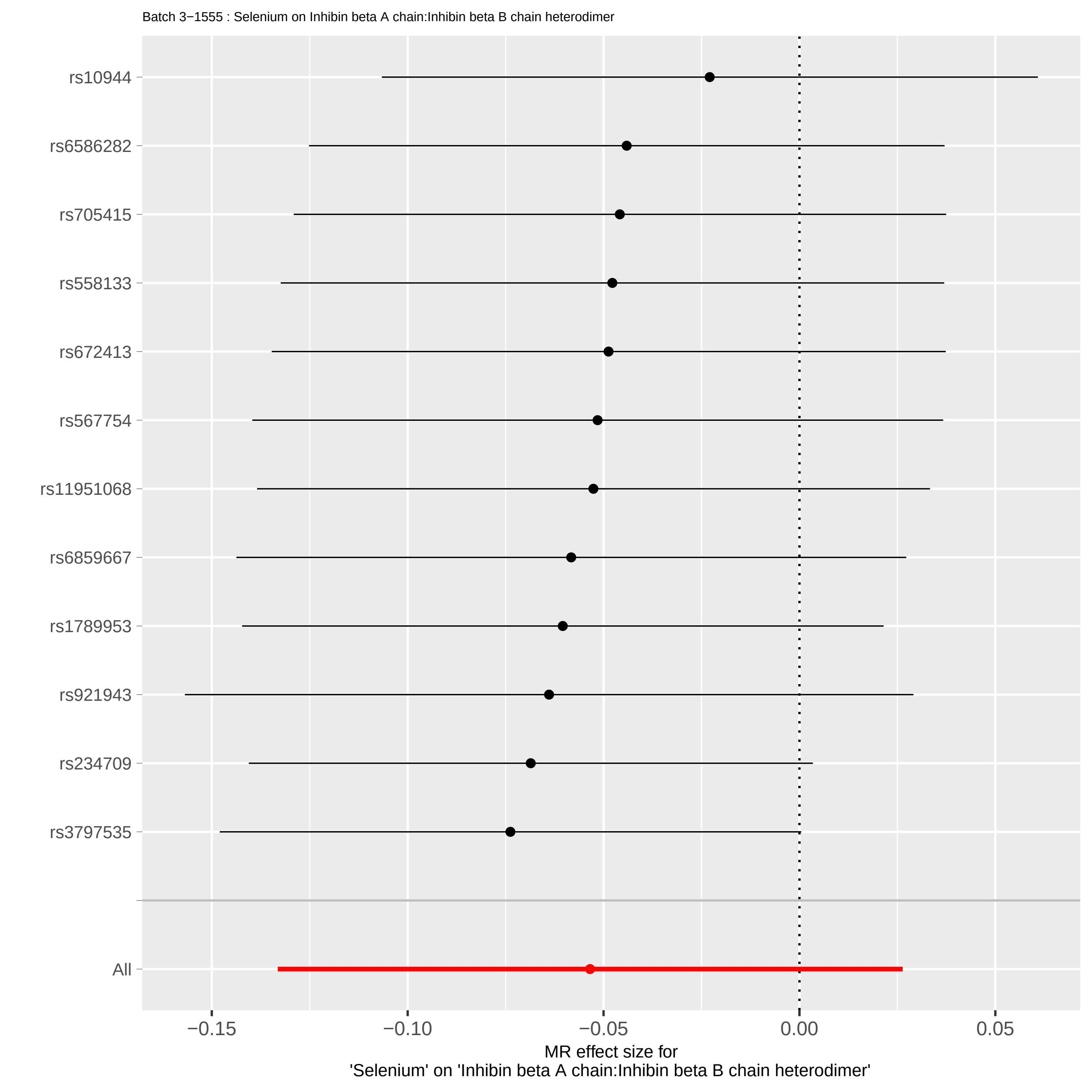

Batch 3−1555 : Selenium on Inhibin beta A chain:Inhibin beta B chain heterodimer
rs10944
rs6586282
rs705415
rs558133
rs672413
rs567754
rs11951068
rs6859667
rs1789953
rs921943
rs234709
rs3797535
All
−0.15
−0.10
−0.05
0.00
0.05
MR effect size for
'Selenium' on 'Inhibin beta A chain:Inhibin beta B chain heterodimer'

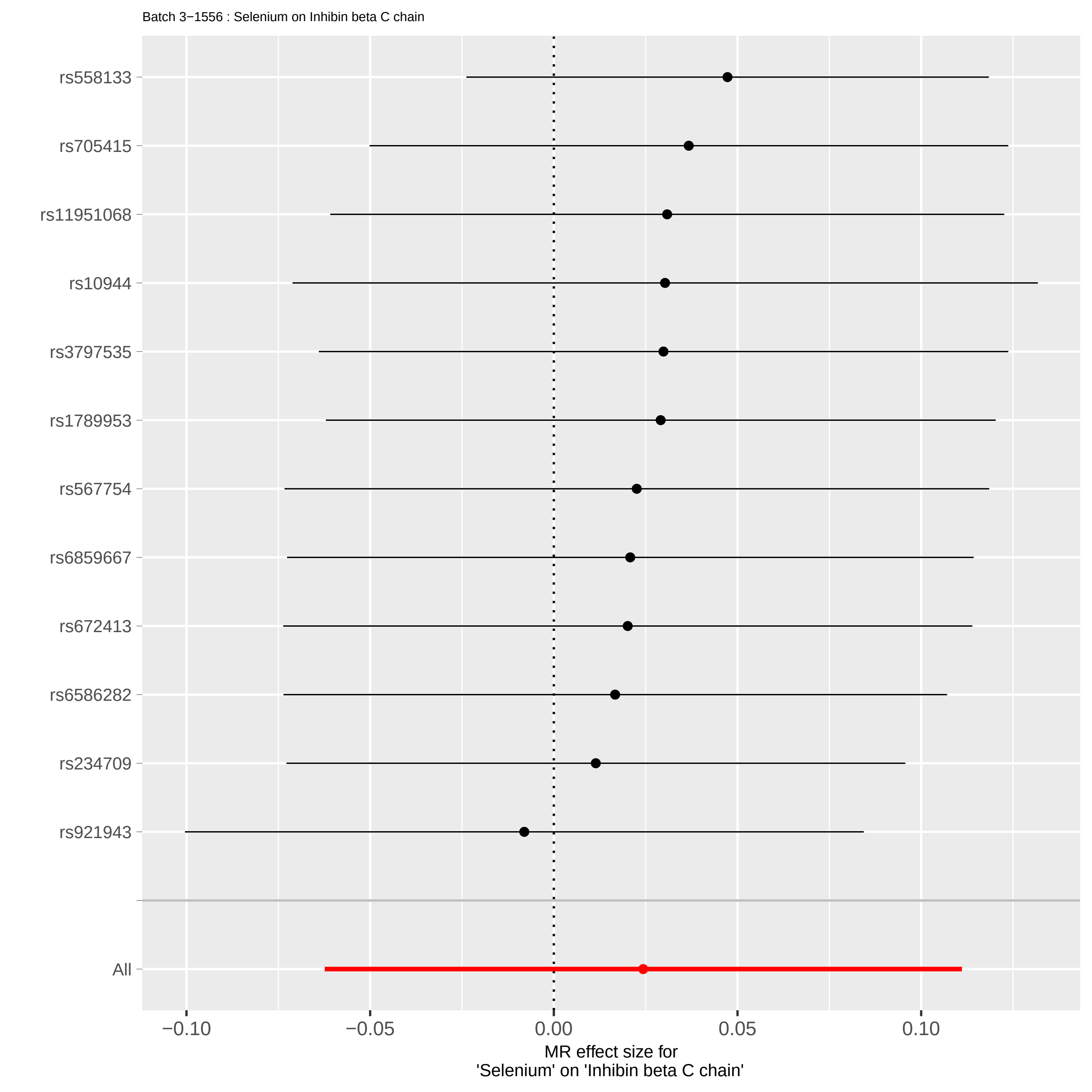

Batch 3−1556 : Selenium on Inhibin beta C chain
rs558133
rs705415
rs11951068
rs10944
rs3797535
rs1789953
rs567754
rs6859667
rs672413
rs6586282
rs234709
rs921943
All
−0.10
−0.05
0.00
0.05
0.10
MR effect size for
'Selenium' on 'Inhibin beta C chain'

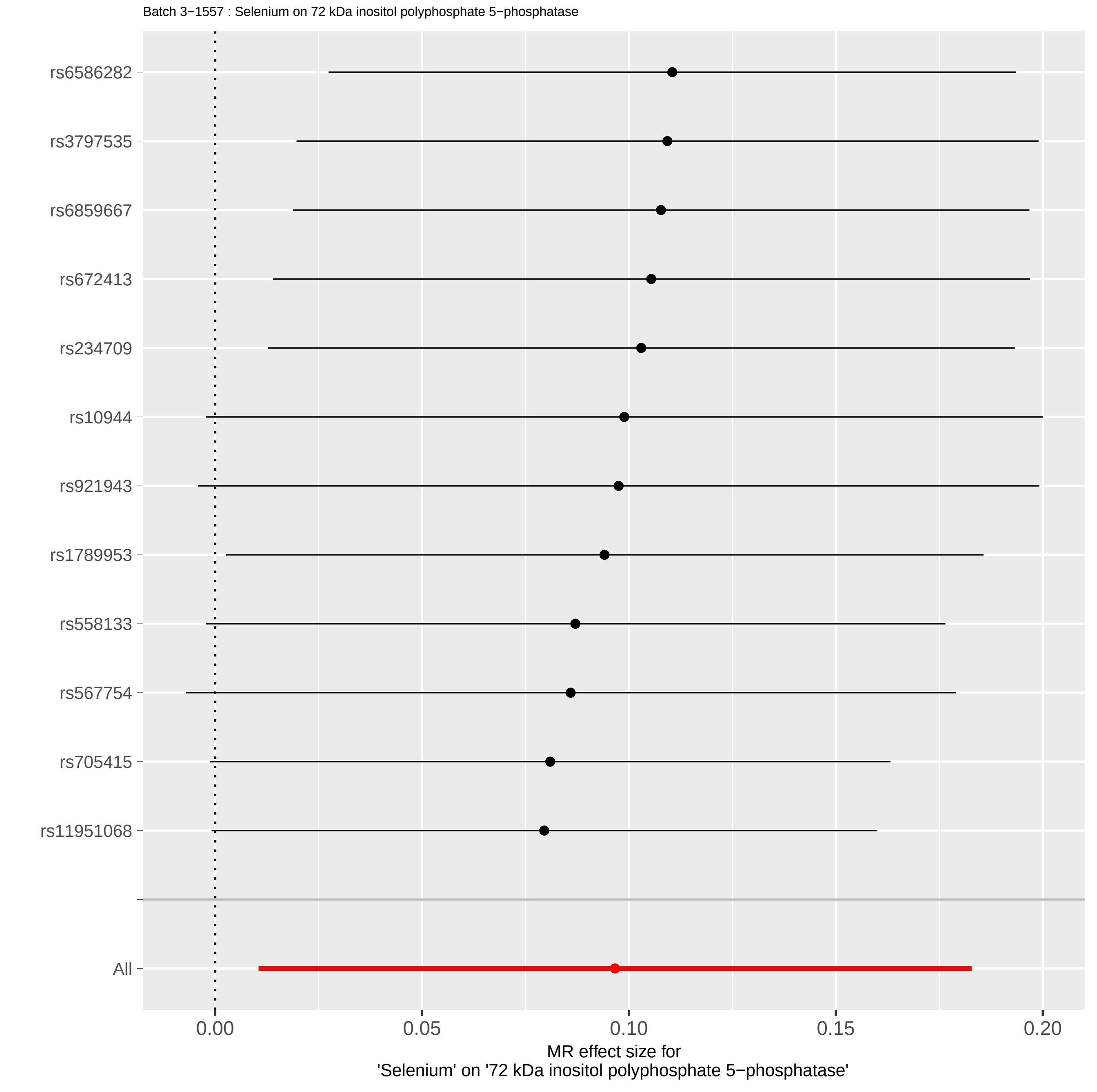

Batch 3−1557 : Selenium on 72 kDa inositol polyphosphate 5−phosphatase
rs6586282
rs3797535
rs6859667
rs672413
rs234709
rs10944
rs921943
rs1789953
rs558133
rs567754
rs705415
rs11951068
All
0.00
0.05
0.10
0.15
0.20
MR effect size for
'Selenium' on '72 kDa inositol polyphosphate 5−phosphatase'

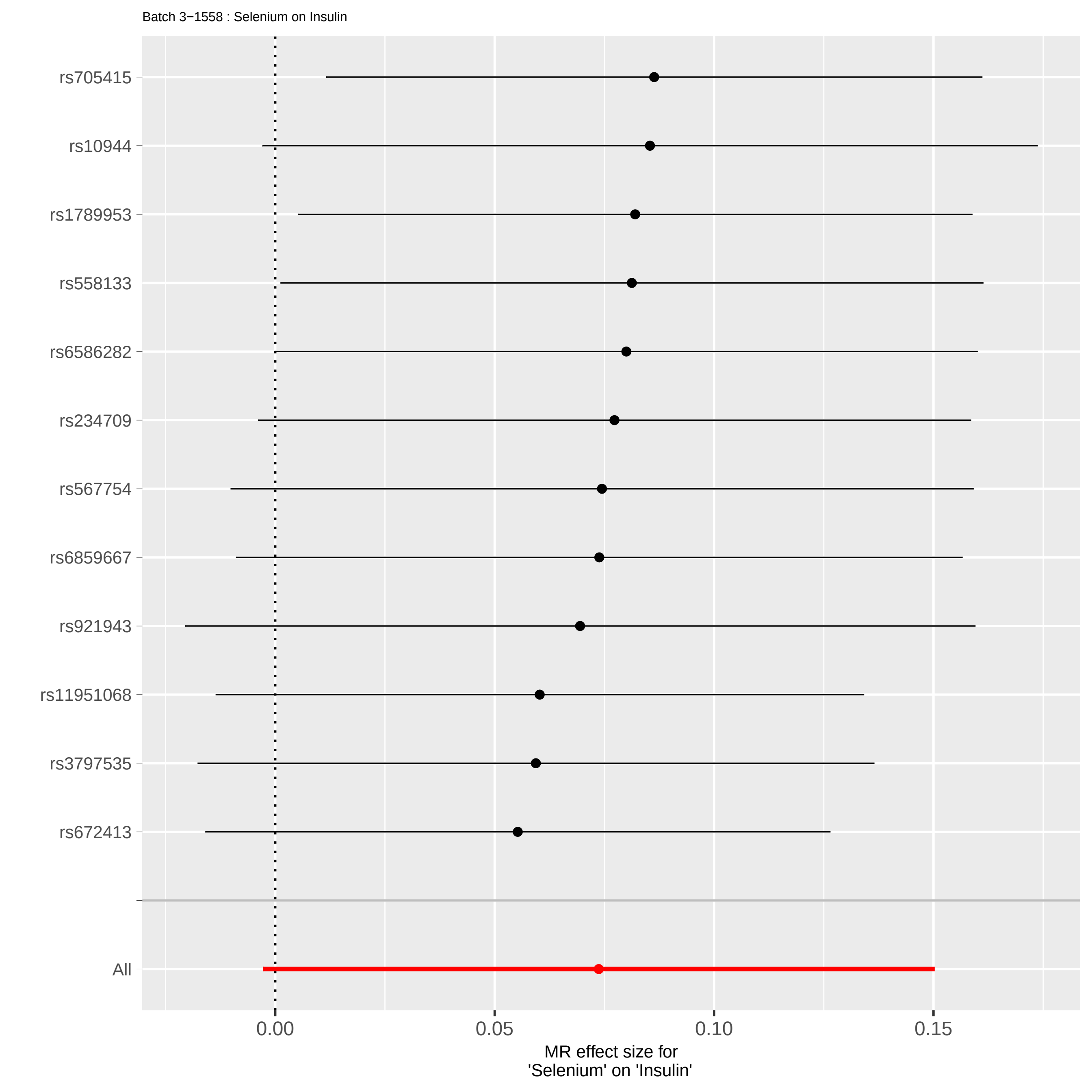

Batch 3−1558 : Selenium on Insulin
rs705415
rs10944
rs1789953
rs558133
rs6586282
rs234709
rs567754
rs6859667
rs921943
rs11951068
rs3797535
rs672413
All
0.00
0.05
0.10
0.15
MR effect size for
'Selenium' on 'Insulin'

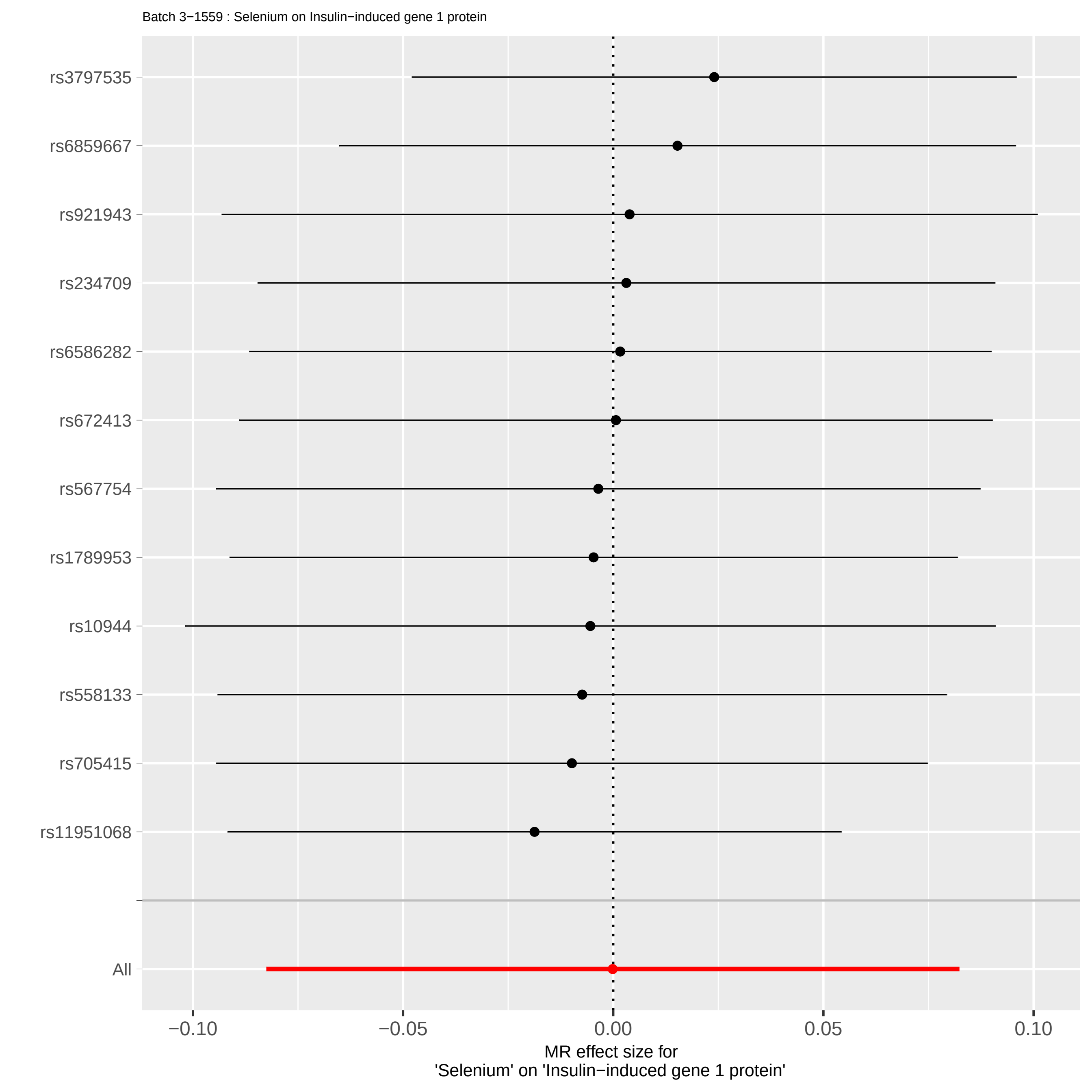

Batch 3−1559 : Selenium on Insulin−induced gene 1 protein
rs3797535
rs6859667
rs921943
rs234709
rs6586282
rs672413
rs567754
rs1789953
rs10944
rs558133
rs705415
rs11951068
All
−0.10
−0.05
0.00
0.05
0.10
MR effect size for
'Selenium' on 'Insulin−induced gene 1 protein'

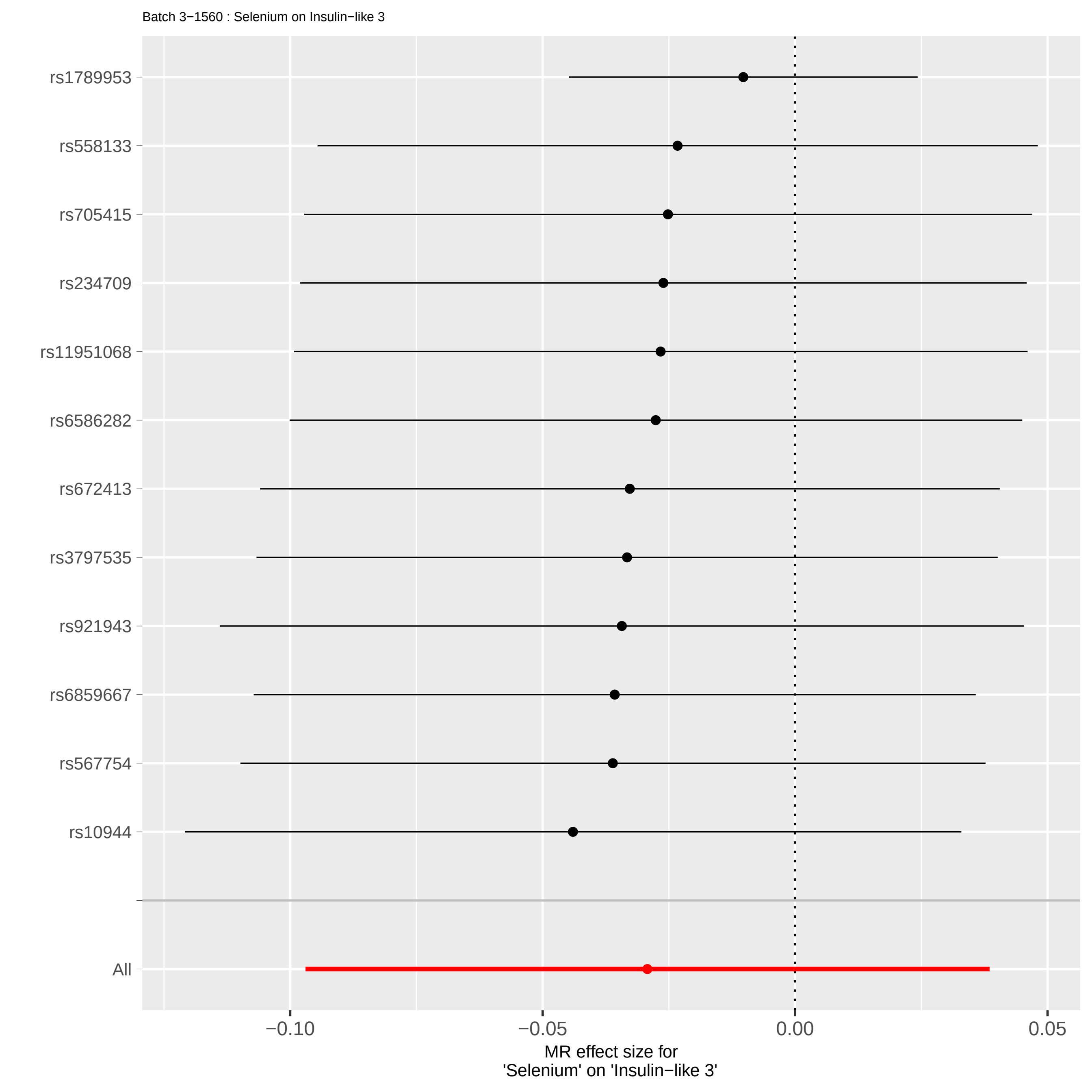

Batch 3−1560 : Selenium on Insulin−like 3
rs1789953
rs558133
rs705415
rs234709
rs11951068
rs6586282
rs672413
rs3797535
rs921943
rs6859667
rs567754
rs10944
All
−0.10
−0.05
0.00
0.05
MR effect size for
'Selenium' on 'Insulin−like 3'

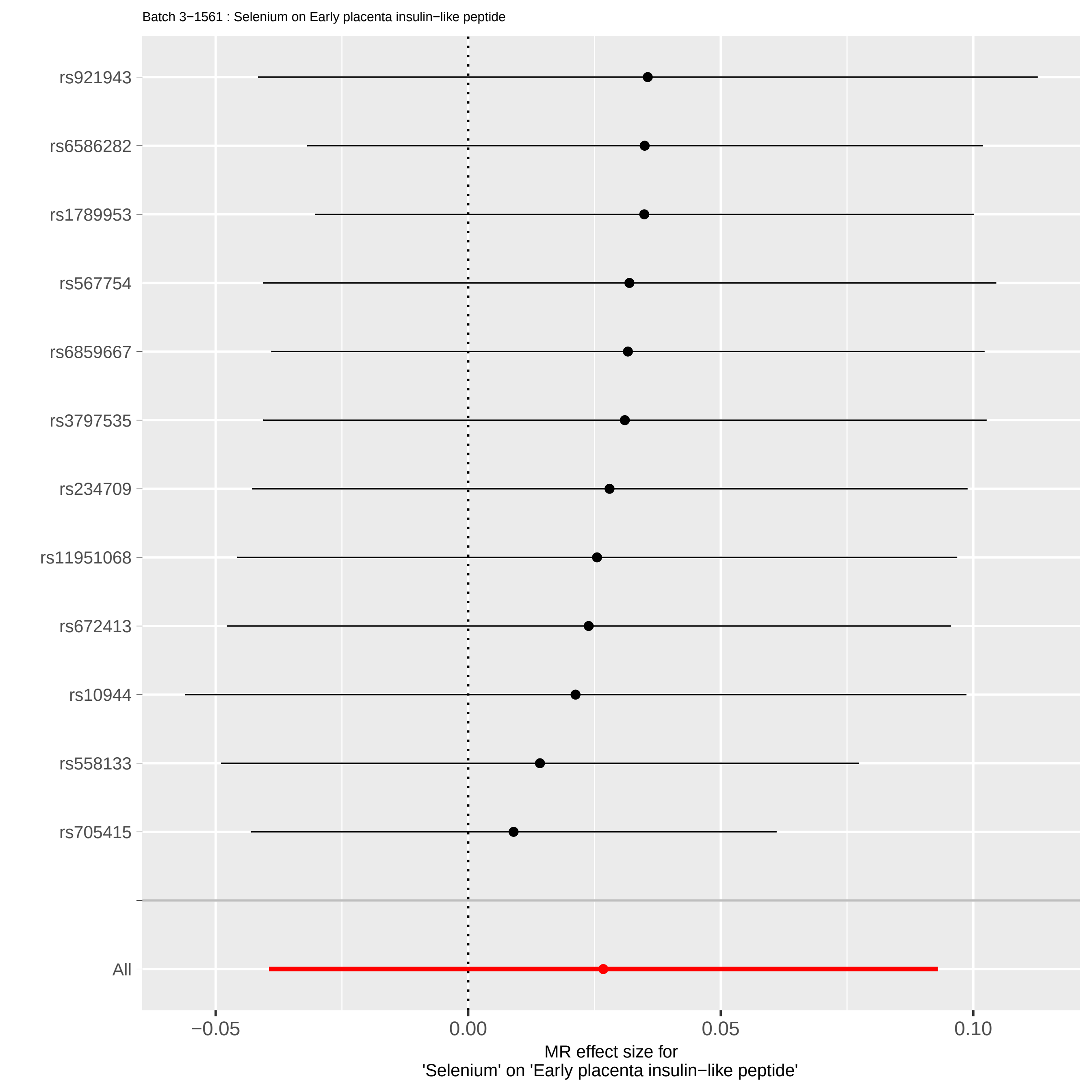

Batch 3−1561 : Selenium on Early placenta insulin−like peptide
rs921943
rs6586282
rs1789953
rs567754
rs6859667
rs3797535
rs234709
rs11951068
rs672413
rs10944
rs558133
rs705415
All
−0.05
0.00
0.05
0.10
MR effect size for
'Selenium' on 'Early placenta insulin−like peptide'

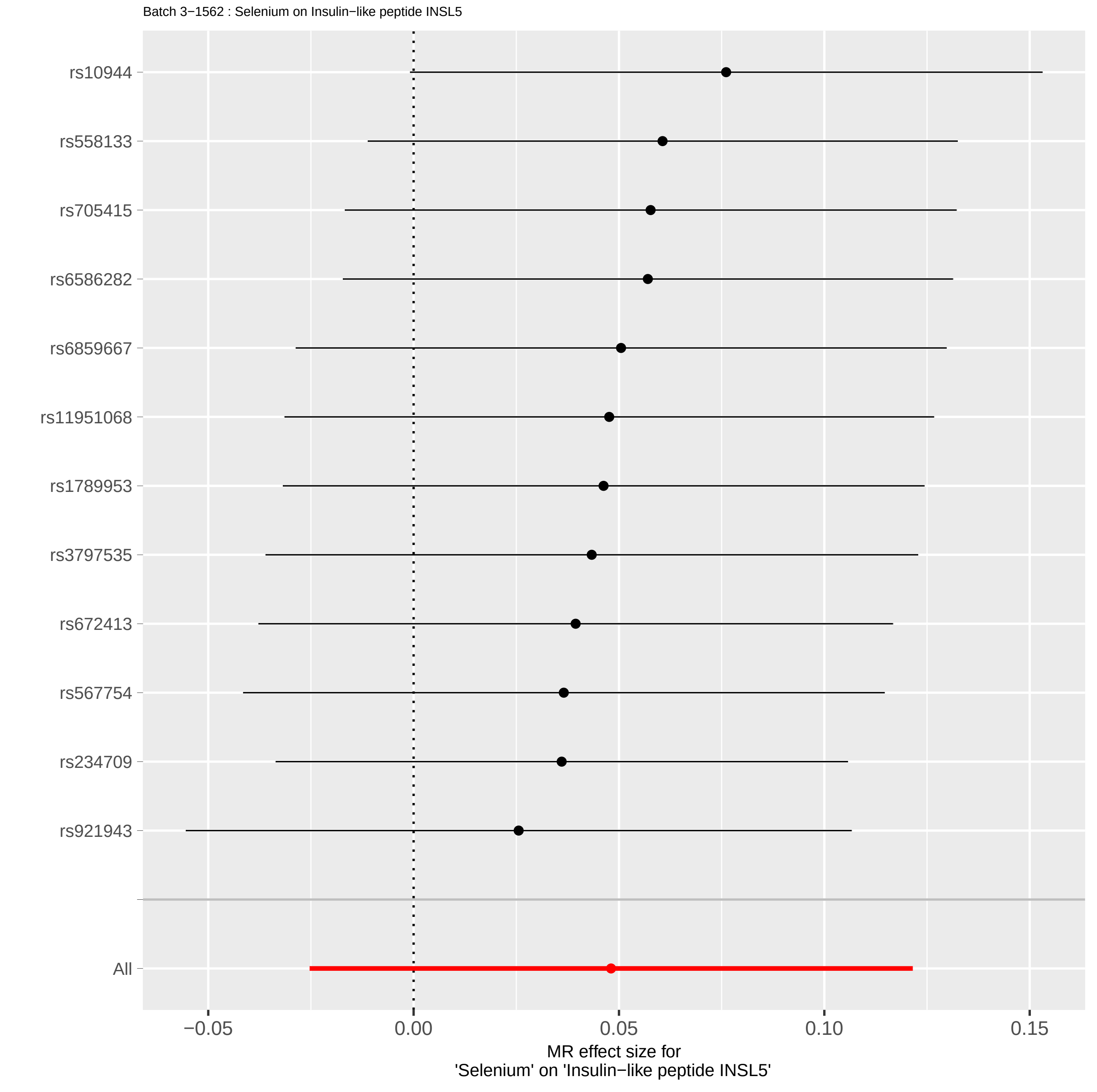
Batch 3−1562 : Selenium on Insulin−like peptide INSL5
rs10944
rs558133
rs705415
rs6586282
rs6859667
rs11951068
rs1789953
rs3797535
rs672413
rs567754
rs234709
rs921943
All
−0.05
0.00
0.05
0.10
0.15
MR effect size for
'Selenium' on 'Insulin−like peptide INSL5'

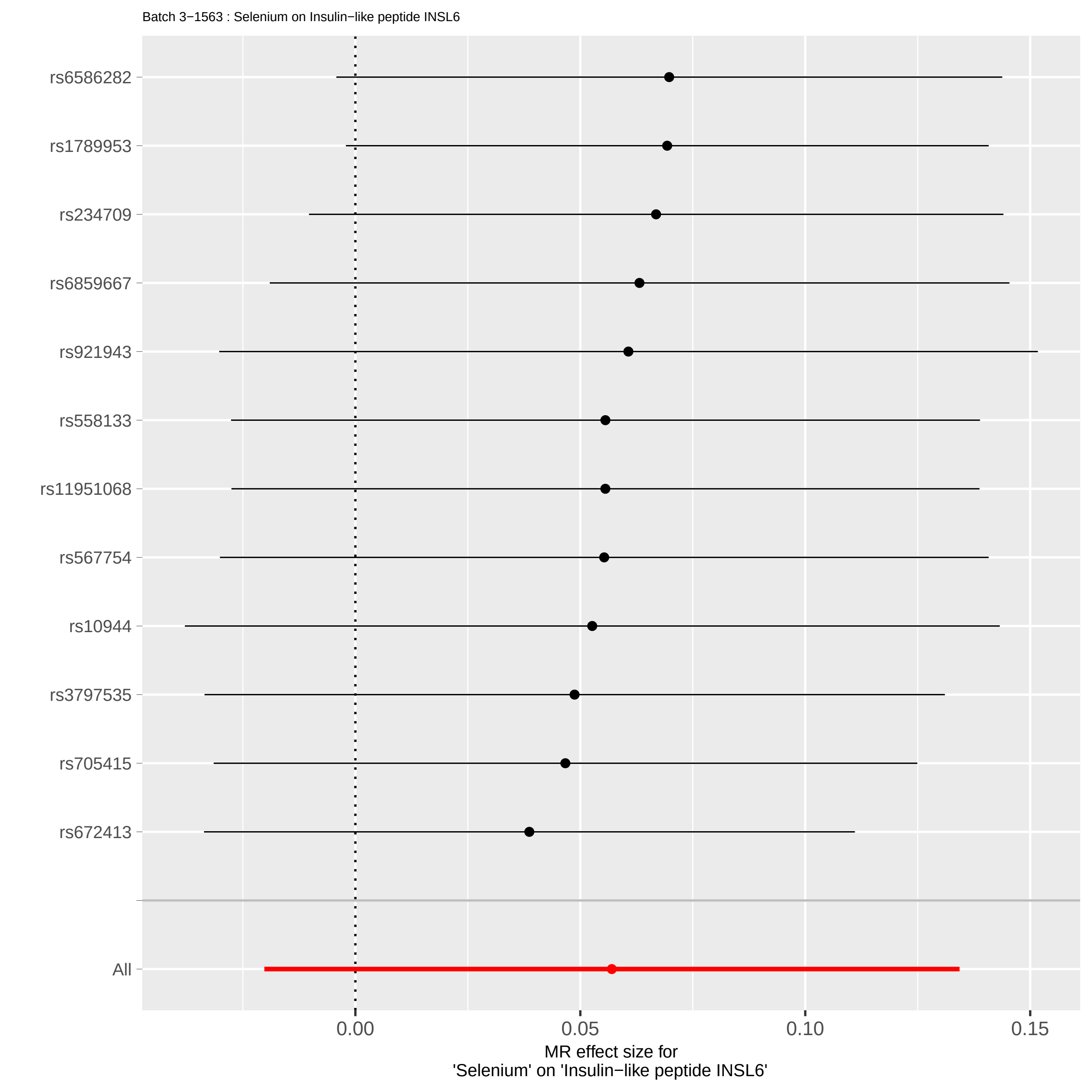

Batch 3−1563 : Selenium on Insulin−like peptide INSL6
rs6586282
rs1789953
rs234709
rs6859667
rs921943
rs558133
rs11951068
rs567754
rs10944
rs3797535
rs705415
rs672413
All
0.00
0.05
0.10
0.15
MR effect size for
'Selenium' on 'Insulin−like peptide INSL6'

Batch 3−1564 : Selenium on Insulin receptor

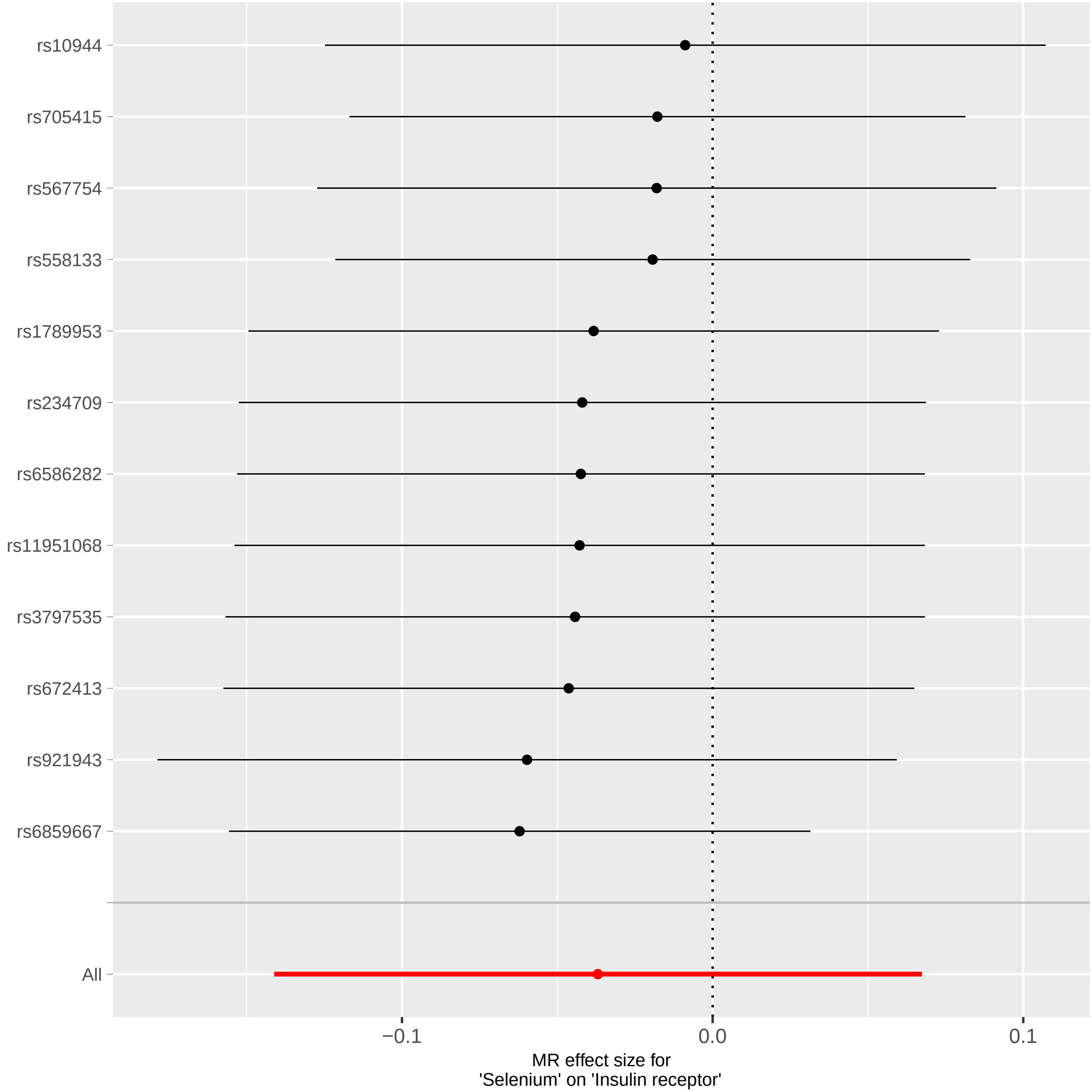

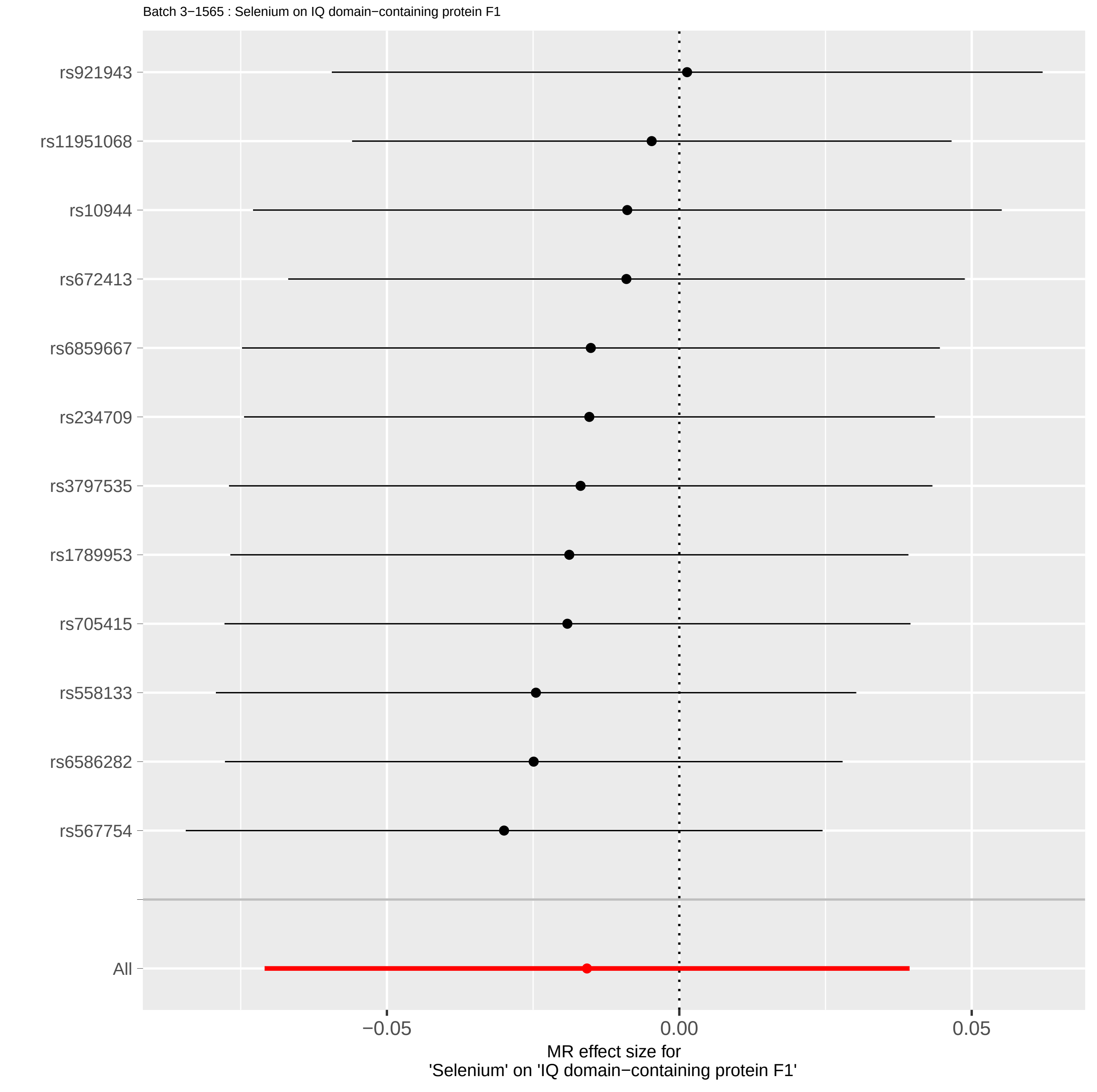
Batch 3−1565 : Selenium on IQ domain−containing protein F1
rs921943
rs11951068
rs10944
rs672413
rs6859667
rs234709
rs3797535
rs1789953
rs705415
rs558133
rs6586282
rs567754
All
−0.05
0.00
0.05
MR effect size for
'Selenium' on 'IQ domain−containing protein F1'

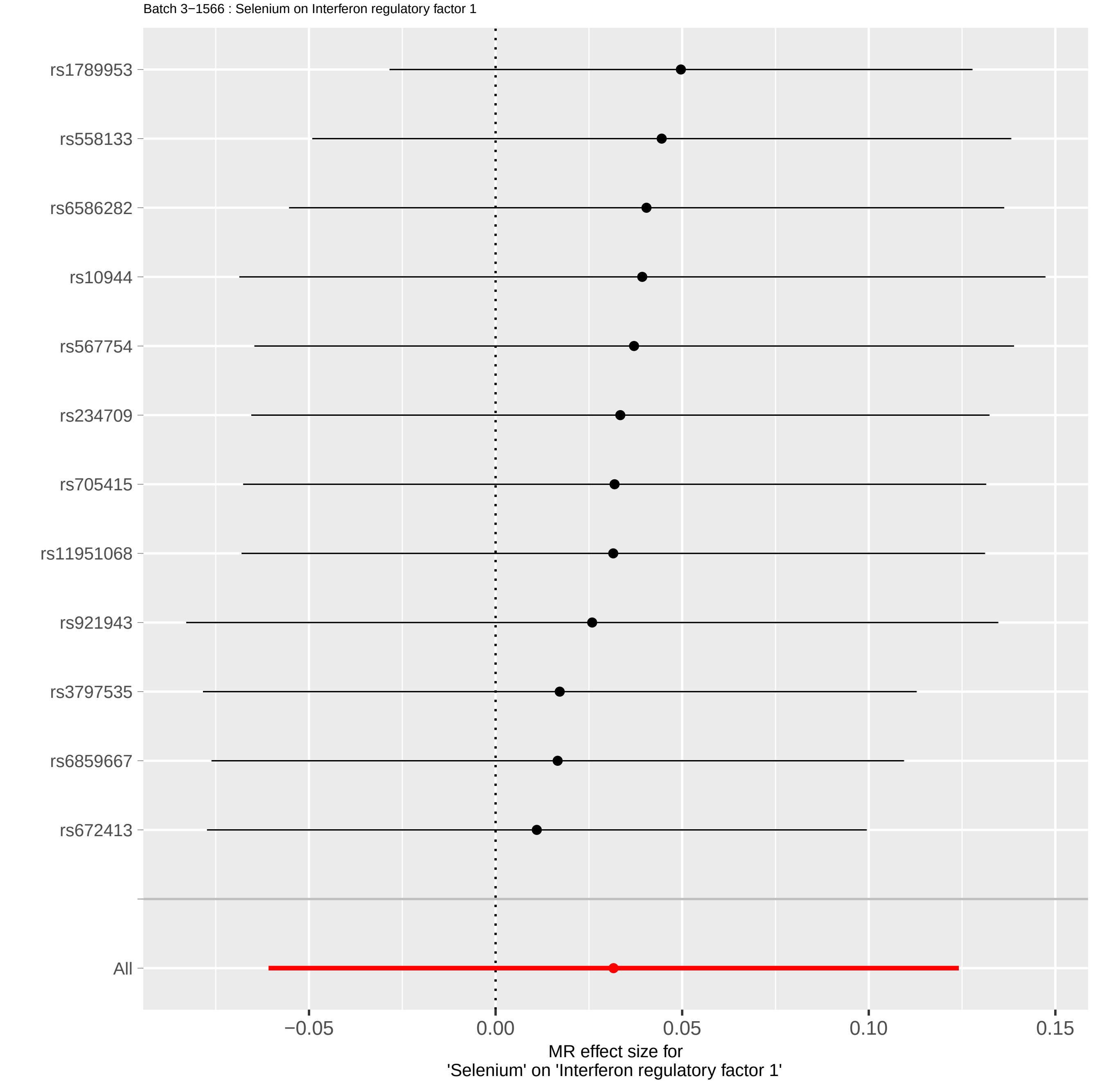

Batch 3−1566 : Selenium on Interferon regulatory factor 1
rs1789953
rs558133
rs6586282
rs10944
rs567754
rs234709
rs705415
rs11951068
rs921943
rs3797535
rs6859667
rs672413
All
−0.05
0.00
0.05
0.10
0.15
MR effect size for
'Selenium' on 'Interferon regulatory factor 1'

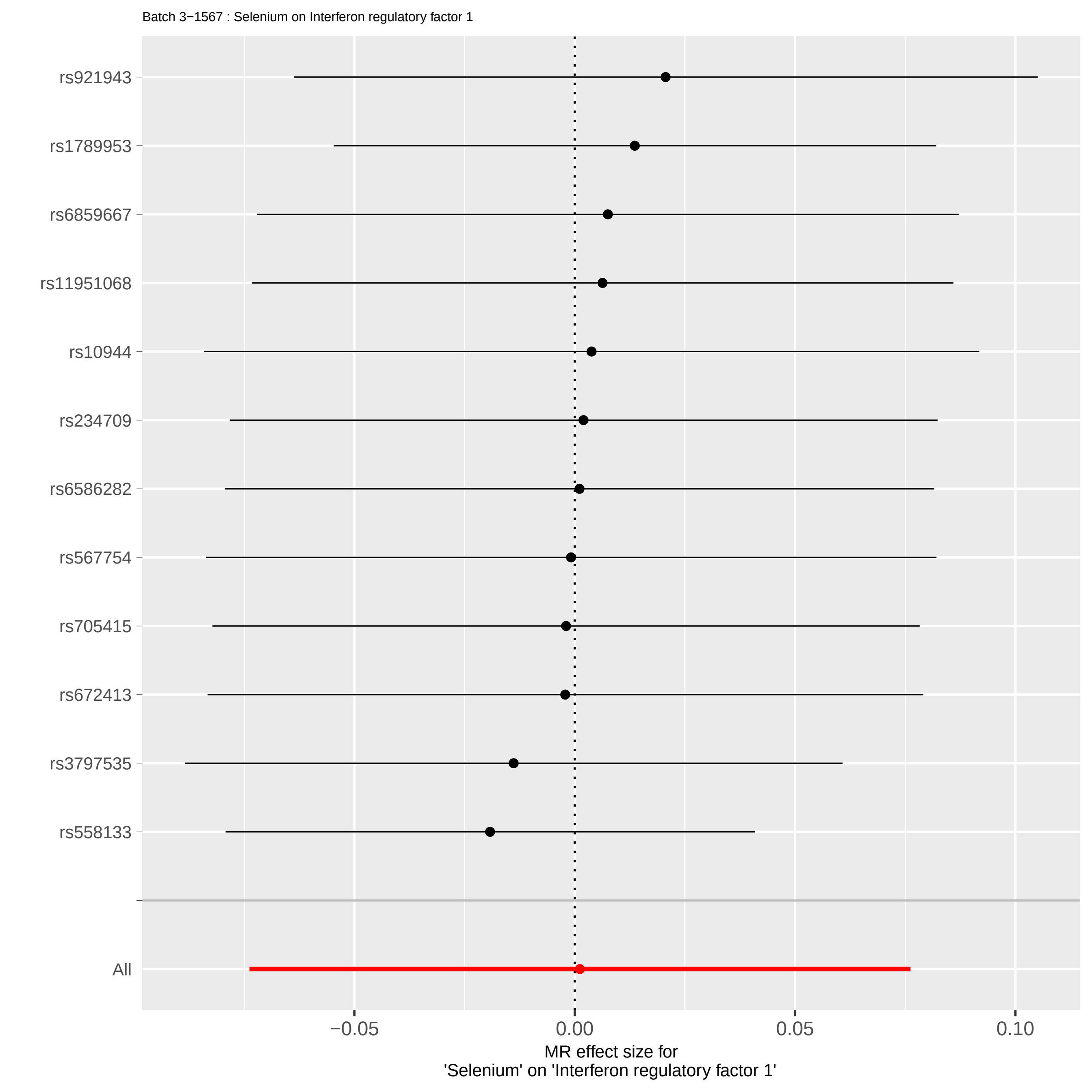
Batch 3−1567 : Selenium on Interferon regulatory factor 1
rs921943
rs1789953
rs6859667
rs11951068
rs10944
rs234709
rs6586282
rs567754
rs705415
rs672413
rs3797535
rs558133
All
−0.05
0.00
0.05
0.10
MR effect size for
'Selenium' on 'Interferon regulatory factor 1'

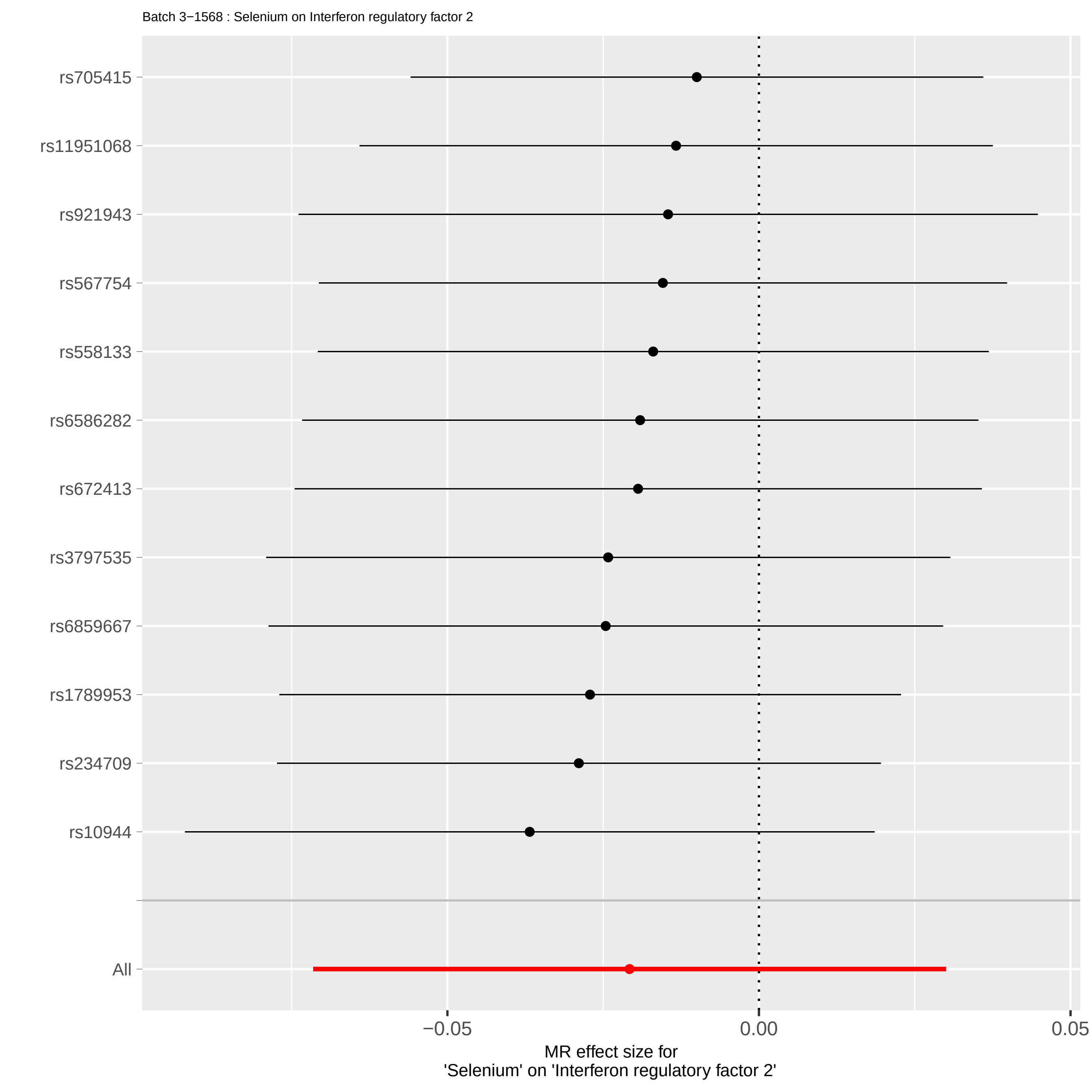

Batch 3−1568 : Selenium on Interferon regulatory factor 2
rs705415
rs11951068
rs921943
rs567754
rs558133
rs6586282
rs672413
rs3797535
rs6859667
rs1789953
rs234709
rs10944
All
−0.05
0.00
0.05
MR effect size for
'Selenium' on 'Interferon regulatory factor 2'

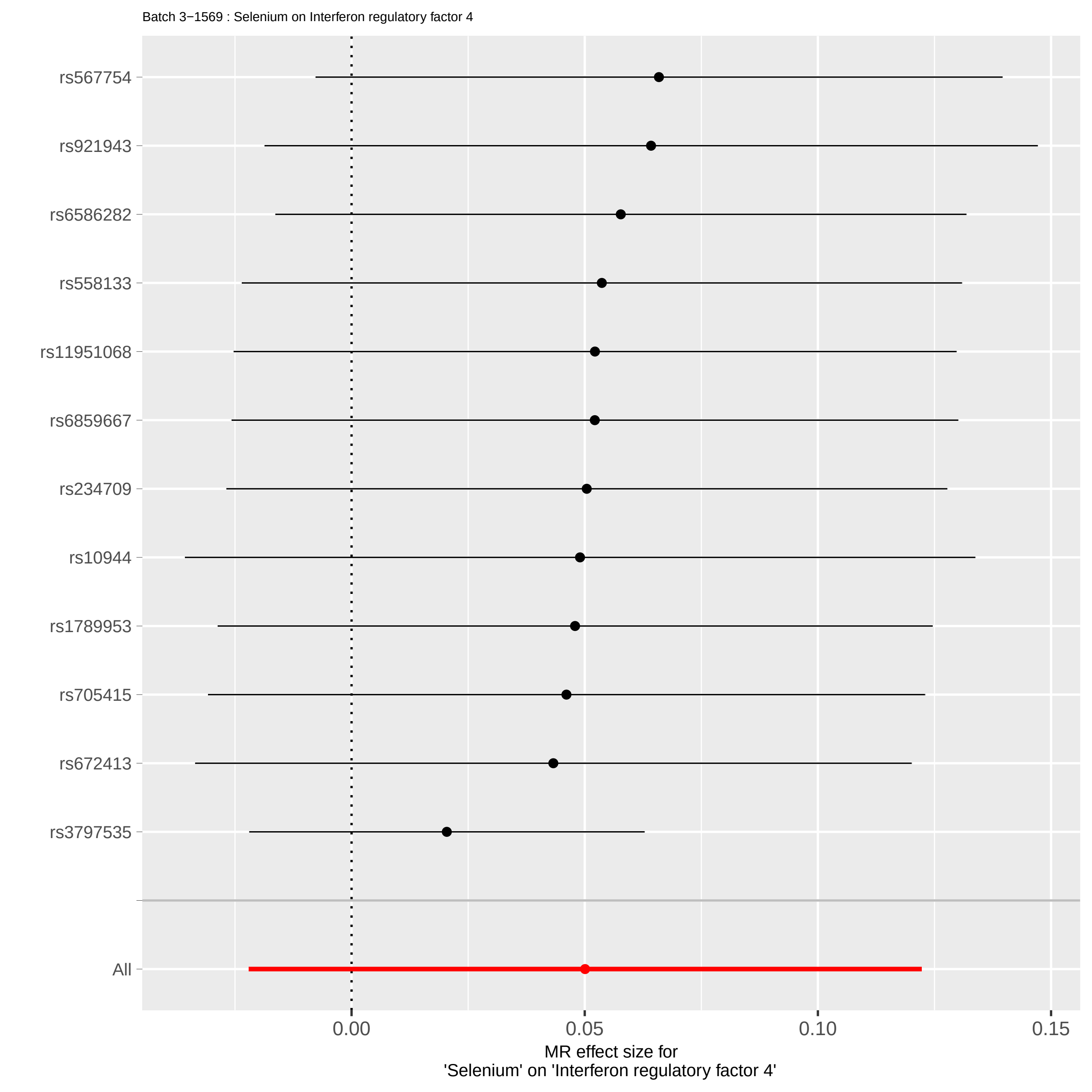
Batch 3−1569 : Selenium on Interferon regulatory factor 4
rs567754
rs921943
rs6586282
rs558133
rs11951068
rs6859667
rs234709
rs10944
rs1789953
rs705415
rs672413
rs3797535
All
0.00
0.05
0.10
0.15
MR effect size for
'Selenium' on 'Interferon regulatory factor 4'

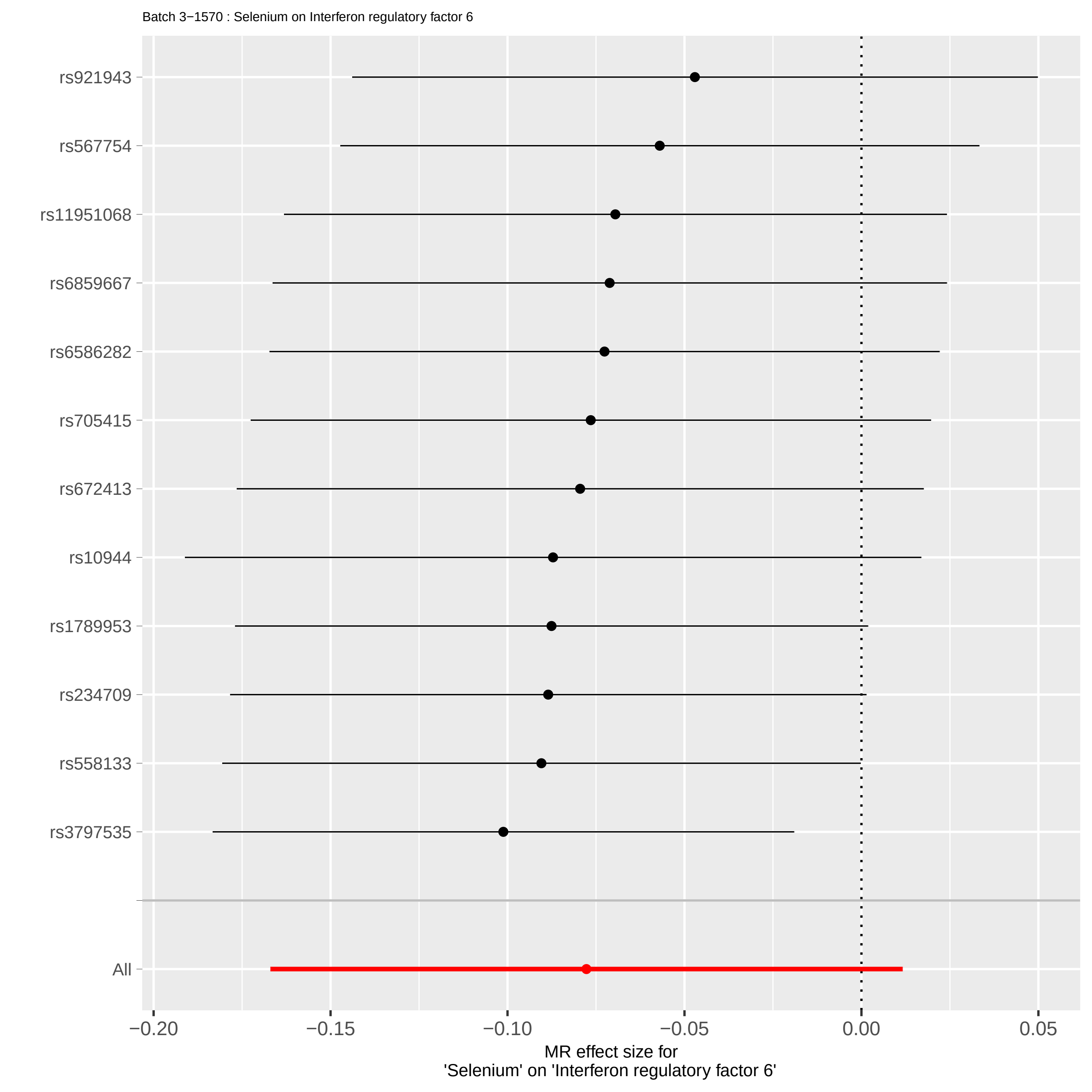
Batch 3−1570 : Selenium on Interferon regulatory factor 6
rs921943
rs567754
rs11951068
rs6859667
rs6586282
rs705415
rs672413
rs10944
rs1789953
rs234709
rs558133
rs3797535
All
−0.20
−0.15
−0.10
−0.05
0.00
0.05
MR effect size for
'Selenium' on 'Interferon regulatory factor 6'

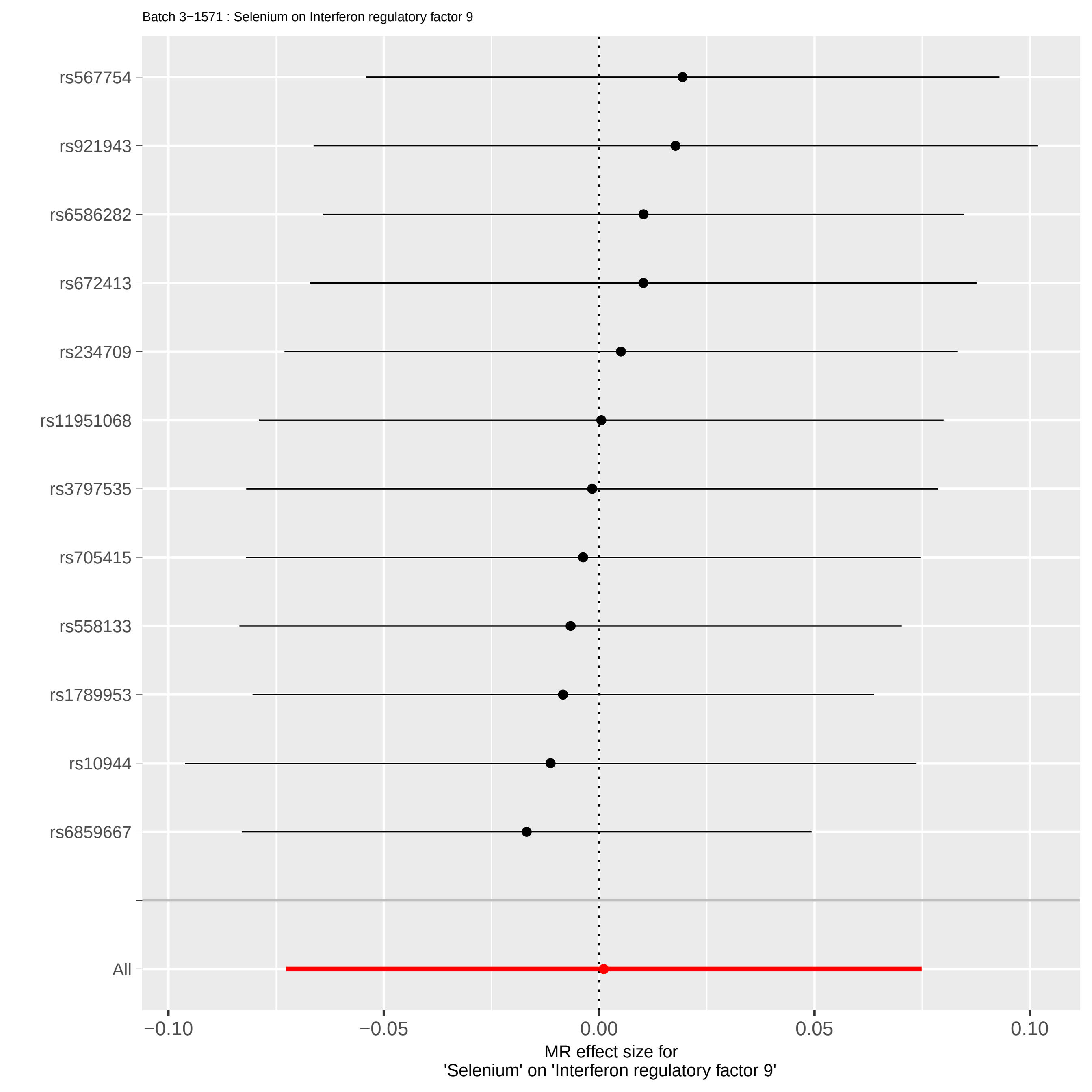
Batch 3−1571 : Selenium on Interferon regulatory factor 9
rs567754
rs921943
rs6586282
rs672413
rs234709
rs11951068
rs3797535
rs705415
rs558133
rs1789953
rs10944
rs6859667
All
−0.10
−0.05
0.00
0.05
0.10
MR effect size for
'Selenium' on 'Interferon regulatory factor 9'

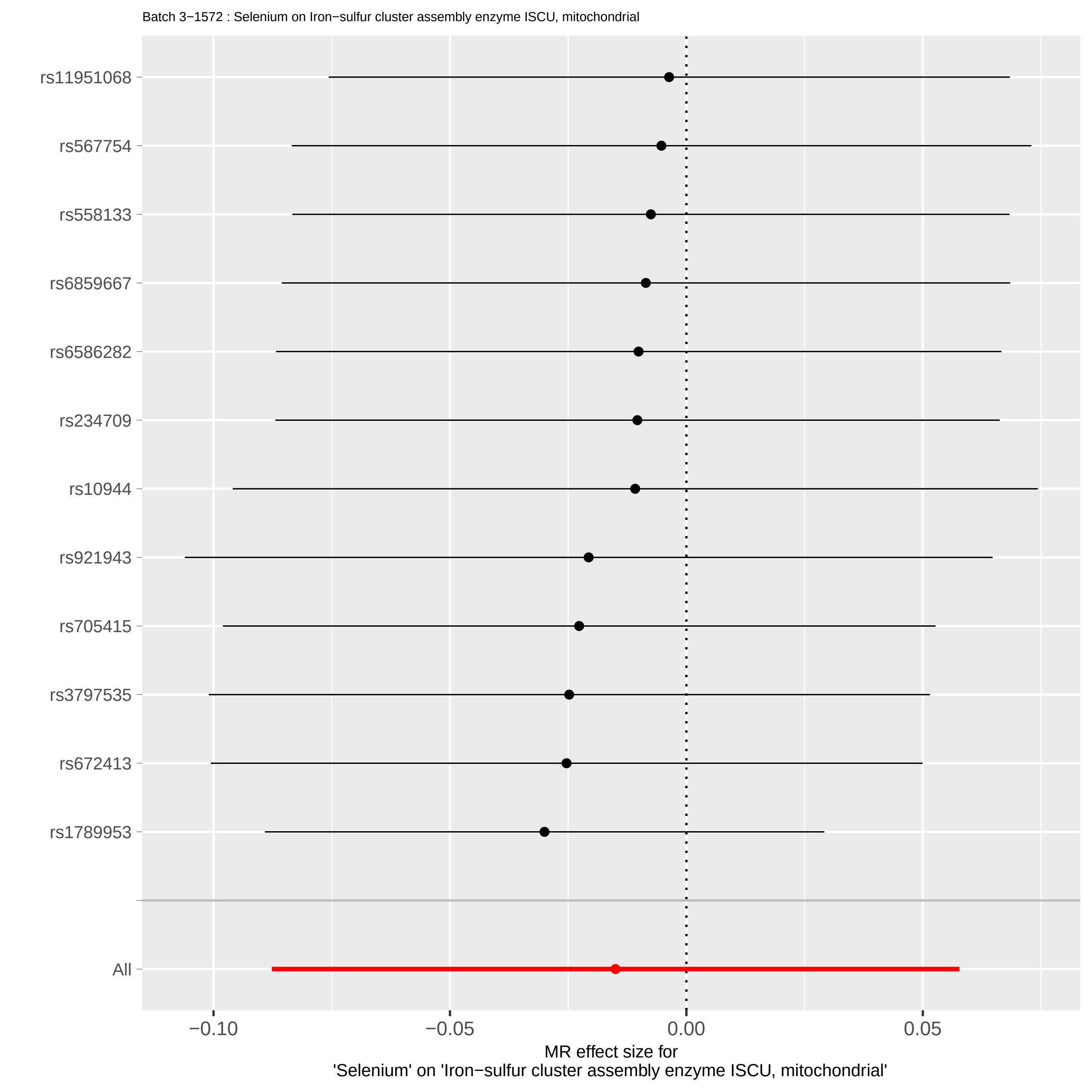

Batch 3−1572 : Selenium on Iron−sulfur cluster assembly enzyme ISCU, mitochondrial
rs11951068
rs567754
rs558133
rs6859667
rs6586282
rs234709
rs10944
rs921943
rs705415
rs3797535
rs672413
rs1789953
All
−0.10
−0.05
0.00
0.05
MR effect size for
'Selenium' on 'Iron−sulfur cluster assembly enzyme ISCU, mitochondrial'

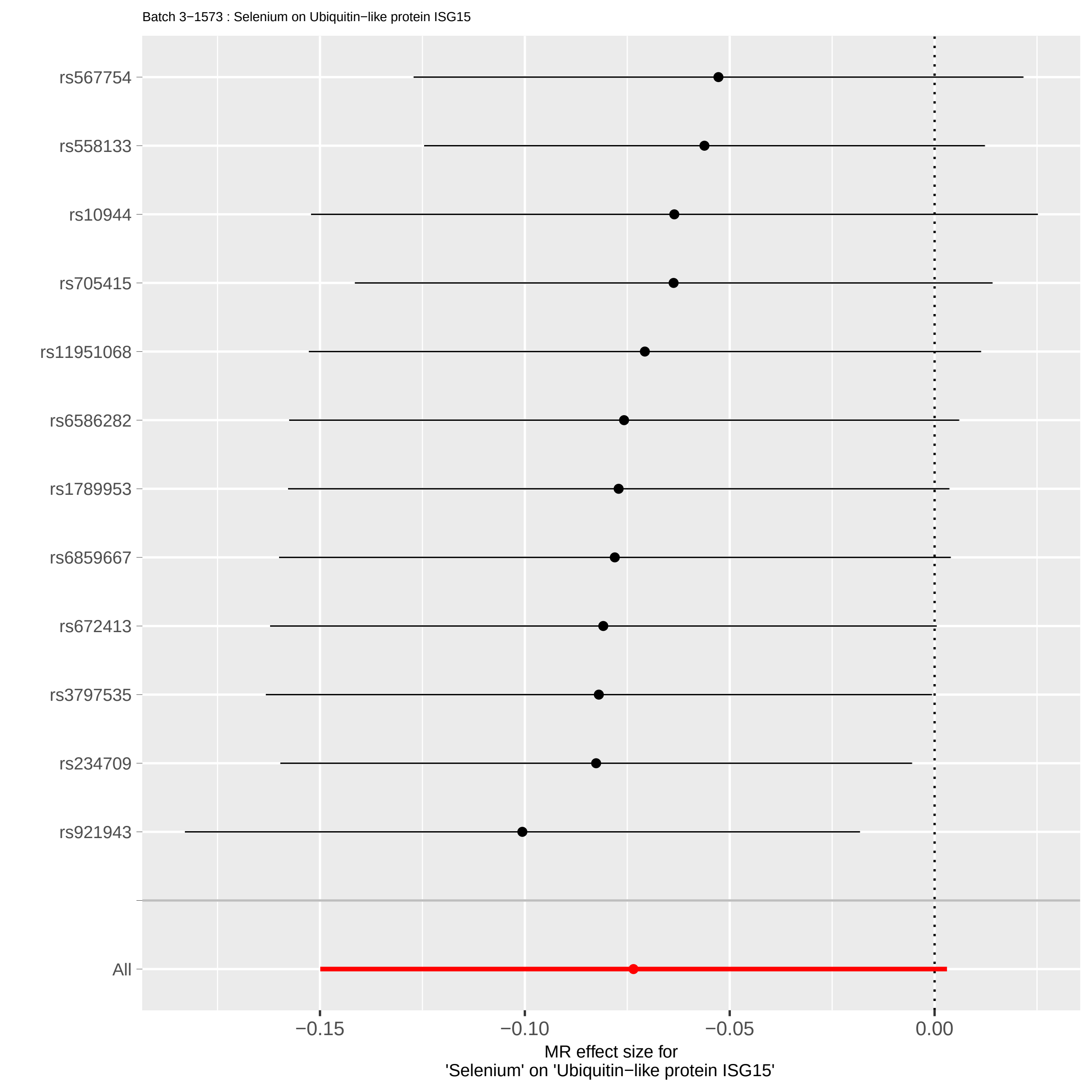
Batch 3−1573 : Selenium on Ubiquitin−like protein ISG15
rs567754
rs558133
rs10944
rs705415
rs11951068
rs6586282
rs1789953
rs6859667
rs672413
rs3797535
rs234709
rs921943
All
−0.15
−0.10
−0.05
0.00
MR effect size for
'Selenium' on 'Ubiquitin−like protein ISG15'

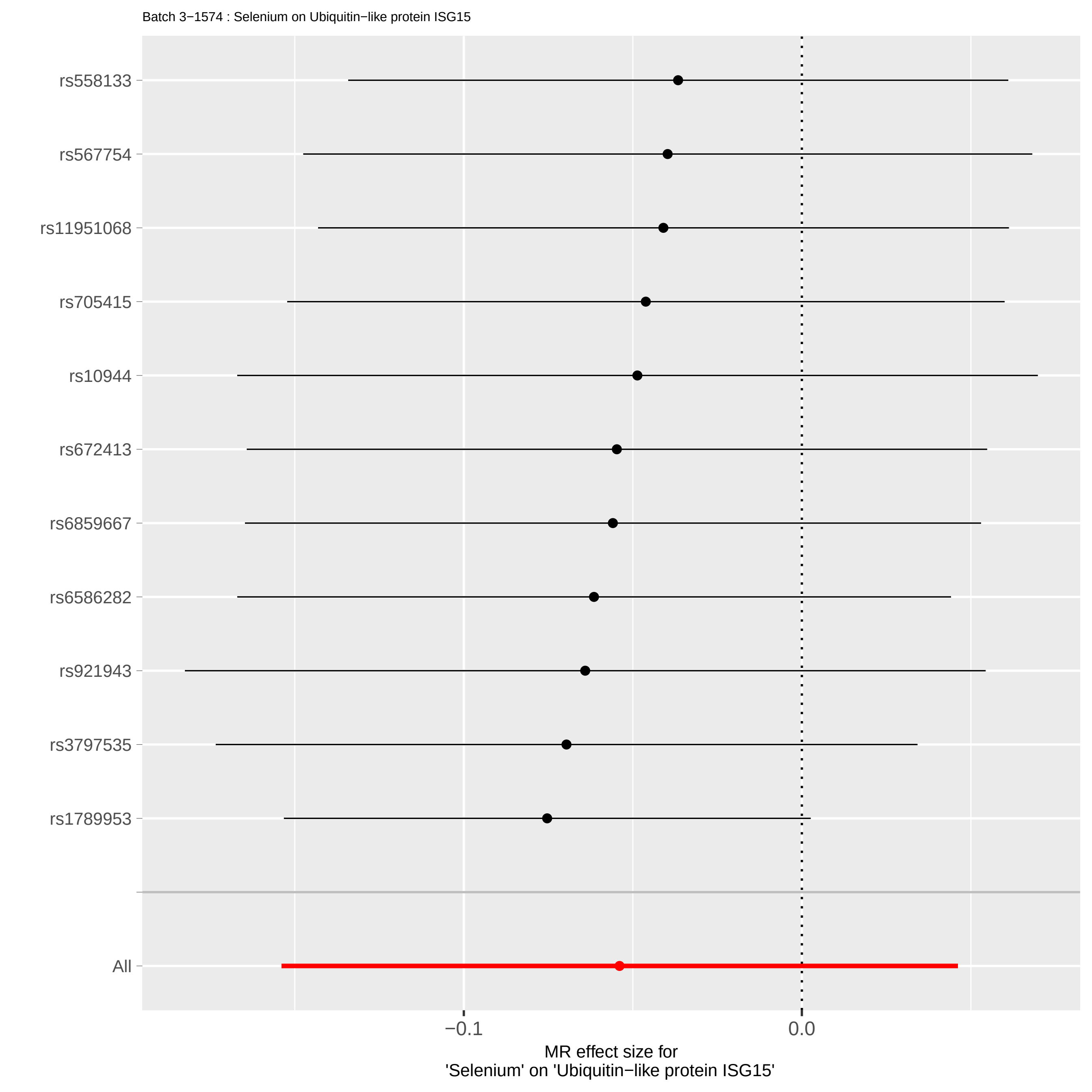

Batch 3−1574 : Selenium on Ubiquitin−like protein ISG15
rs558133
rs567754
rs11951068
rs705415
rs10944
rs672413
rs6859667
rs6586282
rs921943
rs3797535
rs1789953
All
−0.1
0.0
MR effect size for
'Selenium' on 'Ubiquitin−like protein ISG15'

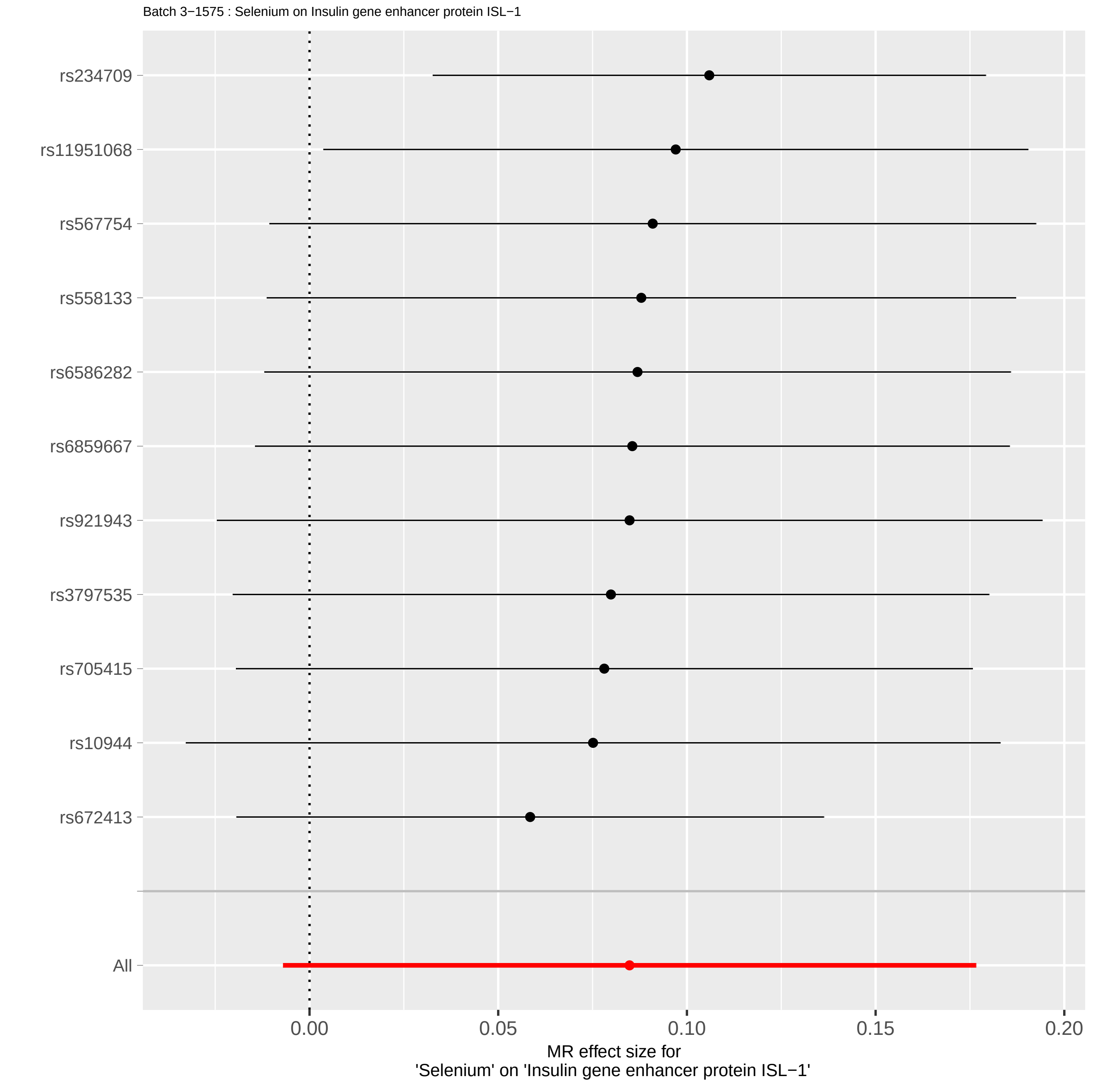

Batch 3−1575 : Selenium on Insulin gene enhancer protein ISL−1
rs234709
rs11951068
rs567754
rs558133
rs6586282
rs6859667
rs921943
rs3797535
rs705415
rs10944
rs672413
All
0.00
0.05
0.10
0.15
0.20
MR effect size for
'Selenium' on 'Insulin gene enhancer protein ISL−1'

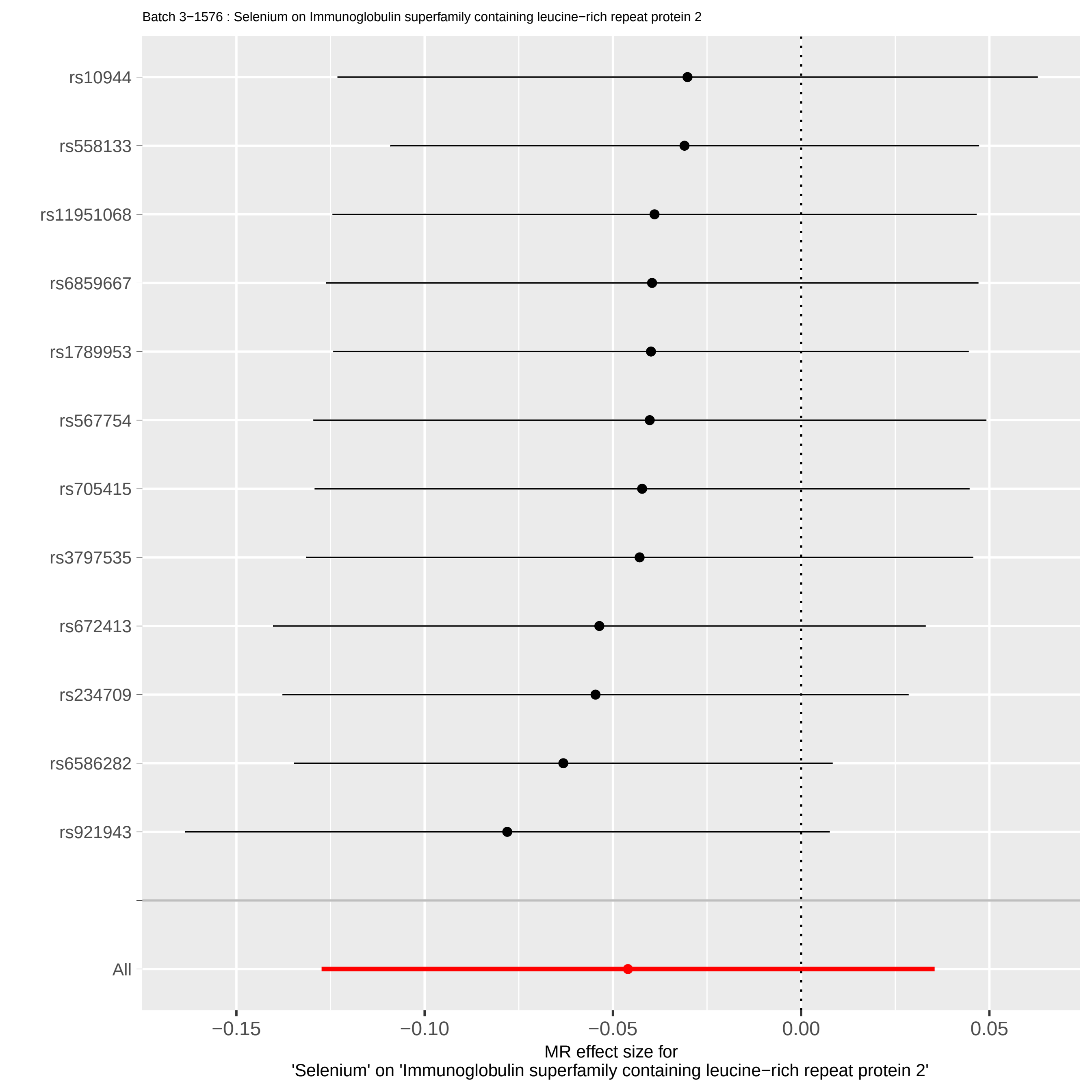
Batch 3−1576 : Selenium on Immunoglobulin superfamily containing leucine−rich repeat protein 2
rs10944
rs558133
rs11951068
rs6859667
rs1789953
rs567754
rs705415
rs3797535
rs672413
rs234709
rs6586282
rs921943
All
−0.15
−0.10
−0.05
0.00
0.05
MR effect size for
'Selenium' on 'Immunoglobulin superfamily containing leucine−rich repeat protein 2'

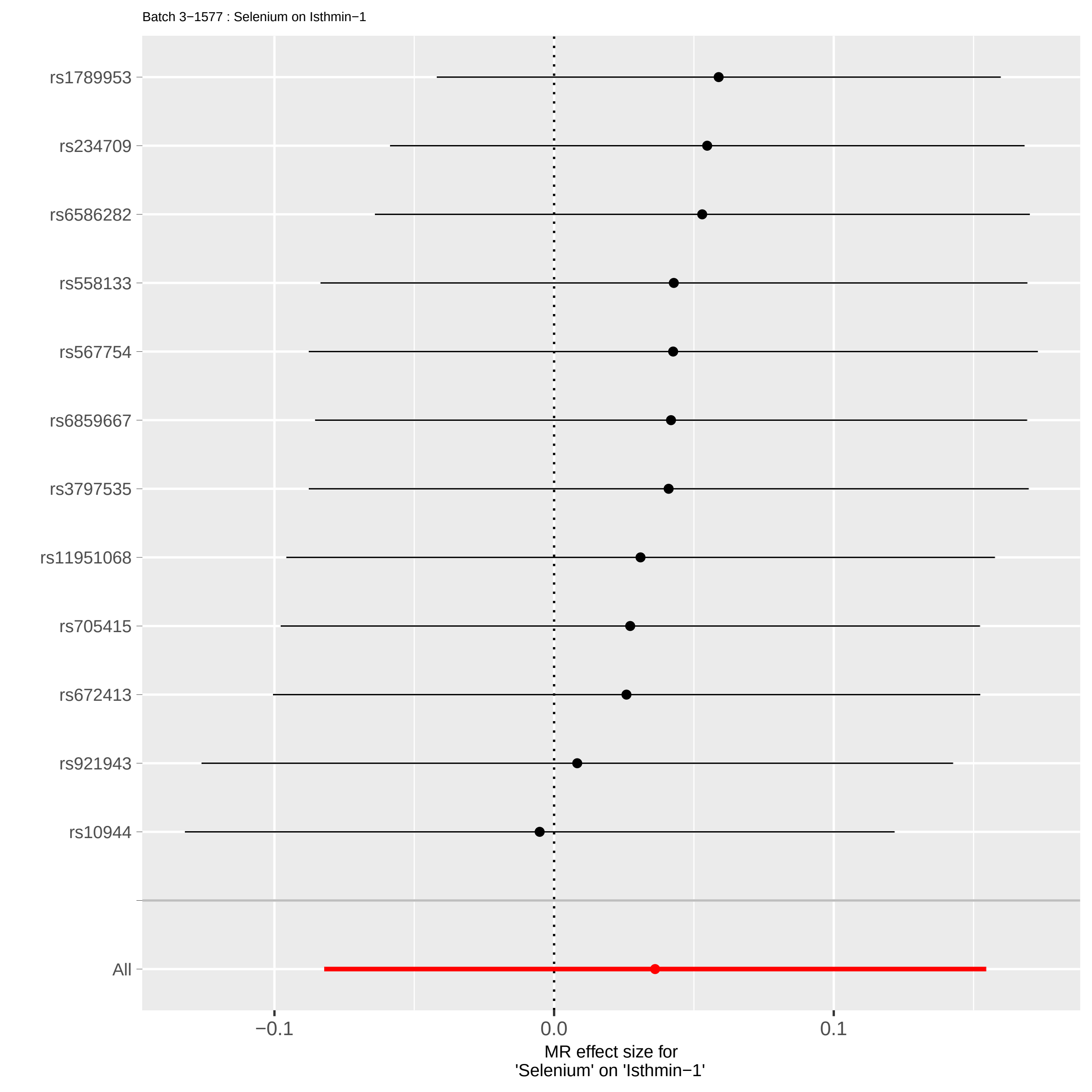

Batch 3−1577 : Selenium on Isthmin−1
rs1789953
rs234709
rs6586282
rs558133
rs567754
rs6859667
rs3797535
rs11951068
rs705415
rs672413
rs921943
rs10944
All
−0.1
0.0
0.1
MR effect size for
'Selenium' on 'Isthmin−1'

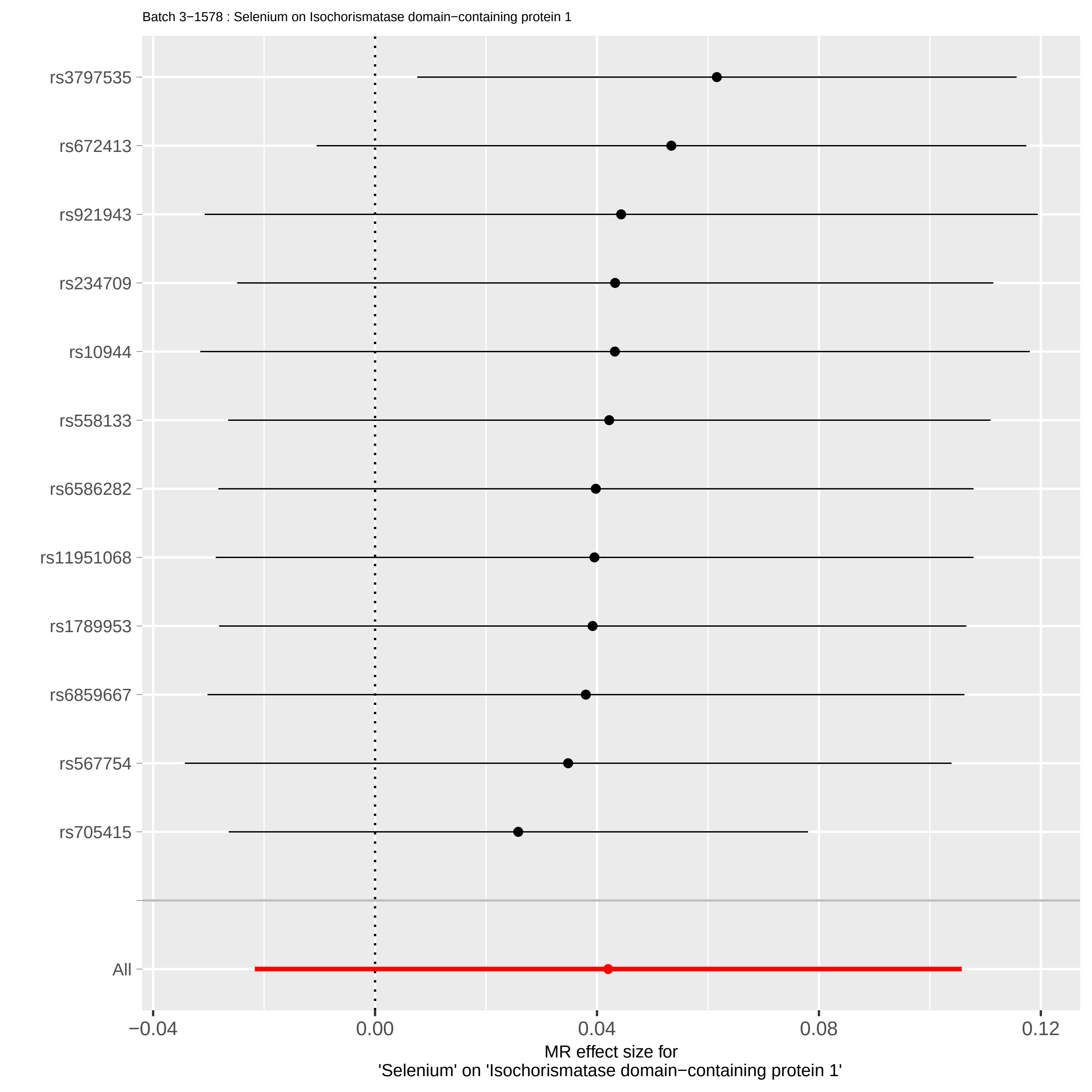

Batch 3−1578 : Selenium on Isochorismatase domain−containing protein 1
rs3797535
rs672413
rs921943
rs234709
rs10944
rs558133
rs6586282
rs11951068
rs1789953
rs6859667
rs567754
rs705415
All
−0.04
0.00
0.04
0.08
0.12
MR effect size for
'Selenium' on 'Isochorismatase domain−containing protein 1'

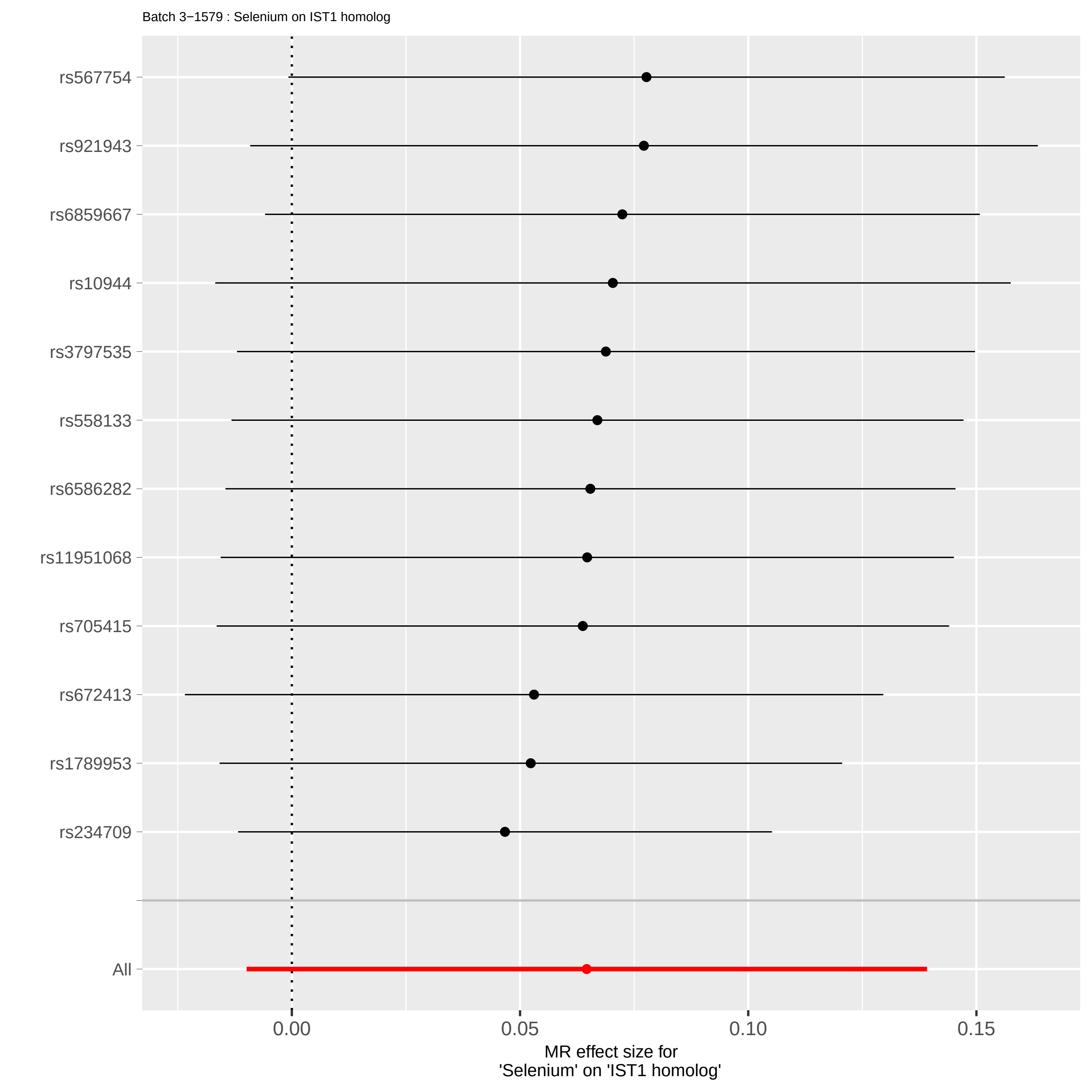

Batch 3−1579 : Selenium on IST1 homolog
rs567754
rs921943
rs6859667
rs10944
rs3797535
rs558133
rs6586282
rs11951068
rs705415
rs672413
rs1789953
rs234709
All
0.00
0.05
0.10
0.15
MR effect size for
'Selenium' on 'IST1 homolog'

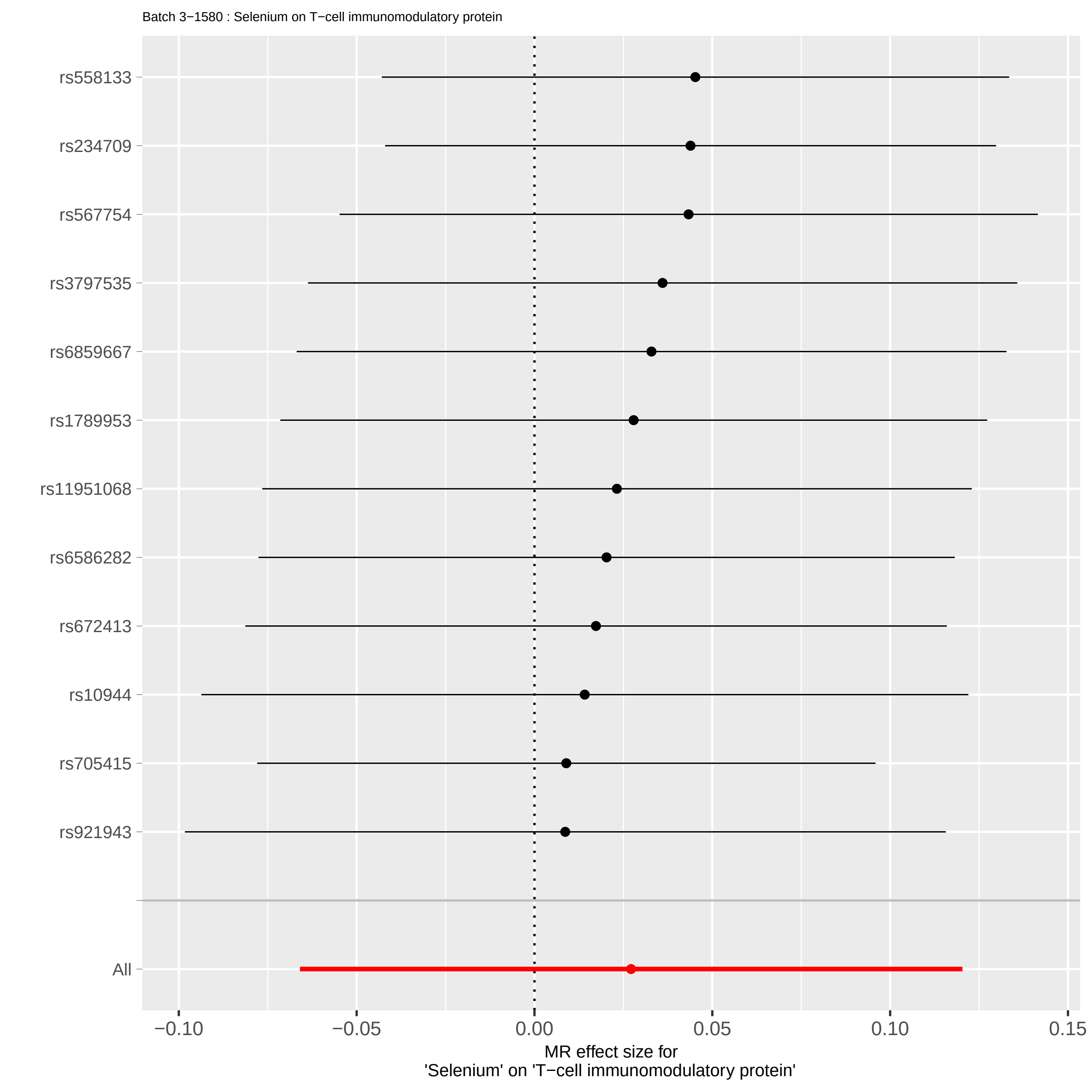

Batch 3−1580 : Selenium on T−cell immunomodulatory protein
rs558133
rs234709
rs567754
rs3797535
rs6859667
rs1789953
rs11951068
rs6586282
rs672413
rs10944
rs705415
rs921943
All
−0.10
−0.05
0.00
0.05
0.10
0.15
MR effect size for
'Selenium' on 'T−cell immunomodulatory protein'

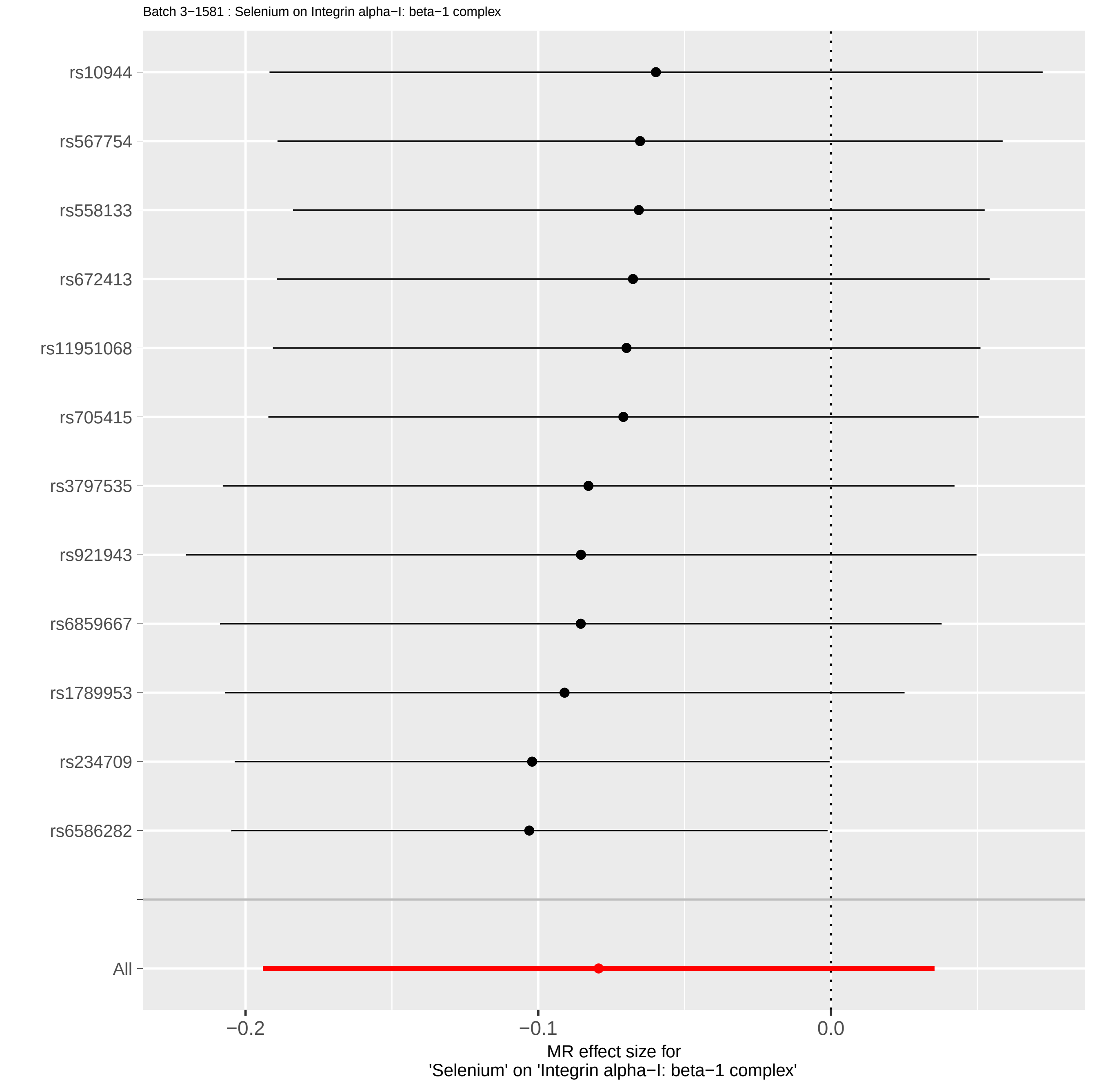

Batch 3−1581 : Selenium on Integrin alpha−I: beta−1 complex
rs10944
rs567754
rs558133
rs672413
rs11951068
rs705415
rs3797535
rs921943
rs6859667
rs1789953
rs234709
rs6586282
All
−0.2
−0.1
0.0
MR effect size for
'Selenium' on 'Integrin alpha−I: beta−1 complex'

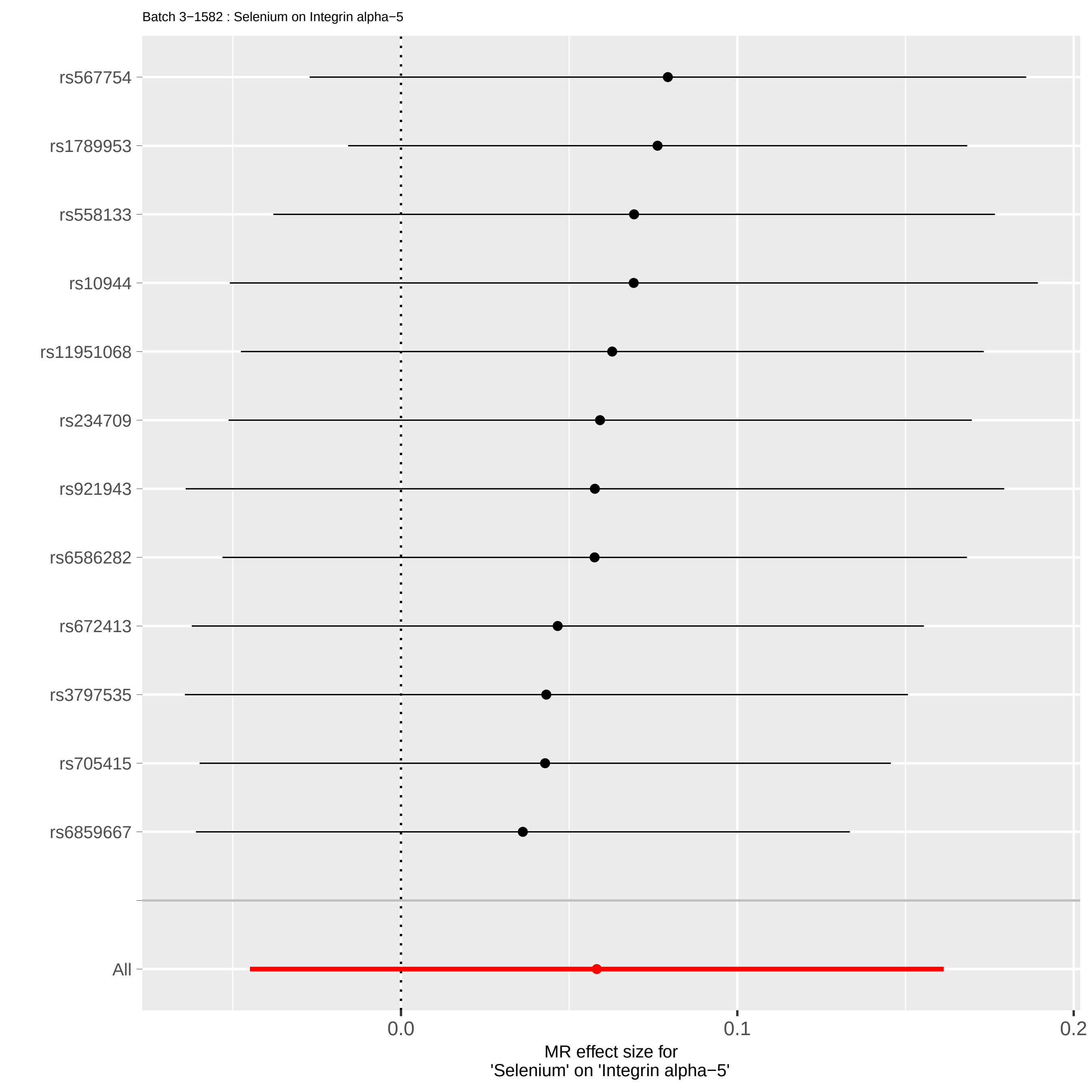

Batch 3−1582 : Selenium on Integrin alpha−5
rs567754
rs1789953
rs558133
rs10944
rs11951068
rs234709
rs921943
rs6586282
rs672413
rs3797535
rs705415
rs6859667
All
0.0
0.1
0.2
MR effect size for
'Selenium' on 'Integrin alpha−5'

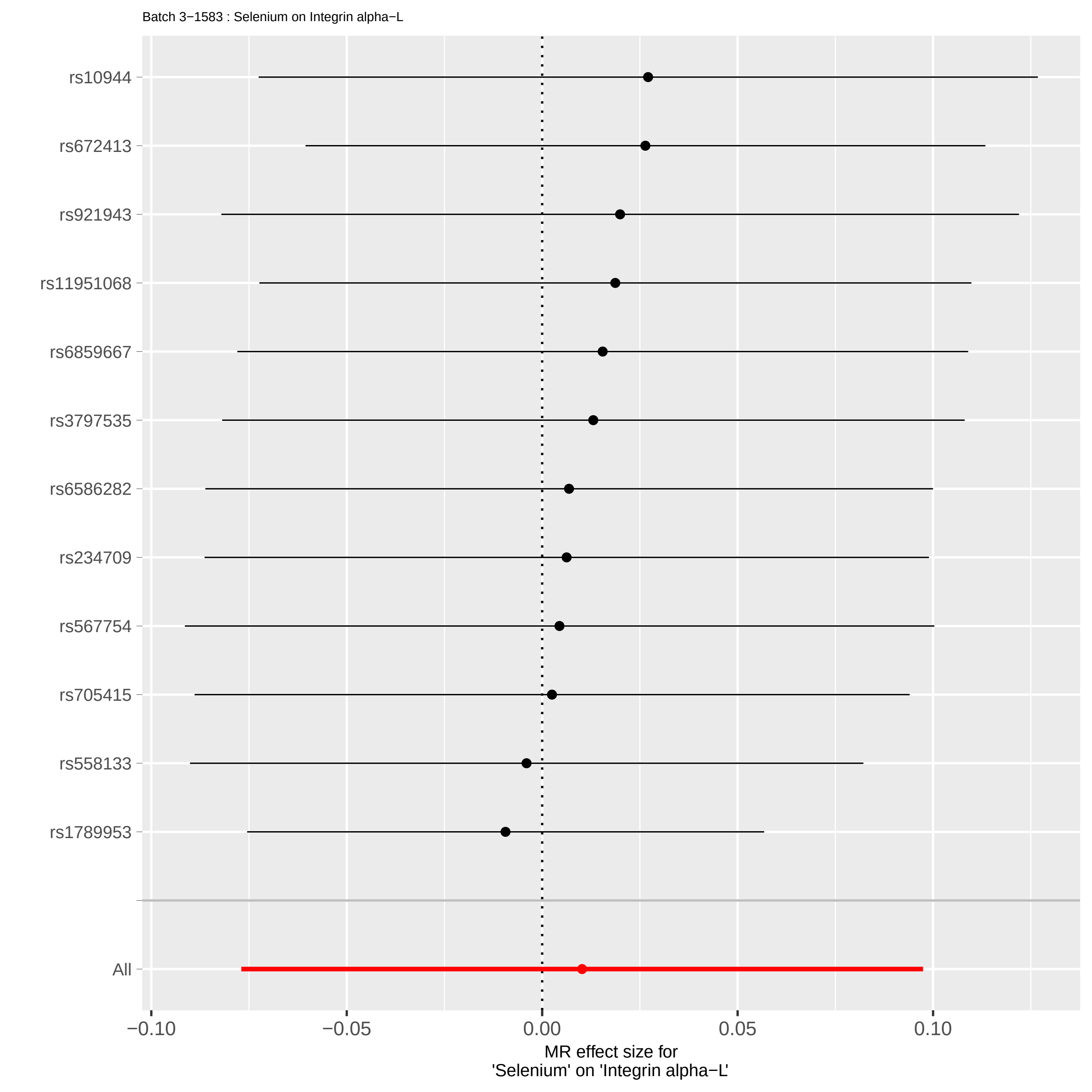

Batch 3−1583 : Selenium on Integrin alpha−L
rs10944
rs672413
rs921943
rs11951068
rs6859667
rs3797535
rs6586282
rs234709
rs567754
rs705415
rs558133
rs1789953
All
−0.10
−0.05
0.00
0.05
0.10
MR effect size for
'Selenium' on 'Integrin alpha−L'

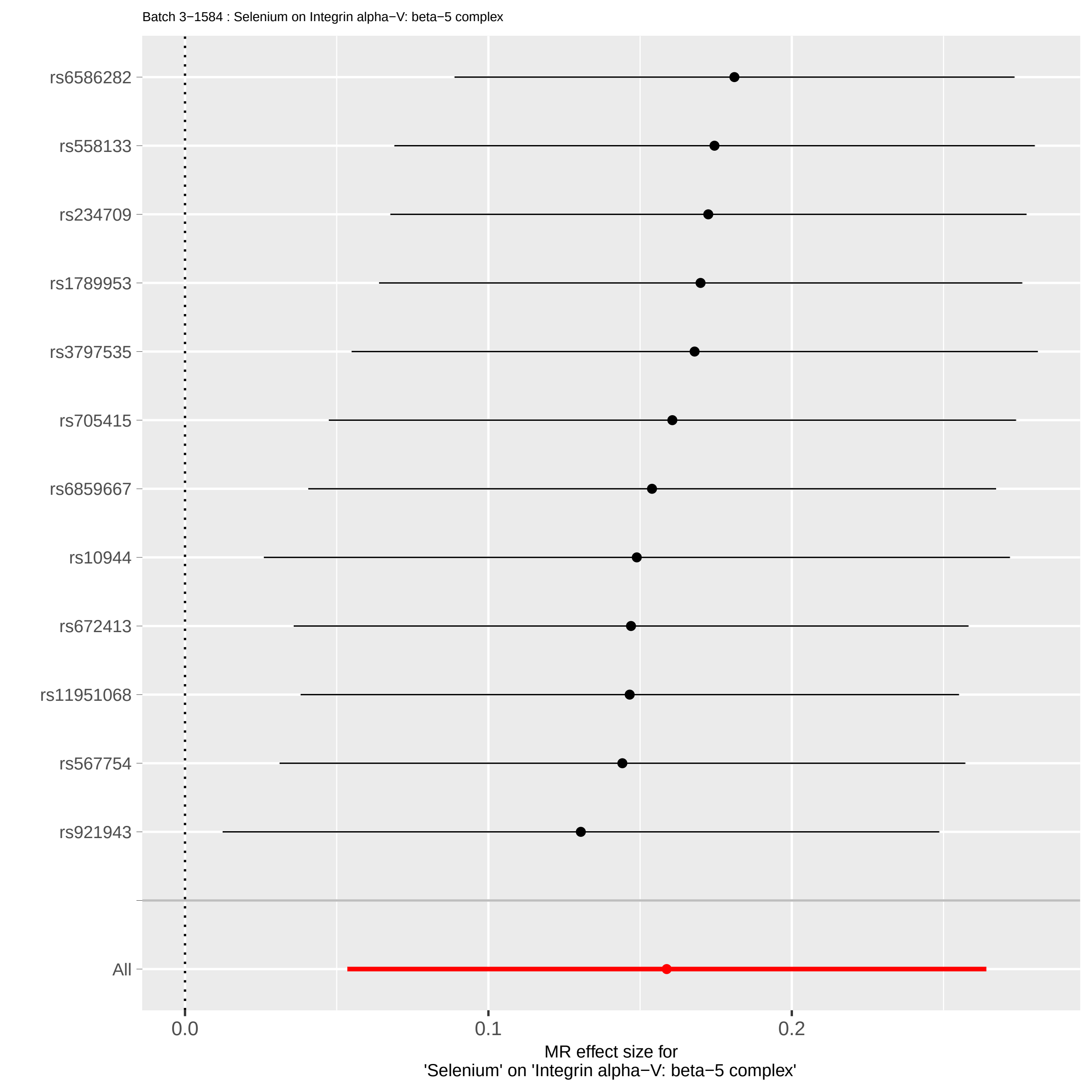
Batch 3−1584 : Selenium on Integrin alpha−V: beta−5 complex
rs6586282
rs558133
rs234709
rs1789953
rs3797535
rs705415
rs6859667
rs10944
rs672413
rs11951068
rs567754
rs921943
All
0.0
0.1
0.2
MR effect size for
'Selenium' on 'Integrin alpha−V: beta−5 complex'

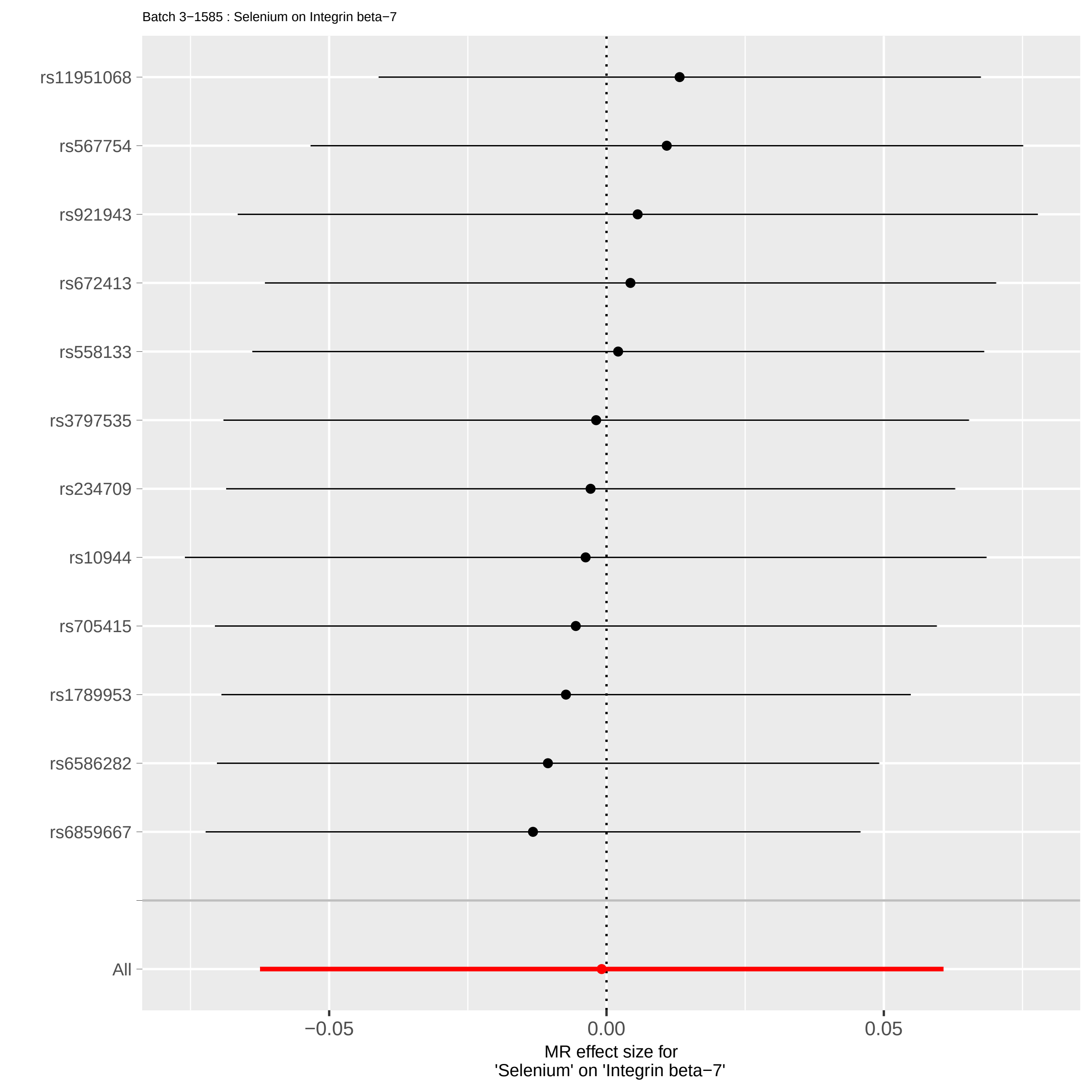
Batch 3−1585 : Selenium on Integrin beta−7
rs11951068
rs567754
rs921943
rs672413
rs558133
rs3797535
rs234709
rs10944
rs705415
rs1789953
rs6586282
rs6859667
All
−0.05
0.00
0.05
MR effect size for
'Selenium' on 'Integrin beta−7'

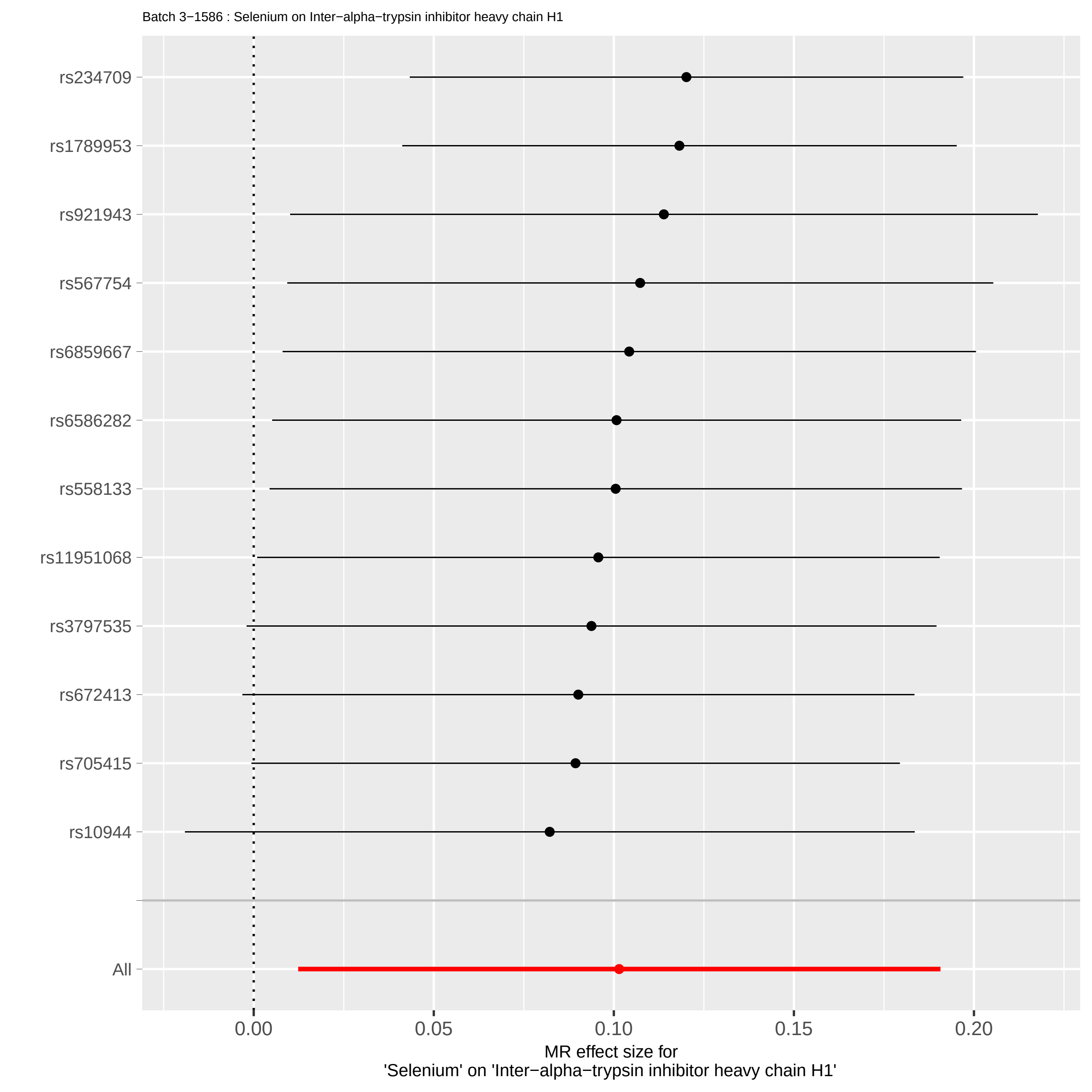

Batch 3−1586 : Selenium on Inter−alpha−trypsin inhibitor heavy chain H1
rs234709
rs1789953
rs921943
rs567754
rs6859667
rs6586282
rs558133
rs11951068
rs3797535
rs672413
rs705415
rs10944
All
0.00
0.05
0.10
0.15
0.20
MR effect size for
'Selenium' on 'Inter−alpha−trypsin inhibitor heavy chain H1'

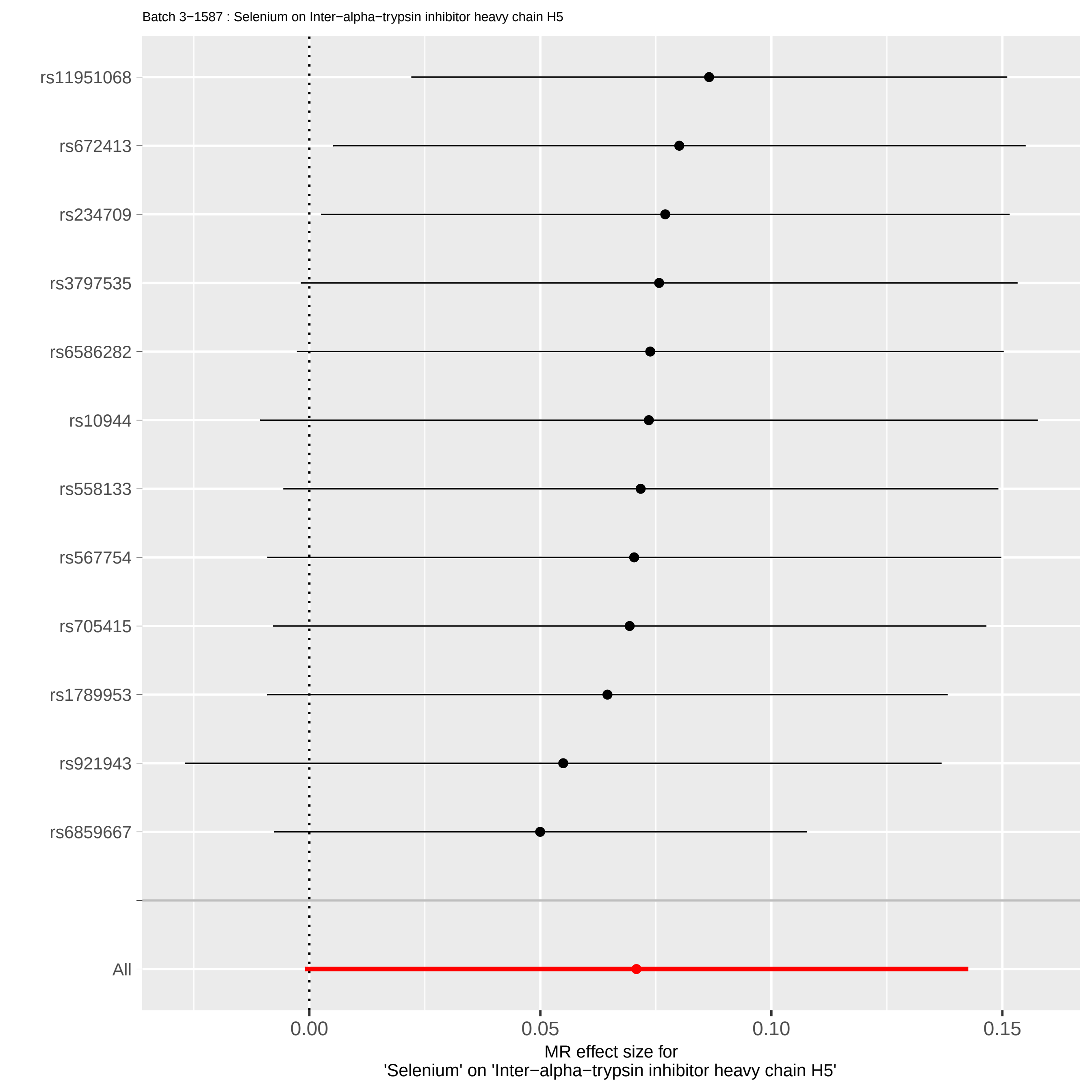

Batch 3−1587 : Selenium on Inter−alpha−trypsin inhibitor heavy chain H5
rs11951068
rs672413
rs234709
rs3797535
rs6586282
rs10944
rs558133
rs567754
rs705415
rs1789953
rs921943
rs6859667
All
0.00
0.05
0.10
0.15
MR effect size for
'Selenium' on 'Inter−alpha−trypsin inhibitor heavy chain H5'

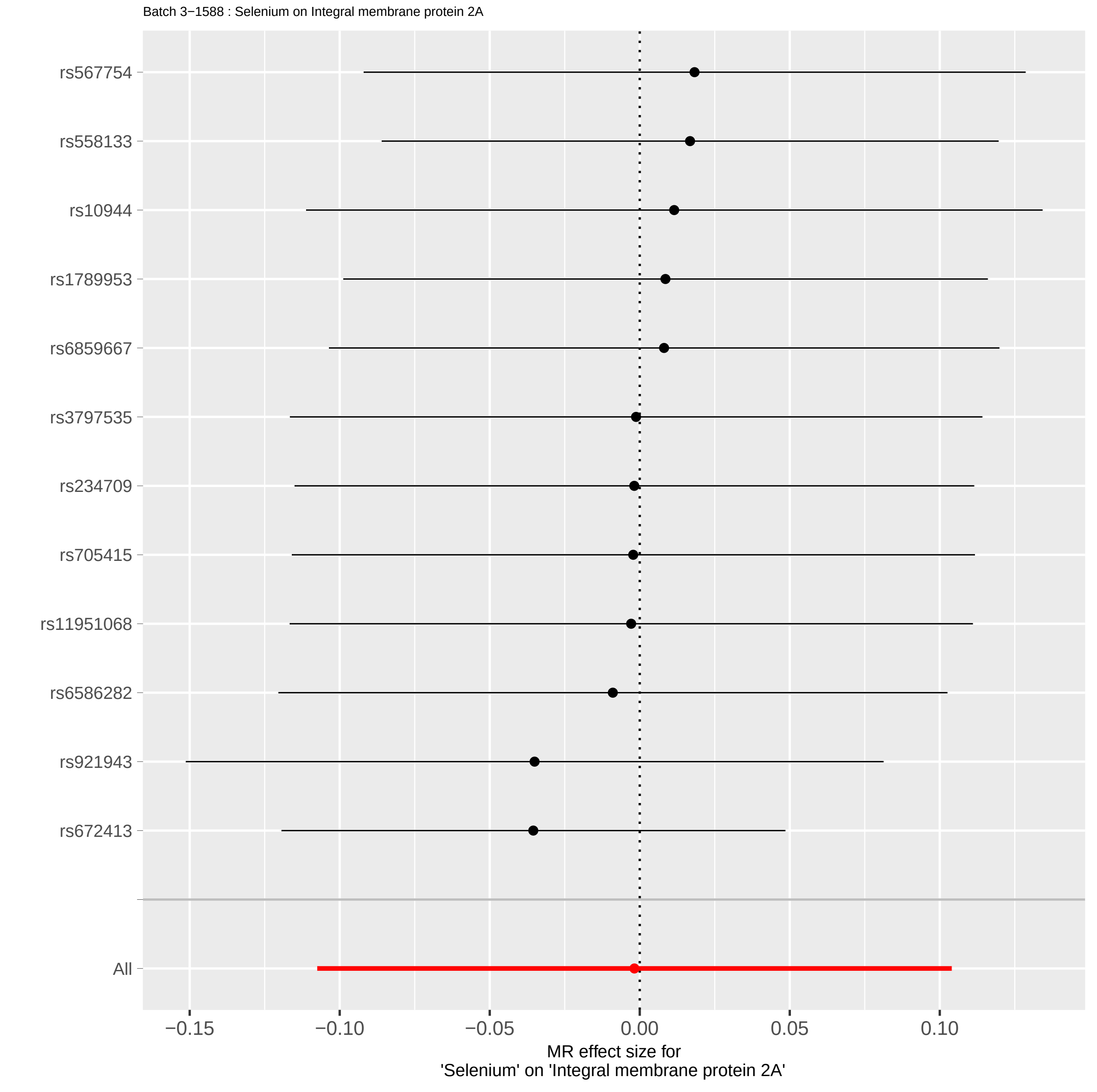

Batch 3−1588 : Selenium on Integral membrane protein 2A
rs567754
rs558133
rs10944
rs1789953
rs6859667
rs3797535
rs234709
rs705415
rs11951068
rs6586282
rs921943
rs672413
All
−0.15
−0.10
−0.05
0.00
0.05
0.10
MR effect size for
'Selenium' on 'Integral membrane protein 2A'

Batch 3−1589 : Selenium on Integral membrane protein 2A

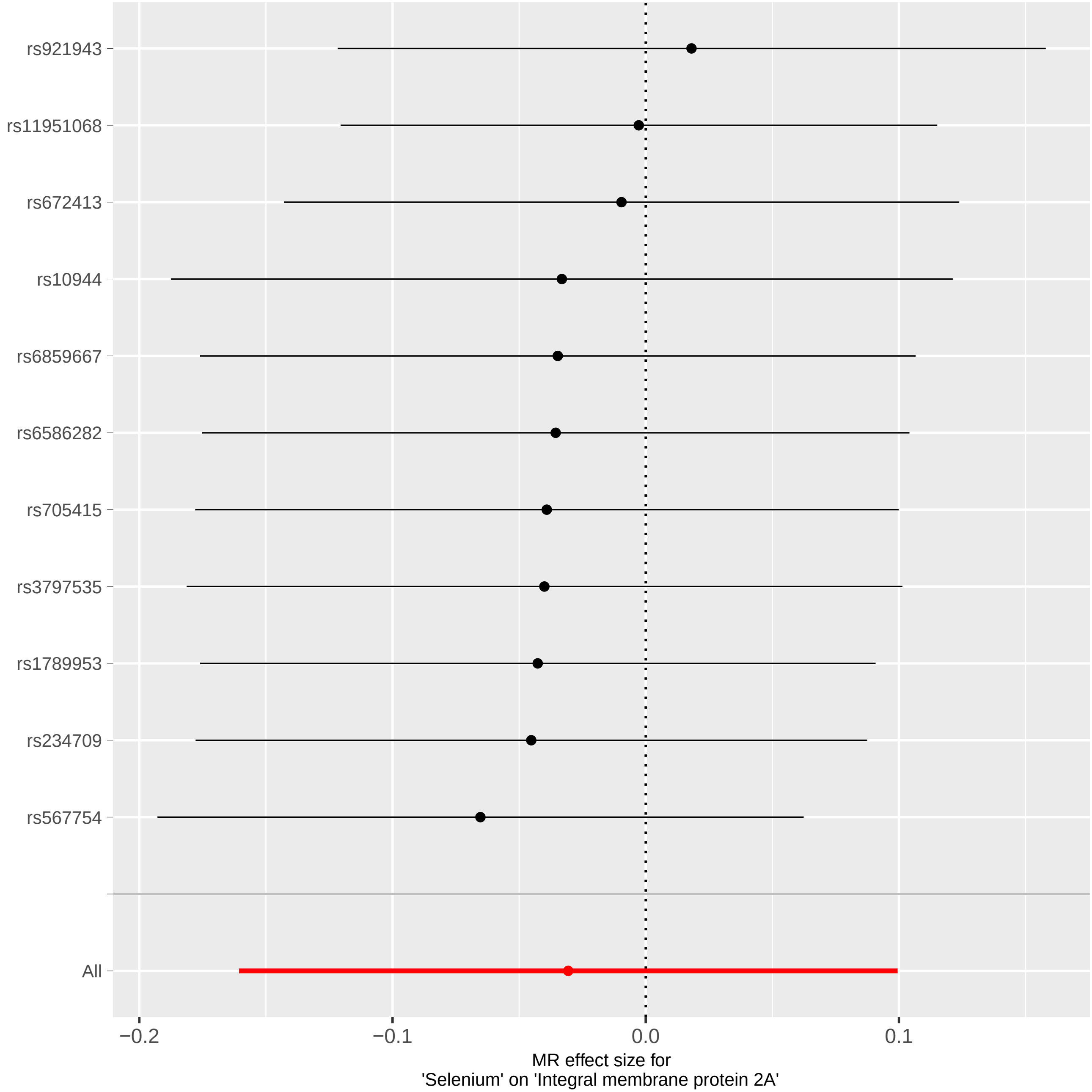

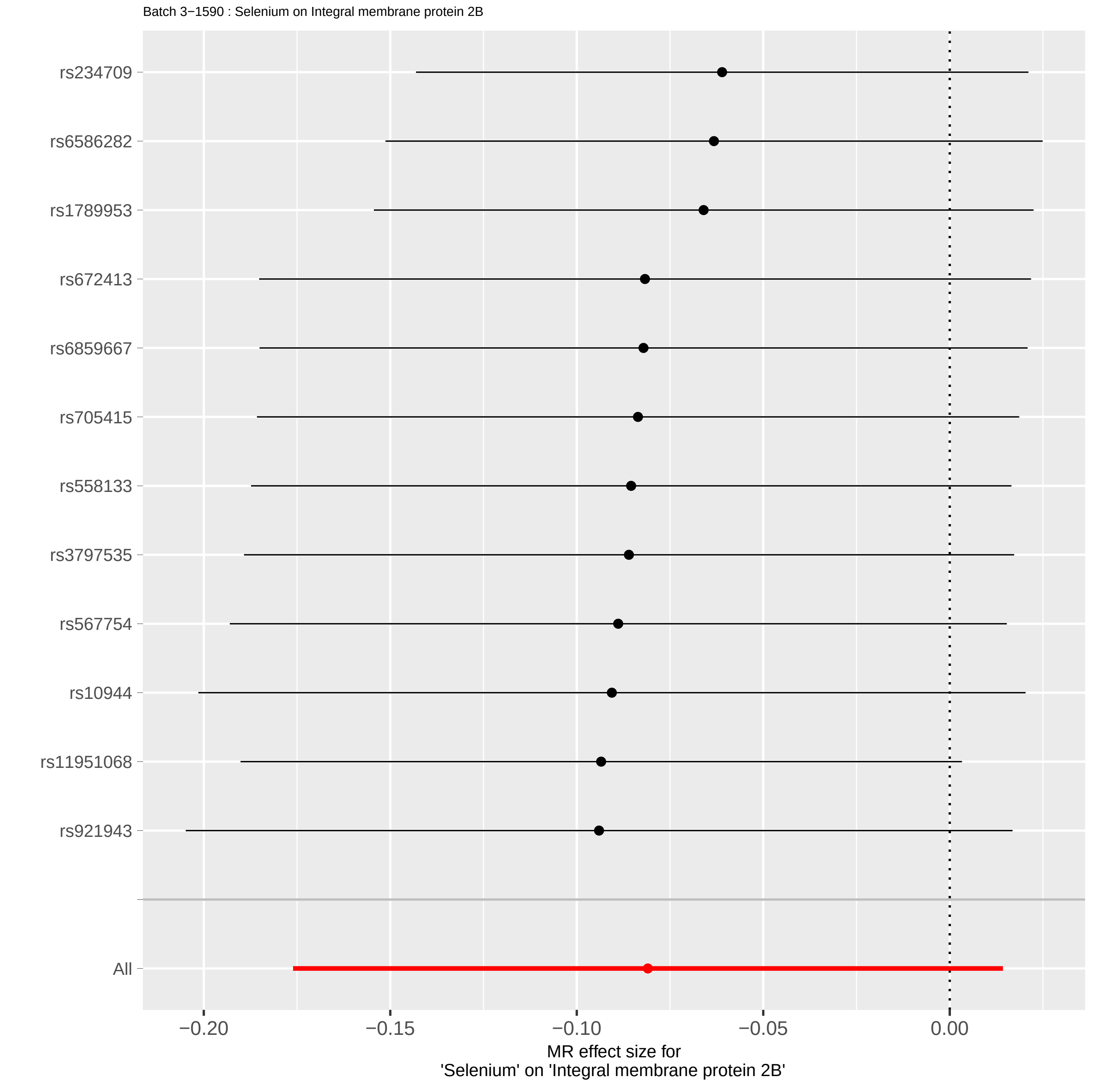
Batch 3−1590 : Selenium on Integral membrane protein 2B
rs234709
rs6586282
rs1789953
rs672413
rs6859667
rs705415
rs558133
rs3797535
rs567754
rs10944
rs11951068
rs921943
All
−0.20
−0.15
−0.10
−0.05
0.00
MR effect size for
'Selenium' on 'Integral membrane protein 2B'

Batch 3−1591 : Selenium on Integral membrane protein 2C

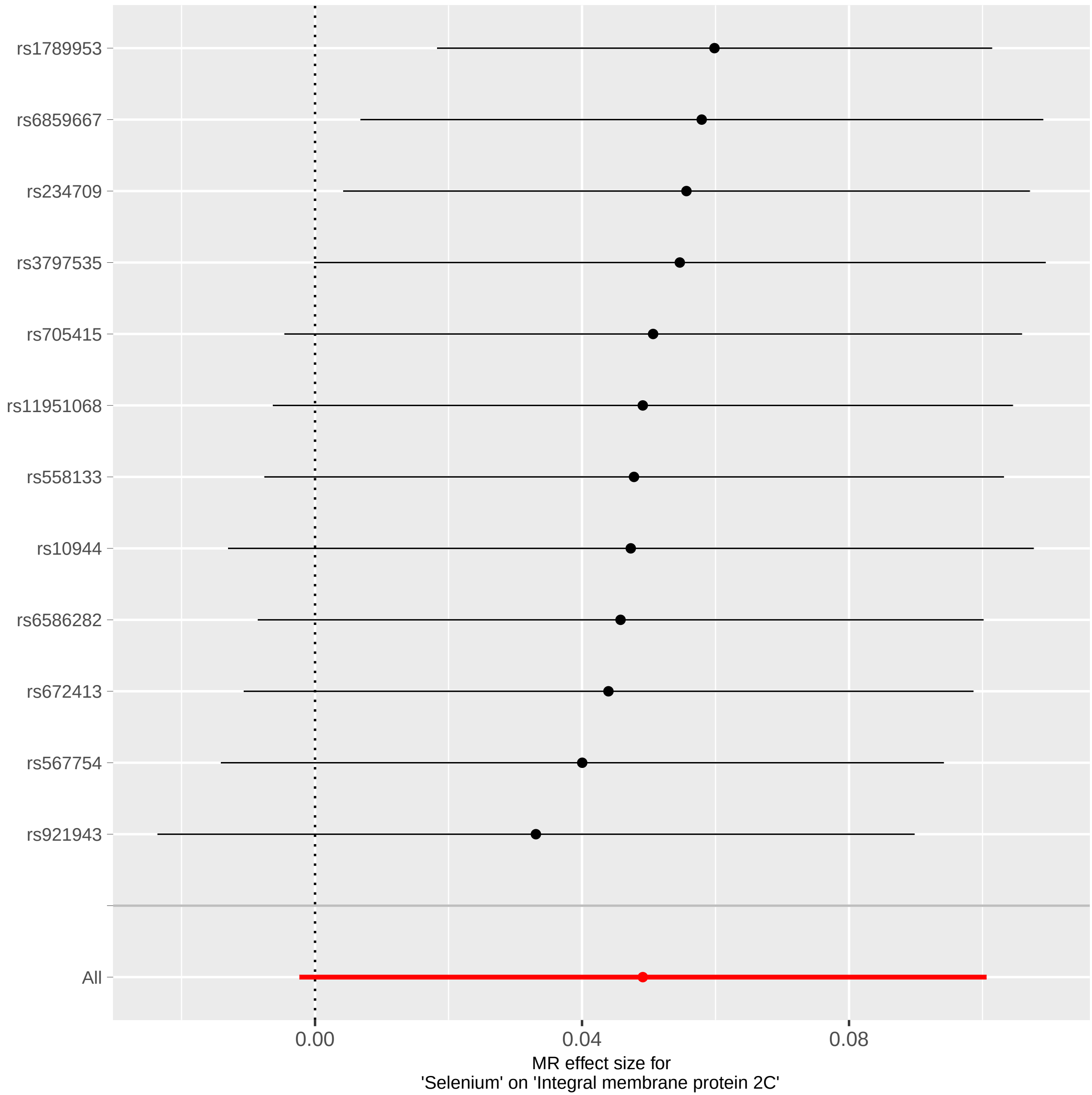

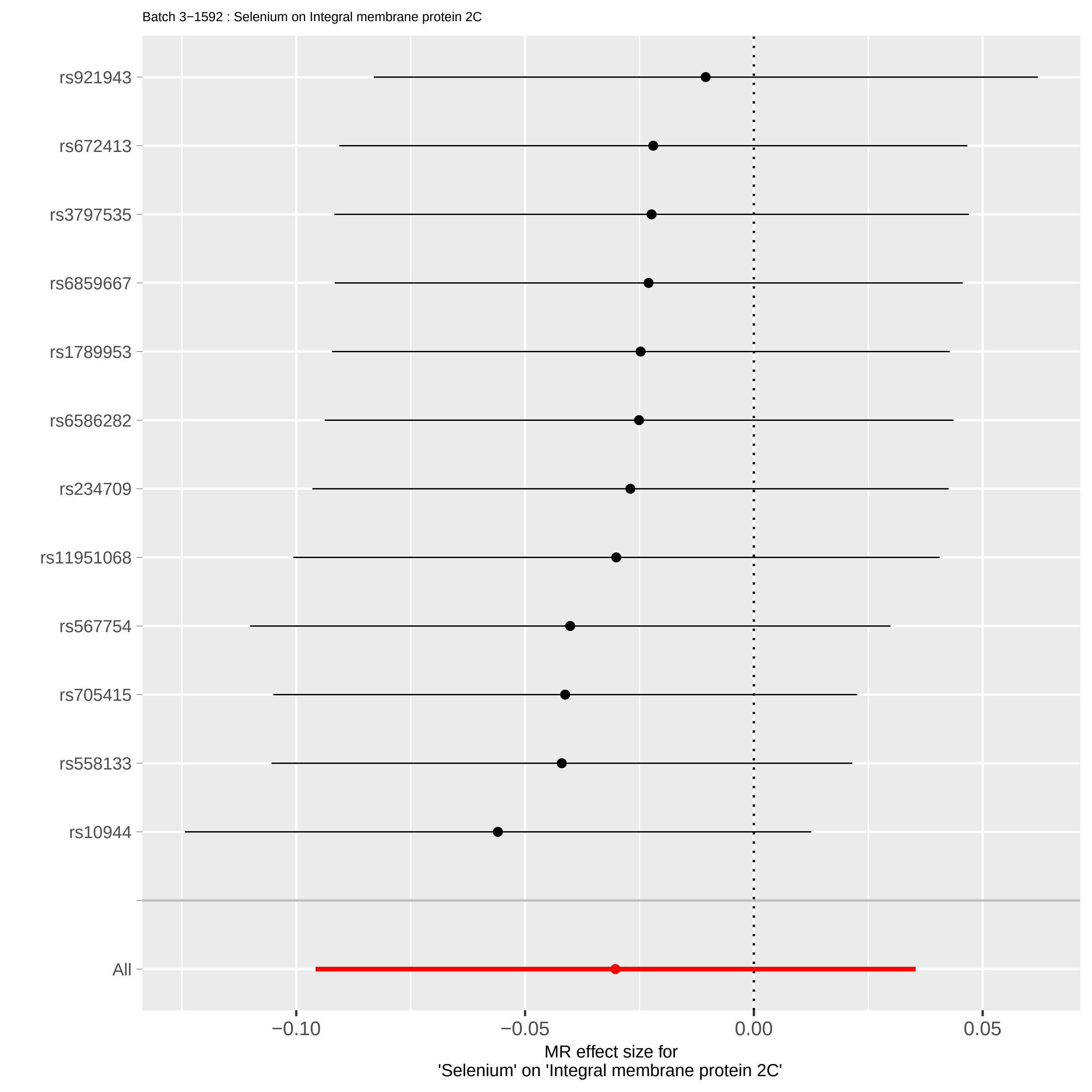
Batch 3−1592 : Selenium on Integral membrane protein 2C
rs921943
rs672413
rs3797535
rs6859667
rs1789953
rs6586282
rs234709
rs11951068
rs567754
rs705415
rs558133
rs10944
All
−0.10
−0.05
0.00
0.05
MR effect size for
'Selenium' on 'Integral membrane protein 2C'

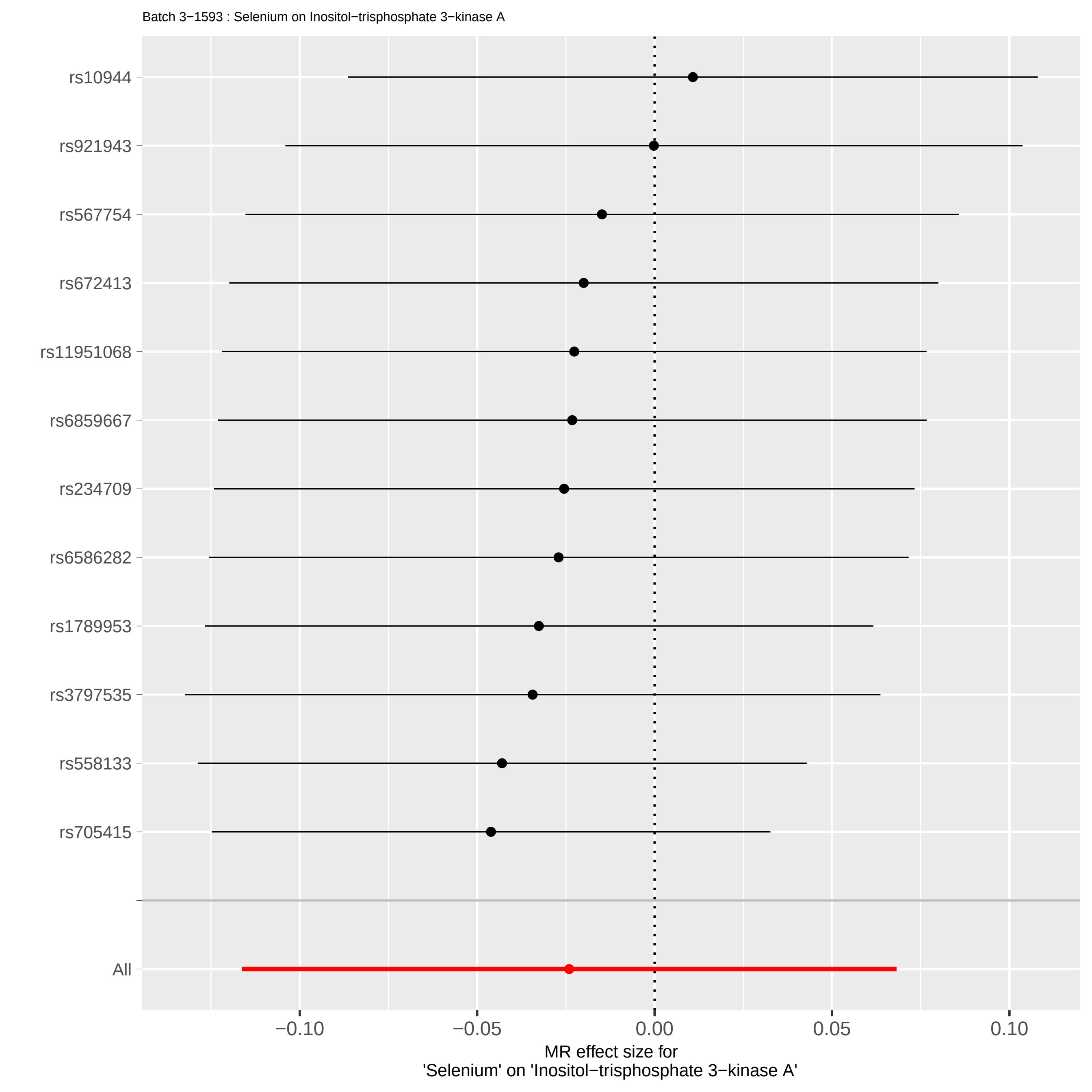
Batch 3−1593 : Selenium on Inositol−trisphosphate 3−kinase A
rs10944
rs921943
rs567754
rs672413
rs11951068
rs6859667
rs234709
rs6586282
rs1789953
rs3797535
rs558133
rs705415
All
−0.10
−0.05
0.00
0.05
0.10
MR effect size for
'Selenium' on 'Inositol−trisphosphate 3−kinase A'

Batch 3−1594 : Selenium on Inositol 1,4,5−trisphosphate receptor−interacting protein−like 1

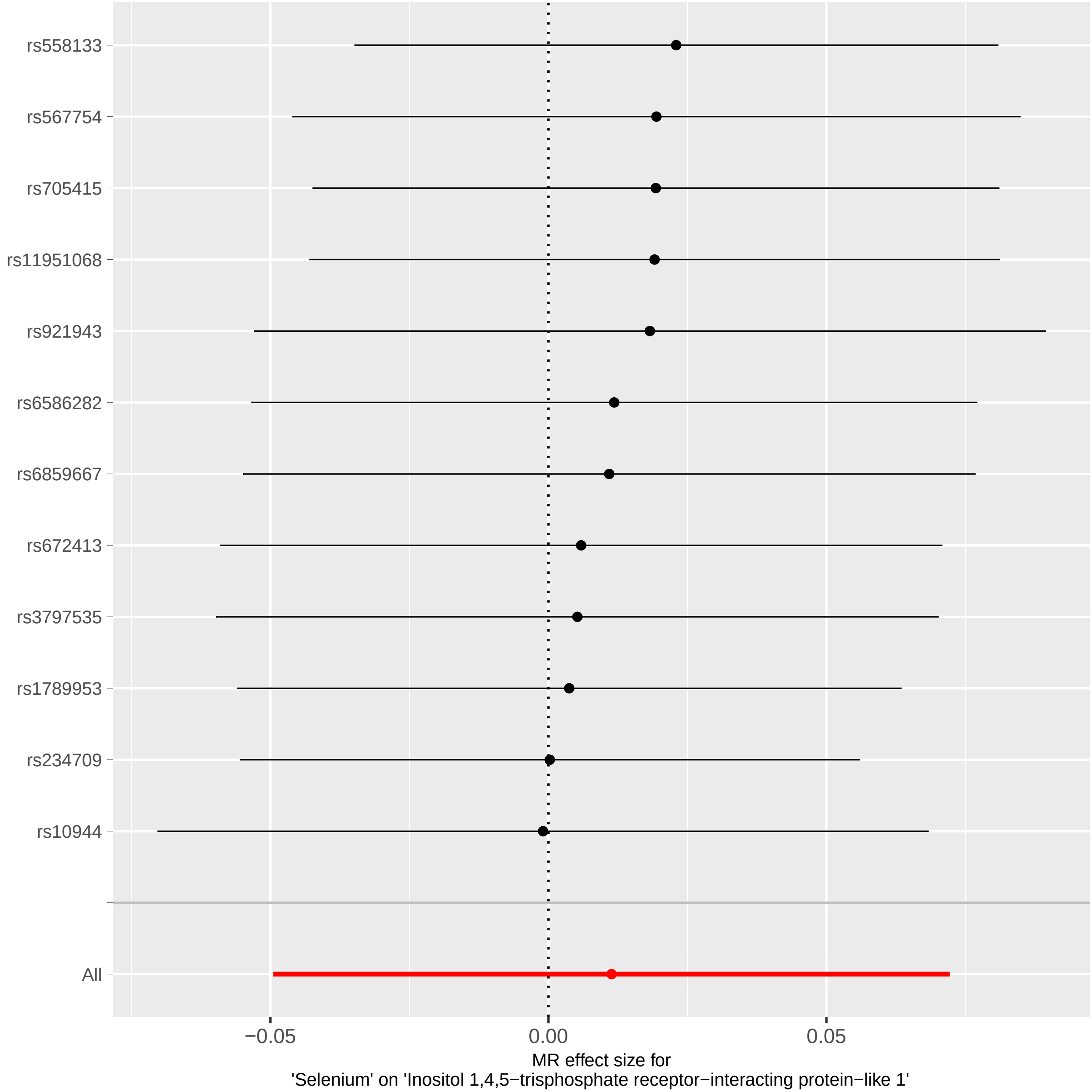

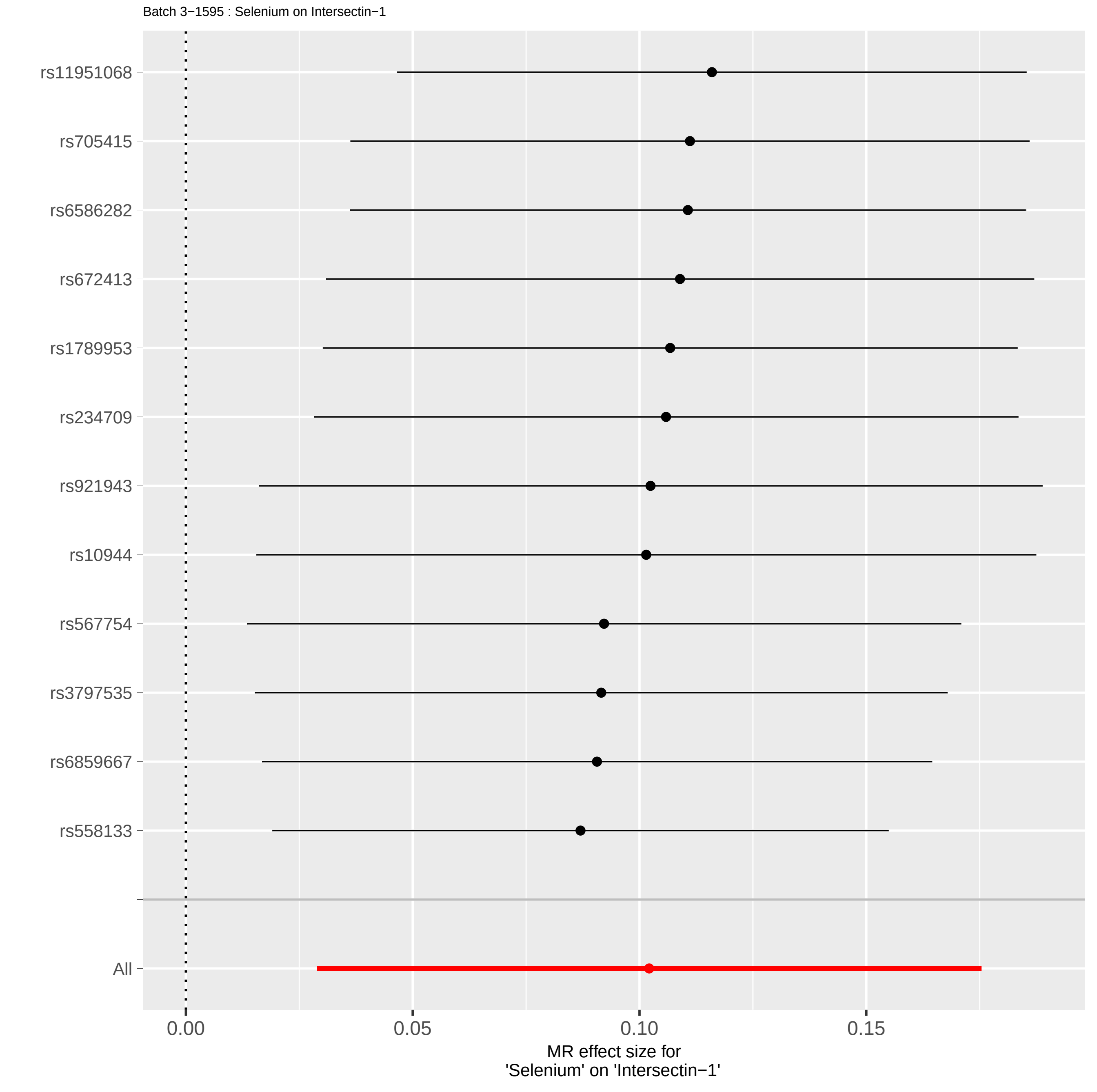

Batch 3−1595 : Selenium on Intersectin−1
rs11951068
rs705415
rs6586282
rs672413
rs1789953
rs234709
rs921943
rs10944
rs567754
rs3797535
rs6859667
rs558133
All
0.00
0.05
0.10
0.15
MR effect size for
'Selenium' on 'Intersectin−1'

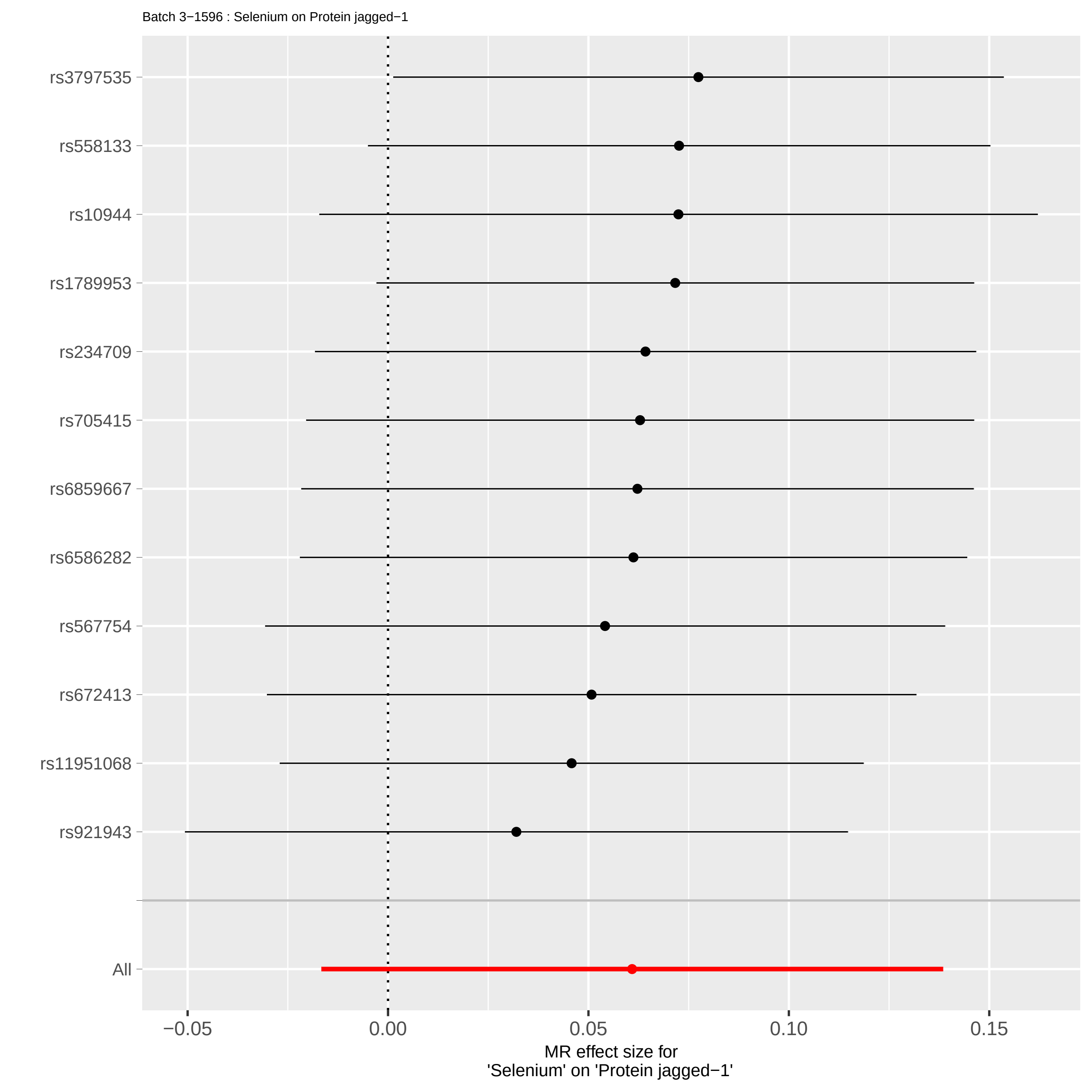

Batch 3−1596 : Selenium on Protein jagged−1
rs3797535
rs558133
rs10944
rs1789953
rs234709
rs705415
rs6859667
rs6586282
rs567754
rs672413
rs11951068
rs921943
All
−0.05
0.00
0.05
0.10
0.15
MR effect size for
'Selenium' on 'Protein jagged−1'

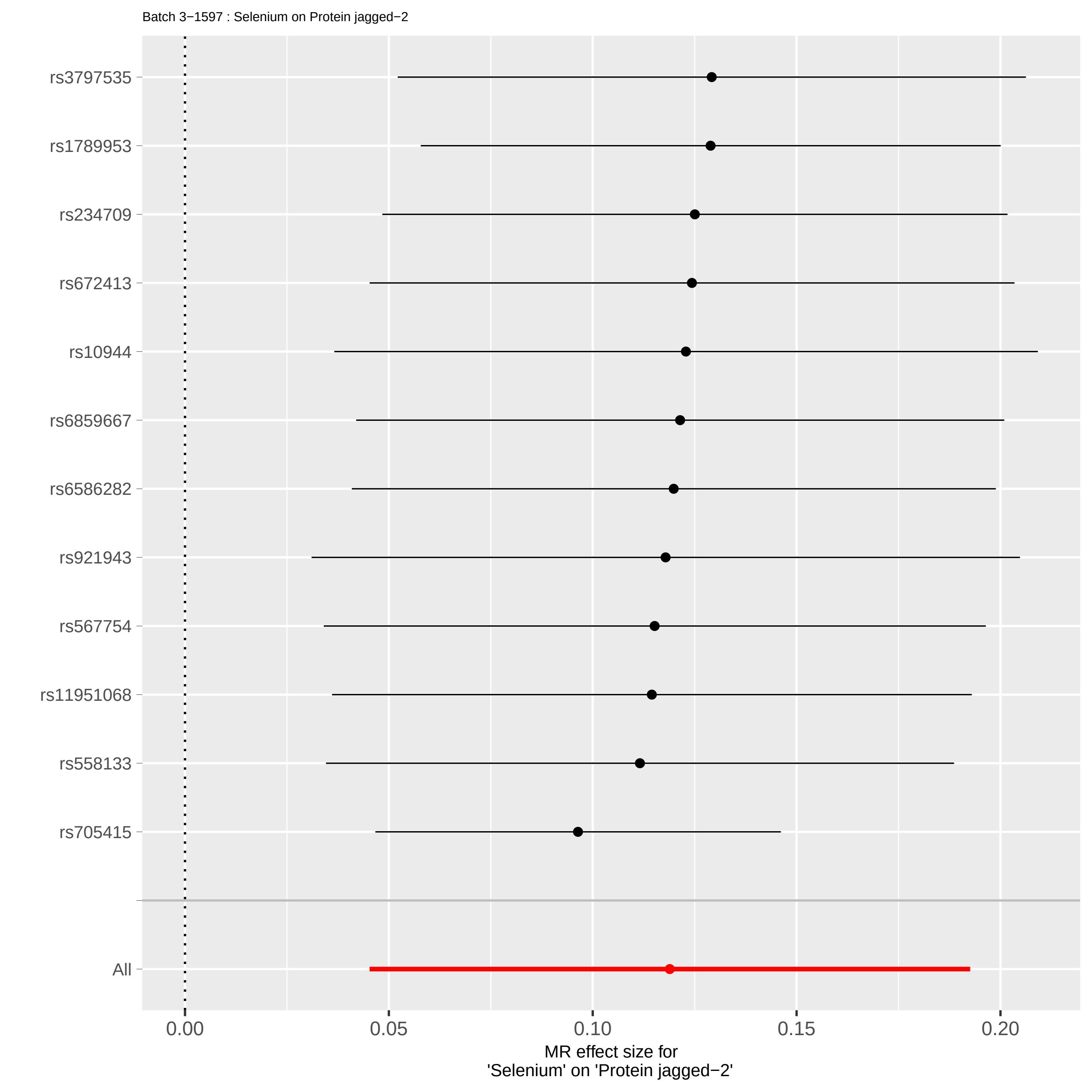
Batch 3−1597 : Selenium on Protein jagged−2
rs3797535
rs1789953
rs234709
rs672413
rs10944
rs6859667
rs6586282
rs921943
rs567754
rs11951068
rs558133
rs705415
All
0.00
0.05
0.10
0.15
0.20
MR effect size for
'Selenium' on 'Protein jagged−2'

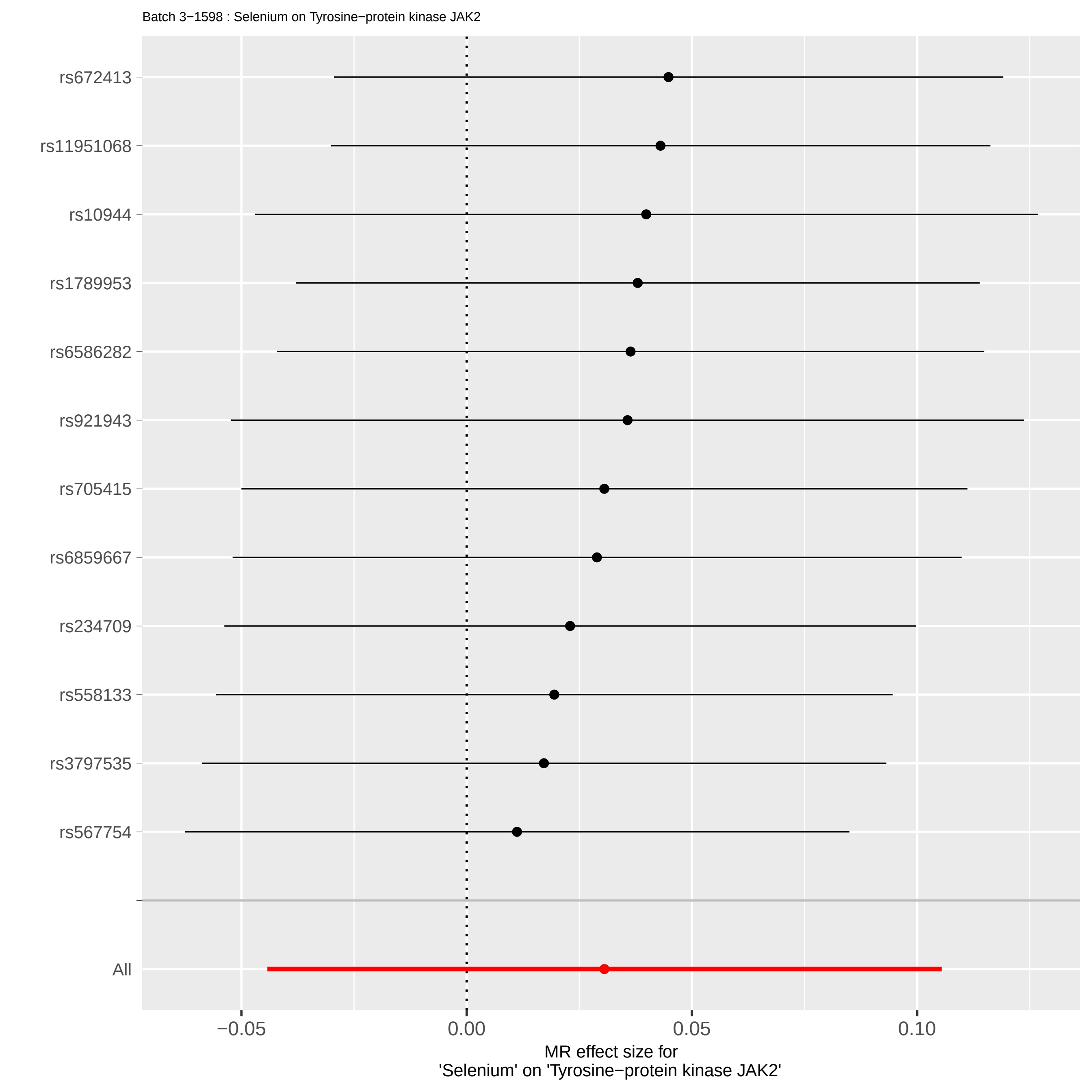

Batch 3−1598 : Selenium on Tyrosine−protein kinase JAK2
rs672413
rs11951068
rs10944
rs1789953
rs6586282
rs921943
rs705415
rs6859667
rs234709
rs558133
rs3797535
rs567754
All
−0.05
0.00
0.05
0.10
MR effect size for
'Selenium' on 'Tyrosine−protein kinase JAK2'

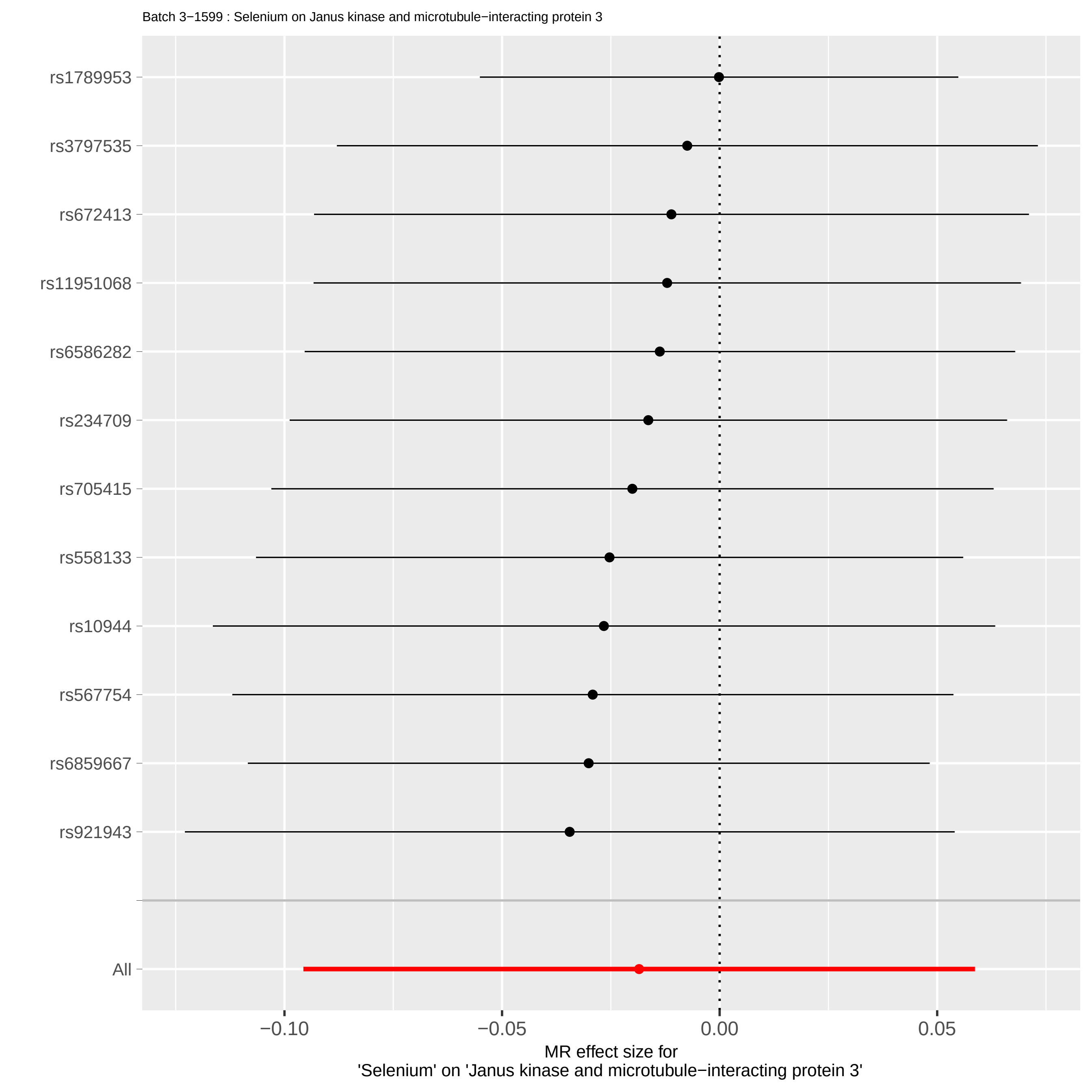

Batch 3−1599 : Selenium on Janus kinase and microtubule−interacting protein 3
rs1789953
rs3797535
rs672413
rs11951068
rs6586282
rs234709
rs705415
rs558133
rs10944
rs567754
rs6859667
rs921943
All
−0.10
−0.05
0.00
0.05
MR effect size for
'Selenium' on 'Janus kinase and microtubule−interacting protein 3'

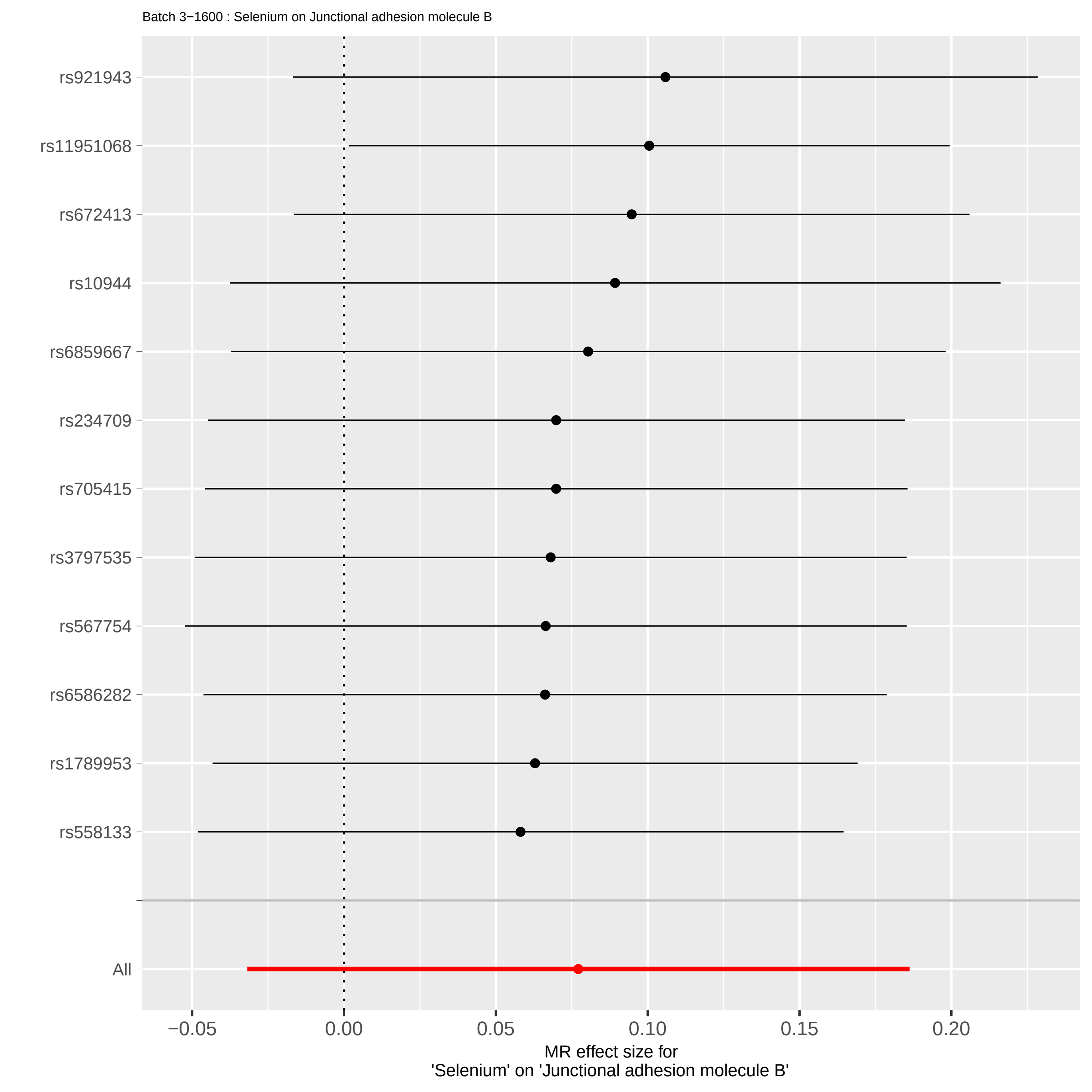

Batch 3−1600 : Selenium on Junctional adhesion molecule B
rs921943
rs11951068
rs672413
rs10944
rs6859667
rs234709
rs705415
rs3797535
rs567754
rs6586282
rs1789953
rs558133
All
−0.05
0.00
0.05
0.10
0.15
0.20
MR effect size for
'Selenium' on 'Junctional adhesion molecule B'

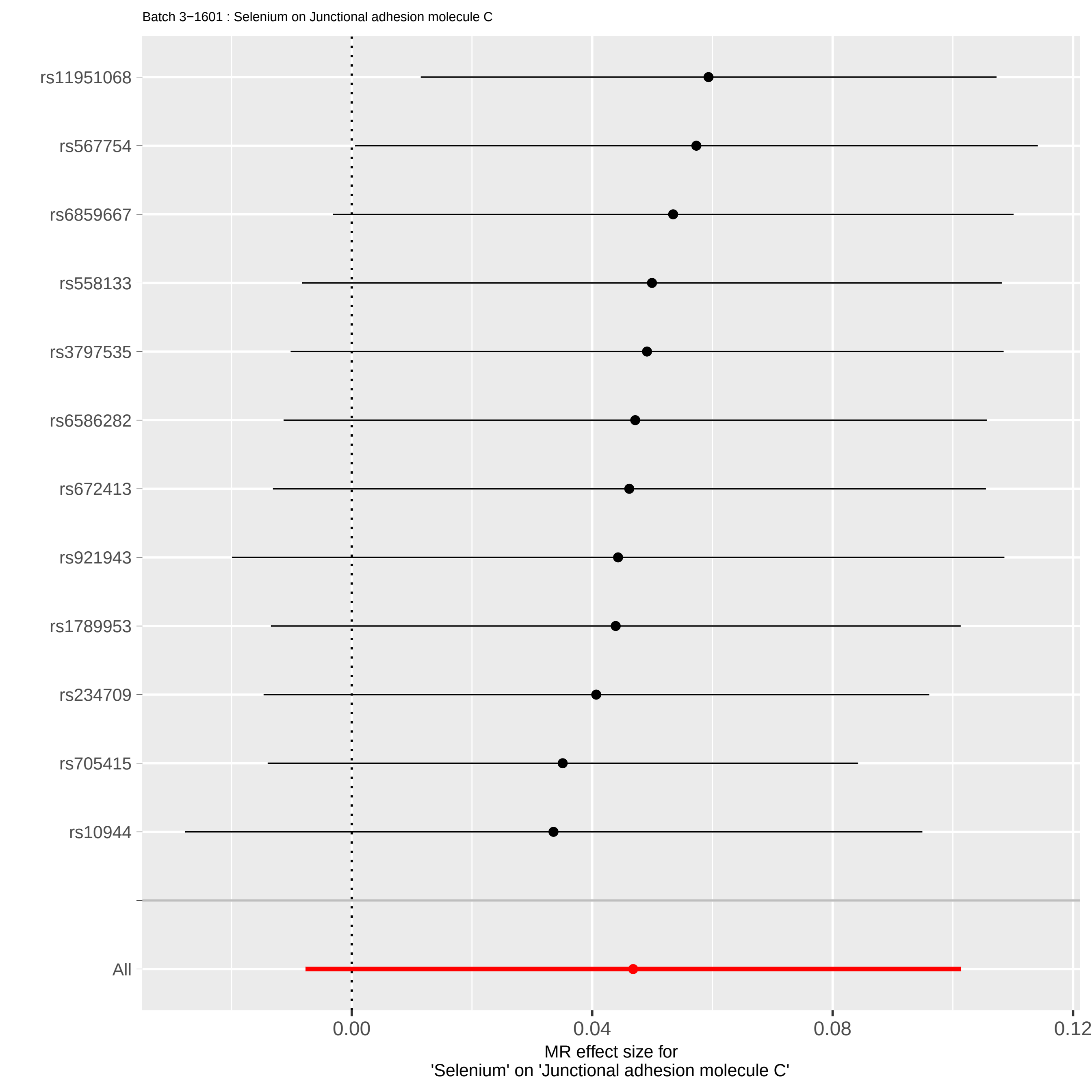

Batch 3−1601 : Selenium on Junctional adhesion molecule C
rs11951068
rs567754
rs6859667
rs558133
rs3797535
rs6586282
rs672413
rs921943
rs1789953
rs234709
rs705415
rs10944
All
0.00
0.04
0.08
0.12
MR effect size for
'Selenium' on 'Junctional adhesion molecule C'

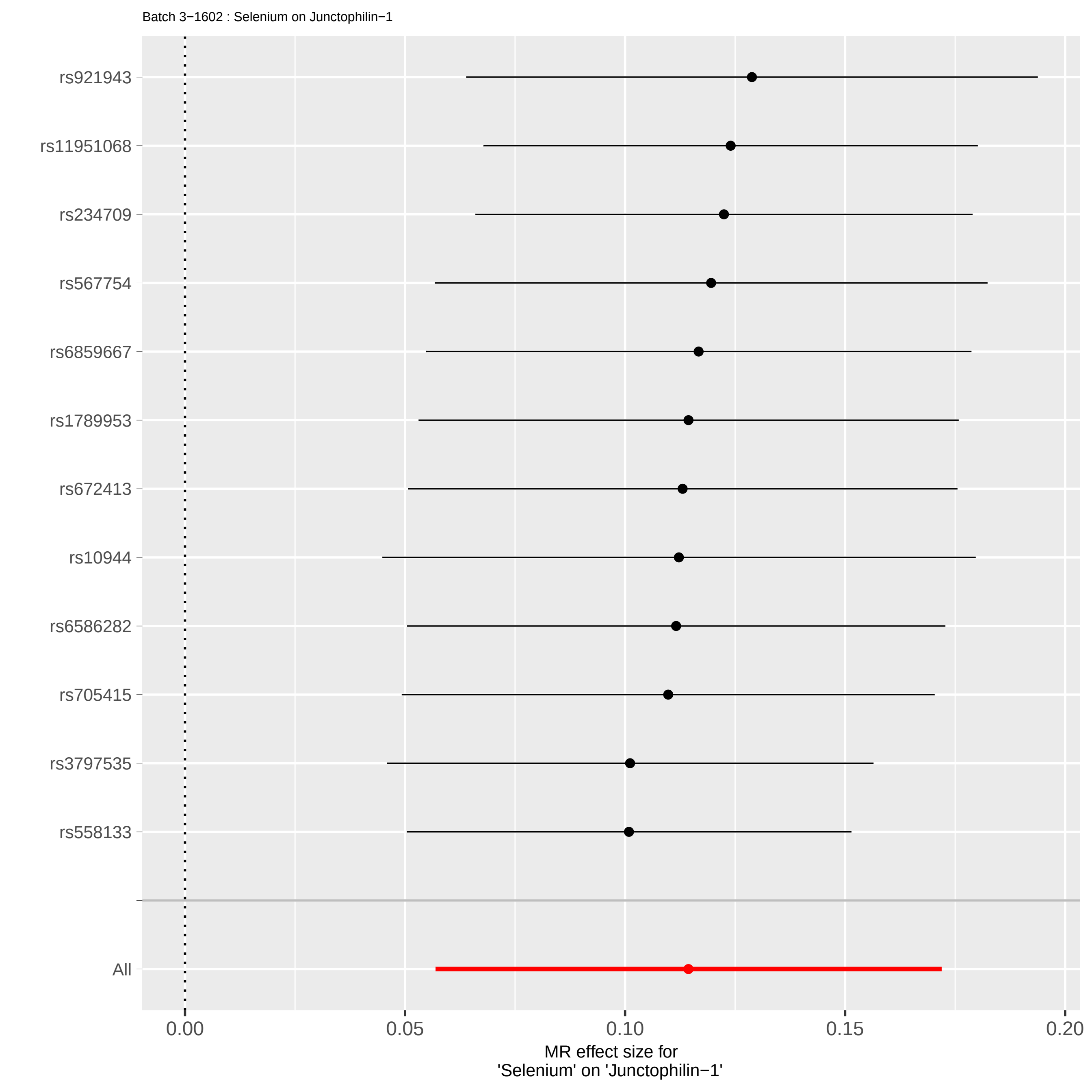

Batch 3−1602 : Selenium on Junctophilin−1
rs921943
rs11951068
rs234709
rs567754
rs6859667
rs1789953
rs672413
rs10944
rs6586282
rs705415
rs3797535
rs558133
All
0.00
0.05
0.10
0.15
0.20
MR effect size for
'Selenium' on 'Junctophilin−1'

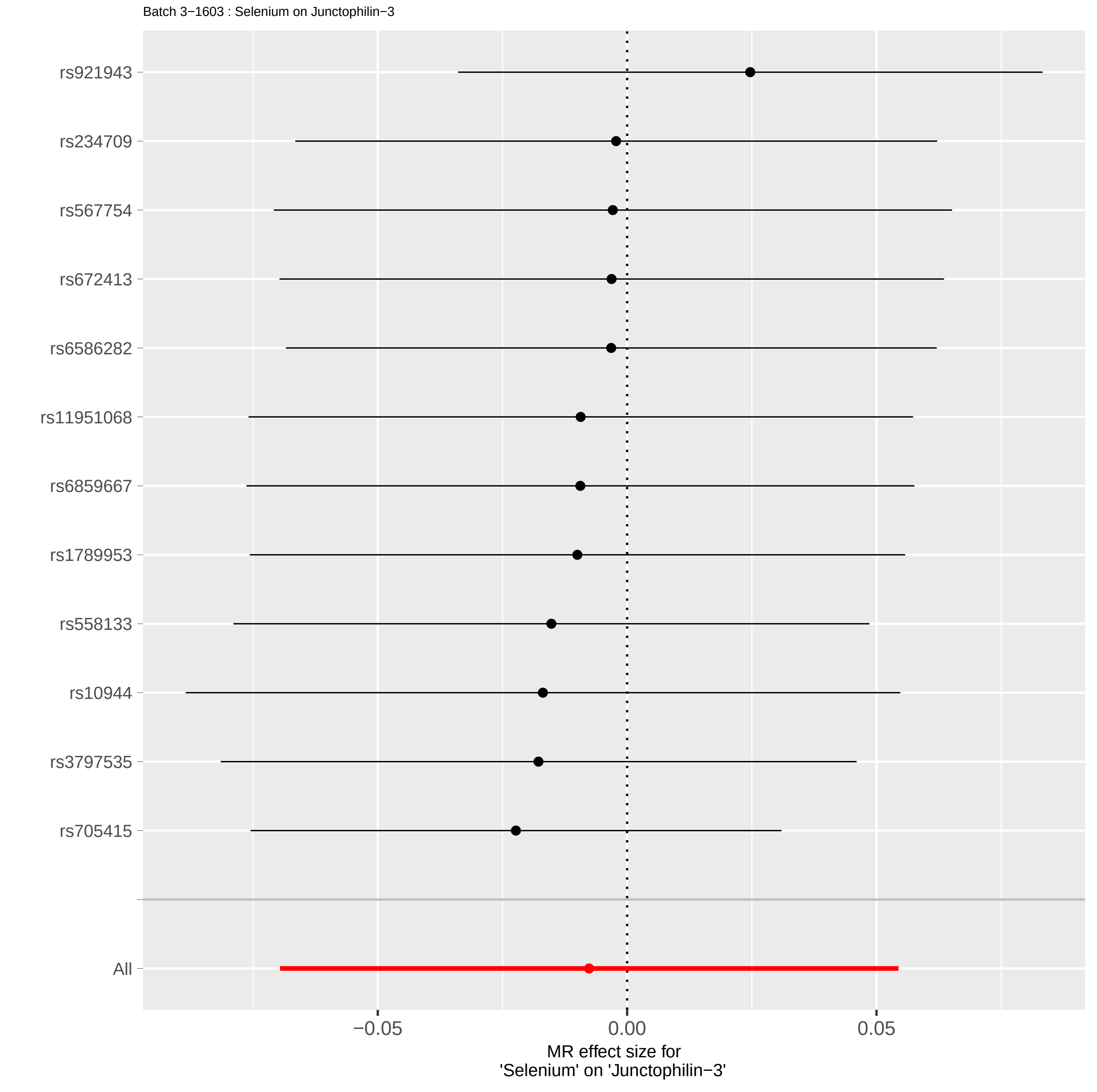
Batch 3−1603 : Selenium on Junctophilin−3
rs921943
rs234709
rs567754
rs672413
rs6586282
rs11951068
rs6859667
rs1789953
rs558133
rs10944
rs3797535
rs705415
All
−0.05
0.00
0.05
MR effect size for
'Selenium' on 'Junctophilin−3'

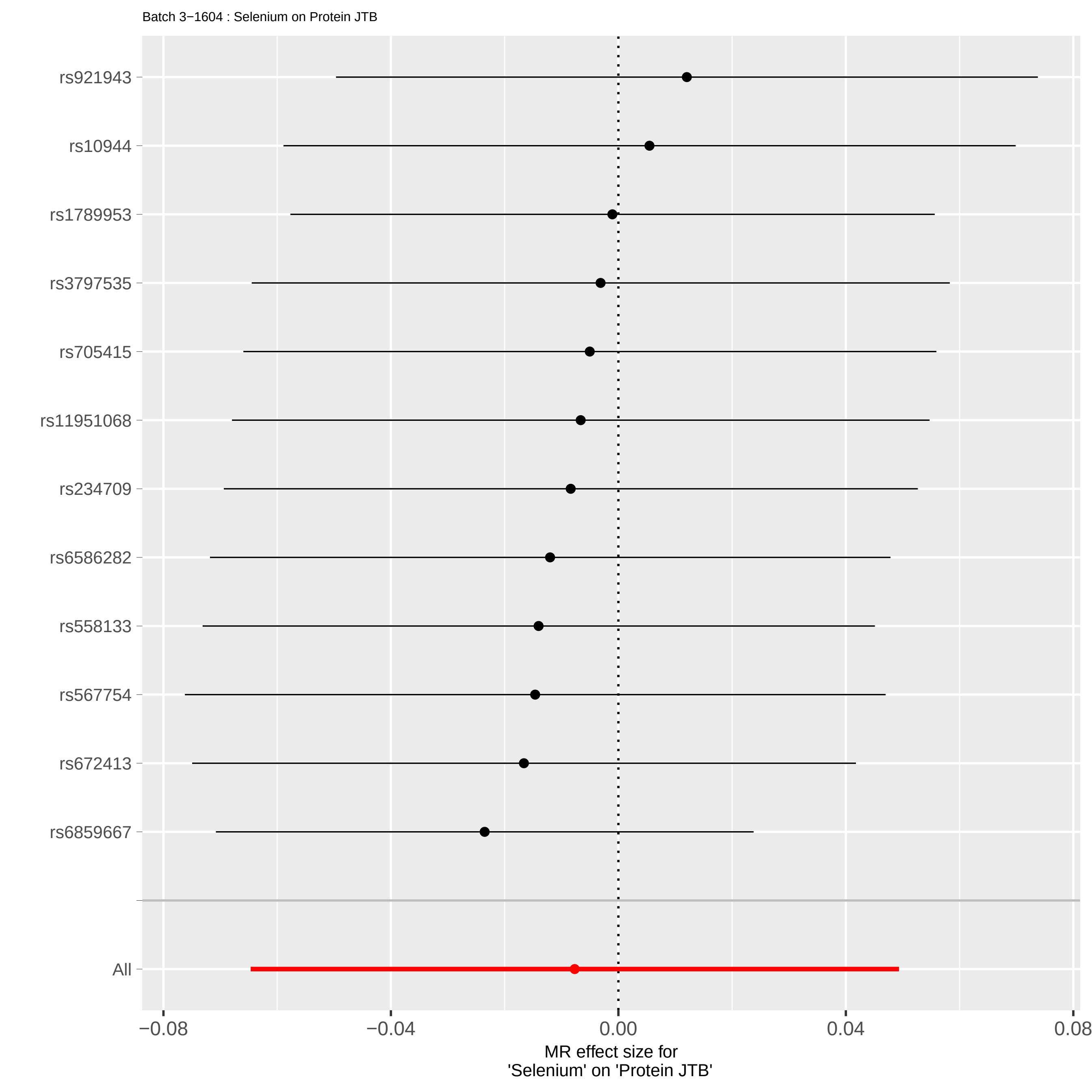

Batch 3−1604 : Selenium on Protein JTB
rs921943
rs10944
rs1789953
rs3797535
rs705415
rs11951068
rs234709
rs6586282
rs558133
rs567754
rs672413
rs6859667
All
−0.08
−0.04
0.00
0.04
0.08
MR effect size for
'Selenium' on 'Protein JTB'

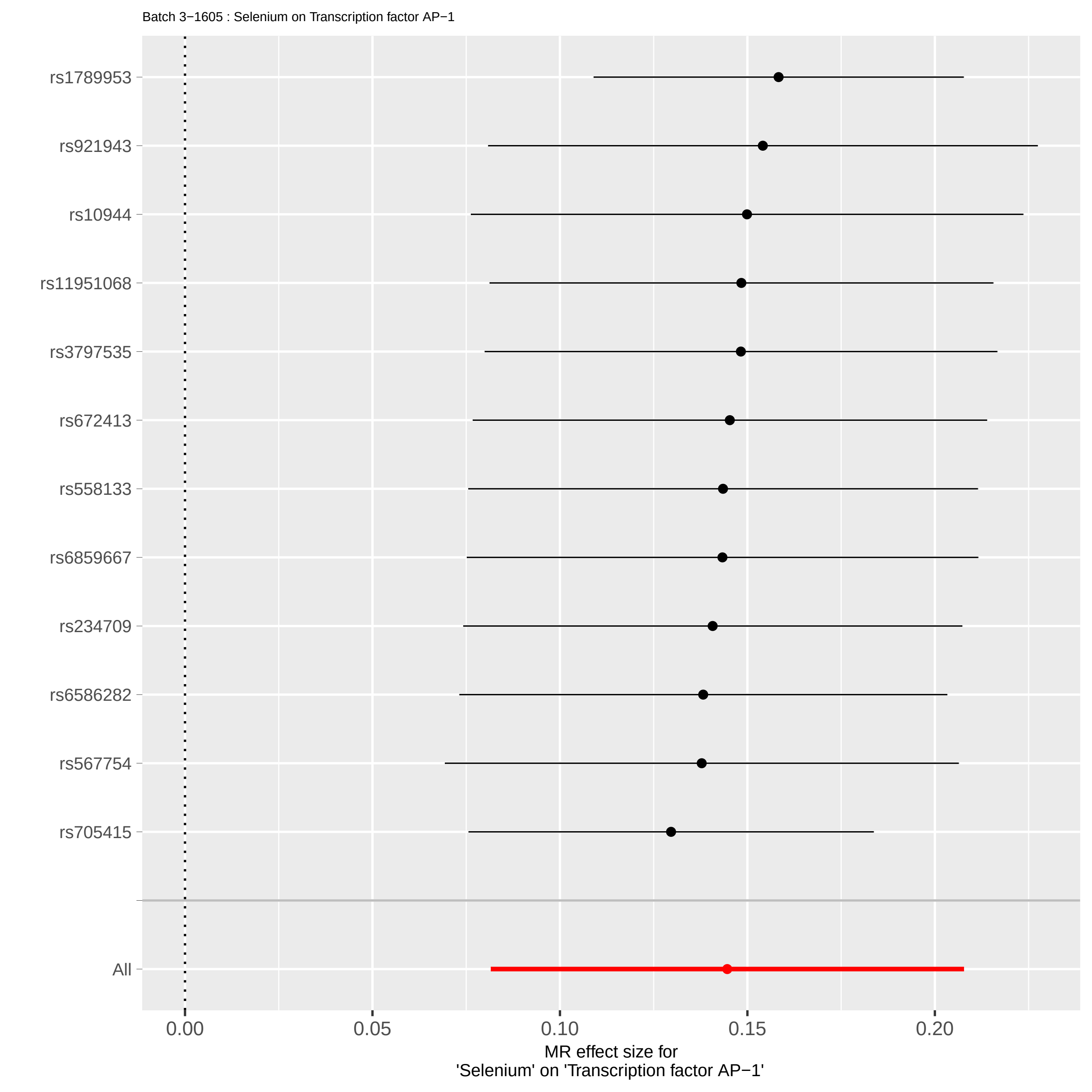

Batch 3−1605 : Selenium on Transcription factor AP−1
rs1789953
rs921943
rs10944
rs11951068
rs3797535
rs672413
rs558133
rs6859667
rs234709
rs6586282
rs567754
rs705415
All
0.00
0.05
0.10
0.15
0.20
MR effect size for
'Selenium' on 'Transcription factor AP−1'

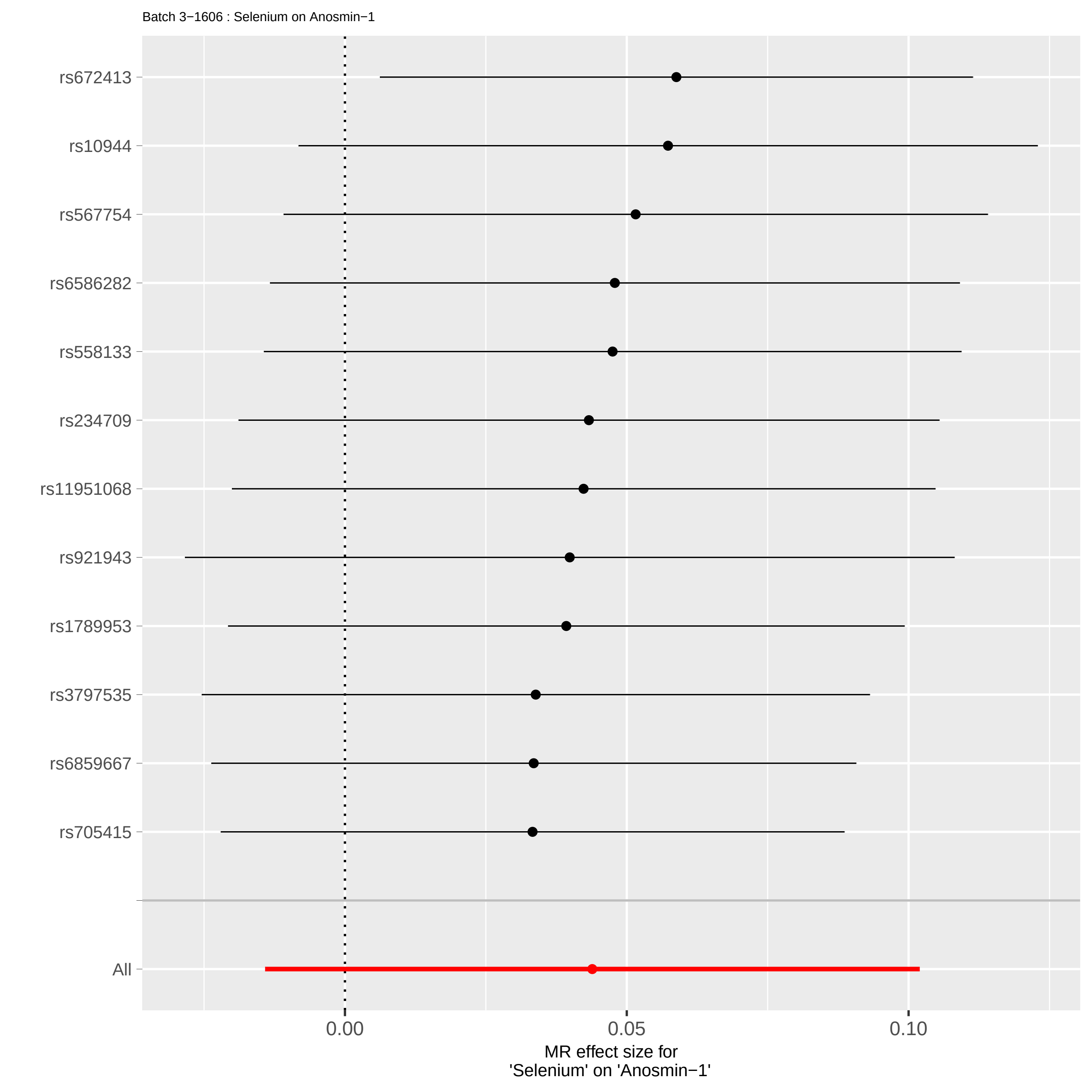

Batch 3−1606 : Selenium on Anosmin−1
rs672413
rs10944
rs567754
rs6586282
rs558133
rs234709
rs11951068
rs921943
rs1789953
rs3797535
rs6859667
rs705415
All
0.00
0.05
0.10
MR effect size for
'Selenium' on 'Anosmin−1'

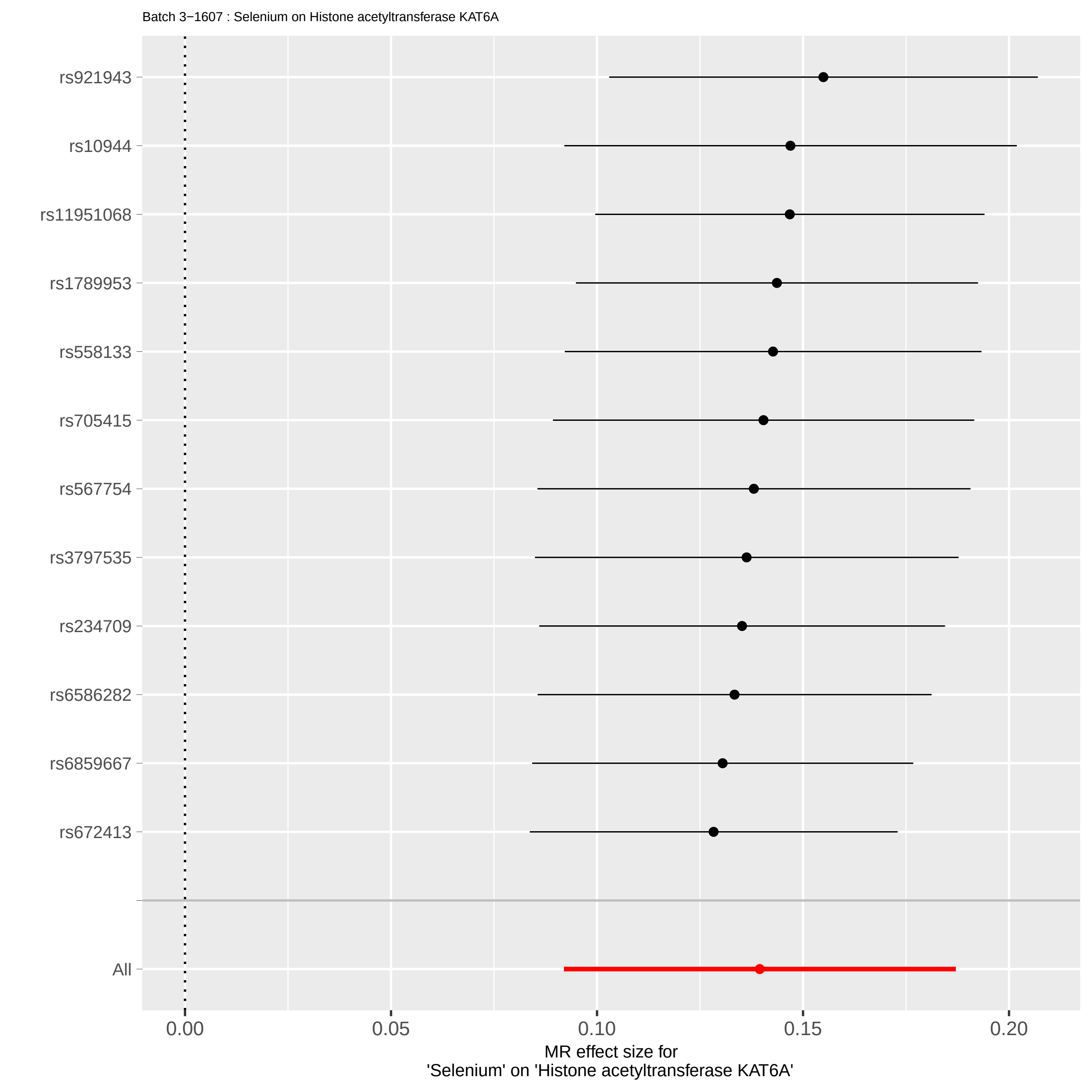

Batch 3−1607 : Selenium on Histone acetyltransferase KAT6A
rs921943
rs10944
rs11951068
rs1789953
rs558133
rs705415
rs567754
rs3797535
rs234709
rs6586282
rs6859667
rs672413
All
0.00
0.05
0.10
0.15
0.20
MR effect size for
'Selenium' on 'Histone acetyltransferase KAT6A'

Batch 3−1608 : Selenium on Potassium voltage−gated channel subfamily A member 10

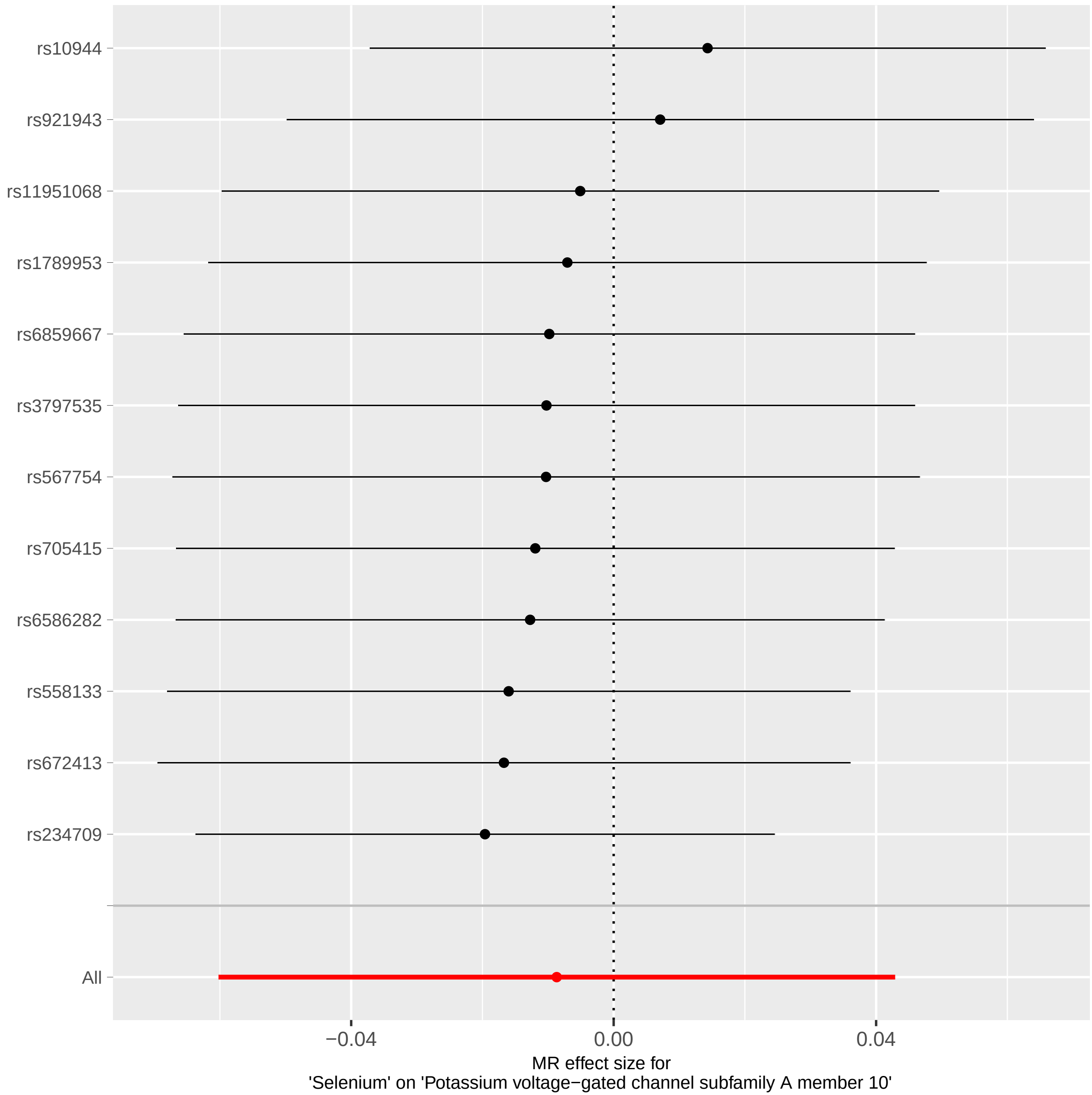

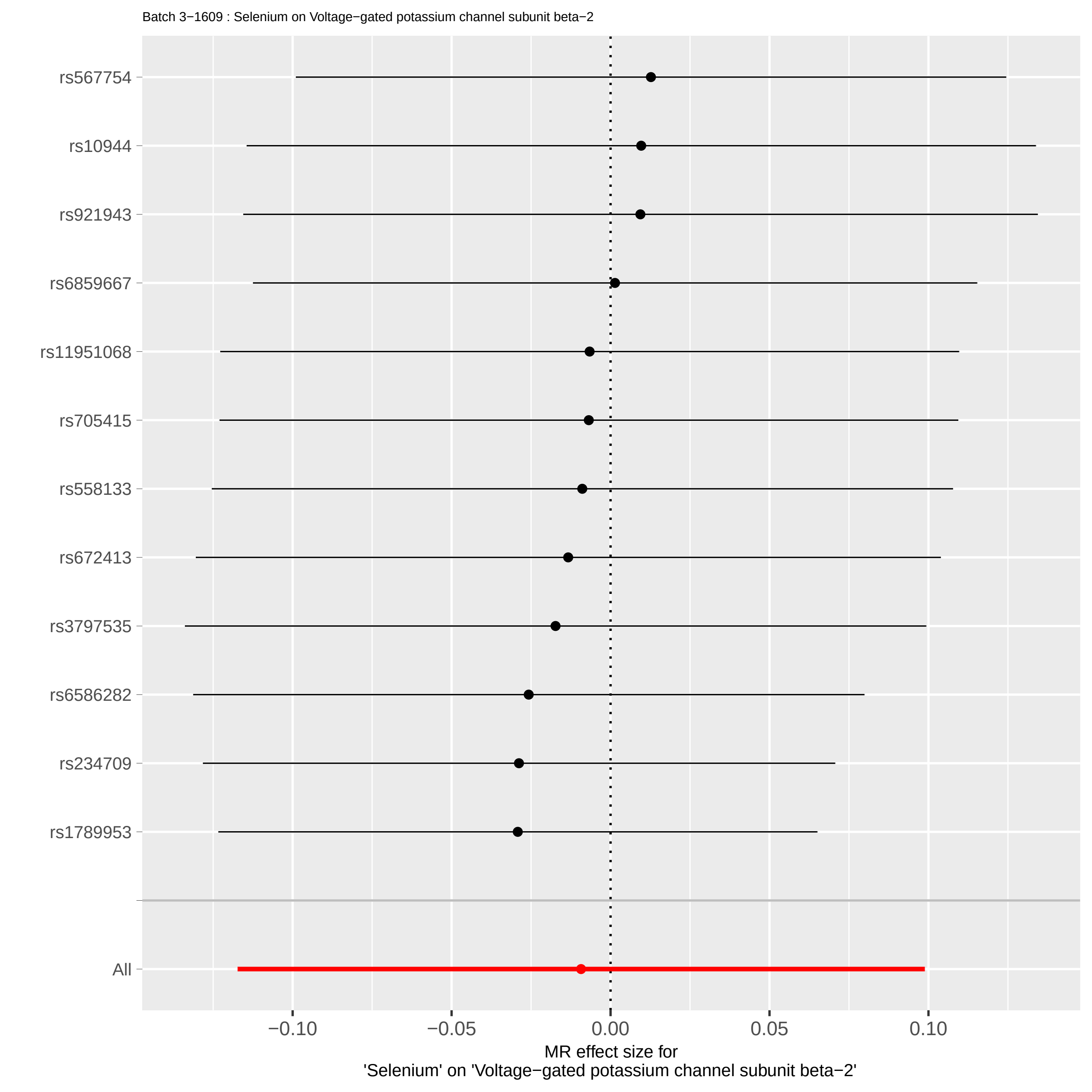
Batch 3−1609 : Selenium on Voltage−gated potassium channel subunit beta−2
rs567754
rs10944
rs921943
rs6859667
rs11951068
rs705415
rs558133
rs672413
rs3797535
rs6586282
rs234709
rs1789953
All
−0.10
−0.05
0.00
0.05
0.10
MR effect size for
'Selenium' on 'Voltage−gated potassium channel subunit beta−2'

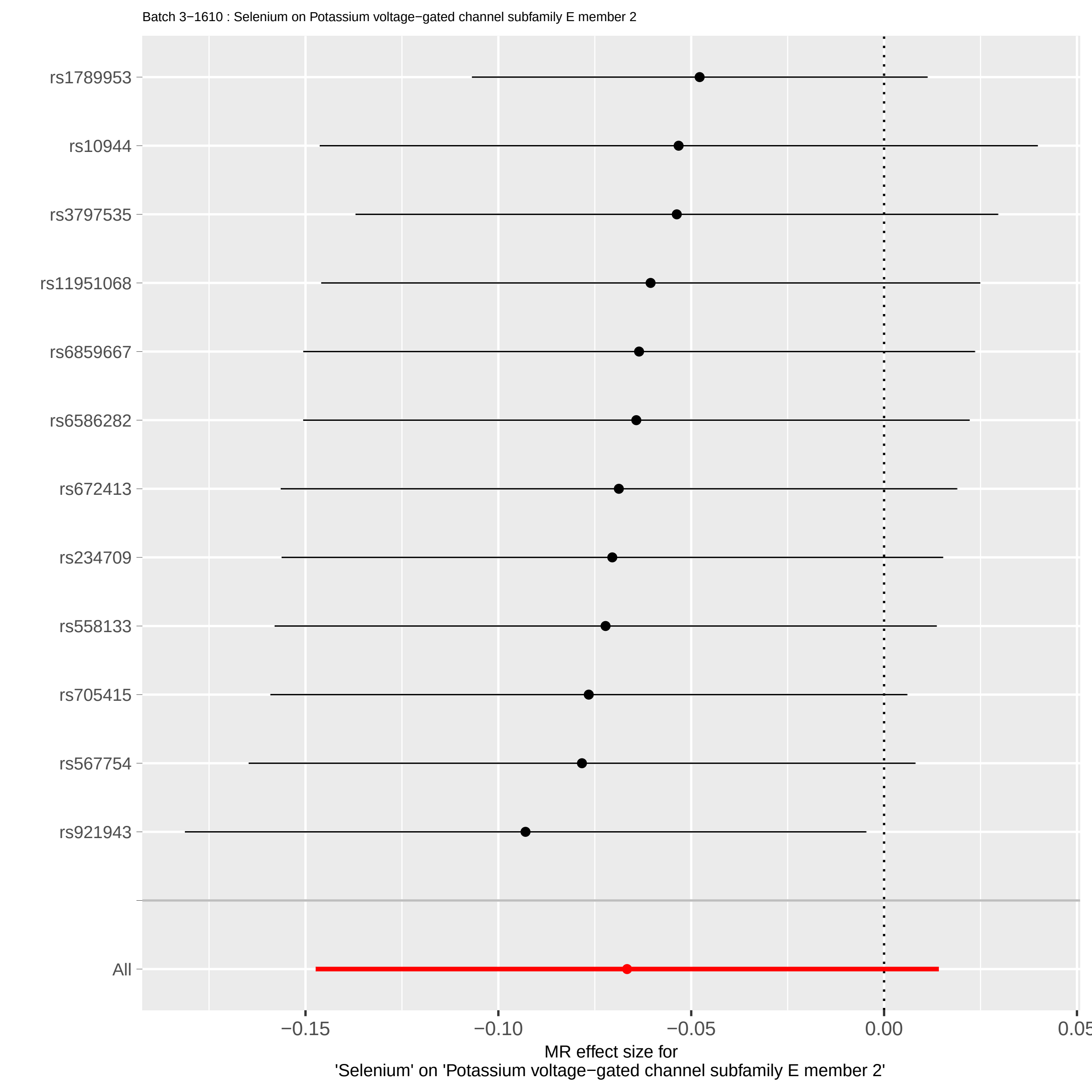

Batch 3−1610 : Selenium on Potassium voltage−gated channel subfamily E member 2
rs1789953
rs10944
rs3797535
rs11951068
rs6859667
rs6586282
rs672413
rs234709
rs558133
rs705415
rs567754
rs921943
All
−0.15
−0.10
−0.05
0.00
0.05
MR effect size for
'Selenium' on 'Potassium voltage−gated channel subfamily E member 2'

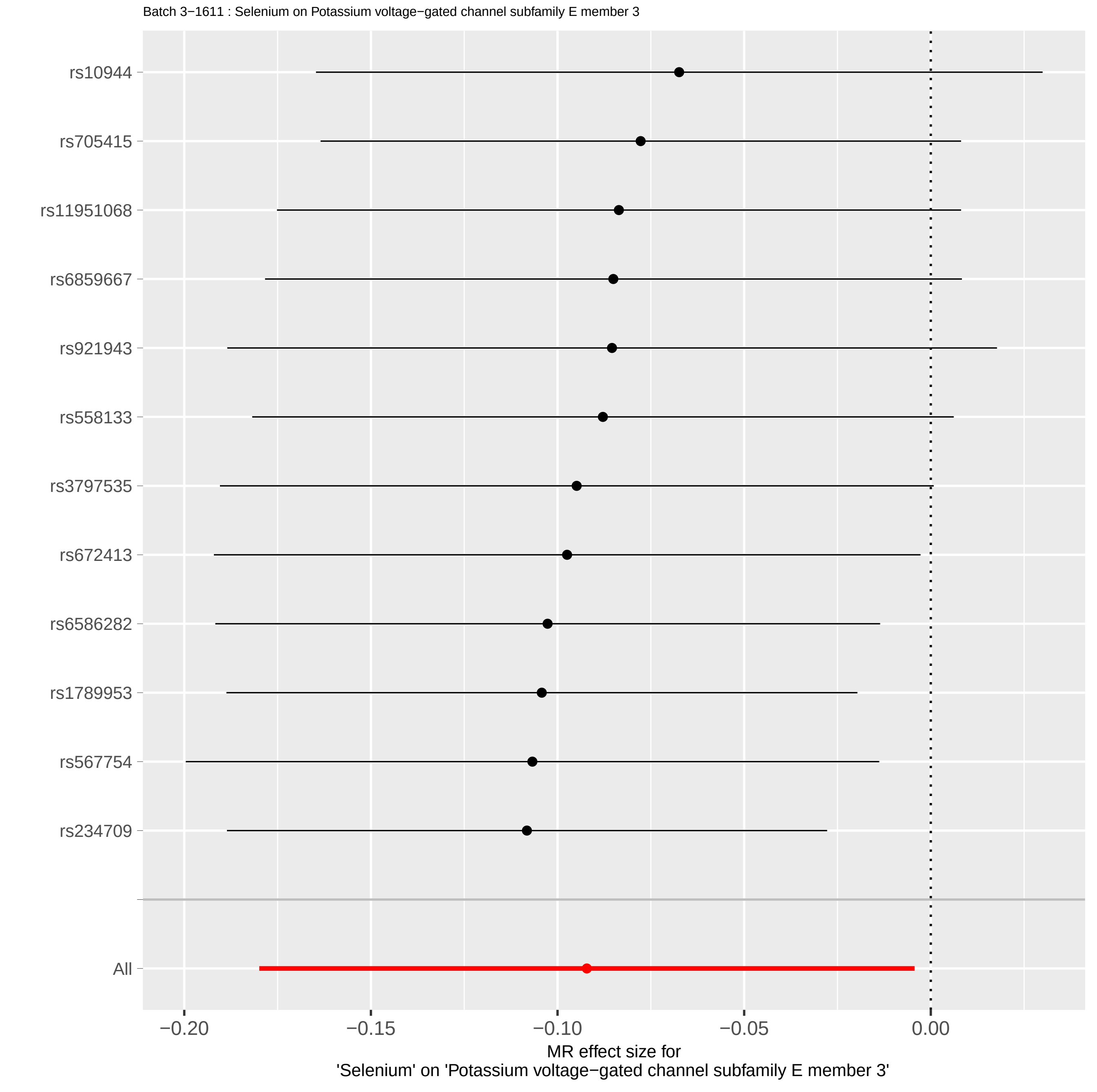

Batch 3−1611 : Selenium on Potassium voltage−gated channel subfamily E member 3
rs10944
rs705415
rs11951068
rs6859667
rs921943
rs558133
rs3797535
rs672413
rs6586282
rs1789953
rs567754
rs234709
All
−0.20
−0.15
−0.10
−0.05
0.00
MR effect size for
'Selenium' on 'Potassium voltage−gated channel subfamily E member 3'

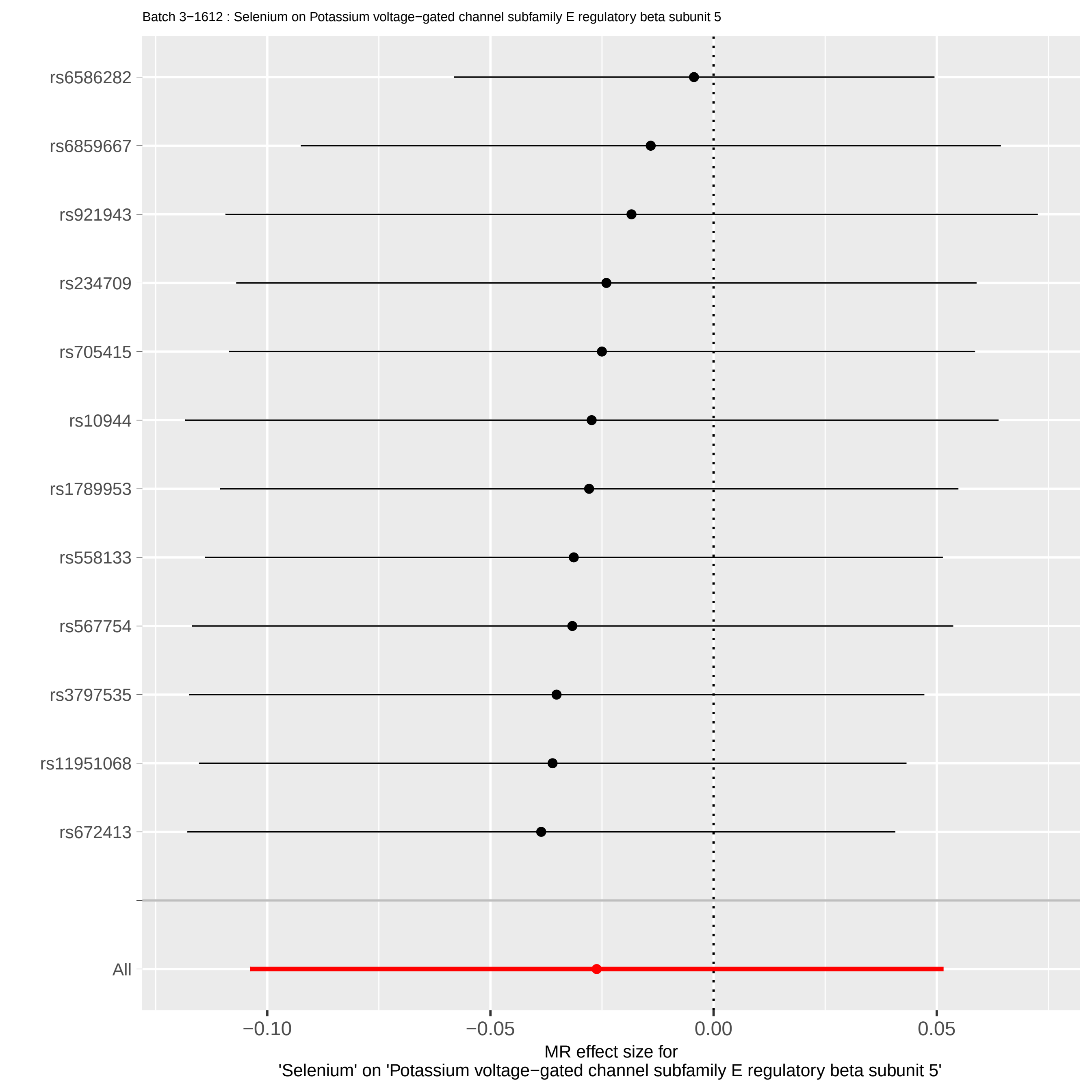

Batch 3−1612 : Selenium on Potassium voltage−gated channel subfamily E regulatory beta subunit 5
rs6586282
rs6859667
rs921943
rs234709
rs705415
rs10944
rs1789953
rs558133
rs567754
rs3797535
rs11951068
rs672413
All
−0.10
−0.05
0.00
0.05
MR effect size for
'Selenium' on 'Potassium voltage−gated channel subfamily E regulatory beta subunit 5'

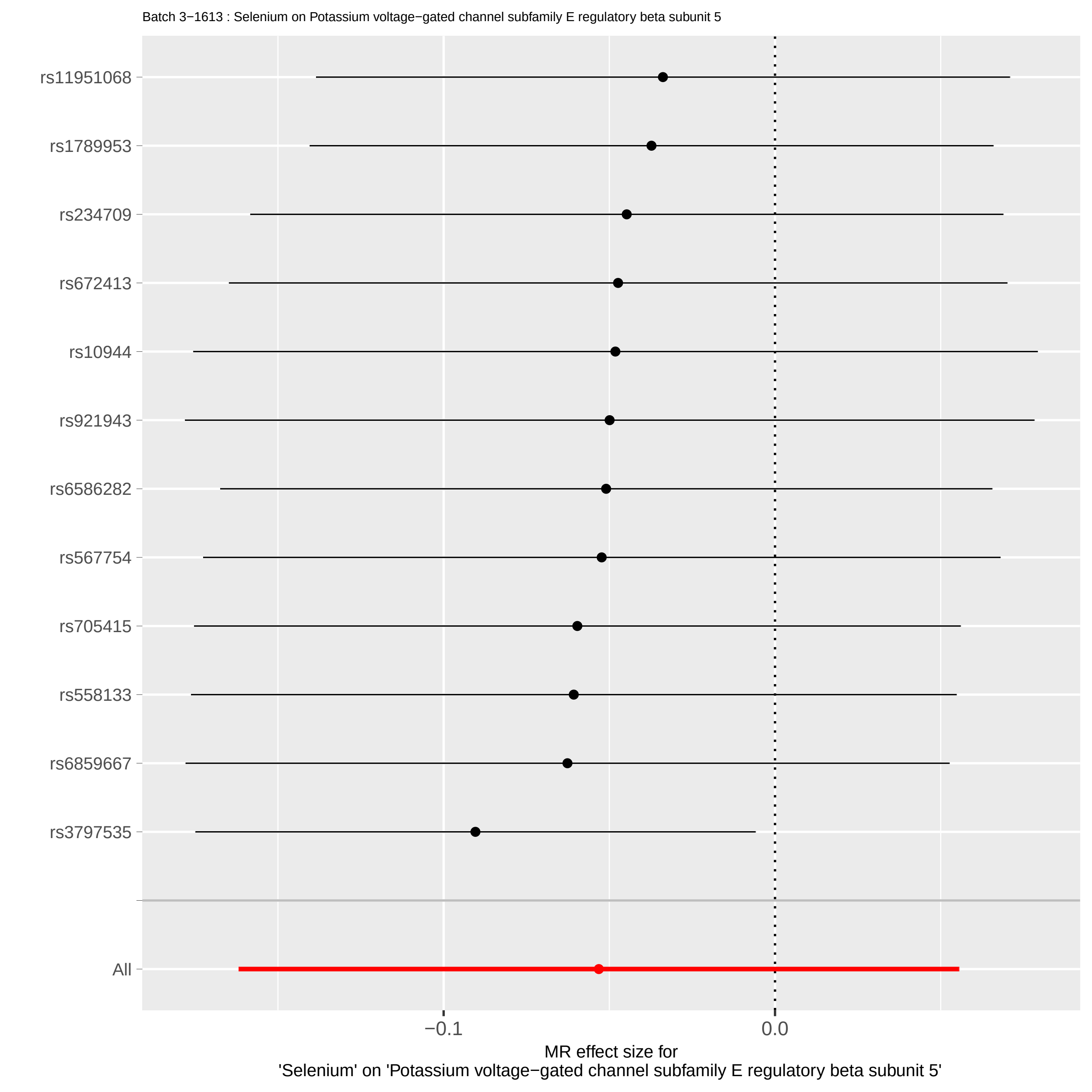
Batch 3−1613 : Selenium on Potassium voltage−gated channel subfamily E regulatory beta subunit 5
rs11951068
rs1789953
rs234709
rs672413
rs10944
rs921943
rs6586282
rs567754
rs705415
rs558133
rs6859667
rs3797535
All
−0.1
0.0
MR effect size for
'Selenium' on 'Potassium voltage−gated channel subfamily E regulatory beta subunit 5'

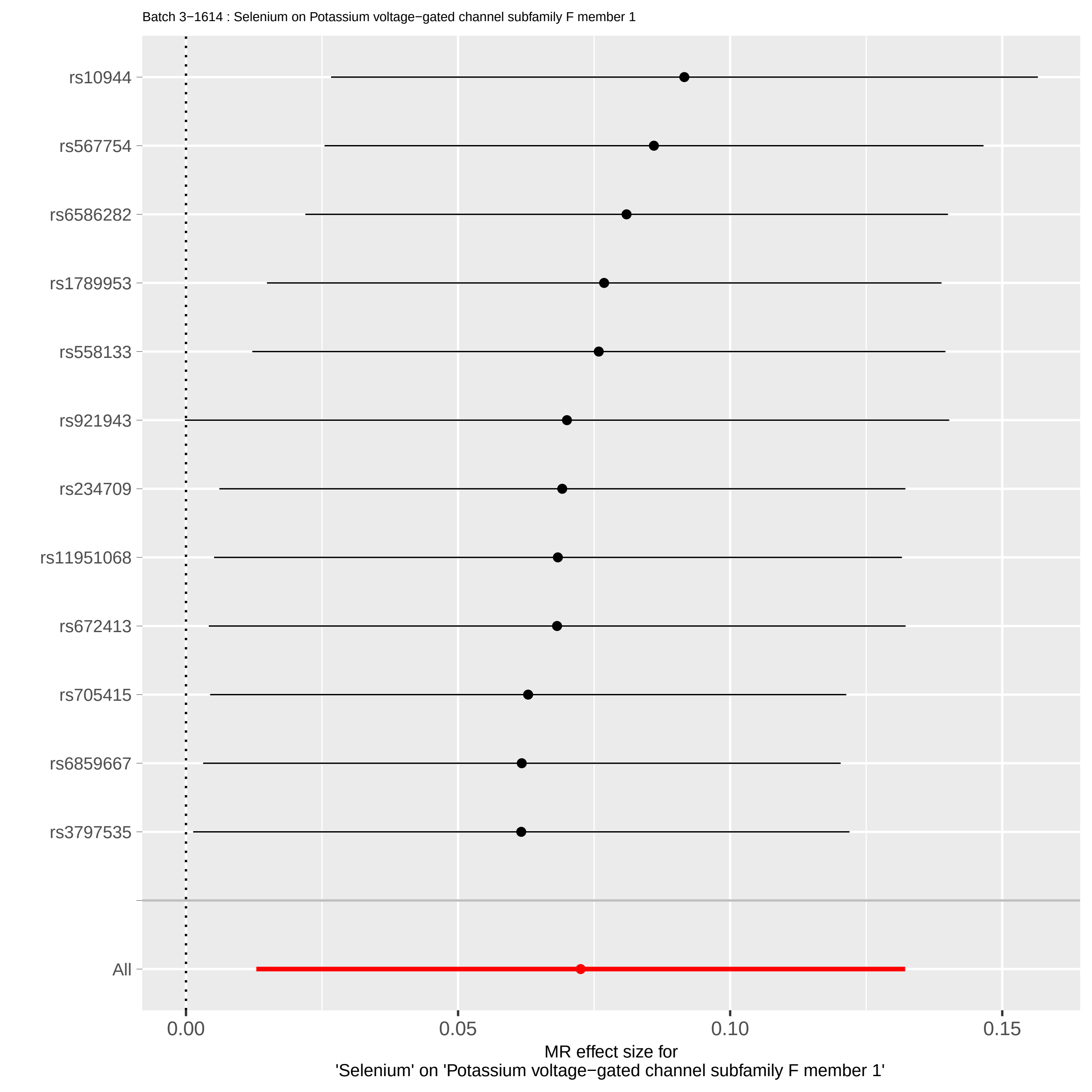

Batch 3−1614 : Selenium on Potassium voltage−gated channel subfamily F member 1
rs10944
rs567754
rs6586282
rs1789953
rs558133
rs921943
rs234709
rs11951068
rs672413
rs705415
rs6859667
rs3797535
All
0.00
0.05
0.10
0.15
MR effect size for
'Selenium' on 'Potassium voltage−gated channel subfamily F member 1'

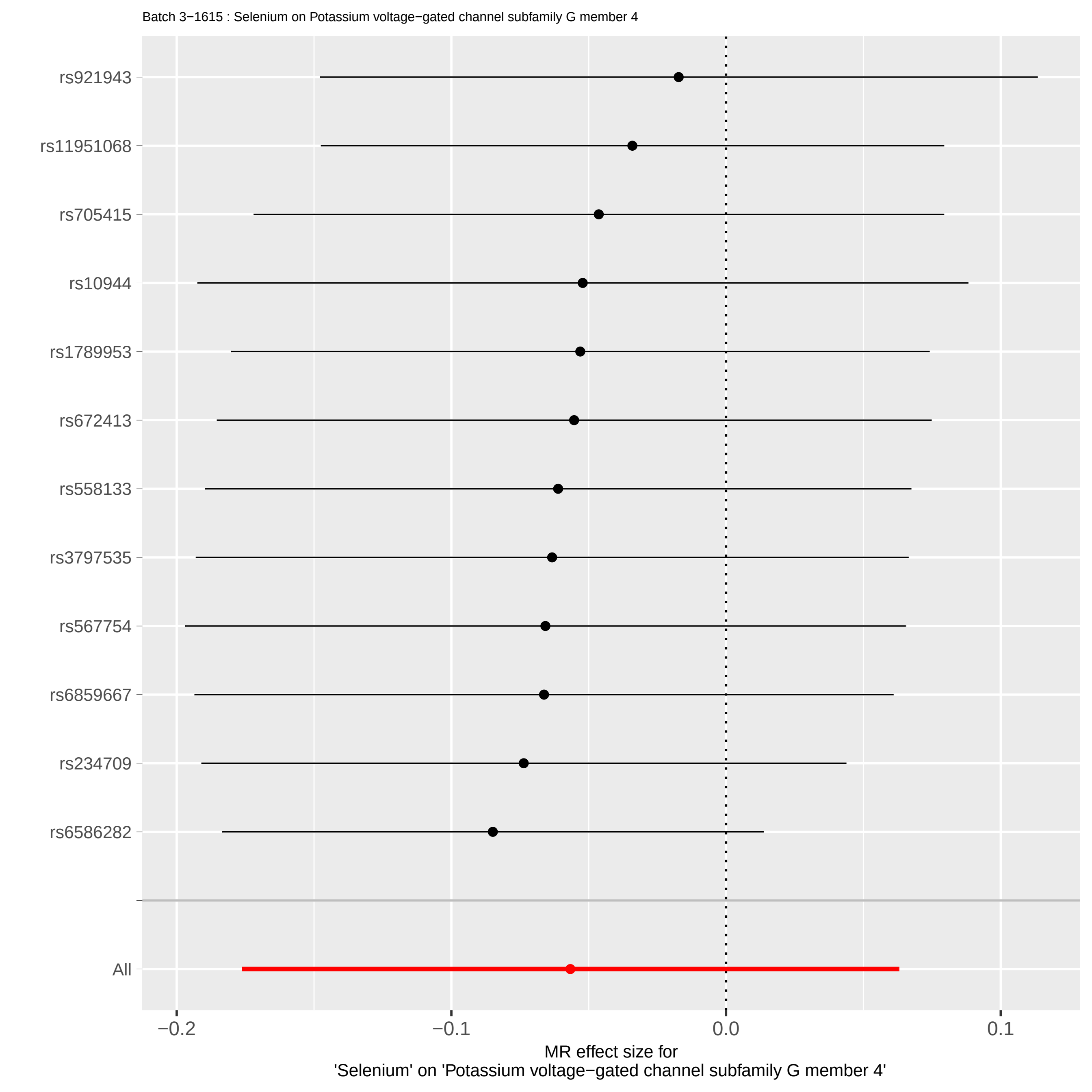

Batch 3−1615 : Selenium on Potassium voltage−gated channel subfamily G member 4
rs921943
rs11951068
rs705415
rs10944
rs1789953
rs672413
rs558133
rs3797535
rs567754
rs6859667
rs234709
rs6586282
All
−0.2
−0.1
0.0
0.1
MR effect size for
'Selenium' on 'Potassium voltage−gated channel subfamily G member 4'

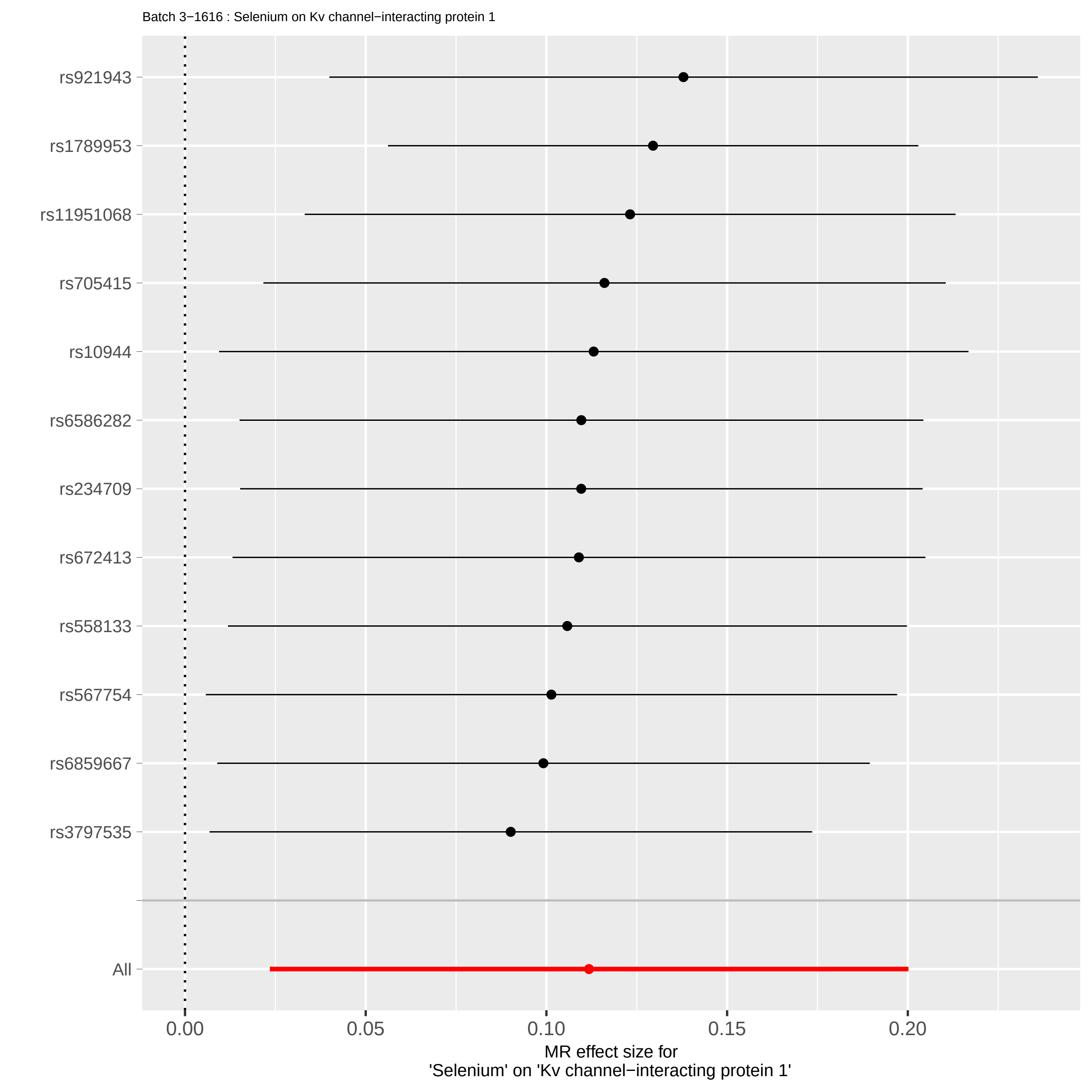

Batch 3−1616 : Selenium on Kv channel−interacting protein 1
rs921943
rs1789953
rs11951068
rs705415
rs10944
rs6586282
rs234709
rs672413
rs558133
rs567754
rs6859667
rs3797535
All
0.00
0.05
0.10
0.15
0.20
MR effect size for
'Selenium' on 'Kv channel−interacting protein 1'

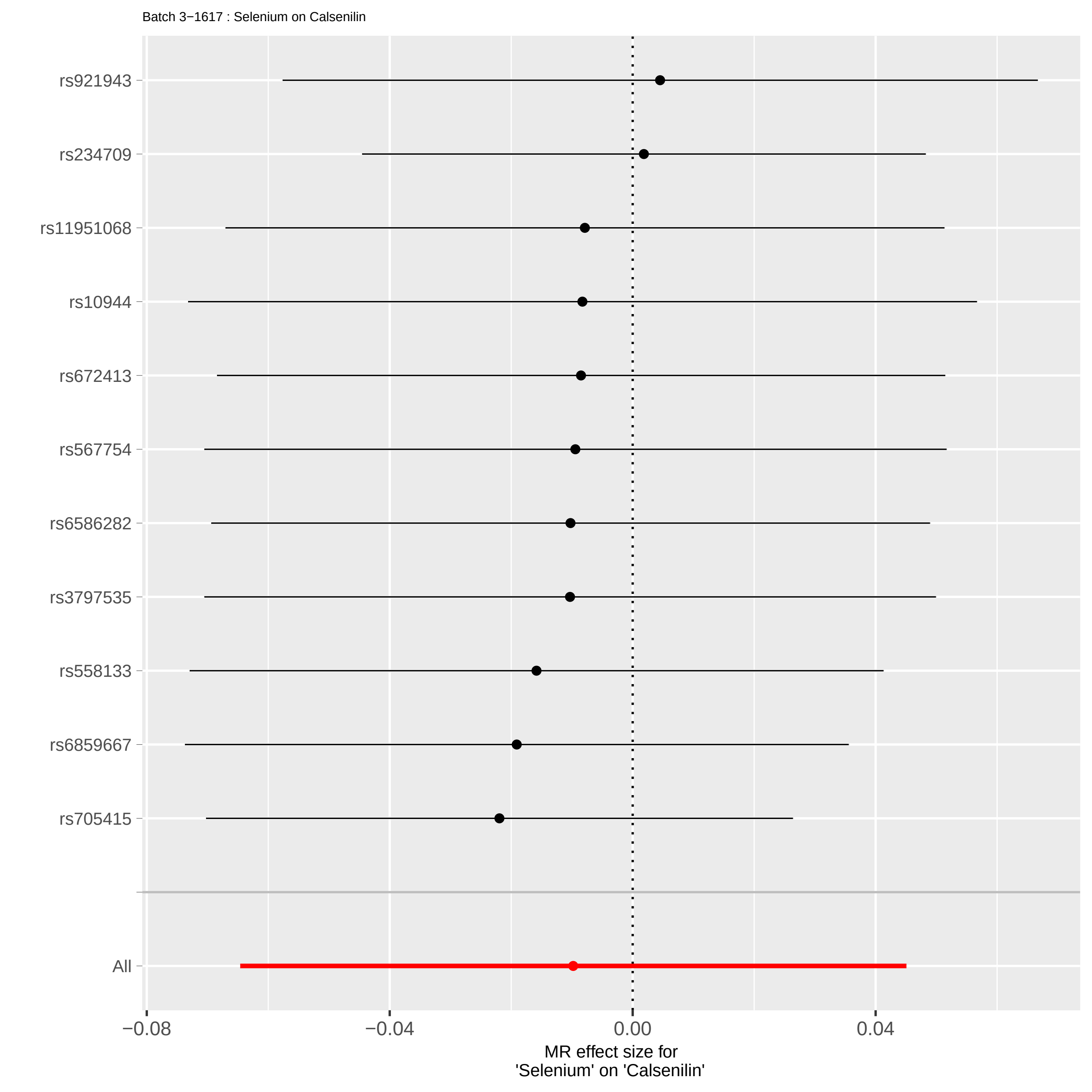

Batch 3−1617 : Selenium on Calsenilin
rs921943
rs234709
rs11951068
rs10944
rs672413
rs567754
rs6586282
rs3797535
rs558133
rs6859667
rs705415
All
−0.08
−0.04
0.00
0.04
MR effect size for
'Selenium' on 'Calsenilin'

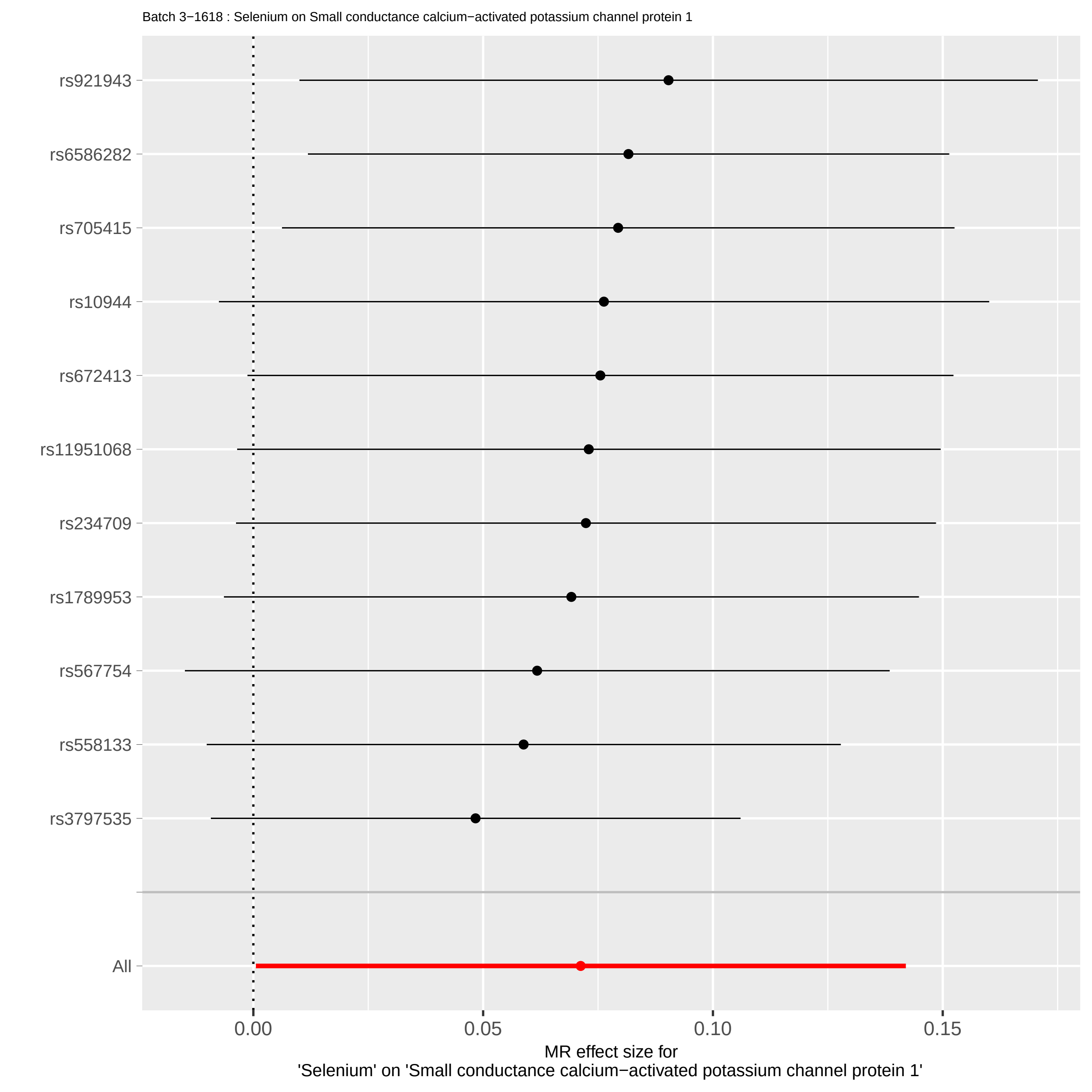

Batch 3−1618 : Selenium on Small conductance calcium−activated potassium channel protein 1
rs921943
rs6586282
rs705415
rs10944
rs672413
rs11951068
rs234709
rs1789953
rs567754
rs558133
rs3797535
All
0.00
0.05
0.10
0.15
MR effect size for
'Selenium' on 'Small conductance calcium−activated potassium channel protein 1'

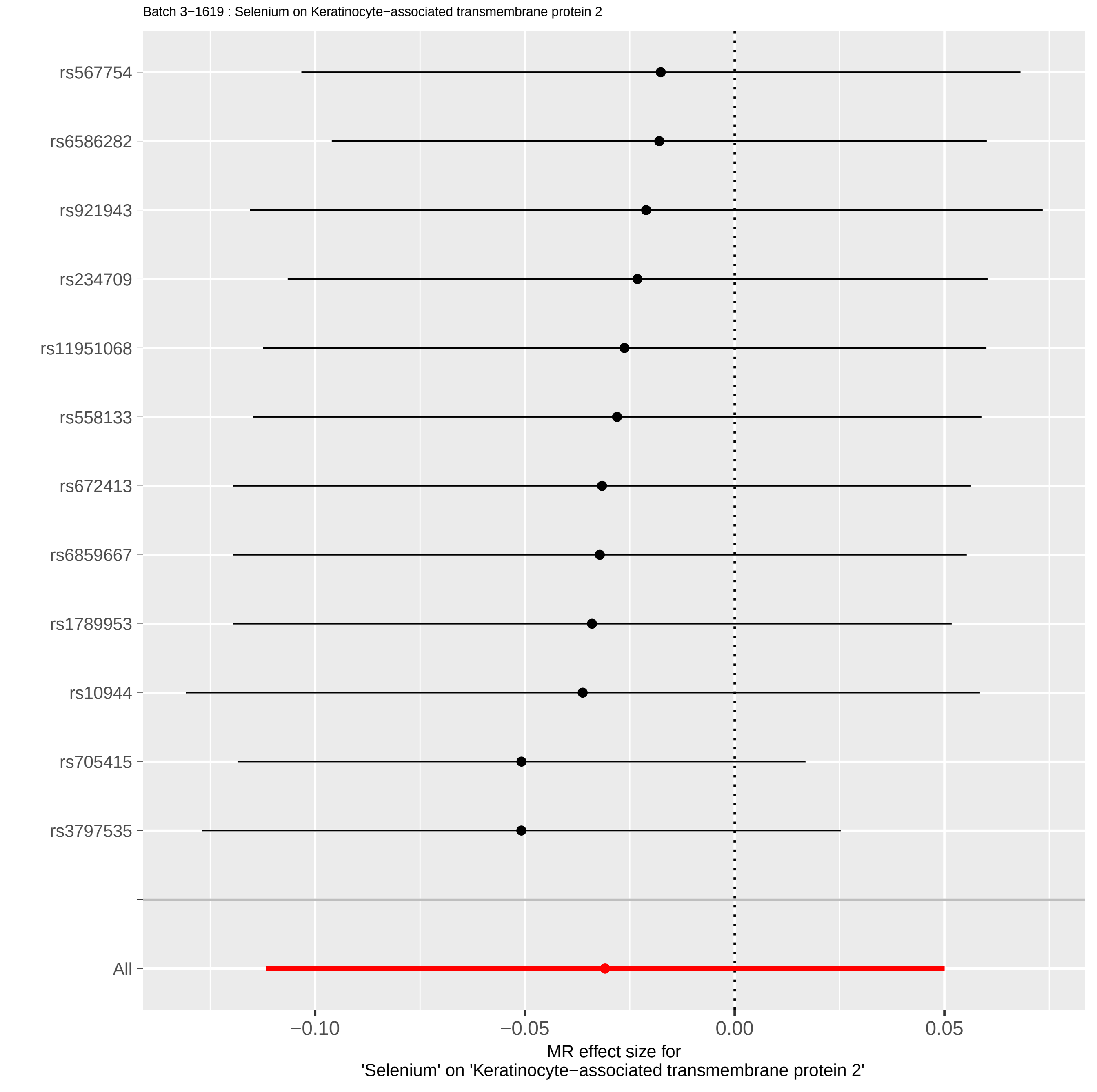

Batch 3−1619 : Selenium on Keratinocyte−associated transmembrane protein 2
rs567754
rs6586282
rs921943
rs234709
rs11951068
rs558133
rs672413
rs6859667
rs1789953
rs10944
rs705415
rs3797535
All
−0.10
−0.05
0.00
0.05
MR effect size for
'Selenium' on 'Keratinocyte−associated transmembrane protein 2'

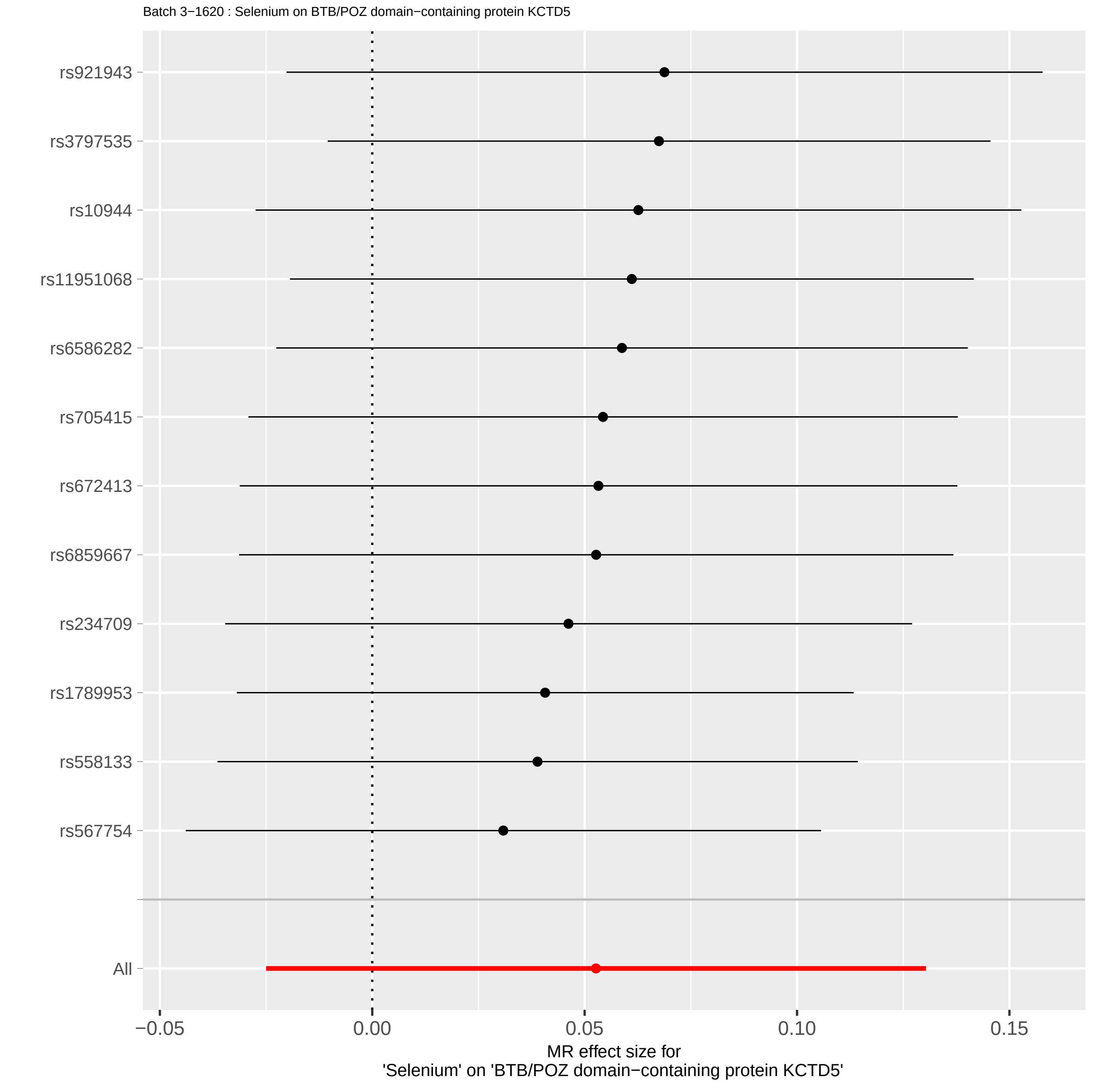
Batch 3−1620 : Selenium on BTB/POZ domain−containing protein KCTD5
rs921943
rs3797535
rs10944
rs11951068
rs6586282
rs705415
rs672413
rs6859667
rs234709
rs1789953
rs558133
rs567754
All
−0.05
0.00
0.05
0.10
0.15
MR effect size for
'Selenium' on 'BTB/POZ domain−containing protein KCTD5'

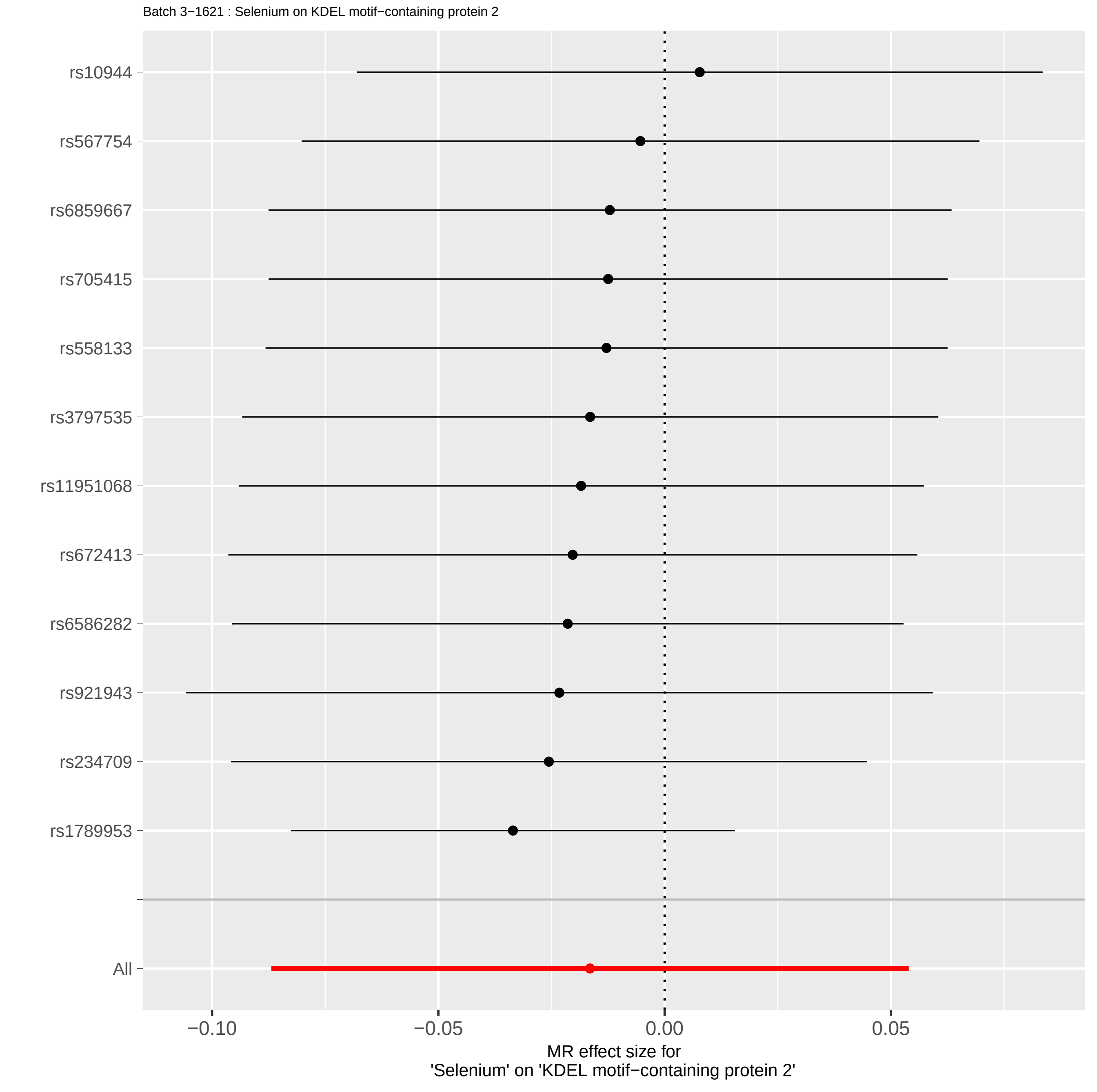

Batch 3−1621 : Selenium on KDEL motif−containing protein 2
rs10944
rs567754
rs6859667
rs705415
rs558133
rs3797535
rs11951068
rs672413
rs6586282
rs921943
rs234709
rs1789953
All
−0.10
−0.05
0.00
0.05
MR effect size for
'Selenium' on 'KDEL motif−containing protein 2'

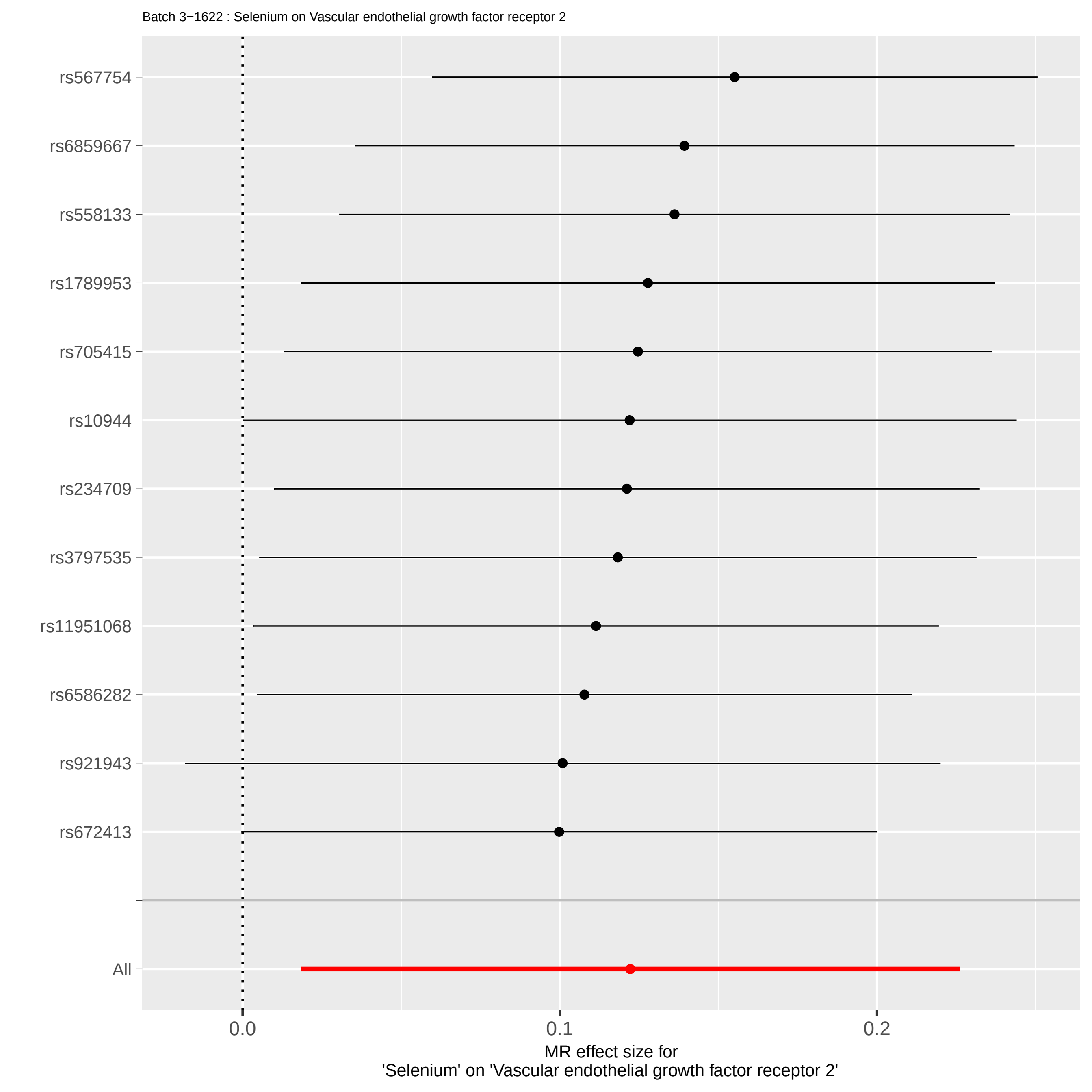

Batch 3−1622 : Selenium on Vascular endothelial growth factor receptor 2
rs567754
rs6859667
rs558133
rs1789953
rs705415
rs10944
rs234709
rs3797535
rs11951068
rs6586282
rs921943
rs672413
All
0.0
0.1
0.2
MR effect size for
'Selenium' on 'Vascular endothelial growth factor receptor 2'

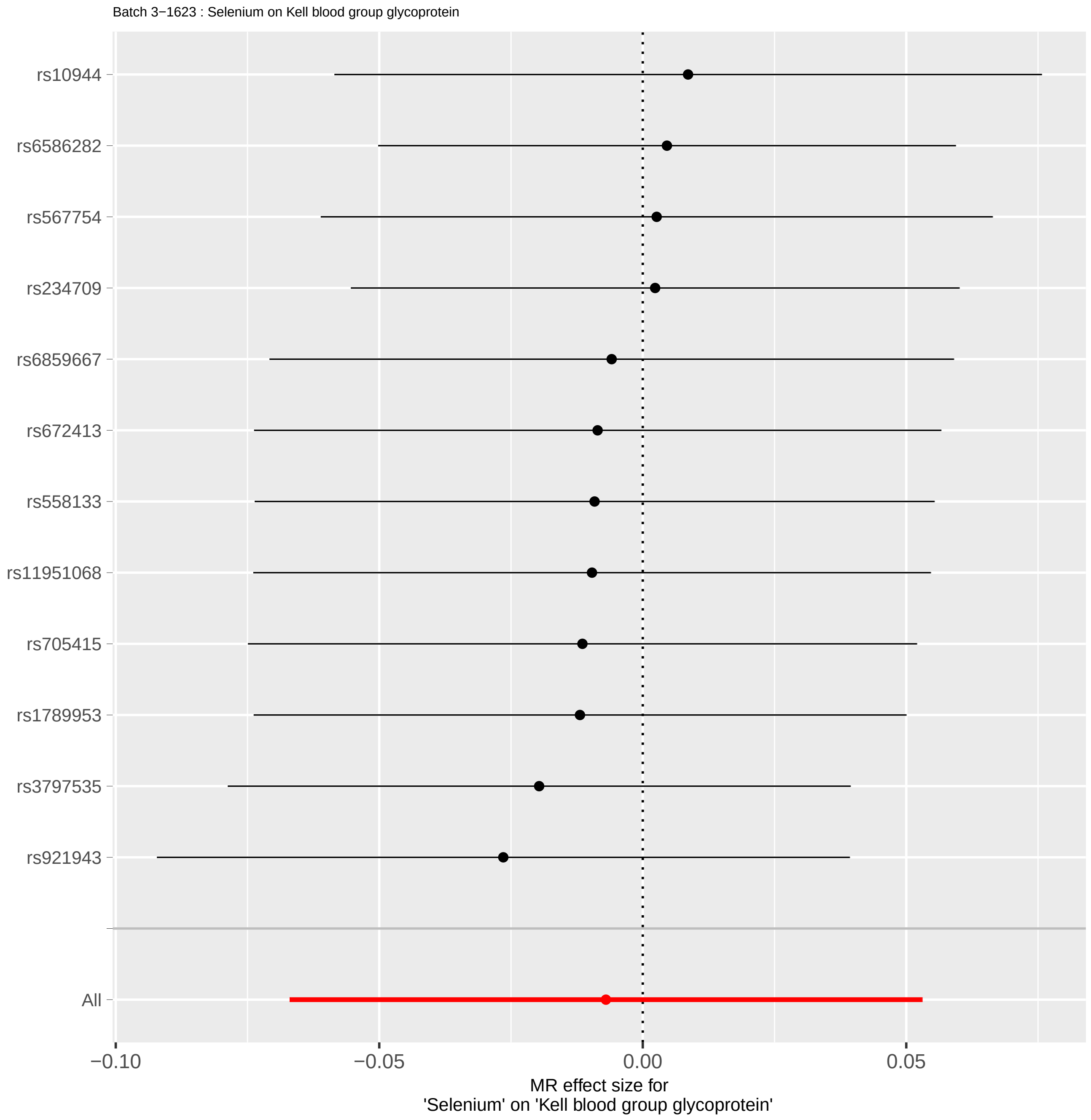

Batch 3−1623 : Selenium on Kell blood group glycoprotein
rs10944
rs6586282
rs567754
rs234709
rs6859667
rs672413
rs558133
rs11951068
rs705415
rs1789953
rs3797535
rs921943
All
−0.10
−0.05
0.00
0.05
MR effect size for
'Selenium' on 'Kell blood group glycoprotein'

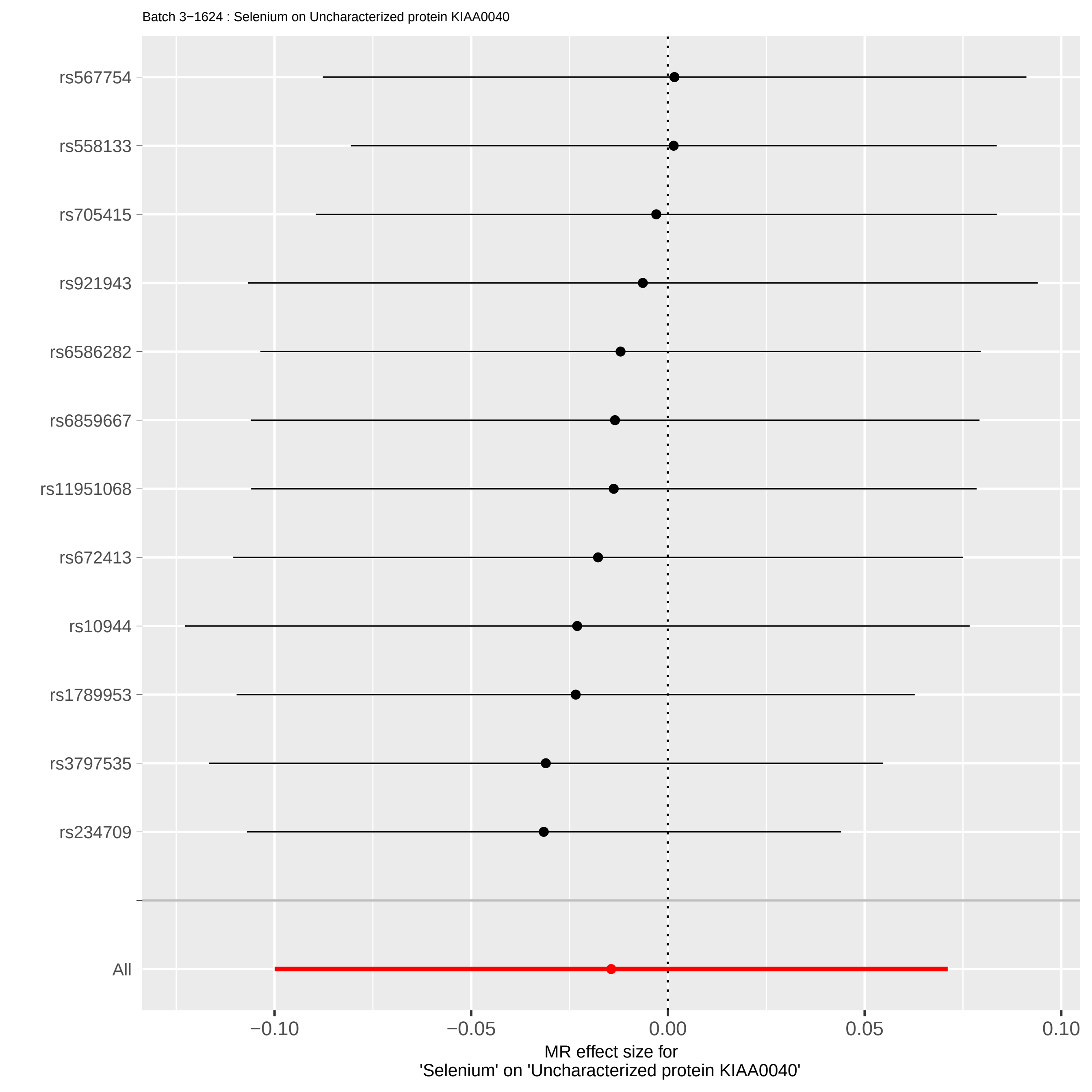
Batch 3−1624 : Selenium on Uncharacterized protein KIAA0040
rs567754
rs558133
rs705415
rs921943
rs6586282
rs6859667
rs11951068
rs672413
rs10944
rs1789953
rs3797535
rs234709
All
−0.10
−0.05
0.00
0.05
0.10
MR effect size for
'Selenium' on 'Uncharacterized protein KIAA0040'

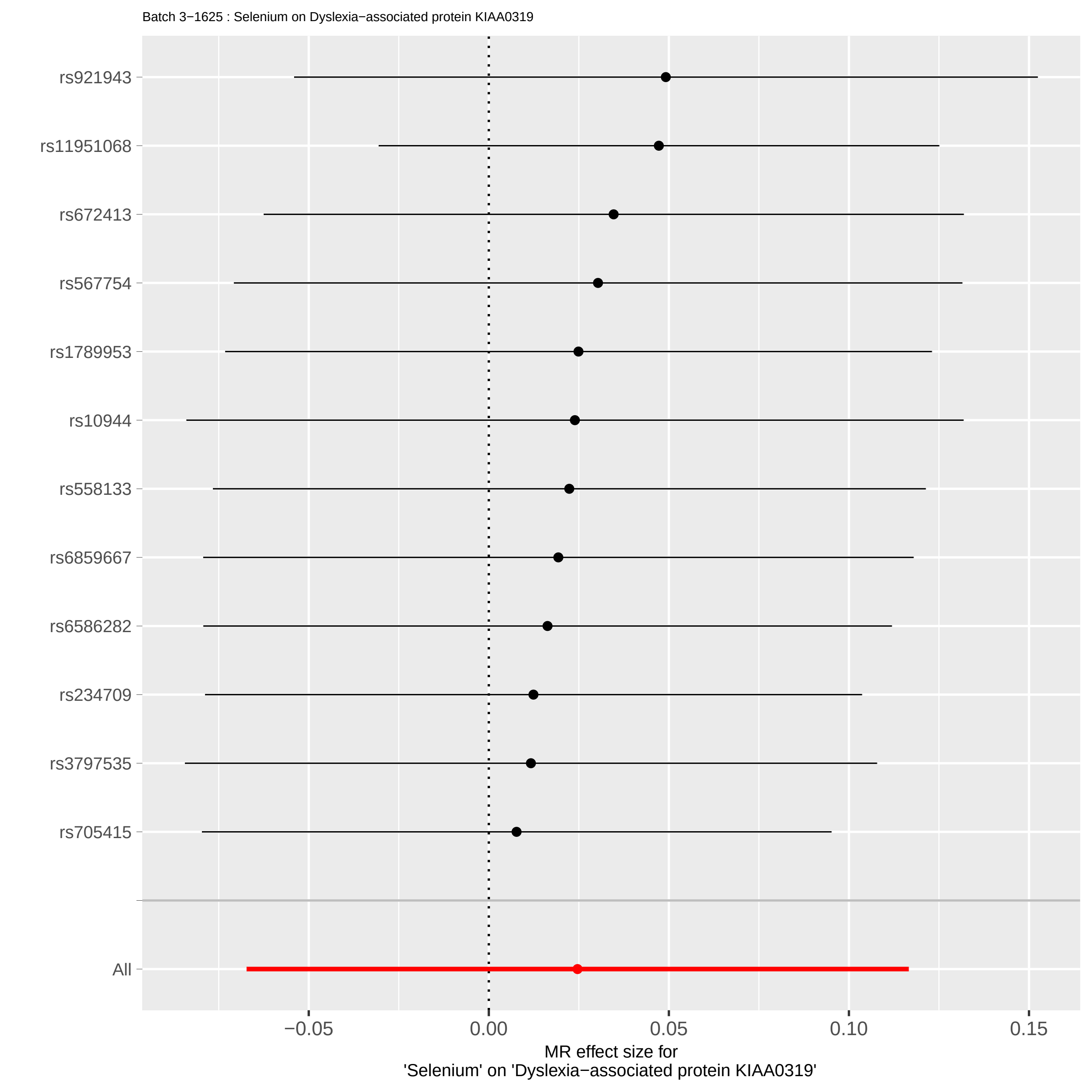

Batch 3−1625 : Selenium on Dyslexia−associated protein KIAA0319
rs921943
rs11951068
rs672413
rs567754
rs1789953
rs10944
rs558133
rs6859667
rs6586282
rs234709
rs3797535
rs705415
All
−0.05
0.00
0.05
0.10
0.15
MR effect size for
'Selenium' on 'Dyslexia−associated protein KIAA0319'

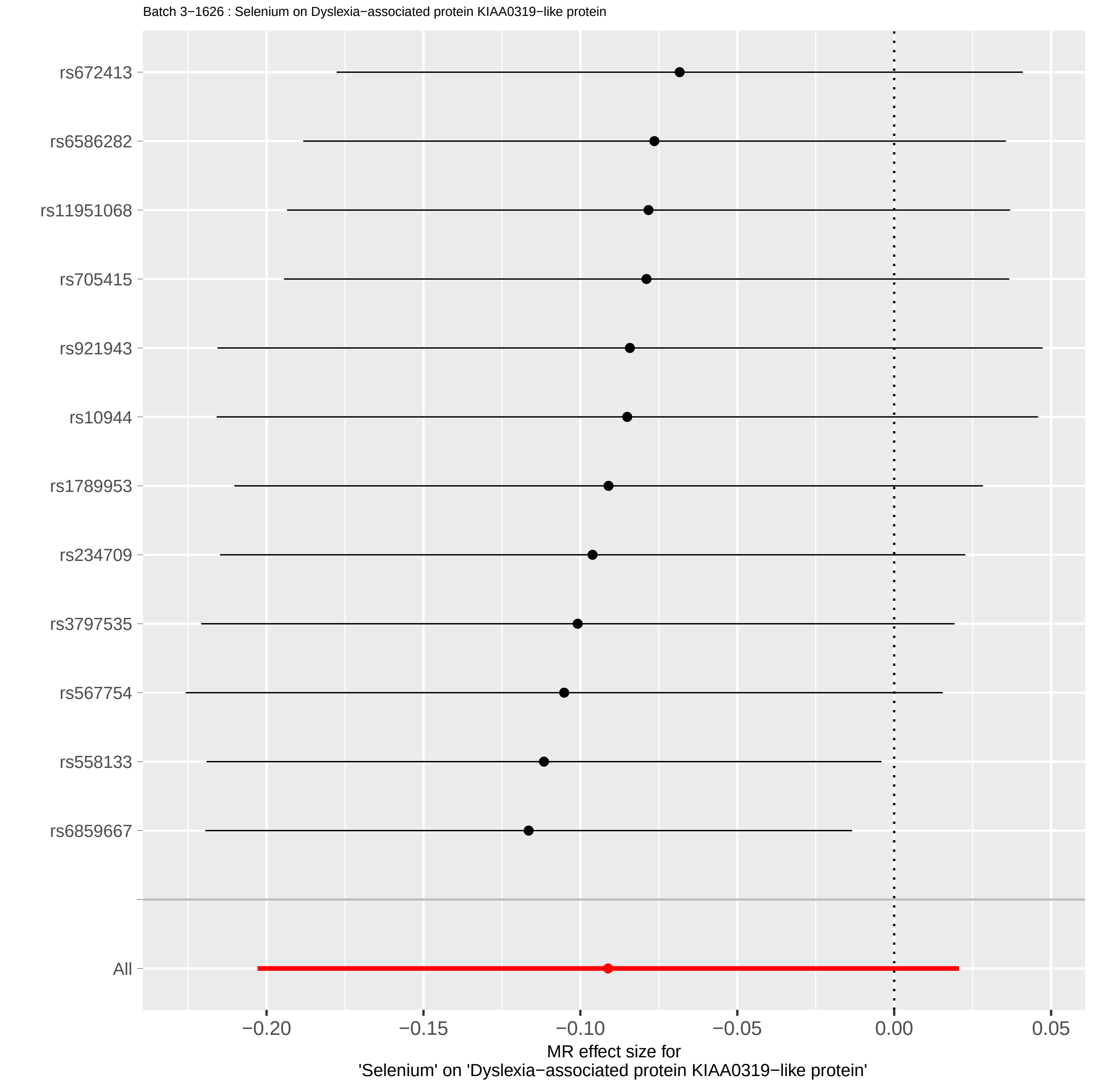

Batch 3−1626 : Selenium on Dyslexia−associated protein KIAA0319−like protein
rs672413
rs6586282
rs11951068
rs705415
rs921943
rs10944
rs1789953
rs234709
rs3797535
rs567754
rs558133
rs6859667
All
−0.20
−0.15
−0.10
−0.05
0.00
0.05
MR effect size for
'Selenium' on 'Dyslexia−associated protein KIAA0319−like protein'

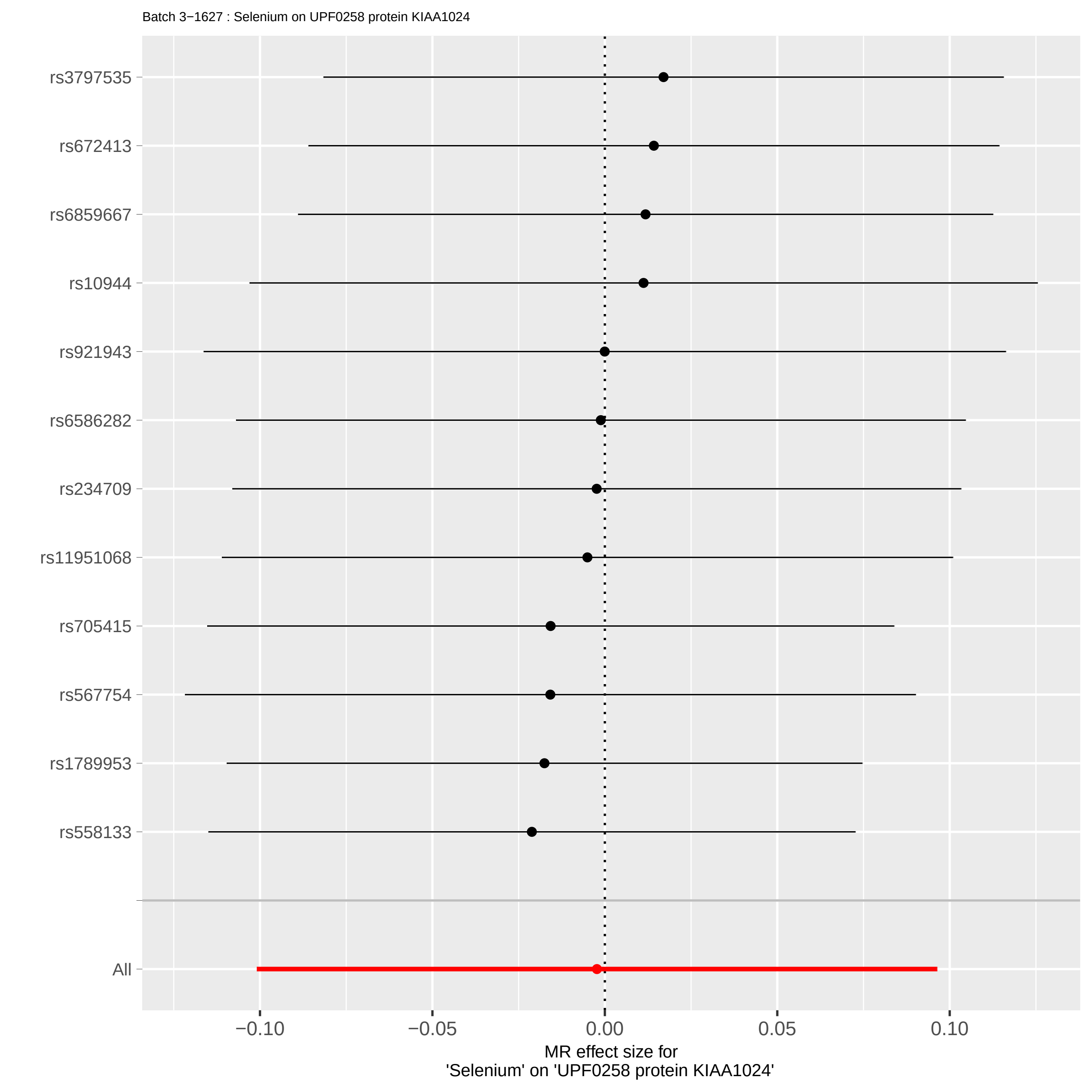

Batch 3−1627 : Selenium on UPF0258 protein KIAA1024
rs3797535
rs672413
rs6859667
rs10944
rs921943
rs6586282
rs234709
rs11951068
rs705415
rs567754
rs1789953
rs558133
All
−0.10
−0.05
0.00
0.05
0.10
MR effect size for
'Selenium' on 'UPF0258 protein KIAA1024'

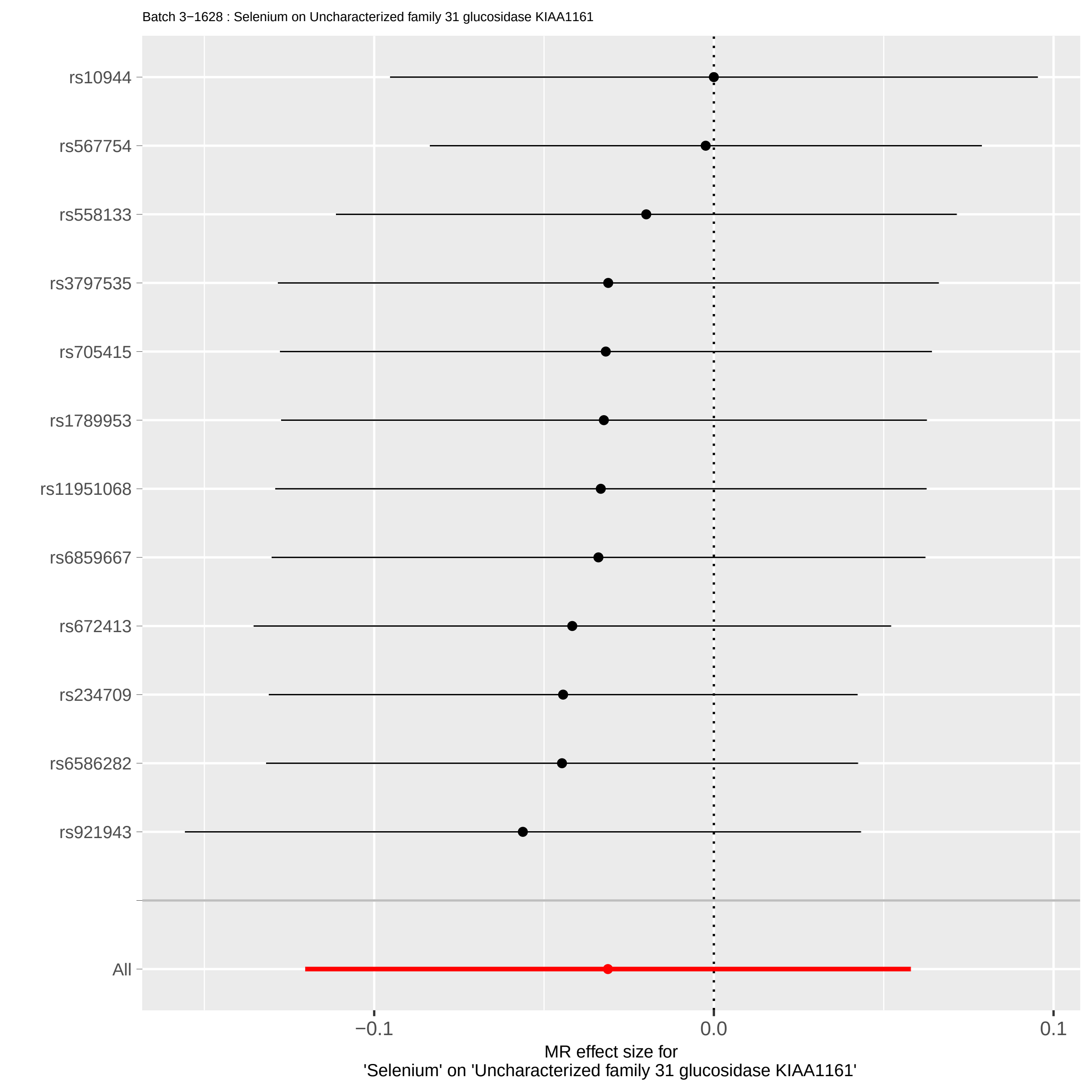
Batch 3−1628 : Selenium on Uncharacterized family 31 glucosidase KIAA1161
rs10944
rs567754
rs558133
rs3797535
rs705415
rs1789953
rs11951068
rs6859667
rs672413
rs234709
rs6586282
rs921943
All
−0.1
0.0
0.1
MR effect size for
'Selenium' on 'Uncharacterized family 31 glucosidase KIAA1161'

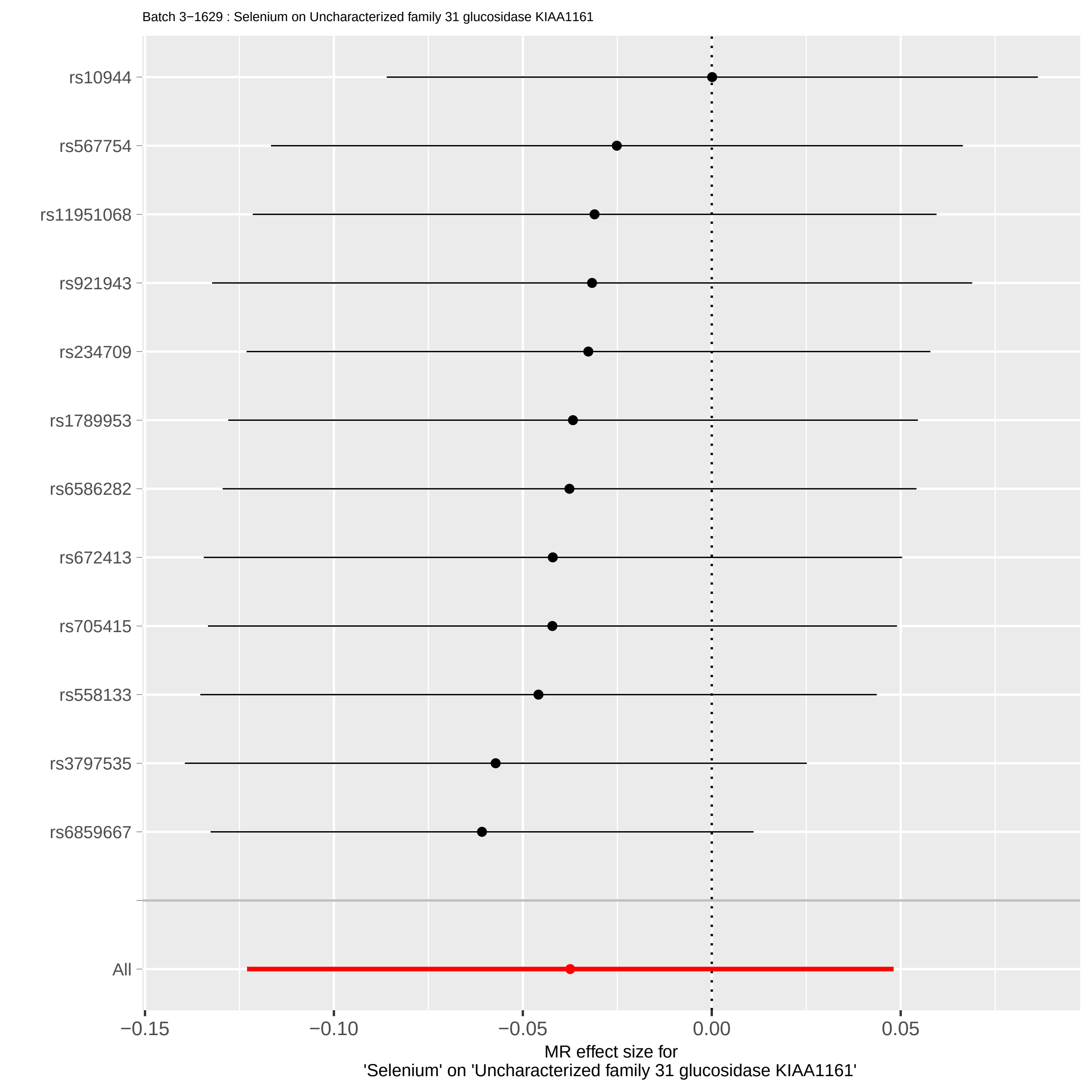

Batch 3−1629 : Selenium on Uncharacterized family 31 glucosidase KIAA1161
rs10944
rs567754
rs11951068
rs921943
rs234709
rs1789953
rs6586282
rs672413
rs705415
rs558133
rs3797535
rs6859667
All
−0.15
−0.10
−0.05
0.00
0.05
MR effect size for
'Selenium' on 'Uncharacterized family 31 glucosidase KIAA1161'

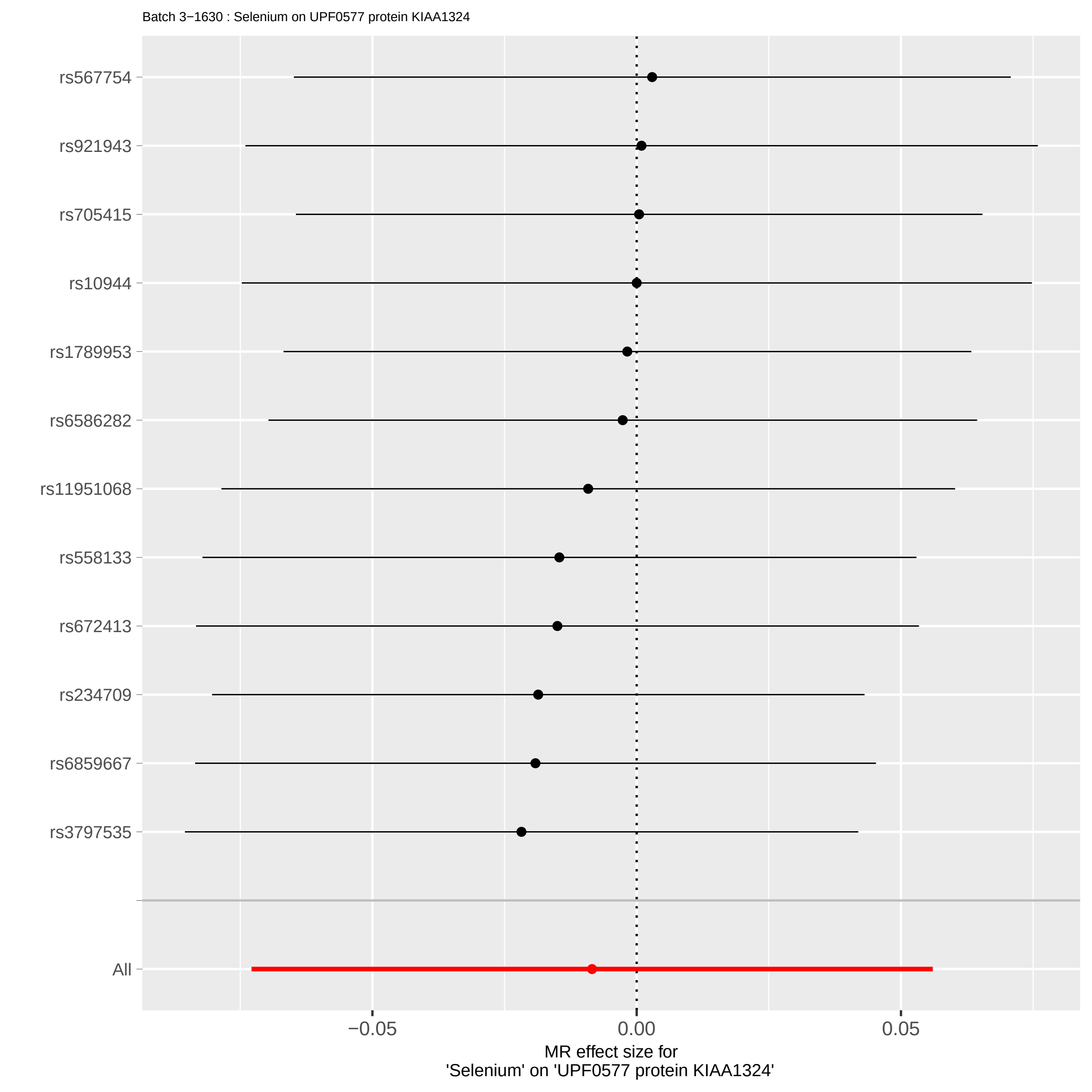

Batch 3−1630 : Selenium on UPF0577 protein KIAA1324
rs567754
rs921943
rs705415
rs10944
rs1789953
rs6586282
rs11951068
rs558133
rs672413
rs234709
rs6859667
rs3797535
All
−0.05
0.00
0.05
MR effect size for
'Selenium' on 'UPF0577 protein KIAA1324'

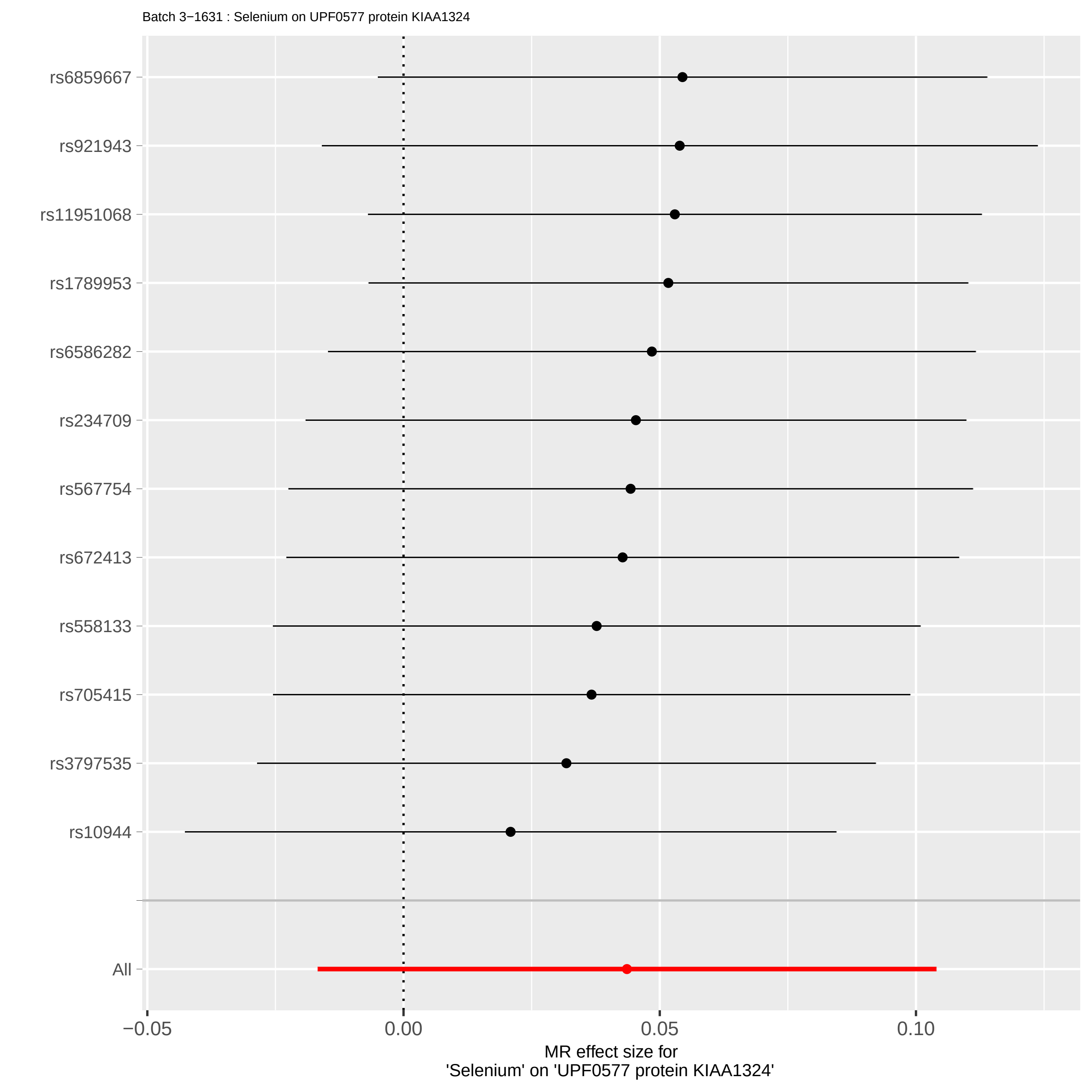

Batch 3−1631 : Selenium on UPF0577 protein KIAA1324
rs6859667
rs921943
rs11951068
rs1789953
rs6586282
rs234709
rs567754
rs672413
rs558133
rs705415
rs3797535
rs10944
All
−0.05
0.00
0.05
0.10
MR effect size for
'Selenium' on 'UPF0577 protein KIAA1324'

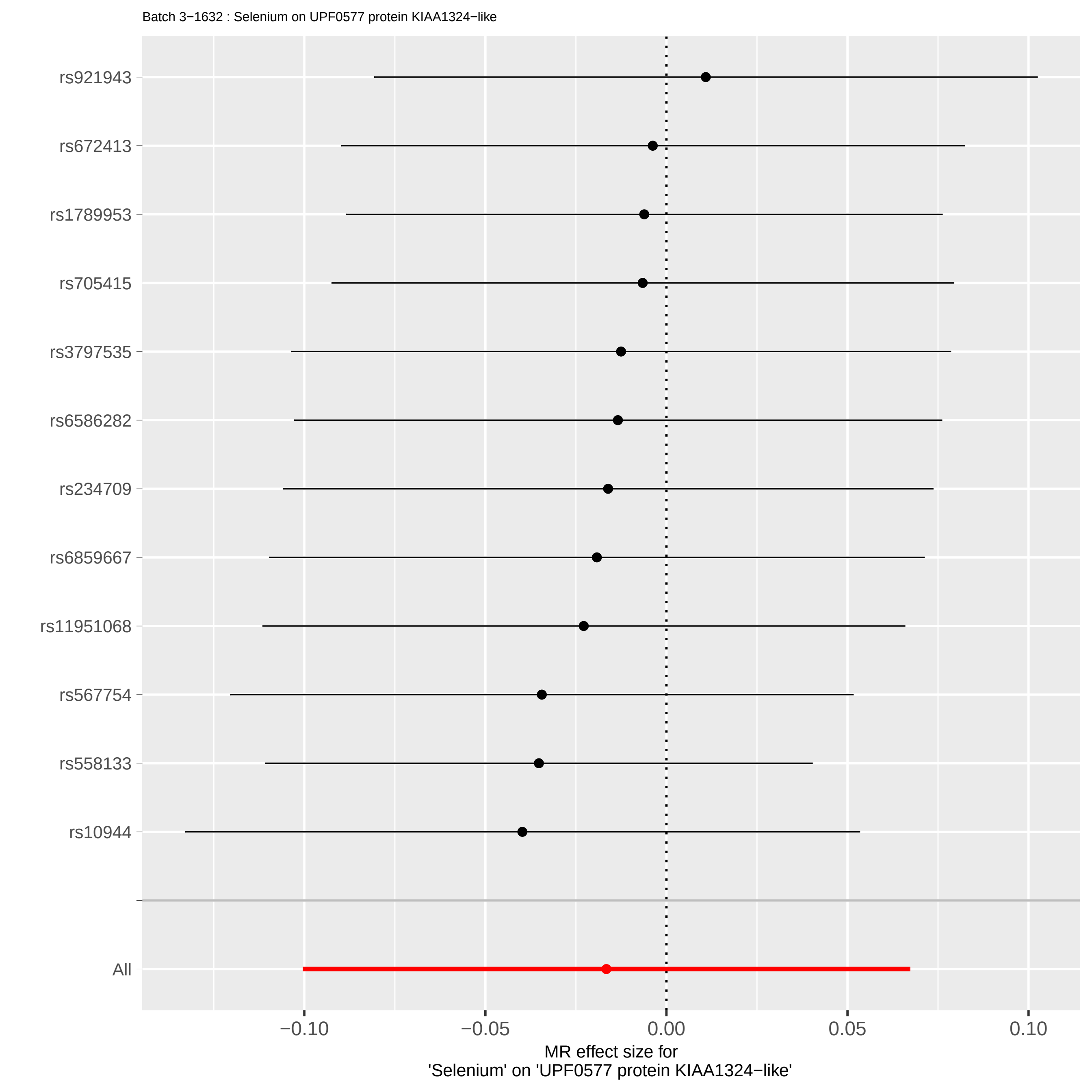

Batch 3−1632 : Selenium on UPF0577 protein KIAA1324−like
rs921943
rs672413
rs1789953
rs705415
rs3797535
rs6586282
rs234709
rs6859667
rs11951068
rs567754
rs558133
rs10944
All
−0.10
−0.05
0.00
0.05
0.10
MR effect size for
'Selenium' on 'UPF0577 protein KIAA1324−like'

Batch 3−1633 : Selenium on Uncharacterized protein KIAA1467

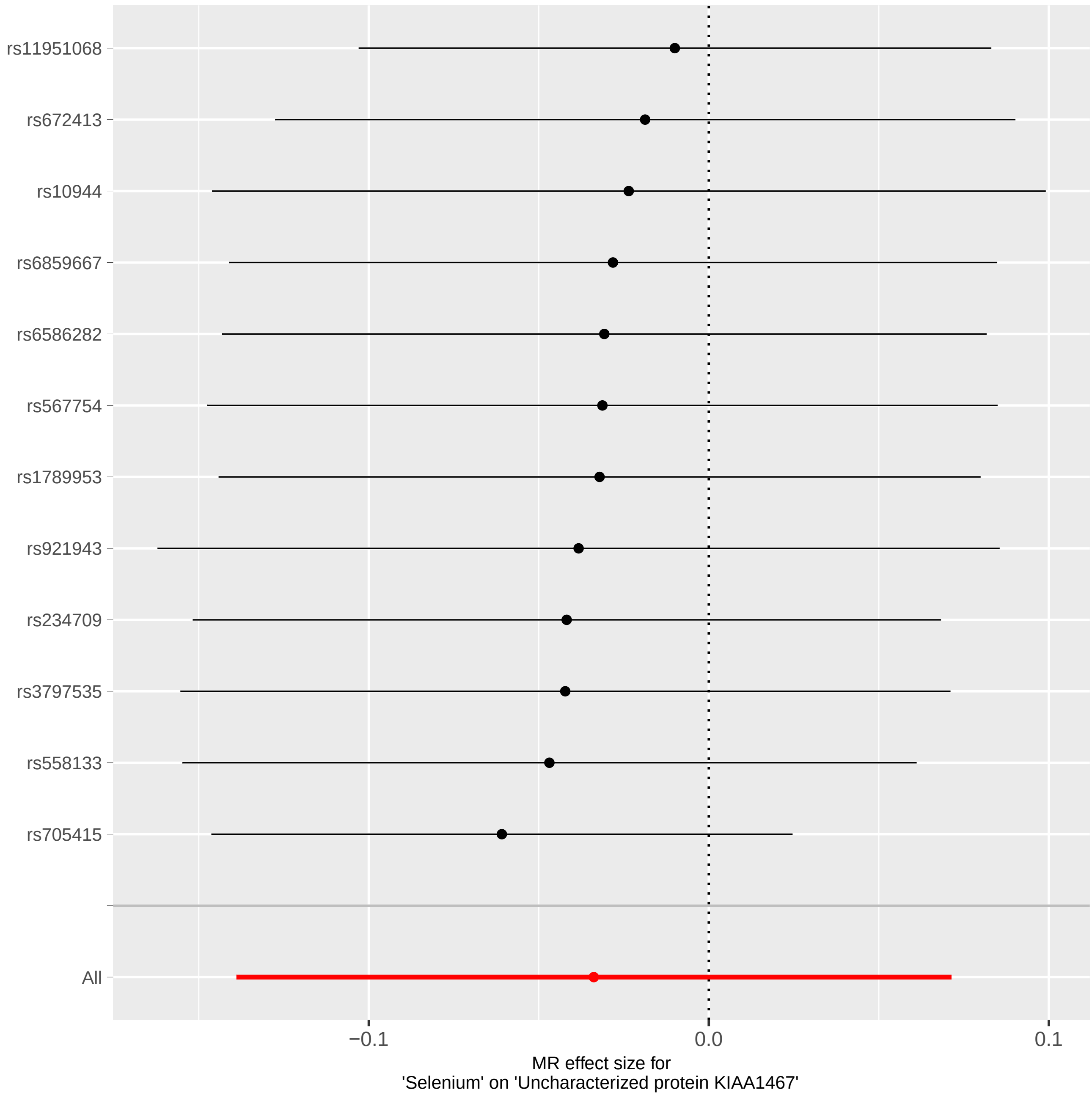

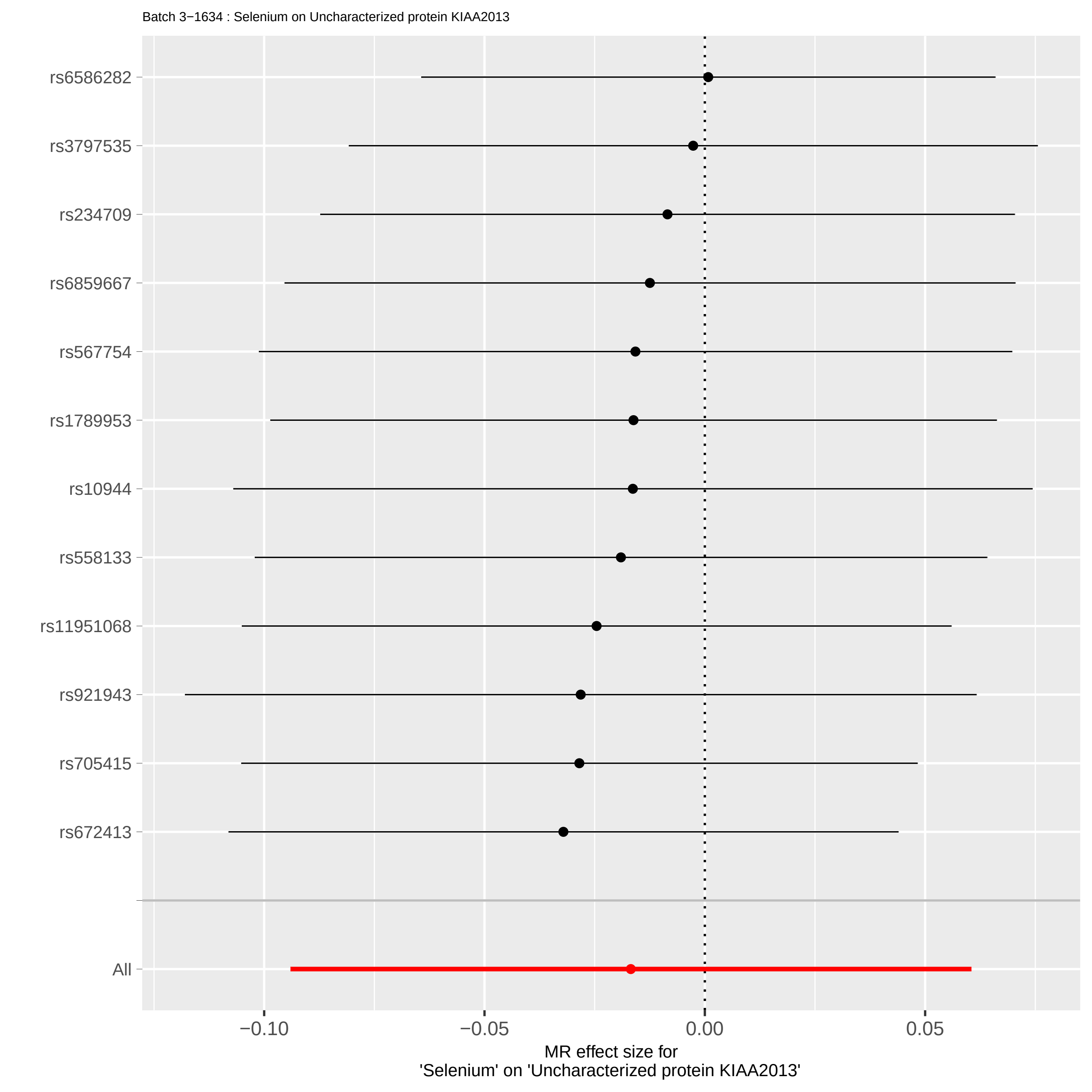

Batch 3−1634 : Selenium on Uncharacterized protein KIAA2013
rs6586282
rs3797535
rs234709
rs6859667
rs567754
rs1789953
rs10944
rs558133
rs11951068
rs921943
rs705415
rs672413
All
−0.10
−0.05
0.00
0.05
MR effect size for
'Selenium' on 'Uncharacterized protein KIAA2013'

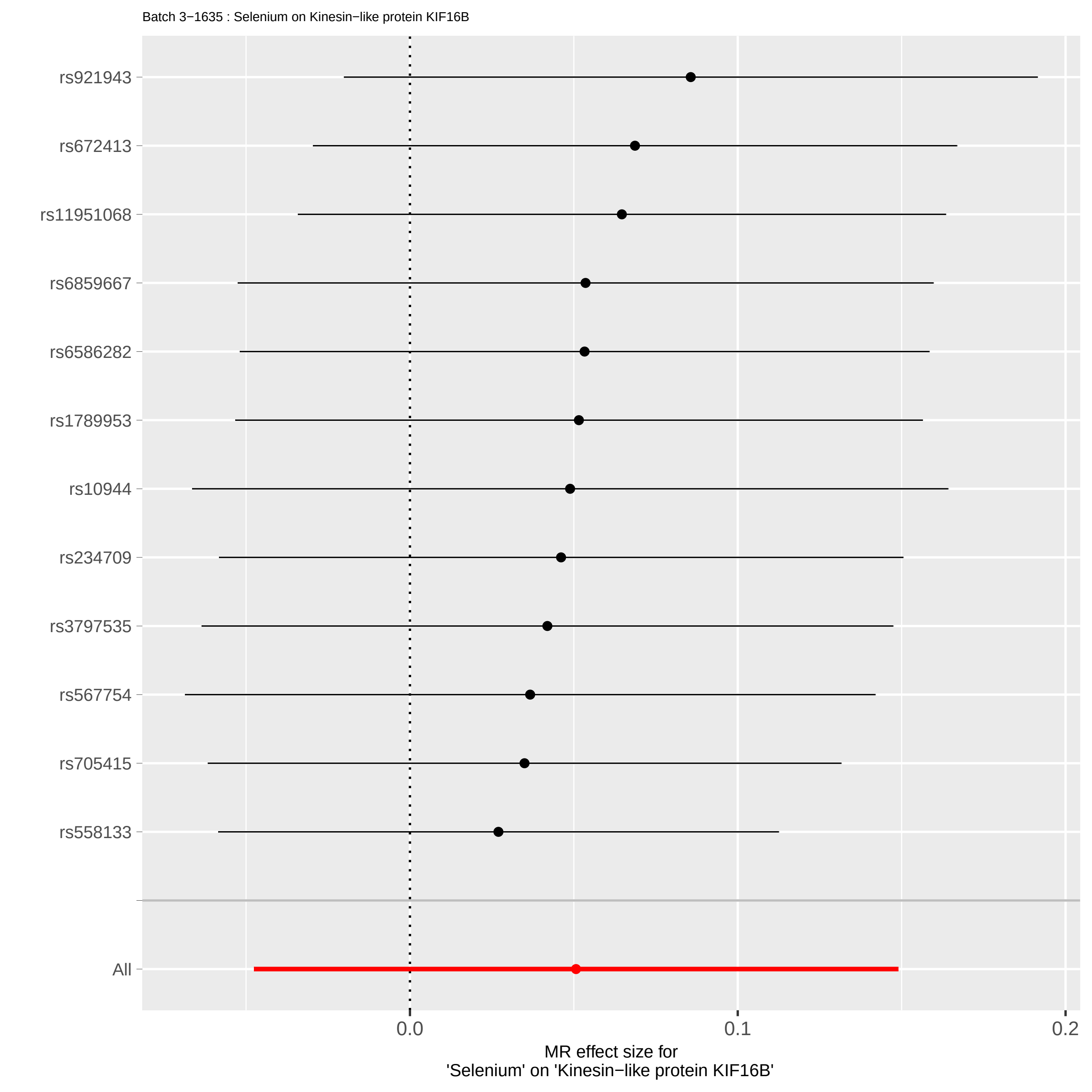

Batch 3−1635 : Selenium on Kinesin−like protein KIF16B
rs921943
rs672413
rs11951068
rs6859667
rs6586282
rs1789953
rs10944
rs234709
rs3797535
rs567754
rs705415
rs558133
All
0.0
0.1
0.2
MR effect size for
'Selenium' on 'Kinesin−like protein KIF16B'

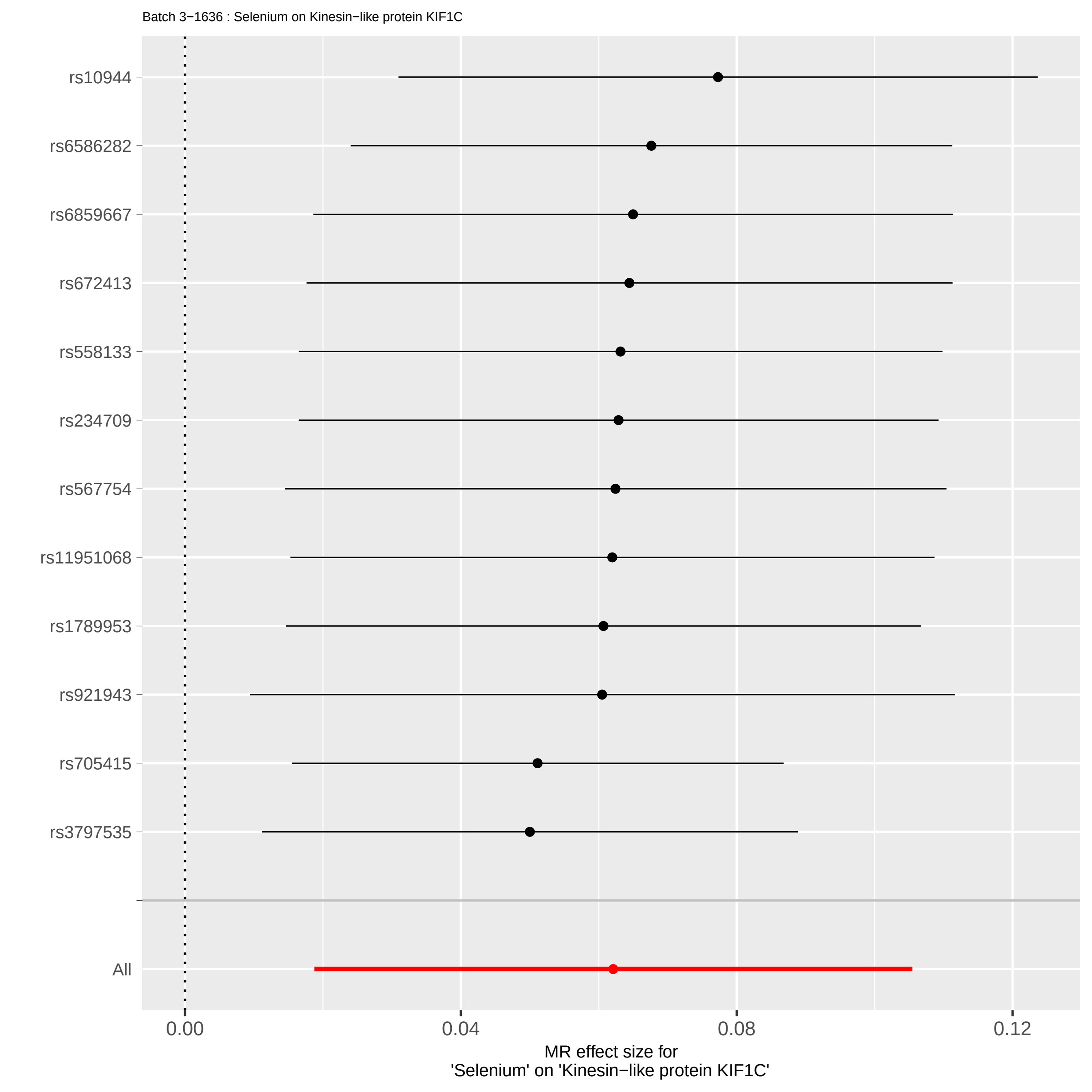

Batch 3−1636 : Selenium on Kinesin−like protein KIF1C
rs10944
rs6586282
rs6859667
rs672413
rs558133
rs234709
rs567754
rs11951068
rs1789953
rs921943
rs705415
rs3797535
All
0.00
0.04
0.08
0.12
MR effect size for
'Selenium' on 'Kinesin−like protein KIF1C'

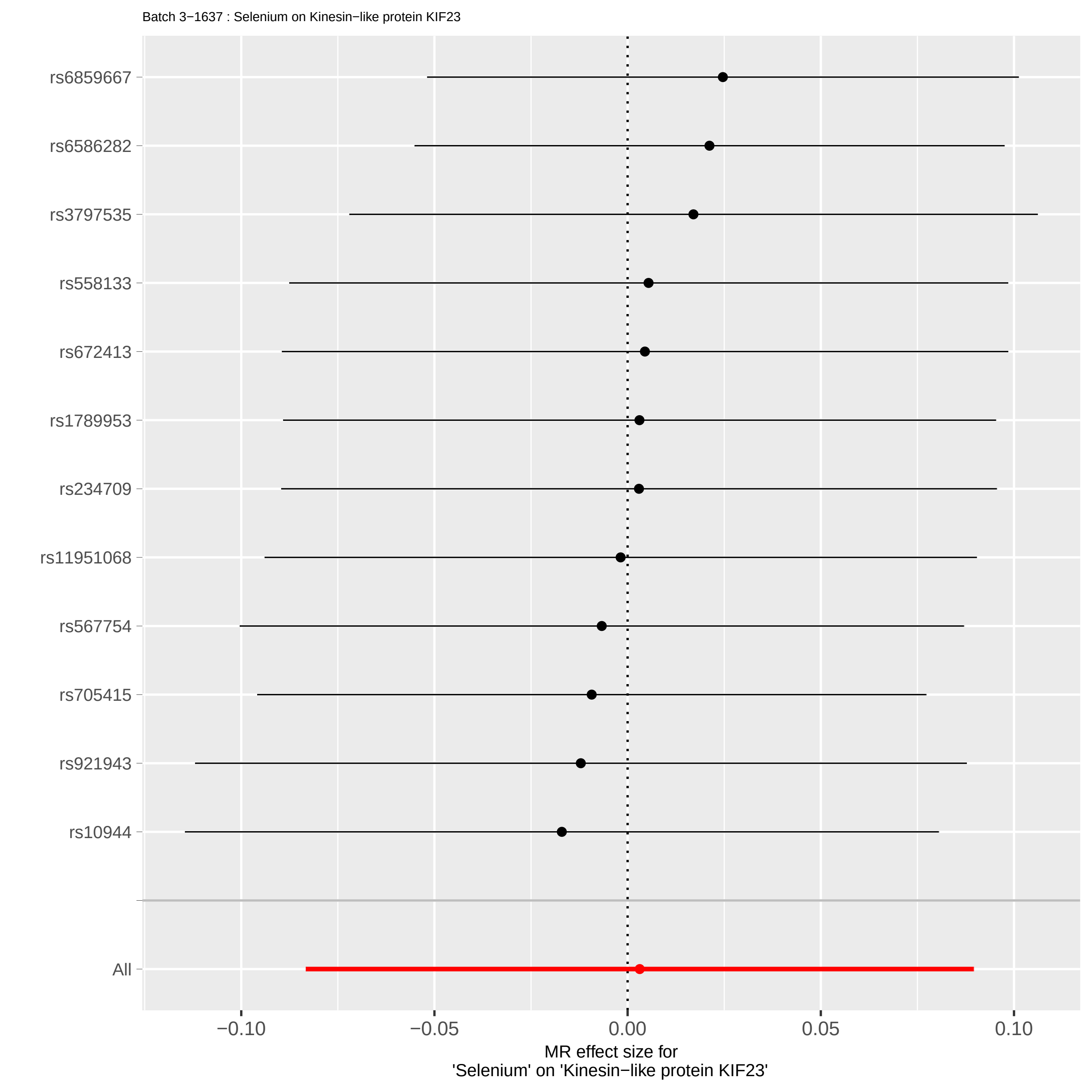
Batch 3−1637 : Selenium on Kinesin−like protein KIF23
rs6859667
rs6586282
rs3797535
rs558133
rs672413
rs1789953
rs234709
rs11951068
rs567754
rs705415
rs921943
rs10944
All
−0.10
−0.05
0.00
0.05
0.10
MR effect size for
'Selenium' on 'Kinesin−like protein KIF23'

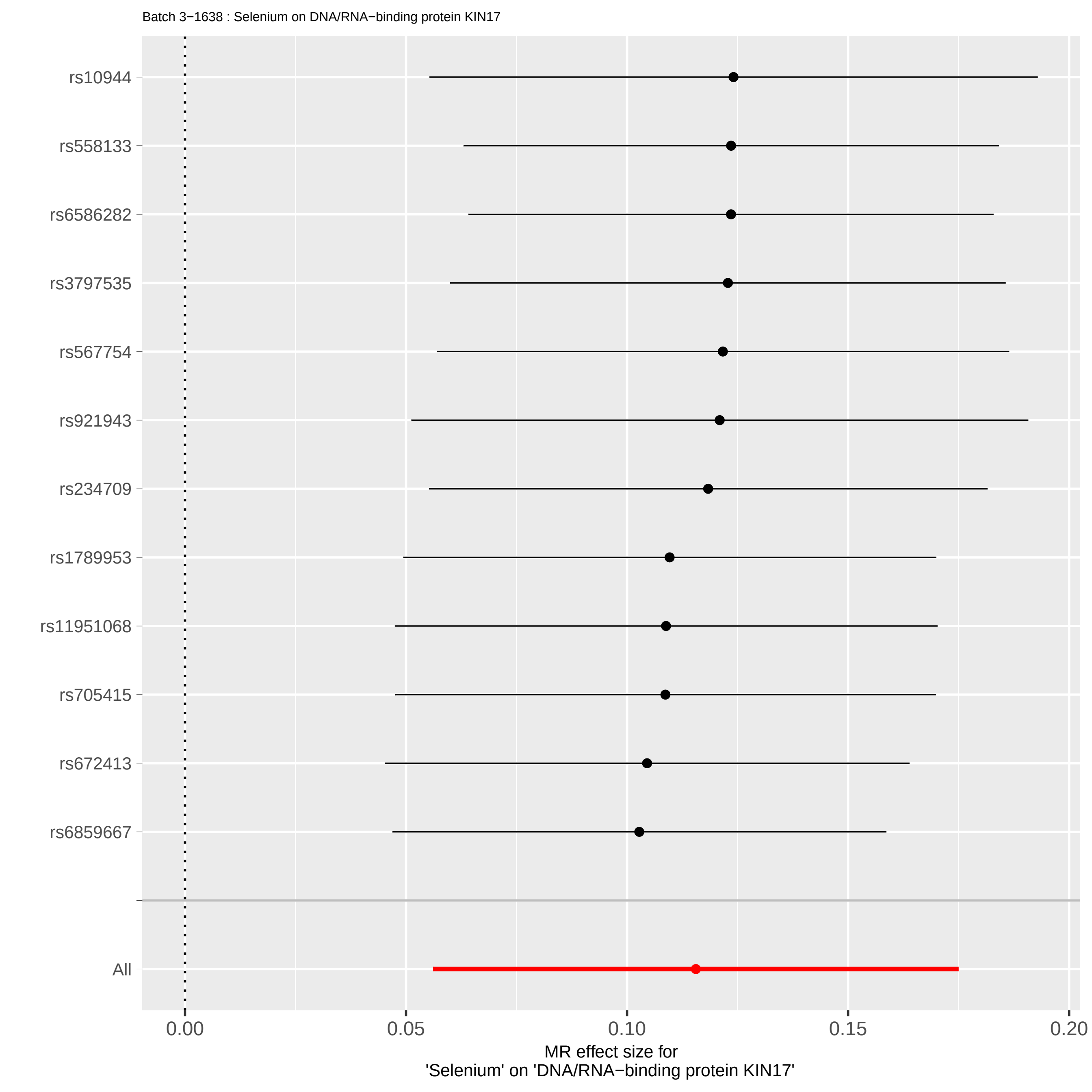
Batch 3−1638 : Selenium on DNA/RNA−binding protein KIN17
rs10944
rs558133
rs6586282
rs3797535
rs567754
rs921943
rs234709
rs1789953
rs11951068
rs705415
rs672413
rs6859667
All
0.00
0.05
0.10
0.15
0.20
MR effect size for
'Selenium' on 'DNA/RNA−binding protein KIN17'

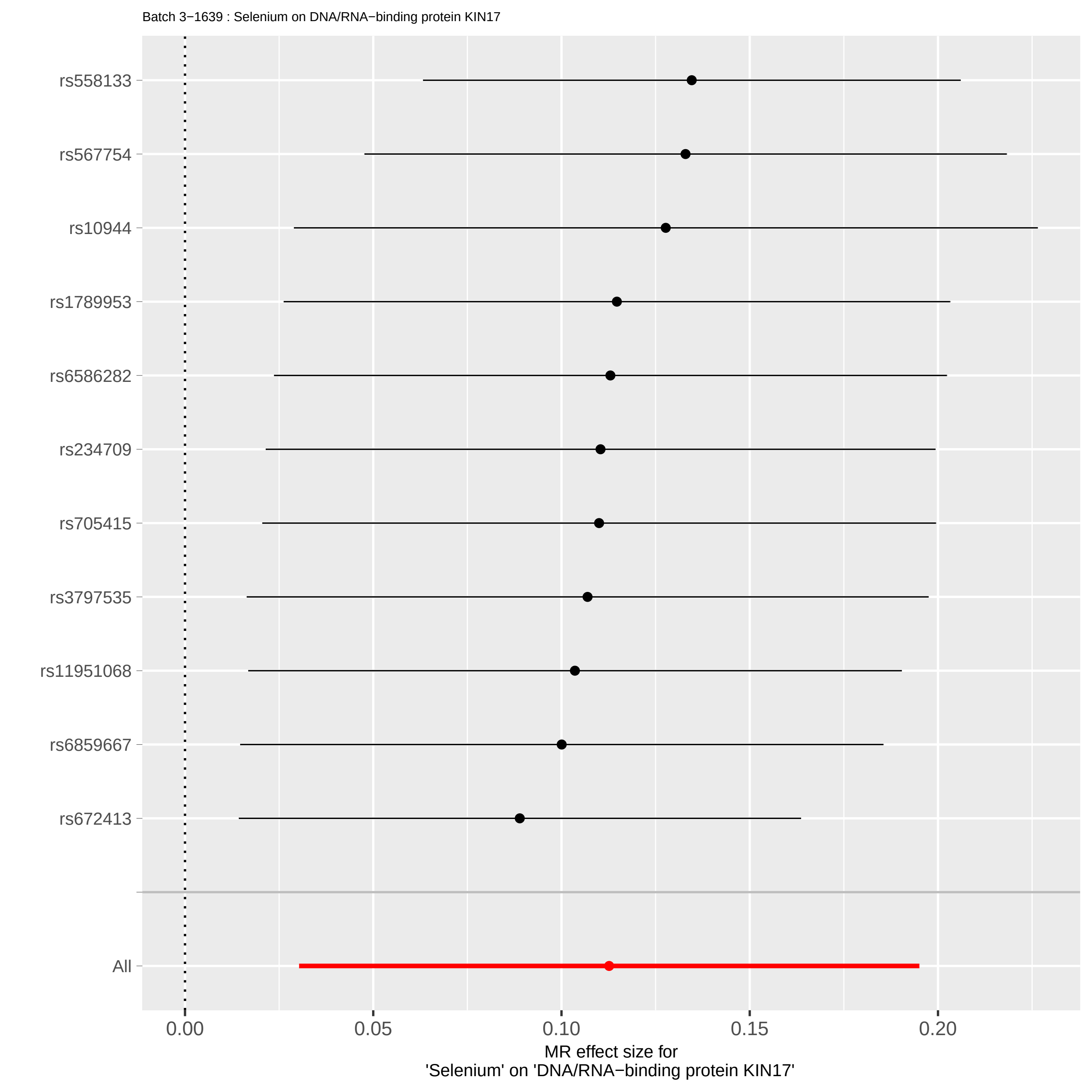

Batch 3−1639 : Selenium on DNA/RNA−binding protein KIN17
rs558133
rs567754
rs10944
rs1789953
rs6586282
rs234709
rs705415
rs3797535
rs11951068
rs6859667
rs672413
All
0.00
0.05
0.10
0.15
0.20
MR effect size for
'Selenium' on 'DNA/RNA−binding protein KIN17'

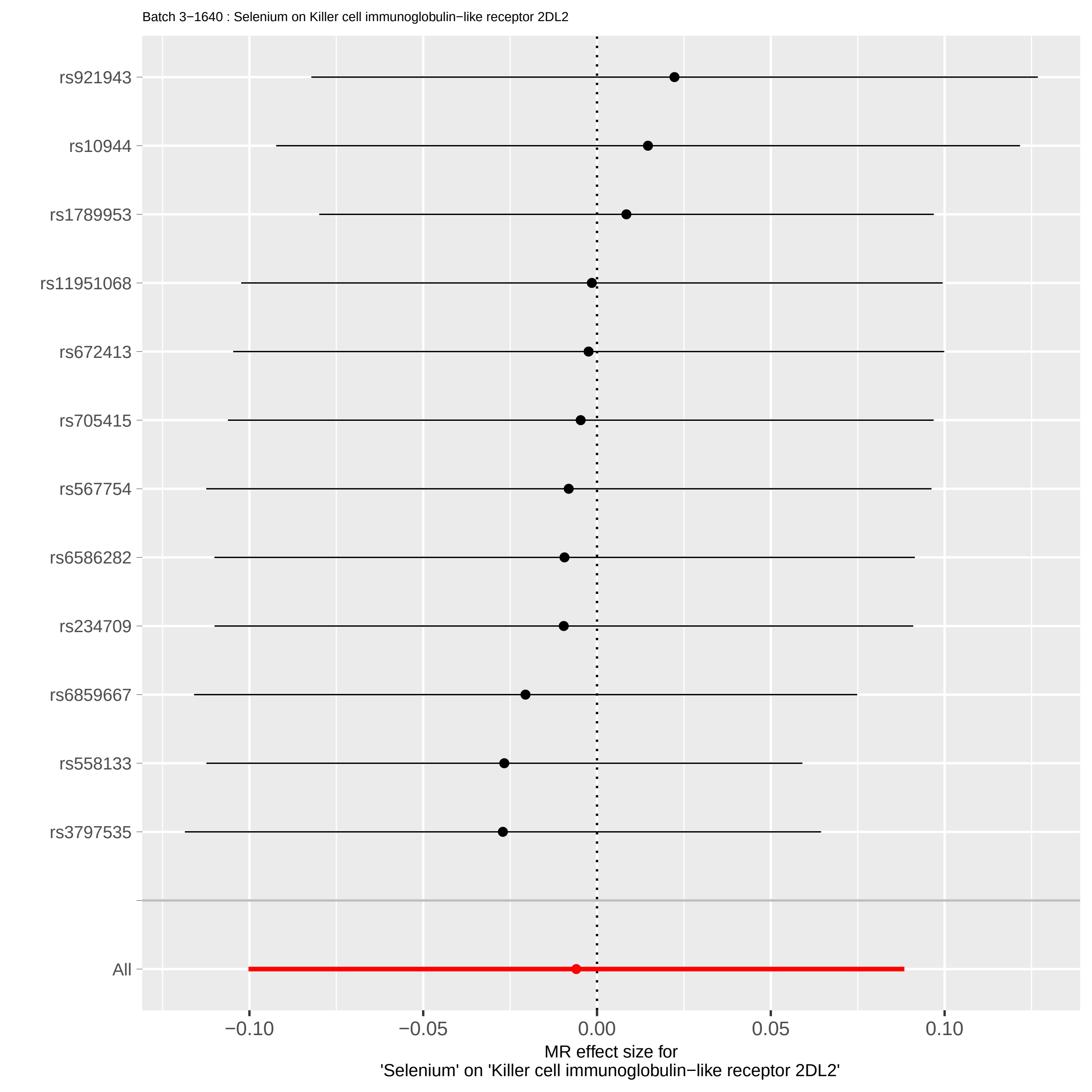

Batch 3−1640 : Selenium on Killer cell immunoglobulin−like receptor 2DL2
rs921943
rs10944
rs1789953
rs11951068
rs672413
rs705415
rs567754
rs6586282
rs234709
rs6859667
rs558133
rs3797535
All
−0.10
−0.05
0.00
0.05
0.10
MR effect size for
'Selenium' on 'Killer cell immunoglobulin−like receptor 2DL2'

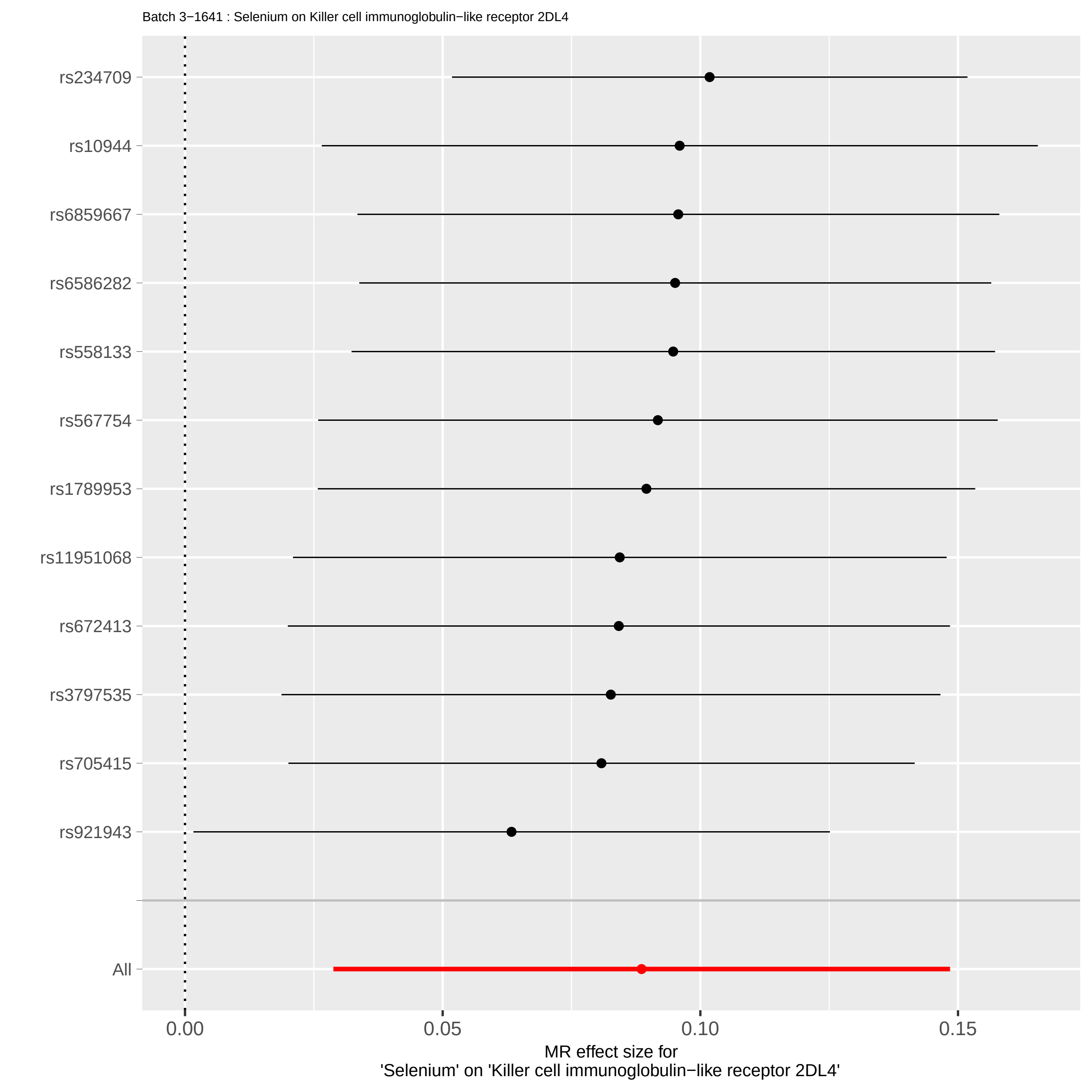

Batch 3−1641 : Selenium on Killer cell immunoglobulin−like receptor 2DL4
rs234709
rs10944
rs6859667
rs6586282
rs558133
rs567754
rs1789953
rs11951068
rs672413
rs3797535
rs705415
rs921943
All
0.00
0.05
0.10
0.15
MR effect size for
'Selenium' on 'Killer cell immunoglobulin−like receptor 2DL4'

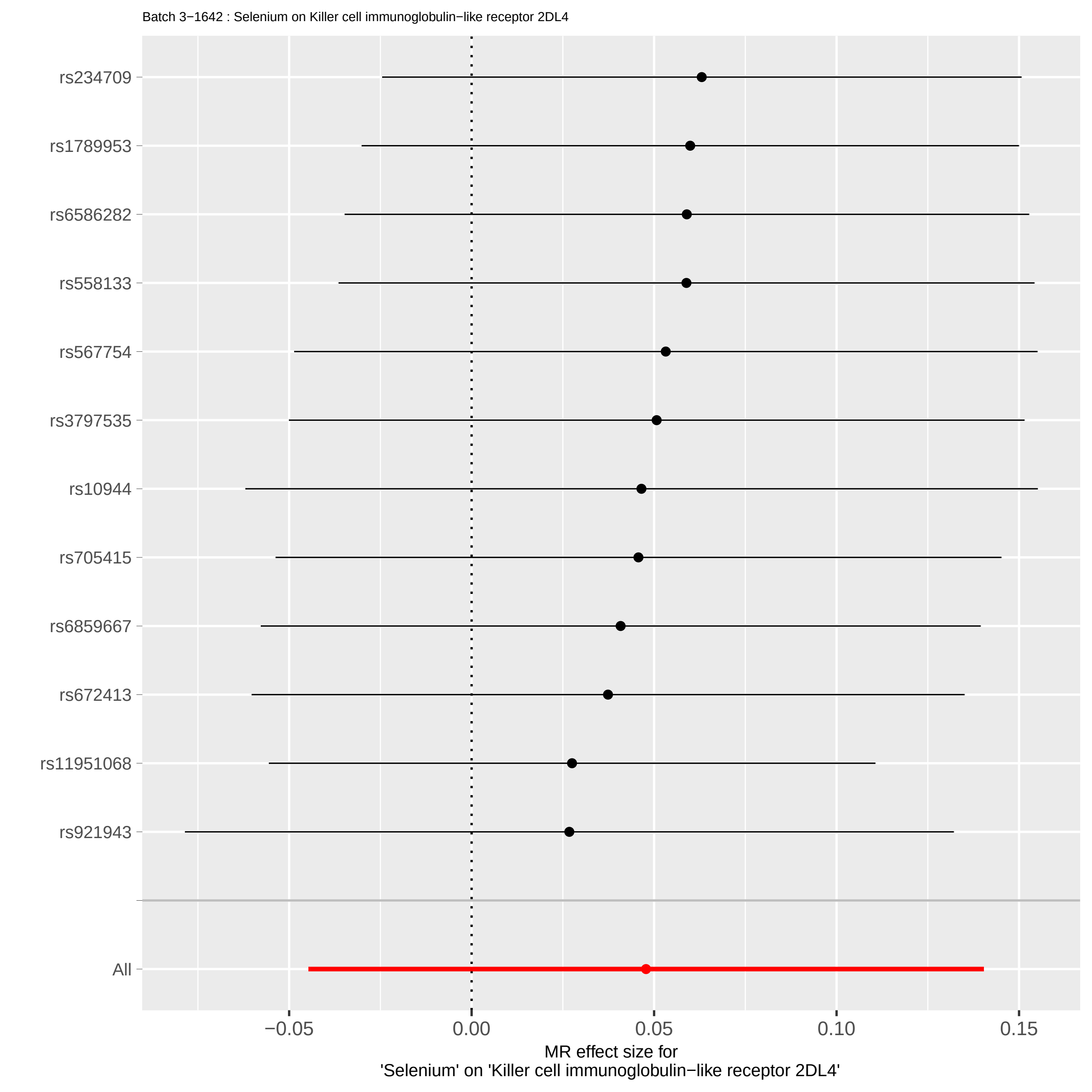

Batch 3−1642 : Selenium on Killer cell immunoglobulin−like receptor 2DL4
rs234709
rs1789953
rs6586282
rs558133
rs567754
rs3797535
rs10944
rs705415
rs6859667
rs672413
rs11951068
rs921943
All
−0.05
0.00
0.05
0.10
0.15
MR effect size for
'Selenium' on 'Killer cell immunoglobulin−like receptor 2DL4'

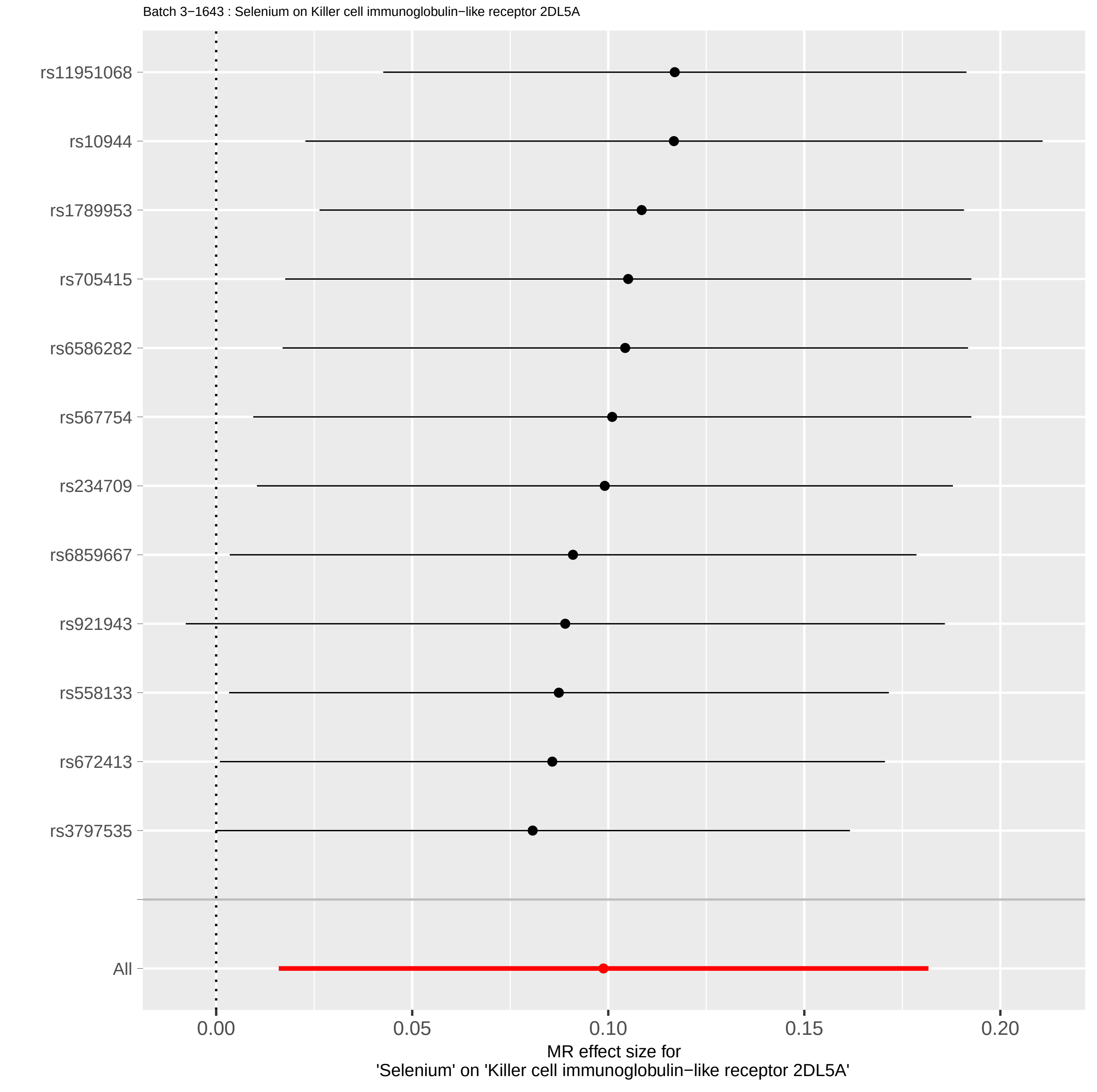

Batch 3−1643 : Selenium on Killer cell immunoglobulin−like receptor 2DL5A
rs11951068
rs10944
rs1789953
rs705415
rs6586282
rs567754
rs234709
rs6859667
rs921943
rs558133
rs672413
rs3797535
All
0.00
0.05
0.10
0.15
0.20
MR effect size for
'Selenium' on 'Killer cell immunoglobulin−like receptor 2DL5A'

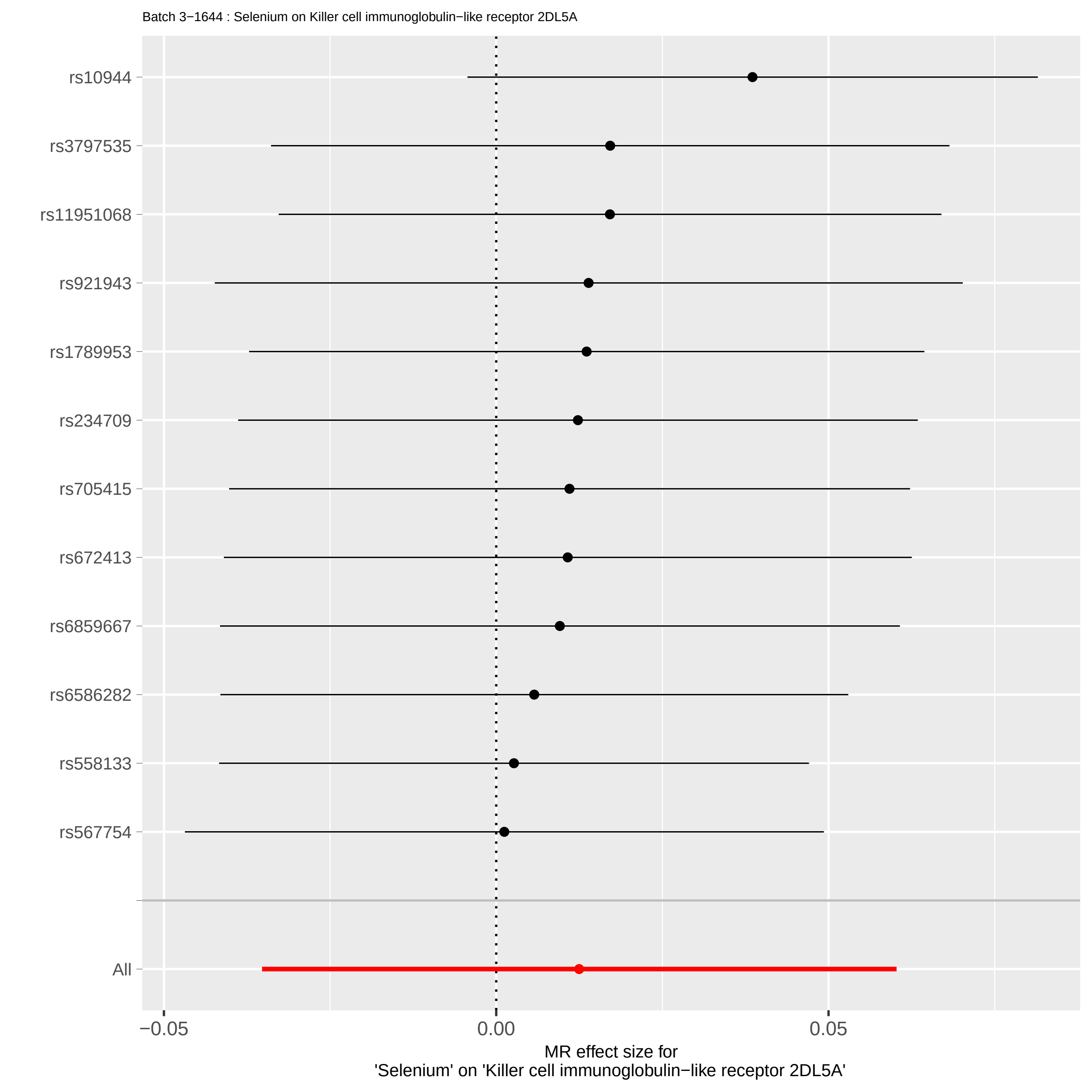

Batch 3−1644 : Selenium on Killer cell immunoglobulin−like receptor 2DL5A
rs10944
rs3797535
rs11951068
rs921943
rs1789953
rs234709
rs705415
rs672413
rs6859667
rs6586282
rs558133
rs567754
All
−0.05
0.00
0.05
MR effect size for
'Selenium' on 'Killer cell immunoglobulin−like receptor 2DL5A'

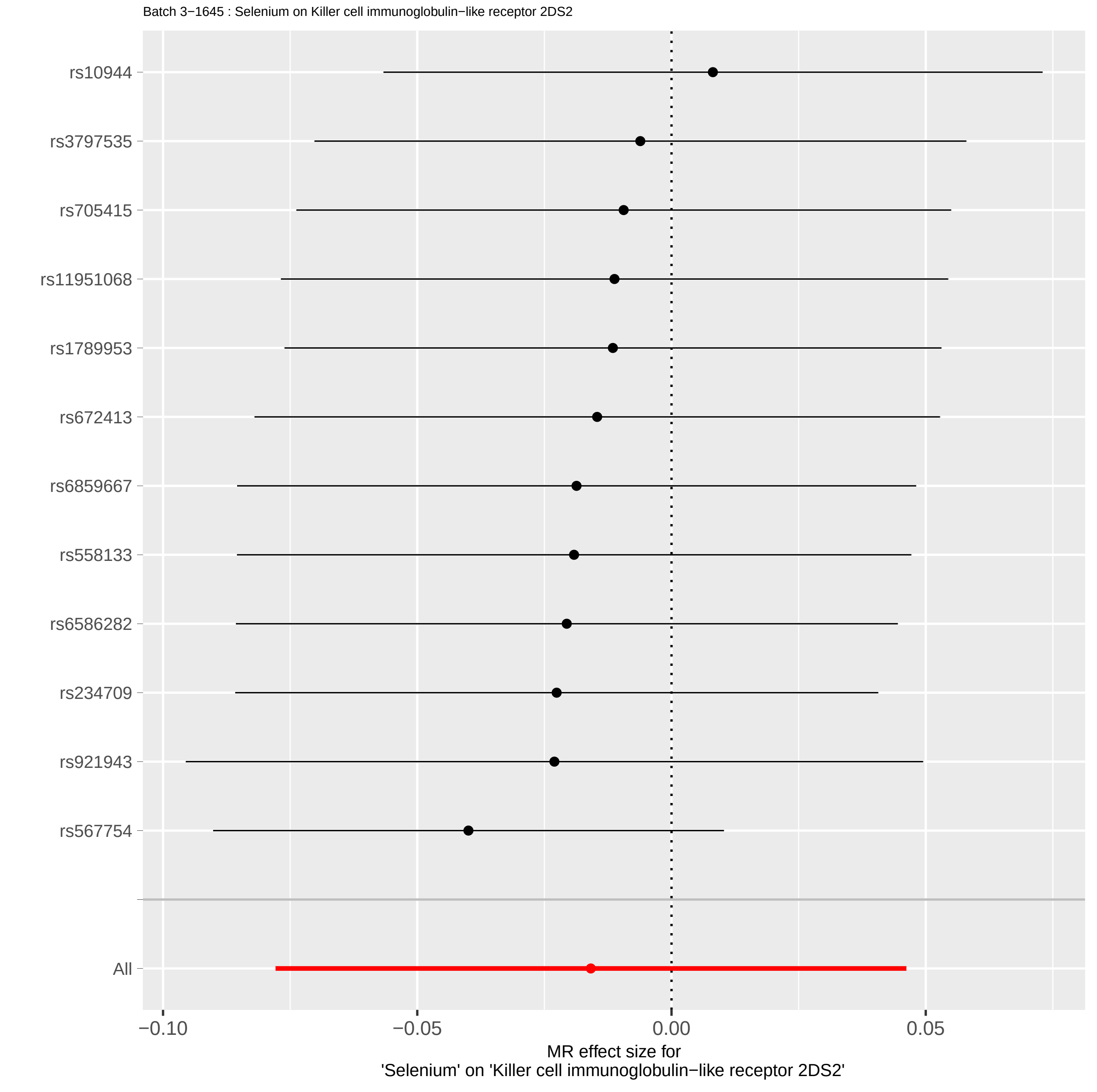

Batch 3−1645 : Selenium on Killer cell immunoglobulin−like receptor 2DS2
rs10944
rs3797535
rs705415
rs11951068
rs1789953
rs672413
rs6859667
rs558133
rs6586282
rs234709
rs921943
rs567754
All
−0.10
−0.05
0.00
0.05
MR effect size for
'Selenium' on 'Killer cell immunoglobulin−like receptor 2DS2'

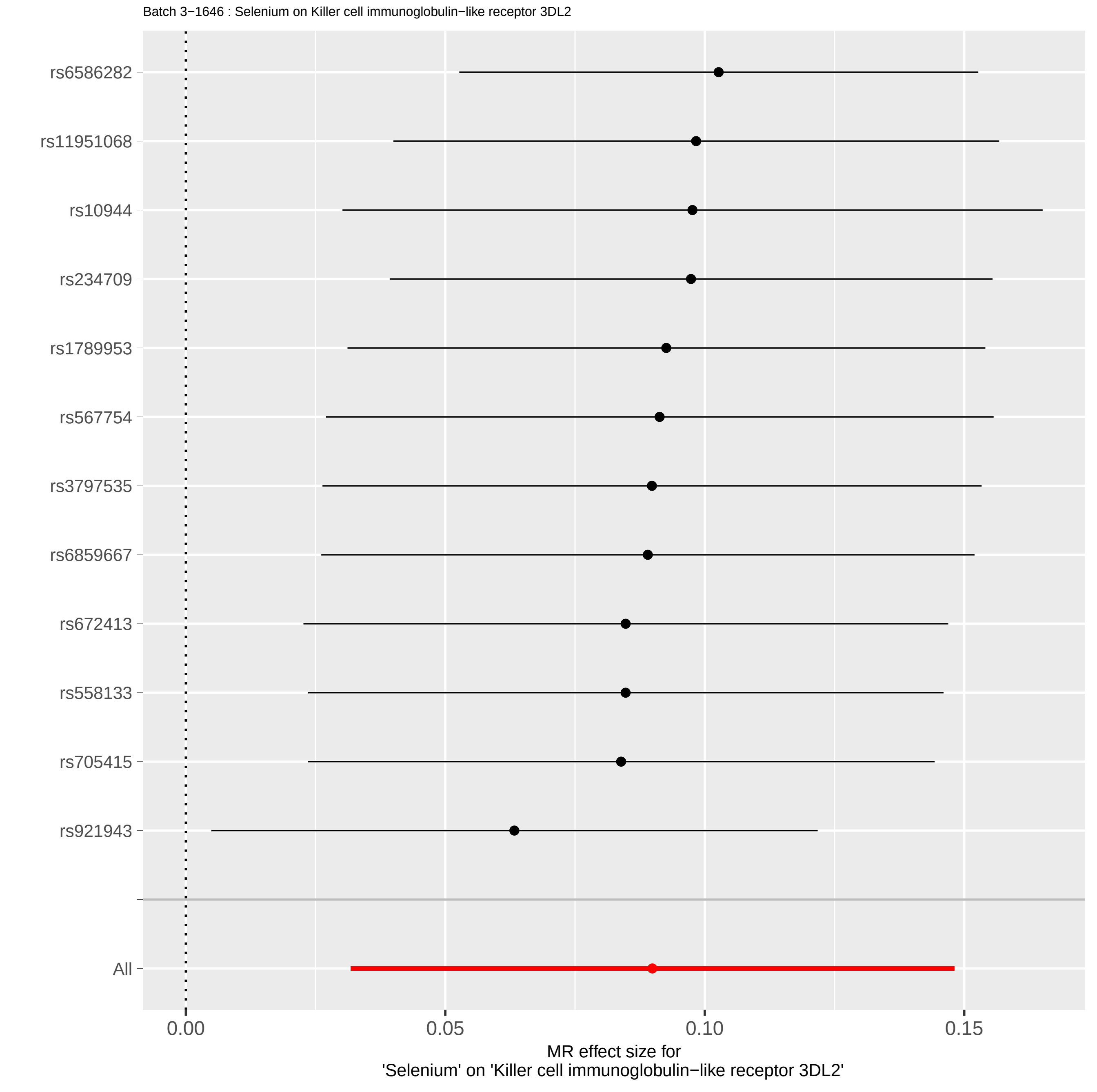

Batch 3−1646 : Selenium on Killer cell immunoglobulin−like receptor 3DL2
rs6586282
rs11951068
rs10944
rs234709
rs1789953
rs567754
rs3797535
rs6859667
rs672413
rs558133
rs705415
rs921943
All
0.00
0.05
0.10
0.15
MR effect size for
'Selenium' on 'Killer cell immunoglobulin−like receptor 3DL2'

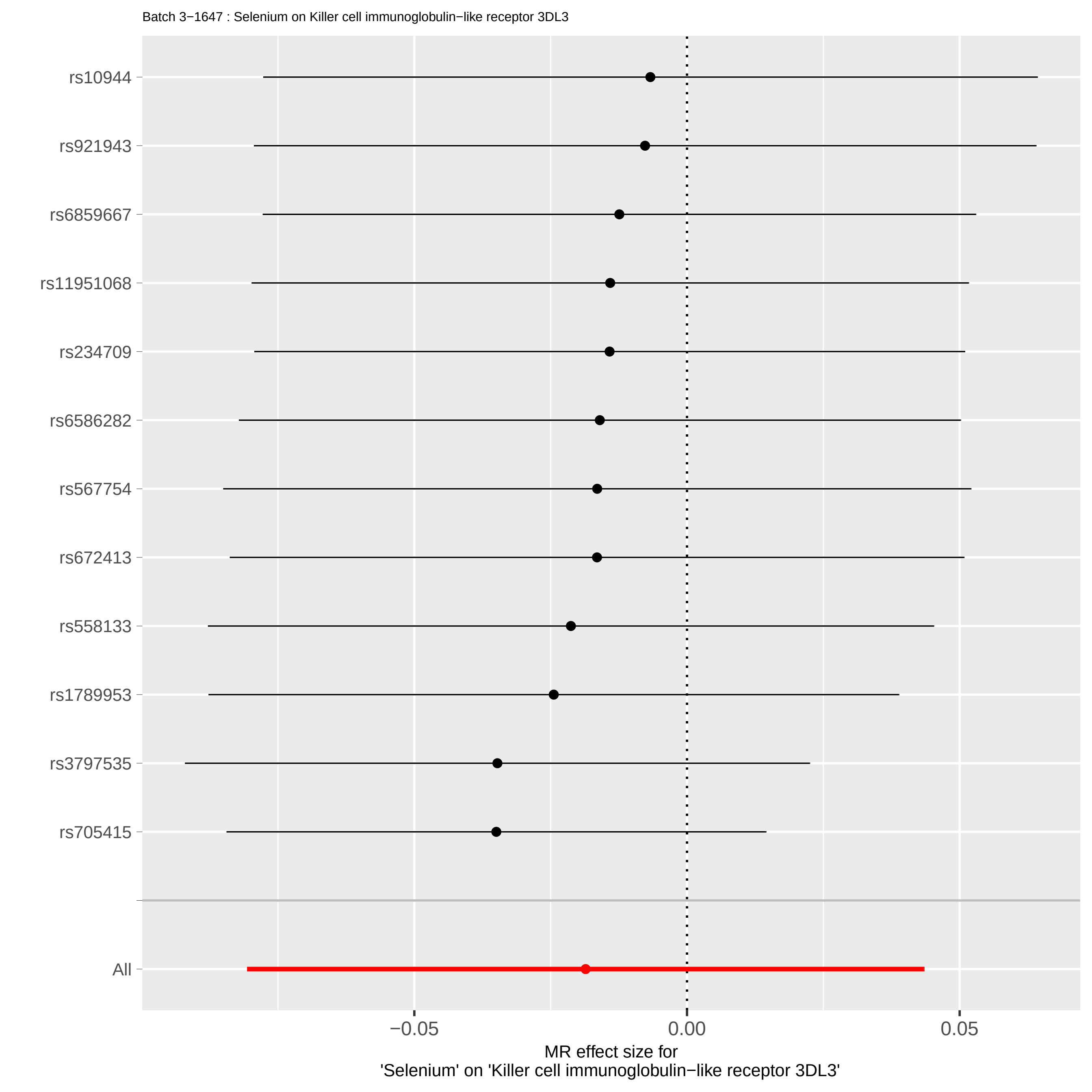
Batch 3−1647 : Selenium on Killer cell immunoglobulin−like receptor 3DL3
rs10944
rs921943
rs6859667
rs11951068
rs234709
rs6586282
rs567754
rs672413
rs558133
rs1789953
rs3797535
rs705415
All
−0.05
0.00
0.05
MR effect size for
'Selenium' on 'Killer cell immunoglobulin−like receptor 3DL3'

Batch 3−1648 : Selenium on Killer cell immunoglobulin−like receptor 3DS1

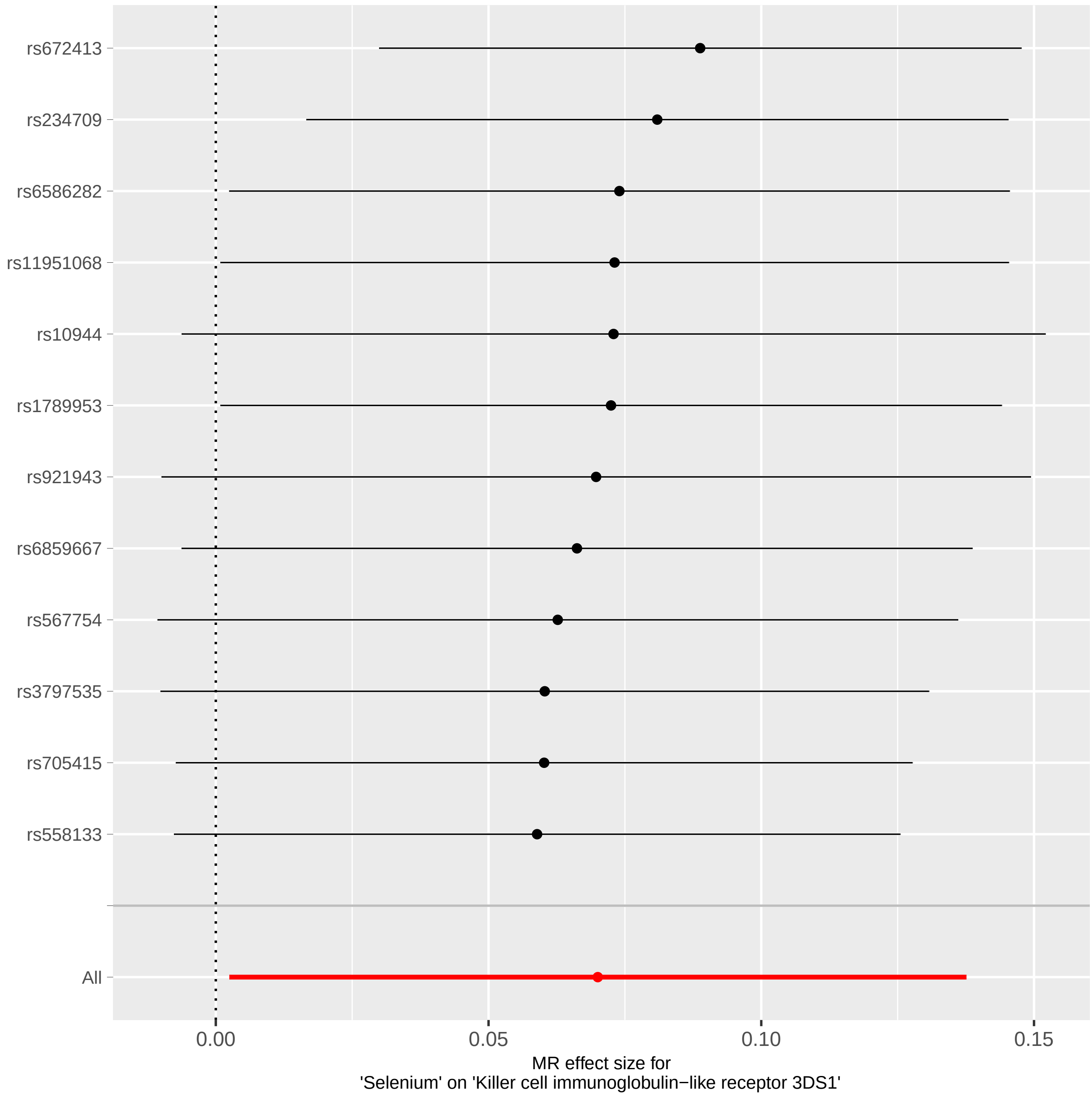

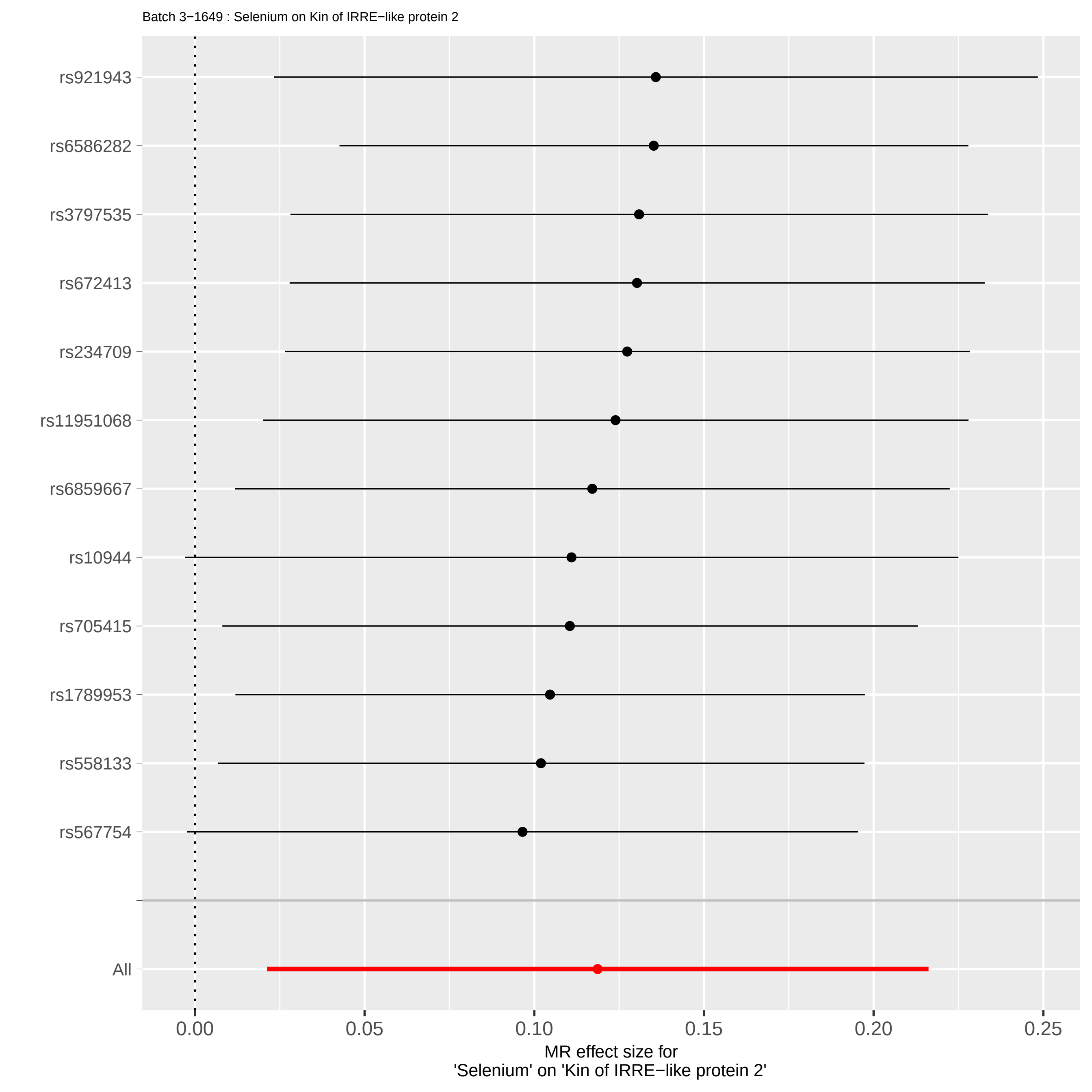

Batch 3−1649 : Selenium on Kin of IRRE−like protein 2
rs921943
rs6586282
rs3797535
rs672413
rs234709
rs11951068
rs6859667
rs10944
rs705415
rs1789953
rs558133
rs567754
All
0.00
0.05
0.10
0.15
0.20
0.25
MR effect size for
'Selenium' on 'Kin of IRRE−like protein 2'

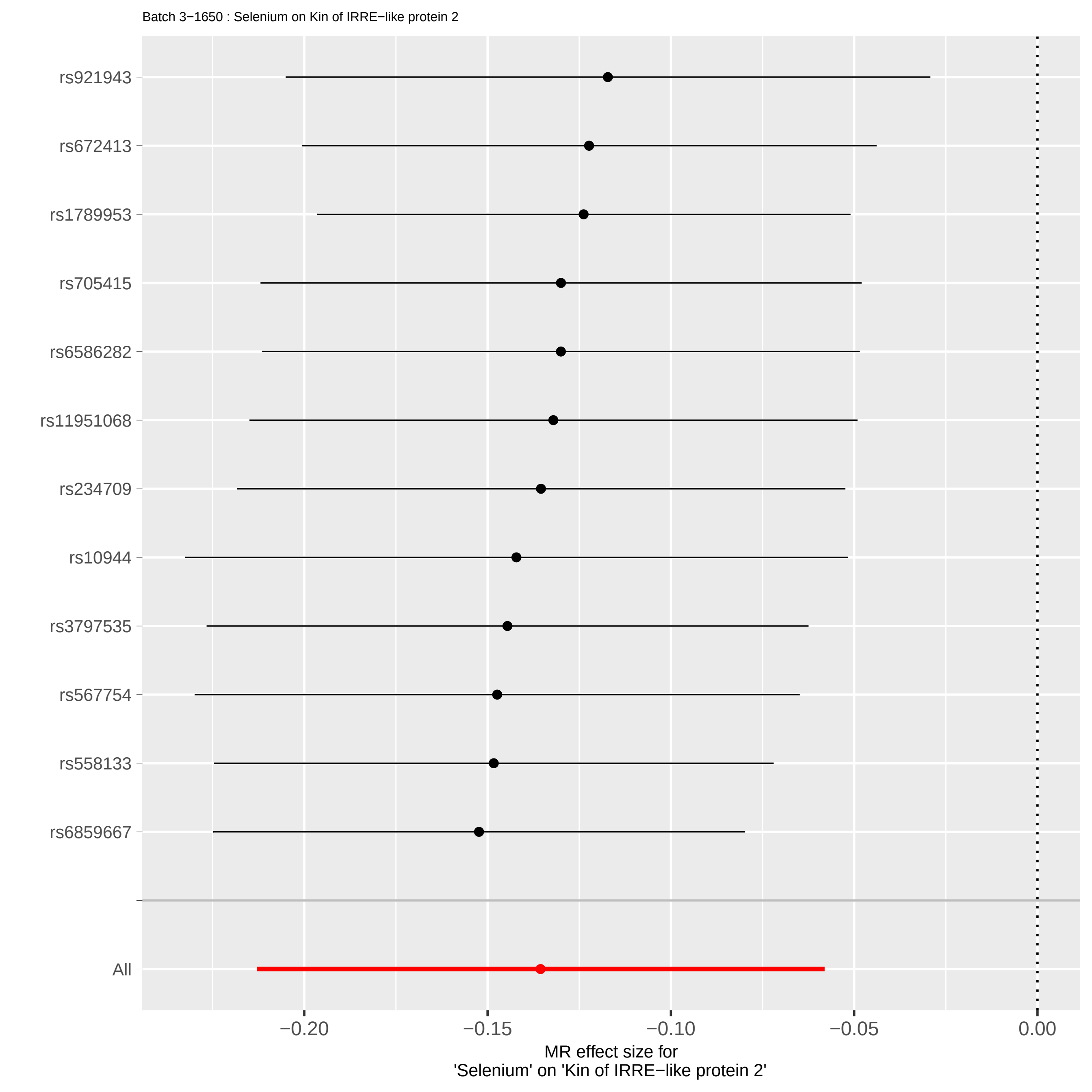

Batch 3−1650 : Selenium on Kin of IRRE−like protein 2
rs921943
rs672413
rs1789953
rs705415
rs6586282
rs11951068
rs234709
rs10944
rs3797535
rs567754
rs558133
rs6859667
All
−0.20
−0.15
−0.10
−0.05
0.00
MR effect size for
'Selenium' on 'Kin of IRRE−like protein 2'

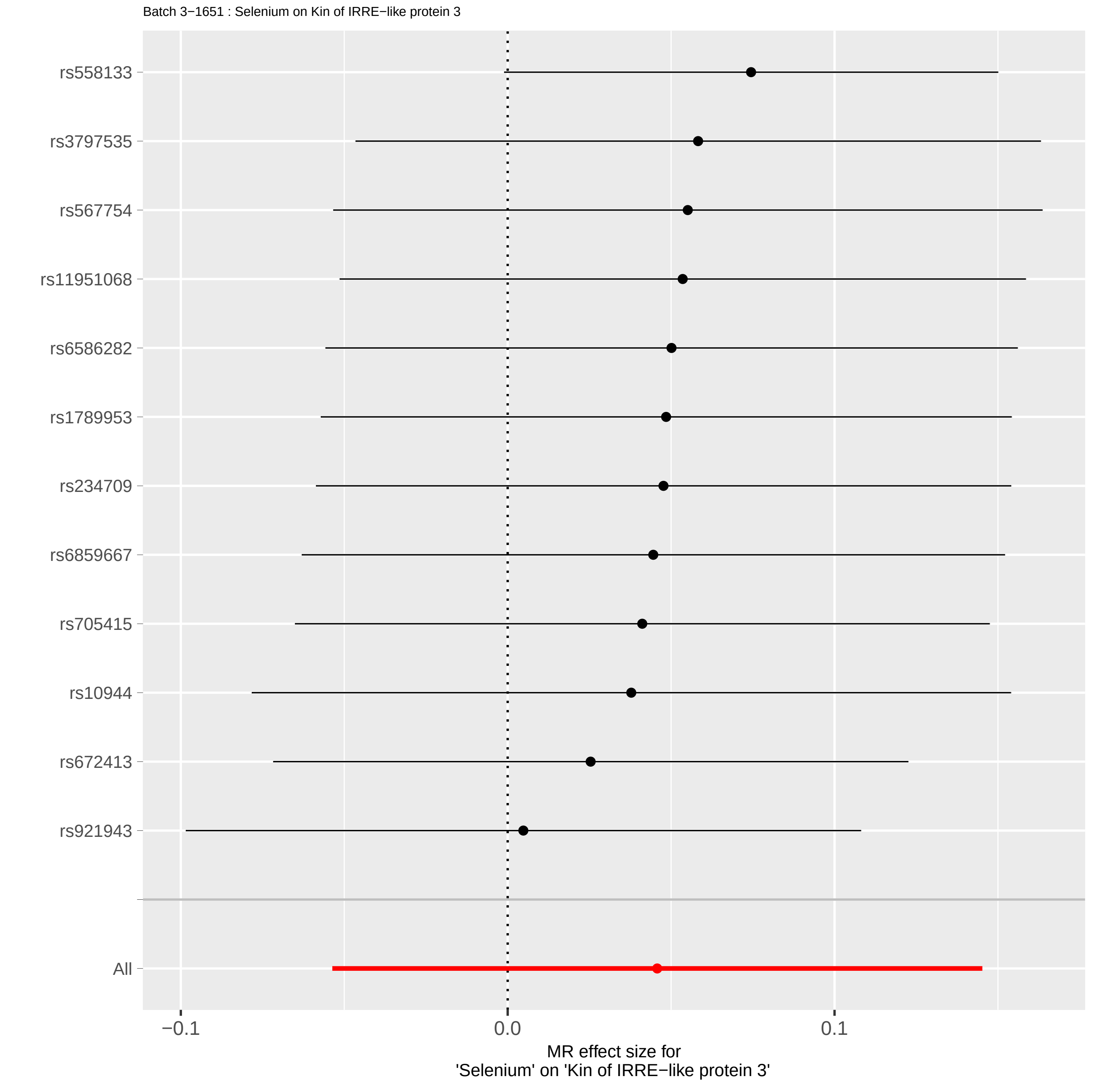

Batch 3−1651 : Selenium on Kin of IRRE−like protein 3
rs558133
rs3797535
rs567754
rs11951068
rs6586282
rs1789953
rs234709
rs6859667
rs705415
rs10944
rs672413
rs921943
All
−0.1
0.0
0.1
MR effect size for
'Selenium' on 'Kin of IRRE−like protein 3'

Batch 3−1652 : Selenium on Mast/stem cell growth factor receptor Kit

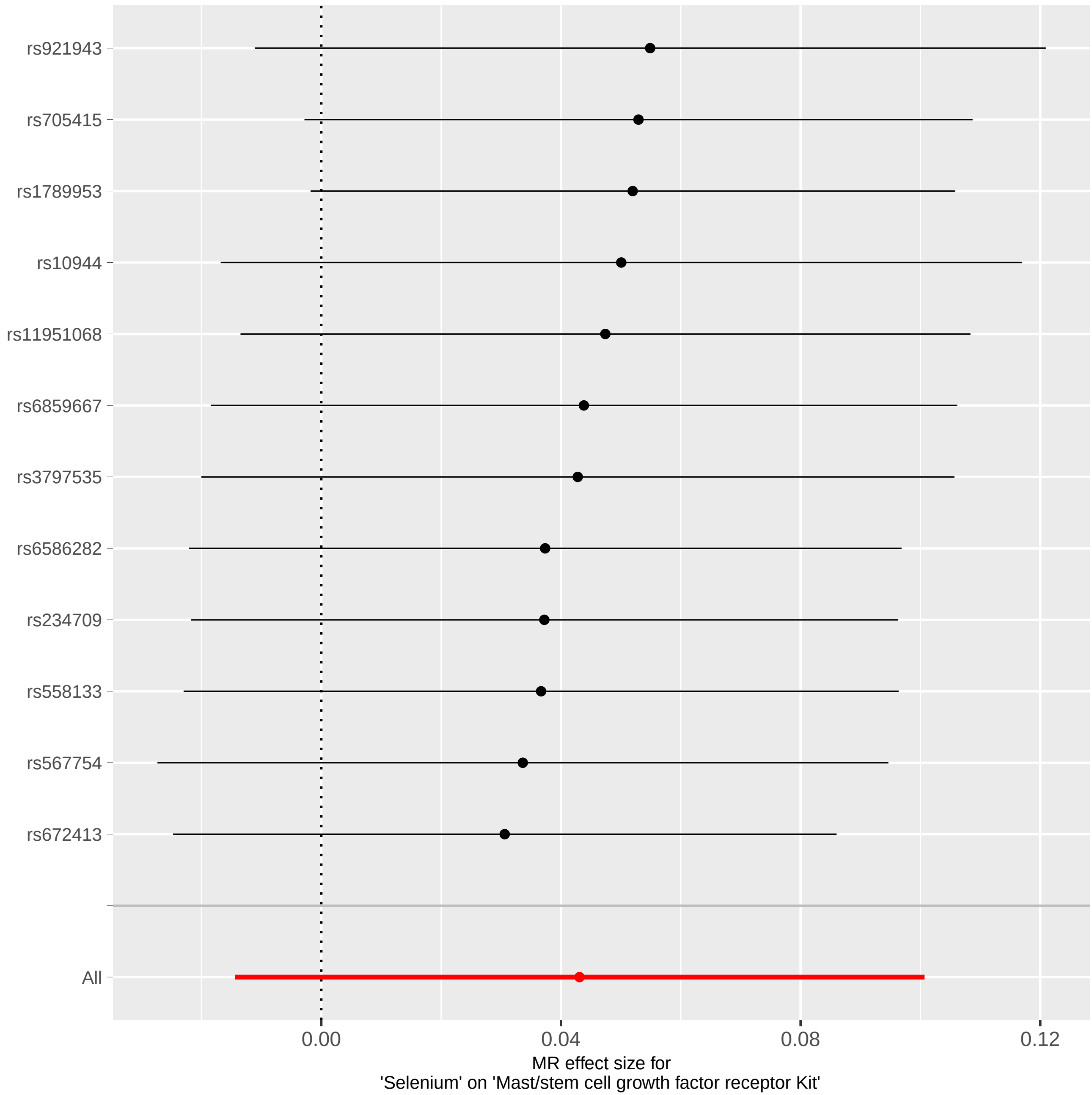

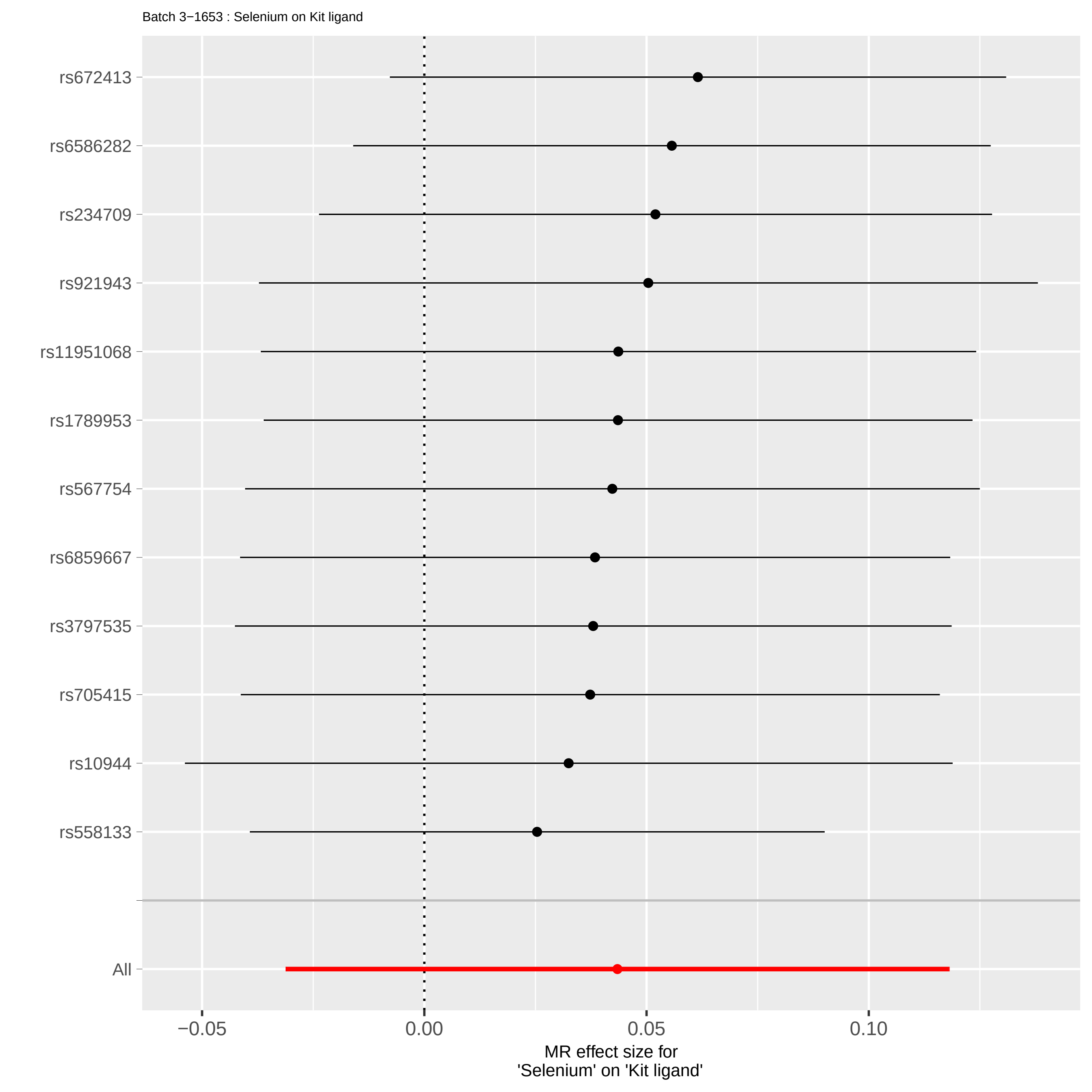
Batch 3−1653 : Selenium on Kit ligand
rs672413
rs6586282
rs234709
rs921943
rs11951068
rs1789953
rs567754
rs6859667
rs3797535
rs705415
rs10944
rs558133
All
−0.05
0.00
0.05
0.10
MR effect size for
'Selenium' on 'Kit ligand'

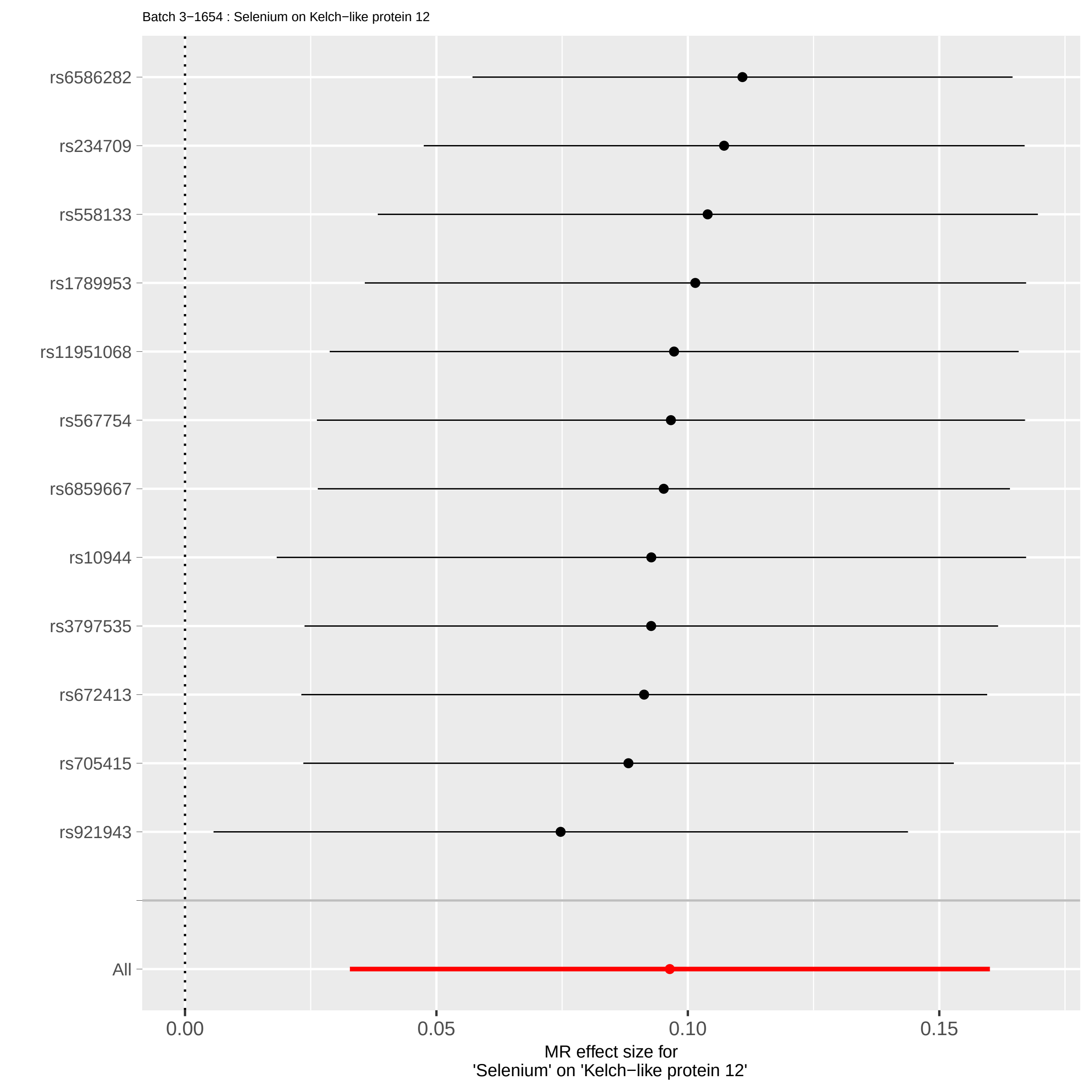

Batch 3−1654 : Selenium on Kelch−like protein 12
rs6586282
rs234709
rs558133
rs1789953
rs11951068
rs567754
rs6859667
rs10944
rs3797535
rs672413
rs705415
rs921943
All
0.00
0.05
0.10
0.15
MR effect size for
'Selenium' on 'Kelch−like protein 12'

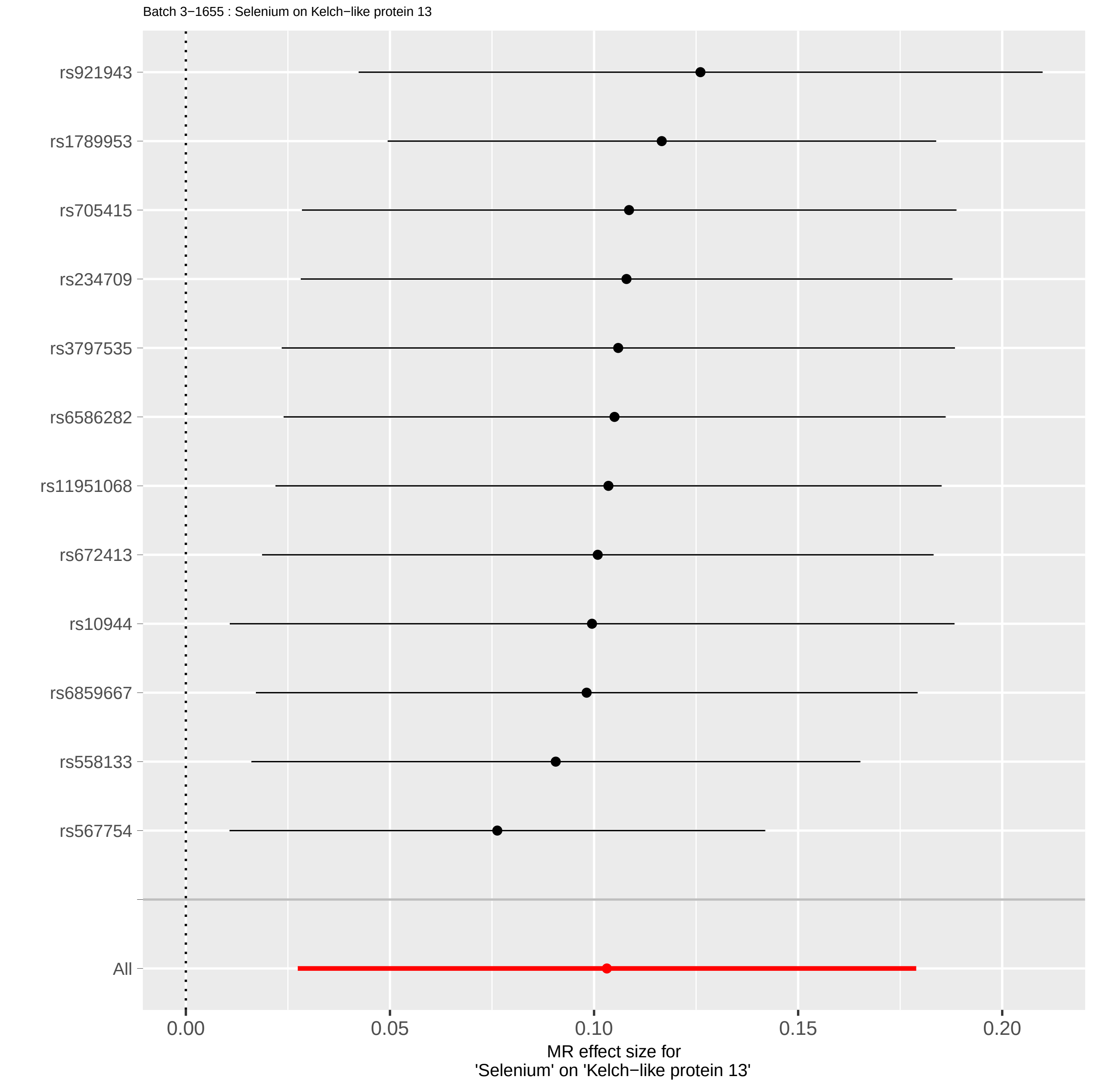
Batch 3−1655 : Selenium on Kelch−like protein 13
rs921943
rs1789953
rs705415
rs234709
rs3797535
rs6586282
rs11951068
rs672413
rs10944
rs6859667
rs558133
rs567754
All
0.00
0.05
0.10
0.15
0.20
MR effect size for
'Selenium' on 'Kelch−like protein 13'

Batch 3−1656 : Selenium on Kallikrein−11

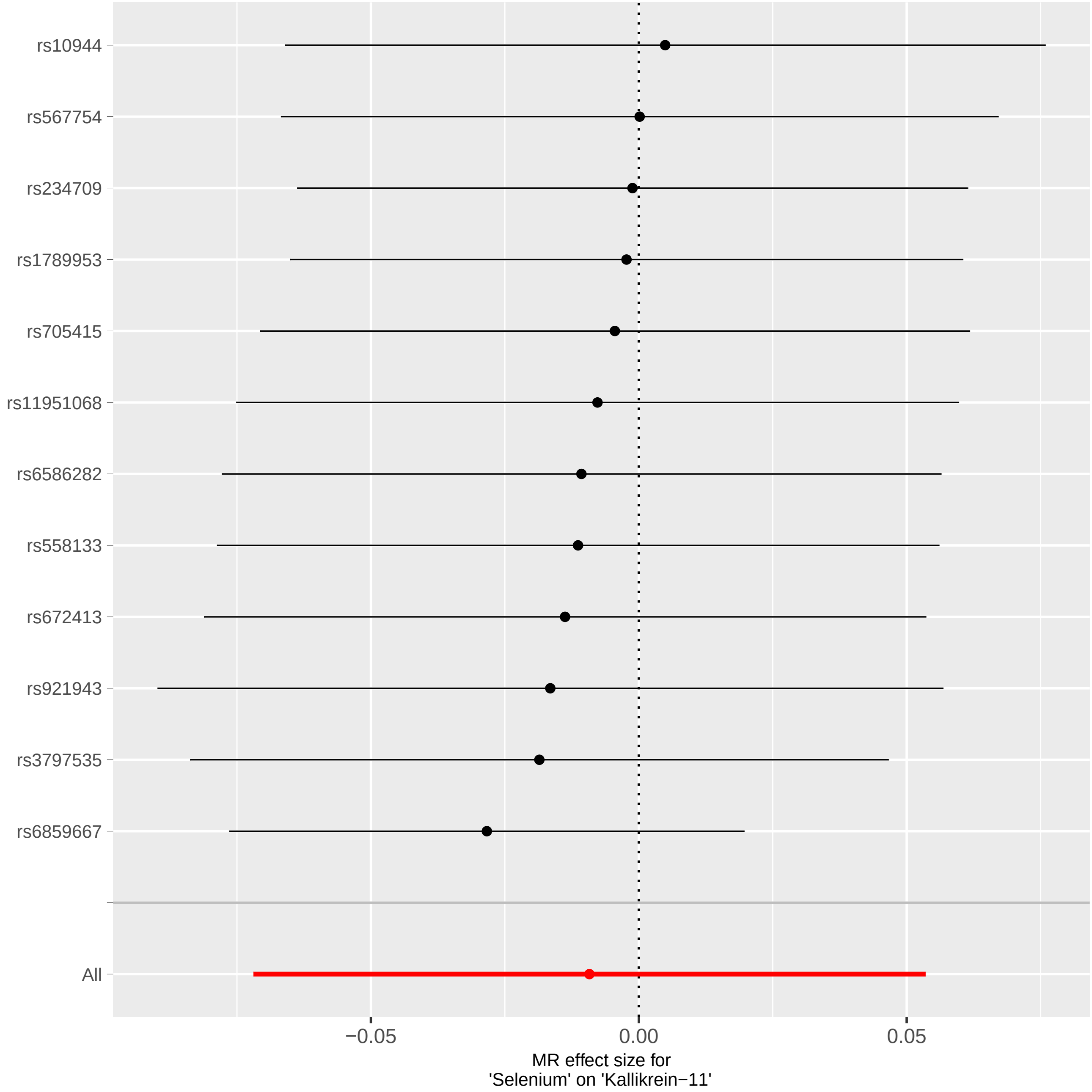

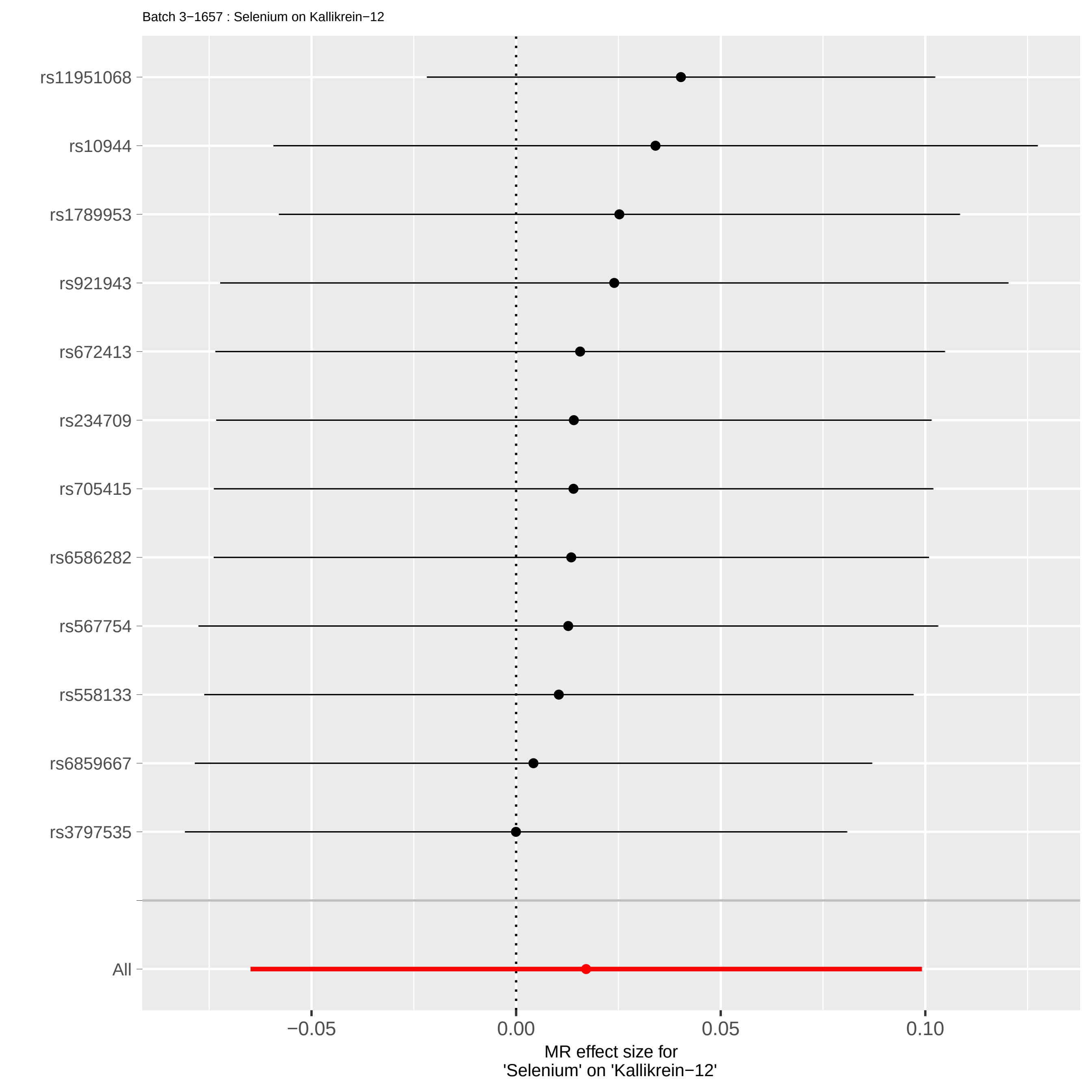

Batch 3−1657 : Selenium on Kallikrein−12
rs11951068
rs10944
rs1789953
rs921943
rs672413
rs234709
rs705415
rs6586282
rs567754
rs558133
rs6859667
rs3797535
All
−0.05
0.00
0.05
0.10
MR effect size for
'Selenium' on 'Kallikrein−12'

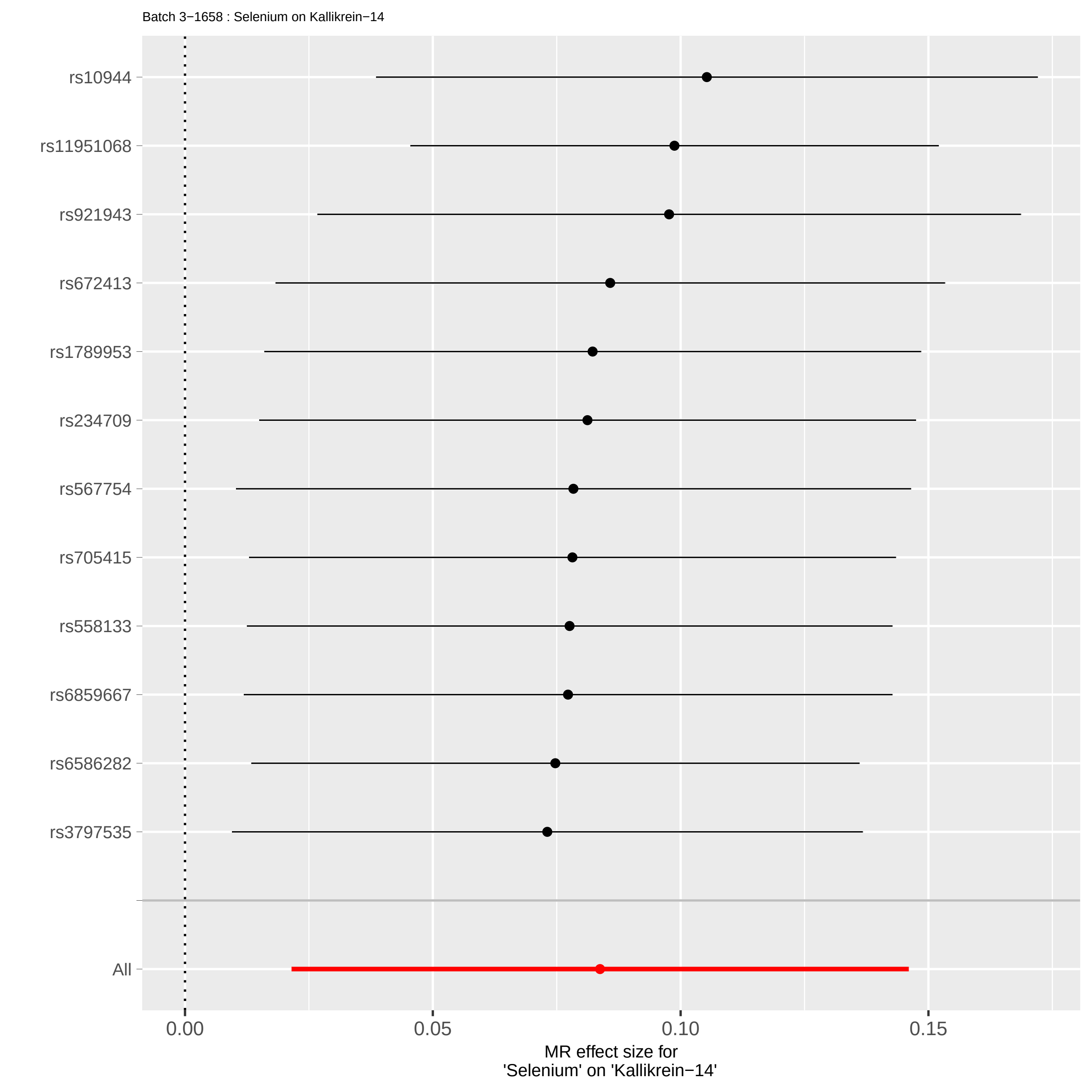

Batch 3−1658 : Selenium on Kallikrein−14
rs10944
rs11951068
rs921943
rs672413
rs1789953
rs234709
rs567754
rs705415
rs558133
rs6859667
rs6586282
rs3797535
All
0.00
0.05
0.10
0.15
MR effect size for
'Selenium' on 'Kallikrein−14'

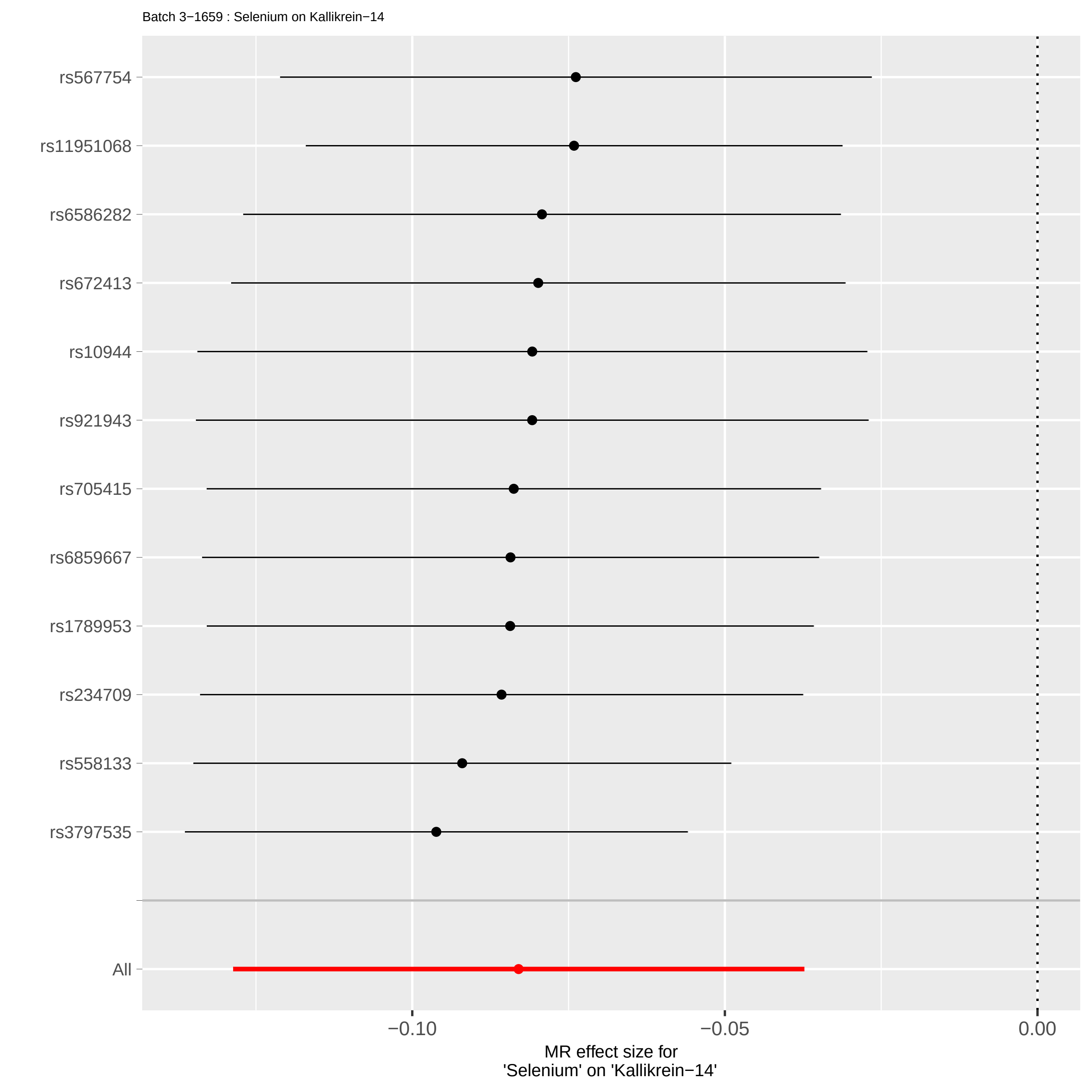

Batch 3−1659 : Selenium on Kallikrein−14
rs567754
rs11951068
rs6586282
rs672413
rs10944
rs921943
rs705415
rs6859667
rs1789953
rs234709
rs558133
rs3797535
All
−0.10
−0.05
0.00
MR effect size for
'Selenium' on 'Kallikrein−14'

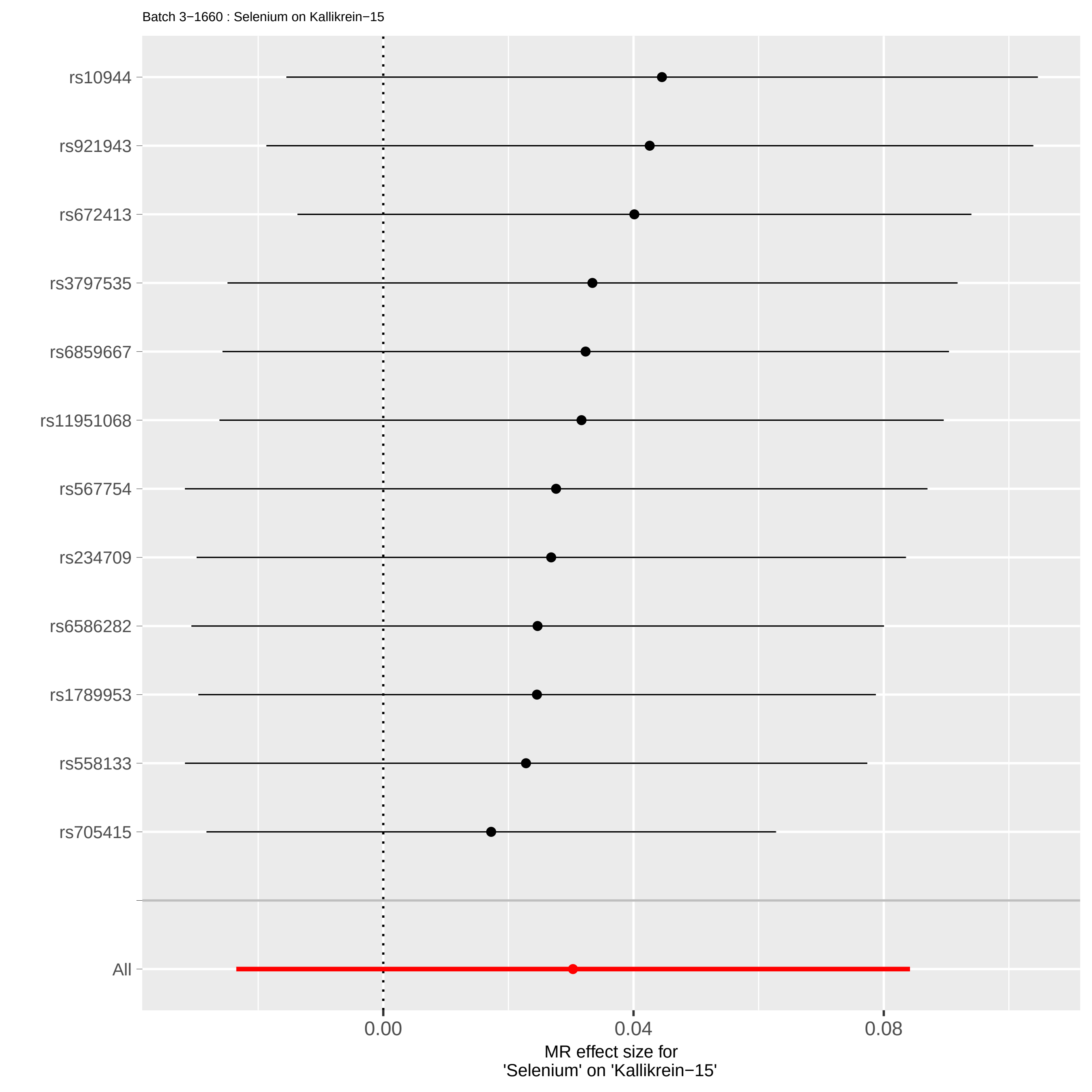

Batch 3−1660 : Selenium on Kallikrein−15
rs10944
rs921943
rs672413
rs3797535
rs6859667
rs11951068
rs567754
rs234709
rs6586282
rs1789953
rs558133
rs705415
All
0.00
0.04
0.08
MR effect size for
'Selenium' on 'Kallikrein−15'

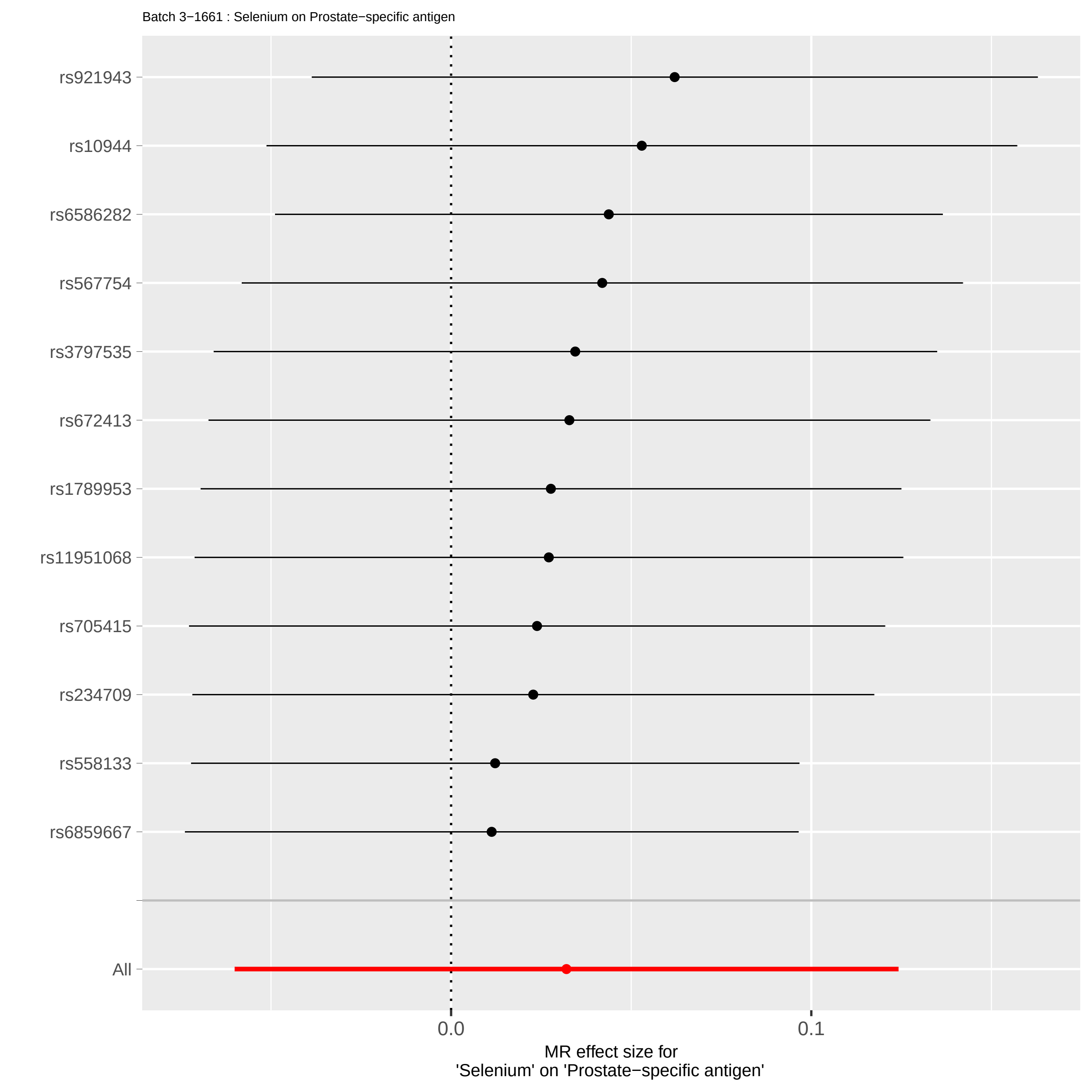
Batch 3−1661 : Selenium on Prostate−specific antigen
rs921943
rs10944
rs6586282
rs567754
rs3797535
rs672413
rs1789953
rs11951068
rs705415
rs234709
rs558133
rs6859667
All
0.0
0.1
MR effect size for
'Selenium' on 'Prostate−specific antigen'

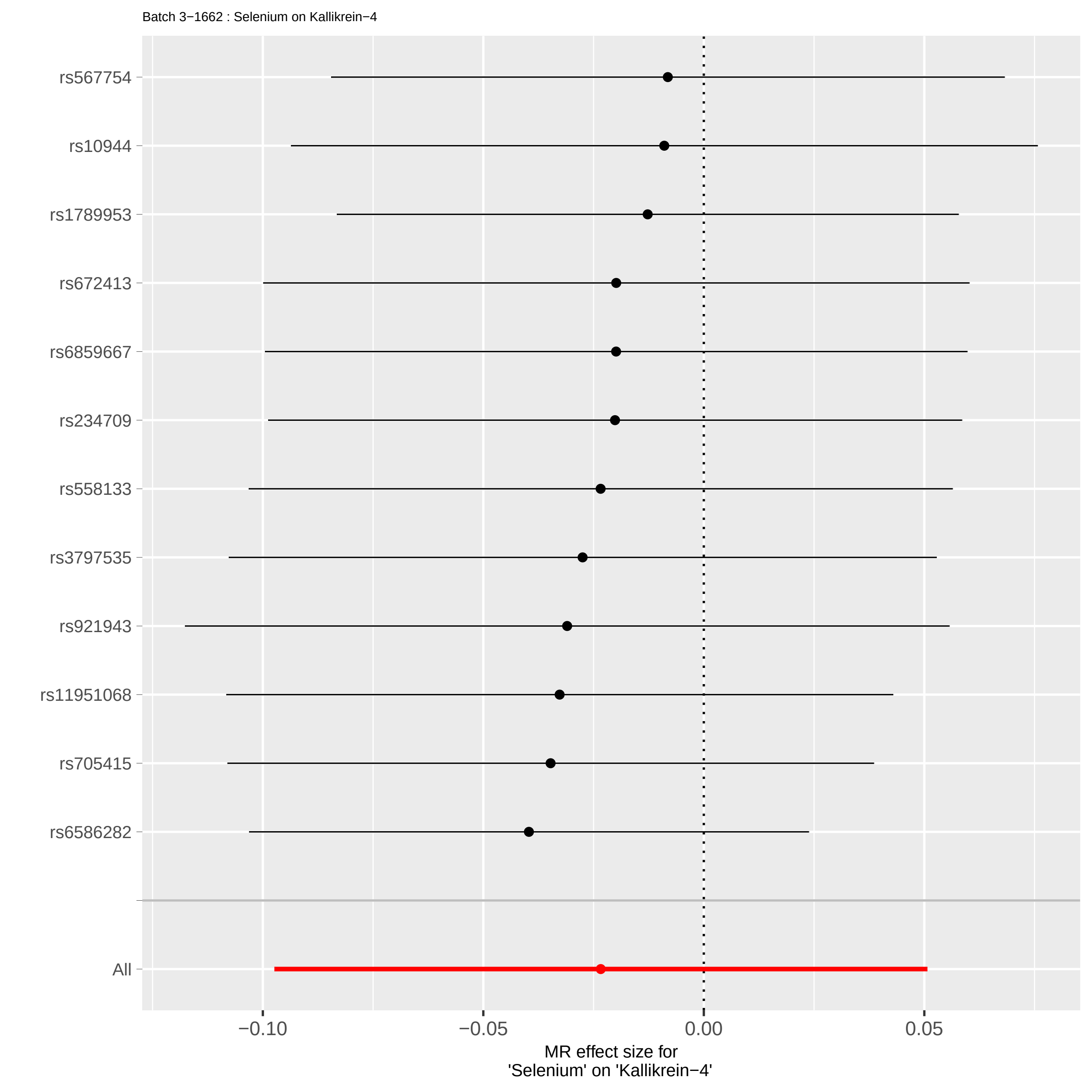
Batch 3−1662 : Selenium on Kallikrein−4
rs567754
rs10944
rs1789953
rs672413
rs6859667
rs234709
rs558133
rs3797535
rs921943
rs11951068
rs705415
rs6586282
All
−0.10
−0.05
0.00
0.05
MR effect size for
'Selenium' on 'Kallikrein−4'

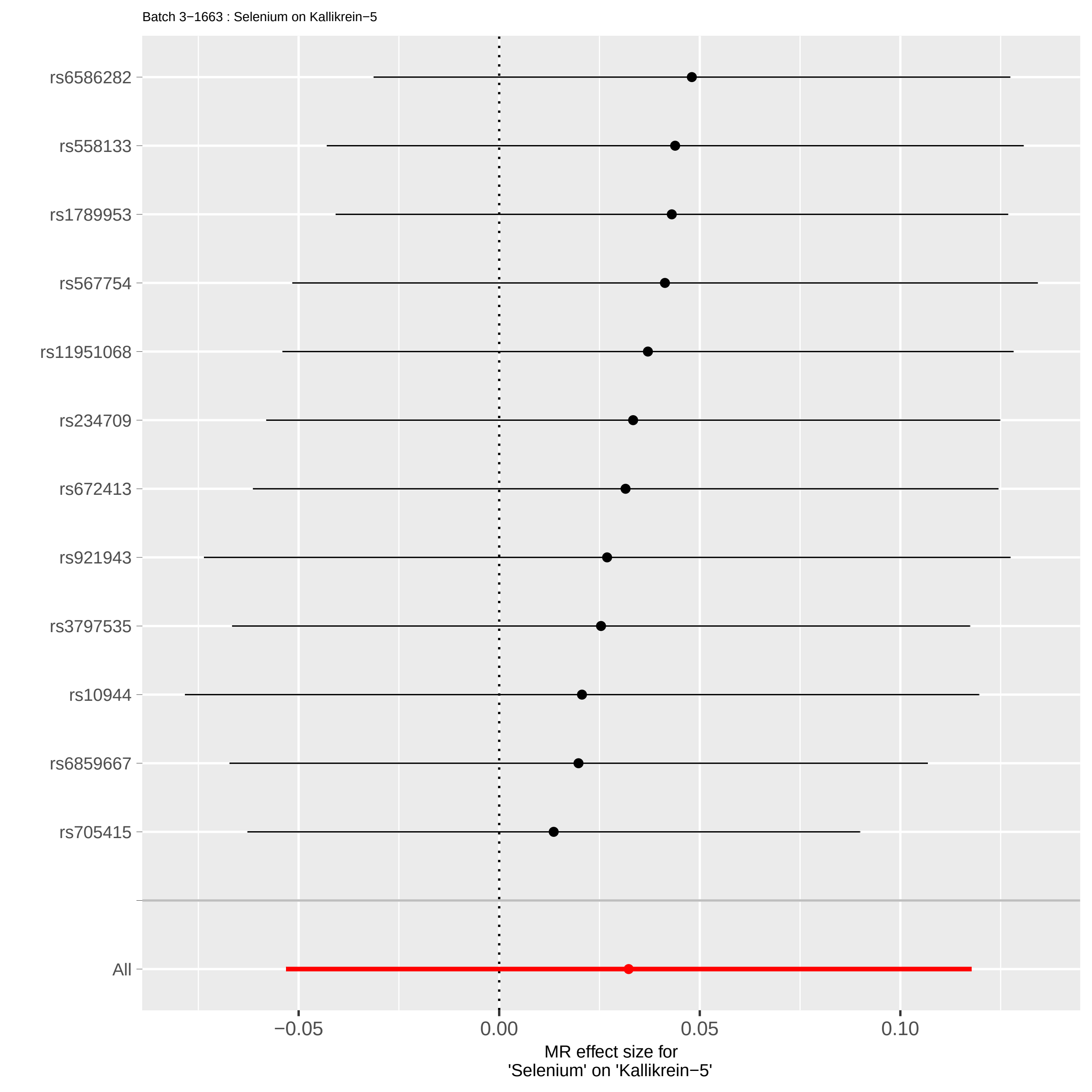
Batch 3−1663 : Selenium on Kallikrein−5
rs6586282
rs558133
rs1789953
rs567754
rs11951068
rs234709
rs672413
rs921943
rs3797535
rs10944
rs6859667
rs705415
All
−0.05
0.00
0.05
0.10
MR effect size for
'Selenium' on 'Kallikrein−5'

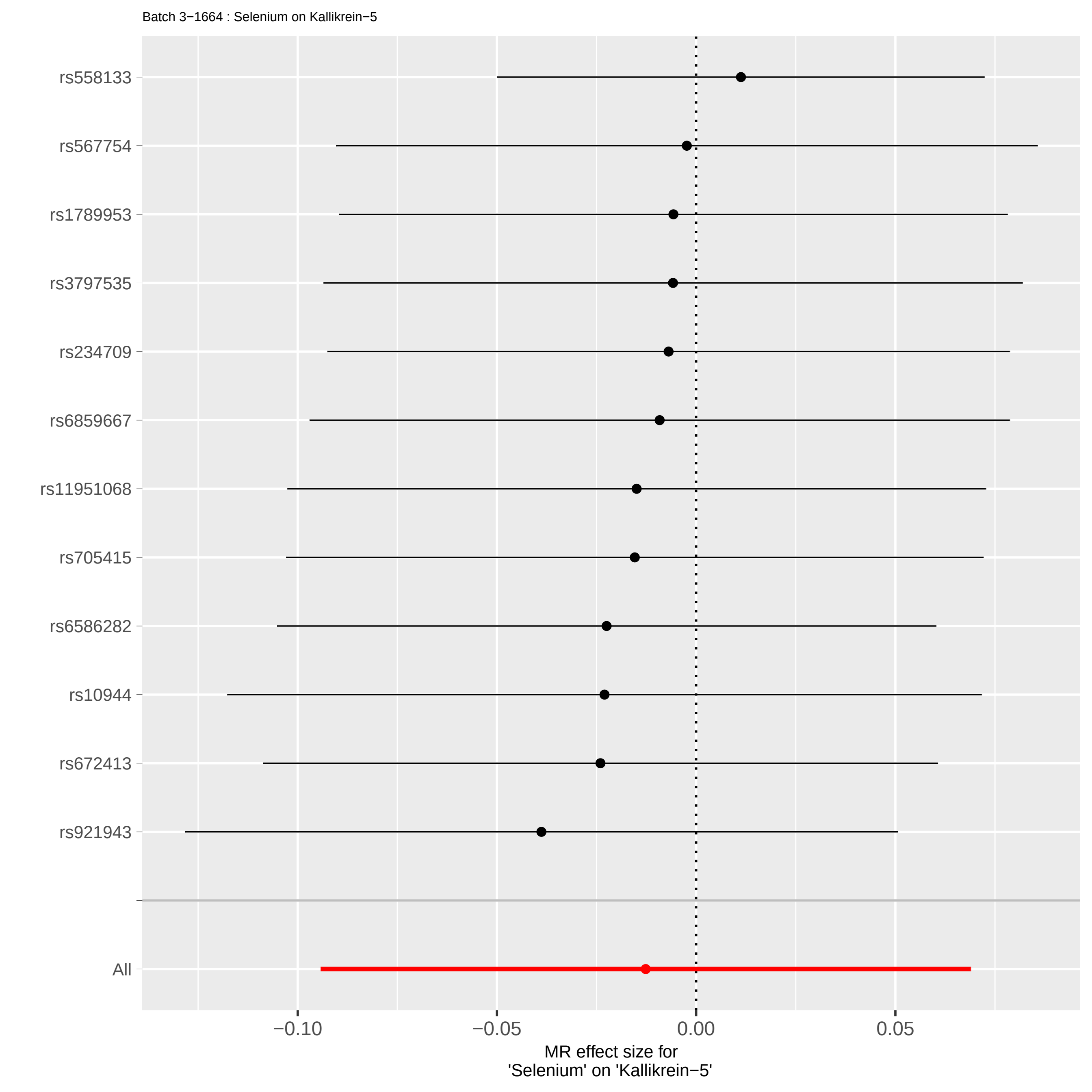

Batch 3−1664 : Selenium on Kallikrein−5
rs558133
rs567754
rs1789953
rs3797535
rs234709
rs6859667
rs11951068
rs705415
rs6586282
rs10944
rs672413
rs921943
All
−0.10
−0.05
0.00
0.05
MR effect size for
'Selenium' on 'Kallikrein−5'

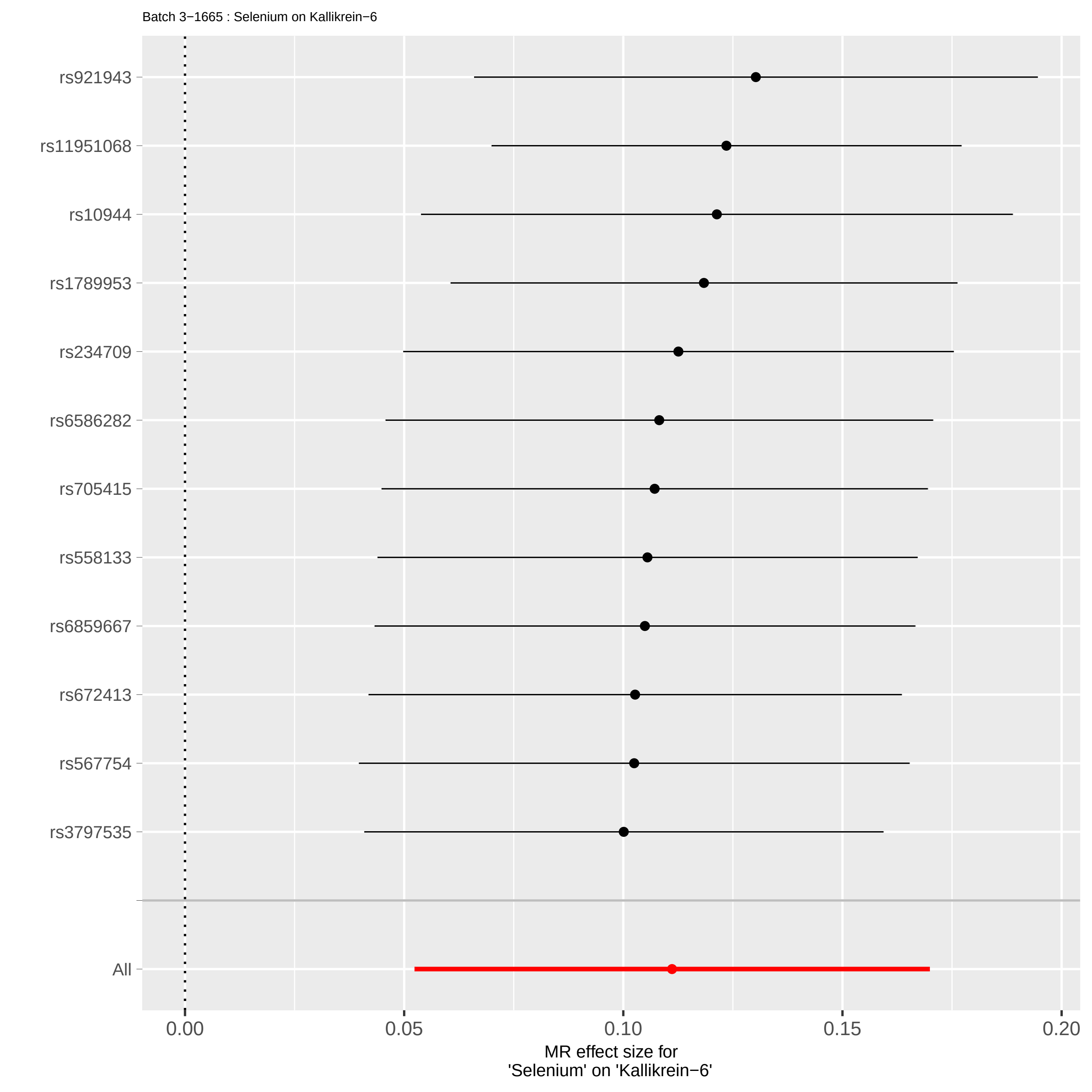

Batch 3−1665 : Selenium on Kallikrein−6
rs921943
rs11951068
rs10944
rs1789953
rs234709
rs6586282
rs705415
rs558133
rs6859667
rs672413
rs567754
rs3797535
All
0.00
0.05
0.10
0.15
0.20
MR effect size for
'Selenium' on 'Kallikrein−6'

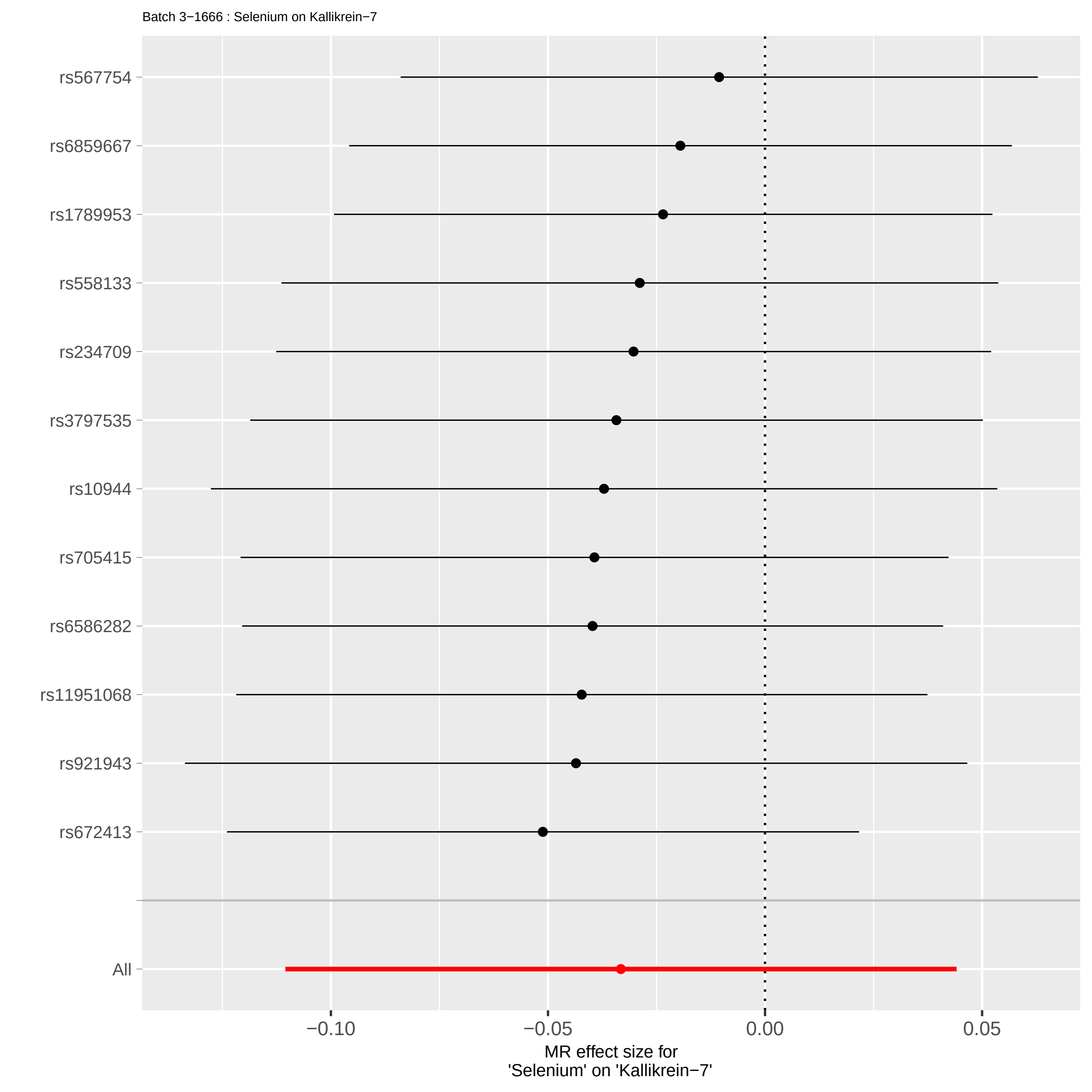
Batch 3−1666 : Selenium on Kallikrein−7
rs567754
rs6859667
rs1789953
rs558133
rs234709
rs3797535
rs10944
rs705415
rs6586282
rs11951068
rs921943
rs672413
All
−0.10
−0.05
0.00
0.05
MR effect size for
'Selenium' on 'Kallikrein−7'

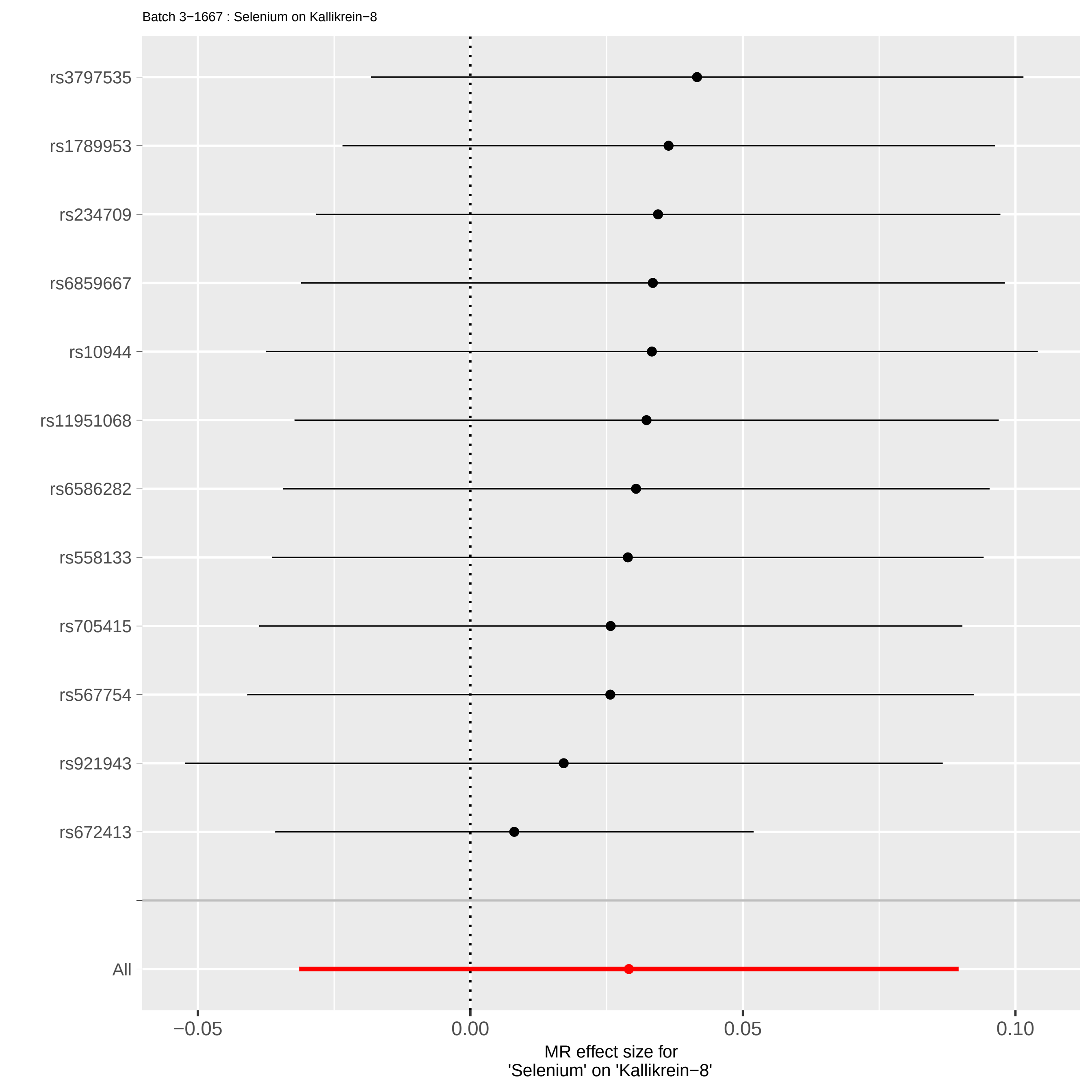

Batch 3−1667 : Selenium on Kallikrein−8
rs3797535
rs1789953
rs234709
rs6859667
rs10944
rs11951068
rs6586282
rs558133
rs705415
rs567754
rs921943
rs672413
All
−0.05
0.00
0.05
0.10
MR effect size for
'Selenium' on 'Kallikrein−8'

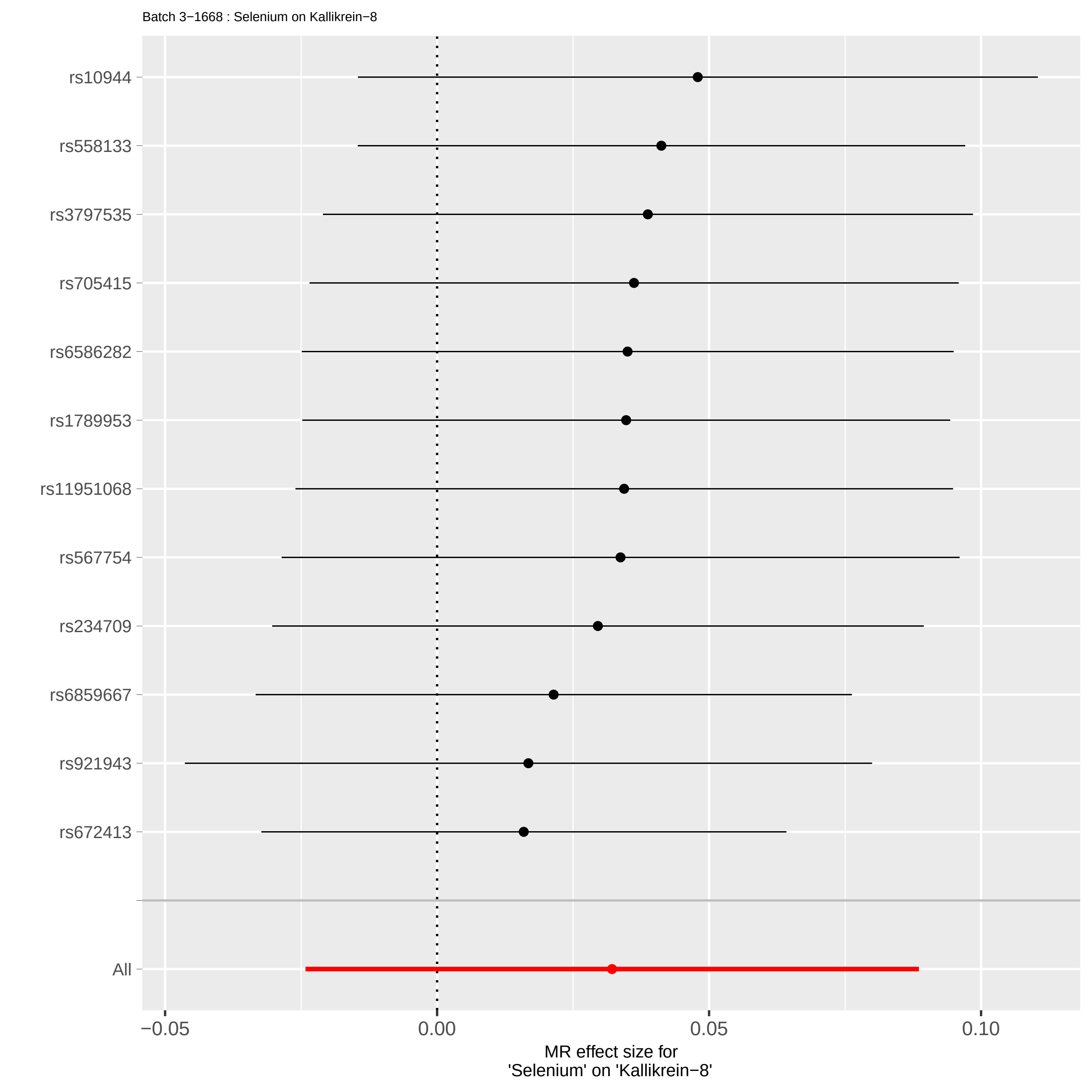

Batch 3−1668 : Selenium on Kallikrein−8
rs10944
rs558133
rs3797535
rs705415
rs6586282
rs1789953
rs11951068
rs567754
rs234709
rs6859667
rs921943
rs672413
All
−0.05
0.00
0.05
0.10
MR effect size for
'Selenium' on 'Kallikrein−8'

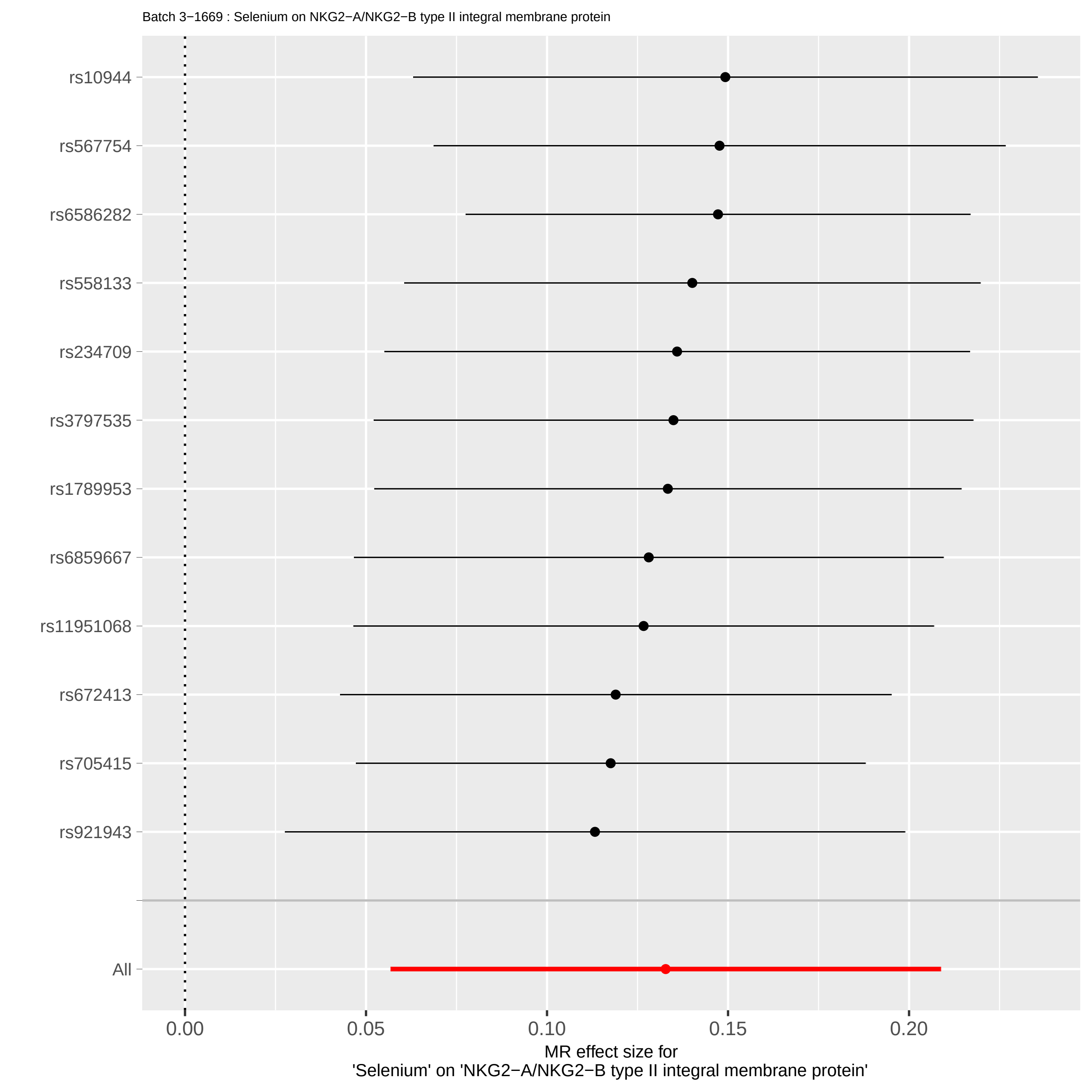

Batch 3−1669 : Selenium on NKG2−A/NKG2−B type II integral membrane protein
rs10944
rs567754
rs6586282
rs558133
rs234709
rs3797535
rs1789953
rs6859667
rs11951068
rs672413
rs705415
rs921943
All
0.00
0.05
0.10
0.15
0.20
MR effect size for
'Selenium' on 'NKG2−A/NKG2−B type II integral membrane protein'

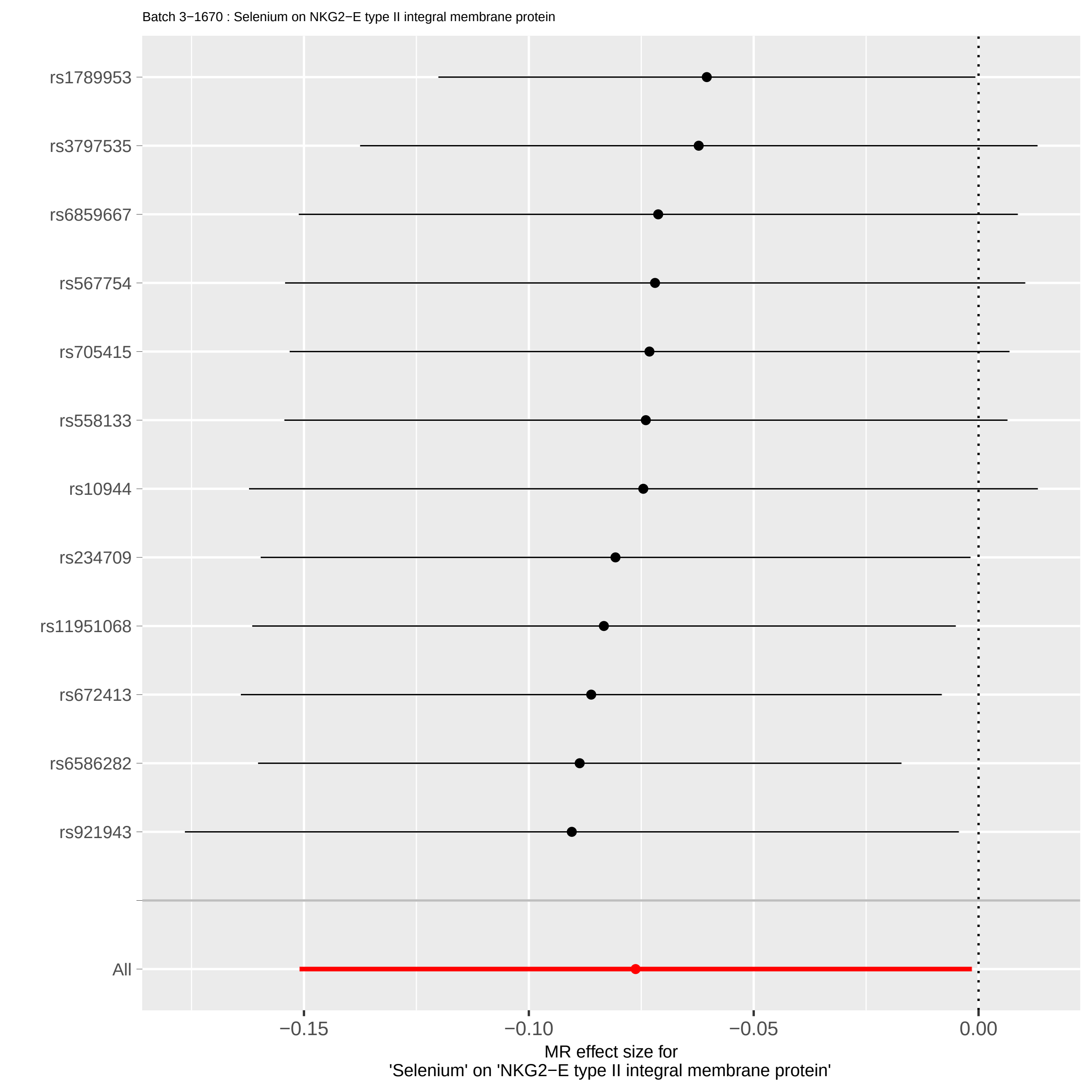

Batch 3−1670 : Selenium on NKG2−E type II integral membrane protein
rs1789953
rs3797535
rs6859667
rs567754
rs705415
rs558133
rs10944
rs234709
rs11951068
rs672413
rs6586282
rs921943
All
−0.15
−0.10
−0.05
0.00
MR effect size for
'Selenium' on 'NKG2−E type II integral membrane protein'

Batch 3−1671 : Selenium on NKG2−E type II integral membrane protein

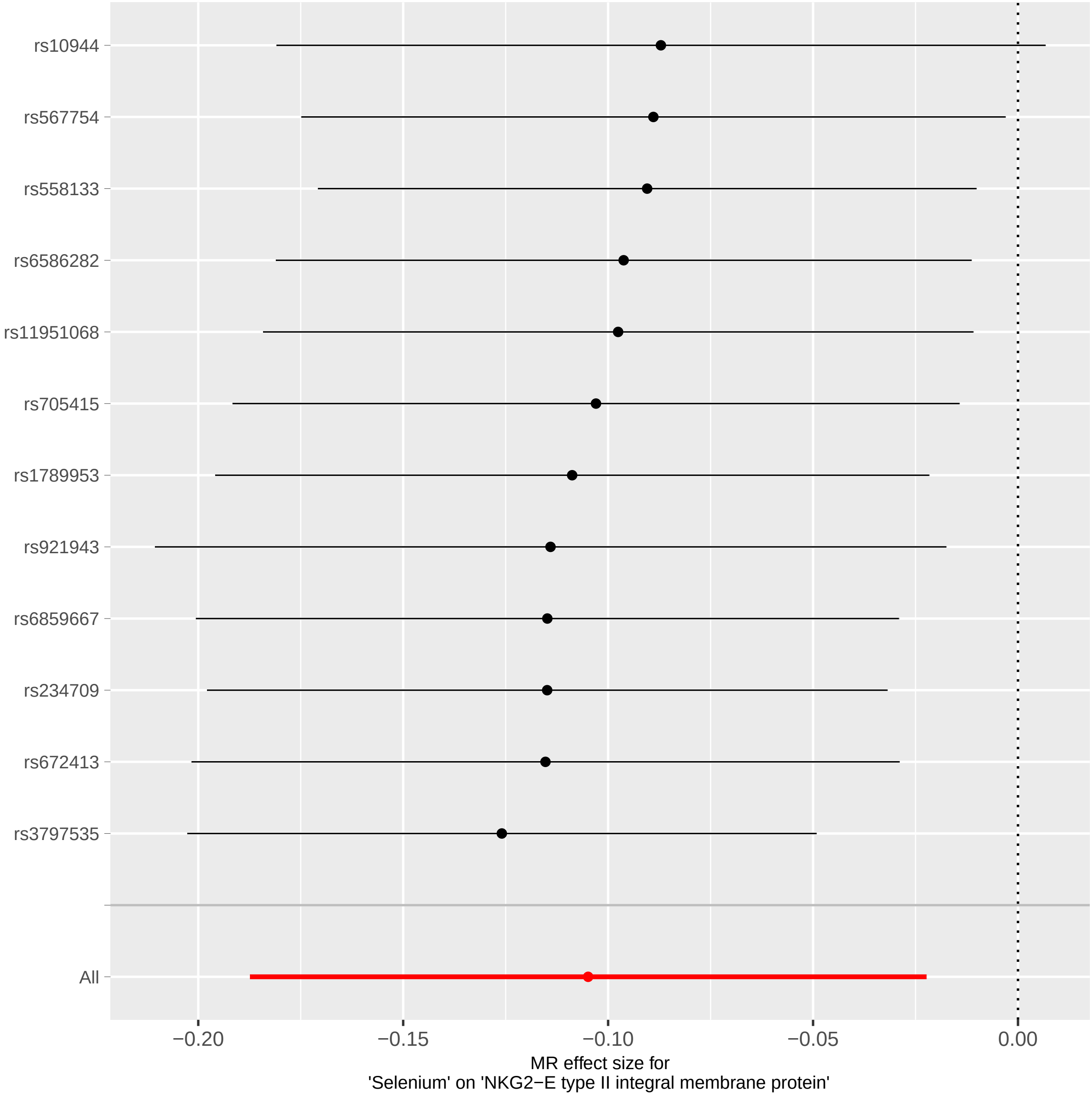

Batch 3−1672 : Selenium on Killer cell lectin−like receptor subfamily F member 1

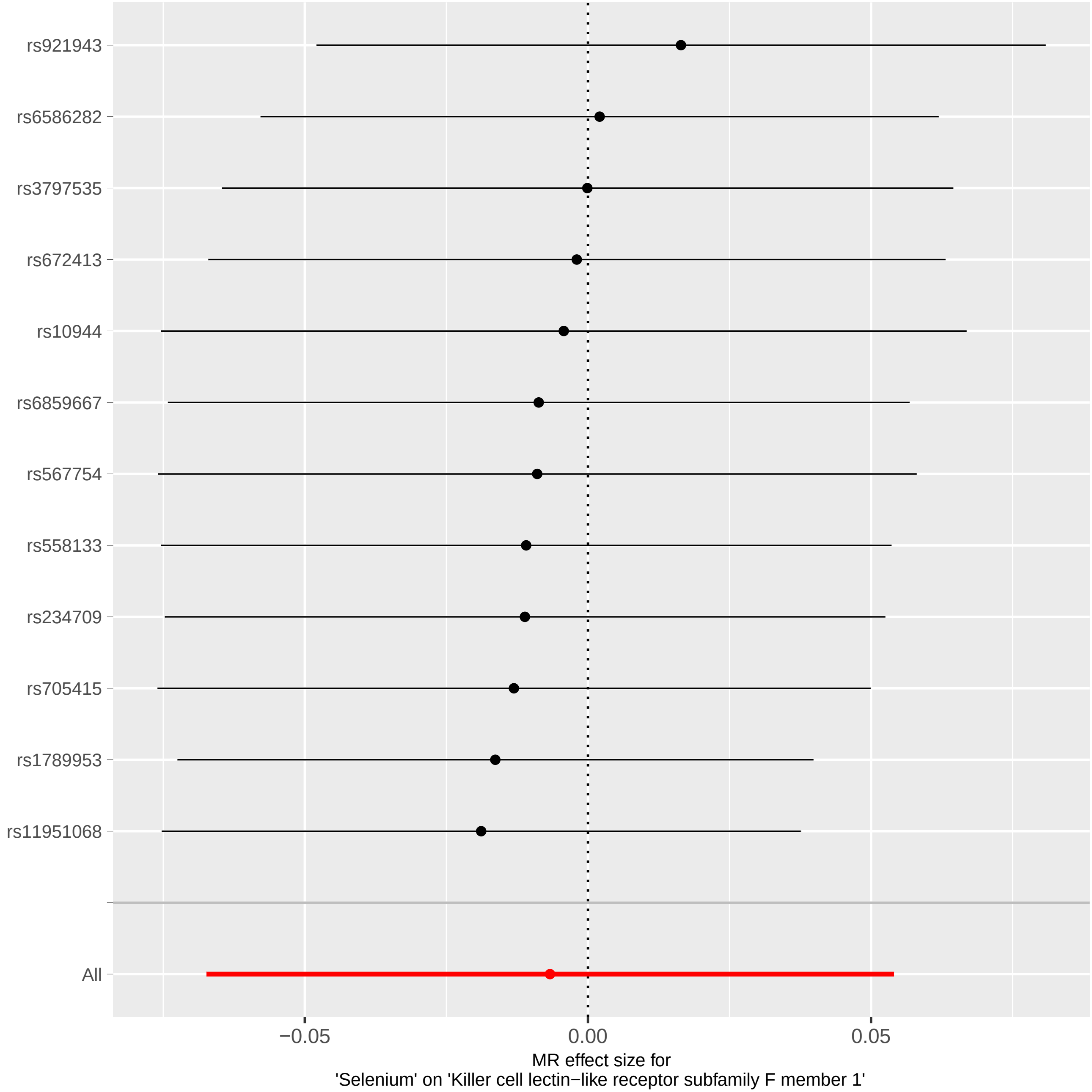

Batch 3−1673 : Selenium on Killer cell lectin−like receptor subfamily F member 1

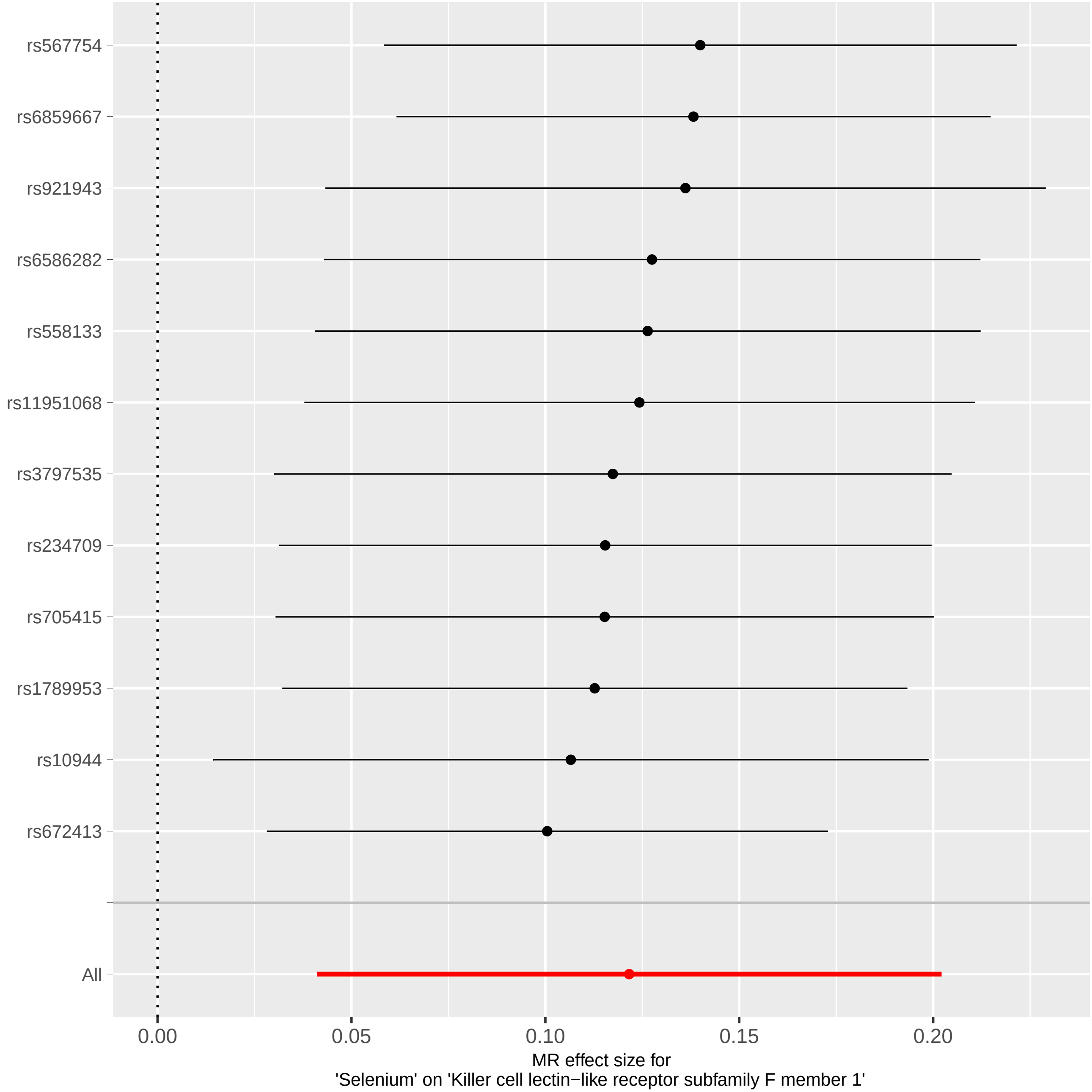

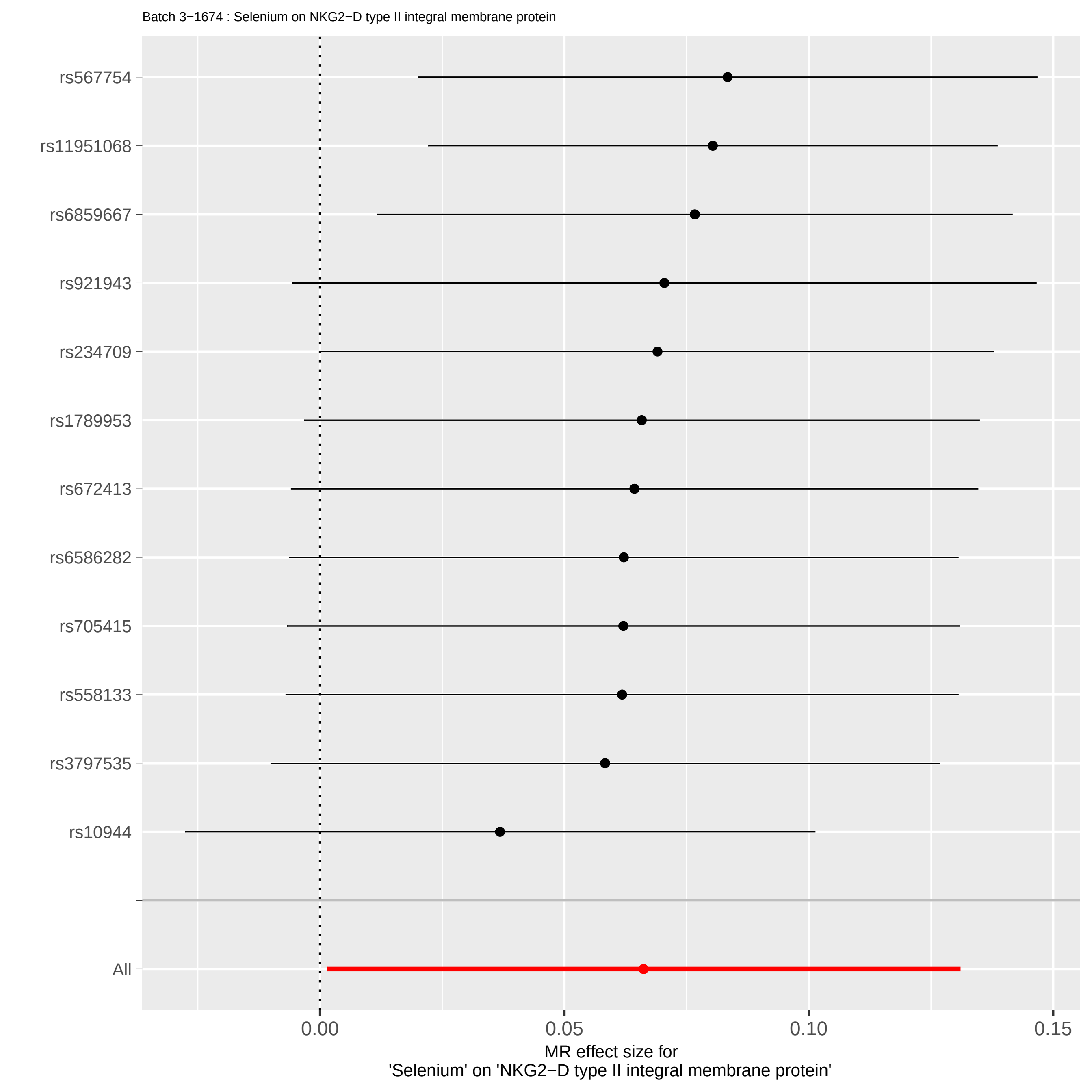

Batch 3−1674 : Selenium on NKG2−D type II integral membrane protein
rs567754
rs11951068
rs6859667
rs921943
rs234709
rs1789953
rs672413
rs6586282
rs705415
rs558133
rs3797535
rs10944
All
0.00
0.05
0.10
0.15
MR effect size for
'Selenium' on 'NKG2−D type II integral membrane protein'

Batch 3−1675 : Selenium on NKG2−D type II integral membrane protein

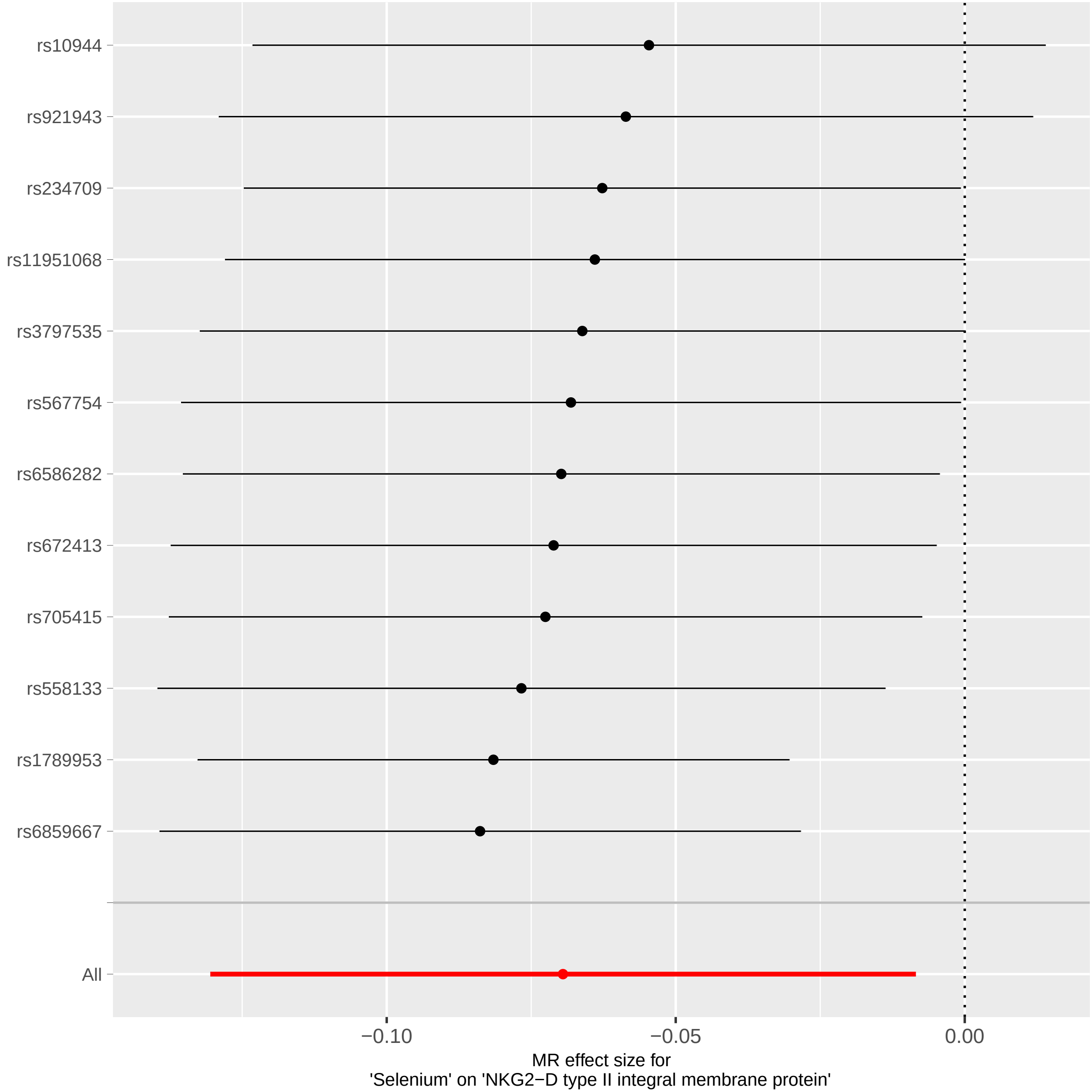

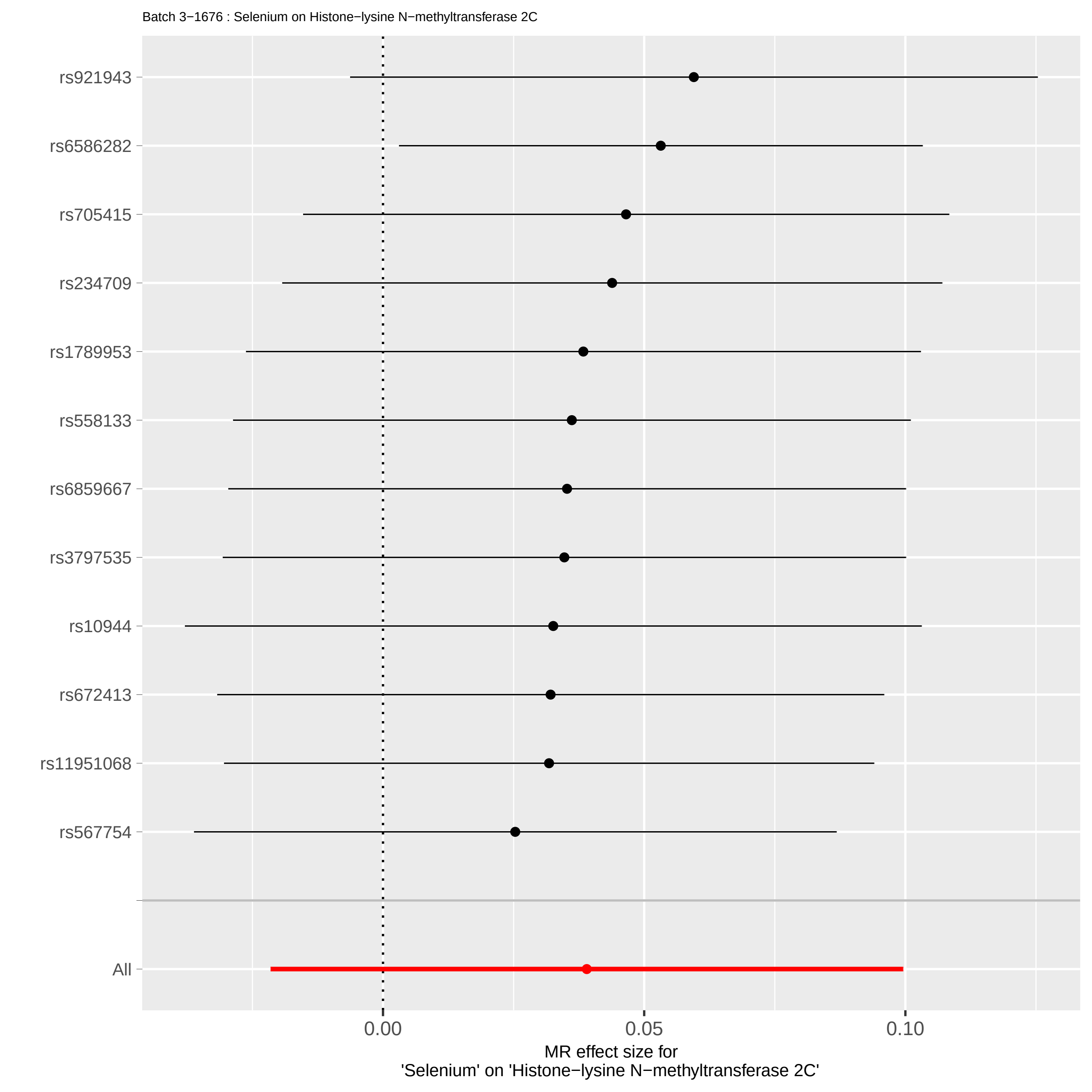
Batch 3−1676 : Selenium on Histone−lysine N−methyltransferase 2C
rs921943
rs6586282
rs705415
rs234709
rs1789953
rs558133
rs6859667
rs3797535
rs10944
rs672413
rs11951068
rs567754
All
0.00
0.05
0.10
MR effect size for
'Selenium' on 'Histone−lysine N−methyltransferase 2C'

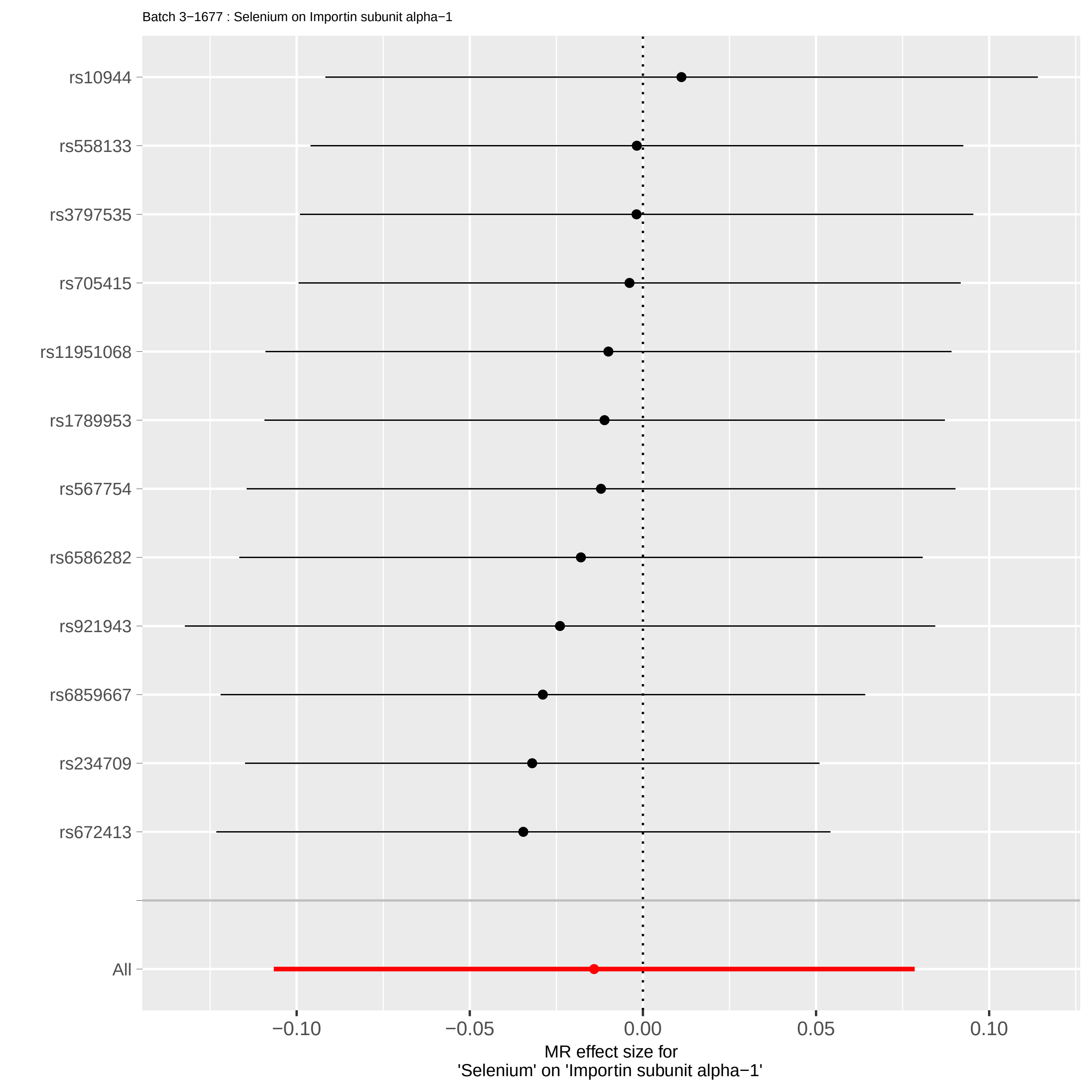

Batch 3−1677 : Selenium on Importin subunit alpha−1
rs10944
rs558133
rs3797535
rs705415
rs11951068
rs1789953
rs567754
rs6586282
rs921943
rs6859667
rs234709
rs672413
All
−0.10
−0.05
0.00
0.05
0.10
MR effect size for
'Selenium' on 'Importin subunit alpha−1'

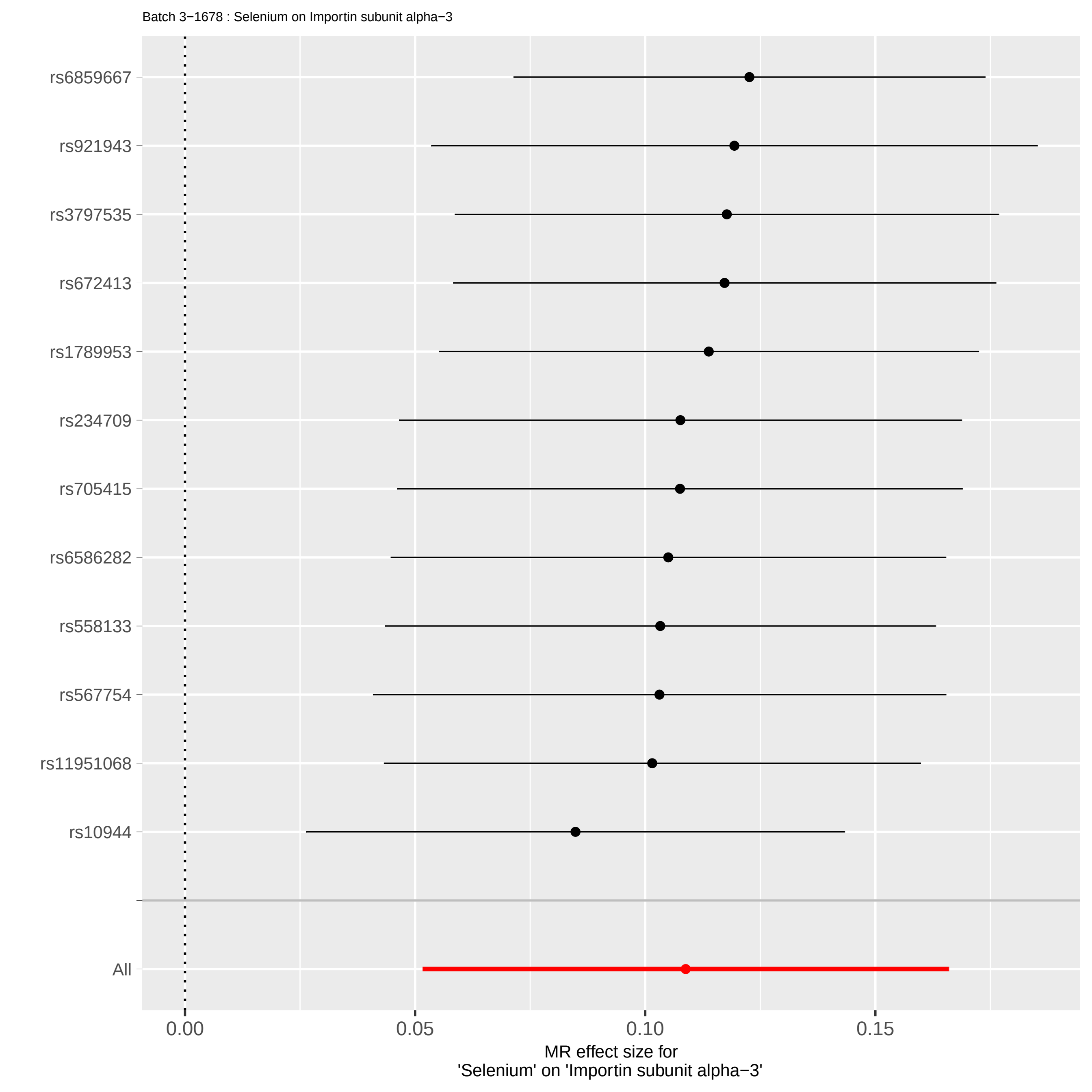

Batch 3−1678 : Selenium on Importin subunit alpha−3
rs6859667
rs921943
rs3797535
rs672413
rs1789953
rs234709
rs705415
rs6586282
rs558133
rs567754
rs11951068
rs10944
All
0.00
0.05
0.10
0.15
MR effect size for
'Selenium' on 'Importin subunit alpha−3'

Batch 3−1679 : Selenium on GTPase KRas

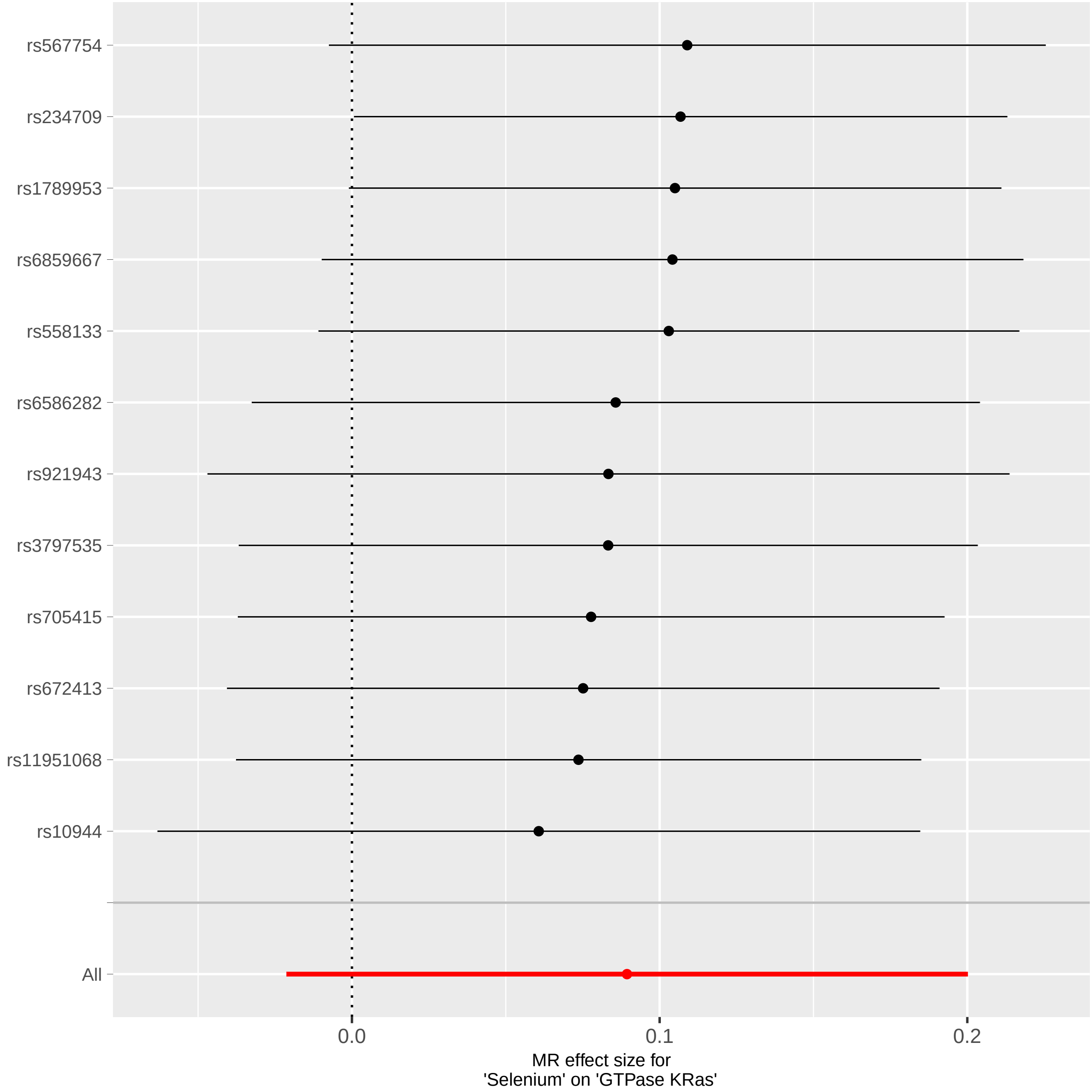

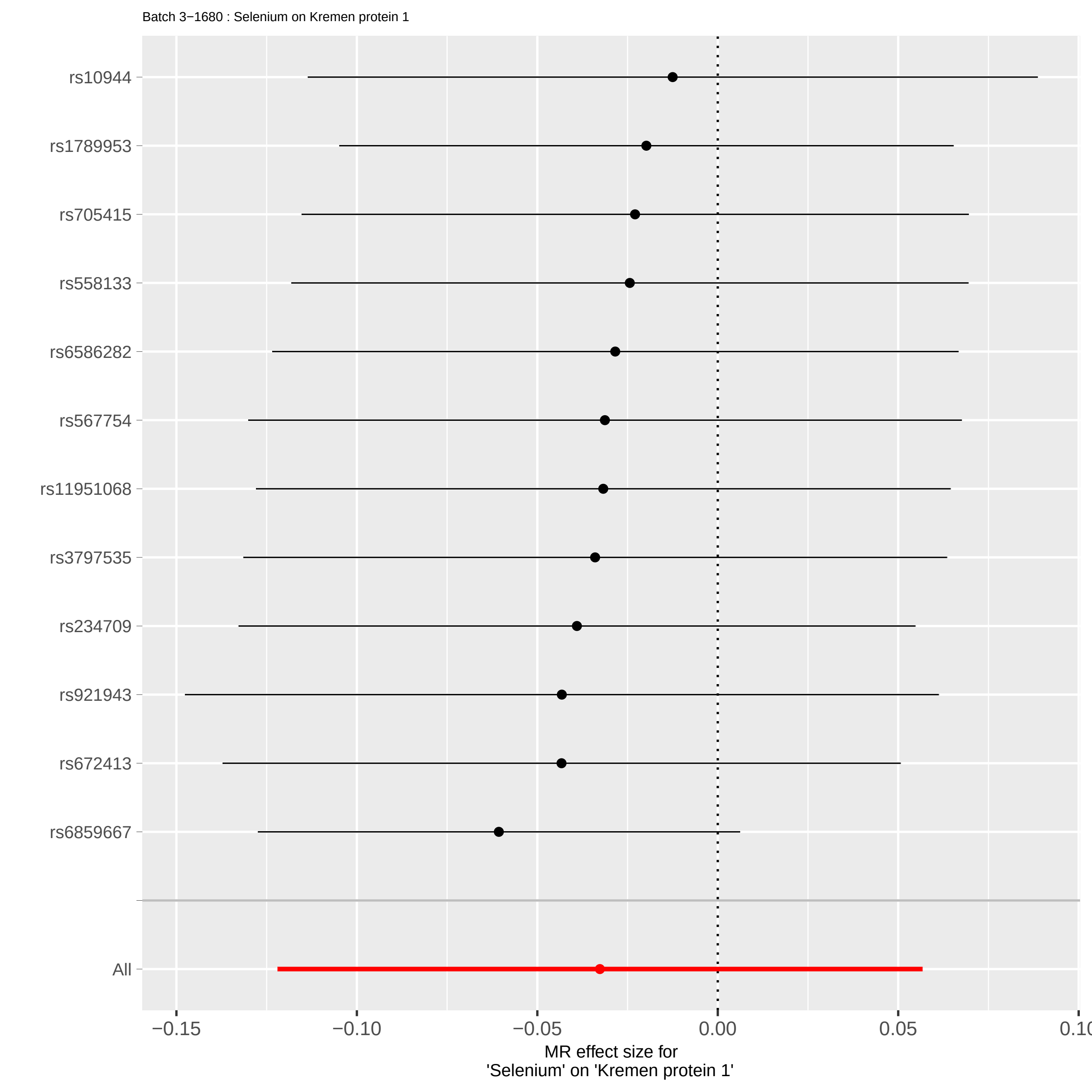
Batch 3−1680 : Selenium on Kremen protein 1
rs10944
rs1789953
rs705415
rs558133
rs6586282
rs567754
rs11951068
rs3797535
rs234709
rs921943
rs672413
rs6859667
All
−0.15
−0.10
−0.05
0.00
0.05
0.10
MR effect size for
'Selenium' on 'Kremen protein 1'

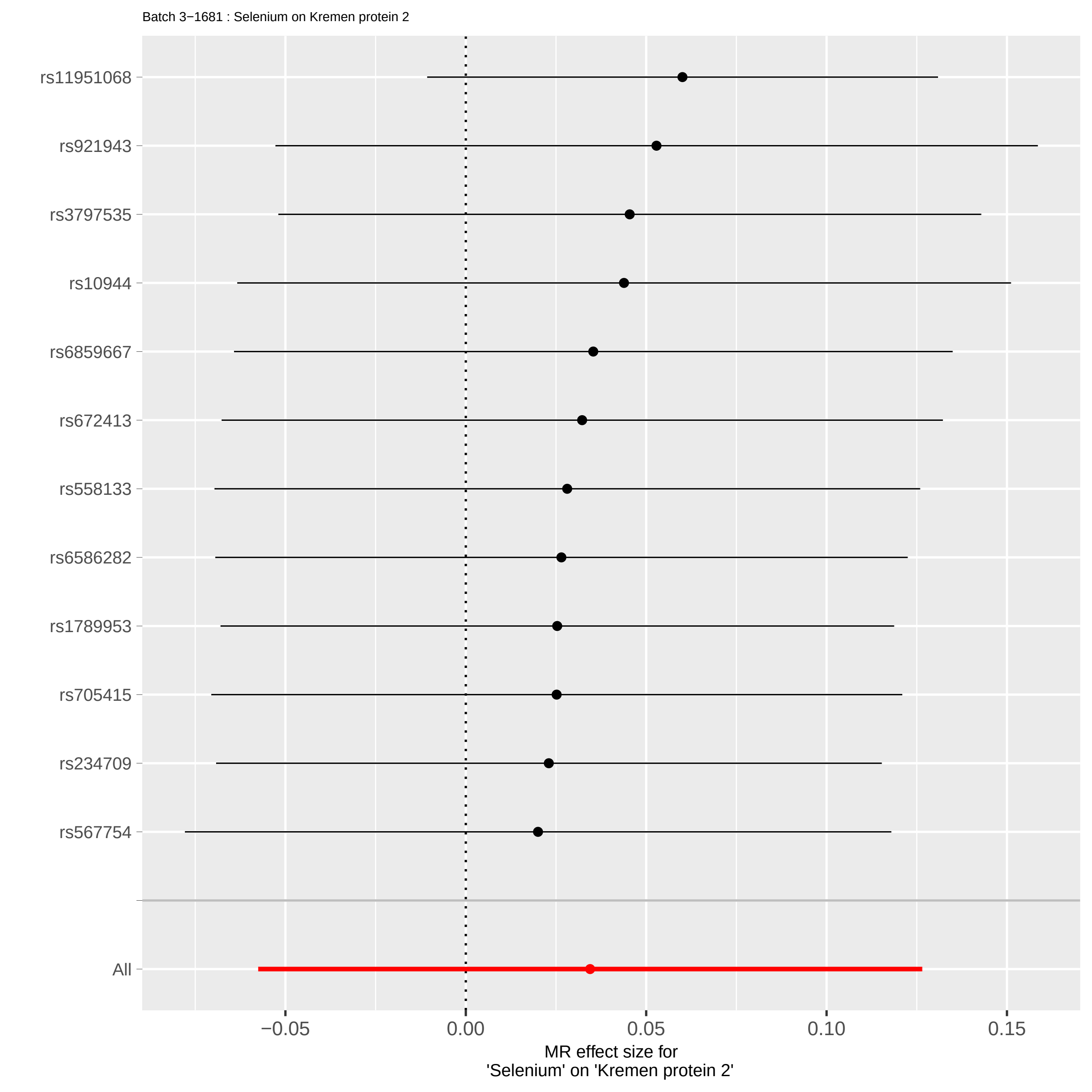

Batch 3−1681 : Selenium on Kremen protein 2
rs11951068
rs921943
rs3797535
rs10944
rs6859667
rs672413
rs558133
rs6586282
rs1789953
rs705415
rs234709
rs567754
All
−0.05
0.00
0.05
0.10
0.15
MR effect size for
'Selenium' on 'Kremen protein 2'

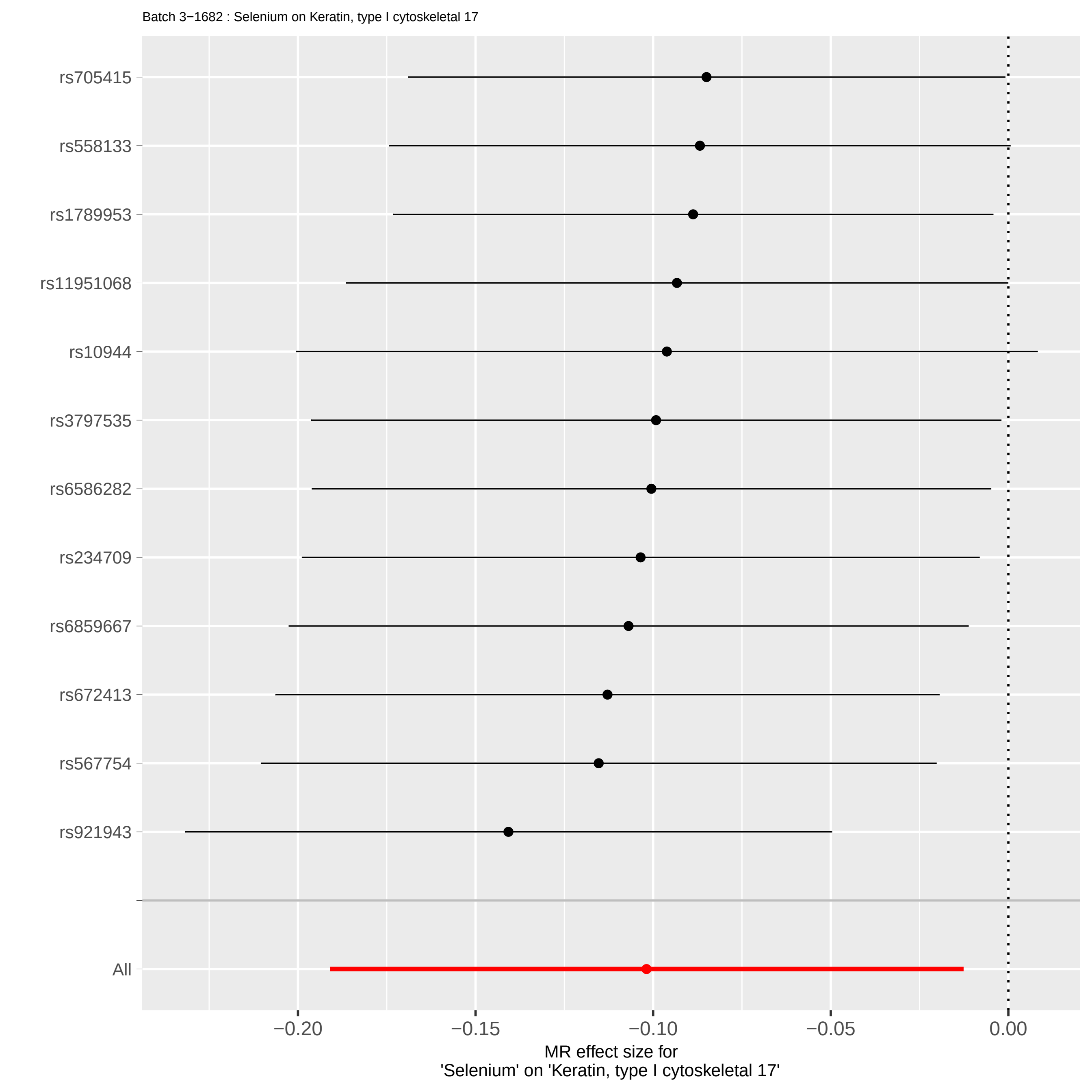

Batch 3−1682 : Selenium on Keratin, type I cytoskeletal 17
rs705415
rs558133
rs1789953
rs11951068
rs10944
rs3797535
rs6586282
rs234709
rs6859667
rs672413
rs567754
rs921943
All
−0.20
−0.15
−0.10
−0.05
0.00
MR effect size for
'Selenium' on 'Keratin, type I cytoskeletal 17'

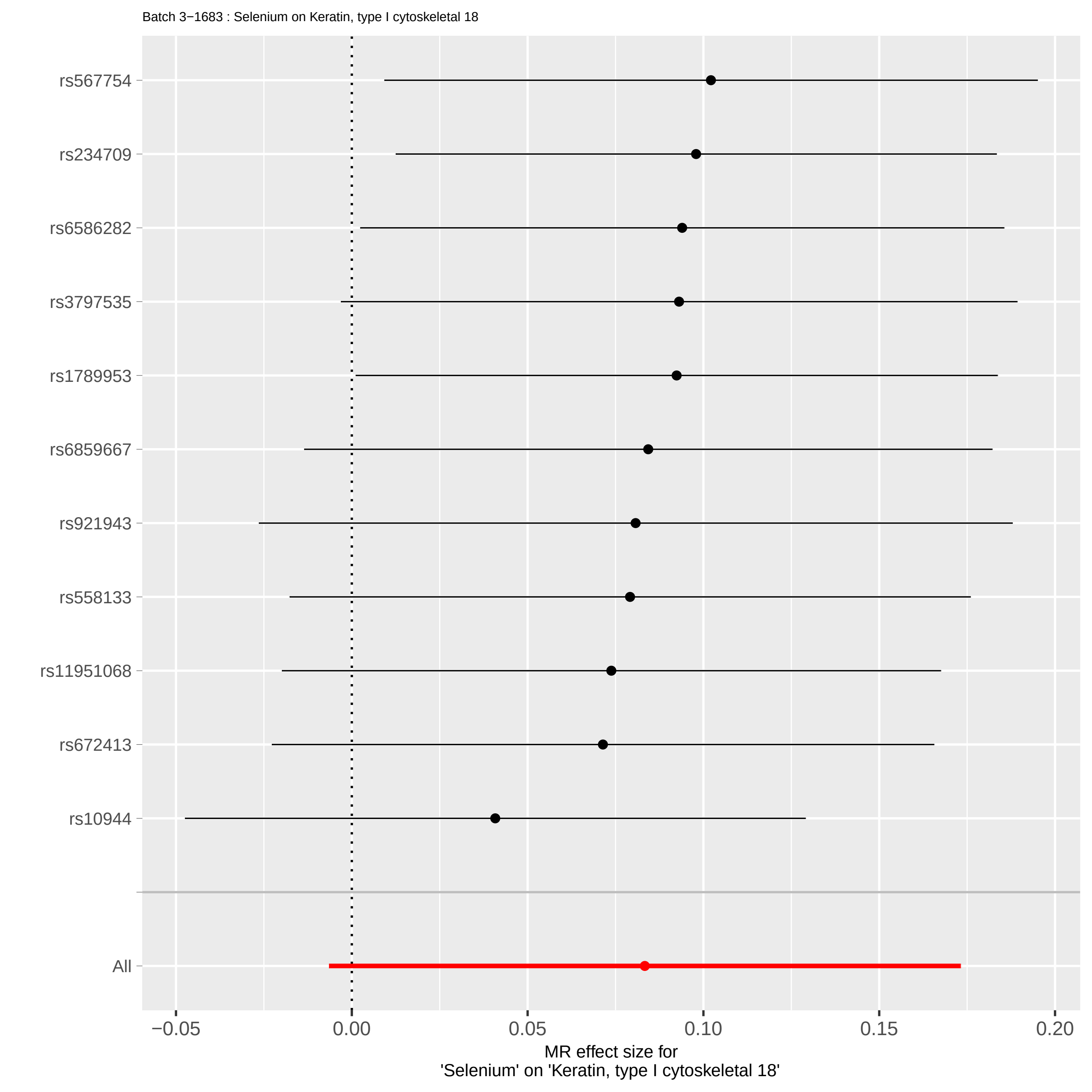

Batch 3−1683 : Selenium on Keratin, type I cytoskeletal 18
rs567754
rs234709
rs6586282
rs3797535
rs1789953
rs6859667
rs921943
rs558133
rs11951068
rs672413
rs10944
All
−0.05
0.00
0.05
0.10
0.15
0.20
MR effect size for
'Selenium' on 'Keratin, type I cytoskeletal 18'

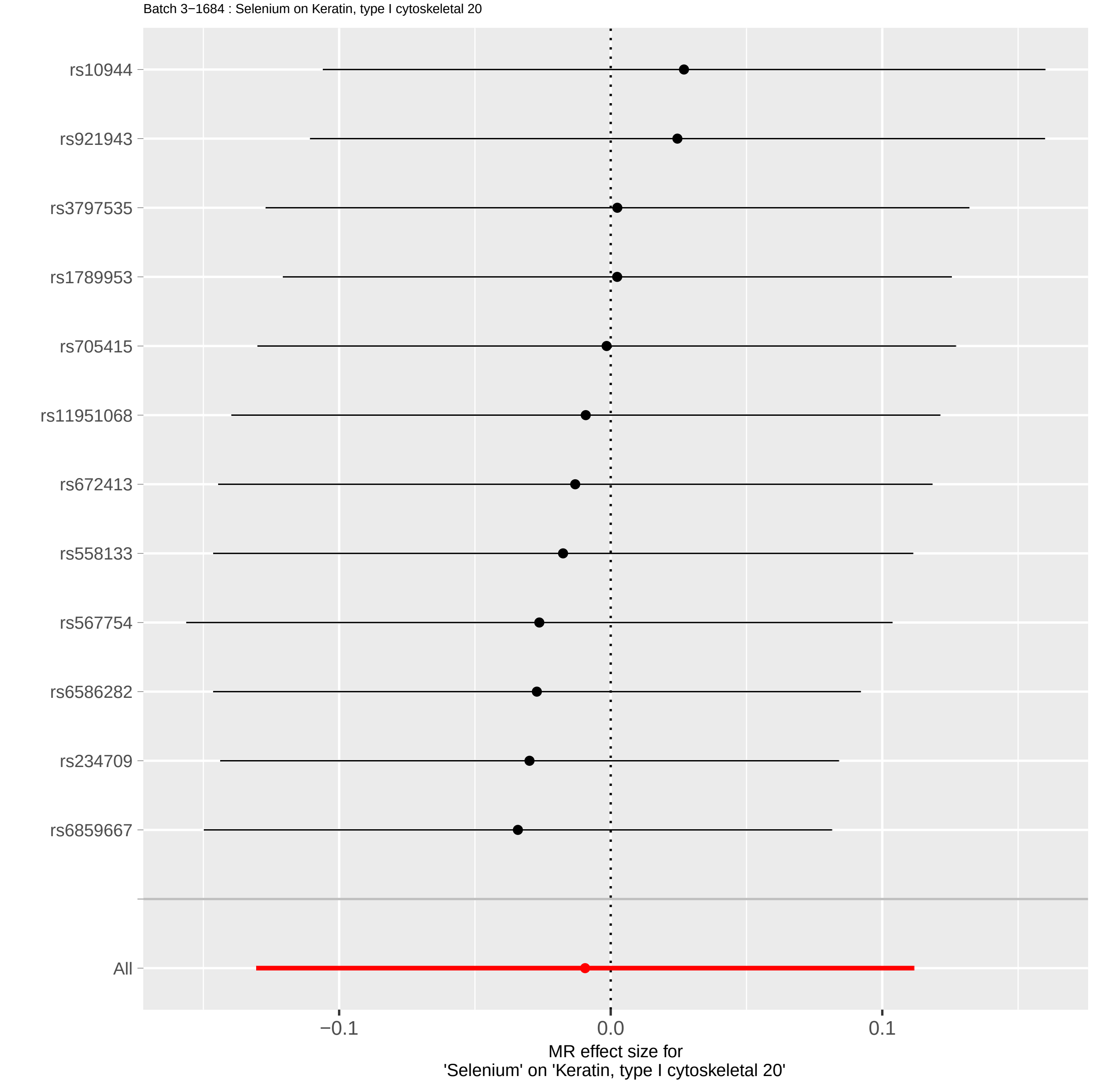

Batch 3−1684 : Selenium on Keratin, type I cytoskeletal 20
rs10944
rs921943
rs3797535
rs1789953
rs705415
rs11951068
rs672413
rs558133
rs567754
rs6586282
rs234709
rs6859667
All
−0.1
0.0
0.1
MR effect size for
'Selenium' on 'Keratin, type I cytoskeletal 20'

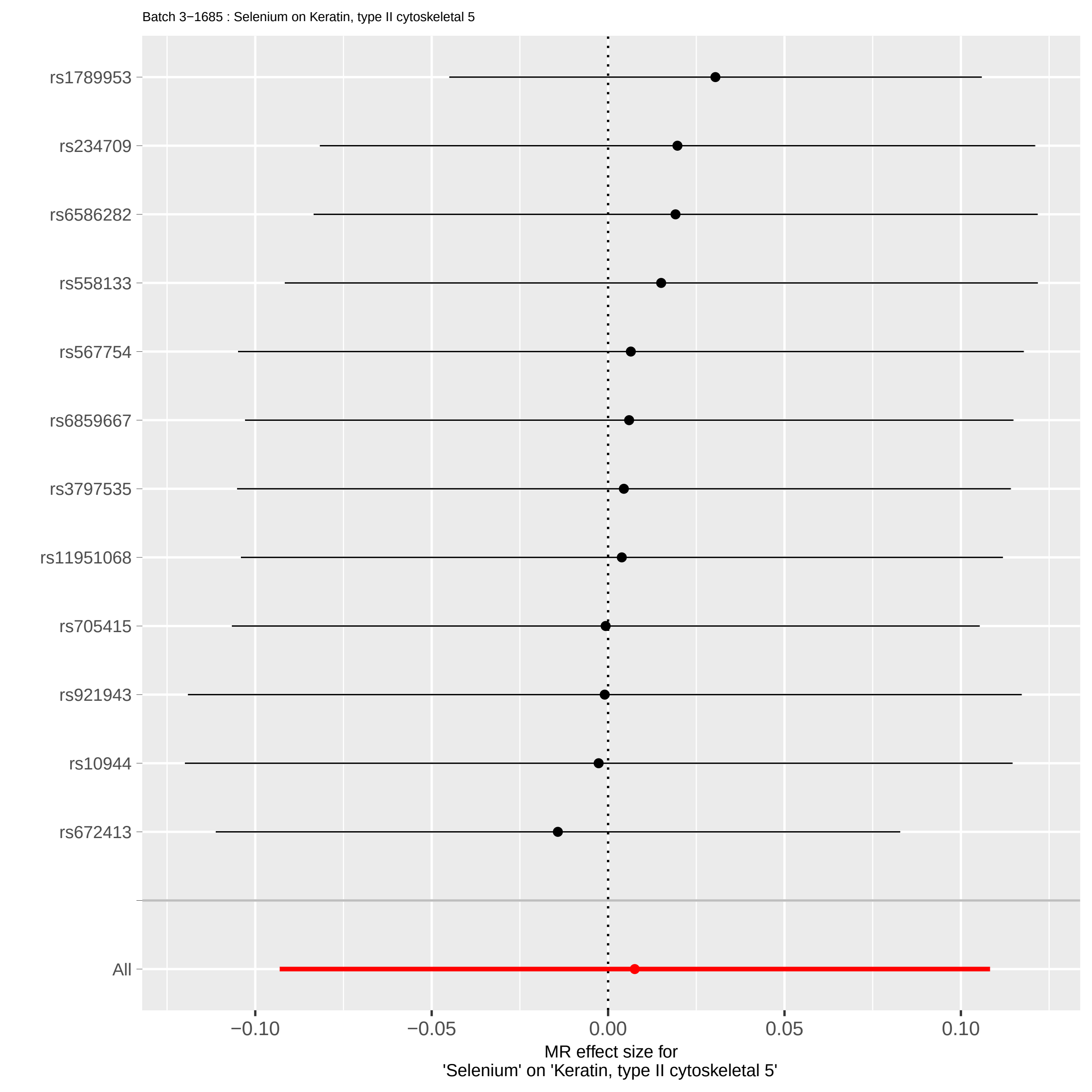

Batch 3−1685 : Selenium on Keratin, type II cytoskeletal 5
rs1789953
rs234709
rs6586282
rs558133
rs567754
rs6859667
rs3797535
rs11951068
rs705415
rs921943
rs10944
rs672413
All
−0.10
−0.05
0.00
0.05
0.10
MR effect size for
'Selenium' on 'Keratin, type II cytoskeletal 5'

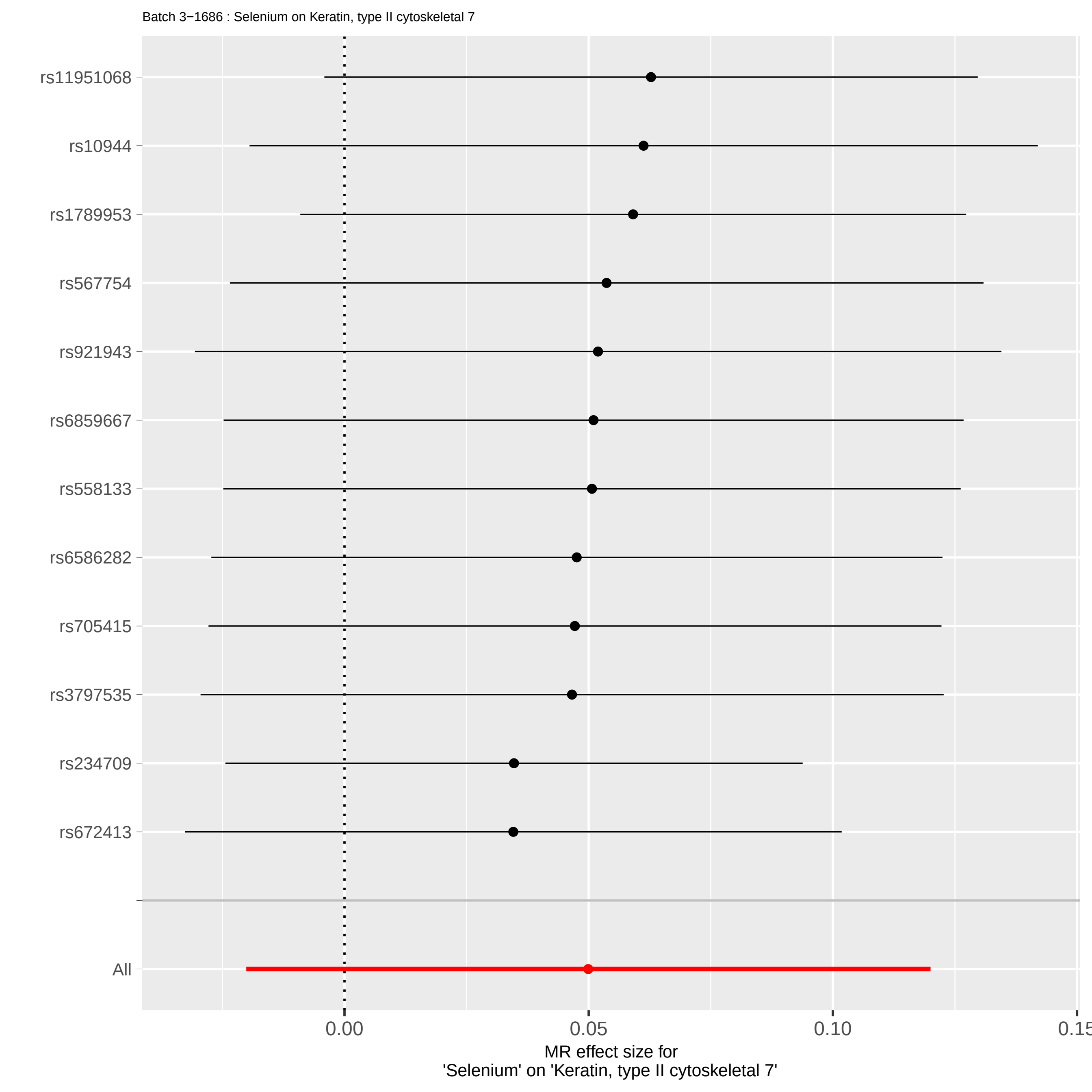

Batch 3−1686 : Selenium on Keratin, type II cytoskeletal 7
rs11951068
rs10944
rs1789953
rs567754
rs921943
rs6859667
rs558133
rs6586282
rs705415
rs3797535
rs234709
rs672413
All
0.00
0.05
0.10
0.15
MR effect size for
'Selenium' on 'Keratin, type II cytoskeletal 7'

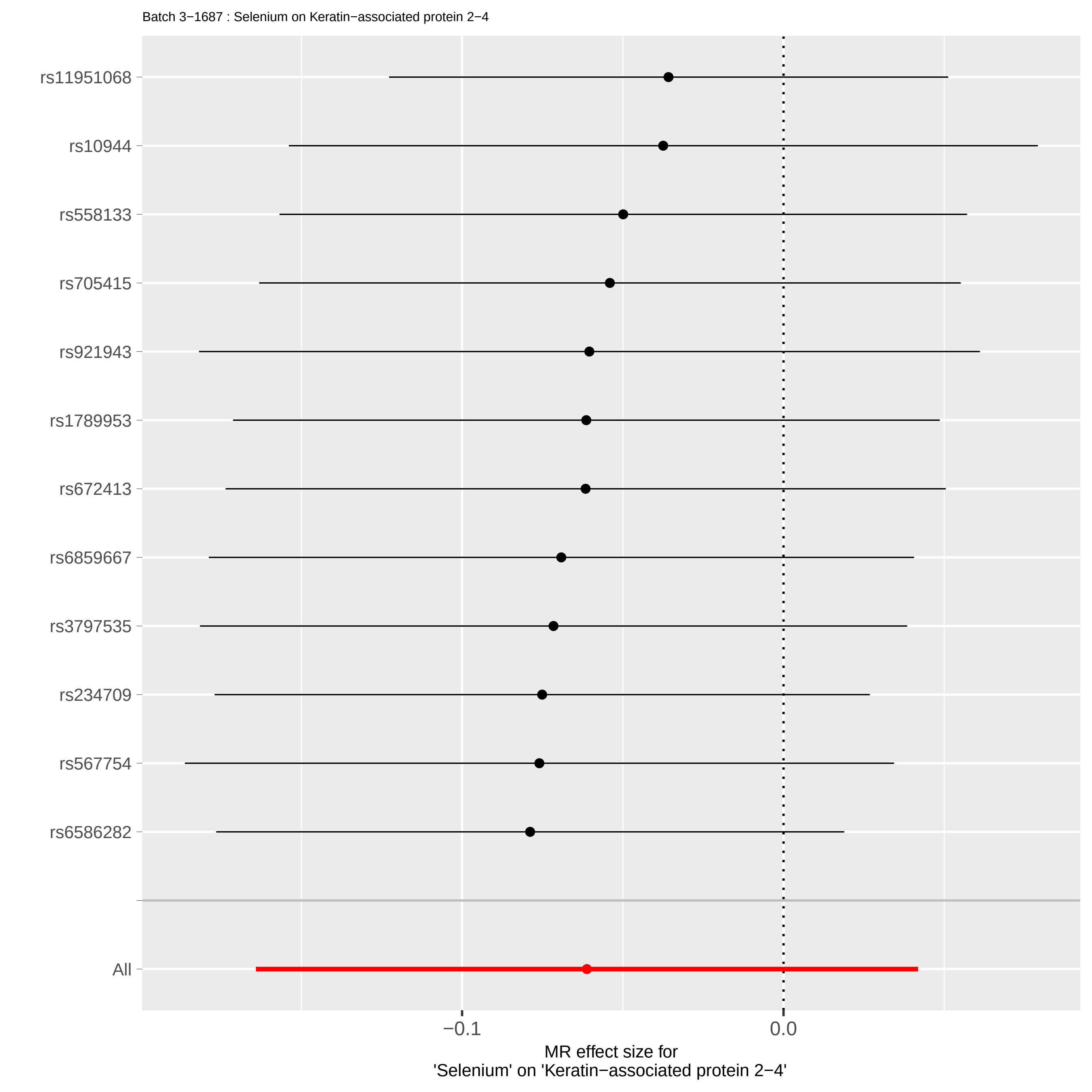
Batch 3−1687 : Selenium on Keratin−associated protein 2−4
rs11951068
rs10944
rs558133
rs705415
rs921943
rs1789953
rs672413
rs6859667
rs3797535
rs234709
rs567754
rs6586282
All
−0.1
0.0
MR effect size for
'Selenium' on 'Keratin−associated protein 2−4'

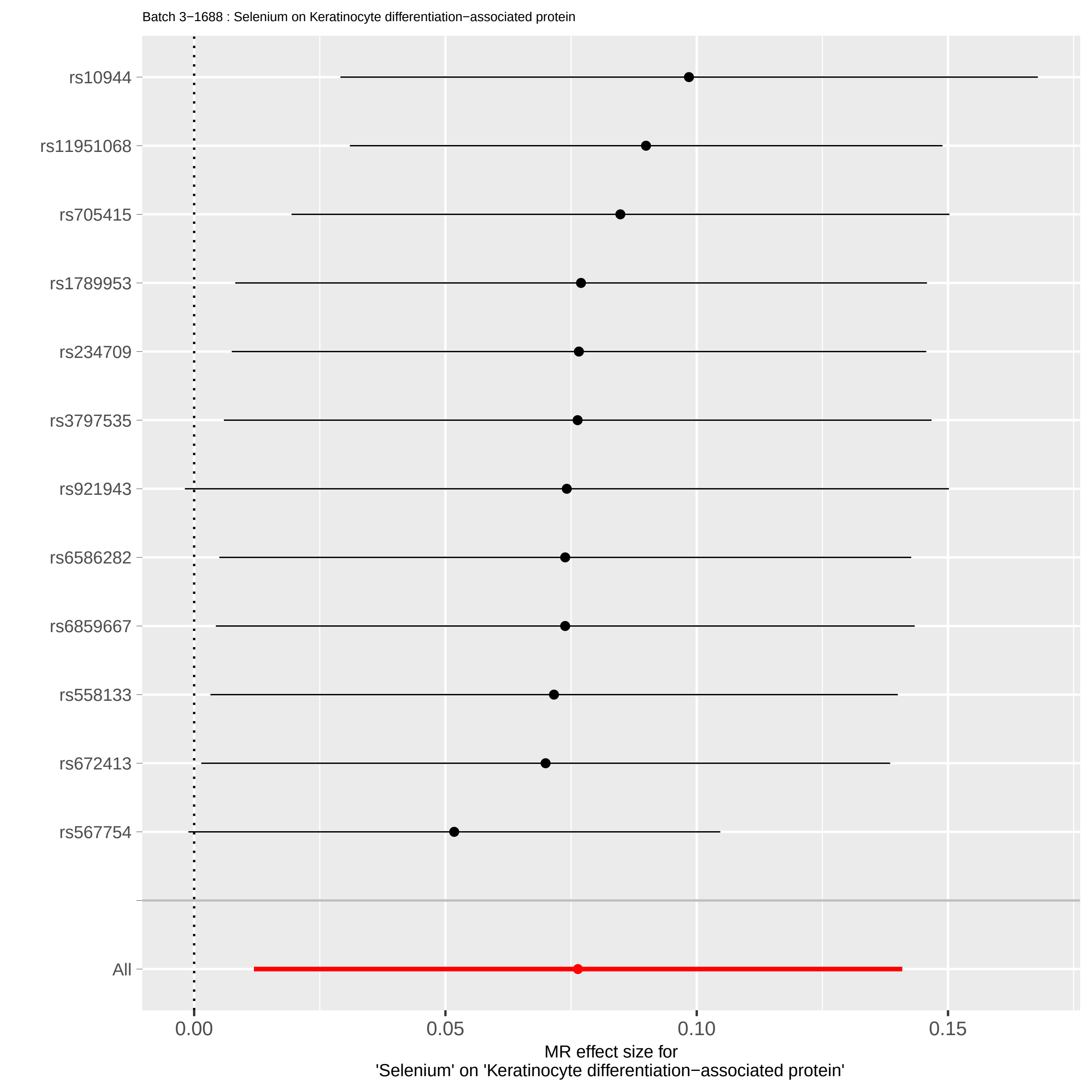
Batch 3−1688 : Selenium on Keratinocyte differentiation−associated protein
rs10944
rs11951068
rs705415
rs1789953
rs234709
rs3797535
rs921943
rs6586282
rs6859667
rs558133
rs672413
rs567754
All
0.00
0.05
0.10
0.15
MR effect size for
'Selenium' on 'Keratinocyte differentiation−associated protein'

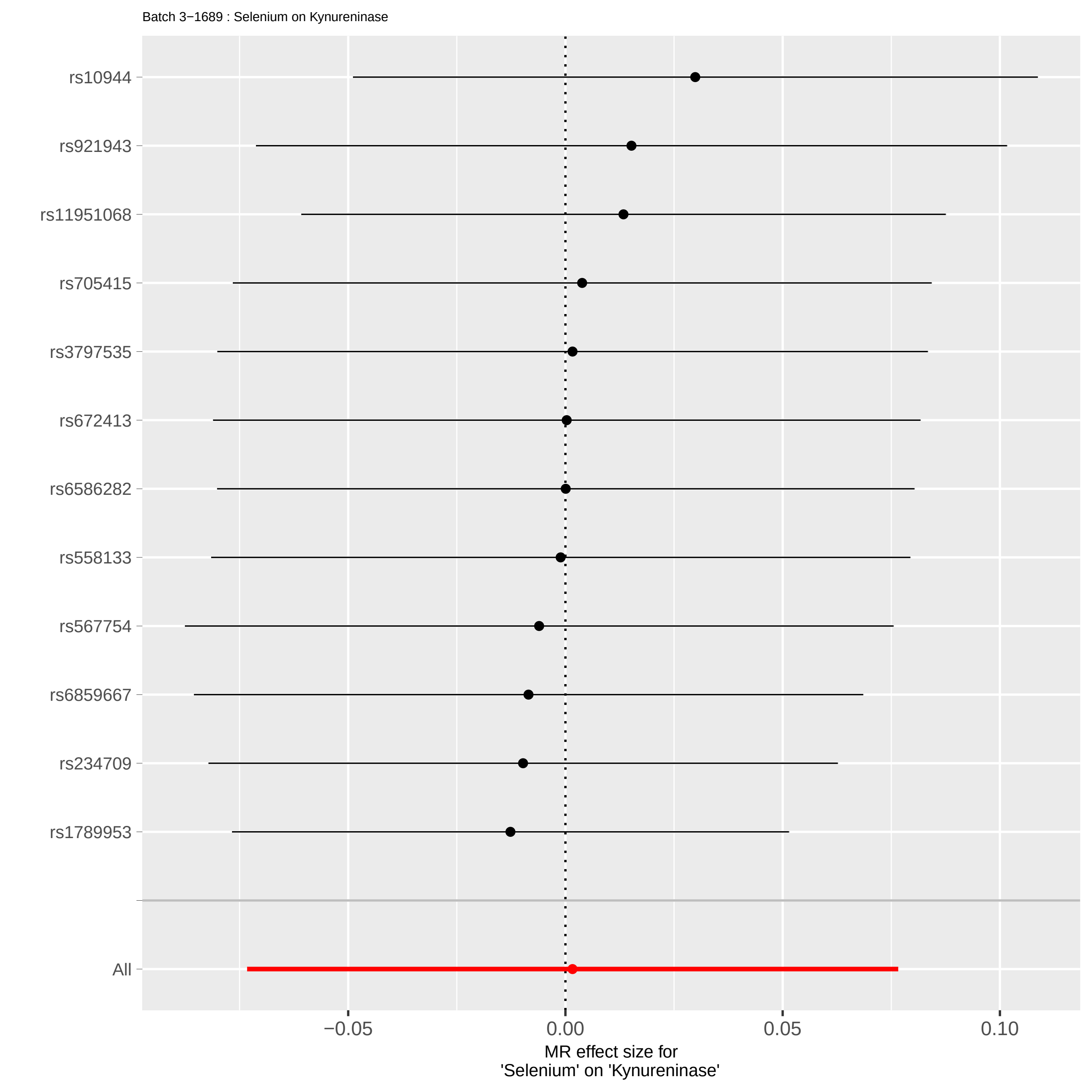

Batch 3−1689 : Selenium on Kynureninase
rs10944
rs921943
rs11951068
rs705415
rs3797535
rs672413
rs6586282
rs558133
rs567754
rs6859667
rs234709
rs1789953
All
−0.05
0.00
0.05
0.10
MR effect size for
'Selenium' on 'Kynureninase'

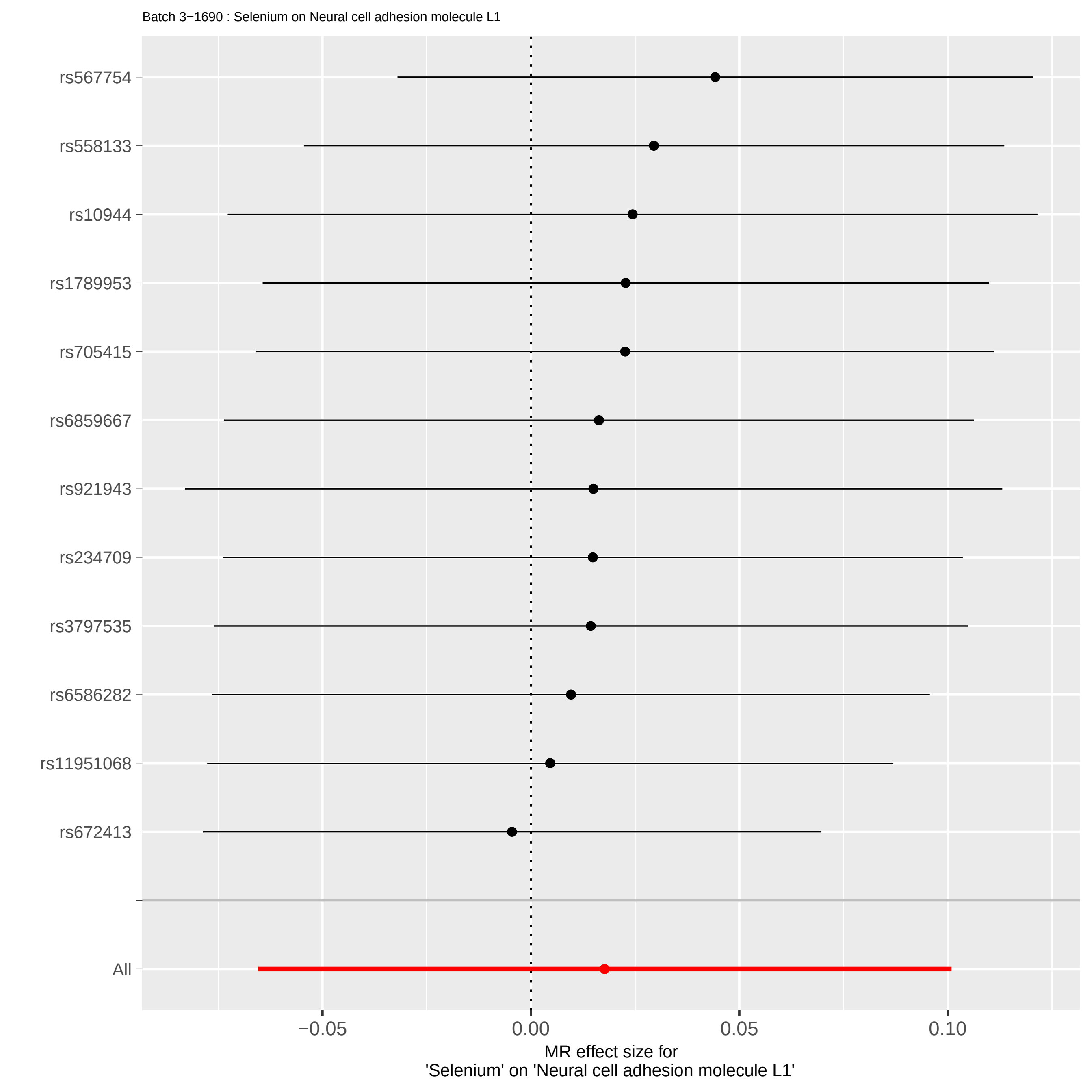

Batch 3−1690 : Selenium on Neural cell adhesion molecule L1
rs567754
rs558133
rs10944
rs1789953
rs705415
rs6859667
rs921943
rs234709
rs3797535
rs6586282
rs11951068
rs672413
All
−0.05
0.00
0.05
0.10
MR effect size for
'Selenium' on 'Neural cell adhesion molecule L1'

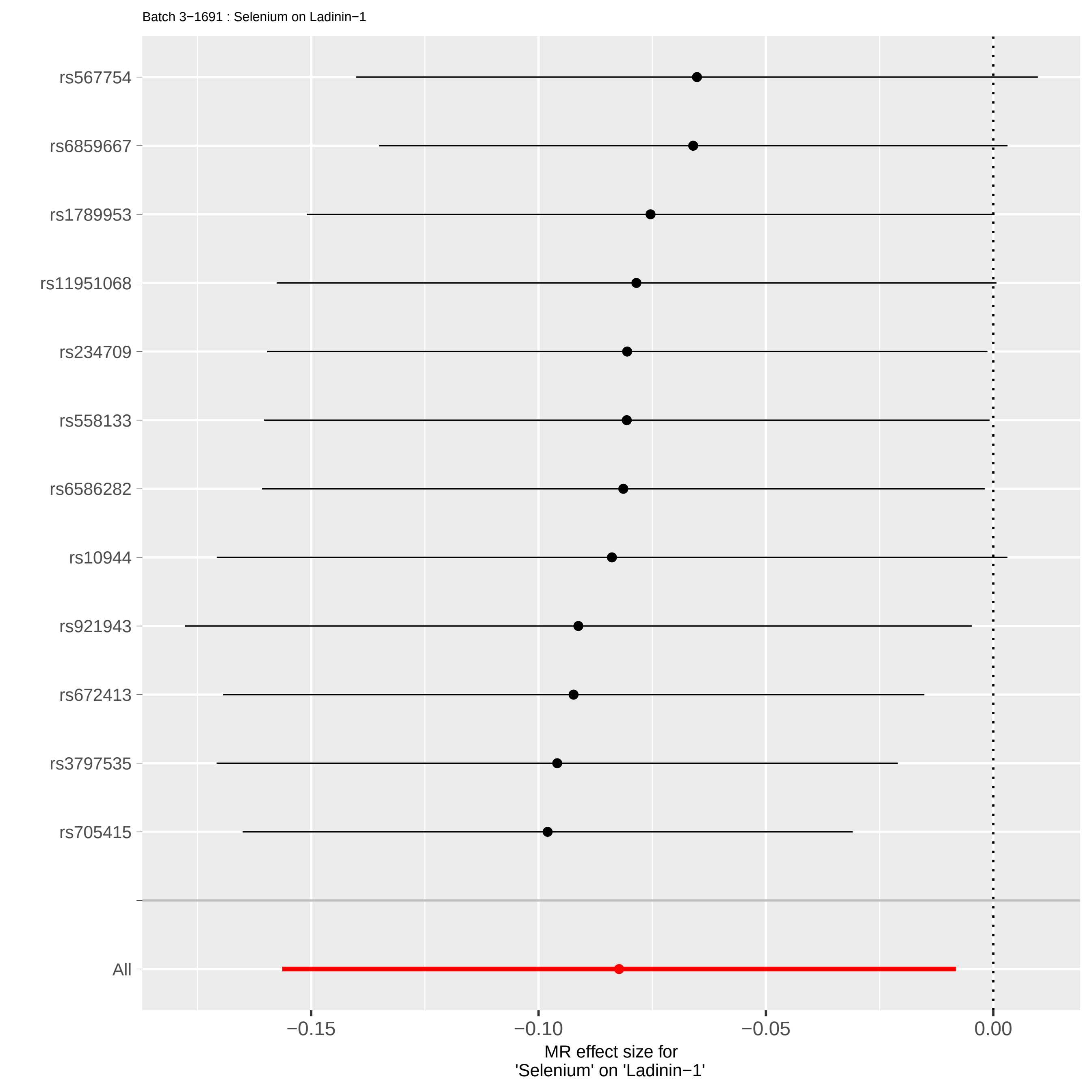

Batch 3−1691 : Selenium on Ladinin−1
rs567754
rs6859667
rs1789953
rs11951068
rs234709
rs558133
rs6586282
rs10944
rs921943
rs672413
rs3797535
rs705415
All
−0.15
−0.10
−0.05
0.00
MR effect size for
'Selenium' on 'Ladinin−1'

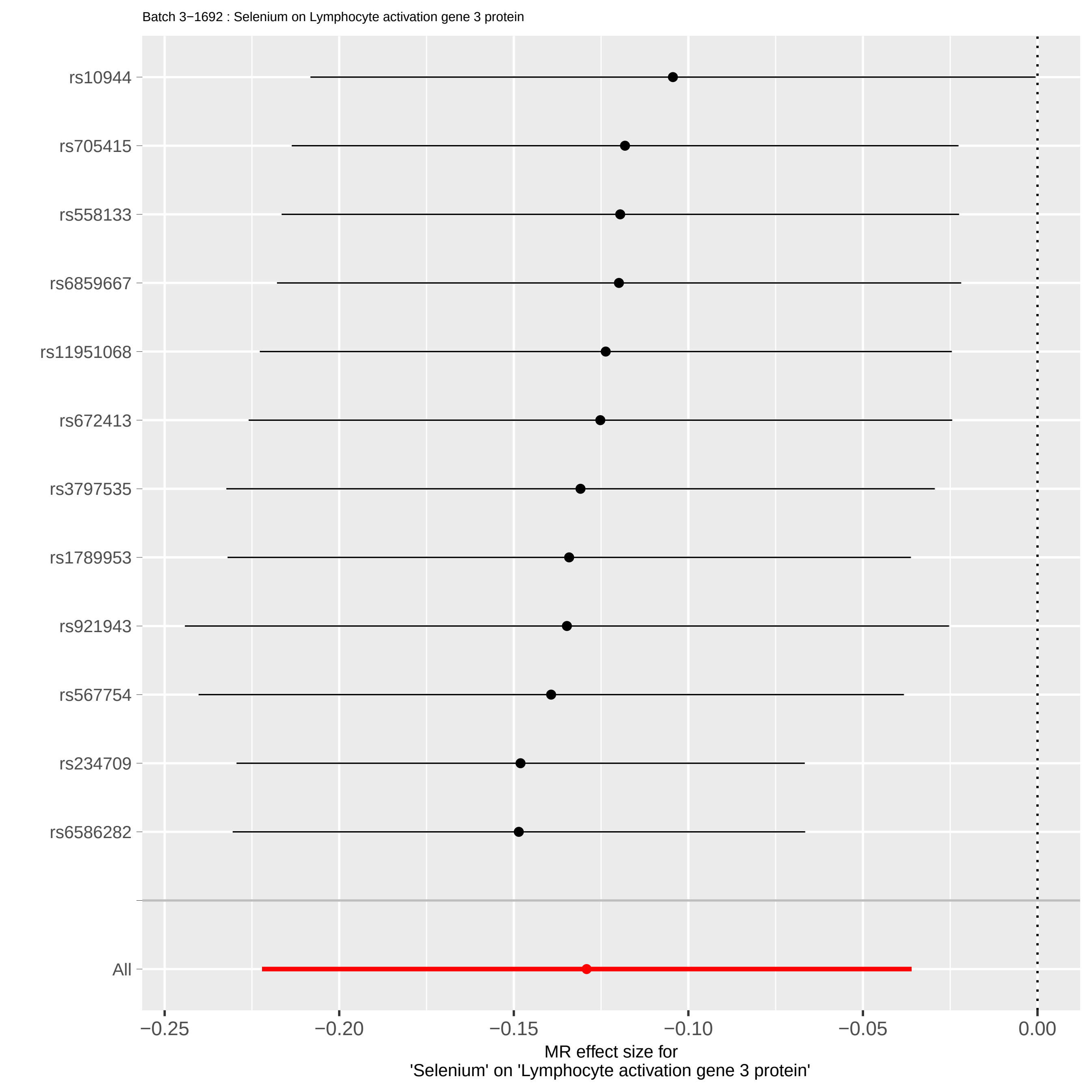

Batch 3−1692 : Selenium on Lymphocyte activation gene 3 protein
rs10944
rs705415
rs558133
rs6859667
rs11951068
rs672413
rs3797535
rs1789953
rs921943
rs567754
rs234709
rs6586282
All
−0.25
−0.20
−0.15
−0.10
−0.05
0.00
MR effect size for
'Selenium' on 'Lymphocyte activation gene 3 protein'

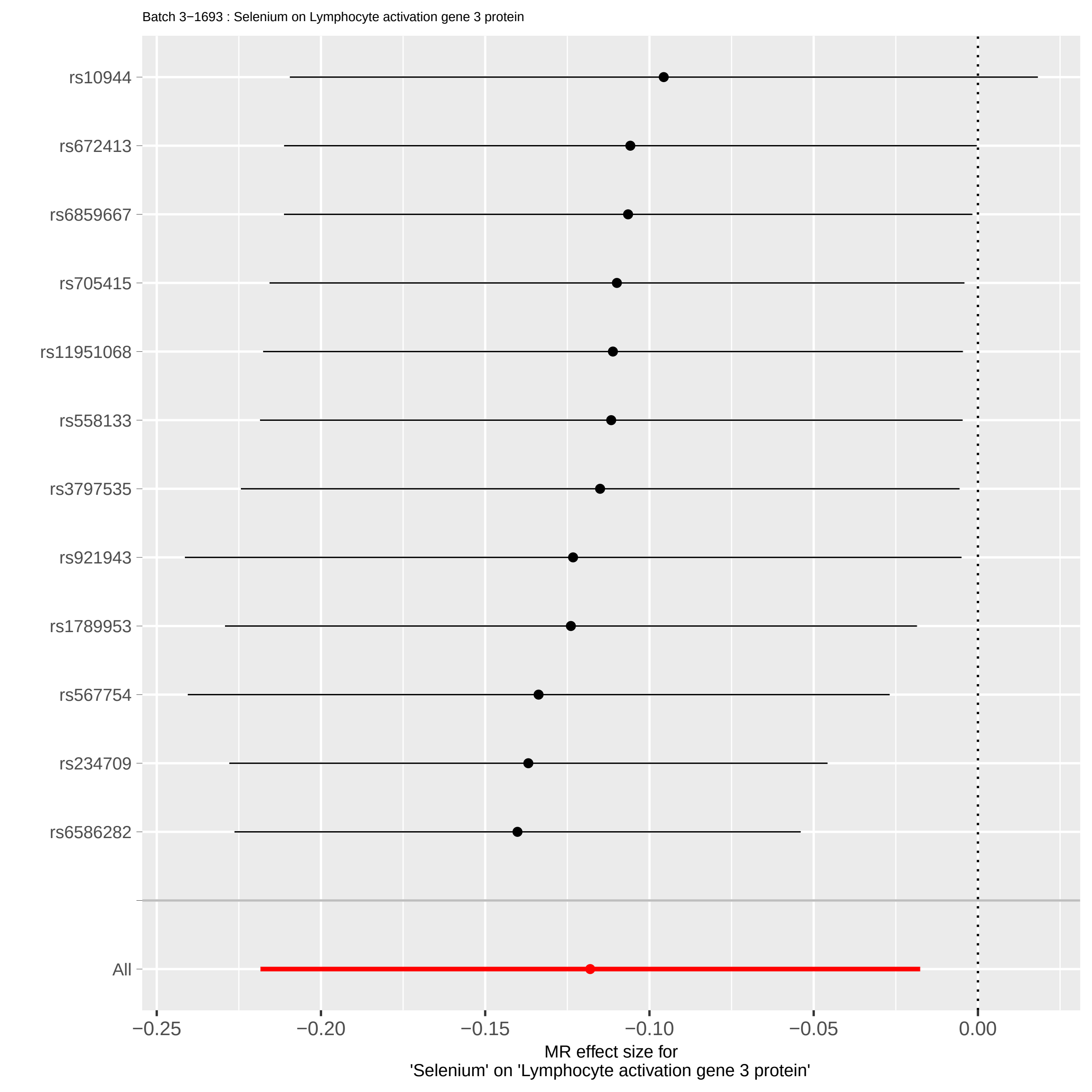

Batch 3−1693 : Selenium on Lymphocyte activation gene 3 protein
rs10944
rs672413
rs6859667
rs705415
rs11951068
rs558133
rs3797535
rs921943
rs1789953
rs567754
rs234709
rs6586282
All
−0.25
−0.20
−0.15
−0.10
−0.05
0.00
MR effect size for
'Selenium' on 'Lymphocyte activation gene 3 protein'

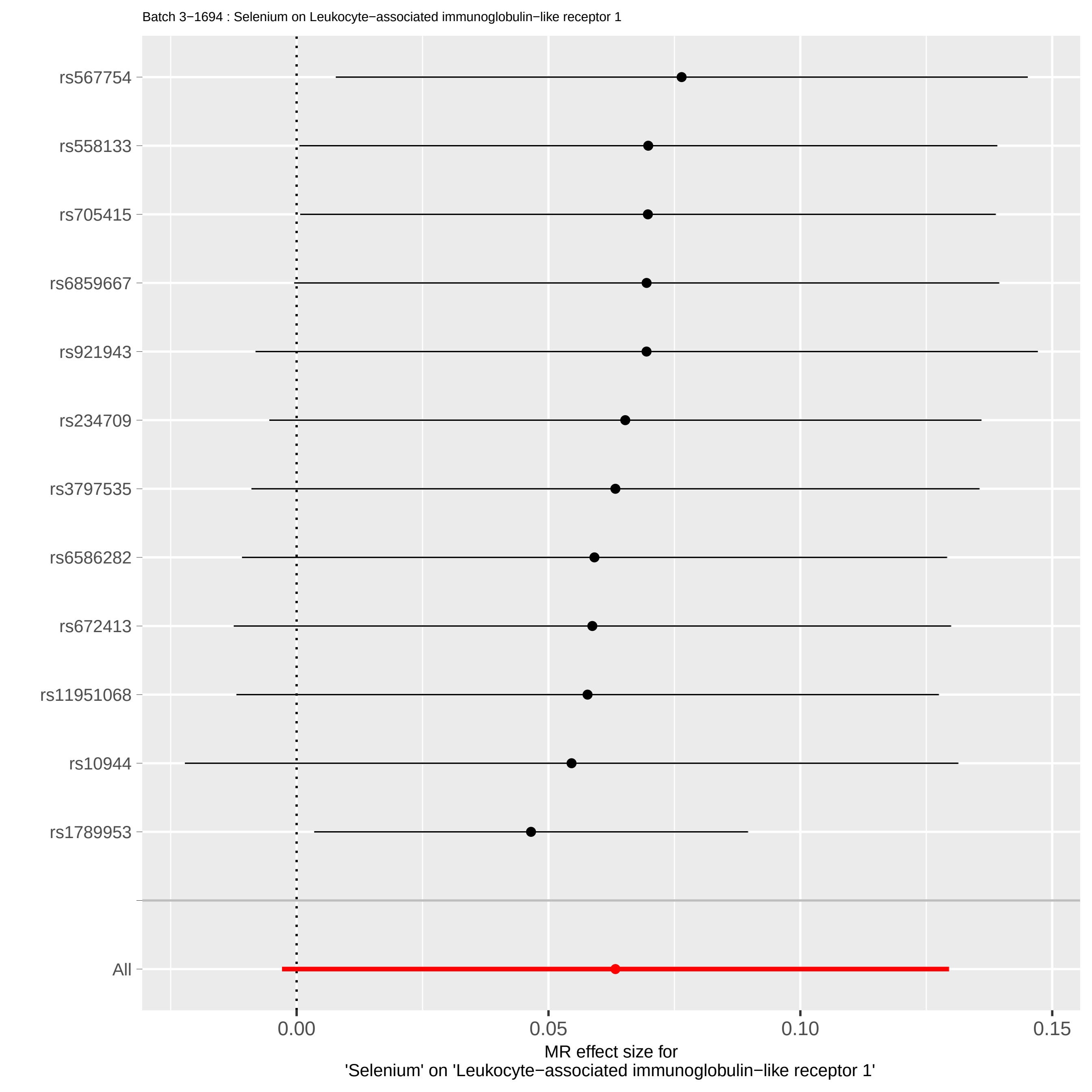
Batch 3−1694 : Selenium on Leukocyte−associated immunoglobulin−like receptor 1
rs567754
rs558133
rs705415
rs6859667
rs921943
rs234709
rs3797535
rs6586282
rs672413
rs11951068
rs10944
rs1789953
All
0.00
0.05
0.10
0.15
MR effect size for
'Selenium' on 'Leukocyte−associated immunoglobulin−like receptor 1'

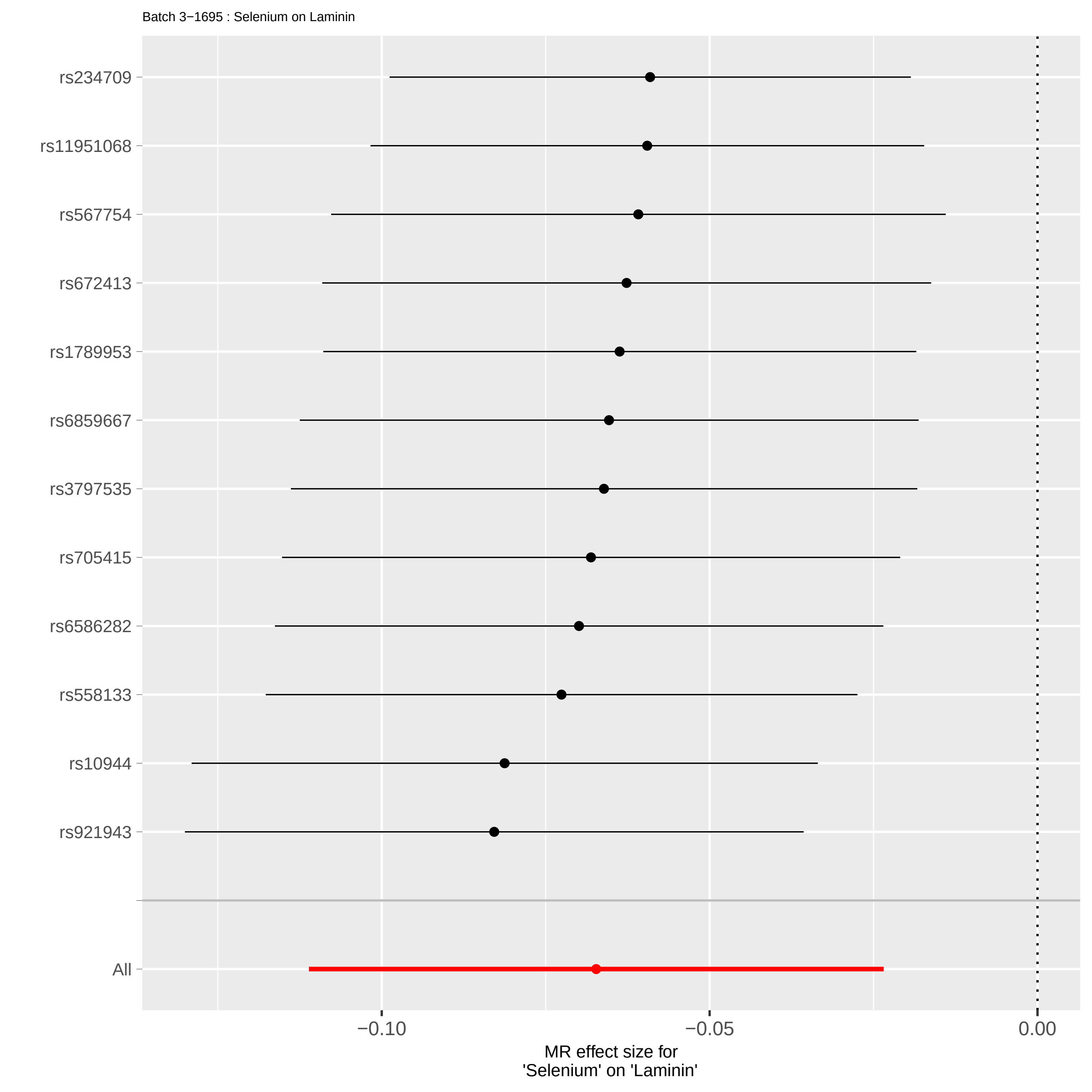
Batch 3−1695 : Selenium on Laminin
rs234709
rs11951068
rs567754
rs672413
rs1789953
rs6859667
rs3797535
rs705415
rs6586282
rs558133
rs10944
rs921943
All
−0.10
−0.05
0.00
MR effect size for
'Selenium' on 'Laminin'

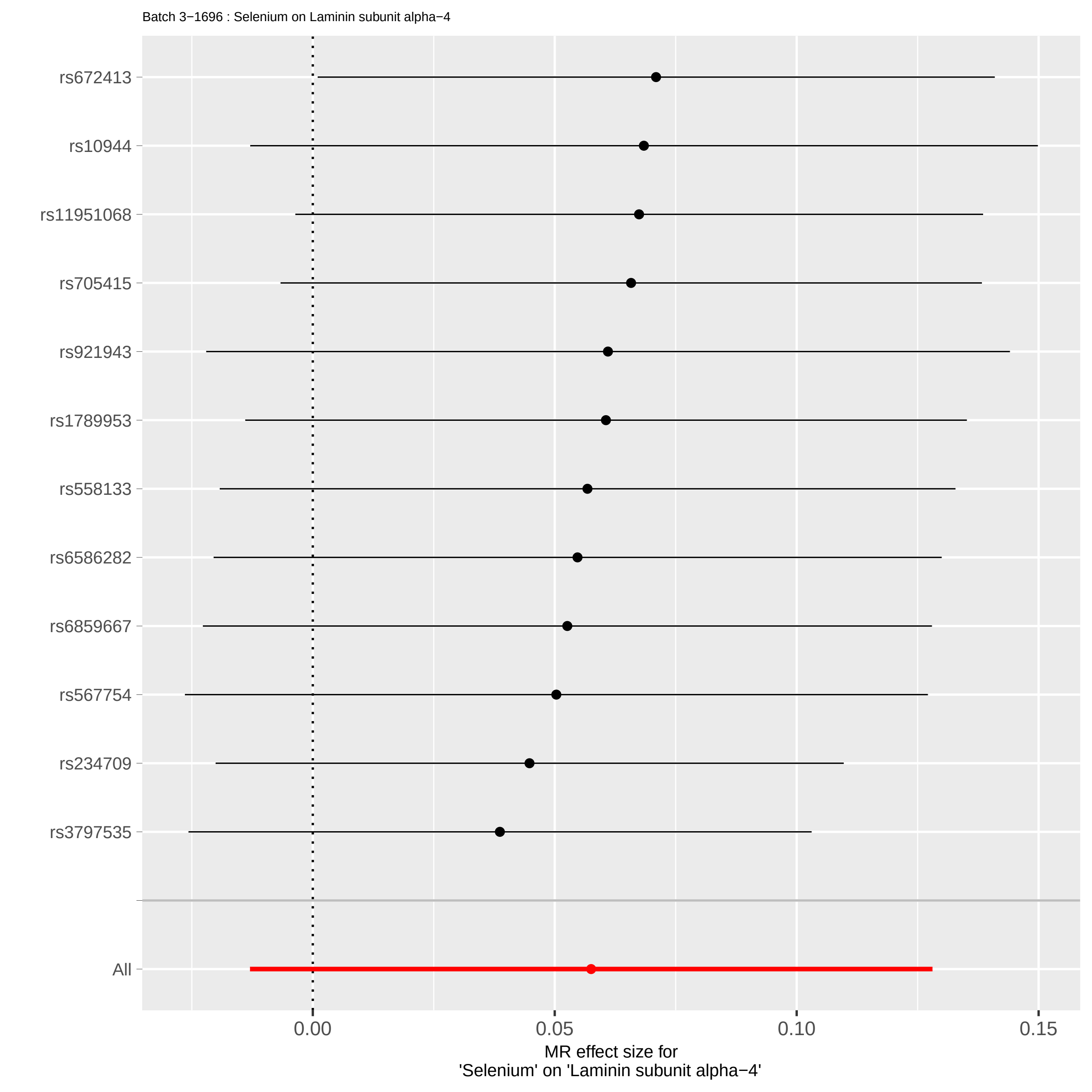

Batch 3−1696 : Selenium on Laminin subunit alpha−4
rs672413
rs10944
rs11951068
rs705415
rs921943
rs1789953
rs558133
rs6586282
rs6859667
rs567754
rs234709
rs3797535
All
0.00
0.05
0.10
0.15
MR effect size for
'Selenium' on 'Laminin subunit alpha−4'

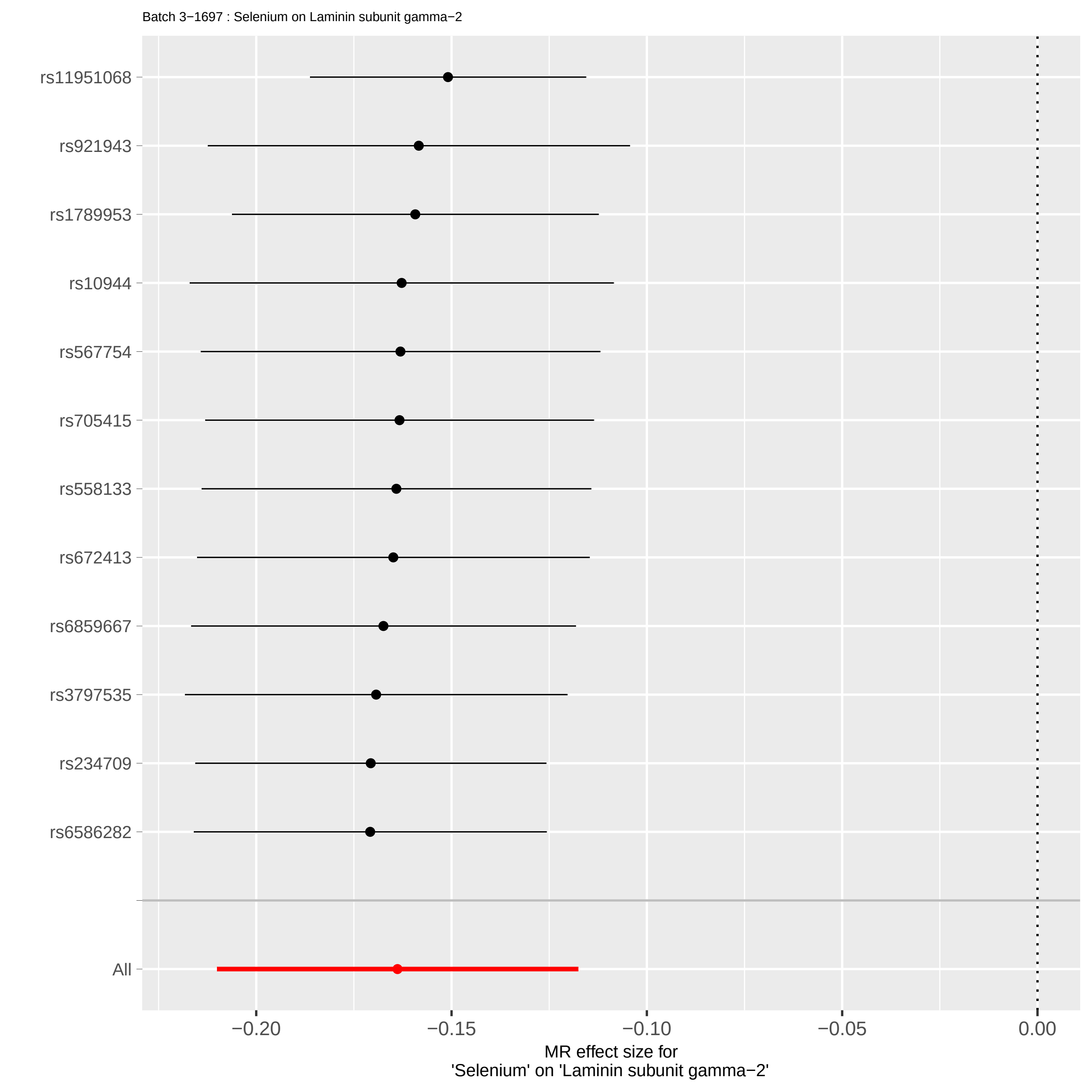

Batch 3−1697 : Selenium on Laminin subunit gamma−2
rs11951068
rs921943
rs1789953
rs10944
rs567754
rs705415
rs558133
rs672413
rs6859667
rs3797535
rs234709
rs6586282
All
−0.20
−0.15
−0.10
−0.05
0.00
MR effect size for
'Selenium' on 'Laminin subunit gamma−2'

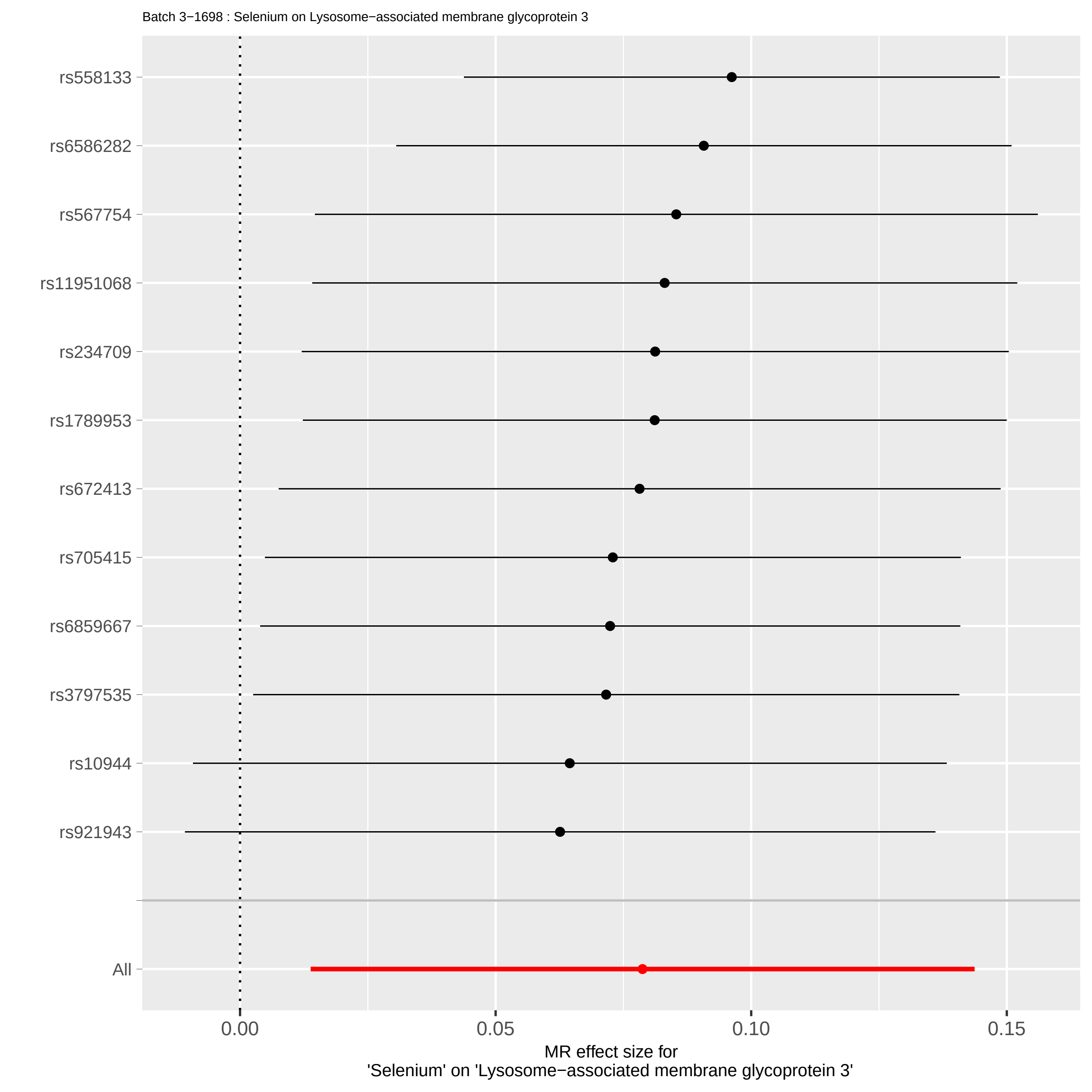

Batch 3−1698 : Selenium on Lysosome−associated membrane glycoprotein 3
rs558133
rs6586282
rs567754
rs11951068
rs234709
rs1789953
rs672413
rs705415
rs6859667
rs3797535
rs10944
rs921943
All
0.00
0.05
0.10
0.15
MR effect size for
'Selenium' on 'Lysosome−associated membrane glycoprotein 3'

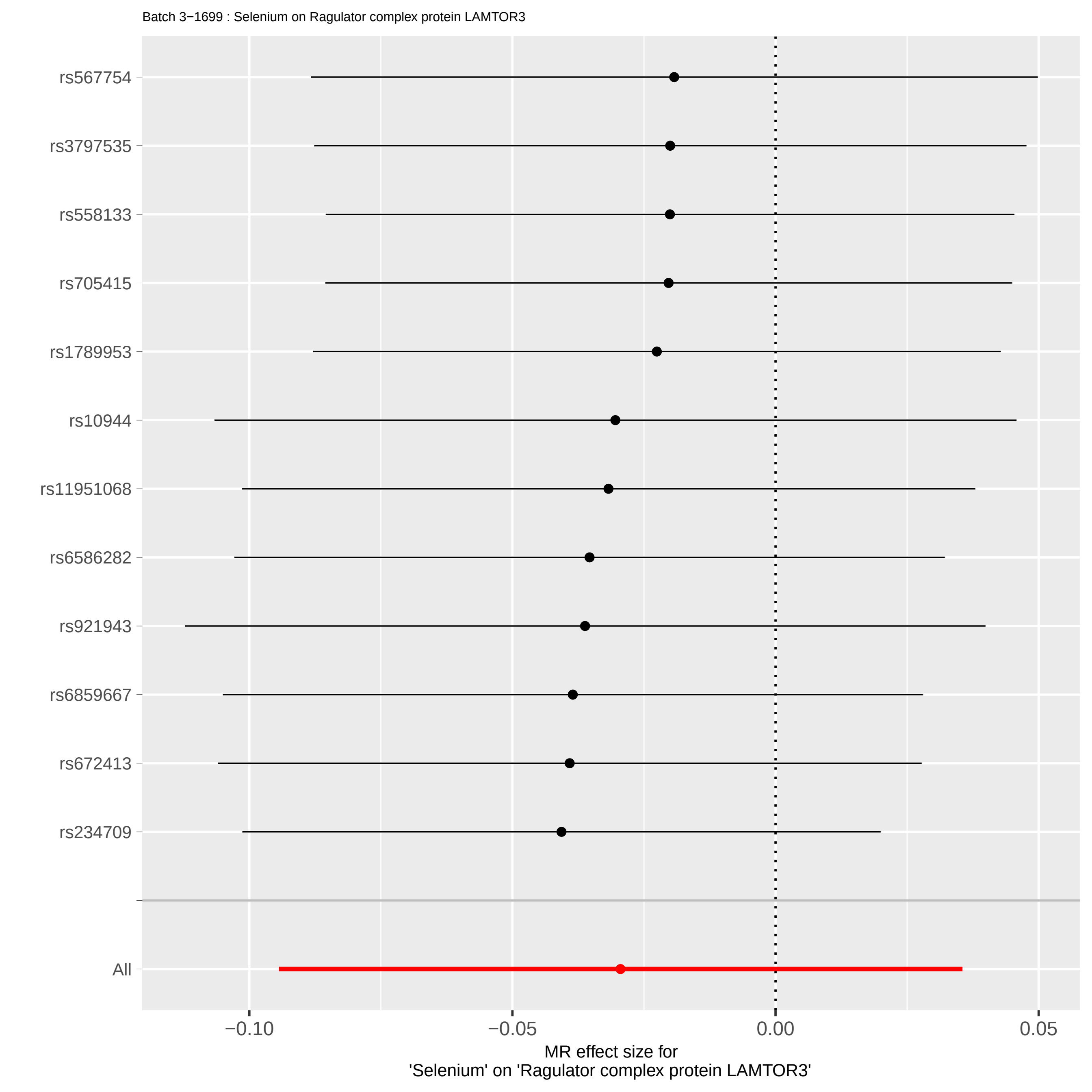
Batch 3−1699 : Selenium on Ragulator complex protein LAMTOR3
rs567754
rs3797535
rs558133
rs705415
rs1789953
rs10944
rs11951068
rs6586282
rs921943
rs6859667
rs672413
rs234709
All
−0.10
−0.05
0.00
0.05
MR effect size for
'Selenium' on 'Ragulator complex protein LAMTOR3'

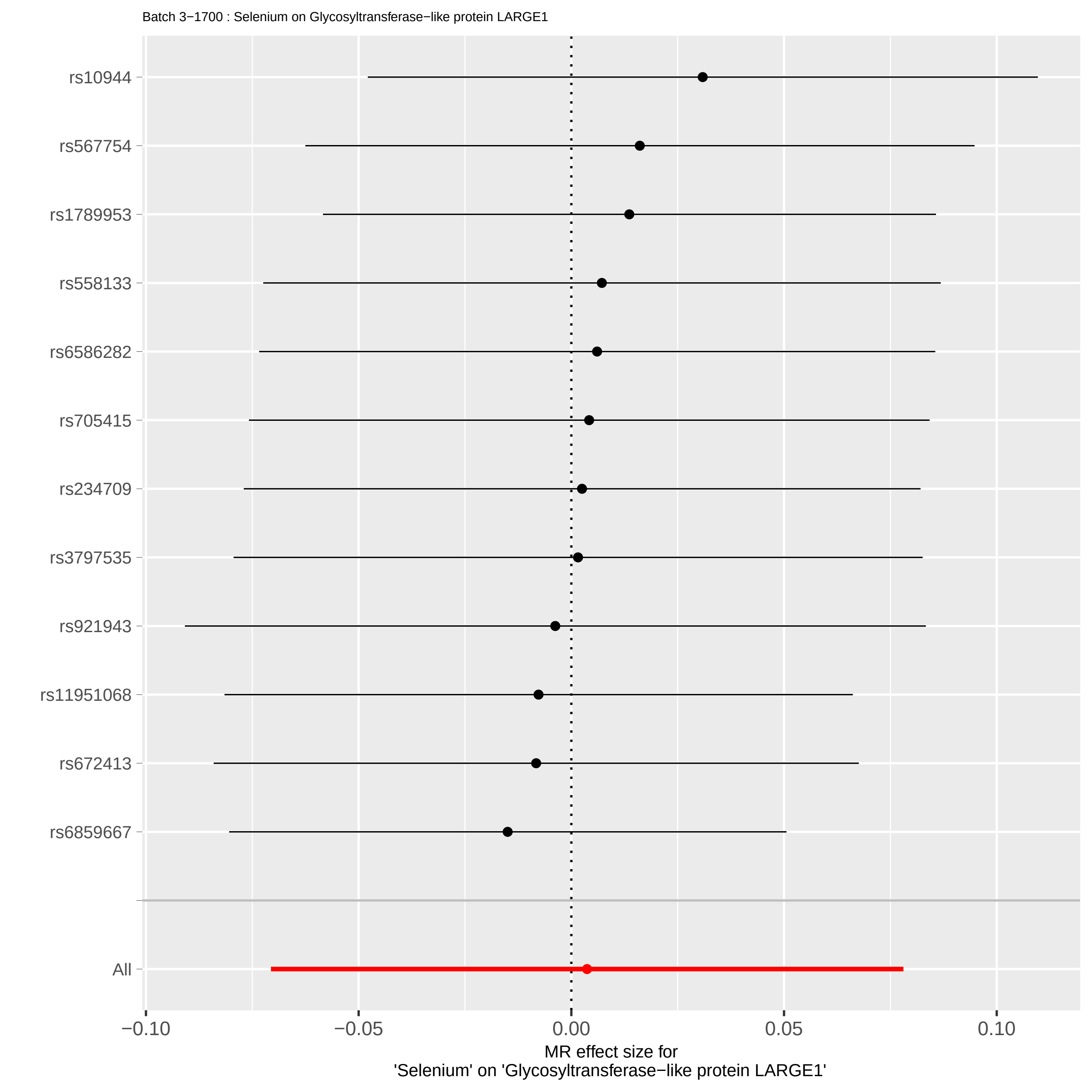

Batch 3−1700 : Selenium on Glycosyltransferase−like protein LARGE1
rs10944
rs567754
rs1789953
rs558133
rs6586282
rs705415
rs234709
rs3797535
rs921943
rs11951068
rs672413
rs6859667
All
−0.10
−0.05
0.00
0.05
0.10
MR effect size for
'Selenium' on 'Glycosyltransferase−like protein LARGE1'

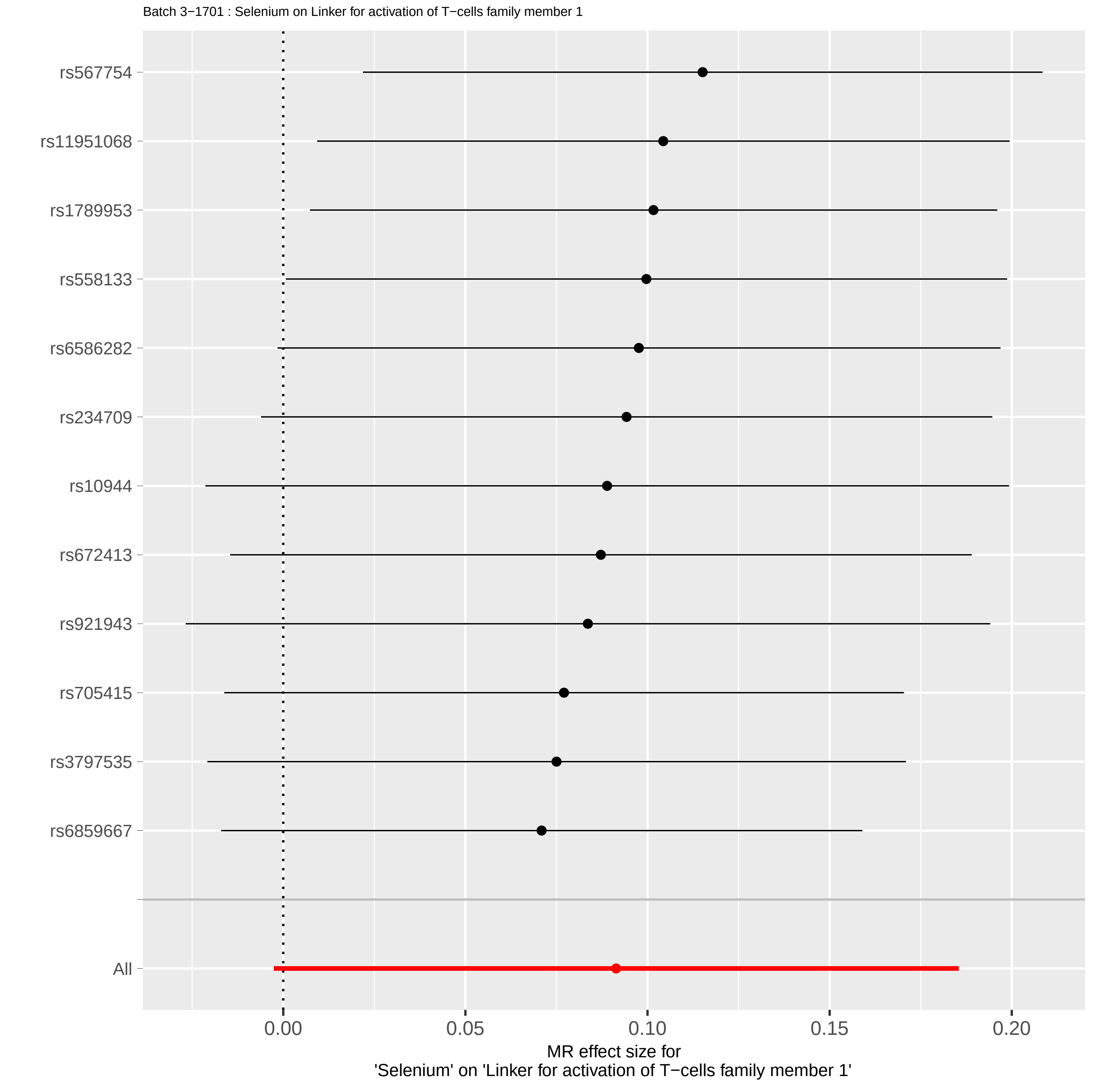

Batch 3−1701 : Selenium on Linker for activation of T−cells family member 1
rs567754
rs11951068
rs1789953
rs558133
rs6586282
rs234709
rs10944
rs672413
rs921943
rs705415
rs3797535
rs6859667
All
0.00
0.05
0.10
0.15
0.20
MR effect size for
'Selenium' on 'Linker for activation of T−cells family member 1'

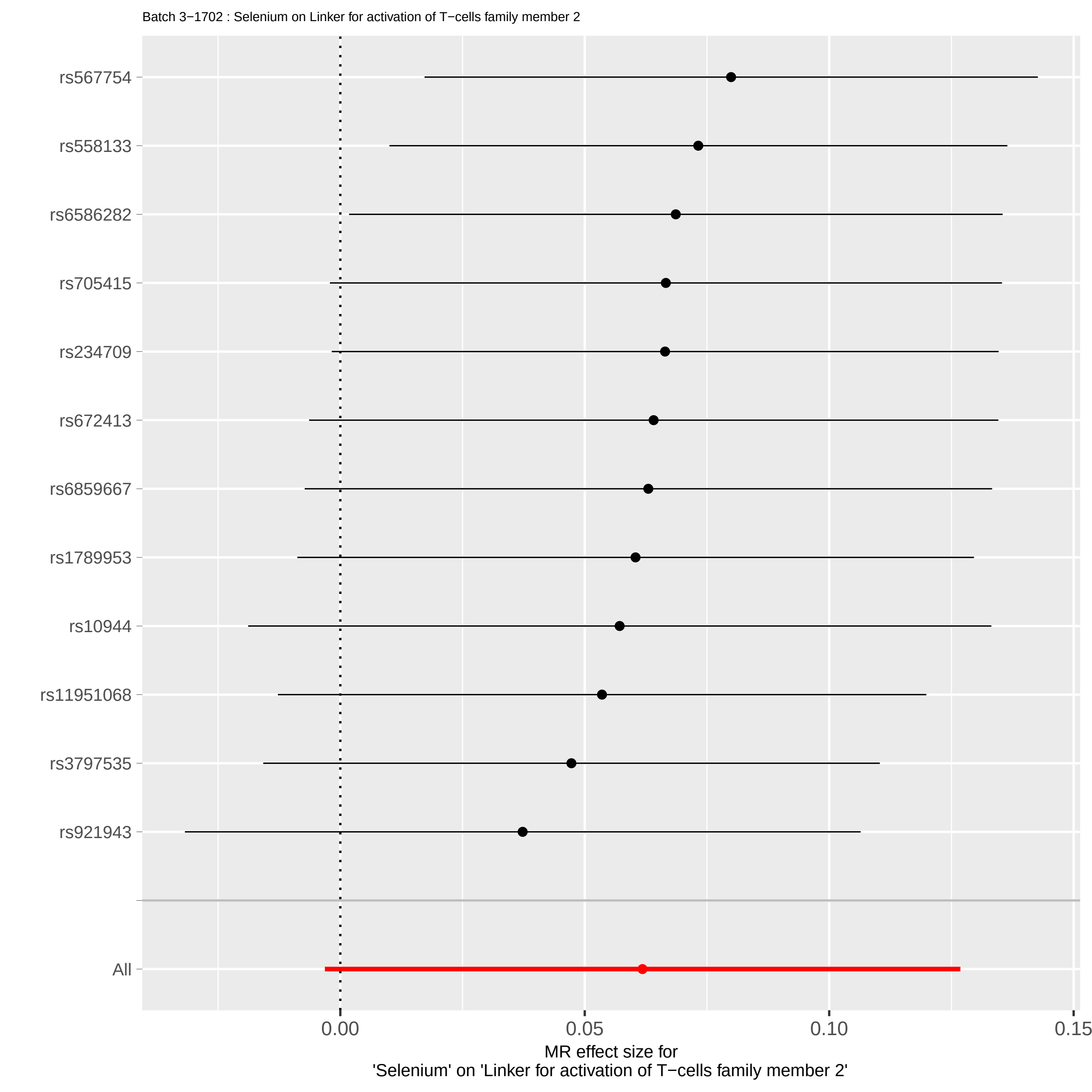

Batch 3−1702 : Selenium on Linker for activation of T−cells family member 2
rs567754
rs558133
rs6586282
rs705415
rs234709
rs672413
rs6859667
rs1789953
rs10944
rs11951068
rs3797535
rs921943
All
0.00
0.05
0.10
0.15
MR effect size for
'Selenium' on 'Linker for activation of T−cells family member 2'

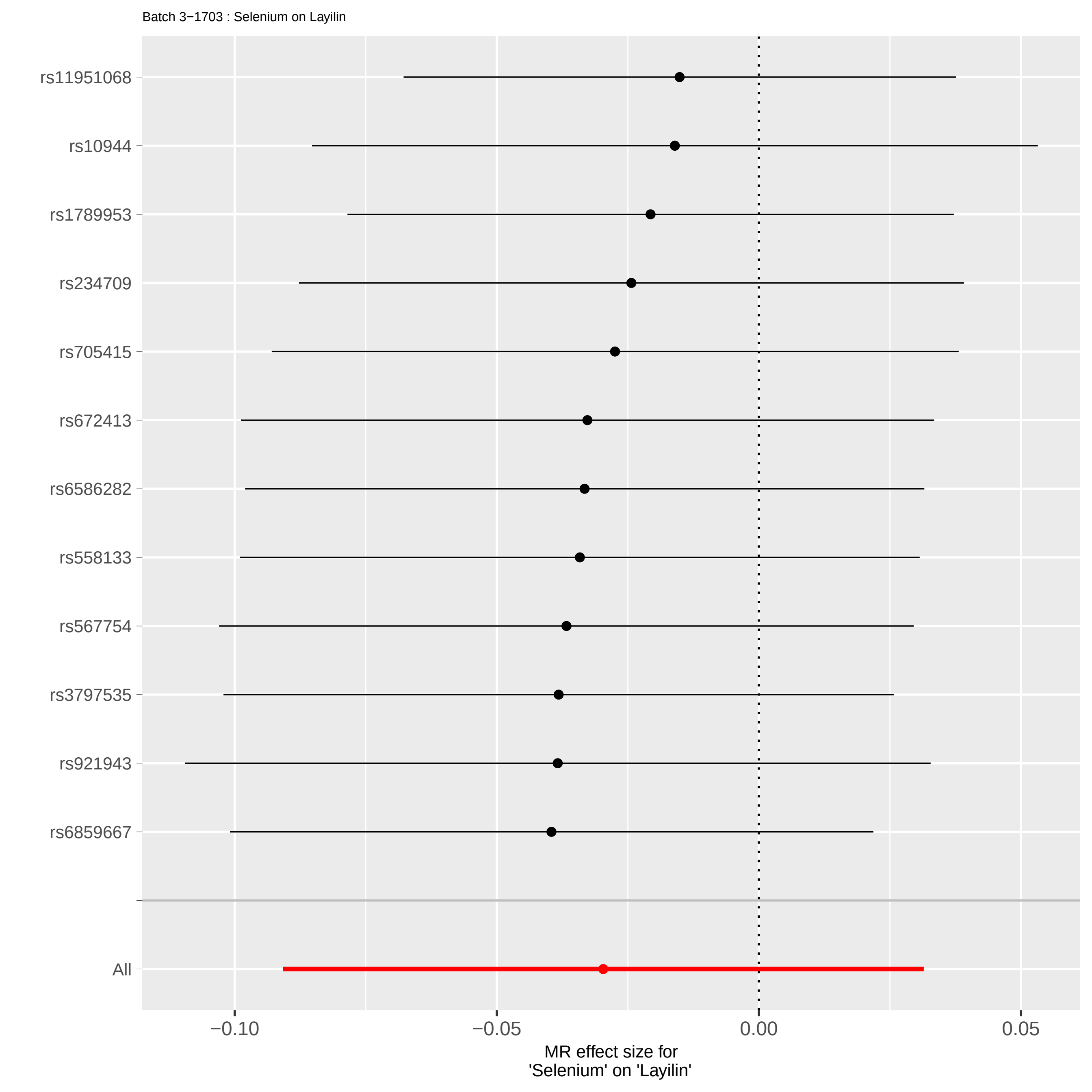
Batch 3−1703 : Selenium on Layilin
rs11951068
rs10944
rs1789953
rs234709
rs705415
rs672413
rs6586282
rs558133
rs567754
rs3797535
rs921943
rs6859667
All
−0.10
−0.05
0.00
0.05
MR effect size for
'Selenium' on 'Layilin'

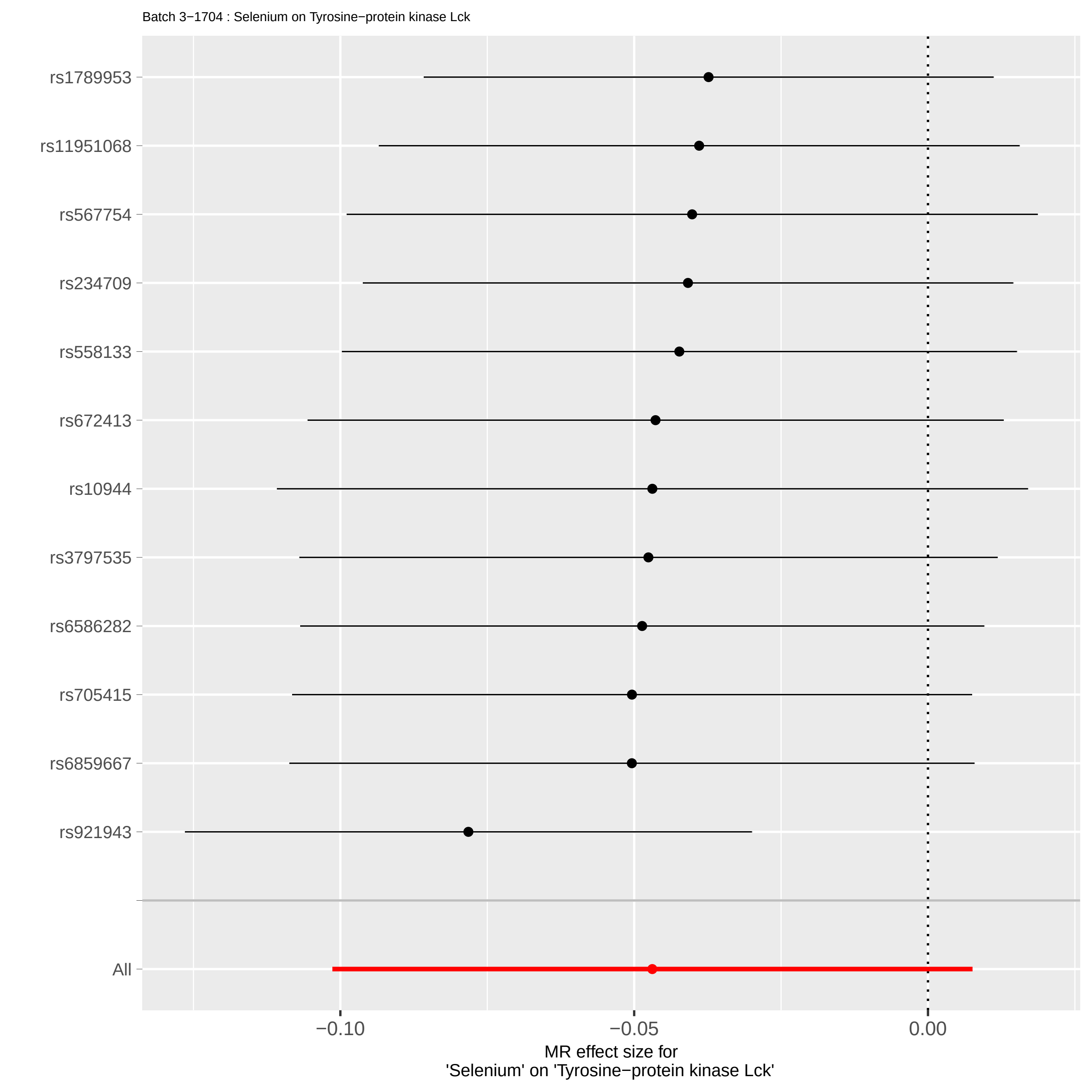

Batch 3−1704 : Selenium on Tyrosine−protein kinase Lck
rs1789953
rs11951068
rs567754
rs234709
rs558133
rs672413
rs10944
rs3797535
rs6586282
rs705415
rs6859667
rs921943
All
−0.10
−0.05
0.00
MR effect size for
'Selenium' on 'Tyrosine−protein kinase Lck'

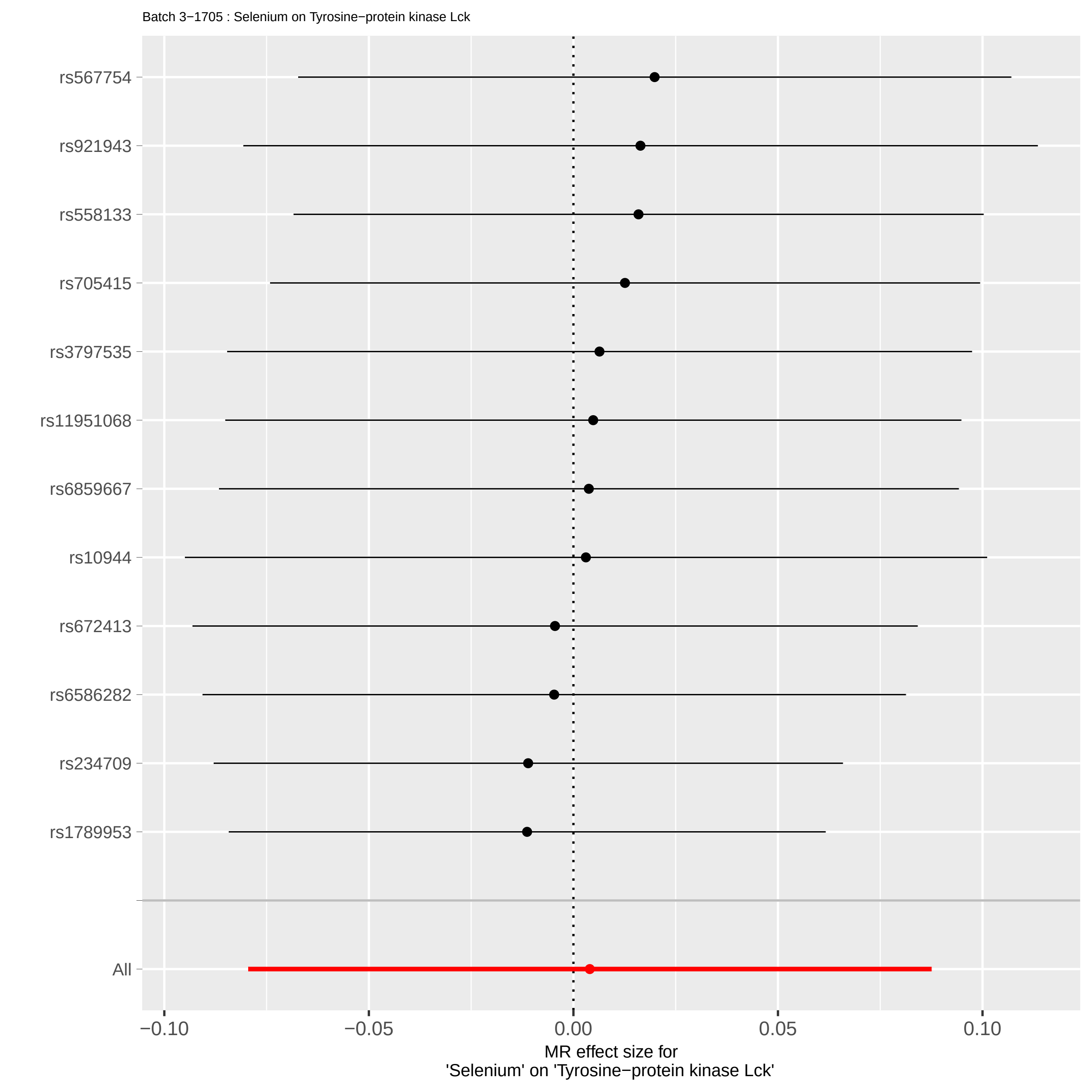

Batch 3−1705 : Selenium on Tyrosine−protein kinase Lck
rs567754
rs921943
rs558133
rs705415
rs3797535
rs11951068
rs6859667
rs10944
rs672413
rs6586282
rs234709
rs1789953
All
−0.10
−0.05
0.00
0.05
0.10
MR effect size for
'Selenium' on 'Tyrosine−protein kinase Lck'

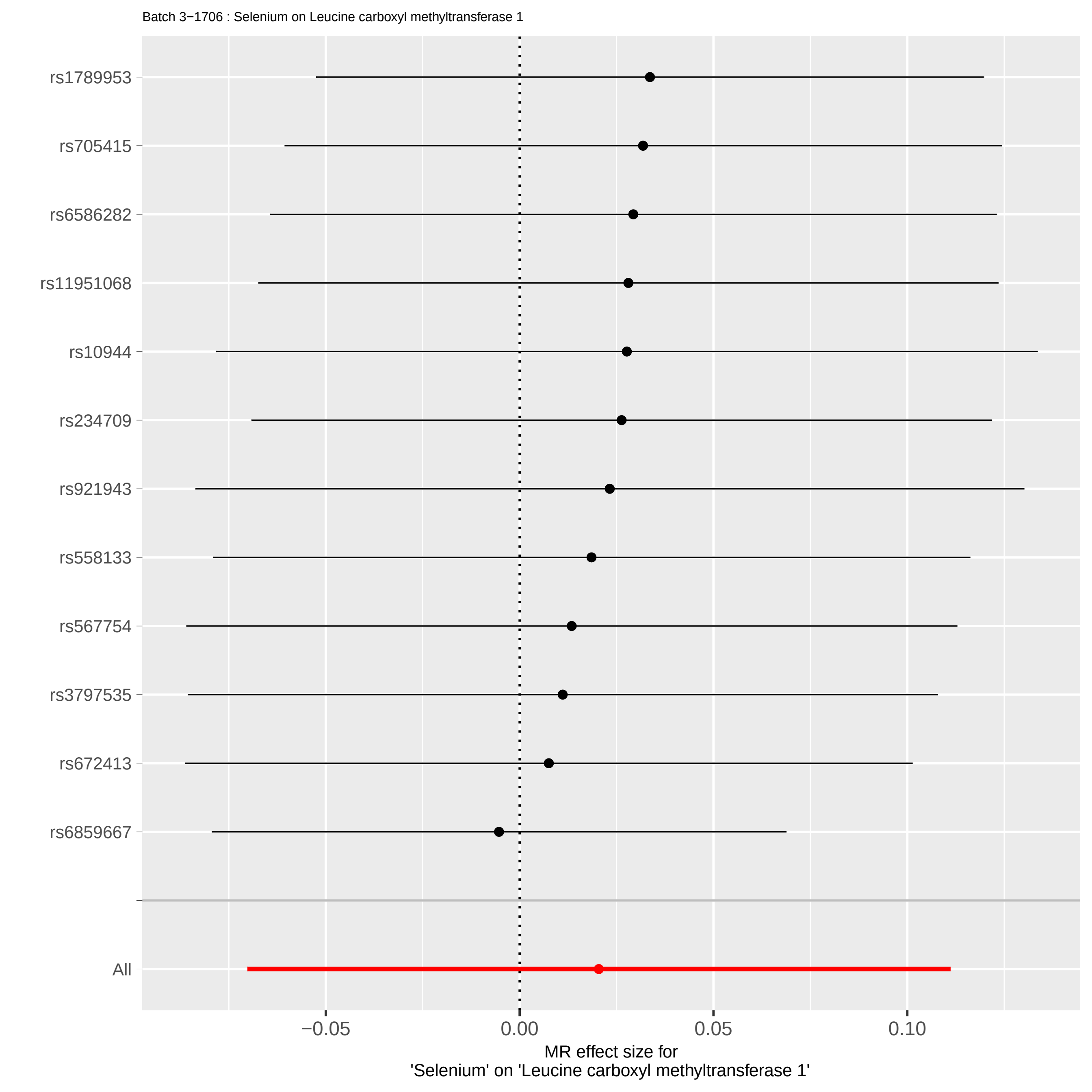

Batch 3−1706 : Selenium on Leucine carboxyl methyltransferase 1
rs1789953
rs705415
rs6586282
rs11951068
rs10944
rs234709
rs921943
rs558133
rs567754
rs3797535
rs672413
rs6859667
All
−0.05
0.00
0.05
0.10
MR effect size for
'Selenium' on 'Leucine carboxyl methyltransferase 1'

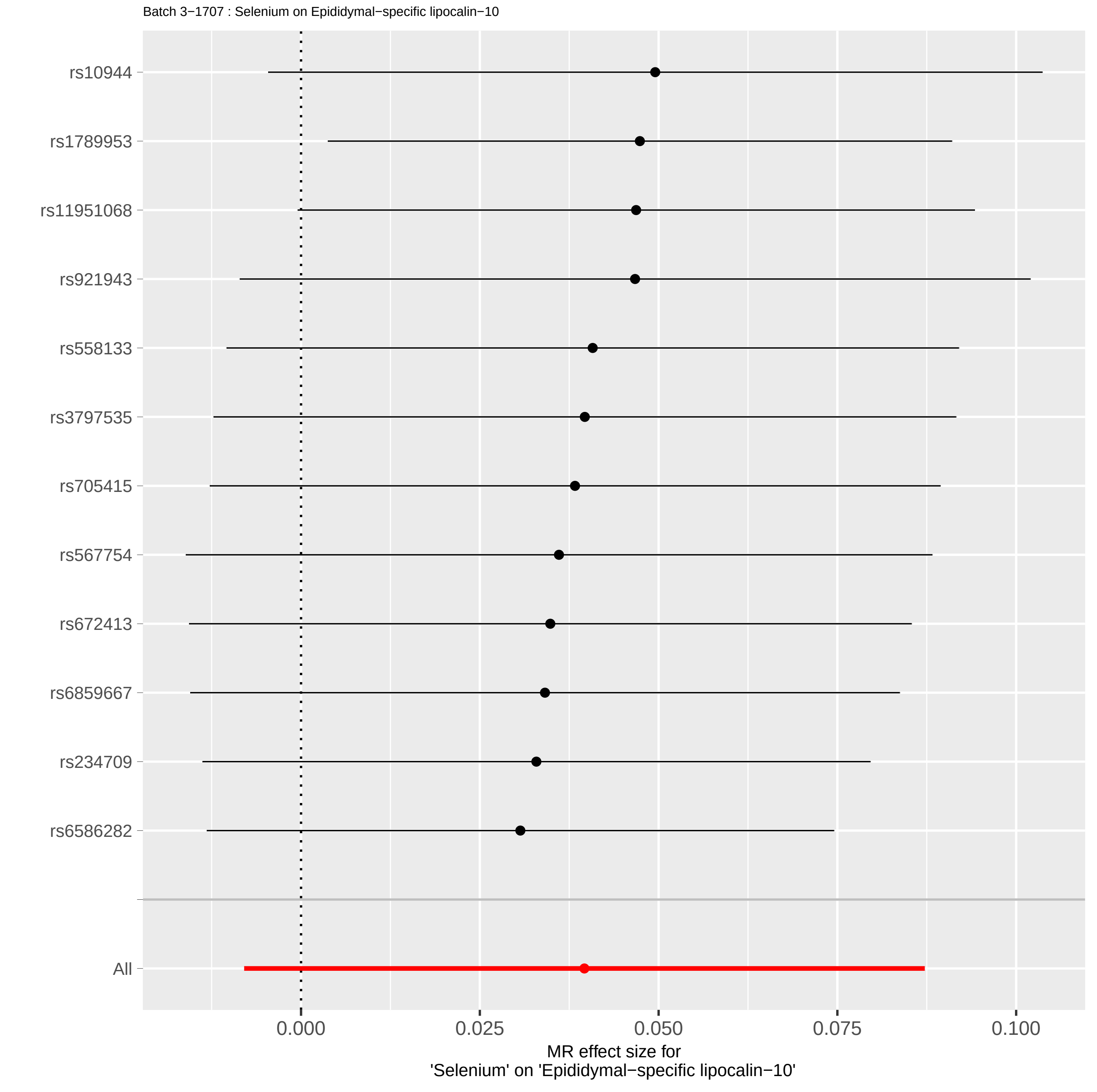
Batch 3−1707 : Selenium on Epididymal−specific lipocalin−10
rs10944
rs1789953
rs11951068
rs921943
rs558133
rs3797535
rs705415
rs567754
rs672413
rs6859667
rs234709
rs6586282
All
0.000
0.025
0.050
0.075
0.100
MR effect size for
'Selenium' on 'Epididymal−specific lipocalin−10'

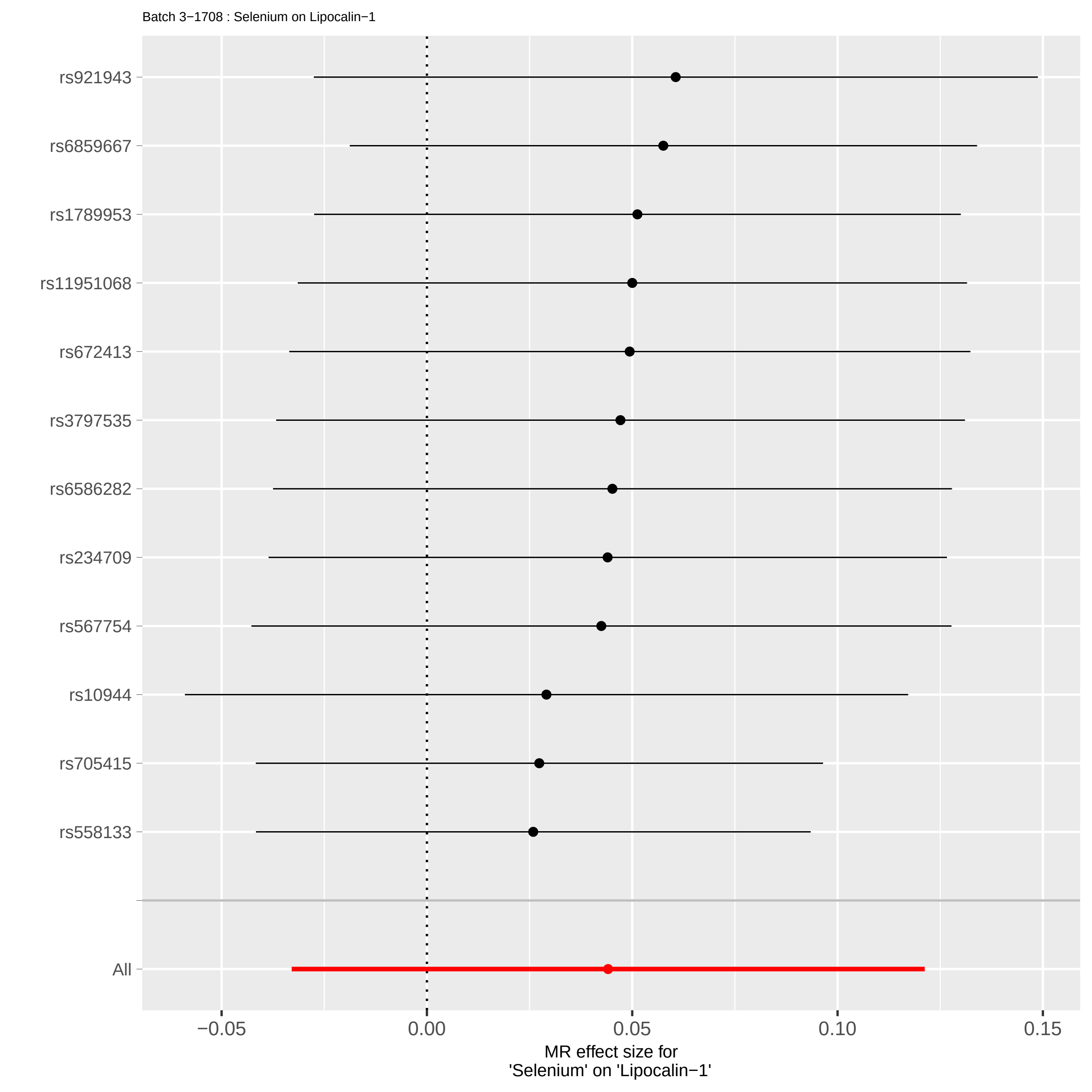

Batch 3−1708 : Selenium on Lipocalin−1
rs921943
rs6859667
rs1789953
rs11951068
rs672413
rs3797535
rs6586282
rs234709
rs567754
rs10944
rs705415
rs558133
All
−0.05
0.00
0.05
0.10
0.15
MR effect size for
'Selenium' on 'Lipocalin−1'

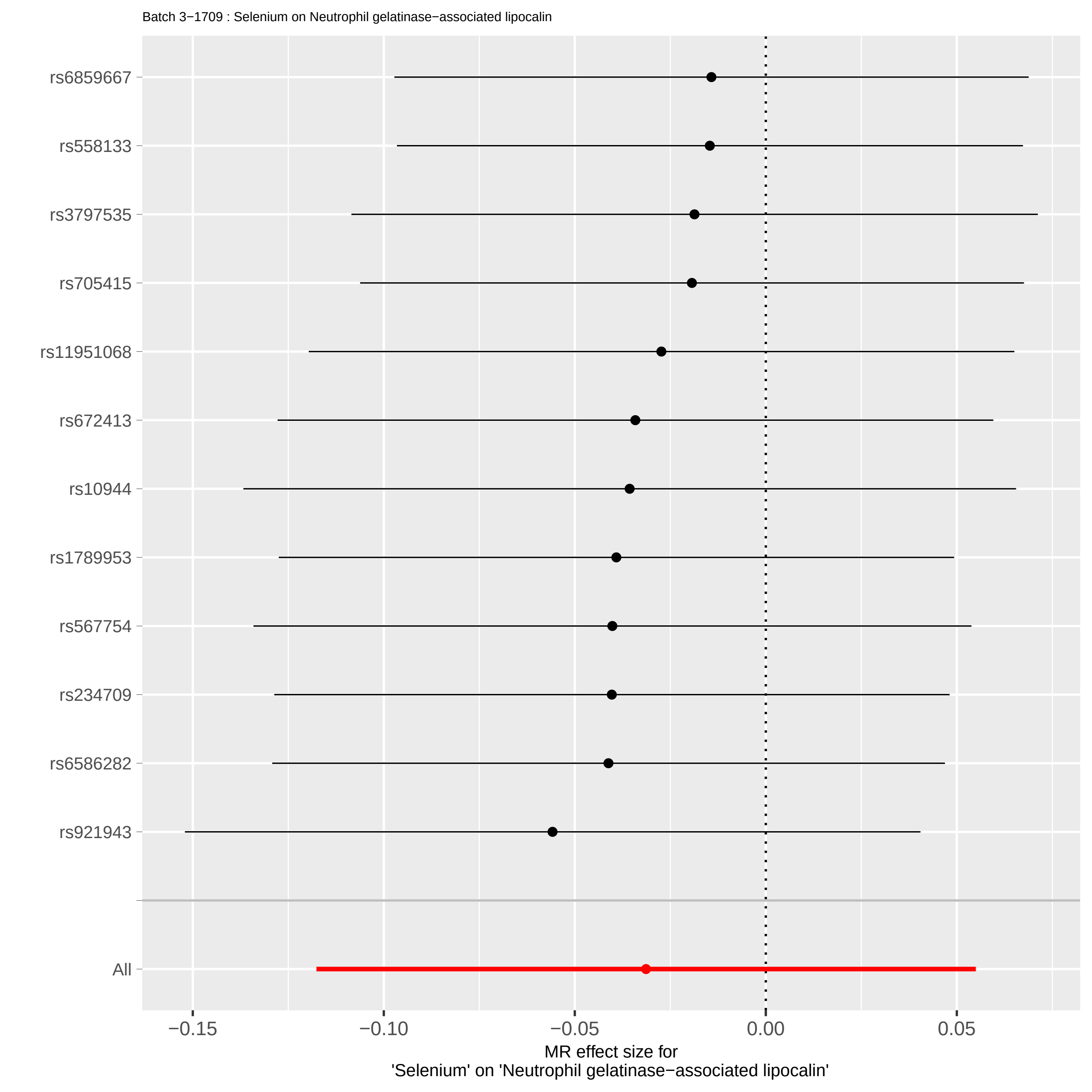

Batch 3−1709 : Selenium on Neutrophil gelatinase−associated lipocalin
rs6859667
rs558133
rs3797535
rs705415
rs11951068
rs672413
rs10944
rs1789953
rs567754
rs234709
rs6586282
rs921943
All
−0.15
−0.10
−0.05
0.00
0.05
MR effect size for
'Selenium' on 'Neutrophil gelatinase−associated lipocalin'

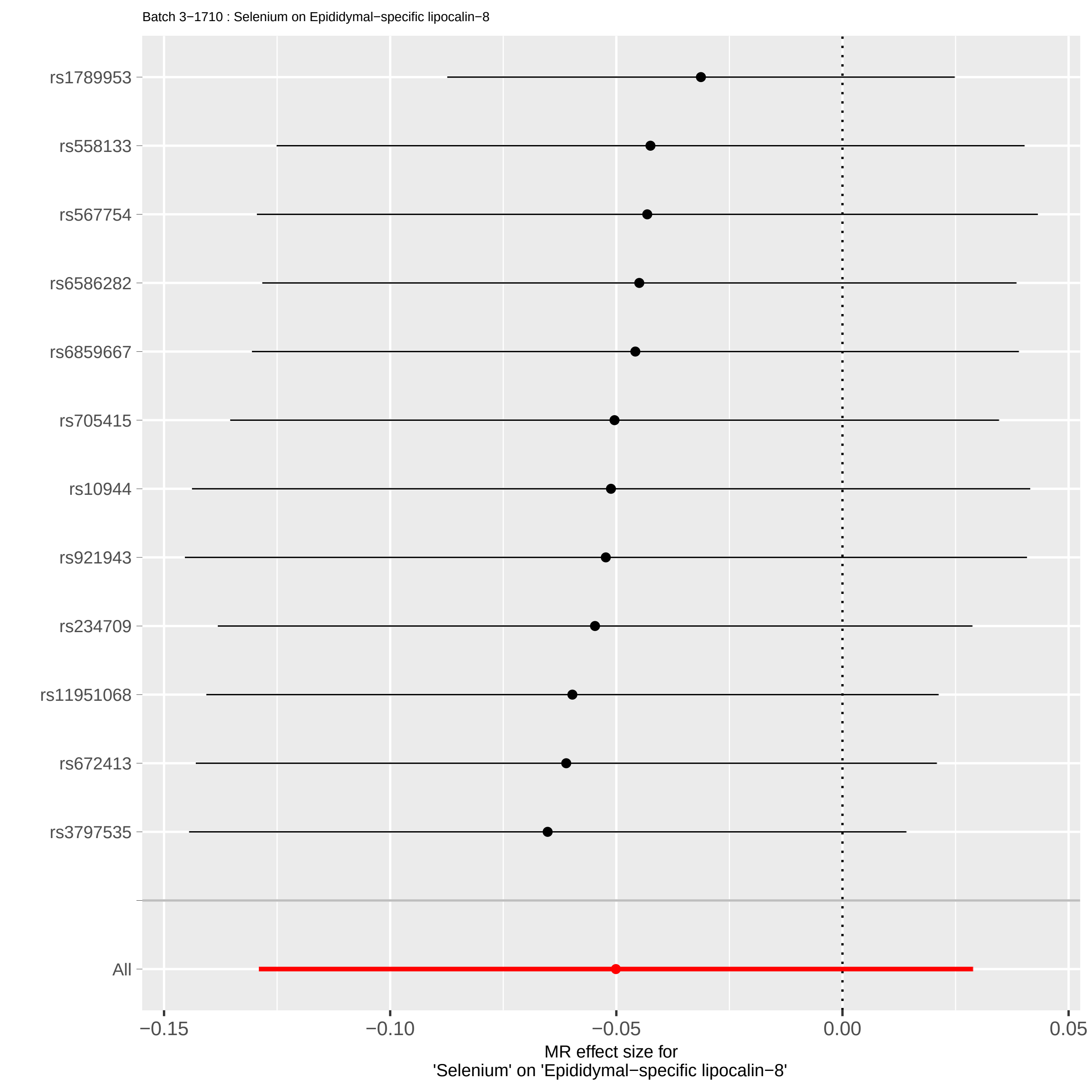

Batch 3−1710 : Selenium on Epididymal−specific lipocalin−8
rs1789953
rs558133
rs567754
rs6586282
rs6859667
rs705415
rs10944
rs921943
rs234709
rs11951068
rs672413
rs3797535
All
−0.15
−0.10
−0.05
0.00
0.05
MR effect size for
'Selenium' on 'Epididymal−specific lipocalin−8'

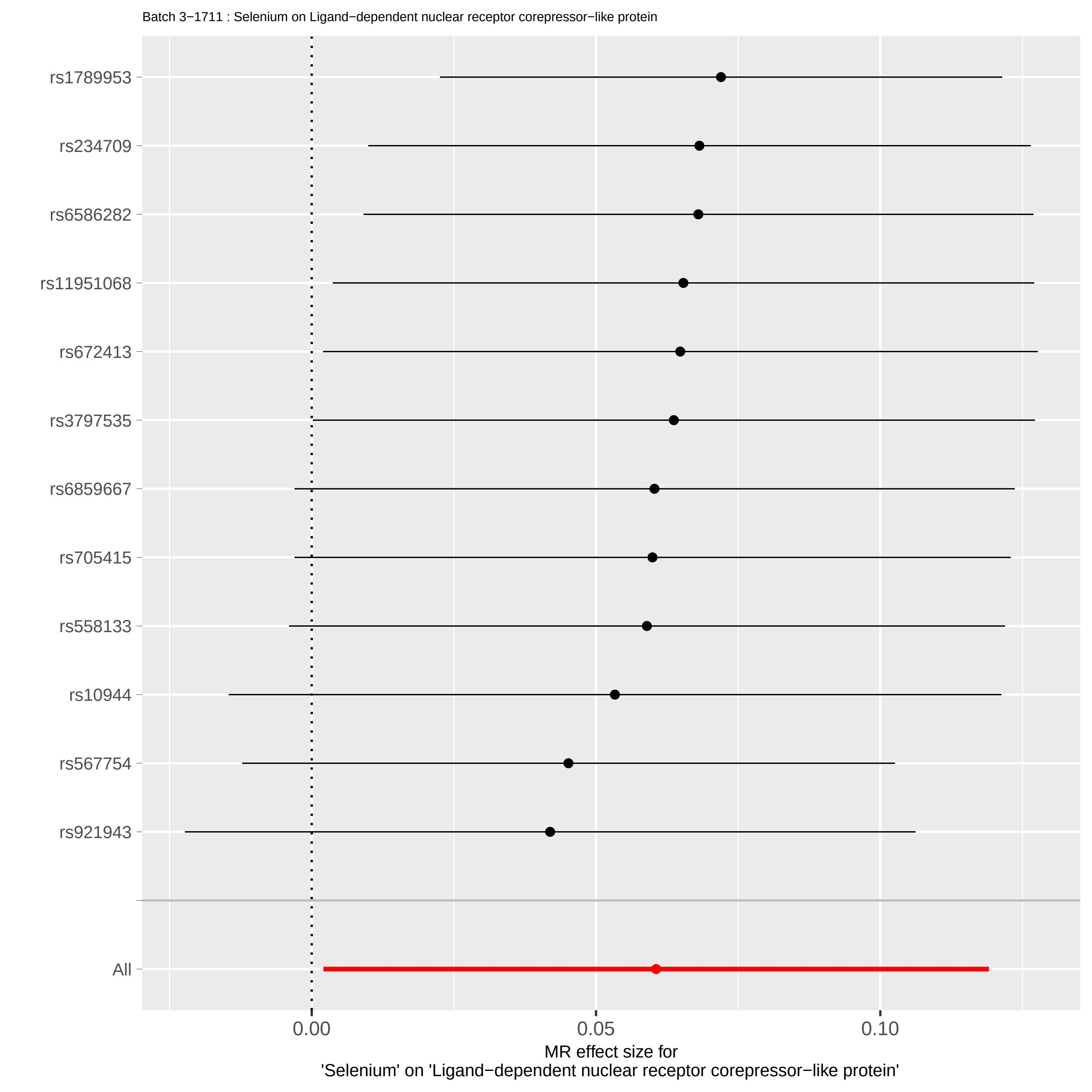
Batch 3−1711 : Selenium on Ligand−dependent nuclear receptor corepressor−like protein
rs1789953
rs234709
rs6586282
rs11951068
rs672413
rs3797535
rs6859667
rs705415
rs558133
rs10944
rs567754
rs921943
All
0.00
0.05
0.10
MR effect size for
'Selenium' on 'Ligand−dependent nuclear receptor corepressor−like protein'

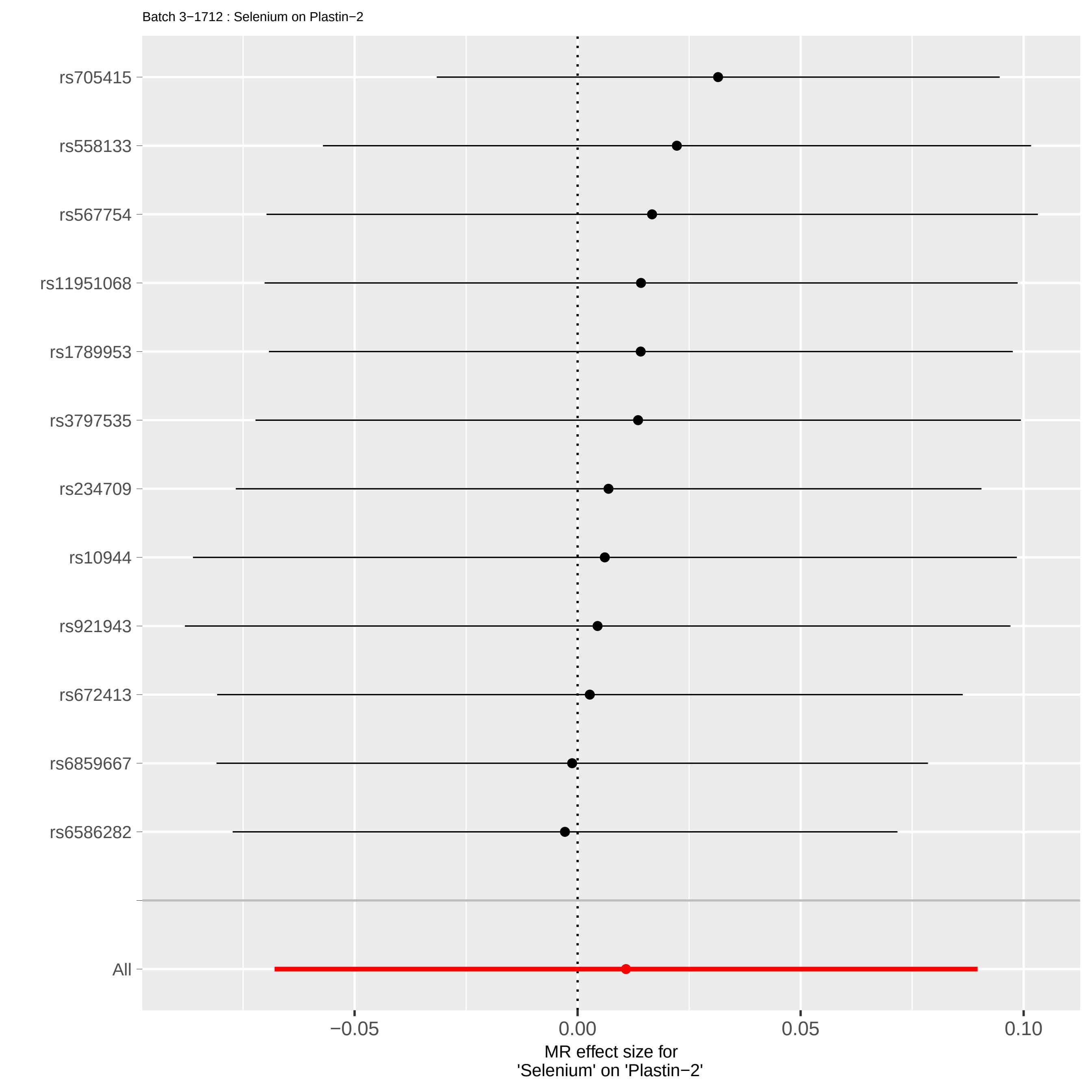
Batch 3−1712 : Selenium on Plastin−2
rs705415
rs558133
rs567754
rs11951068
rs1789953
rs3797535
rs234709
rs10944
rs921943
rs672413
rs6859667
rs6586282
All
−0.05
0.00
0.05
0.10
MR effect size for
'Selenium' on 'Plastin−2'

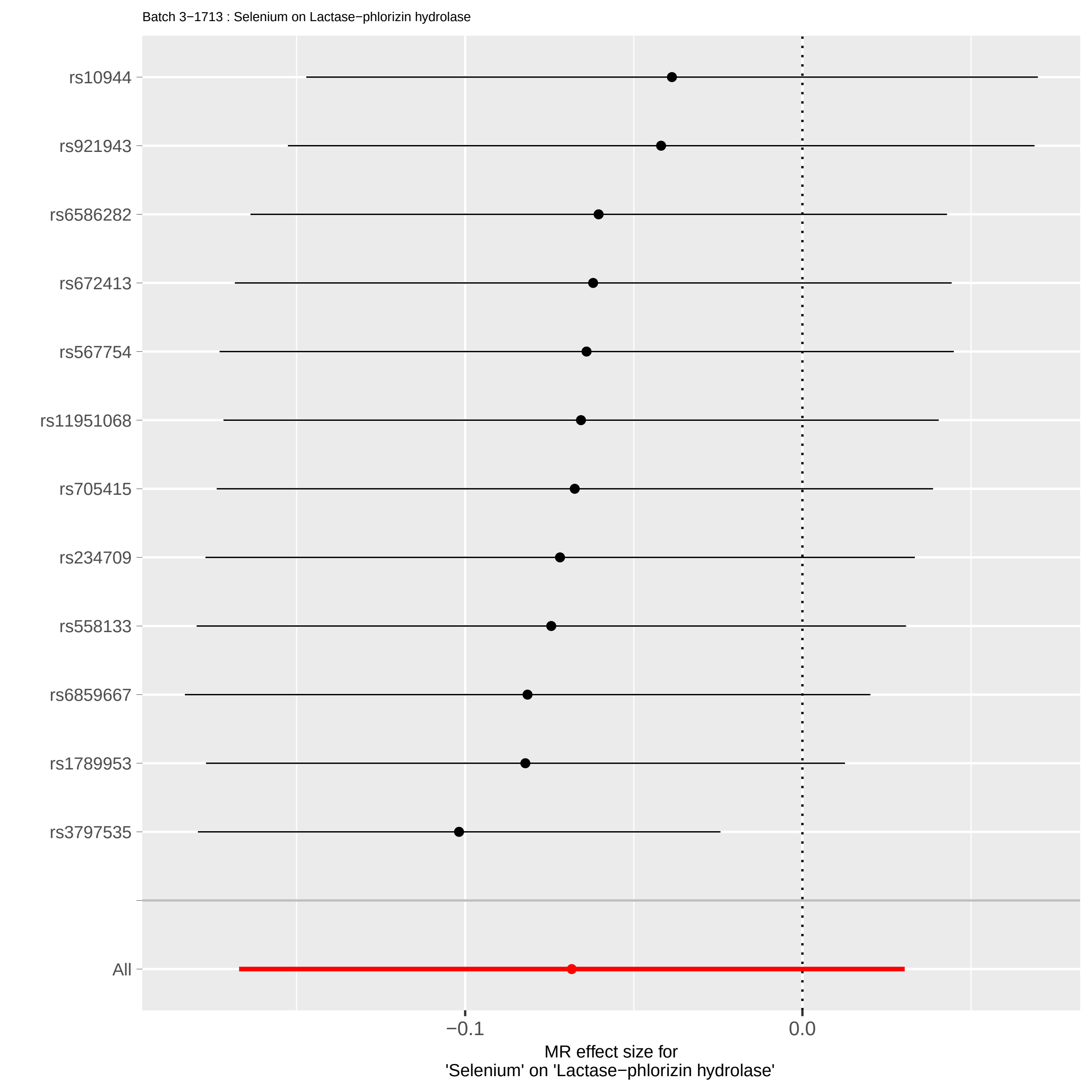

Batch 3−1713 : Selenium on Lactase−phlorizin hydrolase
rs10944
rs921943
rs6586282
rs672413
rs567754
rs11951068
rs705415
rs234709
rs558133
rs6859667
rs1789953
rs3797535
All
−0.1
0.0
MR effect size for
'Selenium' on 'Lactase−phlorizin hydrolase'

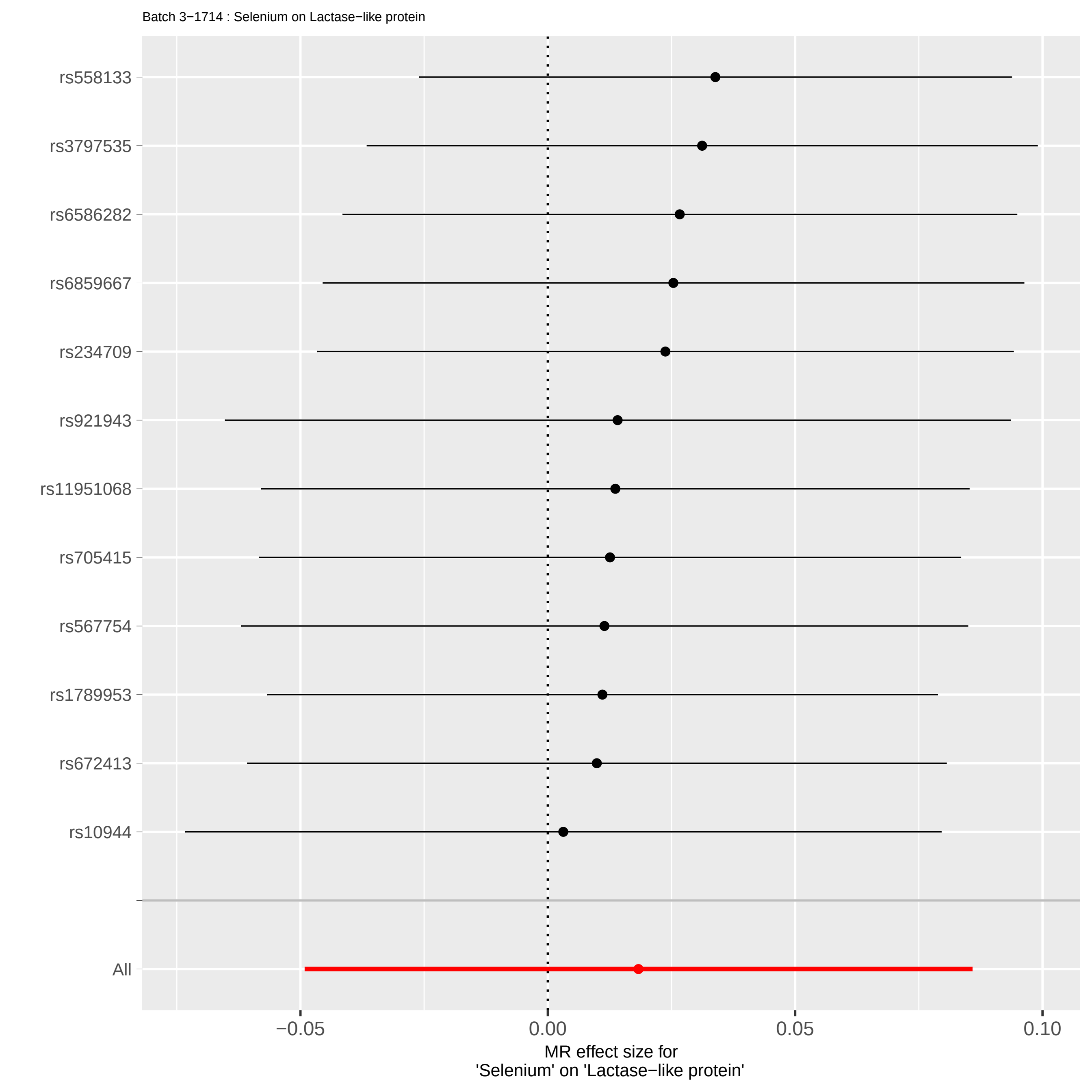

Batch 3−1714 : Selenium on Lactase−like protein
rs558133
rs3797535
rs6586282
rs6859667
rs234709
rs921943
rs11951068
rs705415
rs567754
rs1789953
rs672413
rs10944
All
−0.05
0.00
0.05
0.10
MR effect size for
'Selenium' on 'Lactase−like protein'

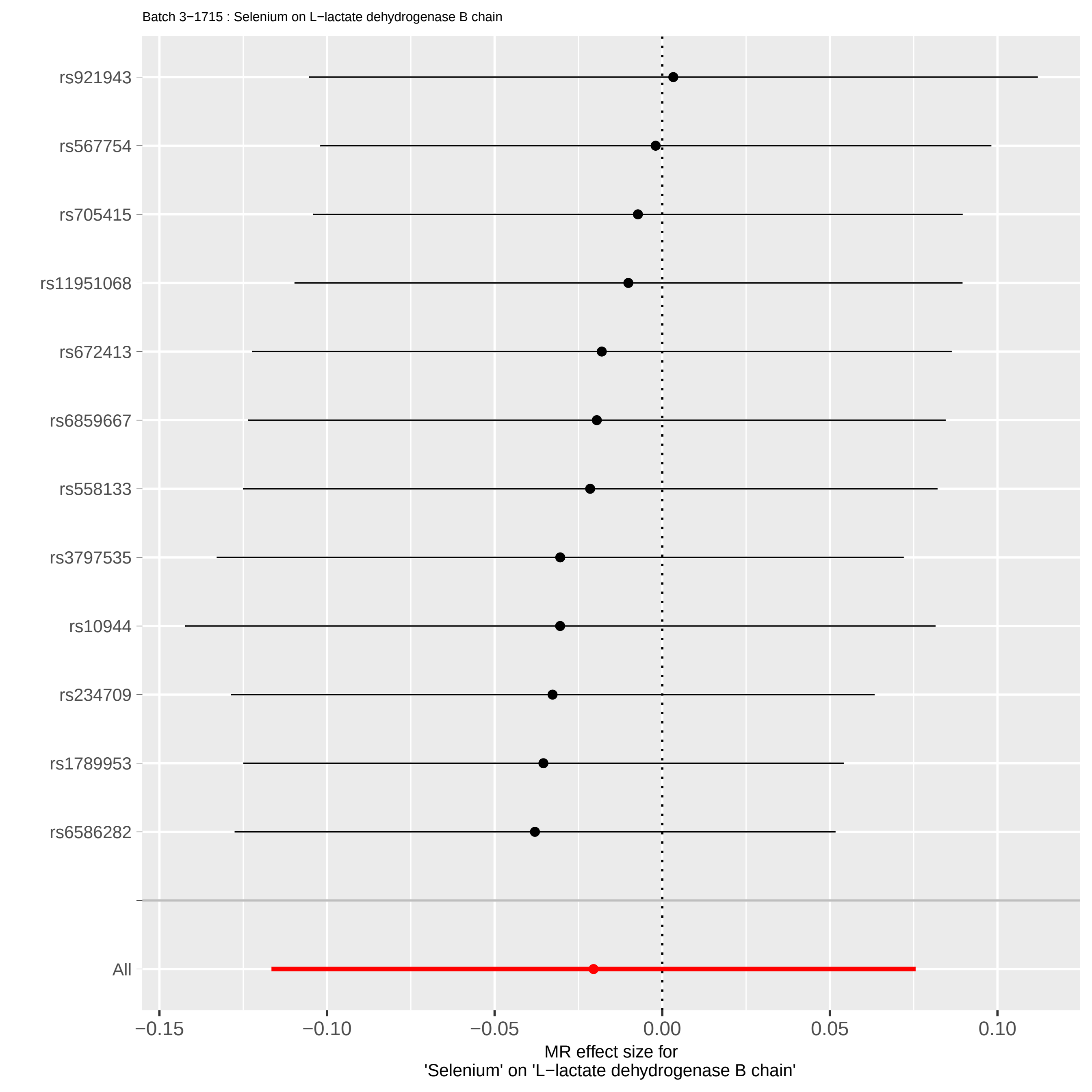

Batch 3−1715 : Selenium on L−lactate dehydrogenase B chain
rs921943
rs567754
rs705415
rs11951068
rs672413
rs6859667
rs558133
rs3797535
rs10944
rs234709
rs1789953
rs6586282
All
−0.15
−0.10
−0.05
0.00
0.05
0.10
MR effect size for
'Selenium' on 'L−lactate dehydrogenase B chain'

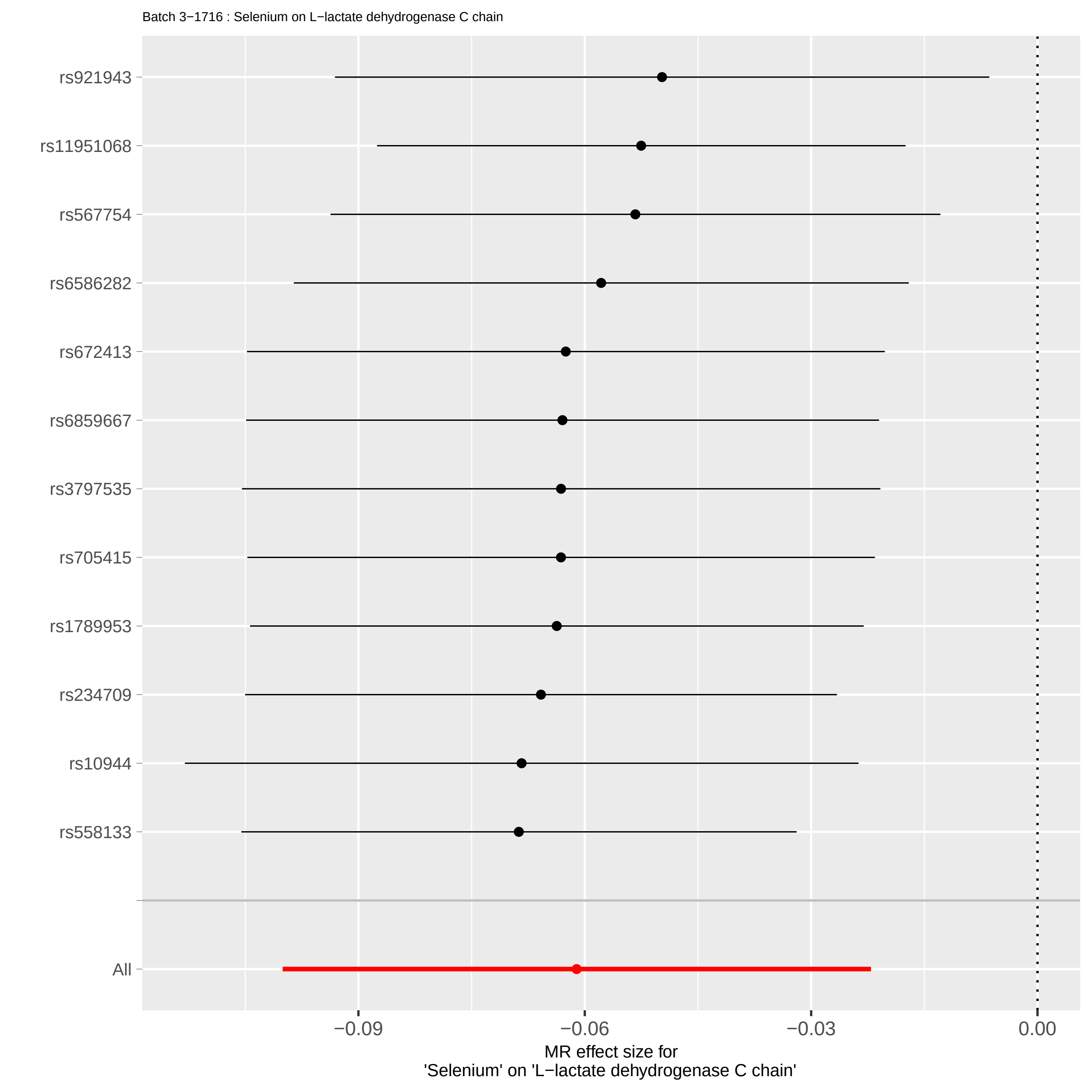

Batch 3−1716 : Selenium on L−lactate dehydrogenase C chain
rs921943
rs11951068
rs567754
rs6586282
rs672413
rs6859667
rs3797535
rs705415
rs1789953
rs234709
rs10944
rs558133
All
−0.09
−0.06
−0.03
0.00
MR effect size for
'Selenium' on 'L−lactate dehydrogenase C chain'

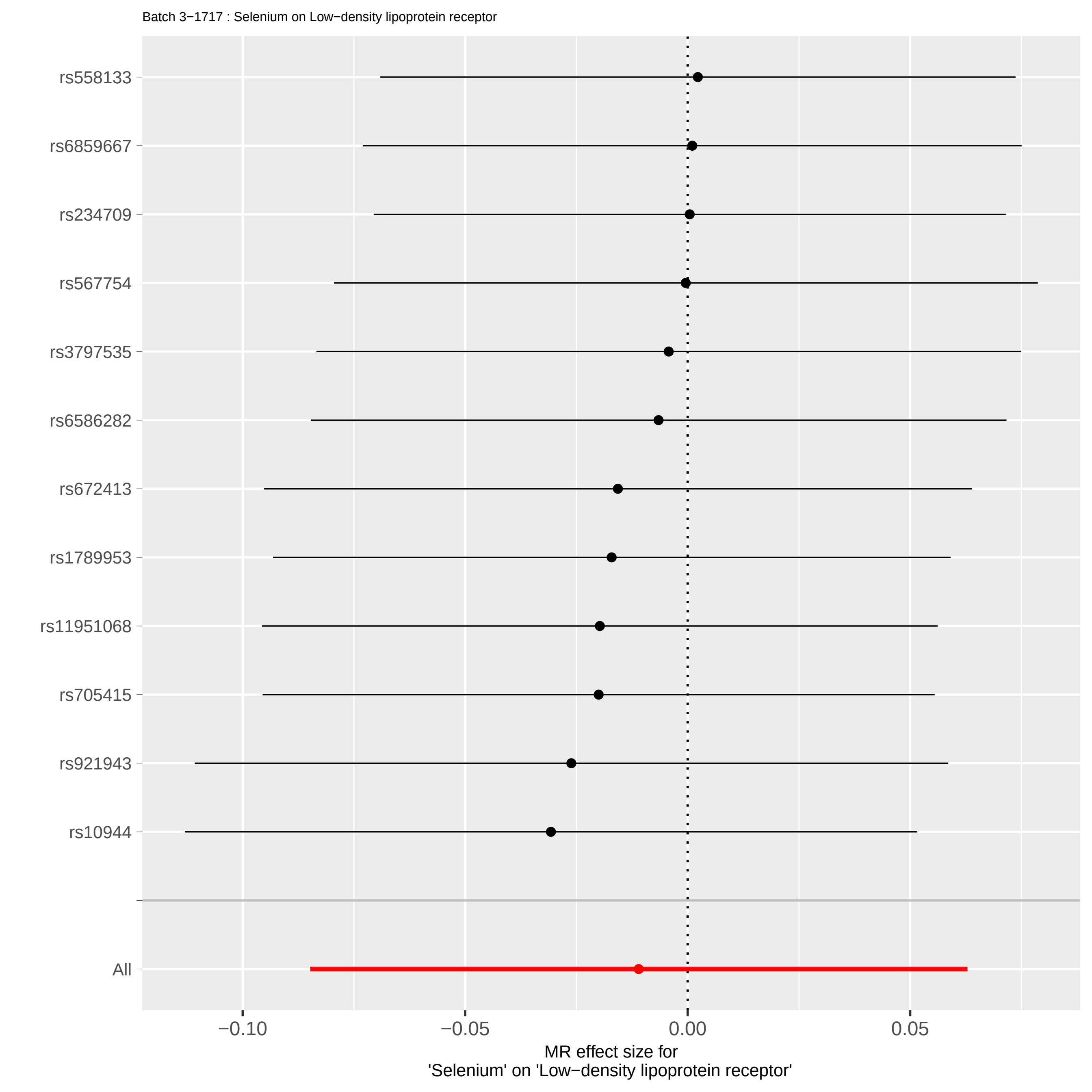

Batch 3−1717 : Selenium on Low−density lipoprotein receptor
rs558133
rs6859667
rs234709
rs567754
rs3797535
rs6586282
rs672413
rs1789953
rs11951068
rs705415
rs921943
rs10944
All
−0.10
−0.05
0.00
0.05
MR effect size for
'Selenium' on 'Low−density lipoprotein receptor'

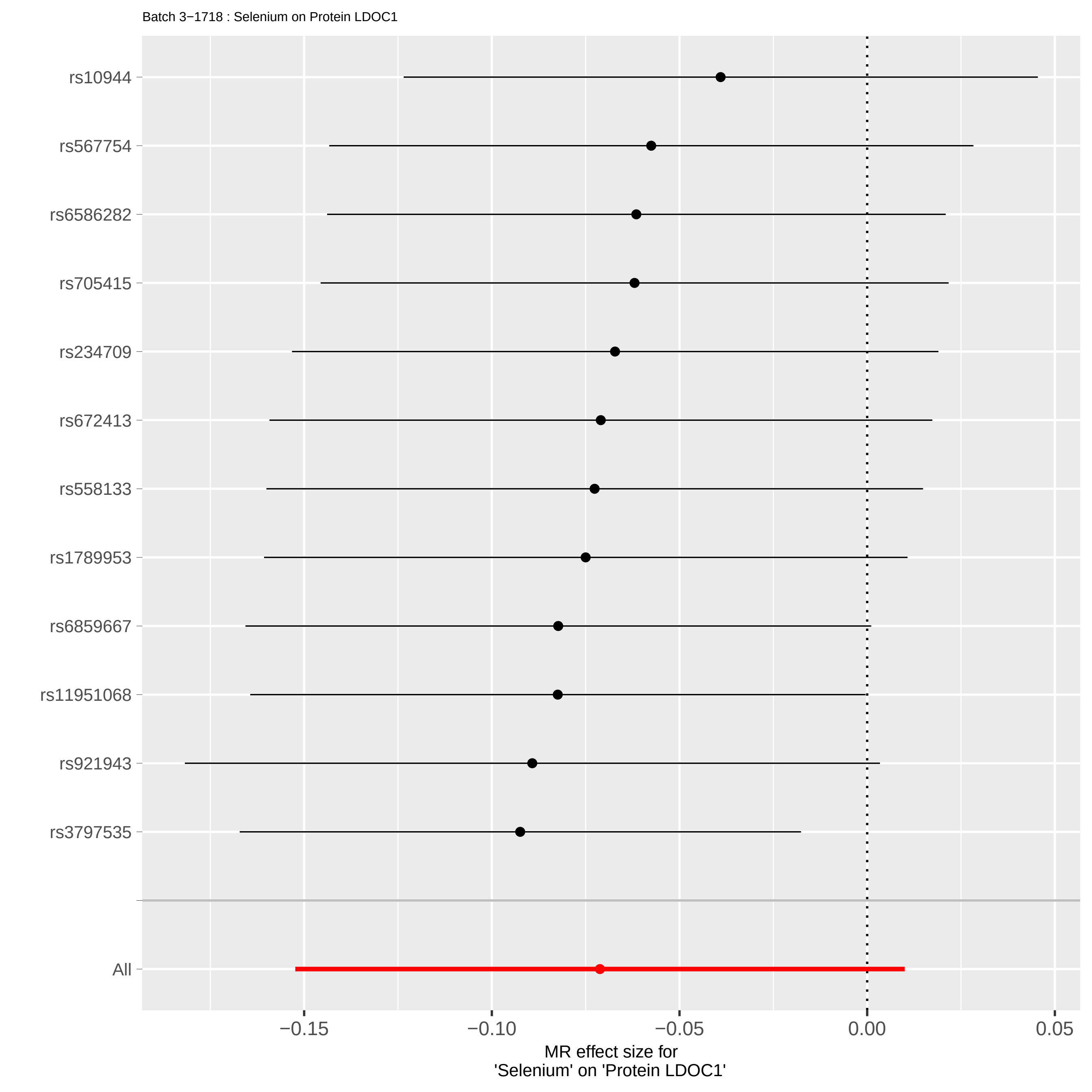

Batch 3−1718 : Selenium on Protein LDOC1
rs10944
rs567754
rs6586282
rs705415
rs234709
rs672413
rs558133
rs1789953
rs6859667
rs11951068
rs921943
rs3797535
All
−0.15
−0.10
−0.05
0.00
0.05
MR effect size for
'Selenium' on 'Protein LDOC1'

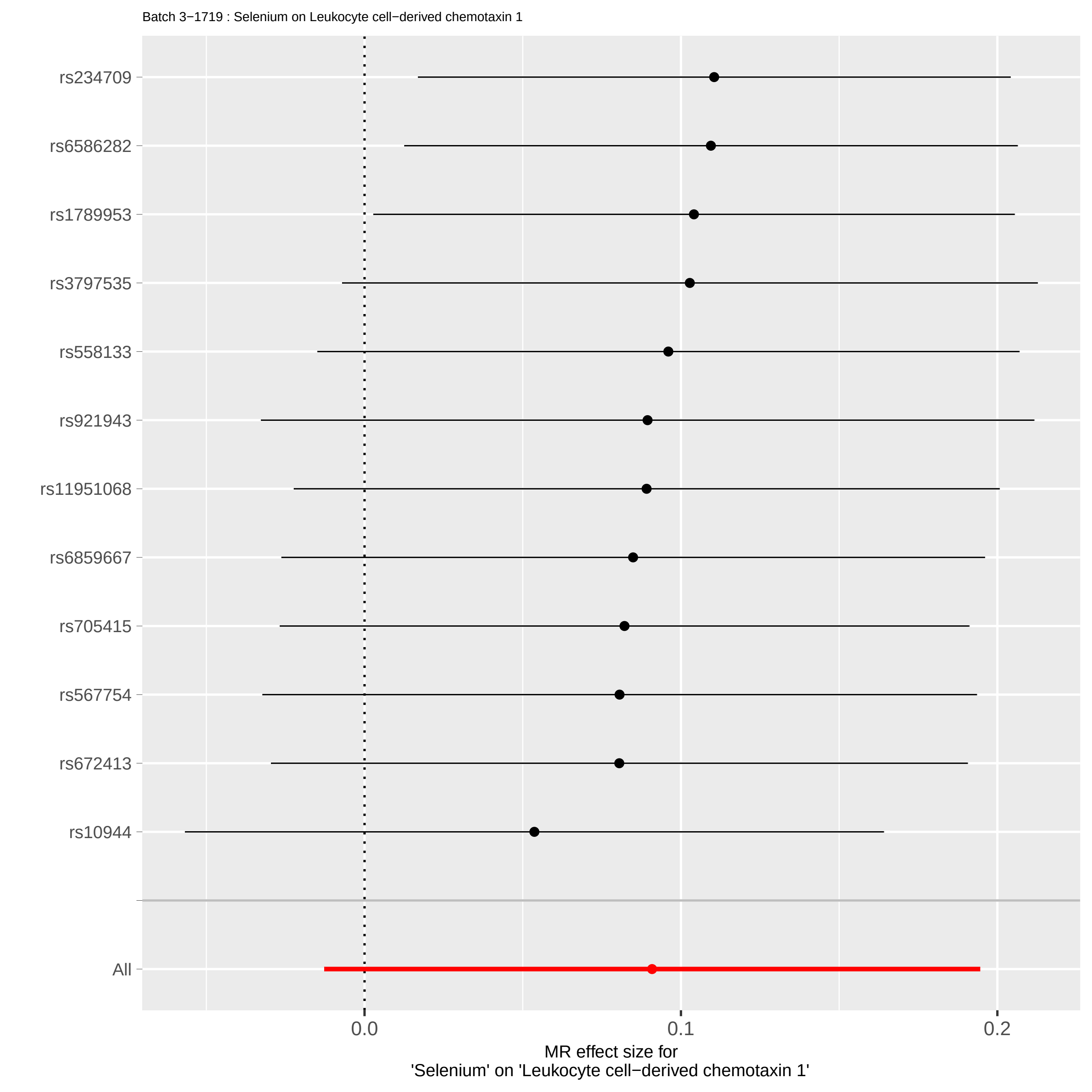

Batch 3−1719 : Selenium on Leukocyte cell−derived chemotaxin 1
rs234709
rs6586282
rs1789953
rs3797535
rs558133
rs921943
rs11951068
rs6859667
rs705415
rs567754
rs672413
rs10944
All
0.0
0.1
0.2
MR effect size for
'Selenium' on 'Leukocyte cell−derived chemotaxin 1'

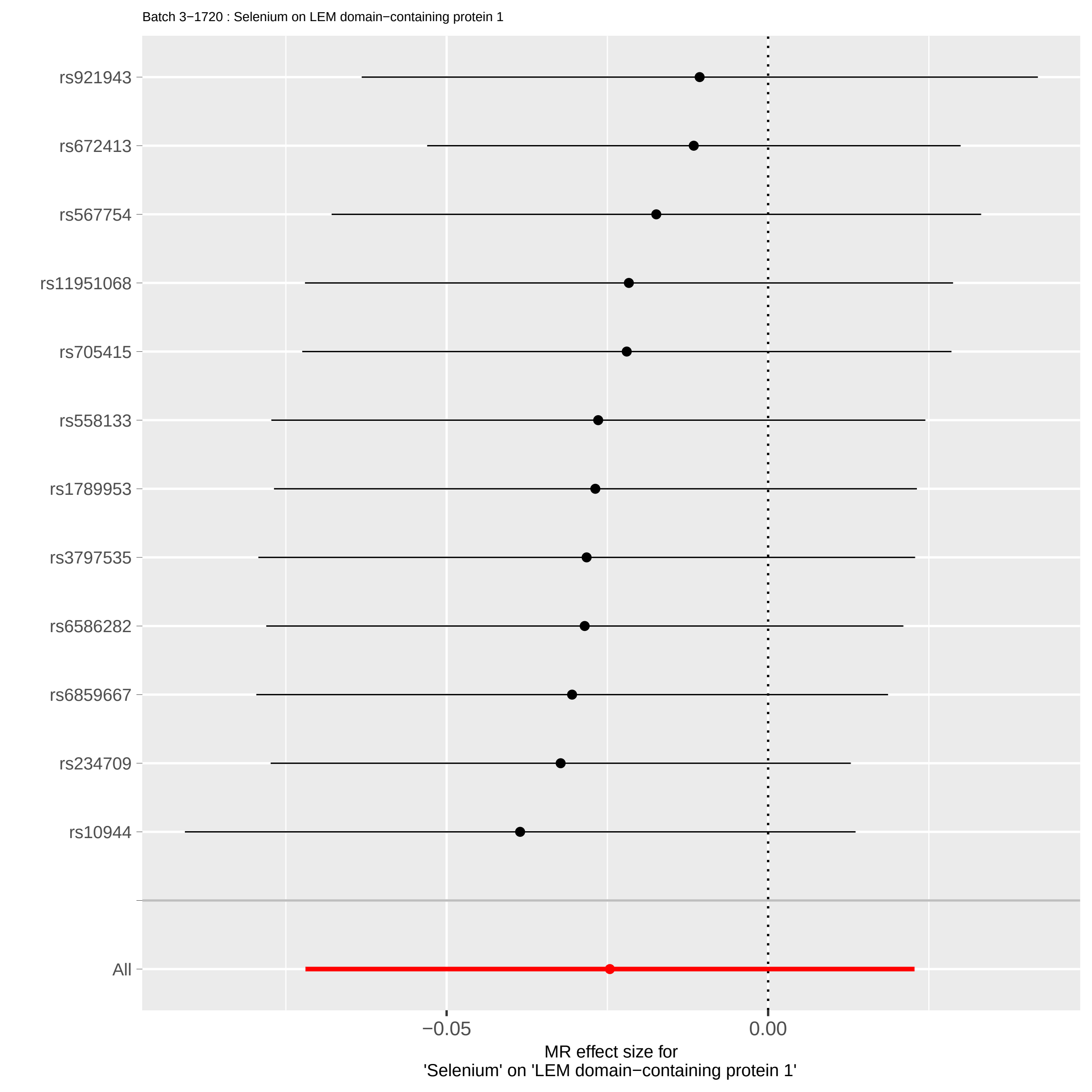
Batch 3−1720 : Selenium on LEM domain−containing protein 1
rs921943
rs672413
rs567754
rs11951068
rs705415
rs558133
rs1789953
rs3797535
rs6586282
rs6859667
rs234709
rs10944
All
−0.05
0.00
MR effect size for
'Selenium' on 'LEM domain−containing protein 1'

Batch 3−1721 : Selenium on Leptin

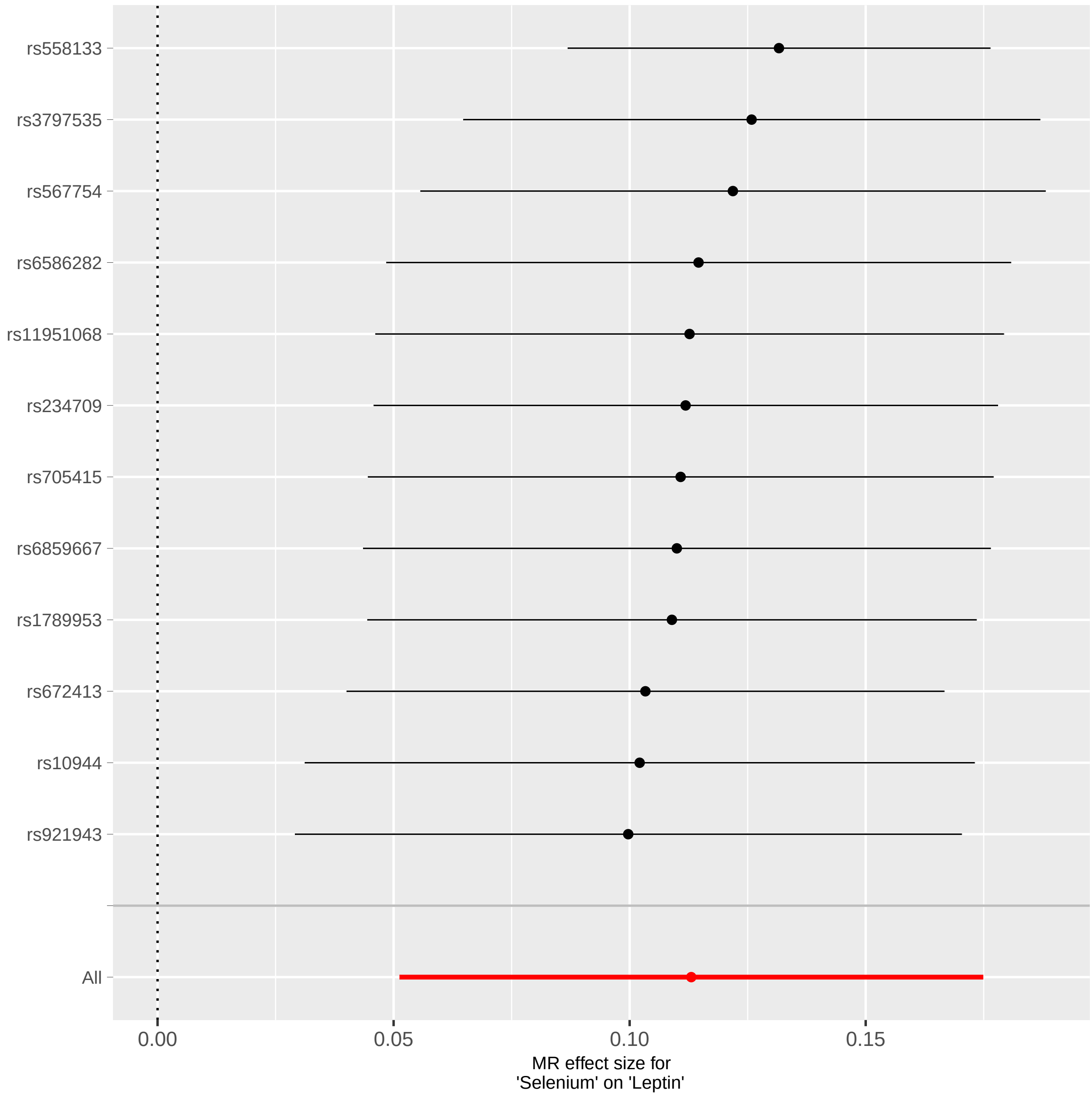

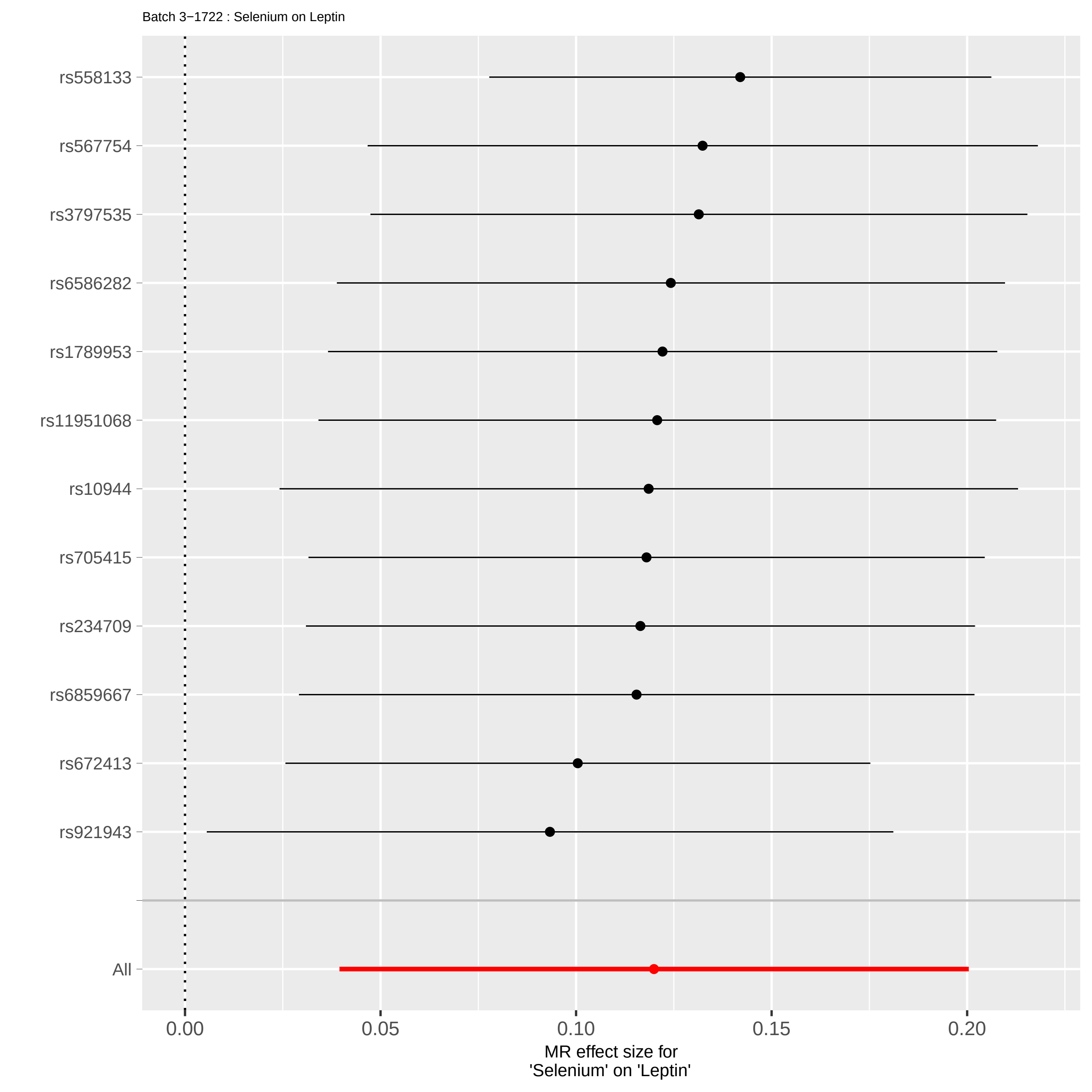
Batch 3−1722 : Selenium on Leptin
rs558133
rs567754
rs3797535
rs6586282
rs1789953
rs11951068
rs10944
rs705415
rs234709
rs6859667
rs672413
rs921943
All
0.00
0.05
0.10
0.15
0.20
MR effect size for
'Selenium' on 'Leptin'

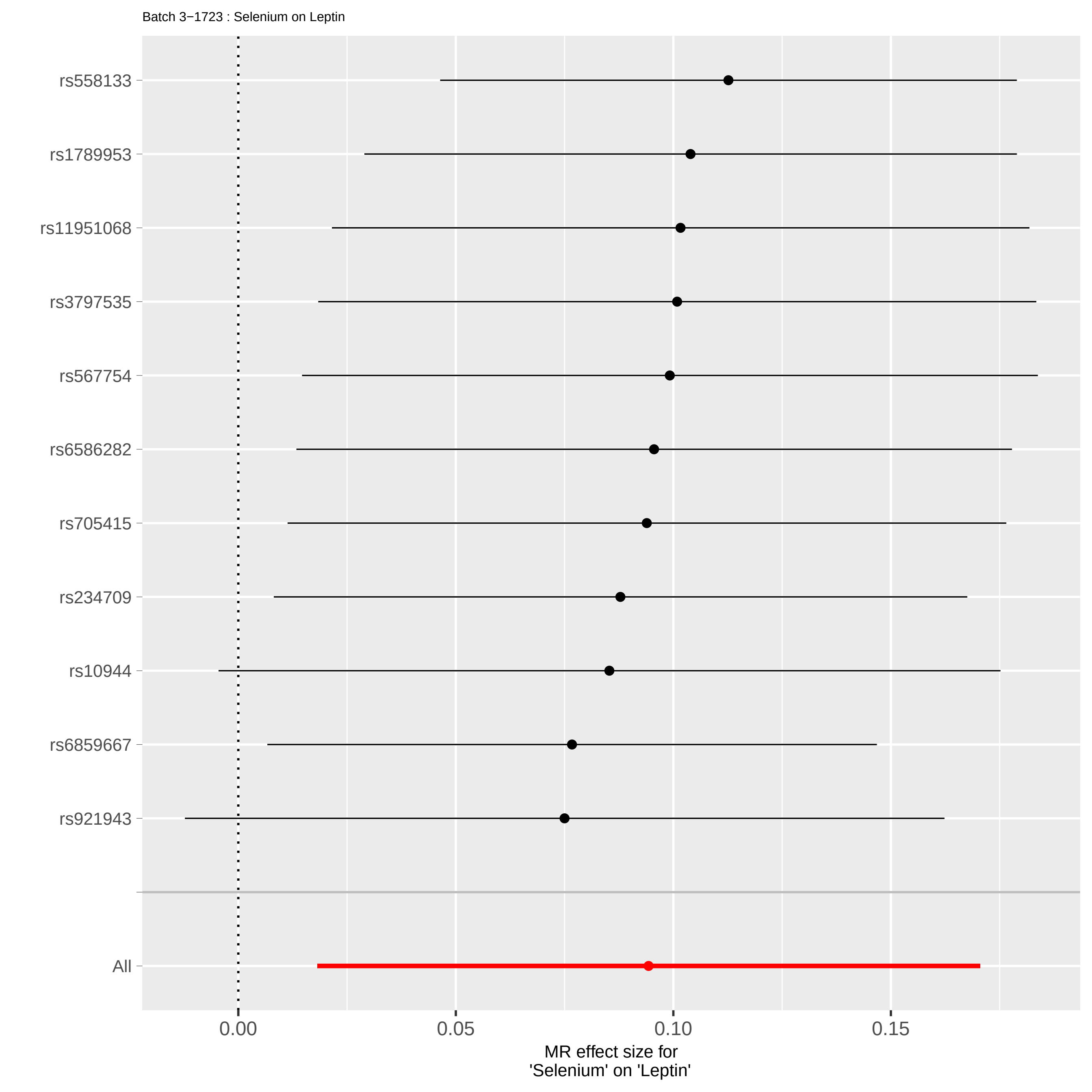

Batch 3−1723 : Selenium on Leptin
rs558133
rs1789953
rs11951068
rs3797535
rs567754
rs6586282
rs705415
rs234709
rs10944
rs6859667
rs921943
All
0.00
0.05
0.10
0.15
MR effect size for
'Selenium' on 'Leptin'

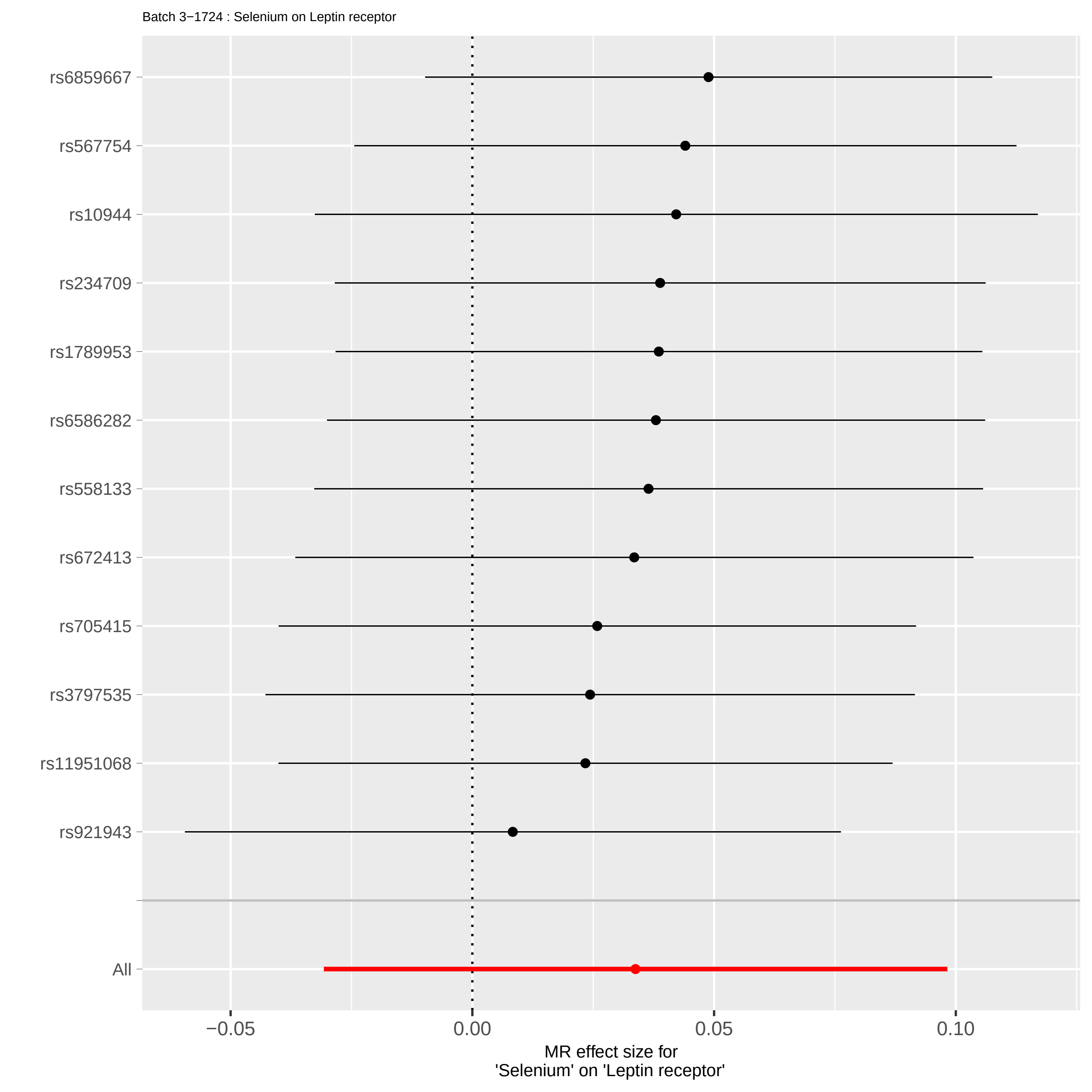
Batch 3−1724 : Selenium on Leptin receptor
rs6859667
rs567754
rs10944
rs234709
rs1789953
rs6586282
rs558133
rs672413
rs705415
rs3797535
rs11951068
rs921943
All
−0.05
0.00
0.05
0.10
MR effect size for
'Selenium' on 'Leptin receptor'

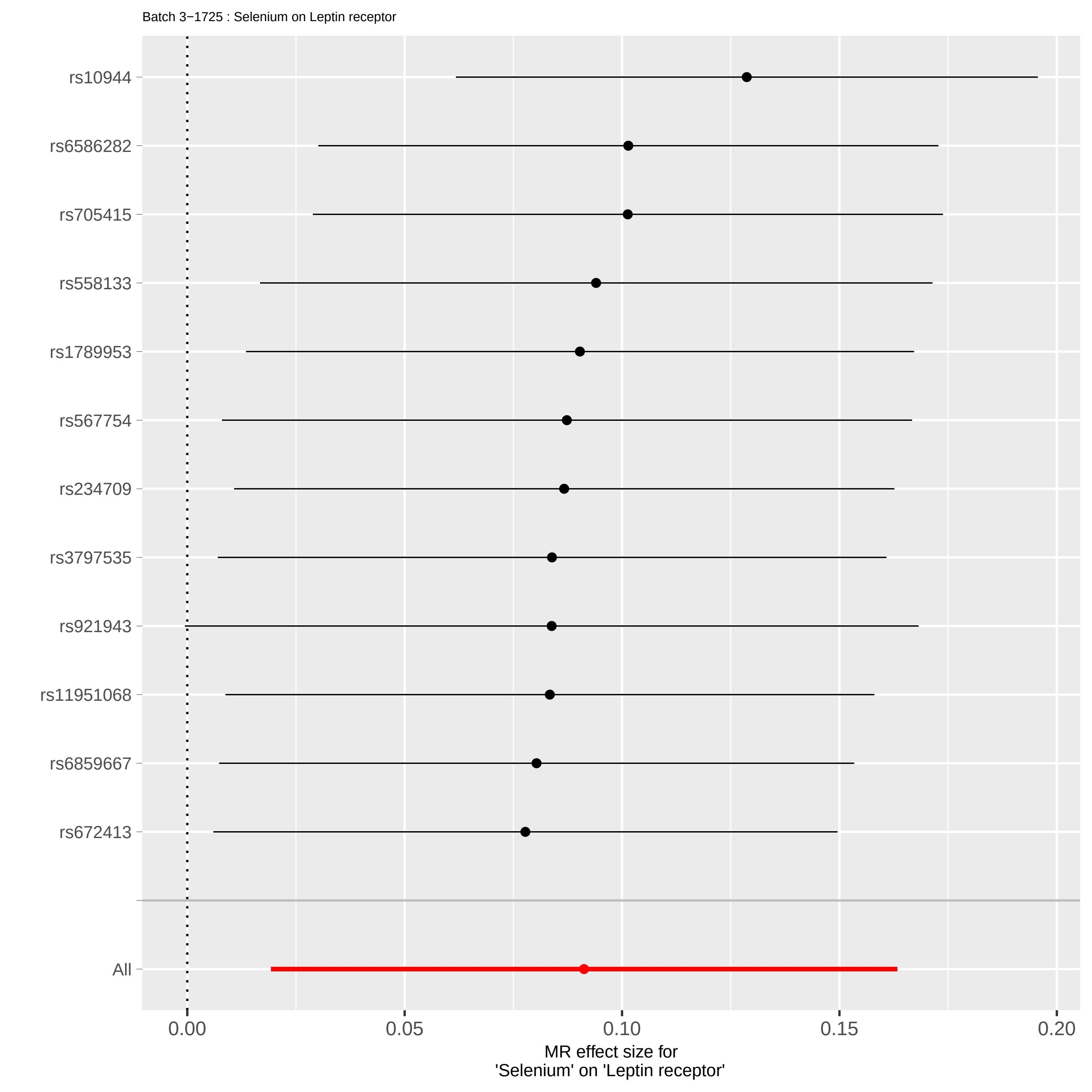

Batch 3−1725 : Selenium on Leptin receptor
rs10944
rs6586282
rs705415
rs558133
rs1789953
rs567754
rs234709
rs3797535
rs921943
rs11951068
rs6859667
rs672413
All
0.00
0.05
0.10
0.15
0.20
MR effect size for
'Selenium' on 'Leptin receptor'

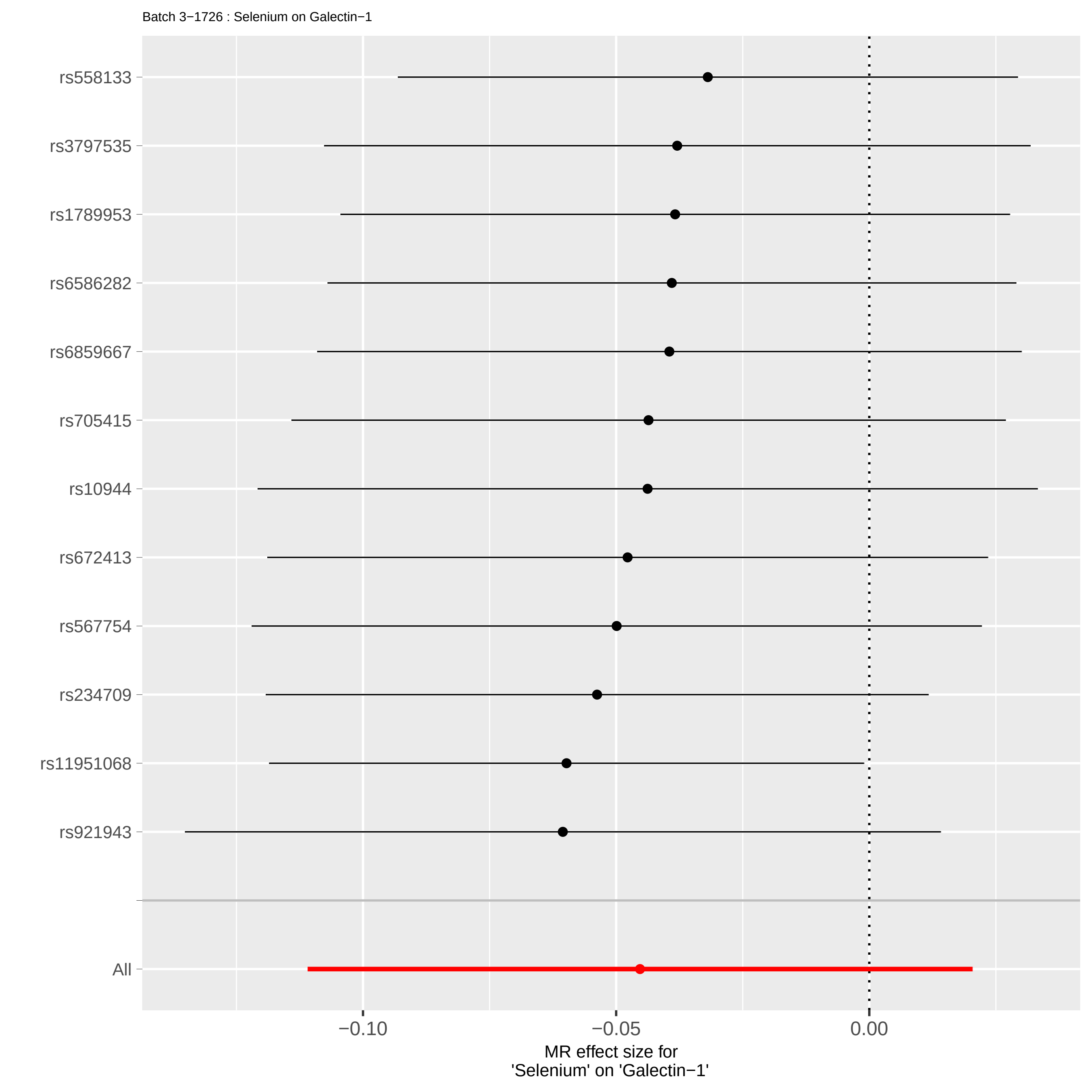

Batch 3−1726 : Selenium on Galectin−1
rs558133
rs3797535
rs1789953
rs6586282
rs6859667
rs705415
rs10944
rs672413
rs567754
rs234709
rs11951068
rs921943
All
−0.10
−0.05
0.00
MR effect size for
'Selenium' on 'Galectin−1'

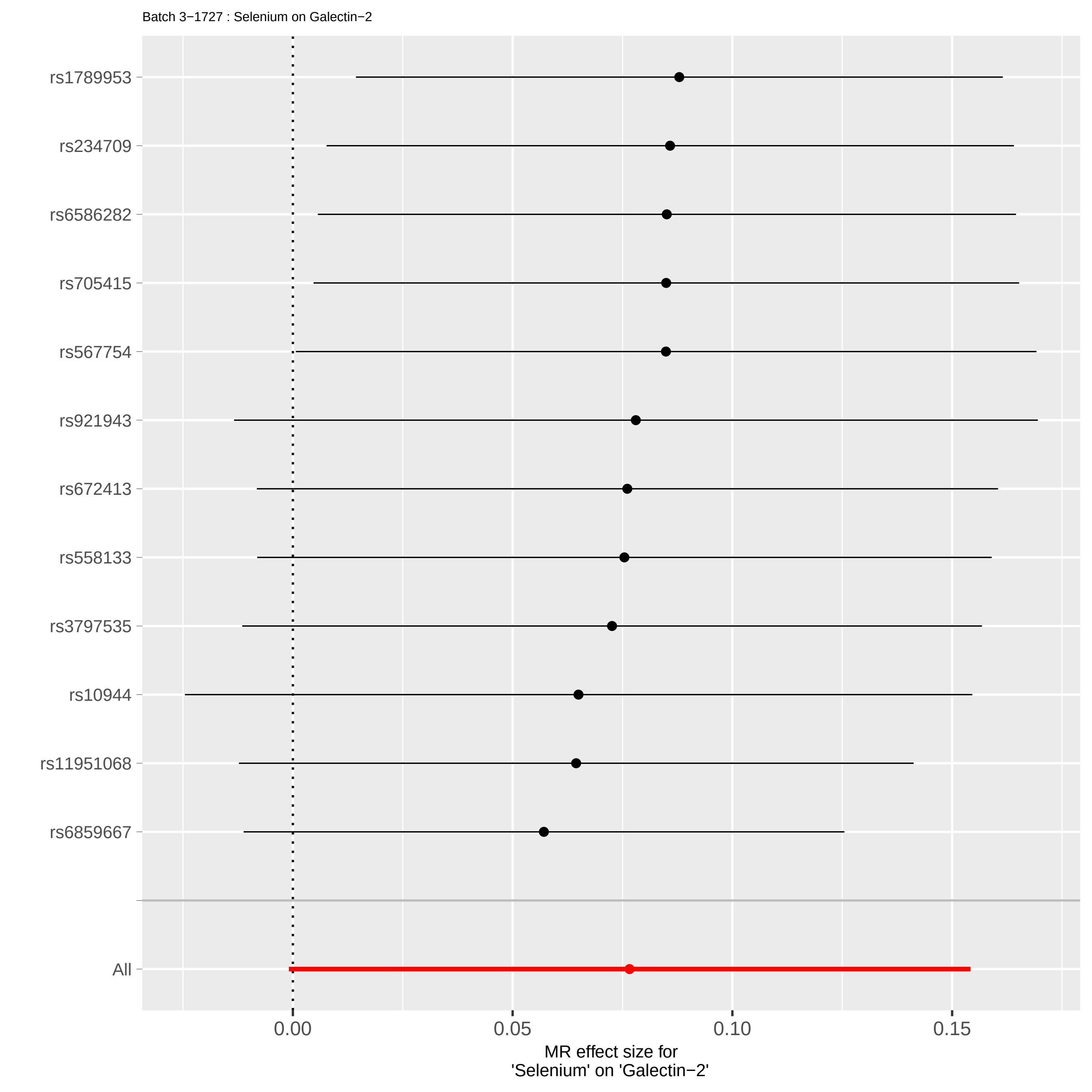

Batch 3−1727 : Selenium on Galectin−2
rs1789953
rs234709
rs6586282
rs705415
rs567754
rs921943
rs672413
rs558133
rs3797535
rs10944
rs11951068
rs6859667
All
0.00
0.05
0.10
0.15
MR effect size for
'Selenium' on 'Galectin−2'

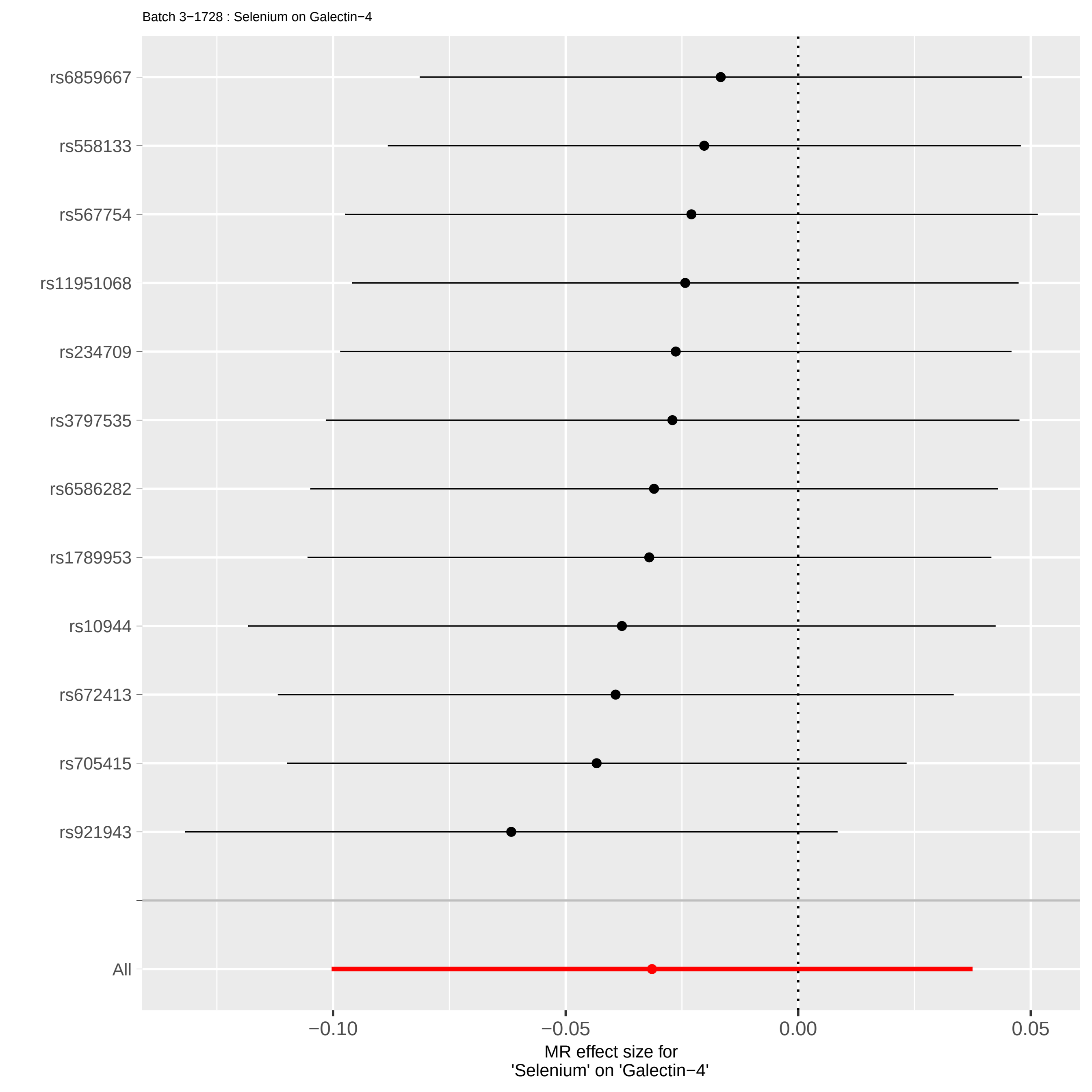
Batch 3−1728 : Selenium on Galectin−4
rs6859667
rs558133
rs567754
rs11951068
rs234709
rs3797535
rs6586282
rs1789953
rs10944
rs672413
rs705415
rs921943
All
−0.10
−0.05
0.00
0.05
MR effect size for
'Selenium' on 'Galectin−4'

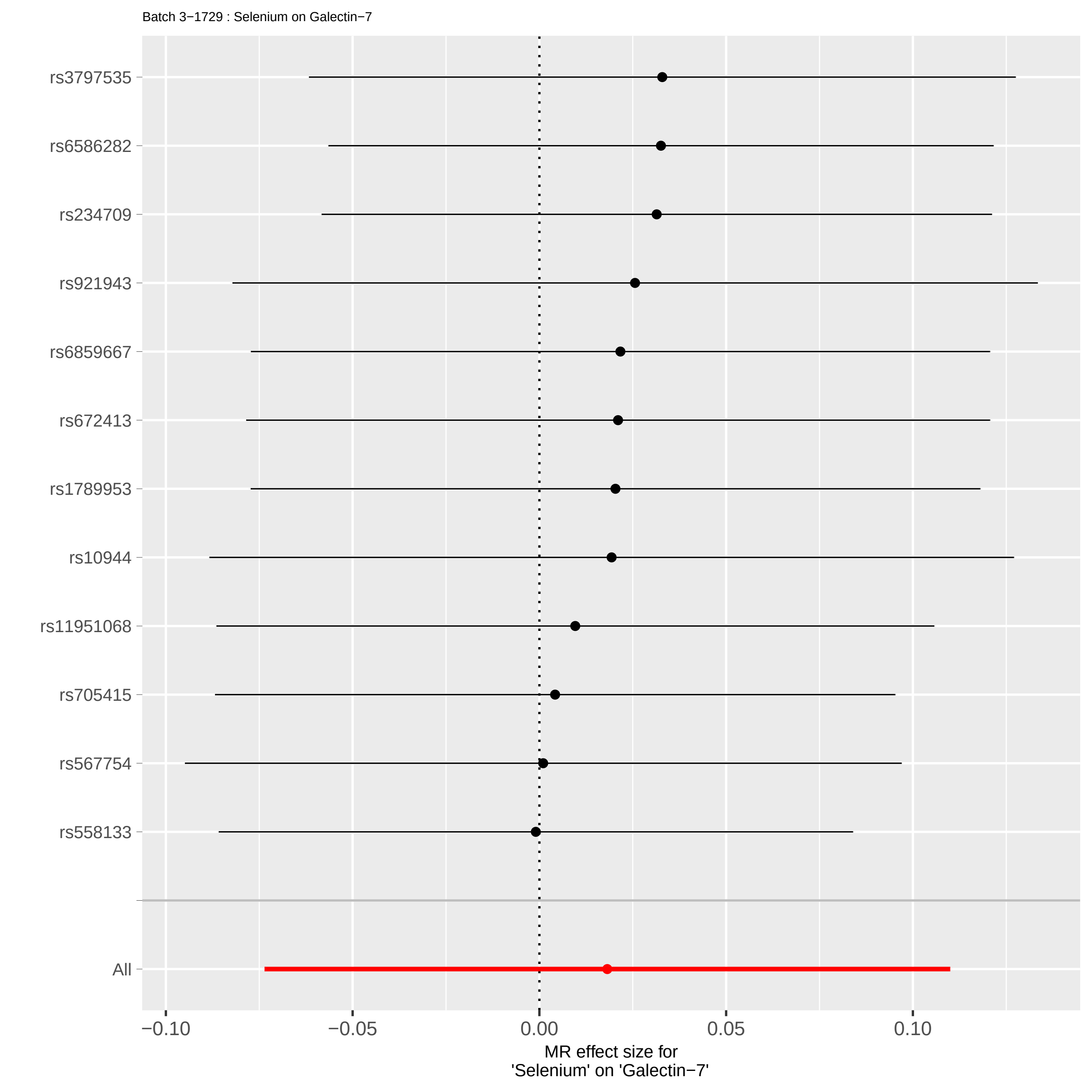

Batch 3−1729 : Selenium on Galectin−7
rs3797535
rs6586282
rs234709
rs921943
rs6859667
rs672413
rs1789953
rs10944
rs11951068
rs705415
rs567754
rs558133
All
−0.10
−0.05
0.00
0.05
0.10
MR effect size for
'Selenium' on 'Galectin−7'

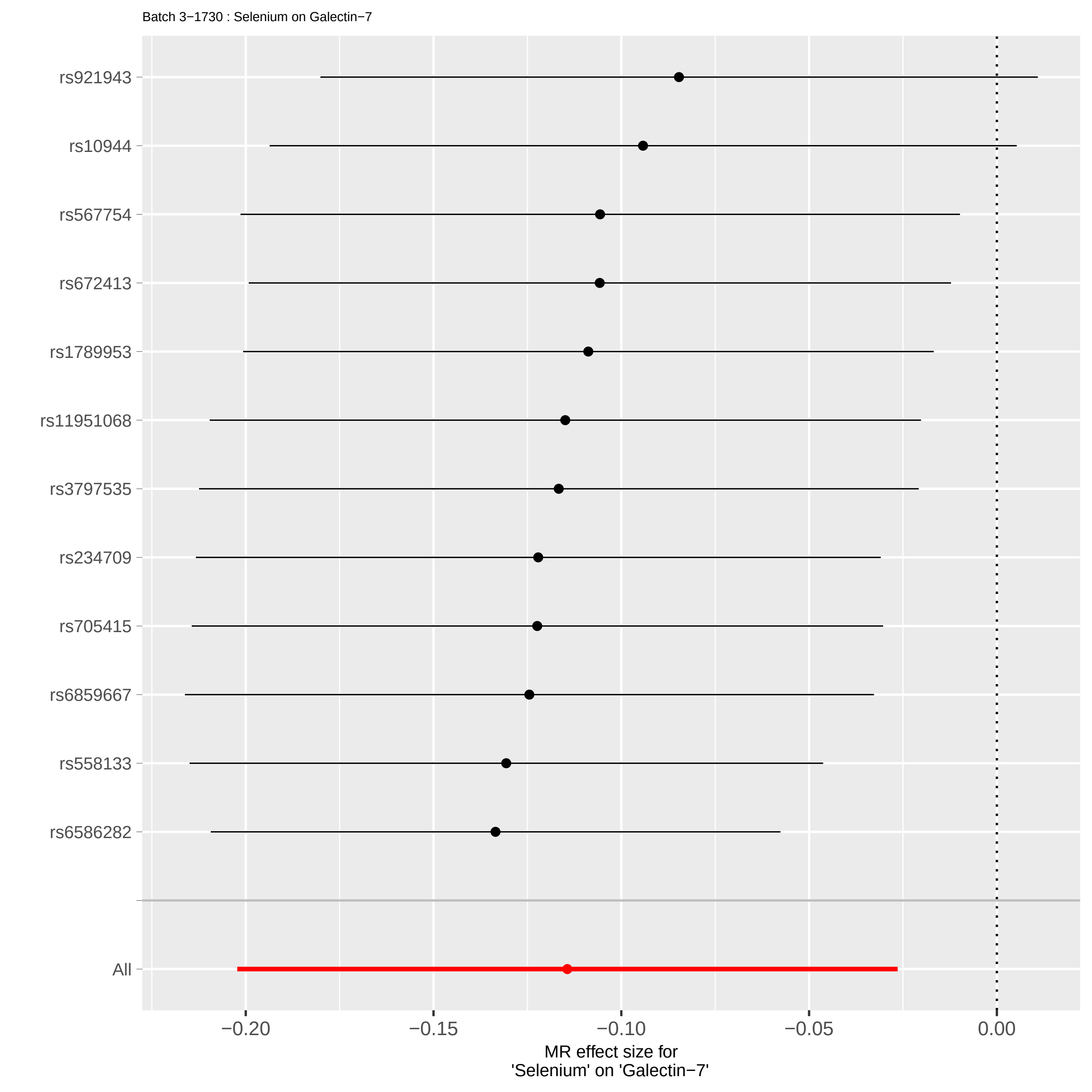

Batch 3−1730 : Selenium on Galectin−7
rs921943
rs10944
rs567754
rs672413
rs1789953
rs11951068
rs3797535
rs234709
rs705415
rs6859667
rs558133
rs6586282
All
−0.20
−0.15
−0.10
−0.05
0.00
MR effect size for
'Selenium' on 'Galectin−7'

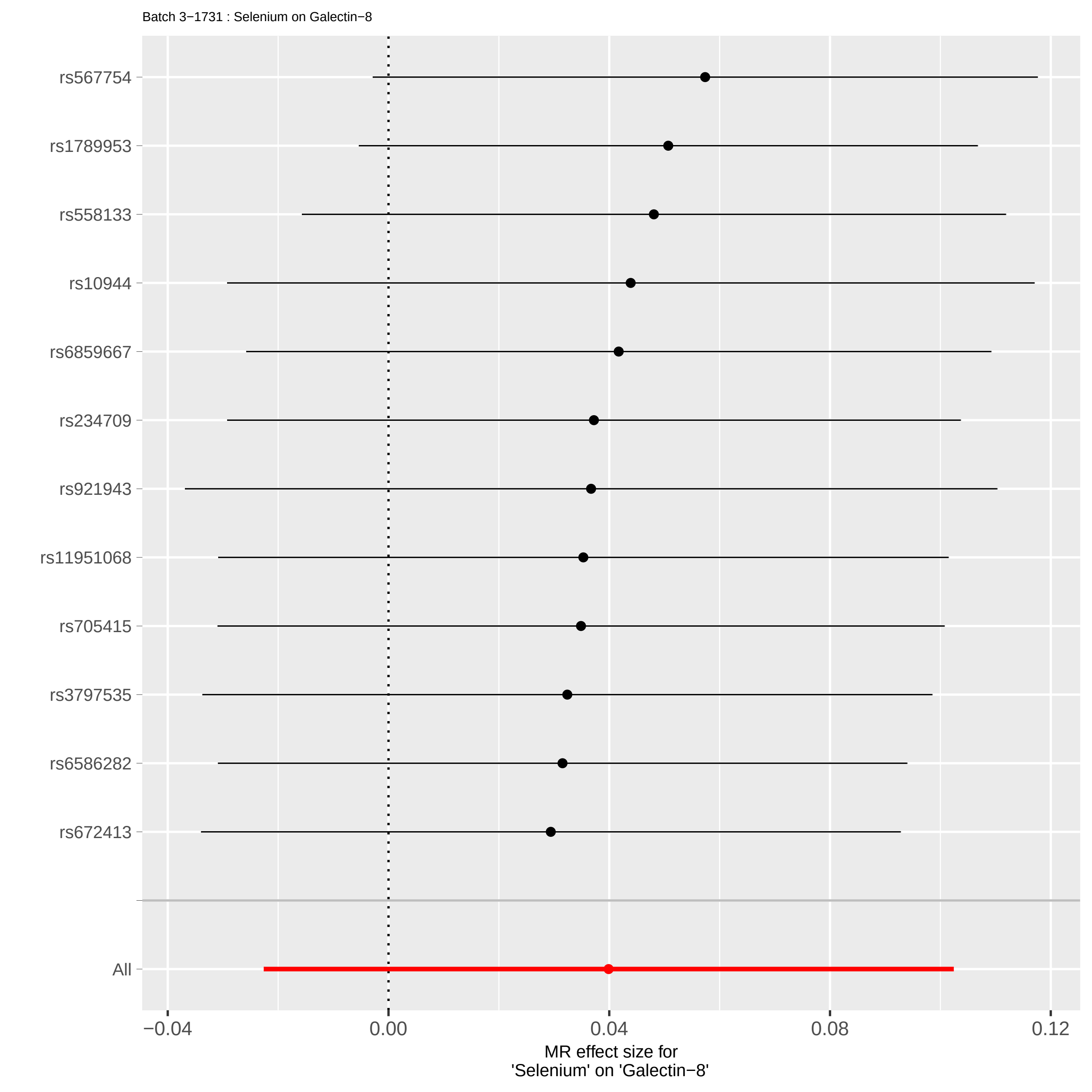

Batch 3−1731 : Selenium on Galectin−8
rs567754
rs1789953
rs558133
rs10944
rs6859667
rs234709
rs921943
rs11951068
rs705415
rs3797535
rs6586282
rs672413
All
−0.04
0.00
0.04
0.08
0.12
MR effect size for
'Selenium' on 'Galectin−8'

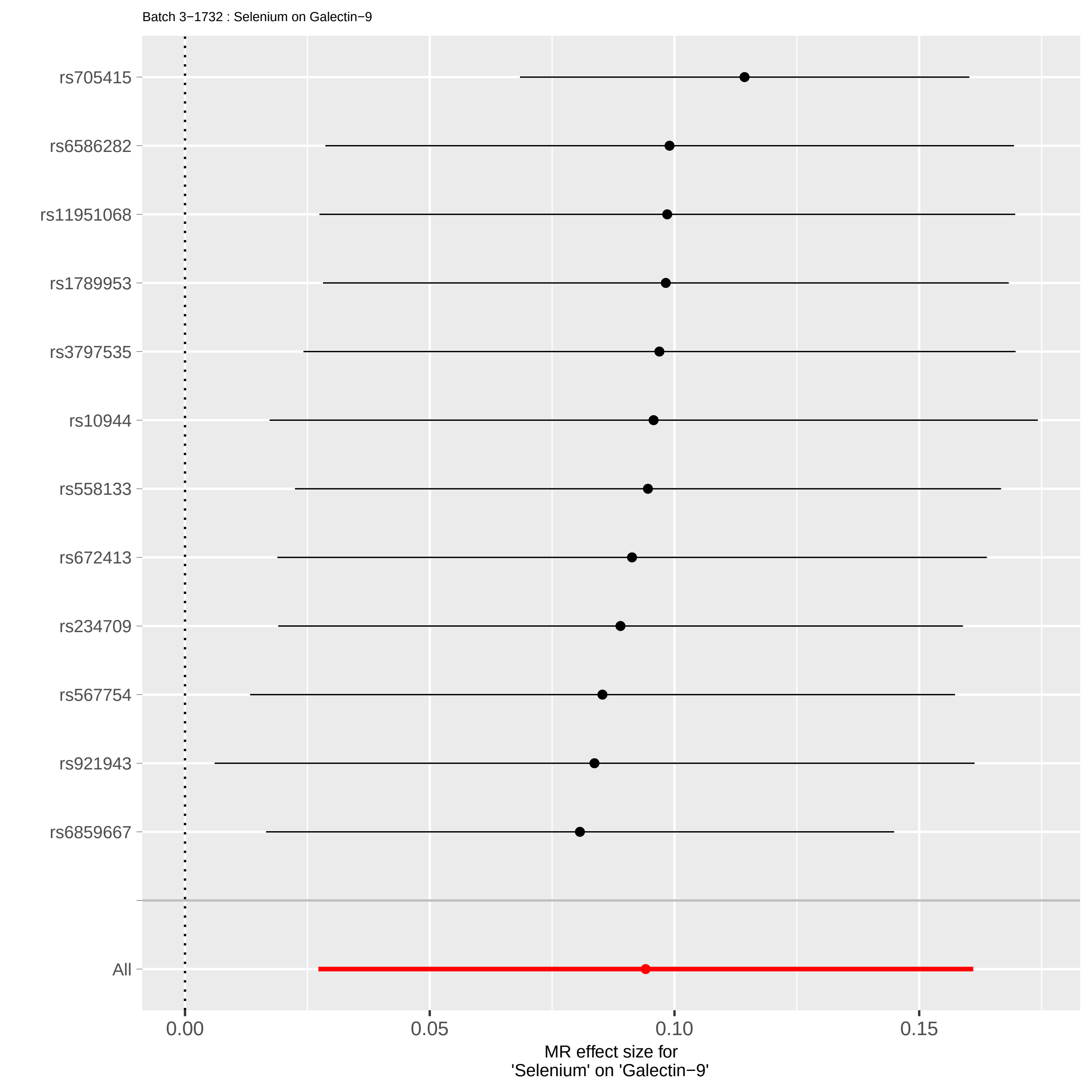
Batch 3−1732 : Selenium on Galectin−9
rs705415
rs6586282
rs11951068
rs1789953
rs3797535
rs10944
rs558133
rs672413
rs234709
rs567754
rs921943
rs6859667
All
0.00
0.05
0.10
0.15
MR effect size for
'Selenium' on 'Galectin−9'

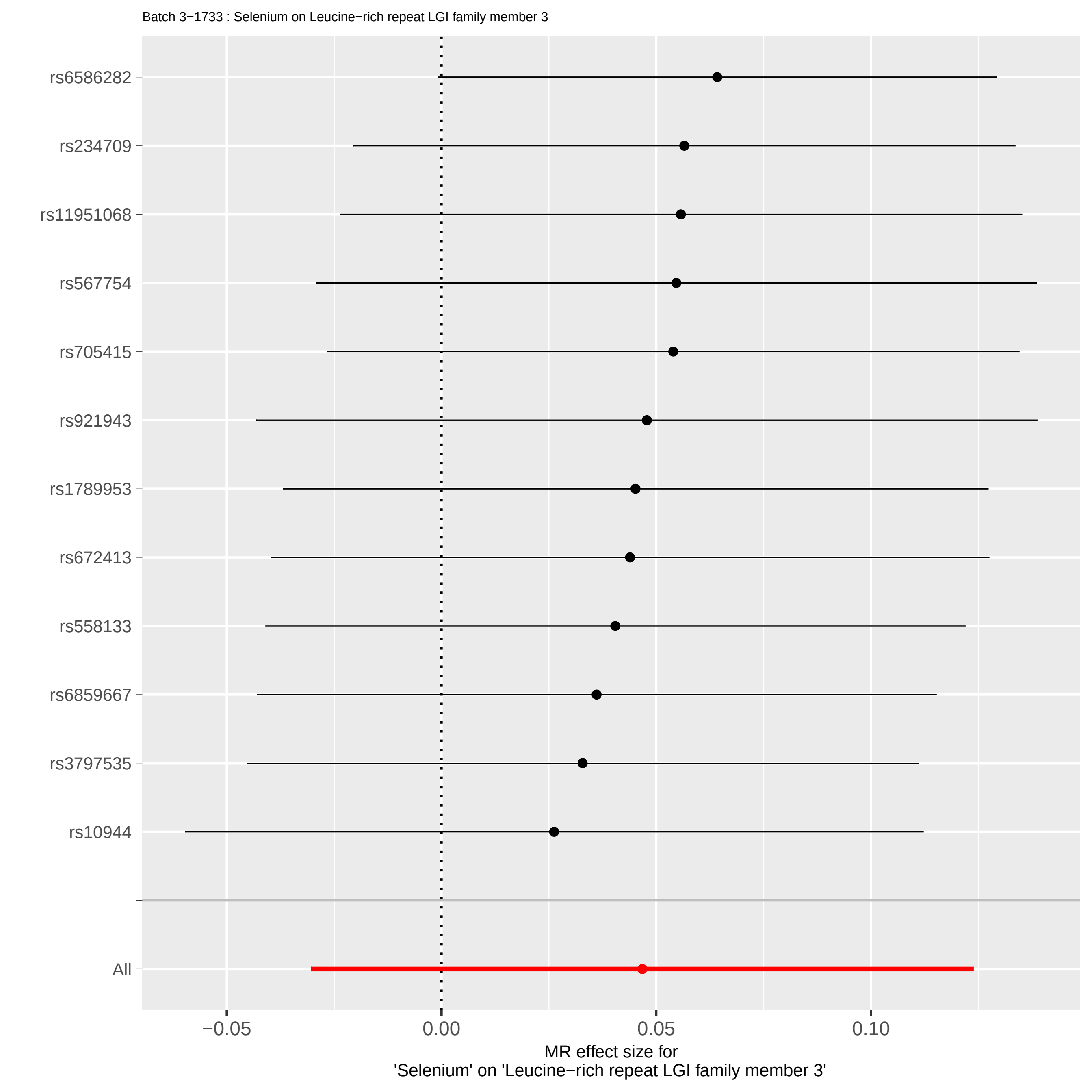
Batch 3−1733 : Selenium on Leucine−rich repeat LGI family member 3
rs6586282
rs234709
rs11951068
rs567754
rs705415
rs921943
rs1789953
rs672413
rs558133
rs6859667
rs3797535
rs10944
All
−0.05
0.00
0.05
0.10
MR effect size for
'Selenium' on 'Leucine−rich repeat LGI family member 3'

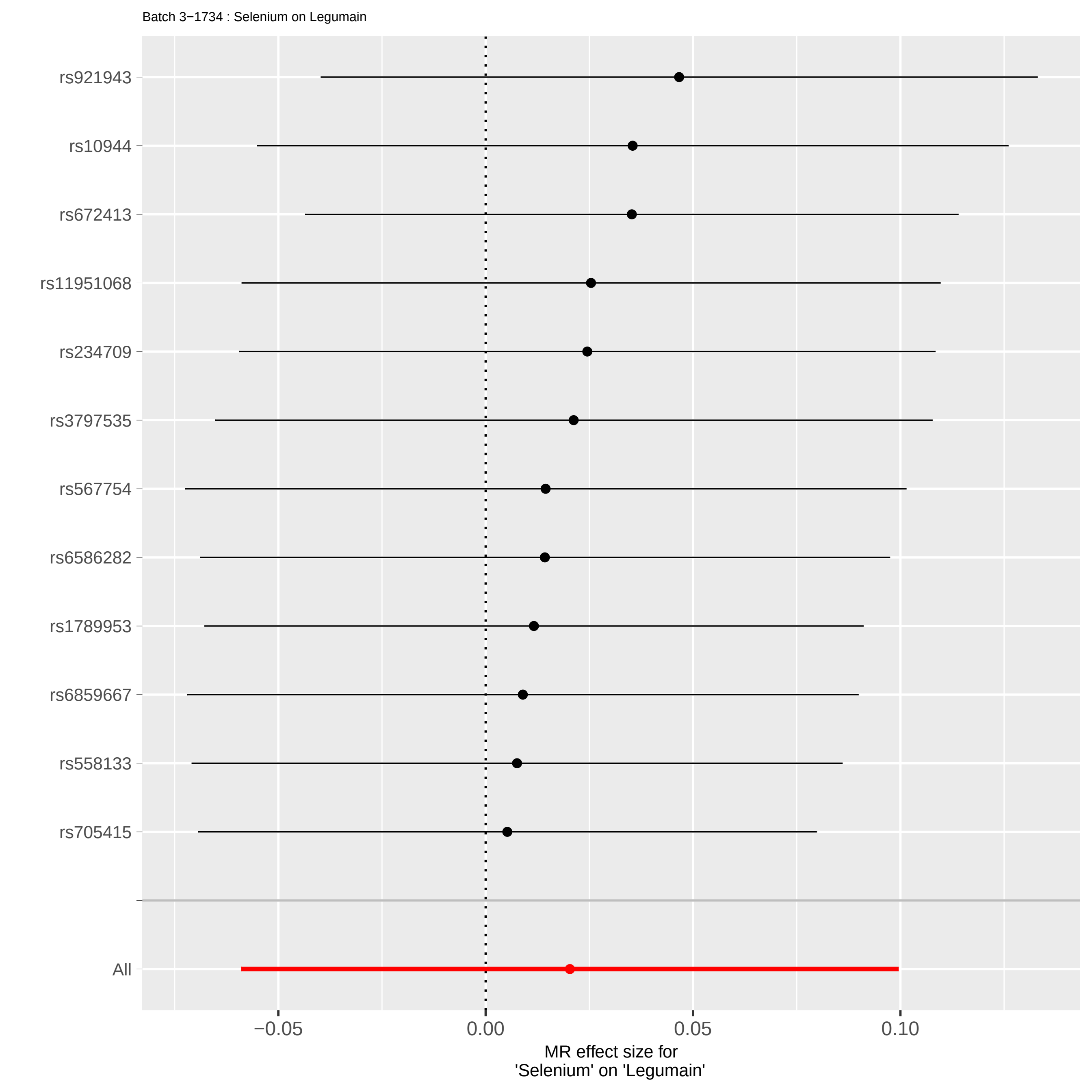

Batch 3−1734 : Selenium on Legumain
rs921943
rs10944
rs672413
rs11951068
rs234709
rs3797535
rs567754
rs6586282
rs1789953
rs6859667
rs558133
rs705415
All
−0.05
0.00
0.05
0.10
MR effect size for
'Selenium' on 'Legumain'

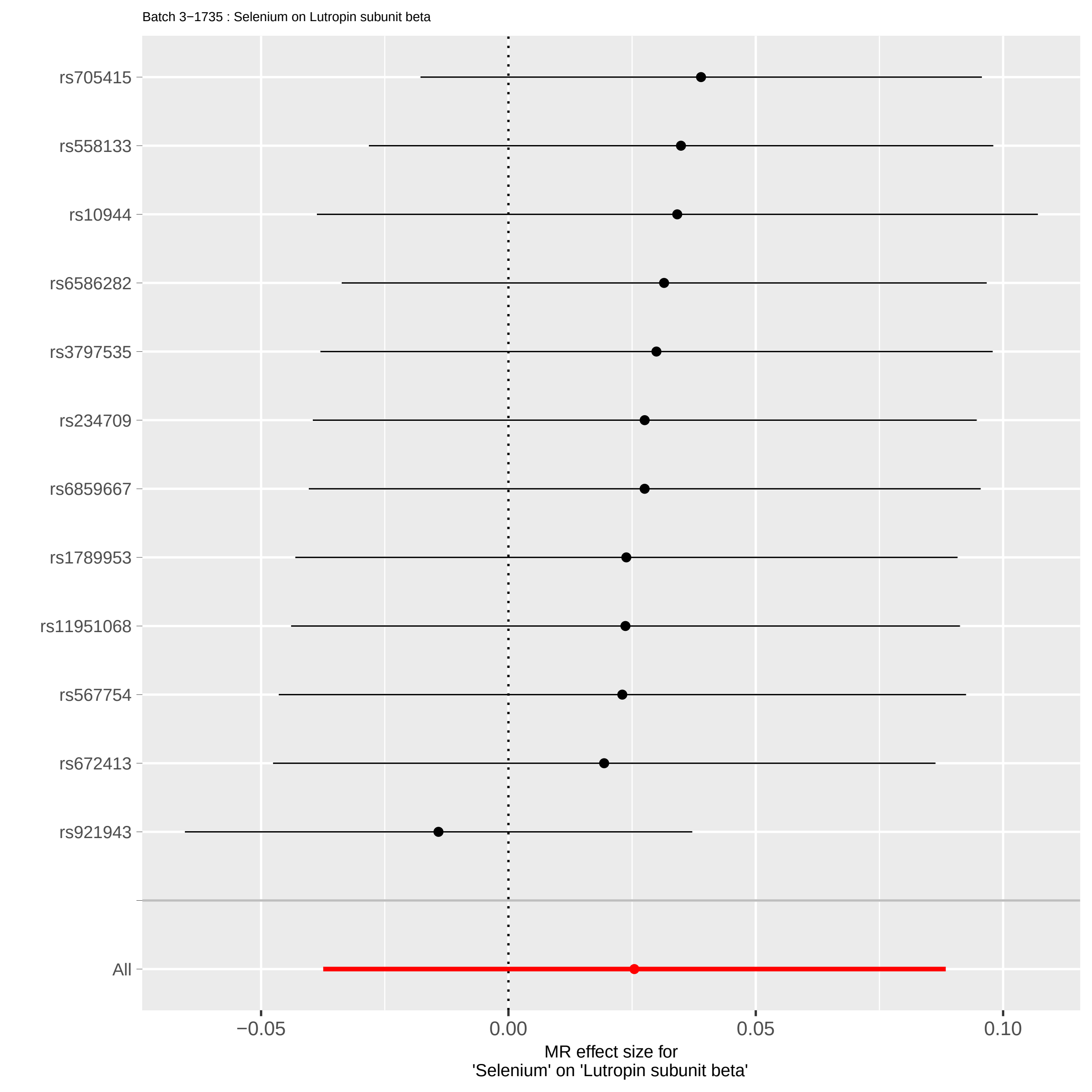

Batch 3−1735 : Selenium on Lutropin subunit beta
rs705415
rs558133
rs10944
rs6586282
rs3797535
rs234709
rs6859667
rs1789953
rs11951068
rs567754
rs672413
rs921943
All
−0.05
0.00
0.05
0.10
MR effect size for
'Selenium' on 'Lutropin subunit beta'

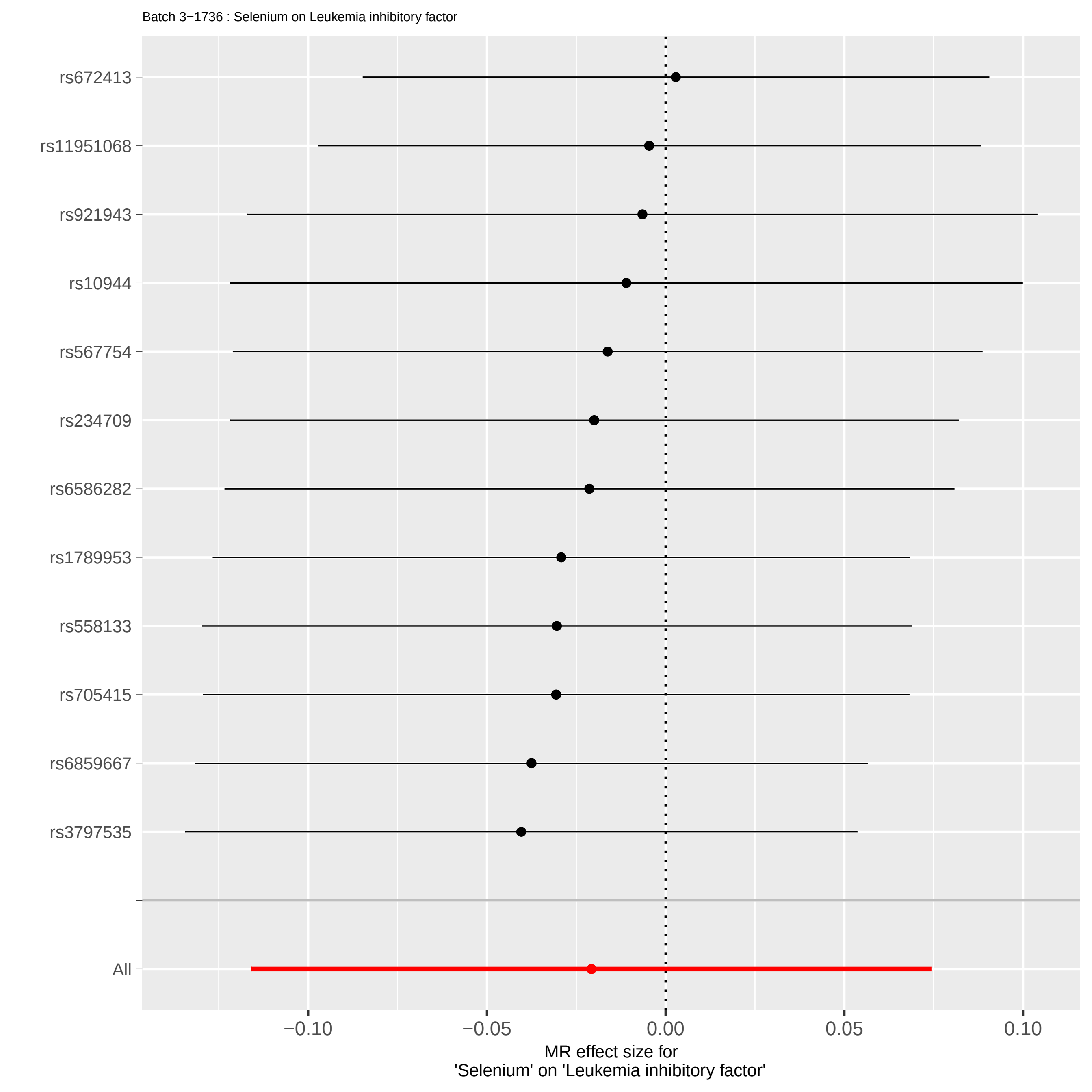

Batch 3−1736 : Selenium on Leukemia inhibitory factor
rs672413
rs11951068
rs921943
rs10944
rs567754
rs234709
rs6586282
rs1789953
rs558133
rs705415
rs6859667
rs3797535
All
−0.10
−0.05
0.00
0.05
0.10
MR effect size for
'Selenium' on 'Leukemia inhibitory factor'

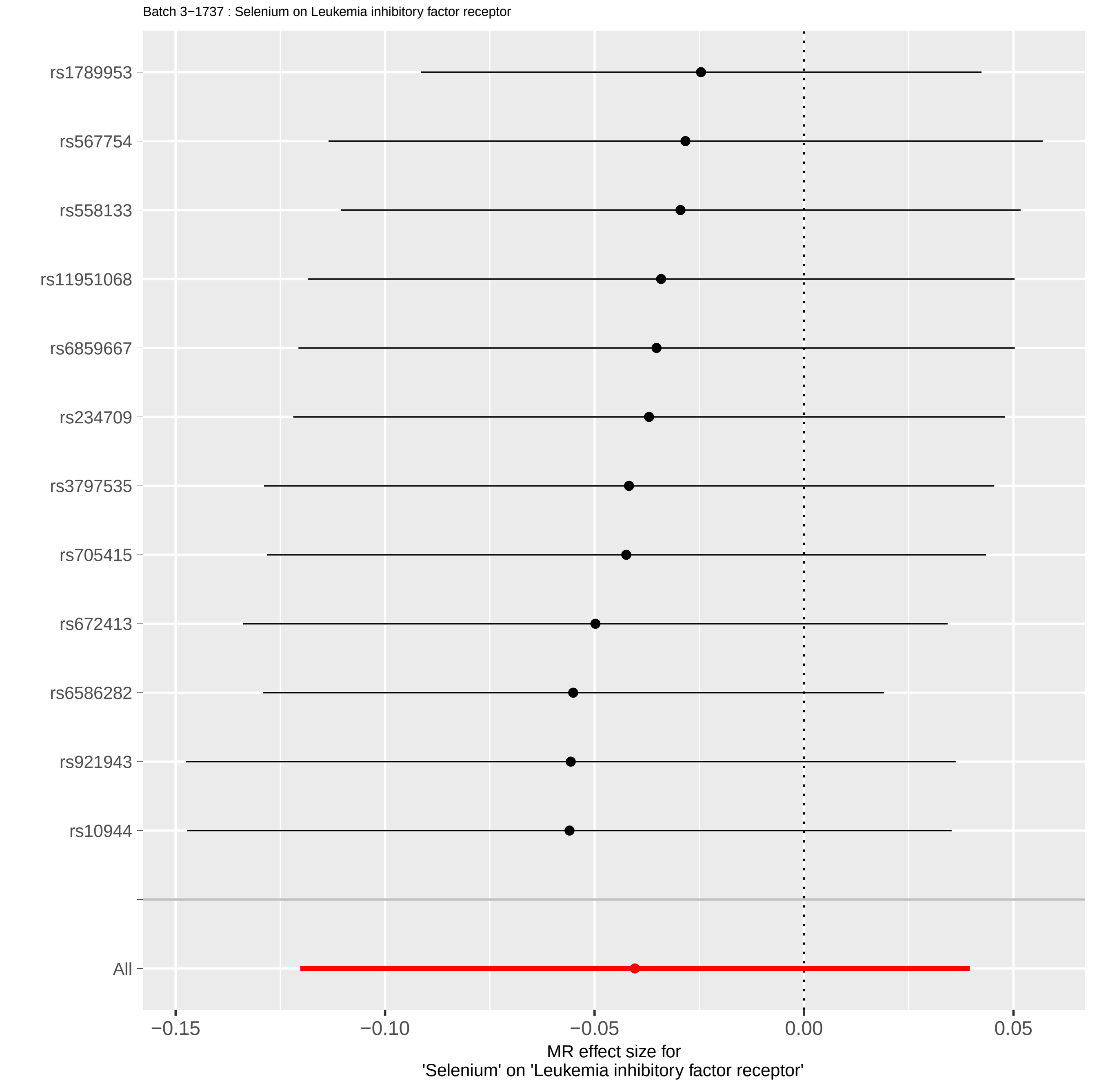

Batch 3−1737 : Selenium on Leukemia inhibitory factor receptor
rs1789953
rs567754
rs558133
rs11951068
rs6859667
rs234709
rs3797535
rs705415
rs672413
rs6586282
rs921943
rs10944
All
−0.15
−0.10
−0.05
0.00
0.05
MR effect size for
'Selenium' on 'Leukemia inhibitory factor receptor'

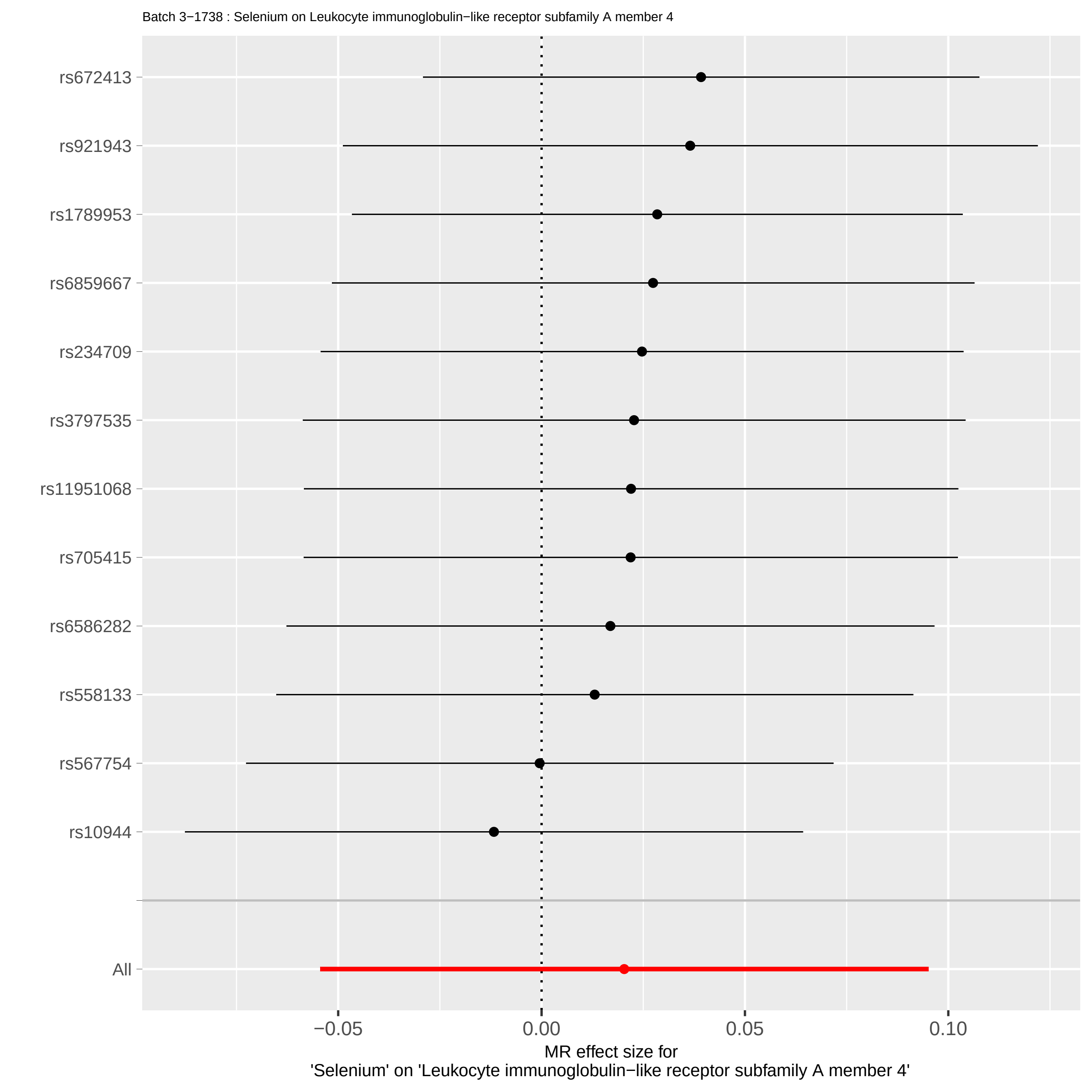
Batch 3−1738 : Selenium on Leukocyte immunoglobulin−like receptor subfamily A member 4
rs672413
rs921943
rs1789953
rs6859667
rs234709
rs3797535
rs11951068
rs705415
rs6586282
rs558133
rs567754
rs10944
All
−0.05
0.00
0.05
0.10
MR effect size for
'Selenium' on 'Leukocyte immunoglobulin−like receptor subfamily A member 4'

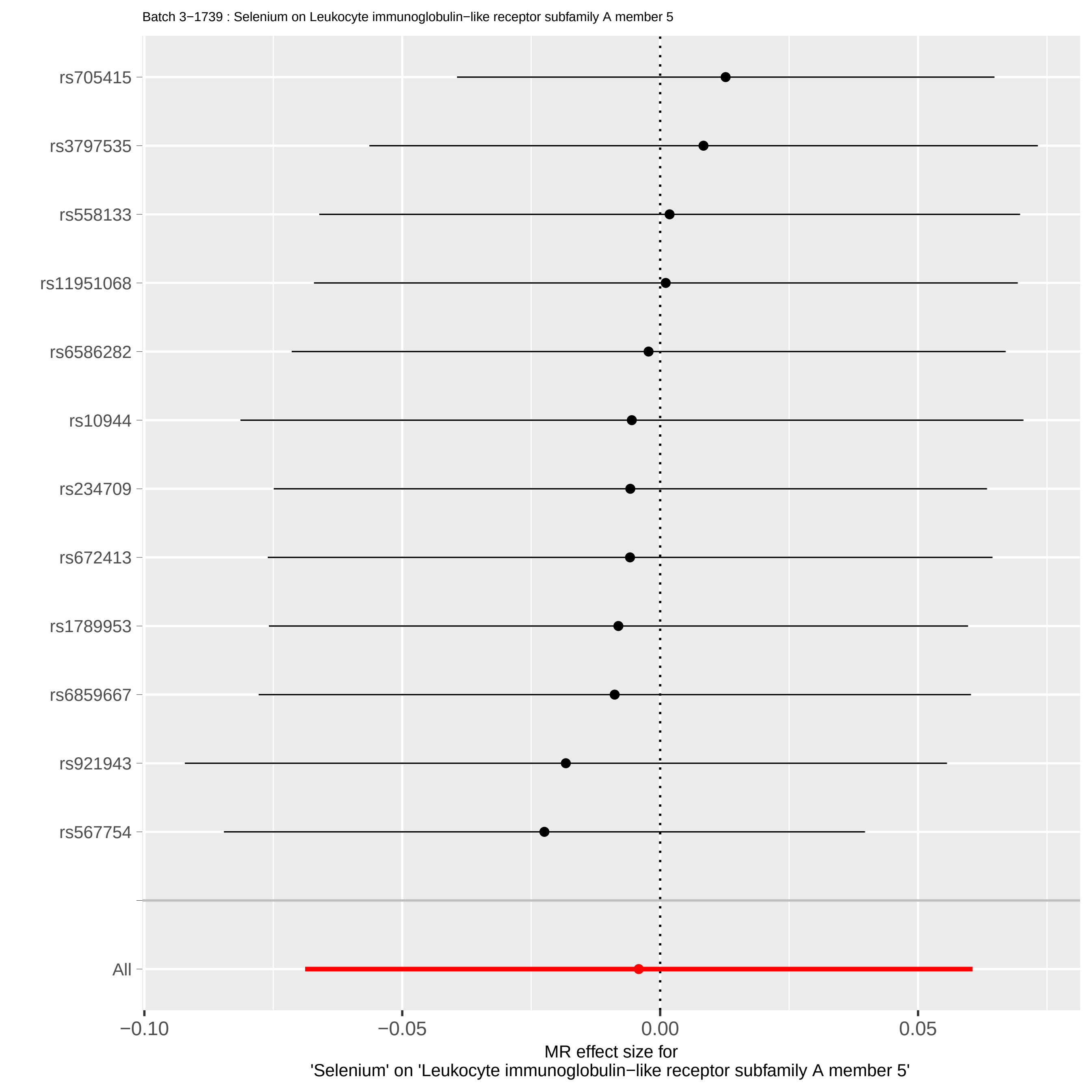
Batch 3−1739 : Selenium on Leukocyte immunoglobulin−like receptor subfamily A member 5
rs705415
rs3797535
rs558133
rs11951068
rs6586282
rs10944
rs234709
rs672413
rs1789953
rs6859667
rs921943
rs567754
All
−0.10
−0.05
0.00
0.05
MR effect size for
'Selenium' on 'Leukocyte immunoglobulin−like receptor subfamily A member 5'

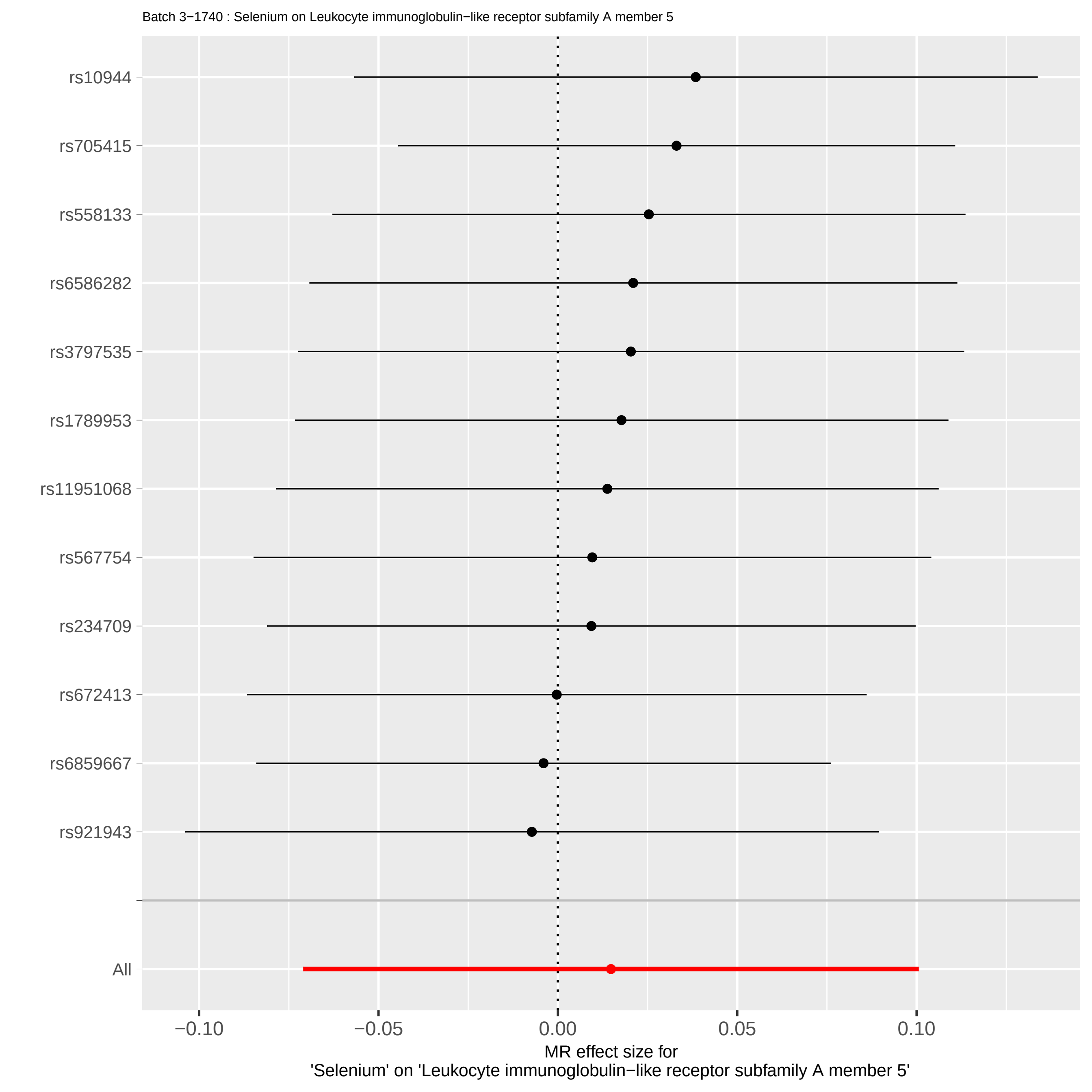

Batch 3−1740 : Selenium on Leukocyte immunoglobulin−like receptor subfamily A member 5
rs10944
rs705415
rs558133
rs6586282
rs3797535
rs1789953
rs11951068
rs567754
rs234709
rs672413
rs6859667
rs921943
All
−0.10
−0.05
0.00
0.05
0.10
MR effect size for
'Selenium' on 'Leukocyte immunoglobulin−like receptor subfamily A member 5'

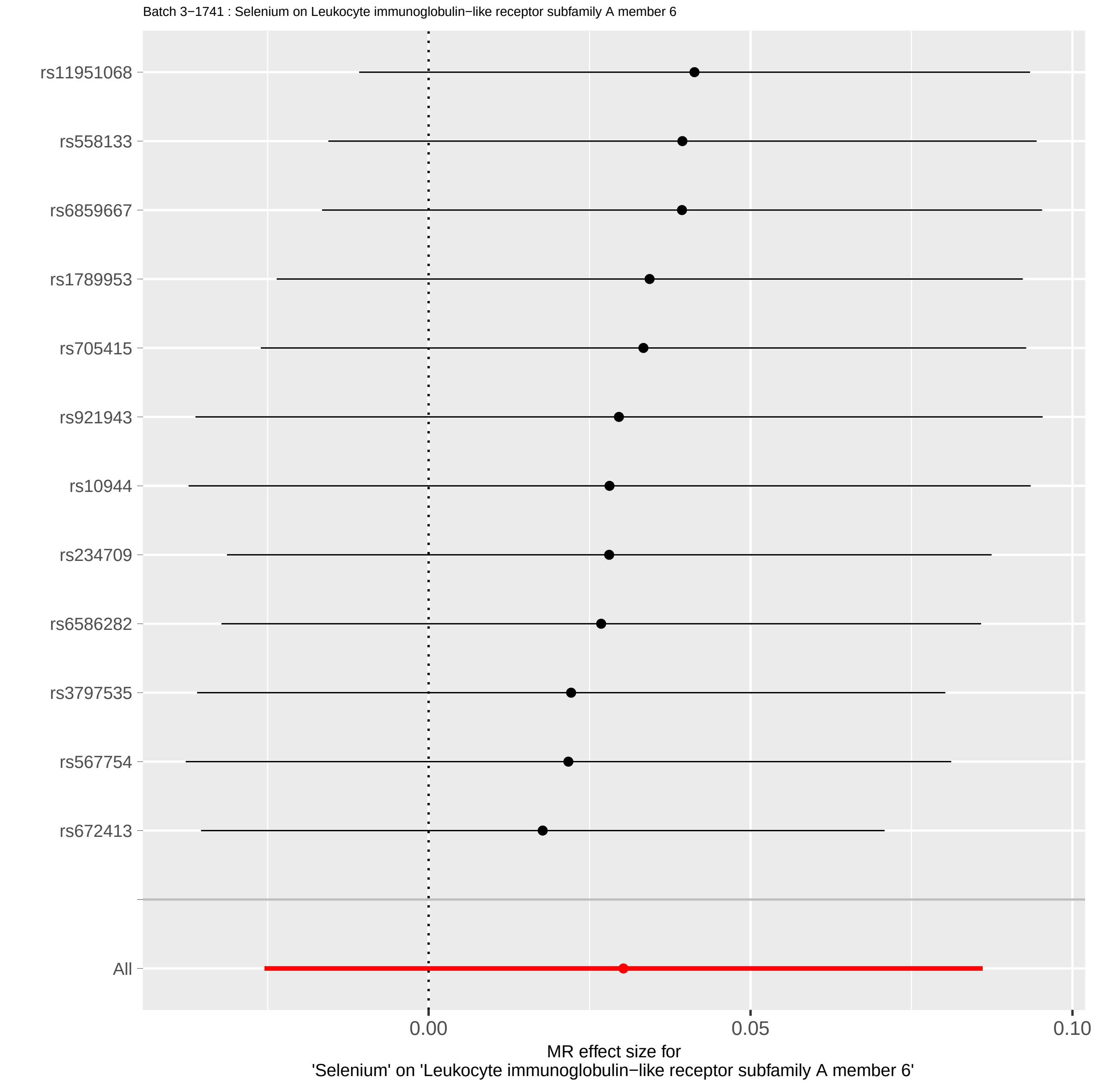

Batch 3−1741 : Selenium on Leukocyte immunoglobulin−like receptor subfamily A member 6
rs11951068
rs558133
rs6859667
rs1789953
rs705415
rs921943
rs10944
rs234709
rs6586282
rs3797535
rs567754
rs672413
All
0.00
0.05
0.10
MR effect size for
'Selenium' on 'Leukocyte immunoglobulin−like receptor subfamily A member 6'

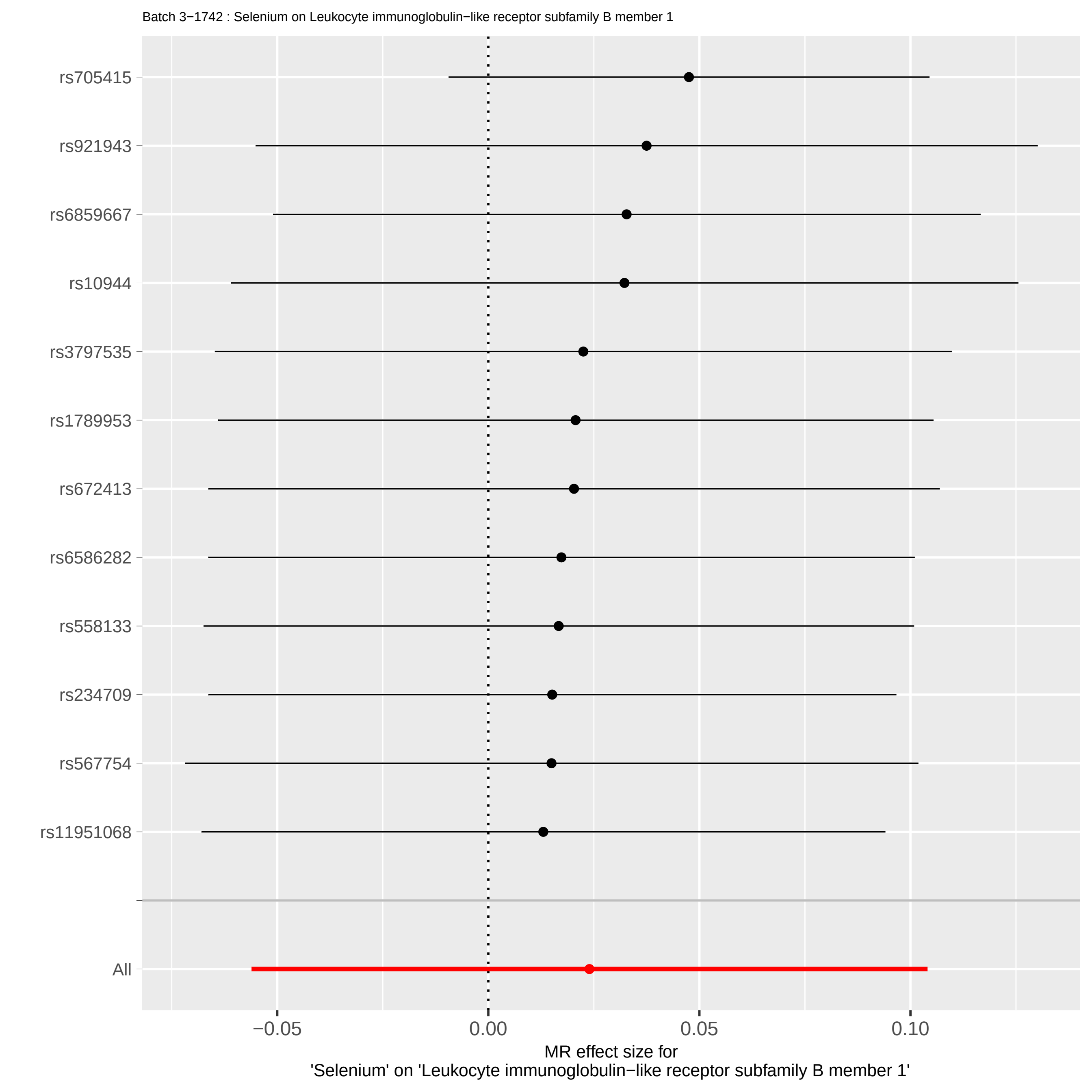

Batch 3−1742 : Selenium on Leukocyte immunoglobulin−like receptor subfamily B member 1
rs705415
rs921943
rs6859667
rs10944
rs3797535
rs1789953
rs672413
rs6586282
rs558133
rs234709
rs567754
rs11951068
All
−0.05
0.00
0.05
0.10
MR effect size for
'Selenium' on 'Leukocyte immunoglobulin−like receptor subfamily B member 1'

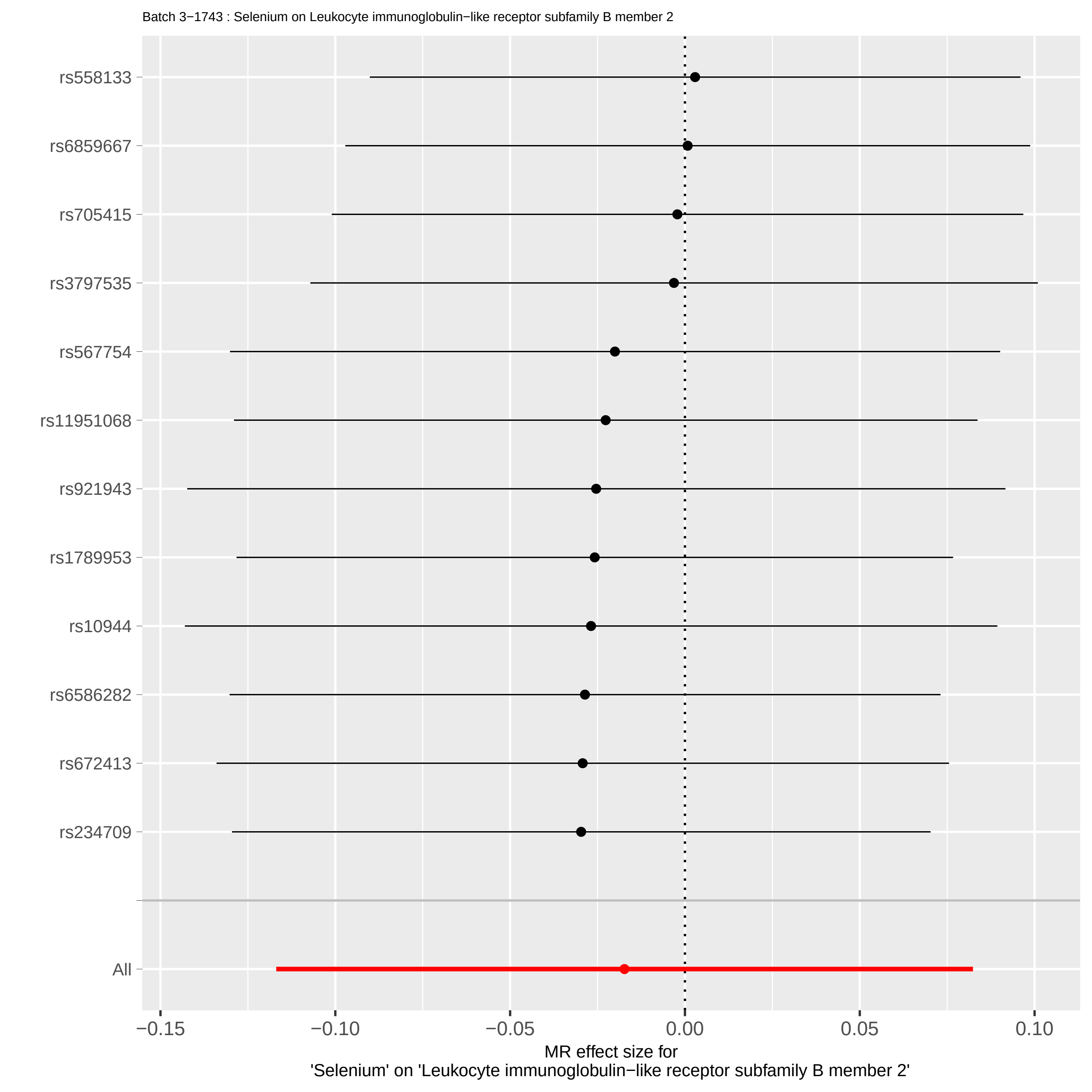

Batch 3−1743 : Selenium on Leukocyte immunoglobulin−like receptor subfamily B member 2
rs558133
rs6859667
rs705415
rs3797535
rs567754
rs11951068
rs921943
rs1789953
rs10944
rs6586282
rs672413
rs234709
All
−0.15
−0.10
−0.05
0.00
0.05
0.10
MR effect size for
'Selenium' on 'Leukocyte immunoglobulin−like receptor subfamily B member 2'

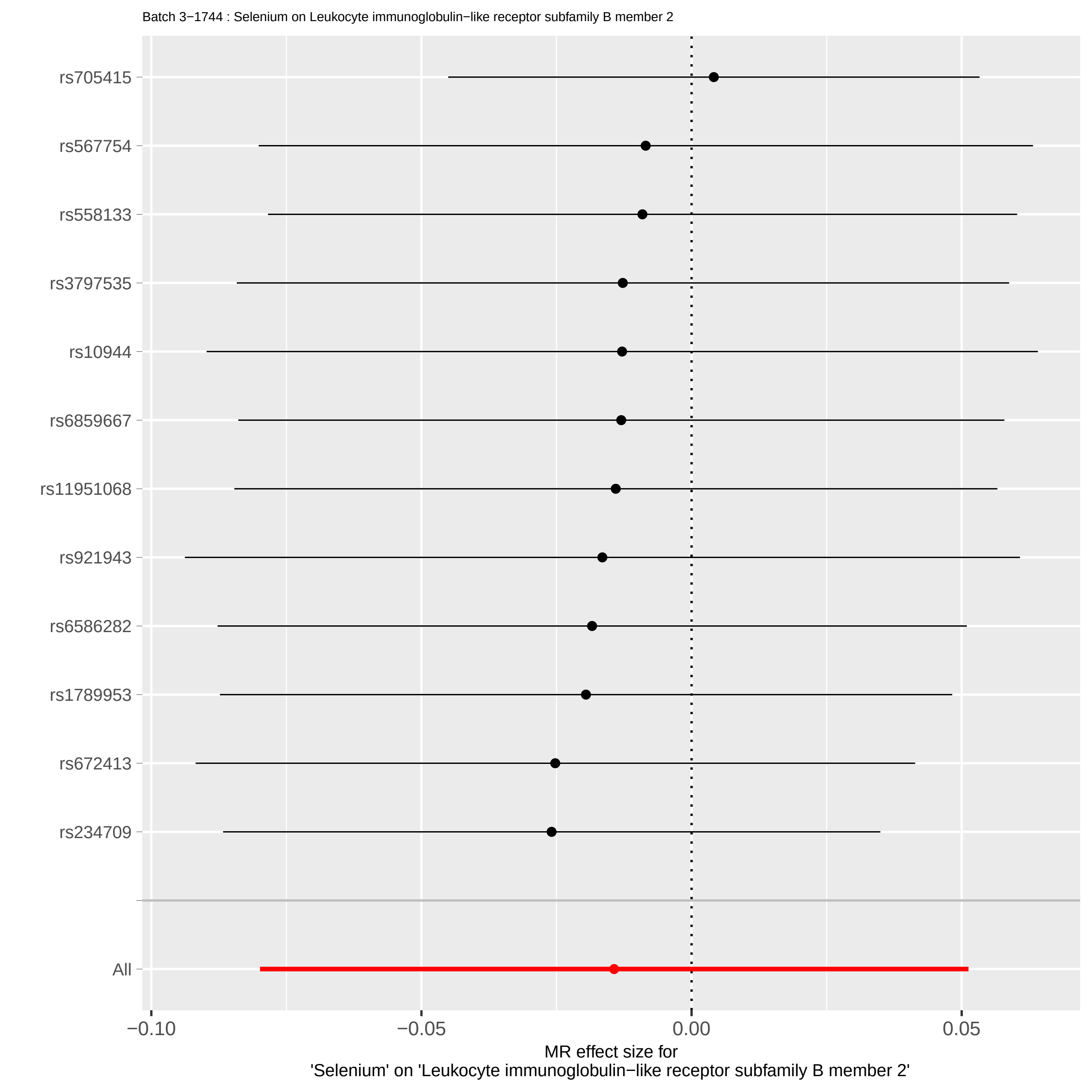

Batch 3−1744 : Selenium on Leukocyte immunoglobulin−like receptor subfamily B member 2
rs705415
rs567754
rs558133
rs3797535
rs10944
rs6859667
rs11951068
rs921943
rs6586282
rs1789953
rs672413
rs234709
All
−0.10
−0.05
0.00
0.05
MR effect size for
'Selenium' on 'Leukocyte immunoglobulin−like receptor subfamily B member 2'

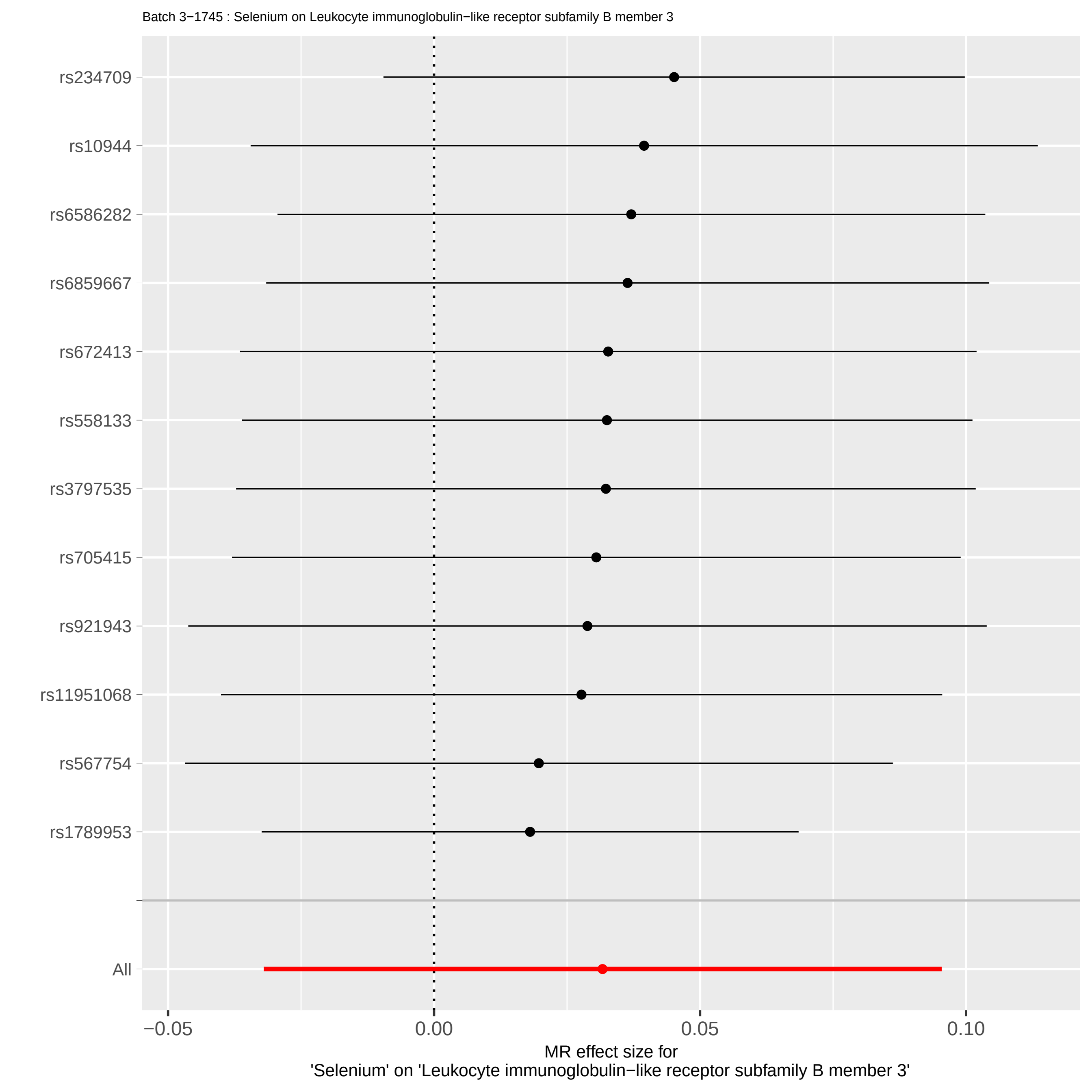
Batch 3−1745 : Selenium on Leukocyte immunoglobulin−like receptor subfamily B member 3
rs234709
rs10944
rs6586282
rs6859667
rs672413
rs558133
rs3797535
rs705415
rs921943
rs11951068
rs567754
rs1789953
All
−0.05
0.00
0.05
0.10
MR effect size for
'Selenium' on 'Leukocyte immunoglobulin−like receptor subfamily B member 3'

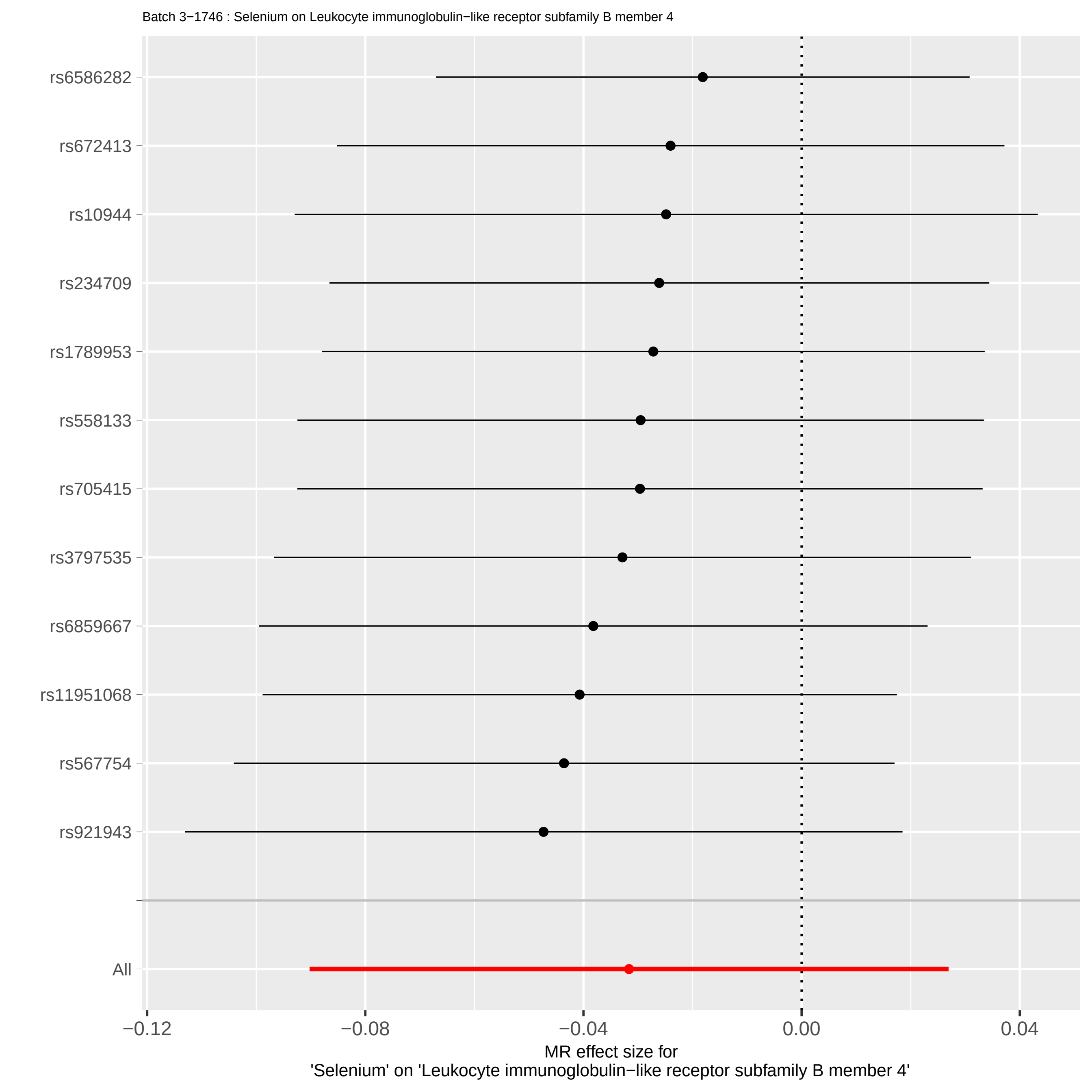
Batch 3−1746 : Selenium on Leukocyte immunoglobulin−like receptor subfamily B member 4
rs6586282
rs672413
rs10944
rs234709
rs1789953
rs558133
rs705415
rs3797535
rs6859667
rs11951068
rs567754
rs921943
All
−0.12
−0.08
−0.04
0.00
0.04
MR effect size for
'Selenium' on 'Leukocyte immunoglobulin−like receptor subfamily B member 4'

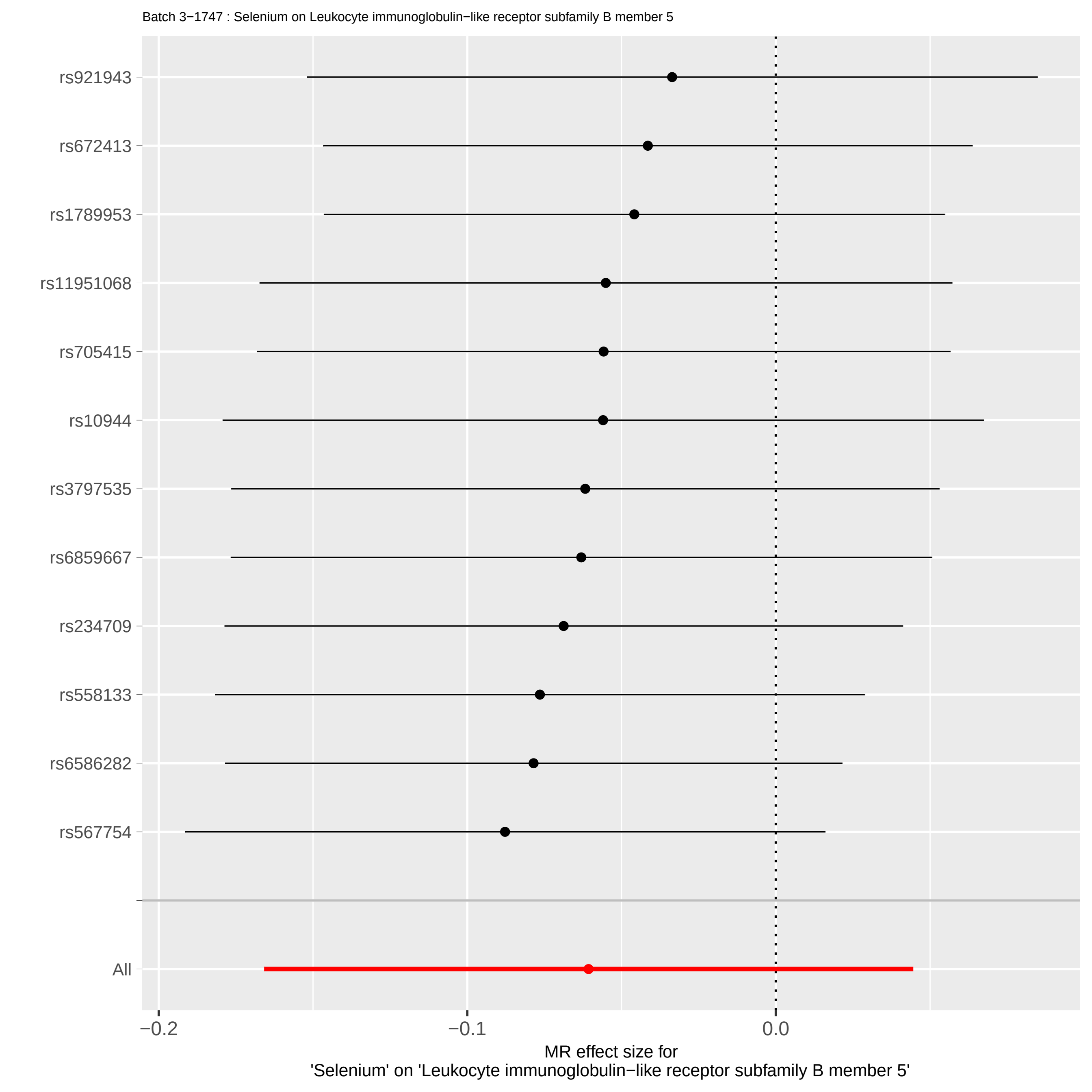
Batch 3−1747 : Selenium on Leukocyte immunoglobulin−like receptor subfamily B member 5
rs921943
rs672413
rs1789953
rs11951068
rs705415
rs10944
rs3797535
rs6859667
rs234709
rs558133
rs6586282
rs567754
All
−0.2
−0.1
0.0
MR effect size for
'Selenium' on 'Leukocyte immunoglobulin−like receptor subfamily B member 5'

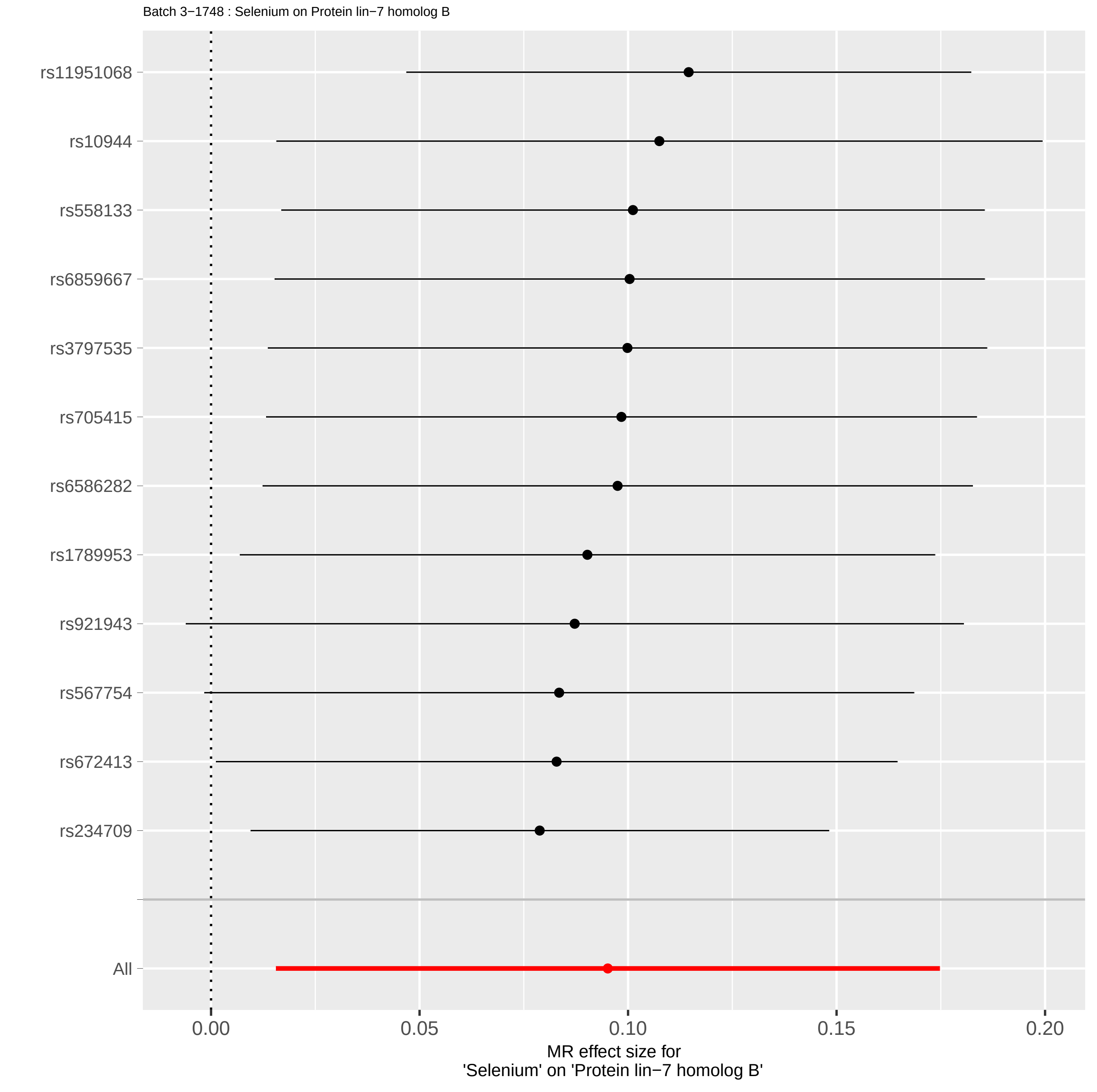
Batch 3−1748 : Selenium on Protein lin−7 homolog B
rs11951068
rs10944
rs558133
rs6859667
rs3797535
rs705415
rs6586282
rs1789953
rs921943
rs567754
rs672413
rs234709
All
0.00
0.05
0.10
0.15
0.20
MR effect size for
'Selenium' on 'Protein lin−7 homolog B'

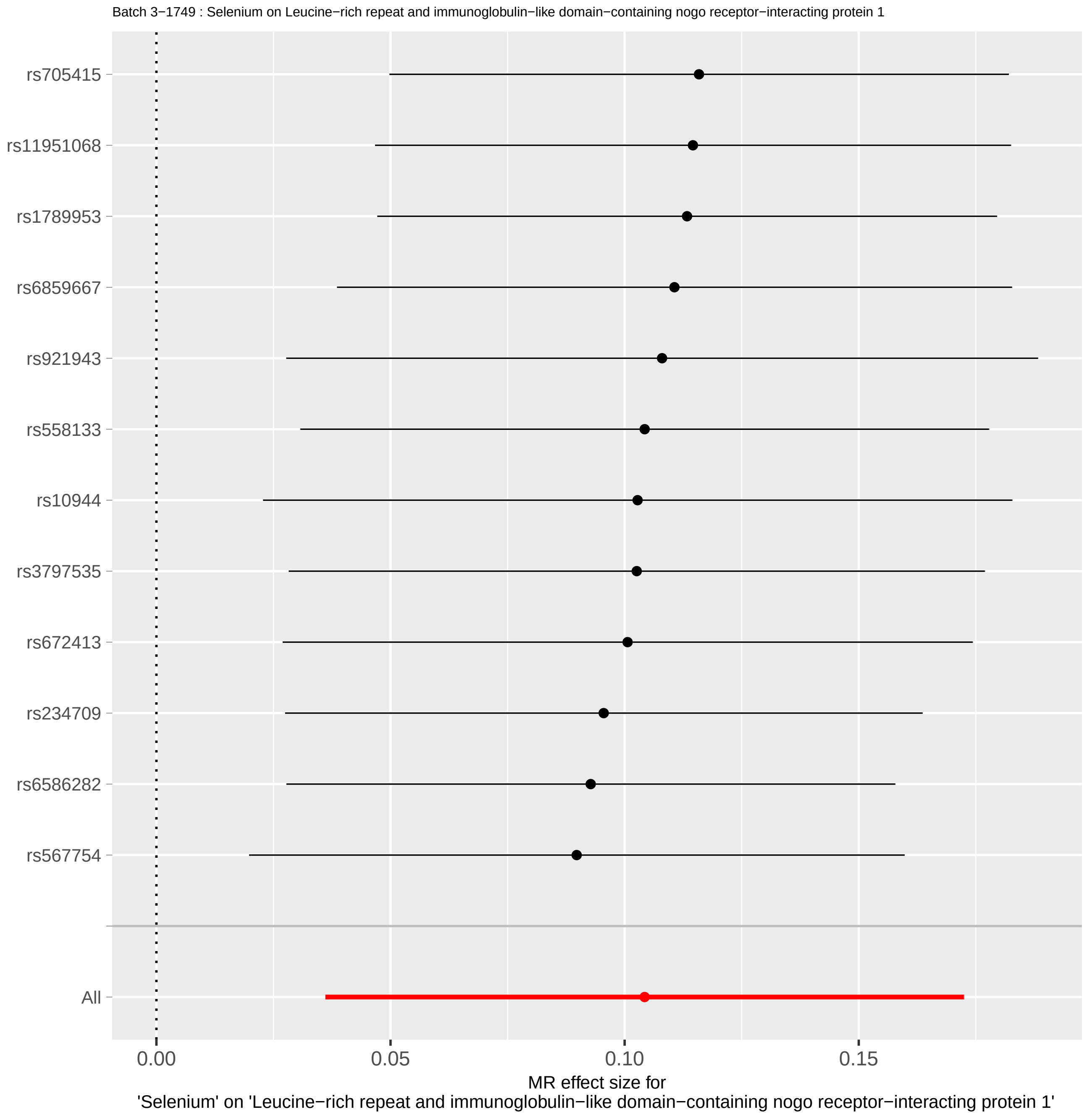
Batch 3−1749 : Selenium on Leucine−rich repeat and immunoglobulin−like domain−containing nogo receptor−interacting protein 1
rs705415
rs11951068
rs1789953
rs6859667
rs921943
rs558133
rs10944
rs3797535
rs672413
rs234709
rs6586282
rs567754
All
0.00
0.05
0.10
0.15
MR effect size for
'Selenium' on 'Leucine−rich repeat and immunoglobulin−like domain−containing nogo receptor−interacting protein 1'

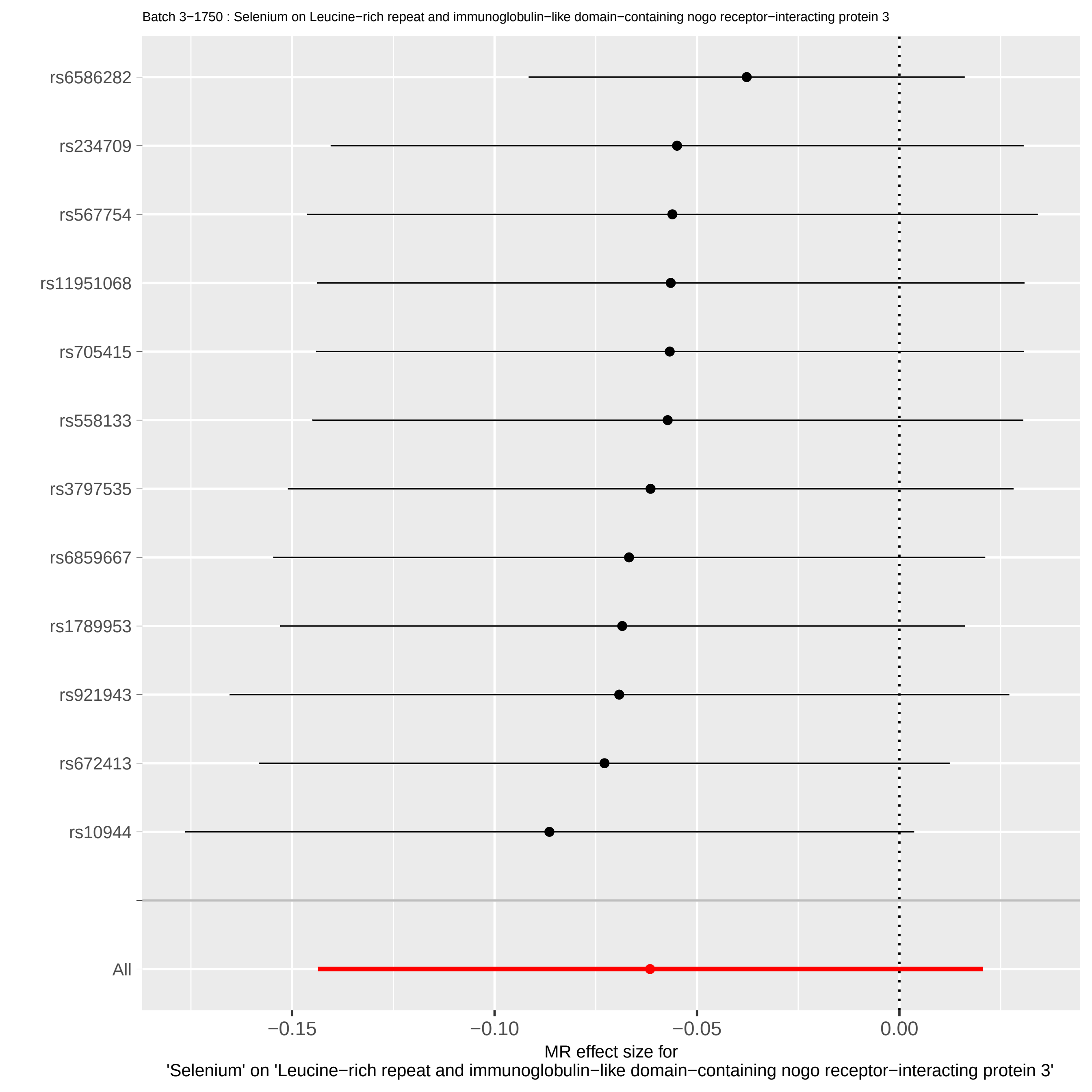

Batch 3−1750 : Selenium on Leucine−rich repeat and immunoglobulin−like domain−containing nogo receptor−interacting protein 3
rs6586282
rs234709
rs567754
rs11951068
rs705415
rs558133
rs3797535
rs6859667
rs1789953
rs921943
rs672413
rs10944
All
−0.15
−0.10
−0.05
0.00
MR effect size for
'Selenium' on 'Leucine−rich repeat and immunoglobulin−like domain−containing nogo receptor−interacting protein 3'

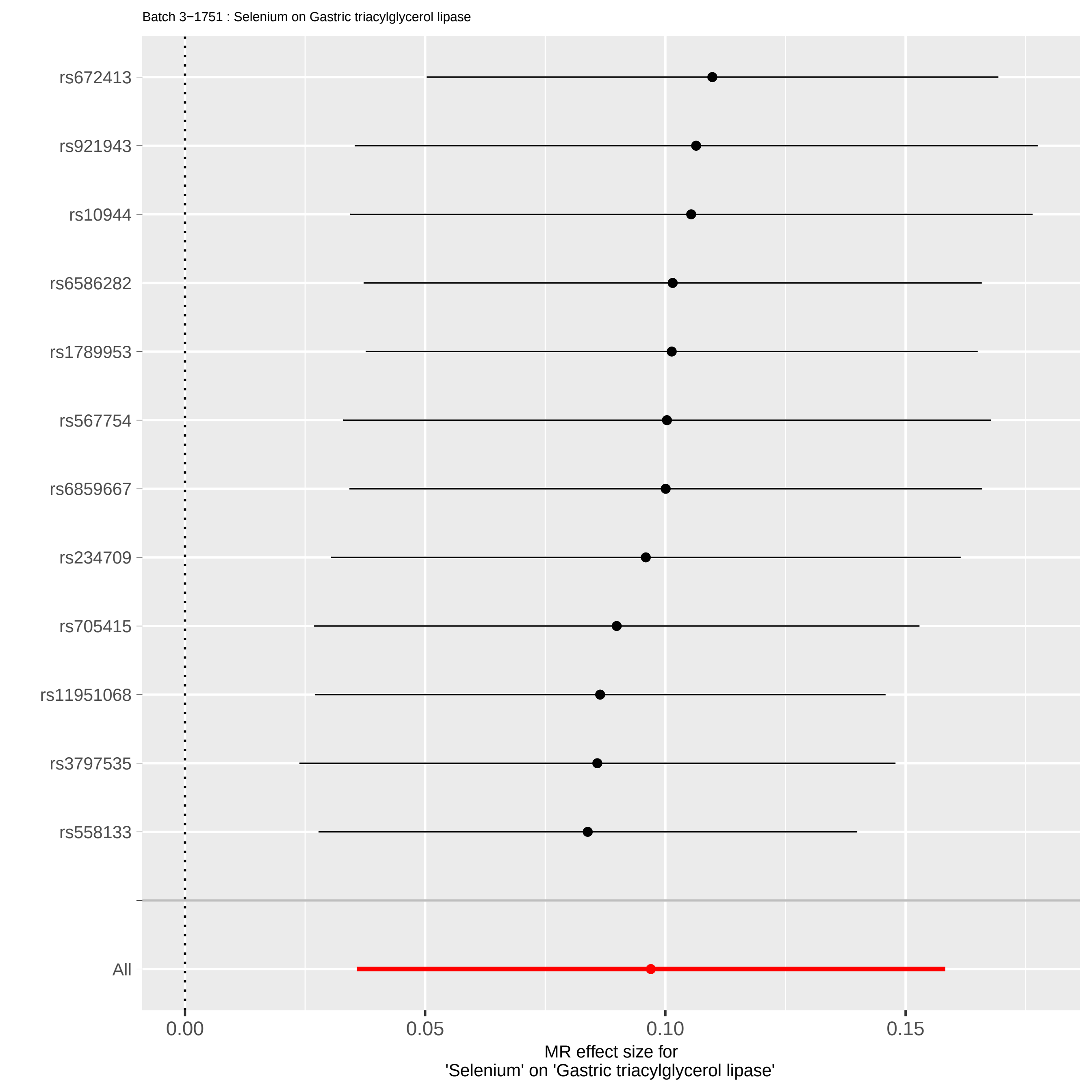
Batch 3−1751 : Selenium on Gastric triacylglycerol lipase
rs672413
rs921943
rs10944
rs6586282
rs1789953
rs567754
rs6859667
rs234709
rs705415
rs11951068
rs3797535
rs558133
All
0.00
0.05
0.10
0.15
MR effect size for
'Selenium' on 'Gastric triacylglycerol lipase'

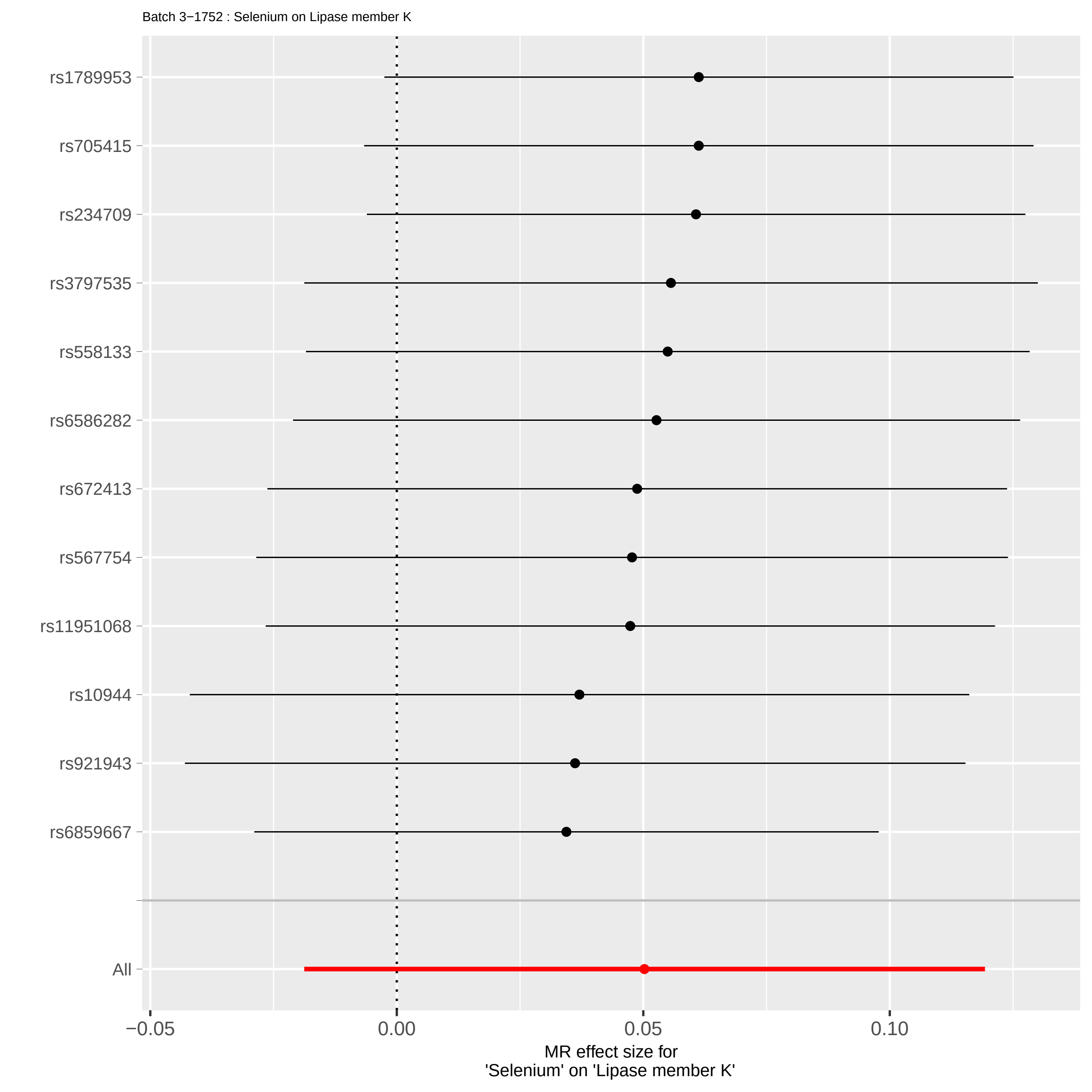
Batch 3−1752 : Selenium on Lipase member K
rs1789953
rs705415
rs234709
rs3797535
rs558133
rs6586282
rs672413
rs567754
rs11951068
rs10944
rs921943
rs6859667
All
−0.05
0.00
0.05
0.10
MR effect size for
'Selenium' on 'Lipase member K'

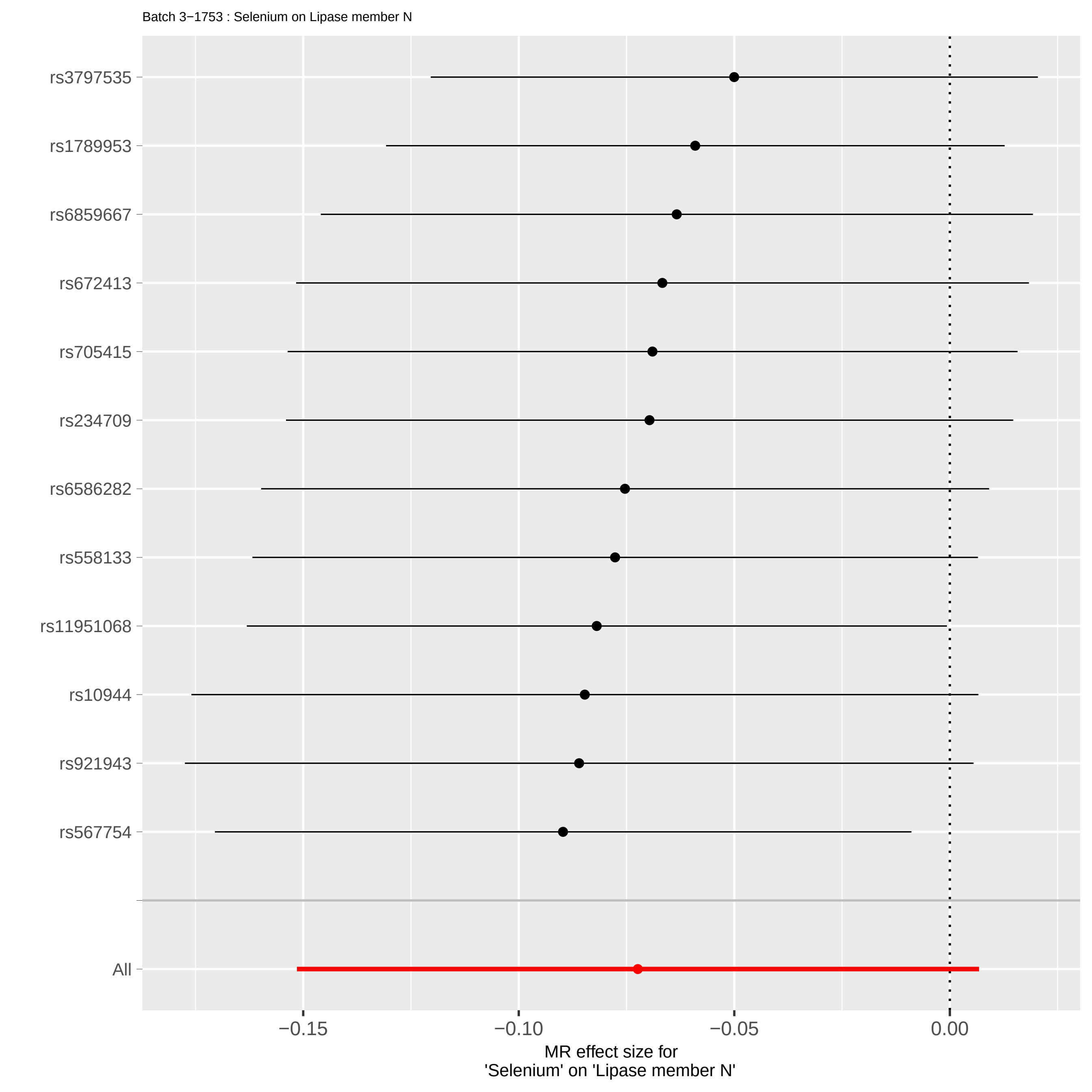

Batch 3−1753 : Selenium on Lipase member N
rs3797535
rs1789953
rs6859667
rs672413
rs705415
rs234709
rs6586282
rs558133
rs11951068
rs10944
rs921943
rs567754
All
−0.15
−0.10
−0.05
0.00
MR effect size for
'Selenium' on 'Lipase member N'

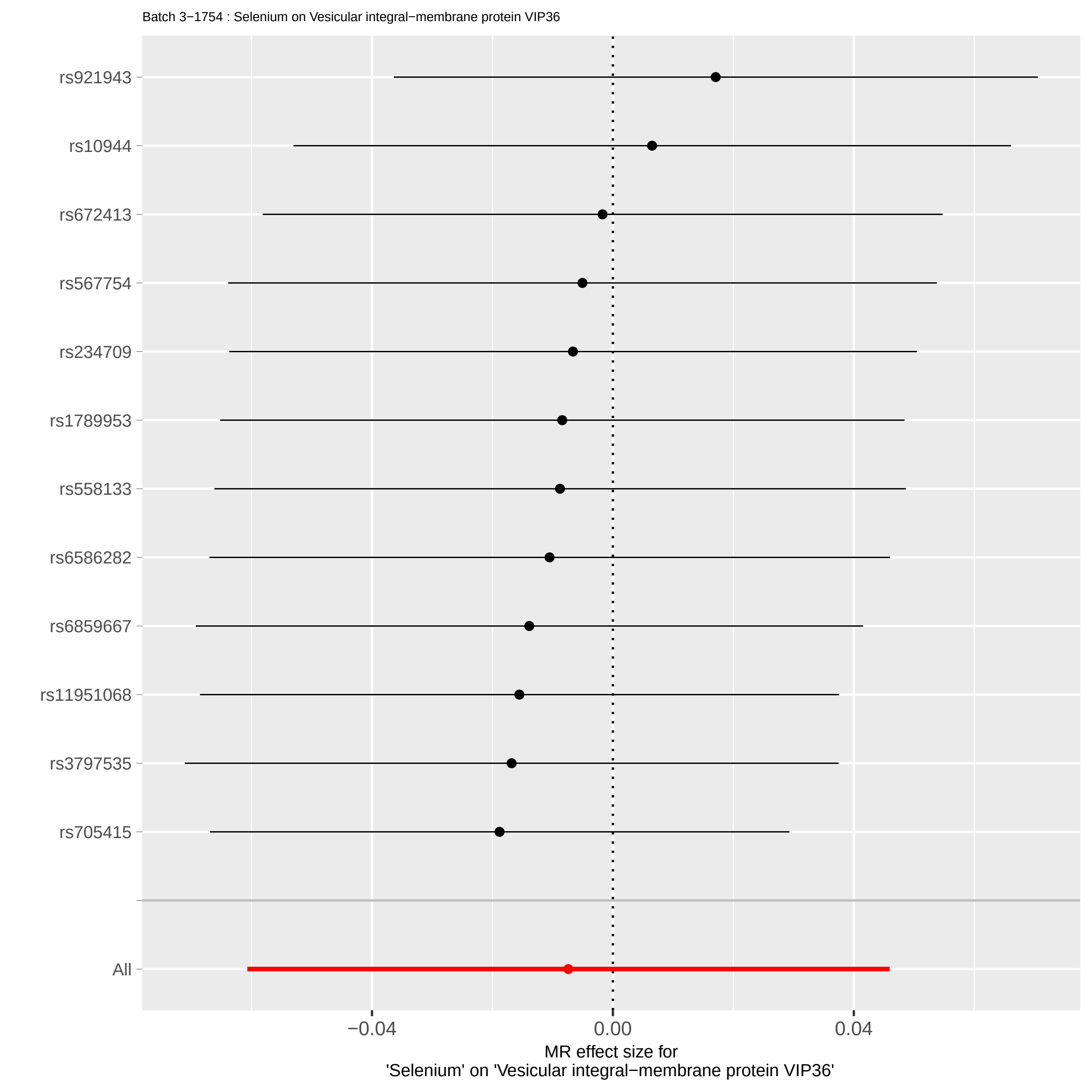
Batch 3−1754 : Selenium on Vesicular integral−membrane protein VIP36
rs921943
rs10944
rs672413
rs567754
rs234709
rs1789953
rs558133
rs6586282
rs6859667
rs11951068
rs3797535
rs705415
All
−0.04
0.00
0.04
MR effect size for
'Selenium' on 'Vesicular integral−membrane protein VIP36'

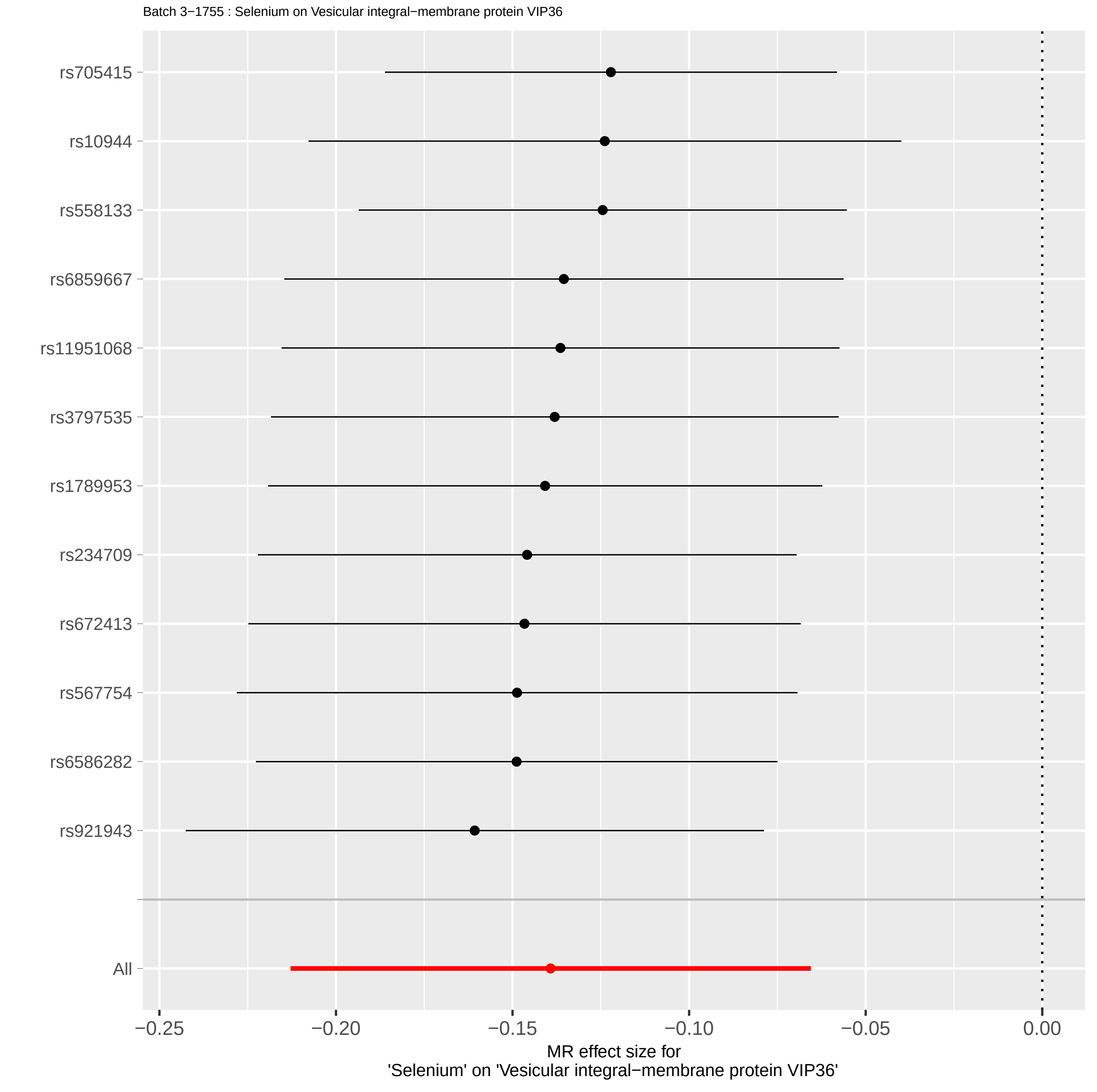

Batch 3−1755 : Selenium on Vesicular integral−membrane protein VIP36
rs705415
rs10944
rs558133
rs6859667
rs11951068
rs3797535
rs1789953
rs234709
rs672413
rs567754
rs6586282
rs921943
All
−0.25
−0.20
−0.15
−0.10
−0.05
0.00
MR effect size for
'Selenium' on 'Vesicular integral−membrane protein VIP36'

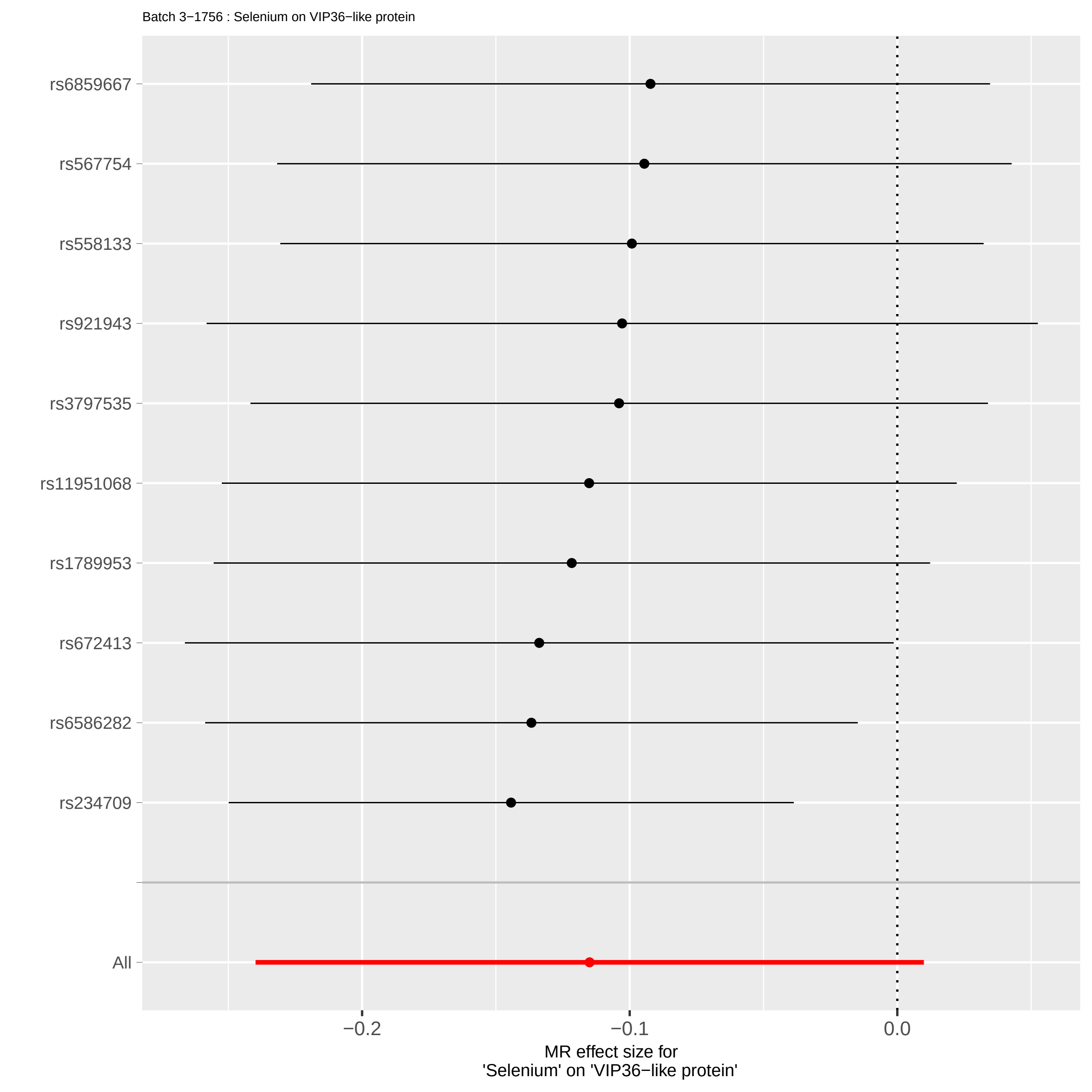

Batch 3−1756 : Selenium on VIP36−like protein
rs6859667
rs567754
rs558133
rs921943
rs3797535
rs11951068
rs1789953
rs672413
rs6586282
rs234709
All
−0.2
−0.1
0.0
MR effect size for
'Selenium' on 'VIP36−like protein'

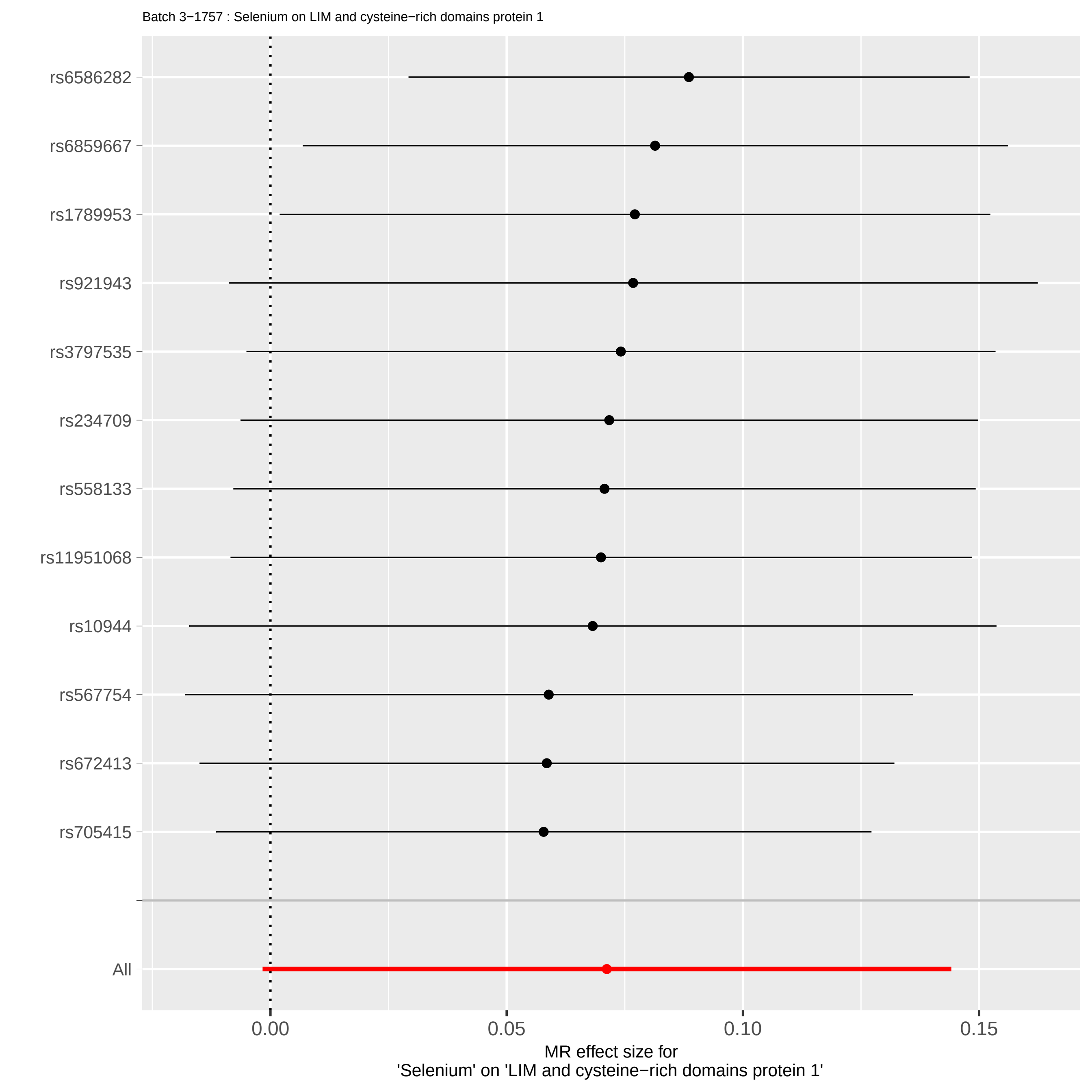

Batch 3−1757 : Selenium on LIM and cysteine−rich domains protein 1
rs6586282
rs6859667
rs1789953
rs921943
rs3797535
rs234709
rs558133
rs11951068
rs10944
rs567754
rs672413
rs705415
All
0.00
0.05
0.10
0.15
MR effect size for
'Selenium' on 'LIM and cysteine−rich domains protein 1'

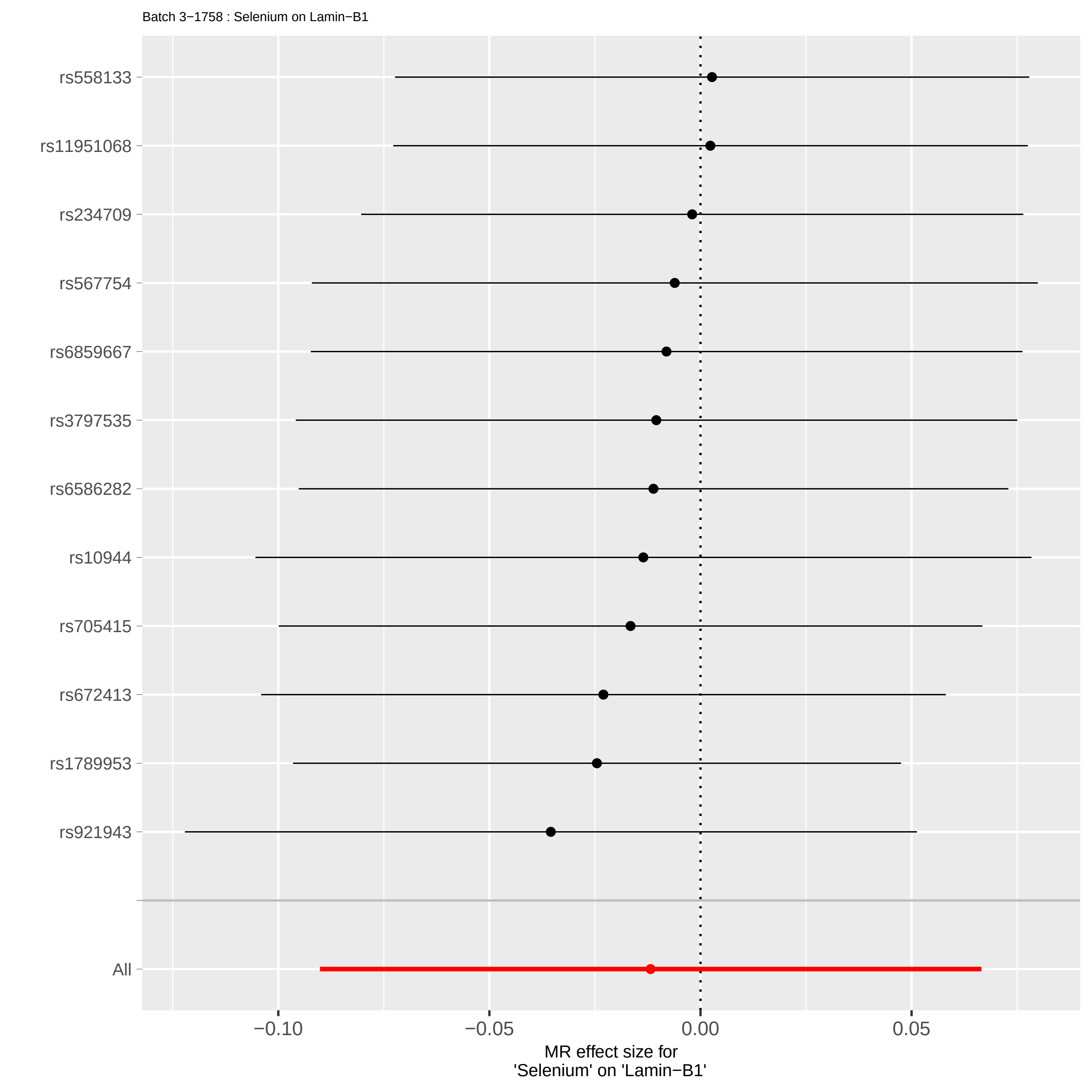

Batch 3−1758 : Selenium on Lamin−B1
rs558133
rs11951068
rs234709
rs567754
rs6859667
rs3797535
rs6586282
rs10944
rs705415
rs672413
rs1789953
rs921943
All
−0.10
−0.05
0.00
0.05
MR effect size for
'Selenium' on 'Lamin−B1'

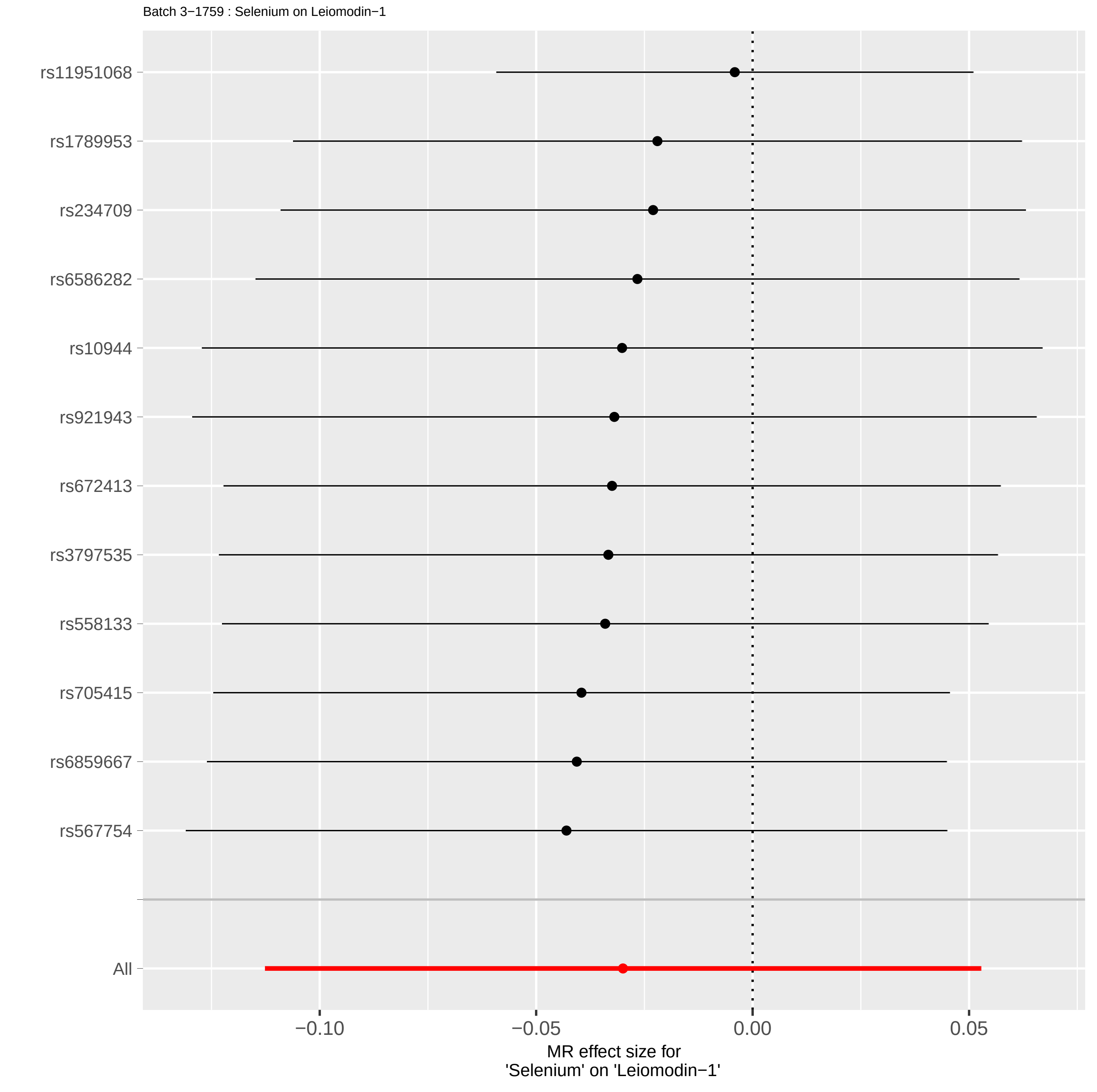

Batch 3−1759 : Selenium on Leiomodin−1
rs11951068
rs1789953
rs234709
rs6586282
rs10944
rs921943
rs672413
rs3797535
rs558133
rs705415
rs6859667
rs567754
All
−0.10
−0.05
0.00
0.05
MR effect size for
'Selenium' on 'Leiomodin−1'

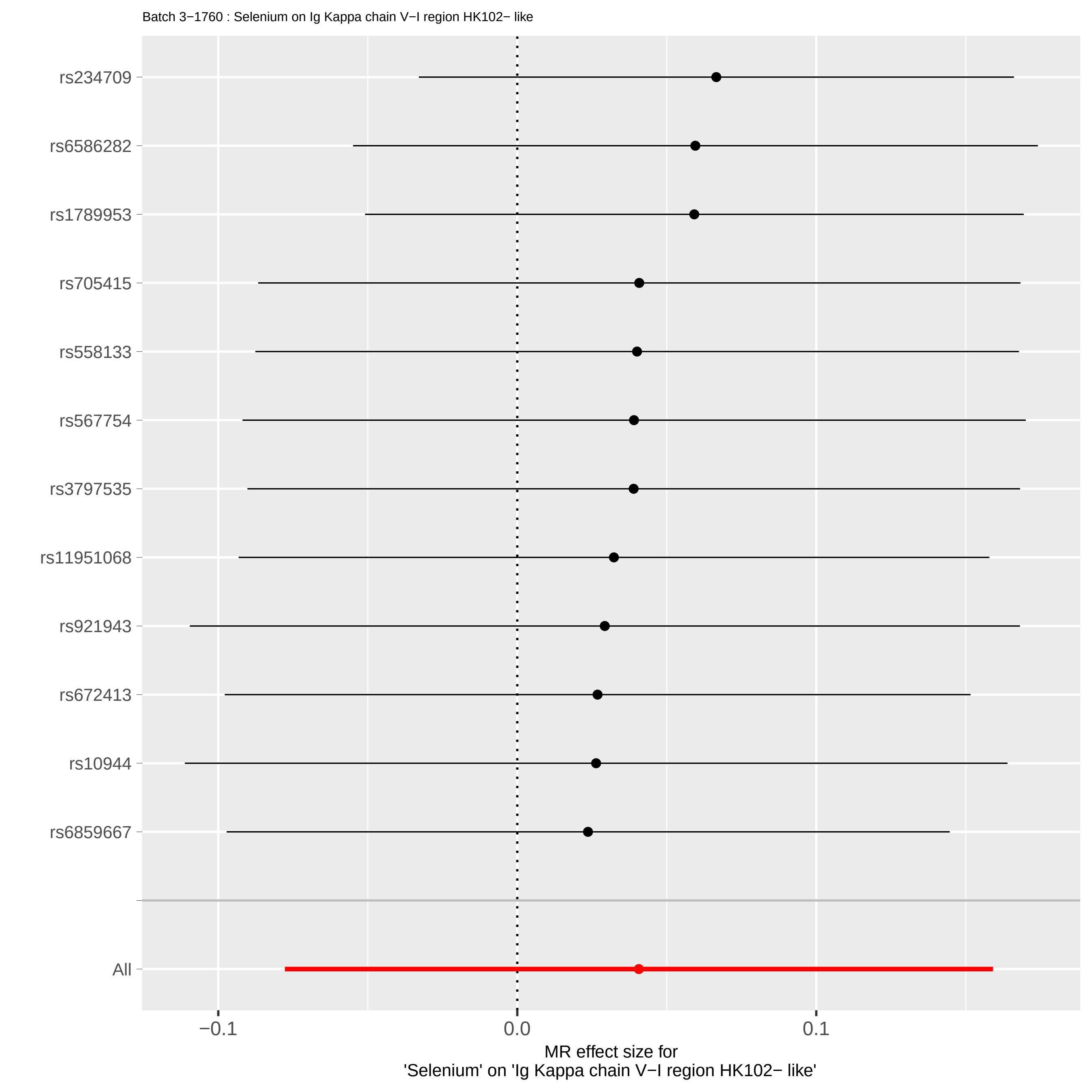
Batch 3−1760 : Selenium on Ig Kappa chain V−I region HK102− like
rs234709
rs6586282
rs1789953
rs705415
rs558133
rs567754
rs3797535
rs11951068
rs921943
rs672413
rs10944
rs6859667
All
−0.1
0.0
0.1
MR effect size for
'Selenium' on 'Ig Kappa chain V−I region HK102− like'

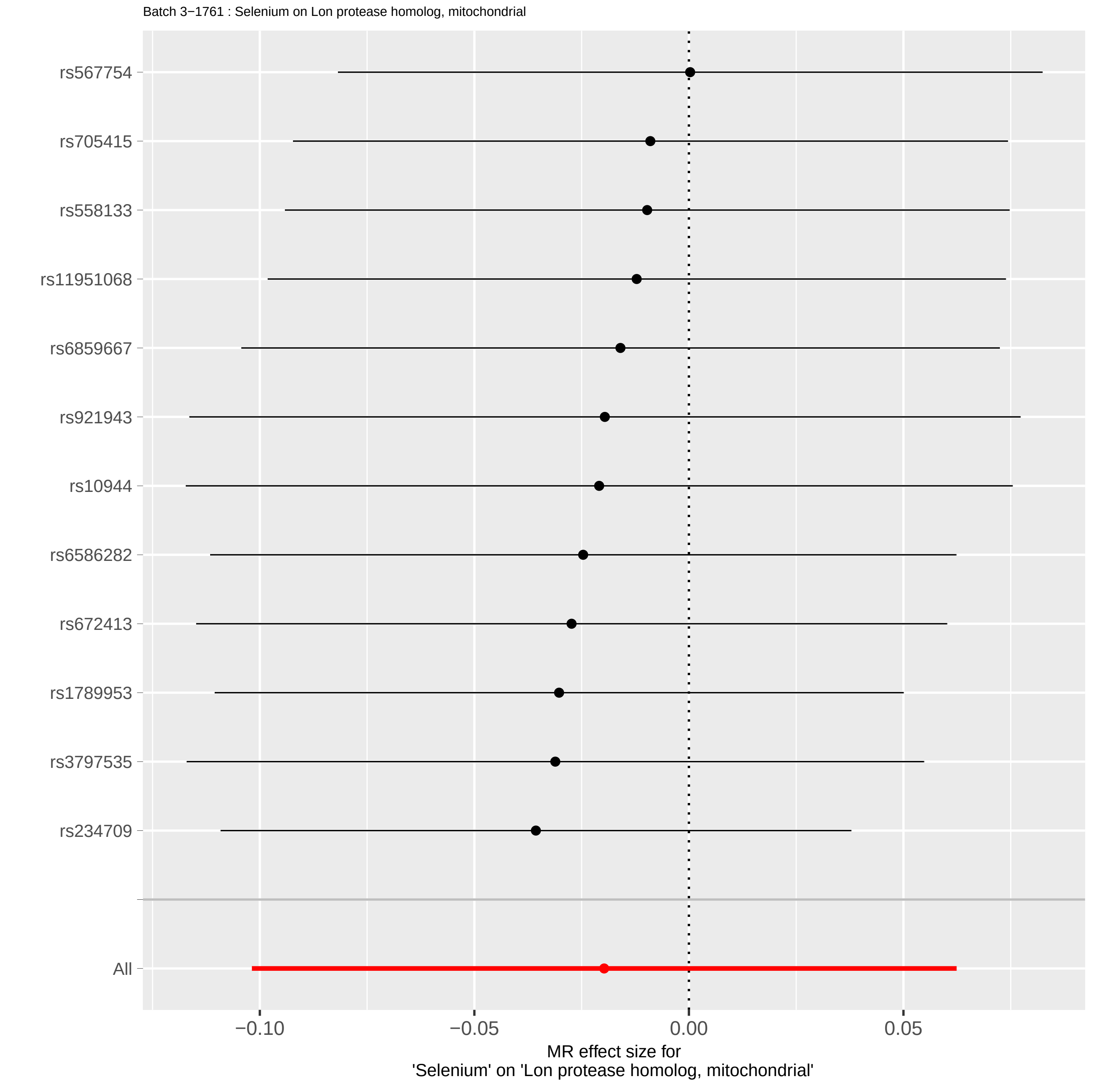

Batch 3−1761 : Selenium on Lon protease homolog, mitochondrial
rs567754
rs705415
rs558133
rs11951068
rs6859667
rs921943
rs10944
rs6586282
rs672413
rs1789953
rs3797535
rs234709
All
−0.10
−0.05
0.00
0.05
MR effect size for
'Selenium' on 'Lon protease homolog, mitochondrial'

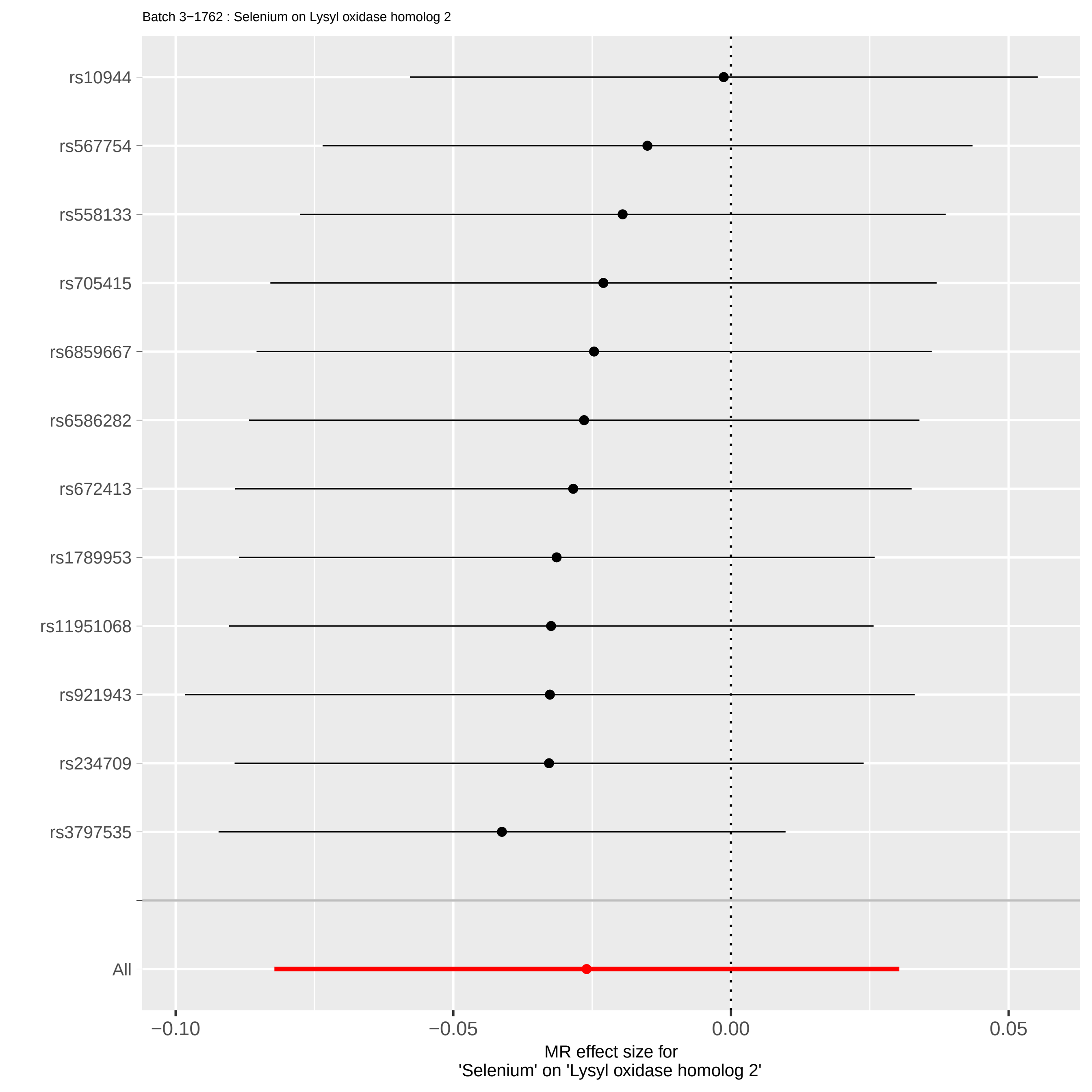
Batch 3−1762 : Selenium on Lysyl oxidase homolog 2
rs10944
rs567754
rs558133
rs705415
rs6859667
rs6586282
rs672413
rs1789953
rs11951068
rs921943
rs234709
rs3797535
All
−0.10
−0.05
0.00
0.05
MR effect size for
'Selenium' on 'Lysyl oxidase homolog 2'

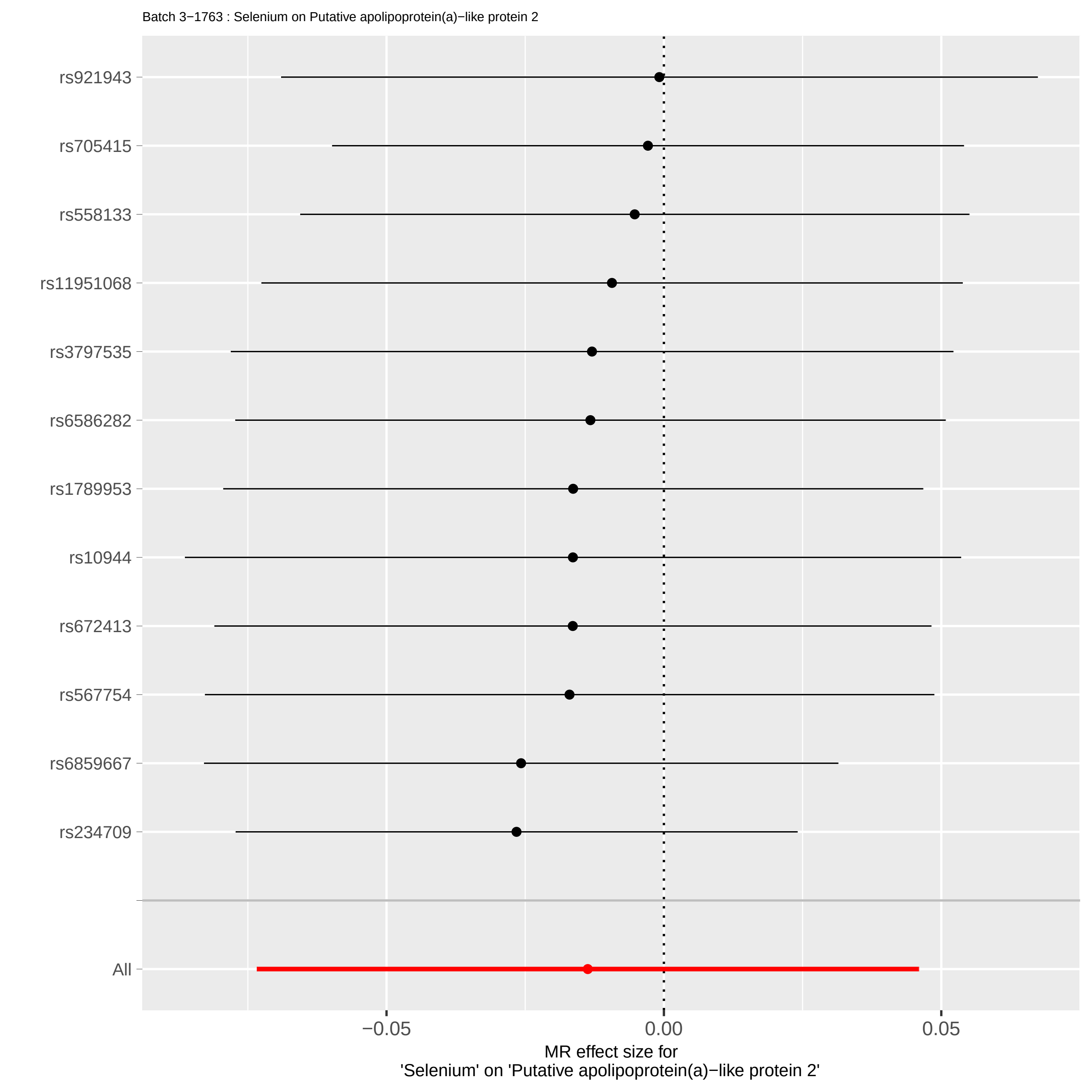
Batch 3−1763 : Selenium on Putative apolipoprotein(a)−like protein 2
rs921943
rs705415
rs558133
rs11951068
rs3797535
rs6586282
rs1789953
rs10944
rs672413
rs567754
rs6859667
rs234709
All
−0.05
0.00
0.05
MR effect size for
'Selenium' on 'Putative apolipoprotein(a)−like protein 2'

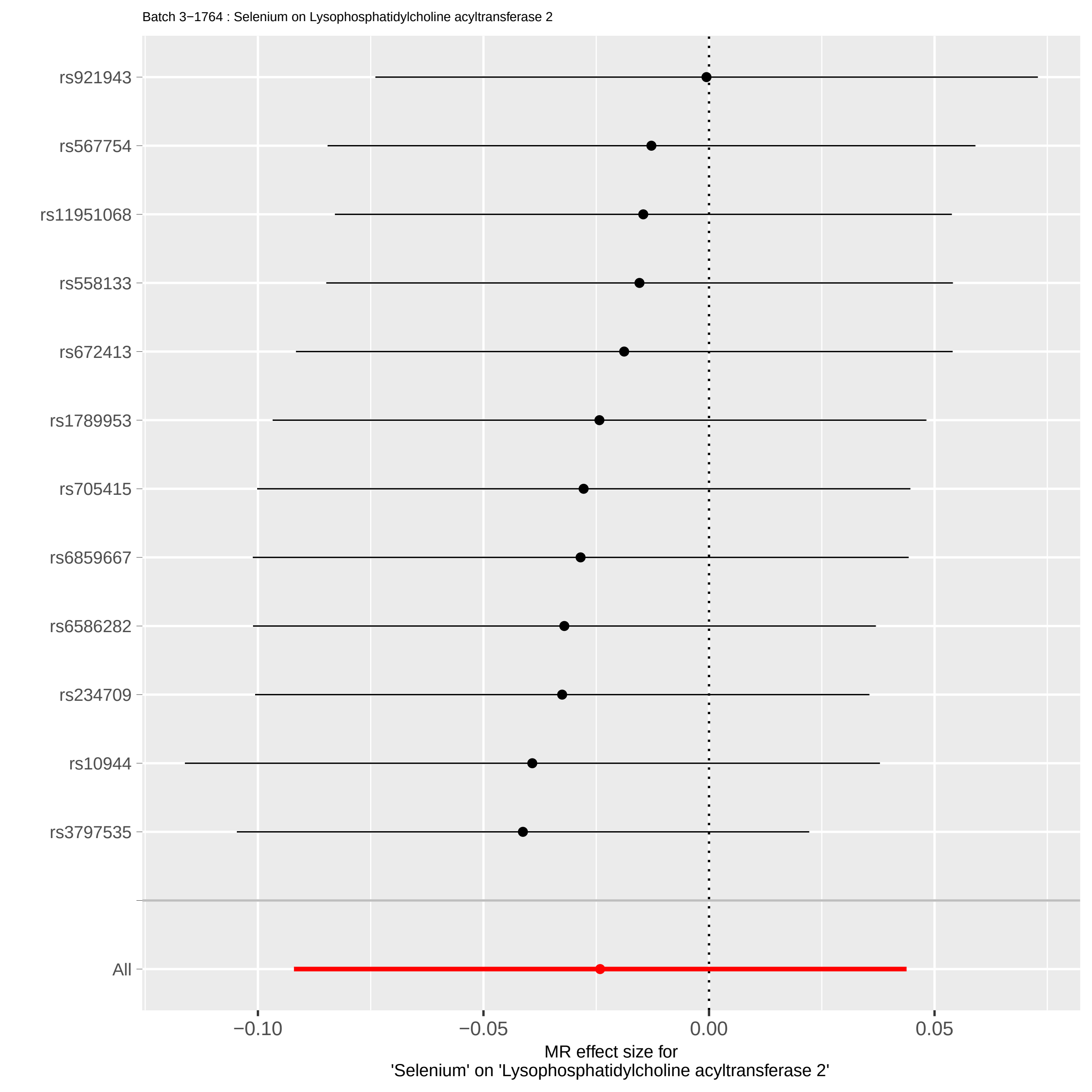
Batch 3−1764 : Selenium on Lysophosphatidylcholine acyltransferase 2
rs921943
rs567754
rs11951068
rs558133
rs672413
rs1789953
rs705415
rs6859667
rs6586282
rs234709
rs10944
rs3797535
All
−0.10
−0.05
0.00
0.05
MR effect size for
'Selenium' on 'Lysophosphatidylcholine acyltransferase 2'

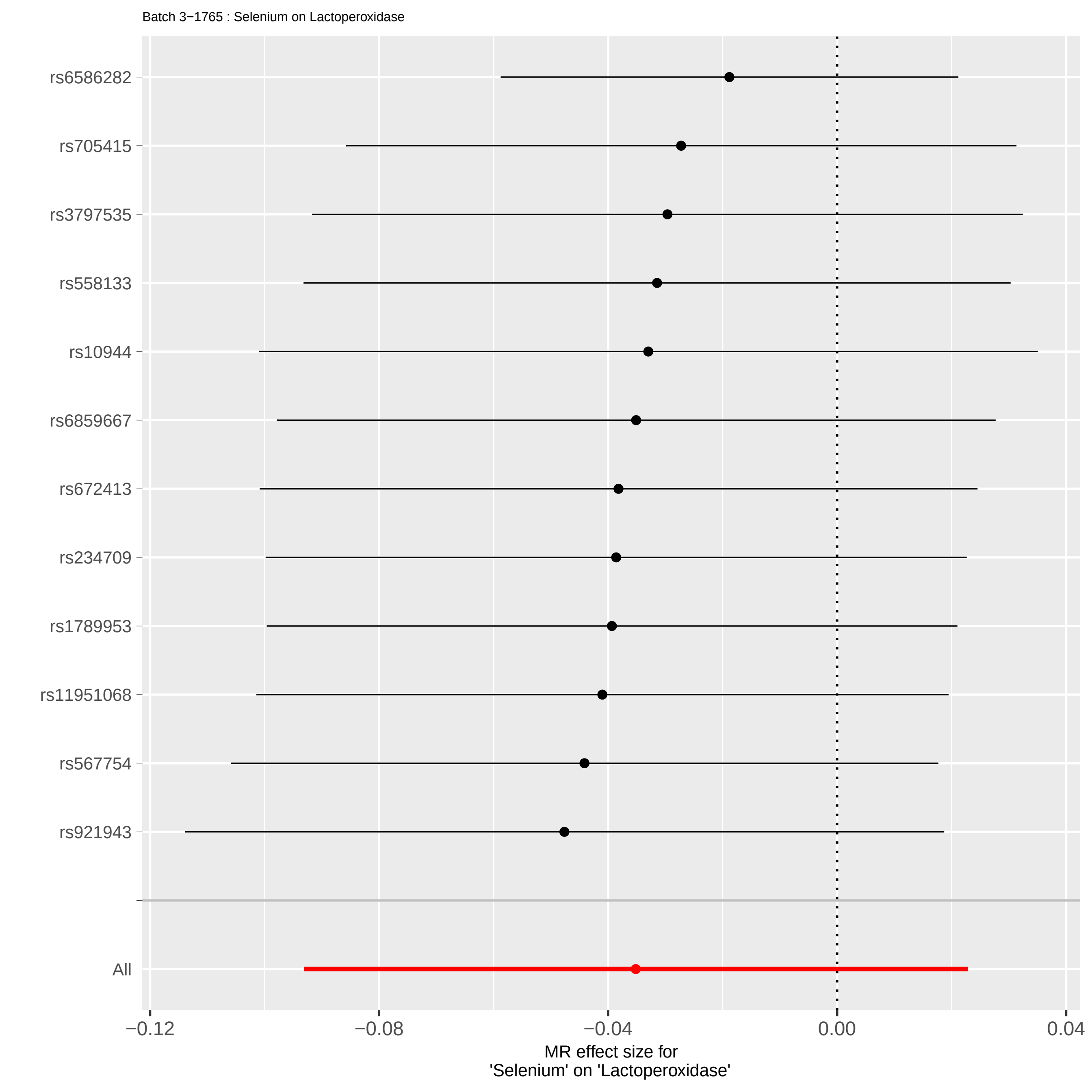

Batch 3−1765 : Selenium on Lactoperoxidase
rs6586282
rs705415
rs3797535
rs558133
rs10944
rs6859667
rs672413
rs234709
rs1789953
rs11951068
rs567754
rs921943
All
−0.12
−0.08
−0.04
0.00
0.04
MR effect size for
'Selenium' on 'Lactoperoxidase'

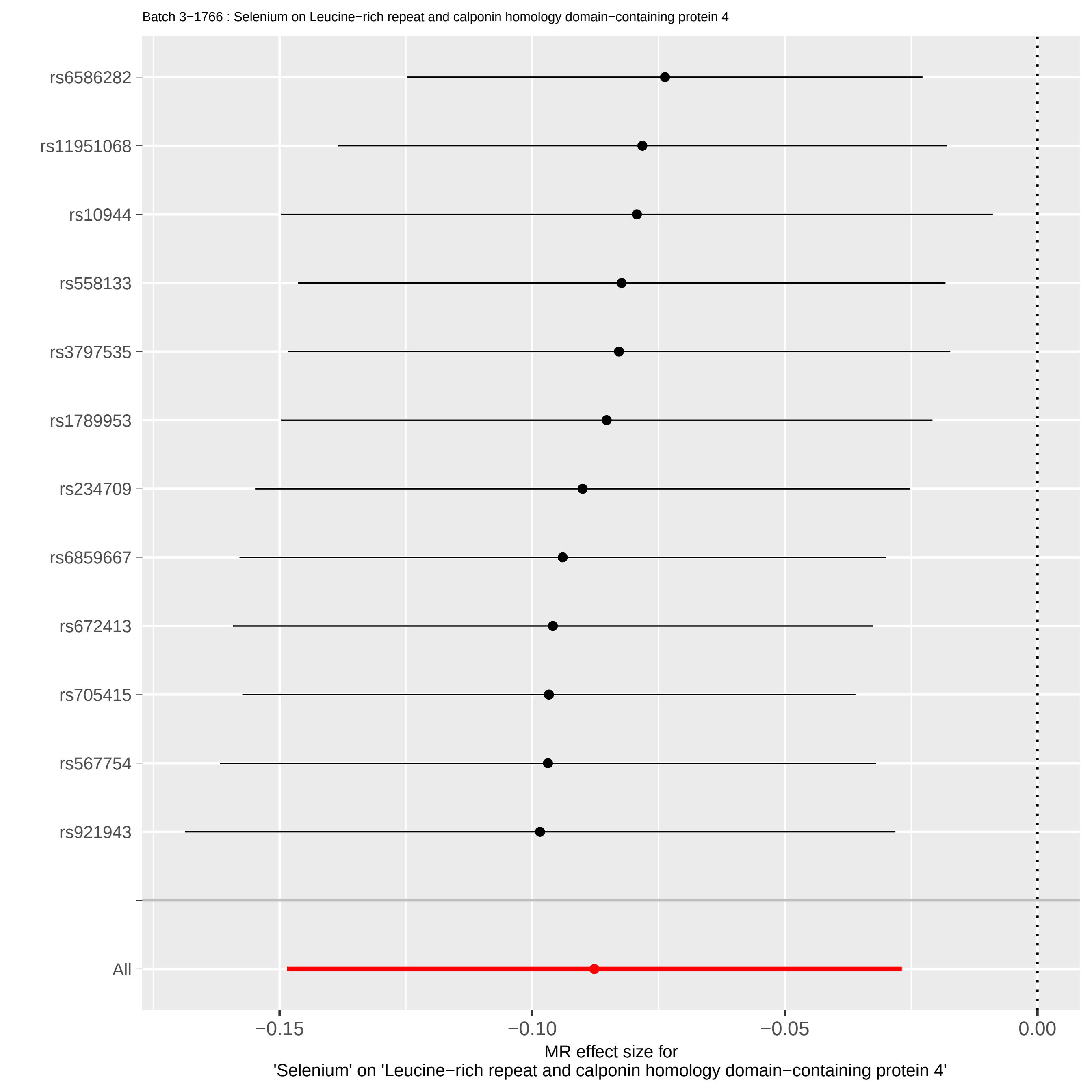

Batch 3−1766 : Selenium on Leucine−rich repeat and calponin homology domain−containing protein 4
rs6586282
rs11951068
rs10944
rs558133
rs3797535
rs1789953
rs234709
rs6859667
rs672413
rs705415
rs567754
rs921943
All
−0.15
−0.10
−0.05
0.00
MR effect size for
'Selenium' on 'Leucine−rich repeat and calponin homology domain−containing protein 4'

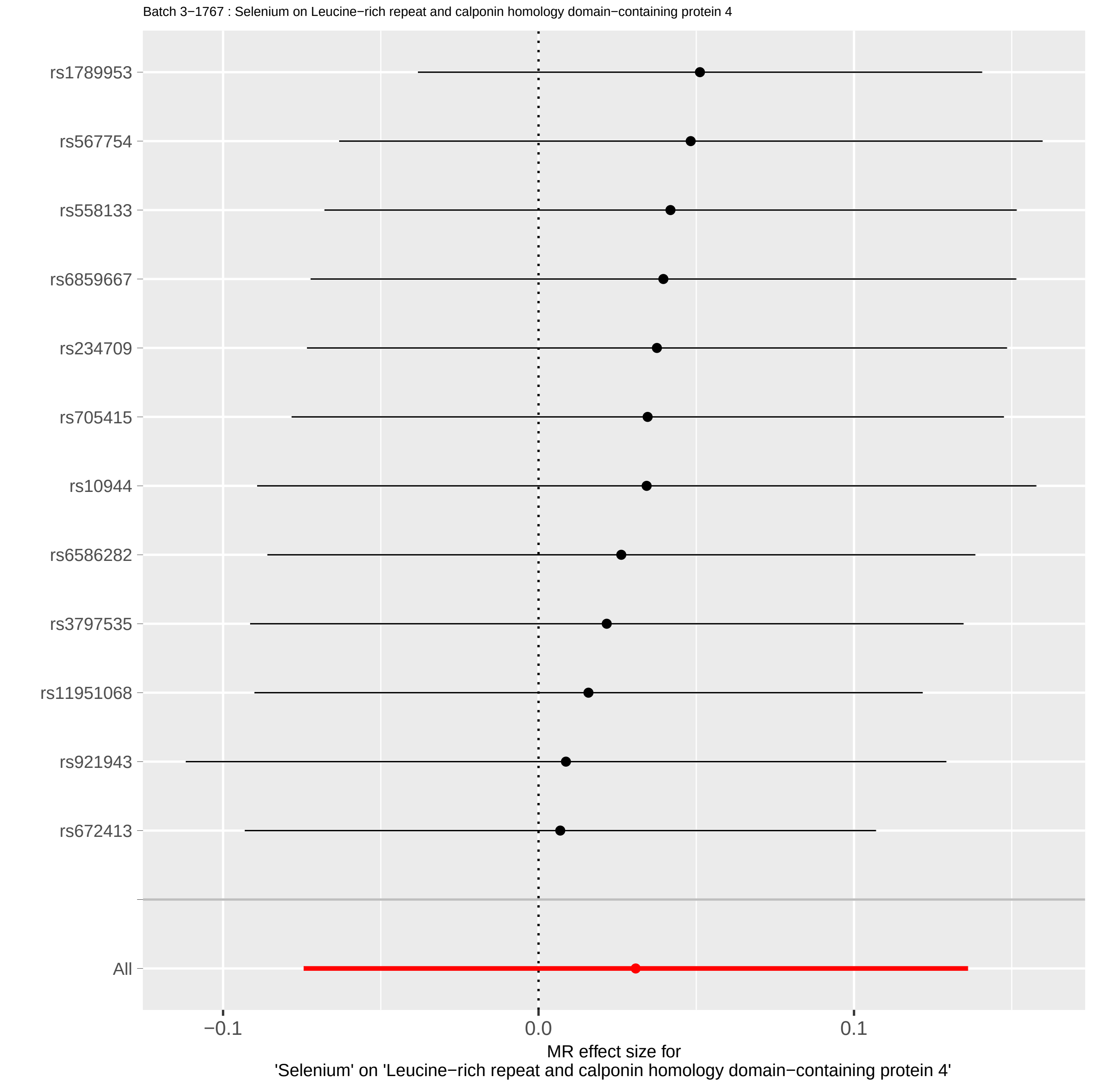

Batch 3−1767 : Selenium on Leucine−rich repeat and calponin homology domain−containing protein 4
rs1789953
rs567754
rs558133
rs6859667
rs234709
rs705415
rs10944
rs6586282
rs3797535
rs11951068
rs921943
rs672413
All
−0.1
0.0
0.1
MR effect size for
'Selenium' on 'Leucine−rich repeat and calponin homology domain−containing protein 4'

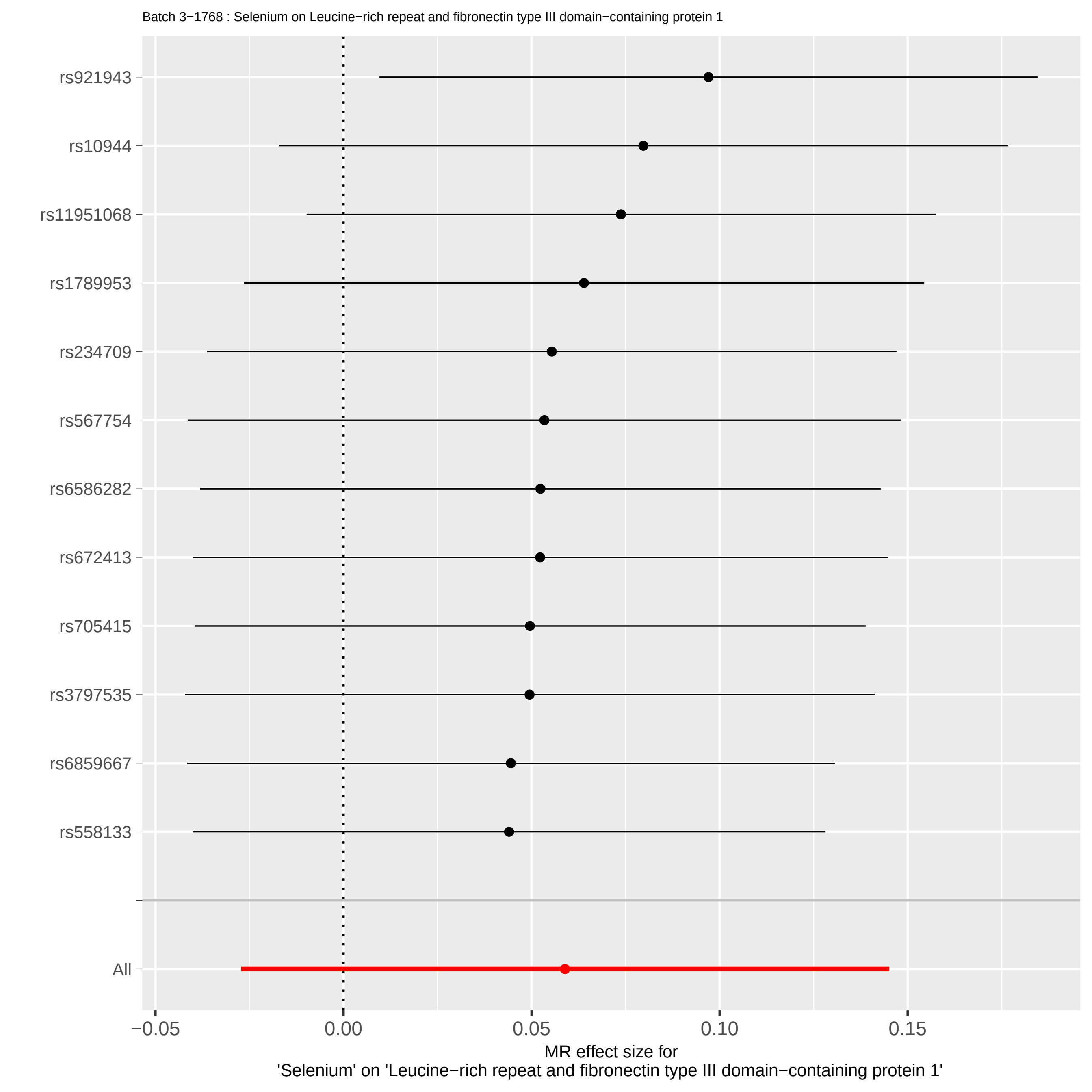

Batch 3−1768 : Selenium on Leucine−rich repeat and fibronectin type III domain−containing protein 1
rs921943
rs10944
rs11951068
rs1789953
rs234709
rs567754
rs6586282
rs672413
rs705415
rs3797535
rs6859667
rs558133
All
−0.05
0.00
0.05
0.10
0.15
MR effect size for
'Selenium' on 'Leucine−rich repeat and fibronectin type III domain−containing protein 1'

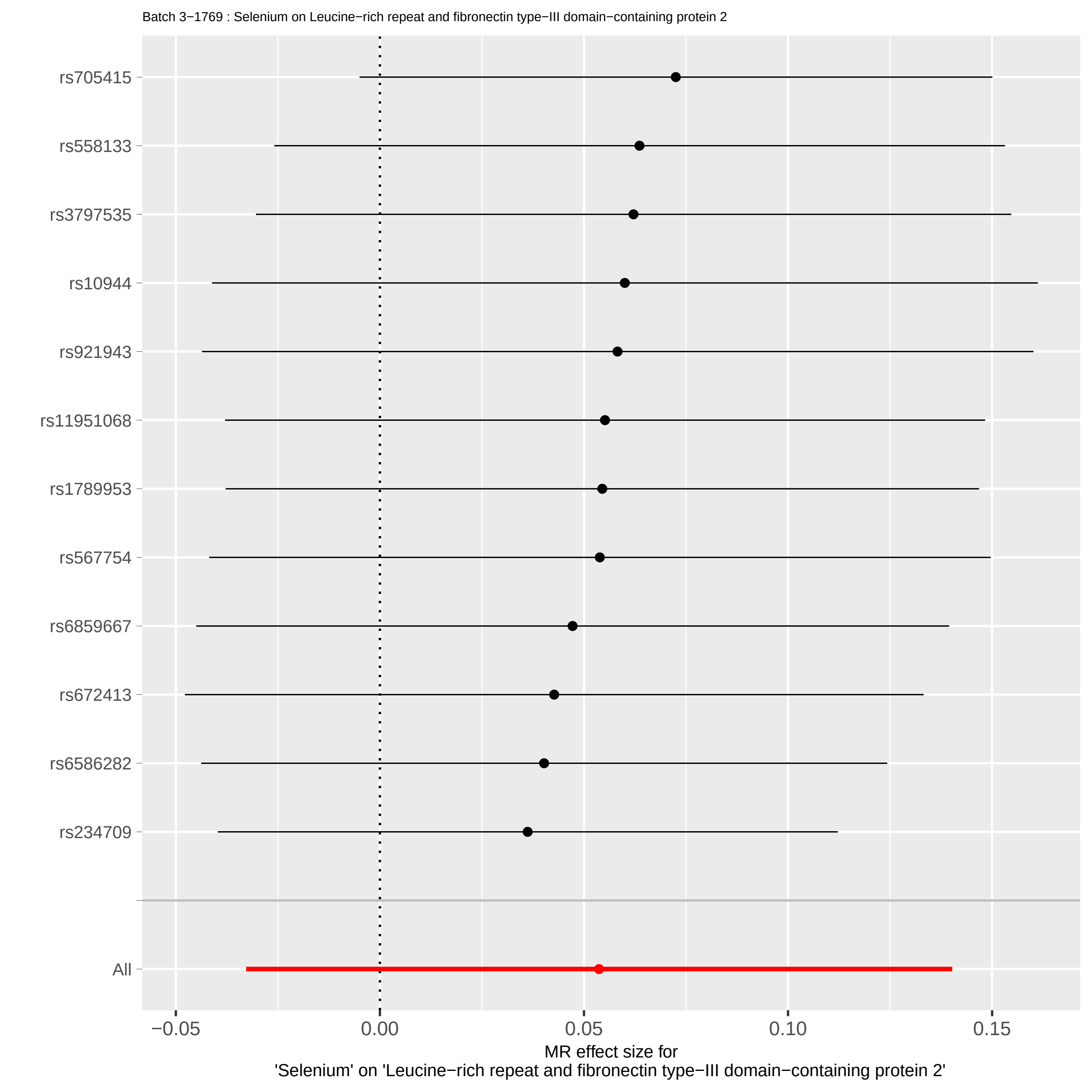
Batch 3−1769 : Selenium on Leucine−rich repeat and fibronectin type−III domain−containing protein 2
rs705415
rs558133
rs3797535
rs10944
rs921943
rs11951068
rs1789953
rs567754
rs6859667
rs672413
rs6586282
rs234709
All
−0.05
0.00
0.05
0.10
0.15
MR effect size for
'Selenium' on 'Leucine−rich repeat and fibronectin type−III domain−containing protein 2'

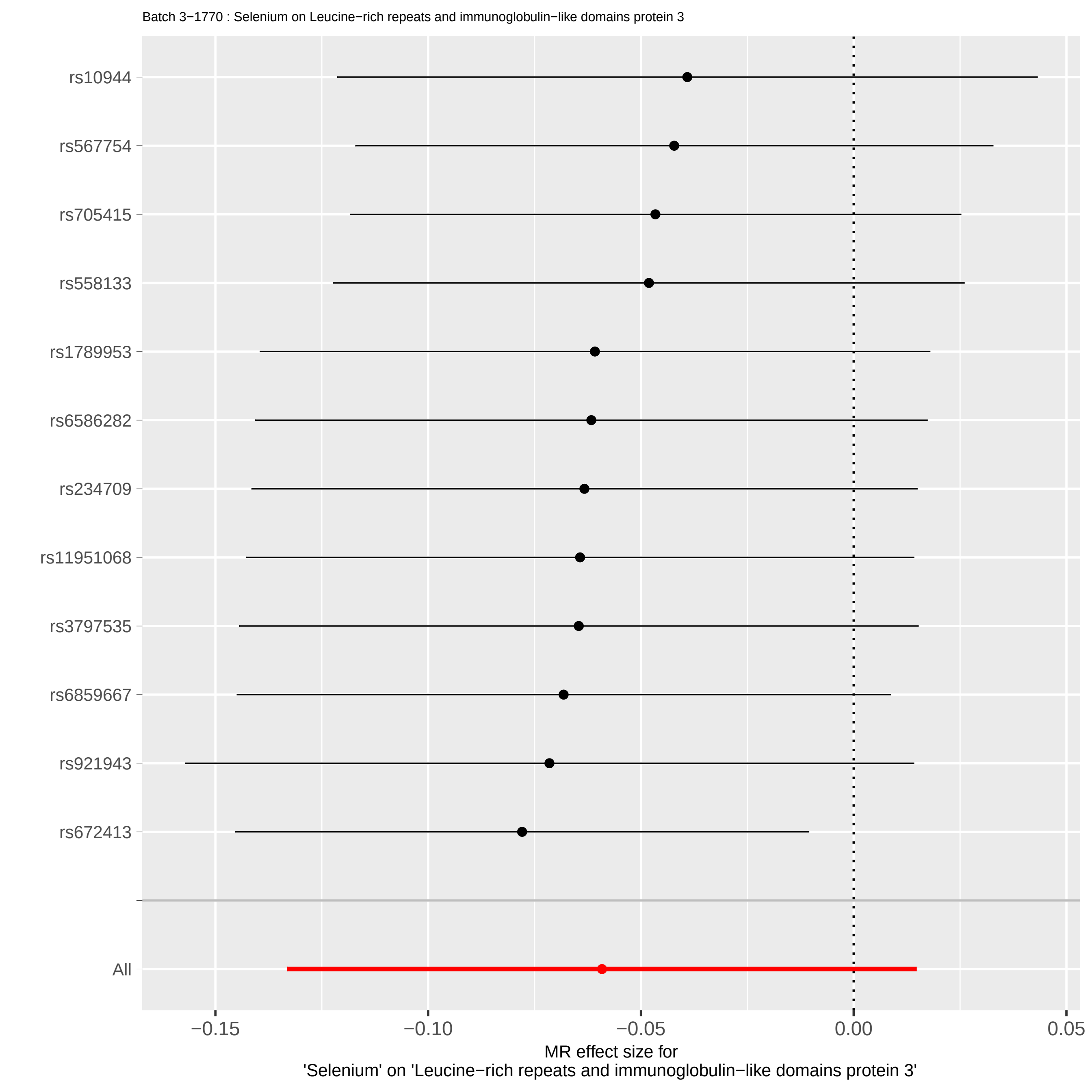

Batch 3−1770 : Selenium on Leucine−rich repeats and immunoglobulin−like domains protein 3
rs10944
rs567754
rs705415
rs558133
rs1789953
rs6586282
rs234709
rs11951068
rs3797535
rs6859667
rs921943
rs672413
All
−0.15
−0.10
−0.05
0.00
0.05
MR effect size for
'Selenium' on 'Leucine−rich repeats and immunoglobulin−like domains protein 3'

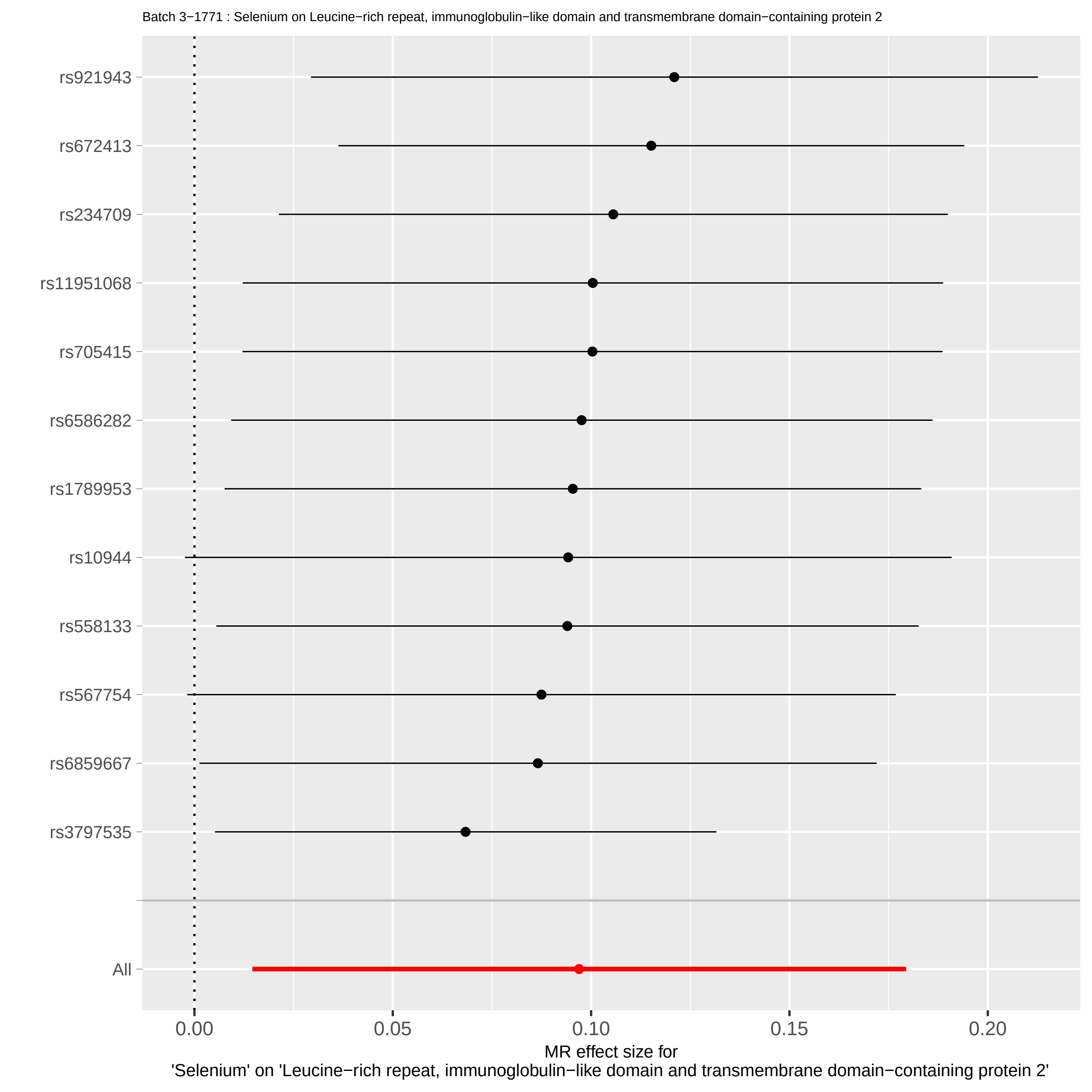

Batch 3−1771 : Selenium on Leucine−rich repeat, immunoglobulin−like domain and transmembrane domain−containing protein 2
rs921943
rs672413
rs234709
rs11951068
rs705415
rs6586282
rs1789953
rs10944
rs558133
rs567754
rs6859667
rs3797535
All
0.00
0.05
0.10
0.15
0.20
MR effect size for
'Selenium' on 'Leucine−rich repeat, immunoglobulin−like domain and transmembrane domain−containing protein 2'

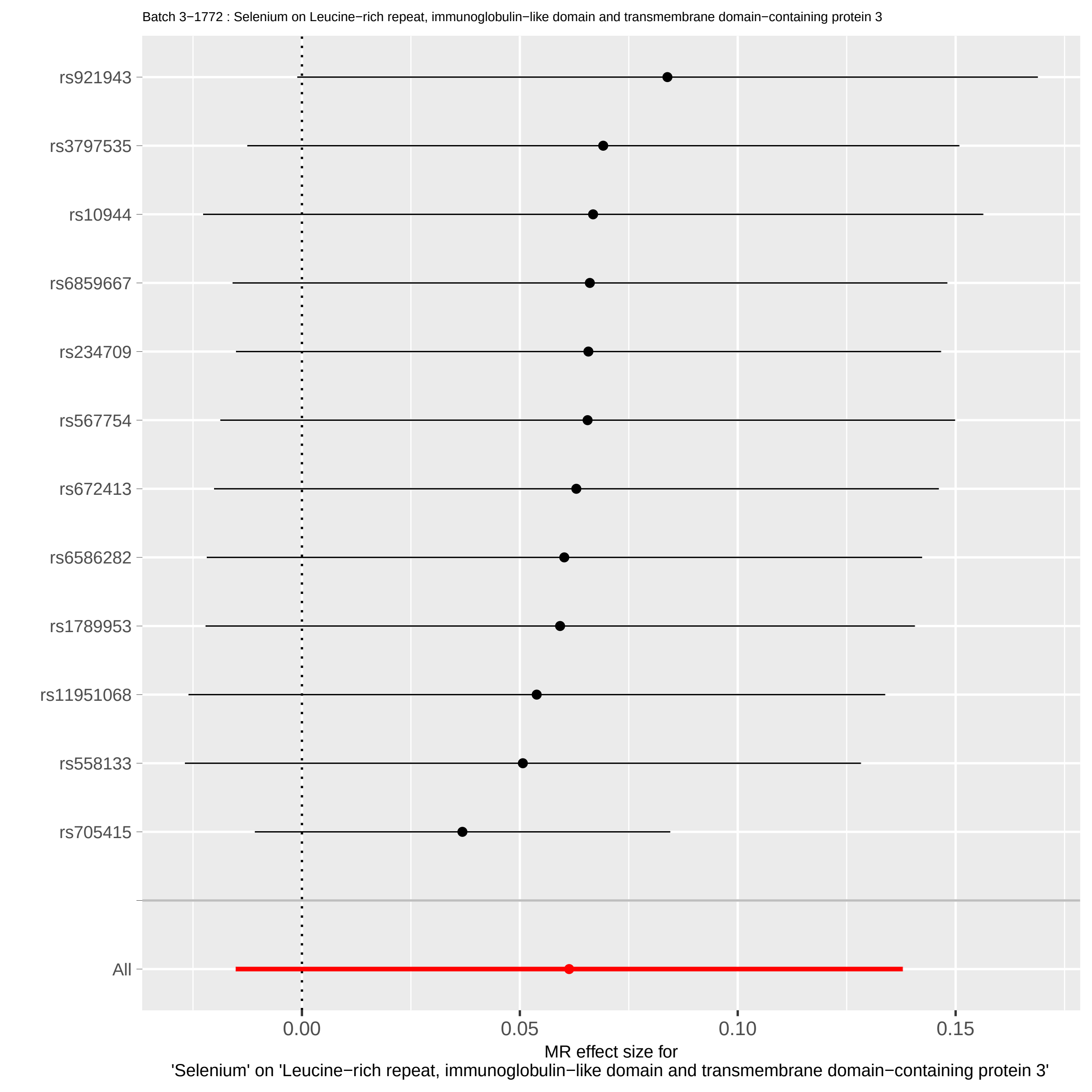

Batch 3−1772 : Selenium on Leucine−rich repeat, immunoglobulin−like domain and transmembrane domain−containing protein 3
rs921943
rs3797535
rs10944
rs6859667
rs234709
rs567754
rs672413
rs6586282
rs1789953
rs11951068
rs558133
rs705415
All
0.00
0.05
0.10
0.15
MR effect size for
'Selenium' on 'Leucine−rich repeat, immunoglobulin−like domain and transmembrane domain−containing protein 3'

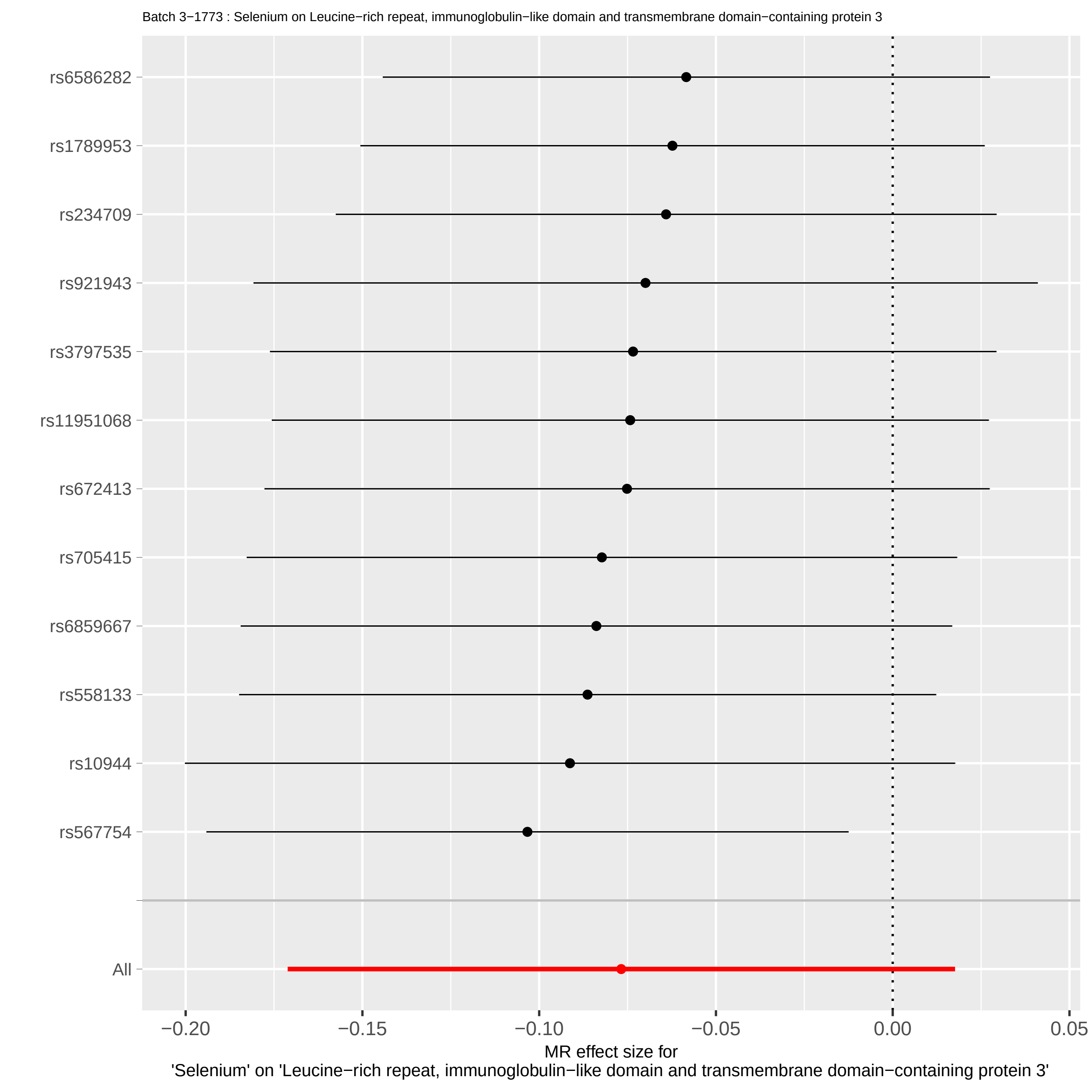
Batch 3−1773 : Selenium on Leucine−rich repeat, immunoglobulin−like domain and transmembrane domain−containing protein 3
rs6586282
rs1789953
rs234709
rs921943
rs3797535
rs11951068
rs672413
rs705415
rs6859667
rs558133
rs10944
rs567754
All
−0.20
−0.15
−0.10
−0.05
0.00
0.05
MR effect size for
'Selenium' on 'Leucine−rich repeat, immunoglobulin−like domain and transmembrane domain−containing protein 3'

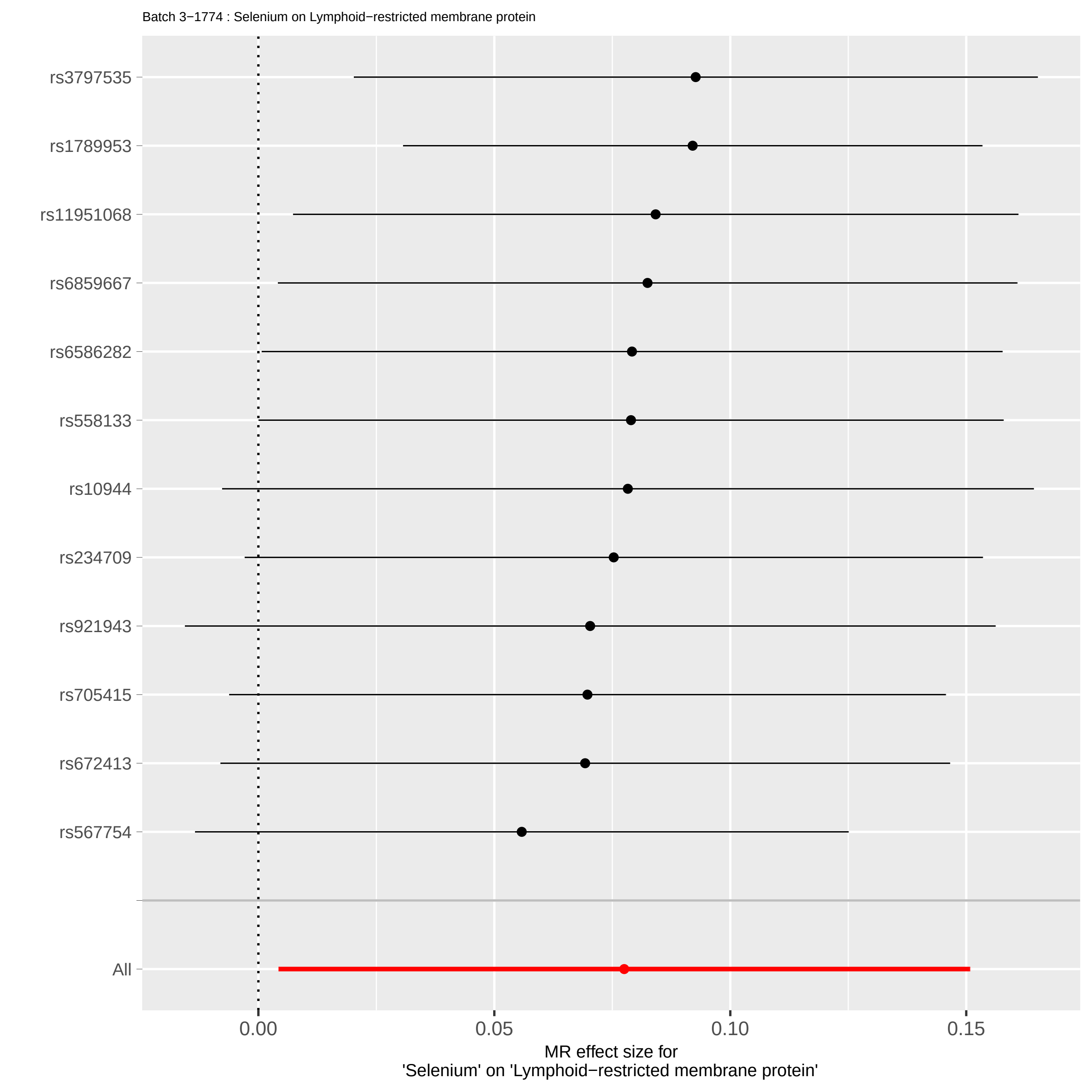
Batch 3−1774 : Selenium on Lymphoid−restricted membrane protein
rs3797535
rs1789953
rs11951068
rs6859667
rs6586282
rs558133
rs10944
rs234709
rs921943
rs705415
rs672413
rs567754
All
0.00
0.05
0.10
0.15
MR effect size for
'Selenium' on 'Lymphoid−restricted membrane protein'

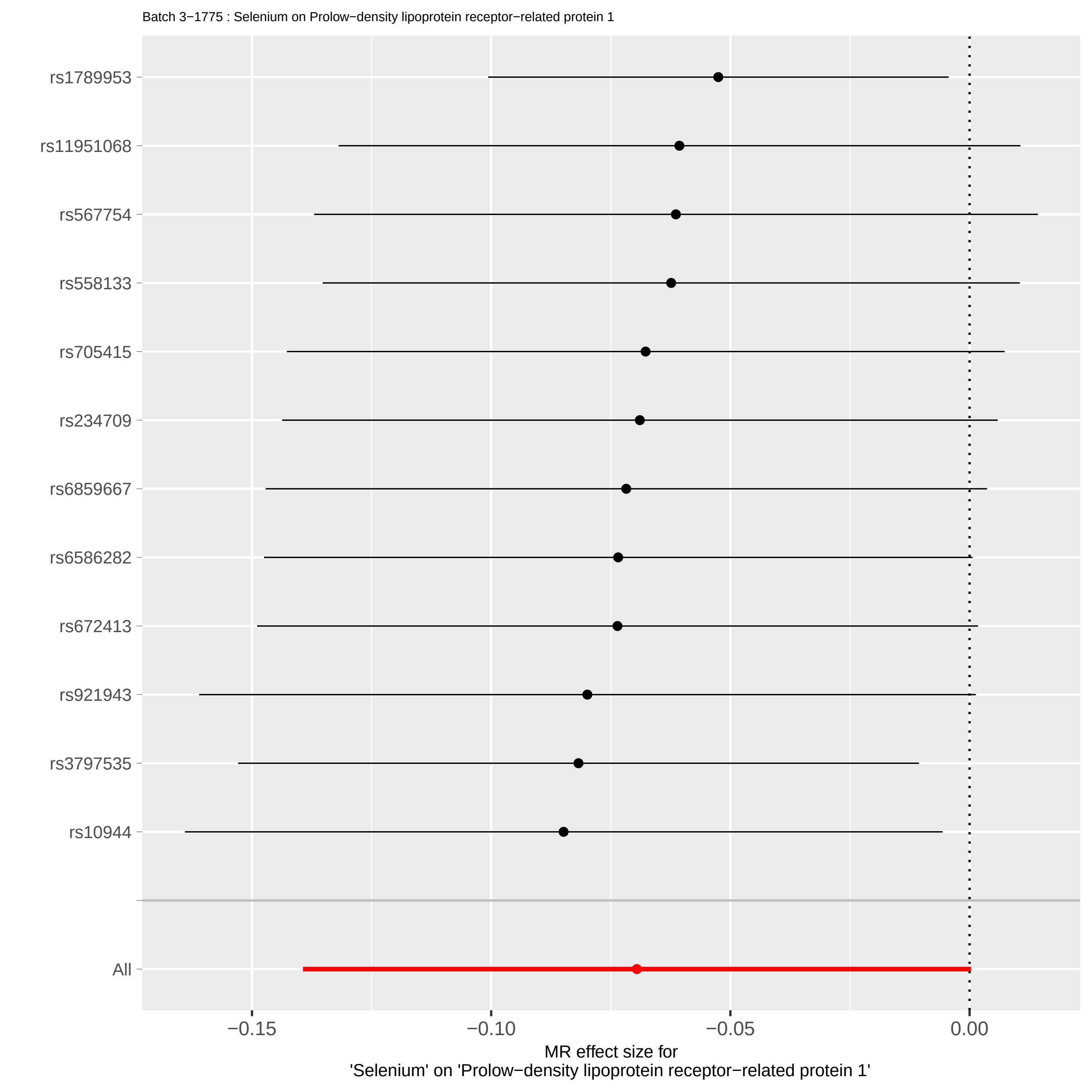
Batch 3−1775 : Selenium on Prolow−density lipoprotein receptor−related protein 1
rs1789953
rs11951068
rs567754
rs558133
rs705415
rs234709
rs6859667
rs6586282
rs672413
rs921943
rs3797535
rs10944
All
−0.15
−0.10
−0.05
0.00
MR effect size for
'Selenium' on 'Prolow−density lipoprotein receptor−related protein 1'

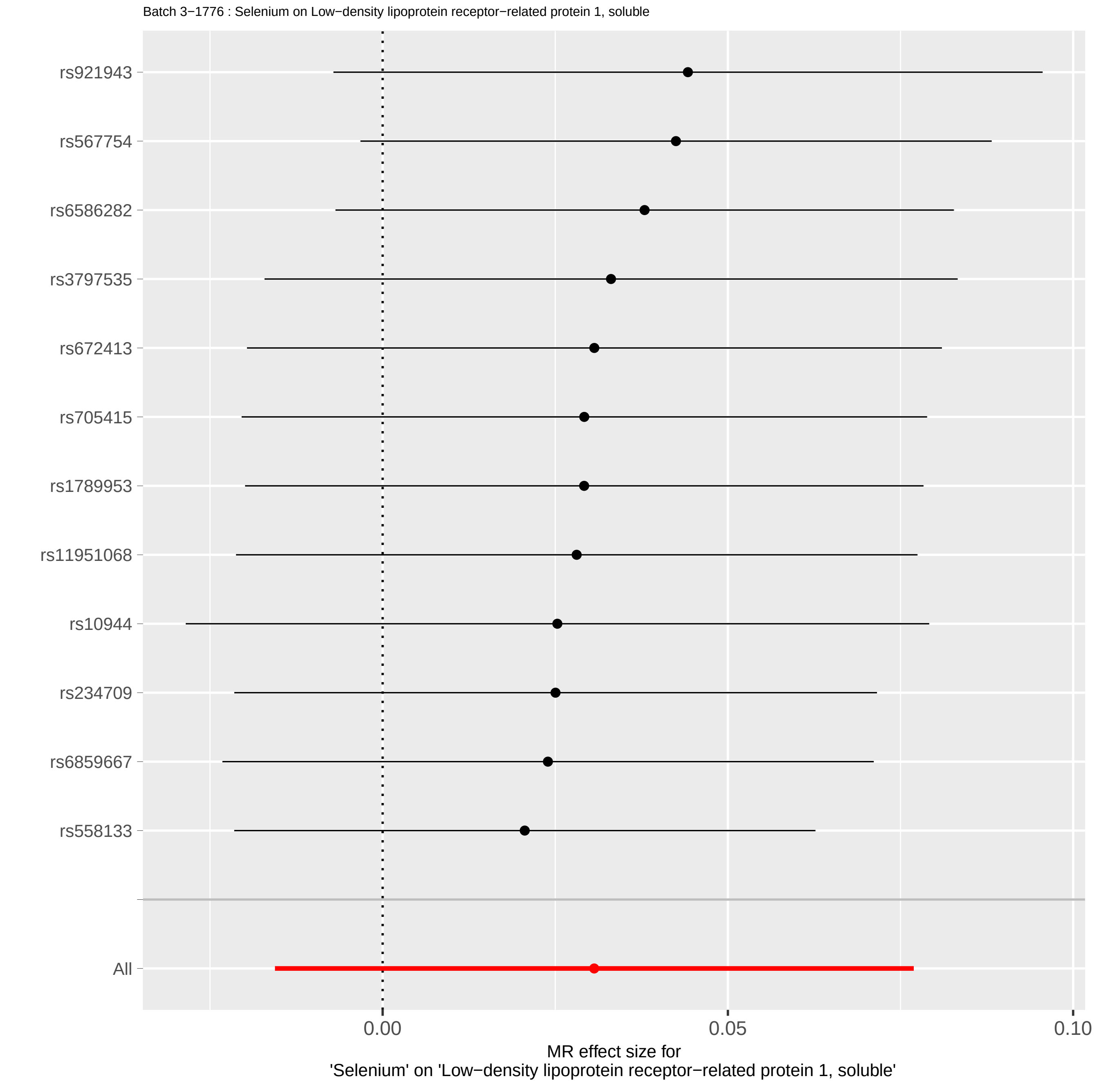
Batch 3−1776 : Selenium on Low−density lipoprotein receptor−related protein 1, soluble
rs921943
rs567754
rs6586282
rs3797535
rs672413
rs705415
rs1789953
rs11951068
rs10944
rs234709
rs6859667
rs558133
All
0.00
0.05
0.10
MR effect size for
'Selenium' on 'Low−density lipoprotein receptor−related protein 1, soluble'

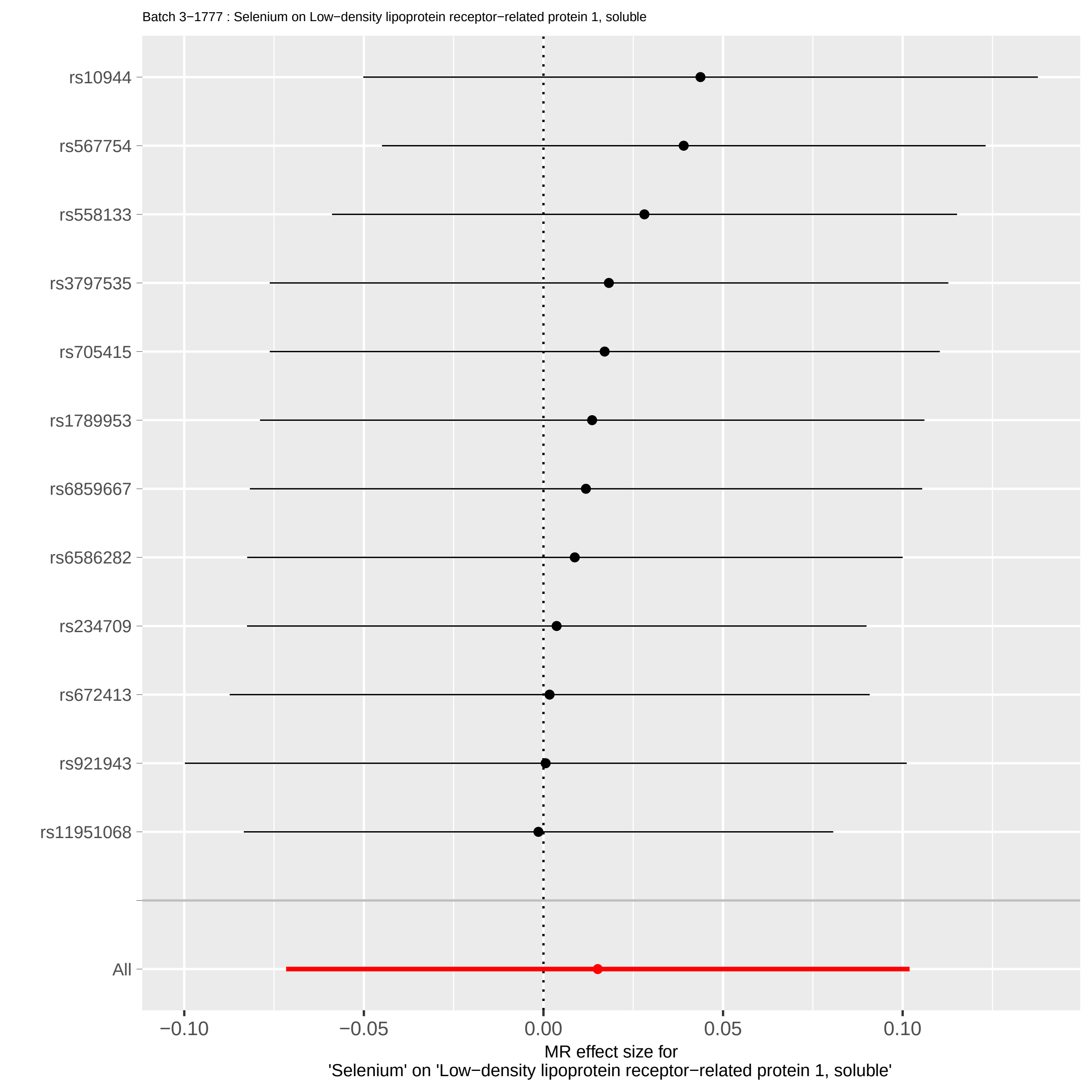

Batch 3−1777 : Selenium on Low−density lipoprotein receptor−related protein 1, soluble
rs10944
rs567754
rs558133
rs3797535
rs705415
rs1789953
rs6859667
rs6586282
rs234709
rs672413
rs921943
rs11951068
All
−0.10
−0.05
0.00
0.05
0.10
MR effect size for
'Selenium' on 'Low−density lipoprotein receptor−related protein 1, soluble'

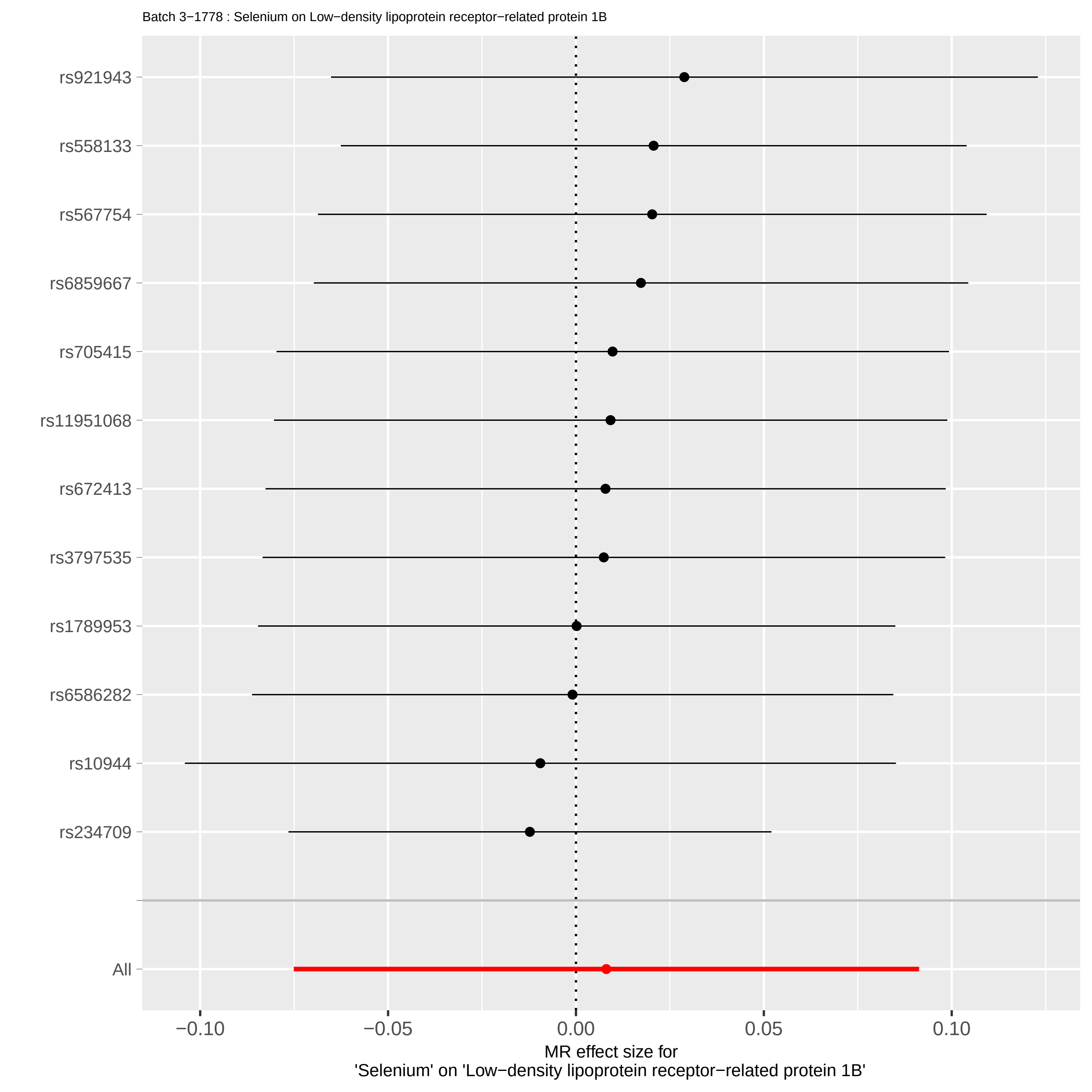

Batch 3−1778 : Selenium on Low−density lipoprotein receptor−related protein 1B
rs921943
rs558133
rs567754
rs6859667
rs705415
rs11951068
rs672413
rs3797535
rs1789953
rs6586282
rs10944
rs234709
All
−0.10
−0.05
0.00
0.05
0.10
MR effect size for
'Selenium' on 'Low−density lipoprotein receptor−related protein 1B'

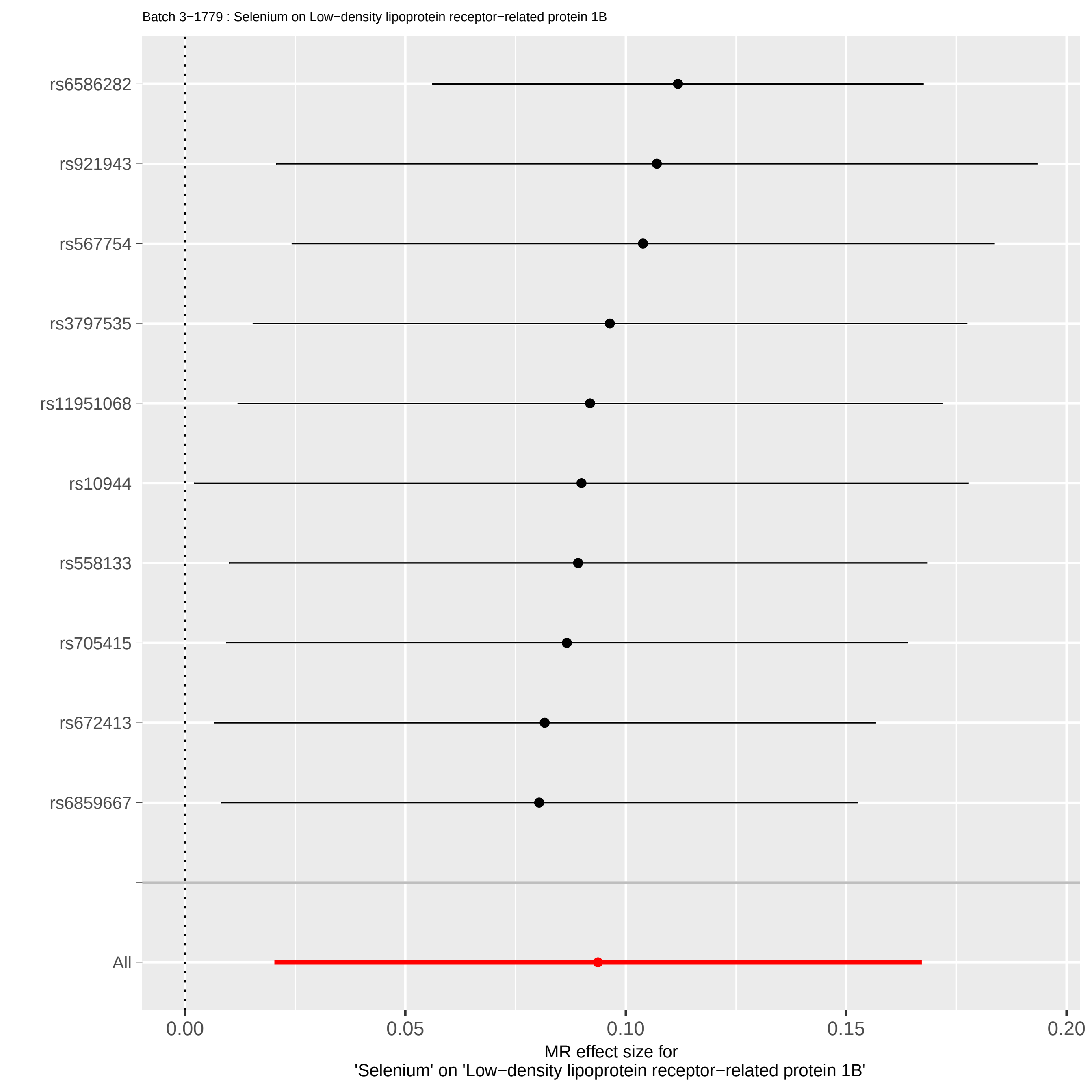

Batch 3−1779 : Selenium on Low−density lipoprotein receptor−related protein 1B
rs6586282
rs921943
rs567754
rs3797535
rs11951068
rs10944
rs558133
rs705415
rs672413
rs6859667
All
0.00
0.05
0.10
0.15
0.20
MR effect size for
'Selenium' on 'Low−density lipoprotein receptor−related protein 1B'

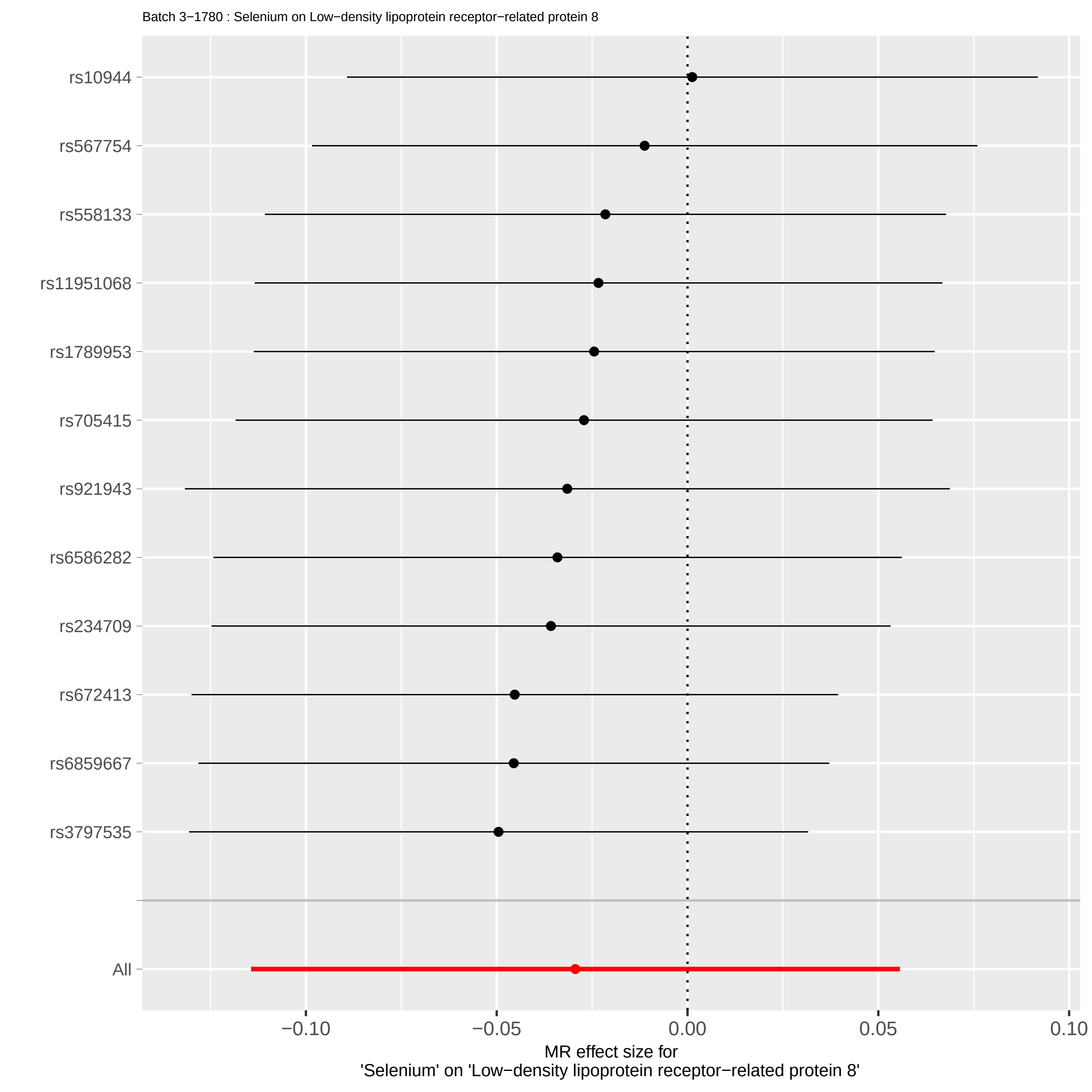

Batch 3−1780 : Selenium on Low−density lipoprotein receptor−related protein 8
rs10944
rs567754
rs558133
rs11951068
rs1789953
rs705415
rs921943
rs6586282
rs234709
rs672413
rs6859667
rs3797535
All
−0.10
−0.05
0.00
0.05
0.10
MR effect size for
'Selenium' on 'Low−density lipoprotein receptor−related protein 8'

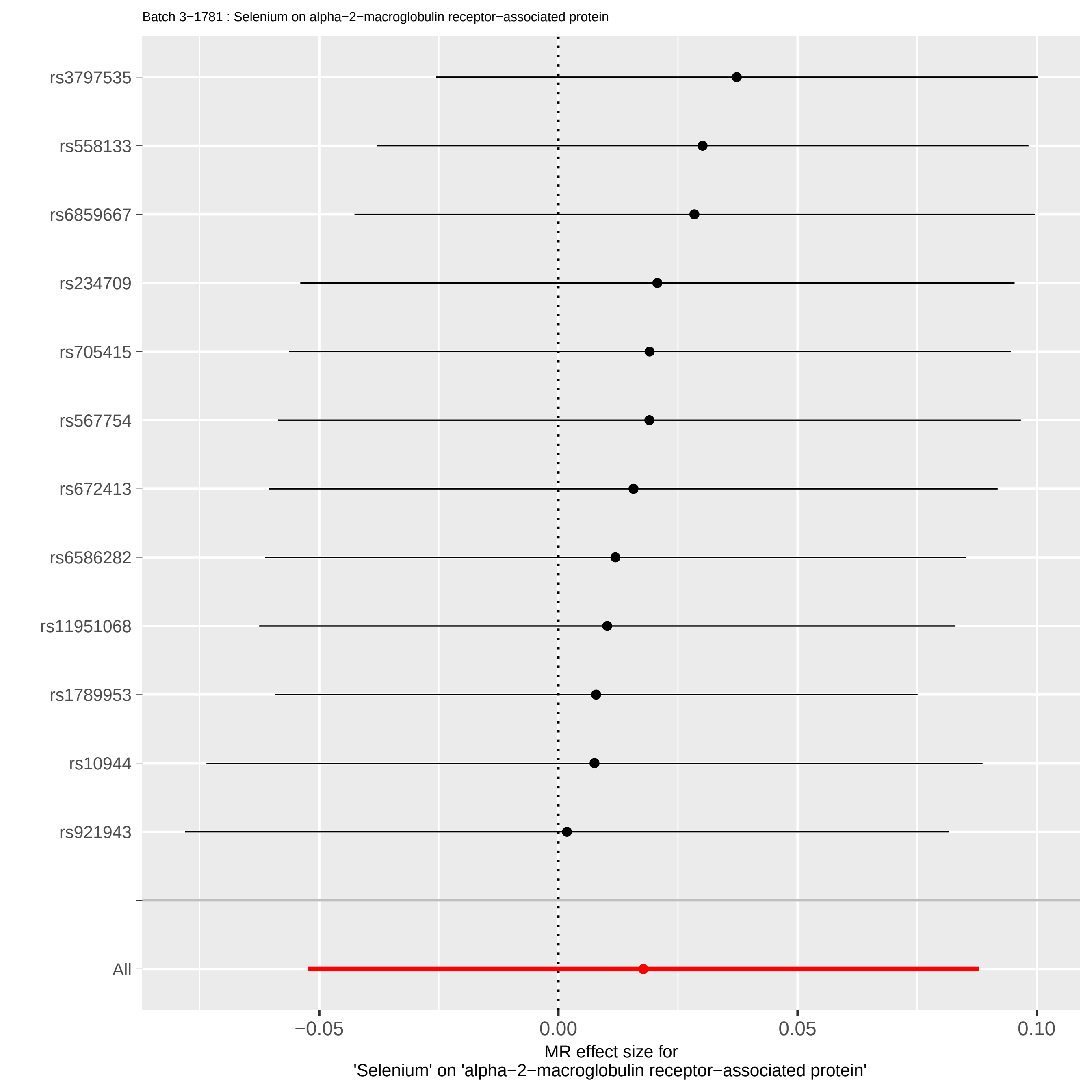

Batch 3−1781 : Selenium on alpha−2−macroglobulin receptor−associated protein
rs3797535
rs558133
rs6859667
rs234709
rs705415
rs567754
rs672413
rs6586282
rs11951068
rs1789953
rs10944
rs921943
All
−0.05
0.00
0.05
0.10
MR effect size for
'Selenium' on 'alpha−2−macroglobulin receptor−associated protein'

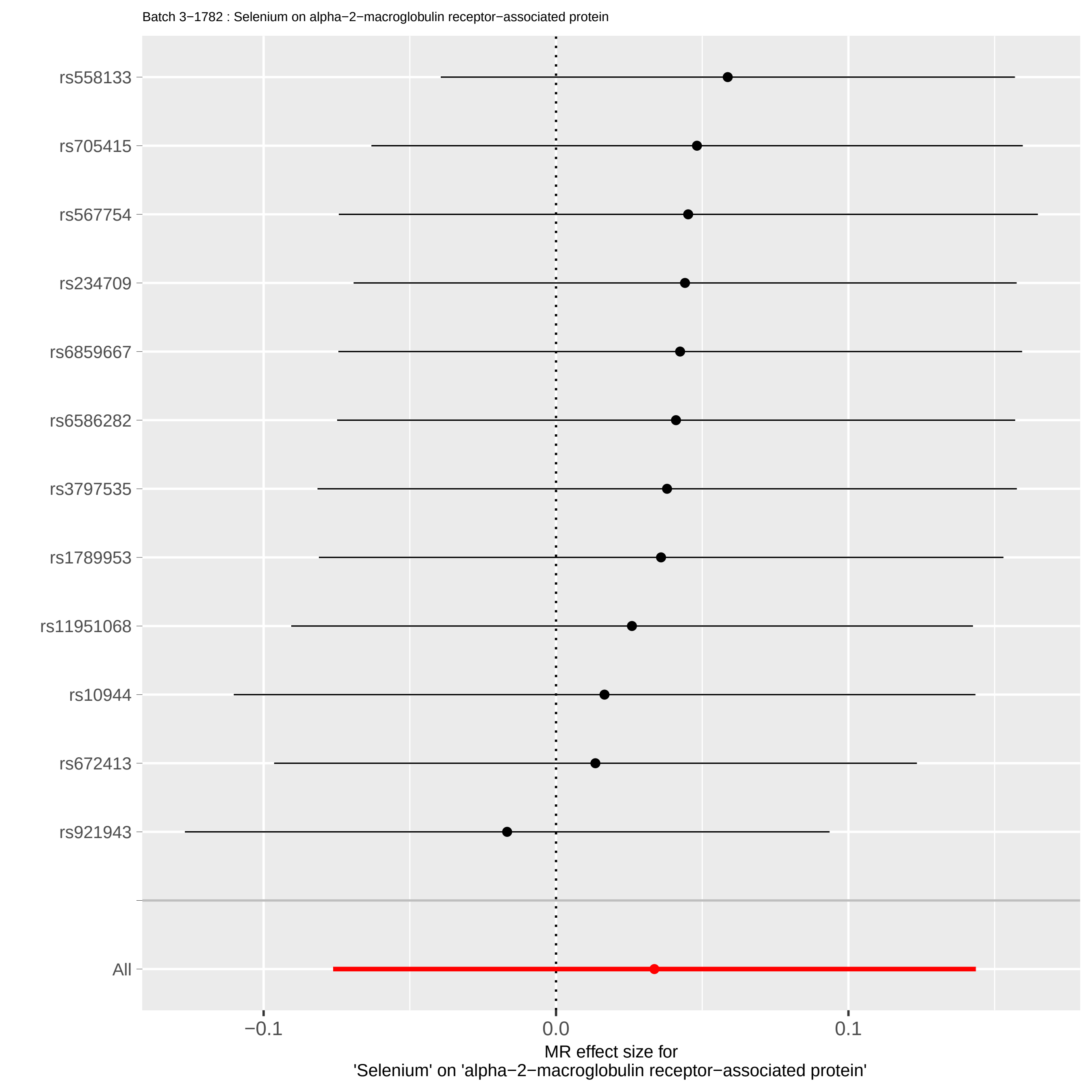

Batch 3−1782 : Selenium on alpha−2−macroglobulin receptor−associated protein
rs558133
rs705415
rs567754
rs234709
rs6859667
rs6586282
rs3797535
rs1789953
rs11951068
rs10944
rs672413
rs921943
All
−0.1
0.0
0.1
MR effect size for
'Selenium' on 'alpha−2−macroglobulin receptor−associated protein'

Batch 3−1783 : Selenium on Leucine−rich PPR motif−containing protein, mitochondrial

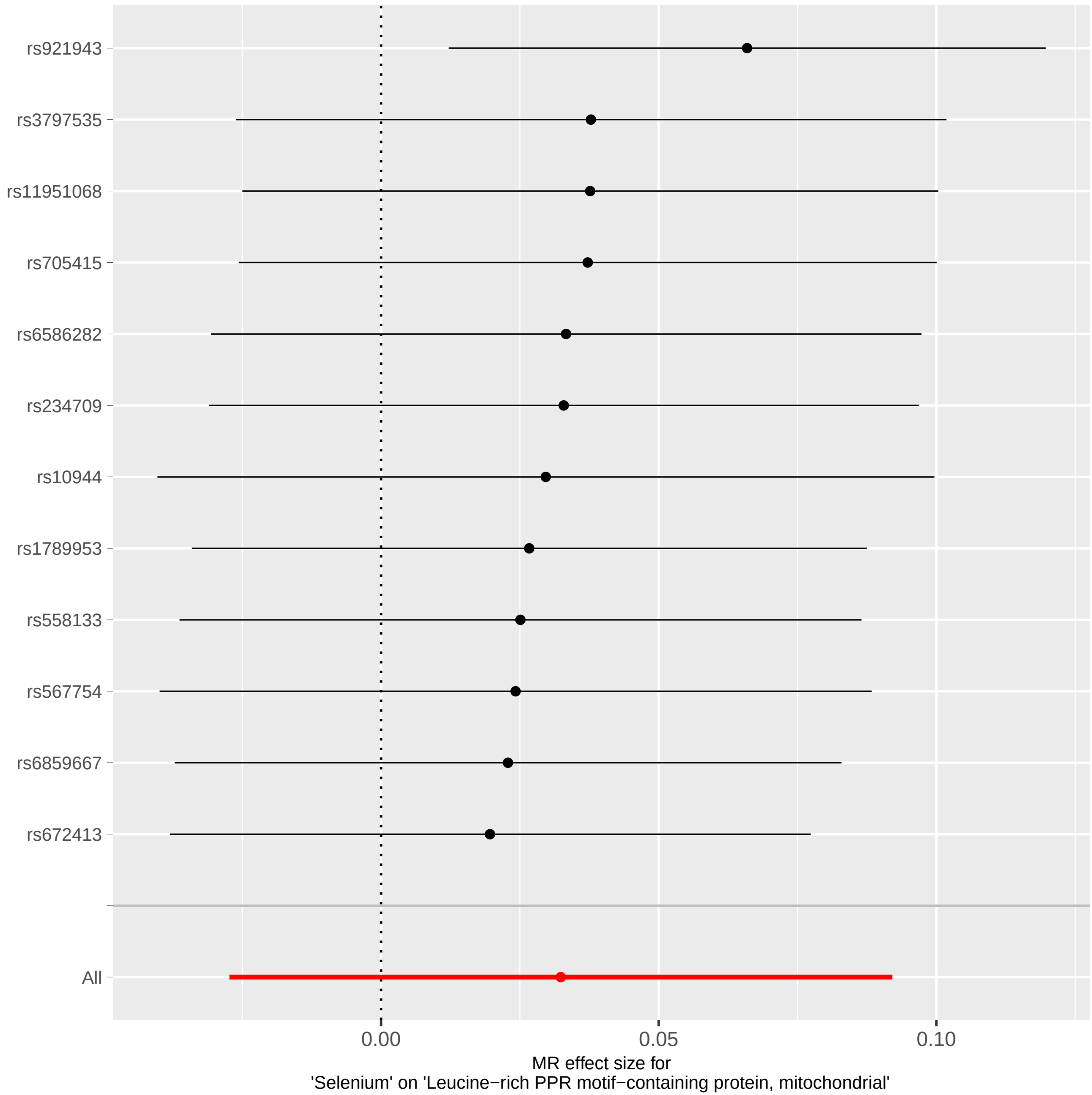

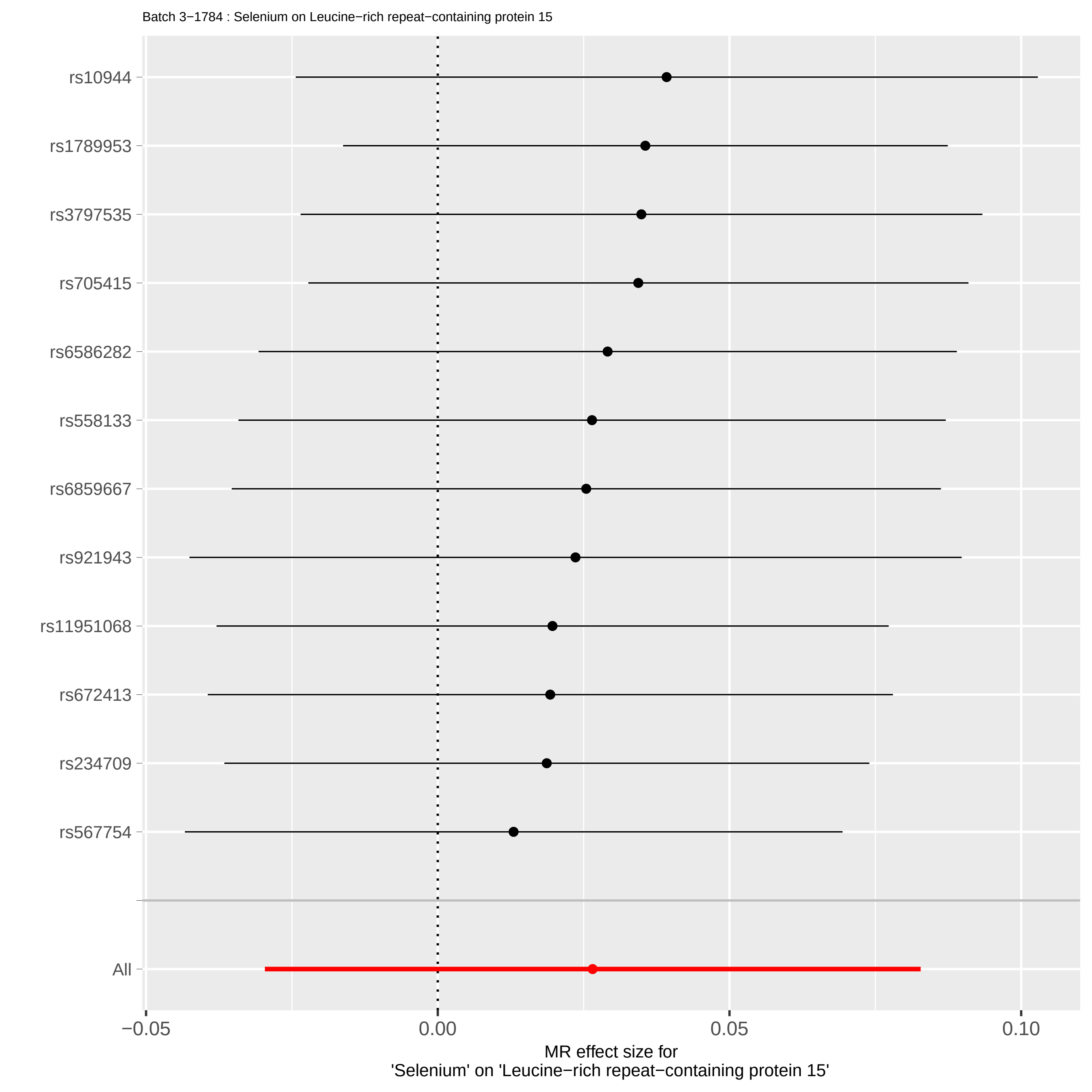

Batch 3−1784 : Selenium on Leucine−rich repeat−containing protein 15
rs10944
rs1789953
rs3797535
rs705415
rs6586282
rs558133
rs6859667
rs921943
rs11951068
rs672413
rs234709
rs567754
All
−0.05
0.00
0.05
0.10
MR effect size for
'Selenium' on 'Leucine−rich repeat−containing protein 15'

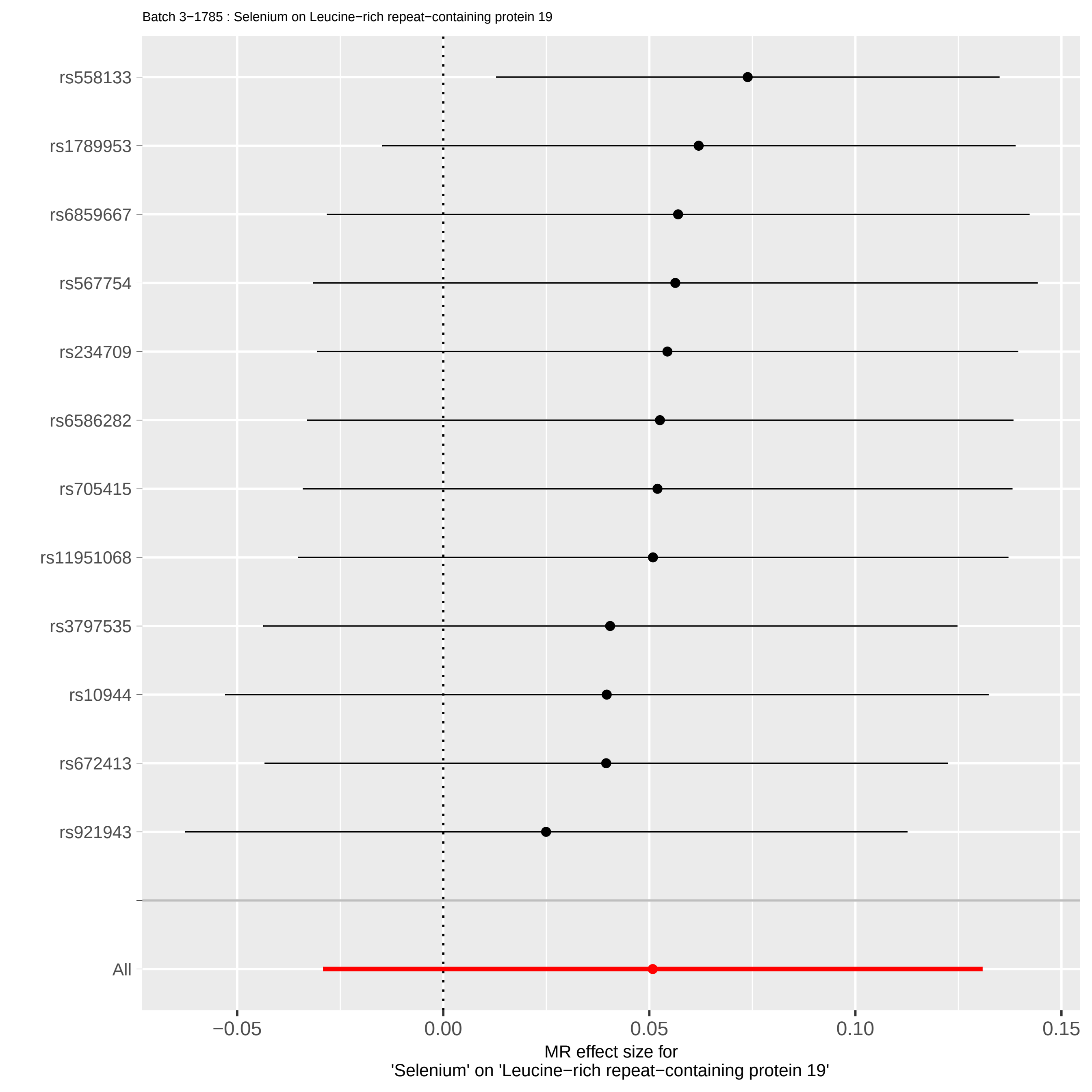

Batch 3−1785 : Selenium on Leucine−rich repeat−containing protein 19
rs558133
rs1789953
rs6859667
rs567754
rs234709
rs6586282
rs705415
rs11951068
rs3797535
rs10944
rs672413
rs921943
All
−0.05
0.00
0.05
0.10
0.15
MR effect size for
'Selenium' on 'Leucine−rich repeat−containing protein 19'

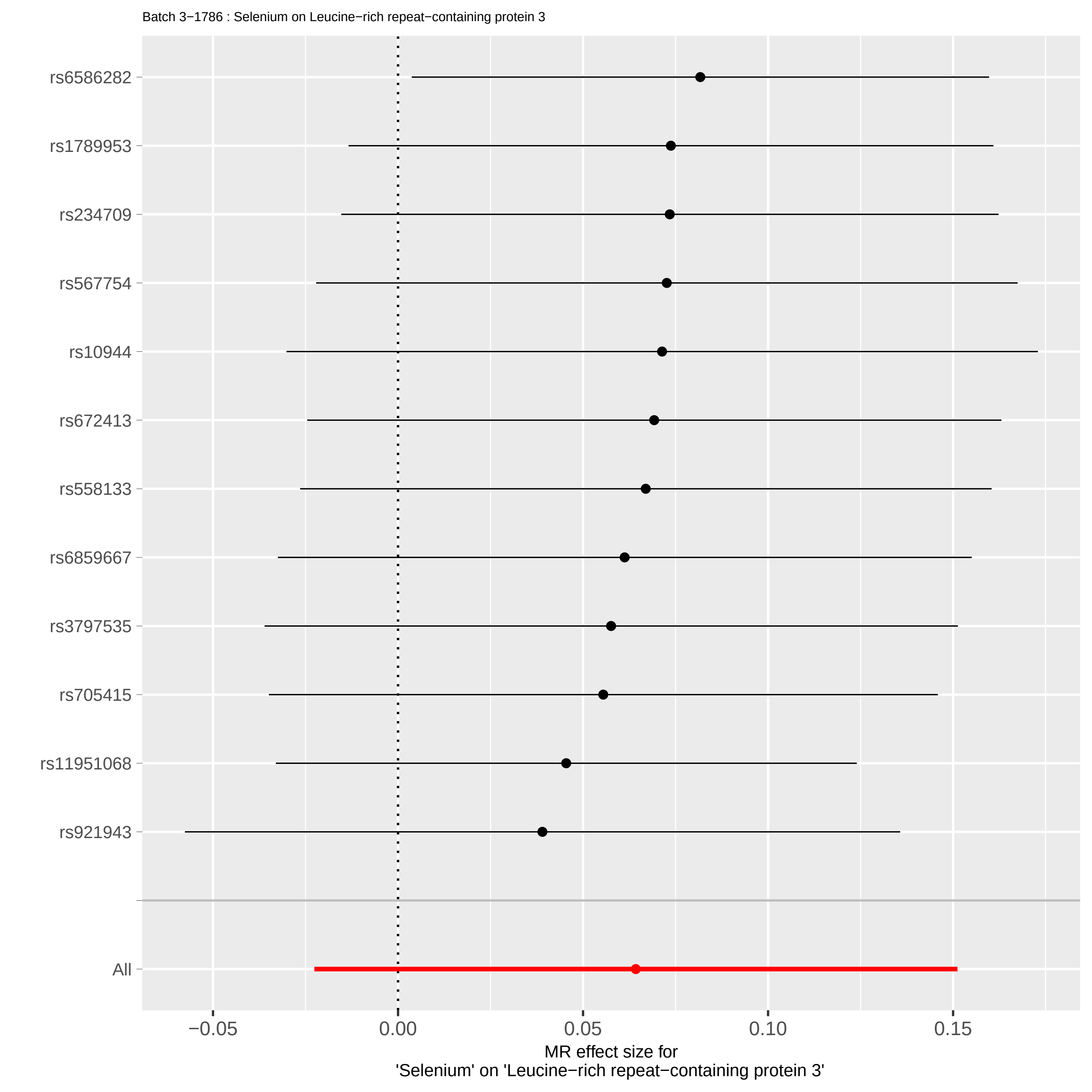
Batch 3−1786 : Selenium on Leucine−rich repeat−containing protein 3
rs6586282
rs1789953
rs234709
rs567754
rs10944
rs672413
rs558133
rs6859667
rs3797535
rs705415
rs11951068
rs921943
All
−0.05
0.00
0.05
0.10
0.15
MR effect size for
'Selenium' on 'Leucine−rich repeat−containing protein 3'

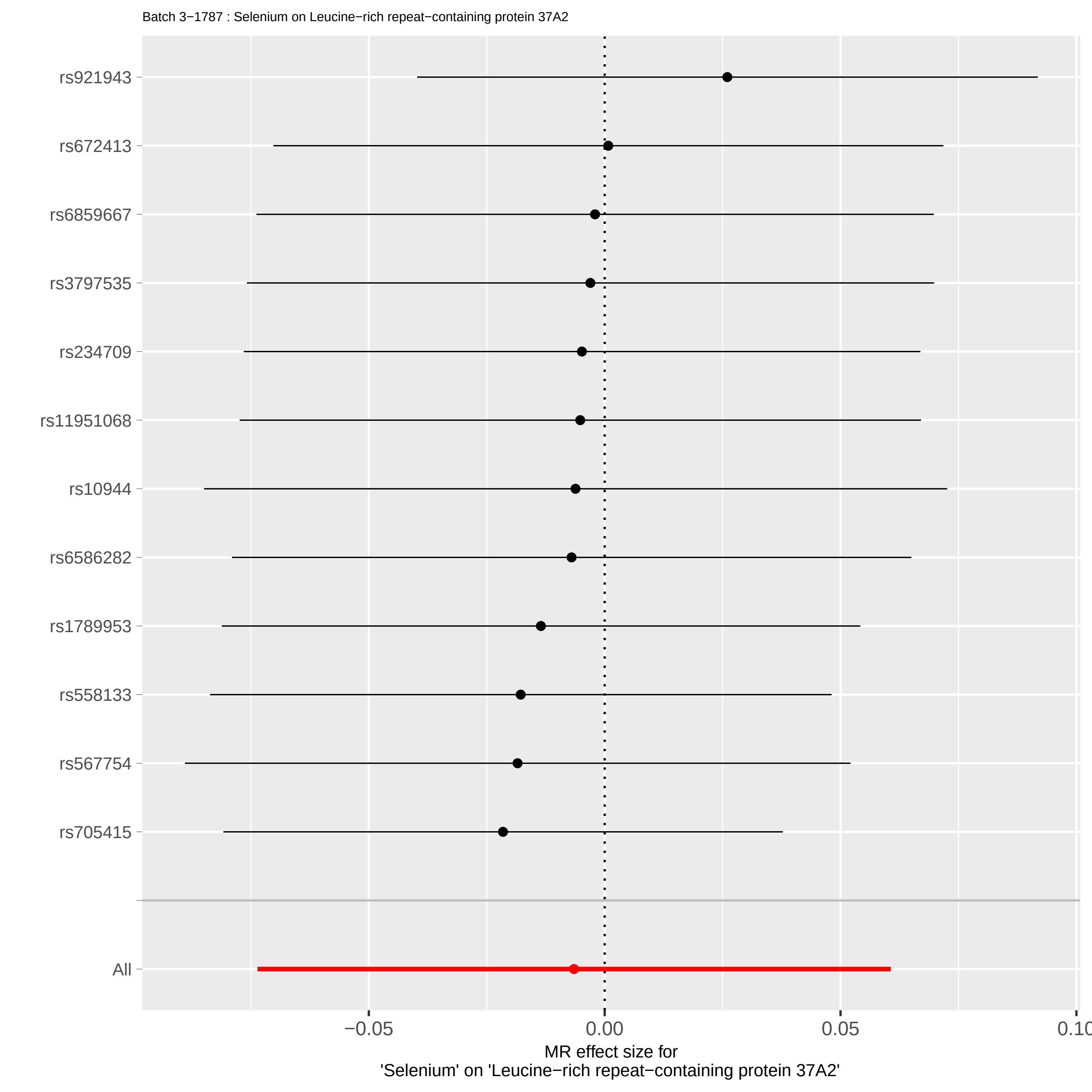

Batch 3−1787 : Selenium on Leucine−rich repeat−containing protein 37A2
rs921943
rs672413
rs6859667
rs3797535
rs234709
rs11951068
rs10944
rs6586282
rs1789953
rs558133
rs567754
rs705415
All
−0.05
0.00
0.05
0.10
MR effect size for
'Selenium' on 'Leucine−rich repeat−containing protein 37A2'

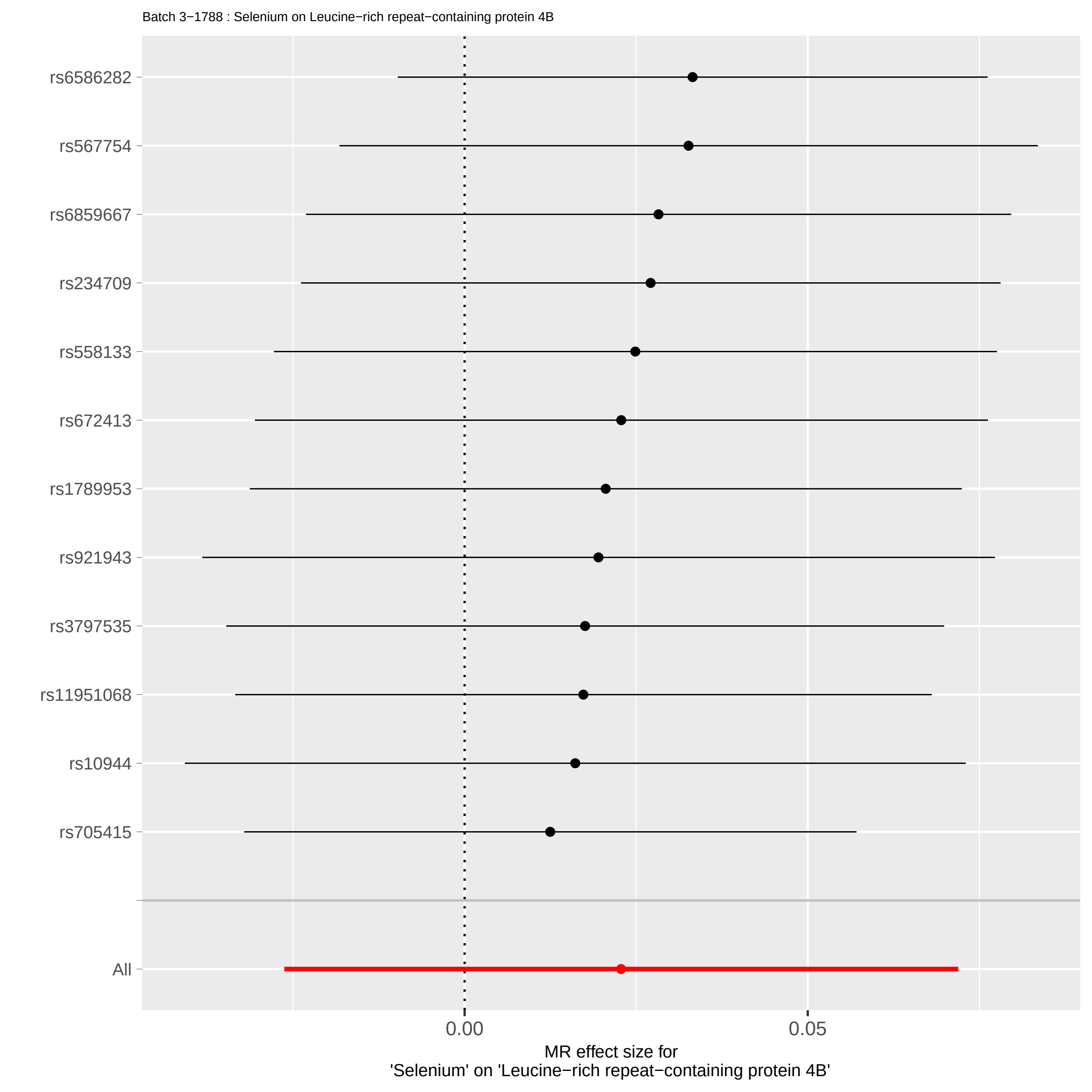

Batch 3−1788 : Selenium on Leucine−rich repeat−containing protein 4B
rs6586282
rs567754
rs6859667
rs234709
rs558133
rs672413
rs1789953
rs921943
rs3797535
rs11951068
rs10944
rs705415
All
0.00
0.05
MR effect size for
'Selenium' on 'Leucine−rich repeat−containing protein 4B'

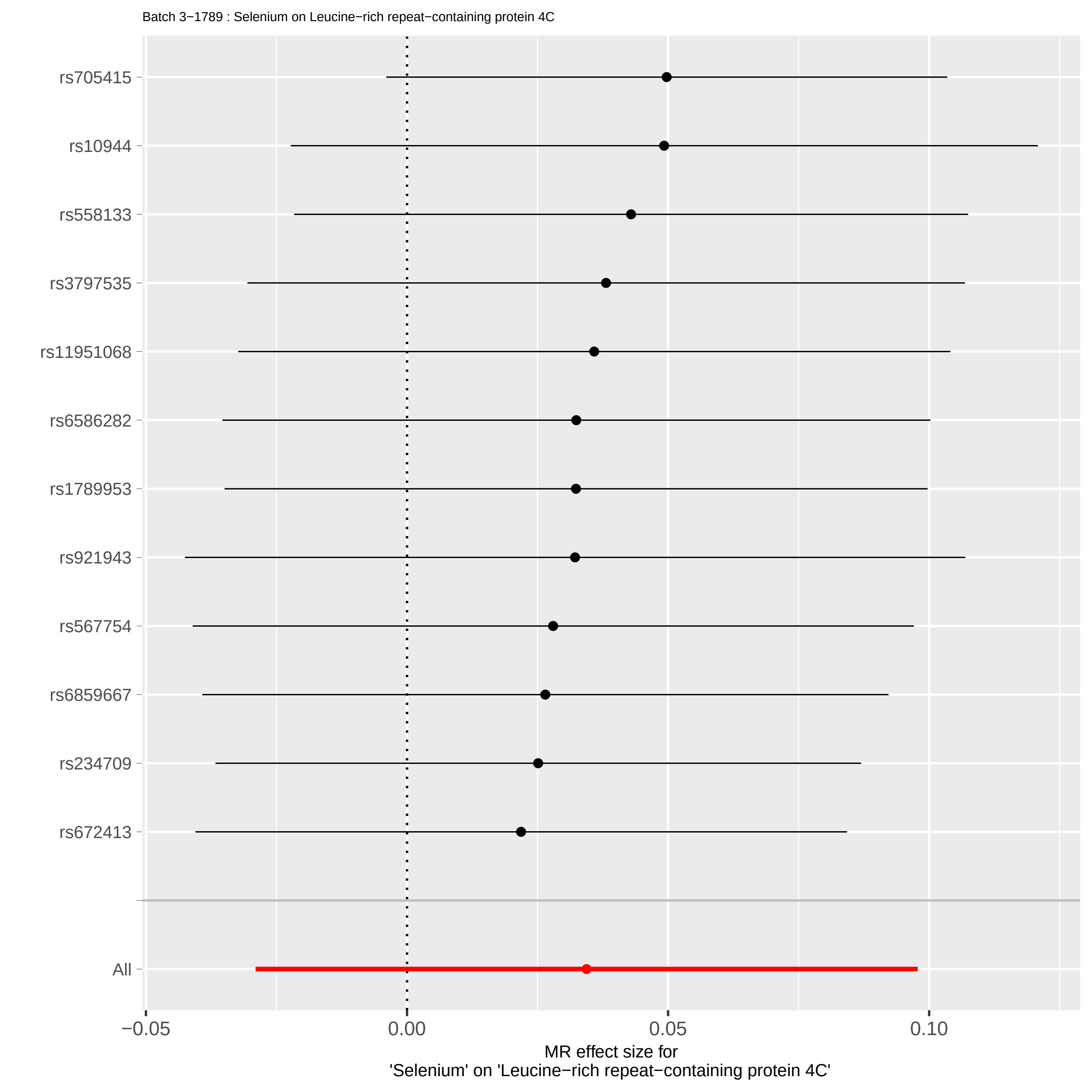
Batch 3−1789 : Selenium on Leucine−rich repeat−containing protein 4C
rs705415
rs10944
rs558133
rs3797535
rs11951068
rs6586282
rs1789953
rs921943
rs567754
rs6859667
rs234709
rs672413
All
−0.05
0.00
0.05
0.10
MR effect size for
'Selenium' on 'Leucine−rich repeat−containing protein 4C'

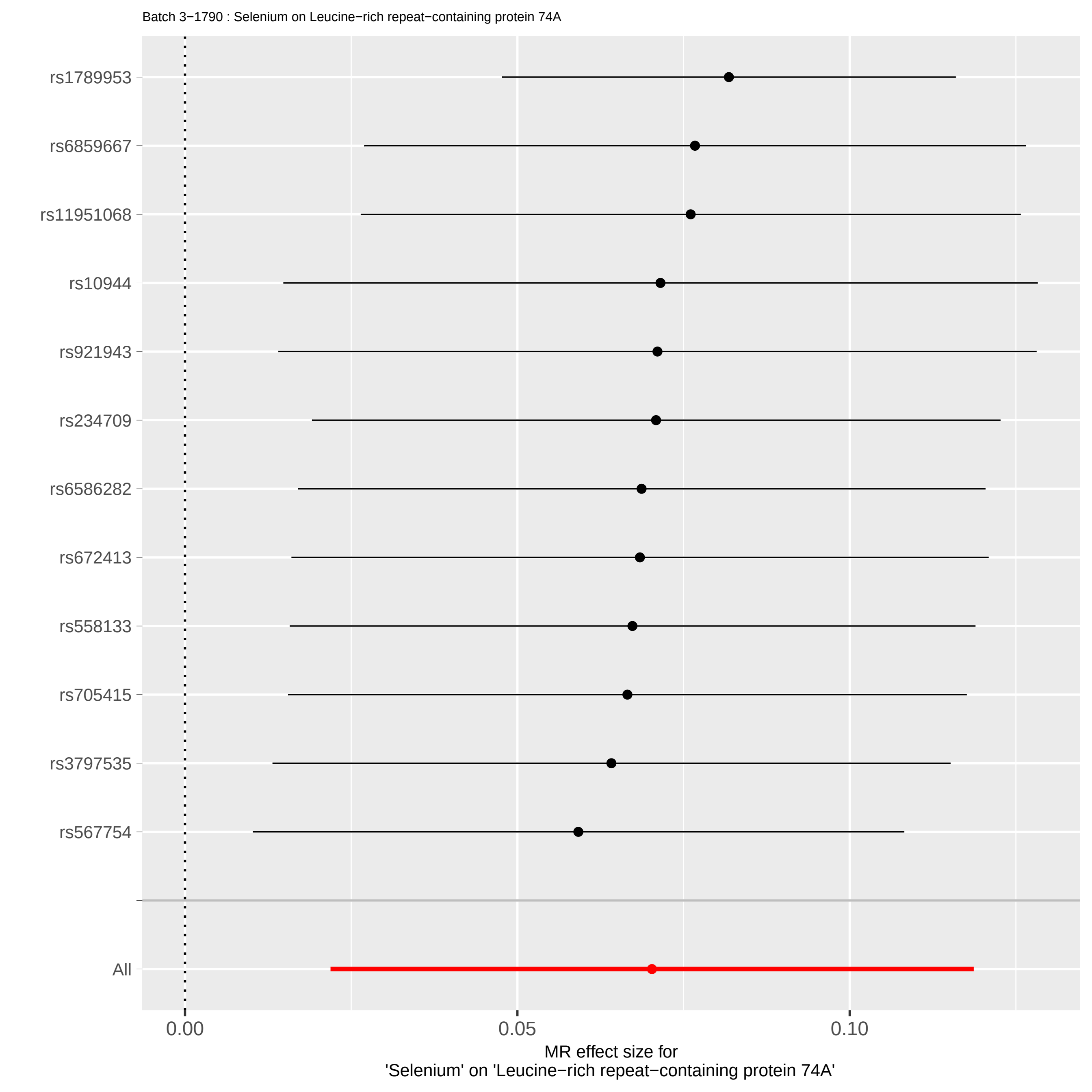
Batch 3−1790 : Selenium on Leucine−rich repeat−containing protein 74A
rs1789953
rs6859667
rs11951068
rs10944
rs921943
rs234709
rs6586282
rs672413
rs558133
rs705415
rs3797535
rs567754
All
0.00
0.05
0.10
MR effect size for
'Selenium' on 'Leucine−rich repeat−containing protein 74A'

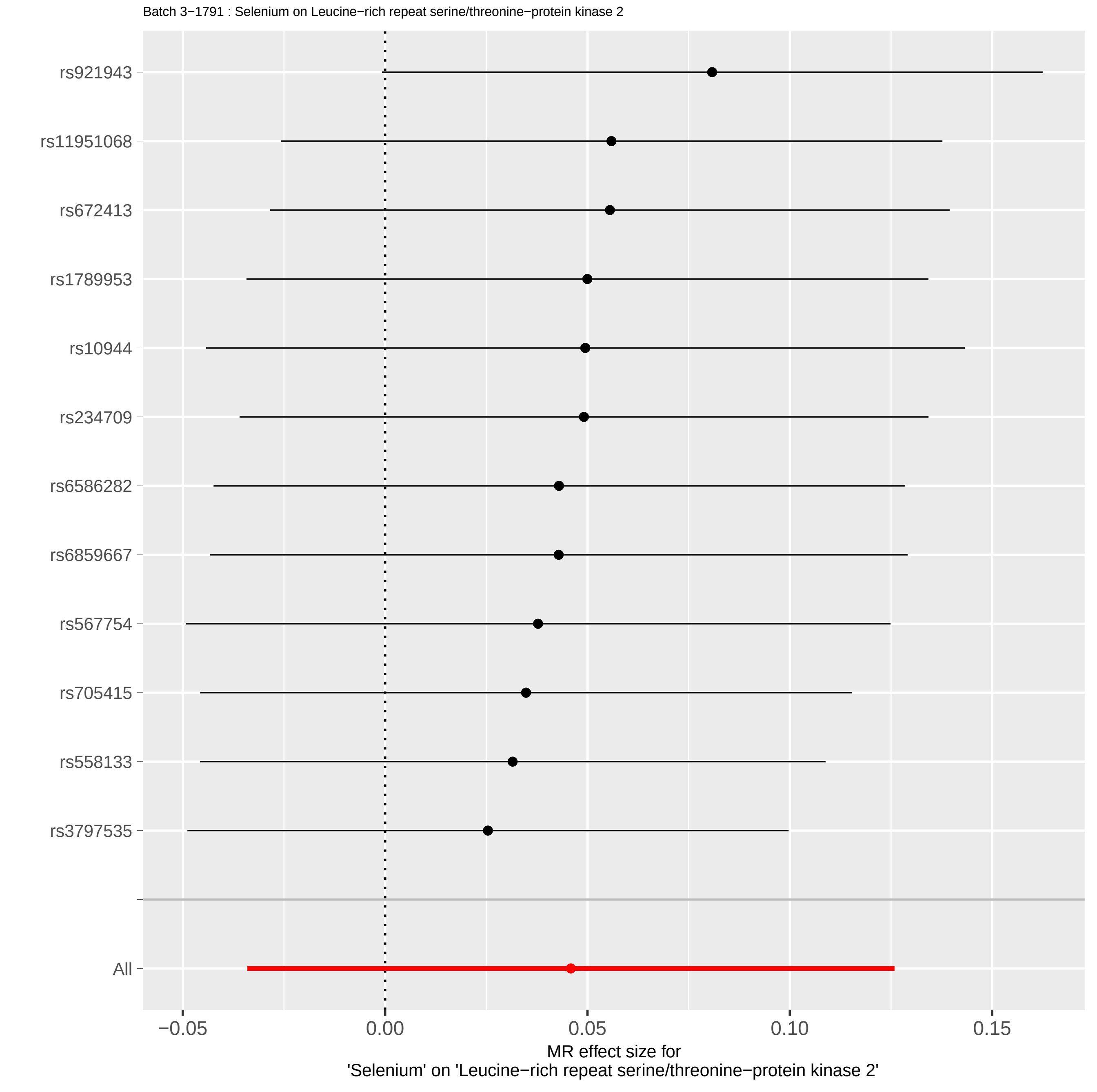

Batch 3−1791 : Selenium on Leucine−rich repeat serine/threonine−protein kinase 2
rs921943
rs11951068
rs672413
rs1789953
rs10944
rs234709
rs6586282
rs6859667
rs567754
rs705415
rs558133
rs3797535
All
−0.05
0.00
0.05
0.10
0.15
MR effect size for
'Selenium' on 'Leucine−rich repeat serine/threonine−protein kinase 2'

Batch 3−1792 : Selenium on Leucine−rich repeat neuronal protein 1

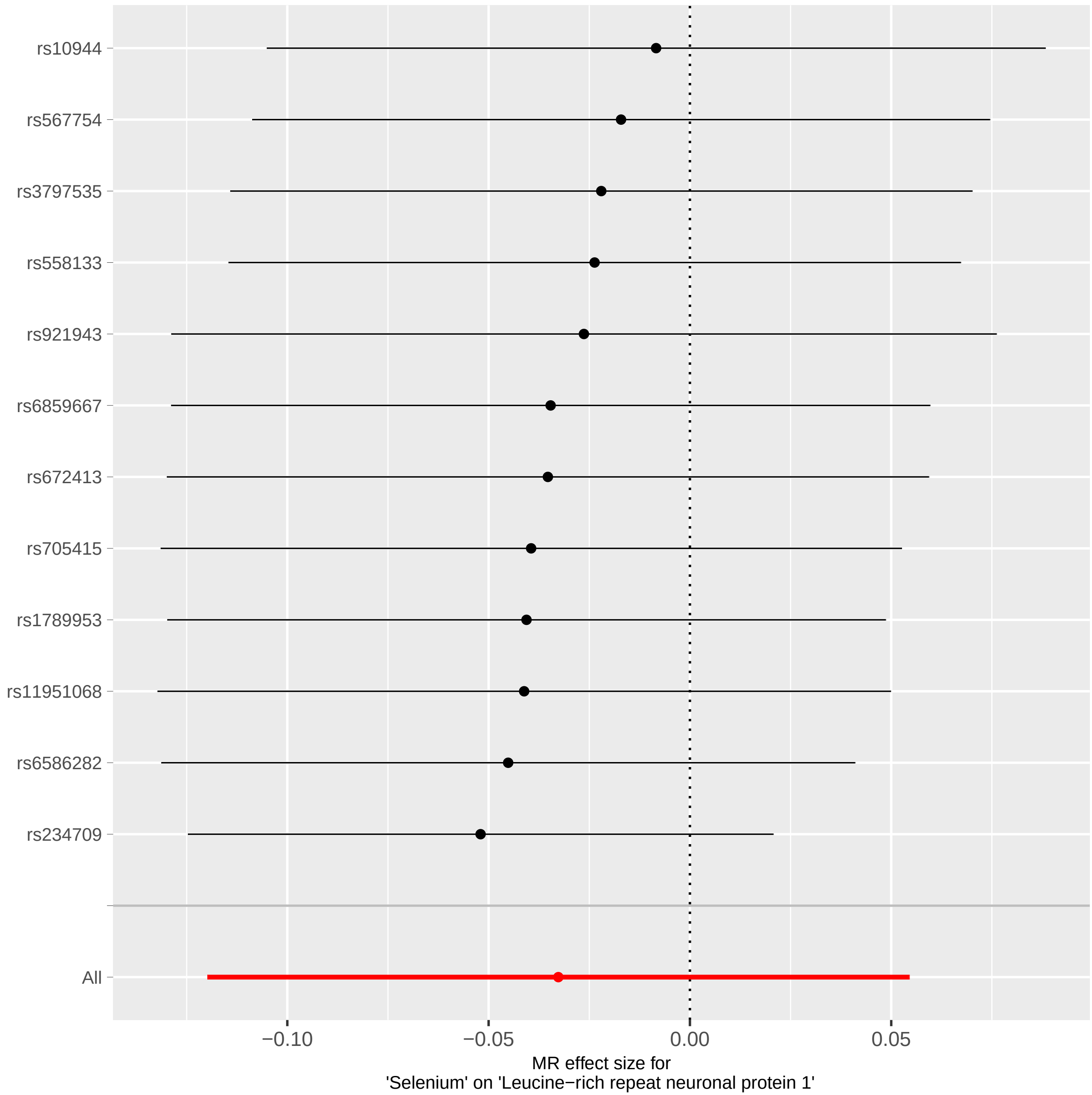

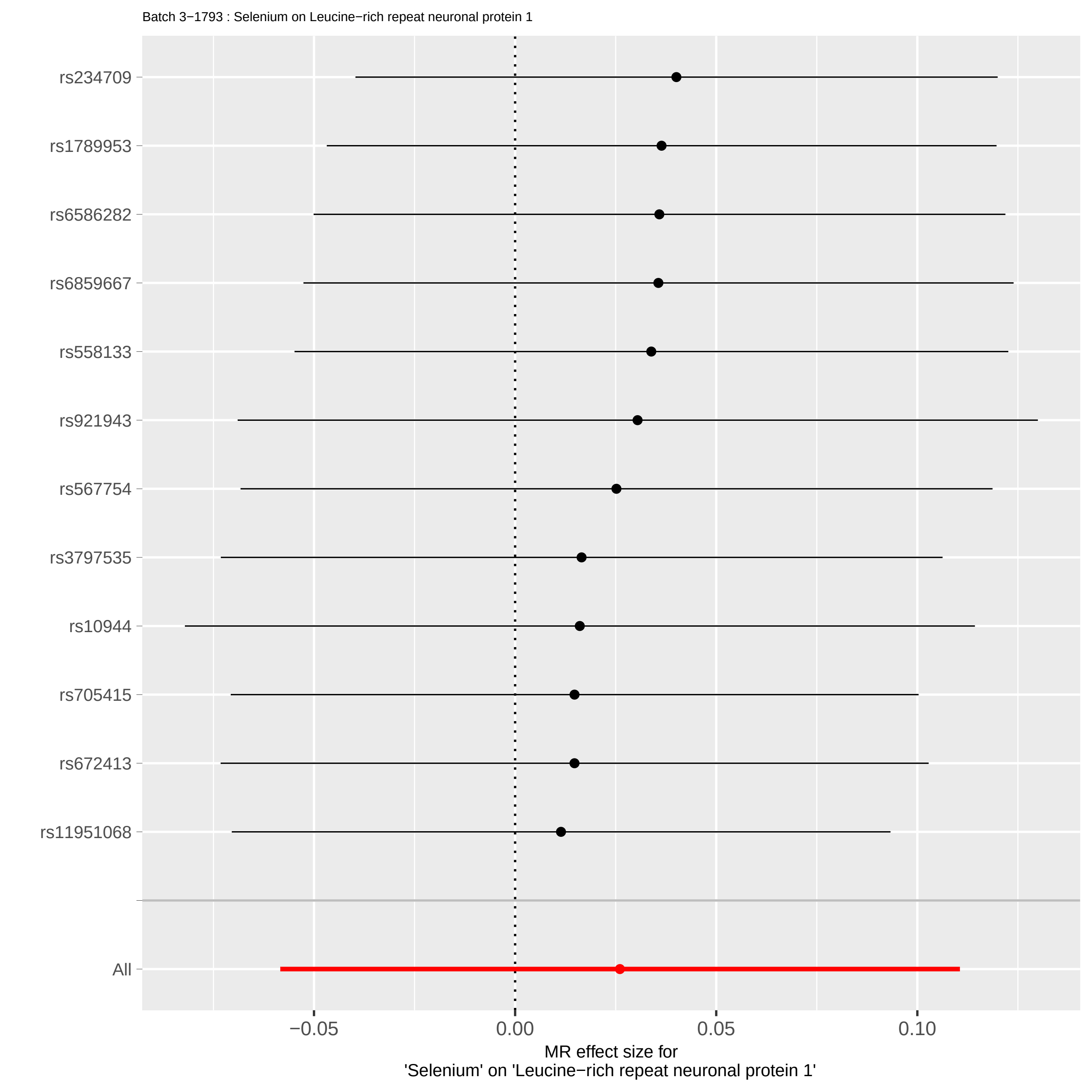
Batch 3−1793 : Selenium on Leucine−rich repeat neuronal protein 1
rs234709
rs1789953
rs6586282
rs6859667
rs558133
rs921943
rs567754
rs3797535
rs10944
rs705415
rs672413
rs11951068
All
−0.05
0.00
0.05
0.10
MR effect size for
'Selenium' on 'Leucine−rich repeat neuronal protein 1'

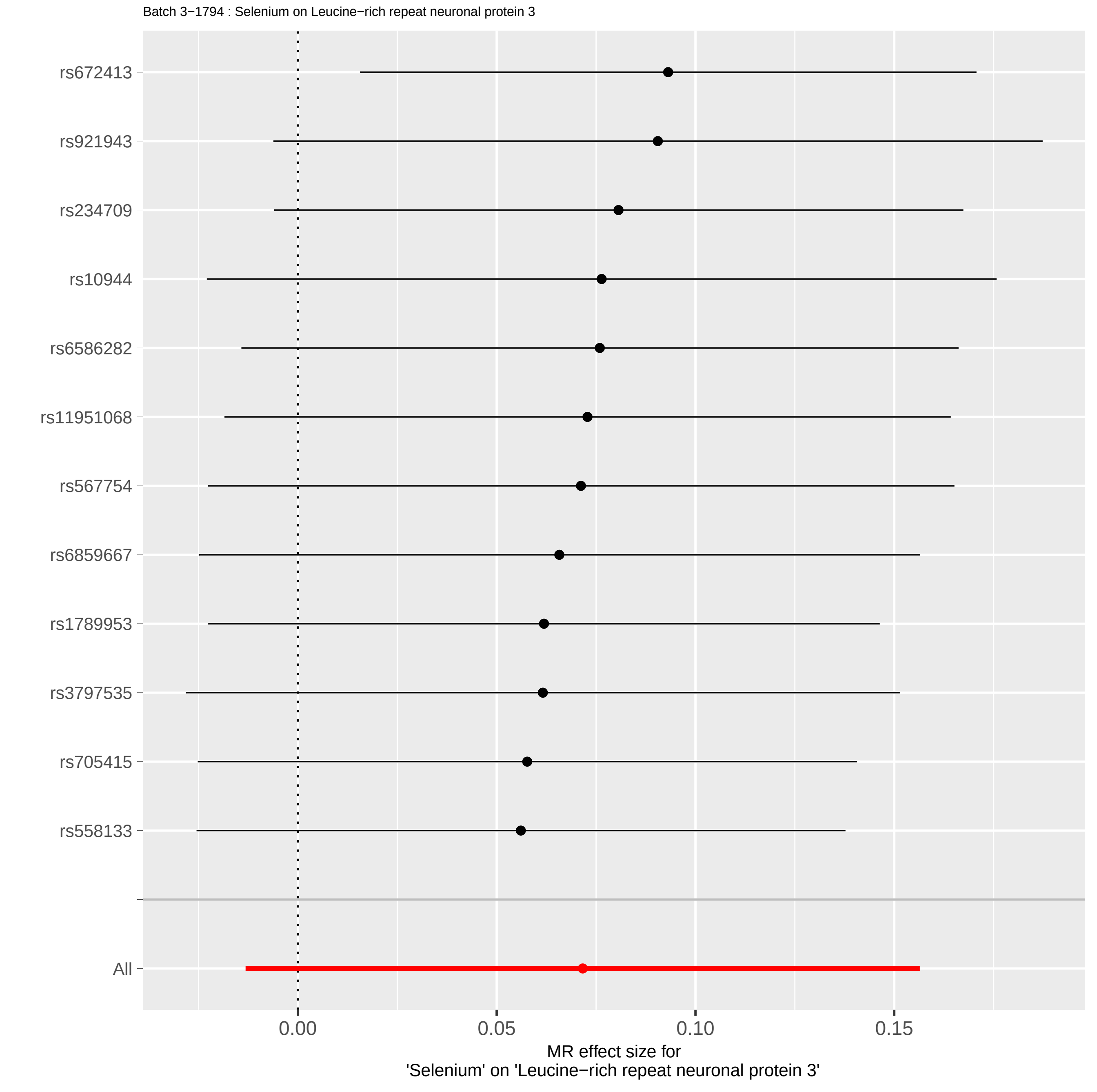

Batch 3−1794 : Selenium on Leucine−rich repeat neuronal protein 3
rs672413
rs921943
rs234709
rs10944
rs6586282
rs11951068
rs567754
rs6859667
rs1789953
rs3797535
rs705415
rs558133
All
0.00
0.05
0.10
0.15
MR effect size for
'Selenium' on 'Leucine−rich repeat neuronal protein 3'

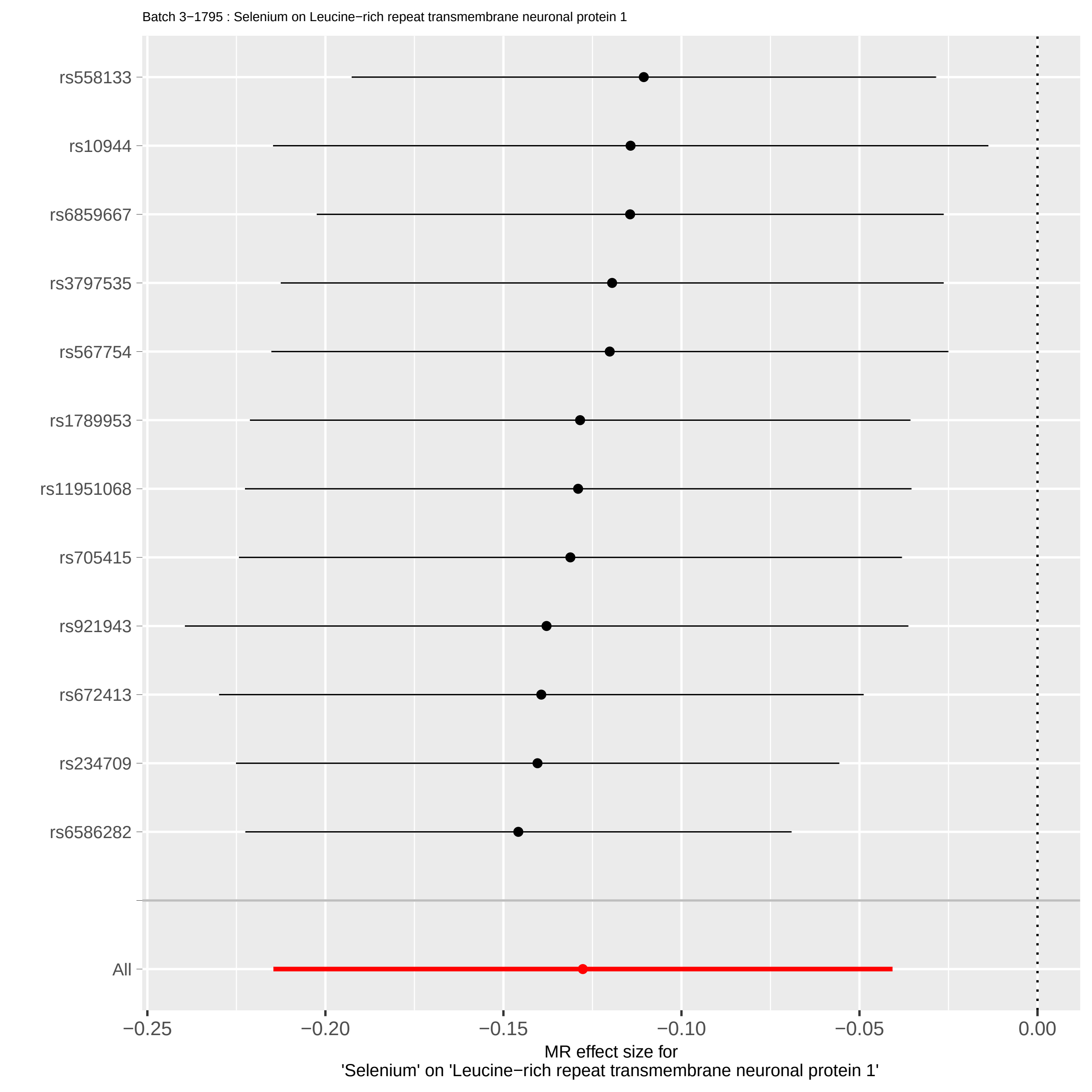

Batch 3−1795 : Selenium on Leucine−rich repeat transmembrane neuronal protein 1
rs558133
rs10944
rs6859667
rs3797535
rs567754
rs1789953
rs11951068
rs705415
rs921943
rs672413
rs234709
rs6586282
All
−0.25
−0.20
−0.15
−0.10
−0.05
0.00
MR effect size for
'Selenium' on 'Leucine−rich repeat transmembrane neuronal protein 1'

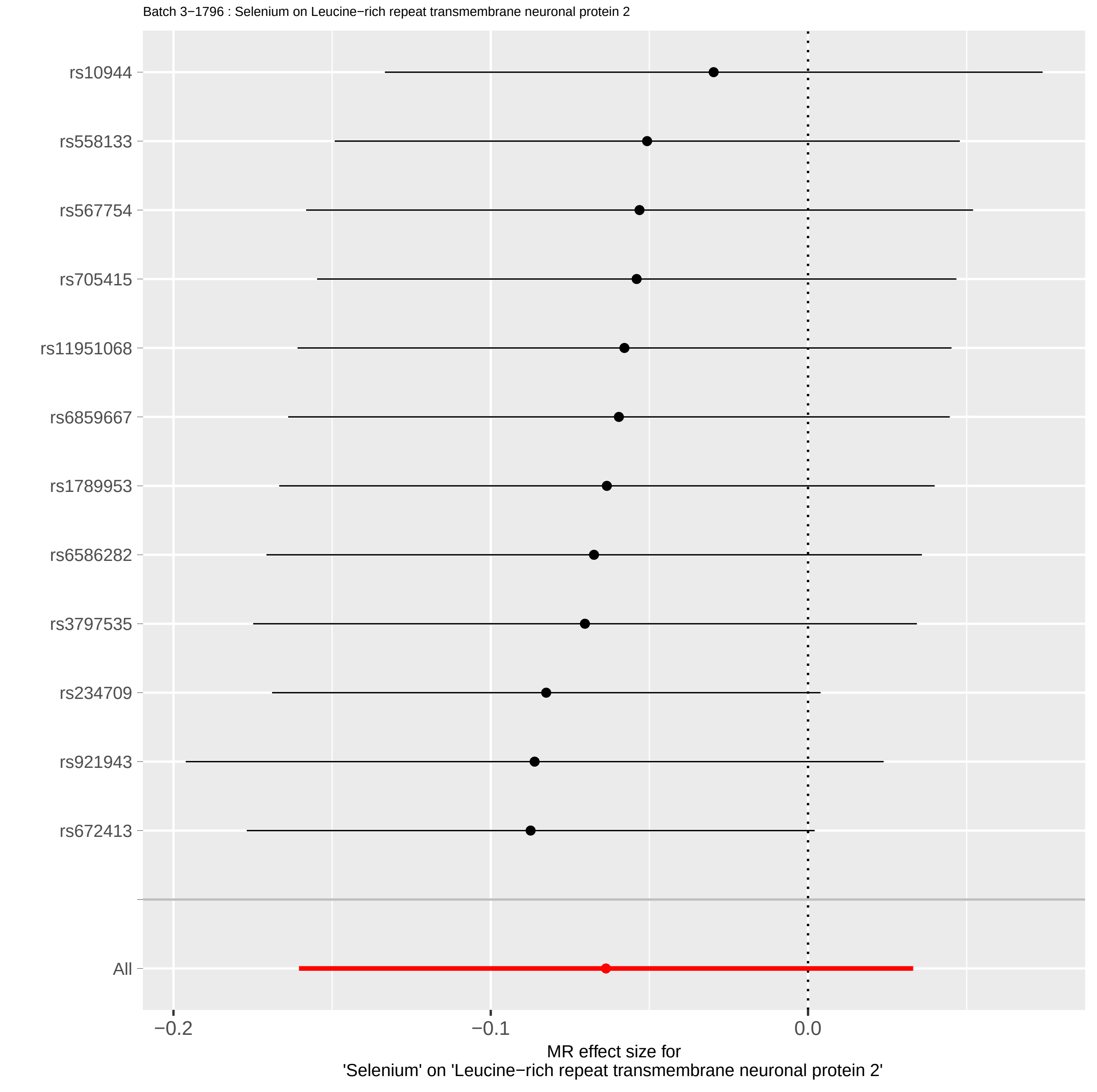

Batch 3−1796 : Selenium on Leucine−rich repeat transmembrane neuronal protein 2
rs10944
rs558133
rs567754
rs705415
rs11951068
rs6859667
rs1789953
rs6586282
rs3797535
rs234709
rs921943
rs672413
All
−0.2
−0.1
0.0
MR effect size for
'Selenium' on 'Leucine−rich repeat transmembrane neuronal protein 2'

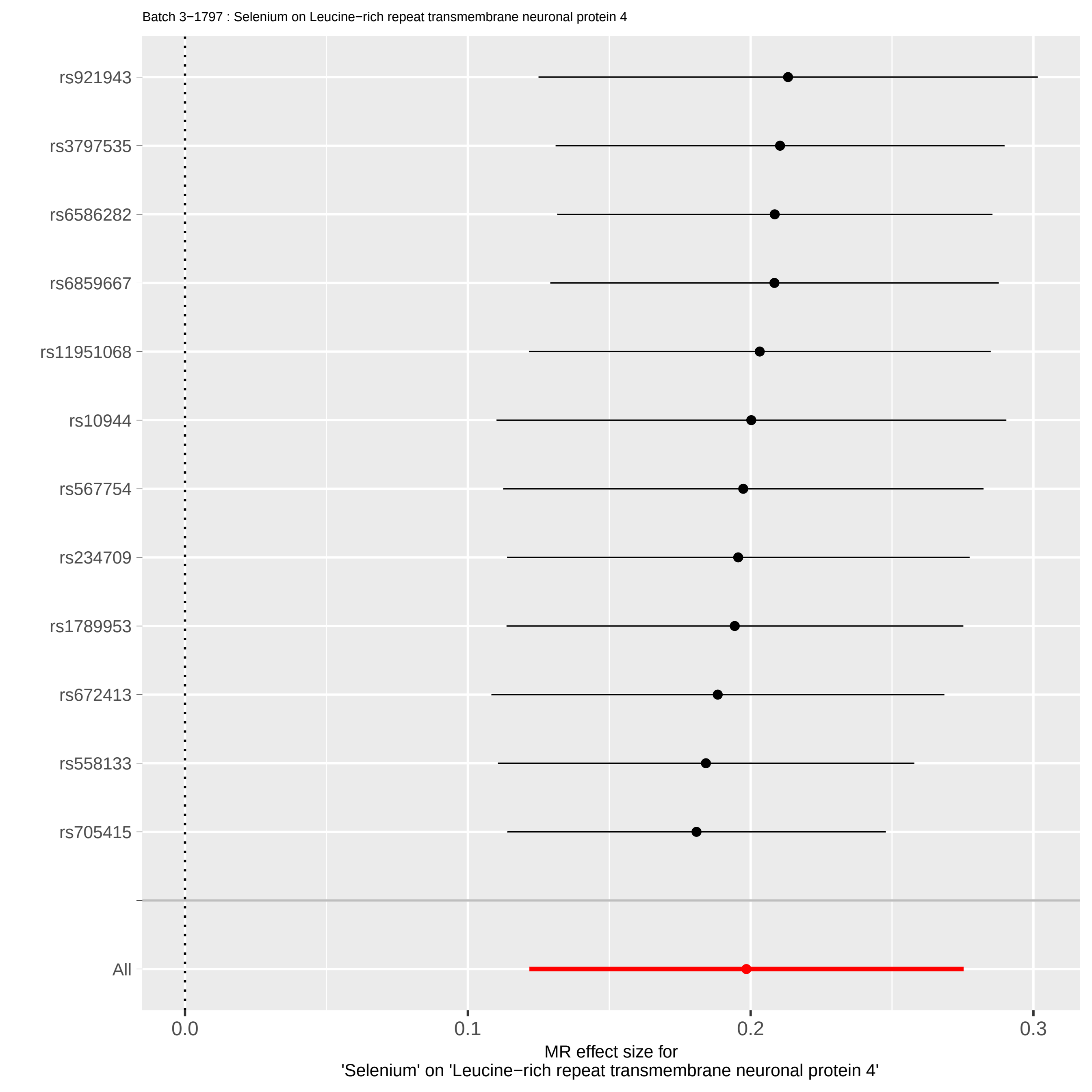
Batch 3−1797 : Selenium on Leucine−rich repeat transmembrane neuronal protein 4
rs921943
rs3797535
rs6586282
rs6859667
rs11951068
rs10944
rs567754
rs234709
rs1789953
rs672413
rs558133
rs705415
All
0.0
0.1
0.2
0.3
MR effect size for
'Selenium' on 'Leucine−rich repeat transmembrane neuronal protein 4'

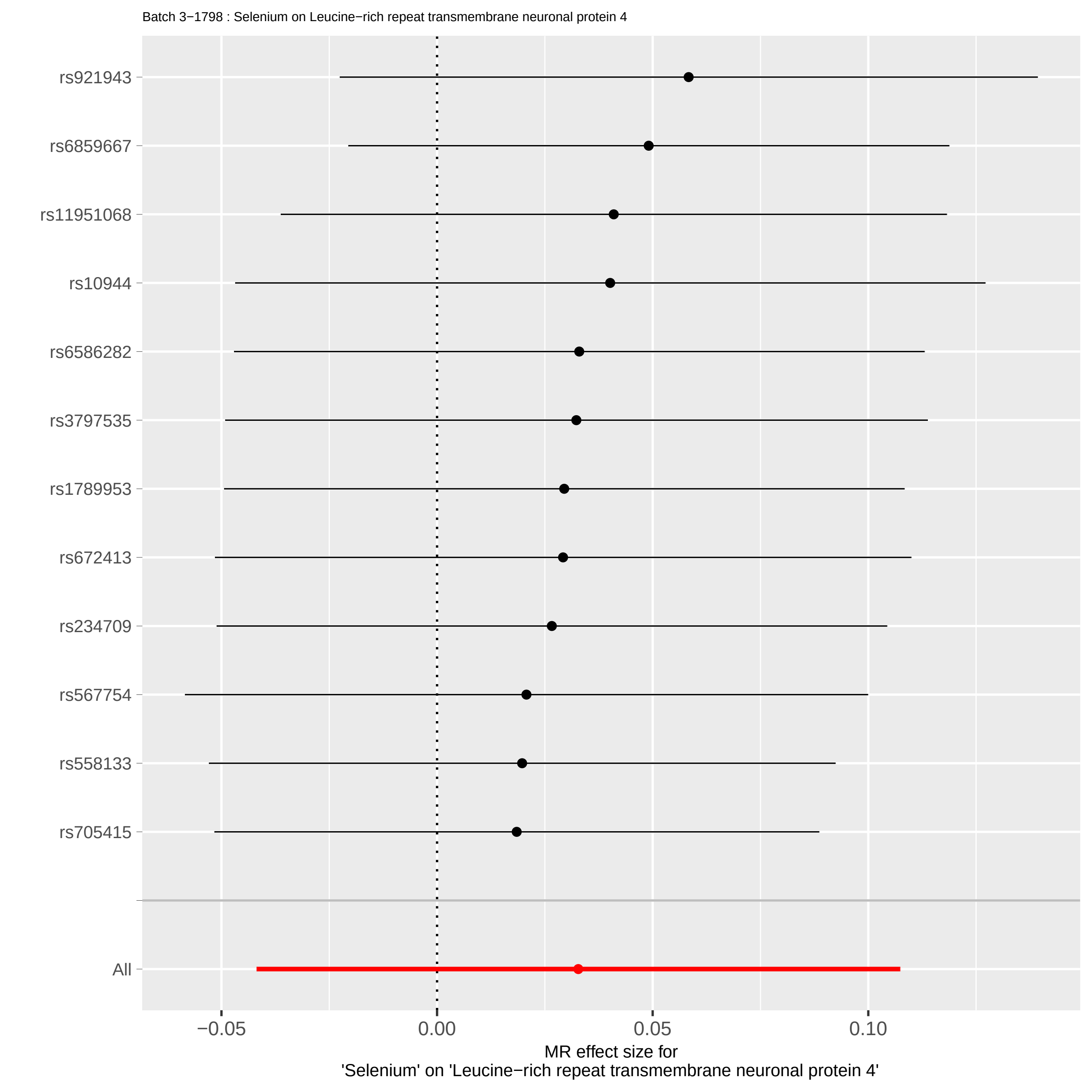

Batch 3−1798 : Selenium on Leucine−rich repeat transmembrane neuronal protein 4
rs921943
rs6859667
rs11951068
rs10944
rs6586282
rs3797535
rs1789953
rs672413
rs234709
rs567754
rs558133
rs705415
All
−0.05
0.00
0.05
0.10
MR effect size for
'Selenium' on 'Leucine−rich repeat transmembrane neuronal protein 4'

Batch 3−1799 : Selenium on Leucine−rich repeat and transmembrane domain−containing protein 1

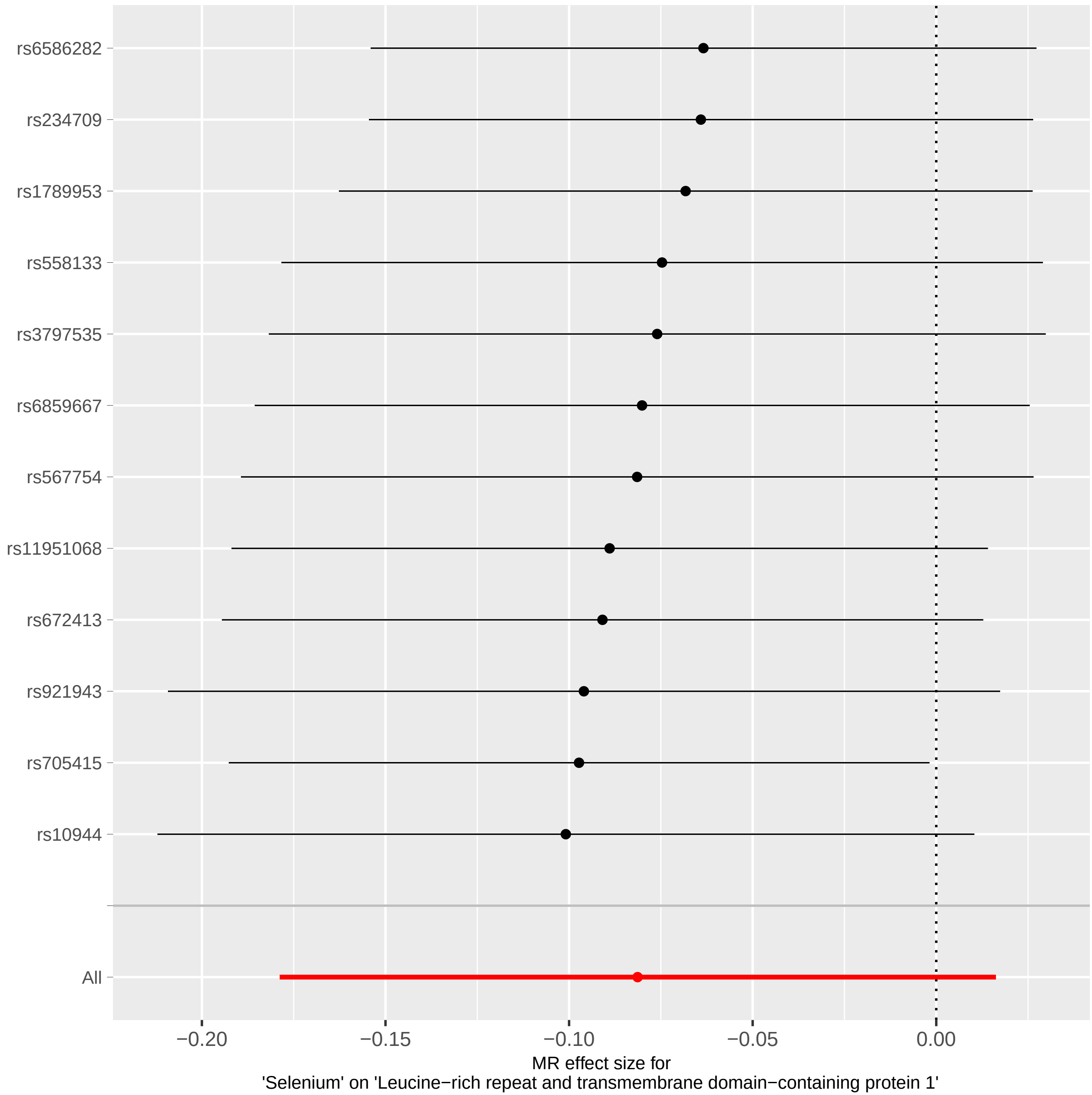

Batch 3−1800 : Selenium on Leucine−rich repeat and transmembrane domain−containing protein 2

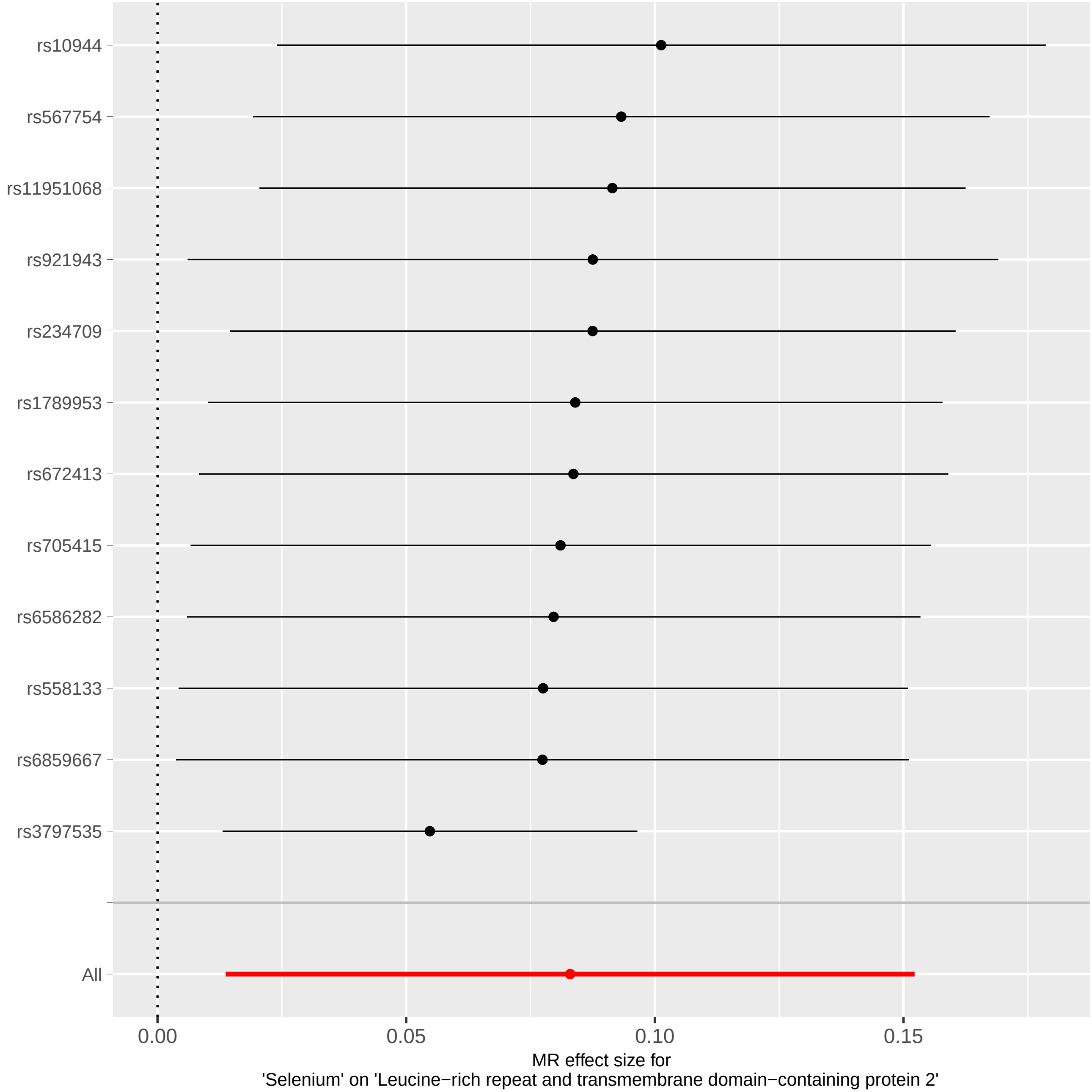

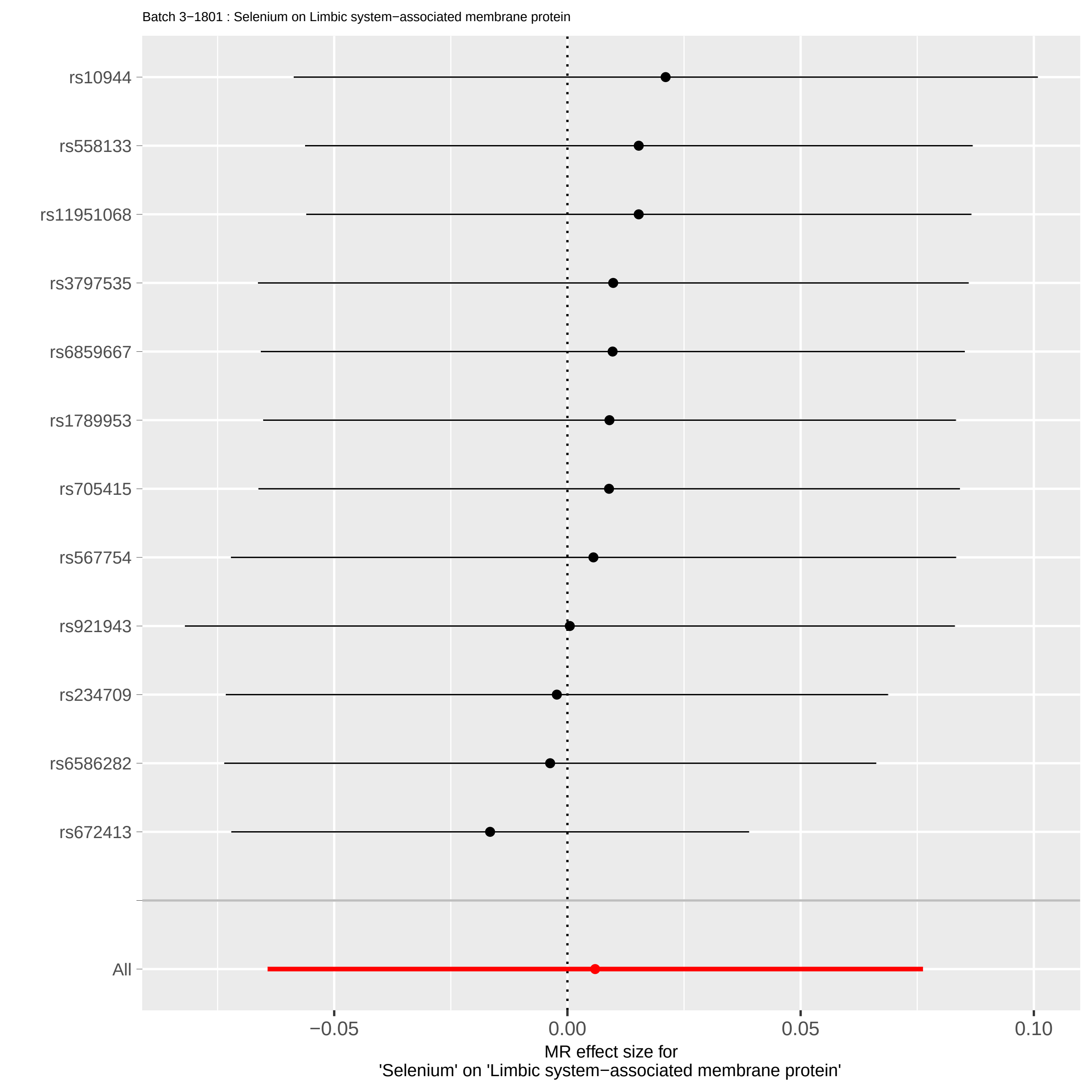
Batch 3−1801 : Selenium on Limbic system−associated membrane protein
rs10944
rs558133
rs11951068
rs3797535
rs6859667
rs1789953
rs705415
rs567754
rs921943
rs234709
rs6586282
rs672413
All
−0.05
0.00
0.05
0.10
MR effect size for
'Selenium' on 'Limbic system−associated membrane protein'

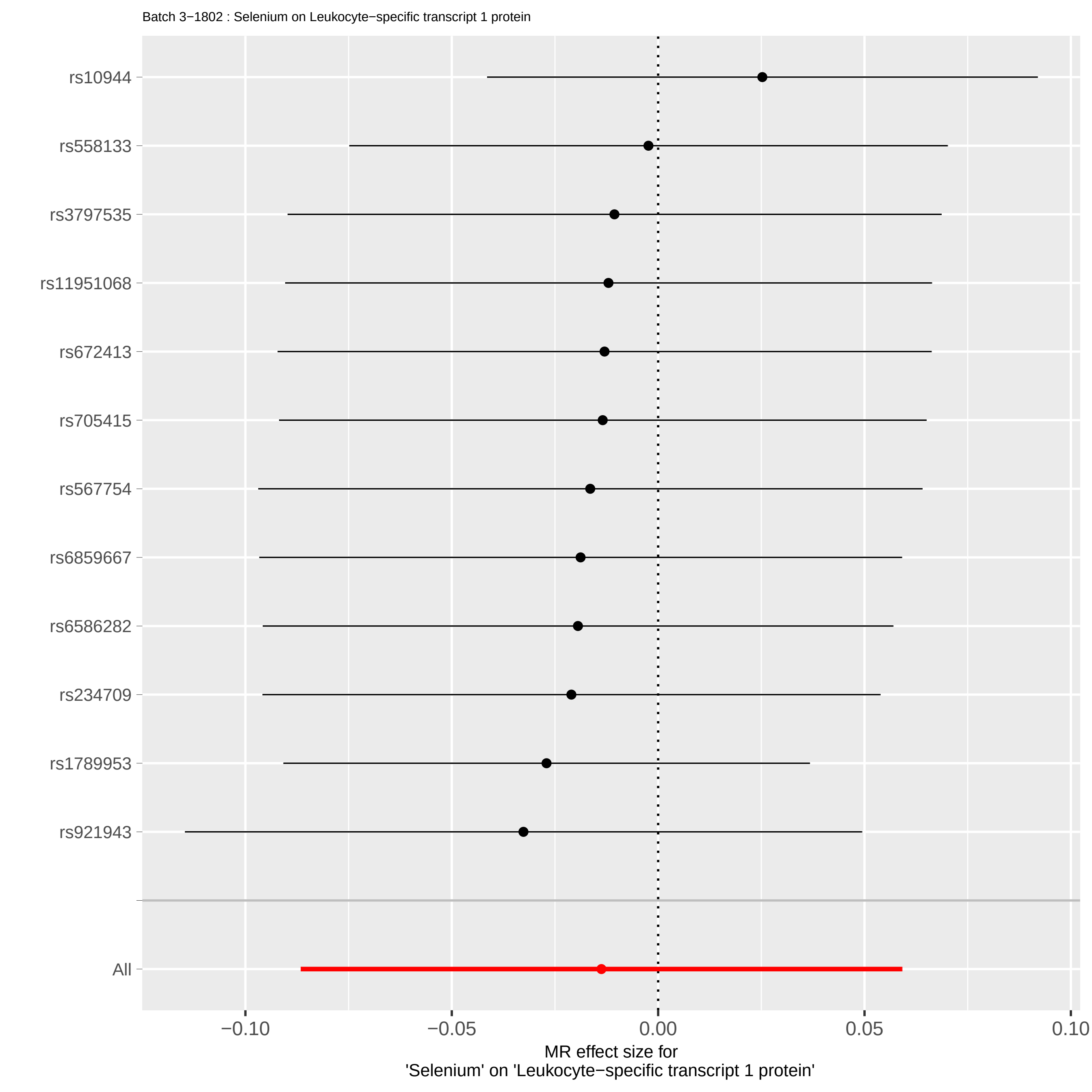
Batch 3−1802 : Selenium on Leukocyte−specific transcript 1 protein
rs10944
rs558133
rs3797535
rs11951068
rs672413
rs705415
rs567754
rs6859667
rs6586282
rs234709
rs1789953
rs921943
All
−0.10
−0.05
0.00
0.05
0.10
MR effect size for
'Selenium' on 'Leukocyte−specific transcript 1 protein'

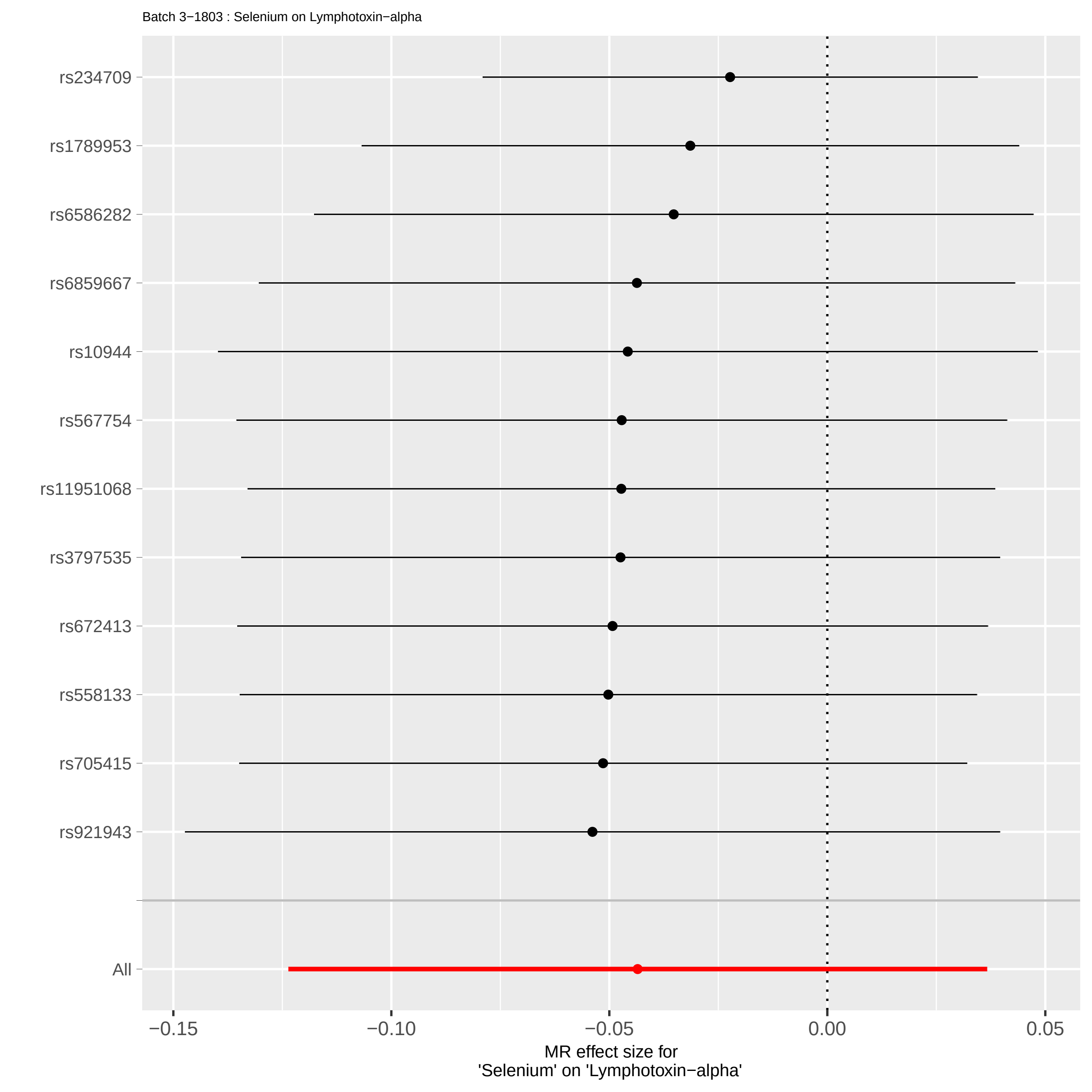

Batch 3−1803 : Selenium on Lymphotoxin−alpha
rs234709
rs1789953
rs6586282
rs6859667
rs10944
rs567754
rs11951068
rs3797535
rs672413
rs558133
rs705415
rs921943
All
−0.15
−0.10
−0.05
0.00
0.05
MR effect size for
'Selenium' on 'Lymphotoxin−alpha'

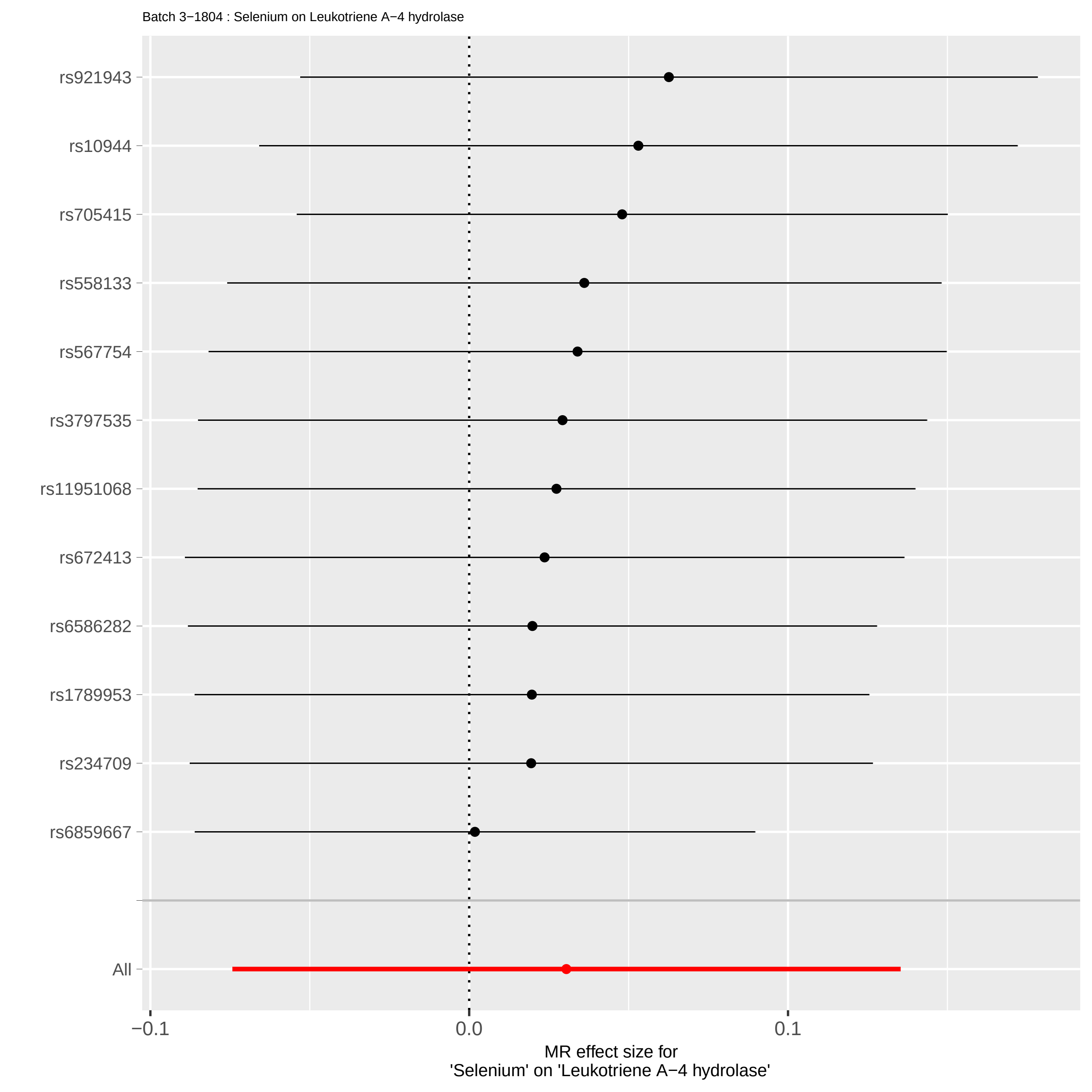

Batch 3−1804 : Selenium on Leukotriene A−4 hydrolase
rs921943
rs10944
rs705415
rs558133
rs567754
rs3797535
rs11951068
rs672413
rs6586282
rs1789953
rs234709
rs6859667
All
−0.1
0.0
0.1
MR effect size for
'Selenium' on 'Leukotriene A−4 hydrolase'

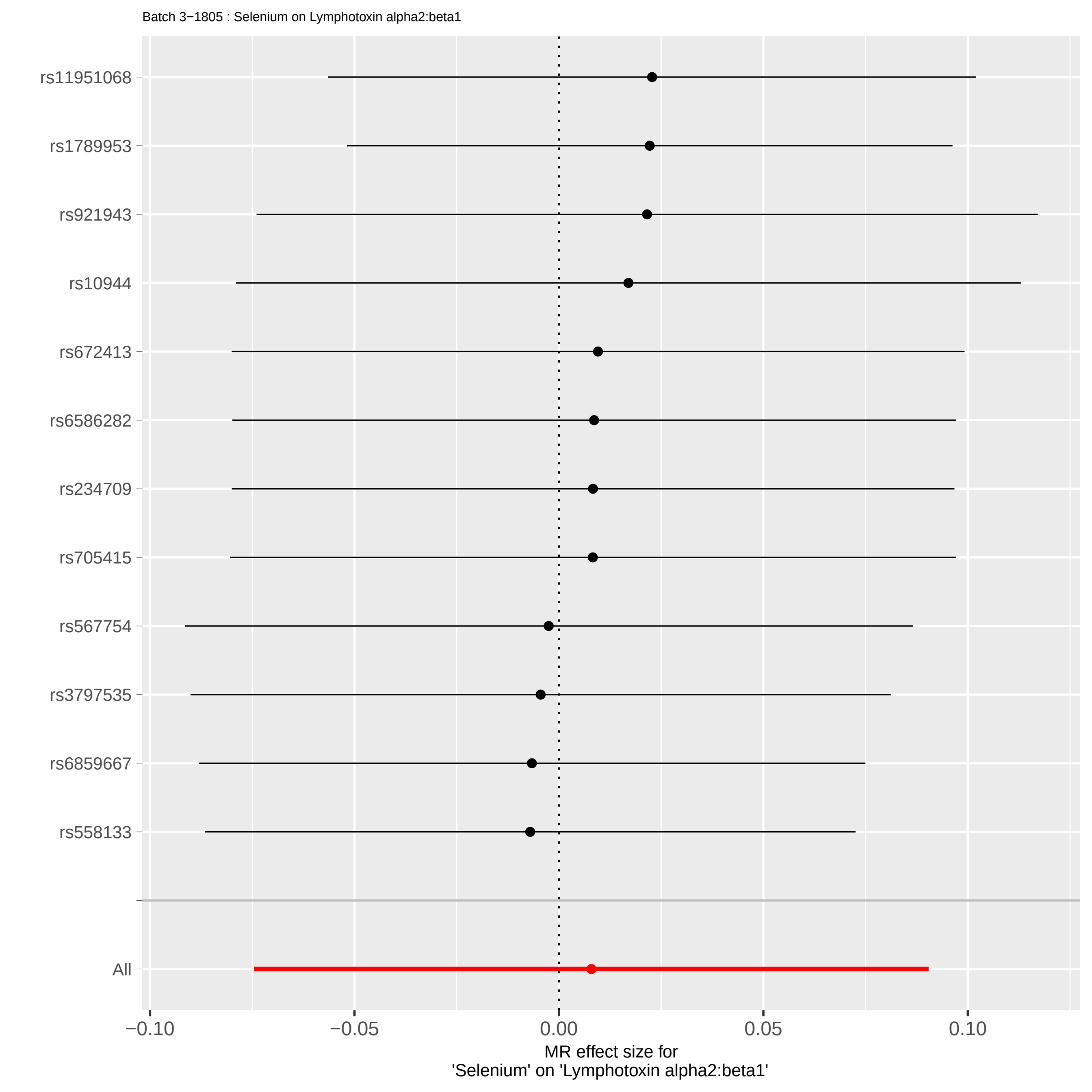

Batch 3−1805 : Selenium on Lymphotoxin alpha2:beta1
rs11951068
rs1789953
rs921943
rs10944
rs672413
rs6586282
rs234709
rs705415
rs567754
rs3797535
rs6859667
rs558133
All
−0.10
−0.05
0.00
0.05
0.10
MR effect size for
'Selenium' on 'Lymphotoxin alpha2:beta1'

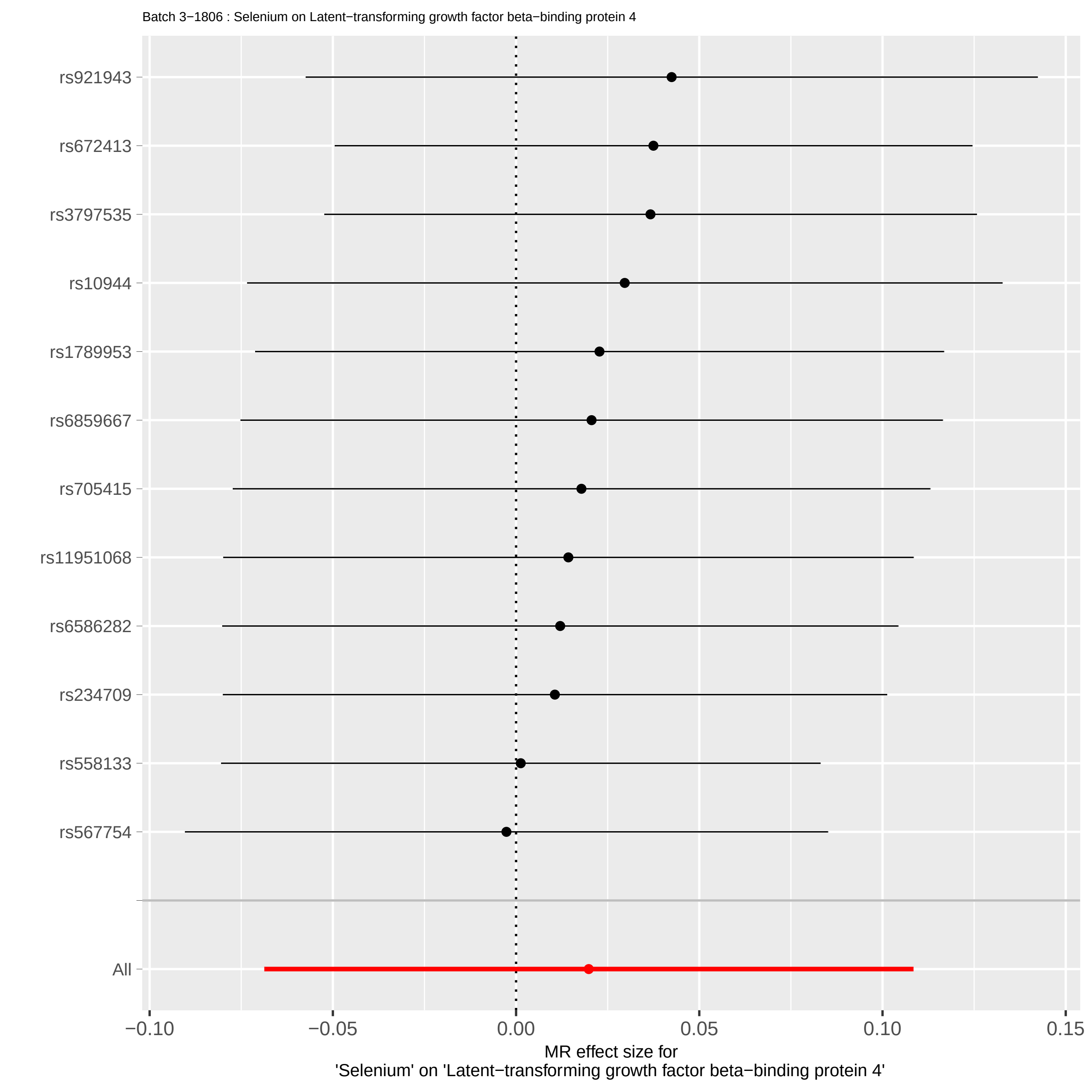
Batch 3−1806 : Selenium on Latent−transforming growth factor beta−binding protein 4
rs921943
rs672413
rs3797535
rs10944
rs1789953
rs6859667
rs705415
rs11951068
rs6586282
rs234709
rs558133
rs567754
All
−0.10
−0.05
0.00
0.05
0.10
0.15
MR effect size for
'Selenium' on 'Latent−transforming growth factor beta−binding protein 4'

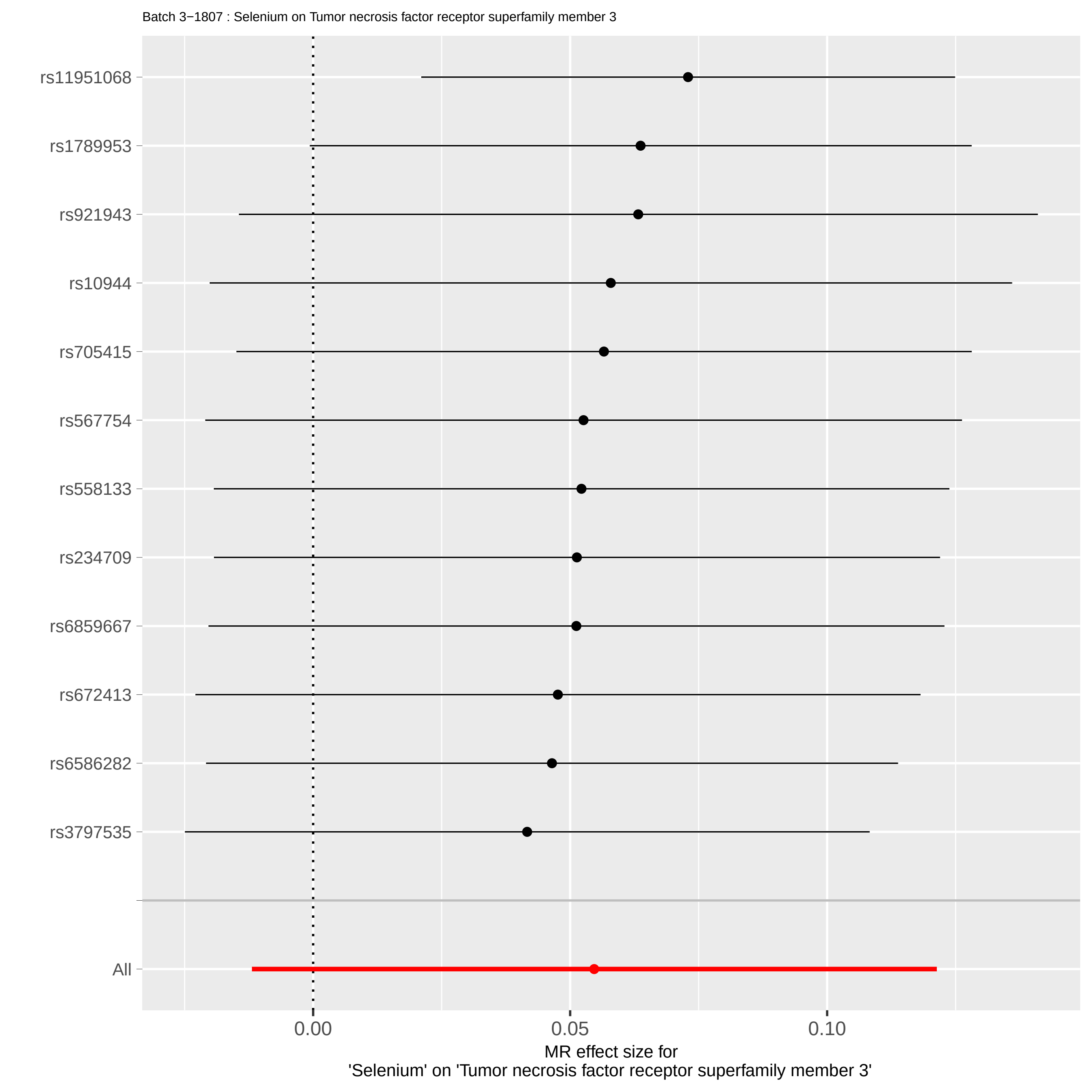

Batch 3−1807 : Selenium on Tumor necrosis factor receptor superfamily member 3
rs11951068
rs1789953
rs921943
rs10944
rs705415
rs567754
rs558133
rs234709
rs6859667
rs672413
rs6586282
rs3797535
All
0.00
0.05
0.10
MR effect size for
'Selenium' on 'Tumor necrosis factor receptor superfamily member 3'

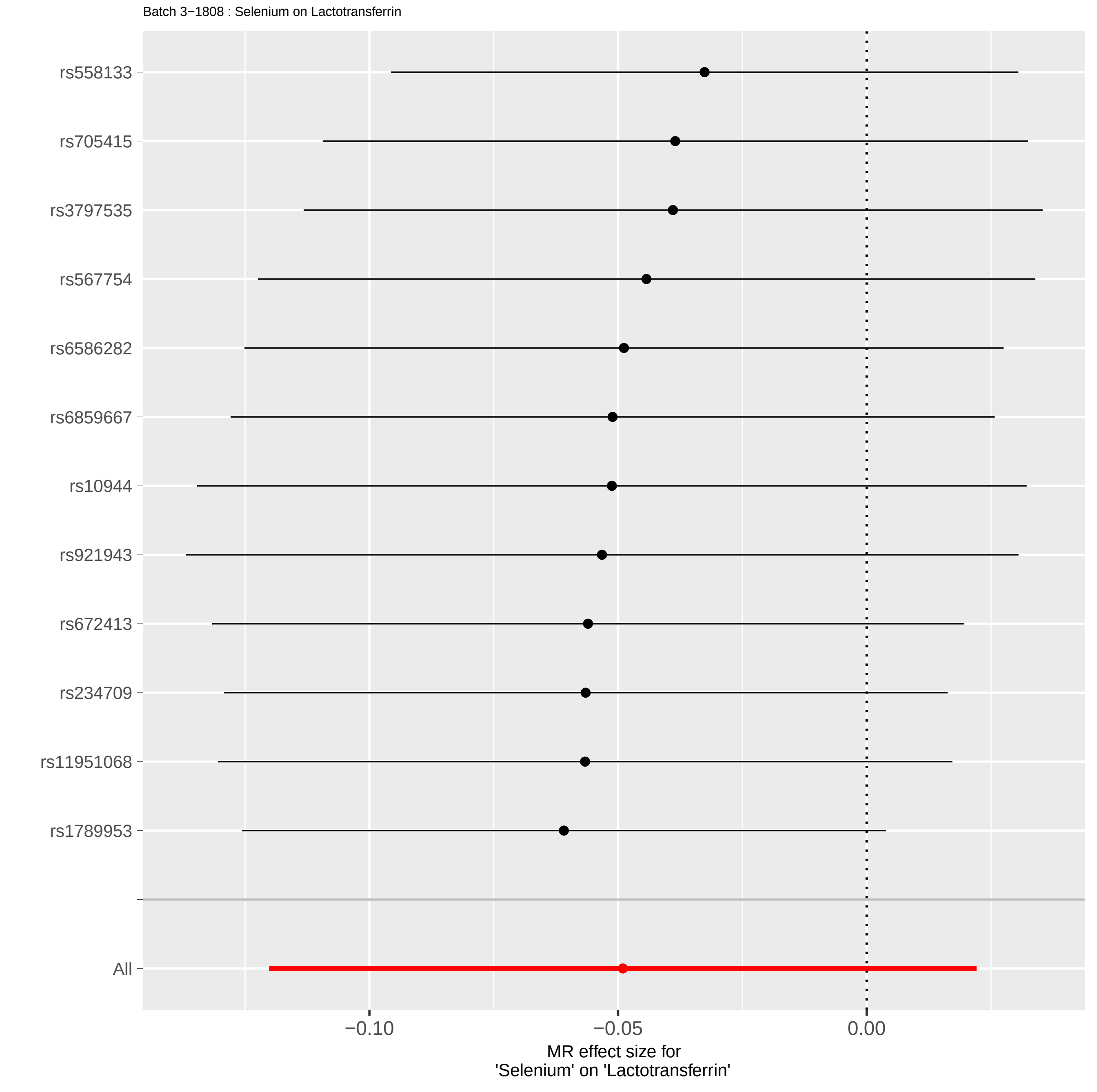
Batch 3−1808 : Selenium on Lactotransferrin
rs558133
rs705415
rs3797535
rs567754
rs6586282
rs6859667
rs10944
rs921943
rs672413
rs234709
rs11951068
rs1789953
All
−0.10
−0.05
0.00
MR effect size for
'Selenium' on 'Lactotransferrin'

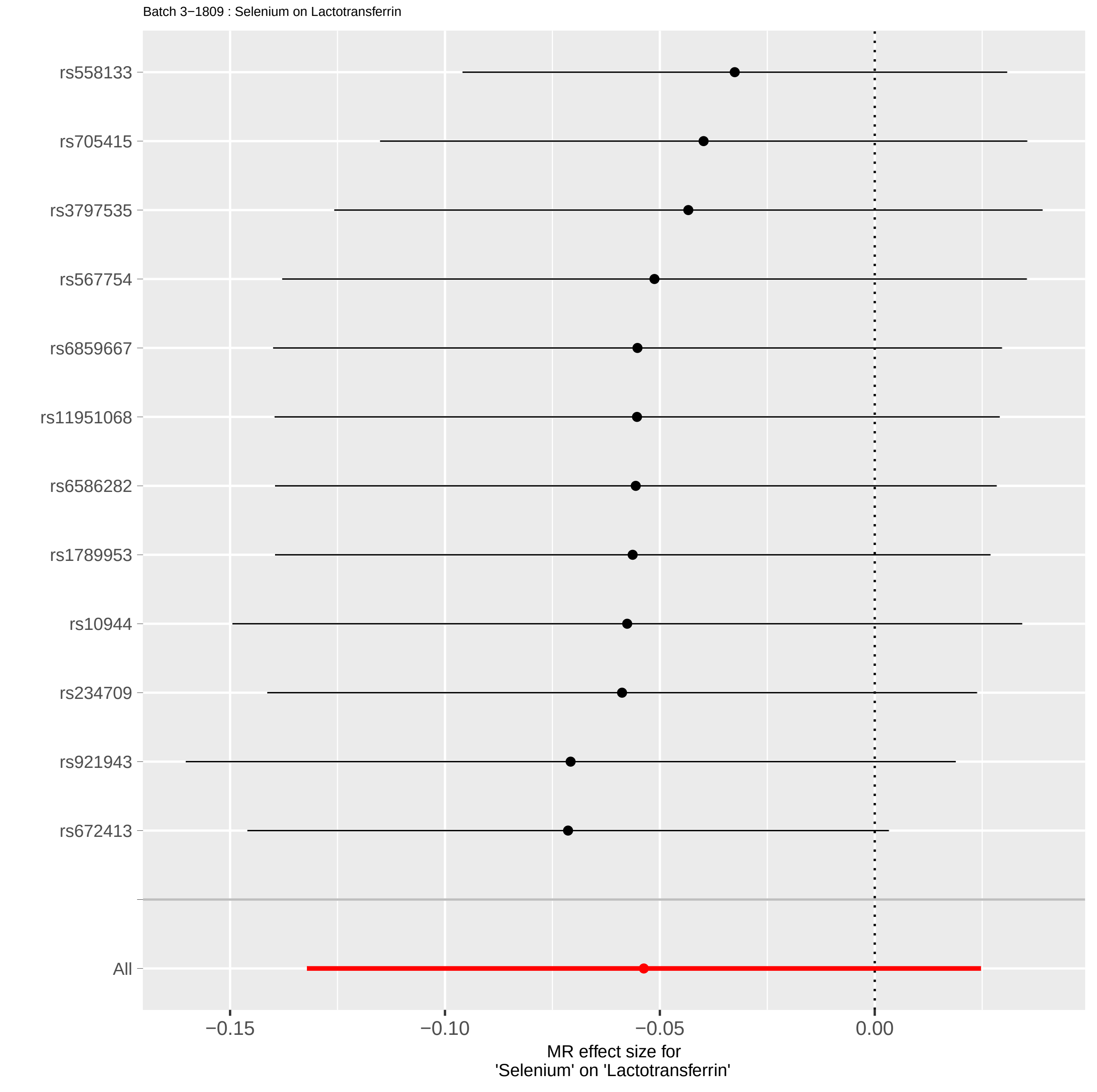

Batch 3−1809 : Selenium on Lactotransferrin
rs558133
rs705415
rs3797535
rs567754
rs6859667
rs11951068
rs6586282
rs1789953
rs10944
rs234709
rs921943
rs672413
All
−0.15
−0.10
−0.05
0.00
MR effect size for
'Selenium' on 'Lactotransferrin'

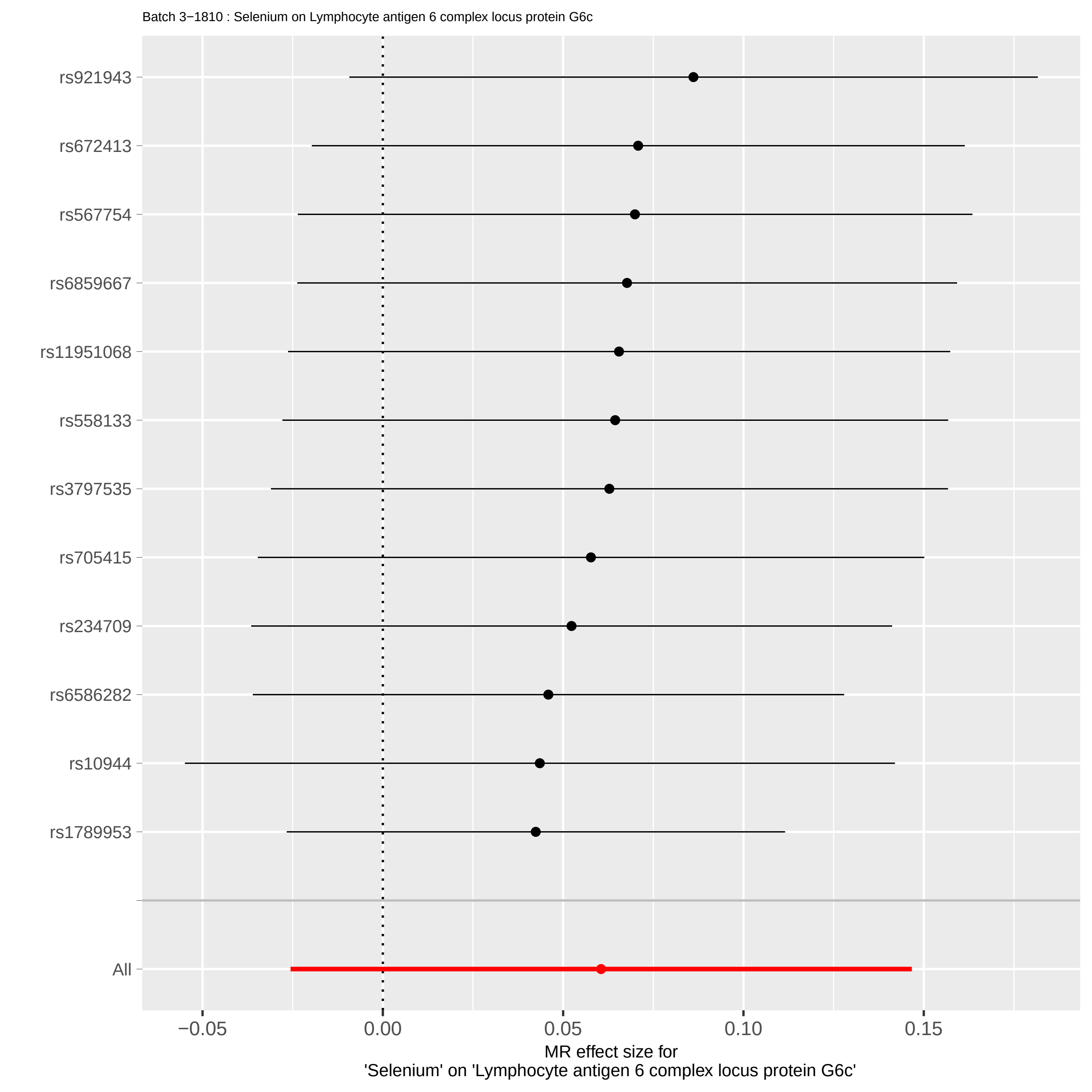

Batch 3−1810 : Selenium on Lymphocyte antigen 6 complex locus protein G6c
rs921943
rs672413
rs567754
rs6859667
rs11951068
rs558133
rs3797535
rs705415
rs234709
rs6586282
rs10944
rs1789953
All
−0.05
0.00
0.05
0.10
0.15
MR effect size for
'Selenium' on 'Lymphocyte antigen 6 complex locus protein G6c'

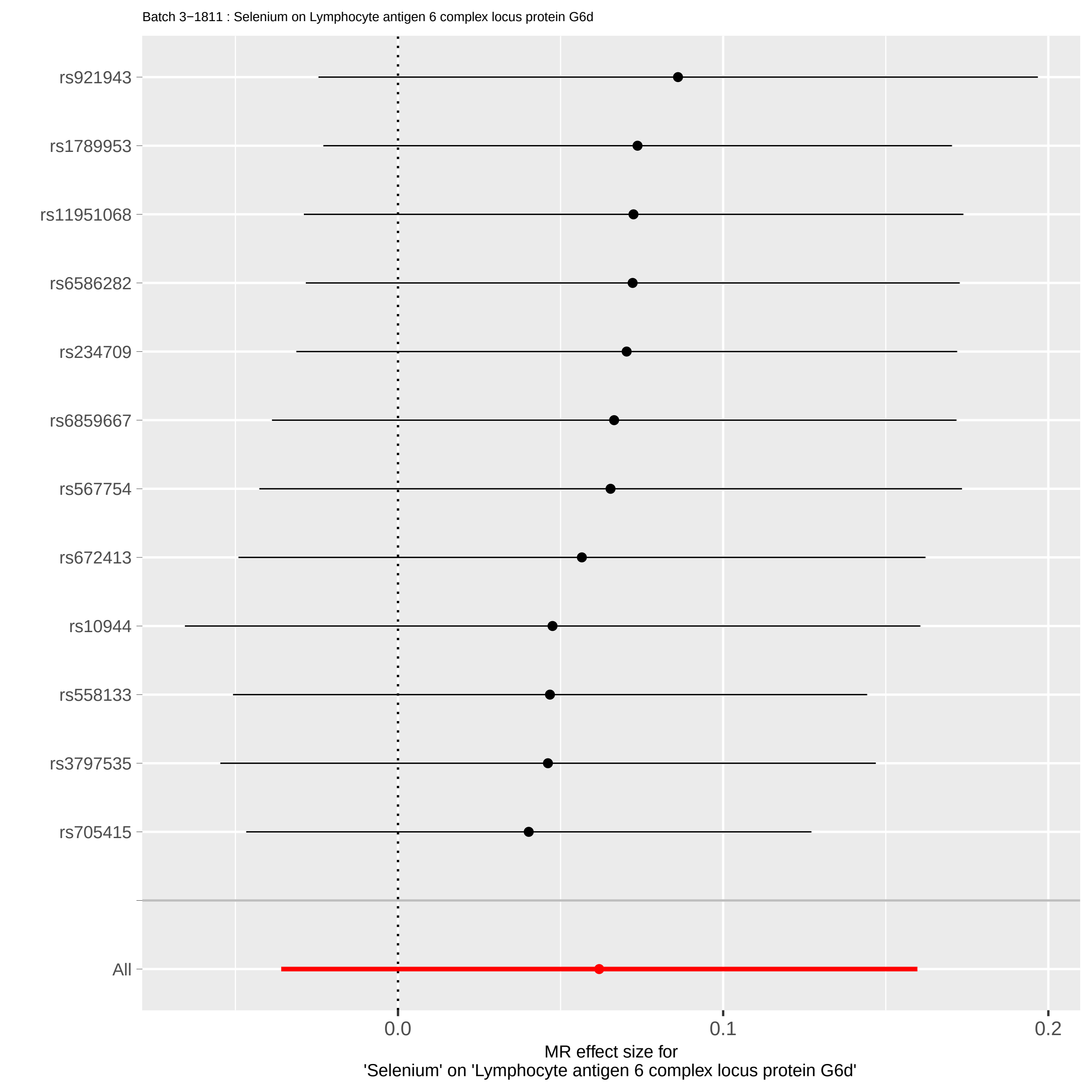

Batch 3−1811 : Selenium on Lymphocyte antigen 6 complex locus protein G6d
rs921943
rs1789953
rs11951068
rs6586282
rs234709
rs6859667
rs567754
rs672413
rs10944
rs558133
rs3797535
rs705415
All
0.0
0.1
0.2
MR effect size for
'Selenium' on 'Lymphocyte antigen 6 complex locus protein G6d'

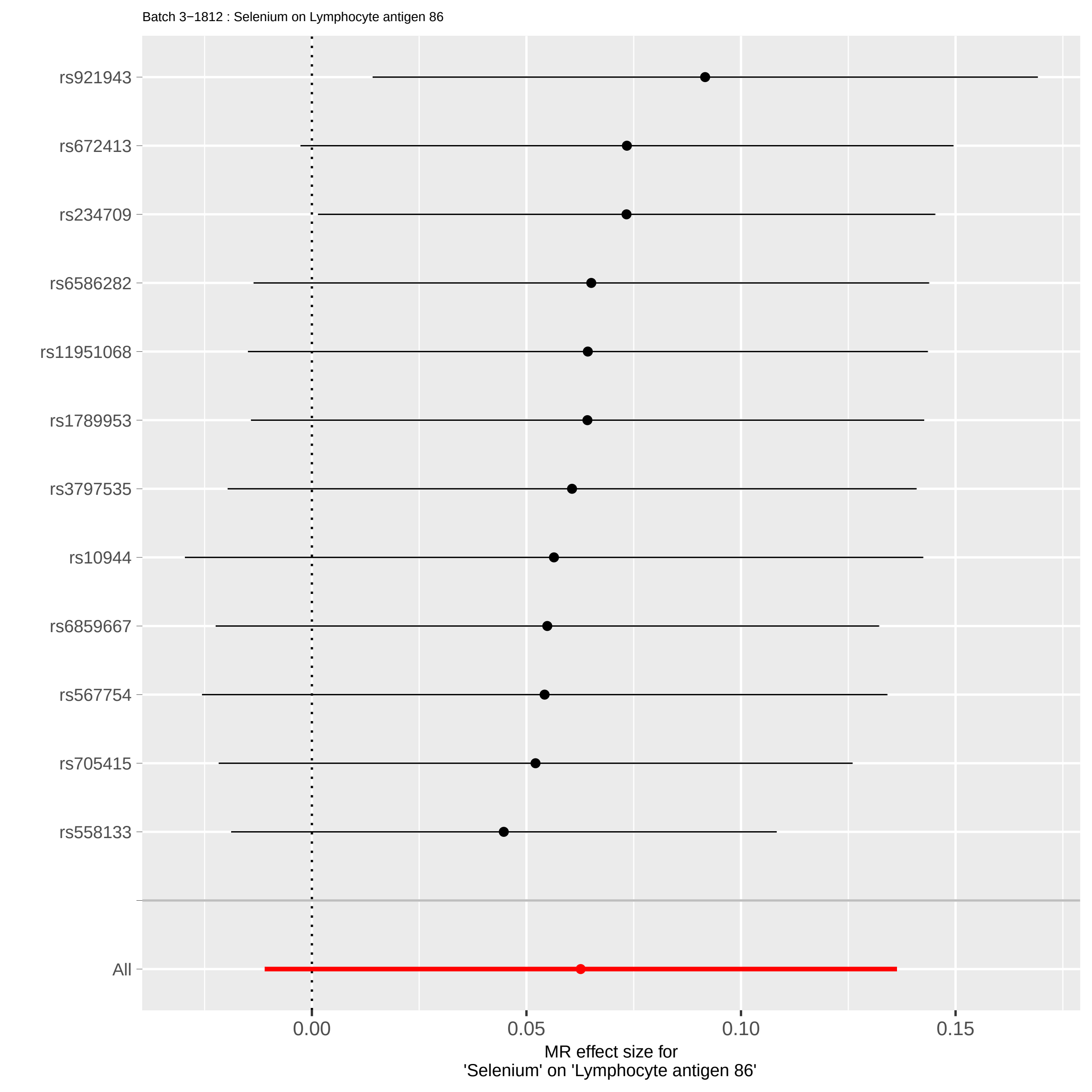

Batch 3−1812 : Selenium on Lymphocyte antigen 86
rs921943
rs672413
rs234709
rs6586282
rs11951068
rs1789953
rs3797535
rs10944
rs6859667
rs567754
rs705415
rs558133
All
0.00
0.05
0.10
0.15
MR effect size for
'Selenium' on 'Lymphocyte antigen 86'

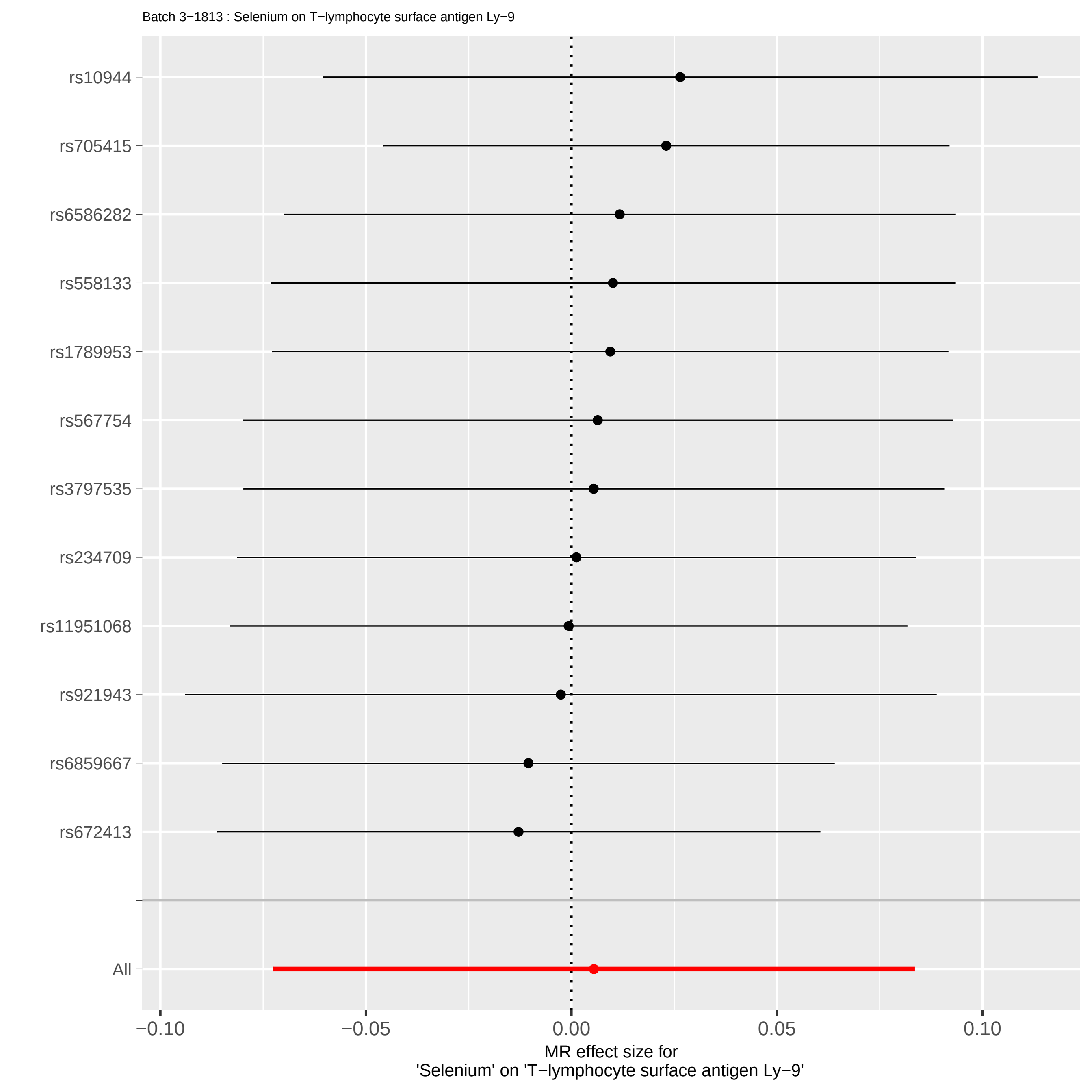

Batch 3−1813 : Selenium on T−lymphocyte surface antigen Ly−9
rs10944
rs705415
rs6586282
rs558133
rs1789953
rs567754
rs3797535
rs234709
rs11951068
rs921943
rs6859667
rs672413
All
−0.10
−0.05
0.00
0.05
0.10
MR effect size for
'Selenium' on 'T−lymphocyte surface antigen Ly−9'

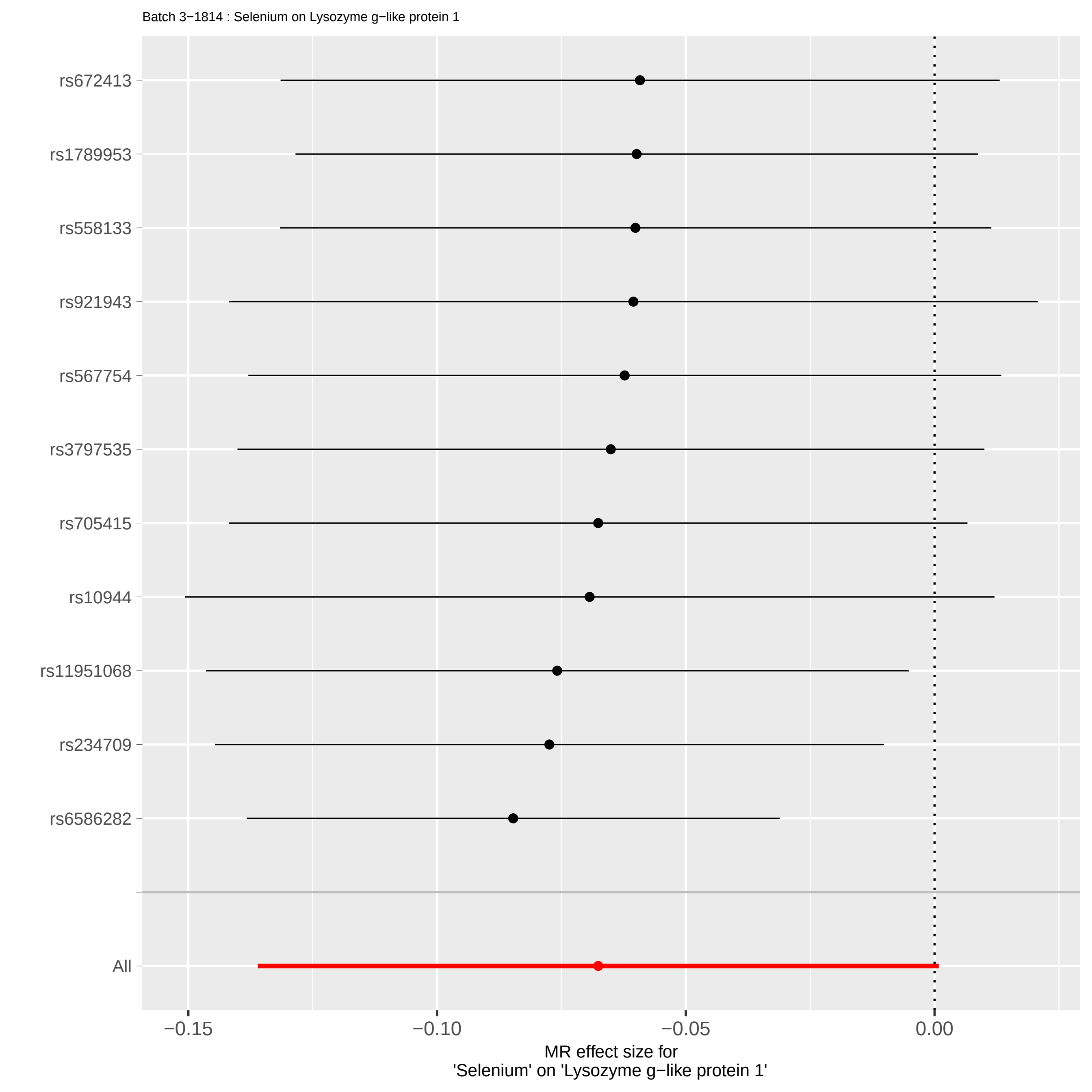

Batch 3−1814 : Selenium on Lysozyme g−like protein 1
rs672413
rs1789953
rs558133
rs921943
rs567754
rs3797535
rs705415
rs10944
rs11951068
rs234709
rs6586282
All
−0.15
−0.10
−0.05
0.00
MR effect size for
'Selenium' on 'Lysozyme g−like protein 1'

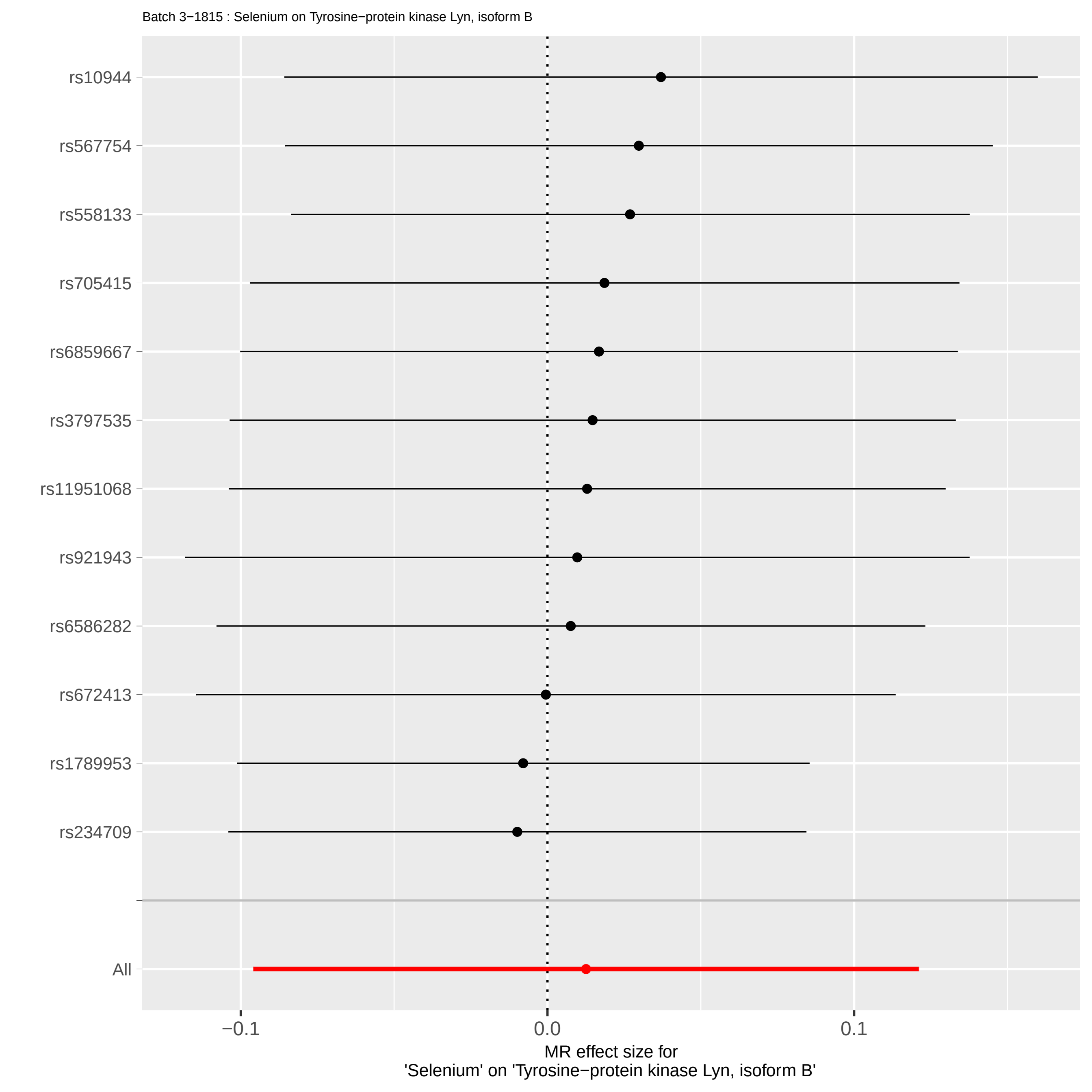
Batch 3−1815 : Selenium on Tyrosine−protein kinase Lyn, isoform B
rs10944
rs567754
rs558133
rs705415
rs6859667
rs3797535
rs11951068
rs921943
rs6586282
rs672413
rs1789953
rs234709
All
−0.1
0.0
0.1
MR effect size for
'Selenium' on 'Tyrosine−protein kinase Lyn, isoform B'

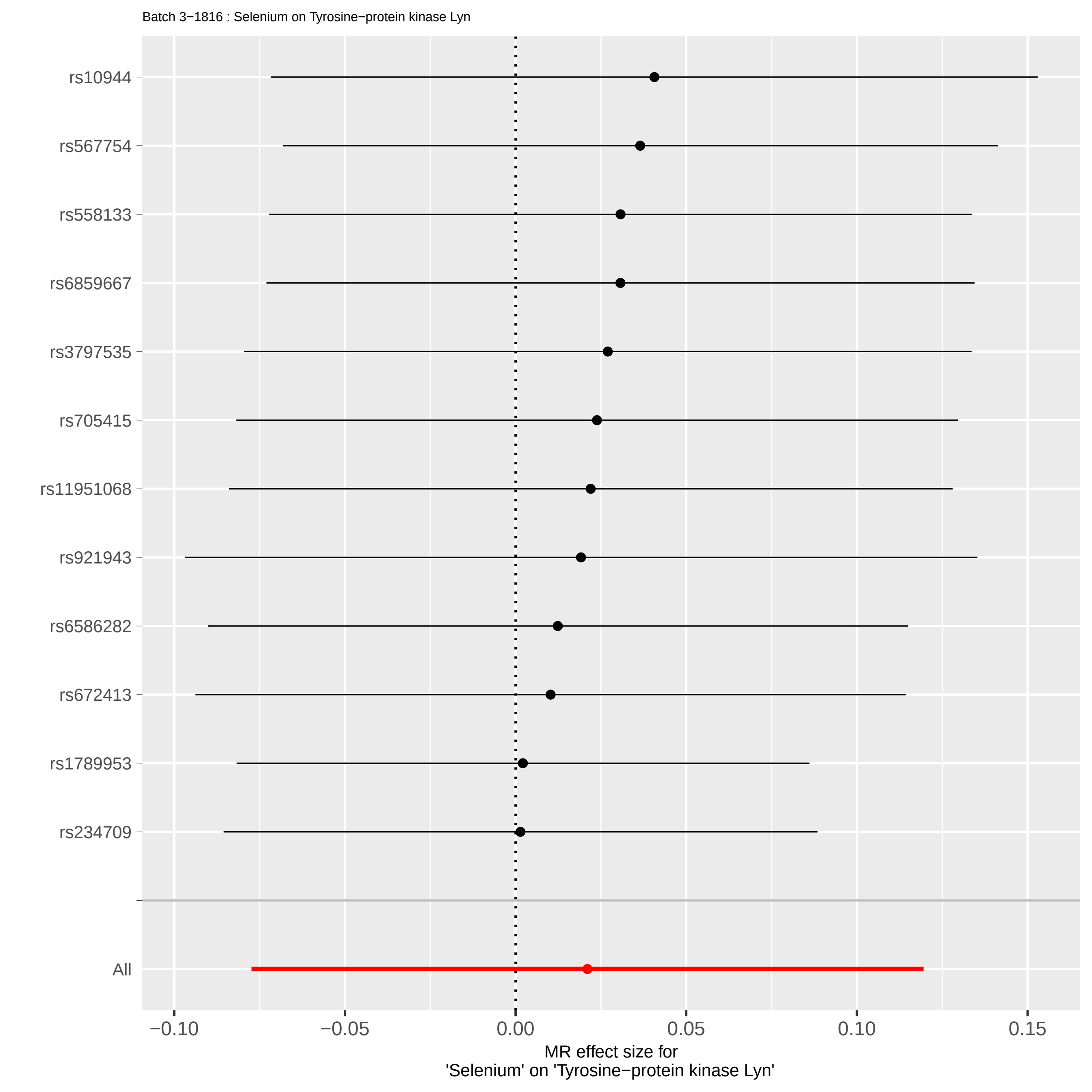

Batch 3−1816 : Selenium on Tyrosine−protein kinase Lyn
rs10944
rs567754
rs558133
rs6859667
rs3797535
rs705415
rs11951068
rs921943
rs6586282
rs672413
rs1789953
rs234709
All
−0.10
−0.05
0.00
0.05
0.10
0.15
MR effect size for
'Selenium' on 'Tyrosine−protein kinase Lyn'

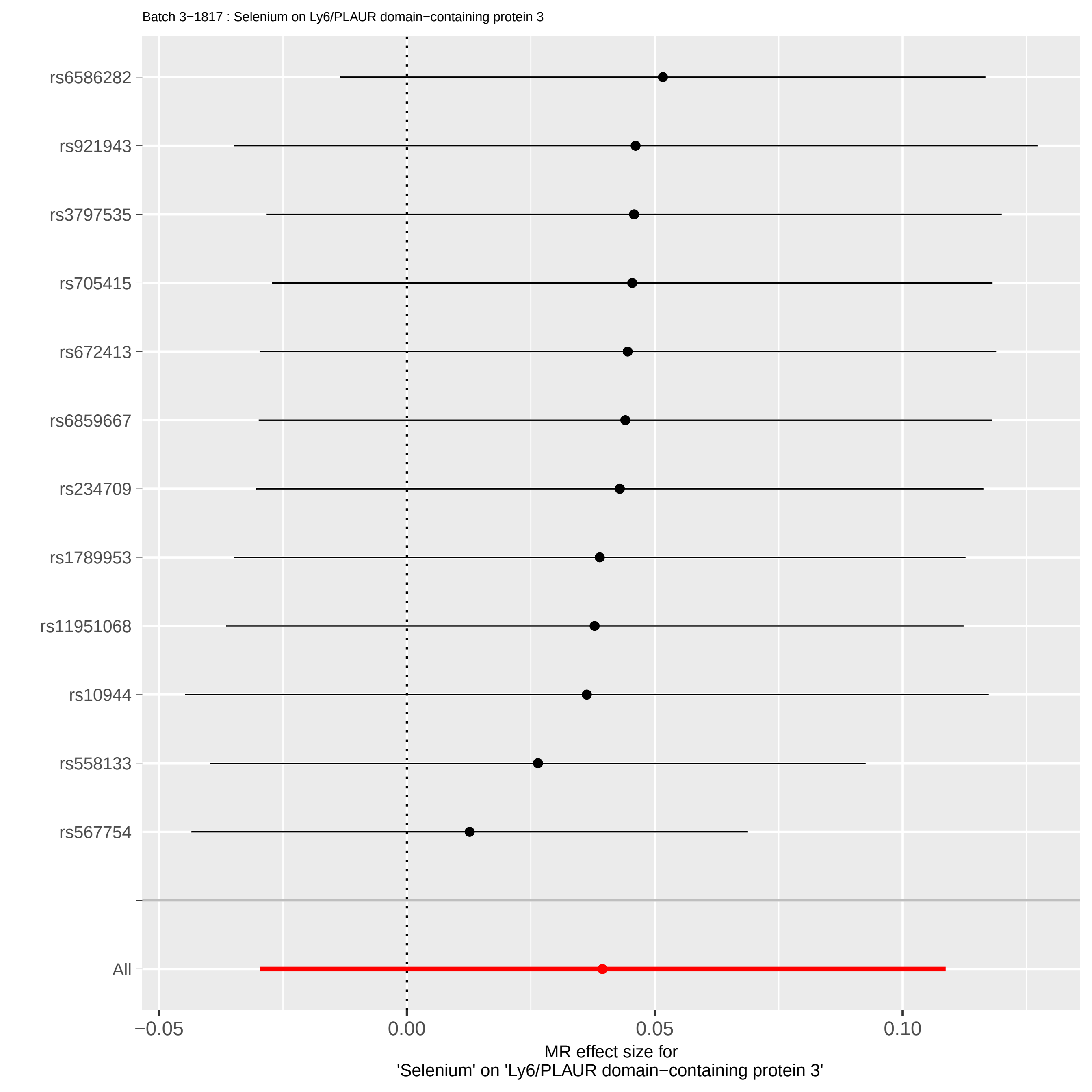
Batch 3−1817 : Selenium on Ly6/PLAUR domain−containing protein 3
rs6586282
rs921943
rs3797535
rs705415
rs672413
rs6859667
rs234709
rs1789953
rs11951068
rs10944
rs558133
rs567754
All
−0.05
0.00
0.05
0.10
MR effect size for
'Selenium' on 'Ly6/PLAUR domain−containing protein 3'

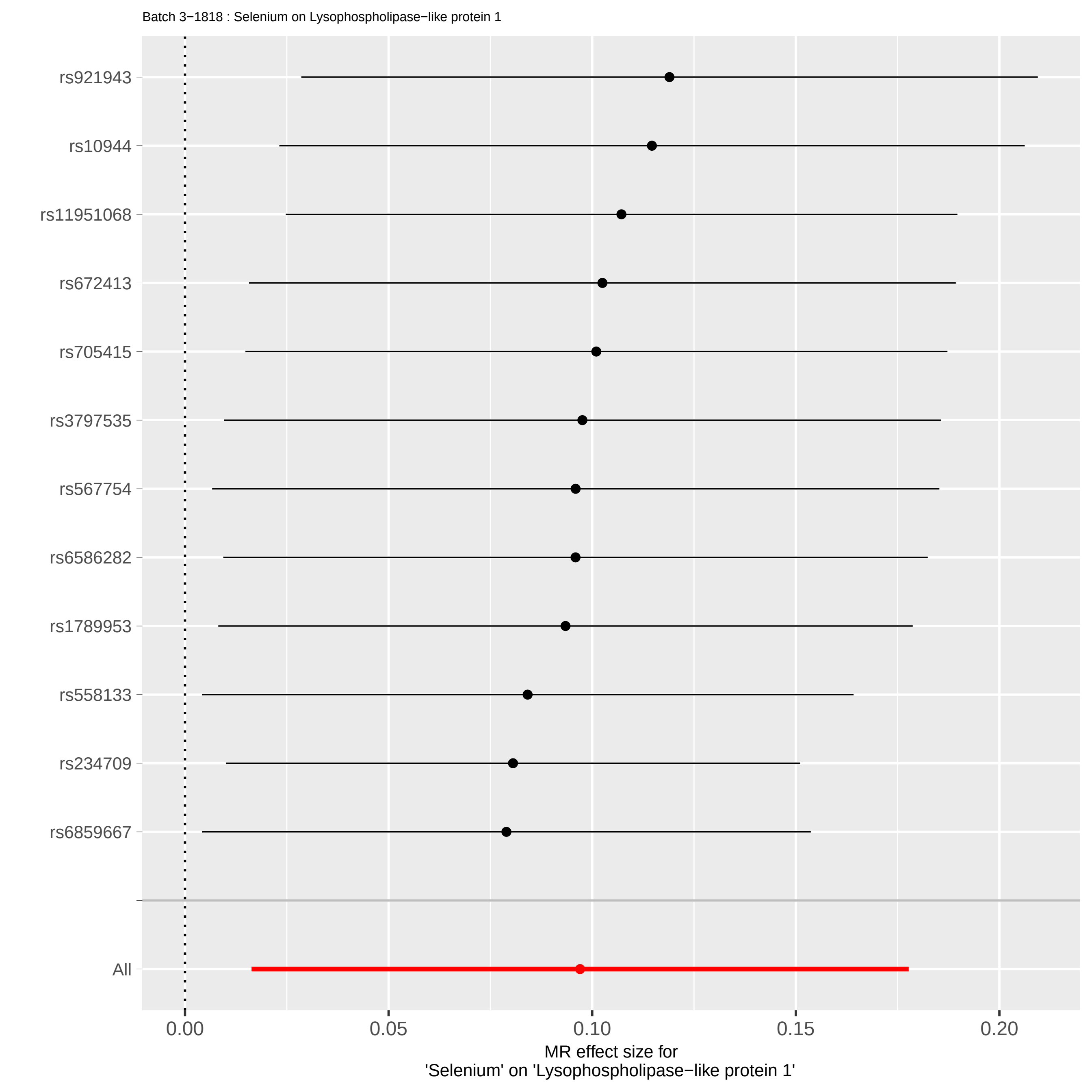

Batch 3−1818 : Selenium on Lysophospholipase−like protein 1
rs921943
rs10944
rs11951068
rs672413
rs705415
rs3797535
rs567754
rs6586282
rs1789953
rs558133
rs234709
rs6859667
All
0.00
0.05
0.10
0.15
0.20
MR effect size for
'Selenium' on 'Lysophospholipase−like protein 1'

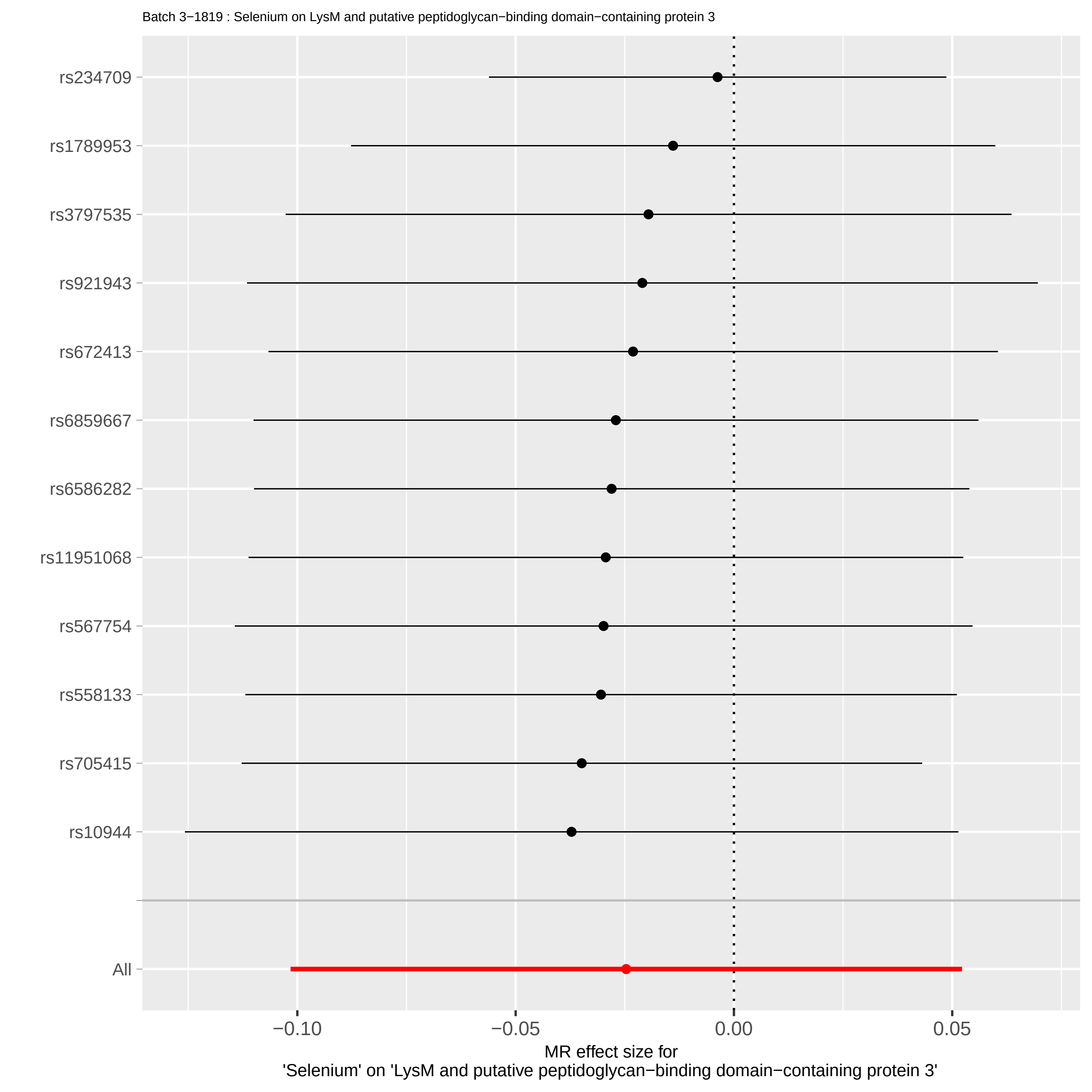

Batch 3−1819 : Selenium on LysM and putative peptidoglycan−binding domain−containing protein 3
rs234709
rs1789953
rs3797535
rs921943
rs672413
rs6859667
rs6586282
rs11951068
rs567754
rs558133
rs705415
rs10944
All
−0.10
−0.05
0.00
0.05
MR effect size for
'Selenium' on 'LysM and putative peptidoglycan−binding domain−containing protein 3'

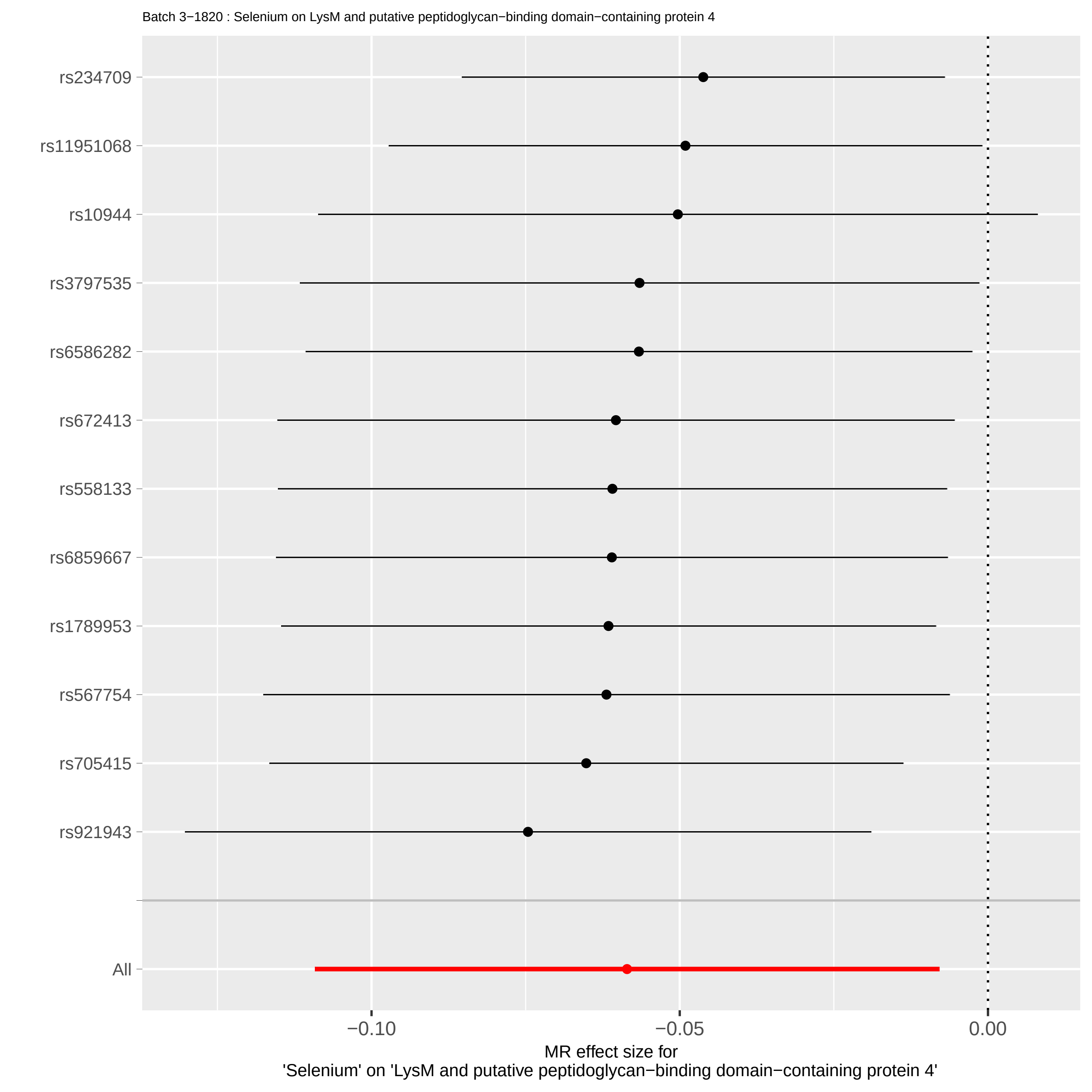

Batch 3−1820 : Selenium on LysM and putative peptidoglycan−binding domain−containing protein 4
rs234709
rs11951068
rs10944
rs3797535
rs6586282
rs672413
rs558133
rs6859667
rs1789953
rs567754
rs705415
rs921943
All
−0.10
−0.05
0.00
MR effect size for
'Selenium' on 'LysM and putative peptidoglycan−binding domain−containing protein 4'

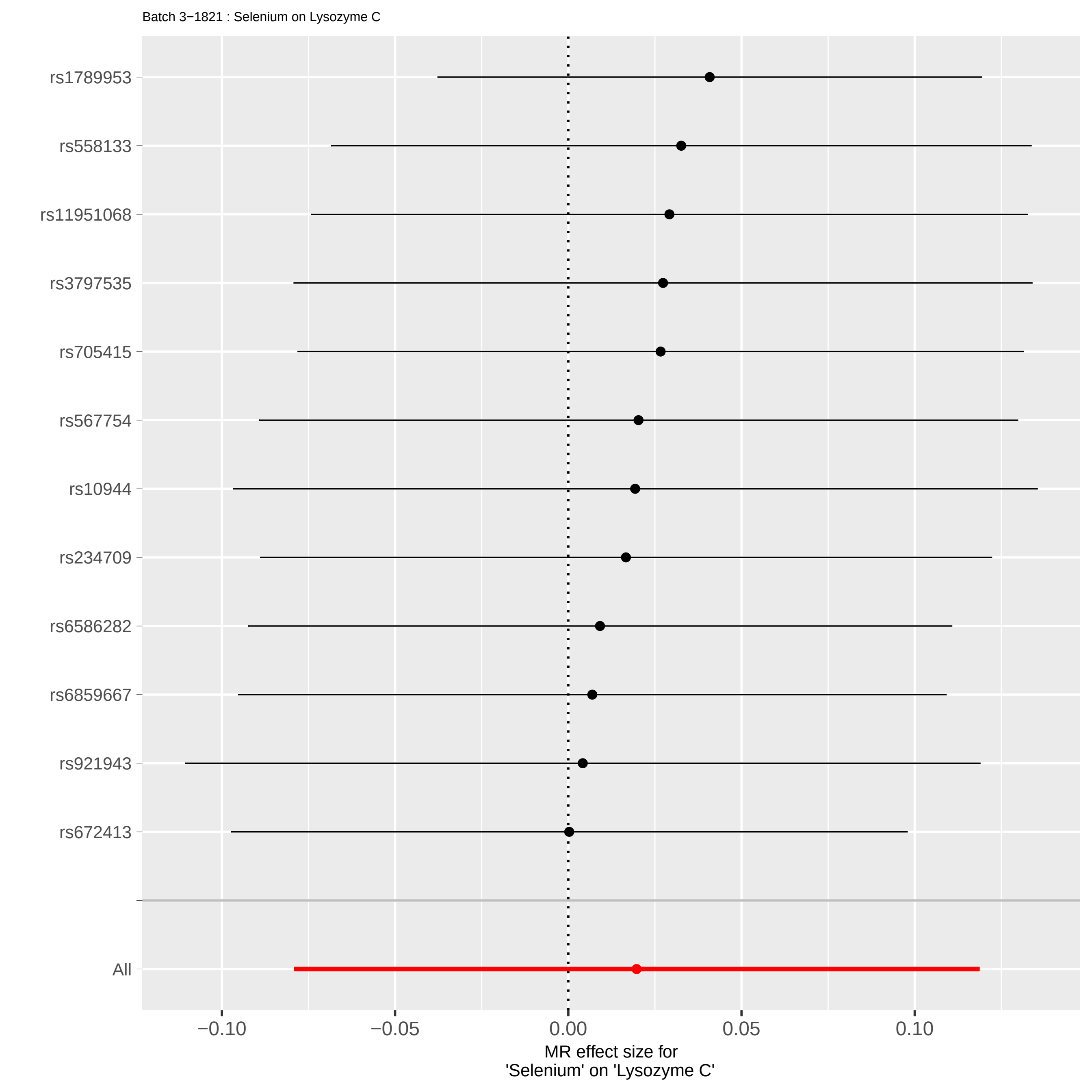

Batch 3−1821 : Selenium on Lysozyme C
rs1789953
rs558133
rs11951068
rs3797535
rs705415
rs567754
rs10944
rs234709
rs6586282
rs6859667
rs921943
rs672413
All
−0.10
−0.05
0.00
0.05
0.10
MR effect size for
'Selenium' on 'Lysozyme C'

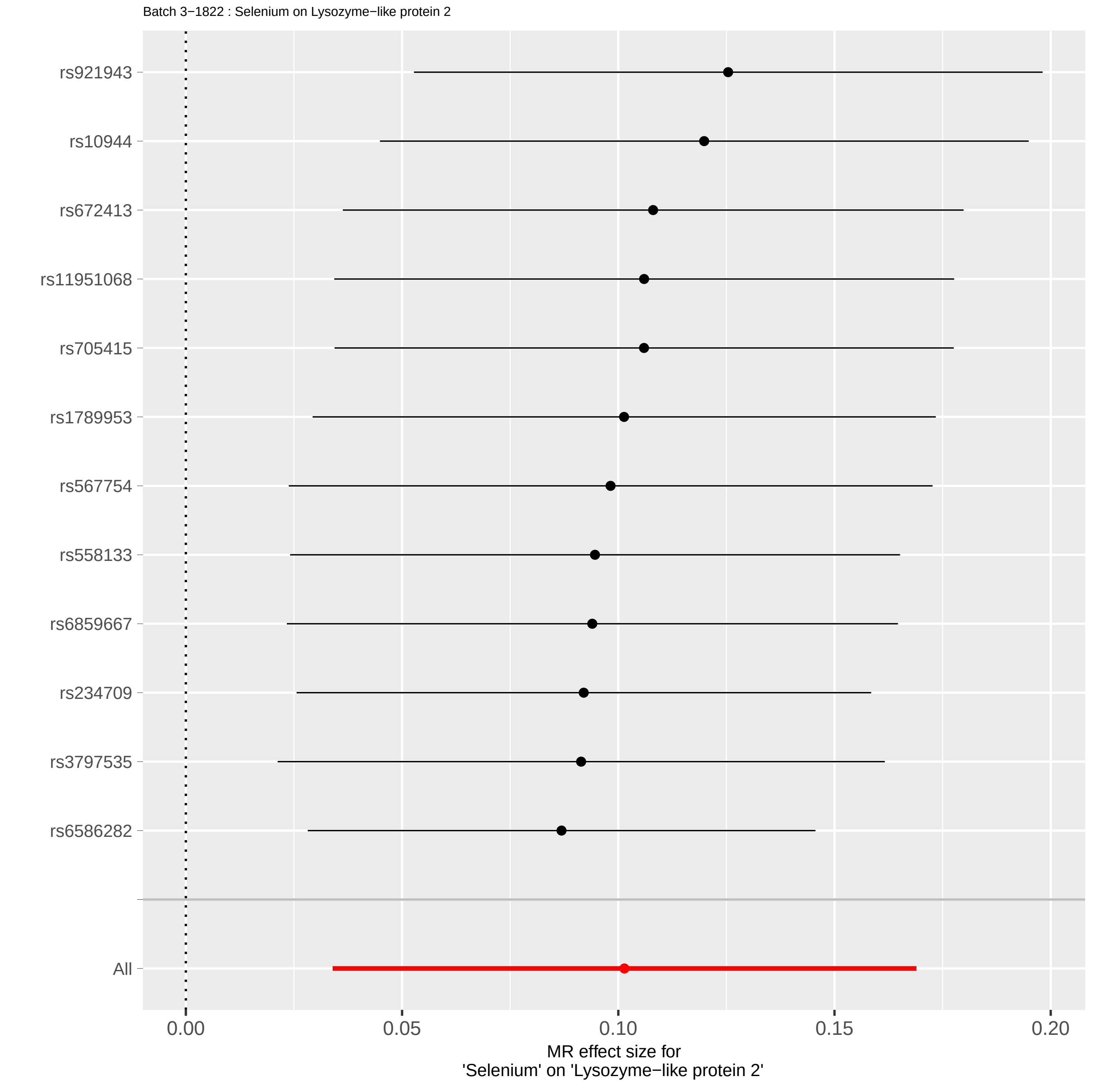

Batch 3−1822 : Selenium on Lysozyme−like protein 2
rs921943
rs10944
rs672413
rs11951068
rs705415
rs1789953
rs567754
rs558133
rs6859667
rs234709
rs3797535
rs6586282
All
0.00
0.05
0.10
0.15
0.20
MR effect size for
'Selenium' on 'Lysozyme−like protein 2'

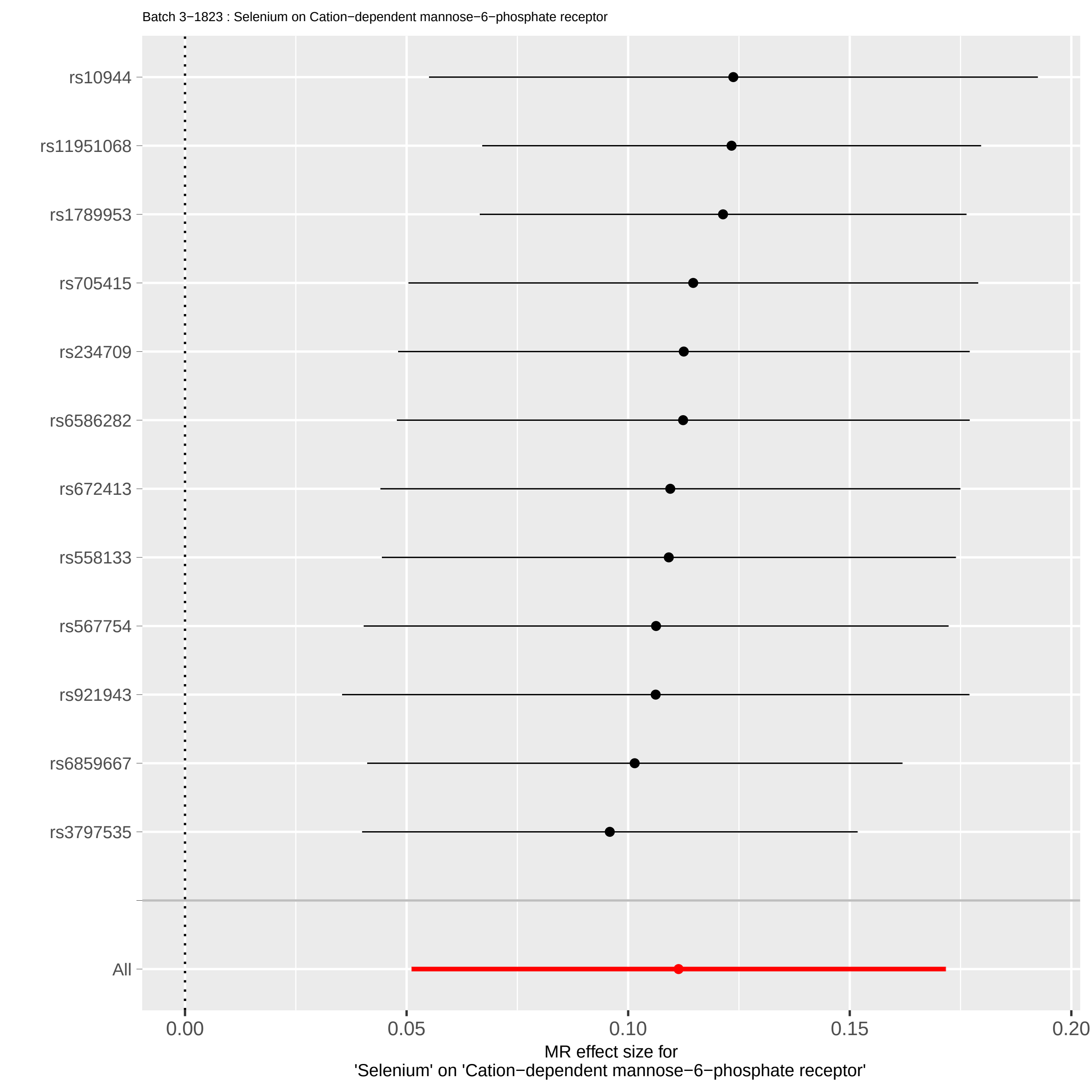
Batch 3−1823 : Selenium on Cation−dependent mannose−6−phosphate receptor
rs10944
rs11951068
rs1789953
rs705415
rs234709
rs6586282
rs672413
rs558133
rs567754
rs921943
rs6859667
rs3797535
All
0.00
0.05
0.10
0.15
0.20
MR effect size for
'Selenium' on 'Cation−dependent mannose−6−phosphate receptor'

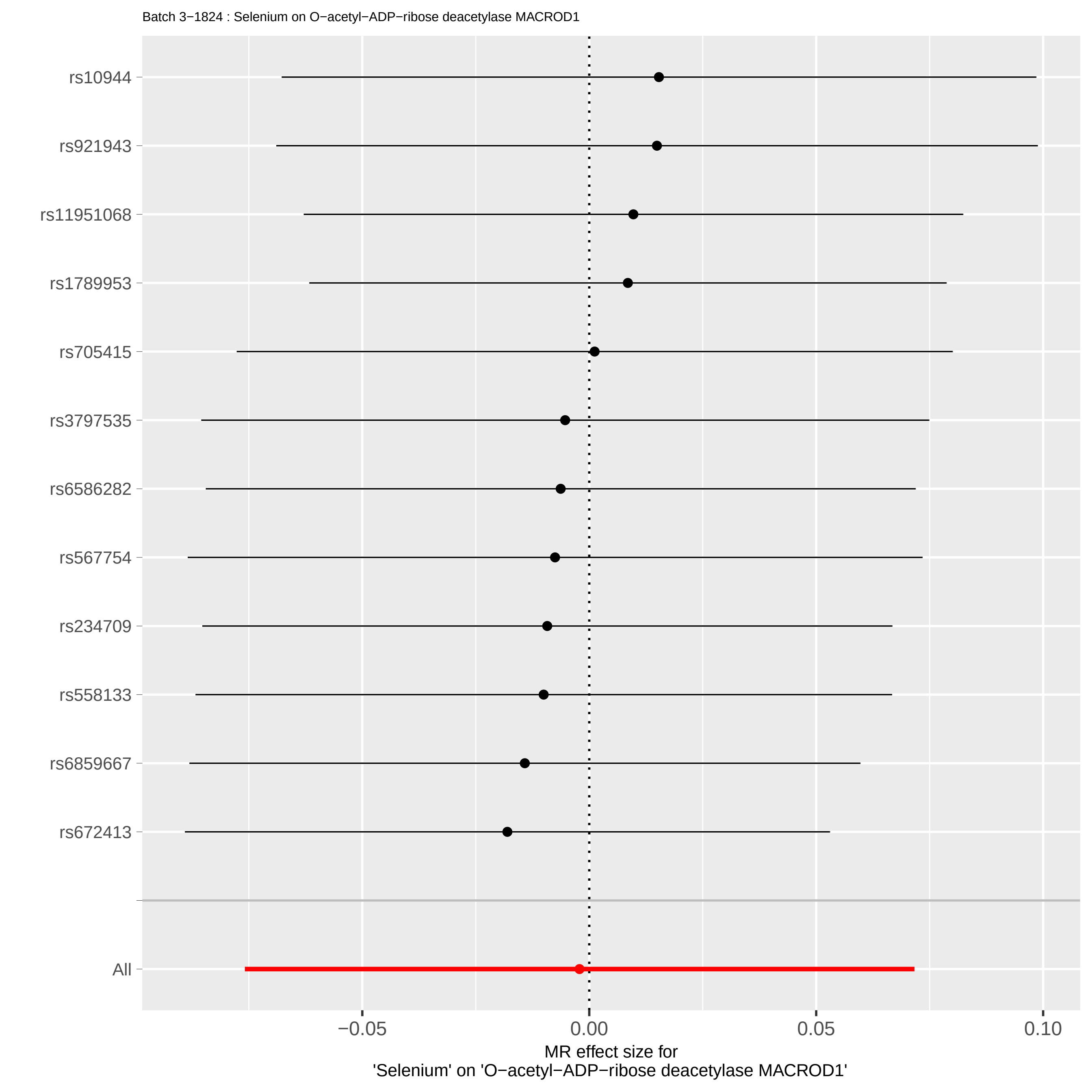

Batch 3−1824 : Selenium on O−acetyl−ADP−ribose deacetylase MACROD1
rs10944
rs921943
rs11951068
rs1789953
rs705415
rs3797535
rs6586282
rs567754
rs234709
rs558133
rs6859667
rs672413
All
−0.05
0.00
0.05
0.10
MR effect size for
'Selenium' on 'O−acetyl−ADP−ribose deacetylase MACROD1'

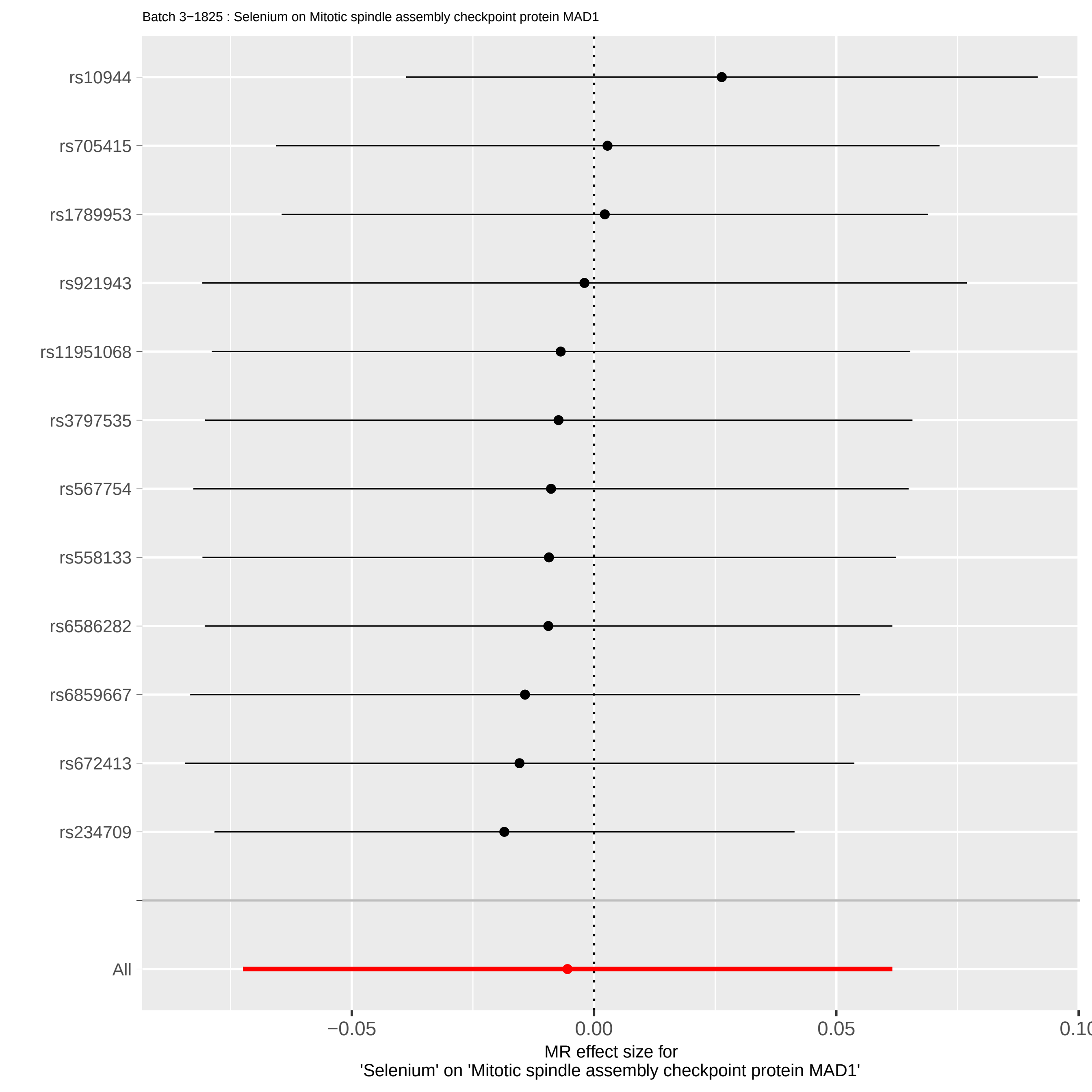

Batch 3−1825 : Selenium on Mitotic spindle assembly checkpoint protein MAD1
rs10944
rs705415
rs1789953
rs921943
rs11951068
rs3797535
rs567754
rs558133
rs6586282
rs6859667
rs672413
rs234709
All
−0.05
0.00
0.05
0.10
MR effect size for
'Selenium' on 'Mitotic spindle assembly checkpoint protein MAD1'

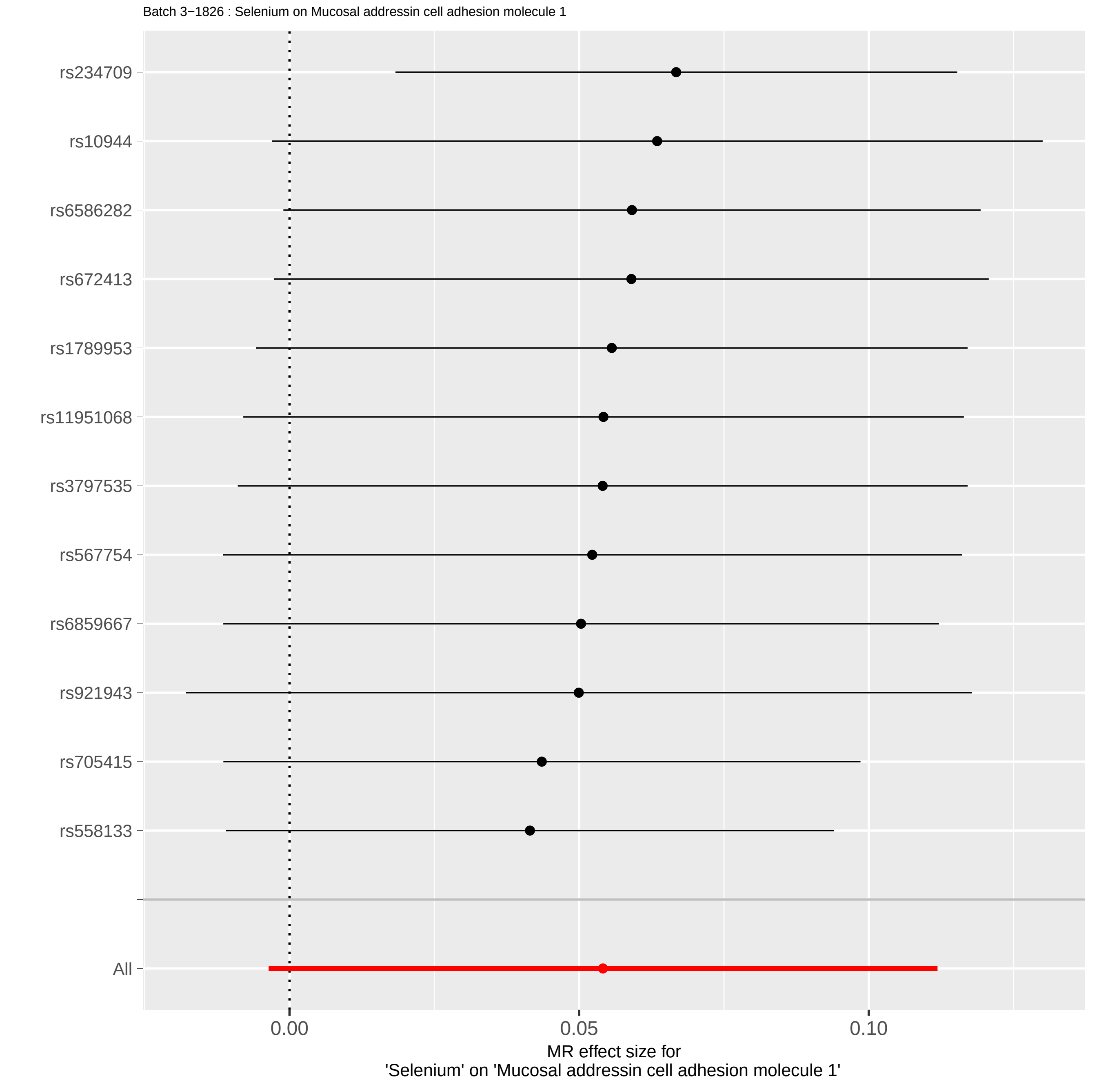

Batch 3−1826 : Selenium on Mucosal addressin cell adhesion molecule 1
rs234709
rs10944
rs6586282
rs672413
rs1789953
rs11951068
rs3797535
rs567754
rs6859667
rs921943
rs705415
rs558133
All
0.00
0.05
0.10
MR effect size for
'Selenium' on 'Mucosal addressin cell adhesion molecule 1'

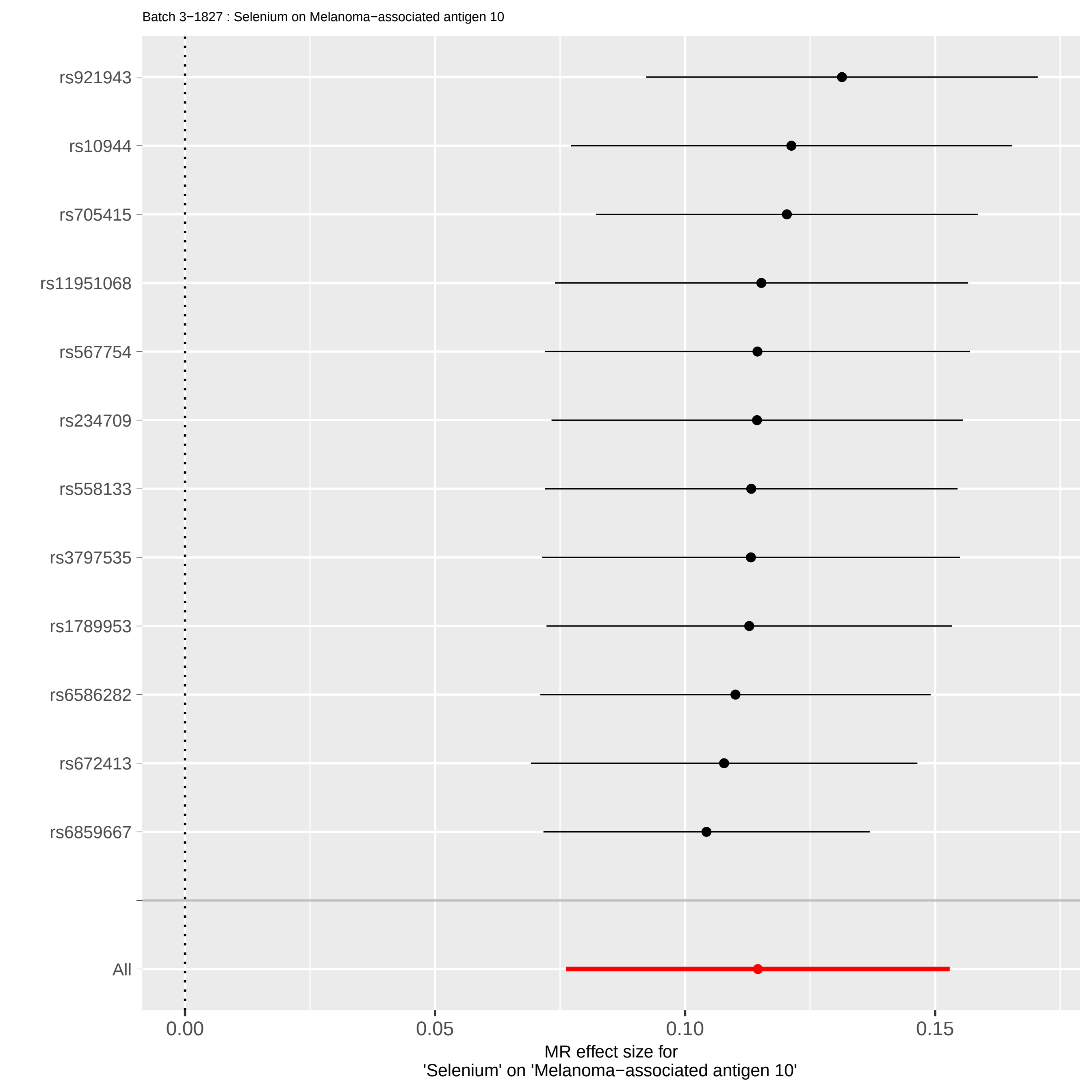
Batch 3−1827 : Selenium on Melanoma−associated antigen 10
rs921943
rs10944
rs705415
rs11951068
rs567754
rs234709
rs558133
rs3797535
rs1789953
rs6586282
rs672413
rs6859667
All
0.00
0.05
0.10
0.15
MR effect size for
'Selenium' on 'Melanoma−associated antigen 10'

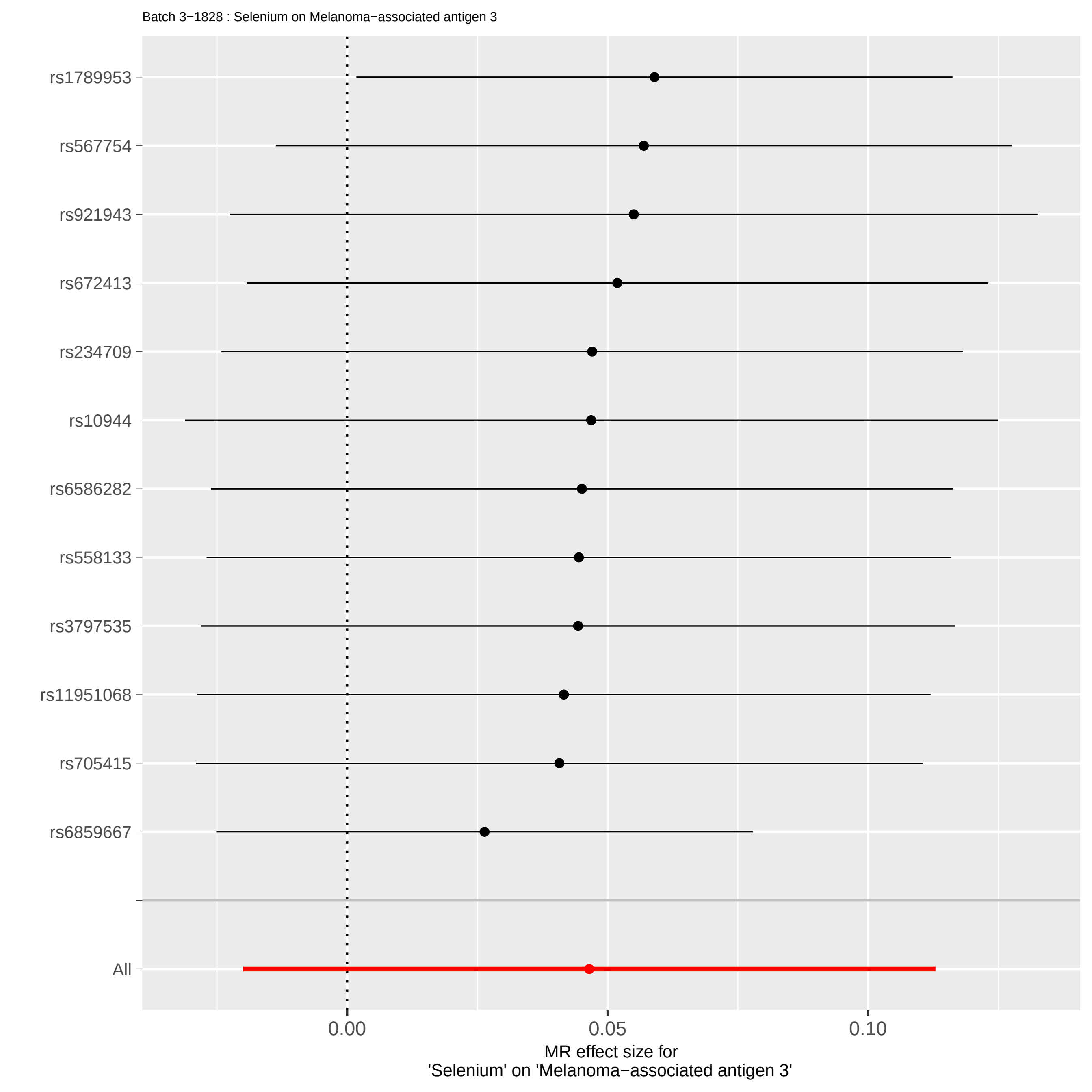

Batch 3−1828 : Selenium on Melanoma−associated antigen 3
rs1789953
rs567754
rs921943
rs672413
rs234709
rs10944
rs6586282
rs558133
rs3797535
rs11951068
rs705415
rs6859667
All
0.00
0.05
0.10
MR effect size for
'Selenium' on 'Melanoma−associated antigen 3'

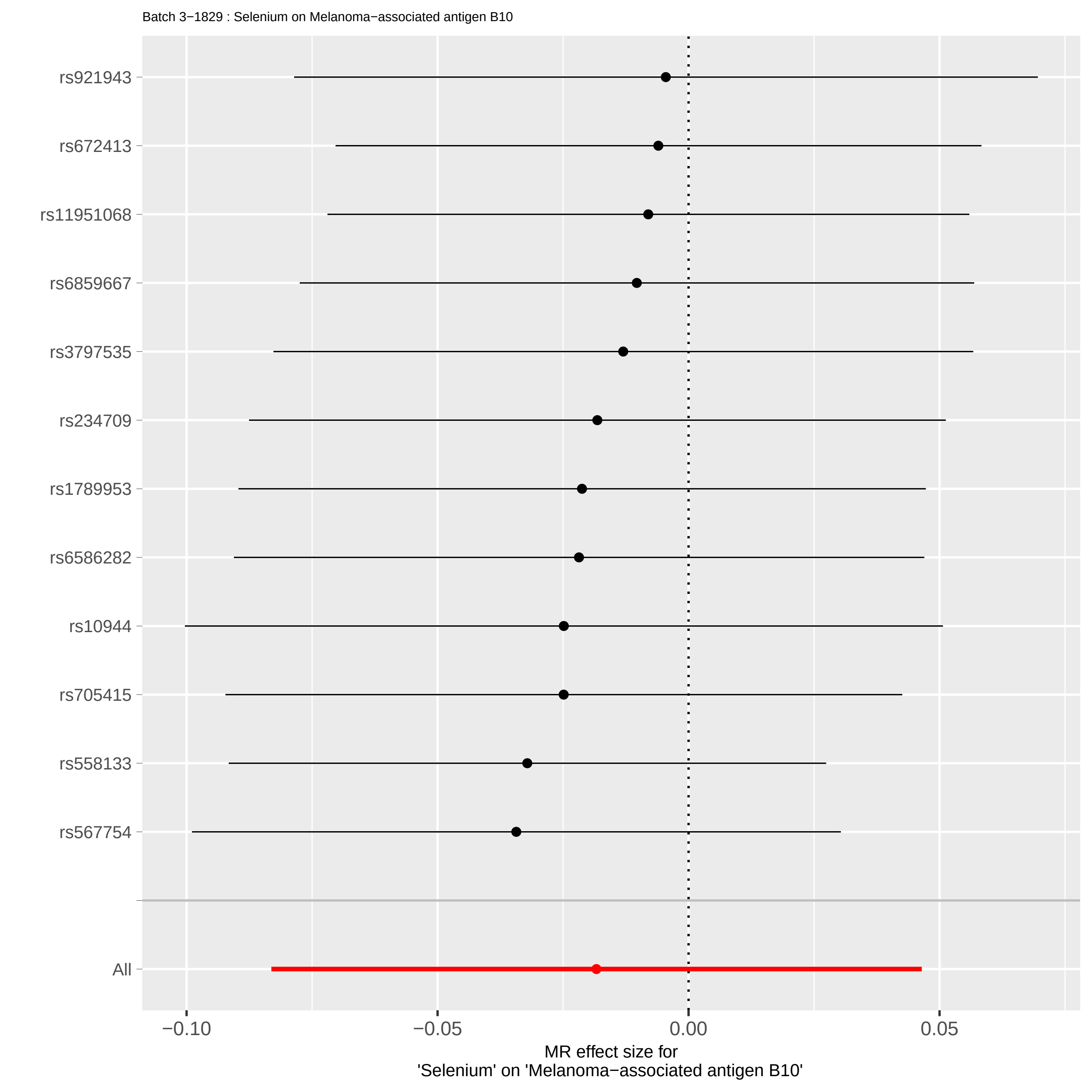

Batch 3−1829 : Selenium on Melanoma−associated antigen B10
rs921943
rs672413
rs11951068
rs6859667
rs3797535
rs234709
rs1789953
rs6586282
rs10944
rs705415
rs558133
rs567754
All
−0.10
−0.05
0.00
0.05
MR effect size for
'Selenium' on 'Melanoma−associated antigen B10'

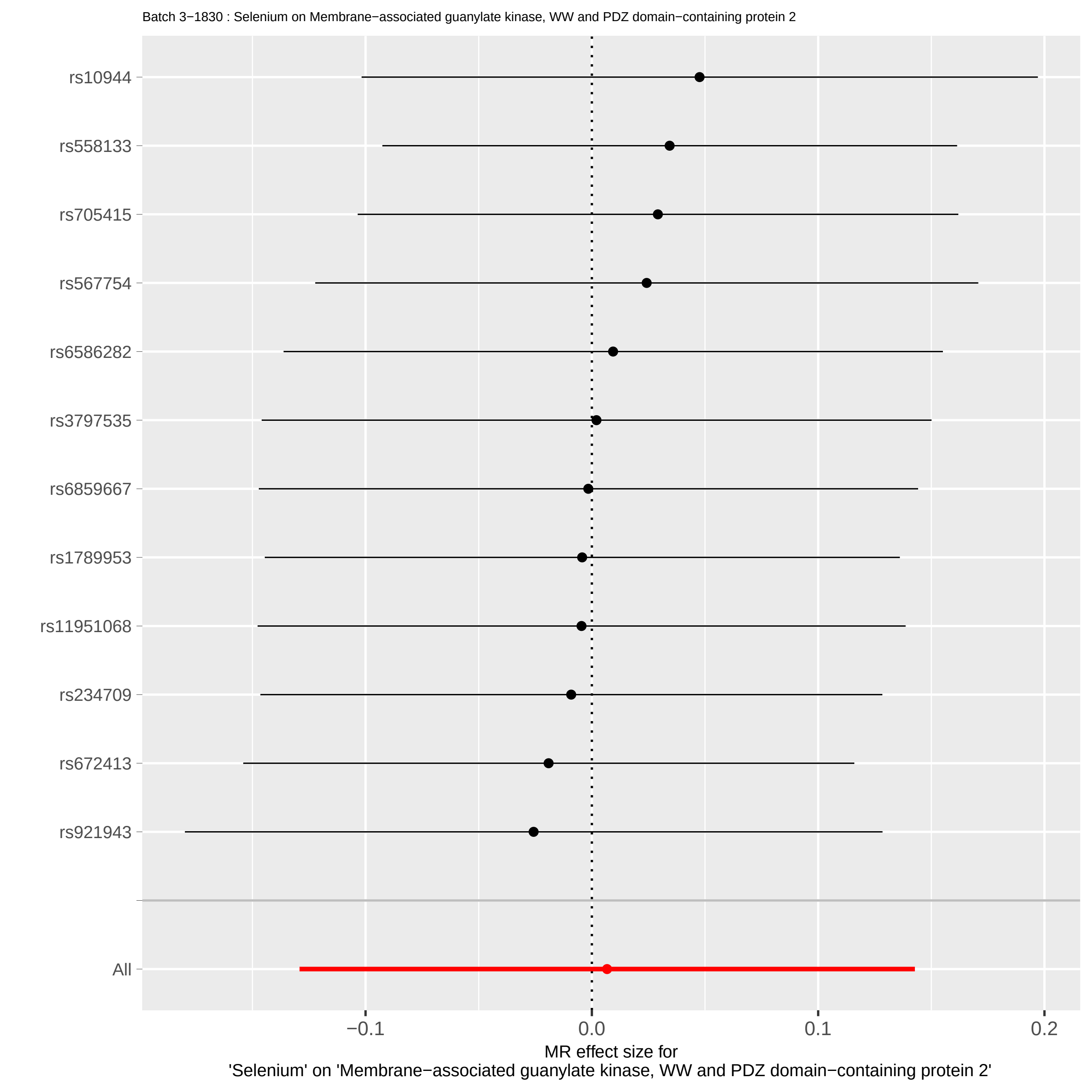
Batch 3−1830 : Selenium on Membrane−associated guanylate kinase, WW and PDZ domain−containing protein 2
rs10944
rs558133
rs705415
rs567754
rs6586282
rs3797535
rs6859667
rs1789953
rs11951068
rs234709
rs672413
rs921943
All
−0.1
0.0
0.1
0.2
MR effect size for
'Selenium' on 'Membrane−associated guanylate kinase, WW and PDZ domain−containing protein 2'

Batch 3−1831 : Selenium on MAM domain−containing protein 2

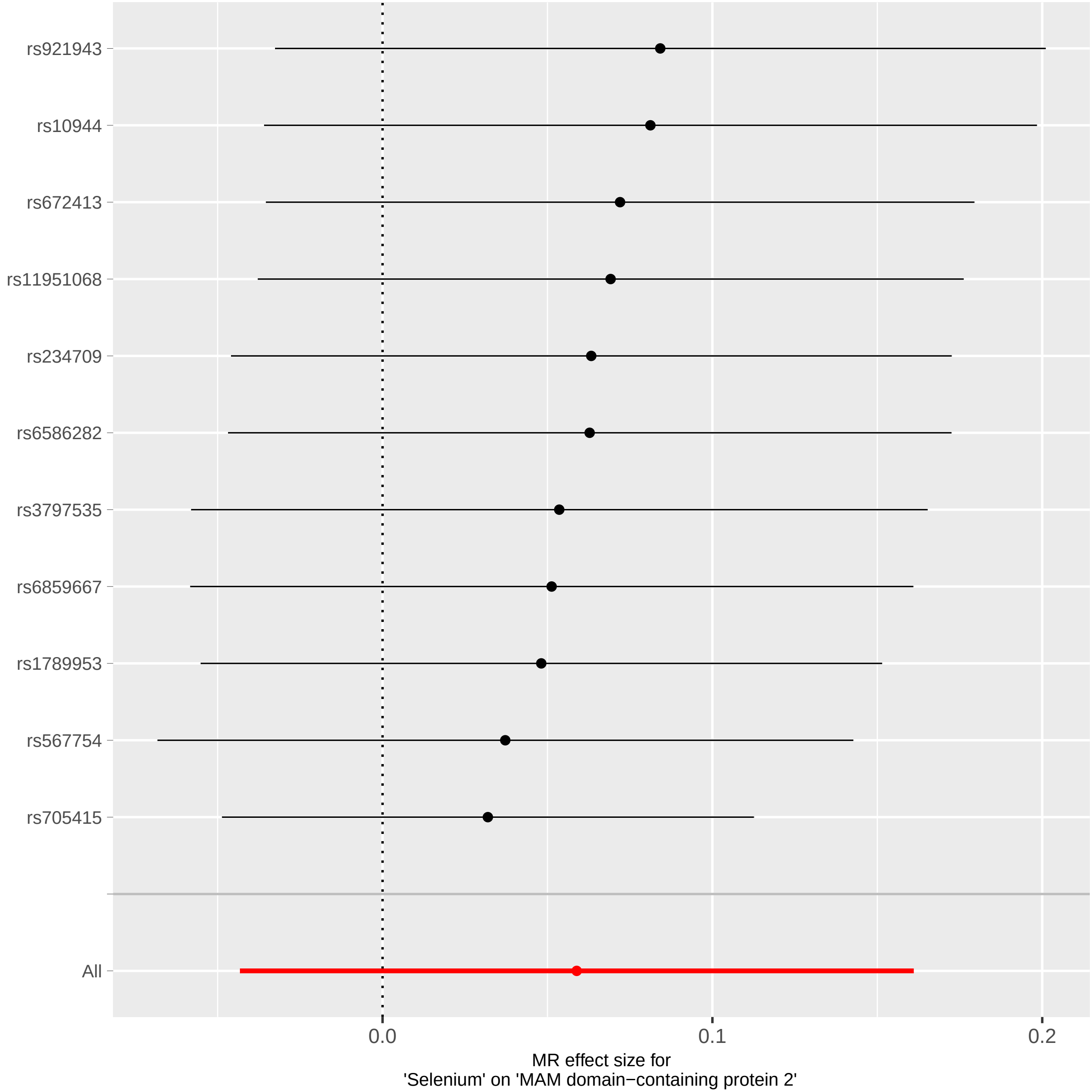

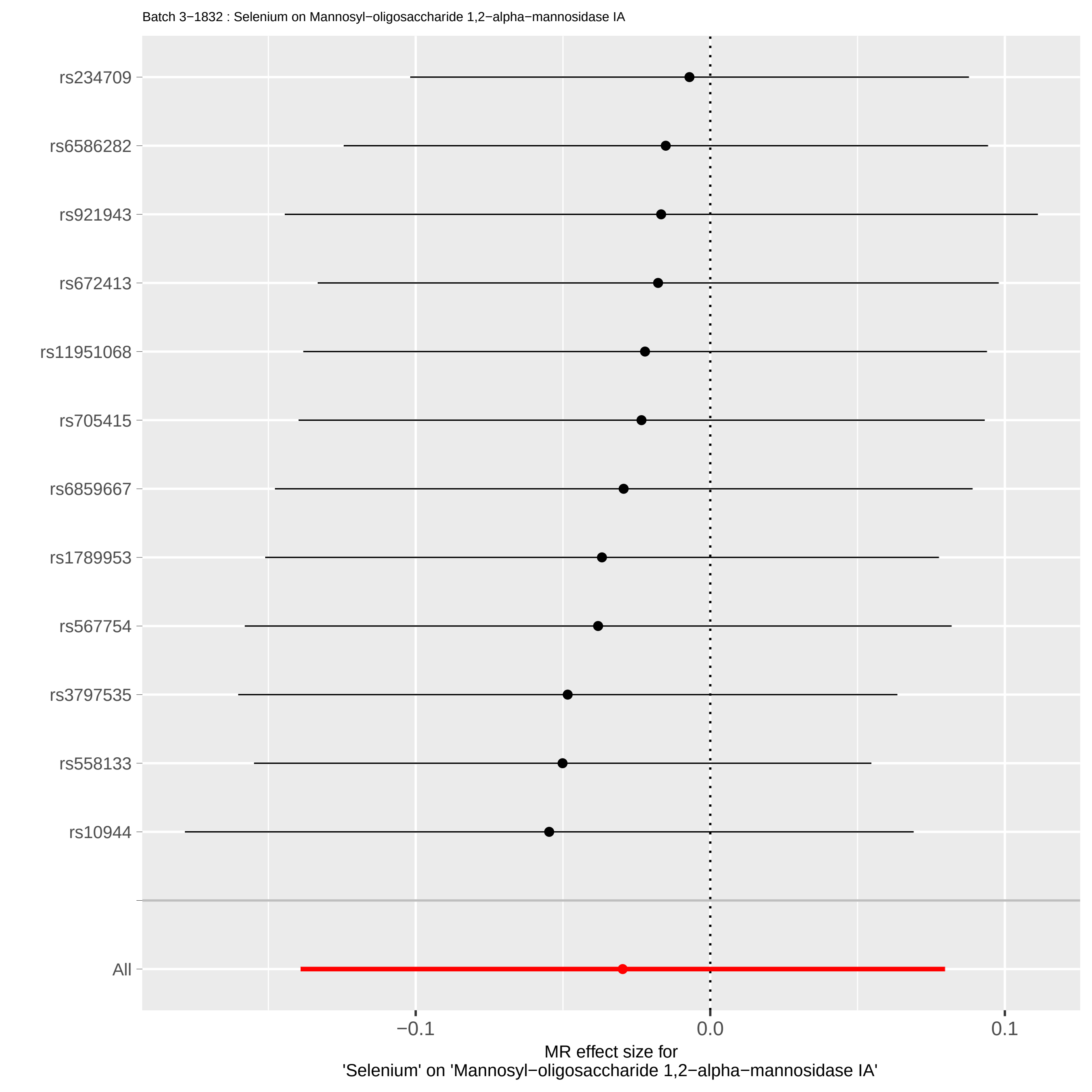

Batch 3−1832 : Selenium on Mannosyl−oligosaccharide 1,2−alpha−mannosidase IA
rs234709
rs6586282
rs921943
rs672413
rs11951068
rs705415
rs6859667
rs1789953
rs567754
rs3797535
rs558133
rs10944
All
−0.1
0.0
0.1
MR effect size for
'Selenium' on 'Mannosyl−oligosaccharide 1,2−alpha−mannosidase IA'

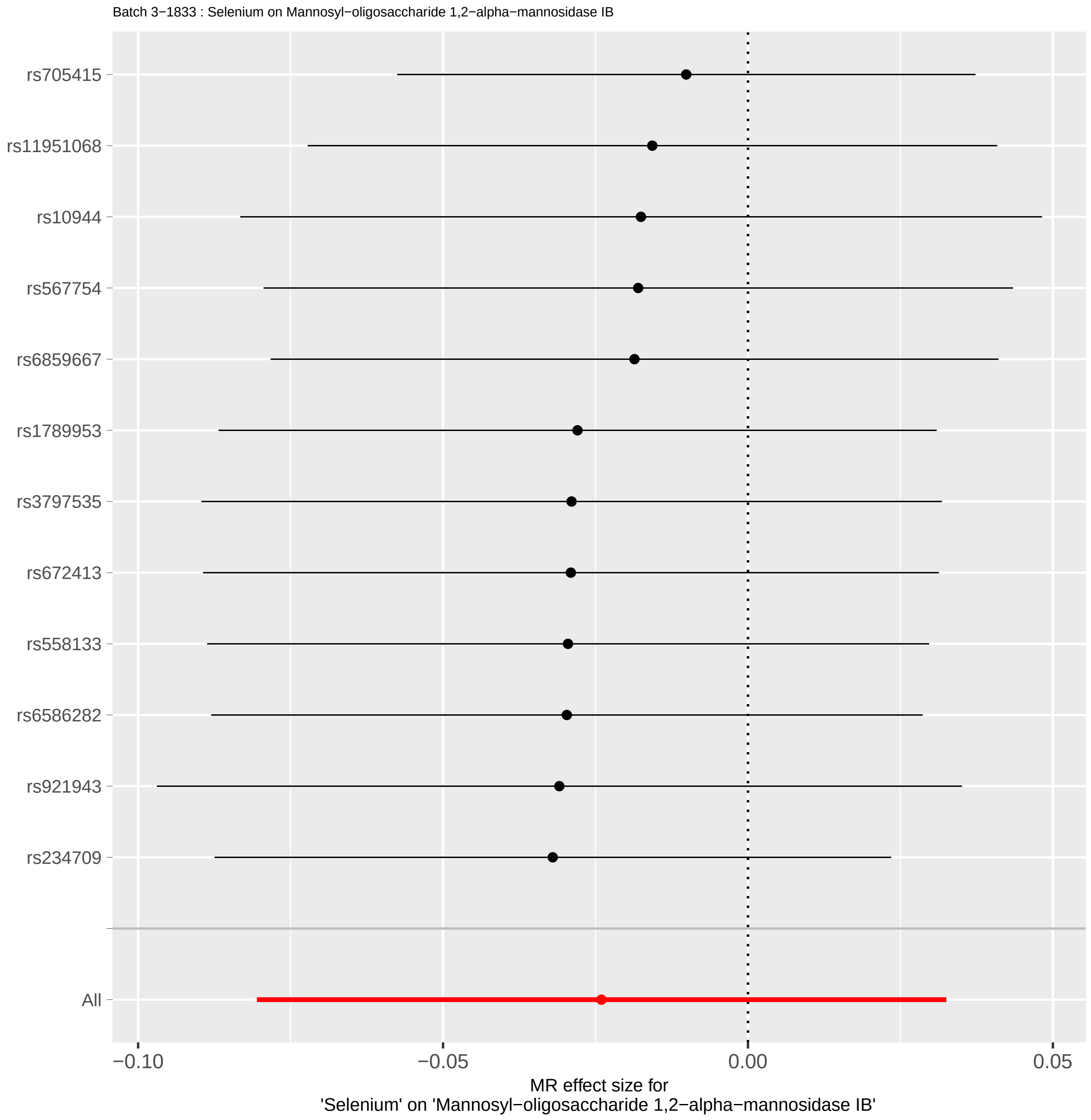
Batch 3−1833 : Selenium on Mannosyl−oligosaccharide 1,2−alpha−mannosidase IB
rs705415
rs11951068
rs10944
rs567754
rs6859667
rs1789953
rs3797535
rs672413
rs558133
rs6586282
rs921943
rs234709
All
−0.10
−0.05
0.00
0.05
MR effect size for
'Selenium' on 'Mannosyl−oligosaccharide 1,2−alpha−mannosidase IB'

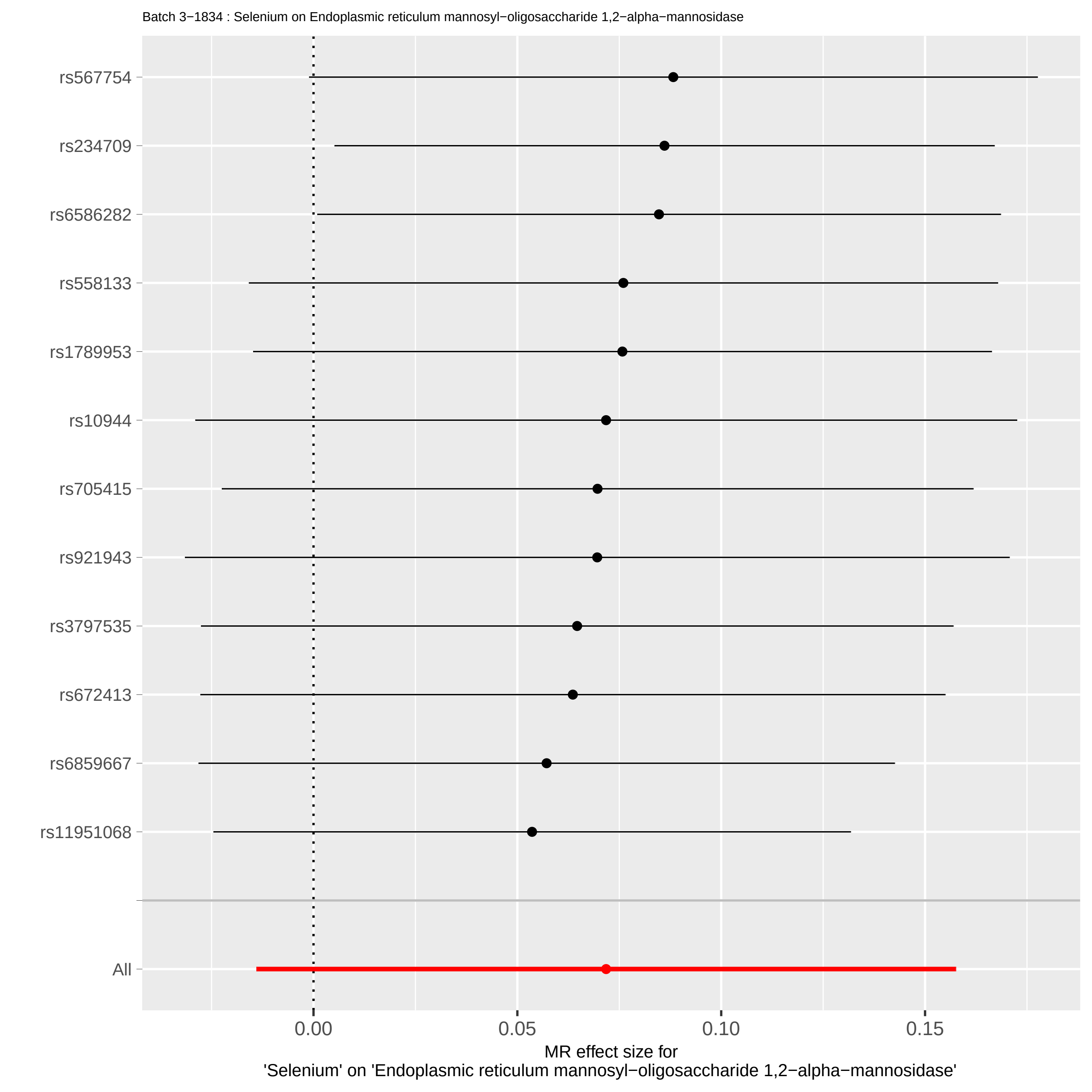

Batch 3−1834 : Selenium on Endoplasmic reticulum mannosyl−oligosaccharide 1,2−alpha−mannosidase
rs567754
rs234709
rs6586282
rs558133
rs1789953
rs10944
rs705415
rs921943
rs3797535
rs672413
rs6859667
rs11951068
All
0.00
0.05
0.10
0.15
MR effect size for
'Selenium' on 'Endoplasmic reticulum mannosyl−oligosaccharide 1,2−alpha−mannosidase'

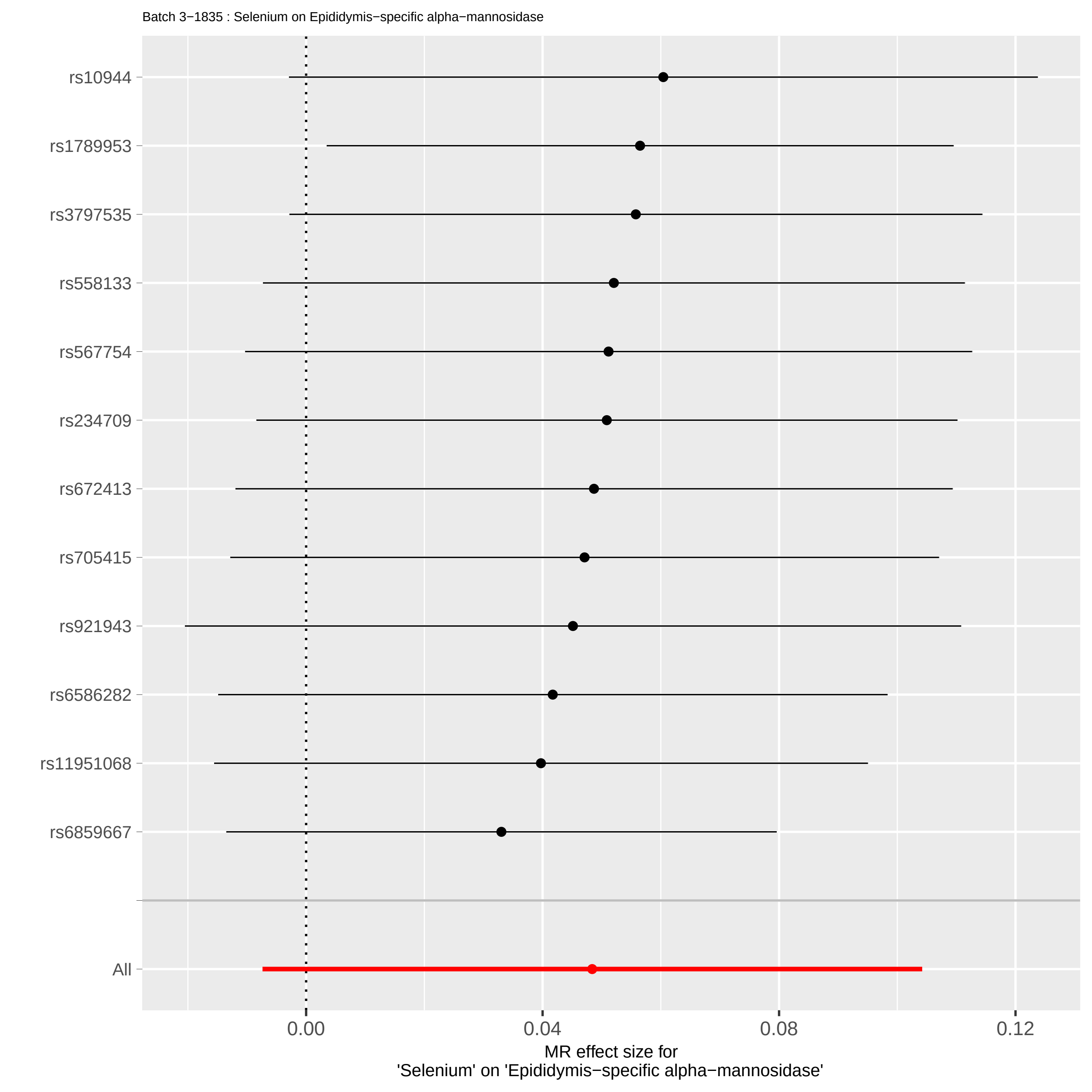
Batch 3−1835 : Selenium on Epididymis−specific alpha−mannosidase
rs10944
rs1789953
rs3797535
rs558133
rs567754
rs234709
rs672413
rs705415
rs921943
rs6586282
rs11951068
rs6859667
All
0.00
0.04
0.08
0.12
MR effect size for
'Selenium' on 'Epididymis−specific alpha−mannosidase'

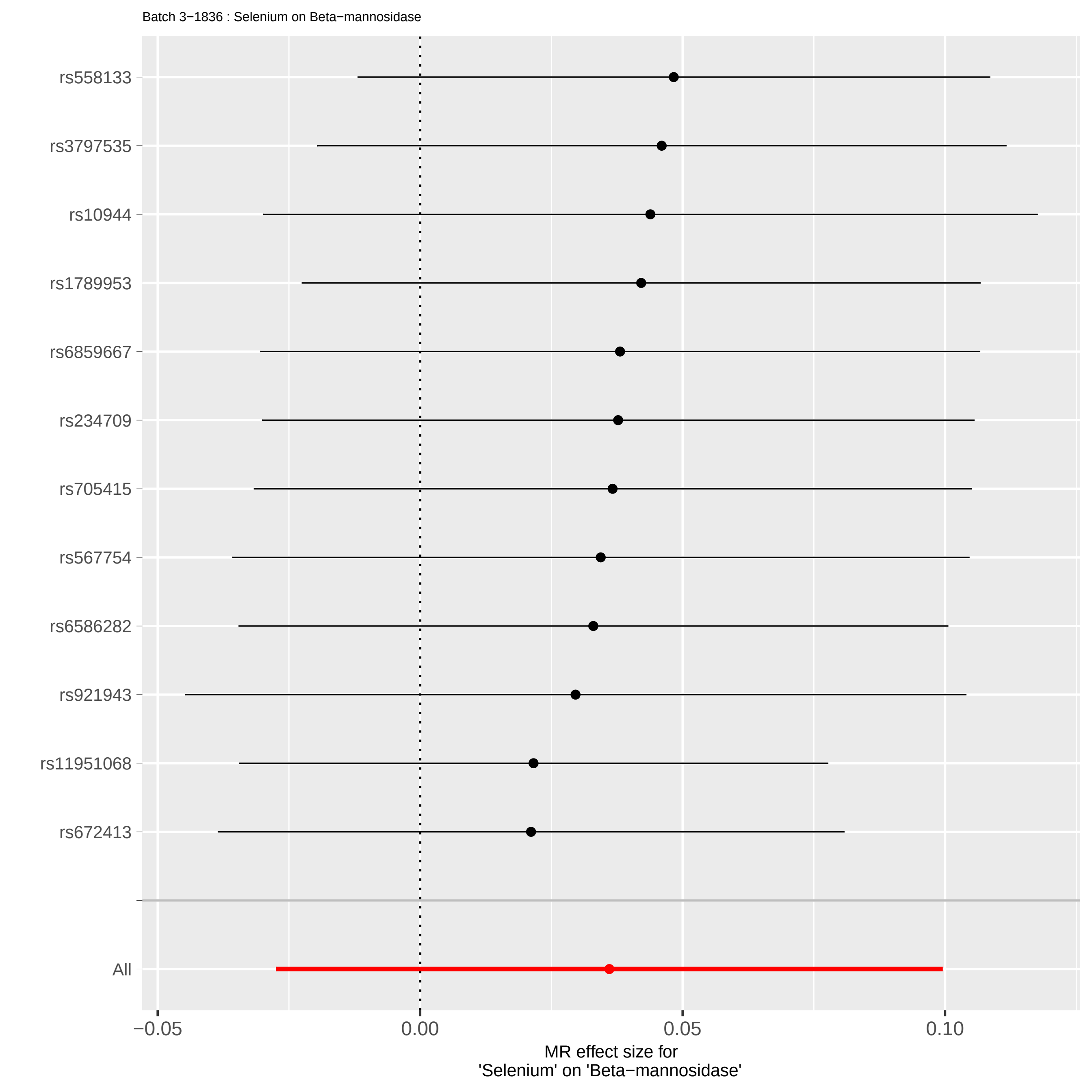
Batch 3−1836 : Selenium on Beta−mannosidase
rs558133
rs3797535
rs10944
rs1789953
rs6859667
rs234709
rs705415
rs567754
rs6586282
rs921943
rs11951068
rs672413
All
−0.05
0.00
0.05
0.10
MR effect size for
'Selenium' on 'Beta−mannosidase'

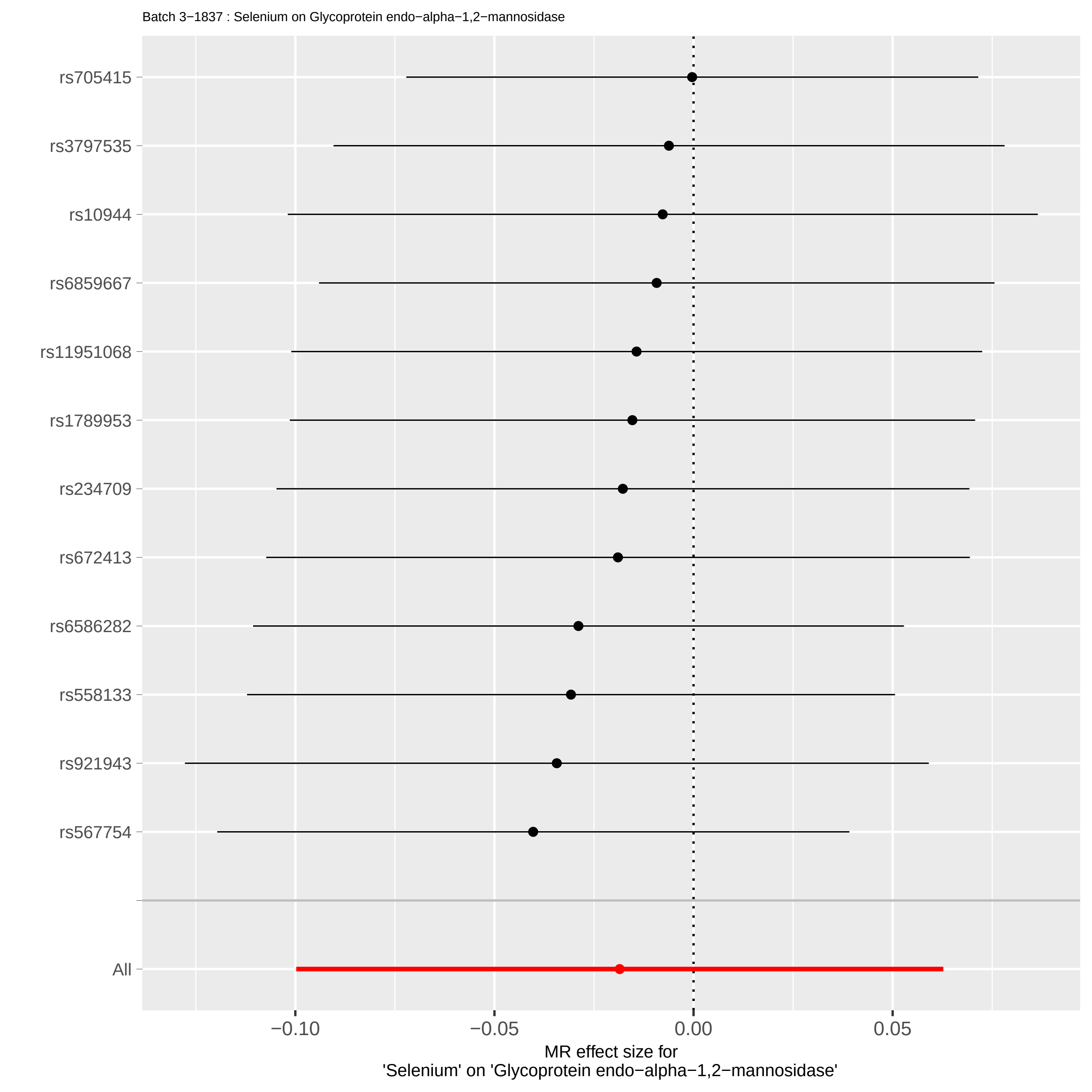

Batch 3−1837 : Selenium on Glycoprotein endo−alpha−1,2−mannosidase
rs705415
rs3797535
rs10944
rs6859667
rs11951068
rs1789953
rs234709
rs672413
rs6586282
rs558133
rs921943
rs567754
All
−0.10
−0.05
0.00
0.05
MR effect size for
'Selenium' on 'Glycoprotein endo−alpha−1,2−mannosidase'

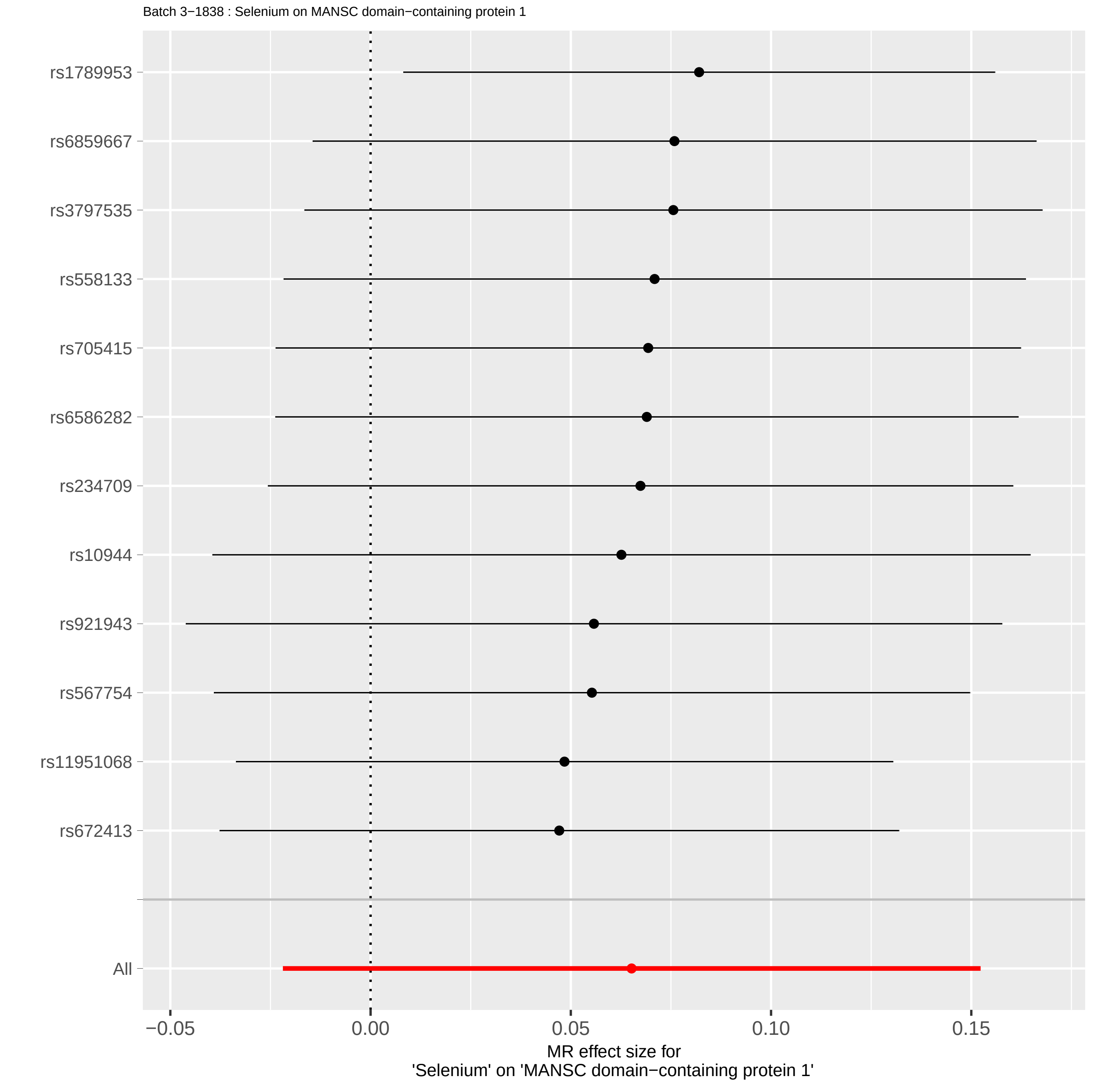

Batch 3−1838 : Selenium on MANSC domain−containing protein 1
rs1789953
rs6859667
rs3797535
rs558133
rs705415
rs6586282
rs234709
rs10944
rs921943
rs567754
rs11951068
rs672413
All
−0.05
0.00
0.05
0.10
0.15
MR effect size for
'Selenium' on 'MANSC domain−containing protein 1'

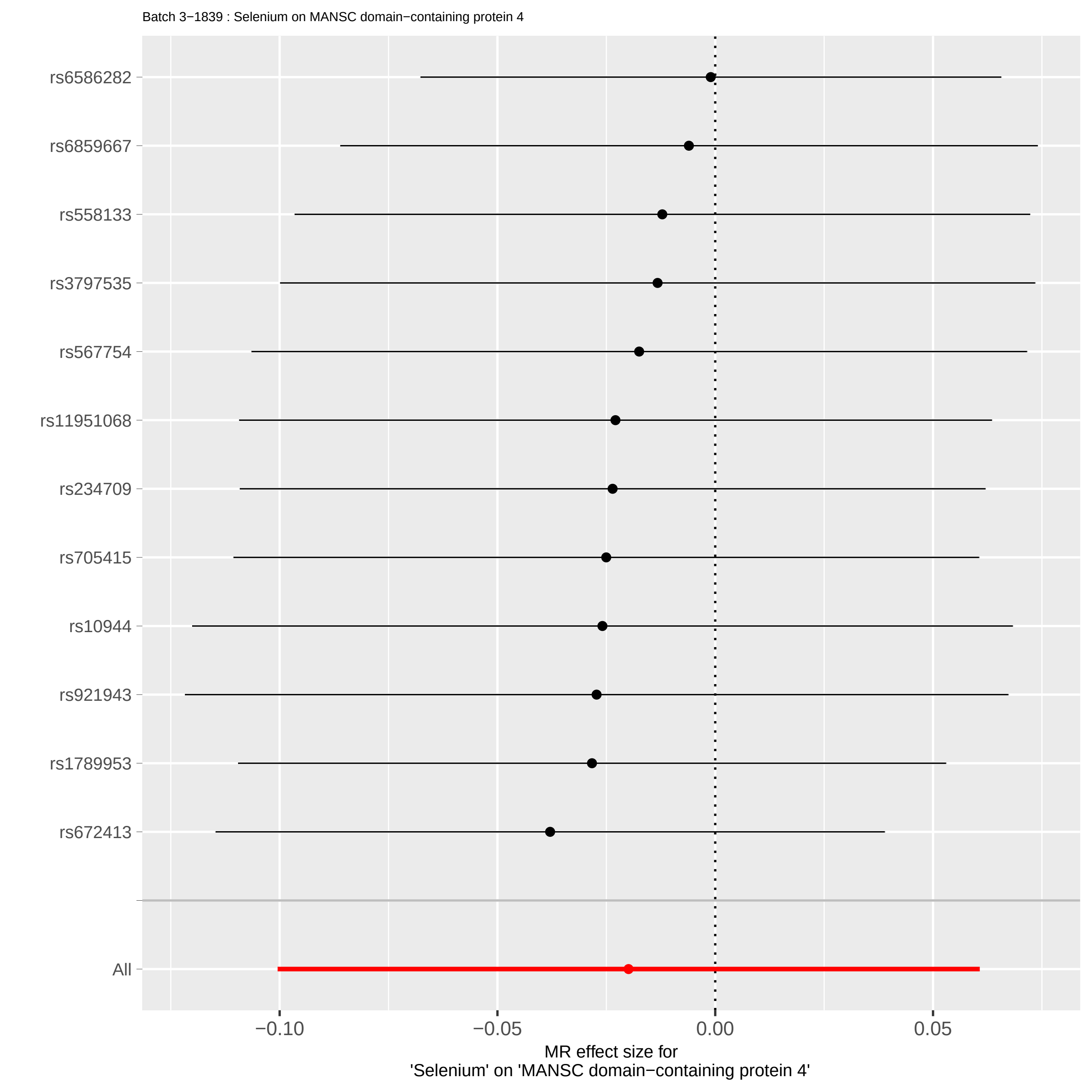

Batch 3−1839 : Selenium on MANSC domain−containing protein 4
rs6586282
rs6859667
rs558133
rs3797535
rs567754
rs11951068
rs234709
rs705415
rs10944
rs921943
rs1789953
rs672413
All
−0.10
−0.05
0.00
0.05
MR effect size for
'Selenium' on 'MANSC domain−containing protein 4'

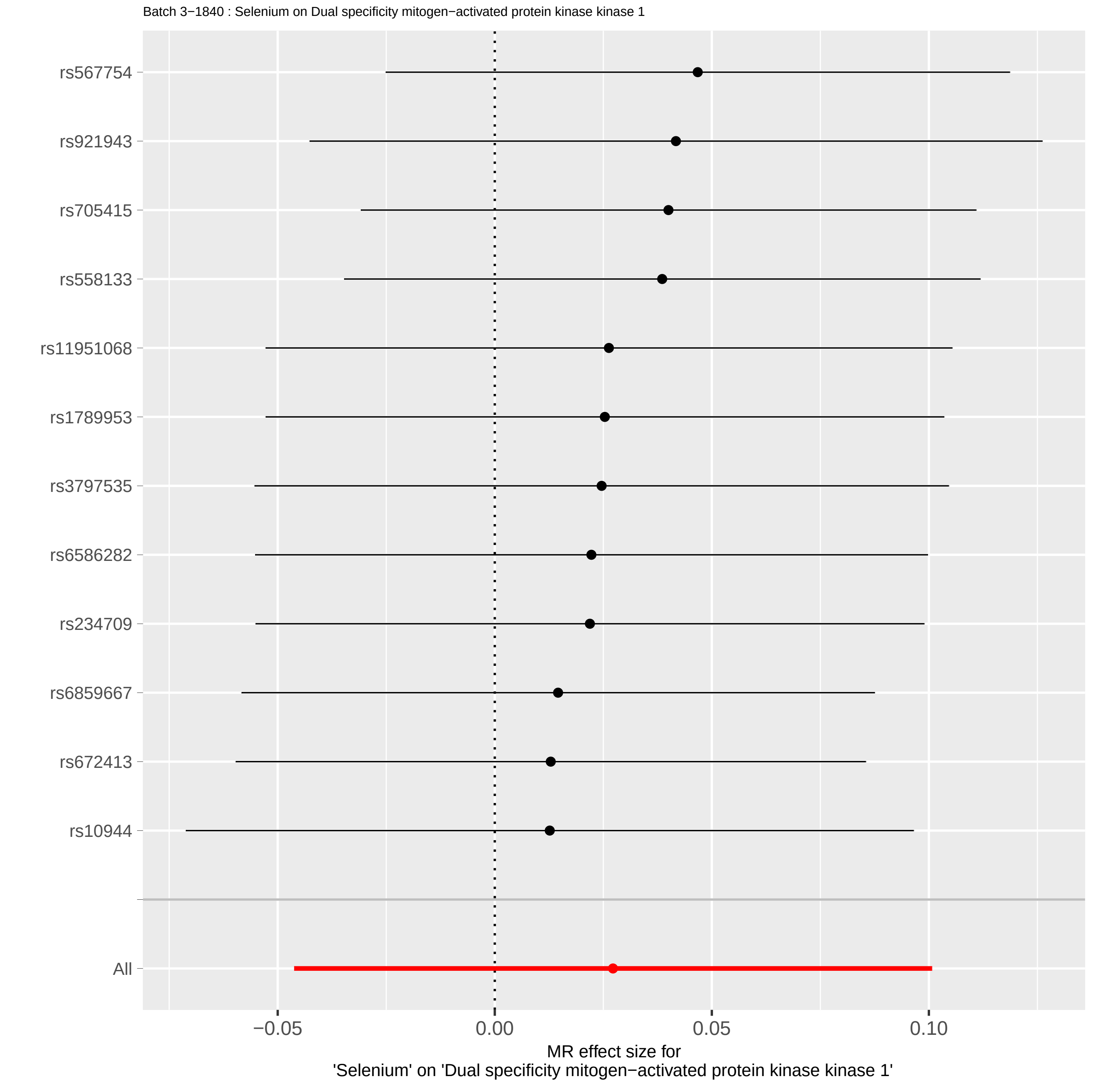

Batch 3−1840 : Selenium on Dual specificity mitogen−activated protein kinase kinase 1
rs567754
rs921943
rs705415
rs558133
rs11951068
rs1789953
rs3797535
rs6586282
rs234709
rs6859667
rs672413
rs10944
All
−0.05
0.00
0.05
0.10
MR effect size for
'Selenium' on 'Dual specificity mitogen−activated protein kinase kinase 1'

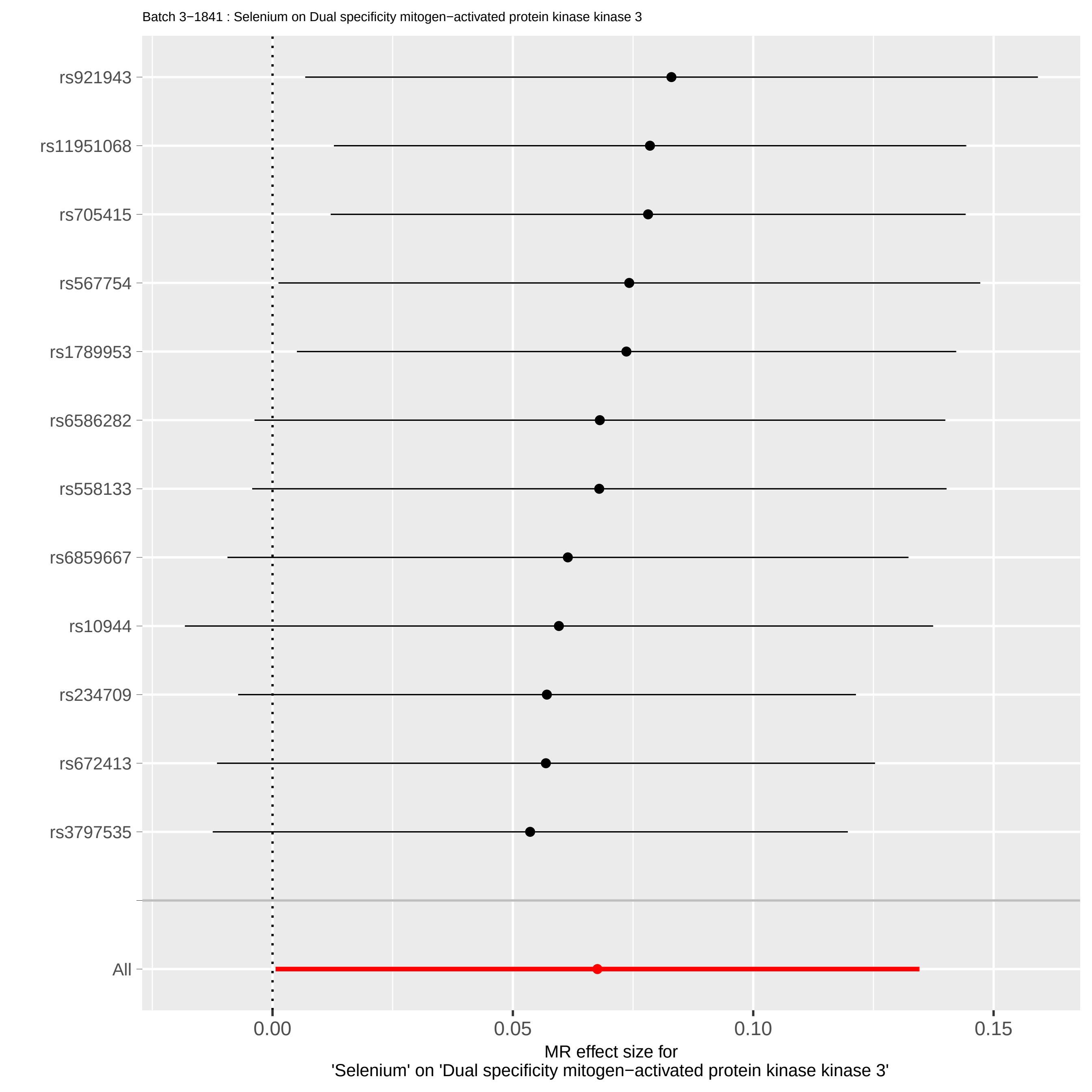

Batch 3−1841 : Selenium on Dual specificity mitogen−activated protein kinase kinase 3
rs921943
rs11951068
rs705415
rs567754
rs1789953
rs6586282
rs558133
rs6859667
rs10944
rs234709
rs672413
rs3797535
All
0.00
0.05
0.10
0.15
MR effect size for
'Selenium' on 'Dual specificity mitogen−activated protein kinase kinase 3'

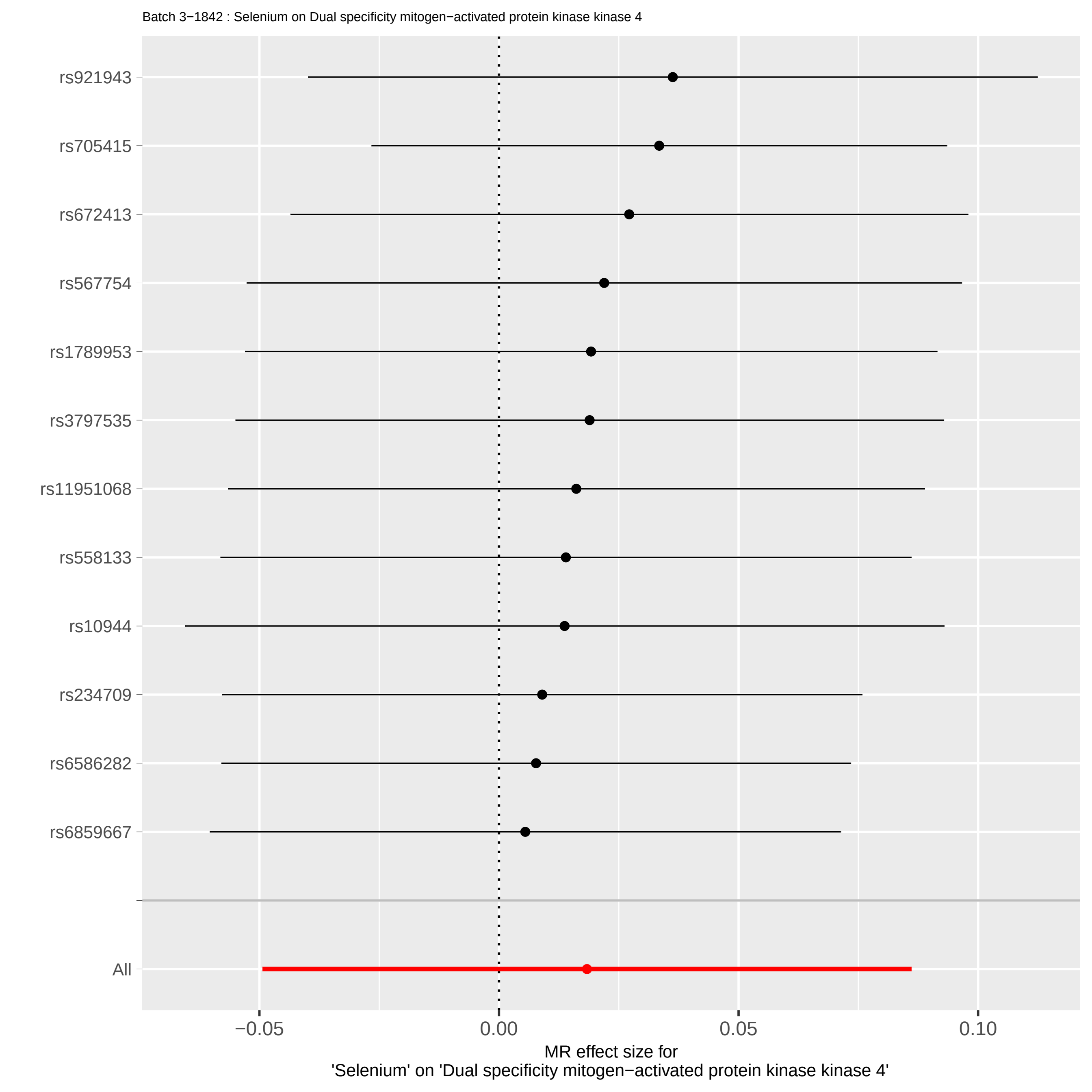

Batch 3−1842 : Selenium on Dual specificity mitogen−activated protein kinase kinase 4
rs921943
rs705415
rs672413
rs567754
rs1789953
rs3797535
rs11951068
rs558133
rs10944
rs234709
rs6586282
rs6859667
All
−0.05
0.00
0.05
0.10
MR effect size for
'Selenium' on 'Dual specificity mitogen−activated protein kinase kinase 4'

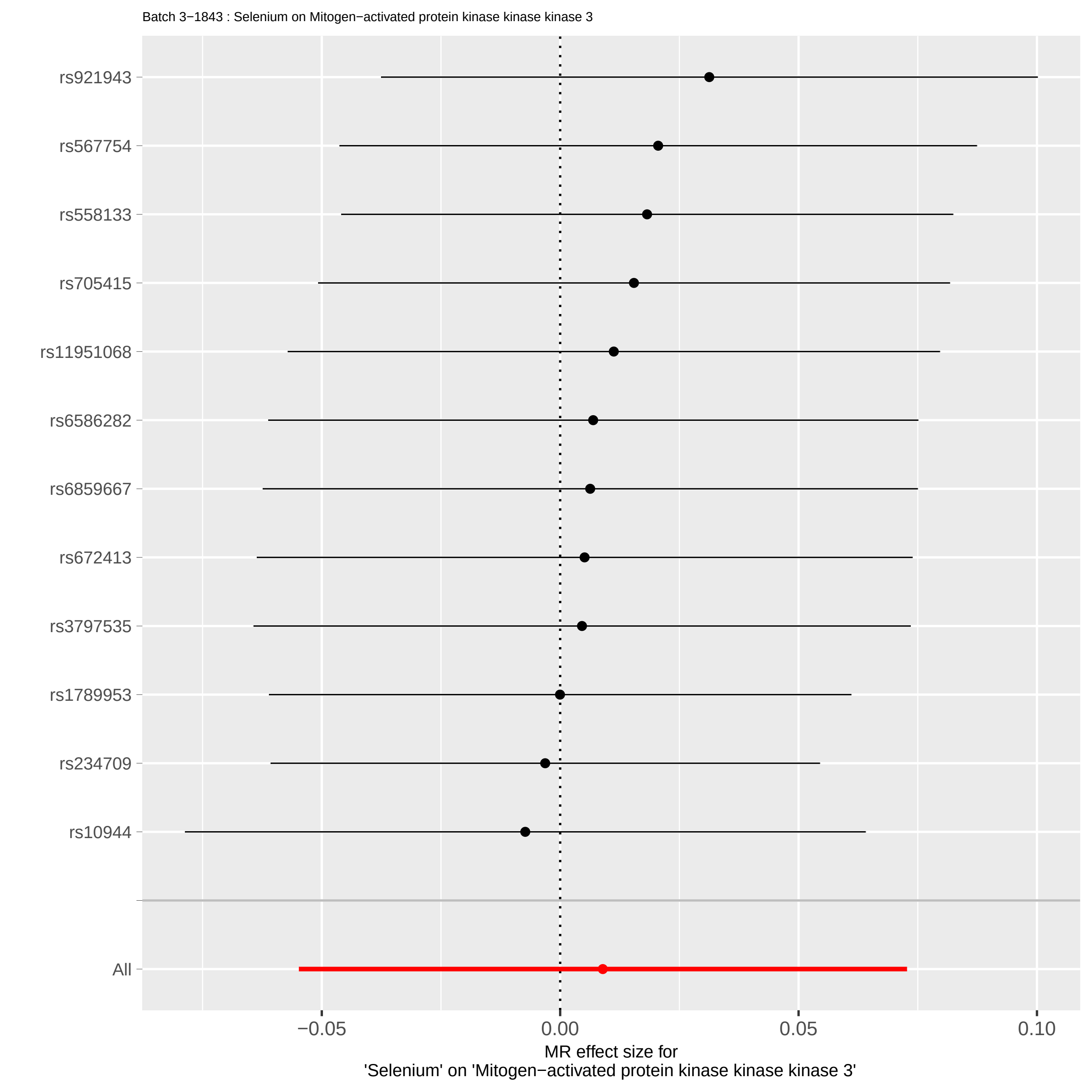

Batch 3−1843 : Selenium on Mitogen−activated protein kinase kinase kinase 3
rs921943
rs567754
rs558133
rs705415
rs11951068
rs6586282
rs6859667
rs672413
rs3797535
rs1789953
rs234709
rs10944
All
−0.05
0.00
0.05
0.10
MR effect size for
'Selenium' on 'Mitogen−activated protein kinase kinase kinase 3'

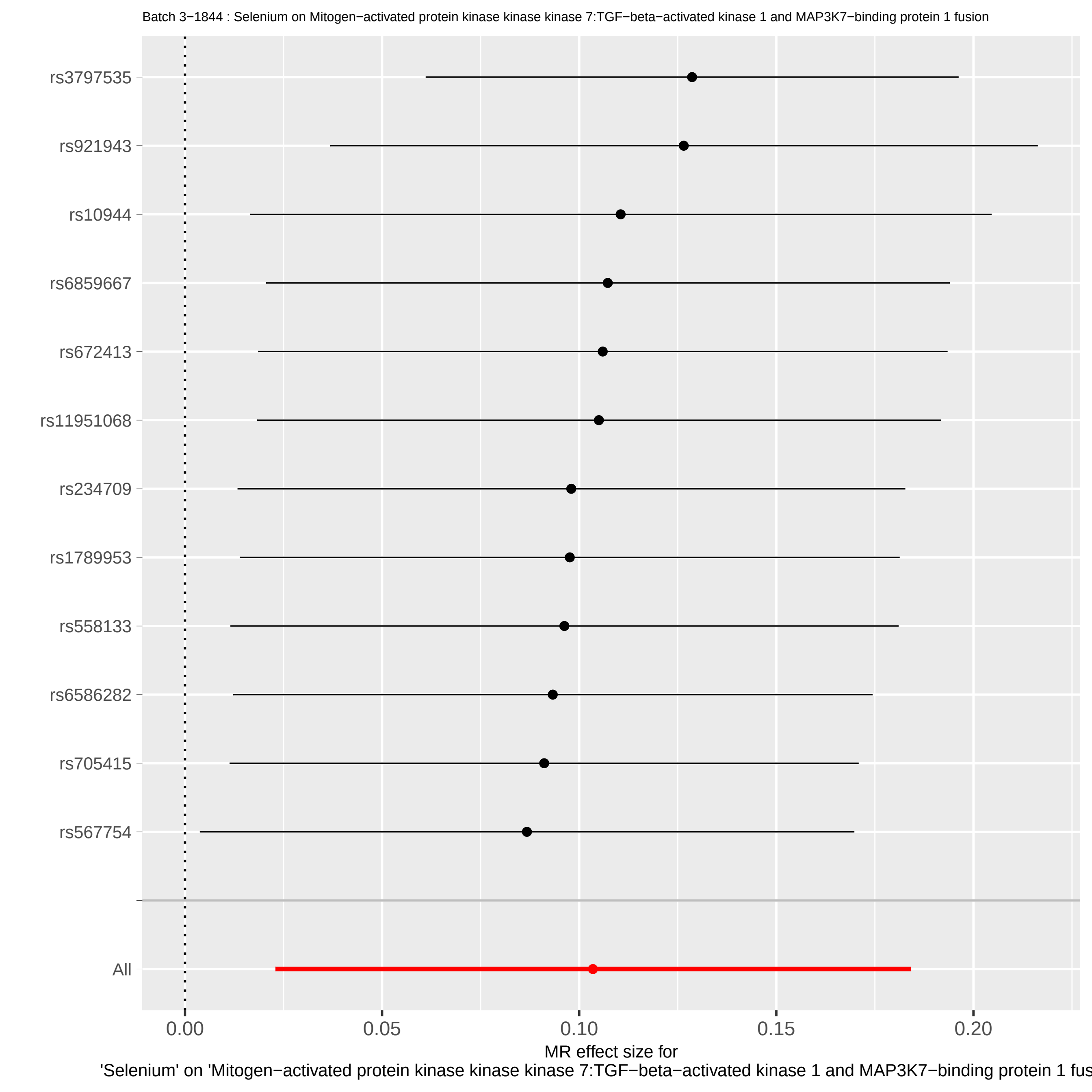

Batch 3−1844 : Selenium on Mitogen−activated protein kinase kinase kinase 7:TGF−beta−activated kinase 1 and MAP3K7−binding protein 1 fusion
rs3797535
rs921943
rs10944
rs6859667
rs672413
rs11951068
rs234709
rs1789953
rs558133
rs6586282
rs705415
rs567754
All
0.00
0.05
0.10
0.15
0.20
MR effect size for
'Selenium' on 'Mitogen−activated protein kinase kinase kinase 7:TGF−beta−activated kinase 1 and MAP3K7−binding protein 1 fus

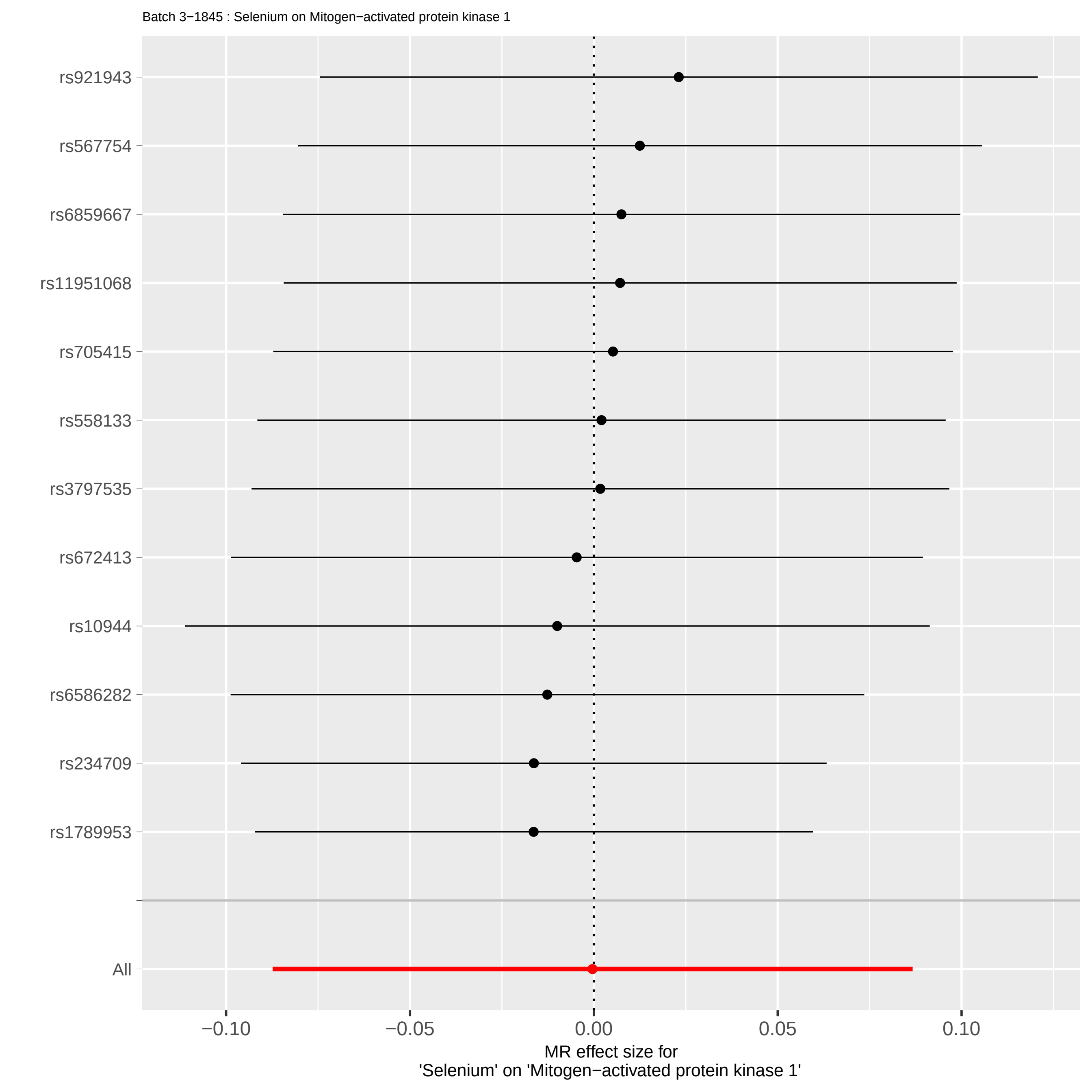
Batch 3−1845 : Selenium on Mitogen−activated protein kinase 1
rs921943
rs567754
rs6859667
rs11951068
rs705415
rs558133
rs3797535
rs672413
rs10944
rs6586282
rs234709
rs1789953
All
−0.10
−0.05
0.00
0.05
0.10
MR effect size for
'Selenium' on 'Mitogen−activated protein kinase 1'

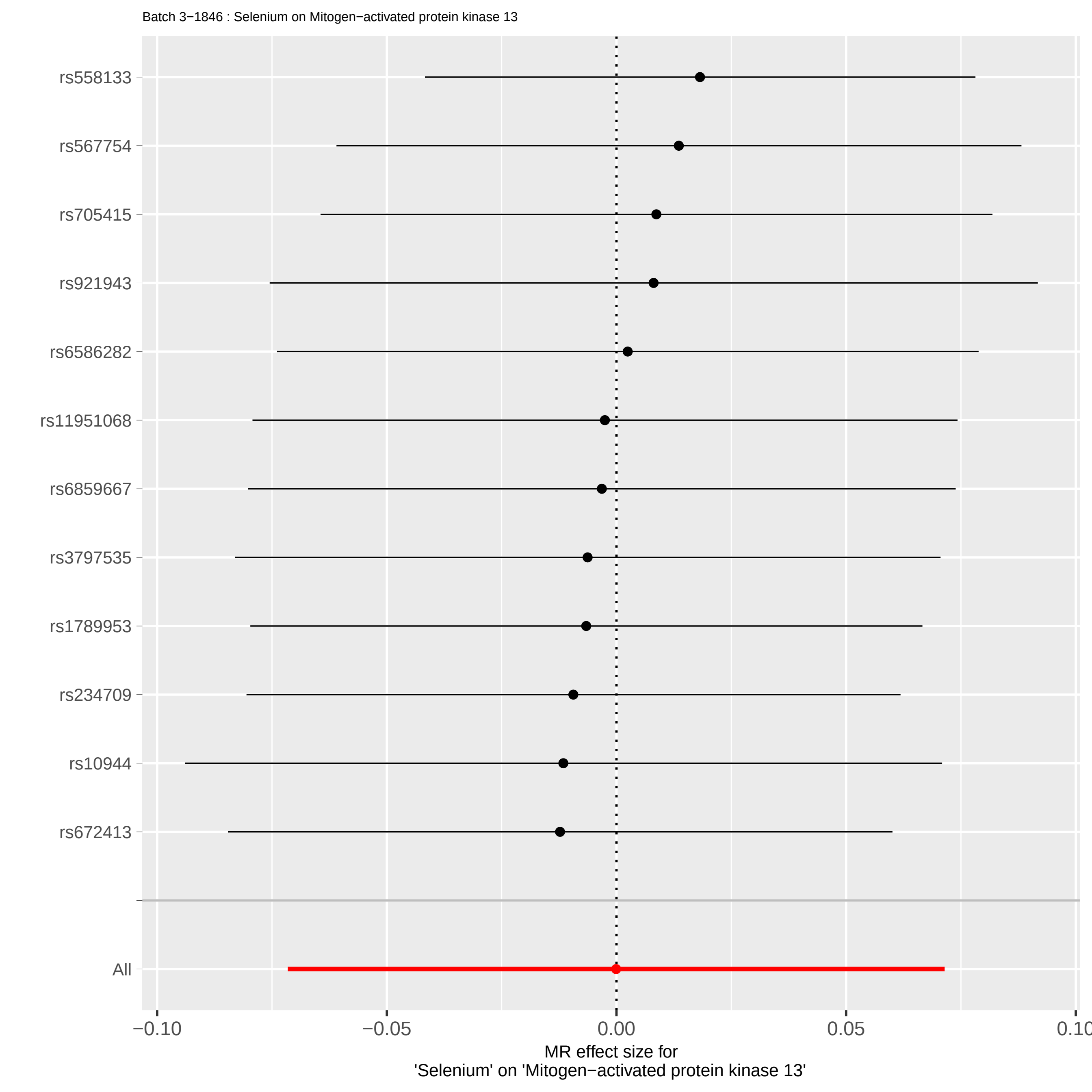
Batch 3−1846 : Selenium on Mitogen−activated protein kinase 13
rs558133
rs567754
rs705415
rs921943
rs6586282
rs11951068
rs6859667
rs3797535
rs1789953
rs234709
rs10944
rs672413
All
−0.10
−0.05
0.00
0.05
0.10
MR effect size for
'Selenium' on 'Mitogen−activated protein kinase 13'

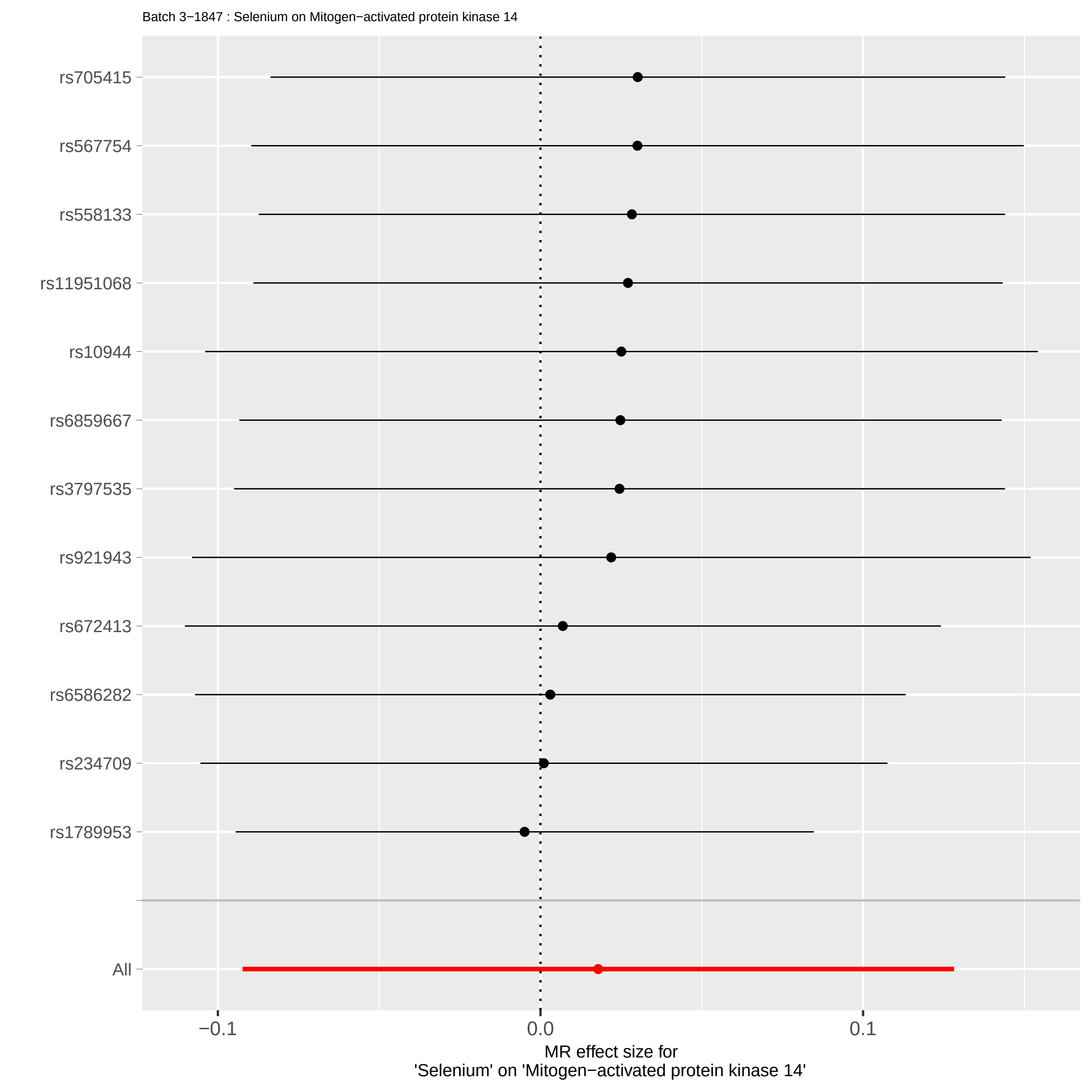

Batch 3−1847 : Selenium on Mitogen−activated protein kinase 14
rs705415
rs567754
rs558133
rs11951068
rs10944
rs6859667
rs3797535
rs921943
rs672413
rs6586282
rs234709
rs1789953
All
−0.1
0.0
0.1
MR effect size for
'Selenium' on 'Mitogen−activated protein kinase 14'

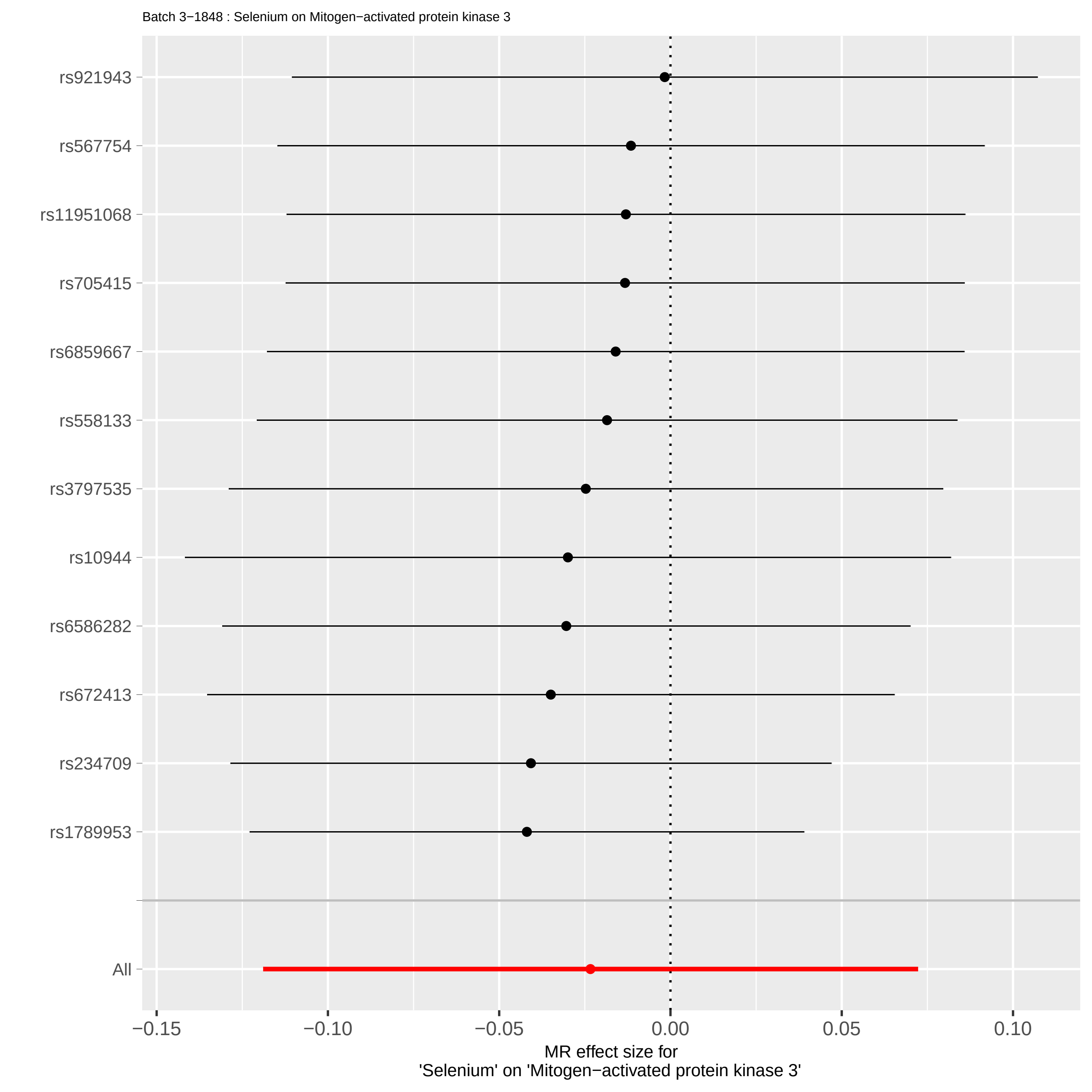
Batch 3−1848 : Selenium on Mitogen−activated protein kinase 3
rs921943
rs567754
rs11951068
rs705415
rs6859667
rs558133
rs3797535
rs10944
rs6586282
rs672413
rs234709
rs1789953
All
−0.15
−0.10
−0.05
0.00
0.05
0.10
MR effect size for
'Selenium' on 'Mitogen−activated protein kinase 3'

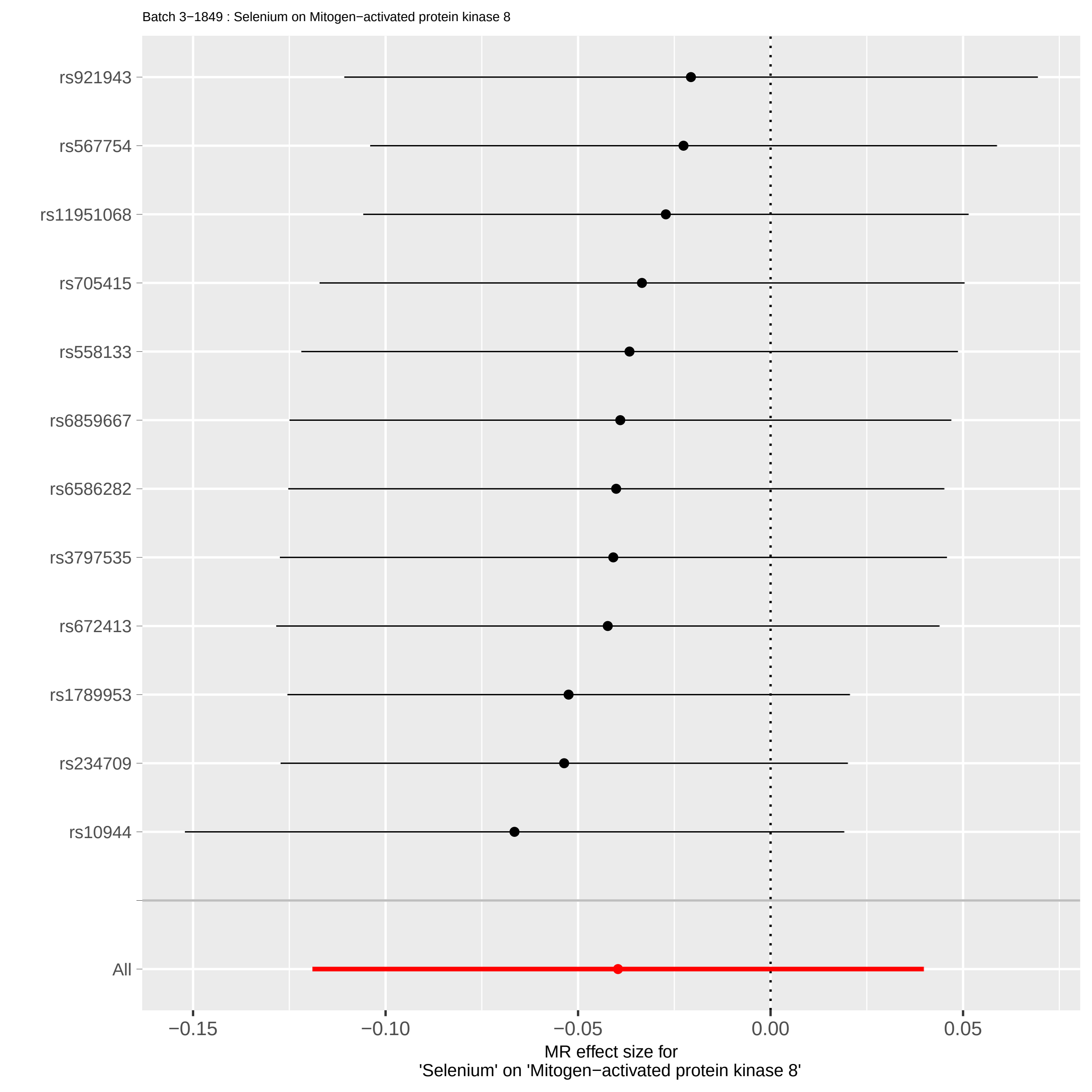

Batch 3−1849 : Selenium on Mitogen−activated protein kinase 8
rs921943
rs567754
rs11951068
rs705415
rs558133
rs6859667
rs6586282
rs3797535
rs672413
rs1789953
rs234709
rs10944
All
−0.15
−0.10
−0.05
0.00
0.05
MR effect size for
'Selenium' on 'Mitogen−activated protein kinase 8'

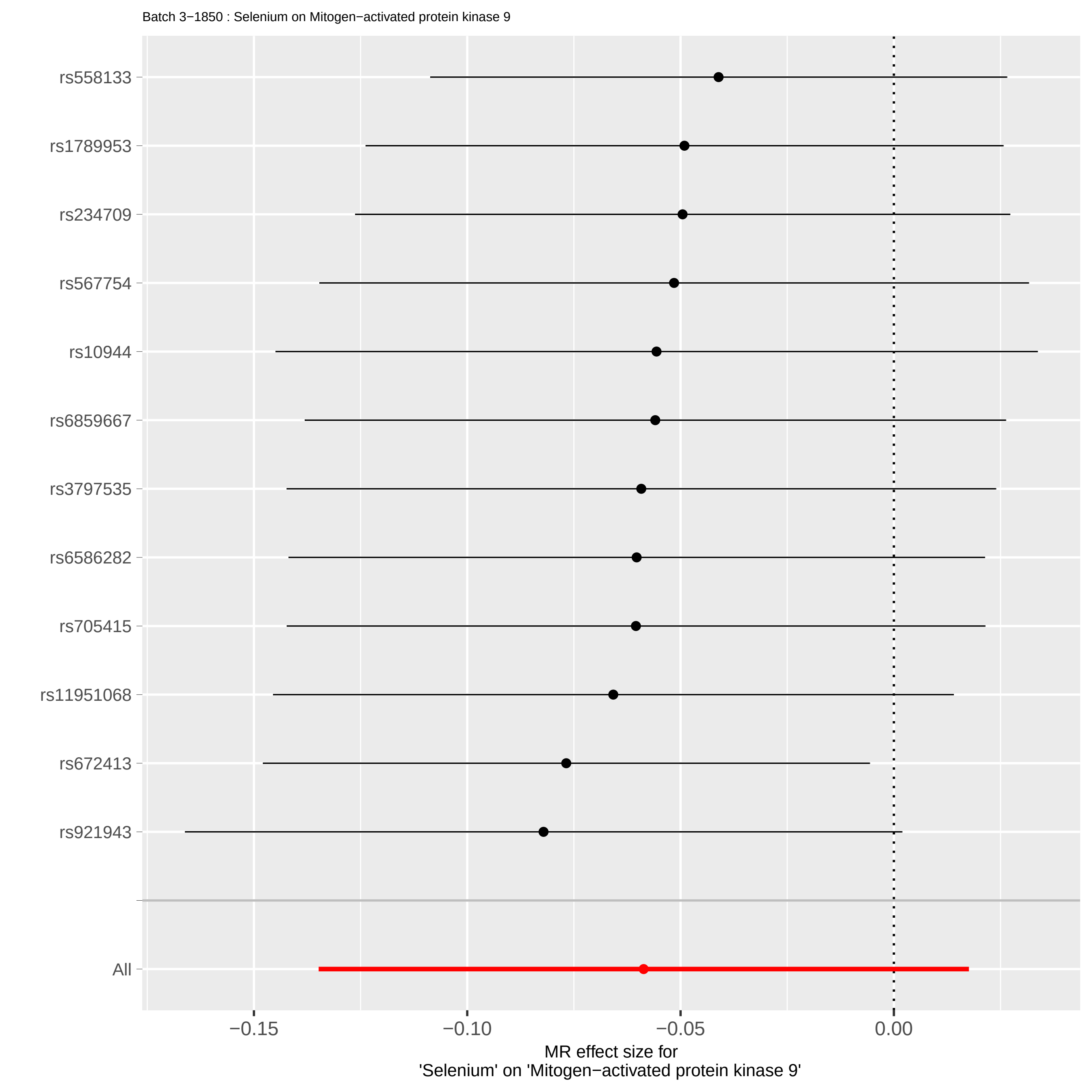

Batch 3−1850 : Selenium on Mitogen−activated protein kinase 9
rs558133
rs1789953
rs234709
rs567754
rs10944
rs6859667
rs3797535
rs6586282
rs705415
rs11951068
rs672413
rs921943
All
−0.15
−0.10
−0.05
0.00
MR effect size for
'Selenium' on 'Mitogen−activated protein kinase 9'

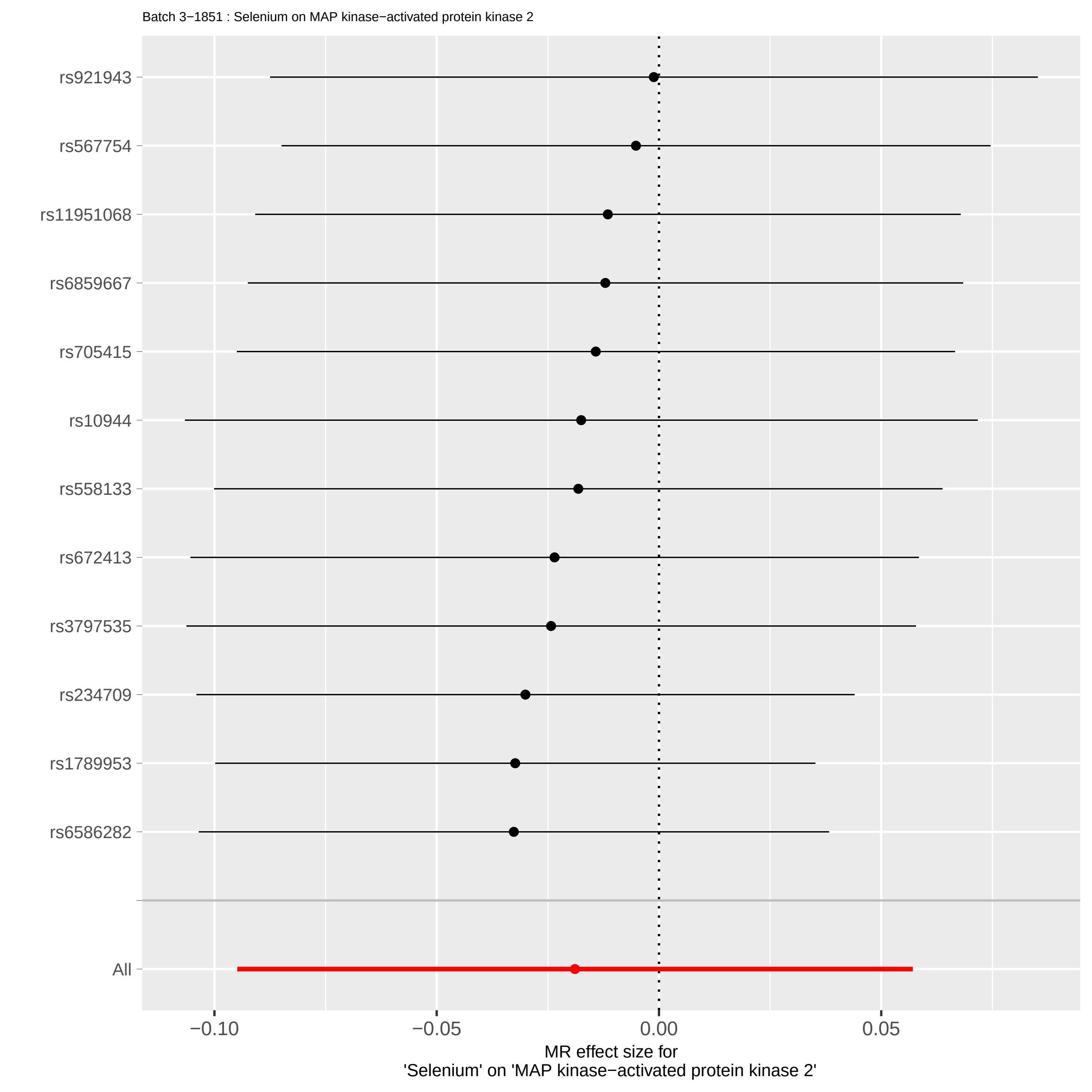

Batch 3−1851 : Selenium on MAP kinase−activated protein kinase 2
rs921943
rs567754
rs11951068
rs6859667
rs705415
rs10944
rs558133
rs672413
rs3797535
rs234709
rs1789953
rs6586282
All
−0.10
−0.05
0.00
0.05
MR effect size for
'Selenium' on 'MAP kinase−activated protein kinase 2'

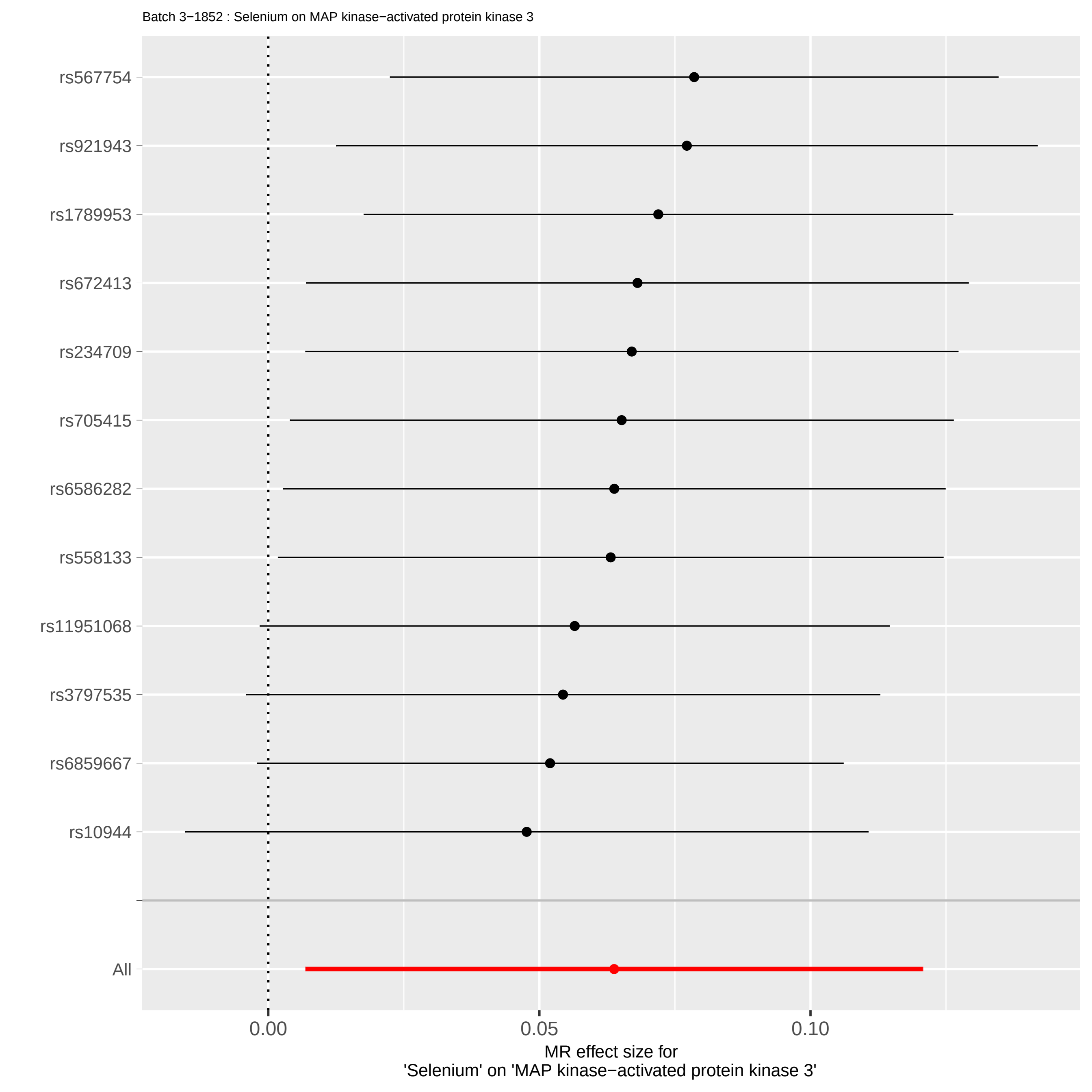

Batch 3−1852 : Selenium on MAP kinase−activated protein kinase 3
rs567754
rs921943
rs1789953
rs672413
rs234709
rs705415
rs6586282
rs558133
rs11951068
rs3797535
rs6859667
rs10944
All
0.00
0.05
0.10
MR effect size for
'Selenium' on 'MAP kinase−activated protein kinase 3'

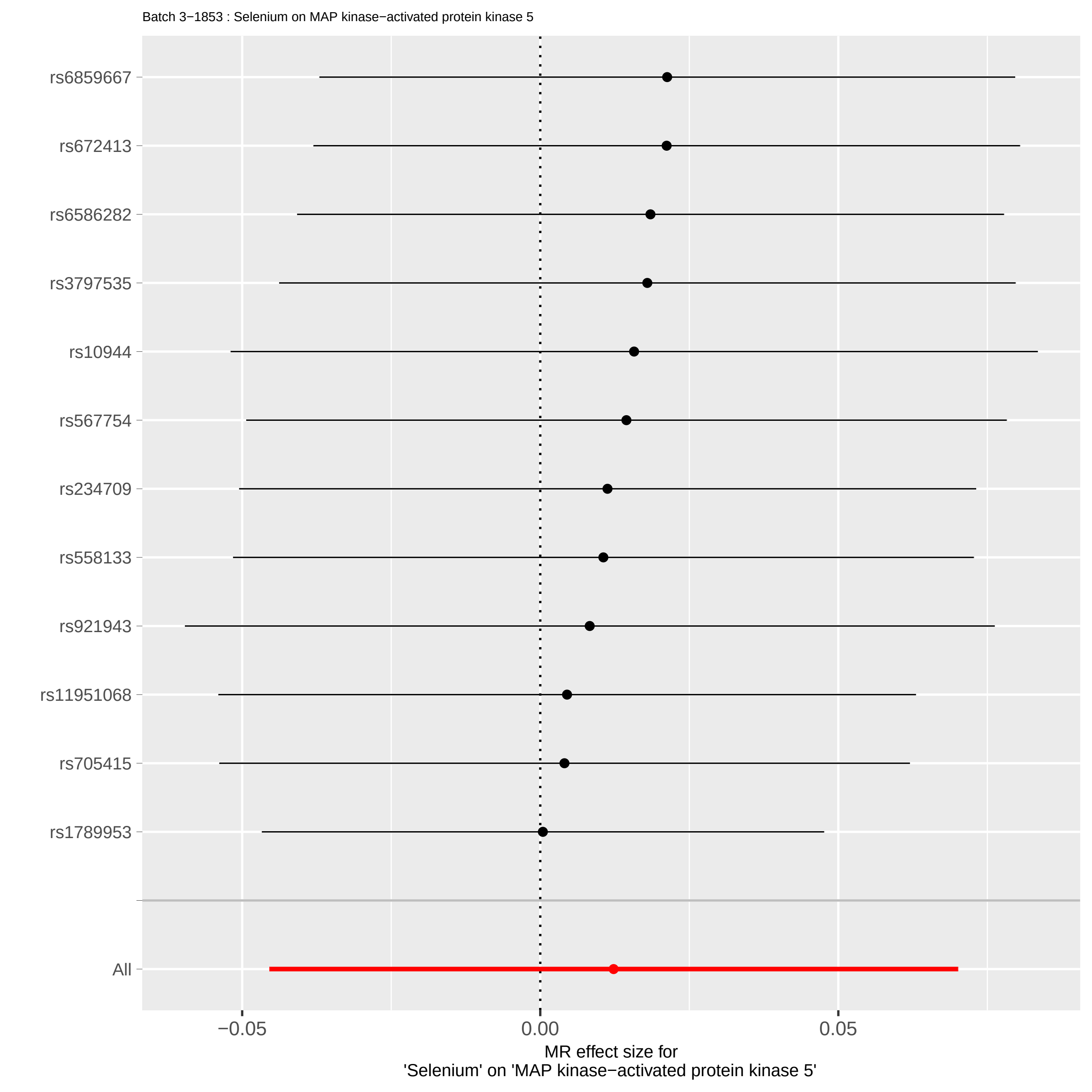

Batch 3−1853 : Selenium on MAP kinase−activated protein kinase 5
rs6859667
rs672413
rs6586282
rs3797535
rs10944
rs567754
rs234709
rs558133
rs921943
rs11951068
rs705415
rs1789953
All
−0.05
0.00
0.05
MR effect size for
'Selenium' on 'MAP kinase−activated protein kinase 5'

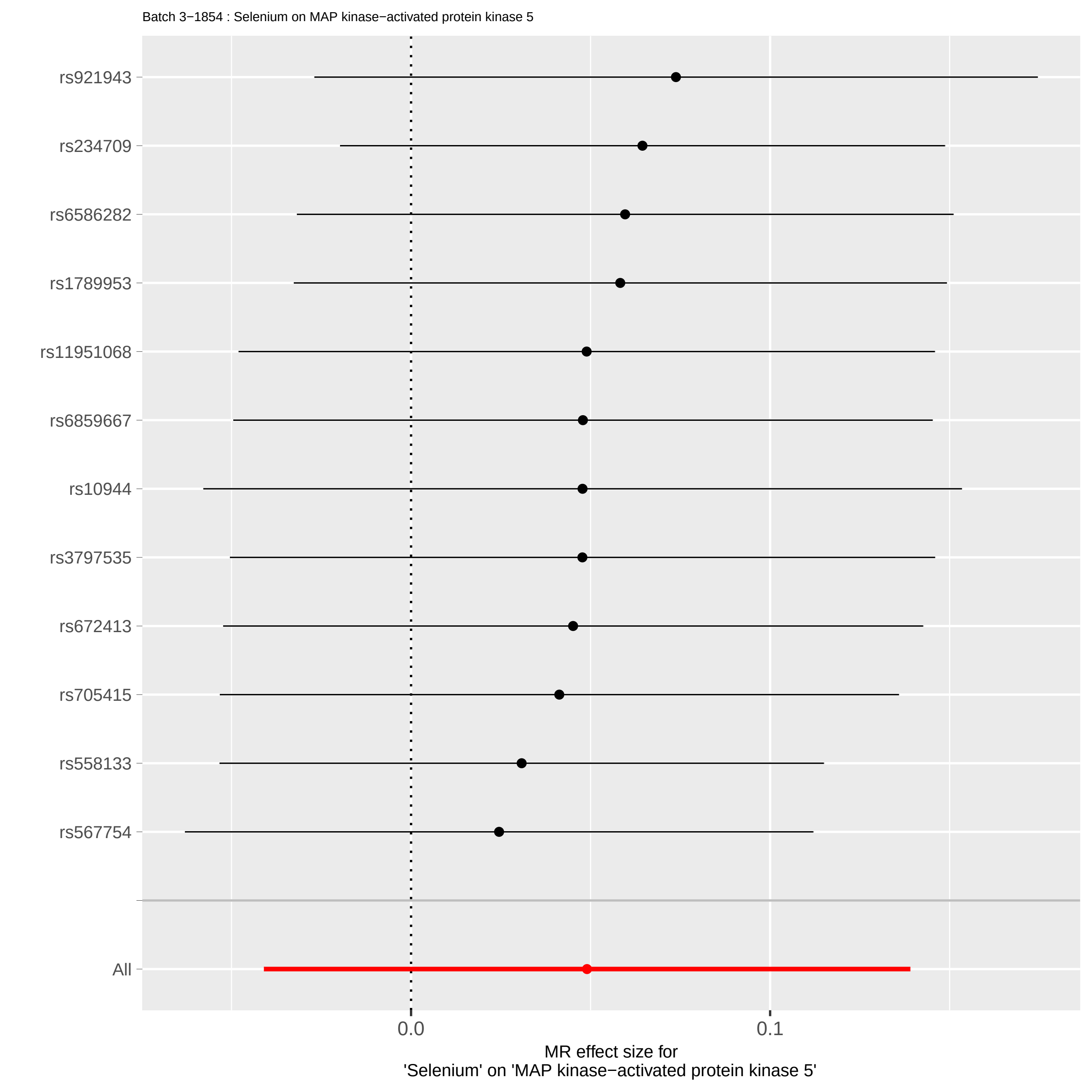

Batch 3−1854 : Selenium on MAP kinase−activated protein kinase 5
rs921943
rs234709
rs6586282
rs1789953
rs11951068
rs6859667
rs10944
rs3797535
rs672413
rs705415
rs558133
rs567754
All
0.0
0.1
MR effect size for
'Selenium' on 'MAP kinase−activated protein kinase 5'

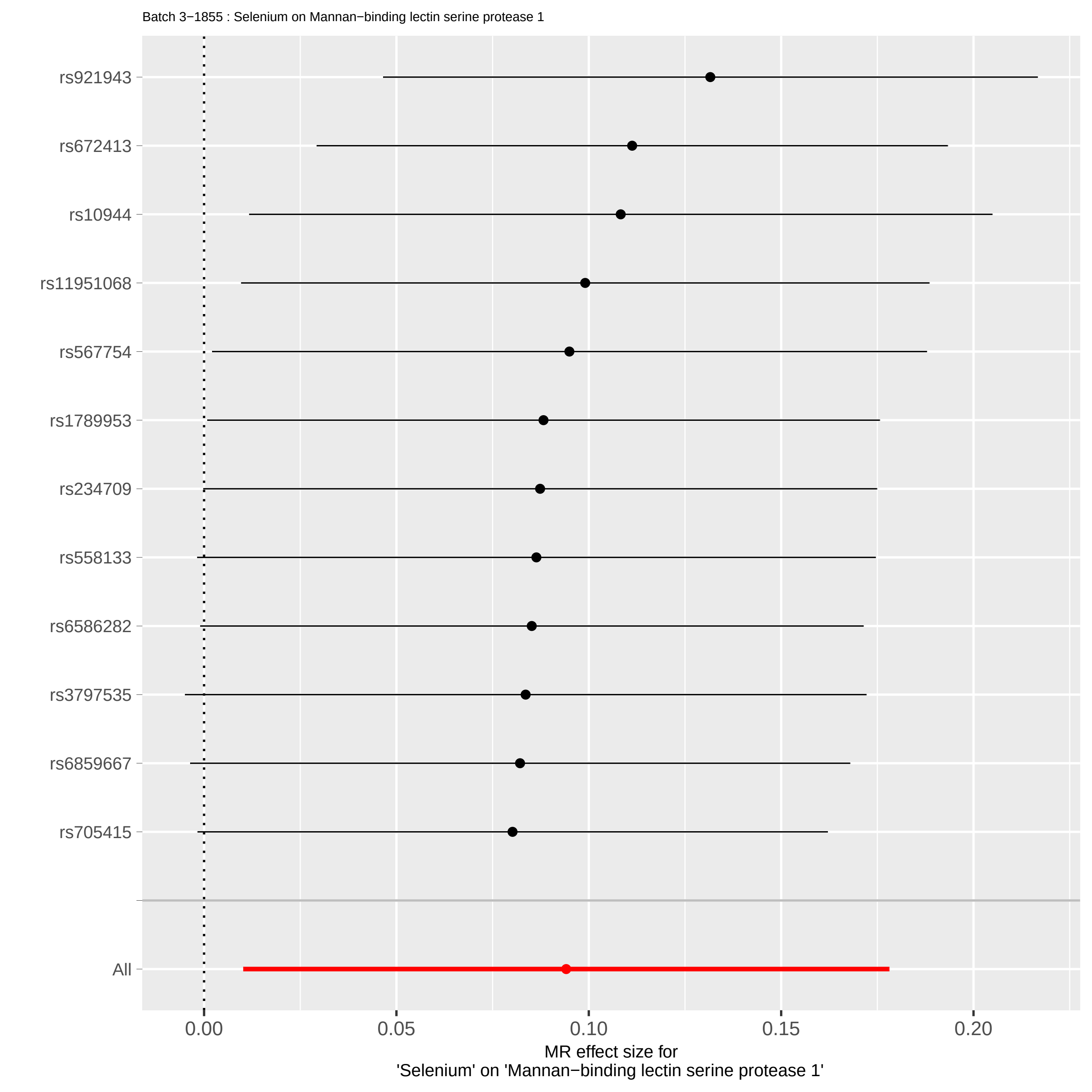
Batch 3−1855 : Selenium on Mannan−binding lectin serine protease 1
rs921943
rs672413
rs10944
rs11951068
rs567754
rs1789953
rs234709
rs558133
rs6586282
rs3797535
rs6859667
rs705415
All
0.00
0.05
0.10
0.15
0.20
MR effect size for
'Selenium' on 'Mannan−binding lectin serine protease 1'

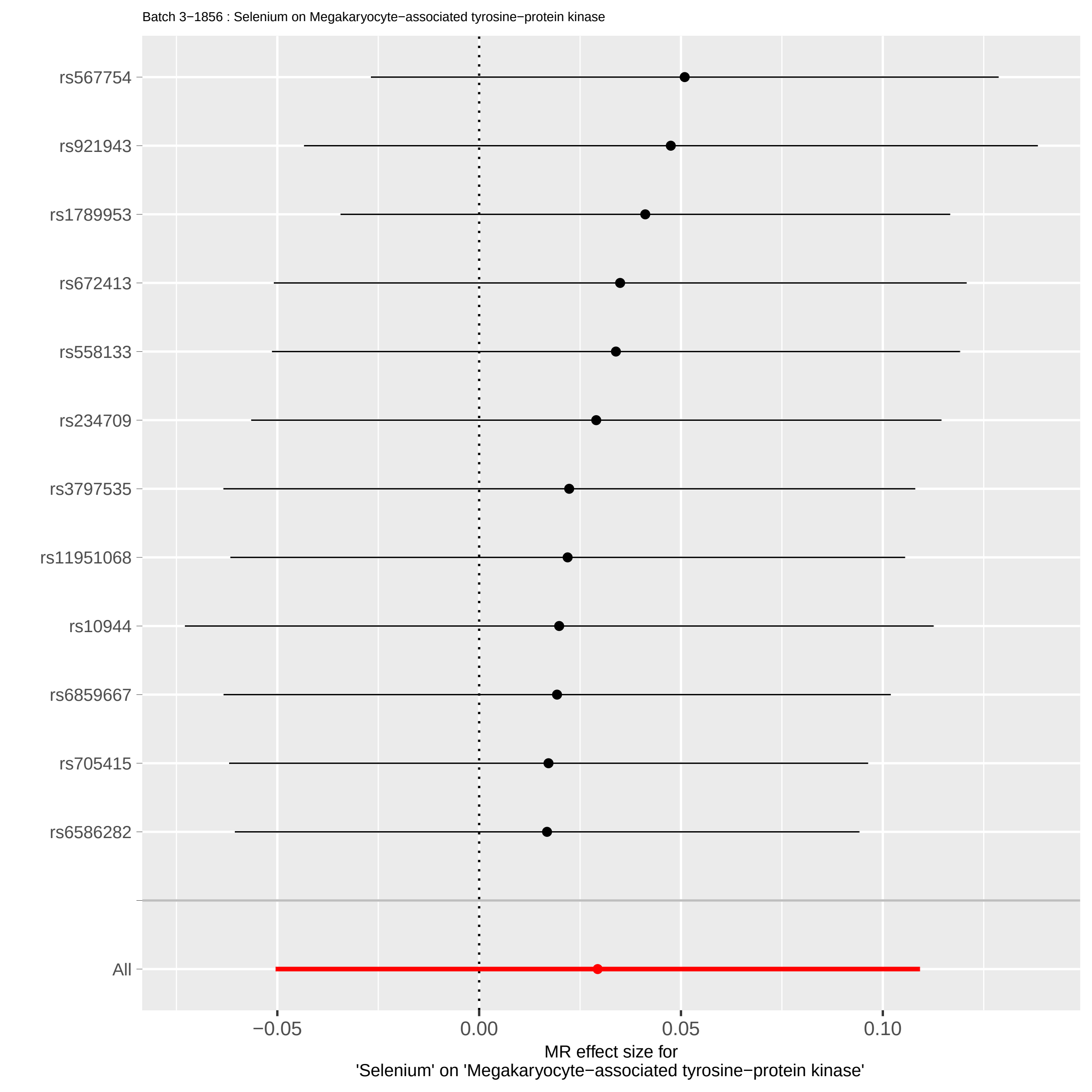

Batch 3−1856 : Selenium on Megakaryocyte−associated tyrosine−protein kinase
rs567754
rs921943
rs1789953
rs672413
rs558133
rs234709
rs3797535
rs11951068
rs10944
rs6859667
rs705415
rs6586282
All
−0.05
0.00
0.05
0.10
MR effect size for
'Selenium' on 'Megakaryocyte−associated tyrosine−protein kinase'

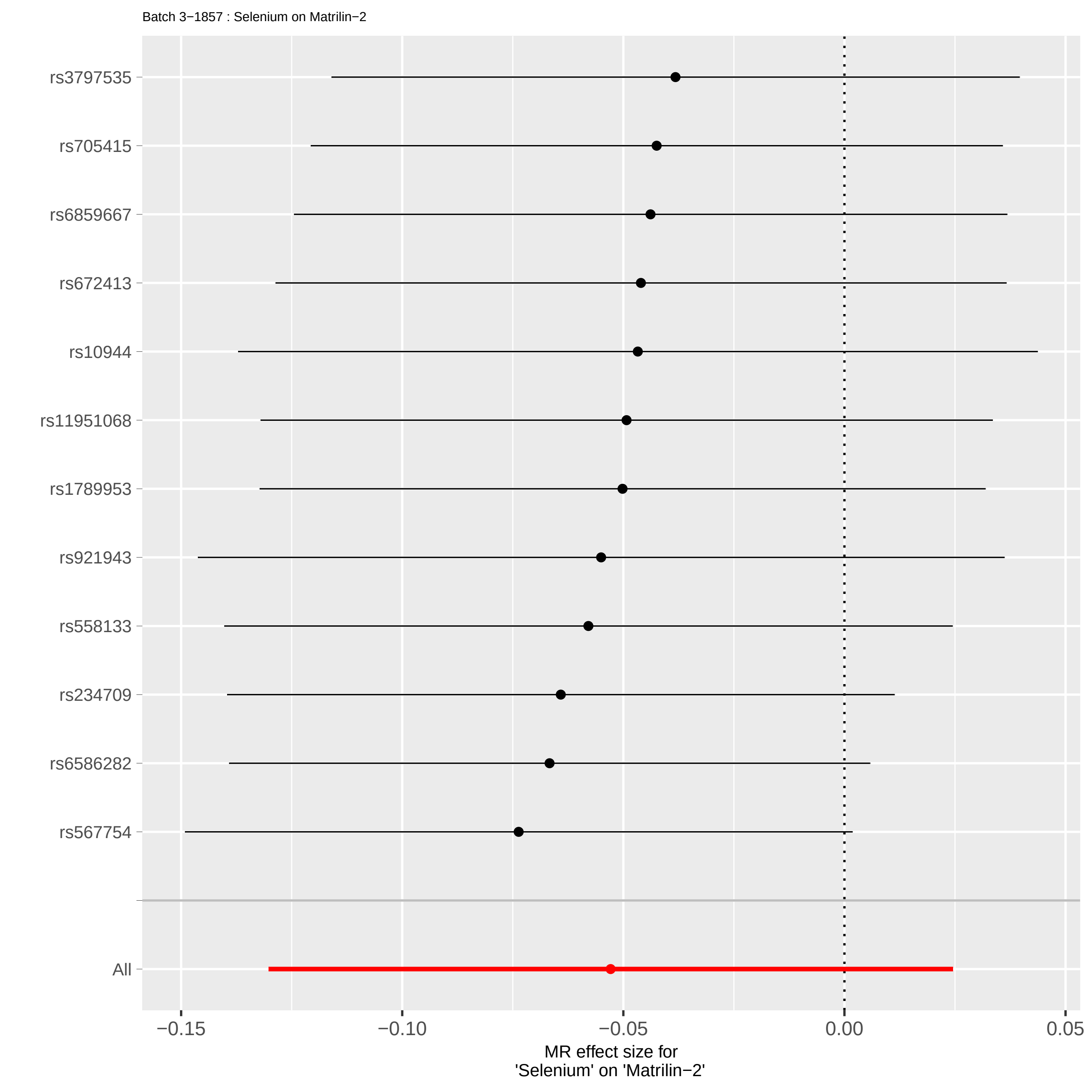

Batch 3−1857 : Selenium on Matrilin−2
rs3797535
rs705415
rs6859667
rs672413
rs10944
rs11951068
rs1789953
rs921943
rs558133
rs234709
rs6586282
rs567754
All
−0.15
−0.10
−0.05
0.00
0.05
MR effect size for
'Selenium' on 'Matrilin−2'

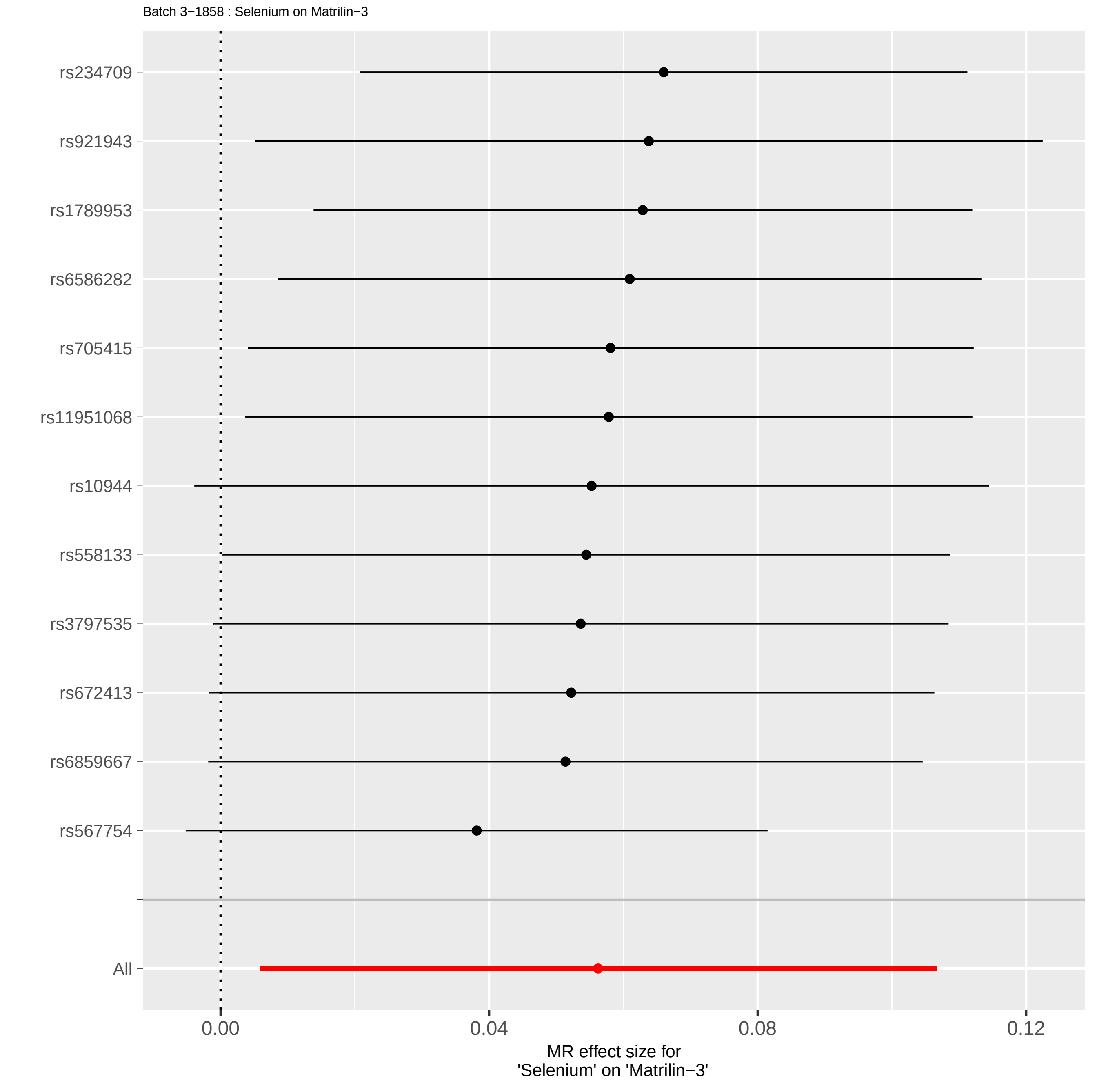

Batch 3−1858 : Selenium on Matrilin−3
rs234709
rs921943
rs1789953
rs6586282
rs705415
rs11951068
rs10944
rs558133
rs3797535
rs672413
rs6859667
rs567754
All
0.00
0.04
0.08
0.12
MR effect size for
'Selenium' on 'Matrilin−3'

Batch 3−1859 : Selenium on Matrilin−4

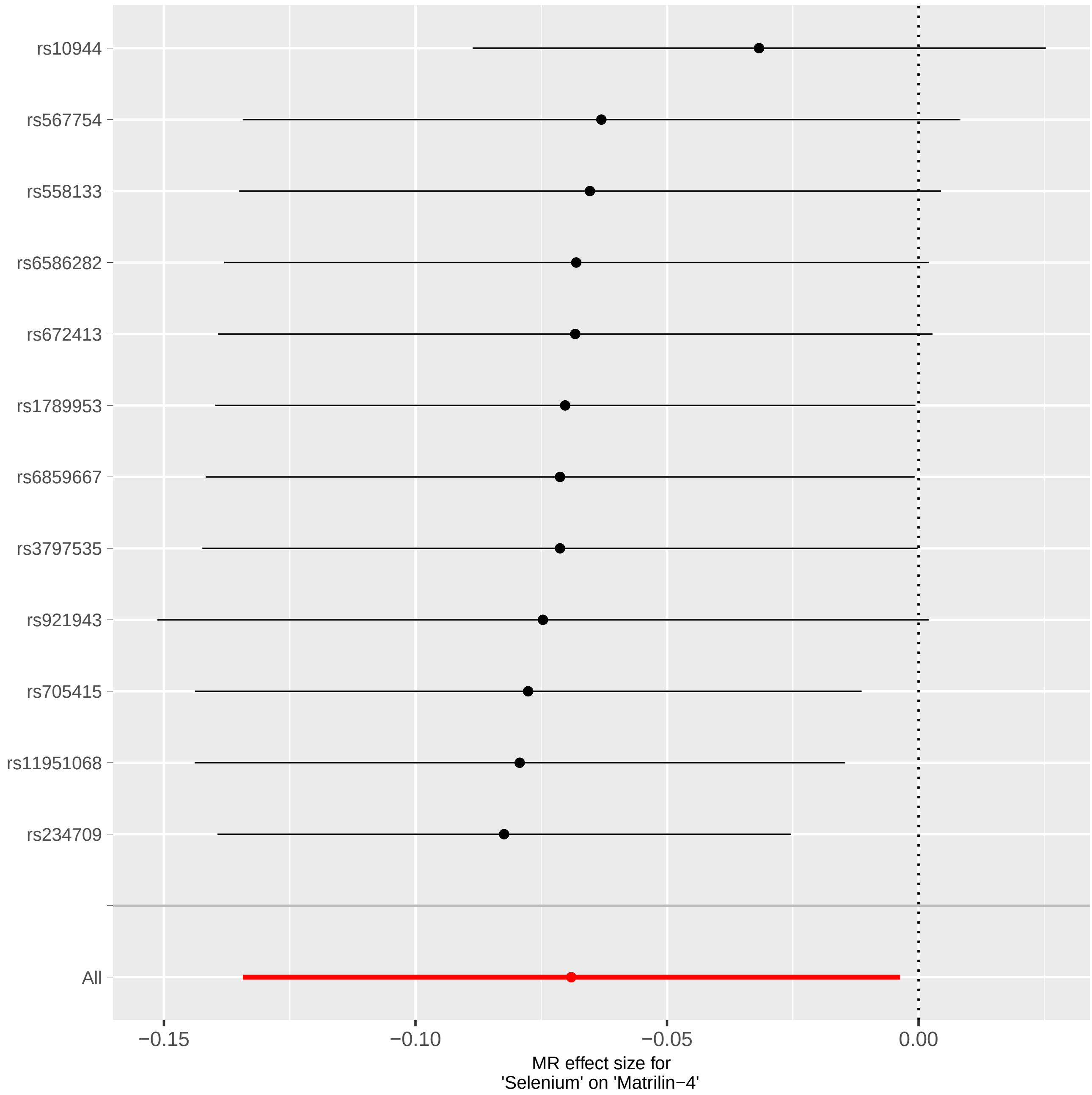

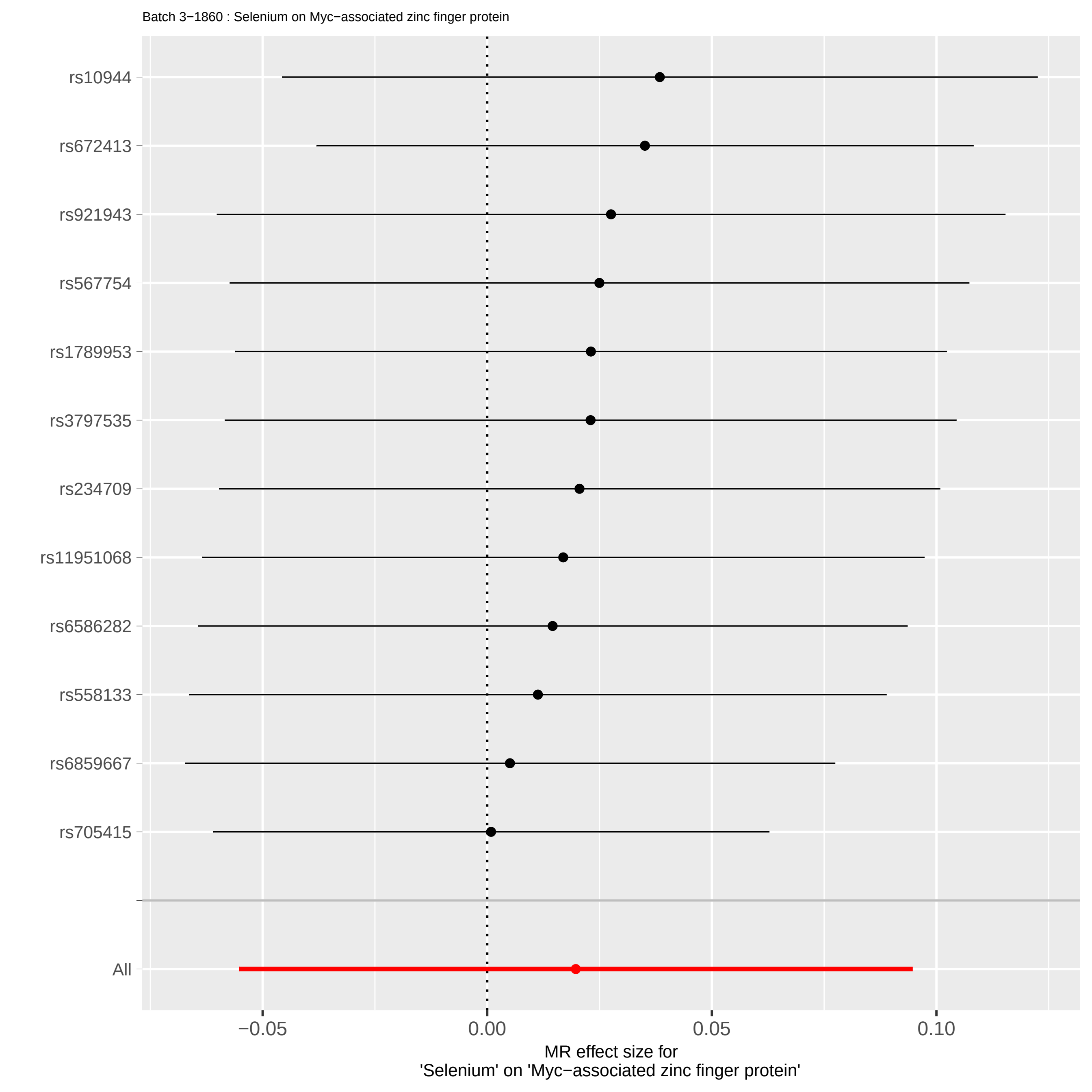

Batch 3−1860 : Selenium on Myc−associated zinc finger protein
rs10944
rs672413
rs921943
rs567754
rs1789953
rs3797535
rs234709
rs11951068
rs6586282
rs558133
rs6859667
rs705415
All
−0.05
0.00
0.05
0.10
MR effect size for
'Selenium' on 'Myc−associated zinc finger protein'

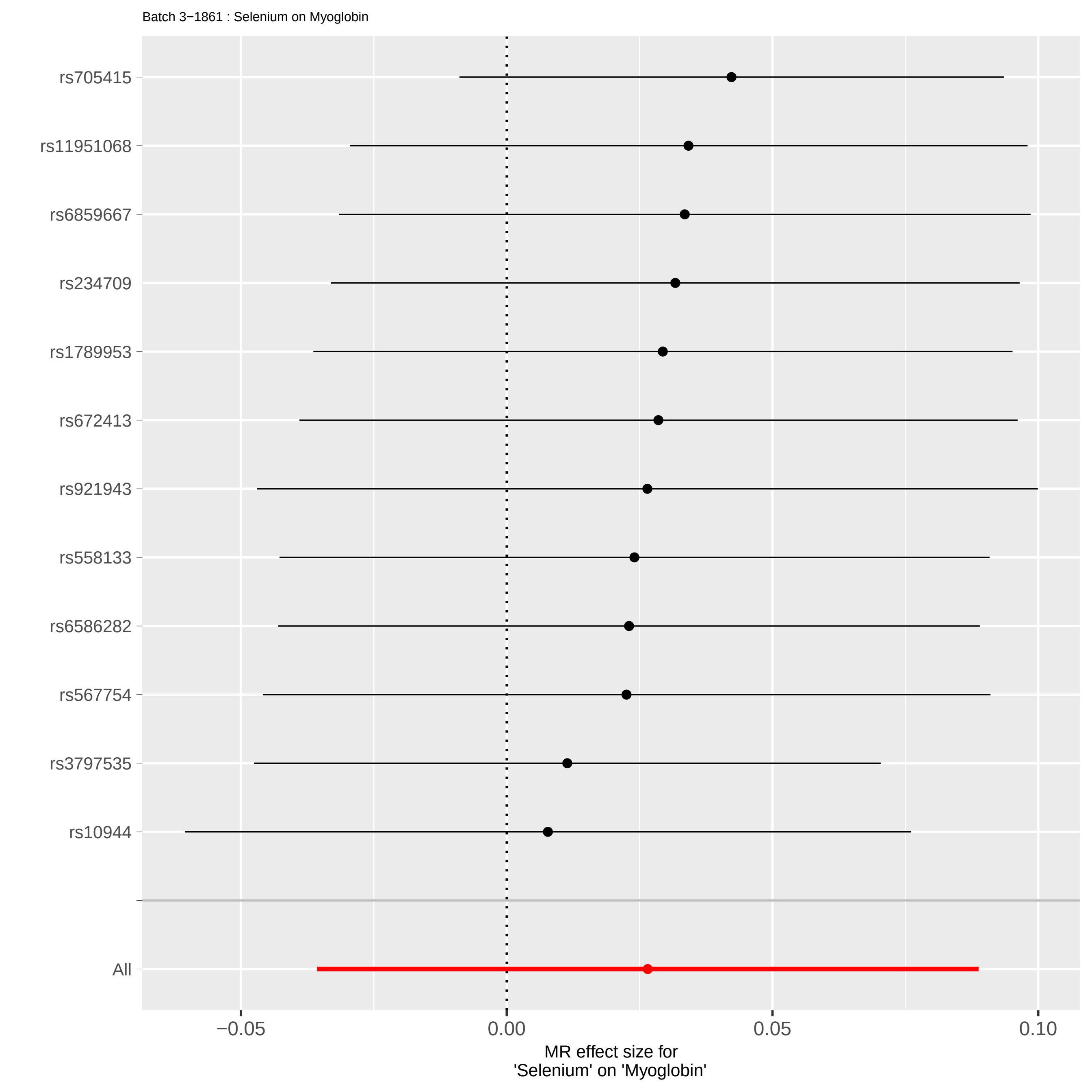

Batch 3−1861 : Selenium on Myoglobin
rs705415
rs11951068
rs6859667
rs234709
rs1789953
rs672413
rs921943
rs558133
rs6586282
rs567754
rs3797535
rs10944
All
−0.05
0.00
0.05
0.10
MR effect size for
'Selenium' on 'Myoglobin'

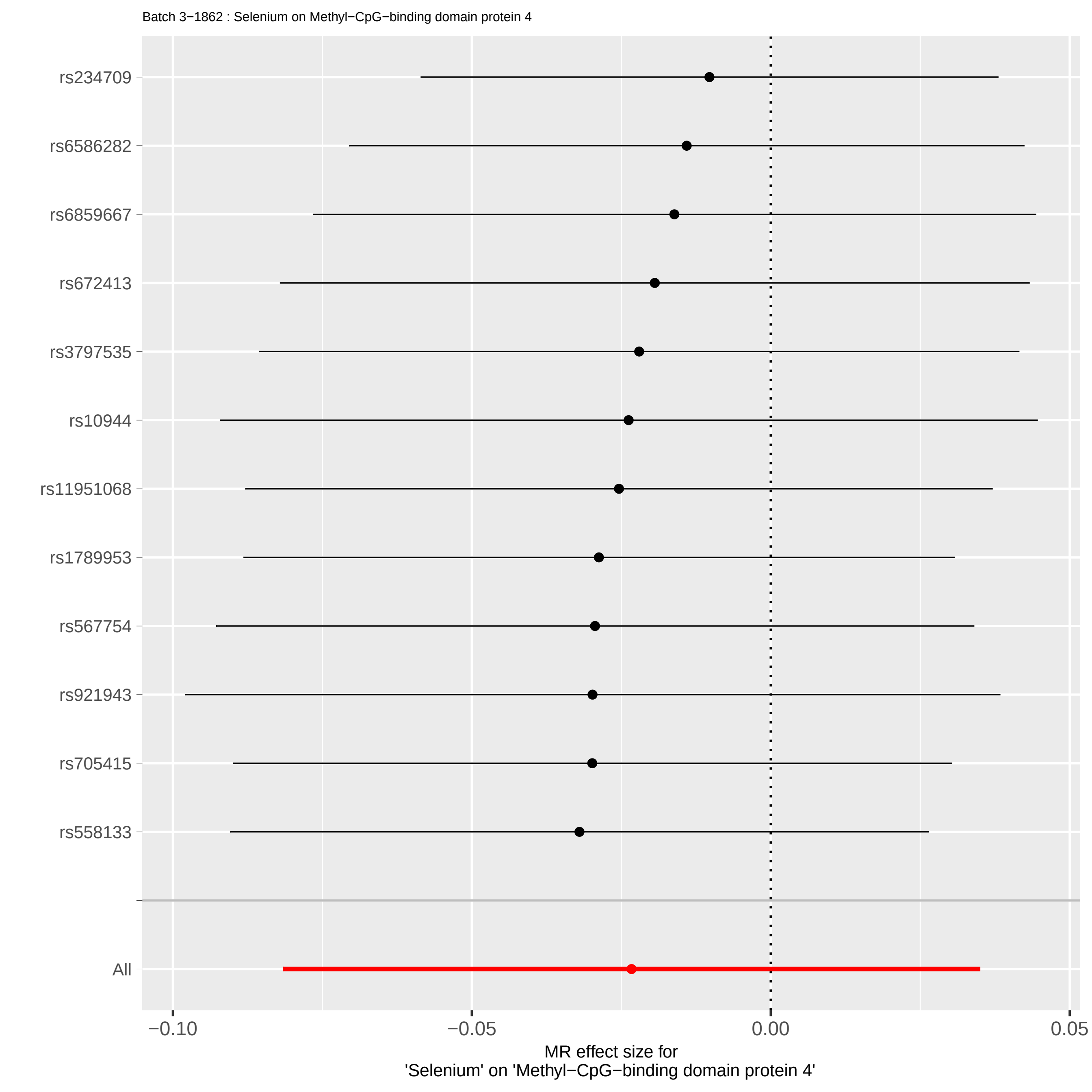
Batch 3−1862 : Selenium on Methyl−CpG−binding domain protein 4
rs234709
rs6586282
rs6859667
rs672413
rs3797535
rs10944
rs11951068
rs1789953
rs567754
rs921943
rs705415
rs558133
All
−0.10
−0.05
0.00
0.05
MR effect size for
'Selenium' on 'Methyl−CpG−binding domain protein 4'

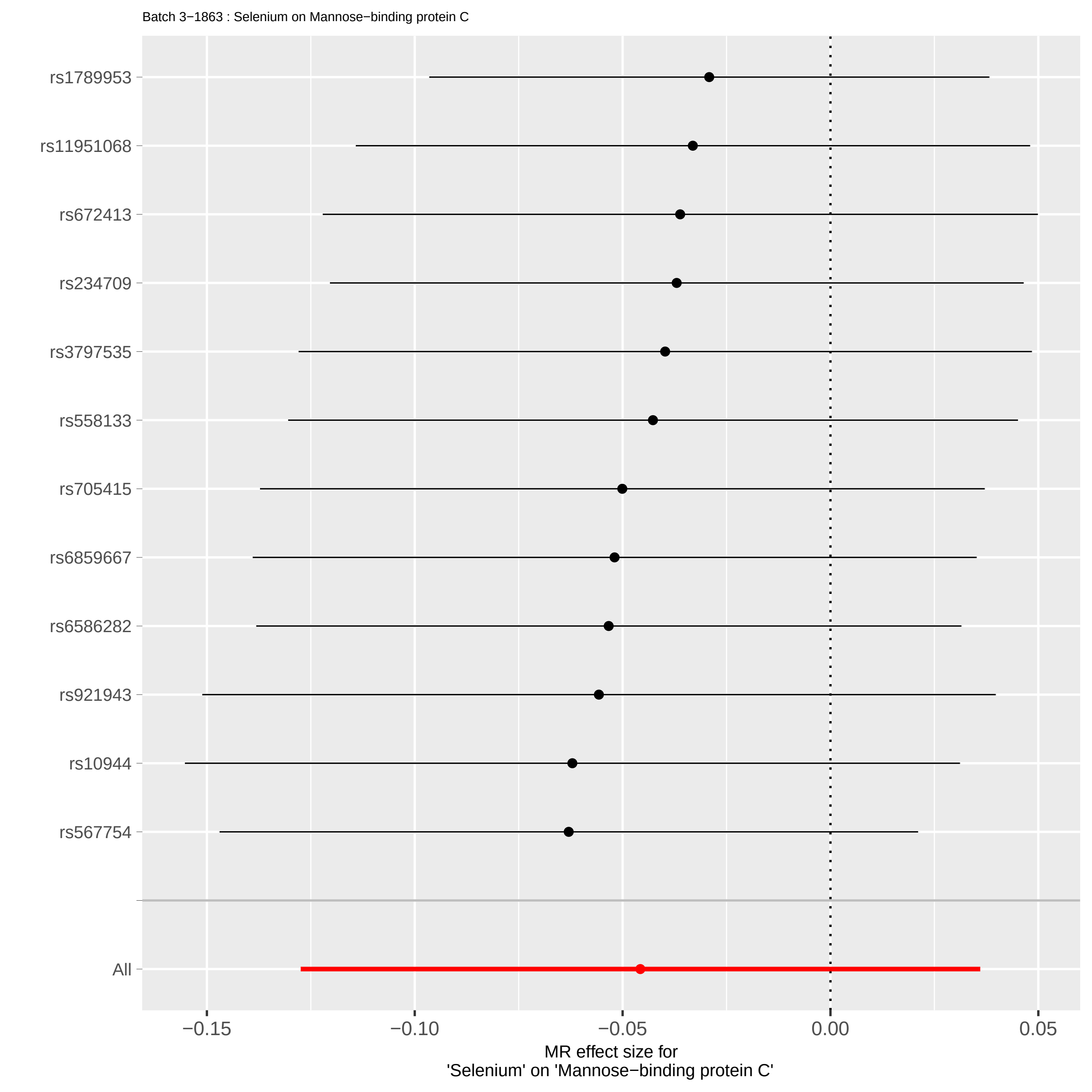

Batch 3−1863 : Selenium on Mannose−binding protein C
rs1789953
rs11951068
rs672413
rs234709
rs3797535
rs558133
rs705415
rs6859667
rs6586282
rs921943
rs10944
rs567754
All
−0.15
−0.10
−0.05
0.00
0.05
MR effect size for
'Selenium' on 'Mannose−binding protein C'

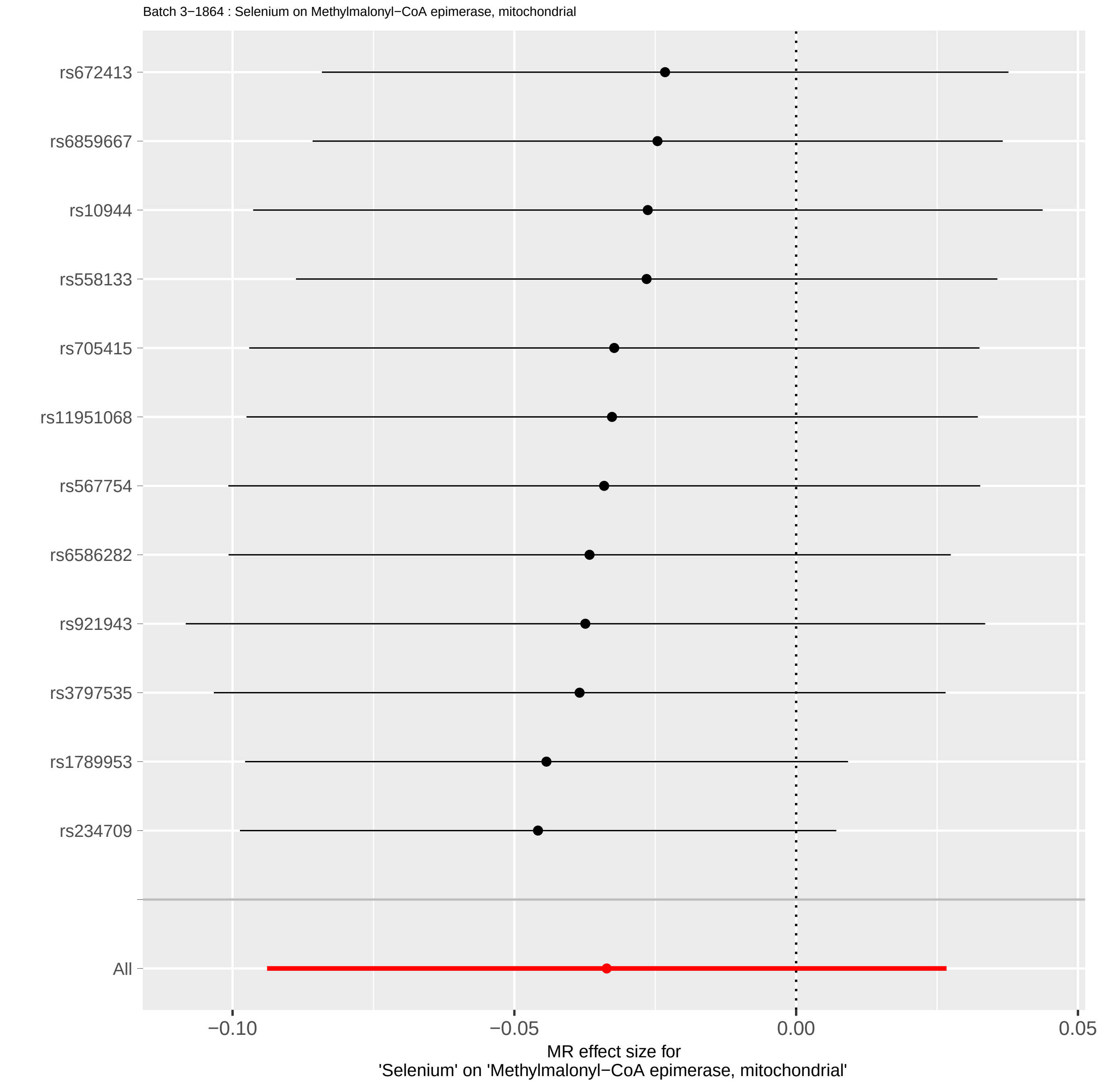
Batch 3−1864 : Selenium on Methylmalonyl−CoA epimerase, mitochondrial
rs672413
rs6859667
rs10944
rs558133
rs705415
rs11951068
rs567754
rs6586282
rs921943
rs3797535
rs1789953
rs234709
All
−0.10
−0.05
0.00
0.05
MR effect size for
'Selenium' on 'Methylmalonyl−CoA epimerase, mitochondrial'

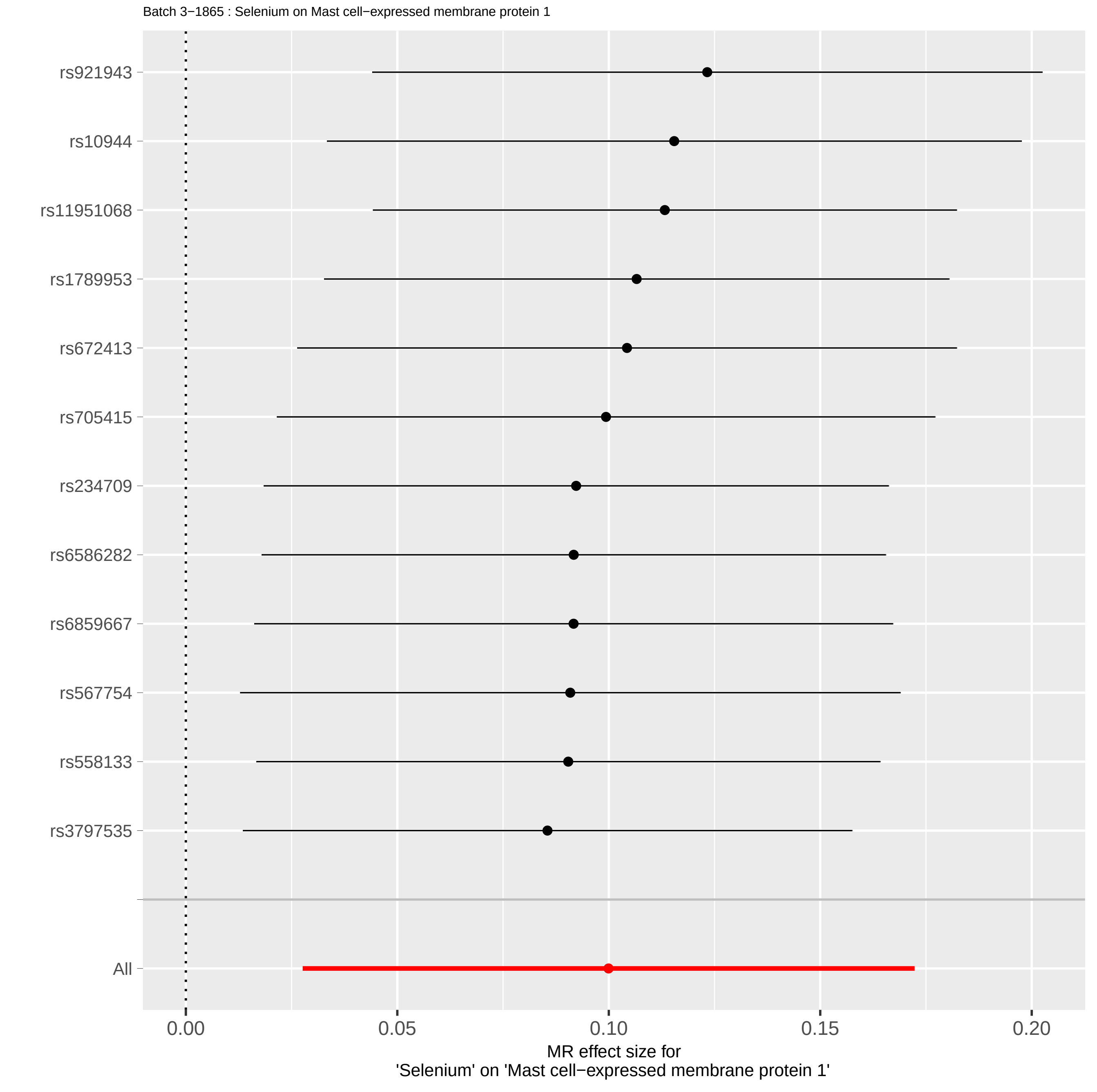
Batch 3−1865 : Selenium on Mast cell−expressed membrane protein 1
rs921943
rs10944
rs11951068
rs1789953
rs672413
rs705415
rs234709
rs6586282
rs6859667
rs567754
rs558133
rs3797535
All
0.00
0.05
0.10
0.15
0.20
MR effect size for
'Selenium' on 'Mast cell−expressed membrane protein 1'

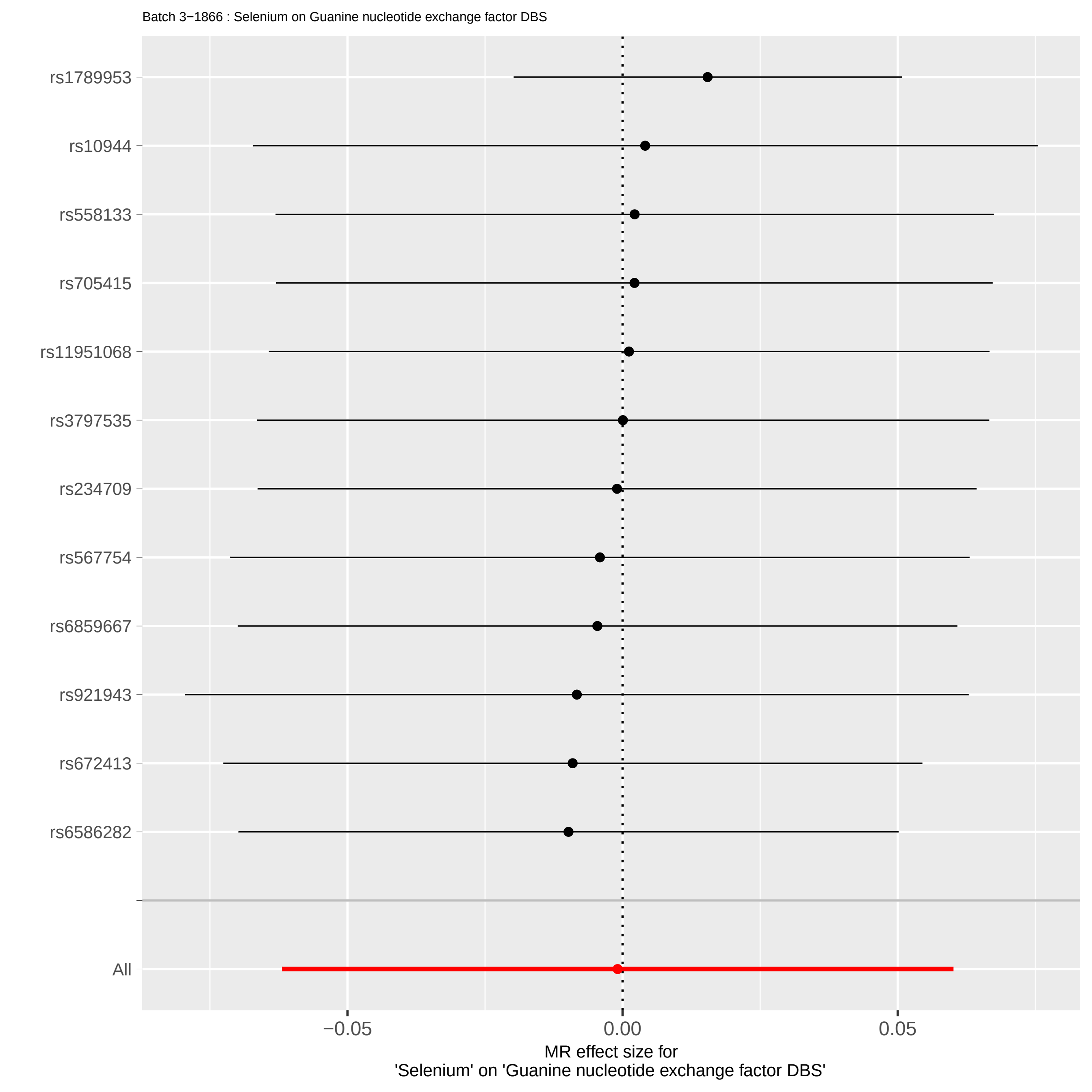

Batch 3−1866 : Selenium on Guanine nucleotide exchange factor DBS
rs1789953
rs10944
rs558133
rs705415
rs11951068
rs3797535
rs234709
rs567754
rs6859667
rs921943
rs672413
rs6586282
All
−0.05
0.00
0.05
MR effect size for
'Selenium' on 'Guanine nucleotide exchange factor DBS'

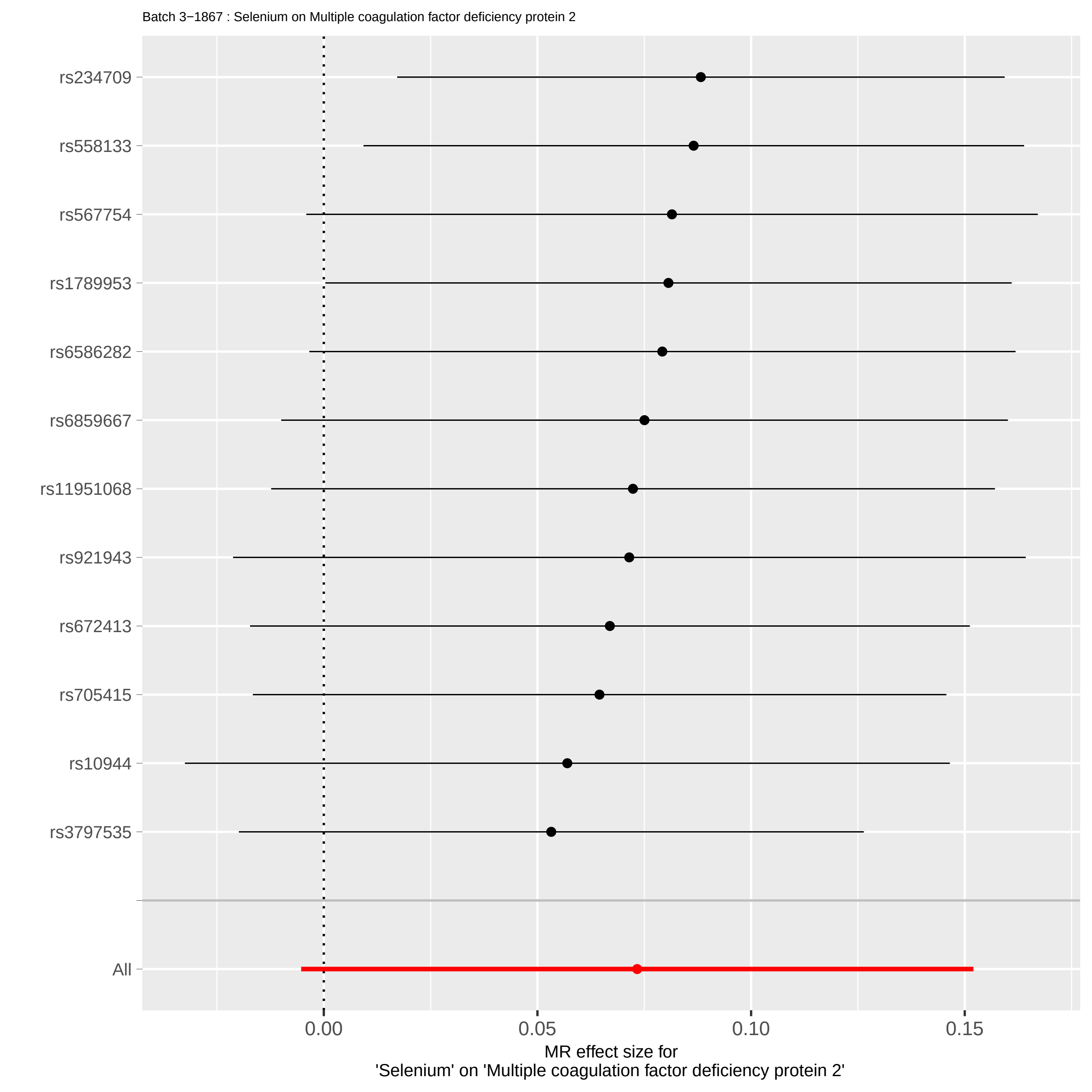

Batch 3−1867 : Selenium on Multiple coagulation factor deficiency protein 2
rs234709
rs558133
rs567754
rs1789953
rs6586282
rs6859667
rs11951068
rs921943
rs672413
rs705415
rs10944
rs3797535
All
0.00
0.05
0.10
0.15
MR effect size for
'Selenium' on 'Multiple coagulation factor deficiency protein 2'

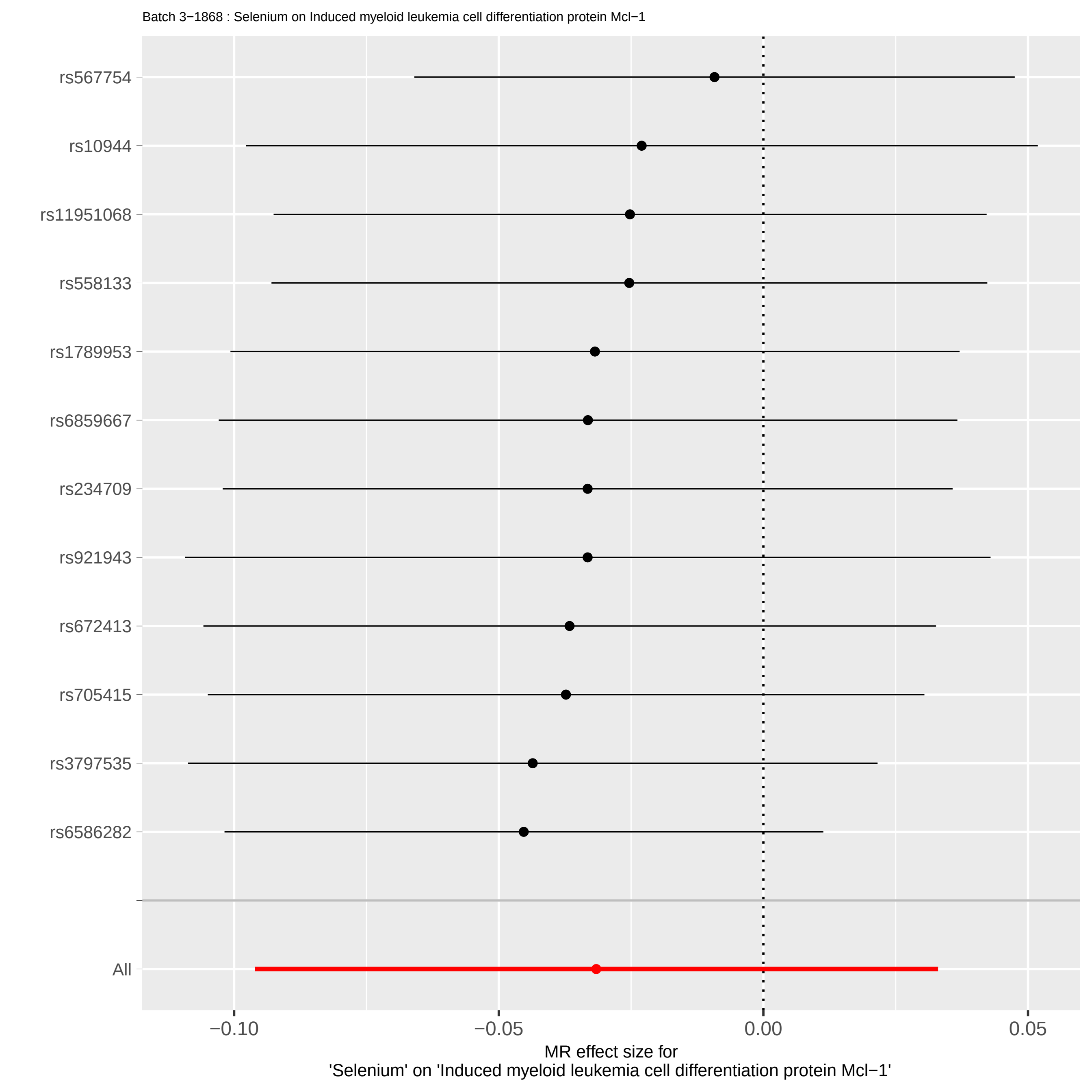

Batch 3−1868 : Selenium on Induced myeloid leukemia cell differentiation protein Mcl−1
rs567754
rs10944
rs11951068
rs558133
rs1789953
rs6859667
rs234709
rs921943
rs672413
rs705415
rs3797535
rs6586282
All
−0.10
−0.05
0.00
0.05
MR effect size for
'Selenium' on 'Induced myeloid leukemia cell differentiation protein Mcl−1'

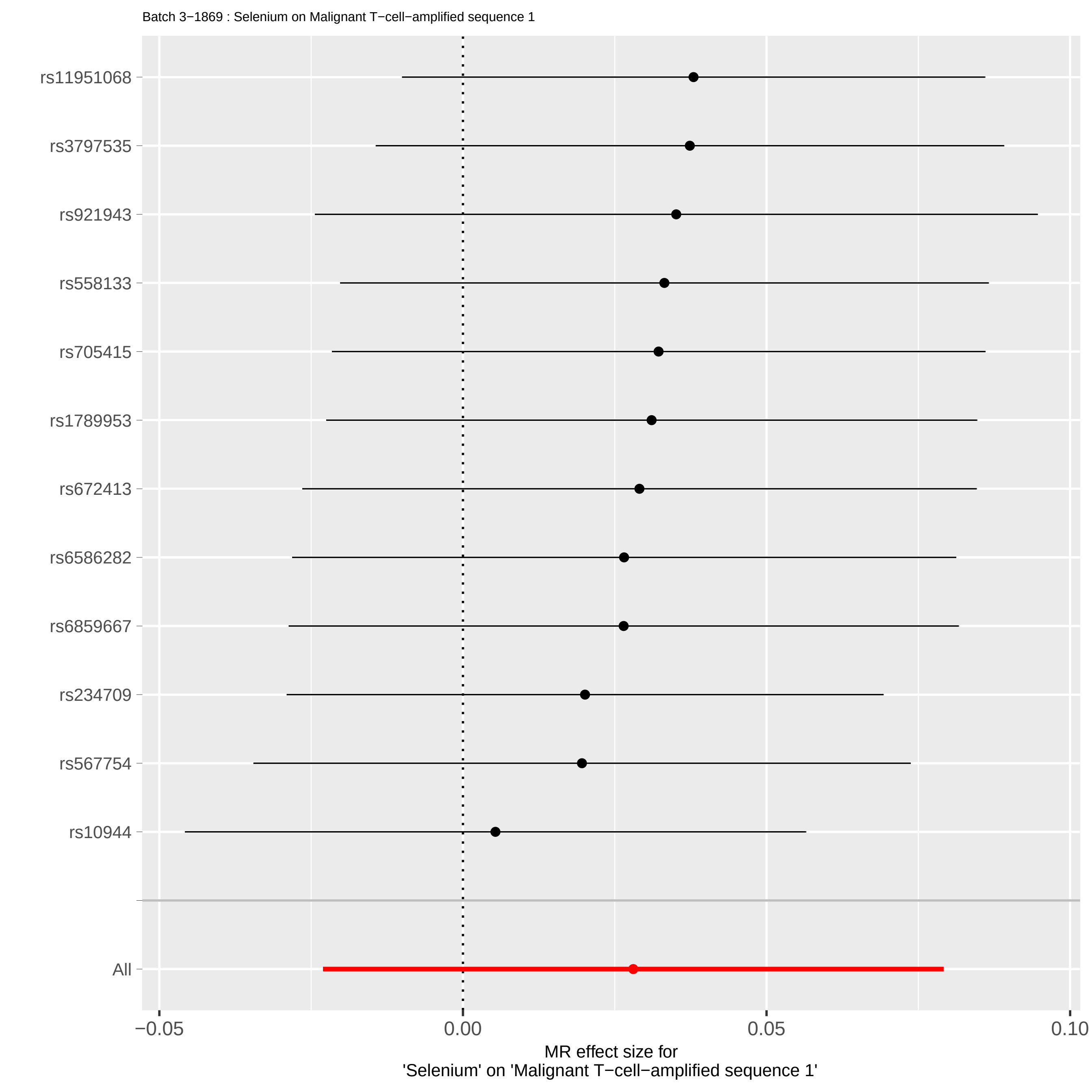
Batch 3−1869 : Selenium on Malignant T−cell−amplified sequence 1
rs11951068
rs3797535
rs921943
rs558133
rs705415
rs1789953
rs672413
rs6586282
rs6859667
rs234709
rs567754
rs10944
All
−0.05
0.00
0.05
0.10
MR effect size for
'Selenium' on 'Malignant T−cell−amplified sequence 1'

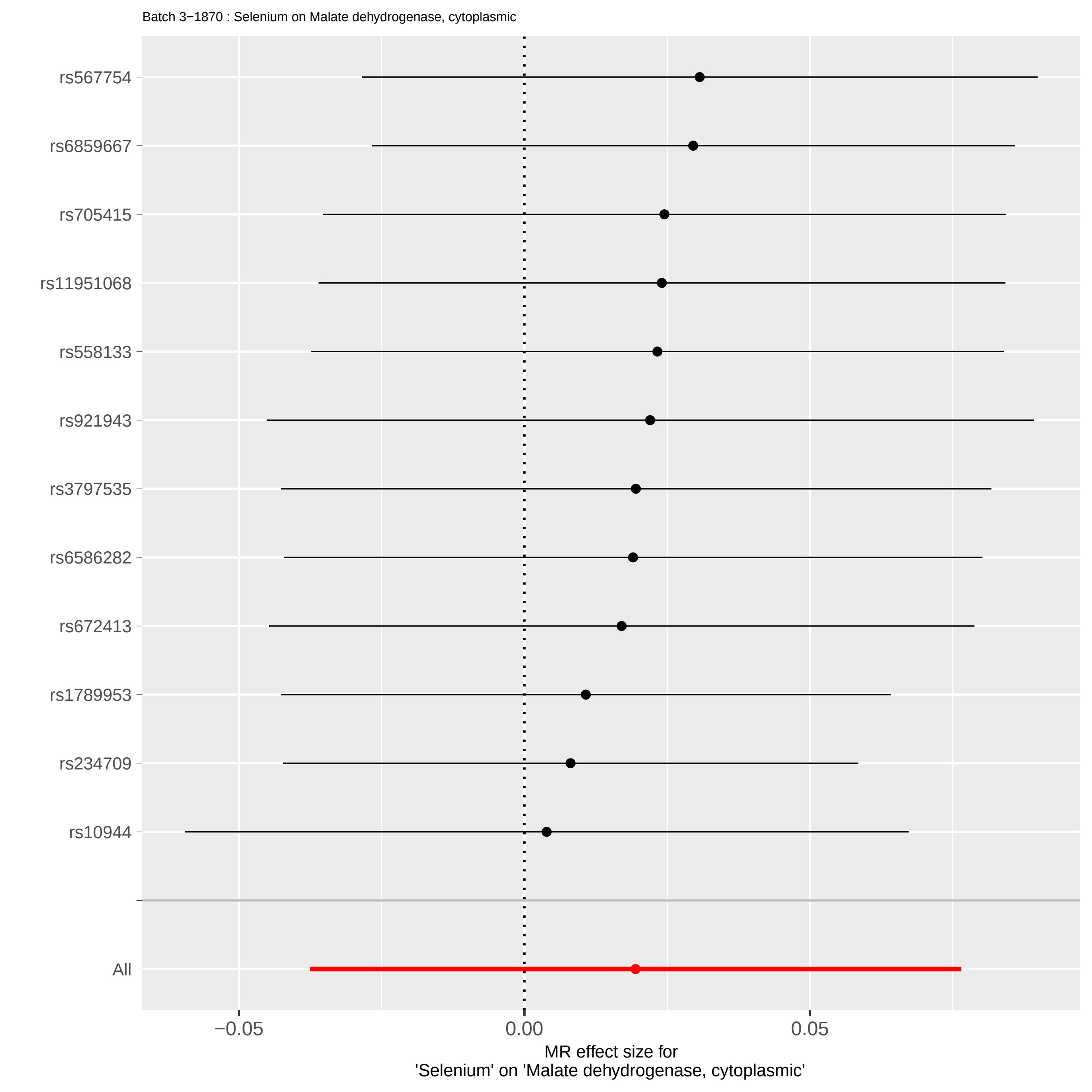

Batch 3−1870 : Selenium on Malate dehydrogenase, cytoplasmic
rs567754
rs6859667
rs705415
rs11951068
rs558133
rs921943
rs3797535
rs6586282
rs672413
rs1789953
rs234709
rs10944
All
−0.05
0.00
0.05
MR effect size for
'Selenium' on 'Malate dehydrogenase, cytoplasmic'

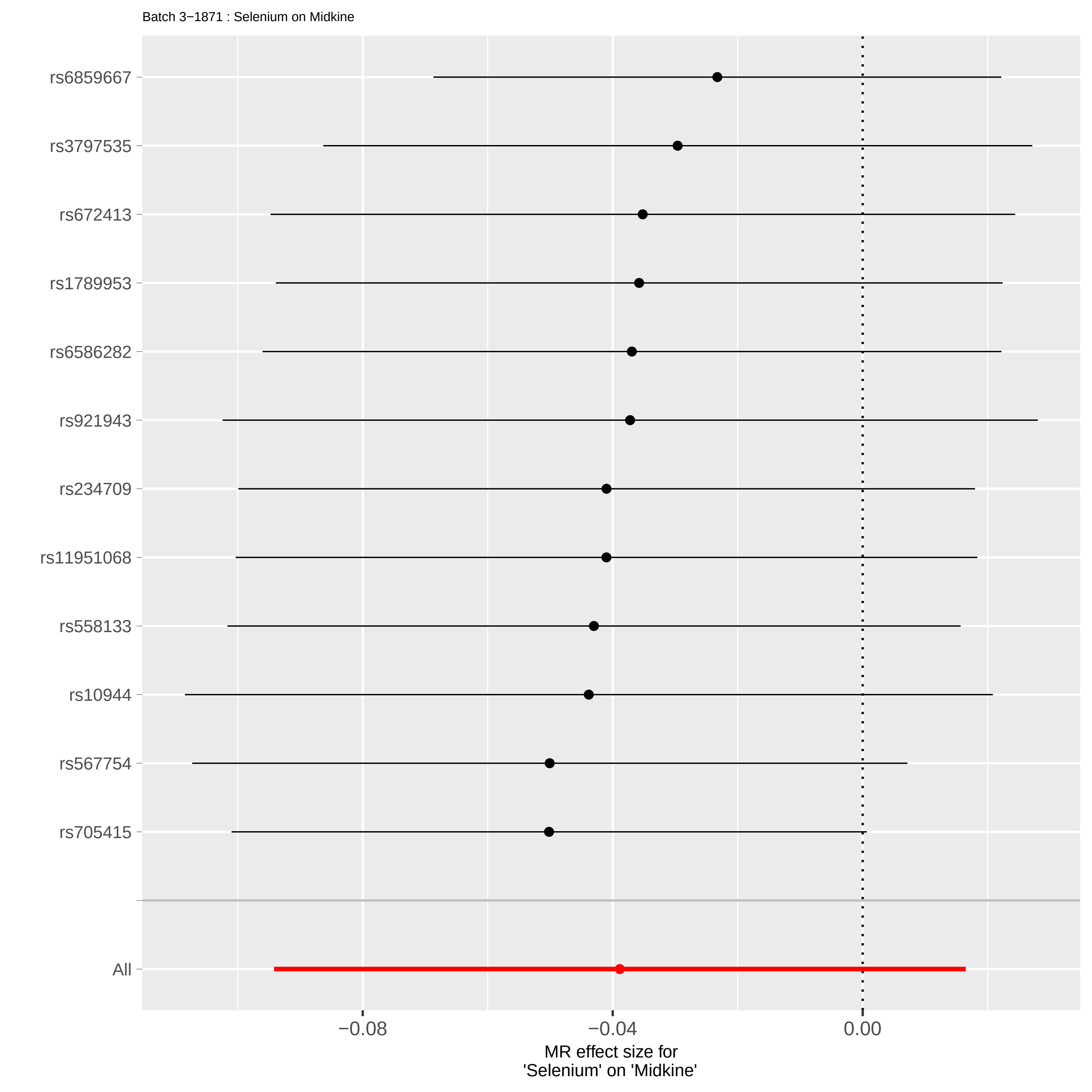

Batch 3−1871 : Selenium on Midkine
rs6859667
rs3797535
rs672413
rs1789953
rs6586282
rs921943
rs234709
rs11951068
rs558133
rs10944
rs567754
rs705415
All
−0.08
−0.04
0.00
MR effect size for
'Selenium' on 'Midkine'

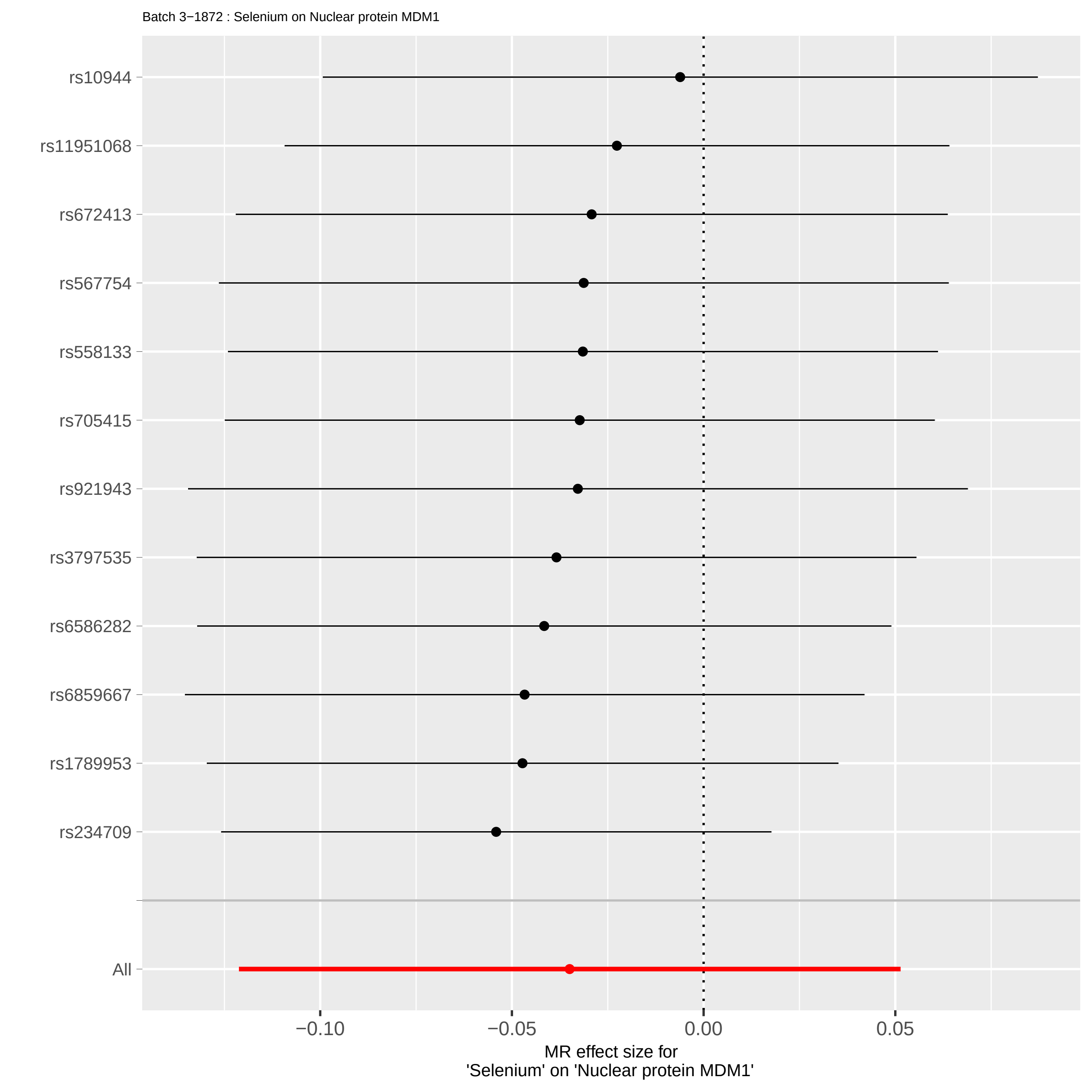
Batch 3−1872 : Selenium on Nuclear protein MDM1
rs10944
rs11951068
rs672413
rs567754
rs558133
rs705415
rs921943
rs3797535
rs6586282
rs6859667
rs1789953
rs234709
All
−0.10
−0.05
0.00
0.05
MR effect size for
'Selenium' on 'Nuclear protein MDM1'

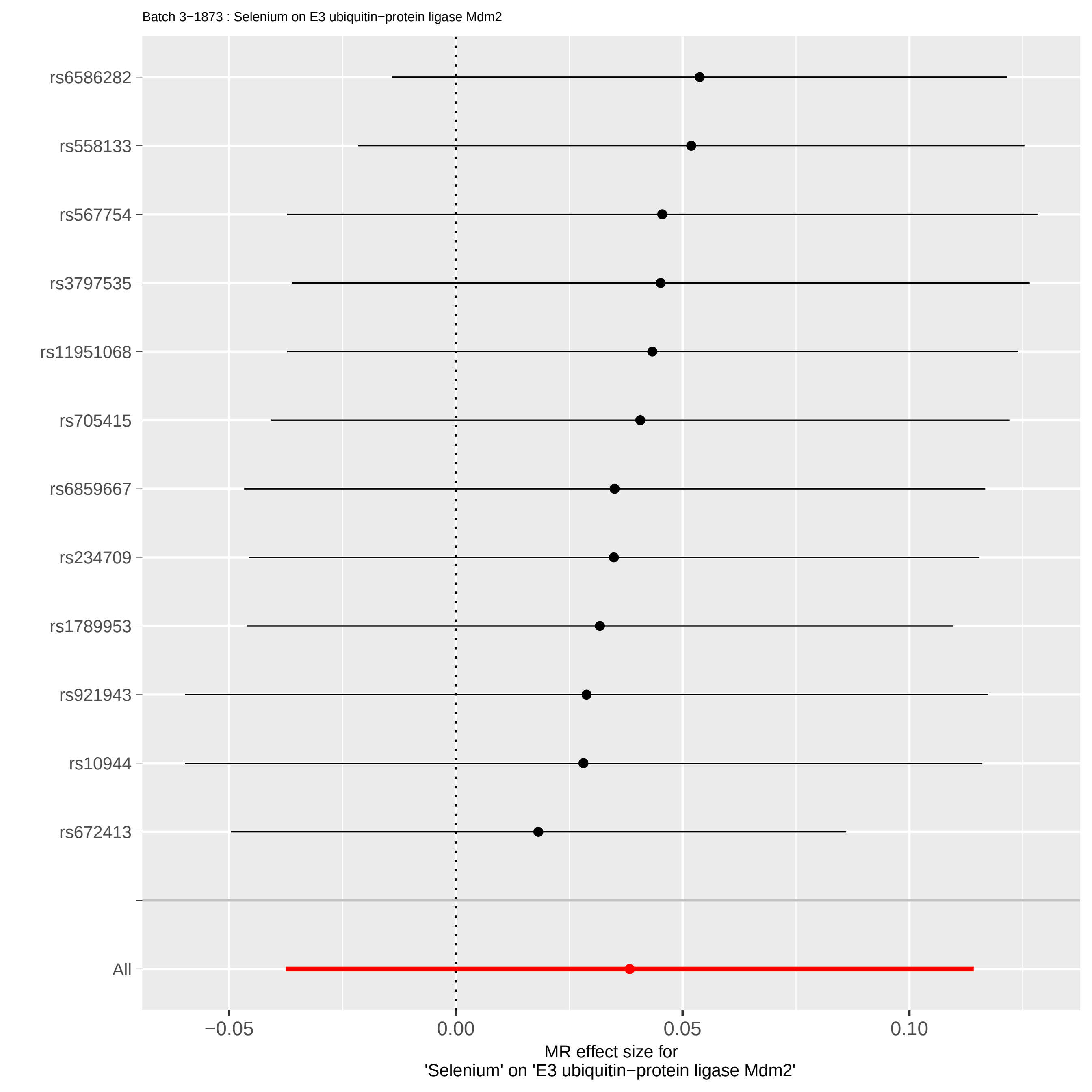

Batch 3−1873 : Selenium on E3 ubiquitin−protein ligase Mdm2
rs6586282
rs558133
rs567754
rs3797535
rs11951068
rs705415
rs6859667
rs234709
rs1789953
rs921943
rs10944
rs672413
All
−0.05
0.00
0.05
0.10
MR effect size for
'Selenium' on 'E3 ubiquitin−protein ligase Mdm2'

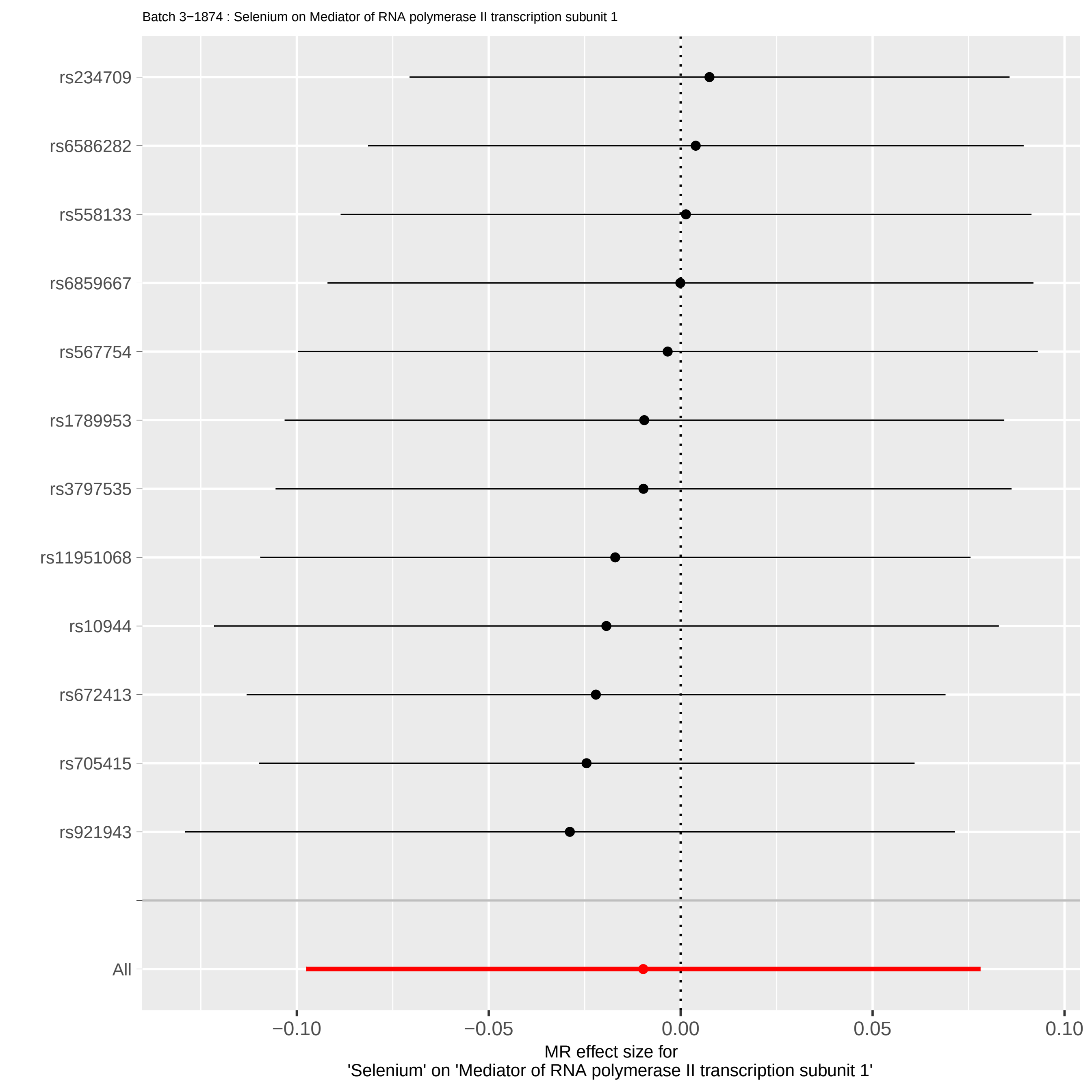

Batch 3−1874 : Selenium on Mediator of RNA polymerase II transcription subunit 1
rs234709
rs6586282
rs558133
rs6859667
rs567754
rs1789953
rs3797535
rs11951068
rs10944
rs672413
rs705415
rs921943
All
−0.10
−0.05
0.00
0.05
0.10
MR effect size for
'Selenium' on 'Mediator of RNA polymerase II transcription subunit 1'

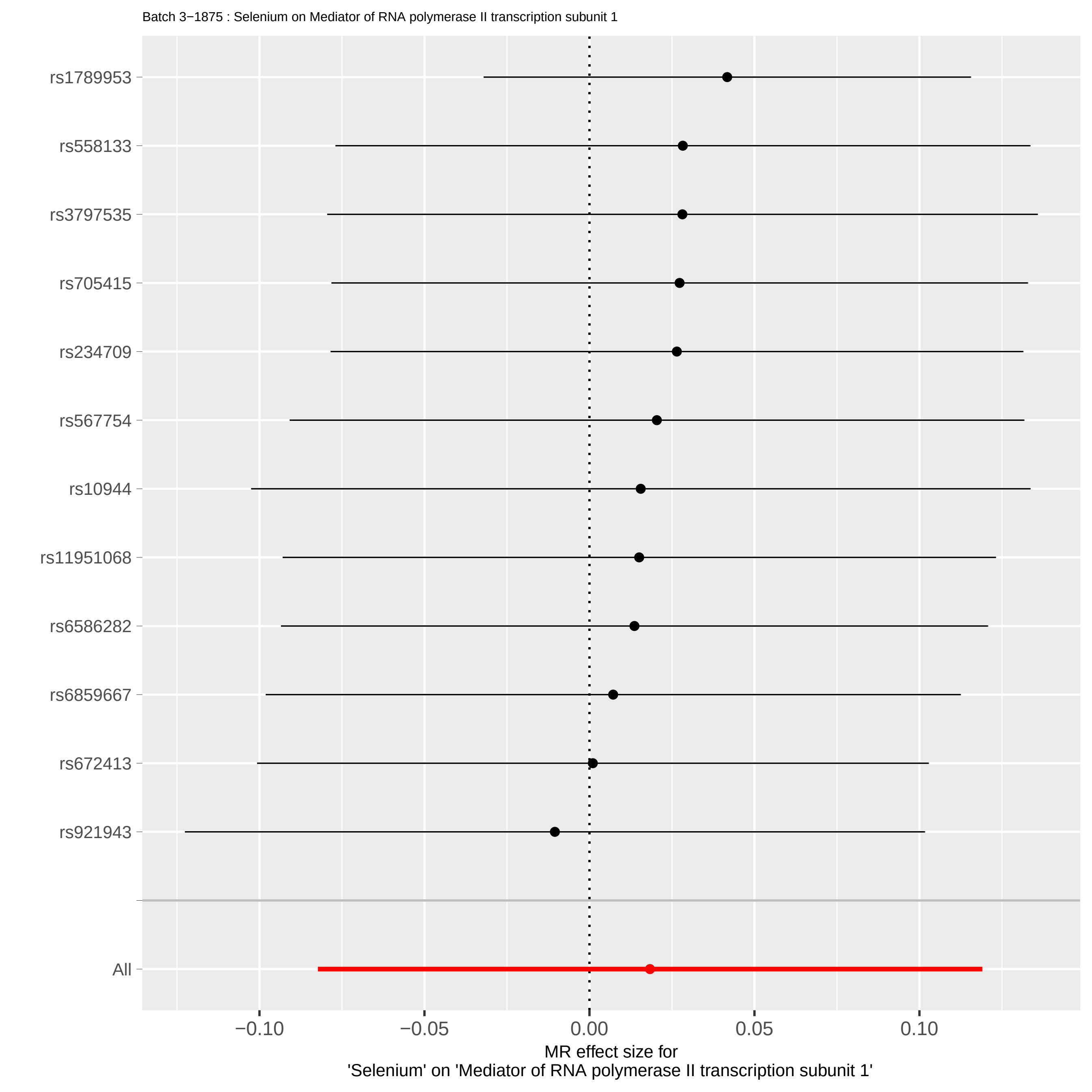

Batch 3−1875 : Selenium on Mediator of RNA polymerase II transcription subunit 1
rs1789953
rs558133
rs3797535
rs705415
rs234709
rs567754
rs10944
rs11951068
rs6586282
rs6859667
rs672413
rs921943
All
−0.10
−0.05
0.00
0.05
0.10
MR effect size for
'Selenium' on 'Mediator of RNA polymerase II transcription subunit 1'

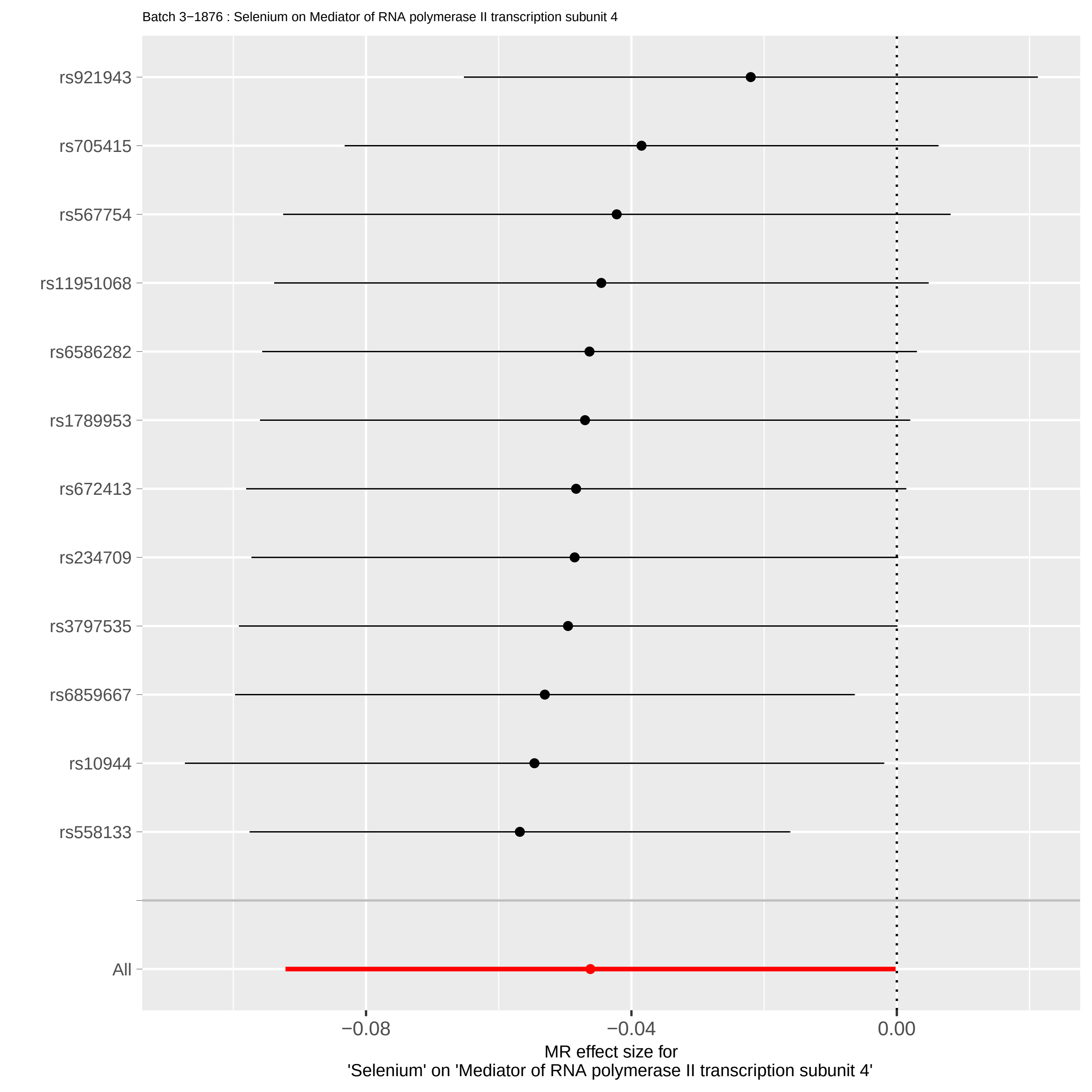

Batch 3−1876 : Selenium on Mediator of RNA polymerase II transcription subunit 4
rs921943
rs705415
rs567754
rs11951068
rs6586282
rs1789953
rs672413
rs234709
rs3797535
rs6859667
rs10944
rs558133
All
−0.08
−0.04
0.00
MR effect size for
'Selenium' on 'Mediator of RNA polymerase II transcription subunit 4'

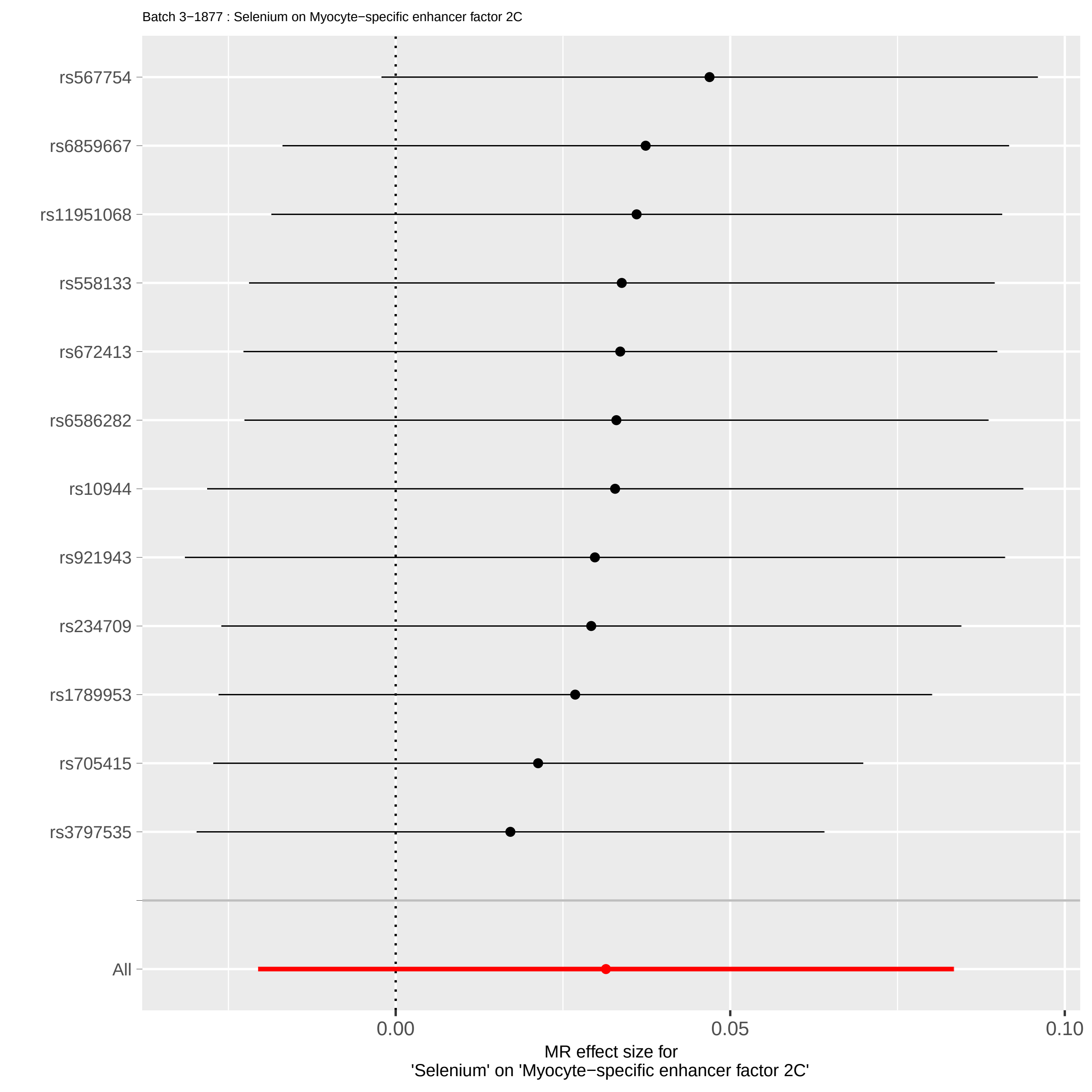

Batch 3−1877 : Selenium on Myocyte−specific enhancer factor 2C
rs567754
rs6859667
rs11951068
rs558133
rs672413
rs6586282
rs10944
rs921943
rs234709
rs1789953
rs705415
rs3797535
All
0.00
0.05
0.10
MR effect size for
'Selenium' on 'Myocyte−specific enhancer factor 2C'

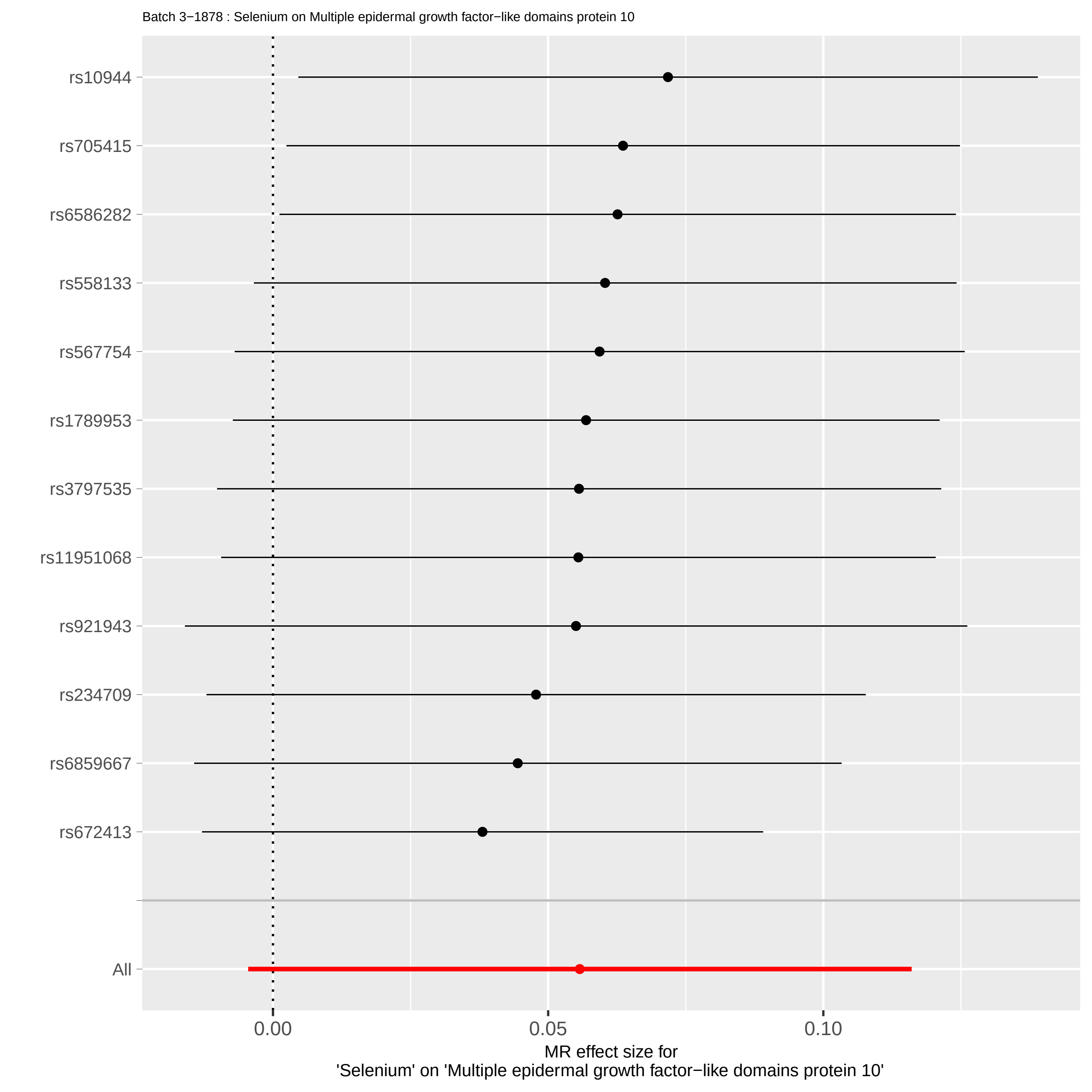
Batch 3−1878 : Selenium on Multiple epidermal growth factor−like domains protein 10
rs10944
rs705415
rs6586282
rs558133
rs567754
rs1789953
rs3797535
rs11951068
rs921943
rs234709
rs6859667
rs672413
All
0.00
0.05
0.10
MR effect size for
'Selenium' on 'Multiple epidermal growth factor−like domains protein 10'

Batch 3−1879 : Selenium on Protein MENT

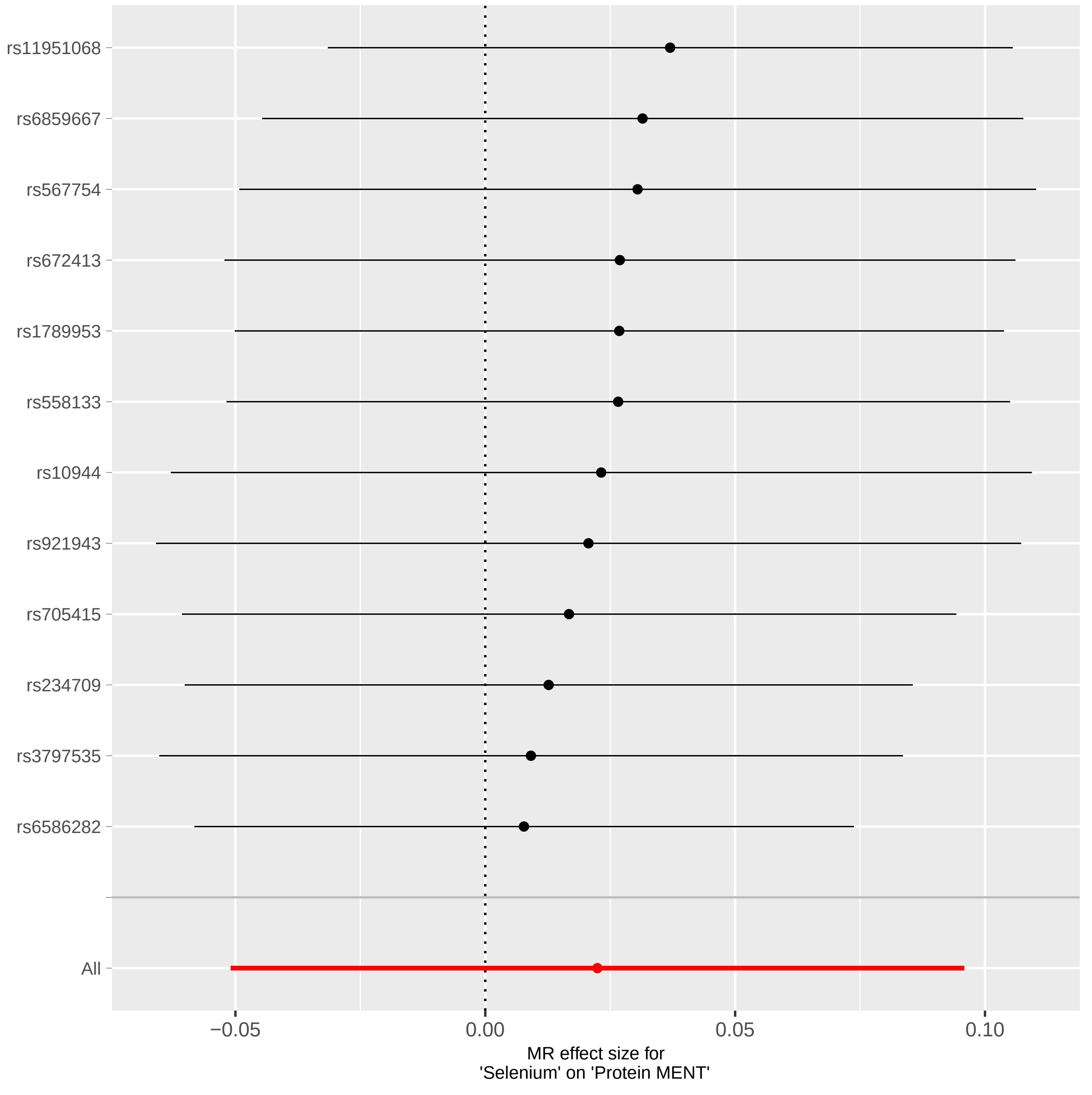

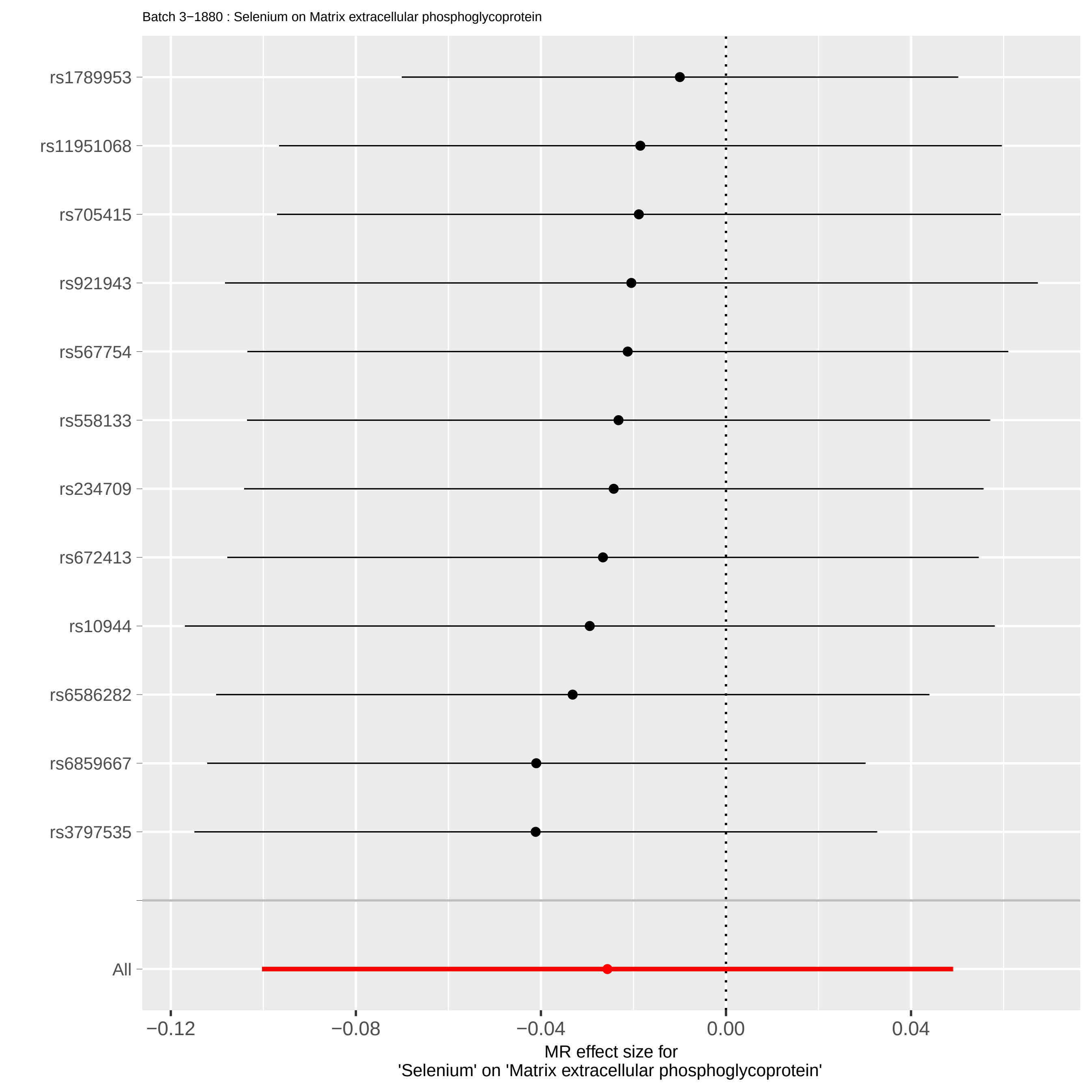
Batch 3−1880 : Selenium on Matrix extracellular phosphoglycoprotein
rs1789953
rs11951068
rs705415
rs921943
rs567754
rs558133
rs234709
rs672413
rs10944
rs6586282
rs6859667
rs3797535
All
−0.12
−0.08
−0.04
0.00
0.04
MR effect size for
'Selenium' on 'Matrix extracellular phosphoglycoprotein'

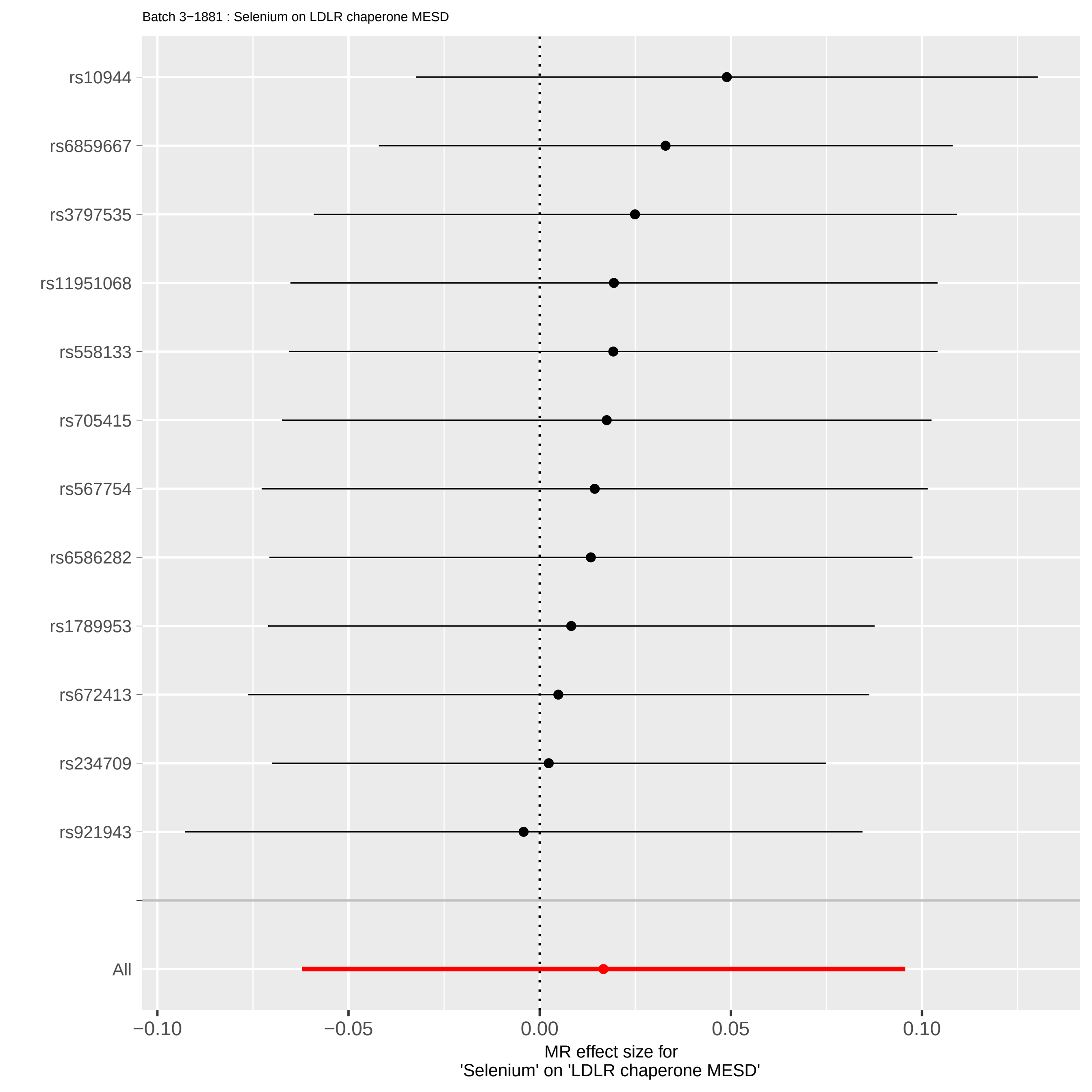
Batch 3−1881 : Selenium on LDLR chaperone MESD
rs10944
rs6859667
rs3797535
rs11951068
rs558133
rs705415
rs567754
rs6586282
rs1789953
rs672413
rs234709
rs921943
All
−0.10
−0.05
0.00
0.05
0.10
MR effect size for
'Selenium' on 'LDLR chaperone MESD'

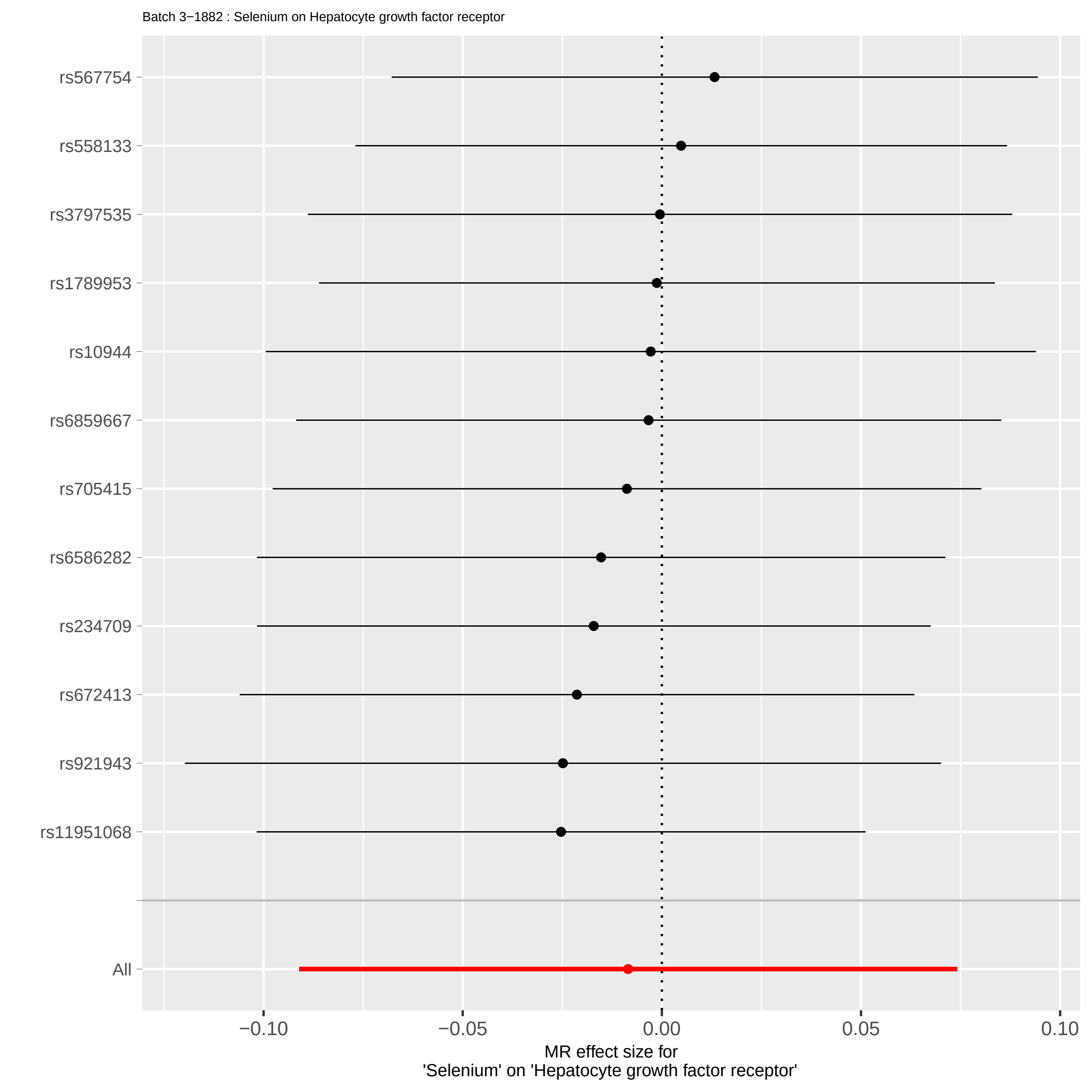

Batch 3−1882 : Selenium on Hepatocyte growth factor receptor
rs567754
rs558133
rs3797535
rs1789953
rs10944
rs6859667
rs705415
rs6586282
rs234709
rs672413
rs921943
rs11951068
All
−0.10
−0.05
0.00
0.05
0.10
MR effect size for
'Selenium' on 'Hepatocyte growth factor receptor'

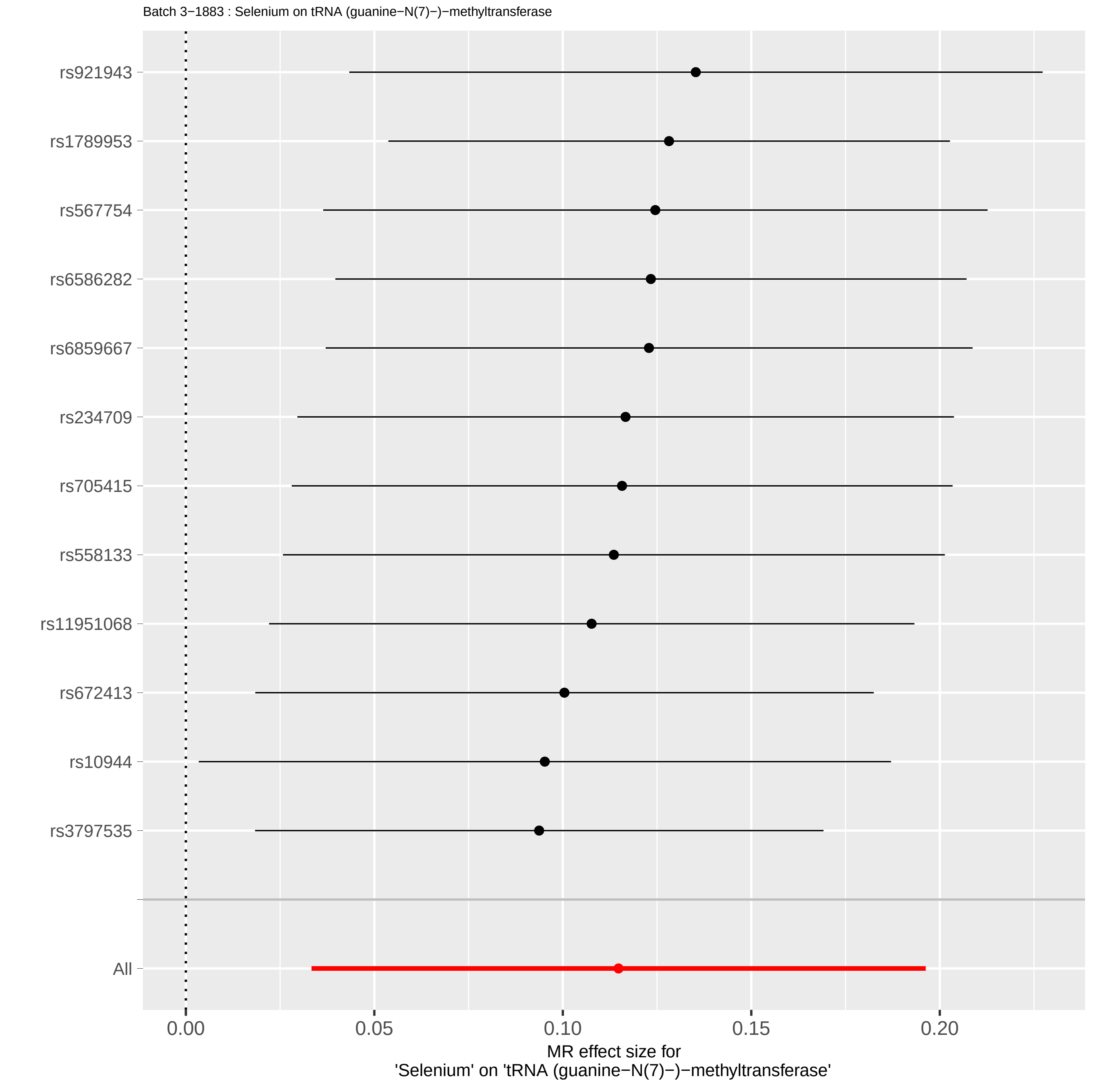

Batch 3−1883 : Selenium on tRNA (guanine−N(7)−)−methyltransferase
rs921943
rs1789953
rs567754
rs6586282
rs6859667
rs234709
rs705415
rs558133
rs11951068
rs672413
rs10944
rs3797535
All
0.00
0.05
0.10
0.15
0.20
MR effect size for
'Selenium' on 'tRNA (guanine−N(7)−)−methyltransferase'

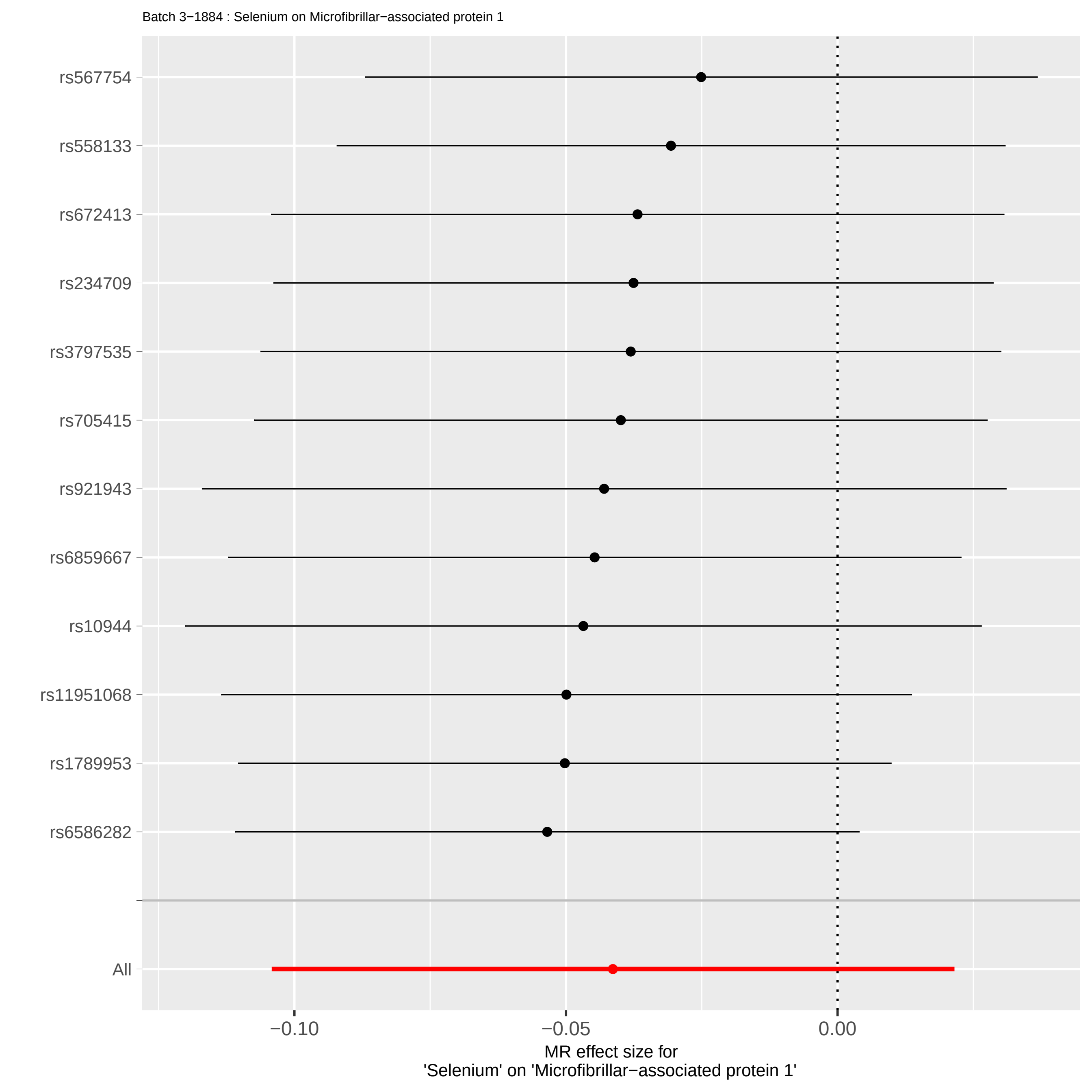

Batch 3−1884 : Selenium on Microfibrillar−associated protein 1
rs567754
rs558133
rs672413
rs234709
rs3797535
rs705415
rs921943
rs6859667
rs10944
rs11951068
rs1789953
rs6586282
All
−0.10
−0.05
0.00
MR effect size for
'Selenium' on 'Microfibrillar−associated protein 1'

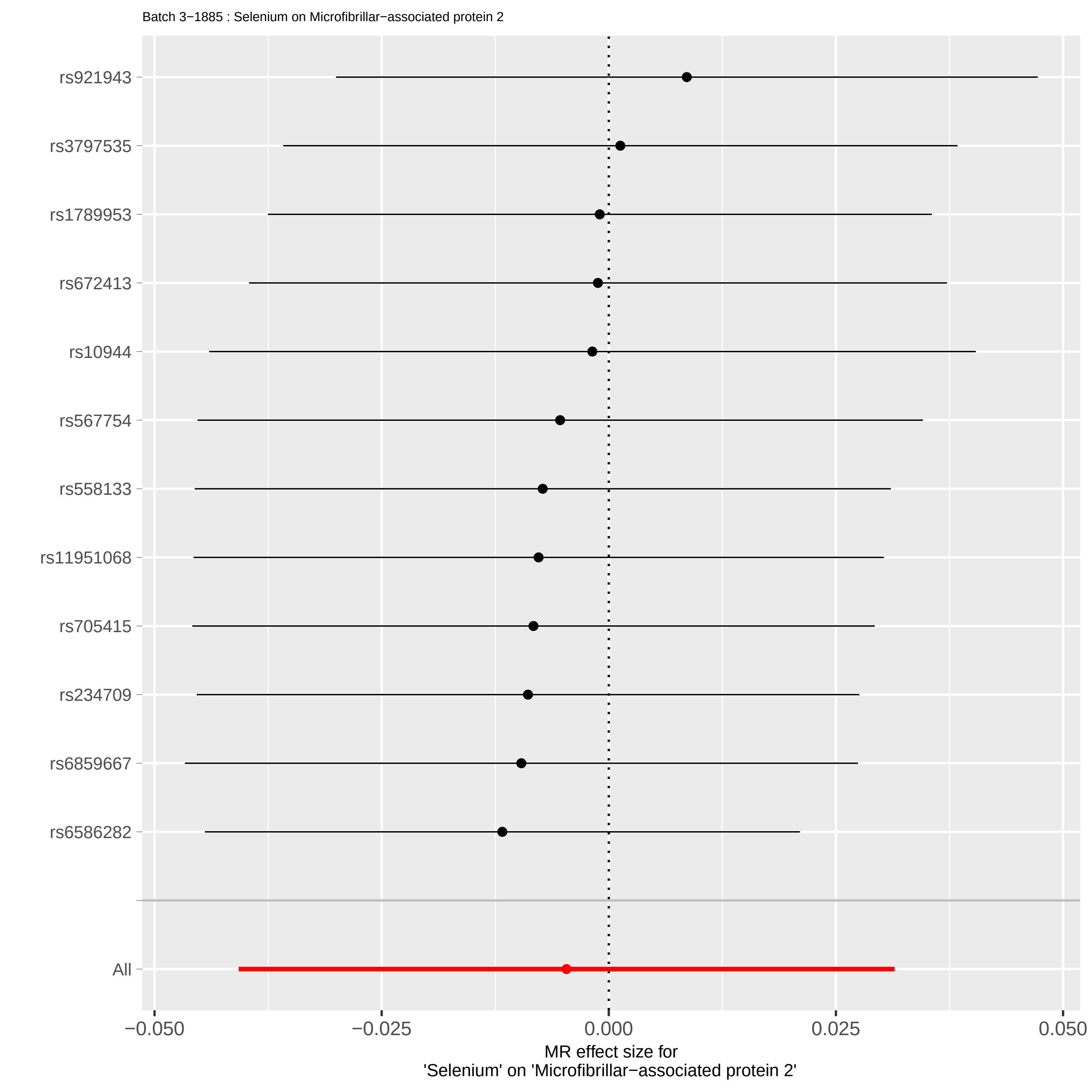

Batch 3−1885 : Selenium on Microfibrillar−associated protein 2
rs921943
rs3797535
rs1789953
rs672413
rs10944
rs567754
rs558133
rs11951068
rs705415
rs234709
rs6859667
rs6586282
All
−0.050
−0.025
0.000
0.025
0.050
MR effect size for
'Selenium' on 'Microfibrillar−associated protein 2'

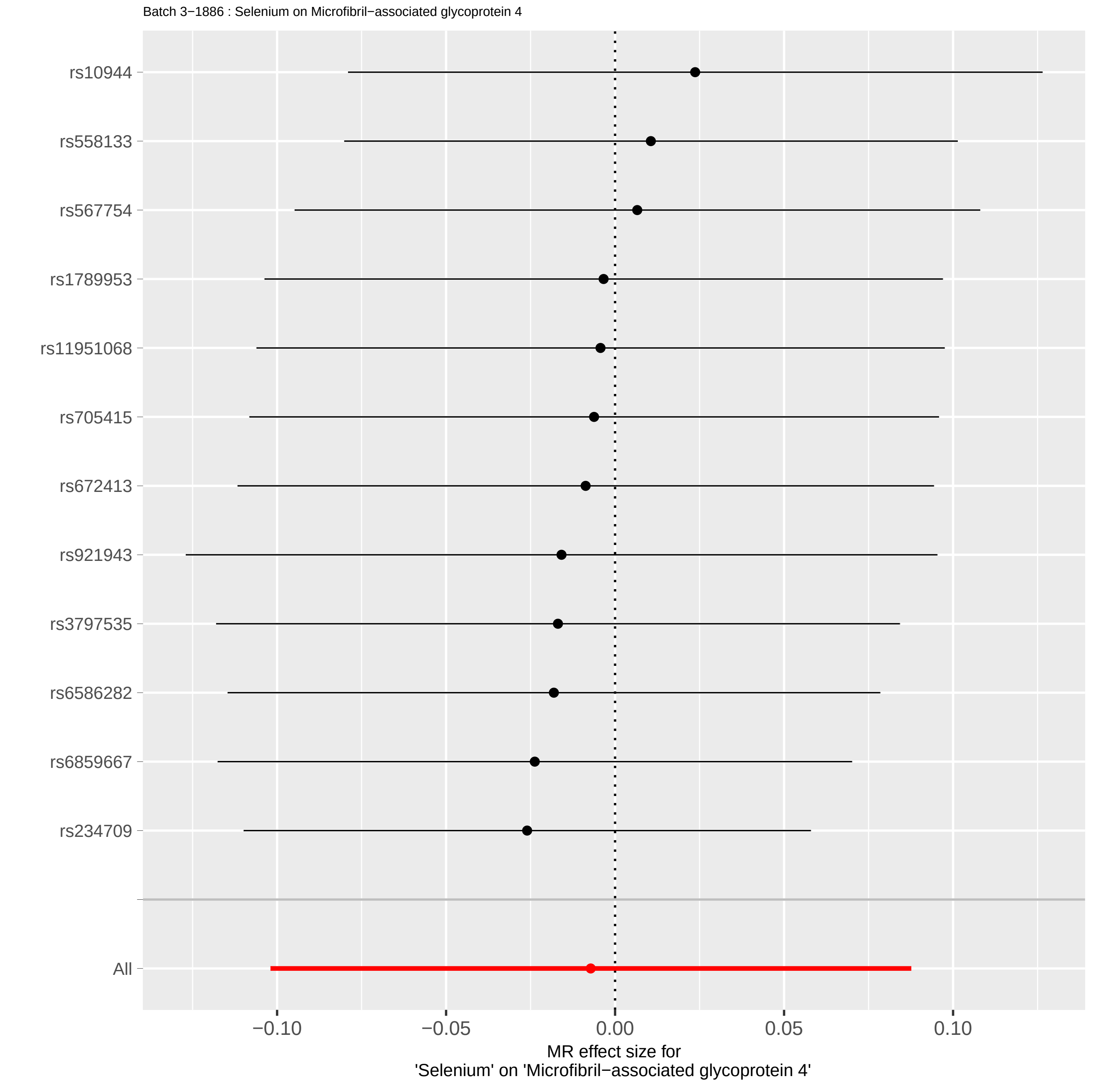

Batch 3−1886 : Selenium on Microfibril−associated glycoprotein 4
rs10944
rs558133
rs567754
rs1789953
rs11951068
rs705415
rs672413
rs921943
rs3797535
rs6586282
rs6859667
rs234709
All
−0.10
−0.05
0.00
0.05
0.10
MR effect size for
'Selenium' on 'Microfibril−associated glycoprotein 4'

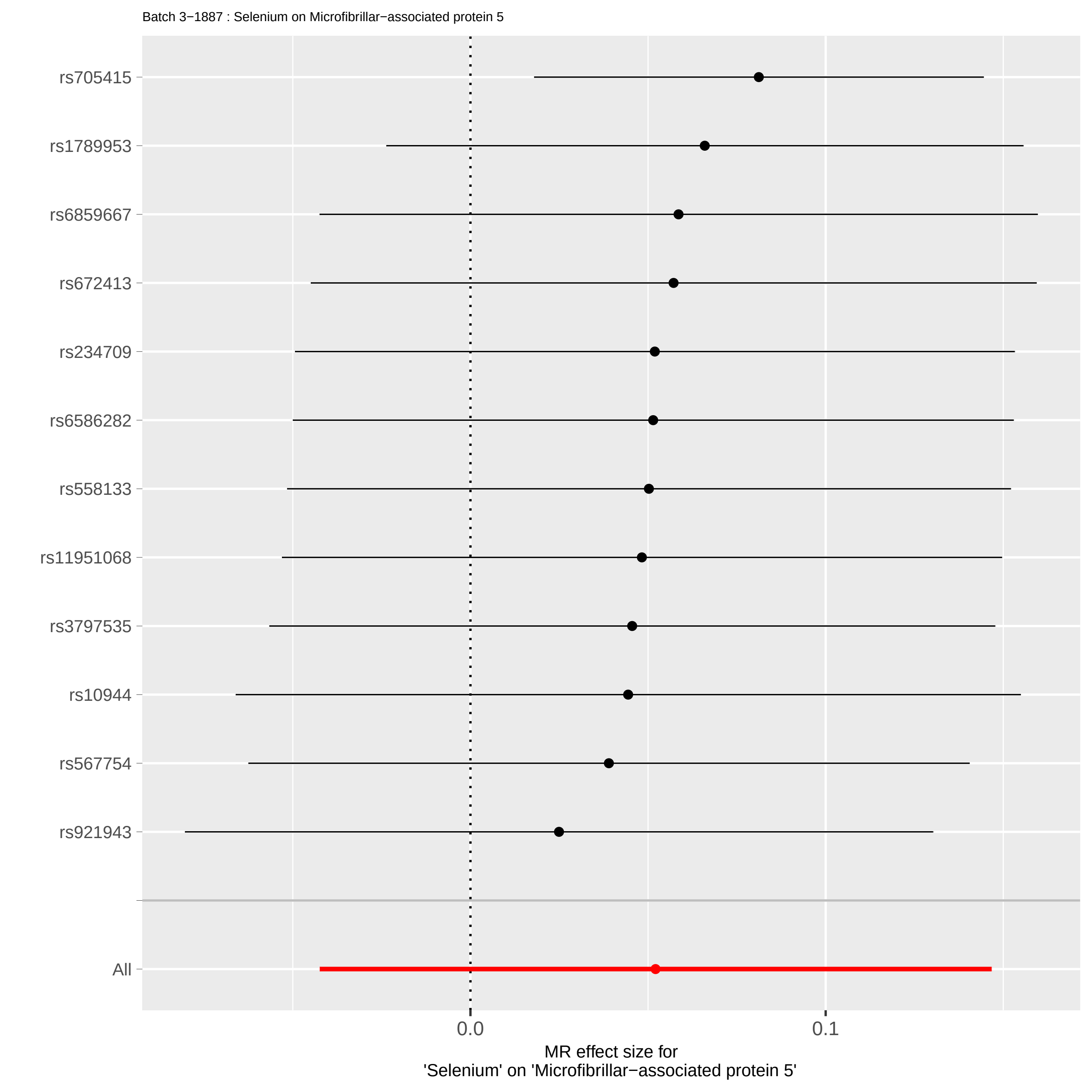

Batch 3−1887 : Selenium on Microfibrillar−associated protein 5
rs705415
rs1789953
rs6859667
rs672413
rs234709
rs6586282
rs558133
rs11951068
rs3797535
rs10944
rs567754
rs921943
All
0.0
0.1
MR effect size for
'Selenium' on 'Microfibrillar−associated protein 5'

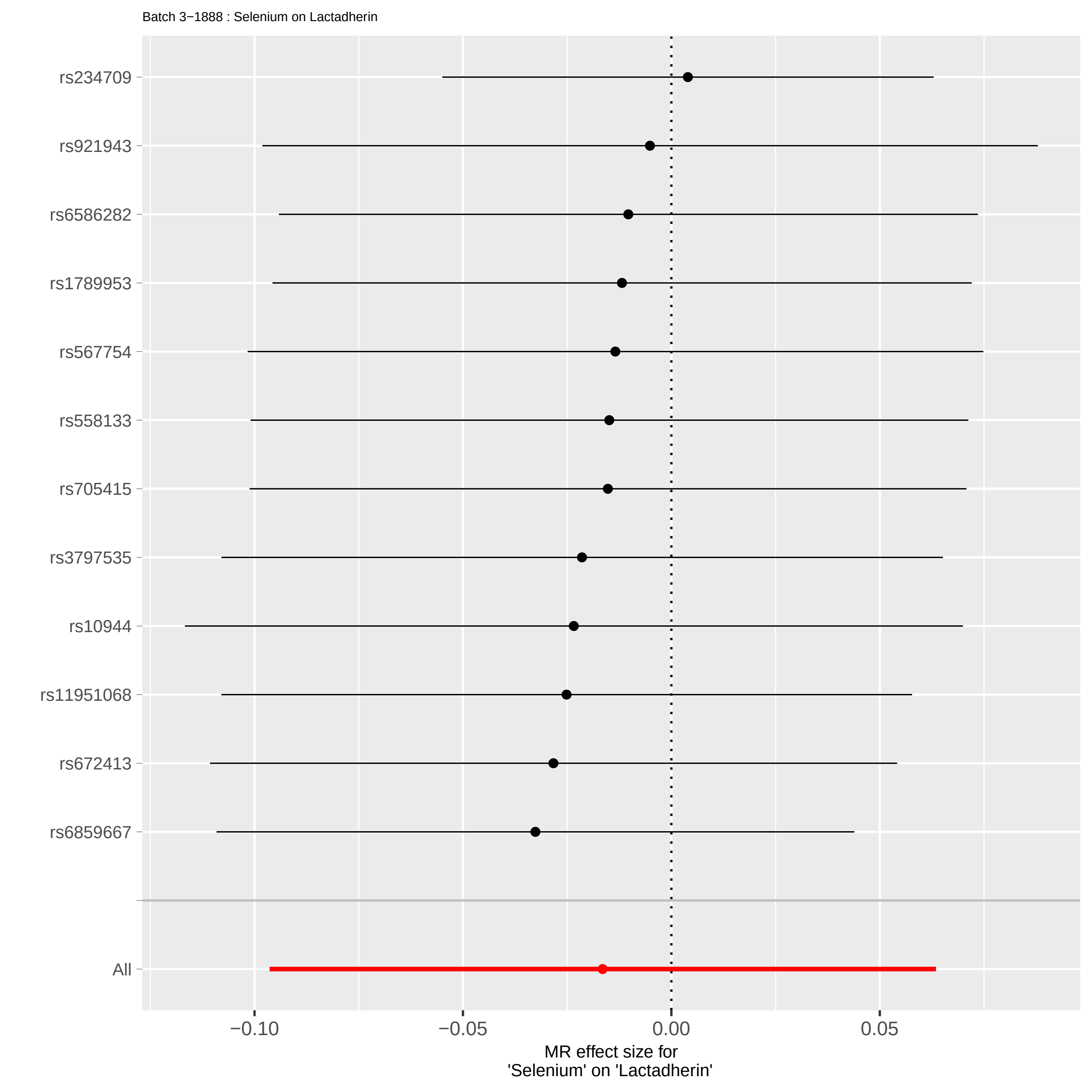

Batch 3−1888 : Selenium on Lactadherin
rs234709
rs921943
rs6586282
rs1789953
rs567754
rs558133
rs705415
rs3797535
rs10944
rs11951068
rs672413
rs6859667
All
−0.10
−0.05
0.00
0.05
MR effect size for
'Selenium' on 'Lactadherin'

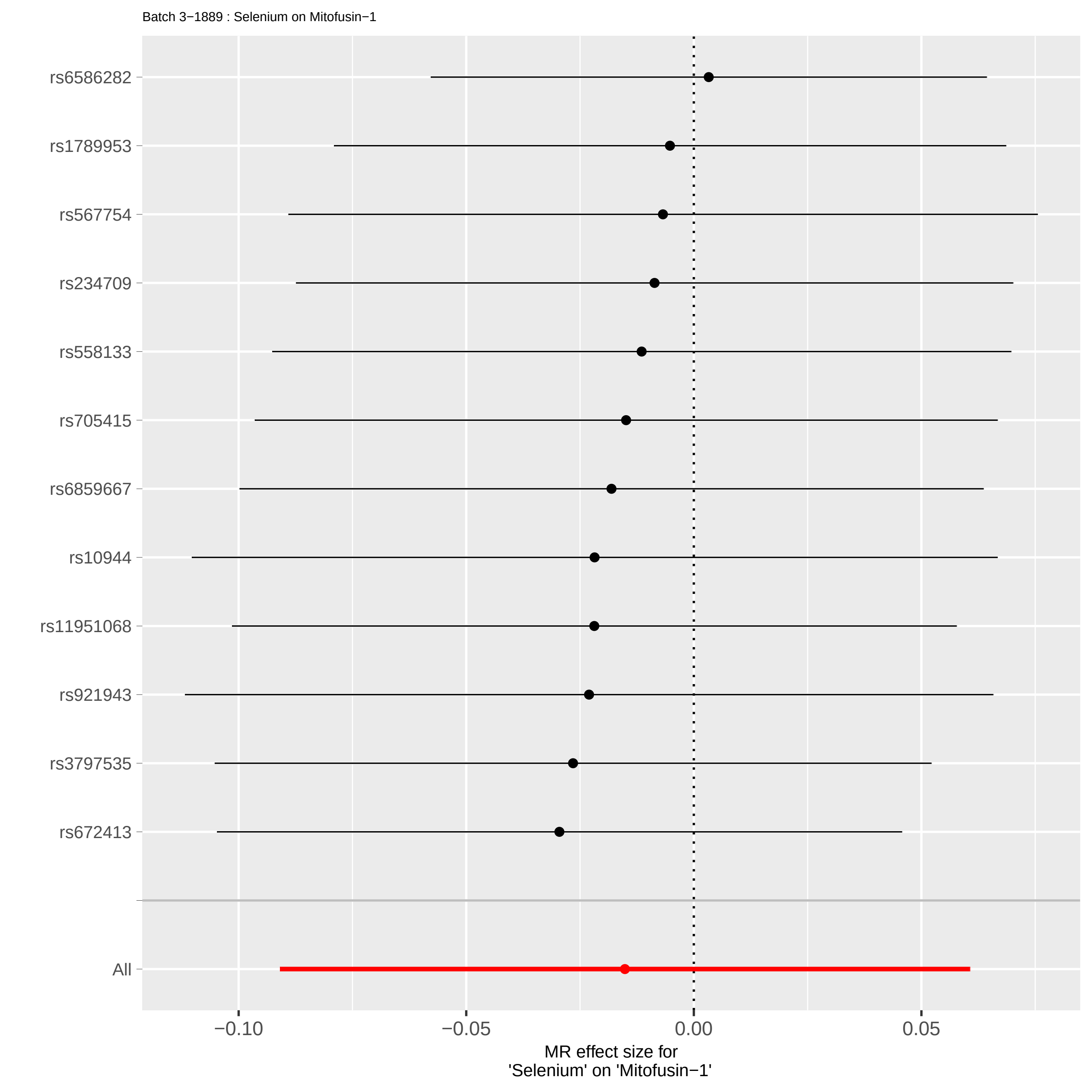
Batch 3−1889 : Selenium on Mitofusin−1
rs6586282
rs1789953
rs567754
rs234709
rs558133
rs705415
rs6859667
rs10944
rs11951068
rs921943
rs3797535
rs672413
All
−0.10
−0.05
0.00
0.05
MR effect size for
'Selenium' on 'Mitofusin−1'

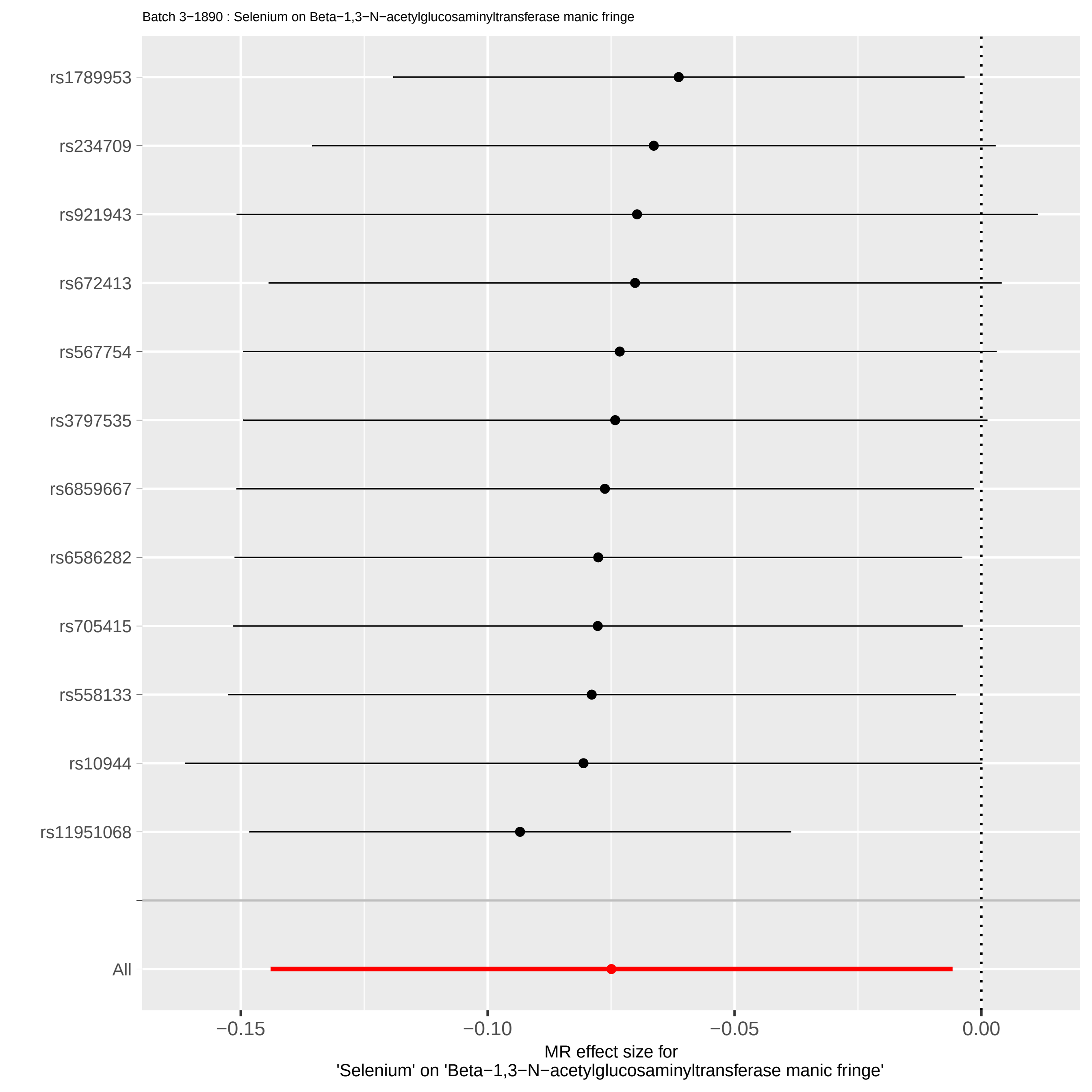

Batch 3−1890 : Selenium on Beta−1,3−N−acetylglucosaminyltransferase manic fringe
rs1789953
rs234709
rs921943
rs672413
rs567754
rs3797535
rs6859667
rs6586282
rs705415
rs558133
rs10944
rs11951068
All
−0.15
−0.10
−0.05
0.00
MR effect size for
'Selenium' on 'Beta−1,3−N−acetylglucosaminyltransferase manic fringe'

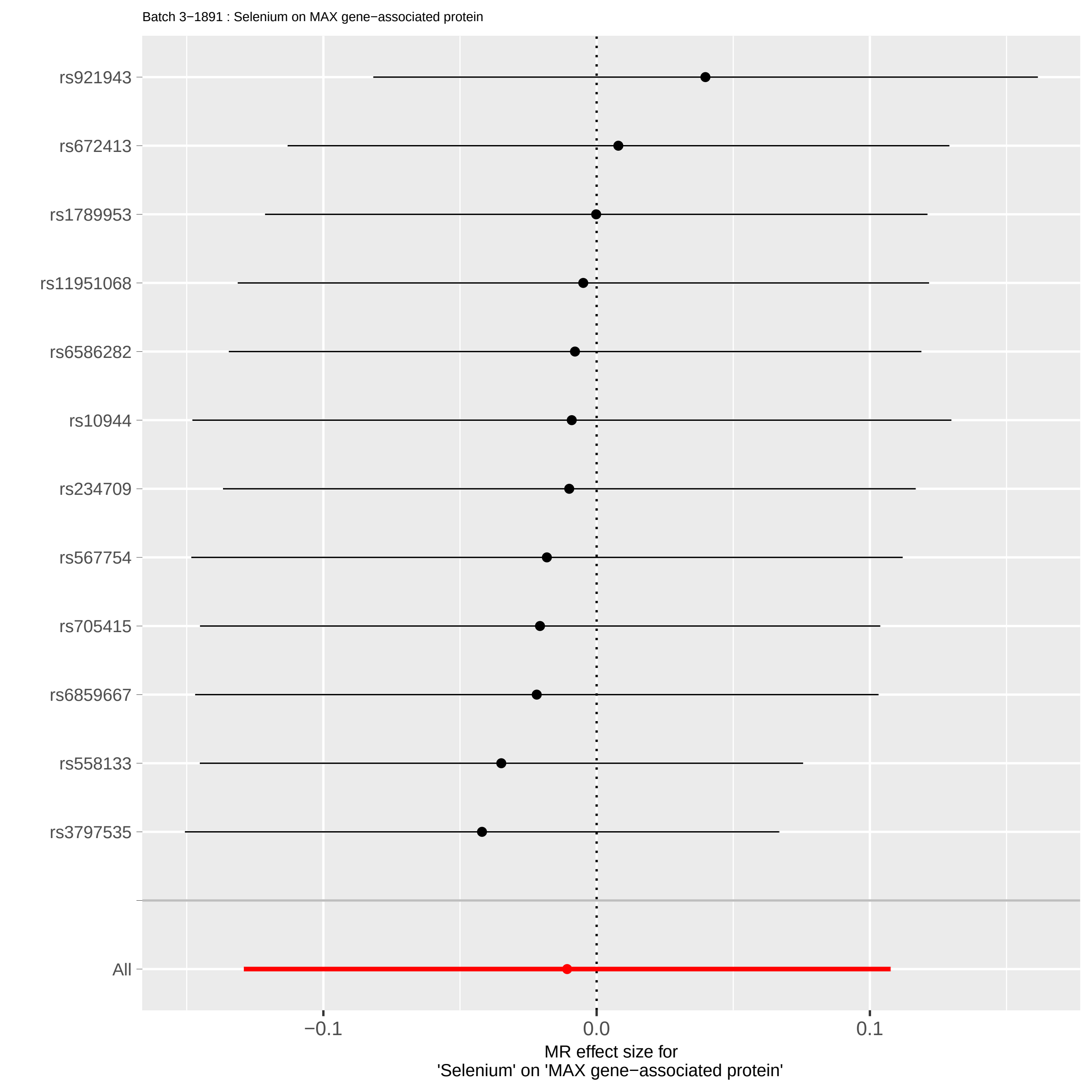
Batch 3−1891 : Selenium on MAX gene−associated protein
rs921943
rs672413
rs1789953
rs11951068
rs6586282
rs10944
rs234709
rs567754
rs705415
rs6859667
rs558133
rs3797535
All
−0.1
0.0
0.1
MR effect size for
'Selenium' on 'MAX gene−associated protein'

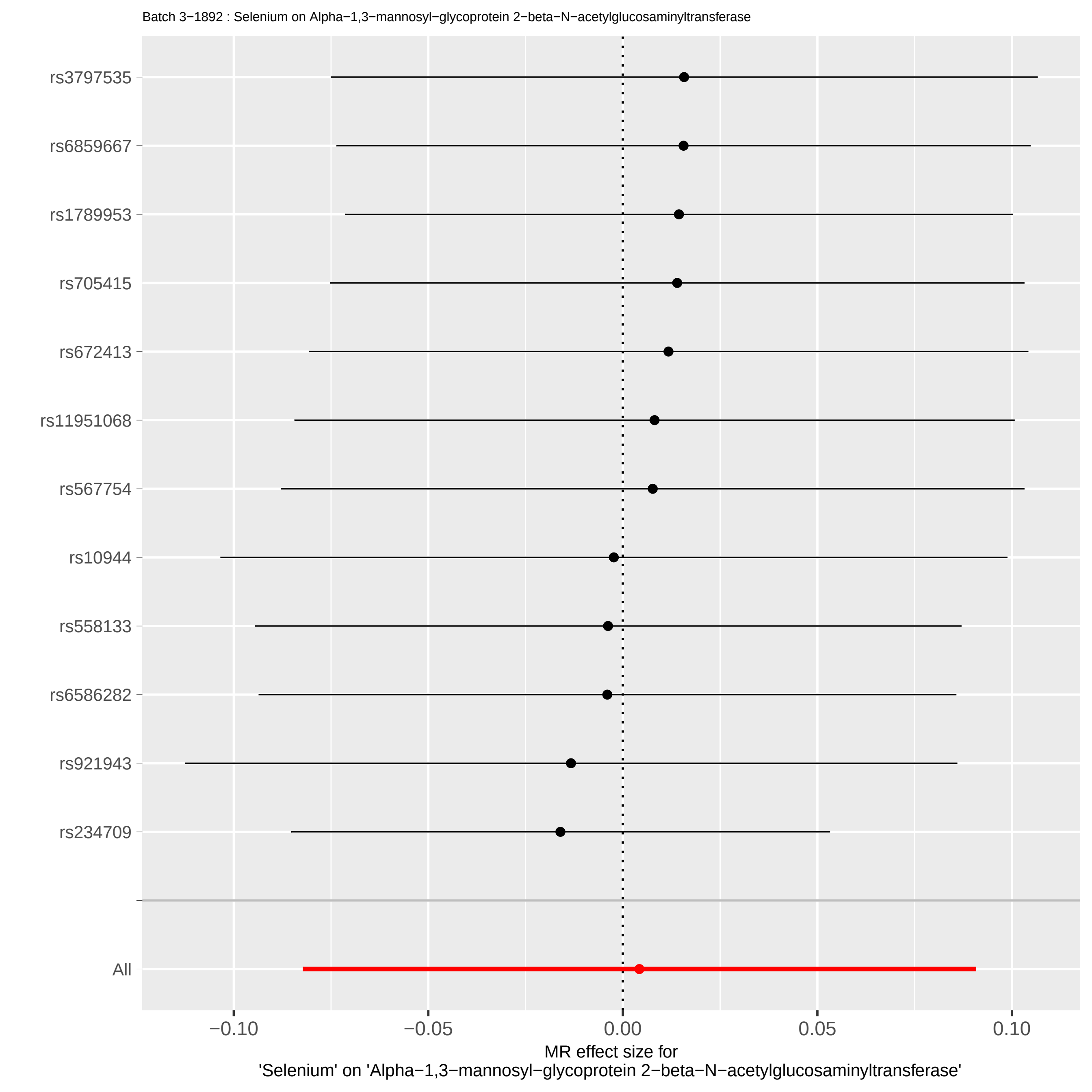

Batch 3−1892 : Selenium on Alpha−1,3−mannosyl−glycoprotein 2−beta−N−acetylglucosaminyltransferase
rs3797535
rs6859667
rs1789953
rs705415
rs672413
rs11951068
rs567754
rs10944
rs558133
rs6586282
rs921943
rs234709
All
−0.10
−0.05
0.00
0.05
0.10
MR effect size for
'Selenium' on 'Alpha−1,3−mannosyl−glycoprotein 2−beta−N−acetylglucosaminyltransferase'

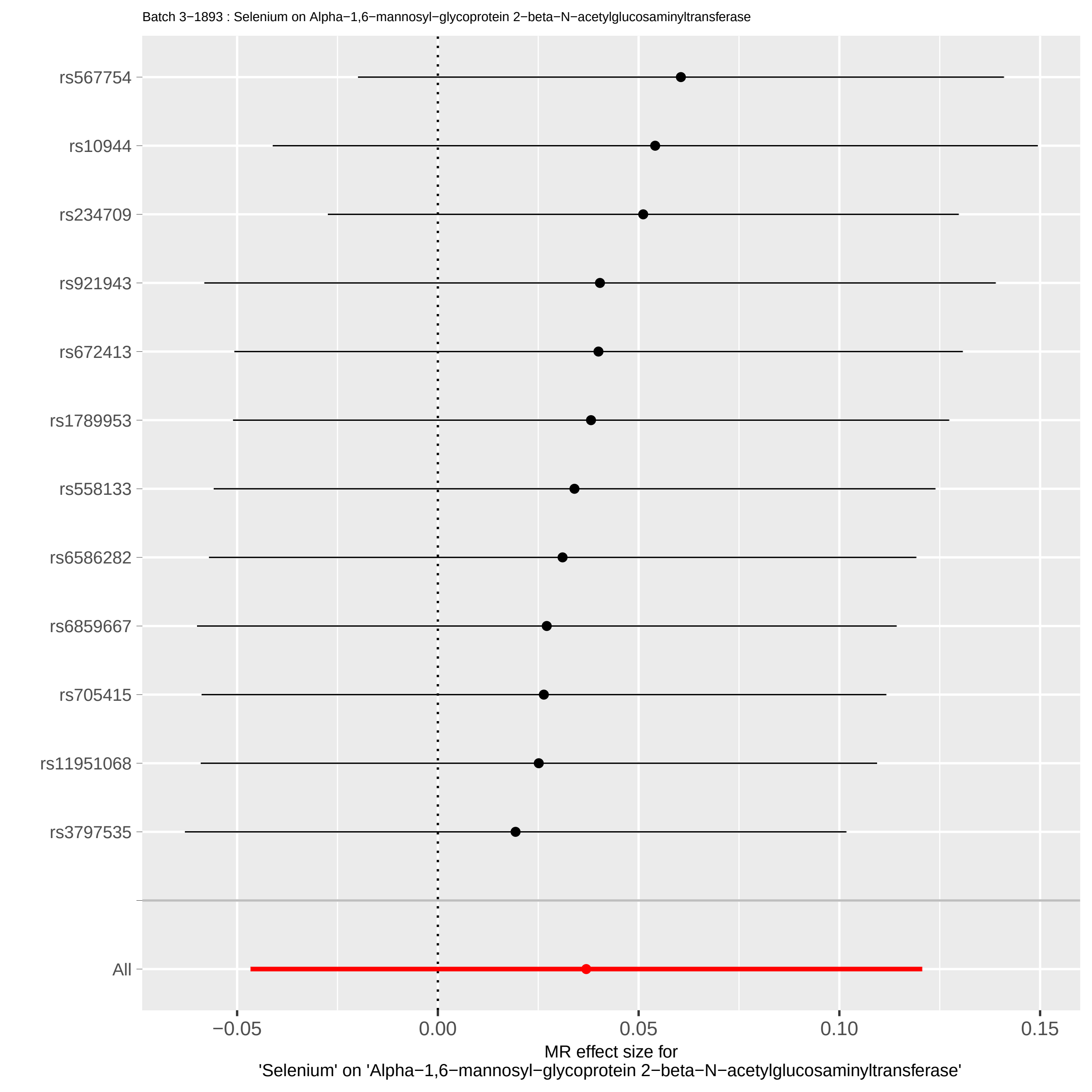

Batch 3−1893 : Selenium on Alpha−1,6−mannosyl−glycoprotein 2−beta−N−acetylglucosaminyltransferase
rs567754
rs10944
rs234709
rs921943
rs672413
rs1789953
rs558133
rs6586282
rs6859667
rs705415
rs11951068
rs3797535
All
−0.05
0.00
0.05
0.10
0.15
MR effect size for
'Selenium' on 'Alpha−1,6−mannosyl−glycoprotein 2−beta−N−acetylglucosaminyltransferase'

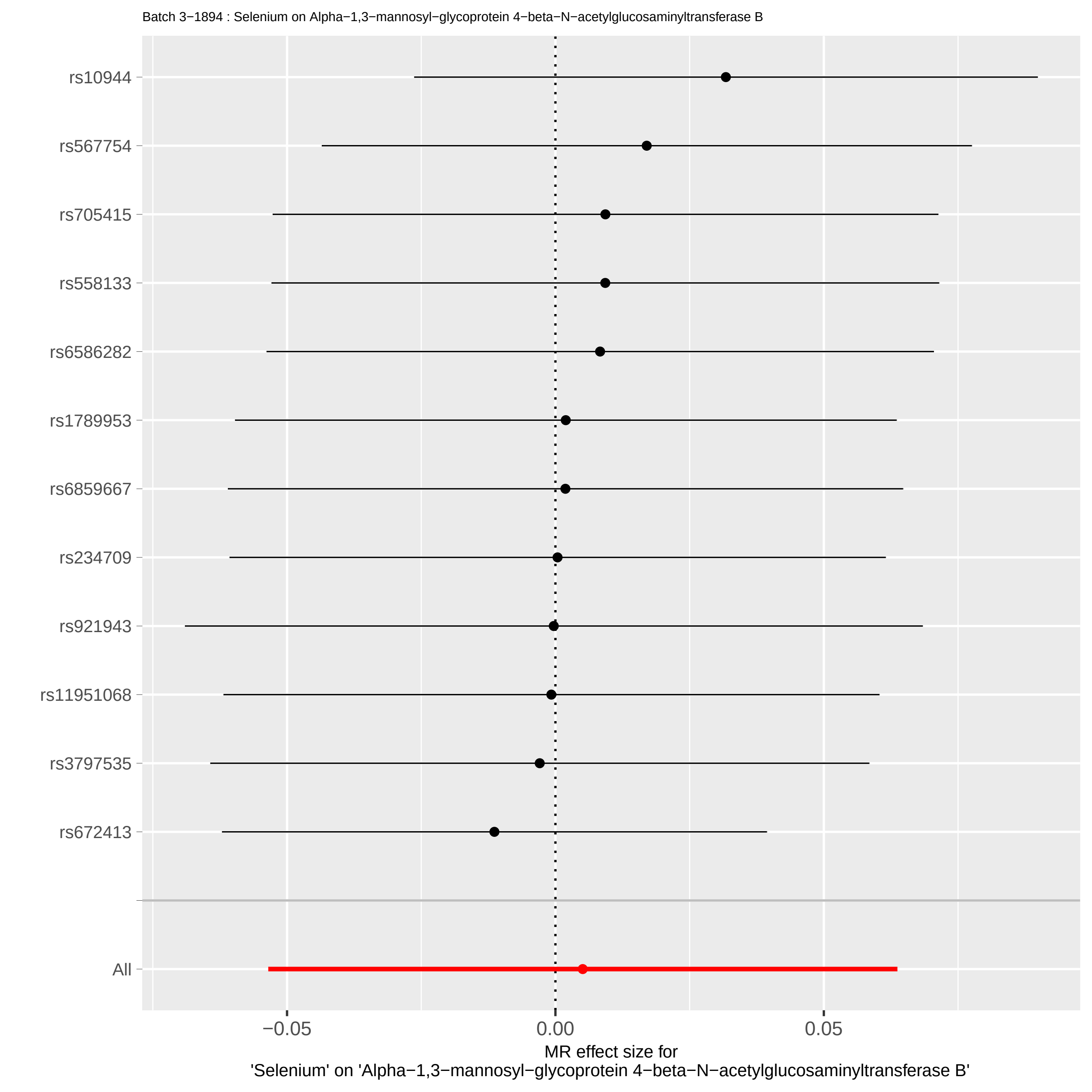

Batch 3−1894 : Selenium on Alpha−1,3−mannosyl−glycoprotein 4−beta−N−acetylglucosaminyltransferase B
rs10944
rs567754
rs705415
rs558133
rs6586282
rs1789953
rs6859667
rs234709
rs921943
rs11951068
rs3797535
rs672413
All
−0.05
0.00
0.05
MR effect size for
'Selenium' on 'Alpha−1,3−mannosyl−glycoprotein 4−beta−N−acetylglucosaminyltransferase B'

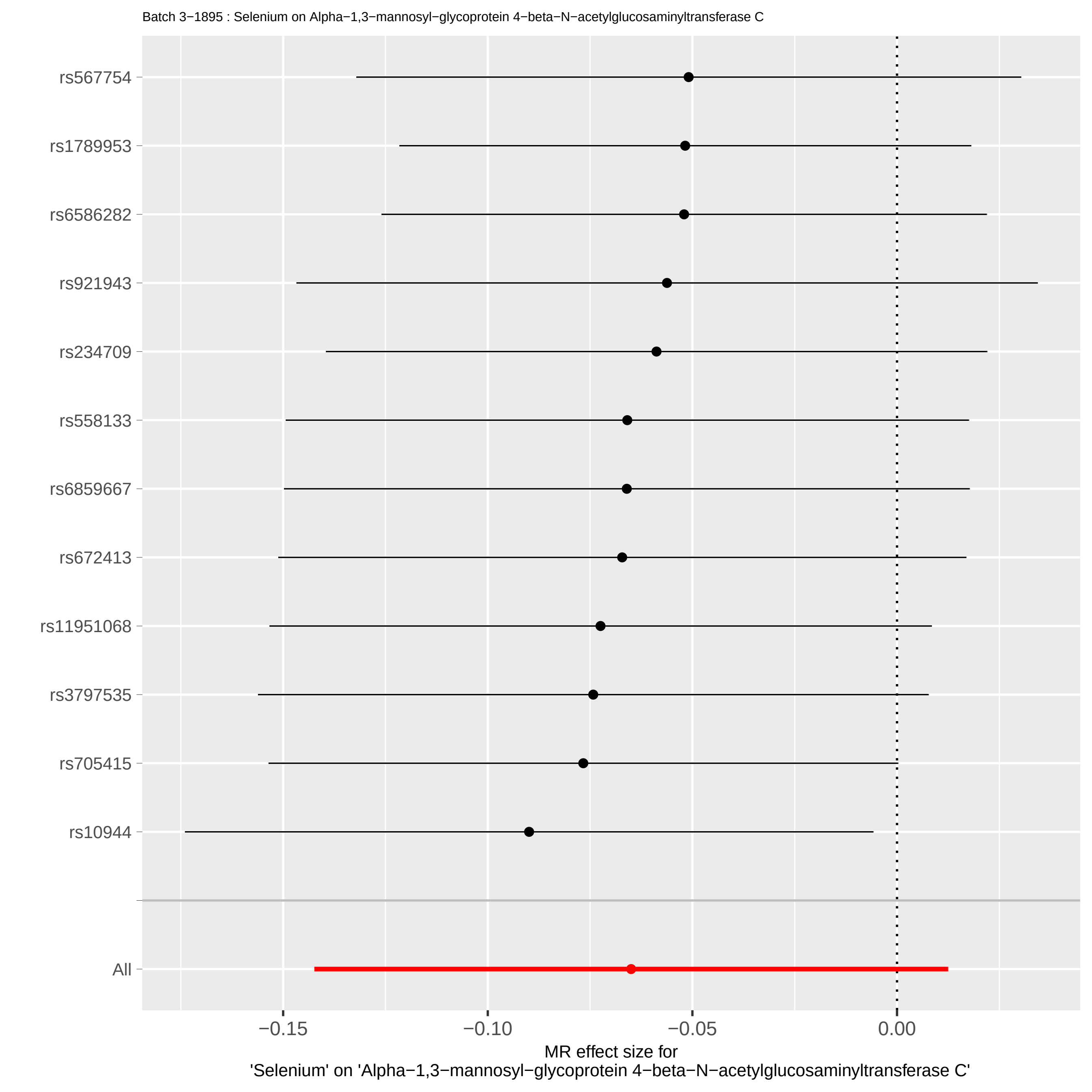

Batch 3−1895 : Selenium on Alpha−1,3−mannosyl−glycoprotein 4−beta−N−acetylglucosaminyltransferase C
rs567754
rs1789953
rs6586282
rs921943
rs234709
rs558133
rs6859667
rs672413
rs11951068
rs3797535
rs705415
rs10944
All
−0.15
−0.10
−0.05
0.00
MR effect size for
'Selenium' on 'Alpha−1,3−mannosyl−glycoprotein 4−beta−N−acetylglucosaminyltransferase C'

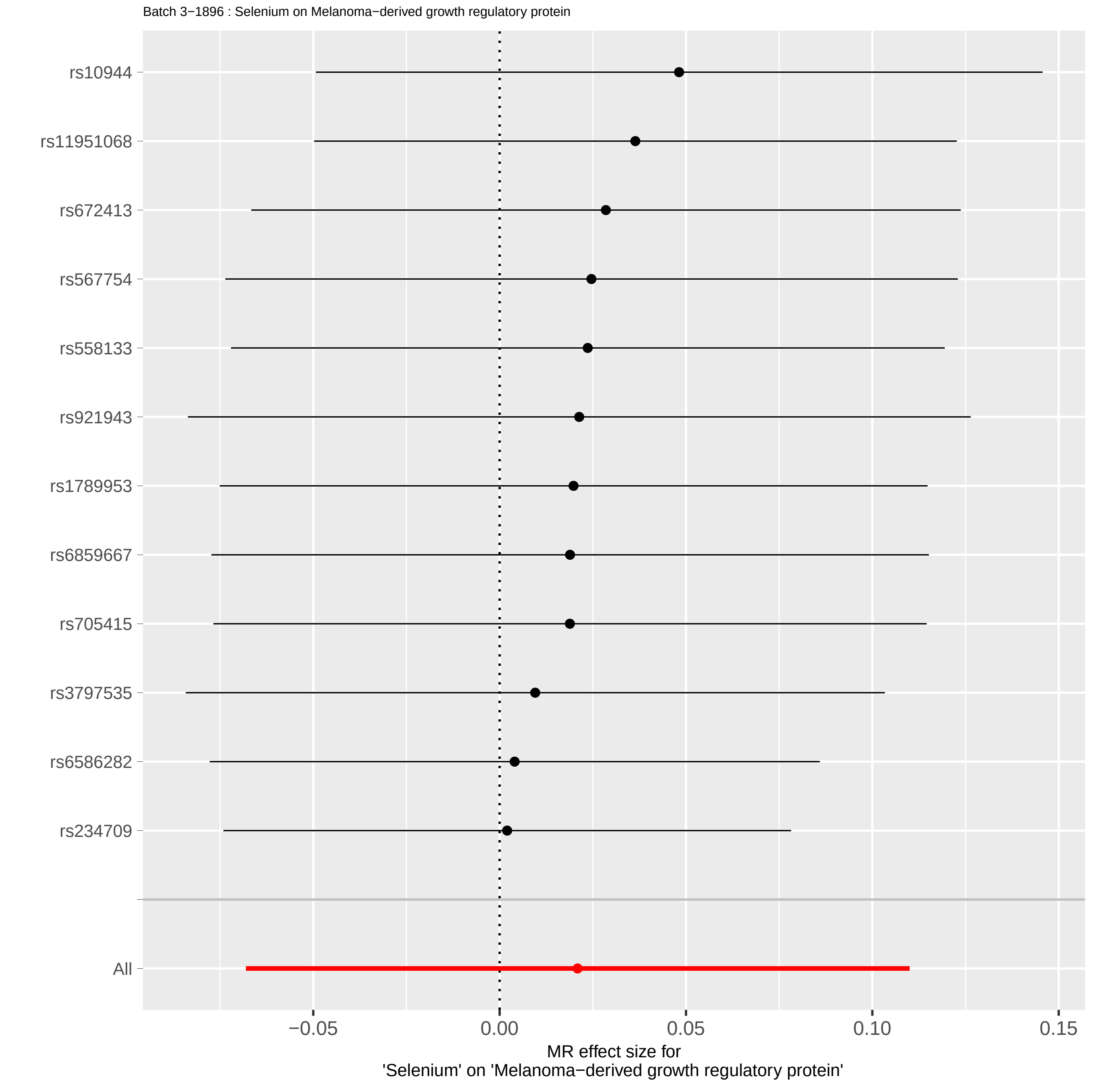
Batch 3−1896 : Selenium on Melanoma−derived growth regulatory protein
rs10944
rs11951068
rs672413
rs567754
rs558133
rs921943
rs1789953
rs6859667
rs705415
rs3797535
rs6586282
rs234709
All
−0.05
0.00
0.05
0.10
0.15
MR effect size for
'Selenium' on 'Melanoma−derived growth regulatory protein'

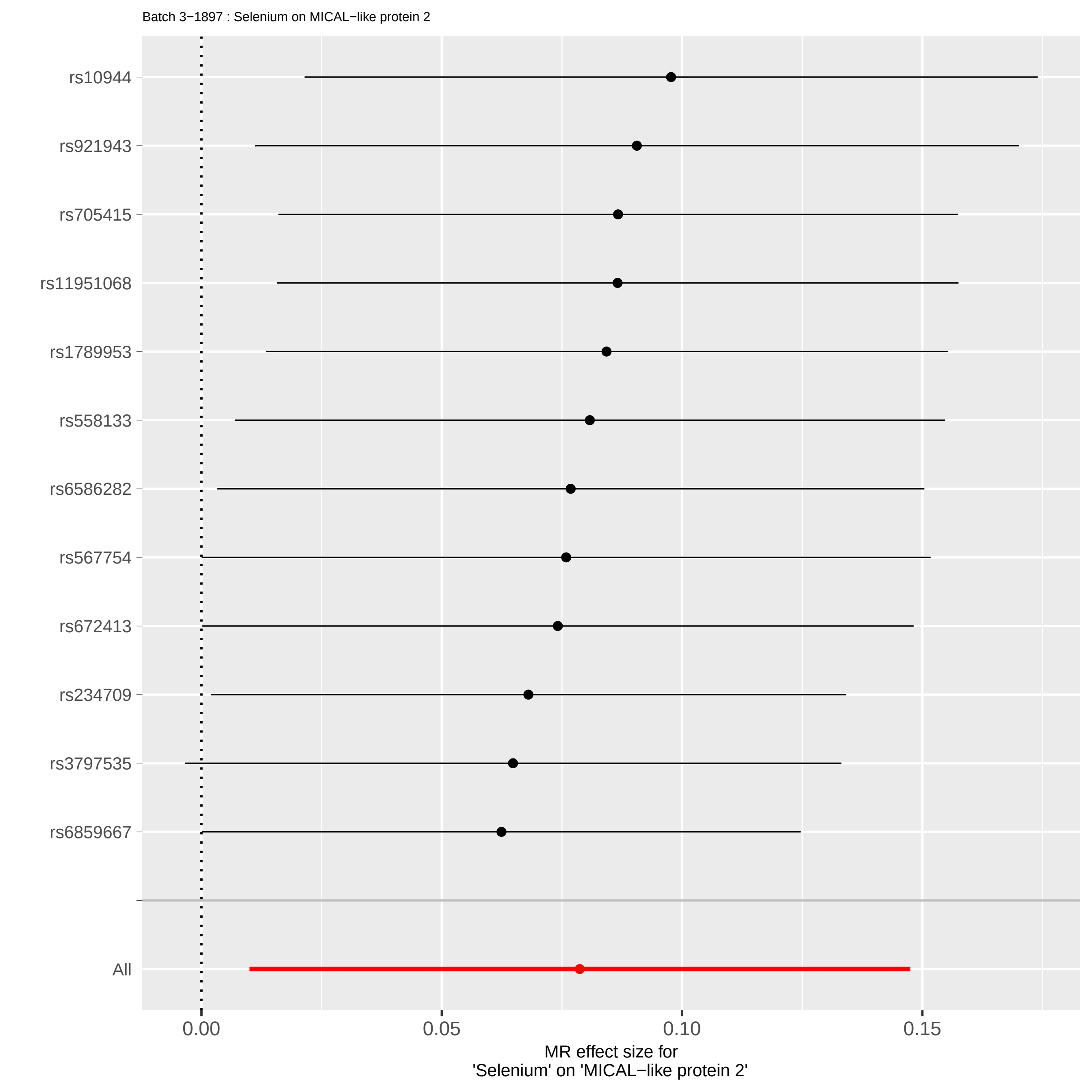

Batch 3−1897 : Selenium on MICAL−like protein 2
rs10944
rs921943
rs705415
rs11951068
rs1789953
rs558133
rs6586282
rs567754
rs672413
rs234709
rs3797535
rs6859667
All
0.00
0.05
0.10
0.15
MR effect size for
'Selenium' on 'MICAL−like protein 2'

Batch 3−1898 : Selenium on MHC class I polypeptide−related sequence B

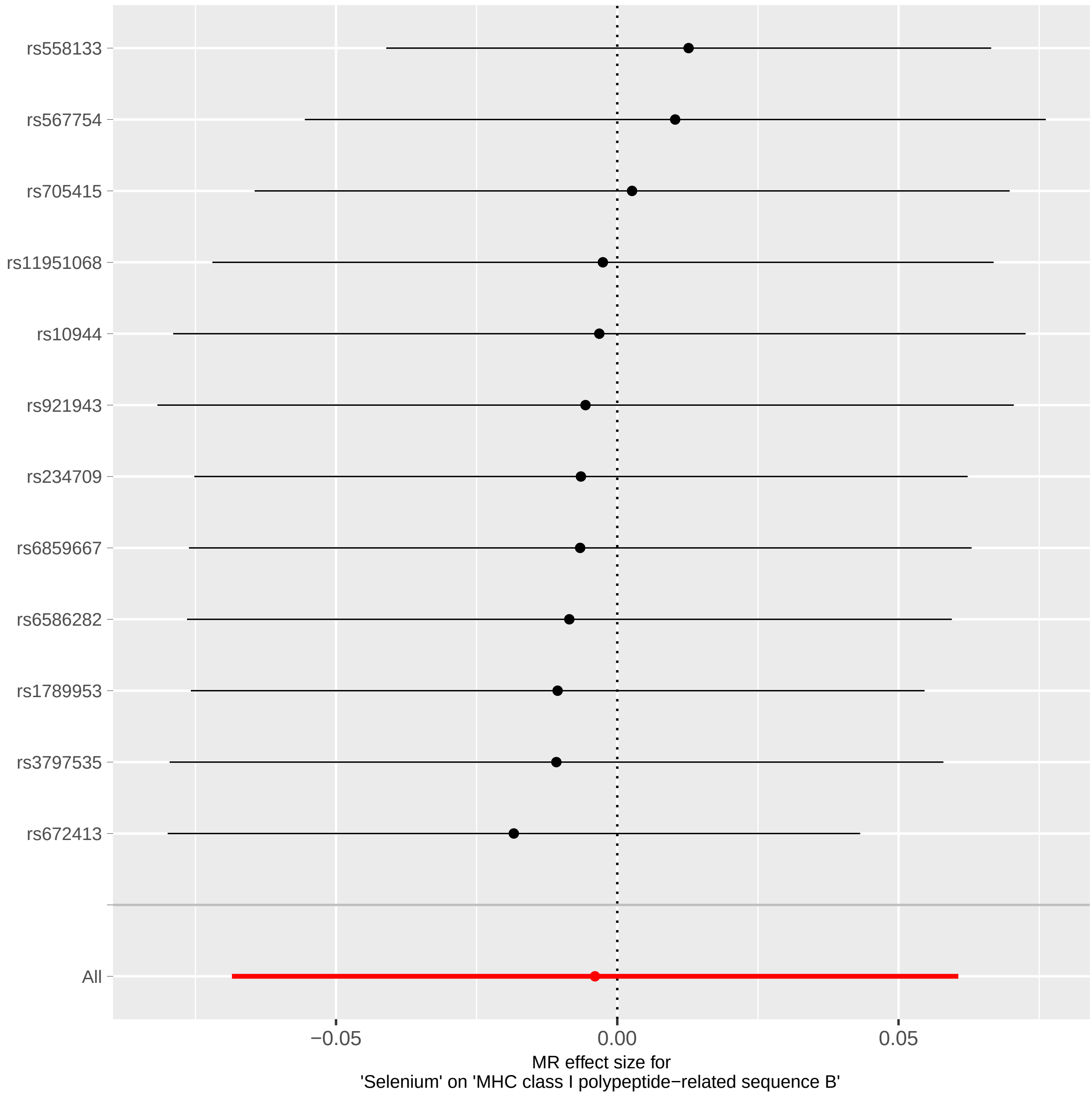

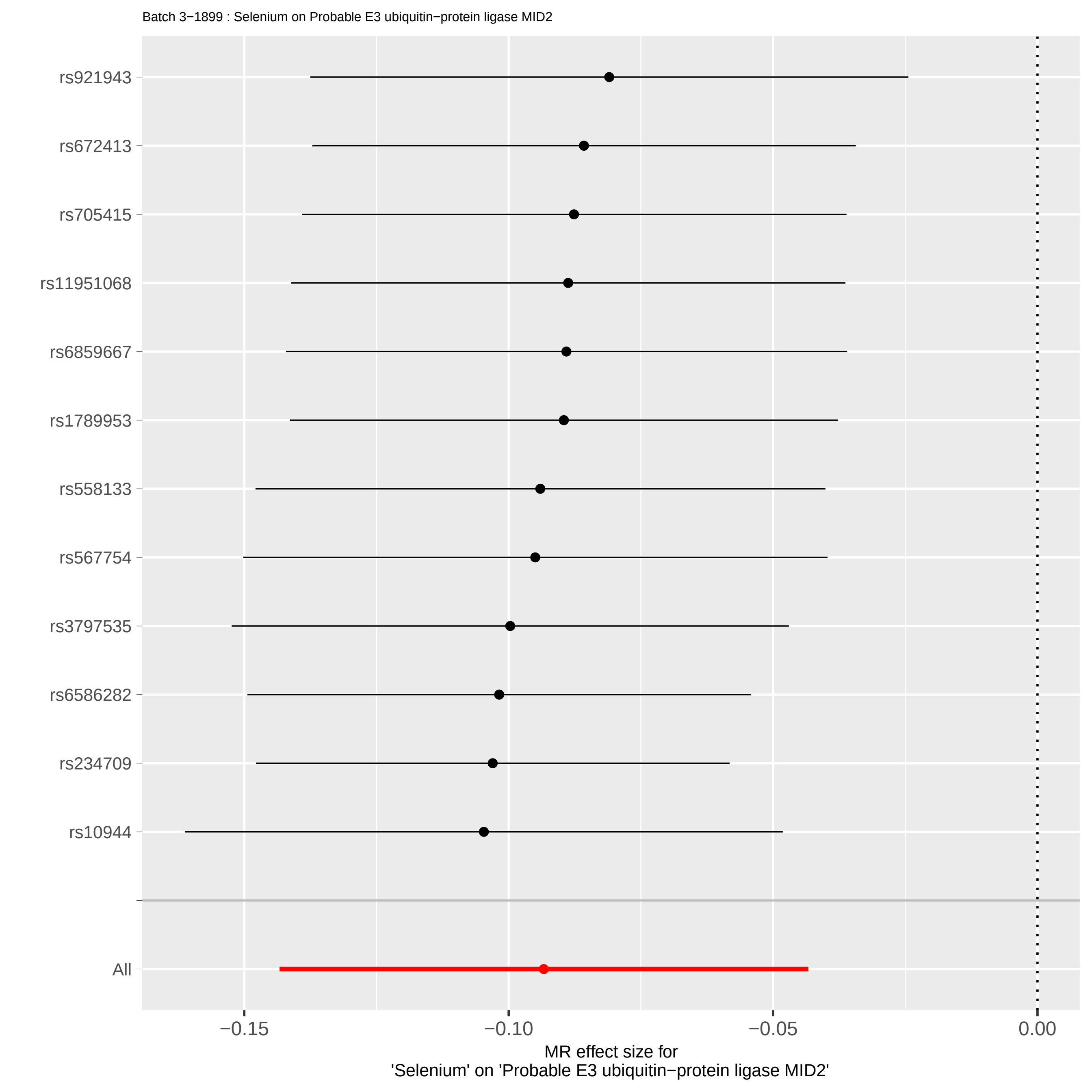

Batch 3−1899 : Selenium on Probable E3 ubiquitin−protein ligase MID2
rs921943
rs672413
rs705415
rs11951068
rs6859667
rs1789953
rs558133
rs567754
rs3797535
rs6586282
rs234709
rs10944
All
−0.15
−0.10
−0.05
0.00
MR effect size for
'Selenium' on 'Probable E3 ubiquitin−protein ligase MID2'

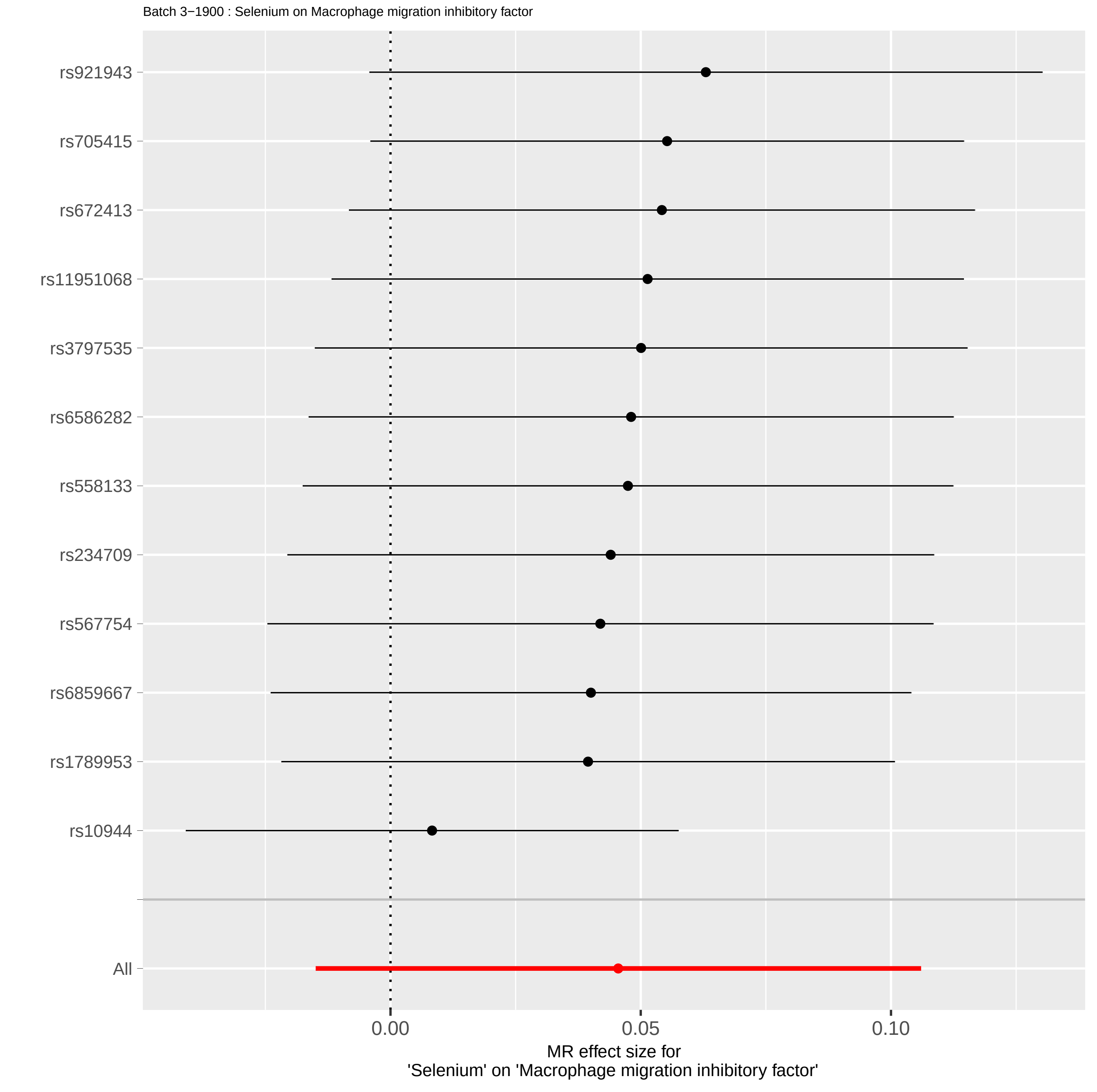
Batch 3−1900 : Selenium on Macrophage migration inhibitory factor
rs921943
rs705415
rs672413
rs11951068
rs3797535
rs6586282
rs558133
rs234709
rs567754
rs6859667
rs1789953
rs10944
All
0.00
0.05
0.10
MR effect size for
'Selenium' on 'Macrophage migration inhibitory factor'

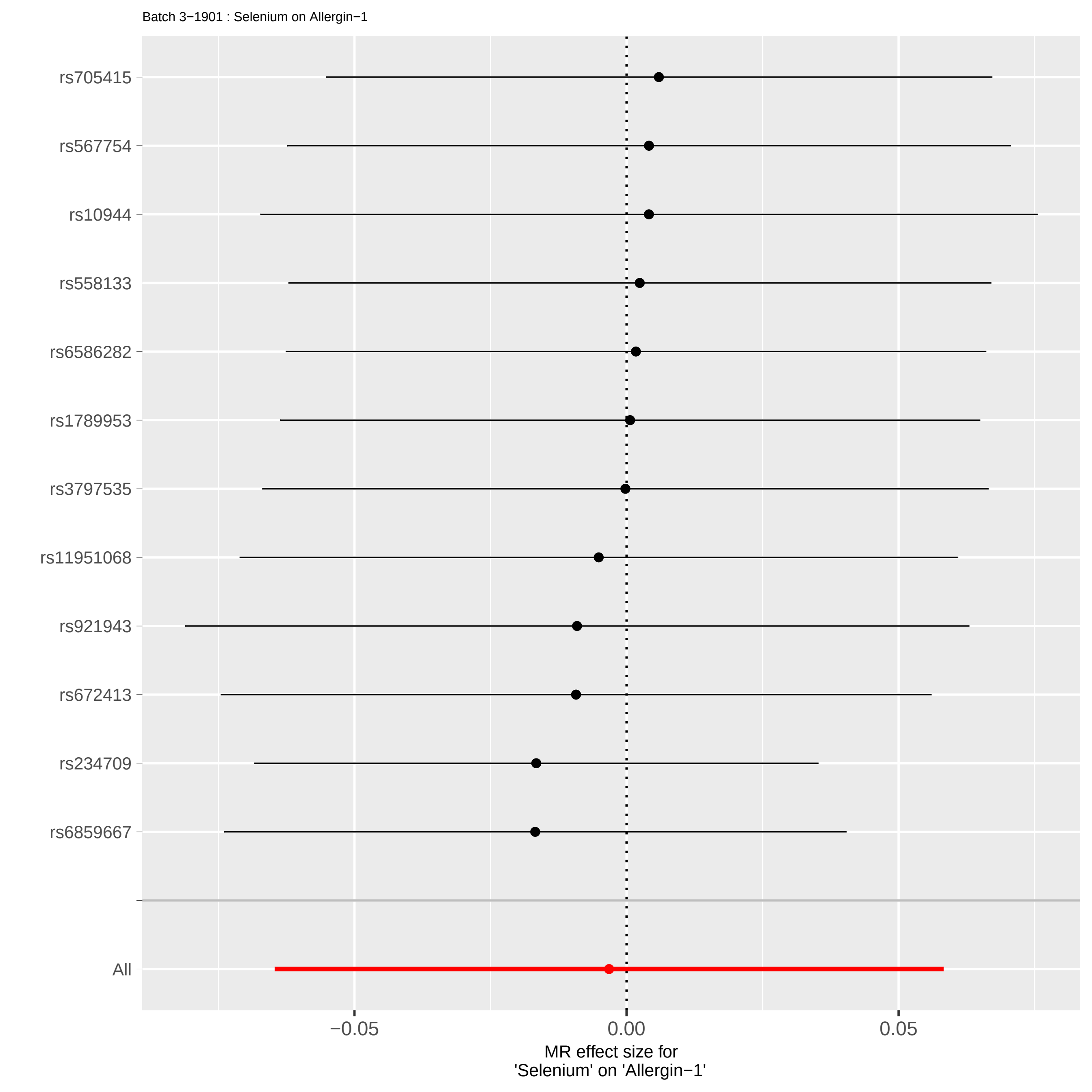

Batch 3−1901 : Selenium on Allergin−1
rs705415
rs567754
rs10944
rs558133
rs6586282
rs1789953
rs3797535
rs11951068
rs921943
rs672413
rs234709
rs6859667
All
−0.05
0.00
0.05
MR effect size for
'Selenium' on 'Allergin−1'

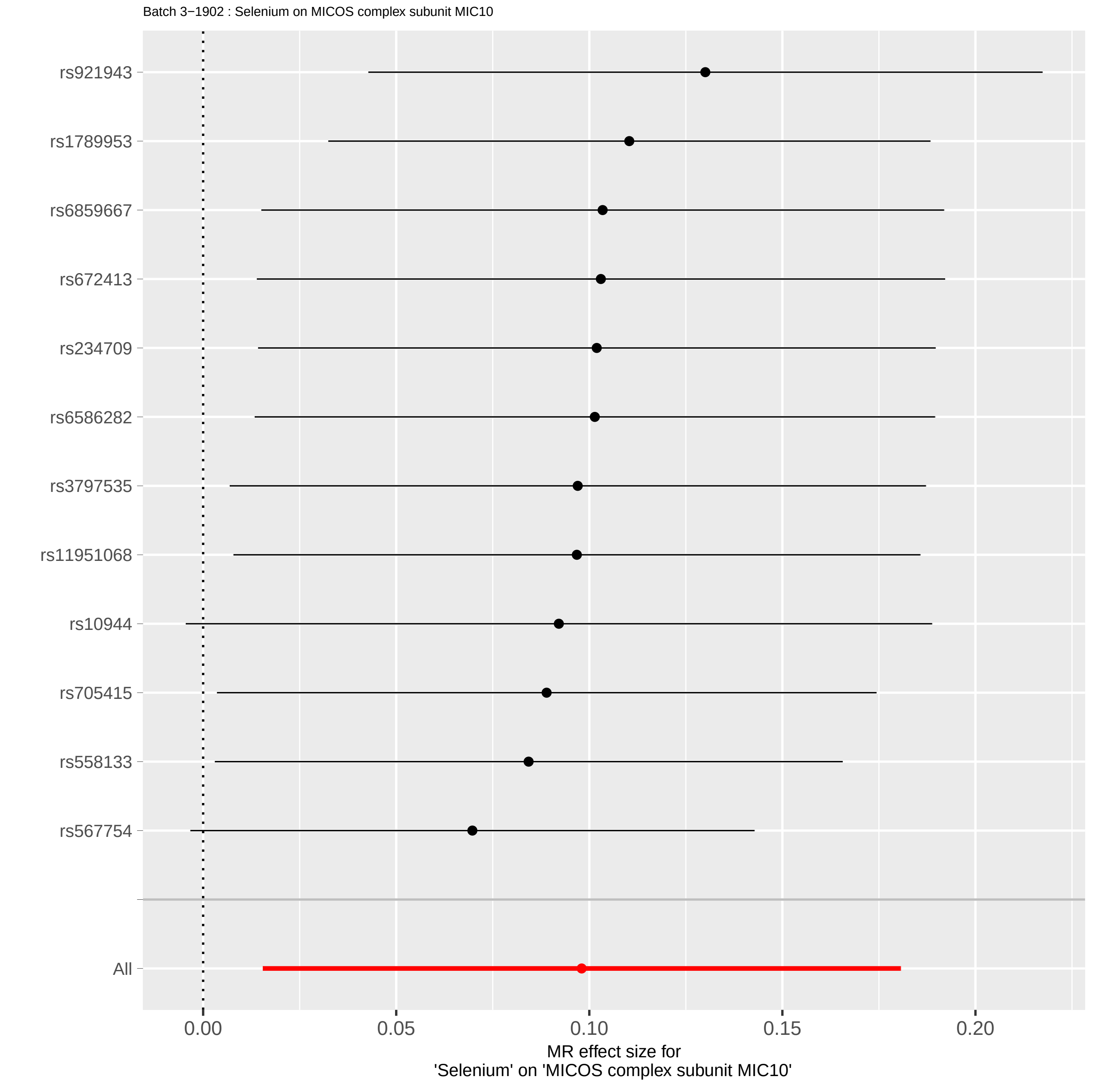

Batch 3−1902 : Selenium on MICOS complex subunit MIC10
rs921943
rs1789953
rs6859667
rs672413
rs234709
rs6586282
rs3797535
rs11951068
rs10944
rs705415
rs558133
rs567754
All
0.00
0.05
0.10
0.15
0.20
MR effect size for
'Selenium' on 'MICOS complex subunit MIC10'

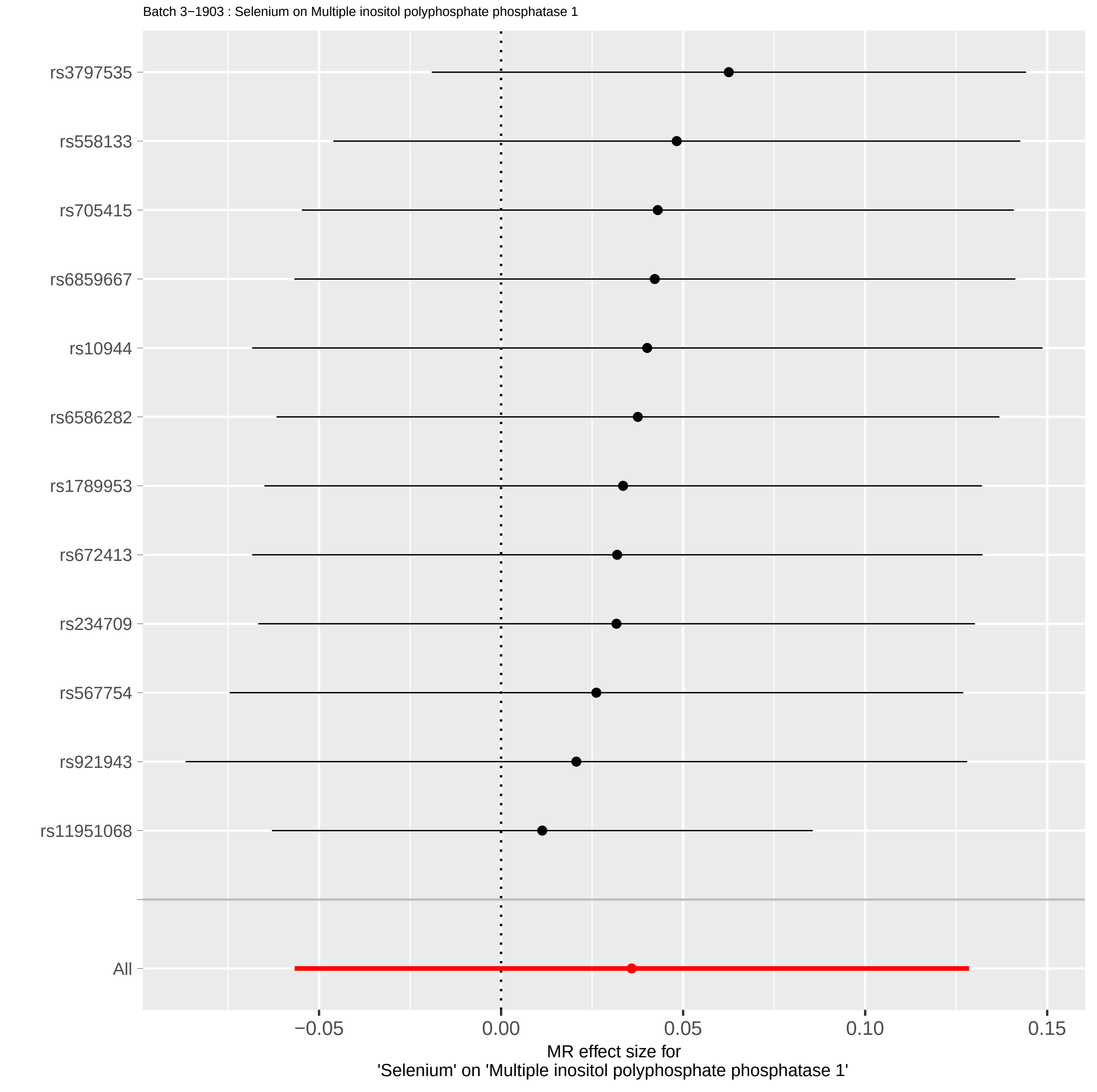

Batch 3−1903 : Selenium on Multiple inositol polyphosphate phosphatase 1
rs3797535
rs558133
rs705415
rs6859667
rs10944
rs6586282
rs1789953
rs672413
rs234709
rs567754
rs921943
rs11951068
All
−0.05
0.00
0.05
0.10
0.15
MR effect size for
'Selenium' on 'Multiple inositol polyphosphate phosphatase 1'

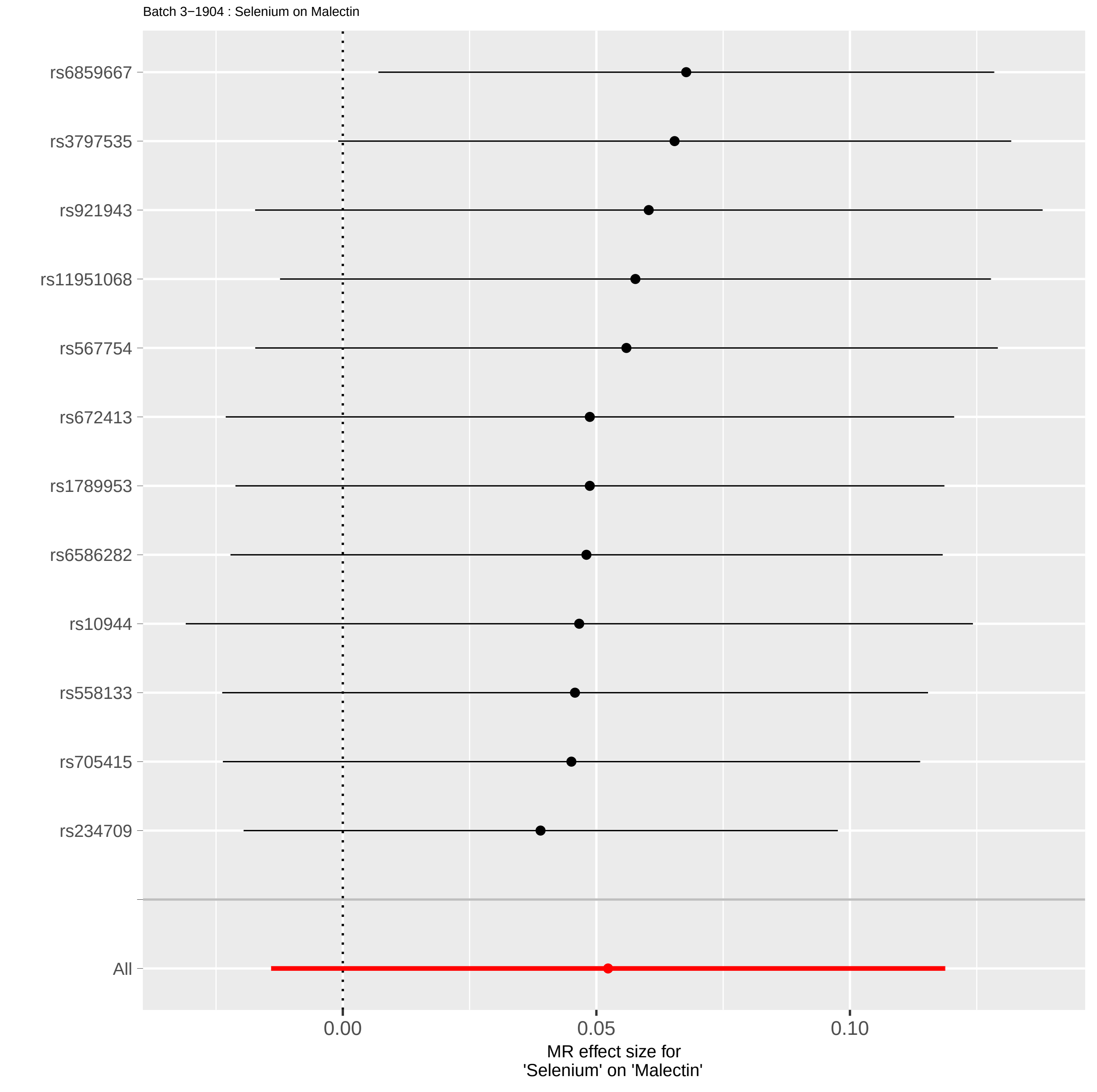

Batch 3−1904 : Selenium on Malectin
rs6859667
rs3797535
rs921943
rs11951068
rs567754
rs672413
rs1789953
rs6586282
rs10944
rs558133
rs705415
rs234709
All
0.00
0.05
0.10
MR effect size for
'Selenium' on 'Malectin'

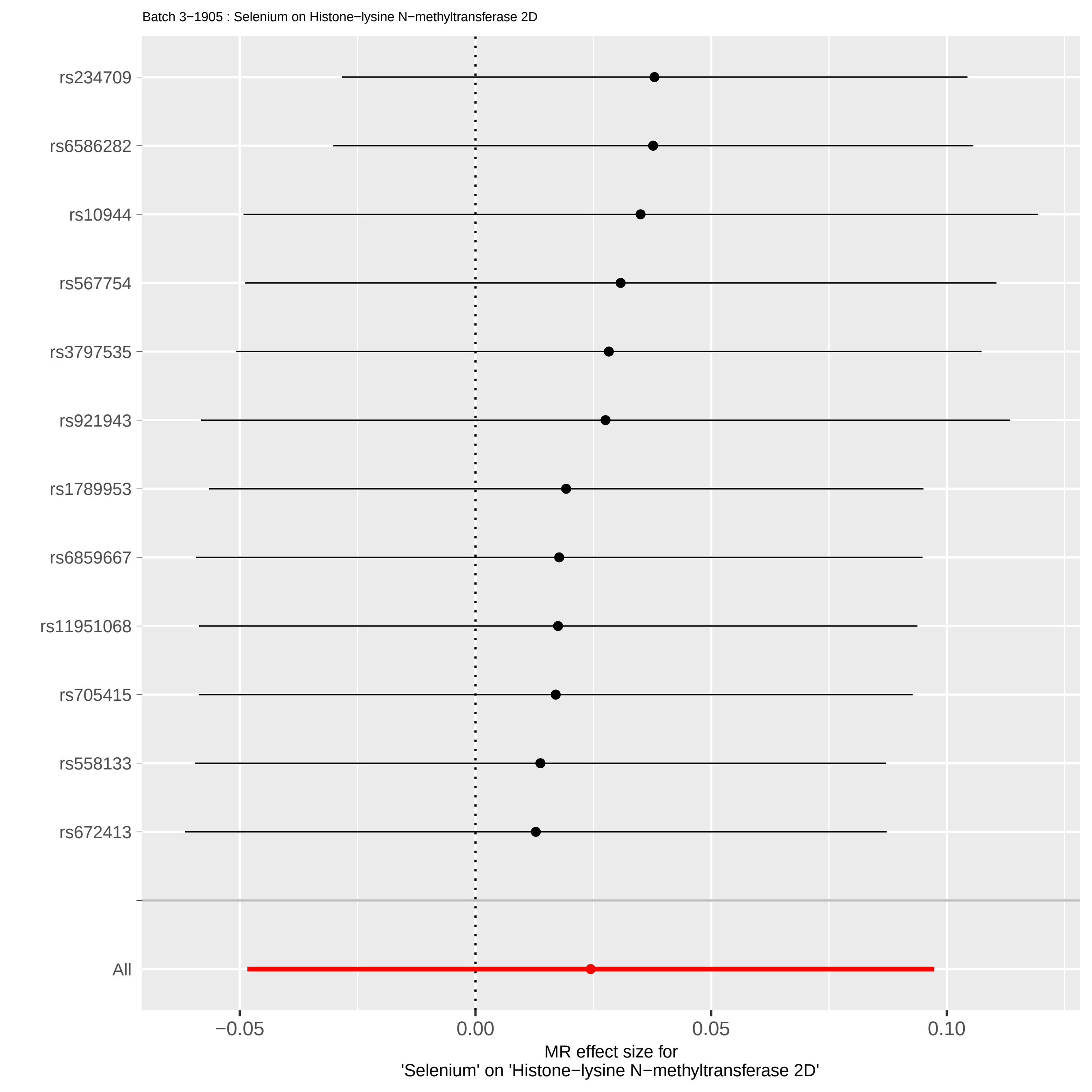

Batch 3−1905 : Selenium on Histone−lysine N−methyltransferase 2D
rs234709
rs6586282
rs10944
rs567754
rs3797535
rs921943
rs1789953
rs6859667
rs11951068
rs705415
rs558133
rs672413
All
−0.05
0.00
0.05
0.10
MR effect size for
'Selenium' on 'Histone−lysine N−methyltransferase 2D'

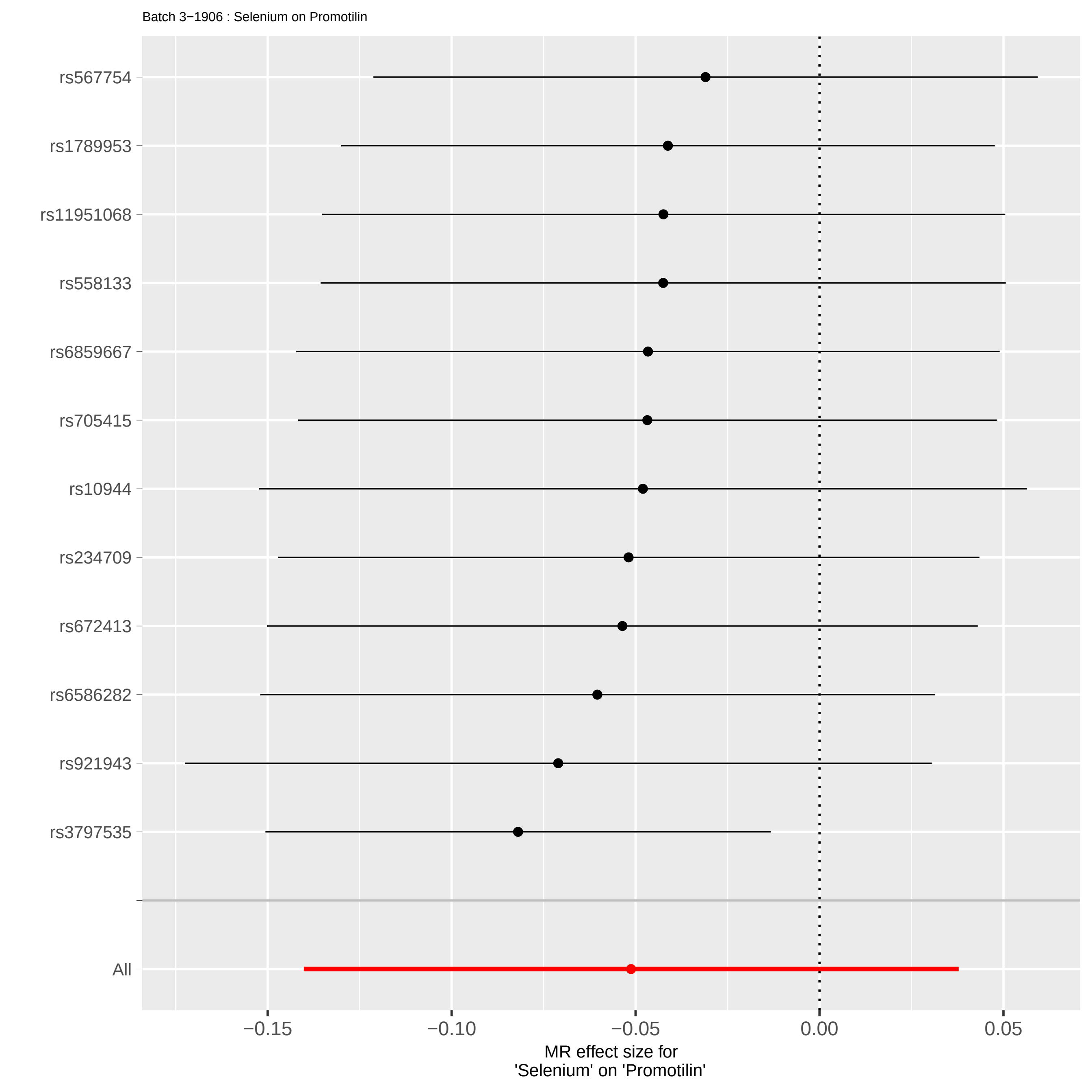

Batch 3−1906 : Selenium on Promotilin
rs567754
rs1789953
rs11951068
rs558133
rs6859667
rs705415
rs10944
rs234709
rs672413
rs6586282
rs921943
rs3797535
All
−0.15
−0.10
−0.05
0.00
0.05
MR effect size for
'Selenium' on 'Promotilin'

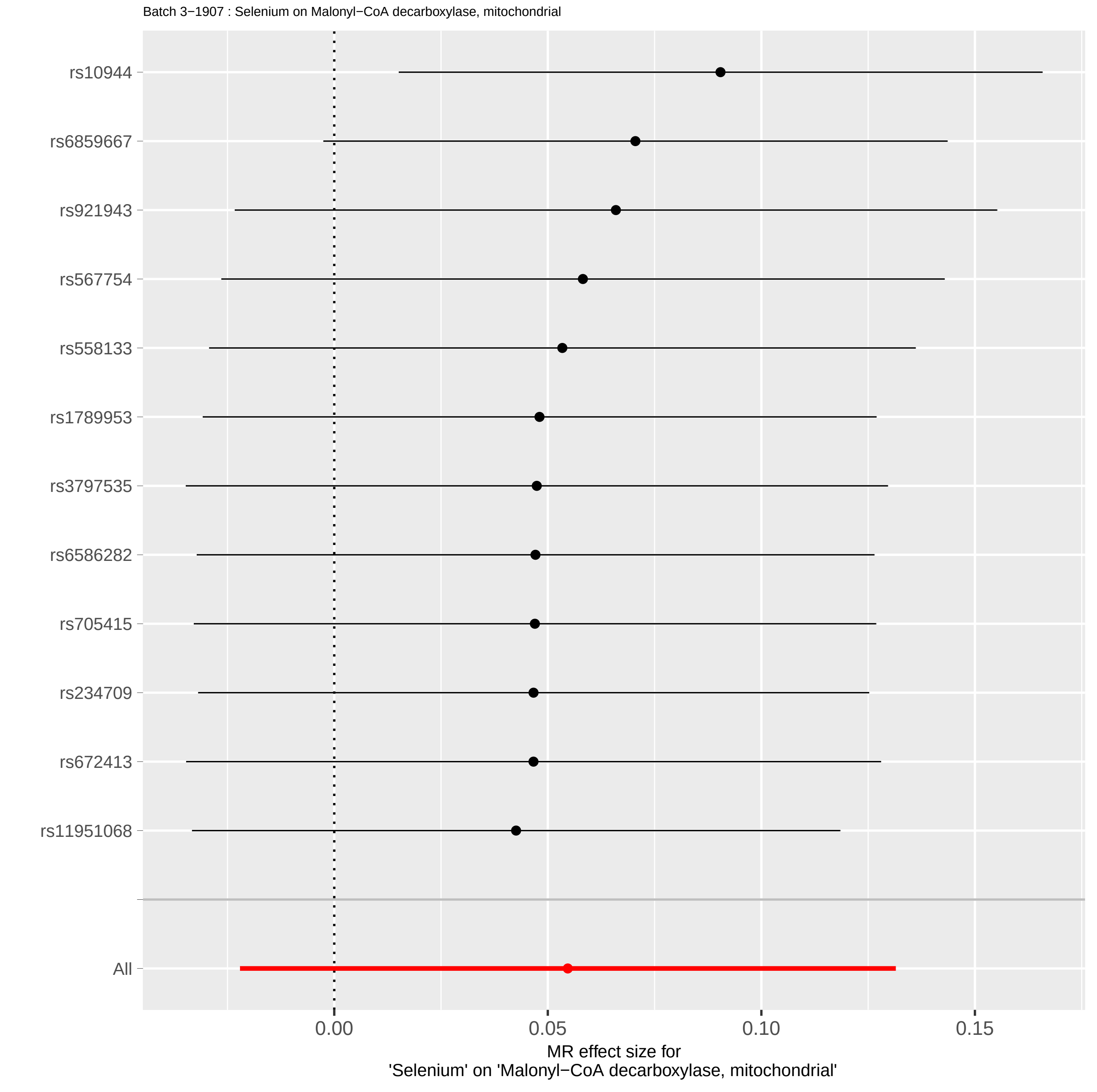

Batch 3−1907 : Selenium on Malonyl−CoA decarboxylase, mitochondrial
rs10944
rs6859667
rs921943
rs567754
rs558133
rs1789953
rs3797535
rs6586282
rs705415
rs234709
rs672413
rs11951068
All
0.00
0.05
0.10
0.15
MR effect size for
'Selenium' on 'Malonyl−CoA decarboxylase, mitochondrial'

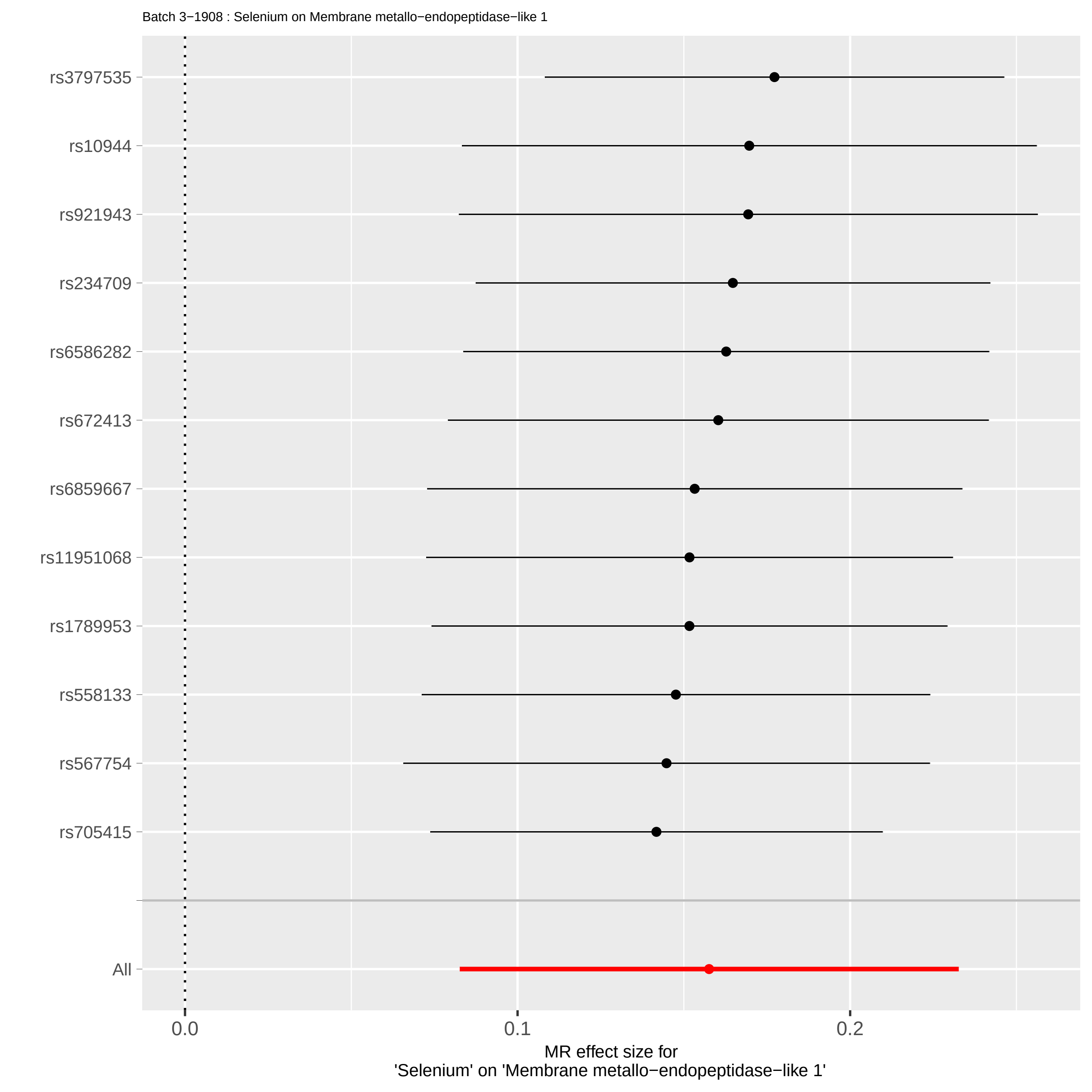
Batch 3−1908 : Selenium on Membrane metallo−endopeptidase−like 1
rs3797535
rs10944
rs921943
rs234709
rs6586282
rs672413
rs6859667
rs11951068
rs1789953
rs558133
rs567754
rs705415
All
0.0
0.1
0.2
MR effect size for
'Selenium' on 'Membrane metallo−endopeptidase−like 1'

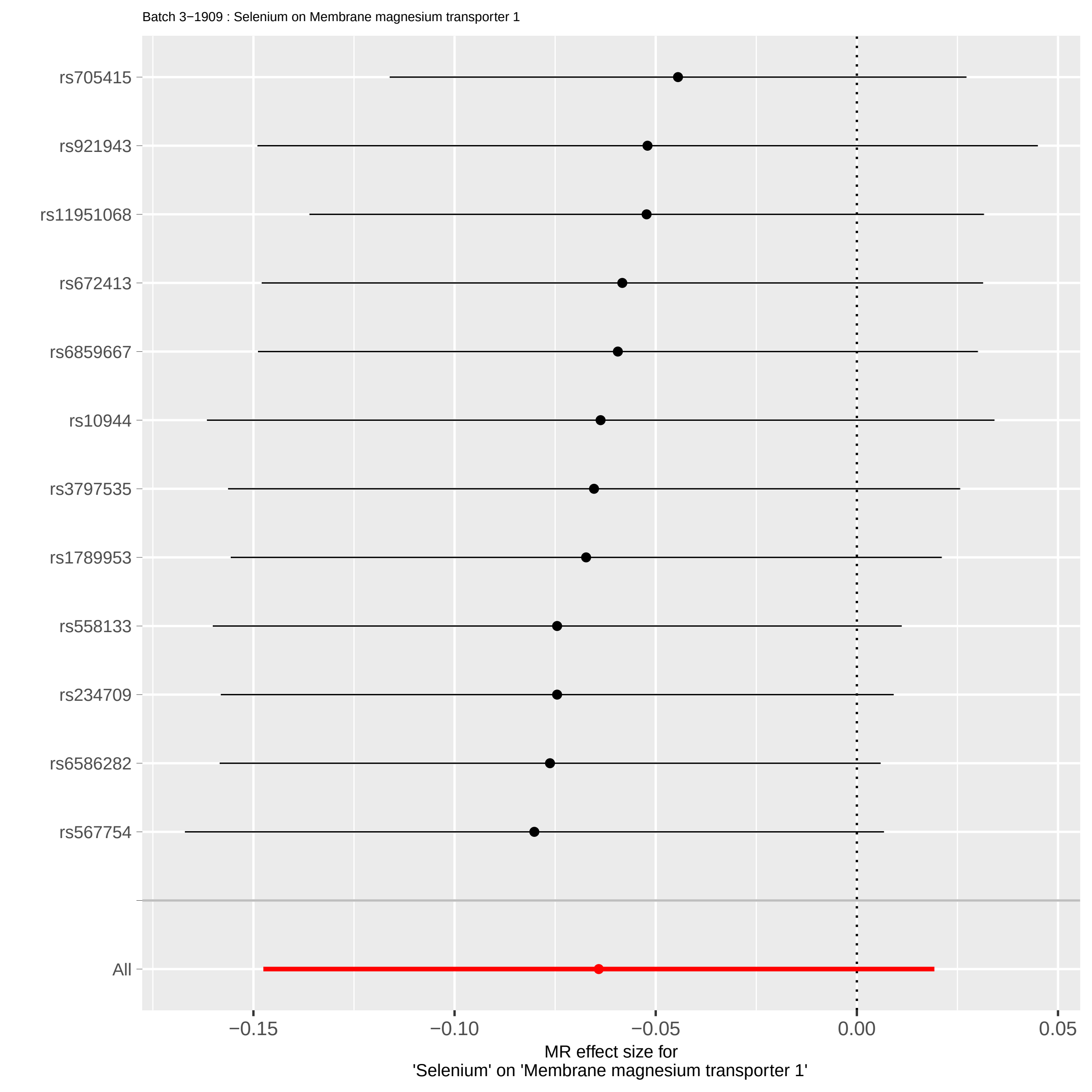

Batch 3−1909 : Selenium on Membrane magnesium transporter 1
rs705415
rs921943
rs11951068
rs672413
rs6859667
rs10944
rs3797535
rs1789953
rs558133
rs234709
rs6586282
rs567754
All
−0.15
−0.10
−0.05
0.00
0.05
MR effect size for
'Selenium' on 'Membrane magnesium transporter 1'

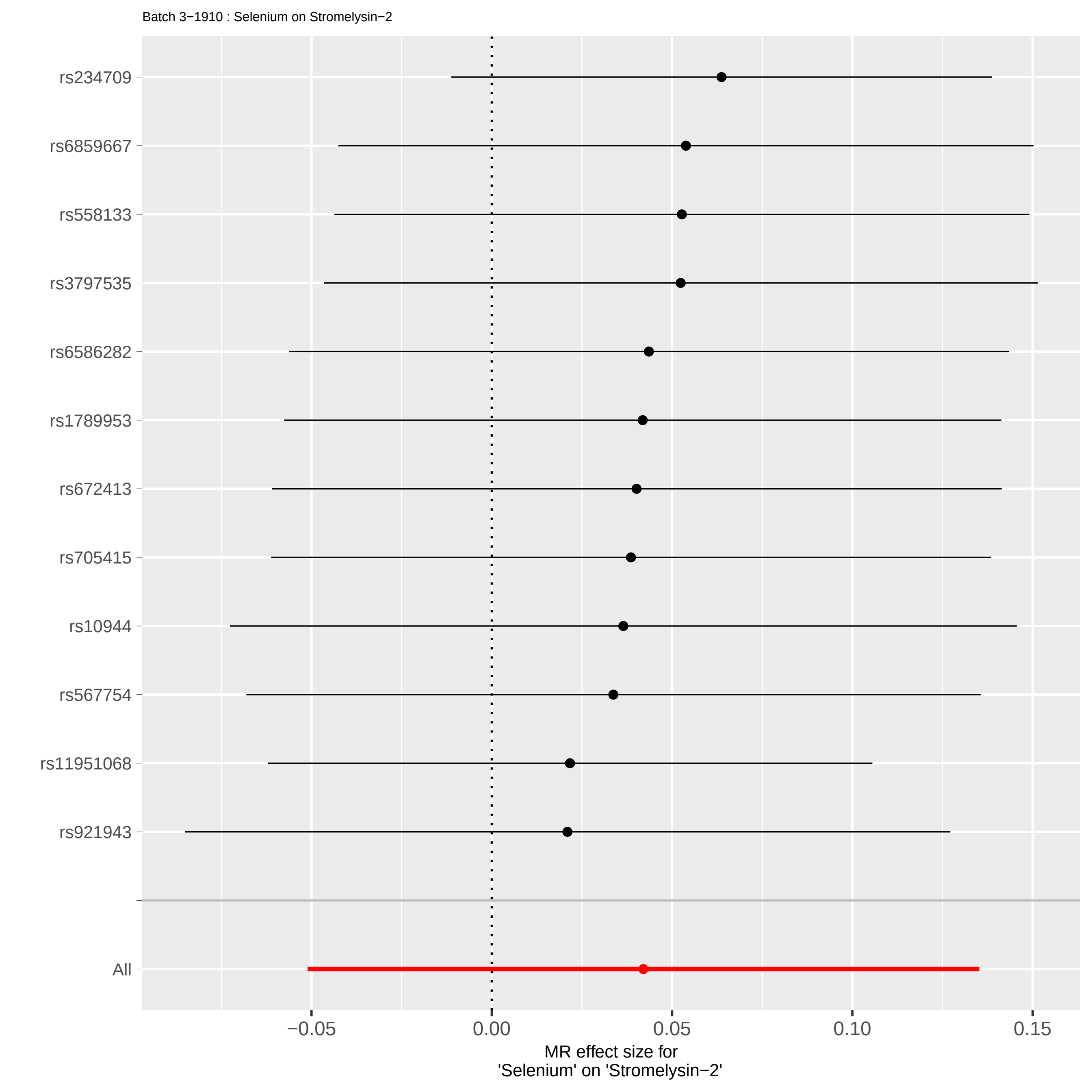

Batch 3−1910 : Selenium on Stromelysin−2
rs234709
rs6859667
rs558133
rs3797535
rs6586282
rs1789953
rs672413
rs705415
rs10944
rs567754
rs11951068
rs921943
All
−0.05
0.00
0.05
0.10
0.15
MR effect size for
'Selenium' on 'Stromelysin−2'

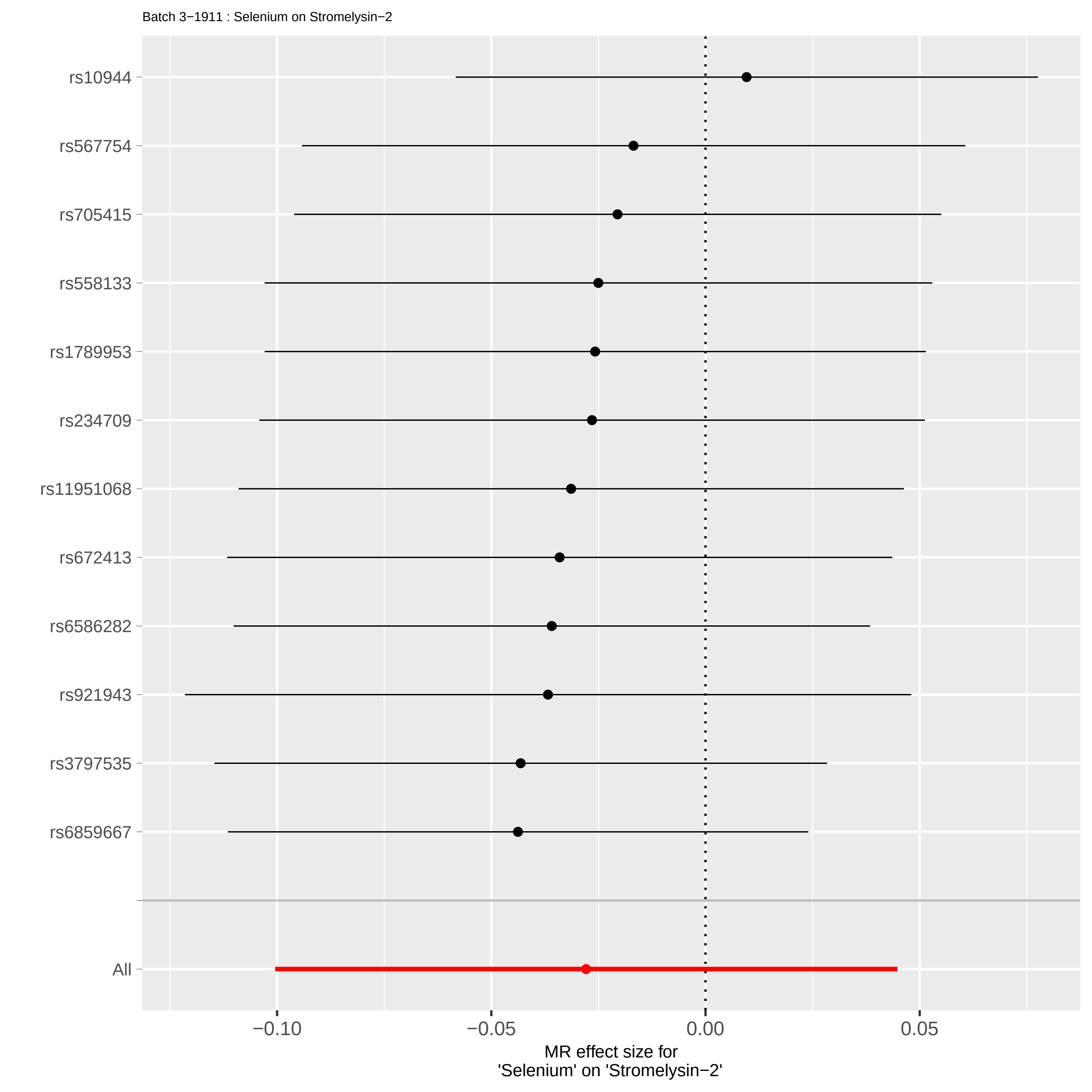

Batch 3−1911 : Selenium on Stromelysin−2
rs10944
rs567754
rs705415
rs558133
rs1789953
rs234709
rs11951068
rs672413
rs6586282
rs921943
rs3797535
rs6859667
All
−0.10
−0.05
0.00
0.05
MR effect size for
'Selenium' on 'Stromelysin−2'

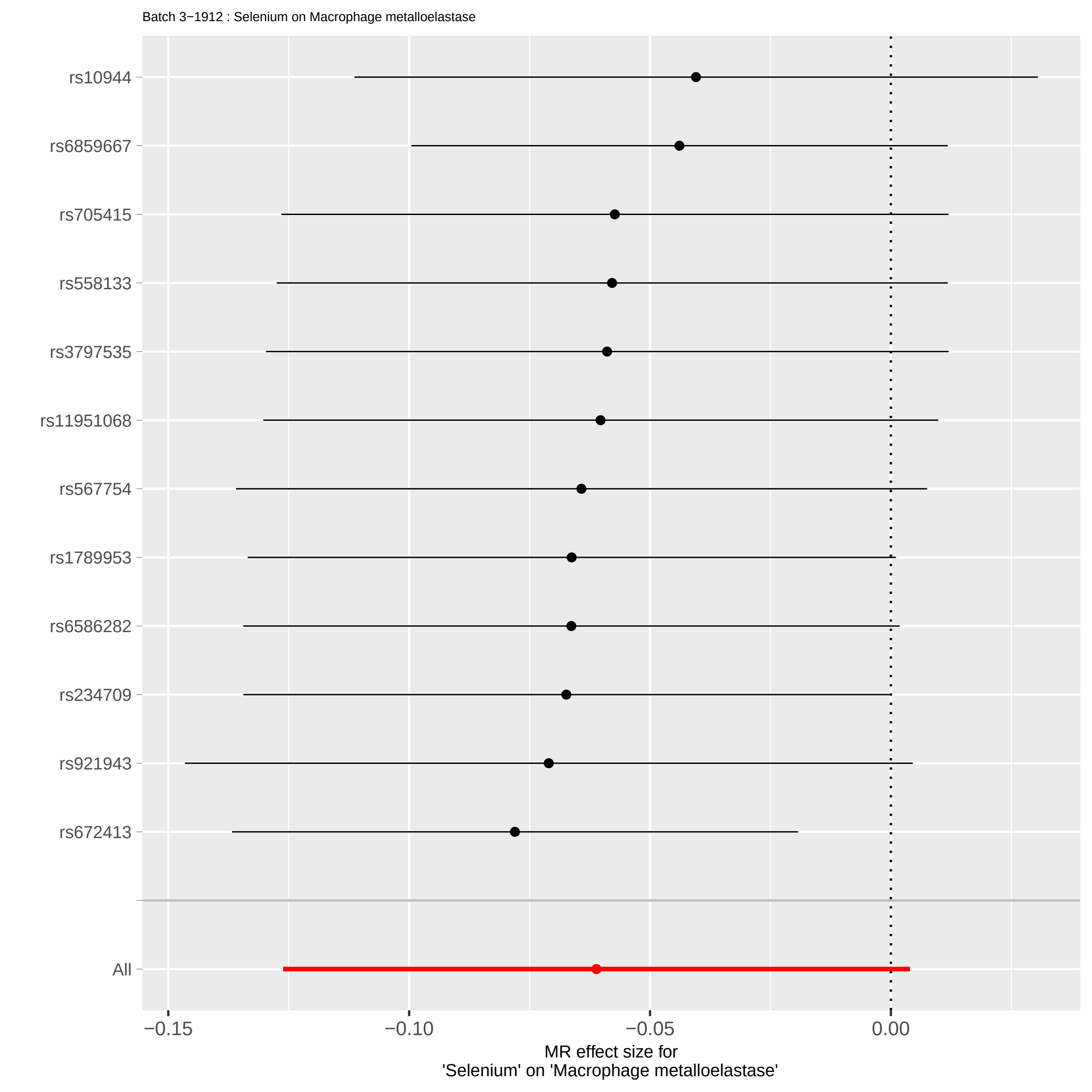

Batch 3−1912 : Selenium on Macrophage metalloelastase
rs10944
rs6859667
rs705415
rs558133
rs3797535
rs11951068
rs567754
rs1789953
rs6586282
rs234709
rs921943
rs672413
All
−0.15
−0.10
−0.05
0.00
MR effect size for
'Selenium' on 'Macrophage metalloelastase'

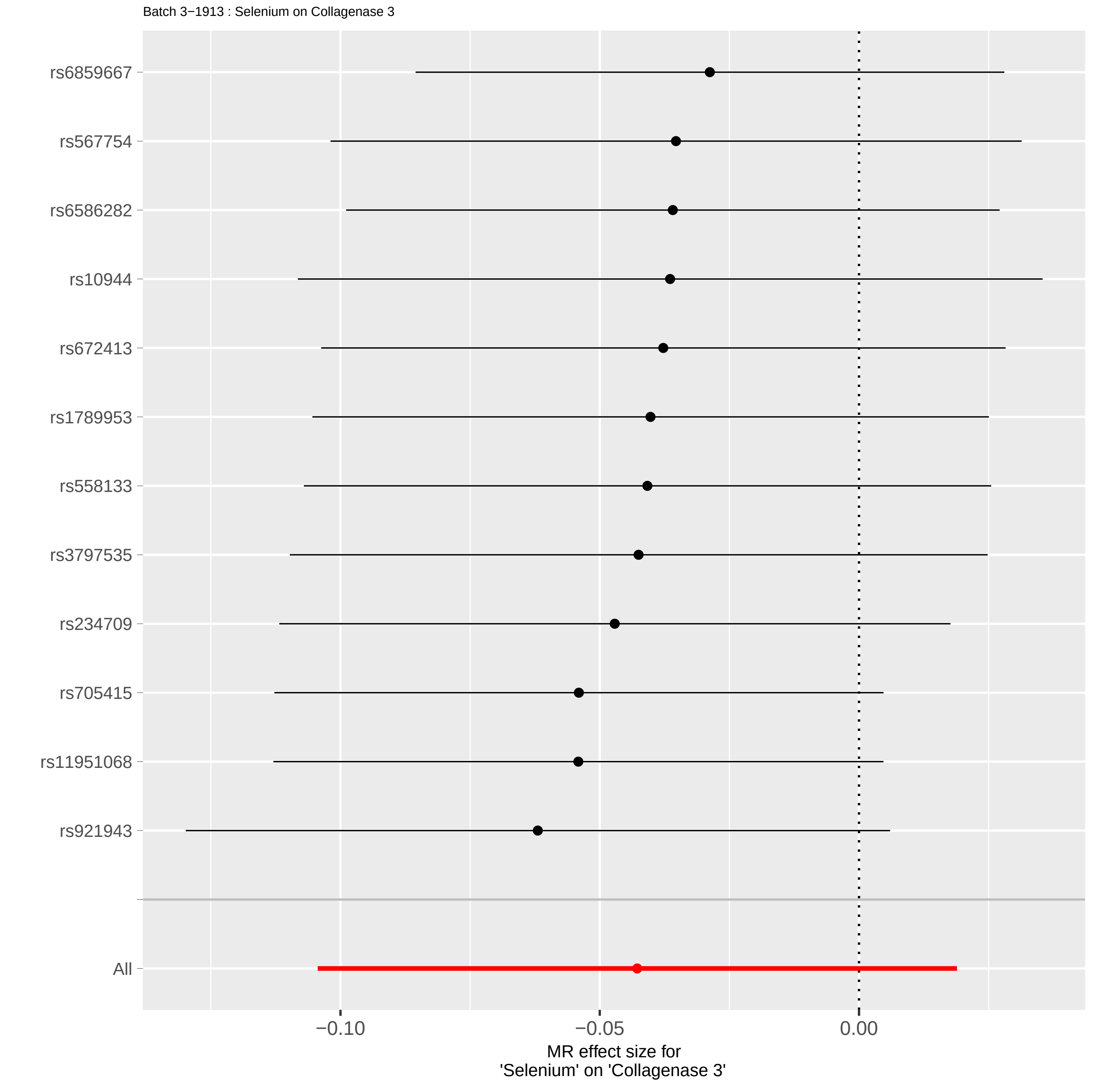

Batch 3−1913 : Selenium on Collagenase 3
rs6859667
rs567754
rs6586282
rs10944
rs672413
rs1789953
rs558133
rs3797535
rs234709
rs705415
rs11951068
rs921943
All
−0.10
−0.05
0.00
MR effect size for
'Selenium' on 'Collagenase 3'

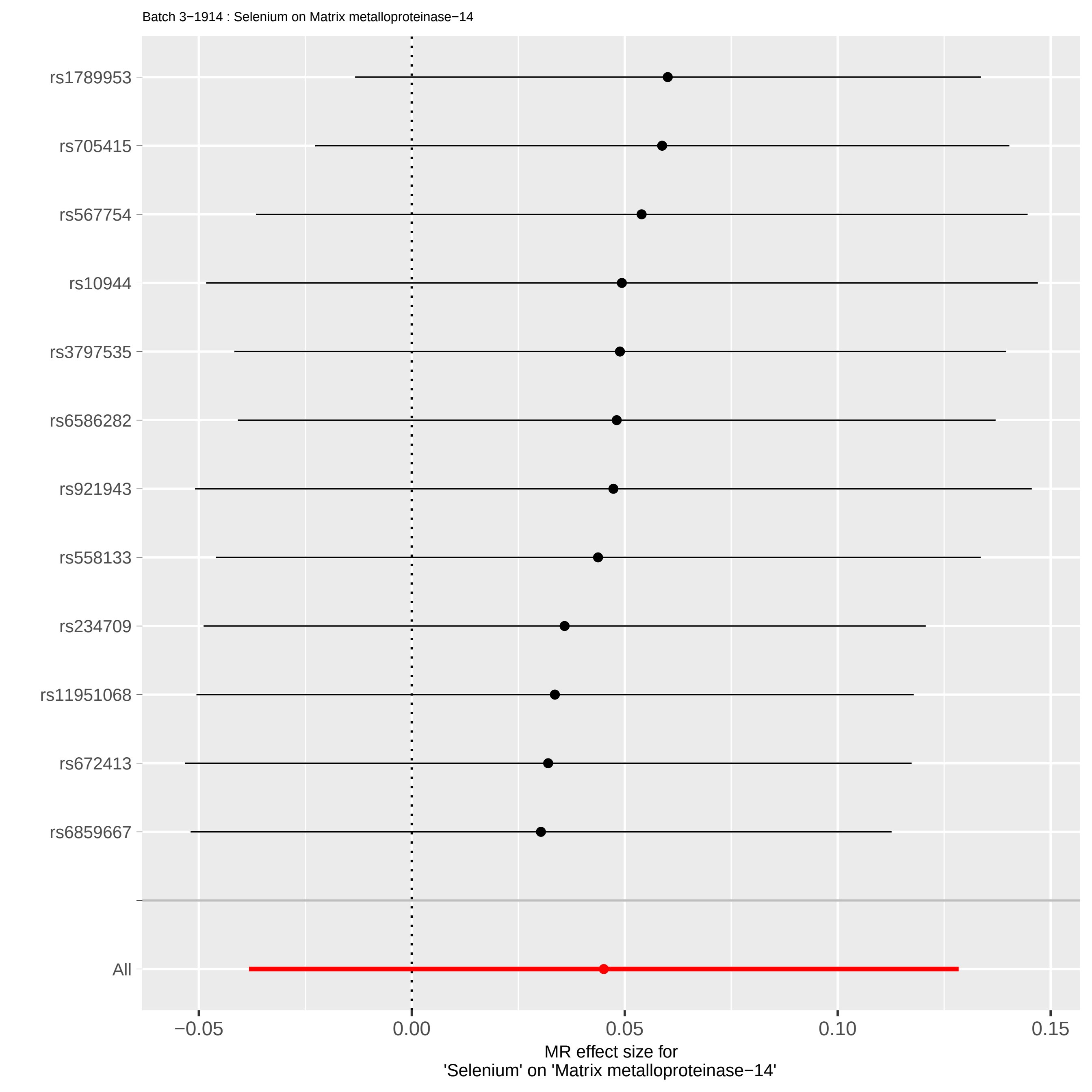

Batch 3−1914 : Selenium on Matrix metalloproteinase−14
rs1789953
rs705415
rs567754
rs10944
rs3797535
rs6586282
rs921943
rs558133
rs234709
rs11951068
rs672413
rs6859667
All
−0.05
0.00
0.05
0.10
0.15
MR effect size for
'Selenium' on 'Matrix metalloproteinase−14'

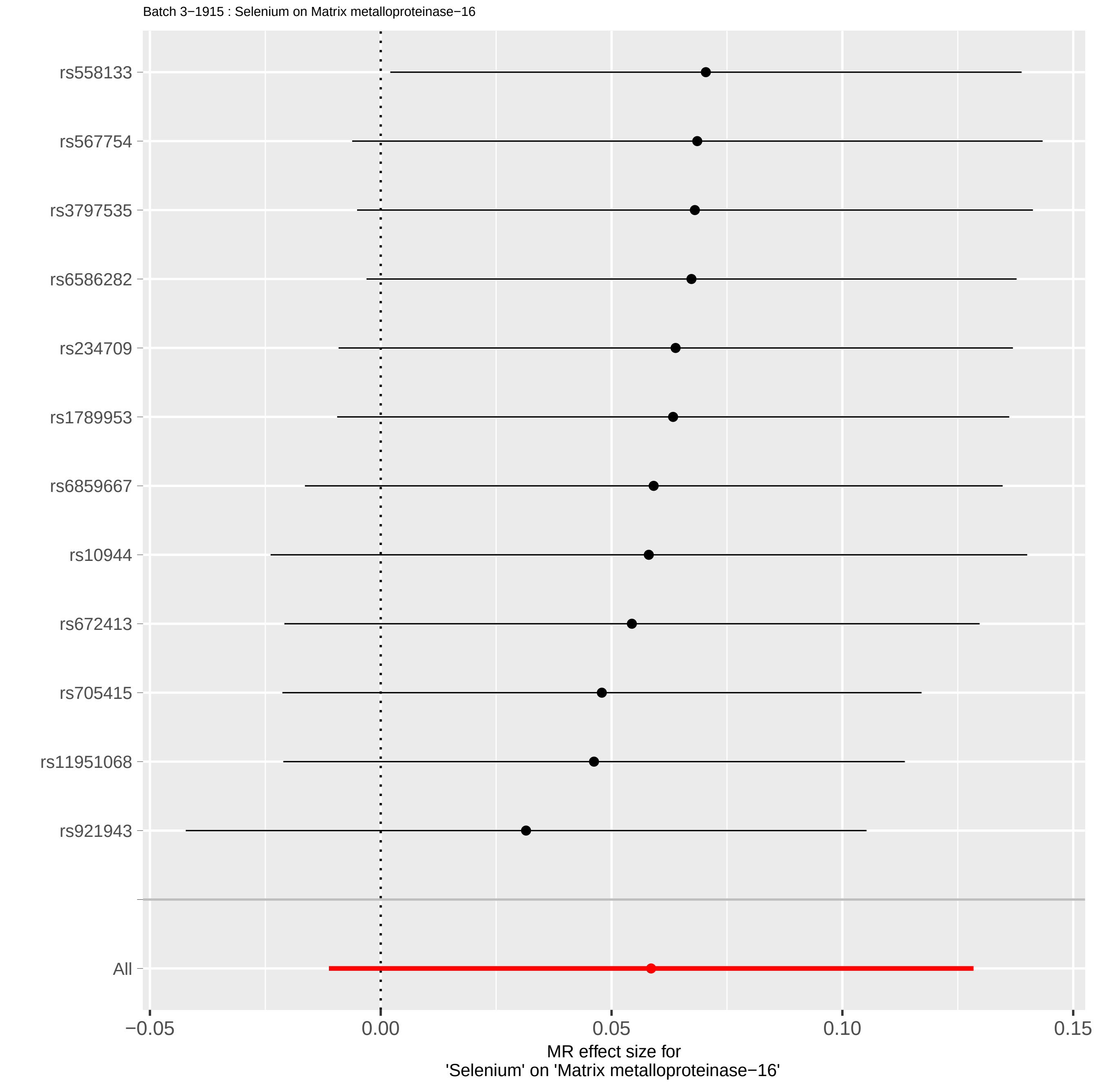

Batch 3−1915 : Selenium on Matrix metalloproteinase−16
rs558133
rs567754
rs3797535
rs6586282
rs234709
rs1789953
rs6859667
rs10944
rs672413
rs705415
rs11951068
rs921943
All
−0.05
0.00
0.05
0.10
0.15
MR effect size for
'Selenium' on 'Matrix metalloproteinase−16'

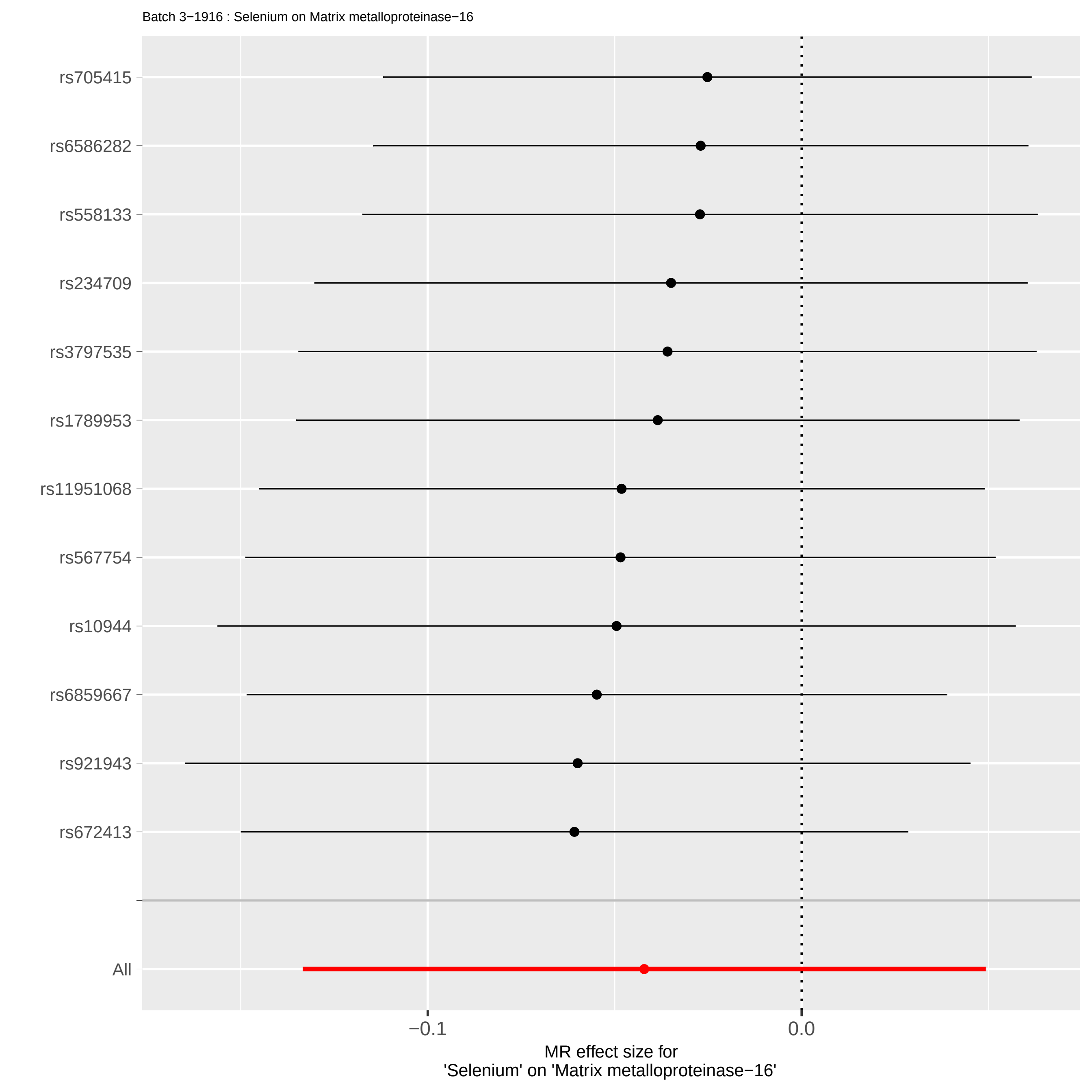
Batch 3−1916 : Selenium on Matrix metalloproteinase−16
rs705415
rs6586282
rs558133
rs234709
rs3797535
rs1789953
rs11951068
rs567754
rs10944
rs6859667
rs921943
rs672413
All
−0.1
0.0
MR effect size for
'Selenium' on 'Matrix metalloproteinase−16'

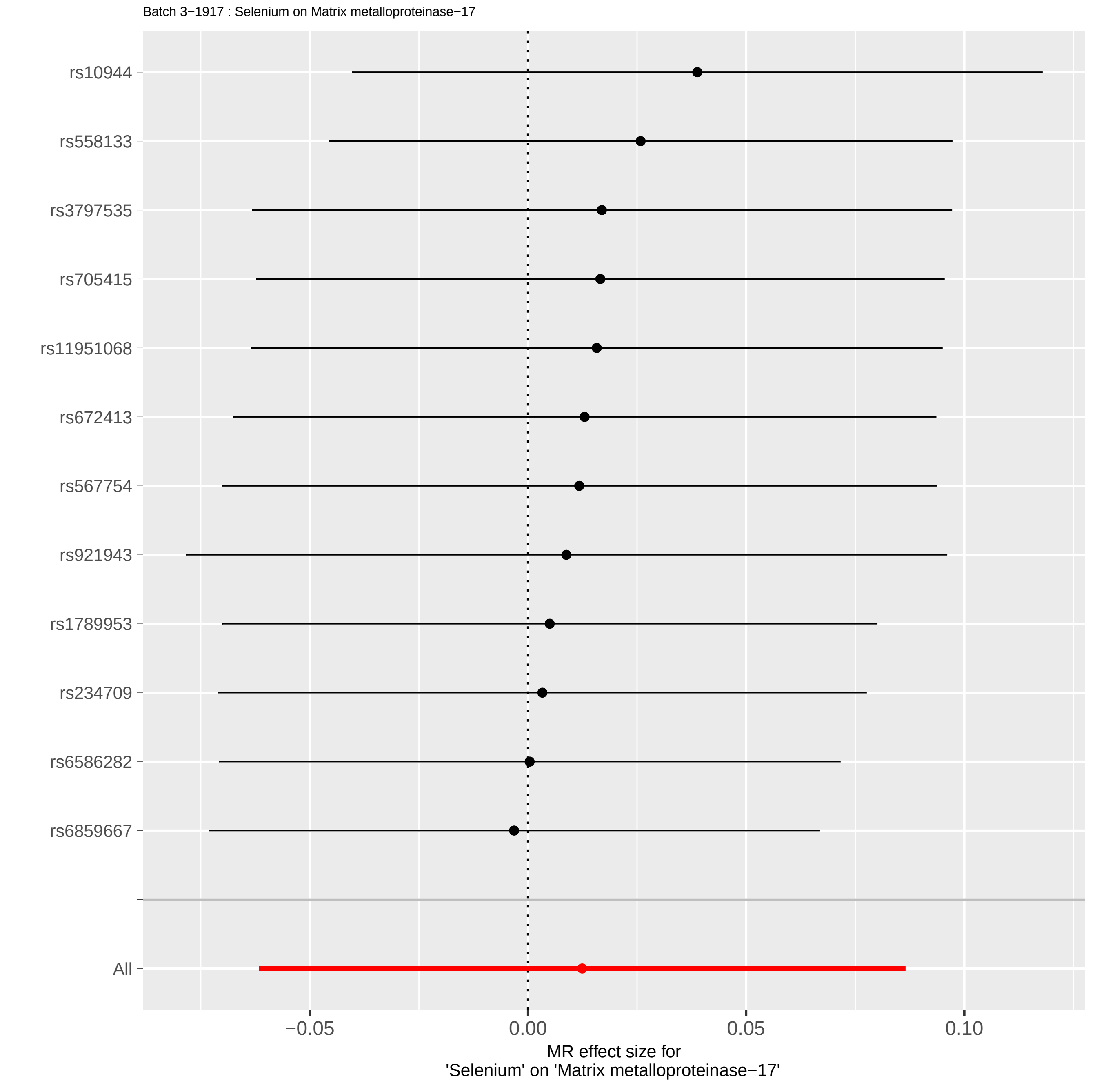

Batch 3−1917 : Selenium on Matrix metalloproteinase−17
rs10944
rs558133
rs3797535
rs705415
rs11951068
rs672413
rs567754
rs921943
rs1789953
rs234709
rs6586282
rs6859667
All
−0.05
0.00
0.05
0.10
MR effect size for
'Selenium' on 'Matrix metalloproteinase−17'

Batch 3−1918 : Selenium on 72 kDa type IV collagenase

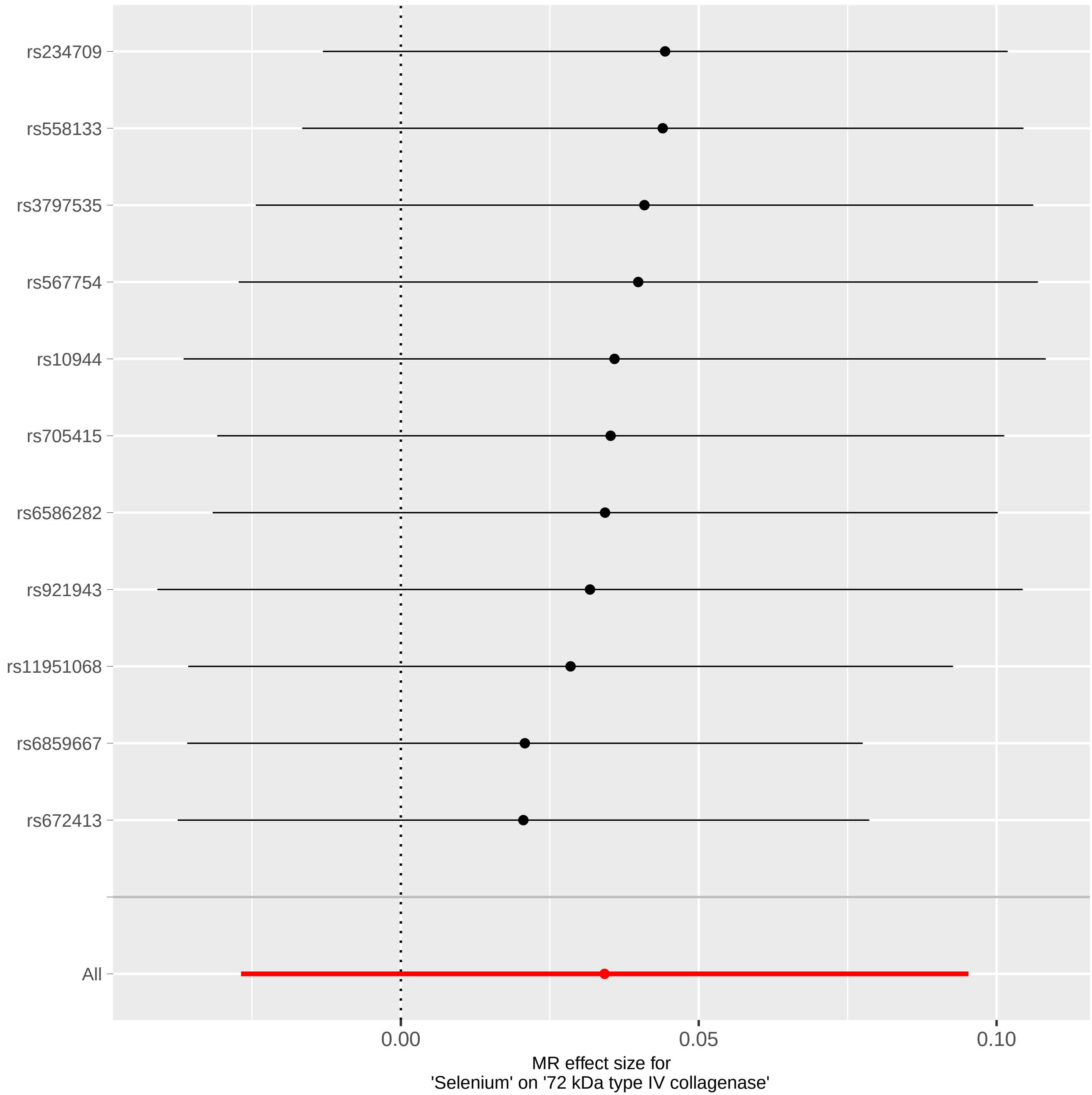

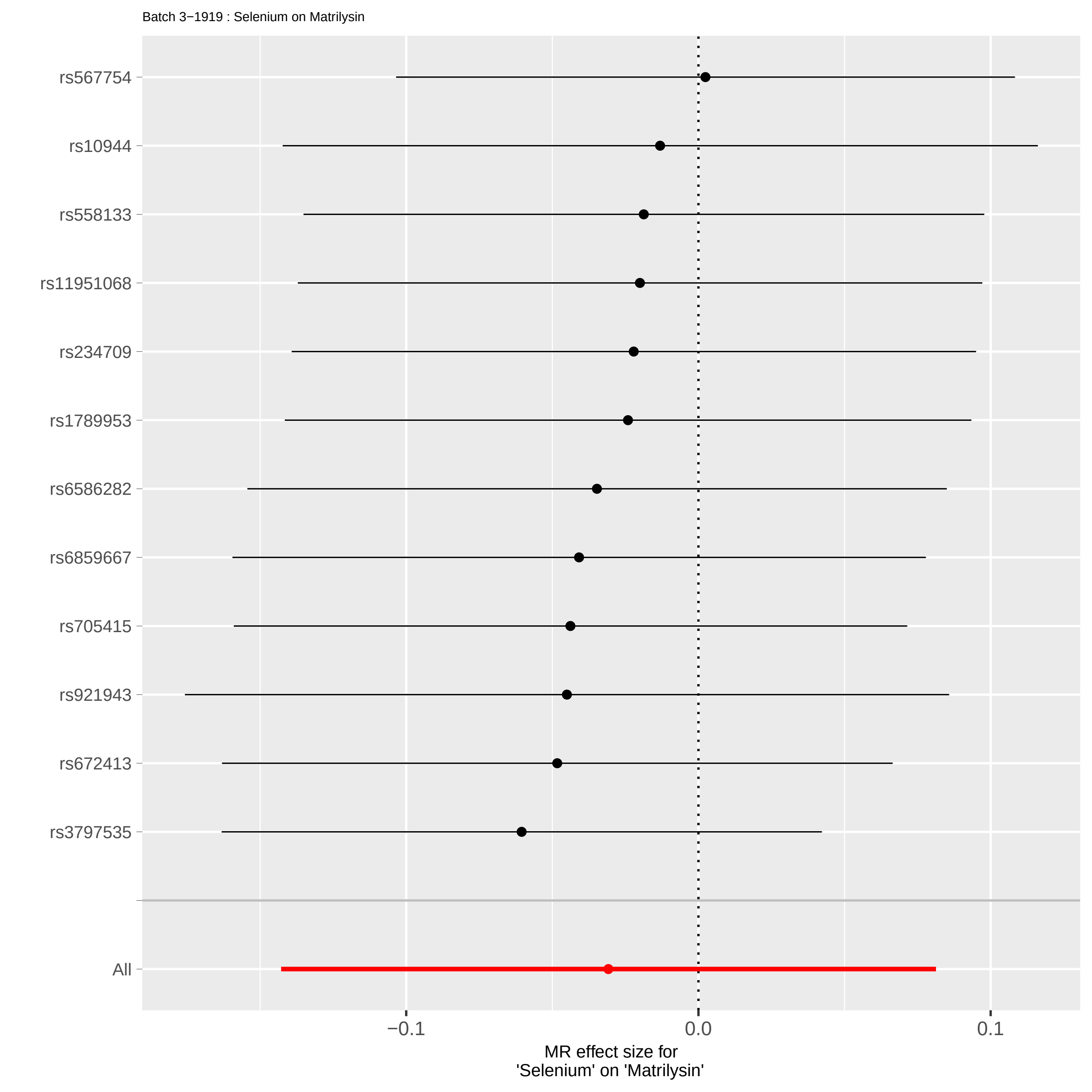

Batch 3−1919 : Selenium on Matrilysin
rs567754
rs10944
rs558133
rs11951068
rs234709
rs1789953
rs6586282
rs6859667
rs705415
rs921943
rs672413
rs3797535
All
−0.1
0.0
0.1
MR effect size for
'Selenium' on 'Matrilysin'

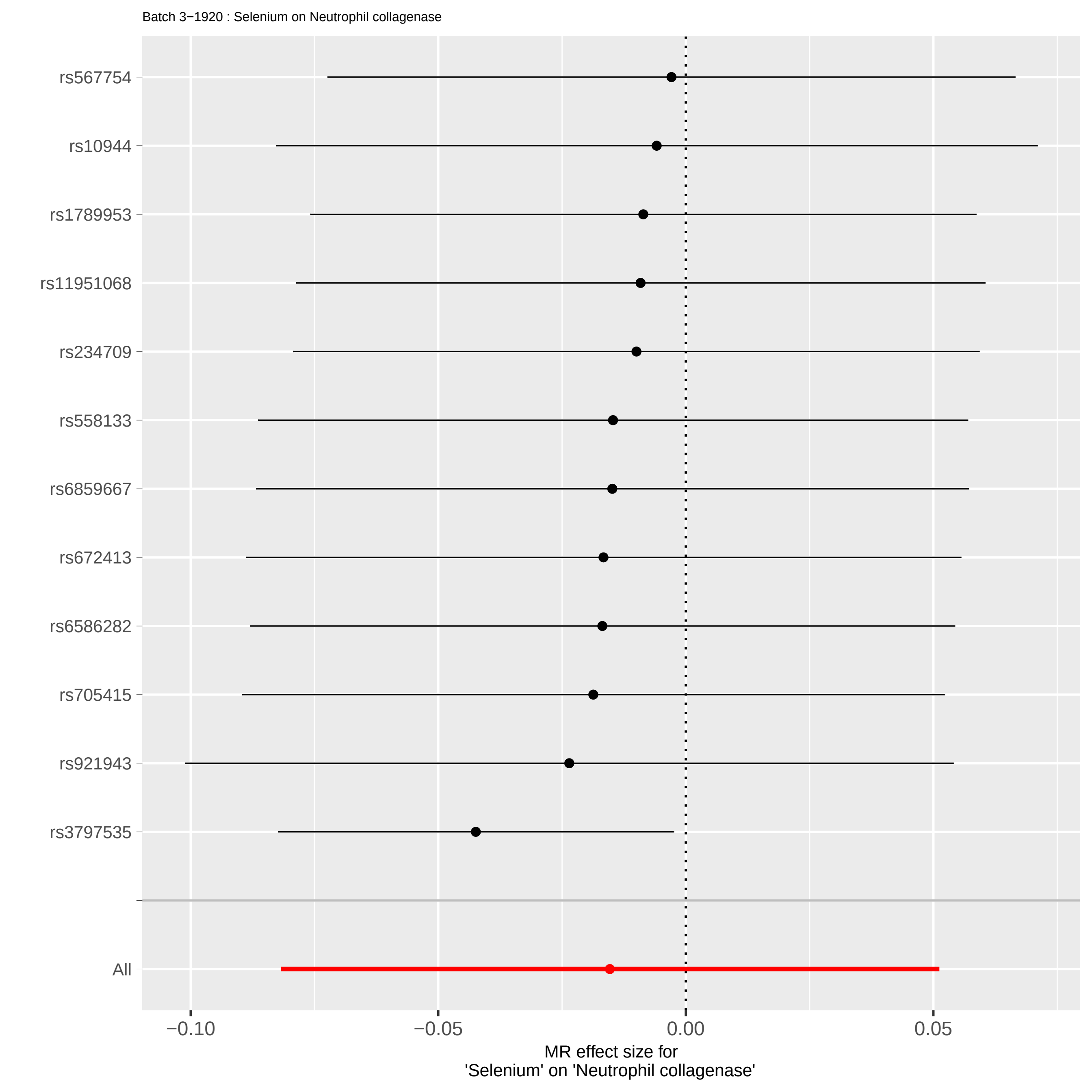

Batch 3−1920 : Selenium on Neutrophil collagenase
rs567754
rs10944
rs1789953
rs11951068
rs234709
rs558133
rs6859667
rs672413
rs6586282
rs705415
rs921943
rs3797535
All
−0.10
−0.05
0.00
0.05
MR effect size for
'Selenium' on 'Neutrophil collagenase'

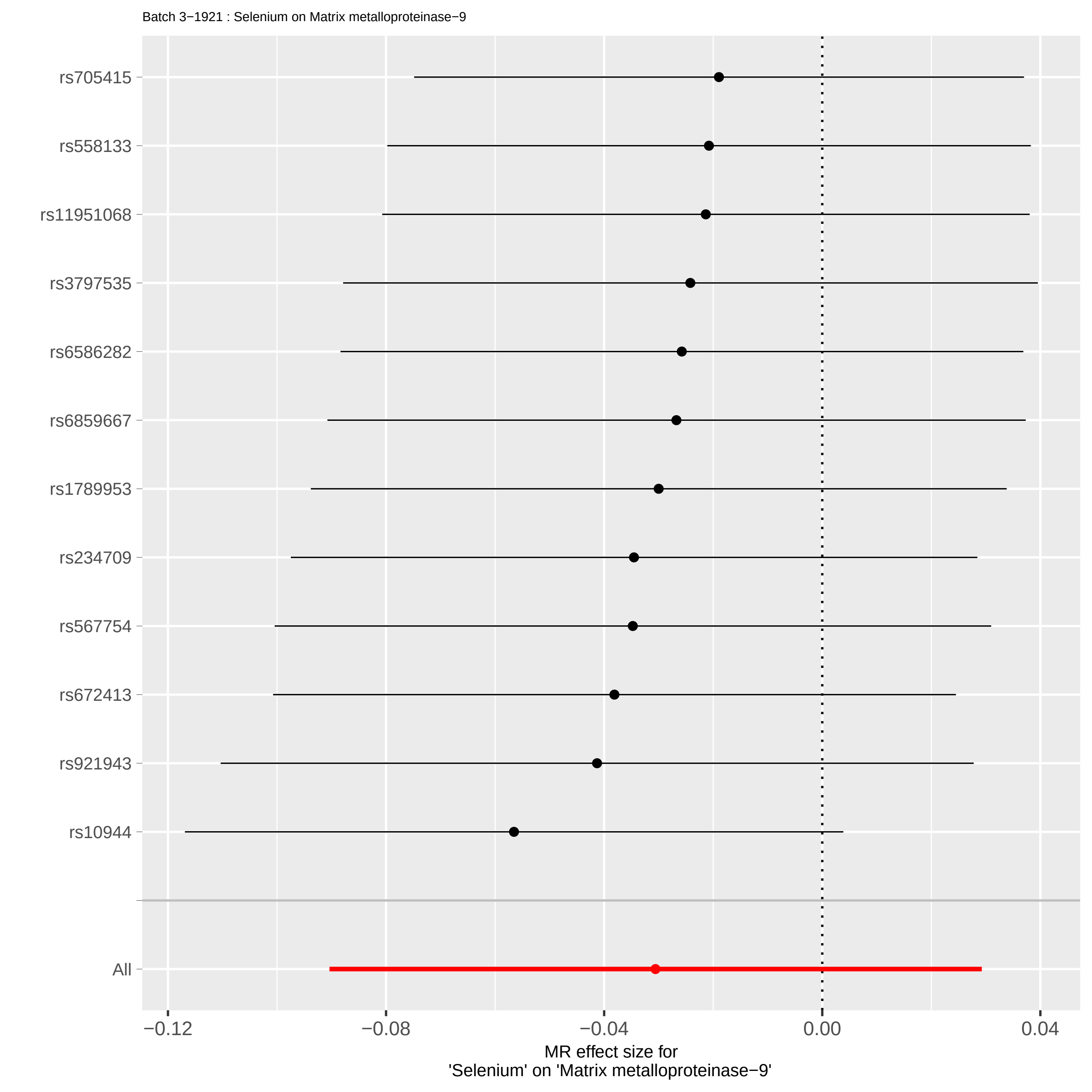

Batch 3−1921 : Selenium on Matrix metalloproteinase−9
rs705415
rs558133
rs11951068
rs3797535
rs6586282
rs6859667
rs1789953
rs234709
rs567754
rs672413
rs921943
rs10944
All
−0.12
−0.08
−0.04
0.00
0.04
MR effect size for
'Selenium' on 'Matrix metalloproteinase−9'

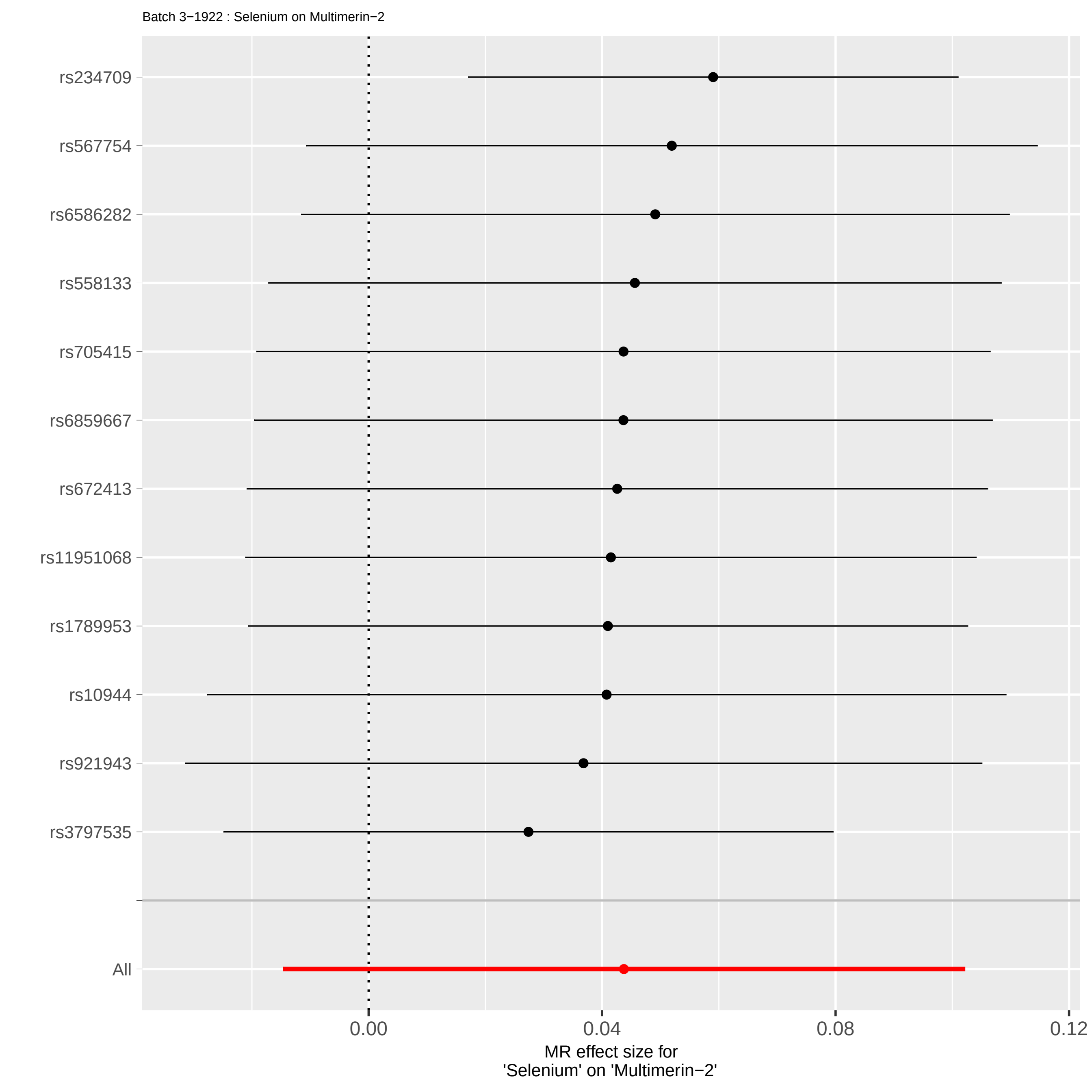
Batch 3−1922 : Selenium on Multimerin−2
rs234709
rs567754
rs6586282
rs558133
rs705415
rs6859667
rs672413
rs11951068
rs1789953
rs10944
rs921943
rs3797535
All
0.00
0.04
0.08
0.12
MR effect size for
'Selenium' on 'Multimerin−2'

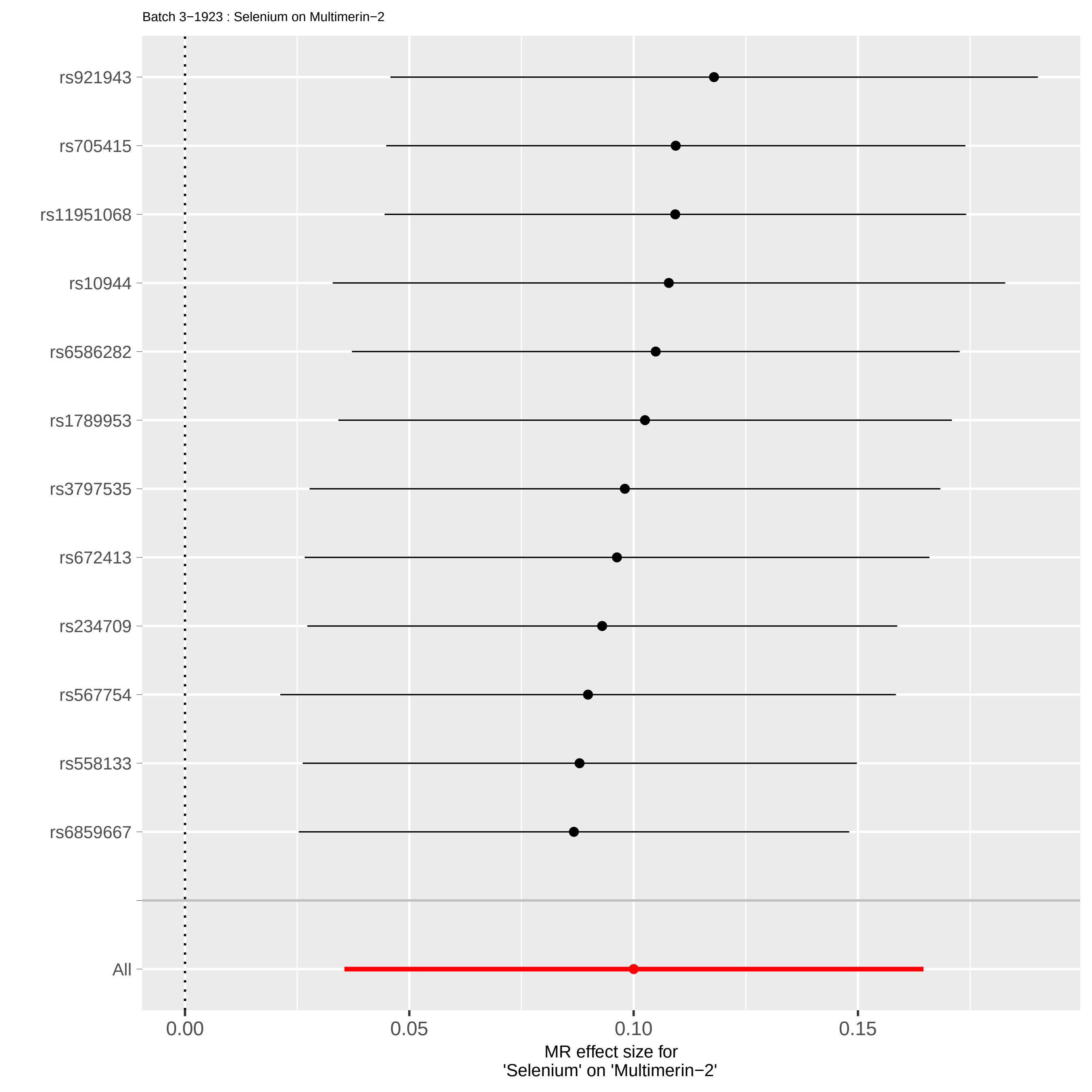

Batch 3−1923 : Selenium on Multimerin−2
rs921943
rs705415
rs11951068
rs10944
rs6586282
rs1789953
rs3797535
rs672413
rs234709
rs567754
rs558133
rs6859667
All
0.00
0.05
0.10
0.15
MR effect size for
'Selenium' on 'Multimerin−2'

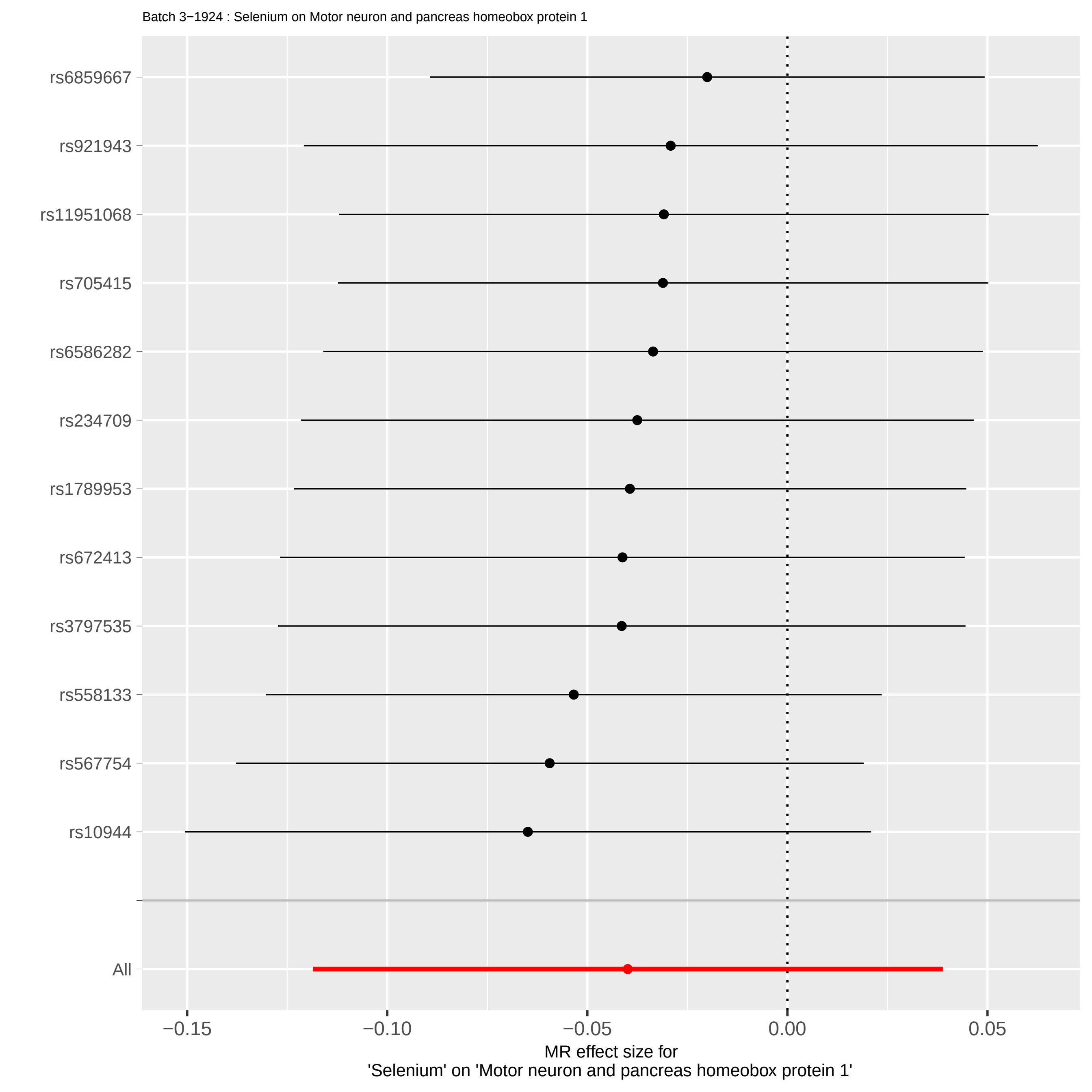

Batch 3−1924 : Selenium on Motor neuron and pancreas homeobox protein 1
rs6859667
rs921943
rs11951068
rs705415
rs6586282
rs234709
rs1789953
rs672413
rs3797535
rs558133
rs567754
rs10944
All
−0.15
−0.10
−0.05
0.00
0.05
MR effect size for
'Selenium' on 'Motor neuron and pancreas homeobox protein 1'

Batch 3−1925 : Selenium on MOB kinase activator 1A

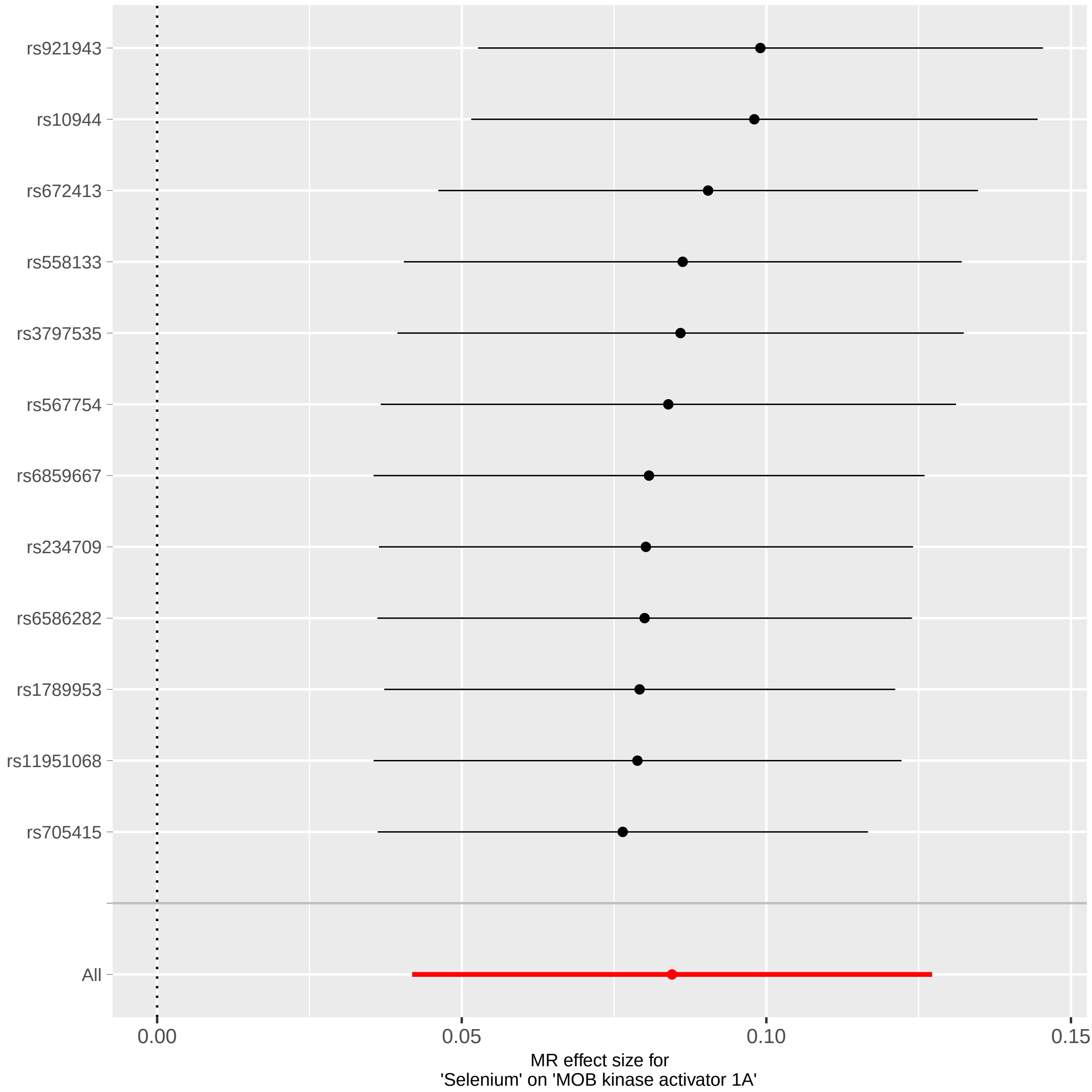

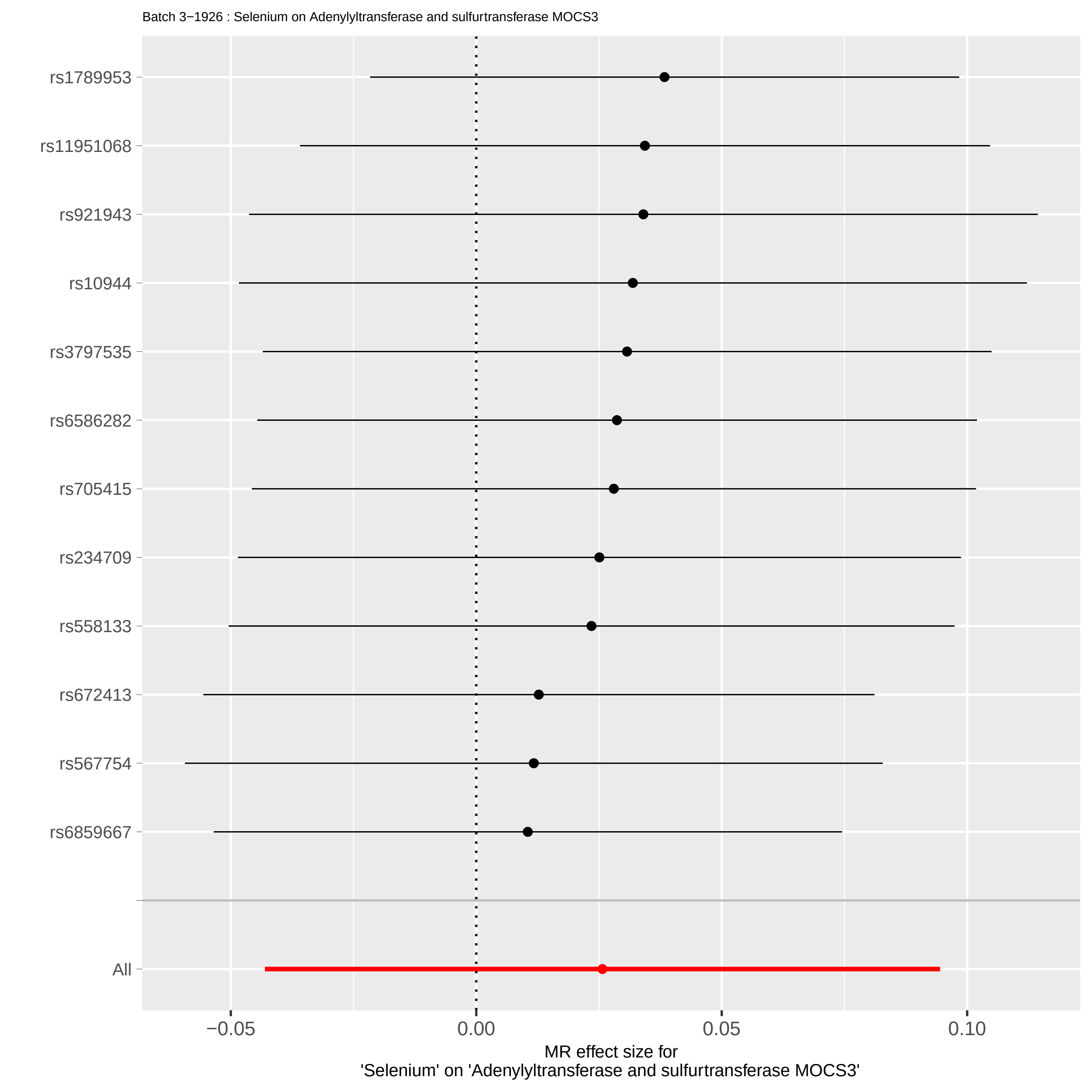

Batch 3−1926 : Selenium on Adenylyltransferase and sulfurtransferase MOCS3
rs1789953
rs11951068
rs921943
rs10944
rs3797535
rs6586282
rs705415
rs234709
rs558133
rs672413
rs567754
rs6859667
All
−0.05
0.00
0.05
0.10
MR effect size for
'Selenium' on 'Adenylyltransferase and sulfurtransferase MOCS3'

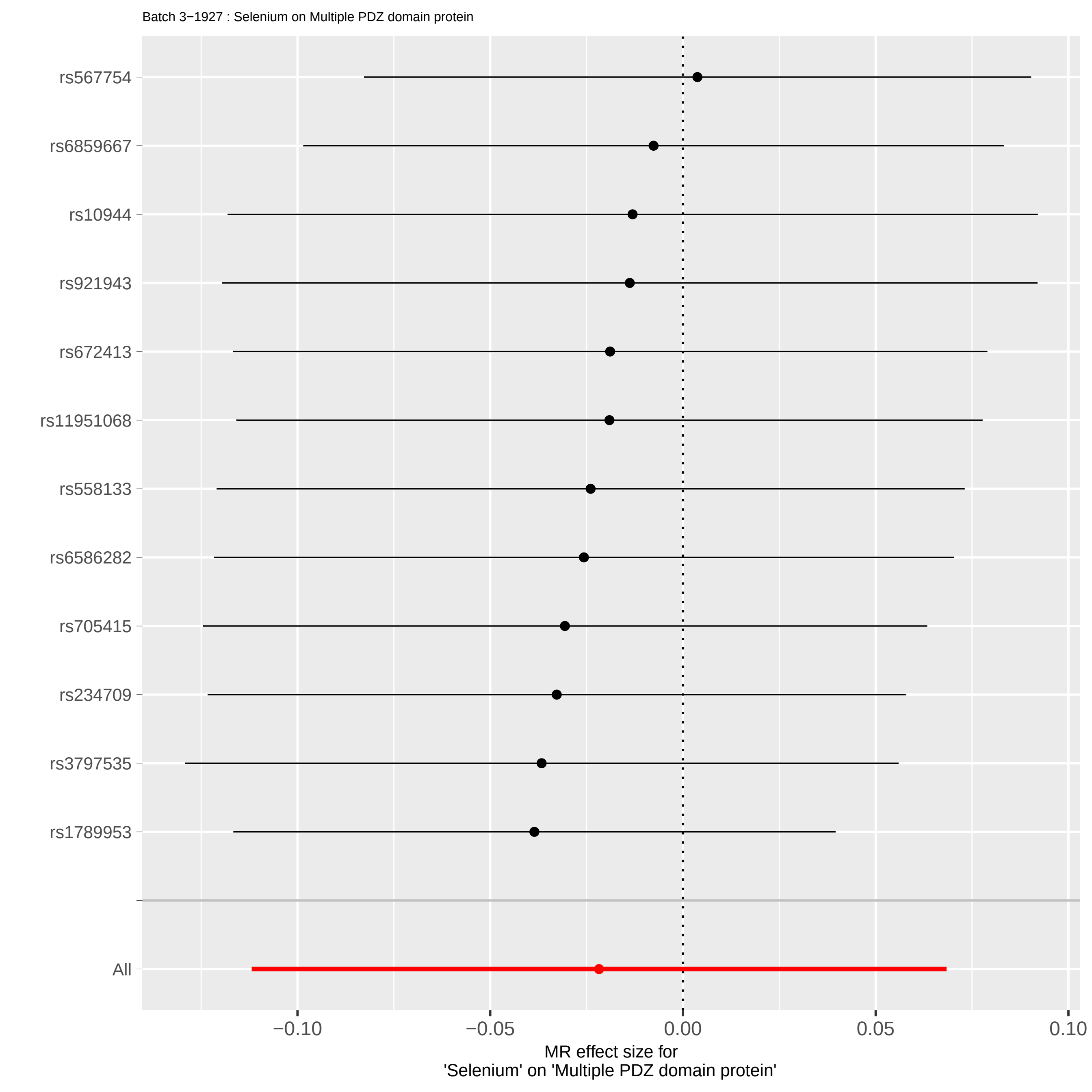

Batch 3−1927 : Selenium on Multiple PDZ domain protein
rs567754
rs6859667
rs10944
rs921943
rs672413
rs11951068
rs558133
rs6586282
rs705415
rs234709
rs3797535
rs1789953
All
−0.10
−0.05
0.00
0.05
0.10
MR effect size for
'Selenium' on 'Multiple PDZ domain protein'

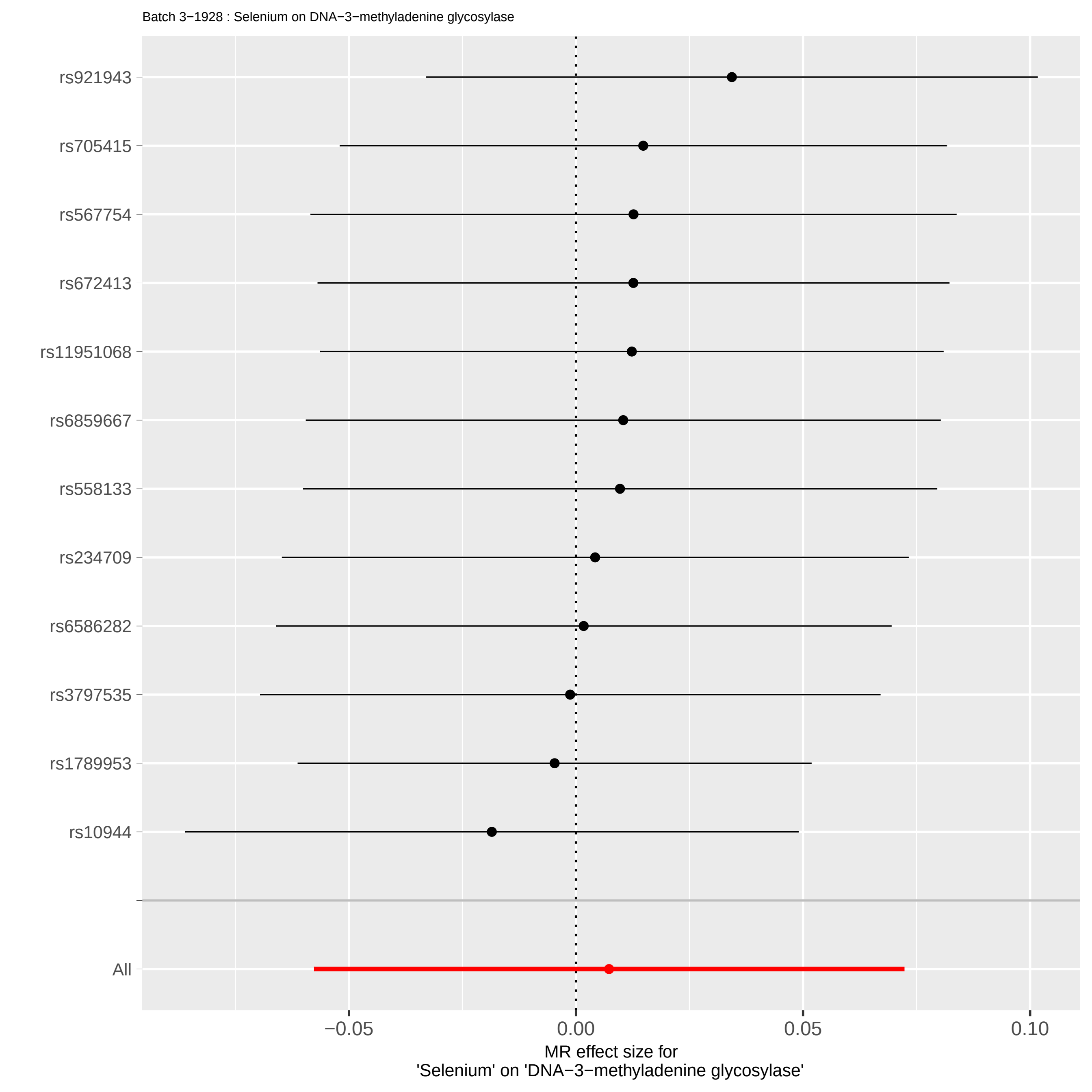

Batch 3−1928 : Selenium on DNA−3−methyladenine glycosylase
rs921943
rs705415
rs567754
rs672413
rs11951068
rs6859667
rs558133
rs234709
rs6586282
rs3797535
rs1789953
rs10944
All
−0.05
0.00
0.05
0.10
MR effect size for
'Selenium' on 'DNA−3−methyladenine glycosylase'

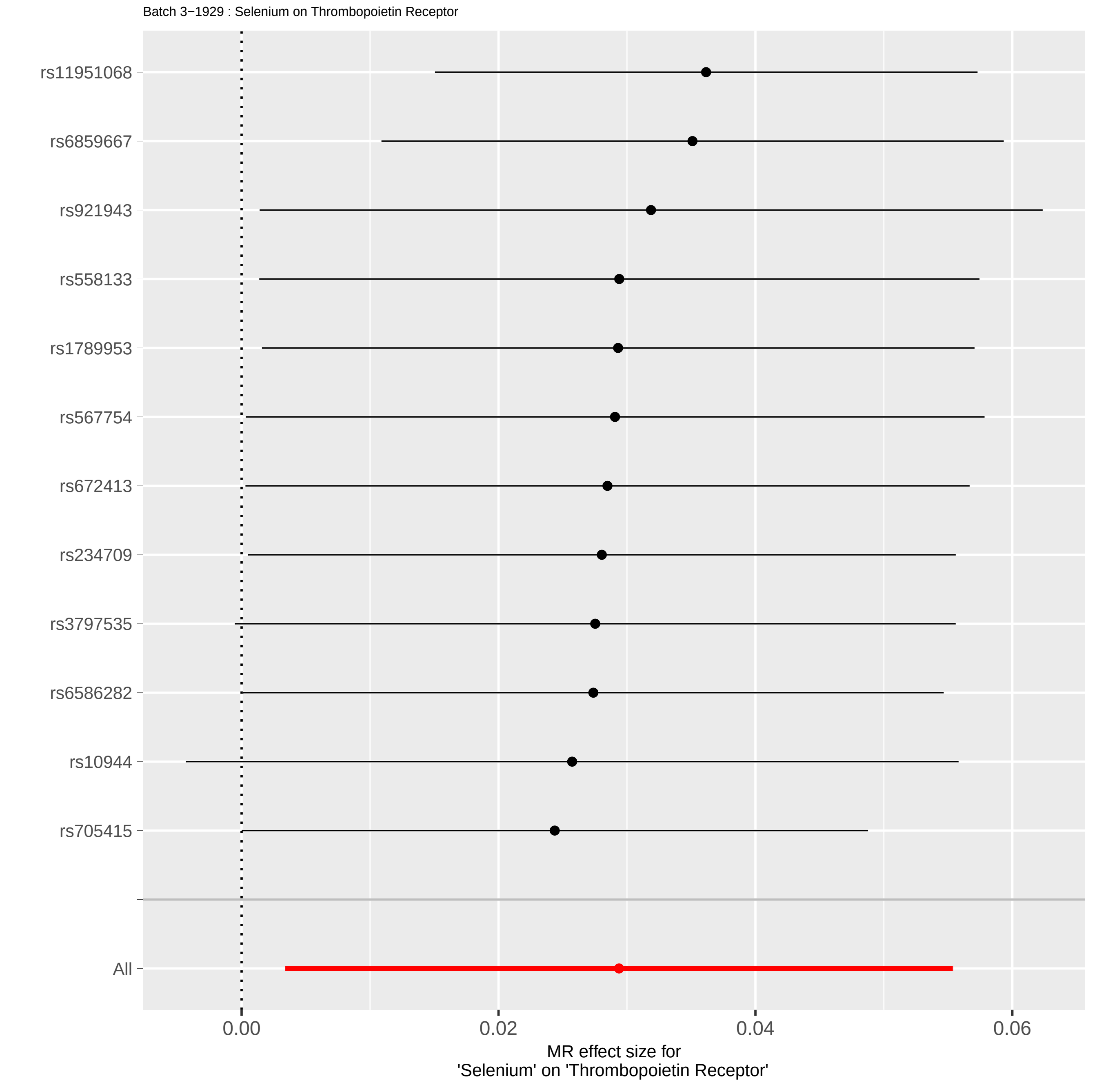

Batch 3−1929 : Selenium on Thrombopoietin Receptor
rs11951068
rs6859667
rs921943
rs558133
rs1789953
rs567754
rs672413
rs234709
rs3797535
rs6586282
rs10944
rs705415
All
0.00
0.02
0.04
0.06
MR effect size for
'Selenium' on 'Thrombopoietin Receptor'

Batch 3−1930 : Selenium on Myeloperoxidase

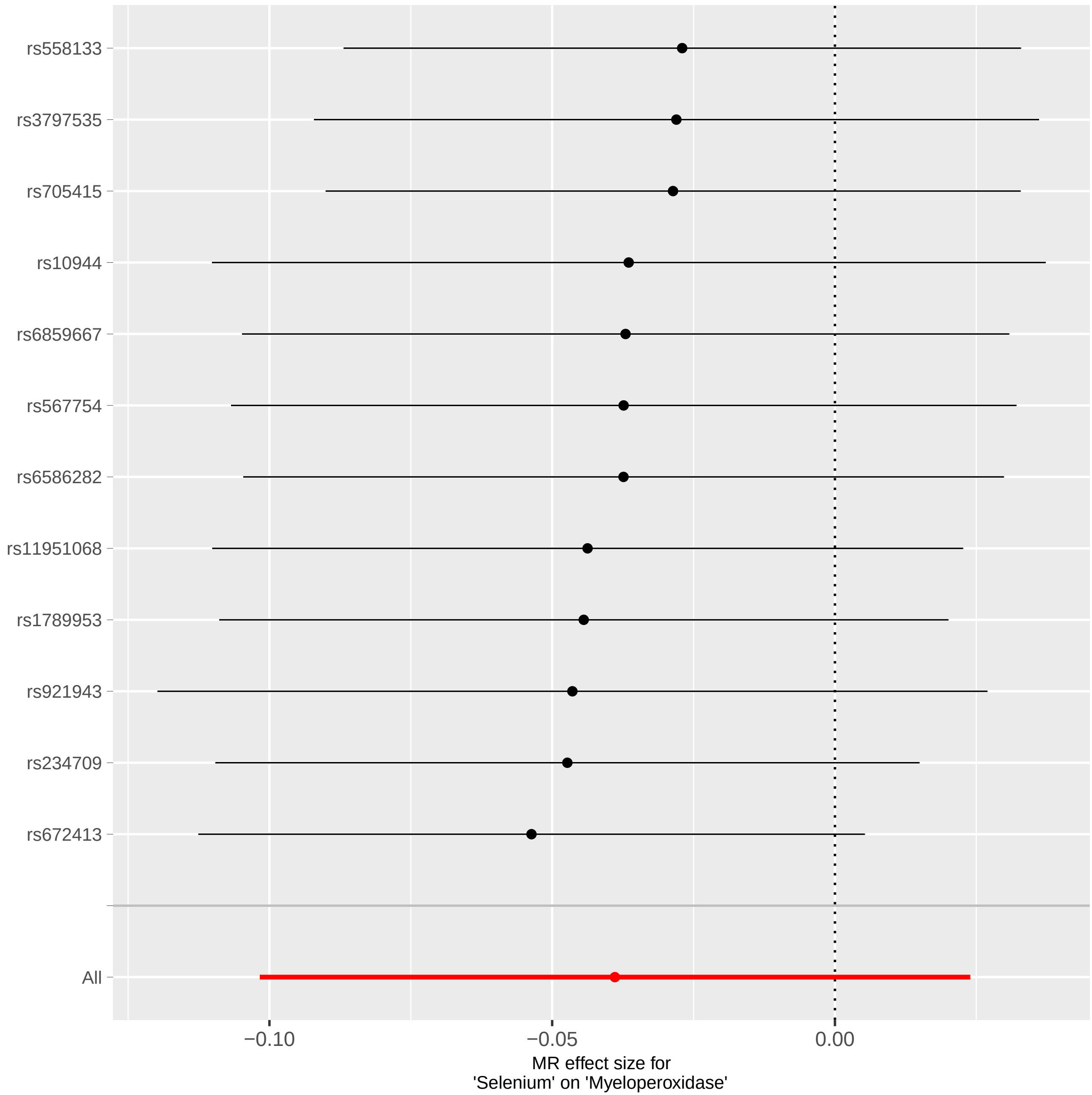

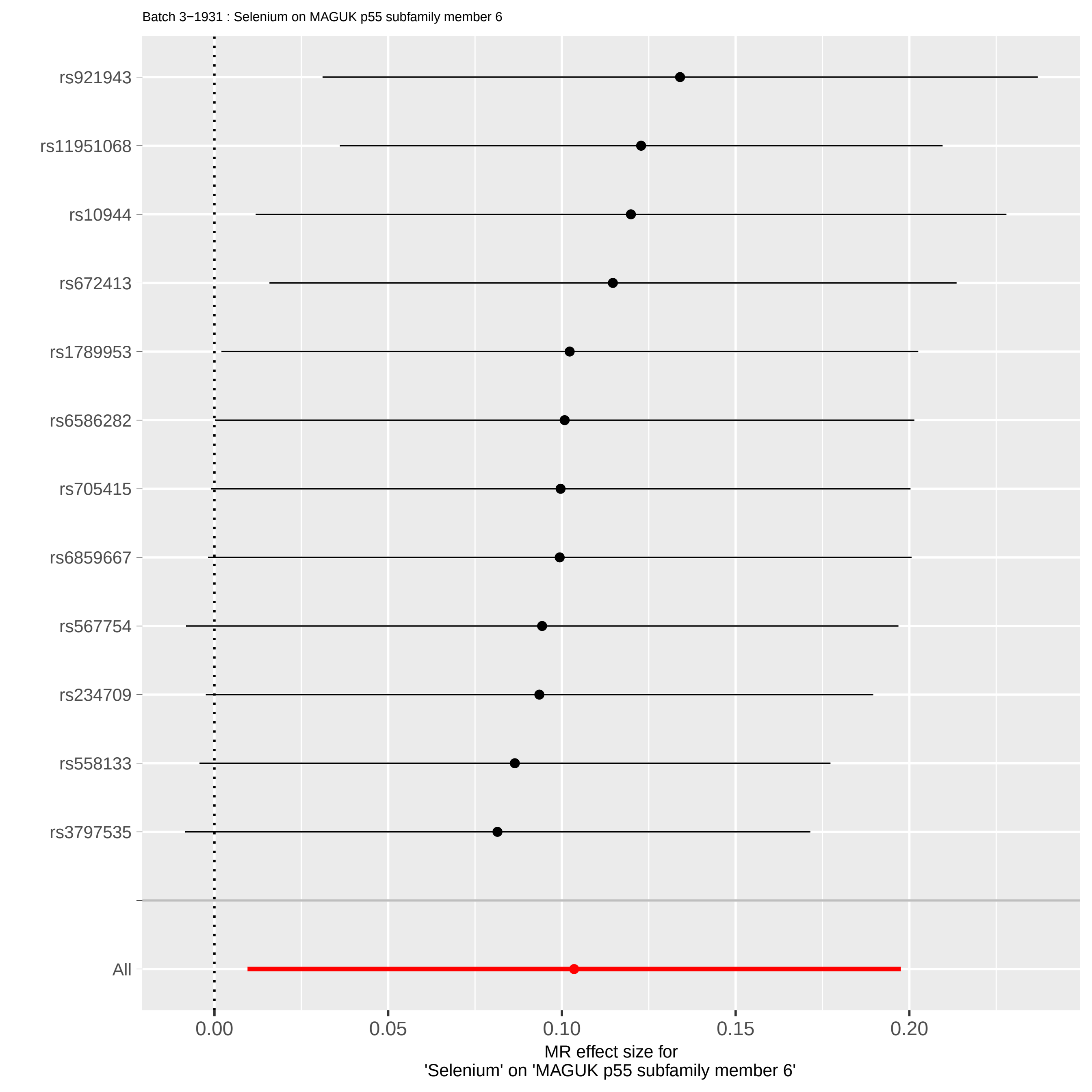

Batch 3−1931 : Selenium on MAGUK p55 subfamily member 6
rs921943
rs11951068
rs10944
rs672413
rs1789953
rs6586282
rs705415
rs6859667
rs567754
rs234709
rs558133
rs3797535
All
0.00
0.05
0.10
0.15
0.20
MR effect size for
'Selenium' on 'MAGUK p55 subfamily member 6'

Batch 3−1932 : Selenium on MAGUK p55 subfamily member 7

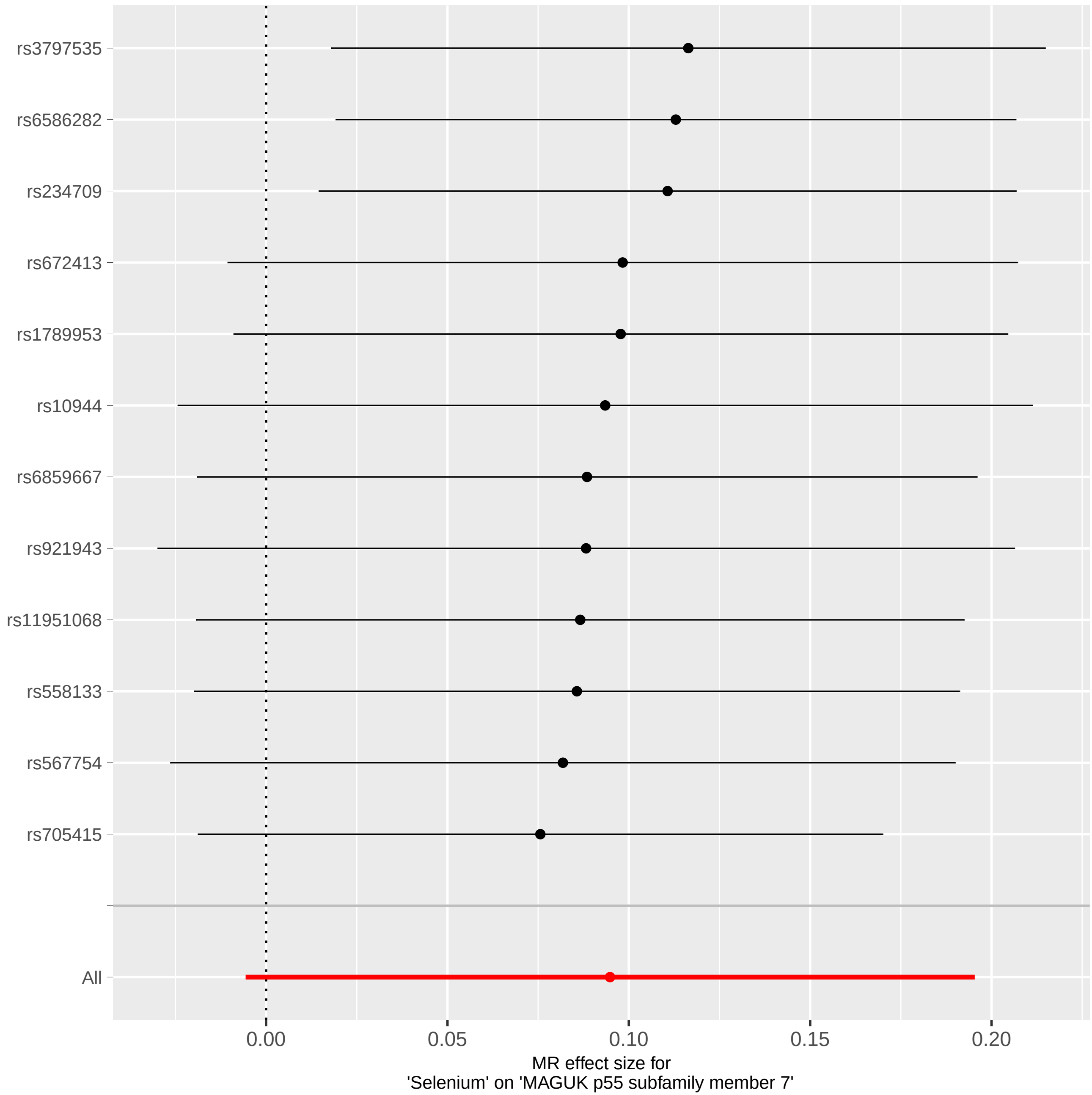

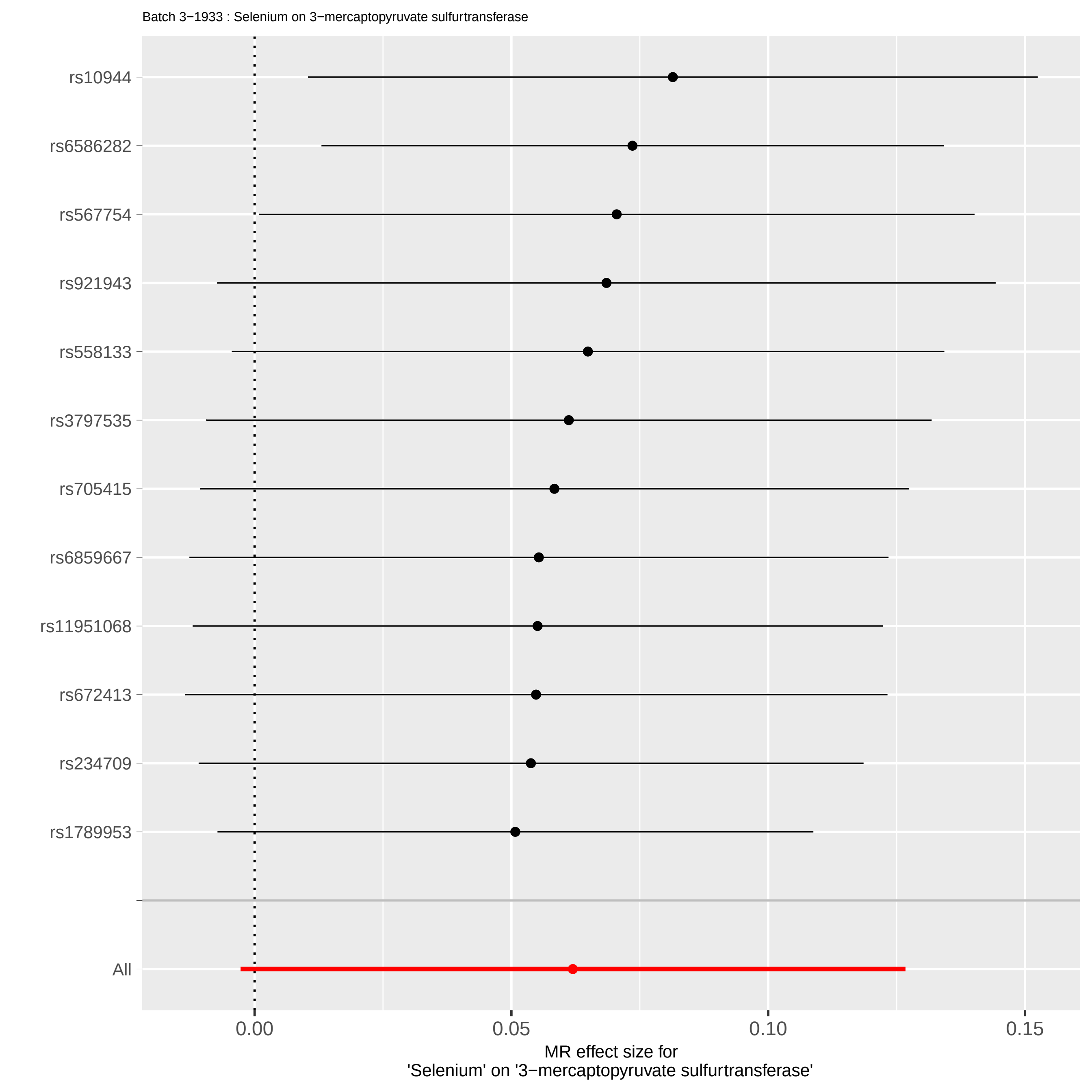

Batch 3−1933 : Selenium on 3−mercaptopyruvate sulfurtransferase
rs10944
rs6586282
rs567754
rs921943
rs558133
rs3797535
rs705415
rs6859667
rs11951068
rs672413
rs234709
rs1789953
All
0.00
0.05
0.10
0.15
MR effect size for
'Selenium' on '3−mercaptopyruvate sulfurtransferase'

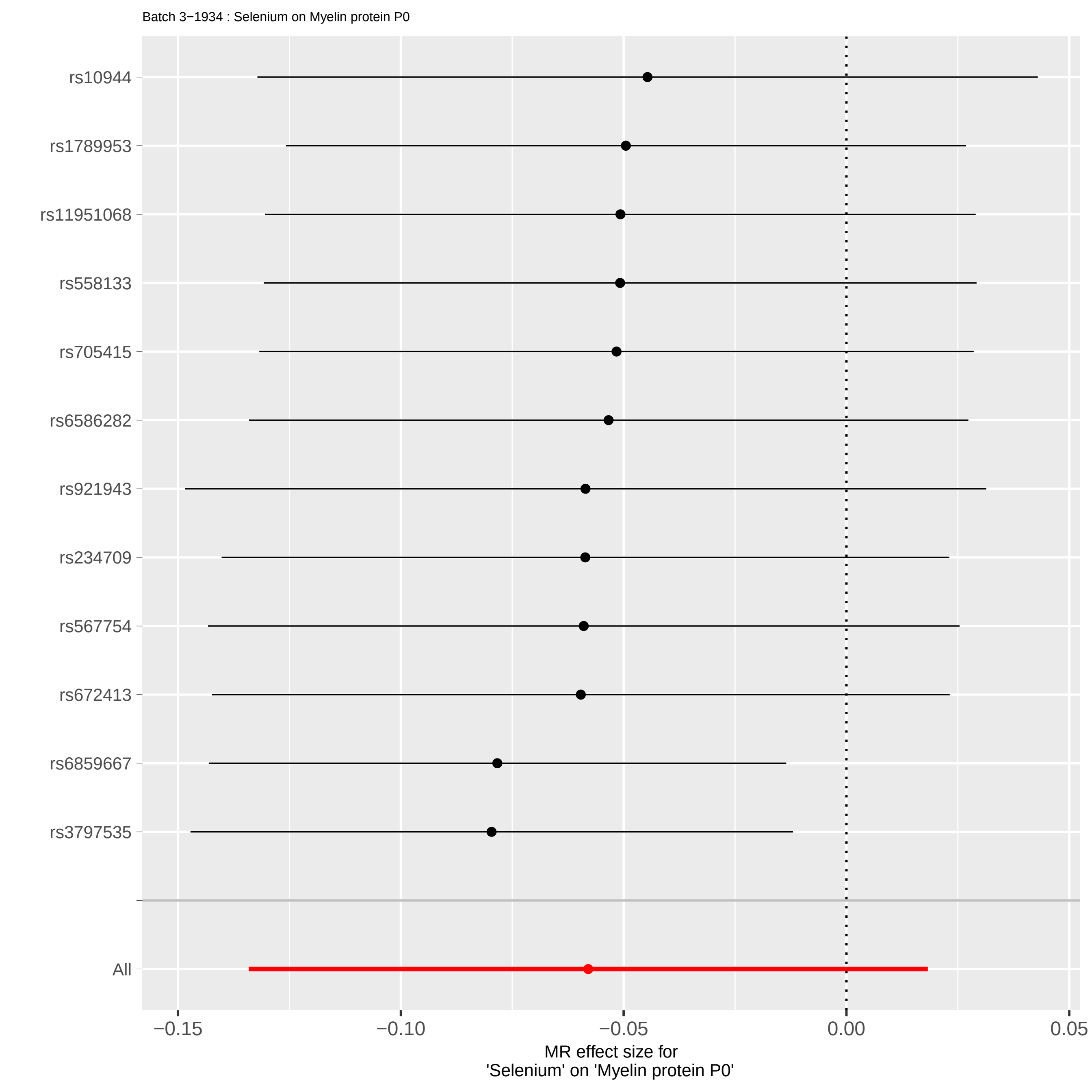

Batch 3−1934 : Selenium on Myelin protein P0
rs10944
rs1789953
rs11951068
rs558133
rs705415
rs6586282
rs921943
rs234709
rs567754
rs672413
rs6859667
rs3797535
All
−0.15
−0.10
−0.05
0.00
0.05
MR effect size for
'Selenium' on 'Myelin protein P0'

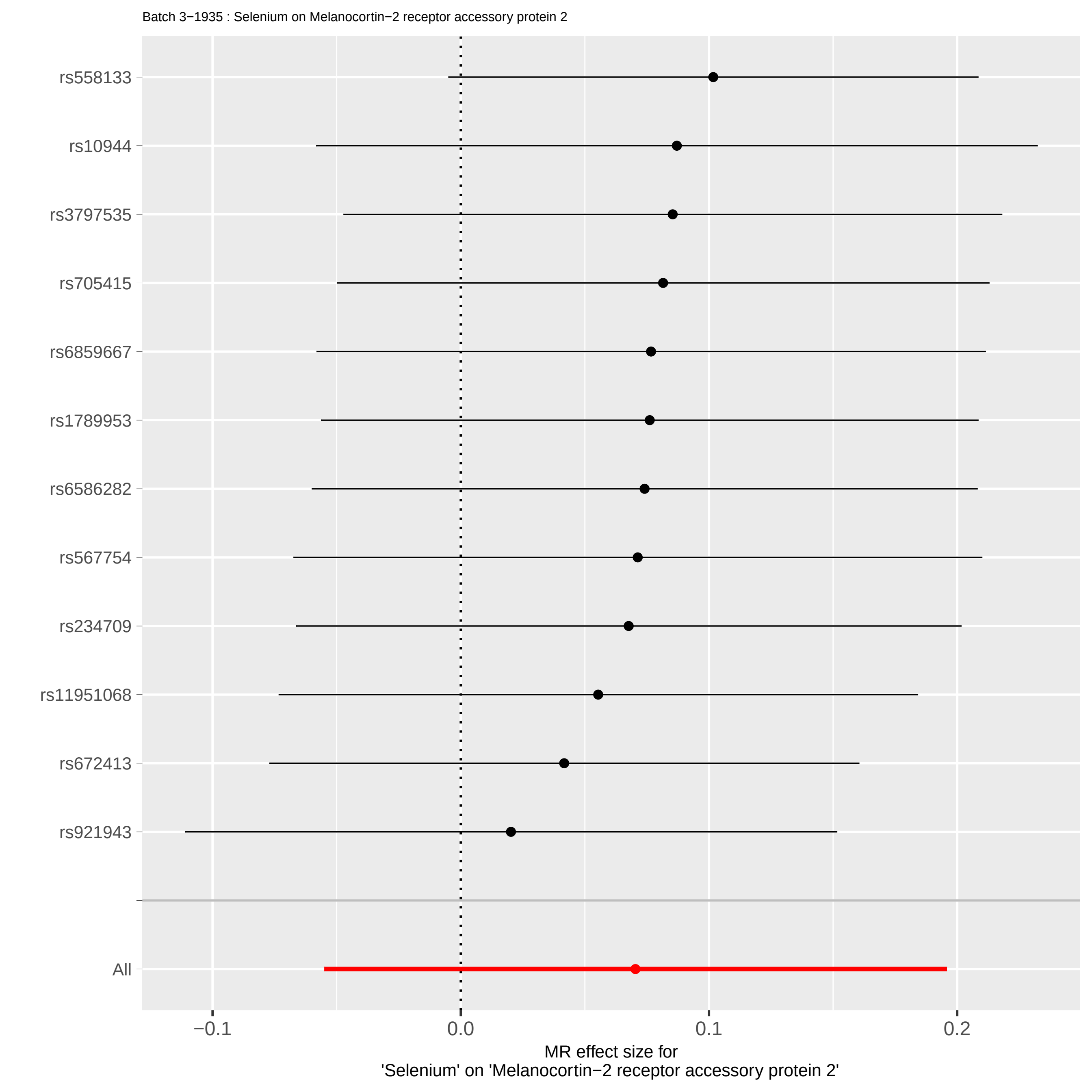

Batch 3−1935 : Selenium on Melanocortin−2 receptor accessory protein 2
rs558133
rs10944
rs3797535
rs705415
rs6859667
rs1789953
rs6586282
rs567754
rs234709
rs11951068
rs672413
rs921943
All
−0.1
0.0
0.1
0.2
MR effect size for
'Selenium' on 'Melanocortin−2 receptor accessory protein 2'

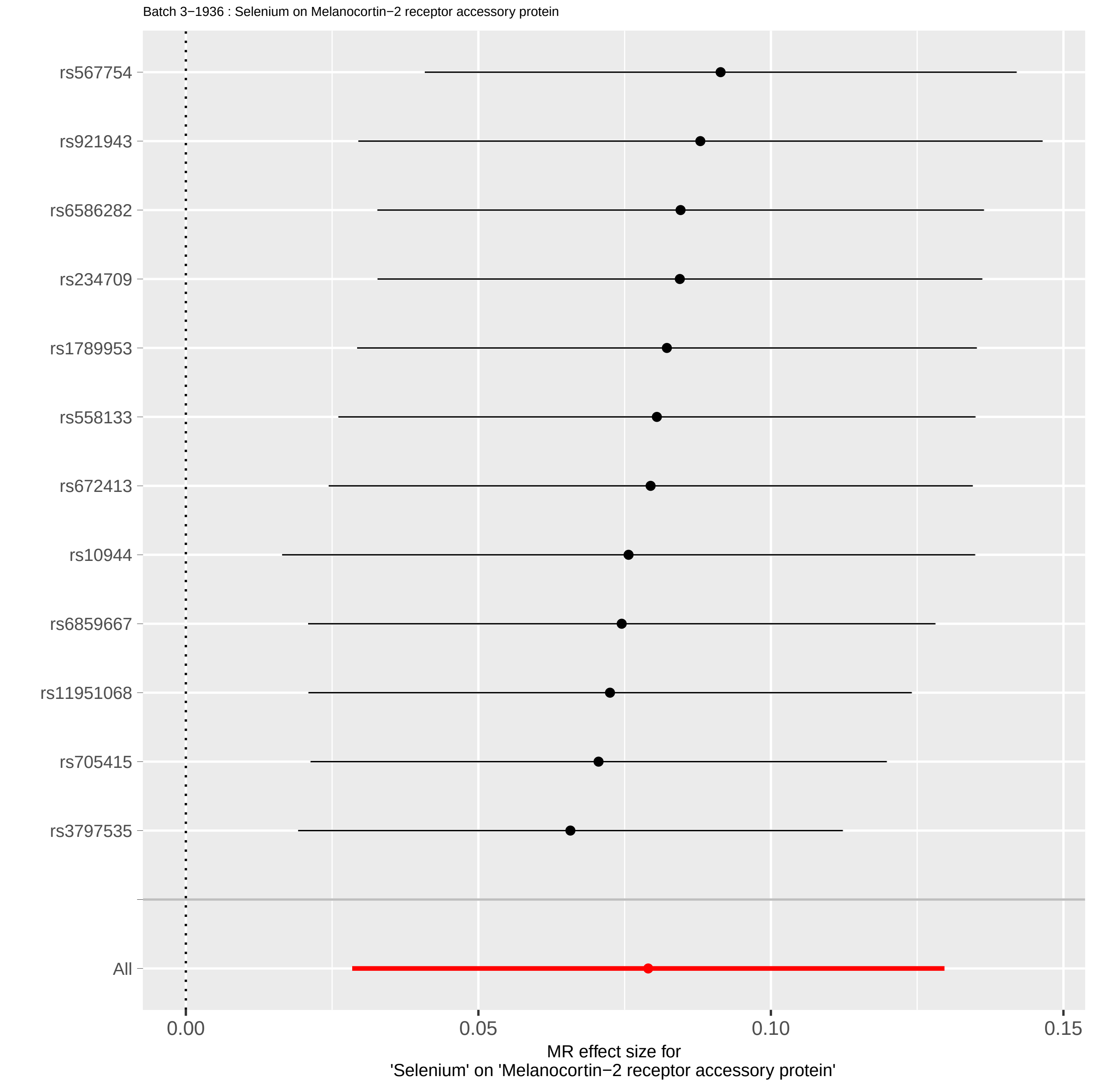
Batch 3−1936 : Selenium on Melanocortin−2 receptor accessory protein
rs567754
rs921943
rs6586282
rs234709
rs1789953
rs558133
rs672413
rs10944
rs6859667
rs11951068
rs705415
rs3797535
All
0.00
0.05
0.10
0.15
MR effect size for
'Selenium' on 'Melanocortin−2 receptor accessory protein'

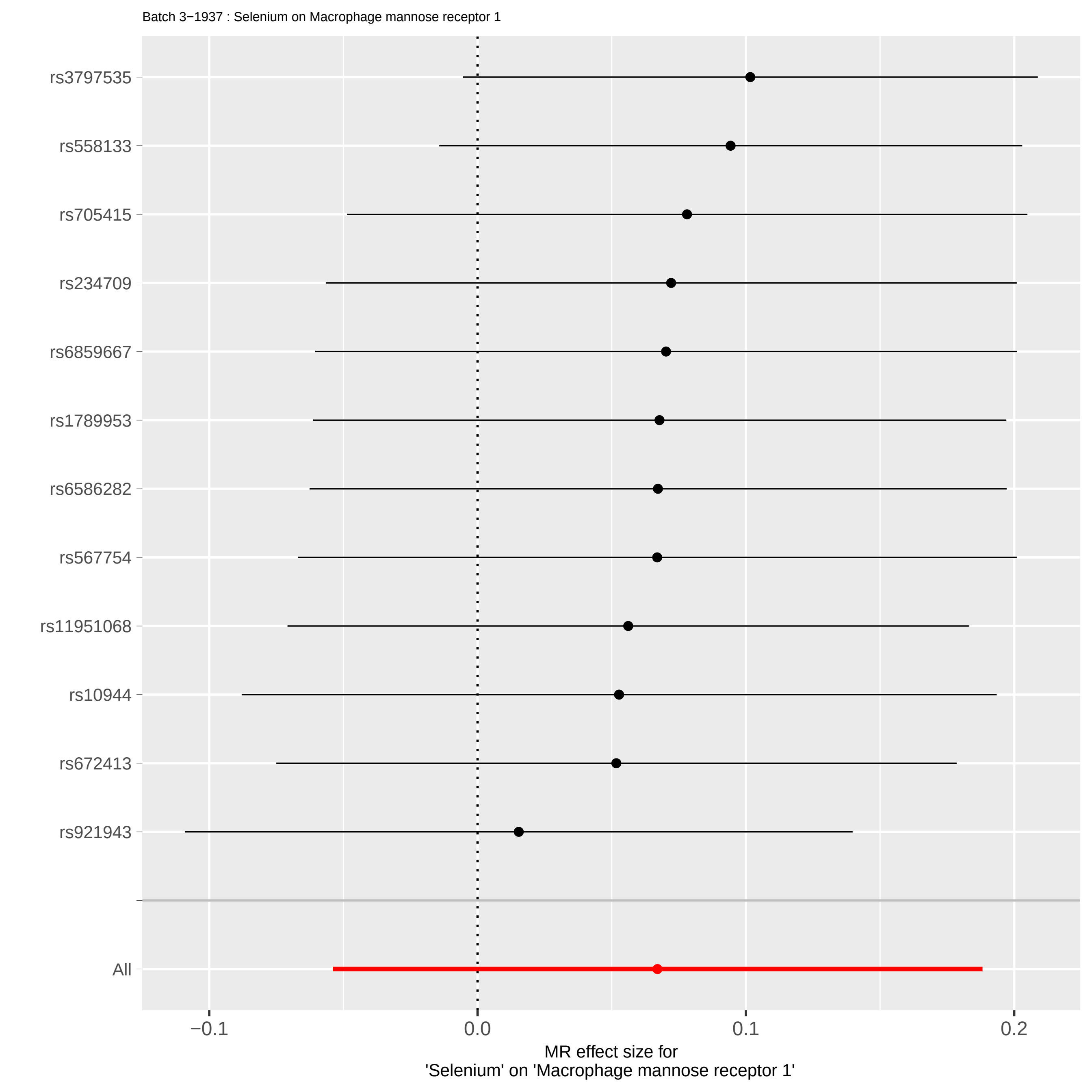

Batch 3−1937 : Selenium on Macrophage mannose receptor 1
rs3797535
rs558133
rs705415
rs234709
rs6859667
rs1789953
rs6586282
rs567754
rs11951068
rs10944
rs672413
rs921943
All
−0.1
0.0
0.1
0.2
MR effect size for
'Selenium' on 'Macrophage mannose receptor 1'

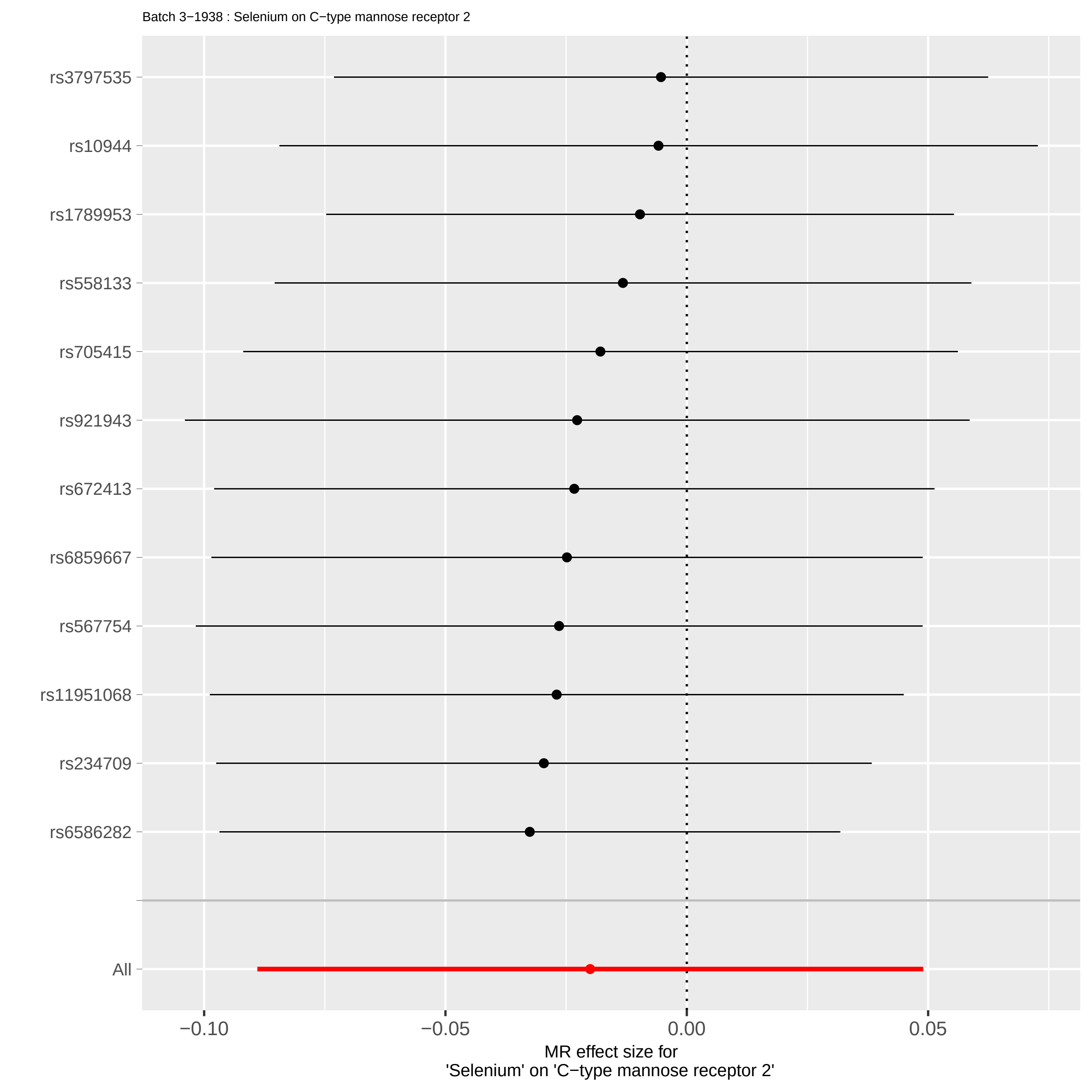
Batch 3−1938 : Selenium on C−type mannose receptor 2
rs3797535
rs10944
rs1789953
rs558133
rs705415
rs921943
rs672413
rs6859667
rs567754
rs11951068
rs234709
rs6586282
All
−0.10
−0.05
0.00
0.05
MR effect size for
'Selenium' on 'C−type mannose receptor 2'

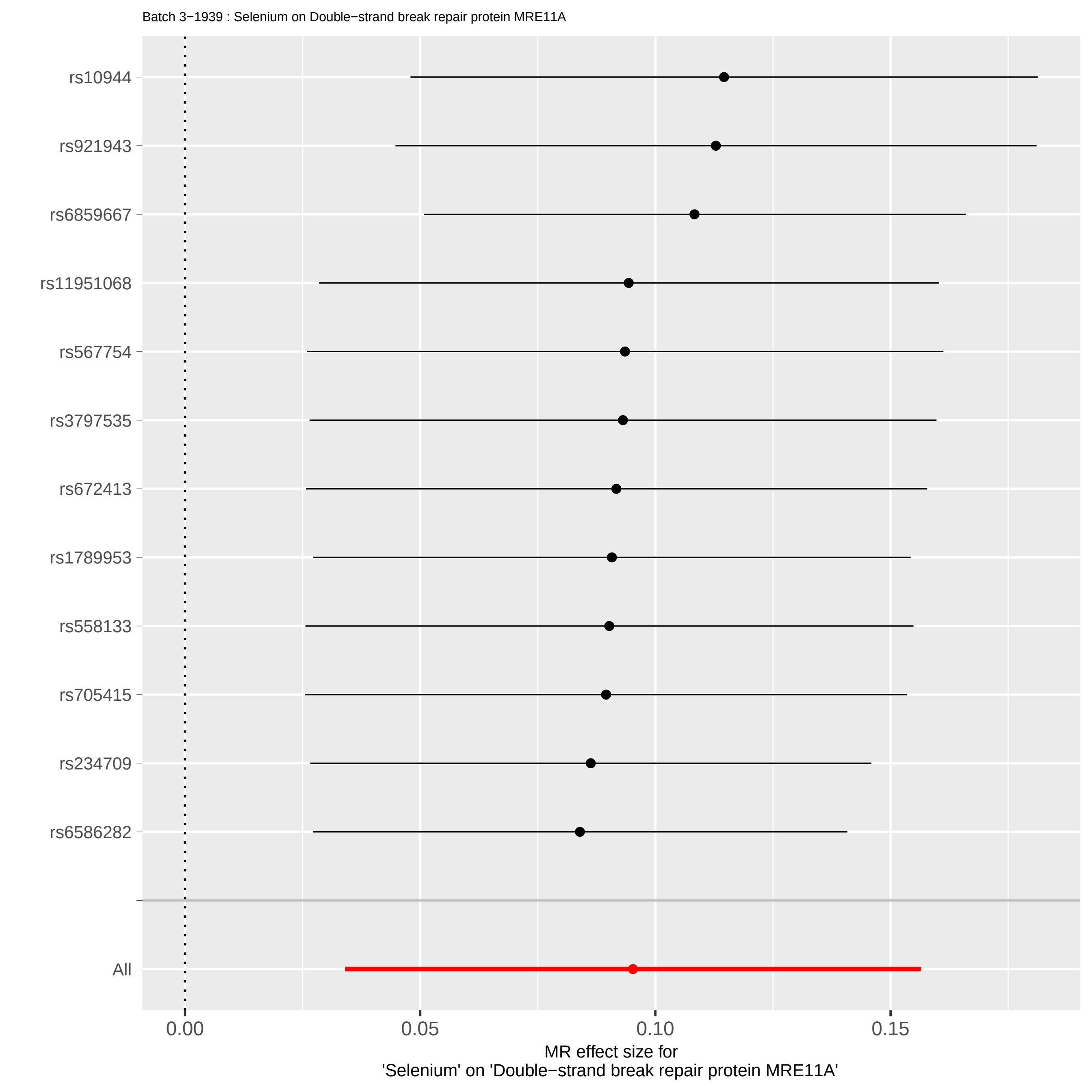

Batch 3−1939 : Selenium on Double−strand break repair protein MRE11A
rs10944
rs921943
rs6859667
rs11951068
rs567754
rs3797535
rs672413
rs1789953
rs558133
rs705415
rs234709
rs6586282
All
0.00
0.05
0.10
0.15
MR effect size for
'Selenium' on 'Double−strand break repair protein MRE11A'

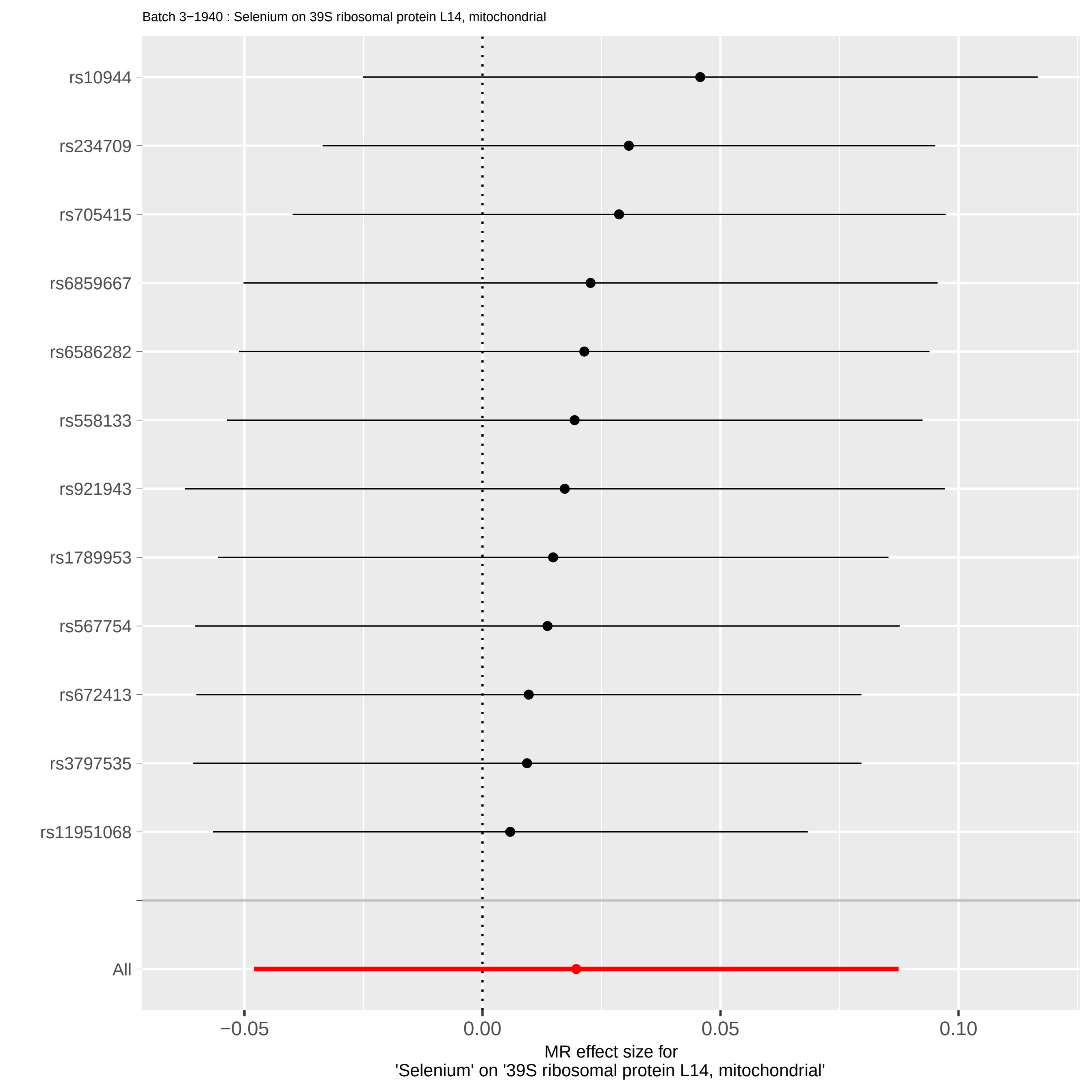

Batch 3−1940 : Selenium on 39S ribosomal protein L14, mitochondrial
rs10944
rs234709
rs705415
rs6859667
rs6586282
rs558133
rs921943
rs1789953
rs567754
rs672413
rs3797535
rs11951068
All
−0.05
0.00
0.05
0.10
MR effect size for
'Selenium' on '39S ribosomal protein L14, mitochondrial'

Batch 3−1941 : Selenium on 39S ribosomal protein L32, mitochondrial

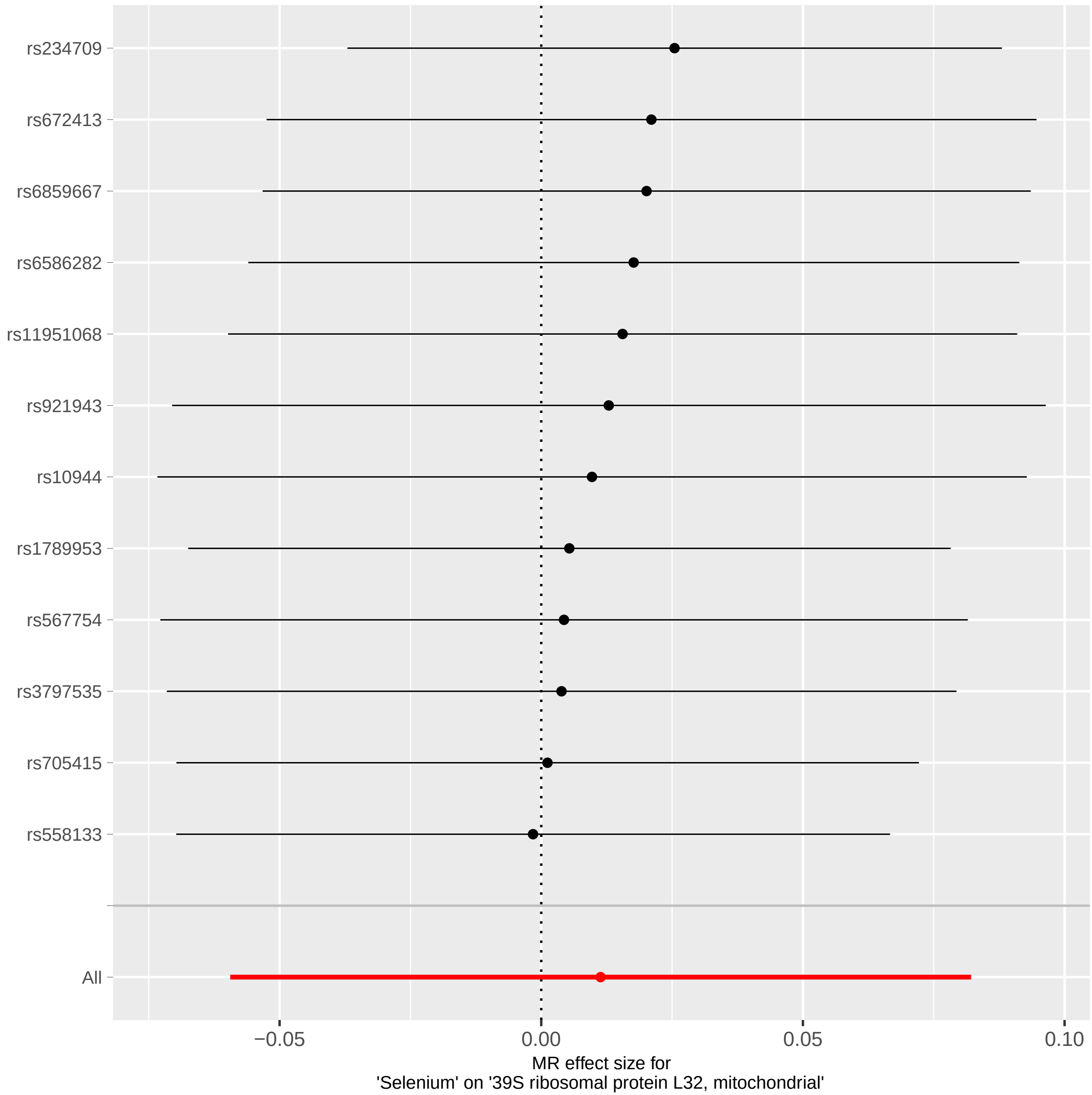

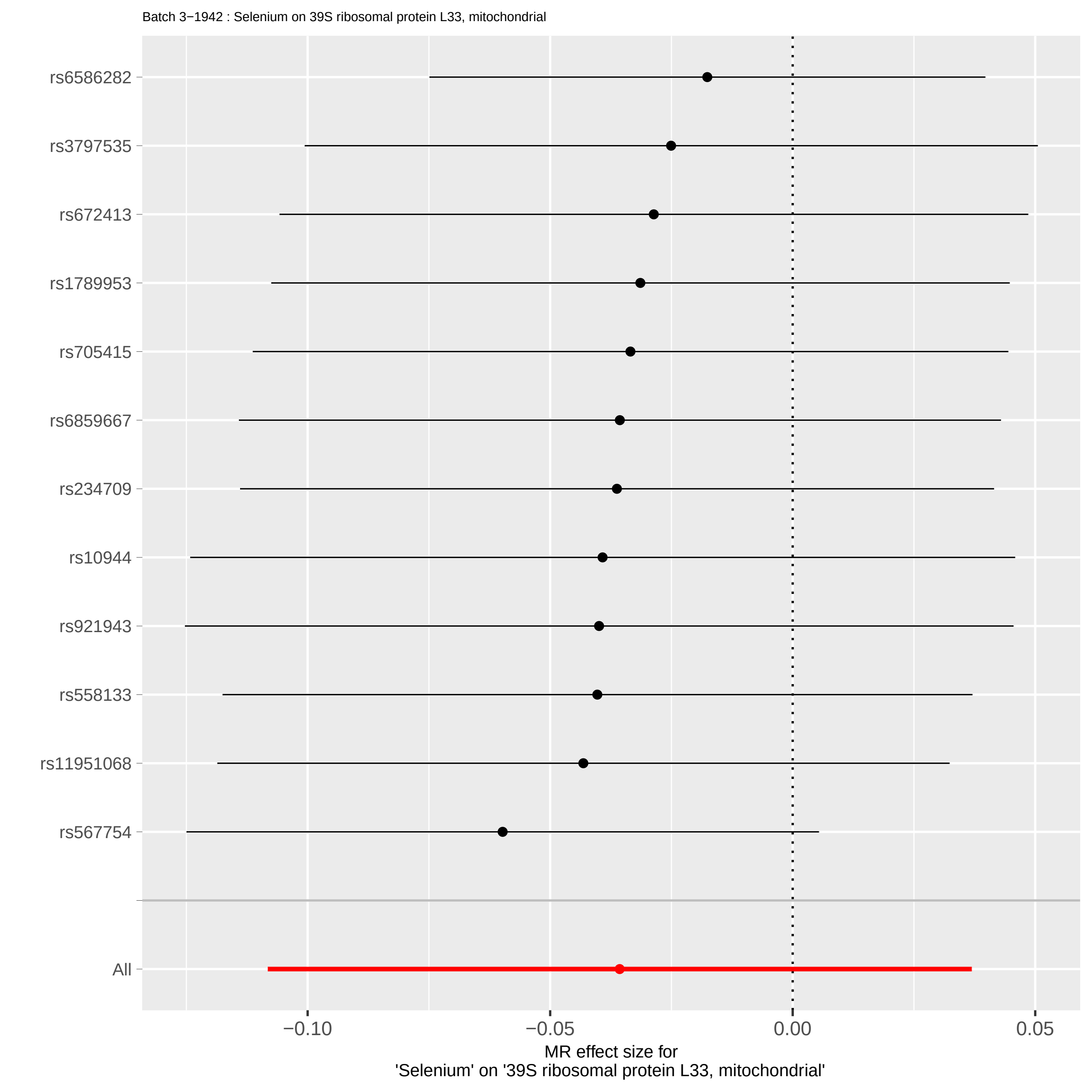

Batch 3−1942 : Selenium on 39S ribosomal protein L33, mitochondrial
rs6586282
rs3797535
rs672413
rs1789953
rs705415
rs6859667
rs234709
rs10944
rs921943
rs558133
rs11951068
rs567754
All
−0.10
−0.05
0.00
0.05
MR effect size for
'Selenium' on '39S ribosomal protein L33, mitochondrial'

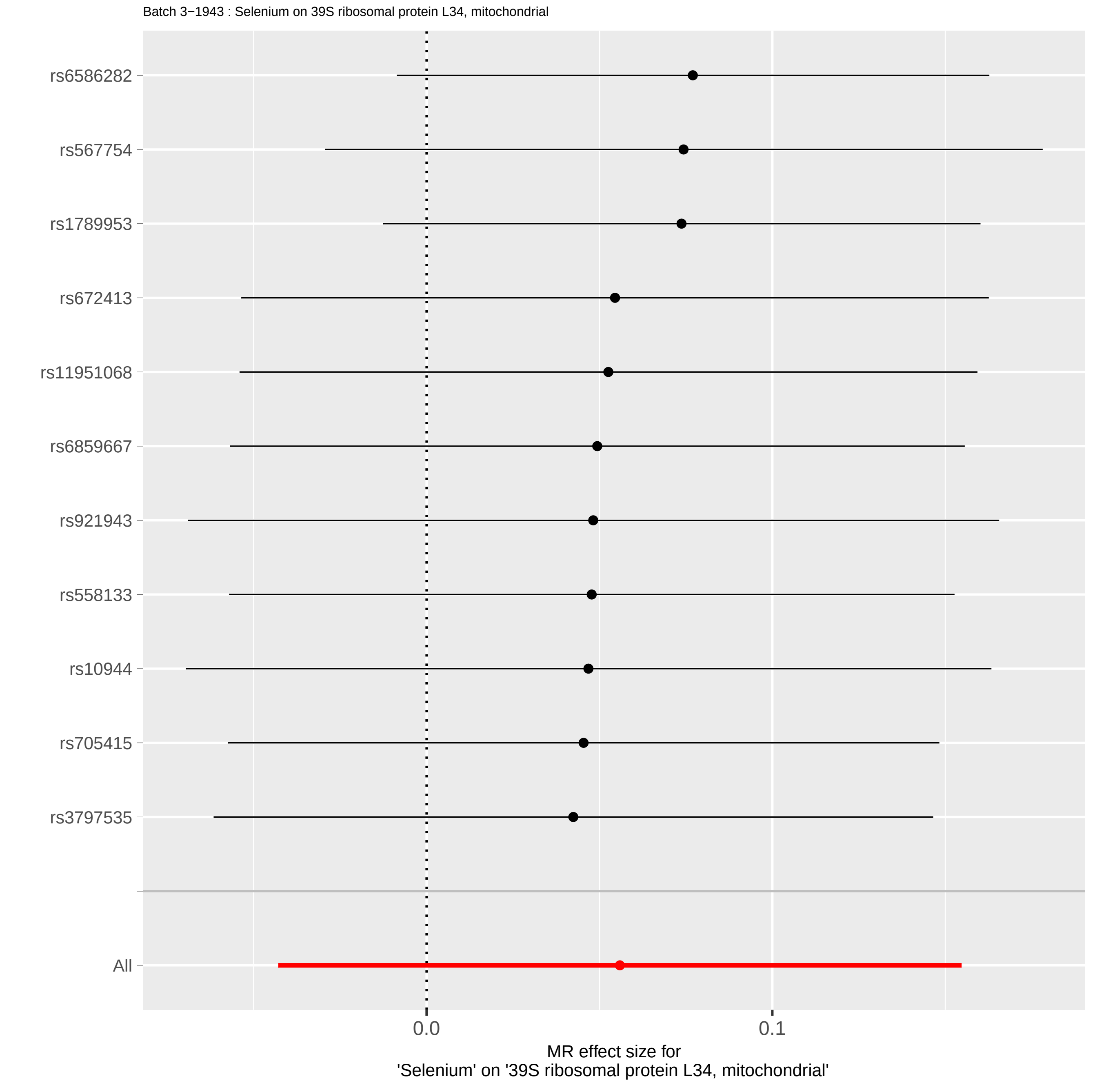

Batch 3−1943 : Selenium on 39S ribosomal protein L34, mitochondrial
rs6586282
rs567754
rs1789953
rs672413
rs11951068
rs6859667
rs921943
rs558133
rs10944
rs705415
rs3797535
All
0.0
0.1
MR effect size for
'Selenium' on '39S ribosomal protein L34, mitochondrial'

Batch 3−1944 : Selenium on 39S ribosomal protein L52, mitochondrial

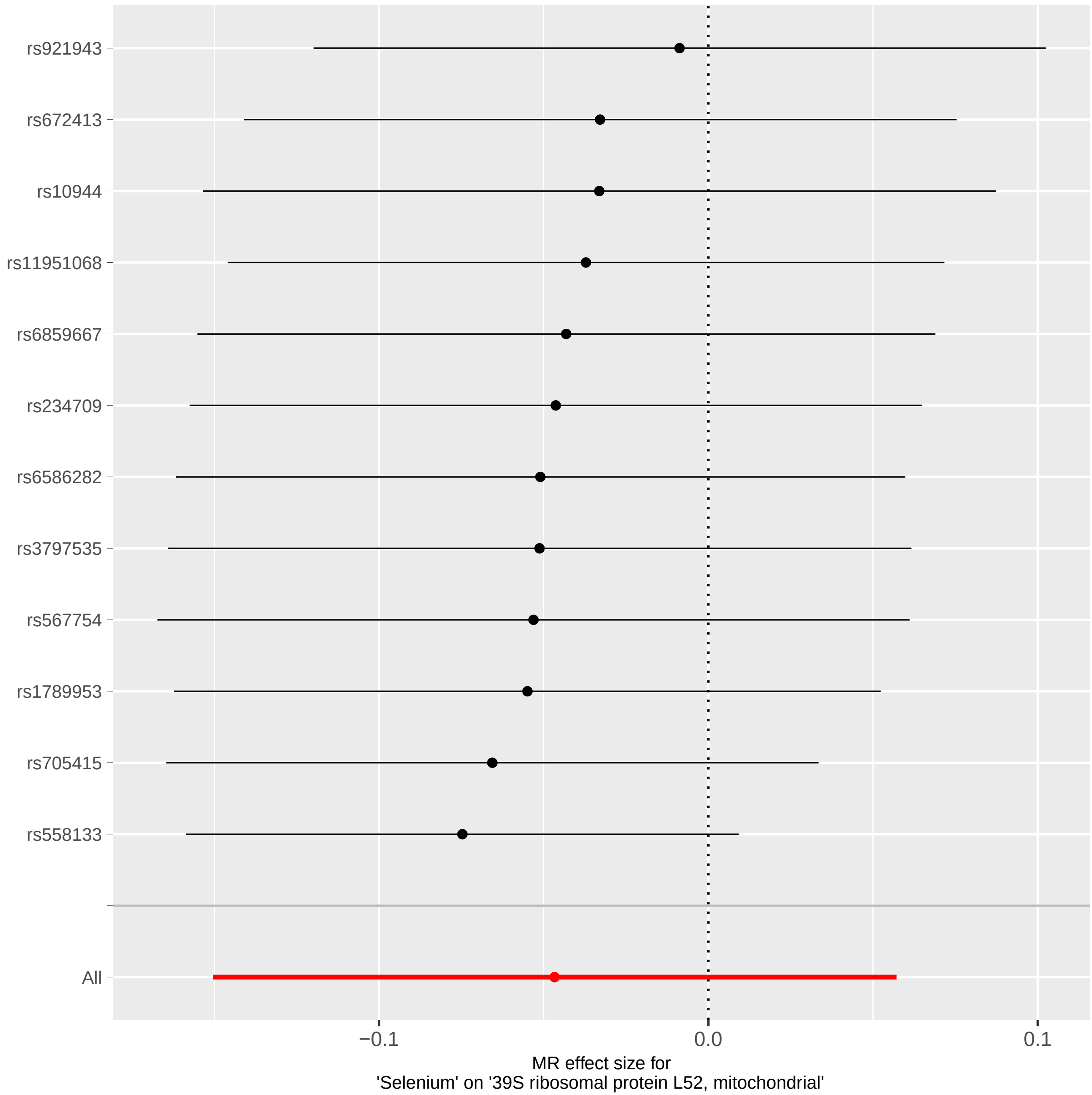

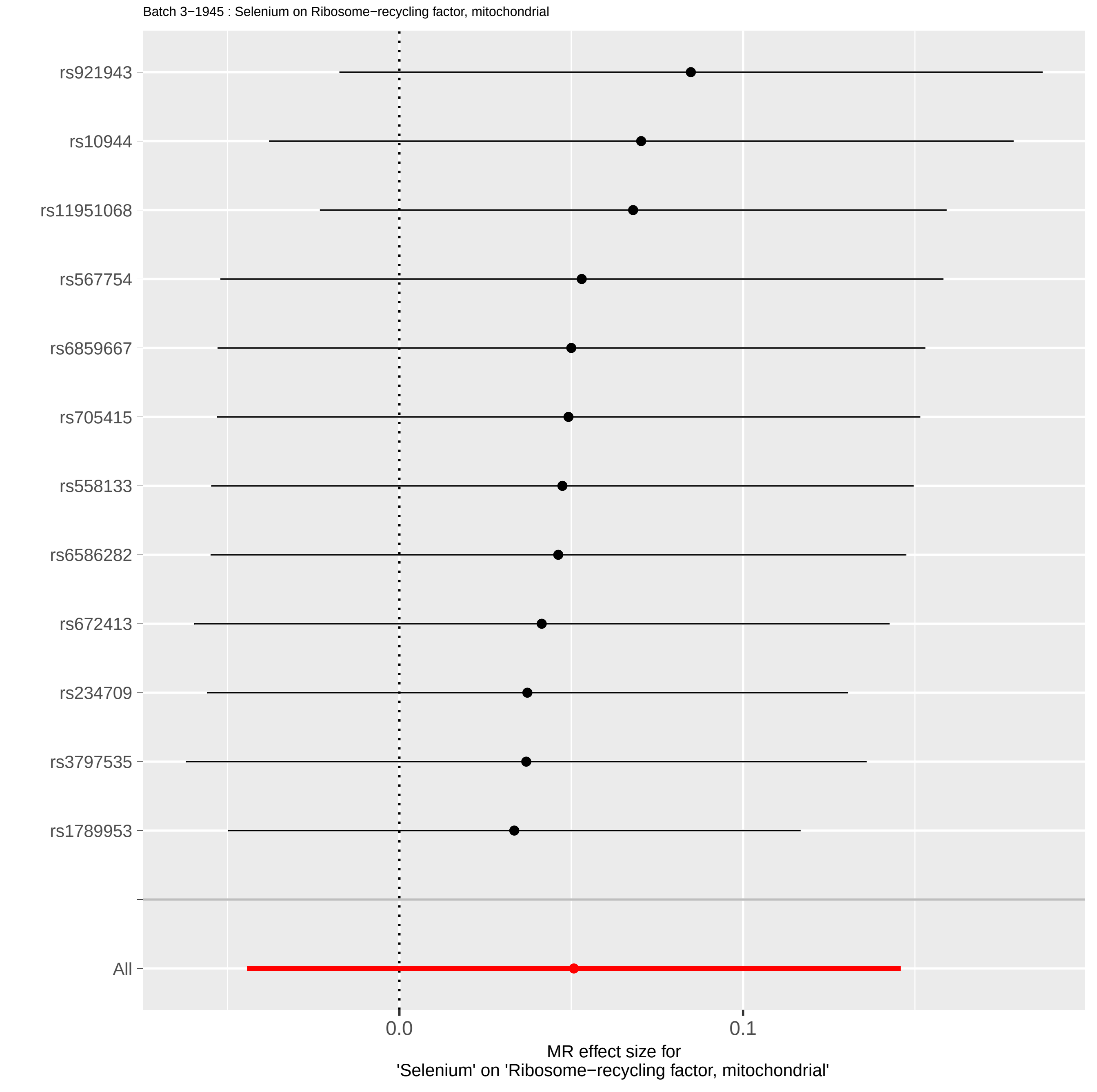

Batch 3−1945 : Selenium on Ribosome−recycling factor, mitochondrial
rs921943
rs10944
rs11951068
rs567754
rs6859667
rs705415
rs558133
rs6586282
rs672413
rs234709
rs3797535
rs1789953
All
0.0
0.1
MR effect size for
'Selenium' on 'Ribosome−recycling factor, mitochondrial'

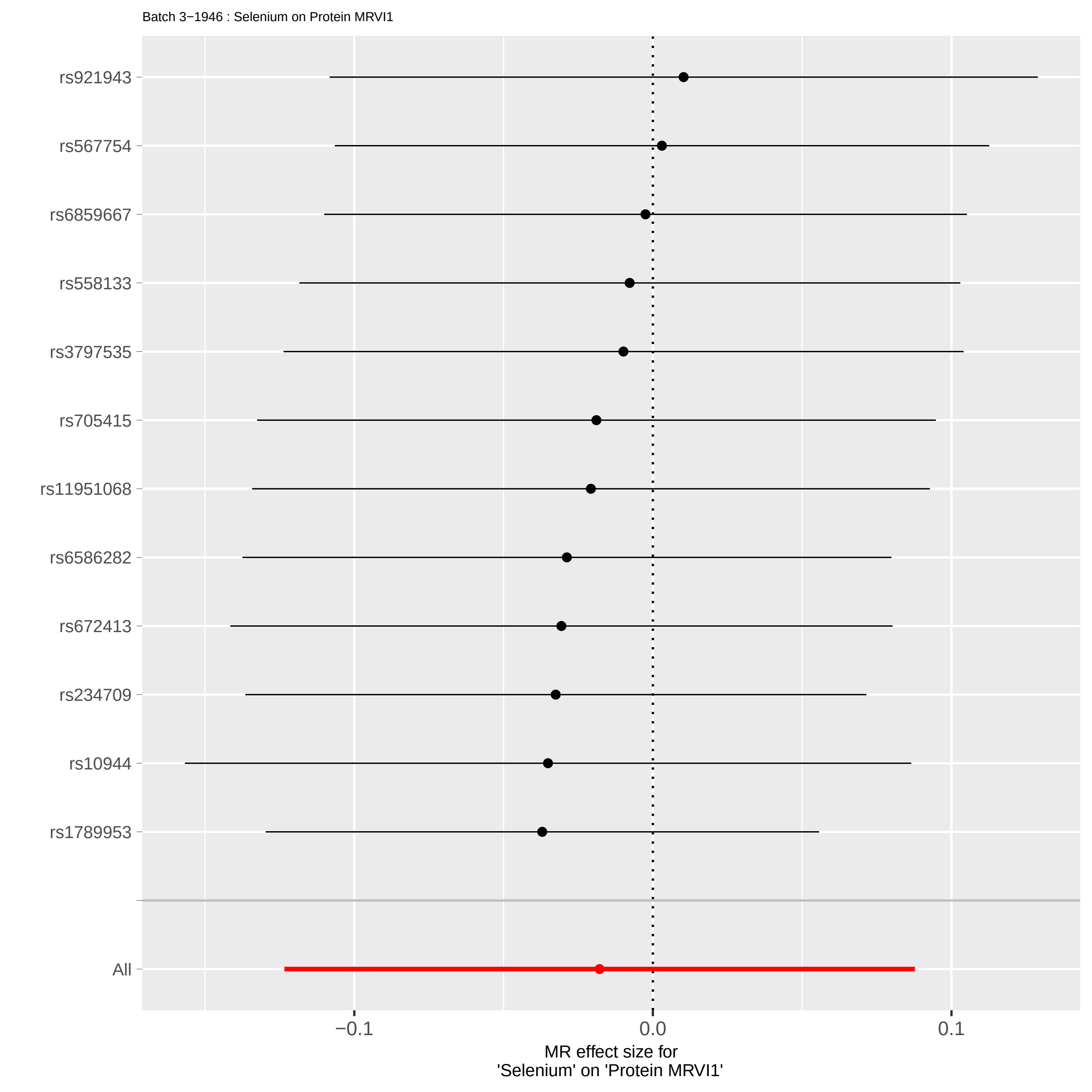

Batch 3−1946 : Selenium on Protein MRVI1
rs921943
rs567754
rs6859667
rs558133
rs3797535
rs705415
rs11951068
rs6586282
rs672413
rs234709
rs10944
rs1789953
All
−0.1
0.0
0.1
MR effect size for
'Selenium' on 'Protein MRVI1'

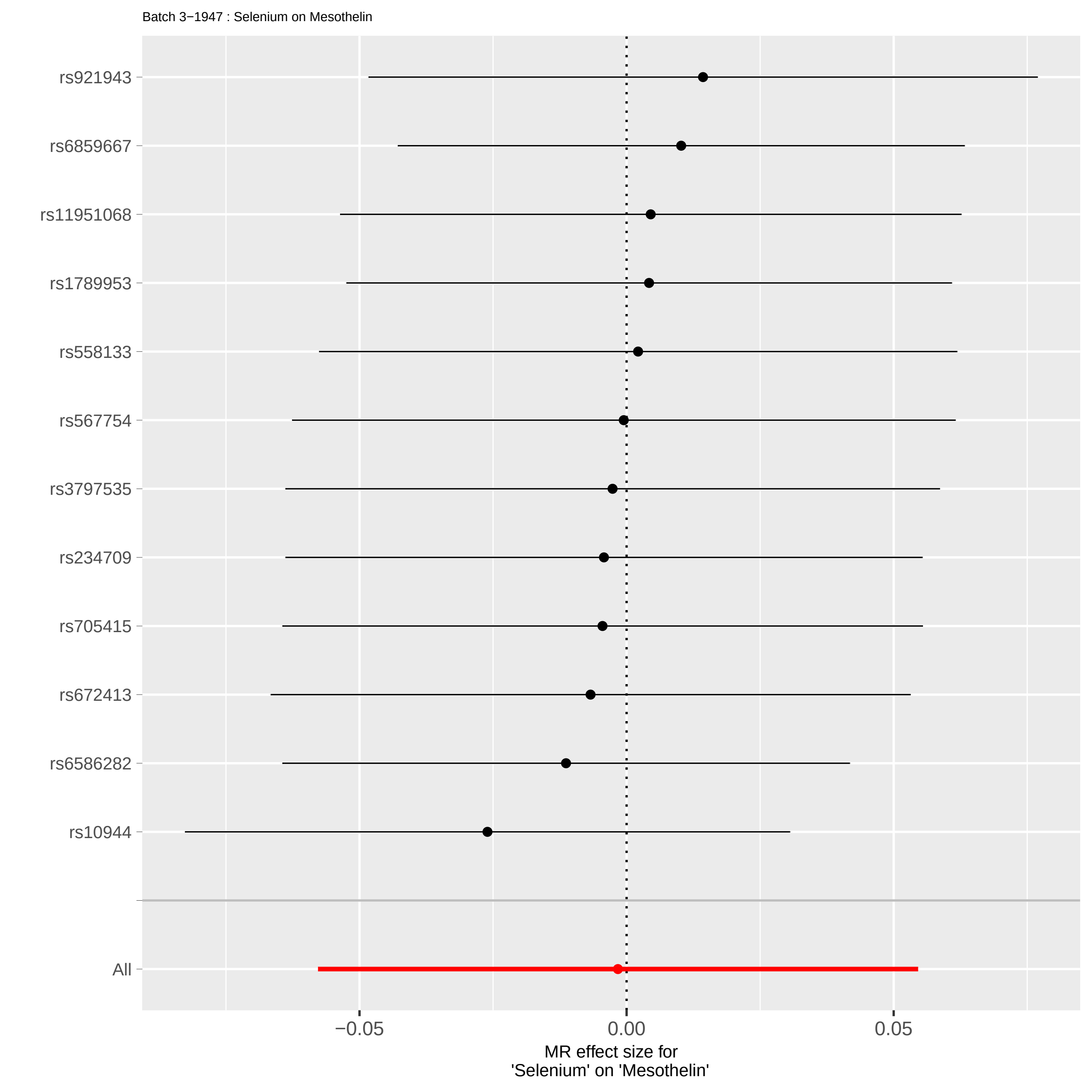

Batch 3−1947 : Selenium on Mesothelin
rs921943
rs6859667
rs11951068
rs1789953
rs558133
rs567754
rs3797535
rs234709
rs705415
rs672413
rs6586282
rs10944
All
−0.05
0.00
0.05
MR effect size for
'Selenium' on 'Mesothelin'

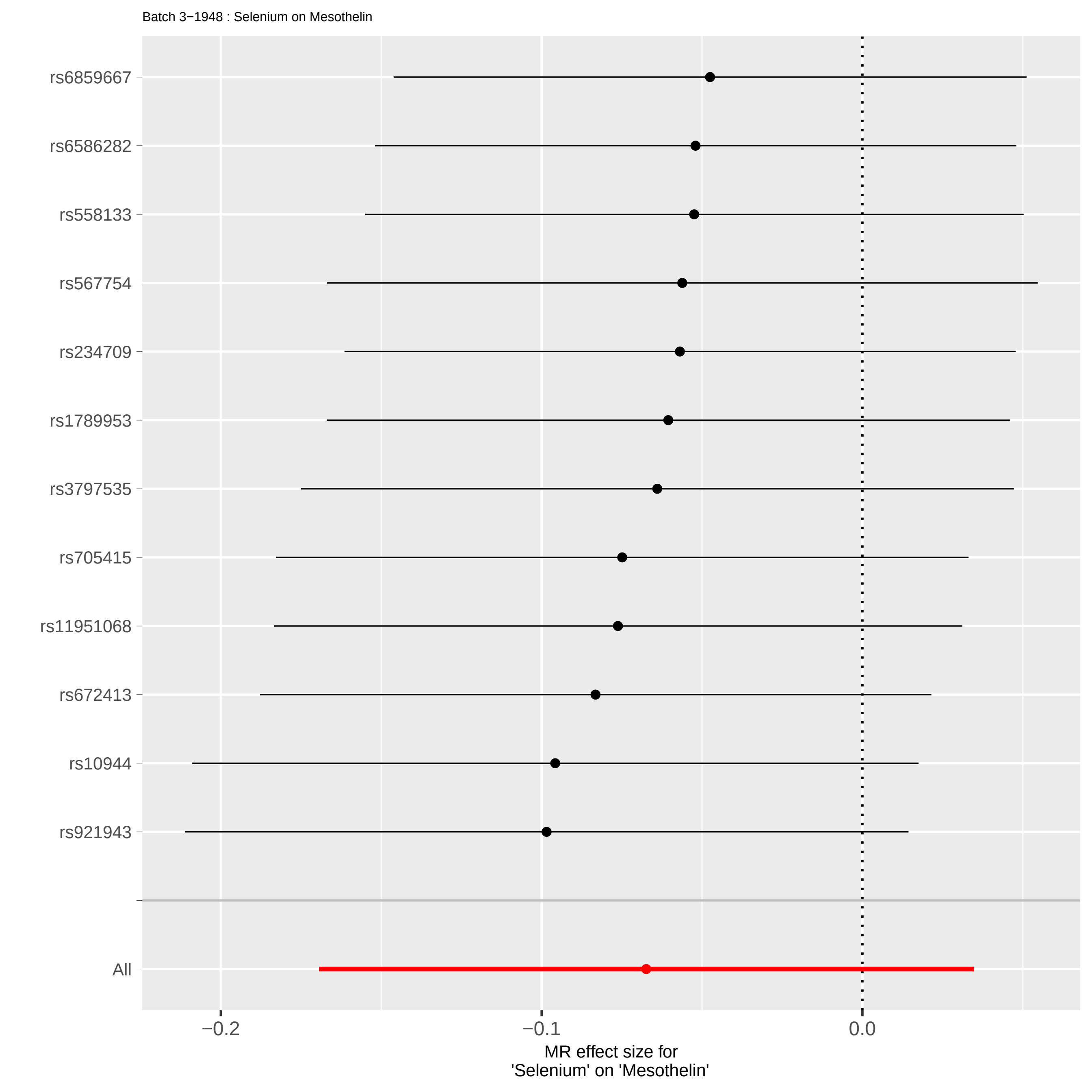
Batch 3−1948 : Selenium on Mesothelin
rs6859667
rs6586282
rs558133
rs567754
rs234709
rs1789953
rs3797535
rs705415
rs11951068
rs672413
rs10944
rs921943
All
−0.2
−0.1
0.0
MR effect size for
'Selenium' on 'Mesothelin'

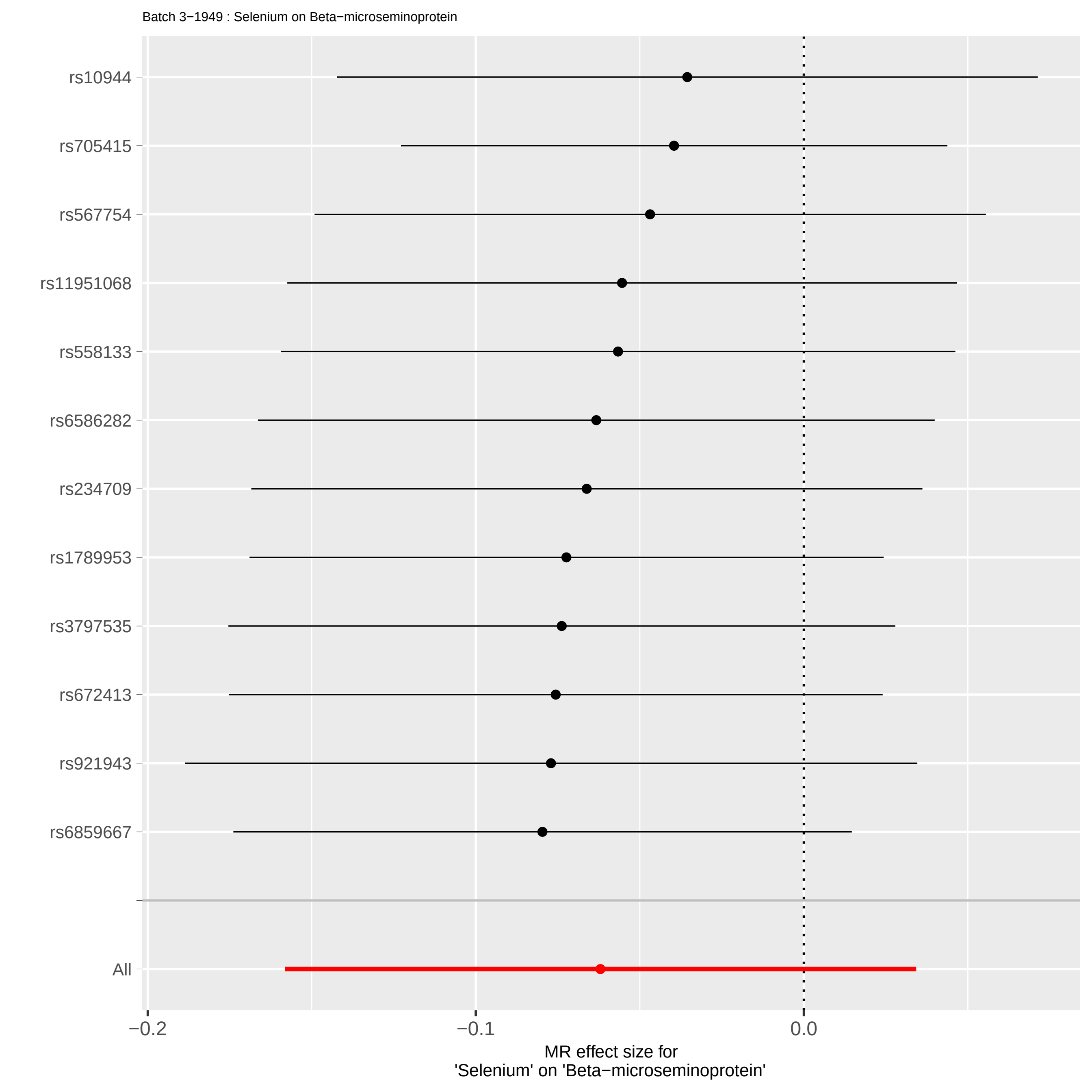

Batch 3−1949 : Selenium on Beta−microseminoprotein
rs10944
rs705415
rs567754
rs11951068
rs558133
rs6586282
rs234709
rs1789953
rs3797535
rs672413
rs921943
rs6859667
All
−0.2
−0.1
0.0
MR effect size for
'Selenium' on 'Beta−microseminoprotein'

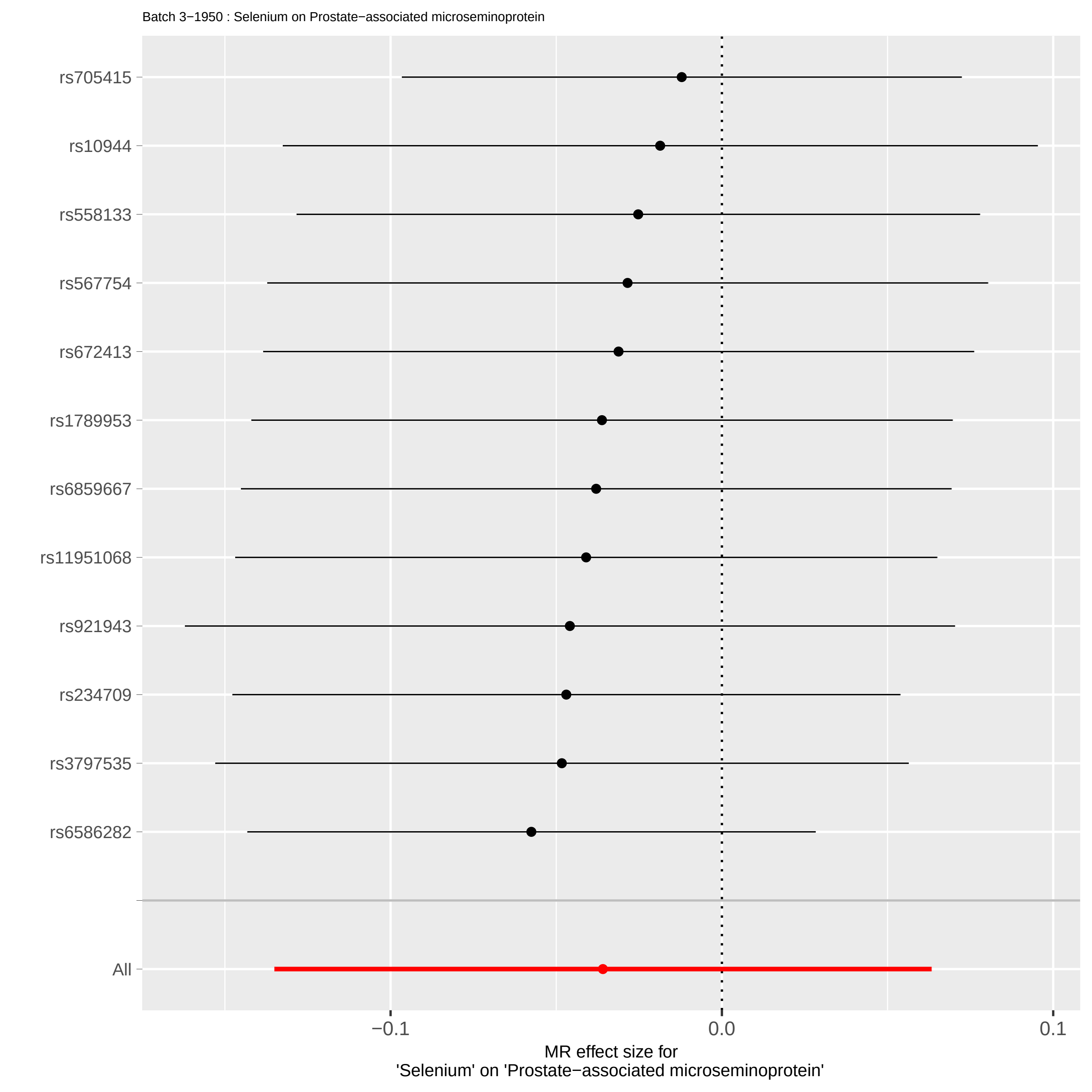
Batch 3−1950 : Selenium on Prostate−associated microseminoprotein
rs705415
rs10944
rs558133
rs567754
rs672413
rs1789953
rs6859667
rs11951068
rs921943
rs234709
rs3797535
rs6586282
All
−0.1
0.0
0.1
MR effect size for
'Selenium' on 'Prostate−associated microseminoprotein'

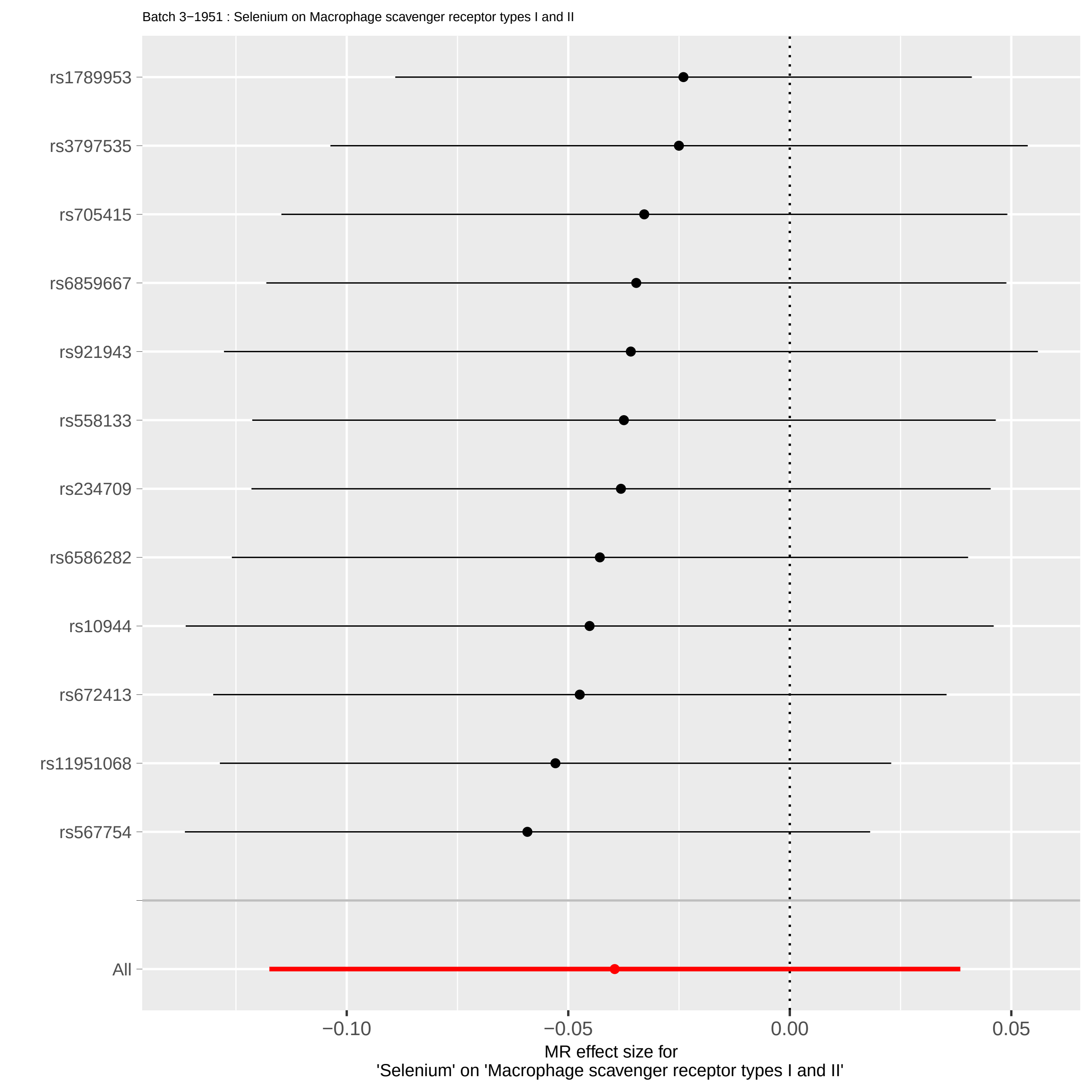

Batch 3−1951 : Selenium on Macrophage scavenger receptor types I and II
rs1789953
rs3797535
rs705415
rs6859667
rs921943
rs558133
rs234709
rs6586282
rs10944
rs672413
rs11951068
rs567754
All
−0.10
−0.05
0.00
0.05
MR effect size for
'Selenium' on 'Macrophage scavenger receptor types I and II'

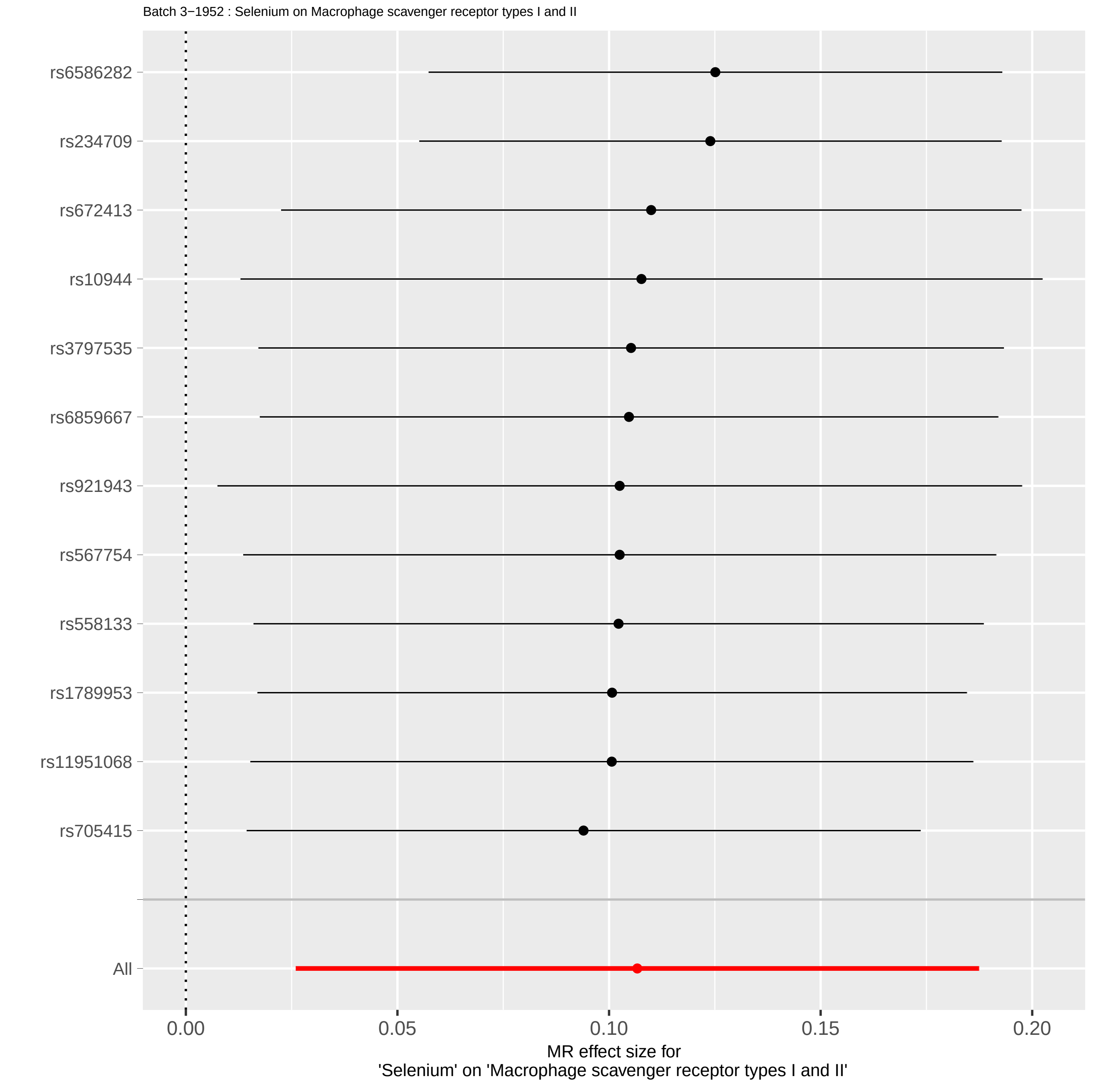

Batch 3−1952 : Selenium on Macrophage scavenger receptor types I and II
rs6586282
rs234709
rs672413
rs10944
rs3797535
rs6859667
rs921943
rs567754
rs558133
rs1789953
rs11951068
rs705415
All
0.00
0.05
0.10
0.15
0.20
MR effect size for
'Selenium' on 'Macrophage scavenger receptor types I and II'

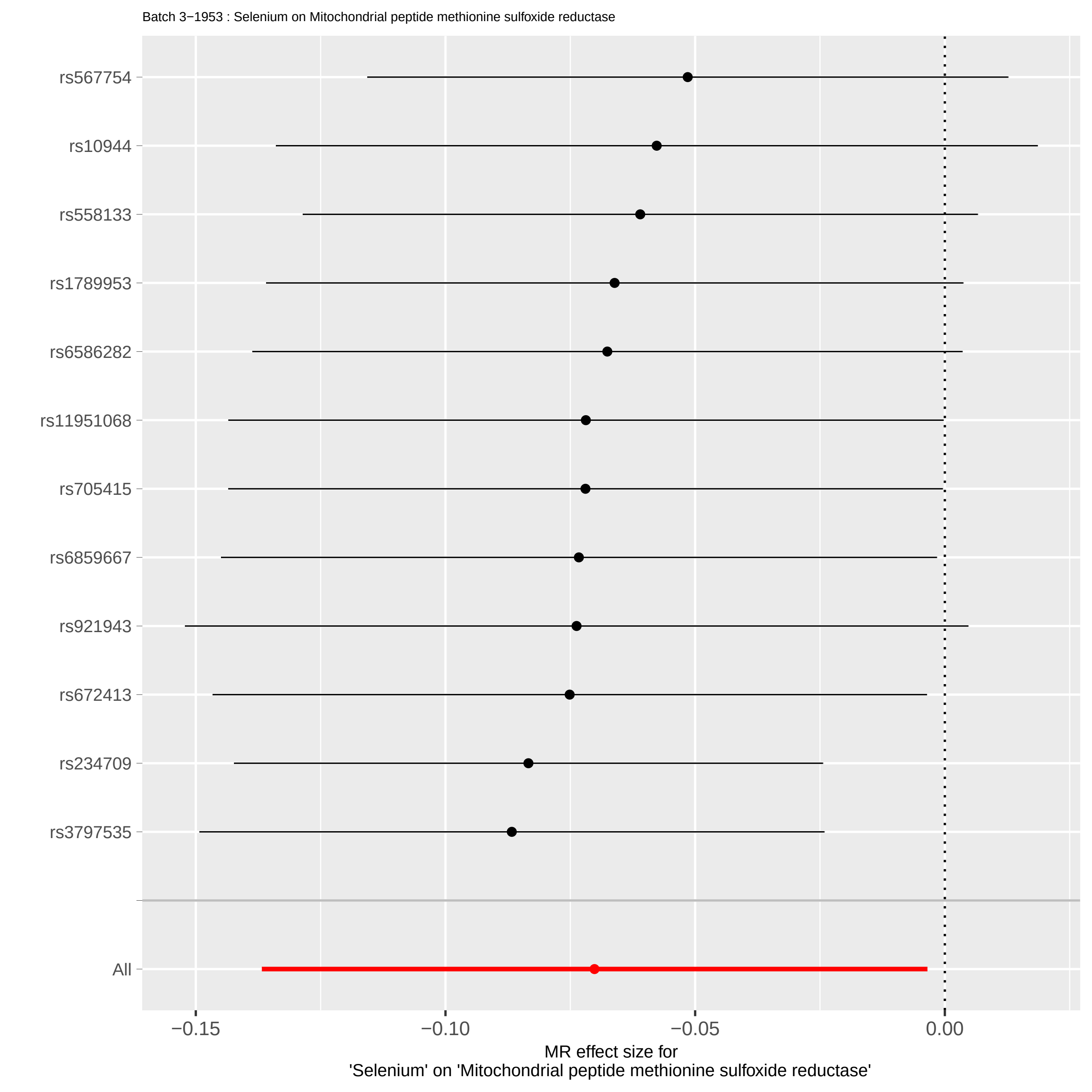

Batch 3−1953 : Selenium on Mitochondrial peptide methionine sulfoxide reductase
rs567754
rs10944
rs558133
rs1789953
rs6586282
rs11951068
rs705415
rs6859667
rs921943
rs672413
rs234709
rs3797535
All
−0.15
−0.10
−0.05
0.00
MR effect size for
'Selenium' on 'Mitochondrial peptide methionine sulfoxide reductase'

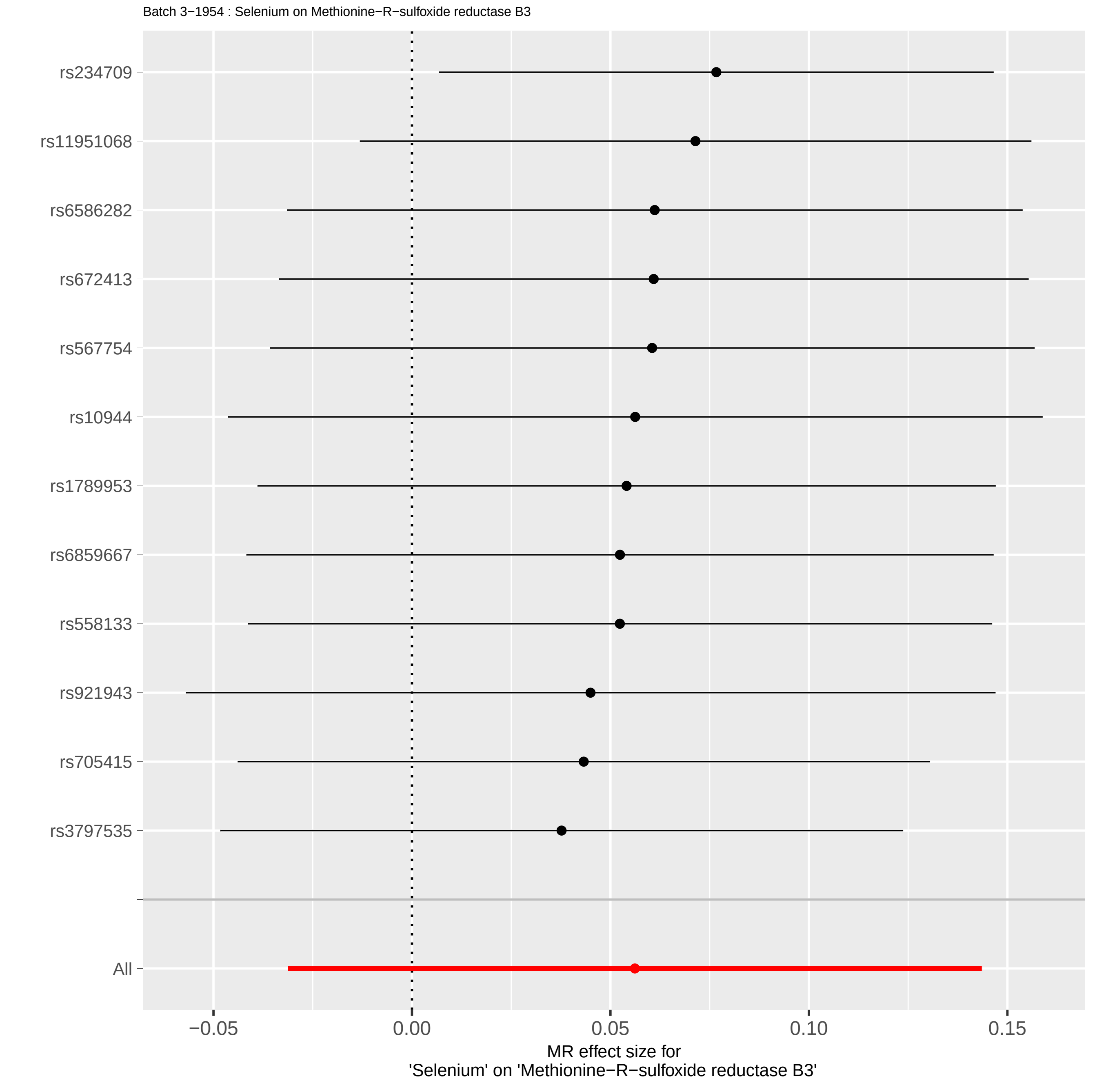

Batch 3−1954 : Selenium on Methionine−R−sulfoxide reductase B3
rs234709
rs11951068
rs6586282
rs672413
rs567754
rs10944
rs1789953
rs6859667
rs558133
rs921943
rs705415
rs3797535
All
−0.05
0.00
0.05
0.10
0.15
MR effect size for
'Selenium' on 'Methionine−R−sulfoxide reductase B3'

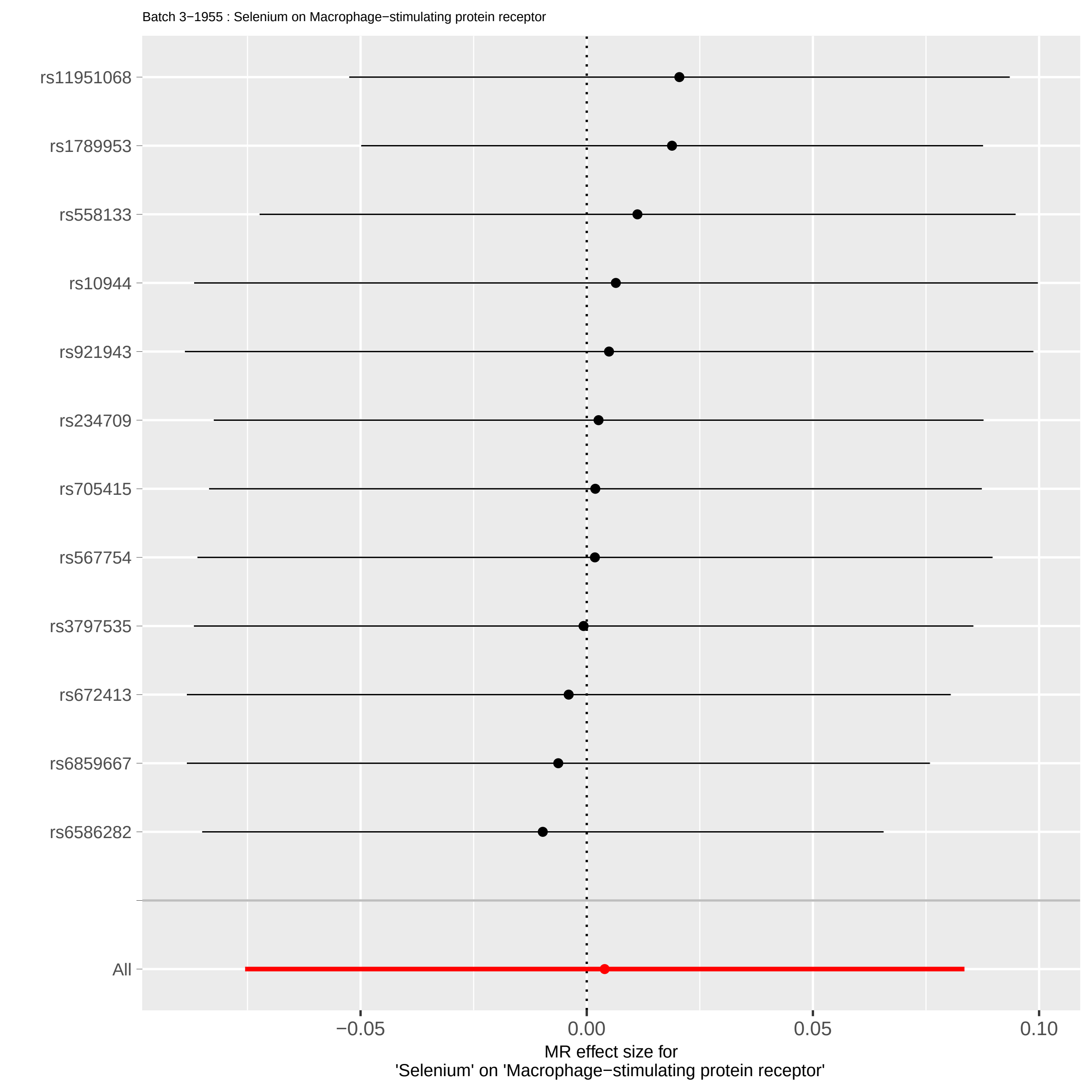

Batch 3−1955 : Selenium on Macrophage−stimulating protein receptor
rs11951068
rs1789953
rs558133
rs10944
rs921943
rs234709
rs705415
rs567754
rs3797535
rs672413
rs6859667
rs6586282
All
−0.05
0.00
0.05
0.10
MR effect size for
'Selenium' on 'Macrophage−stimulating protein receptor'

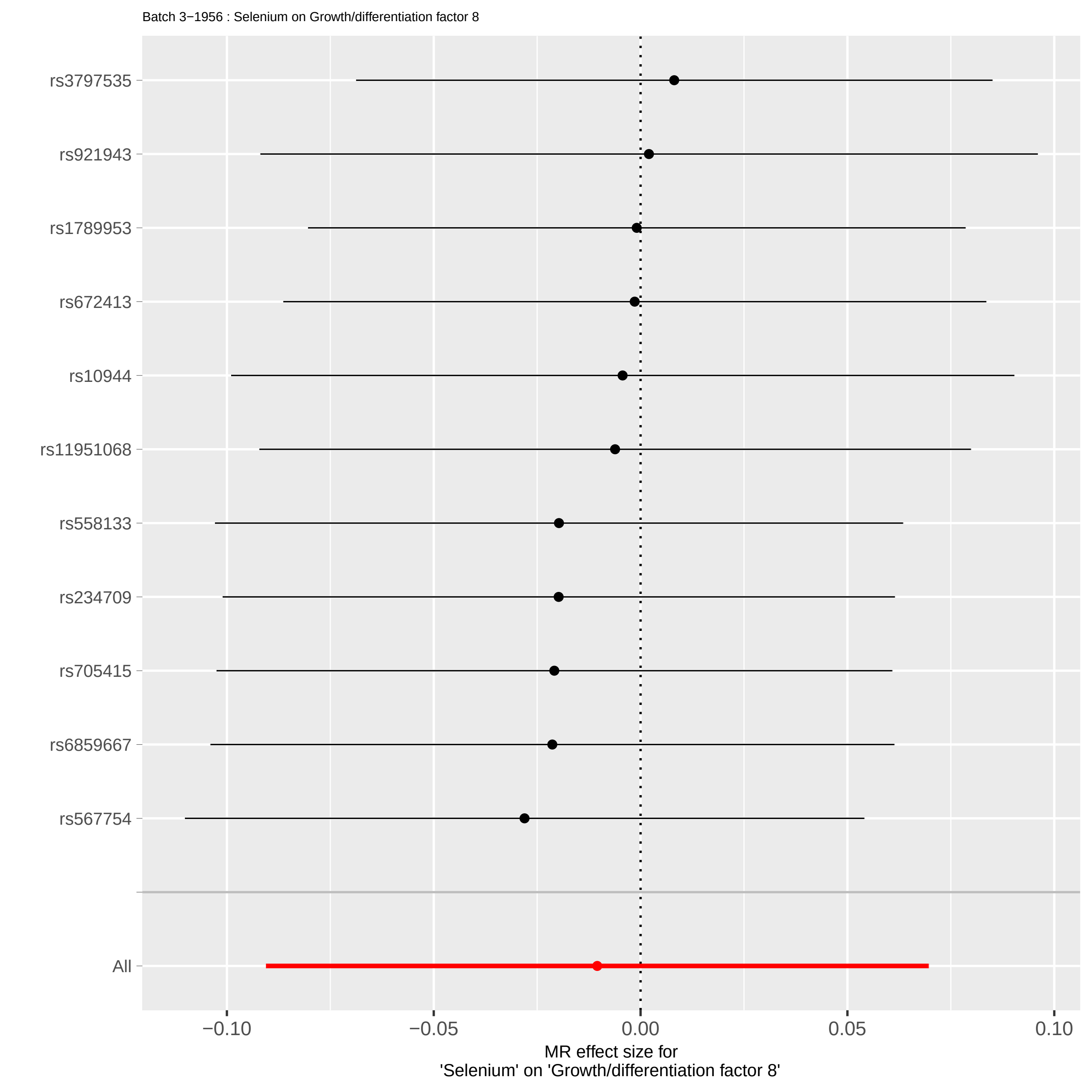
Batch 3−1956 : Selenium on Growth/differentiation factor 8
rs3797535
rs921943
rs1789953
rs672413
rs10944
rs11951068
rs558133
rs234709
rs705415
rs6859667
rs567754
All
−0.10
−0.05
0.00
0.05
0.10
MR effect size for
'Selenium' on 'Growth/differentiation factor 8'

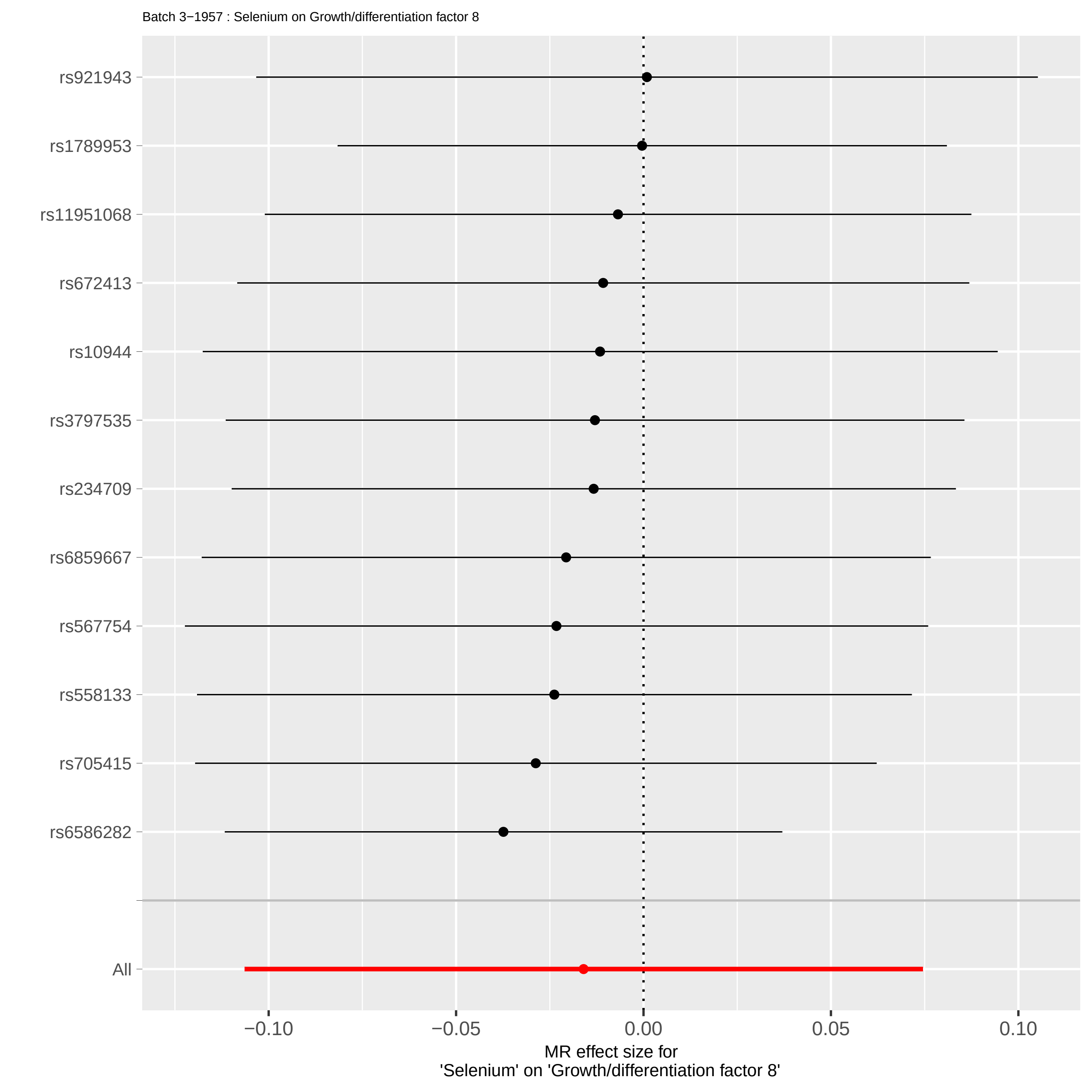

Batch 3−1957 : Selenium on Growth/differentiation factor 8
rs921943
rs1789953
rs11951068
rs672413
rs10944
rs3797535
rs234709
rs6859667
rs567754
rs558133
rs705415
rs6586282
All
−0.10
−0.05
0.00
0.05
0.10
MR effect size for
'Selenium' on 'Growth/differentiation factor 8'

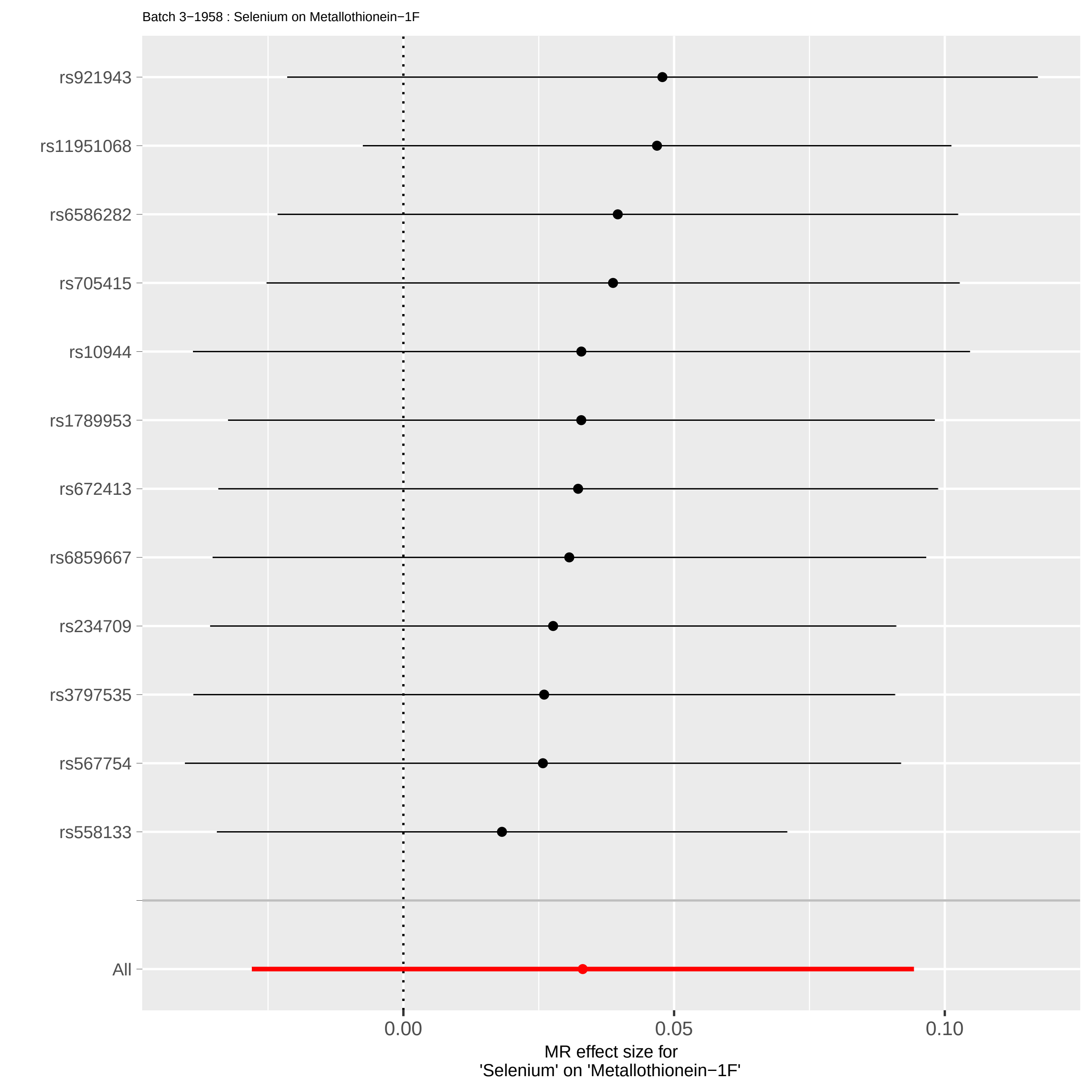

Batch 3−1958 : Selenium on Metallothionein−1F
rs921943
rs11951068
rs6586282
rs705415
rs10944
rs1789953
rs672413
rs6859667
rs234709
rs3797535
rs567754
rs558133
All
0.00
0.05
0.10
MR effect size for
'Selenium' on 'Metallothionein−1F'

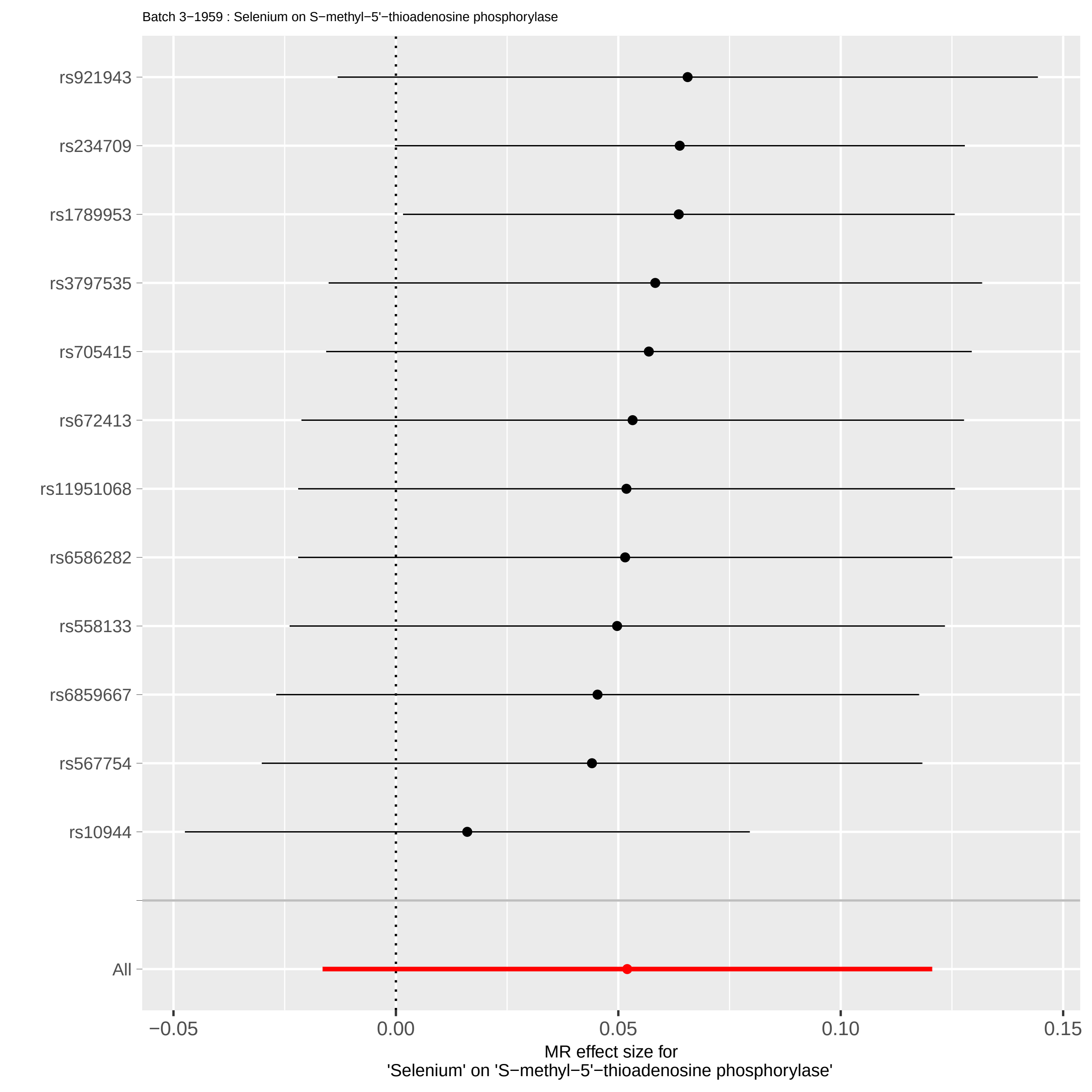
Batch 3−1959 : Selenium on S−methyl−5'−thioadenosine phosphorylase
rs921943
rs234709
rs1789953
rs3797535
rs705415
rs672413
rs11951068
rs6586282
rs558133
rs6859667
rs567754
rs10944
All
−0.05
0.00
0.05
0.10
0.15
MR effect size for
'Selenium' on 'S−methyl−5'−thioadenosine phosphorylase'

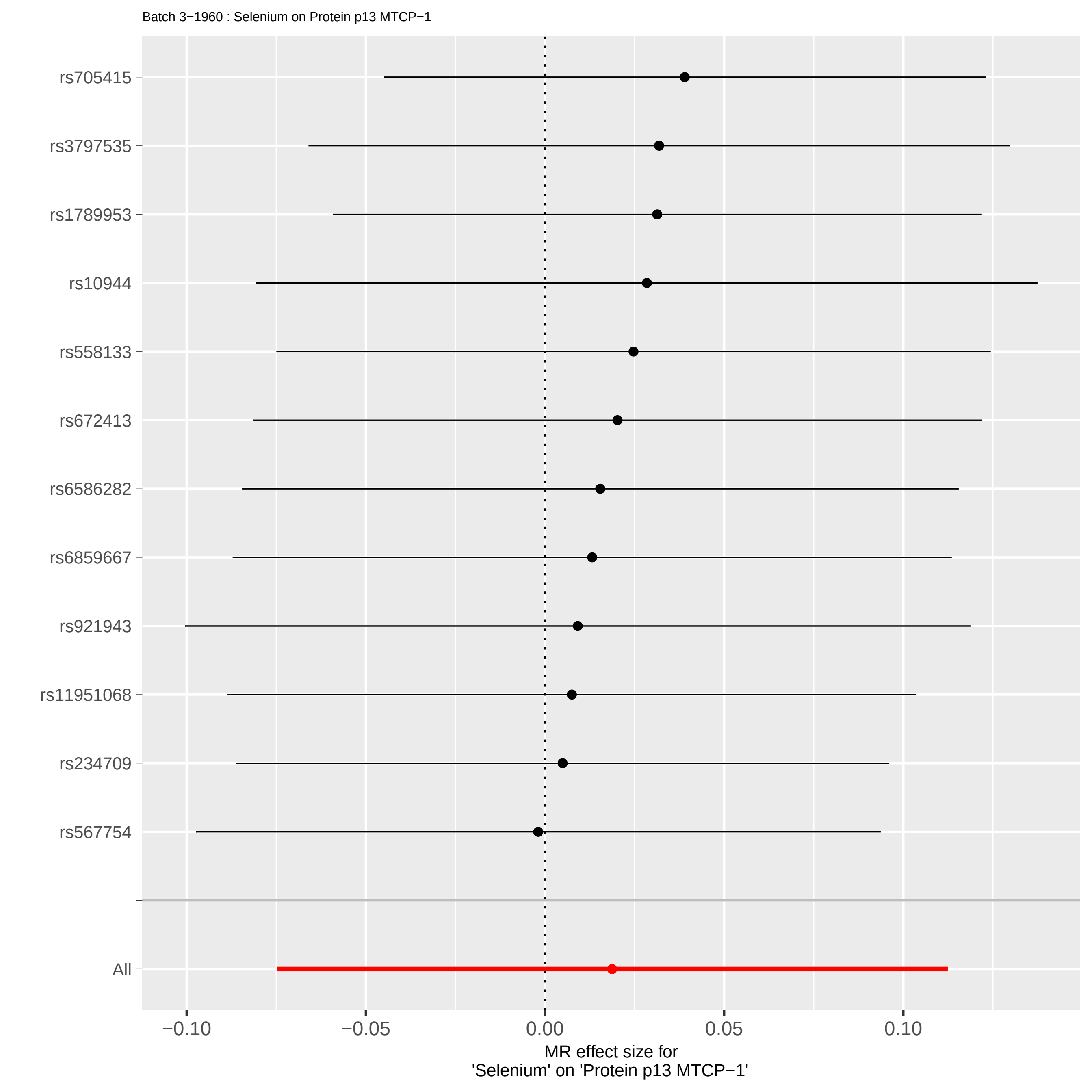

Batch 3−1960 : Selenium on Protein p13 MTCP−1
rs705415
rs3797535
rs1789953
rs10944
rs558133
rs672413
rs6586282
rs6859667
rs921943
rs11951068
rs234709
rs567754
All
−0.10
−0.05
0.00
0.05
0.10
MR effect size for
'Selenium' on 'Protein p13 MTCP−1'

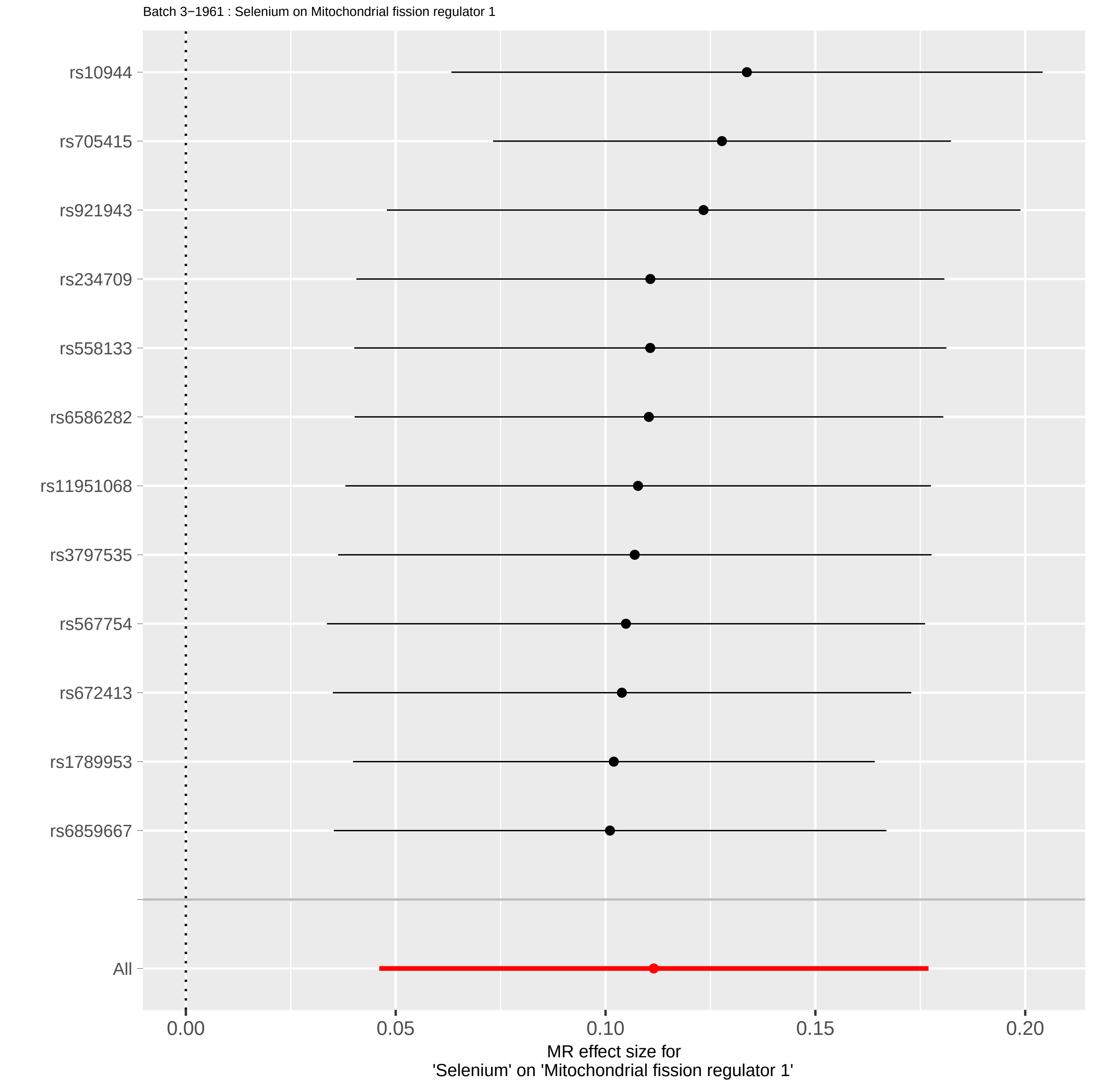

Batch 3−1961 : Selenium on Mitochondrial fission regulator 1
rs10944
rs705415
rs921943
rs234709
rs558133
rs6586282
rs11951068
rs3797535
rs567754
rs672413
rs1789953
rs6859667
All
0.00
0.05
0.10
0.15
0.20
MR effect size for
'Selenium' on 'Mitochondrial fission regulator 1'

Batch 3−1962 : Selenium on 5−formyltetrahydrofolate cyclo−ligase

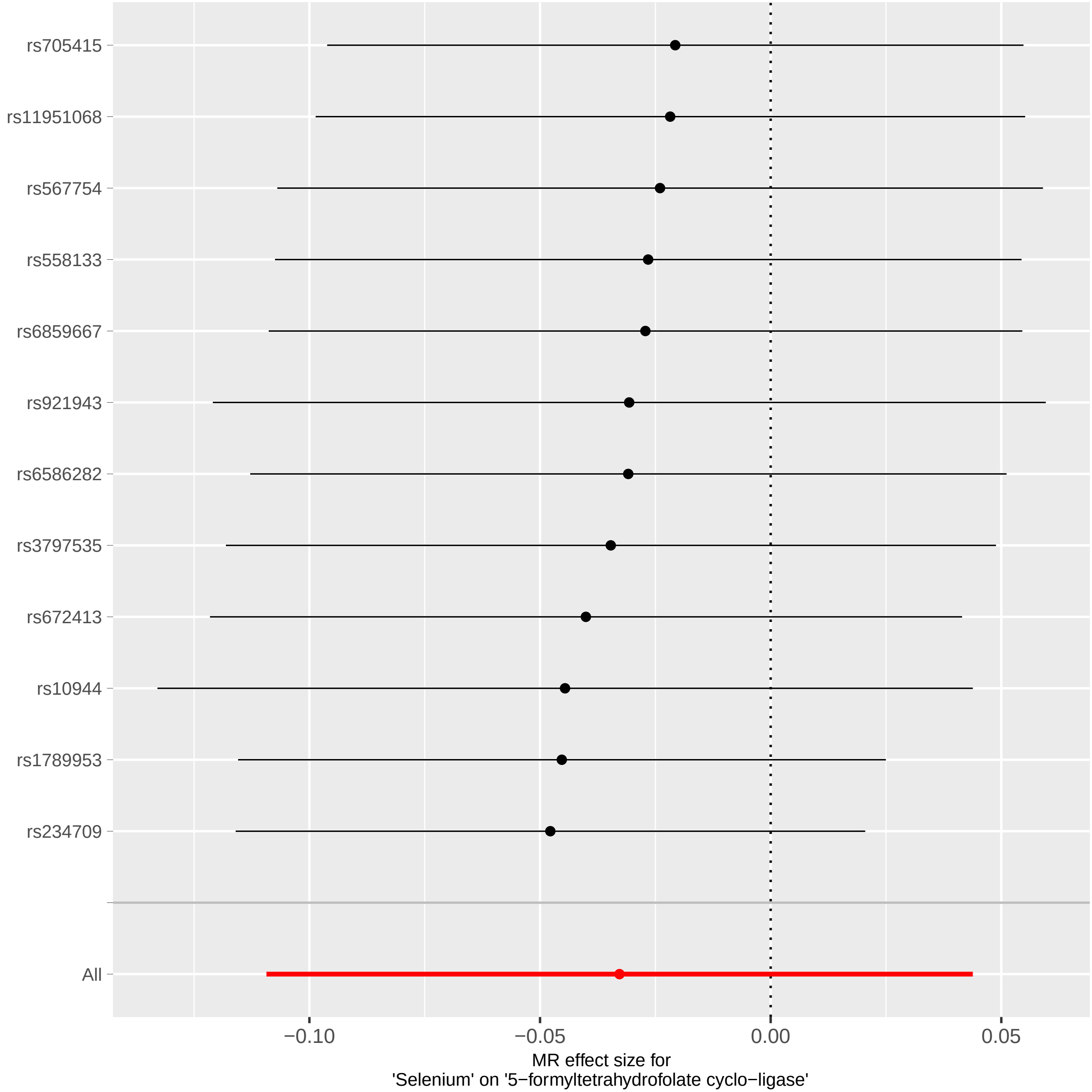

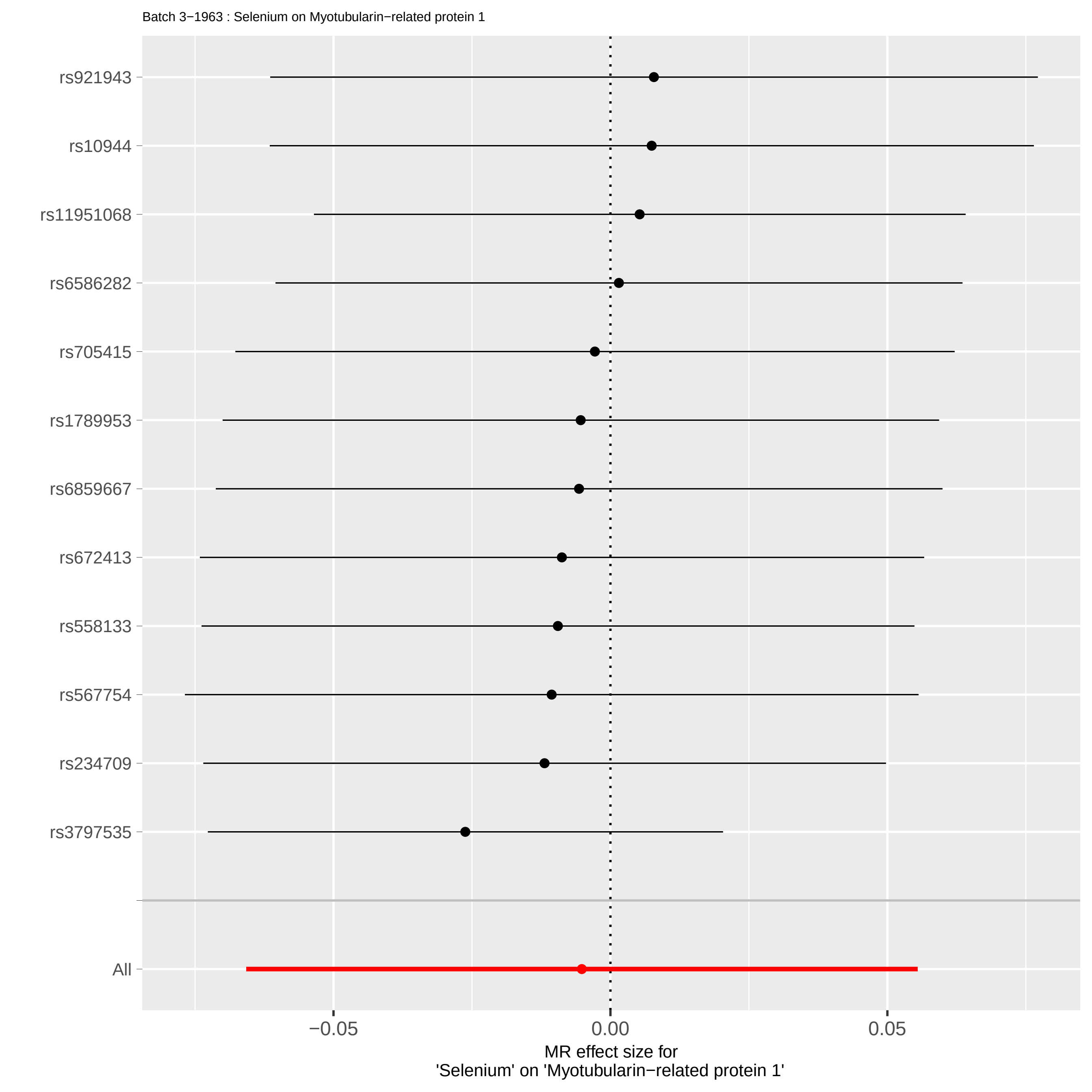

Batch 3−1963 : Selenium on Myotubularin−related protein 1
rs921943
rs10944
rs11951068
rs6586282
rs705415
rs1789953
rs6859667
rs672413
rs558133
rs567754
rs234709
rs3797535
All
−0.05
0.00
0.05
MR effect size for
'Selenium' on 'Myotubularin−related protein 1'

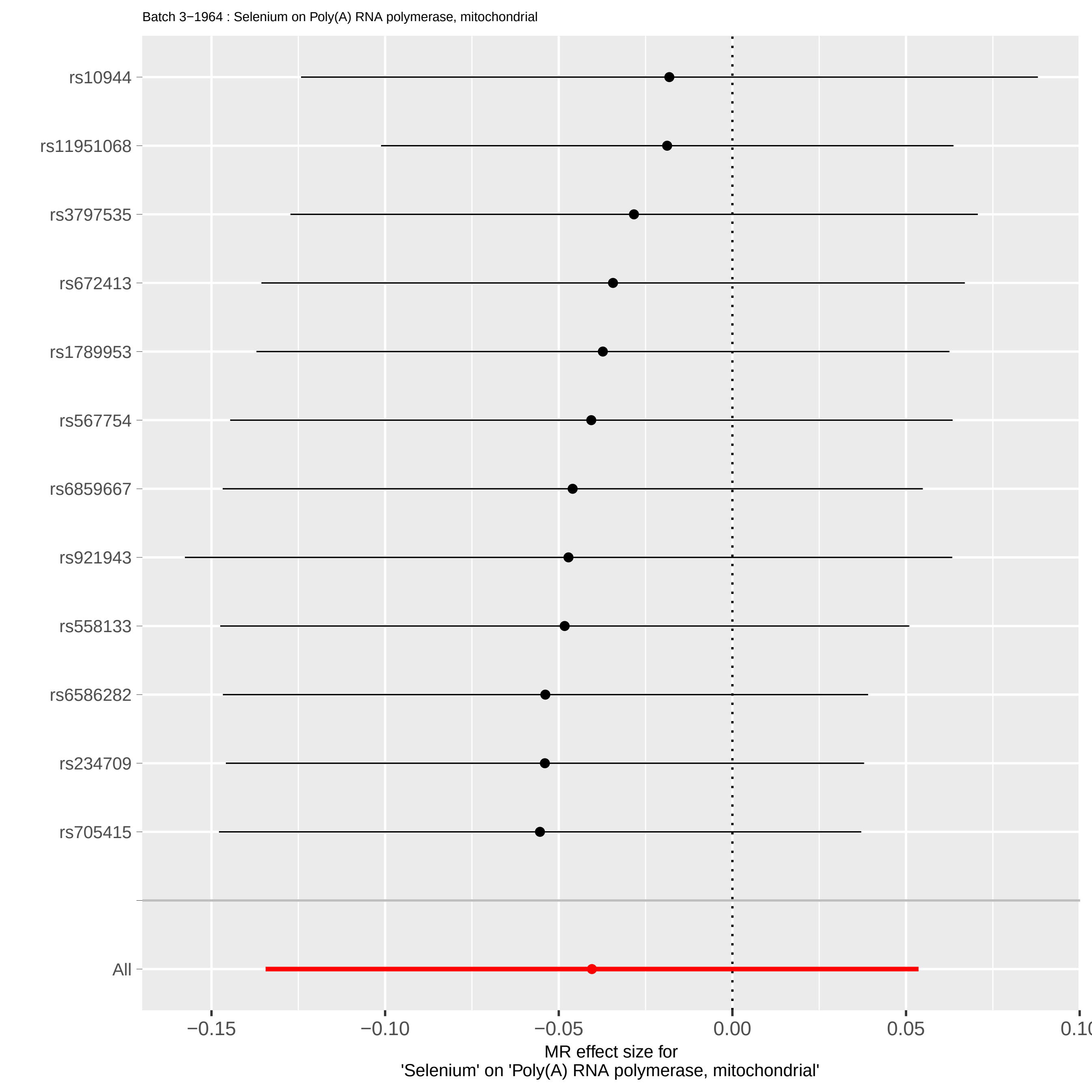

Batch 3−1964 : Selenium on Poly(A) RNA polymerase, mitochondrial
rs10944
rs11951068
rs3797535
rs672413
rs1789953
rs567754
rs6859667
rs921943
rs558133
rs6586282
rs234709
rs705415
All
−0.15
−0.10
−0.05
0.00
0.05
0.10
MR effect size for
'Selenium' on 'Poly(A) RNA polymerase, mitochondrial'

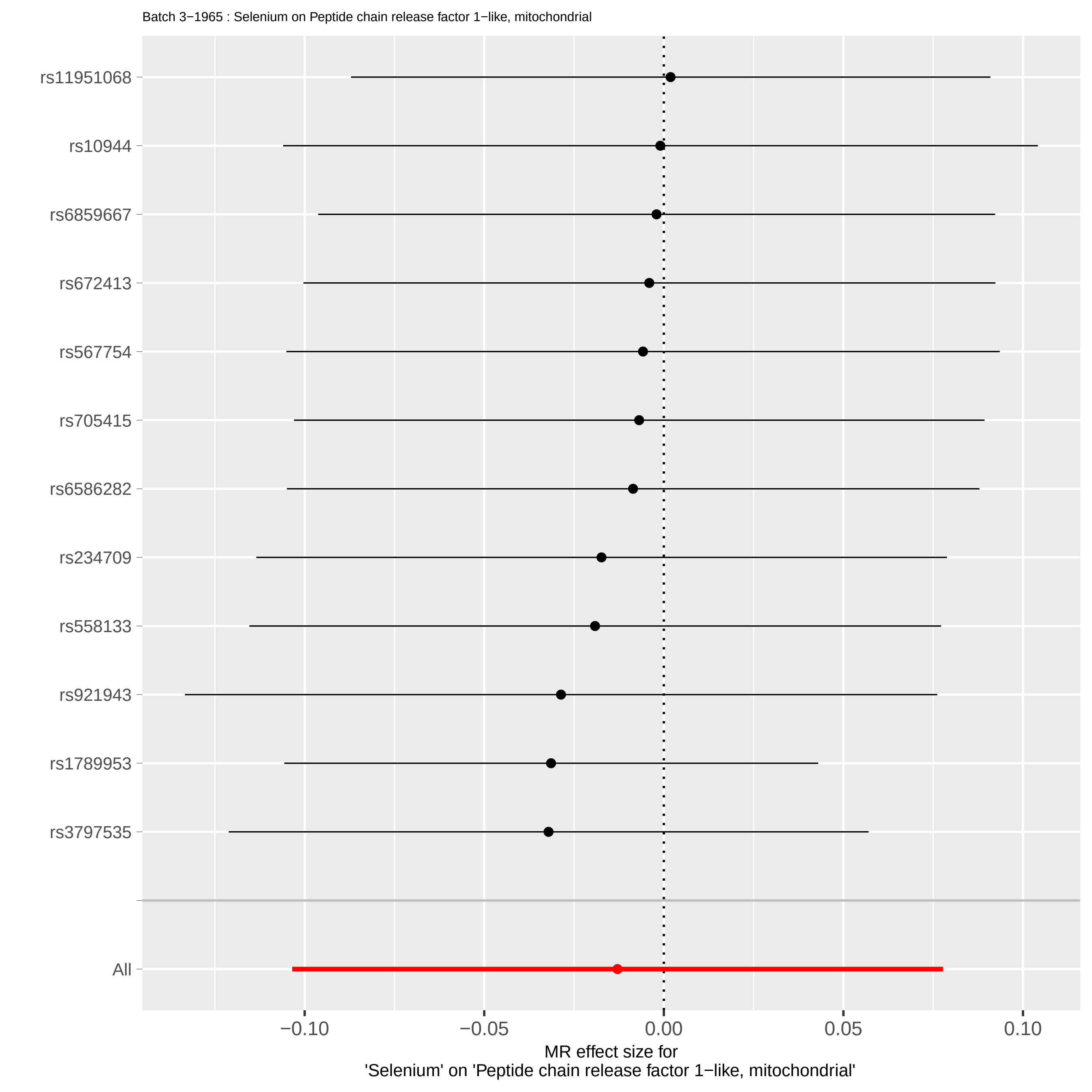

Batch 3−1965 : Selenium on Peptide chain release factor 1−like, mitochondrial
rs11951068
rs10944
rs6859667
rs672413
rs567754
rs705415
rs6586282
rs234709
rs558133
rs921943
rs1789953
rs3797535
All
−0.10
−0.05
0.00
0.05
0.10
MR effect size for
'Selenium' on 'Peptide chain release factor 1−like, mitochondrial'

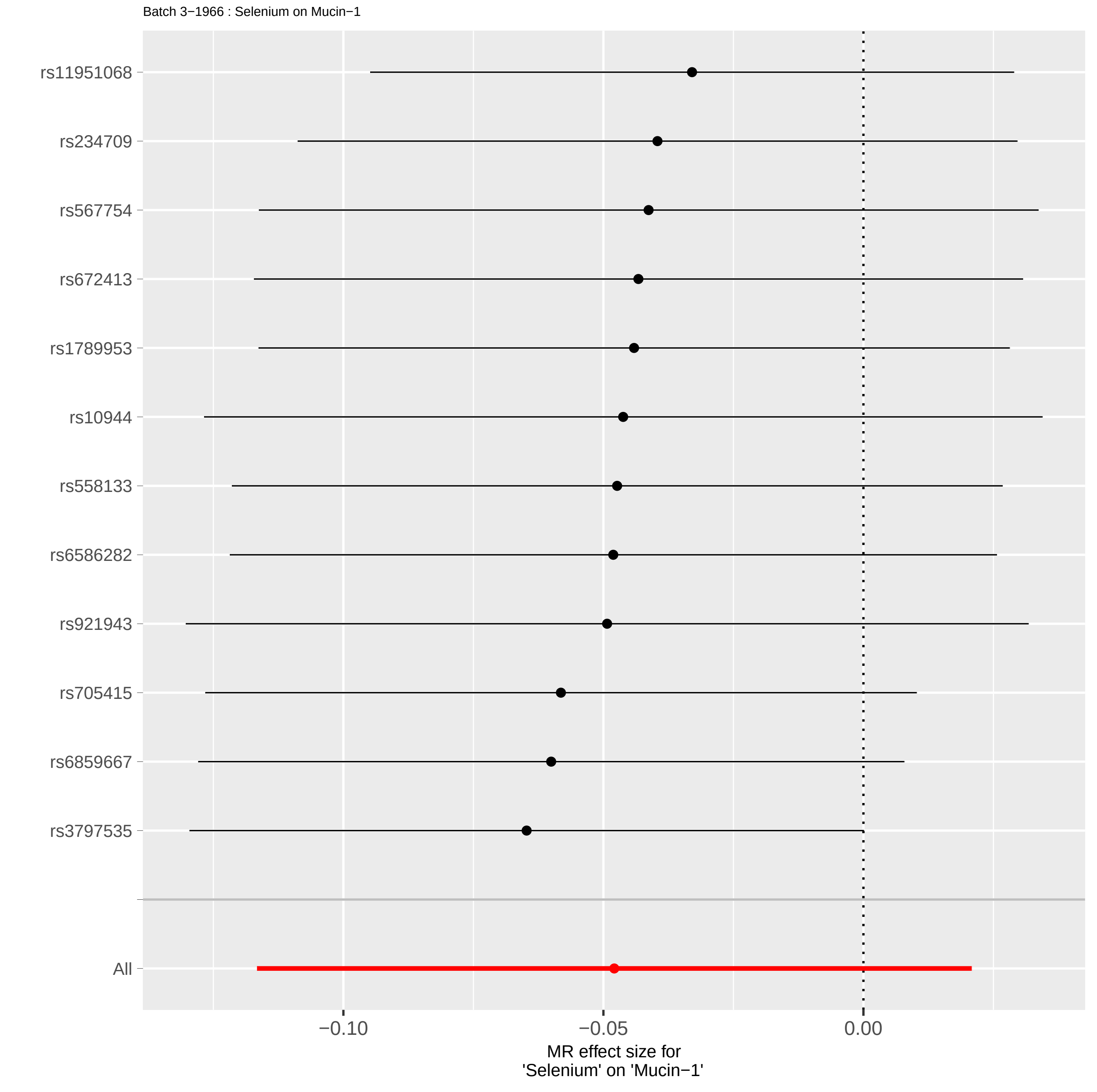
Batch 3−1966 : Selenium on Mucin−1
rs11951068
rs234709
rs567754
rs672413
rs1789953
rs10944
rs558133
rs6586282
rs921943
rs705415
rs6859667
rs3797535
All
−0.10
−0.05
0.00
MR effect size for
'Selenium' on 'Mucin−1'

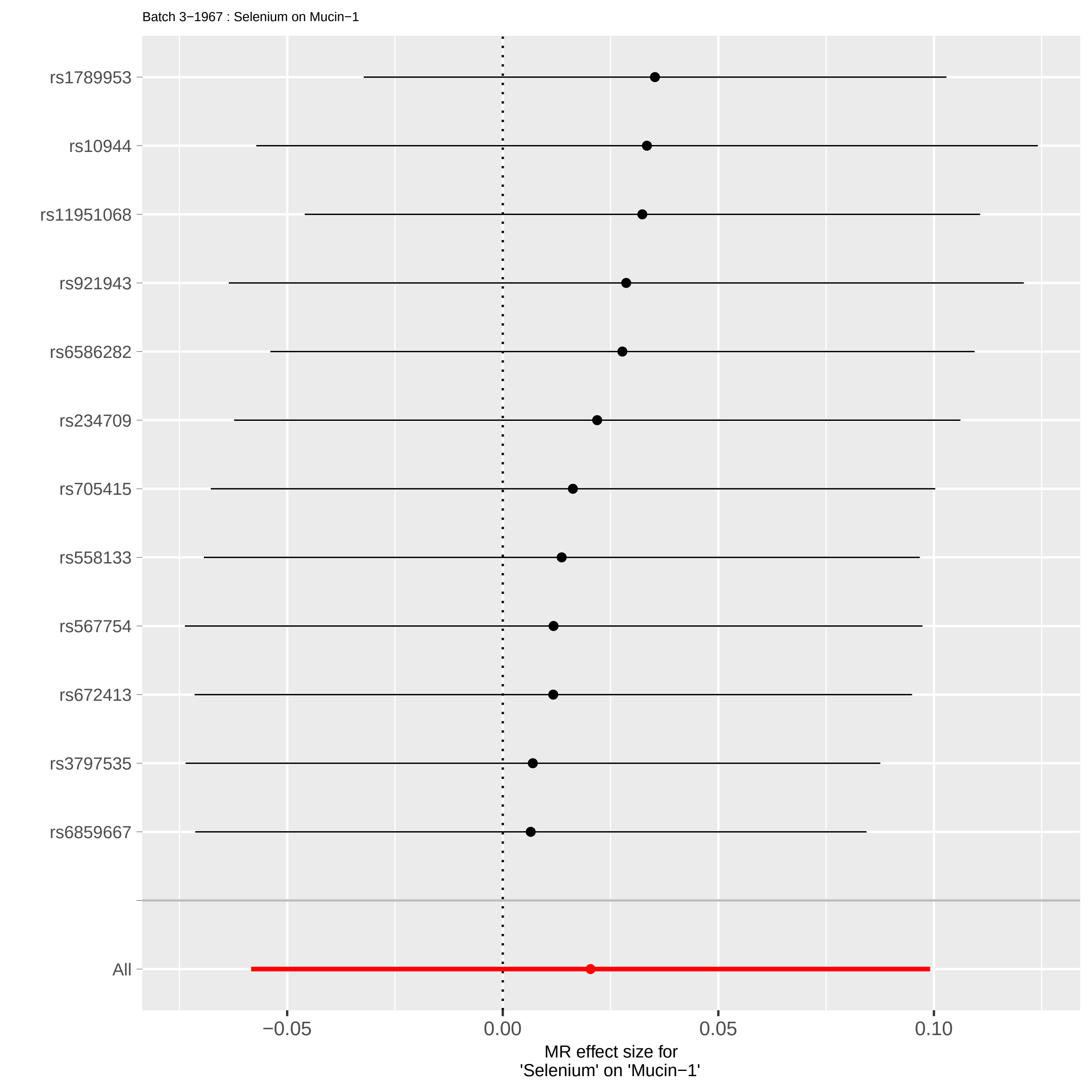

Batch 3−1967 : Selenium on Mucin−1
rs1789953
rs10944
rs11951068
rs921943
rs6586282
rs234709
rs705415
rs558133
rs567754
rs672413
rs3797535
rs6859667
All
−0.05
0.00
0.05
0.10
MR effect size for
'Selenium' on 'Mucin−1'

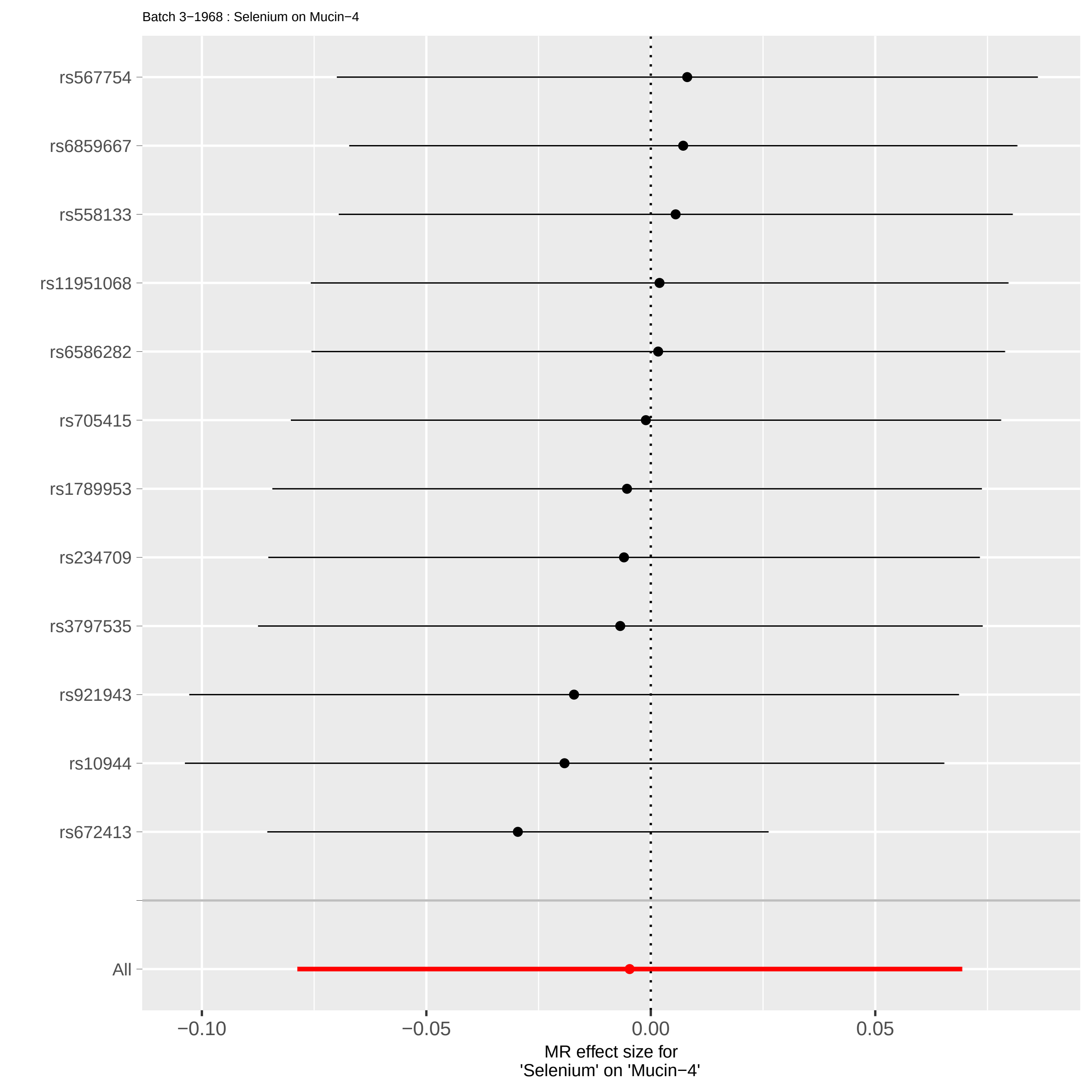
Batch 3−1968 : Selenium on Mucin−4
rs567754
rs6859667
rs558133
rs11951068
rs6586282
rs705415
rs1789953
rs234709
rs3797535
rs921943
rs10944
rs672413
All
−0.10
−0.05
0.00
0.05
MR effect size for
'Selenium' on 'Mucin−4'

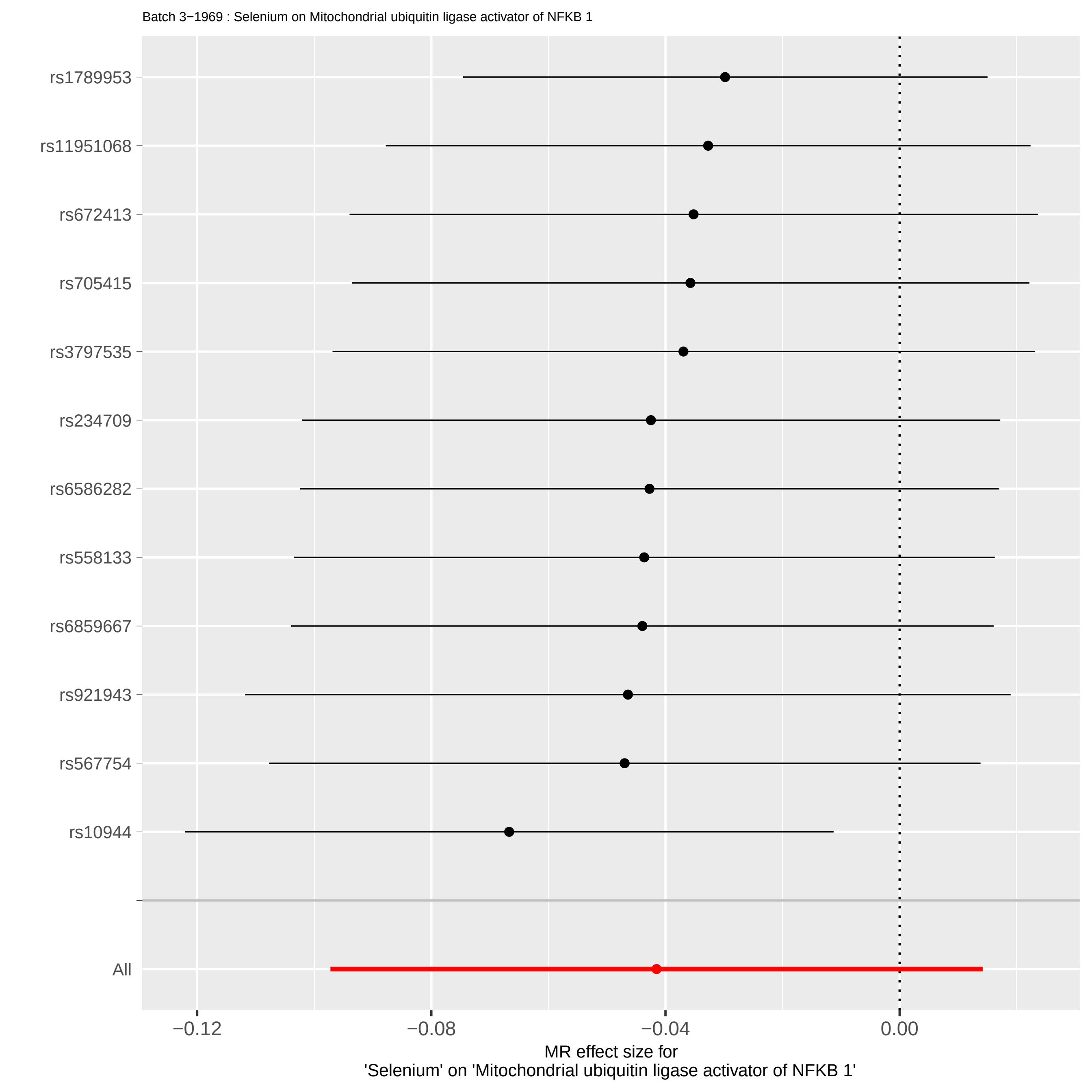

Batch 3−1969 : Selenium on Mitochondrial ubiquitin ligase activator of NFKB 1
rs1789953
rs11951068
rs672413
rs705415
rs3797535
rs234709
rs6586282
rs558133
rs6859667
rs921943
rs567754
rs10944
All
−0.12
−0.08
−0.04
0.00
MR effect size for
'Selenium' on 'Mitochondrial ubiquitin ligase activator of NFKB 1'

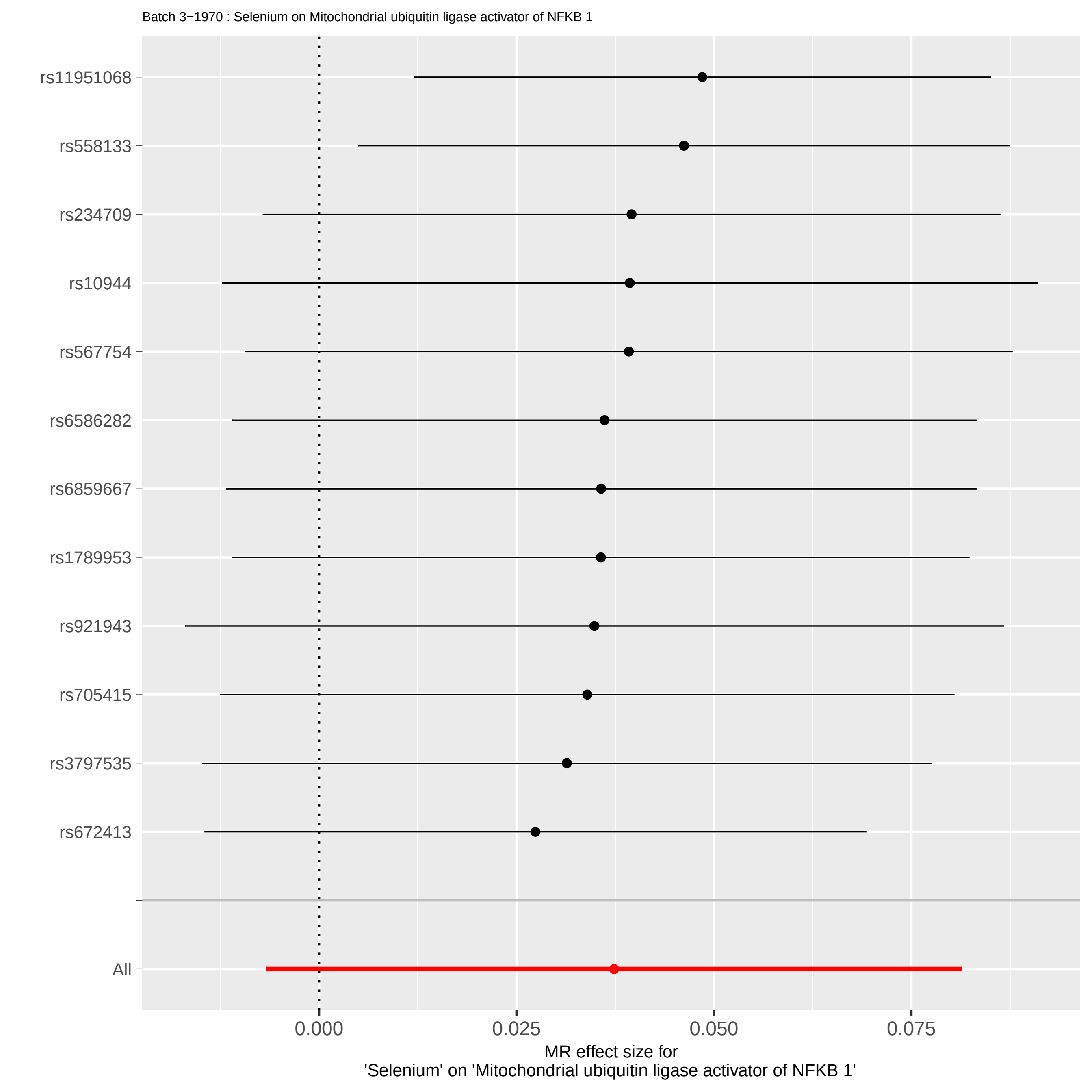

Batch 3−1970 : Selenium on Mitochondrial ubiquitin ligase activator of NFKB 1
rs11951068
rs558133
rs234709
rs10944
rs567754
rs6586282
rs6859667
rs1789953
rs921943
rs705415
rs3797535
rs672413
All
0.000
0.025
0.050
0.075
MR effect size for
'Selenium' on 'Mitochondrial ubiquitin ligase activator of NFKB 1'

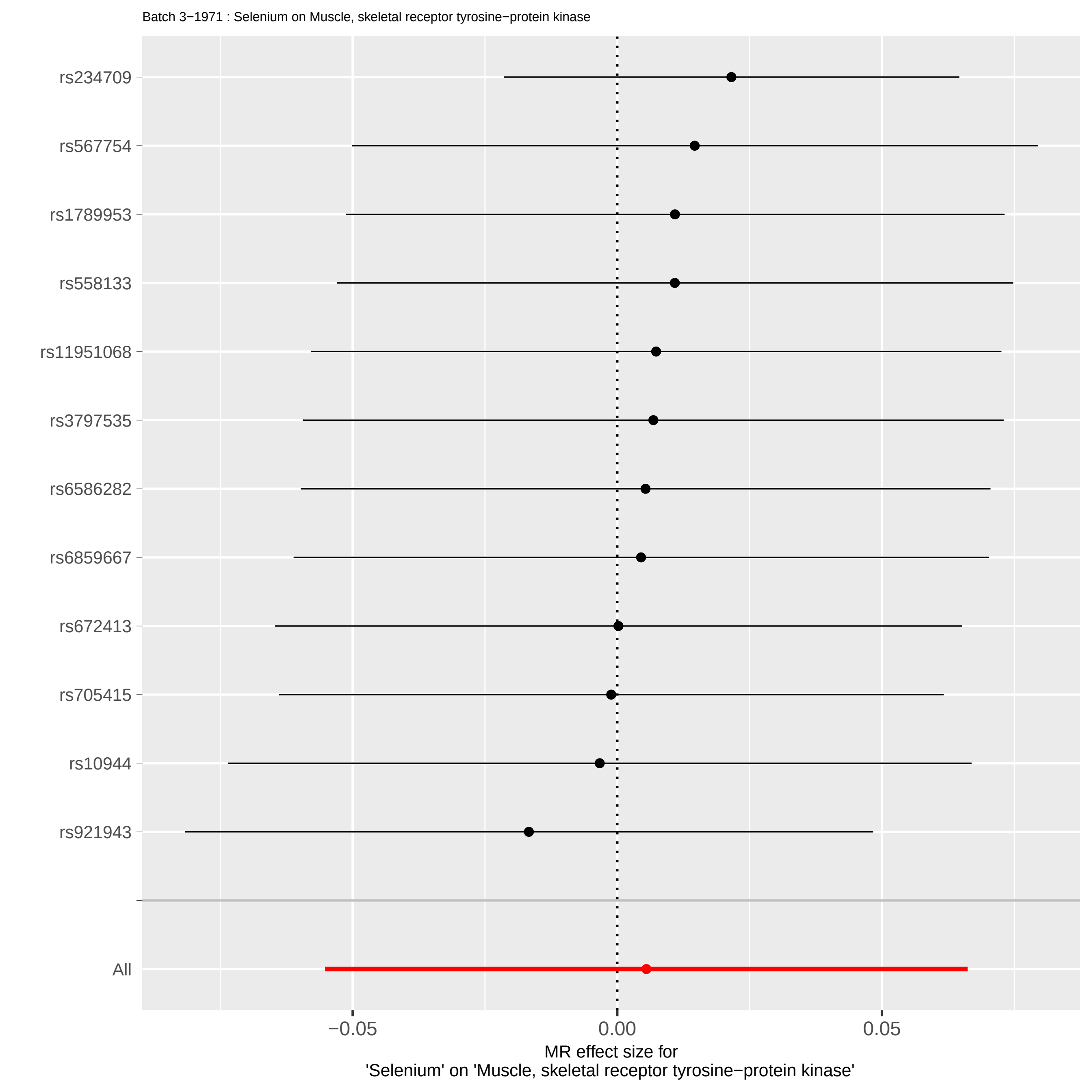
Batch 3−1971 : Selenium on Muscle, skeletal receptor tyrosine−protein kinase
rs234709
rs567754
rs1789953
rs558133
rs11951068
rs3797535
rs6586282
rs6859667
rs672413
rs705415
rs10944
rs921943
All
−0.05
0.00
0.05
MR effect size for
'Selenium' on 'Muscle, skeletal receptor tyrosine−protein kinase'

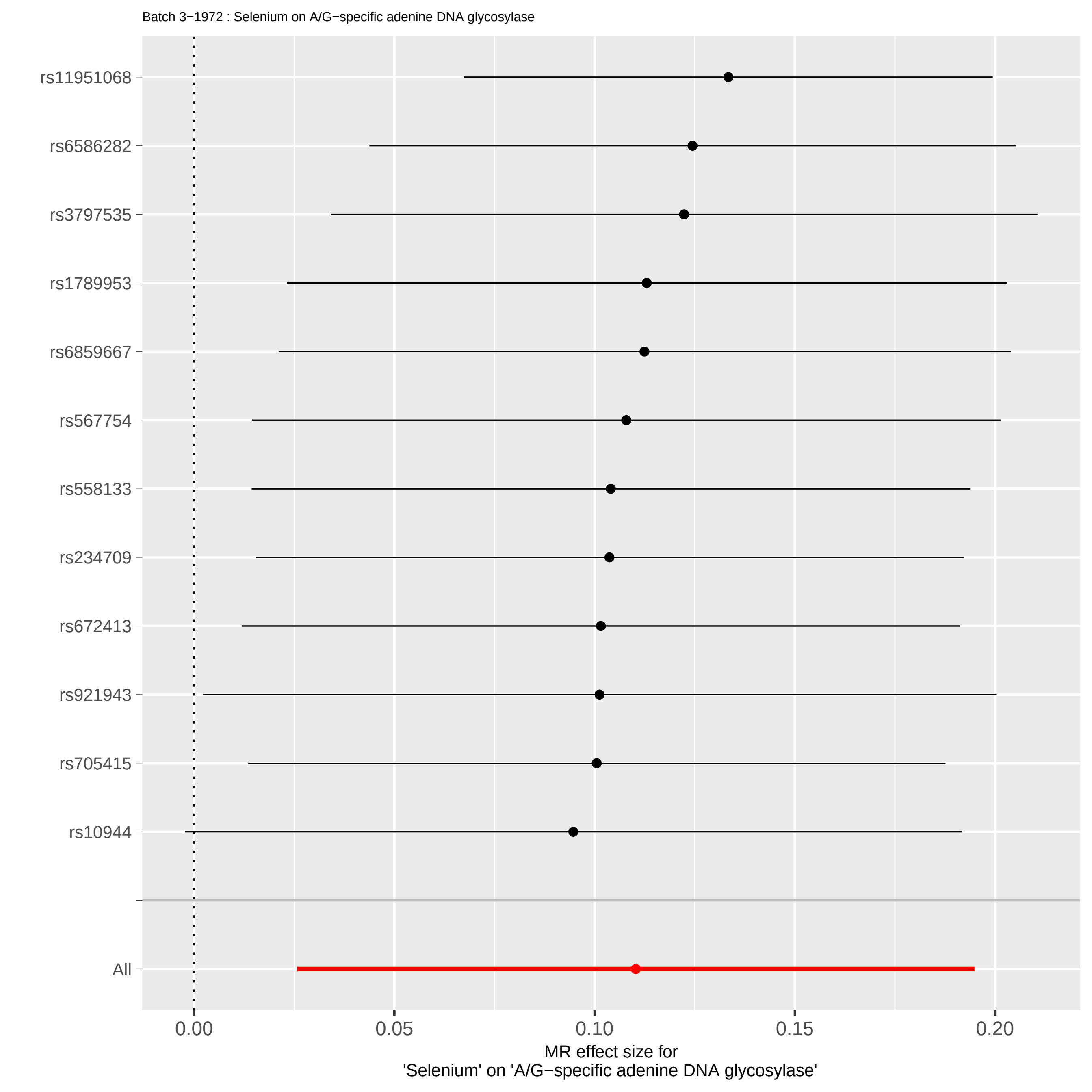
Batch 3−1972 : Selenium on A/G−specific adenine DNA glycosylase
rs11951068
rs6586282
rs3797535
rs1789953
rs6859667
rs567754
rs558133
rs234709
rs672413
rs921943
rs705415
rs10944
All
0.00
0.05
0.10
0.15
0.20
MR effect size for
'Selenium' on 'A/G−specific adenine DNA glycosylase'

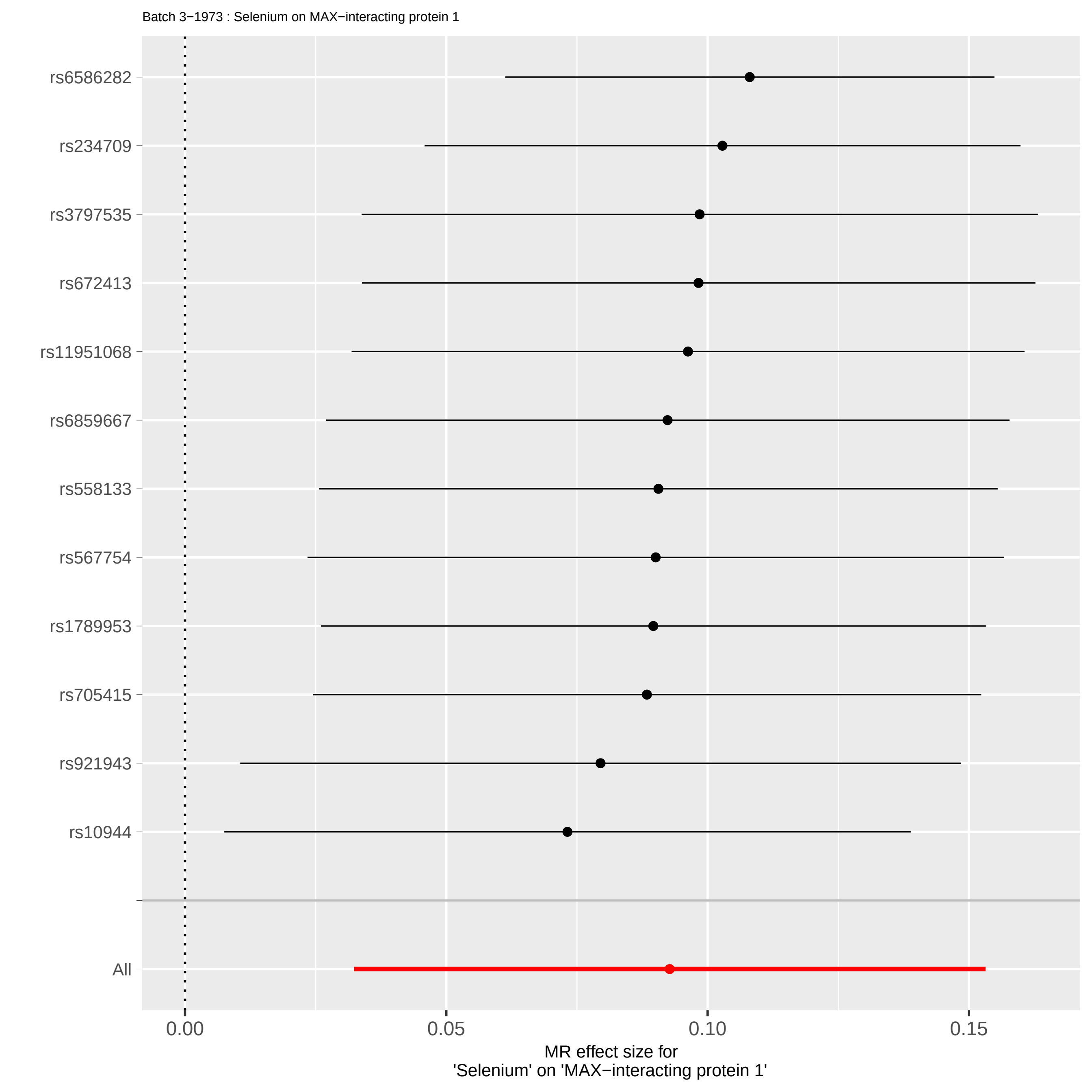

Batch 3−1973 : Selenium on MAX−interacting protein 1
rs6586282
rs234709
rs3797535
rs672413
rs11951068
rs6859667
rs558133
rs567754
rs1789953
rs705415
rs921943
rs10944
All
0.00
0.05
0.10
0.15
MR effect size for
'Selenium' on 'MAX−interacting protein 1'

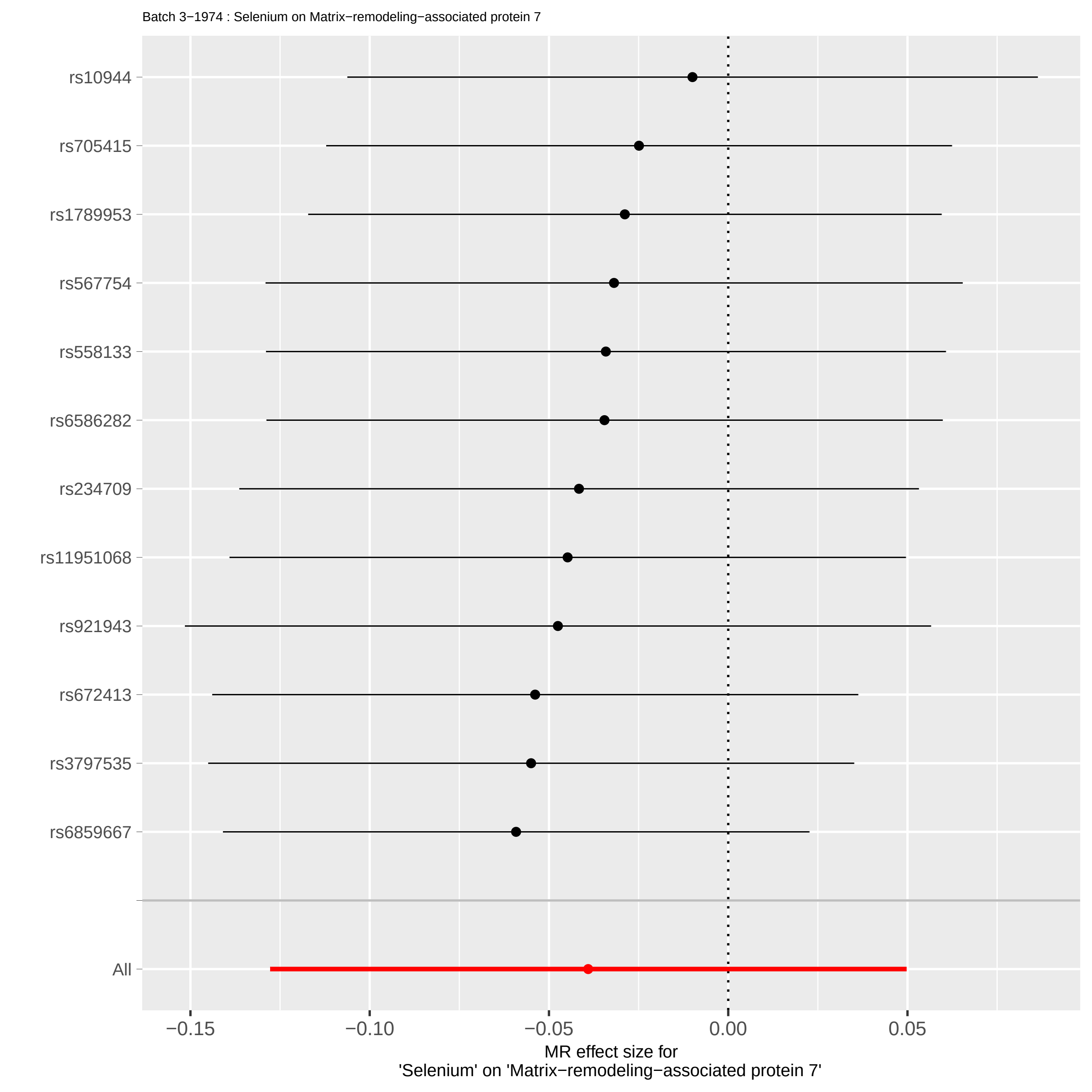

Batch 3−1974 : Selenium on Matrix−remodeling−associated protein 7
rs10944
rs705415
rs1789953
rs567754
rs558133
rs6586282
rs234709
rs11951068
rs921943
rs672413
rs3797535
rs6859667
All
−0.15
−0.10
−0.05
0.00
0.05
MR effect size for
'Selenium' on 'Matrix−remodeling−associated protein 7'

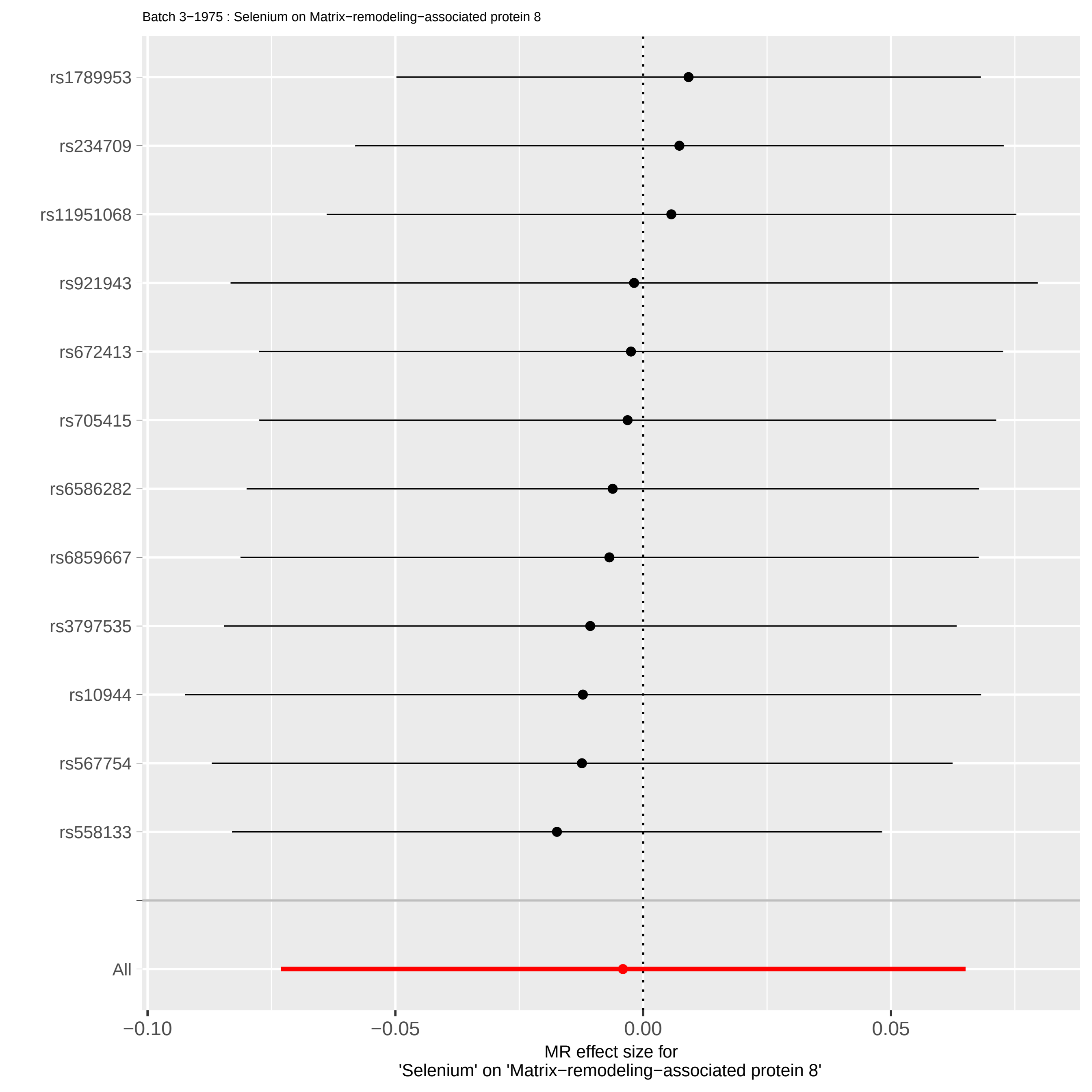

Batch 3−1975 : Selenium on Matrix−remodeling−associated protein 8
rs1789953
rs234709
rs11951068
rs921943
rs672413
rs705415
rs6586282
rs6859667
rs3797535
rs10944
rs567754
rs558133
All
−0.10
−0.05
0.00
0.05
MR effect size for
'Selenium' on 'Matrix−remodeling−associated protein 8'

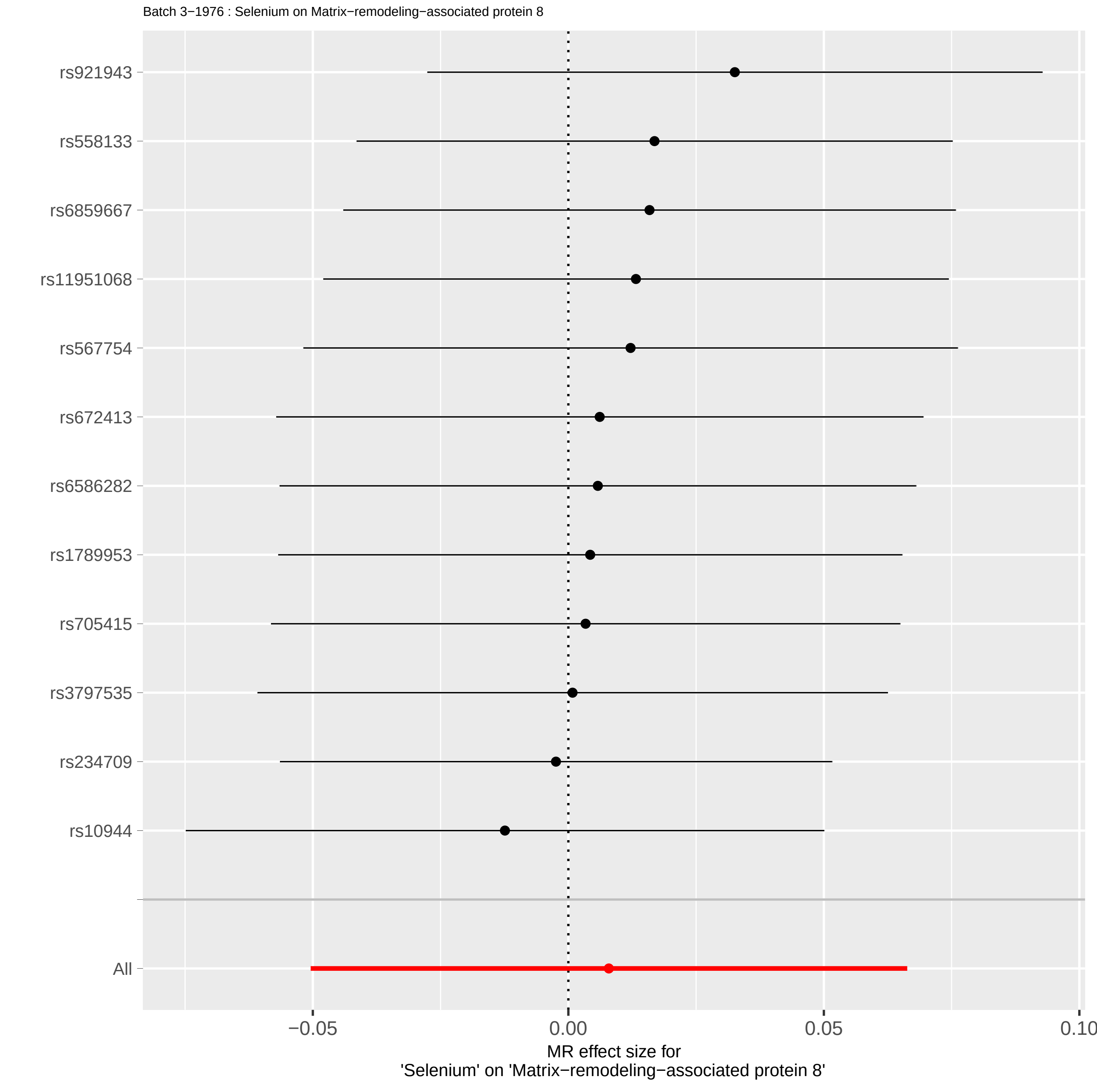

Batch 3−1976 : Selenium on Matrix−remodeling−associated protein 8
rs921943
rs558133
rs6859667
rs11951068
rs567754
rs672413
rs6586282
rs1789953
rs705415
rs3797535
rs234709
rs10944
All
−0.05
0.00
0.05
0.10
MR effect size for
'Selenium' on 'Matrix−remodeling−associated protein 8'

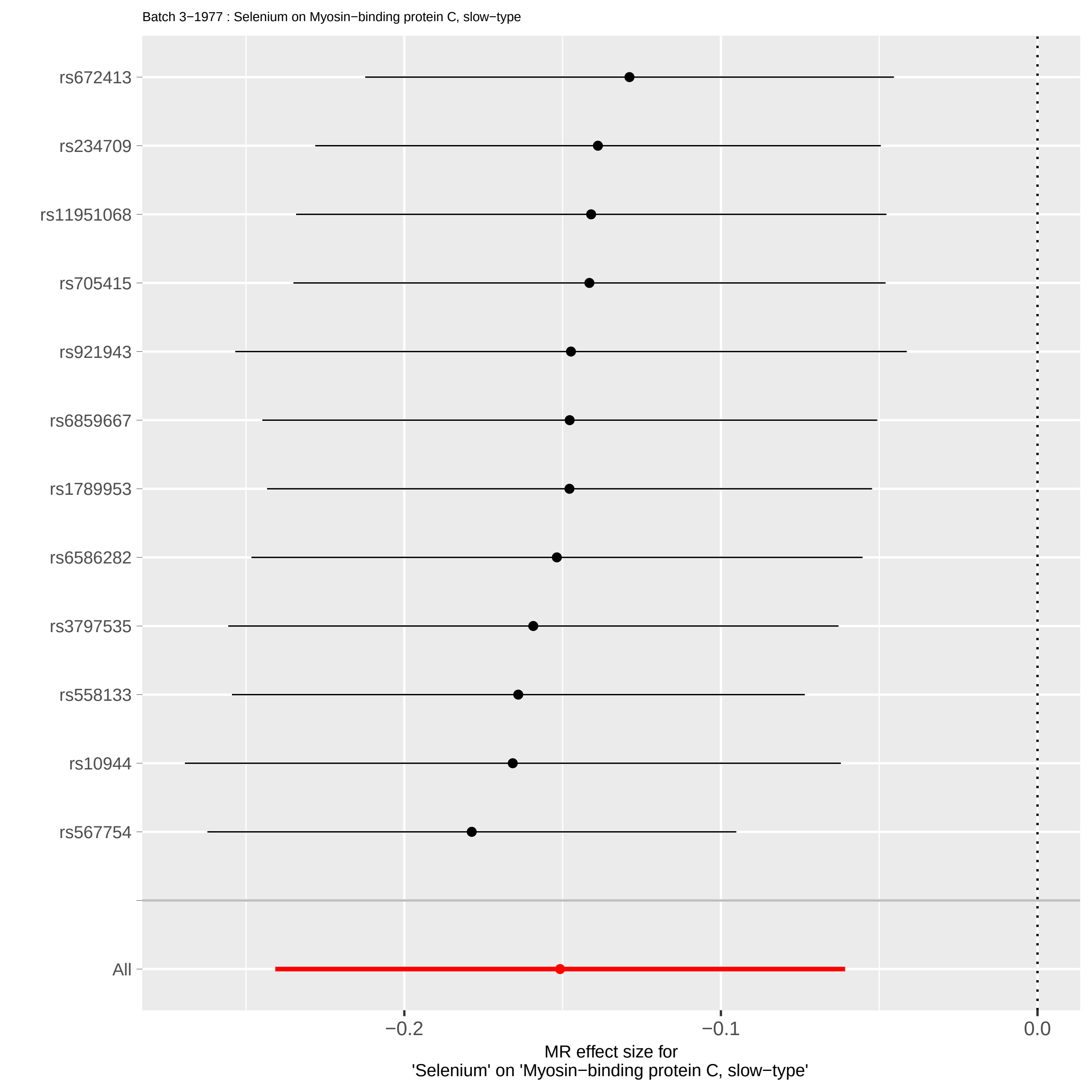
Batch 3−1977 : Selenium on Myosin−binding protein C, slow−type
rs672413
rs234709
rs11951068
rs705415
rs921943
rs6859667
rs1789953
rs6586282
rs3797535
rs558133
rs10944
rs567754
All
−0.2
−0.1
0.0
MR effect size for
'Selenium' on 'Myosin−binding protein C, slow−type'

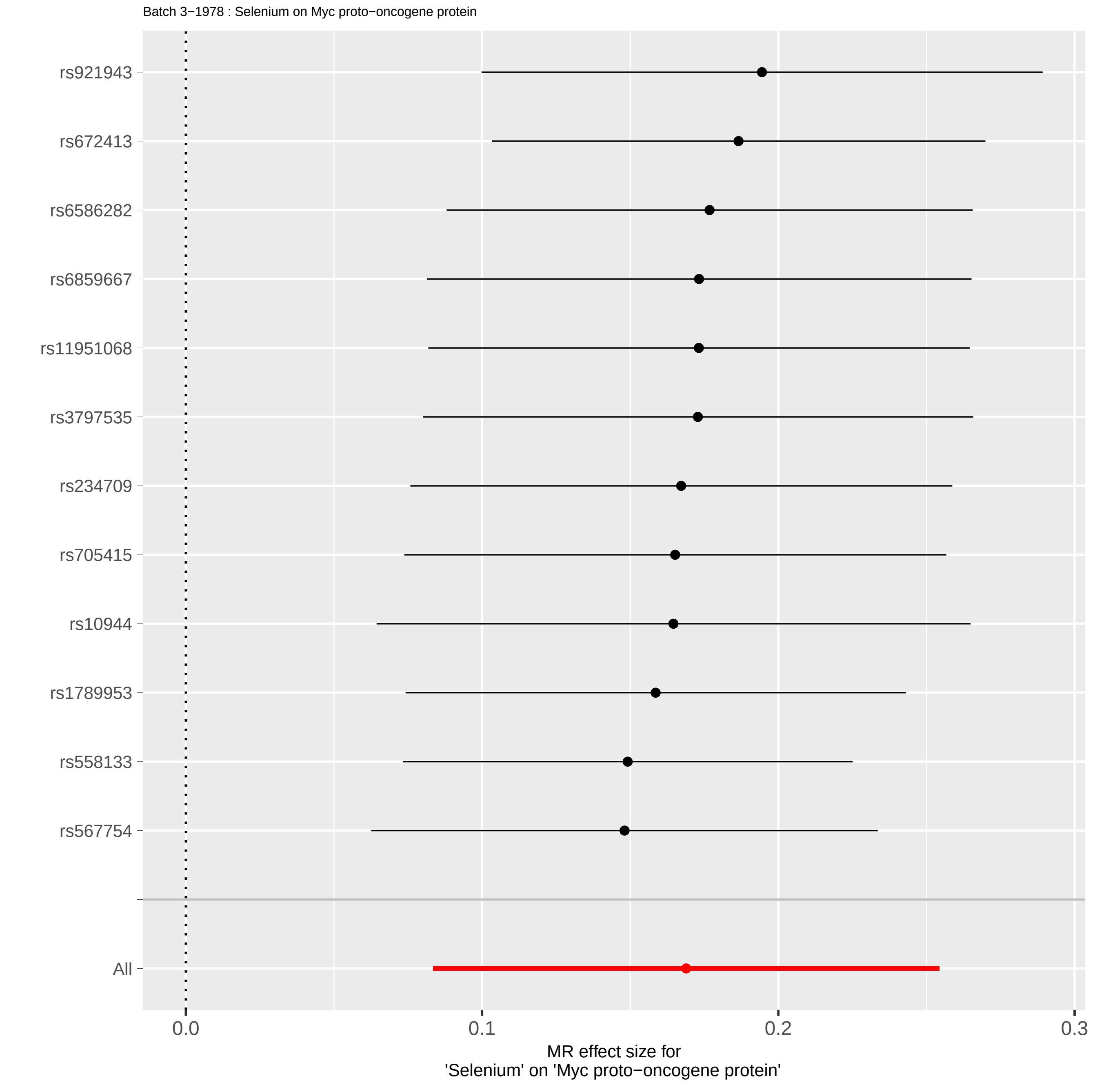
Batch 3−1978 : Selenium on Myc proto−oncogene protein
rs921943
rs672413
rs6586282
rs6859667
rs11951068
rs3797535
rs234709
rs705415
rs10944
rs1789953
rs558133
rs567754
All
0.0
0.1
0.2
0.3
MR effect size for
'Selenium' on 'Myc proto−oncogene protein'

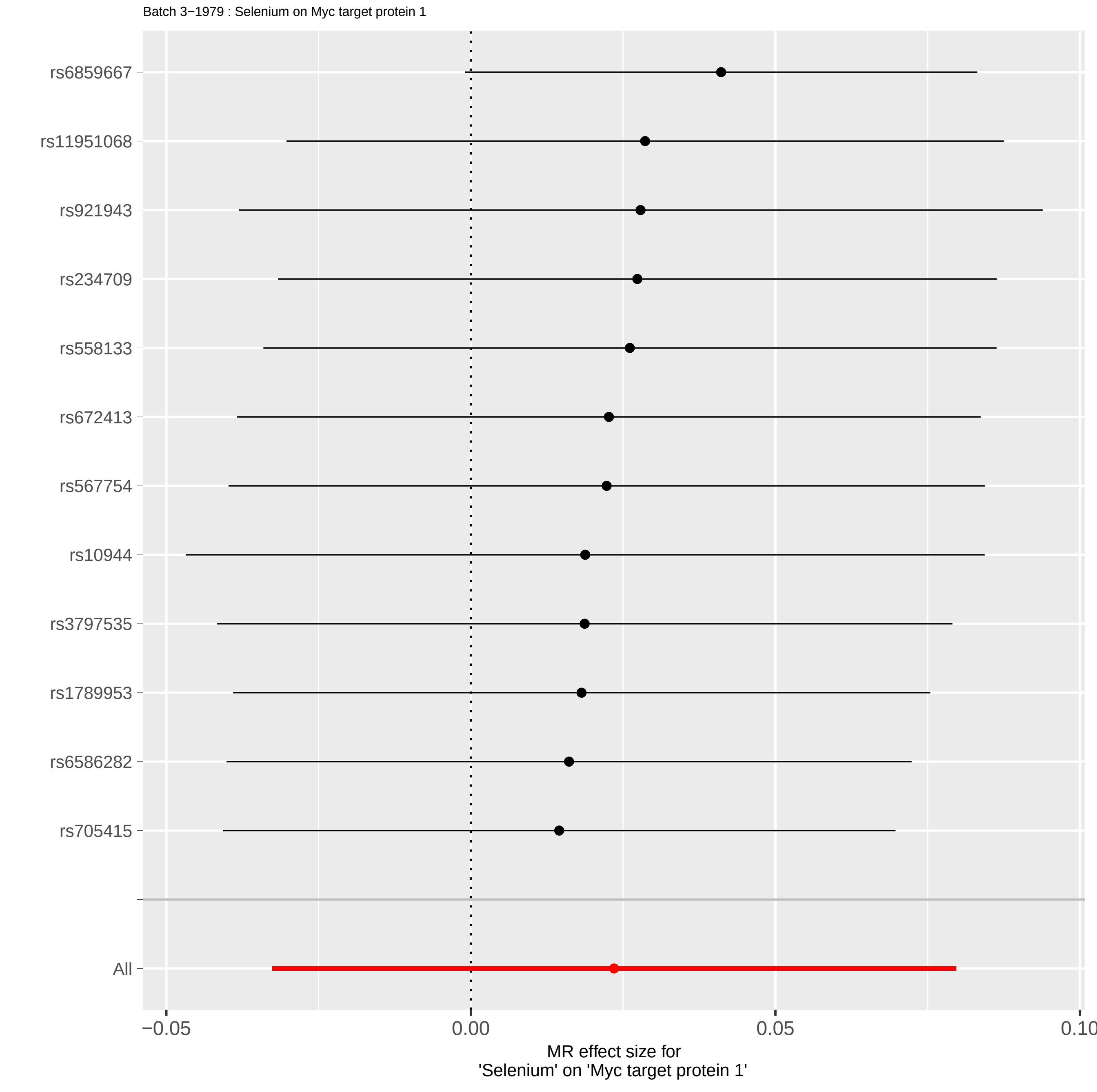

Batch 3−1979 : Selenium on Myc target protein 1
rs6859667
rs11951068
rs921943
rs234709
rs558133
rs672413
rs567754
rs10944
rs3797535
rs1789953
rs6586282
rs705415
All
−0.05
0.00
0.05
0.10
MR effect size for
'Selenium' on 'Myc target protein 1'

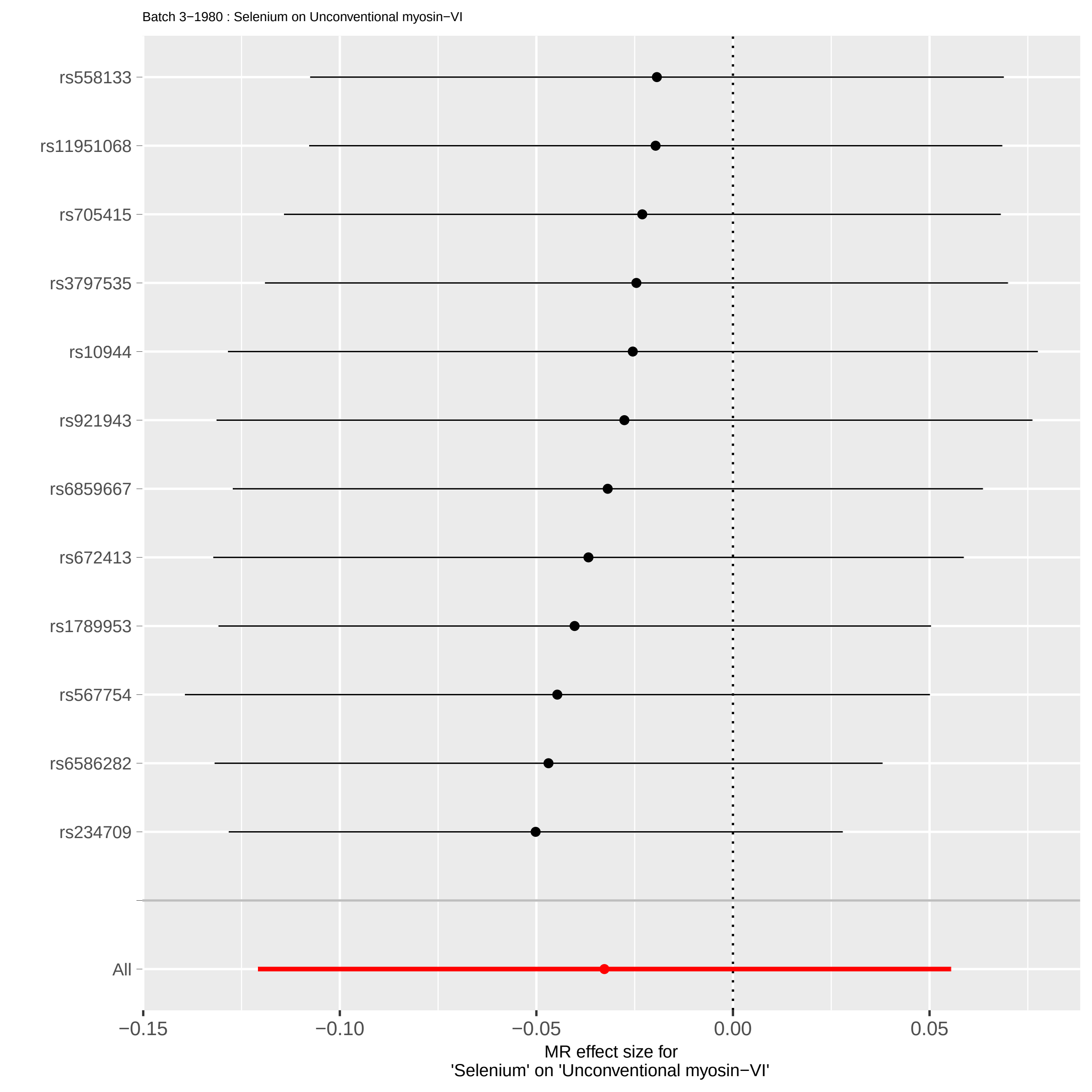

Batch 3−1980 : Selenium on Unconventional myosin−VI
rs558133
rs11951068
rs705415
rs3797535
rs10944
rs921943
rs6859667
rs672413
rs1789953
rs567754
rs6586282
rs234709
All
−0.15
−0.10
−0.05
0.00
0.05
MR effect size for
'Selenium' on 'Unconventional myosin−VI'

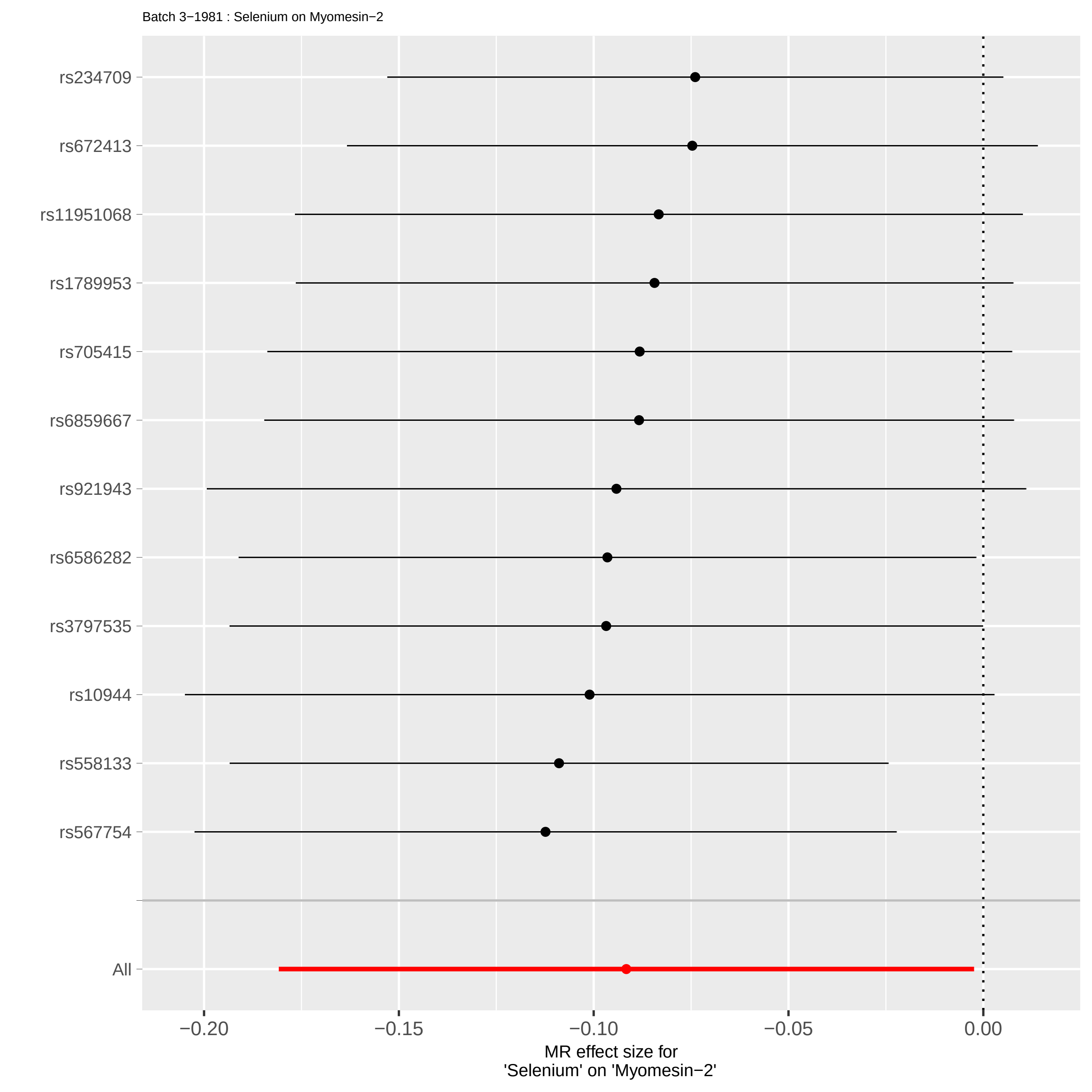

Batch 3−1981 : Selenium on Myomesin−2
rs234709
rs672413
rs11951068
rs1789953
rs705415
rs6859667
rs921943
rs6586282
rs3797535
rs10944
rs558133
rs567754
All
−0.20
−0.15
−0.10
−0.05
0.00
MR effect size for
'Selenium' on 'Myomesin−2'

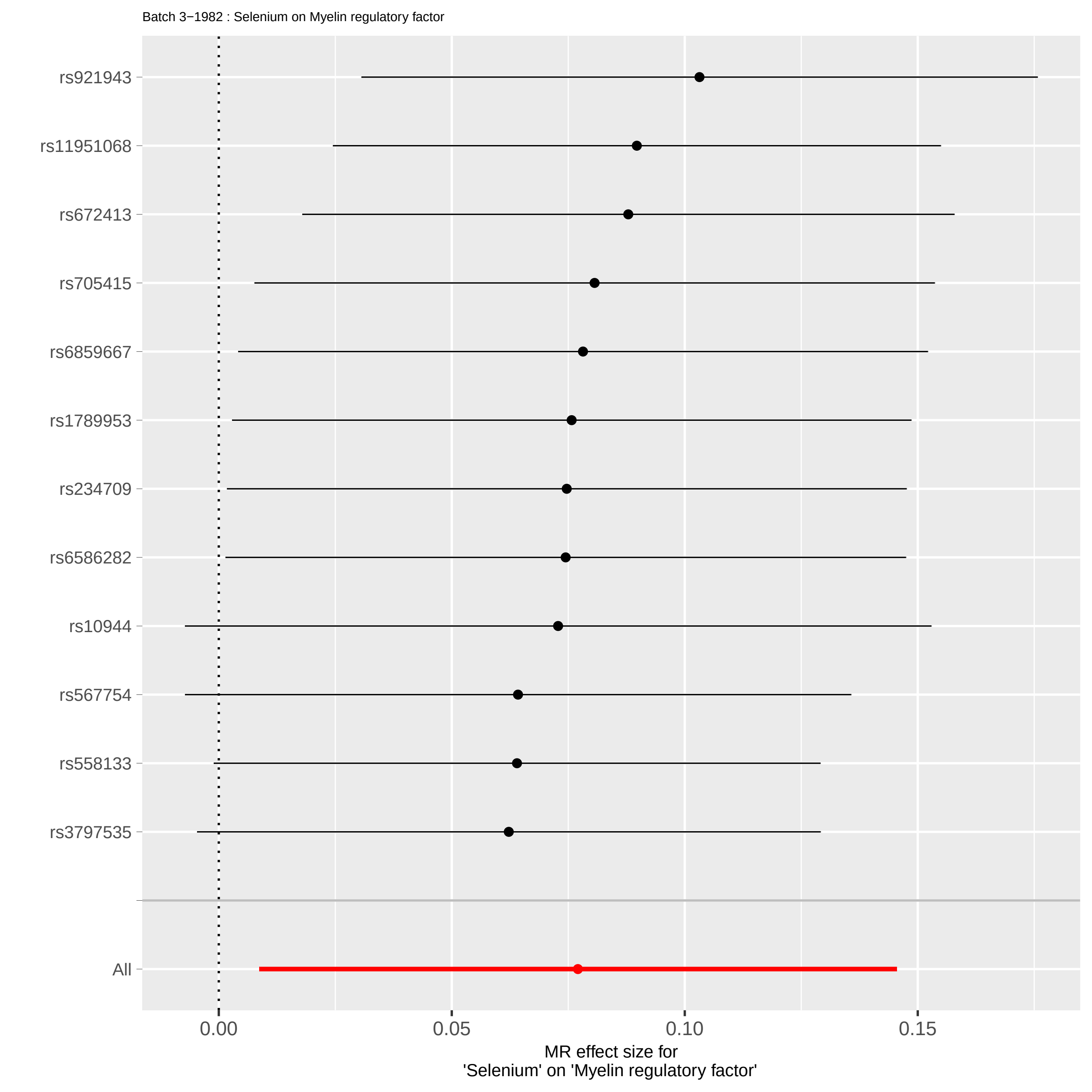

Batch 3−1982 : Selenium on Myelin regulatory factor
rs921943
rs11951068
rs672413
rs705415
rs6859667
rs1789953
rs234709
rs6586282
rs10944
rs567754
rs558133
rs3797535
All
0.00
0.05
0.10
0.15
MR effect size for
'Selenium' on 'Myelin regulatory factor'

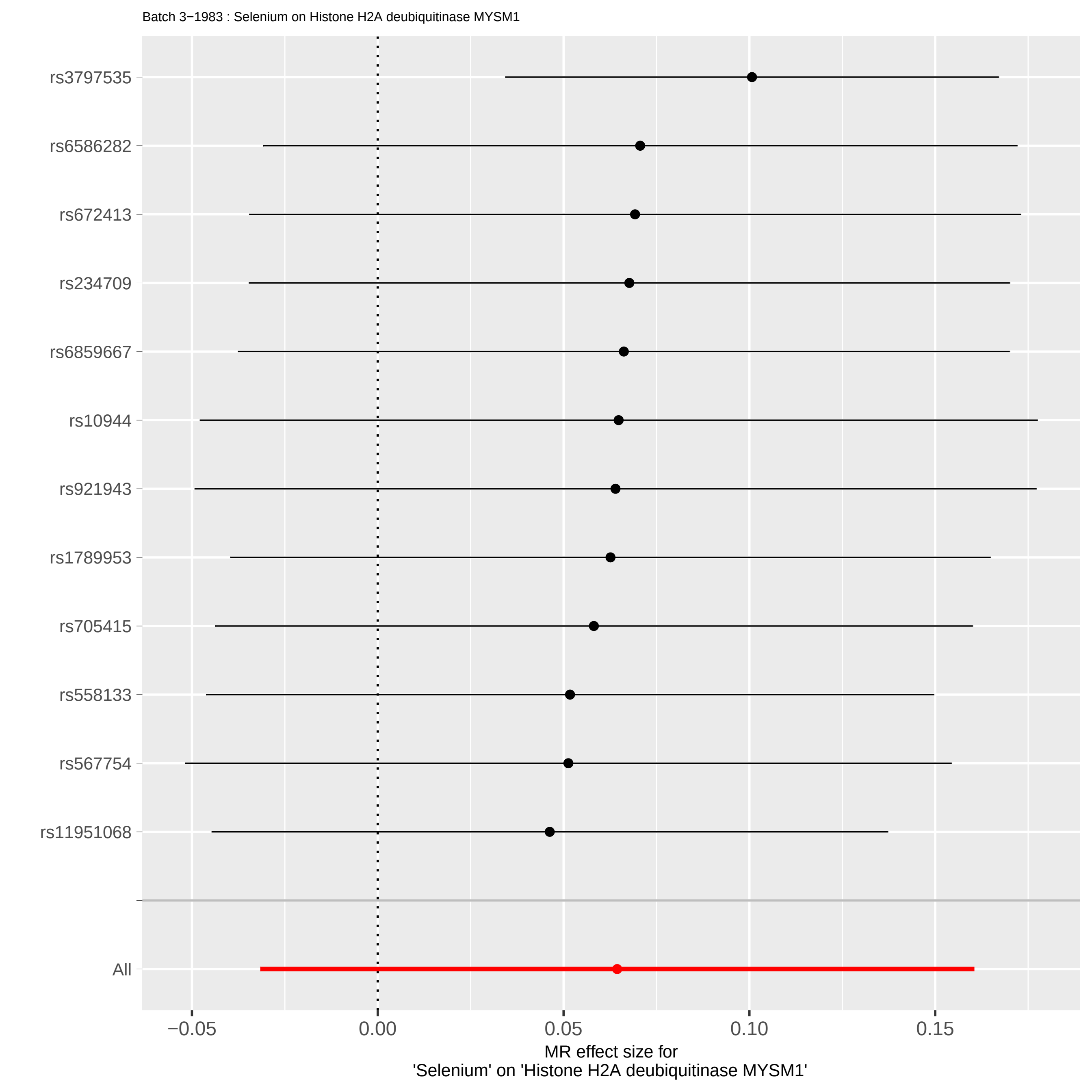

Batch 3−1983 : Selenium on Histone H2A deubiquitinase MYSM1
rs3797535
rs6586282
rs672413
rs234709
rs6859667
rs10944
rs921943
rs1789953
rs705415
rs558133
rs567754
rs11951068
All
−0.05
0.00
0.05
0.10
0.15
MR effect size for
'Selenium' on 'Histone H2A deubiquitinase MYSM1'

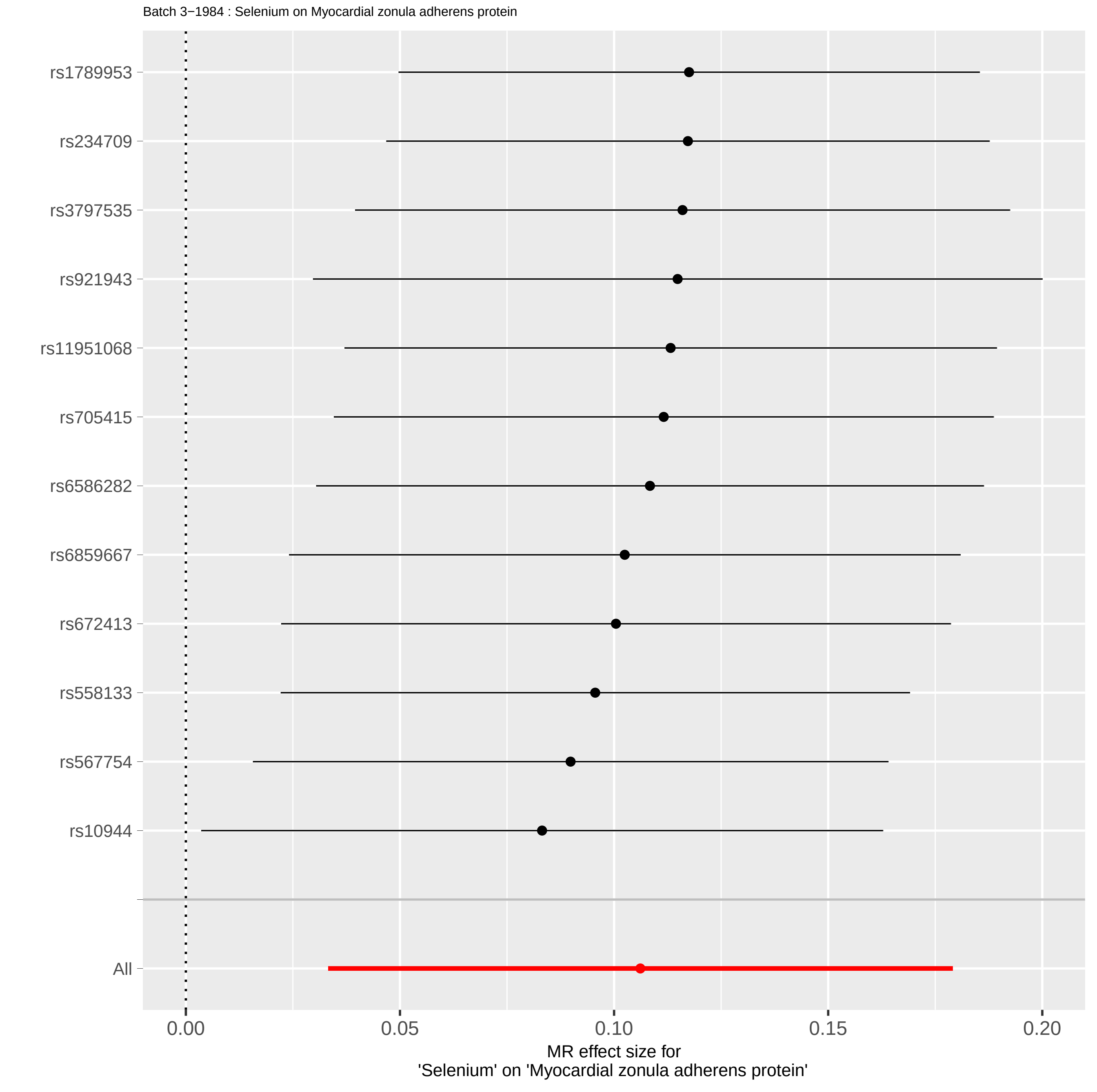

Batch 3−1984 : Selenium on Myocardial zonula adherens protein
rs1789953
rs234709
rs3797535
rs921943
rs11951068
rs705415
rs6586282
rs6859667
rs672413
rs558133
rs567754
rs10944
All
0.00
0.05
0.10
0.15
0.20
MR effect size for
'Selenium' on 'Myocardial zonula adherens protein'

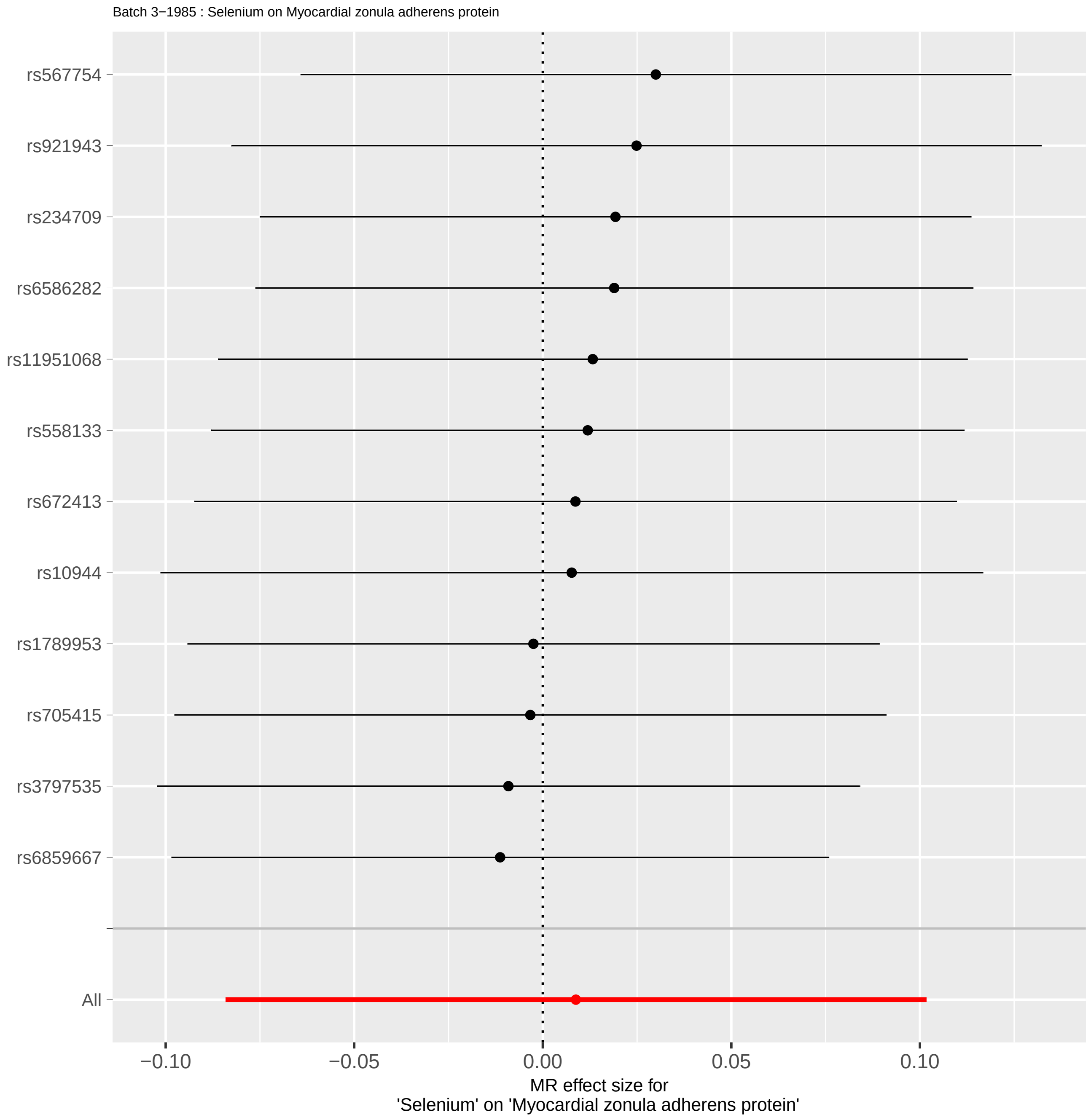

Batch 3−1985 : Selenium on Myocardial zonula adherens protein
rs567754
rs921943
rs234709
rs6586282
rs11951068
rs558133
rs672413
rs10944
rs1789953
rs705415
rs3797535
rs6859667
All
−0.10
−0.05
0.00
0.05
0.10
MR effect size for
'Selenium' on 'Myocardial zonula adherens protein'

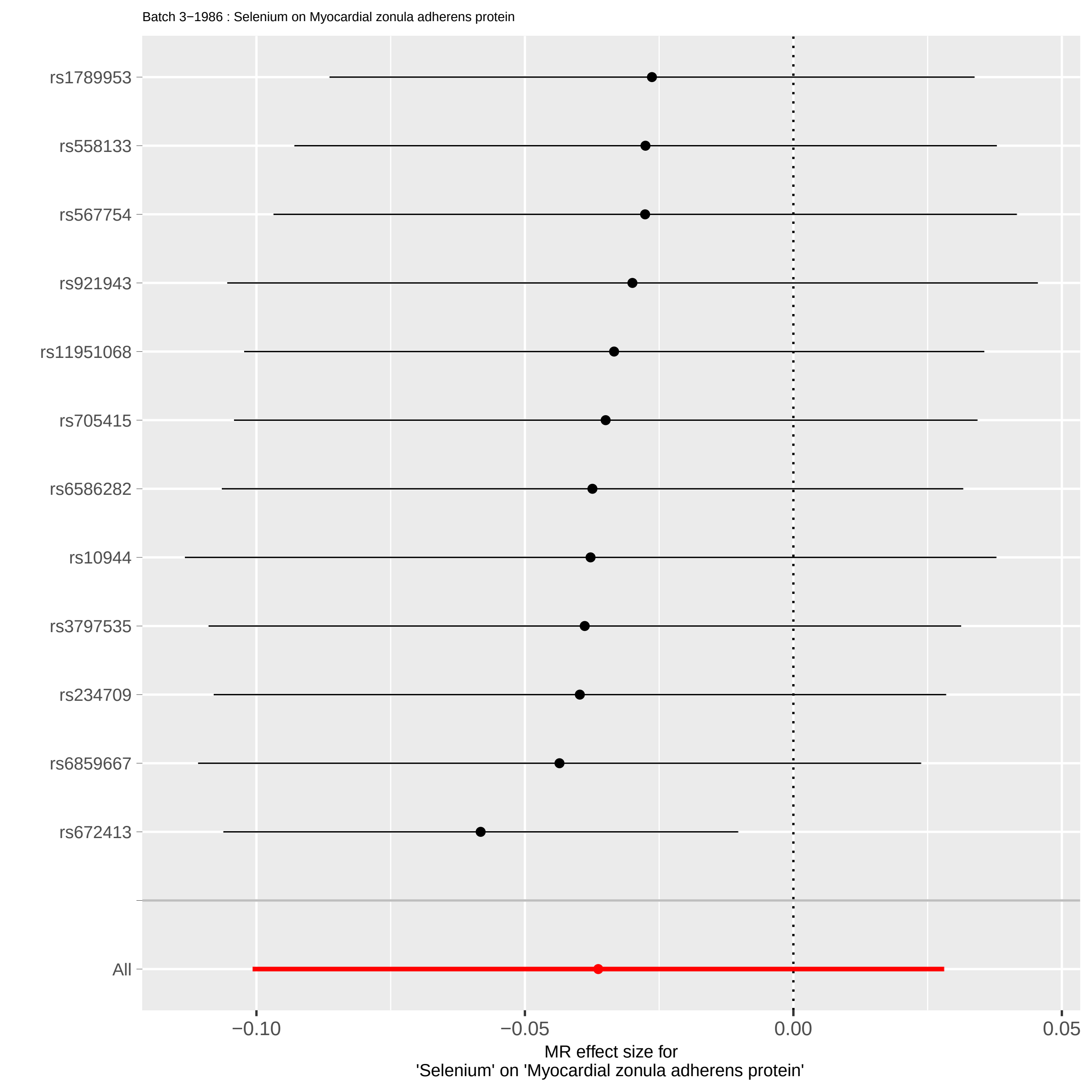

Batch 3−1986 : Selenium on Myocardial zonula adherens protein
rs1789953
rs558133
rs567754
rs921943
rs11951068
rs705415
rs6586282
rs10944
rs3797535
rs234709
rs6859667
rs672413
All
−0.10
−0.05
0.00
0.05
MR effect size for
'Selenium' on 'Myocardial zonula adherens protein'

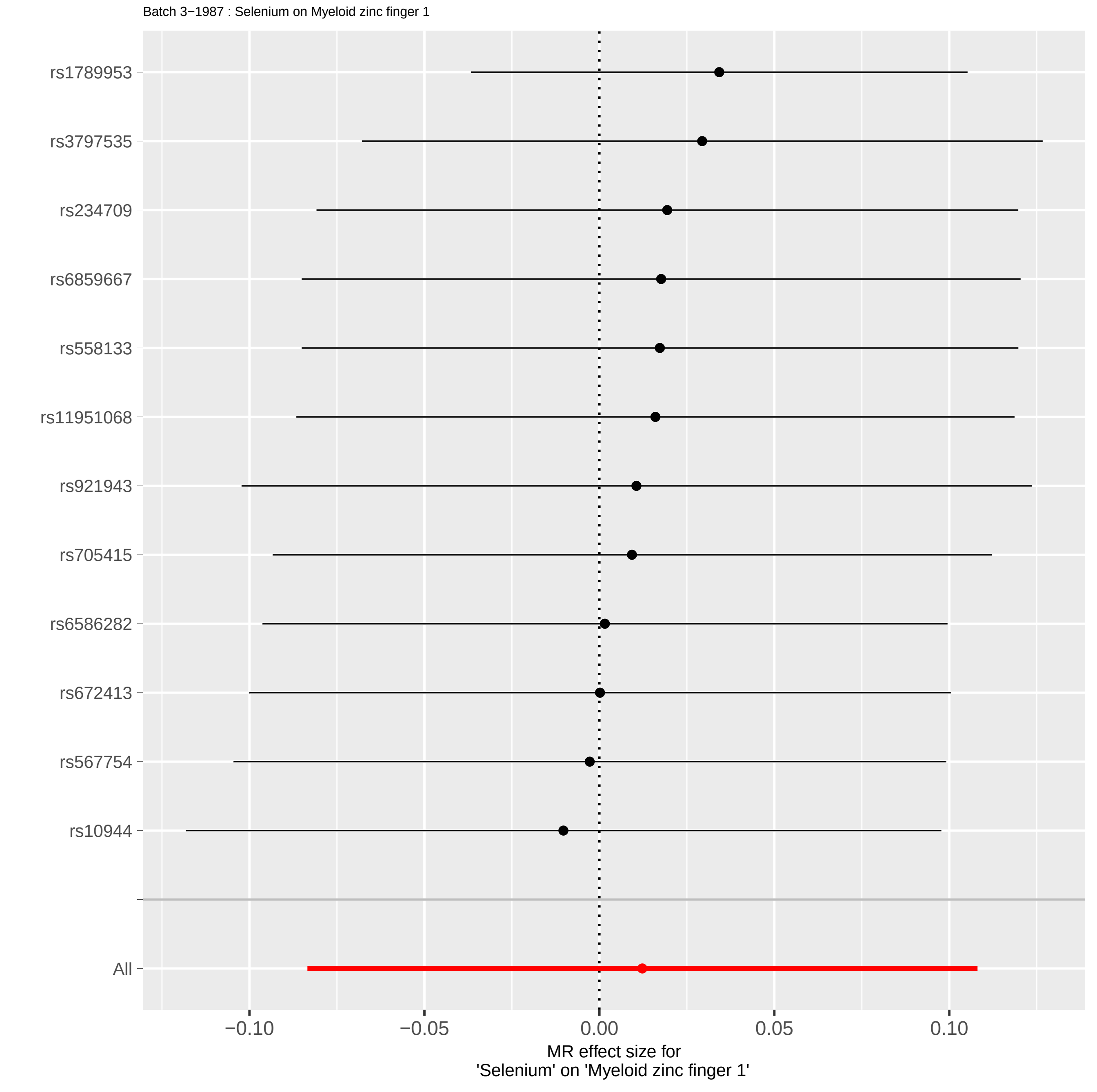

Batch 3−1987 : Selenium on Myeloid zinc finger 1
rs1789953
rs3797535
rs234709
rs6859667
rs558133
rs11951068
rs921943
rs705415
rs6586282
rs672413
rs567754
rs10944
All
−0.10
−0.05
0.00
0.05
0.10
MR effect size for
'Selenium' on 'Myeloid zinc finger 1'

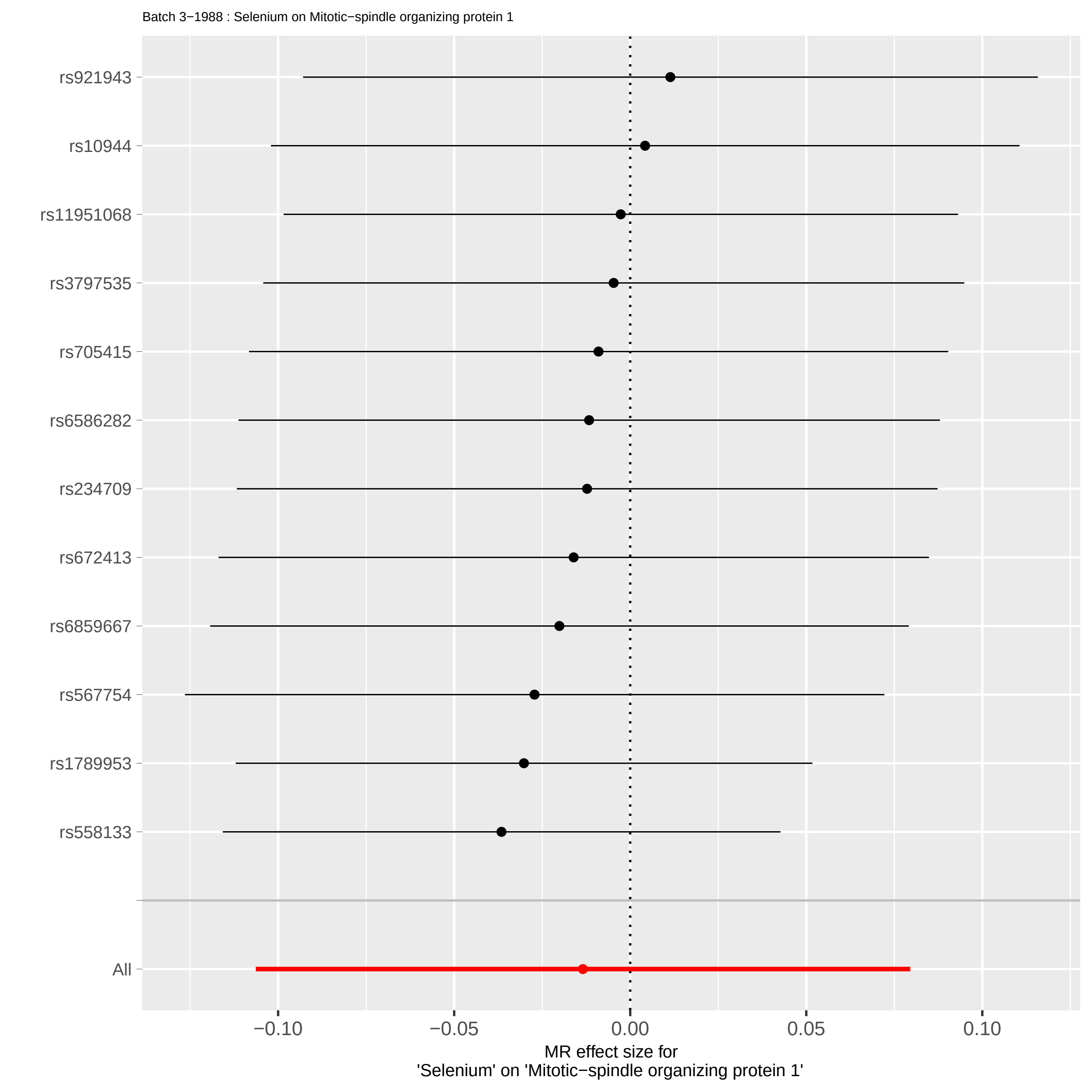
Batch 3−1988 : Selenium on Mitotic−spindle organizing protein 1
rs921943
rs10944
rs11951068
rs3797535
rs705415
rs6586282
rs234709
rs672413
rs6859667
rs567754
rs1789953
rs558133
All
−0.10
−0.05
0.00
0.05
0.10
MR effect size for
'Selenium' on 'Mitotic−spindle organizing protein 1'

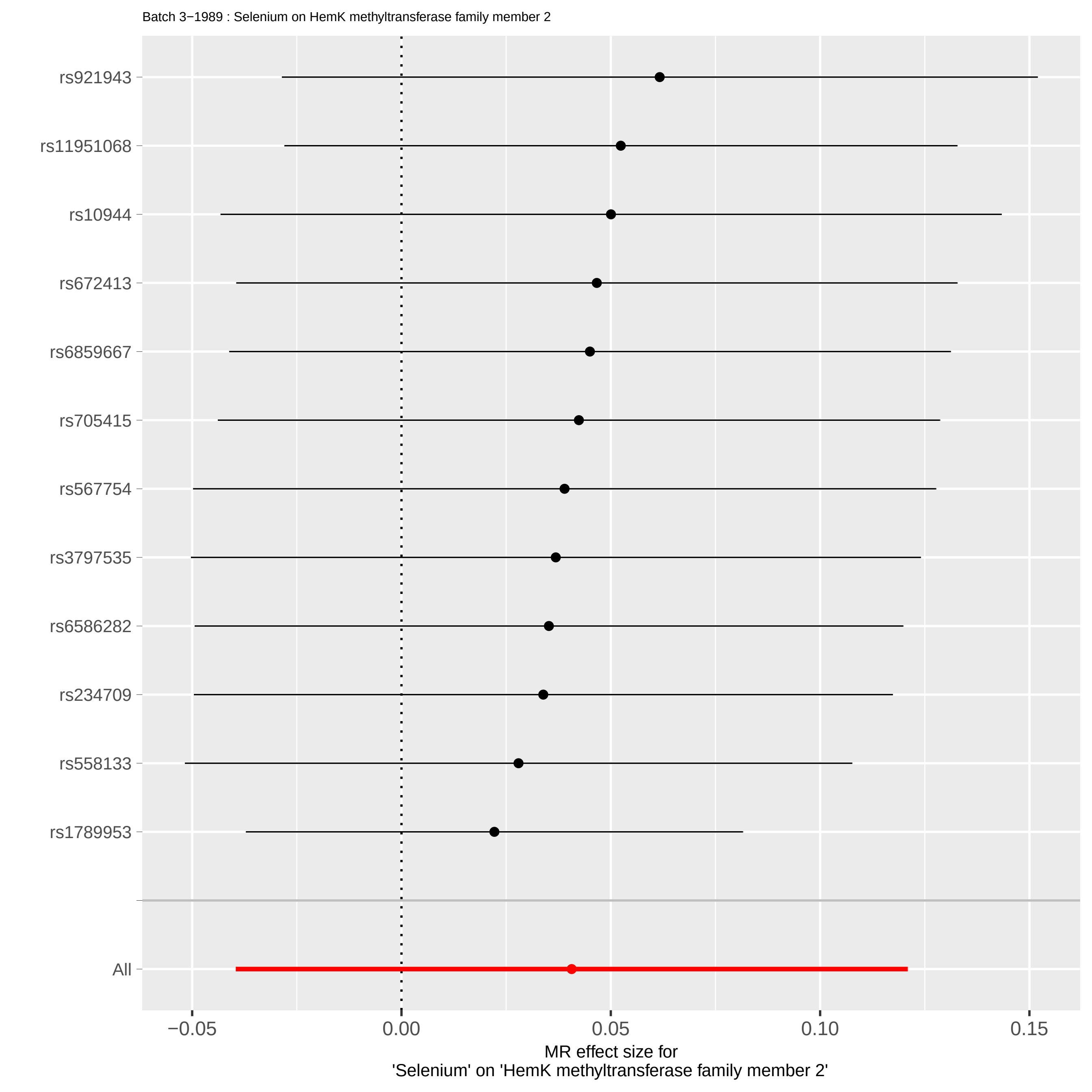

Batch 3−1989 : Selenium on HemK methyltransferase family member 2
rs921943
rs11951068
rs10944
rs672413
rs6859667
rs705415
rs567754
rs3797535
rs6586282
rs234709
rs558133
rs1789953
All
−0.05
0.00
0.05
0.10
0.15
MR effect size for
'Selenium' on 'HemK methyltransferase family member 2'

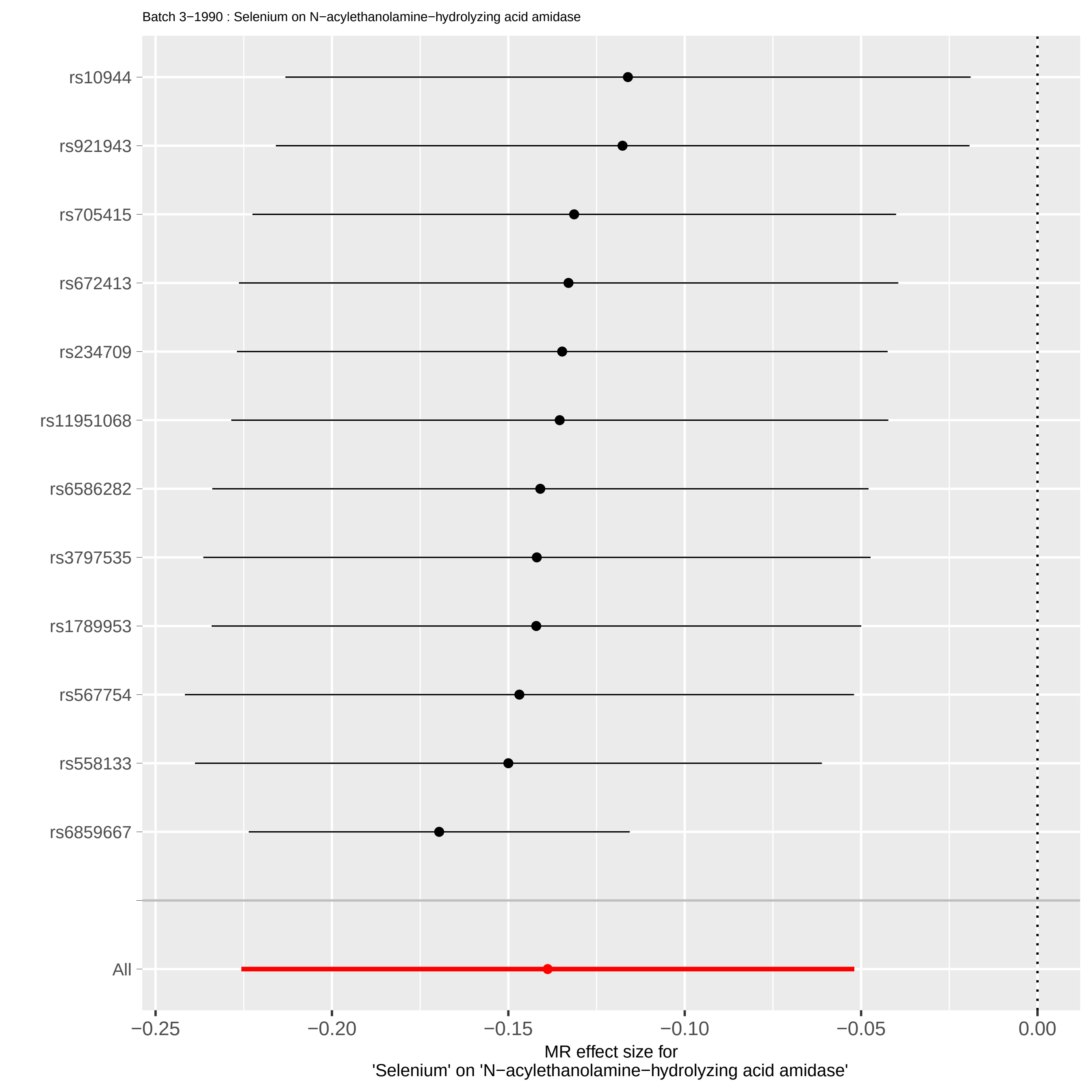

Batch 3−1990 : Selenium on N−acylethanolamine−hydrolyzing acid amidase
rs10944
rs921943
rs705415
rs672413
rs234709
rs11951068
rs6586282
rs3797535
rs1789953
rs567754
rs558133
rs6859667
All
−0.25
−0.20
−0.15
−0.10
−0.05
0.00
MR effect size for
'Selenium' on 'N−acylethanolamine−hydrolyzing acid amidase'

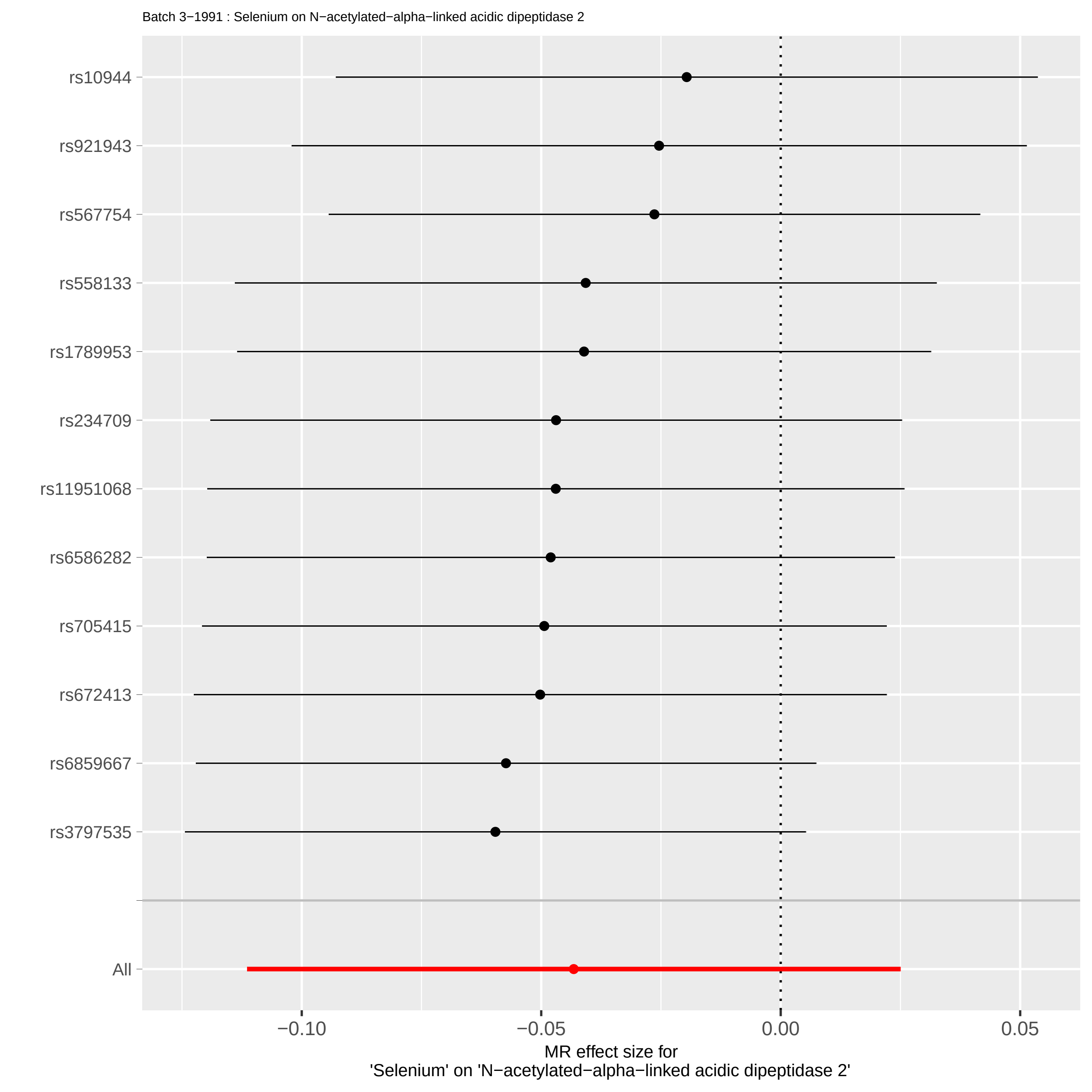
Batch 3−1991 : Selenium on N−acetylated−alpha−linked acidic dipeptidase 2
rs10944
rs921943
rs567754
rs558133
rs1789953
rs234709
rs11951068
rs6586282
rs705415
rs672413
rs6859667
rs3797535
All
−0.10
−0.05
0.00
0.05
MR effect size for
'Selenium' on 'N−acetylated−alpha−linked acidic dipeptidase 2'

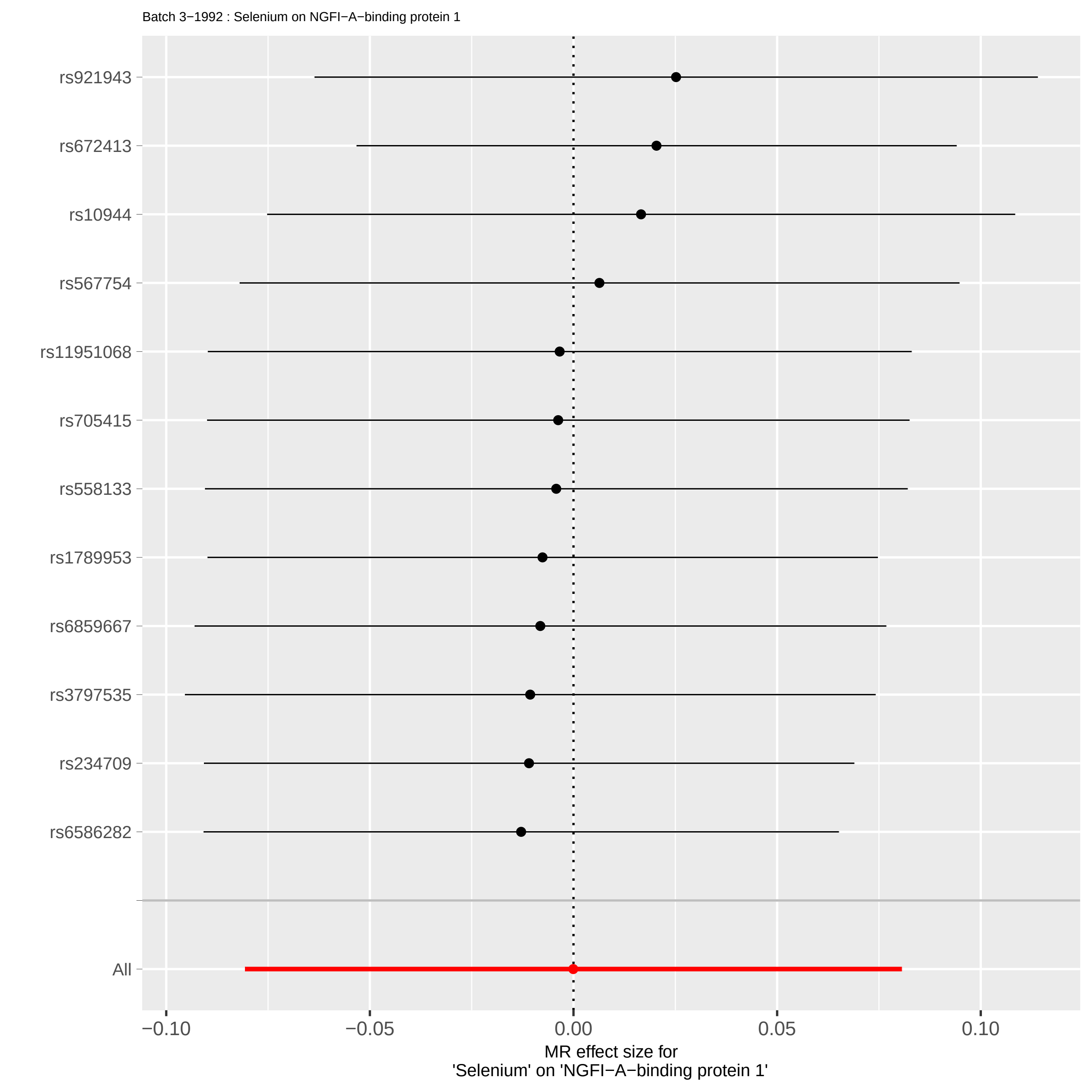
Batch 3−1992 : Selenium on NGFI−A−binding protein 1
rs921943
rs672413
rs10944
rs567754
rs11951068
rs705415
rs558133
rs1789953
rs6859667
rs3797535
rs234709
rs6586282
All
−0.10
−0.05
0.00
0.05
0.10
MR effect size for
'Selenium' on 'NGFI−A−binding protein 1'

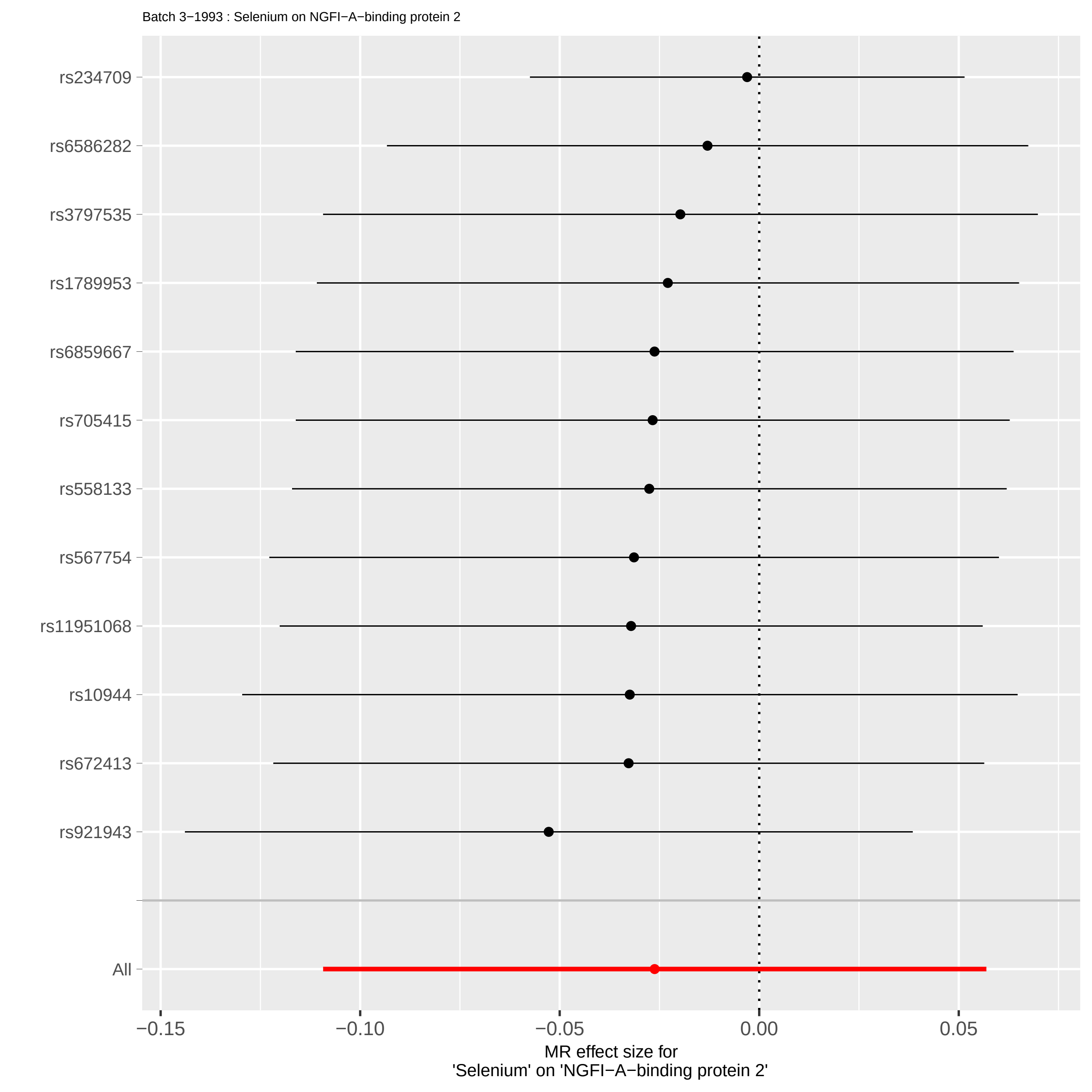
Batch 3−1993 : Selenium on NGFI−A−binding protein 2
rs234709
rs6586282
rs3797535
rs1789953
rs6859667
rs705415
rs558133
rs567754
rs11951068
rs10944
rs672413
rs921943
All
−0.15
−0.10
−0.05
0.00
0.05
MR effect size for
'Selenium' on 'NGFI−A−binding protein 2'

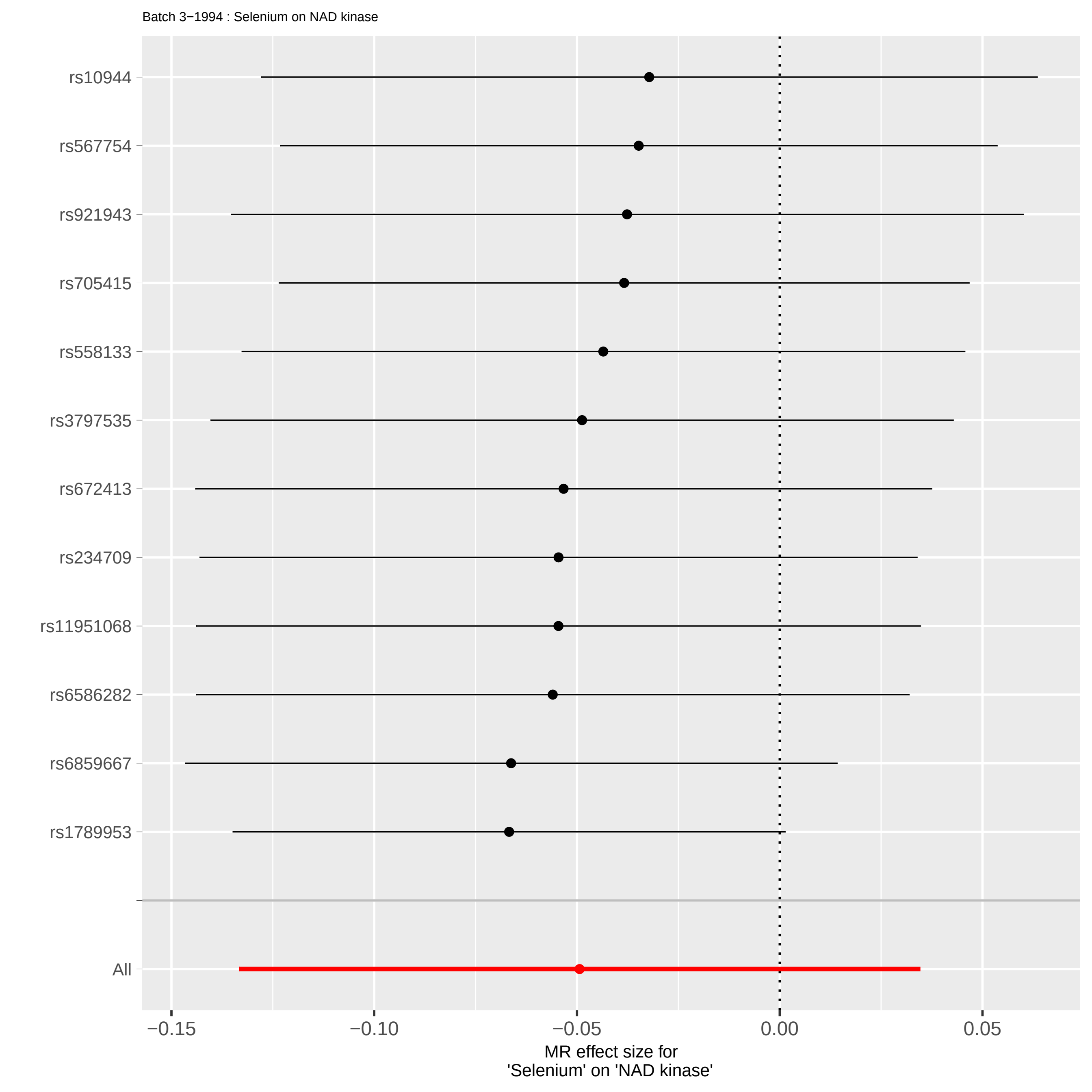

Batch 3−1994 : Selenium on NAD kinase
rs10944
rs567754
rs921943
rs705415
rs558133
rs3797535
rs672413
rs234709
rs11951068
rs6586282
rs6859667
rs1789953
All
−0.15
−0.10
−0.05
0.00
0.05
MR effect size for
'Selenium' on 'NAD kinase'

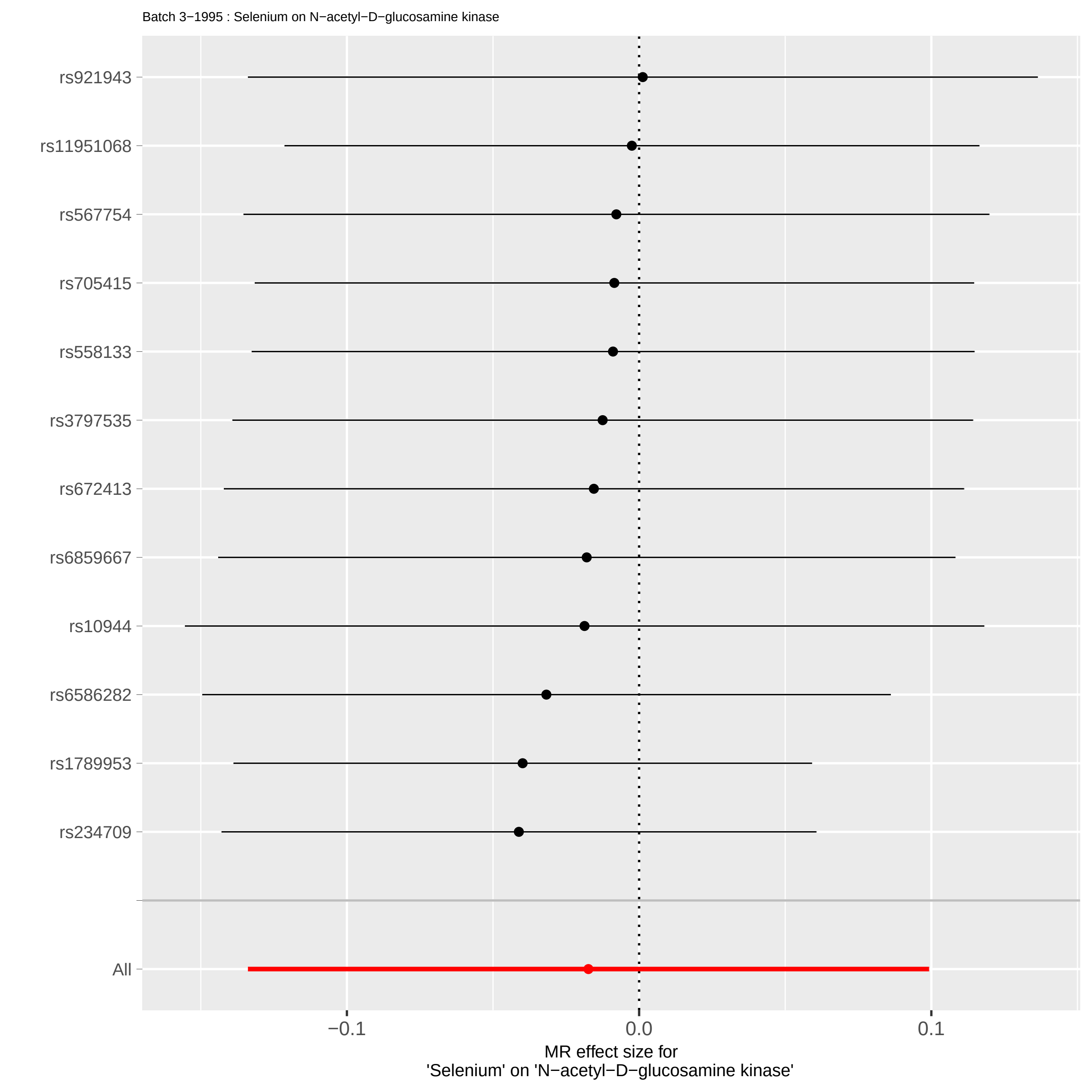

Batch 3−1995 : Selenium on N−acetyl−D−glucosamine kinase
rs921943
rs11951068
rs567754
rs705415
rs558133
rs3797535
rs672413
rs6859667
rs10944
rs6586282
rs1789953
rs234709
All
−0.1
0.0
0.1
MR effect size for
'Selenium' on 'N−acetyl−D−glucosamine kinase'

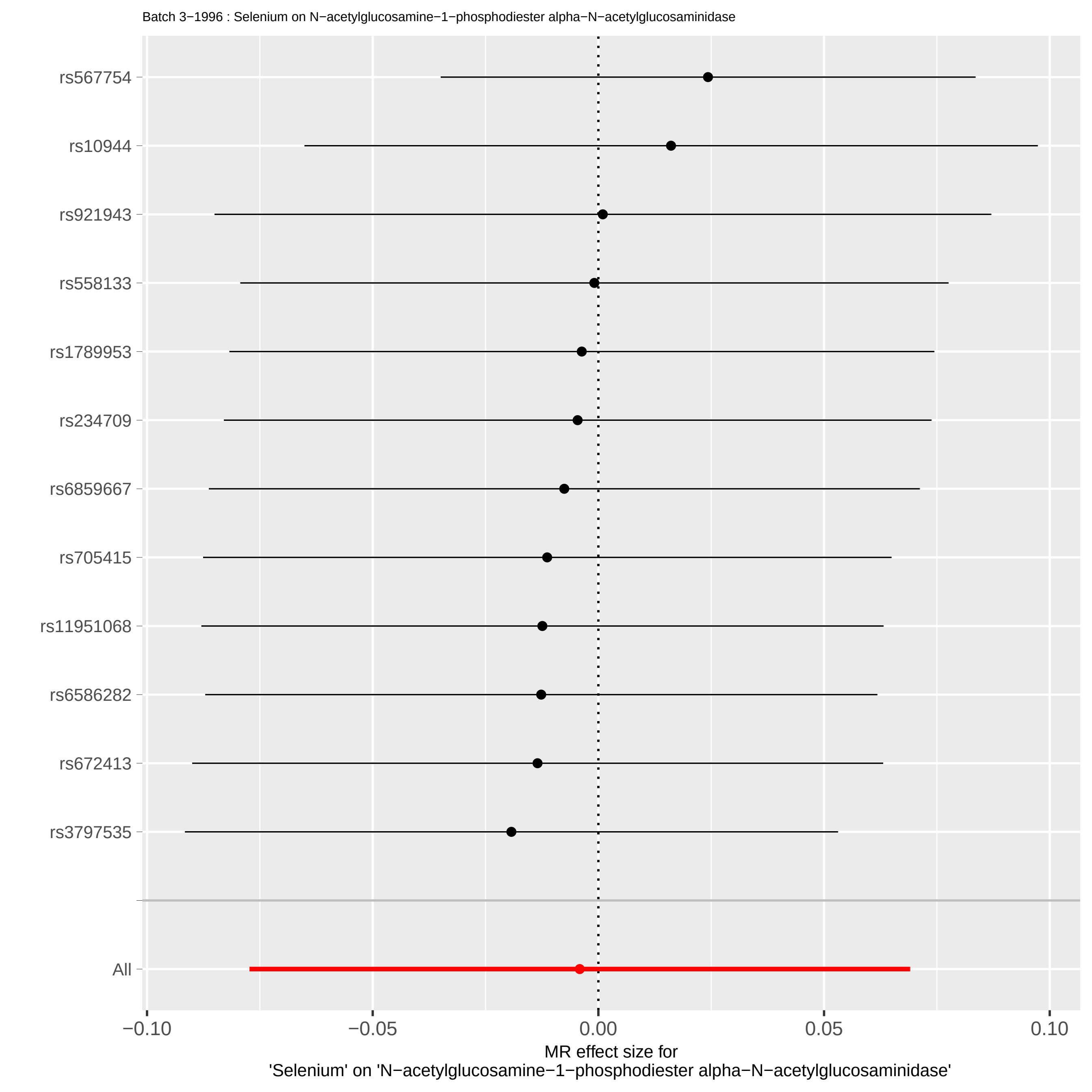

Batch 3−1996 : Selenium on N−acetylglucosamine−1−phosphodiester alpha−N−acetylglucosaminidase
rs567754
rs10944
rs921943
rs558133
rs1789953
rs234709
rs6859667
rs705415
rs11951068
rs6586282
rs672413
rs3797535
All
−0.10
−0.05
0.00
0.05
0.10
MR effect size for
'Selenium' on 'N−acetylglucosamine−1−phosphodiester alpha−N−acetylglucosaminidase'

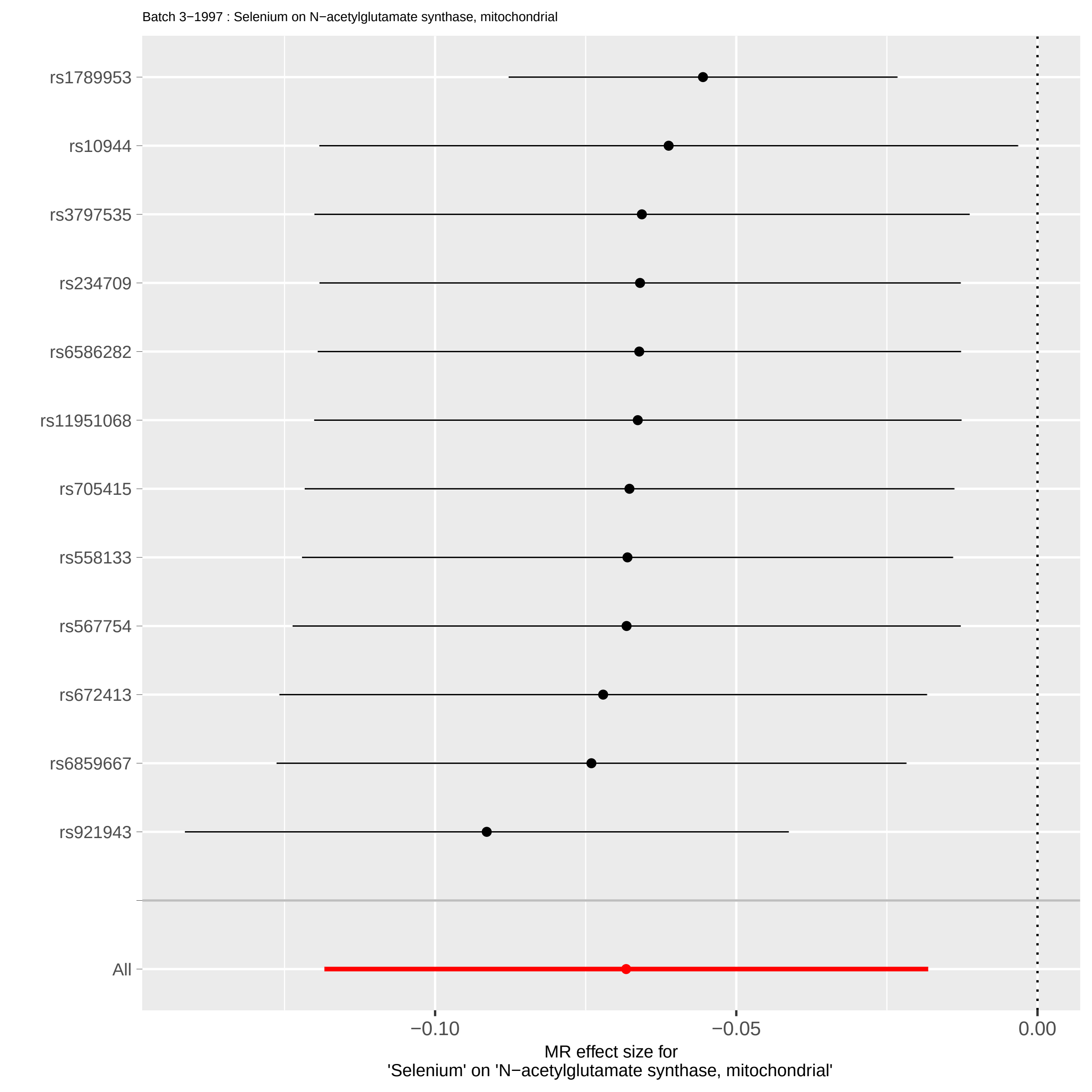

Batch 3−1997 : Selenium on N−acetylglutamate synthase, mitochondrial
rs1789953
rs10944
rs3797535
rs234709
rs6586282
rs11951068
rs705415
rs558133
rs567754
rs672413
rs6859667
rs921943
All
−0.10
−0.05
0.00
MR effect size for
'Selenium' on 'N−acetylglutamate synthase, mitochondrial'

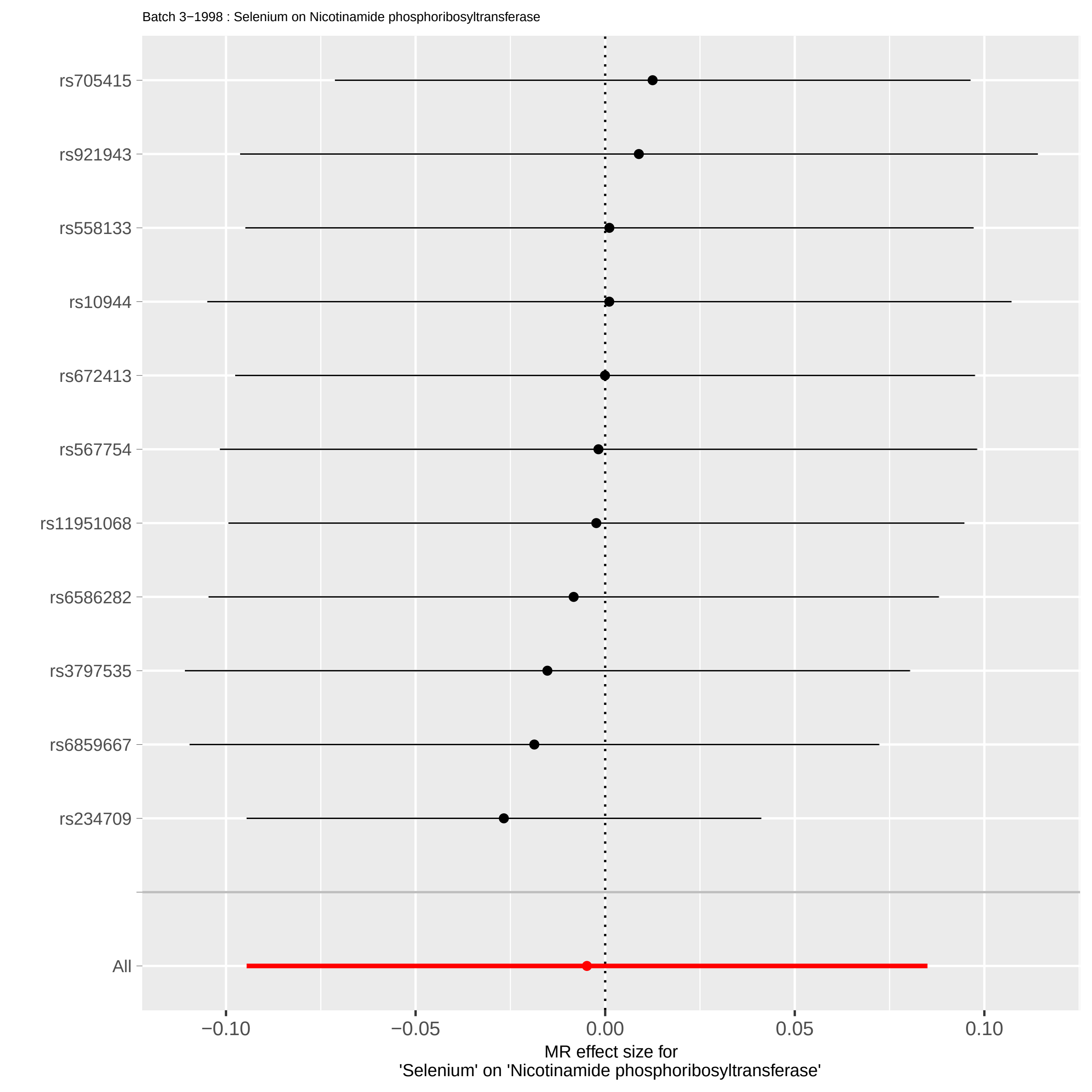

Batch 3−1998 : Selenium on Nicotinamide phosphoribosyltransferase
rs705415
rs921943
rs558133
rs10944
rs672413
rs567754
rs11951068
rs6586282
rs3797535
rs6859667
rs234709
All
−0.10
−0.05
0.00
0.05
0.10
MR effect size for
'Selenium' on 'Nicotinamide phosphoribosyltransferase'

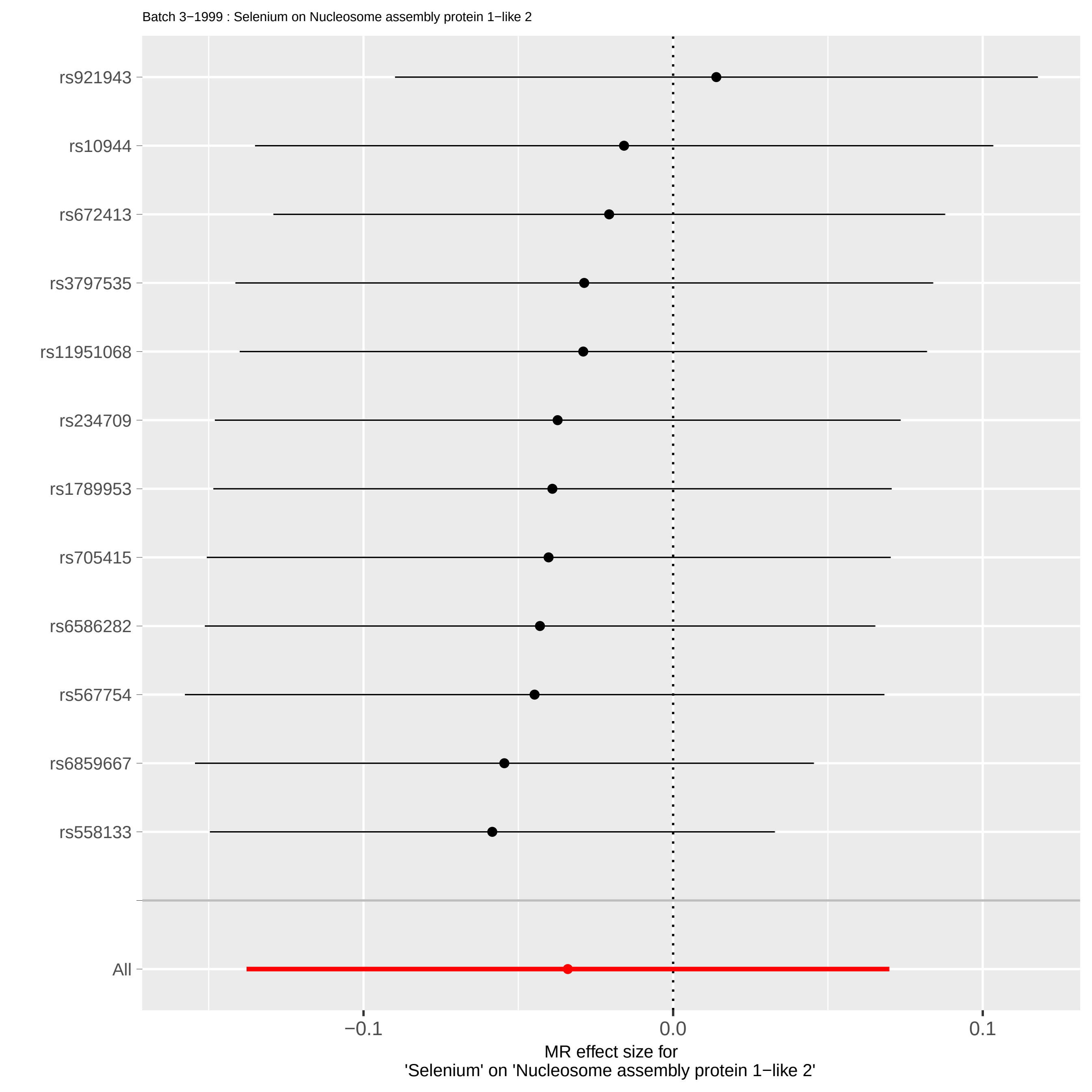
Batch 3−1999 : Selenium on Nucleosome assembly protein 1−like 2
rs921943
rs10944
rs672413
rs3797535
rs11951068
rs234709
rs1789953
rs705415
rs6586282
rs567754
rs6859667
rs558133
All
−0.1
0.0
0.1
MR effect size for
'Selenium' on 'Nucleosome assembly protein 1−like 2'

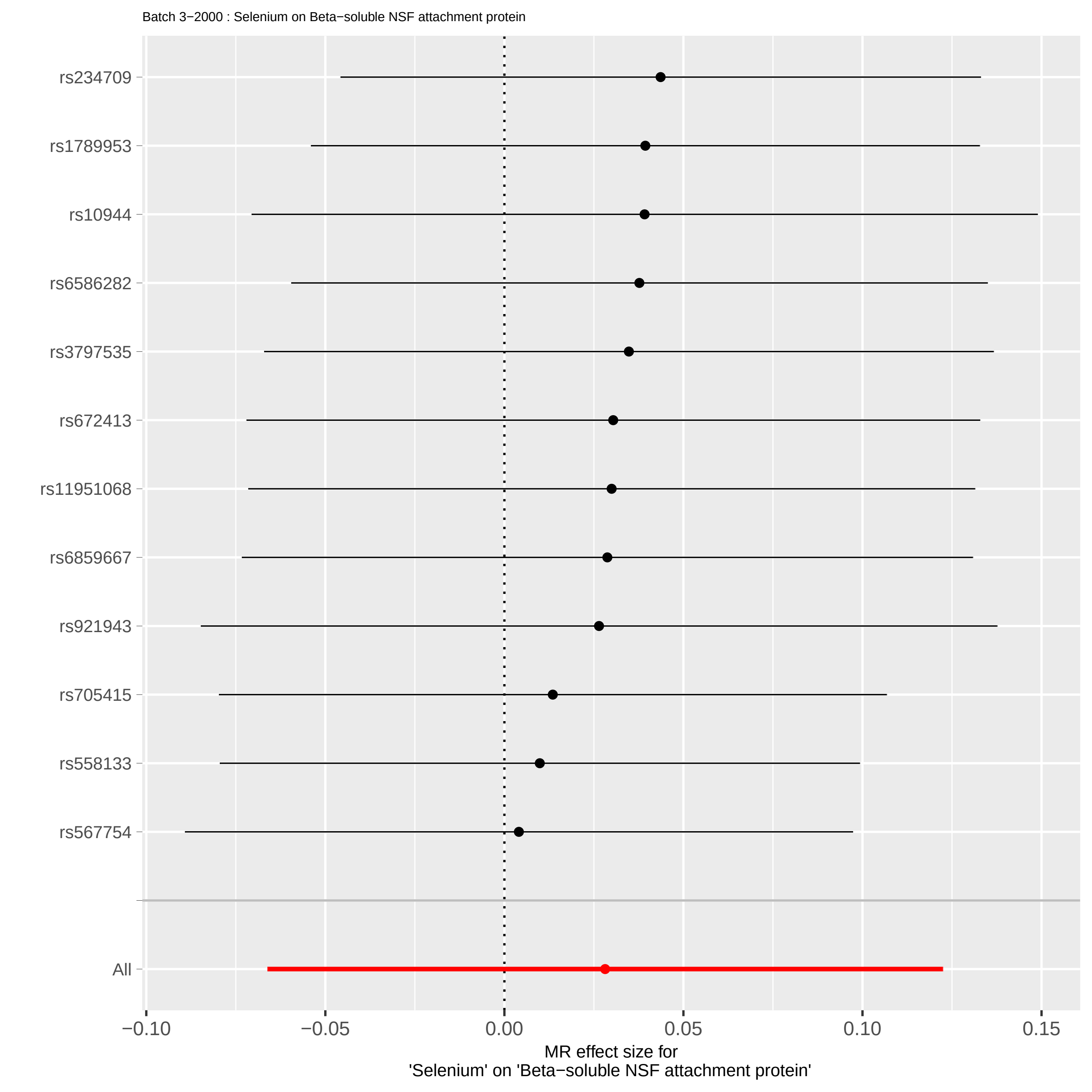

Batch 3−2000 : Selenium on Beta−soluble NSF attachment protein
rs234709
rs1789953
rs10944
rs6586282
rs3797535
rs672413
rs11951068
rs6859667
rs921943
rs705415
rs558133
rs567754
All
−0.10
−0.05
0.00
0.05
0.10
0.15
MR effect size for
'Selenium' on 'Beta−soluble NSF attachment protein'

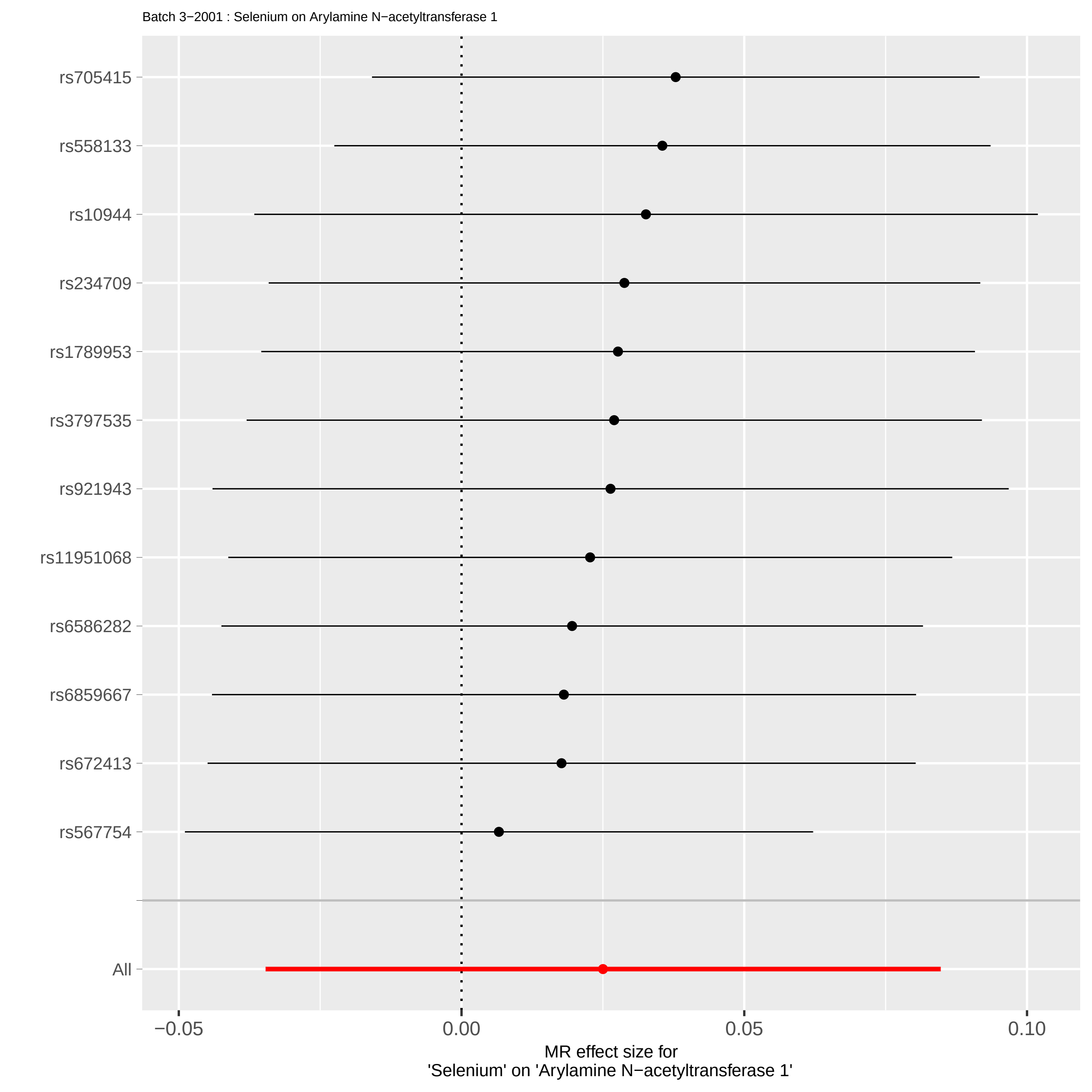

Batch 3−2001 : Selenium on Arylamine N−acetyltransferase 1
rs705415
rs558133
rs10944
rs234709
rs1789953
rs3797535
rs921943
rs11951068
rs6586282
rs6859667
rs672413
rs567754
All
−0.05
0.00
0.05
0.10
MR effect size for
'Selenium' on 'Arylamine N−acetyltransferase 1'

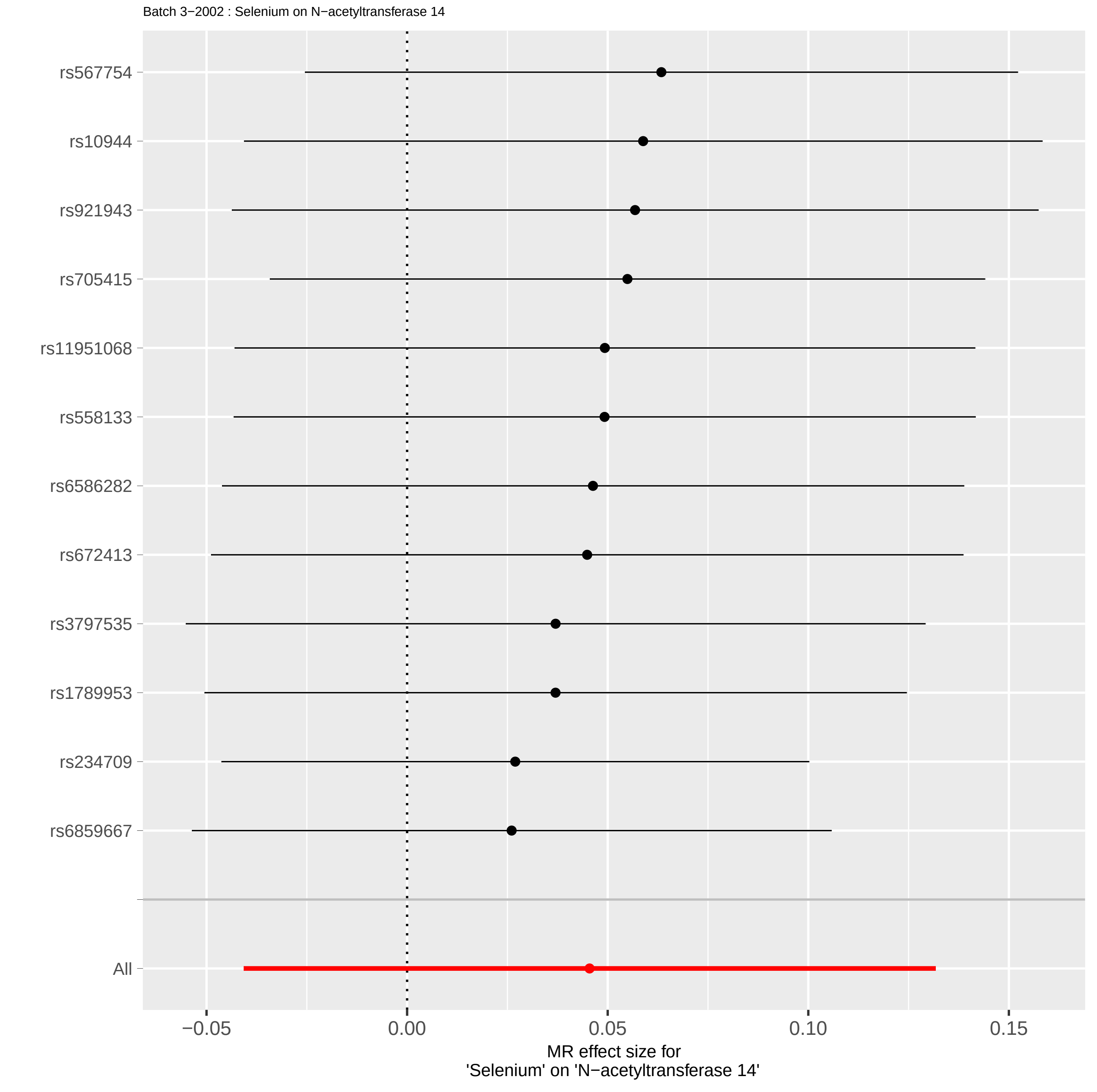

Batch 3−2002 : Selenium on N−acetyltransferase 14
rs567754
rs10944
rs921943
rs705415
rs11951068
rs558133
rs6586282
rs672413
rs3797535
rs1789953
rs234709
rs6859667
All
−0.05
0.00
0.05
0.10
0.15
MR effect size for
'Selenium' on 'N−acetyltransferase 14'

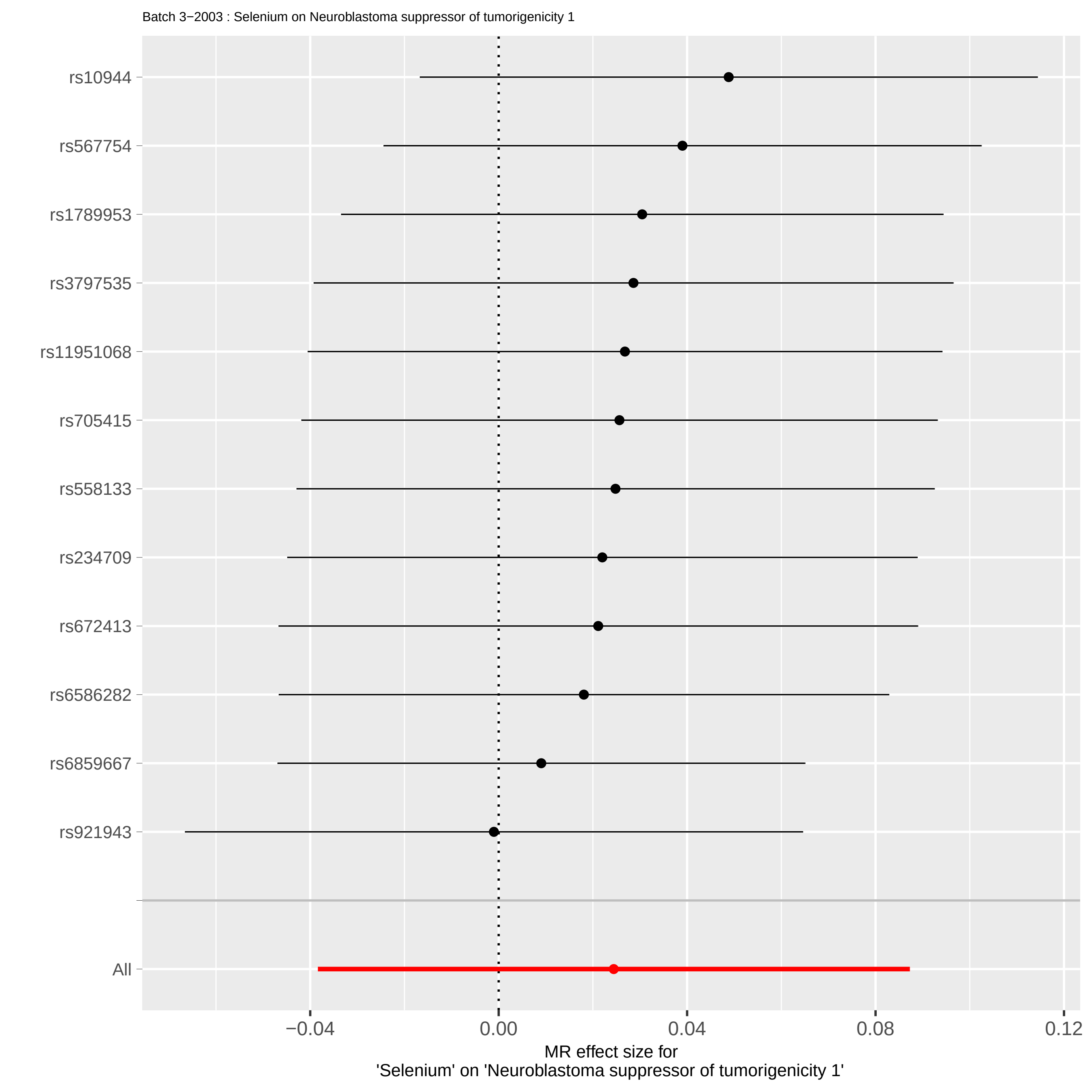

Batch 3−2003 : Selenium on Neuroblastoma suppressor of tumorigenicity 1
rs10944
rs567754
rs1789953
rs3797535
rs11951068
rs705415
rs558133
rs234709
rs672413
rs6586282
rs6859667
rs921943
All
−0.04
0.00
0.04
0.08
0.12
MR effect size for
'Selenium' on 'Neuroblastoma suppressor of tumorigenicity 1'

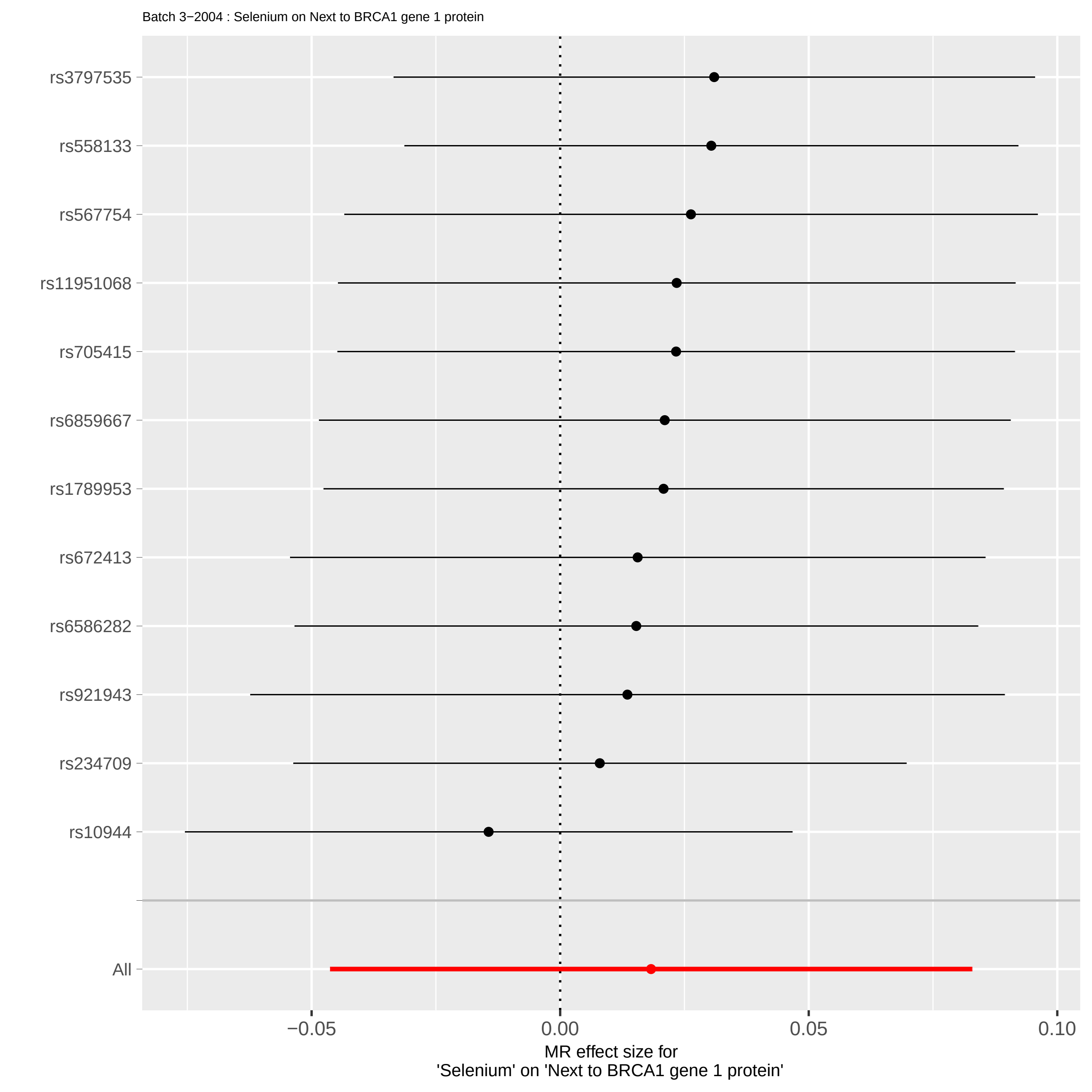
Batch 3−2004 : Selenium on Next to BRCA1 gene 1 protein
rs3797535
rs558133
rs567754
rs11951068
rs705415
rs6859667
rs1789953
rs672413
rs6586282
rs921943
rs234709
rs10944
All
−0.05
0.00
0.05
0.10
MR effect size for
'Selenium' on 'Next to BRCA1 gene 1 protein'

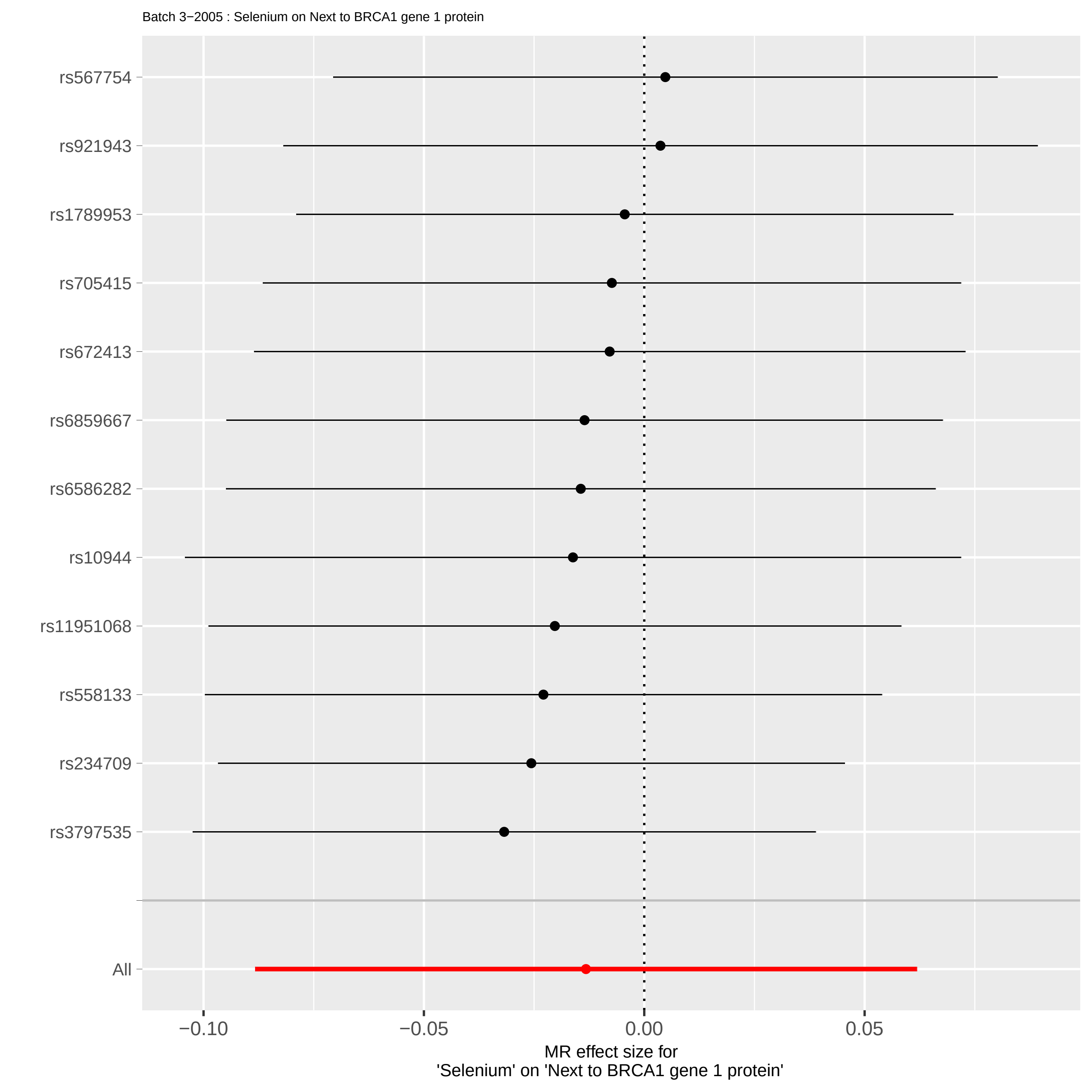
Batch 3−2005 : Selenium on Next to BRCA1 gene 1 protein
rs567754
rs921943
rs1789953
rs705415
rs672413
rs6859667
rs6586282
rs10944
rs11951068
rs558133
rs234709
rs3797535
All
−0.10
−0.05
0.00
0.05
MR effect size for
'Selenium' on 'Next to BRCA1 gene 1 protein'

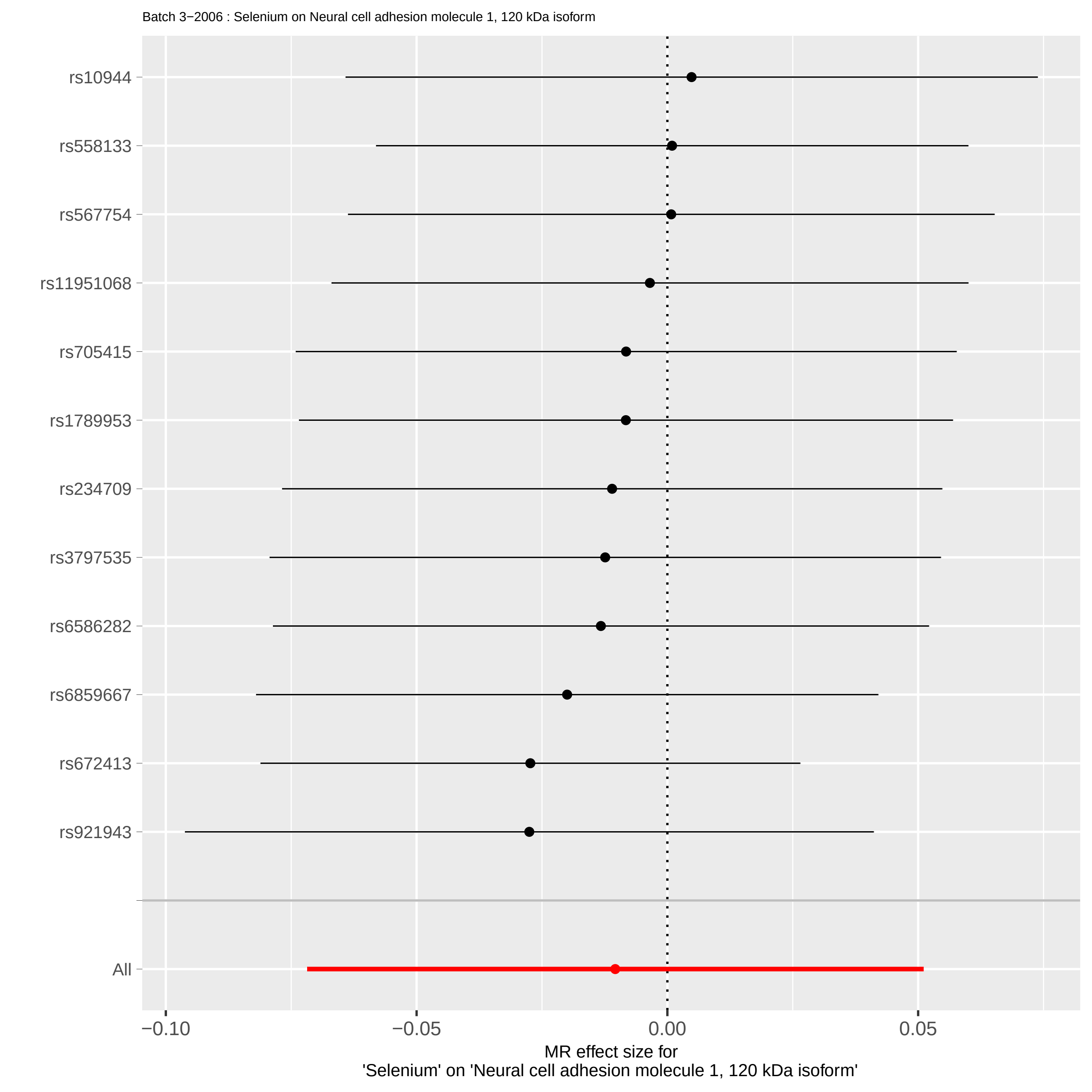

Batch 3−2006 : Selenium on Neural cell adhesion molecule 1, 120 kDa isoform
rs10944
rs558133
rs567754
rs11951068
rs705415
rs1789953
rs234709
rs3797535
rs6586282
rs6859667
rs672413
rs921943
All
−0.10
−0.05
0.00
0.05
MR effect size for
'Selenium' on 'Neural cell adhesion molecule 1, 120 kDa isoform'

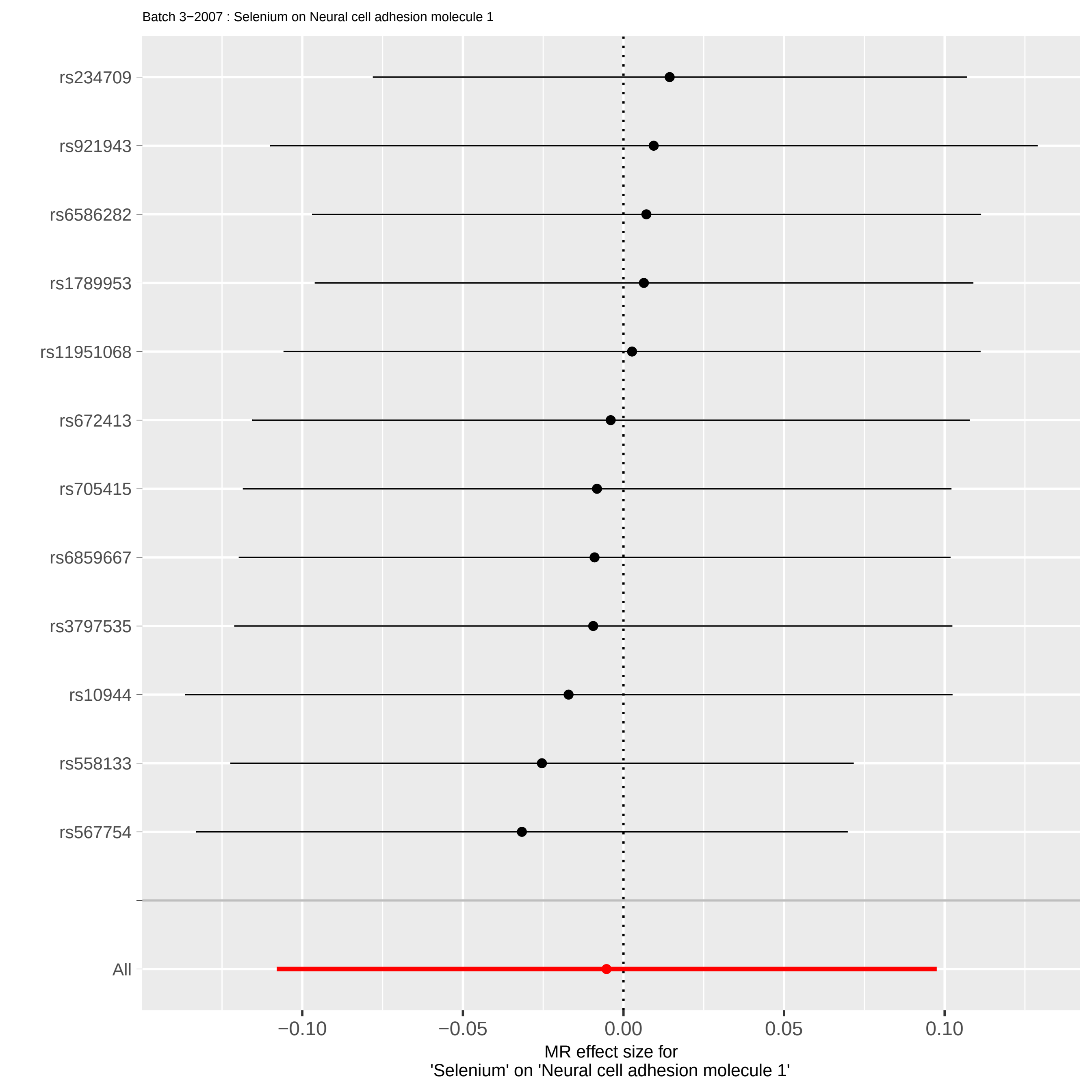
Batch 3−2007 : Selenium on Neural cell adhesion molecule 1
rs234709
rs921943
rs6586282
rs1789953
rs11951068
rs672413
rs705415
rs6859667
rs3797535
rs10944
rs558133
rs567754
All
−0.10
−0.05
0.00
0.05
0.10
MR effect size for
'Selenium' on 'Neural cell adhesion molecule 1'

Batch 3−2008 : Selenium on Neural cell adhesion molecule 2

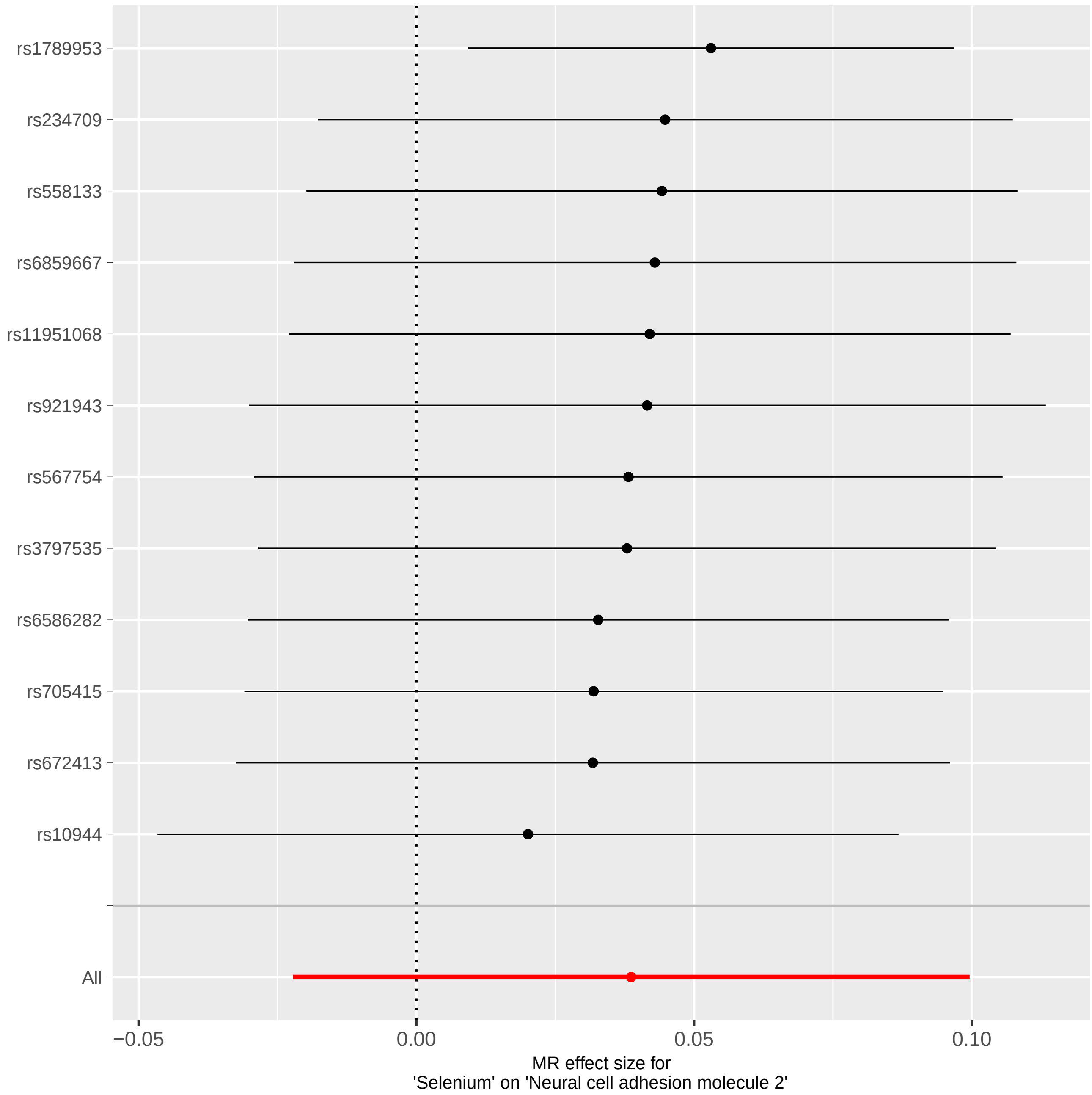

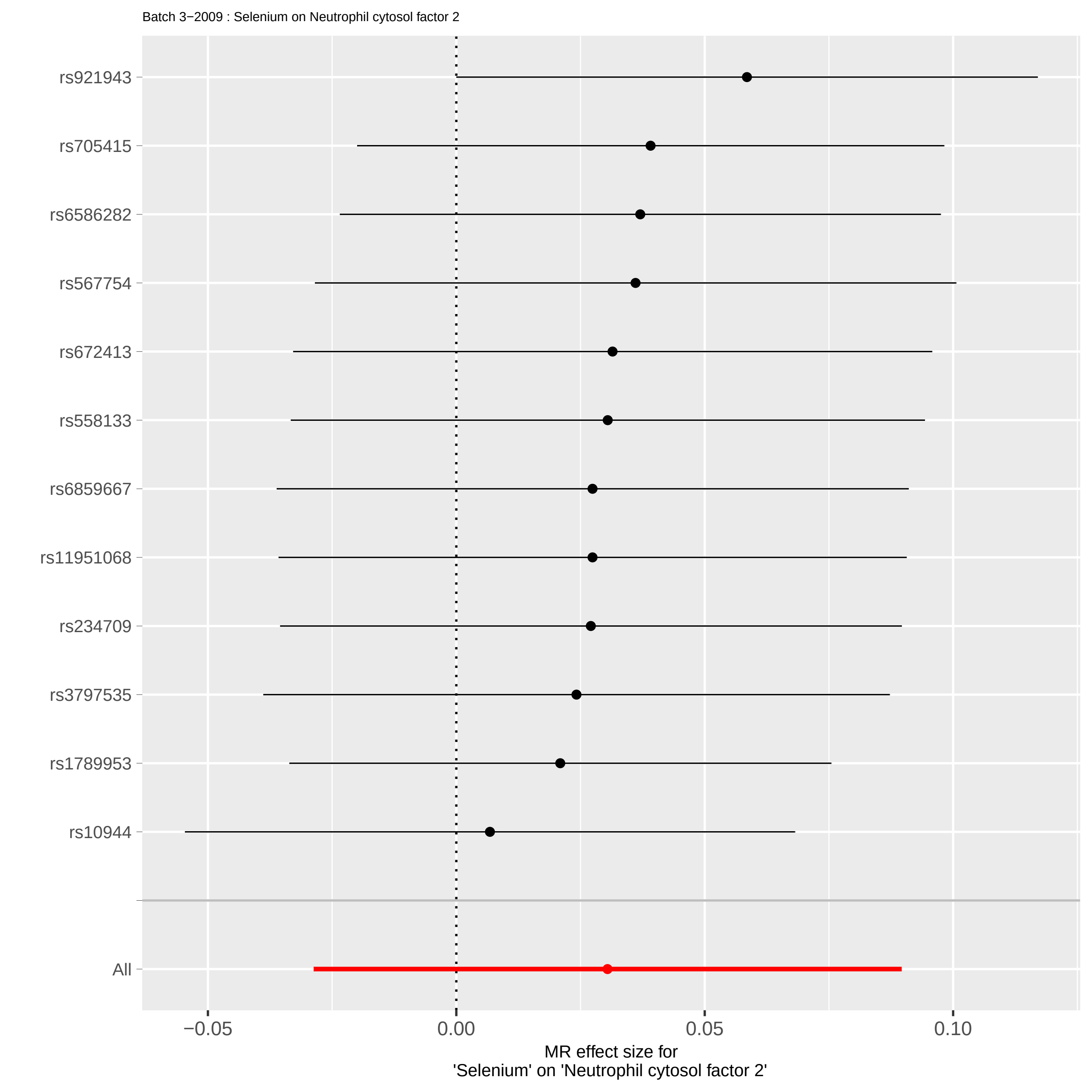

Batch 3−2009 : Selenium on Neutrophil cytosol factor 2
rs921943
rs705415
rs6586282
rs567754
rs672413
rs558133
rs6859667
rs11951068
rs234709
rs3797535
rs1789953
rs10944
All
−0.05
0.00
0.05
0.10
MR effect size for
'Selenium' on 'Neutrophil cytosol factor 2'

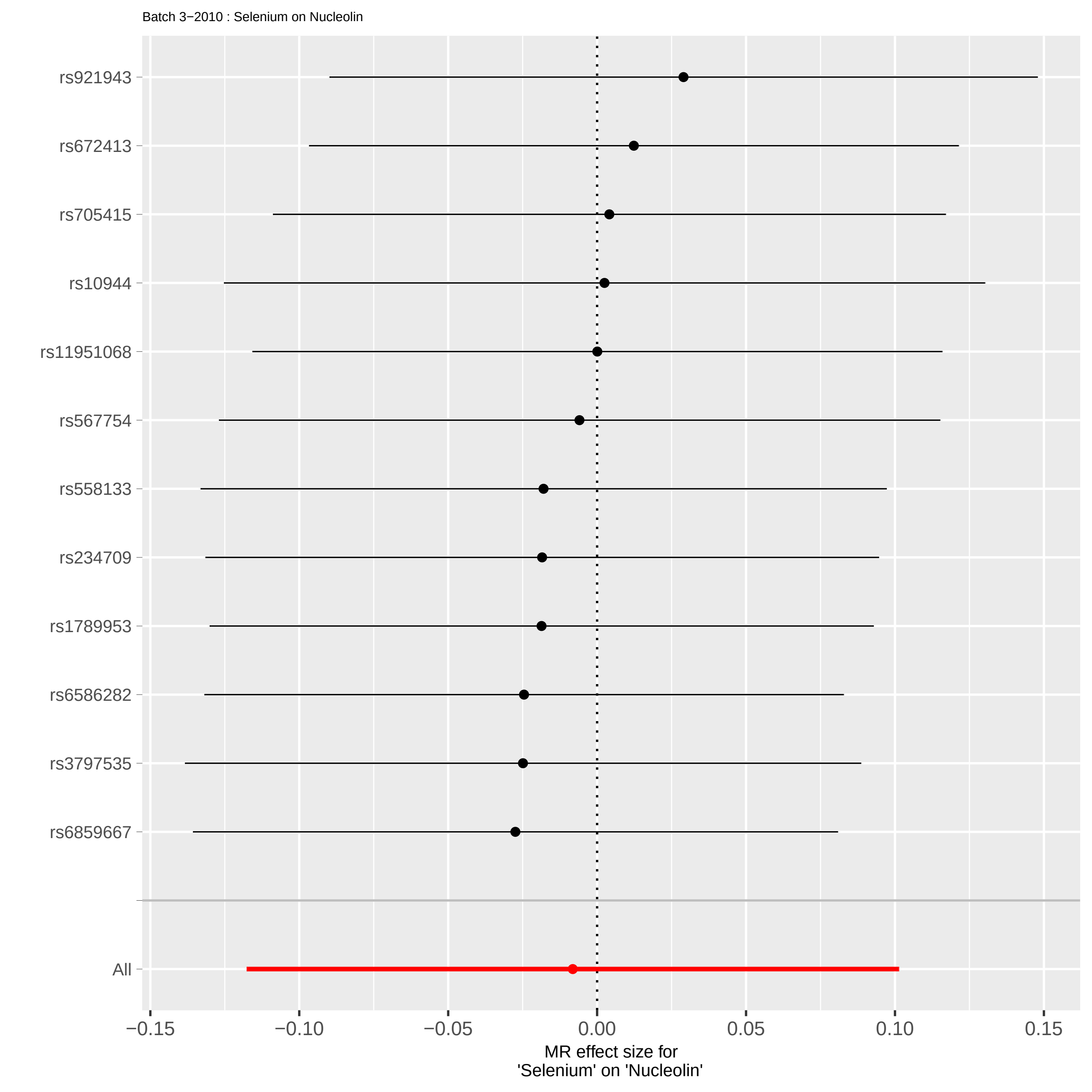
Batch 3−2010 : Selenium on Nucleolin
rs921943
rs672413
rs705415
rs10944
rs11951068
rs567754
rs558133
rs234709
rs1789953
rs6586282
rs3797535
rs6859667
All
−0.15
−0.10
−0.05
0.00
0.05
0.10
0.15
MR effect size for
'Selenium' on 'Nucleolin'

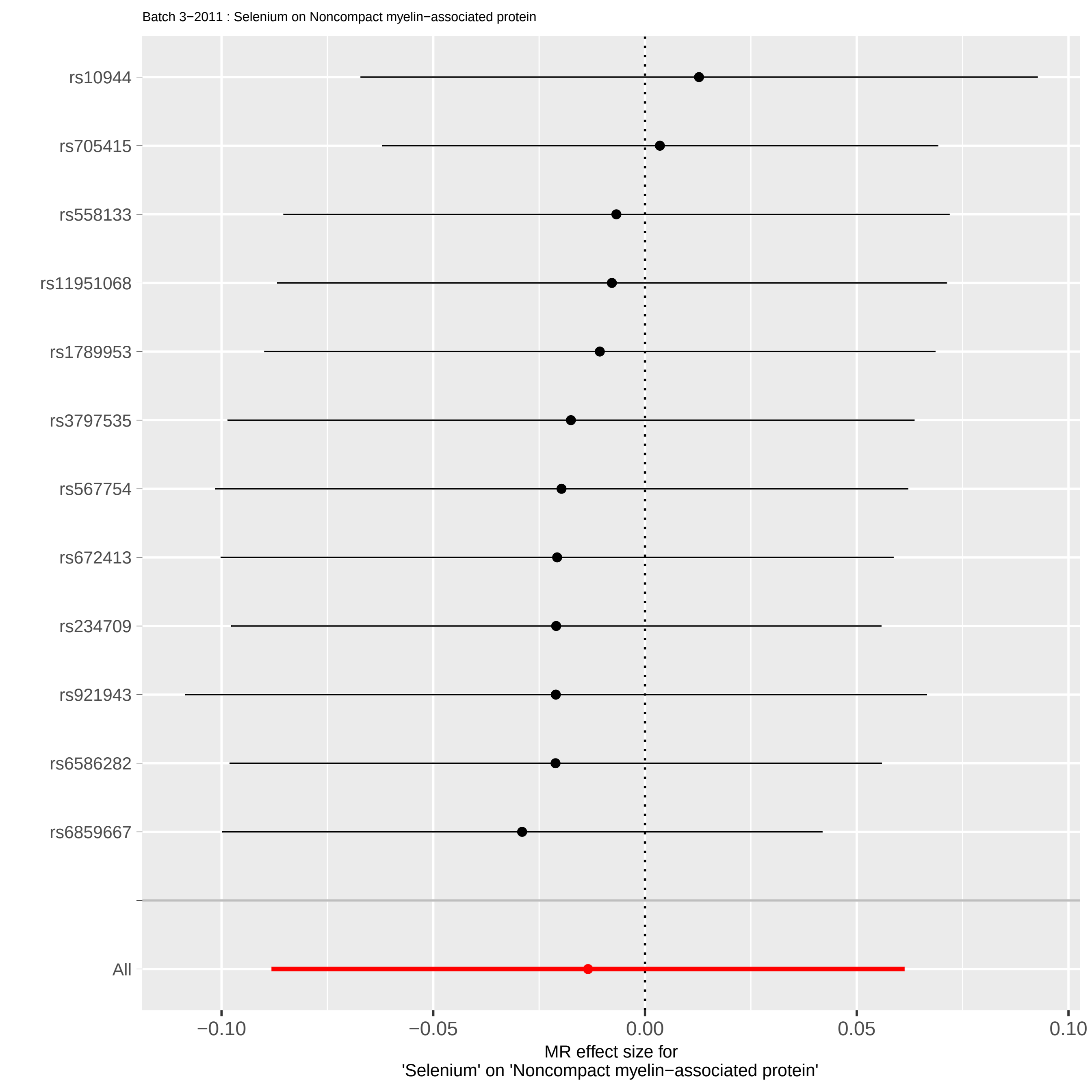

Batch 3−2011 : Selenium on Noncompact myelin−associated protein
rs10944
rs705415
rs558133
rs11951068
rs1789953
rs3797535
rs567754
rs672413
rs234709
rs921943
rs6586282
rs6859667
All
−0.10
−0.05
0.00
0.05
0.10
MR effect size for
'Selenium' on 'Noncompact myelin−associated protein'

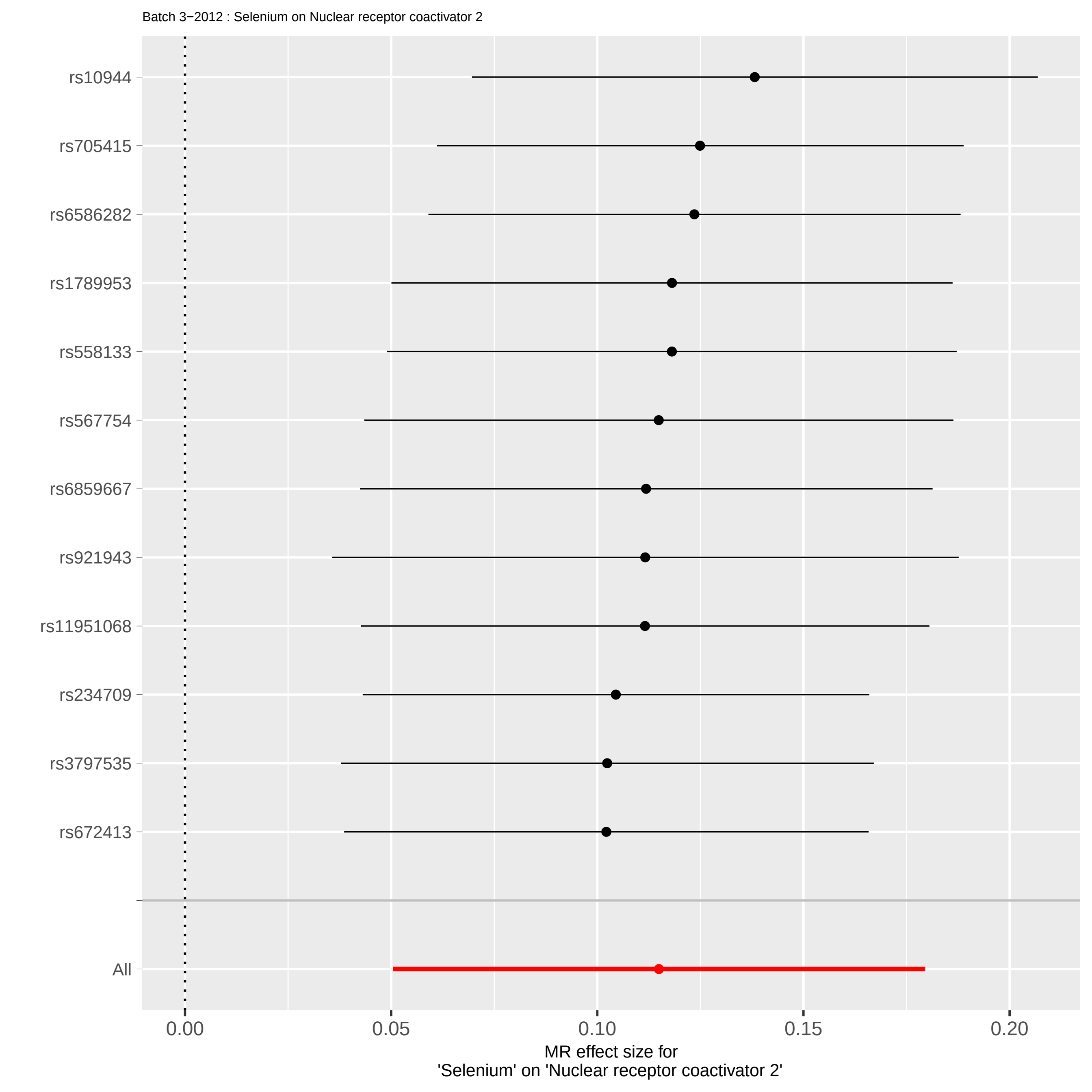

Batch 3−2012 : Selenium on Nuclear receptor coactivator 2
rs10944
rs705415
rs6586282
rs1789953
rs558133
rs567754
rs6859667
rs921943
rs11951068
rs234709
rs3797535
rs672413
All
0.00
0.05
0.10
0.15
0.20
MR effect size for
'Selenium' on 'Nuclear receptor coactivator 2'

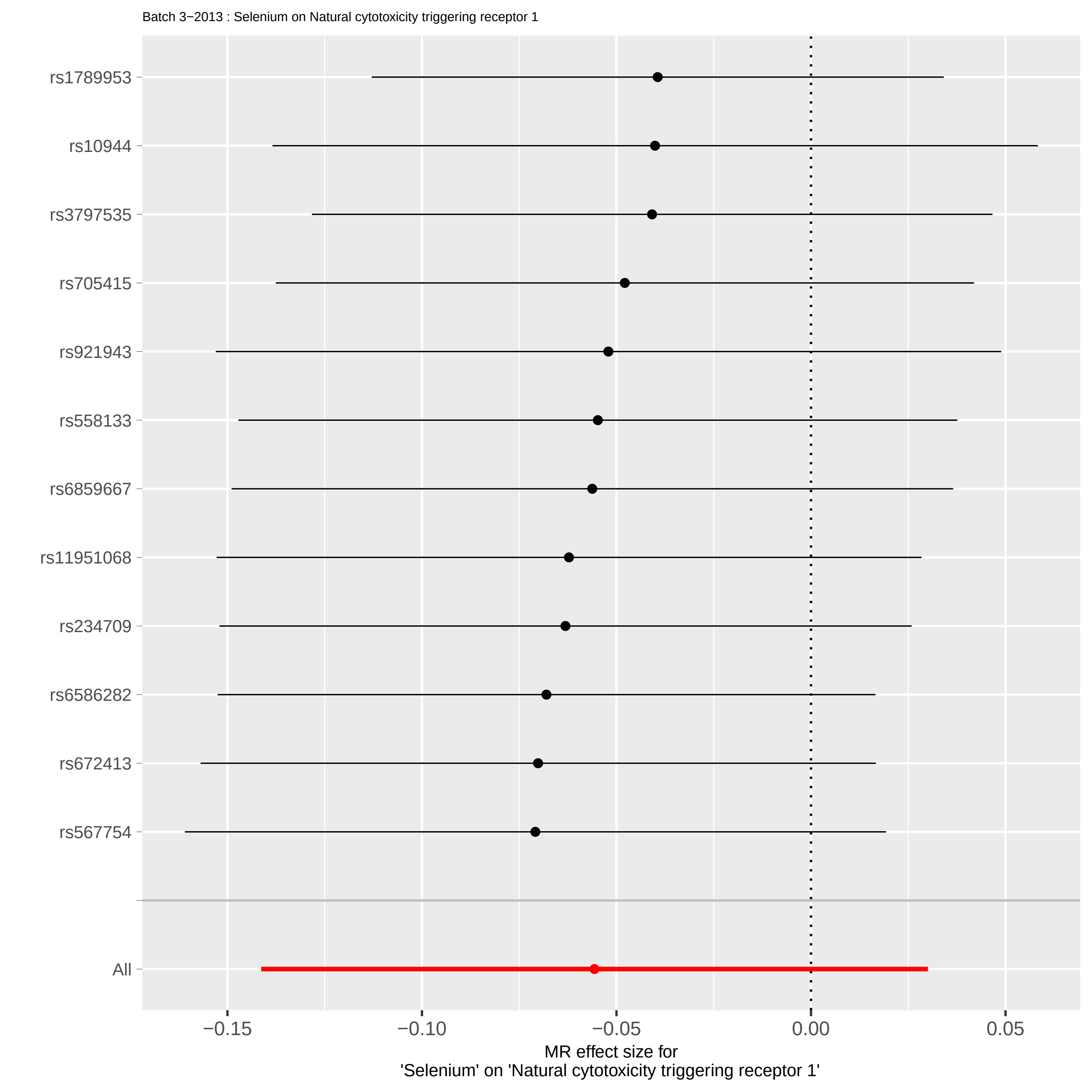
Batch 3−2013 : Selenium on Natural cytotoxicity triggering receptor 1
rs1789953
rs10944
rs3797535
rs705415
rs921943
rs558133
rs6859667
rs11951068
rs234709
rs6586282
rs672413
rs567754
All
−0.15
−0.10
−0.05
0.00
0.05
MR effect size for
'Selenium' on 'Natural cytotoxicity triggering receptor 1'

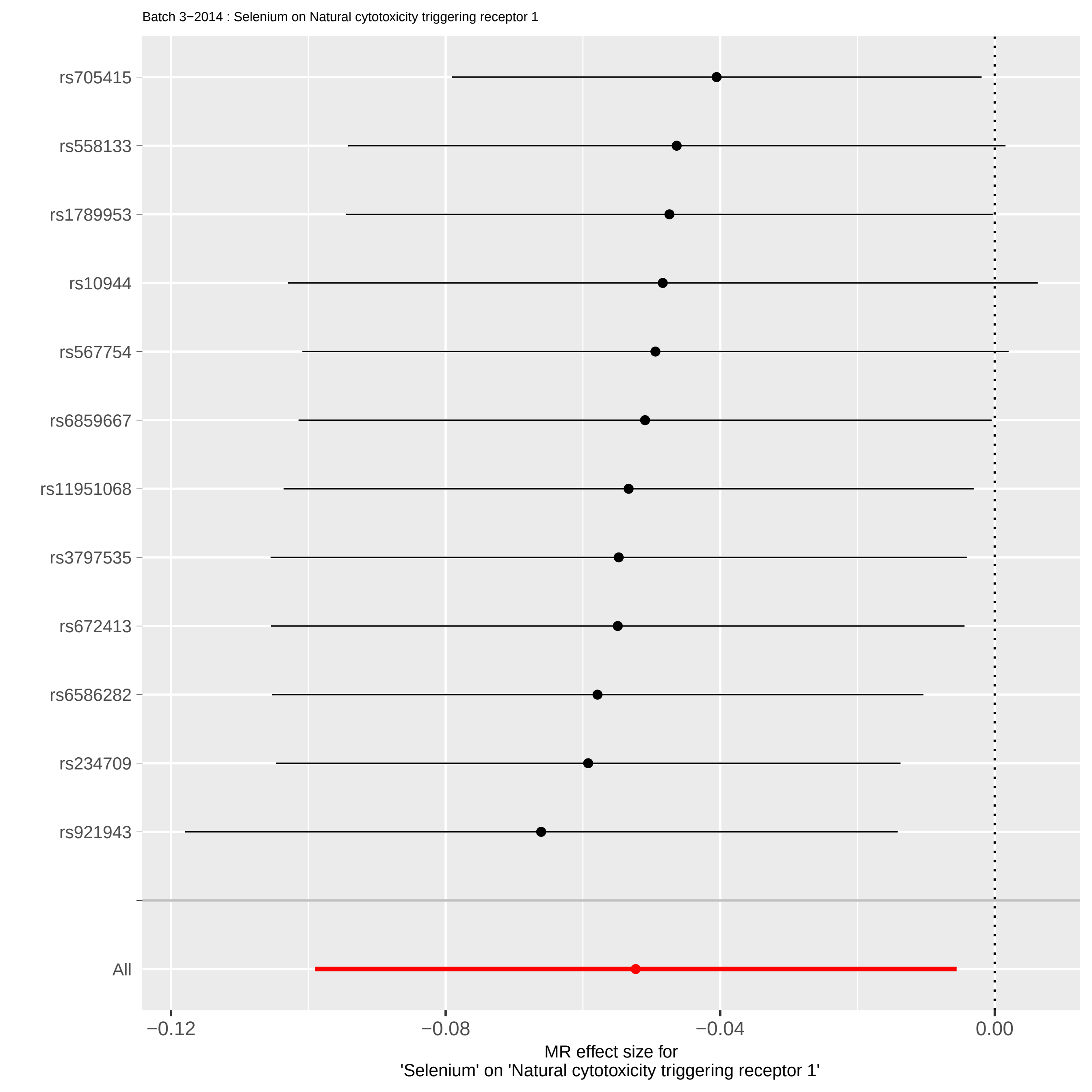
Batch 3−2014 : Selenium on Natural cytotoxicity triggering receptor 1
rs705415
rs558133
rs1789953
rs10944
rs567754
rs6859667
rs11951068
rs3797535
rs672413
rs6586282
rs234709
rs921943
All
−0.12
−0.08
−0.04
0.00
MR effect size for
'Selenium' on 'Natural cytotoxicity triggering receptor 1'

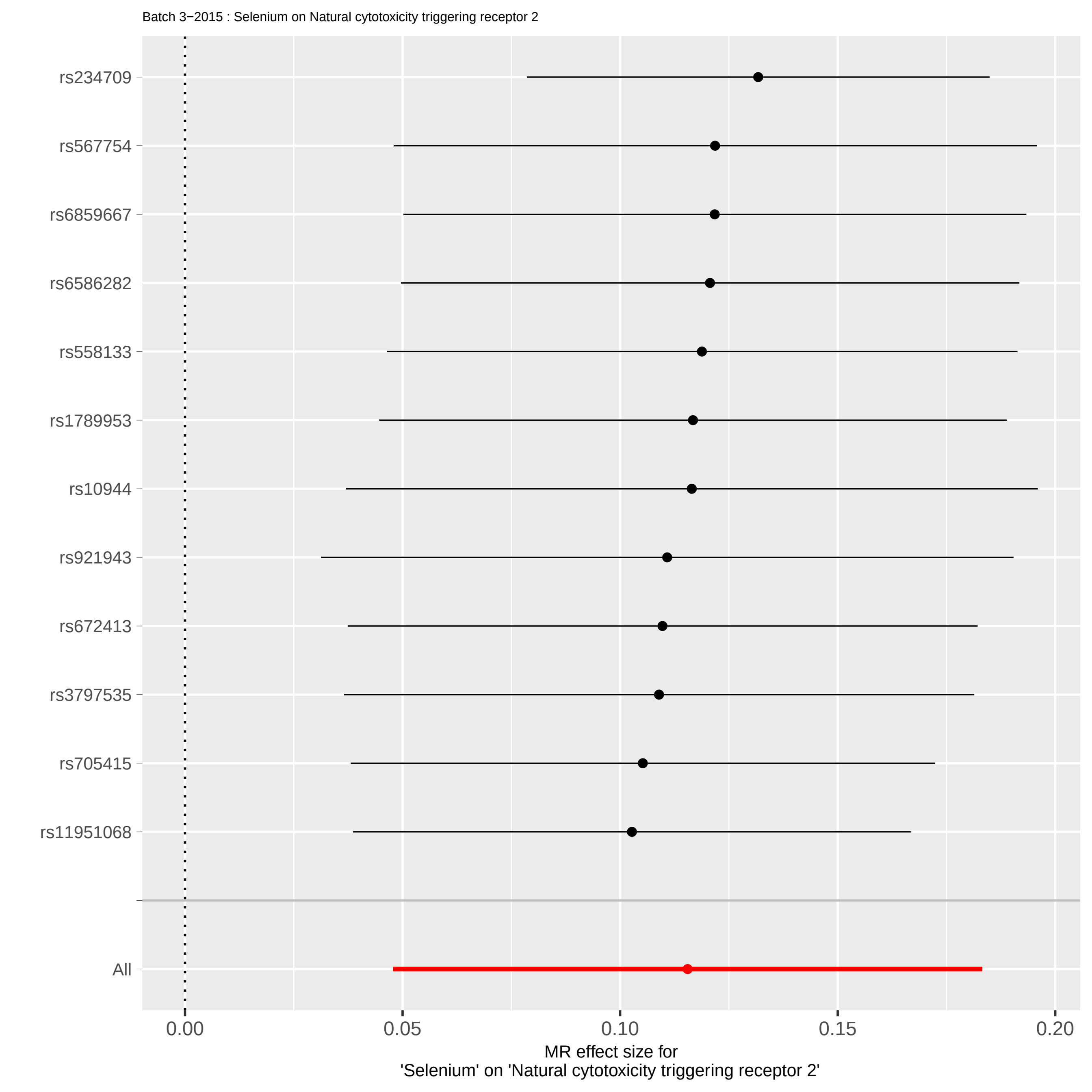

Batch 3−2015 : Selenium on Natural cytotoxicity triggering receptor 2
rs234709
rs567754
rs6859667
rs6586282
rs558133
rs1789953
rs10944
rs921943
rs672413
rs3797535
rs705415
rs11951068
All
0.00
0.05
0.10
0.15
0.20
MR effect size for
'Selenium' on 'Natural cytotoxicity triggering receptor 2'

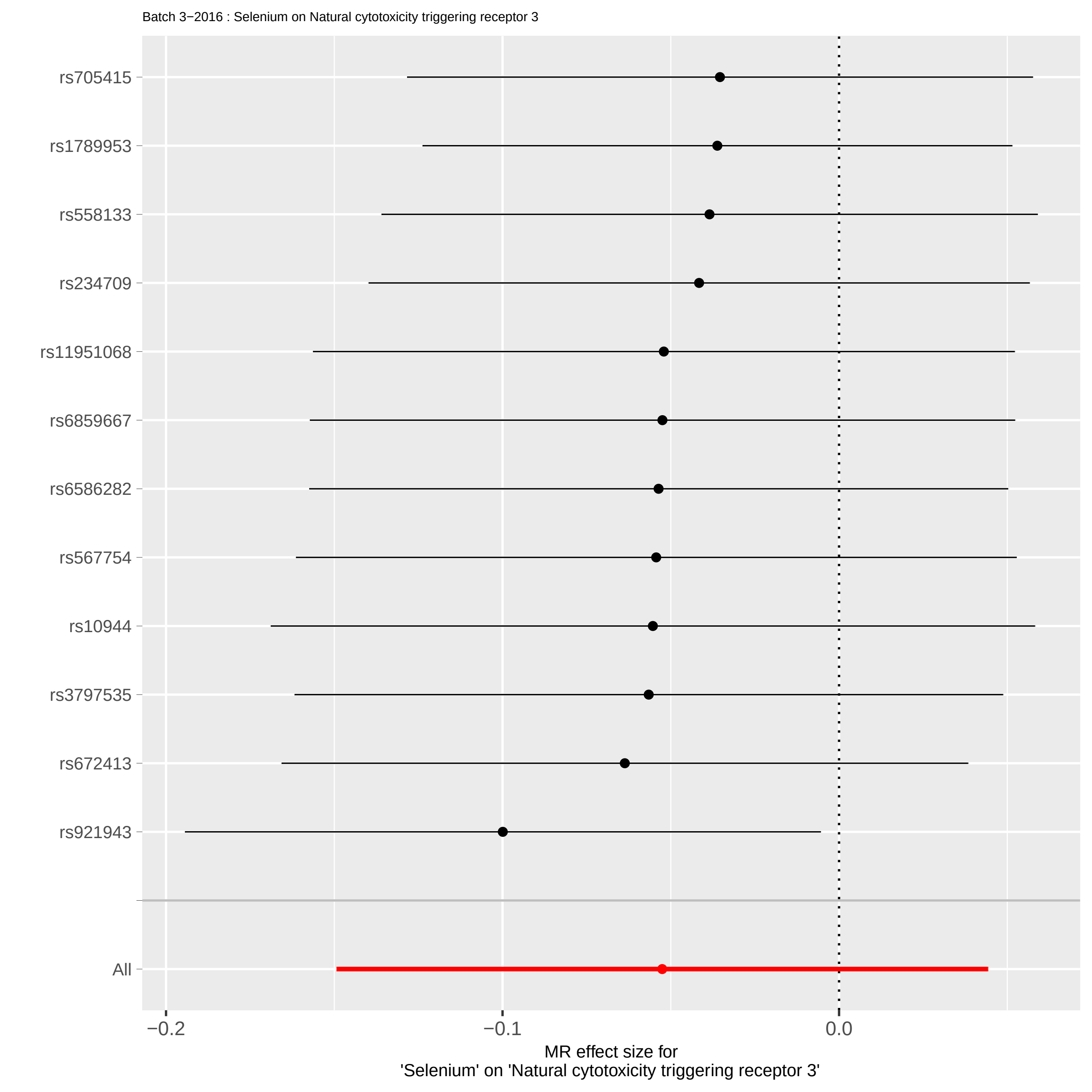

Batch 3−2016 : Selenium on Natural cytotoxicity triggering receptor 3
rs705415
rs1789953
rs558133
rs234709
rs11951068
rs6859667
rs6586282
rs567754
rs10944
rs3797535
rs672413
rs921943
All
−0.2
−0.1
0.0
MR effect size for
'Selenium' on 'Natural cytotoxicity triggering receptor 3'

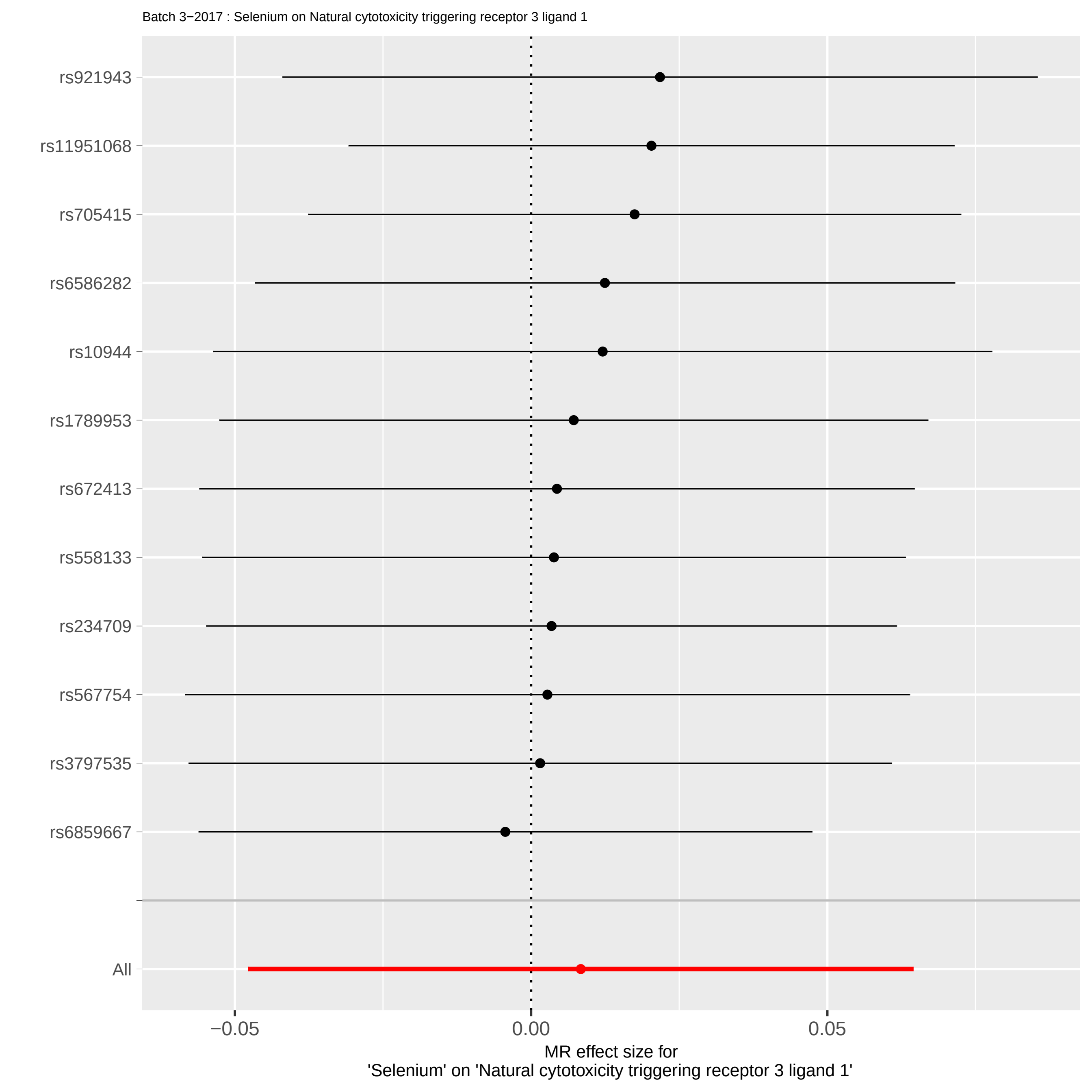

Batch 3−2017 : Selenium on Natural cytotoxicity triggering receptor 3 ligand 1
rs921943
rs11951068
rs705415
rs6586282
rs10944
rs1789953
rs672413
rs558133
rs234709
rs567754
rs3797535
rs6859667
All
−0.05
0.00
0.05
MR effect size for
'Selenium' on 'Natural cytotoxicity triggering receptor 3 ligand 1'

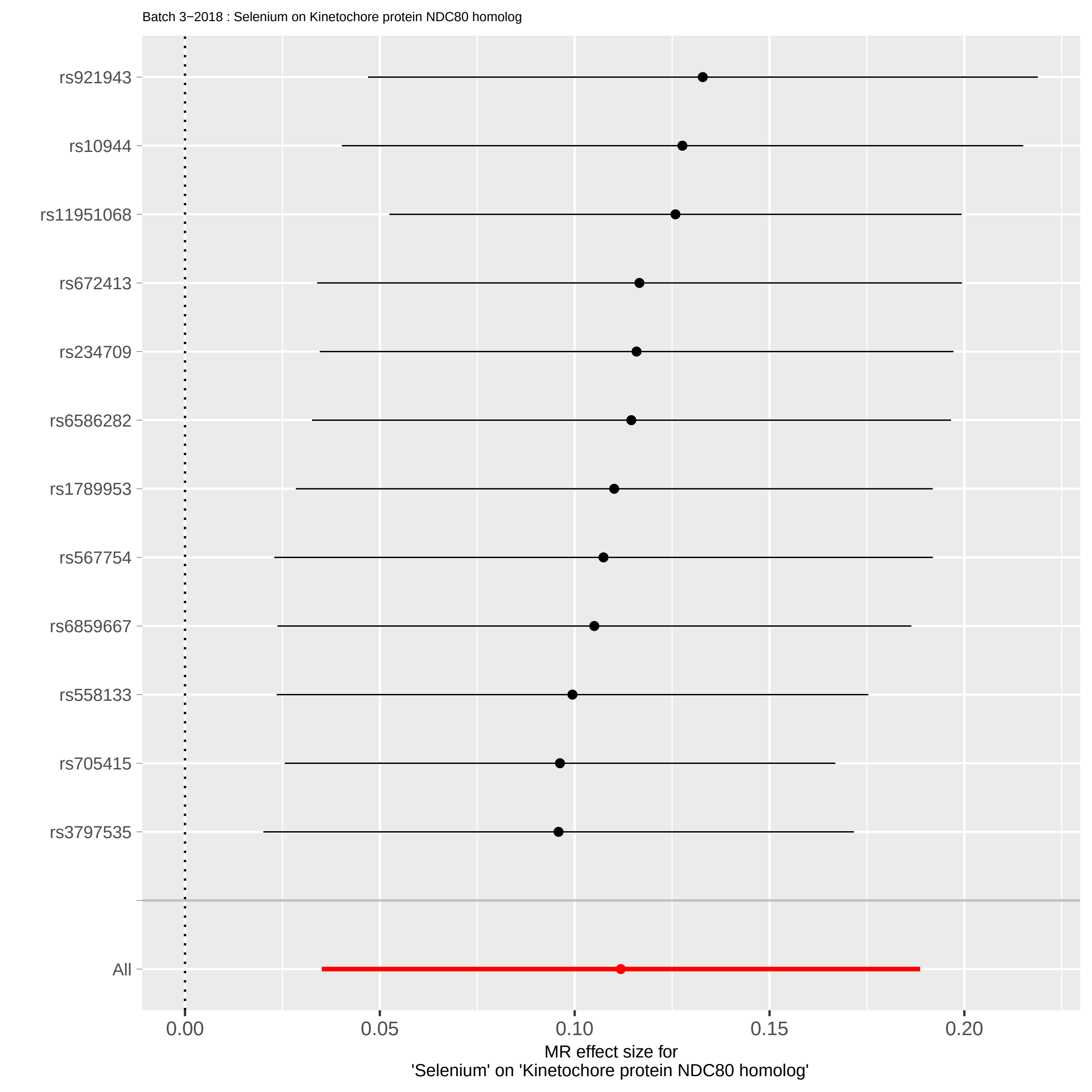
Batch 3−2018 : Selenium on Kinetochore protein NDC80 homolog
rs921943
rs10944
rs11951068
rs672413
rs234709
rs6586282
rs1789953
rs567754
rs6859667
rs558133
rs705415
rs3797535
All
0.00
0.05
0.10
0.15
0.20
MR effect size for
'Selenium' on 'Kinetochore protein NDC80 homolog'

Batch 3−2019 : Selenium on Protein NDNF

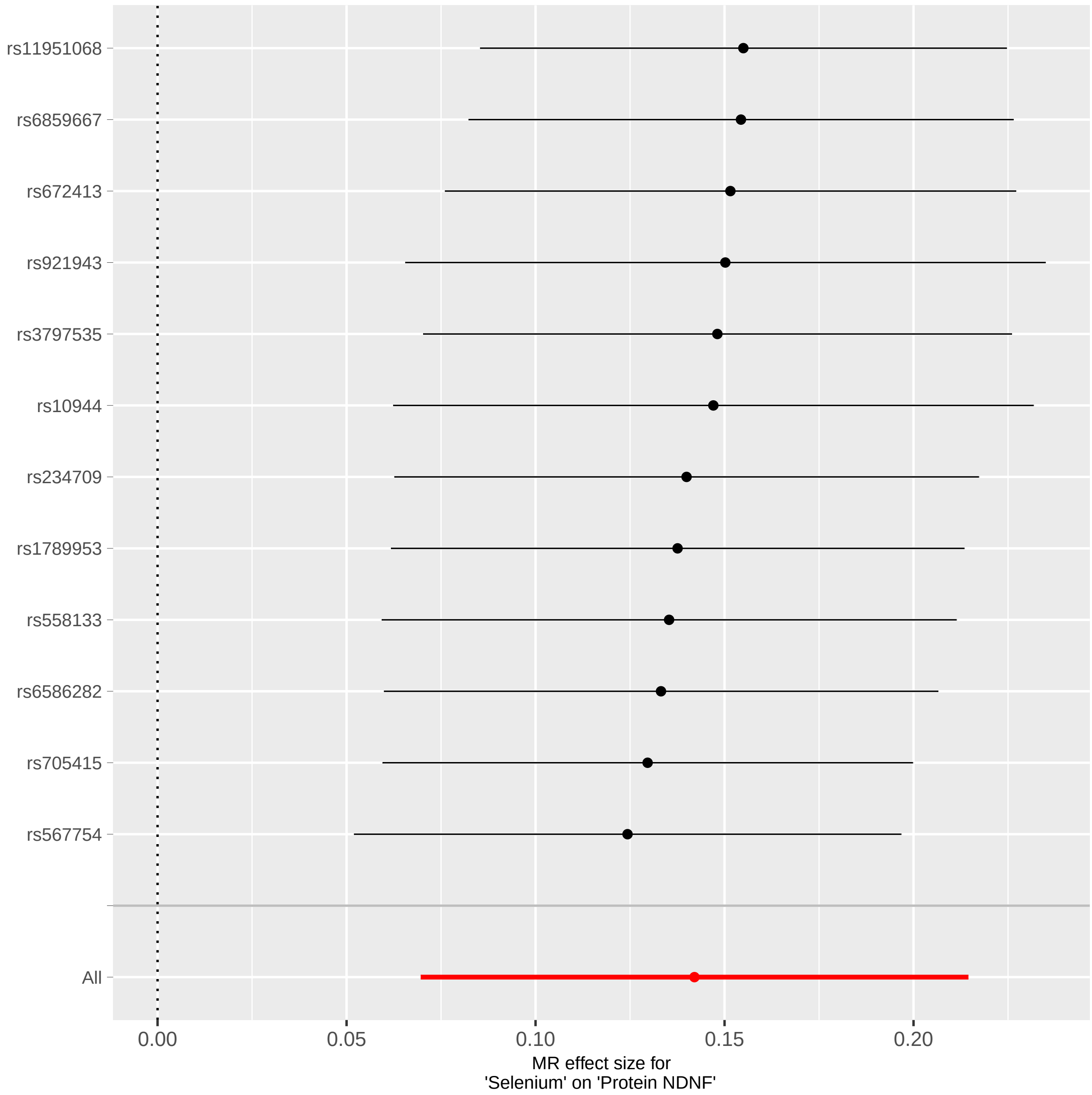

Batch 3−2020 : Selenium on Protein NDRG4

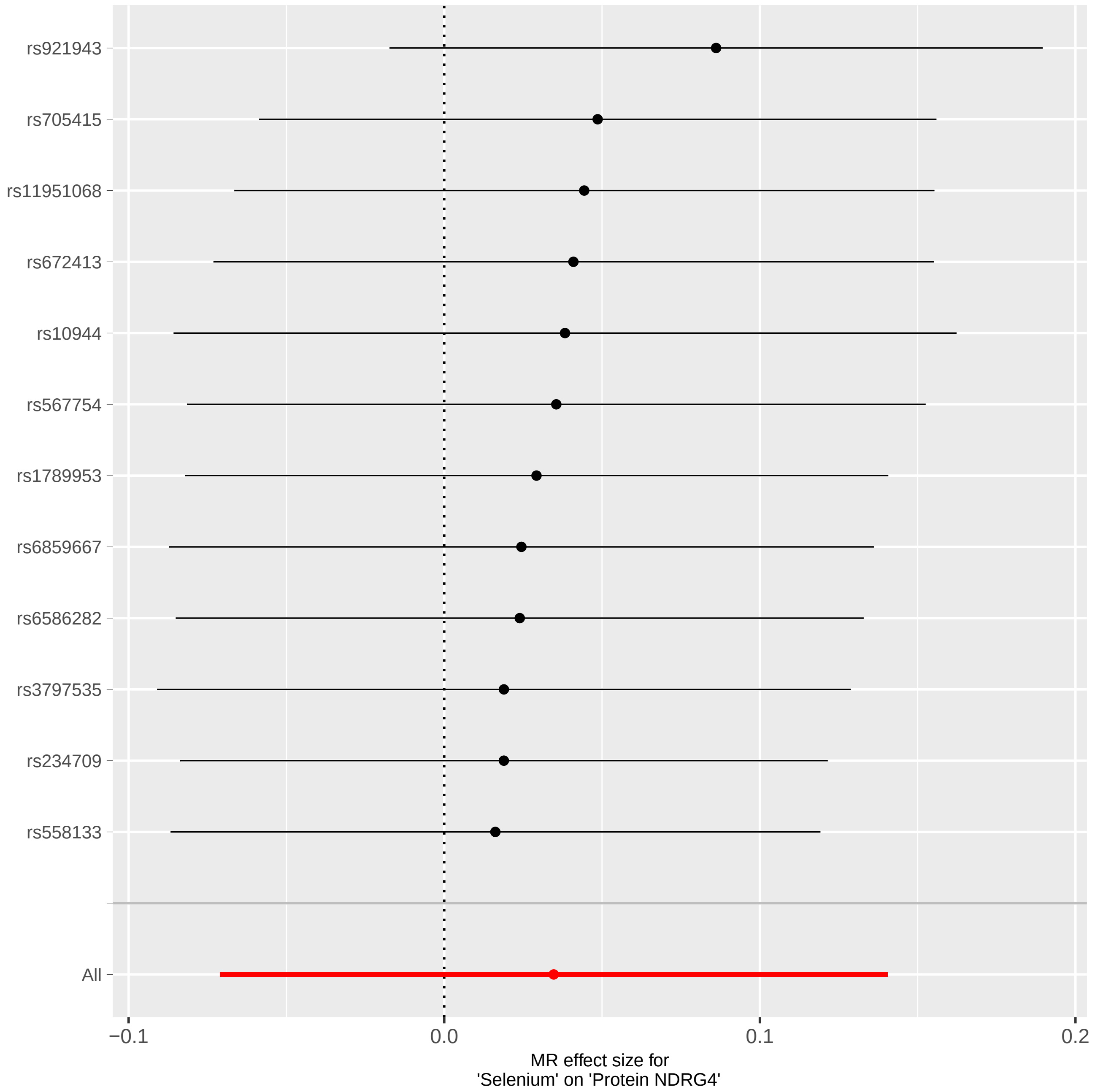

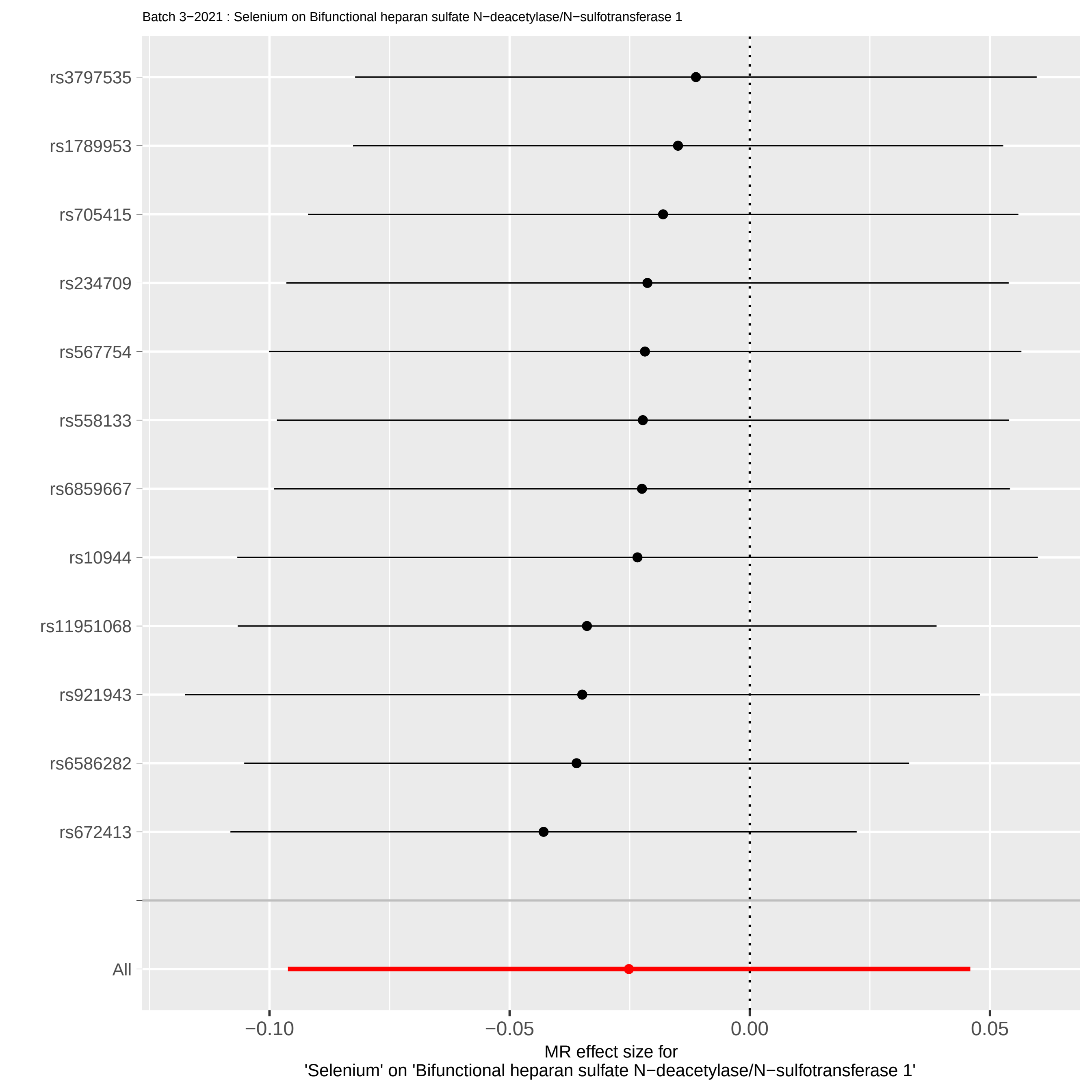
Batch 3−2021 : Selenium on Bifunctional heparan sulfate N−deacetylase/N−sulfotransferase 1
rs3797535
rs1789953
rs705415
rs234709
rs567754
rs558133
rs6859667
rs10944
rs11951068
rs921943
rs6586282
rs672413
All
−0.10
−0.05
0.00
0.05
MR effect size for
'Selenium' on 'Bifunctional heparan sulfate N−deacetylase/N−sulfotransferase 1'

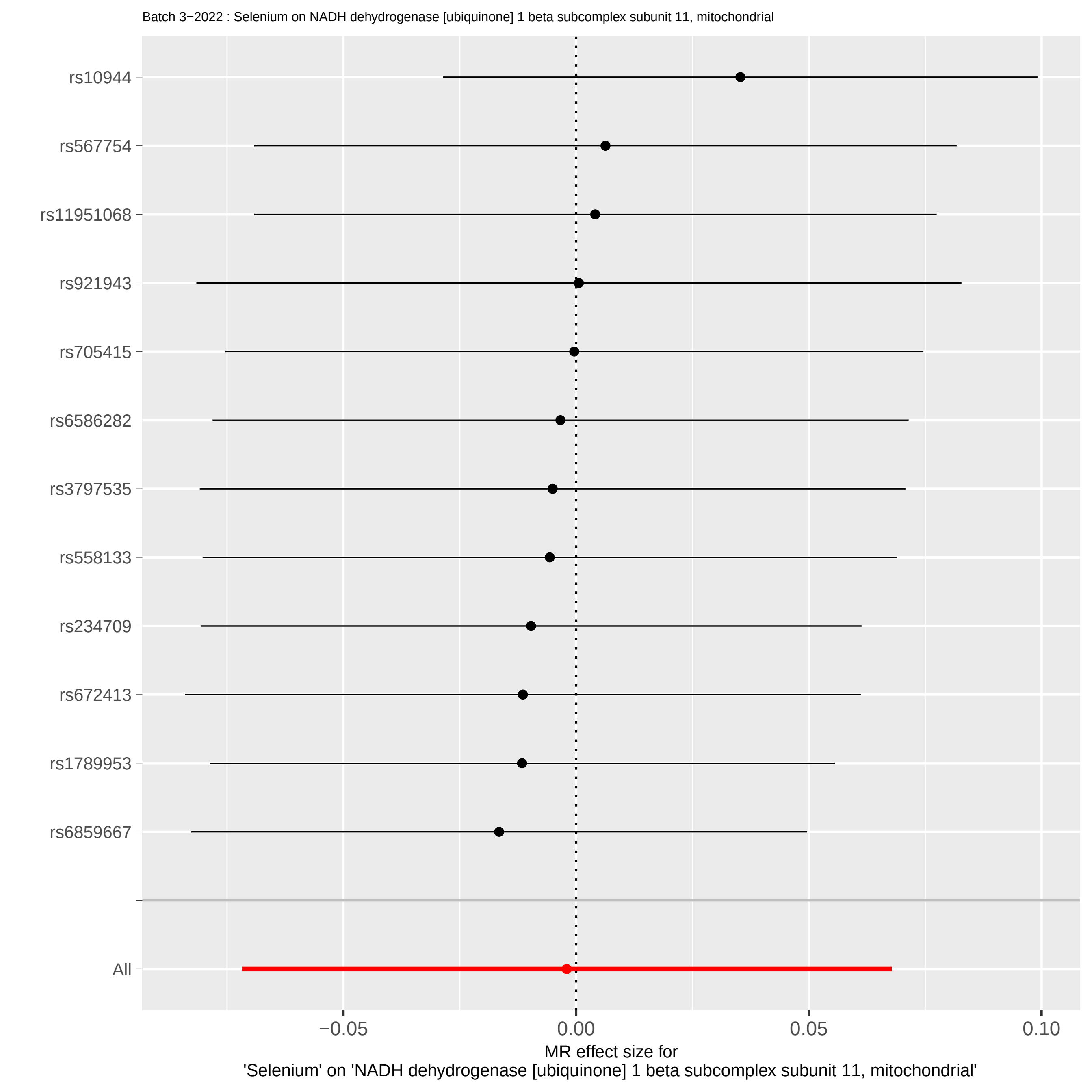
Batch 3−2022 : Selenium on NADH dehydrogenase [ubiquinone] 1 beta subcomplex subunit 11, mitochondrial
rs10944
rs567754
rs11951068
rs921943
rs705415
rs6586282
rs3797535
rs558133
rs234709
rs672413
rs1789953
rs6859667
All
−0.05
0.00
0.05
0.10
MR effect size for
'Selenium' on 'NADH dehydrogenase [ubiquinone] 1 beta subcomplex subunit 11, mitochondrial'

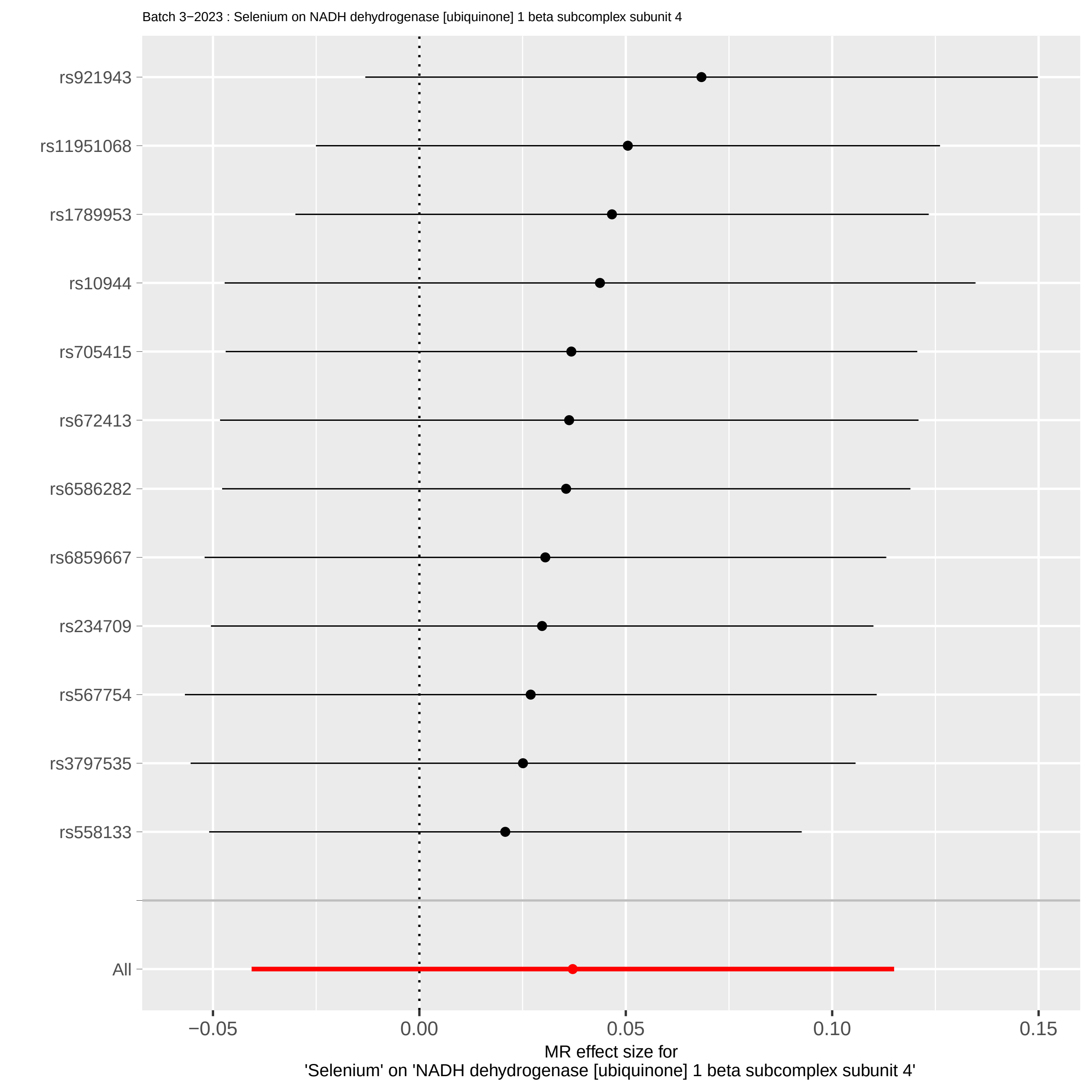

Batch 3−2023 : Selenium on NADH dehydrogenase [ubiquinone] 1 beta subcomplex subunit 4
rs921943
rs11951068
rs1789953
rs10944
rs705415
rs672413
rs6586282
rs6859667
rs234709
rs567754
rs3797535
rs558133
All
−0.05
0.00
0.05
0.10
0.15
MR effect size for
'Selenium' on 'NADH dehydrogenase [ubiquinone] 1 beta subcomplex subunit 4'

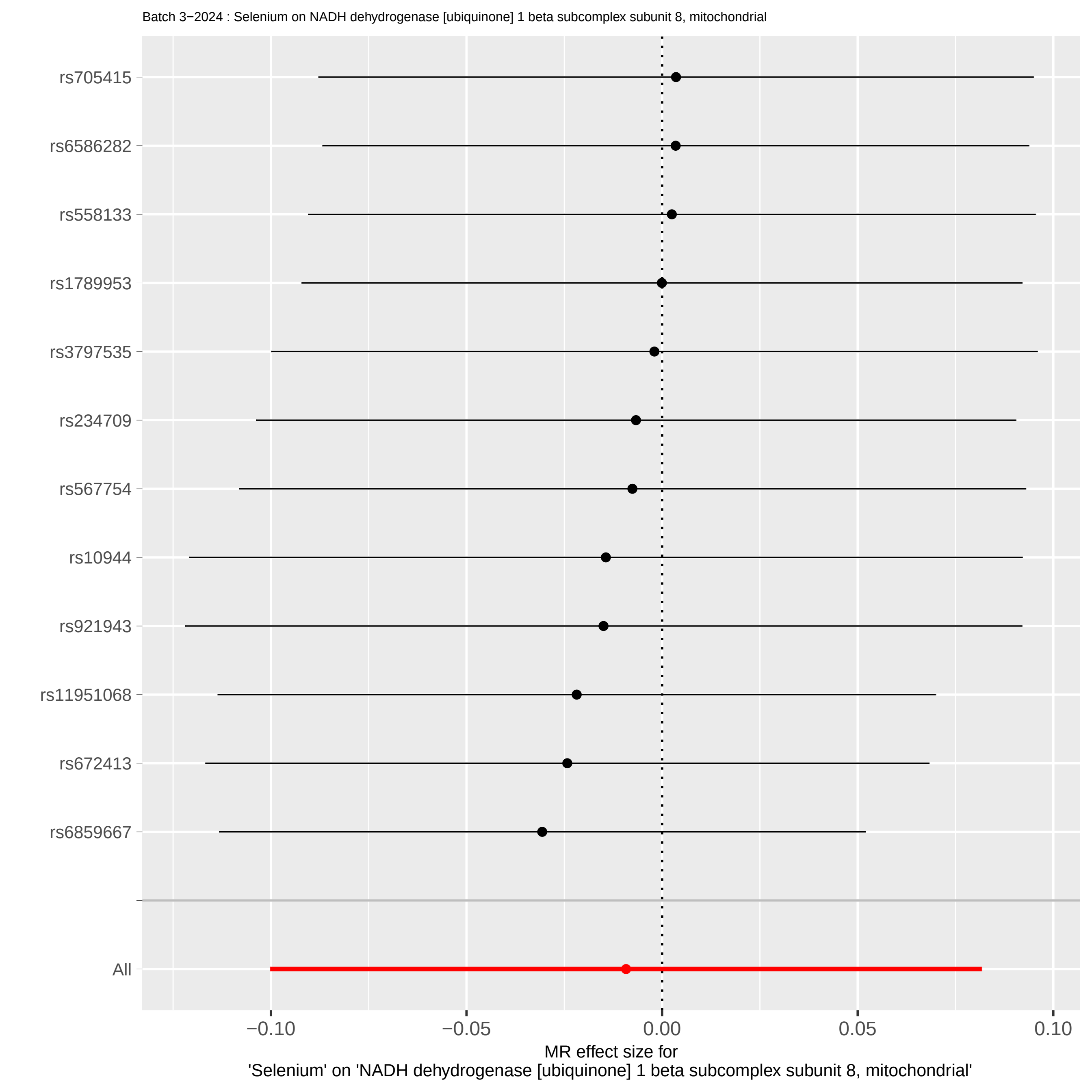

Batch 3−2024 : Selenium on NADH dehydrogenase [ubiquinone] 1 beta subcomplex subunit 8, mitochondrial
rs705415
rs6586282
rs558133
rs1789953
rs3797535
rs234709
rs567754
rs10944
rs921943
rs11951068
rs672413
rs6859667
All
−0.10
−0.05
0.00
0.05
0.10
MR effect size for
'Selenium' on 'NADH dehydrogenase [ubiquinone] 1 beta subcomplex subunit 8, mitochondrial'

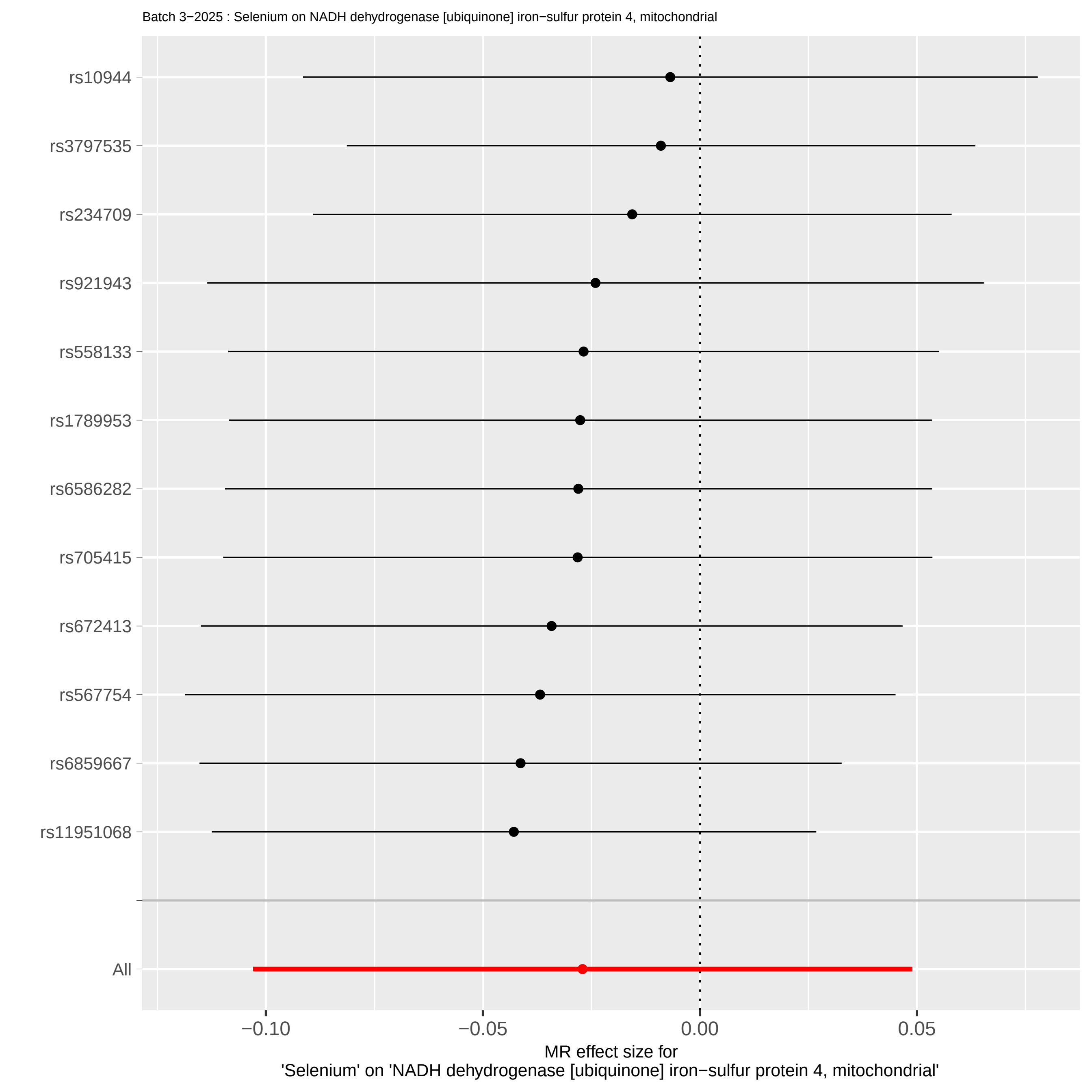

Batch 3−2025 : Selenium on NADH dehydrogenase [ubiquinone] iron−sulfur protein 4, mitochondrial
rs10944
rs3797535
rs234709
rs921943
rs558133
rs1789953
rs6586282
rs705415
rs672413
rs567754
rs6859667
rs11951068
All
−0.10
−0.05
0.00
0.05
MR effect size for
'Selenium' on 'NADH dehydrogenase [ubiquinone] iron−sulfur protein 4, mitochondrial'

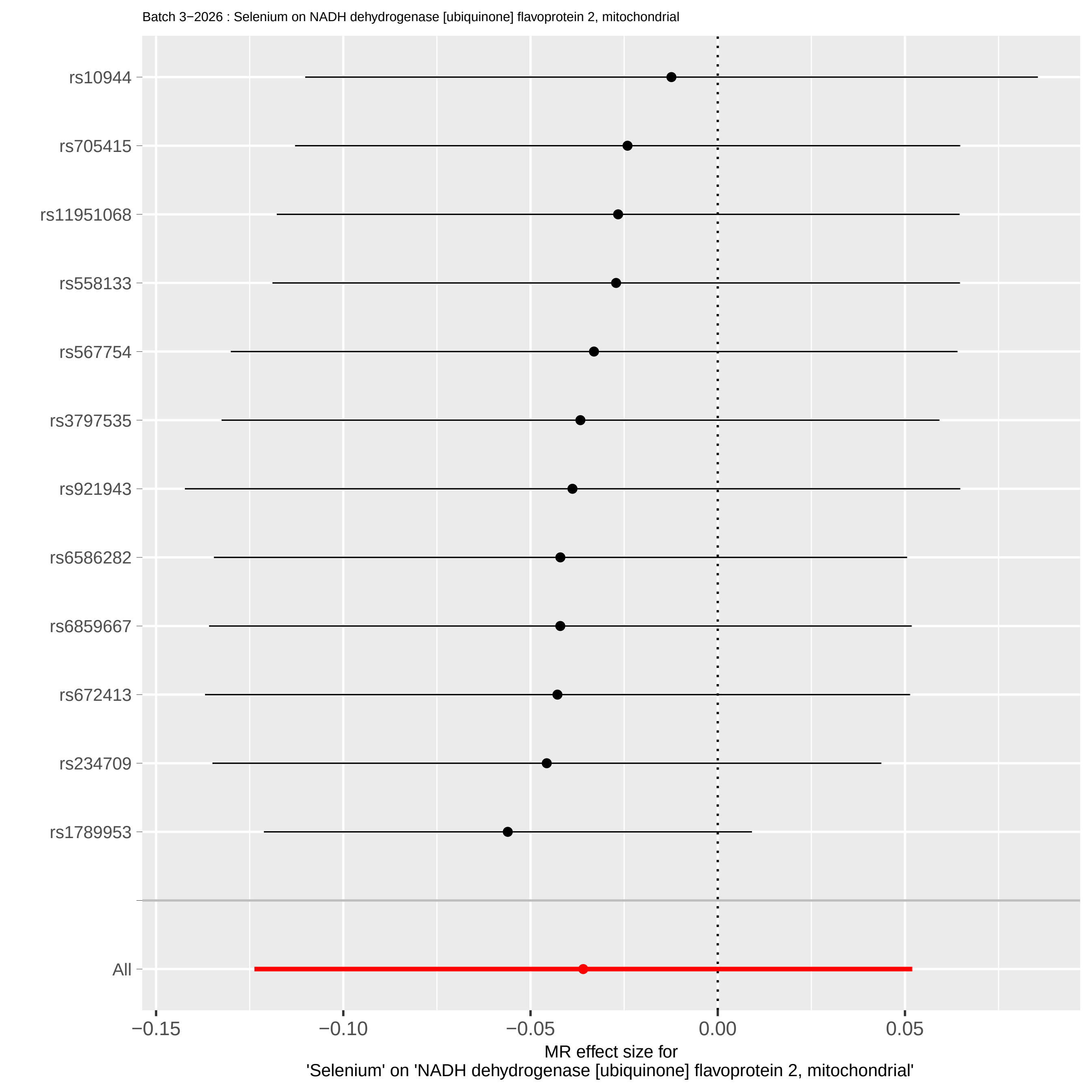

Batch 3−2026 : Selenium on NADH dehydrogenase [ubiquinone] flavoprotein 2, mitochondrial
rs10944
rs705415
rs11951068
rs558133
rs567754
rs3797535
rs921943
rs6586282
rs6859667
rs672413
rs234709
rs1789953
All
−0.15
−0.10
−0.05
0.00
0.05
MR effect size for
'Selenium' on 'NADH dehydrogenase [ubiquinone] flavoprotein 2, mitochondrial'

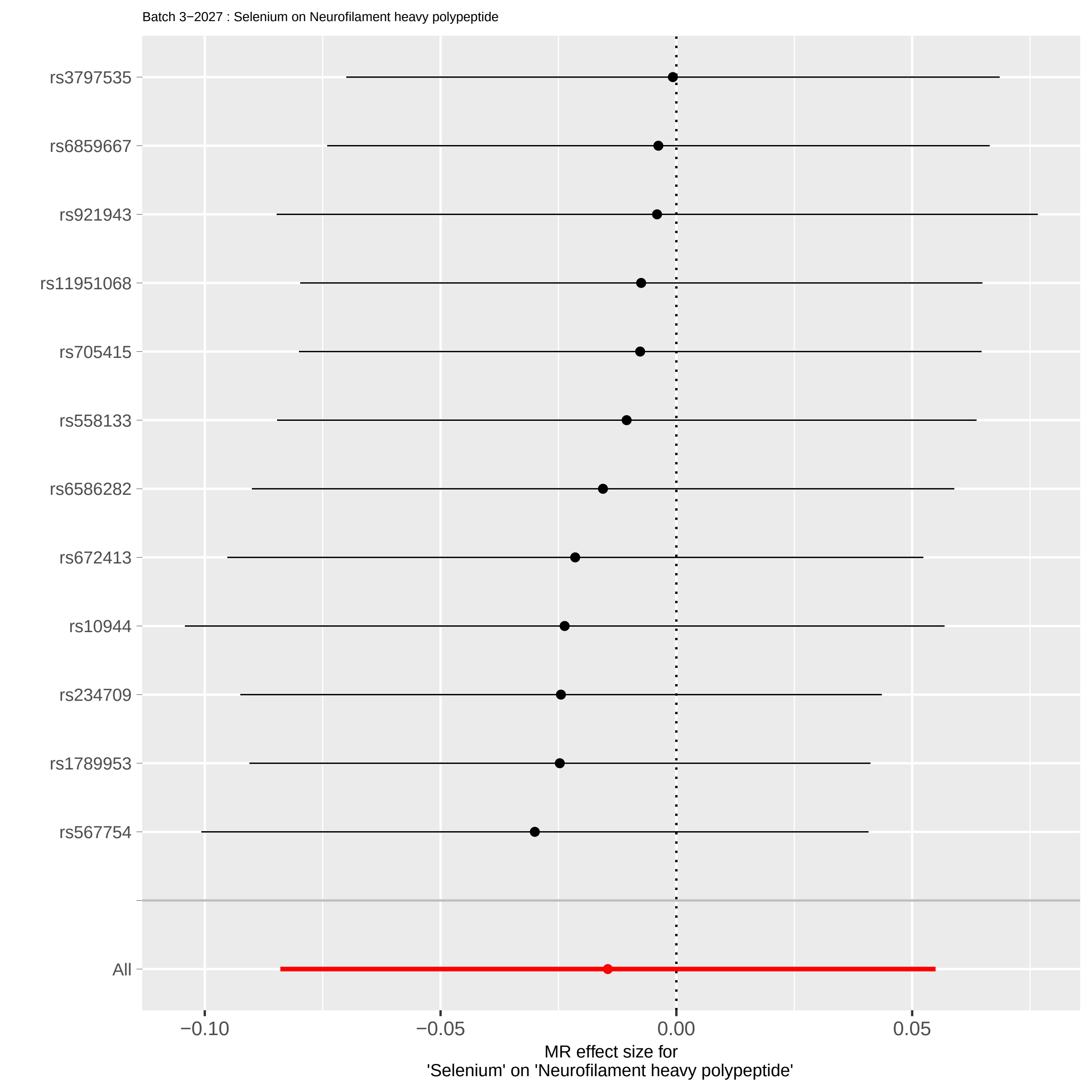

Batch 3−2027 : Selenium on Neurofilament heavy polypeptide
rs3797535
rs6859667
rs921943
rs11951068
rs705415
rs558133
rs6586282
rs672413
rs10944
rs234709
rs1789953
rs567754
All
−0.10
−0.05
0.00
0.05
MR effect size for
'Selenium' on 'Neurofilament heavy polypeptide'

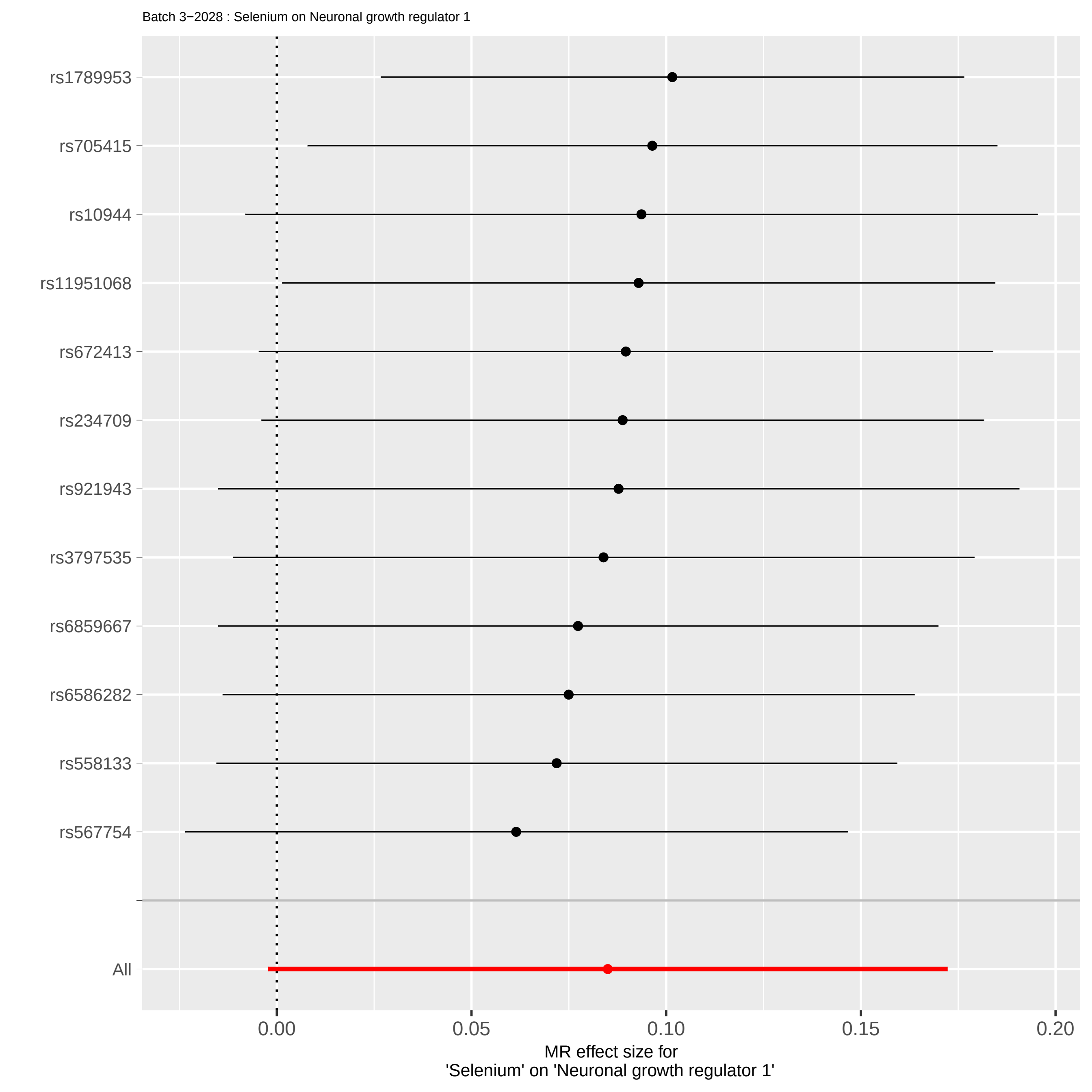

Batch 3−2028 : Selenium on Neuronal growth regulator 1
rs1789953
rs705415
rs10944
rs11951068
rs672413
rs234709
rs921943
rs3797535
rs6859667
rs6586282
rs558133
rs567754
All
0.00
0.05
0.10
0.15
0.20
MR effect size for
'Selenium' on 'Neuronal growth regulator 1'

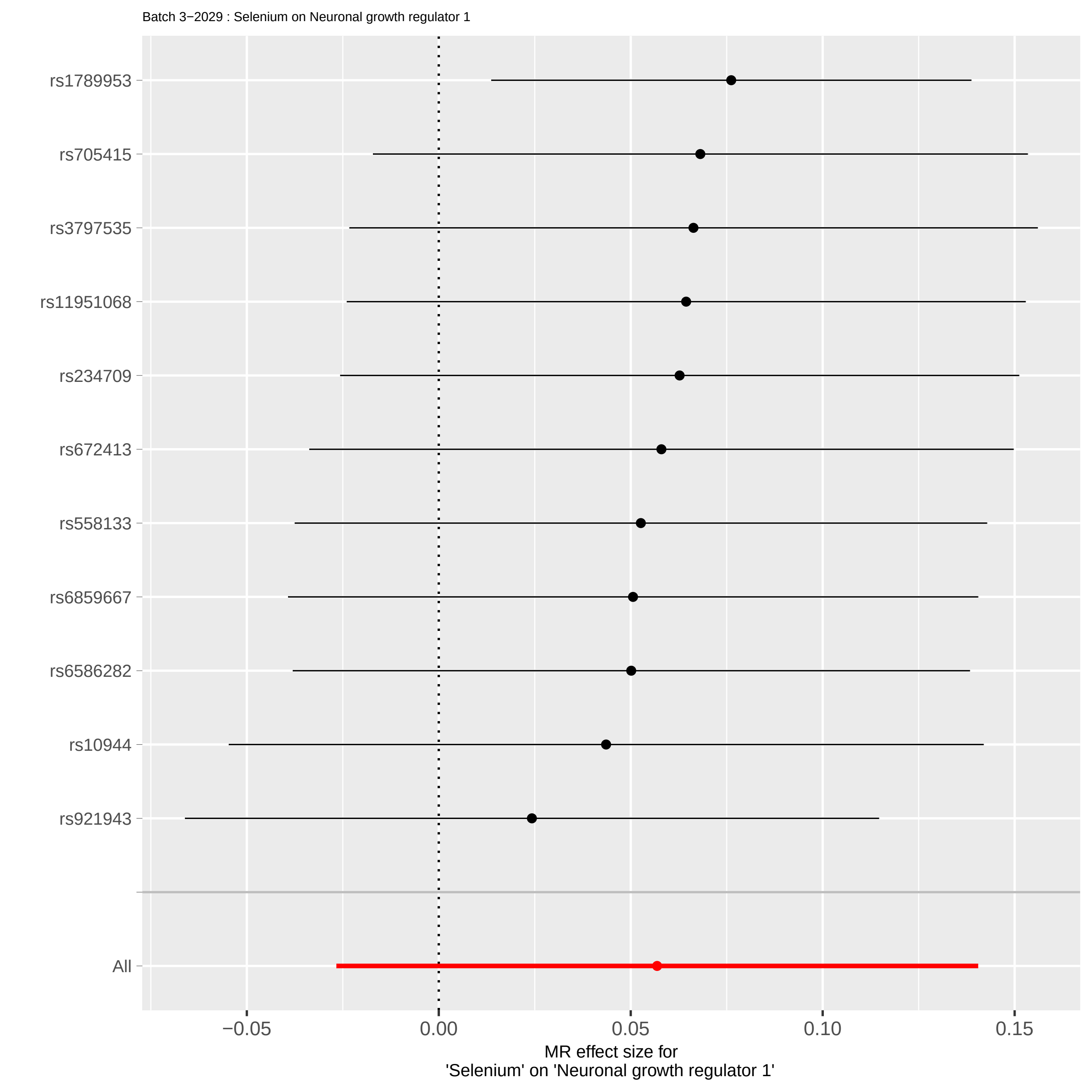

Batch 3−2029 : Selenium on Neuronal growth regulator 1
rs1789953
rs705415
rs3797535
rs11951068
rs234709
rs672413
rs558133
rs6859667
rs6586282
rs10944
rs921943
All
−0.05
0.00
0.05
0.10
0.15
MR effect size for
'Selenium' on 'Neuronal growth regulator 1'

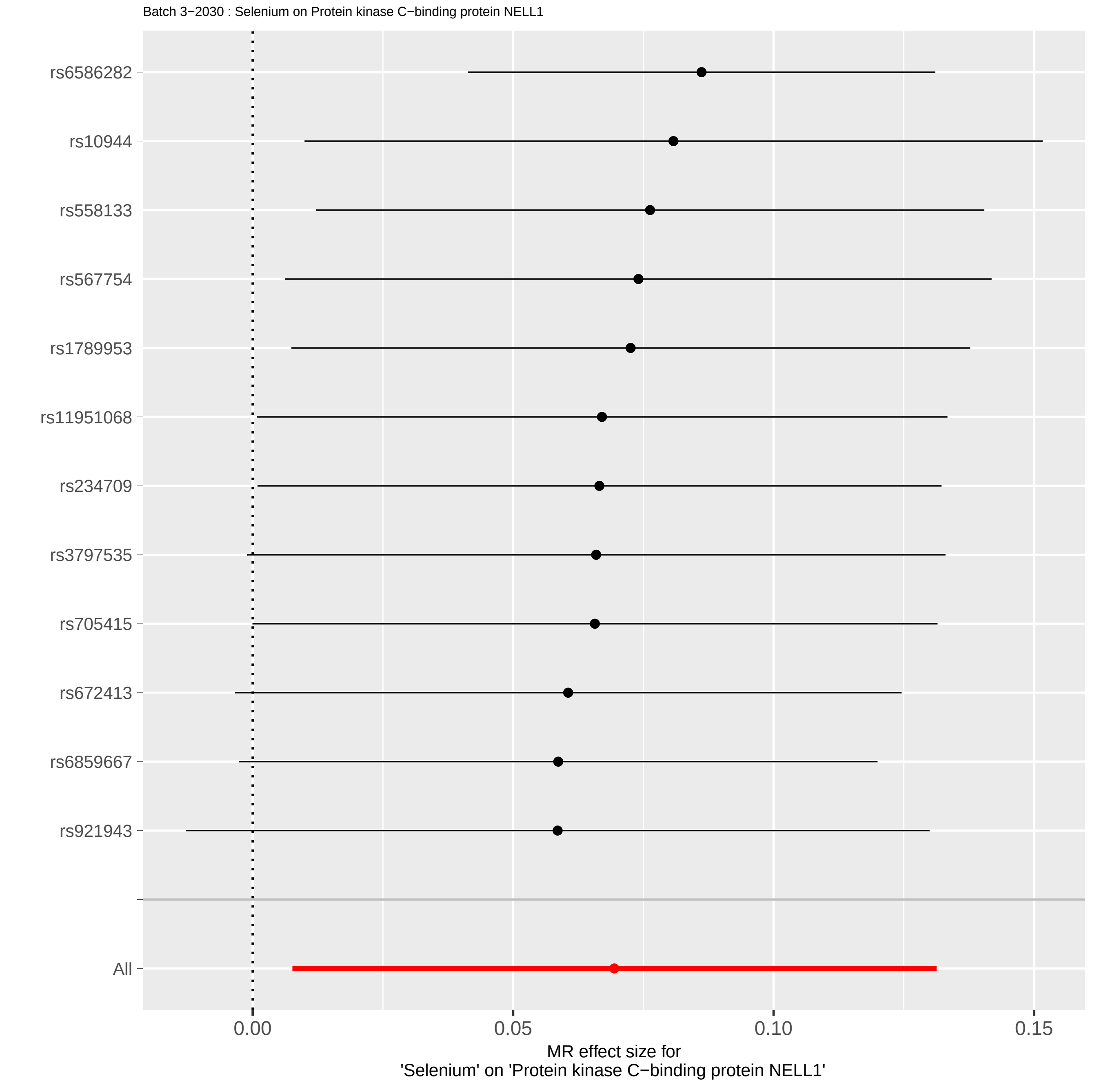
Batch 3−2030 : Selenium on Protein kinase C−binding protein NELL1
rs6586282
rs10944
rs558133
rs567754
rs1789953
rs11951068
rs234709
rs3797535
rs705415
rs672413
rs6859667
rs921943
All
0.00
0.05
0.10
0.15
MR effect size for
'Selenium' on 'Protein kinase C−binding protein NELL1'

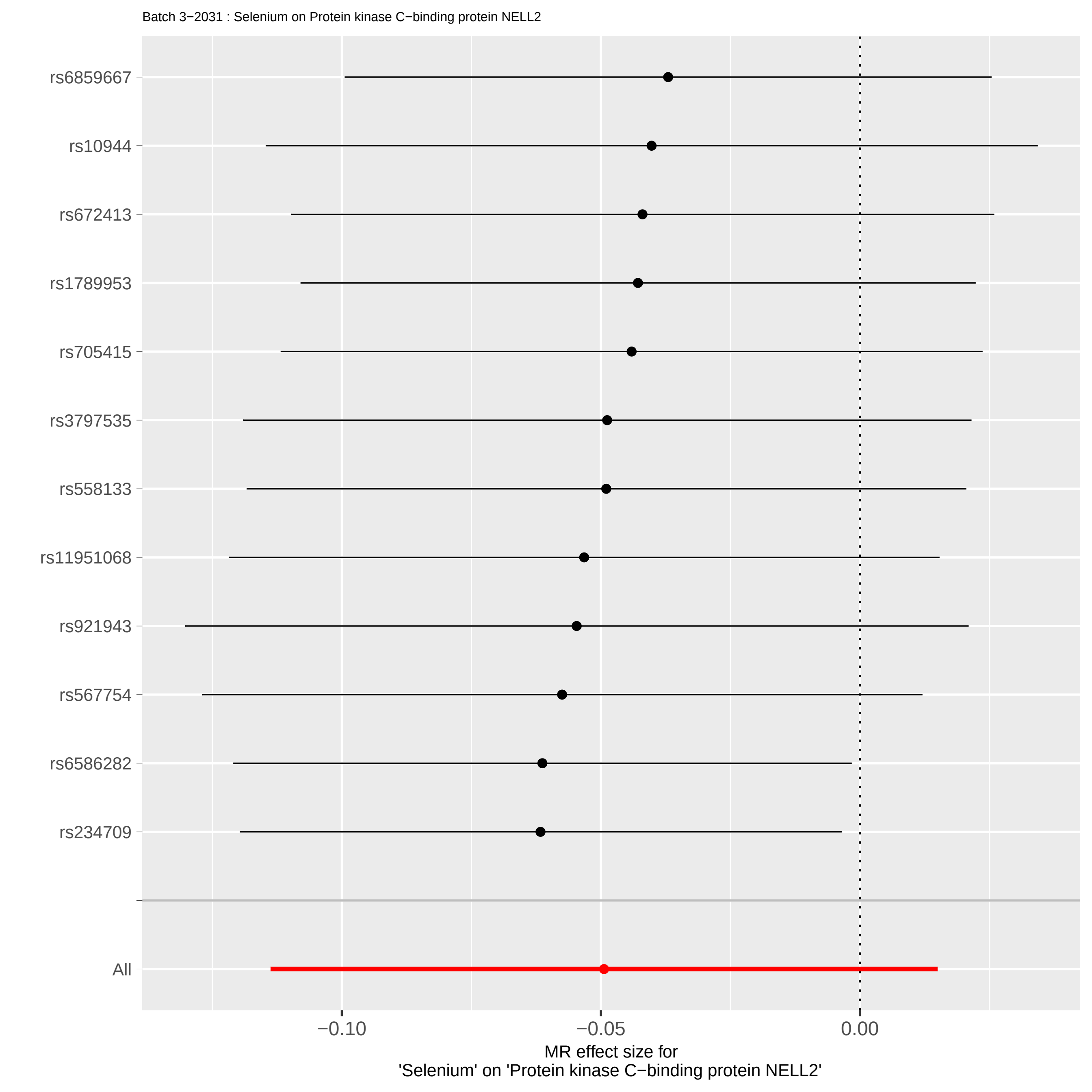

Batch 3−2031 : Selenium on Protein kinase C−binding protein NELL2
rs6859667
rs10944
rs672413
rs1789953
rs705415
rs3797535
rs558133
rs11951068
rs921943
rs567754
rs6586282
rs234709
All
−0.10
−0.05
0.00
MR effect size for
'Selenium' on 'Protein kinase C−binding protein NELL2'

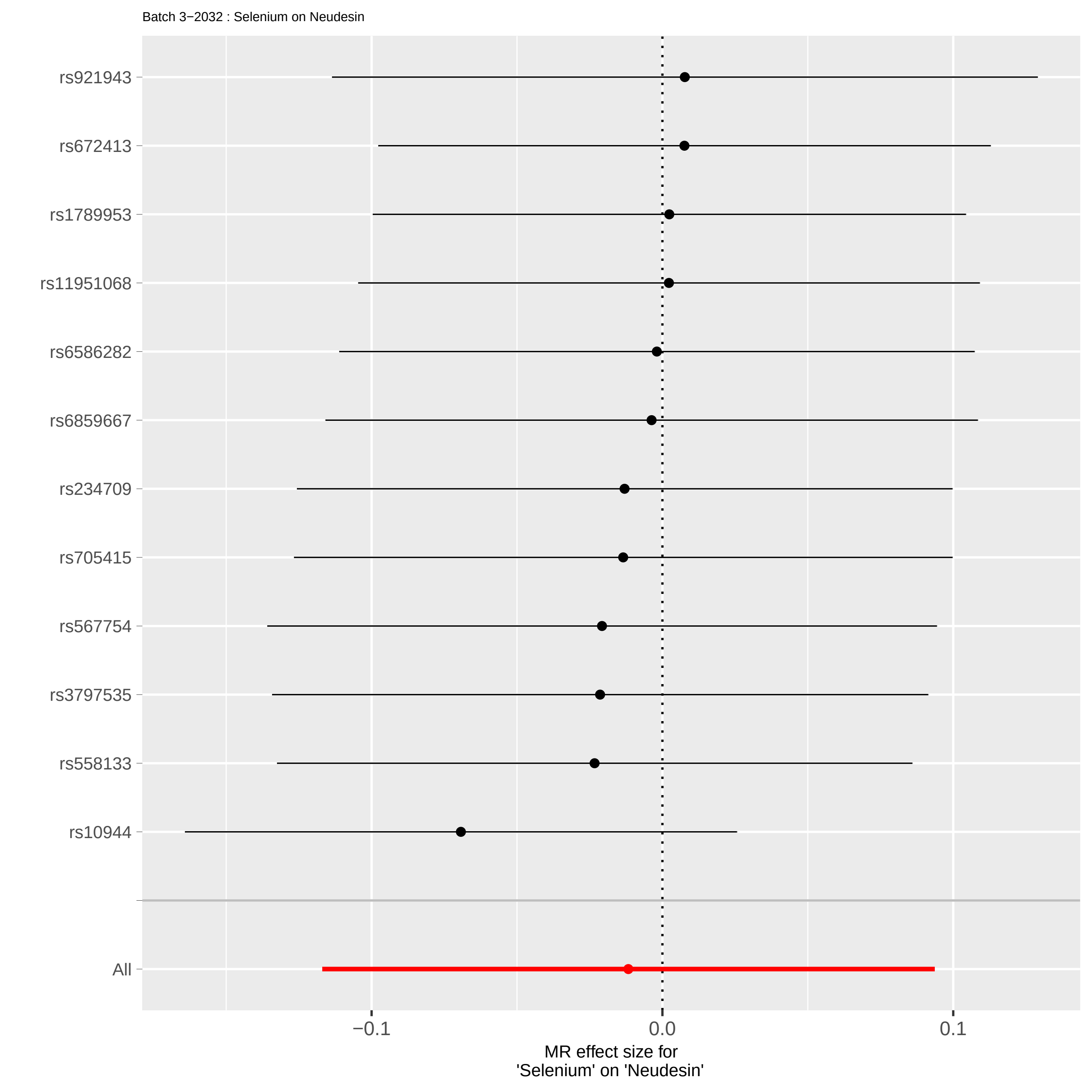

Batch 3−2032 : Selenium on Neudesin
rs921943
rs672413
rs1789953
rs11951068
rs6586282
rs6859667
rs234709
rs705415
rs567754
rs3797535
rs558133
rs10944
All
−0.1
0.0
0.1
MR effect size for
'Selenium' on 'Neudesin'

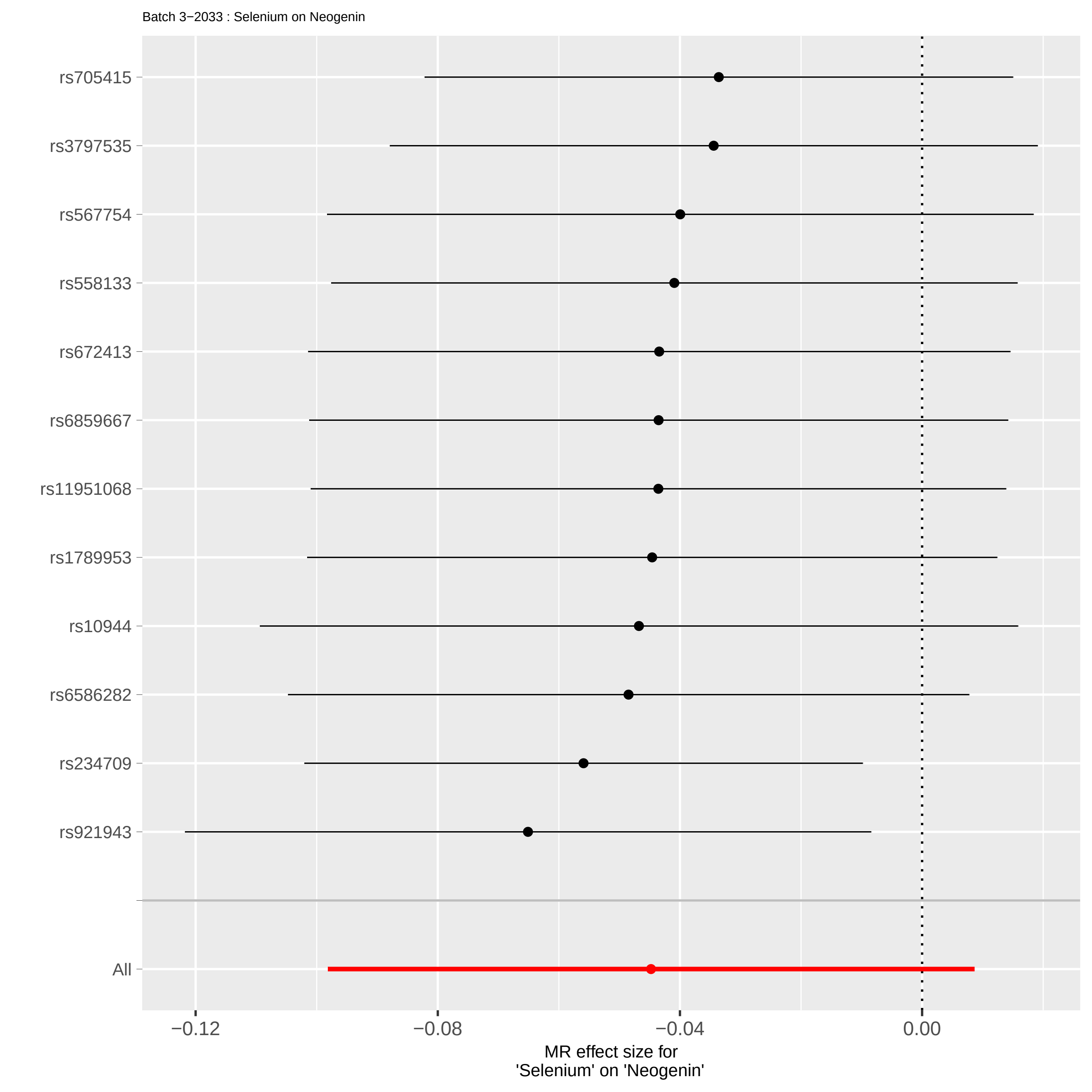

Batch 3−2033 : Selenium on Neogenin
rs705415
rs3797535
rs567754
rs558133
rs672413
rs6859667
rs11951068
rs1789953
rs10944
rs6586282
rs234709
rs921943
All
−0.12
−0.08
−0.04
0.00
MR effect size for
'Selenium' on 'Neogenin'

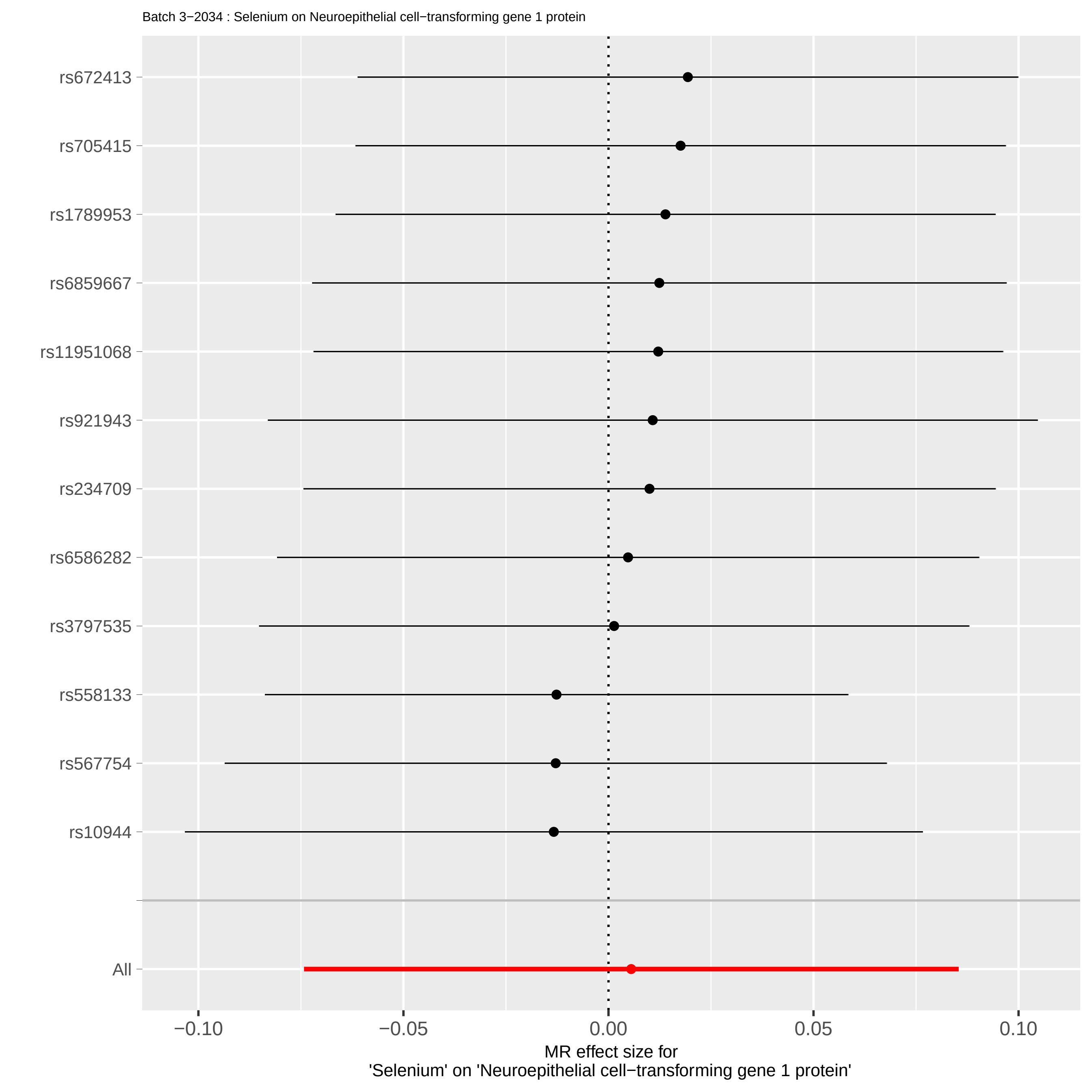

Batch 3−2034 : Selenium on Neuroepithelial cell−transforming gene 1 protein
rs672413
rs705415
rs1789953
rs6859667
rs11951068
rs921943
rs234709
rs6586282
rs3797535
rs558133
rs567754
rs10944
All
−0.10
−0.05
0.00
0.05
0.10
MR effect size for
'Selenium' on 'Neuroepithelial cell−transforming gene 1 protein'

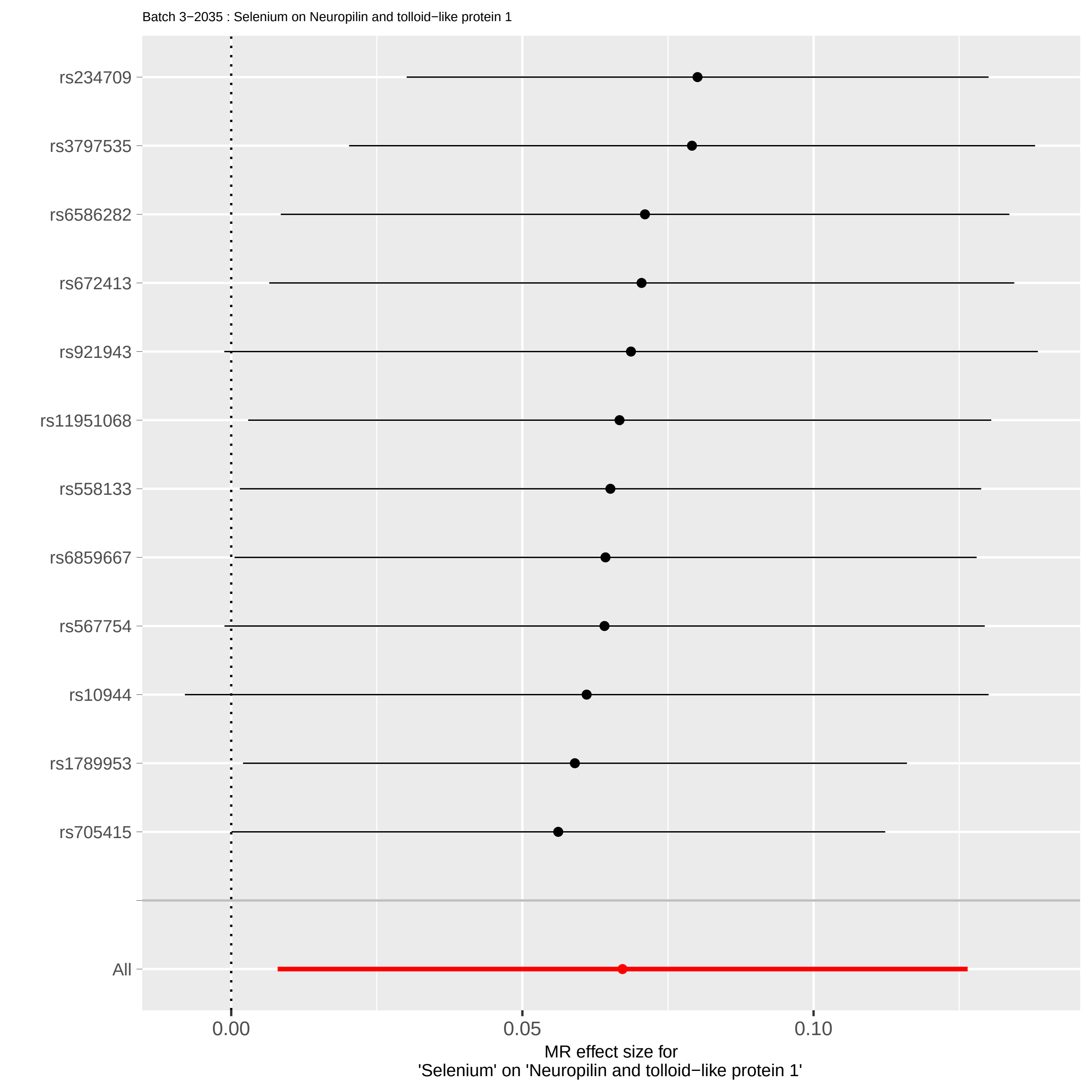

Batch 3−2035 : Selenium on Neuropilin and tolloid−like protein 1
rs234709
rs3797535
rs6586282
rs672413
rs921943
rs11951068
rs558133
rs6859667
rs567754
rs10944
rs1789953
rs705415
All
0.00
0.05
0.10
MR effect size for
'Selenium' on 'Neuropilin and tolloid−like protein 1'

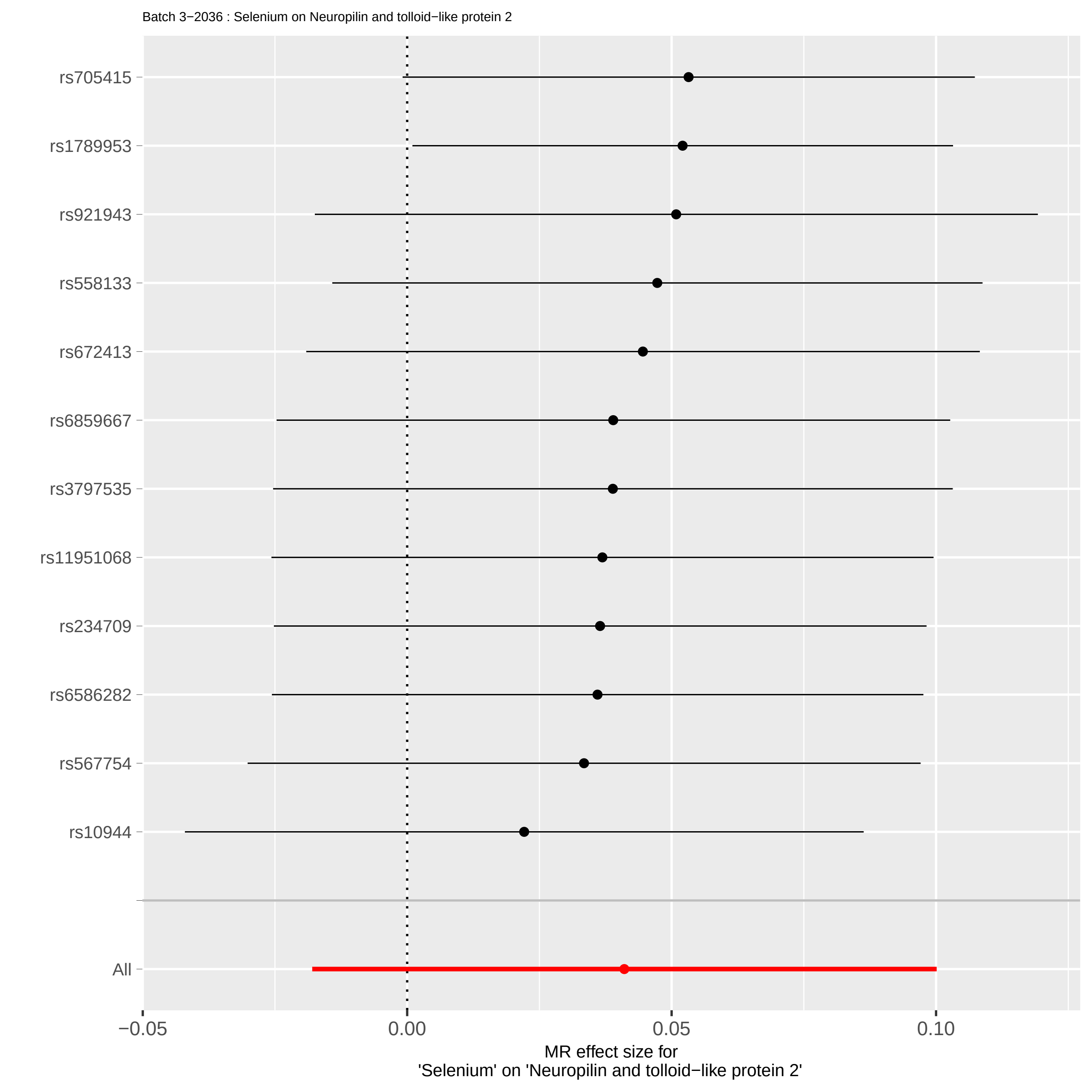
Batch 3−2036 : Selenium on Neuropilin and tolloid−like protein 2
rs705415
rs1789953
rs921943
rs558133
rs672413
rs6859667
rs3797535
rs11951068
rs234709
rs6586282
rs567754
rs10944
All
−0.05
0.00
0.05
0.10
MR effect size for
'Selenium' on 'Neuropilin and tolloid−like protein 2'

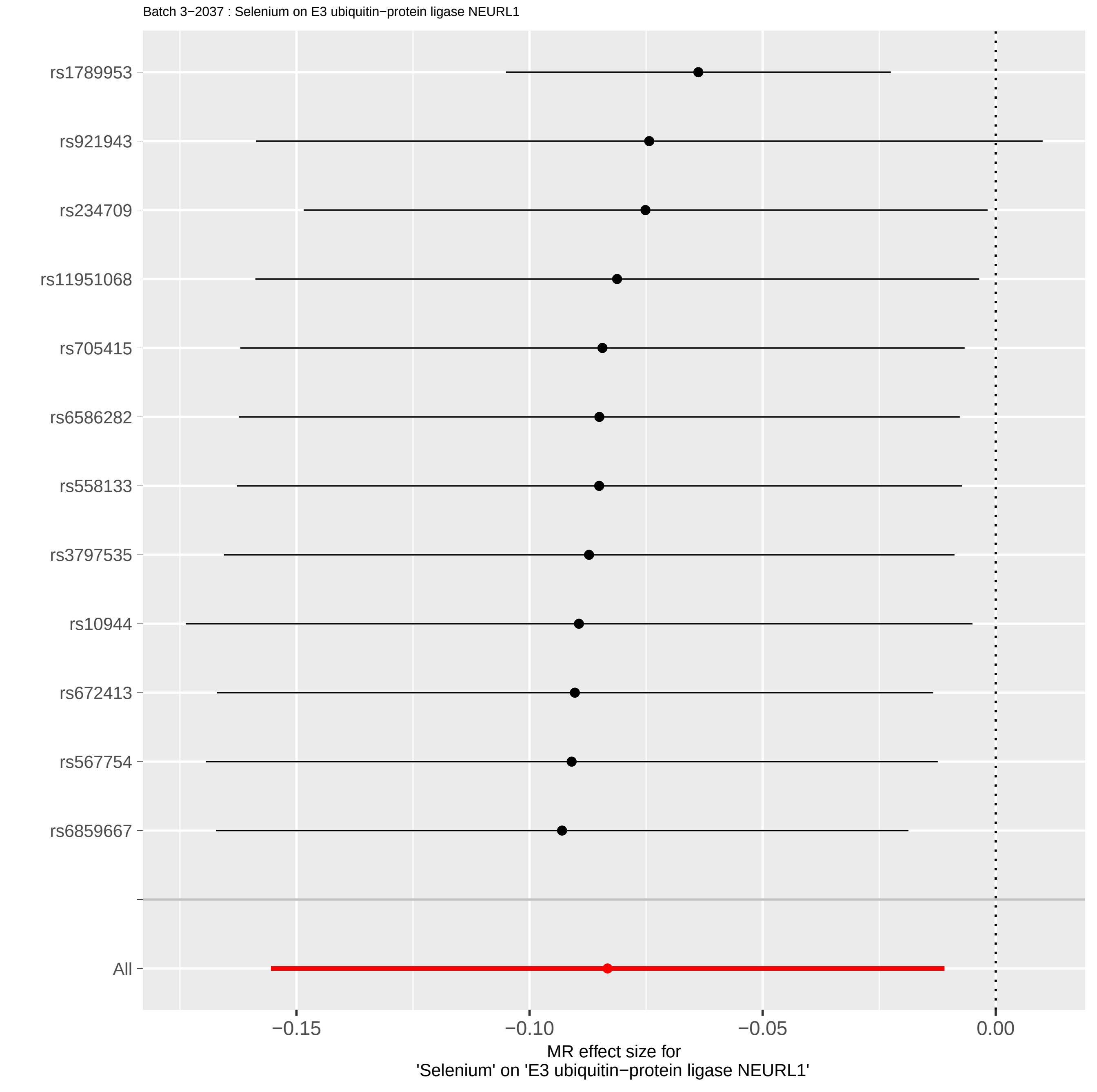
Batch 3−2037 : Selenium on E3 ubiquitin−protein ligase NEURL1
rs1789953
rs921943
rs234709
rs11951068
rs705415
rs6586282
rs558133
rs3797535
rs10944
rs672413
rs567754
rs6859667
All
−0.15
−0.10
−0.05
0.00
MR effect size for
'Selenium' on 'E3 ubiquitin−protein ligase NEURL1'

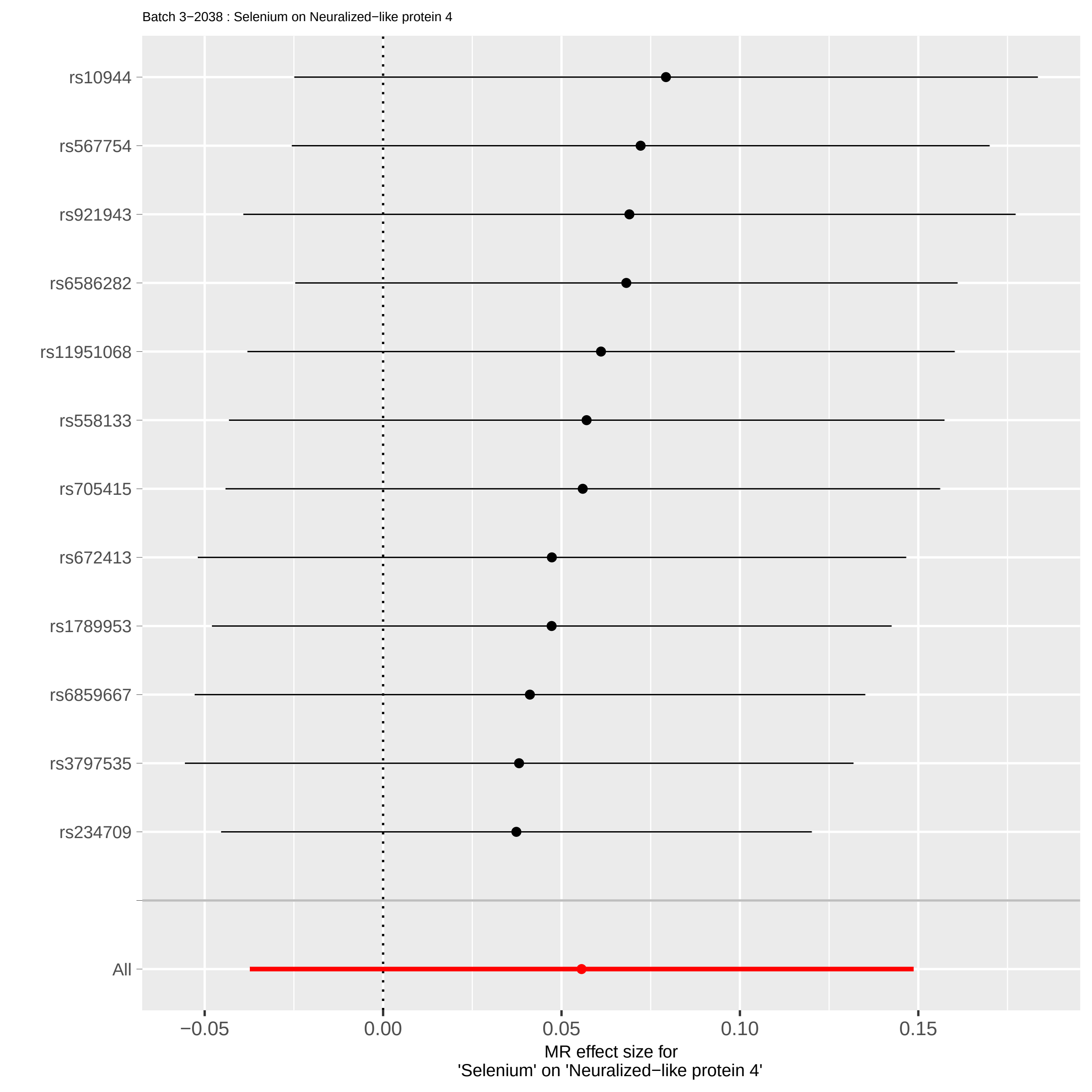

Batch 3−2038 : Selenium on Neuralized−like protein 4
rs10944
rs567754
rs921943
rs6586282
rs11951068
rs558133
rs705415
rs672413
rs1789953
rs6859667
rs3797535
rs234709
All
−0.05
0.00
0.05
0.10
0.15
MR effect size for
'Selenium' on 'Neuralized−like protein 4'

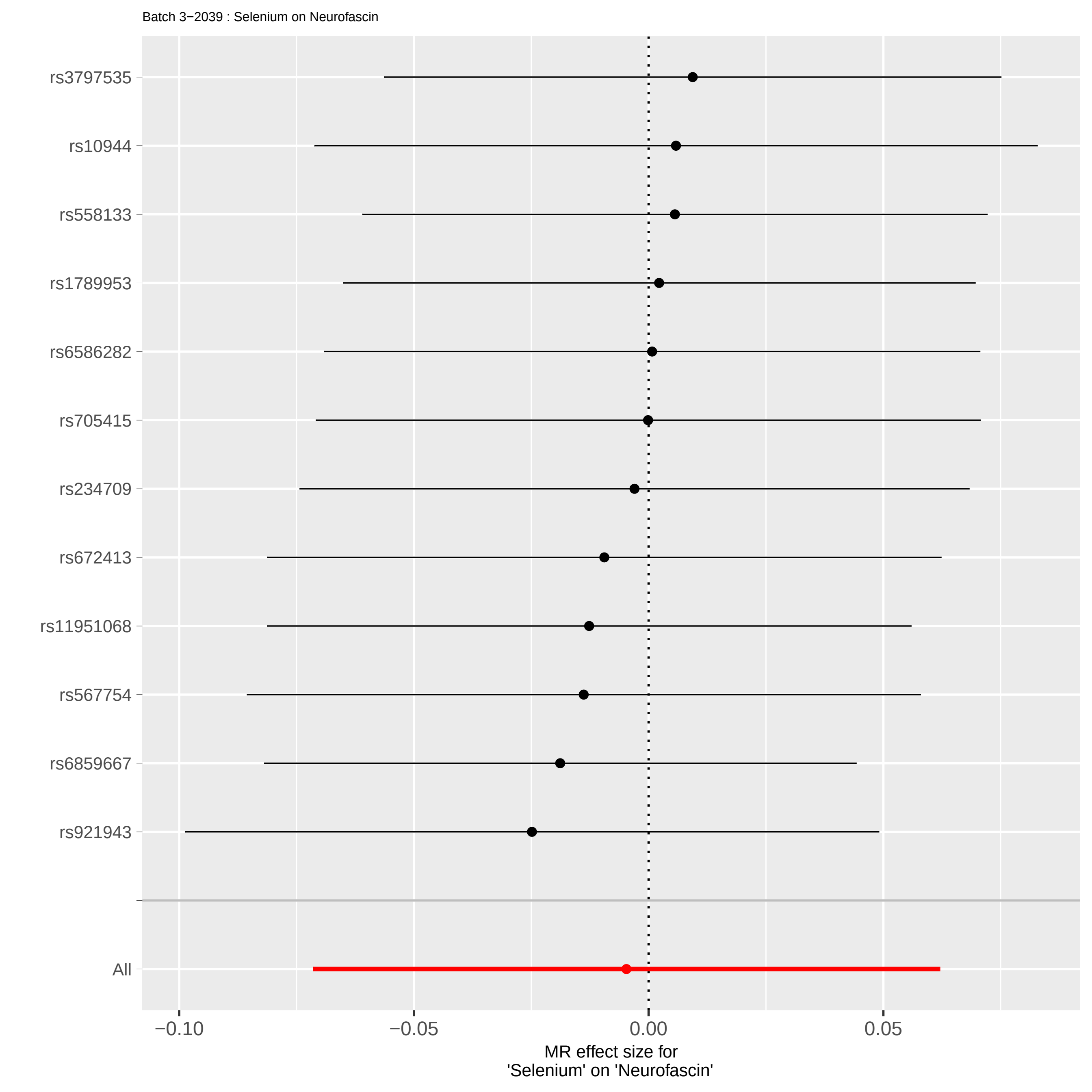
Batch 3−2039 : Selenium on Neurofascin
rs3797535
rs10944
rs558133
rs1789953
rs6586282
rs705415
rs234709
rs672413
rs11951068
rs567754
rs6859667
rs921943
All
−0.10
−0.05
0.00
0.05
MR effect size for
'Selenium' on 'Neurofascin'

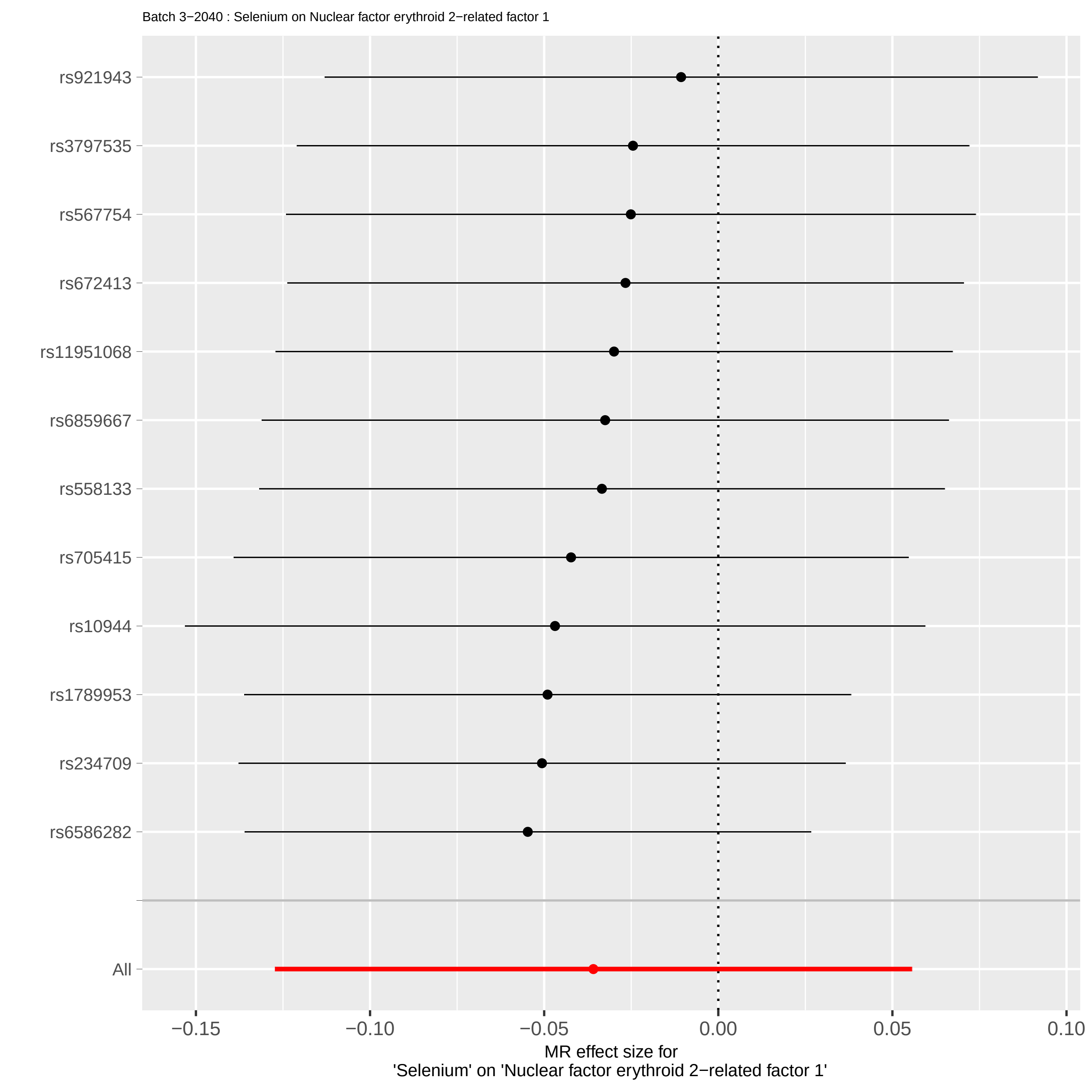

Batch 3−2040 : Selenium on Nuclear factor erythroid 2−related factor 1
rs921943
rs3797535
rs567754
rs672413
rs11951068
rs6859667
rs558133
rs705415
rs10944
rs1789953
rs234709
rs6586282
All
−0.15
−0.10
−0.05
0.00
0.05
0.10
MR effect size for
'Selenium' on 'Nuclear factor erythroid 2−related factor 1'

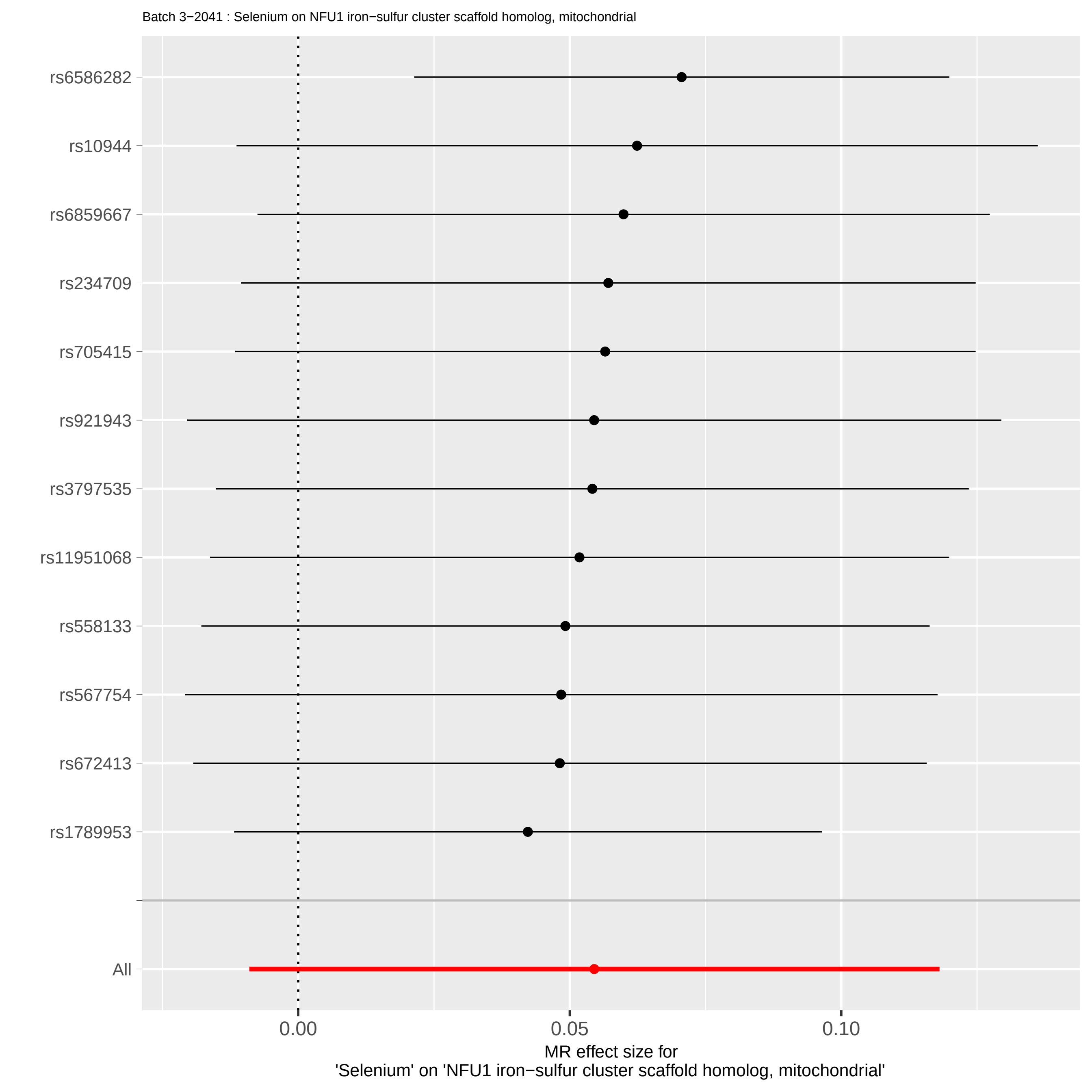

Batch 3−2041 : Selenium on NFU1 iron−sulfur cluster scaffold homolog, mitochondrial
rs6586282
rs10944
rs6859667
rs234709
rs705415
rs921943
rs3797535
rs11951068
rs558133
rs567754
rs672413
rs1789953
All
0.00
0.05
0.10
MR effect size for
'Selenium' on 'NFU1 iron−sulfur cluster scaffold homolog, mitochondrial'

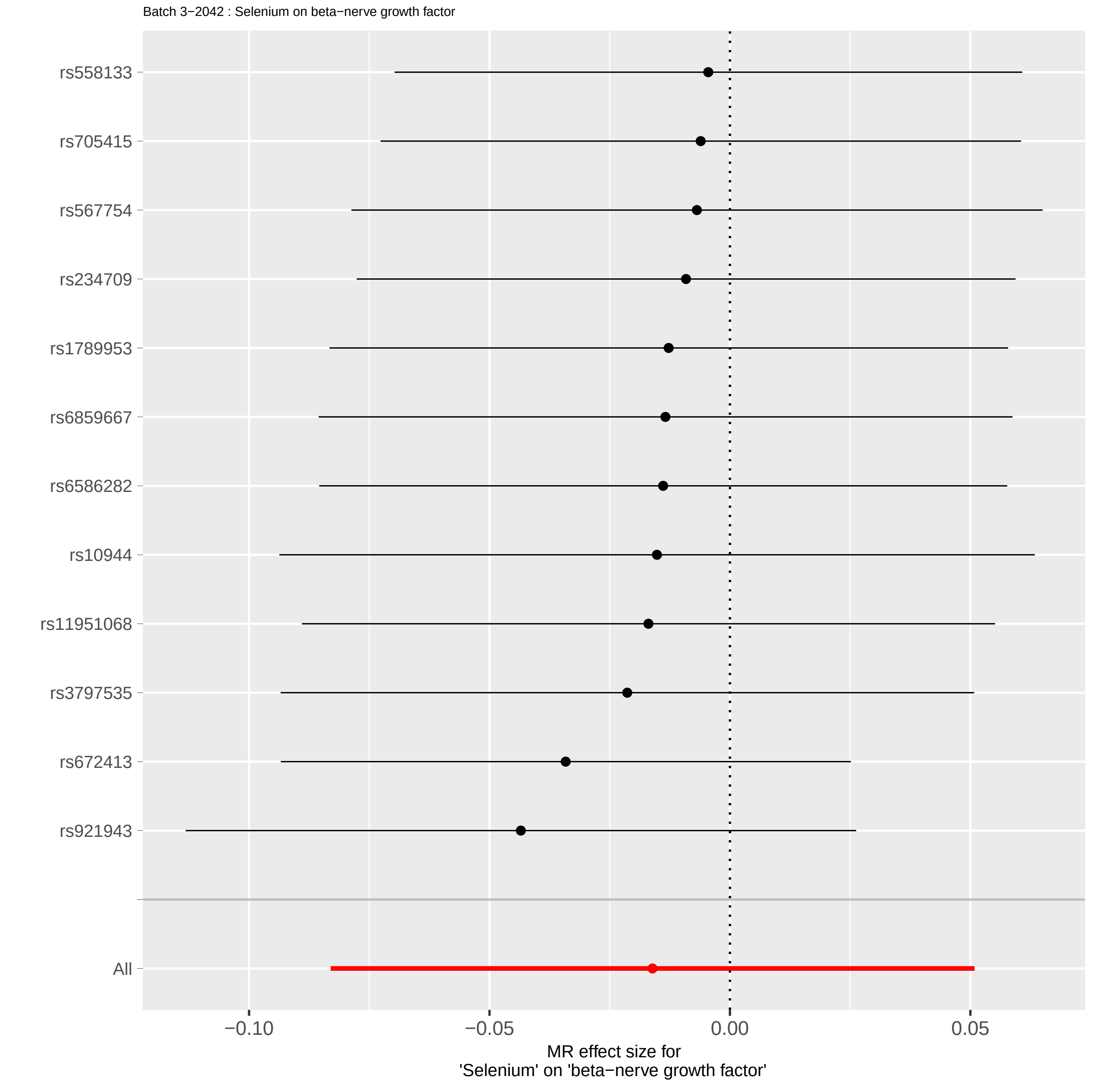

Batch 3−2042 : Selenium on beta−nerve growth factor
rs558133
rs705415
rs567754
rs234709
rs1789953
rs6859667
rs6586282
rs10944
rs11951068
rs3797535
rs672413
rs921943
All
−0.10
−0.05
0.00
0.05
MR effect size for
'Selenium' on 'beta−nerve growth factor'

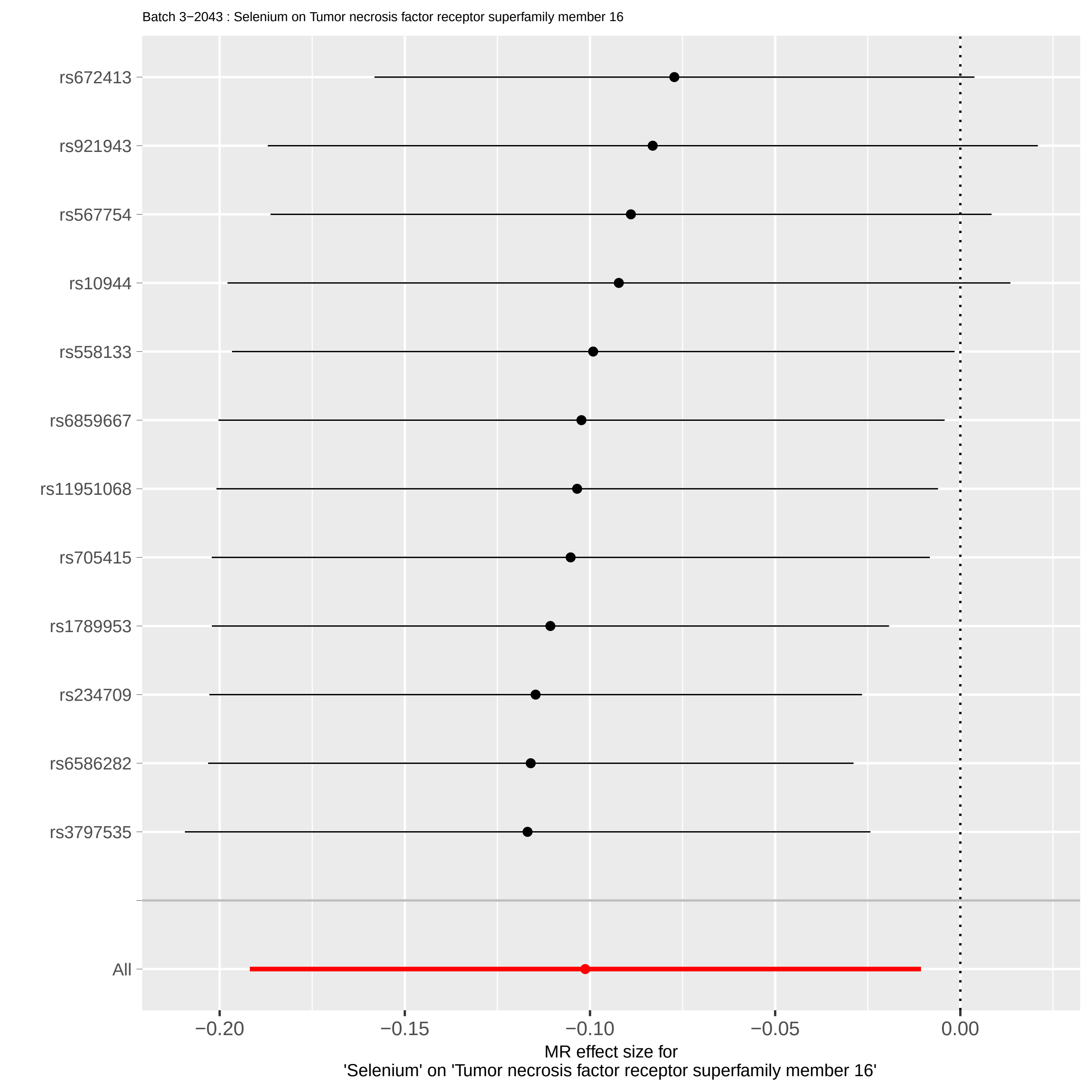
Batch 3−2043 : Selenium on Tumor necrosis factor receptor superfamily member 16
rs672413
rs921943
rs567754
rs10944
rs558133
rs6859667
rs11951068
rs705415
rs1789953
rs234709
rs6586282
rs3797535
All
−0.20
−0.15
−0.10
−0.05
0.00
MR effect size for
'Selenium' on 'Tumor necrosis factor receptor superfamily member 16'

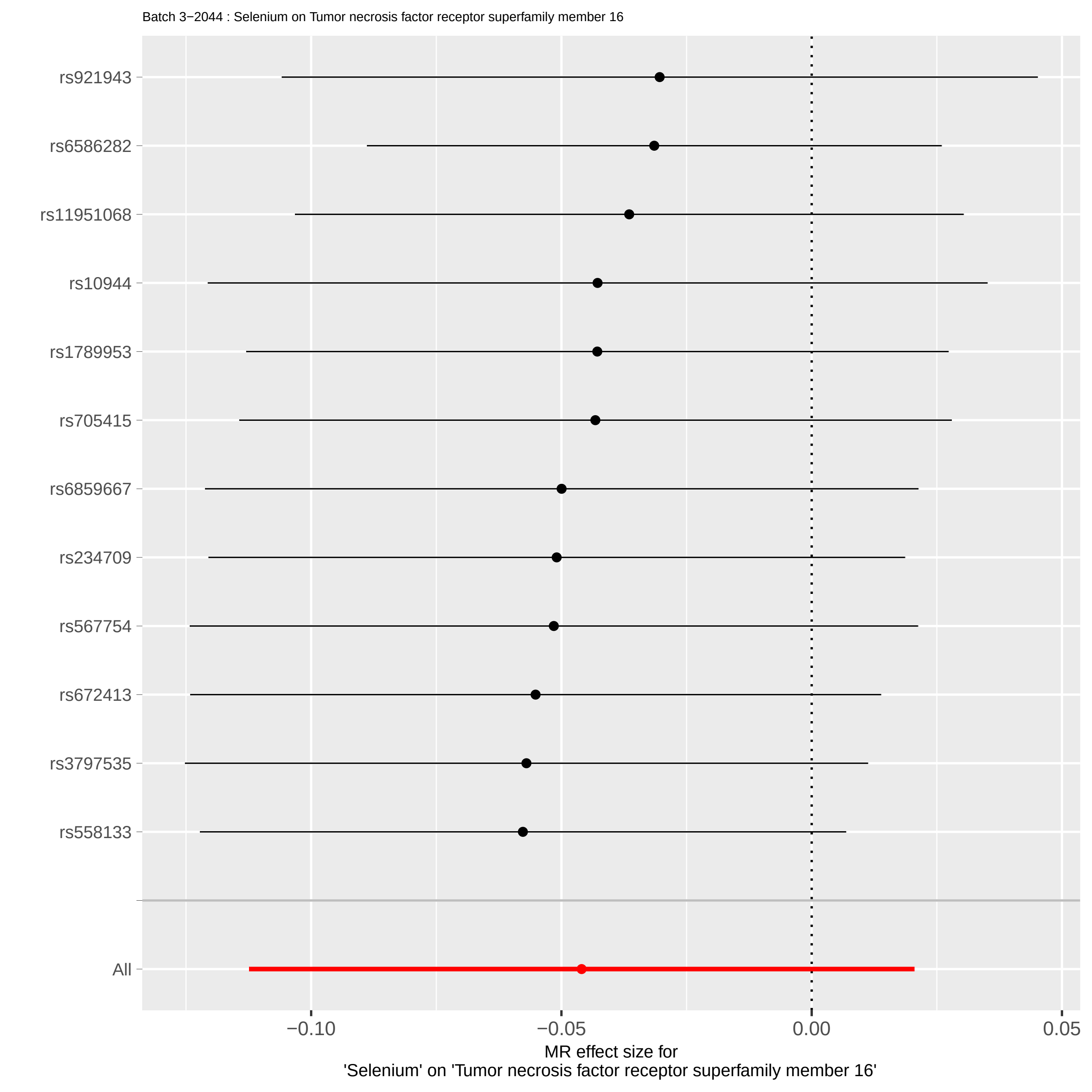
Batch 3−2044 : Selenium on Tumor necrosis factor receptor superfamily member 16
rs921943
rs6586282
rs11951068
rs10944
rs1789953
rs705415
rs6859667
rs234709
rs567754
rs672413
rs3797535
rs558133
All
−0.10
−0.05
0.00
0.05
MR effect size for
'Selenium' on 'Tumor necrosis factor receptor superfamily member 16'

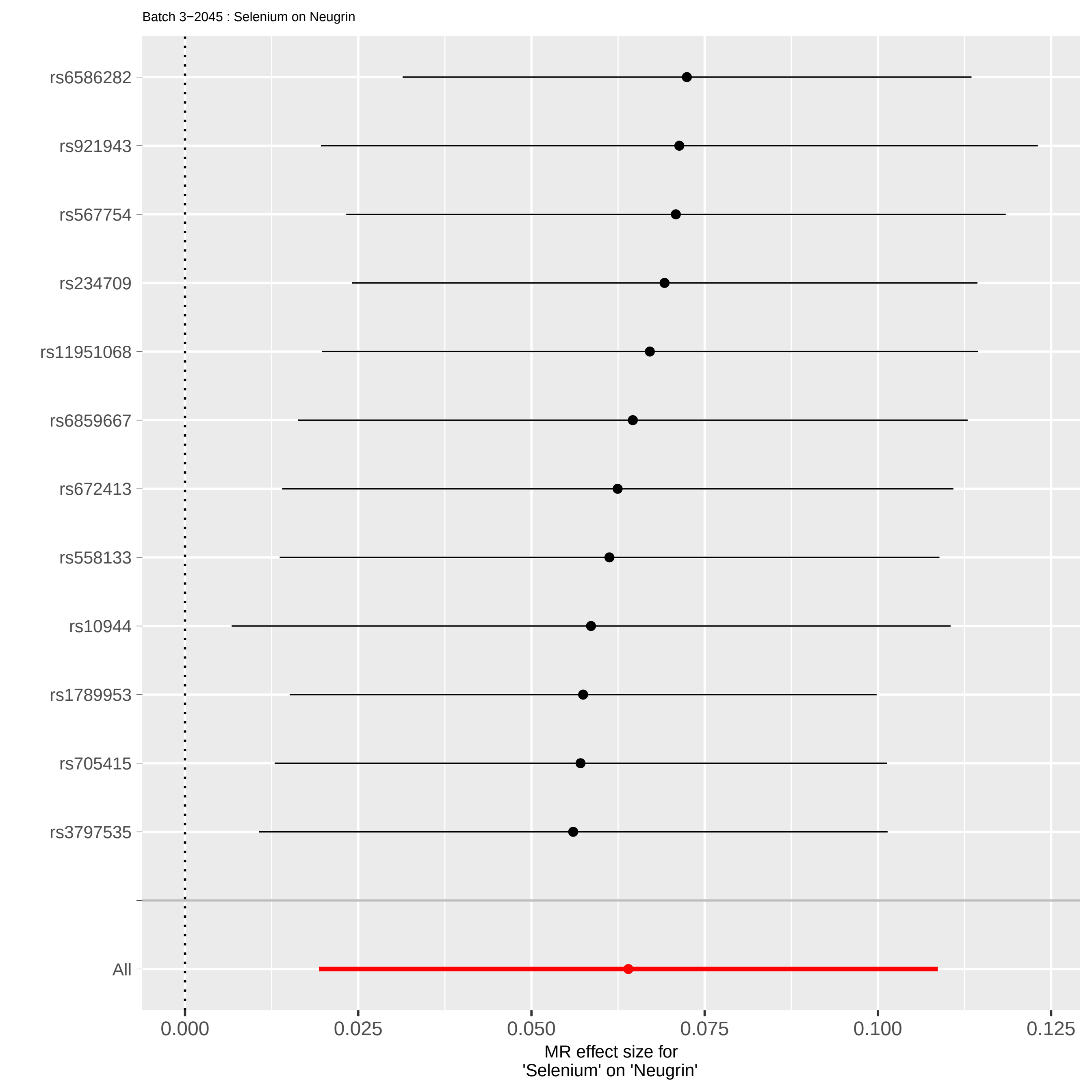

Batch 3−2045 : Selenium on Neugrin
rs6586282
rs921943
rs567754
rs234709
rs11951068
rs6859667
rs672413
rs558133
rs10944
rs1789953
rs705415
rs3797535
All
0.000
0.025
0.050
0.075
0.100
0.125
MR effect size for
'Selenium' on 'Neugrin'

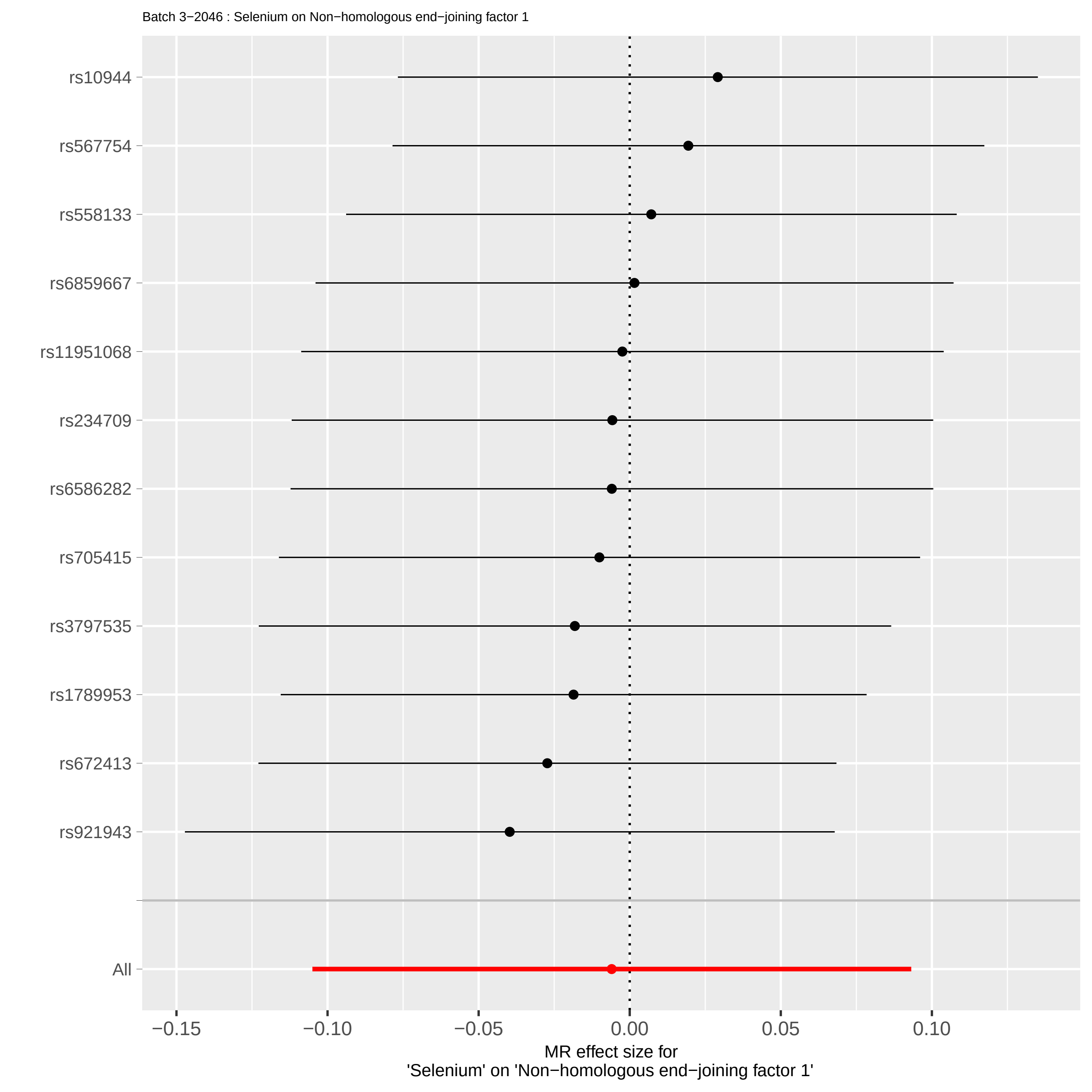

Batch 3−2046 : Selenium on Non−homologous end−joining factor 1
rs10944
rs567754
rs558133
rs6859667
rs11951068
rs234709
rs6586282
rs705415
rs3797535
rs1789953
rs672413
rs921943
All
−0.15
−0.10
−0.05
0.00
0.05
0.10
MR effect size for
'Selenium' on 'Non−homologous end−joining factor 1'

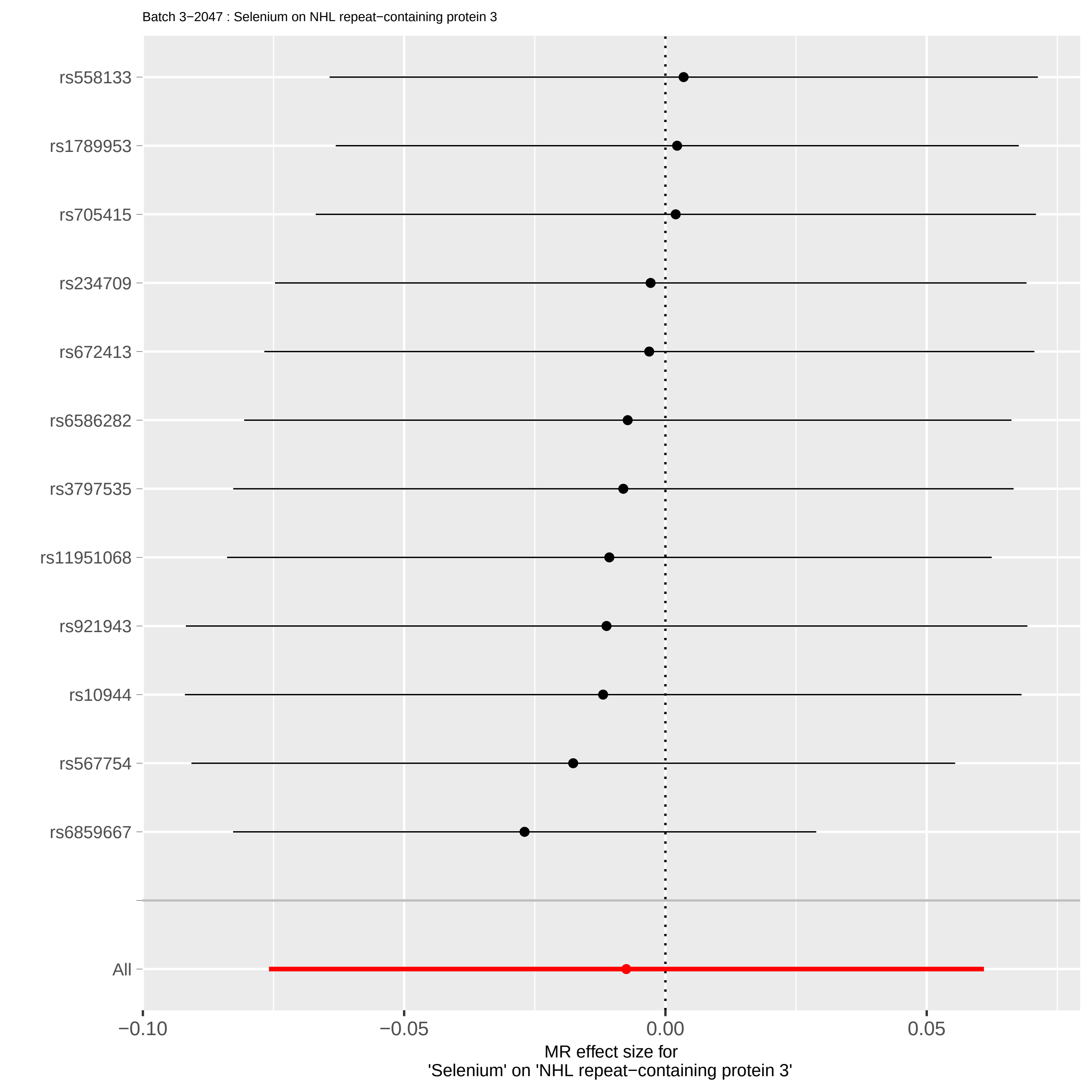

Batch 3−2047 : Selenium on NHL repeat−containing protein 3
rs558133
rs1789953
rs705415
rs234709
rs672413
rs6586282
rs3797535
rs11951068
rs921943
rs10944
rs567754
rs6859667
All
−0.10
−0.05
0.00
0.05
MR effect size for
'Selenium' on 'NHL repeat−containing protein 3'

Batch 3−2048 : Selenium on NHP2−like protein 1

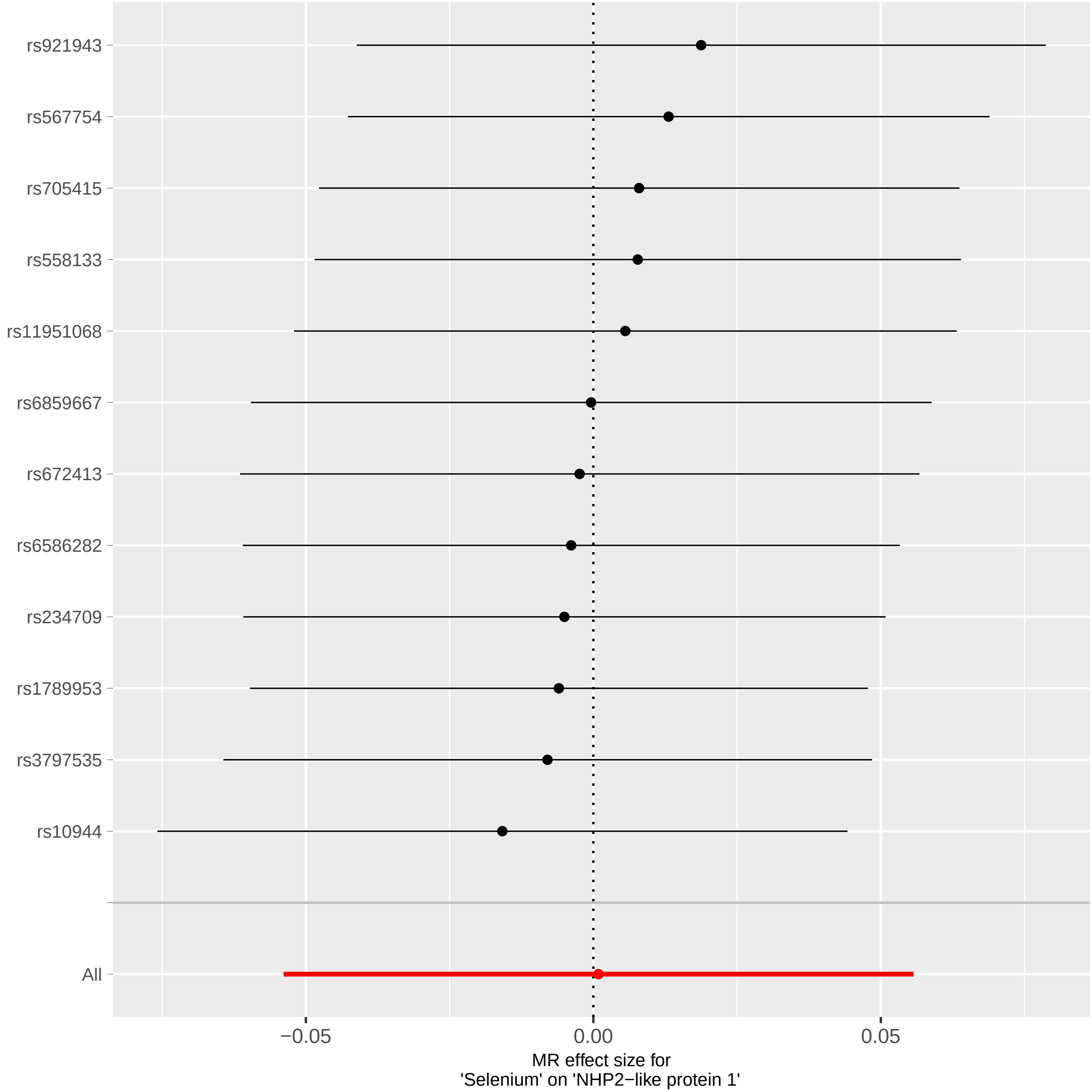

Batch 3−2049 : Selenium on Nidogen−1

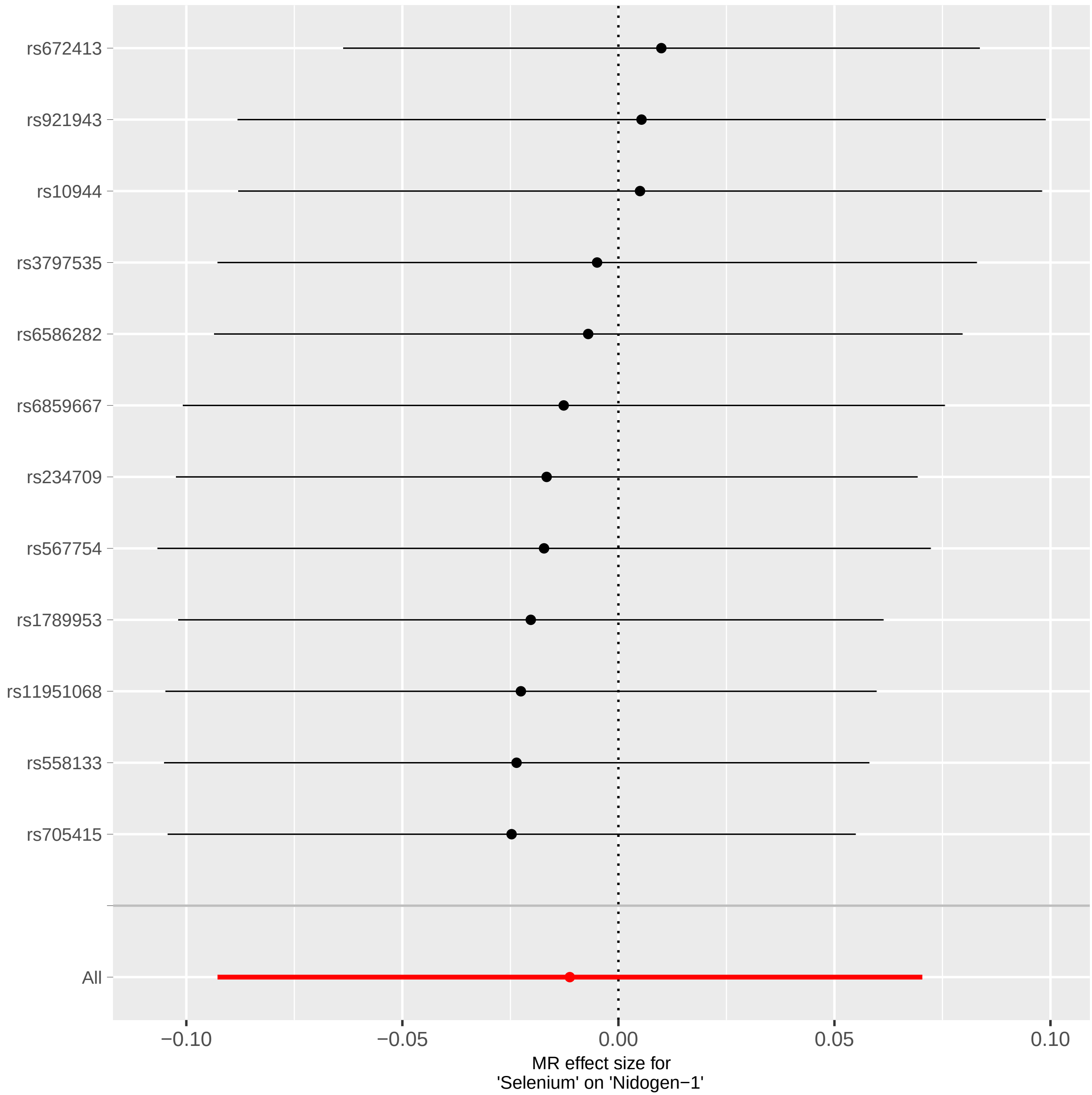

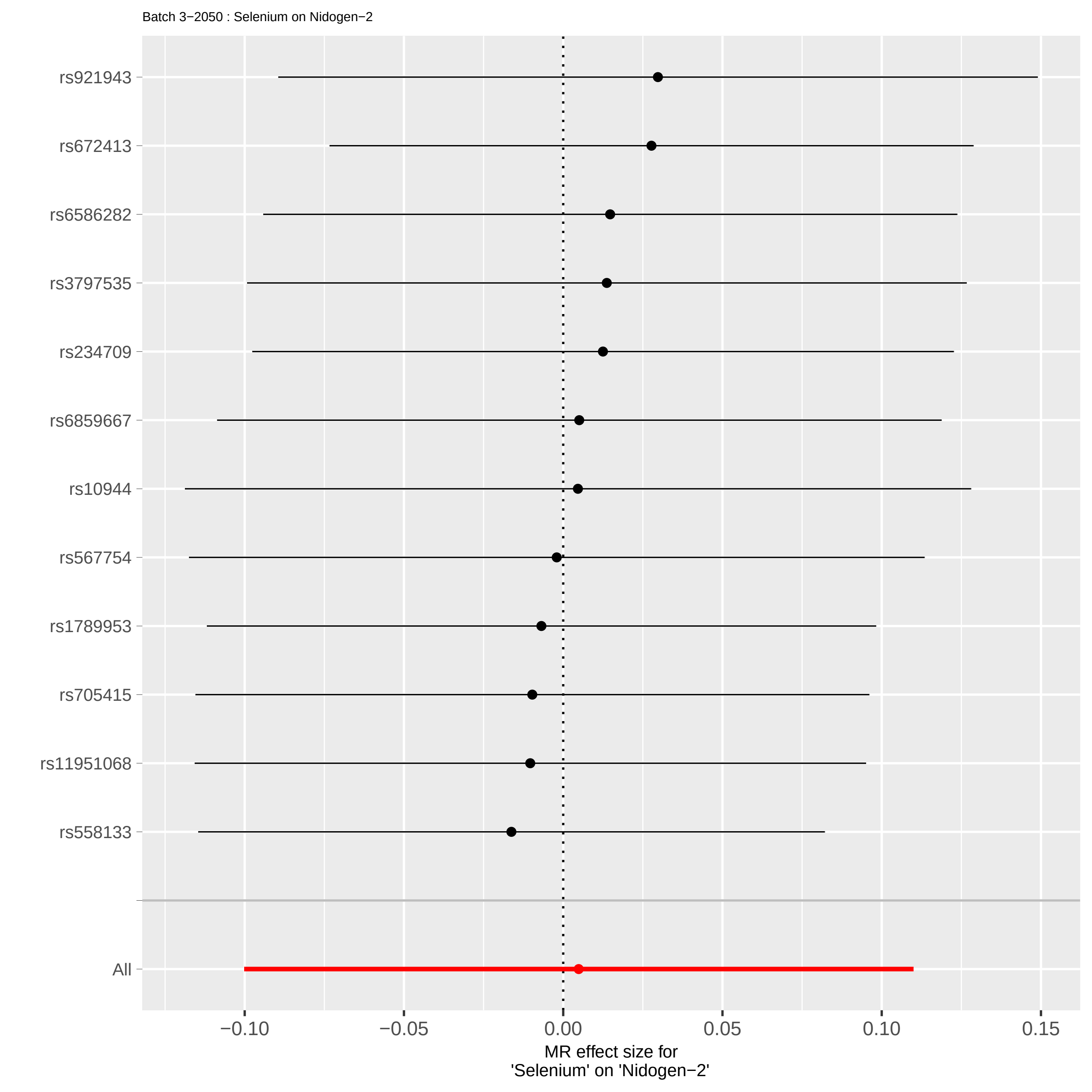
Batch 3−2050 : Selenium on Nidogen−2
rs921943
rs672413
rs6586282
rs3797535
rs234709
rs6859667
rs10944
rs567754
rs1789953
rs705415
rs11951068
rs558133
All
−0.10
−0.05
0.00
0.05
0.10
0.15
MR effect size for
'Selenium' on 'Nidogen−2'

Batch 3−2051 : Selenium on Magnesium transporter NIPA4

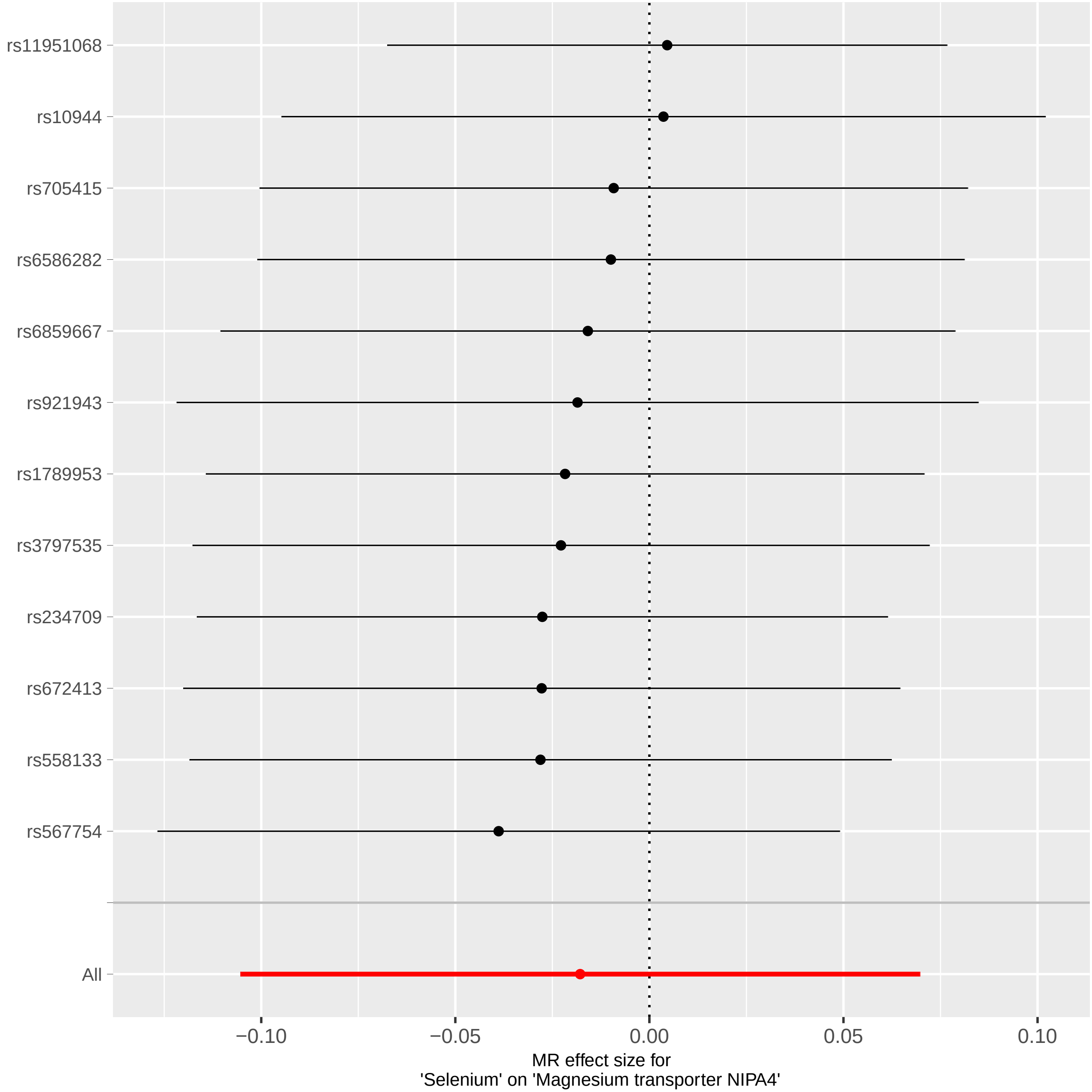

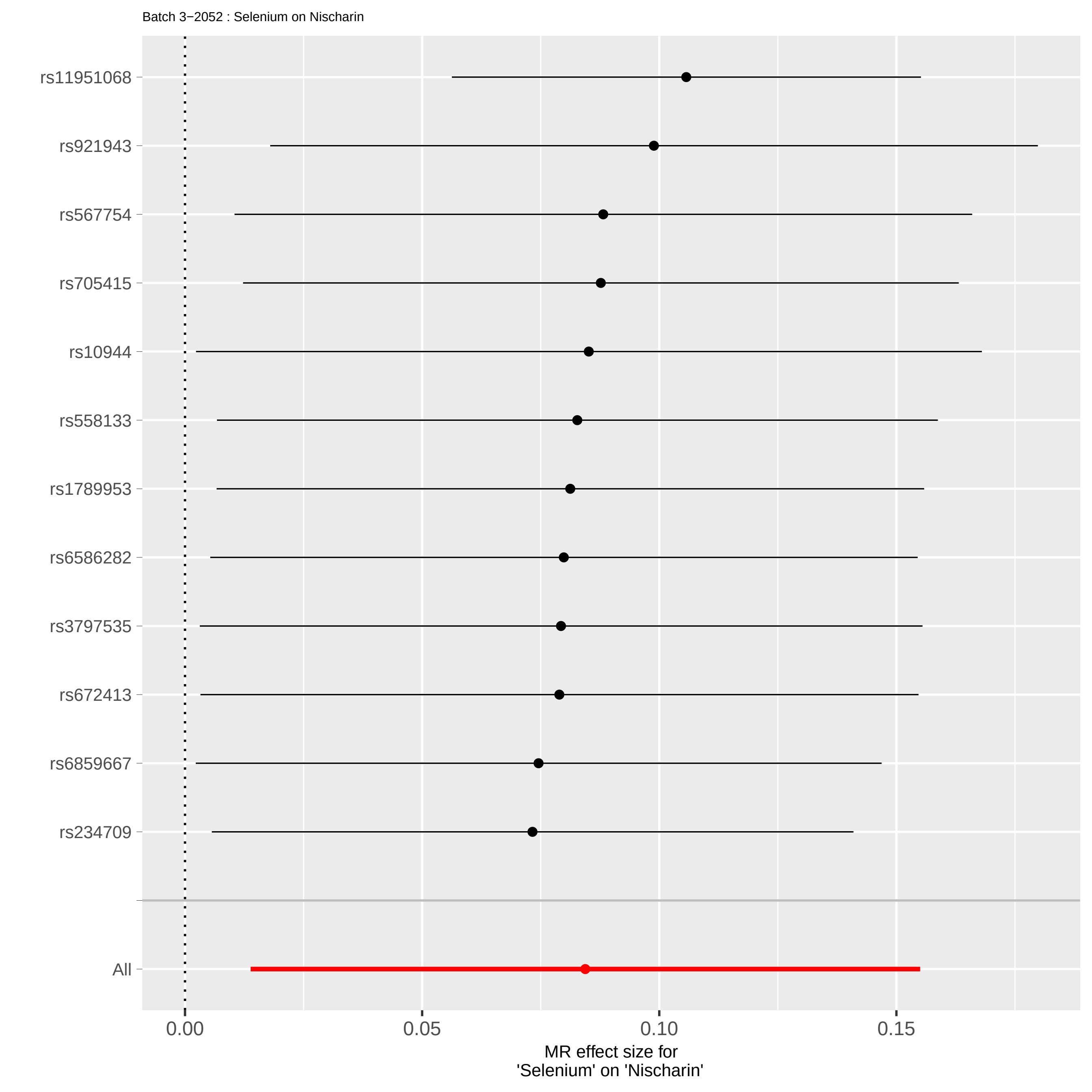

Batch 3−2052 : Selenium on Nischarin
rs11951068
rs921943
rs567754
rs705415
rs10944
rs558133
rs1789953
rs6586282
rs3797535
rs672413
rs6859667
rs234709
All
0.00
0.05
0.10
0.15
MR effect size for
'Selenium' on 'Nischarin'

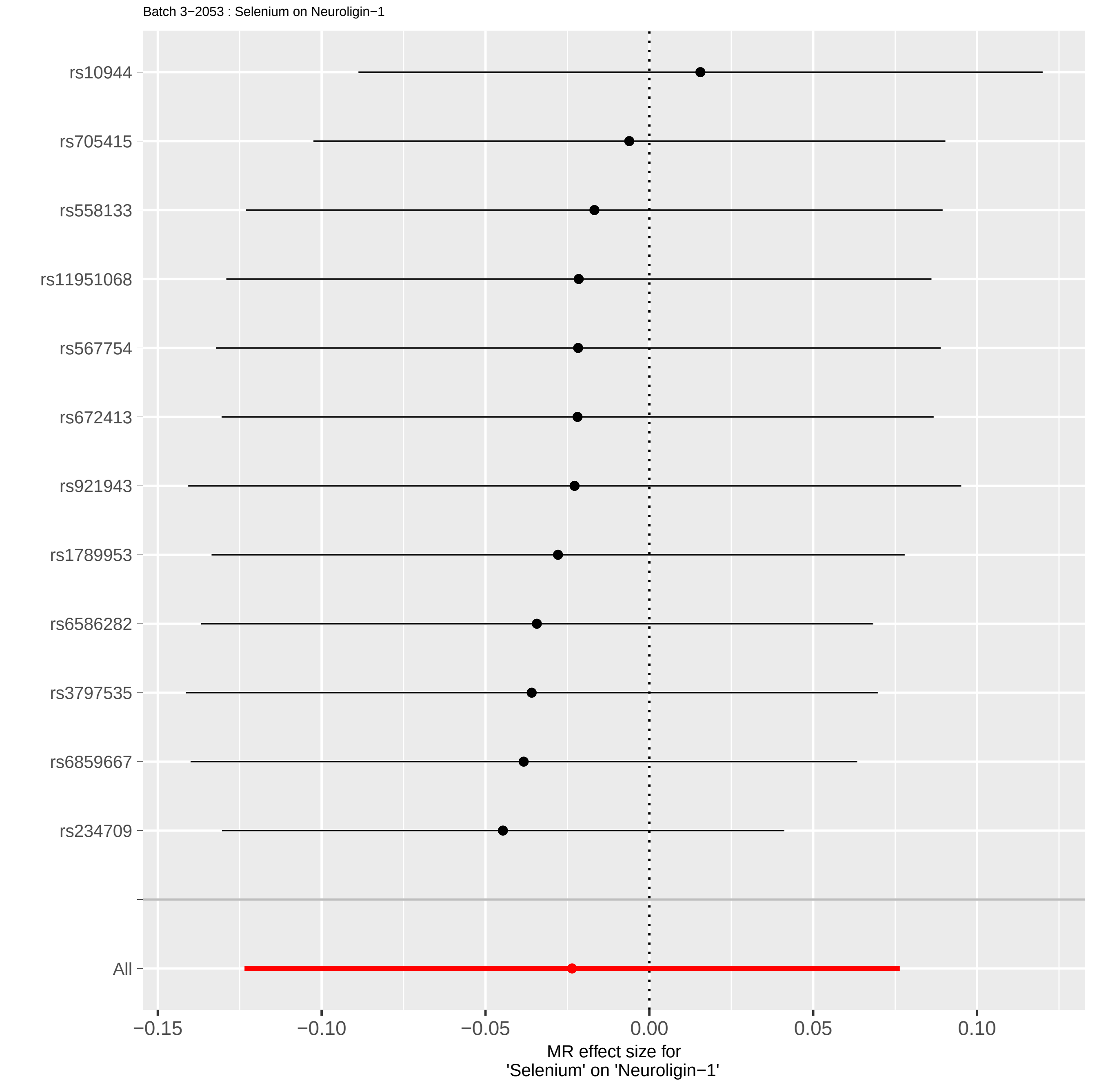

Batch 3−2053 : Selenium on Neuroligin−1
rs10944
rs705415
rs558133
rs11951068
rs567754
rs672413
rs921943
rs1789953
rs6586282
rs3797535
rs6859667
rs234709
All
−0.15
−0.10
−0.05
0.00
0.05
0.10
MR effect size for
'Selenium' on 'Neuroligin−1'

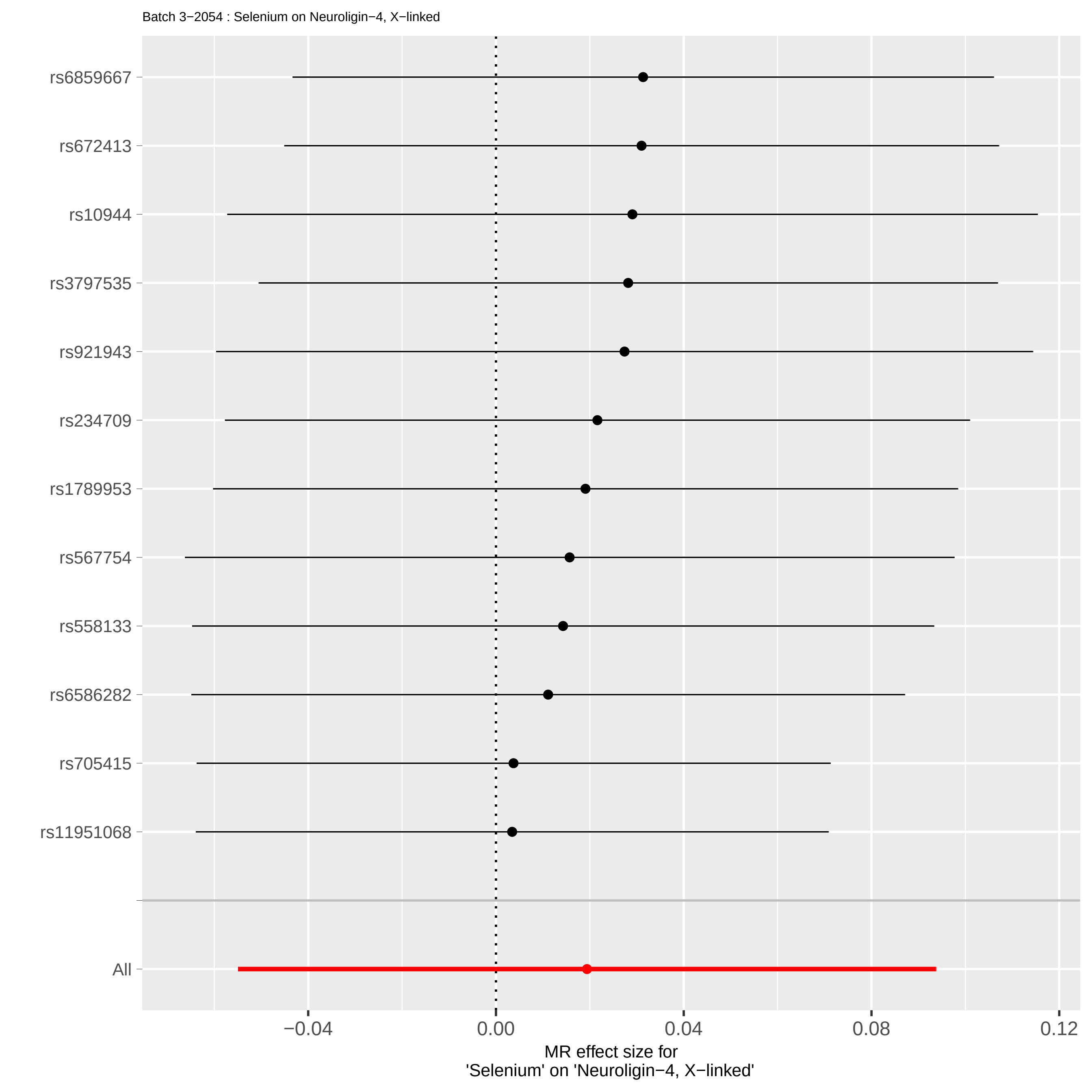

Batch 3−2054 : Selenium on Neuroligin−4, X−linked
rs6859667
rs672413
rs10944
rs3797535
rs921943
rs234709
rs1789953
rs567754
rs558133
rs6586282
rs705415
rs11951068
All
−0.04
0.00
0.04
0.08
0.12
MR effect size for
'Selenium' on 'Neuroligin−4, X−linked'

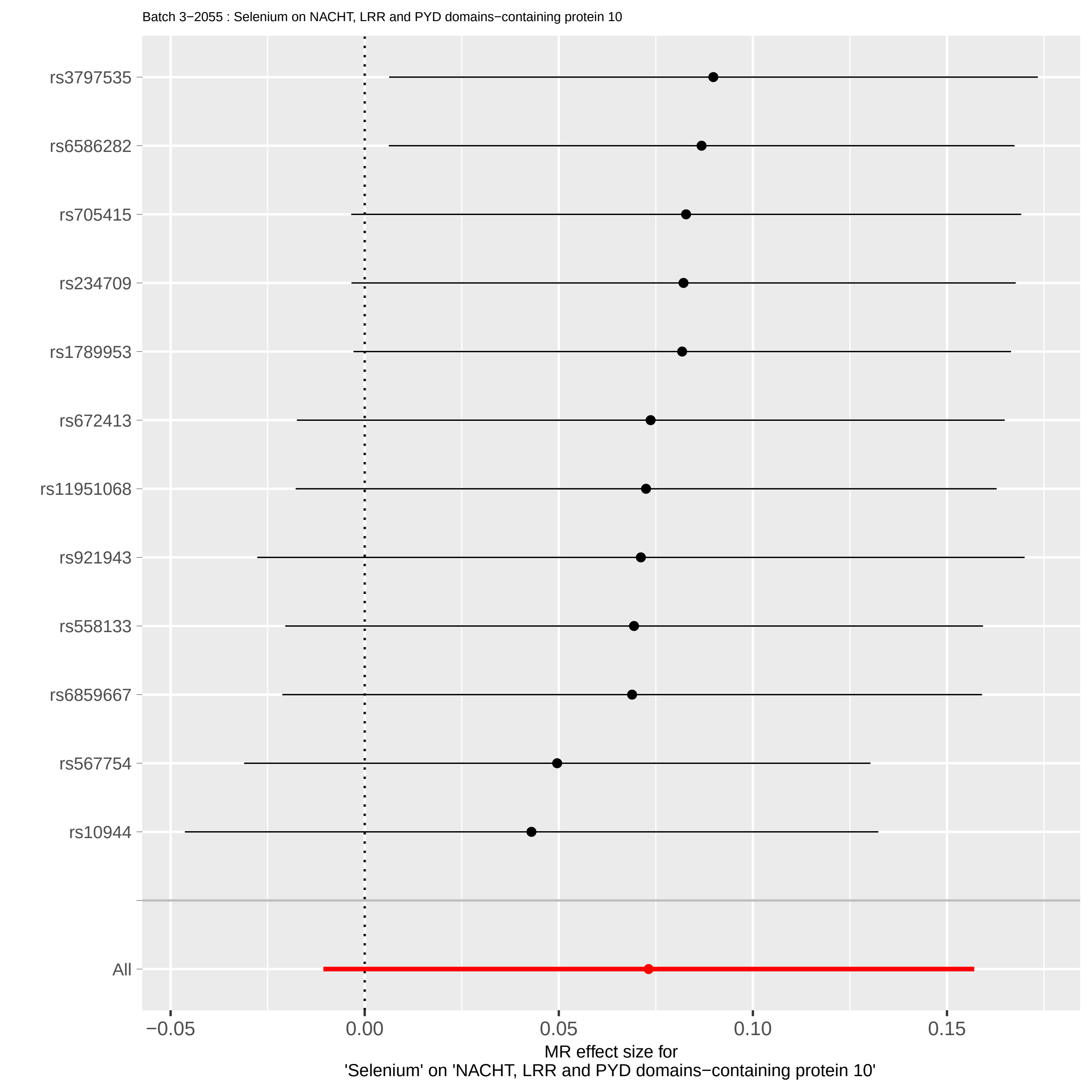

Batch 3−2055 : Selenium on NACHT, LRR and PYD domains−containing protein 10
rs3797535
rs6586282
rs705415
rs234709
rs1789953
rs672413
rs11951068
rs921943
rs558133
rs6859667
rs567754
rs10944
All
−0.05
0.00
0.05
0.10
0.15
MR effect size for
'Selenium' on 'NACHT, LRR and PYD domains−containing protein 10'

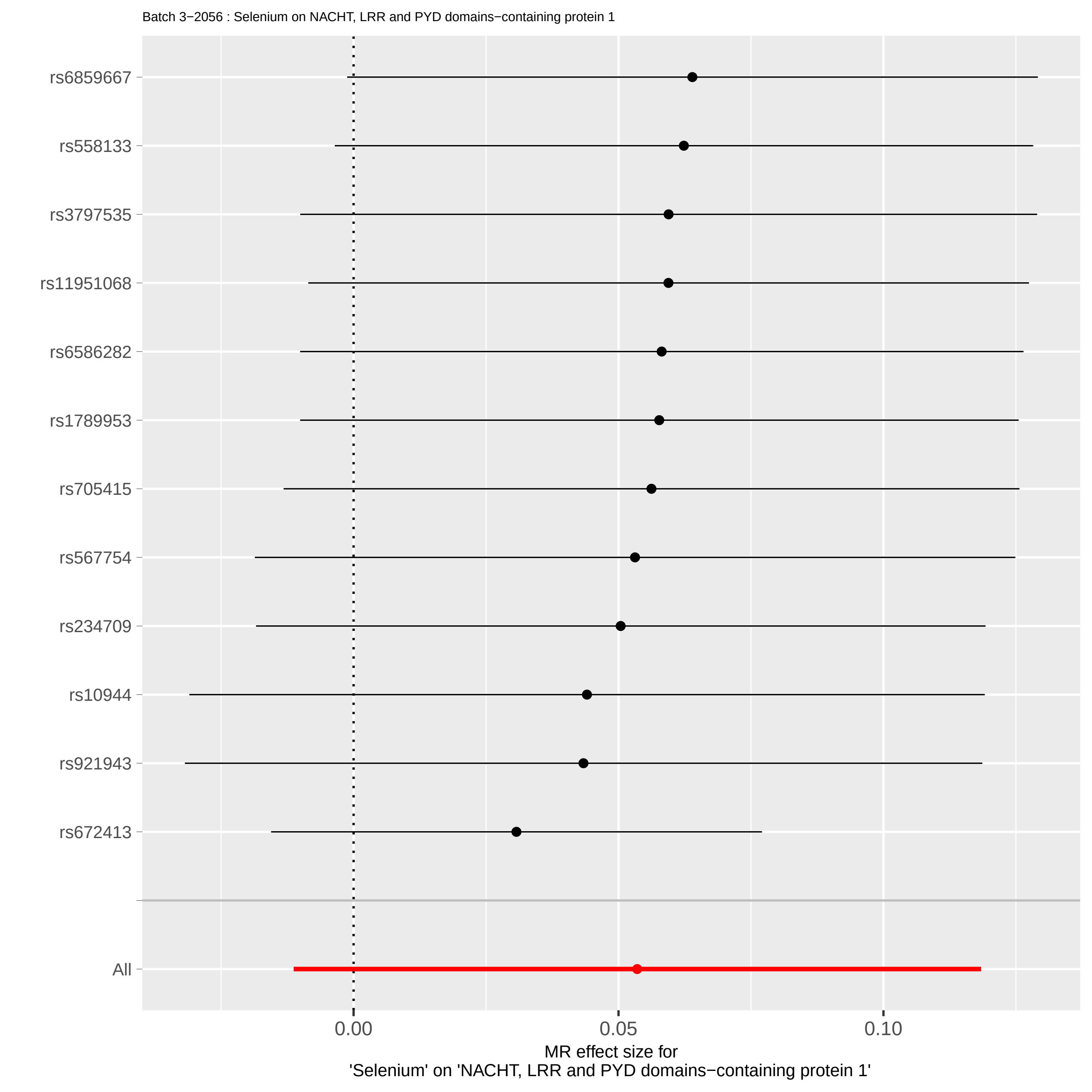

Batch 3−2056 : Selenium on NACHT, LRR and PYD domains−containing protein 1
rs6859667
rs558133
rs3797535
rs11951068
rs6586282
rs1789953
rs705415
rs567754
rs234709
rs10944
rs921943
rs672413
All
0.00
0.05
0.10
MR effect size for
'Selenium' on 'NACHT, LRR and PYD domains−containing protein 1'

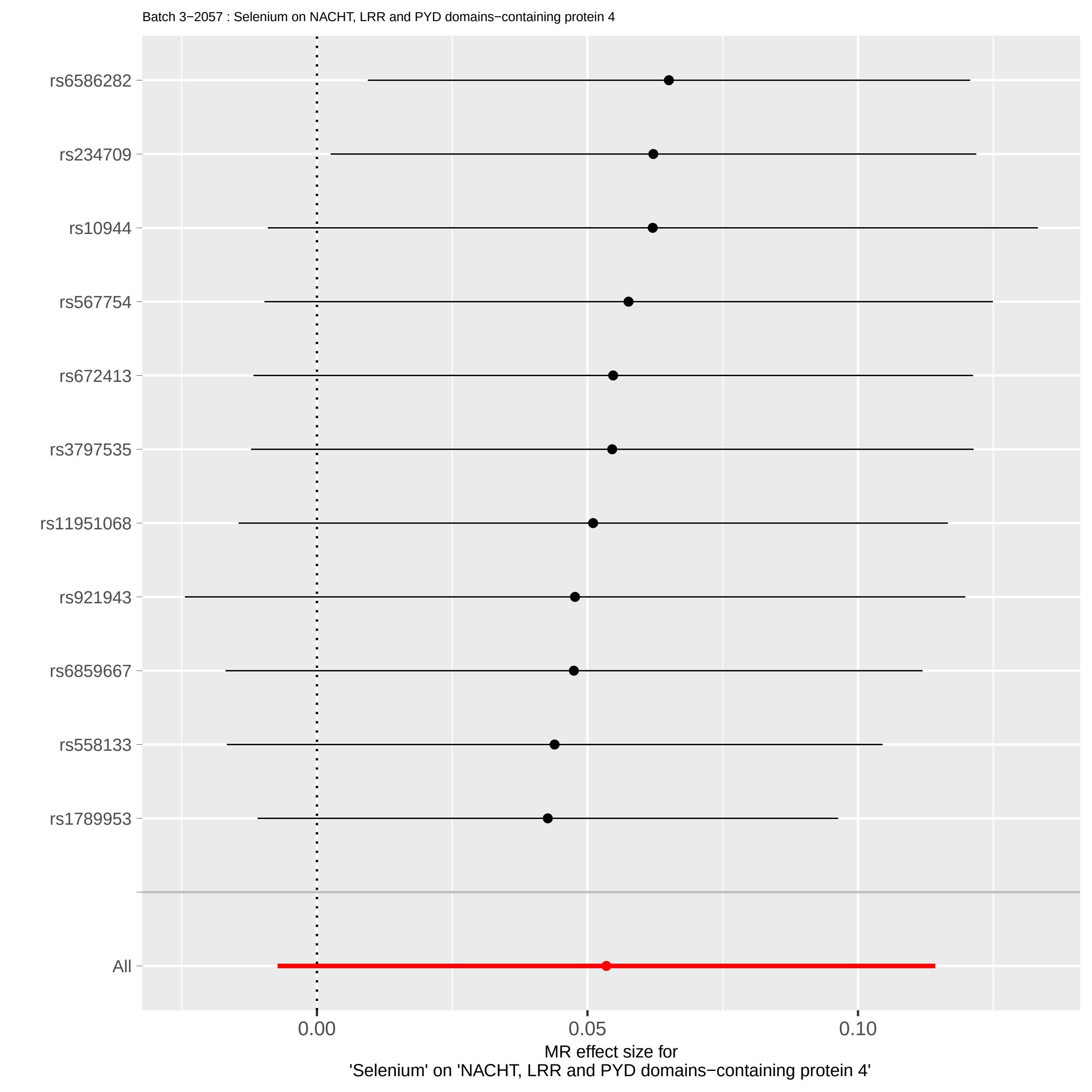

Batch 3−2057 : Selenium on NACHT, LRR and PYD domains−containing protein 4
rs6586282
rs234709
rs10944
rs567754
rs672413
rs3797535
rs11951068
rs921943
rs6859667
rs558133
rs1789953
All
0.00
0.05
0.10
MR effect size for
'Selenium' on 'NACHT, LRR and PYD domains−containing protein 4'

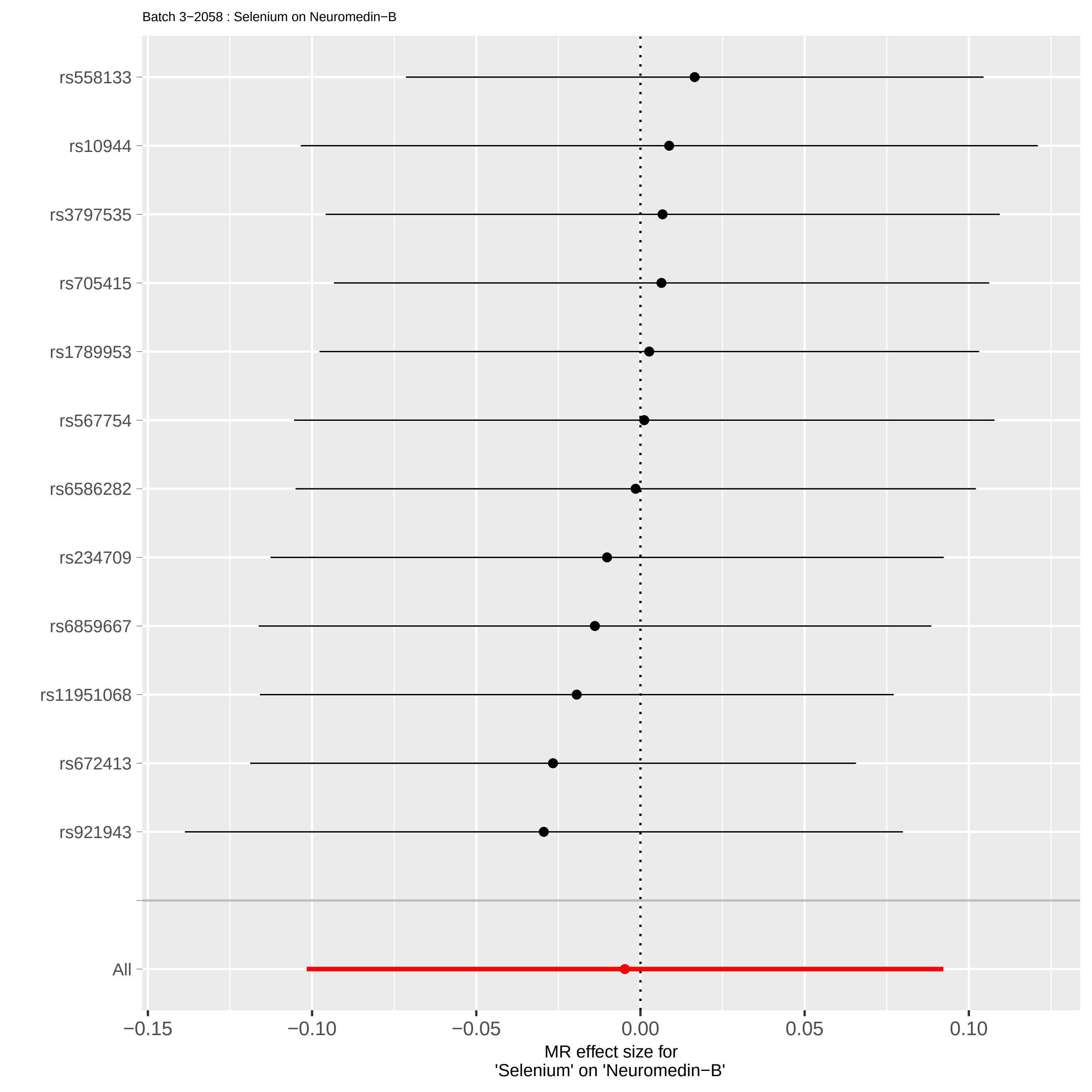

Batch 3−2058 : Selenium on Neuromedin−B
rs558133
rs10944
rs3797535
rs705415
rs1789953
rs567754
rs6586282
rs234709
rs6859667
rs11951068
rs672413
rs921943
All
−0.15
−0.10
−0.05
0.00
0.05
0.10
MR effect size for
'Selenium' on 'Neuromedin−B'

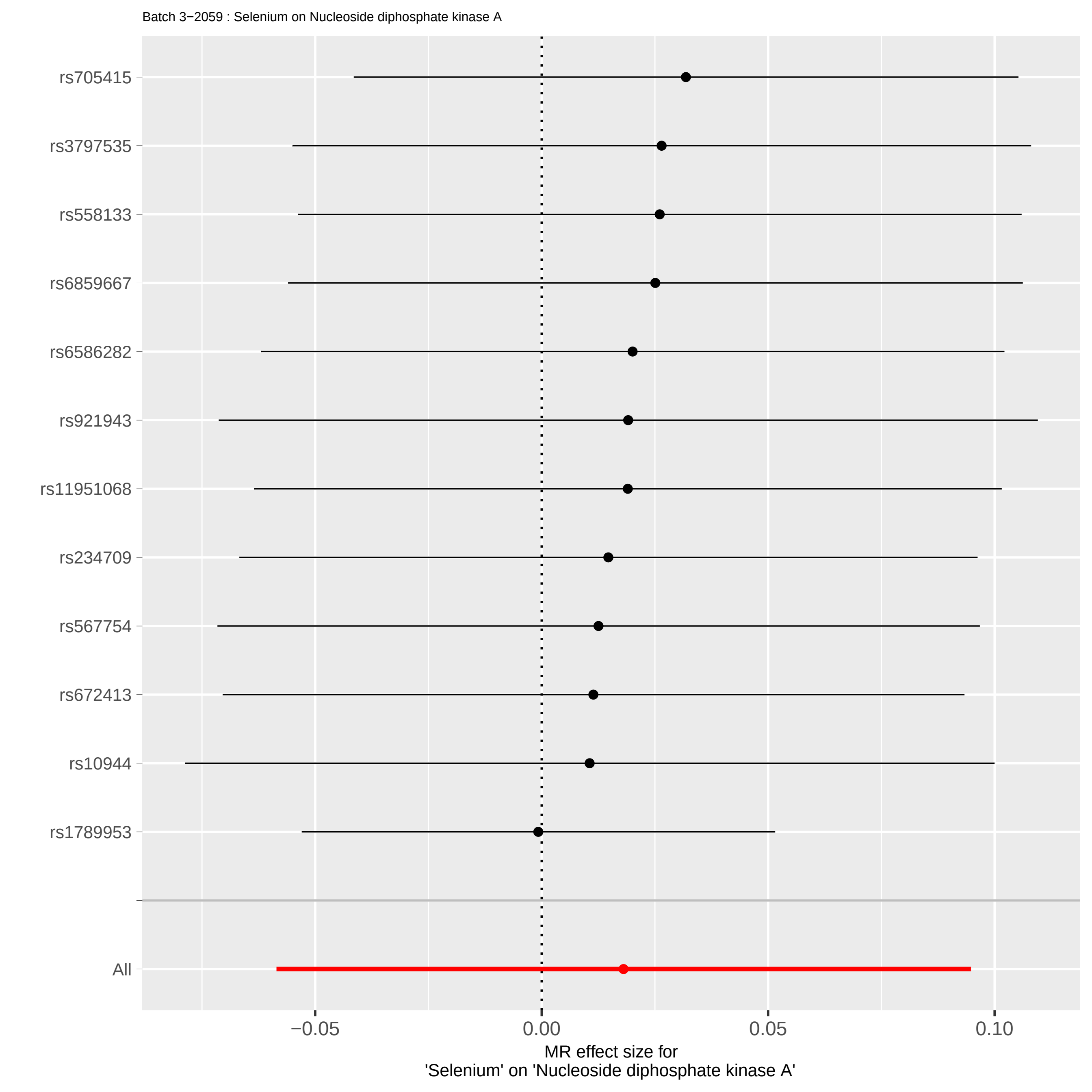

Batch 3−2059 : Selenium on Nucleoside diphosphate kinase A
rs705415
rs3797535
rs558133
rs6859667
rs6586282
rs921943
rs11951068
rs234709
rs567754
rs672413
rs10944
rs1789953
All
−0.05
0.00
0.05
0.10
MR effect size for
'Selenium' on 'Nucleoside diphosphate kinase A'

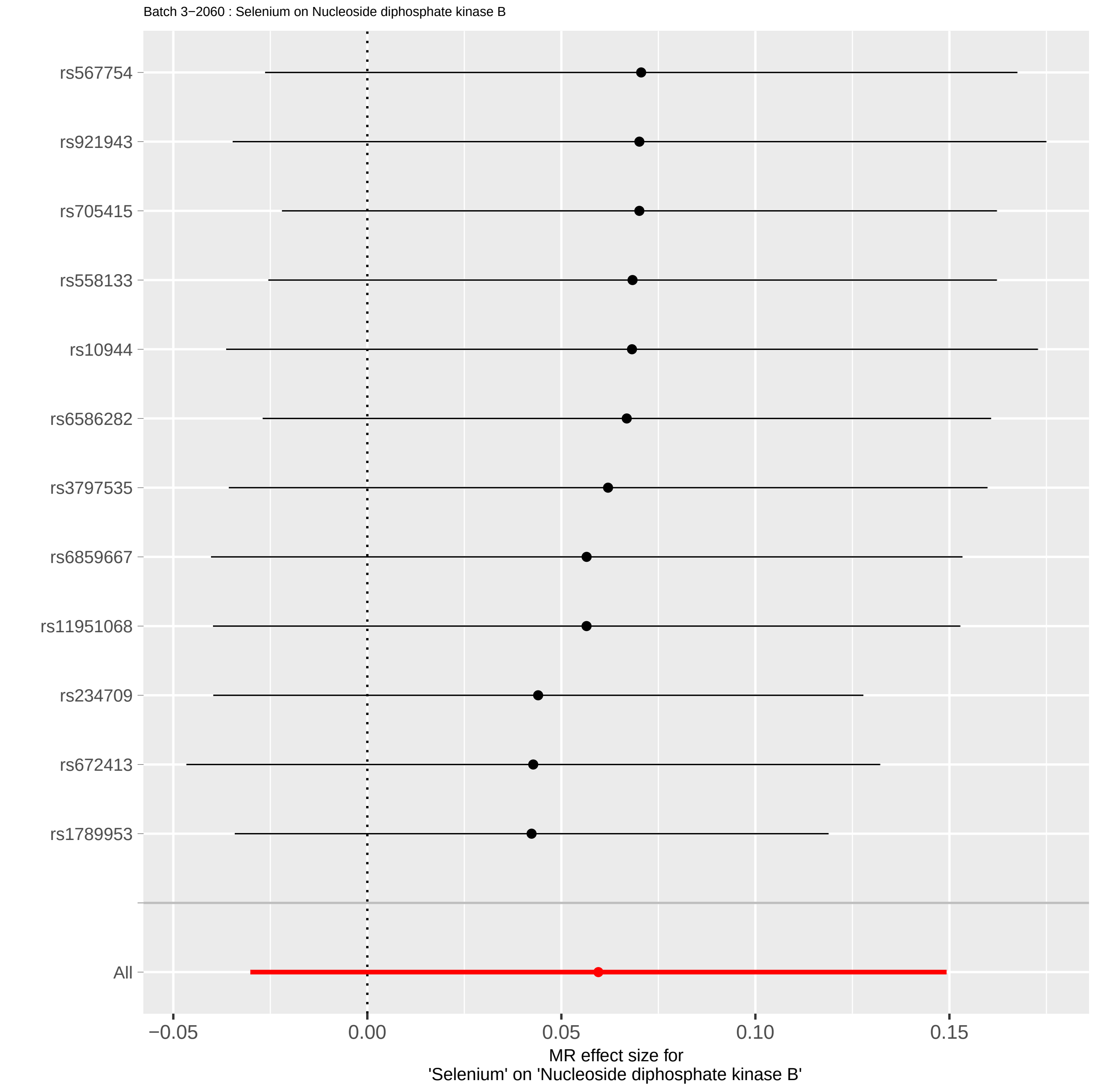

Batch 3−2060 : Selenium on Nucleoside diphosphate kinase B
rs567754
rs921943
rs705415
rs558133
rs10944
rs6586282
rs3797535
rs6859667
rs11951068
rs234709
rs672413
rs1789953
All
−0.05
0.00
0.05
0.10
0.15
MR effect size for
'Selenium' on 'Nucleoside diphosphate kinase B'

Batch 3−2061 : Selenium on Normal mucosa of esophagus−specific gene 1 protein

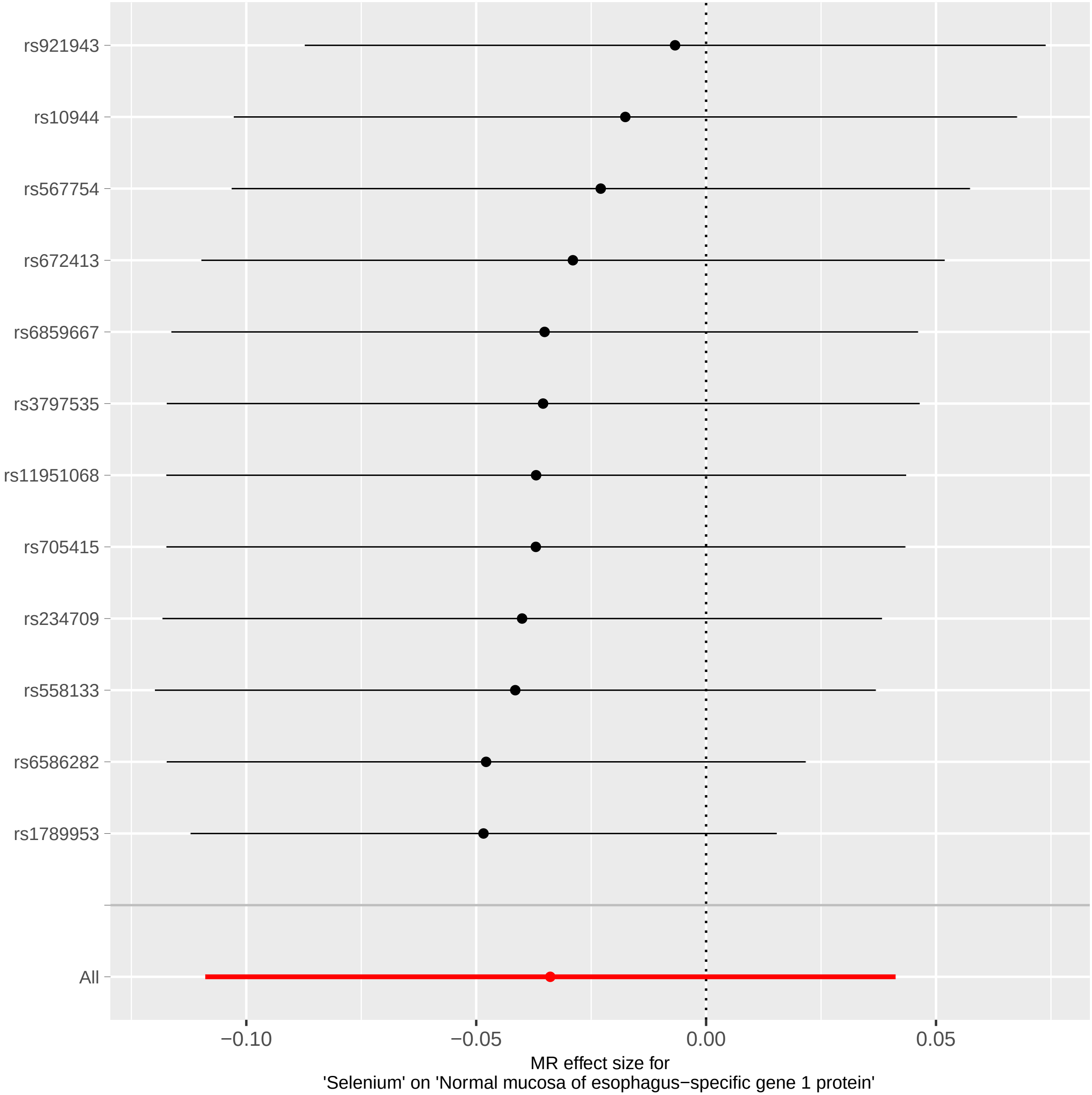

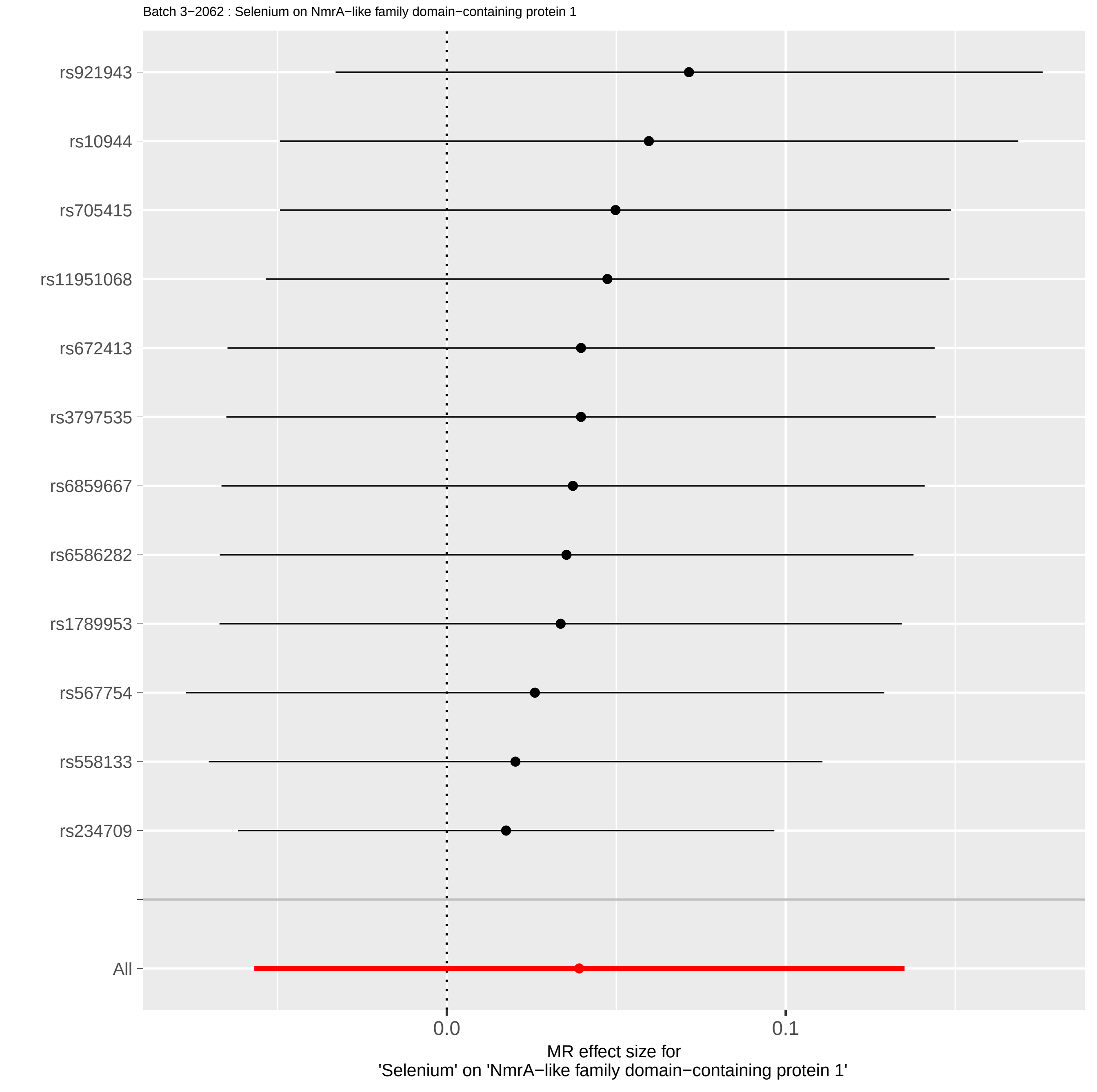

Batch 3−2062 : Selenium on NmrA−like family domain−containing protein 1
rs921943
rs10944
rs705415
rs11951068
rs672413
rs3797535
rs6859667
rs6586282
rs1789953
rs567754
rs558133
rs234709
All
0.0
0.1
MR effect size for
'Selenium' on 'NmrA−like family domain−containing protein 1'

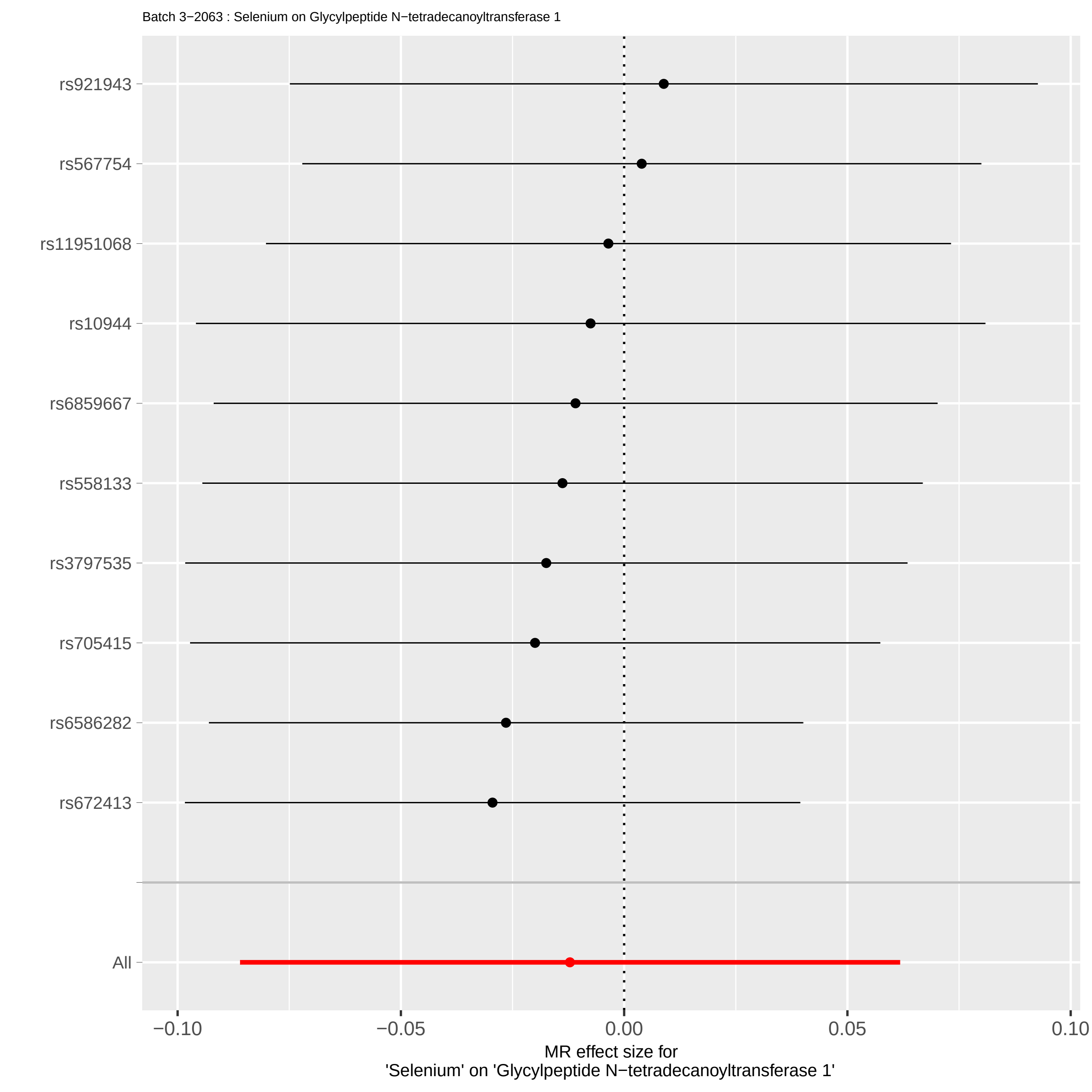
Batch 3−2063 : Selenium on Glycylpeptide N−tetradecanoyltransferase 1
rs921943
rs567754
rs11951068
rs10944
rs6859667
rs558133
rs3797535
rs705415
rs6586282
rs672413
All
−0.10
−0.05
0.00
0.05
0.10
MR effect size for
'Selenium' on 'Glycylpeptide N−tetradecanoyltransferase 1'

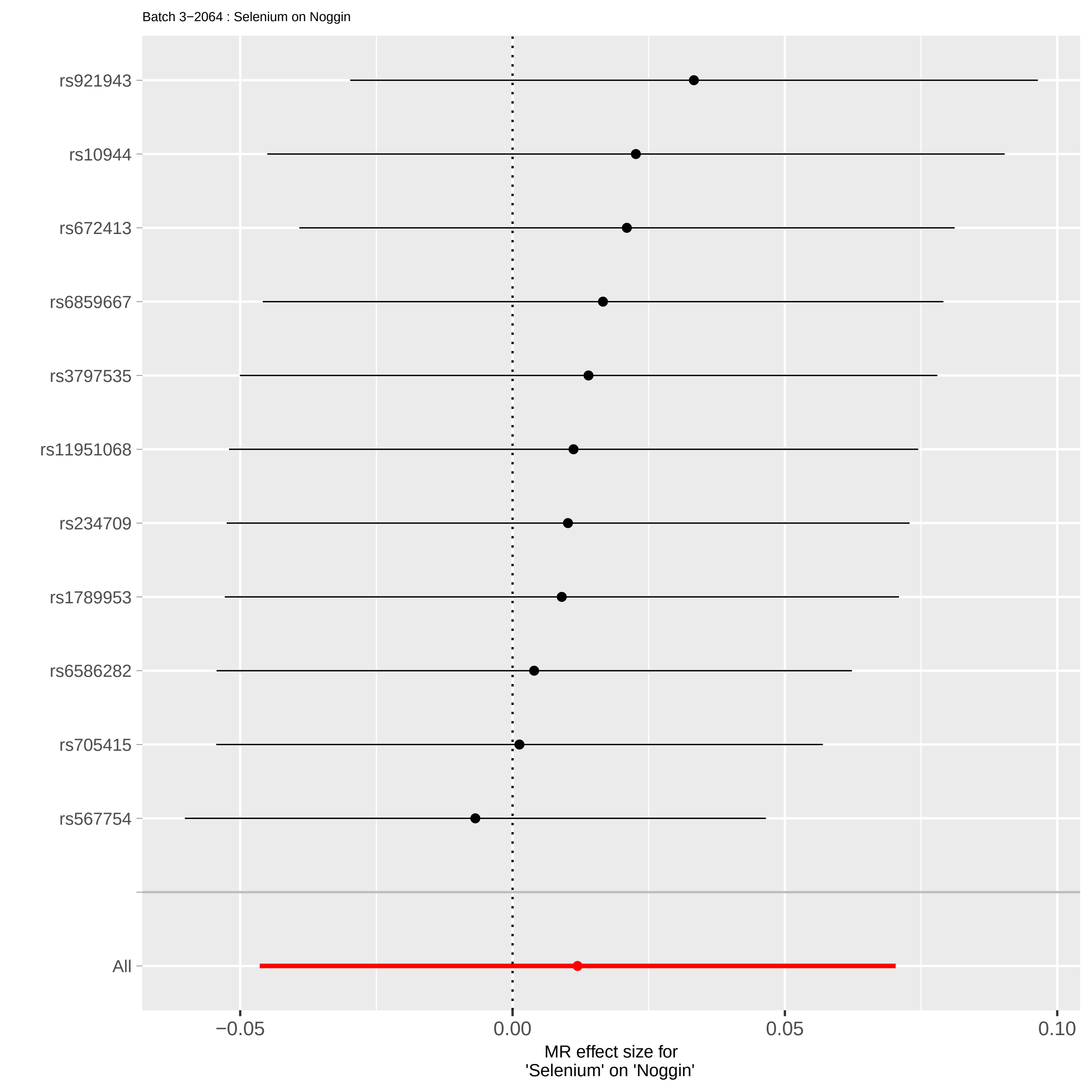

Batch 3−2064 : Selenium on Noggin
rs921943
rs10944
rs672413
rs6859667
rs3797535
rs11951068
rs234709
rs1789953
rs6586282
rs705415
rs567754
All
−0.05
0.00
0.05
0.10
MR effect size for
'Selenium' on 'Noggin'

Batch 3−2065 : Selenium on NoneX

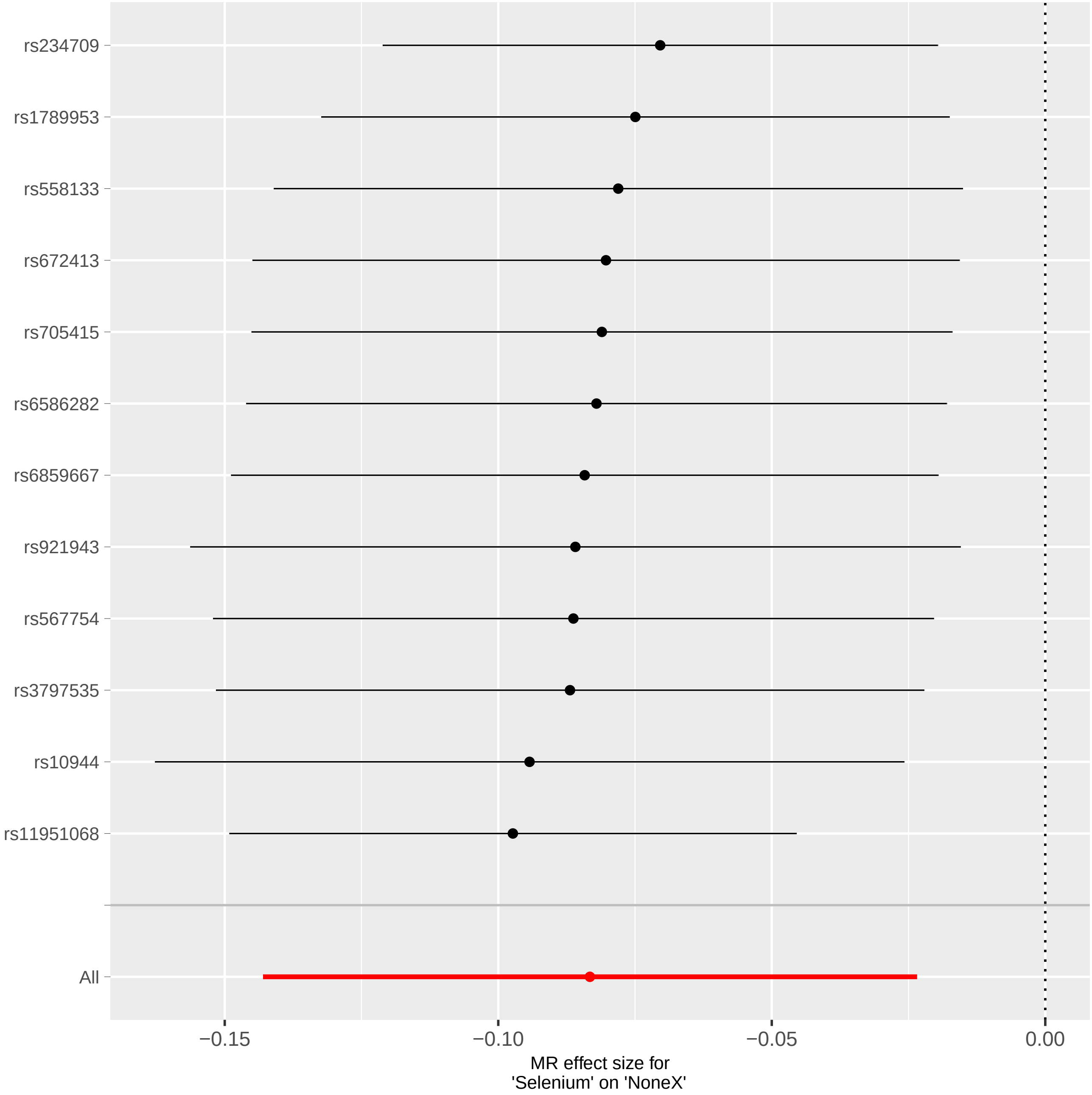

Batch 3−2066 : Selenium on NoneX

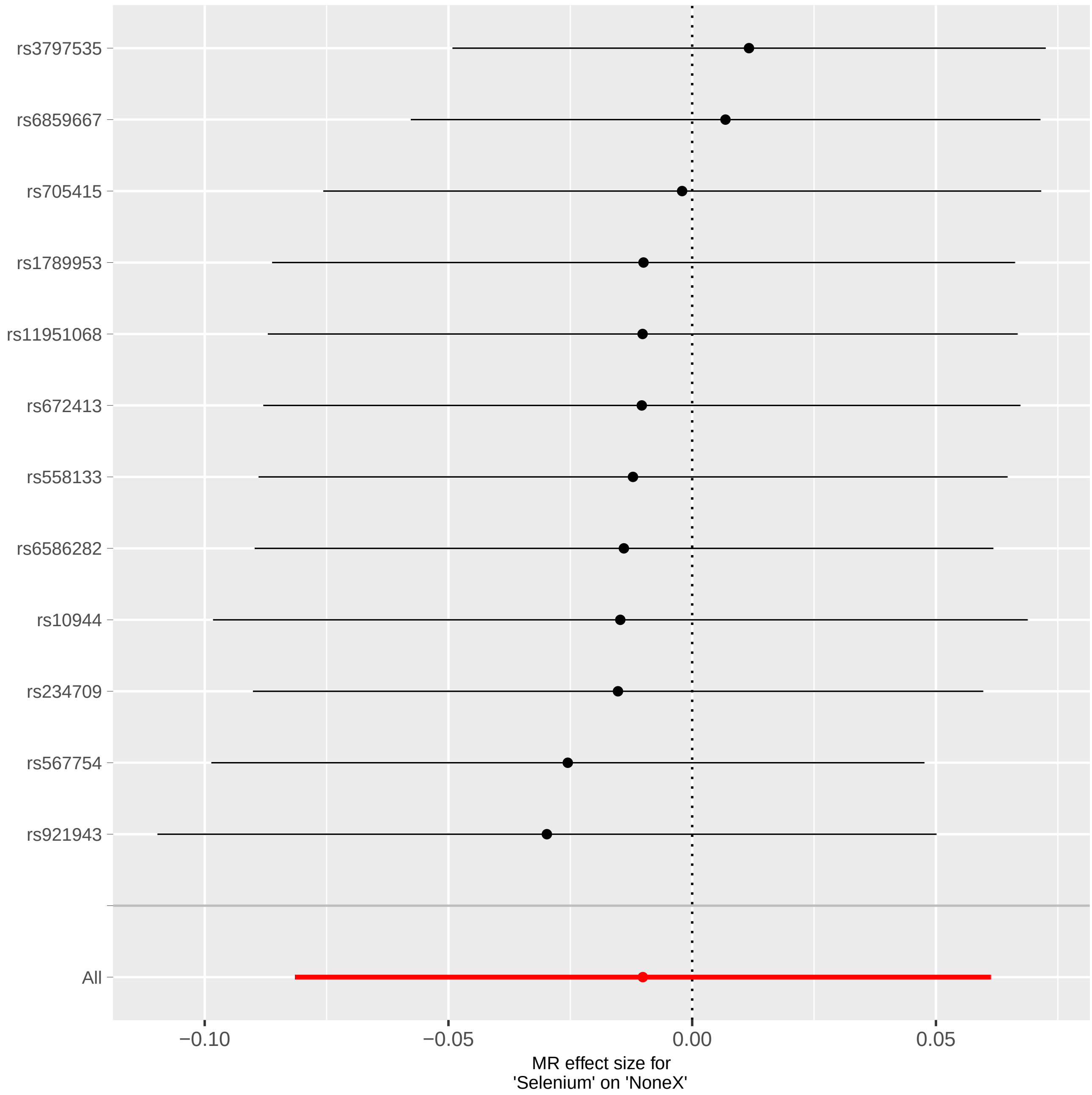

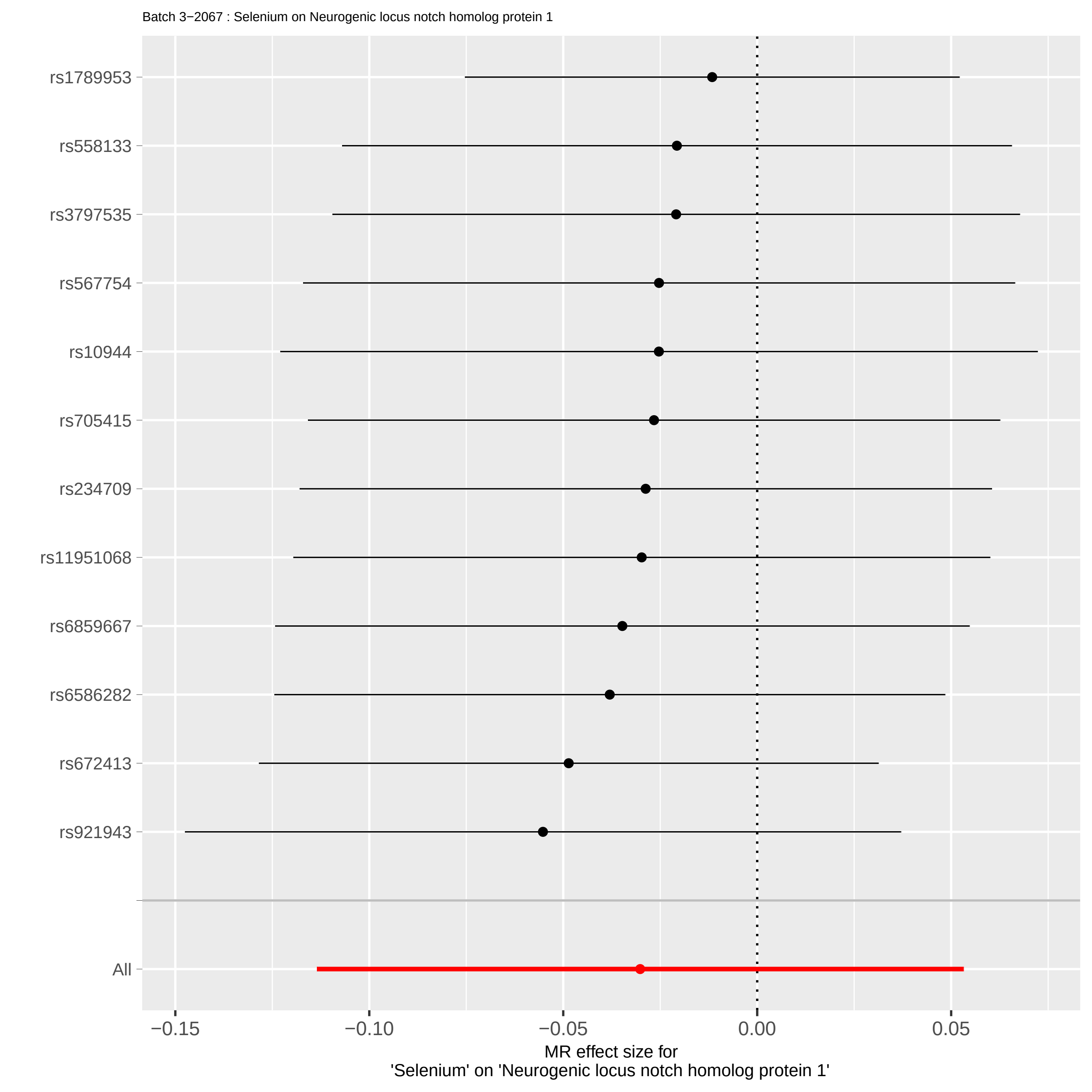

Batch 3−2067 : Selenium on Neurogenic locus notch homolog protein 1
rs1789953
rs558133
rs3797535
rs567754
rs10944
rs705415
rs234709
rs11951068
rs6859667
rs6586282
rs672413
rs921943
All
−0.15
−0.10
−0.05
0.00
0.05
MR effect size for
'Selenium' on 'Neurogenic locus notch homolog protein 1'

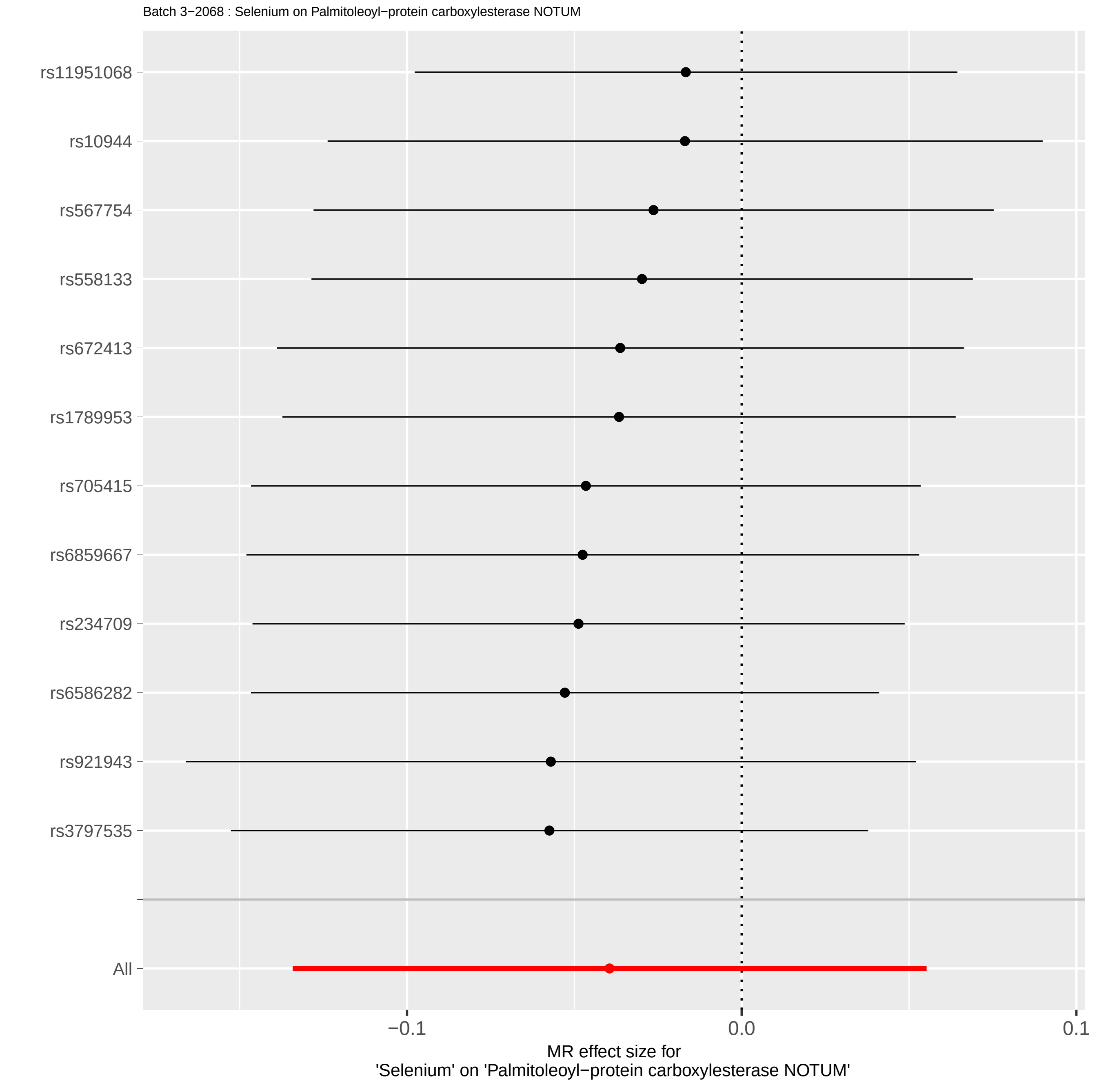

Batch 3−2068 : Selenium on Palmitoleoyl−protein carboxylesterase NOTUM
rs11951068
rs10944
rs567754
rs558133
rs672413
rs1789953
rs705415
rs6859667
rs234709
rs6586282
rs921943
rs3797535
All
−0.1
0.0
0.1
MR effect size for
'Selenium' on 'Palmitoleoyl−protein carboxylesterase NOTUM'

Batch 3−2069 : Selenium on Protein NOV homolog

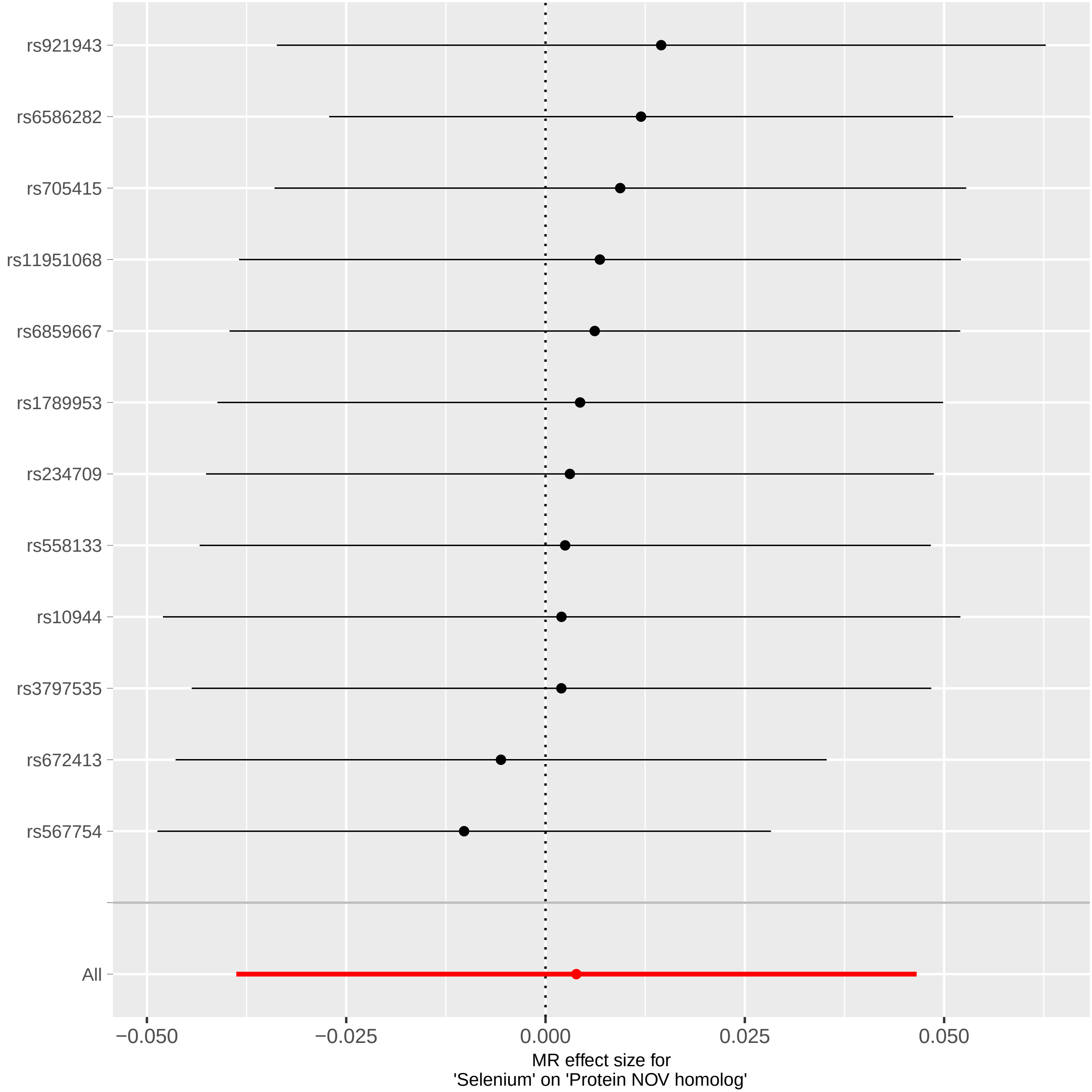

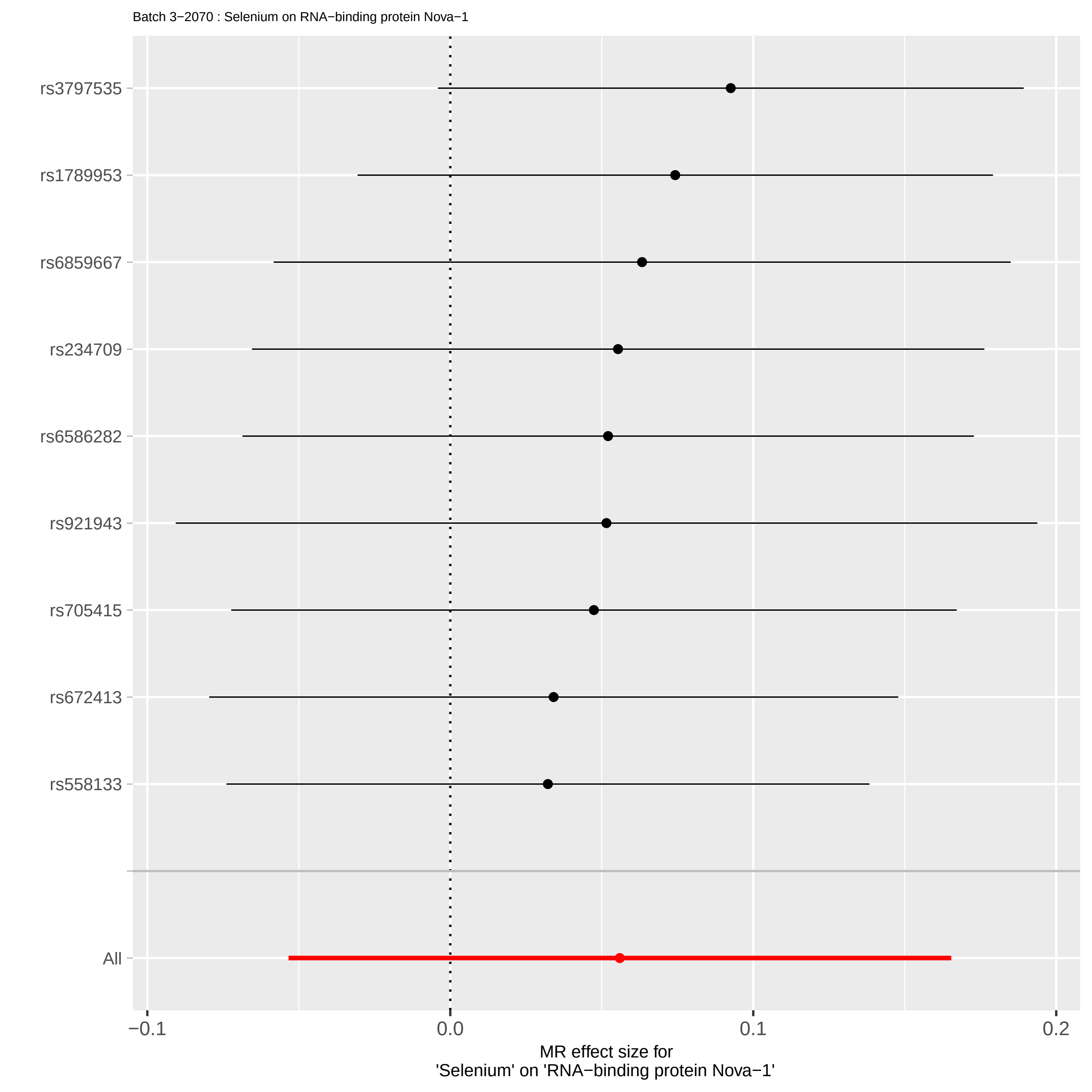
Batch 3−2070 : Selenium on RNA−binding protein Nova−1
rs3797535
rs1789953
rs6859667
rs234709
rs6586282
rs921943
rs705415
rs672413
rs558133
All
−0.1
0.0
0.1
0.2
MR effect size for
'Selenium' on 'RNA−binding protein Nova−1'

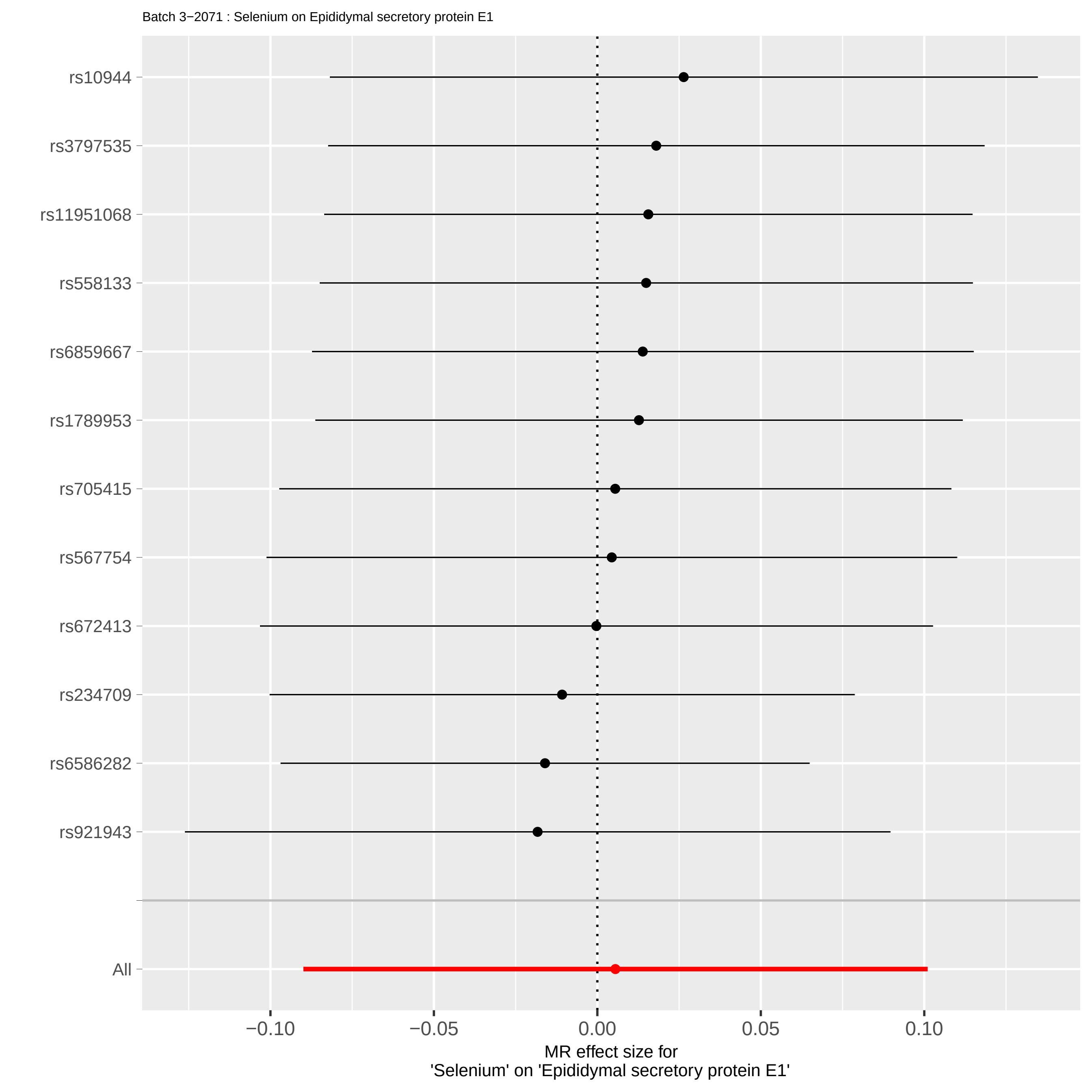

Batch 3−2071 : Selenium on Epididymal secretory protein E1
rs10944
rs3797535
rs11951068
rs558133
rs6859667
rs1789953
rs705415
rs567754
rs672413
rs234709
rs6586282
rs921943
All
−0.10
−0.05
0.00
0.05
0.10
MR effect size for
'Selenium' on 'Epididymal secretory protein E1'

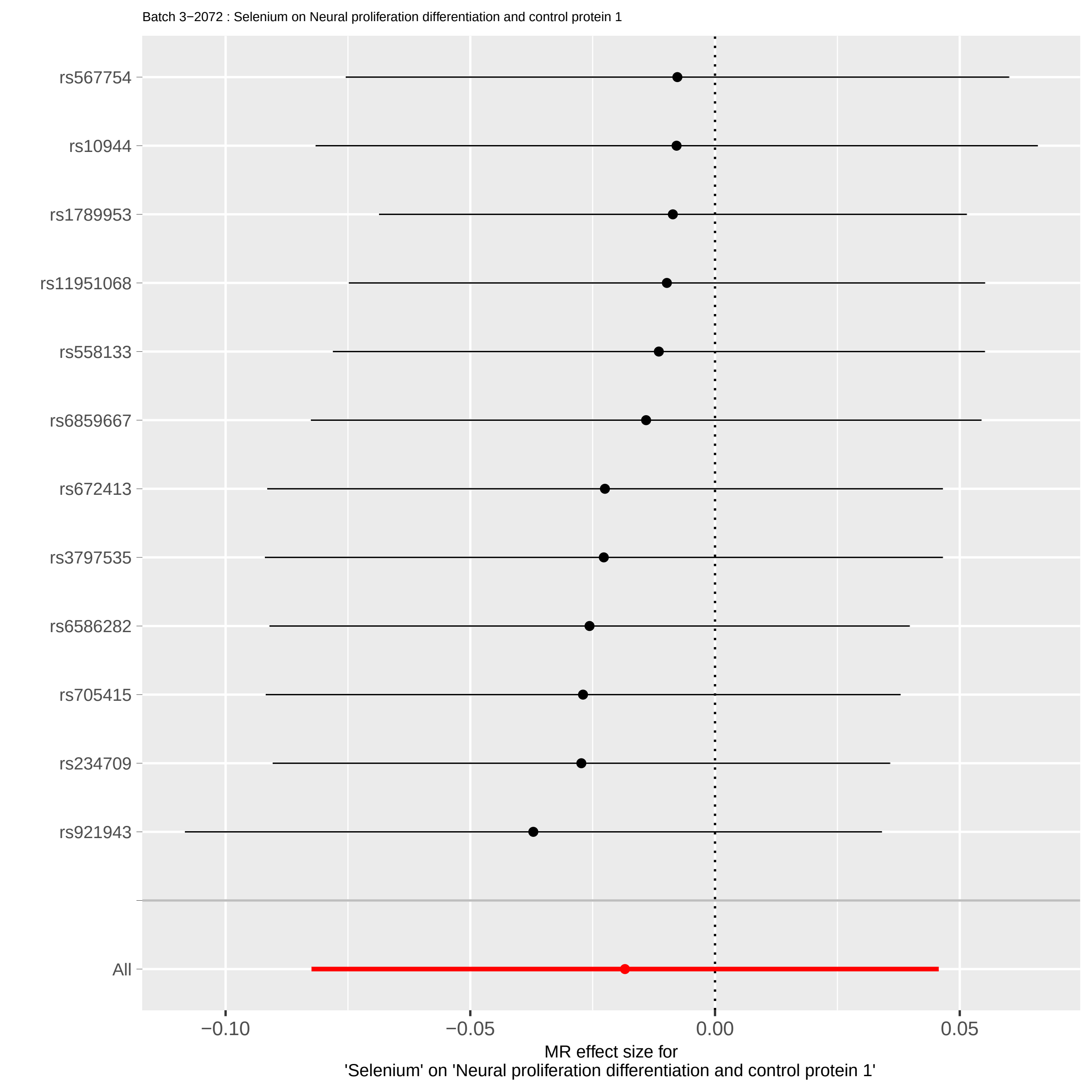

Batch 3−2072 : Selenium on Neural proliferation differentiation and control protein 1
rs567754
rs10944
rs1789953
rs11951068
rs558133
rs6859667
rs672413
rs3797535
rs6586282
rs705415
rs234709
rs921943
All
−0.10
−0.05
0.00
0.05
MR effect size for
'Selenium' on 'Neural proliferation differentiation and control protein 1'

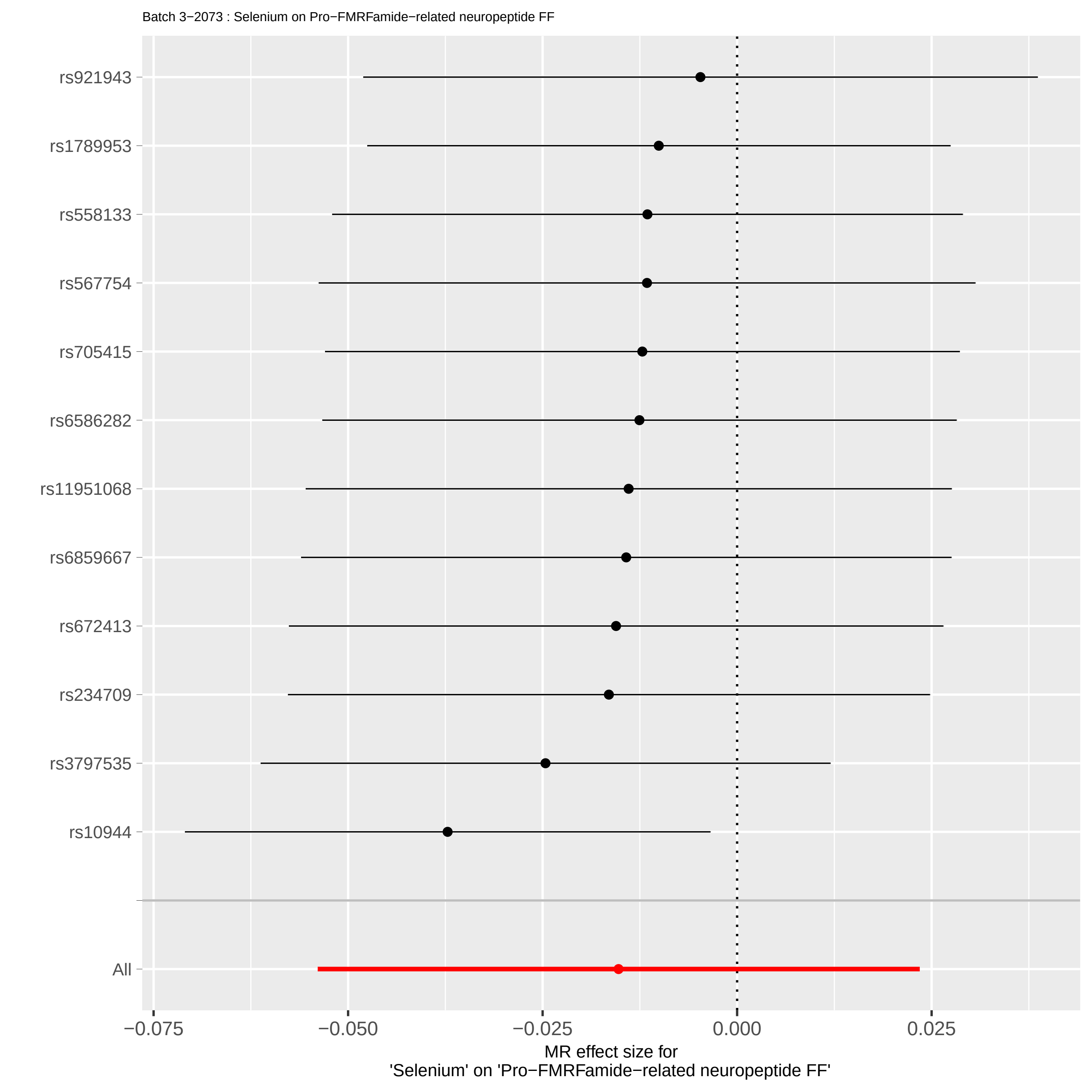

Batch 3−2073 : Selenium on Pro−FMRFamide−related neuropeptide FF
rs921943
rs1789953
rs558133
rs567754
rs705415
rs6586282
rs11951068
rs6859667
rs672413
rs234709
rs3797535
rs10944
All
−0.075
−0.050
−0.025
0.000
0.025
MR effect size for
'Selenium' on 'Pro−FMRFamide−related neuropeptide FF'

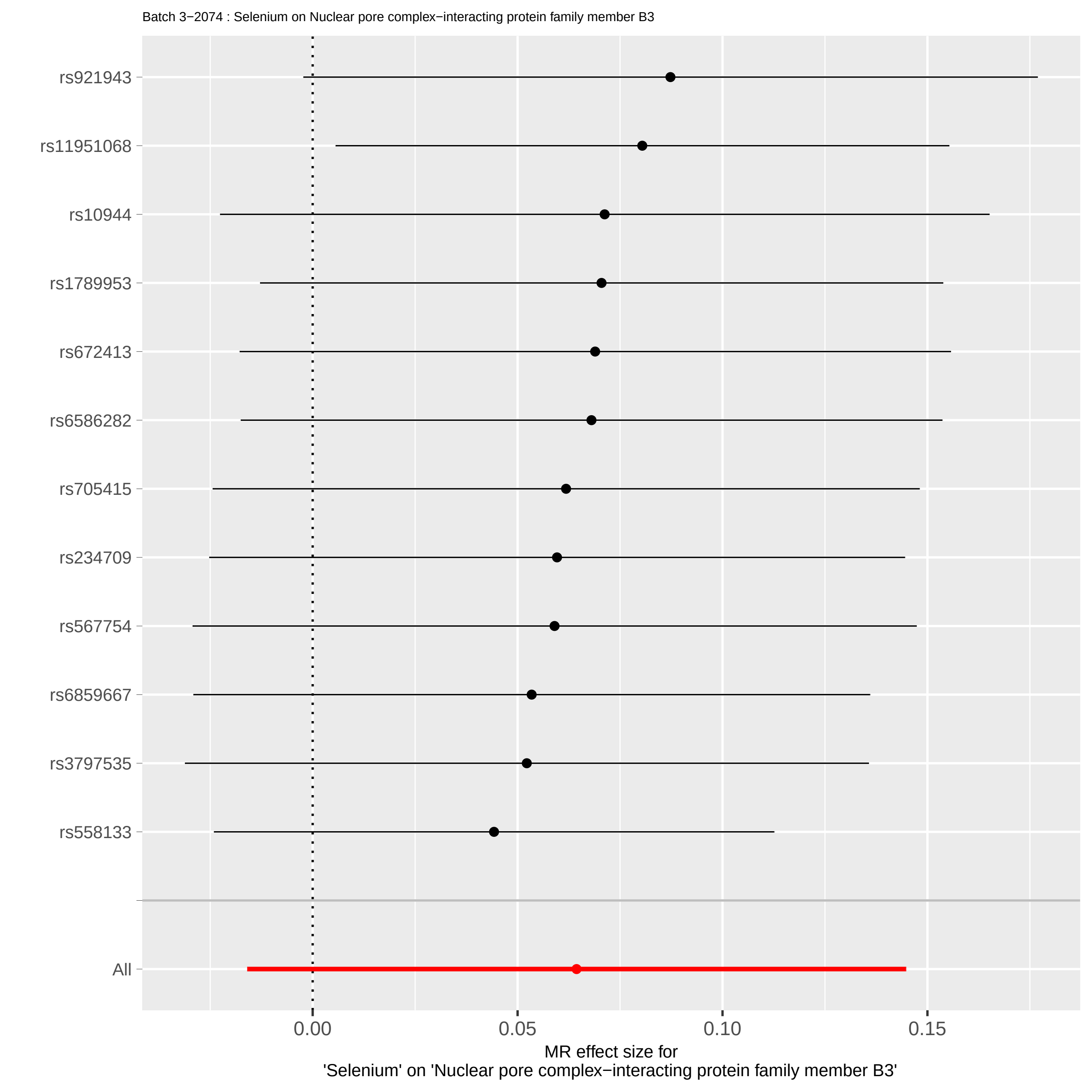

Batch 3−2074 : Selenium on Nuclear pore complex−interacting protein family member B3
rs921943
rs11951068
rs10944
rs1789953
rs672413
rs6586282
rs705415
rs234709
rs567754
rs6859667
rs3797535
rs558133
All
0.00
0.05
0.10
0.15
MR effect size for
'Selenium' on 'Nuclear pore complex−interacting protein family member B3'

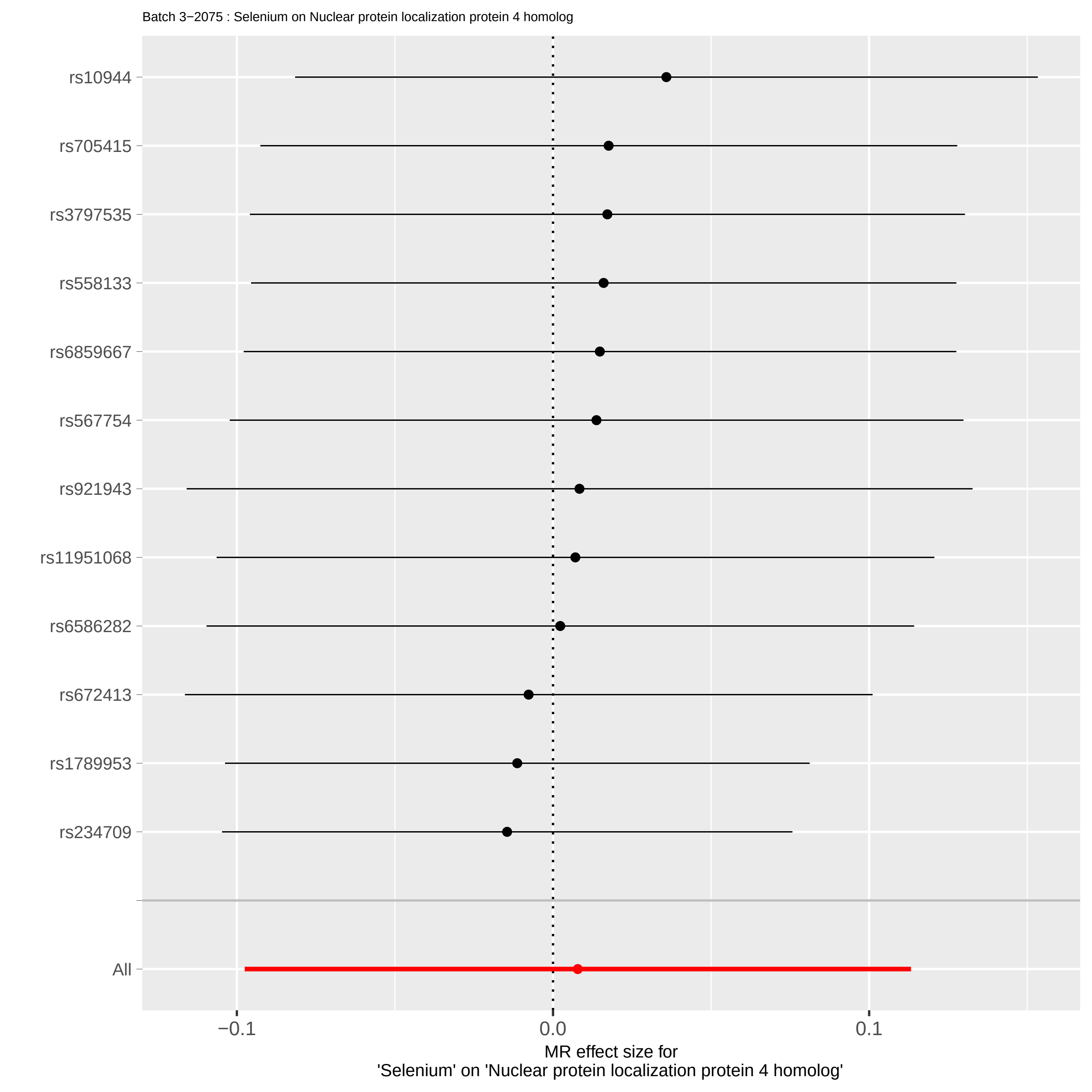

Batch 3−2075 : Selenium on Nuclear protein localization protein 4 homolog
rs10944
rs705415
rs3797535
rs558133
rs6859667
rs567754
rs921943
rs11951068
rs6586282
rs672413
rs1789953
rs234709
All
−0.1
0.0
0.1
MR effect size for
'Selenium' on 'Nuclear protein localization protein 4 homolog'

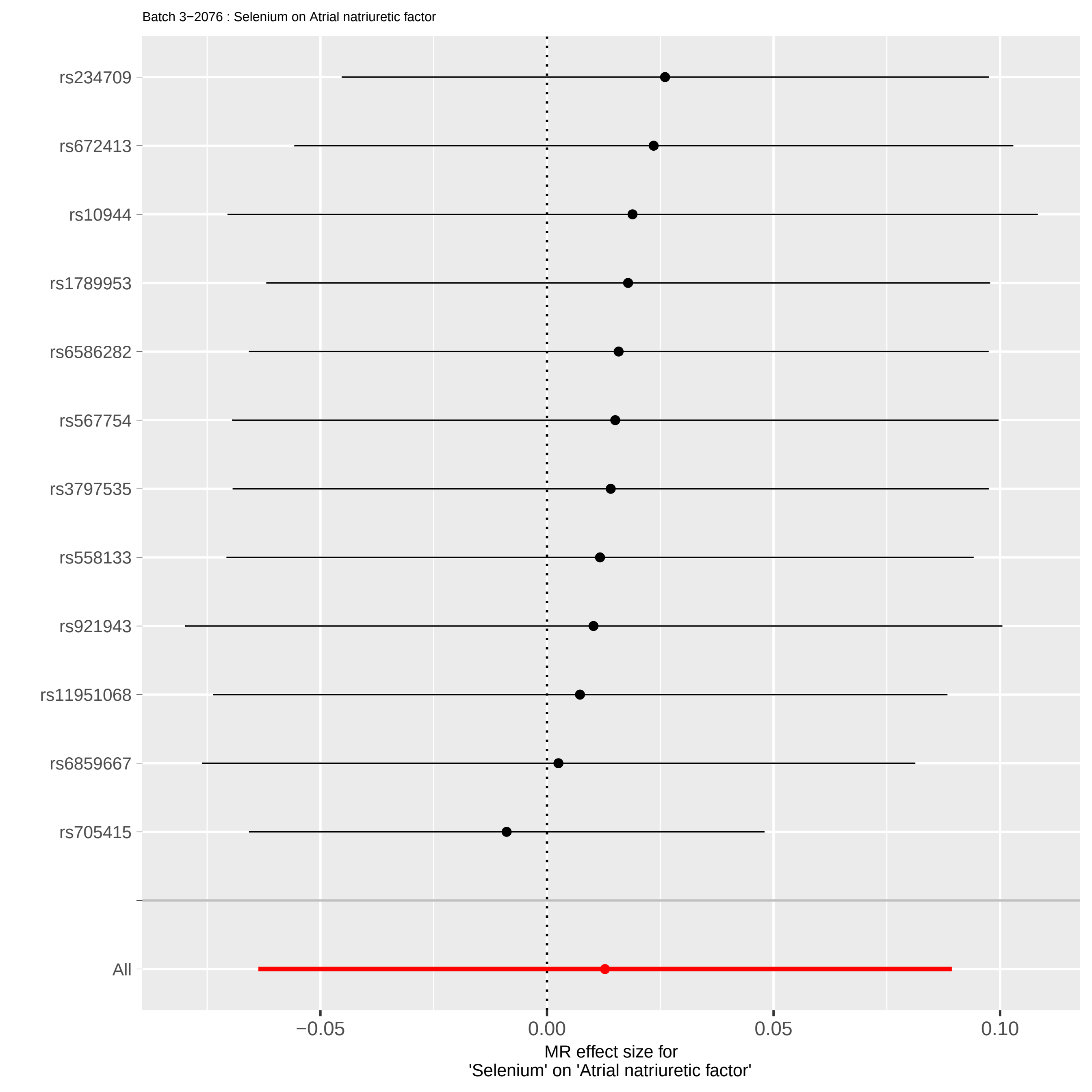

Batch 3−2076 : Selenium on Atrial natriuretic factor
rs234709
rs672413
rs10944
rs1789953
rs6586282
rs567754
rs3797535
rs558133
rs921943
rs11951068
rs6859667
rs705415
All
−0.05
0.00
0.05
0.10
MR effect size for
'Selenium' on 'Atrial natriuretic factor'

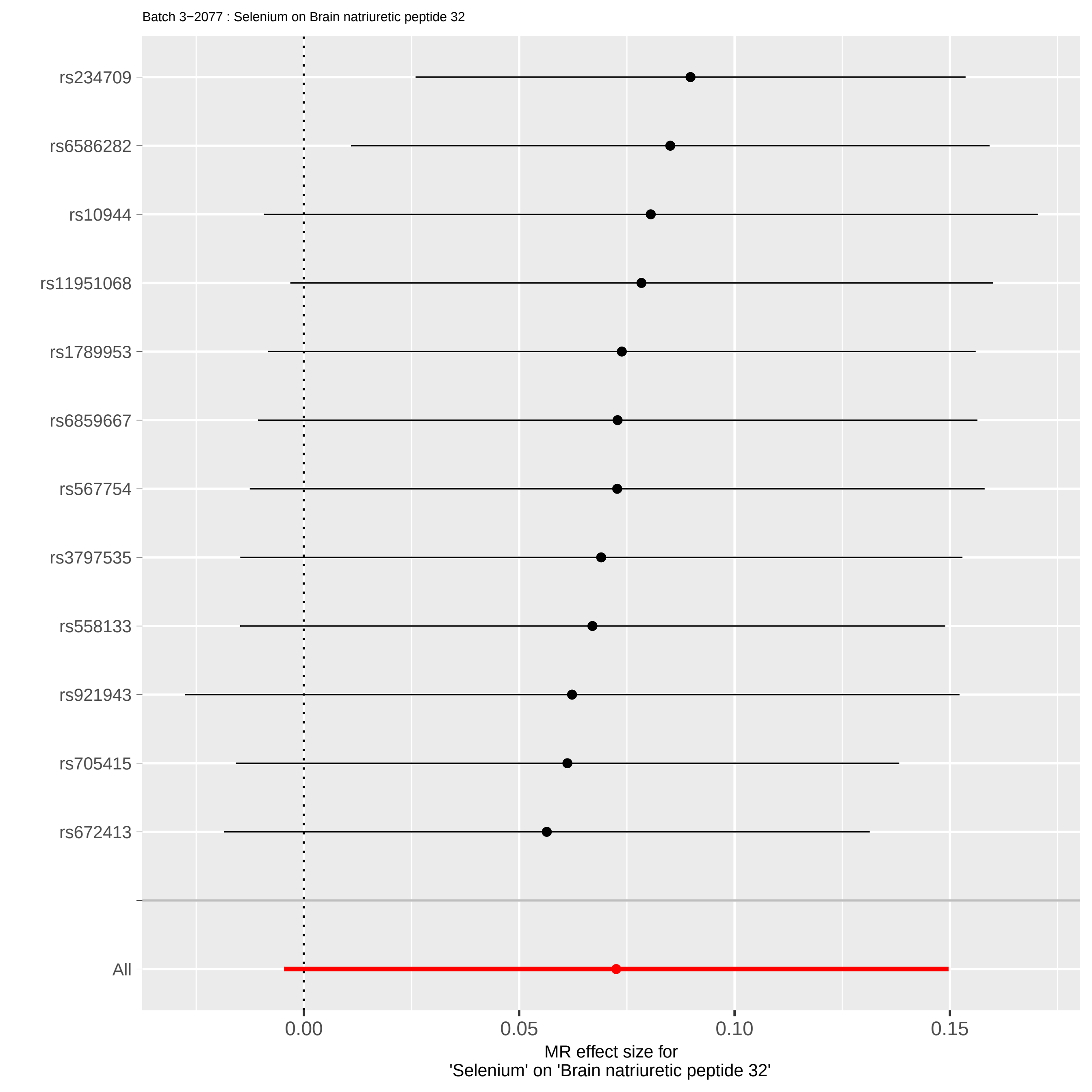

Batch 3−2077 : Selenium on Brain natriuretic peptide 32
rs234709
rs6586282
rs10944
rs11951068
rs1789953
rs6859667
rs567754
rs3797535
rs558133
rs921943
rs705415
rs672413
All
0.00
0.05
0.10
0.15
MR effect size for
'Selenium' on 'Brain natriuretic peptide 32'

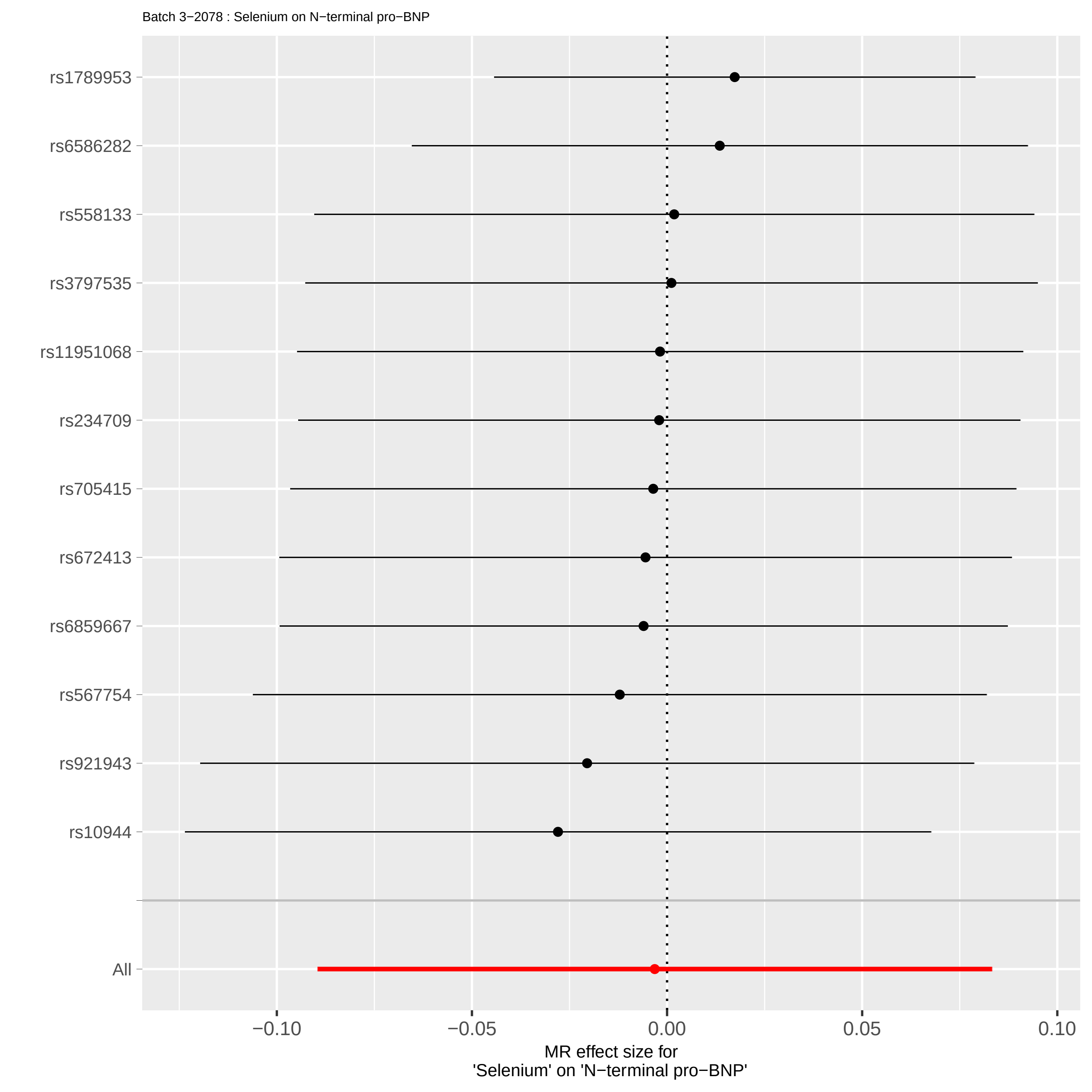

Batch 3−2078 : Selenium on N−terminal pro−BNP
rs1789953
rs6586282
rs558133
rs3797535
rs11951068
rs234709
rs705415
rs672413
rs6859667
rs567754
rs921943
rs10944
All
−0.10
−0.05
0.00
0.05
0.10
MR effect size for
'Selenium' on 'N−terminal pro−BNP'

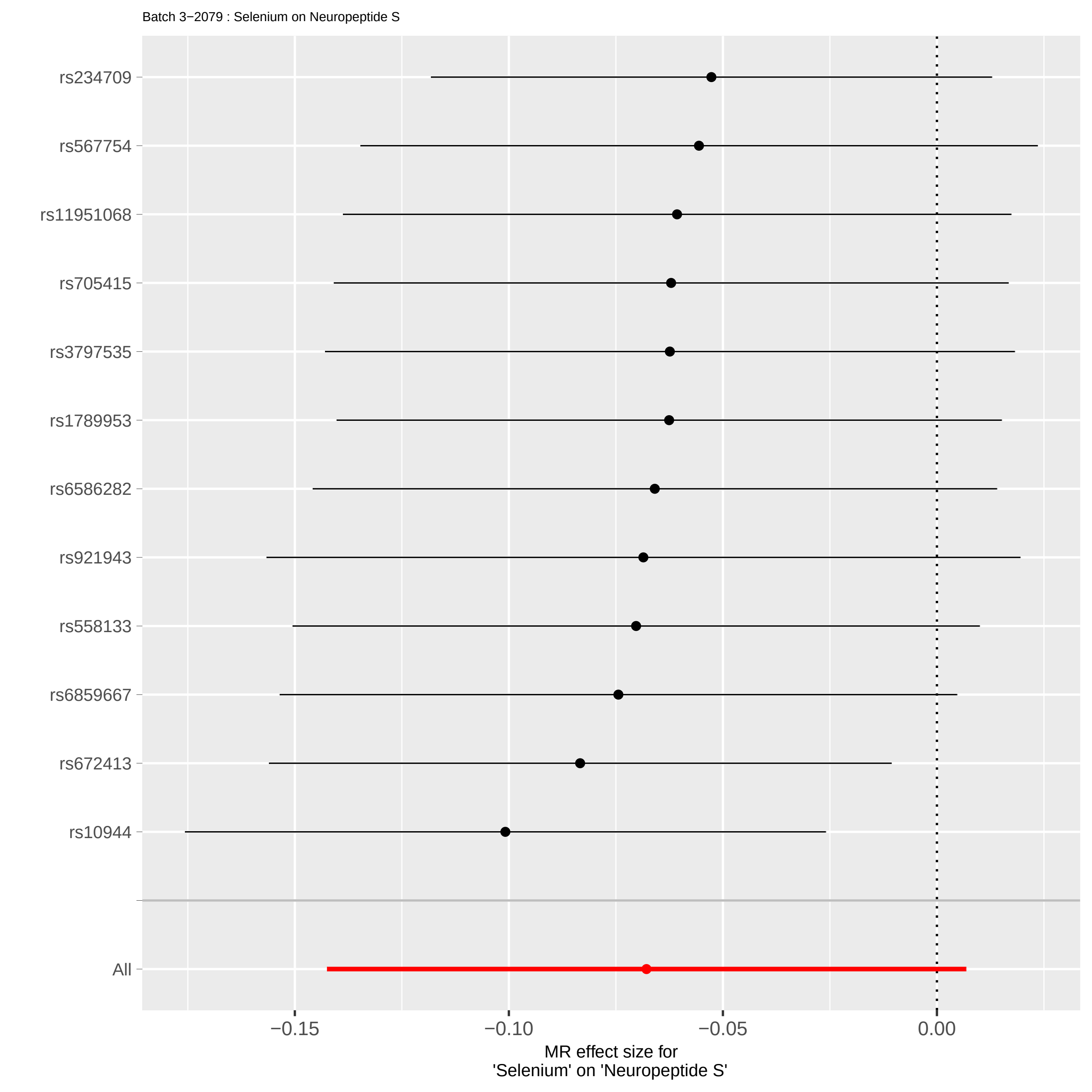
Batch 3−2079 : Selenium on Neuropeptide S
rs234709
rs567754
rs11951068
rs705415
rs3797535
rs1789953
rs6586282
rs921943
rs558133
rs6859667
rs672413
rs10944
All
−0.15
−0.10
−0.05
0.00
MR effect size for
'Selenium' on 'Neuropeptide S'

Batch 3−2080 : Selenium on Neuronal pentraxin−1

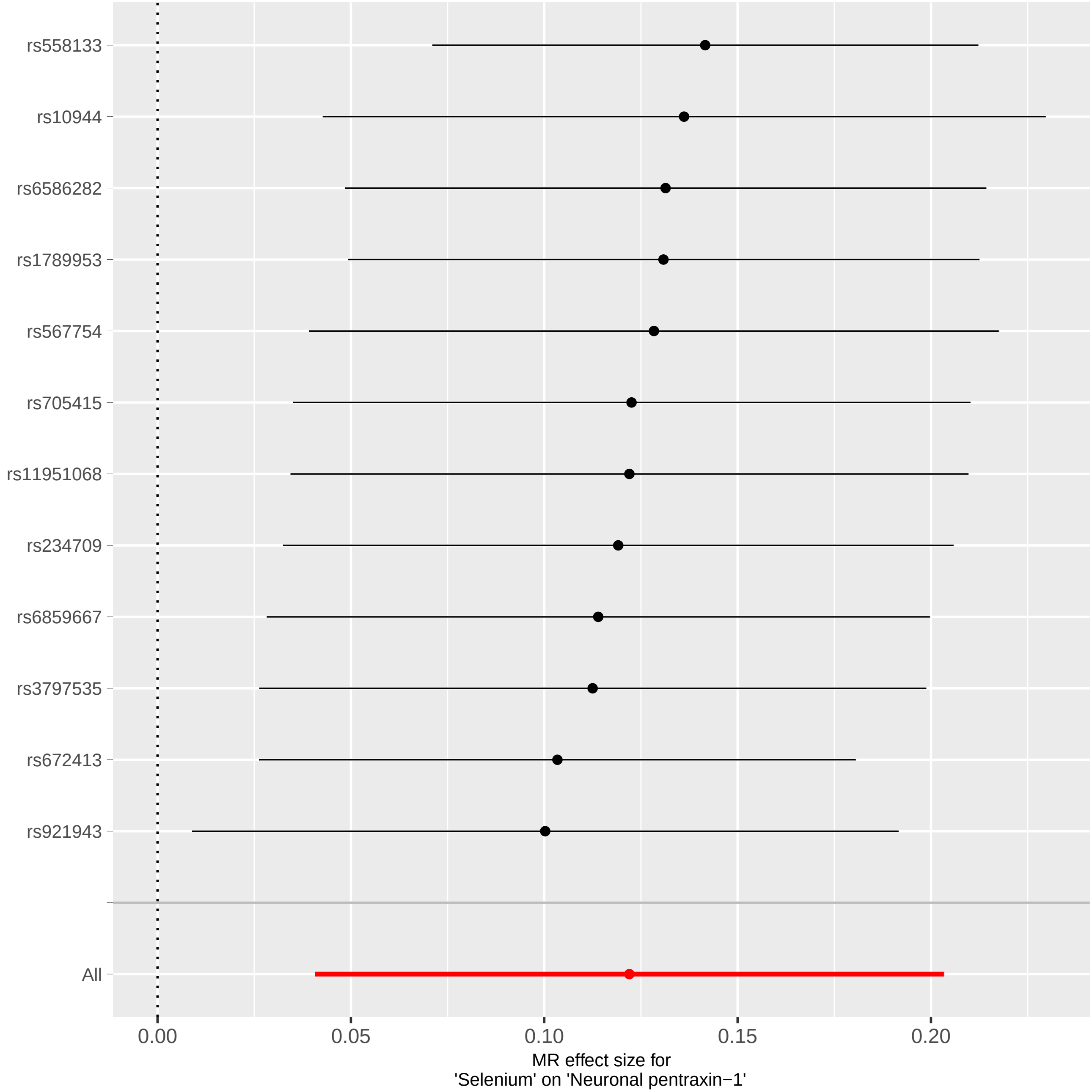

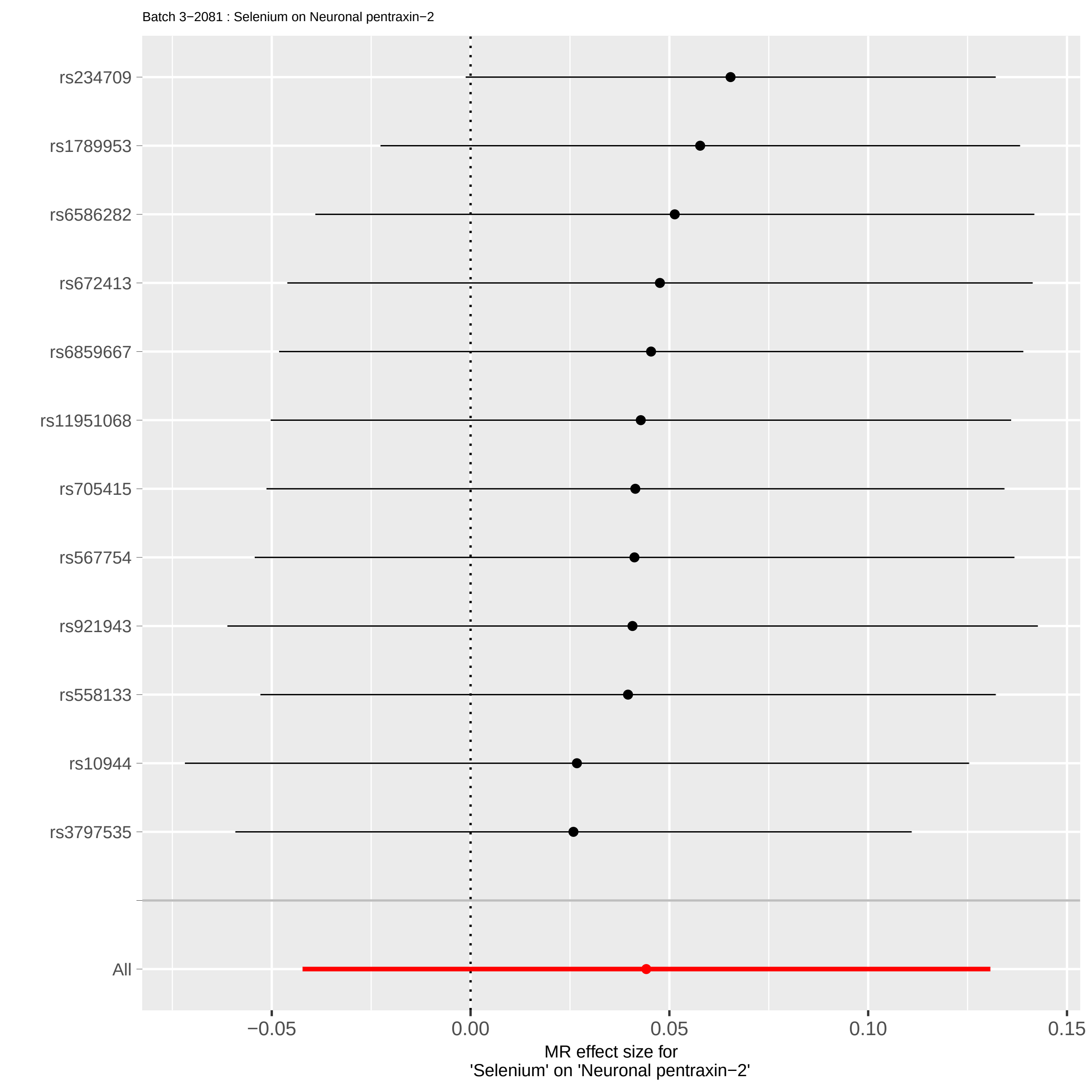

Batch 3−2081 : Selenium on Neuronal pentraxin−2
rs234709
rs1789953
rs6586282
rs672413
rs6859667
rs11951068
rs705415
rs567754
rs921943
rs558133
rs10944
rs3797535
All
−0.05
0.00
0.05
0.10
0.15
MR effect size for
'Selenium' on 'Neuronal pentraxin−2'

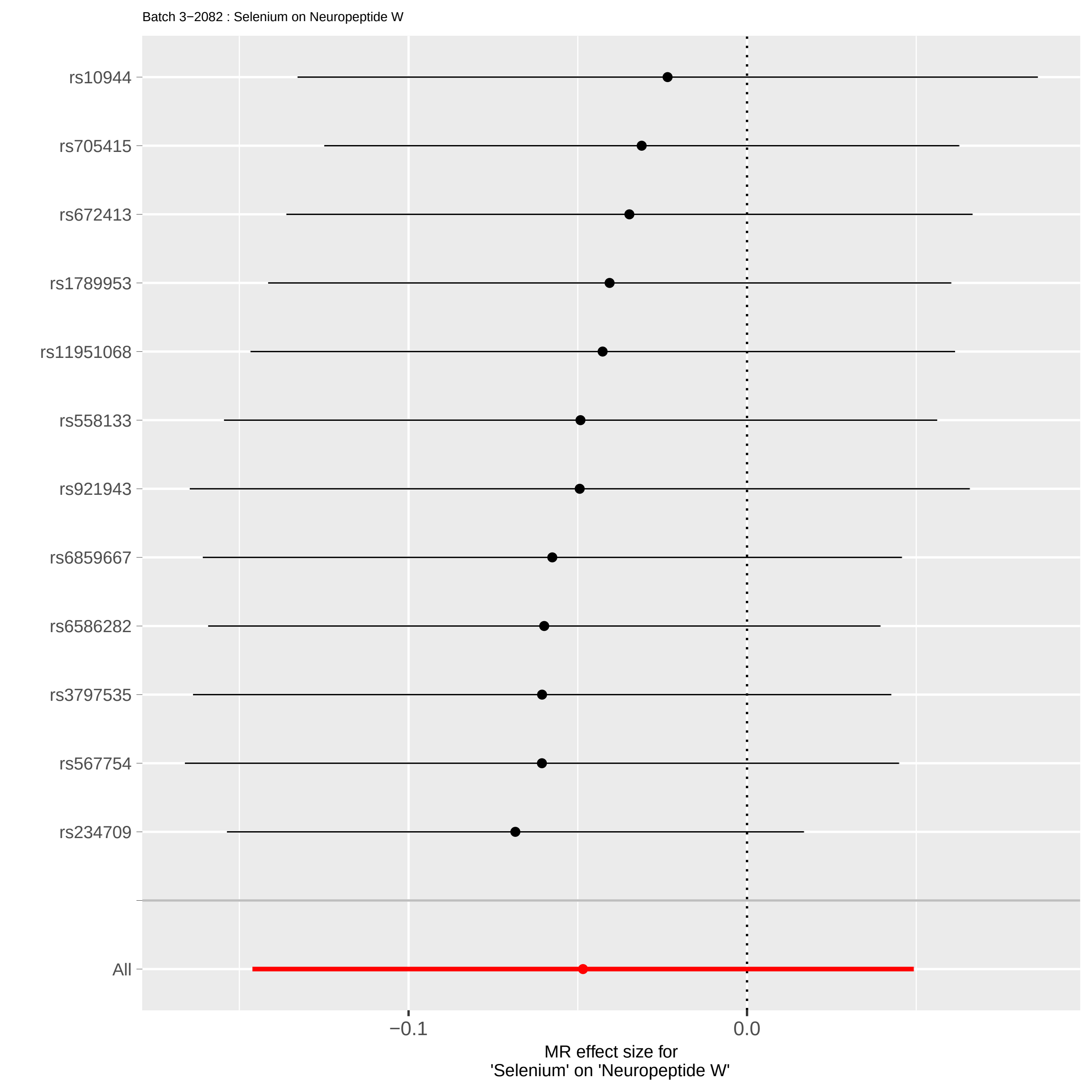

Batch 3−2082 : Selenium on Neuropeptide W
rs10944
rs705415
rs672413
rs1789953
rs11951068
rs558133
rs921943
rs6859667
rs6586282
rs3797535
rs567754
rs234709
All
−0.1
0.0
MR effect size for
'Selenium' on 'Neuropeptide W'

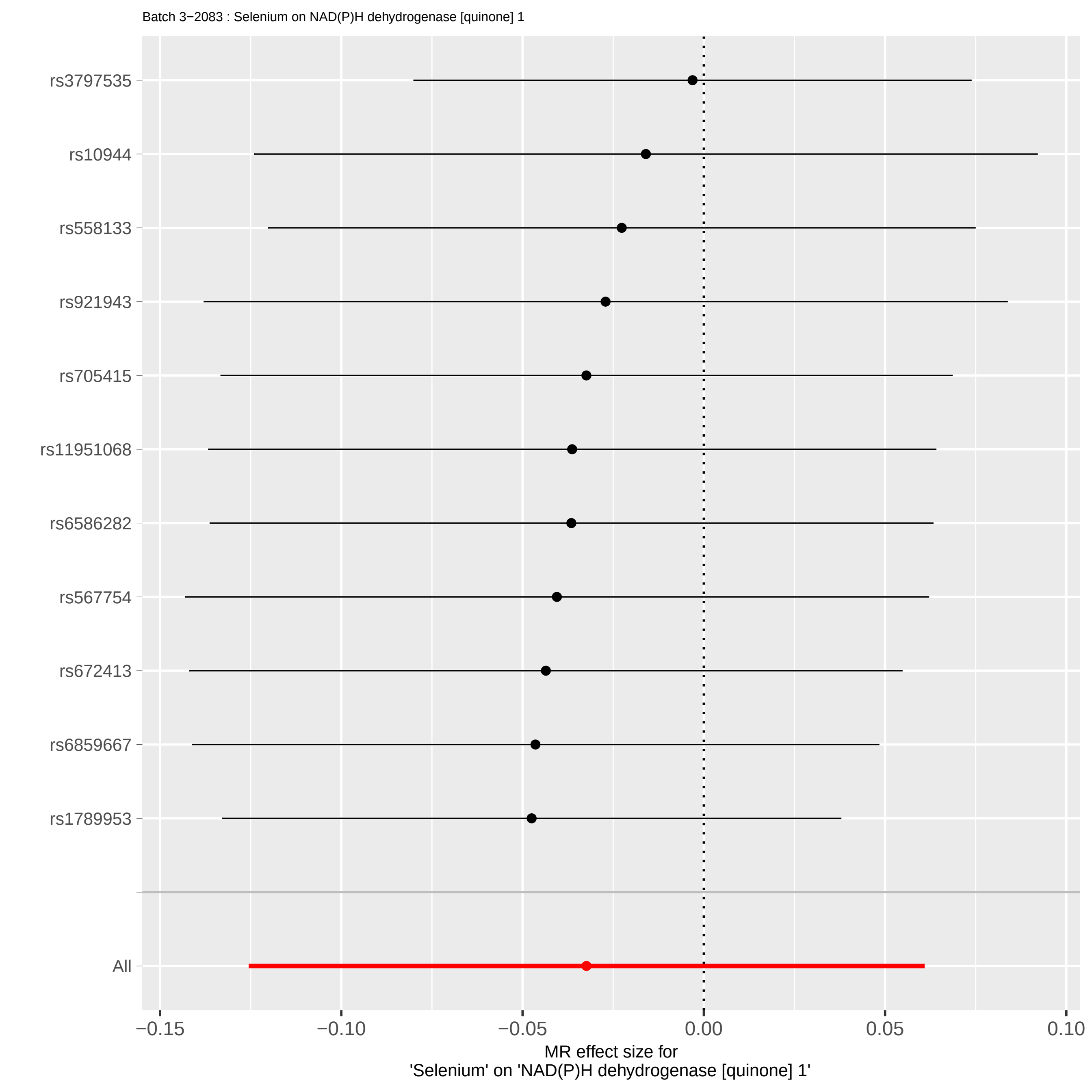

Batch 3−2083 : Selenium on NAD(P)H dehydrogenase [quinone] 1
rs3797535
rs10944
rs558133
rs921943
rs705415
rs11951068
rs6586282
rs567754
rs672413
rs6859667
rs1789953
All
−0.15
−0.10
−0.05
0.00
0.05
0.10
MR effect size for
'Selenium' on 'NAD(P)H dehydrogenase [quinone] 1'

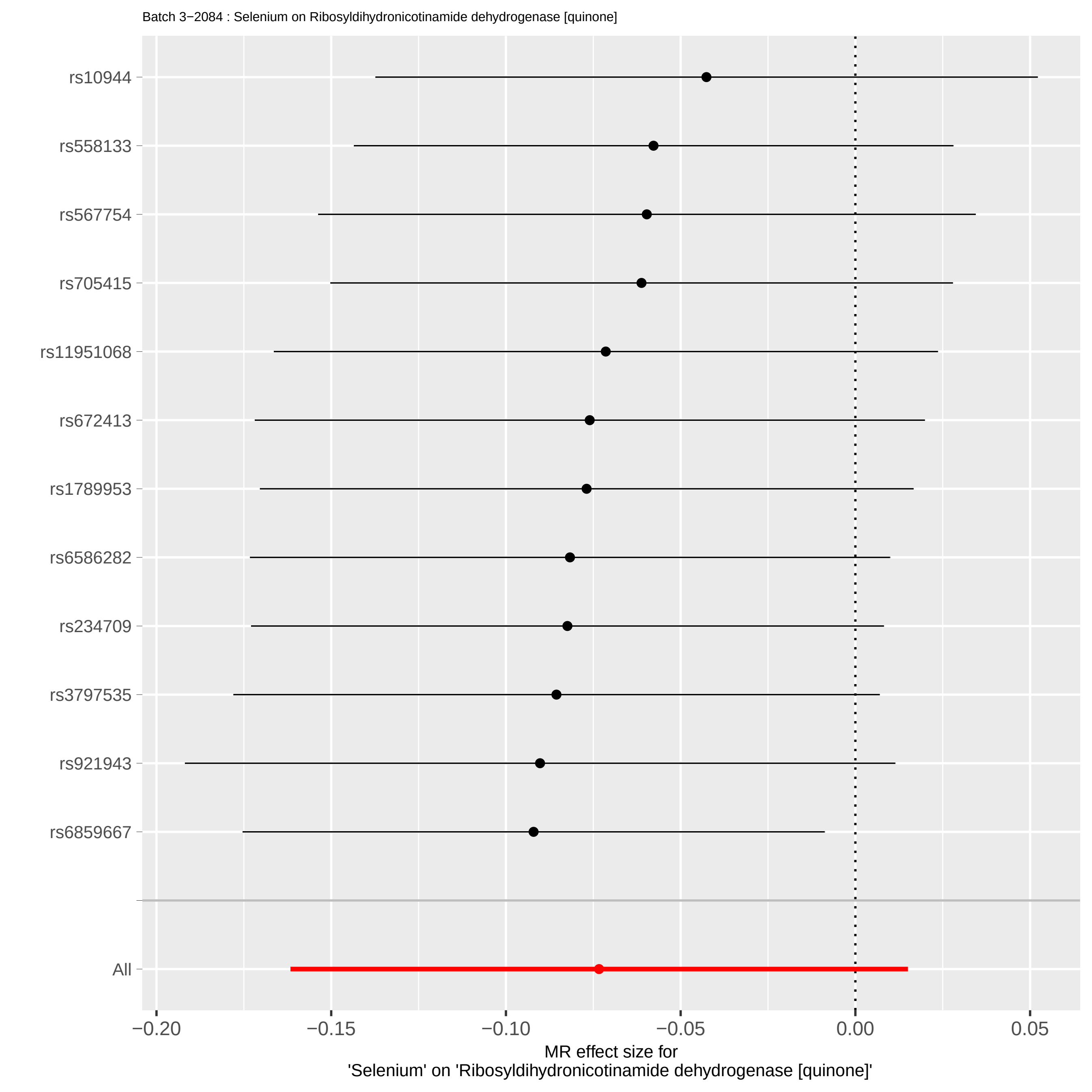

Batch 3−2084 : Selenium on Ribosyldihydronicotinamide dehydrogenase [quinone]
rs10944
rs558133
rs567754
rs705415
rs11951068
rs672413
rs1789953
rs6586282
rs234709
rs3797535
rs921943
rs6859667
All
−0.20
−0.15
−0.10
−0.05
0.00
0.05
MR effect size for
'Selenium' on 'Ribosyldihydronicotinamide dehydrogenase [quinone]'

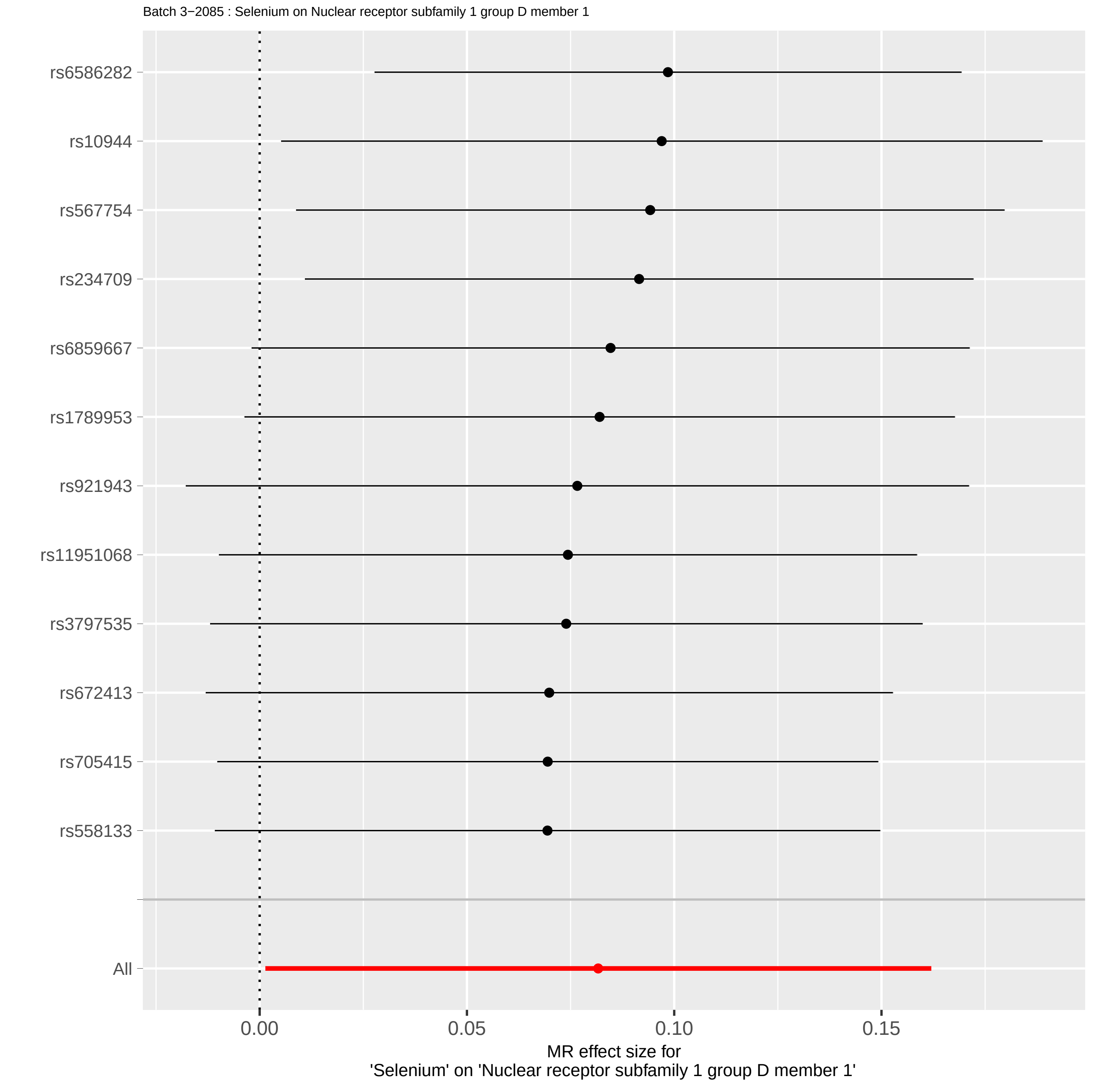

Batch 3−2085 : Selenium on Nuclear receptor subfamily 1 group D member 1
rs6586282
rs10944
rs567754
rs234709
rs6859667
rs1789953
rs921943
rs11951068
rs3797535
rs672413
rs705415
rs558133
All
0.00
0.05
0.10
0.15
MR effect size for
'Selenium' on 'Nuclear receptor subfamily 1 group D member 1'

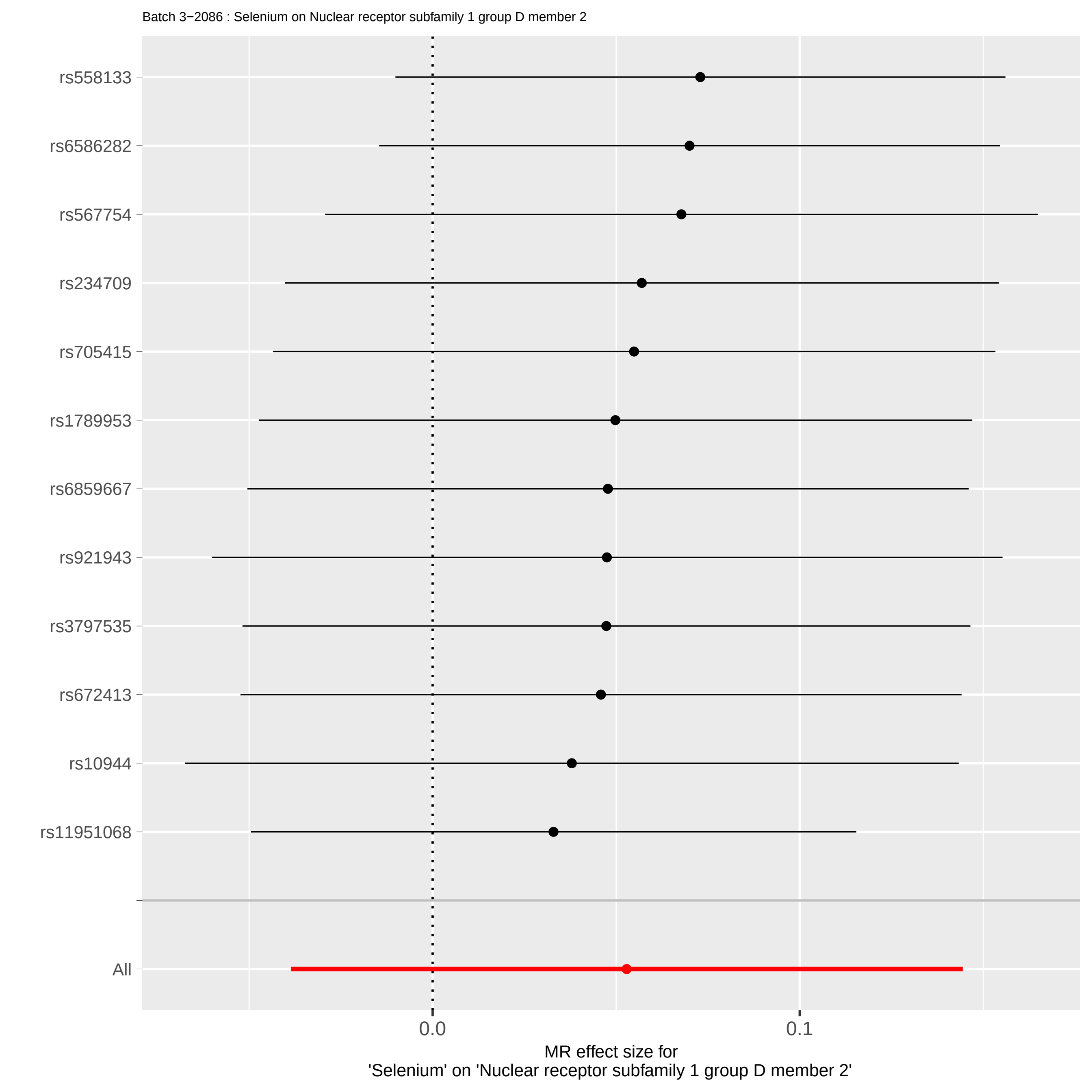

Batch 3−2086 : Selenium on Nuclear receptor subfamily 1 group D member 2
rs558133
rs6586282
rs567754
rs234709
rs705415
rs1789953
rs6859667
rs921943
rs3797535
rs672413
rs10944
rs11951068
All
0.0
0.1
MR effect size for
'Selenium' on 'Nuclear receptor subfamily 1 group D member 2'

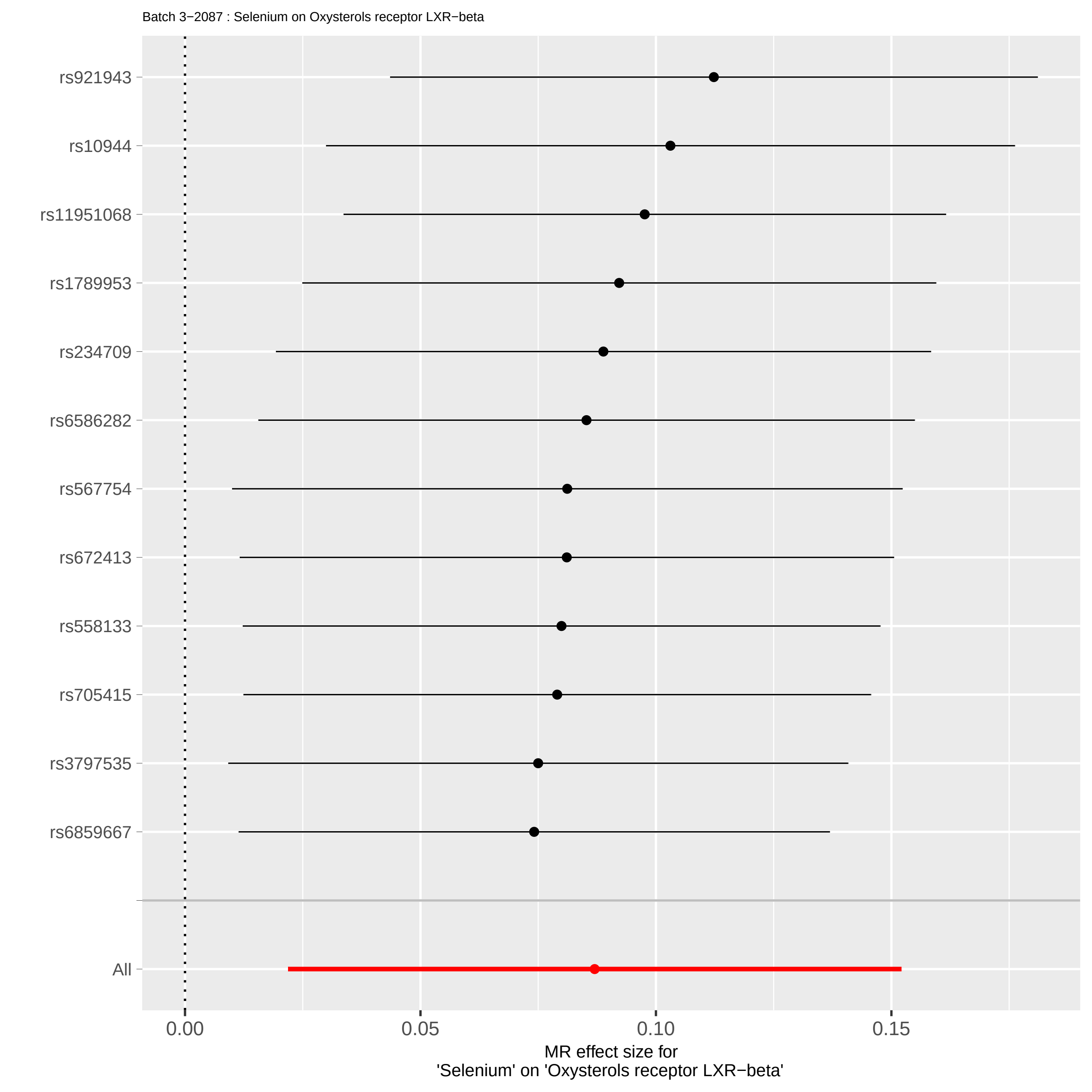

Batch 3−2087 : Selenium on Oxysterols receptor LXR−beta
rs921943
rs10944
rs11951068
rs1789953
rs234709
rs6586282
rs567754
rs672413
rs558133
rs705415
rs3797535
rs6859667
All
0.00
0.05
0.10
0.15
MR effect size for
'Selenium' on 'Oxysterols receptor LXR−beta'

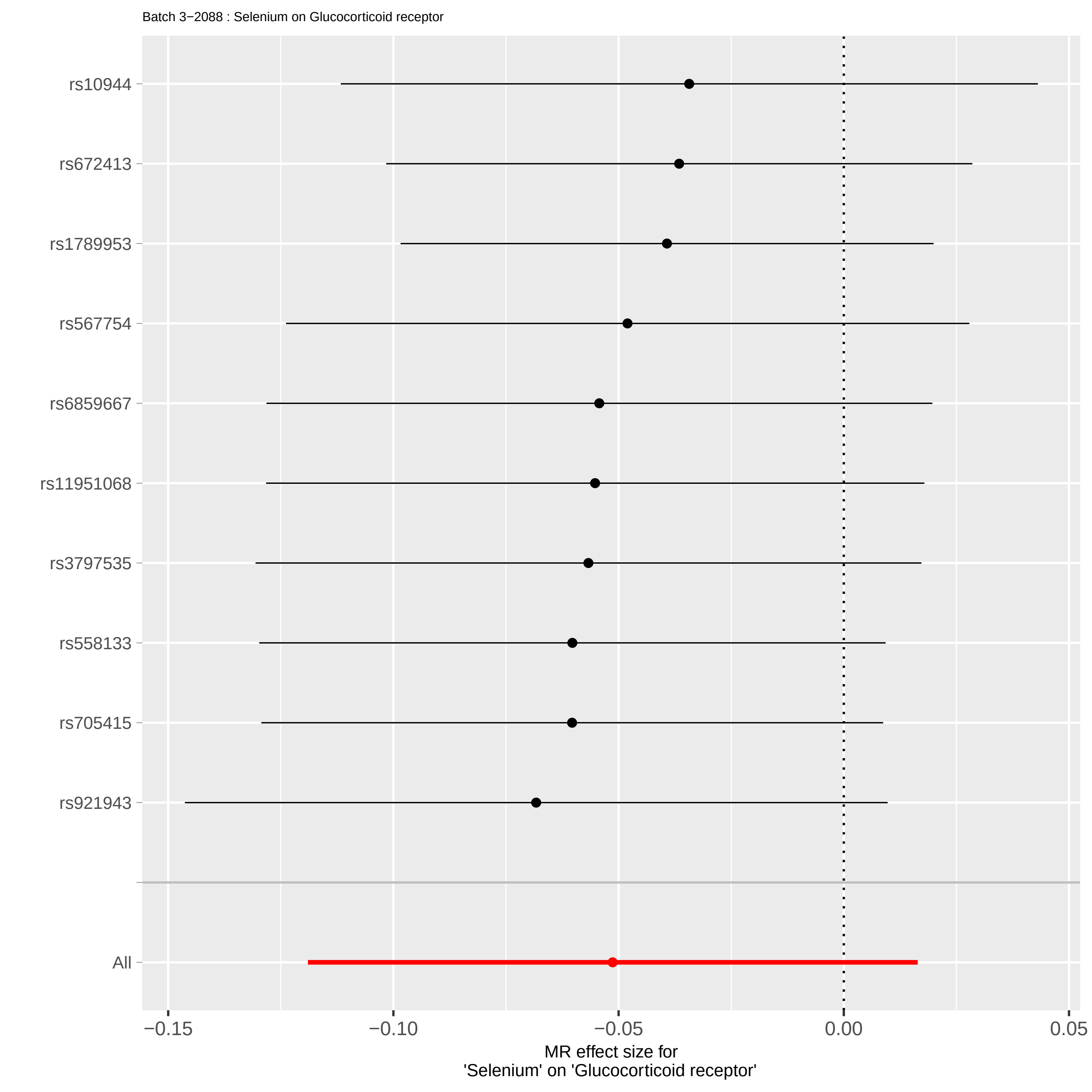

Batch 3−2088 : Selenium on Glucocorticoid receptor
rs10944
rs672413
rs1789953
rs567754
rs6859667
rs11951068
rs3797535
rs558133
rs705415
rs921943
All
−0.15
−0.10
−0.05
0.00
0.05
MR effect size for
'Selenium' on 'Glucocorticoid receptor'

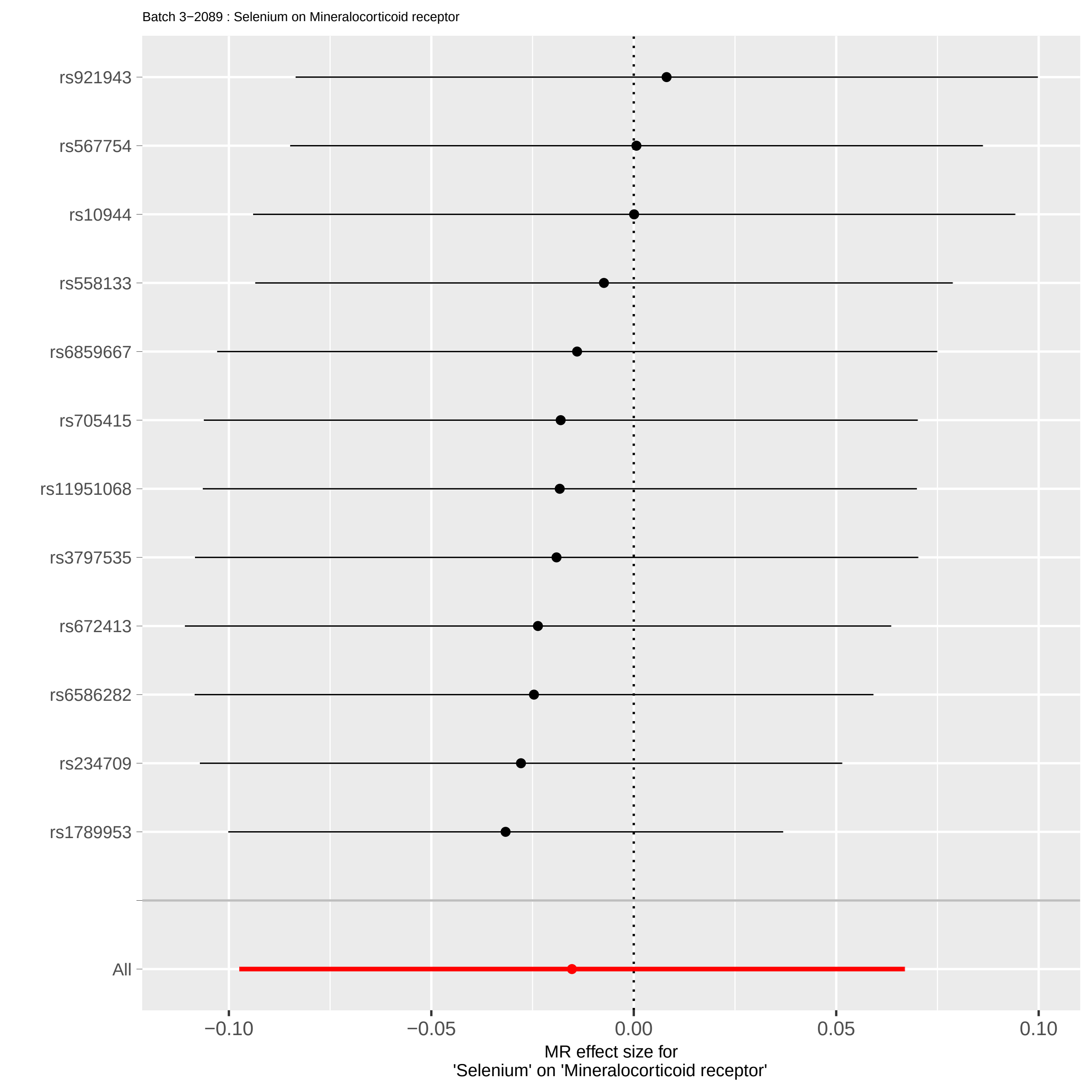

Batch 3−2089 : Selenium on Mineralocorticoid receptor
rs921943
rs567754
rs10944
rs558133
rs6859667
rs705415
rs11951068
rs3797535
rs672413
rs6586282
rs234709
rs1789953
All
−0.10
−0.05
0.00
0.05
0.10
MR effect size for
'Selenium' on 'Mineralocorticoid receptor'

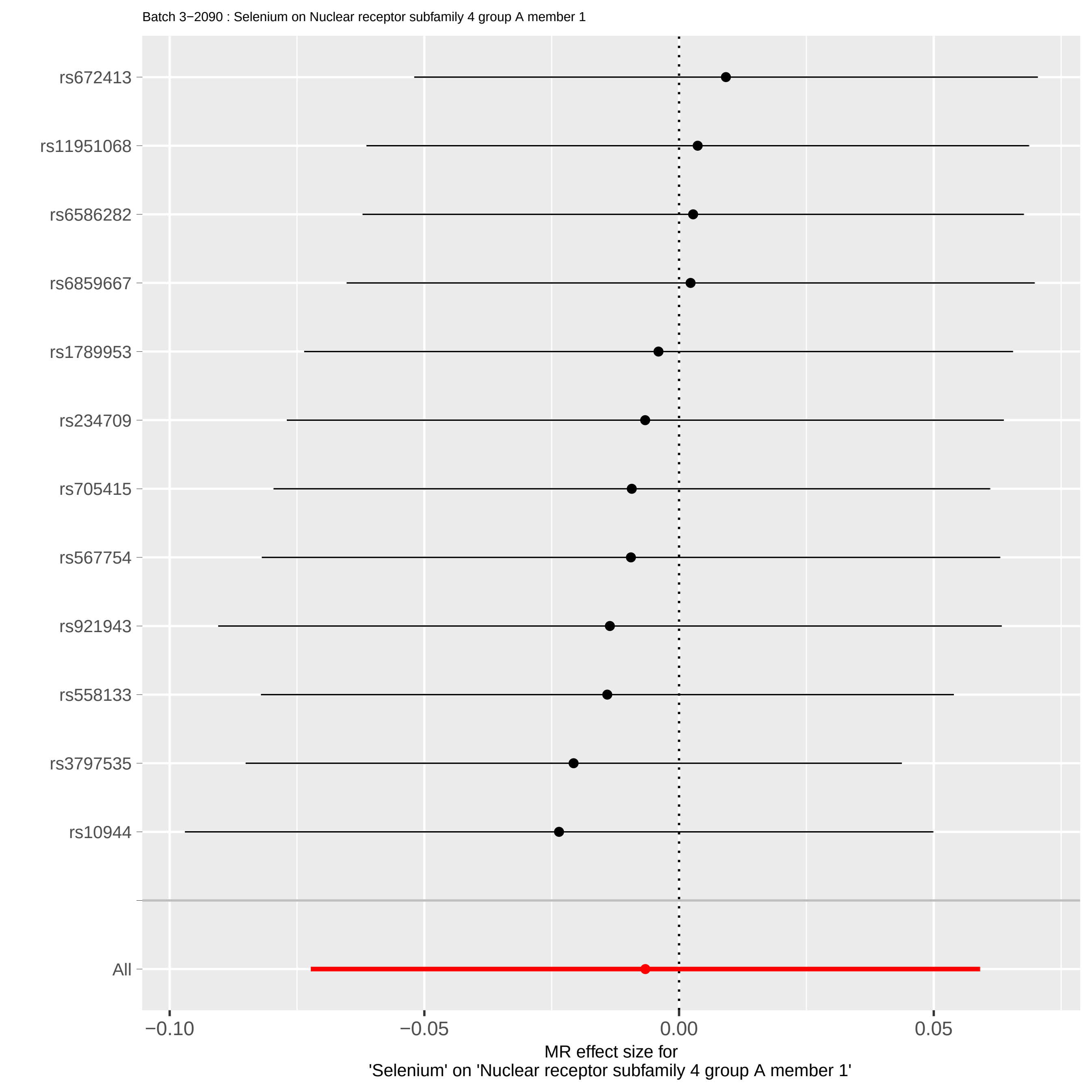

Batch 3−2090 : Selenium on Nuclear receptor subfamily 4 group A member 1
rs672413
rs11951068
rs6586282
rs6859667
rs1789953
rs234709
rs705415
rs567754
rs921943
rs558133
rs3797535
rs10944
All
−0.10
−0.05
0.00
0.05
MR effect size for
'Selenium' on 'Nuclear receptor subfamily 4 group A member 1'

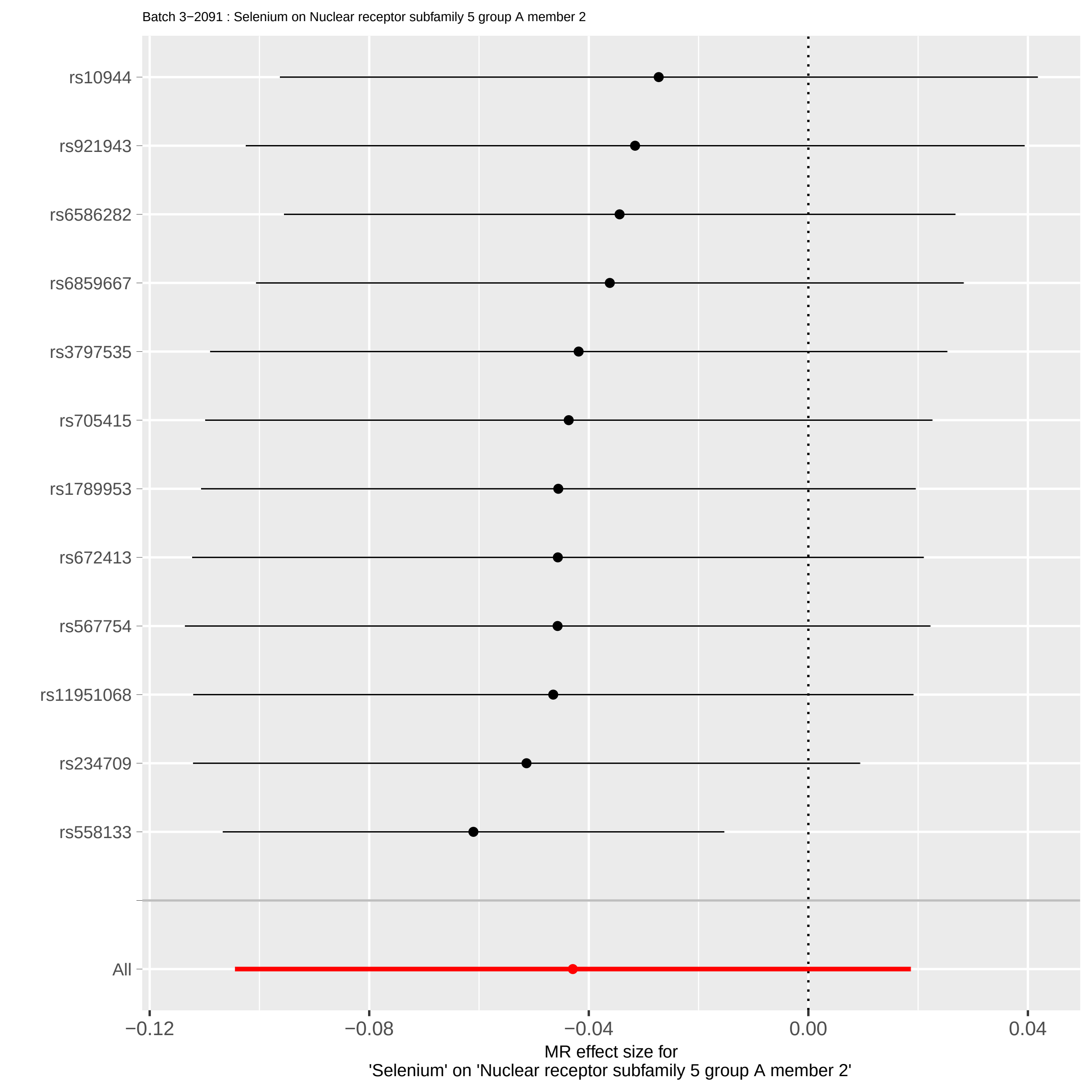
Batch 3−2091 : Selenium on Nuclear receptor subfamily 5 group A member 2
rs10944
rs921943
rs6586282
rs6859667
rs3797535
rs705415
rs1789953
rs672413
rs567754
rs11951068
rs234709
rs558133
All
−0.12
−0.08
−0.04
0.00
0.04
MR effect size for
'Selenium' on 'Nuclear receptor subfamily 5 group A member 2'

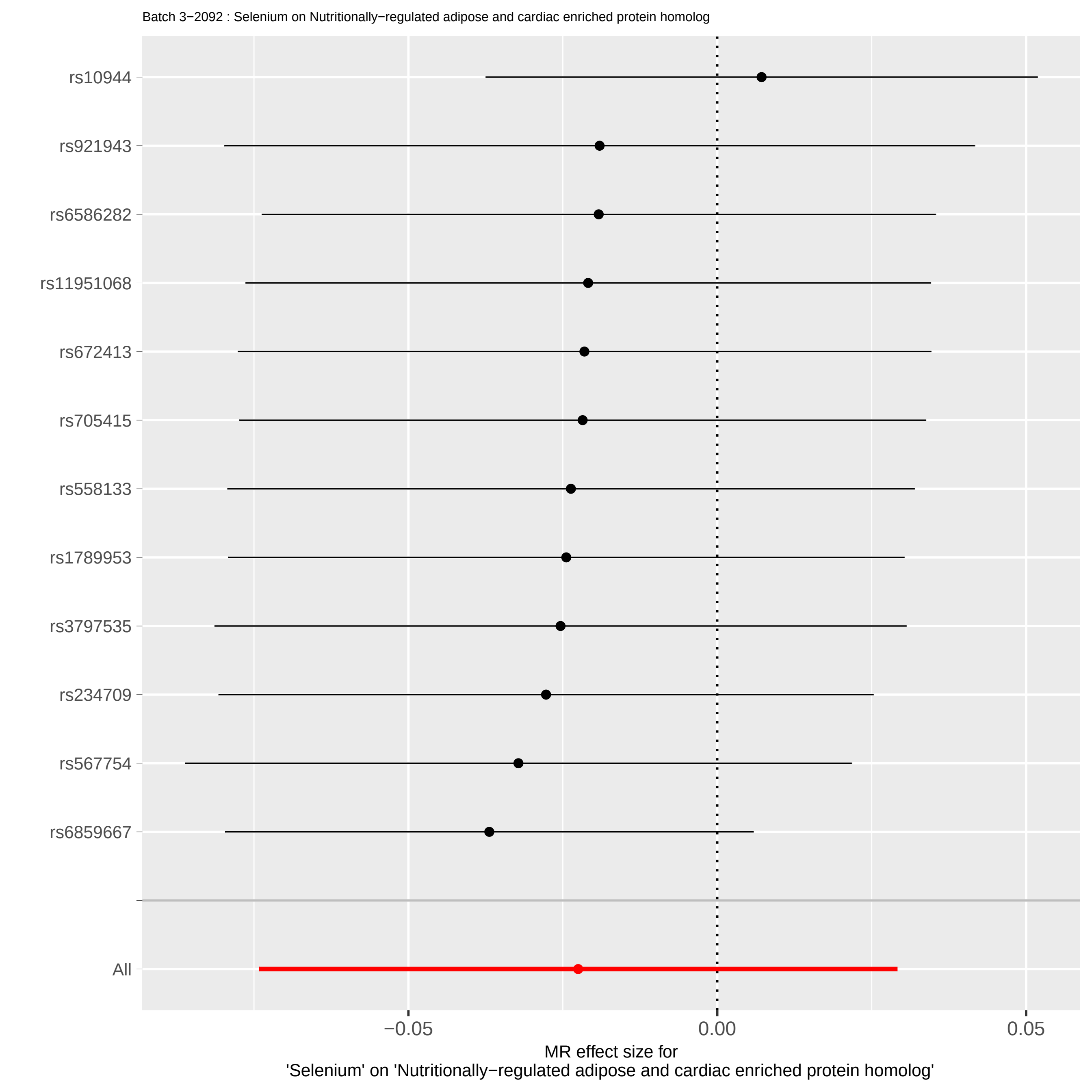
Batch 3−2092 : Selenium on Nutritionally−regulated adipose and cardiac enriched protein homolog
rs10944
rs921943
rs6586282
rs11951068
rs672413
rs705415
rs558133
rs1789953
rs3797535
rs234709
rs567754
rs6859667
All
−0.05
0.00
0.05
MR effect size for
'Selenium' on 'Nutritionally−regulated adipose and cardiac enriched protein homolog'

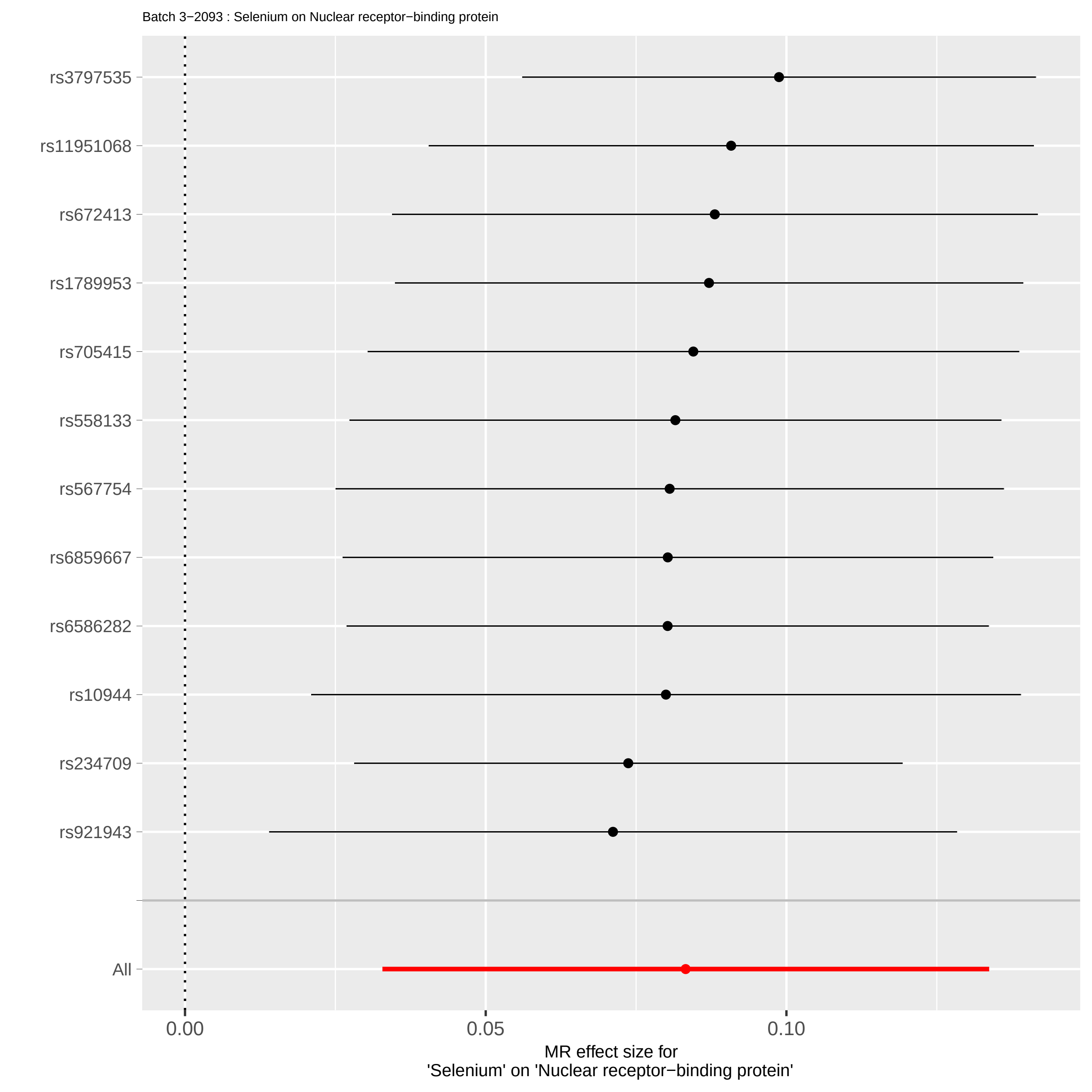
Batch 3−2093 : Selenium on Nuclear receptor−binding protein
rs3797535
rs11951068
rs672413
rs1789953
rs705415
rs558133
rs567754
rs6859667
rs6586282
rs10944
rs234709
rs921943
All
0.00
0.05
0.10
MR effect size for
'Selenium' on 'Nuclear receptor−binding protein'

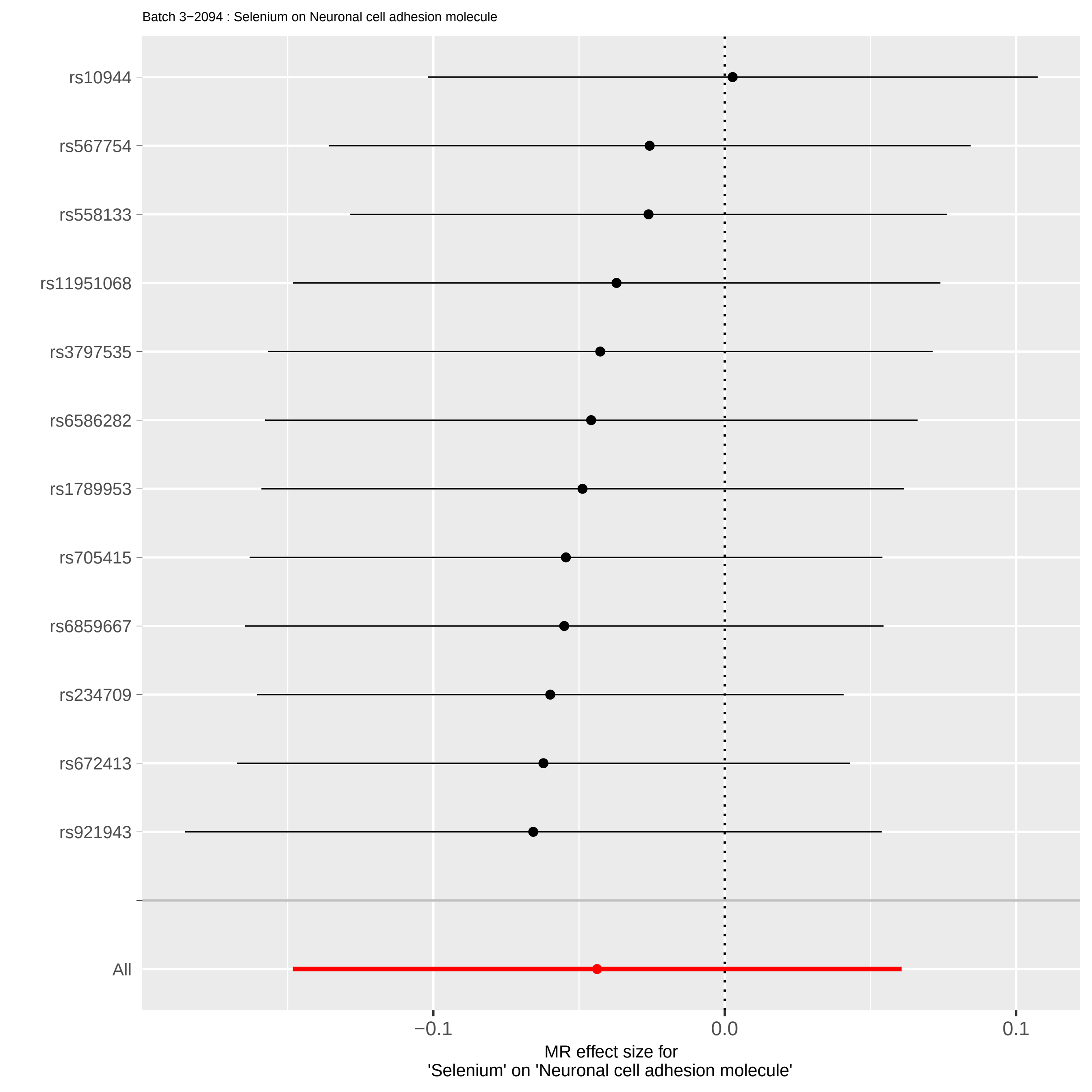
Batch 3−2094 : Selenium on Neuronal cell adhesion molecule
rs10944
rs567754
rs558133
rs11951068
rs3797535
rs6586282
rs1789953
rs705415
rs6859667
rs234709
rs672413
rs921943
All
−0.1
0.0
0.1
MR effect size for
'Selenium' on 'Neuronal cell adhesion molecule'

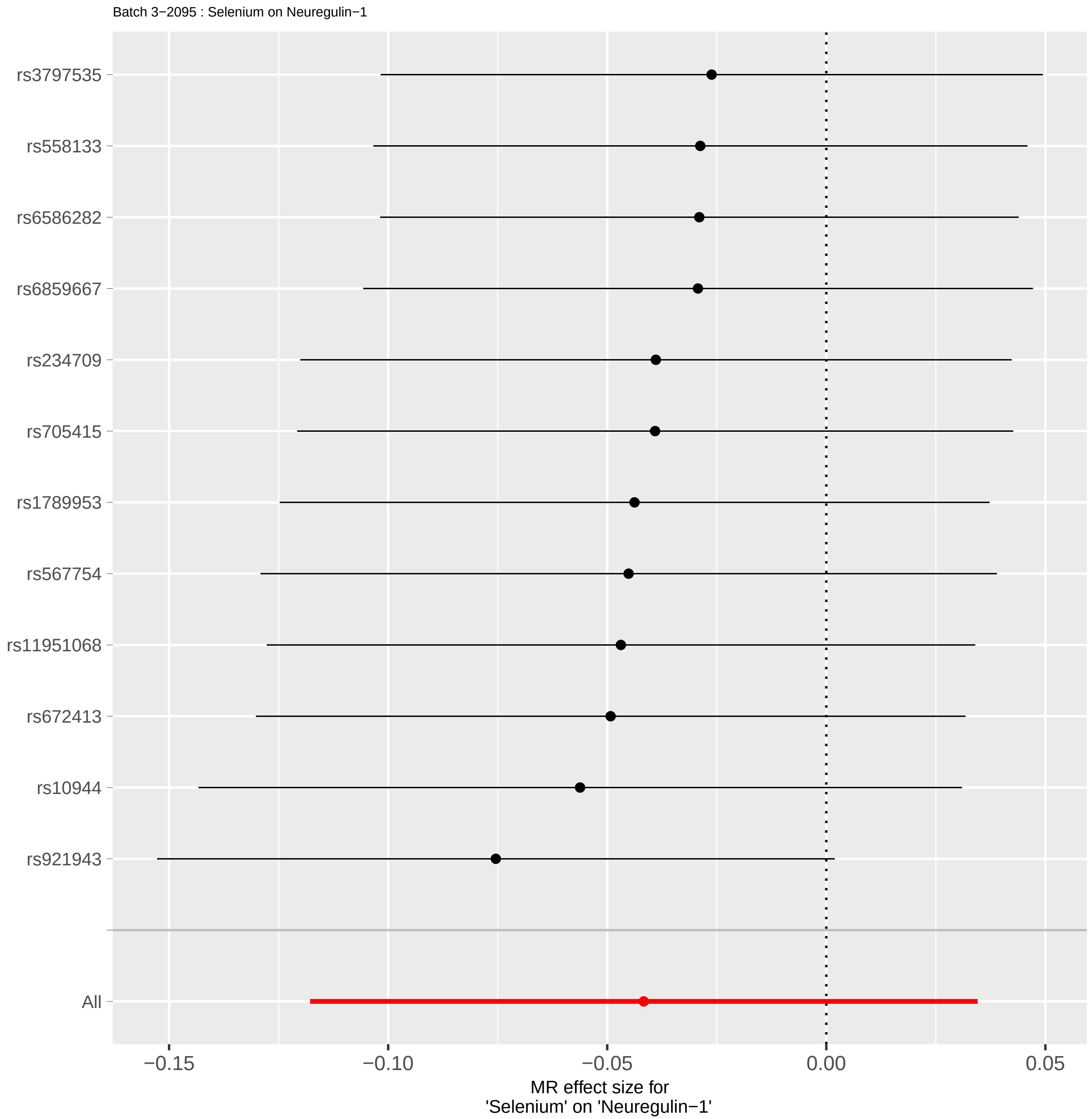

Batch 3−2095 : Selenium on Neuregulin−1
rs3797535
rs558133
rs6586282
rs6859667
rs234709
rs705415
rs1789953
rs567754
rs11951068
rs672413
rs10944
rs921943
All
−0.15
−0.10
−0.05
0.00
0.05
MR effect size for
'Selenium' on 'Neuregulin−1'

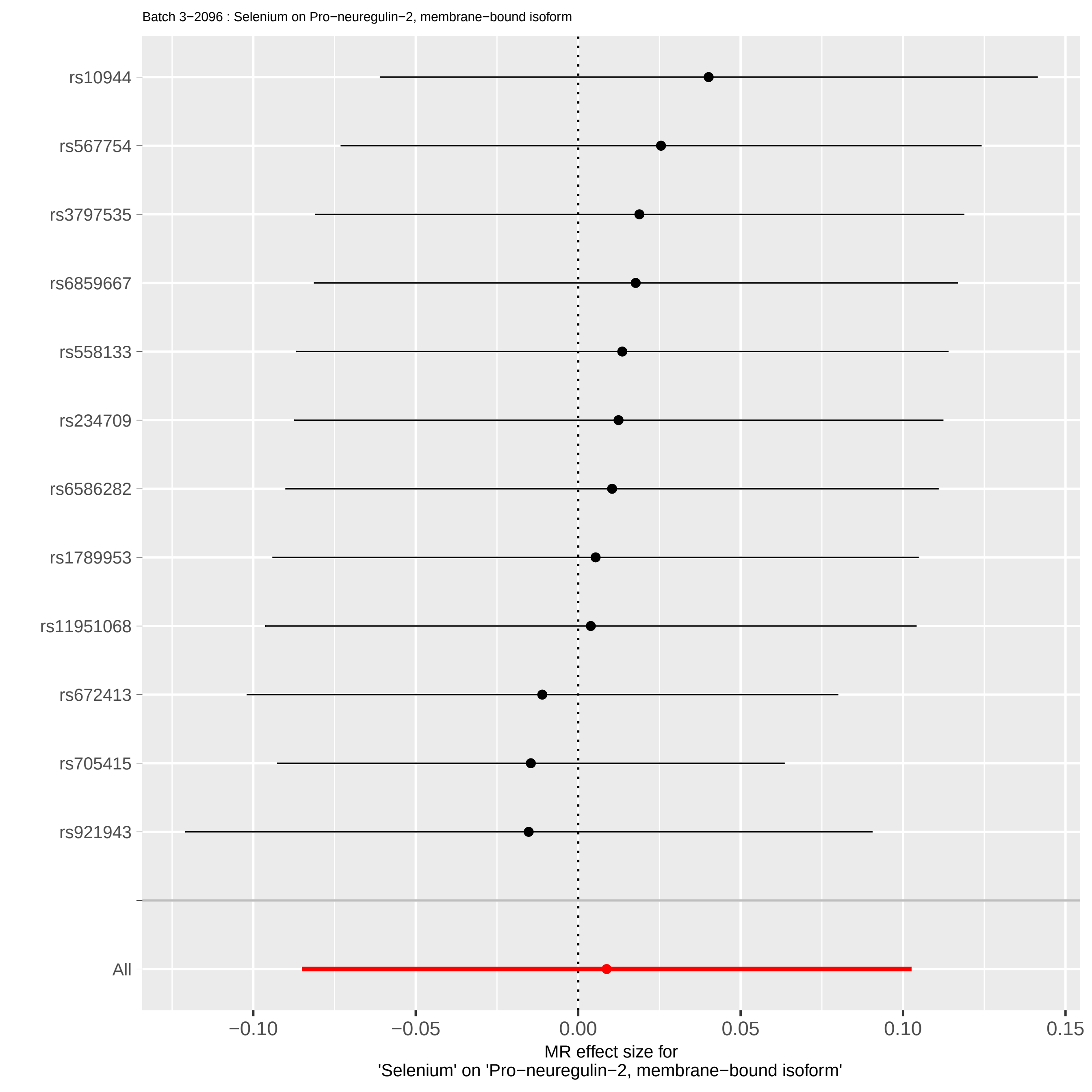

Batch 3−2096 : Selenium on Pro−neuregulin−2, membrane−bound isoform
rs10944
rs567754
rs3797535
rs6859667
rs558133
rs234709
rs6586282
rs1789953
rs11951068
rs672413
rs705415
rs921943
All
−0.10
−0.05
0.00
0.05
0.10
0.15
MR effect size for
'Selenium' on 'Pro−neuregulin−2, membrane−bound isoform'

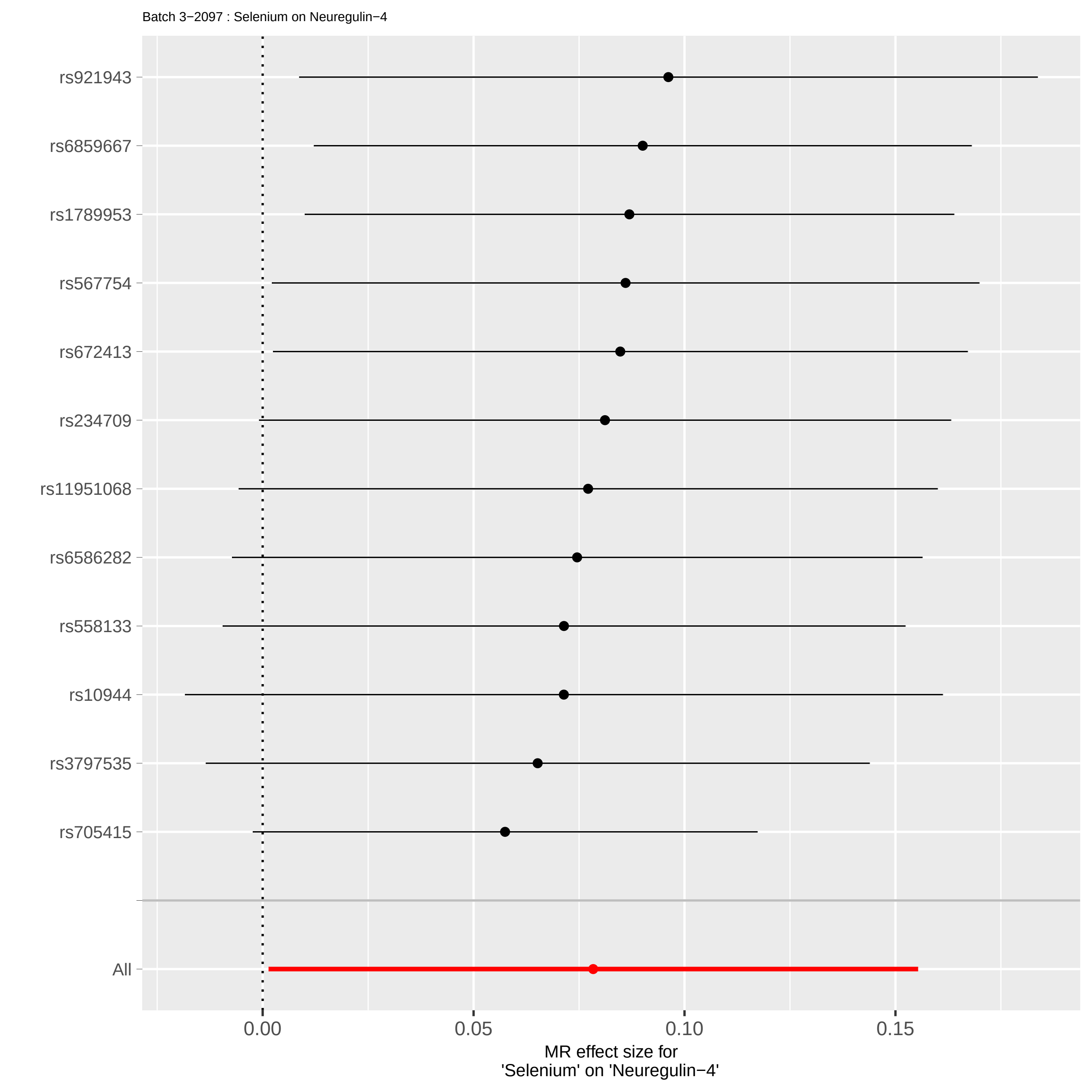

Batch 3−2097 : Selenium on Neuregulin−4
rs921943
rs6859667
rs1789953
rs567754
rs672413
rs234709
rs11951068
rs6586282
rs558133
rs10944
rs3797535
rs705415
All
0.00
0.05
0.10
0.15
MR effect size for
'Selenium' on 'Neuregulin−4'

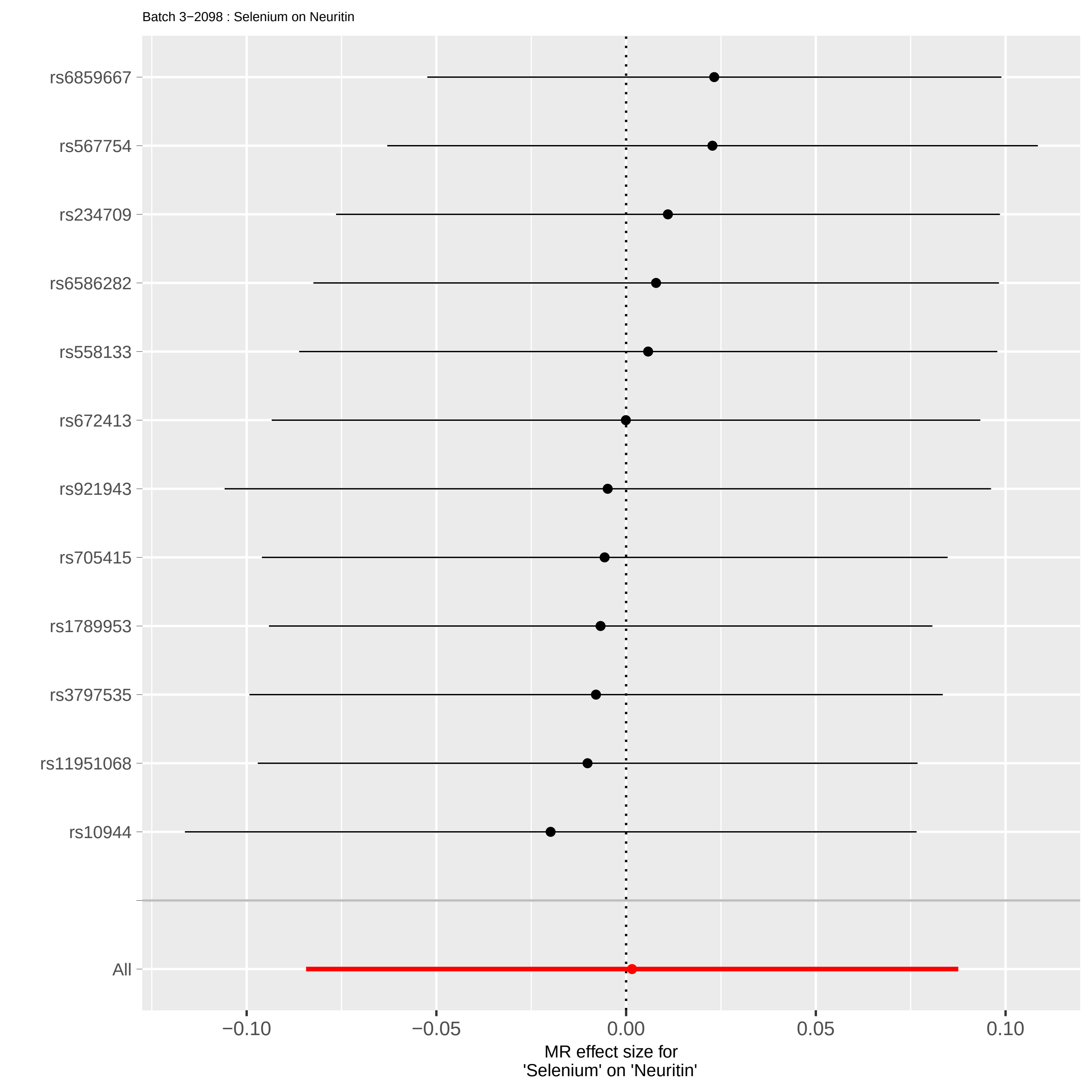

Batch 3−2098 : Selenium on Neuritin
rs6859667
rs567754
rs234709
rs6586282
rs558133
rs672413
rs921943
rs705415
rs1789953
rs3797535
rs11951068
rs10944
All
−0.10
−0.05
0.00
0.05
0.10
MR effect size for
'Selenium' on 'Neuritin'

Batch 3−2099 : Selenium on Neuropilin−1

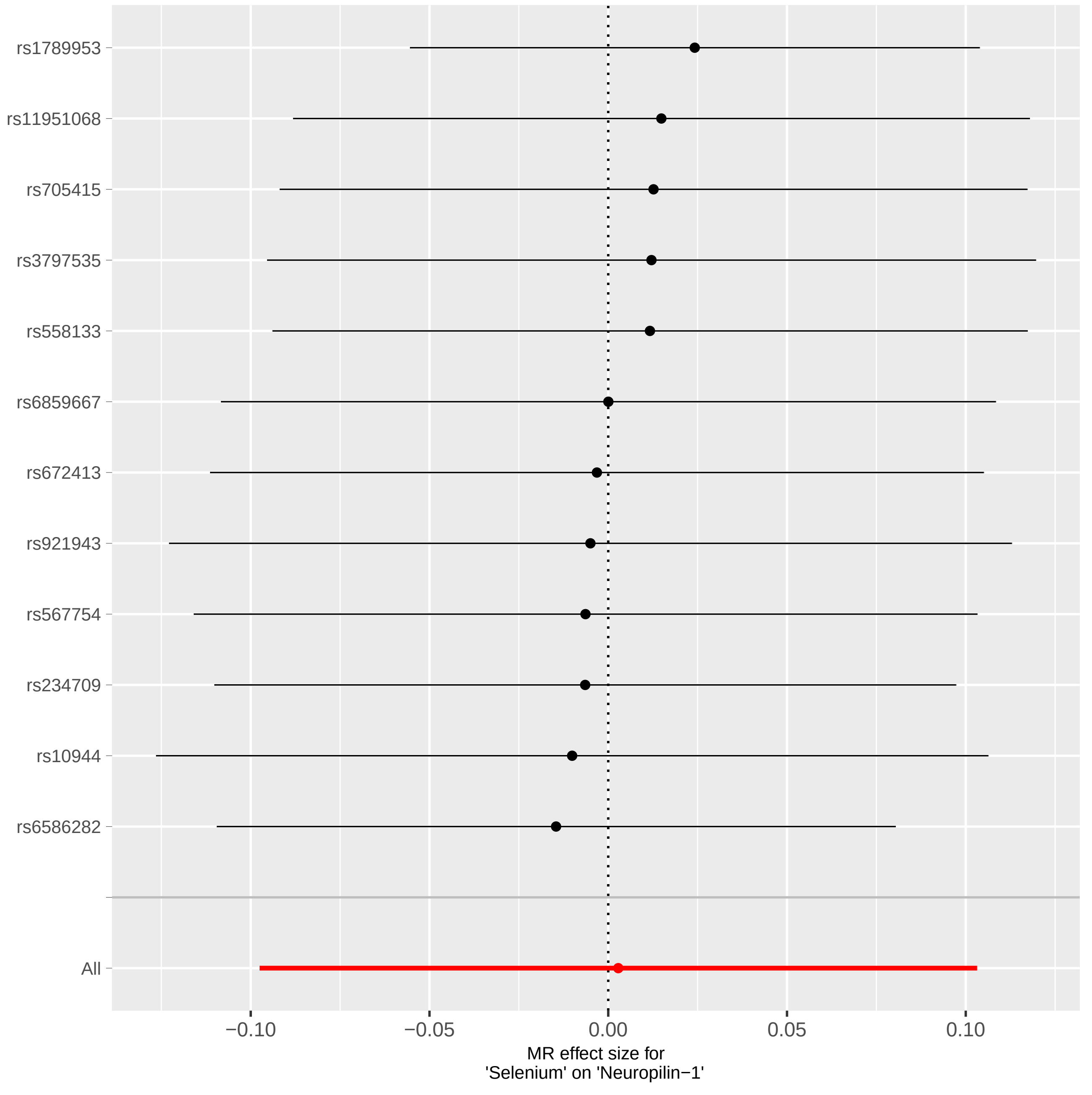

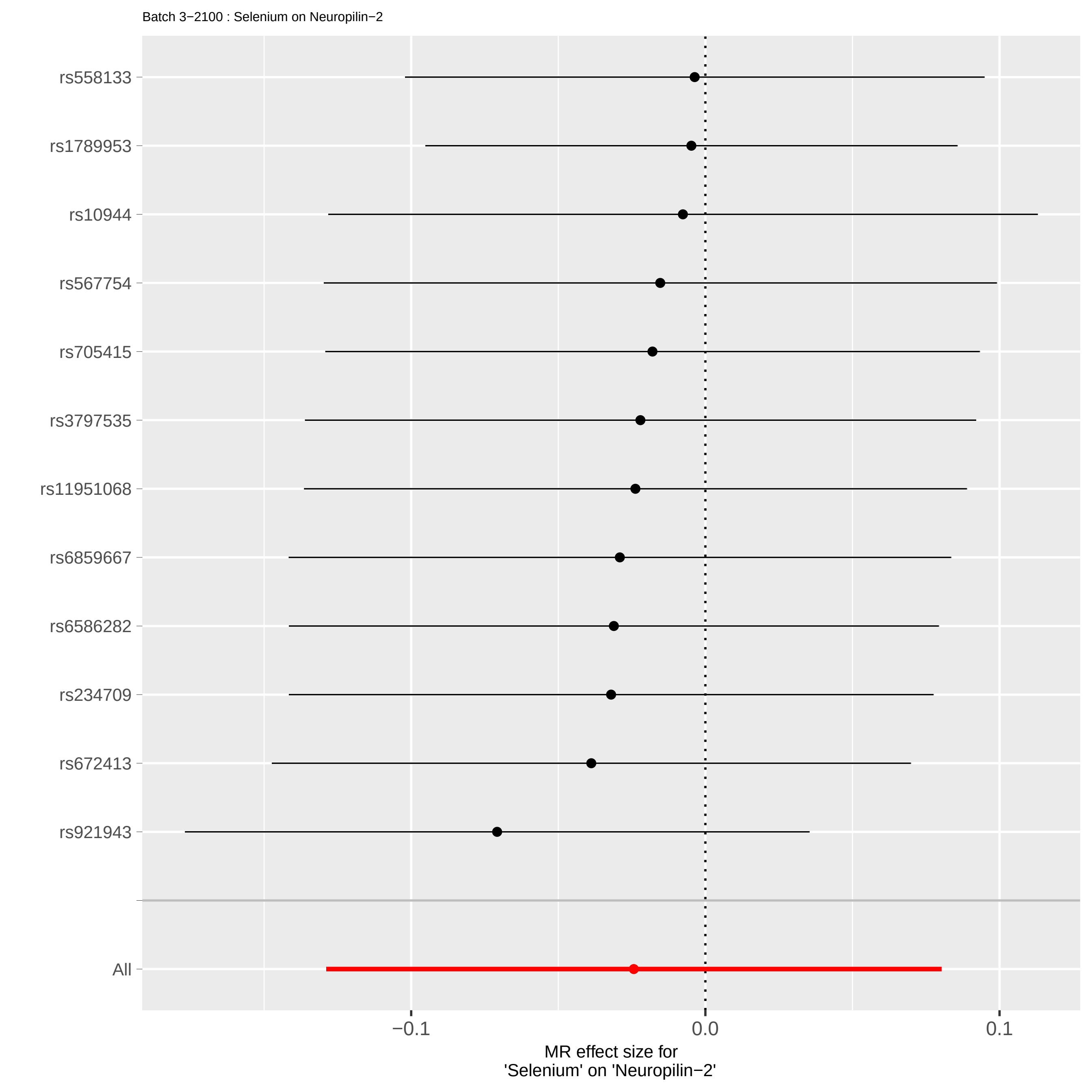

Batch 3−2100 : Selenium on Neuropilin−2
rs558133
rs1789953
rs10944
rs567754
rs705415
rs3797535
rs11951068
rs6859667
rs6586282
rs234709
rs672413
rs921943
All
−0.1
0.0
0.1
MR effect size for
'Selenium' on 'Neuropilin−2'

Batch 3−2101 : Selenium on Neurensin−1

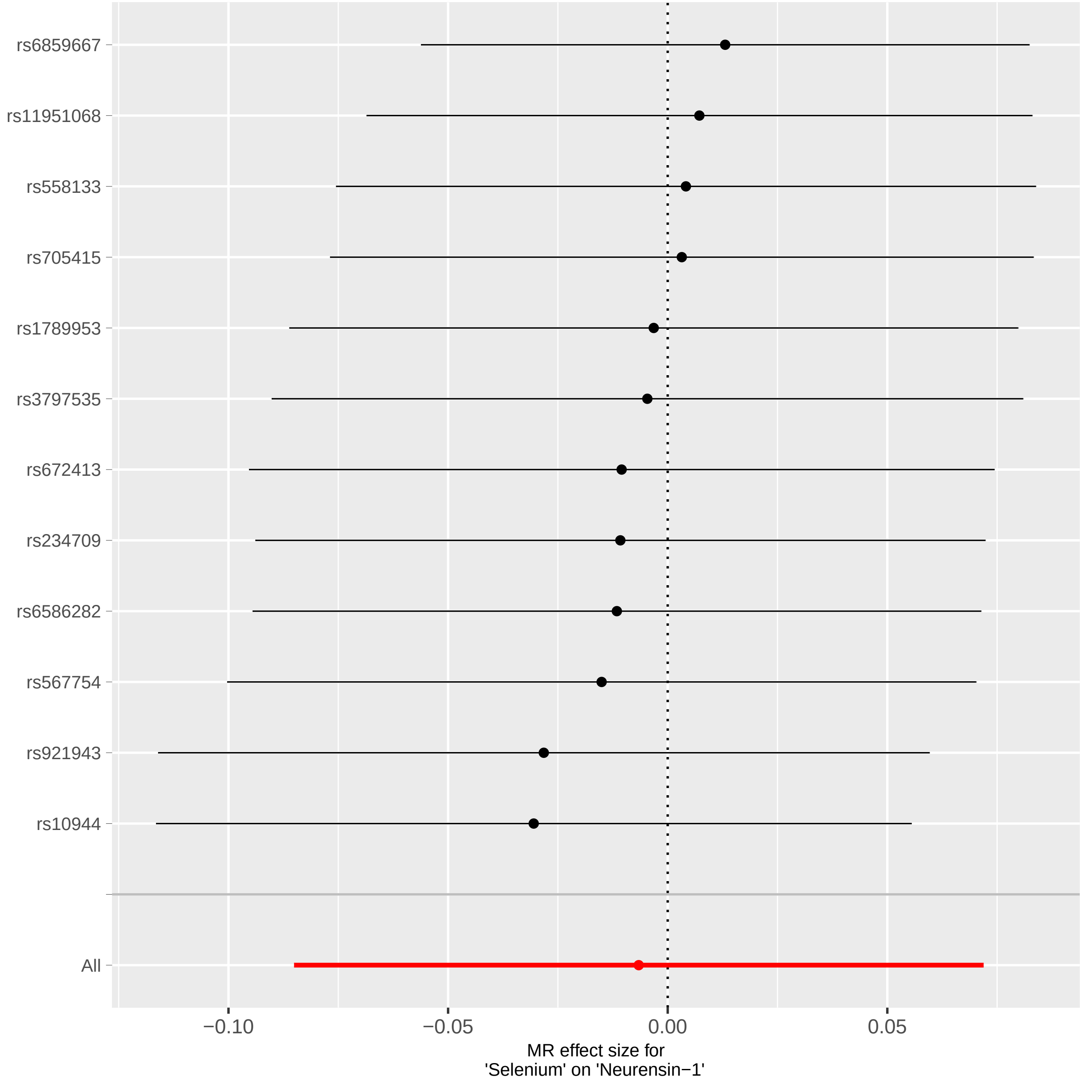

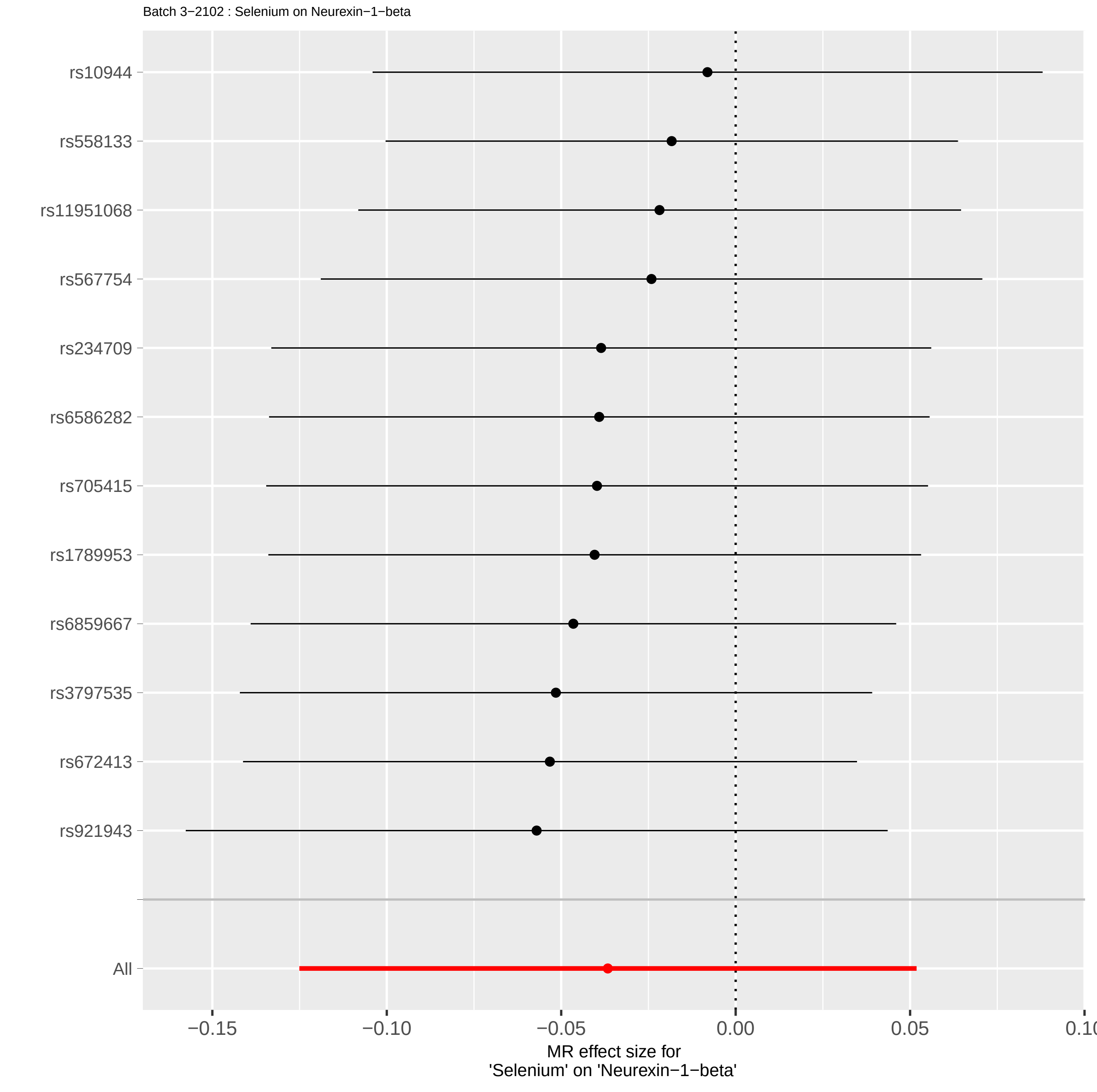

Batch 3−2102 : Selenium on Neurexin−1−beta
rs10944
rs558133
rs11951068
rs567754
rs234709
rs6586282
rs705415
rs1789953
rs6859667
rs3797535
rs672413
rs921943
All
−0.15
−0.10
−0.05
0.00
0.05
MR effect size for
'Selenium' on 'Neurexin−1−beta'

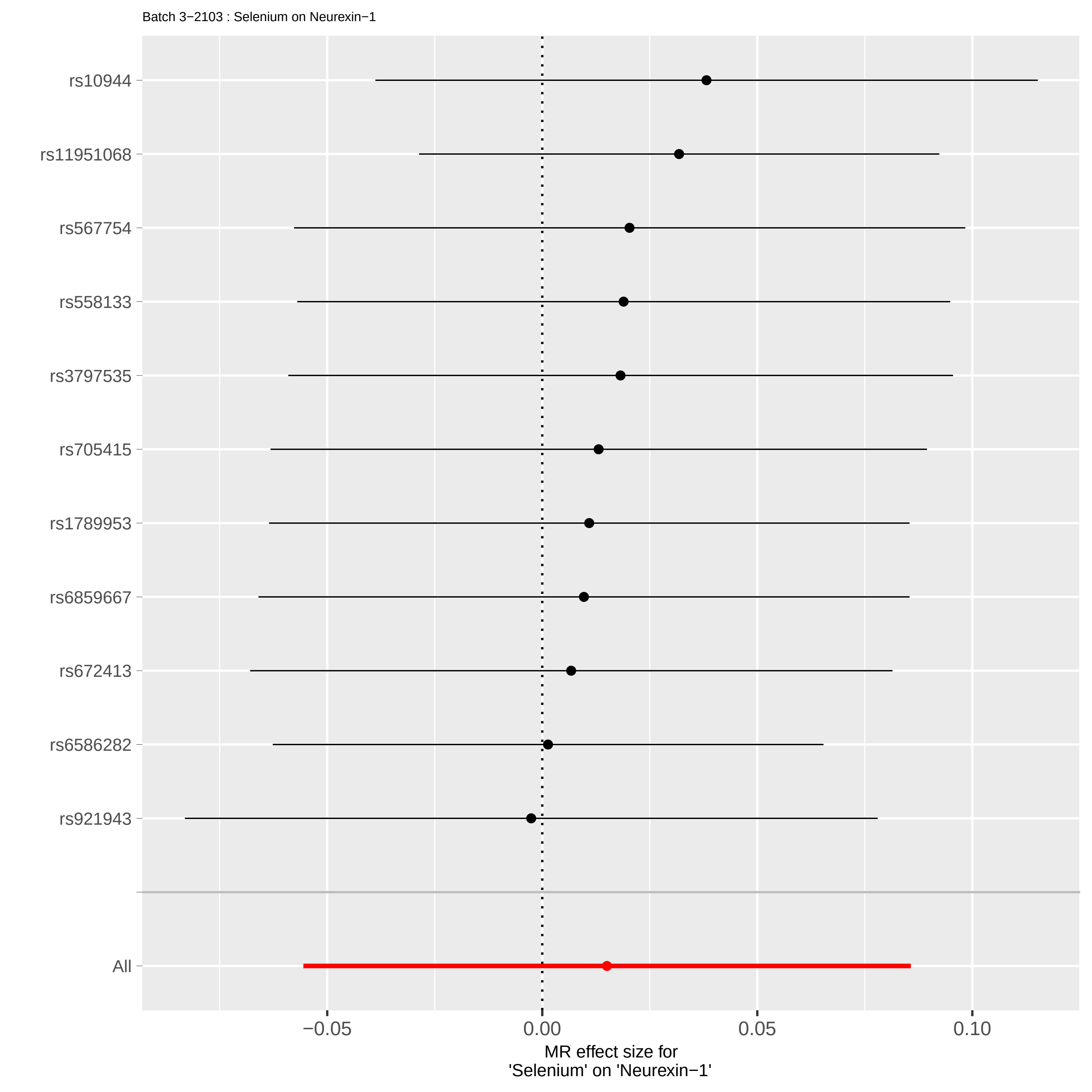

Batch 3−2103 : Selenium on Neurexin−1
rs10944
rs11951068
rs567754
rs558133
rs3797535
rs705415
rs1789953
rs6859667
rs672413
rs6586282
rs921943
All
−0.05
0.00
0.05
0.10
MR effect size for
'Selenium' on 'Neurexin−1'

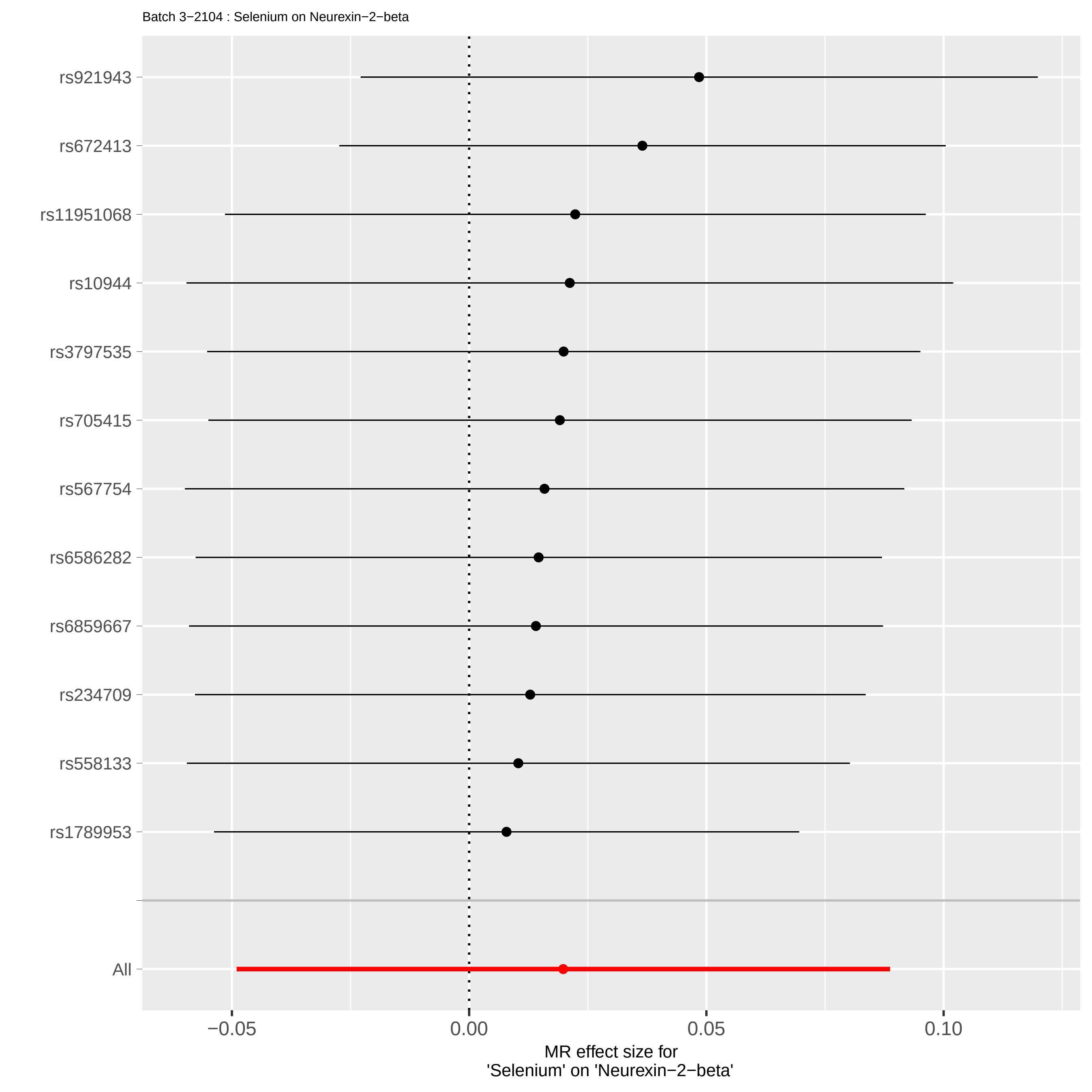

Batch 3−2104 : Selenium on Neurexin−2−beta
rs921943
rs672413
rs11951068
rs10944
rs3797535
rs705415
rs567754
rs6586282
rs6859667
rs234709
rs558133
rs1789953
All
−0.05
0.00
0.05
0.10
MR effect size for
'Selenium' on 'Neurexin−2−beta'

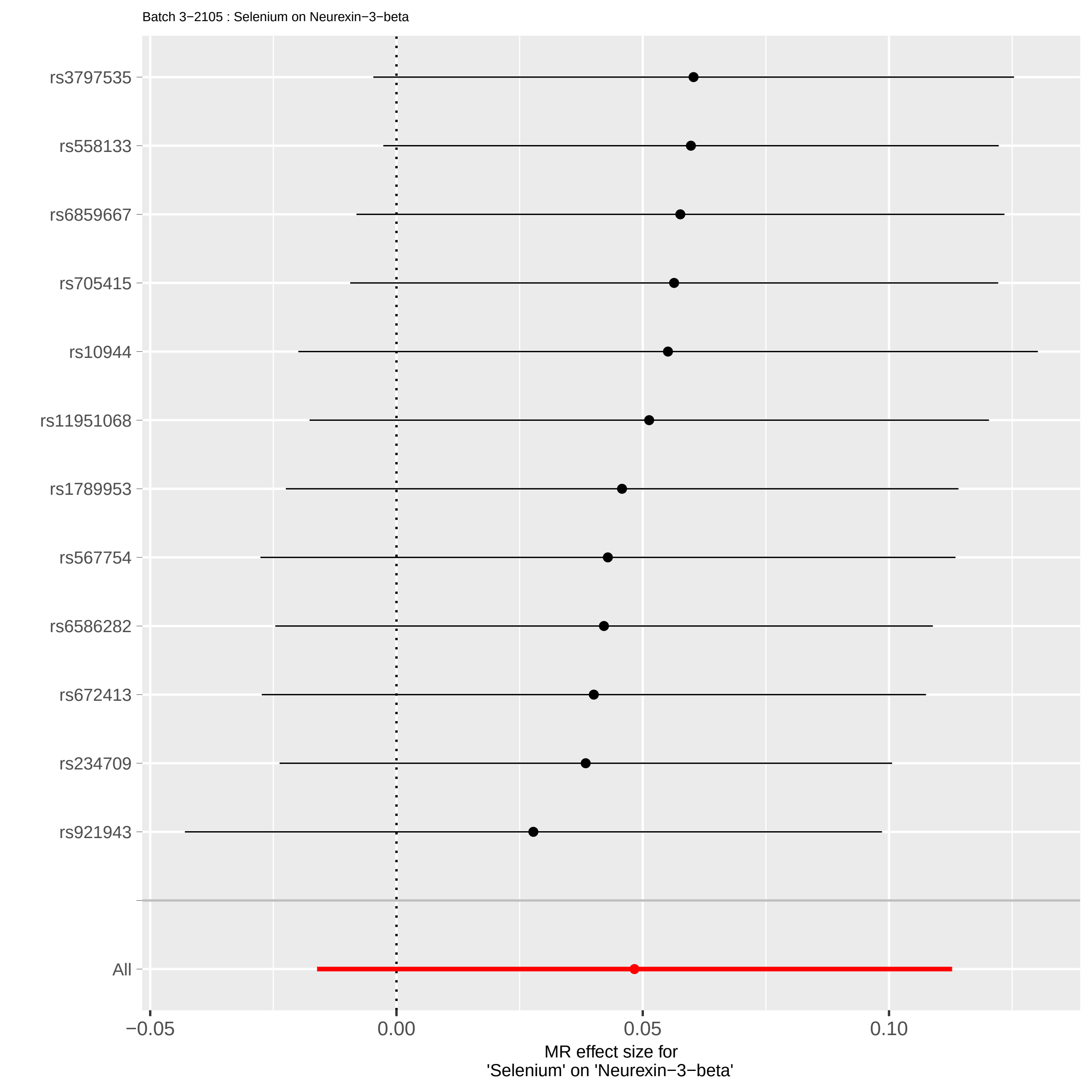

Batch 3−2105 : Selenium on Neurexin−3−beta
rs3797535
rs558133
rs6859667
rs705415
rs10944
rs11951068
rs1789953
rs567754
rs6586282
rs672413
rs234709
rs921943
All
−0.05
0.00
0.05
0.10
MR effect size for
'Selenium' on 'Neurexin−3−beta'

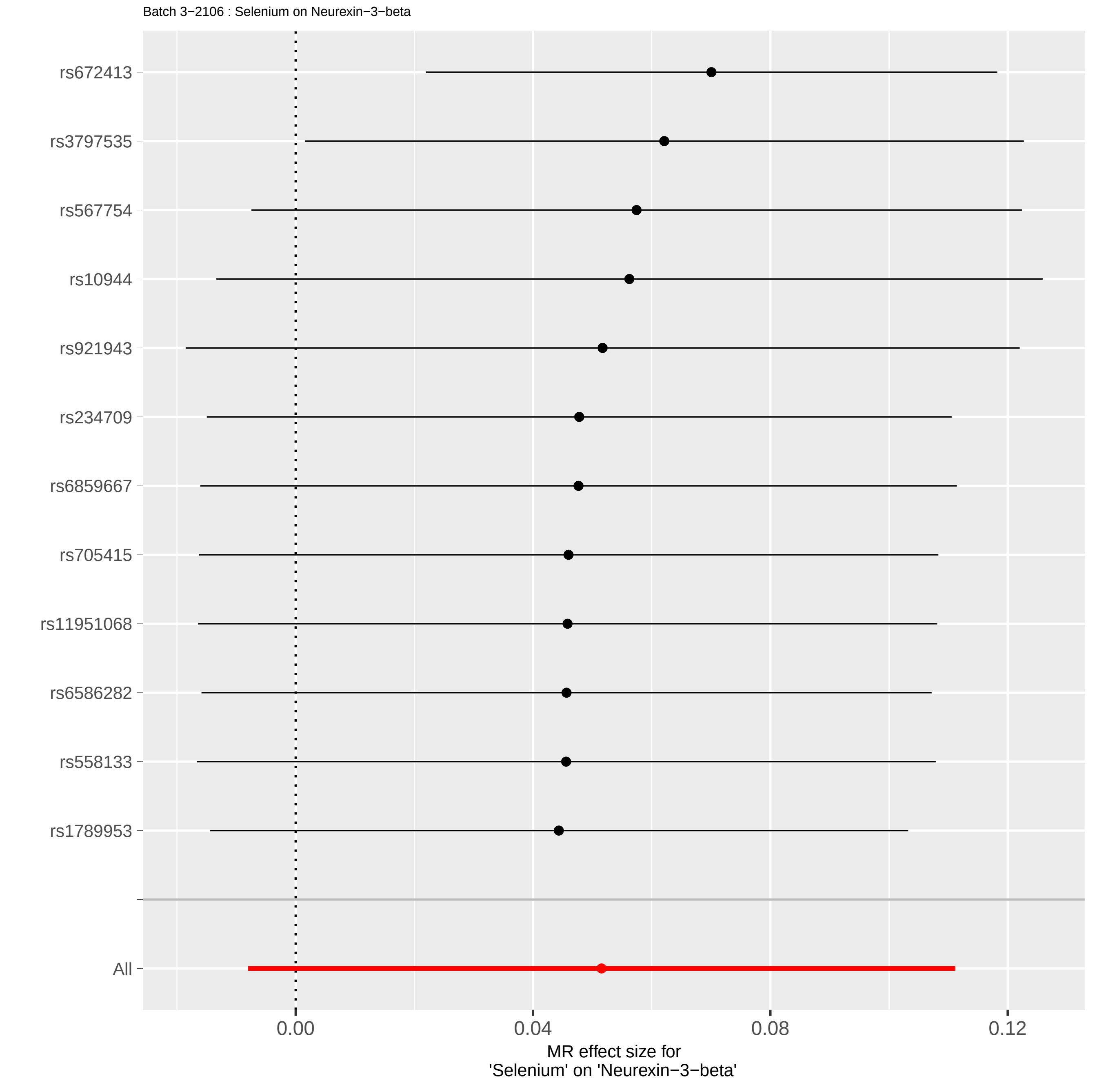

Batch 3−2106 : Selenium on Neurexin−3−beta
rs672413
rs3797535
rs567754
rs10944
rs921943
rs234709
rs6859667
rs705415
rs11951068
rs6586282
rs558133
rs1789953
All
0.00
0.04
0.08
0.12
MR effect size for
'Selenium' on 'Neurexin−3−beta'

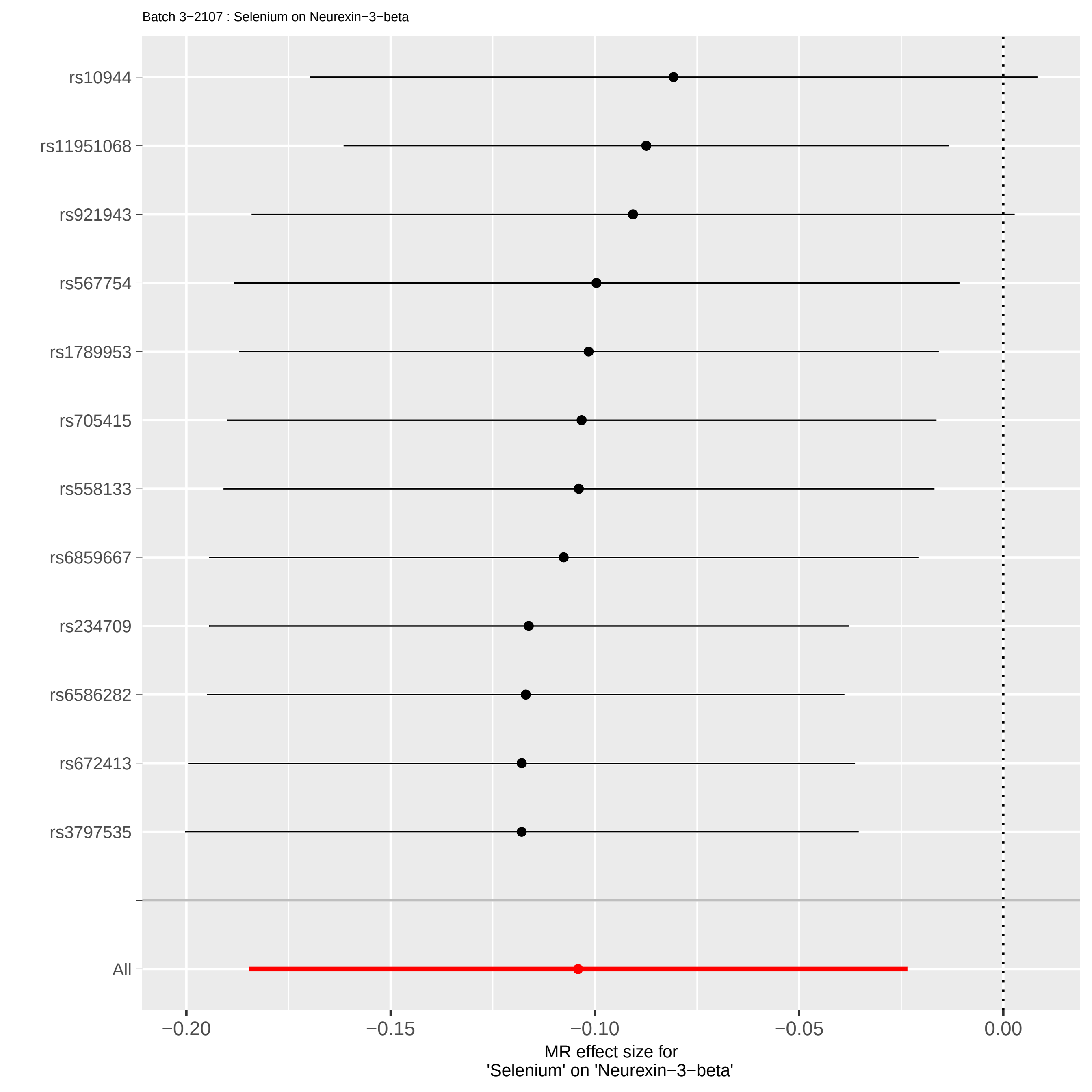

Batch 3−2107 : Selenium on Neurexin−3−beta
rs10944
rs11951068
rs921943
rs567754
rs1789953
rs705415
rs558133
rs6859667
rs234709
rs6586282
rs672413
rs3797535
All
−0.20
−0.15
−0.10
−0.05
0.00
MR effect size for
'Selenium' on 'Neurexin−3−beta'

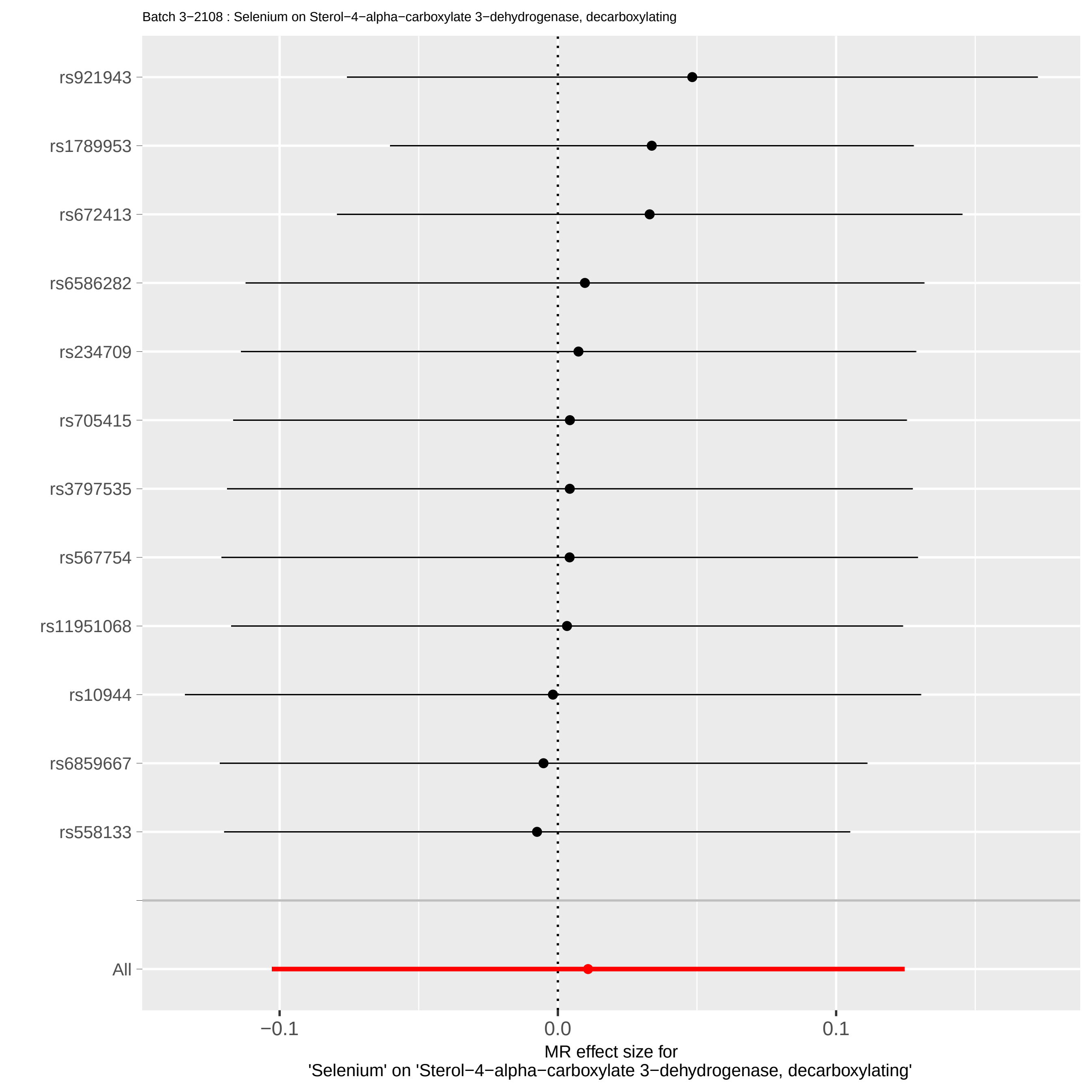

Batch 3−2108 : Selenium on Sterol−4−alpha−carboxylate 3−dehydrogenase, decarboxylating
rs921943
rs1789953
rs672413
rs6586282
rs234709
rs705415
rs3797535
rs567754
rs11951068
rs10944
rs6859667
rs558133
All
−0.1
0.0
0.1
MR effect size for
'Selenium' on 'Sterol−4−alpha−carboxylate 3−dehydrogenase, decarboxylating'

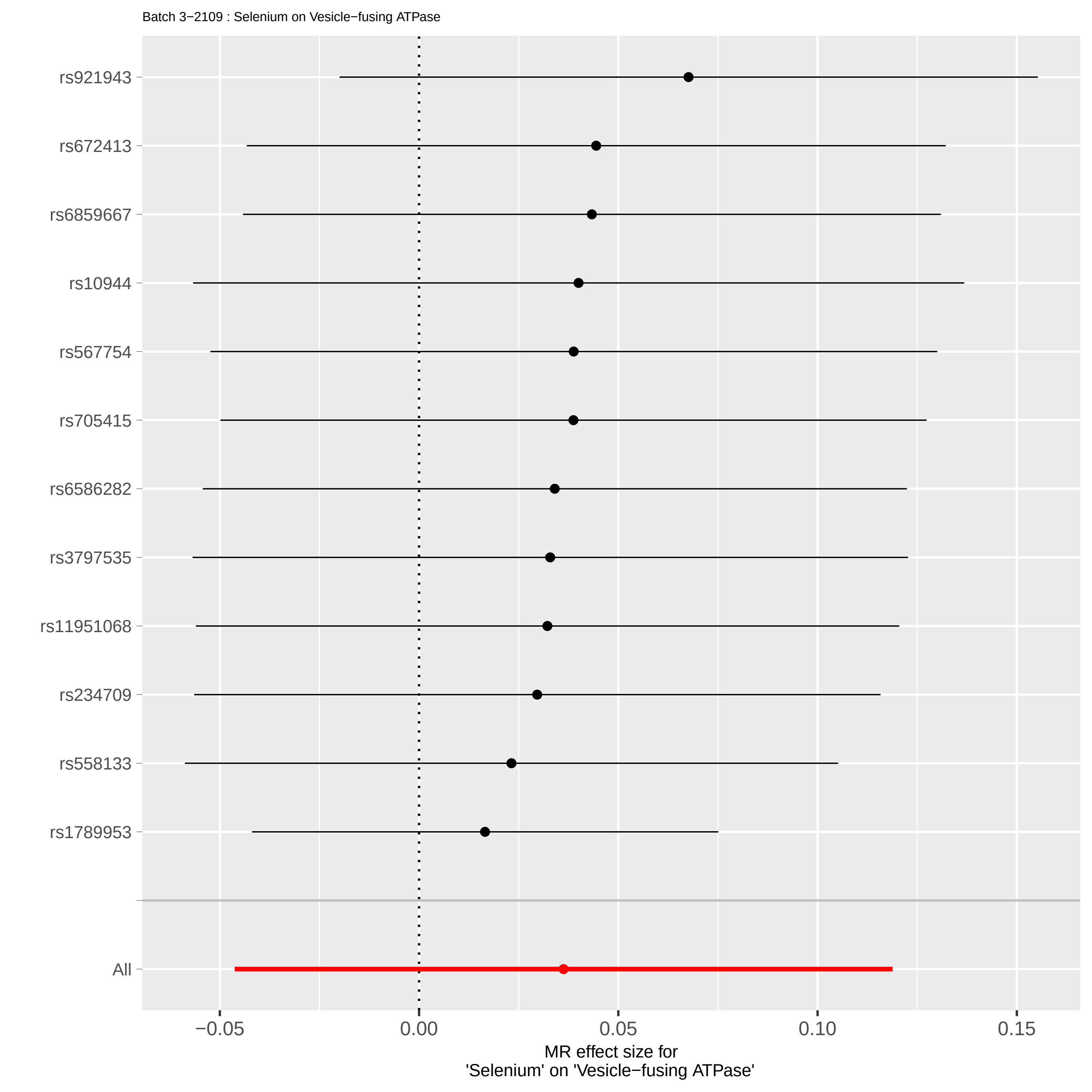

Batch 3−2109 : Selenium on Vesicle−fusing ATPase
rs921943
rs672413
rs6859667
rs10944
rs567754
rs705415
rs6586282
rs3797535
rs11951068
rs234709
rs558133
rs1789953
All
−0.05
0.00
0.05
0.10
0.15
MR effect size for
'Selenium' on 'Vesicle−fusing ATPase'

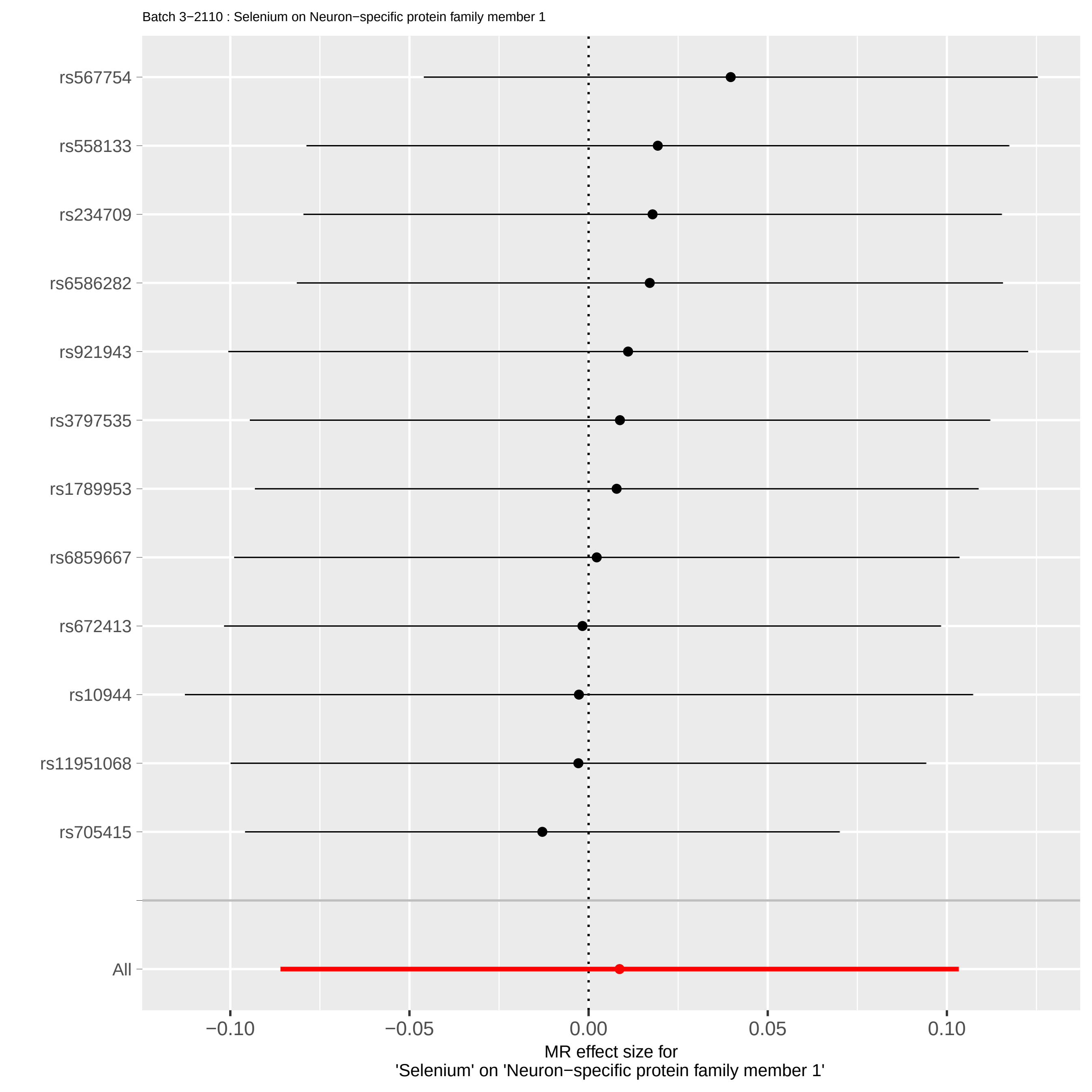
Batch 3−2110 : Selenium on Neuron−specific protein family member 1
rs567754
rs558133
rs234709
rs6586282
rs921943
rs3797535
rs1789953
rs6859667
rs672413
rs10944
rs11951068
rs705415
All
−0.10
−0.05
0.00
0.05
0.10
MR effect size for
'Selenium' on 'Neuron−specific protein family member 1'

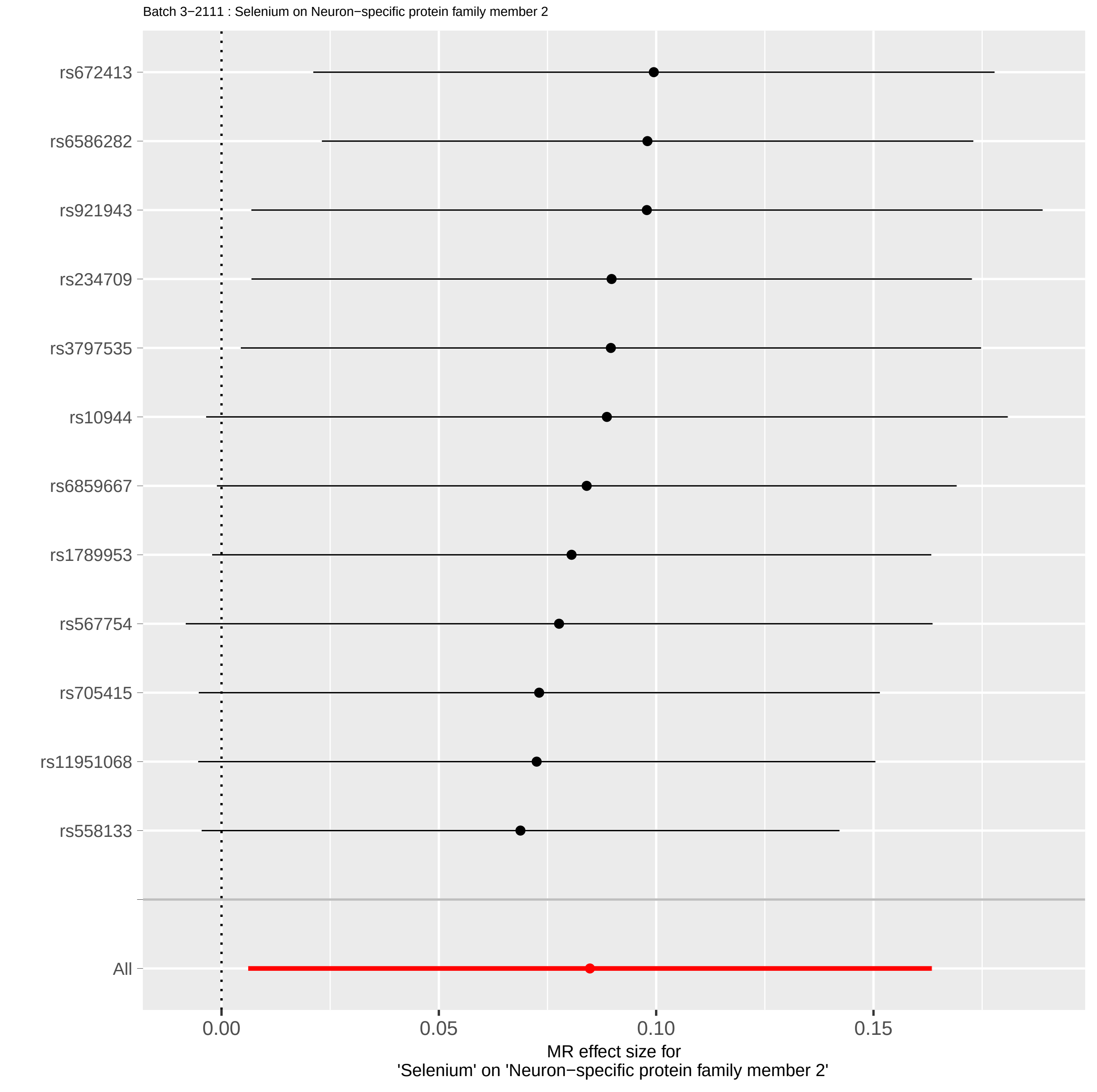
Batch 3−2111 : Selenium on Neuron−specific protein family member 2
rs672413
rs6586282
rs921943
rs234709
rs3797535
rs10944
rs6859667
rs1789953
rs567754
rs705415
rs11951068
rs558133
All
0.00
0.05
0.10
0.15
MR effect size for
'Selenium' on 'Neuron−specific protein family member 2'

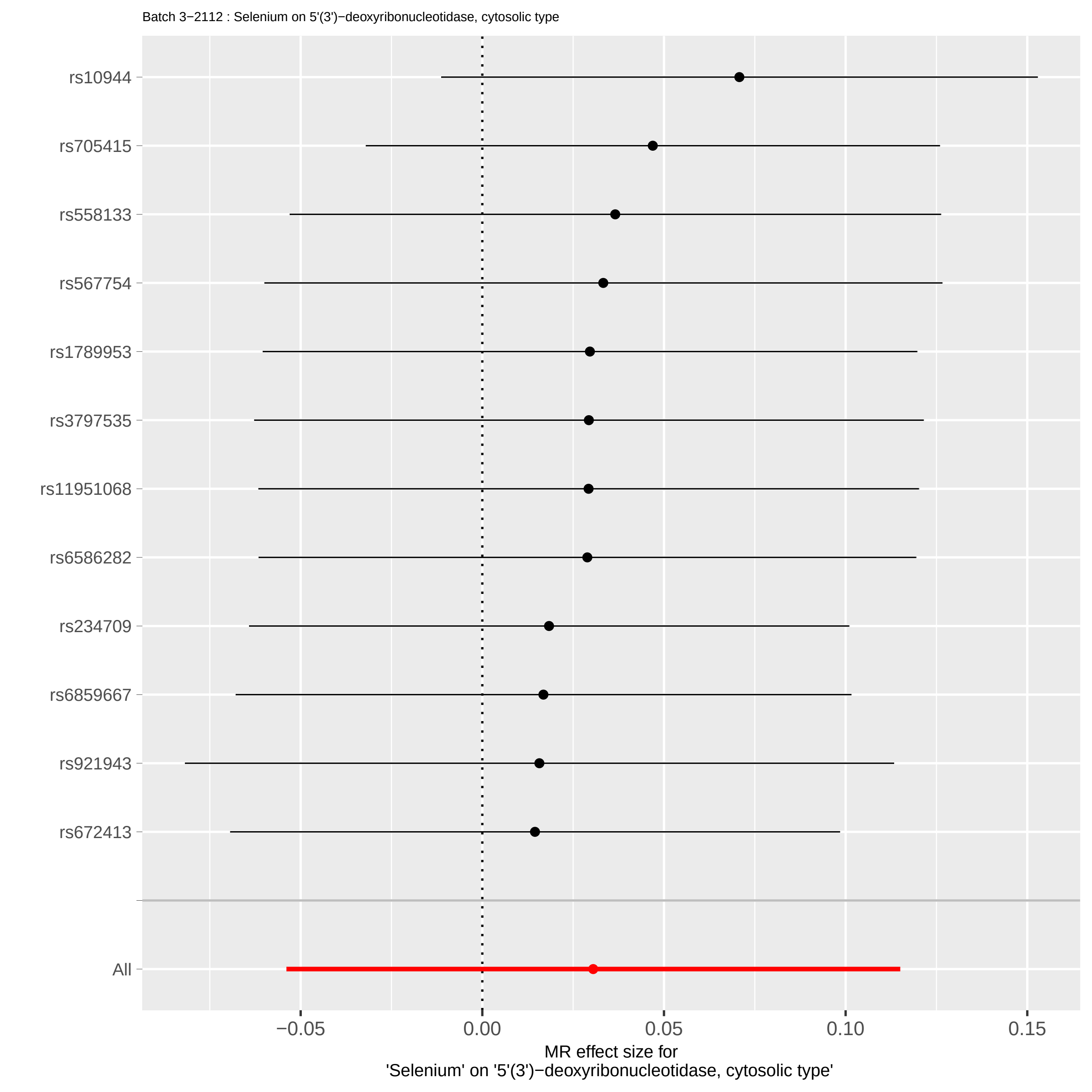

Batch 3−2112 : Selenium on 5'(3')−deoxyribonucleotidase, cytosolic type
rs10944
rs705415
rs558133
rs567754
rs1789953
rs3797535
rs11951068
rs6586282
rs234709
rs6859667
rs921943
rs672413
All
−0.05
0.00
0.05
0.10
0.15
MR effect size for
'Selenium' on '5'(3')−deoxyribonucleotidase, cytosolic type'

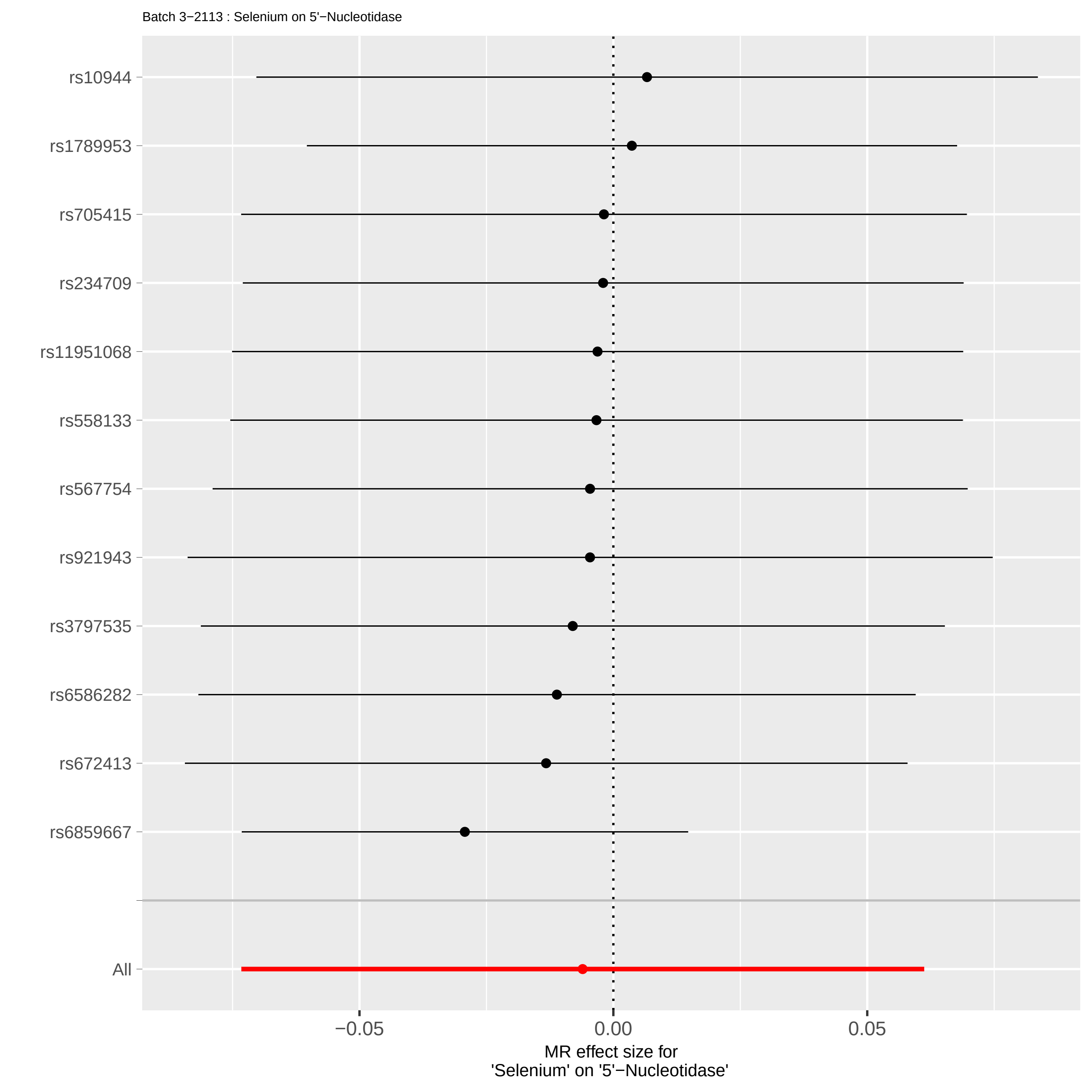
Batch 3−2113 : Selenium on 5'−Nucleotidase
rs10944
rs1789953
rs705415
rs234709
rs11951068
rs558133
rs567754
rs921943
rs3797535
rs6586282
rs672413
rs6859667
All
−0.05
0.00
0.05
MR effect size for
'Selenium' on '5'−Nucleotidase'

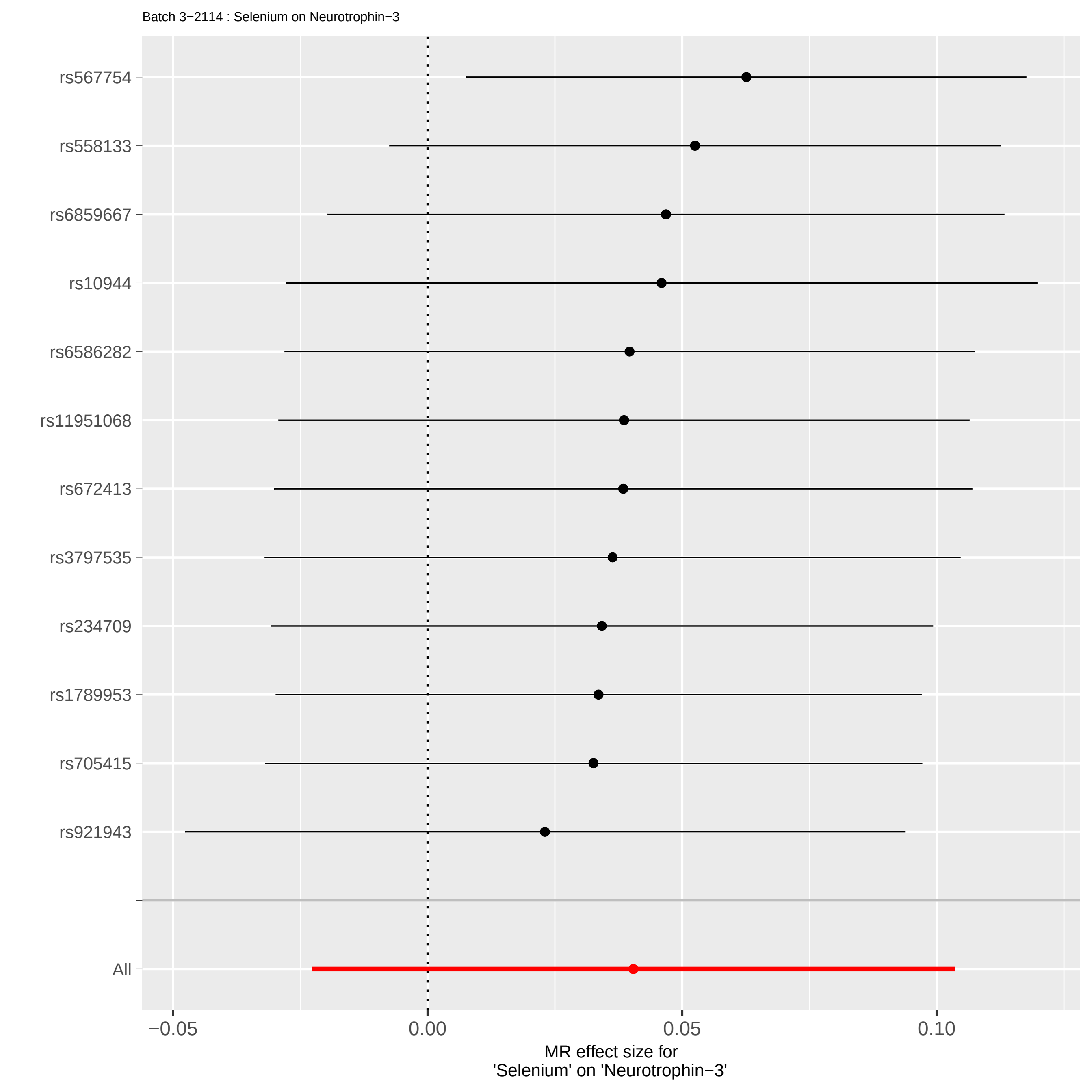

Batch 3−2114 : Selenium on Neurotrophin−3
rs567754
rs558133
rs6859667
rs10944
rs6586282
rs11951068
rs672413
rs3797535
rs234709
rs1789953
rs705415
rs921943
All
−0.05
0.00
0.05
0.10
MR effect size for
'Selenium' on 'Neurotrophin−3'

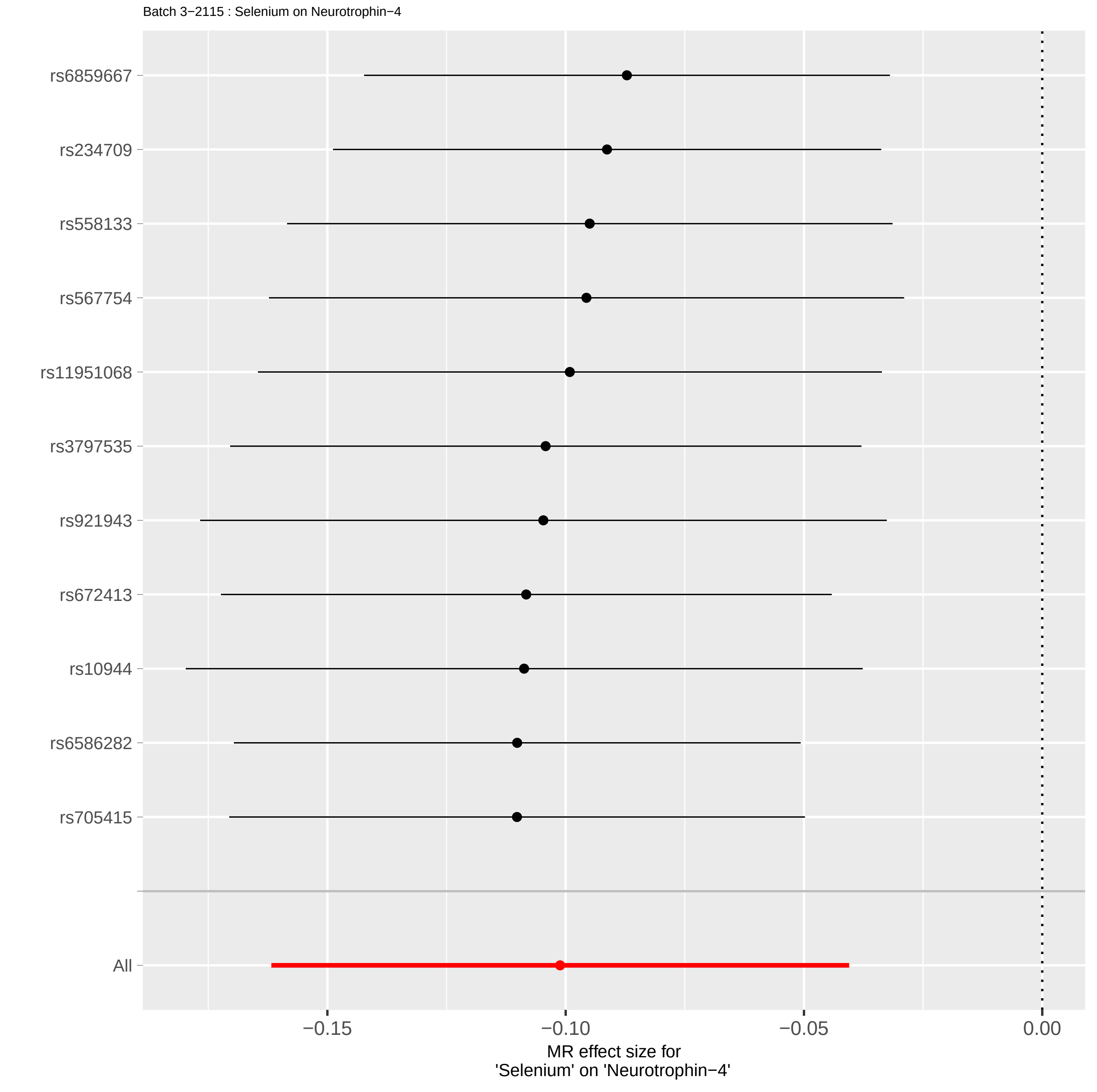

Batch 3−2115 : Selenium on Neurotrophin−4
rs6859667
rs234709
rs558133
rs567754
rs11951068
rs3797535
rs921943
rs672413
rs10944
rs6586282
rs705415
All
−0.15
−0.10
−0.05
0.00
MR effect size for
'Selenium' on 'Neurotrophin−4'

Batch 3−2116 : Selenium on N−terminal Xaa−Pro−Lys N−methyltransferase 1

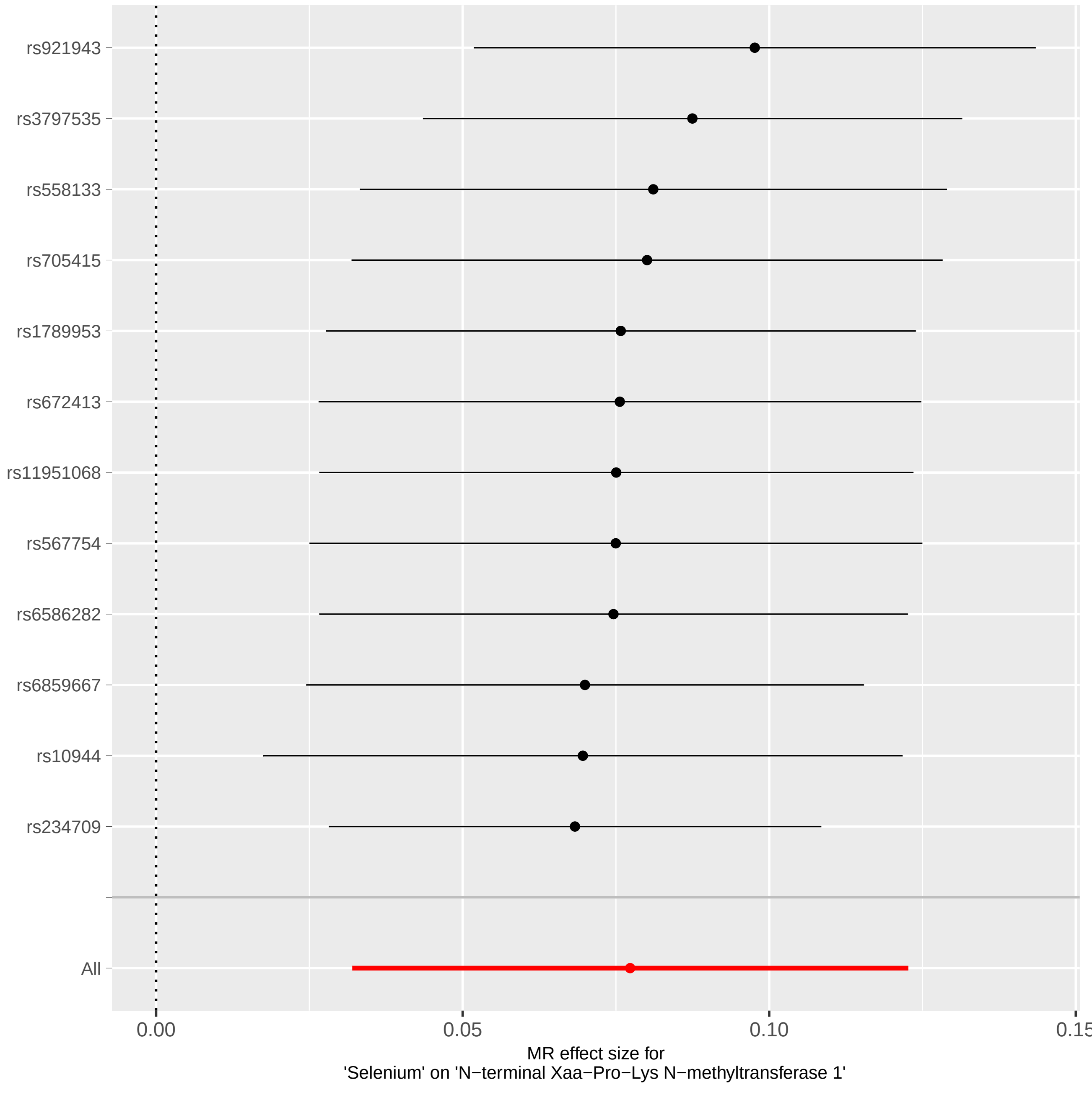

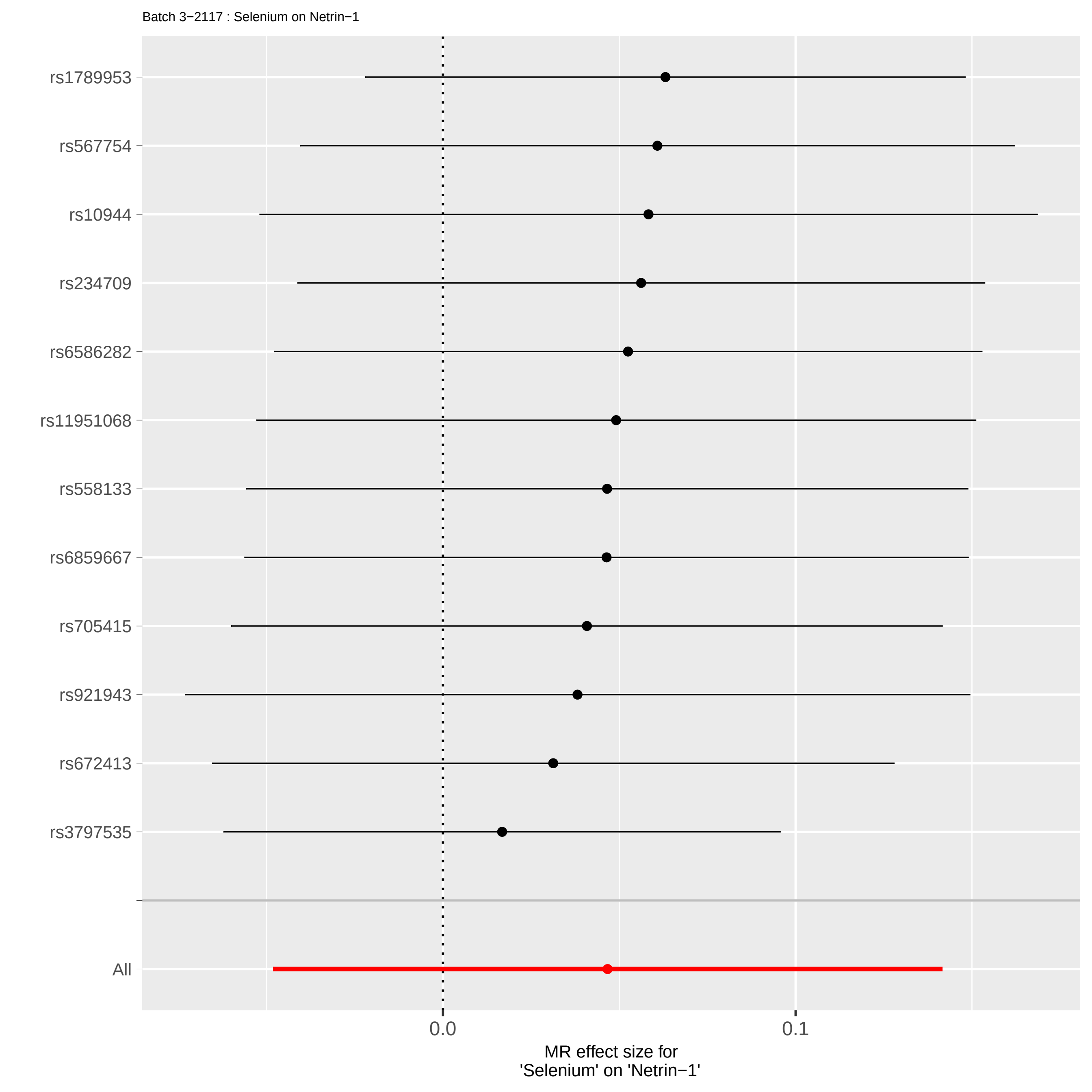

Batch 3−2117 : Selenium on Netrin−1
rs1789953
rs567754
rs10944
rs234709
rs6586282
rs11951068
rs558133
rs6859667
rs705415
rs921943
rs672413
rs3797535
All
0.0
0.1
MR effect size for
'Selenium' on 'Netrin−1'

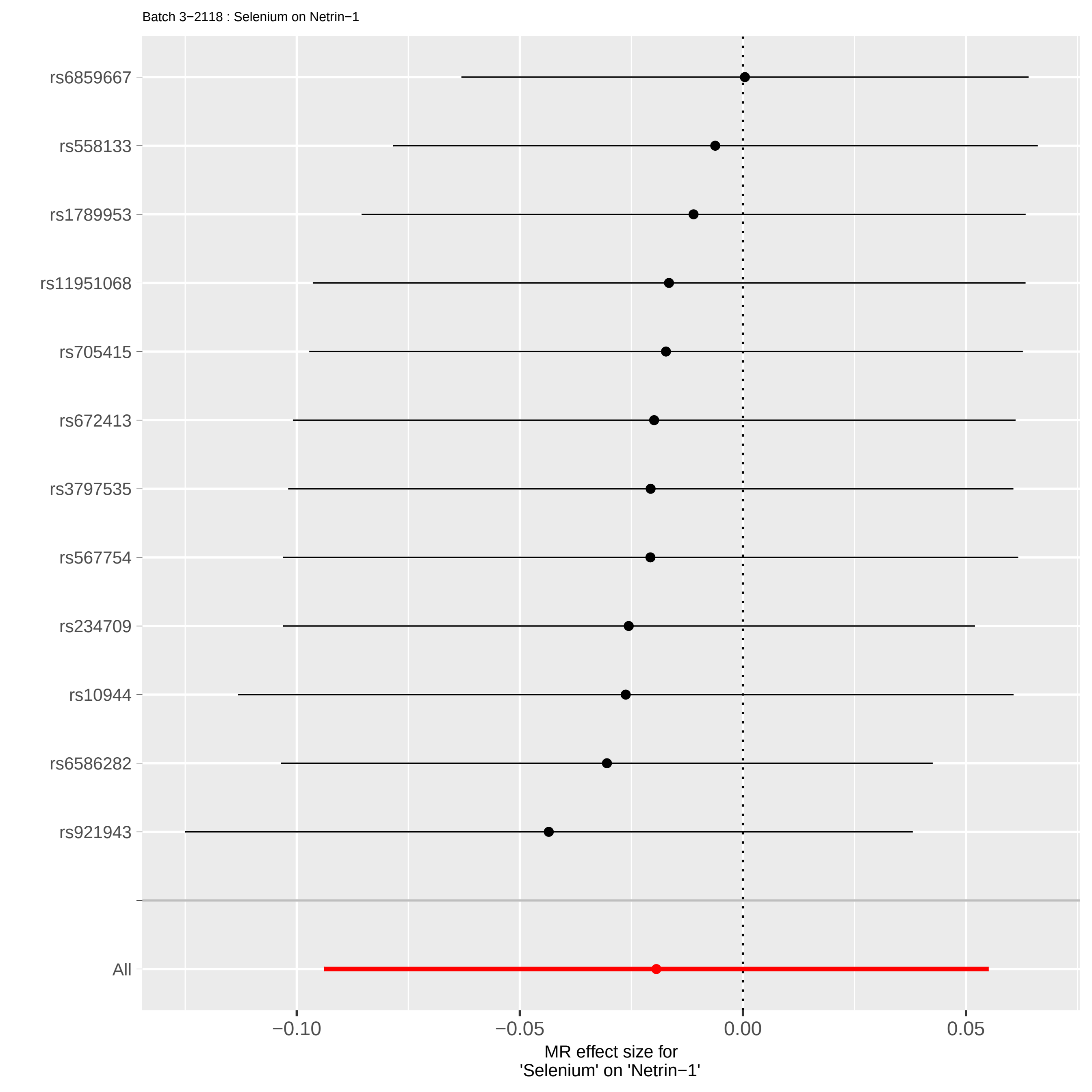

Batch 3−2118 : Selenium on Netrin−1
rs6859667
rs558133
rs1789953
rs11951068
rs705415
rs672413
rs3797535
rs567754
rs234709
rs10944
rs6586282
rs921943
All
−0.10
−0.05
0.00
0.05
MR effect size for
'Selenium' on 'Netrin−1'

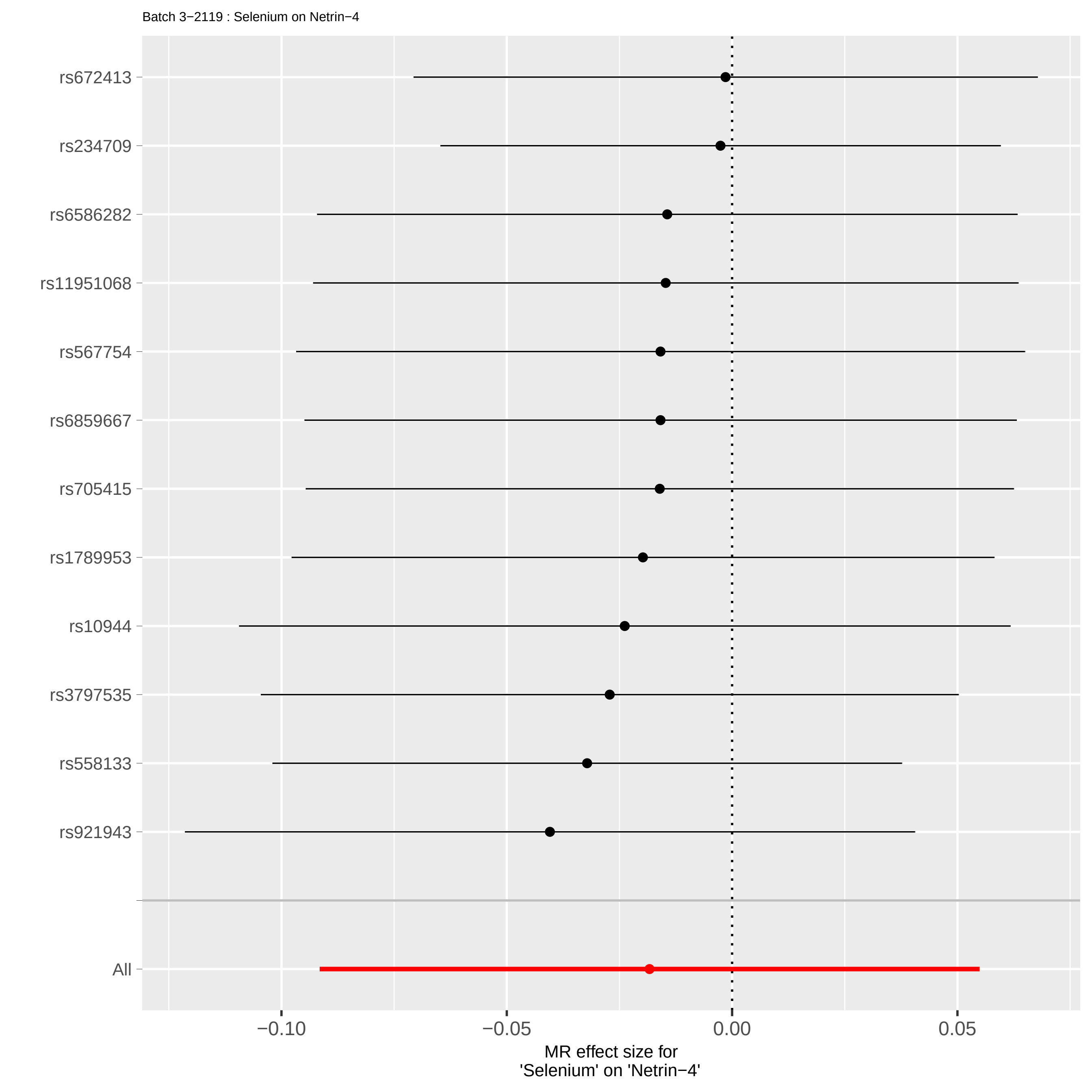

Batch 3−2119 : Selenium on Netrin−4
rs672413
rs234709
rs6586282
rs11951068
rs567754
rs6859667
rs705415
rs1789953
rs10944
rs3797535
rs558133
rs921943
All
−0.10
−0.05
0.00
0.05
MR effect size for
'Selenium' on 'Netrin−4'

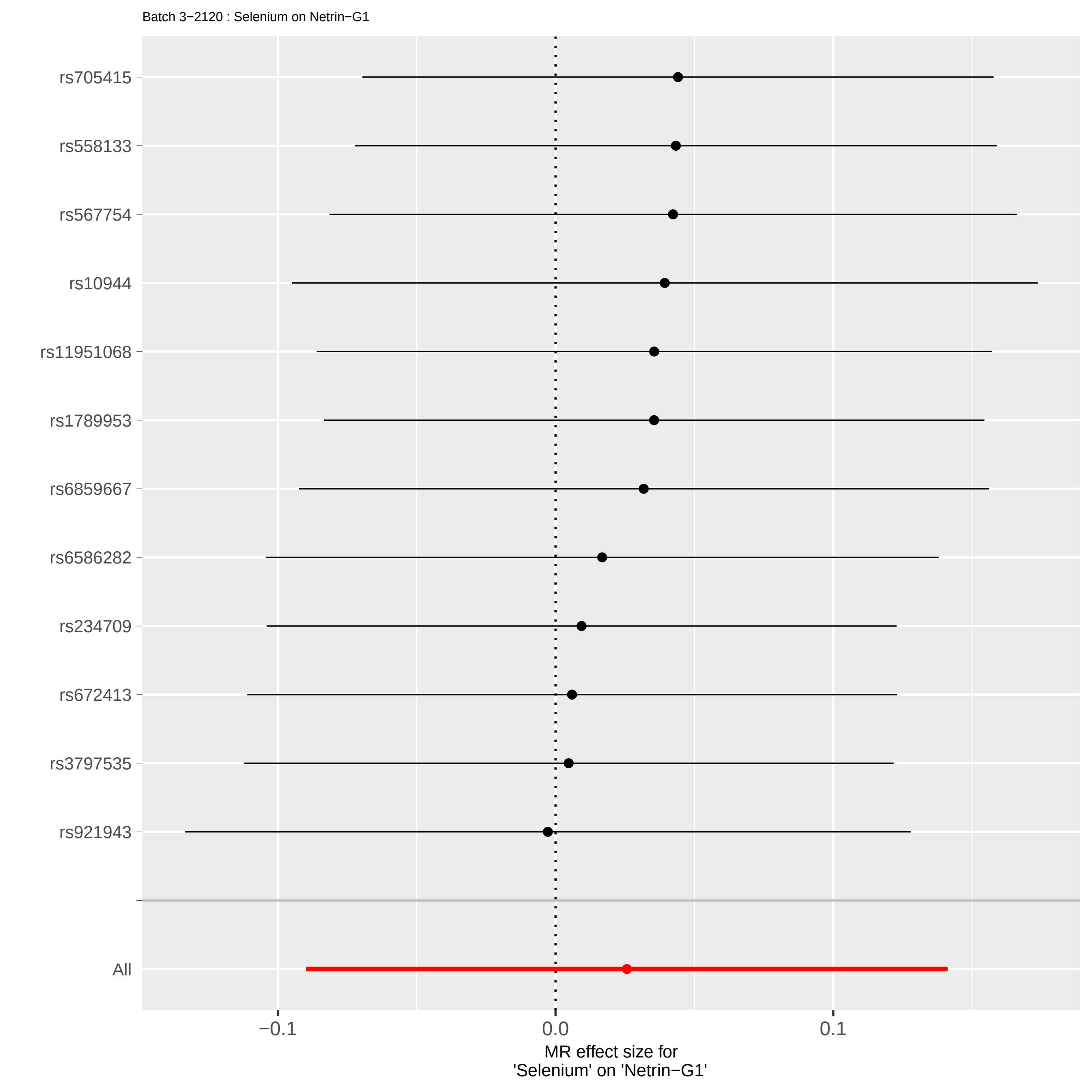
Batch 3−2120 : Selenium on Netrin−G1
rs705415
rs558133
rs567754
rs10944
rs11951068
rs1789953
rs6859667
rs6586282
rs234709
rs672413
rs3797535
rs921943
All
−0.1
0.0
0.1
MR effect size for
'Selenium' on 'Netrin−G1'

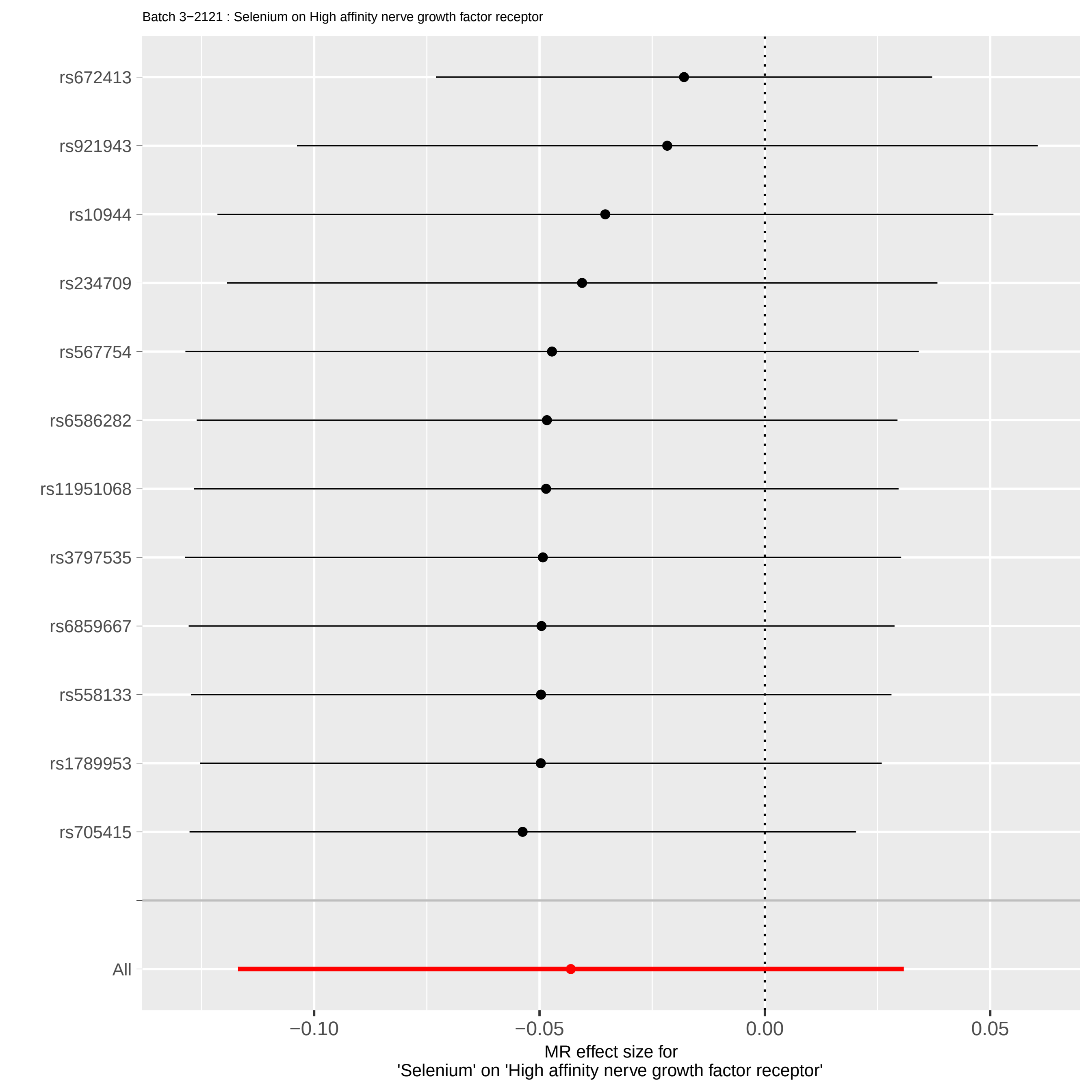
Batch 3−2121 : Selenium on High affinity nerve growth factor receptor
rs672413
rs921943
rs10944
rs234709
rs567754
rs6586282
rs11951068
rs3797535
rs6859667
rs558133
rs1789953
rs705415
All
−0.10
−0.05
0.00
0.05
MR effect size for
'Selenium' on 'High affinity nerve growth factor receptor'

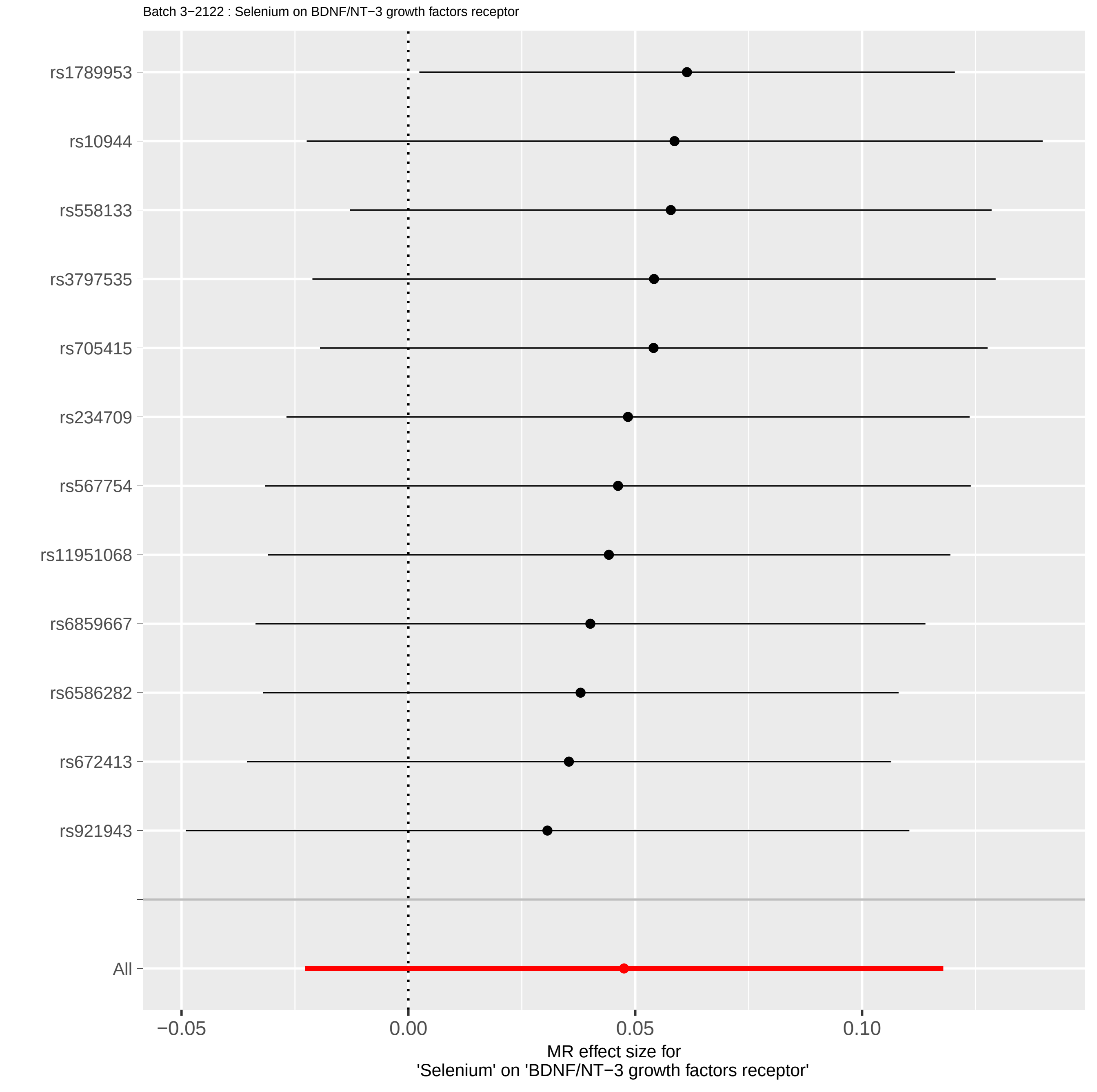

Batch 3−2122 : Selenium on BDNF/NT−3 growth factors receptor
rs1789953
rs10944
rs558133
rs3797535
rs705415
rs234709
rs567754
rs11951068
rs6859667
rs6586282
rs672413
rs921943
All
−0.05
0.00
0.05
0.10
MR effect size for
'Selenium' on 'BDNF/NT−3 growth factors receptor'

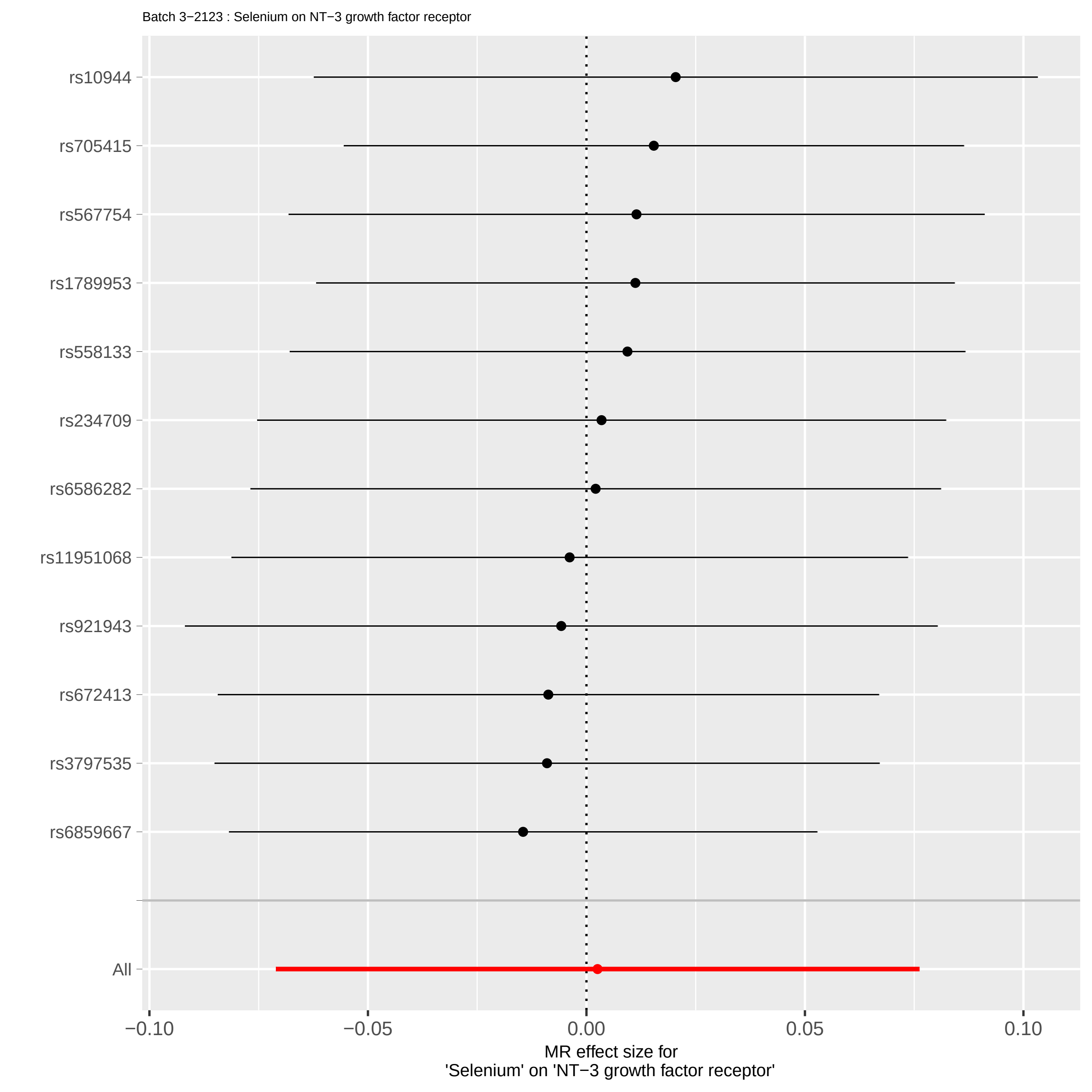

Batch 3−2123 : Selenium on NT−3 growth factor receptor
rs10944
rs705415
rs567754
rs1789953
rs558133
rs234709
rs6586282
rs11951068
rs921943
rs672413
rs3797535
rs6859667
All
−0.10
−0.05
0.00
0.05
0.10
MR effect size for
'Selenium' on 'NT−3 growth factor receptor'

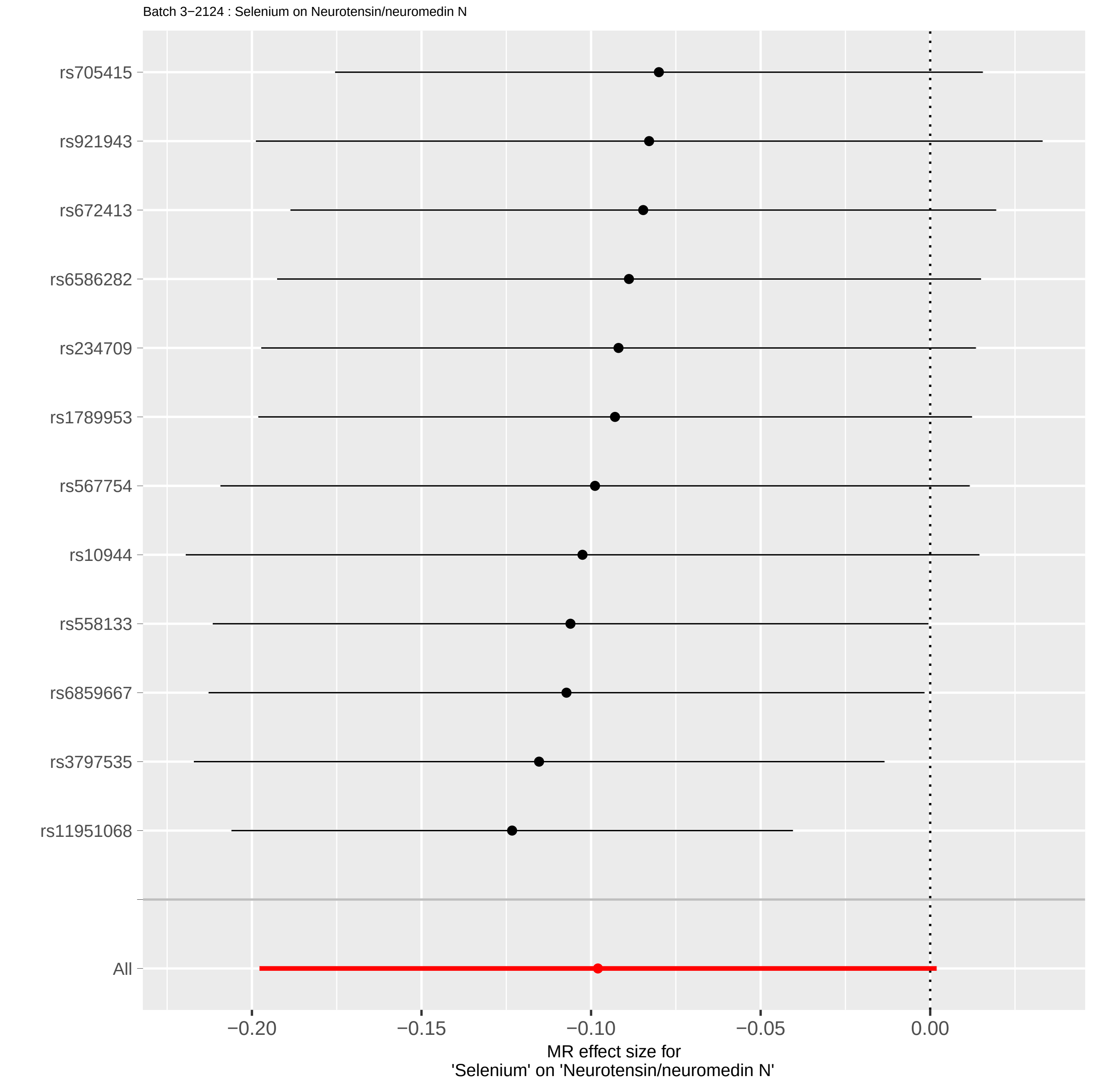
Batch 3−2124 : Selenium on Neurotensin/neuromedin N
rs705415
rs921943
rs672413
rs6586282
rs234709
rs1789953
rs567754
rs10944
rs558133
rs6859667
rs3797535
rs11951068
All
−0.20
−0.15
−0.10
−0.05
0.00
MR effect size for
'Selenium' on 'Neurotensin/neuromedin N'

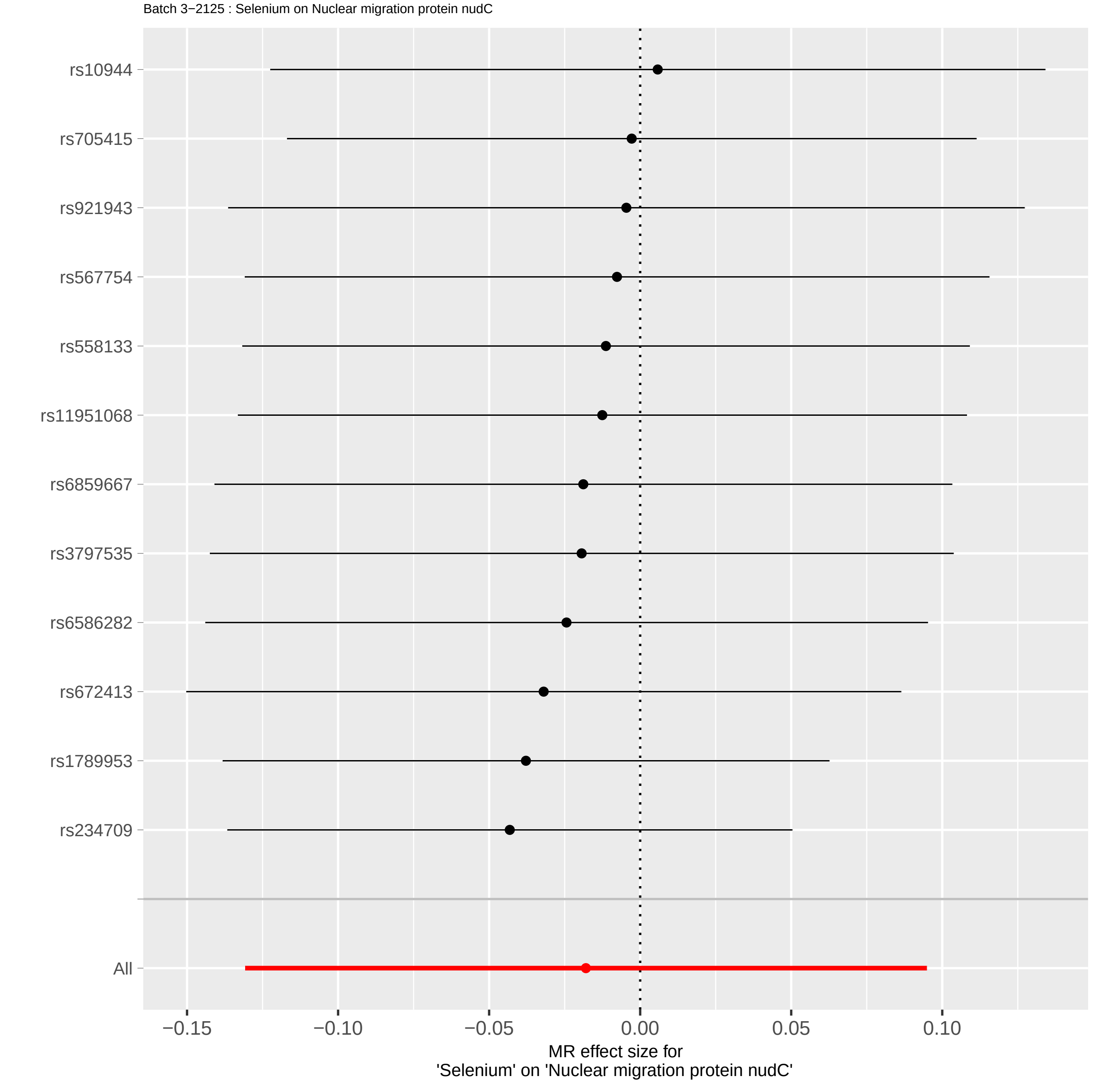

Batch 3−2125 : Selenium on Nuclear migration protein nudC
rs10944
rs705415
rs921943
rs567754
rs558133
rs11951068
rs6859667
rs3797535
rs6586282
rs672413
rs1789953
rs234709
All
−0.15
−0.10
−0.05
0.00
0.05
0.10
MR effect size for
'Selenium' on 'Nuclear migration protein nudC'

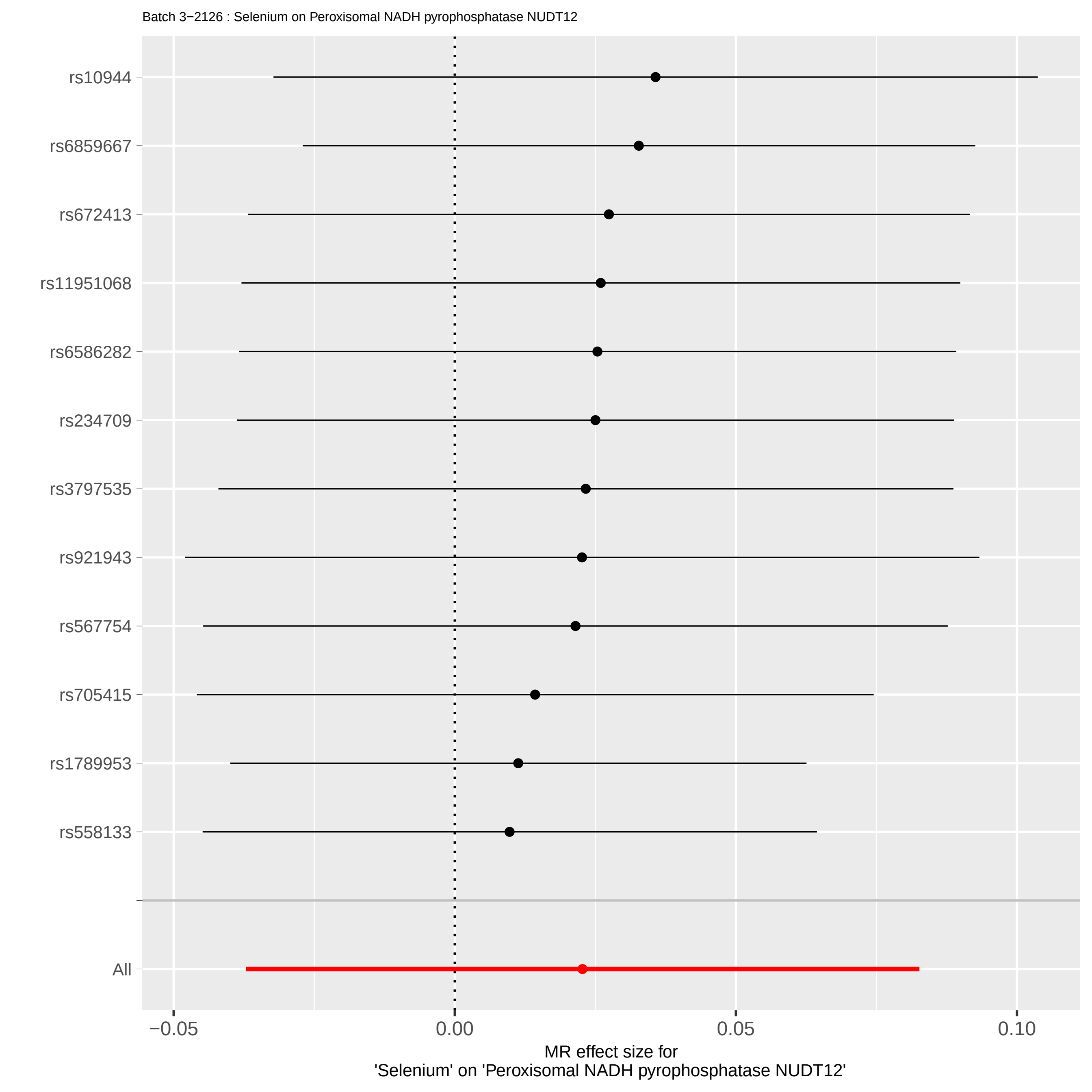

Batch 3−2126 : Selenium on Peroxisomal NADH pyrophosphatase NUDT12
rs10944
rs6859667
rs672413
rs11951068
rs6586282
rs234709
rs3797535
rs921943
rs567754
rs705415
rs1789953
rs558133
All
−0.05
0.00
0.05
0.10
MR effect size for
'Selenium' on 'Peroxisomal NADH pyrophosphatase NUDT12'

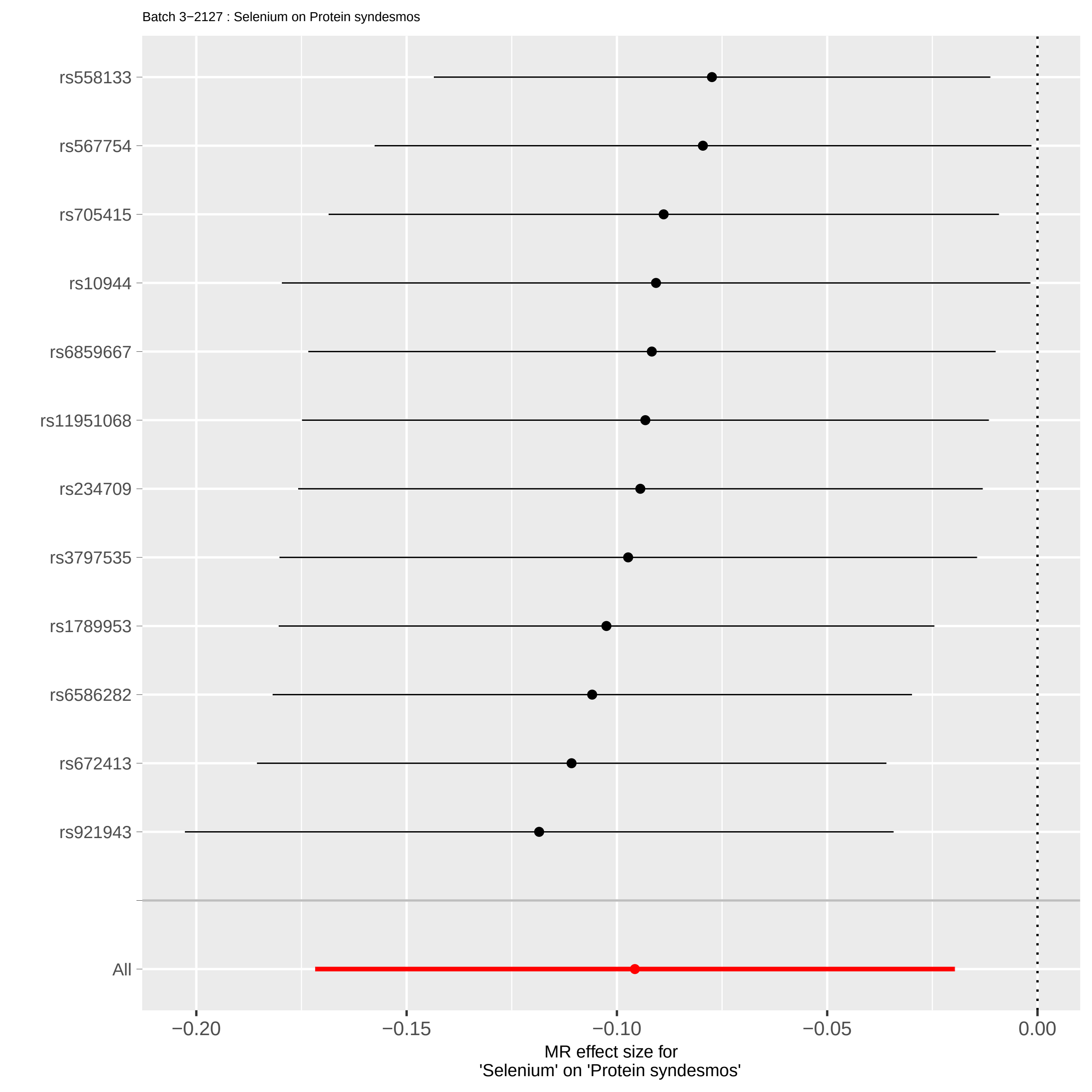

Batch 3−2127 : Selenium on Protein syndesmos
rs558133
rs567754
rs705415
rs10944
rs6859667
rs11951068
rs234709
rs3797535
rs1789953
rs6586282
rs672413
rs921943
All
−0.20
−0.15
−0.10
−0.05
0.00
MR effect size for
'Selenium' on 'Protein syndesmos'

Batch 3−2128 : Selenium on Nucleoside diphosphate−linked moiety X motif 8, mitochondrial

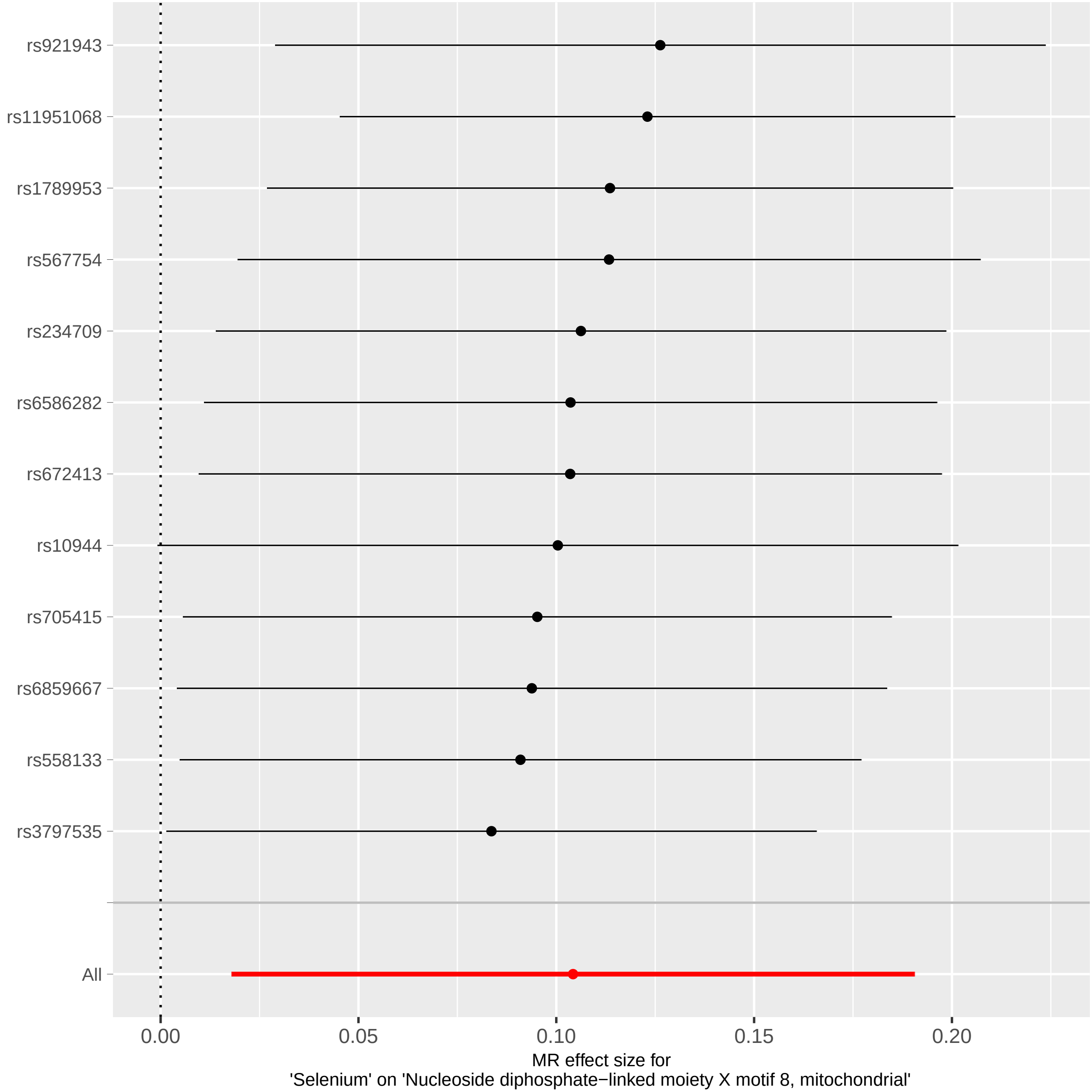

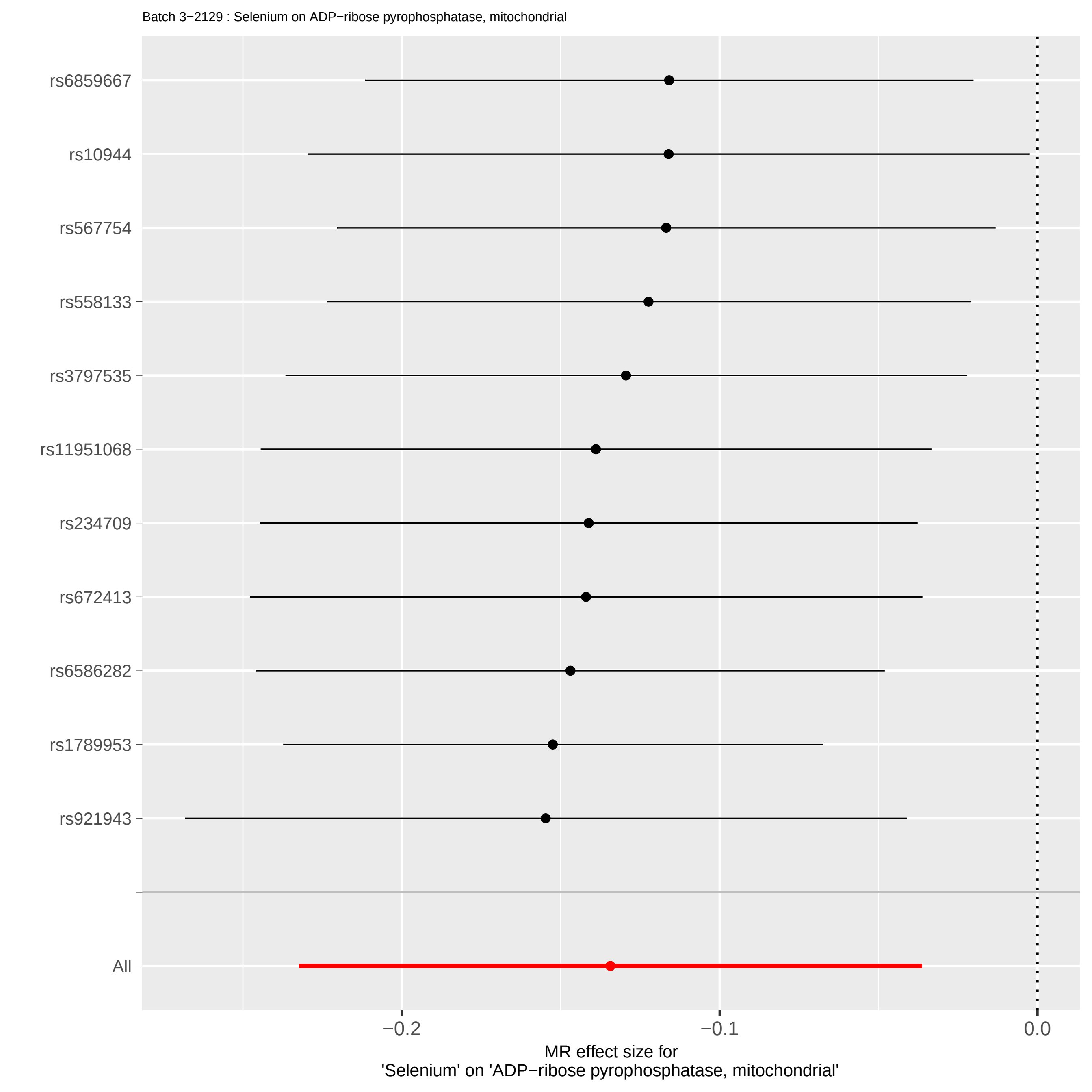
Batch 3−2129 : Selenium on ADP−ribose pyrophosphatase, mitochondrial
rs6859667
rs10944
rs567754
rs558133
rs3797535
rs11951068
rs234709
rs672413
rs6586282
rs1789953
rs921943
All
−0.2
−0.1
0.0
MR effect size for
'Selenium' on 'ADP−ribose pyrophosphatase, mitochondrial'

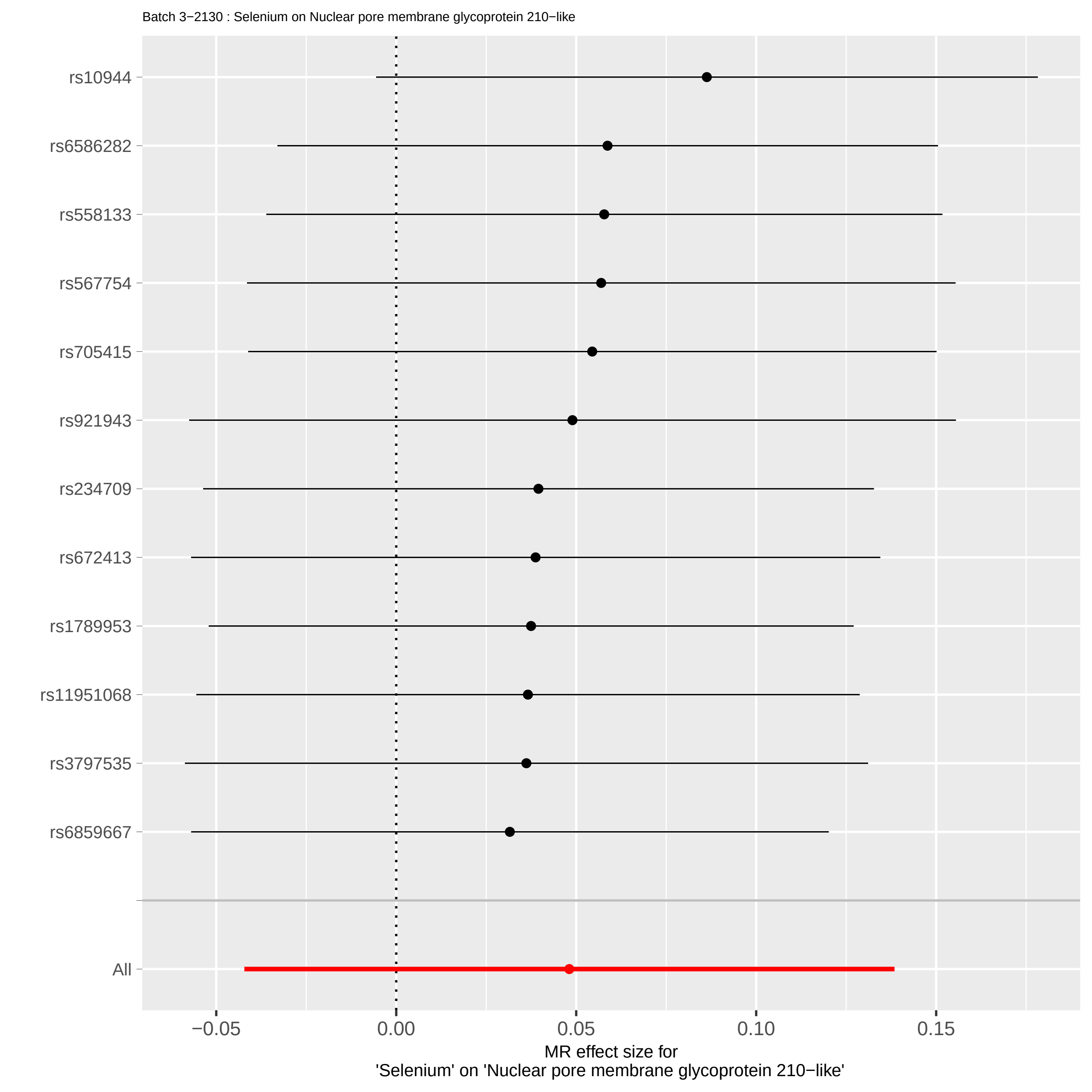

Batch 3−2130 : Selenium on Nuclear pore membrane glycoprotein 210−like
rs10944
rs6586282
rs558133
rs567754
rs705415
rs921943
rs234709
rs672413
rs1789953
rs11951068
rs3797535
rs6859667
All
−0.05
0.00
0.05
0.10
0.15
MR effect size for
'Selenium' on 'Nuclear pore membrane glycoprotein 210−like'

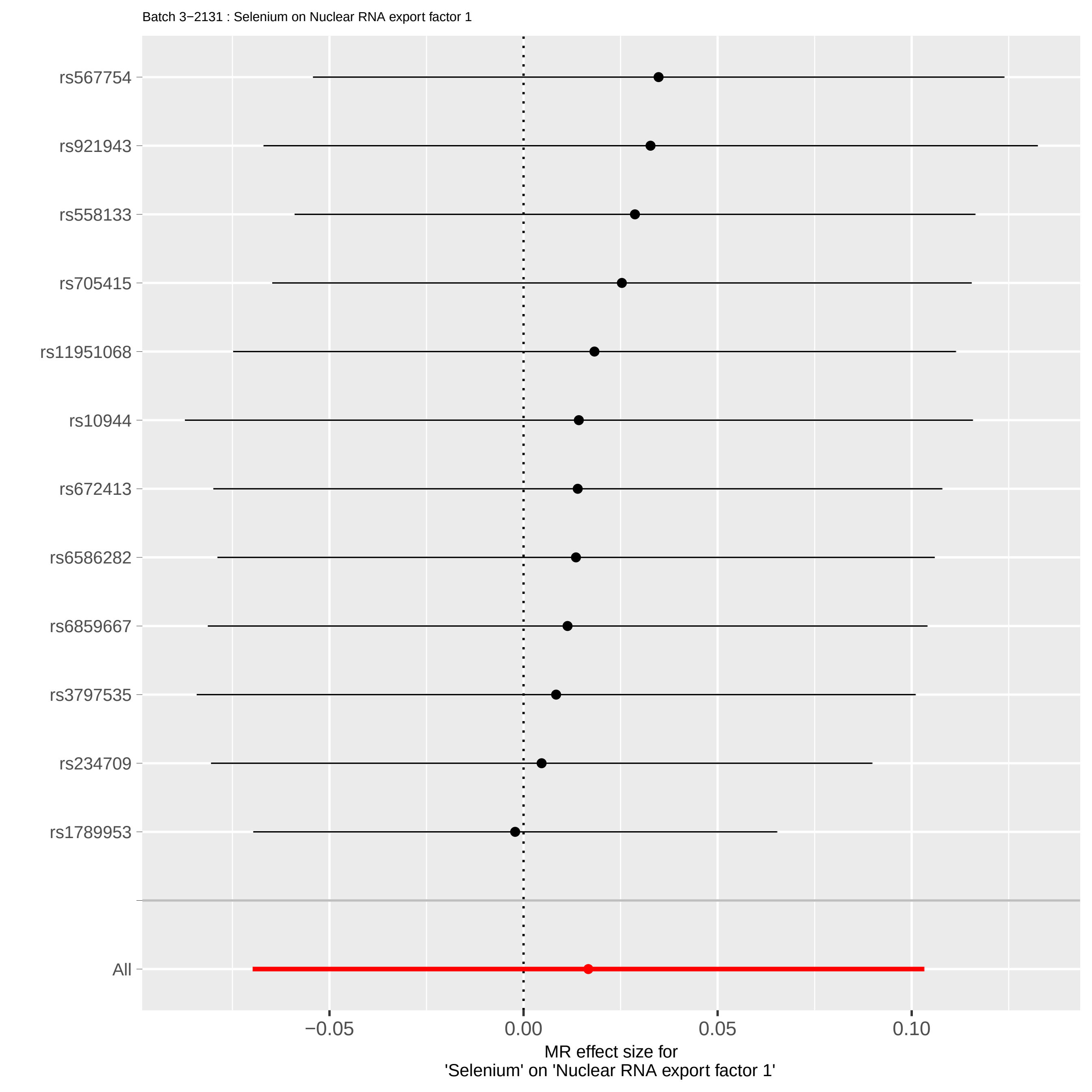

Batch 3−2131 : Selenium on Nuclear RNA export factor 1
rs567754
rs921943
rs558133
rs705415
rs11951068
rs10944
rs672413
rs6586282
rs6859667
rs3797535
rs234709
rs1789953
All
−0.05
0.00
0.05
0.10
MR effect size for
'Selenium' on 'Nuclear RNA export factor 1'

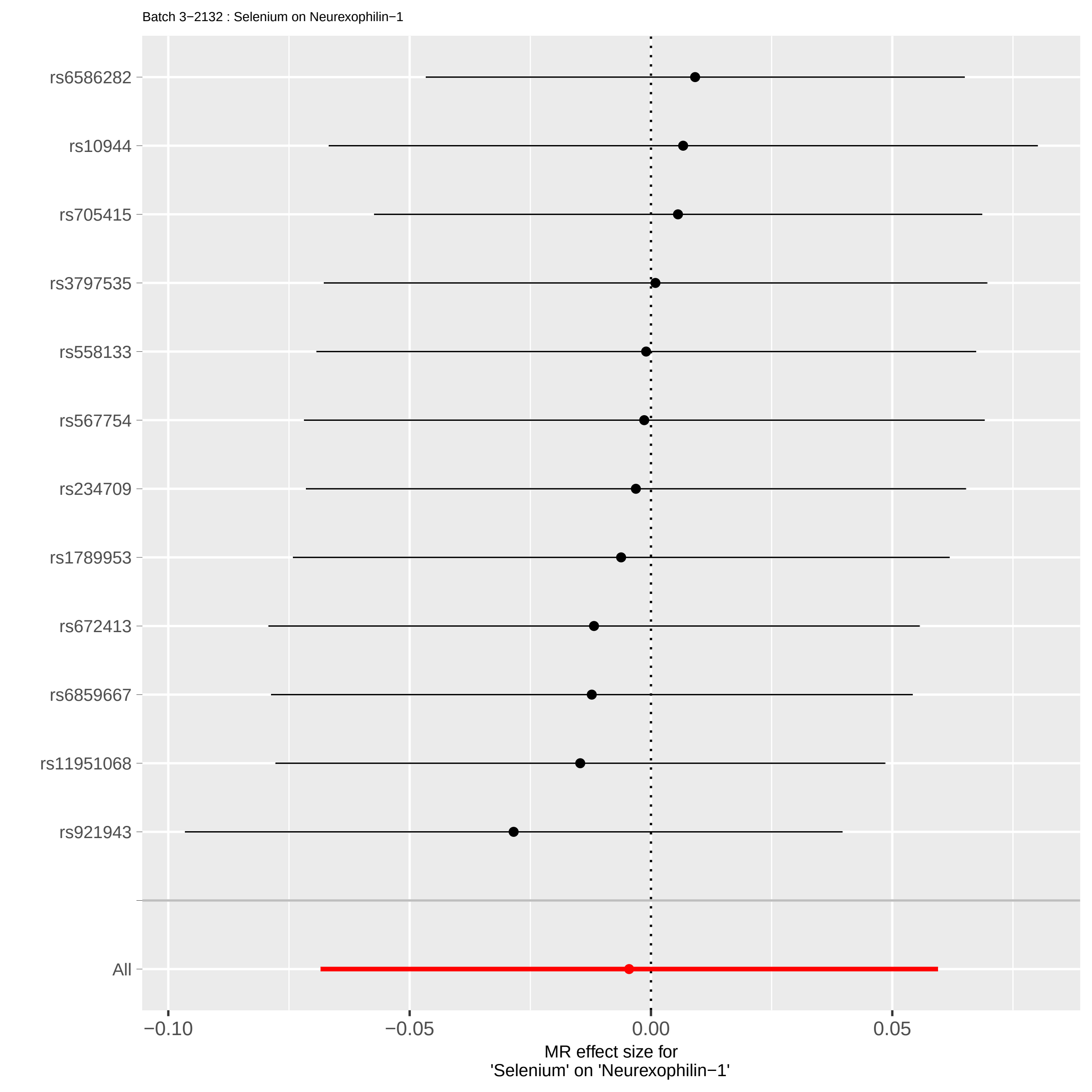

Batch 3−2132 : Selenium on Neurexophilin−1
rs6586282
rs10944
rs705415
rs3797535
rs558133
rs567754
rs234709
rs1789953
rs672413
rs6859667
rs11951068
rs921943
All
−0.10
−0.05
0.00
0.05
MR effect size for
'Selenium' on 'Neurexophilin−1'

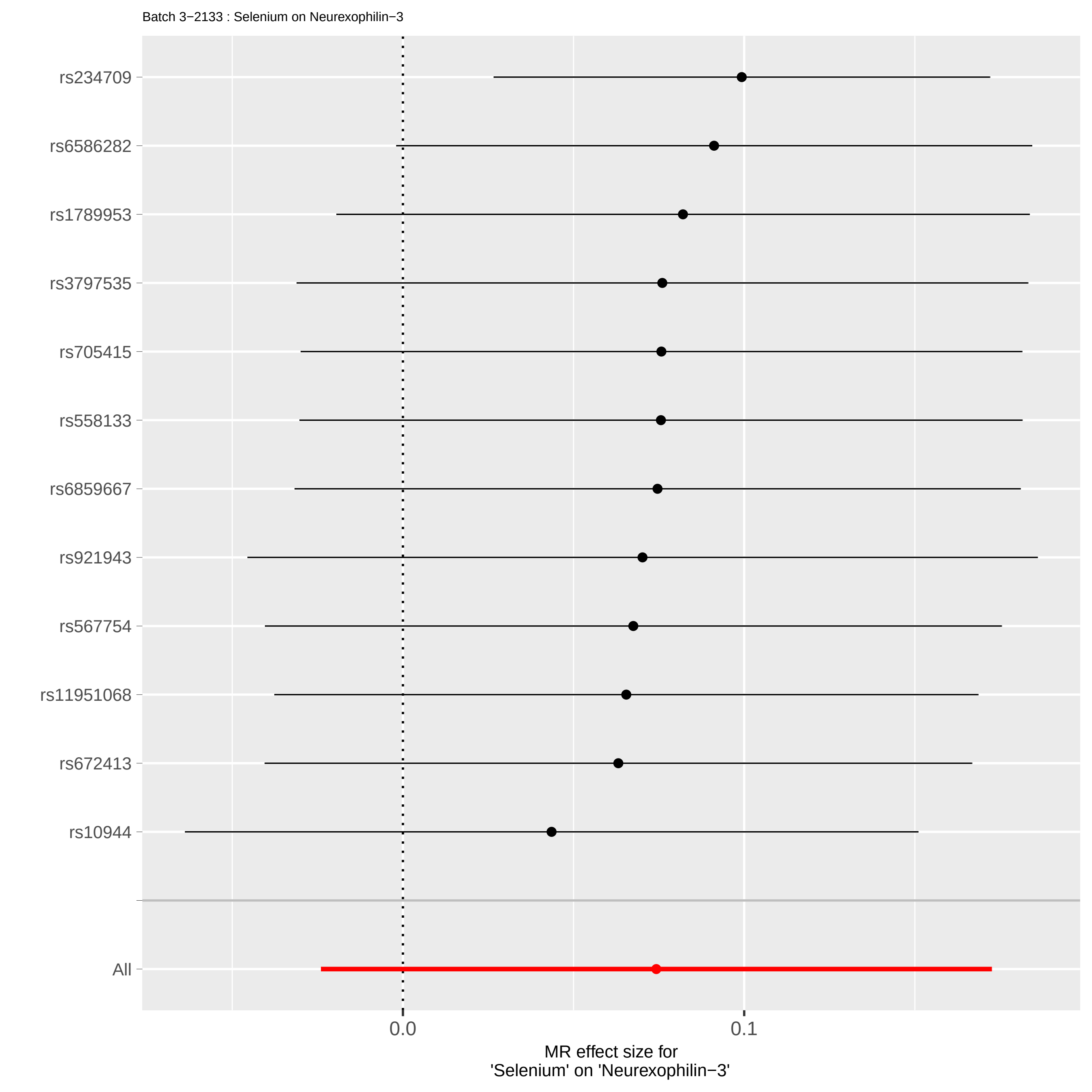
Batch 3−2133 : Selenium on Neurexophilin−3
rs234709
rs6586282
rs1789953
rs3797535
rs705415
rs558133
rs6859667
rs921943
rs567754
rs11951068
rs672413
rs10944
All
0.0
0.1
MR effect size for
'Selenium' on 'Neurexophilin−3'

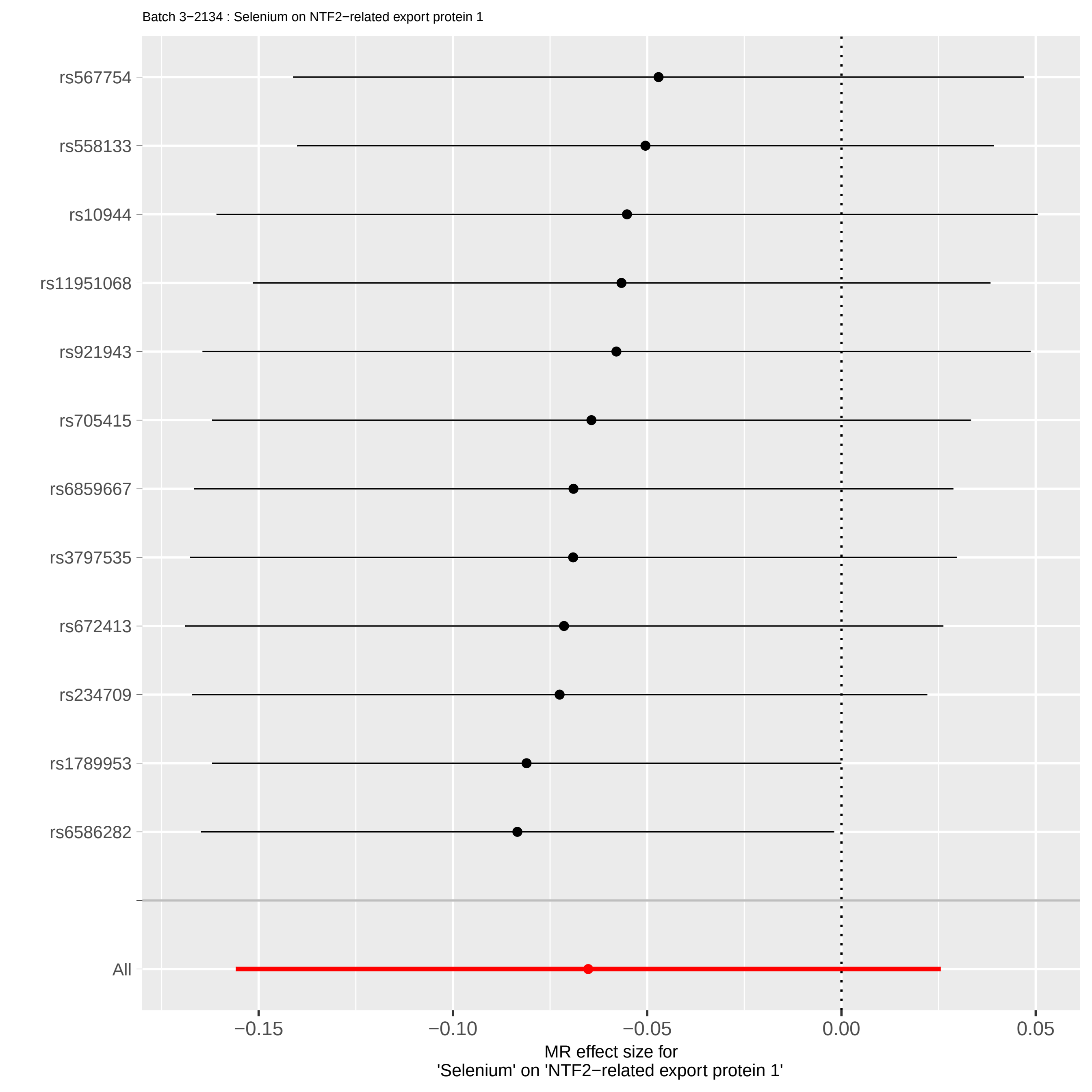

Batch 3−2134 : Selenium on NTF2−related export protein 1
rs567754
rs558133
rs10944
rs11951068
rs921943
rs705415
rs6859667
rs3797535
rs672413
rs234709
rs1789953
rs6586282
All
−0.15
−0.10
−0.05
0.00
0.05
MR effect size for
'Selenium' on 'NTF2−related export protein 1'

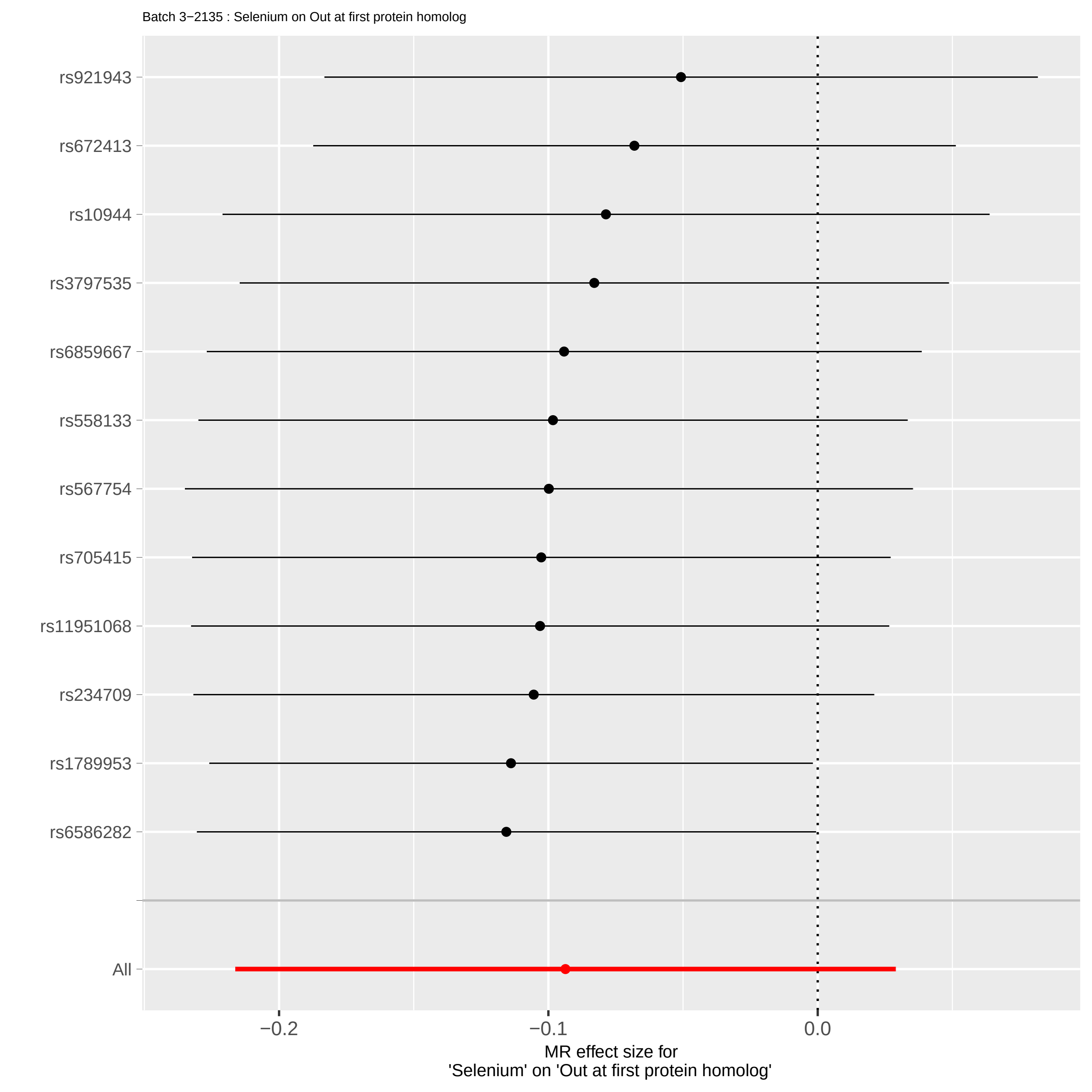

Batch 3−2135 : Selenium on Out at first protein homolog
rs921943
rs672413
rs10944
rs3797535
rs6859667
rs558133
rs567754
rs705415
rs11951068
rs234709
rs1789953
rs6586282
All
−0.2
−0.1
0.0
MR effect size for
'Selenium' on 'Out at first protein homolog'

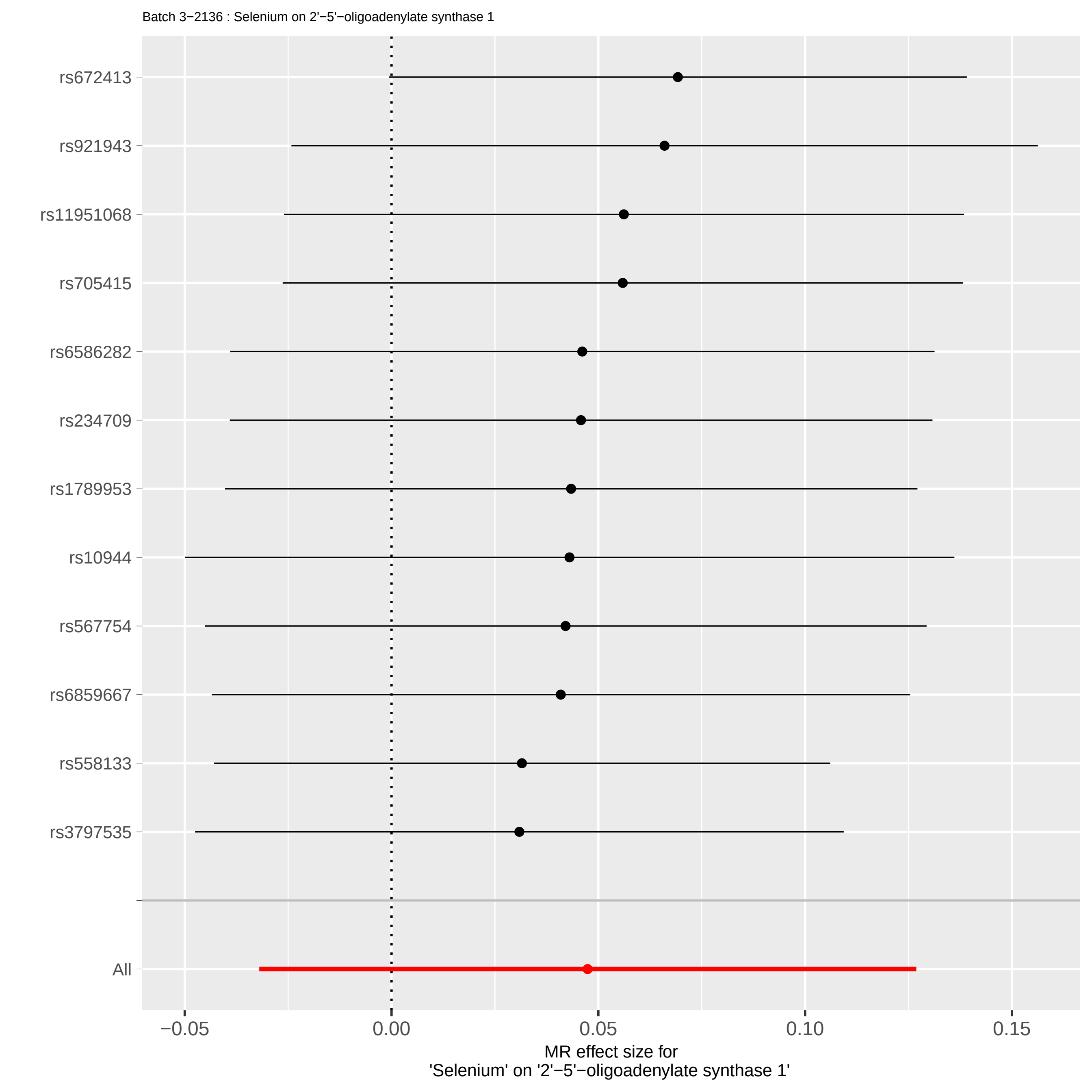

Batch 3−2136 : Selenium on 2'−5'−oligoadenylate synthase 1
rs672413
rs921943
rs11951068
rs705415
rs6586282
rs234709
rs1789953
rs10944
rs567754
rs6859667
rs558133
rs3797535
All
−0.05
0.00
0.05
0.10
0.15
MR effect size for
'Selenium' on '2'−5'−oligoadenylate synthase 1'

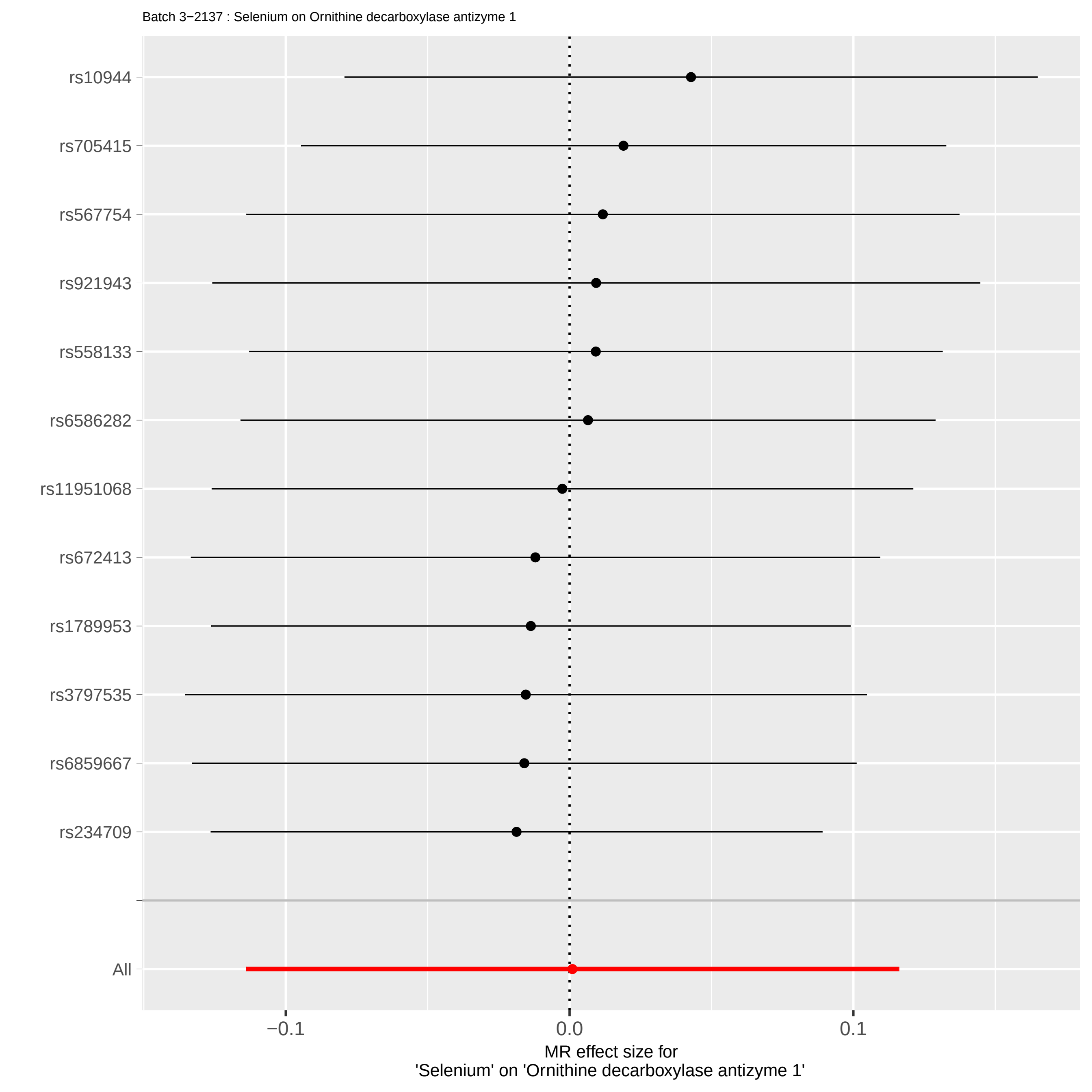

Batch 3−2137 : Selenium on Ornithine decarboxylase antizyme 1
rs10944
rs705415
rs567754
rs921943
rs558133
rs6586282
rs11951068
rs672413
rs1789953
rs3797535
rs6859667
rs234709
All
−0.1
0.0
0.1
MR effect size for
'Selenium' on 'Ornithine decarboxylase antizyme 1'

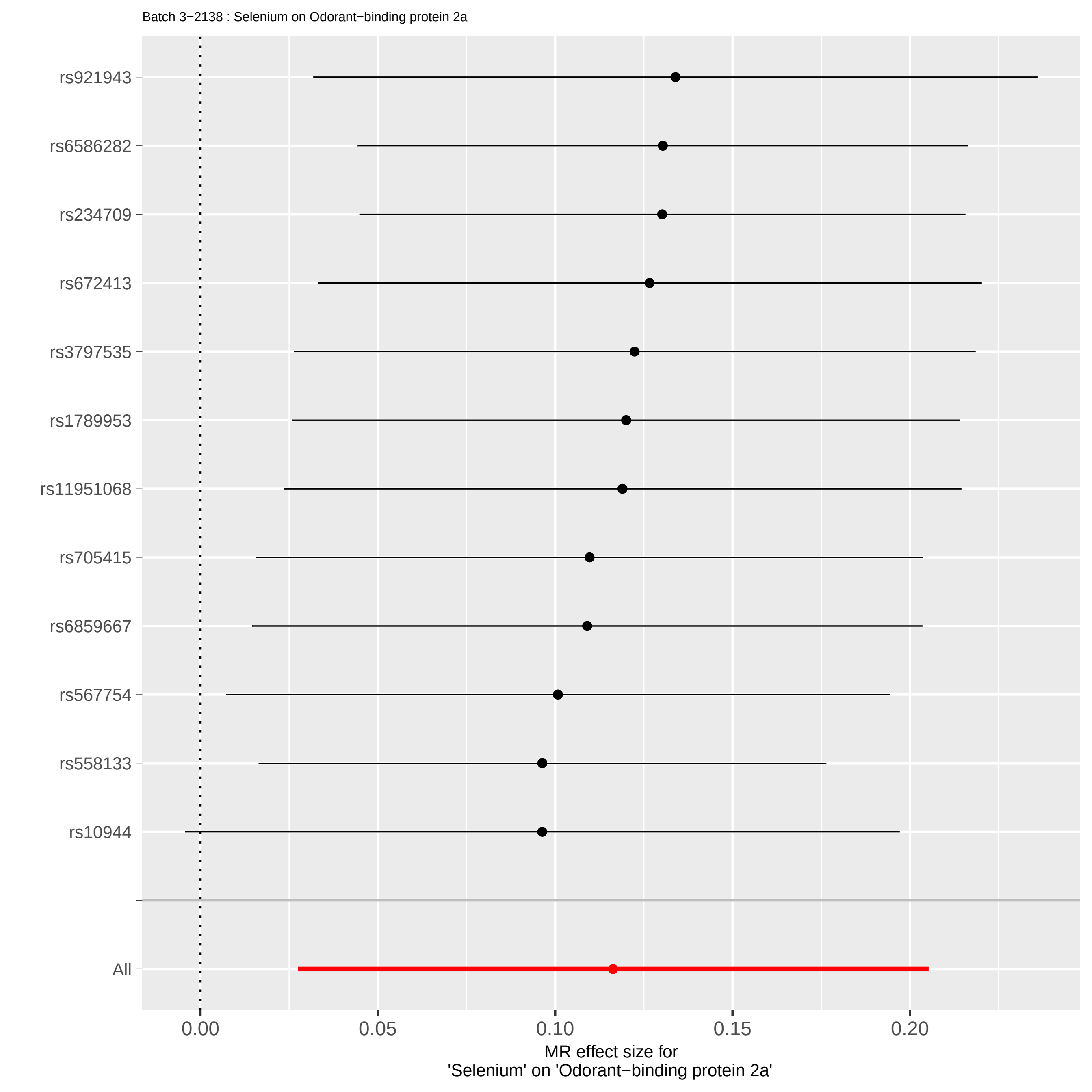

Batch 3−2138 : Selenium on Odorant−binding protein 2a
rs921943
rs6586282
rs234709
rs672413
rs3797535
rs1789953
rs11951068
rs705415
rs6859667
rs567754
rs558133
rs10944
All
0.00
0.05
0.10
0.15
0.20
MR effect size for
'Selenium' on 'Odorant−binding protein 2a'

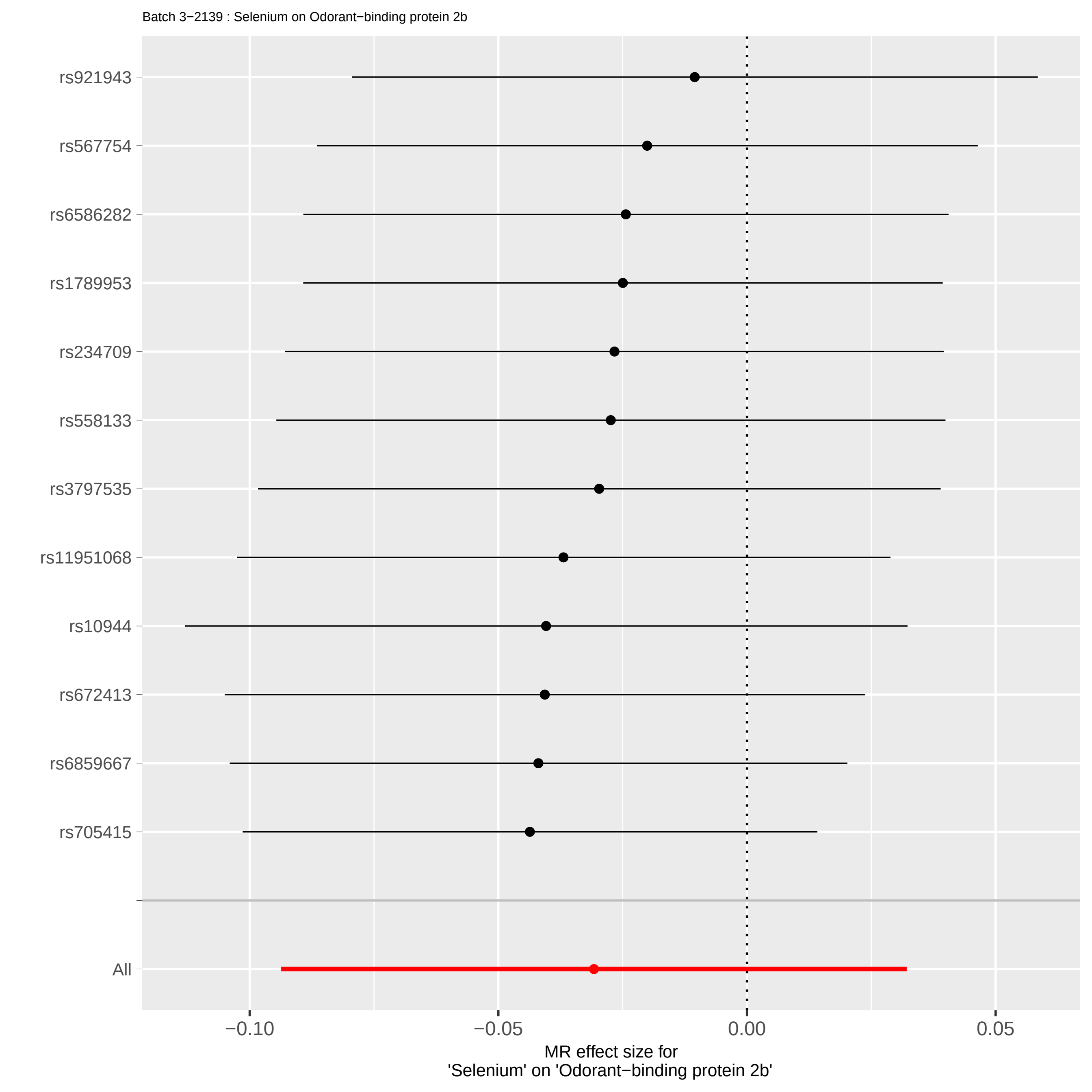

Batch 3−2139 : Selenium on Odorant−binding protein 2b
rs921943
rs567754
rs6586282
rs1789953
rs234709
rs558133
rs3797535
rs11951068
rs10944
rs672413
rs6859667
rs705415
All
−0.10
−0.05
0.00
0.05
MR effect size for
'Selenium' on 'Odorant−binding protein 2b'

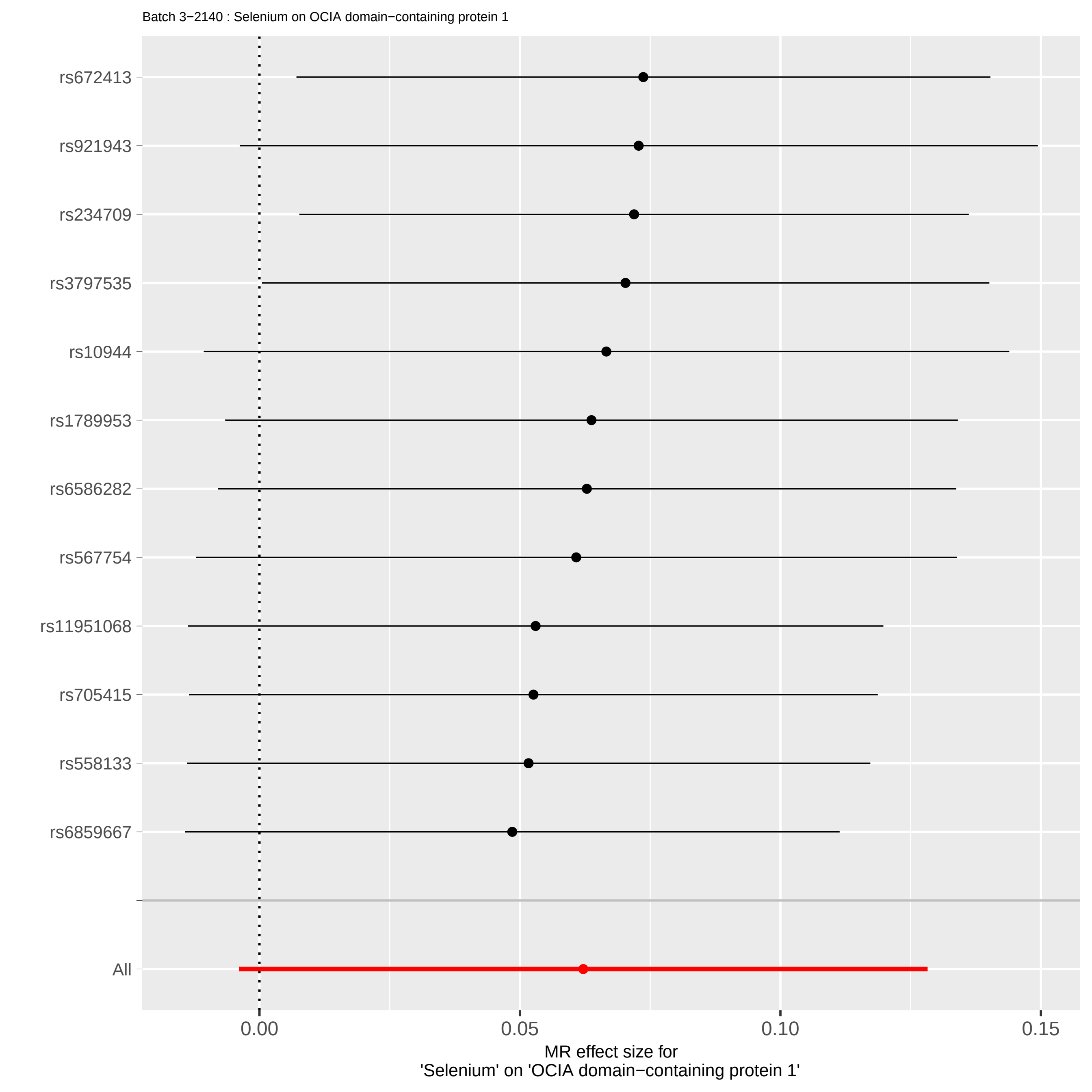

Batch 3−2140 : Selenium on OCIA domain−containing protein 1
rs672413
rs921943
rs234709
rs3797535
rs10944
rs1789953
rs6586282
rs567754
rs11951068
rs705415
rs558133
rs6859667
All
0.00
0.05
0.10
0.15
MR effect size for
'Selenium' on 'OCIA domain−containing protein 1'

Batch 3−2141 : Selenium on Inositol polyphosphate 5−phosphatase OCRL−1

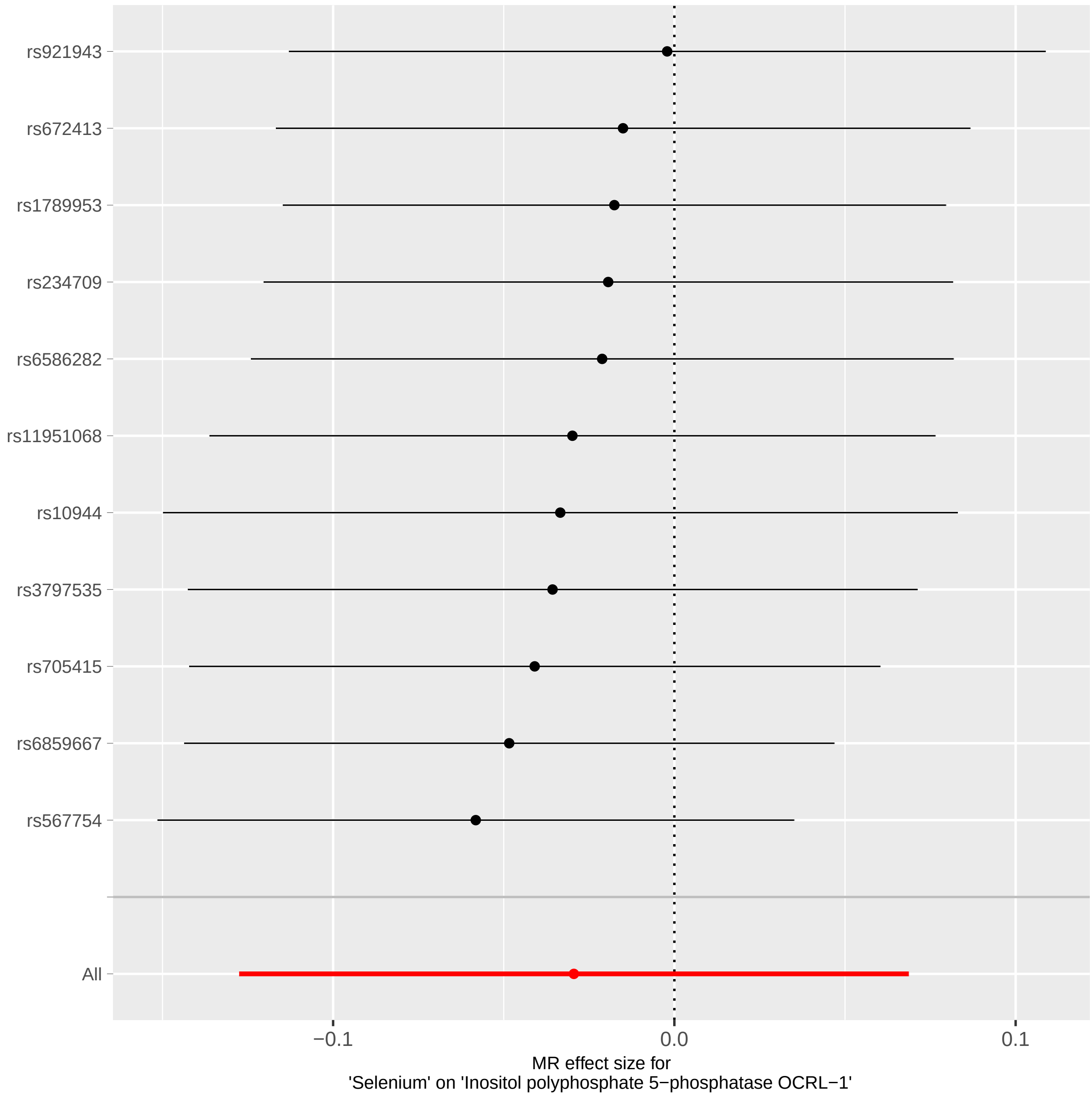

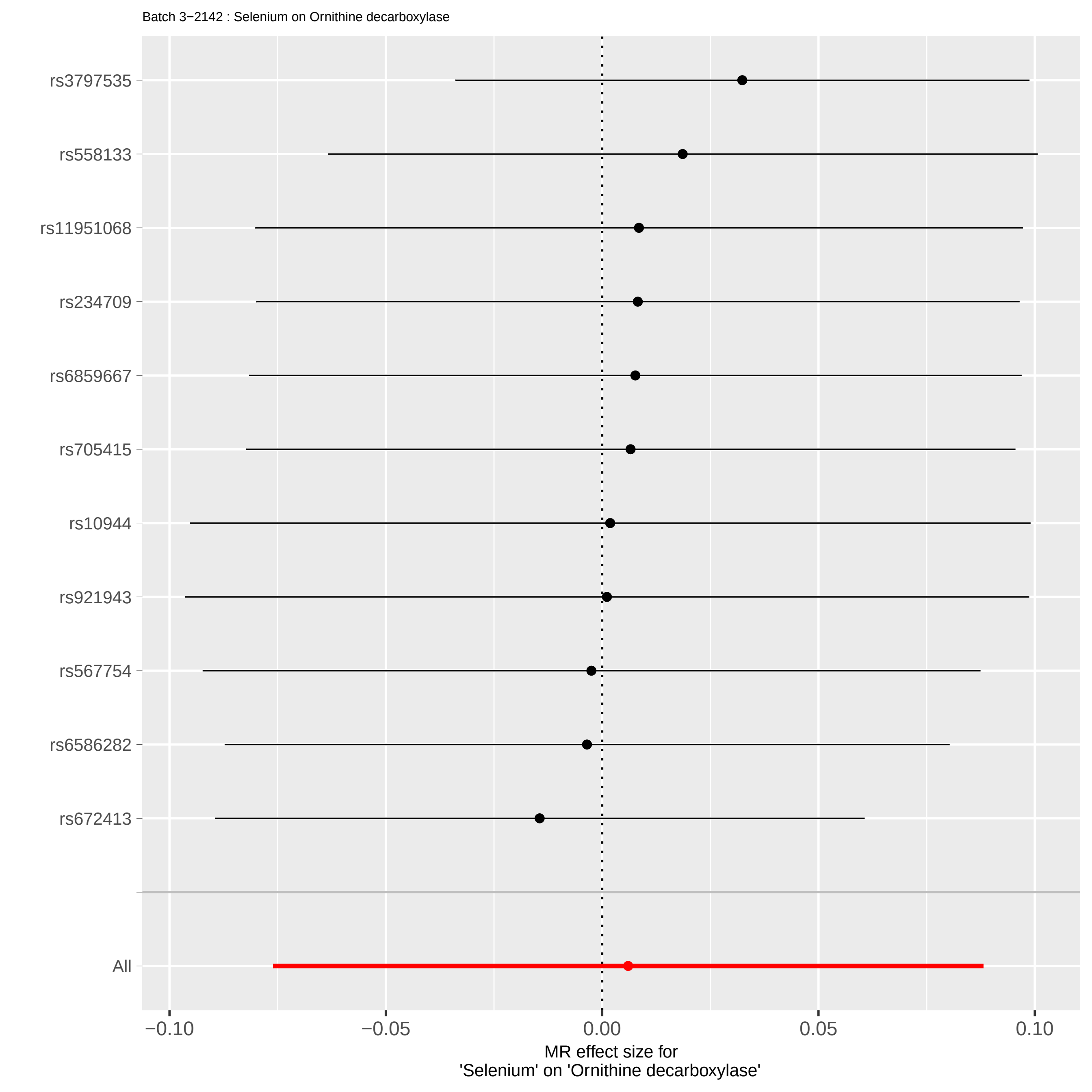

Batch 3−2142 : Selenium on Ornithine decarboxylase
rs3797535
rs558133
rs11951068
rs234709
rs6859667
rs705415
rs10944
rs921943
rs567754
rs6586282
rs672413
All
−0.10
−0.05
0.00
0.05
0.10
MR effect size for
'Selenium' on 'Ornithine decarboxylase'

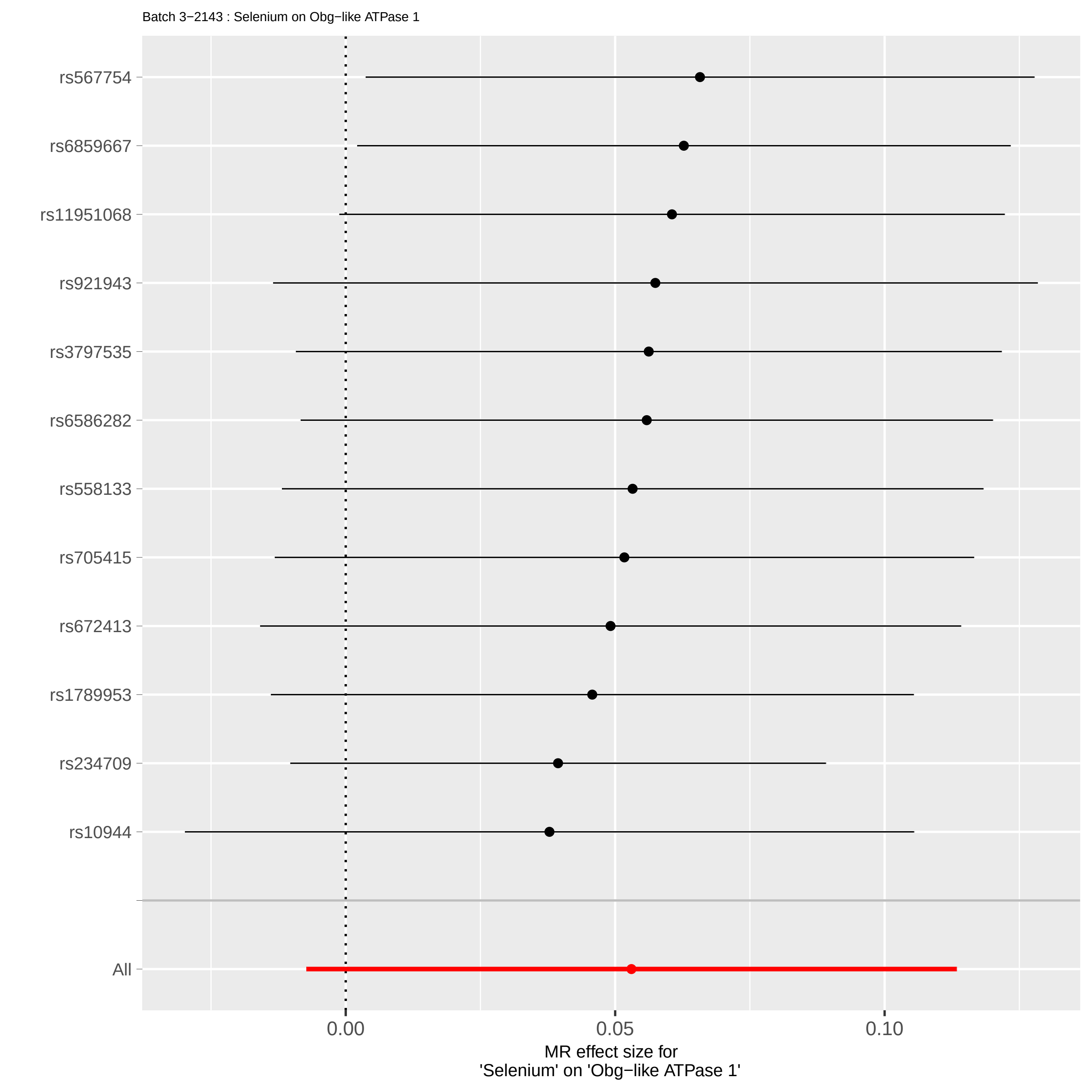

Batch 3−2143 : Selenium on Obg−like ATPase 1
rs567754
rs6859667
rs11951068
rs921943
rs3797535
rs6586282
rs558133
rs705415
rs672413
rs1789953
rs234709
rs10944
All
0.00
0.05
0.10
MR effect size for
'Selenium' on 'Obg−like ATPase 1'

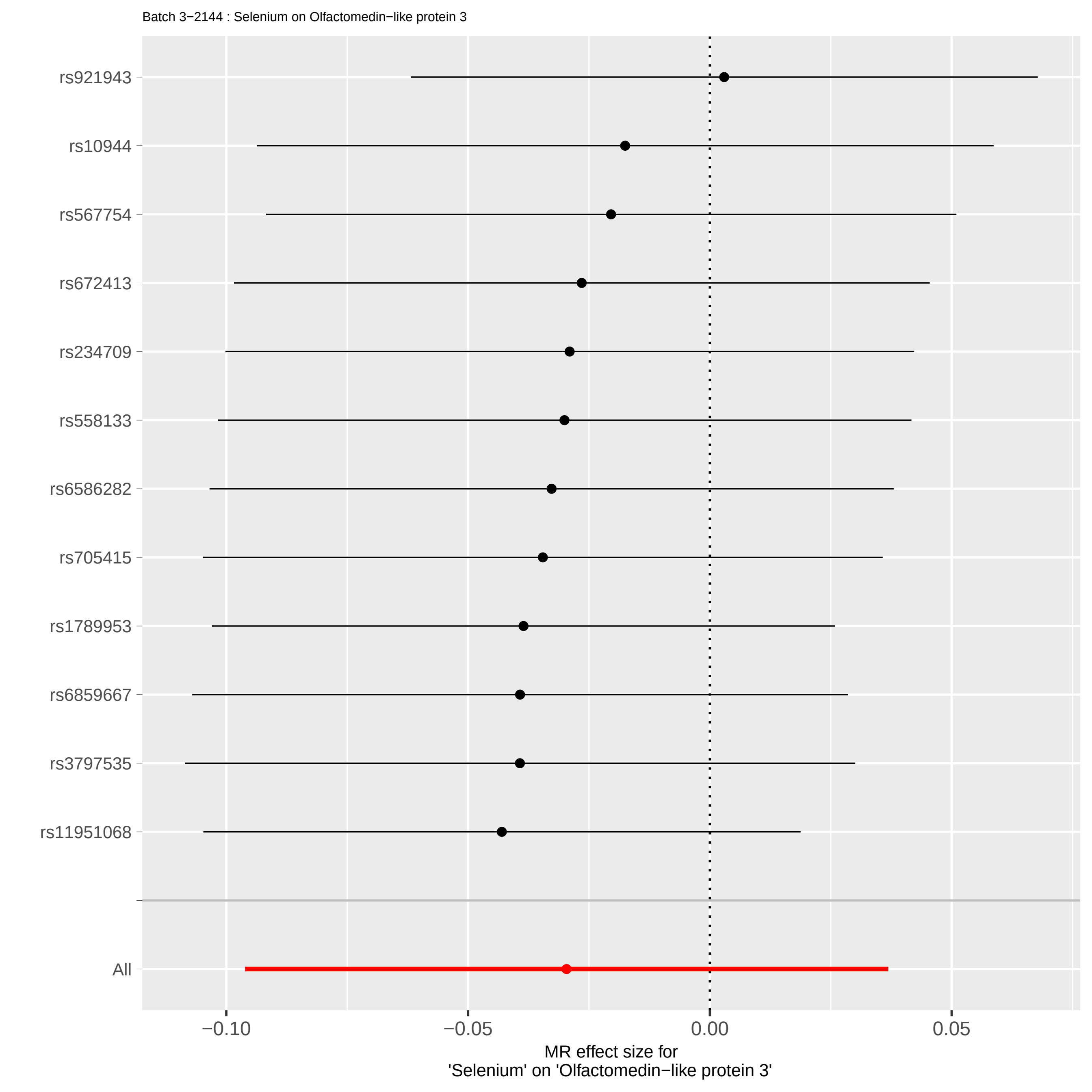

Batch 3−2144 : Selenium on Olfactomedin−like protein 3
rs921943
rs10944
rs567754
rs672413
rs234709
rs558133
rs6586282
rs705415
rs1789953
rs6859667
rs3797535
rs11951068
All
−0.10
−0.05
0.00
0.05
MR effect size for
'Selenium' on 'Olfactomedin−like protein 3'

Batch 3−2145 : Selenium on Olfactomedin−like protein 3

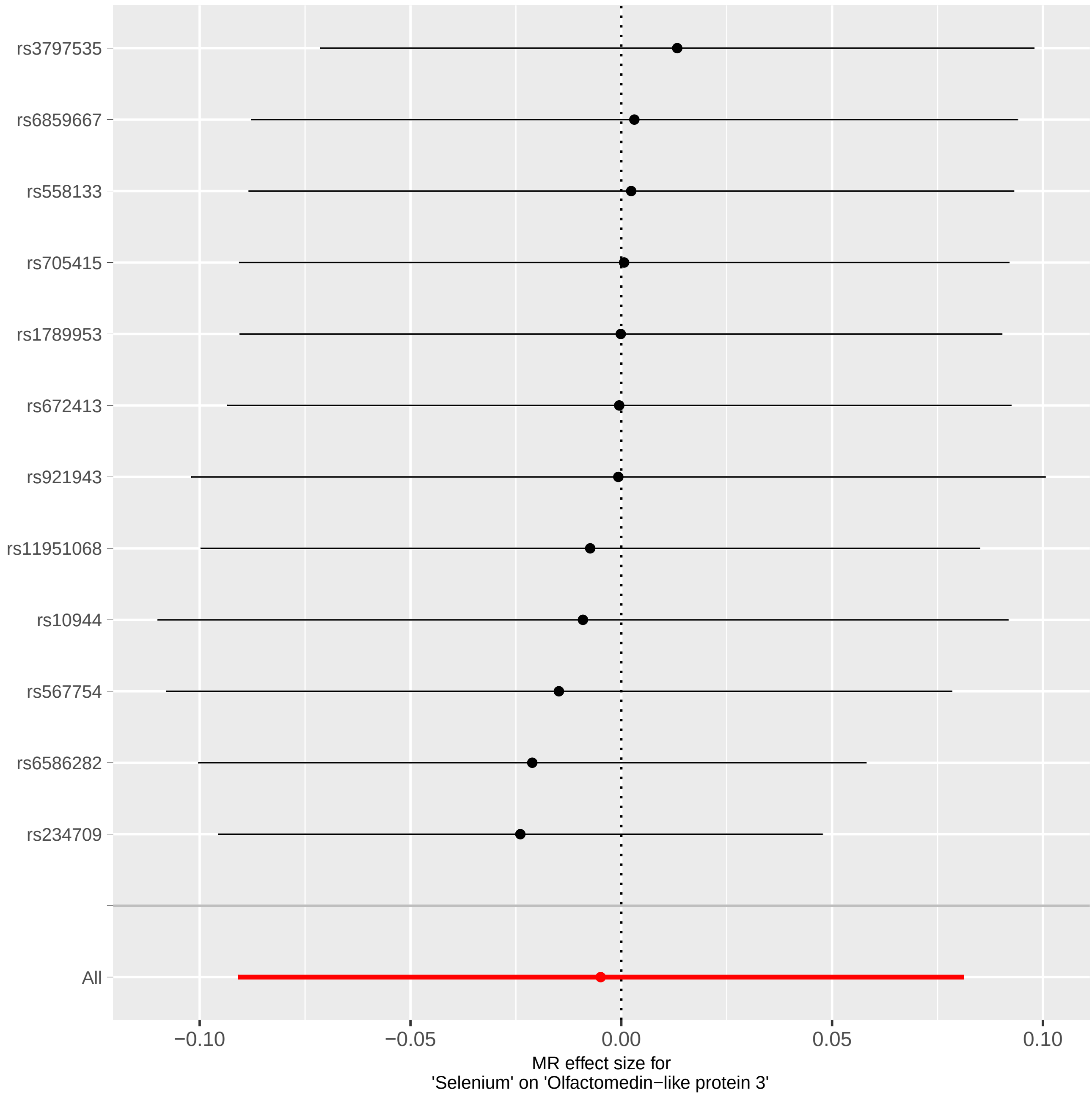

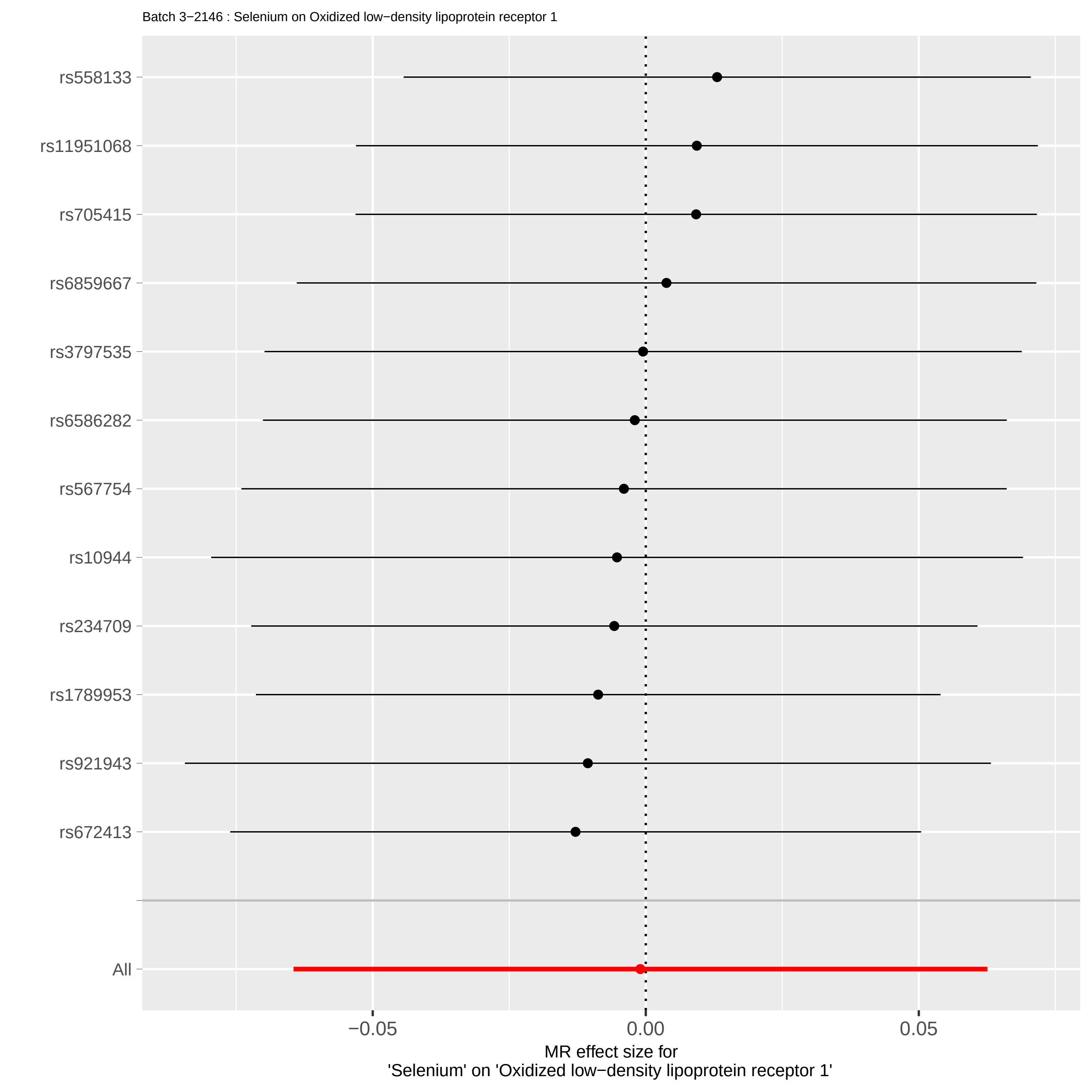
Batch 3−2146 : Selenium on Oxidized low−density lipoprotein receptor 1
rs558133
rs11951068
rs705415
rs6859667
rs3797535
rs6586282
rs567754
rs10944
rs234709
rs1789953
rs921943
rs672413
All
−0.05
0.00
0.05
MR effect size for
'Selenium' on 'Oxidized low−density lipoprotein receptor 1'

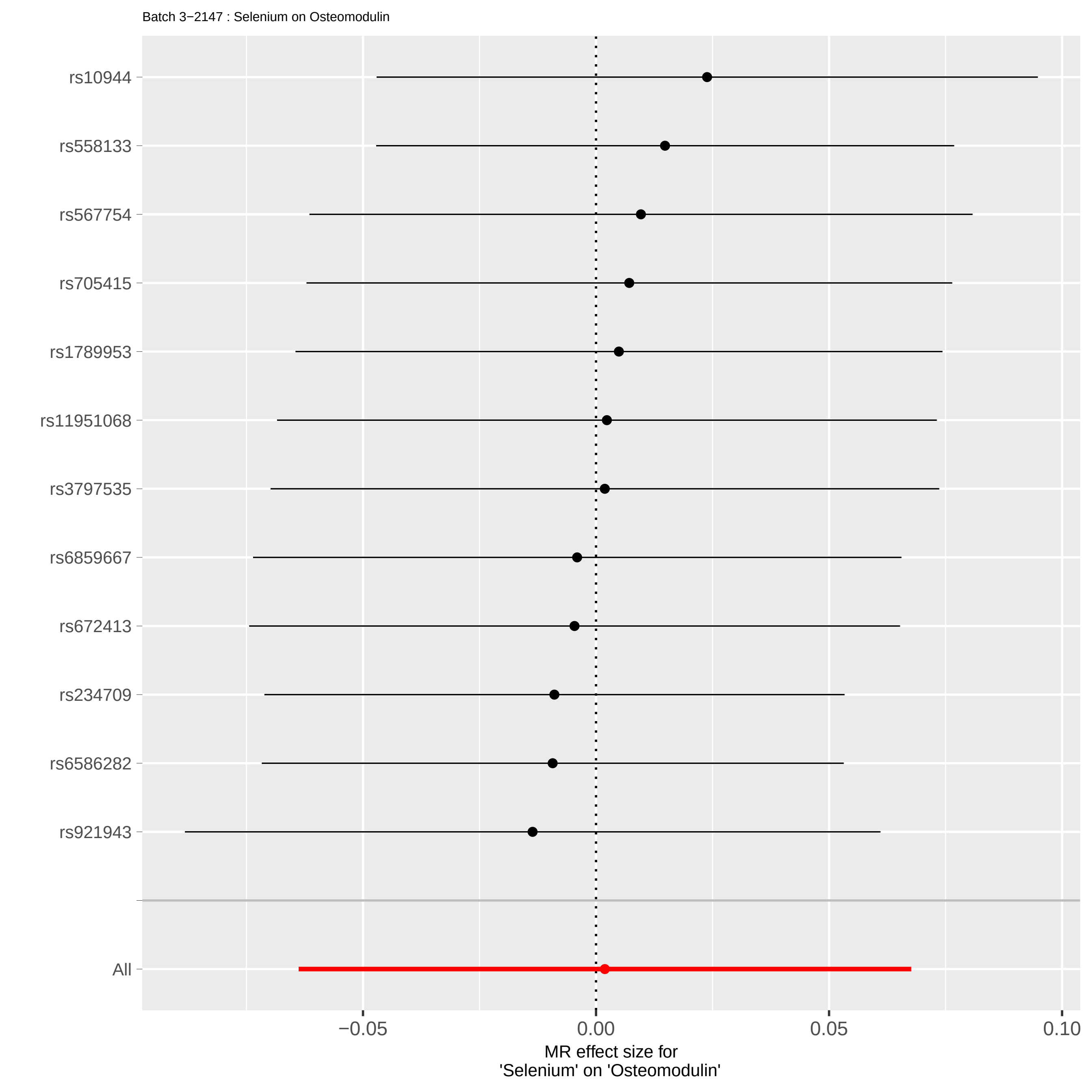
Batch 3−2147 : Selenium on Osteomodulin
rs10944
rs558133
rs567754
rs705415
rs1789953
rs11951068
rs3797535
rs6859667
rs672413
rs234709
rs6586282
rs921943
All
−0.05
0.00
0.05
0.10
MR effect size for
'Selenium' on 'Osteomodulin'

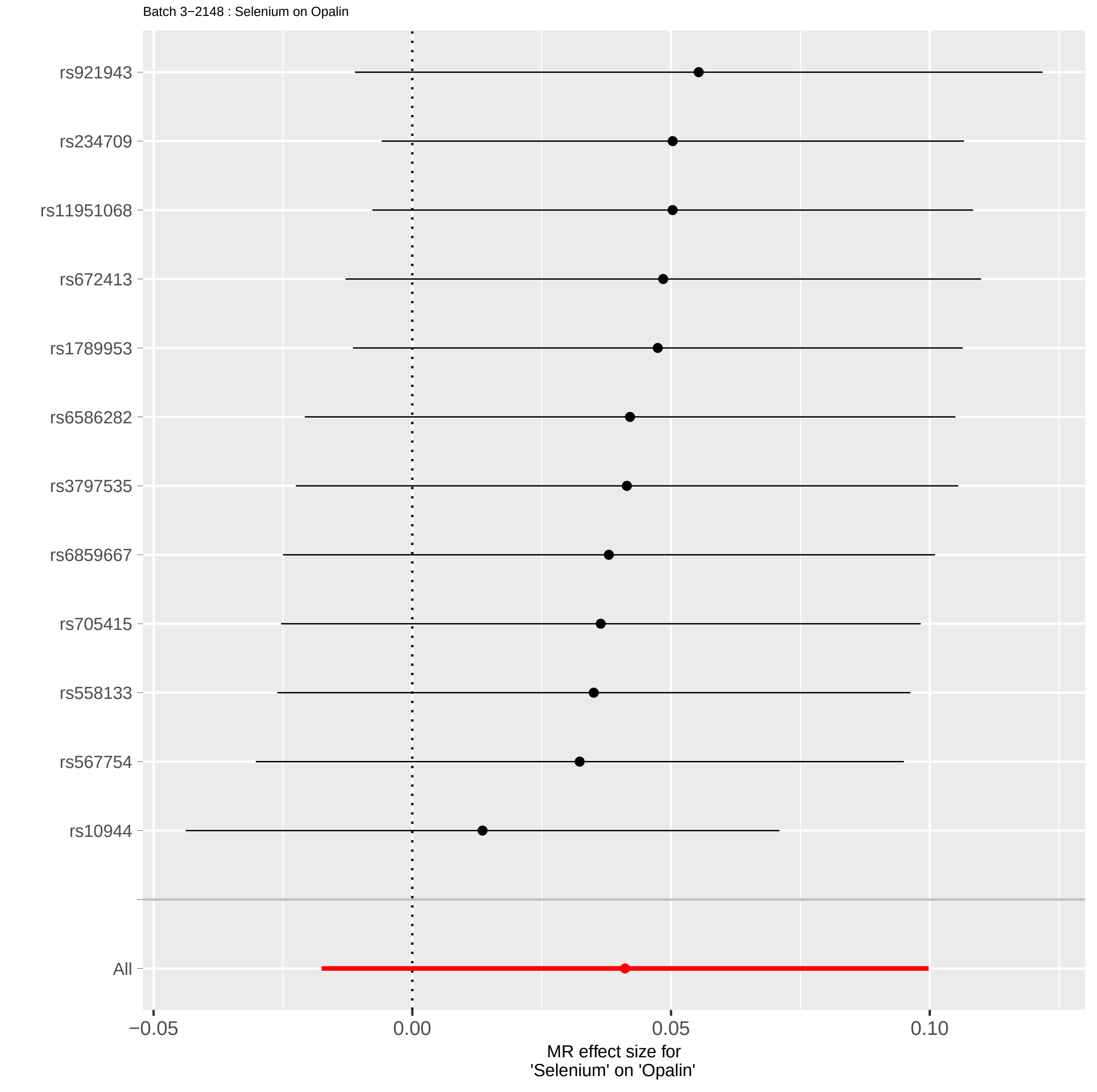

Batch 3−2148 : Selenium on Opalin
rs921943
rs234709
rs11951068
rs672413
rs1789953
rs6586282
rs3797535
rs6859667
rs705415
rs558133
rs567754
rs10944
All
−0.05
0.00
0.05
0.10
MR effect size for
'Selenium' on 'Opalin'

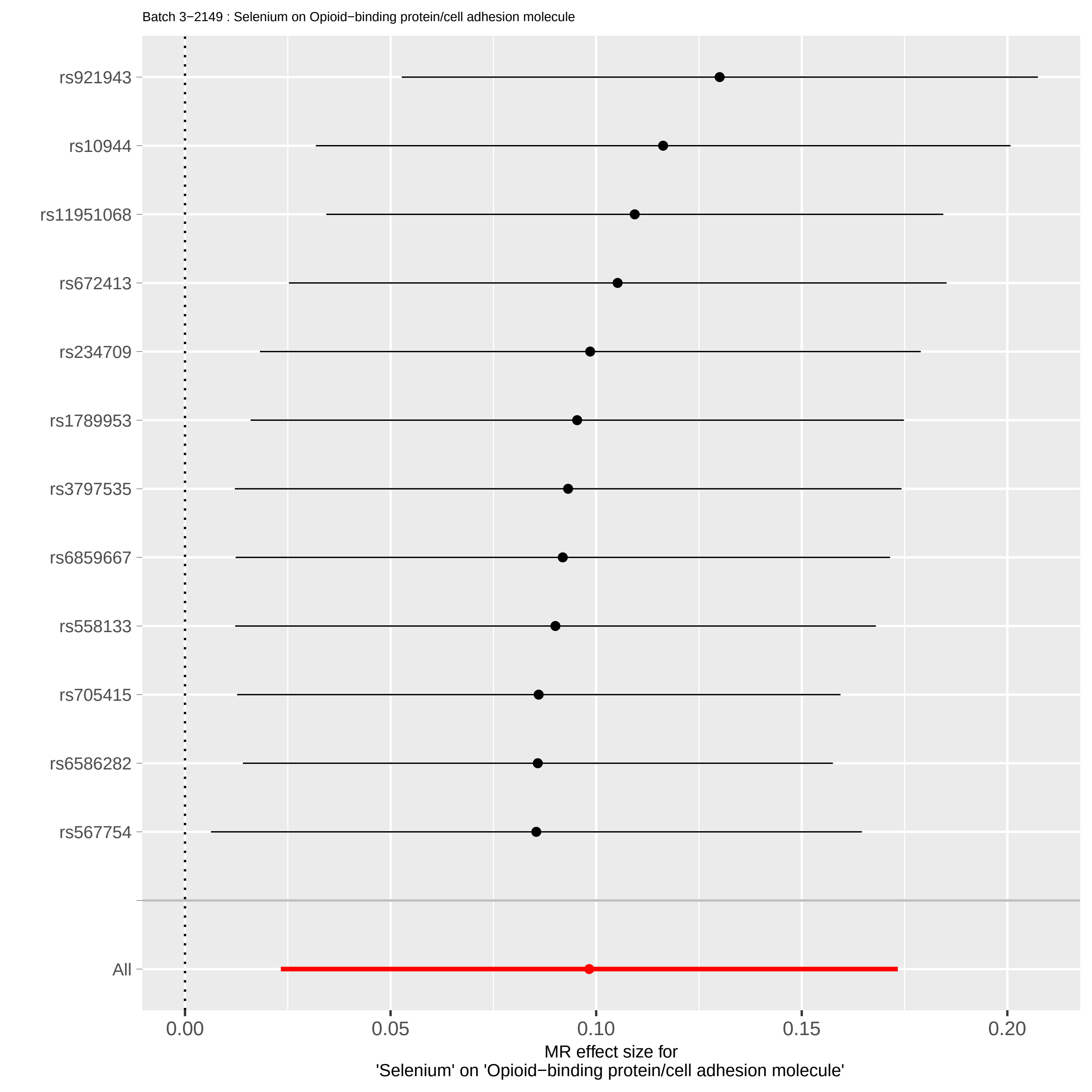
Batch 3−2149 : Selenium on Opioid−binding protein/cell adhesion molecule
rs921943
rs10944
rs11951068
rs672413
rs234709
rs1789953
rs3797535
rs6859667
rs558133
rs705415
rs6586282
rs567754
All
0.00
0.05
0.10
0.15
0.20
MR effect size for
'Selenium' on 'Opioid−binding protein/cell adhesion molecule'

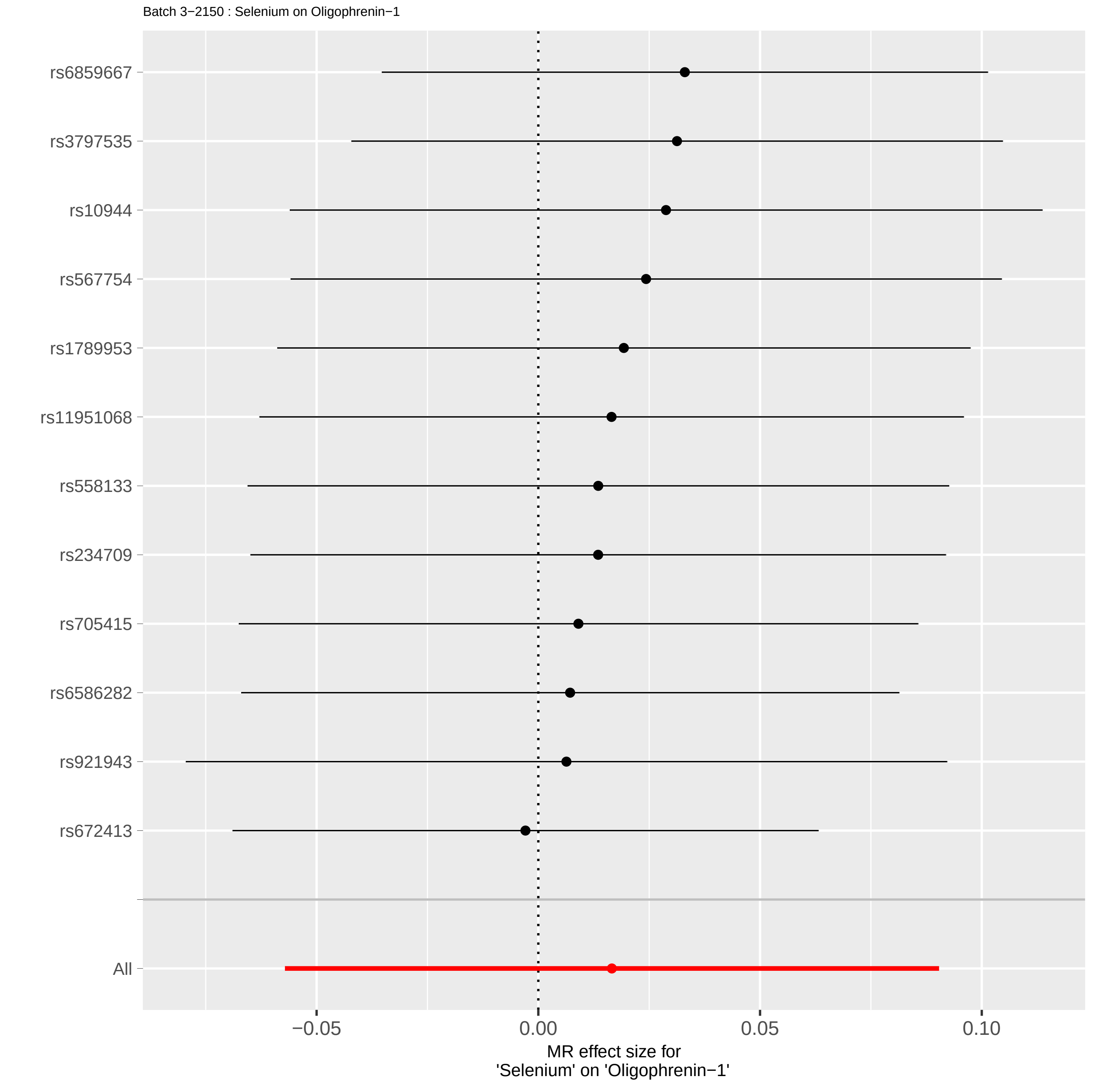
Batch 3−2150 : Selenium on Oligophrenin−1
rs6859667
rs3797535
rs10944
rs567754
rs1789953
rs11951068
rs558133
rs234709
rs705415
rs6586282
rs921943
rs672413
All
−0.05
0.00
0.05
0.10
MR effect size for
'Selenium' on 'Oligophrenin−1'

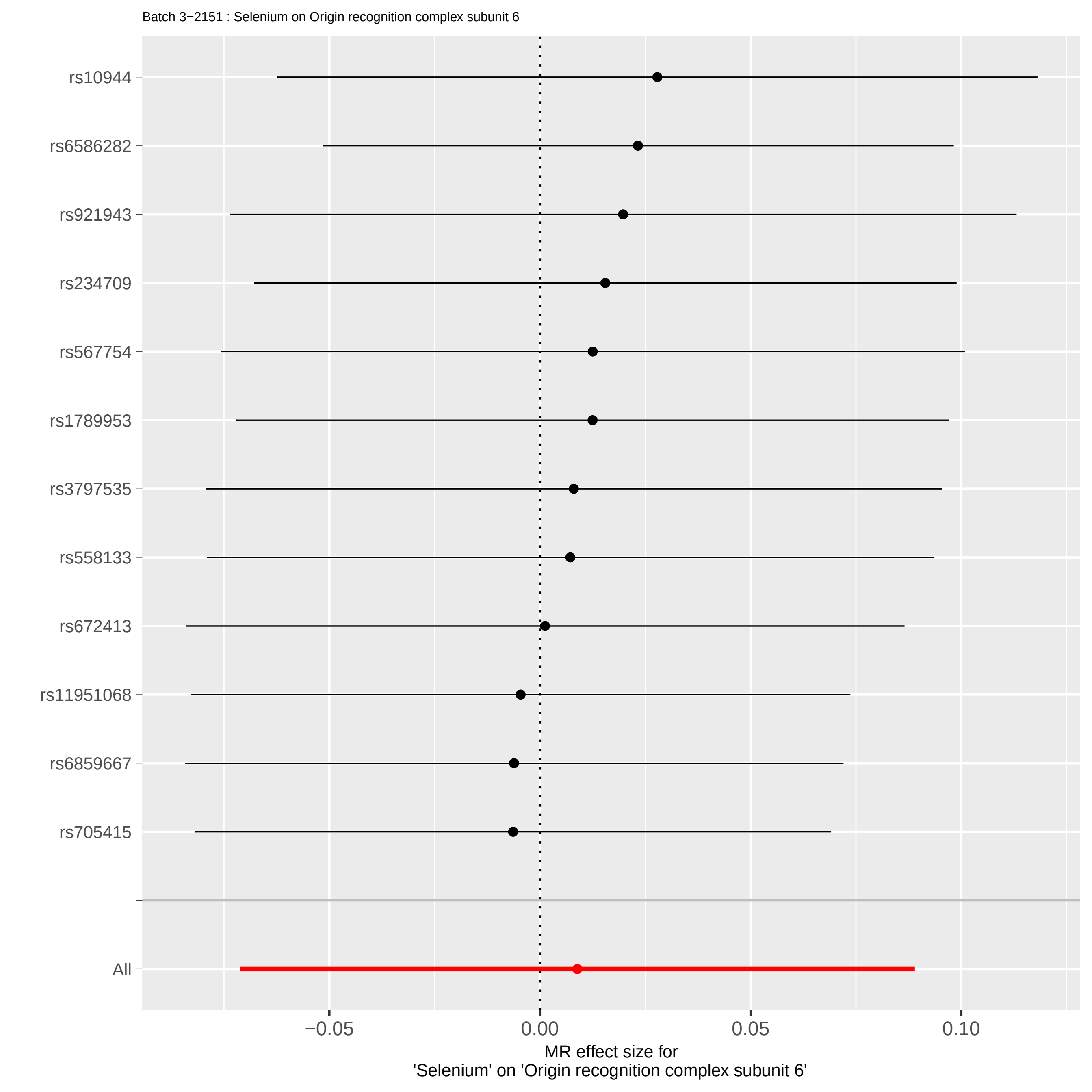
Batch 3−2151 : Selenium on Origin recognition complex subunit 6
rs10944
rs6586282
rs921943
rs234709
rs567754
rs1789953
rs3797535
rs558133
rs672413
rs11951068
rs6859667
rs705415
All
−0.05
0.00
0.05
0.10
MR effect size for
'Selenium' on 'Origin recognition complex subunit 6'

Batch 3−2152 : Selenium on Oxysterol−binding protein−related protein 11

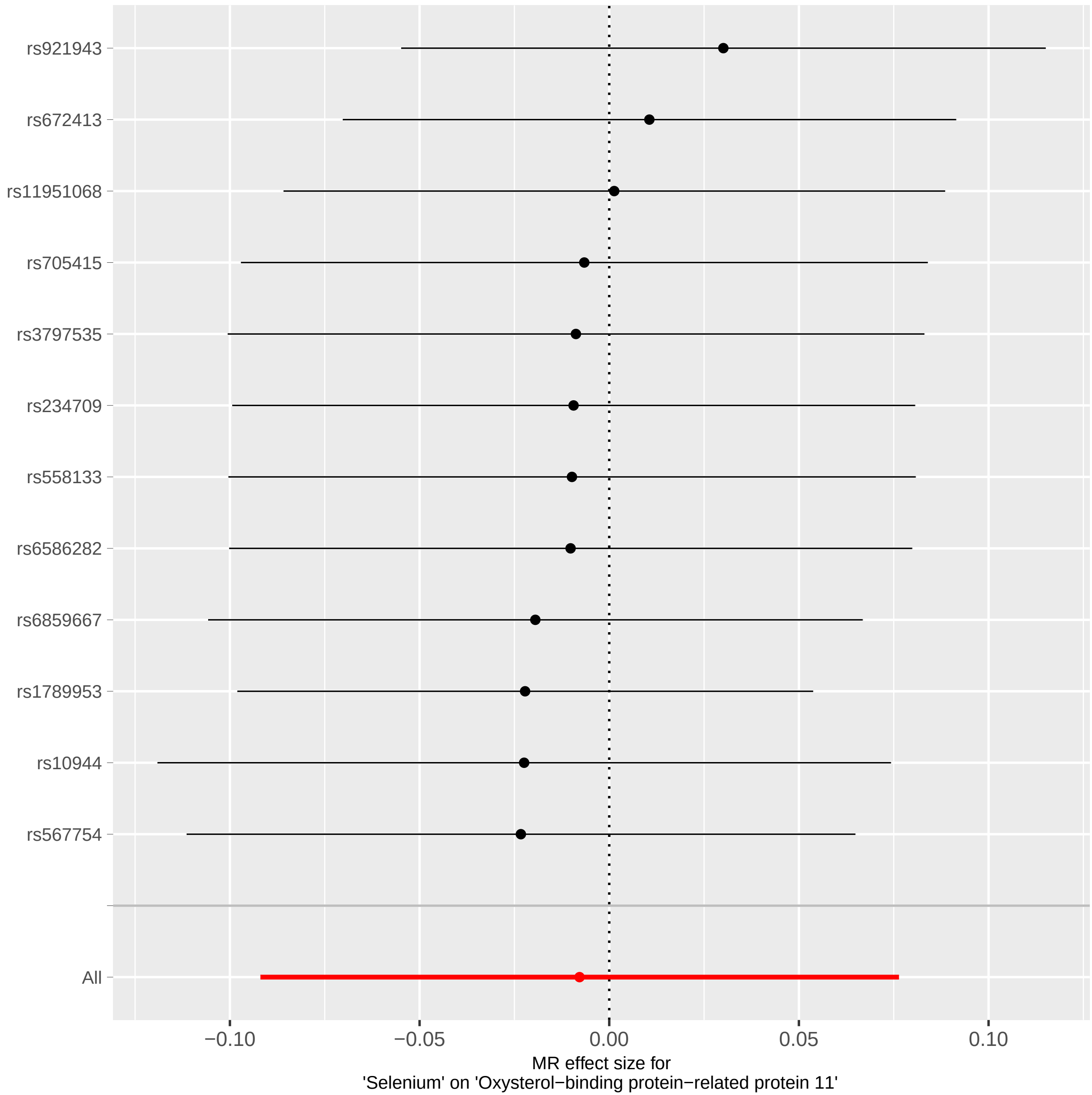

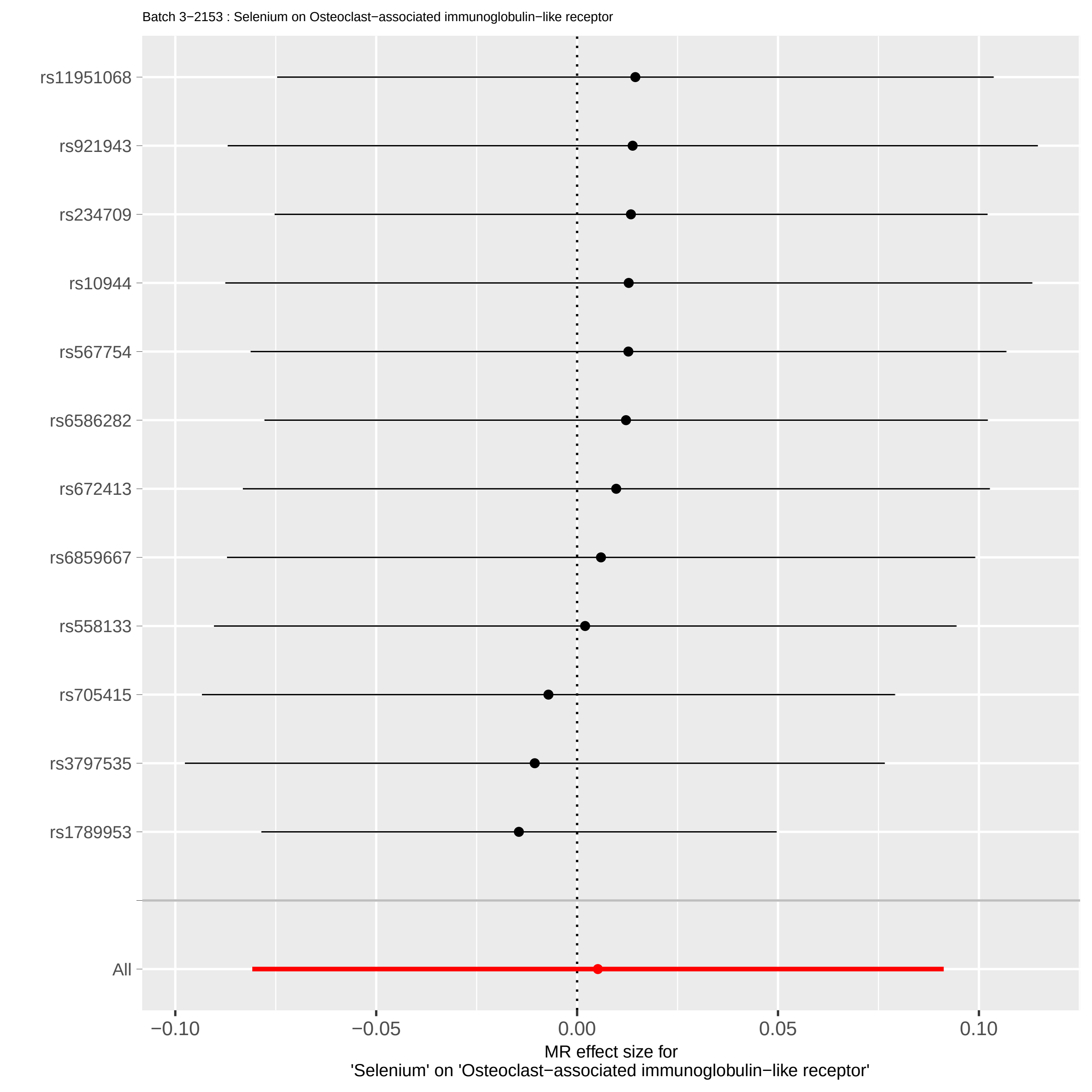
Batch 3−2153 : Selenium on Osteoclast−associated immunoglobulin−like receptor
rs11951068
rs921943
rs234709
rs10944
rs567754
rs6586282
rs672413
rs6859667
rs558133
rs705415
rs3797535
rs1789953
All
−0.10
−0.05
0.00
0.05
0.10
MR effect size for
'Selenium' on 'Osteoclast−associated immunoglobulin−like receptor'

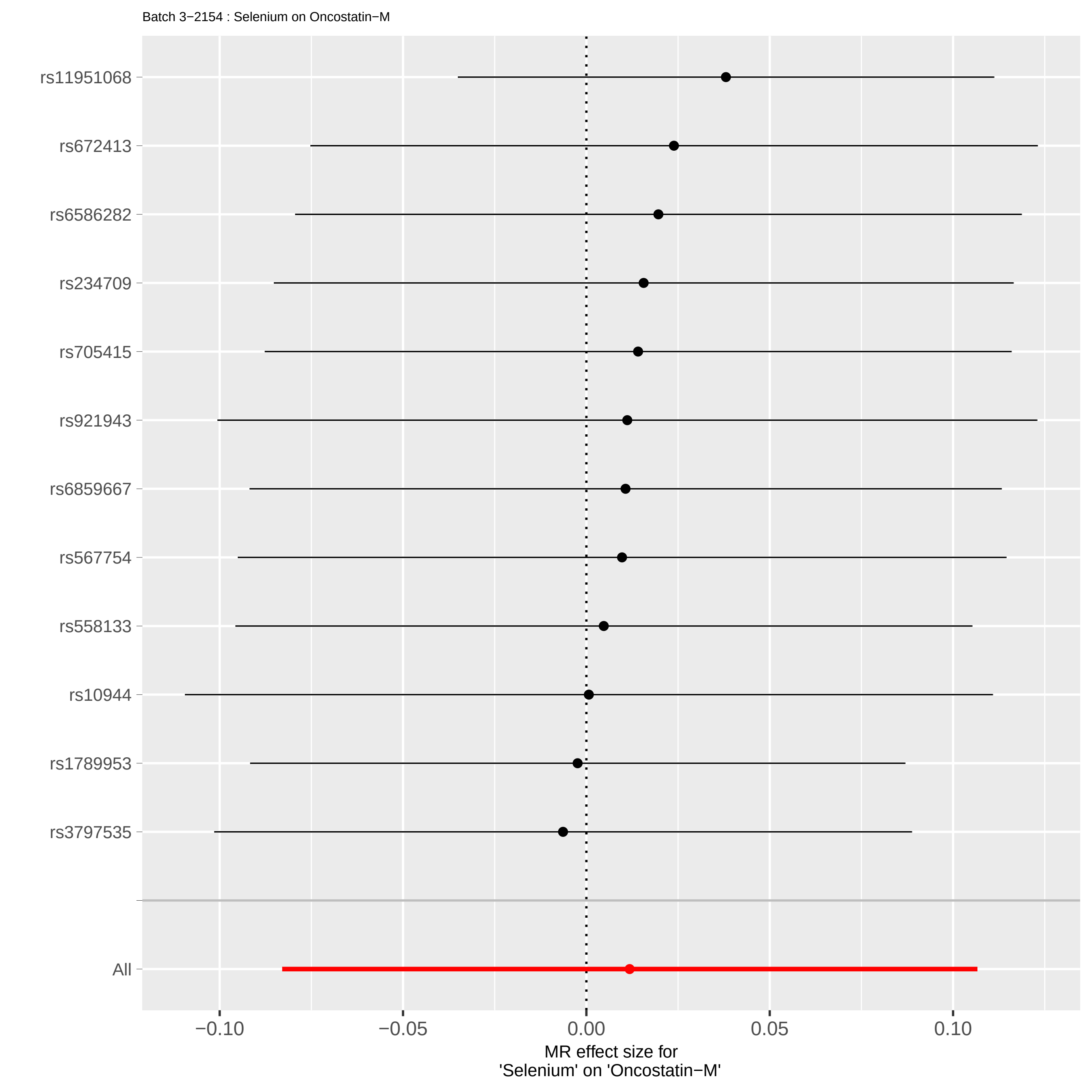

Batch 3−2154 : Selenium on Oncostatin−M
rs11951068
rs672413
rs6586282
rs234709
rs705415
rs921943
rs6859667
rs567754
rs558133
rs10944
rs1789953
rs3797535
All
−0.10
−0.05
0.00
0.05
0.10
MR effect size for
'Selenium' on 'Oncostatin−M'

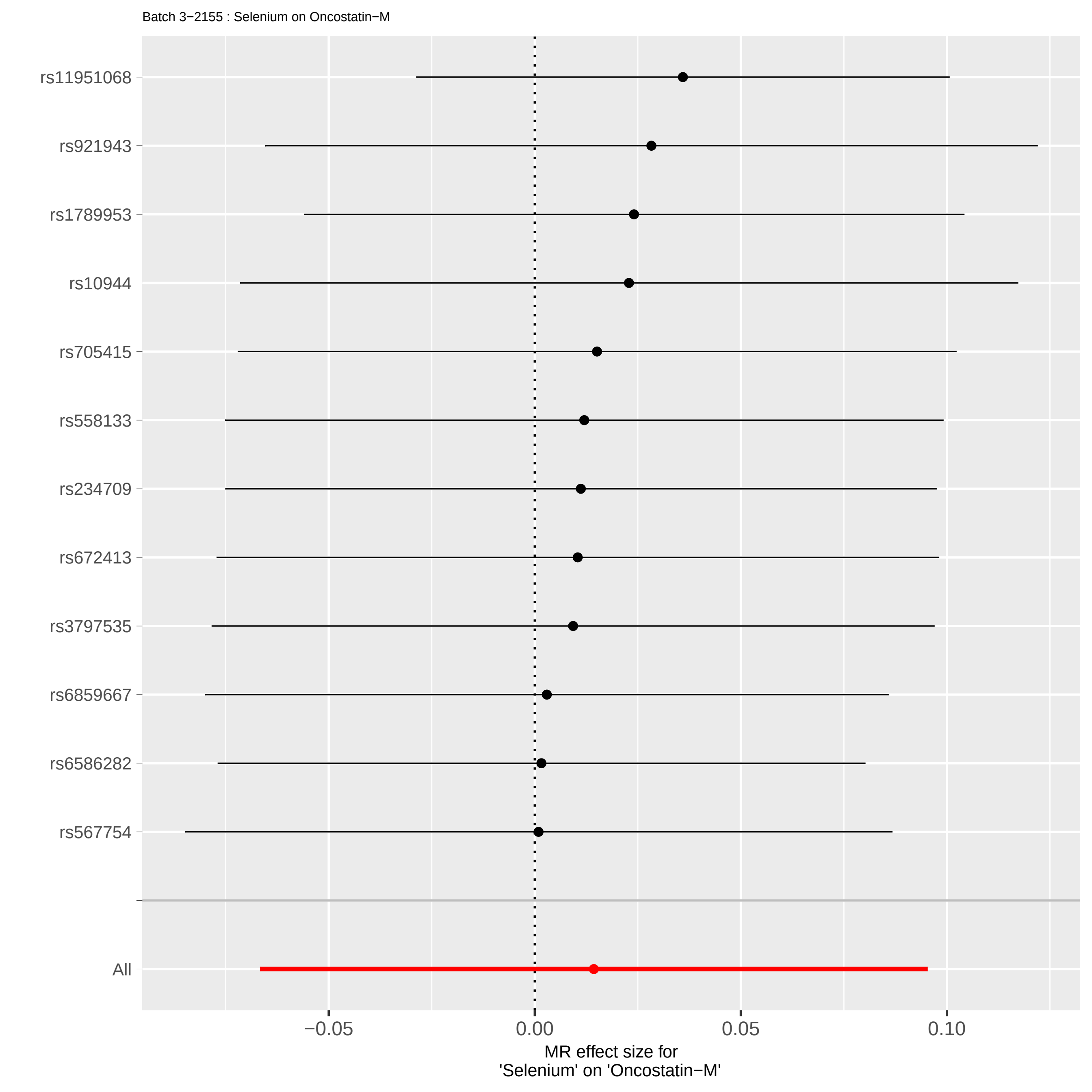
Batch 3−2155 : Selenium on Oncostatin−M
rs11951068
rs921943
rs1789953
rs10944
rs705415
rs558133
rs234709
rs672413
rs3797535
rs6859667
rs6586282
rs567754
All
−0.05
0.00
0.05
0.10
MR effect size for
'Selenium' on 'Oncostatin−M'

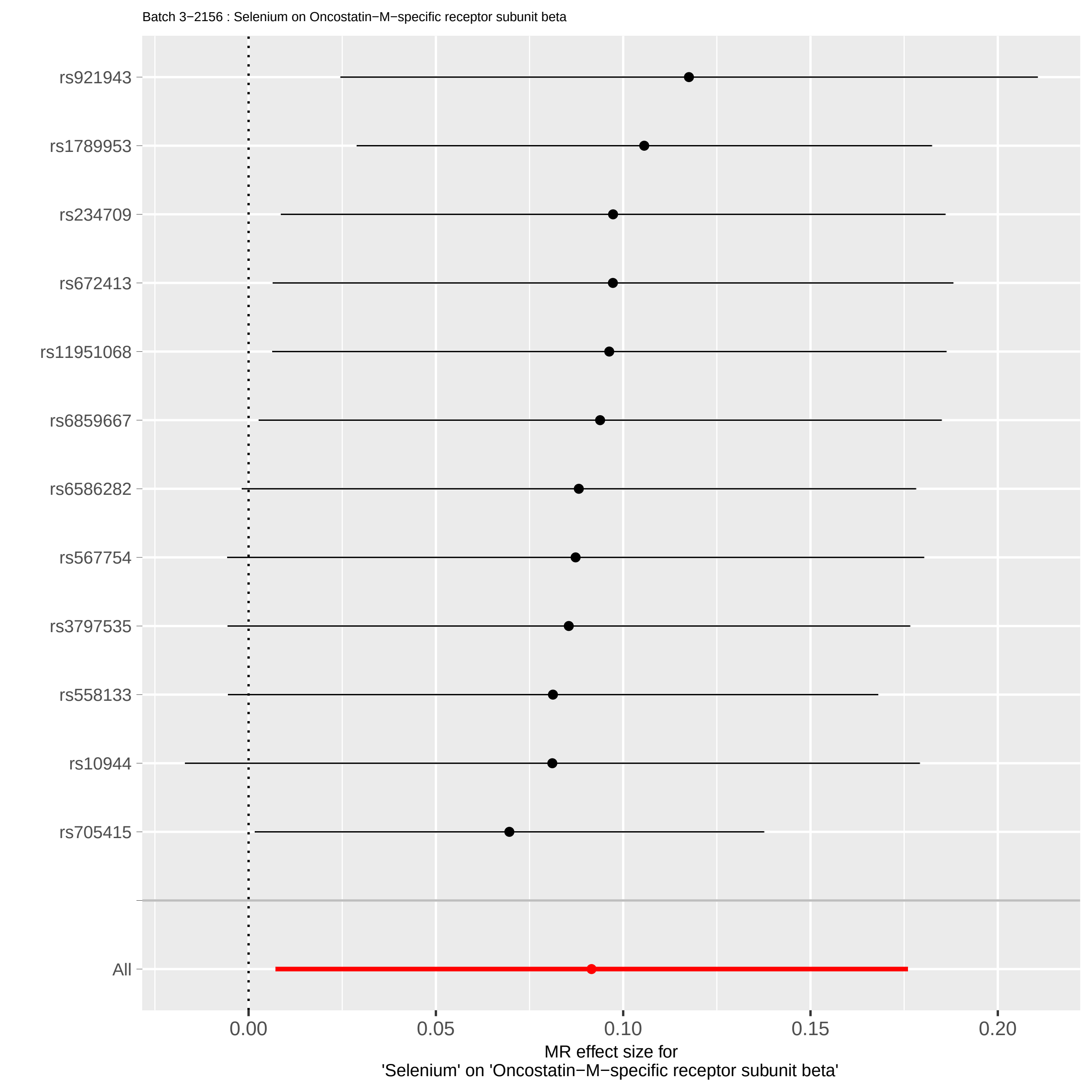

Batch 3−2156 : Selenium on Oncostatin−M−specific receptor subunit beta
rs921943
rs1789953
rs234709
rs672413
rs11951068
rs6859667
rs6586282
rs567754
rs3797535
rs558133
rs10944
rs705415
All
0.00
0.05
0.10
0.15
0.20
MR effect size for
'Selenium' on 'Oncostatin−M−specific receptor subunit beta'

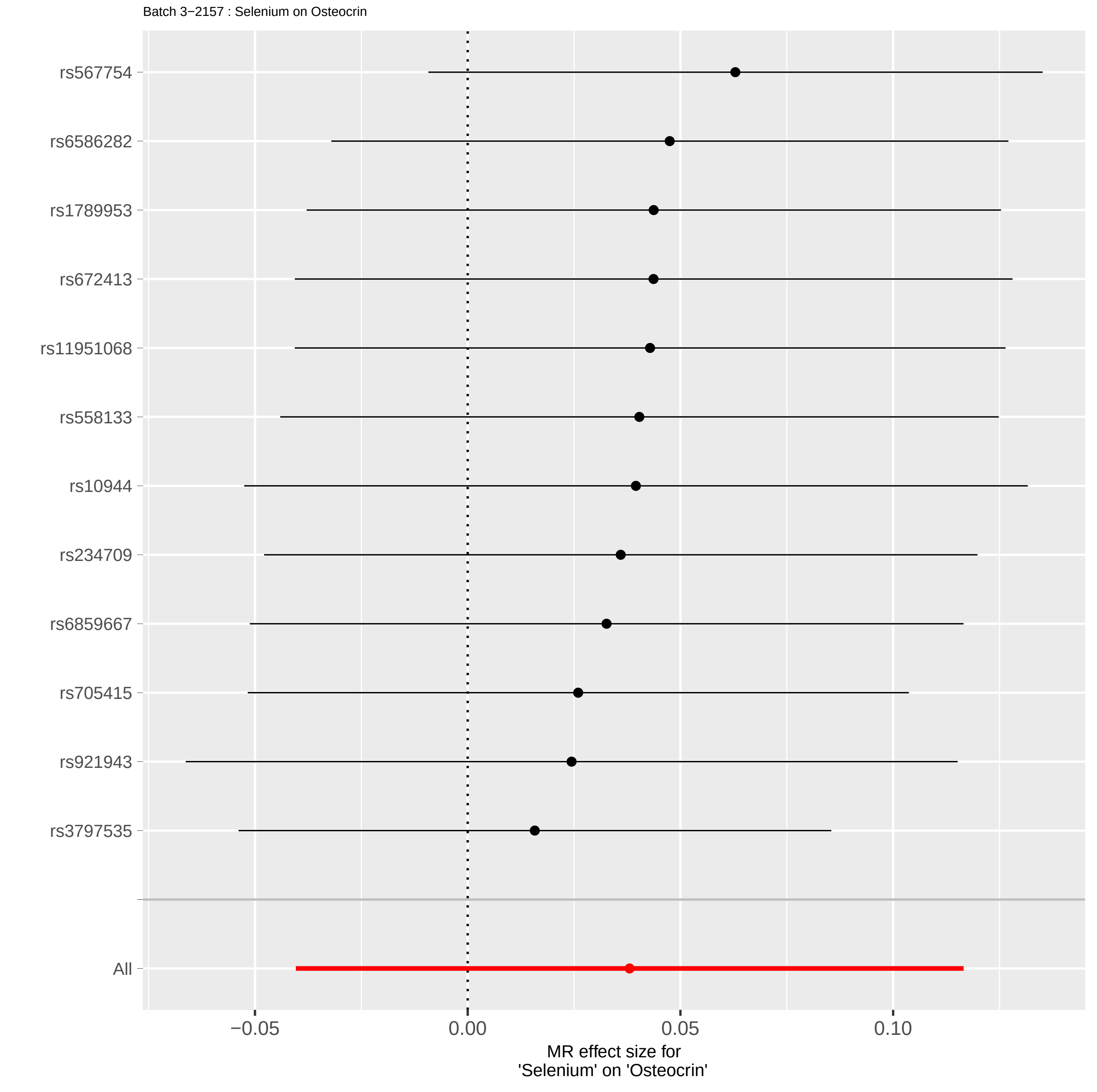

Batch 3−2157 : Selenium on Osteocrin
rs567754
rs6586282
rs1789953
rs672413
rs11951068
rs558133
rs10944
rs234709
rs6859667
rs705415
rs921943
rs3797535
All
−0.05
0.00
0.05
0.10
MR effect size for
'Selenium' on 'Osteocrin'

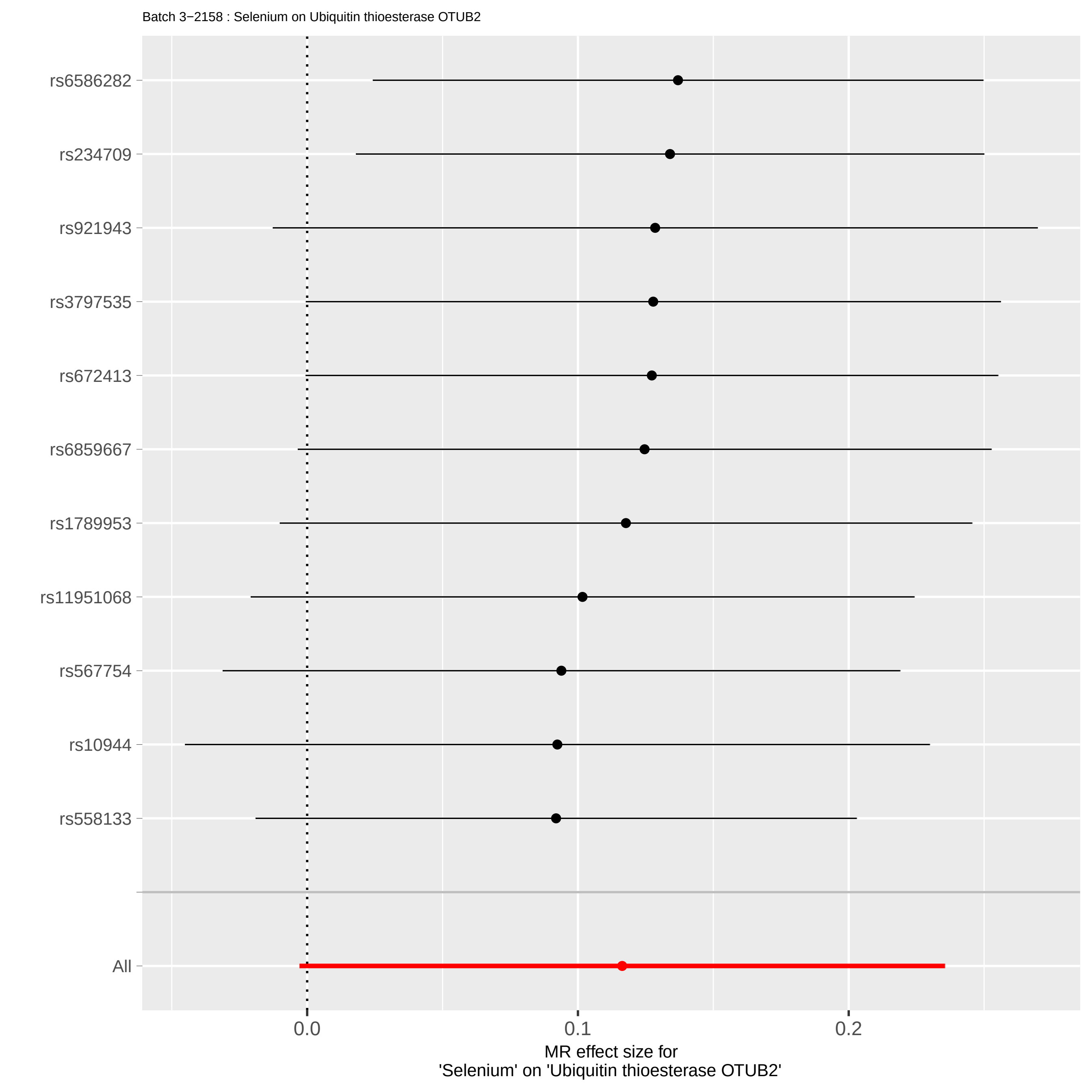

Batch 3−2158 : Selenium on Ubiquitin thioesterase OTUB2
rs6586282
rs234709
rs921943
rs3797535
rs672413
rs6859667
rs1789953
rs11951068
rs567754
rs10944
rs558133
All
0.0
0.1
0.2
MR effect size for
'Selenium' on 'Ubiquitin thioesterase OTUB2'

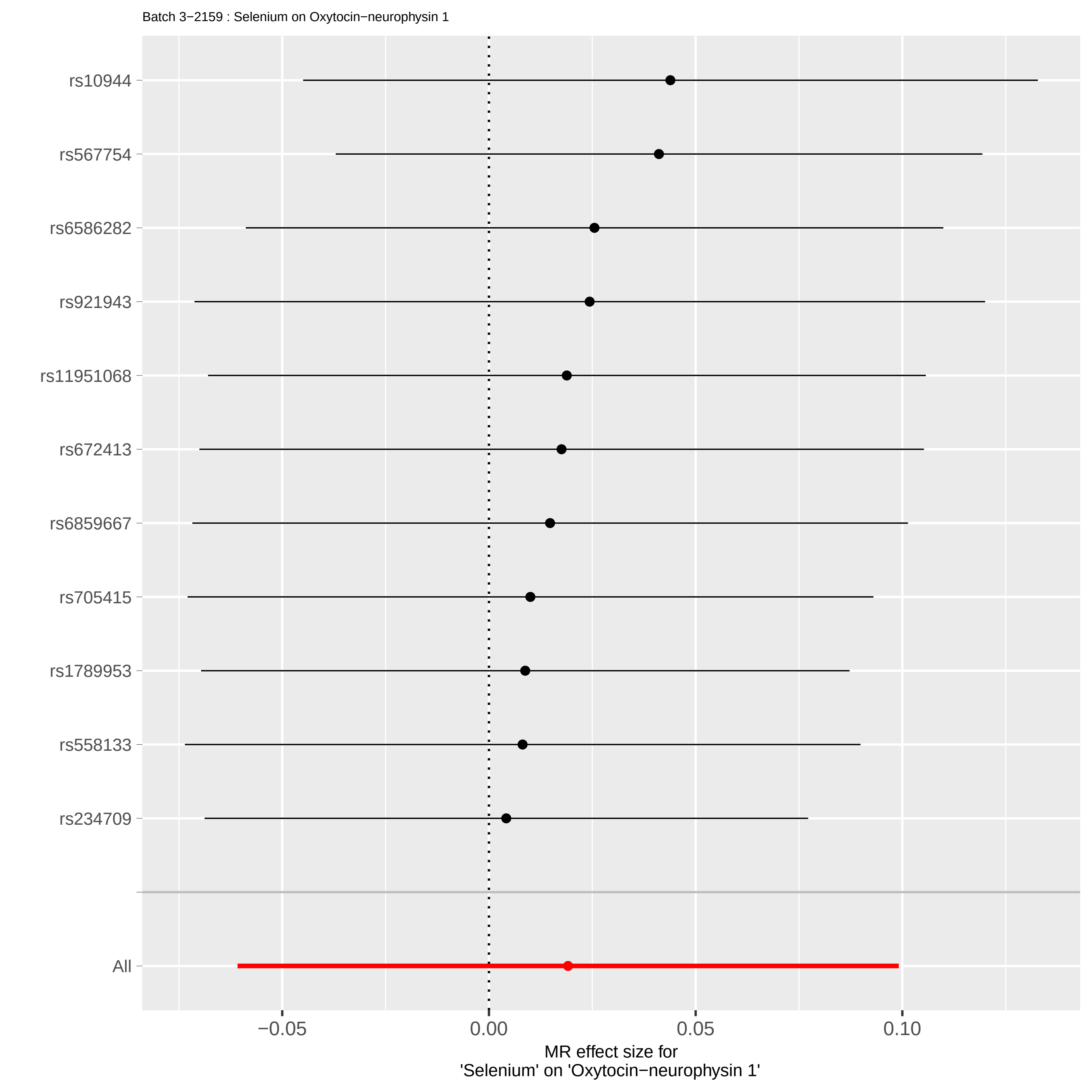

Batch 3−2159 : Selenium on Oxytocin−neurophysin 1
rs10944
rs567754
rs6586282
rs921943
rs11951068
rs672413
rs6859667
rs705415
rs1789953
rs558133
rs234709
All
−0.05
0.00
0.05
0.10
MR effect size for
'Selenium' on 'Oxytocin−neurophysin 1'

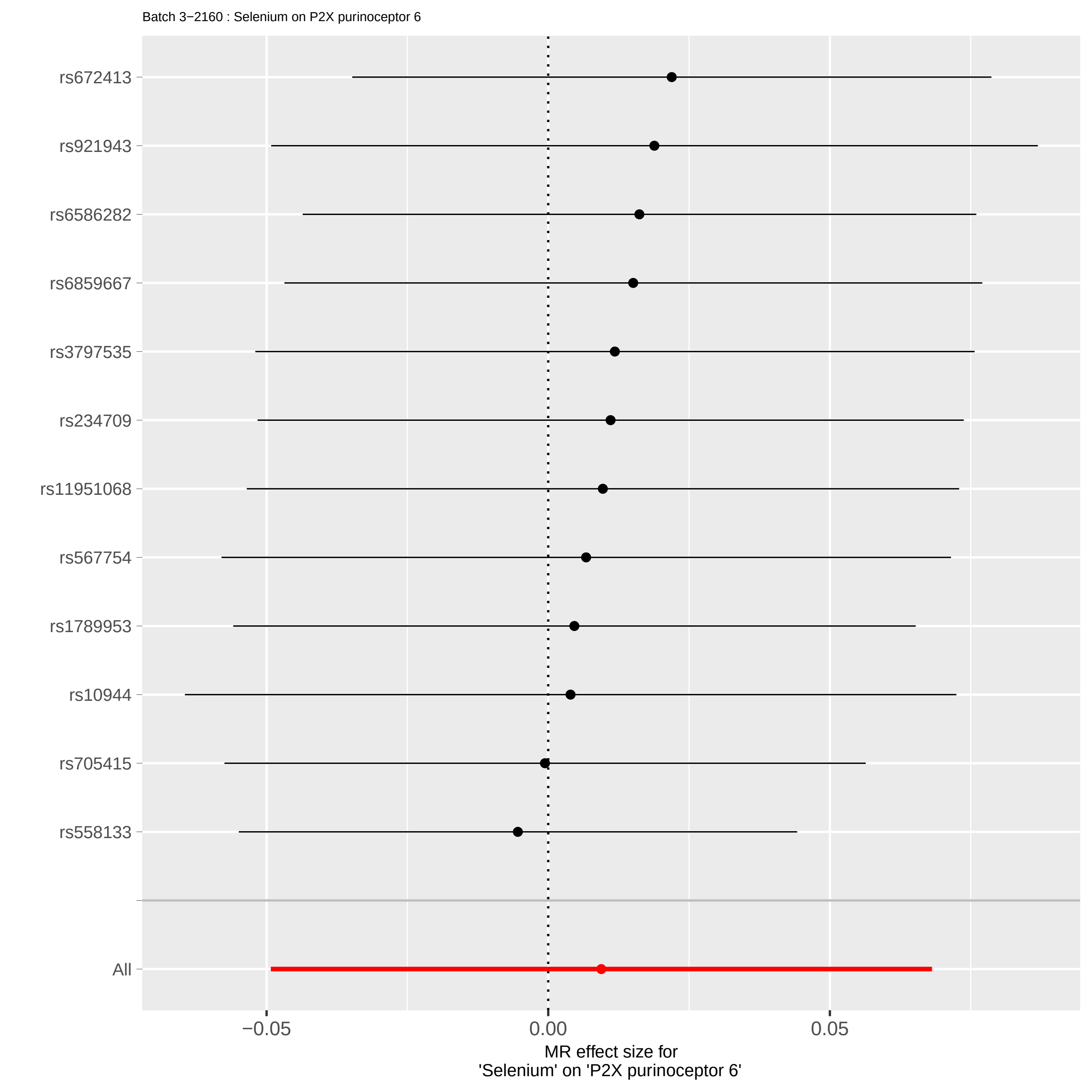
Batch 3−2160 : Selenium on P2X purinoceptor 6
rs672413
rs921943
rs6586282
rs6859667
rs3797535
rs234709
rs11951068
rs567754
rs1789953
rs10944
rs705415
rs558133
All
−0.05
0.00
0.05
MR effect size for
'Selenium' on 'P2X purinoceptor 6'

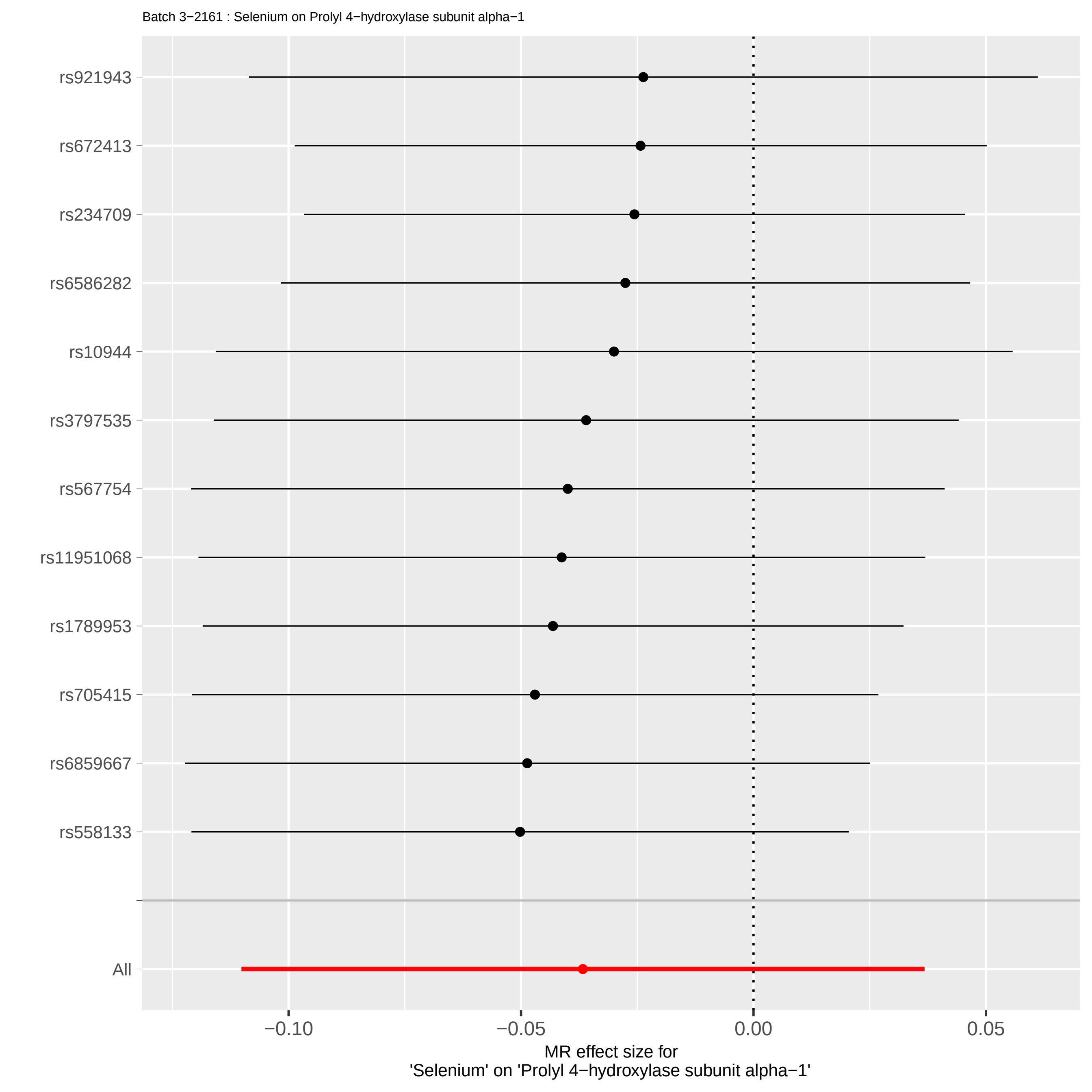

Batch 3−2161 : Selenium on Prolyl 4−hydroxylase subunit alpha−1
rs921943
rs672413
rs234709
rs6586282
rs10944
rs3797535
rs567754
rs11951068
rs1789953
rs705415
rs6859667
rs558133
All
−0.10
−0.05
0.00
0.05
MR effect size for
'Selenium' on 'Prolyl 4−hydroxylase subunit alpha−1'

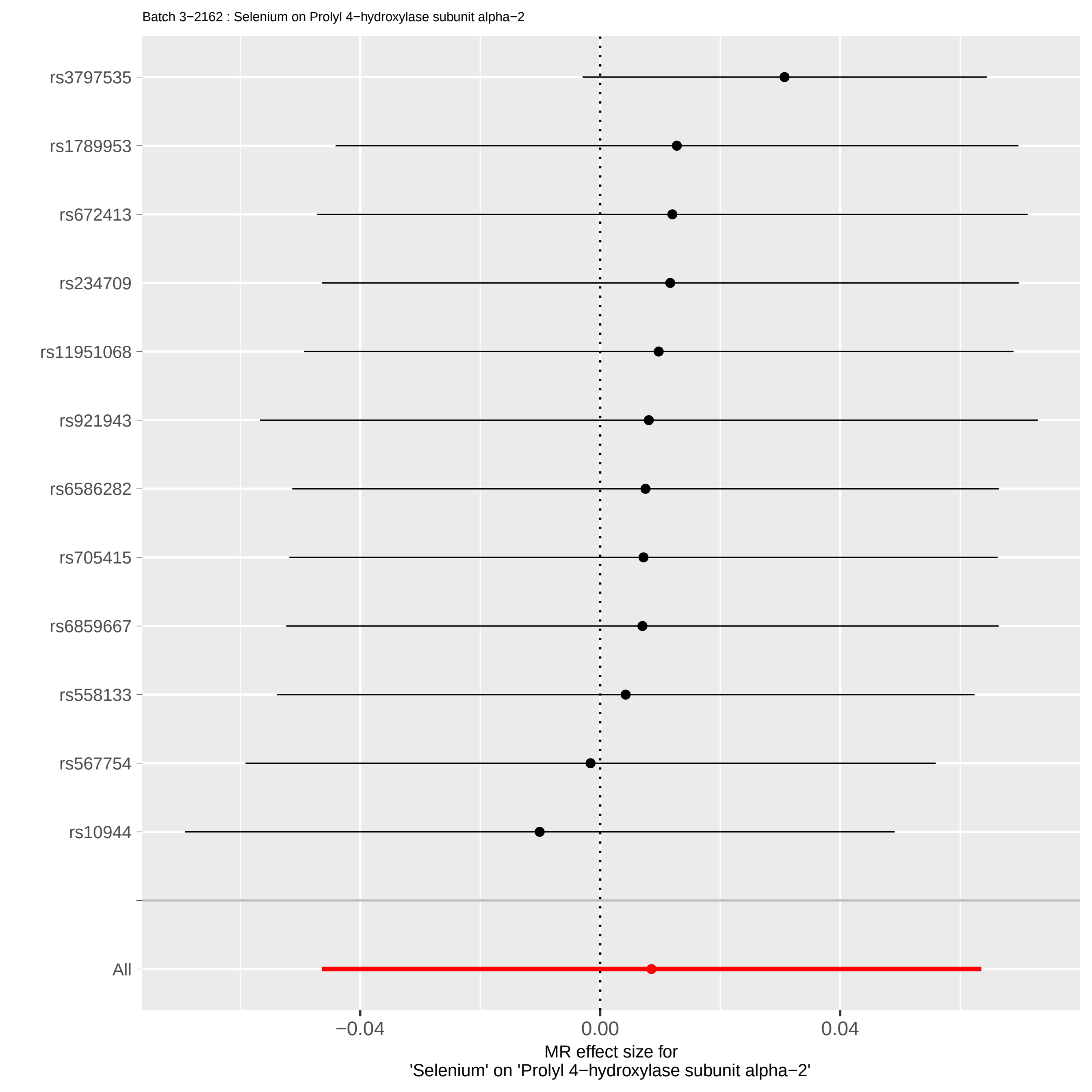
Batch 3−2162 : Selenium on Prolyl 4−hydroxylase subunit alpha−2
rs3797535
rs1789953
rs672413
rs234709
rs11951068
rs921943
rs6586282
rs705415
rs6859667
rs558133
rs567754
rs10944
All
−0.04
0.00
0.04
MR effect size for
'Selenium' on 'Prolyl 4−hydroxylase subunit alpha−2'

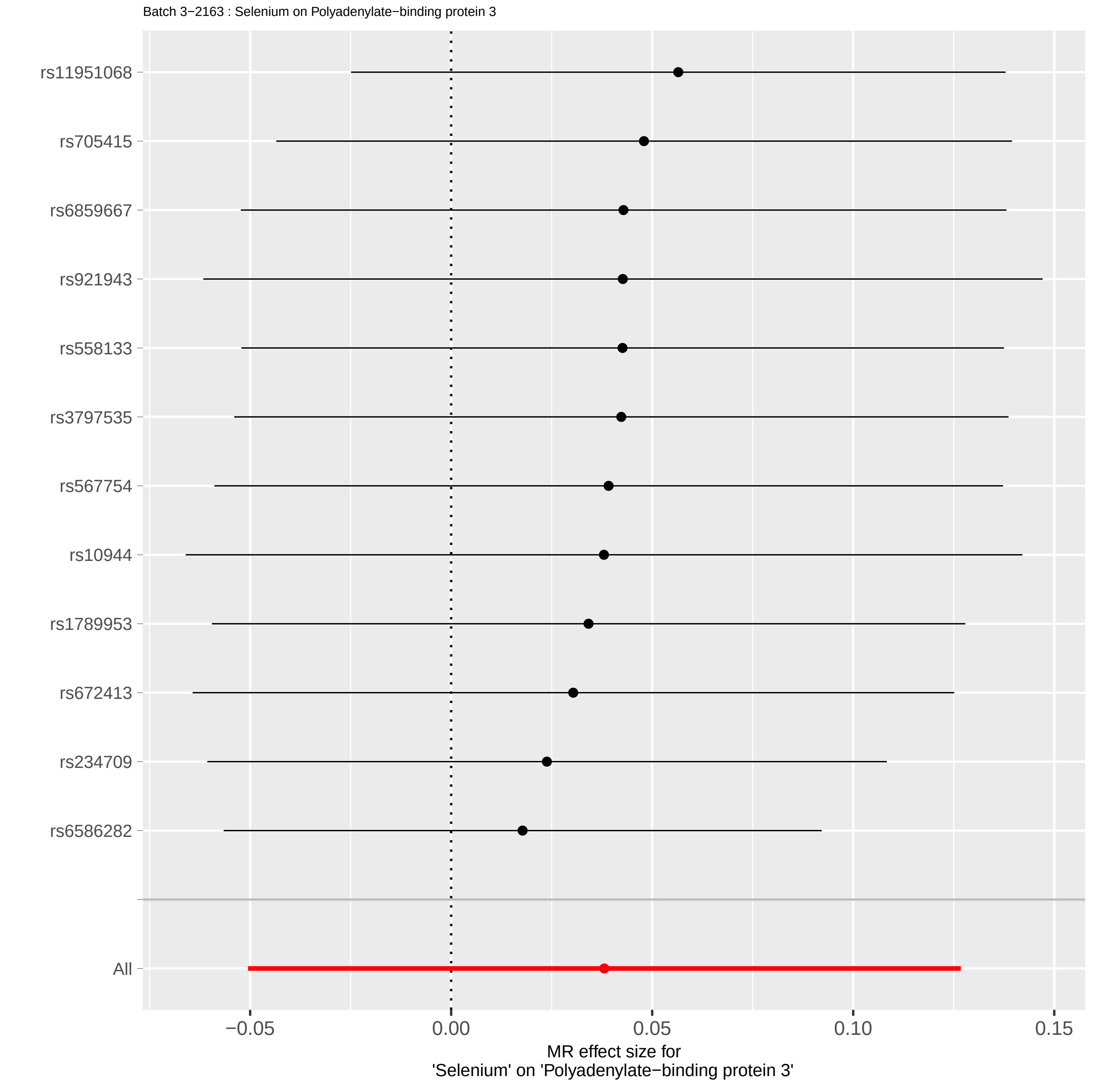

Batch 3−2163 : Selenium on Polyadenylate−binding protein 3
rs11951068
rs705415
rs6859667
rs921943
rs558133
rs3797535
rs567754
rs10944
rs1789953
rs672413
rs234709
rs6586282
All
−0.05
0.00
0.05
0.10
0.15
MR effect size for
'Selenium' on 'Polyadenylate−binding protein 3'

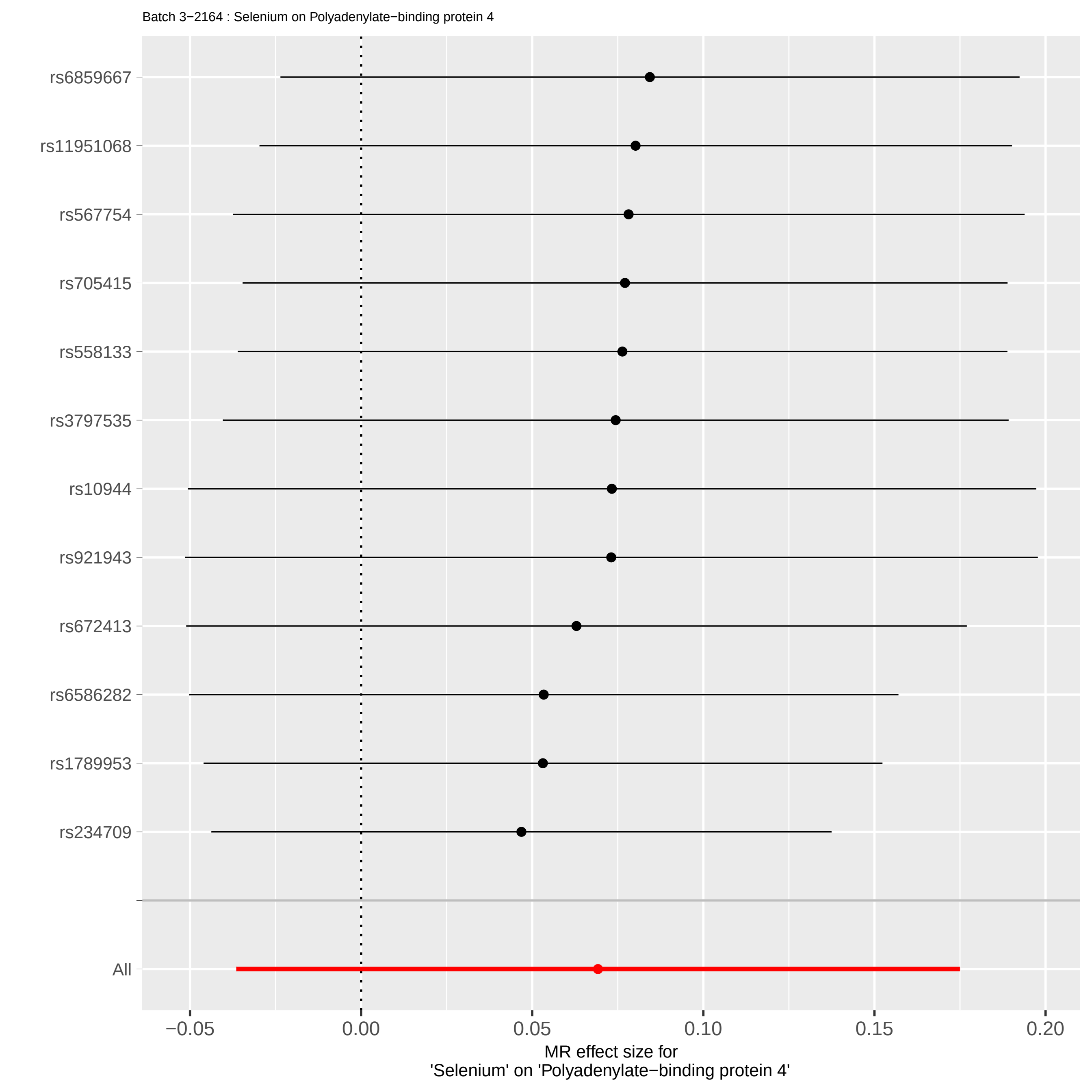

Batch 3−2164 : Selenium on Polyadenylate−binding protein 4
rs6859667
rs11951068
rs567754
rs705415
rs558133
rs3797535
rs10944
rs921943
rs672413
rs6586282
rs1789953
rs234709
All
−0.05
0.00
0.05
0.10
0.15
0.20
MR effect size for
'Selenium' on 'Polyadenylate−binding protein 4'

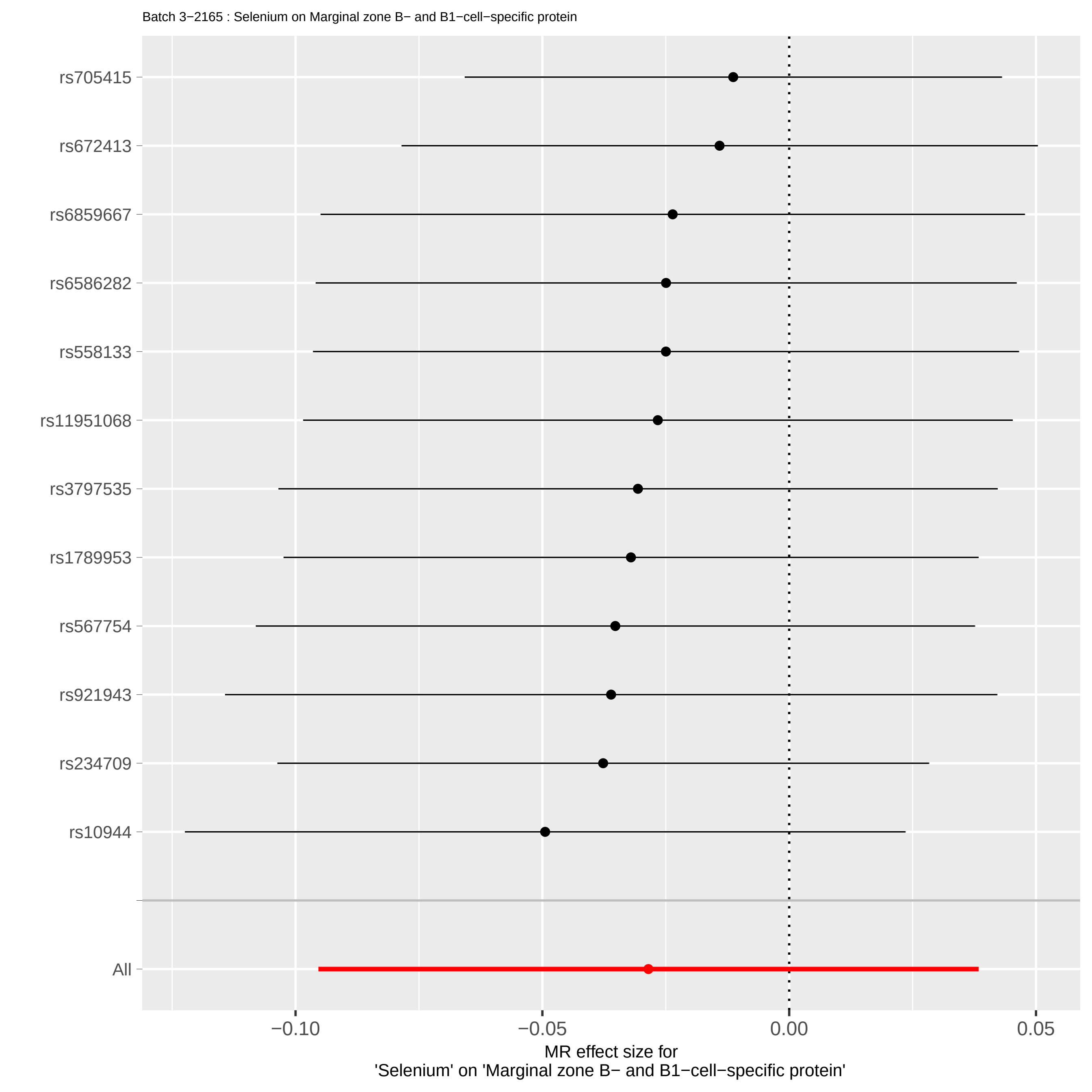

Batch 3−2165 : Selenium on Marginal zone B− and B1−cell−specific protein
rs705415
rs672413
rs6859667
rs6586282
rs558133
rs11951068
rs3797535
rs1789953
rs567754
rs921943
rs234709
rs10944
All
−0.10
−0.05
0.00
0.05
MR effect size for
'Selenium' on 'Marginal zone B− and B1−cell−specific protein'

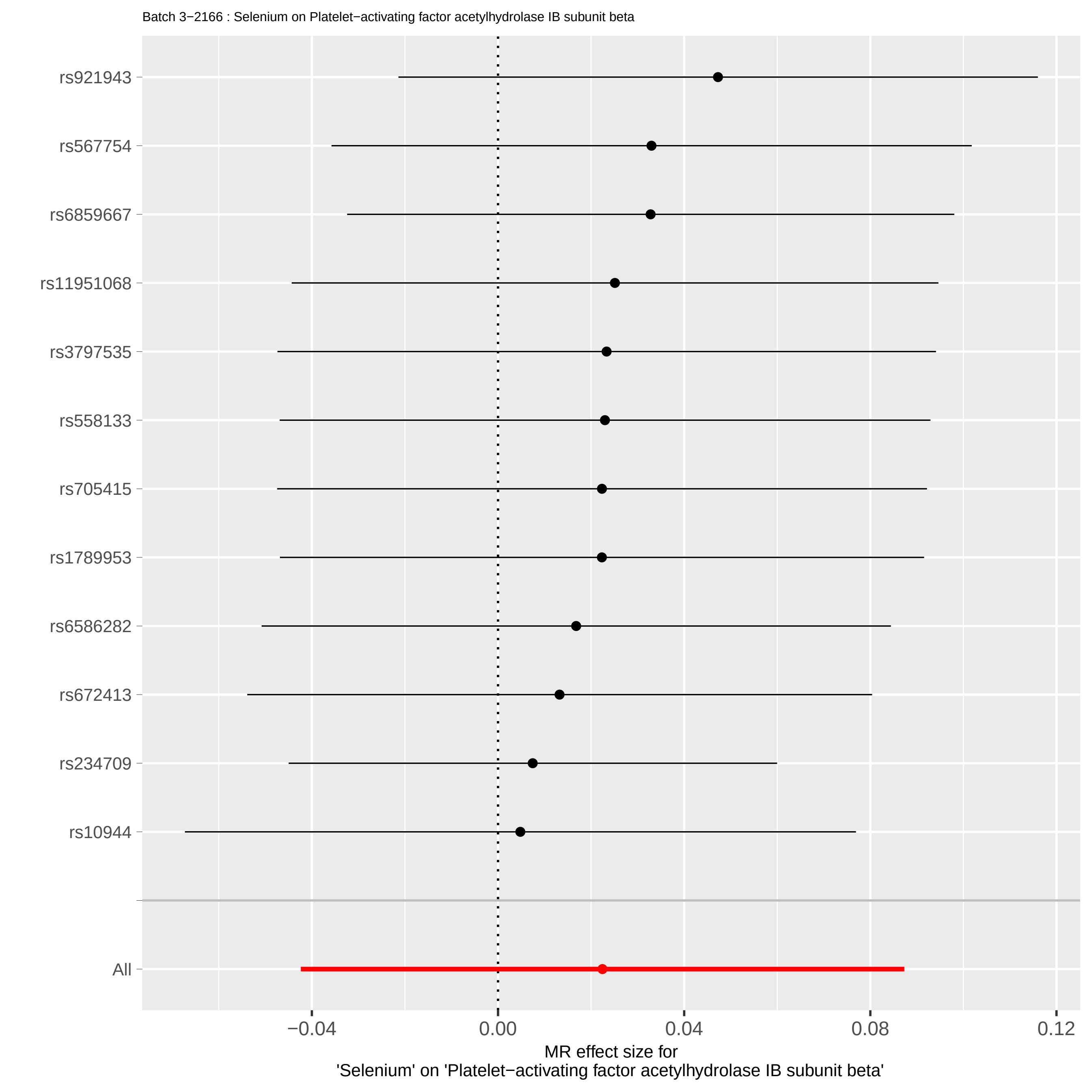

Batch 3−2166 : Selenium on Platelet−activating factor acetylhydrolase IB subunit beta
rs921943
rs567754
rs6859667
rs11951068
rs3797535
rs558133
rs705415
rs1789953
rs6586282
rs672413
rs234709
rs10944
All
−0.04
0.00
0.04
0.08
0.12
MR effect size for
'Selenium' on 'Platelet−activating factor acetylhydrolase IB subunit beta'

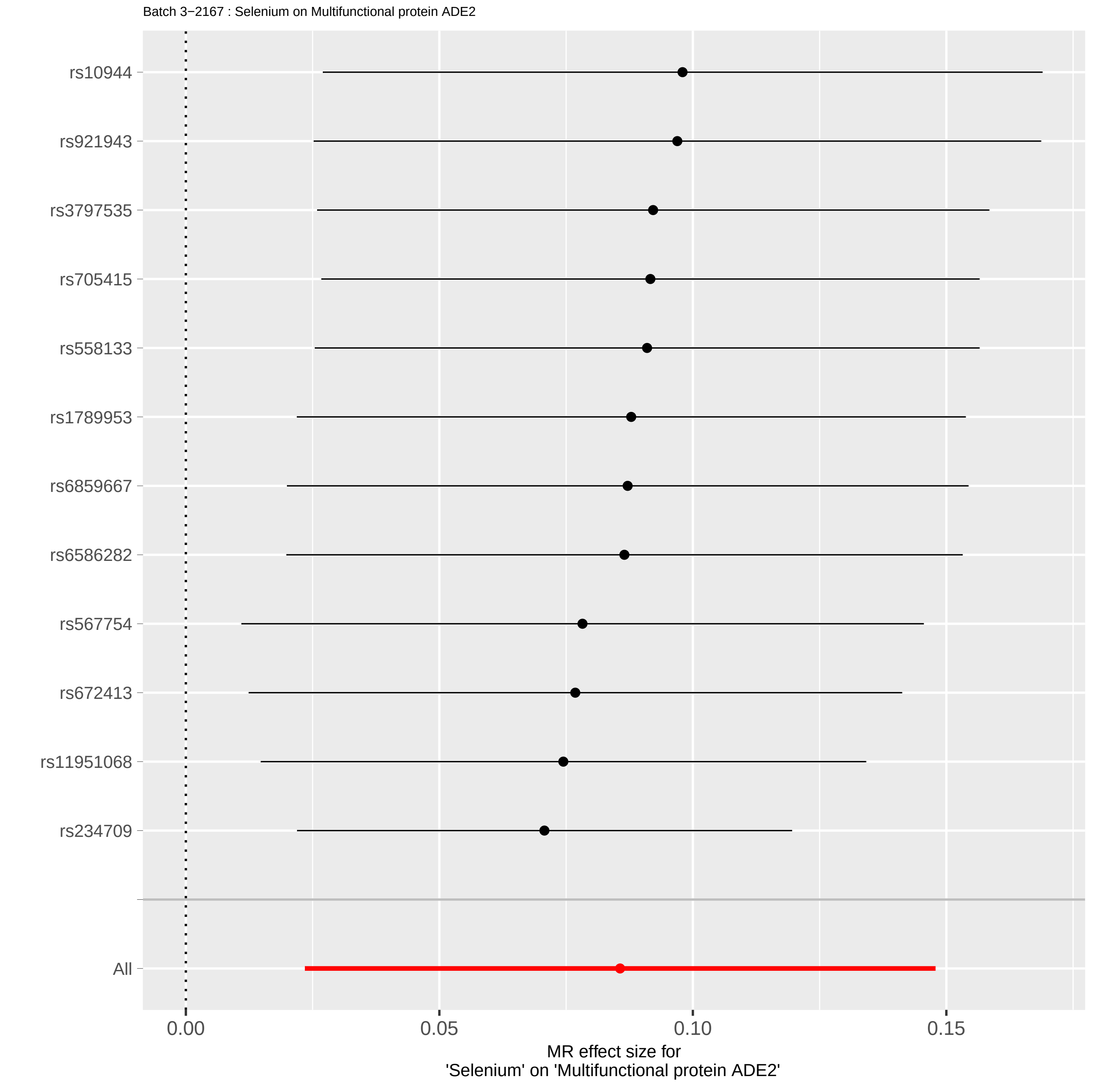

Batch 3−2167 : Selenium on Multifunctional protein ADE2
rs10944
rs921943
rs3797535
rs705415
rs558133
rs1789953
rs6859667
rs6586282
rs567754
rs672413
rs11951068
rs234709
All
0.00
0.05
0.10
0.15
MR effect size for
'Selenium' on 'Multifunctional protein ADE2'

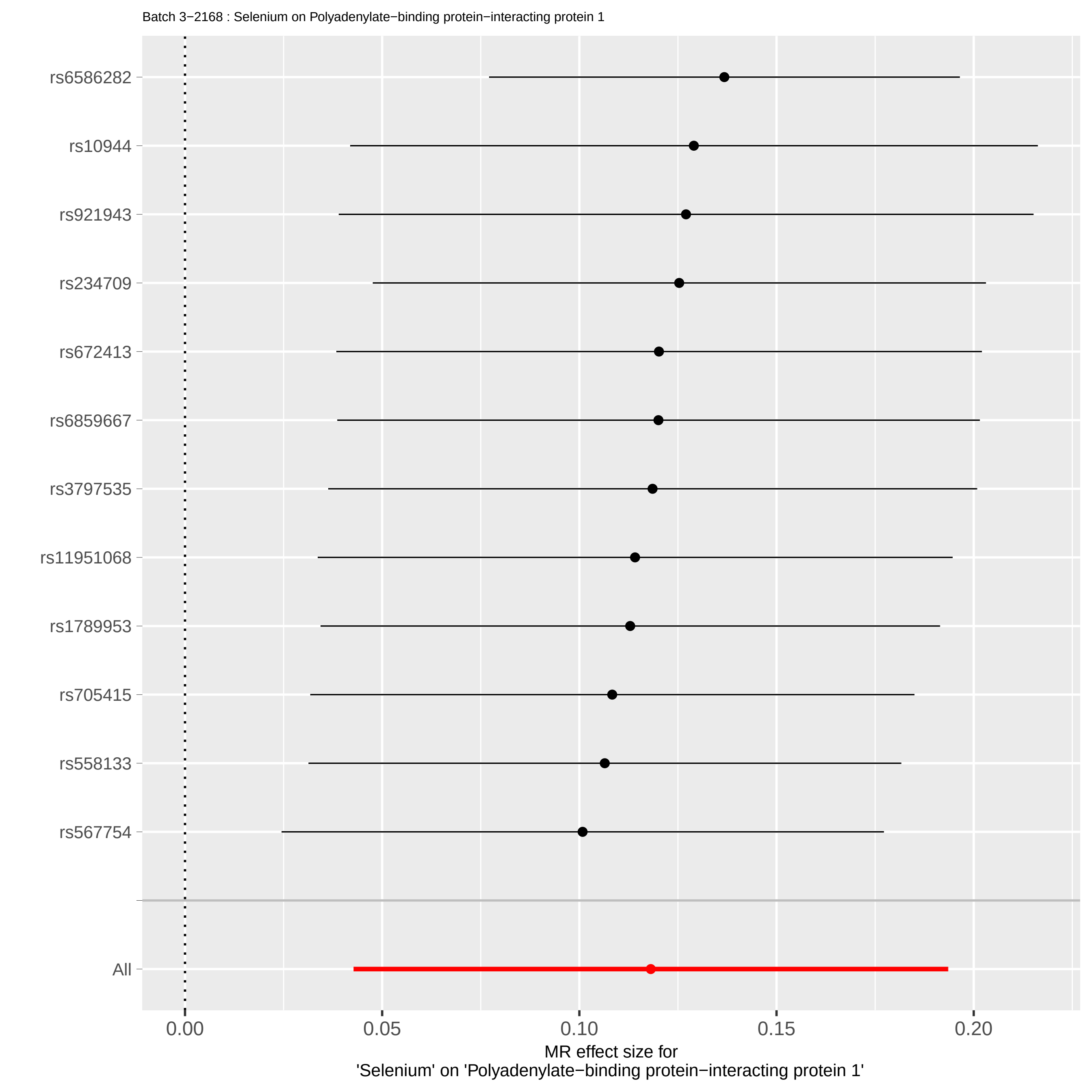

Batch 3−2168 : Selenium on Polyadenylate−binding protein−interacting protein 1
rs6586282
rs10944
rs921943
rs234709
rs672413
rs6859667
rs3797535
rs11951068
rs1789953
rs705415
rs558133
rs567754
All
0.00
0.05
0.10
0.15
0.20
MR effect size for
'Selenium' on 'Polyadenylate−binding protein−interacting protein 1'

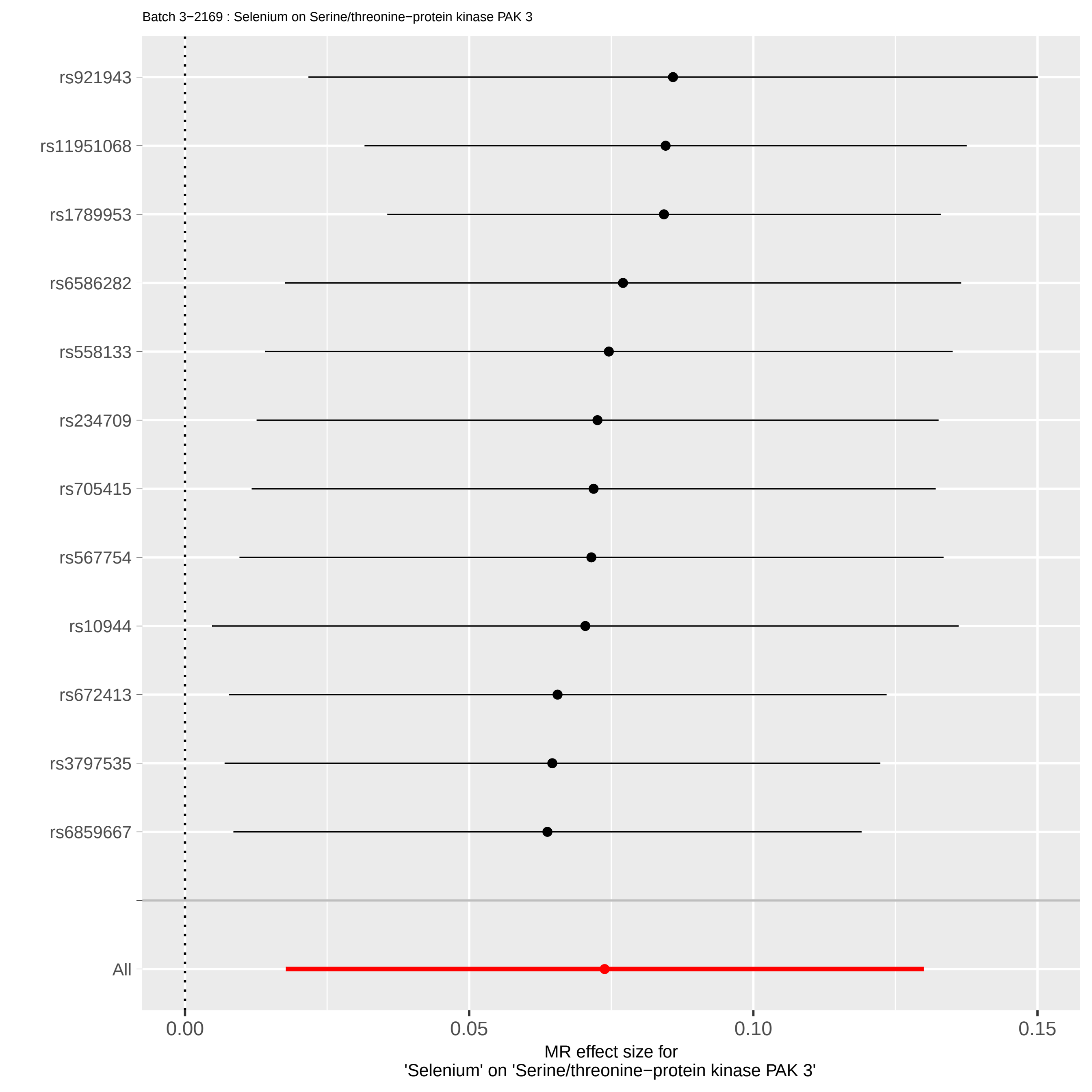
Batch 3−2169 : Selenium on Serine/threonine−protein kinase PAK 3
rs921943
rs11951068
rs1789953
rs6586282
rs558133
rs234709
rs705415
rs567754
rs10944
rs672413
rs3797535
rs6859667
All
0.00
0.05
0.10
0.15
MR effect size for
'Selenium' on 'Serine/threonine−protein kinase PAK 3'

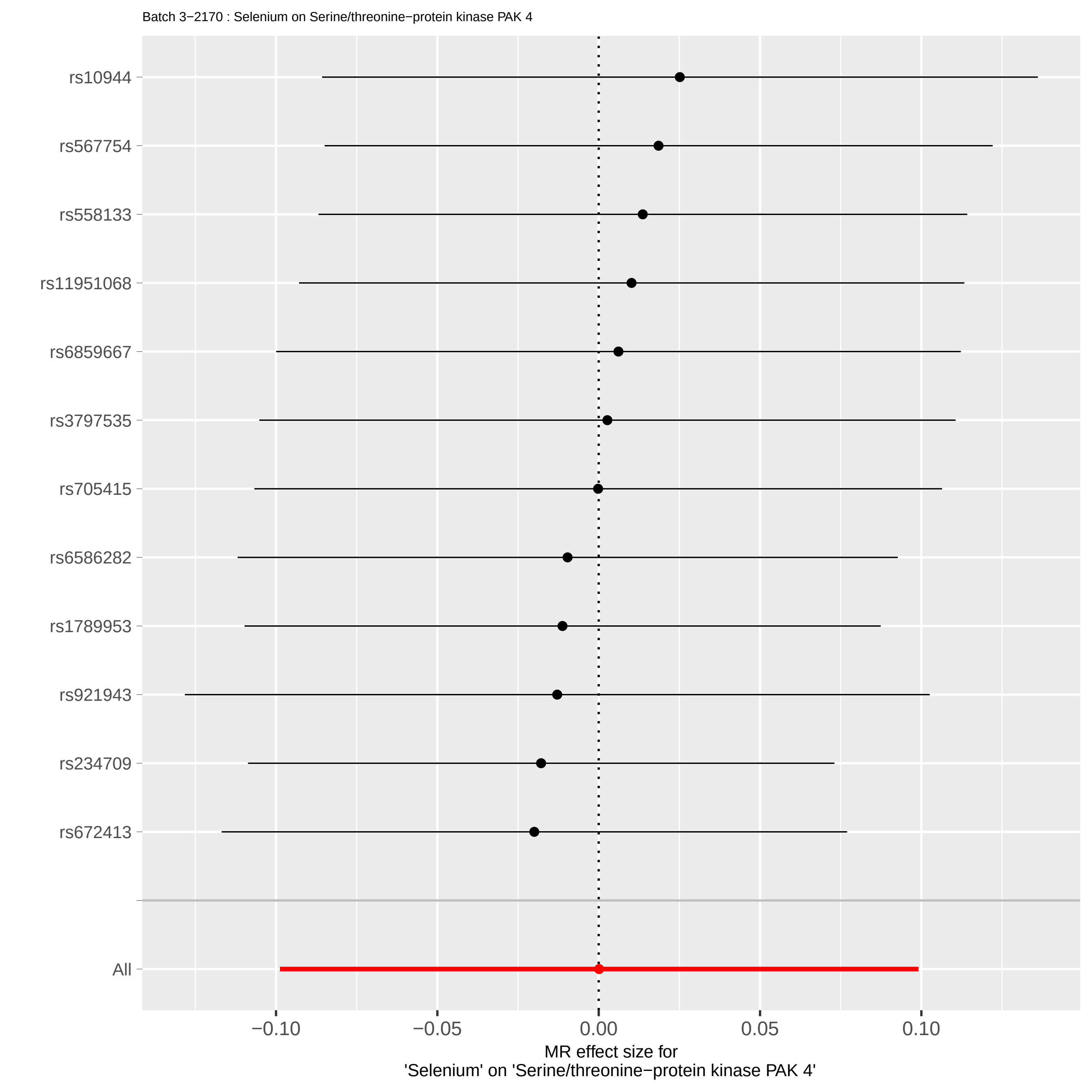

Batch 3−2170 : Selenium on Serine/threonine−protein kinase PAK 4
rs10944
rs567754
rs558133
rs11951068
rs6859667
rs3797535
rs705415
rs6586282
rs1789953
rs921943
rs234709
rs672413
All
−0.10
−0.05
0.00
0.05
0.10
MR effect size for
'Selenium' on 'Serine/threonine−protein kinase PAK 4'

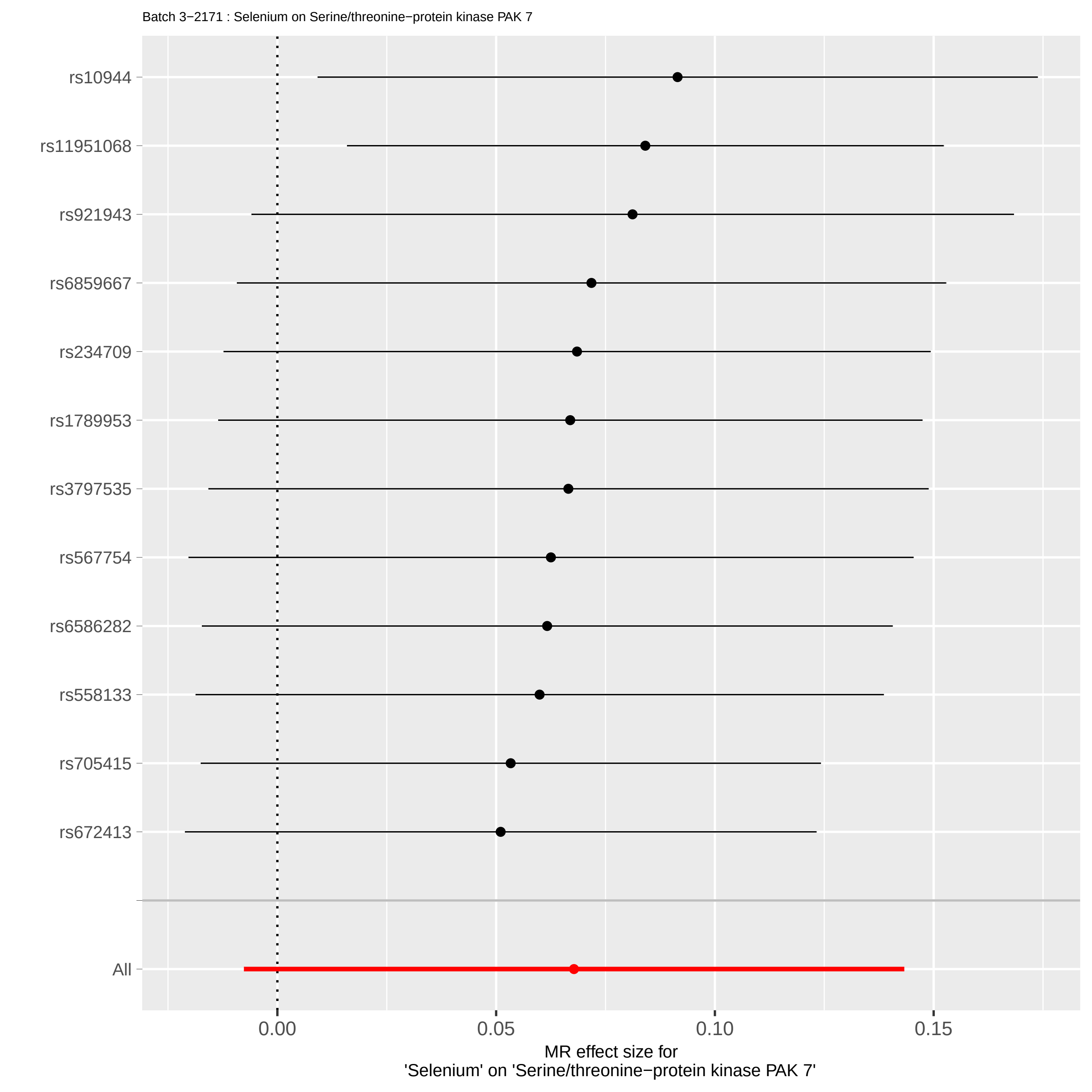
Batch 3−2171 : Selenium on Serine/threonine−protein kinase PAK 7
rs10944
rs11951068
rs921943
rs6859667
rs234709
rs1789953
rs3797535
rs567754
rs6586282
rs558133
rs705415
rs672413
All
0.00
0.05
0.10
0.15
MR effect size for
'Selenium' on 'Serine/threonine−protein kinase PAK 7'

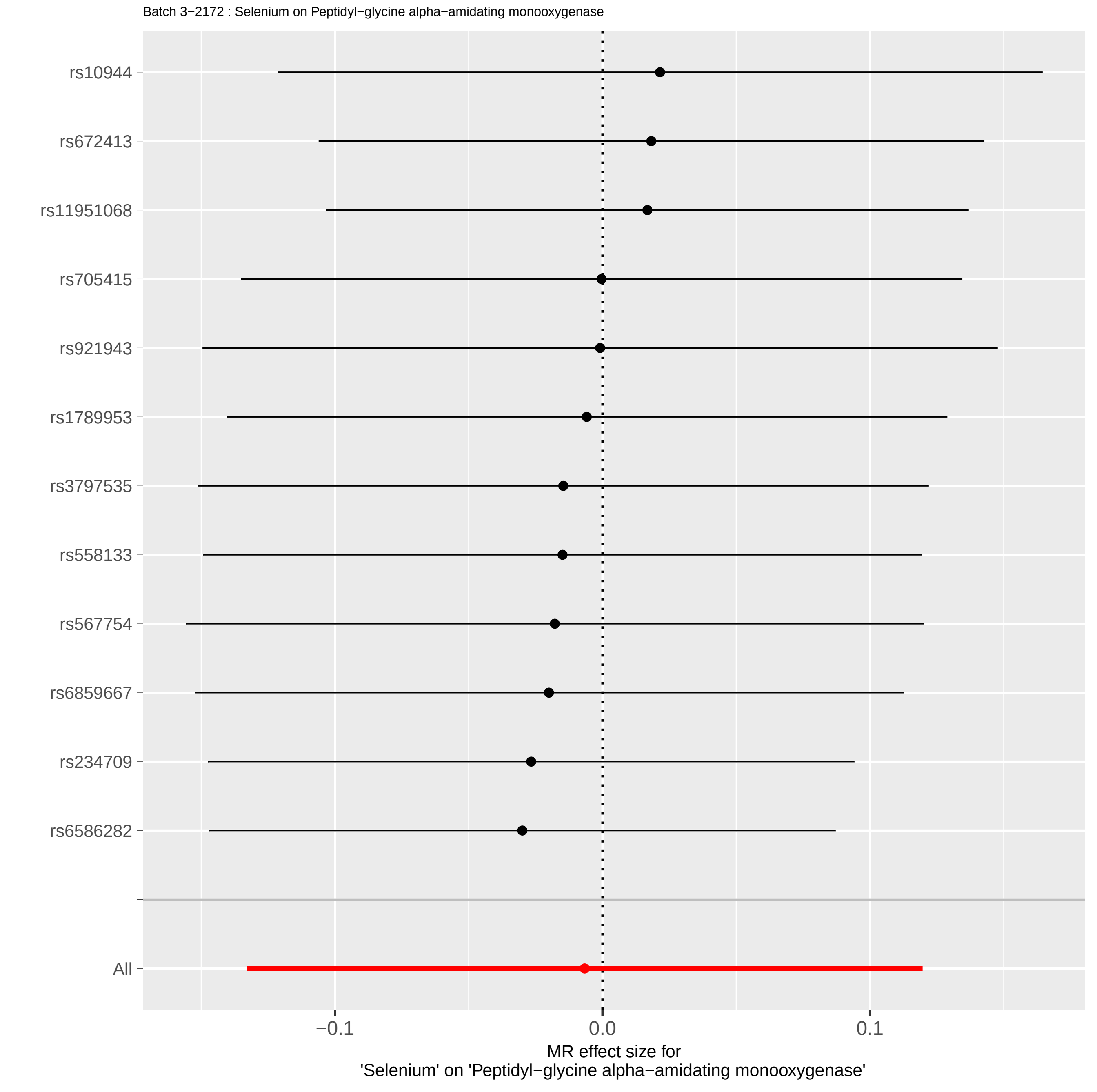
Batch 3−2172 : Selenium on Peptidyl−glycine alpha−amidating monooxygenase
rs10944
rs672413
rs11951068
rs705415
rs921943
rs1789953
rs3797535
rs558133
rs567754
rs6859667
rs234709
rs6586282
All
−0.1
0.0
0.1
MR effect size for
'Selenium' on 'Peptidyl−glycine alpha−amidating monooxygenase'

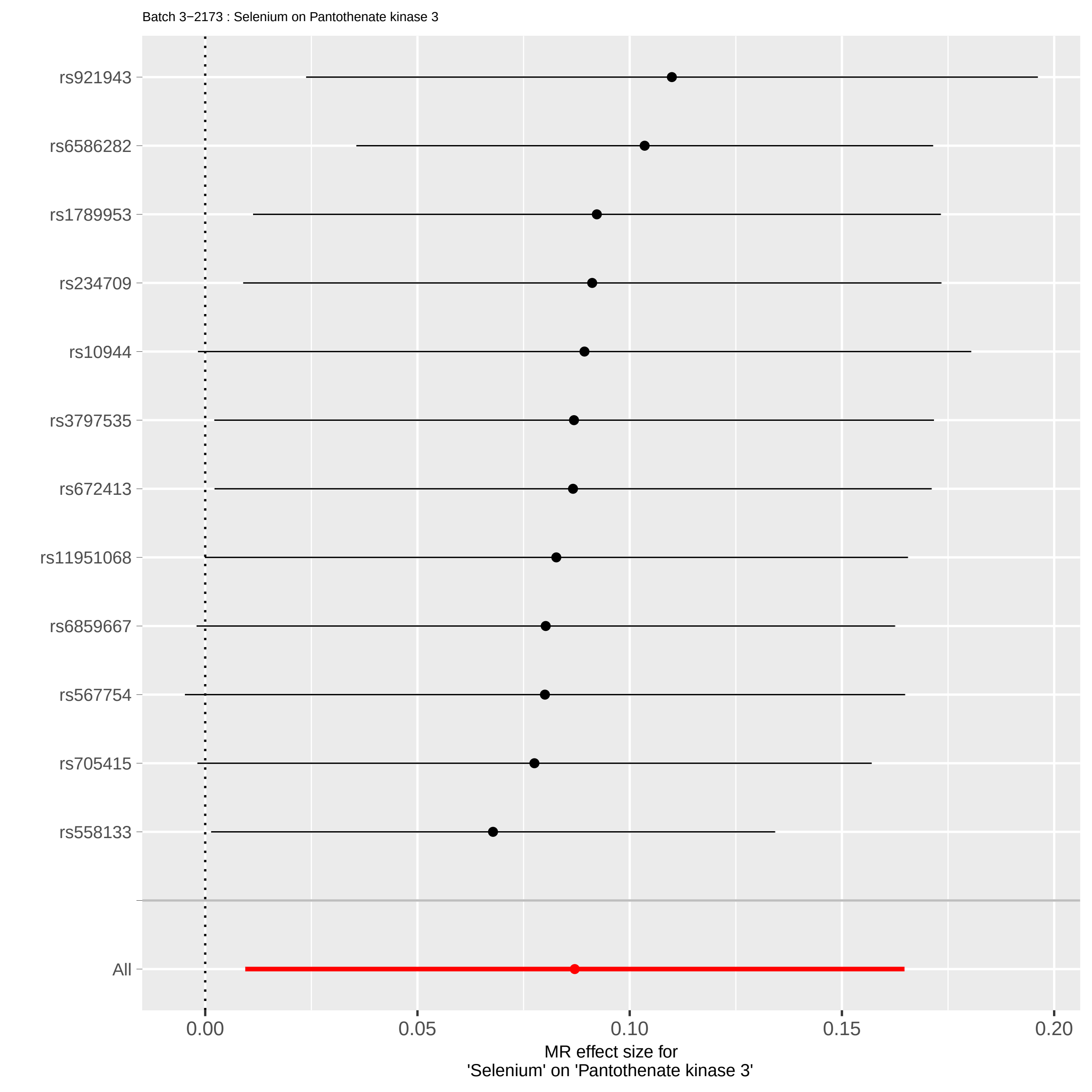

Batch 3−2173 : Selenium on Pantothenate kinase 3
rs921943
rs6586282
rs1789953
rs234709
rs10944
rs3797535
rs672413
rs11951068
rs6859667
rs567754
rs705415
rs558133
All
0.00
0.05
0.10
0.15
0.20
MR effect size for
'Selenium' on 'Pantothenate kinase 3'

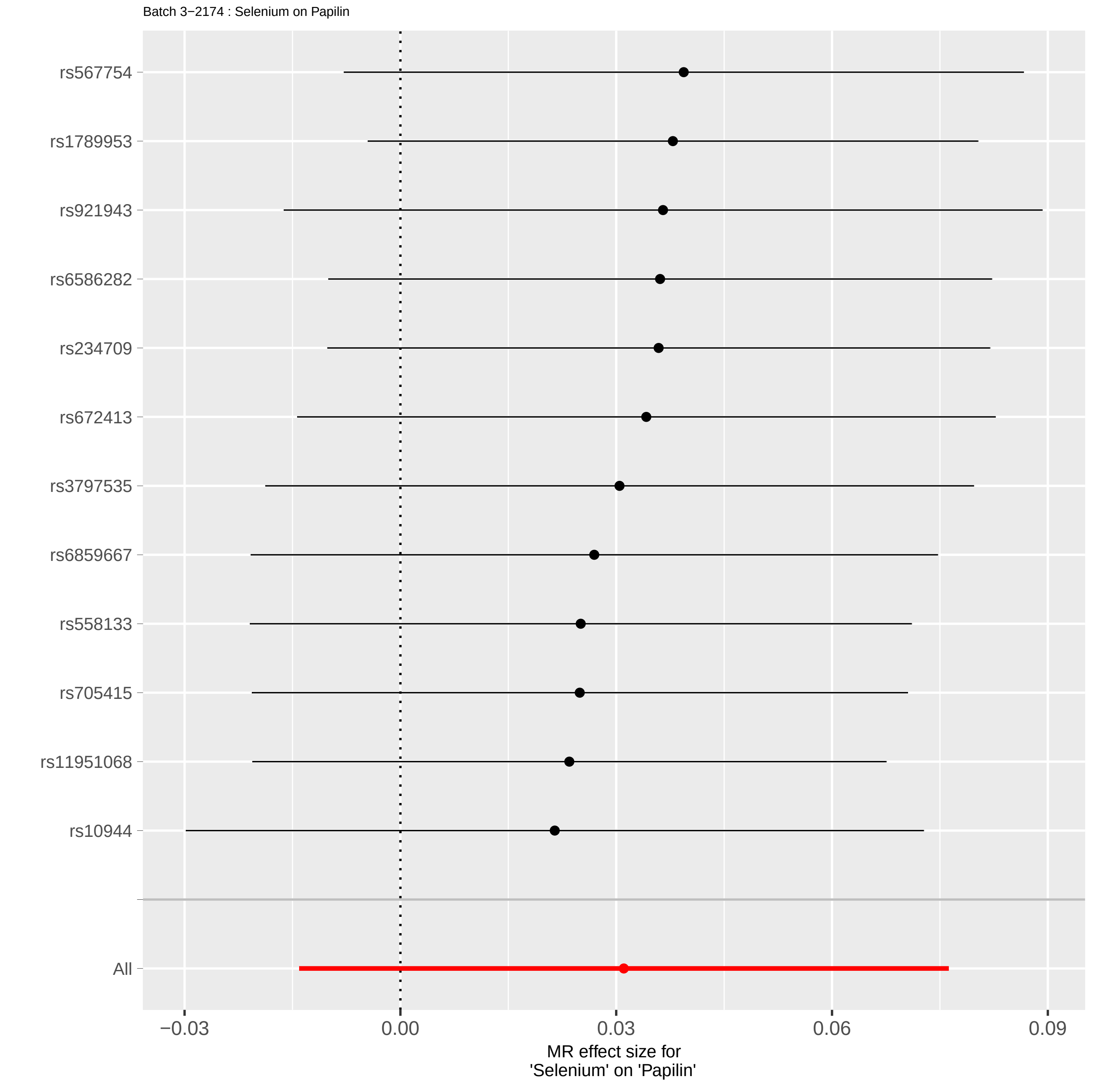

Batch 3−2174 : Selenium on Papilin
rs567754
rs1789953
rs921943
rs6586282
rs234709
rs672413
rs3797535
rs6859667
rs558133
rs705415
rs11951068
rs10944
All
−0.03
0.00
0.03
0.06
0.09
MR effect size for
'Selenium' on 'Papilin'

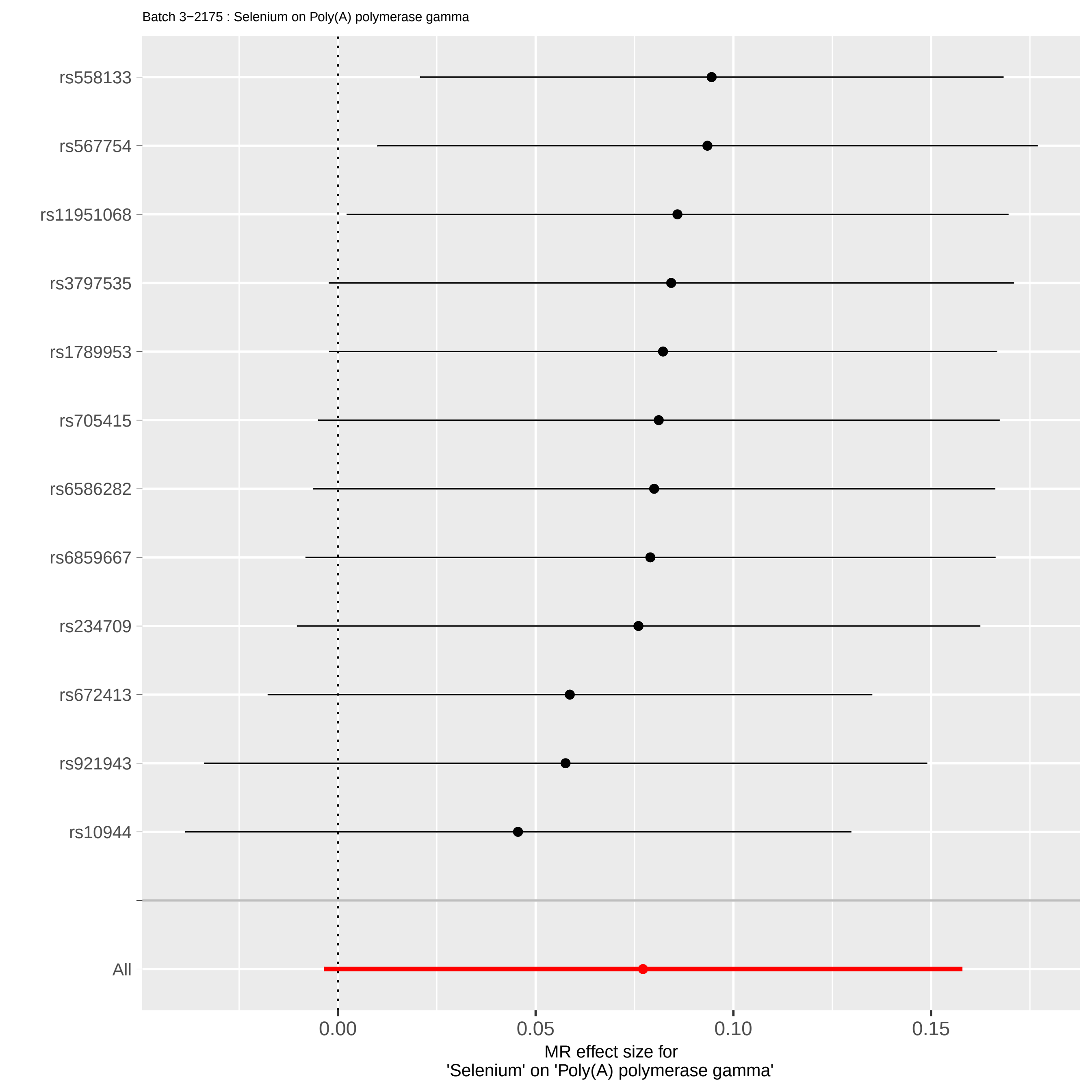
Batch 3−2175 : Selenium on Poly(A) polymerase gamma
rs558133
rs567754
rs11951068
rs3797535
rs1789953
rs705415
rs6586282
rs6859667
rs234709
rs672413
rs921943
rs10944
All
0.00
0.05
0.10
0.15
MR effect size for
'Selenium' on 'Poly(A) polymerase gamma'

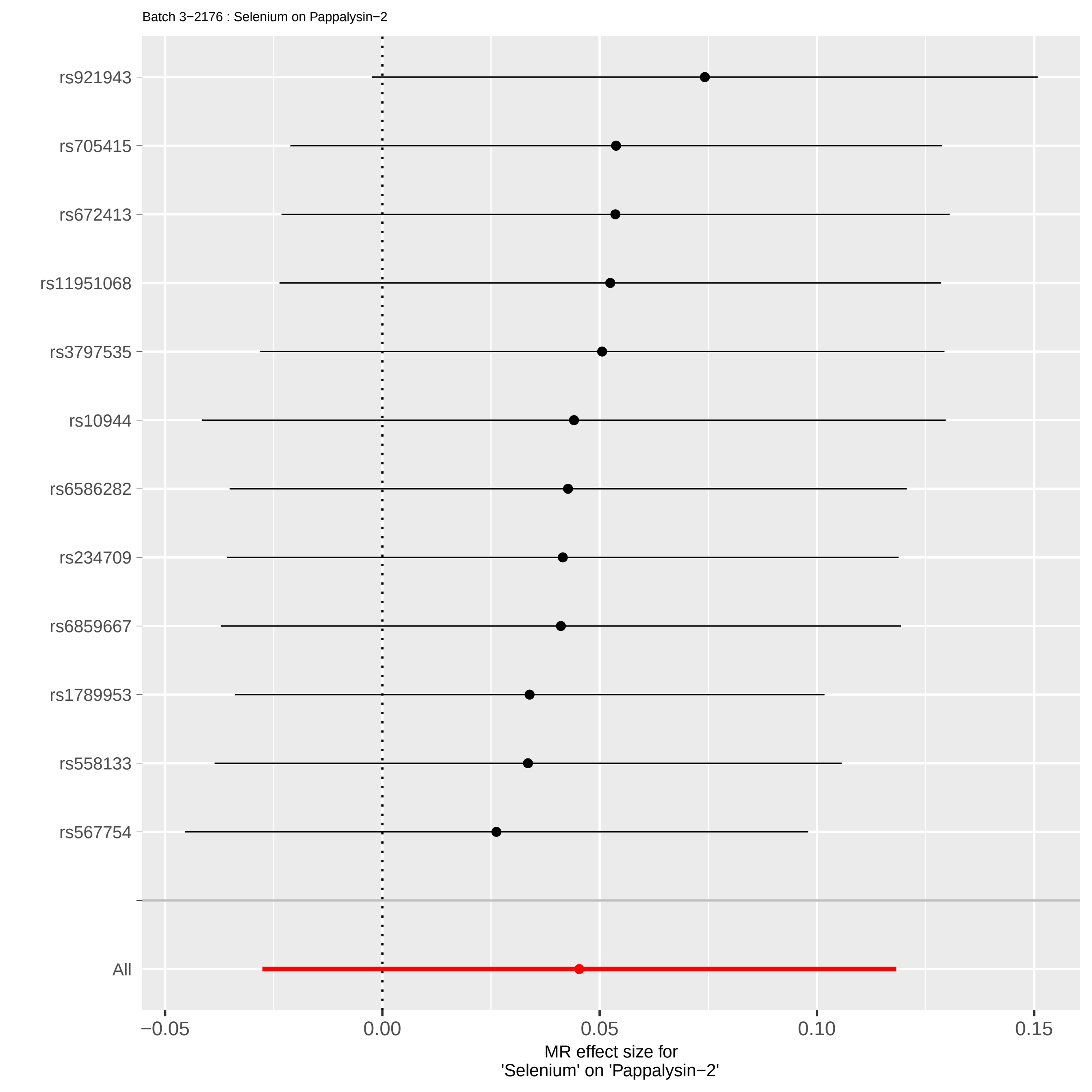

Batch 3−2176 : Selenium on Pappalysin−2
rs921943
rs705415
rs672413
rs11951068
rs3797535
rs10944
rs6586282
rs234709
rs6859667
rs1789953
rs558133
rs567754
All
−0.05
0.00
0.05
0.10
0.15
MR effect size for
'Selenium' on 'Pappalysin−2'

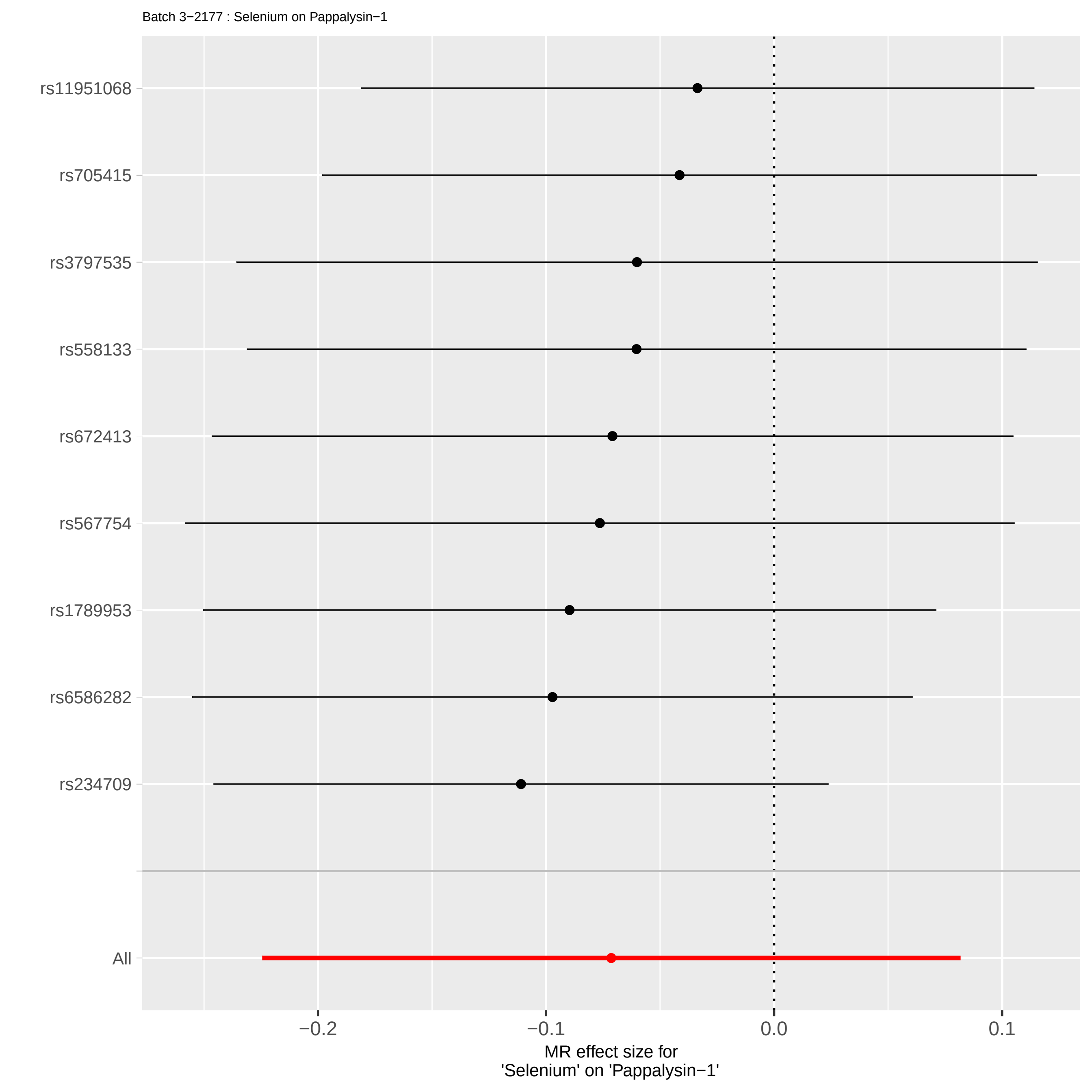

Batch 3−2177 : Selenium on Pappalysin−1
rs11951068
rs705415
rs3797535
rs558133
rs672413
rs567754
rs1789953
rs6586282
rs234709
All
−0.2
−0.1
0.0
0.1
MR effect size for
'Selenium' on 'Pappalysin−1'

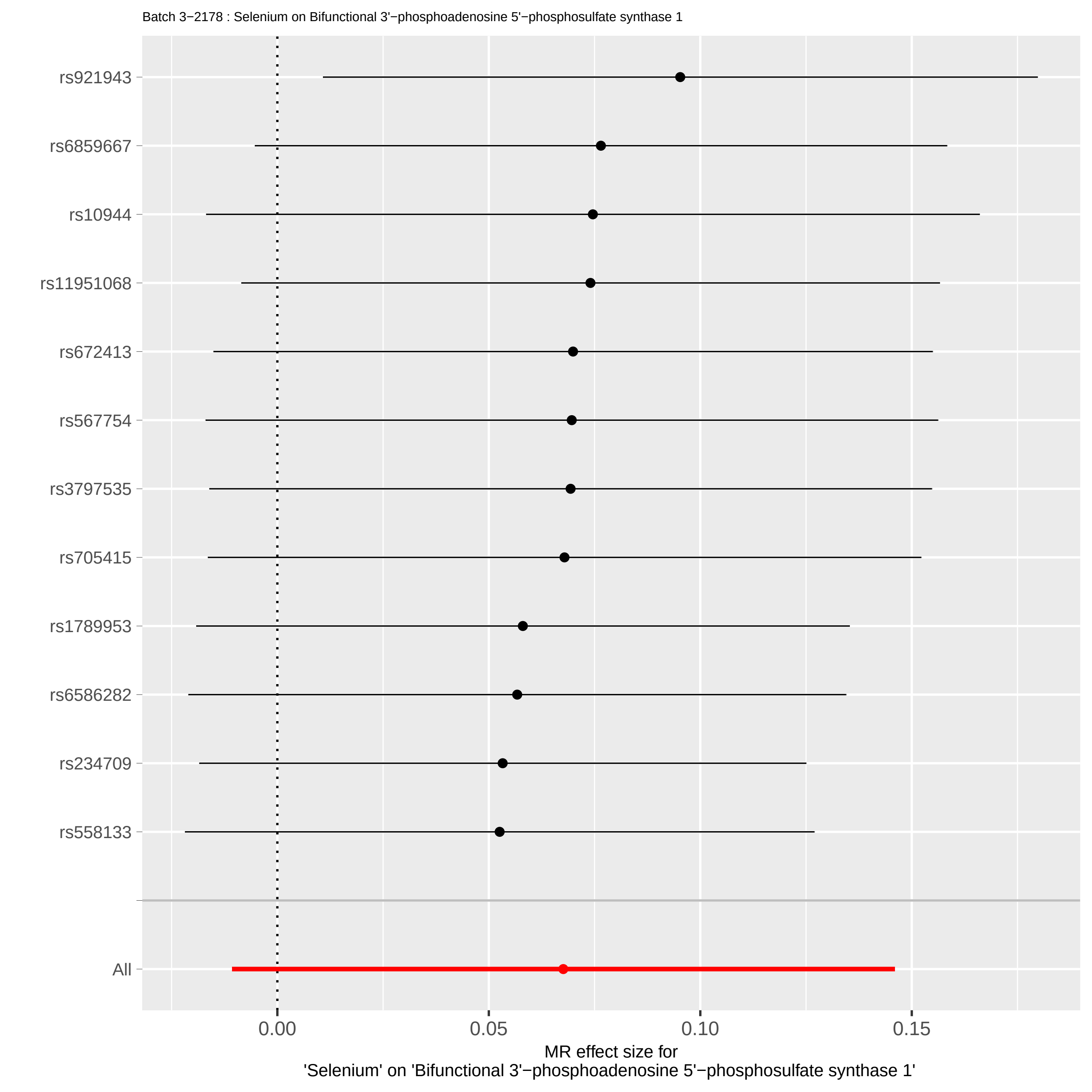

Batch 3−2178 : Selenium on Bifunctional 3'−phosphoadenosine 5'−phosphosulfate synthase 1
rs921943
rs6859667
rs10944
rs11951068
rs672413
rs567754
rs3797535
rs705415
rs1789953
rs6586282
rs234709
rs558133
All
0.00
0.05
0.10
0.15
MR effect size for
'Selenium' on 'Bifunctional 3'−phosphoadenosine 5'−phosphosulfate synthase 1'

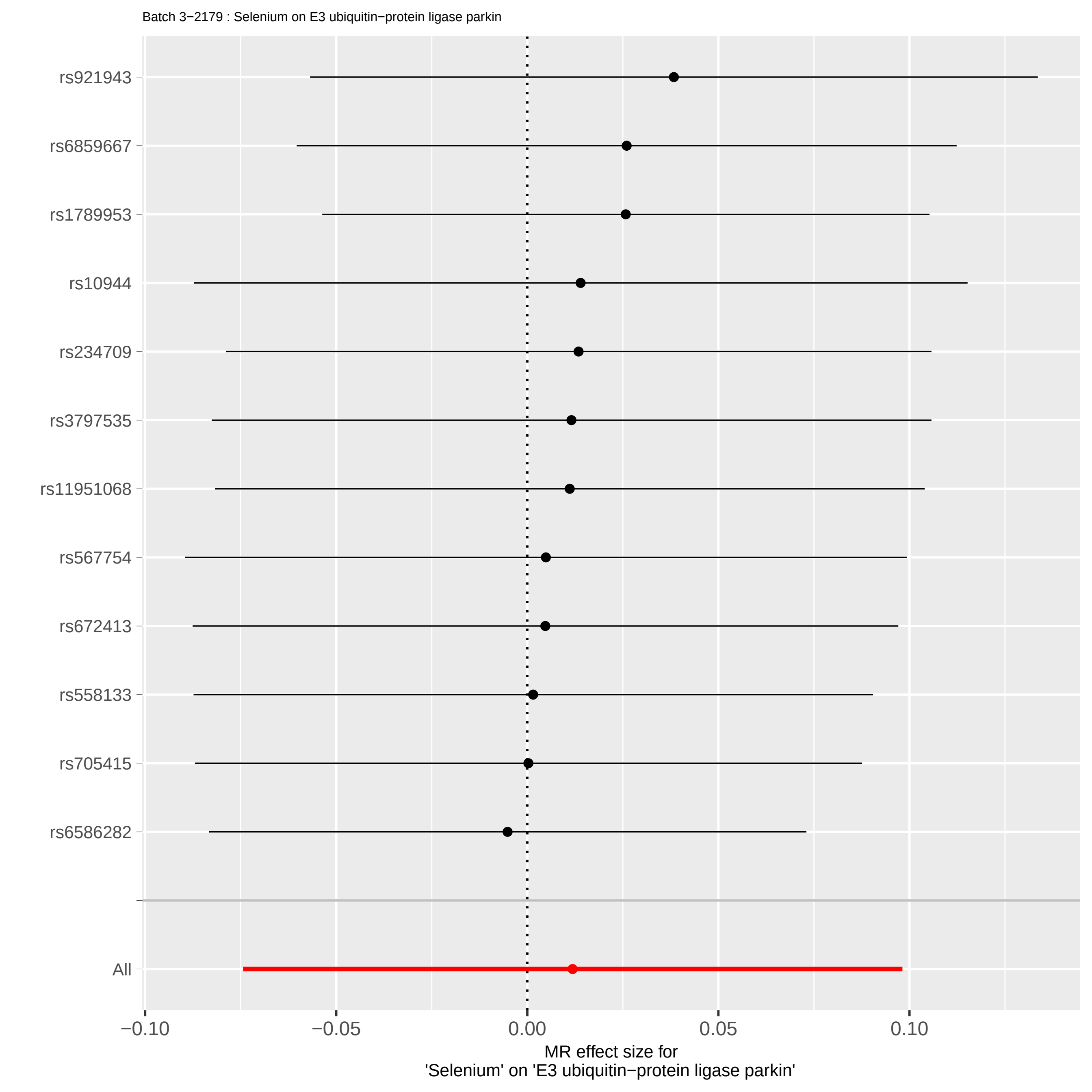

Batch 3−2179 : Selenium on E3 ubiquitin−protein ligase parkin
rs921943
rs6859667
rs1789953
rs10944
rs234709
rs3797535
rs11951068
rs567754
rs672413
rs558133
rs705415
rs6586282
All
−0.10
−0.05
0.00
0.05
0.10
MR effect size for
'Selenium' on 'E3 ubiquitin−protein ligase parkin'

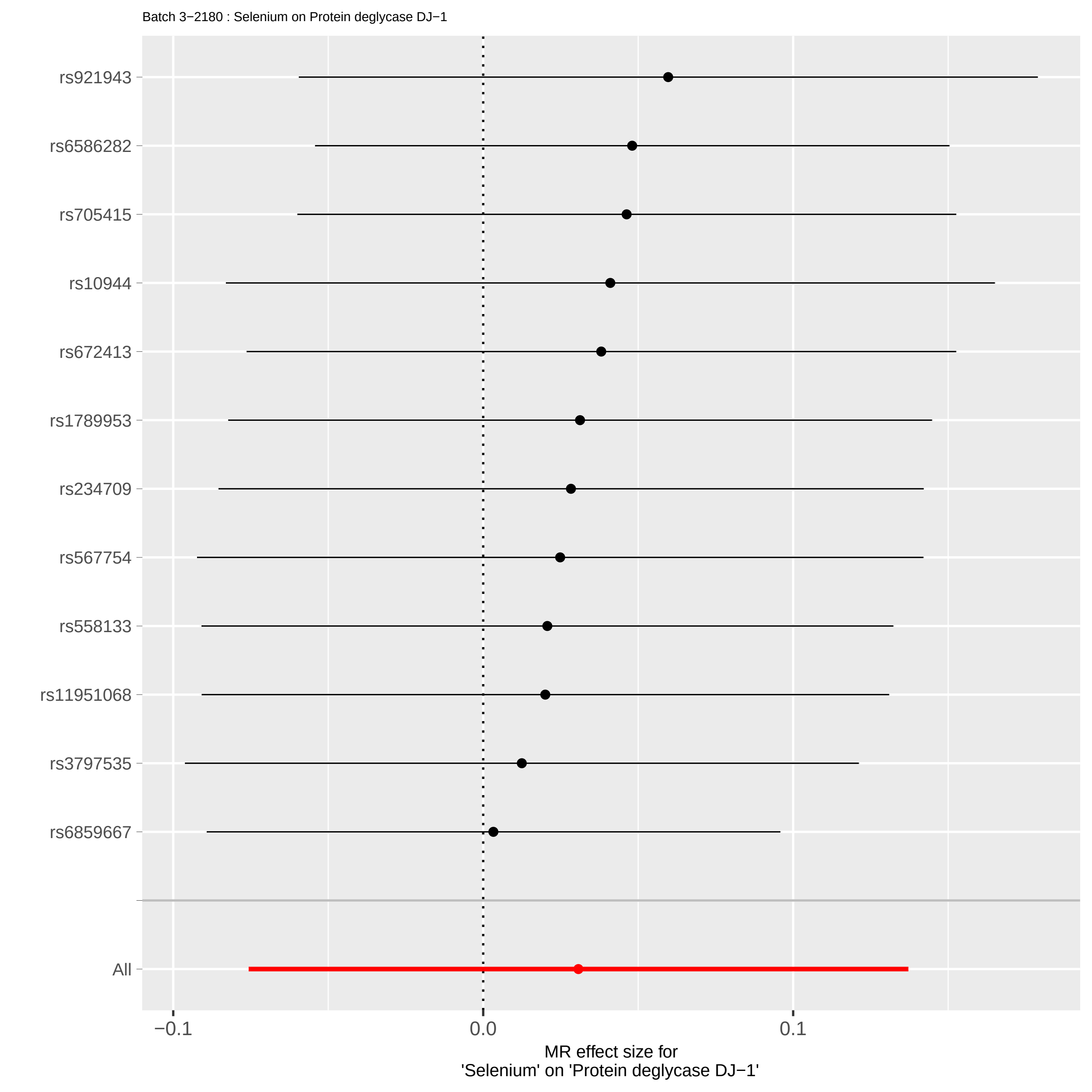

Batch 3−2180 : Selenium on Protein deglycase DJ−1
rs921943
rs6586282
rs705415
rs10944
rs672413
rs1789953
rs234709
rs567754
rs558133
rs11951068
rs3797535
rs6859667
All
−0.1
0.0
0.1
MR effect size for
'Selenium' on 'Protein deglycase DJ−1'

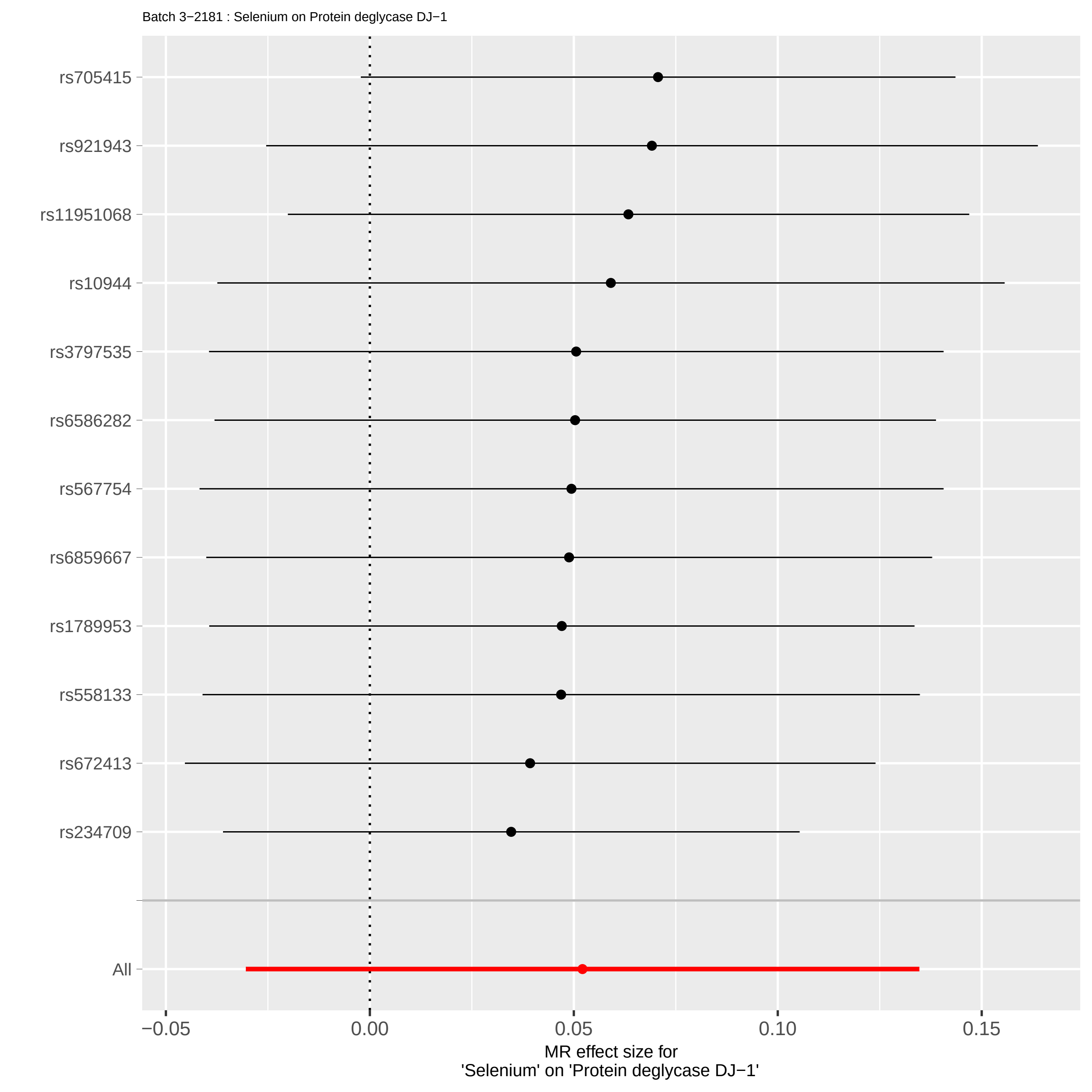

Batch 3−2181 : Selenium on Protein deglycase DJ−1
rs705415
rs921943
rs11951068
rs10944
rs3797535
rs6586282
rs567754
rs6859667
rs1789953
rs558133
rs672413
rs234709
All
−0.05
0.00
0.05
0.10
0.15
MR effect size for
'Selenium' on 'Protein deglycase DJ−1'

Batch 3−2182 : Selenium on Poly [ADP−ribose] polymerase 1

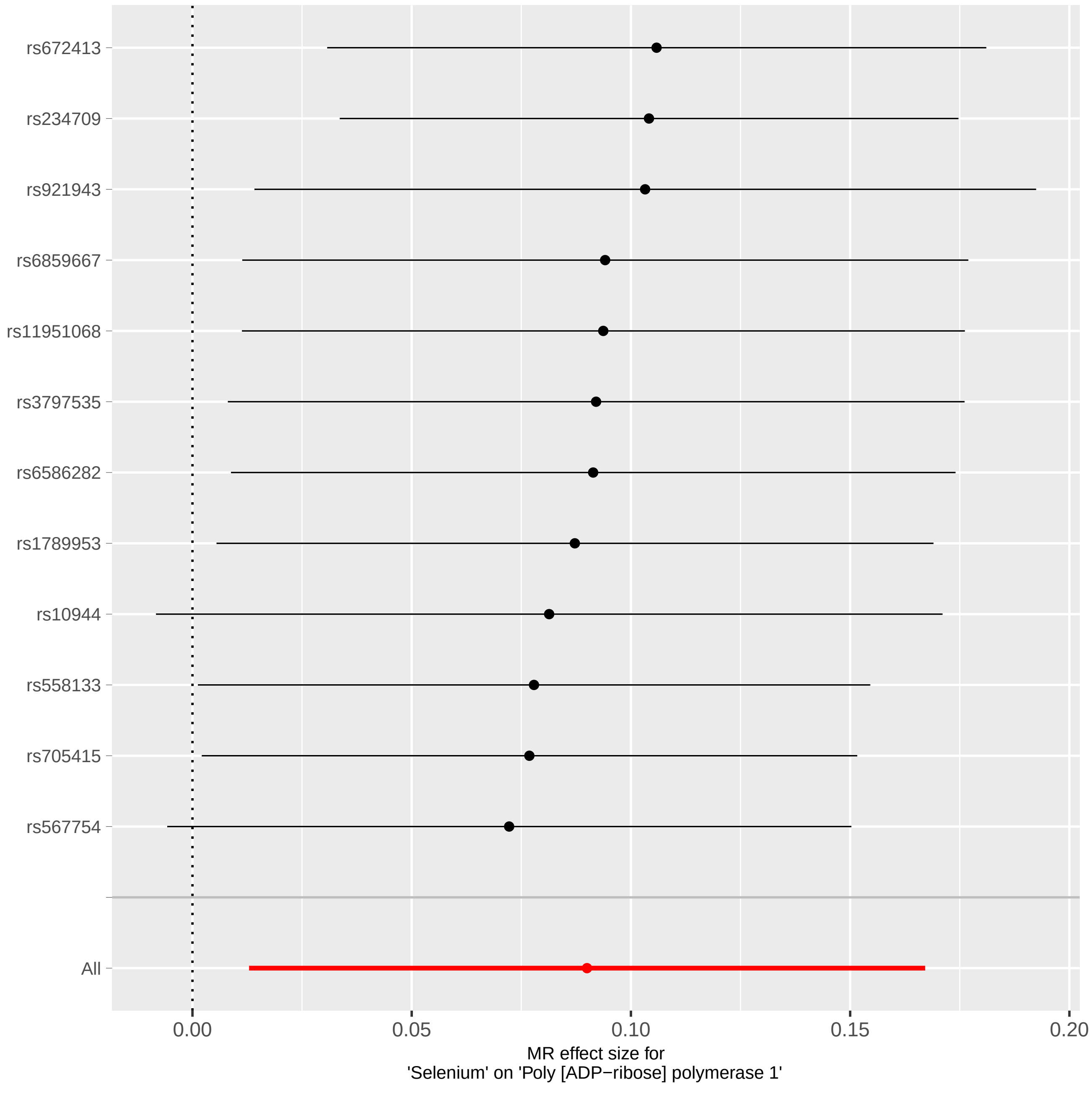

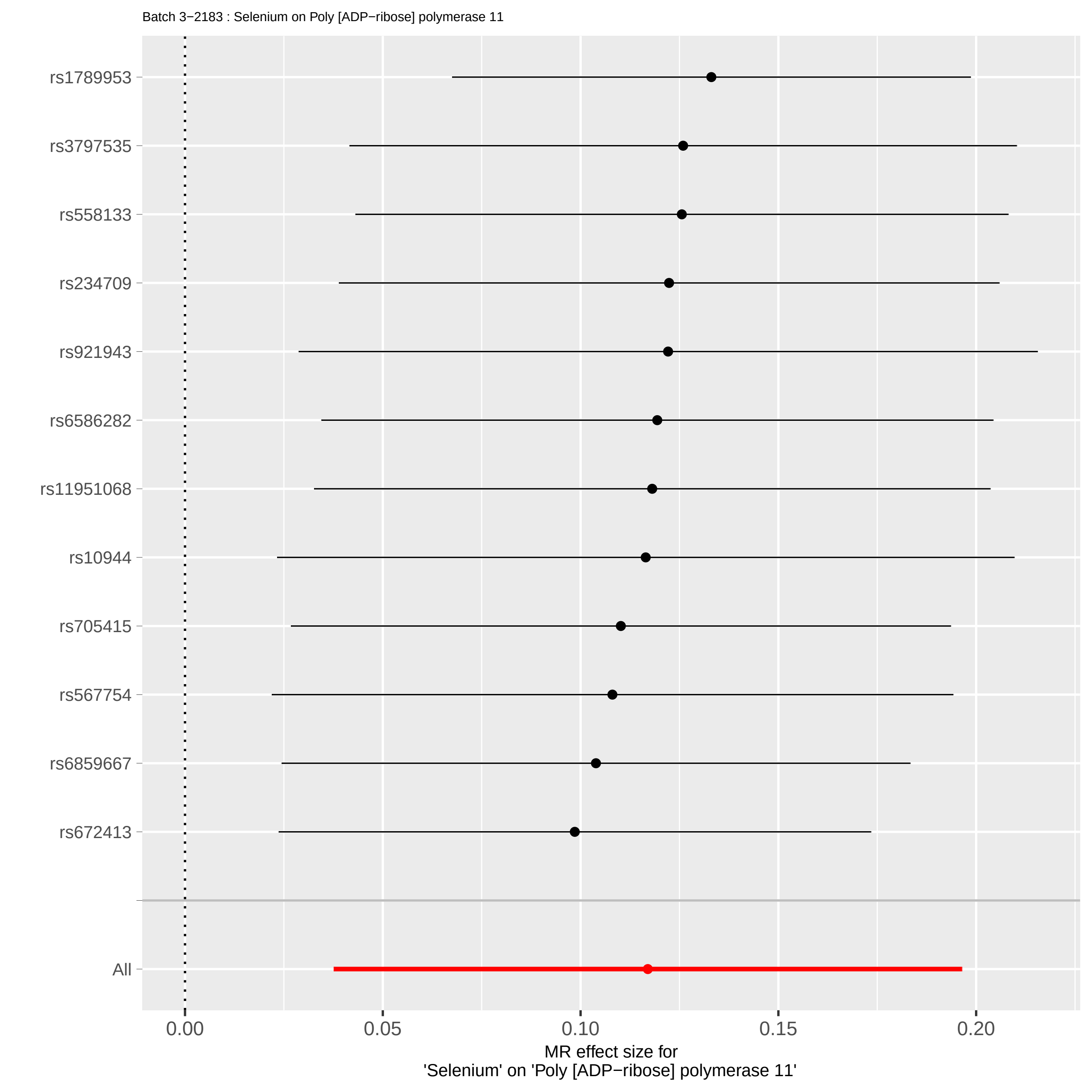

Batch 3−2183 : Selenium on Poly [ADP−ribose] polymerase 11
rs1789953
rs3797535
rs558133
rs234709
rs921943
rs6586282
rs11951068
rs10944
rs705415
rs567754
rs6859667
rs672413
All
0.00
0.05
0.10
0.15
0.20
MR effect size for
'Selenium' on 'Poly [ADP−ribose] polymerase 11'

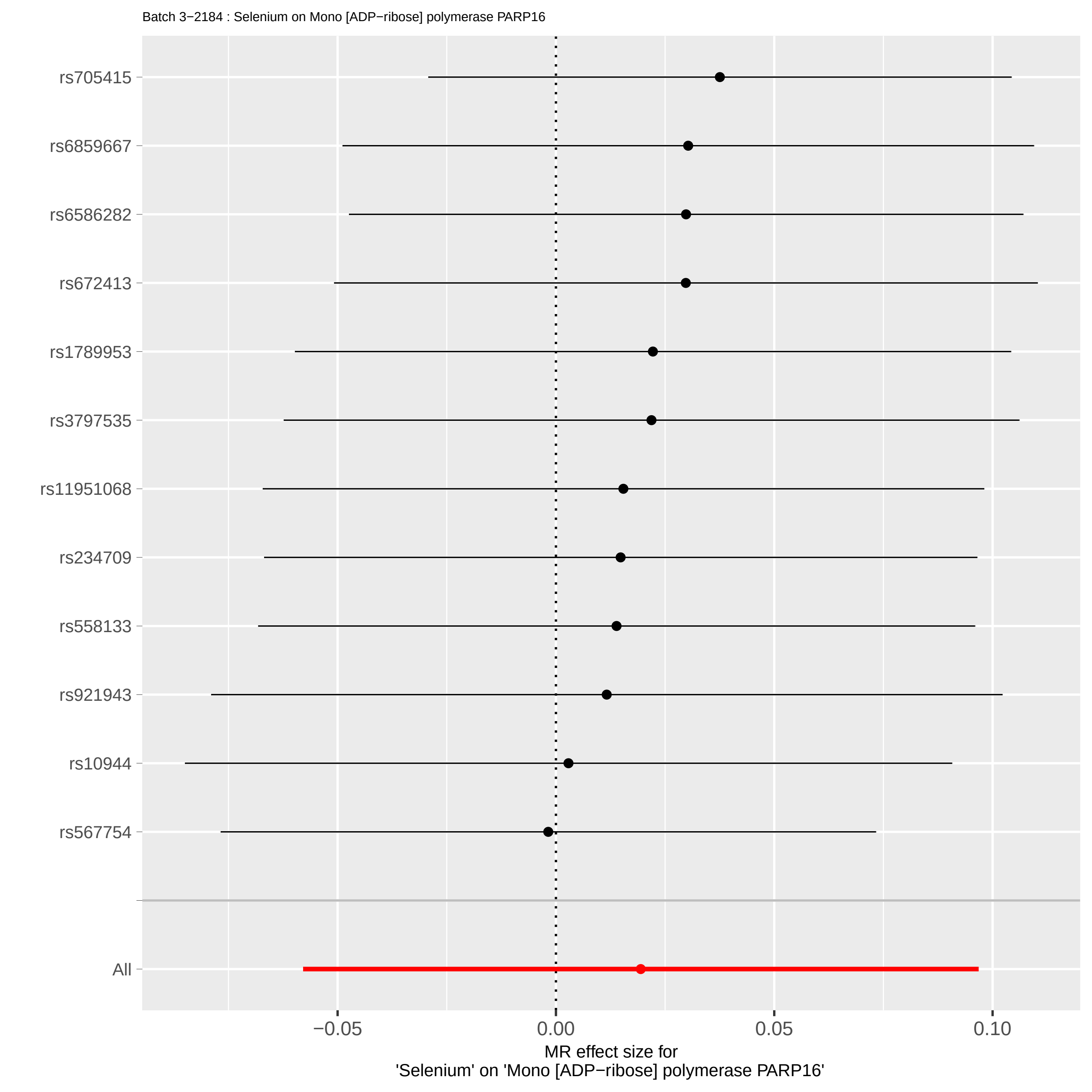

Batch 3−2184 : Selenium on Mono [ADP−ribose] polymerase PARP16
rs705415
rs6859667
rs6586282
rs672413
rs1789953
rs3797535
rs11951068
rs234709
rs558133
rs921943
rs10944
rs567754
All
−0.05
0.00
0.05
0.10
MR effect size for
'Selenium' on 'Mono [ADP−ribose] polymerase PARP16'

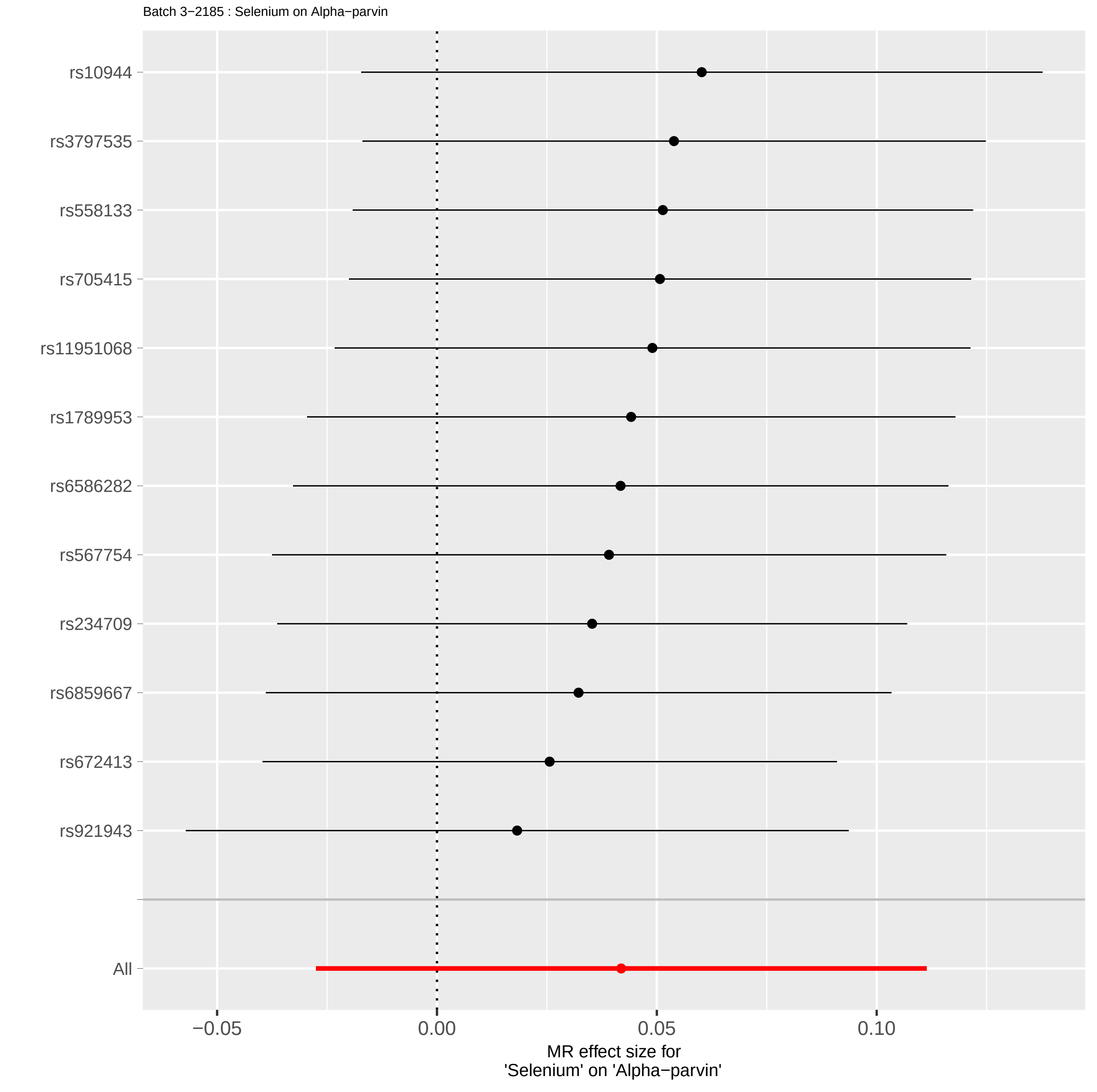

Batch 3−2185 : Selenium on Alpha−parvin
rs10944
rs3797535
rs558133
rs705415
rs11951068
rs1789953
rs6586282
rs567754
rs234709
rs6859667
rs672413
rs921943
All
−0.05
0.00
0.05
0.10
MR effect size for
'Selenium' on 'Alpha−parvin'

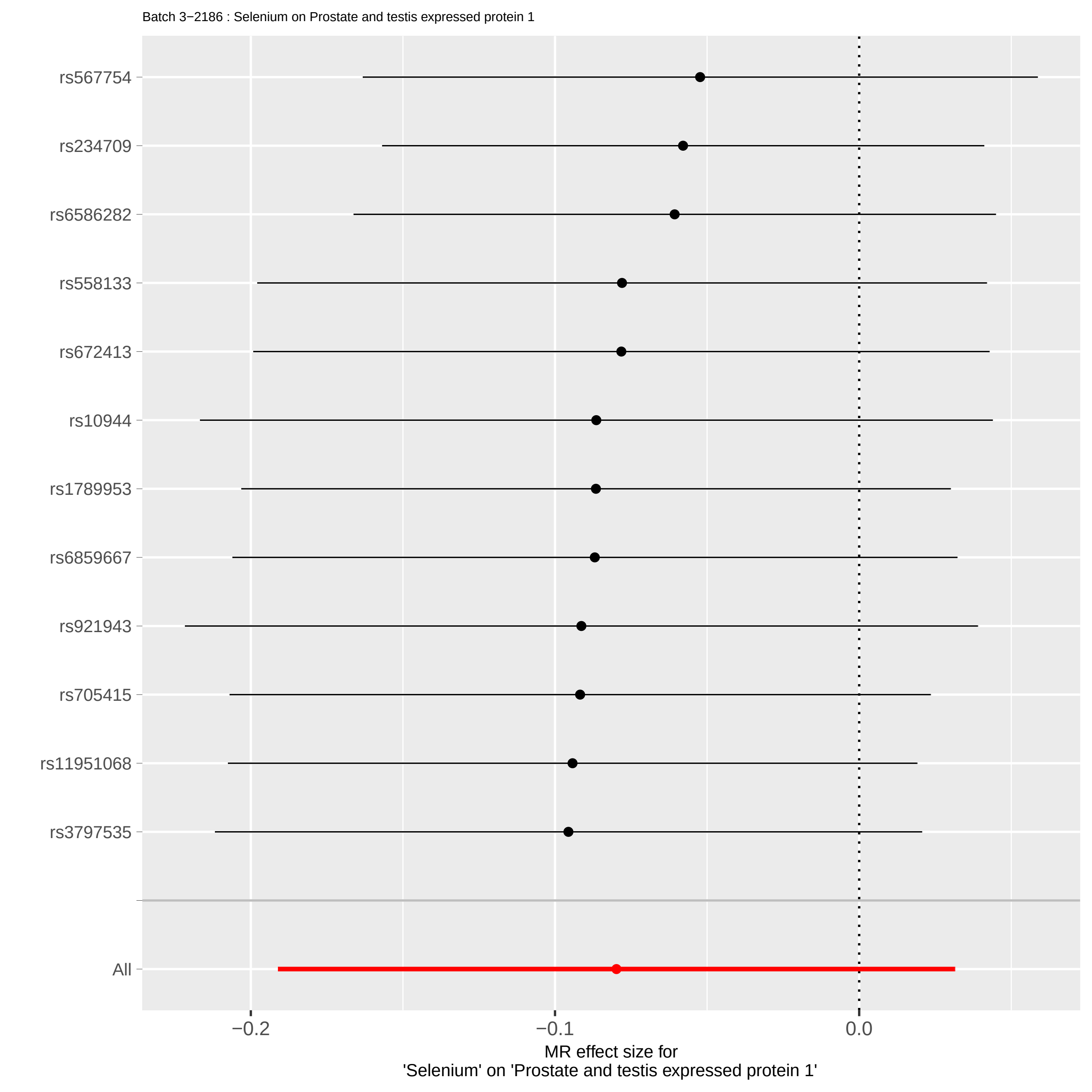
Batch 3−2186 : Selenium on Prostate and testis expressed protein 1
rs567754
rs234709
rs6586282
rs558133
rs672413
rs10944
rs1789953
rs6859667
rs921943
rs705415
rs11951068
rs3797535
All
−0.2
−0.1
0.0
MR effect size for
'Selenium' on 'Prostate and testis expressed protein 1'

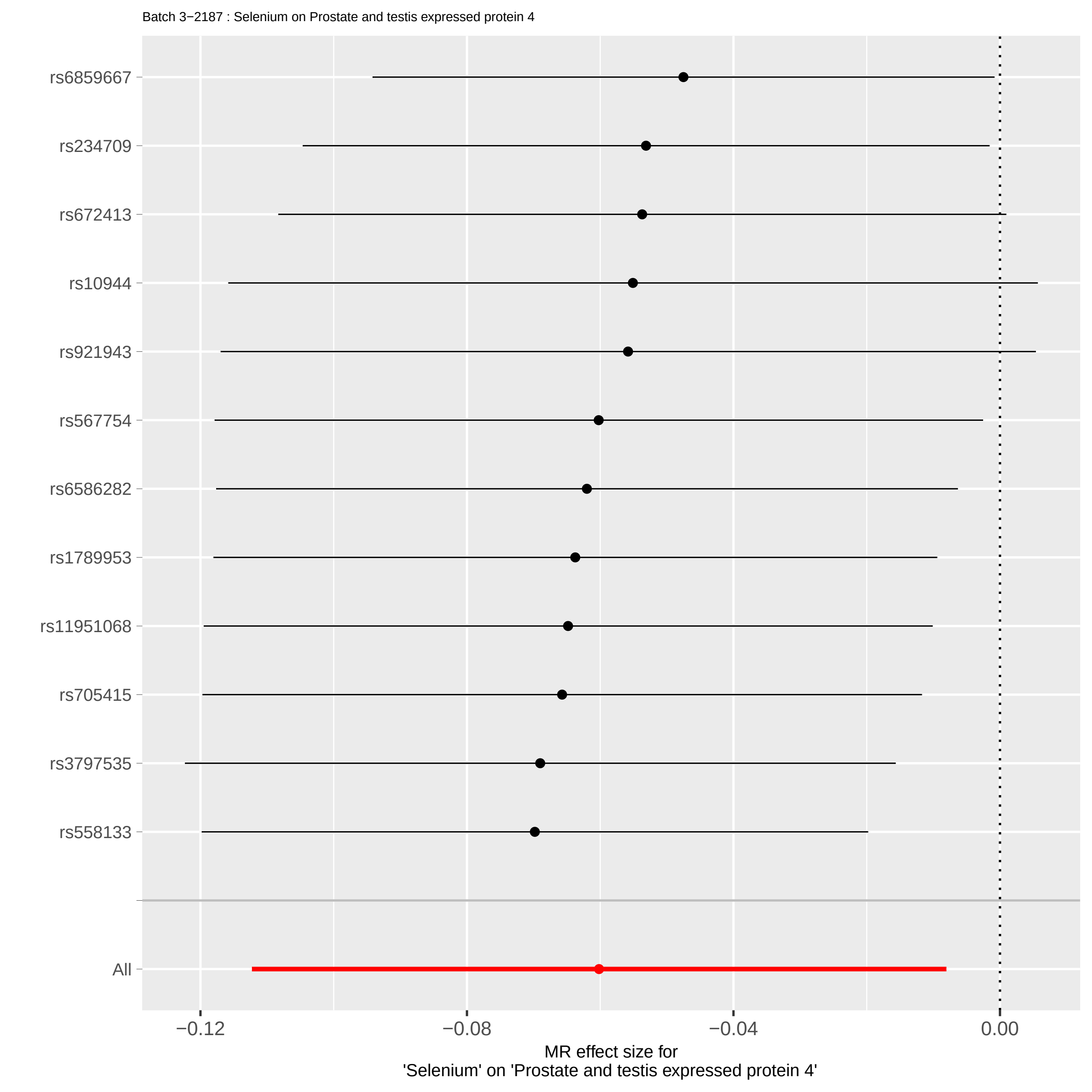

Batch 3−2187 : Selenium on Prostate and testis expressed protein 4
rs6859667
rs234709
rs672413
rs10944
rs921943
rs567754
rs6586282
rs1789953
rs11951068
rs705415
rs3797535
rs558133
All
−0.12
−0.08
−0.04
0.00
MR effect size for
'Selenium' on 'Prostate and testis expressed protein 4'

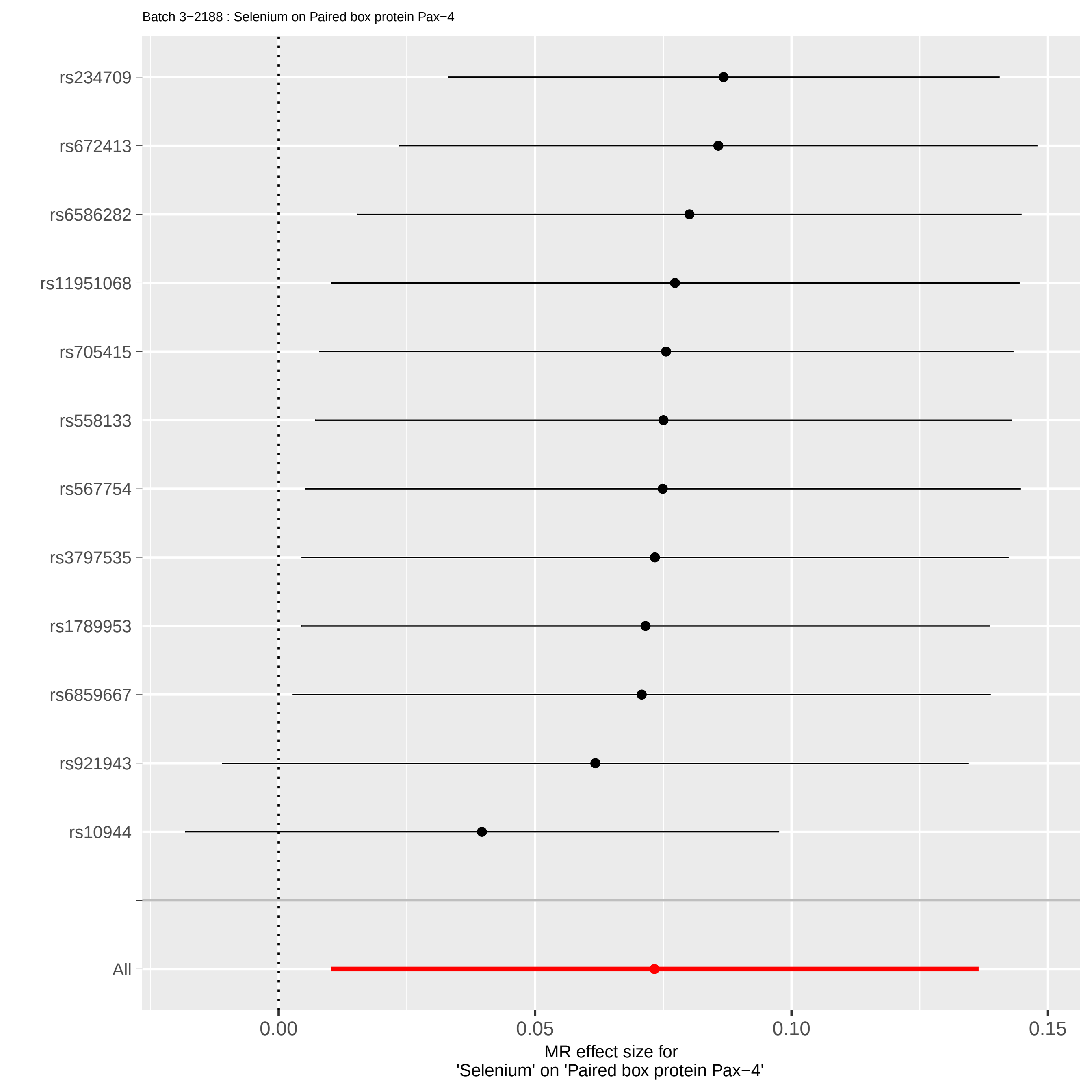

Batch 3−2188 : Selenium on Paired box protein Pax−4
rs234709
rs672413
rs6586282
rs11951068
rs705415
rs558133
rs567754
rs3797535
rs1789953
rs6859667
rs921943
rs10944
All
0.00
0.05
0.10
0.15
MR effect size for
'Selenium' on 'Paired box protein Pax−4'

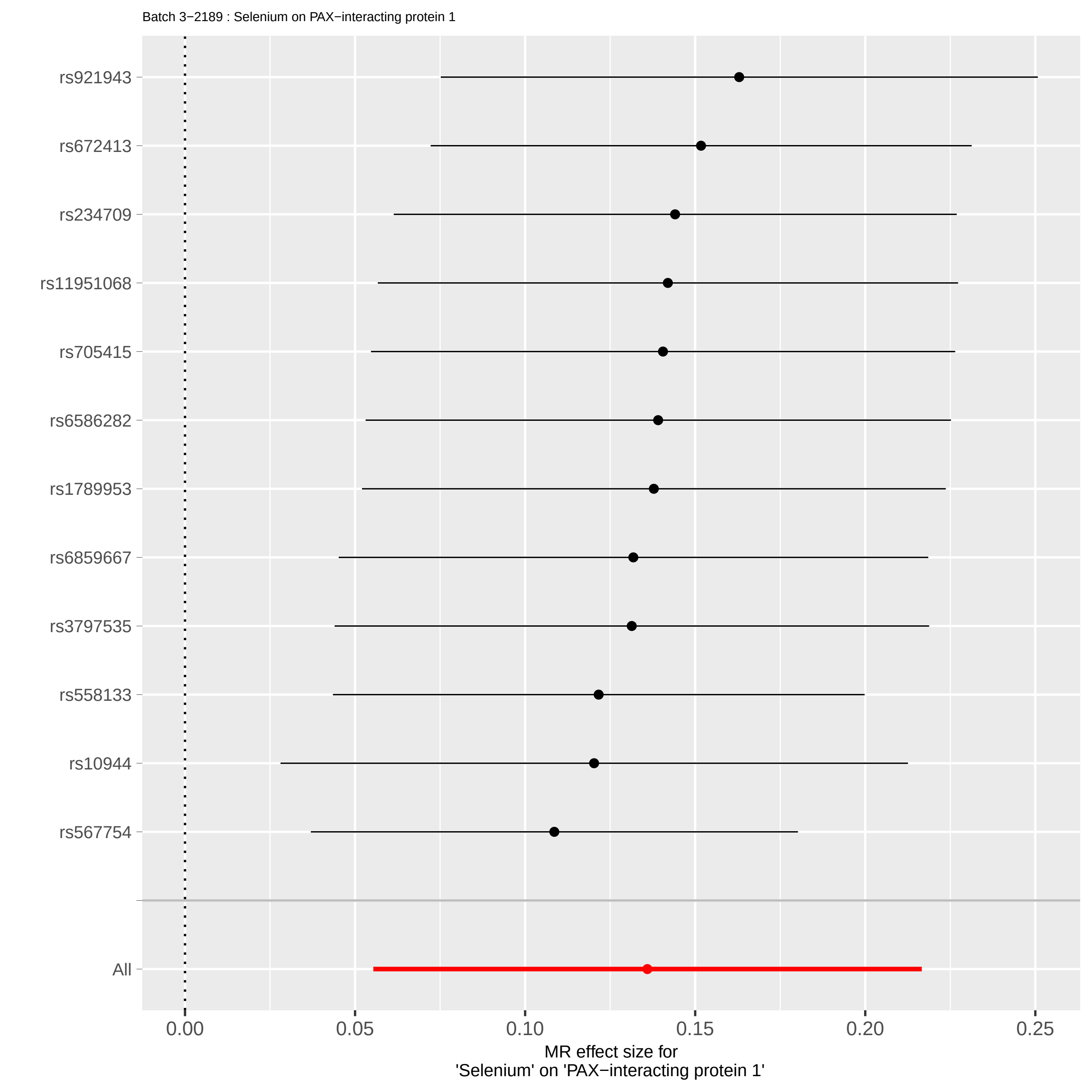
Batch 3−2189 : Selenium on PAX−interacting protein 1
rs921943
rs672413
rs234709
rs11951068
rs705415
rs6586282
rs1789953
rs6859667
rs3797535
rs558133
rs10944
rs567754
All
0.00
0.05
0.10
0.15
0.20
0.25
MR effect size for
'Selenium' on 'PAX−interacting protein 1'

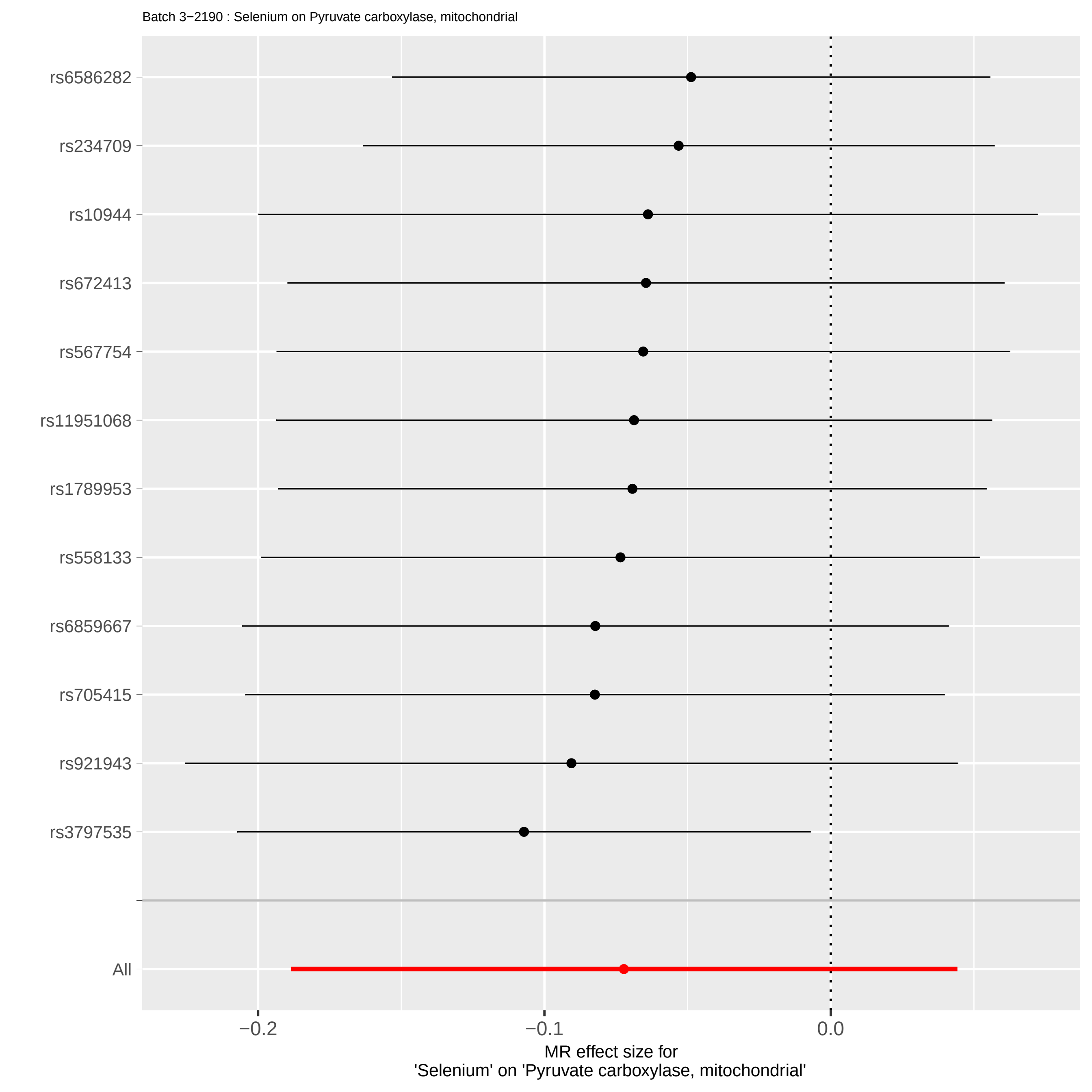

Batch 3−2190 : Selenium on Pyruvate carboxylase, mitochondrial
rs6586282
rs234709
rs10944
rs672413
rs567754
rs11951068
rs1789953
rs558133
rs6859667
rs705415
rs921943
rs3797535
All
−0.2
−0.1
0.0
MR effect size for
'Selenium' on 'Pyruvate carboxylase, mitochondrial'

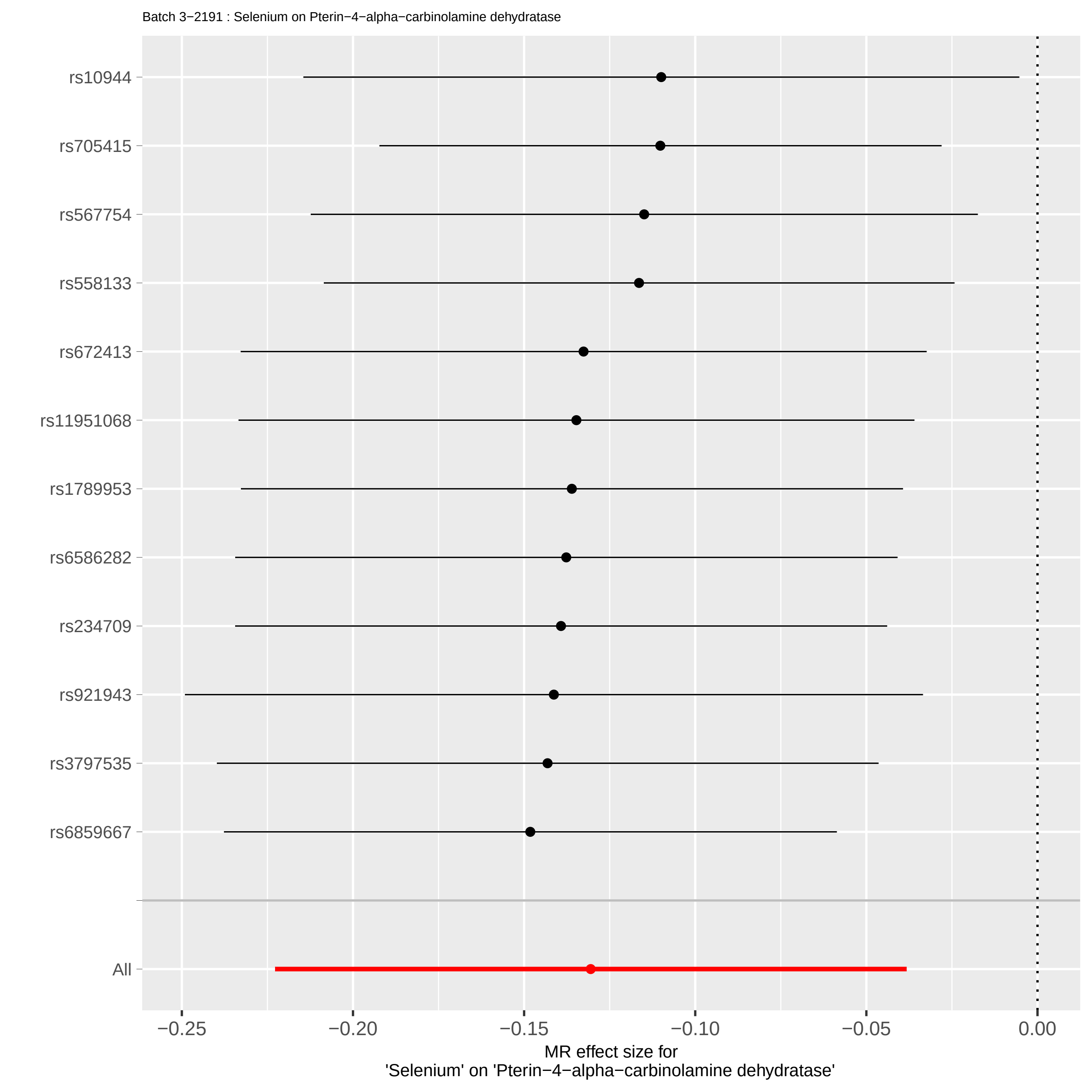

Batch 3−2191 : Selenium on Pterin−4−alpha−carbinolamine dehydratase
rs10944
rs705415
rs567754
rs558133
rs672413
rs11951068
rs1789953
rs6586282
rs234709
rs921943
rs3797535
rs6859667
All
−0.25
−0.20
−0.15
−0.10
−0.05
0.00
MR effect size for
'Selenium' on 'Pterin−4−alpha−carbinolamine dehydratase'

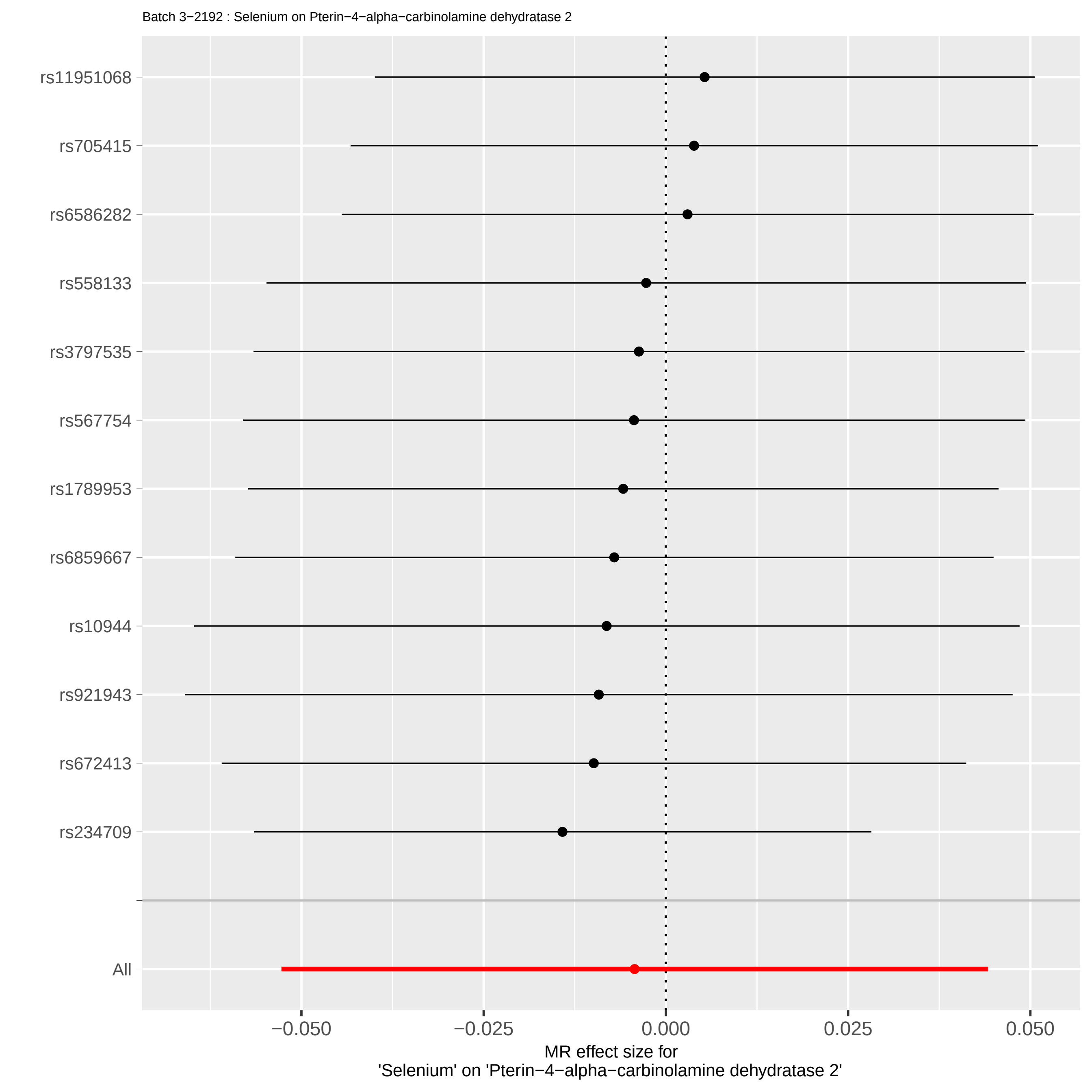

Batch 3−2192 : Selenium on Pterin−4−alpha−carbinolamine dehydratase 2
rs11951068
rs705415
rs6586282
rs558133
rs3797535
rs567754
rs1789953
rs6859667
rs10944
rs921943
rs672413
rs234709
All
−0.050
−0.025
0.000
0.025
0.050
MR effect size for
'Selenium' on 'Pterin−4−alpha−carbinolamine dehydratase 2'

Batch 3−2193 : Selenium on Poly(rC)−binding protein 1

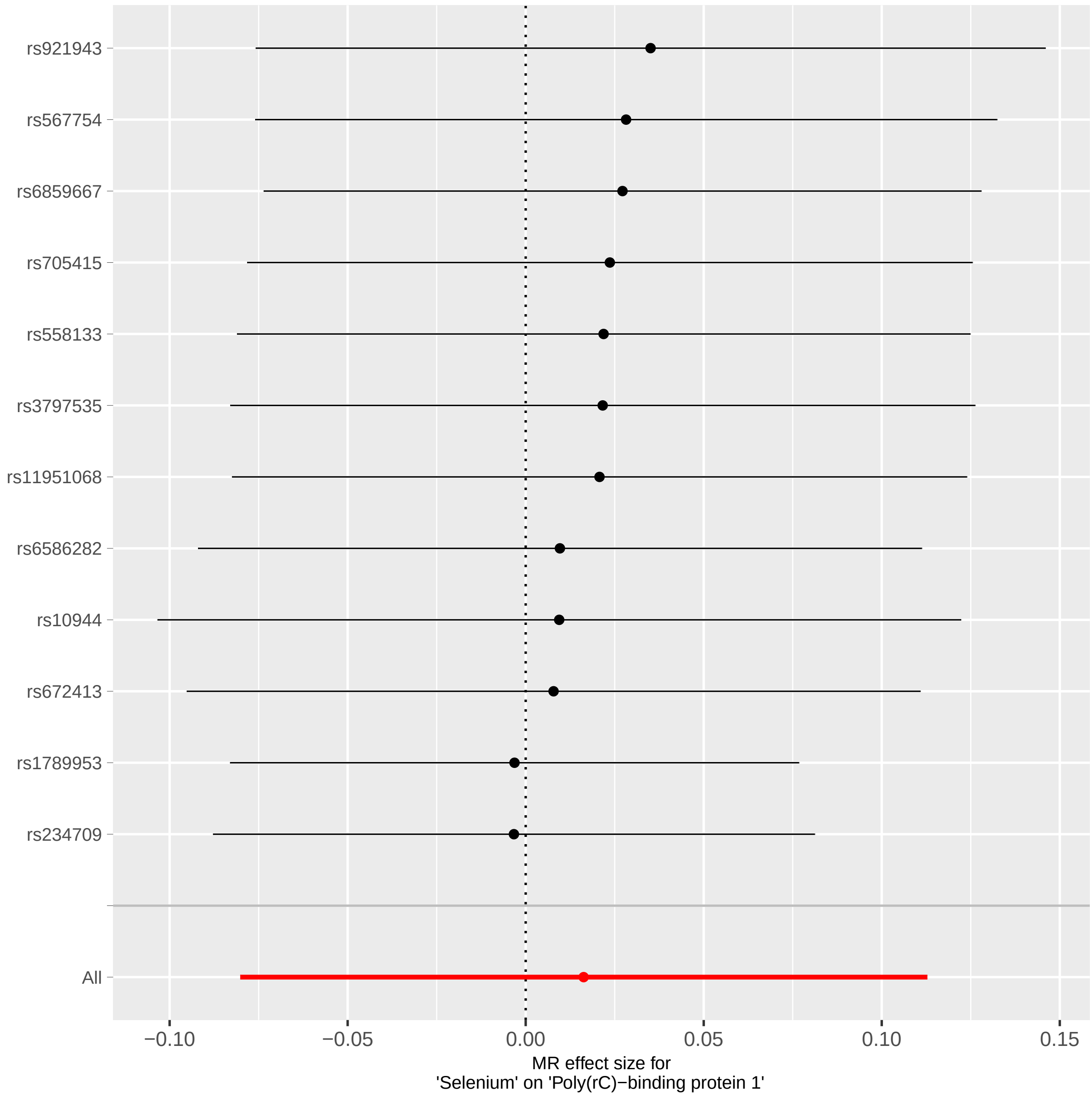

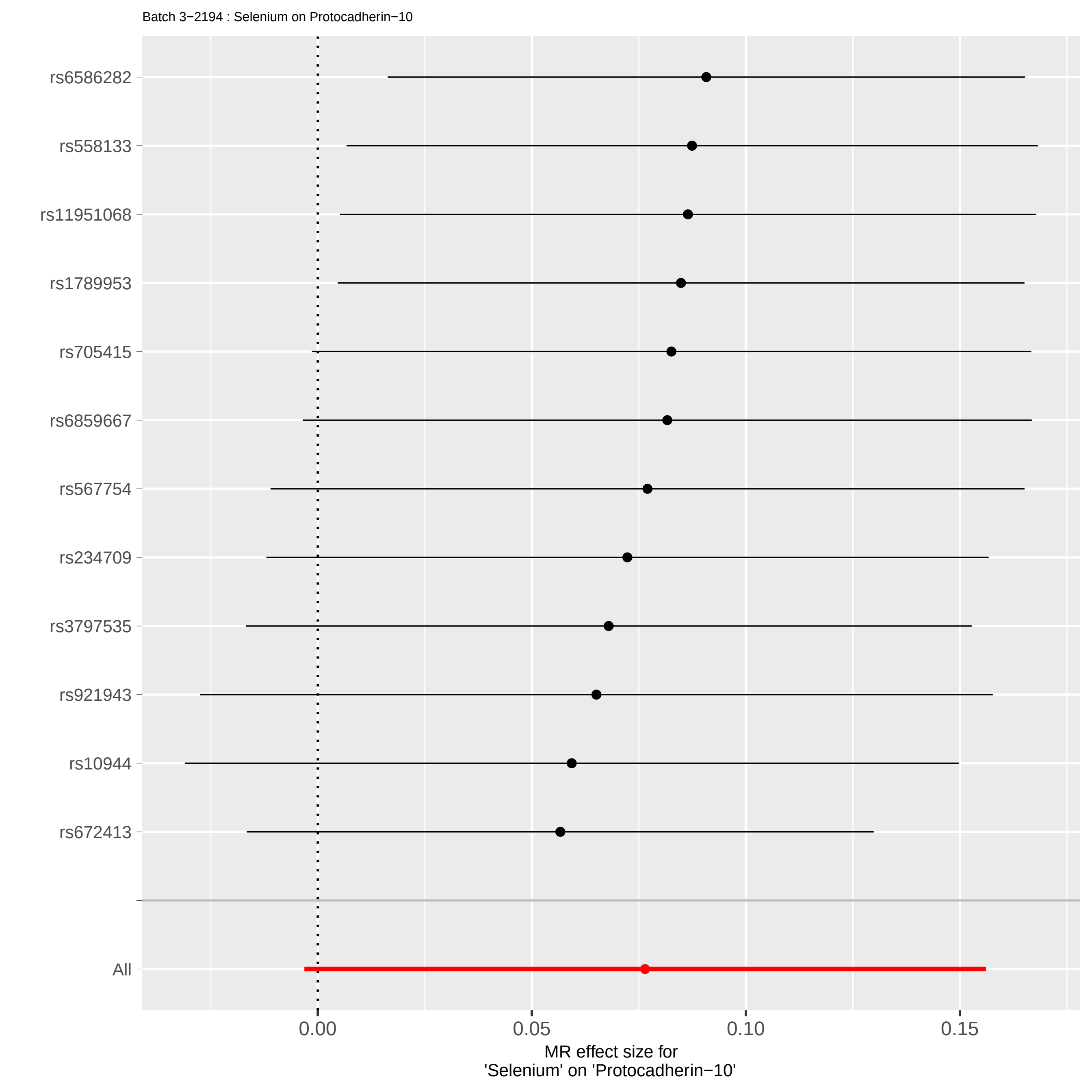

Batch 3−2194 : Selenium on Protocadherin−10
rs6586282
rs558133
rs11951068
rs1789953
rs705415
rs6859667
rs567754
rs234709
rs3797535
rs921943
rs10944
rs672413
All
0.00
0.05
0.10
0.15
MR effect size for
'Selenium' on 'Protocadherin−10'

Batch 3−2195 : Selenium on Protocadherin−10

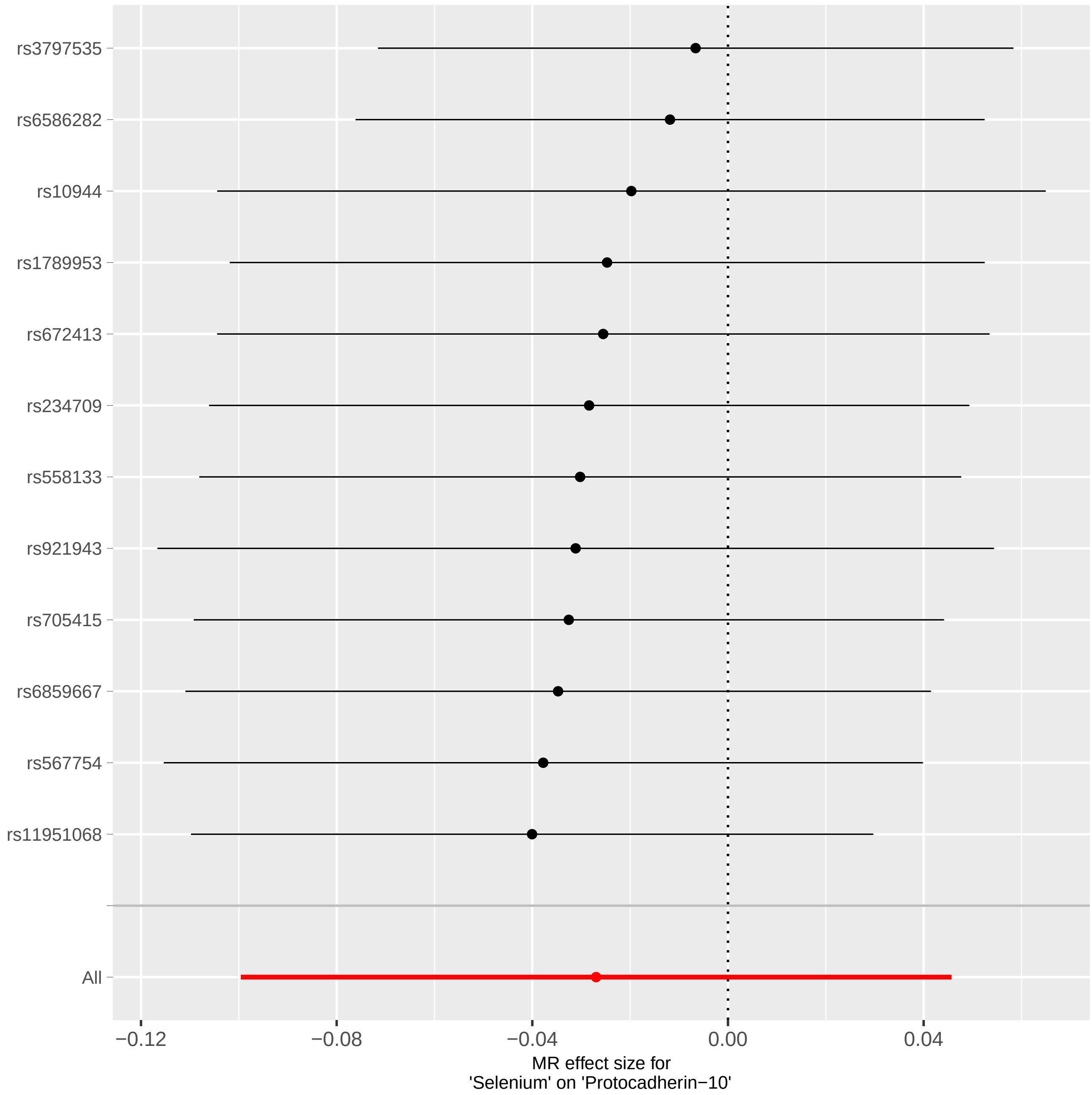

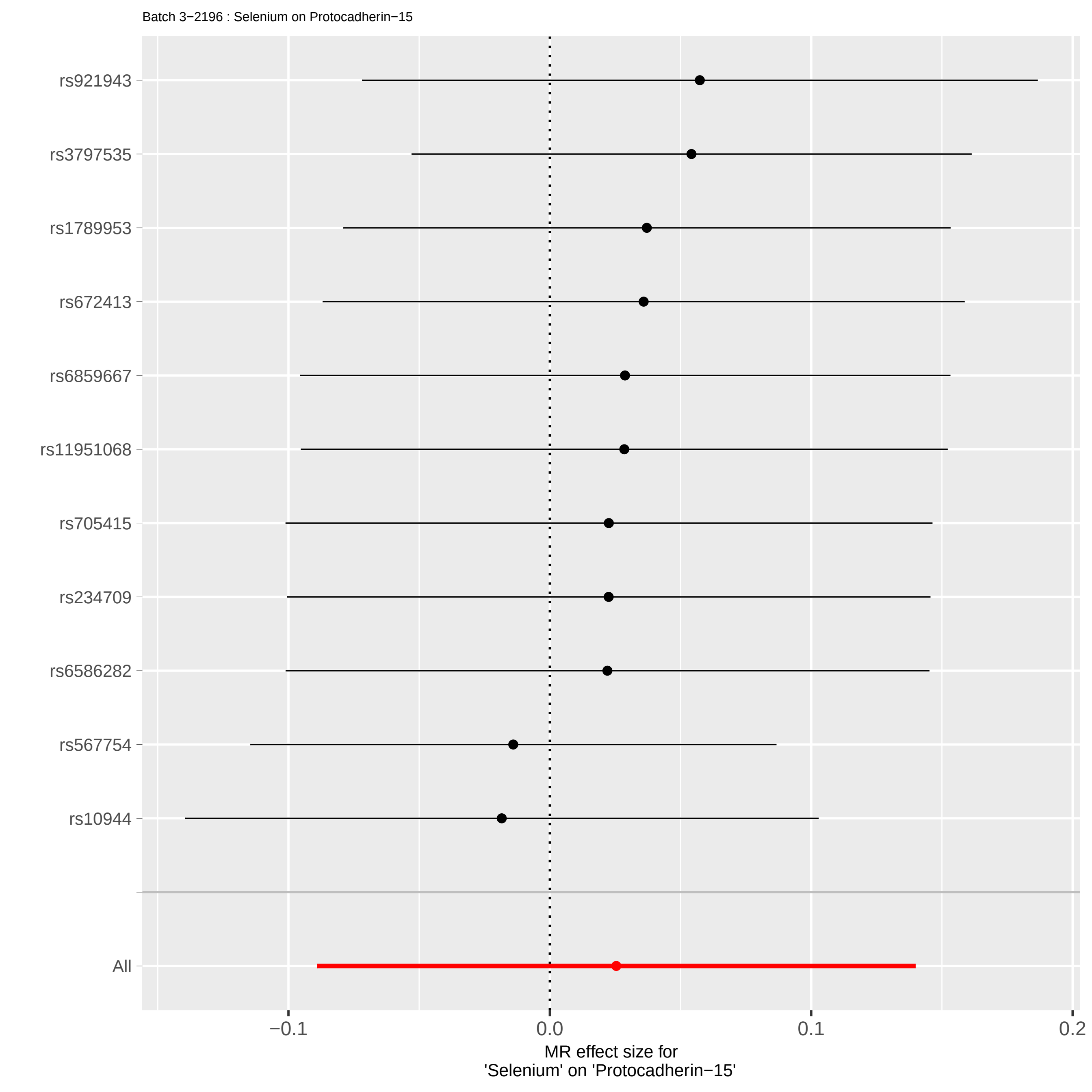

Batch 3−2196 : Selenium on Protocadherin−15
rs921943
rs3797535
rs1789953
rs672413
rs6859667
rs11951068
rs705415
rs234709
rs6586282
rs567754
rs10944
All
−0.1
0.0
0.1
0.2
MR effect size for
'Selenium' on 'Protocadherin−15'

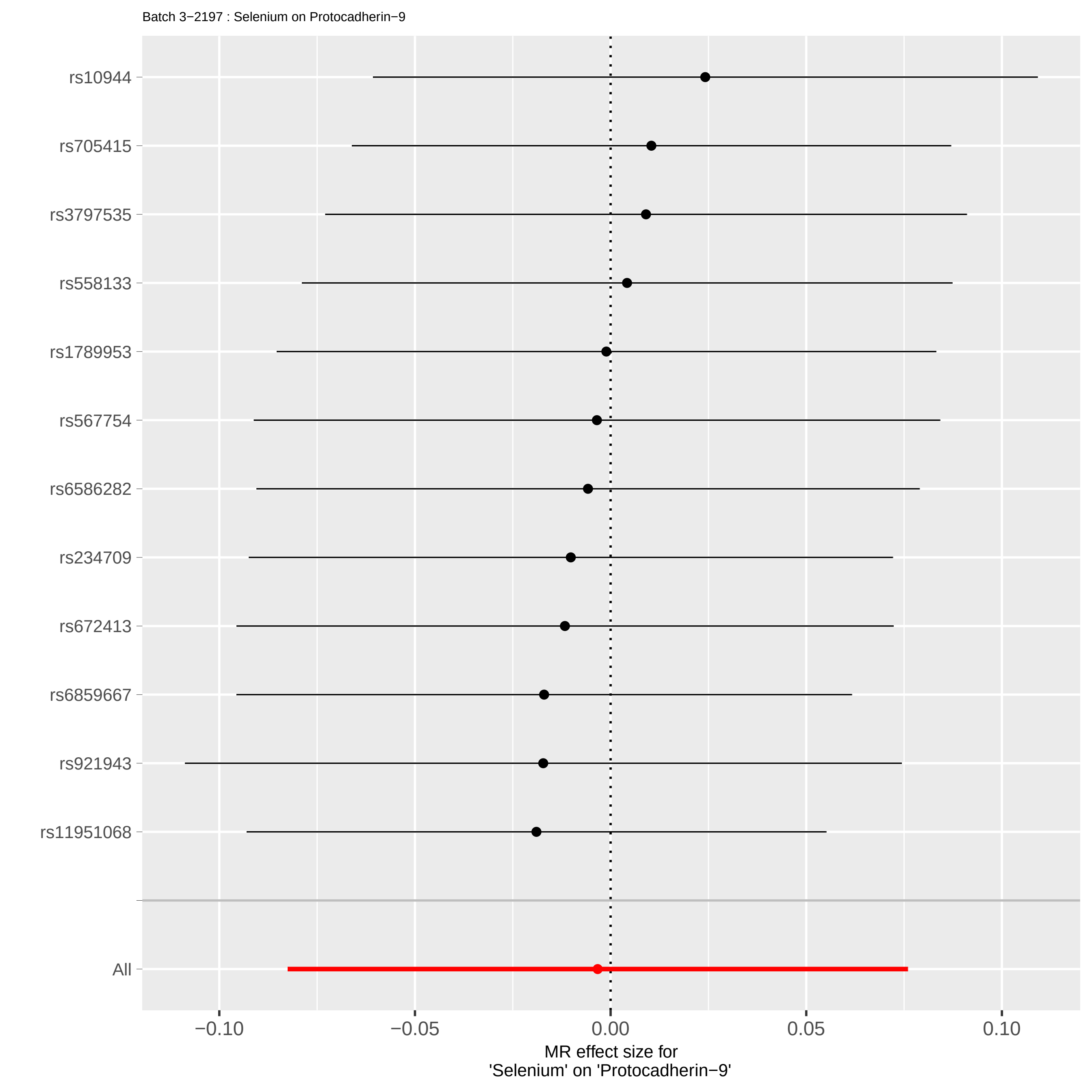

Batch 3−2197 : Selenium on Protocadherin−9
rs10944
rs705415
rs3797535
rs558133
rs1789953
rs567754
rs6586282
rs234709
rs672413
rs6859667
rs921943
rs11951068
All
−0.10
−0.05
0.00
0.05
0.10
MR effect size for
'Selenium' on 'Protocadherin−9'

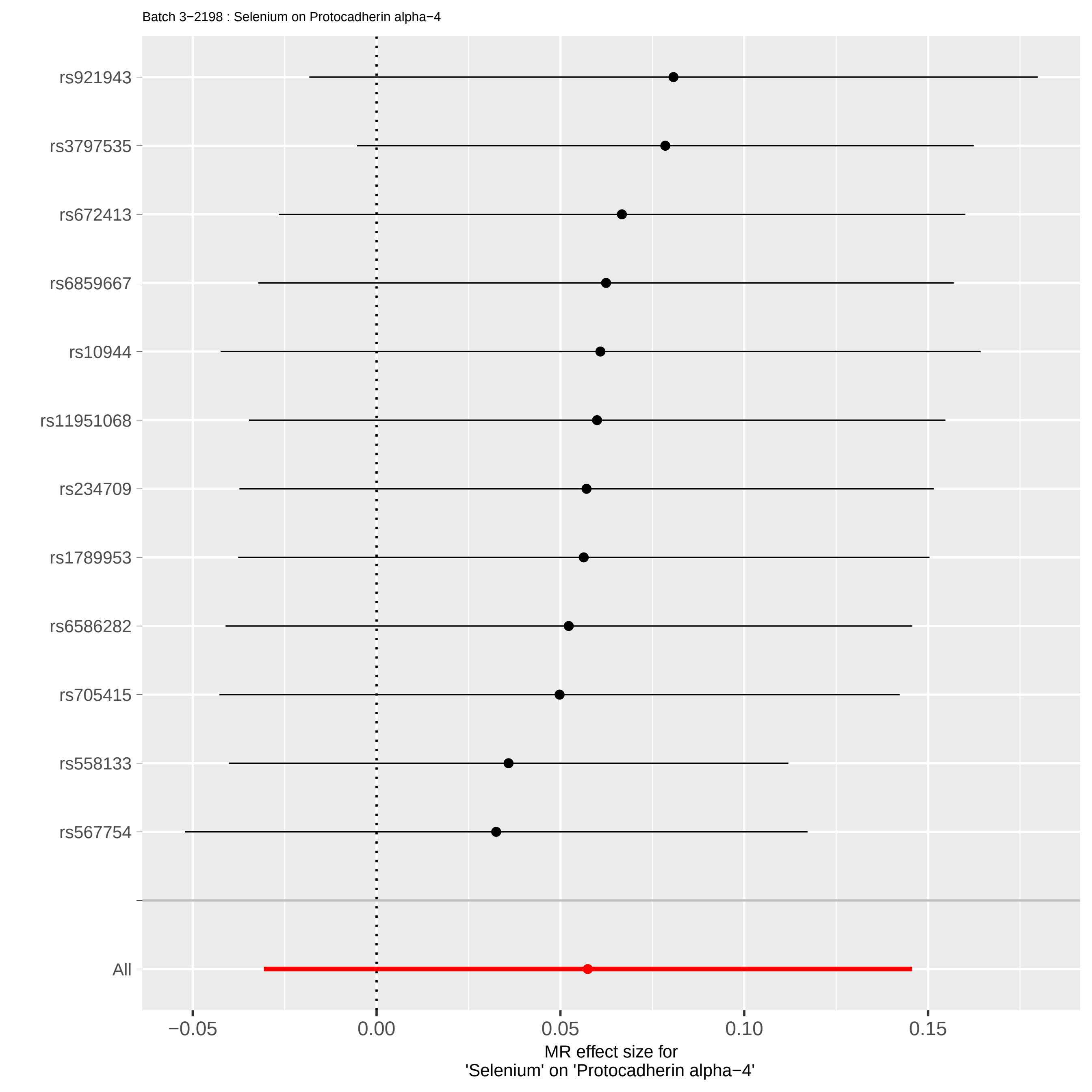

Batch 3−2198 : Selenium on Protocadherin alpha−4
rs921943
rs3797535
rs672413
rs6859667
rs10944
rs11951068
rs234709
rs1789953
rs6586282
rs705415
rs558133
rs567754
All
−0.05
0.00
0.05
0.10
0.15
MR effect size for
'Selenium' on 'Protocadherin alpha−4'

Batch 3−2199 : Selenium on Protocadherin alpha−4

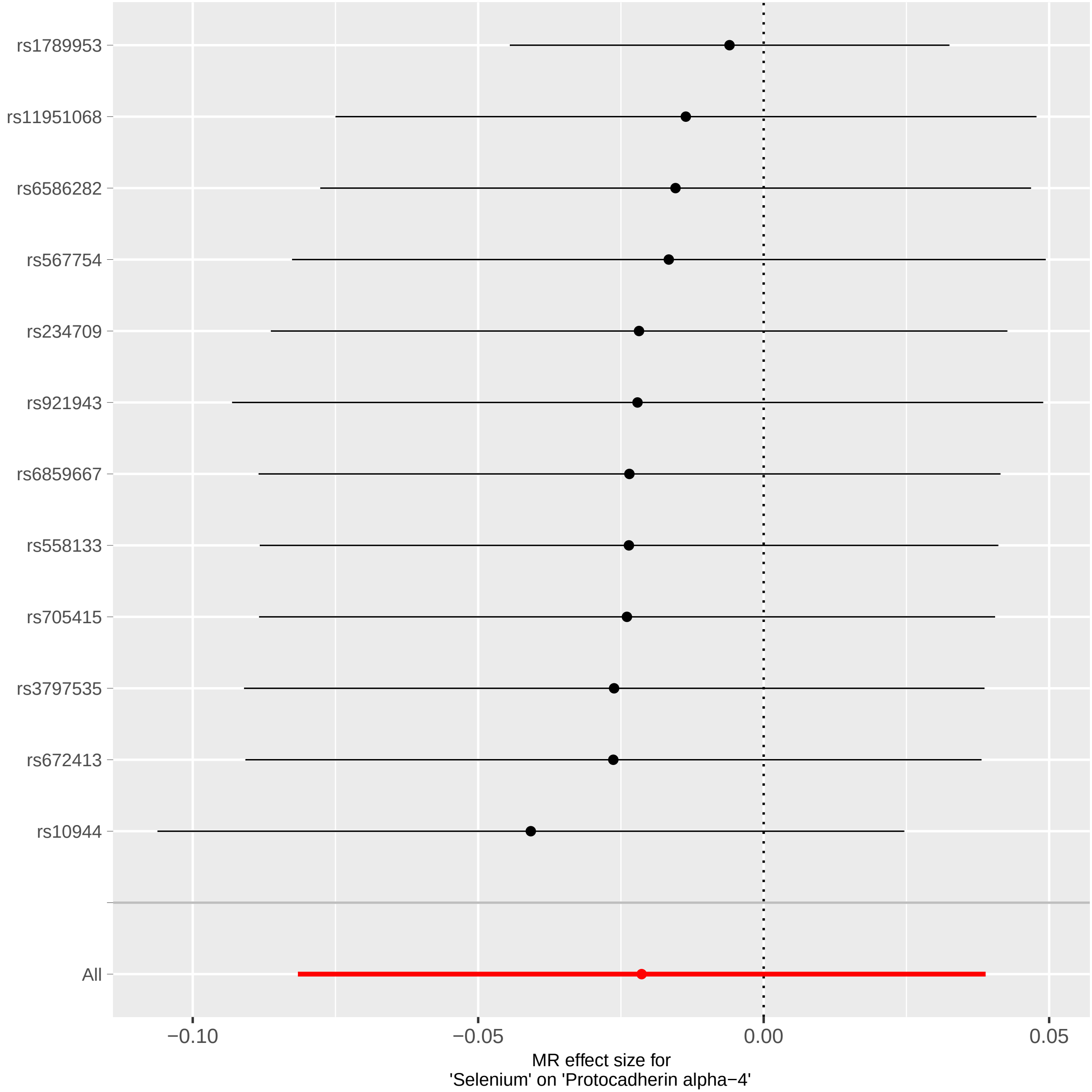

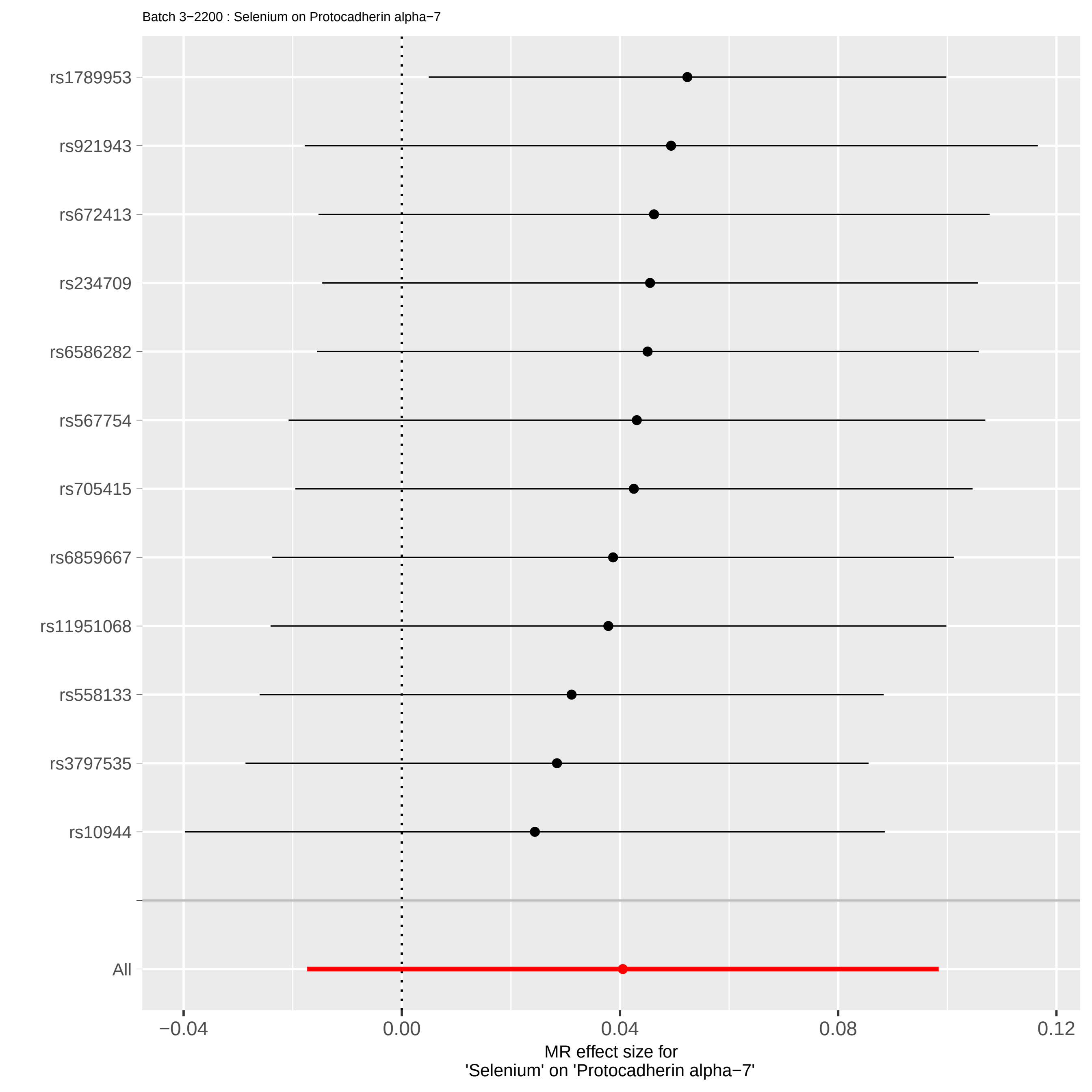

Batch 3−2200 : Selenium on Protocadherin alpha−7
rs1789953
rs921943
rs672413
rs234709
rs6586282
rs567754
rs705415
rs6859667
rs11951068
rs558133
rs3797535
rs10944
All
−0.04
0.00
0.04
0.08
0.12
MR effect size for
'Selenium' on 'Protocadherin alpha−7'

Batch 3−2201 : Selenium on Protocadherin alpha−C2

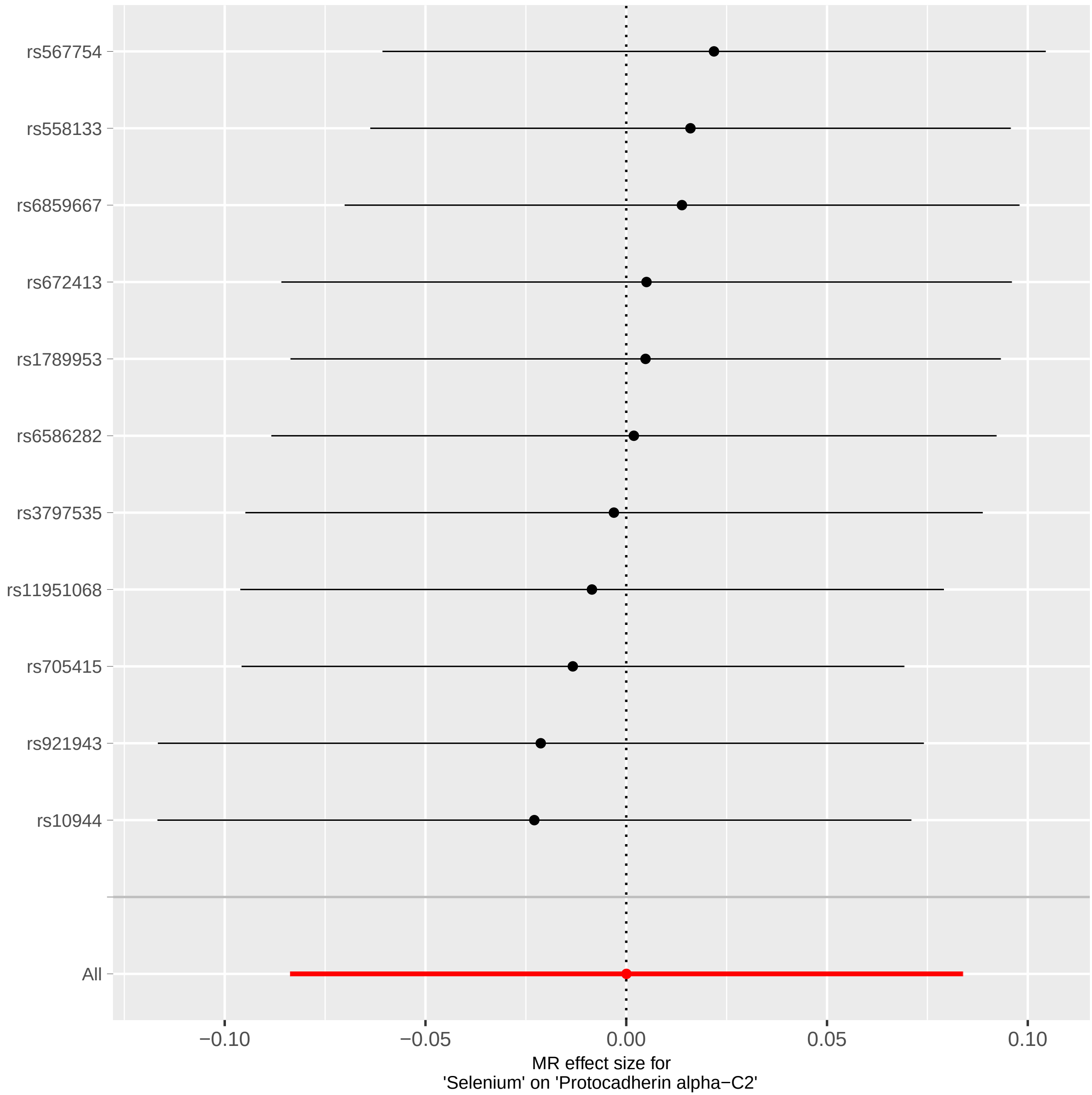

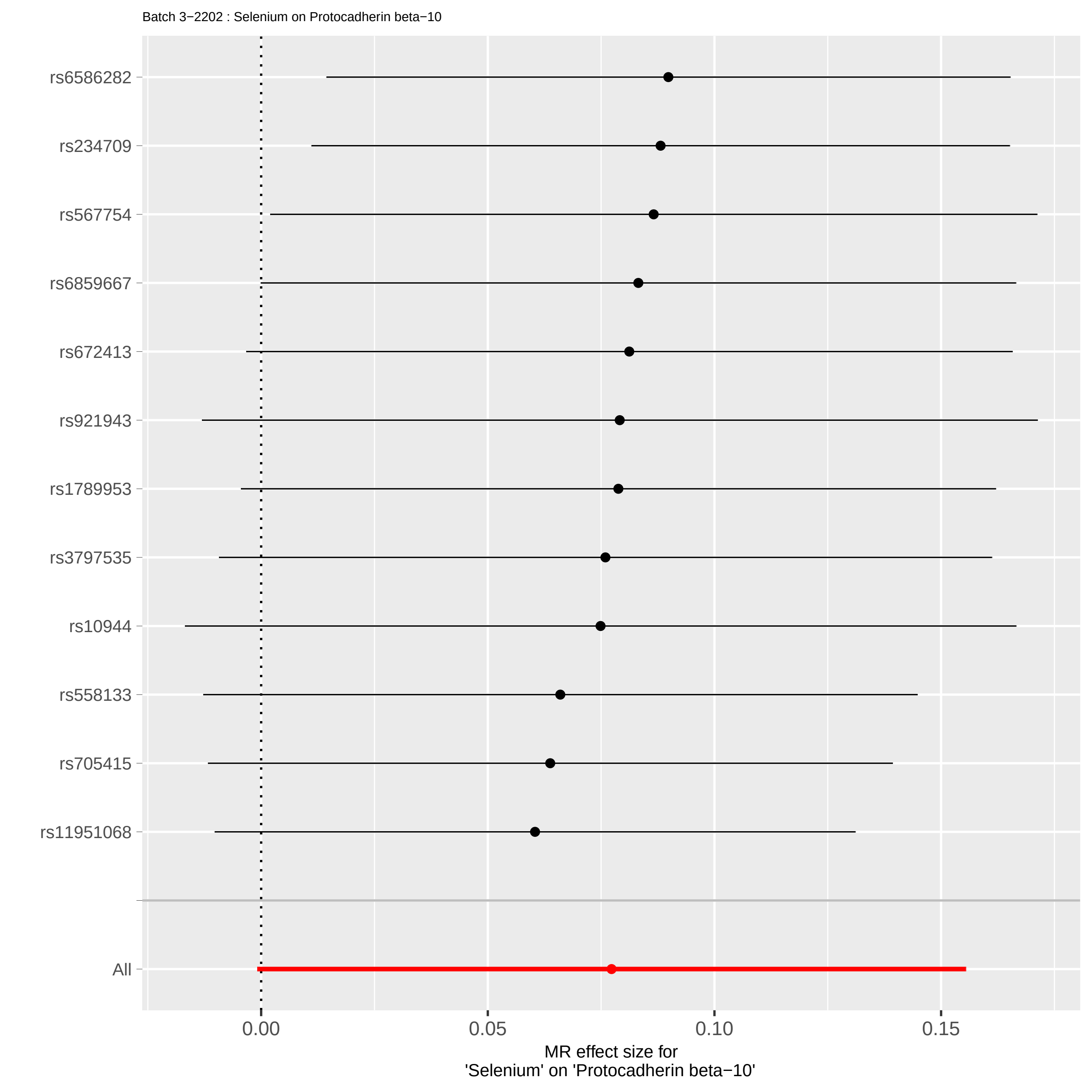
Batch 3−2202 : Selenium on Protocadherin beta−10
rs6586282
rs234709
rs567754
rs6859667
rs672413
rs921943
rs1789953
rs3797535
rs10944
rs558133
rs705415
rs11951068
All
0.00
0.05
0.10
0.15
MR effect size for
'Selenium' on 'Protocadherin beta−10'

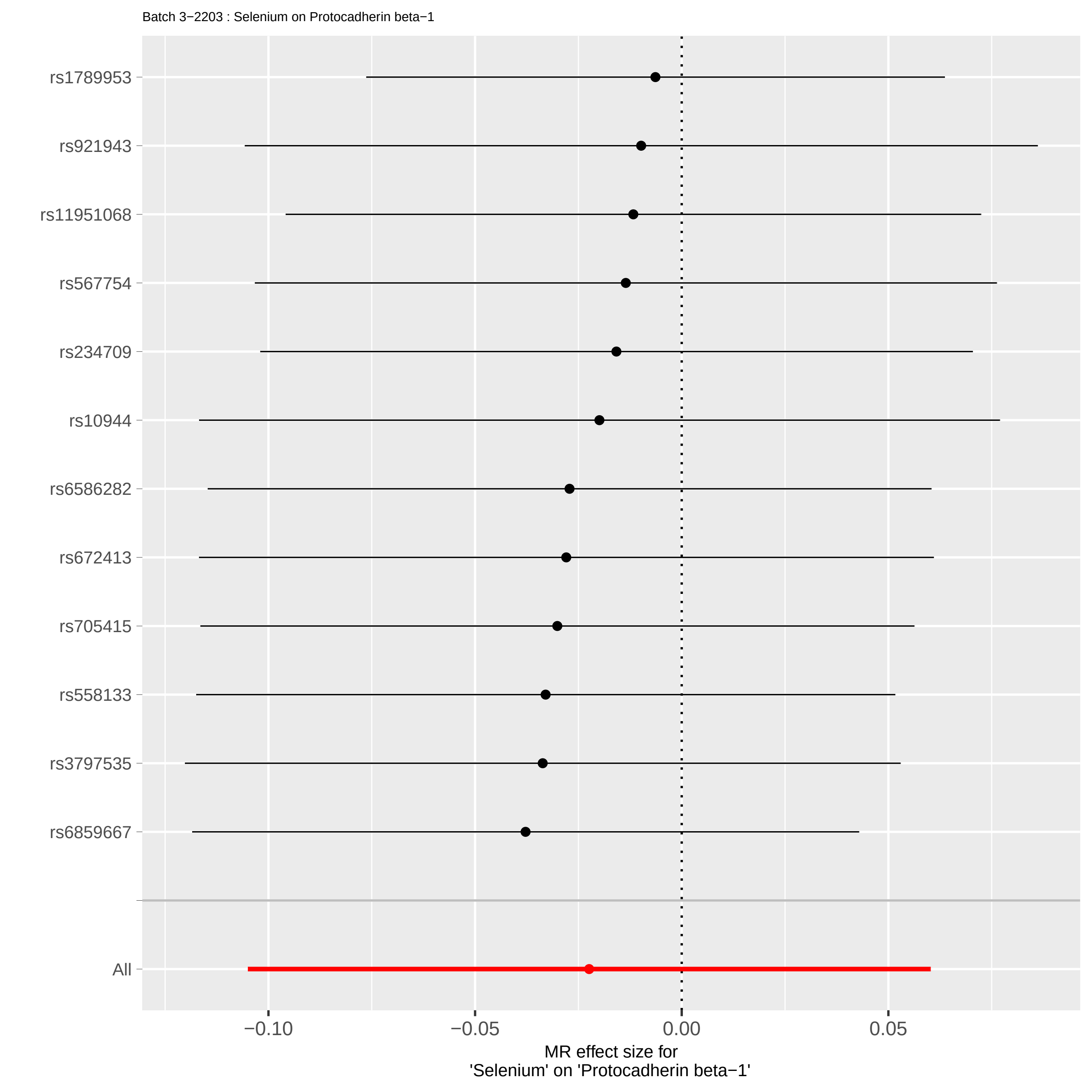

Batch 3−2203 : Selenium on Protocadherin beta−1
rs1789953
rs921943
rs11951068
rs567754
rs234709
rs10944
rs6586282
rs672413
rs705415
rs558133
rs3797535
rs6859667
All
−0.10
−0.05
0.00
0.05
MR effect size for
'Selenium' on 'Protocadherin beta−1'

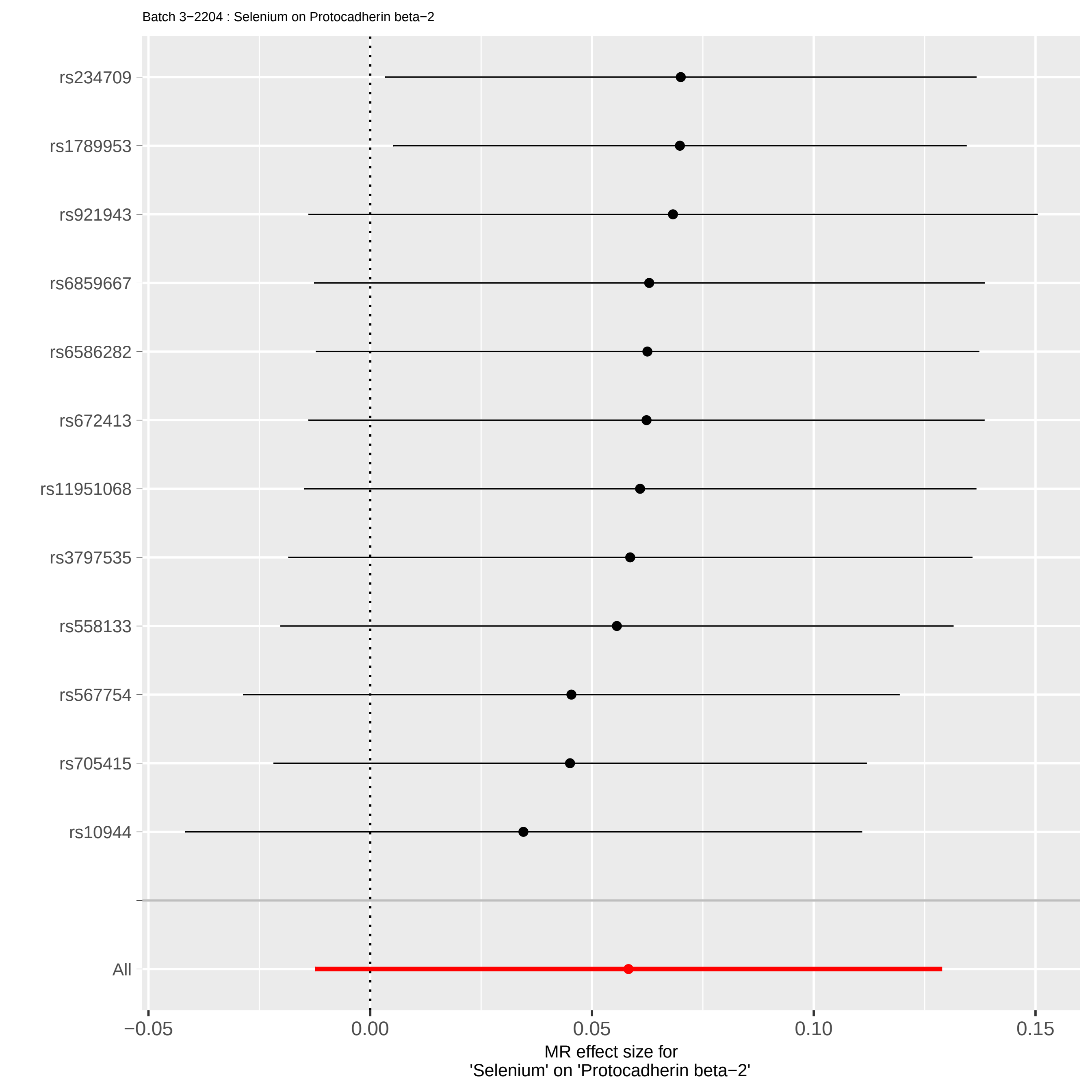
Batch 3−2204 : Selenium on Protocadherin beta−2
rs234709
rs1789953
rs921943
rs6859667
rs6586282
rs672413
rs11951068
rs3797535
rs558133
rs567754
rs705415
rs10944
All
−0.05
0.00
0.05
0.10
0.15
MR effect size for
'Selenium' on 'Protocadherin beta−2'

Batch 3−2205 : Selenium on Protocadherin beta−4

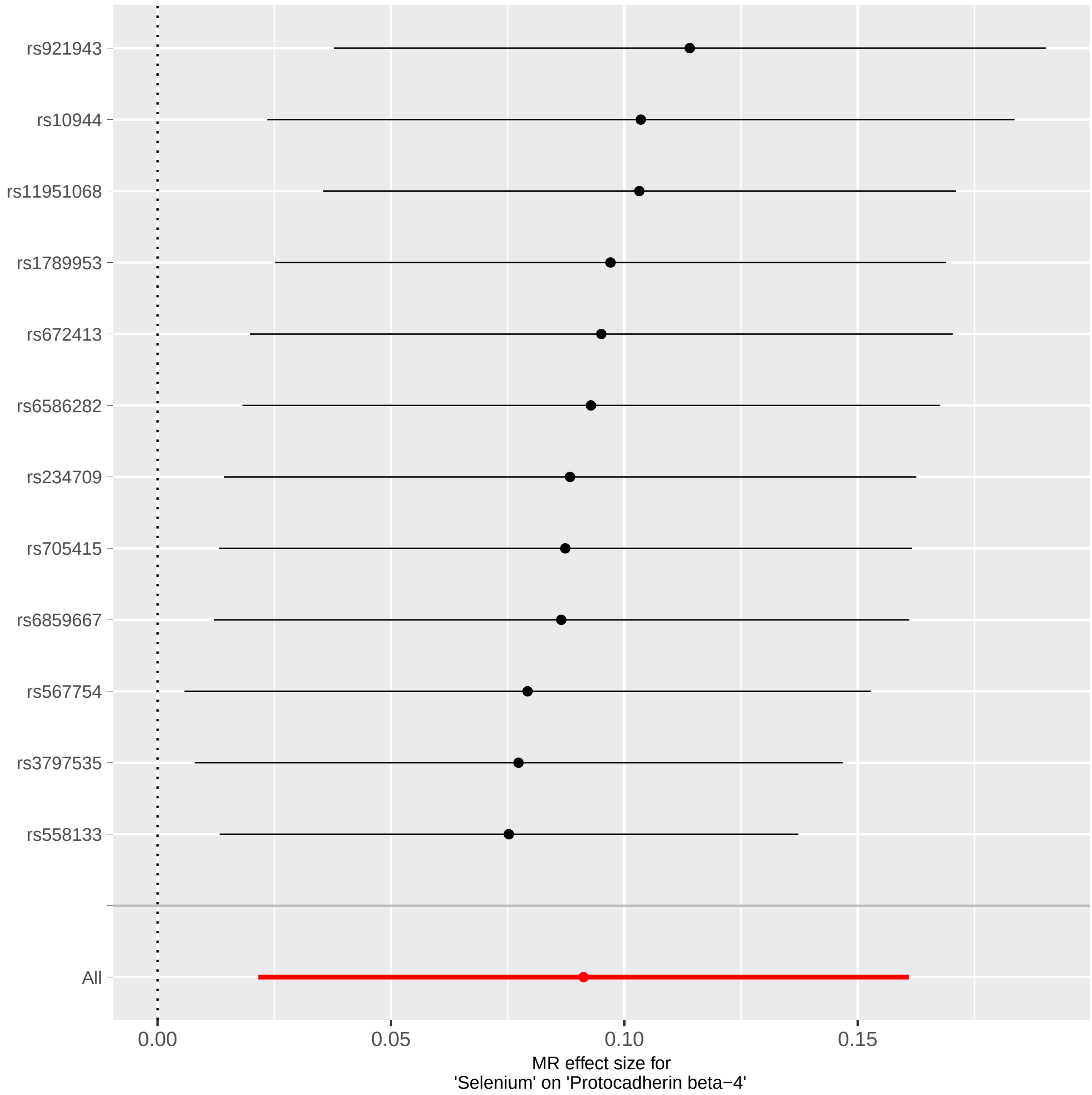

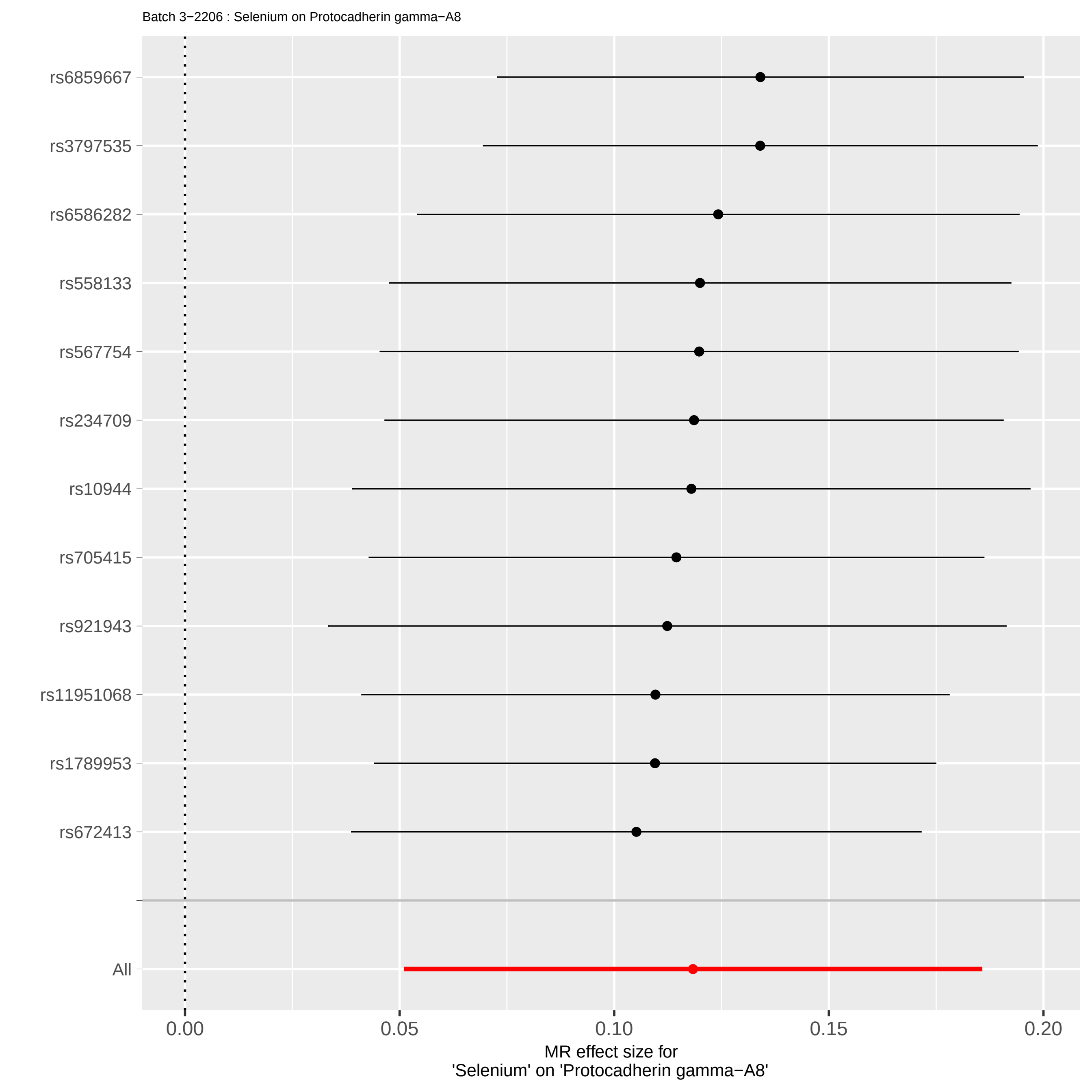
Batch 3−2206 : Selenium on Protocadherin gamma−A8
rs6859667
rs3797535
rs6586282
rs558133
rs567754
rs234709
rs10944
rs705415
rs921943
rs11951068
rs1789953
rs672413
All
0.00
0.05
0.10
0.15
0.20
MR effect size for
'Selenium' on 'Protocadherin gamma−A8'

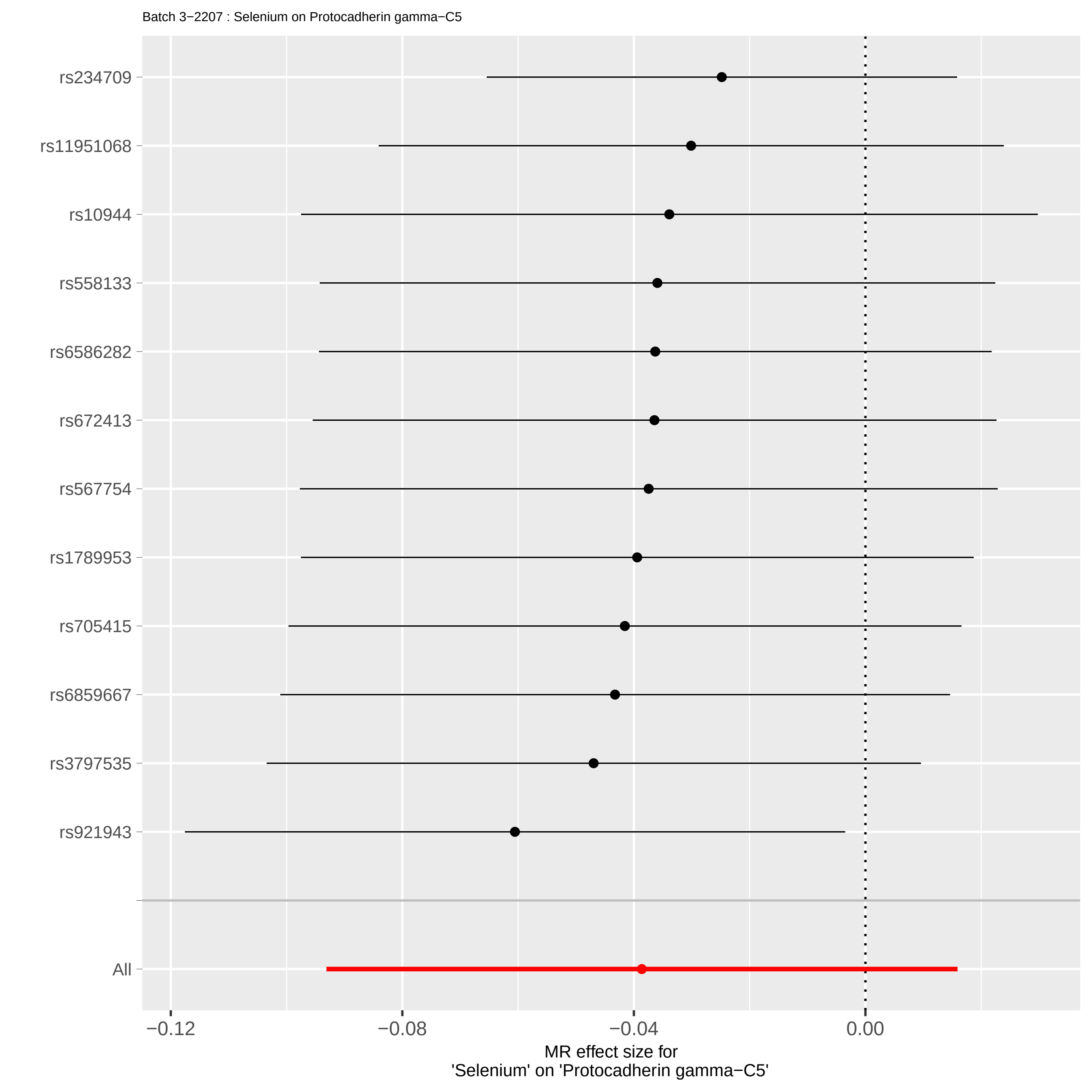

Batch 3−2207 : Selenium on Protocadherin gamma−C5
rs234709
rs11951068
rs10944
rs558133
rs6586282
rs672413
rs567754
rs1789953
rs705415
rs6859667
rs3797535
rs921943
All
−0.12
−0.08
−0.04
0.00
MR effect size for
'Selenium' on 'Protocadherin gamma−C5'

Batch 3−2208 : Selenium on Proliferating cell nuclear antigen

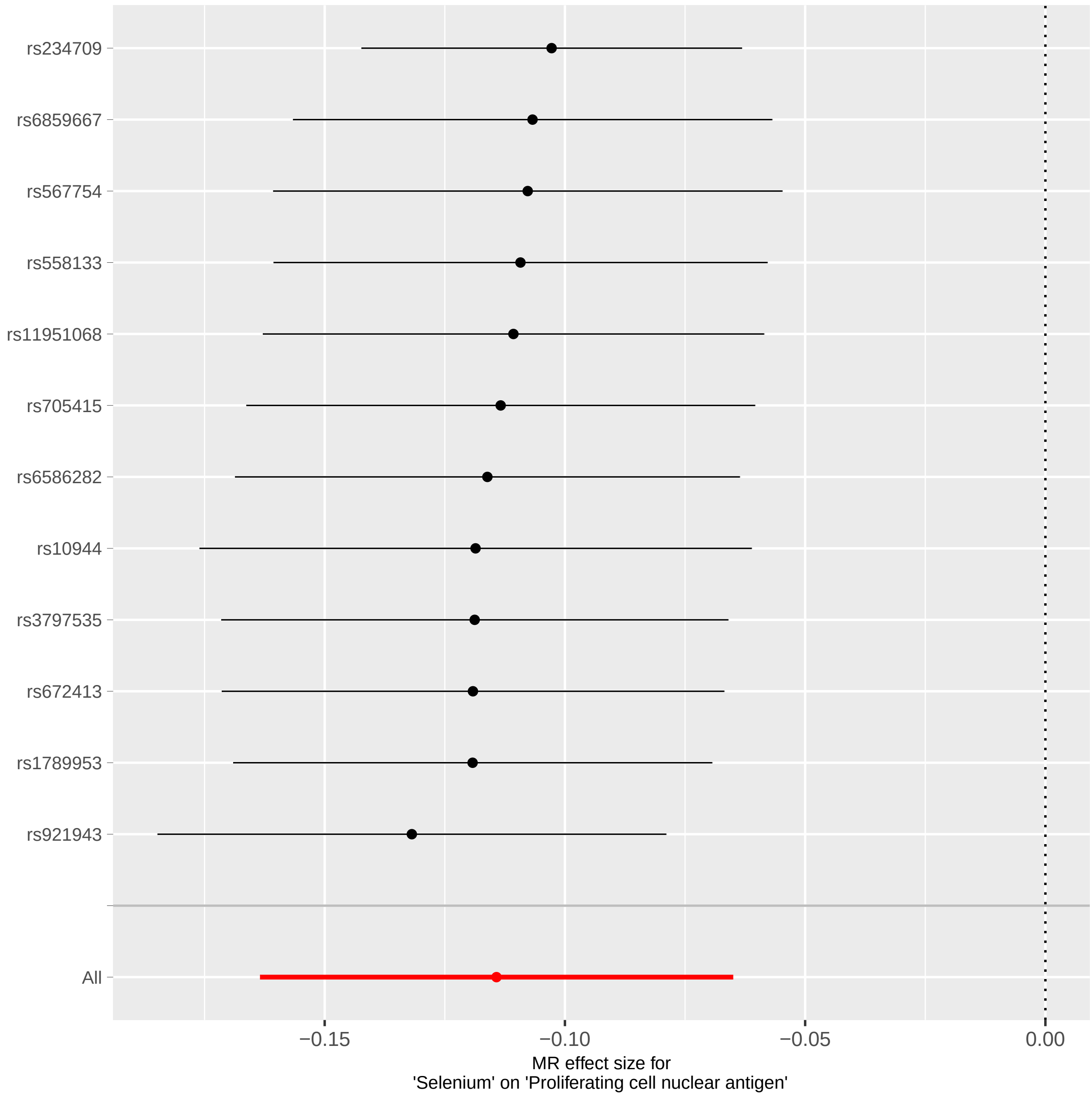

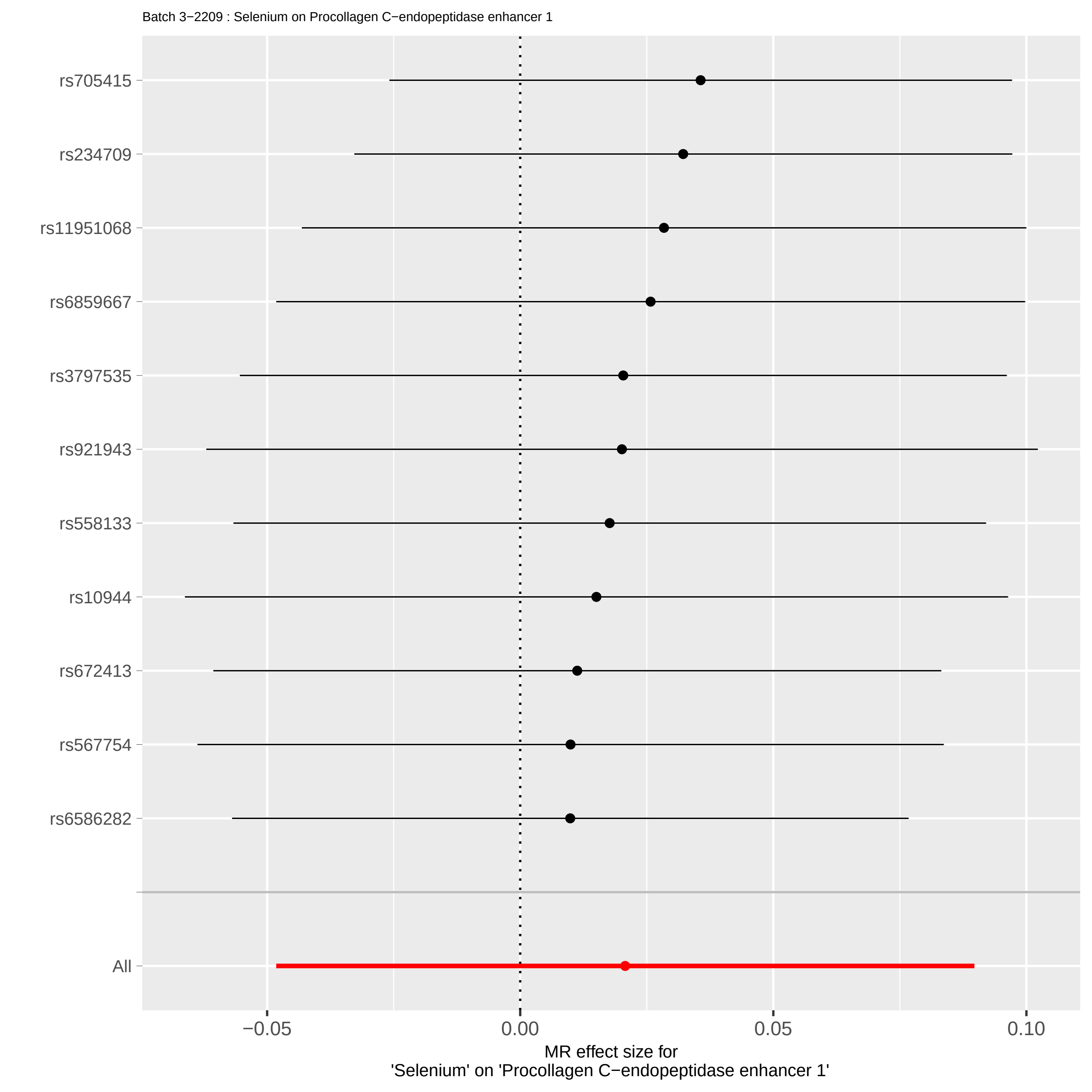
Batch 3−2209 : Selenium on Procollagen C−endopeptidase enhancer 1
rs705415
rs234709
rs11951068
rs6859667
rs3797535
rs921943
rs558133
rs10944
rs672413
rs567754
rs6586282
All
−0.05
0.00
0.05
0.10
MR effect size for
'Selenium' on 'Procollagen C−endopeptidase enhancer 1'

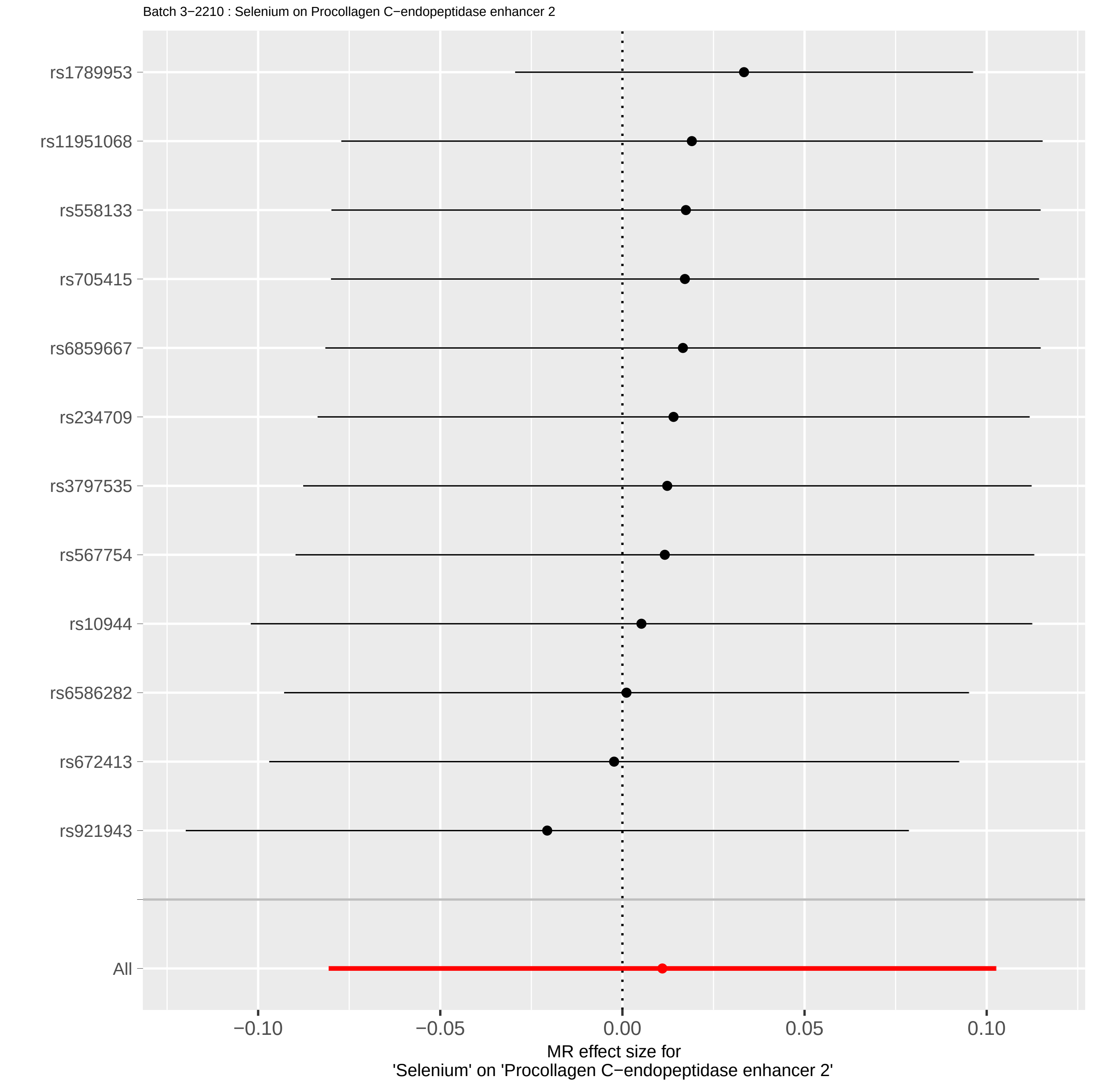

Batch 3−2210 : Selenium on Procollagen C−endopeptidase enhancer 2
rs1789953
rs11951068
rs558133
rs705415
rs6859667
rs234709
rs3797535
rs567754
rs10944
rs6586282
rs672413
rs921943
All
−0.10
−0.05
0.00
0.05
0.10
MR effect size for
'Selenium' on 'Procollagen C−endopeptidase enhancer 2'

Batch 3−2211 : Selenium on Neuroendocrine convertase 1

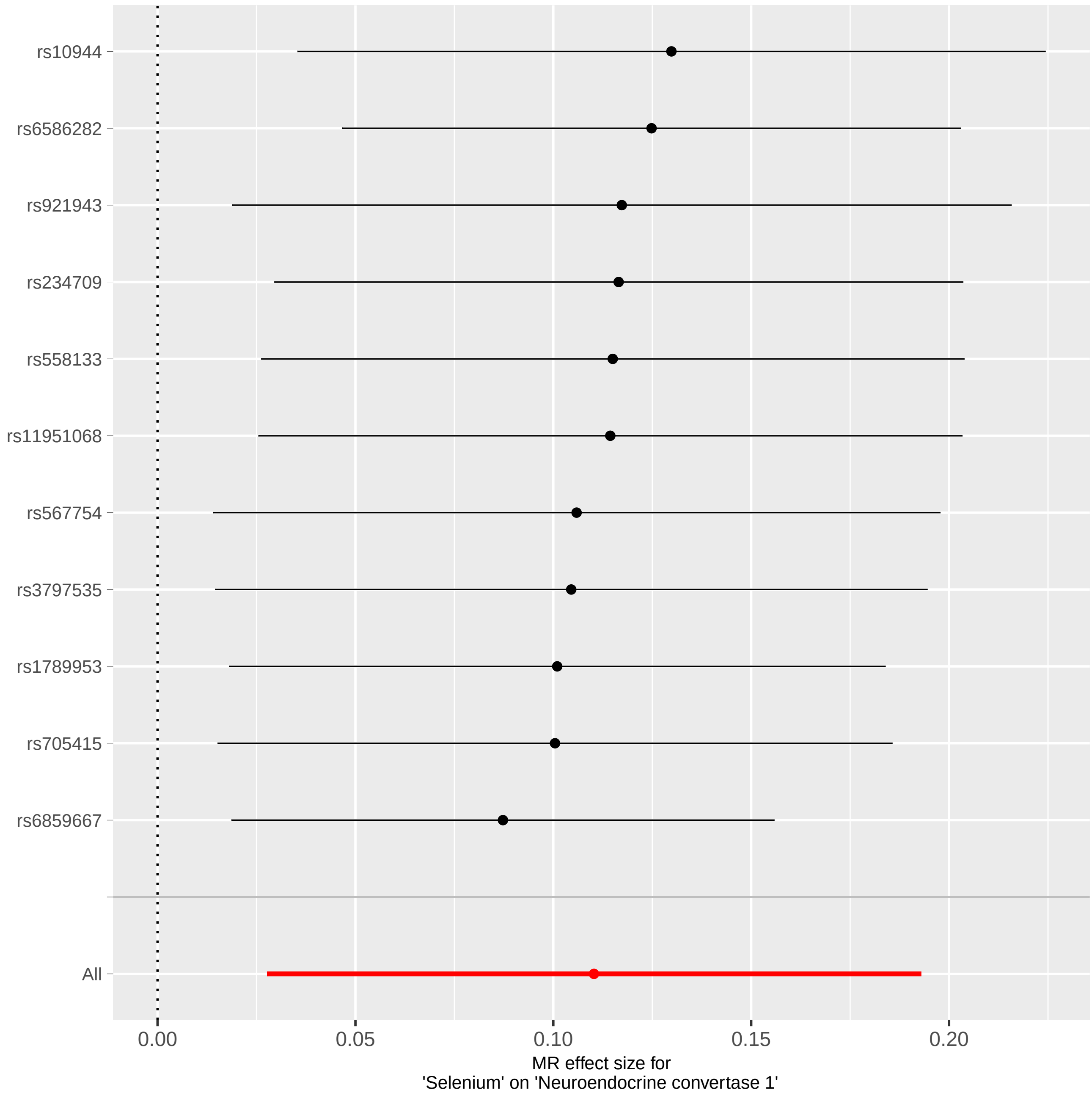

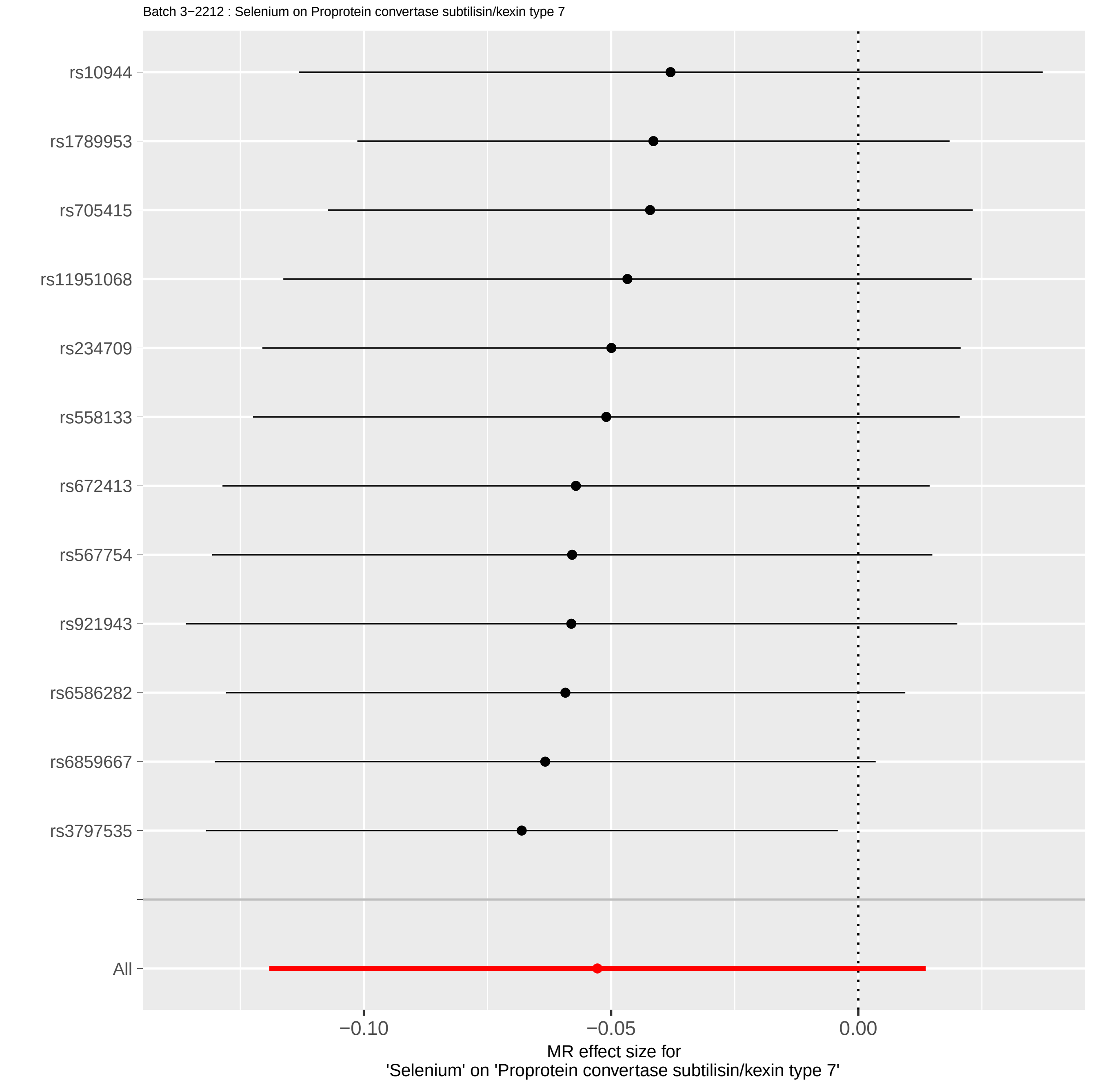

Batch 3−2212 : Selenium on Proprotein convertase subtilisin/kexin type 7
rs10944
rs1789953
rs705415
rs11951068
rs234709
rs558133
rs672413
rs567754
rs921943
rs6586282
rs6859667
rs3797535
All
−0.10
−0.05
0.00
MR effect size for
'Selenium' on 'Proprotein convertase subtilisin/kexin type 7'

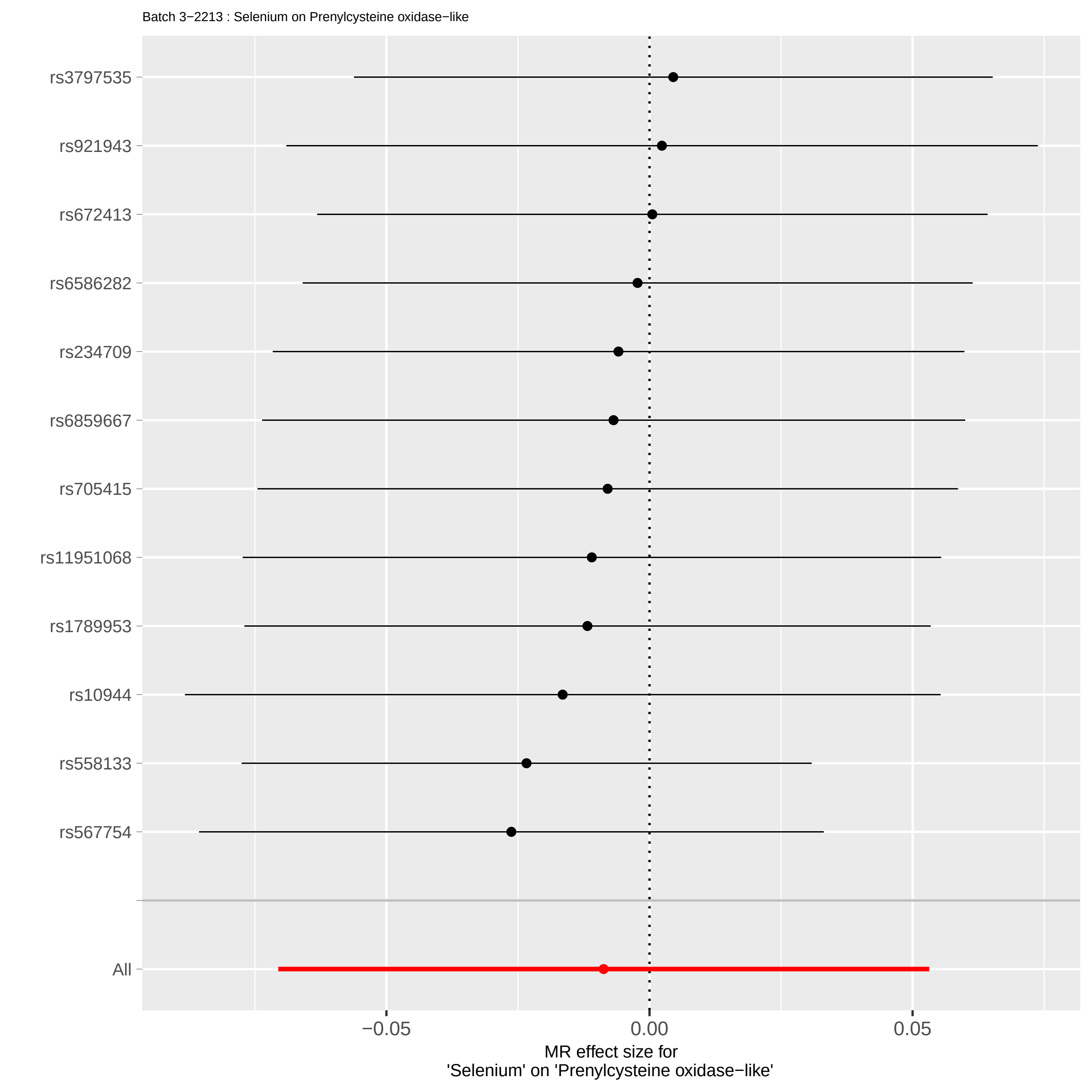

Batch 3−2213 : Selenium on Prenylcysteine oxidase−like
rs3797535
rs921943
rs672413
rs6586282
rs234709
rs6859667
rs705415
rs11951068
rs1789953
rs10944
rs558133
rs567754
All
−0.05
0.00
0.05
MR effect size for
'Selenium' on 'Prenylcysteine oxidase−like'

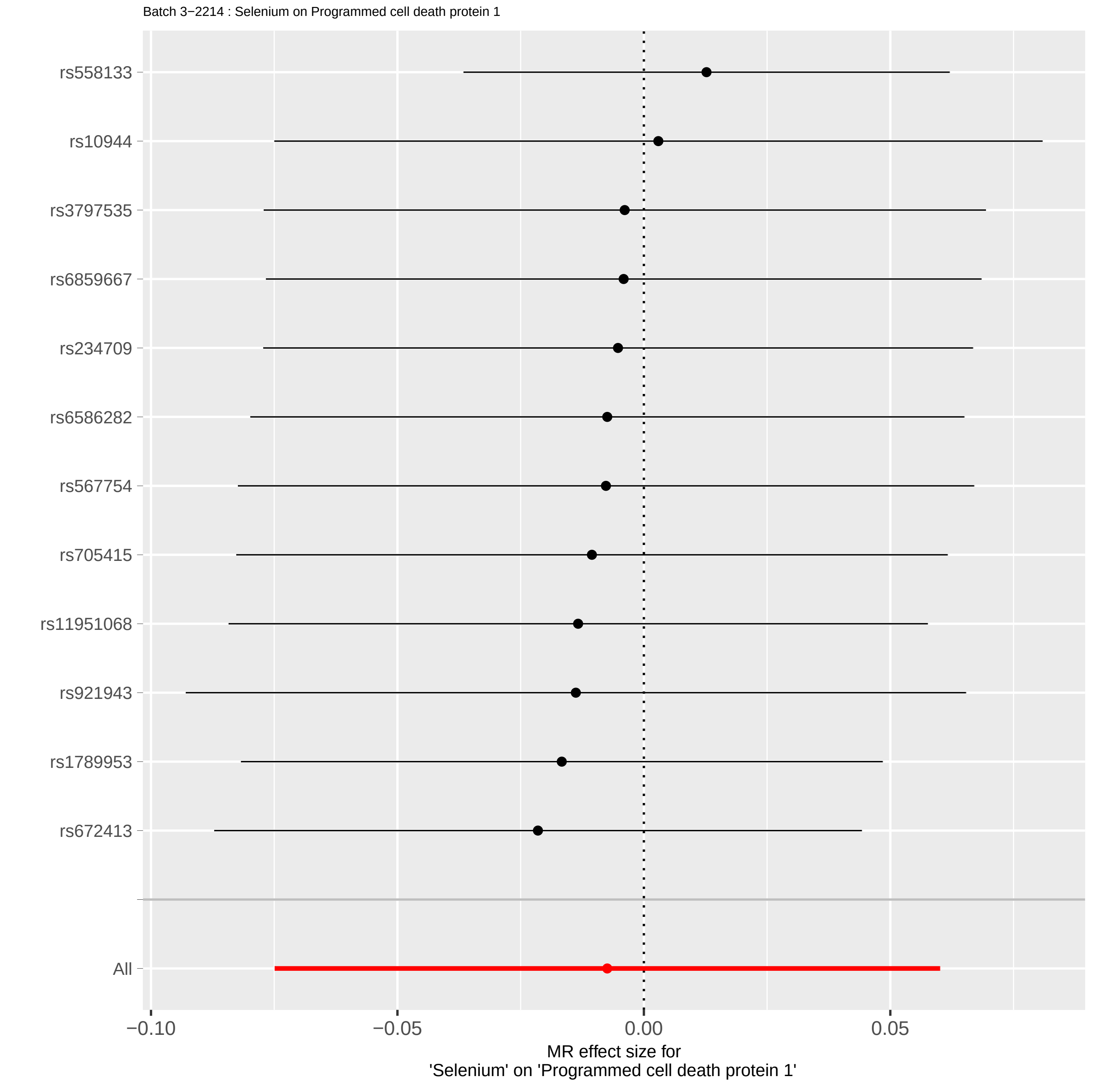
Batch 3−2214 : Selenium on Programmed cell death protein 1
rs558133
rs10944
rs3797535
rs6859667
rs234709
rs6586282
rs567754
rs705415
rs11951068
rs921943
rs1789953
rs672413
All
−0.10
−0.05
0.00
0.05
MR effect size for
'Selenium' on 'Programmed cell death protein 1'

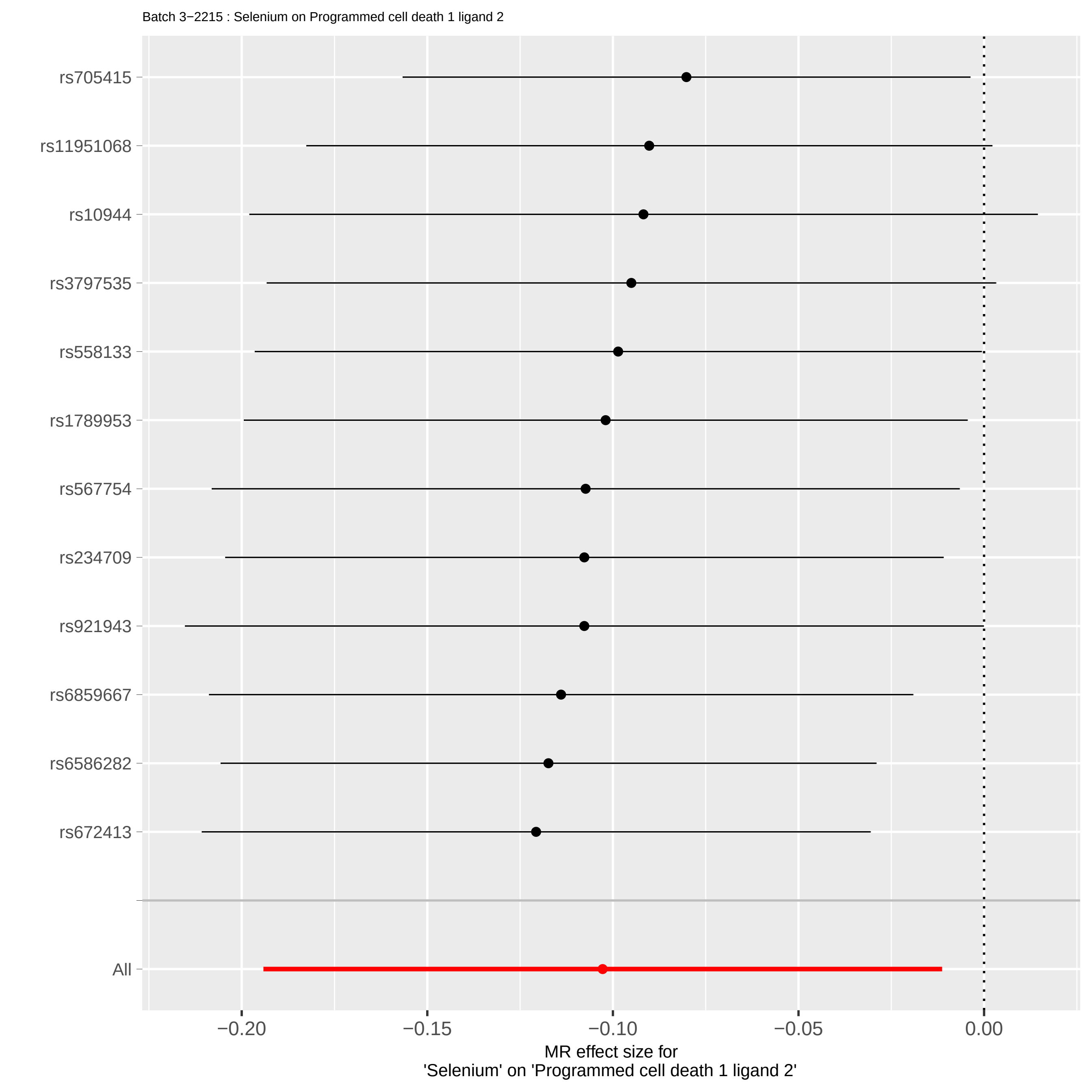

Batch 3−2215 : Selenium on Programmed cell death 1 ligand 2
rs705415
rs11951068
rs10944
rs3797535
rs558133
rs1789953
rs567754
rs234709
rs921943
rs6859667
rs6586282
rs672413
All
−0.20
−0.15
−0.10
−0.05
0.00
MR effect size for
'Selenium' on 'Programmed cell death 1 ligand 2'

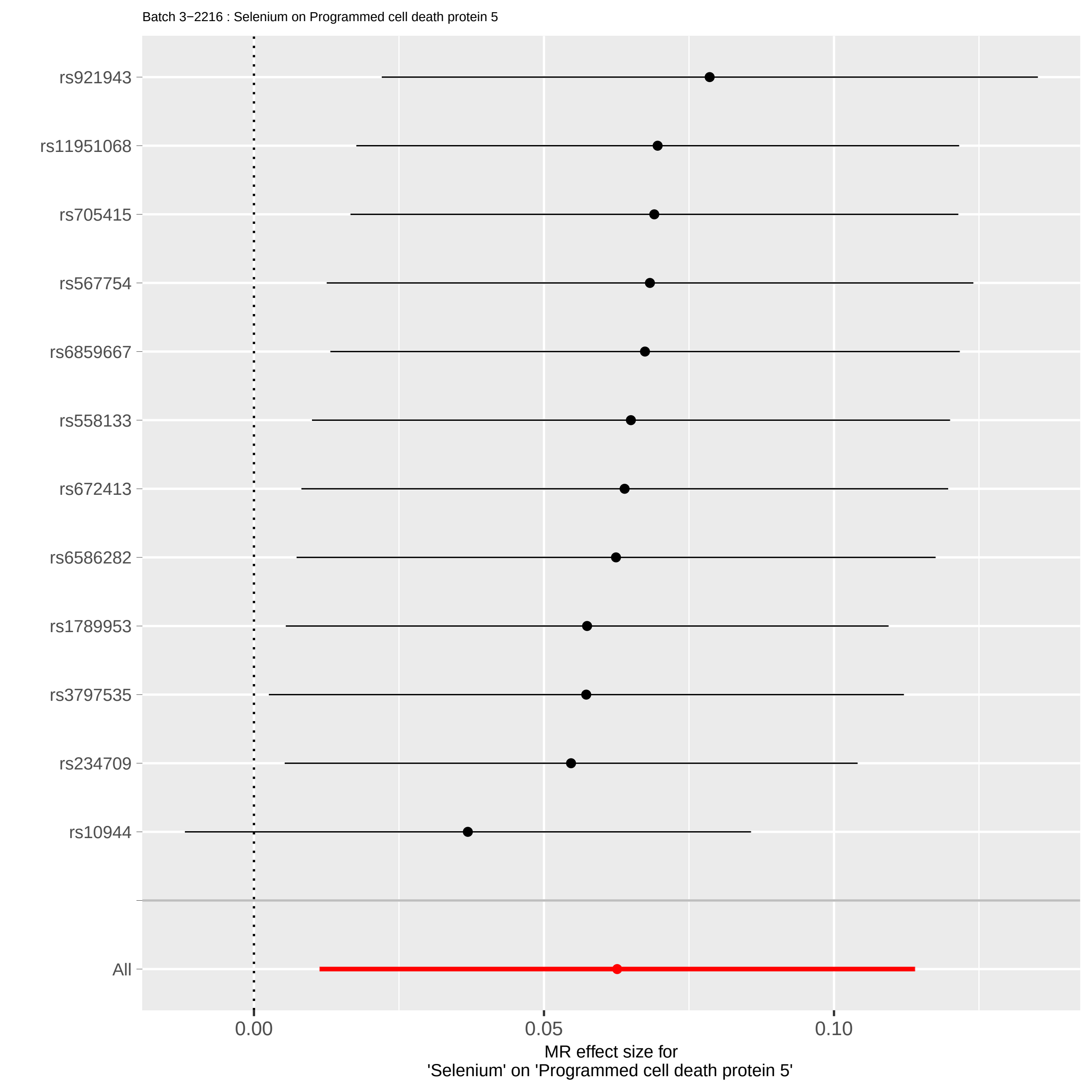

Batch 3−2216 : Selenium on Programmed cell death protein 5
rs921943
rs11951068
rs705415
rs567754
rs6859667
rs558133
rs672413
rs6586282
rs1789953
rs3797535
rs234709
rs10944
All
0.00
0.05
0.10
MR effect size for
'Selenium' on 'Programmed cell death protein 5'

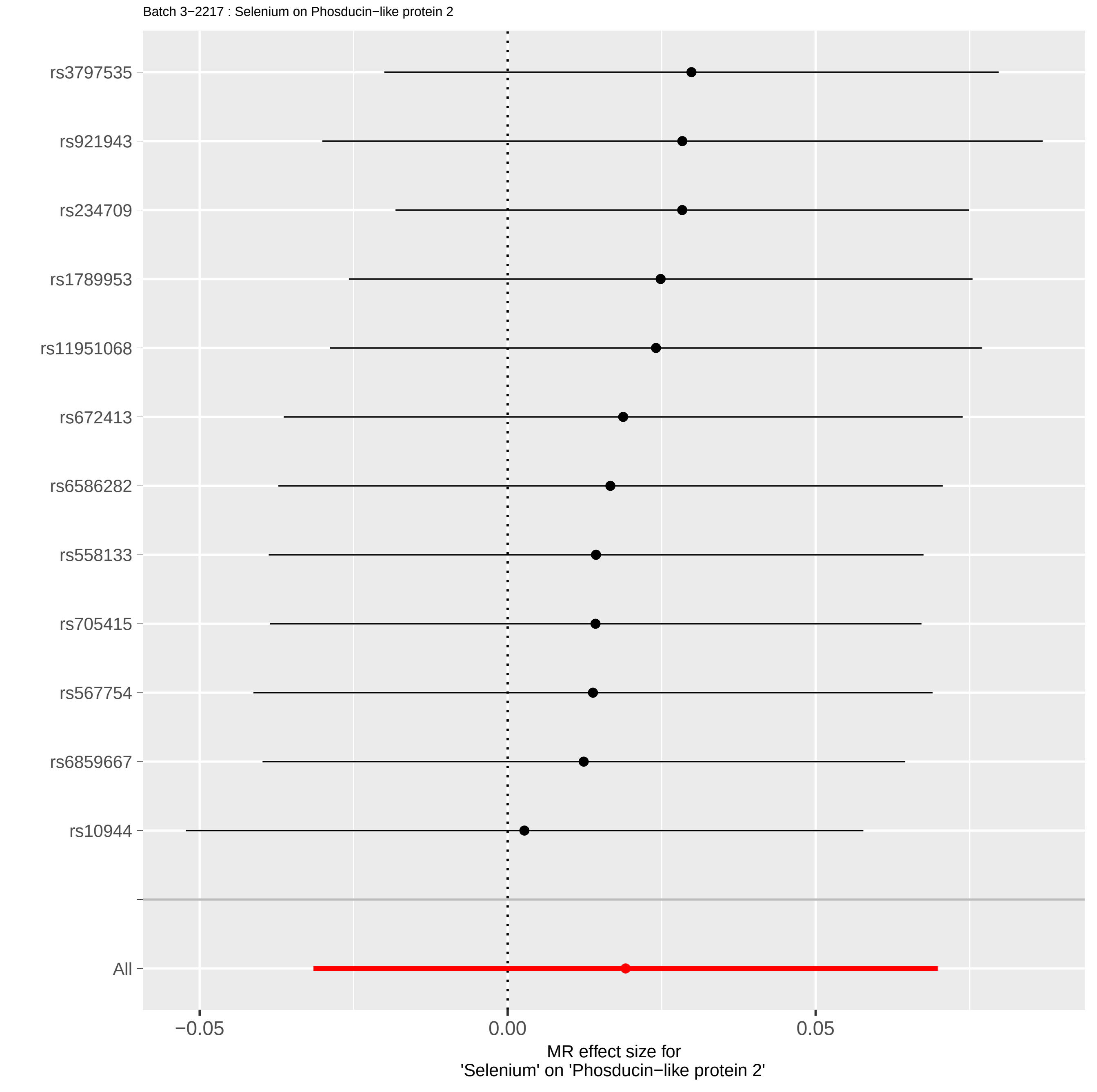
Batch 3−2217 : Selenium on Phosducin−like protein 2
rs3797535
rs921943
rs234709
rs1789953
rs11951068
rs672413
rs6586282
rs558133
rs705415
rs567754
rs6859667
rs10944
All
−0.05
0.00
0.05
MR effect size for
'Selenium' on 'Phosducin−like protein 2'

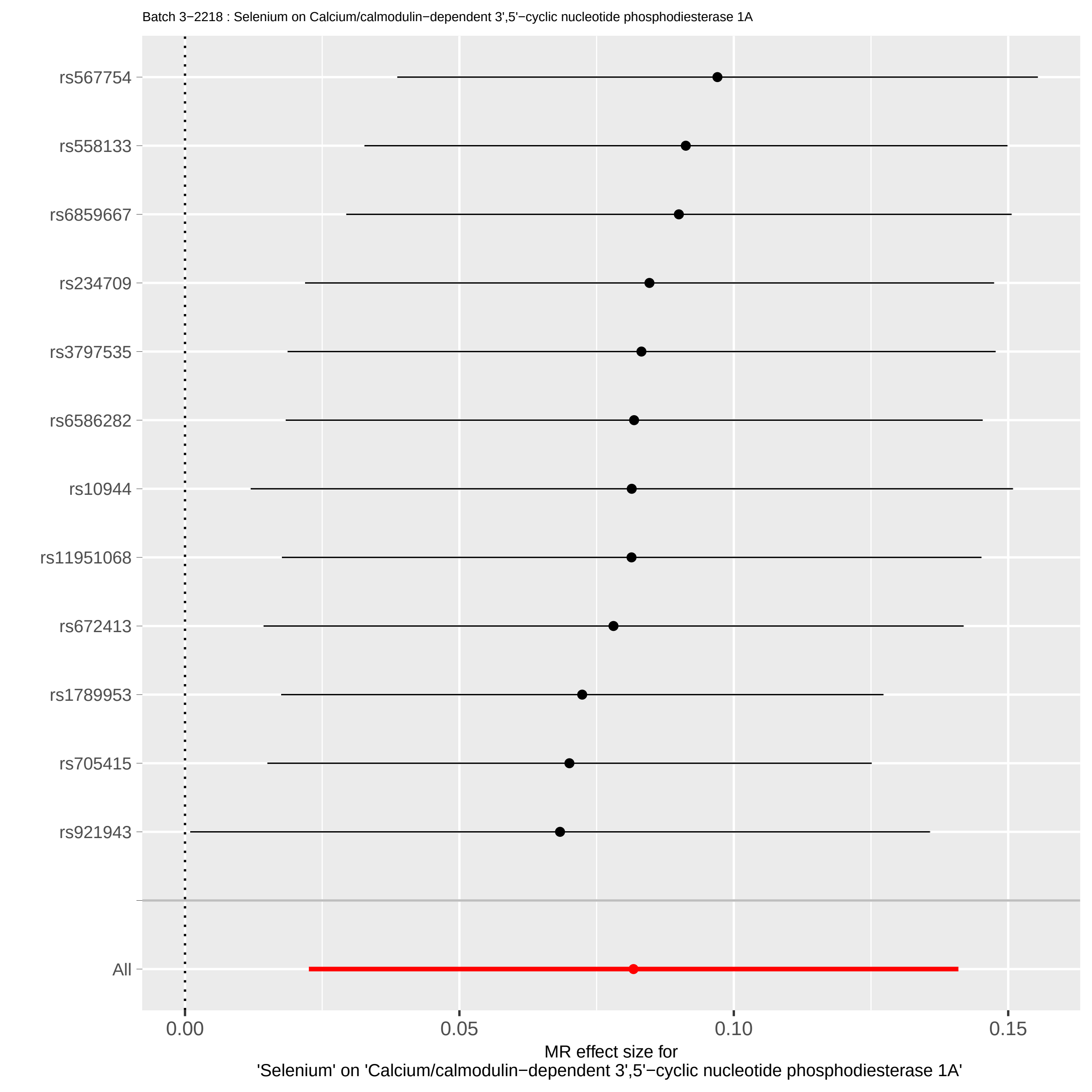

Batch 3−2218 : Selenium on Calcium/calmodulin−dependent 3',5'−cyclic nucleotide phosphodiesterase 1A
rs567754
rs558133
rs6859667
rs234709
rs3797535
rs6586282
rs10944
rs11951068
rs672413
rs1789953
rs705415
rs921943
All
0.00
0.05
0.10
0.15
MR effect size for
'Selenium' on 'Calcium/calmodulin−dependent 3',5'−cyclic nucleotide phosphodiesterase 1A'

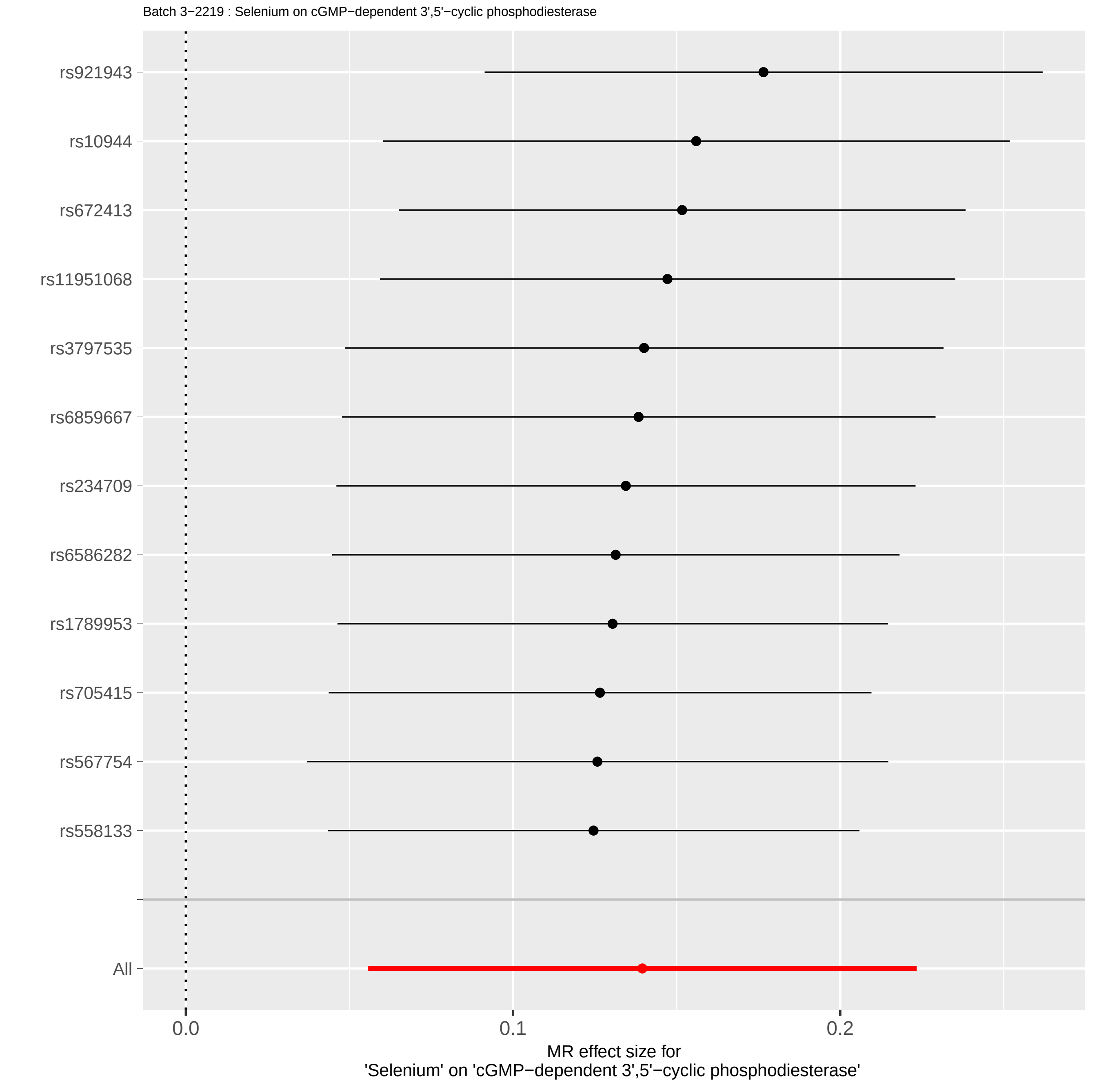
Batch 3−2219 : Selenium on cGMP−dependent 3',5'−cyclic phosphodiesterase
rs921943
rs10944
rs672413
rs11951068
rs3797535
rs6859667
rs234709
rs6586282
rs1789953
rs705415
rs567754
rs558133
All
0.0
0.1
0.2
MR effect size for
'Selenium' on 'cGMP−dependent 3',5'−cyclic phosphodiesterase'

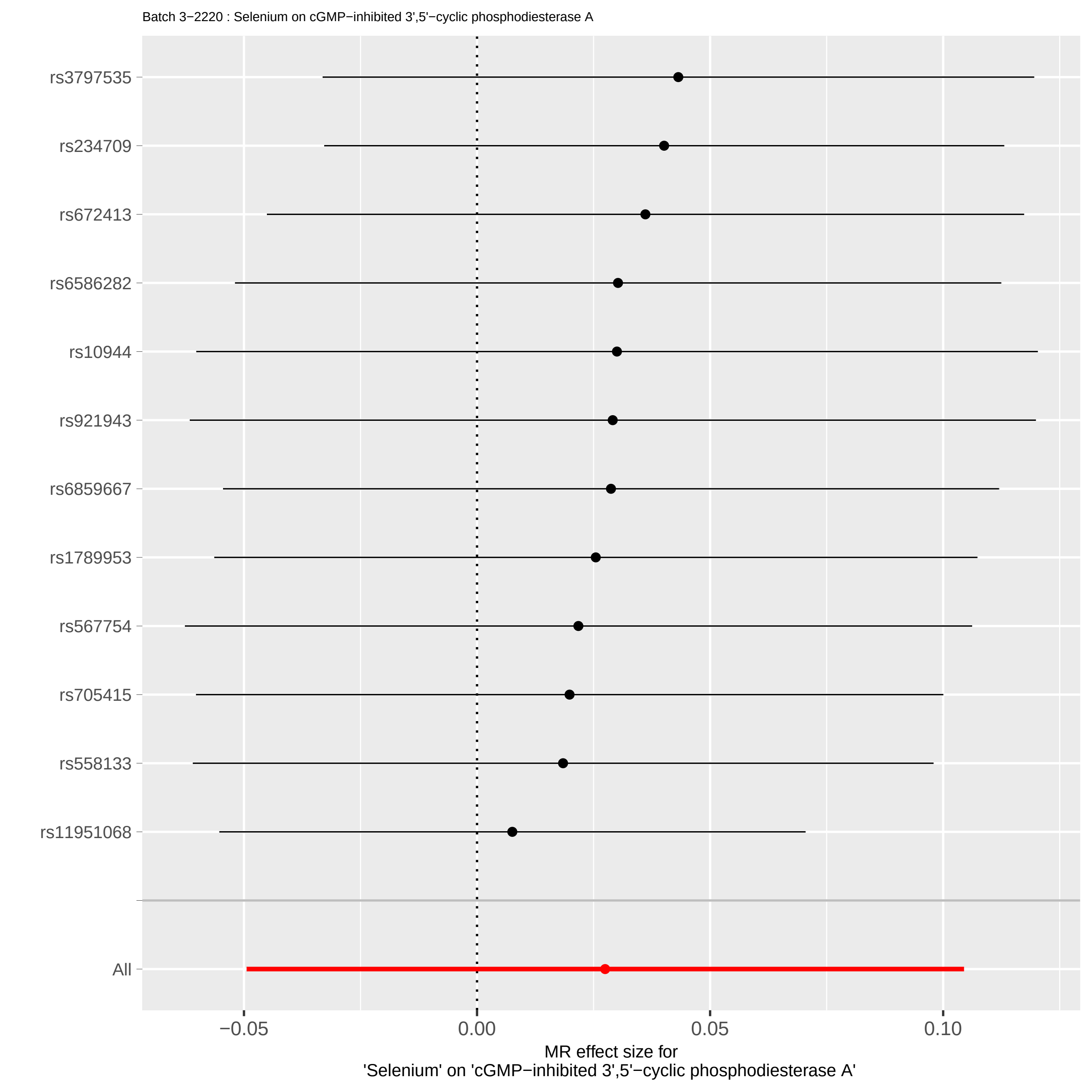

Batch 3−2220 : Selenium on cGMP−inhibited 3',5'−cyclic phosphodiesterase A
rs3797535
rs234709
rs672413
rs6586282
rs10944
rs921943
rs6859667
rs1789953
rs567754
rs705415
rs558133
rs11951068
All
−0.05
0.00
0.05
0.10
MR effect size for
'Selenium' on 'cGMP−inhibited 3',5'−cyclic phosphodiesterase A'

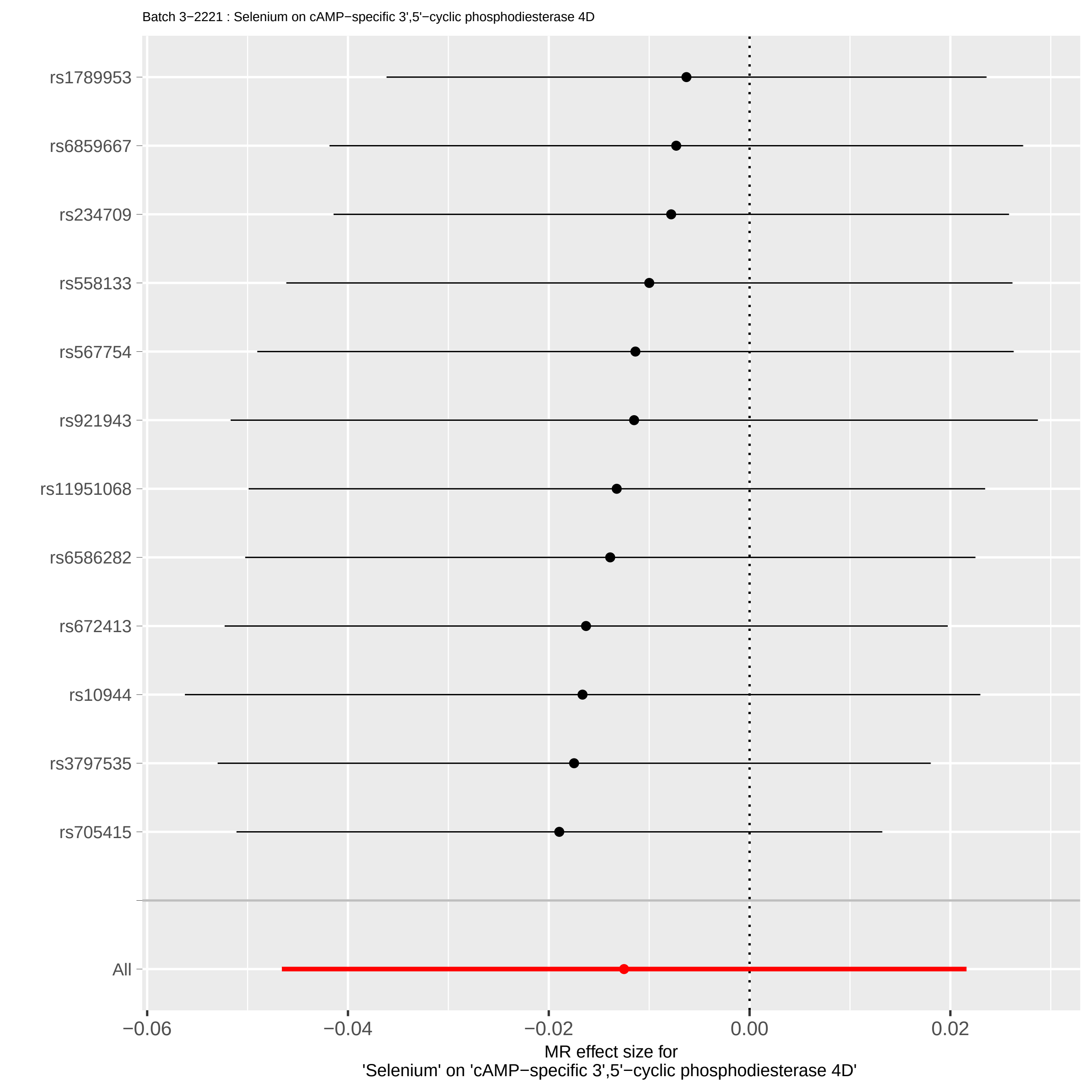

Batch 3−2221 : Selenium on cAMP−specific 3',5'−cyclic phosphodiesterase 4D
rs1789953
rs6859667
rs234709
rs558133
rs567754
rs921943
rs11951068
rs6586282
rs672413
rs10944
rs3797535
rs705415
All
−0.06
−0.04
−0.02
0.00
0.02
MR effect size for
'Selenium' on 'cAMP−specific 3',5'−cyclic phosphodiesterase 4D'

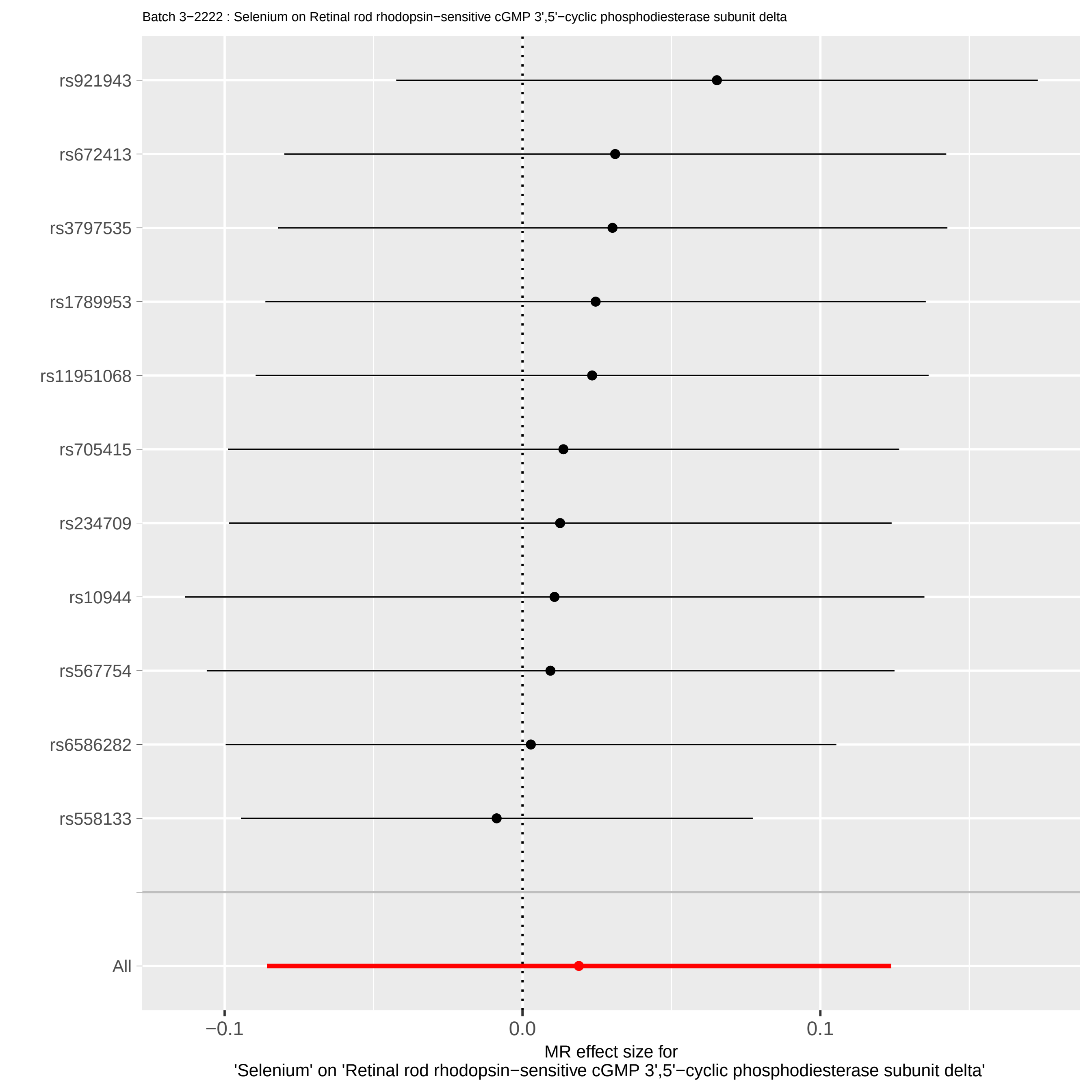

Batch 3−2222 : Selenium on Retinal rod rhodopsin−sensitive cGMP 3',5'−cyclic phosphodiesterase subunit delta
rs921943
rs672413
rs3797535
rs1789953
rs11951068
rs705415
rs234709
rs10944
rs567754
rs6586282
rs558133
All
−0.1
0.0
0.1
MR effect size for
'Selenium' on 'Retinal rod rhodopsin−sensitive cGMP 3',5'−cyclic phosphodiesterase subunit delta'

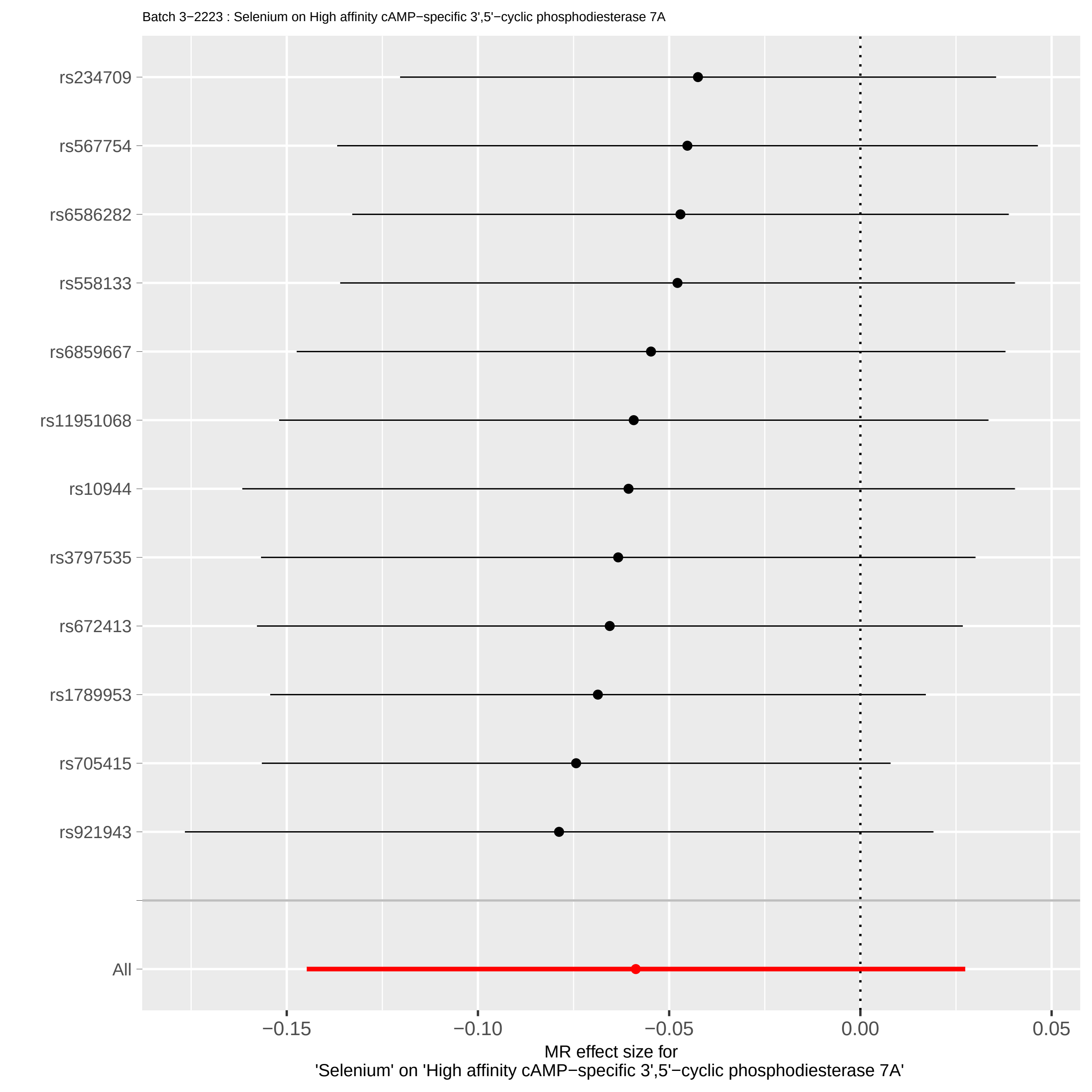

Batch 3−2223 : Selenium on High affinity cAMP−specific 3',5'−cyclic phosphodiesterase 7A
rs234709
rs567754
rs6586282
rs558133
rs6859667
rs11951068
rs10944
rs3797535
rs672413
rs1789953
rs705415
rs921943
All
−0.15
−0.10
−0.05
0.00
0.05
MR effect size for
'Selenium' on 'High affinity cAMP−specific 3',5'−cyclic phosphodiesterase 7A'

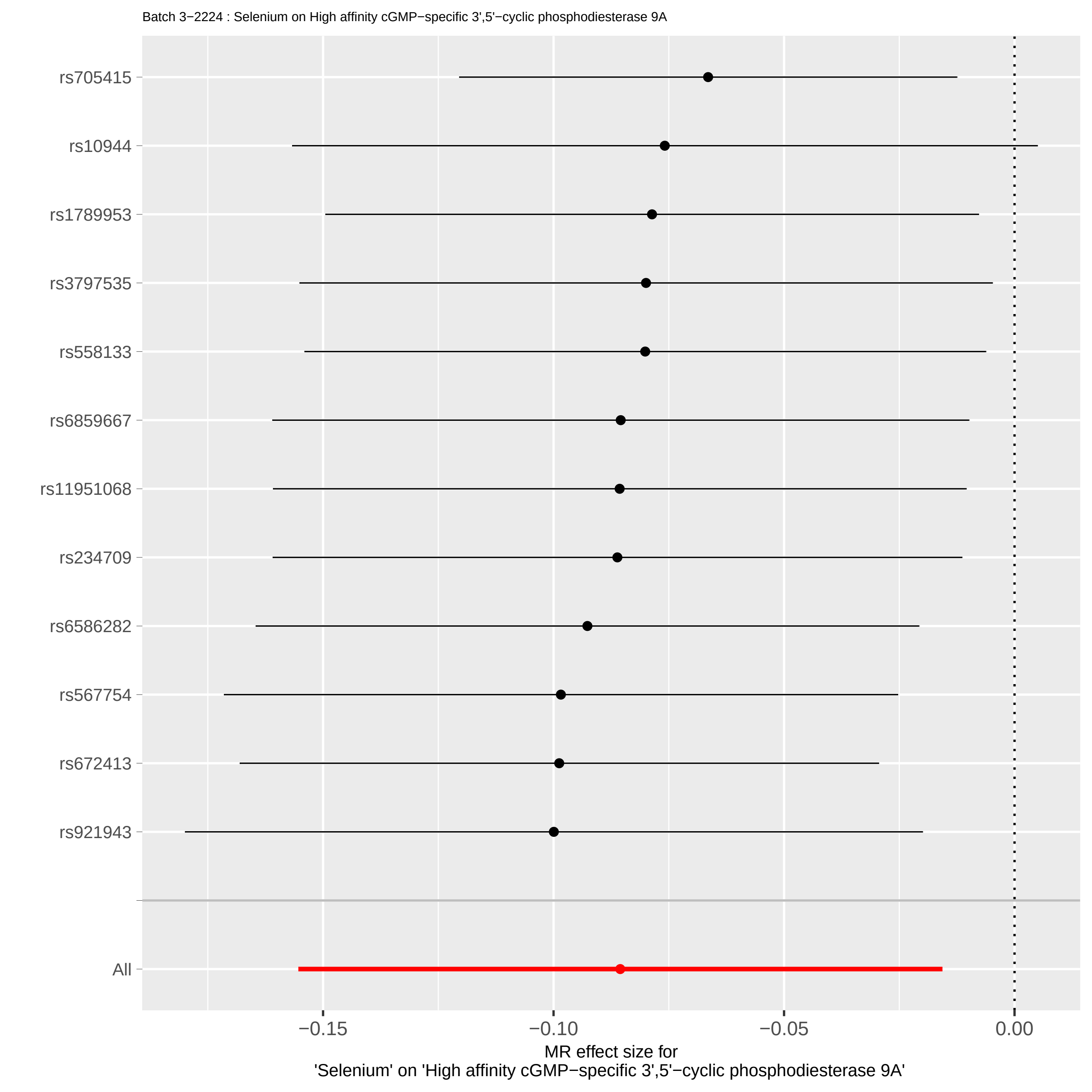

Batch 3−2224 : Selenium on High affinity cGMP−specific 3',5'−cyclic phosphodiesterase 9A
rs705415
rs10944
rs1789953
rs3797535
rs558133
rs6859667
rs11951068
rs234709
rs6586282
rs567754
rs672413
rs921943
All
−0.15
−0.10
−0.05
0.00
MR effect size for
'Selenium' on 'High affinity cGMP−specific 3',5'−cyclic phosphodiesterase 9A'

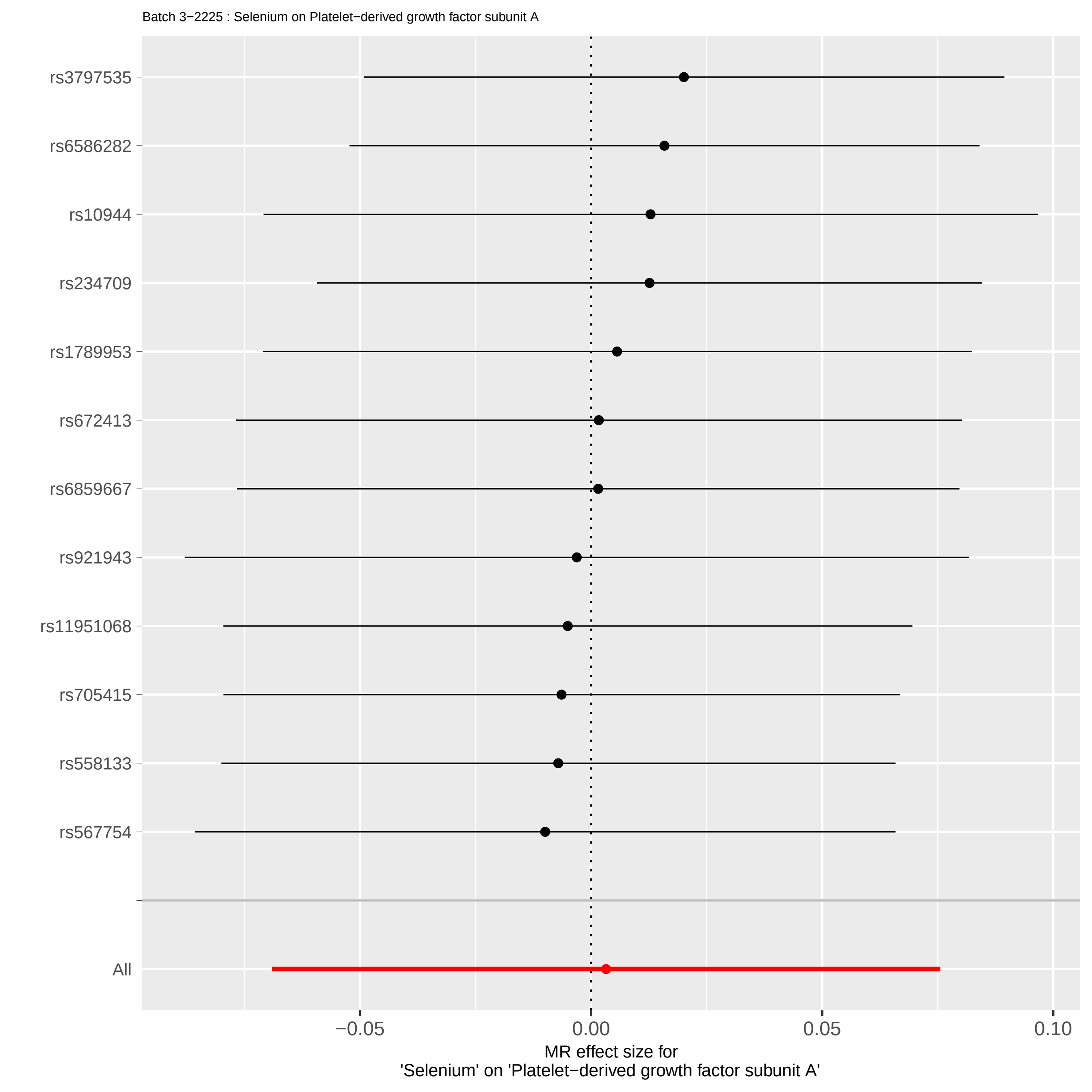
Batch 3−2225 : Selenium on Platelet−derived growth factor subunit A
rs3797535
rs6586282
rs10944
rs234709
rs1789953
rs672413
rs6859667
rs921943
rs11951068
rs705415
rs558133
rs567754
All
−0.05
0.00
0.05
0.10
MR effect size for
'Selenium' on 'Platelet−derived growth factor subunit A'

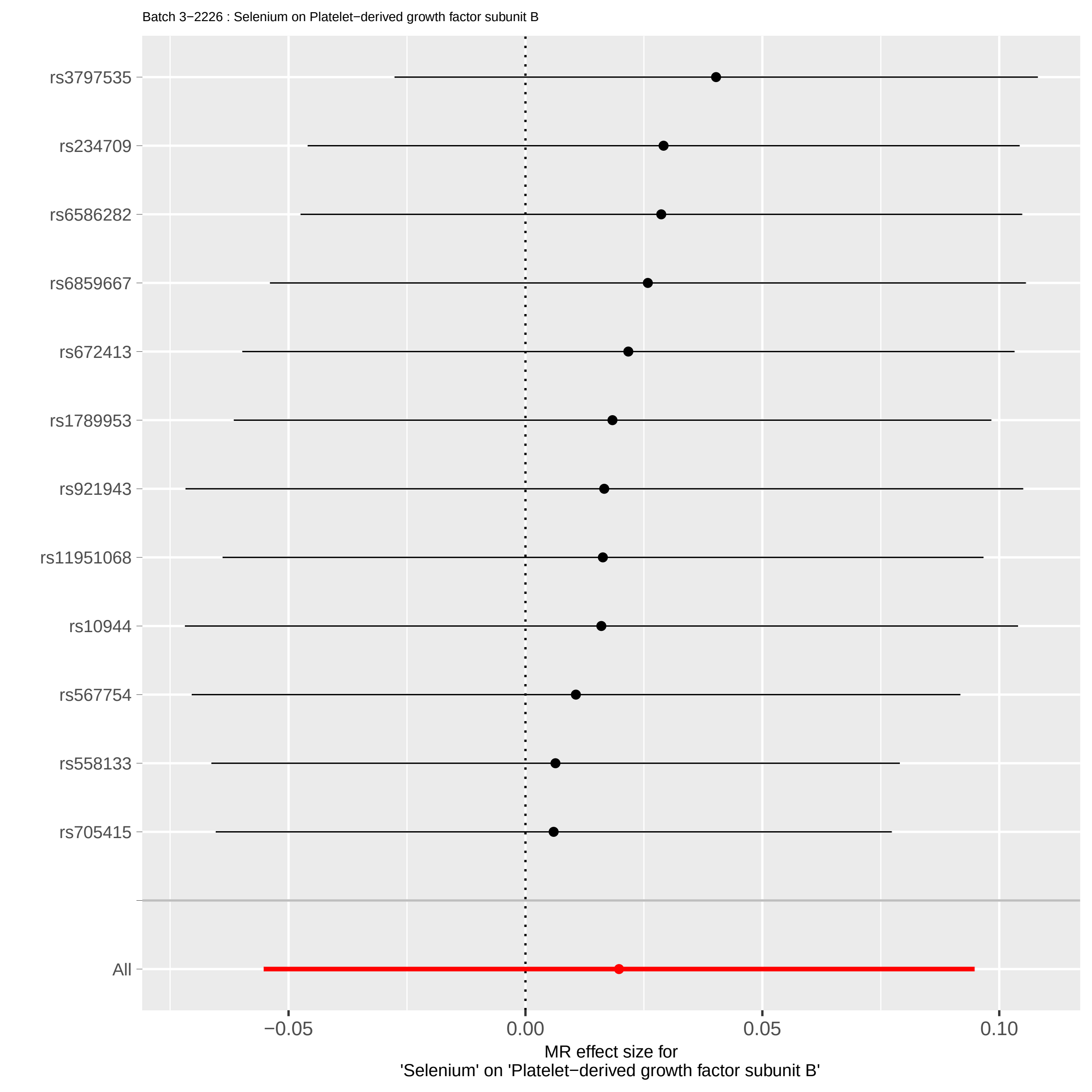

Batch 3−2226 : Selenium on Platelet−derived growth factor subunit B
rs3797535
rs234709
rs6586282
rs6859667
rs672413
rs1789953
rs921943
rs11951068
rs10944
rs567754
rs558133
rs705415
All
−0.05
0.00
0.05
0.10
MR effect size for
'Selenium' on 'Platelet−derived growth factor subunit B'

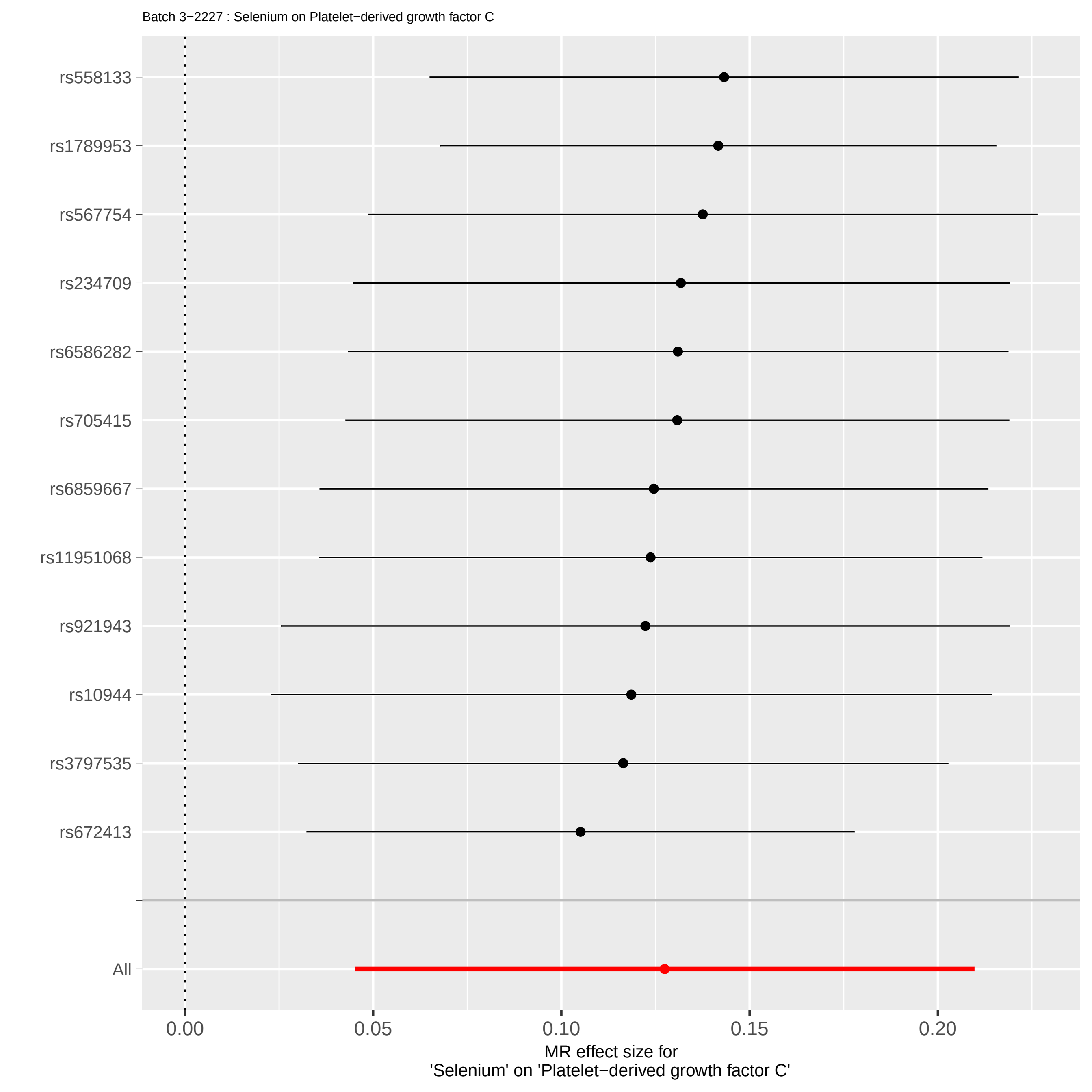

Batch 3−2227 : Selenium on Platelet−derived growth factor C
rs558133
rs1789953
rs567754
rs234709
rs6586282
rs705415
rs6859667
rs11951068
rs921943
rs10944
rs3797535
rs672413
All
0.00
0.05
0.10
0.15
0.20
MR effect size for
'Selenium' on 'Platelet−derived growth factor C'

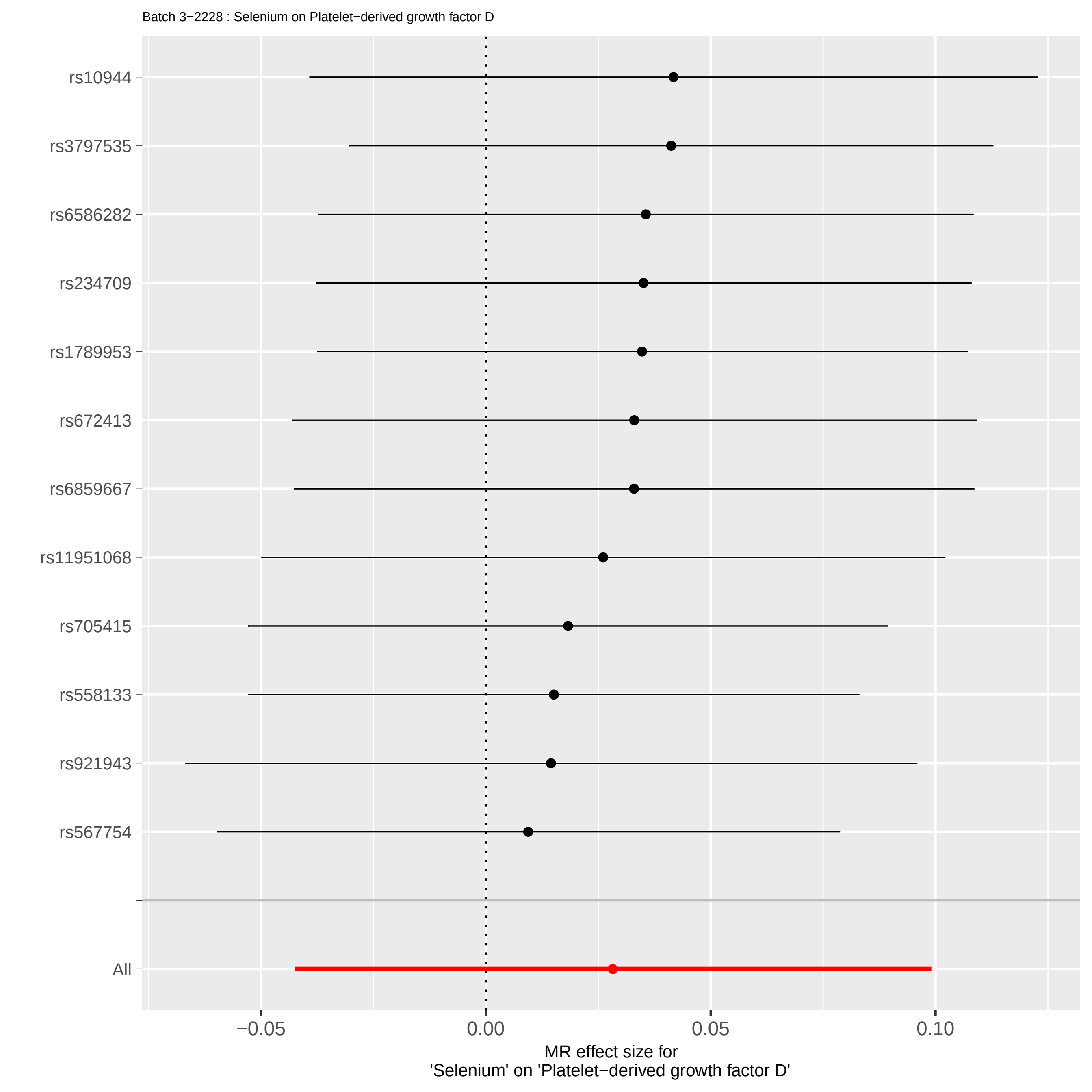

Batch 3−2228 : Selenium on Platelet−derived growth factor D
rs10944
rs3797535
rs6586282
rs234709
rs1789953
rs672413
rs6859667
rs11951068
rs705415
rs558133
rs921943
rs567754
All
−0.05
0.00
0.05
0.10
MR effect size for
'Selenium' on 'Platelet−derived growth factor D'

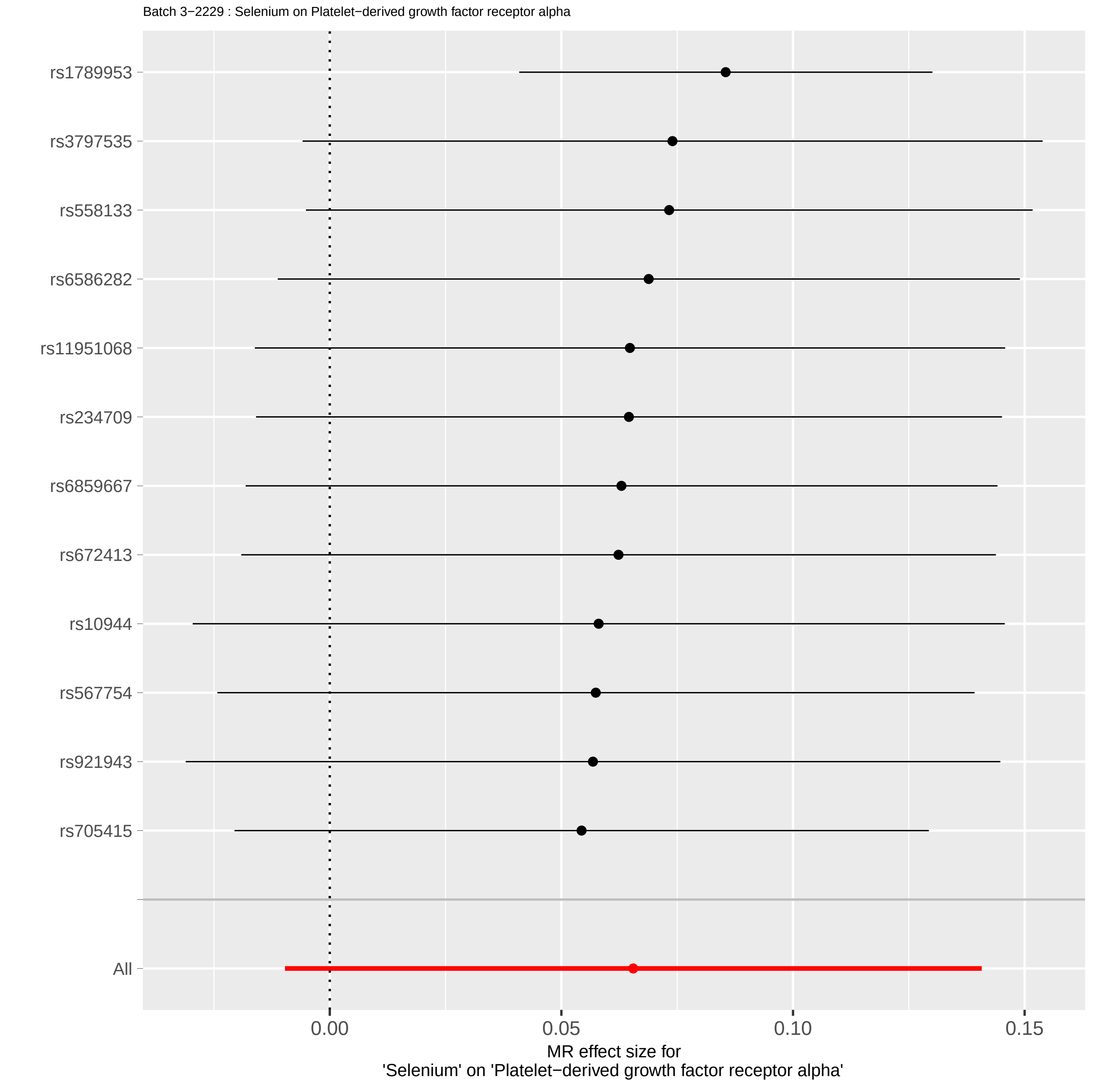

Batch 3−2229 : Selenium on Platelet−derived growth factor receptor alpha
rs1789953
rs3797535
rs558133
rs6586282
rs11951068
rs234709
rs6859667
rs672413
rs10944
rs567754
rs921943
rs705415
All
0.00
0.05
0.10
0.15
MR effect size for
'Selenium' on 'Platelet−derived growth factor receptor alpha'

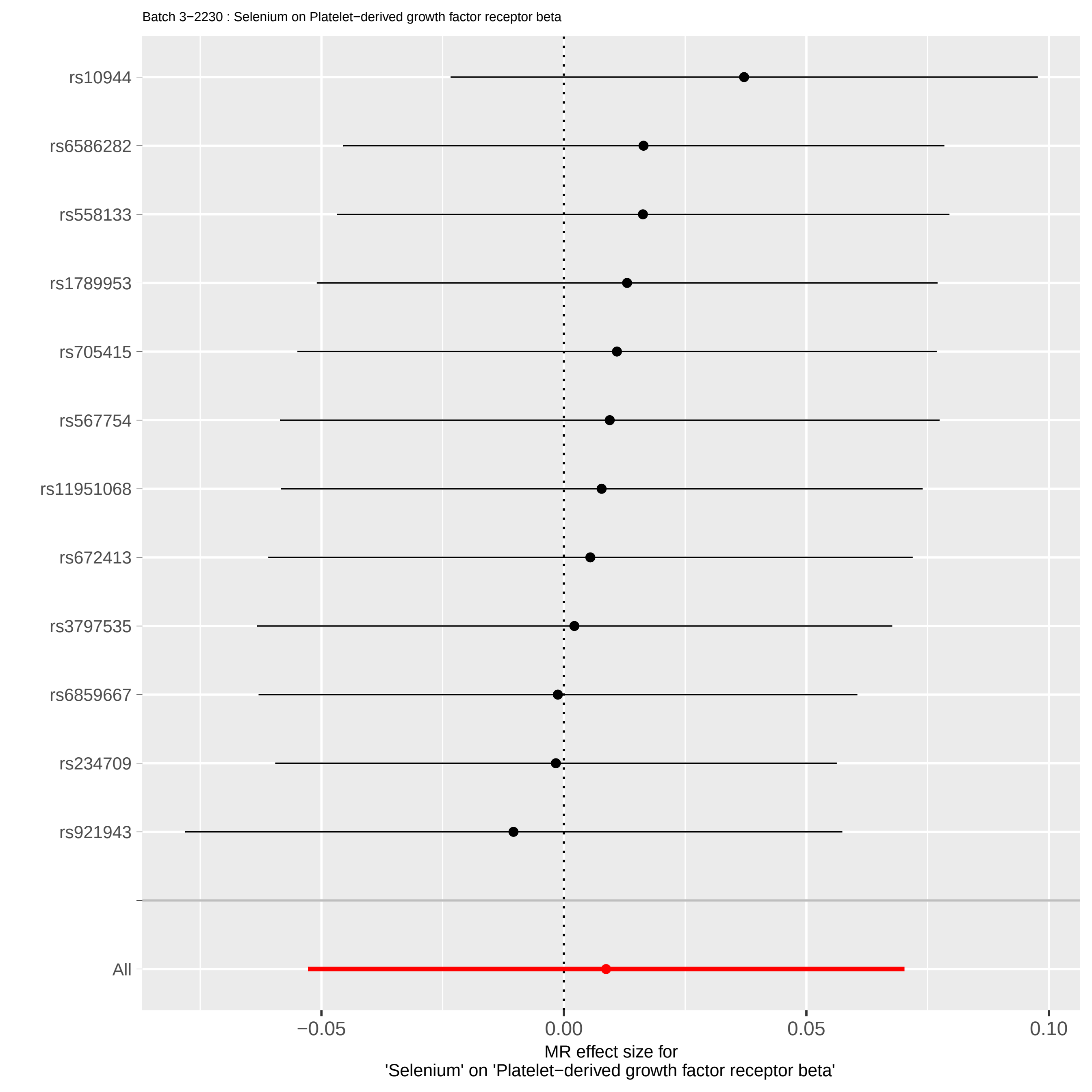

Batch 3−2230 : Selenium on Platelet−derived growth factor receptor beta
rs10944
rs6586282
rs558133
rs1789953
rs705415
rs567754
rs11951068
rs672413
rs3797535
rs6859667
rs234709
rs921943
All
−0.05
0.00
0.05
0.10
MR effect size for
'Selenium' on 'Platelet−derived growth factor receptor beta'

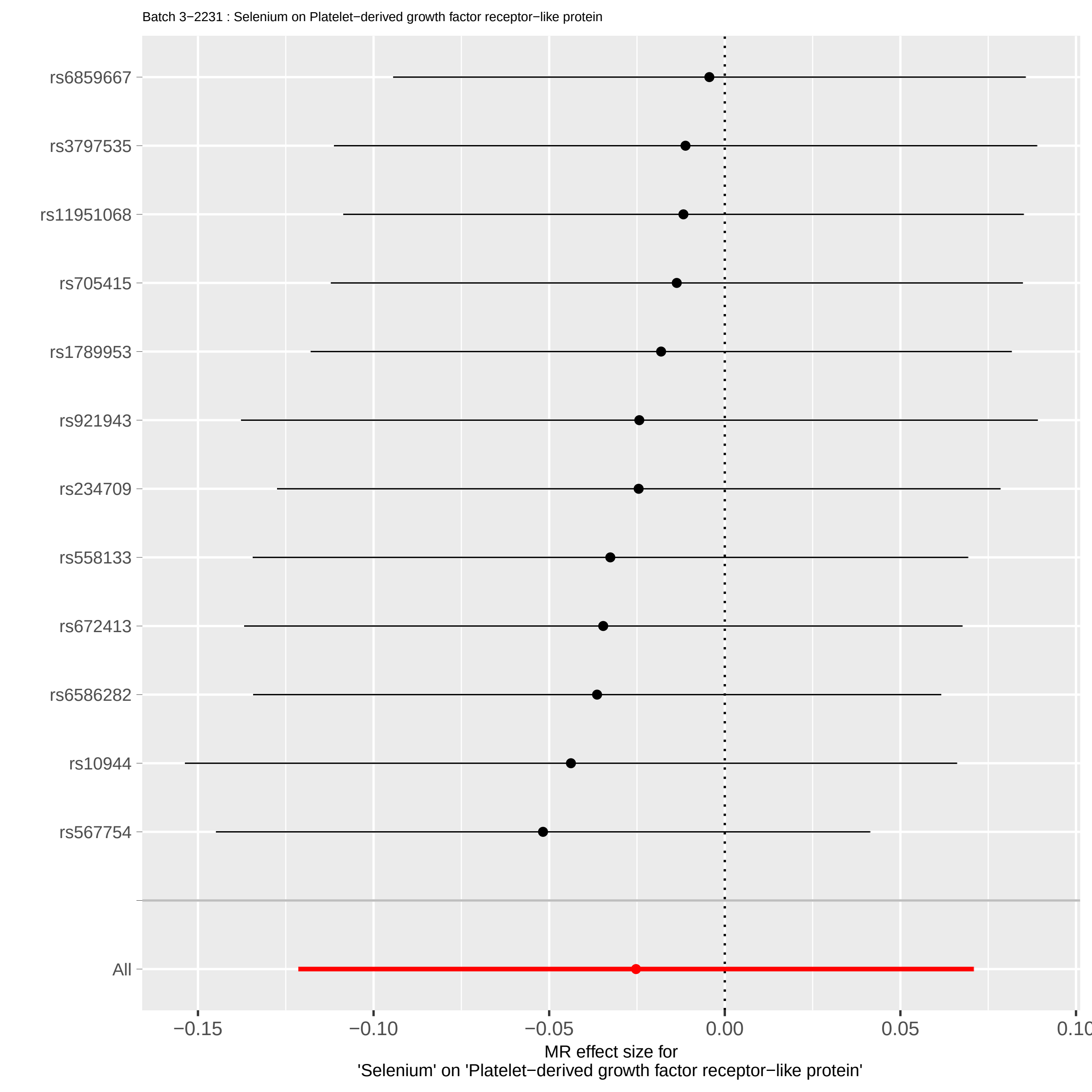

Batch 3−2231 : Selenium on Platelet−derived growth factor receptor−like protein
rs6859667
rs3797535
rs11951068
rs705415
rs1789953
rs921943
rs234709
rs558133
rs672413
rs6586282
rs10944
rs567754
All
−0.15
−0.10
−0.05
0.00
0.05
0.10
MR effect size for
'Selenium' on 'Platelet−derived growth factor receptor−like protein'

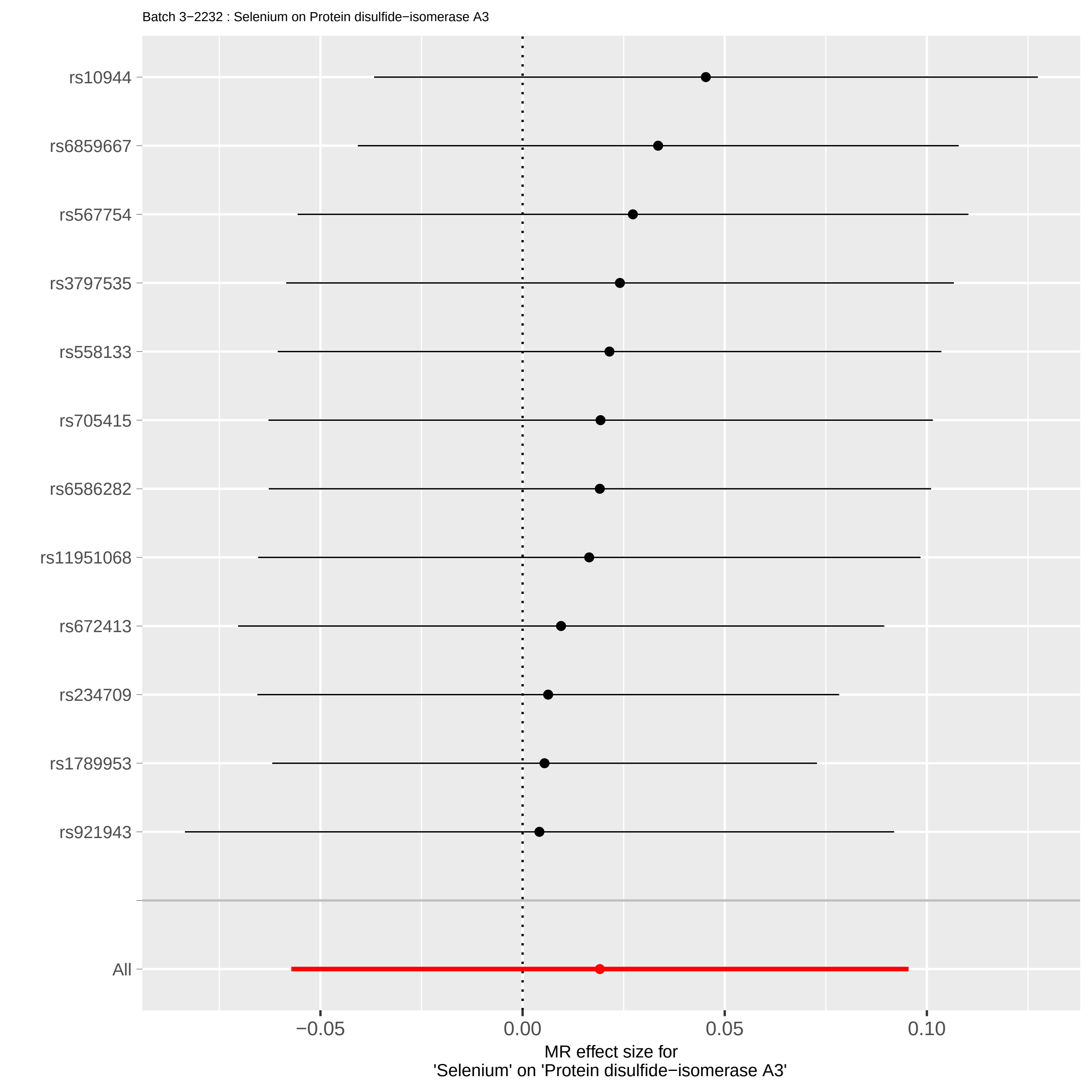

Batch 3−2232 : Selenium on Protein disulfide−isomerase A3
rs10944
rs6859667
rs567754
rs3797535
rs558133
rs705415
rs6586282
rs11951068
rs672413
rs234709
rs1789953
rs921943
All
−0.05
0.00
0.05
0.10
MR effect size for
'Selenium' on 'Protein disulfide−isomerase A3'

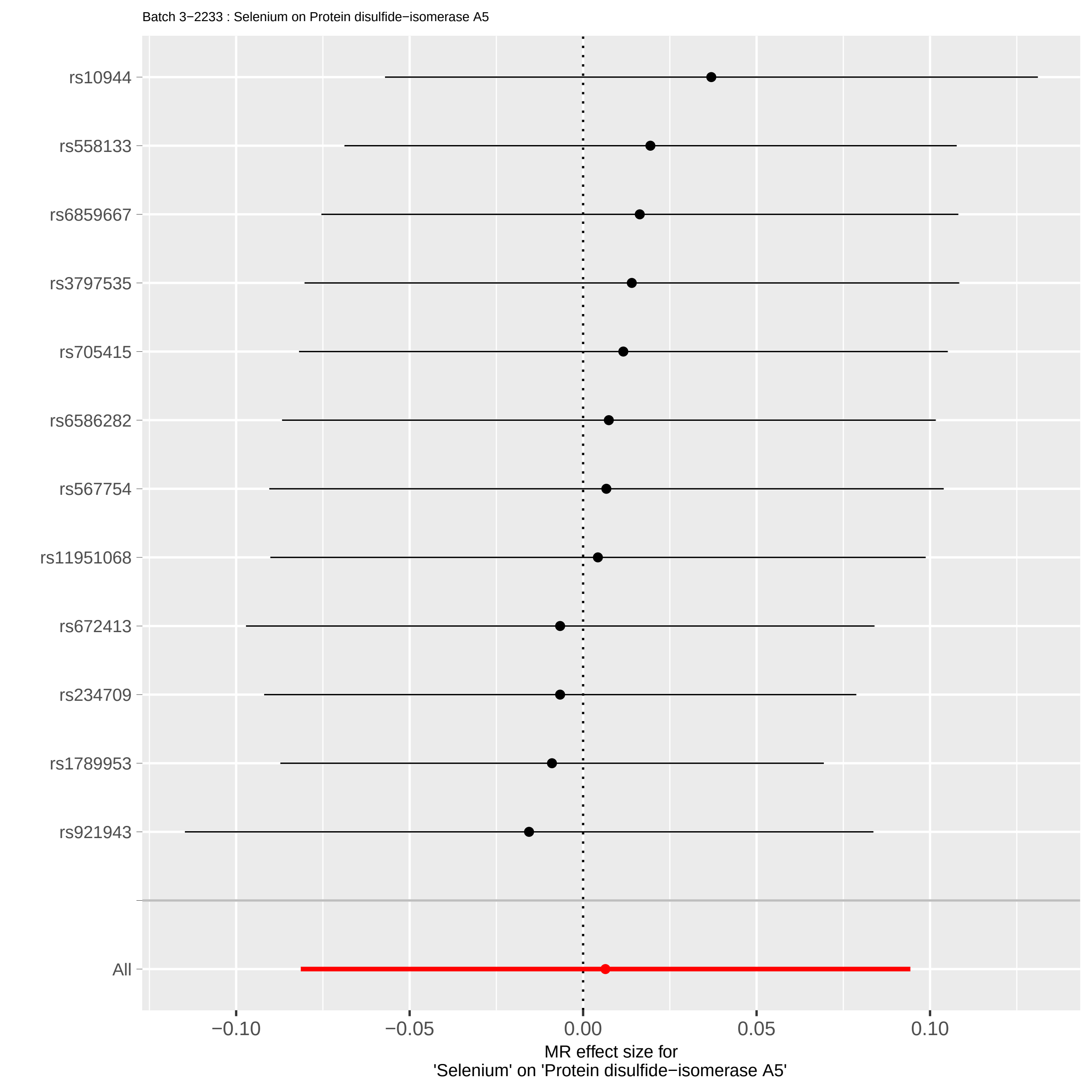
Batch 3−2233 : Selenium on Protein disulfide−isomerase A5
rs10944
rs558133
rs6859667
rs3797535
rs705415
rs6586282
rs567754
rs11951068
rs672413
rs234709
rs1789953
rs921943
All
−0.10
−0.05
0.00
0.05
0.10
MR effect size for
'Selenium' on 'Protein disulfide−isomerase A5'

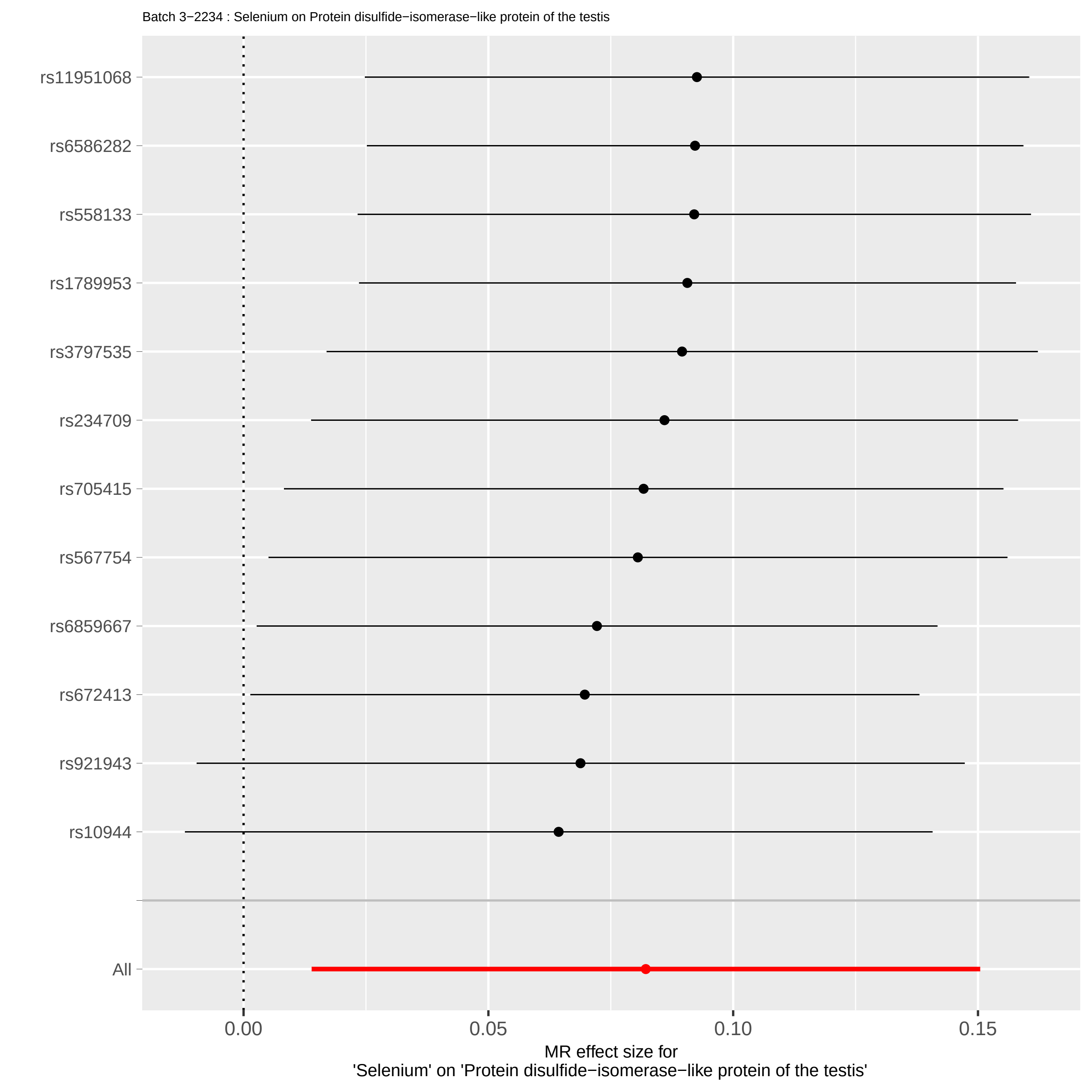

Batch 3−2234 : Selenium on Protein disulfide−isomerase−like protein of the testis
rs11951068
rs6586282
rs558133
rs1789953
rs3797535
rs234709
rs705415
rs567754
rs6859667
rs672413
rs921943
rs10944
All
0.00
0.05
0.10
0.15
MR effect size for
'Selenium' on 'Protein disulfide−isomerase−like protein of the testis'

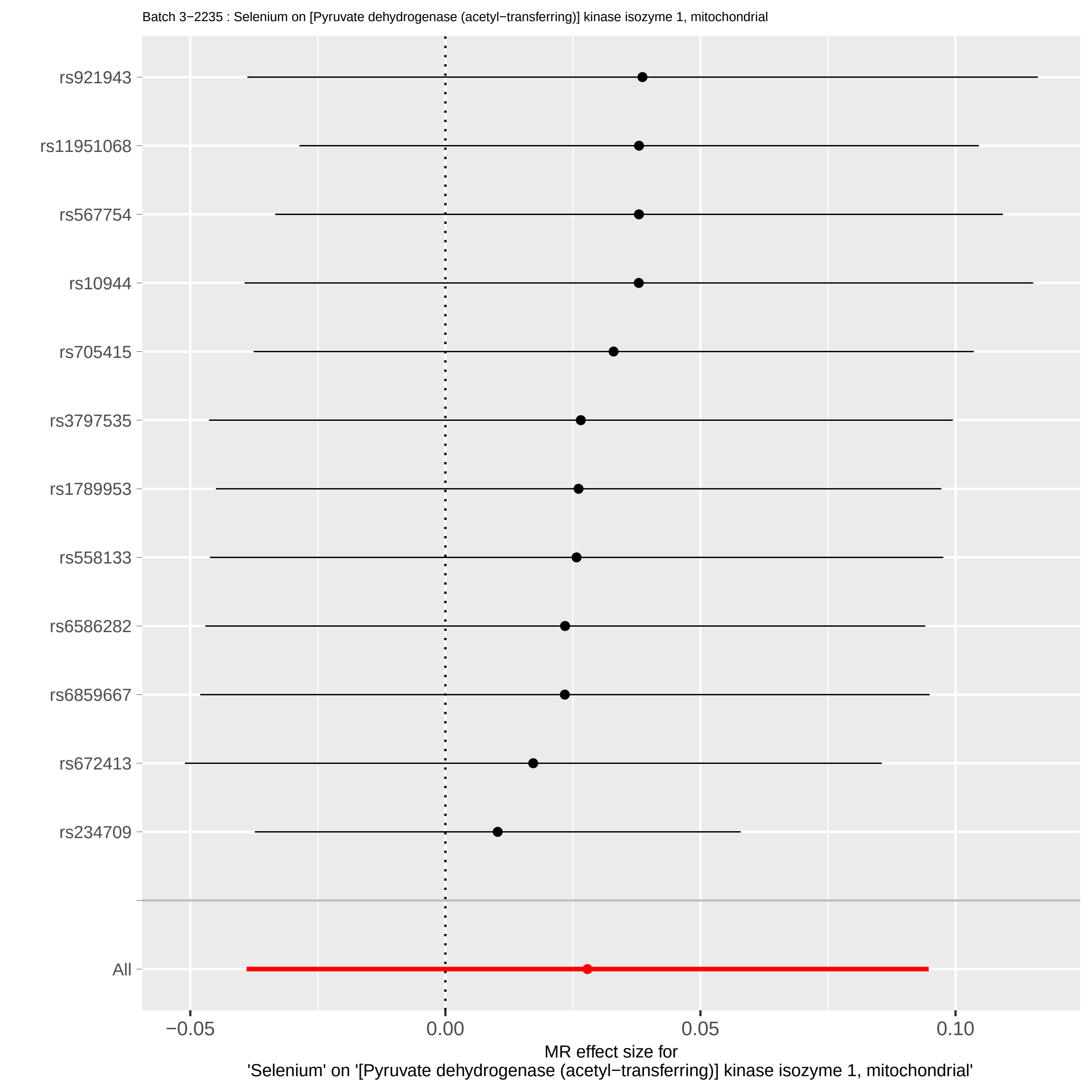
Batch 3−2235 : Selenium on [Pyruvate dehydrogenase (acetyl−transferring)] kinase isozyme 1, mitochondrial
rs921943
rs11951068
rs567754
rs10944
rs705415
rs3797535
rs1789953
rs558133
rs6586282
rs6859667
rs672413
rs234709
All
−0.05
0.00
0.05
0.10
MR effect size for
'Selenium' on '[Pyruvate dehydrogenase (acetyl−transferring)] kinase isozyme 1, mitochondrial'

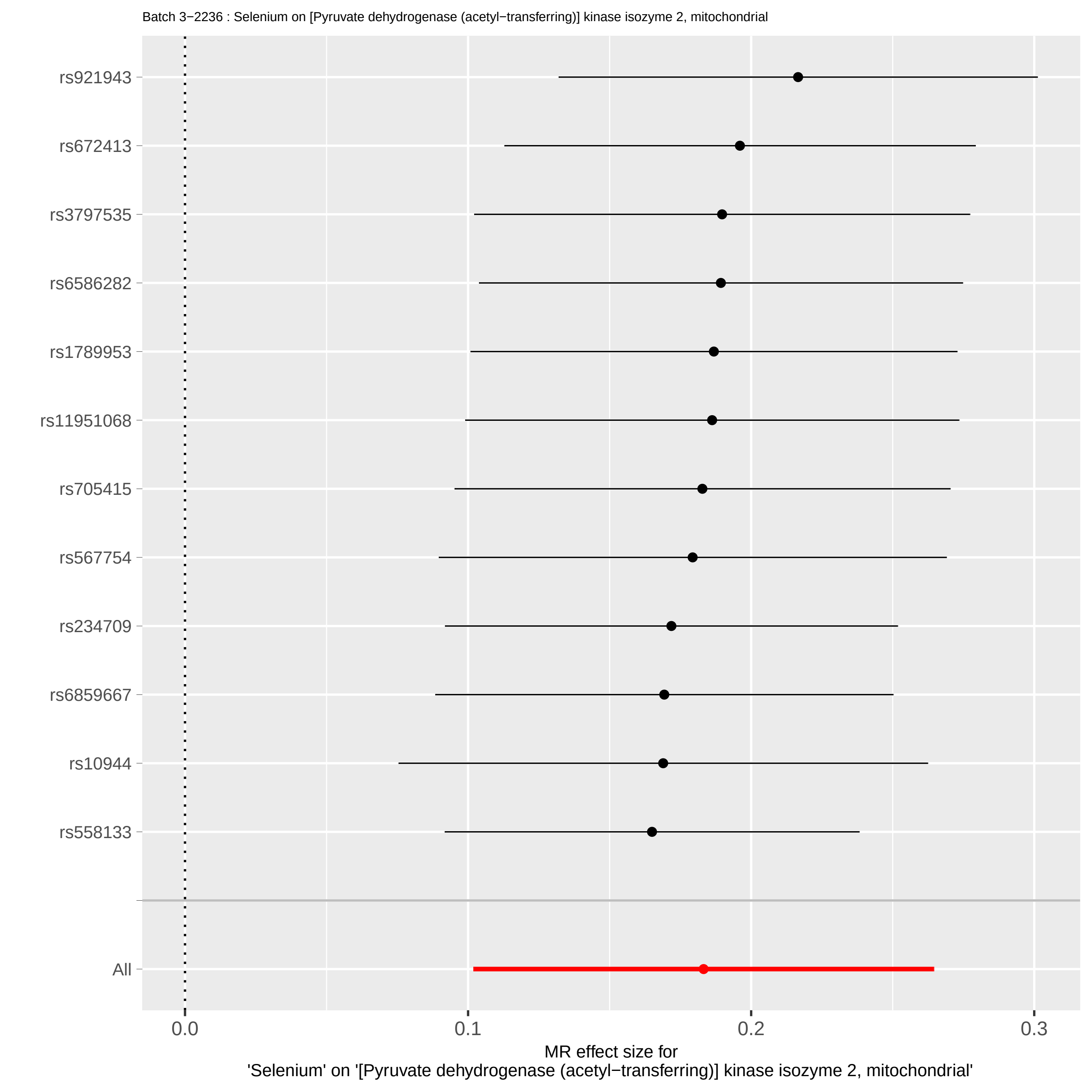
Batch 3−2236 : Selenium on [Pyruvate dehydrogenase (acetyl−transferring)] kinase isozyme 2, mitochondrial
rs921943
rs672413
rs3797535
rs6586282
rs1789953
rs11951068
rs705415
rs567754
rs234709
rs6859667
rs10944
rs558133
All
0.0
0.1
0.2
0.3
MR effect size for
'Selenium' on '[Pyruvate dehydrogenase (acetyl−transferring)] kinase isozyme 2, mitochondrial'

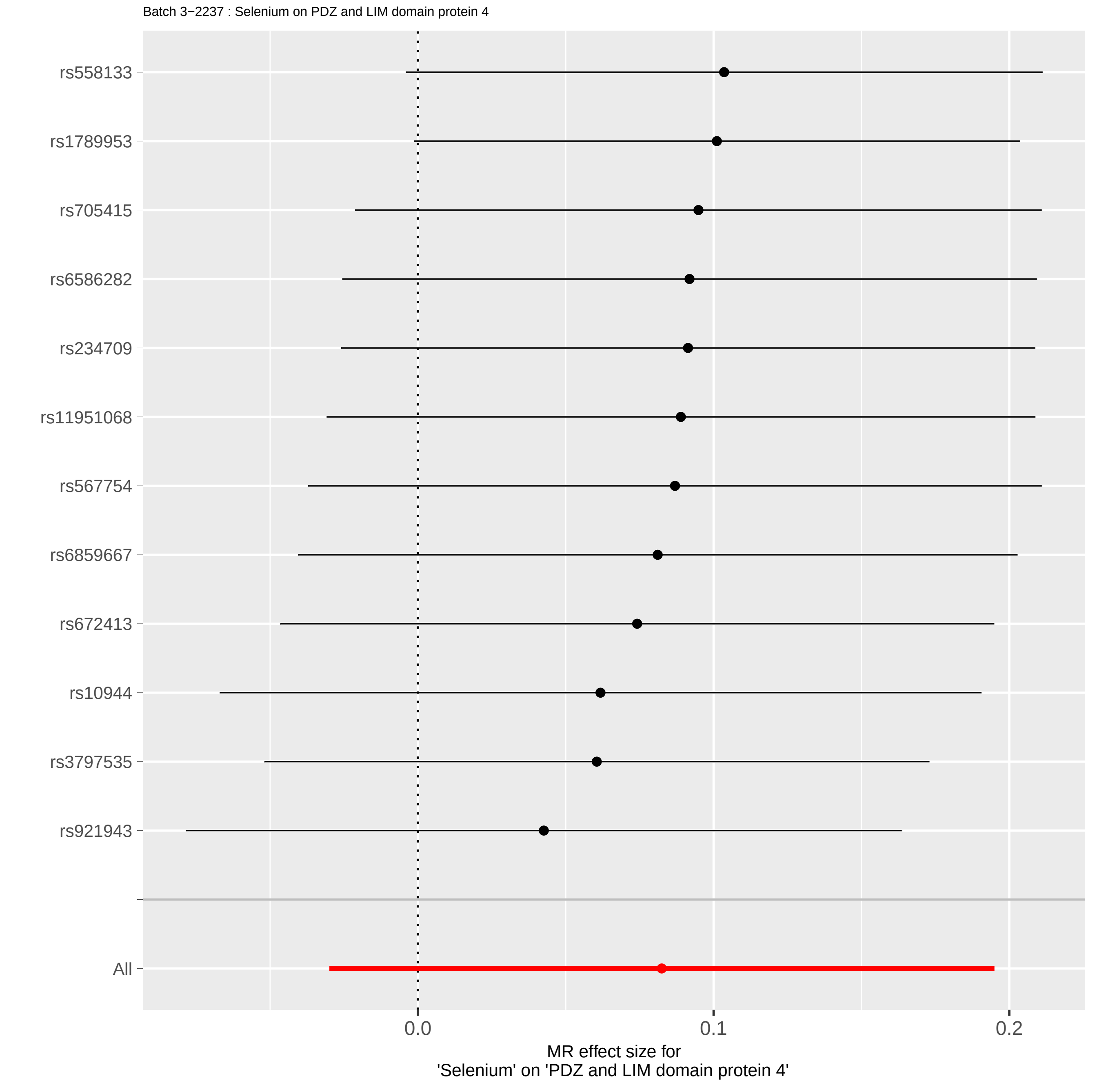
Batch 3−2237 : Selenium on PDZ and LIM domain protein 4
rs558133
rs1789953
rs705415
rs6586282
rs234709
rs11951068
rs567754
rs6859667
rs672413
rs10944
rs3797535
rs921943
All
0.0
0.1
0.2
MR effect size for
'Selenium' on 'PDZ and LIM domain protein 4'

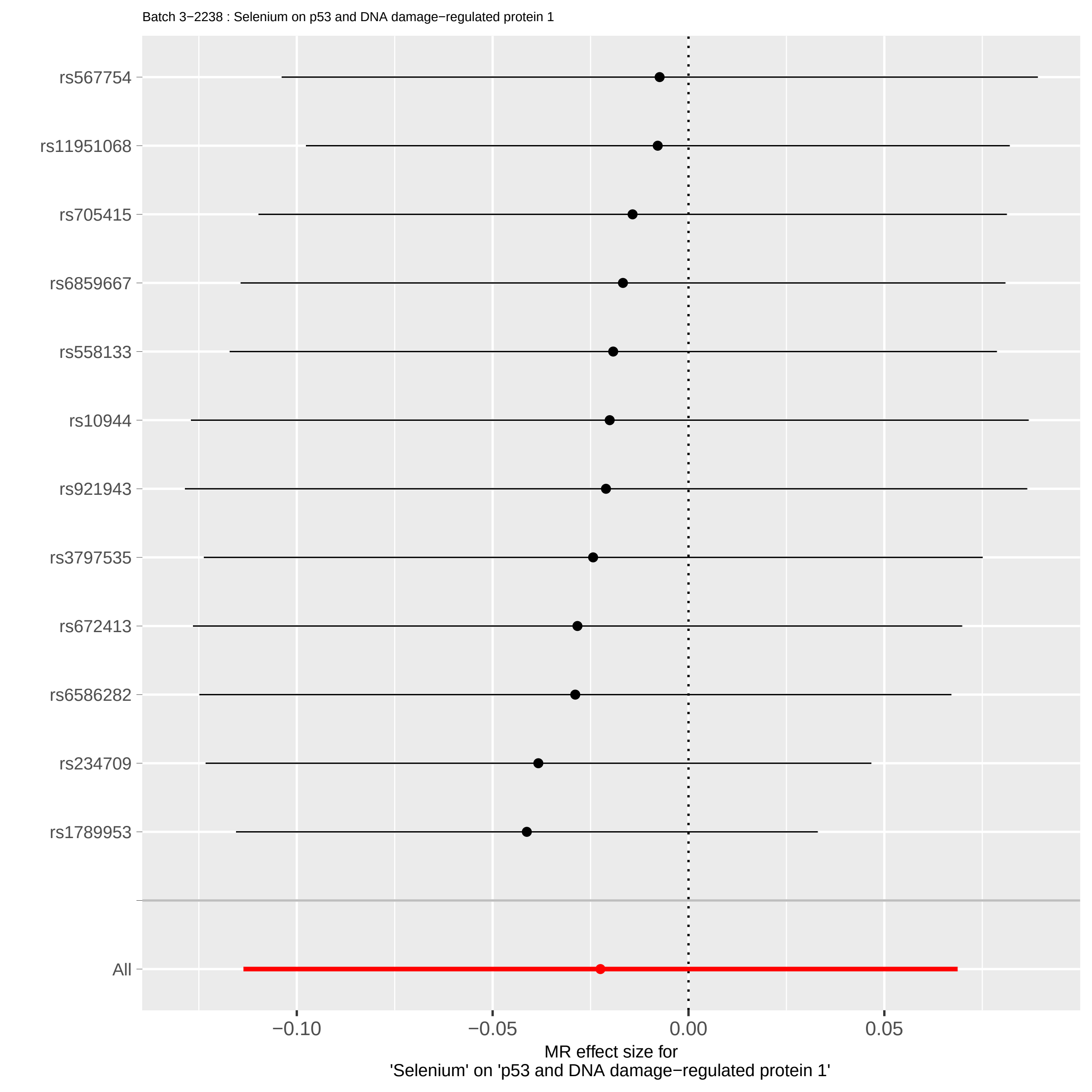

Batch 3−2238 : Selenium on p53 and DNA damage−regulated protein 1
rs567754
rs11951068
rs705415
rs6859667
rs558133
rs10944
rs921943
rs3797535
rs672413
rs6586282
rs234709
rs1789953
All
−0.10
−0.05
0.00
0.05
MR effect size for
'Selenium' on 'p53 and DNA damage−regulated protein 1'

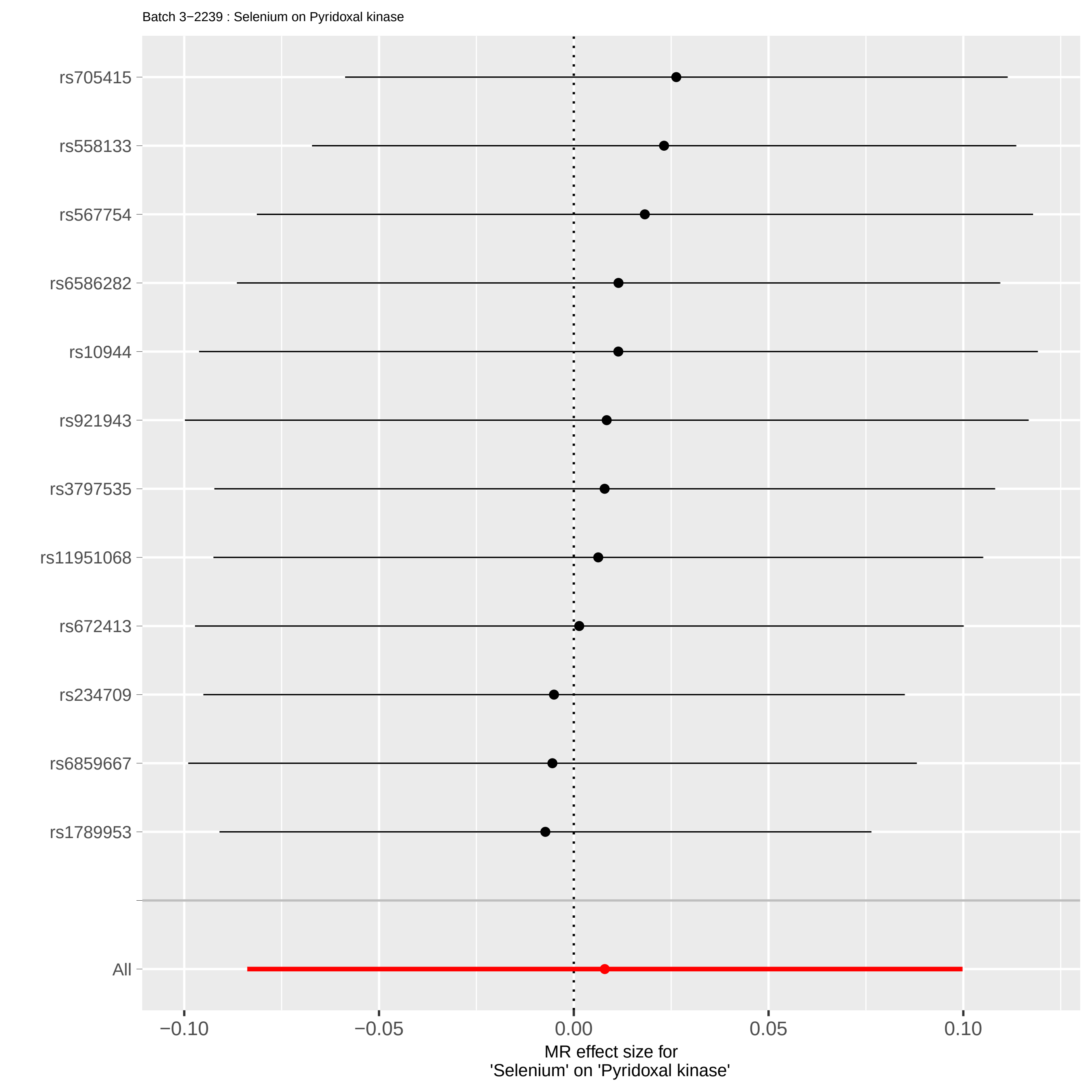

Batch 3−2239 : Selenium on Pyridoxal kinase
rs705415
rs558133
rs567754
rs6586282
rs10944
rs921943
rs3797535
rs11951068
rs672413
rs234709
rs6859667
rs1789953
All
−0.10
−0.05
0.00
0.05
0.10
MR effect size for
'Selenium' on 'Pyridoxal kinase'

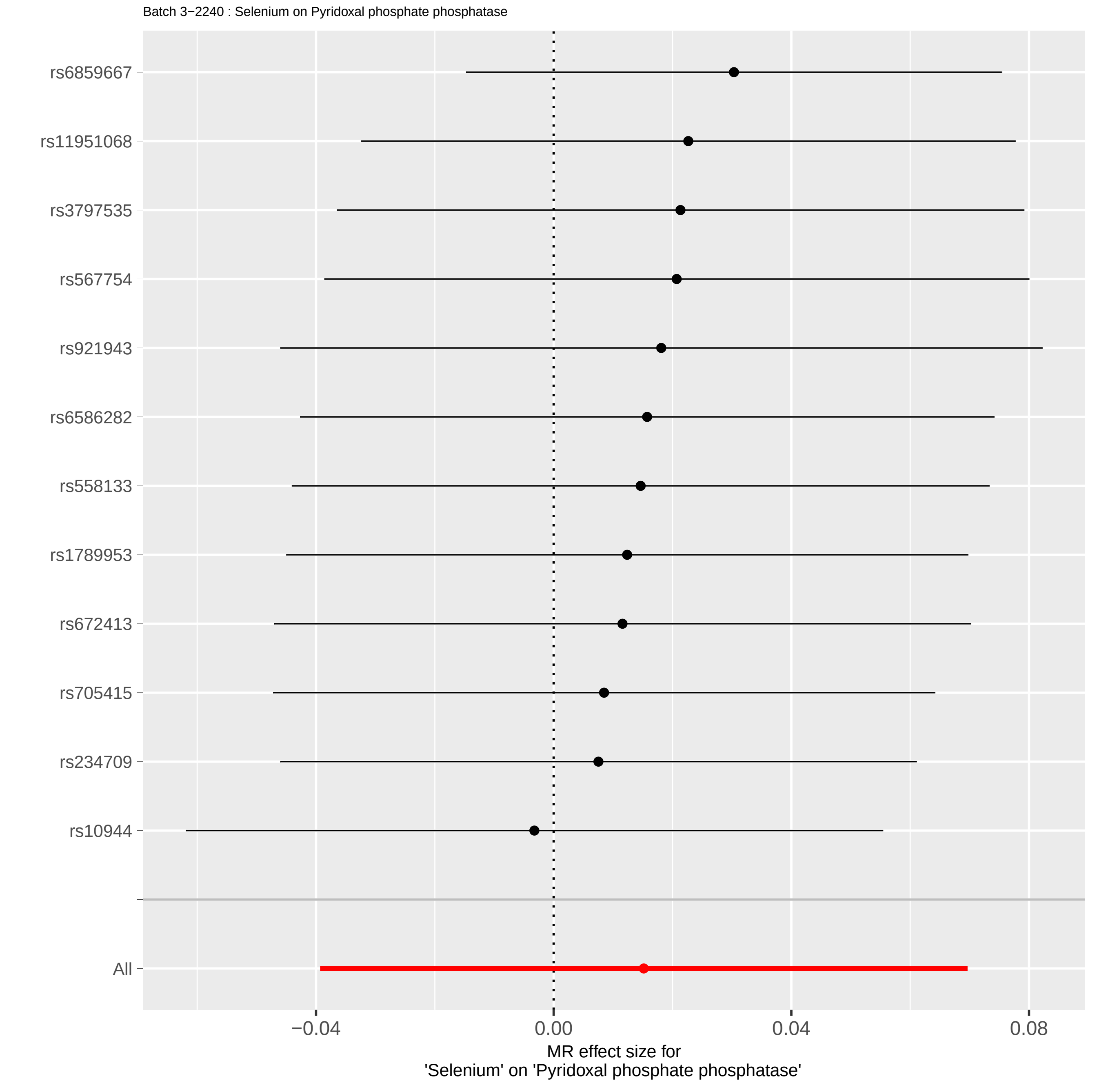
Batch 3−2240 : Selenium on Pyridoxal phosphate phosphatase
rs6859667
rs11951068
rs3797535
rs567754
rs921943
rs6586282
rs558133
rs1789953
rs672413
rs705415
rs234709
rs10944
All
−0.04
0.00
0.04
0.08
MR effect size for
'Selenium' on 'Pyridoxal phosphate phosphatase'

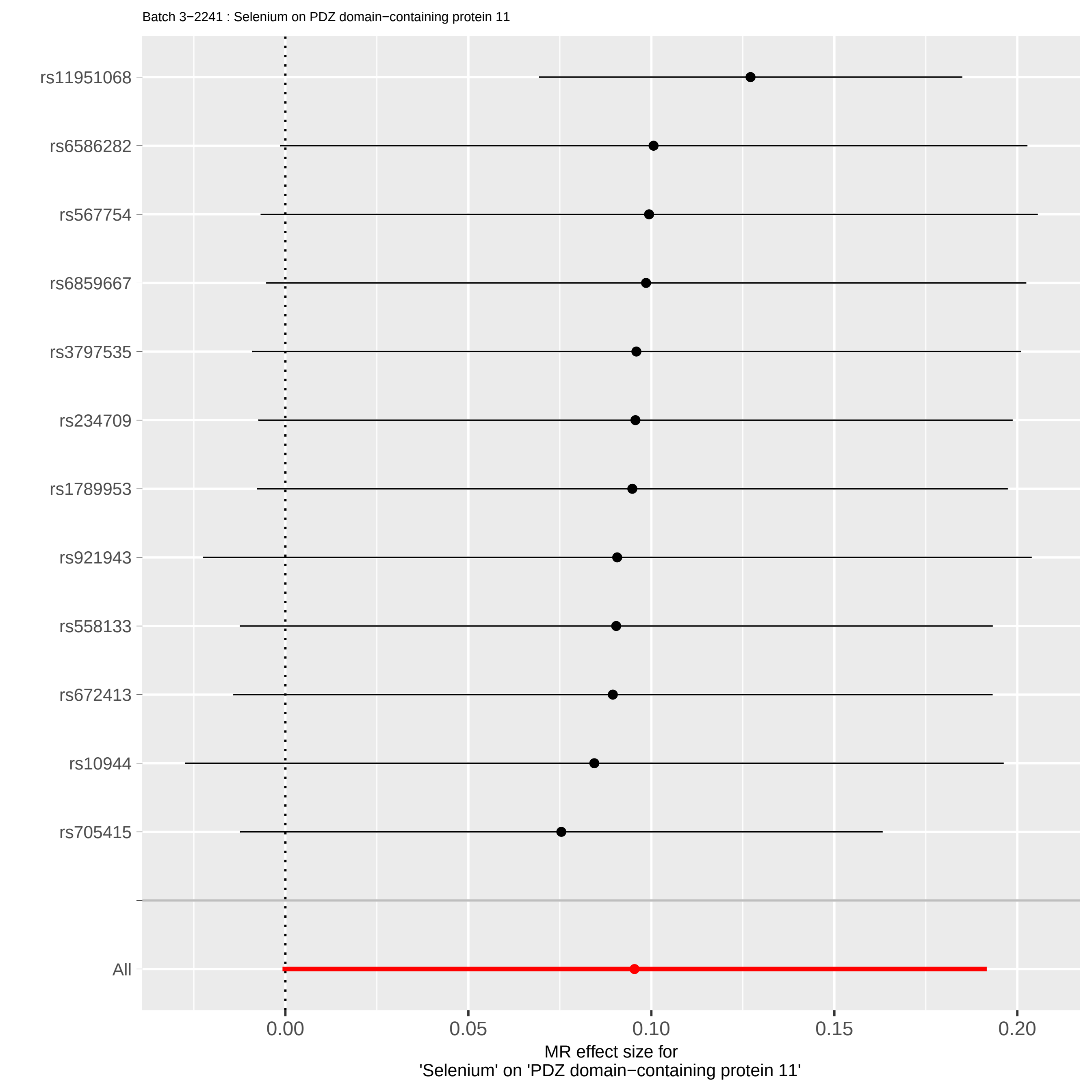

Batch 3−2241 : Selenium on PDZ domain−containing protein 11
rs11951068
rs6586282
rs567754
rs6859667
rs3797535
rs234709
rs1789953
rs921943
rs558133
rs672413
rs10944
rs705415
All
0.00
0.05
0.10
0.15
0.20
MR effect size for
'Selenium' on 'PDZ domain−containing protein 11'

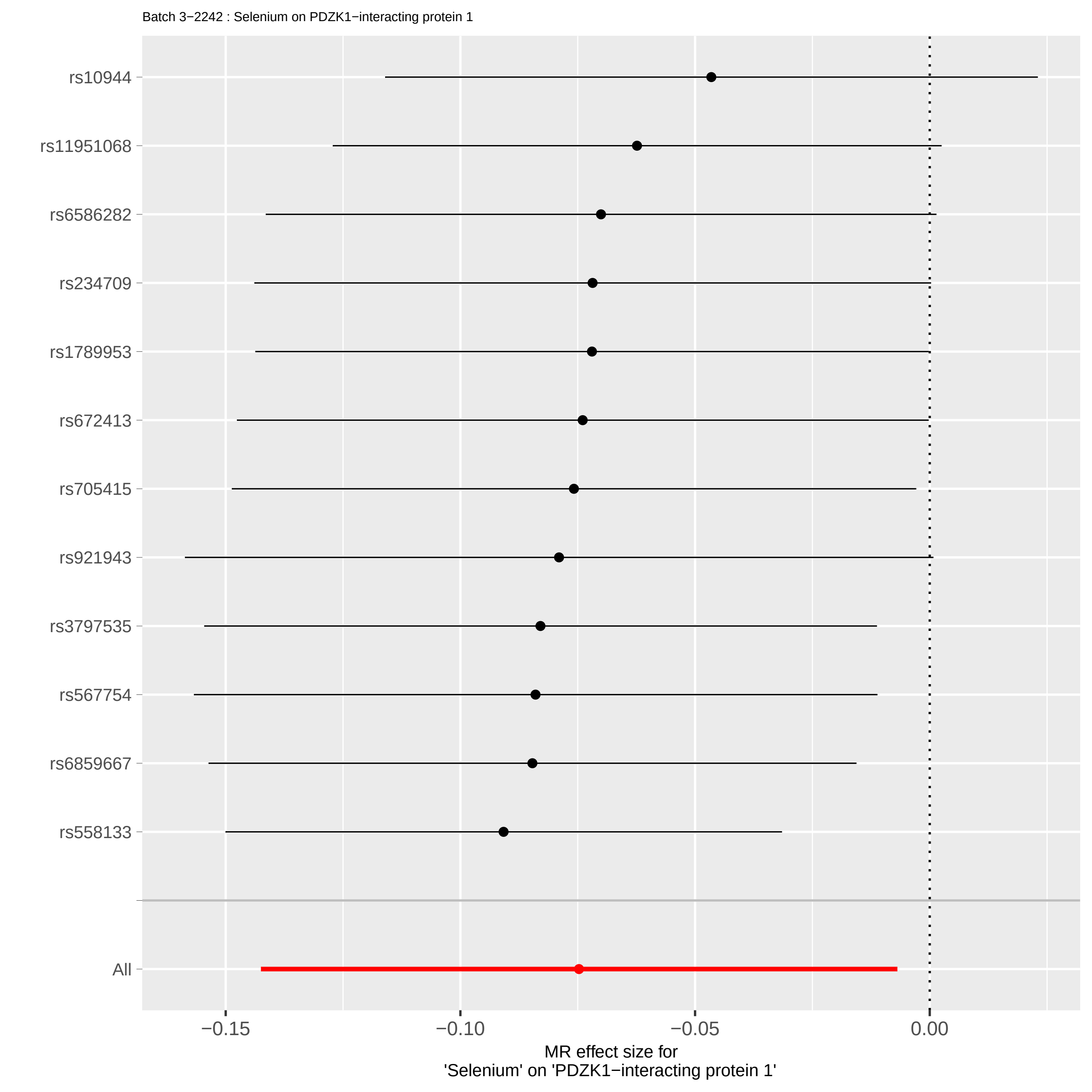

Batch 3−2242 : Selenium on PDZK1−interacting protein 1
rs10944
rs11951068
rs6586282
rs234709
rs1789953
rs672413
rs705415
rs921943
rs3797535
rs567754
rs6859667
rs558133
All
−0.15
−0.10
−0.05
0.00
MR effect size for
'Selenium' on 'PDZK1−interacting protein 1'

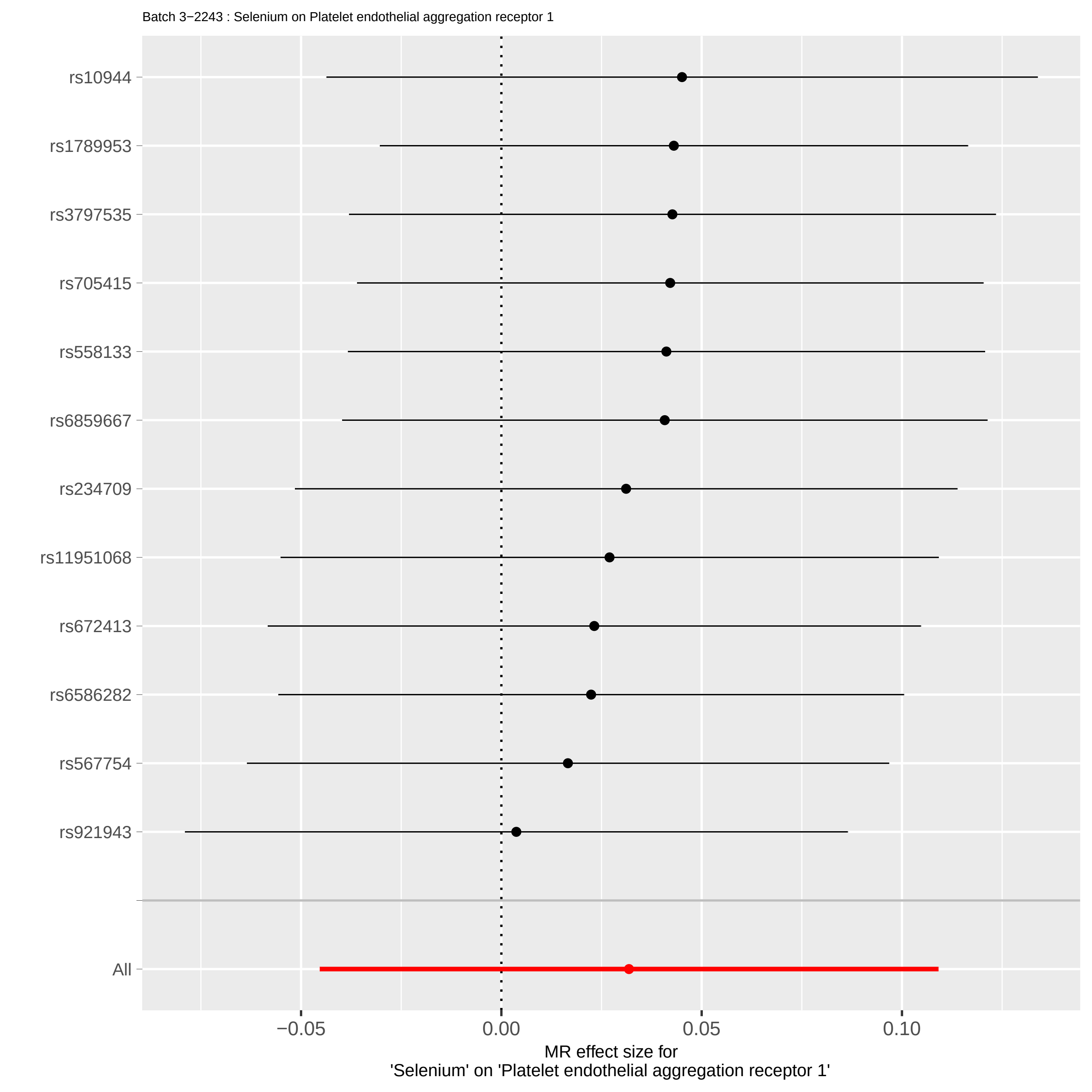

Batch 3−2243 : Selenium on Platelet endothelial aggregation receptor 1
rs10944
rs1789953
rs3797535
rs705415
rs558133
rs6859667
rs234709
rs11951068
rs672413
rs6586282
rs567754
rs921943
All
−0.05
0.00
0.05
0.10
MR effect size for
'Selenium' on 'Platelet endothelial aggregation receptor 1'

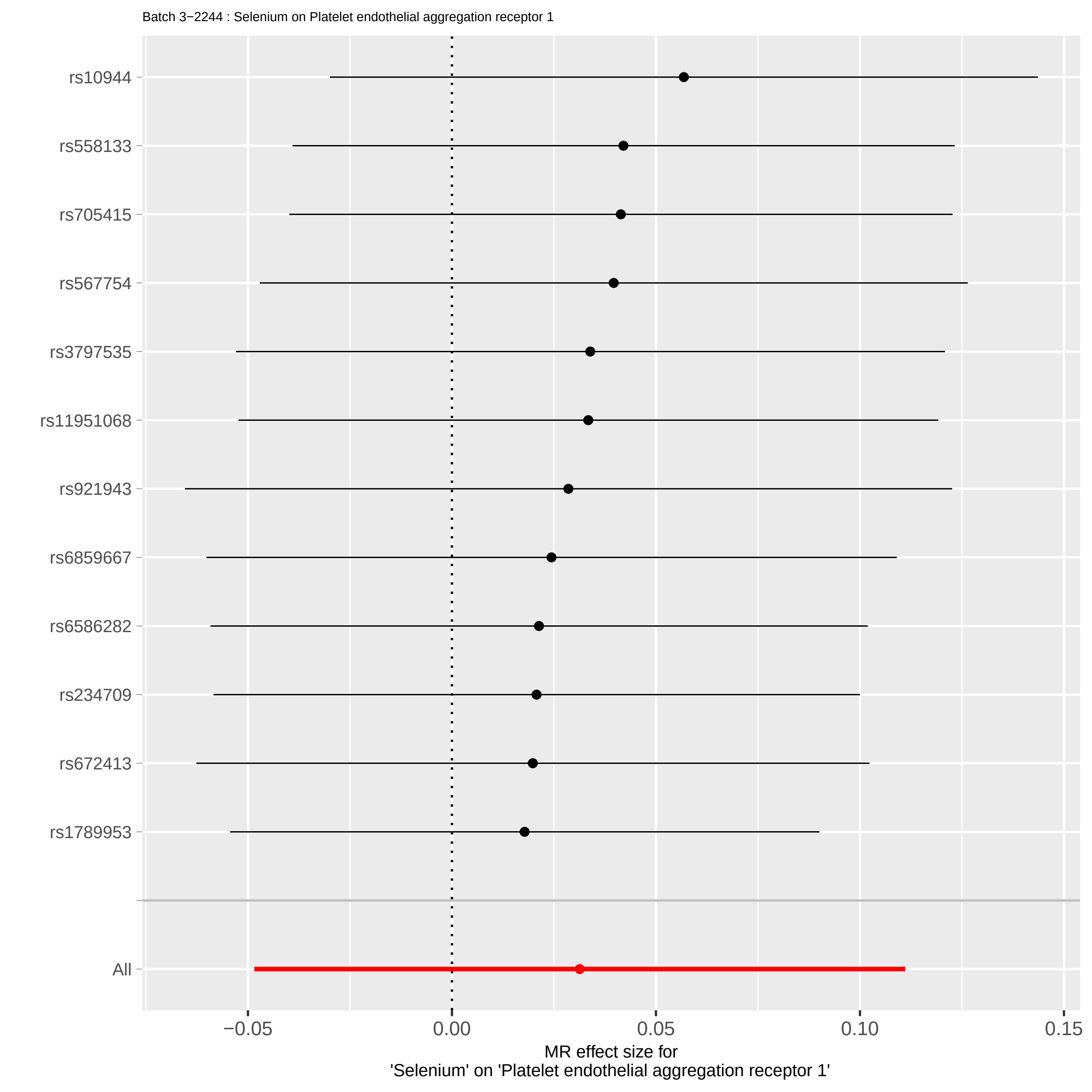

Batch 3−2244 : Selenium on Platelet endothelial aggregation receptor 1
rs10944
rs558133
rs705415
rs567754
rs3797535
rs11951068
rs921943
rs6859667
rs6586282
rs234709
rs672413
rs1789953
All
−0.05
0.00
0.05
0.10
0.15
MR effect size for
'Selenium' on 'Platelet endothelial aggregation receptor 1'

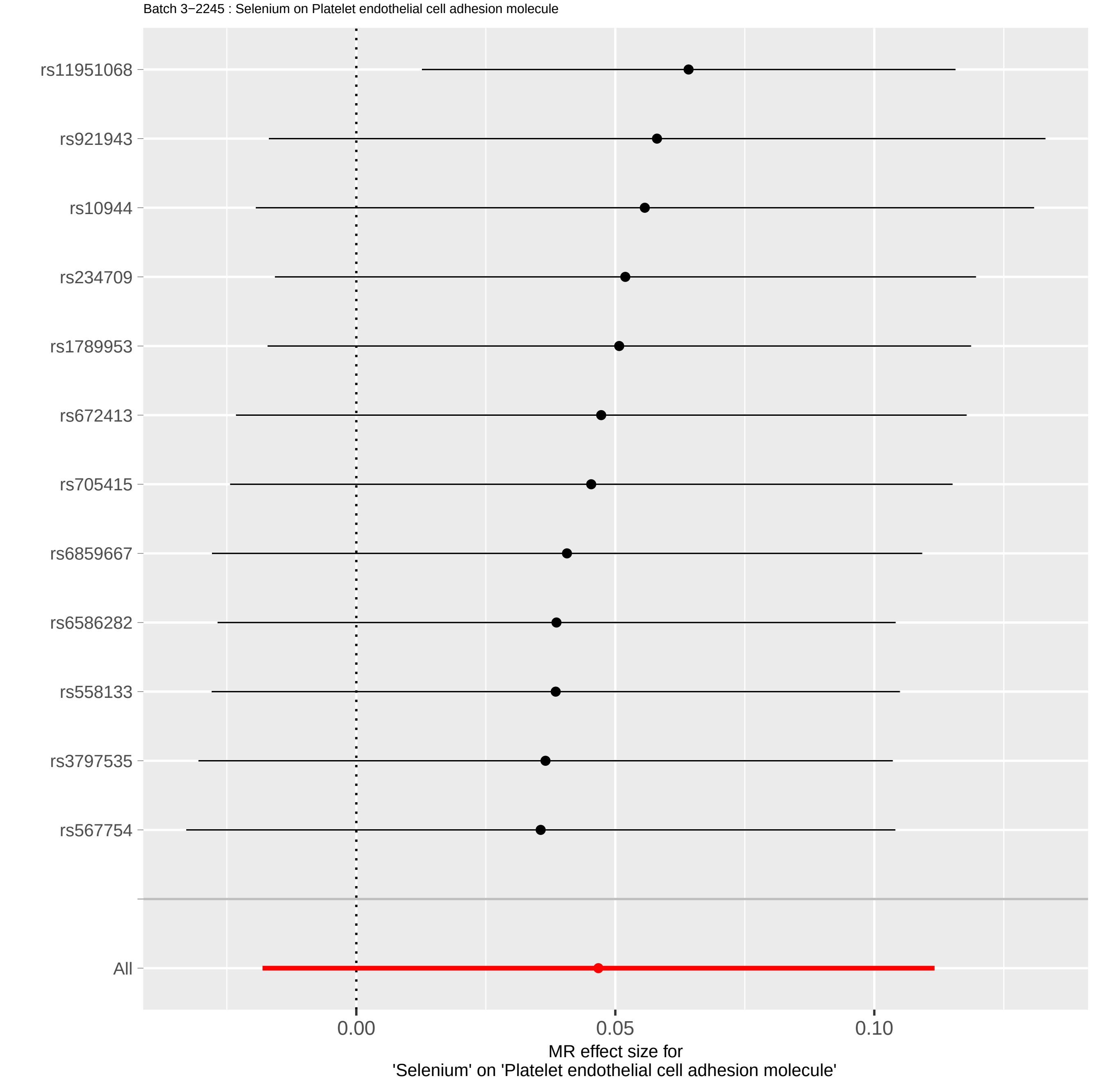

Batch 3−2245 : Selenium on Platelet endothelial cell adhesion molecule
rs11951068
rs921943
rs10944
rs234709
rs1789953
rs672413
rs705415
rs6859667
rs6586282
rs558133
rs3797535
rs567754
All
0.00
0.05
0.10
MR effect size for
'Selenium' on 'Platelet endothelial cell adhesion molecule'

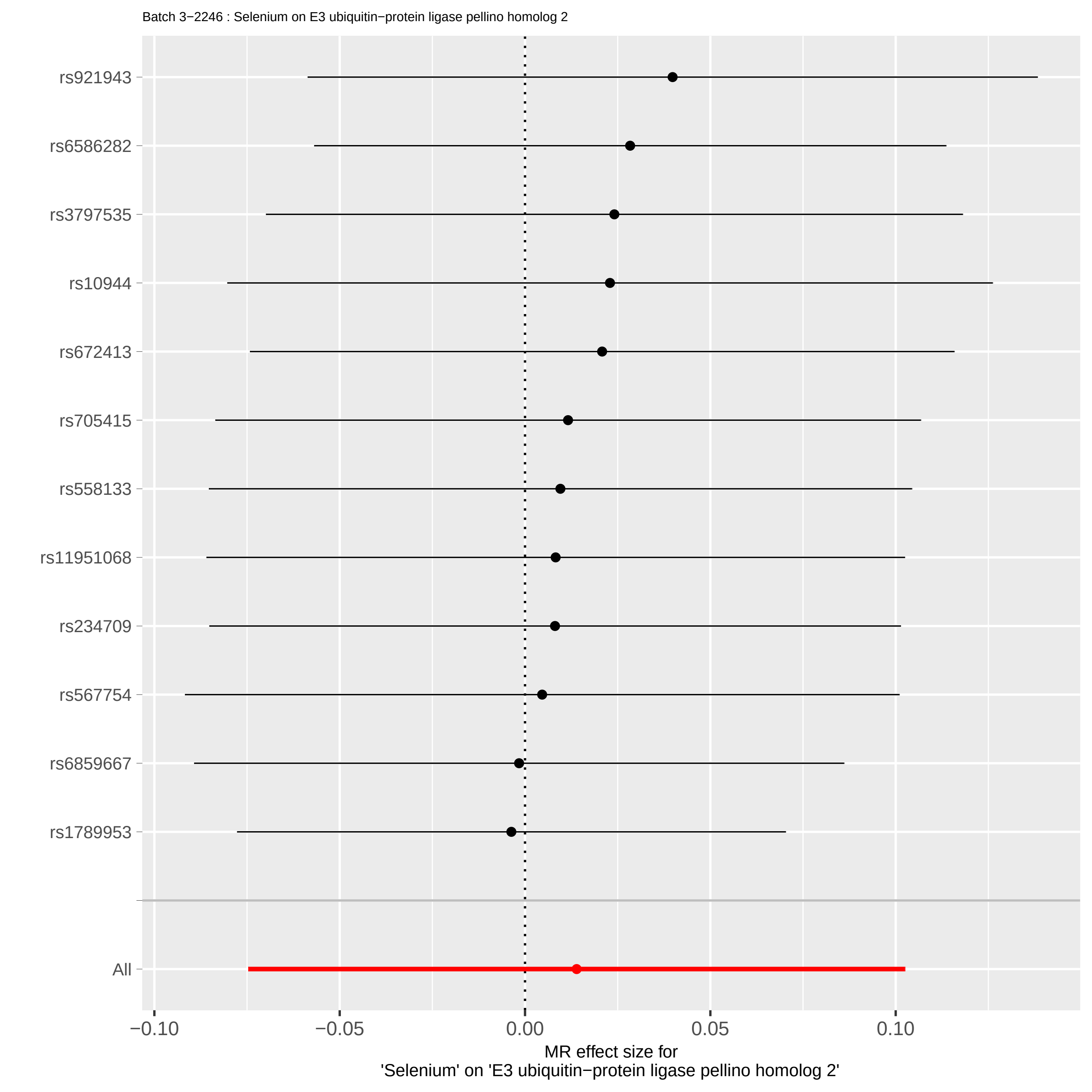

Batch 3−2246 : Selenium on E3 ubiquitin−protein ligase pellino homolog 2
rs921943
rs6586282
rs3797535
rs10944
rs672413
rs705415
rs558133
rs11951068
rs234709
rs567754
rs6859667
rs1789953
All
−0.10
−0.05
0.00
0.05
0.10
MR effect size for
'Selenium' on 'E3 ubiquitin−protein ligase pellino homolog 2'

Batch 3−2247 : Selenium on Proenkephalin−A

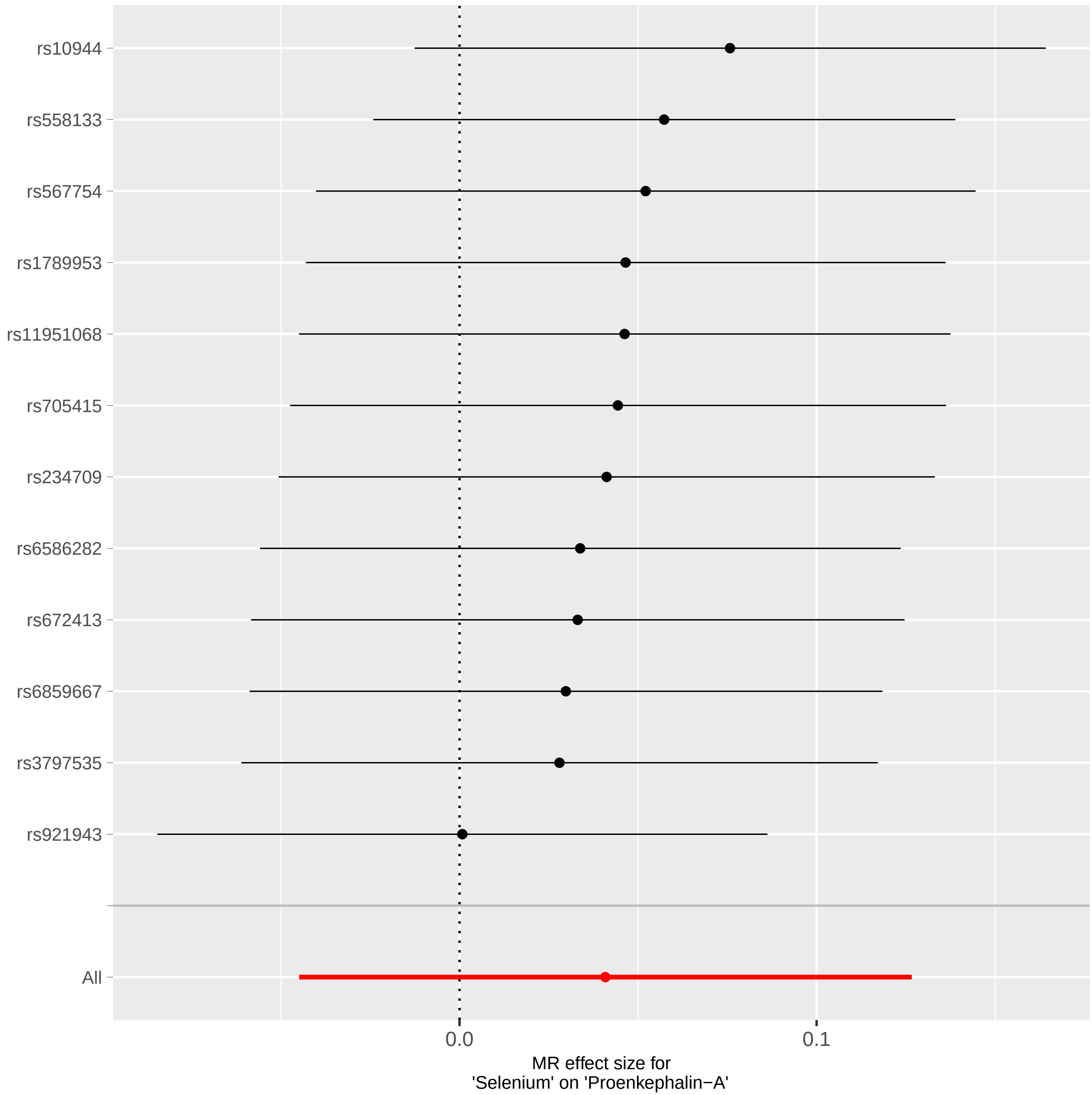

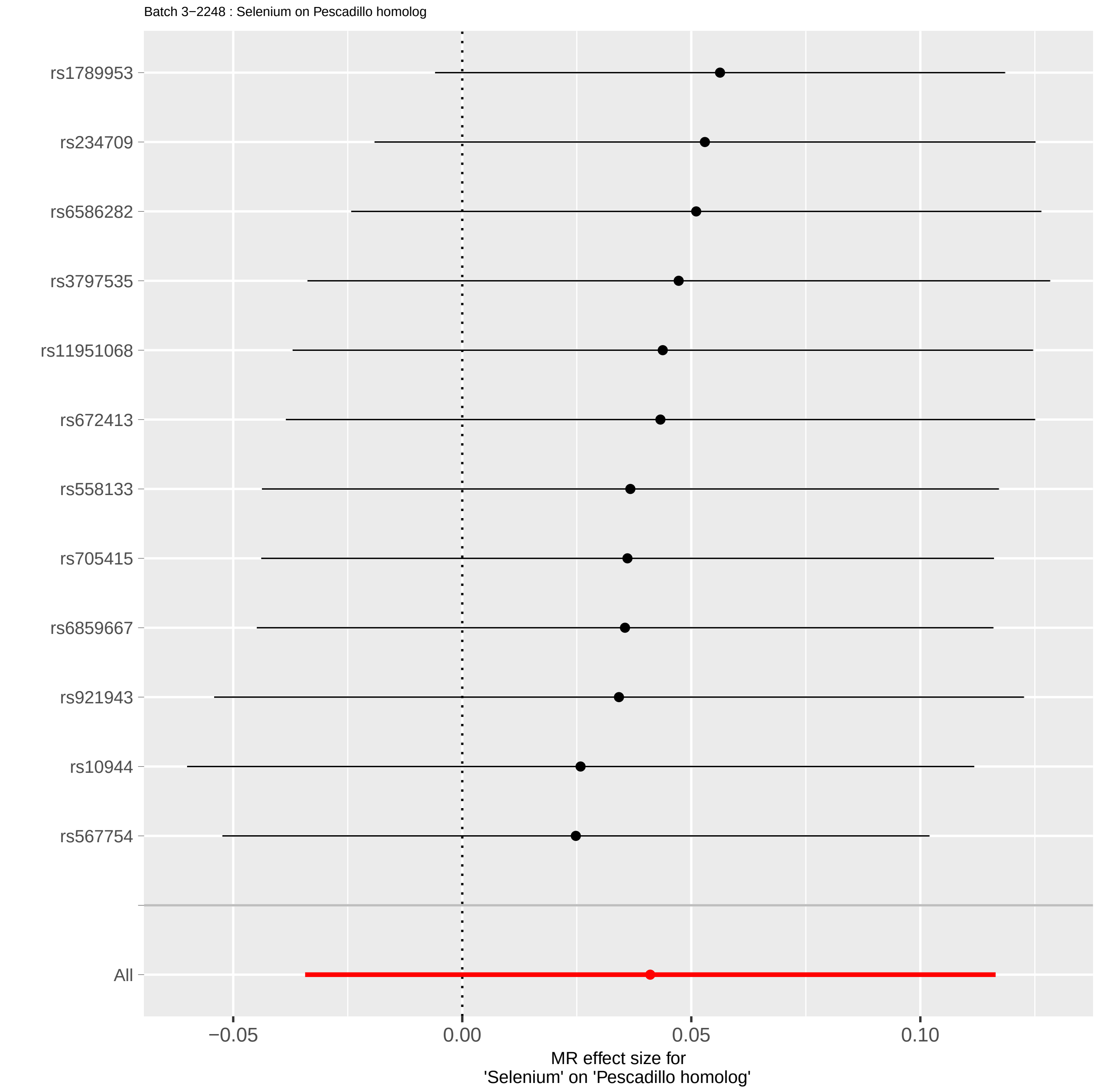
Batch 3−2248 : Selenium on Pescadillo homolog
rs1789953
rs234709
rs6586282
rs3797535
rs11951068
rs672413
rs558133
rs705415
rs6859667
rs921943
rs10944
rs567754
All
−0.05
0.00
0.05
0.10
MR effect size for
'Selenium' on 'Pescadillo homolog'

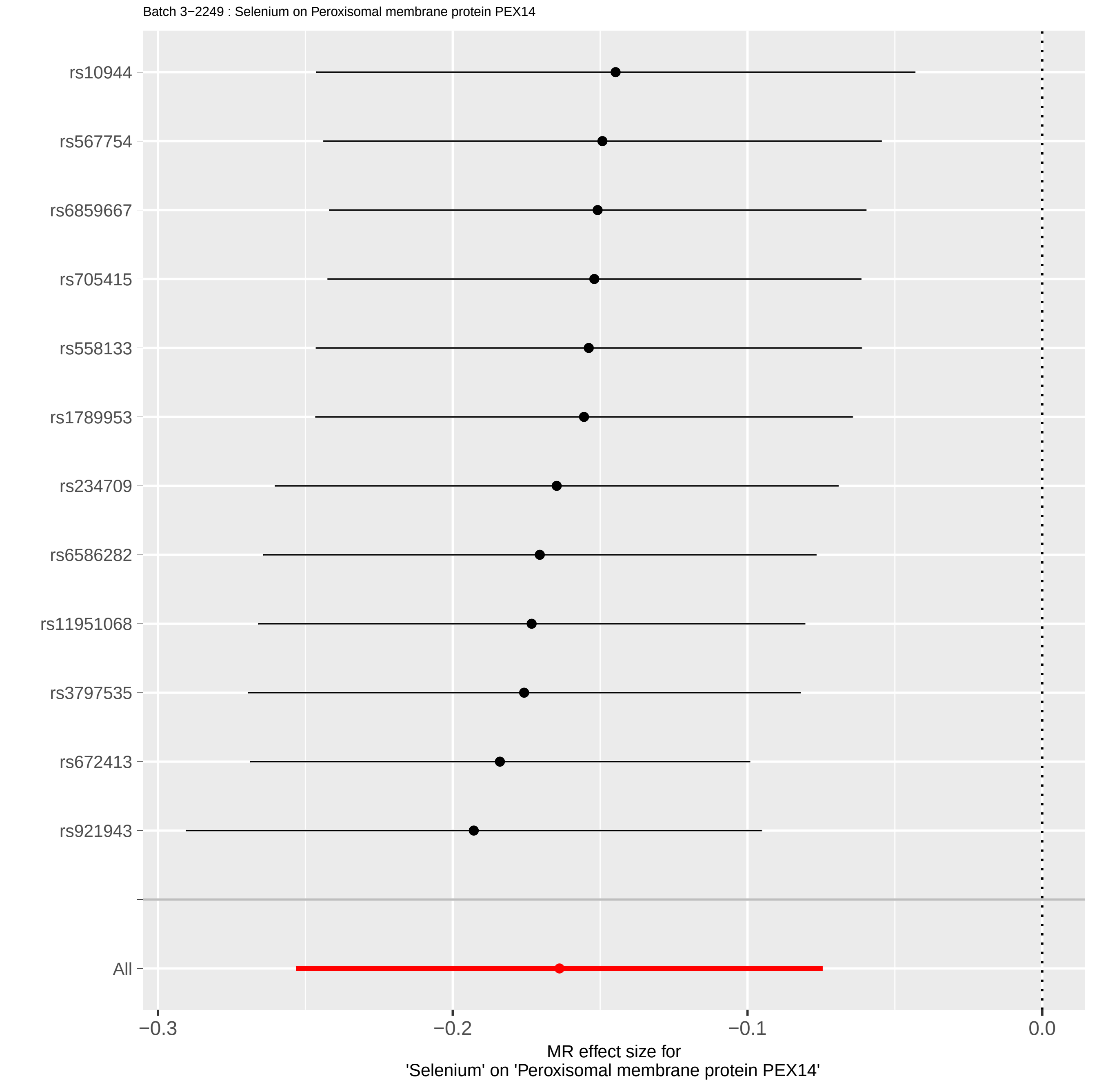

Batch 3−2249 : Selenium on Peroxisomal membrane protein PEX14
rs10944
rs567754
rs6859667
rs705415
rs558133
rs1789953
rs234709
rs6586282
rs11951068
rs3797535
rs672413
rs921943
All
−0.3
−0.2
−0.1
0.0
MR effect size for
'Selenium' on 'Peroxisomal membrane protein PEX14'

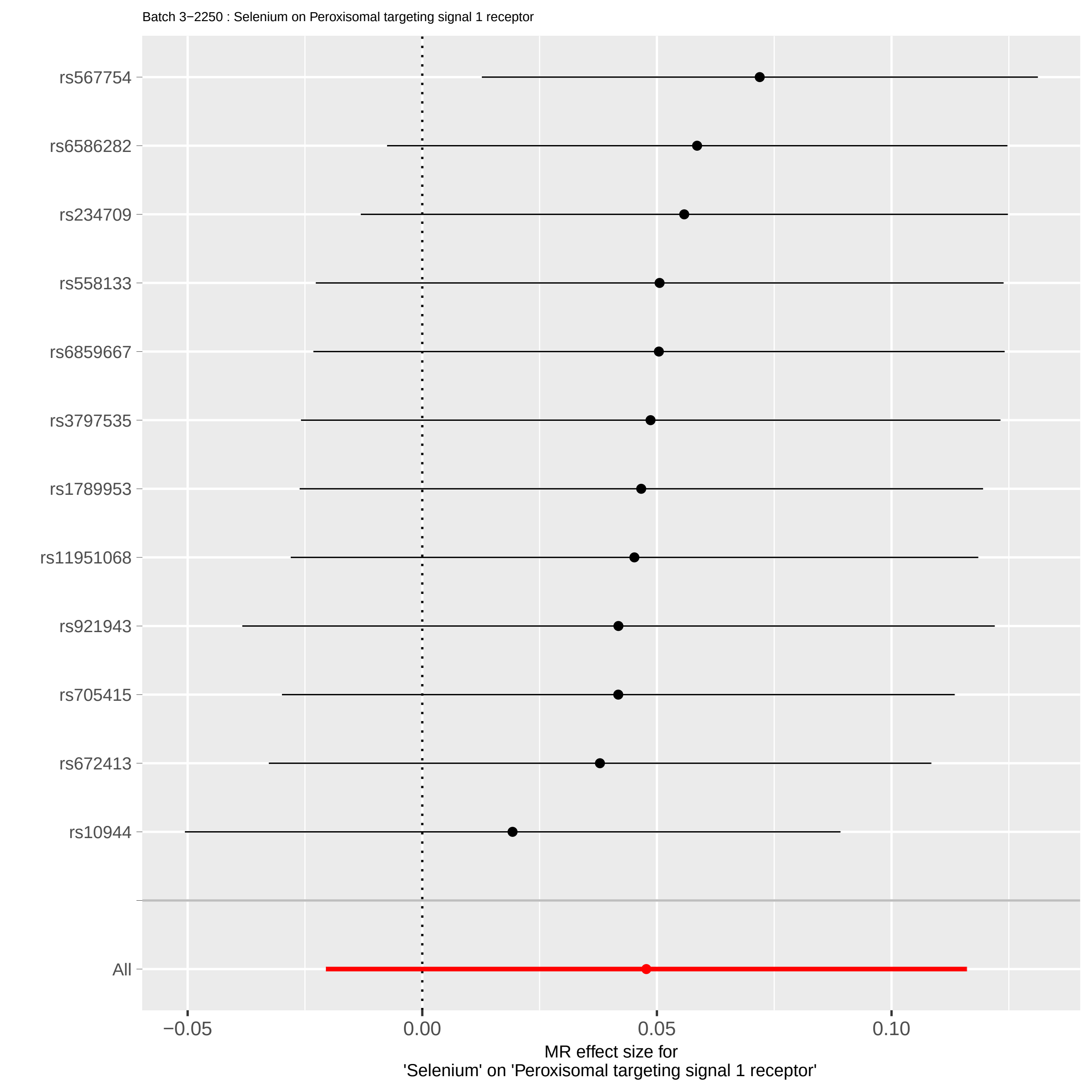

Batch 3−2250 : Selenium on Peroxisomal targeting signal 1 receptor
rs567754
rs6586282
rs234709
rs558133
rs6859667
rs3797535
rs1789953
rs11951068
rs921943
rs705415
rs672413
rs10944
All
−0.05
0.00
0.05
0.10
MR effect size for
'Selenium' on 'Peroxisomal targeting signal 1 receptor'

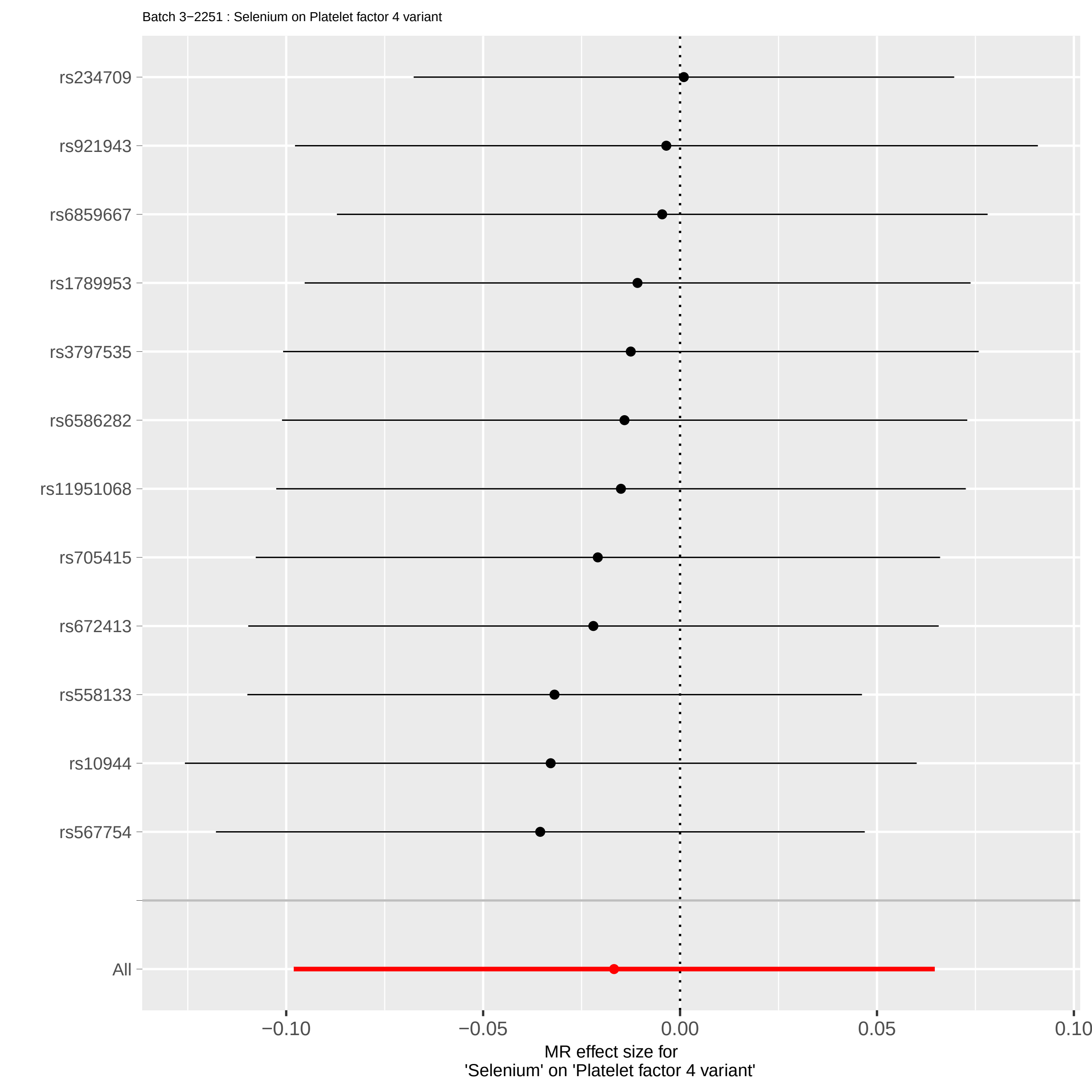

Batch 3−2251 : Selenium on Platelet factor 4 variant
rs234709
rs921943
rs6859667
rs1789953
rs3797535
rs6586282
rs11951068
rs705415
rs672413
rs558133
rs10944
rs567754
All
−0.10
−0.05
0.00
0.05
0.10
MR effect size for
'Selenium' on 'Platelet factor 4 variant'

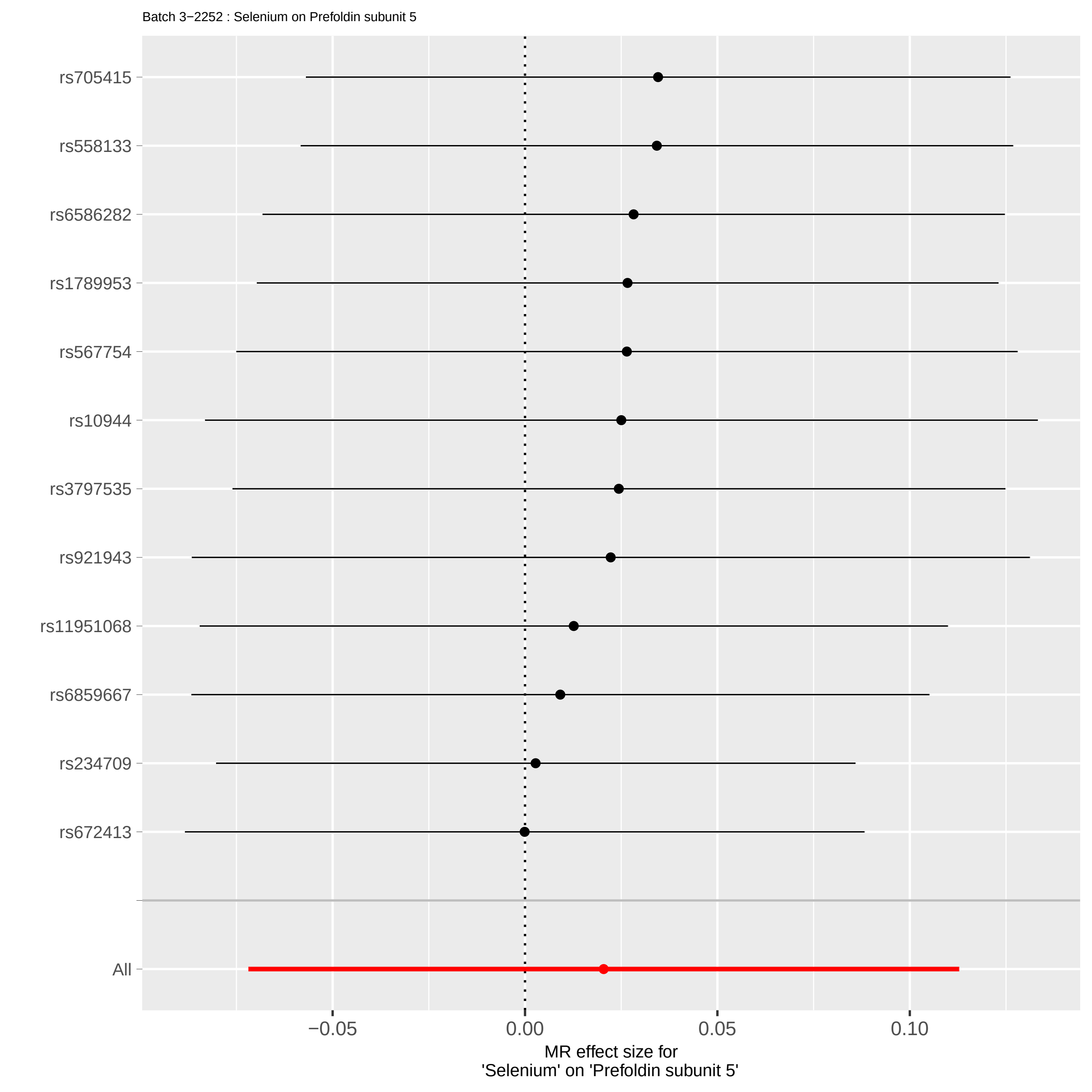

Batch 3−2252 : Selenium on Prefoldin subunit 5
rs705415
rs558133
rs6586282
rs1789953
rs567754
rs10944
rs3797535
rs921943
rs11951068
rs6859667
rs234709
rs672413
All
−0.05
0.00
0.05
0.10
MR effect size for
'Selenium' on 'Prefoldin subunit 5'

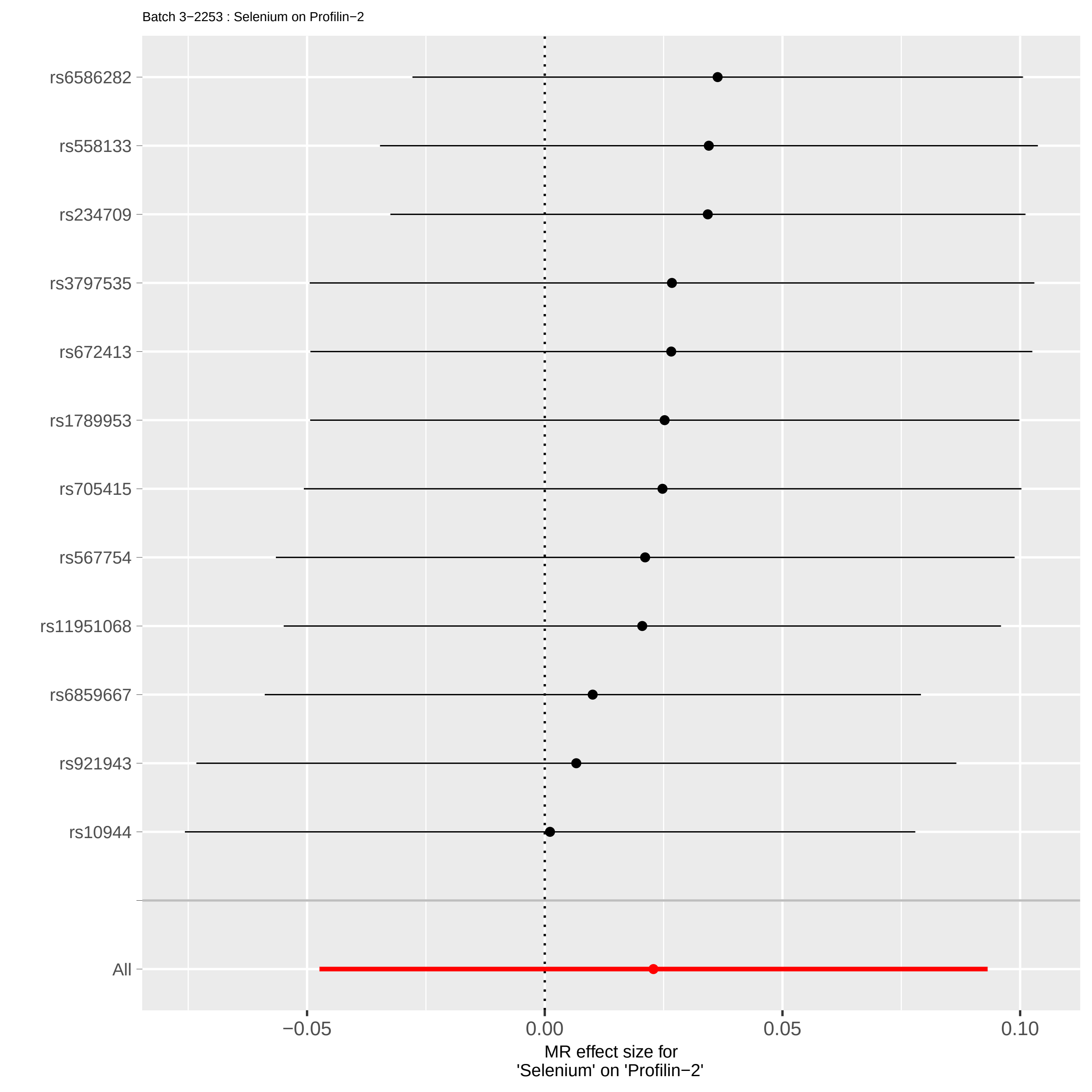

Batch 3−2253 : Selenium on Profilin−2
rs6586282
rs558133
rs234709
rs3797535
rs672413
rs1789953
rs705415
rs567754
rs11951068
rs6859667
rs921943
rs10944
All
−0.05
0.00
0.05
0.10
MR effect size for
'Selenium' on 'Profilin−2'

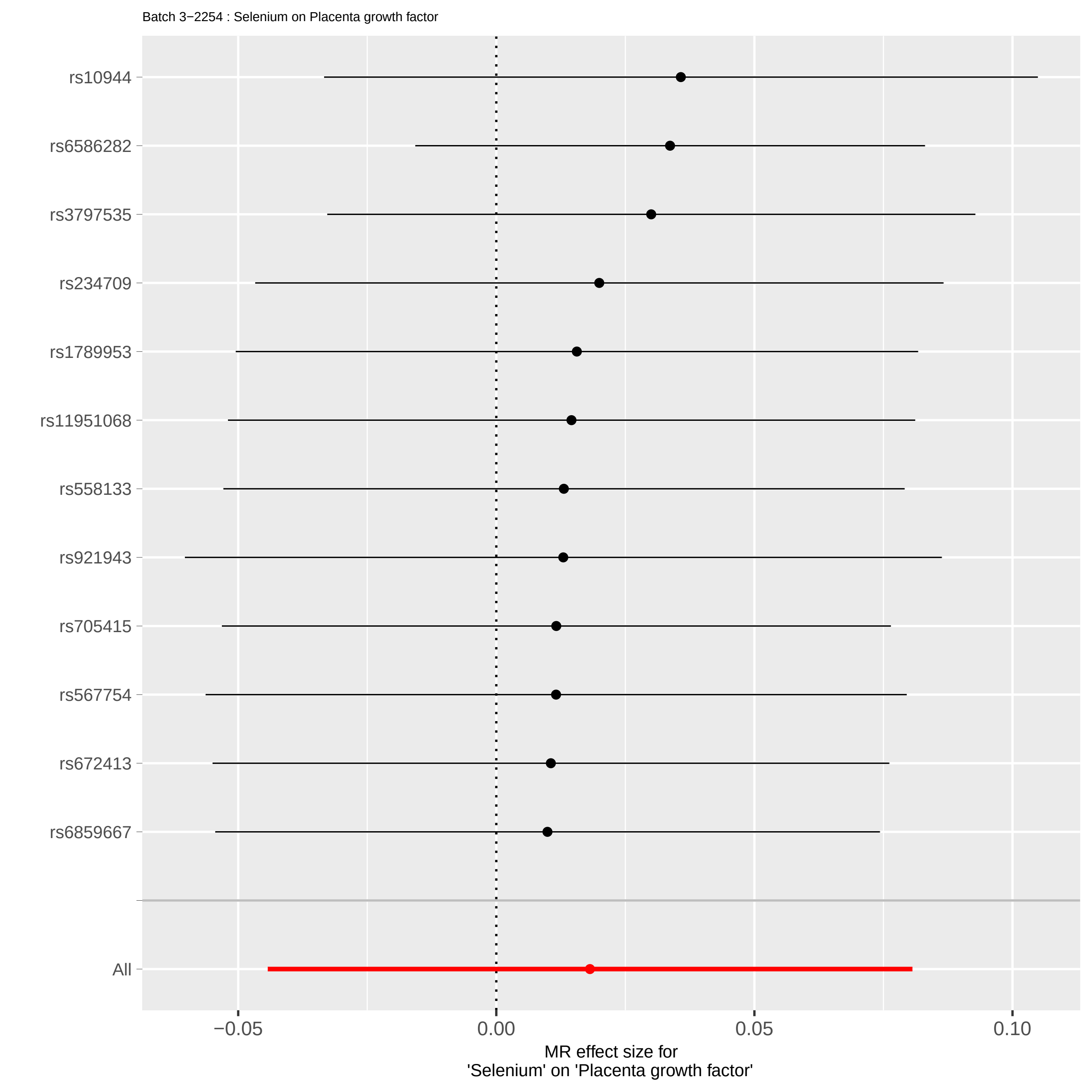

Batch 3−2254 : Selenium on Placenta growth factor
rs10944
rs6586282
rs3797535
rs234709
rs1789953
rs11951068
rs558133
rs921943
rs705415
rs567754
rs672413
rs6859667
All
−0.05
0.00
0.05
0.10
MR effect size for
'Selenium' on 'Placenta growth factor'

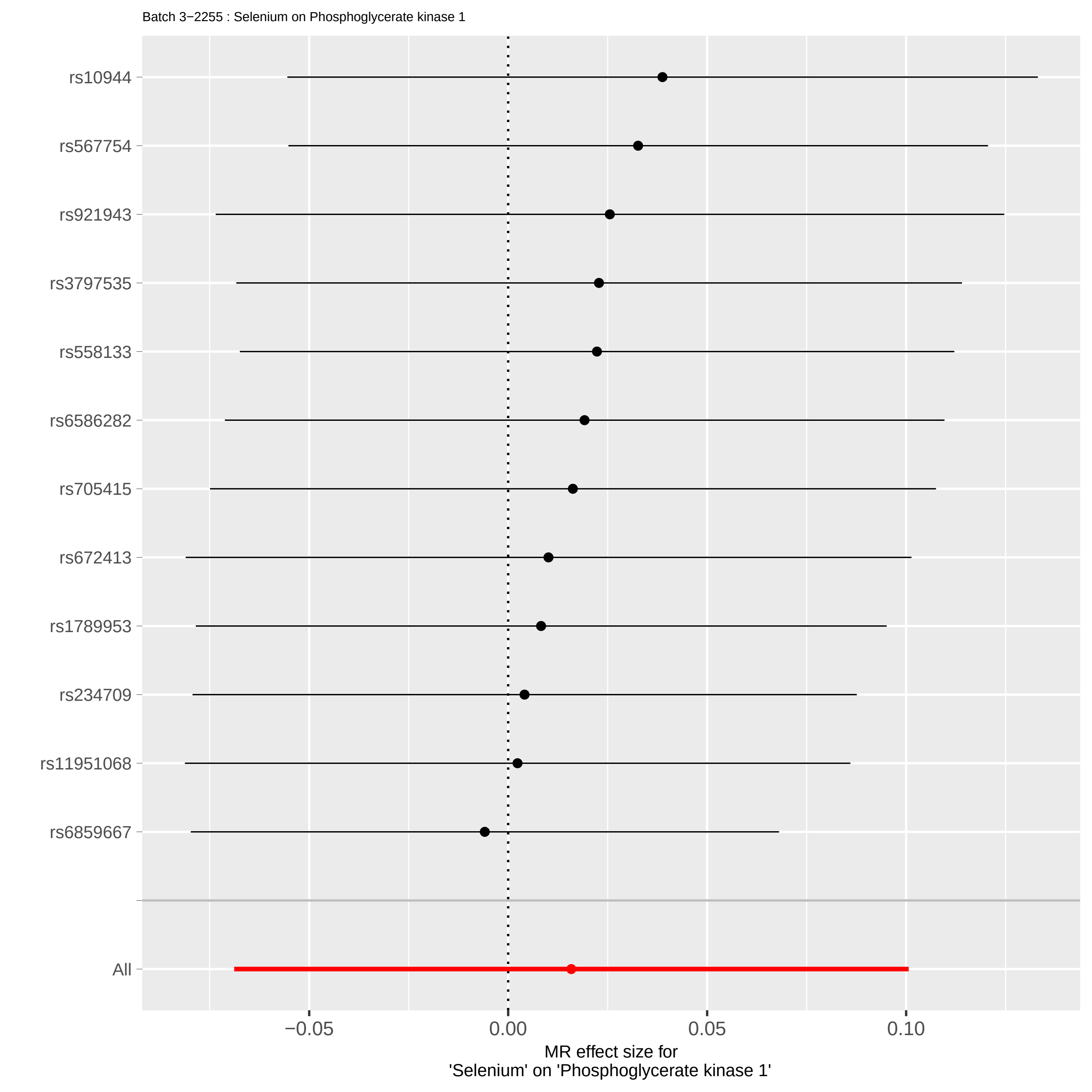
Batch 3−2255 : Selenium on Phosphoglycerate kinase 1
rs10944
rs567754
rs921943
rs3797535
rs558133
rs6586282
rs705415
rs672413
rs1789953
rs234709
rs11951068
rs6859667
All
−0.05
0.00
0.05
0.10
MR effect size for
'Selenium' on 'Phosphoglycerate kinase 1'

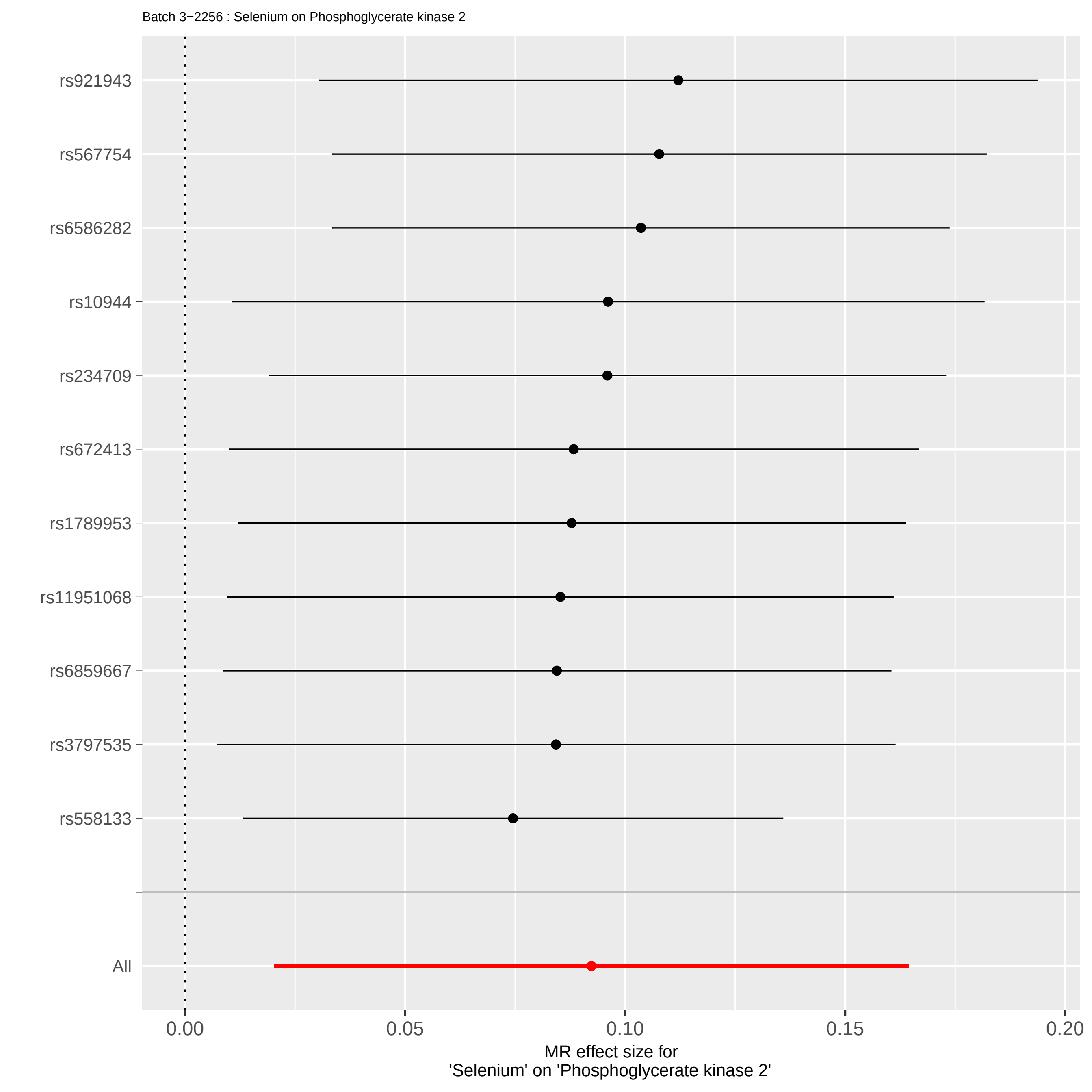
Batch 3−2256 : Selenium on Phosphoglycerate kinase 2
rs921943
rs567754
rs6586282
rs10944
rs234709
rs672413
rs1789953
rs11951068
rs6859667
rs3797535
rs558133
All
0.00
0.05
0.10
0.15
0.20
MR effect size for
'Selenium' on 'Phosphoglycerate kinase 2'

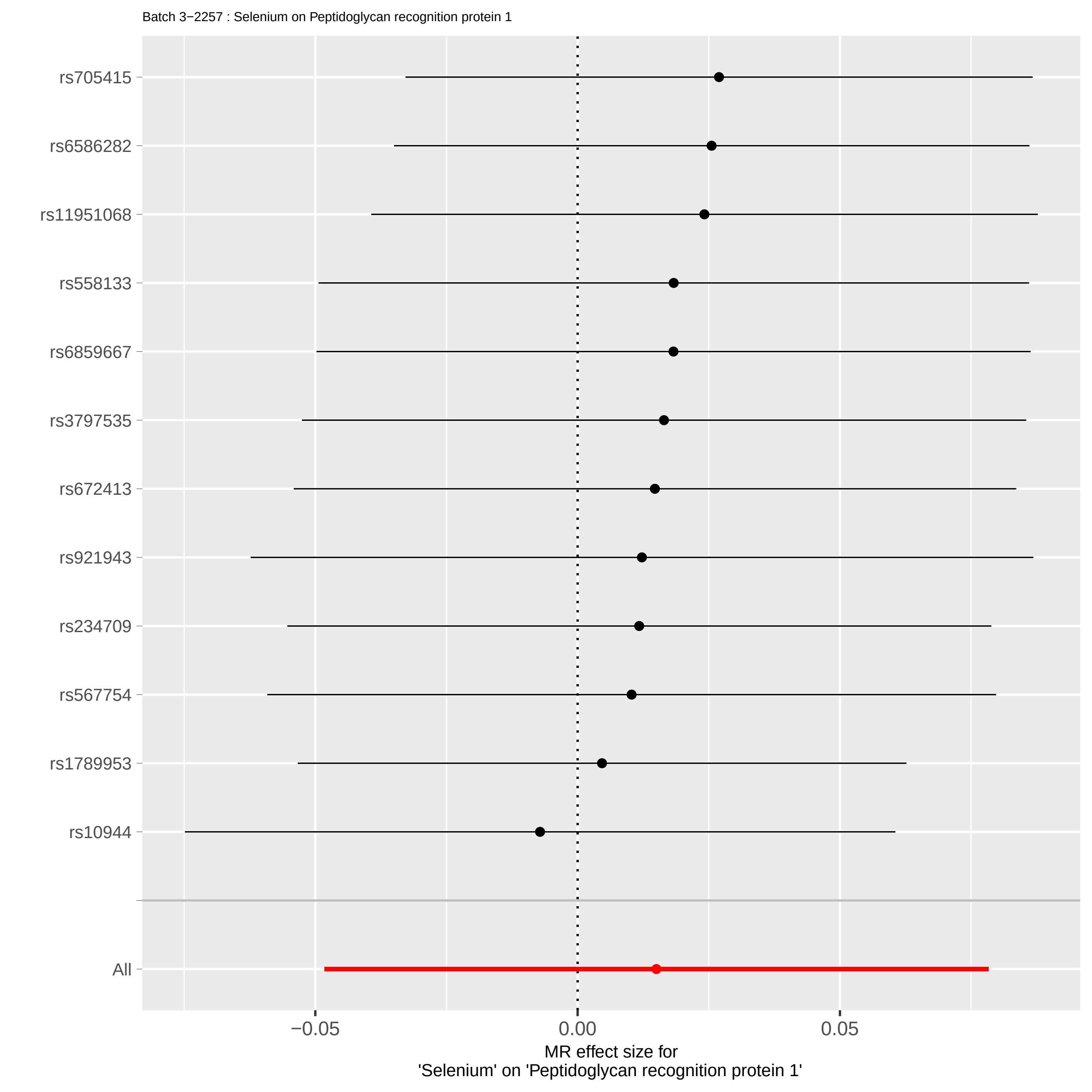
Batch 3−2257 : Selenium on Peptidoglycan recognition protein 1
rs705415
rs6586282
rs11951068
rs558133
rs6859667
rs3797535
rs672413
rs921943
rs234709
rs567754
rs1789953
rs10944
All
−0.05
0.00
0.05
MR effect size for
'Selenium' on 'Peptidoglycan recognition protein 1'

Batch 3−2258 : Selenium on Phosphoglucomutase−1

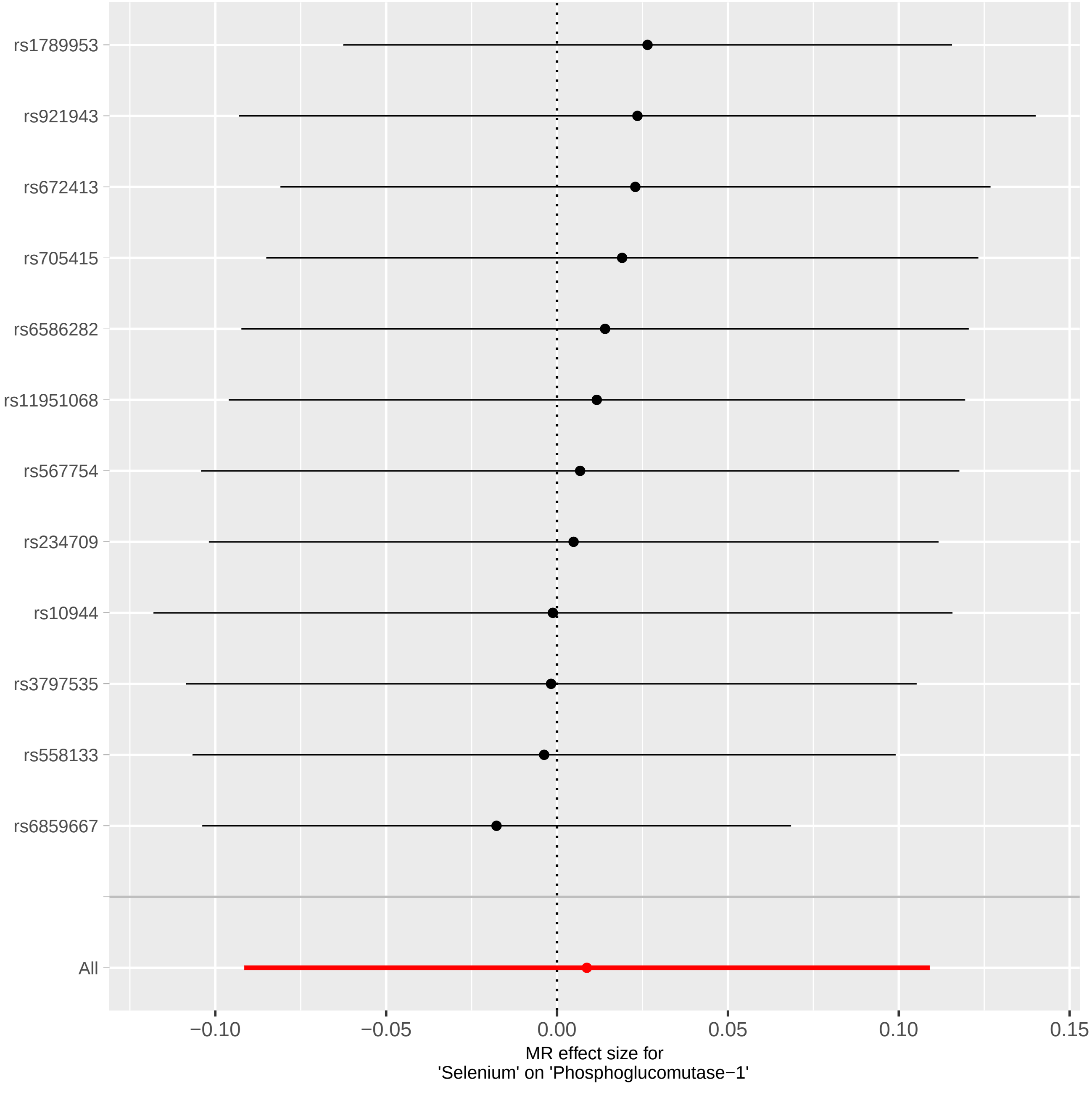

Batch 3−2259 : Selenium on Membrane−associated progesterone receptor component 1

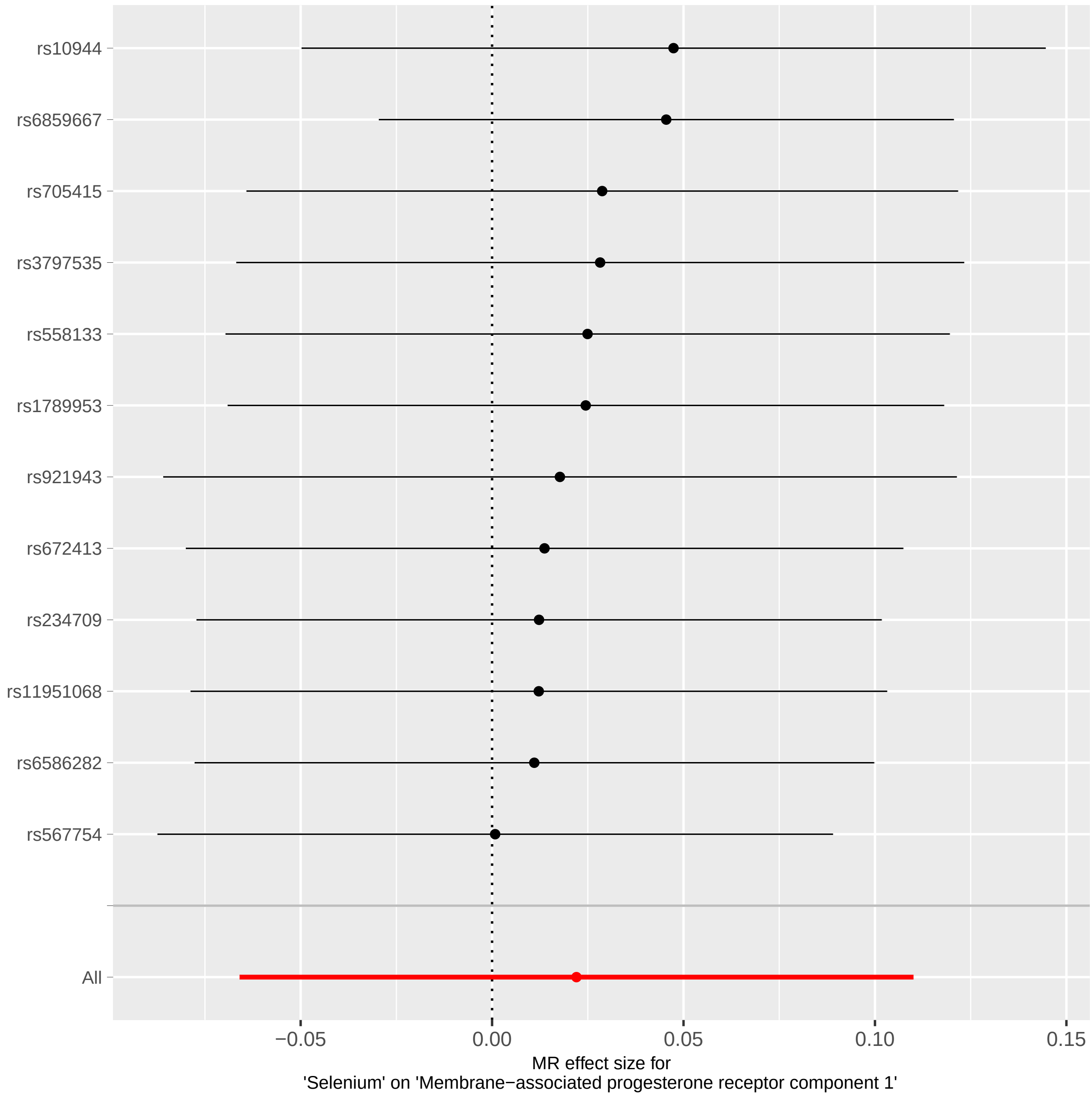

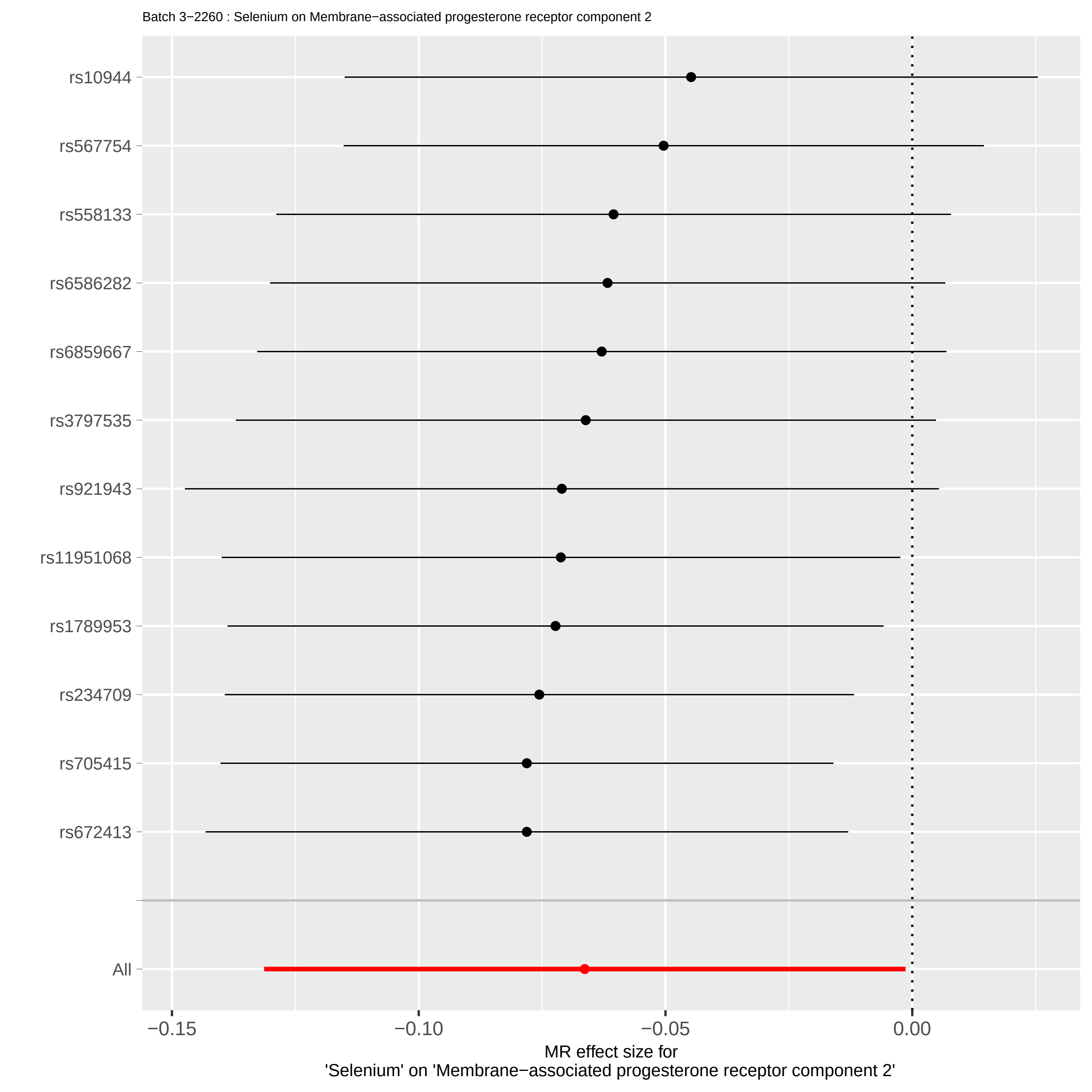

Batch 3−2260 : Selenium on Membrane−associated progesterone receptor component 2
rs10944
rs567754
rs558133
rs6586282
rs6859667
rs3797535
rs921943
rs11951068
rs1789953
rs234709
rs705415
rs672413
All
−0.15
−0.10
−0.05
0.00
MR effect size for
'Selenium' on 'Membrane−associated progesterone receptor component 2'

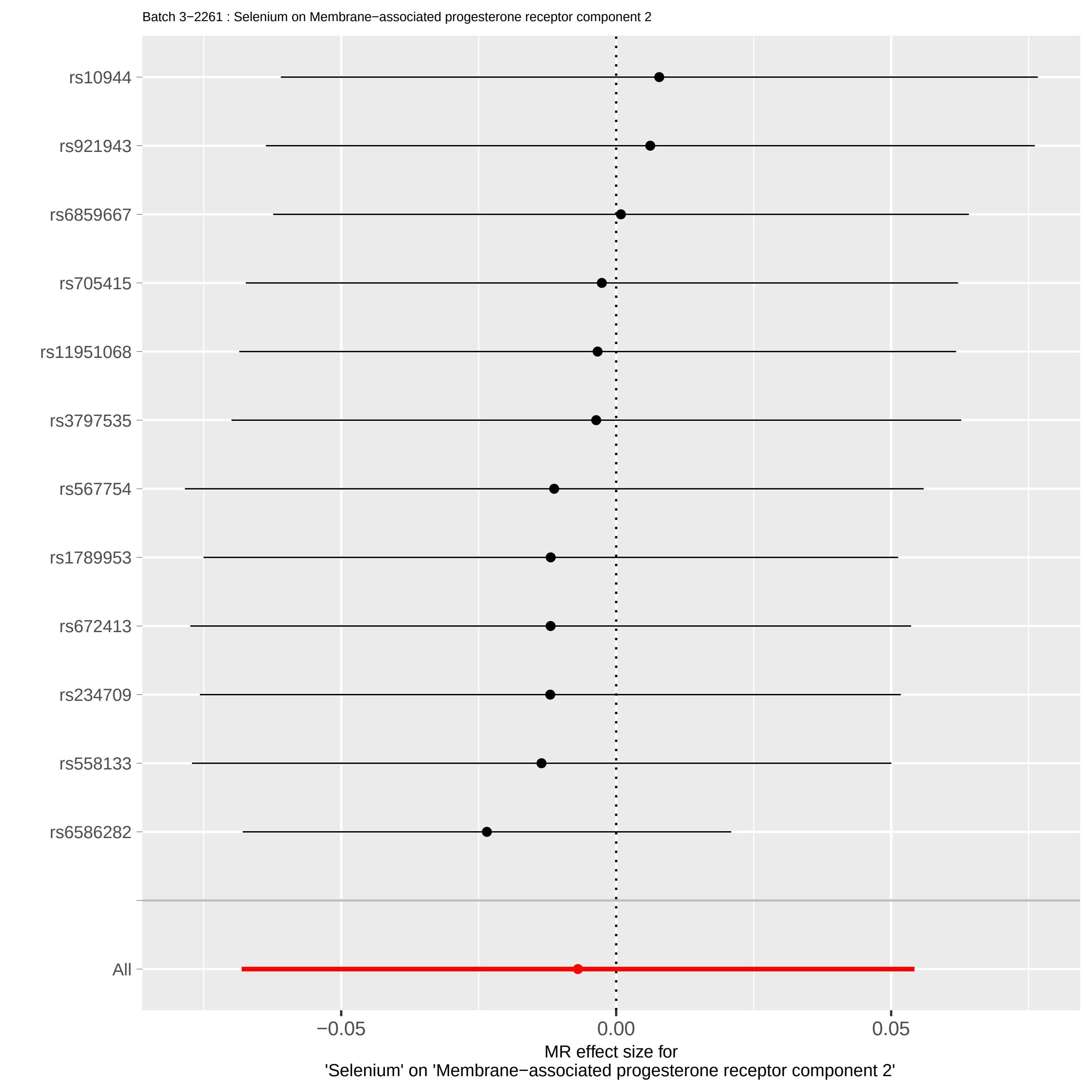
Batch 3−2261 : Selenium on Membrane−associated progesterone receptor component 2
rs10944
rs921943
rs6859667
rs705415
rs11951068
rs3797535
rs567754
rs1789953
rs672413
rs234709
rs558133
rs6586282
All
−0.05
0.00
0.05
MR effect size for
'Selenium' on 'Membrane−associated progesterone receptor component 2'

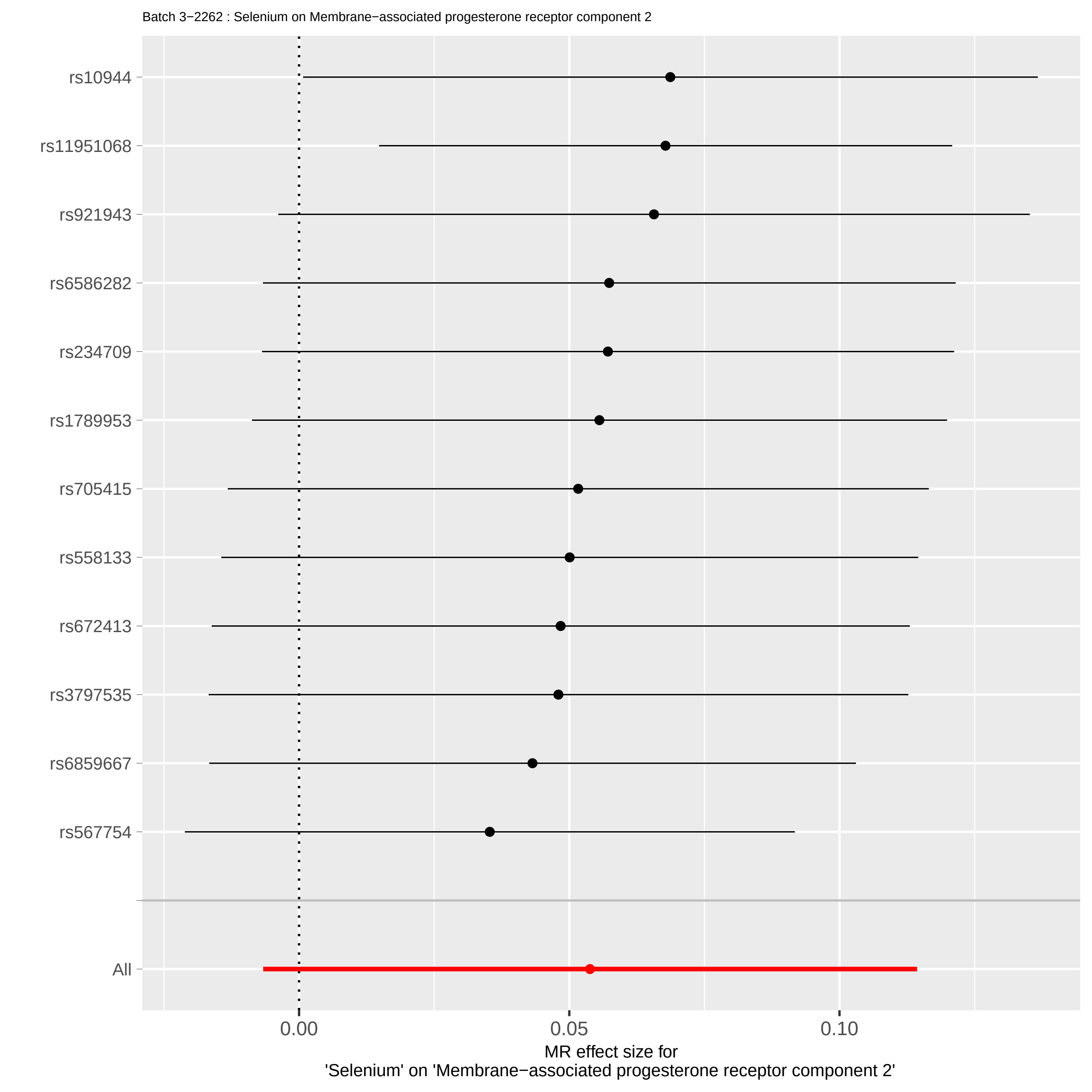

Batch 3−2262 : Selenium on Membrane−associated progesterone receptor component 2
rs10944
rs11951068
rs921943
rs6586282
rs234709
rs1789953
rs705415
rs558133
rs672413
rs3797535
rs6859667
rs567754
All
0.00
0.05
0.10
MR effect size for
'Selenium' on 'Membrane−associated progesterone receptor component 2'

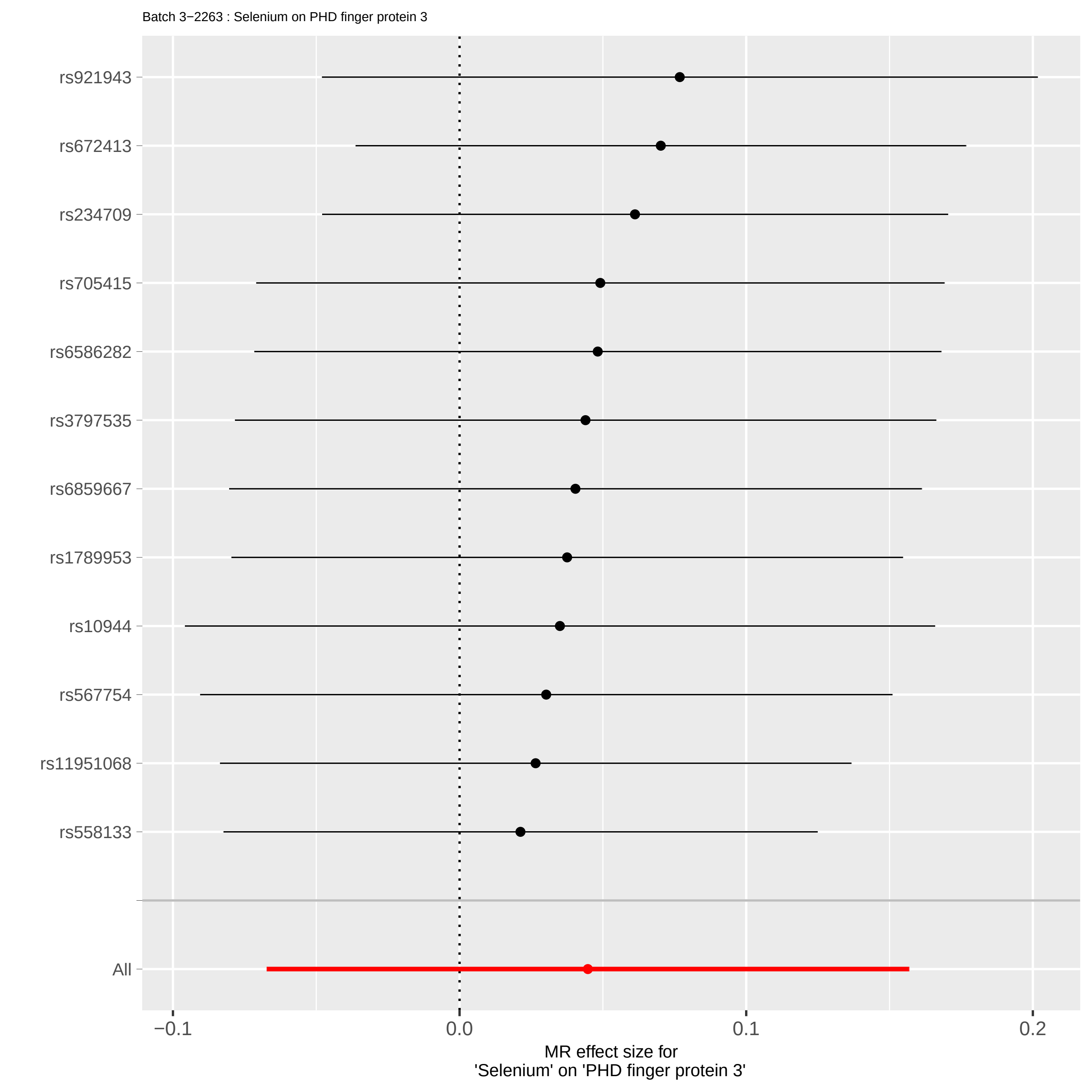

Batch 3−2263 : Selenium on PHD finger protein 3
rs921943
rs672413
rs234709
rs705415
rs6586282
rs3797535
rs6859667
rs1789953
rs10944
rs567754
rs11951068
rs558133
All
−0.1
0.0
0.1
0.2
MR effect size for
'Selenium' on 'PHD finger protein 3'

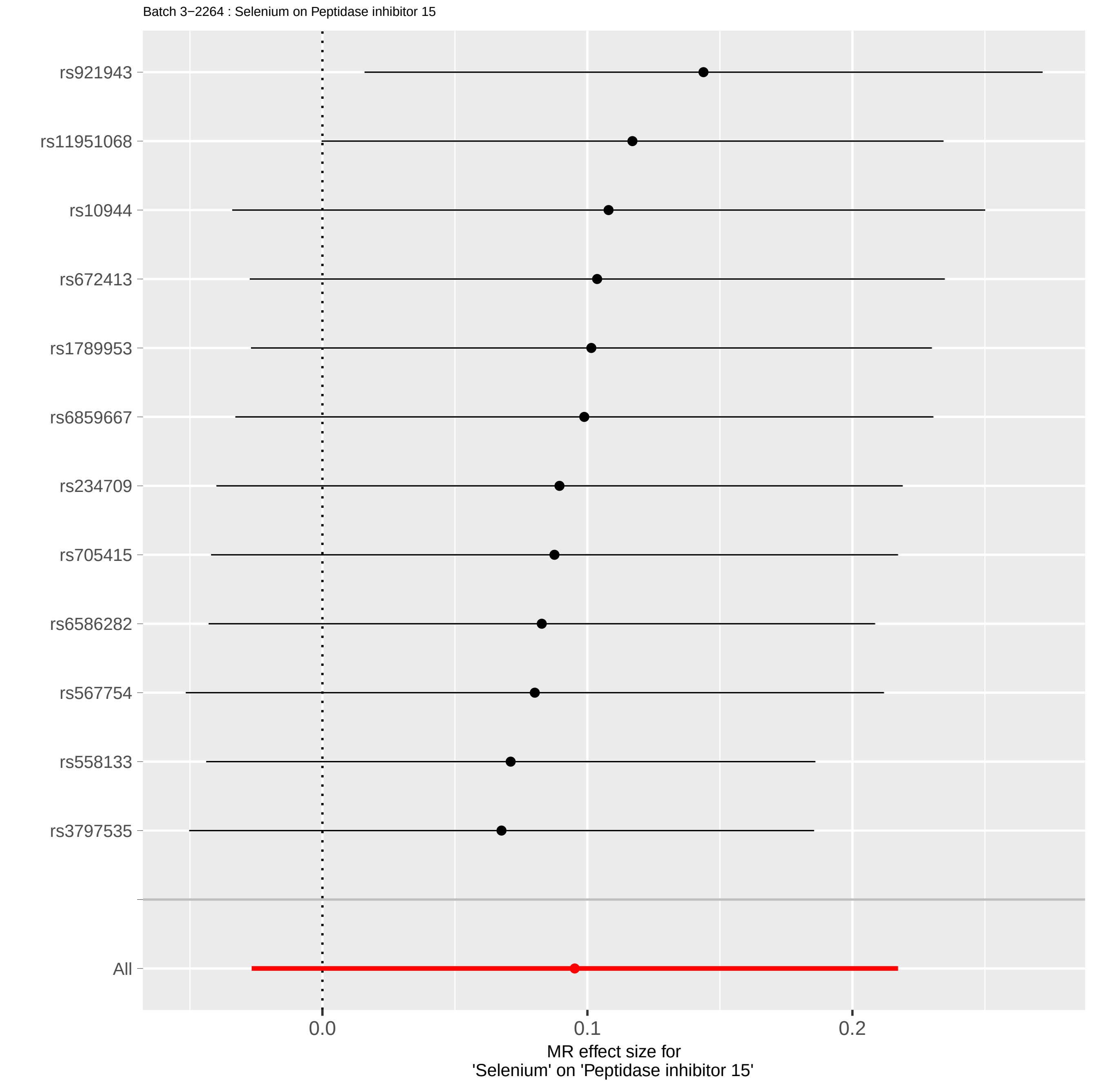

Batch 3−2264 : Selenium on Peptidase inhibitor 15
rs921943
rs11951068
rs10944
rs672413
rs1789953
rs6859667
rs234709
rs705415
rs6586282
rs567754
rs558133
rs3797535
All
0.0
0.1
0.2
MR effect size for
'Selenium' on 'Peptidase inhibitor 15'

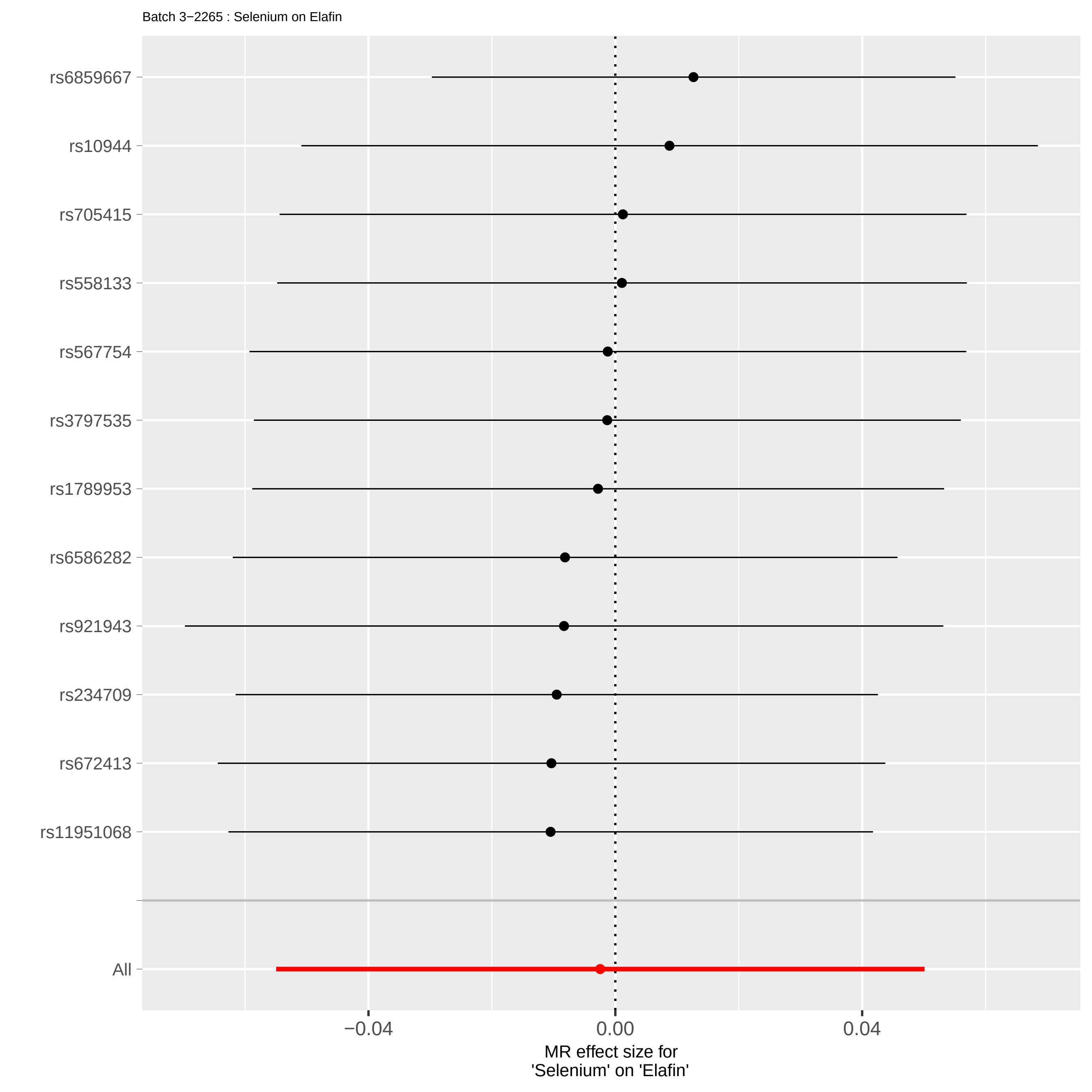

Batch 3−2265 : Selenium on Elafin
rs6859667
rs10944
rs705415
rs558133
rs567754
rs3797535
rs1789953
rs6586282
rs921943
rs234709
rs672413
rs11951068
All
−0.04
0.00
0.04
MR effect size for
'Selenium' on 'Elafin'

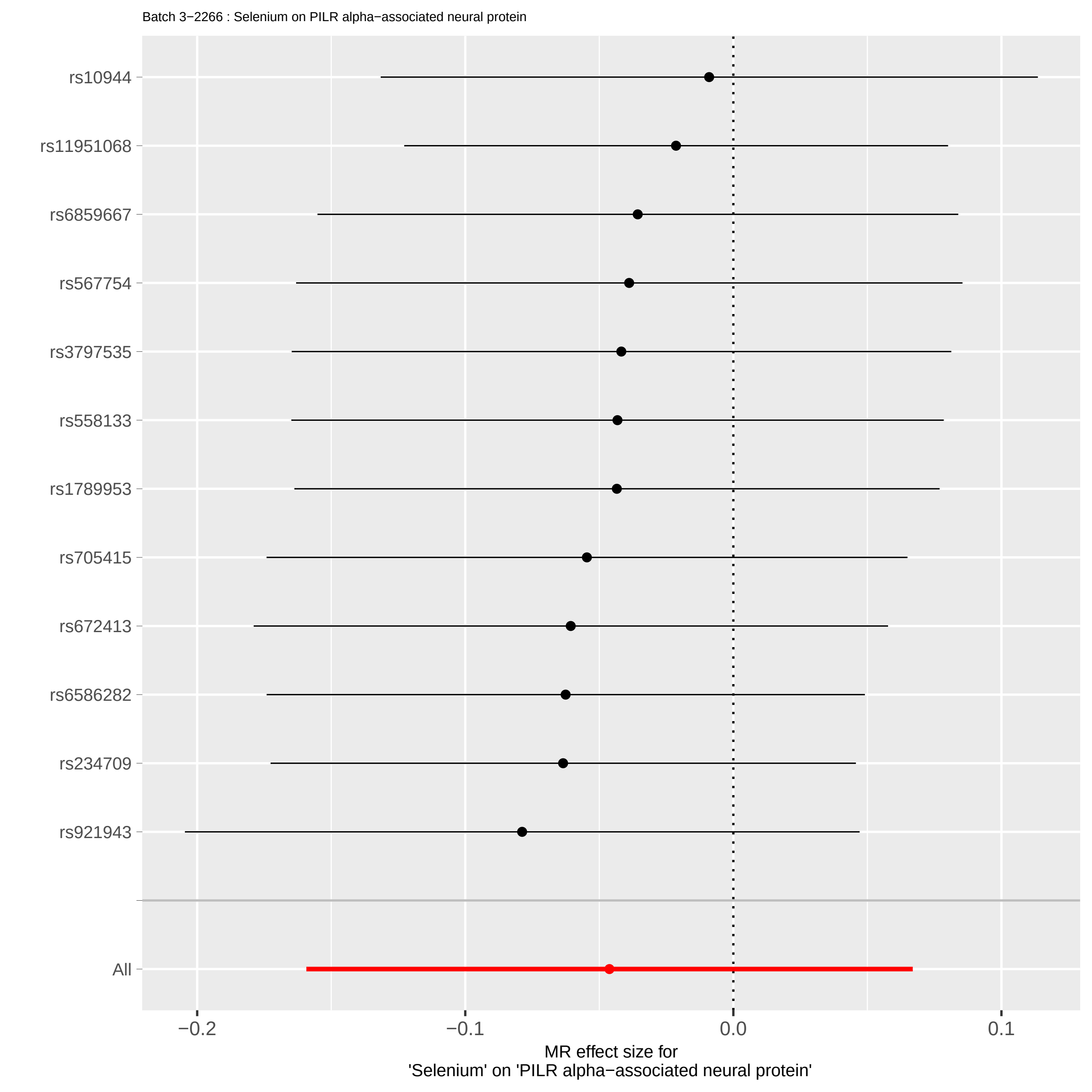
Batch 3−2266 : Selenium on PILR alpha−associated neural protein
rs10944
rs11951068
rs6859667
rs567754
rs3797535
rs558133
rs1789953
rs705415
rs672413
rs6586282
rs234709
rs921943
All
−0.2
−0.1
0.0
0.1
MR effect size for
'Selenium' on 'PILR alpha−associated neural protein'

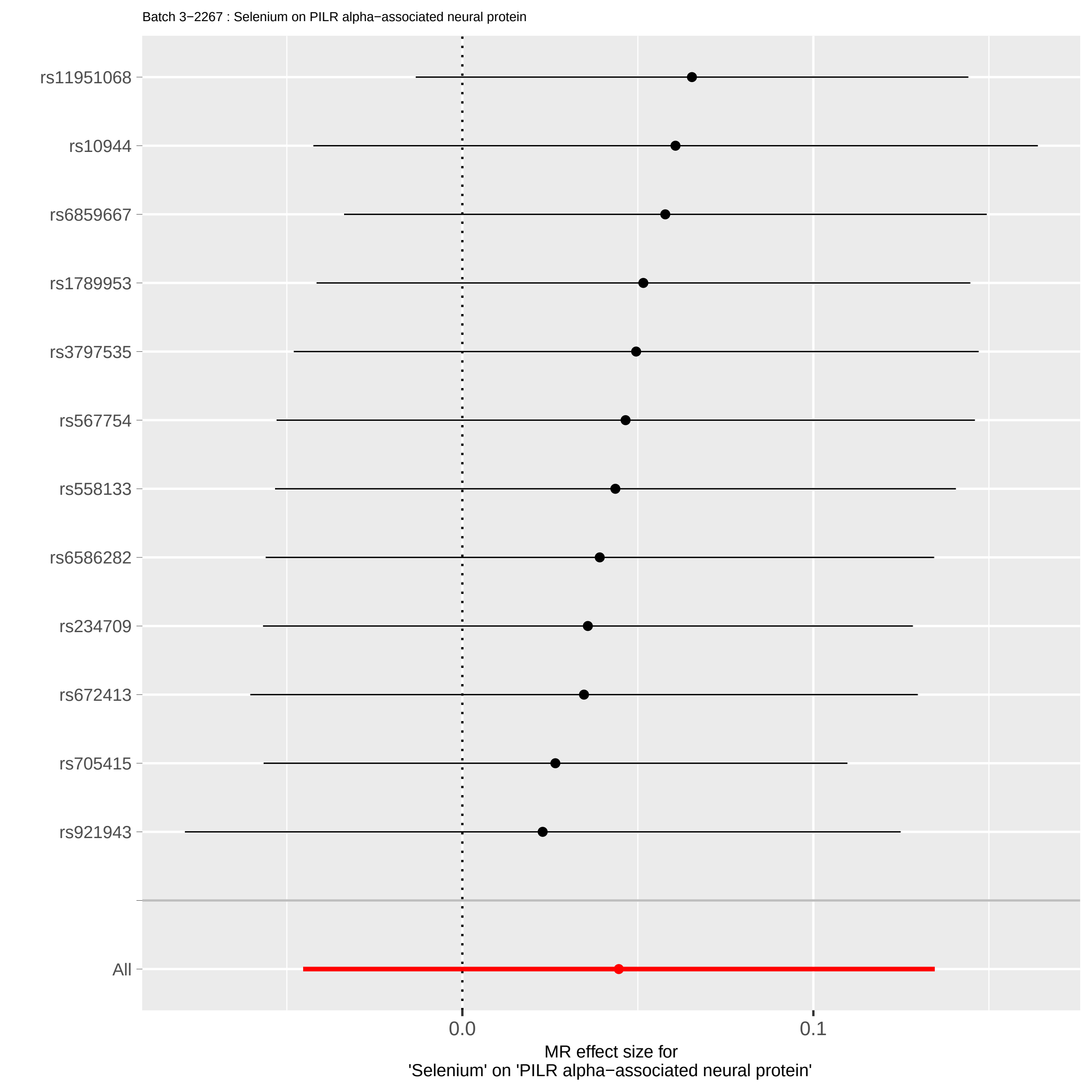
Batch 3−2267 : Selenium on PILR alpha−associated neural protein
rs11951068
rs10944
rs6859667
rs1789953
rs3797535
rs567754
rs558133
rs6586282
rs234709
rs672413
rs705415
rs921943
All
0.0
0.1
MR effect size for
'Selenium' on 'PILR alpha−associated neural protein'

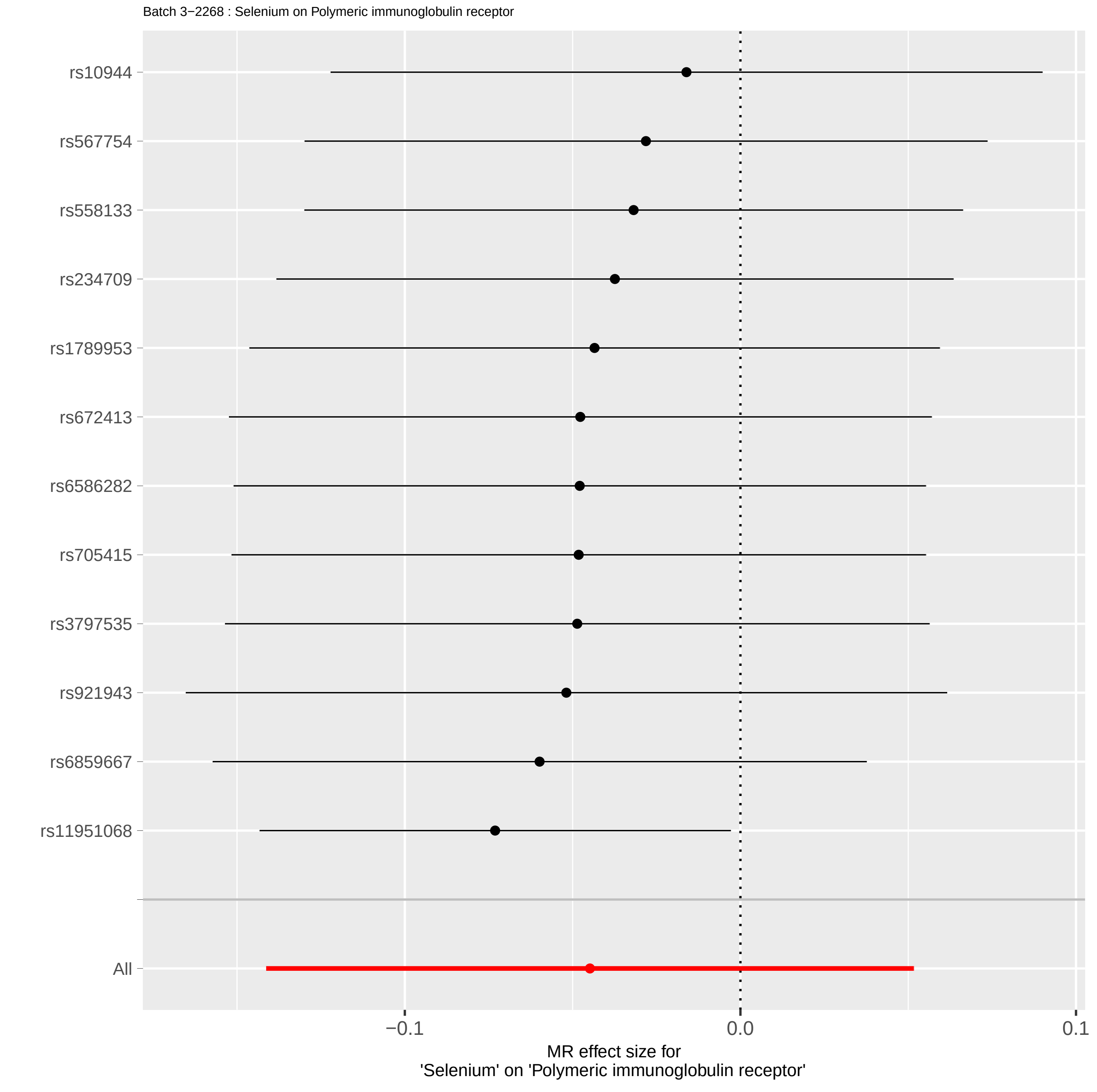

Batch 3−2268 : Selenium on Polymeric immunoglobulin receptor
rs10944
rs567754
rs558133
rs234709
rs1789953
rs672413
rs6586282
rs705415
rs3797535
rs921943
rs6859667
rs11951068
All
−0.1
0.0
0.1
MR effect size for
'Selenium' on 'Polymeric immunoglobulin receptor'

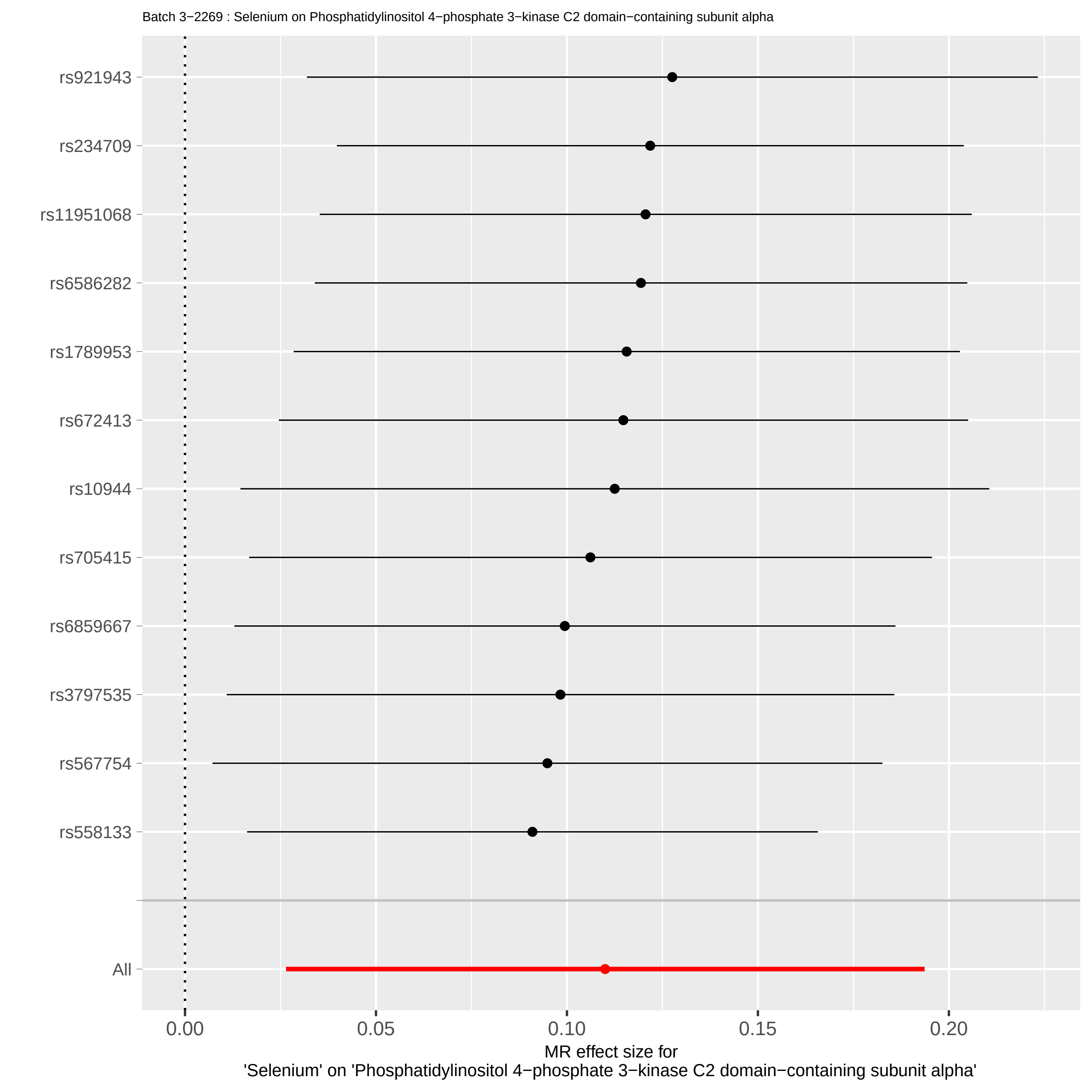
Batch 3−2269 : Selenium on Phosphatidylinositol 4−phosphate 3−kinase C2 domain−containing subunit alpha
rs921943
rs234709
rs11951068
rs6586282
rs1789953
rs672413
rs10944
rs705415
rs6859667
rs3797535
rs567754
rs558133
All
0.00
0.05
0.10
0.15
0.20
MR effect size for
'Selenium' on 'Phosphatidylinositol 4−phosphate 3−kinase C2 domain−containing subunit alpha'

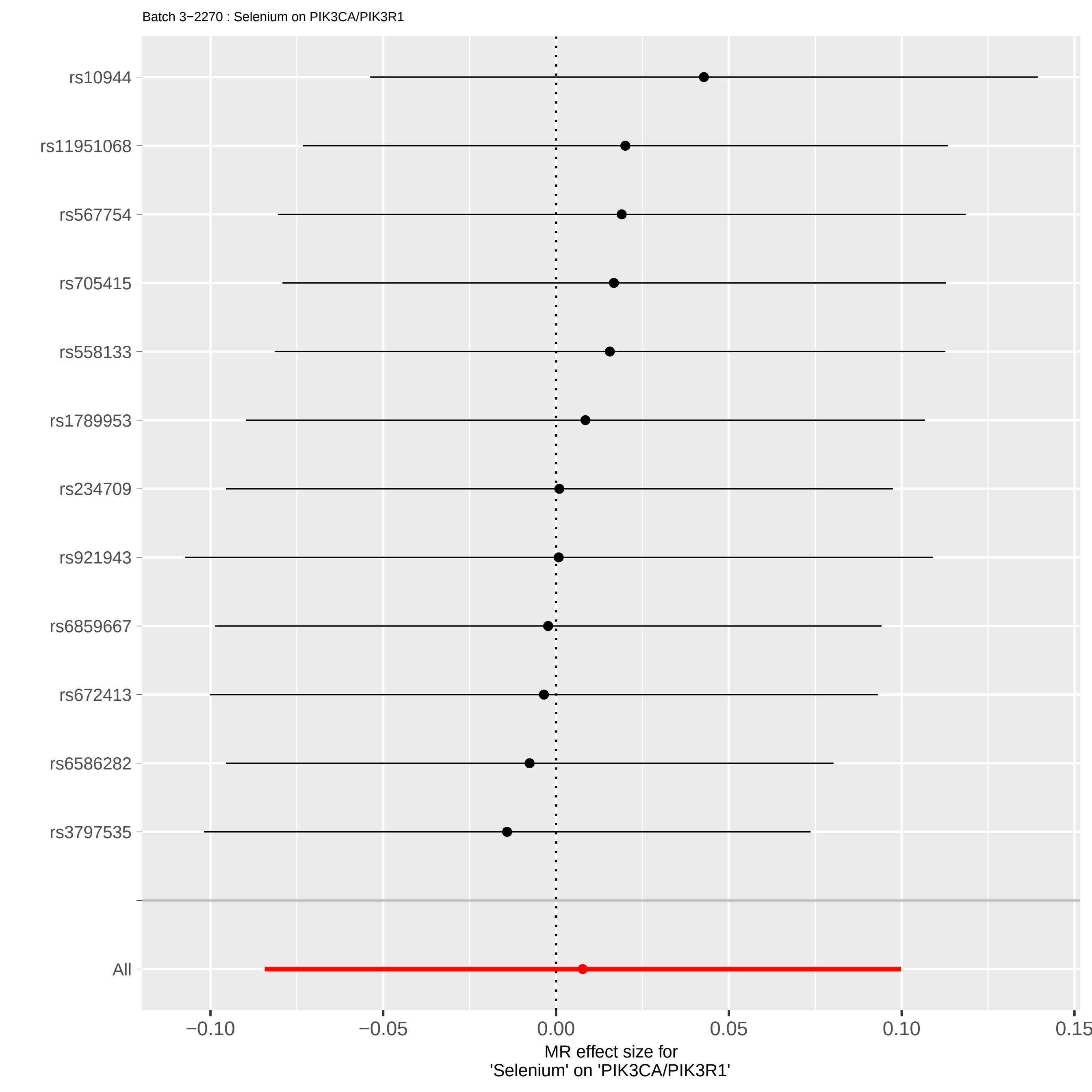
Batch 3−2270 : Selenium on PIK3CA/PIK3R1
rs10944
rs11951068
rs567754
rs705415
rs558133
rs1789953
rs234709
rs921943
rs6859667
rs672413
rs6586282
rs3797535
All
−0.10
−0.05
0.00
0.05
0.10
0.15
MR effect size for
'Selenium' on 'PIK3CA/PIK3R1'

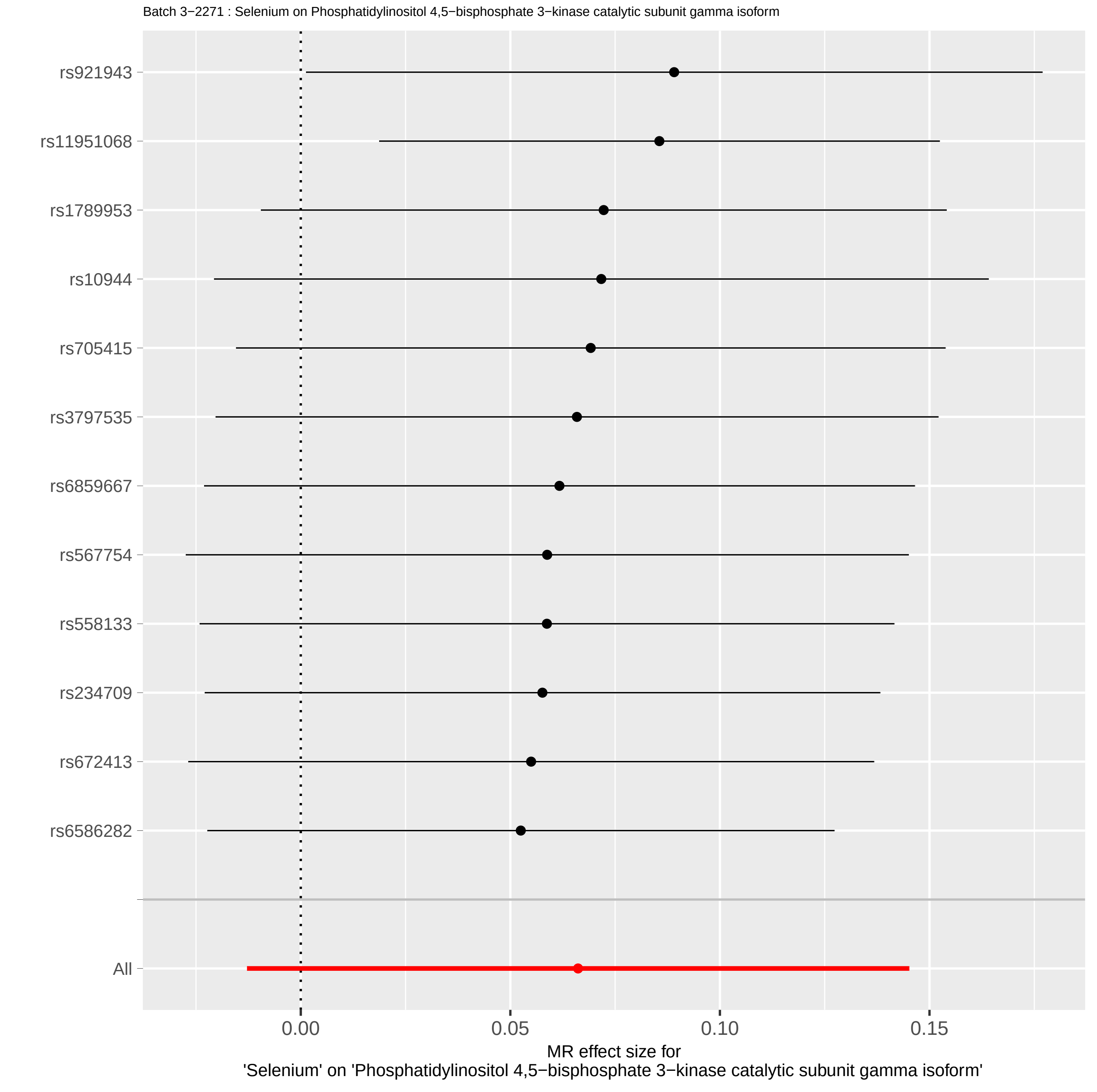

Batch 3−2271 : Selenium on Phosphatidylinositol 4,5−bisphosphate 3−kinase catalytic subunit gamma isoform
rs921943
rs11951068
rs1789953
rs10944
rs705415
rs3797535
rs6859667
rs567754
rs558133
rs234709
rs672413
rs6586282
All
0.00
0.05
0.10
0.15
MR effect size for
'Selenium' on 'Phosphatidylinositol 4,5−bisphosphate 3−kinase catalytic subunit gamma isoform'

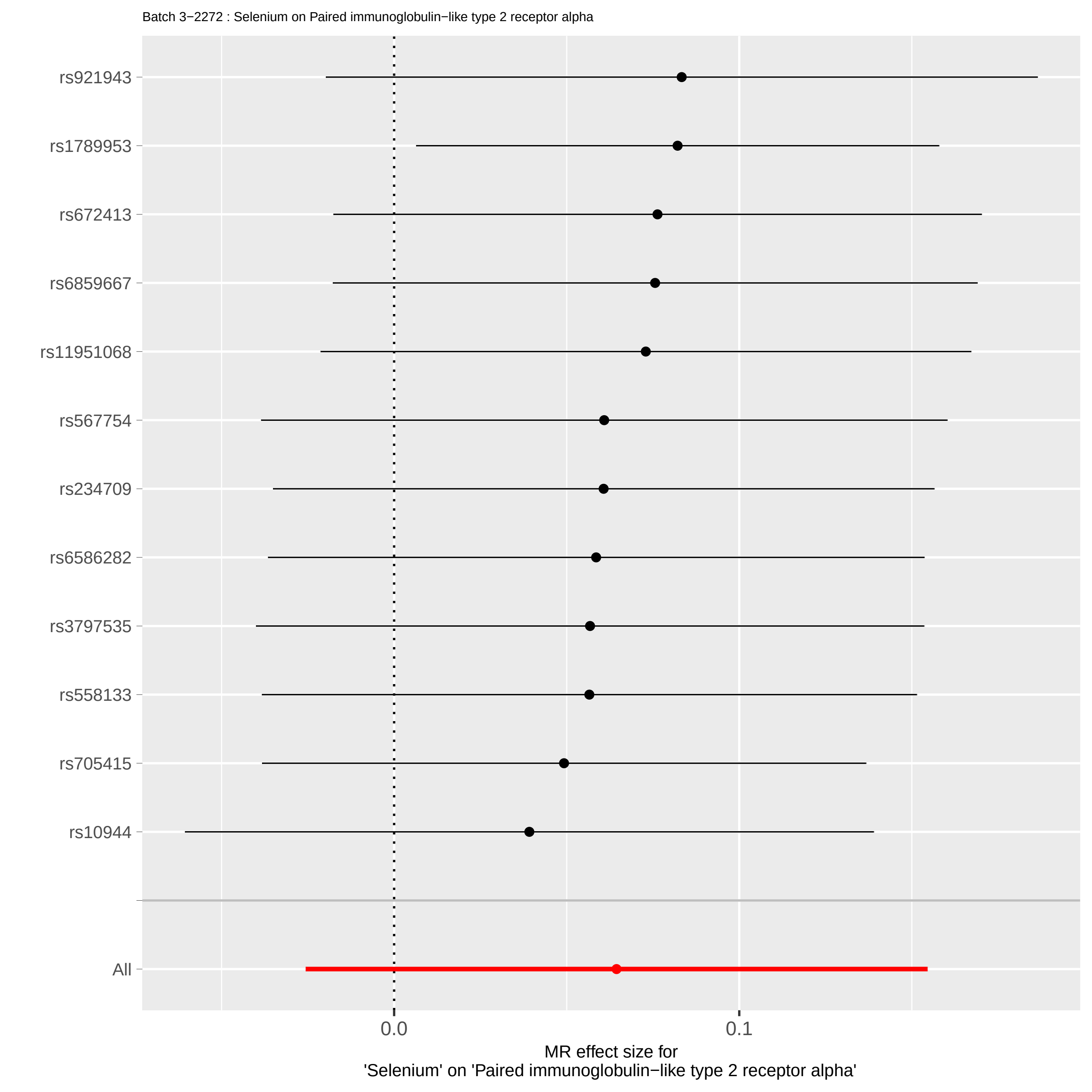
Batch 3−2272 : Selenium on Paired immunoglobulin−like type 2 receptor alpha
rs921943
rs1789953
rs672413
rs6859667
rs11951068
rs567754
rs234709
rs6586282
rs3797535
rs558133
rs705415
rs10944
All
0.0
0.1
MR effect size for
'Selenium' on 'Paired immunoglobulin−like type 2 receptor alpha'

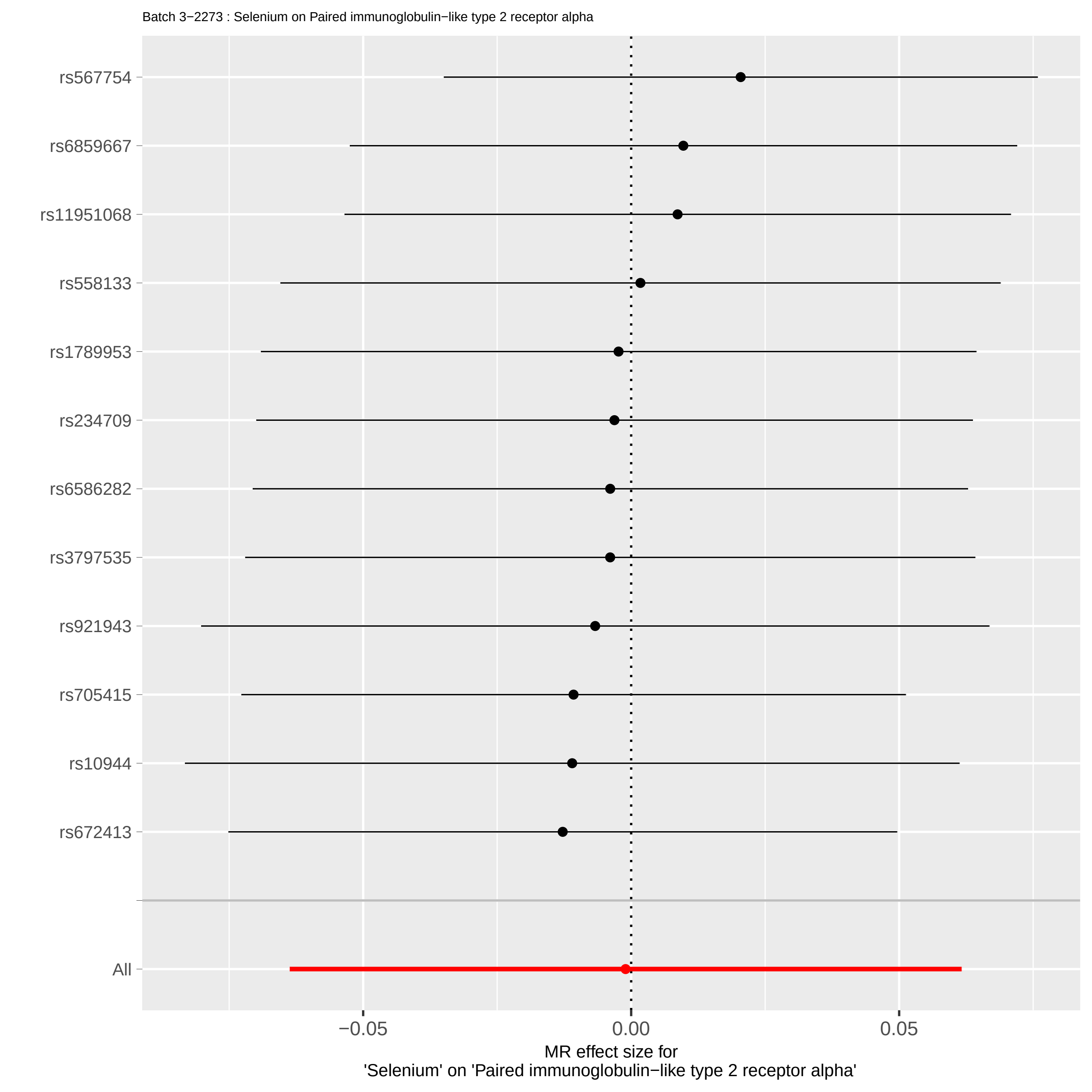
Batch 3−2273 : Selenium on Paired immunoglobulin−like type 2 receptor alpha
rs567754
rs6859667
rs11951068
rs558133
rs1789953
rs234709
rs6586282
rs3797535
rs921943
rs705415
rs10944
rs672413
All
−0.05
0.00
0.05
MR effect size for
'Selenium' on 'Paired immunoglobulin−like type 2 receptor alpha'

Batch 3−2274 : Selenium on Serine/threonine−protein kinase pim−1

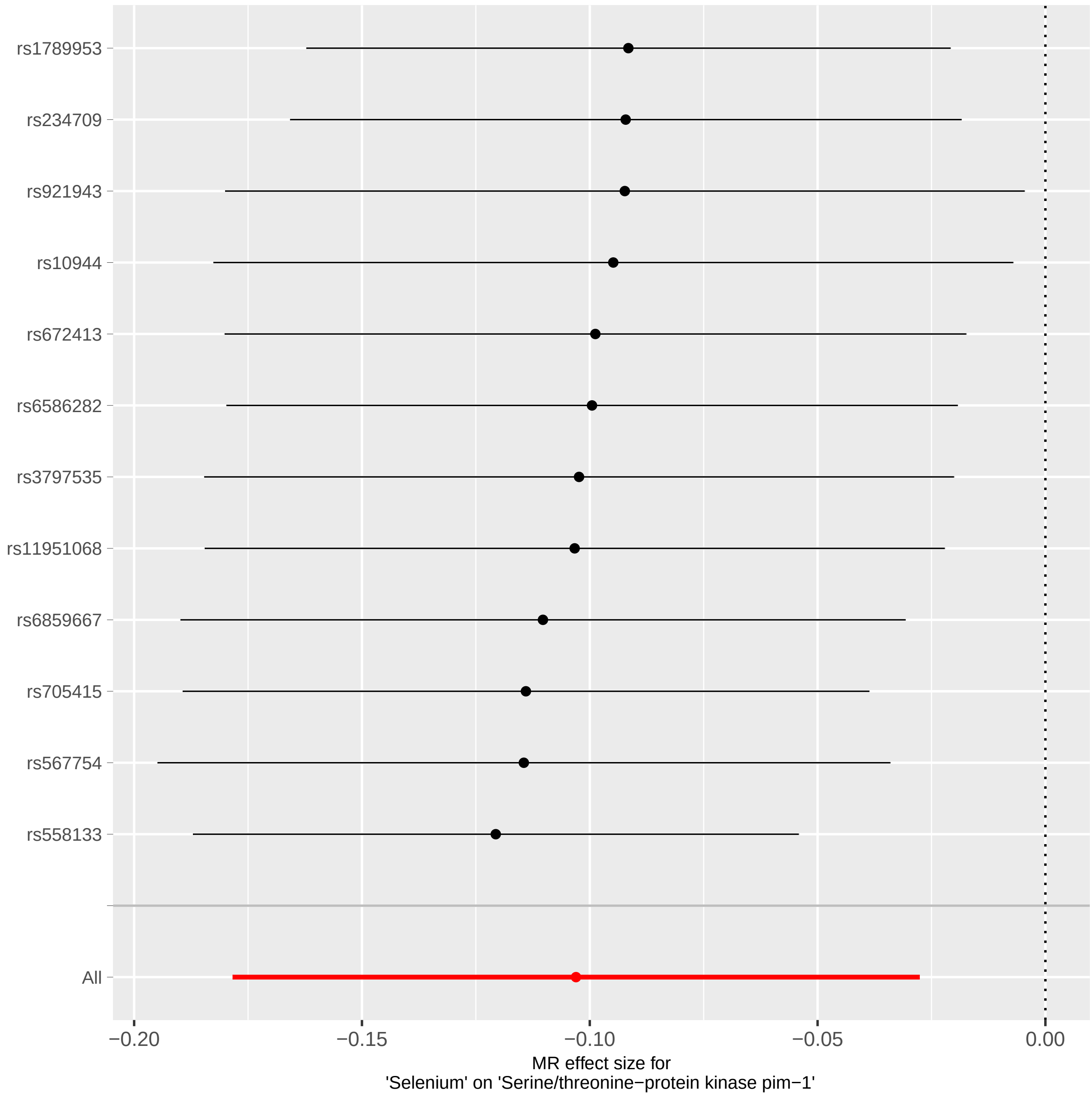

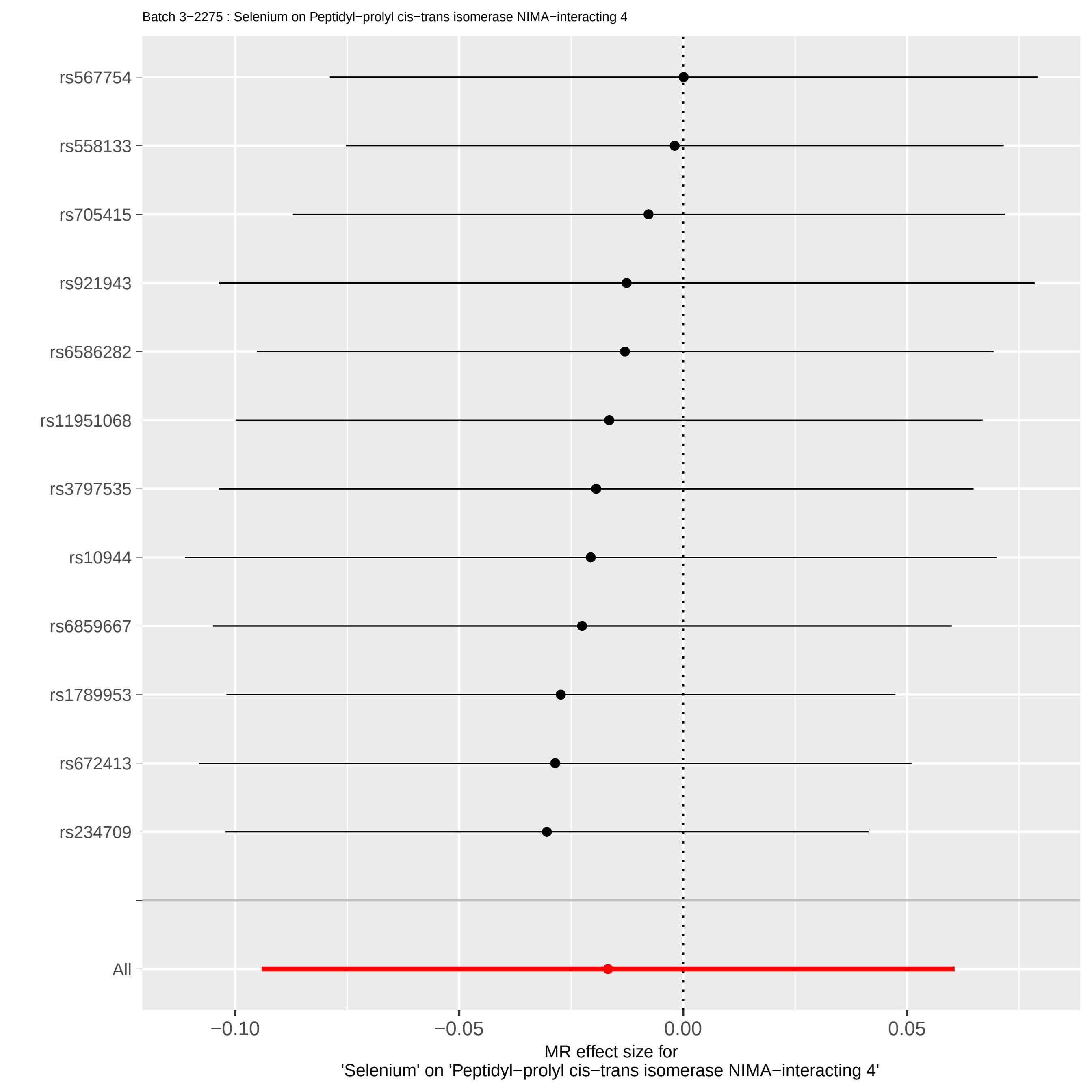

Batch 3−2275 : Selenium on Peptidyl−prolyl cis−trans isomerase NIMA−interacting 4
rs567754
rs558133
rs705415
rs921943
rs6586282
rs11951068
rs3797535
rs10944
rs6859667
rs1789953
rs672413
rs234709
All
−0.10
−0.05
0.00
0.05
MR effect size for
'Selenium' on 'Peptidyl−prolyl cis−trans isomerase NIMA−interacting 4'

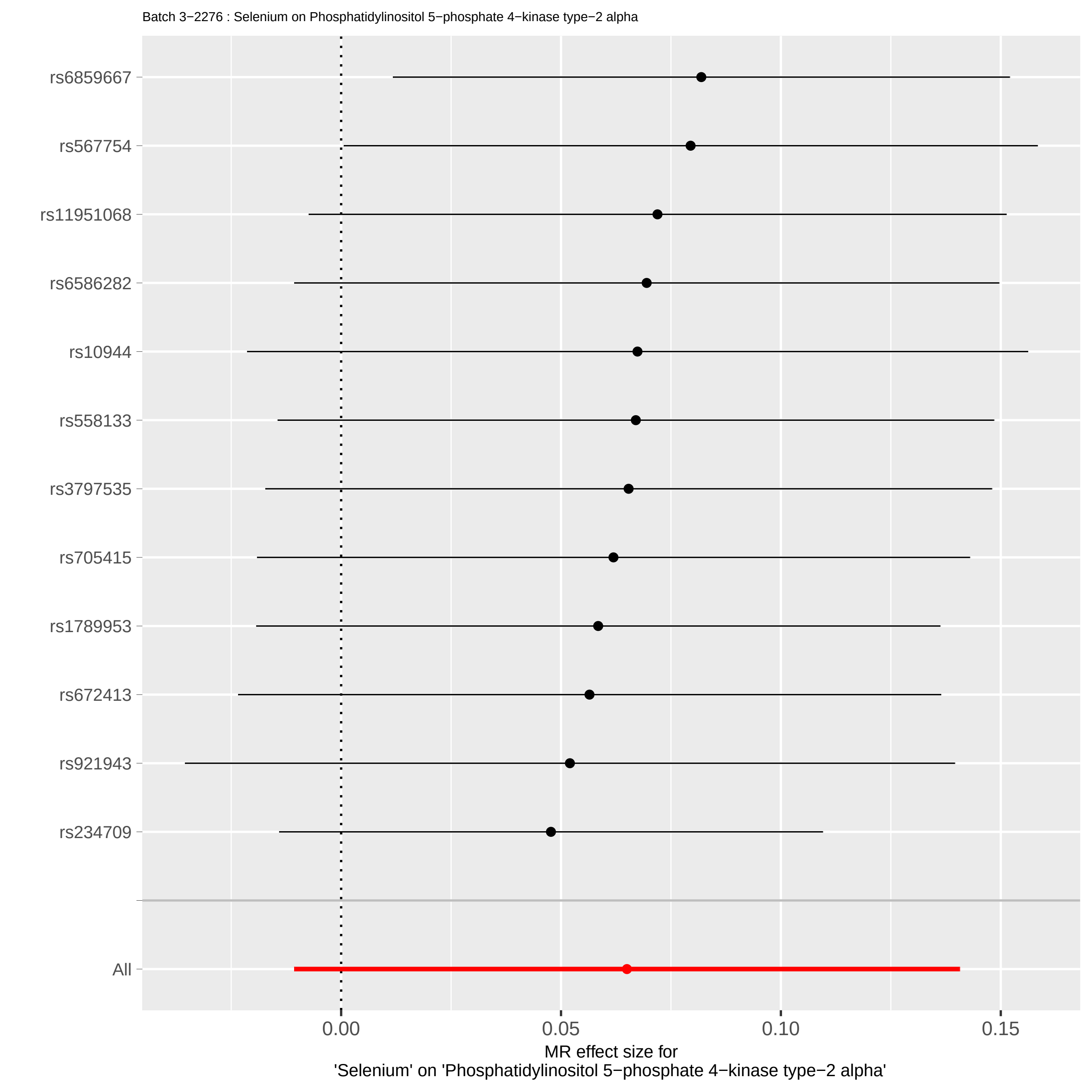

Batch 3−2276 : Selenium on Phosphatidylinositol 5−phosphate 4−kinase type−2 alpha
rs6859667
rs567754
rs11951068
rs6586282
rs10944
rs558133
rs3797535
rs705415
rs1789953
rs672413
rs921943
rs234709
All
0.00
0.05
0.10
0.15
MR effect size for
'Selenium' on 'Phosphatidylinositol 5−phosphate 4−kinase type−2 alpha'

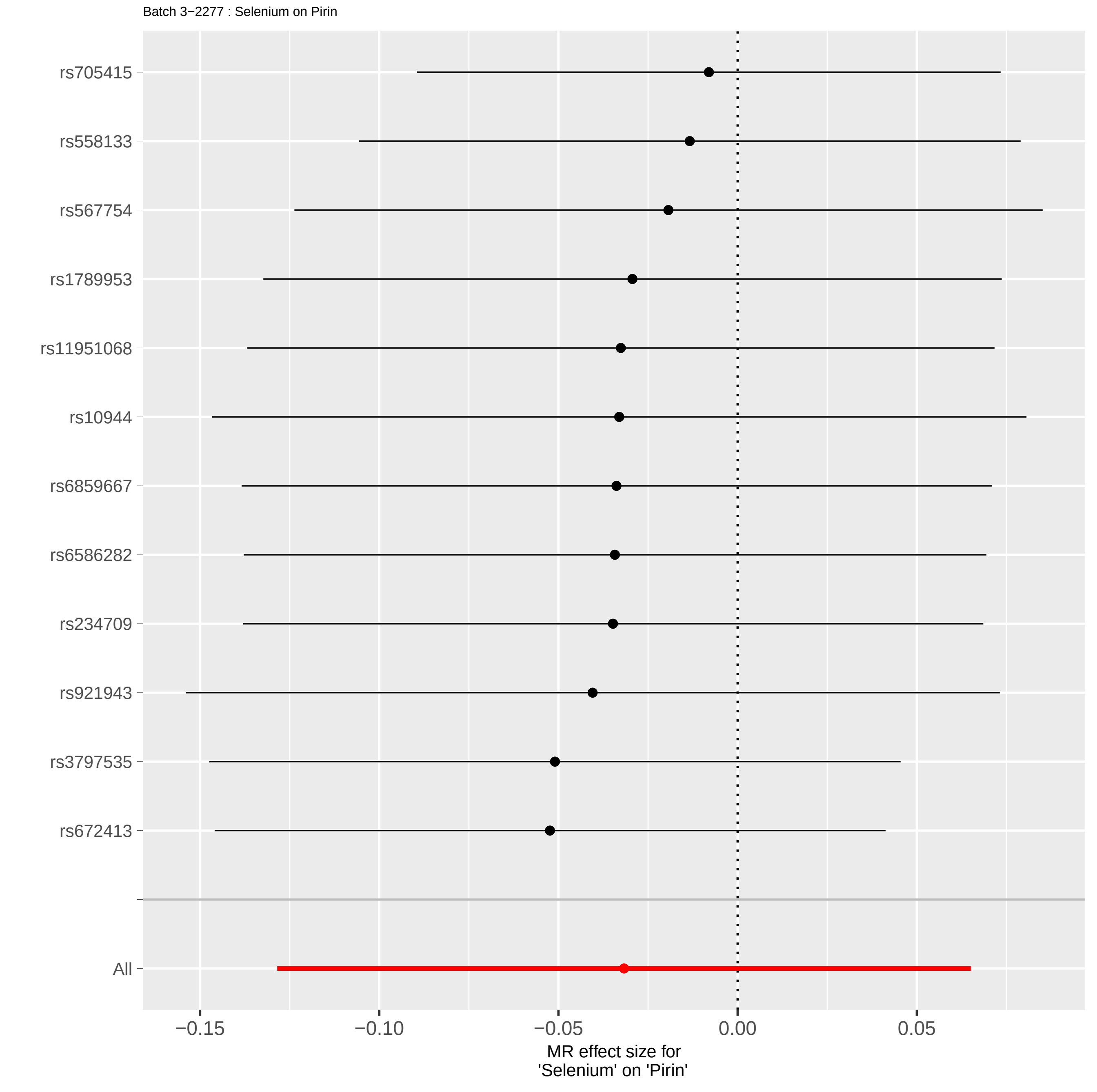
Batch 3−2277 : Selenium on Pirin
rs705415
rs558133
rs567754
rs1789953
rs11951068
rs10944
rs6859667
rs6586282
rs234709
rs921943
rs3797535
rs672413
All
−0.15
−0.10
−0.05
0.00
0.05
MR effect size for
'Selenium' on 'Pirin'

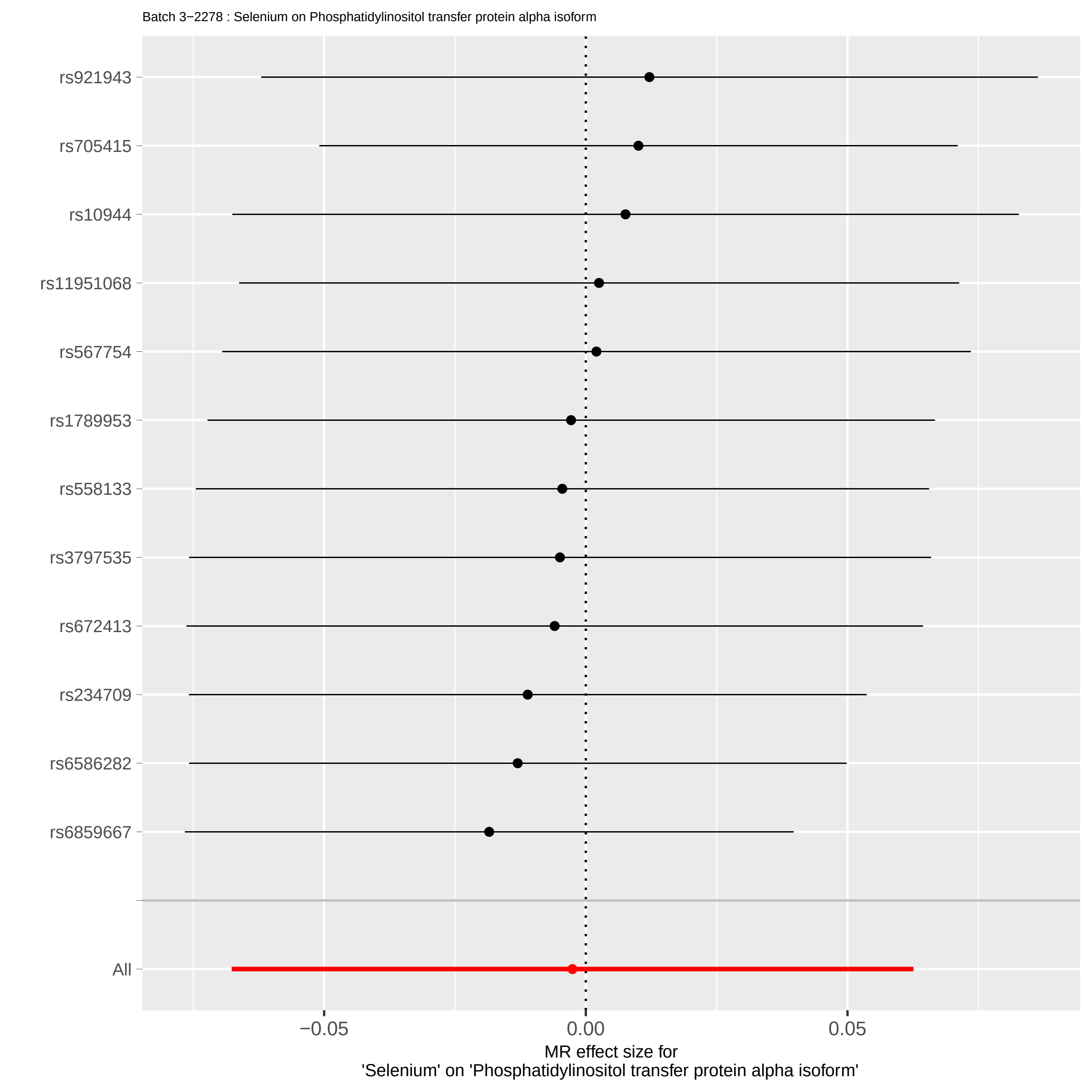

Batch 3−2278 : Selenium on Phosphatidylinositol transfer protein alpha isoform
rs921943
rs705415
rs10944
rs11951068
rs567754
rs1789953
rs558133
rs3797535
rs672413
rs234709
rs6586282
rs6859667
All
−0.05
0.00
0.05
MR effect size for
'Selenium' on 'Phosphatidylinositol transfer protein alpha isoform'

Batch 3−2279 : Selenium on Phosphatidylinositol transfer protein beta isoform

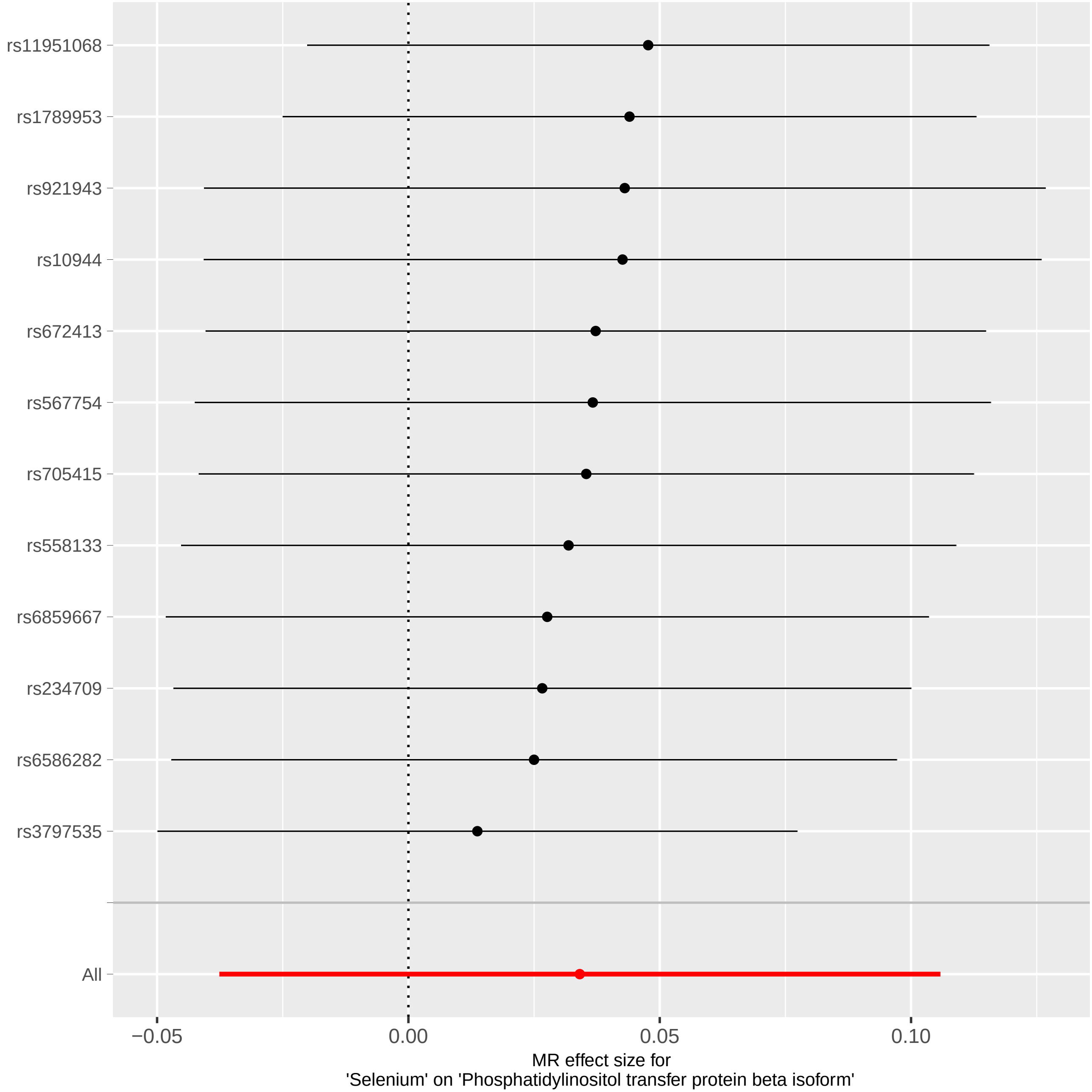

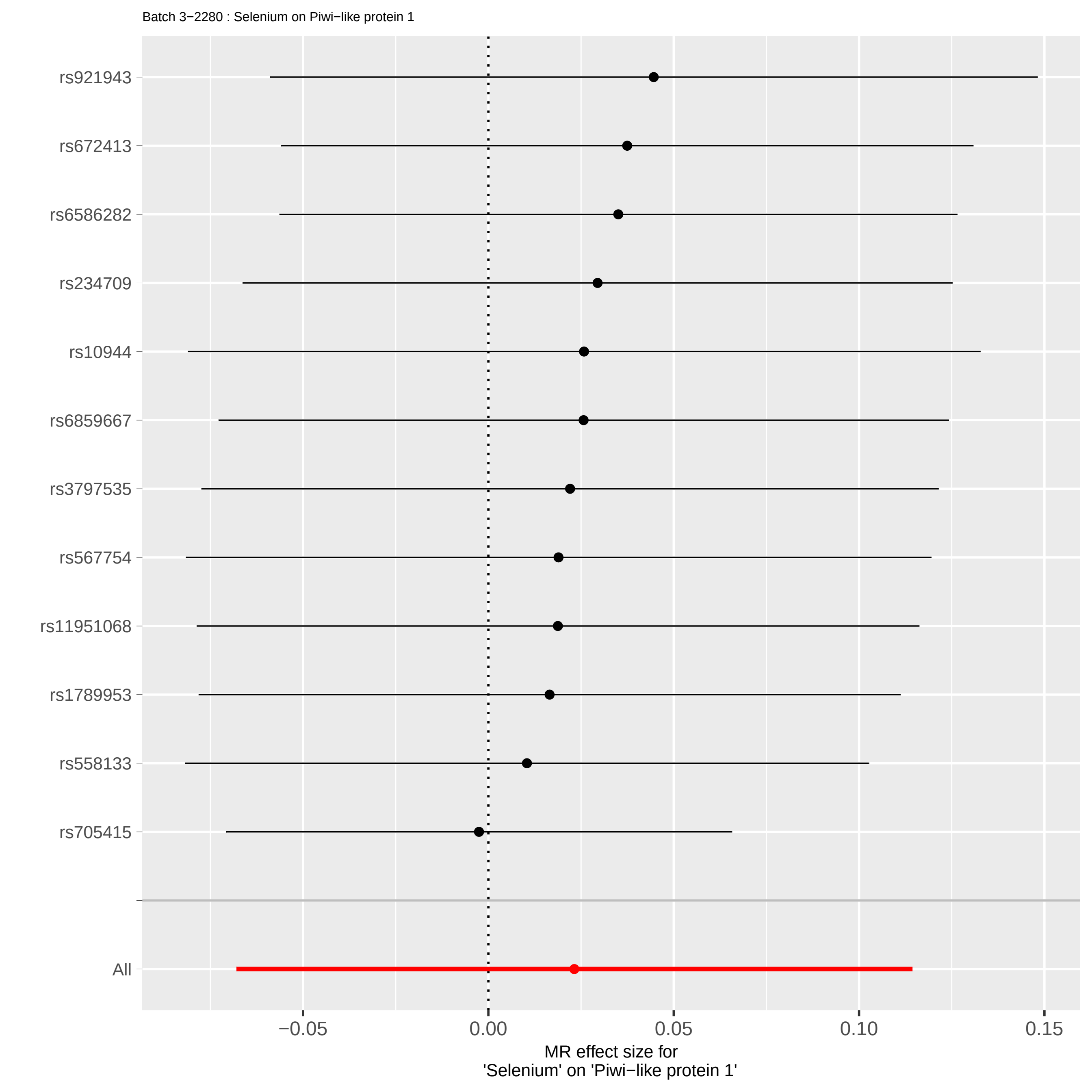

Batch 3−2280 : Selenium on Piwi−like protein 1
rs921943
rs672413
rs6586282
rs234709
rs10944
rs6859667
rs3797535
rs567754
rs11951068
rs1789953
rs558133
rs705415
All
−0.05
0.00
0.05
0.10
0.15
MR effect size for
'Selenium' on 'Piwi−like protein 1'

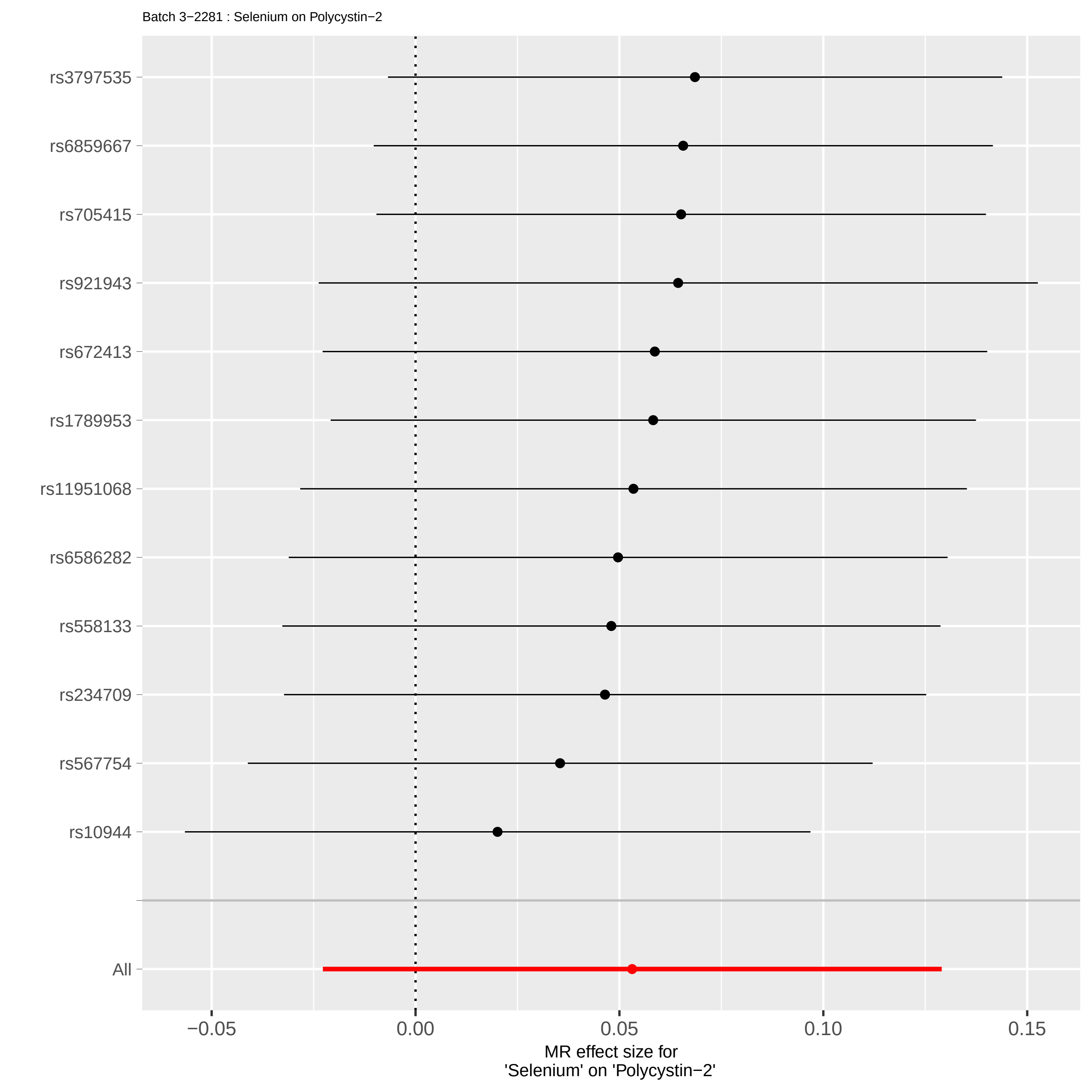
Batch 3−2281 : Selenium on Polycystin−2
rs3797535
rs6859667
rs705415
rs921943
rs672413
rs1789953
rs11951068
rs6586282
rs558133
rs234709
rs567754
rs10944
All
−0.05
0.00
0.05
0.10
0.15
MR effect size for
'Selenium' on 'Polycystin−2'

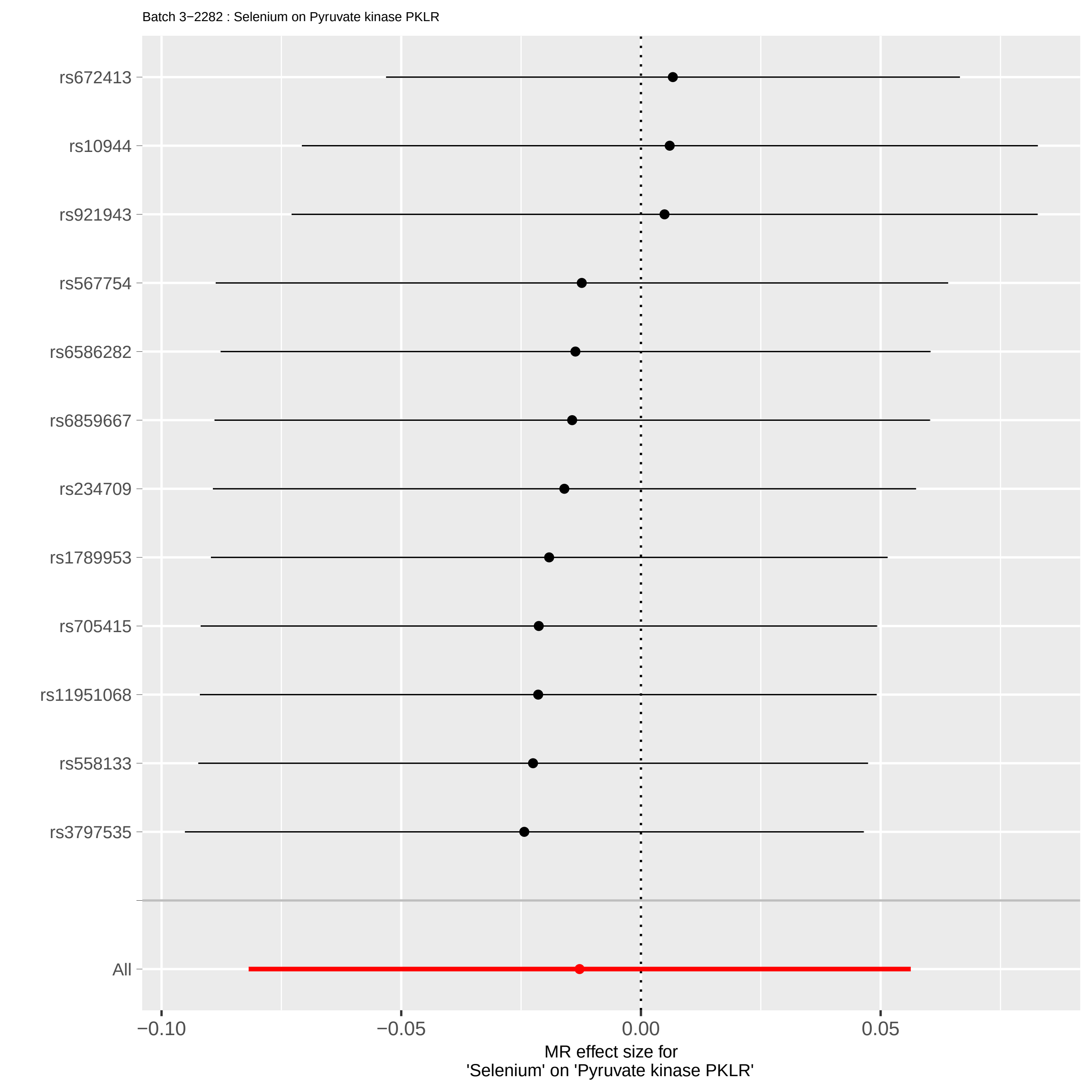

Batch 3−2282 : Selenium on Pyruvate kinase PKLR
rs672413
rs10944
rs921943
rs567754
rs6586282
rs6859667
rs234709
rs1789953
rs705415
rs11951068
rs558133
rs3797535
All
−0.10
−0.05
0.00
0.05
MR effect size for
'Selenium' on 'Pyruvate kinase PKLR'

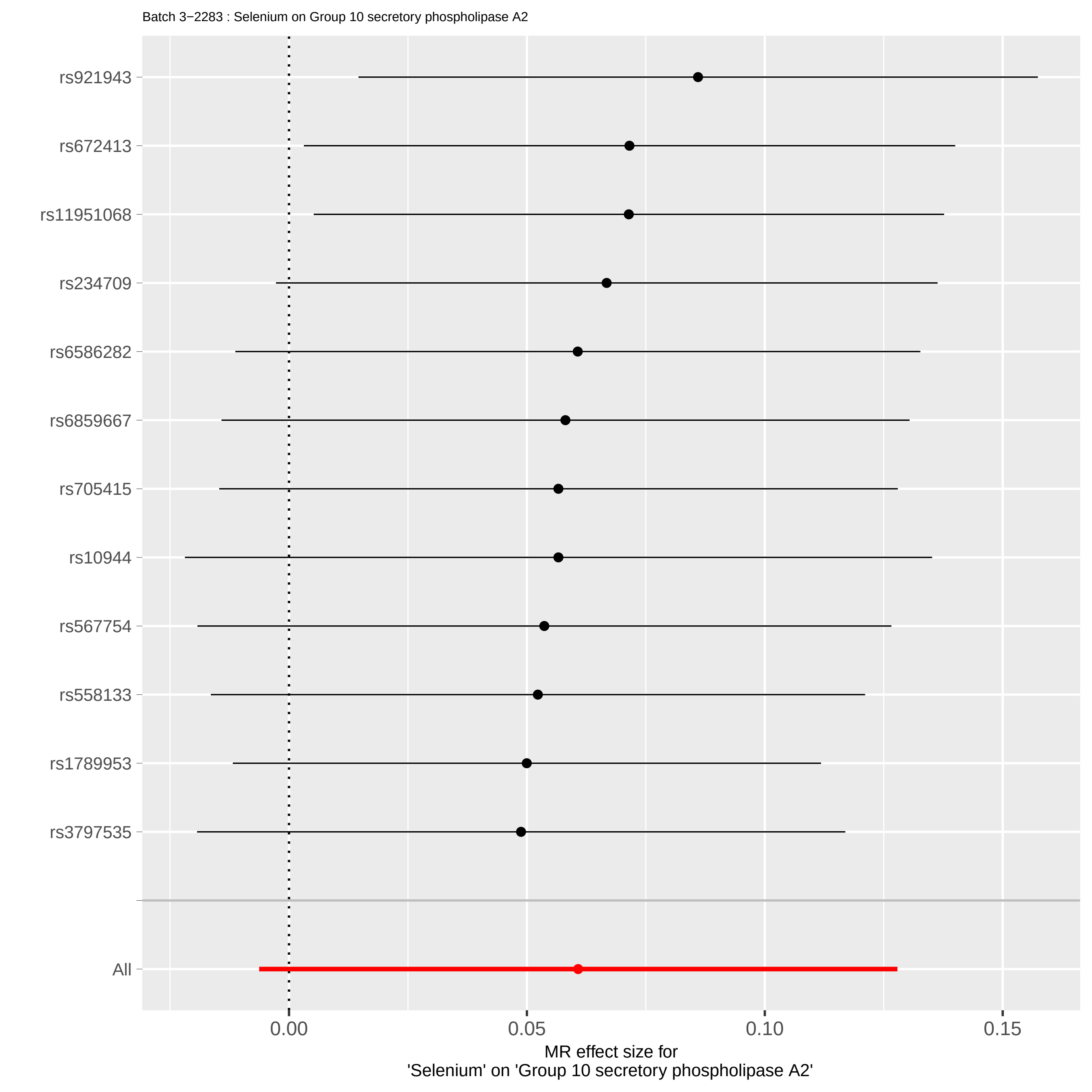

Batch 3−2283 : Selenium on Group 10 secretory phospholipase A2
rs921943
rs672413
rs11951068
rs234709
rs6586282
rs6859667
rs705415
rs10944
rs567754
rs558133
rs1789953
rs3797535
All
0.00
0.05
0.10
0.15
MR effect size for
'Selenium' on 'Group 10 secretory phospholipase A2'

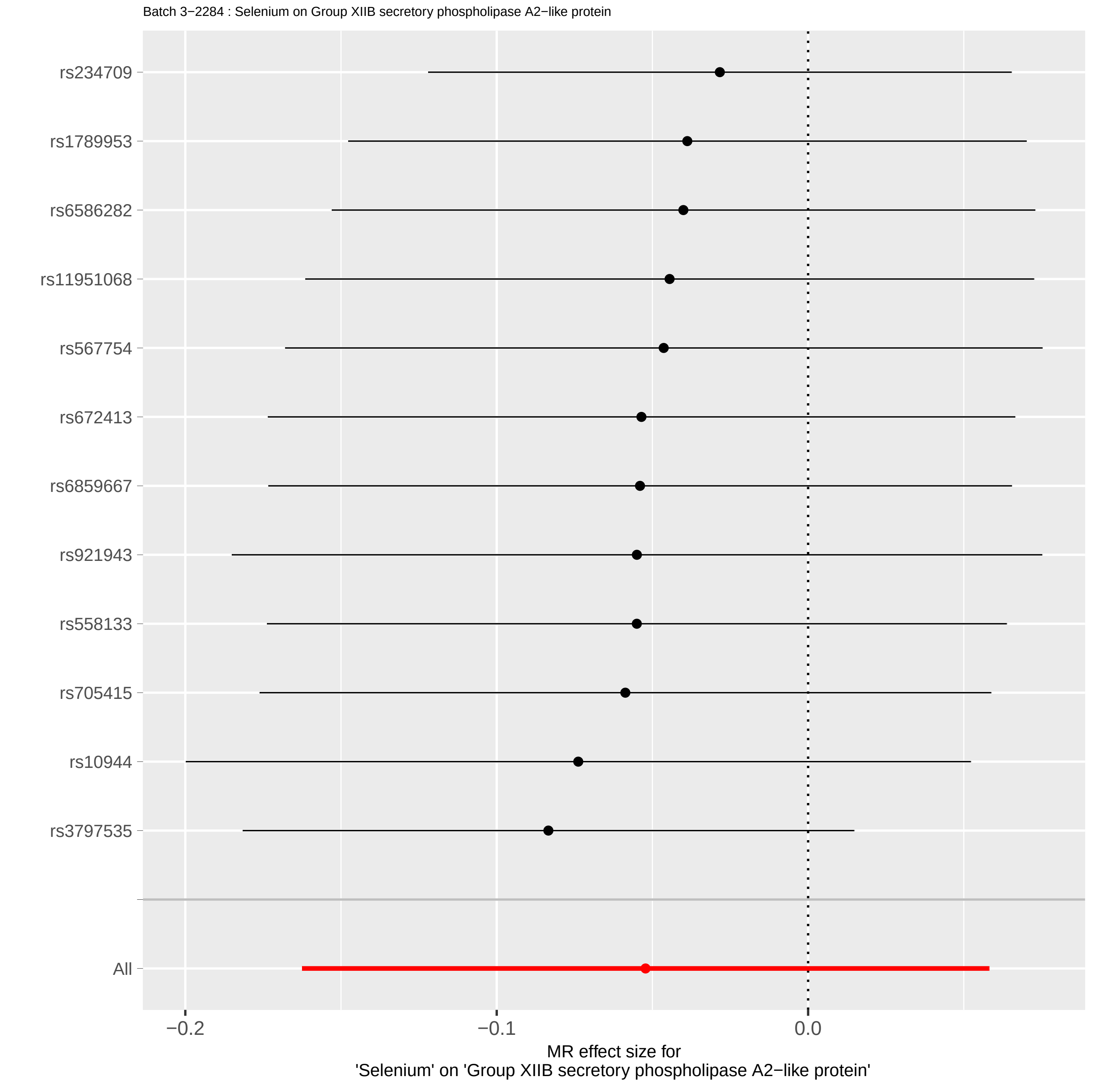

Batch 3−2284 : Selenium on Group XIIB secretory phospholipase A2−like protein
rs234709
rs1789953
rs6586282
rs11951068
rs567754
rs672413
rs6859667
rs921943
rs558133
rs705415
rs10944
rs3797535
All
−0.2
−0.1
0.0
MR effect size for
'Selenium' on 'Group XIIB secretory phospholipase A2−like protein'

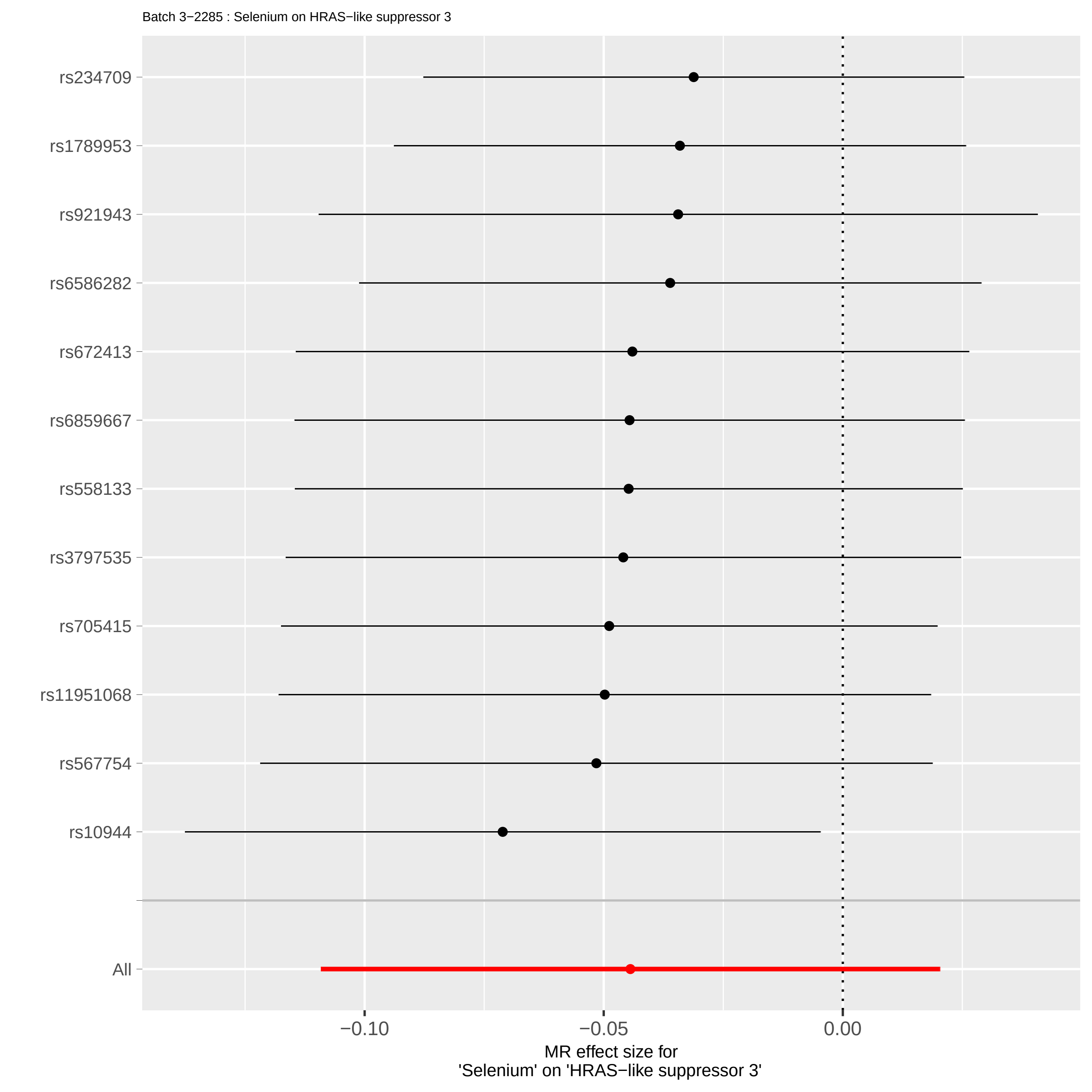

Batch 3−2285 : Selenium on HRAS−like suppressor 3
rs234709
rs1789953
rs921943
rs6586282
rs672413
rs6859667
rs558133
rs3797535
rs705415
rs11951068
rs567754
rs10944
All
−0.10
−0.05
0.00
MR effect size for
'Selenium' on 'HRAS−like suppressor 3'

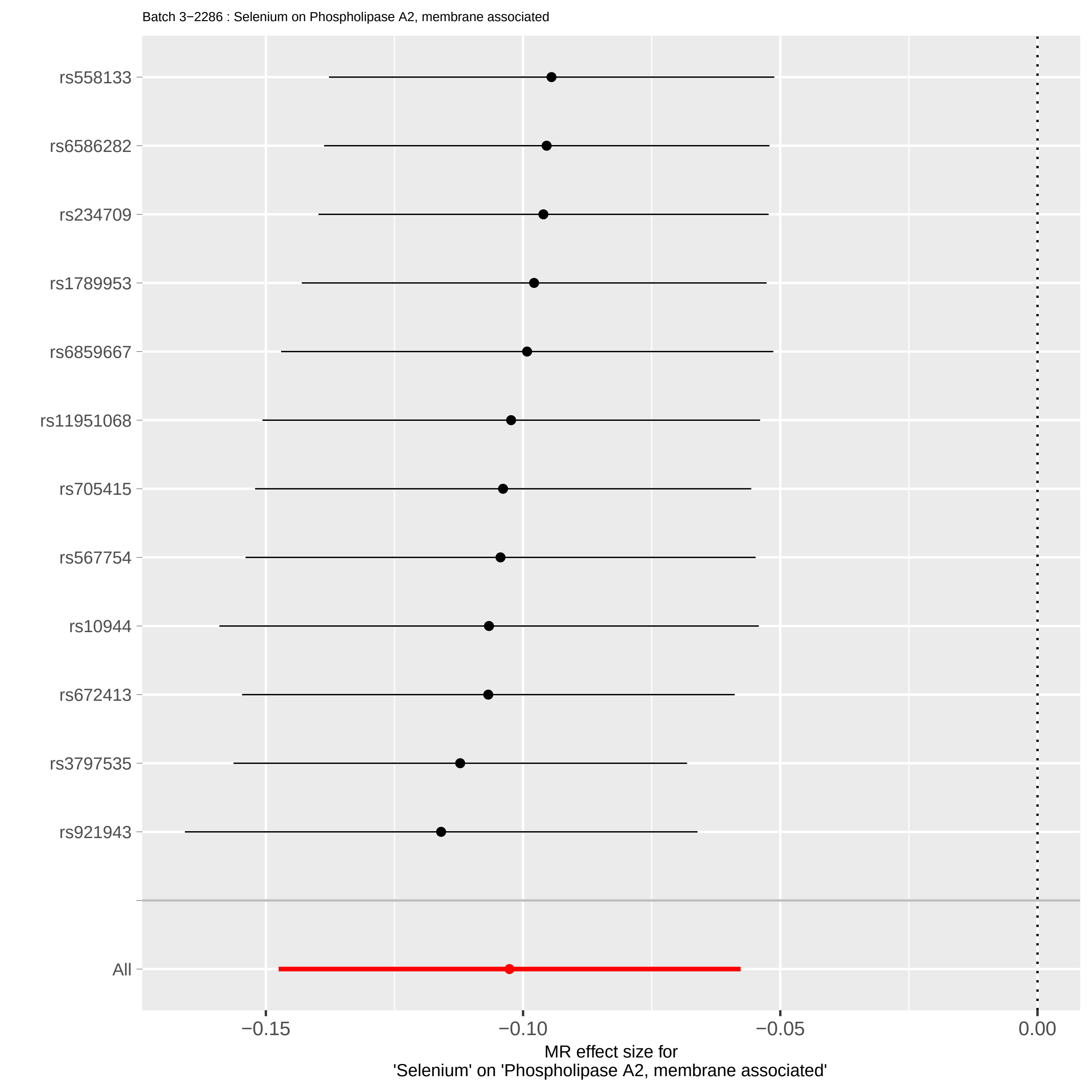

Batch 3−2286 : Selenium on Phospholipase A2, membrane associated
rs558133
rs6586282
rs234709
rs1789953
rs6859667
rs11951068
rs705415
rs567754
rs10944
rs672413
rs3797535
rs921943
All
−0.15
−0.10
−0.05
0.00
MR effect size for
'Selenium' on 'Phospholipase A2, membrane associated'

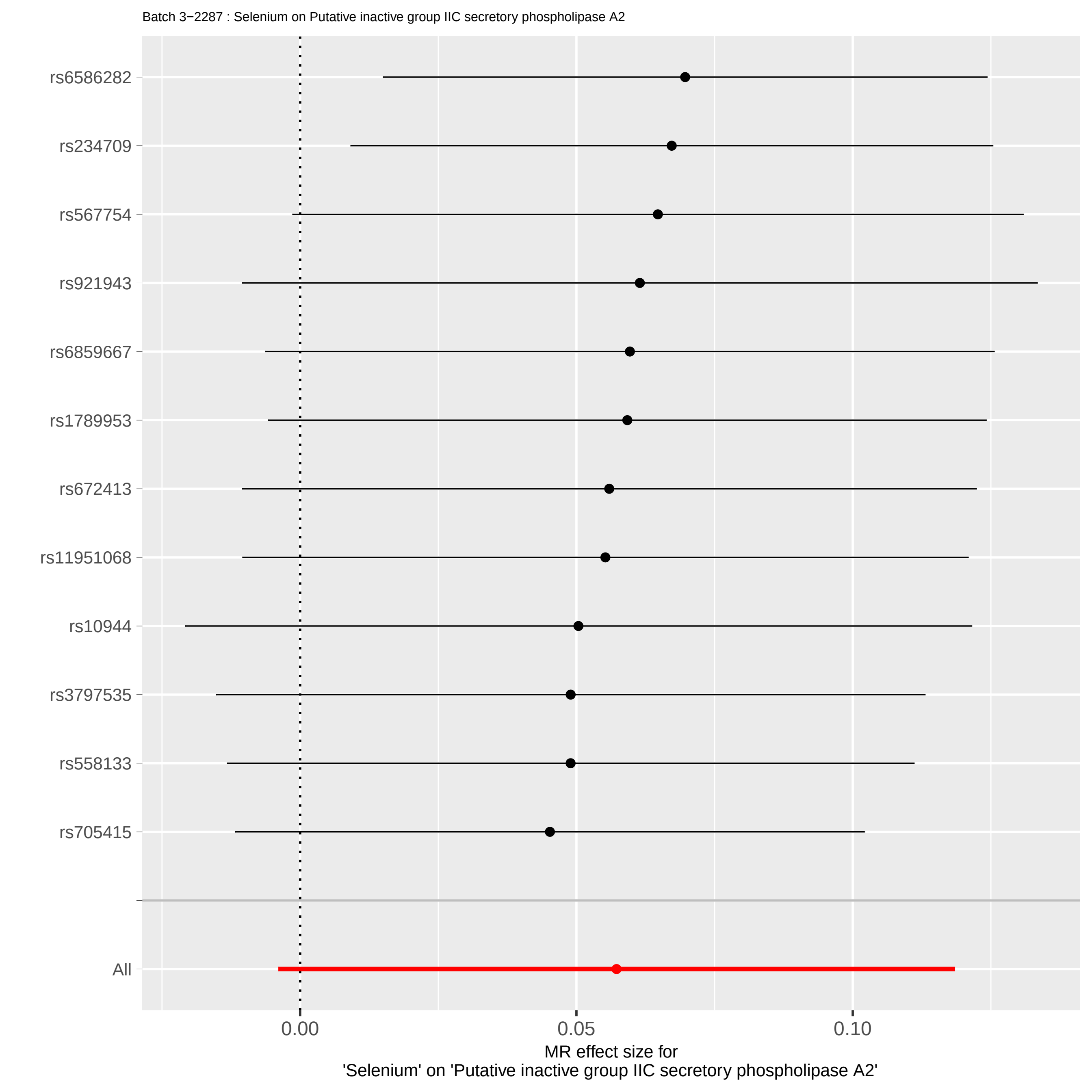
Batch 3−2287 : Selenium on Putative inactive group IIC secretory phospholipase A2
rs6586282
rs234709
rs567754
rs921943
rs6859667
rs1789953
rs672413
rs11951068
rs10944
rs3797535
rs558133
rs705415
All
0.00
0.05
0.10
MR effect size for
'Selenium' on 'Putative inactive group IIC secretory phospholipase A2'

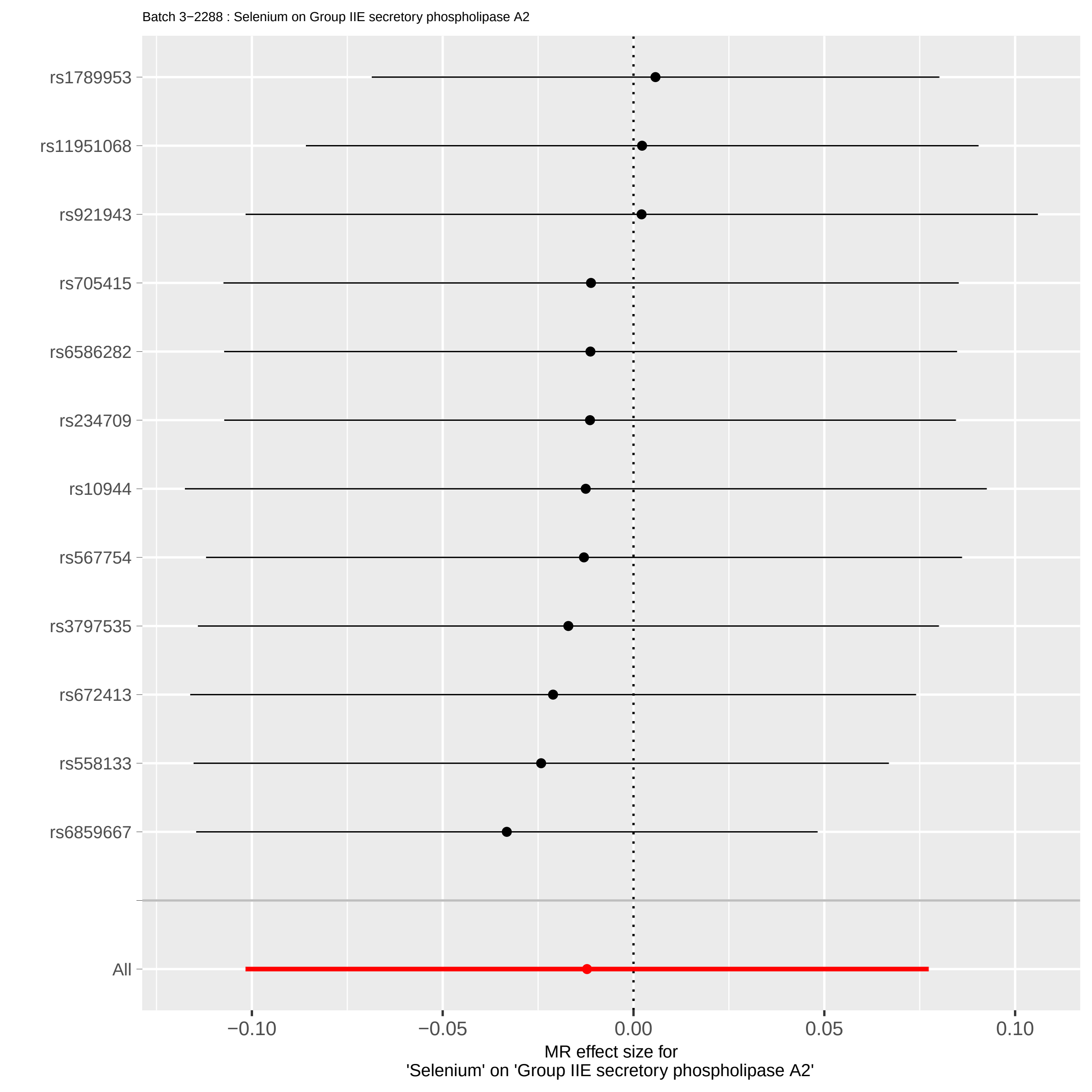

Batch 3−2288 : Selenium on Group IIE secretory phospholipase A2
rs1789953
rs11951068
rs921943
rs705415
rs6586282
rs234709
rs10944
rs567754
rs3797535
rs672413
rs558133
rs6859667
All
−0.10
−0.05
0.00
0.05
0.10
MR effect size for
'Selenium' on 'Group IIE secretory phospholipase A2'

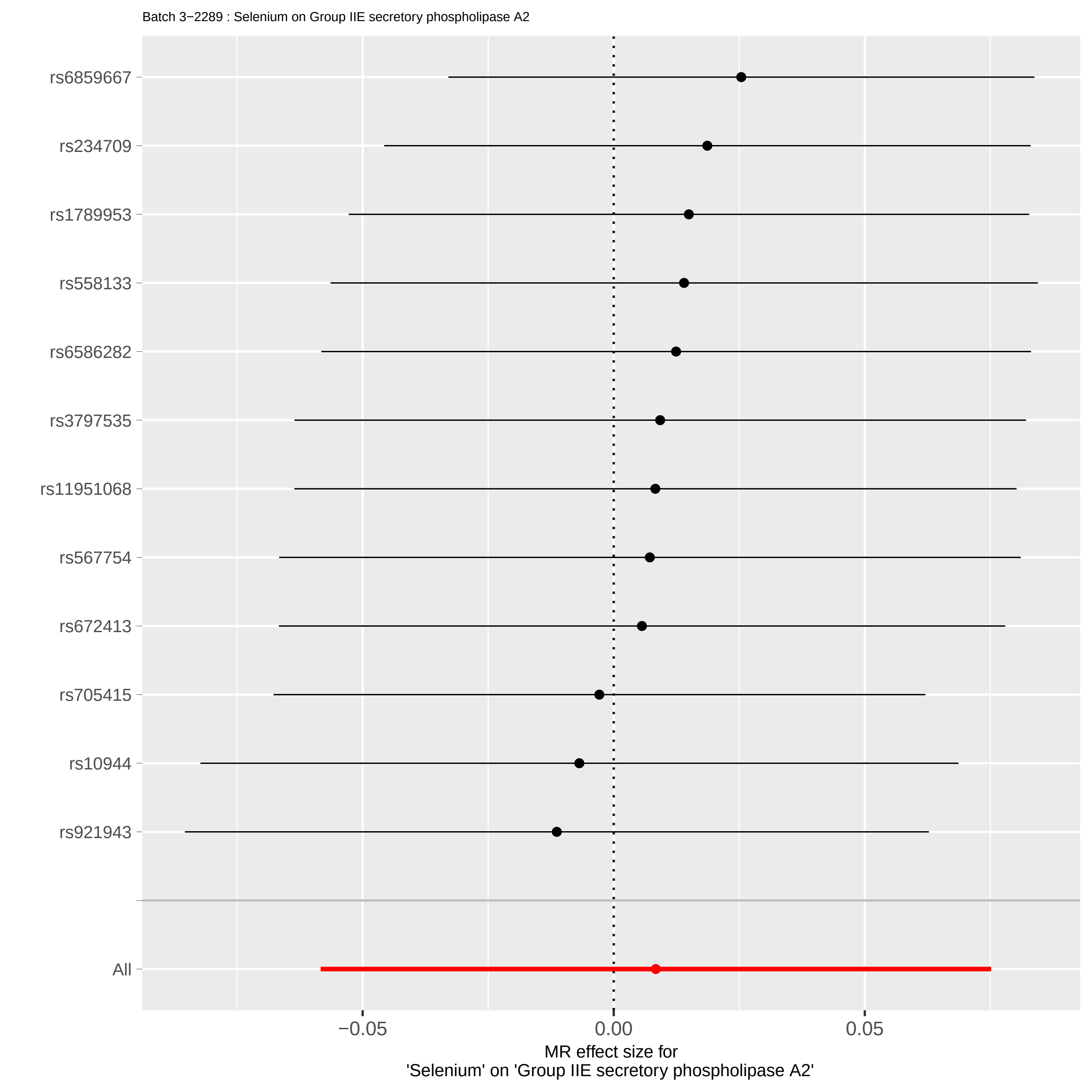

Batch 3−2289 : Selenium on Group IIE secretory phospholipase A2
rs6859667
rs234709
rs1789953
rs558133
rs6586282
rs3797535
rs11951068
rs567754
rs672413
rs705415
rs10944
rs921943
All
−0.05
0.00
0.05
MR effect size for
'Selenium' on 'Group IIE secretory phospholipase A2'

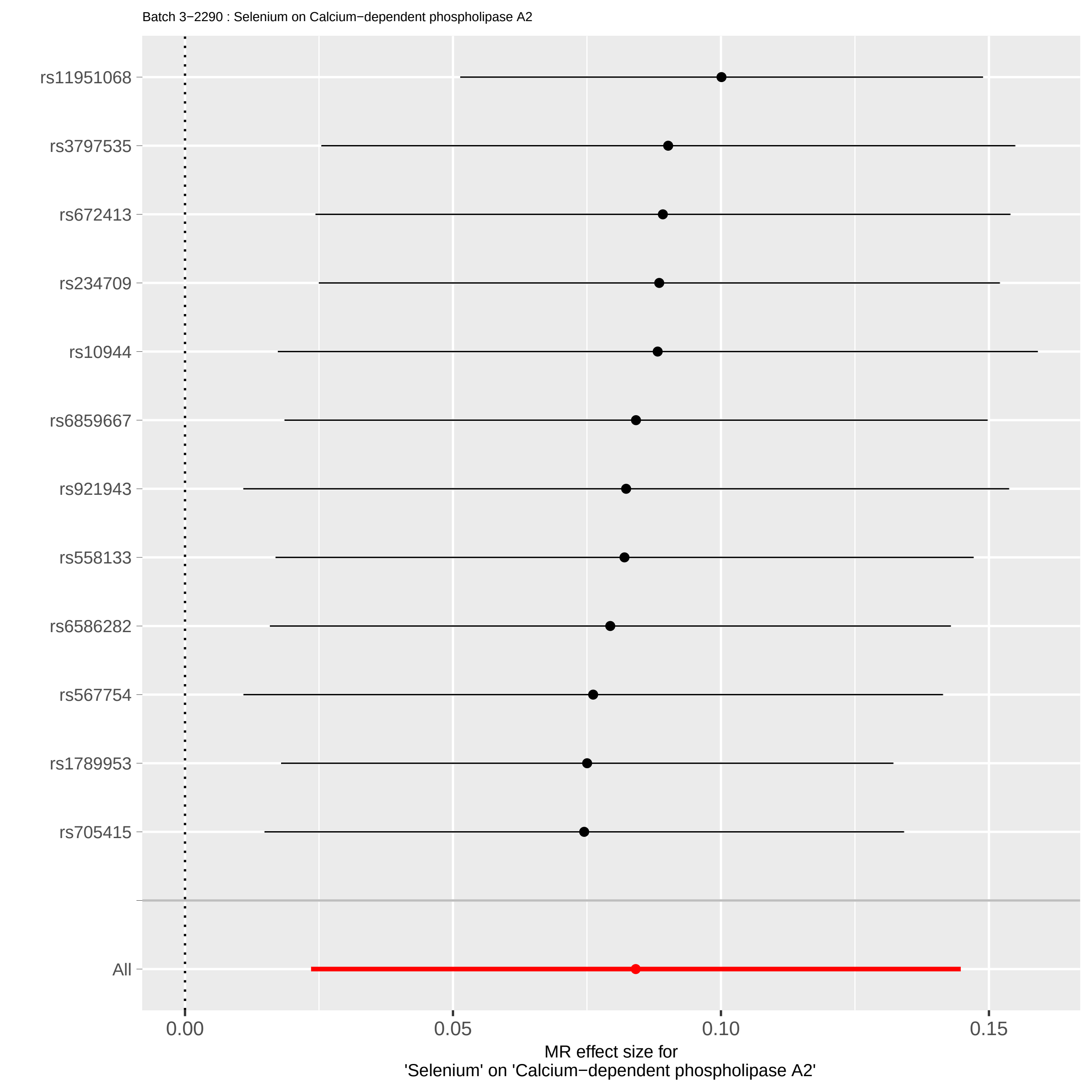

Batch 3−2290 : Selenium on Calcium−dependent phospholipase A2
rs11951068
rs3797535
rs672413
rs234709
rs10944
rs6859667
rs921943
rs558133
rs6586282
rs567754
rs1789953
rs705415
All
0.00
0.05
0.10
0.15
MR effect size for
'Selenium' on 'Calcium−dependent phospholipase A2'

Batch 3−2291 : Selenium on Secretory phospholipase A2 receptor

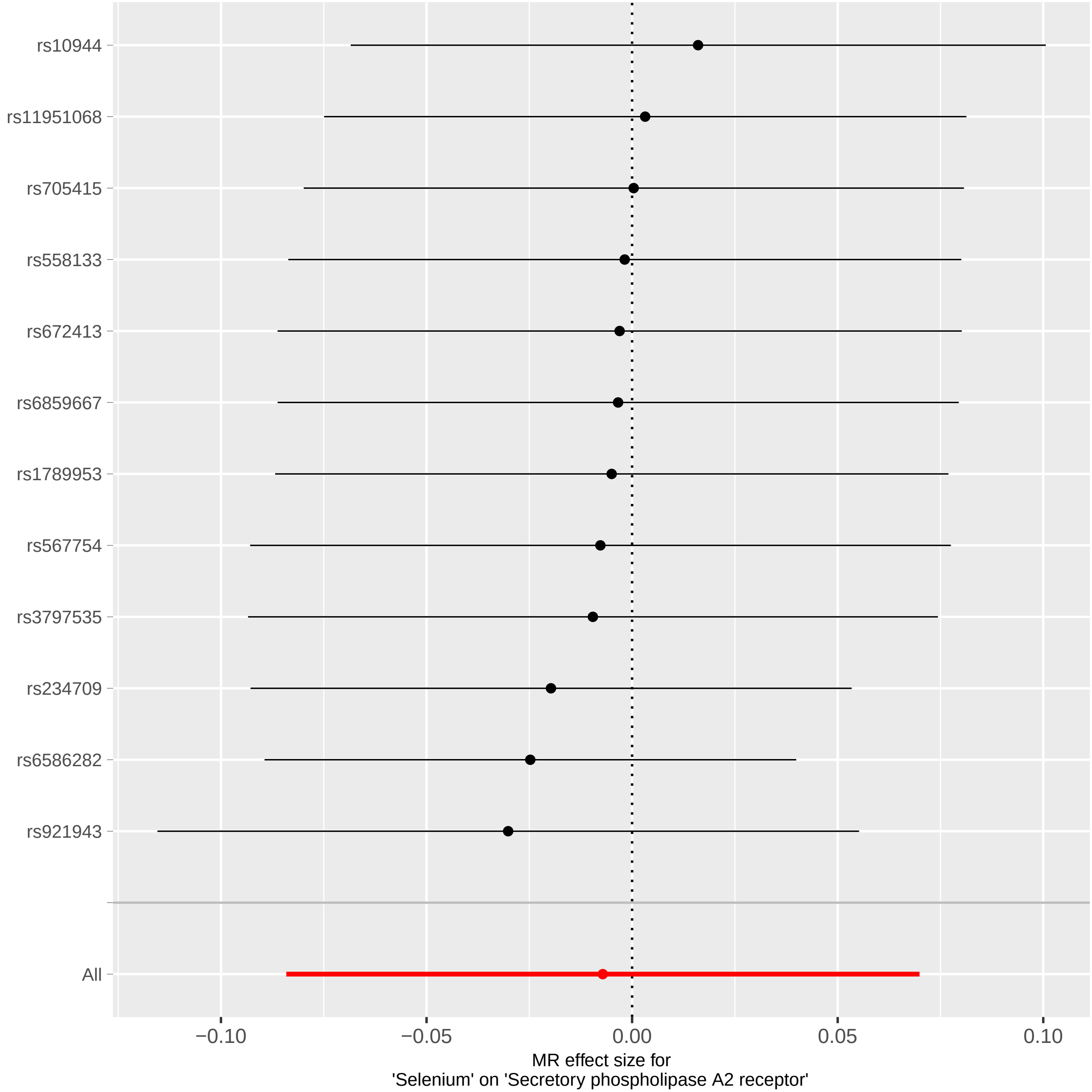

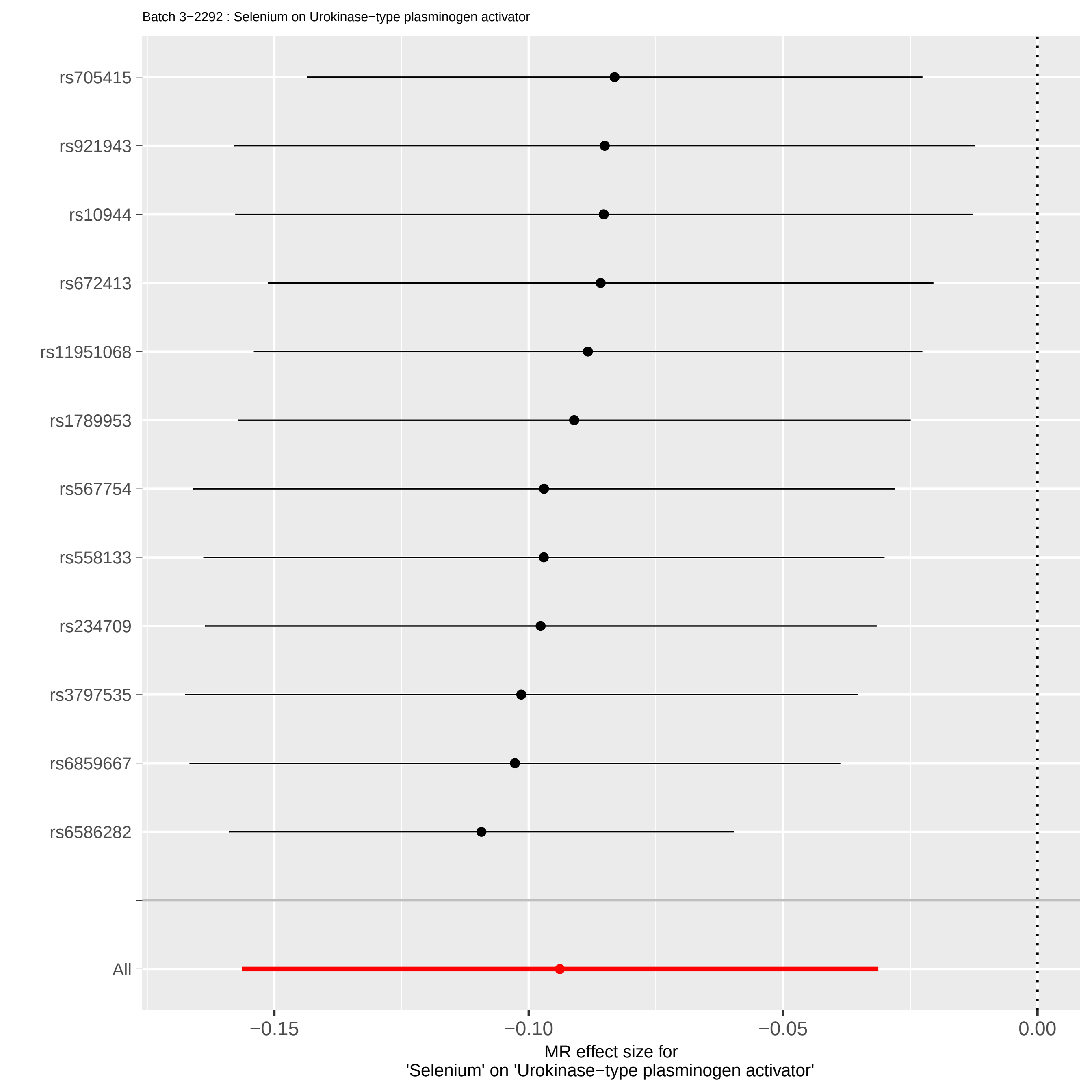
Batch 3−2292 : Selenium on Urokinase−type plasminogen activator
rs705415
rs921943
rs10944
rs672413
rs11951068
rs1789953
rs567754
rs558133
rs234709
rs3797535
rs6859667
rs6586282
All
−0.15
−0.10
−0.05
0.00
MR effect size for
'Selenium' on 'Urokinase−type plasminogen activator'

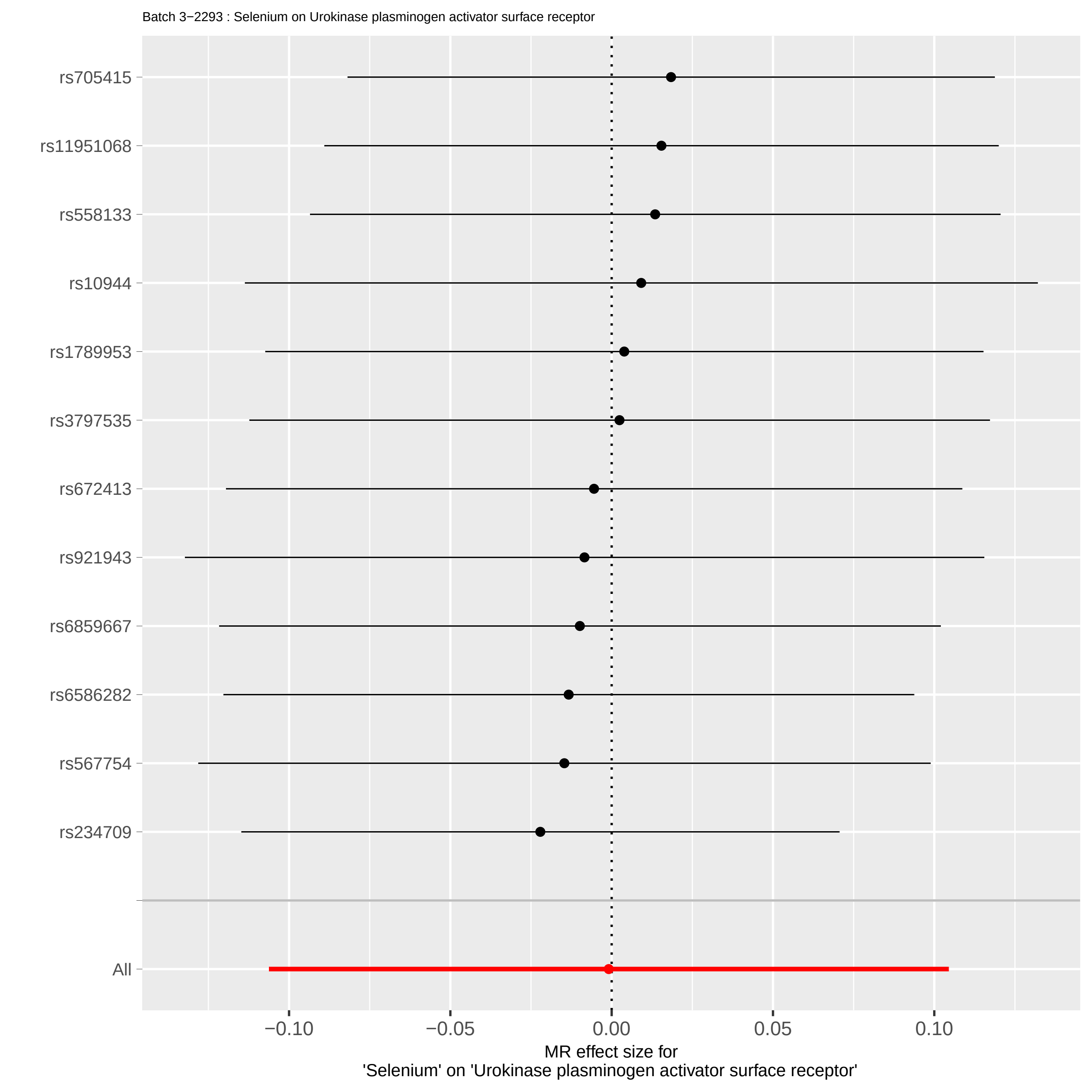

Batch 3−2293 : Selenium on Urokinase plasminogen activator surface receptor
rs705415
rs11951068
rs558133
rs10944
rs1789953
rs3797535
rs672413
rs921943
rs6859667
rs6586282
rs567754
rs234709
All
−0.10
−0.05
0.00
0.05
0.10
MR effect size for
'Selenium' on 'Urokinase plasminogen activator surface receptor'

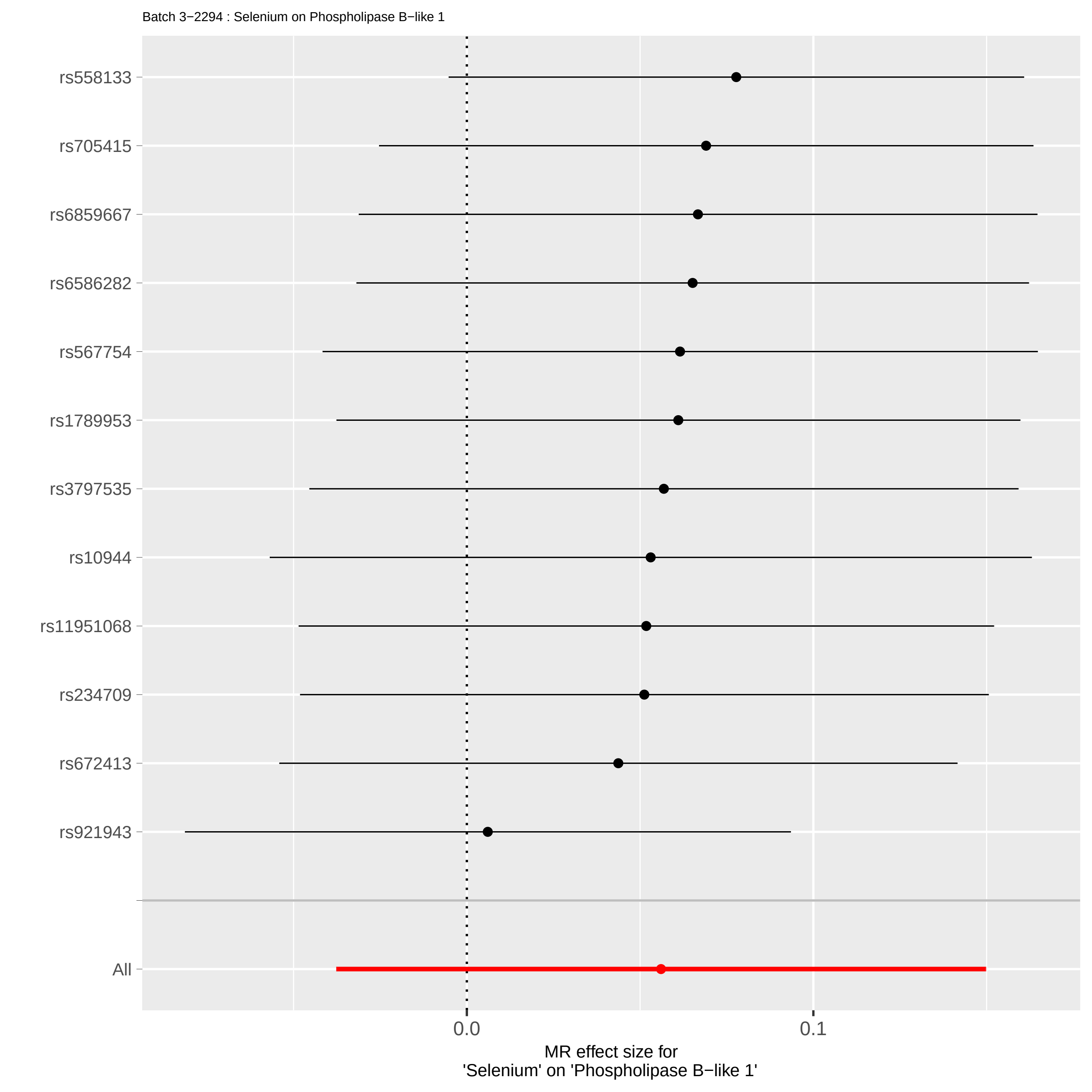

Batch 3−2294 : Selenium on Phospholipase B−like 1
rs558133
rs705415
rs6859667
rs6586282
rs567754
rs1789953
rs3797535
rs10944
rs11951068
rs234709
rs672413
rs921943
All
0.0
0.1
MR effect size for
'Selenium' on 'Phospholipase B−like 1'

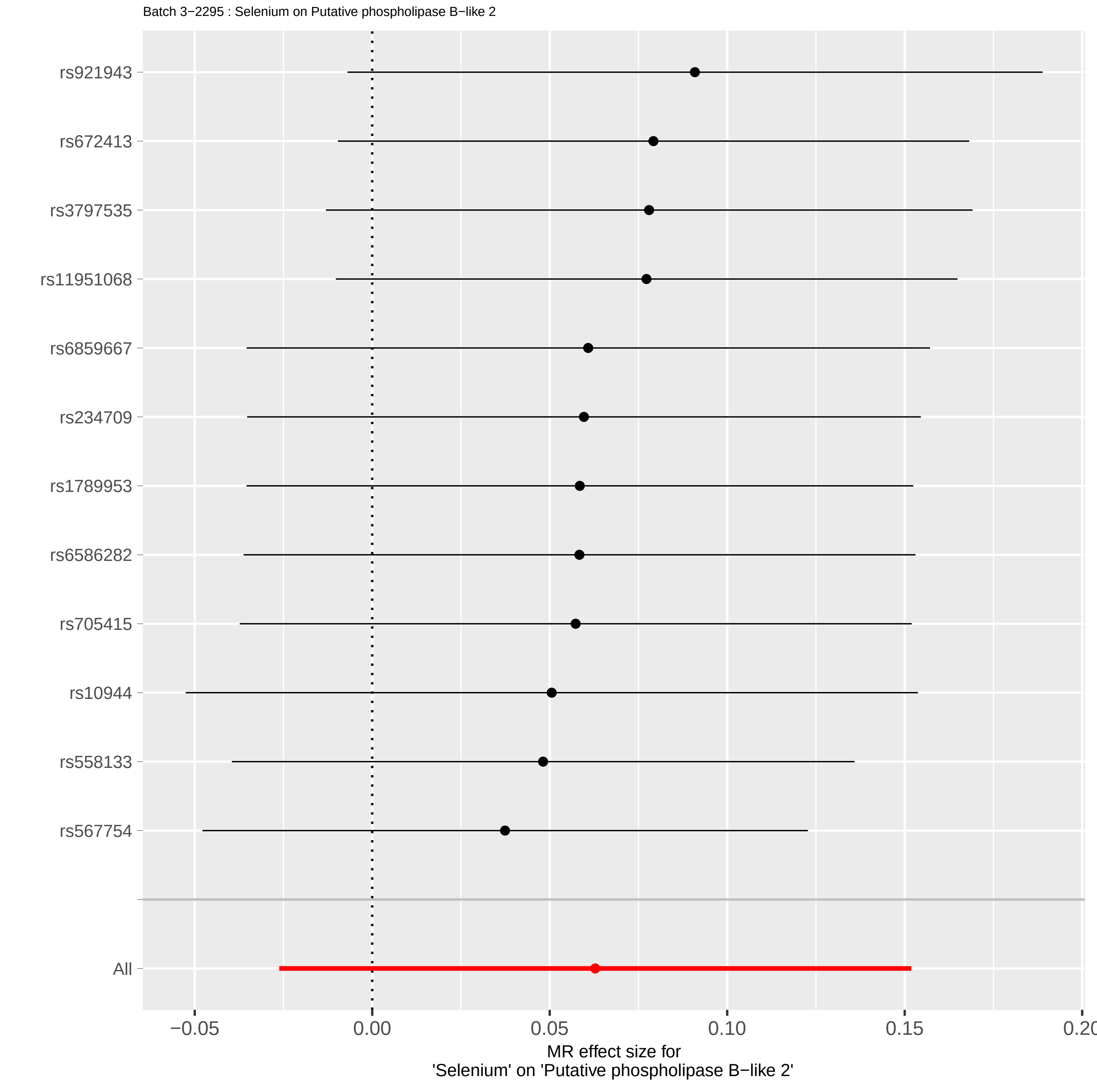

Batch 3−2295 : Selenium on Putative phospholipase B−like 2
rs921943
rs672413
rs3797535
rs11951068
rs6859667
rs234709
rs1789953
rs6586282
rs705415
rs10944
rs558133
rs567754
All
−0.05
0.00
0.05
0.10
0.15
0.20
MR effect size for
'Selenium' on 'Putative phospholipase B−like 2'

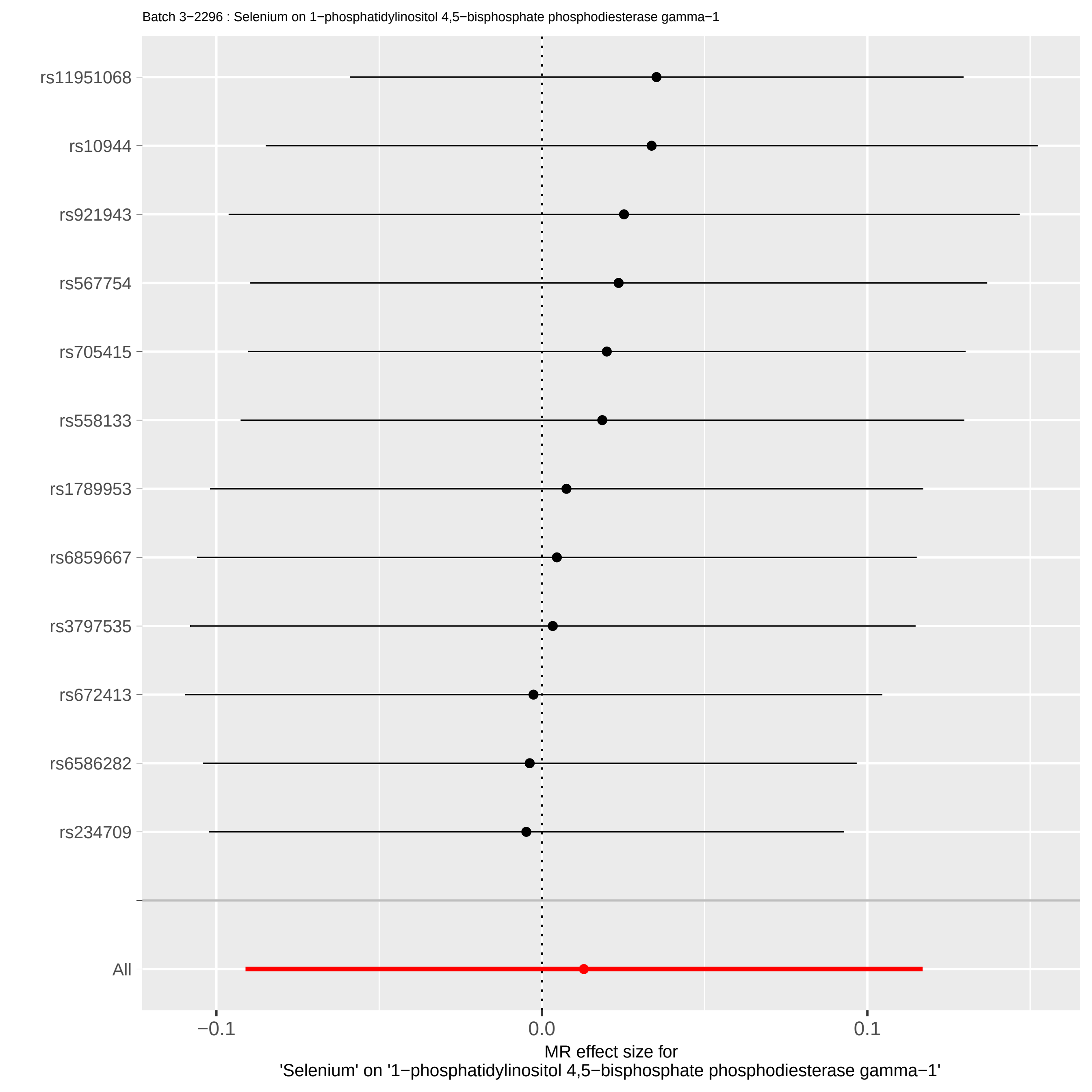

Batch 3−2296 : Selenium on 1−phosphatidylinositol 4,5−bisphosphate phosphodiesterase gamma−1
rs11951068
rs10944
rs921943
rs567754
rs705415
rs558133
rs1789953
rs6859667
rs3797535
rs672413
rs6586282
rs234709
All
−0.1
0.0
0.1
MR effect size for
'Selenium' on '1−phosphatidylinositol 4,5−bisphosphate phosphodiesterase gamma−1'

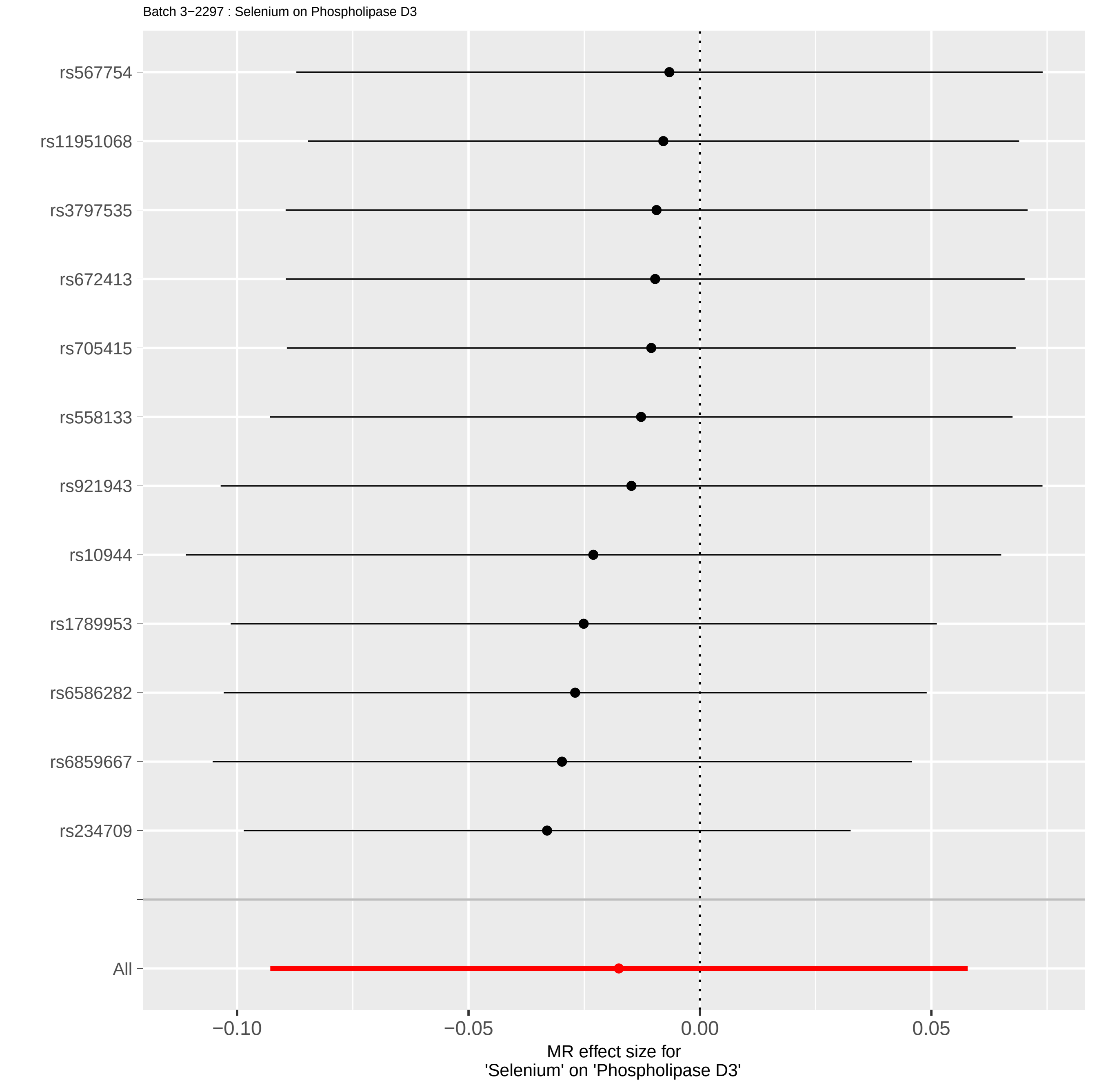

Batch 3−2297 : Selenium on Phospholipase D3
rs567754
rs11951068
rs3797535
rs672413
rs705415
rs558133
rs921943
rs10944
rs1789953
rs6586282
rs6859667
rs234709
All
−0.10
−0.05
0.00
0.05
MR effect size for
'Selenium' on 'Phospholipase D3'

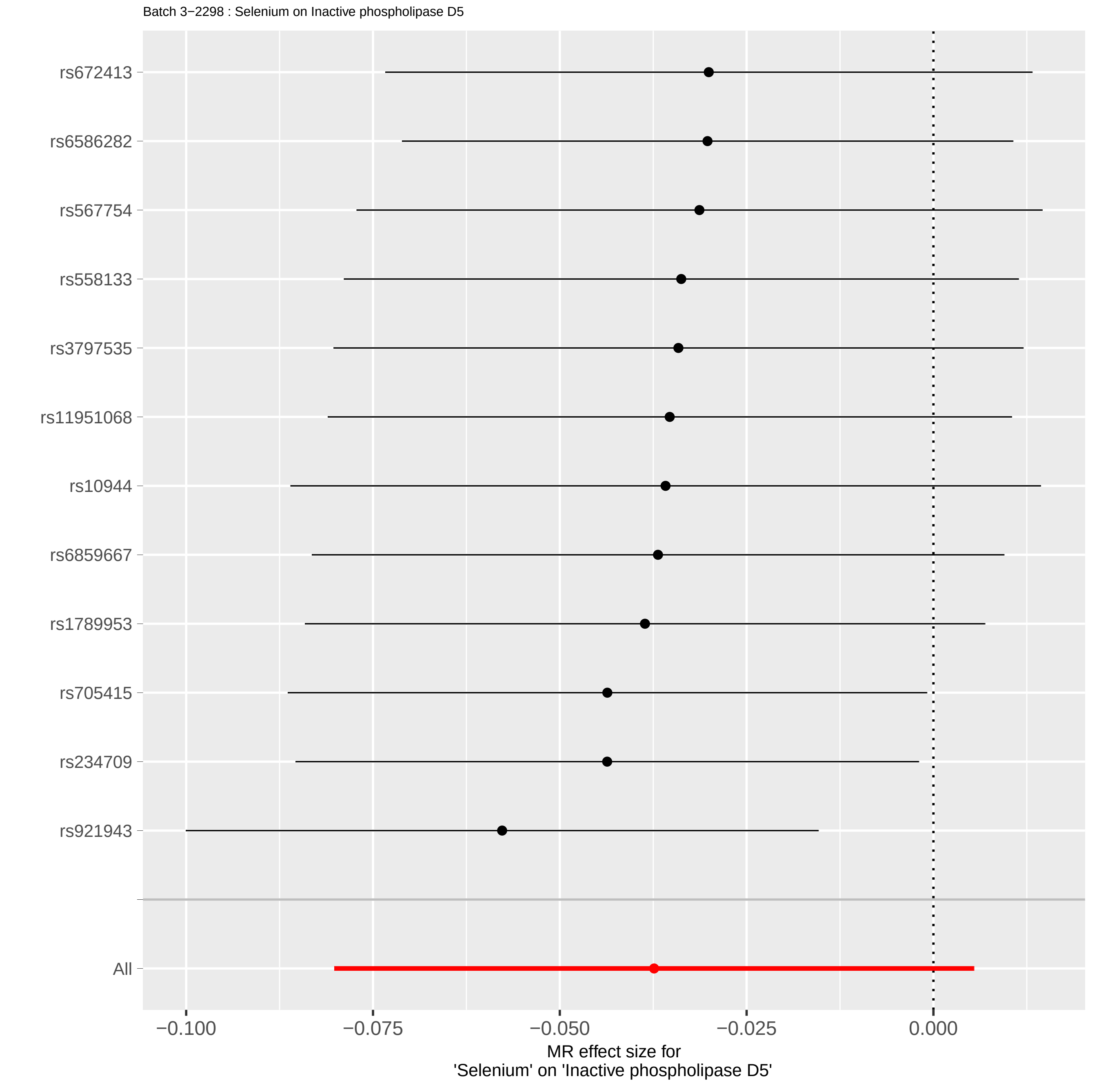

Batch 3−2298 : Selenium on Inactive phospholipase D5
rs672413
rs6586282
rs567754
rs558133
rs3797535
rs11951068
rs10944
rs6859667
rs1789953
rs705415
rs234709
rs921943
All
−0.100
−0.075
−0.050
−0.025
0.000
MR effect size for
'Selenium' on 'Inactive phospholipase D5'

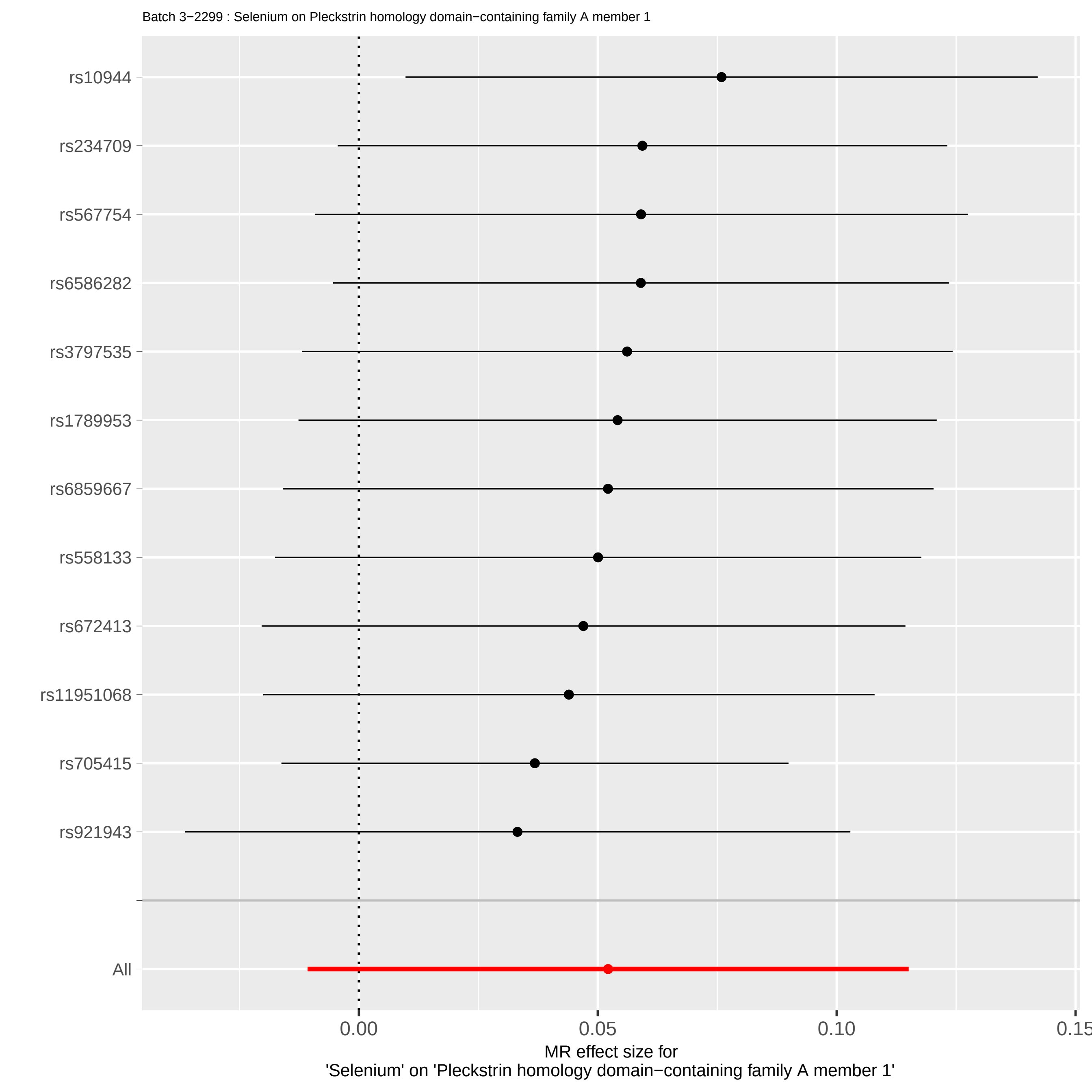

Batch 3−2299 : Selenium on Pleckstrin homology domain−containing family A member 1
rs10944
rs234709
rs567754
rs6586282
rs3797535
rs1789953
rs6859667
rs558133
rs672413
rs11951068
rs705415
rs921943
All
0.00
0.05
0.10
0.15
MR effect size for
'Selenium' on 'Pleckstrin homology domain−containing family A member 1'

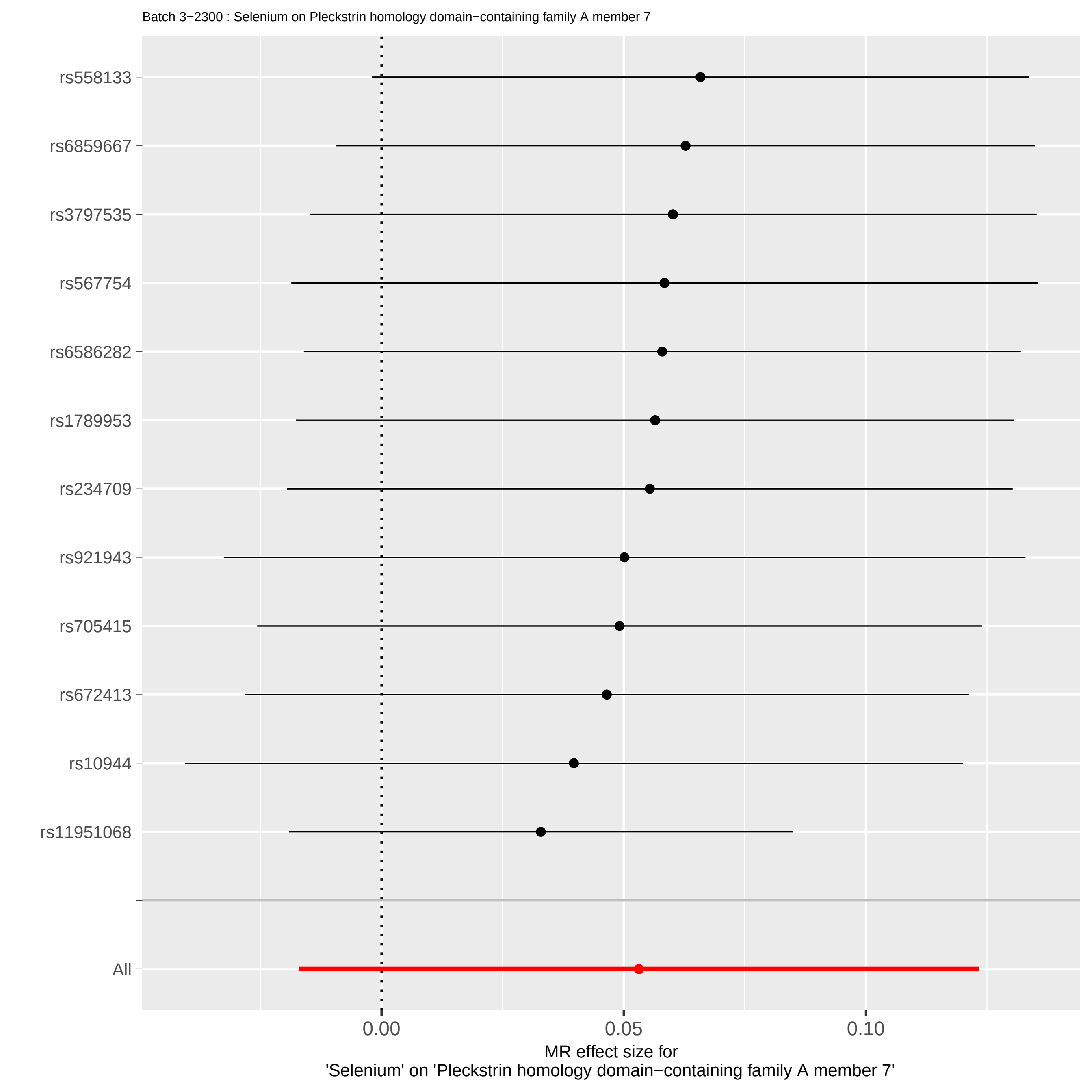

Batch 3−2300 : Selenium on Pleckstrin homology domain−containing family A member 7
rs558133
rs6859667
rs3797535
rs567754
rs6586282
rs1789953
rs234709
rs921943
rs705415
rs672413
rs10944
rs11951068
All
0.00
0.05
0.10
MR effect size for
'Selenium' on 'Pleckstrin homology domain−containing family A member 7'

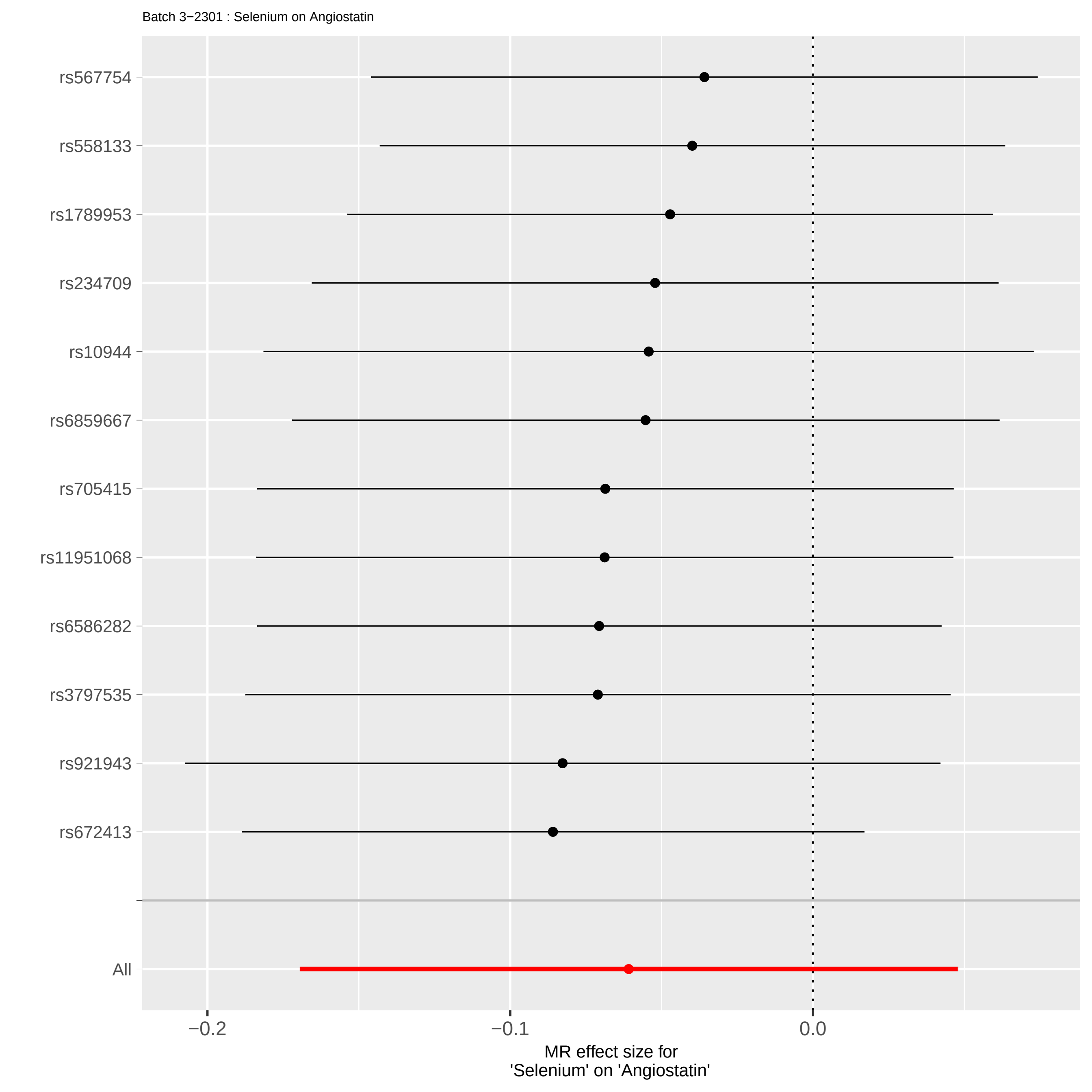

Batch 3−2301 : Selenium on Angiostatin
rs567754
rs558133
rs1789953
rs234709
rs10944
rs6859667
rs705415
rs11951068
rs6586282
rs3797535
rs921943
rs672413
All
−0.2
−0.1
0.0
MR effect size for
'Selenium' on 'Angiostatin'

Batch 3−2302 : Selenium on Plasmin

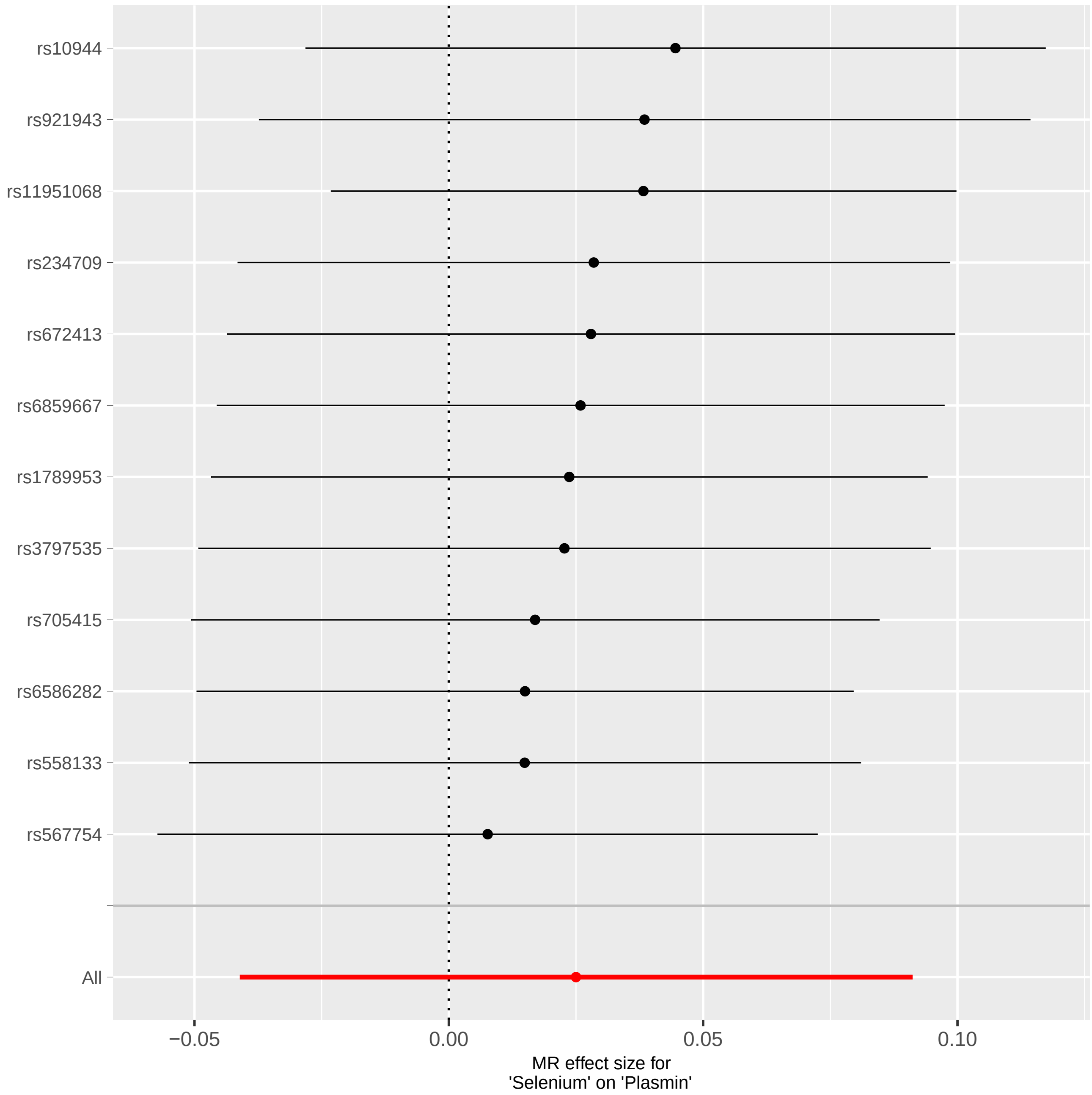

Batch 3−2303 : Selenium on Serine/threonine−protein kinase PLK1

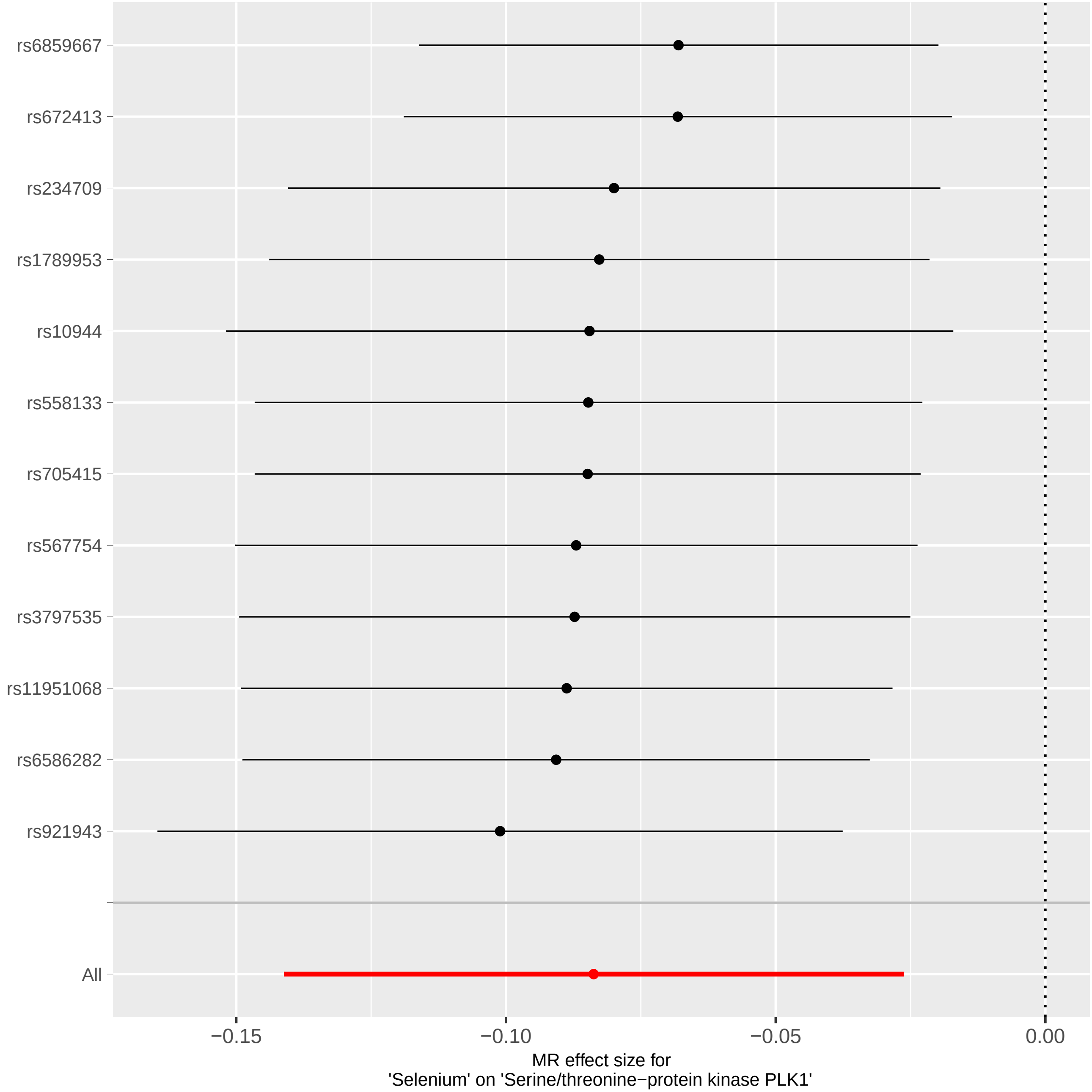

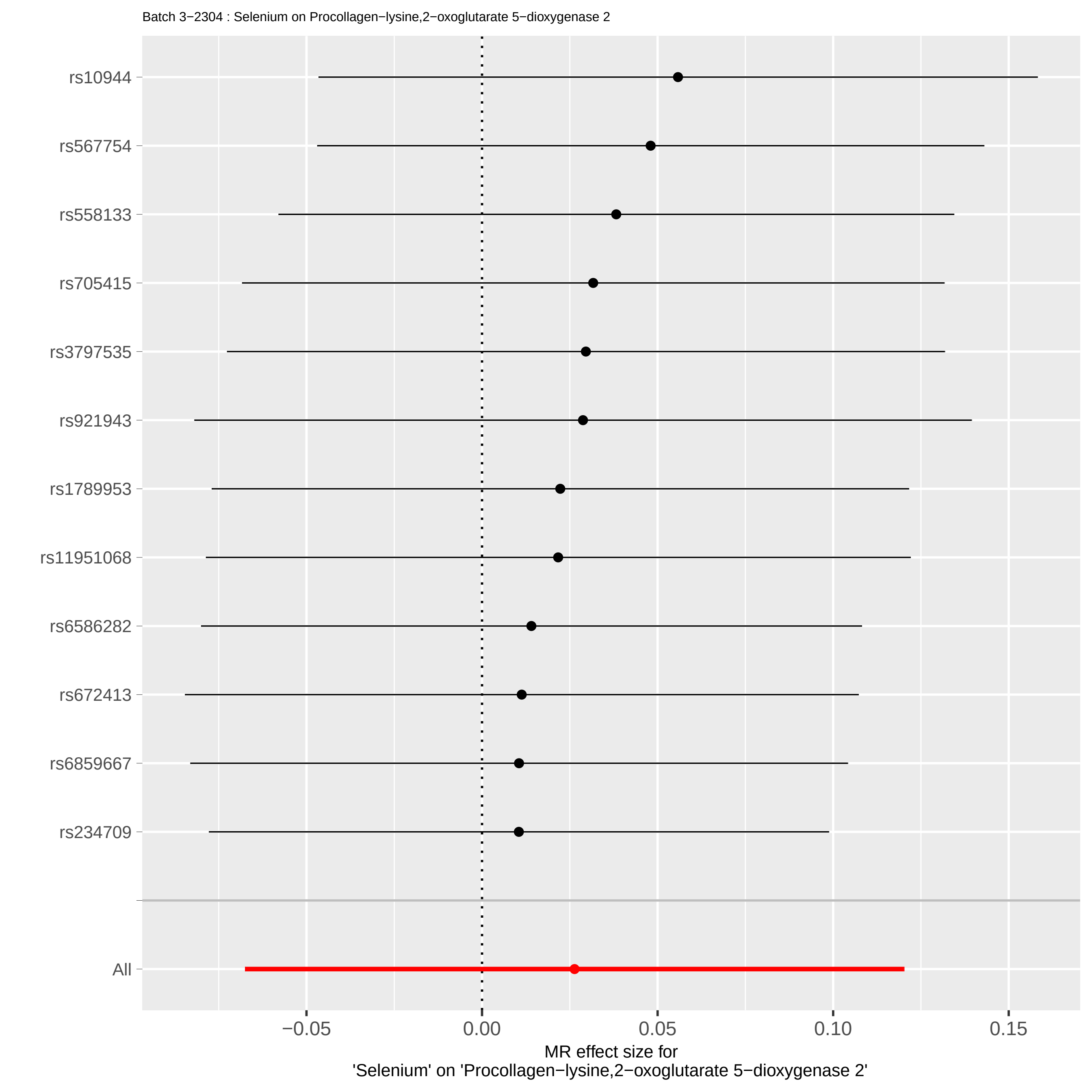

Batch 3−2304 : Selenium on Procollagen−lysine,2−oxoglutarate 5−dioxygenase 2
rs10944
rs567754
rs558133
rs705415
rs3797535
rs921943
rs1789953
rs11951068
rs6586282
rs672413
rs6859667
rs234709
All
−0.05
0.00
0.05
0.10
0.15
MR effect size for
'Selenium' on 'Procollagen−lysine,2−oxoglutarate 5−dioxygenase 2'

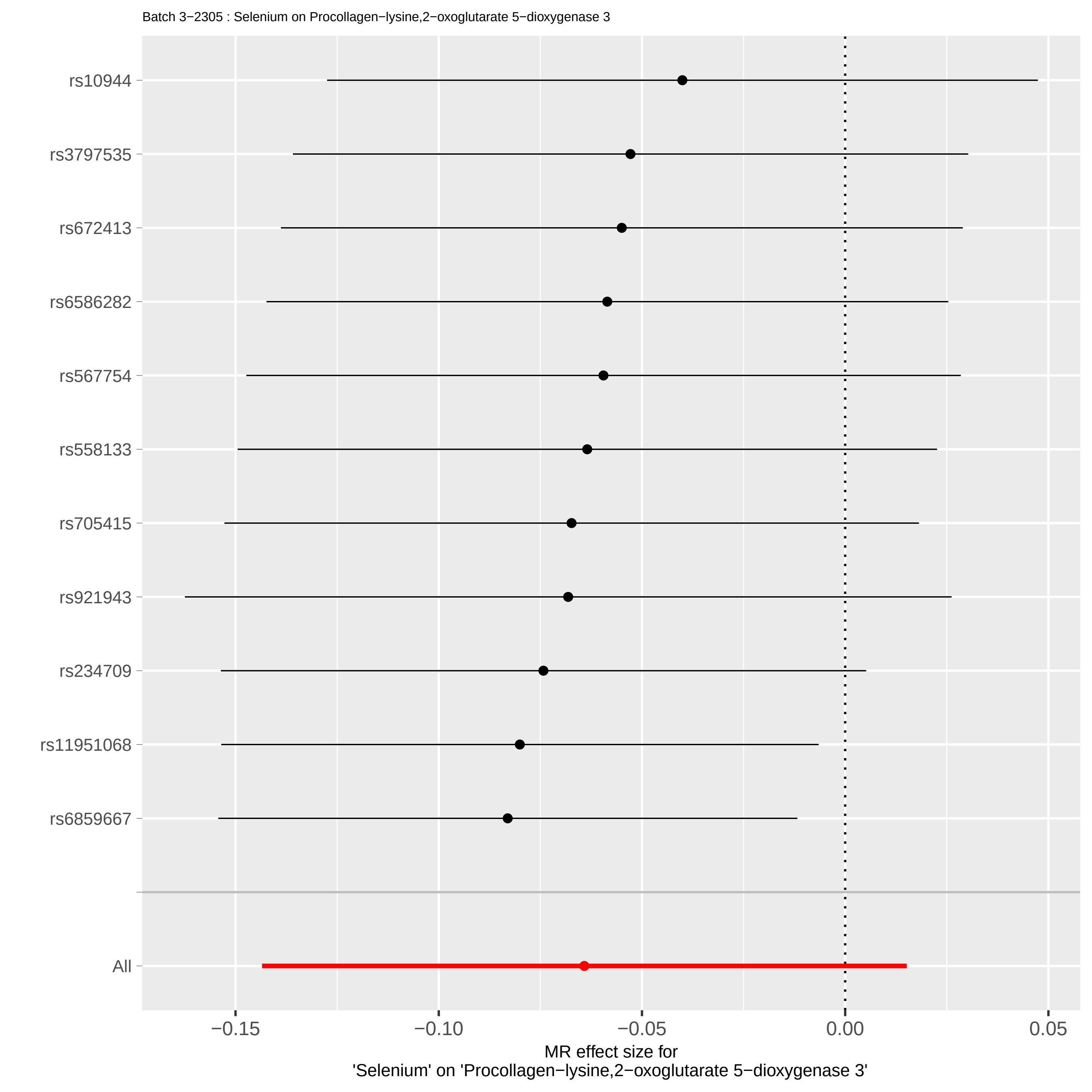
Batch 3−2305 : Selenium on Procollagen−lysine,2−oxoglutarate 5−dioxygenase 3
rs10944
rs3797535
rs672413
rs6586282
rs567754
rs558133
rs705415
rs921943
rs234709
rs11951068
rs6859667
All
−0.15
−0.10
−0.05
0.00
0.05
MR effect size for
'Selenium' on 'Procollagen−lysine,2−oxoglutarate 5−dioxygenase 3'

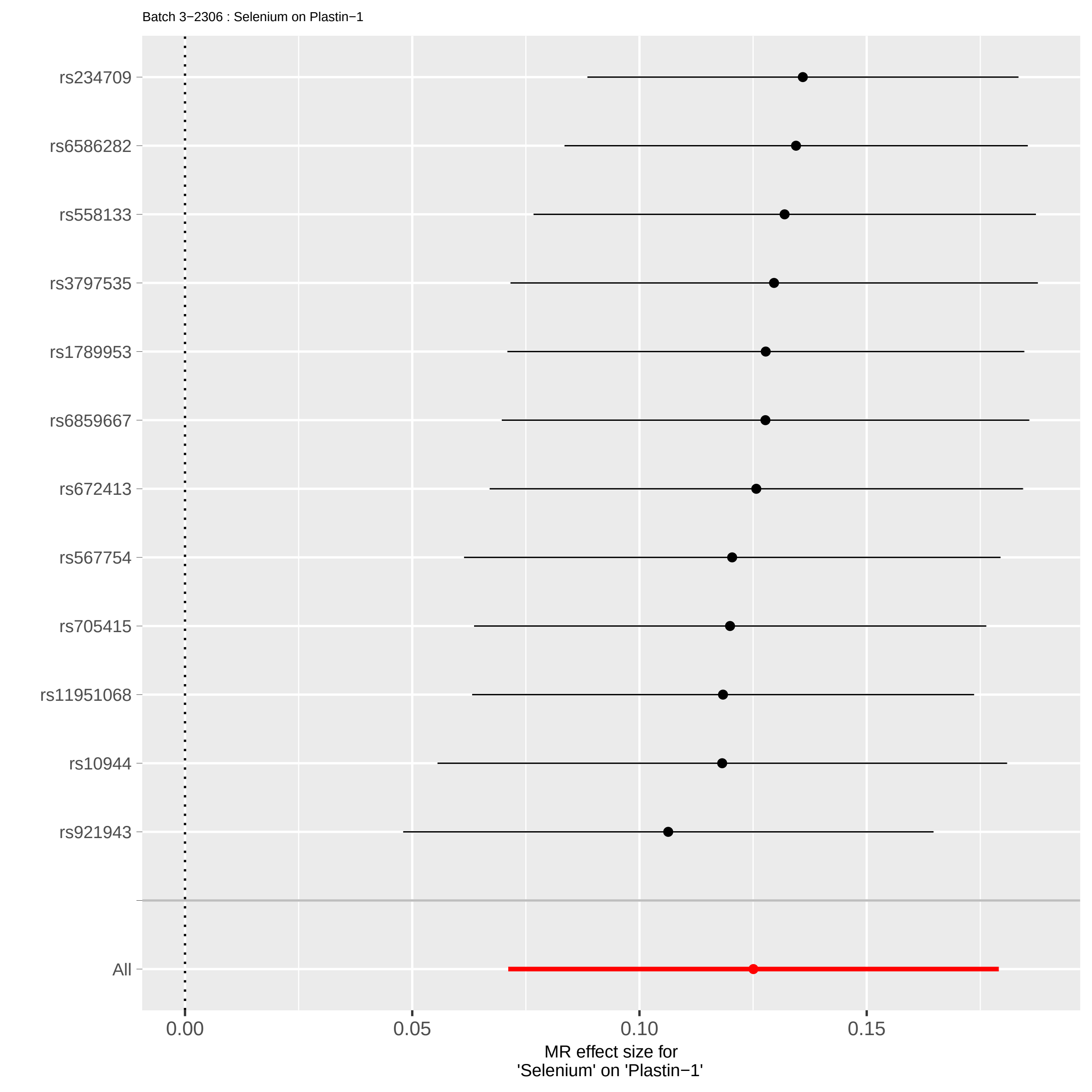

Batch 3−2306 : Selenium on Plastin−1
rs234709
rs6586282
rs558133
rs3797535
rs1789953
rs6859667
rs672413
rs567754
rs705415
rs11951068
rs10944
rs921943
All
0.00
0.05
0.10
0.15
MR effect size for
'Selenium' on 'Plastin−1'

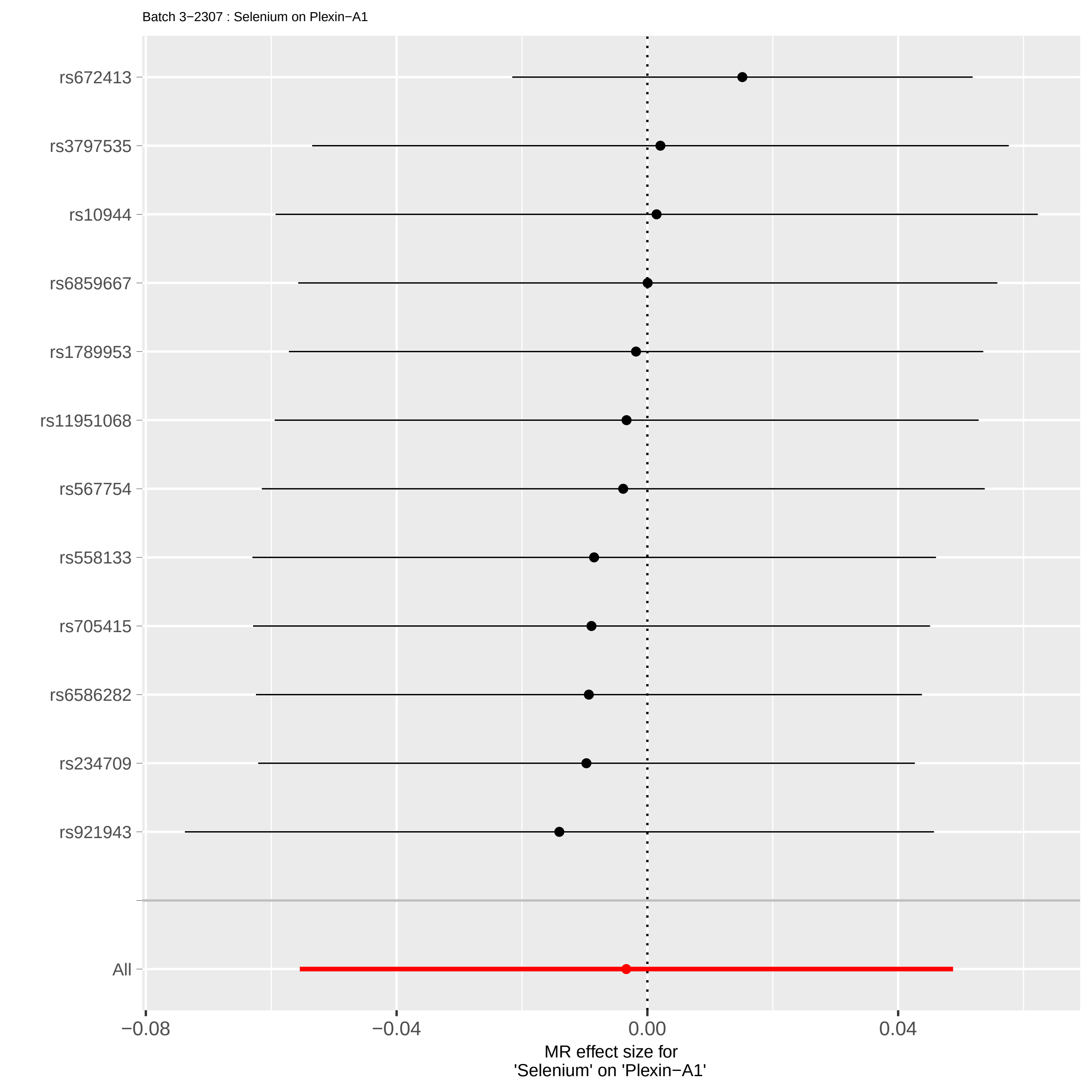
Batch 3−2307 : Selenium on Plexin−A1
rs672413
rs3797535
rs10944
rs6859667
rs1789953
rs11951068
rs567754
rs558133
rs705415
rs6586282
rs234709
rs921943
All
−0.08
−0.04
0.00
0.04
MR effect size for
'Selenium' on 'Plexin−A1'

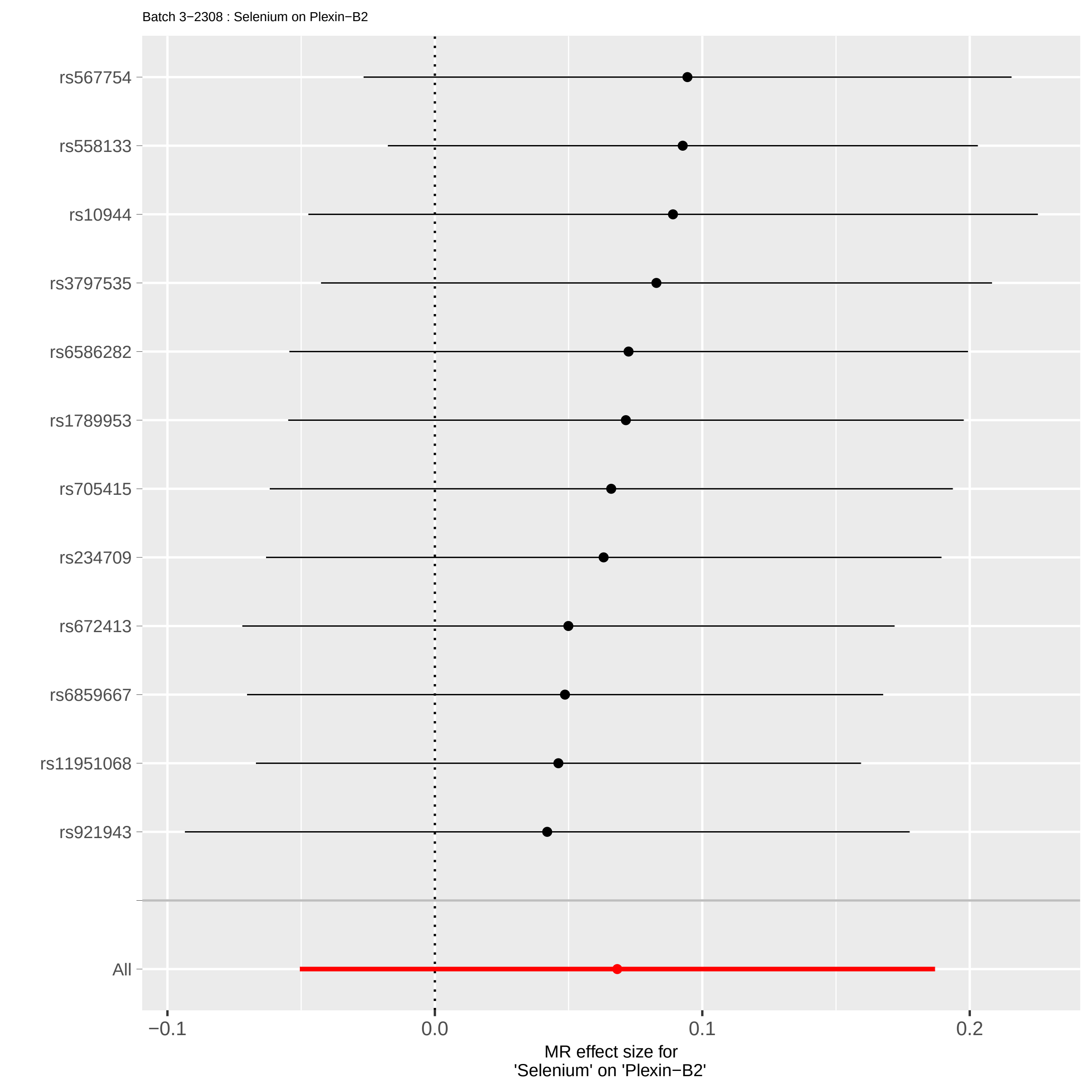

Batch 3−2308 : Selenium on Plexin−B2
rs567754
rs558133
rs10944
rs3797535
rs6586282
rs1789953
rs705415
rs234709
rs672413
rs6859667
rs11951068
rs921943
All
−0.1
0.0
0.1
0.2
MR effect size for
'Selenium' on 'Plexin−B2'

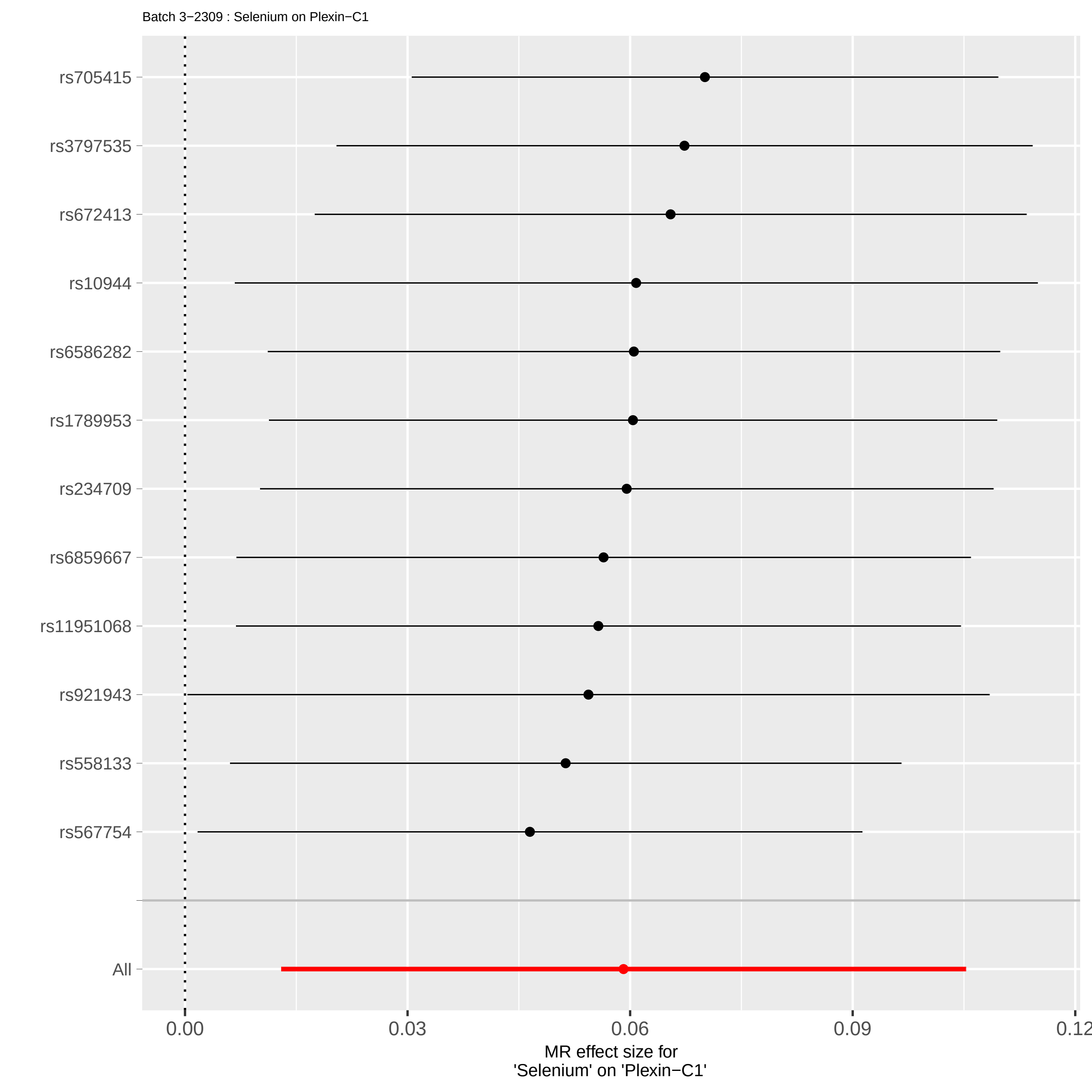
Batch 3−2309 : Selenium on Plexin−C1
rs705415
rs3797535
rs672413
rs10944
rs6586282
rs1789953
rs234709
rs6859667
rs11951068
rs921943
rs558133
rs567754
All
0.00
0.03
0.06
0.09
0.12
MR effect size for
'Selenium' on 'Plexin−C1'

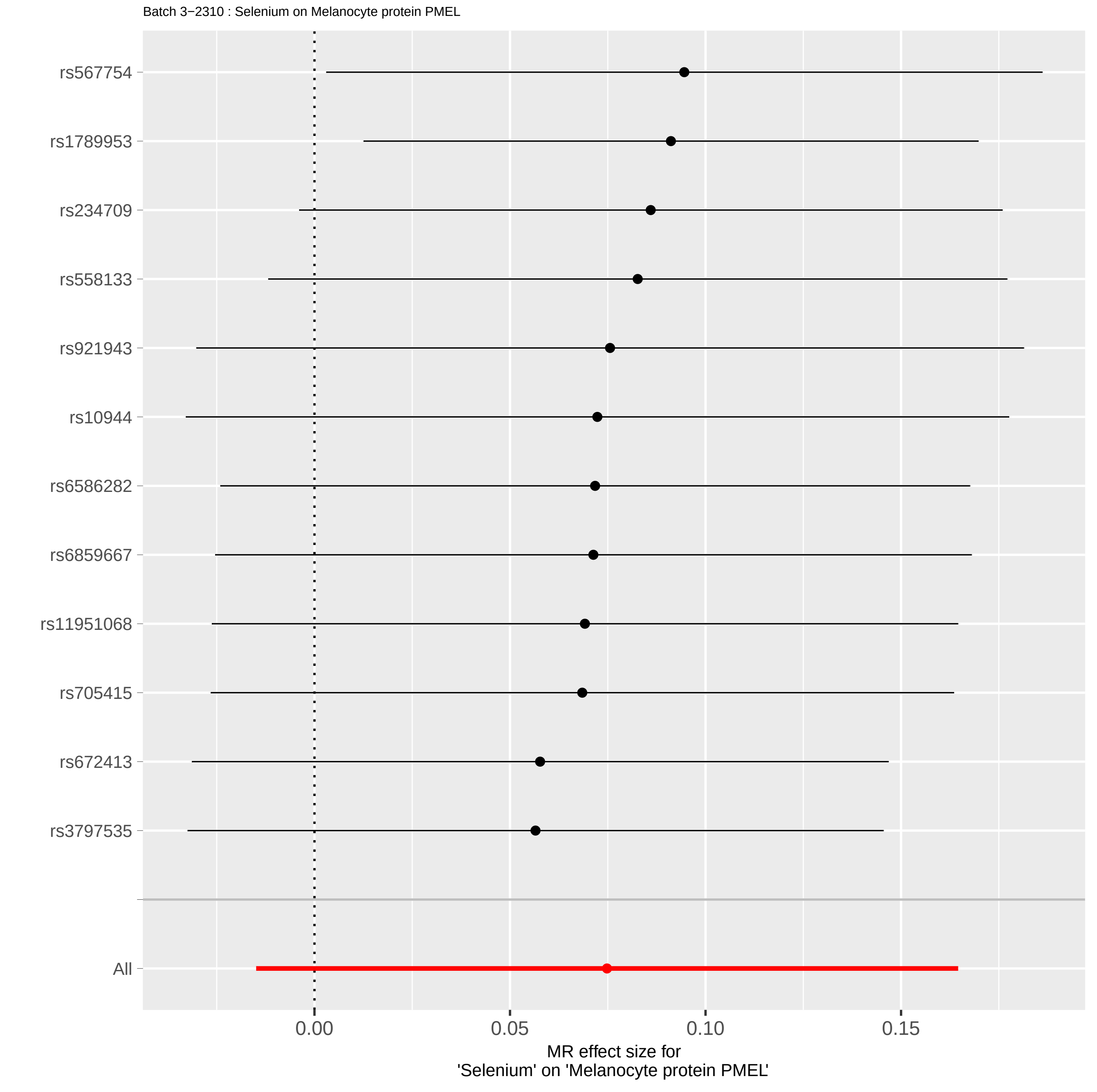

Batch 3−2310 : Selenium on Melanocyte protein PMEL
rs567754
rs1789953
rs234709
rs558133
rs921943
rs10944
rs6586282
rs6859667
rs11951068
rs705415
rs672413
rs3797535
All
0.00
0.05
0.10
0.15
MR effect size for
'Selenium' on 'Melanocyte protein PMEL'

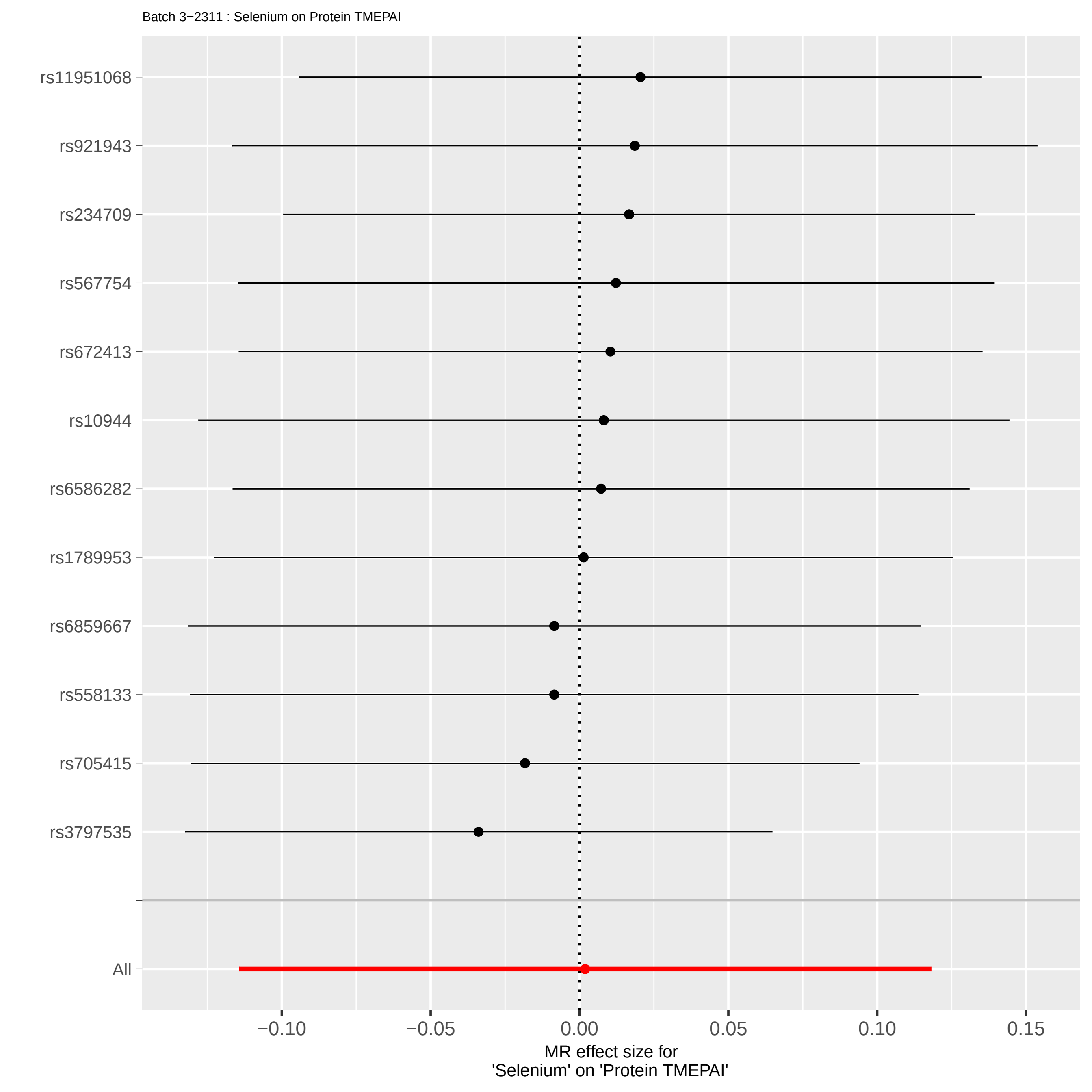

Batch 3−2311 : Selenium on Protein TMEPAI
rs11951068
rs921943
rs234709
rs567754
rs672413
rs10944
rs6586282
rs1789953
rs6859667
rs558133
rs705415
rs3797535
All
−0.10
−0.05
0.00
0.05
0.10
0.15
MR effect size for
'Selenium' on 'Protein TMEPAI'

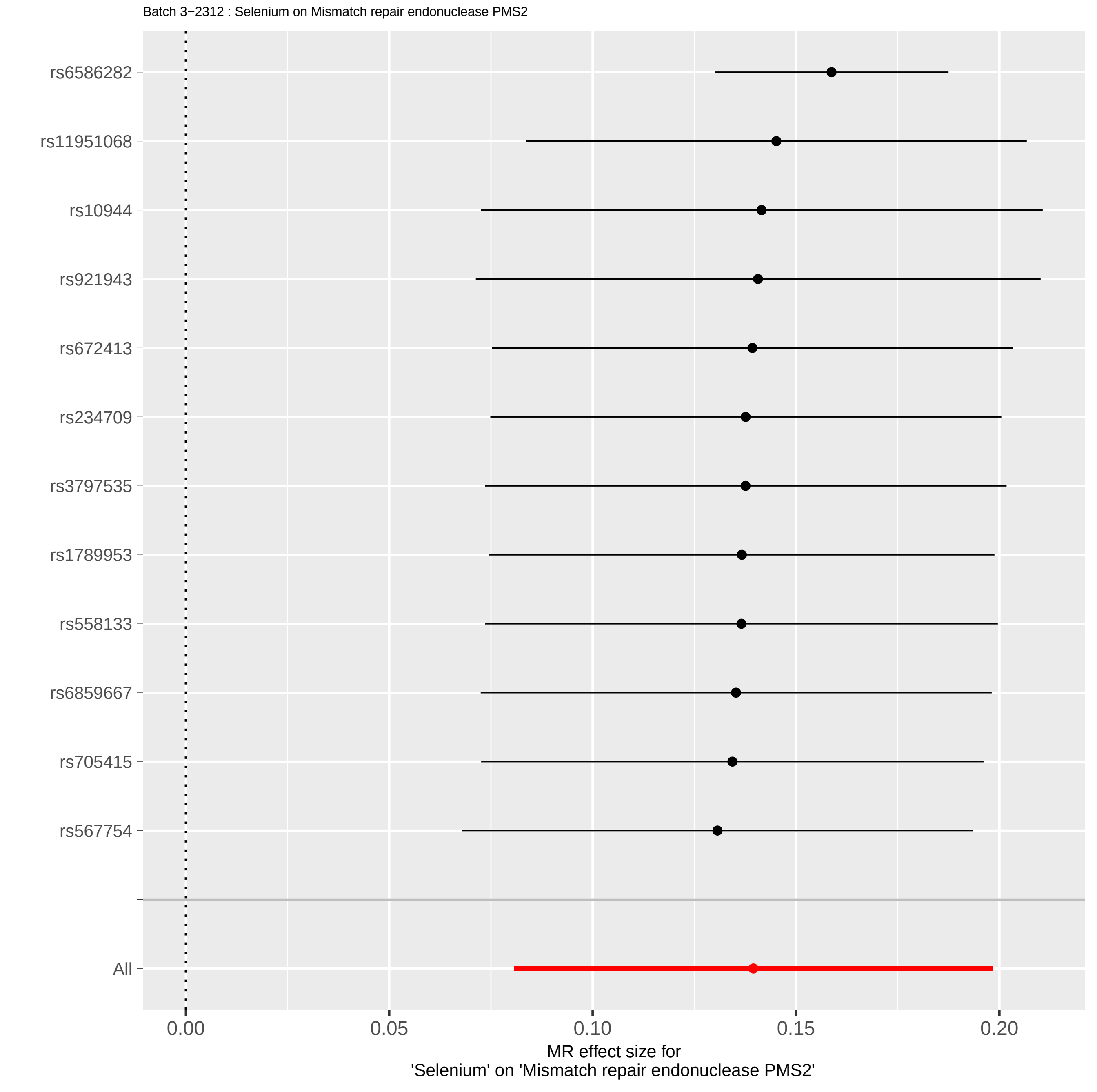
Batch 3−2312 : Selenium on Mismatch repair endonuclease PMS2
rs6586282
rs11951068
rs10944
rs921943
rs672413
rs234709
rs3797535
rs1789953
rs558133
rs6859667
rs705415
rs567754
All
0.00
0.05
0.10
0.15
0.20
MR effect size for
'Selenium' on 'Mismatch repair endonuclease PMS2'

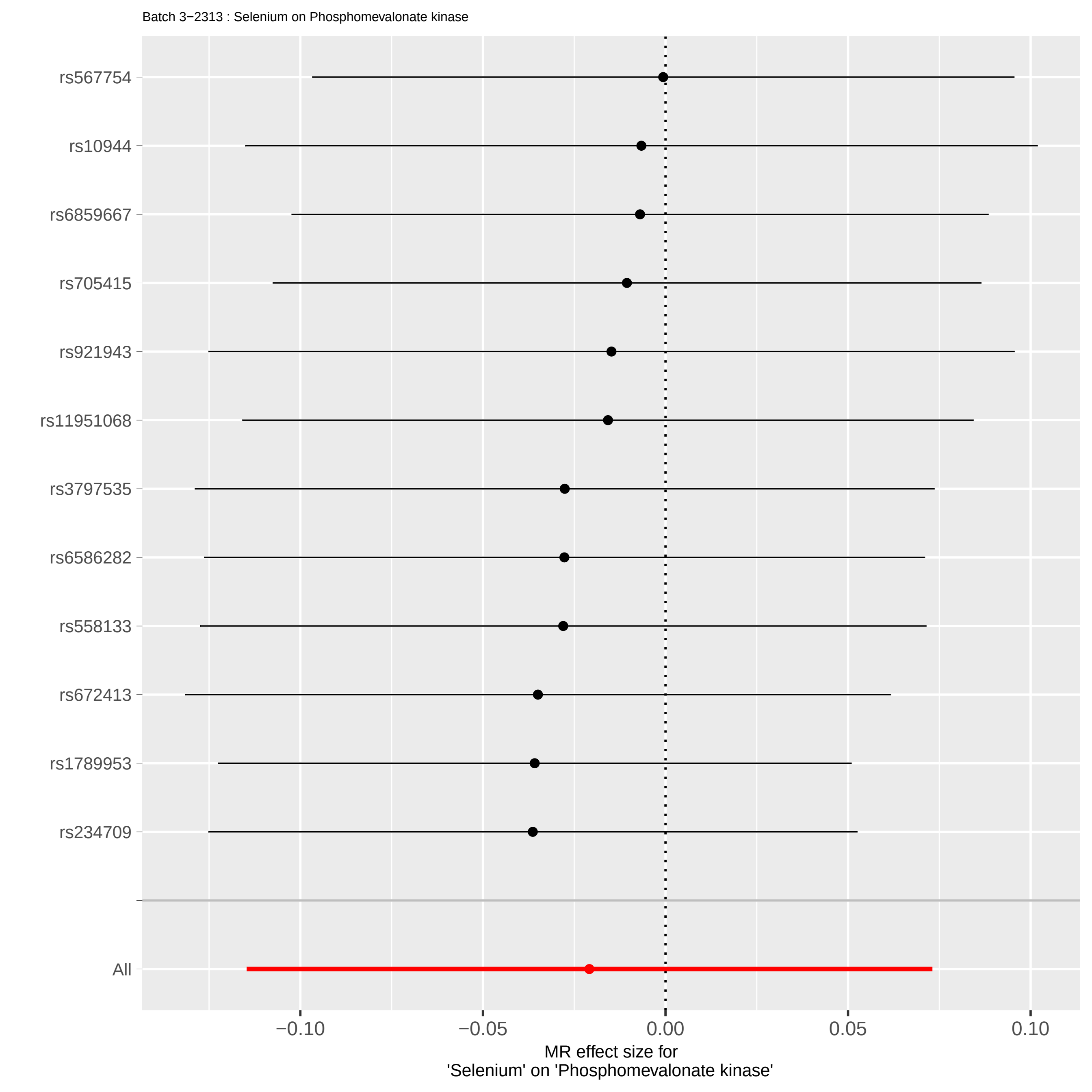
Batch 3−2313 : Selenium on Phosphomevalonate kinase
rs567754
rs10944
rs6859667
rs705415
rs921943
rs11951068
rs3797535
rs6586282
rs558133
rs672413
rs1789953
rs234709
All
−0.10
−0.05
0.00
0.05
0.10
MR effect size for
'Selenium' on 'Phosphomevalonate kinase'

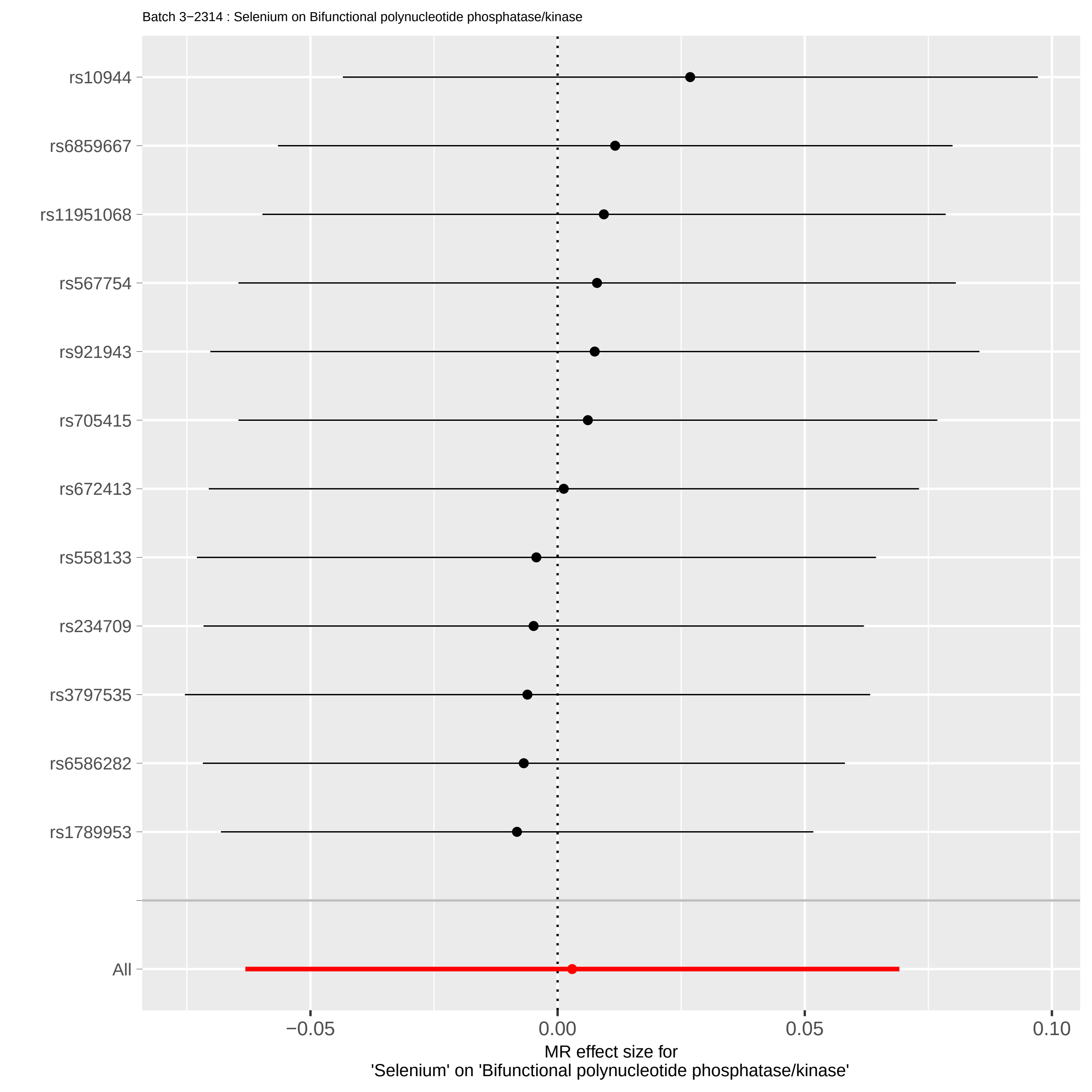

Batch 3−2314 : Selenium on Bifunctional polynucleotide phosphatase/kinase
rs10944
rs6859667
rs11951068
rs567754
rs921943
rs705415
rs672413
rs558133
rs234709
rs3797535
rs6586282
rs1789953
All
−0.05
0.00
0.05
0.10
MR effect size for
'Selenium' on 'Bifunctional polynucleotide phosphatase/kinase'

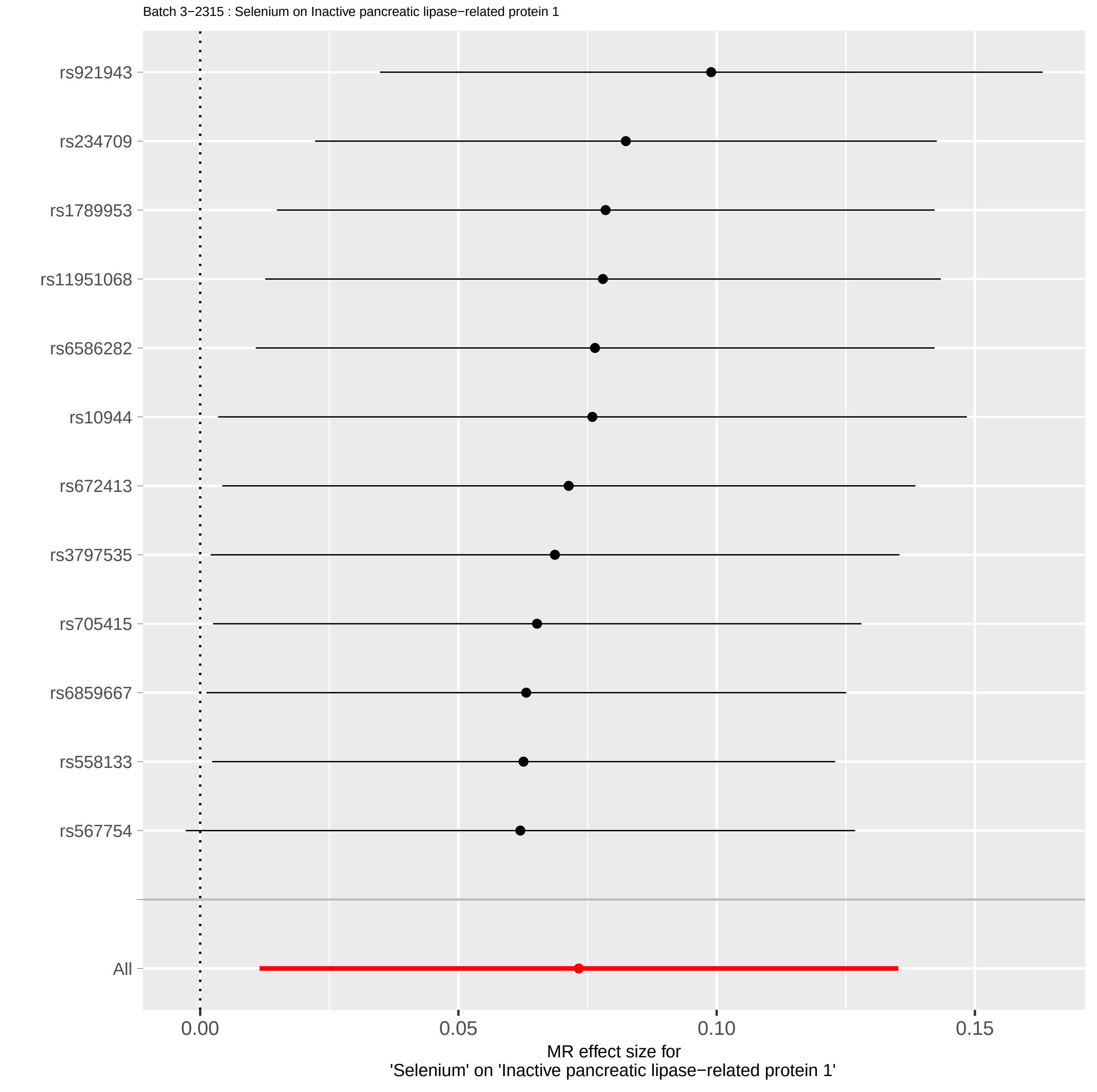

Batch 3−2315 : Selenium on Inactive pancreatic lipase−related protein 1
rs921943
rs234709
rs1789953
rs11951068
rs6586282
rs10944
rs672413
rs3797535
rs705415
rs6859667
rs558133
rs567754
All
0.00
0.05
0.10
0.15
MR effect size for
'Selenium' on 'Inactive pancreatic lipase−related protein 1'

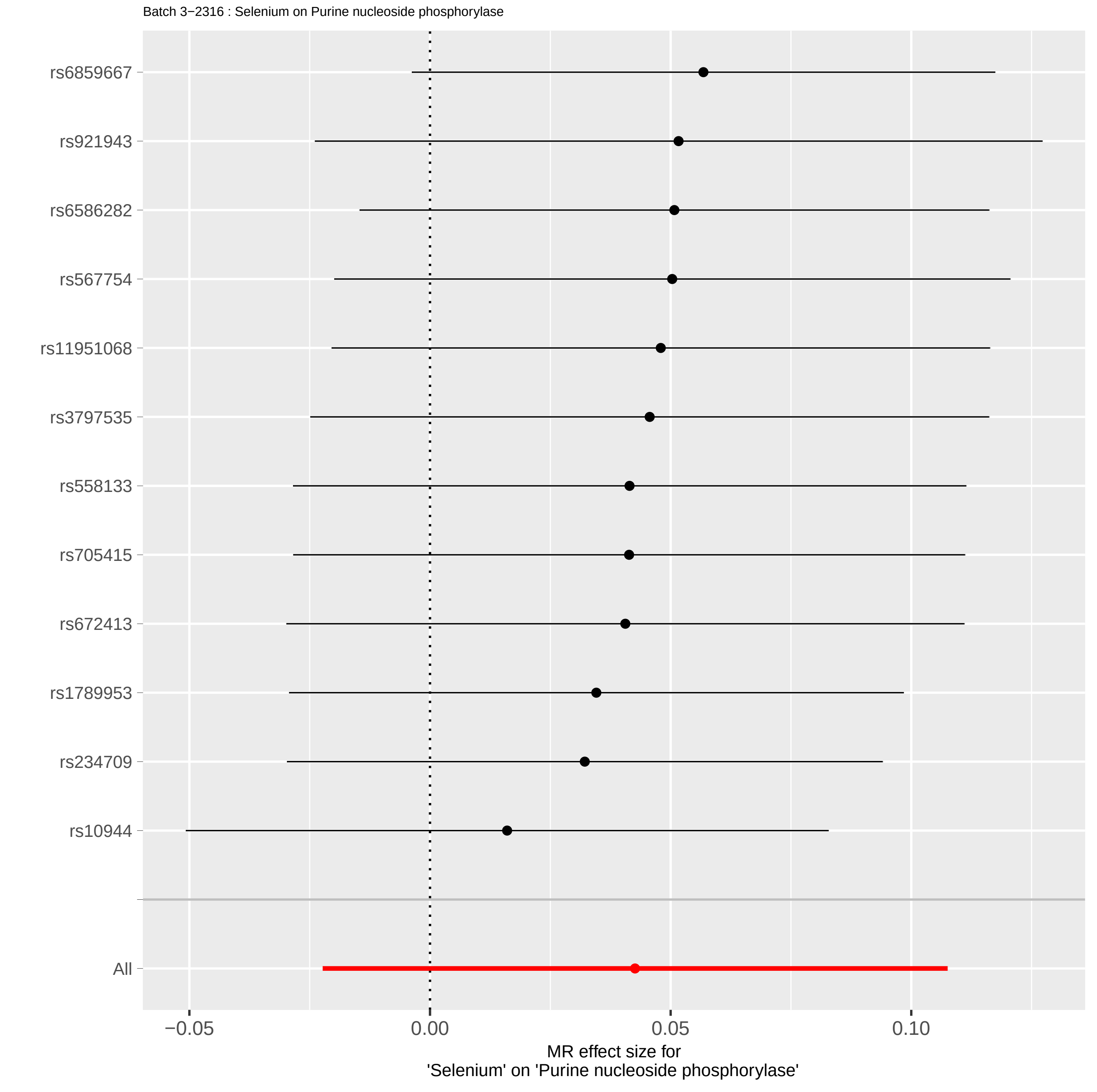

Batch 3−2316 : Selenium on Purine nucleoside phosphorylase
rs6859667
rs921943
rs6586282
rs567754
rs11951068
rs3797535
rs558133
rs705415
rs672413
rs1789953
rs234709
rs10944
All
−0.05
0.00
0.05
0.10
MR effect size for
'Selenium' on 'Purine nucleoside phosphorylase'

Batch 3−2317 : Selenium on Podocalyxin

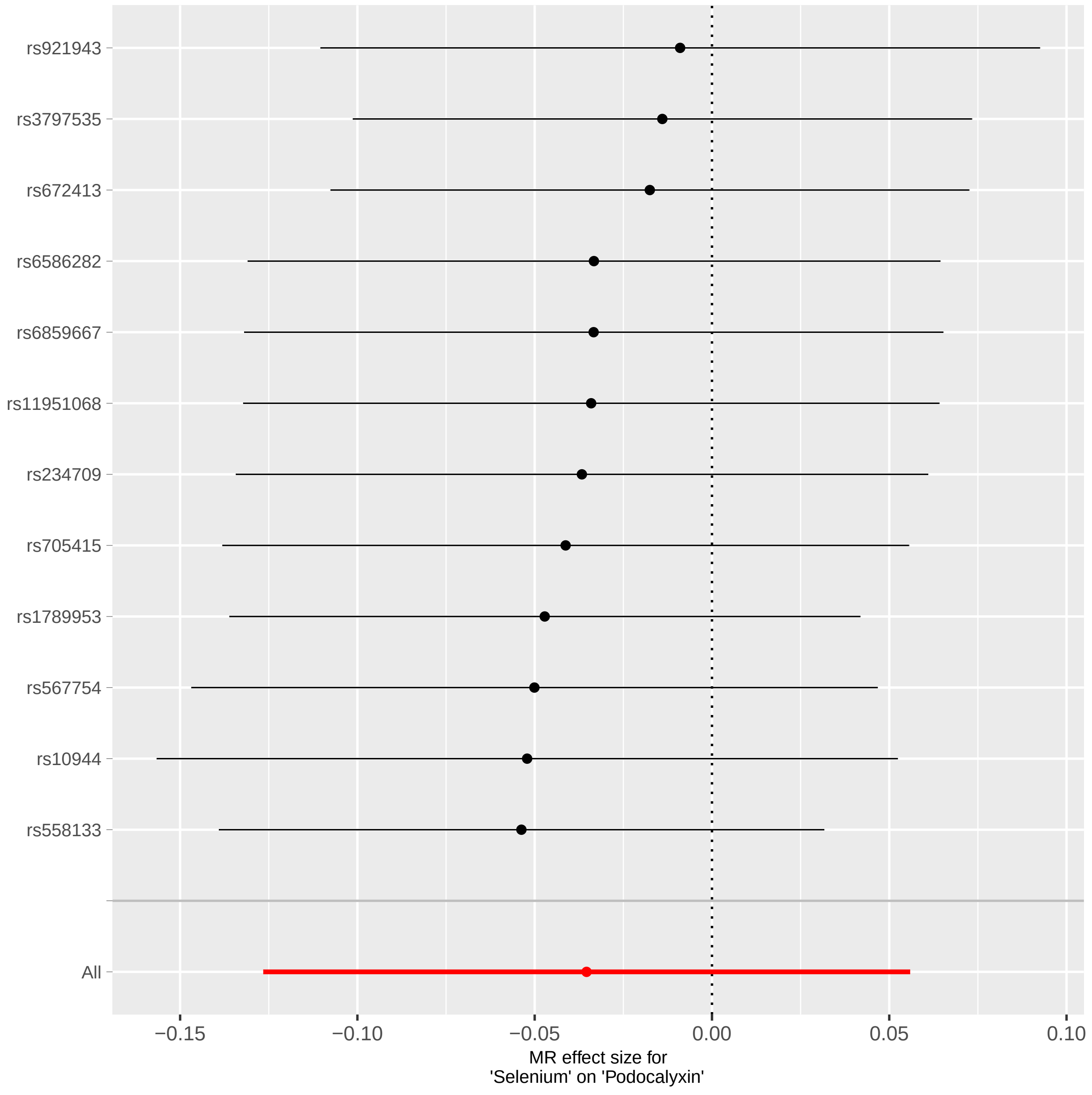

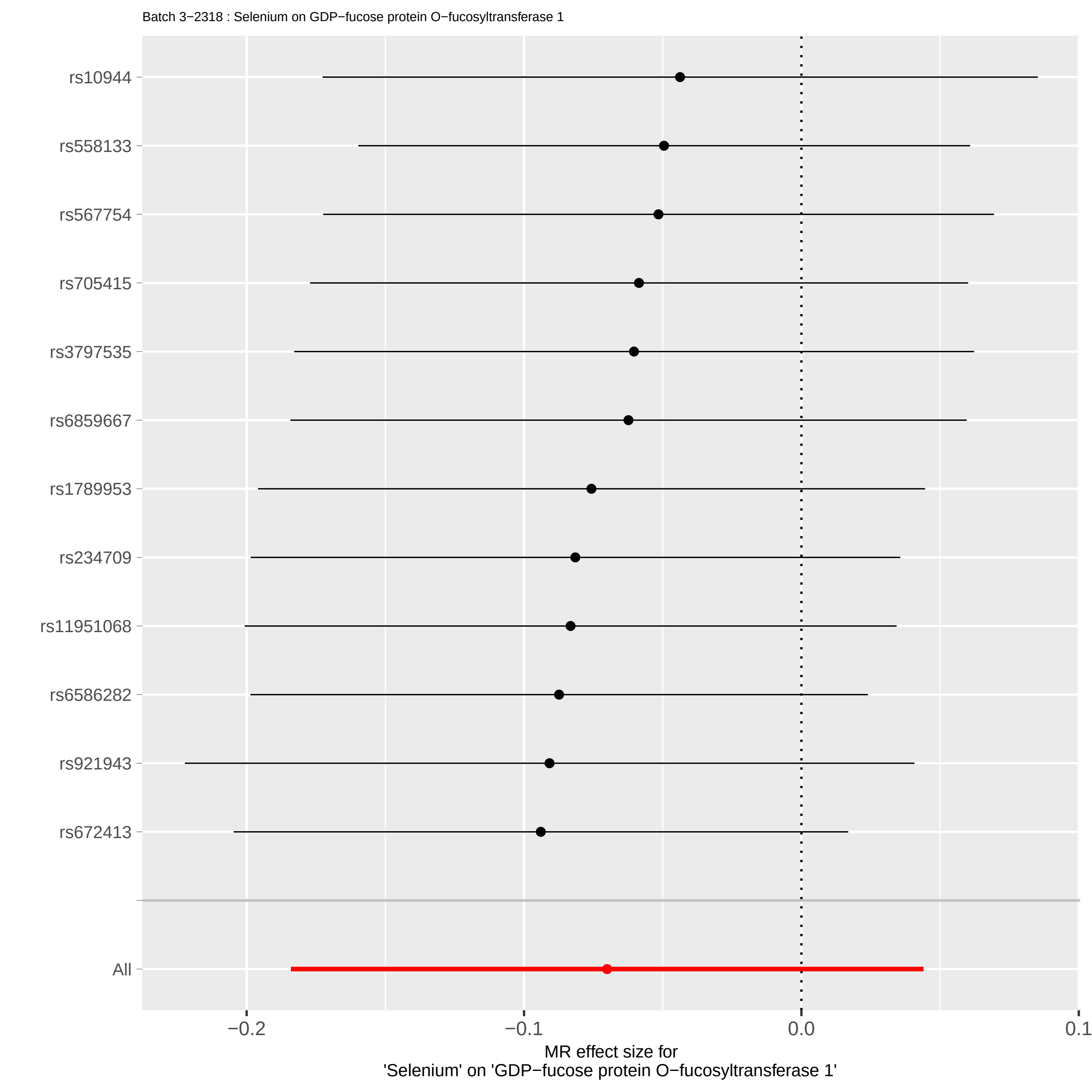
Batch 3−2318 : Selenium on GDP−fucose protein O−fucosyltransferase 1
rs10944
rs558133
rs567754
rs705415
rs3797535
rs6859667
rs1789953
rs234709
rs11951068
rs6586282
rs921943
rs672413
All
−0.2
−0.1
0.0
0.1
MR effect size for
'Selenium' on 'GDP−fucose protein O−fucosyltransferase 1'

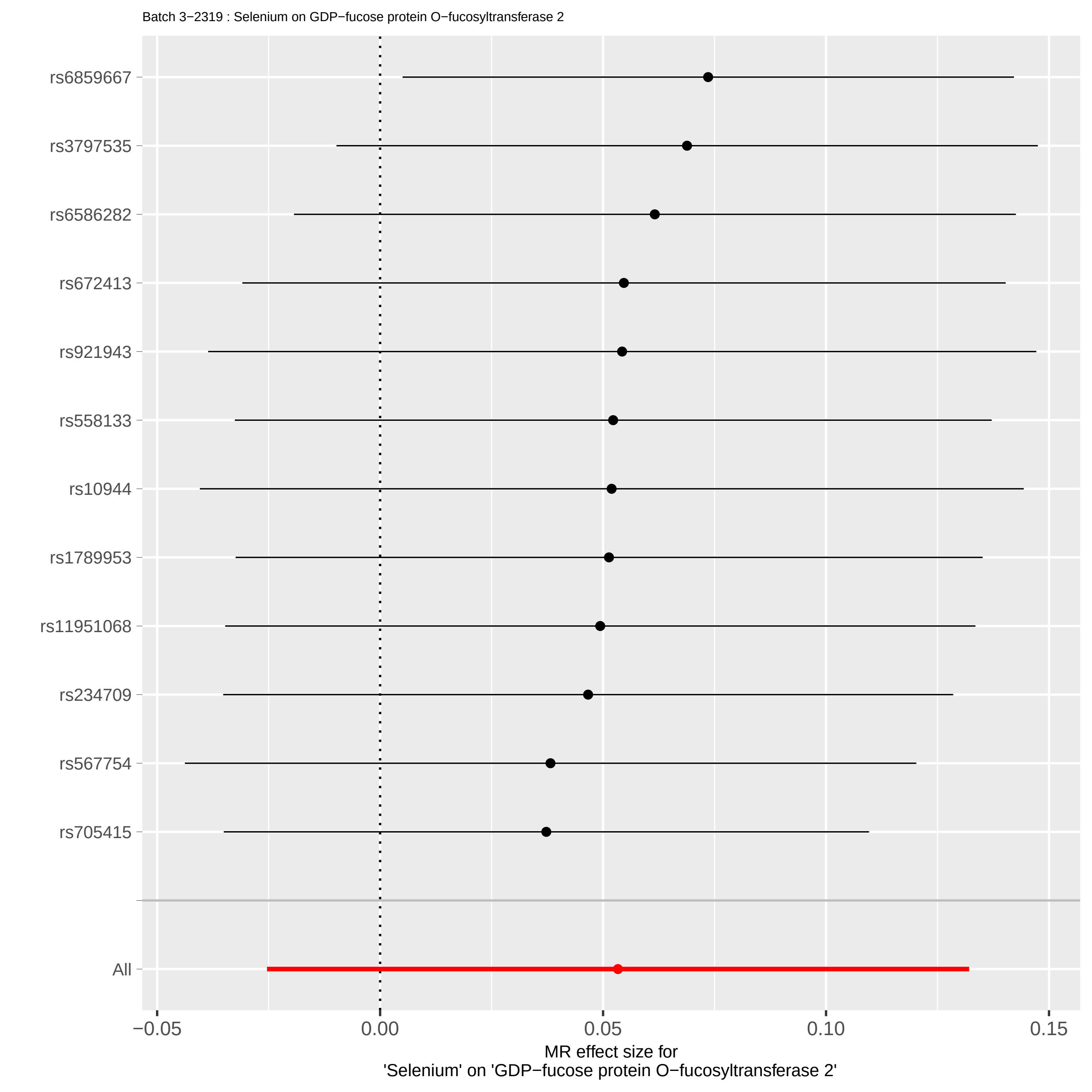

Batch 3−2319 : Selenium on GDP−fucose protein O−fucosyltransferase 2
rs6859667
rs3797535
rs6586282
rs672413
rs921943
rs558133
rs10944
rs1789953
rs11951068
rs234709
rs567754
rs705415
All
−0.05
0.00
0.05
0.10
0.15
MR effect size for
'Selenium' on 'GDP−fucose protein O−fucosyltransferase 2'

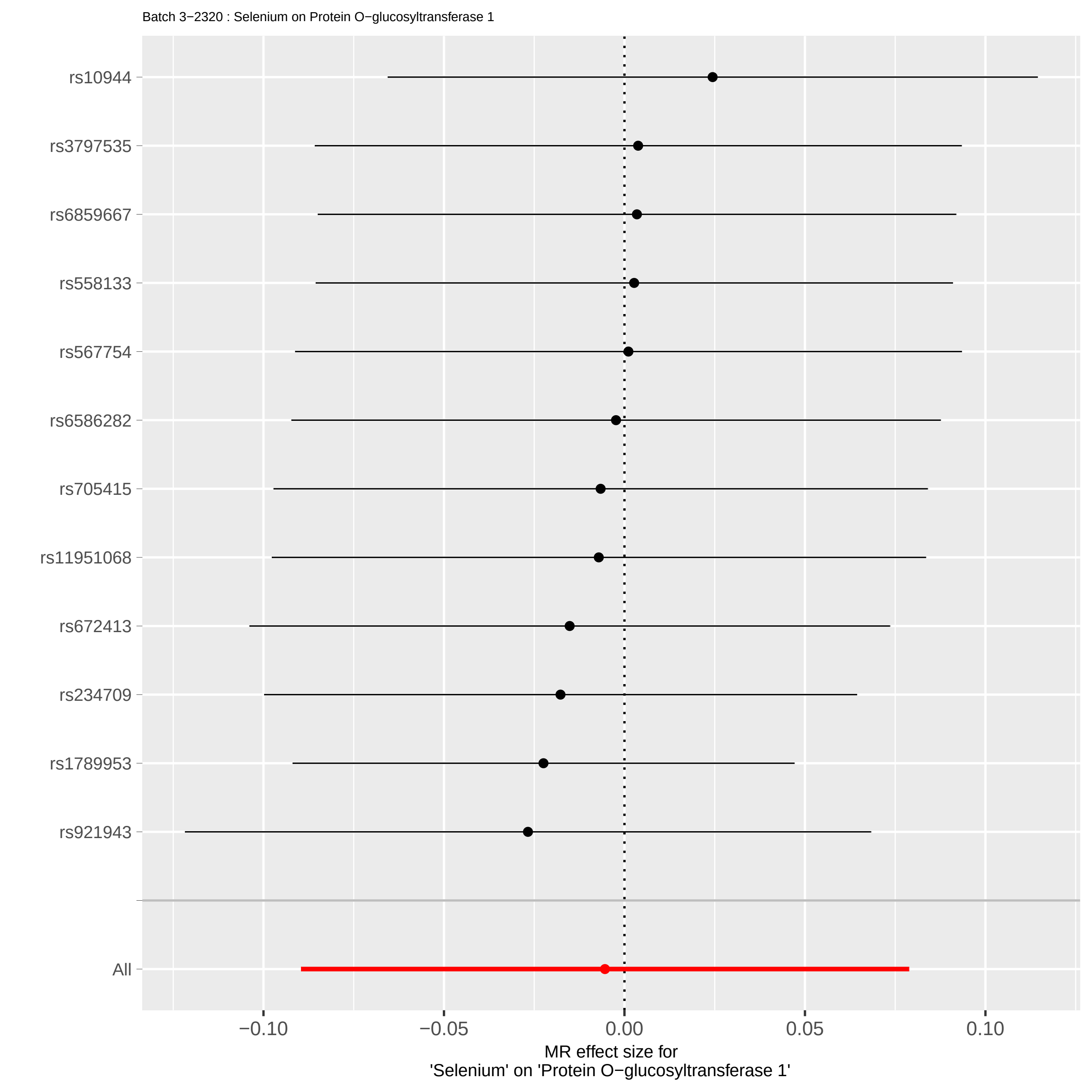

Batch 3−2320 : Selenium on Protein O−glucosyltransferase 1
rs10944
rs3797535
rs6859667
rs558133
rs567754
rs6586282
rs705415
rs11951068
rs672413
rs234709
rs1789953
rs921943
All
−0.10
−0.05
0.00
0.05
0.10
MR effect size for
'Selenium' on 'Protein O−glucosyltransferase 1'

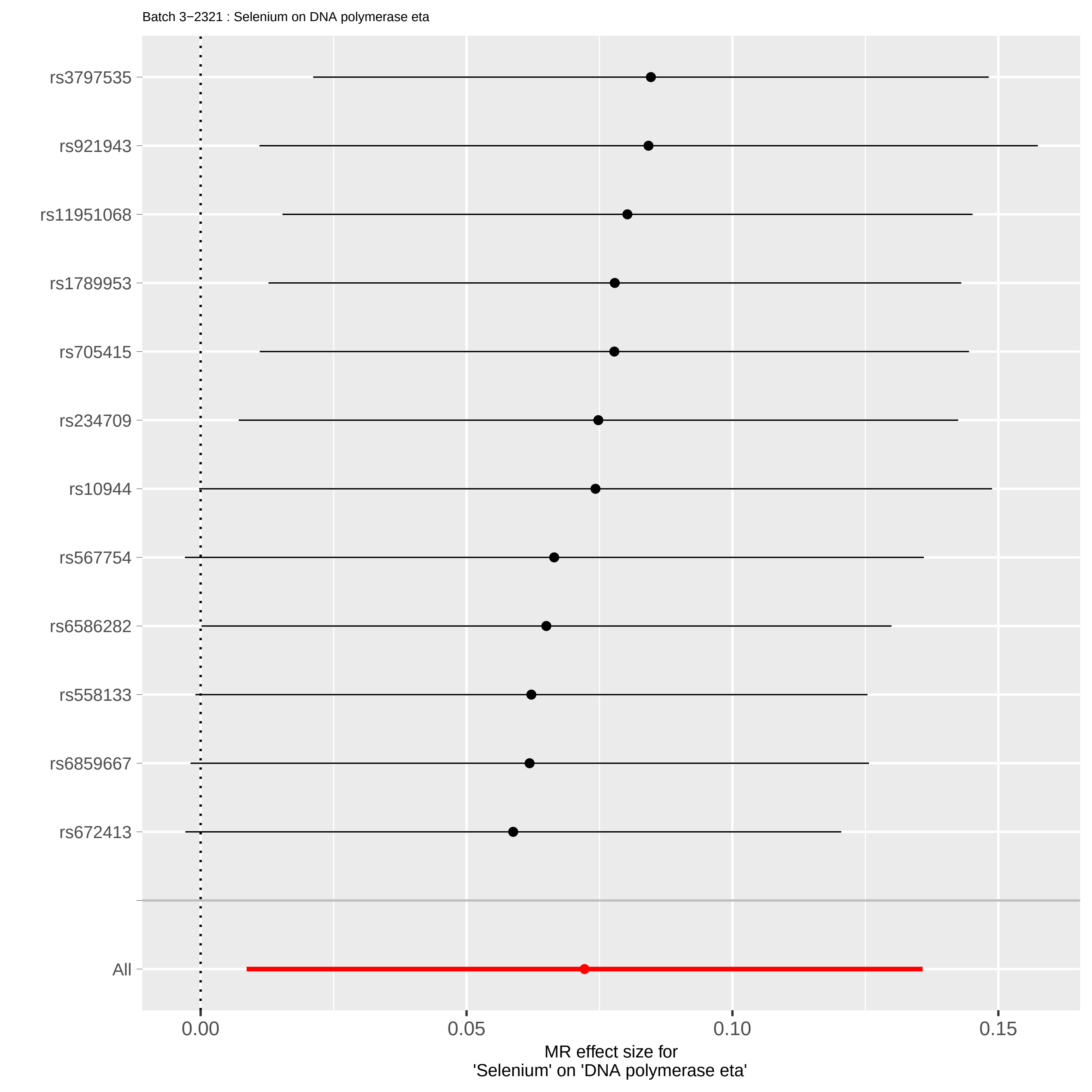

Batch 3−2321 : Selenium on DNA polymerase eta
rs3797535
rs921943
rs11951068
rs1789953
rs705415
rs234709
rs10944
rs567754
rs6586282
rs558133
rs6859667
rs672413
All
0.00
0.05
0.10
0.15
MR effect size for
'Selenium' on 'DNA polymerase eta'

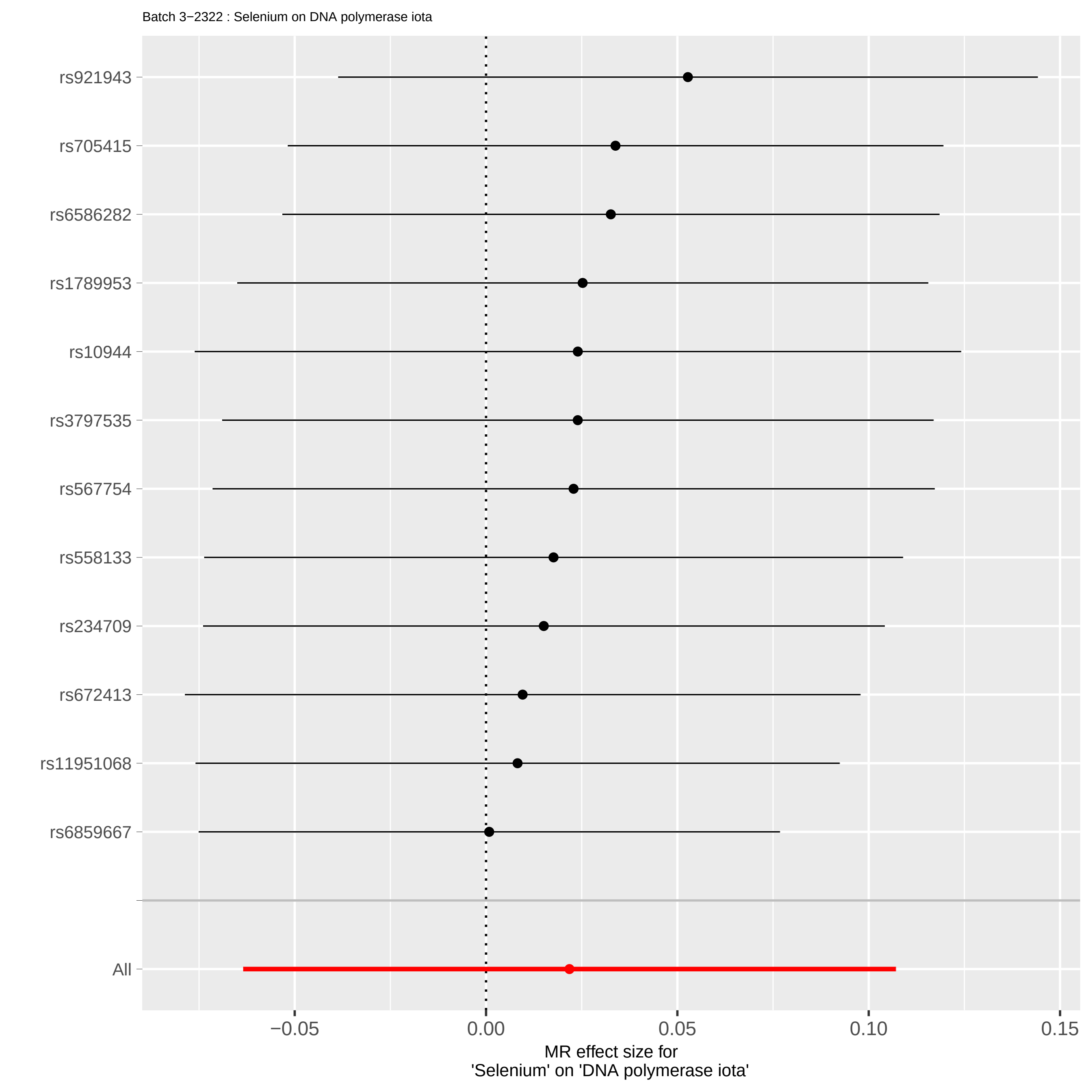
Batch 3−2322 : Selenium on DNA polymerase iota
rs921943
rs705415
rs6586282
rs1789953
rs10944
rs3797535
rs567754
rs558133
rs234709
rs672413
rs11951068
rs6859667
All
−0.05
0.00
0.05
0.10
0.15
MR effect size for
'Selenium' on 'DNA polymerase iota'

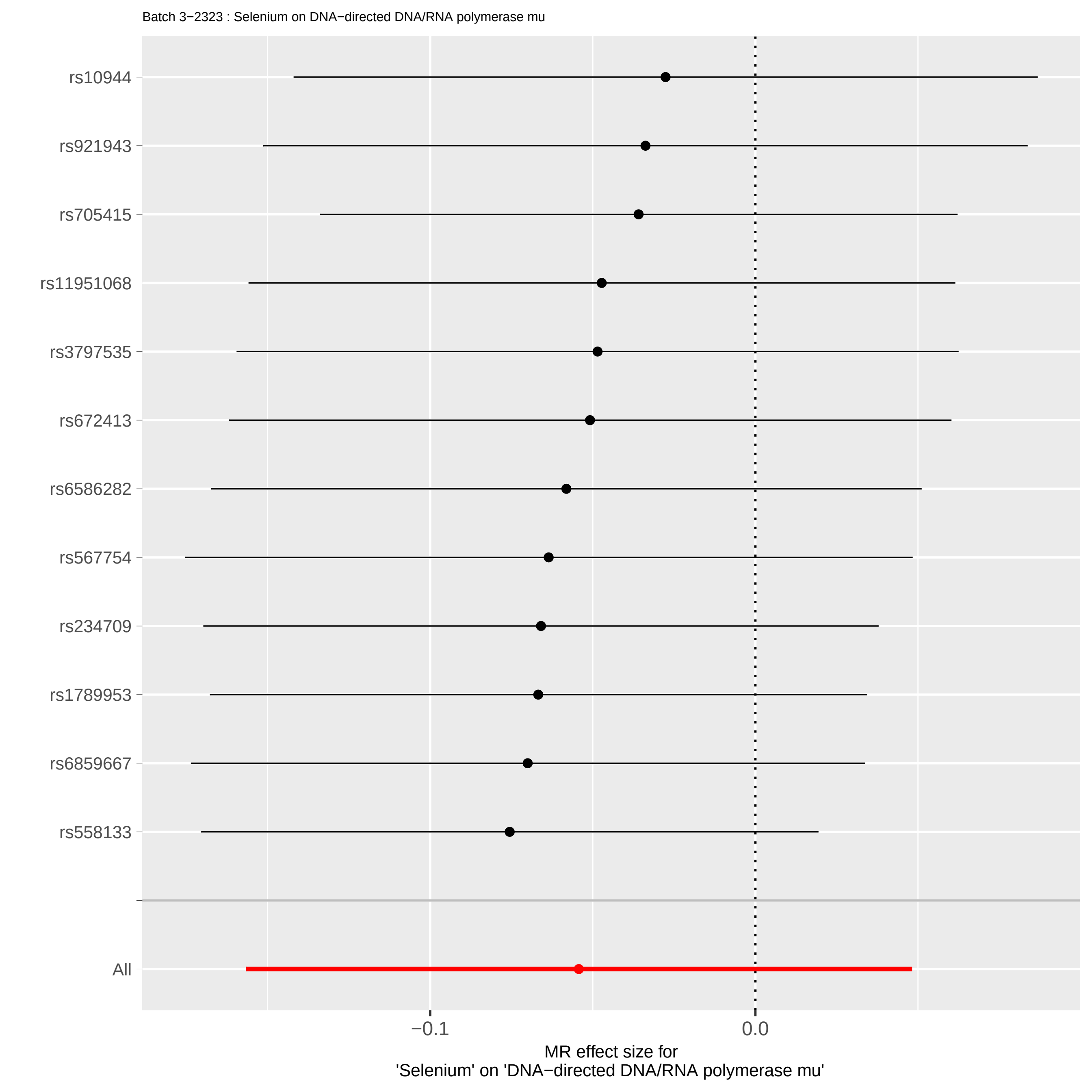

Batch 3−2323 : Selenium on DNA−directed DNA/RNA polymerase mu
rs10944
rs921943
rs705415
rs11951068
rs3797535
rs672413
rs6586282
rs567754
rs234709
rs1789953
rs6859667
rs558133
All
−0.1
0.0
MR effect size for
'Selenium' on 'DNA−directed DNA/RNA polymerase mu'

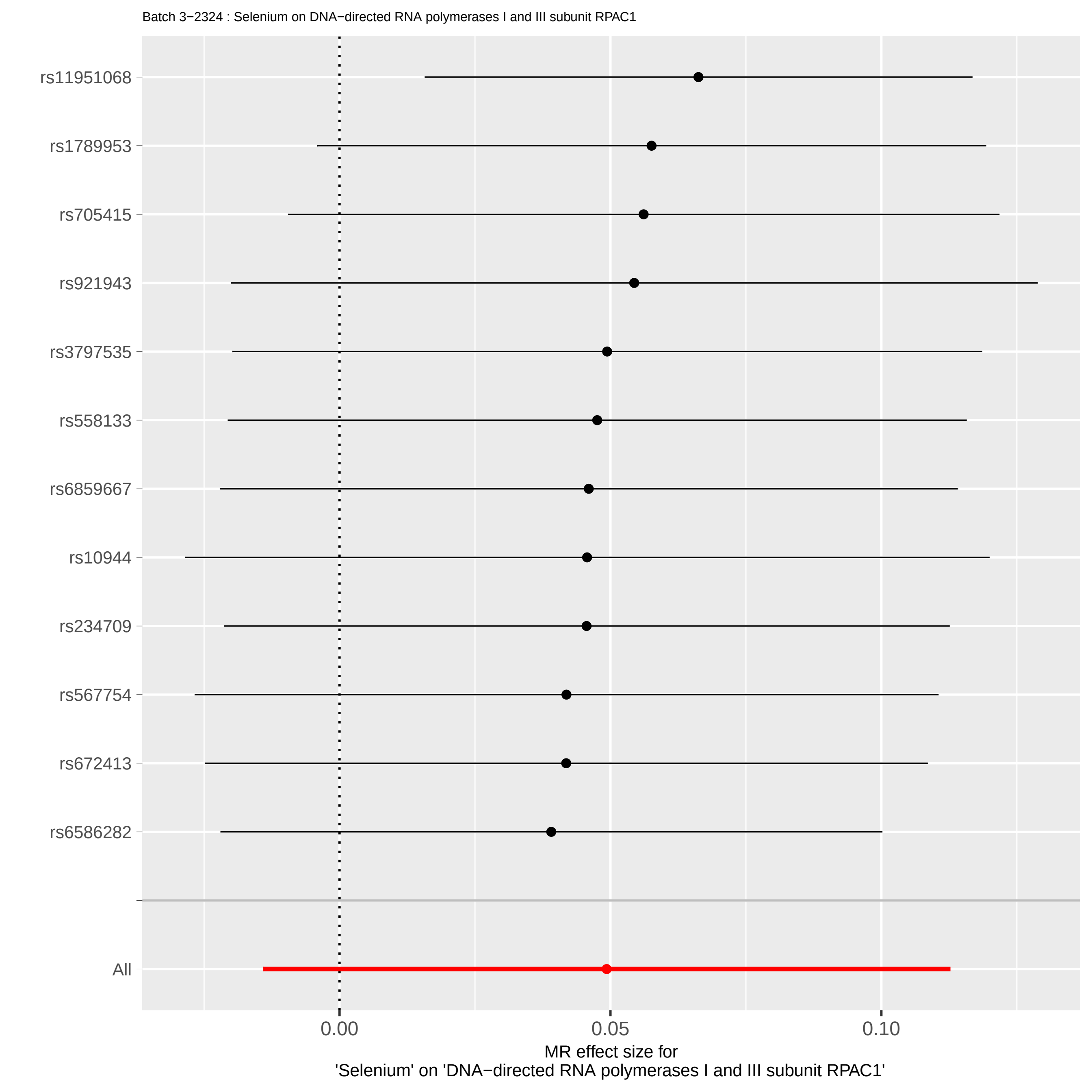
Batch 3−2324 : Selenium on DNA−directed RNA polymerases I and III subunit RPAC1
rs11951068
rs1789953
rs705415
rs921943
rs3797535
rs558133
rs6859667
rs10944
rs234709
rs567754
rs672413
rs6586282
All
0.00
0.05
0.10
MR effect size for
'Selenium' on 'DNA−directed RNA polymerases I and III subunit RPAC1'

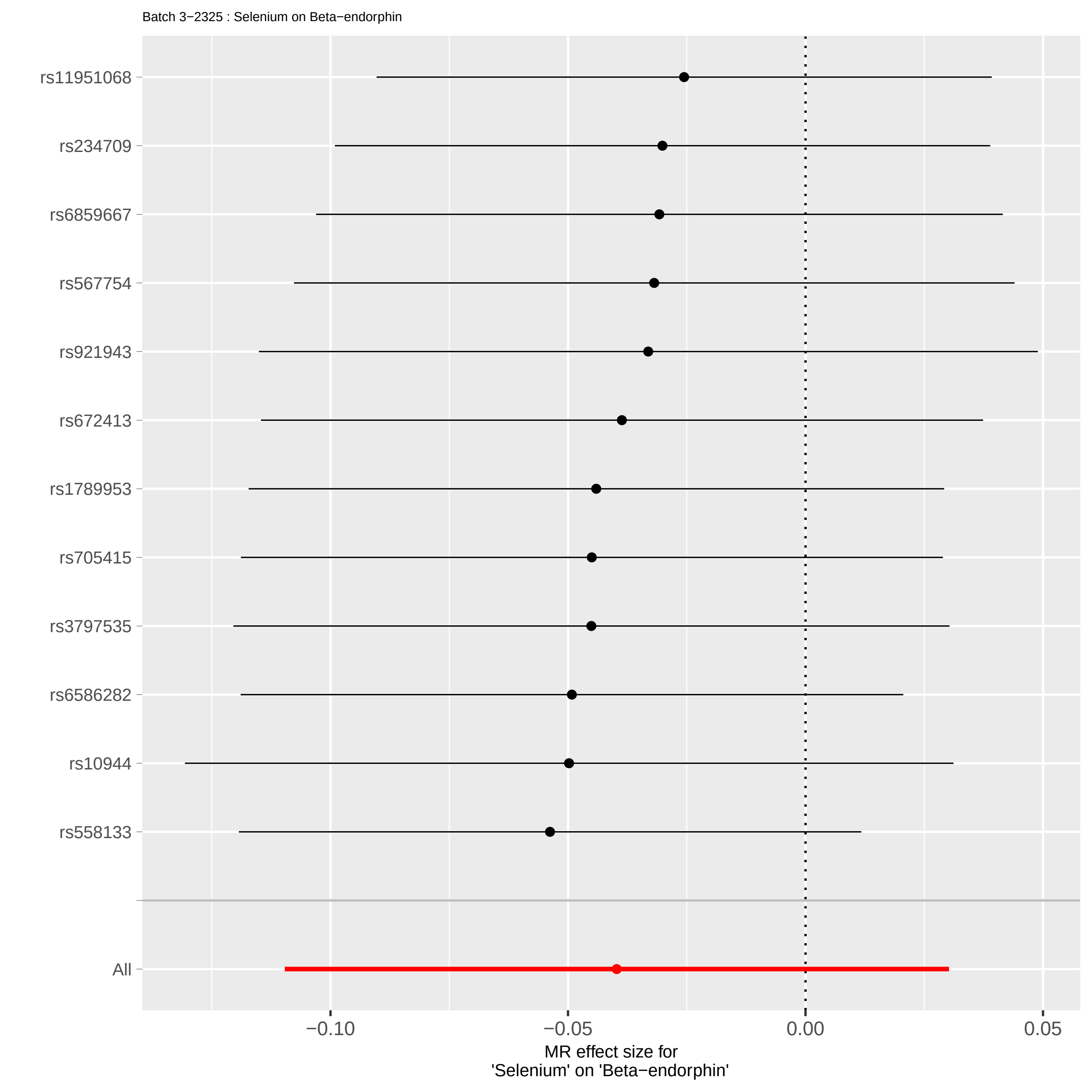

Batch 3−2325 : Selenium on Beta−endorphin
rs11951068
rs234709
rs6859667
rs567754
rs921943
rs672413
rs1789953
rs705415
rs3797535
rs6586282
rs10944
rs558133
All
−0.10
−0.05
0.00
0.05
MR effect size for
'Selenium' on 'Beta−endorphin'

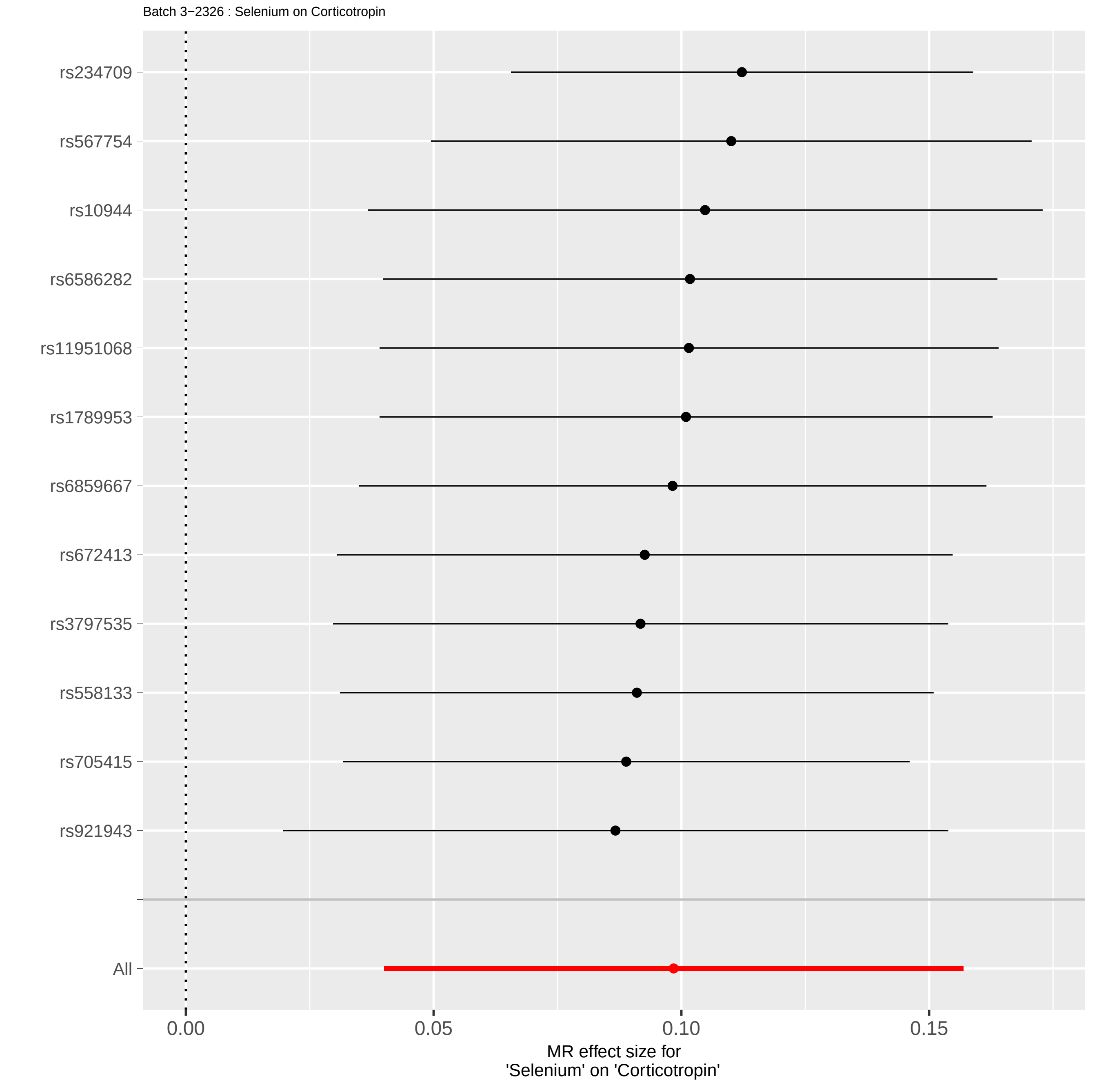

Batch 3−2326 : Selenium on Corticotropin
rs234709
rs567754
rs10944
rs6586282
rs11951068
rs1789953
rs6859667
rs672413
rs3797535
rs558133
rs705415
rs921943
All
0.00
0.05
0.10
0.15
MR effect size for
'Selenium' on 'Corticotropin'

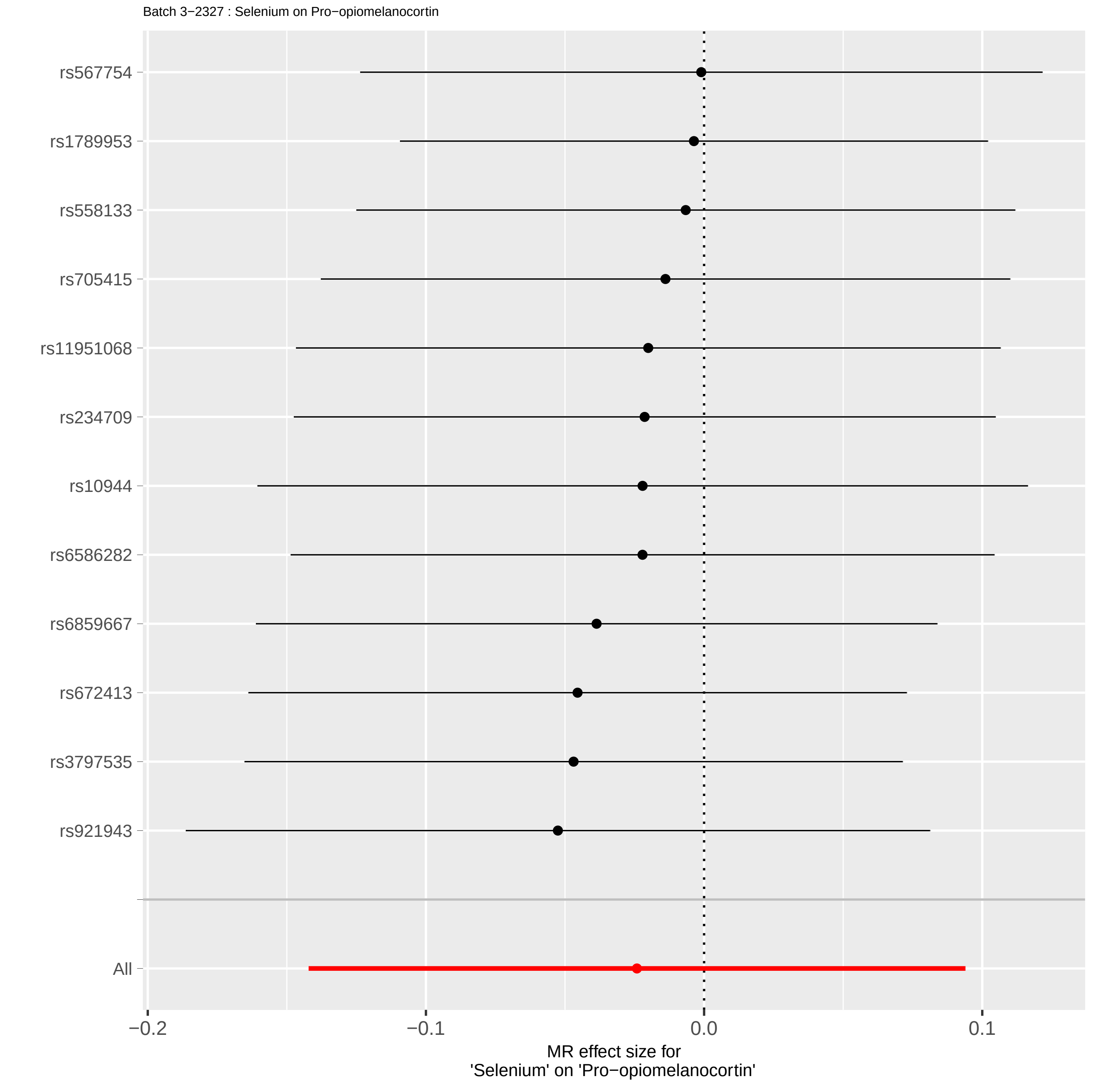
Batch 3−2327 : Selenium on Pro−opiomelanocortin
rs567754
rs1789953
rs558133
rs705415
rs11951068
rs234709
rs10944
rs6586282
rs6859667
rs672413
rs3797535
rs921943
All
−0.2
−0.1
0.0
0.1
MR effect size for
'Selenium' on 'Pro−opiomelanocortin'

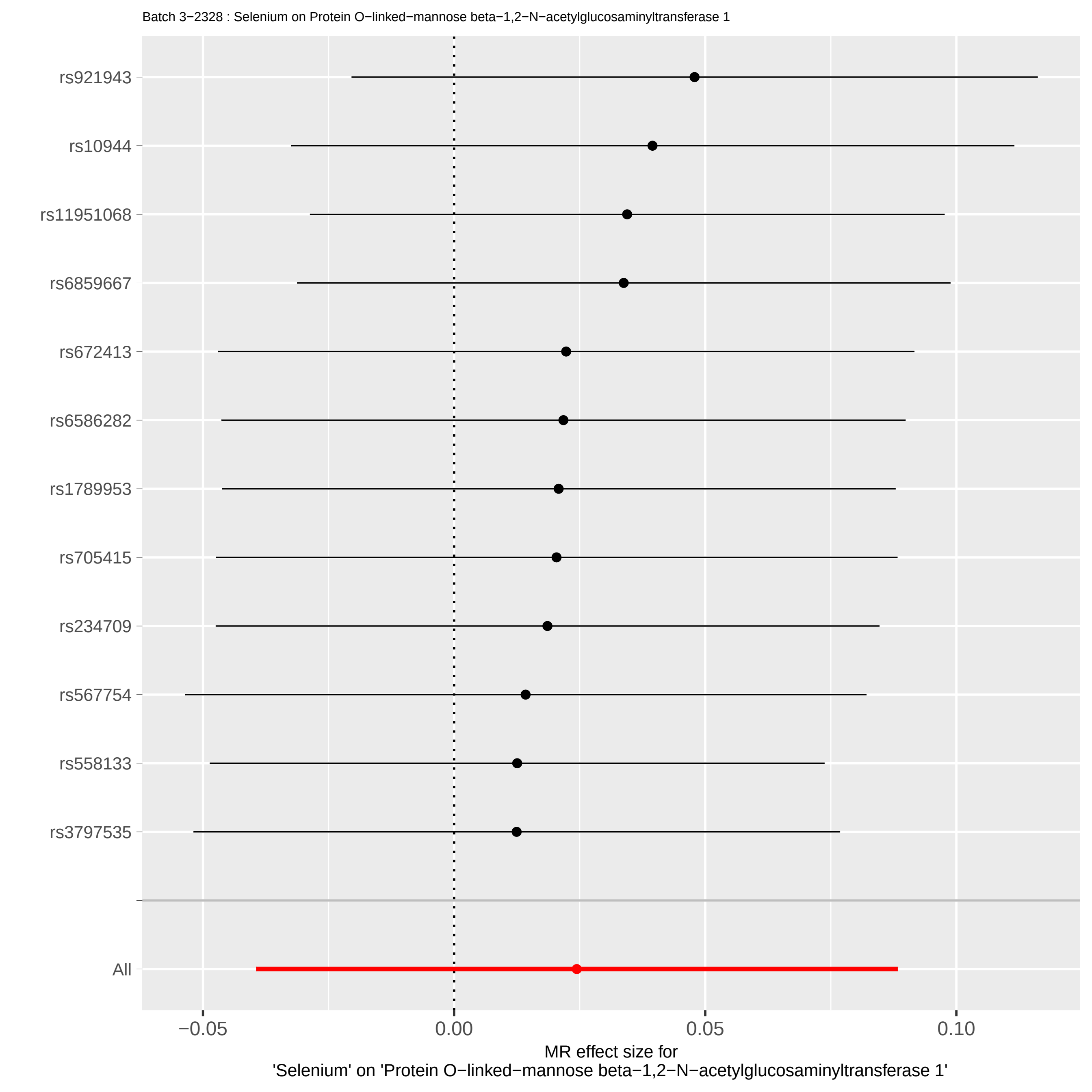
Batch 3−2328 : Selenium on Protein O−linked−mannose beta−1,2−N−acetylglucosaminyltransferase 1
rs921943
rs10944
rs11951068
rs6859667
rs672413
rs6586282
rs1789953
rs705415
rs234709
rs567754
rs558133
rs3797535
All
−0.05
0.00
0.05
0.10
MR effect size for
'Selenium' on 'Protein O−linked−mannose beta−1,2−N−acetylglucosaminyltransferase 1'

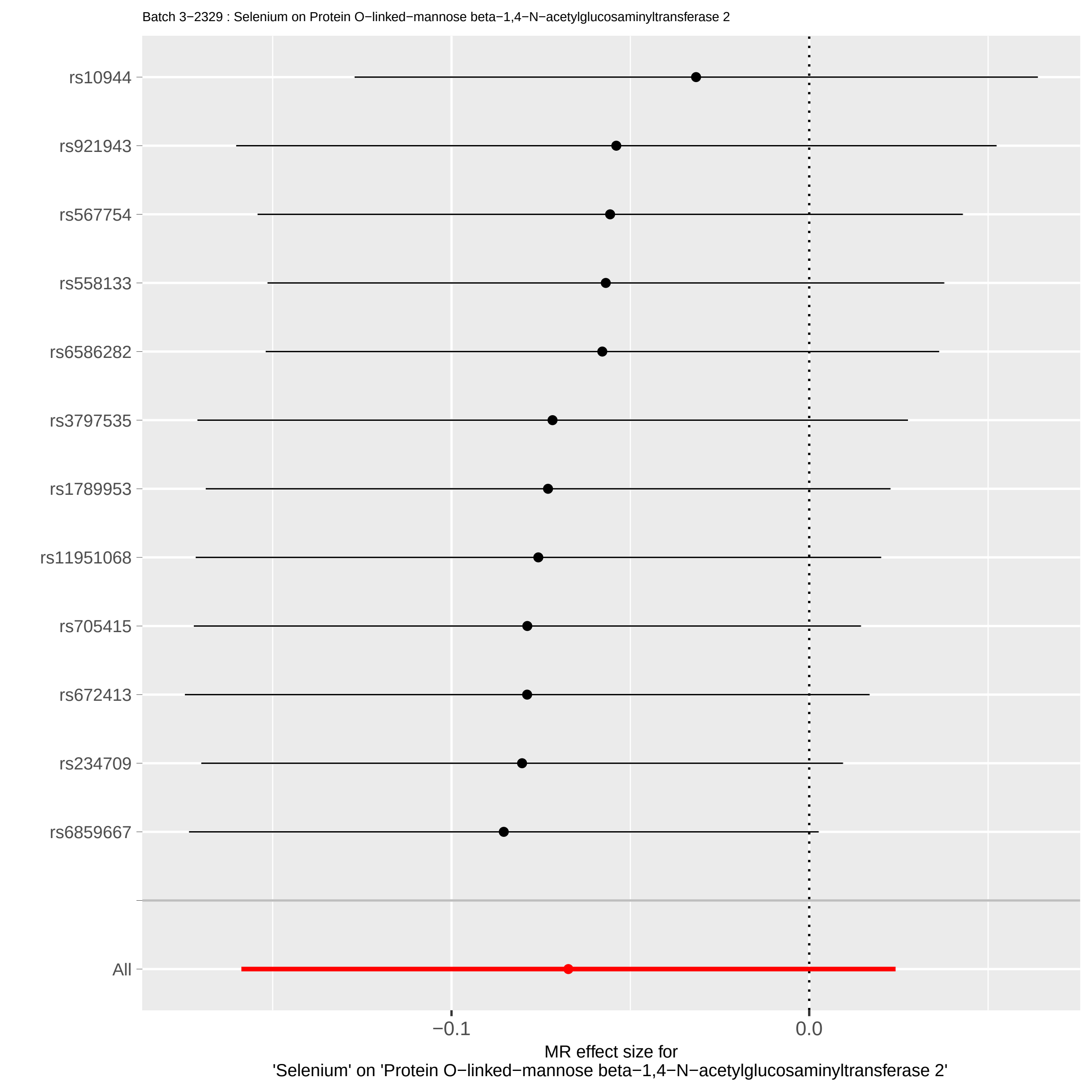

Batch 3−2329 : Selenium on Protein O−linked−mannose beta−1,4−N−acetylglucosaminyltransferase 2
rs10944
rs921943
rs567754
rs558133
rs6586282
rs3797535
rs1789953
rs11951068
rs705415
rs672413
rs234709
rs6859667
All
−0.1
0.0
MR effect size for
'Selenium' on 'Protein O−linked−mannose beta−1,4−N−acetylglucosaminyltransferase 2'

Batch 3−2330 : Selenium on NADPH−−cytochrome P450 reductase

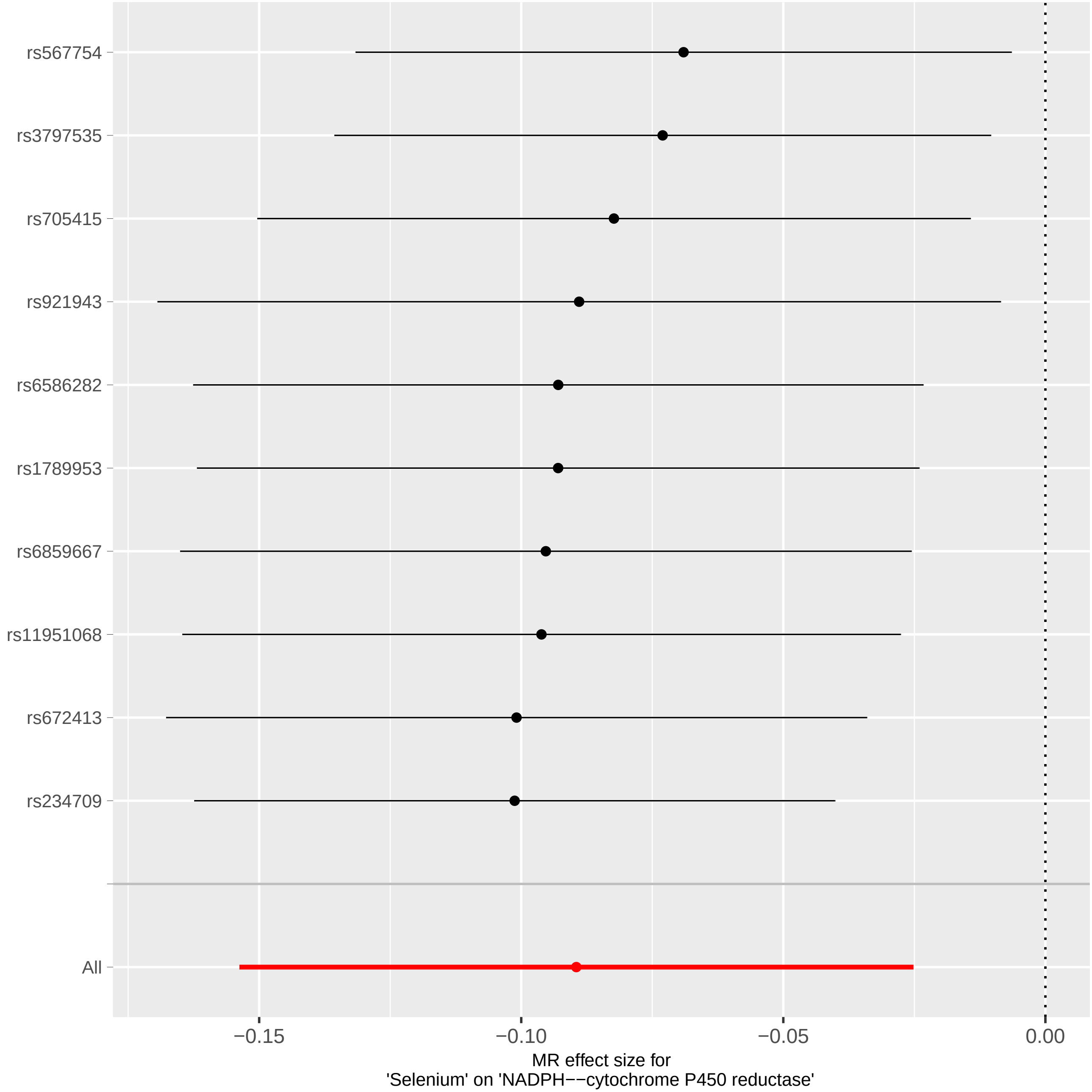

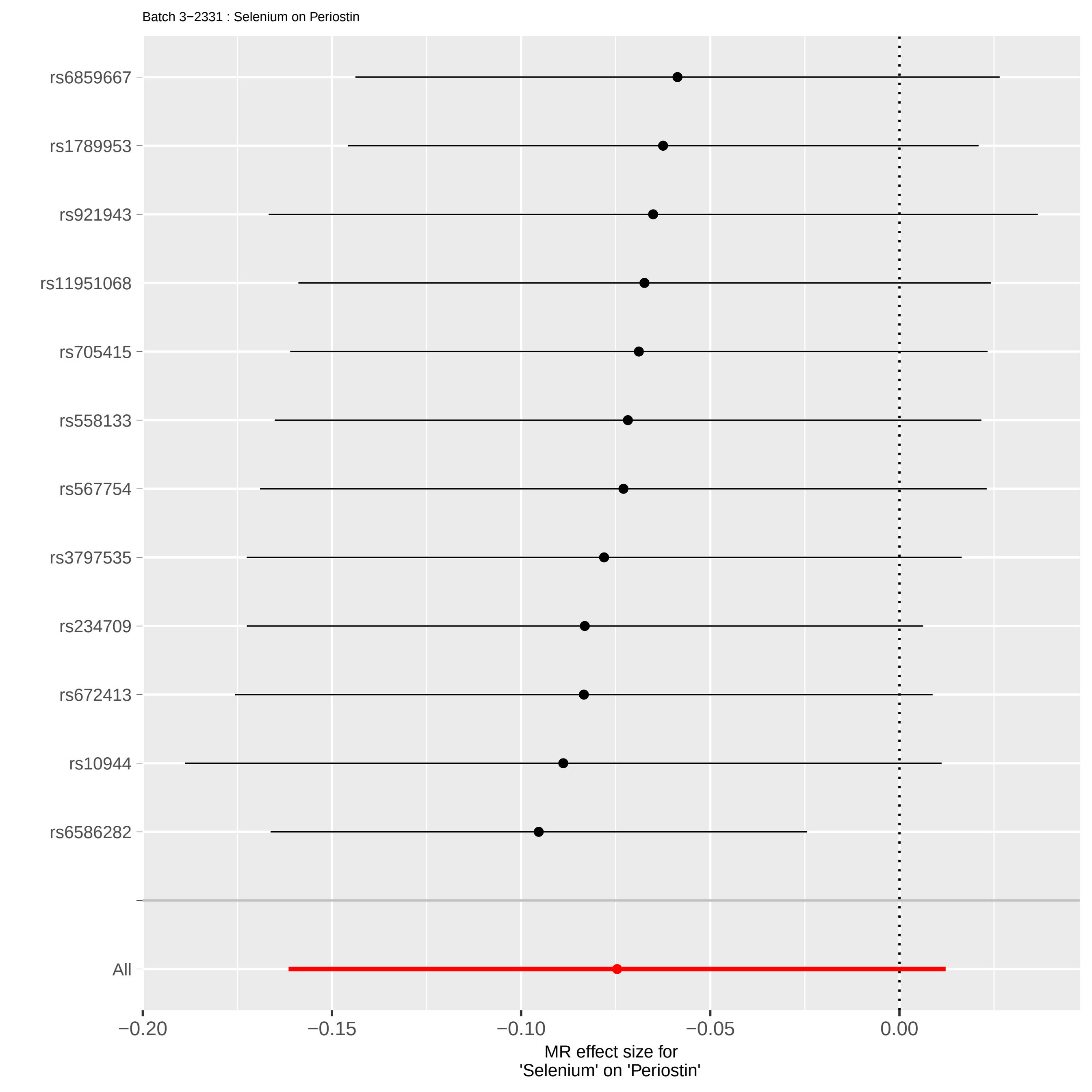

Batch 3−2331 : Selenium on Periostin
rs6859667
rs1789953
rs921943
rs11951068
rs705415
rs558133
rs567754
rs3797535
rs234709
rs672413
rs10944
rs6586282
All
−0.20
−0.15
−0.10
−0.05
0.00
MR effect size for
'Selenium' on 'Periostin'

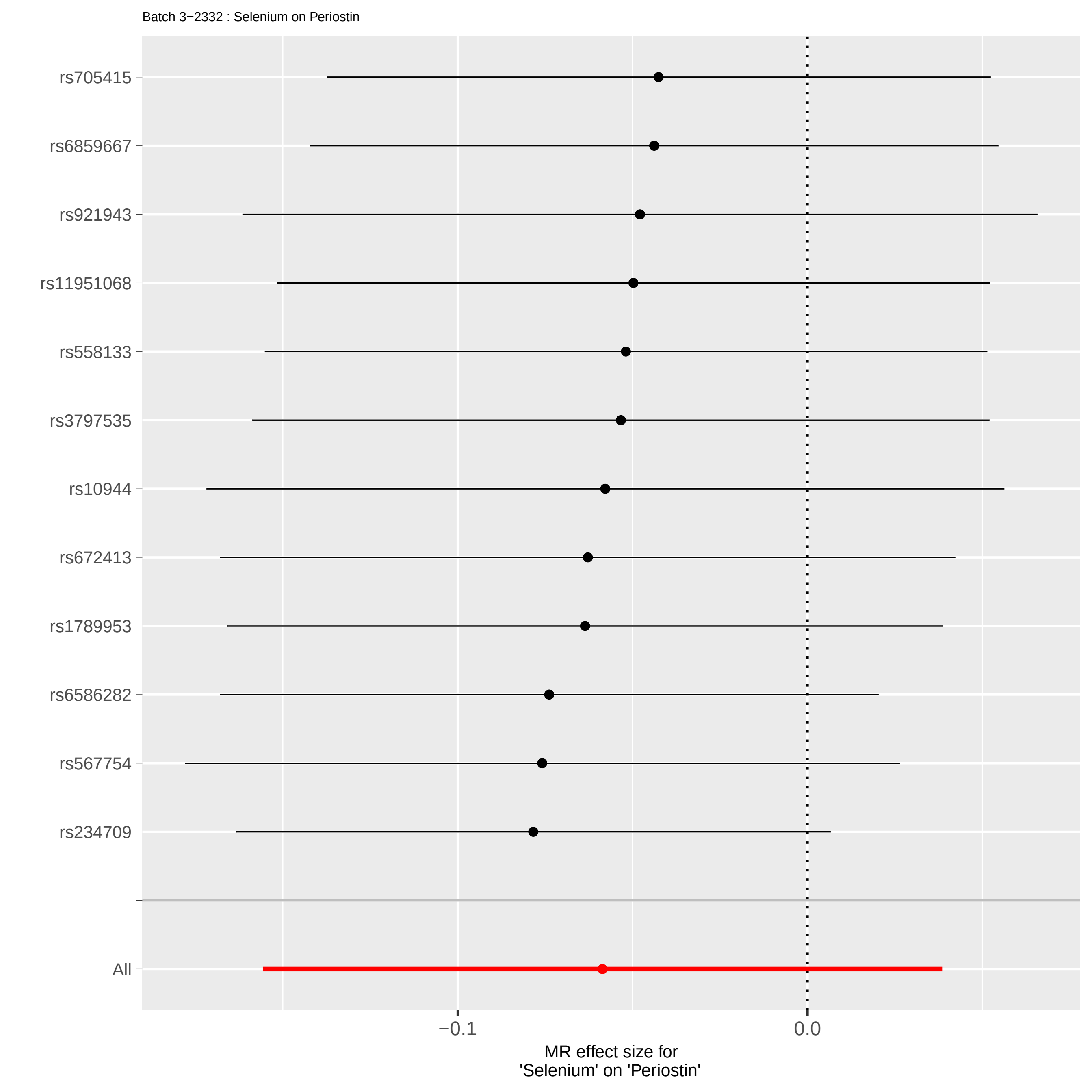
Batch 3−2332 : Selenium on Periostin
rs705415
rs6859667
rs921943
rs11951068
rs558133
rs3797535
rs10944
rs672413
rs1789953
rs6586282
rs567754
rs234709
All
−0.1
0.0
MR effect size for
'Selenium' on 'Periostin'

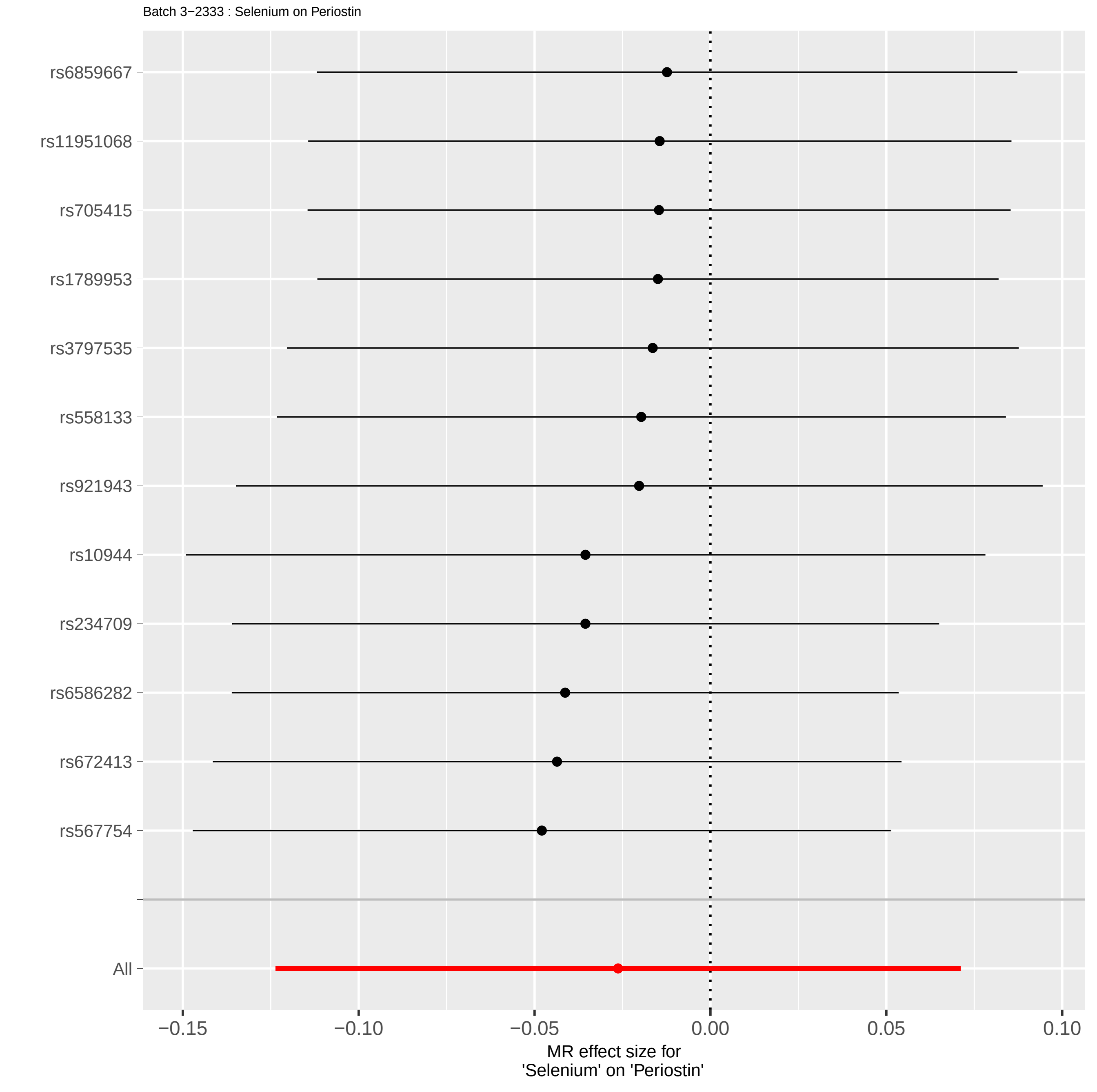

Batch 3−2333 : Selenium on Periostin
rs6859667
rs11951068
rs705415
rs1789953
rs3797535
rs558133
rs921943
rs10944
rs234709
rs6586282
rs672413
rs567754
All
−0.15
−0.10
−0.05
0.00
0.05
0.10
MR effect size for
'Selenium' on 'Periostin'

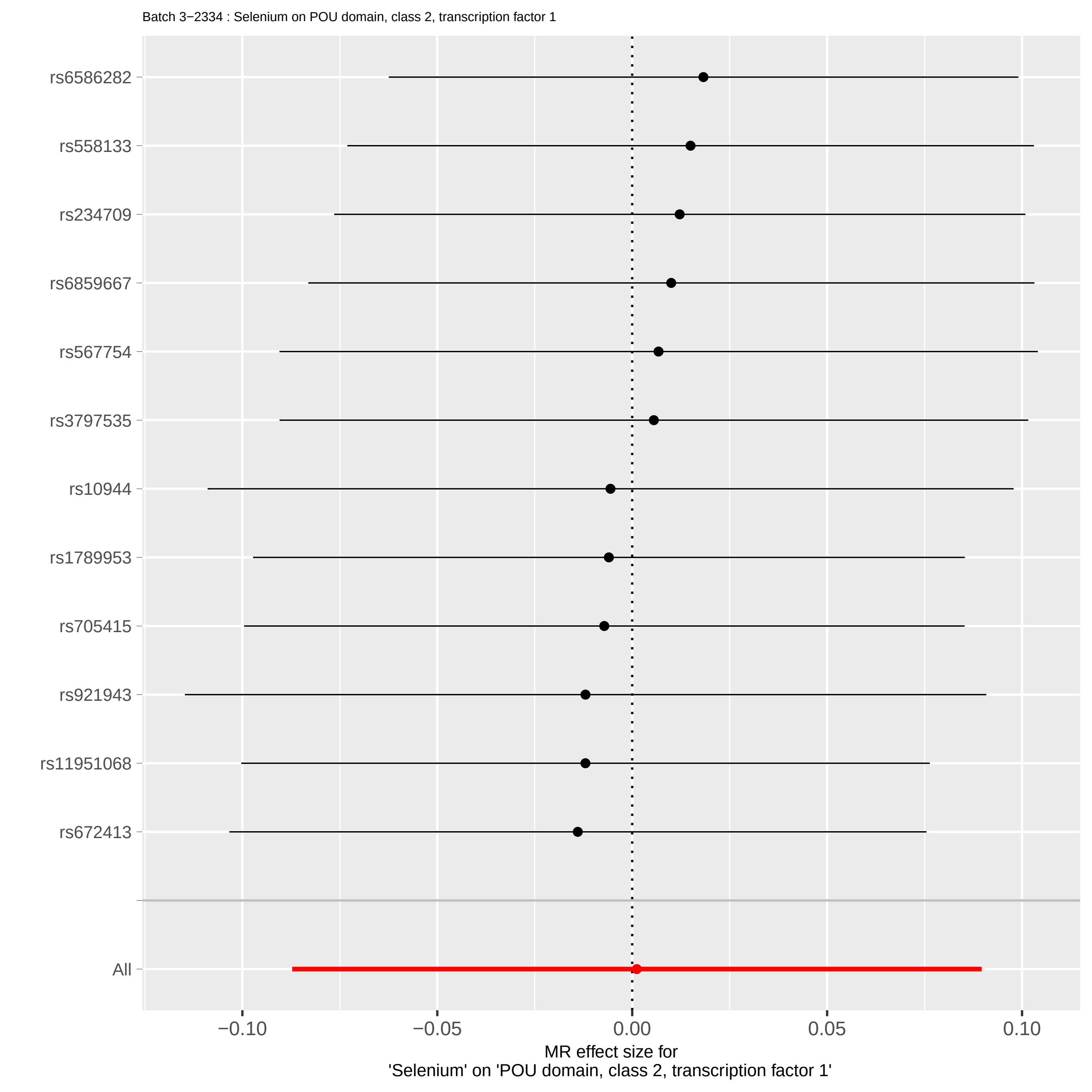

Batch 3−2334 : Selenium on POU domain, class 2, transcription factor 1
rs6586282
rs558133
rs234709
rs6859667
rs567754
rs3797535
rs10944
rs1789953
rs705415
rs921943
rs11951068
rs672413
All
−0.10
−0.05
0.00
0.05
0.10
MR effect size for
'Selenium' on 'POU domain, class 2, transcription factor 1'

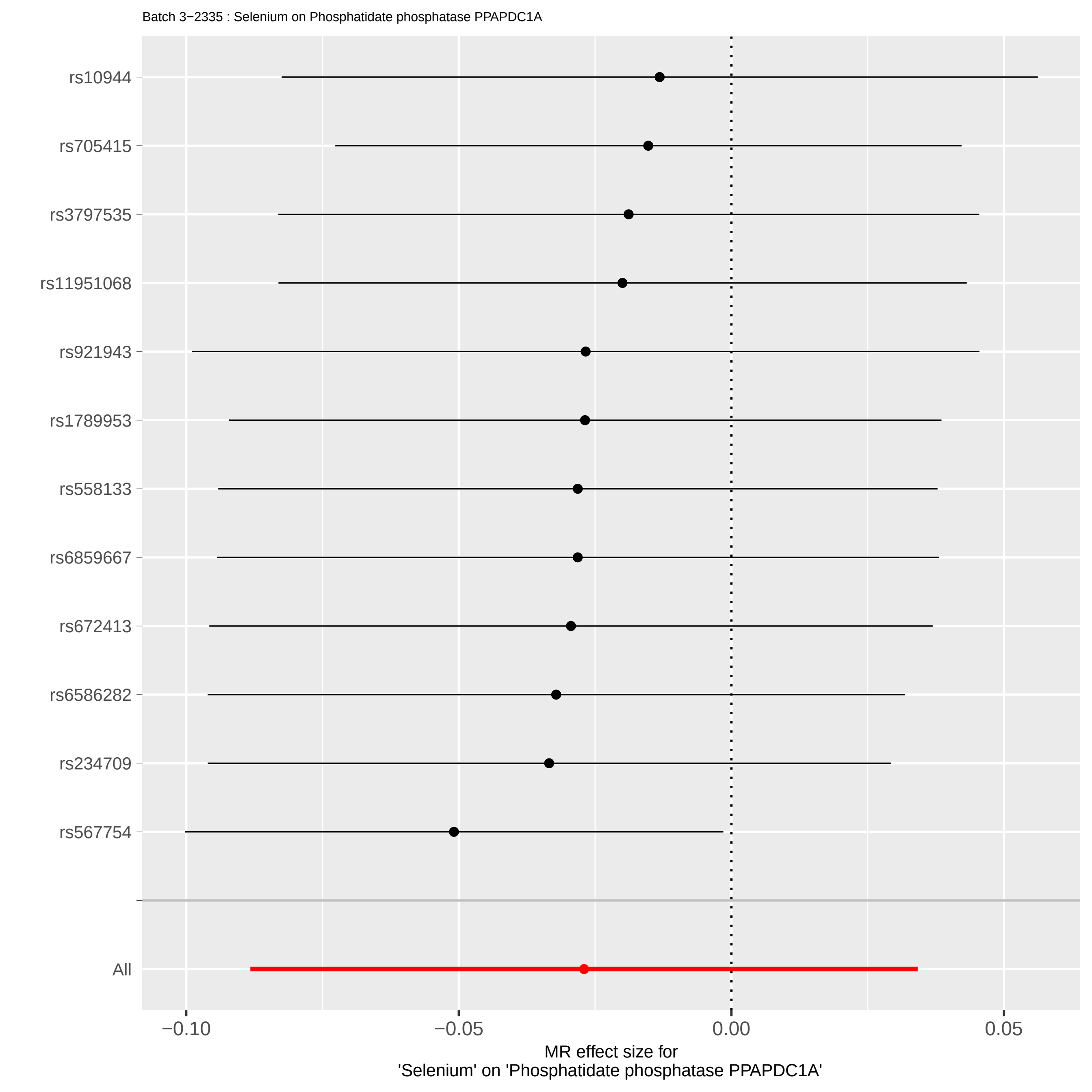
Batch 3−2335 : Selenium on Phosphatidate phosphatase PPAPDC1A
rs10944
rs705415
rs3797535
rs11951068
rs921943
rs1789953
rs558133
rs6859667
rs672413
rs6586282
rs234709
rs567754
All
−0.10
−0.05
0.00
0.05
MR effect size for
'Selenium' on 'Phosphatidate phosphatase PPAPDC1A'

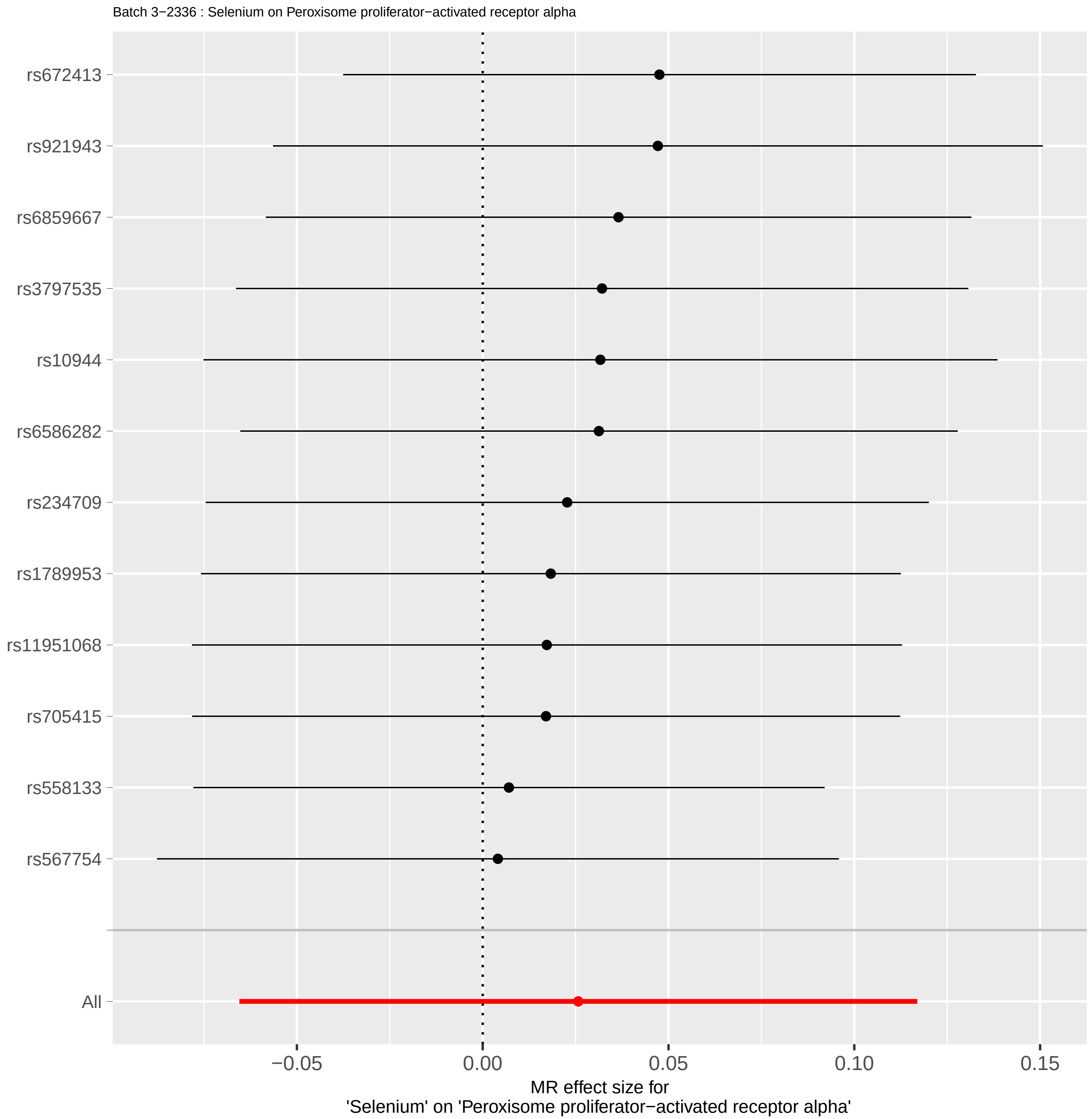
Batch 3−2336 : Selenium on Peroxisome proliferator−activated receptor alpha
rs672413
rs921943
rs6859667
rs3797535
rs10944
rs6586282
rs234709
rs1789953
rs11951068
rs705415
rs558133
rs567754
All
−0.05
0.00
0.05
0.10
0.15
MR effect size for
'Selenium' on 'Peroxisome proliferator−activated receptor alpha'

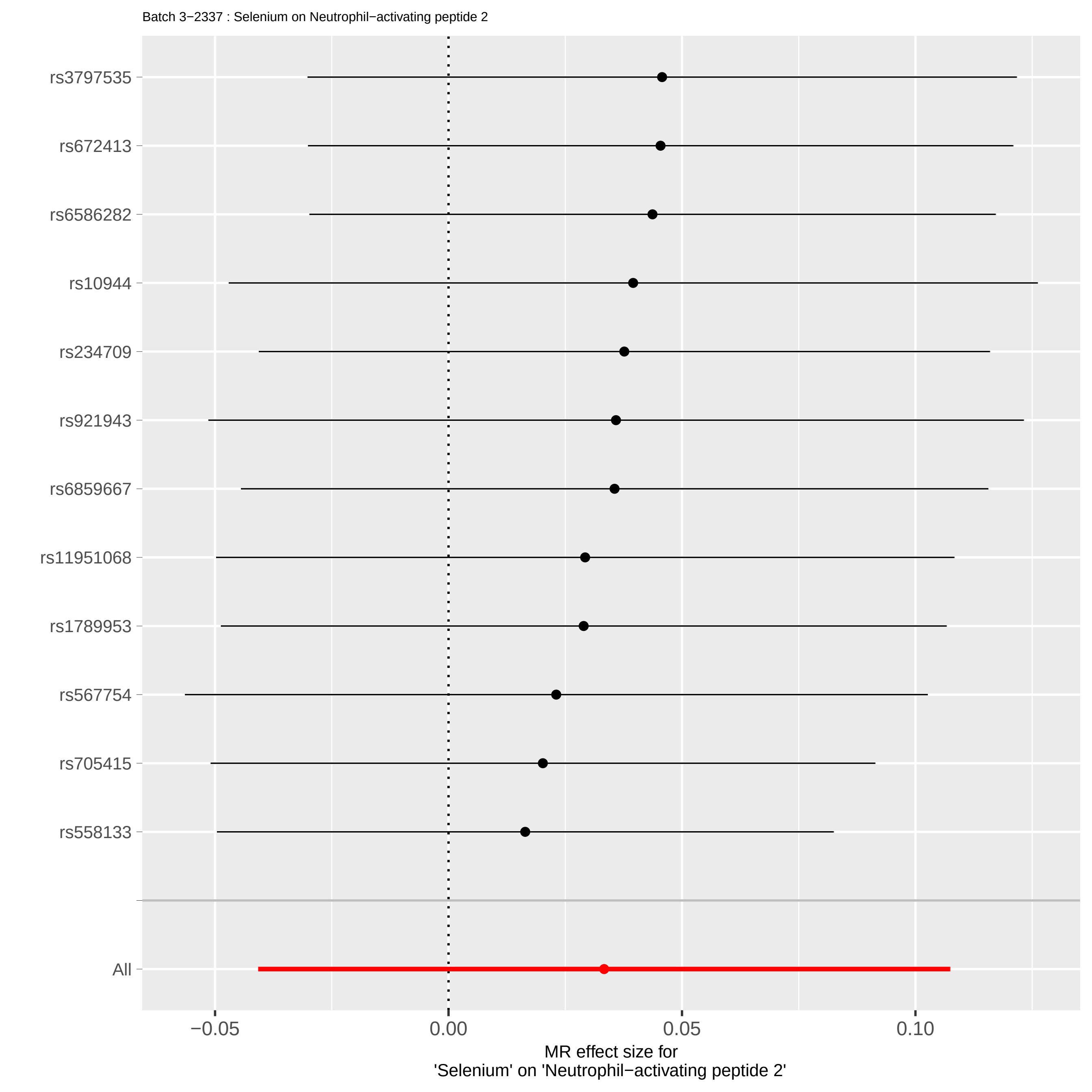

Batch 3−2337 : Selenium on Neutrophil−activating peptide 2
rs3797535
rs672413
rs6586282
rs10944
rs234709
rs921943
rs6859667
rs11951068
rs1789953
rs567754
rs705415
rs558133
All
−0.05
0.00
0.05
0.10
MR effect size for
'Selenium' on 'Neutrophil−activating peptide 2'

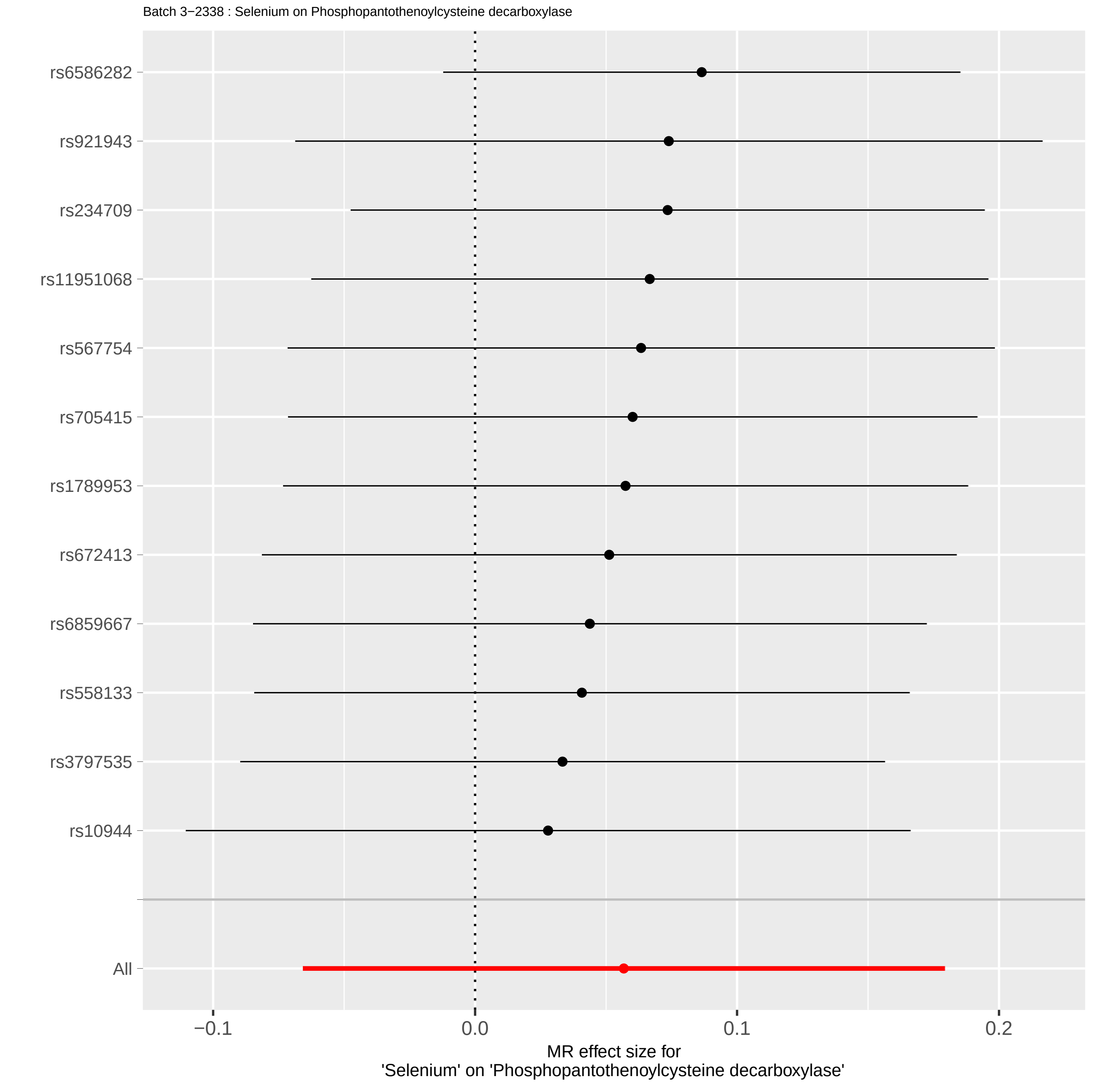

Batch 3−2338 : Selenium on Phosphopantothenoylcysteine decarboxylase
rs6586282
rs921943
rs234709
rs11951068
rs567754
rs705415
rs1789953
rs672413
rs6859667
rs558133
rs3797535
rs10944
All
−0.1
0.0
0.1
0.2
MR effect size for
'Selenium' on 'Phosphopantothenoylcysteine decarboxylase'

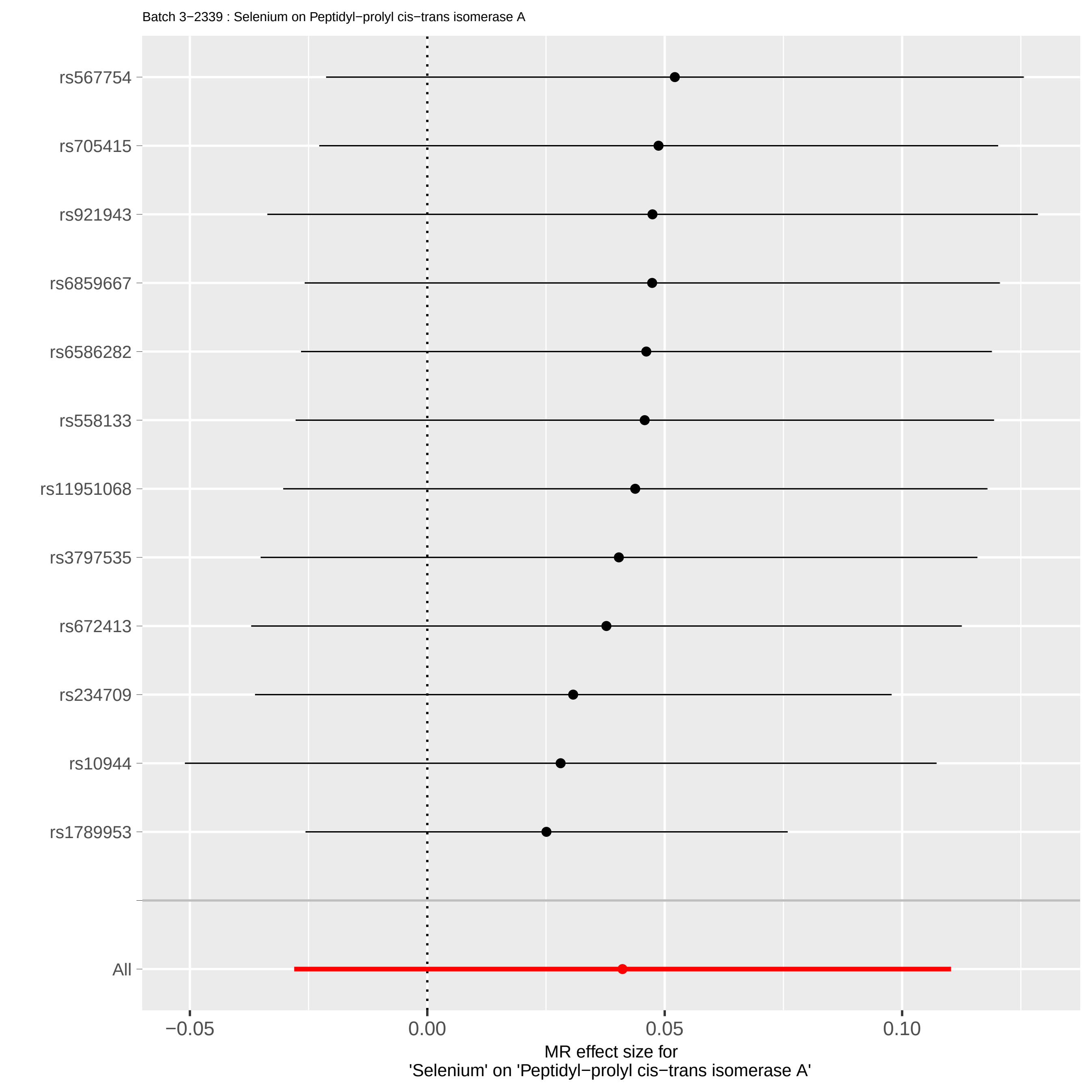

Batch 3−2339 : Selenium on Peptidyl−prolyl cis−trans isomerase A
rs567754
rs705415
rs921943
rs6859667
rs6586282
rs558133
rs11951068
rs3797535
rs672413
rs234709
rs10944
rs1789953
All
−0.05
0.00
0.05
0.10
MR effect size for
'Selenium' on 'Peptidyl−prolyl cis−trans isomerase A'

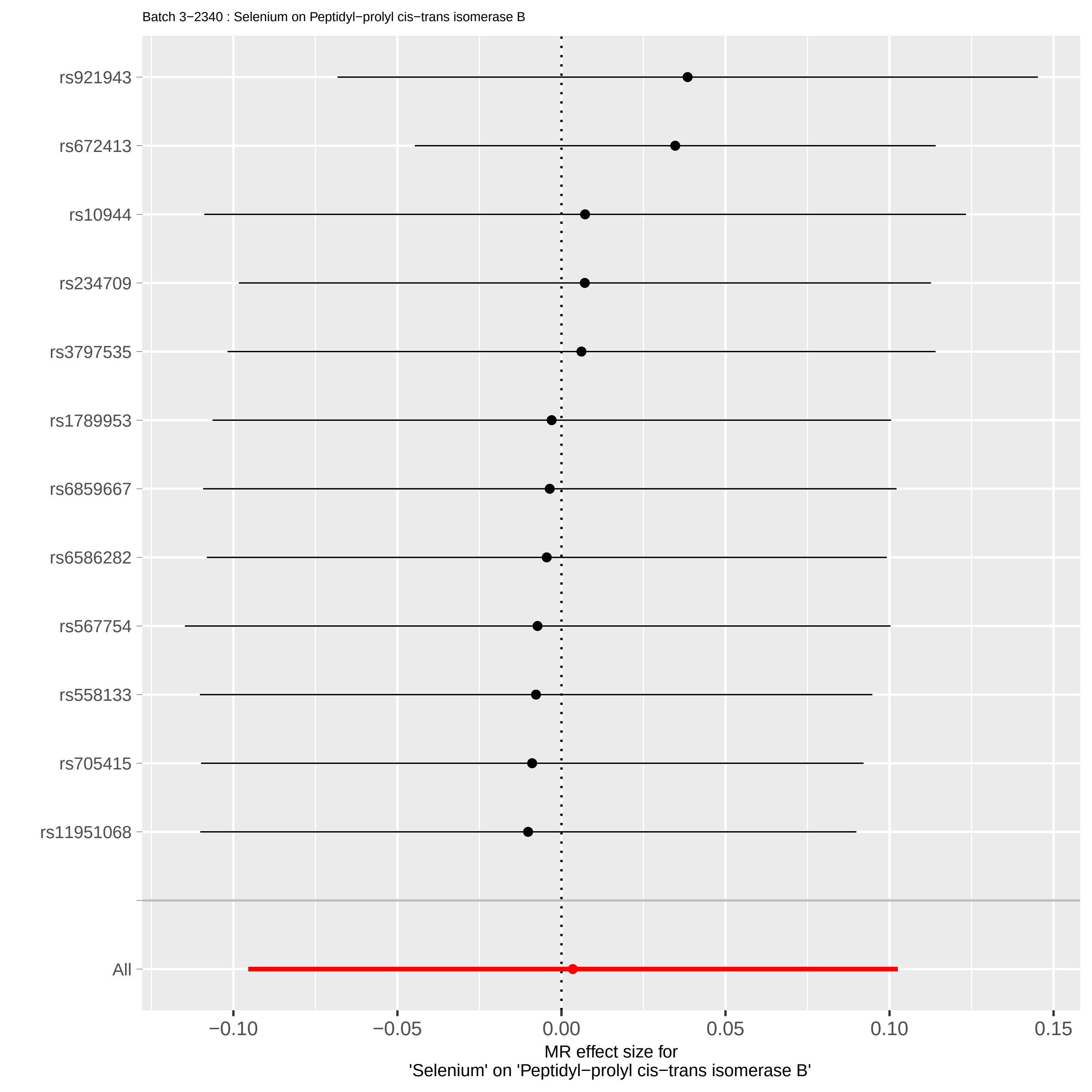

Batch 3−2340 : Selenium on Peptidyl−prolyl cis−trans isomerase B
rs921943
rs672413
rs10944
rs234709
rs3797535
rs1789953
rs6859667
rs6586282
rs567754
rs558133
rs705415
rs11951068
All
−0.10
−0.05
0.00
0.05
0.10
0.15
MR effect size for
'Selenium' on 'Peptidyl−prolyl cis−trans isomerase B'

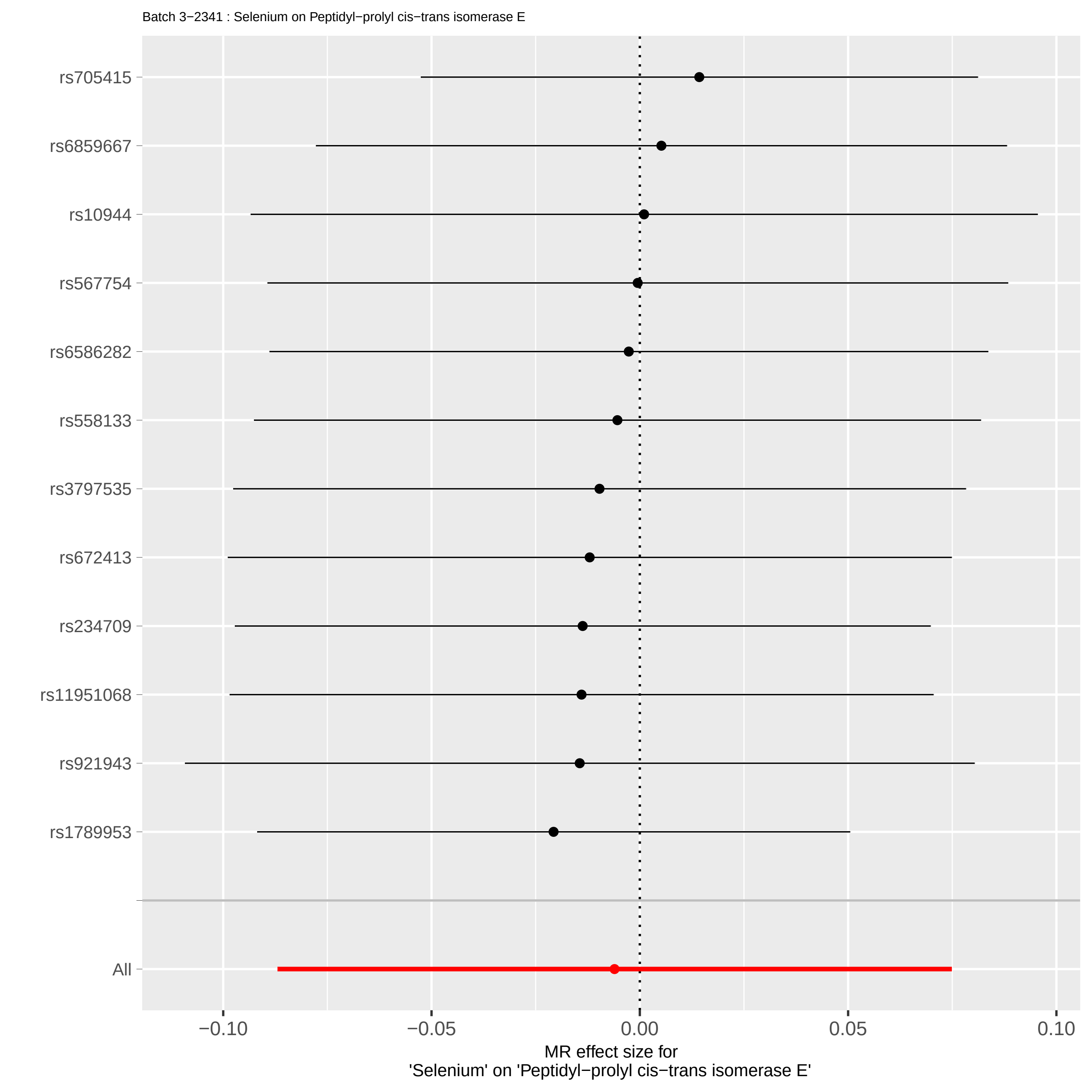

Batch 3−2341 : Selenium on Peptidyl−prolyl cis−trans isomerase E
rs705415
rs6859667
rs10944
rs567754
rs6586282
rs558133
rs3797535
rs672413
rs234709
rs11951068
rs921943
rs1789953
All
−0.10
−0.05
0.00
0.05
0.10
MR effect size for
'Selenium' on 'Peptidyl−prolyl cis−trans isomerase E'

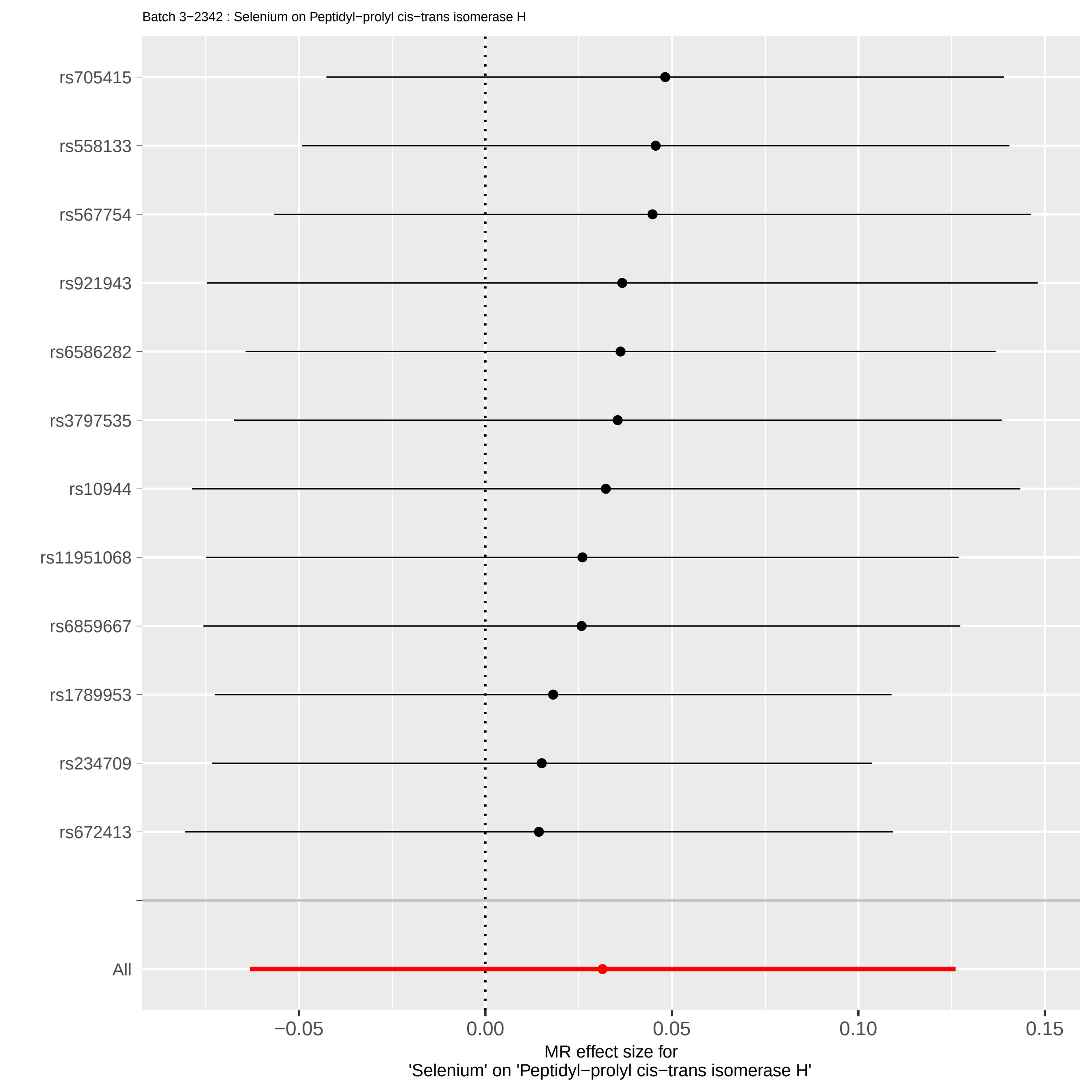

Batch 3−2342 : Selenium on Peptidyl−prolyl cis−trans isomerase H
rs705415
rs558133
rs567754
rs921943
rs6586282
rs3797535
rs10944
rs11951068
rs6859667
rs1789953
rs234709
rs672413
All
−0.05
0.00
0.05
0.10
0.15
MR effect size for
'Selenium' on 'Peptidyl−prolyl cis−trans isomerase H'

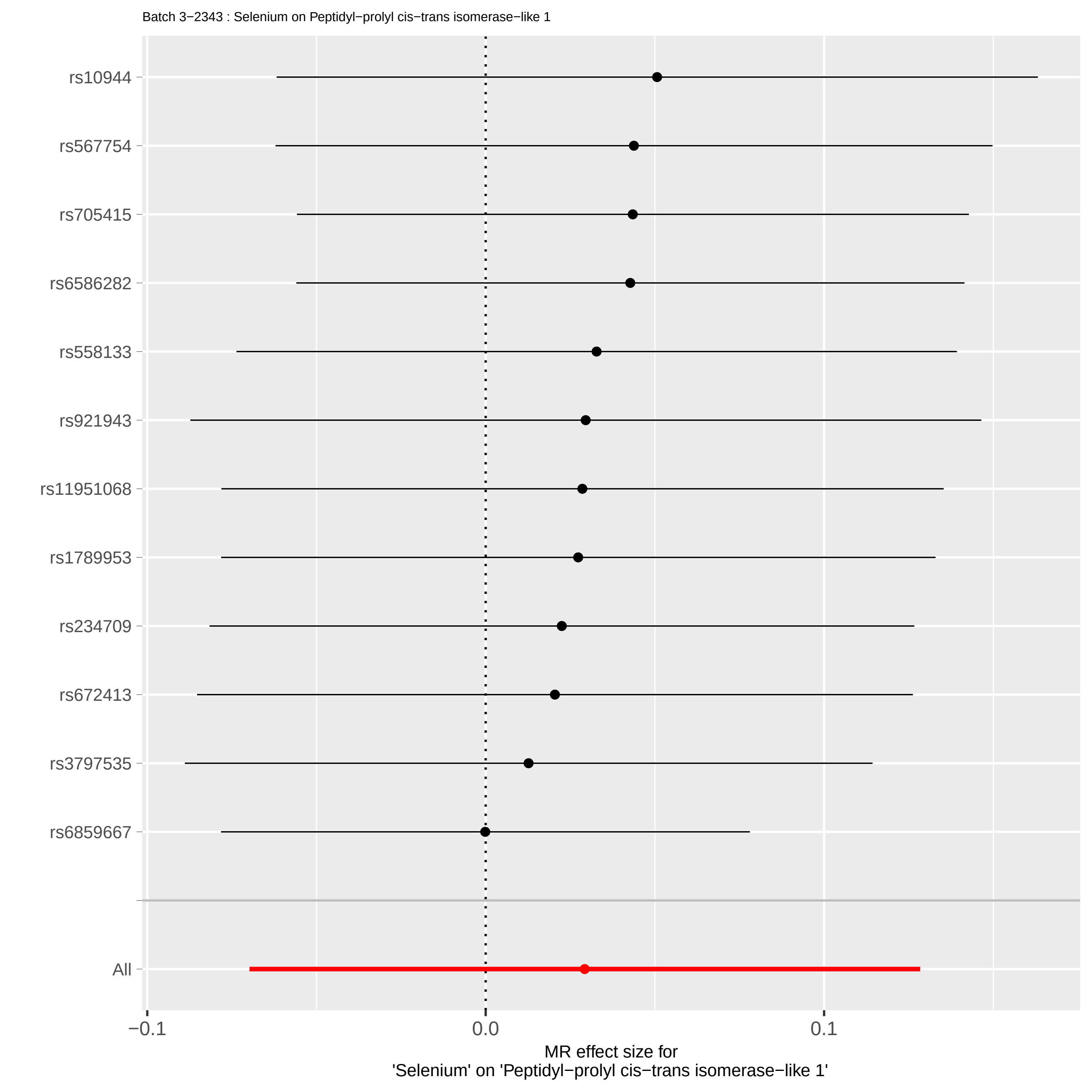

Batch 3−2343 : Selenium on Peptidyl−prolyl cis−trans isomerase−like 1
rs10944
rs567754
rs705415
rs6586282
rs558133
rs921943
rs11951068
rs1789953
rs234709
rs672413
rs3797535
rs6859667
All
−0.1
0.0
0.1
MR effect size for
'Selenium' on 'Peptidyl−prolyl cis−trans isomerase−like 1'

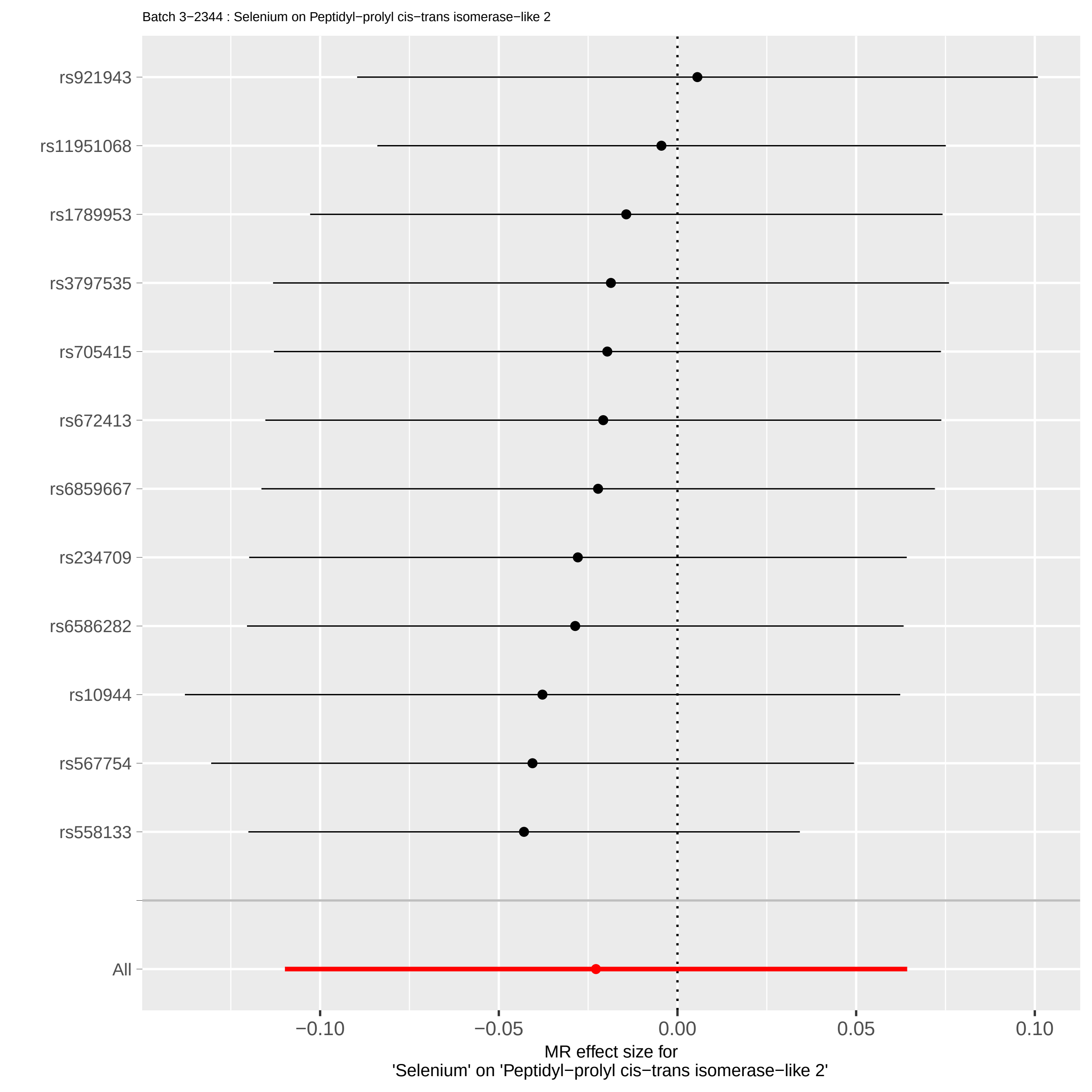

Batch 3−2344 : Selenium on Peptidyl−prolyl cis−trans isomerase−like 2
rs921943
rs11951068
rs1789953
rs3797535
rs705415
rs672413
rs6859667
rs234709
rs6586282
rs10944
rs567754
rs558133
All
−0.10
−0.05
0.00
0.05
0.10
MR effect size for
'Selenium' on 'Peptidyl−prolyl cis−trans isomerase−like 2'

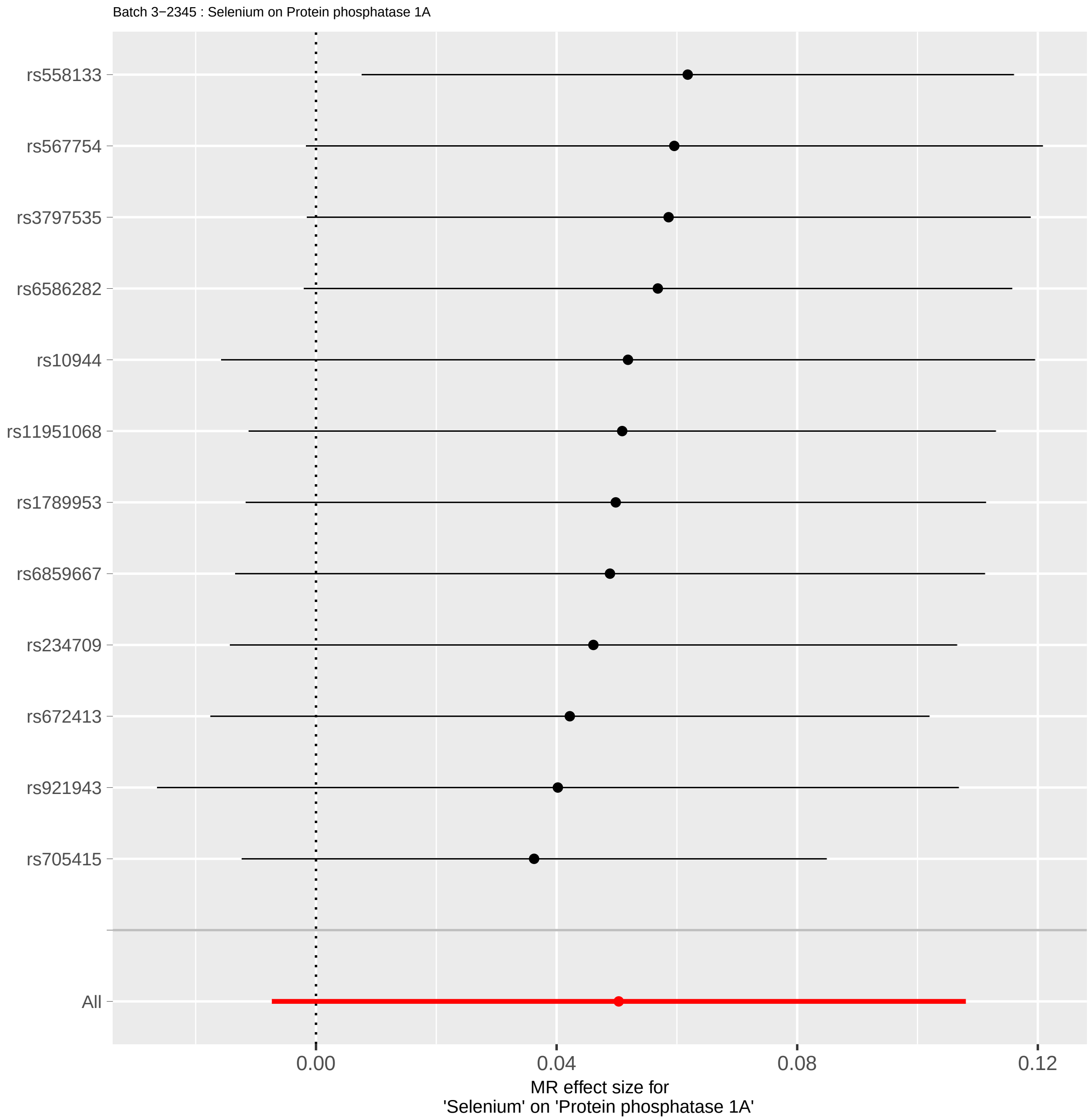
Batch 3−2345 : Selenium on Protein phosphatase 1A
rs558133
rs567754
rs3797535
rs6586282
rs10944
rs11951068
rs1789953
rs6859667
rs234709
rs672413
rs921943
rs705415
All
0.00
0.04
0.08
0.12
MR effect size for
'Selenium' on 'Protein phosphatase 1A'

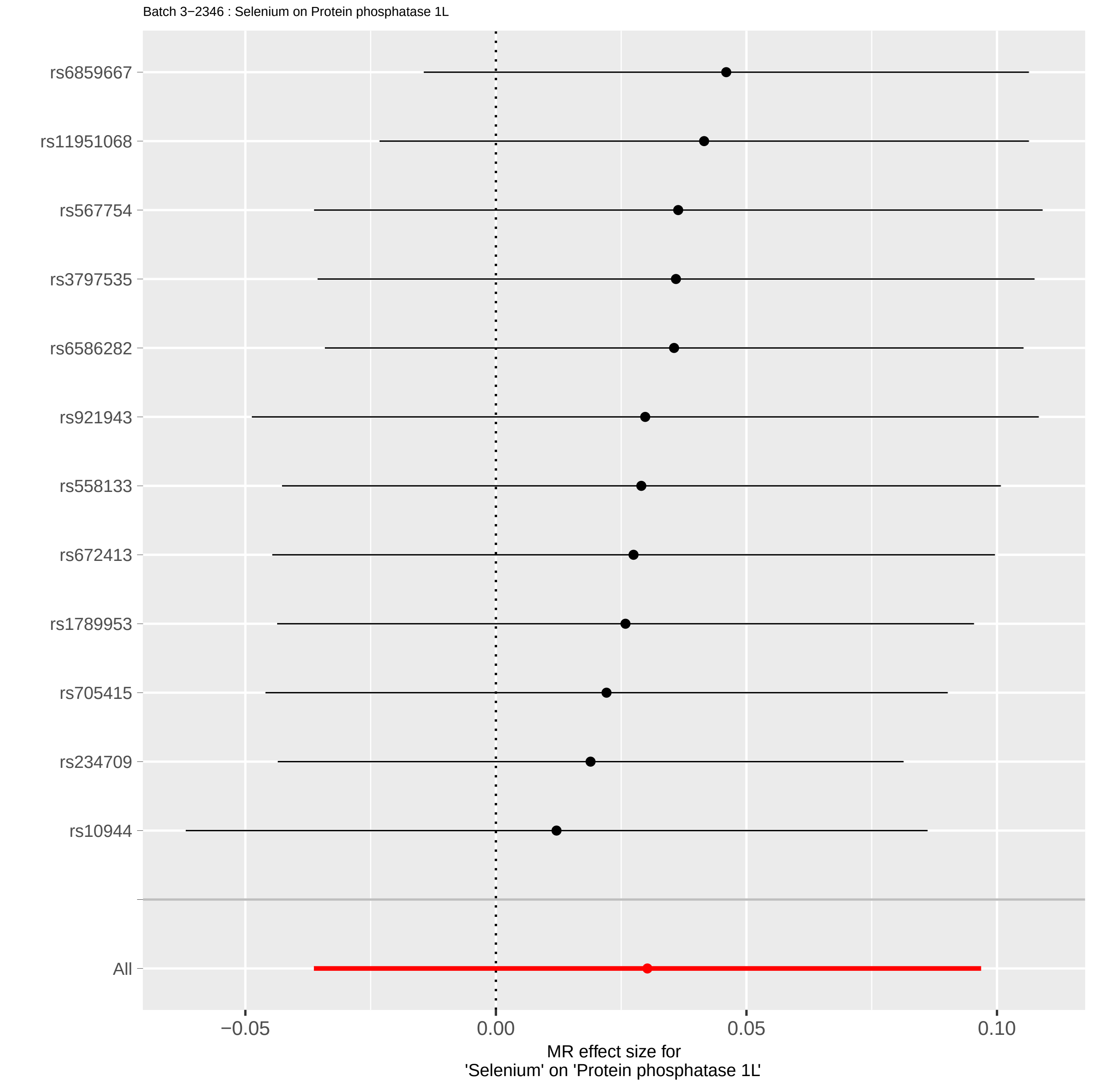

Batch 3−2346 : Selenium on Protein phosphatase 1L
rs6859667
rs11951068
rs567754
rs3797535
rs6586282
rs921943
rs558133
rs672413
rs1789953
rs705415
rs234709
rs10944
All
−0.05
0.00
0.05
0.10
MR effect size for
'Selenium' on 'Protein phosphatase 1L'

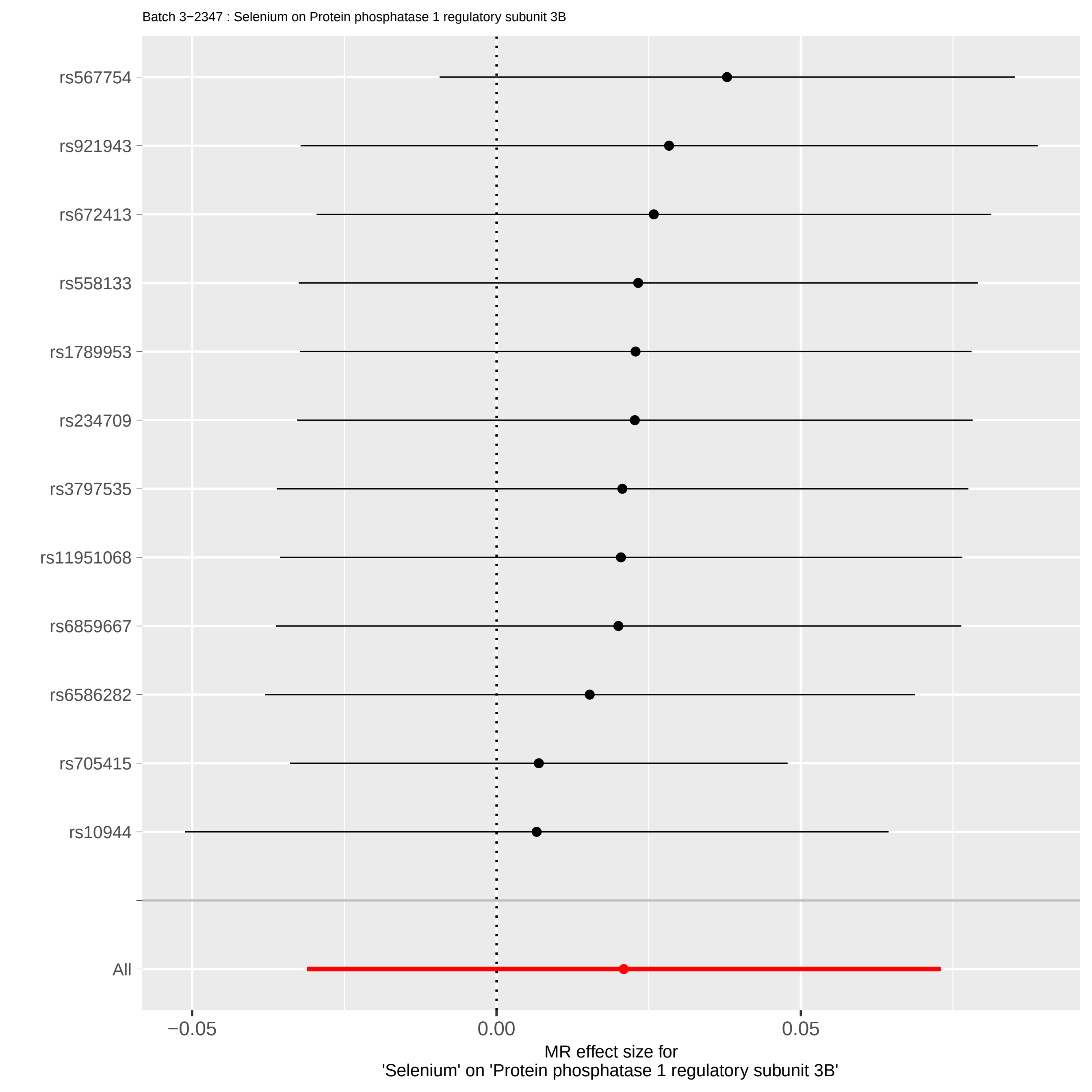

Batch 3−2347 : Selenium on Protein phosphatase 1 regulatory subunit 3B
rs567754
rs921943
rs672413
rs558133
rs1789953
rs234709
rs3797535
rs11951068
rs6859667
rs6586282
rs705415
rs10944
All
−0.05
0.00
0.05
MR effect size for
'Selenium' on 'Protein phosphatase 1 regulatory subunit 3B'

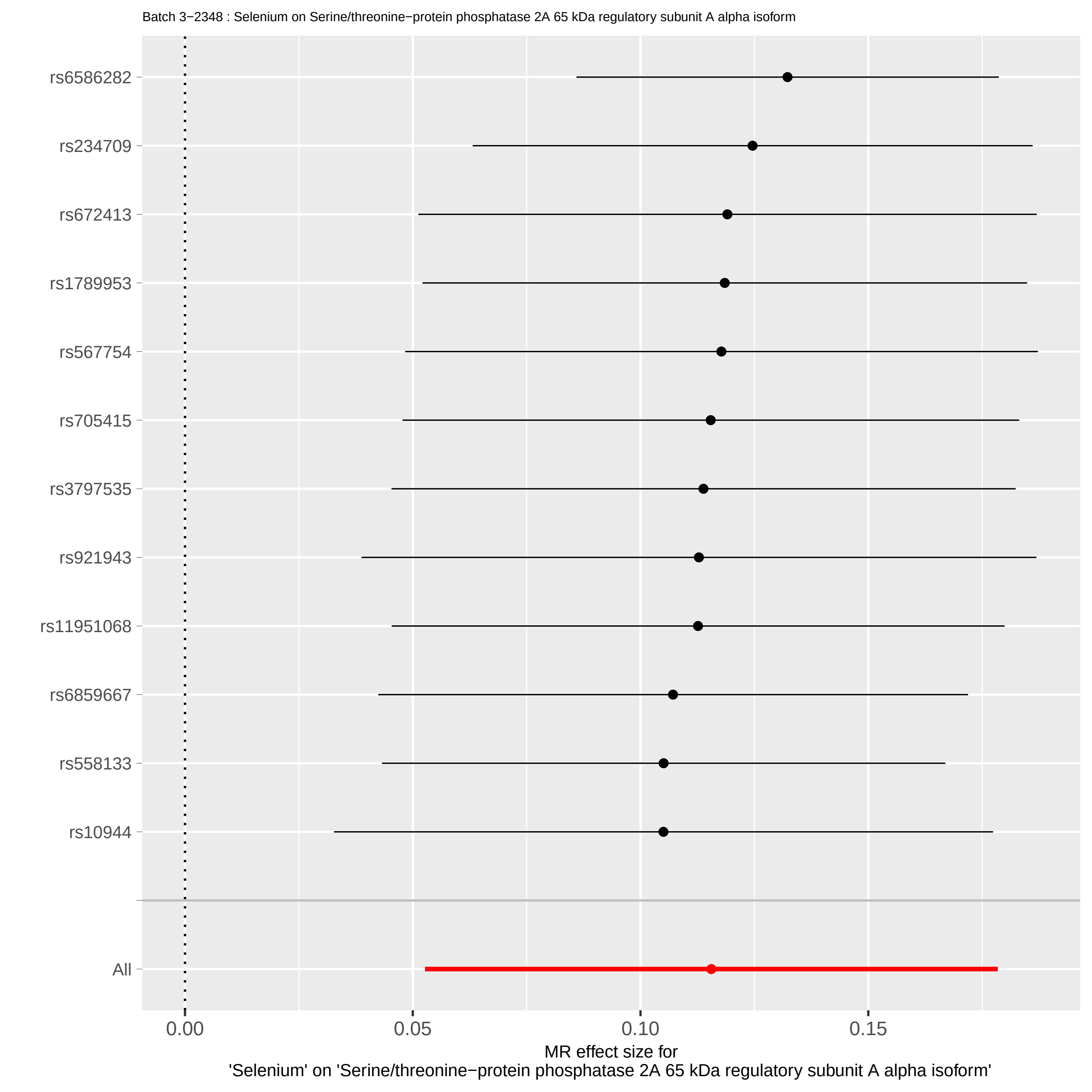

Batch 3−2348 : Selenium on Serine/threonine−protein phosphatase 2A 65 kDa regulatory subunit A alpha isoform
rs6586282
rs234709
rs672413
rs1789953
rs567754
rs705415
rs3797535
rs921943
rs11951068
rs6859667
rs558133
rs10944
All
0.00
0.05
0.10
0.15
MR effect size for
'Selenium' on 'Serine/threonine−protein phosphatase 2A 65 kDa regulatory subunit A alpha isoform'

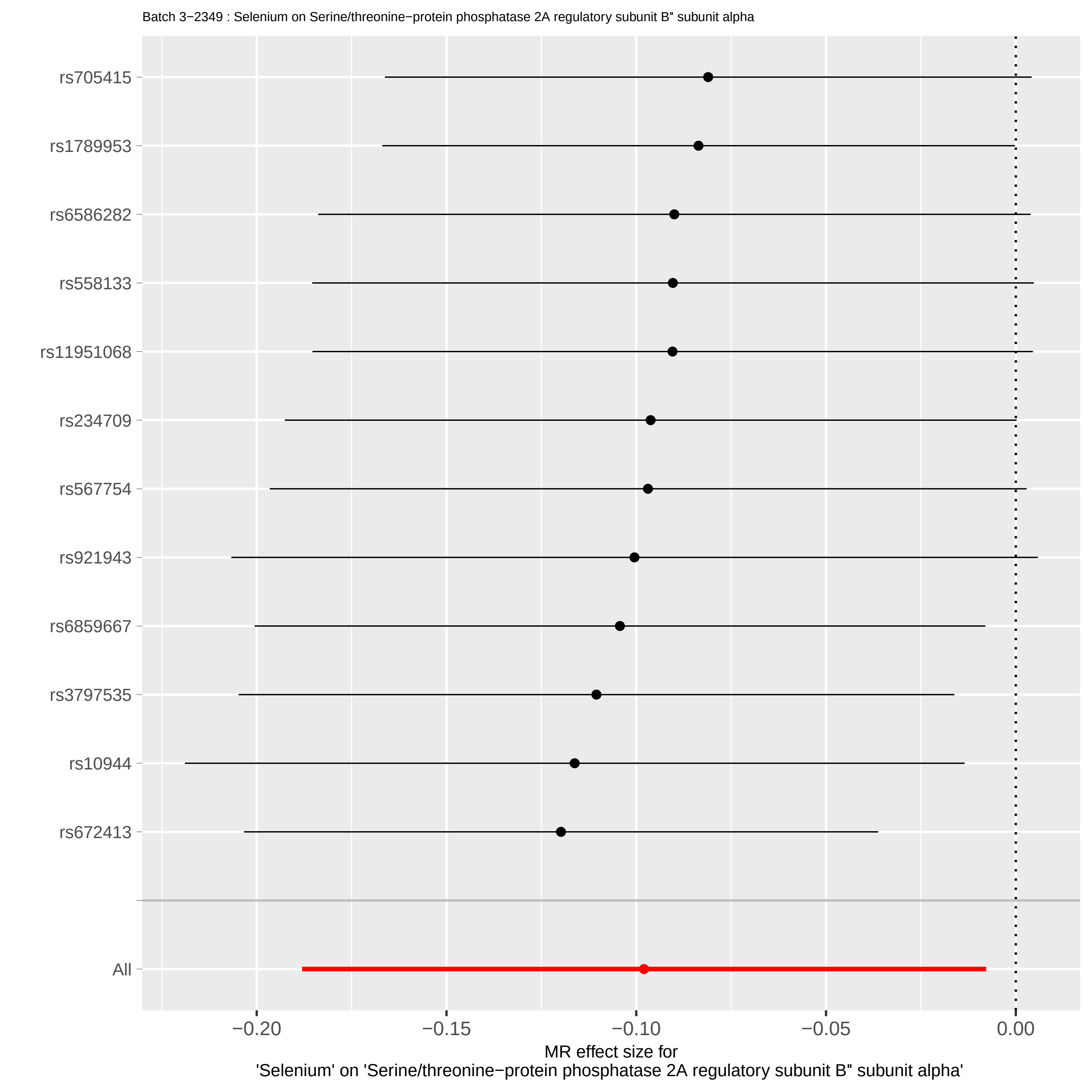

Batch 3−2349 : Selenium on Serine/threonine−protein phosphatase 2A regulatory subunit B" subunit alpha
rs705415
rs1789953
rs6586282
rs558133
rs11951068
rs234709
rs567754
rs921943
rs6859667
rs3797535
rs10944
rs672413
All
−0.20
−0.15
−0.10
−0.05
0.00
MR effect size for
'Selenium' on 'Serine/threonine−protein phosphatase 2A regulatory subunit B" subunit alpha'

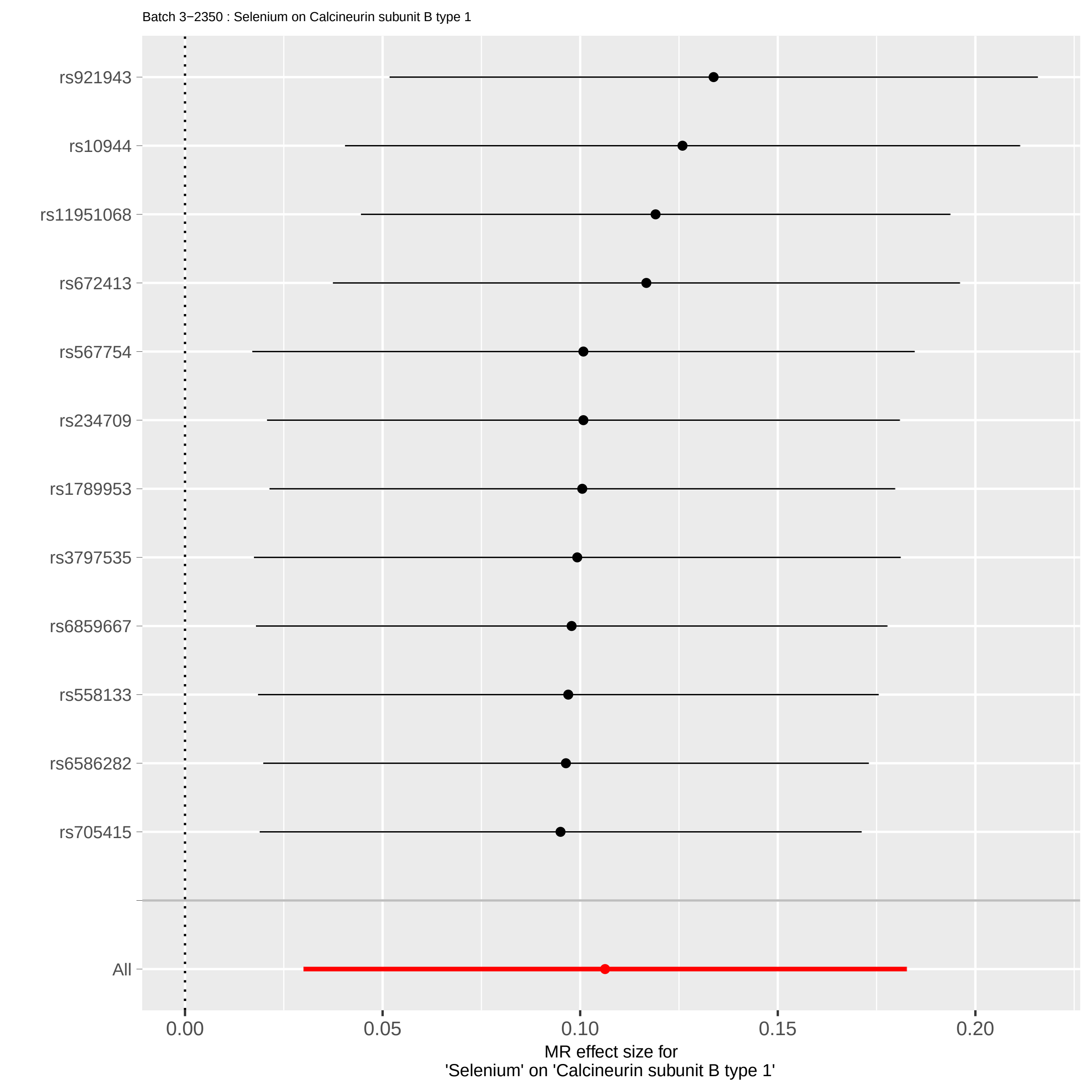

Batch 3−2350 : Selenium on Calcineurin subunit B type 1
rs921943
rs10944
rs11951068
rs672413
rs567754
rs234709
rs1789953
rs3797535
rs6859667
rs558133
rs6586282
rs705415
All
0.00
0.05
0.10
0.15
0.20
MR effect size for
'Selenium' on 'Calcineurin subunit B type 1'

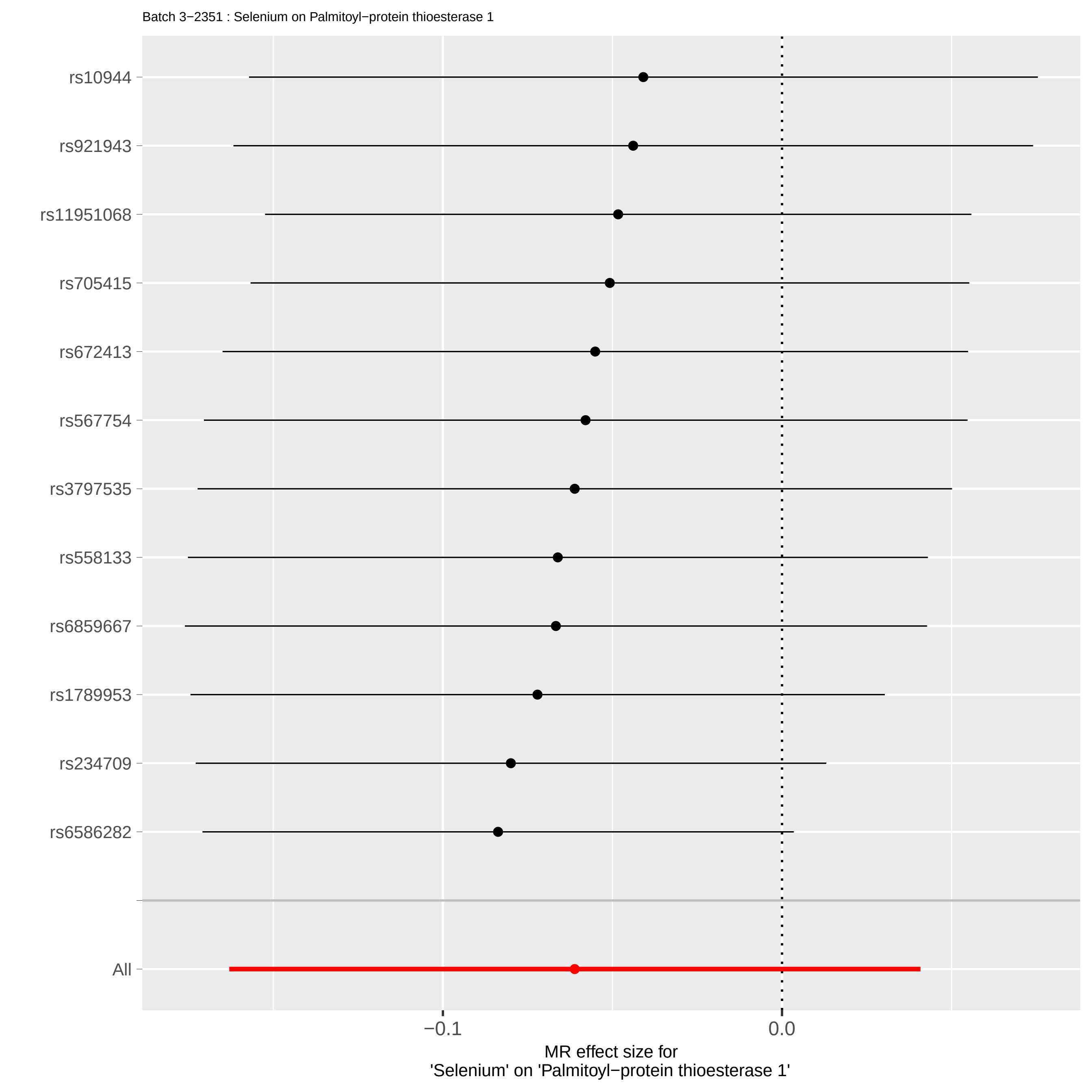

Batch 3−2351 : Selenium on Palmitoyl−protein thioesterase 1
rs10944
rs921943
rs11951068
rs705415
rs672413
rs567754
rs3797535
rs558133
rs6859667
rs1789953
rs234709
rs6586282
All
−0.1
0.0
MR effect size for
'Selenium' on 'Palmitoyl−protein thioesterase 1'

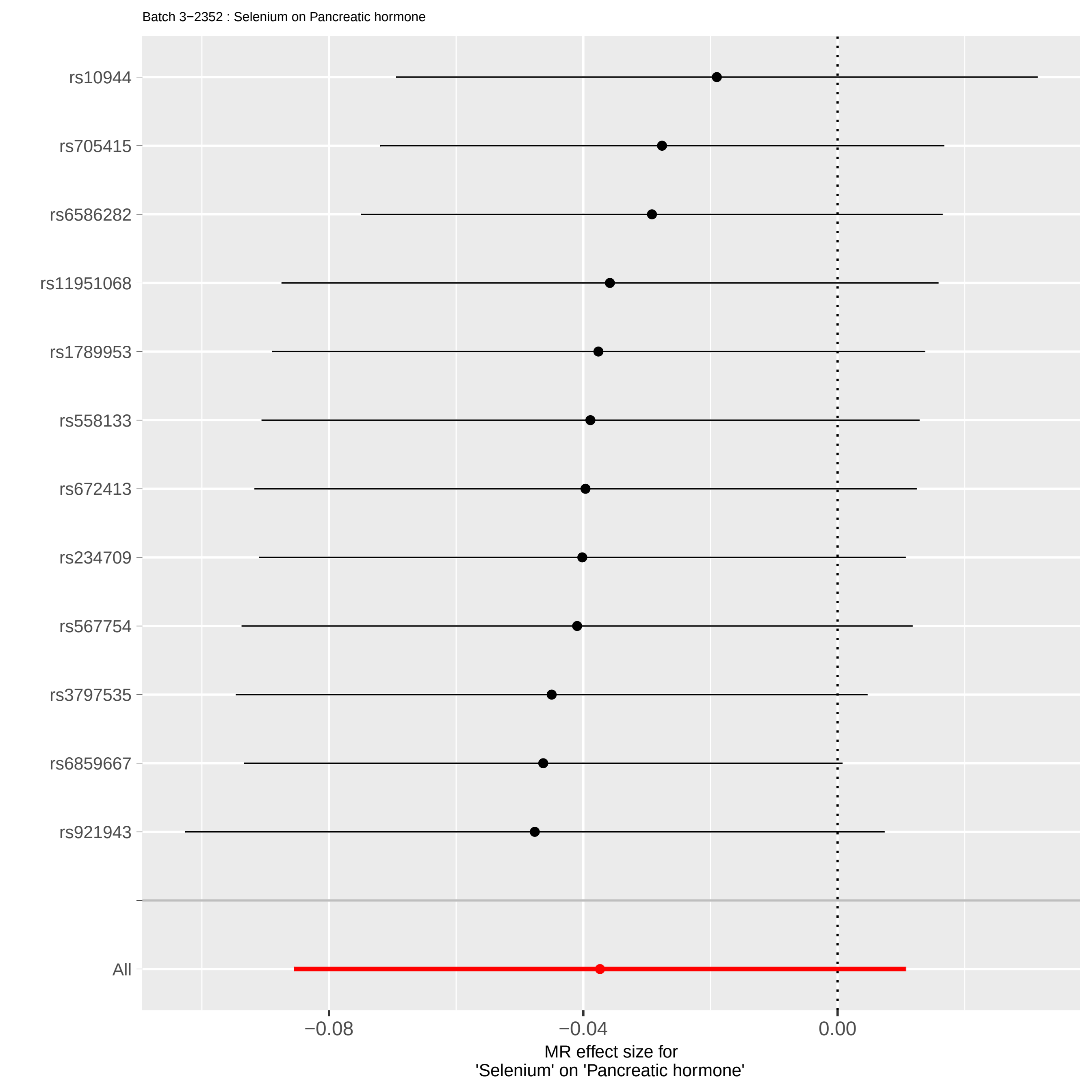
Batch 3−2352 : Selenium on Pancreatic hormone
rs10944
rs705415
rs6586282
rs11951068
rs1789953
rs558133
rs672413
rs234709
rs567754
rs3797535
rs6859667
rs921943
All
−0.08
−0.04
0.00
MR effect size for
'Selenium' on 'Pancreatic hormone'

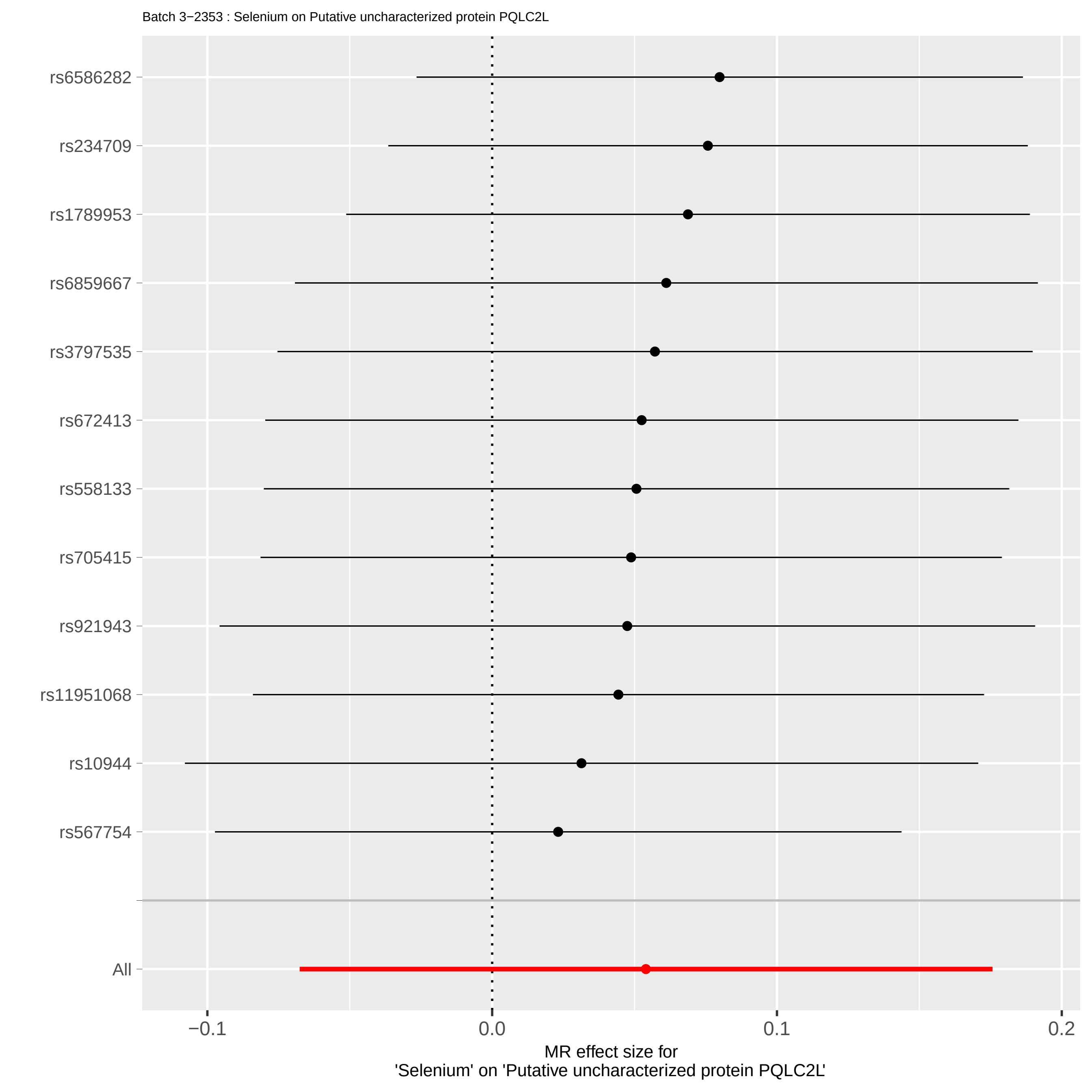
Batch 3−2353 : Selenium on Putative uncharacterized protein PQLC2L
rs6586282
rs234709
rs1789953
rs6859667
rs3797535
rs672413
rs558133
rs705415
rs921943
rs11951068
rs10944
rs567754
All
−0.1
0.0
0.1
0.2
MR effect size for
'Selenium' on 'Putative uncharacterized protein PQLC2L'

Batch 3−2354 : Selenium on Basic salivary proline−rich protein 4

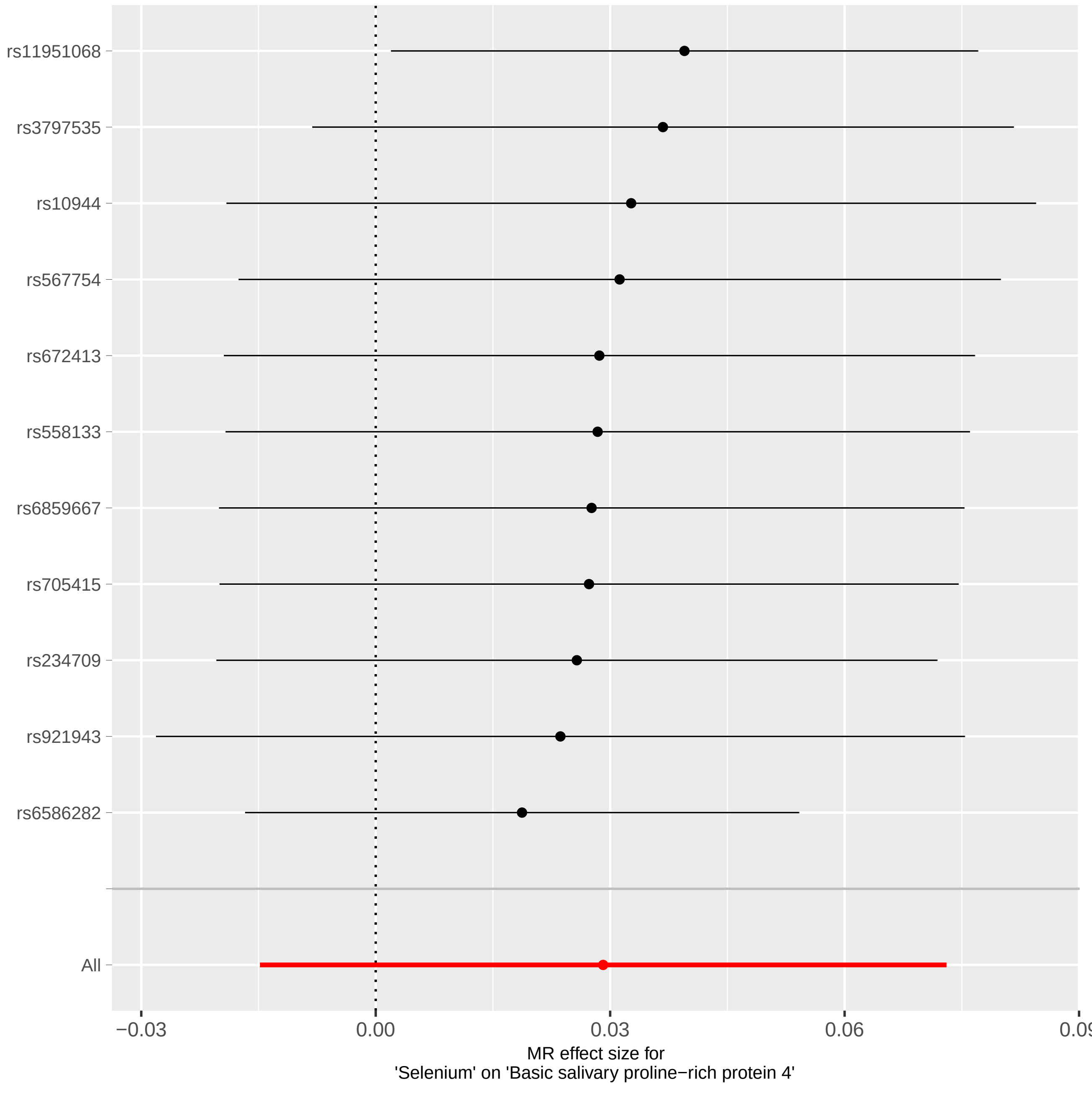

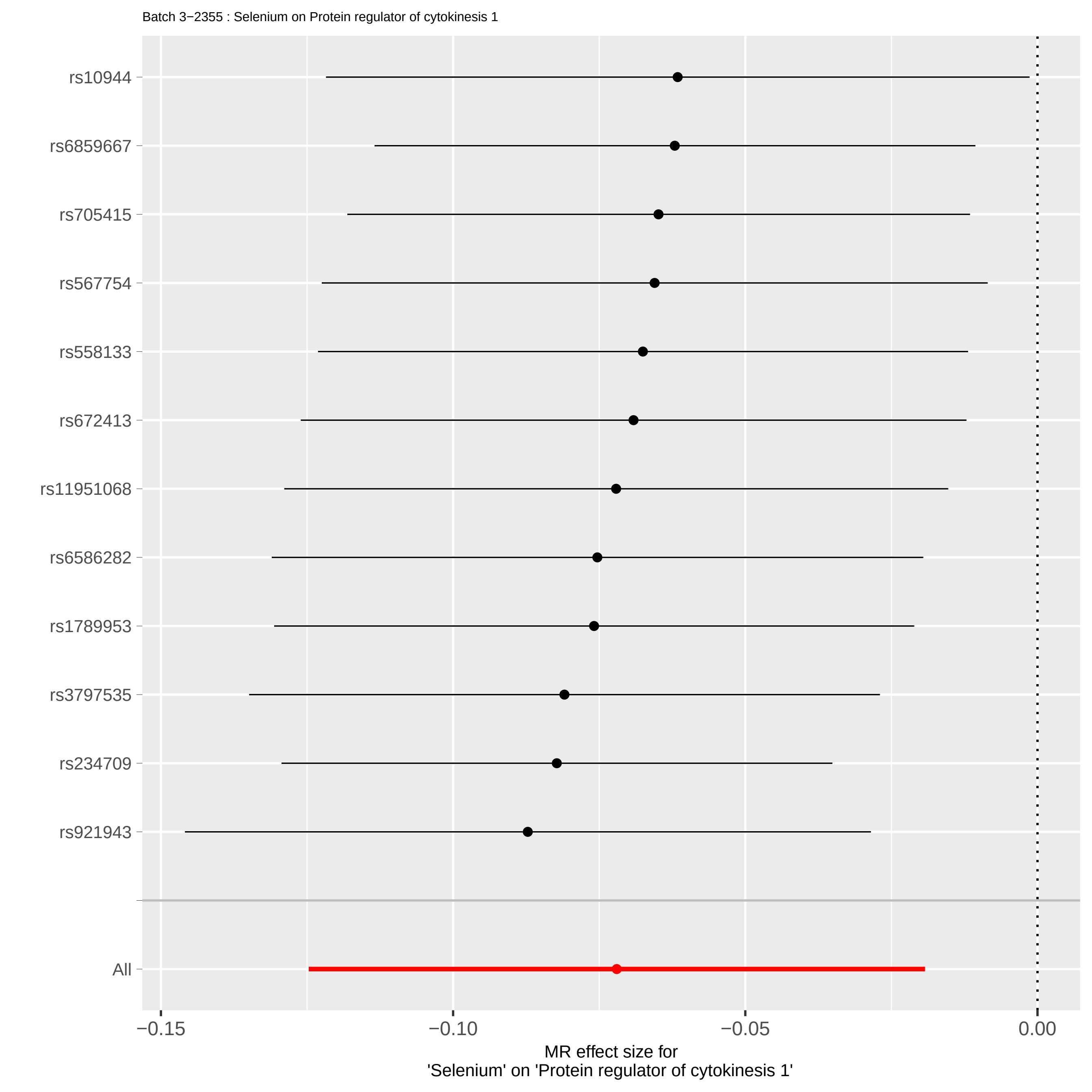

Batch 3−2355 : Selenium on Protein regulator of cytokinesis 1
rs10944
rs6859667
rs705415
rs567754
rs558133
rs672413
rs11951068
rs6586282
rs1789953
rs3797535
rs234709
rs921943
All
−0.15
−0.10
−0.05
0.00
MR effect size for
'Selenium' on 'Protein regulator of cytokinesis 1'

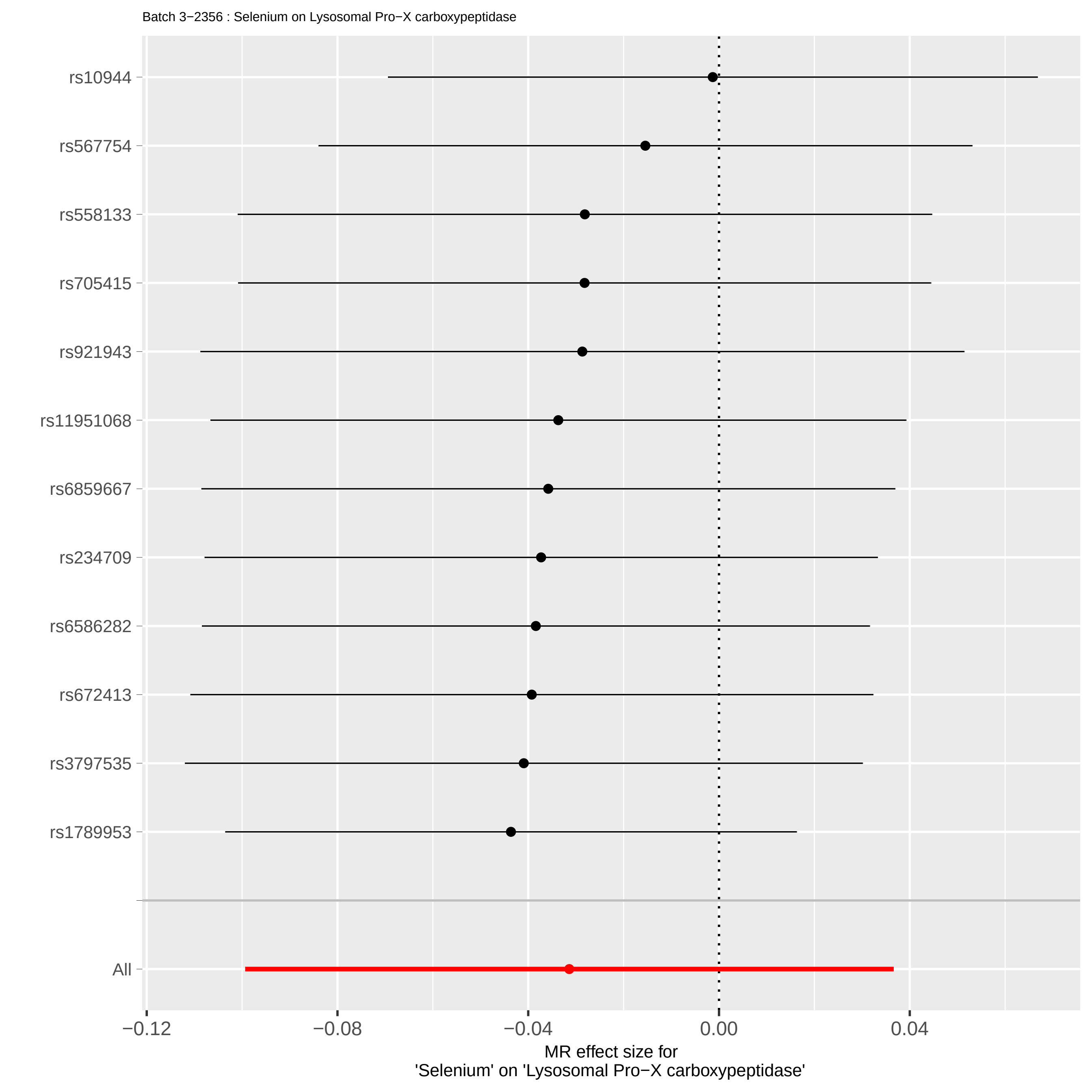

Batch 3−2356 : Selenium on Lysosomal Pro−X carboxypeptidase
rs10944
rs567754
rs558133
rs705415
rs921943
rs11951068
rs6859667
rs234709
rs6586282
rs672413
rs3797535
rs1789953
All
−0.12
−0.08
−0.04
0.00
0.04
MR effect size for
'Selenium' on 'Lysosomal Pro−X carboxypeptidase'

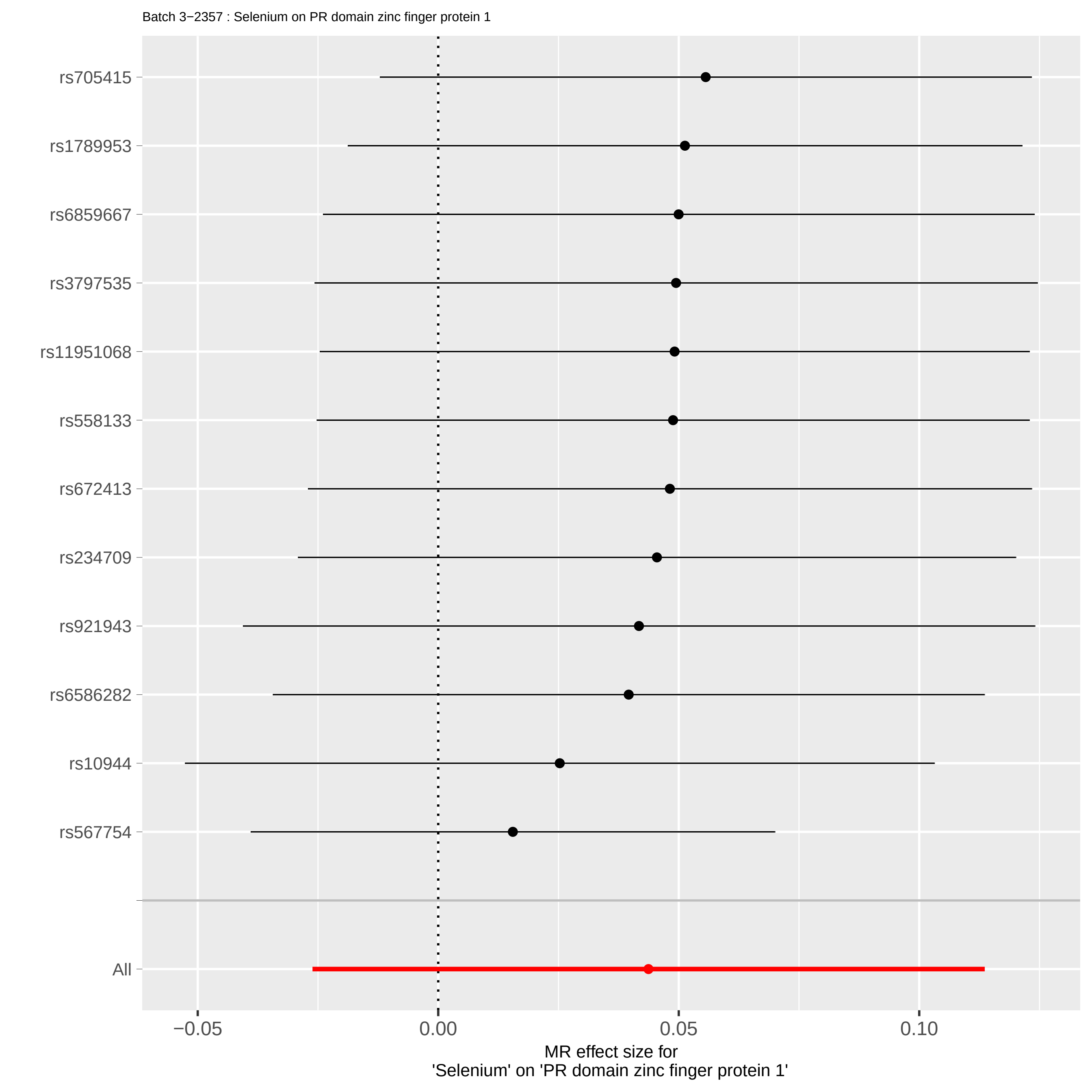

Batch 3−2357 : Selenium on PR domain zinc finger protein 1
rs705415
rs1789953
rs6859667
rs3797535
rs11951068
rs558133
rs672413
rs234709
rs921943
rs6586282
rs10944
rs567754
All
−0.05
0.00
0.05
0.10
MR effect size for
'Selenium' on 'PR domain zinc finger protein 1'

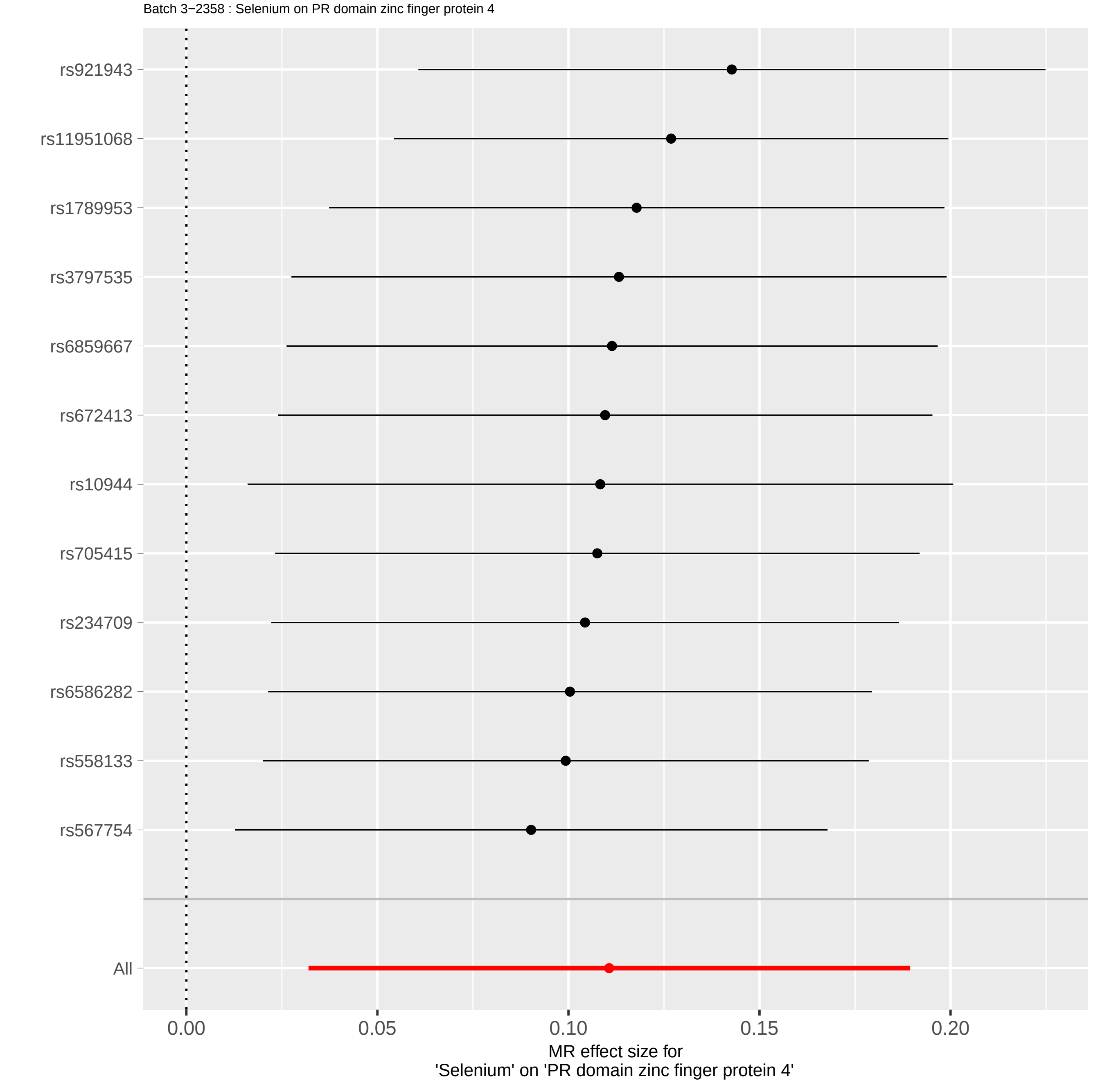

Batch 3−2358 : Selenium on PR domain zinc finger protein 4
rs921943
rs11951068
rs1789953
rs3797535
rs6859667
rs672413
rs10944
rs705415
rs234709
rs6586282
rs558133
rs567754
All
0.00
0.05
0.10
0.15
0.20
MR effect size for
'Selenium' on 'PR domain zinc finger protein 4'

Batch 3−2359 : Selenium on Peroxiredoxin−1

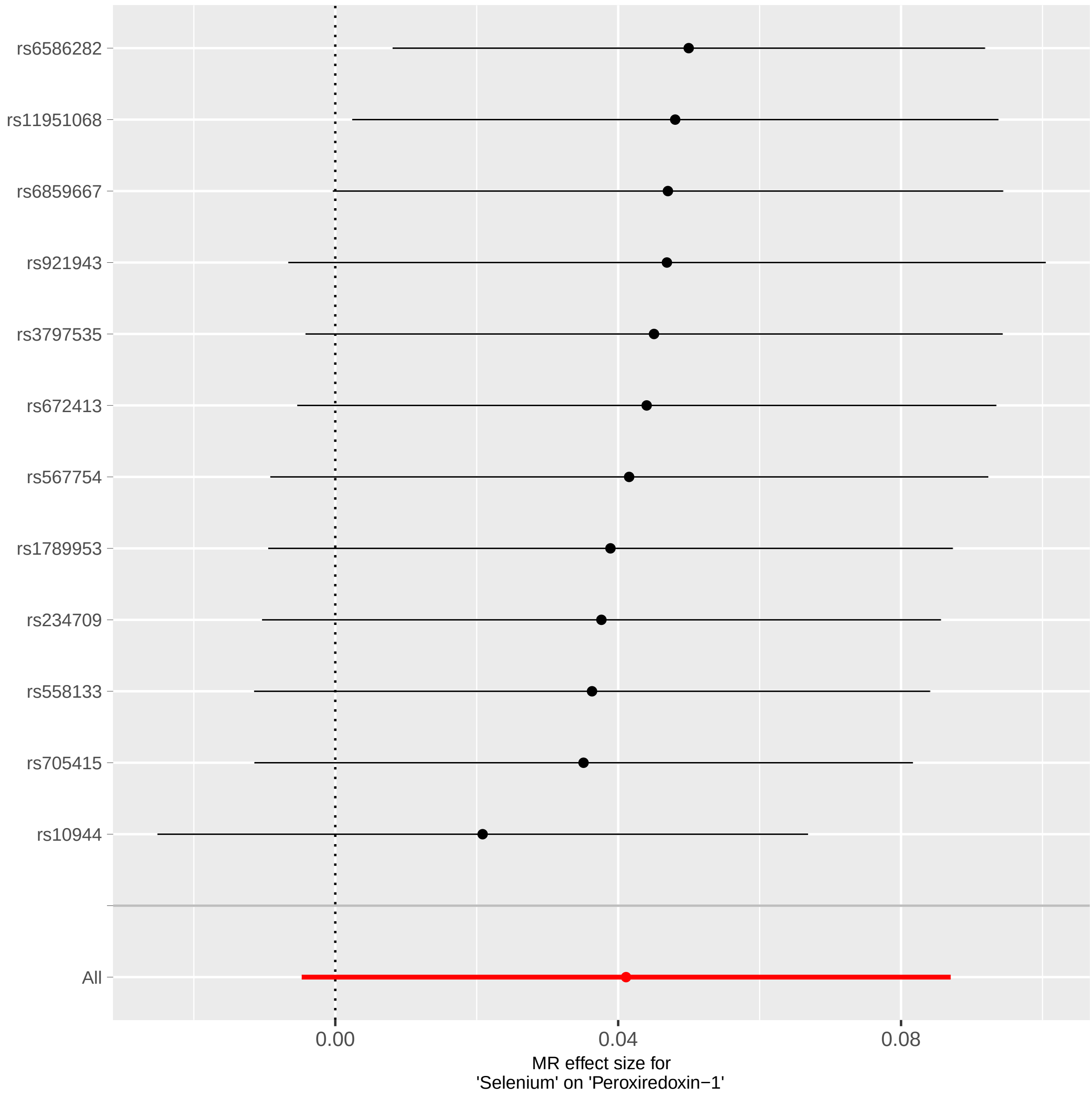

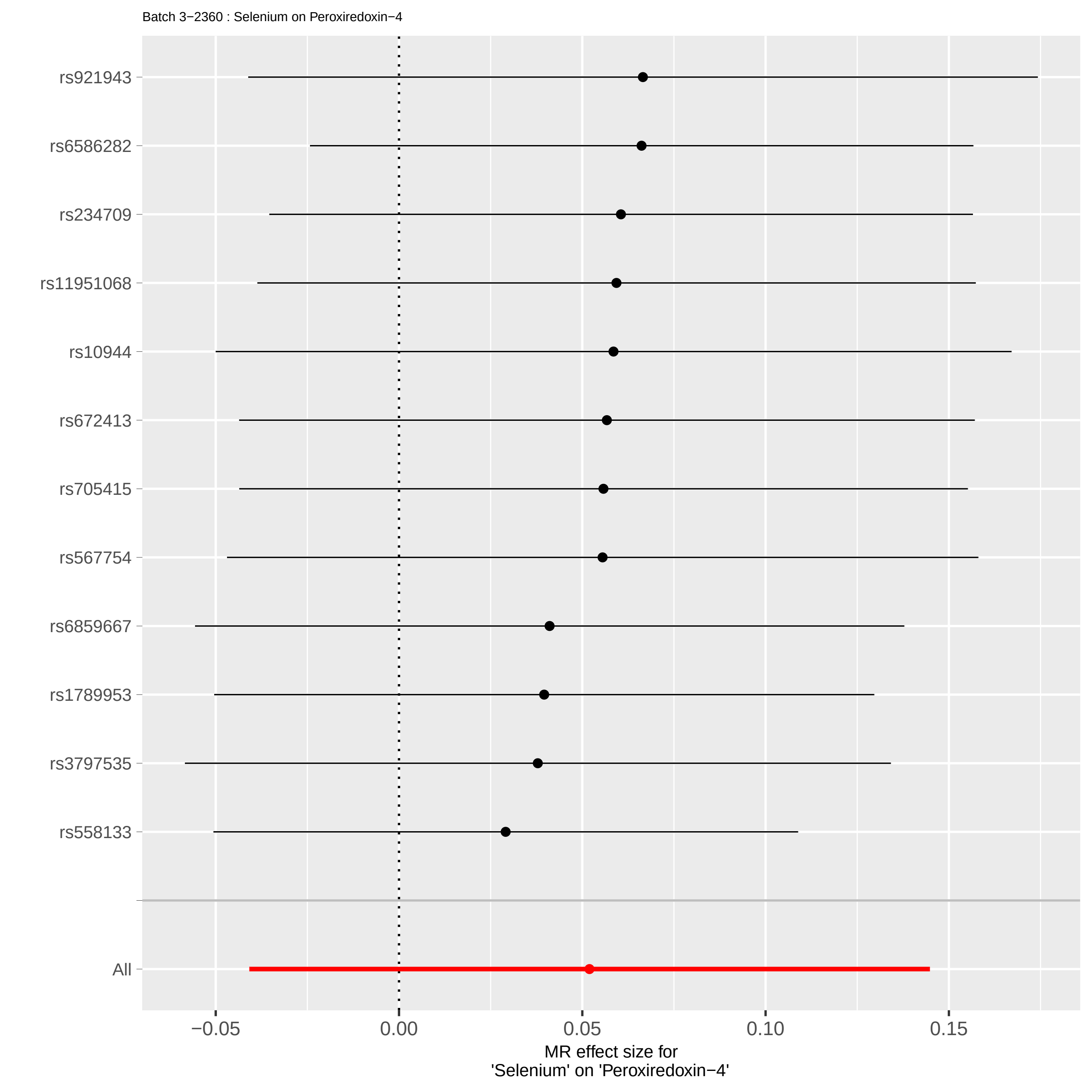

Batch 3−2360 : Selenium on Peroxiredoxin−4
rs921943
rs6586282
rs234709
rs11951068
rs10944
rs672413
rs705415
rs567754
rs6859667
rs1789953
rs3797535
rs558133
All
−0.05
0.00
0.05
0.10
0.15
MR effect size for
'Selenium' on 'Peroxiredoxin−4'

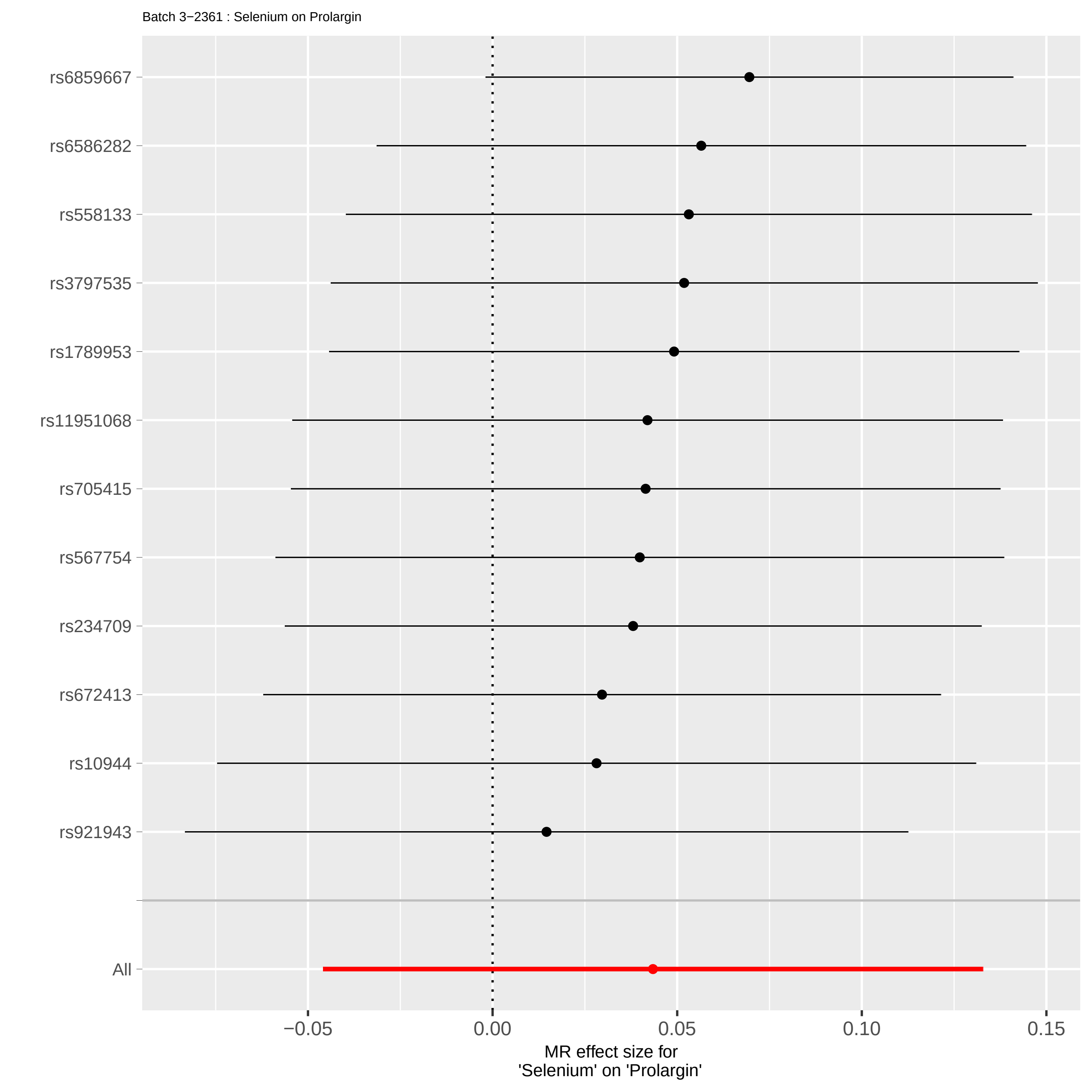

Batch 3−2361 : Selenium on Prolargin
rs6859667
rs6586282
rs558133
rs3797535
rs1789953
rs11951068
rs705415
rs567754
rs234709
rs672413
rs10944
rs921943
All
−0.05
0.00
0.05
0.10
0.15
MR effect size for
'Selenium' on 'Prolargin'

Batch 3−2362 : Selenium on Proteoglycan 3

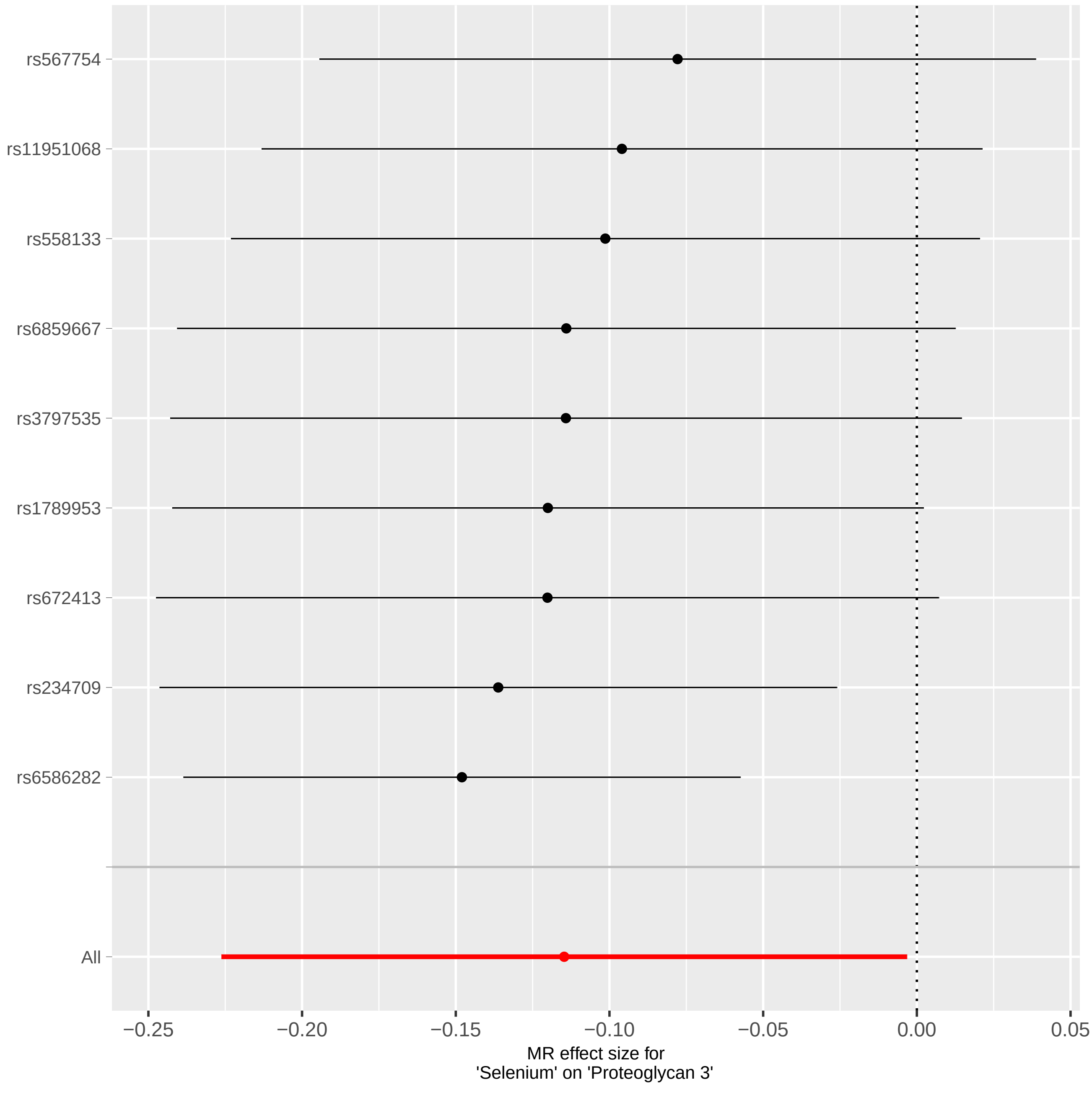

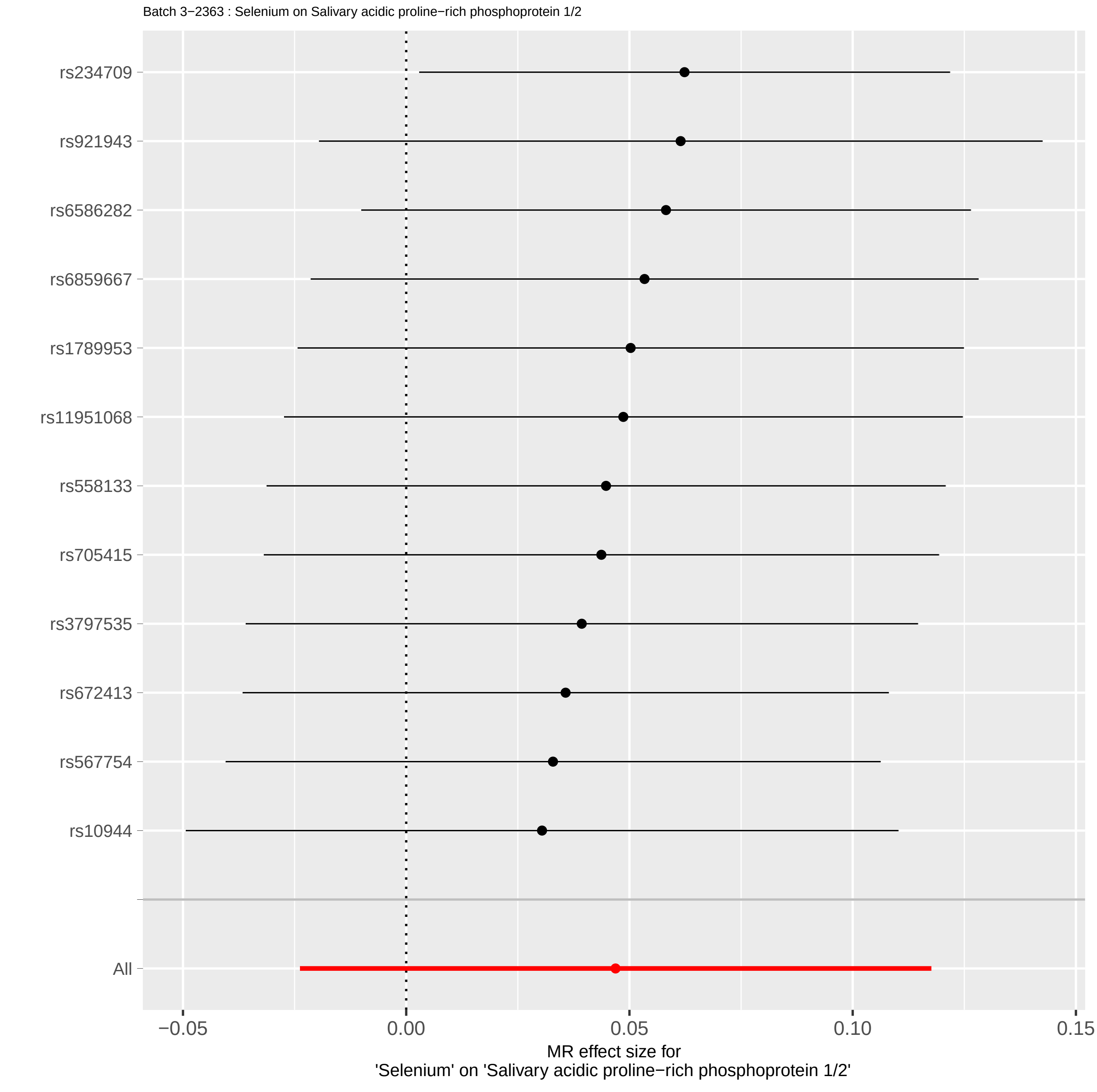

Batch 3−2363 : Selenium on Salivary acidic proline−rich phosphoprotein 1/2
rs234709
rs921943
rs6586282
rs6859667
rs1789953
rs11951068
rs558133
rs705415
rs3797535
rs672413
rs567754
rs10944
All
−0.05
0.00
0.05
0.10
0.15
MR effect size for
'Selenium' on 'Salivary acidic proline−rich phosphoprotein 1/2'

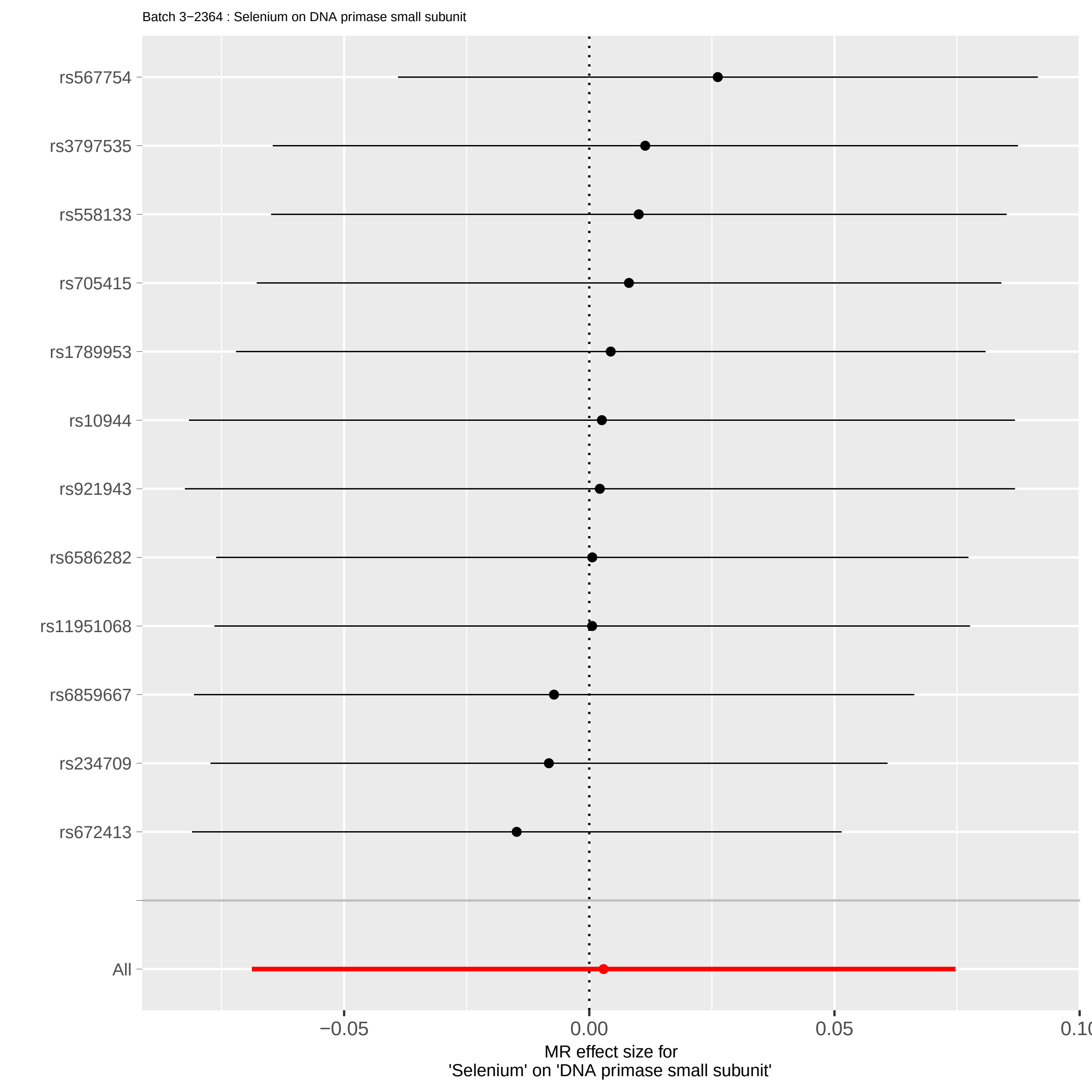
Batch 3−2364 : Selenium on DNA primase small subunit
rs567754
rs3797535
rs558133
rs705415
rs1789953
rs10944
rs921943
rs6586282
rs11951068
rs6859667
rs234709
rs672413
All
−0.05
0.00
0.05
0.10
MR effect size for
'Selenium' on 'DNA primase small subunit'

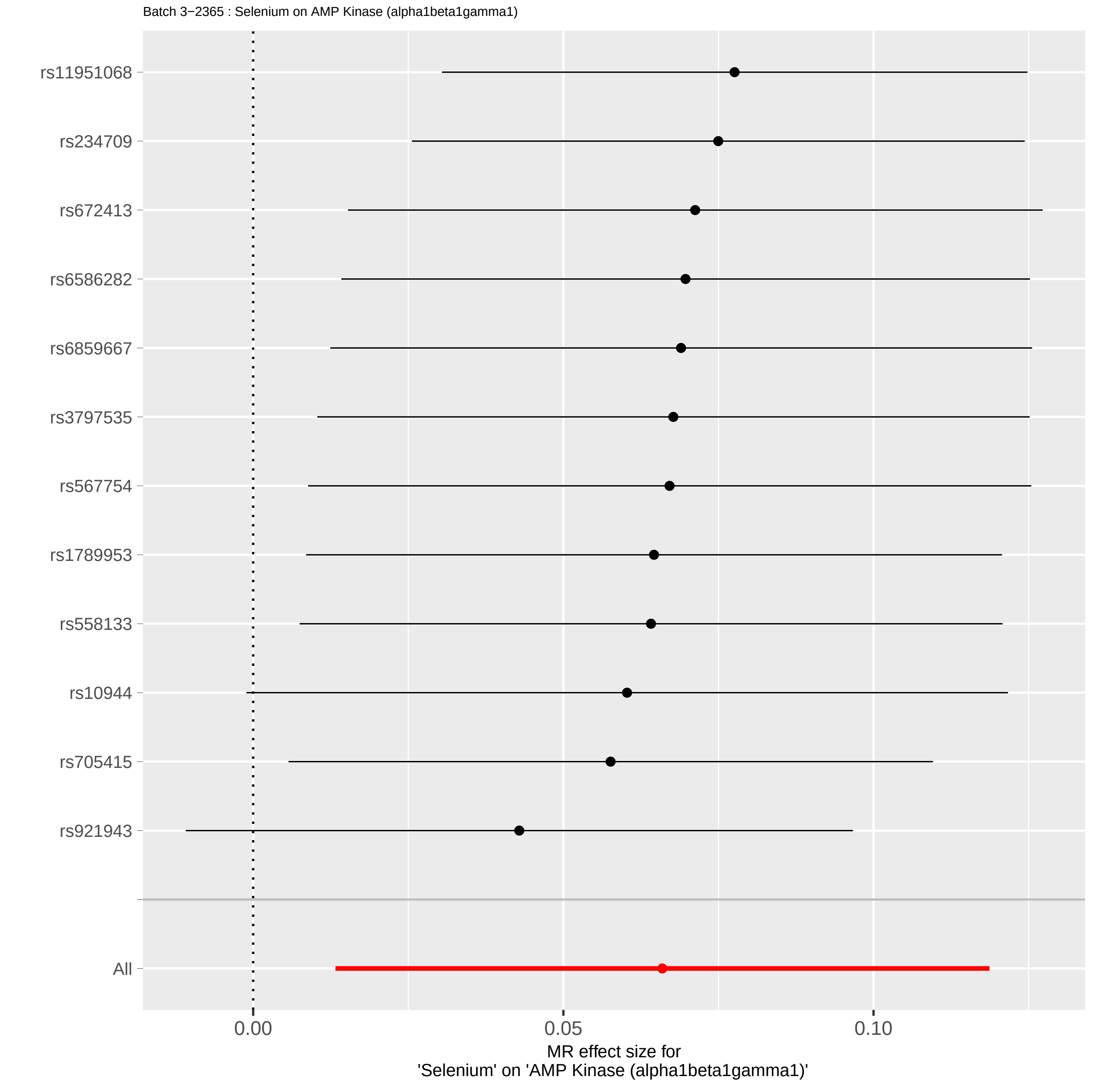

Batch 3−2365 : Selenium on AMP Kinase (alpha1beta1gamma1)
rs11951068
rs234709
rs672413
rs6586282
rs6859667
rs3797535
rs567754
rs1789953
rs558133
rs10944
rs705415
rs921943
All
0.00
0.05
0.10
MR effect size for
'Selenium' on 'AMP Kinase (alpha1beta1gamma1)'

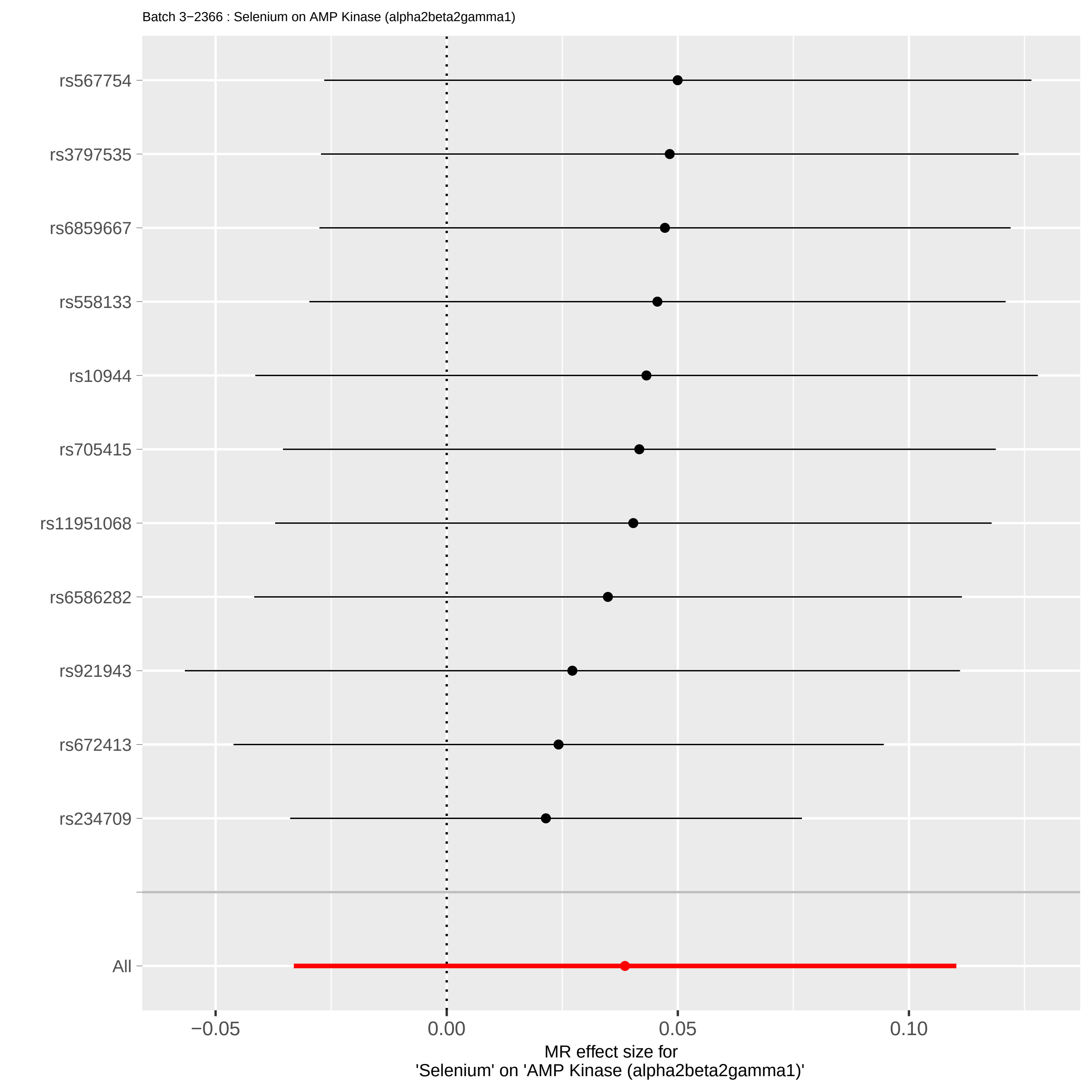

Batch 3−2366 : Selenium on AMP Kinase (alpha2beta2gamma1)
rs567754
rs3797535
rs6859667
rs558133
rs10944
rs705415
rs11951068
rs6586282
rs921943
rs672413
rs234709
All
−0.05
0.00
0.05
0.10
MR effect size for
'Selenium' on 'AMP Kinase (alpha2beta2gamma1)'

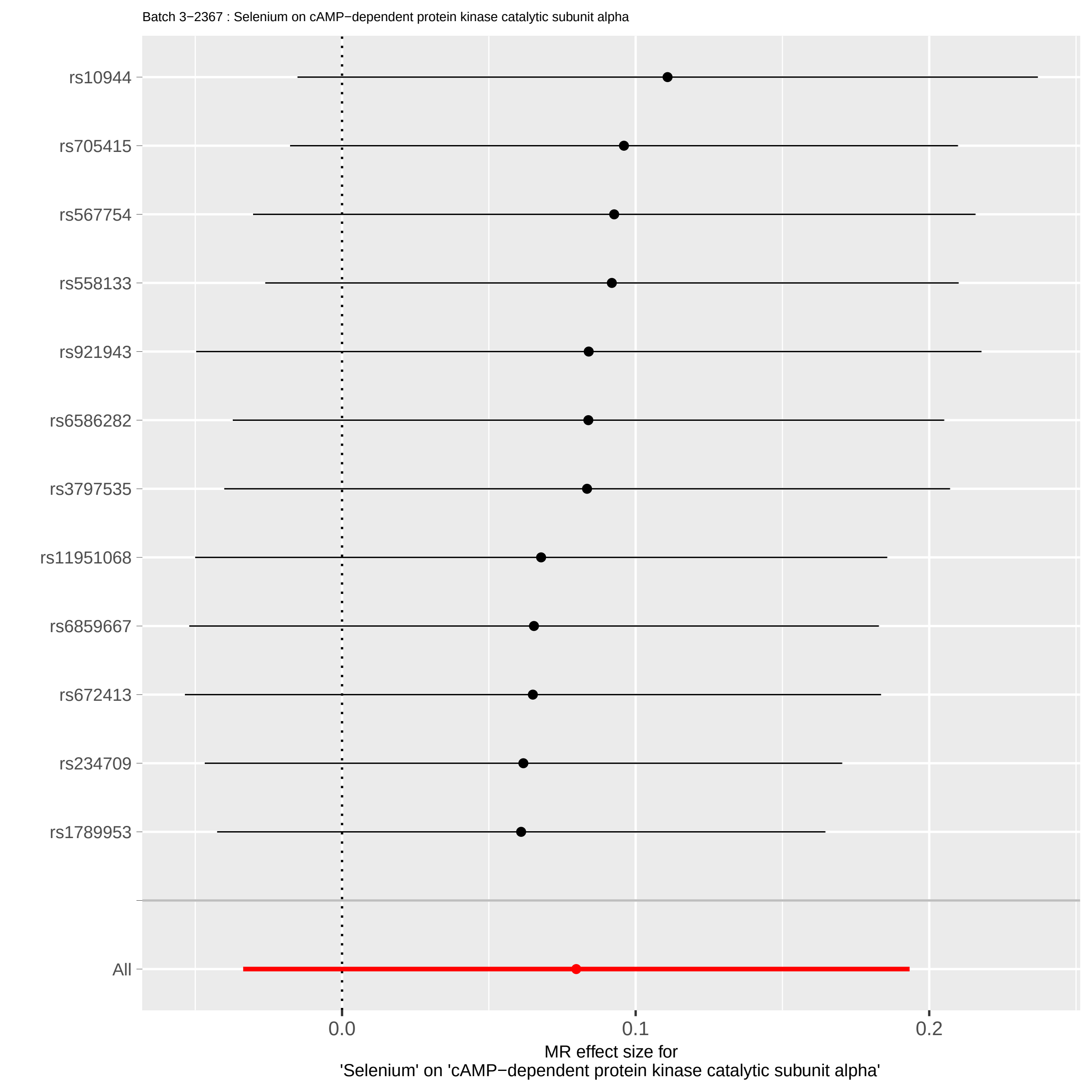

Batch 3−2367 : Selenium on cAMP−dependent protein kinase catalytic subunit alpha
rs10944
rs705415
rs567754
rs558133
rs921943
rs6586282
rs3797535
rs11951068
rs6859667
rs672413
rs234709
rs1789953
All
0.0
0.1
0.2
MR effect size for
'Selenium' on 'cAMP−dependent protein kinase catalytic subunit alpha'

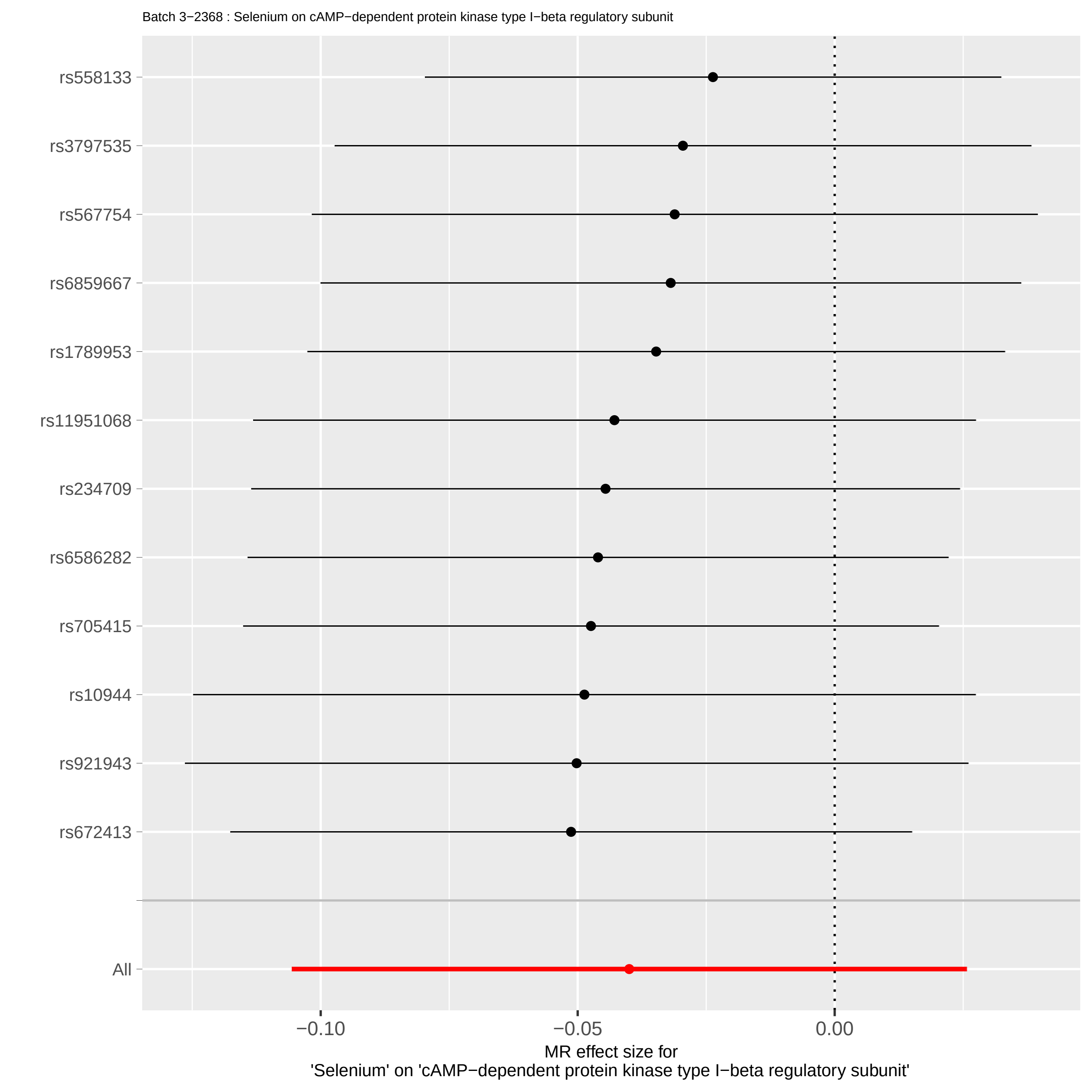

Batch 3−2368 : Selenium on cAMP−dependent protein kinase type I−beta regulatory subunit
rs558133
rs3797535
rs567754
rs6859667
rs1789953
rs11951068
rs234709
rs6586282
rs705415
rs10944
rs921943
rs672413
All
−0.10
−0.05
0.00
MR effect size for
'Selenium' on 'cAMP−dependent protein kinase type I−beta regulatory subunit'

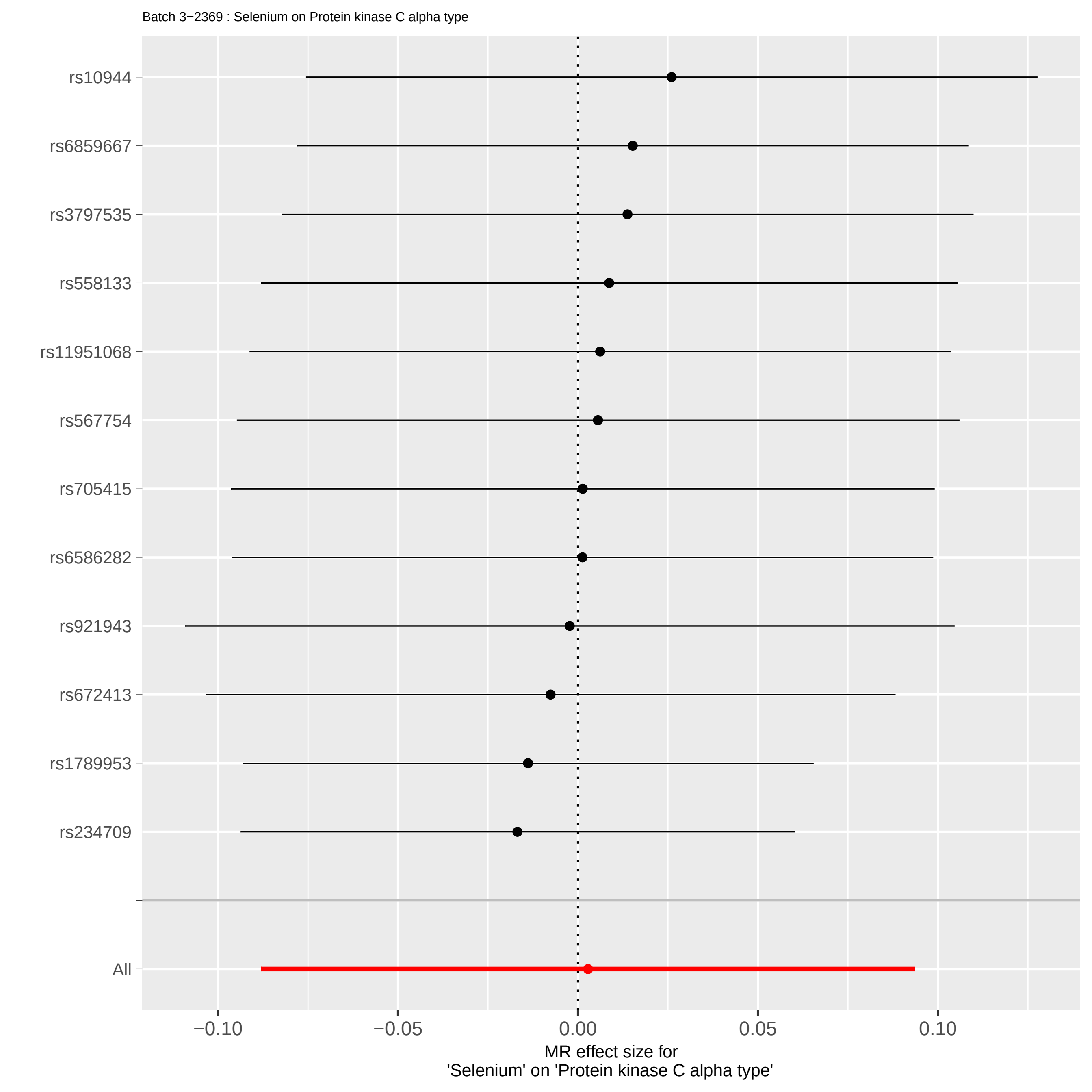

Batch 3−2369 : Selenium on Protein kinase C alpha type
rs10944
rs6859667
rs3797535
rs558133
rs11951068
rs567754
rs705415
rs6586282
rs921943
rs672413
rs1789953
rs234709
All
−0.10
−0.05
0.00
0.05
0.10
MR effect size for
'Selenium' on 'Protein kinase C alpha type'

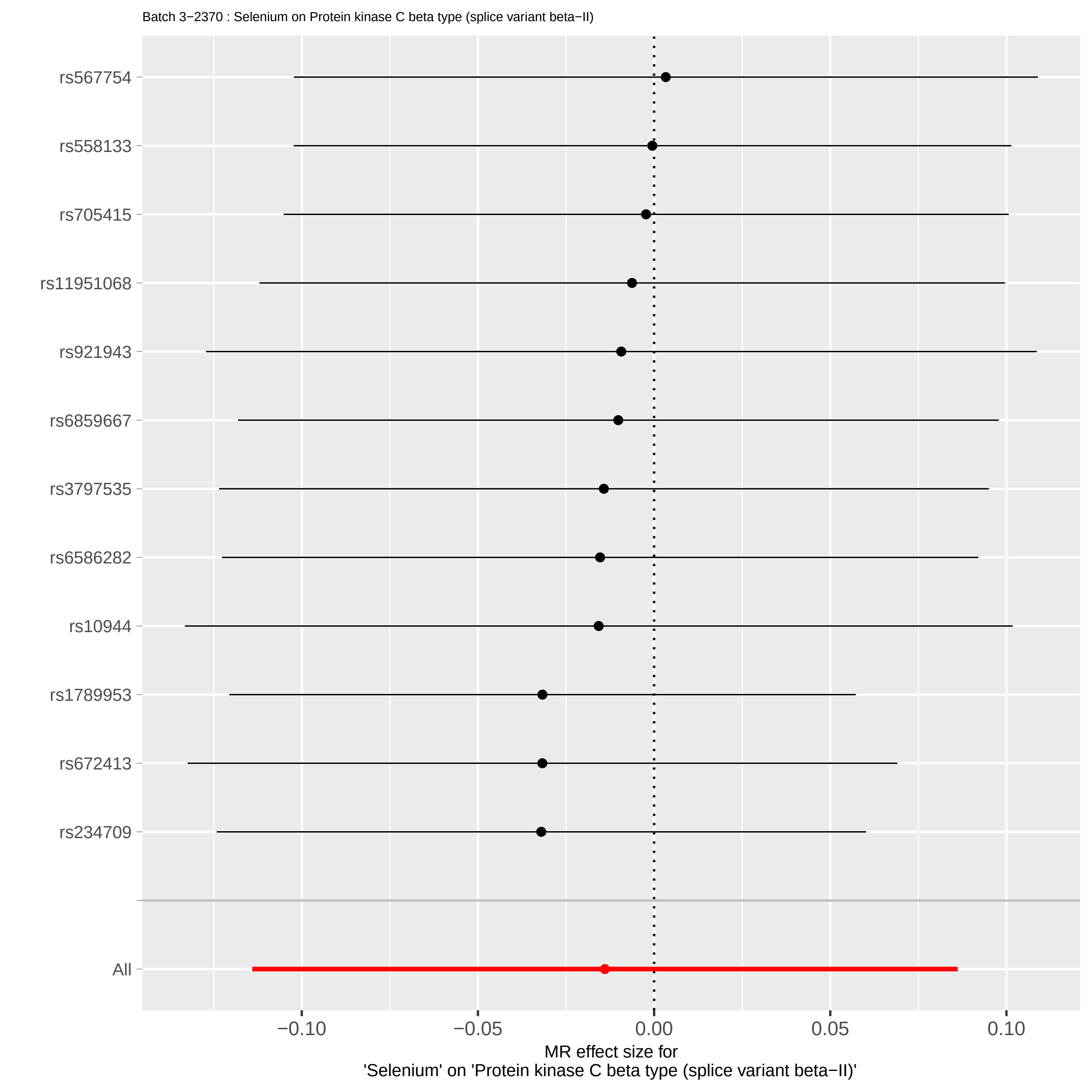

Batch 3−2370 : Selenium on Protein kinase C beta type (splice variant beta−II)
rs567754
rs558133
rs705415
rs11951068
rs921943
rs6859667
rs3797535
rs6586282
rs10944
rs1789953
rs672413
rs234709
All
−0.10
−0.05
0.00
0.05
0.10
MR effect size for
'Selenium' on 'Protein kinase C beta type (splice variant beta−II)'

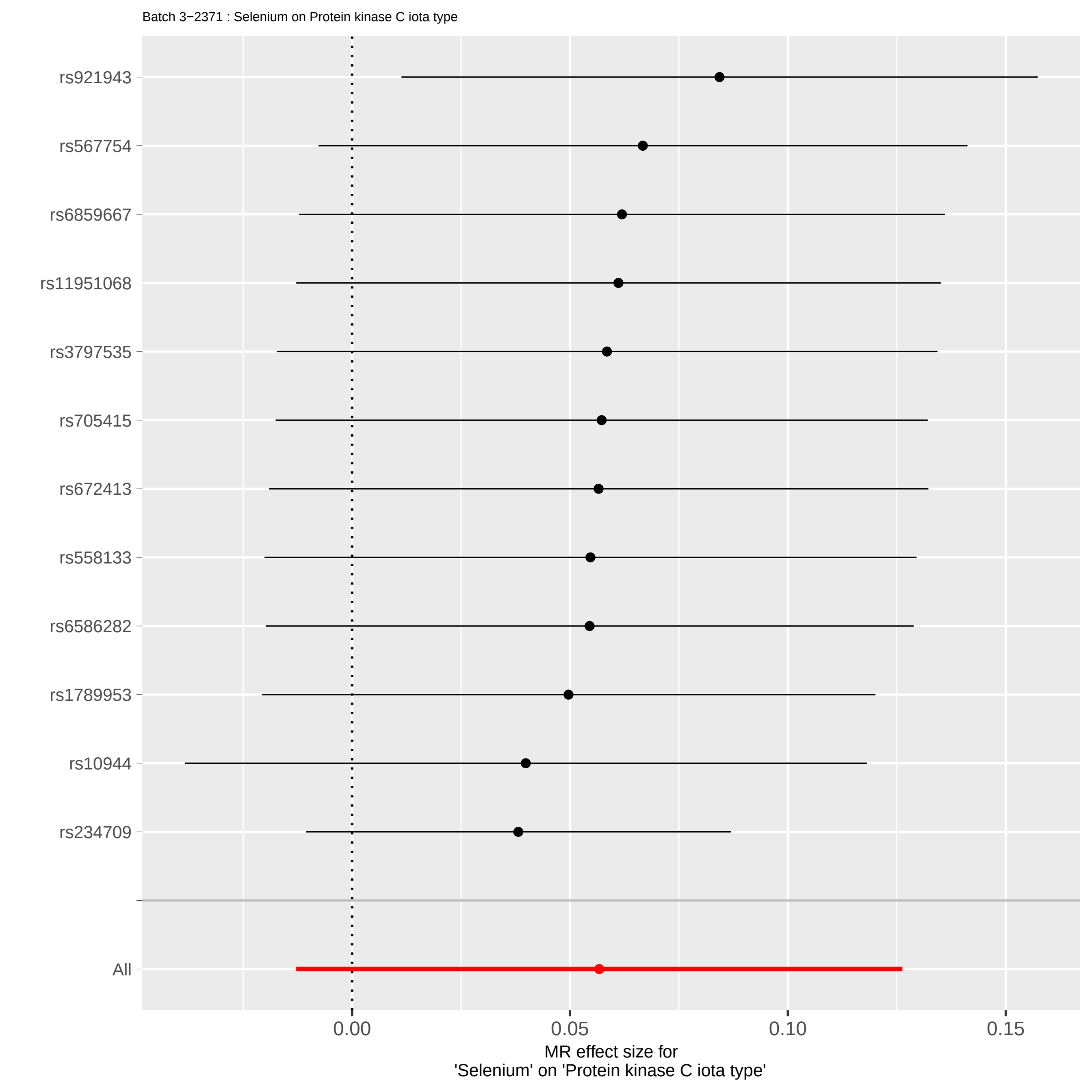
Batch 3−2371 : Selenium on Protein kinase C iota type
rs921943
rs567754
rs6859667
rs11951068
rs3797535
rs705415
rs672413
rs558133
rs6586282
rs1789953
rs10944
rs234709
All
0.00
0.05
0.10
0.15
MR effect size for
'Selenium' on 'Protein kinase C iota type'

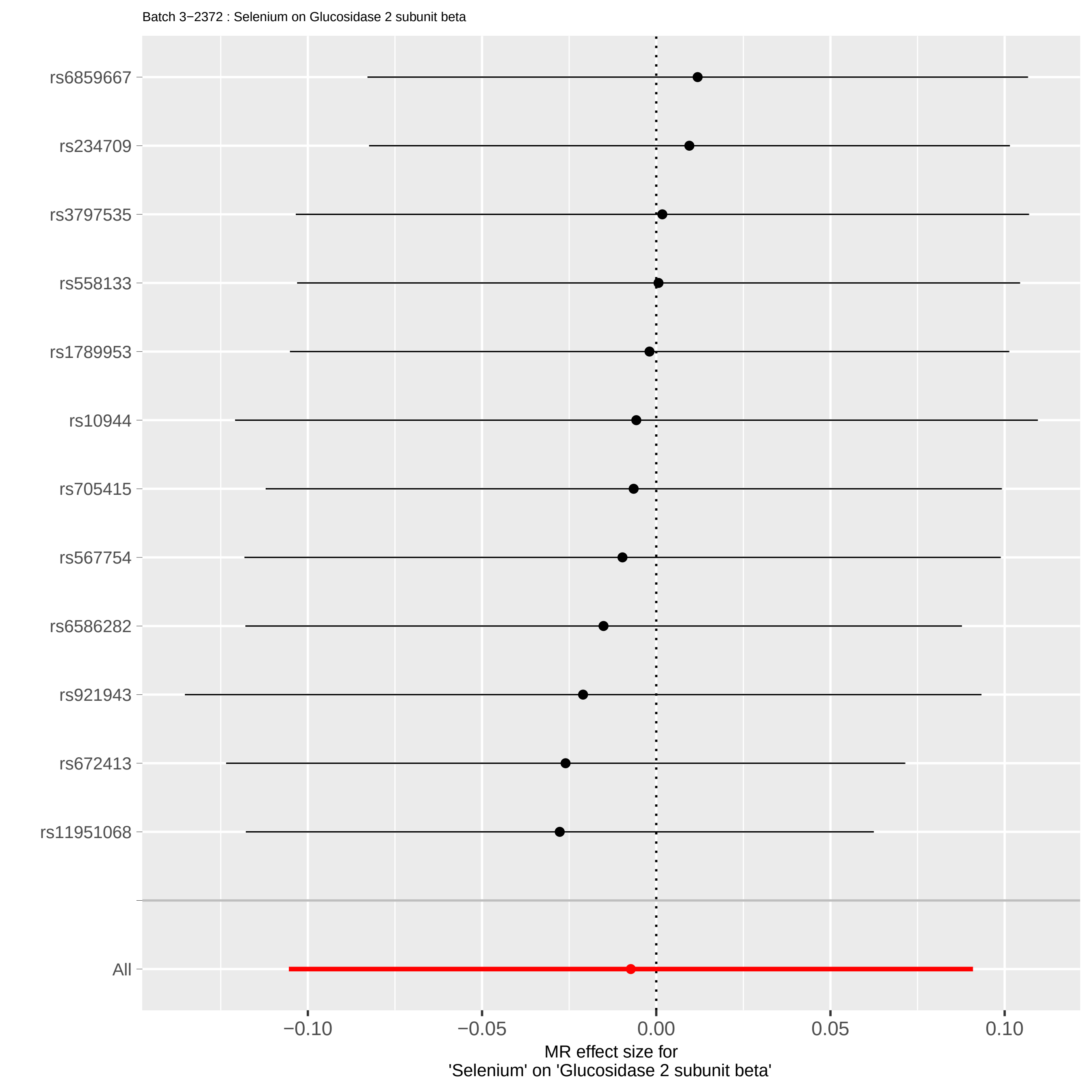

Batch 3−2372 : Selenium on Glucosidase 2 subunit beta
rs6859667
rs234709
rs3797535
rs558133
rs1789953
rs10944
rs705415
rs567754
rs6586282
rs921943
rs672413
rs11951068
All
−0.10
−0.05
0.00
0.05
0.10
MR effect size for
'Selenium' on 'Glucosidase 2 subunit beta'

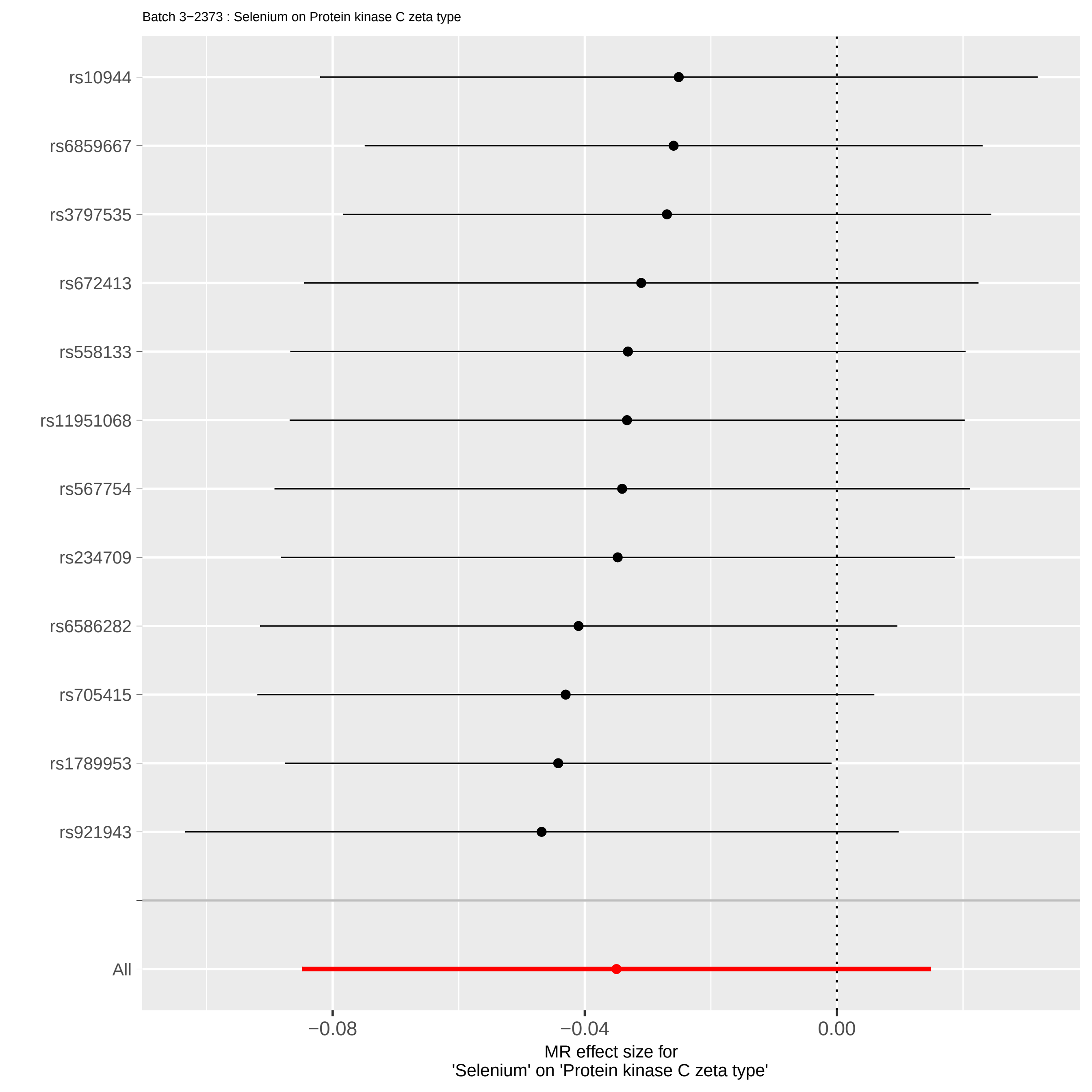

Batch 3−2373 : Selenium on Protein kinase C zeta type
rs10944
rs6859667
rs3797535
rs672413
rs558133
rs11951068
rs567754
rs234709
rs6586282
rs705415
rs1789953
rs921943
All
−0.08
−0.04
0.00
MR effect size for
'Selenium' on 'Protein kinase C zeta type'

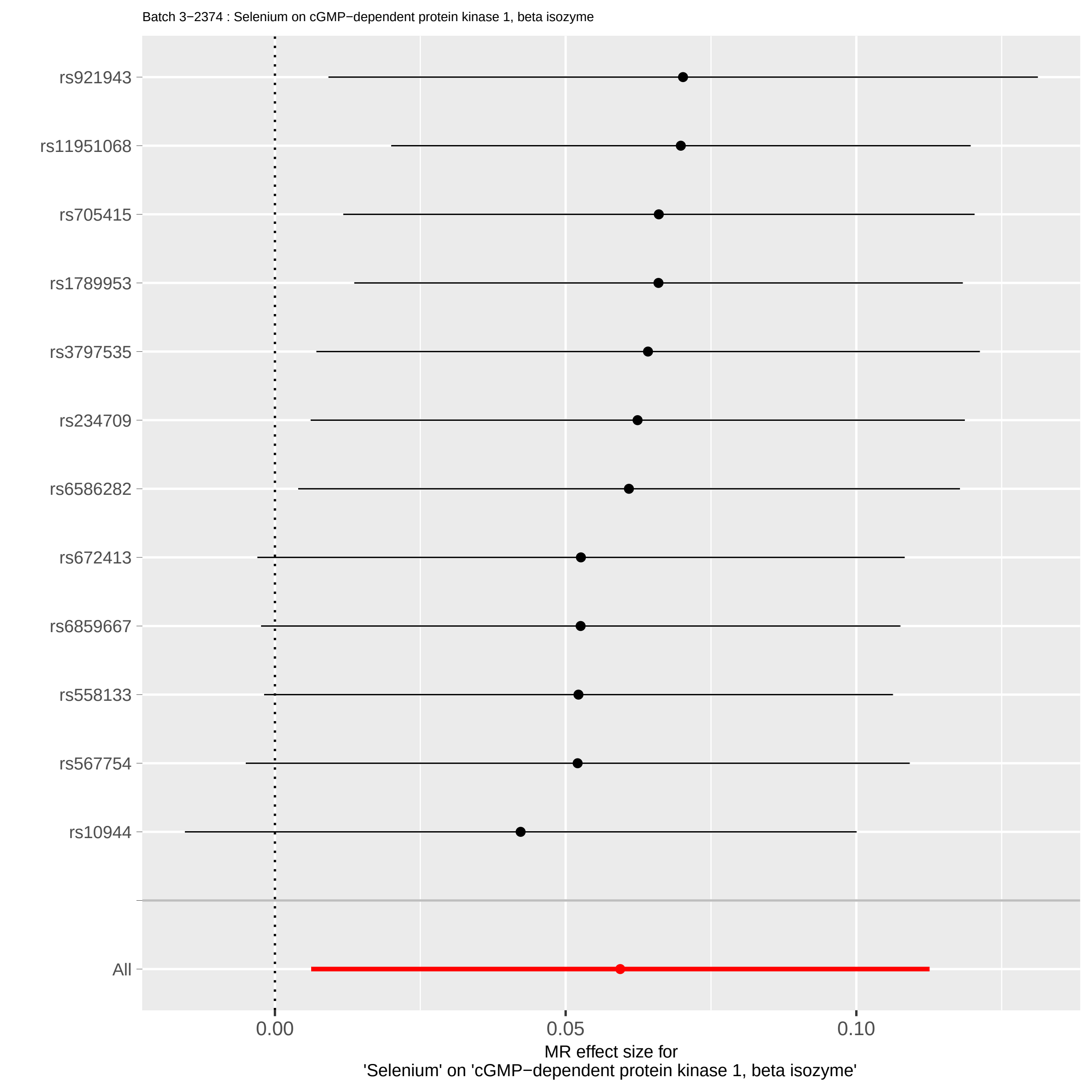

Batch 3−2374 : Selenium on cGMP−dependent protein kinase 1, beta isozyme
rs921943
rs11951068
rs705415
rs1789953
rs3797535
rs234709
rs6586282
rs672413
rs6859667
rs558133
rs567754
rs10944
All
0.00
0.05
0.10
MR effect size for
'Selenium' on 'cGMP−dependent protein kinase 1, beta isozyme'

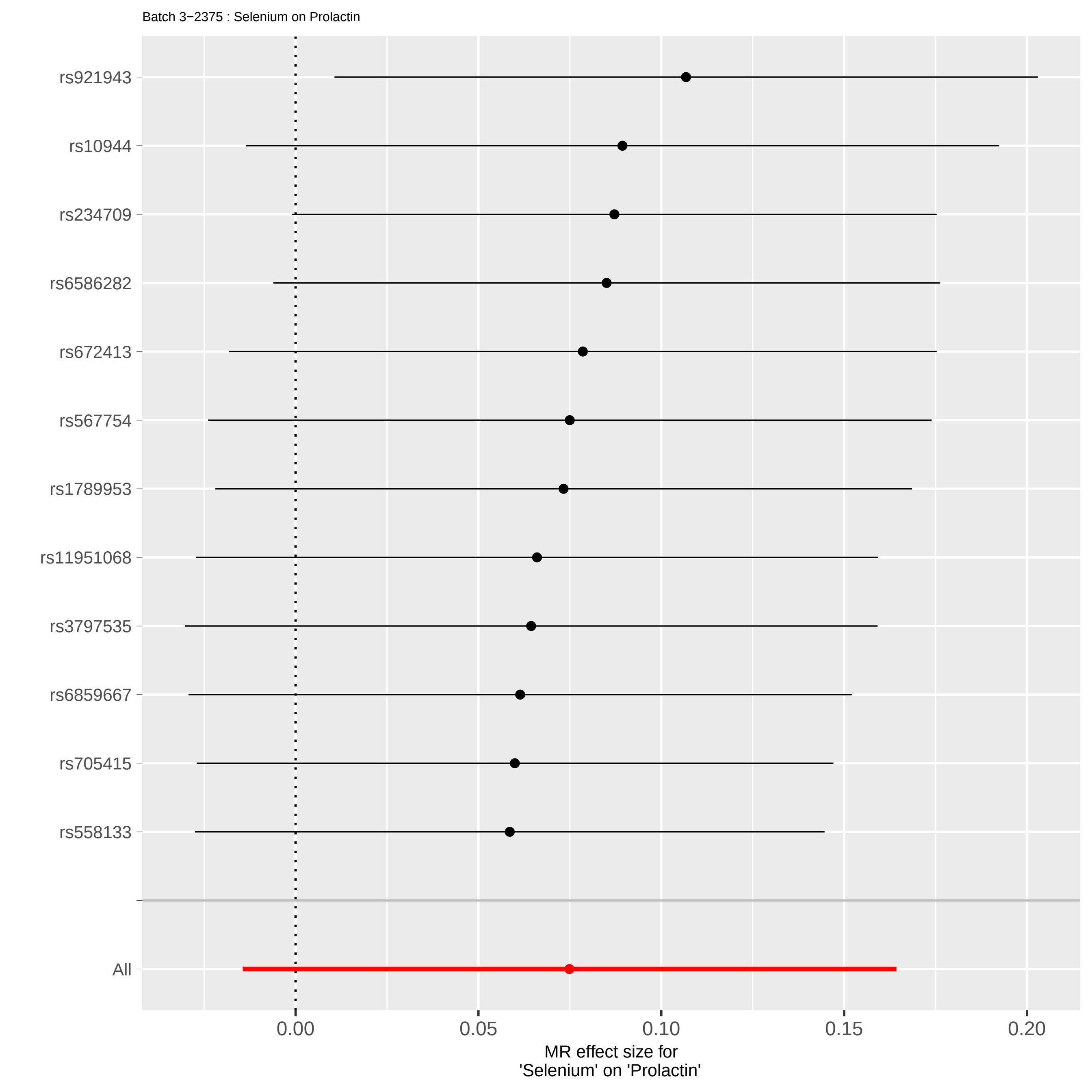

Batch 3−2375 : Selenium on Prolactin
rs921943
rs10944
rs234709
rs6586282
rs672413
rs567754
rs1789953
rs11951068
rs3797535
rs6859667
rs705415
rs558133
All
0.00
0.05
0.10
0.15
0.20
MR effect size for
'Selenium' on 'Prolactin'

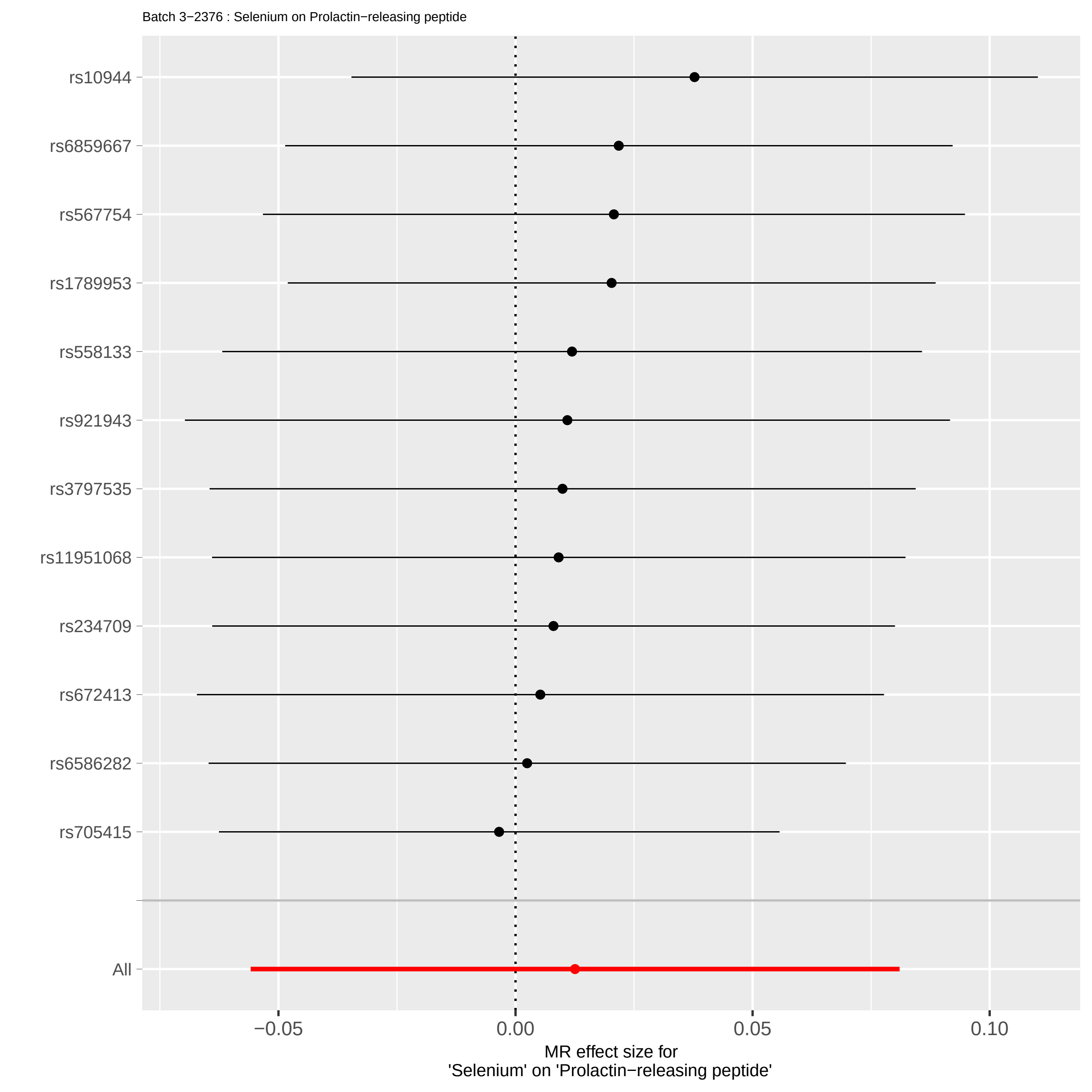

Batch 3−2376 : Selenium on Prolactin−releasing peptide
rs10944
rs6859667
rs567754
rs1789953
rs558133
rs921943
rs3797535
rs11951068
rs234709
rs672413
rs6586282
rs705415
All
−0.05
0.00
0.05
0.10
MR effect size for
'Selenium' on 'Prolactin−releasing peptide'

Batch 3−2377 : Selenium on Prolactin receptor

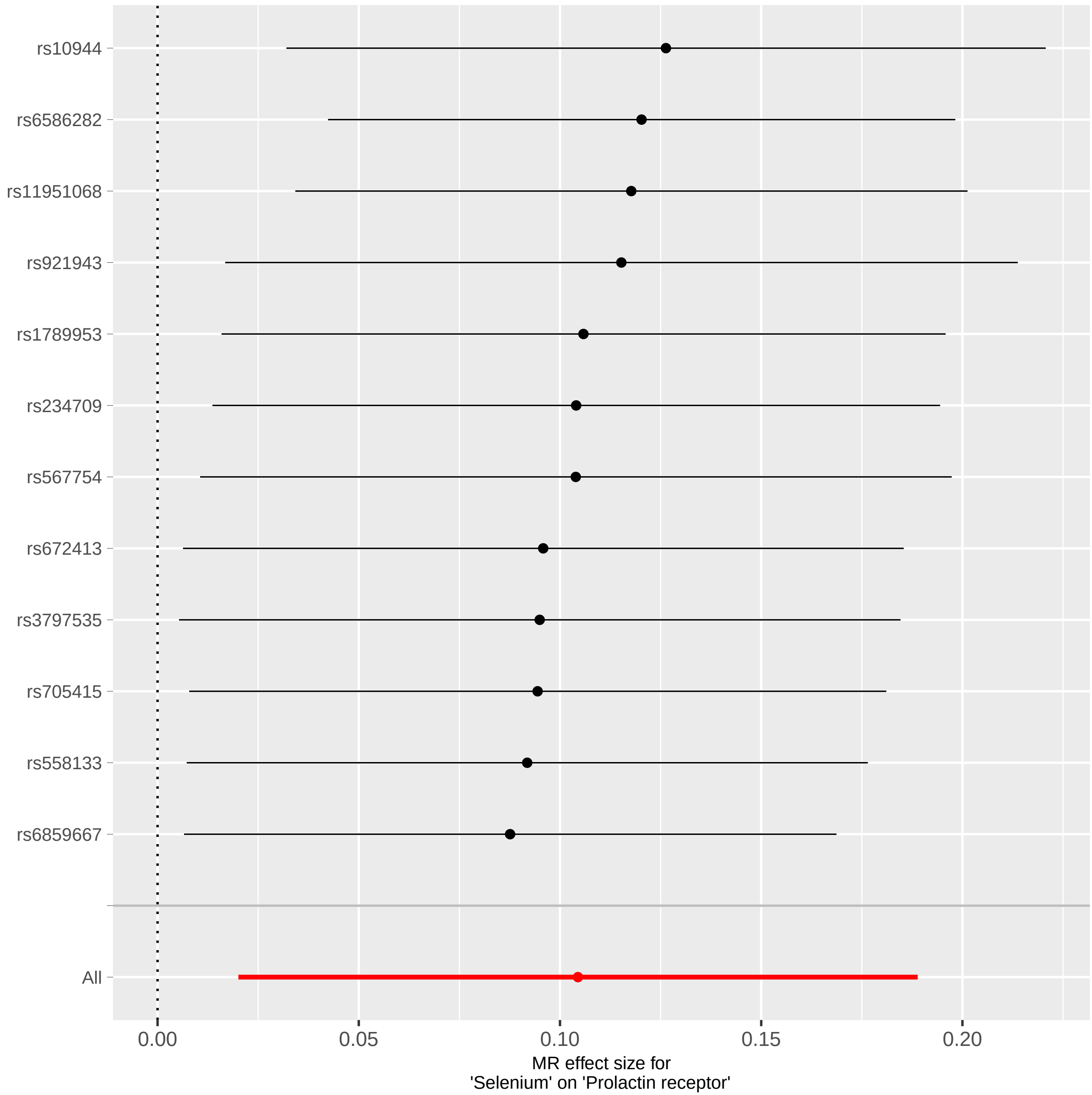

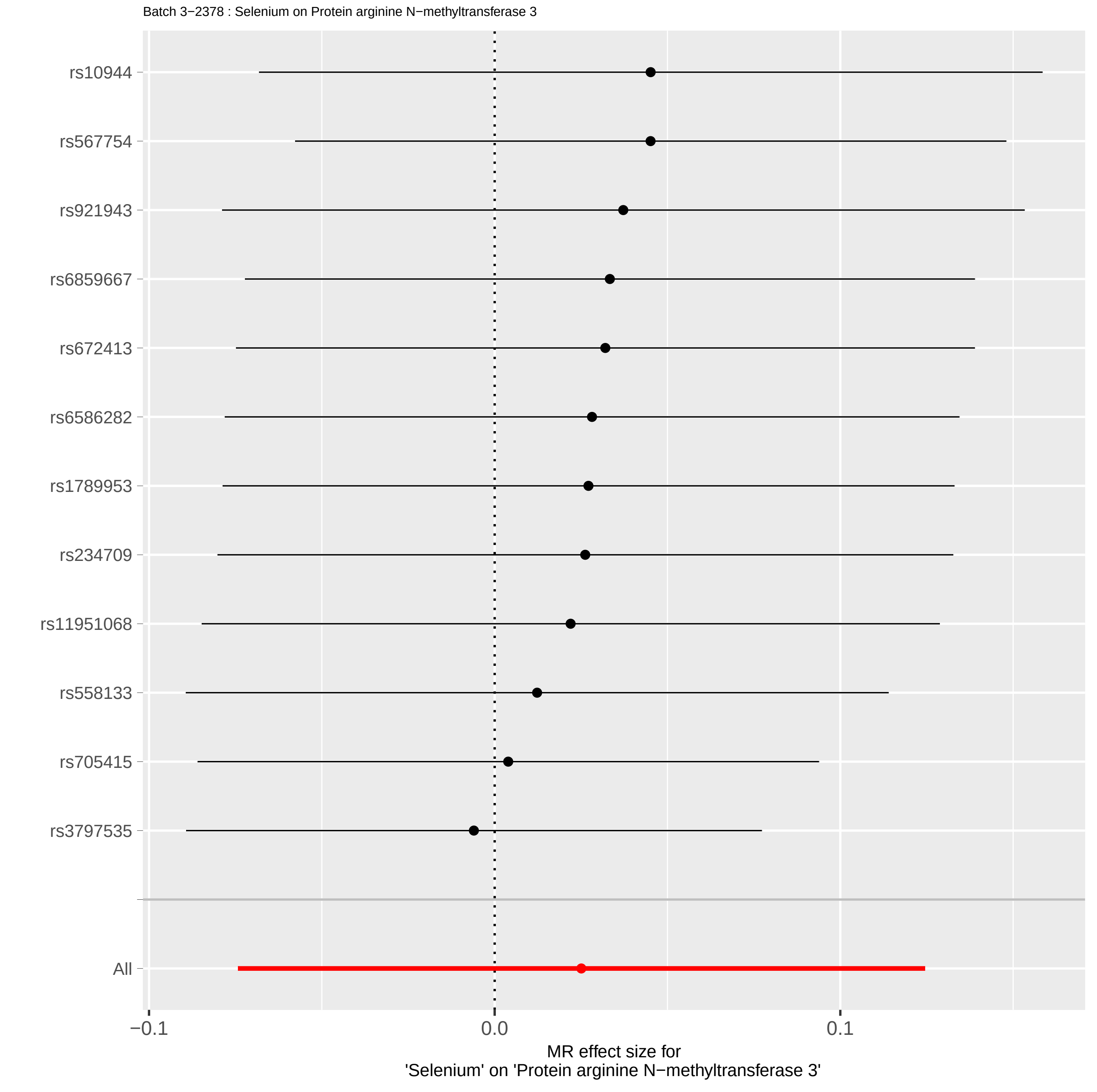

Batch 3−2378 : Selenium on Protein arginine N−methyltransferase 3
rs10944
rs567754
rs921943
rs6859667
rs672413
rs6586282
rs1789953
rs234709
rs11951068
rs558133
rs705415
rs3797535
All
−0.1
0.0
0.1
MR effect size for
'Selenium' on 'Protein arginine N−methyltransferase 3'

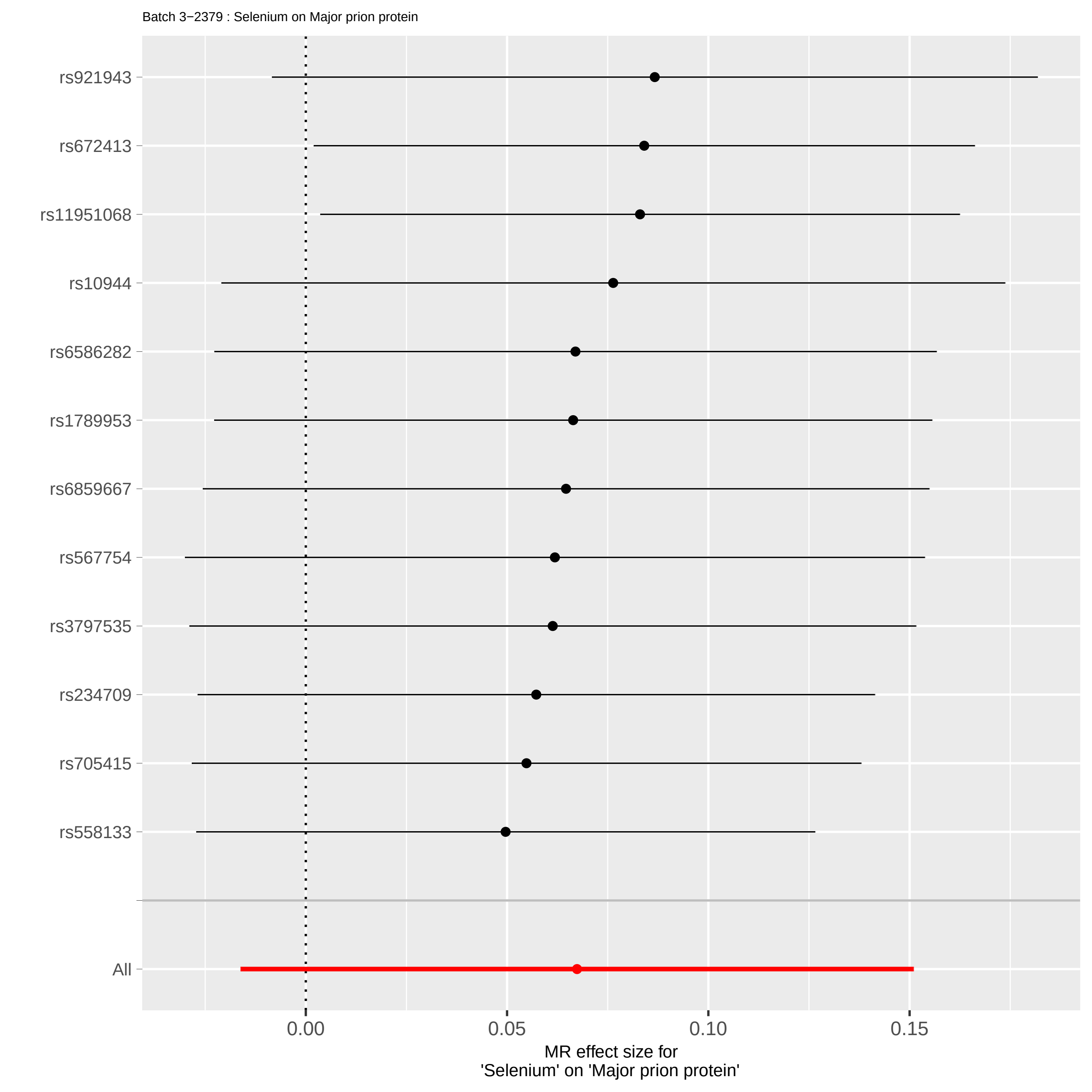

Batch 3−2379 : Selenium on Major prion protein
rs921943
rs672413
rs11951068
rs10944
rs6586282
rs1789953
rs6859667
rs567754
rs3797535
rs234709
rs705415
rs558133
All
0.00
0.05
0.10
0.15
MR effect size for
'Selenium' on 'Major prion protein'

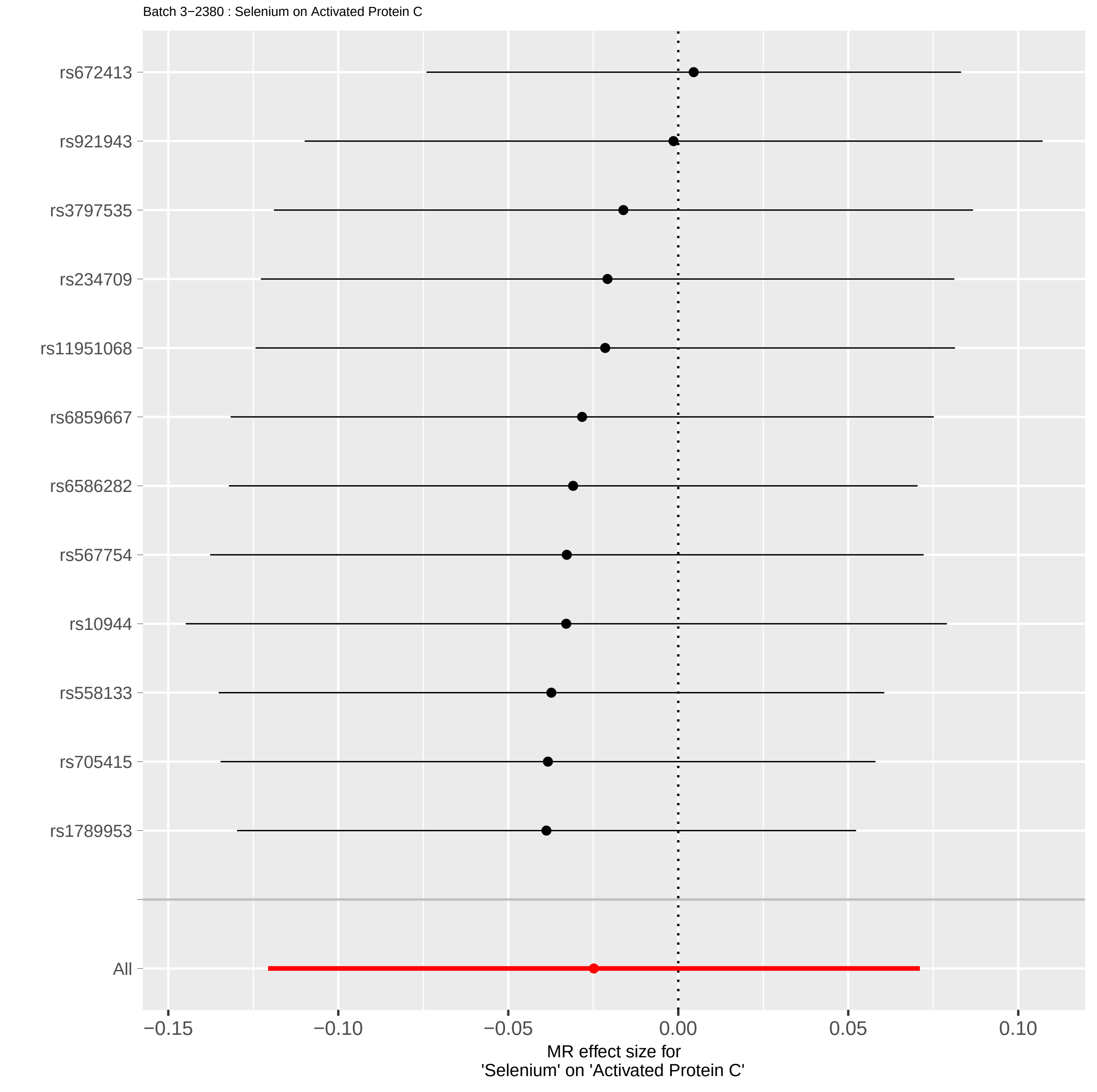

Batch 3−2380 : Selenium on Activated Protein C
rs672413
rs921943
rs3797535
rs234709
rs11951068
rs6859667
rs6586282
rs567754
rs10944
rs558133
rs705415
rs1789953
All
−0.15
−0.10
−0.05
0.00
0.05
0.10
MR effect size for
'Selenium' on 'Activated Protein C'

Batch 3−2381 : Selenium on Activated Protein C

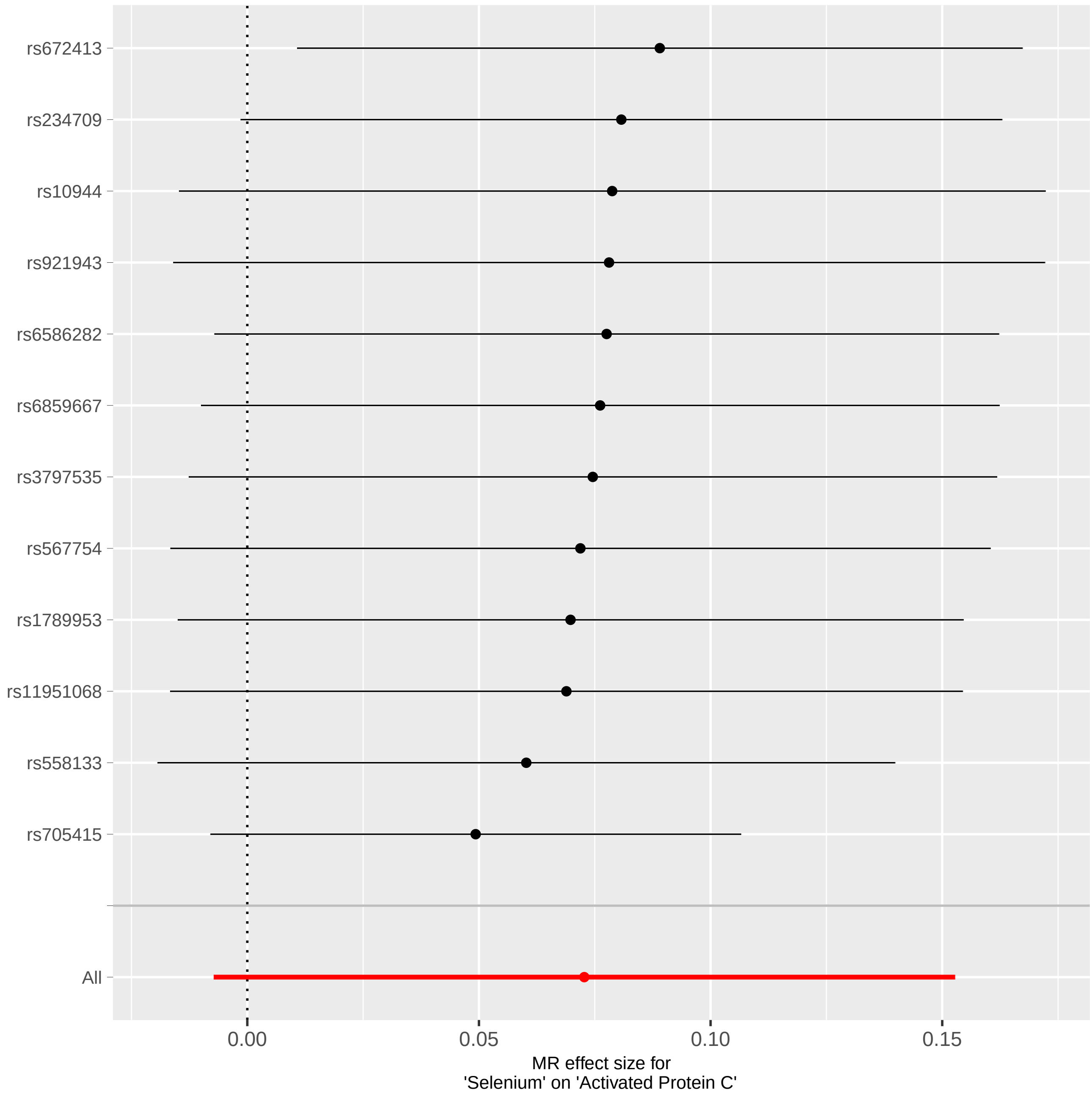

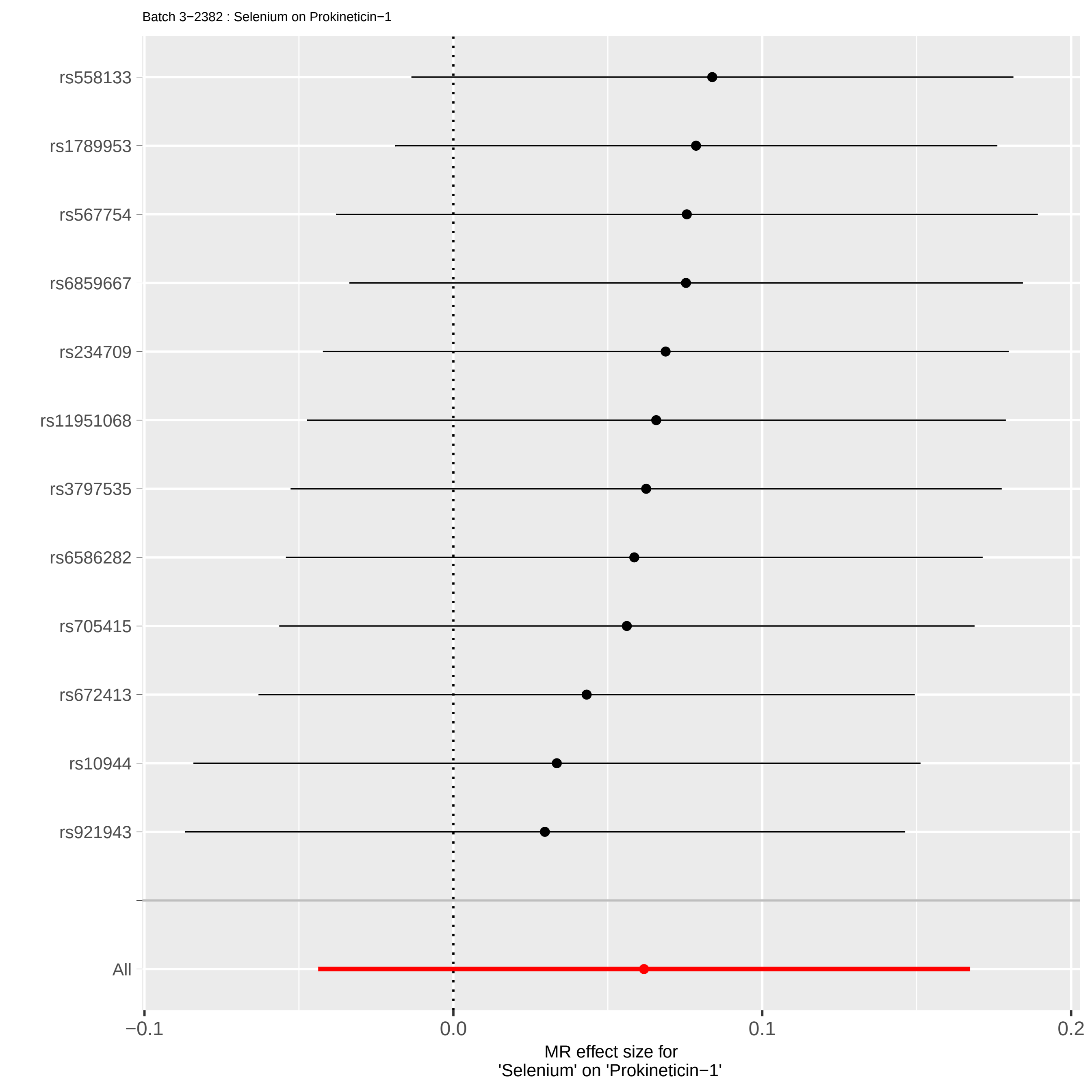

Batch 3−2382 : Selenium on Prokineticin−1
rs558133
rs1789953
rs567754
rs6859667
rs234709
rs11951068
rs3797535
rs6586282
rs705415
rs672413
rs10944
rs921943
All
−0.1
0.0
0.1
0.2
MR effect size for
'Selenium' on 'Prokineticin−1'

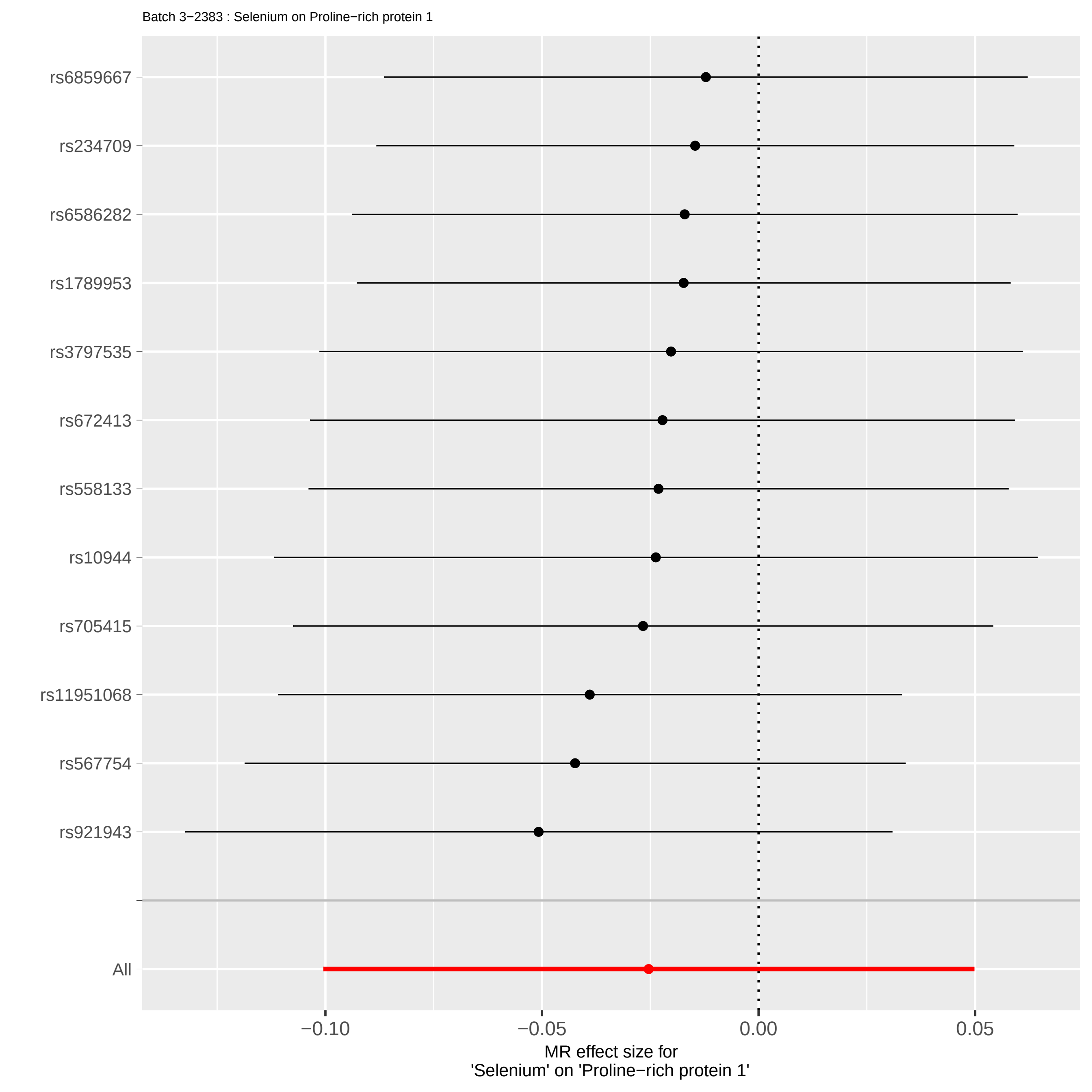

Batch 3−2383 : Selenium on Proline−rich protein 1
rs6859667
rs234709
rs6586282
rs1789953
rs3797535
rs672413
rs558133
rs10944
rs705415
rs11951068
rs567754
rs921943
All
−0.10
−0.05
0.00
0.05
MR effect size for
'Selenium' on 'Proline−rich protein 1'

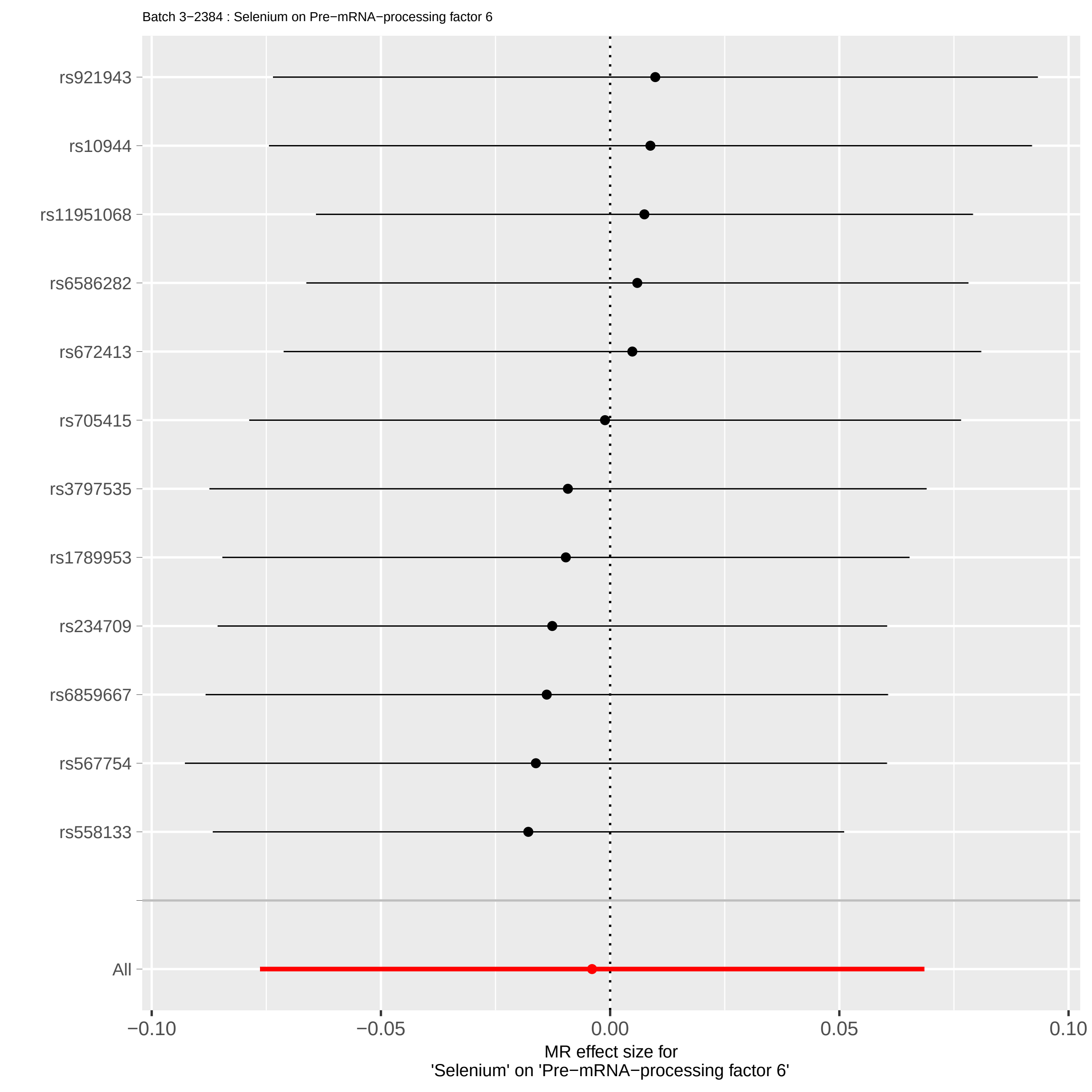

Batch 3−2384 : Selenium on Pre−mRNA−processing factor 6
rs921943
rs10944
rs11951068
rs6586282
rs672413
rs705415
rs3797535
rs1789953
rs234709
rs6859667
rs567754
rs558133
All
−0.10
−0.05
0.00
0.05
0.10
MR effect size for
'Selenium' on 'Pre−mRNA−processing factor 6'

Batch 3−2385 : Selenium on Phosphoribosyl pyrophosphate synthase−associated protein 1

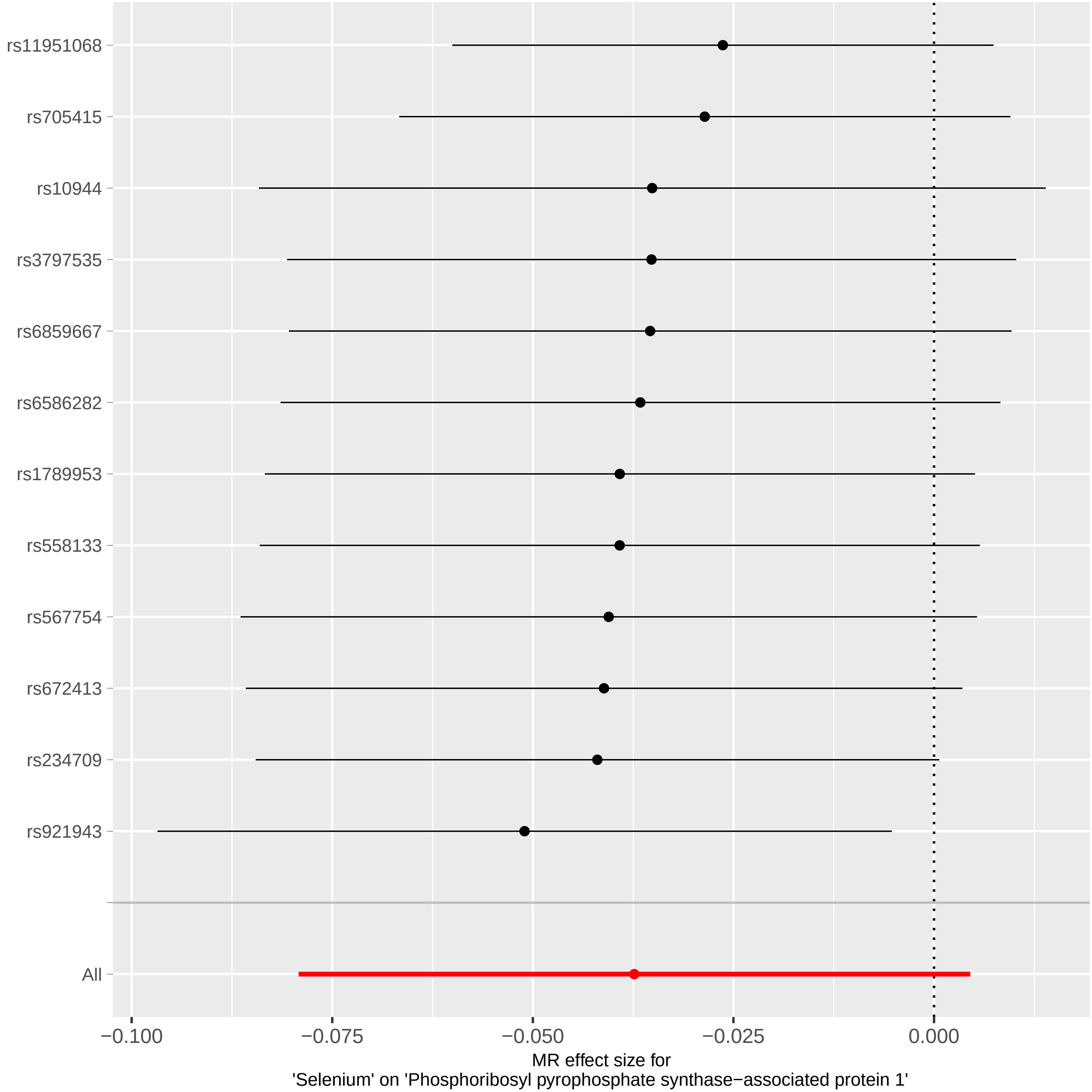

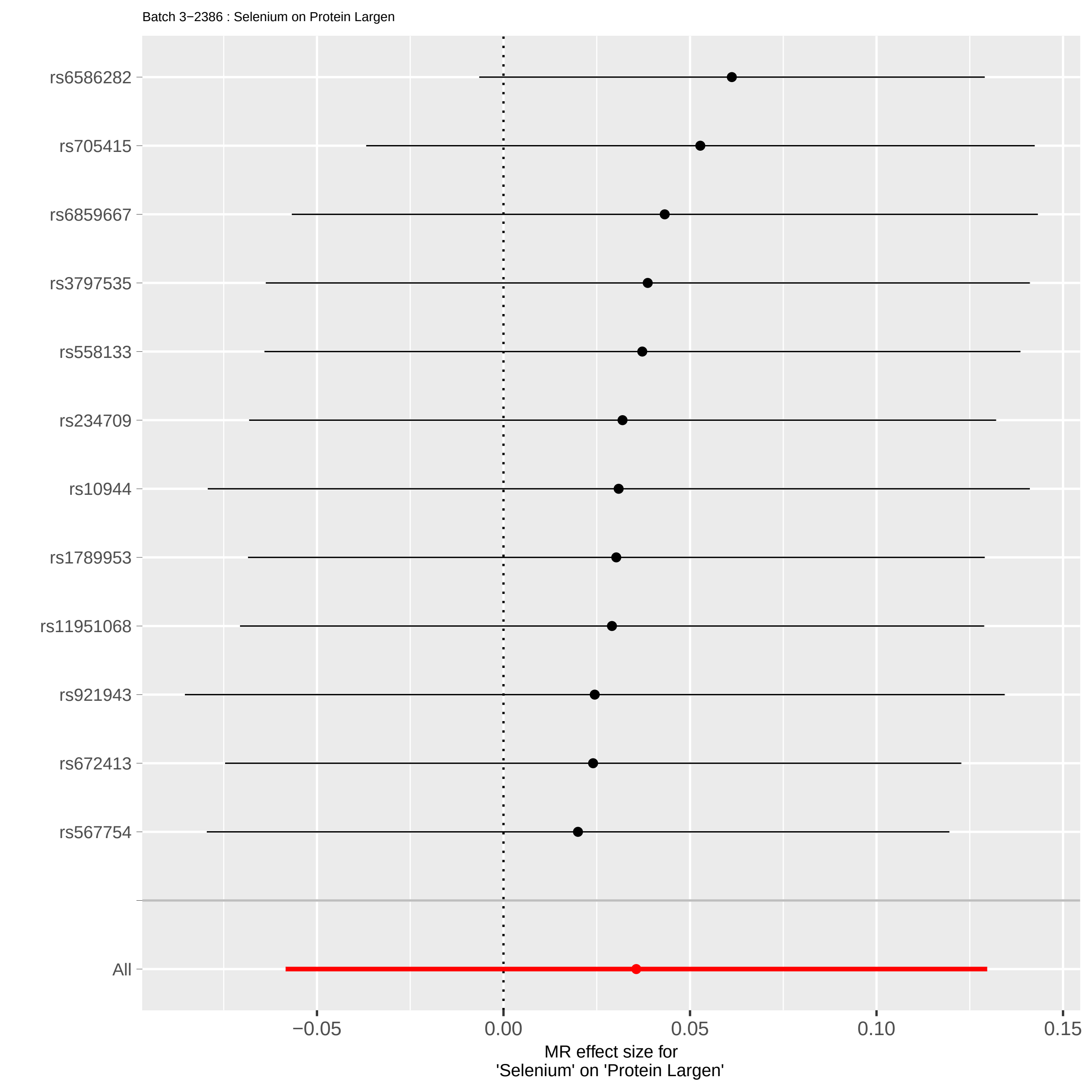

Batch 3−2386 : Selenium on Protein Largen
rs6586282
rs705415
rs6859667
rs3797535
rs558133
rs234709
rs10944
rs1789953
rs11951068
rs921943
rs672413
rs567754
All
−0.05
0.00
0.05
0.10
0.15
MR effect size for
'Selenium' on 'Protein Largen'

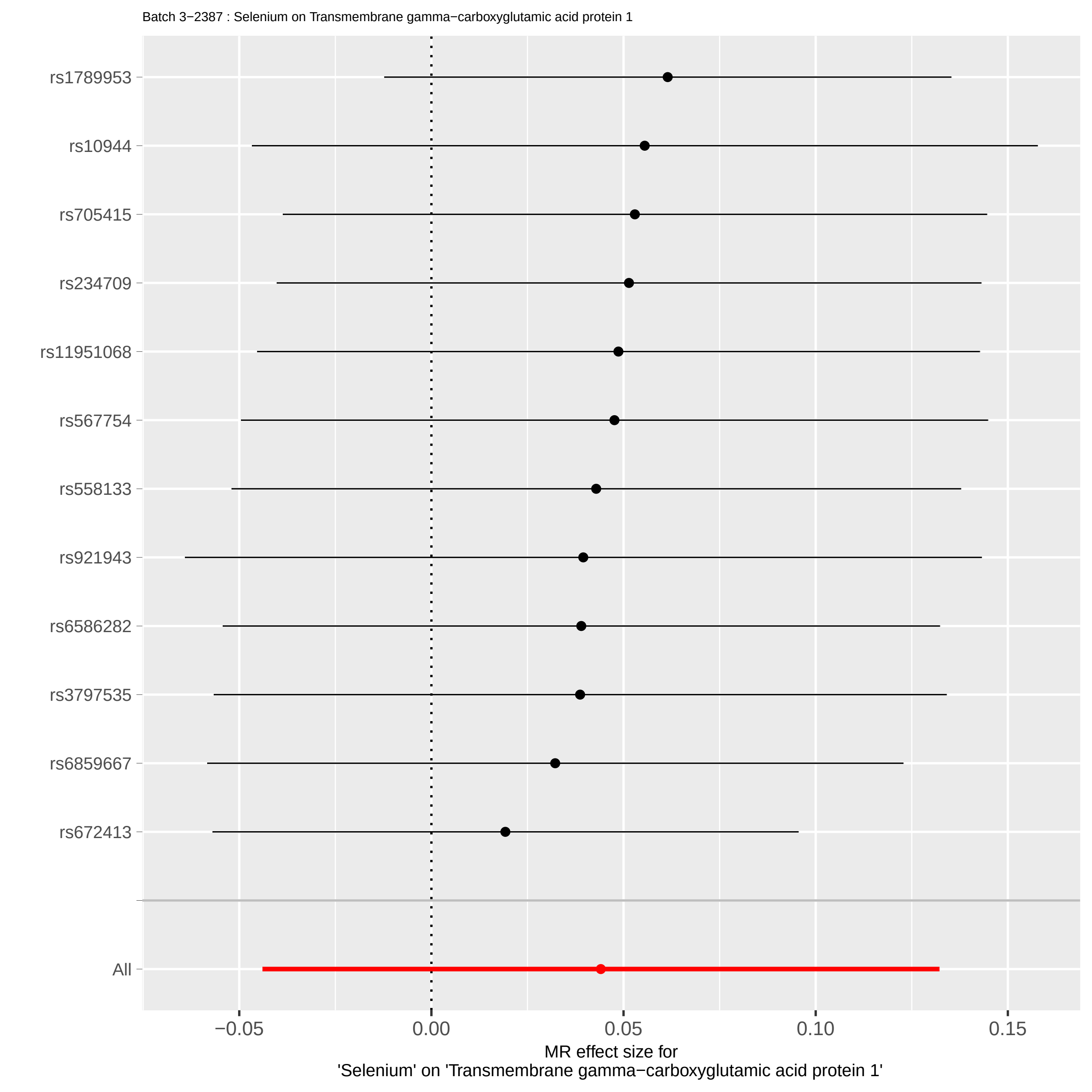
Batch 3−2387 : Selenium on Transmembrane gamma−carboxyglutamic acid protein 1
rs1789953
rs10944
rs705415
rs234709
rs11951068
rs567754
rs558133
rs921943
rs6586282
rs3797535
rs6859667
rs672413
All
−0.05
0.00
0.05
0.10
0.15
MR effect size for
'Selenium' on 'Transmembrane gamma−carboxyglutamic acid protein 1'

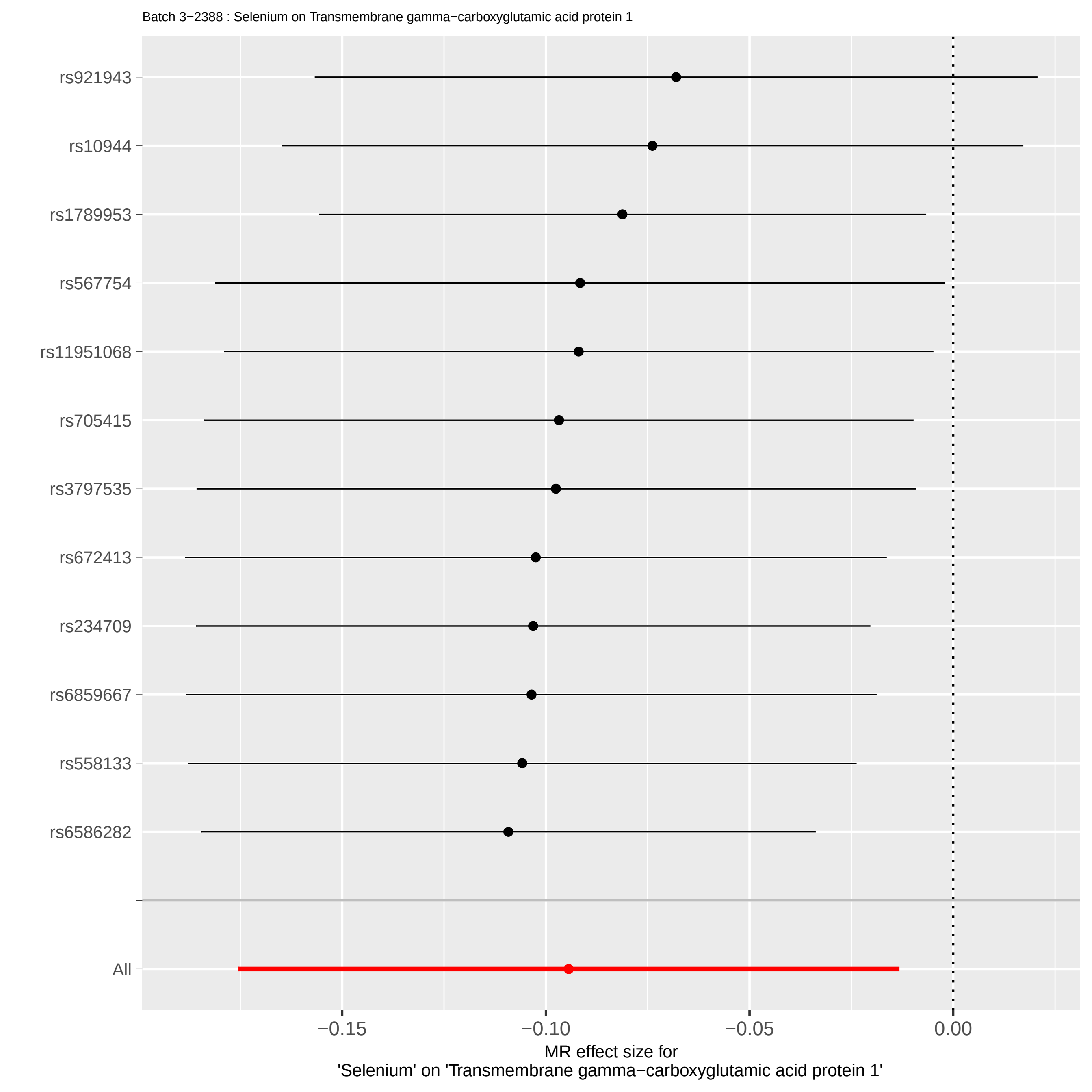

Batch 3−2388 : Selenium on Transmembrane gamma−carboxyglutamic acid protein 1
rs921943
rs10944
rs1789953
rs567754
rs11951068
rs705415
rs3797535
rs672413
rs234709
rs6859667
rs558133
rs6586282
All
−0.15
−0.10
−0.05
0.00
MR effect size for
'Selenium' on 'Transmembrane gamma−carboxyglutamic acid protein 1'

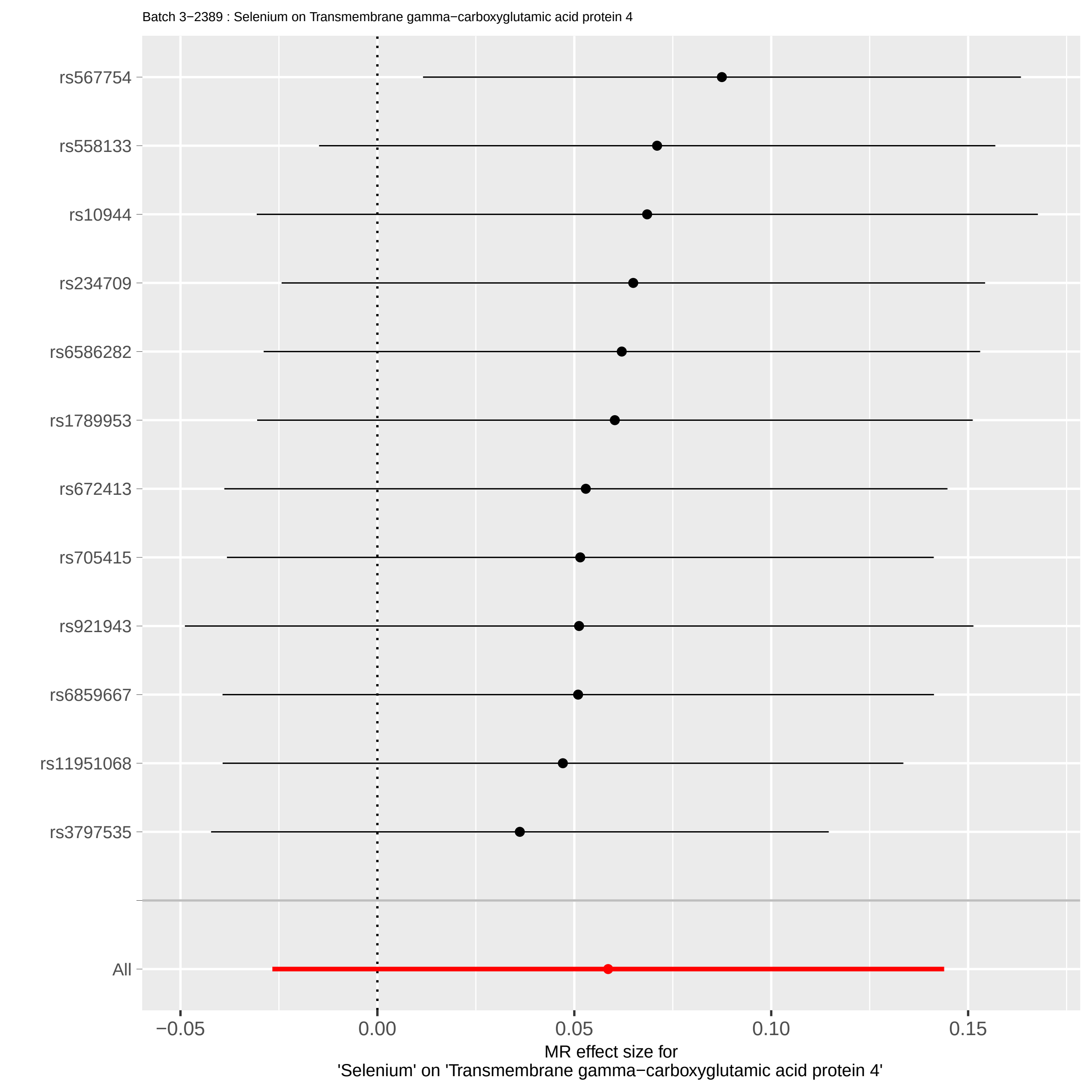
Batch 3−2389 : Selenium on Transmembrane gamma−carboxyglutamic acid protein 4
rs567754
rs558133
rs10944
rs234709
rs6586282
rs1789953
rs672413
rs705415
rs921943
rs6859667
rs11951068
rs3797535
All
−0.05
0.00
0.05
0.10
0.15
MR effect size for
'Selenium' on 'Transmembrane gamma−carboxyglutamic acid protein 4'

Batch 3−2390 : Selenium on Trypsin−1

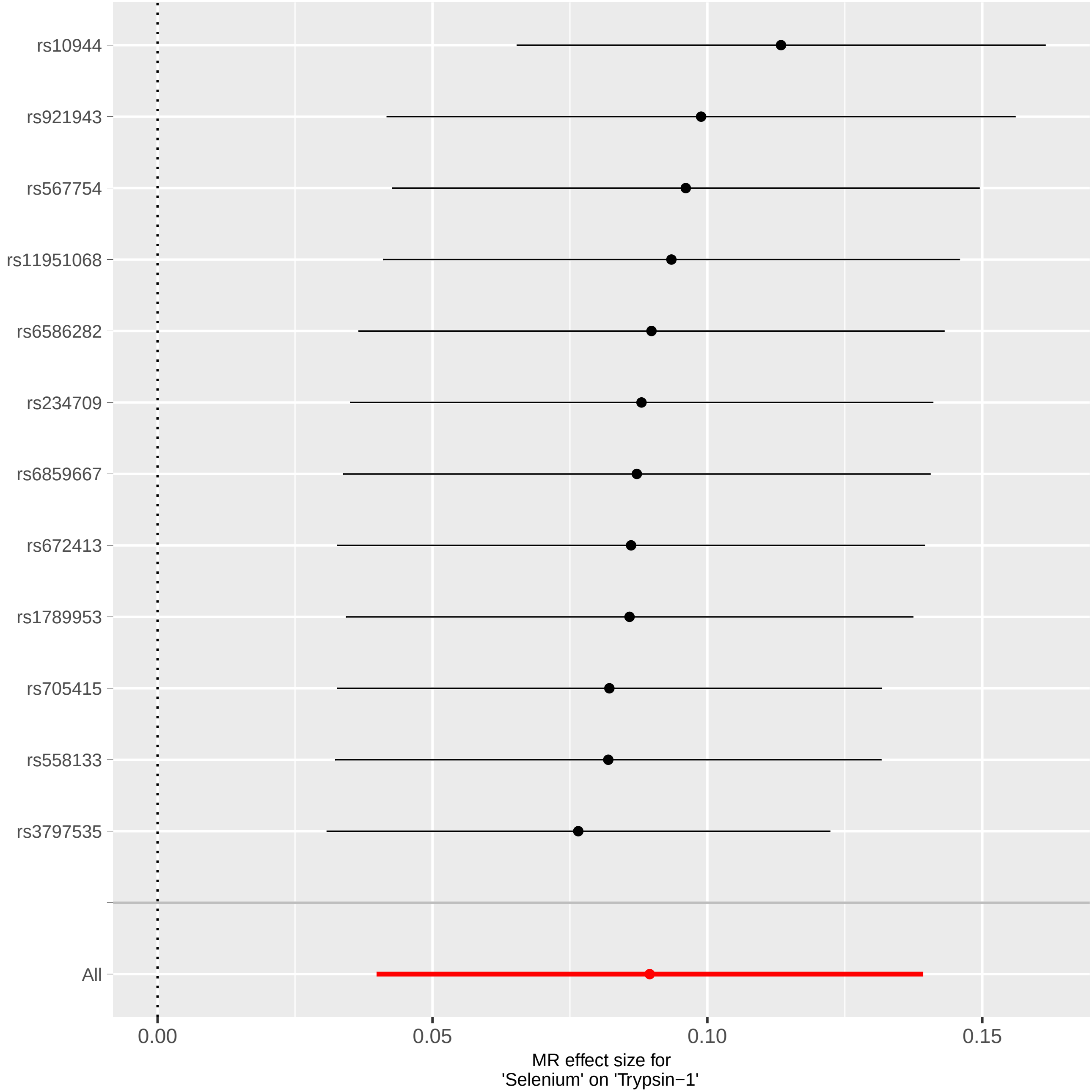

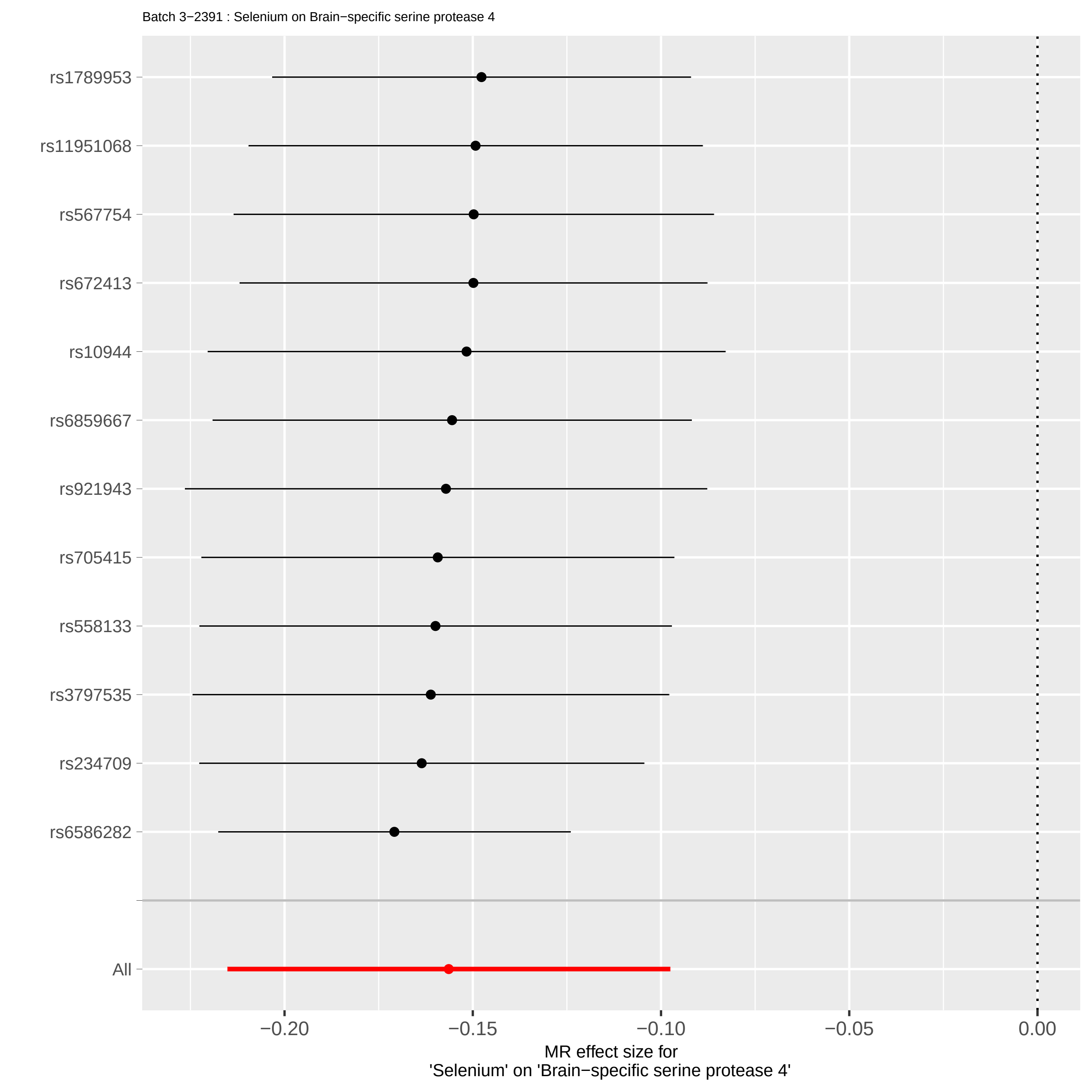

Batch 3−2391 : Selenium on Brain−specific serine protease 4
rs1789953
rs11951068
rs567754
rs672413
rs10944
rs6859667
rs921943
rs705415
rs558133
rs3797535
rs234709
rs6586282
All
−0.20
−0.15
−0.10
−0.05
0.00
MR effect size for
'Selenium' on 'Brain−specific serine protease 4'

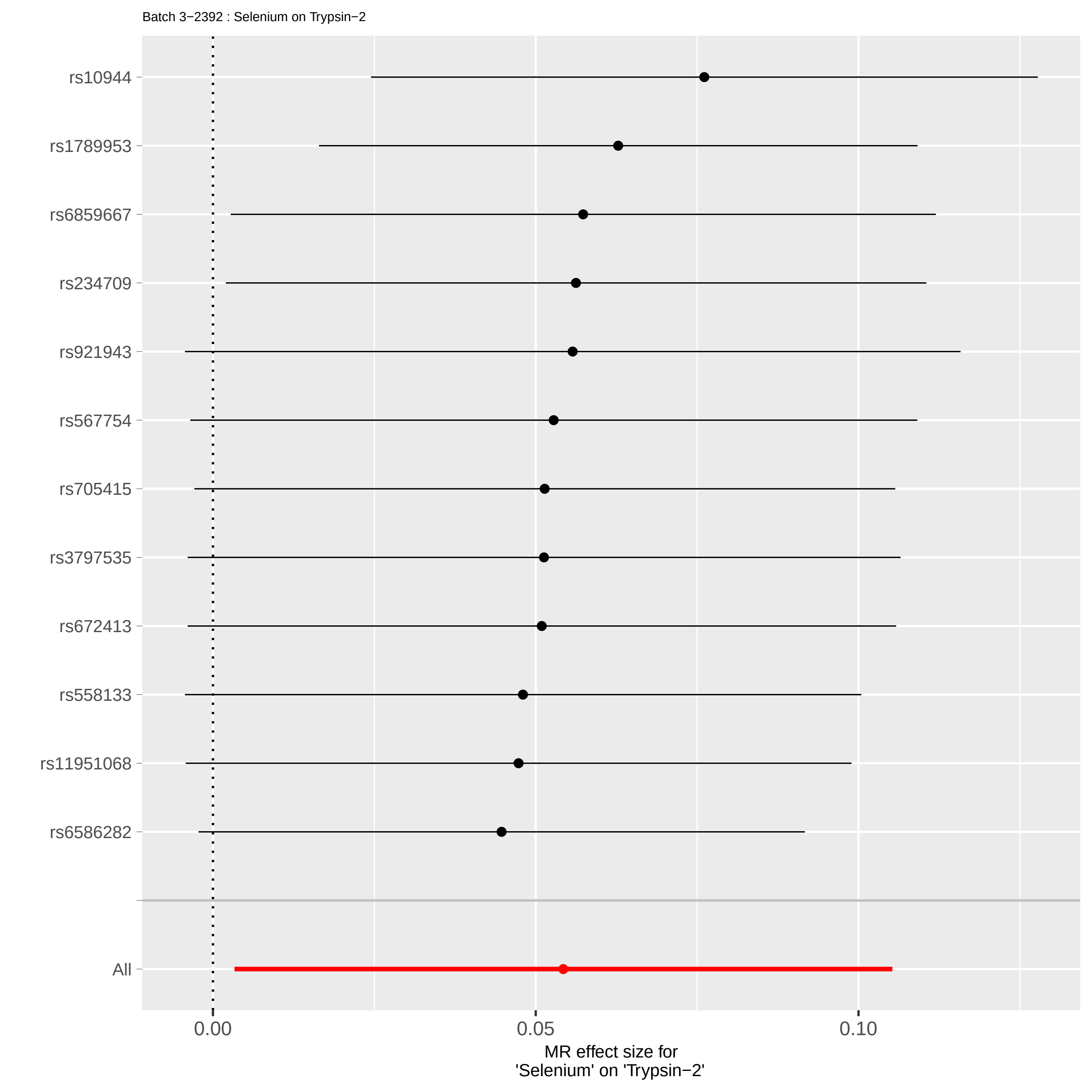

Batch 3−2392 : Selenium on Trypsin−2
rs10944
rs1789953
rs6859667
rs234709
rs921943
rs567754
rs705415
rs3797535
rs672413
rs558133
rs11951068
rs6586282
All
0.00
0.05
0.10
MR effect size for
'Selenium' on 'Trypsin−2'

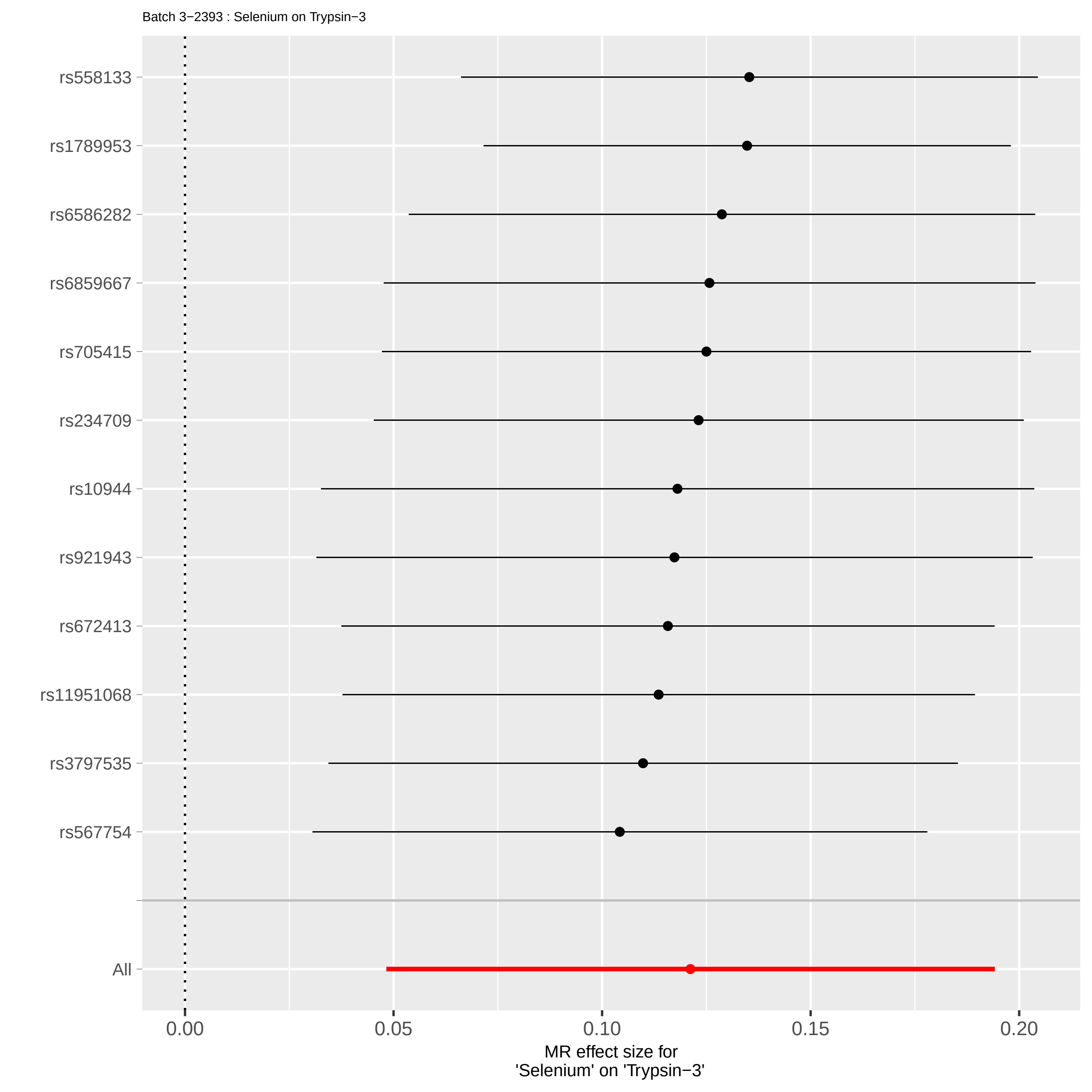

Batch 3−2393 : Selenium on Trypsin−3
rs558133
rs1789953
rs6586282
rs6859667
rs705415
rs234709
rs10944
rs921943
rs672413
rs11951068
rs3797535
rs567754
All
0.00
0.05
0.10
0.15
0.20
MR effect size for
'Selenium' on 'Trypsin−3'

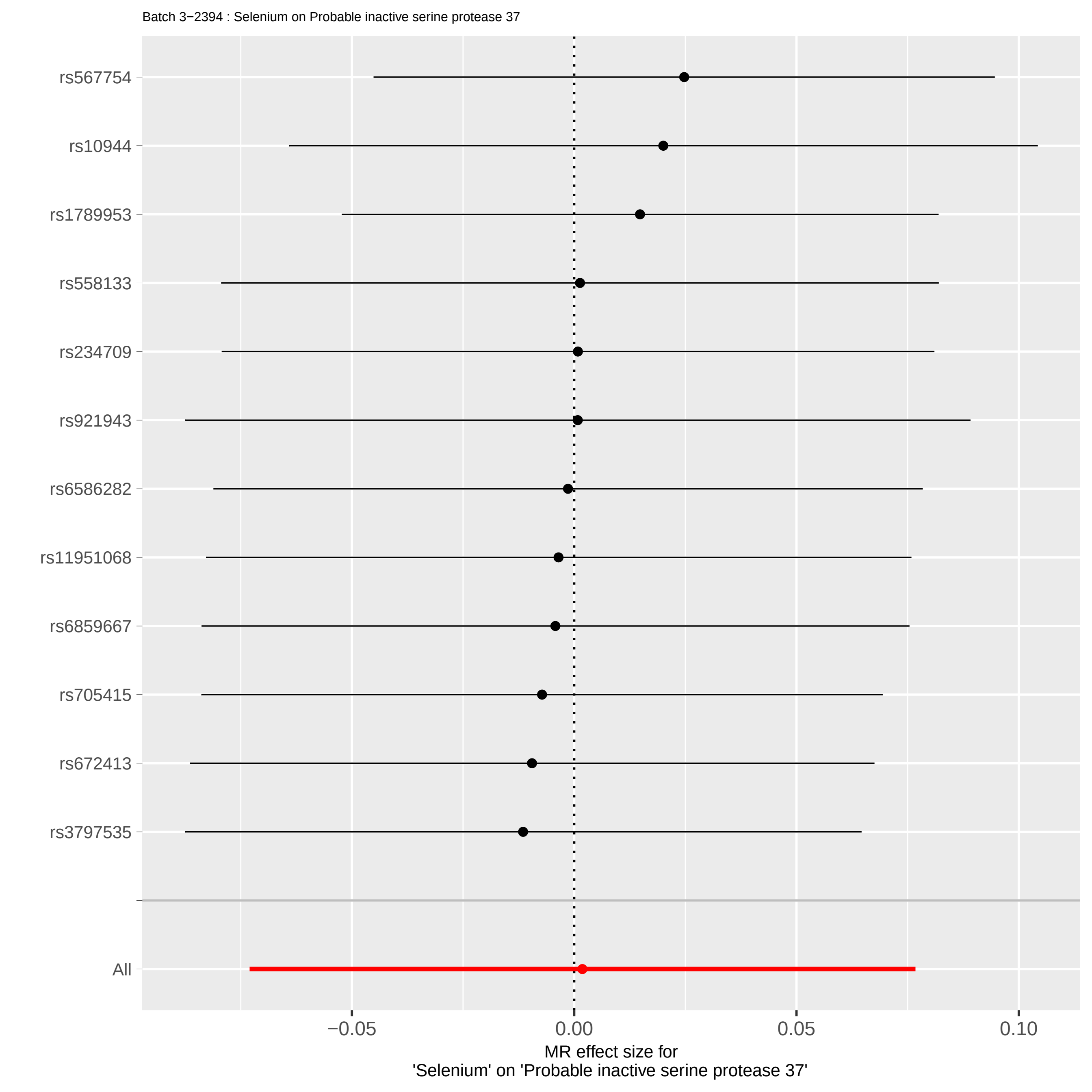

Batch 3−2394 : Selenium on Probable inactive serine protease 37
rs567754
rs10944
rs1789953
rs558133
rs234709
rs921943
rs6586282
rs11951068
rs6859667
rs705415
rs672413
rs3797535
All
−0.05
0.00
0.05
0.10
MR effect size for
'Selenium' on 'Probable inactive serine protease 37'

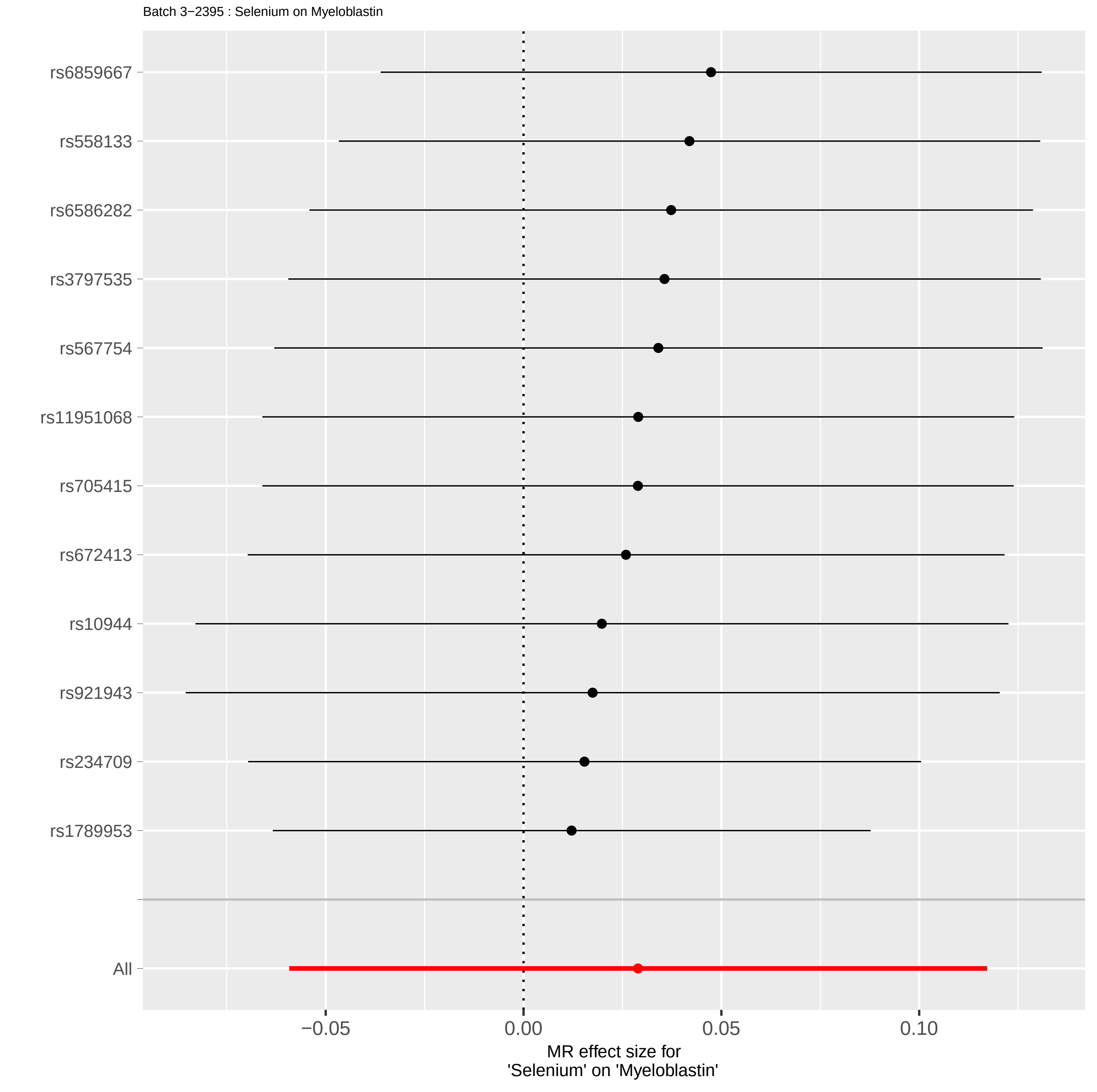
Batch 3−2395 : Selenium on Myeloblastin
rs6859667
rs558133
rs6586282
rs3797535
rs567754
rs11951068
rs705415
rs672413
rs10944
rs921943
rs234709
rs1789953
All
−0.05
0.00
0.05
0.10
MR effect size for
'Selenium' on 'Myeloblastin'

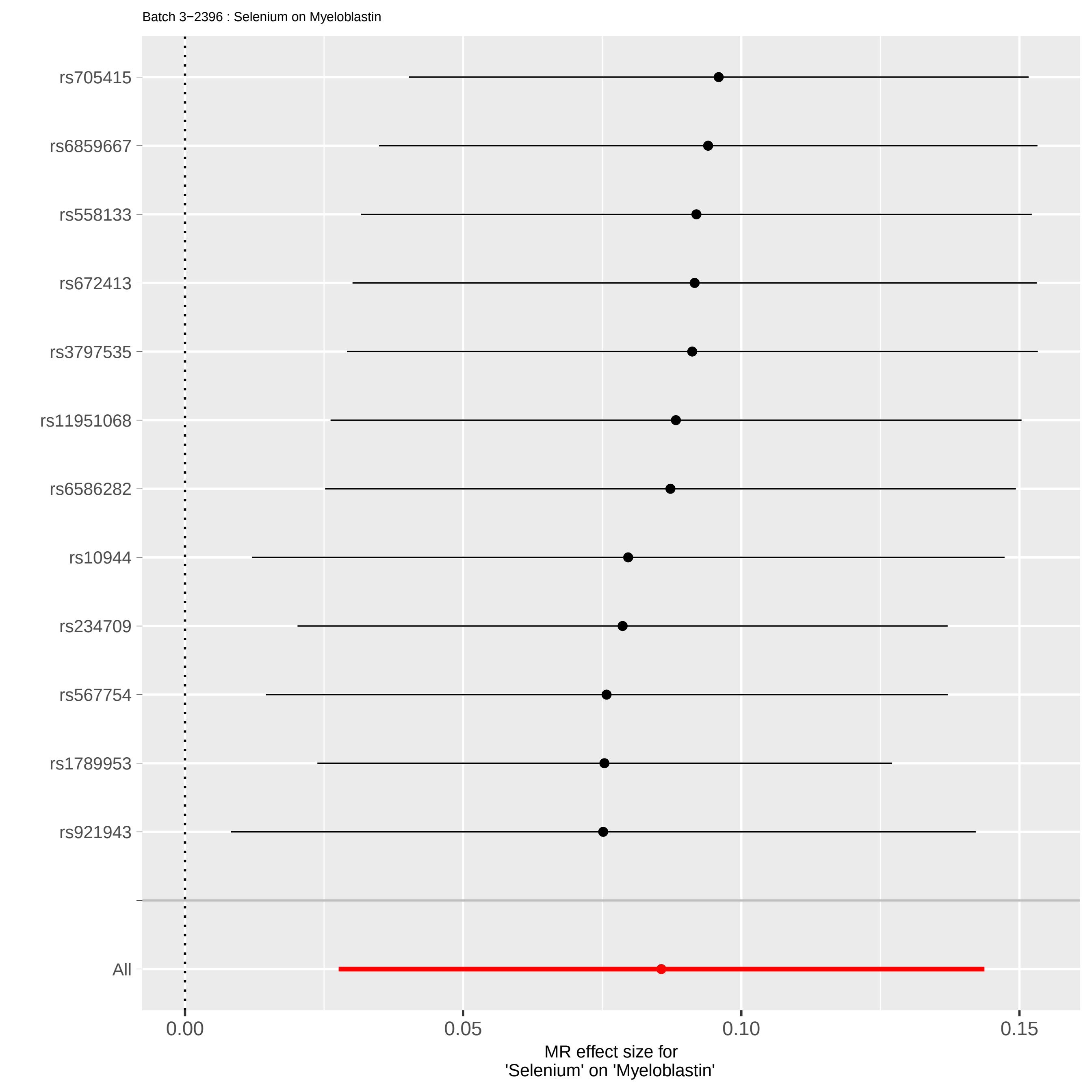

Batch 3−2396 : Selenium on Myeloblastin
rs705415
rs6859667
rs558133
rs672413
rs3797535
rs11951068
rs6586282
rs10944
rs234709
rs567754
rs1789953
rs921943
All
0.00
0.05
0.10
0.15
MR effect size for
'Selenium' on 'Myeloblastin'

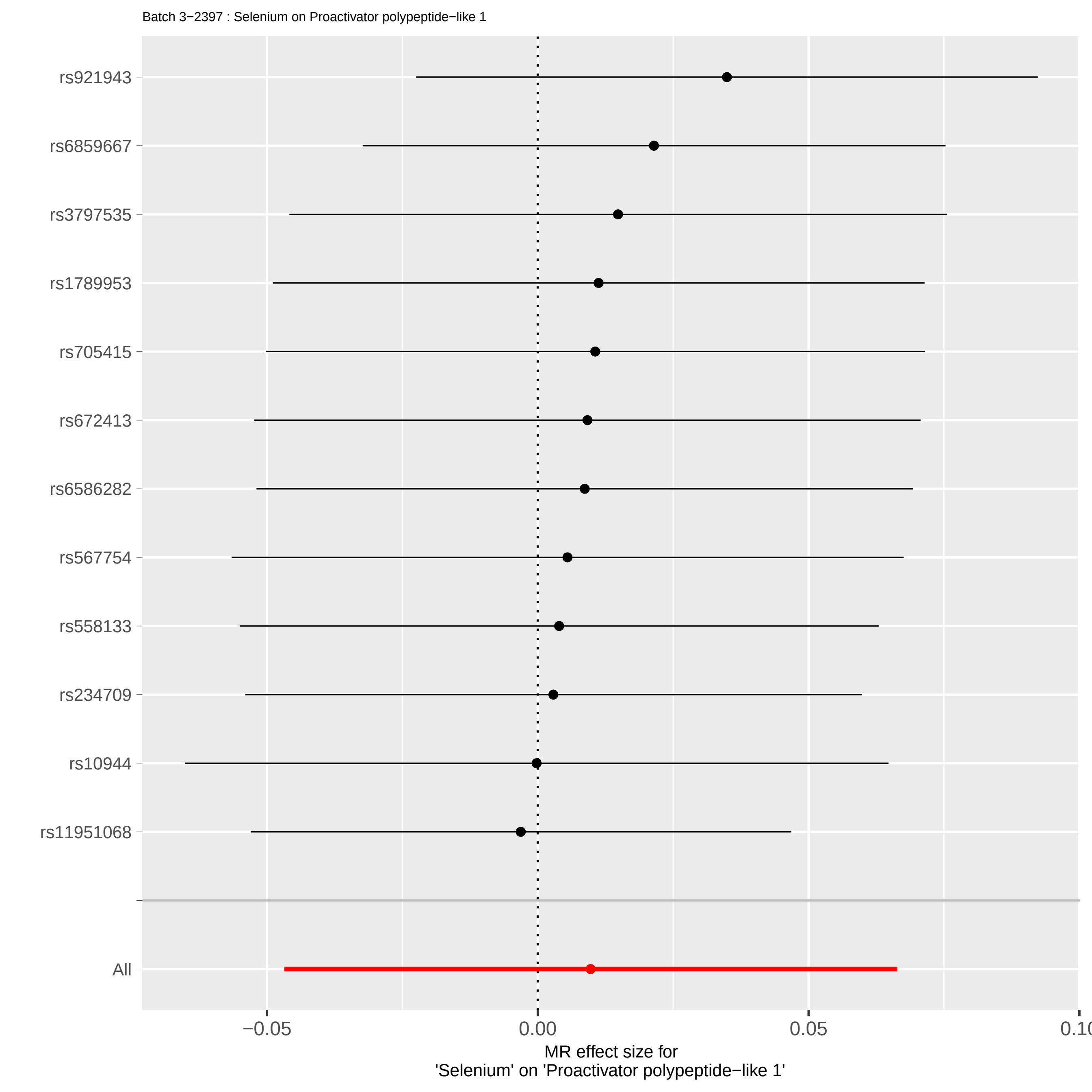

Batch 3−2397 : Selenium on Proactivator polypeptide−like 1
rs921943
rs6859667
rs3797535
rs1789953
rs705415
rs672413
rs6586282
rs567754
rs558133
rs234709
rs10944
rs11951068
All
−0.05
0.00
0.05
0.10
MR effect size for
'Selenium' on 'Proactivator polypeptide−like 1'

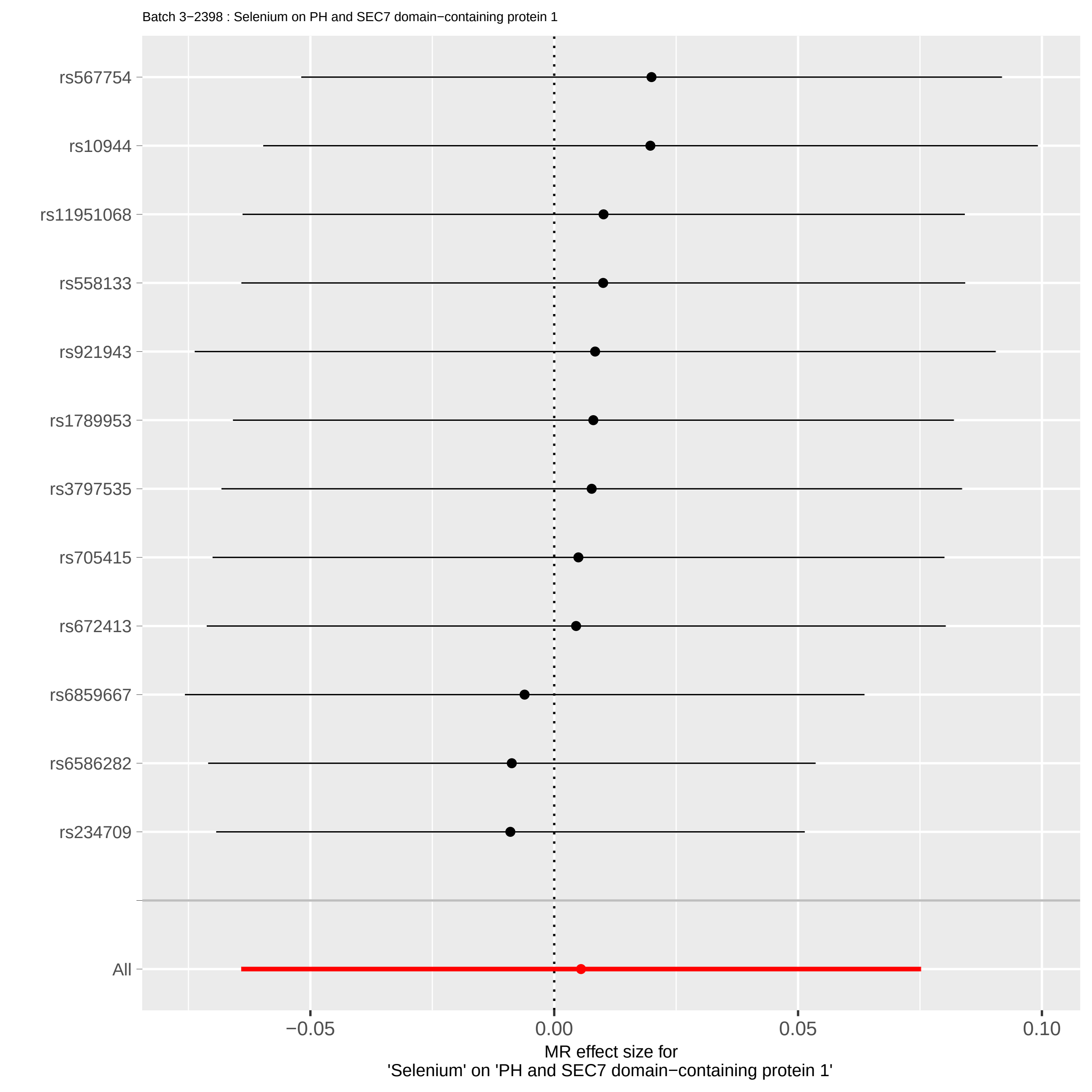
Batch 3−2398 : Selenium on PH and SEC7 domain−containing protein 1
rs567754
rs10944
rs11951068
rs558133
rs921943
rs1789953
rs3797535
rs705415
rs672413
rs6859667
rs6586282
rs234709
All
−0.05
0.00
0.05
0.10
MR effect size for
'Selenium' on 'PH and SEC7 domain−containing protein 1'

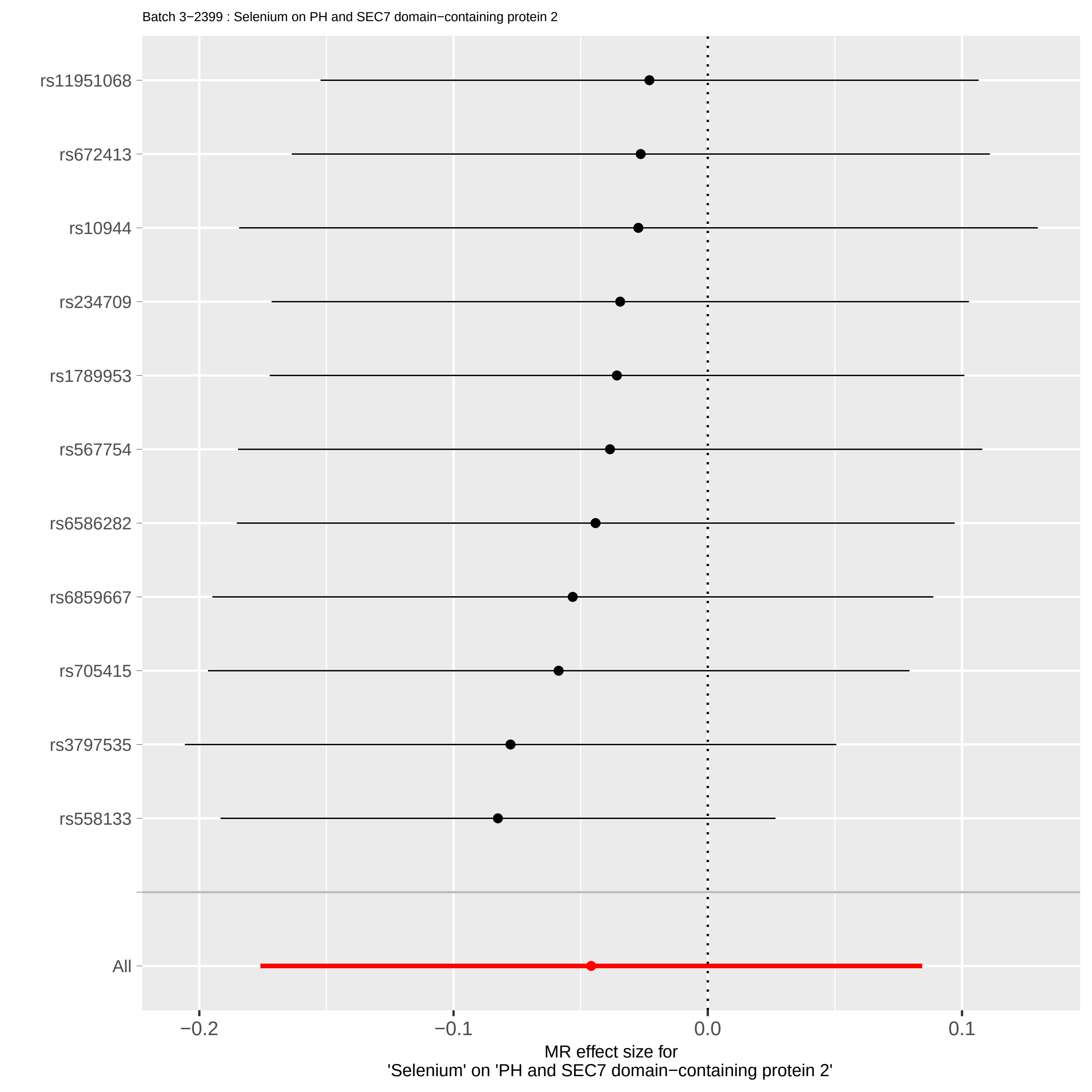

Batch 3−2399 : Selenium on PH and SEC7 domain−containing protein 2
rs11951068
rs672413
rs10944
rs234709
rs1789953
rs567754
rs6586282
rs6859667
rs705415
rs3797535
rs558133
All
−0.2
−0.1
0.0
0.1
MR effect size for
'Selenium' on 'PH and SEC7 domain−containing protein 2'

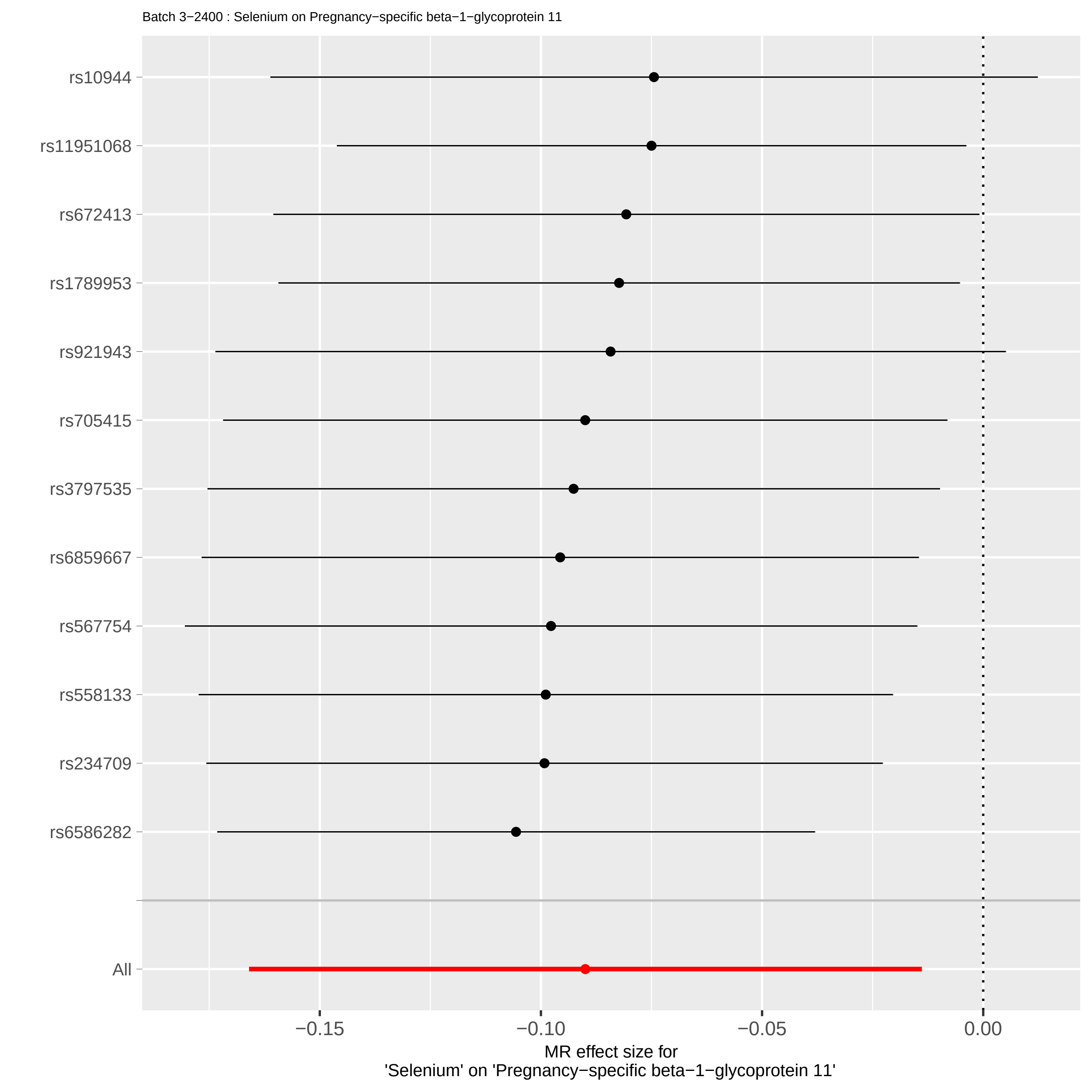

Batch 3−2400 : Selenium on Pregnancy−specific beta−1−glycoprotein 11
rs10944
rs11951068
rs672413
rs1789953
rs921943
rs705415
rs3797535
rs6859667
rs567754
rs558133
rs234709
rs6586282
All
−0.15
−0.10
−0.05
0.00
MR effect size for
'Selenium' on 'Pregnancy−specific beta−1−glycoprotein 11'

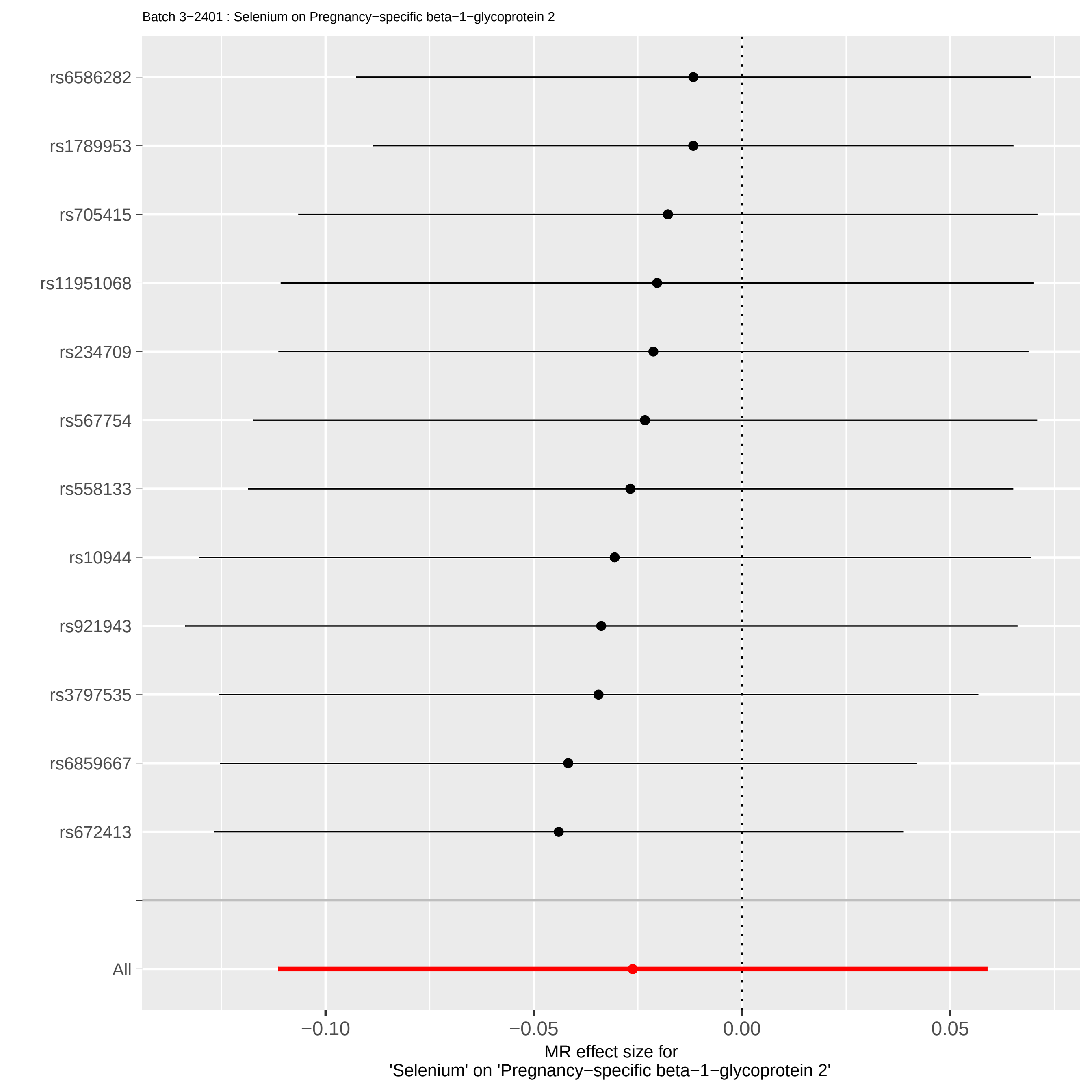

Batch 3−2401 : Selenium on Pregnancy−specific beta−1−glycoprotein 2
rs6586282
rs1789953
rs705415
rs11951068
rs234709
rs567754
rs558133
rs10944
rs921943
rs3797535
rs6859667
rs672413
All
−0.10
−0.05
0.00
0.05
MR effect size for
'Selenium' on 'Pregnancy−specific beta−1−glycoprotein 2'

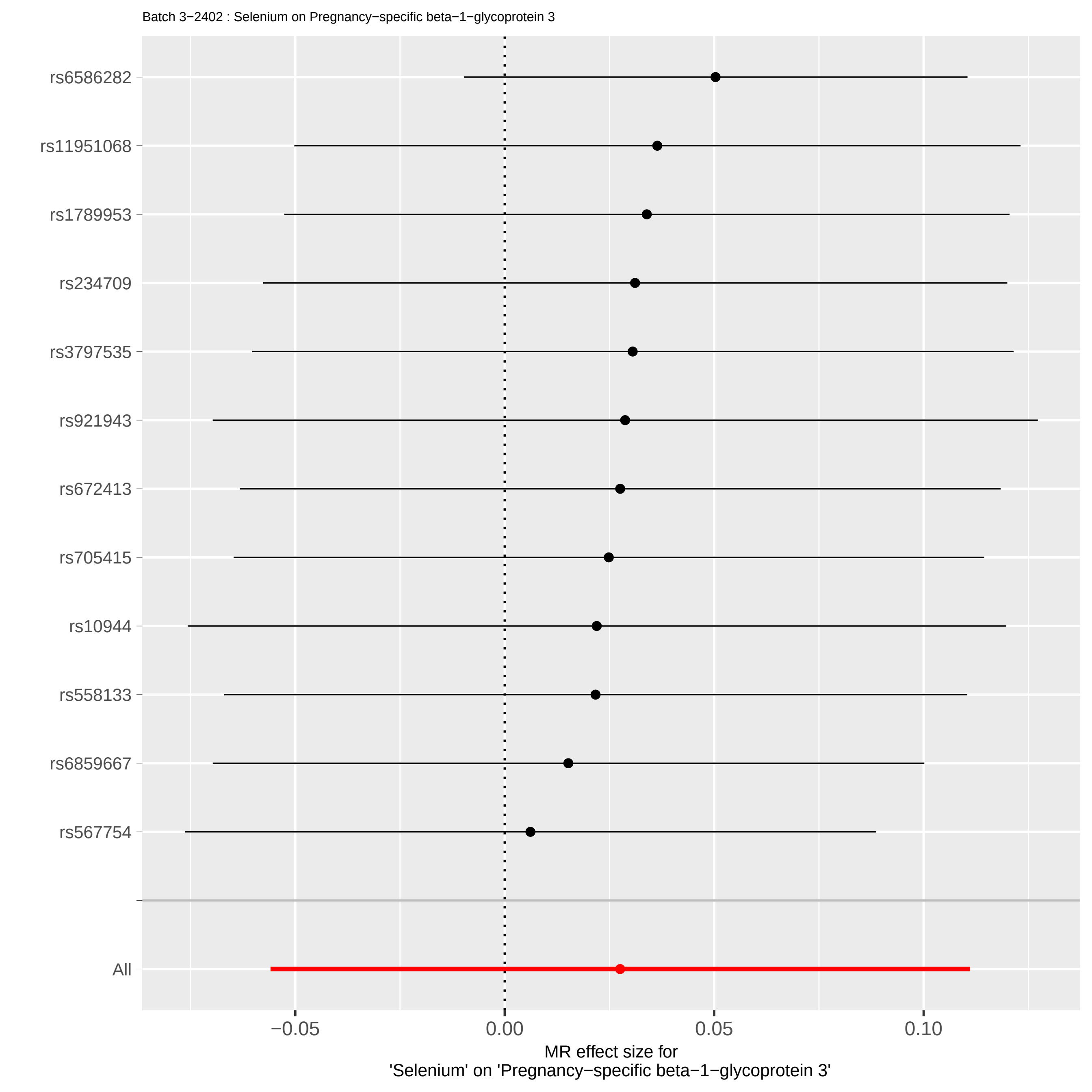

Batch 3−2402 : Selenium on Pregnancy−specific beta−1−glycoprotein 3
rs6586282
rs11951068
rs1789953
rs234709
rs3797535
rs921943
rs672413
rs705415
rs10944
rs558133
rs6859667
rs567754
All
−0.05
0.00
0.05
0.10
MR effect size for
'Selenium' on 'Pregnancy−specific beta−1−glycoprotein 3'

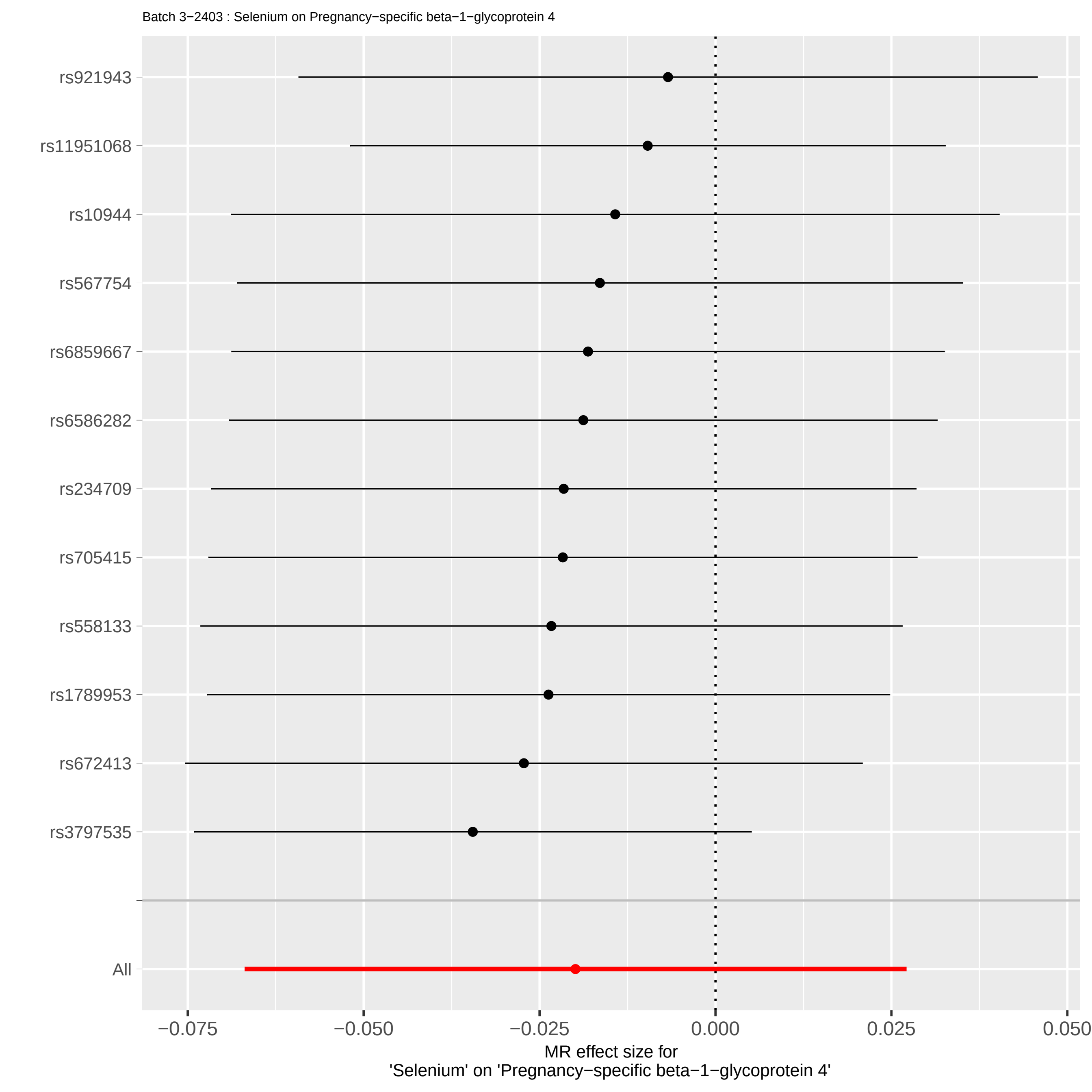

Batch 3−2403 : Selenium on Pregnancy−specific beta−1−glycoprotein 4
rs921943
rs11951068
rs10944
rs567754
rs6859667
rs6586282
rs234709
rs705415
rs558133
rs1789953
rs672413
rs3797535
All
−0.075
−0.050
−0.025
0.000
0.025
0.050
MR effect size for
'Selenium' on 'Pregnancy−specific beta−1−glycoprotein 4'

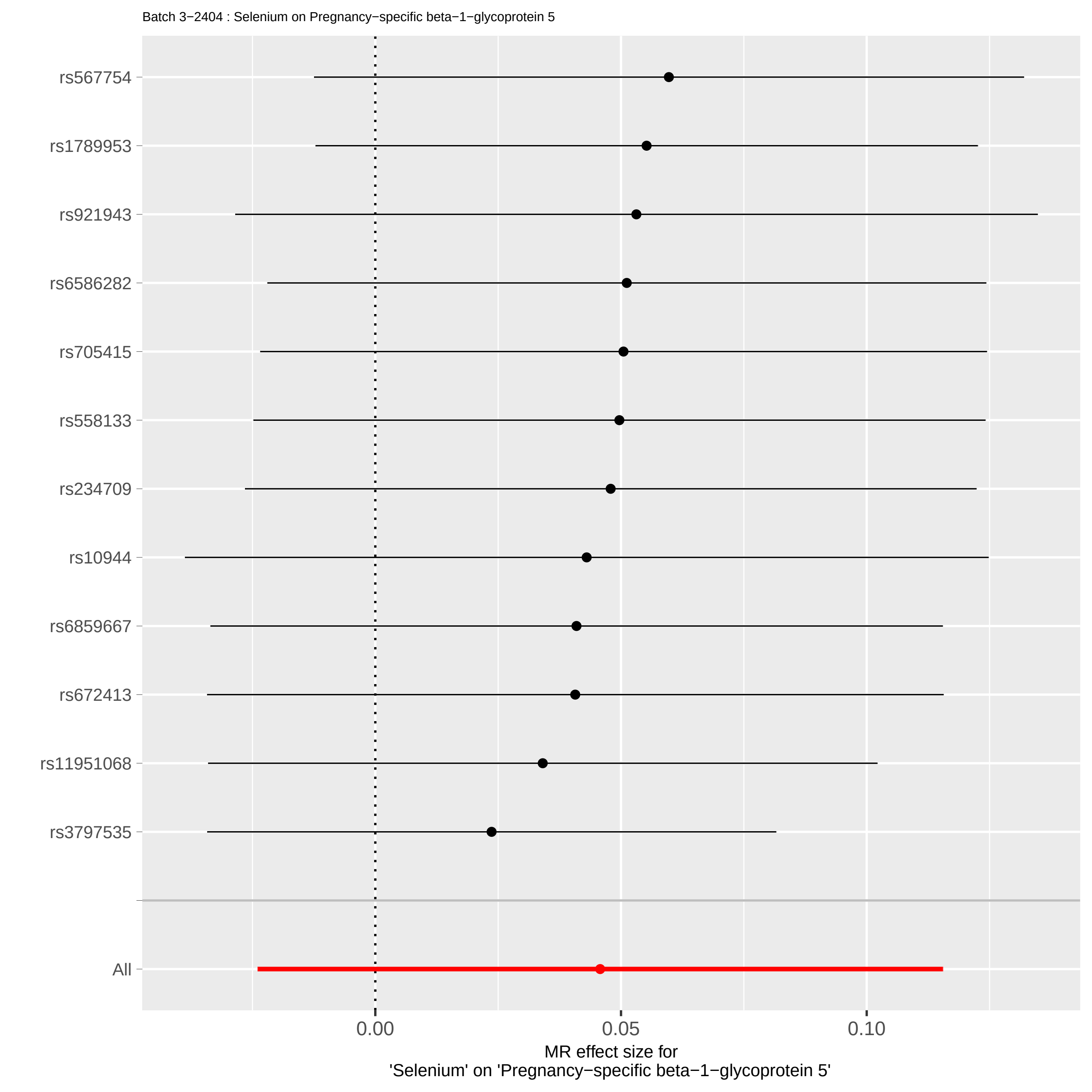

Batch 3−2404 : Selenium on Pregnancy−specific beta−1−glycoprotein 5
rs567754
rs1789953
rs921943
rs6586282
rs705415
rs558133
rs234709
rs10944
rs6859667
rs672413
rs11951068
rs3797535
All
0.00
0.05
0.10
MR effect size for
'Selenium' on 'Pregnancy−specific beta−1−glycoprotein 5'

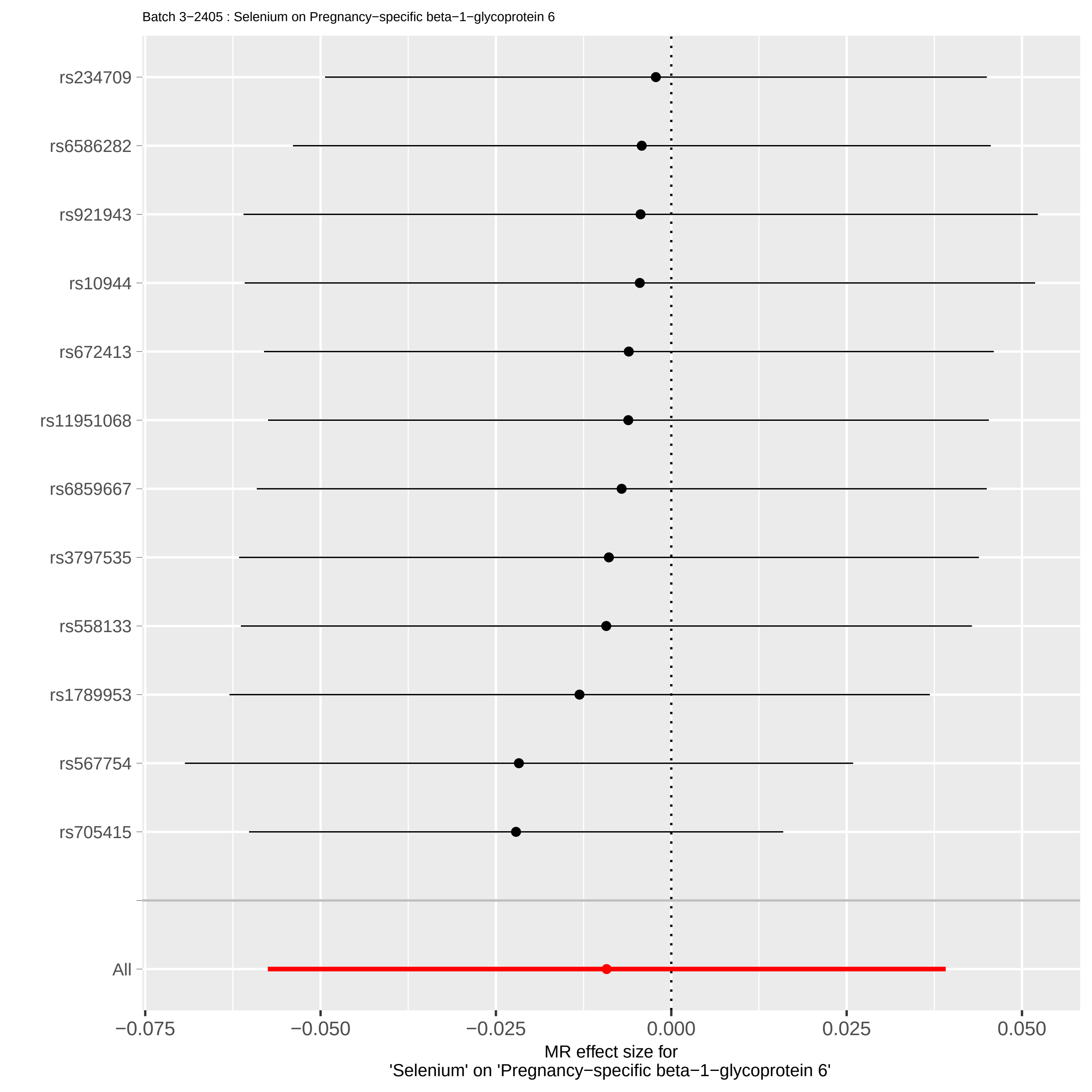
Batch 3−2405 : Selenium on Pregnancy−specific beta−1−glycoprotein 6
rs234709
rs6586282
rs921943
rs10944
rs672413
rs11951068
rs6859667
rs3797535
rs558133
rs1789953
rs567754
rs705415
All
−0.075
−0.050
−0.025
0.000
0.025
0.050
MR effect size for
'Selenium' on 'Pregnancy−specific beta−1−glycoprotein 6'

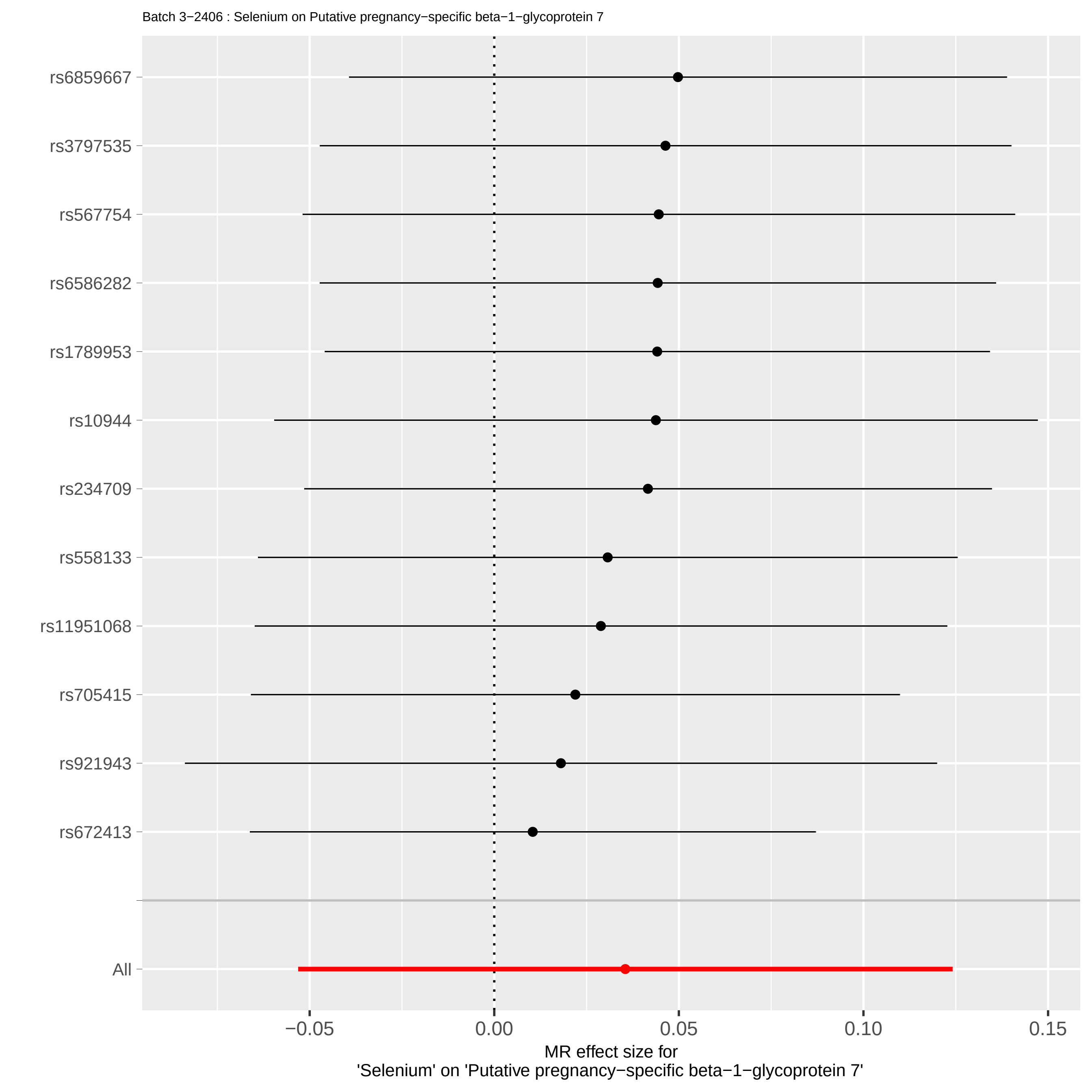

Batch 3−2406 : Selenium on Putative pregnancy−specific beta−1−glycoprotein 7
rs6859667
rs3797535
rs567754
rs6586282
rs1789953
rs10944
rs234709
rs558133
rs11951068
rs705415
rs921943
rs672413
All
−0.05
0.00
0.05
0.10
0.15
MR effect size for
'Selenium' on 'Putative pregnancy−specific beta−1−glycoprotein 7'

Batch 3−2407 : Selenium on Pregnancy−specific beta−1−glycoprotein 8

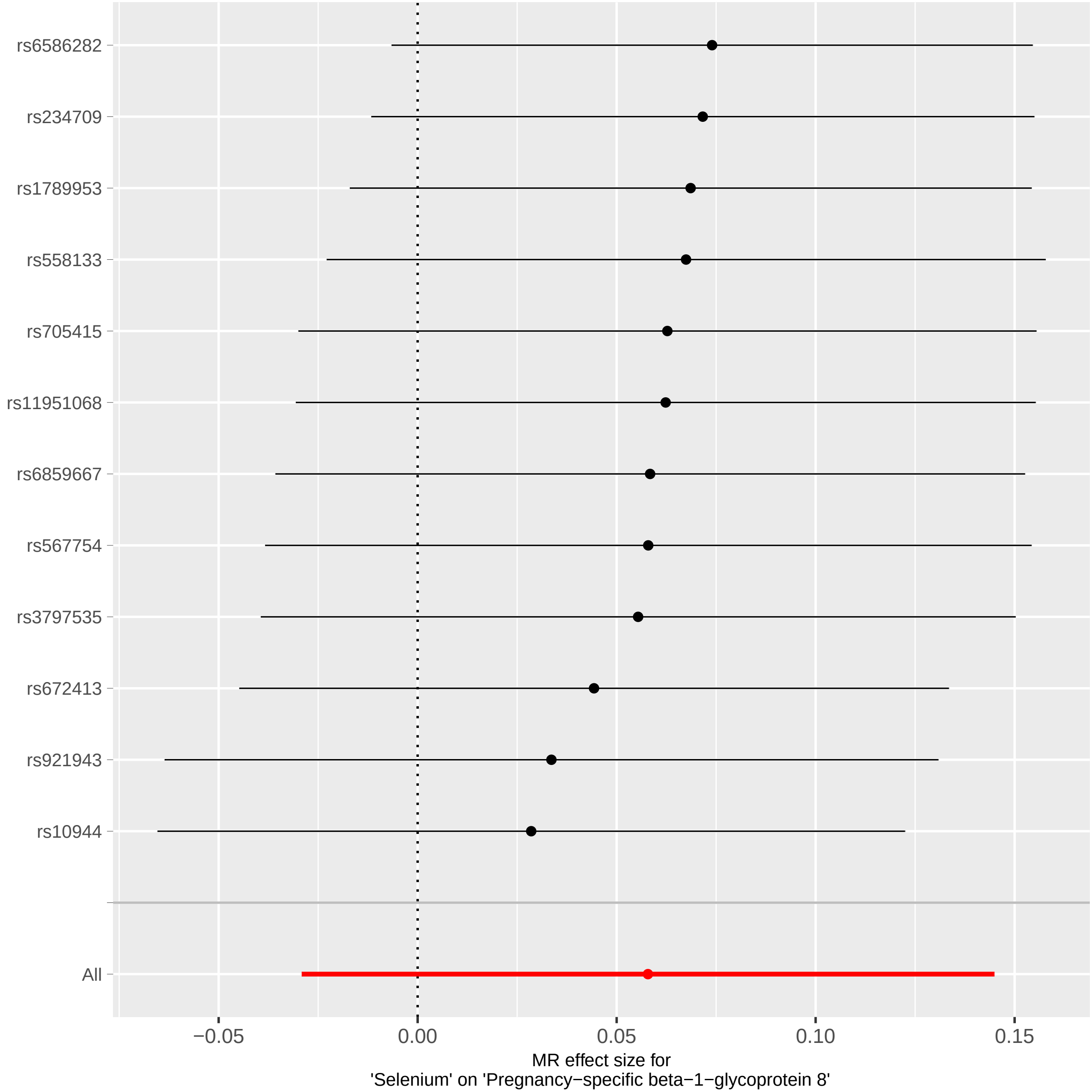

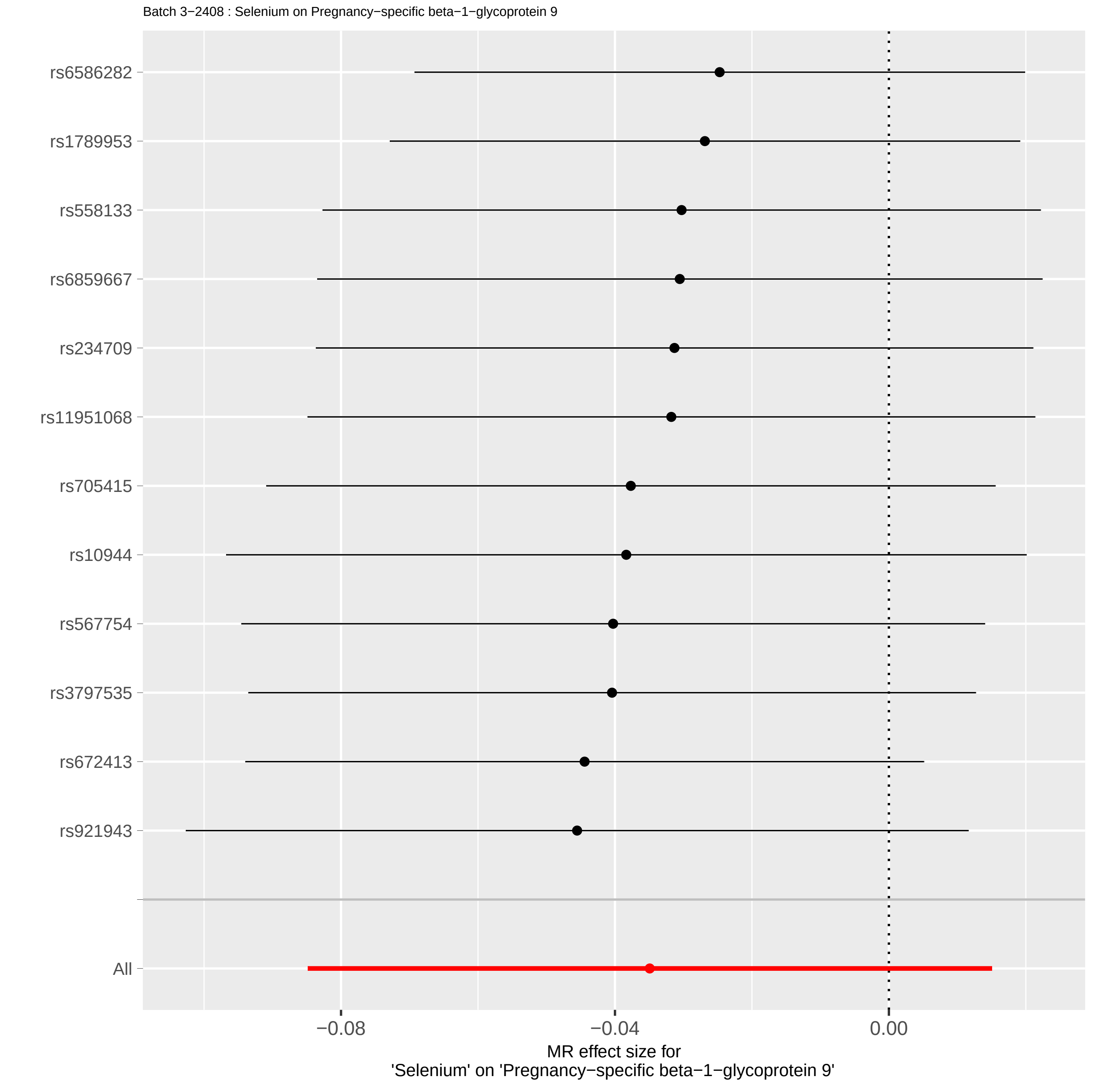
Batch 3−2408 : Selenium on Pregnancy−specific beta−1−glycoprotein 9
rs6586282
rs1789953
rs558133
rs6859667
rs234709
rs11951068
rs705415
rs10944
rs567754
rs3797535
rs672413
rs921943
All
−0.08
−0.04
0.00
MR effect size for
'Selenium' on 'Pregnancy−specific beta−1−glycoprotein 9'

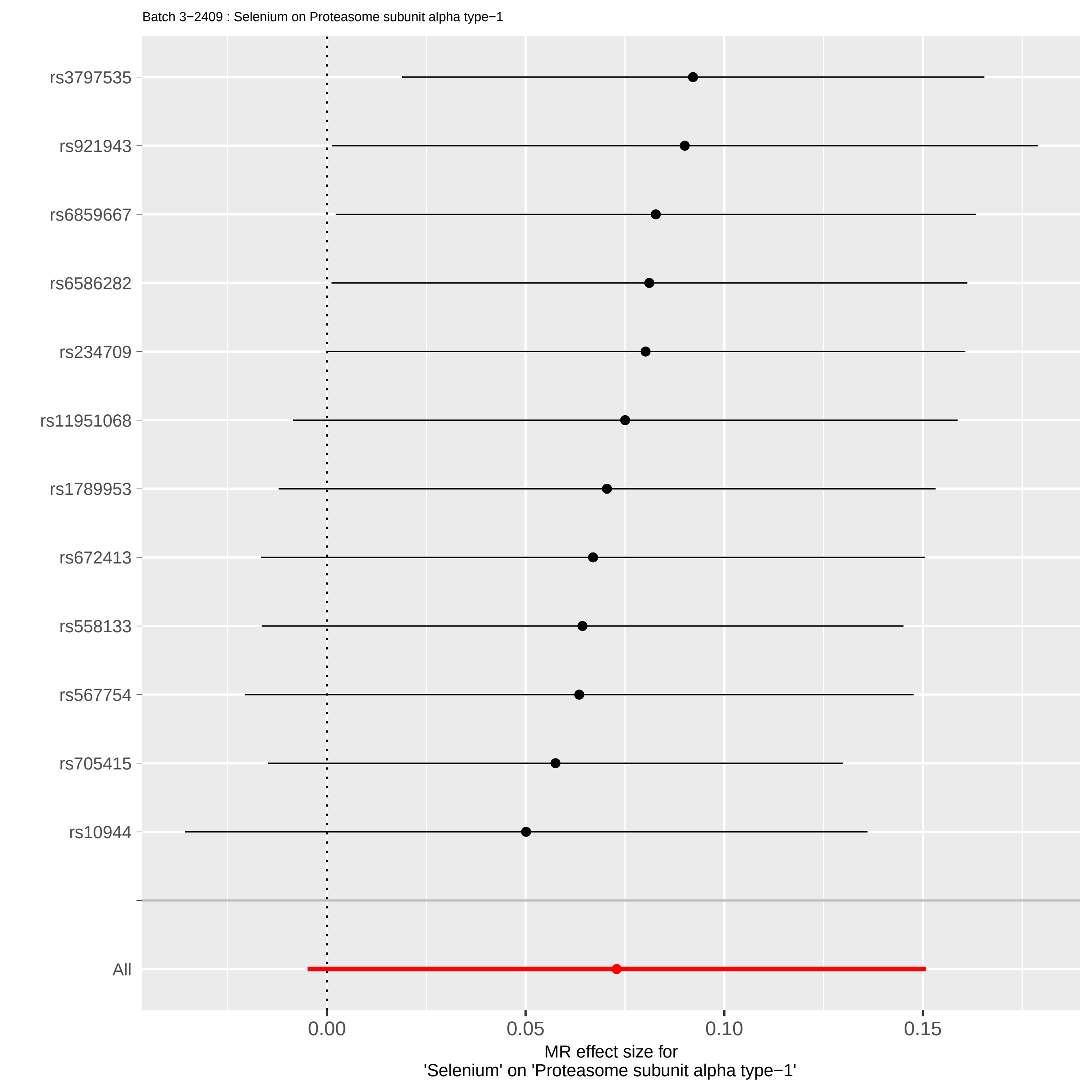

Batch 3−2409 : Selenium on Proteasome subunit alpha type−1
rs3797535
rs921943
rs6859667
rs6586282
rs234709
rs11951068
rs1789953
rs672413
rs558133
rs567754
rs705415
rs10944
All
0.00
0.05
0.10
0.15
MR effect size for
'Selenium' on 'Proteasome subunit alpha type−1'

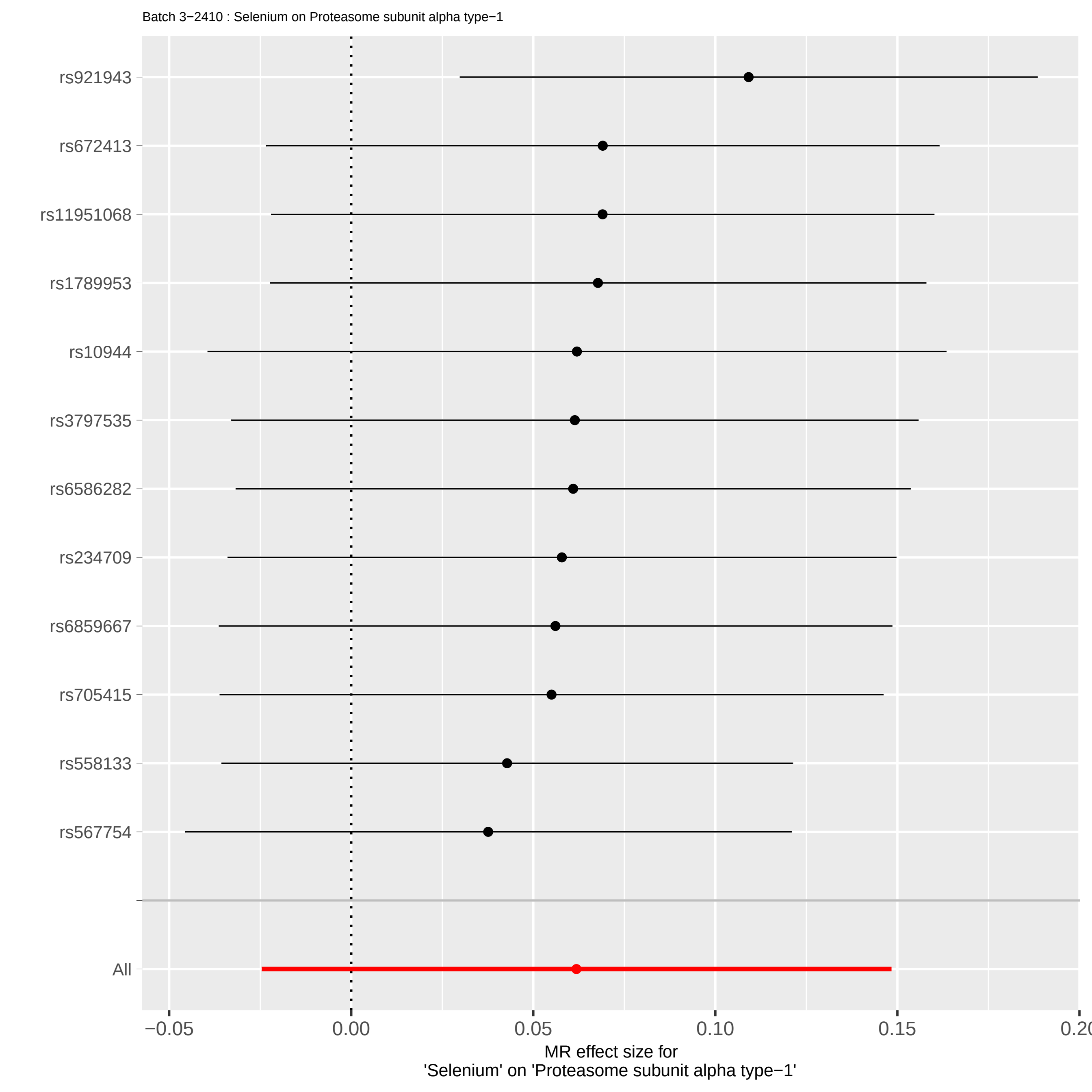

Batch 3−2410 : Selenium on Proteasome subunit alpha type−1
rs921943
rs672413
rs11951068
rs1789953
rs10944
rs3797535
rs6586282
rs234709
rs6859667
rs705415
rs558133
rs567754
All
−0.05
0.00
0.05
0.10
0.15
0.20
MR effect size for
'Selenium' on 'Proteasome subunit alpha type−1'

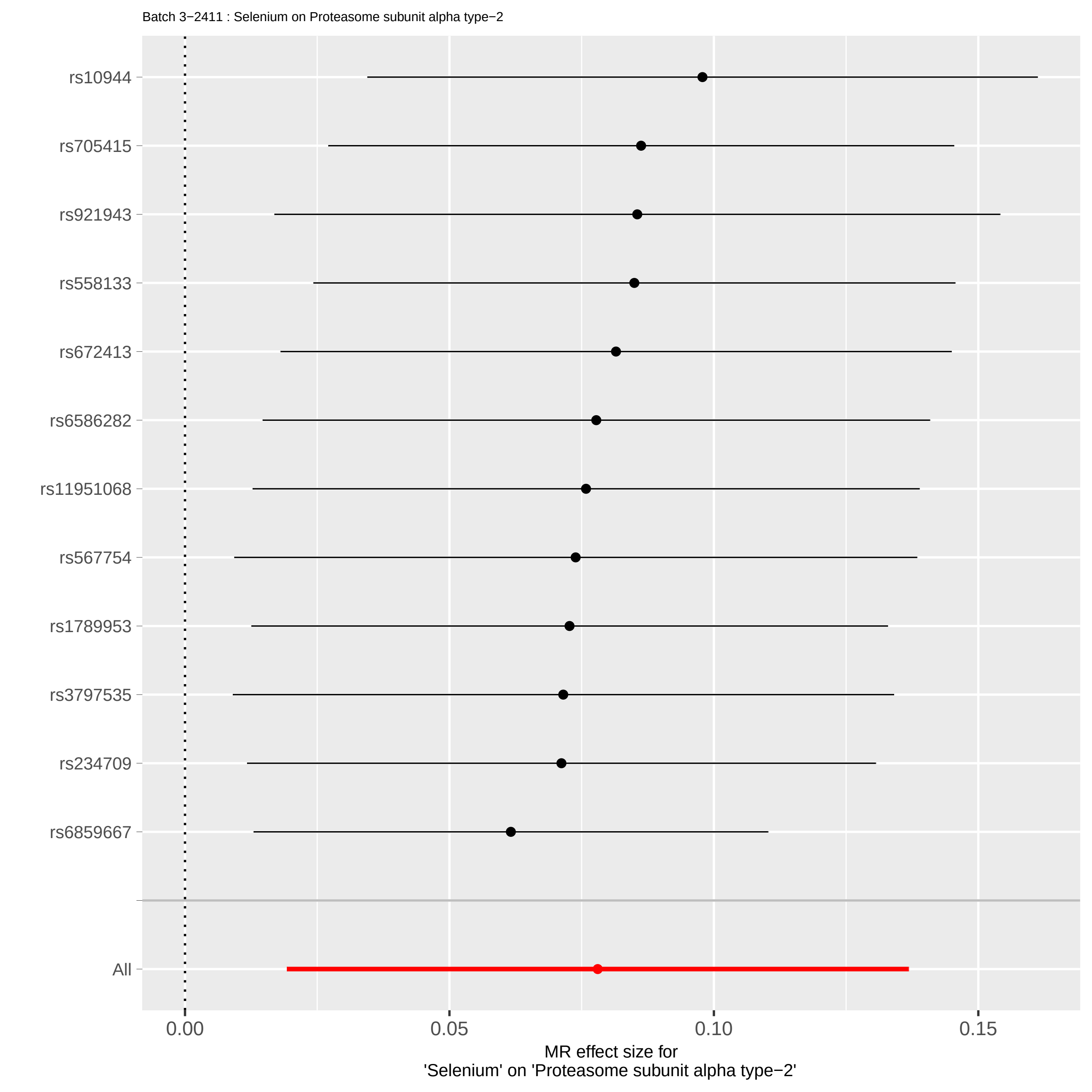

Batch 3−2411 : Selenium on Proteasome subunit alpha type−2
rs10944
rs705415
rs921943
rs558133
rs672413
rs6586282
rs11951068
rs567754
rs1789953
rs3797535
rs234709
rs6859667
All
0.00
0.05
0.10
0.15
MR effect size for
'Selenium' on 'Proteasome subunit alpha type−2'

Batch 3−2412 : Selenium on Proteasome subunit alpha type−4

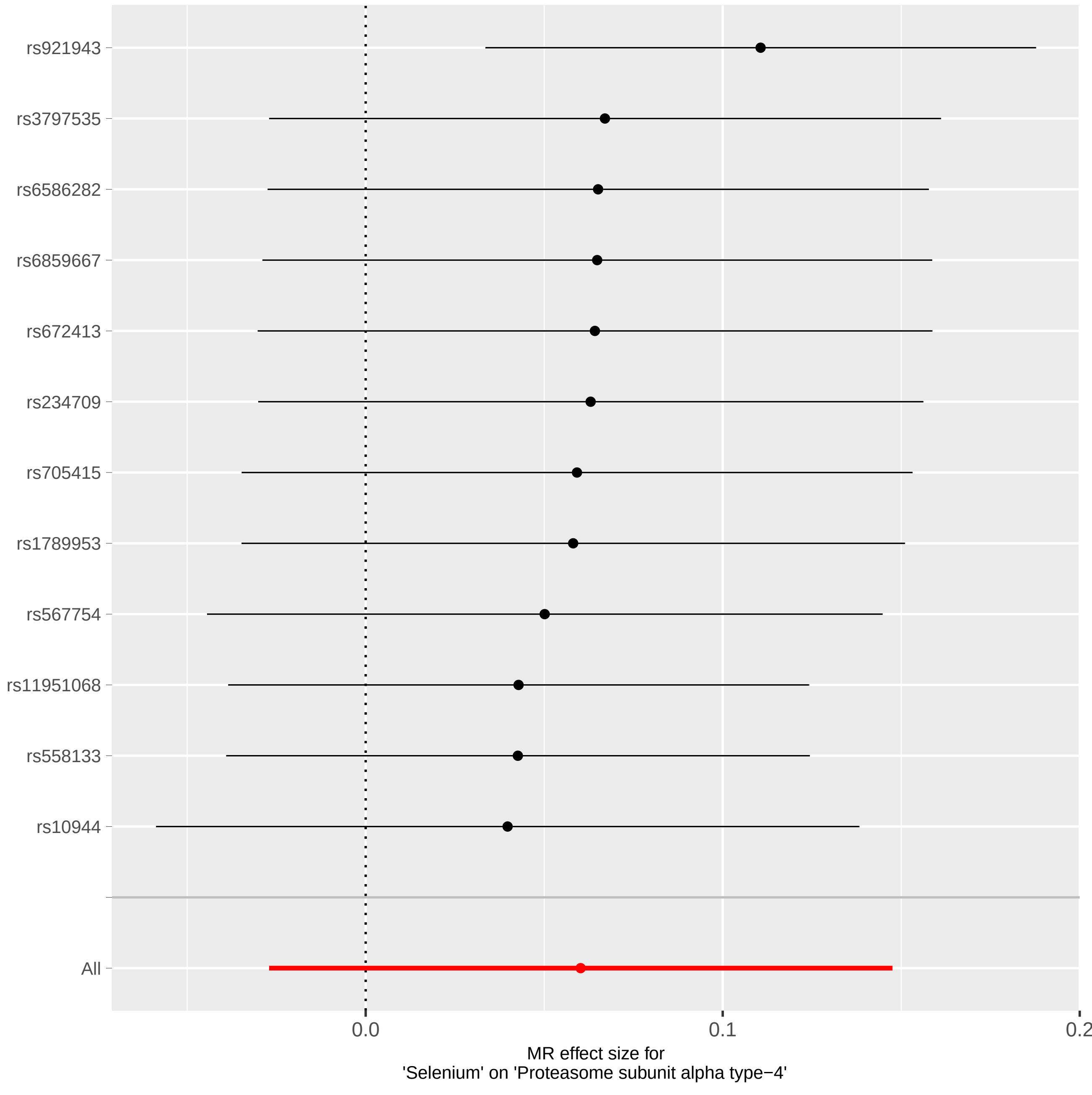

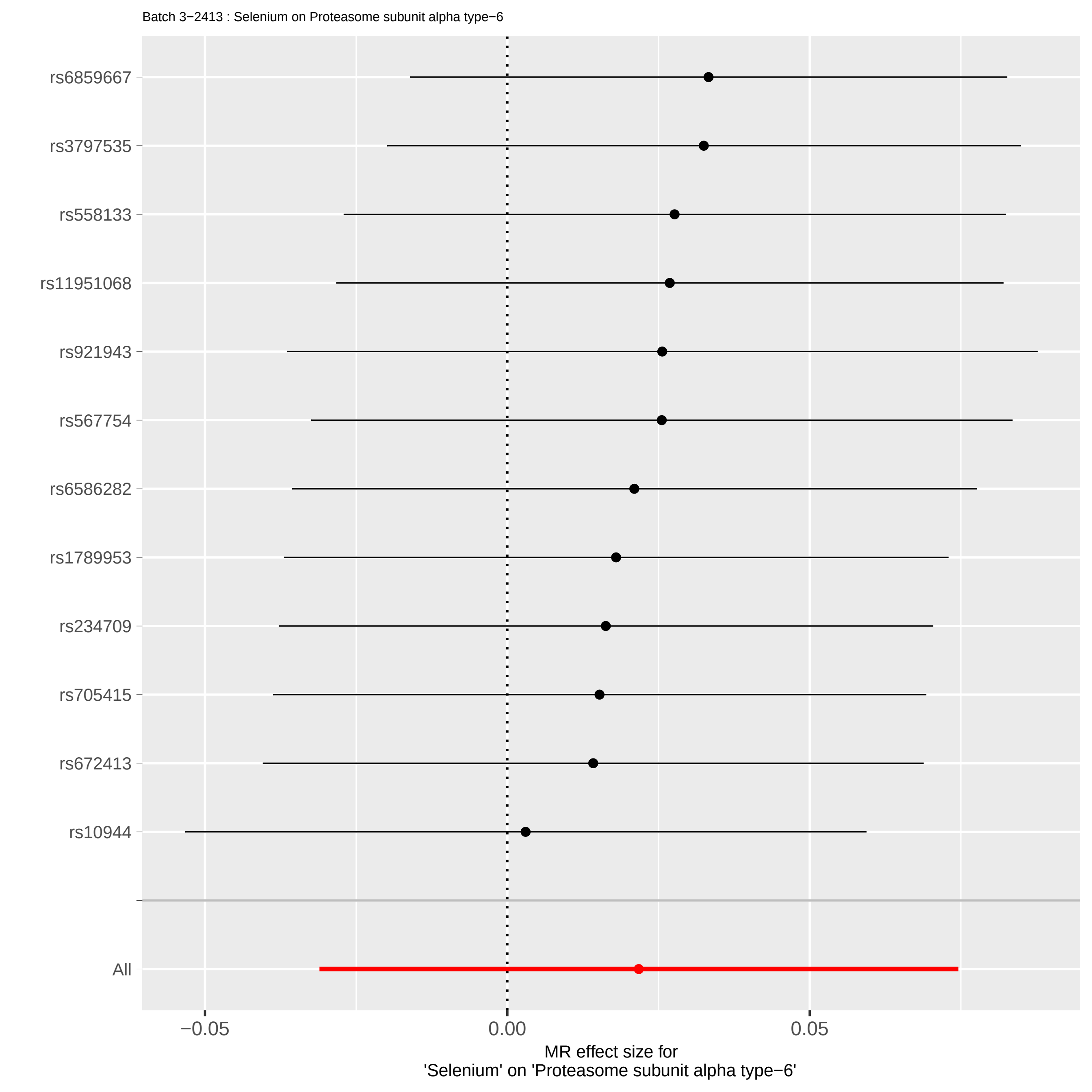

Batch 3−2413 : Selenium on Proteasome subunit alpha type−6
rs6859667
rs3797535
rs558133
rs11951068
rs921943
rs567754
rs6586282
rs1789953
rs234709
rs705415
rs672413
rs10944
All
−0.05
0.00
0.05
MR effect size for
'Selenium' on 'Proteasome subunit alpha type−6'

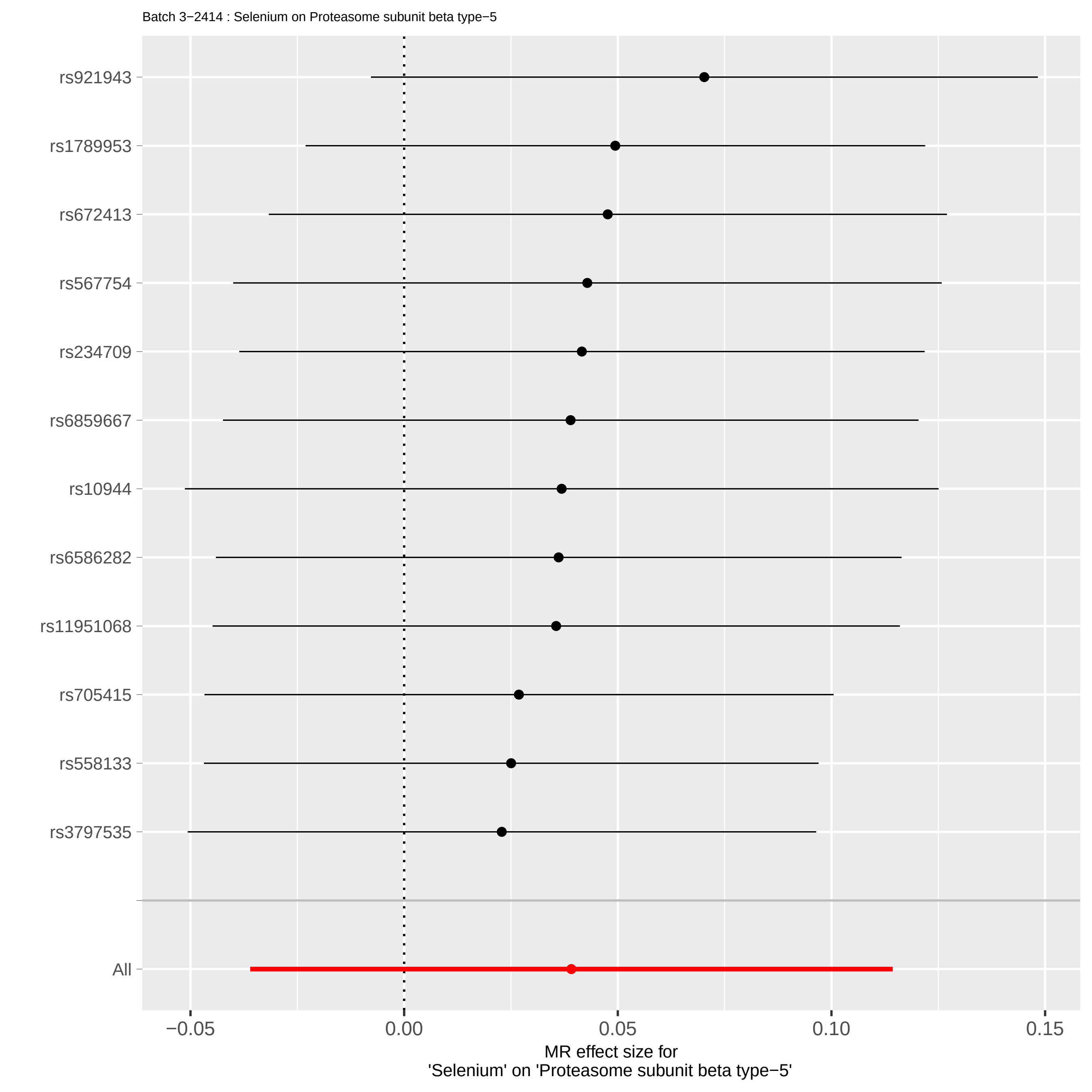

Batch 3−2414 : Selenium on Proteasome subunit beta type−5
rs921943
rs1789953
rs672413
rs567754
rs234709
rs6859667
rs10944
rs6586282
rs11951068
rs705415
rs558133
rs3797535
All
−0.05
0.00
0.05
0.10
0.15
MR effect size for
'Selenium' on 'Proteasome subunit beta type−5'

Batch 3−2415 : Selenium on 26S proteasome non−ATPase regulatory subunit 11

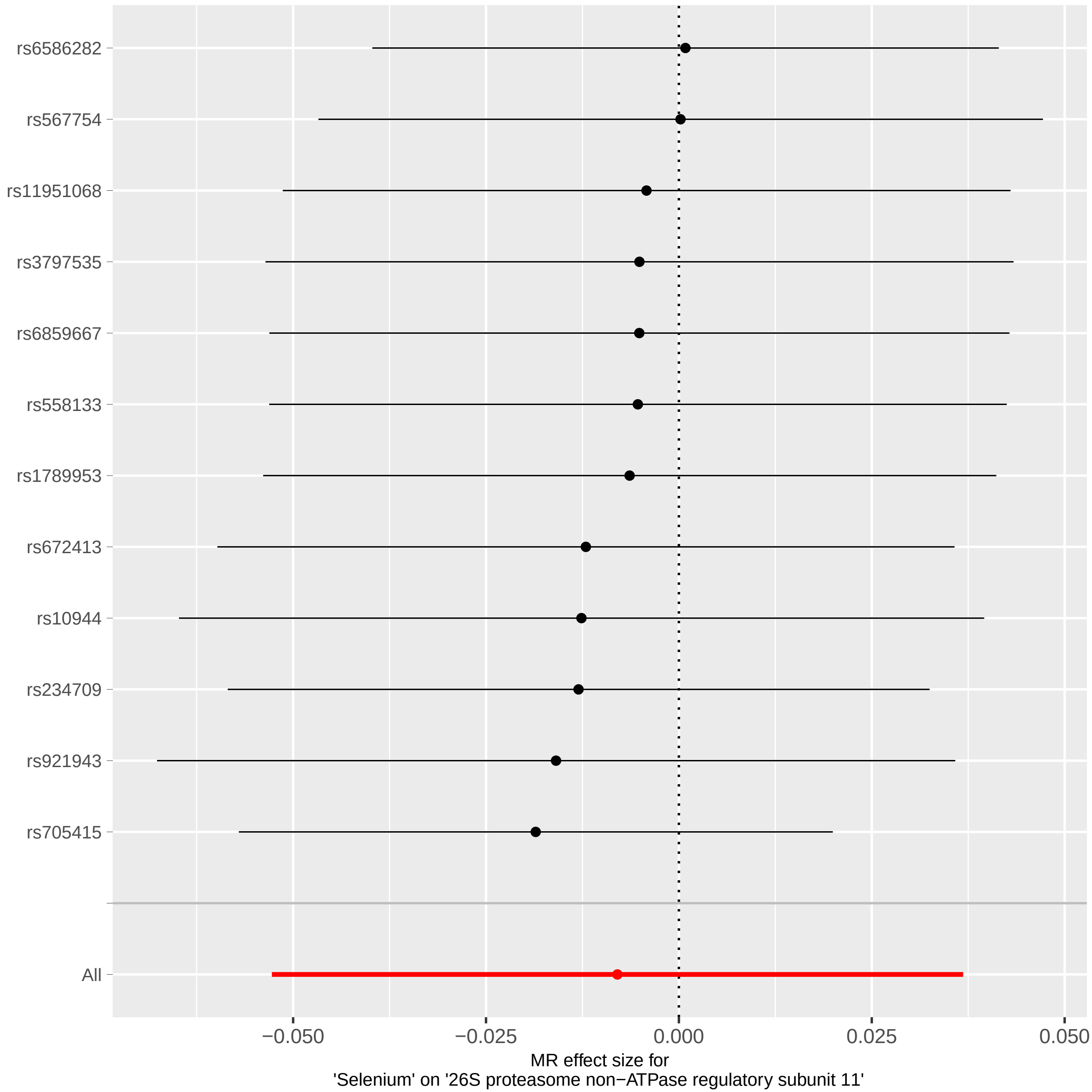

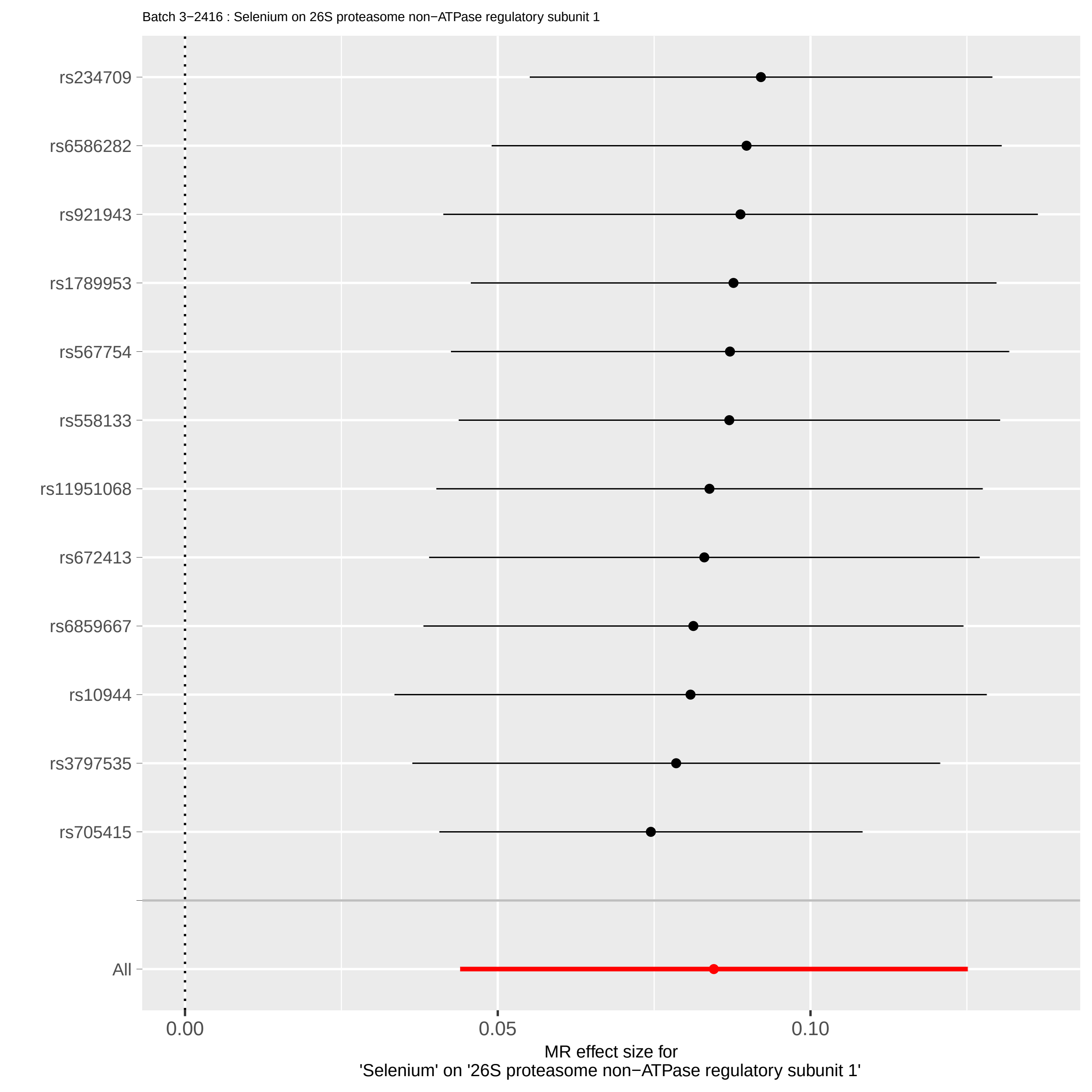

Batch 3−2416 : Selenium on 26S proteasome non−ATPase regulatory subunit 1
rs234709
rs6586282
rs921943
rs1789953
rs567754
rs558133
rs11951068
rs672413
rs6859667
rs10944
rs3797535
rs705415
All
0.00
0.05
0.10
MR effect size for
'Selenium' on '26S proteasome non−ATPase regulatory subunit 1'

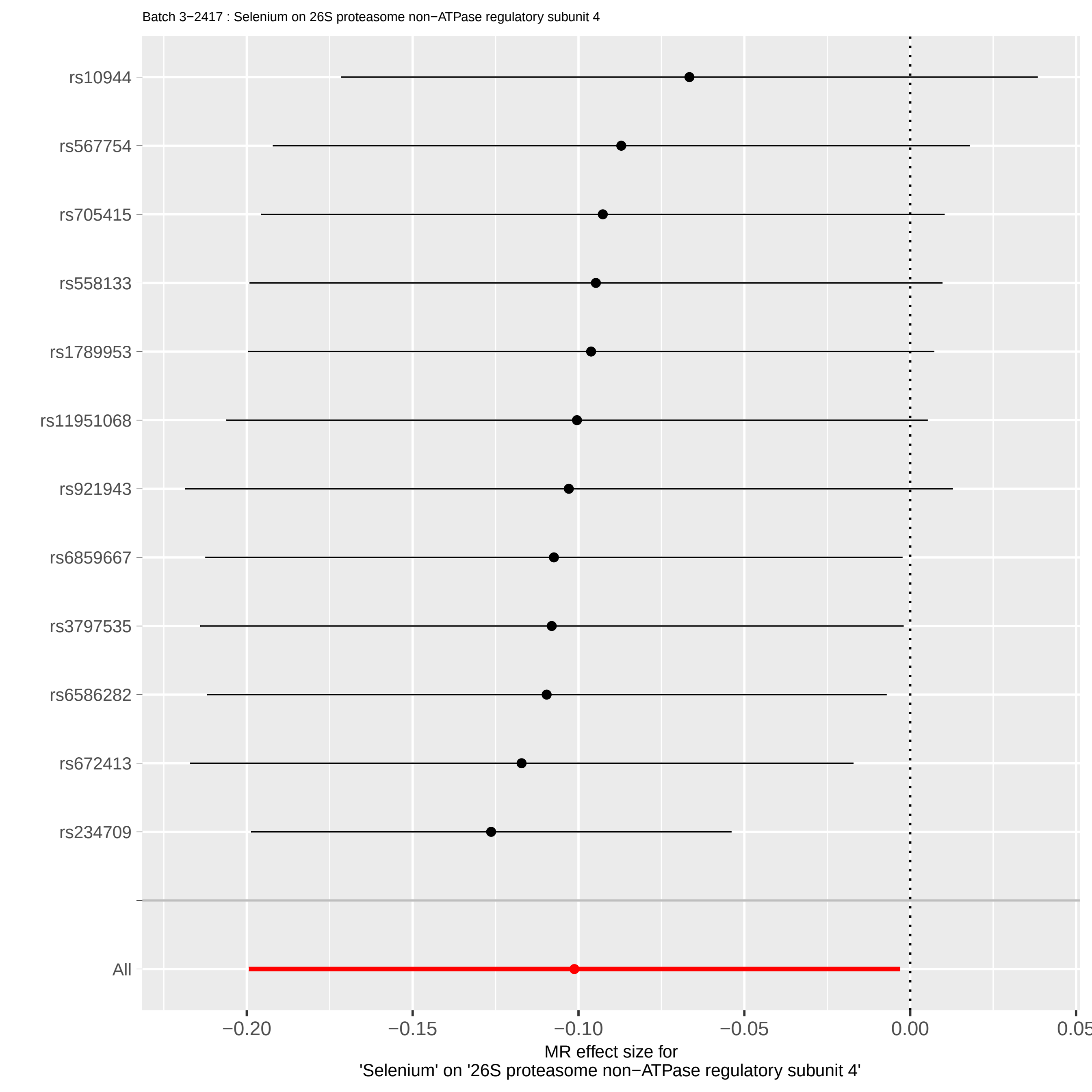

Batch 3−2417 : Selenium on 26S proteasome non−ATPase regulatory subunit 4
rs10944
rs567754
rs705415
rs558133
rs1789953
rs11951068
rs921943
rs6859667
rs3797535
rs6586282
rs672413
rs234709
All
−0.20
−0.15
−0.10
−0.05
0.00
0.05
MR effect size for
'Selenium' on '26S proteasome non−ATPase regulatory subunit 4'

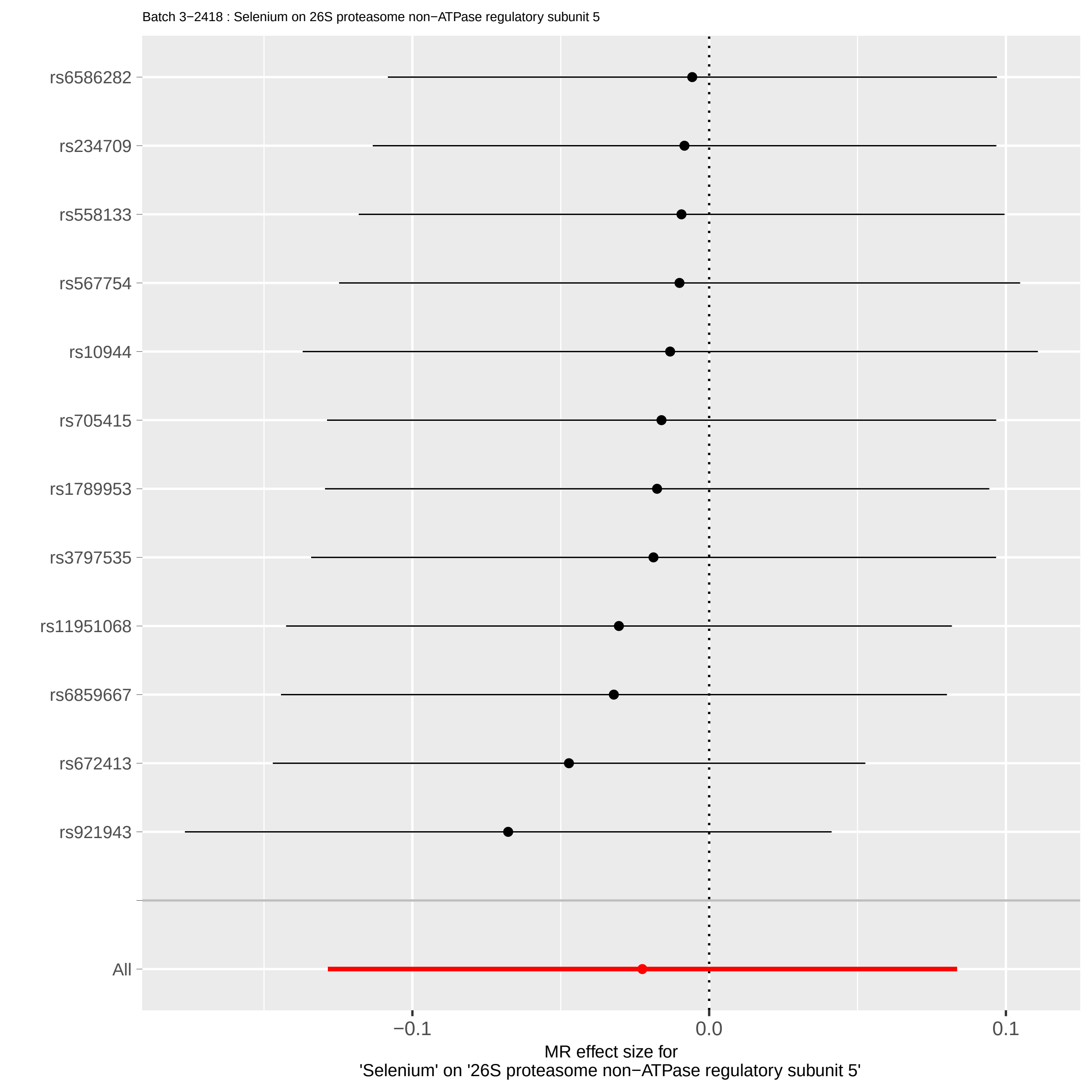
Batch 3−2418 : Selenium on 26S proteasome non−ATPase regulatory subunit 5
rs6586282
rs234709
rs558133
rs567754
rs10944
rs705415
rs1789953
rs3797535
rs11951068
rs6859667
rs672413
rs921943
All
−0.1
0.0
0.1
MR effect size for
'Selenium' on '26S proteasome non−ATPase regulatory subunit 5'

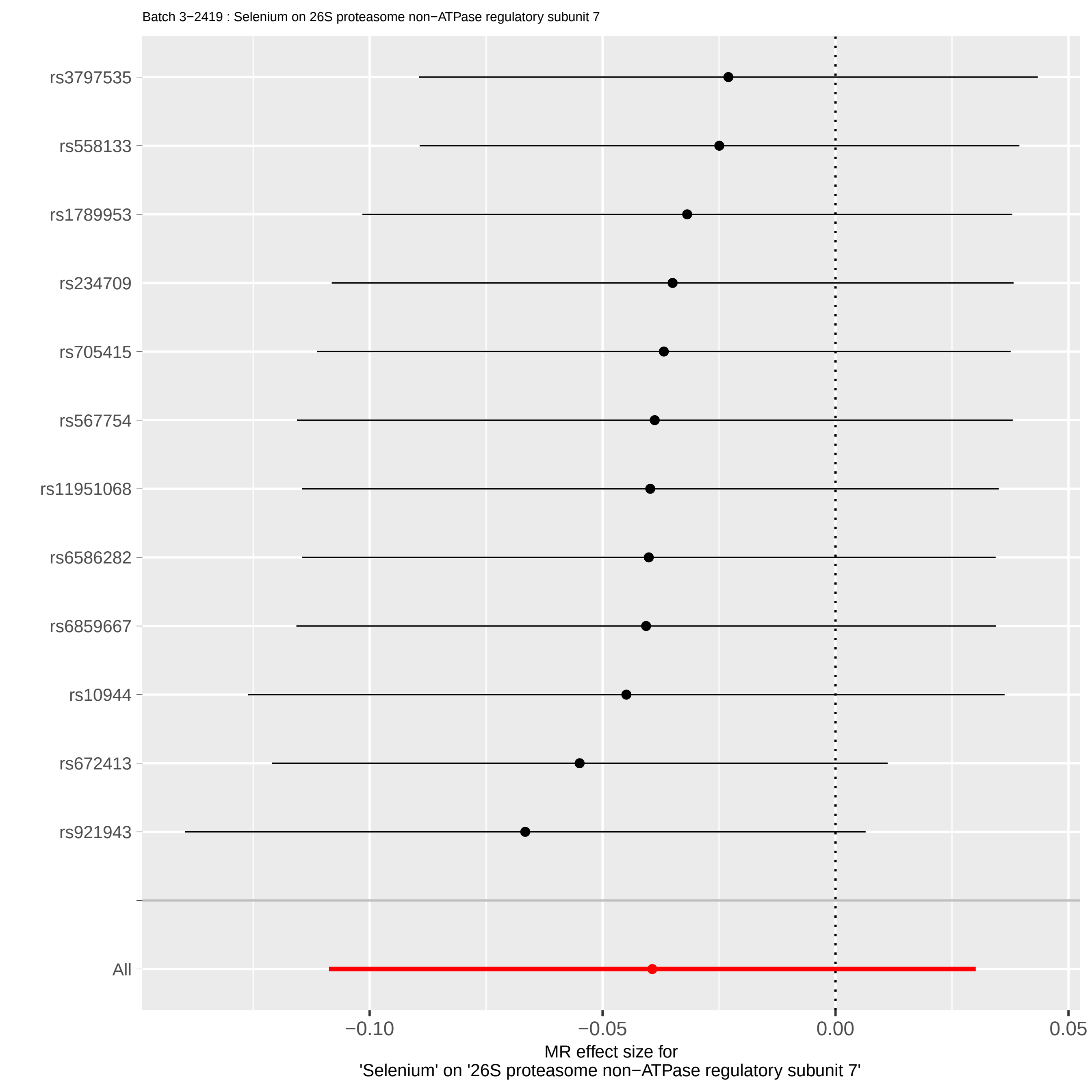

Batch 3−2419 : Selenium on 26S proteasome non−ATPase regulatory subunit 7
rs3797535
rs558133
rs1789953
rs234709
rs705415
rs567754
rs11951068
rs6586282
rs6859667
rs10944
rs672413
rs921943
All
−0.10
−0.05
0.00
0.05
MR effect size for
'Selenium' on '26S proteasome non−ATPase regulatory subunit 7'

Batch 3−2420 : Selenium on Proteasome activator complex subunit 1

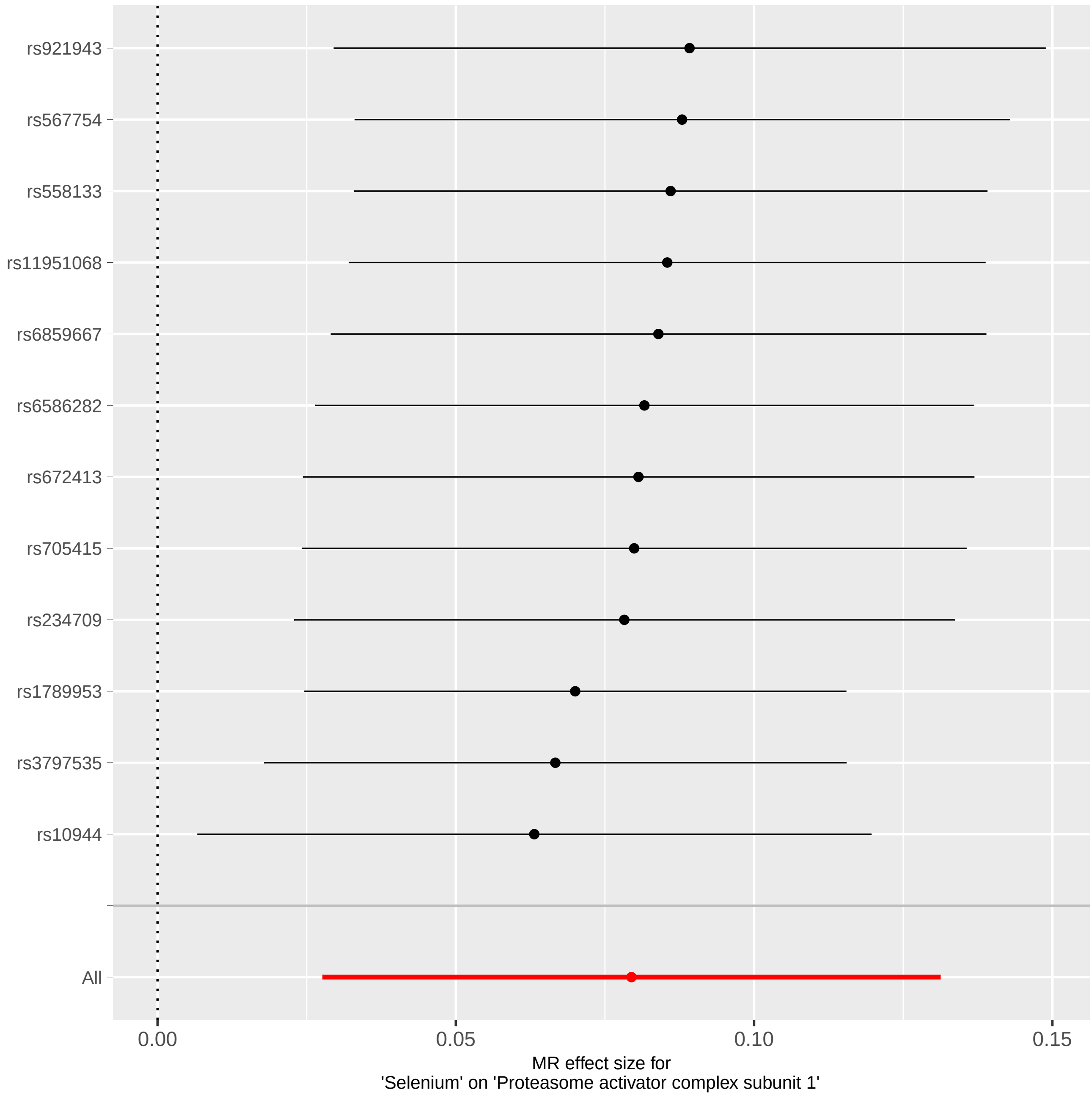

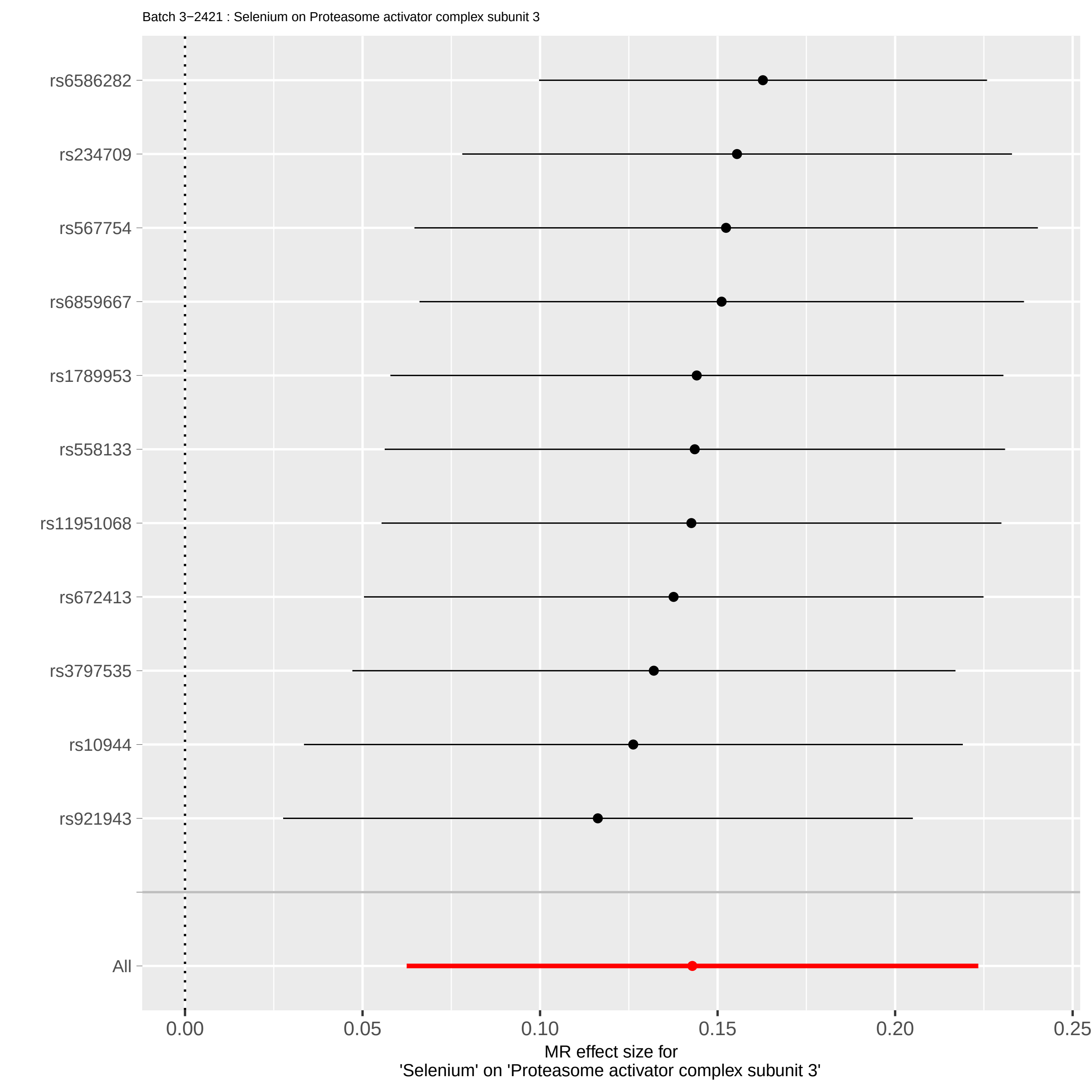

Batch 3−2421 : Selenium on Proteasome activator complex subunit 3
rs6586282
rs234709
rs567754
rs6859667
rs1789953
rs558133
rs11951068
rs672413
rs3797535
rs10944
rs921943
All
0.00
0.05
0.10
0.15
0.20
0.25
MR effect size for
'Selenium' on 'Proteasome activator complex subunit 3'

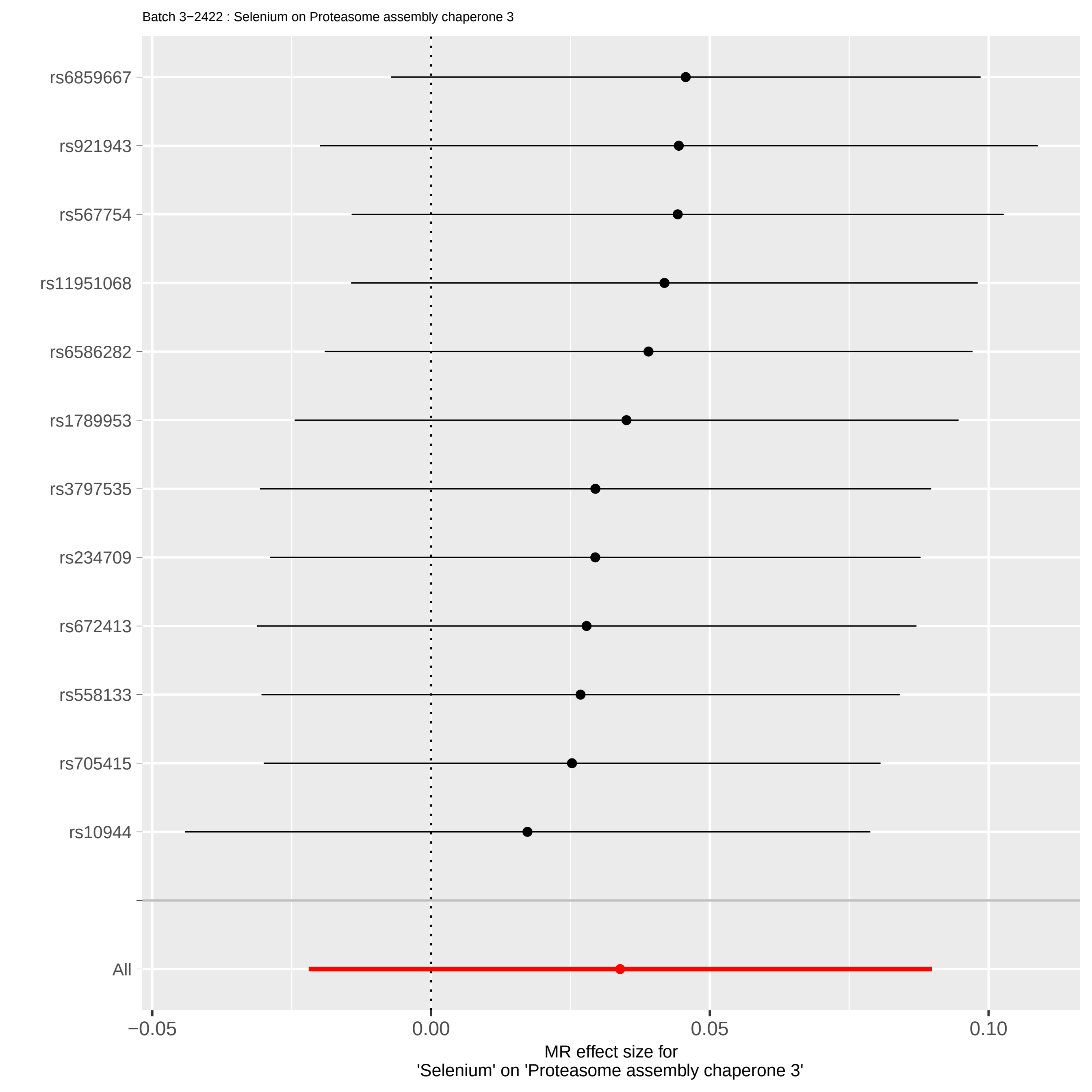
Batch 3−2422 : Selenium on Proteasome assembly chaperone 3
rs6859667
rs921943
rs567754
rs11951068
rs6586282
rs1789953
rs3797535
rs234709
rs672413
rs558133
rs705415
rs10944
All
−0.05
0.00
0.05
0.10
MR effect size for
'Selenium' on 'Proteasome assembly chaperone 3'

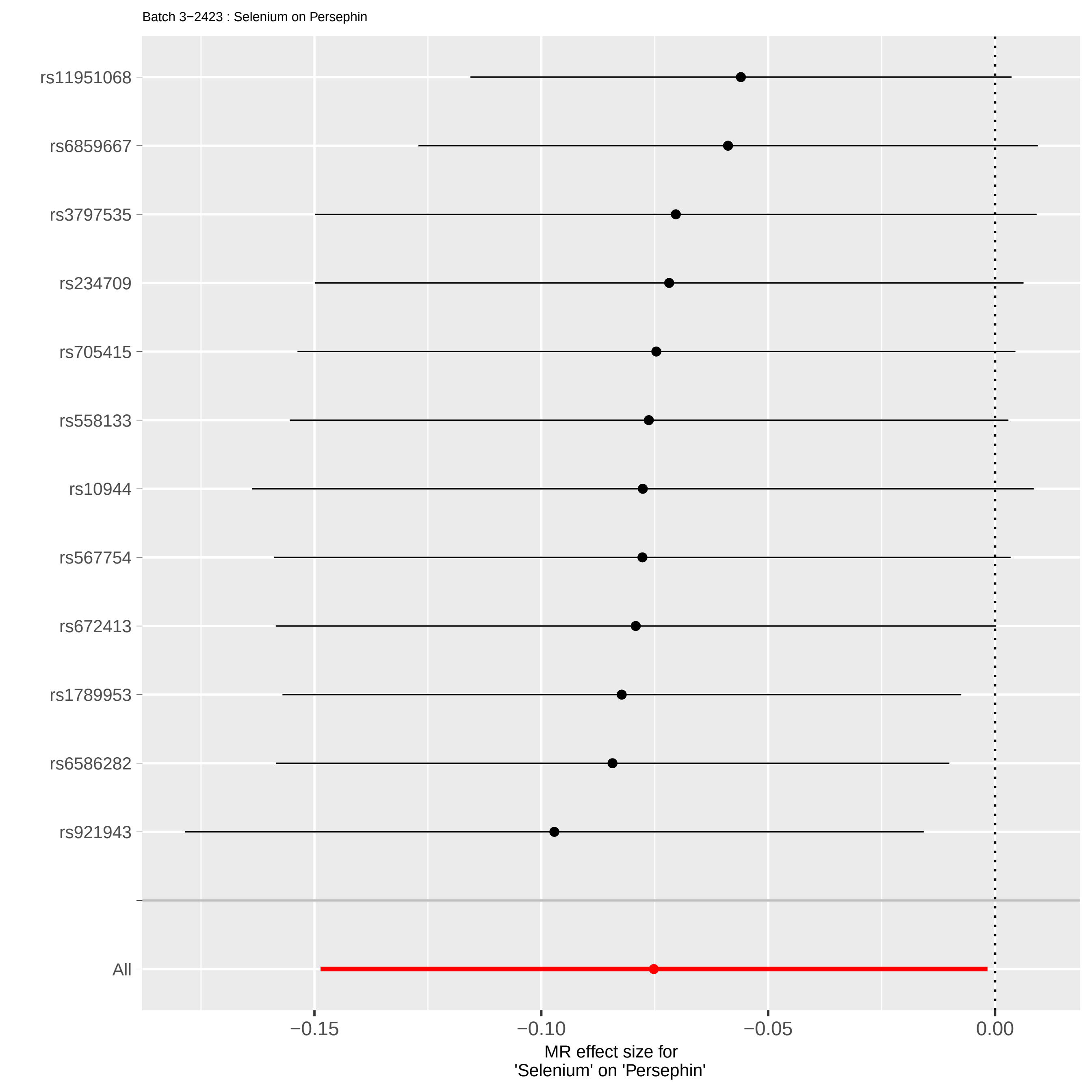

Batch 3−2423 : Selenium on Persephin
rs11951068
rs6859667
rs3797535
rs234709
rs705415
rs558133
rs10944
rs567754
rs672413
rs1789953
rs6586282
rs921943
All
−0.15
−0.10
−0.05
0.00
MR effect size for
'Selenium' on 'Persephin'

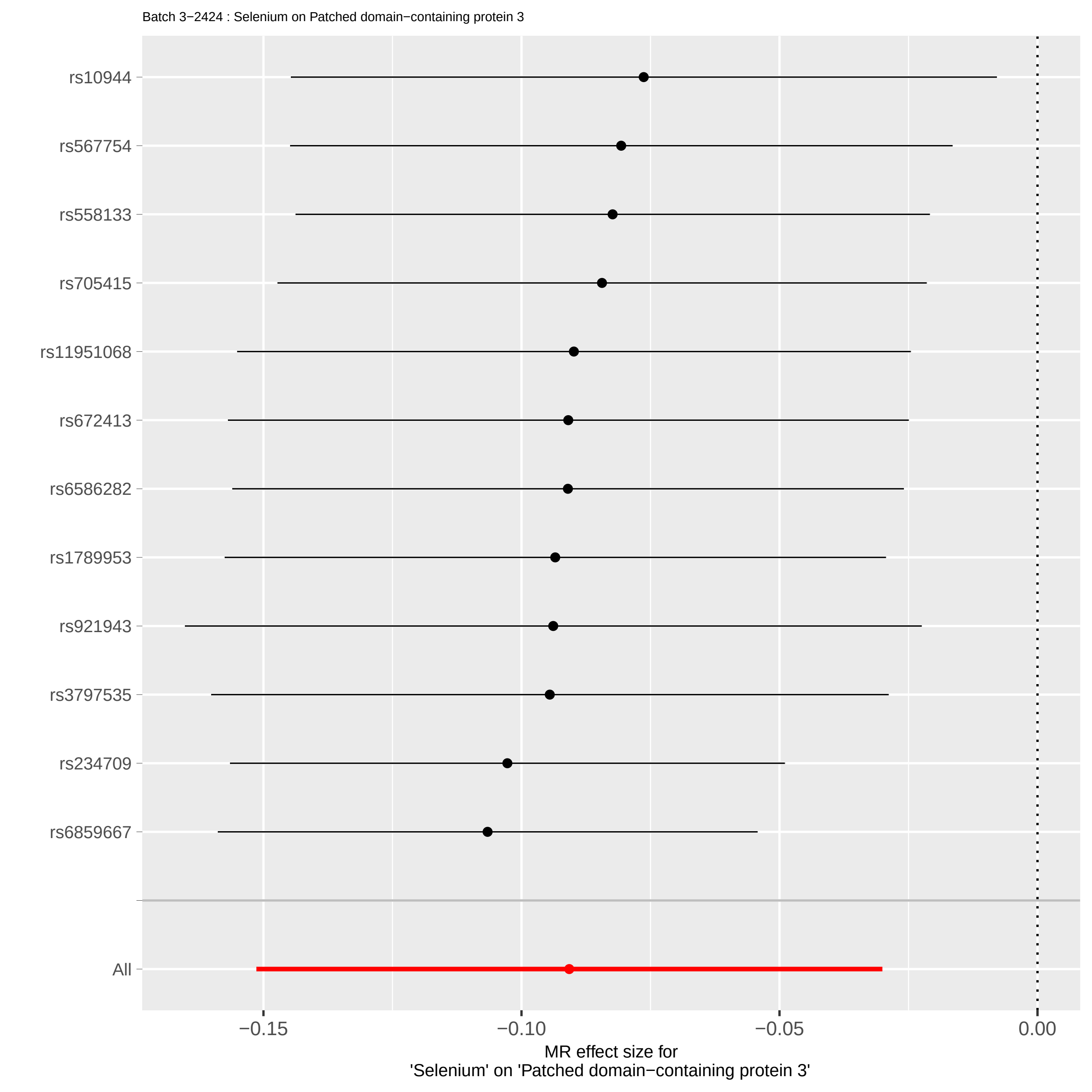

Batch 3−2424 : Selenium on Patched domain−containing protein 3
rs10944
rs567754
rs558133
rs705415
rs11951068
rs672413
rs6586282
rs1789953
rs921943
rs3797535
rs234709
rs6859667
All
−0.15
−0.10
−0.05
0.00
MR effect size for
'Selenium' on 'Patched domain−containing protein 3'

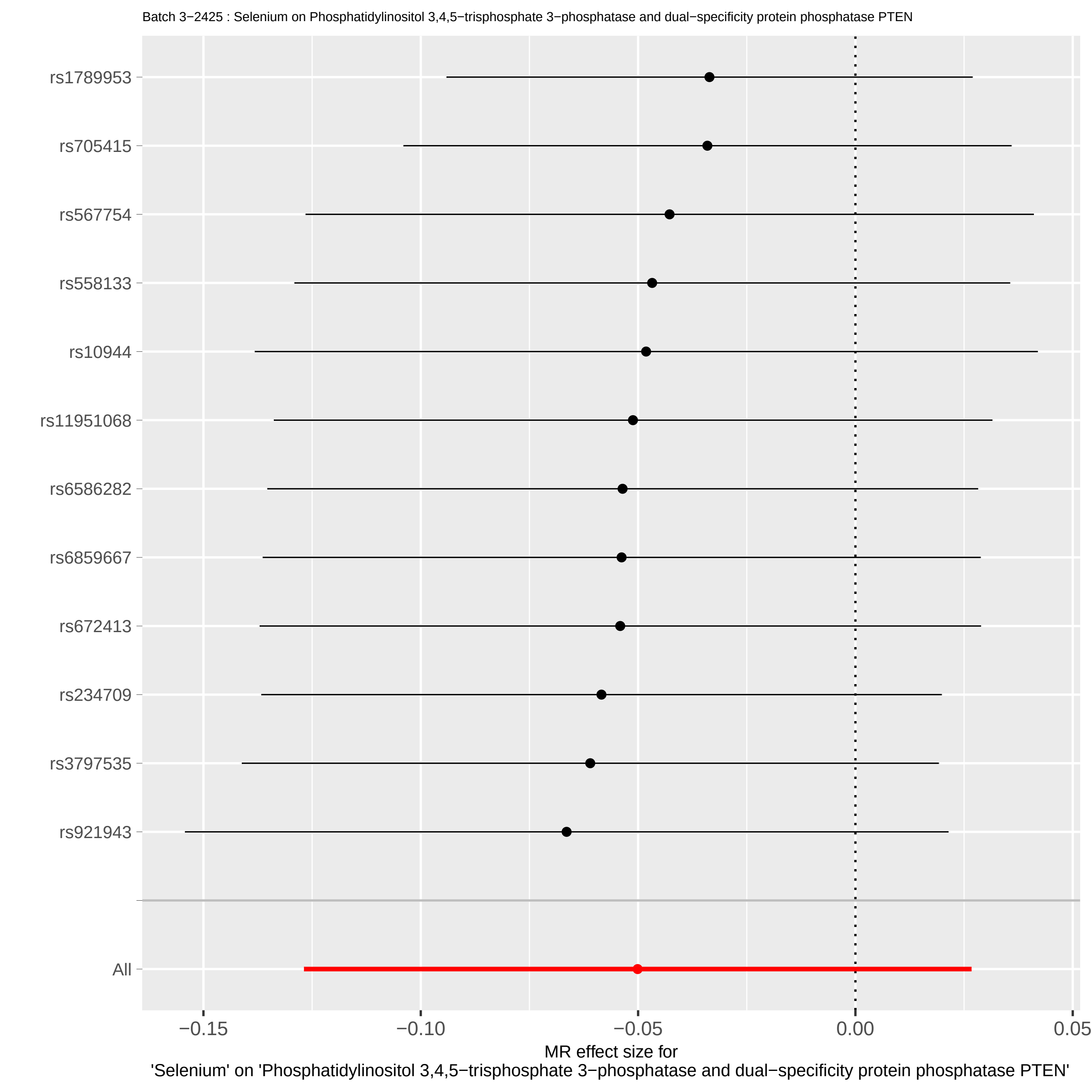

Batch 3−2425 : Selenium on Phosphatidylinositol 3,4,5−trisphosphate 3−phosphatase and dual−specificity protein phosphatase PTEN
rs1789953
rs705415
rs567754
rs558133
rs10944
rs11951068
rs6586282
rs6859667
rs672413
rs234709
rs3797535
rs921943
All
−0.15
−0.10
−0.05
0.00
0.05
MR effect size for
'Selenium' on 'Phosphatidylinositol 3,4,5−trisphosphate 3−phosphatase and dual−specificity protein phosphatase PTEN'

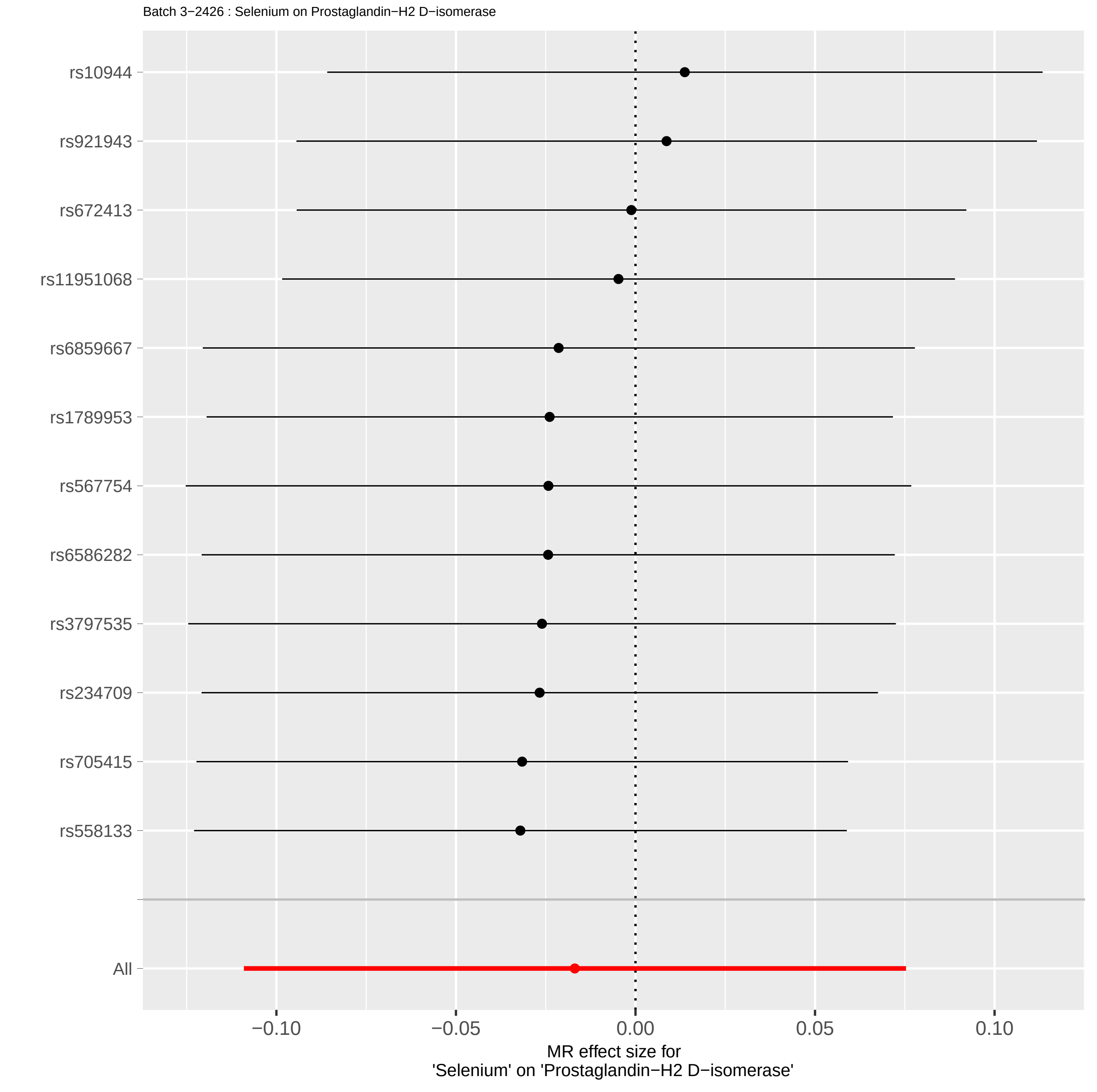
Batch 3−2426 : Selenium on Prostaglandin−H2 D−isomerase
rs10944
rs921943
rs672413
rs11951068
rs6859667
rs1789953
rs567754
rs6586282
rs3797535
rs234709
rs705415
rs558133
All
−0.10
−0.05
0.00
0.05
0.10
MR effect size for
'Selenium' on 'Prostaglandin−H2 D−isomerase'

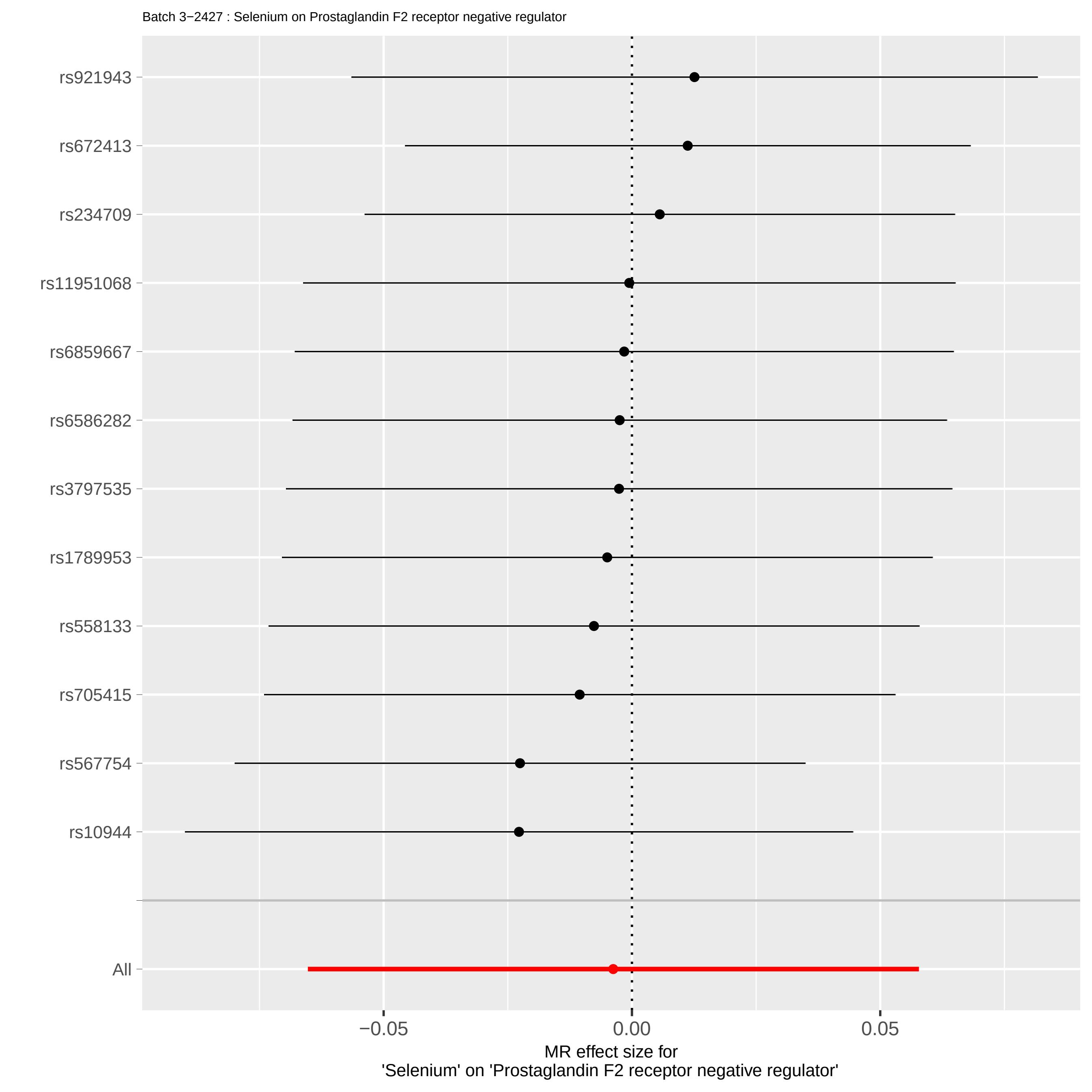

Batch 3−2427 : Selenium on Prostaglandin F2 receptor negative regulator
rs921943
rs672413
rs234709
rs11951068
rs6859667
rs6586282
rs3797535
rs1789953
rs558133
rs705415
rs567754
rs10944
All
−0.05
0.00
0.05
MR effect size for
'Selenium' on 'Prostaglandin F2 receptor negative regulator'

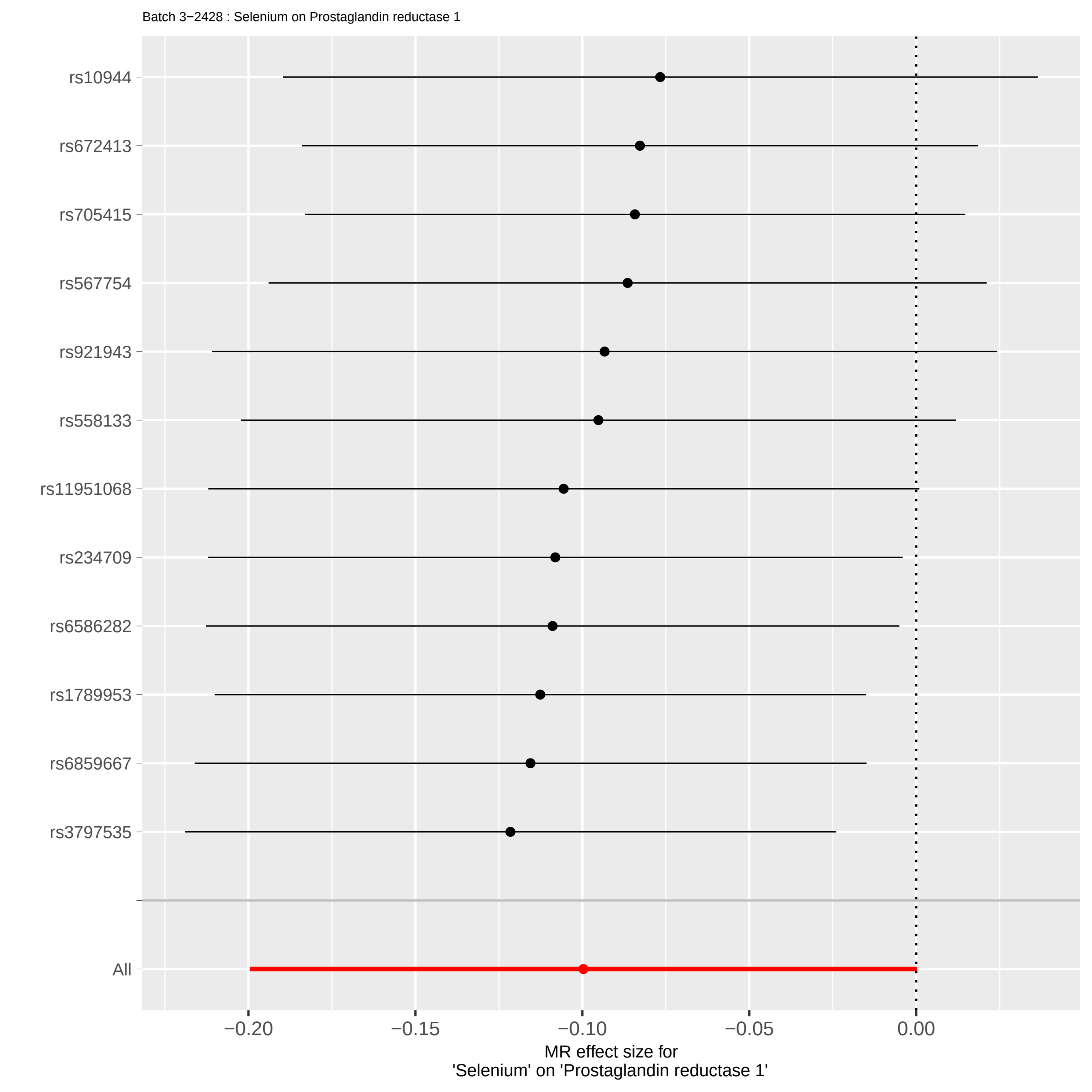

Batch 3−2428 : Selenium on Prostaglandin reductase 1
rs10944
rs672413
rs705415
rs567754
rs921943
rs558133
rs11951068
rs234709
rs6586282
rs1789953
rs6859667
rs3797535
All
−0.20
−0.15
−0.10
−0.05
0.00
MR effect size for
'Selenium' on 'Prostaglandin reductase 1'

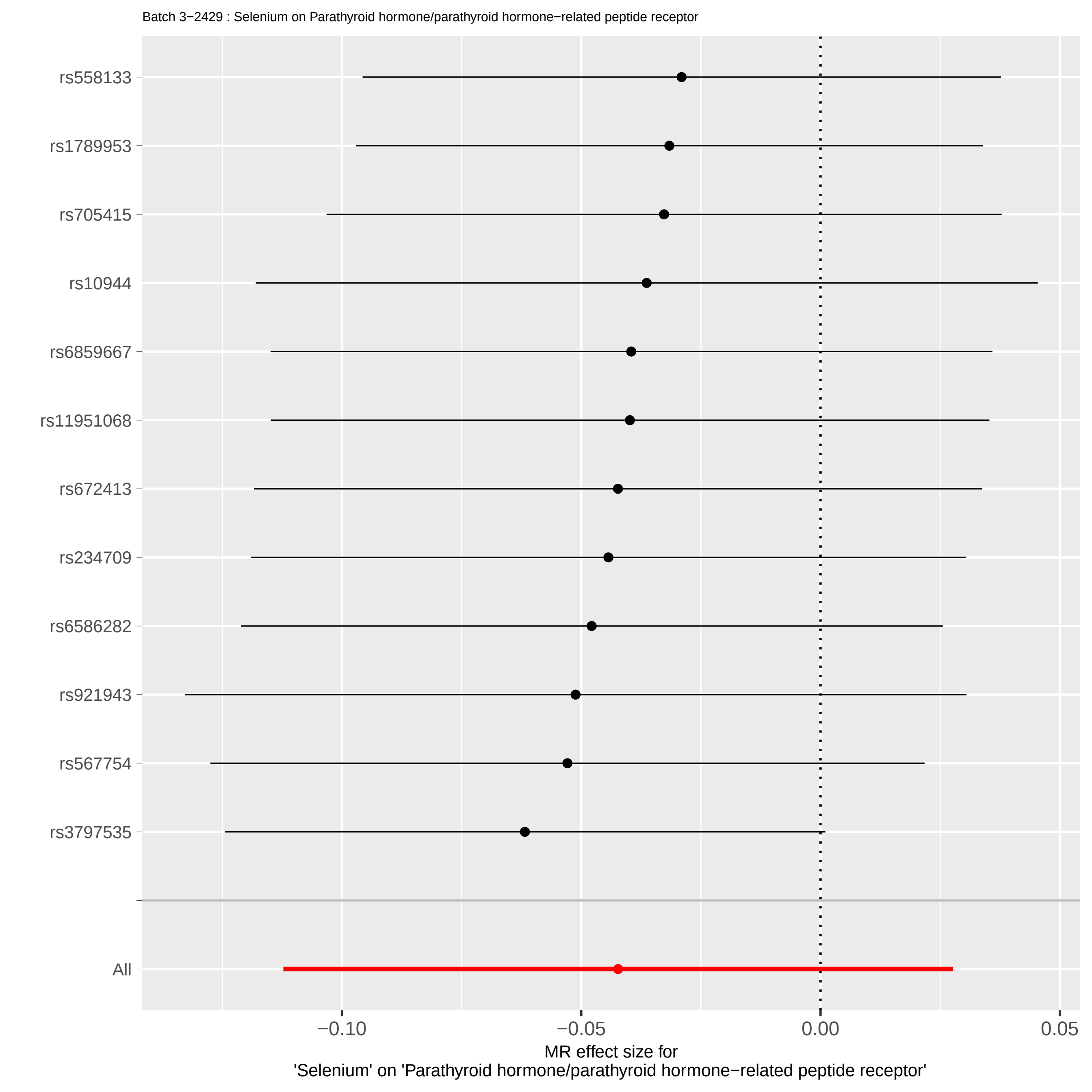

Batch 3−2429 : Selenium on Parathyroid hormone/parathyroid hormone−related peptide receptor
rs558133
rs1789953
rs705415
rs10944
rs6859667
rs11951068
rs672413
rs234709
rs6586282
rs921943
rs567754
rs3797535
All
−0.10
−0.05
0.00
0.05
MR effect size for
'Selenium' on 'Parathyroid hormone/parathyroid hormone−related peptide receptor'

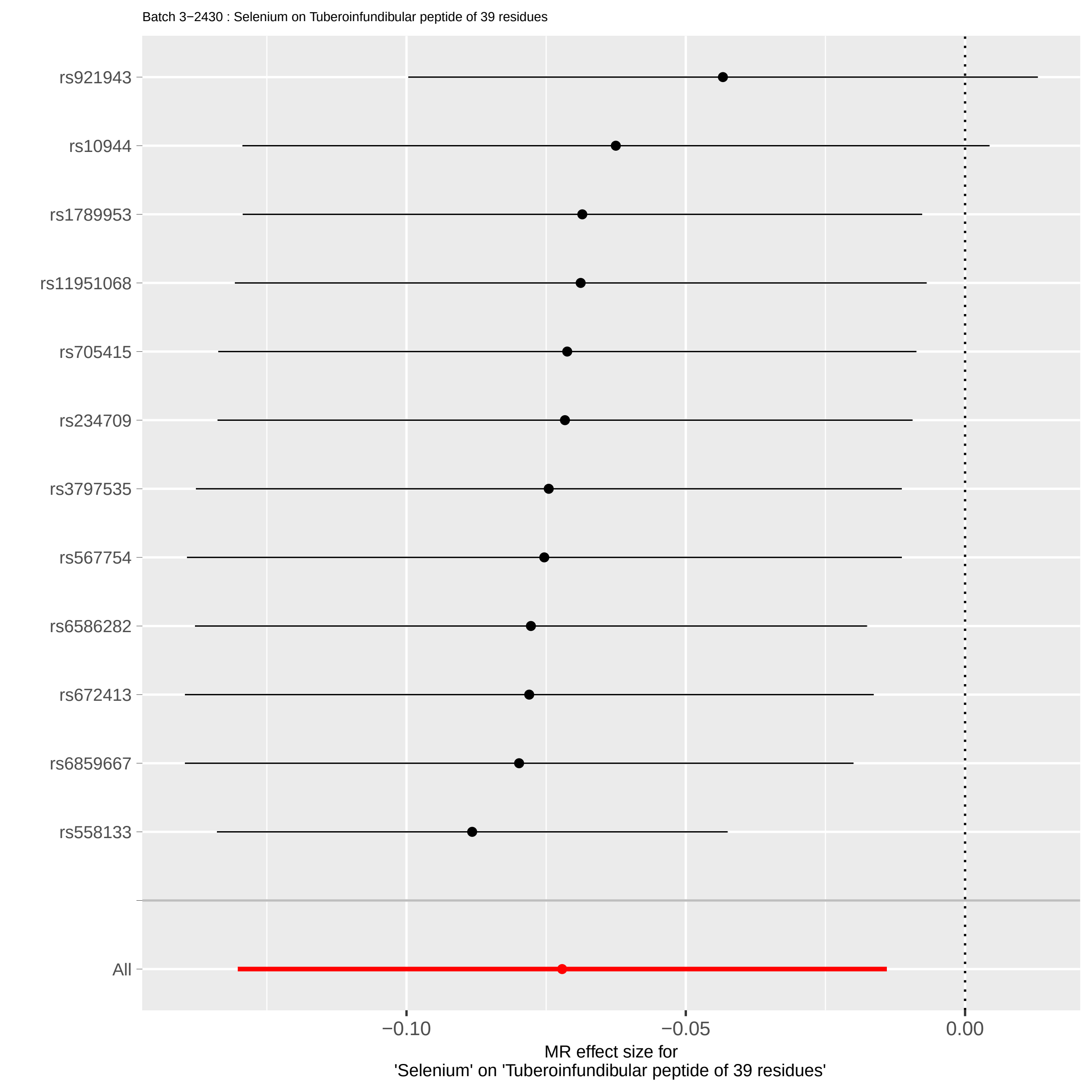

Batch 3−2430 : Selenium on Tuberoinfundibular peptide of 39 residues
rs921943
rs10944
rs1789953
rs11951068
rs705415
rs234709
rs3797535
rs567754
rs6586282
rs672413
rs6859667
rs558133
All
−0.10
−0.05
0.00
MR effect size for
'Selenium' on 'Tuberoinfundibular peptide of 39 residues'

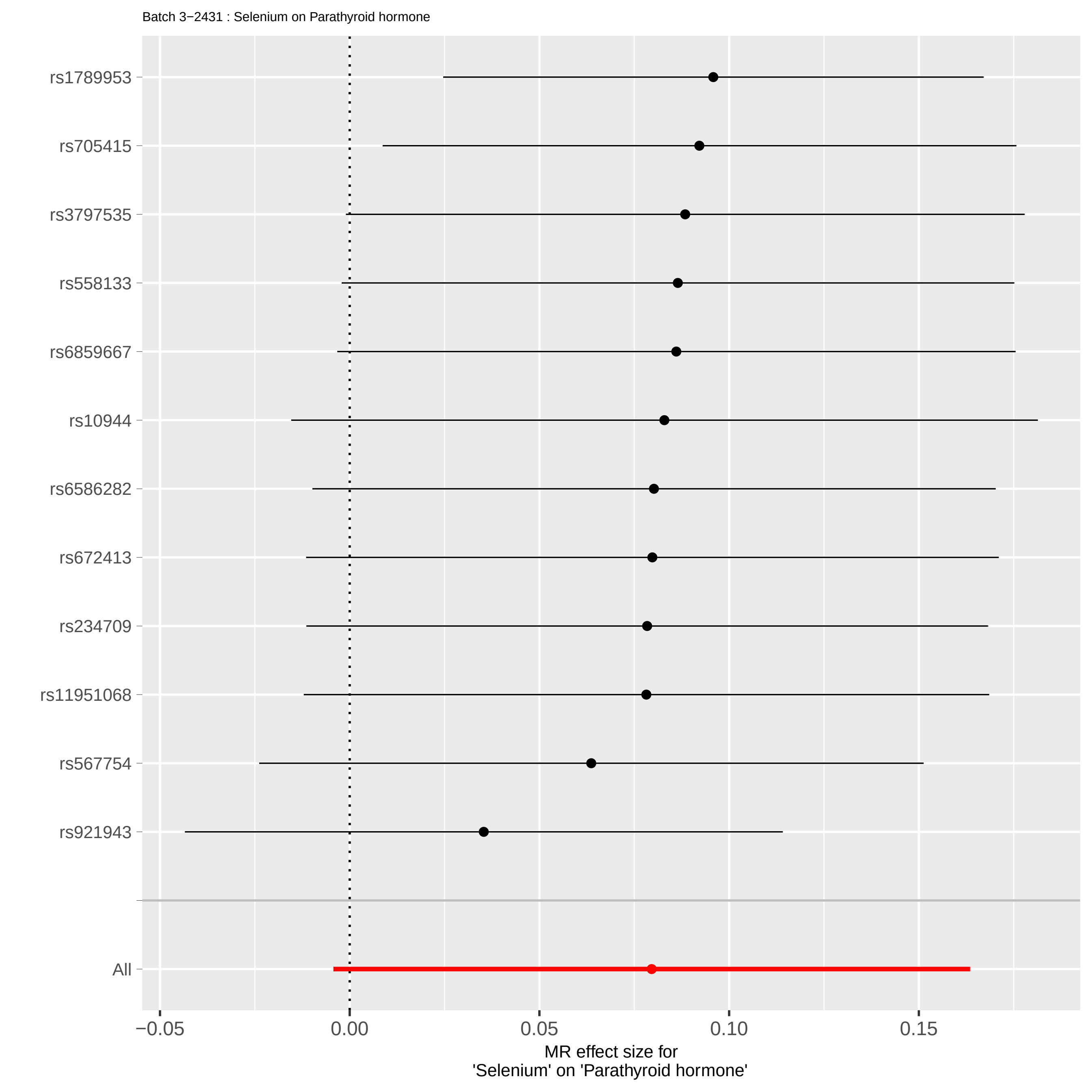

Batch 3−2431 : Selenium on Parathyroid hormone
rs1789953
rs705415
rs3797535
rs558133
rs6859667
rs10944
rs6586282
rs672413
rs234709
rs11951068
rs567754
rs921943
All
−0.05
0.00
0.05
0.10
0.15
MR effect size for
'Selenium' on 'Parathyroid hormone'

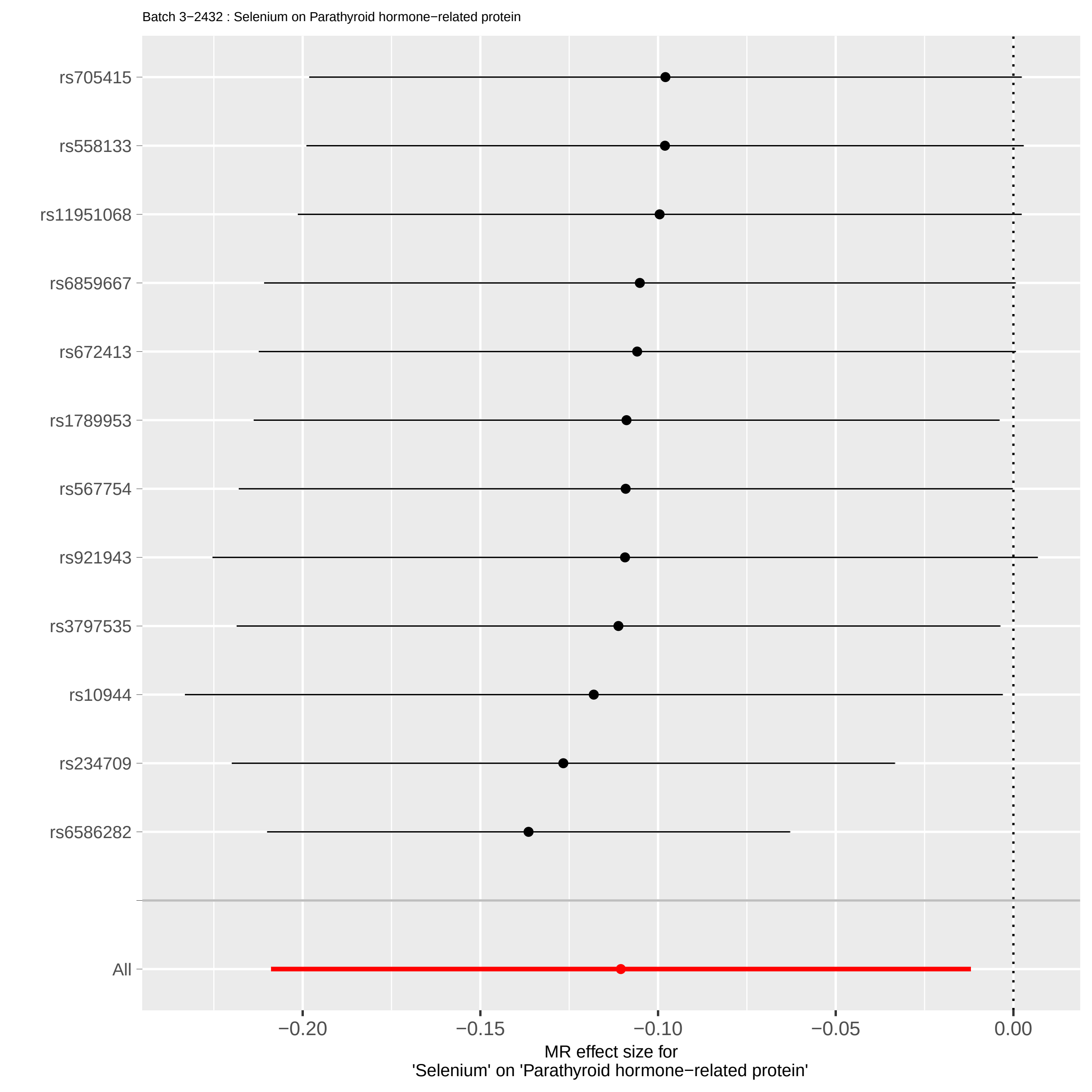

Batch 3−2432 : Selenium on Parathyroid hormone−related protein
rs705415
rs558133
rs11951068
rs6859667
rs672413
rs1789953
rs567754
rs921943
rs3797535
rs10944
rs234709
rs6586282
All
−0.20
−0.15
−0.10
−0.05
0.00
MR effect size for
'Selenium' on 'Parathyroid hormone−related protein'

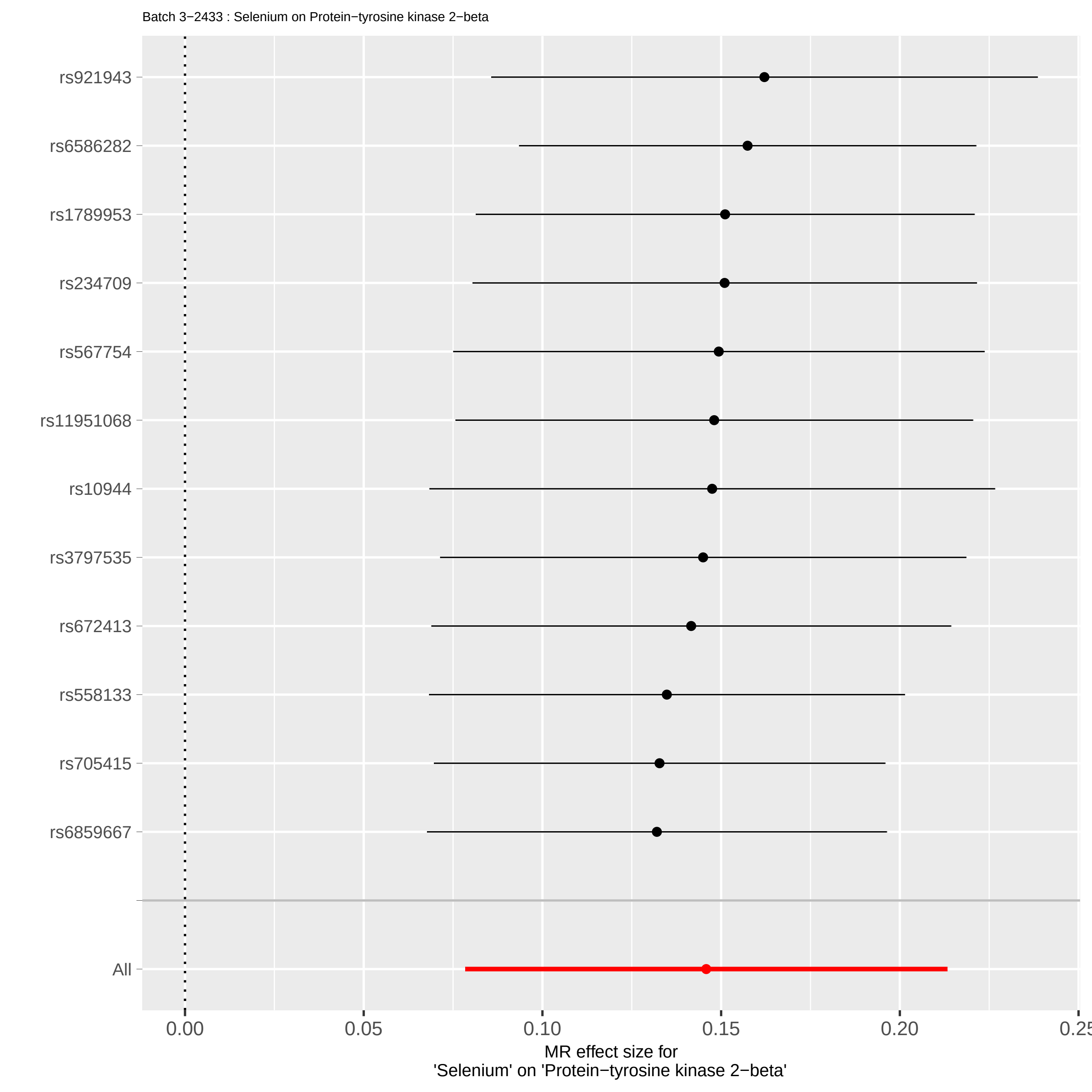
Batch 3−2433 : Selenium on Protein−tyrosine kinase 2−beta
rs921943
rs6586282
rs1789953
rs234709
rs567754
rs11951068
rs10944
rs3797535
rs672413
rs558133
rs705415
rs6859667
All
0.00
0.05
0.10
0.15
0.20
MR effect size for
'Selenium' on 'Protein−tyrosine kinase 2−beta'

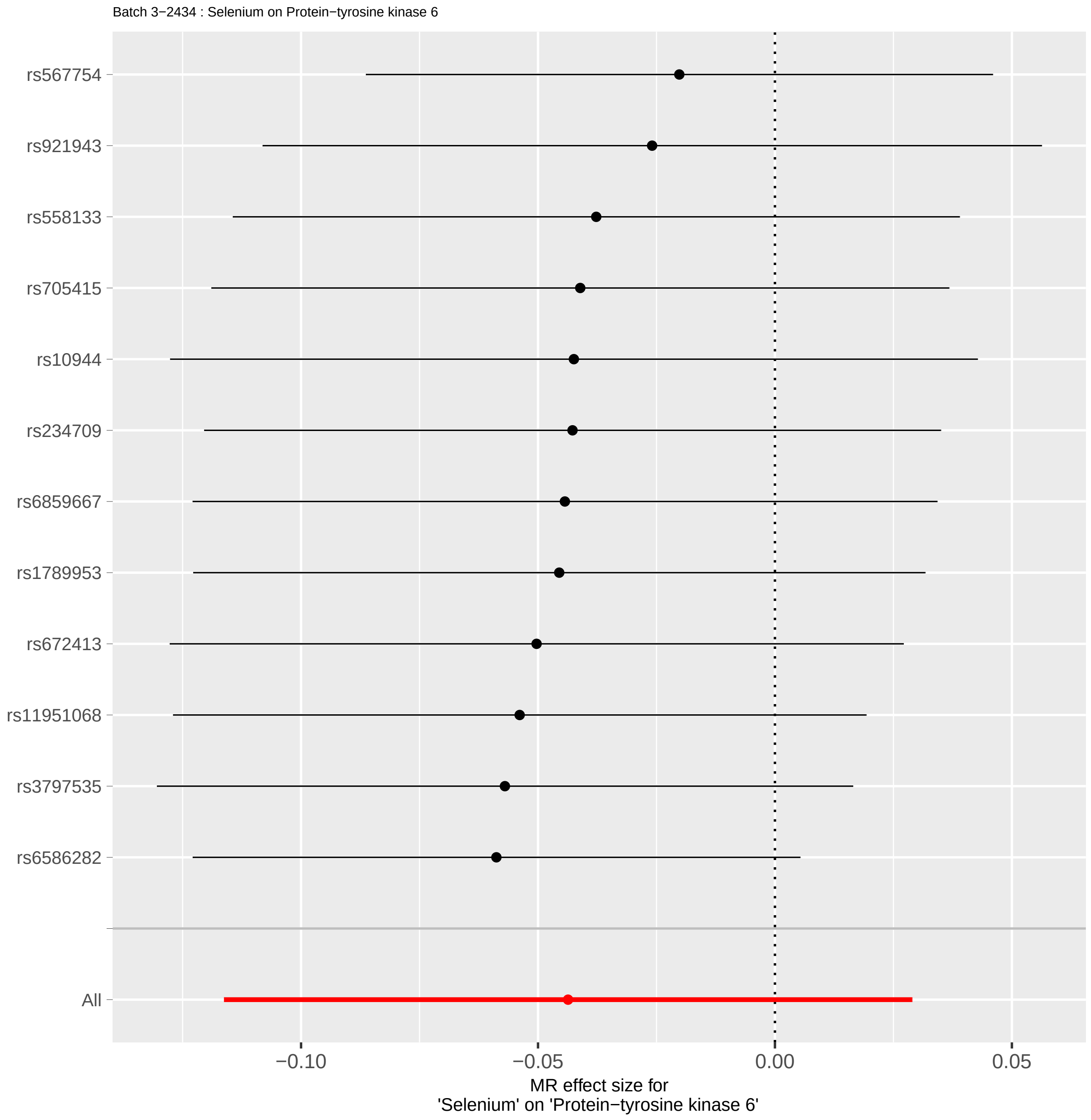

Batch 3−2434 : Selenium on Protein−tyrosine kinase 6
rs567754
rs921943
rs558133
rs705415
rs10944
rs234709
rs6859667
rs1789953
rs672413
rs11951068
rs3797535
rs6586282
All
−0.10
−0.05
0.00
0.05
MR effect size for
'Selenium' on 'Protein−tyrosine kinase 6'

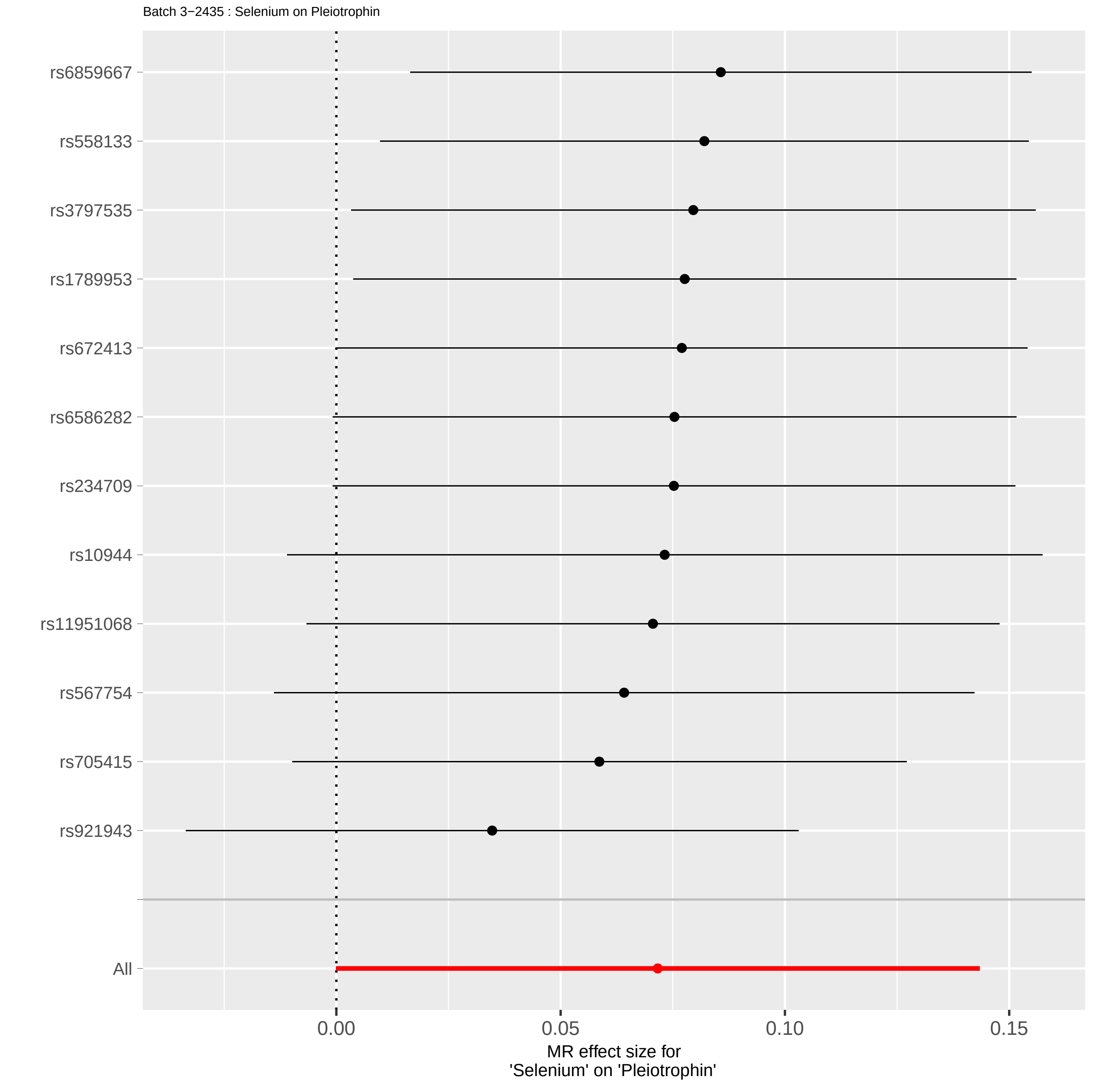

Batch 3−2435 : Selenium on Pleiotrophin
rs6859667
rs558133
rs3797535
rs1789953
rs672413
rs6586282
rs234709
rs10944
rs11951068
rs567754
rs705415
rs921943
All
0.00
0.05
0.10
0.15
MR effect size for
'Selenium' on 'Pleiotrophin'

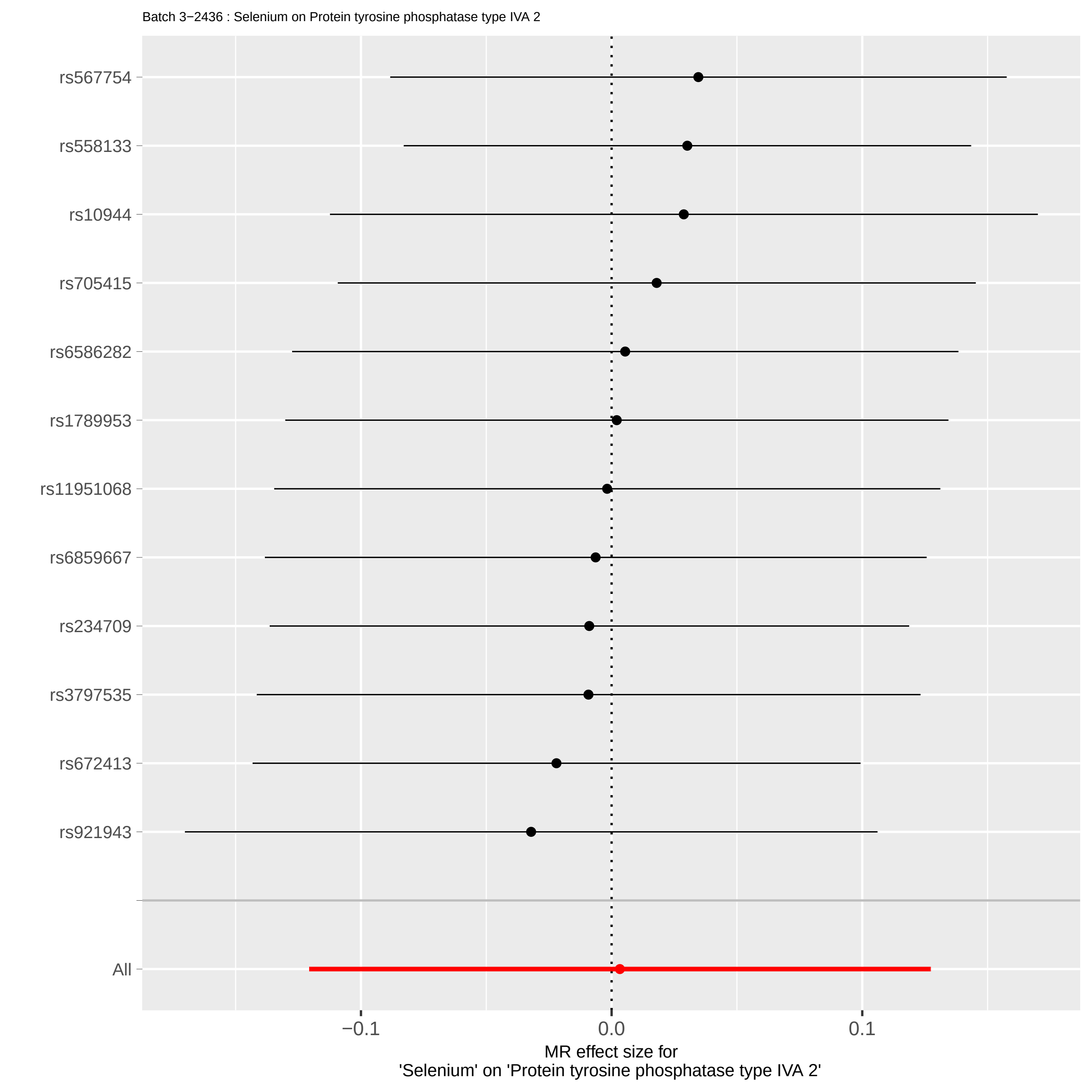
Batch 3−2436 : Selenium on Protein tyrosine phosphatase type IVA 2
rs567754
rs558133
rs10944
rs705415
rs6586282
rs1789953
rs11951068
rs6859667
rs234709
rs3797535
rs672413
rs921943
All
−0.1
0.0
0.1
MR effect size for
'Selenium' on 'Protein tyrosine phosphatase type IVA 2'

Batch 3−2437 : Selenium on Tyrosine−protein phosphatase non−receptor type 11

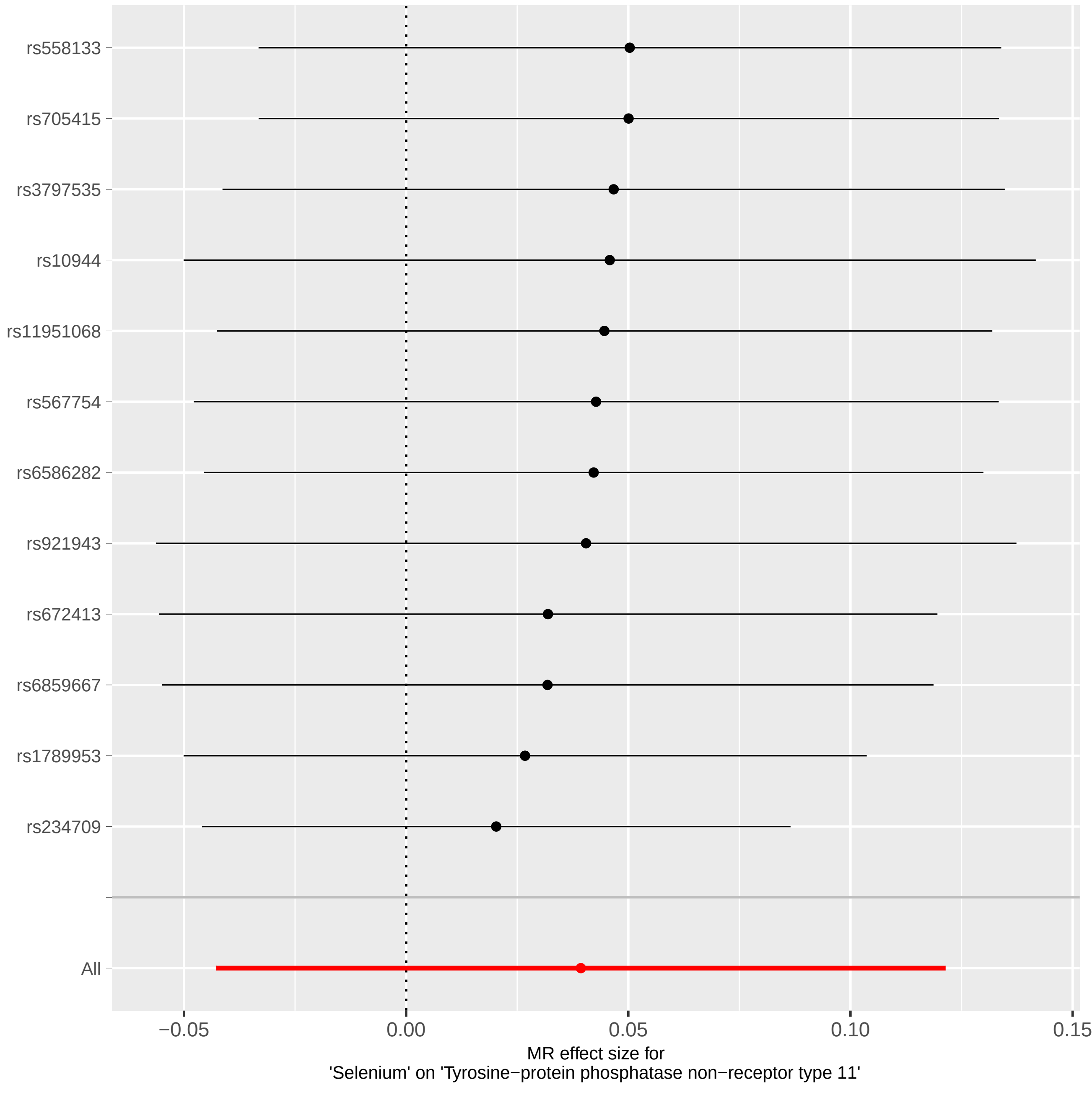

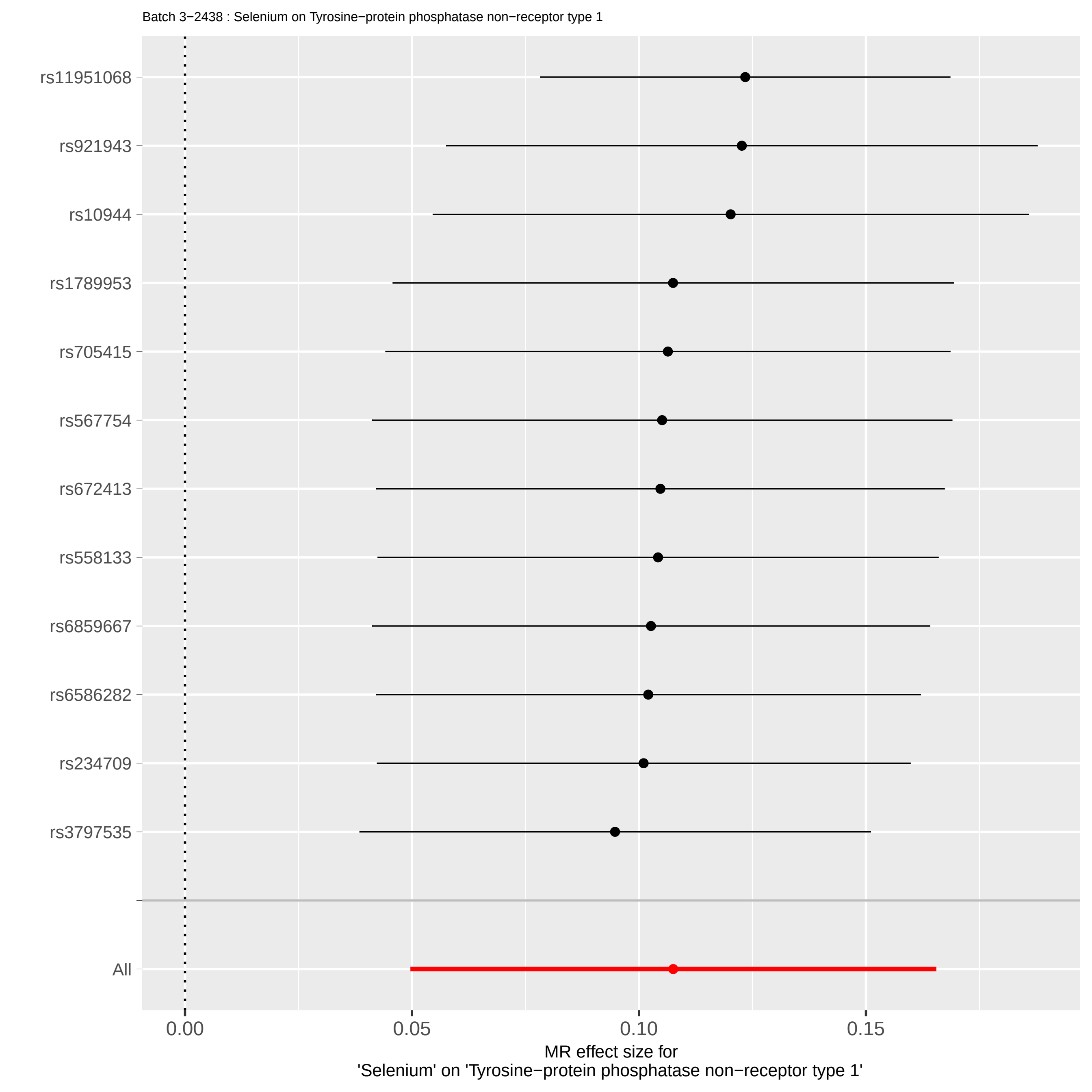

Batch 3−2438 : Selenium on Tyrosine−protein phosphatase non−receptor type 1
rs11951068
rs921943
rs10944
rs1789953
rs705415
rs567754
rs672413
rs558133
rs6859667
rs6586282
rs234709
rs3797535
All
0.00
0.05
0.10
0.15
MR effect size for
'Selenium' on 'Tyrosine−protein phosphatase non−receptor type 1'

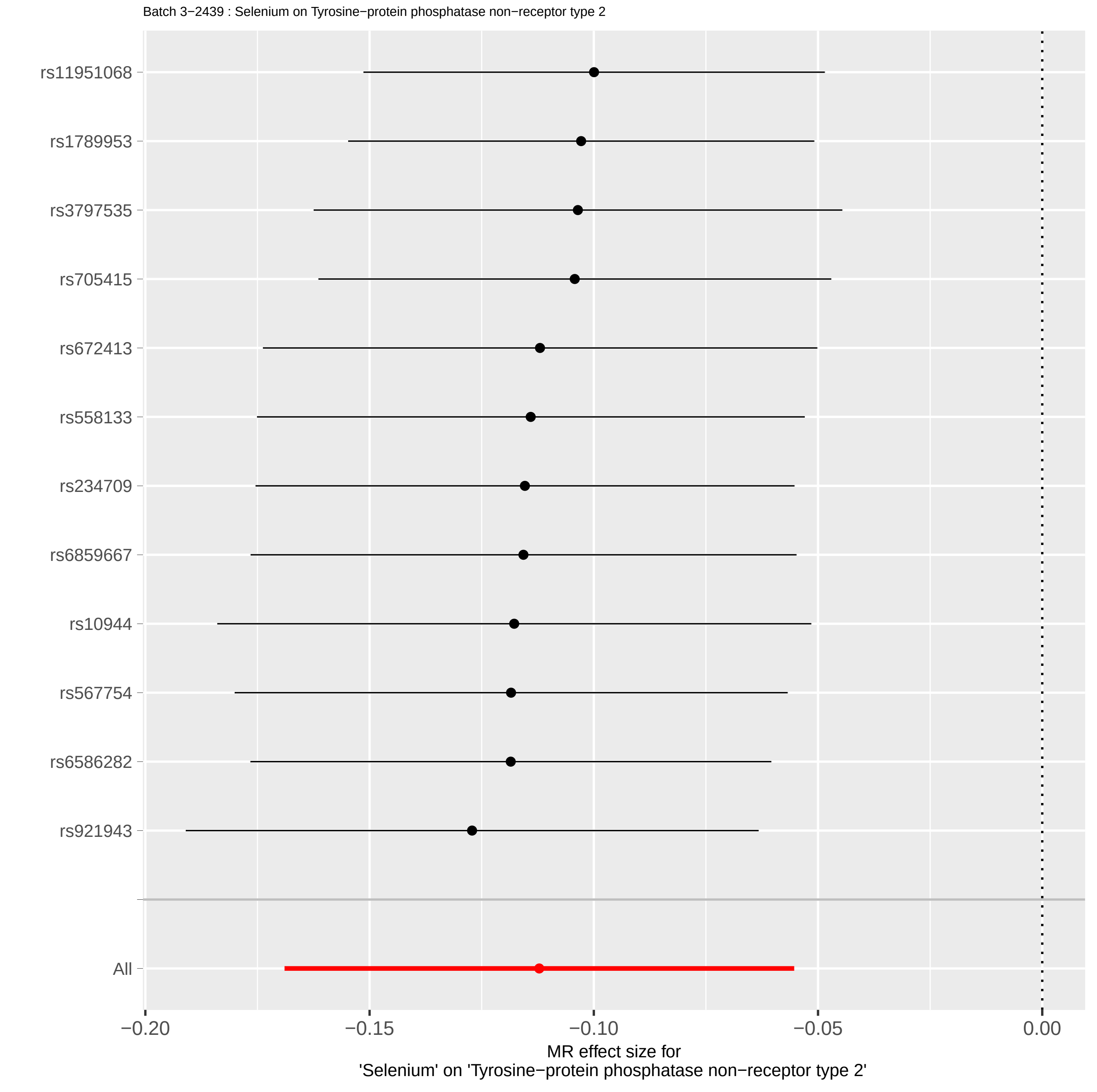
Batch 3−2439 : Selenium on Tyrosine−protein phosphatase non−receptor type 2
rs11951068
rs1789953
rs3797535
rs705415
rs672413
rs558133
rs234709
rs6859667
rs10944
rs567754
rs6586282
rs921943
All
−0.20
−0.15
−0.10
−0.05
0.00
MR effect size for
'Selenium' on 'Tyrosine−protein phosphatase non−receptor type 2'

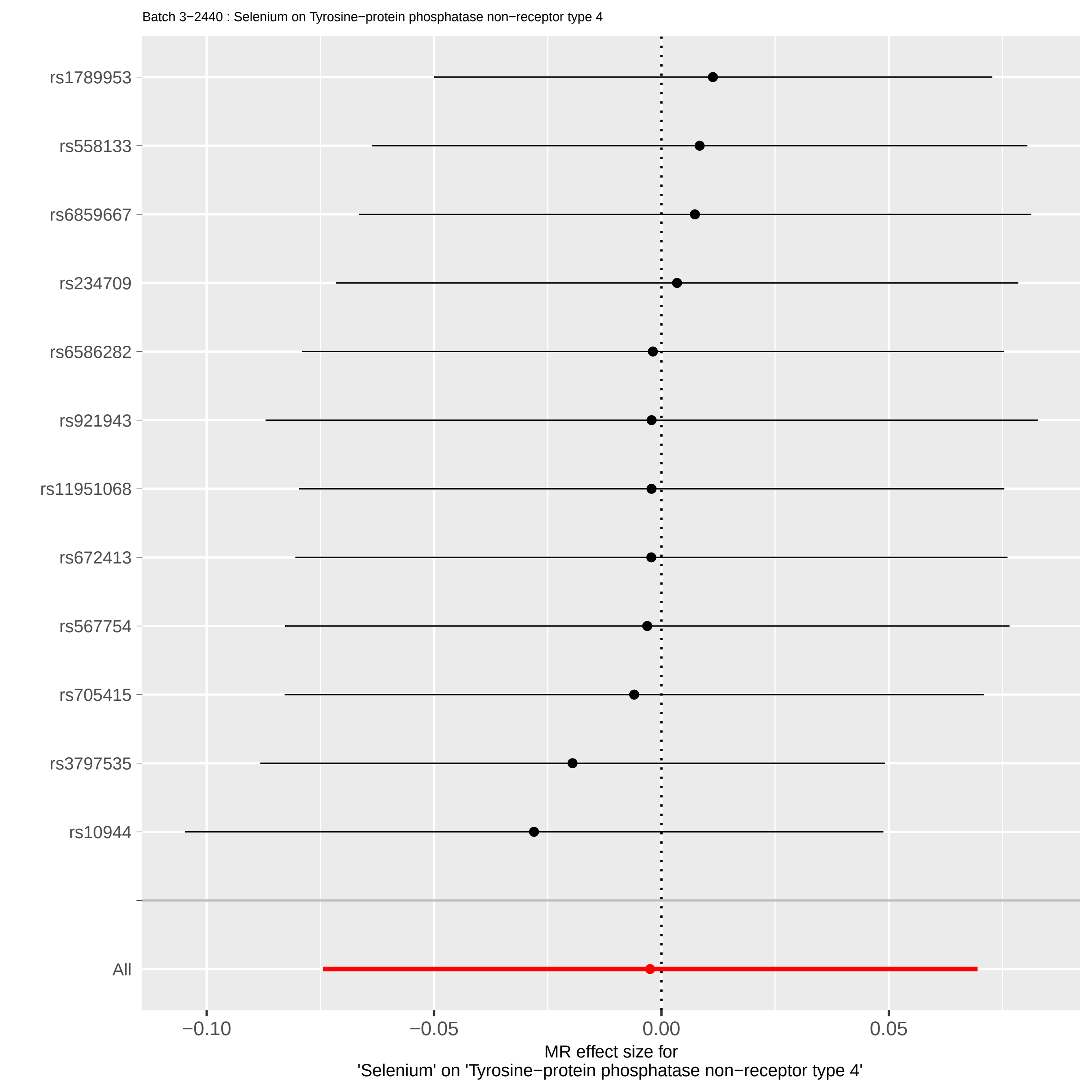

Batch 3−2440 : Selenium on Tyrosine−protein phosphatase non−receptor type 4
rs1789953
rs558133
rs6859667
rs234709
rs6586282
rs921943
rs11951068
rs672413
rs567754
rs705415
rs3797535
rs10944
All
−0.10
−0.05
0.00
0.05
MR effect size for
'Selenium' on 'Tyrosine−protein phosphatase non−receptor type 4'

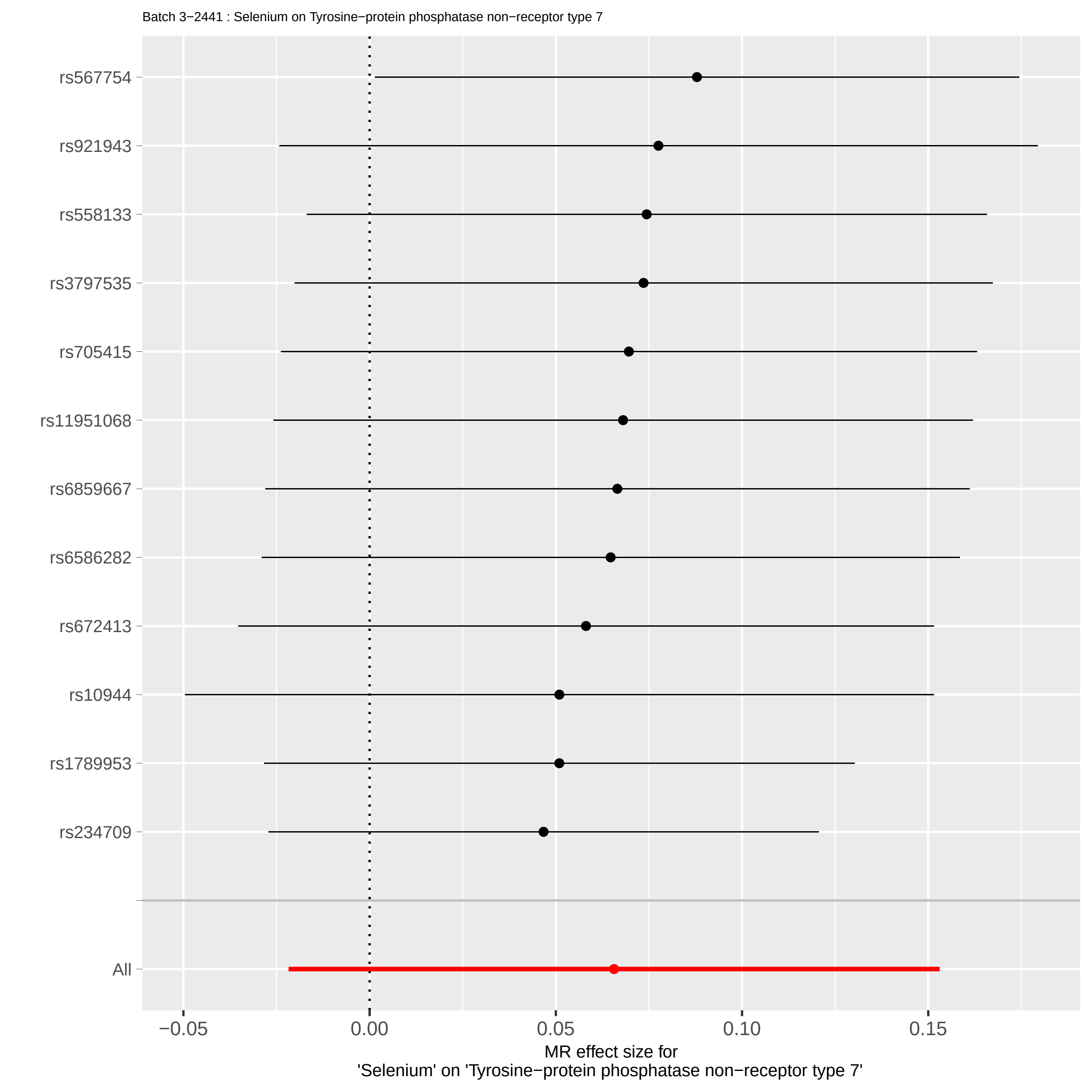

Batch 3−2441 : Selenium on Tyrosine−protein phosphatase non−receptor type 7
rs567754
rs921943
rs558133
rs3797535
rs705415
rs11951068
rs6859667
rs6586282
rs672413
rs10944
rs1789953
rs234709
All
−0.05
0.00
0.05
0.10
0.15
MR effect size for
'Selenium' on 'Tyrosine−protein phosphatase non−receptor type 7'

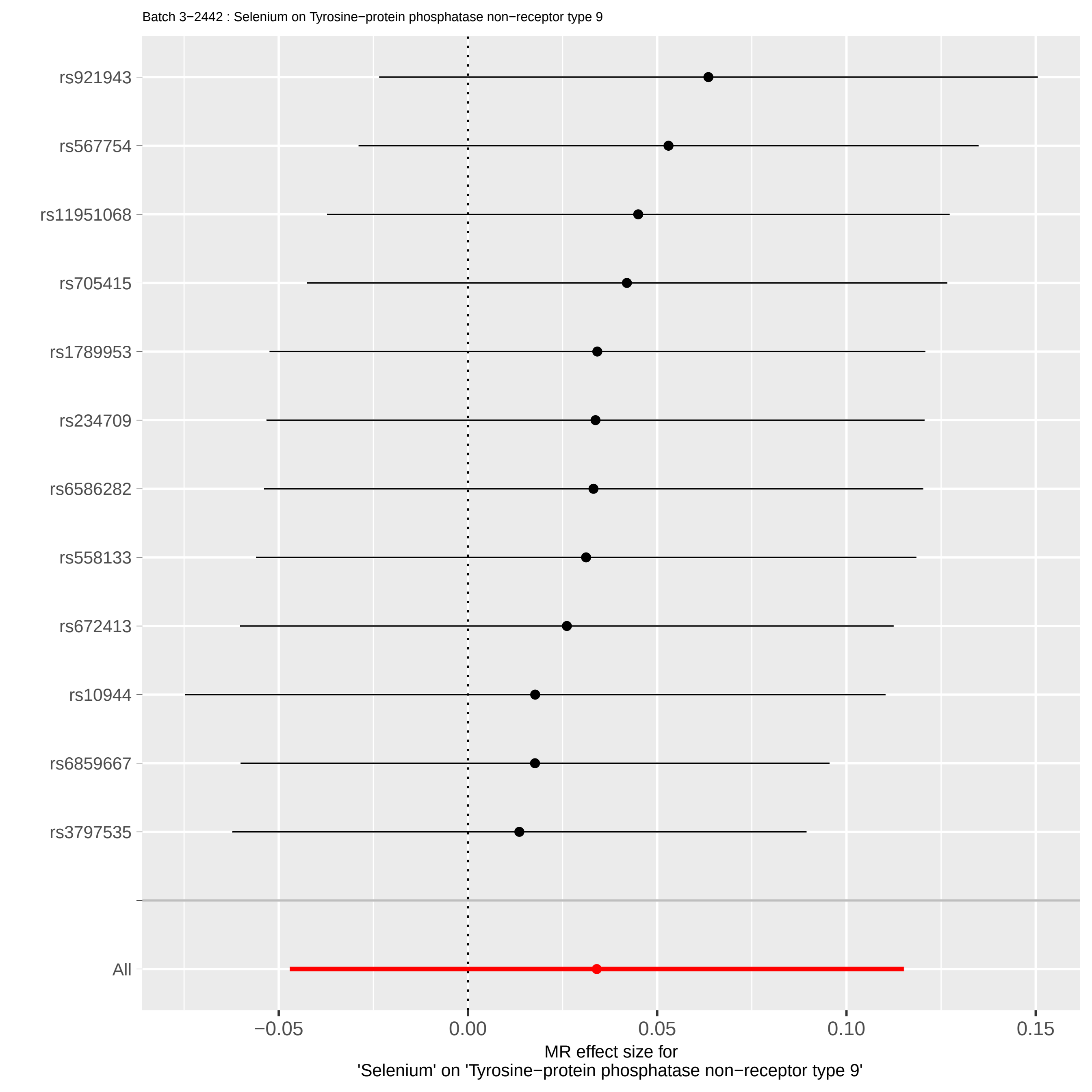

Batch 3−2442 : Selenium on Tyrosine−protein phosphatase non−receptor type 9
rs921943
rs567754
rs11951068
rs705415
rs1789953
rs234709
rs6586282
rs558133
rs672413
rs10944
rs6859667
rs3797535
All
−0.05
0.00
0.05
0.10
0.15
MR effect size for
'Selenium' on 'Tyrosine−protein phosphatase non−receptor type 9'

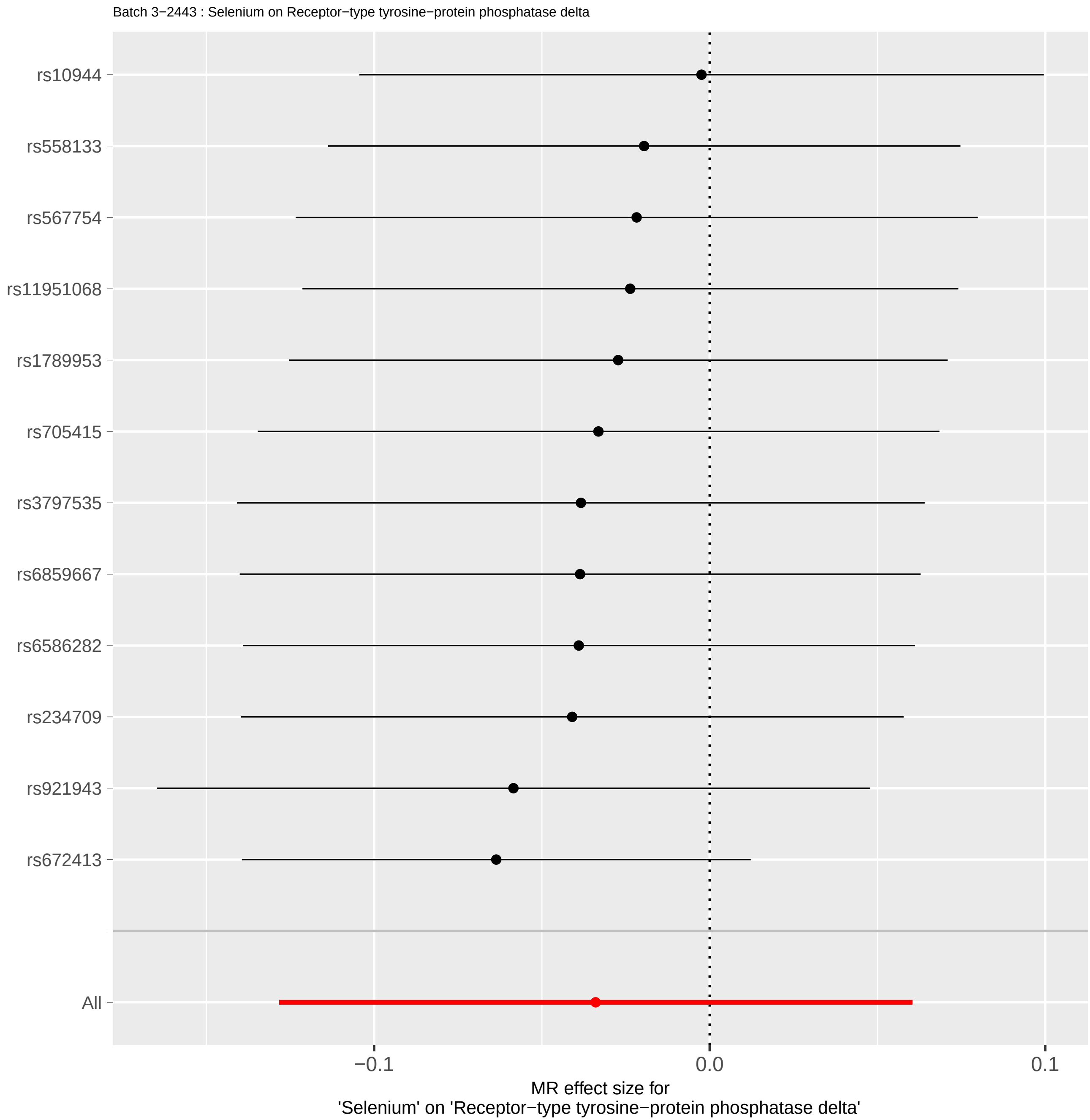

Batch 3−2443 : Selenium on Receptor−type tyrosine−protein phosphatase delta
rs10944
rs558133
rs567754
rs11951068
rs1789953
rs705415
rs3797535
rs6859667
rs6586282
rs234709
rs921943
rs672413
All
−0.1
0.0
0.1
MR effect size for
'Selenium' on 'Receptor−type tyrosine−protein phosphatase delta'

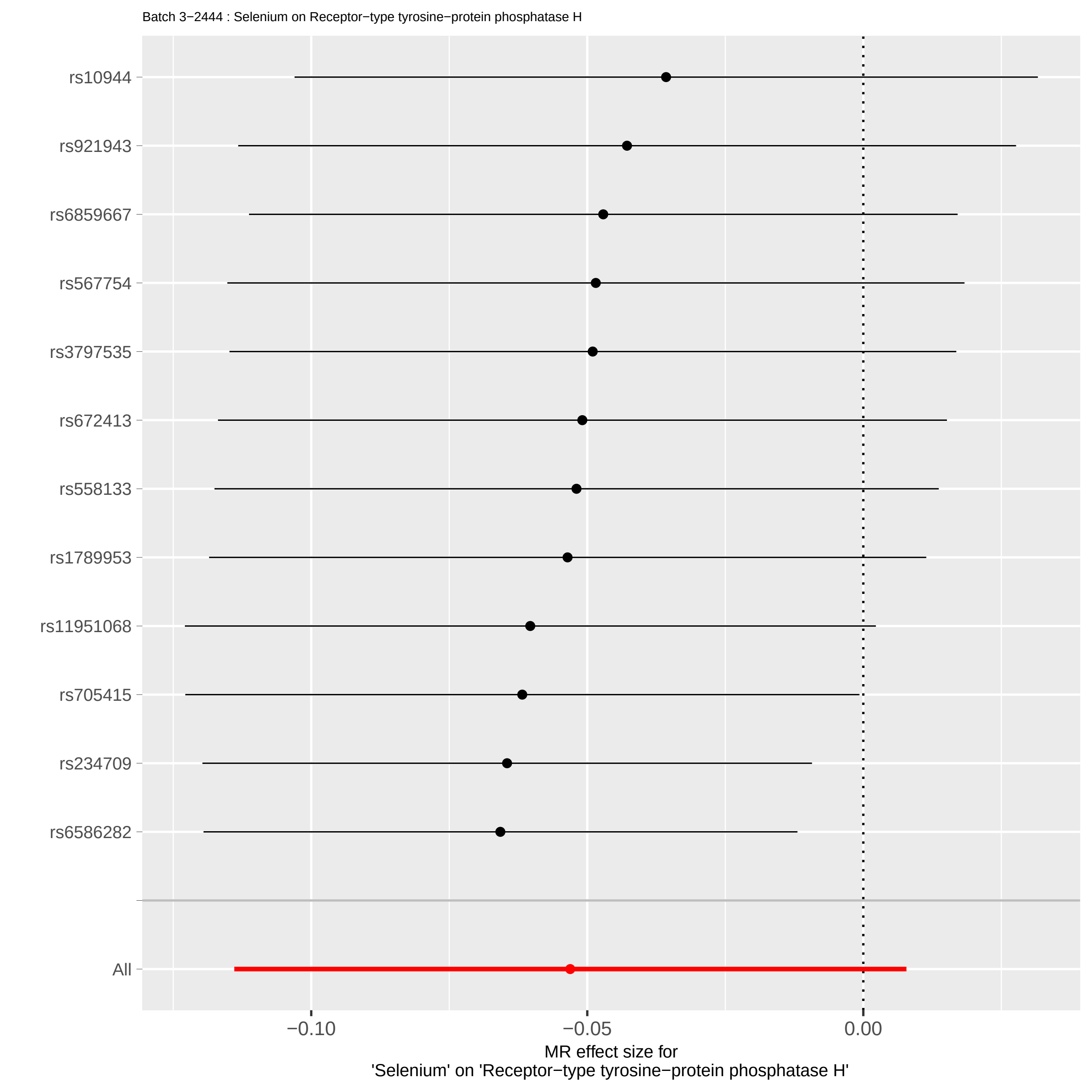

Batch 3−2444 : Selenium on Receptor−type tyrosine−protein phosphatase H
rs10944
rs921943
rs6859667
rs567754
rs3797535
rs672413
rs558133
rs1789953
rs11951068
rs705415
rs234709
rs6586282
All
−0.10
−0.05
0.00
MR effect size for
'Selenium' on 'Receptor−type tyrosine−protein phosphatase H'

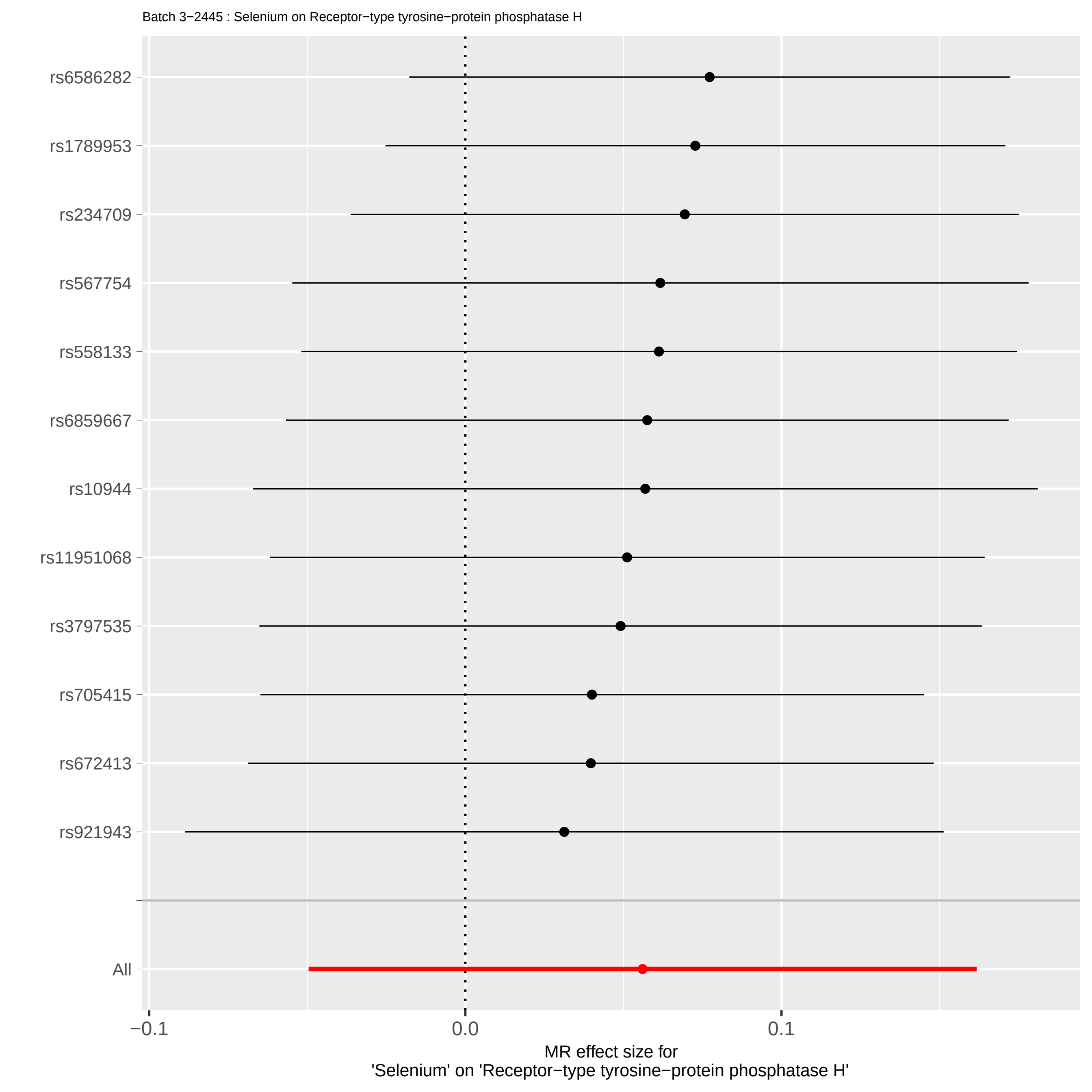
Batch 3−2445 : Selenium on Receptor−type tyrosine−protein phosphatase H
rs6586282
rs1789953
rs234709
rs567754
rs558133
rs6859667
rs10944
rs11951068
rs3797535
rs705415
rs672413
rs921943
All
−0.1
0.0
0.1
MR effect size for
'Selenium' on 'Receptor−type tyrosine−protein phosphatase H'

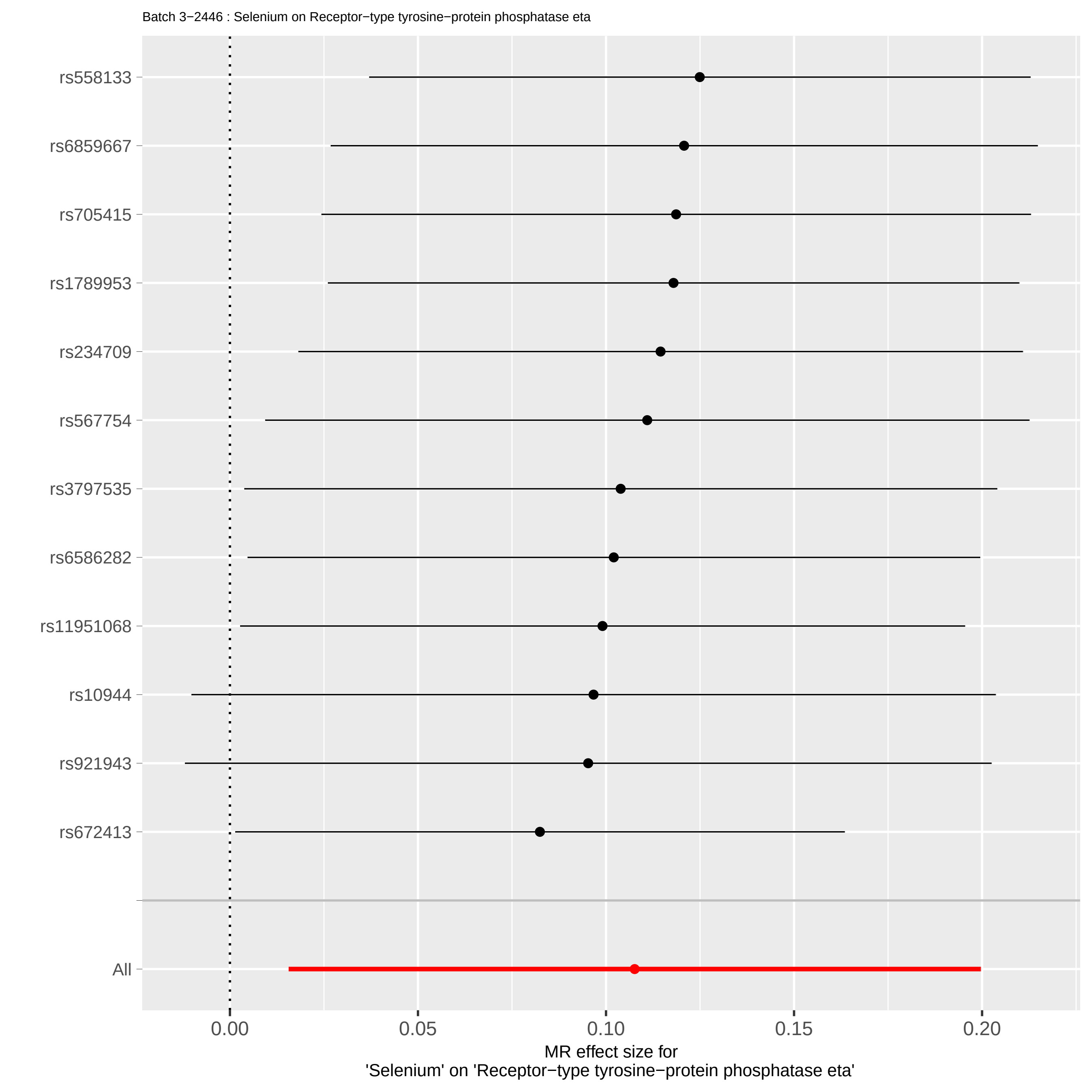

Batch 3−2446 : Selenium on Receptor−type tyrosine−protein phosphatase eta
rs558133
rs6859667
rs705415
rs1789953
rs234709
rs567754
rs3797535
rs6586282
rs11951068
rs10944
rs921943
rs672413
All
0.00
0.05
0.10
0.15
0.20
MR effect size for
'Selenium' on 'Receptor−type tyrosine−protein phosphatase eta'

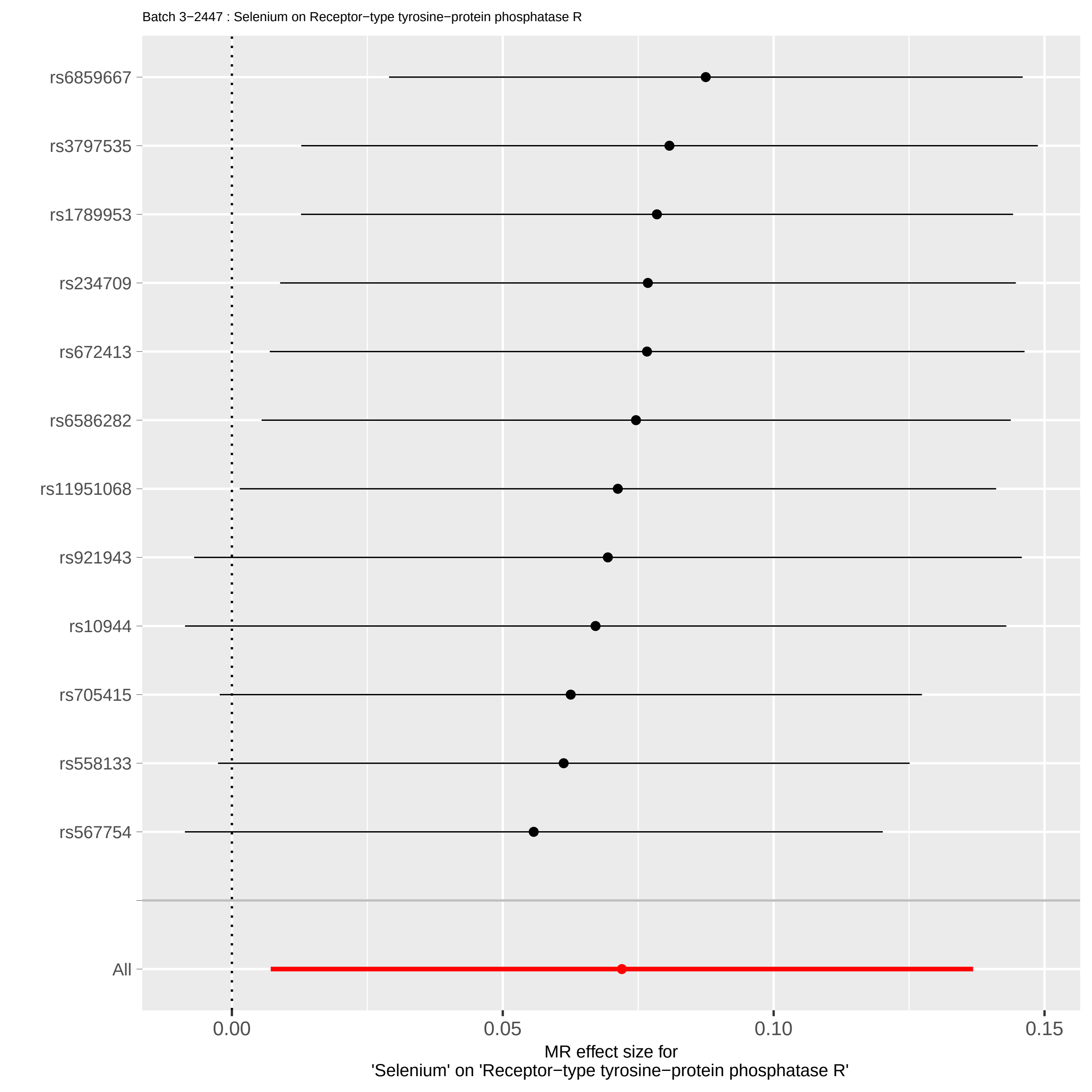
Batch 3−2447 : Selenium on Receptor−type tyrosine−protein phosphatase R
rs6859667
rs3797535
rs1789953
rs234709
rs672413
rs6586282
rs11951068
rs921943
rs10944
rs705415
rs558133
rs567754
All
0.00
0.05
0.10
0.15
MR effect size for
'Selenium' on 'Receptor−type tyrosine−protein phosphatase R'

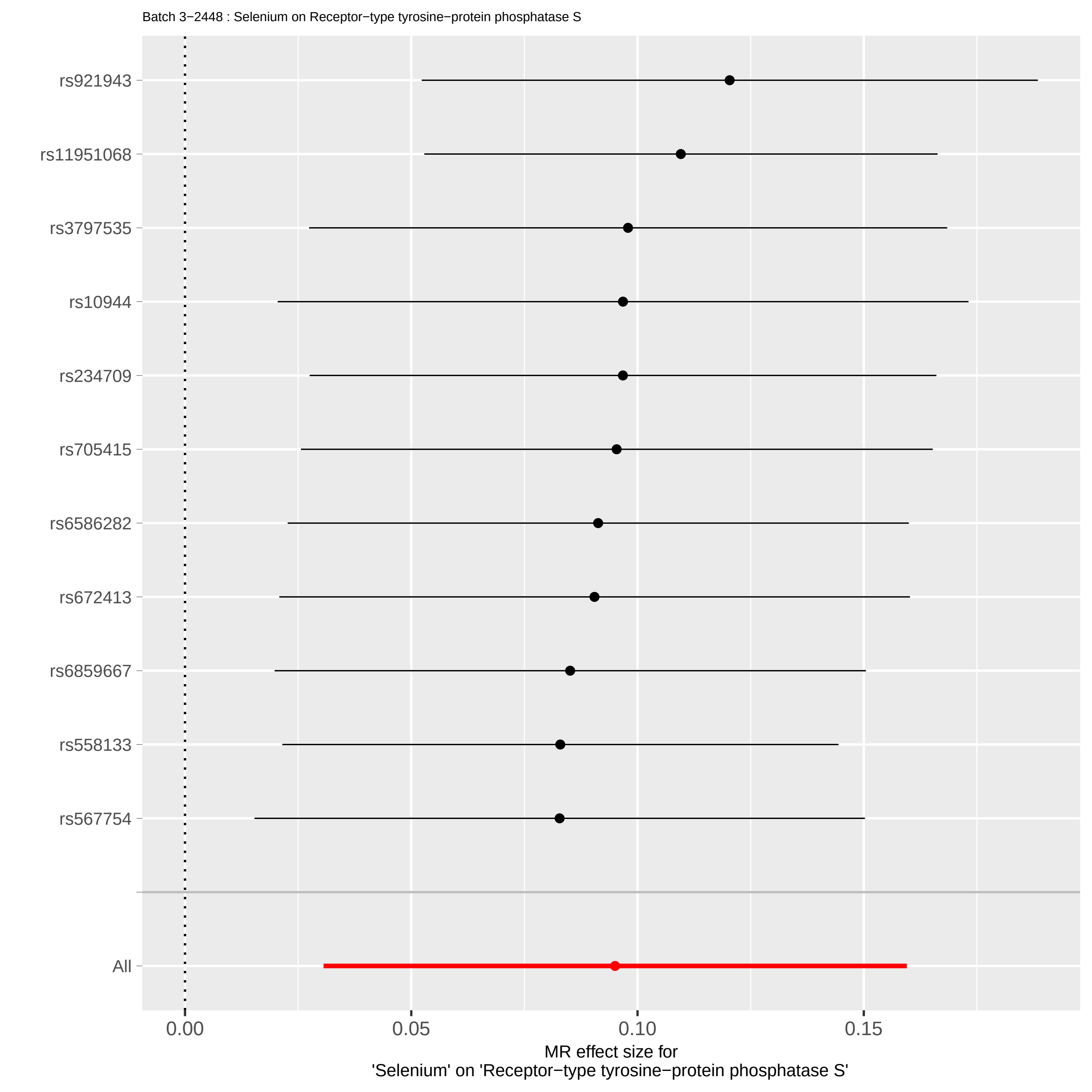
Batch 3−2448 : Selenium on Receptor−type tyrosine−protein phosphatase S
rs921943
rs11951068
rs3797535
rs10944
rs234709
rs705415
rs6586282
rs672413
rs6859667
rs558133
rs567754
All
0.00
0.05
0.10
0.15
MR effect size for
'Selenium' on 'Receptor−type tyrosine−protein phosphatase S'

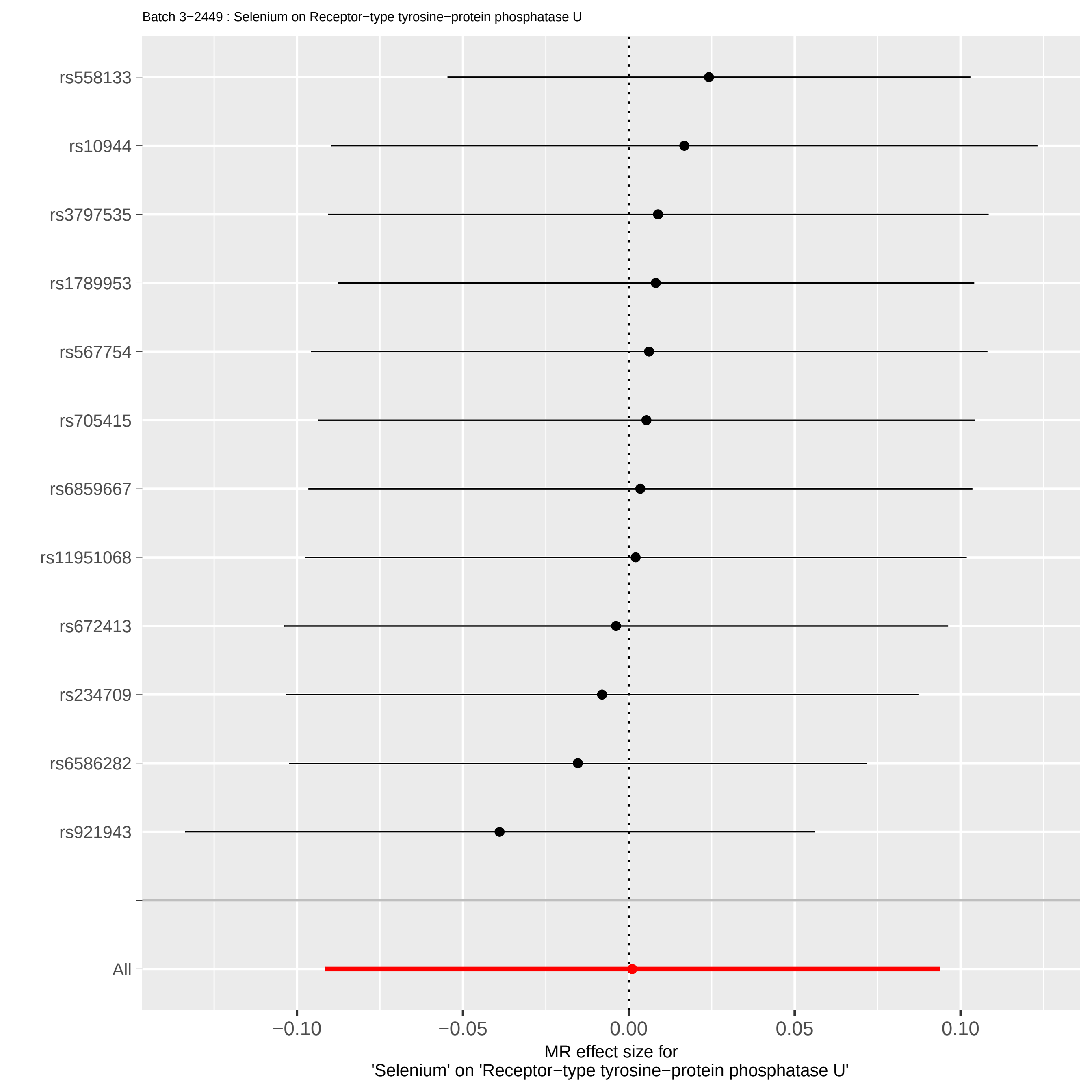

Batch 3−2449 : Selenium on Receptor−type tyrosine−protein phosphatase U
rs558133
rs10944
rs3797535
rs1789953
rs567754
rs705415
rs6859667
rs11951068
rs672413
rs234709
rs6586282
rs921943
All
−0.10
−0.05
0.00
0.05
0.10
MR effect size for
'Selenium' on 'Receptor−type tyrosine−protein phosphatase U'

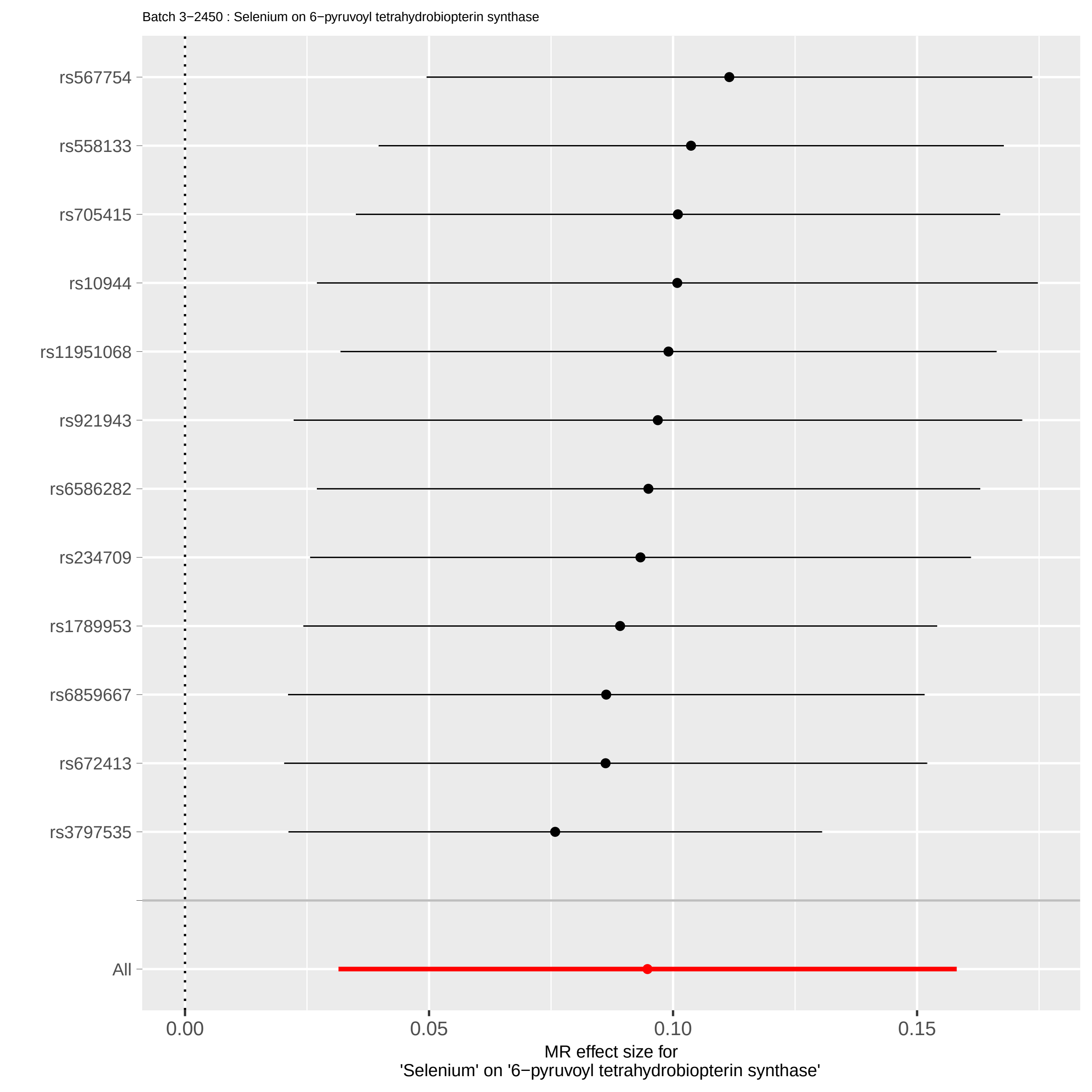

Batch 3−2450 : Selenium on 6−pyruvoyl tetrahydrobiopterin synthase
rs567754
rs558133
rs705415
rs10944
rs11951068
rs921943
rs6586282
rs234709
rs1789953
rs6859667
rs672413
rs3797535
All
0.00
0.05
0.10
0.15
MR effect size for
'Selenium' on '6−pyruvoyl tetrahydrobiopterin synthase'

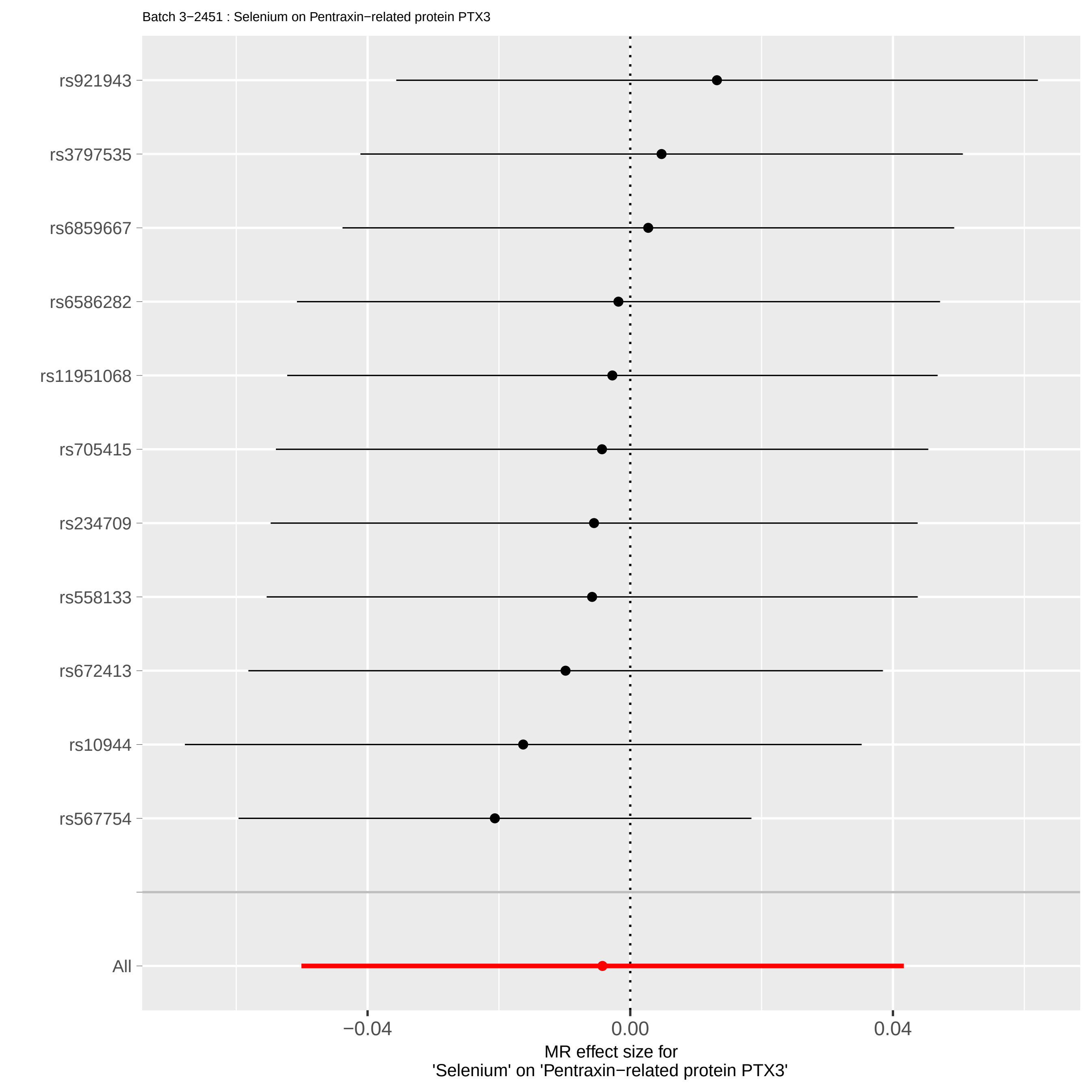

Batch 3−2451 : Selenium on Pentraxin−related protein PTX3
rs921943
rs3797535
rs6859667
rs6586282
rs11951068
rs705415
rs234709
rs558133
rs672413
rs10944
rs567754
All
−0.04
0.00
0.04
MR effect size for
'Selenium' on 'Pentraxin−related protein PTX3'

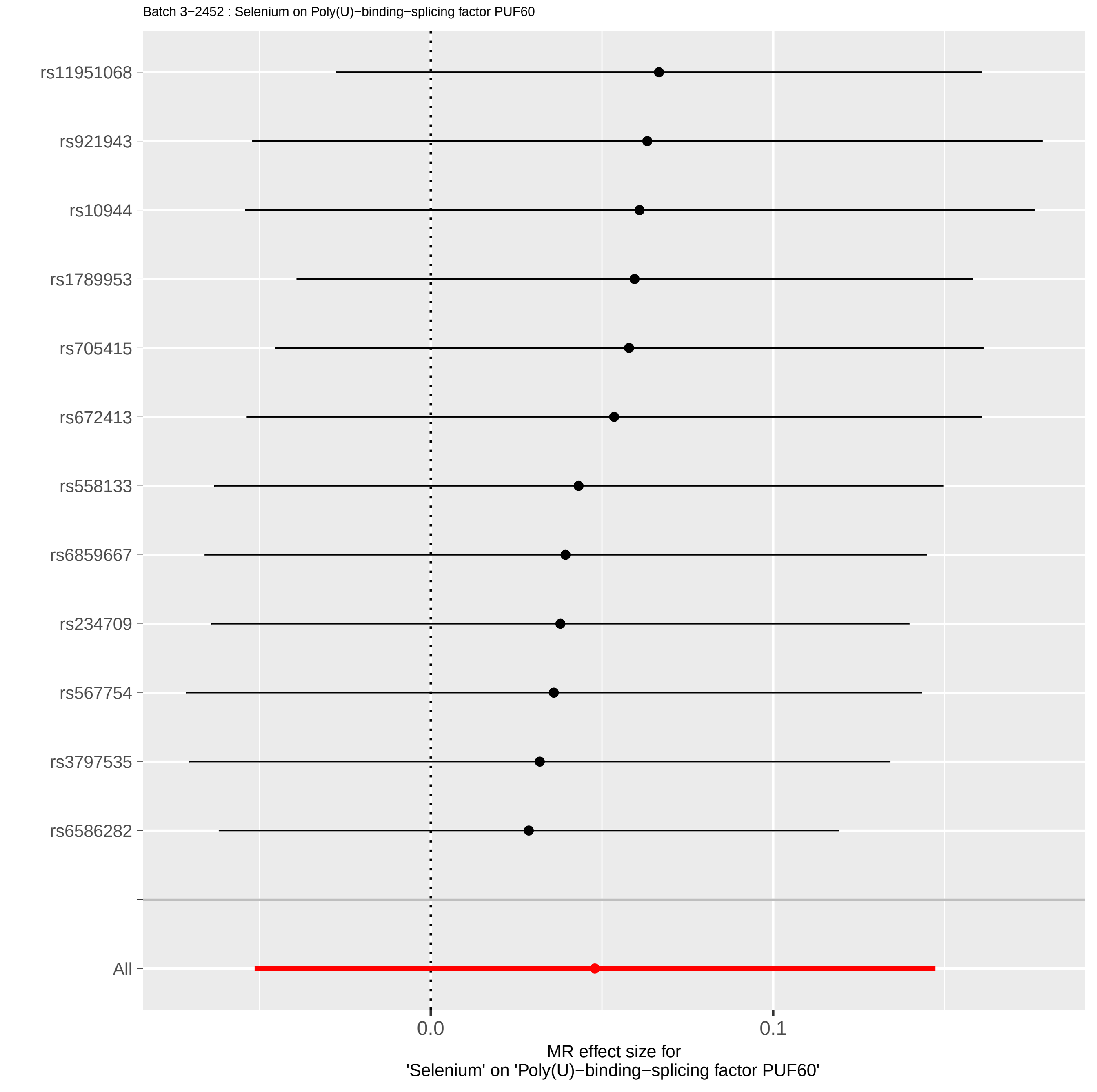

Batch 3−2452 : Selenium on Poly(U)−binding−splicing factor PUF60
rs11951068
rs921943
rs10944
rs1789953
rs705415
rs672413
rs558133
rs6859667
rs234709
rs567754
rs3797535
rs6586282
All
0.0
0.1
MR effect size for
'Selenium' on 'Poly(U)−binding−splicing factor PUF60'

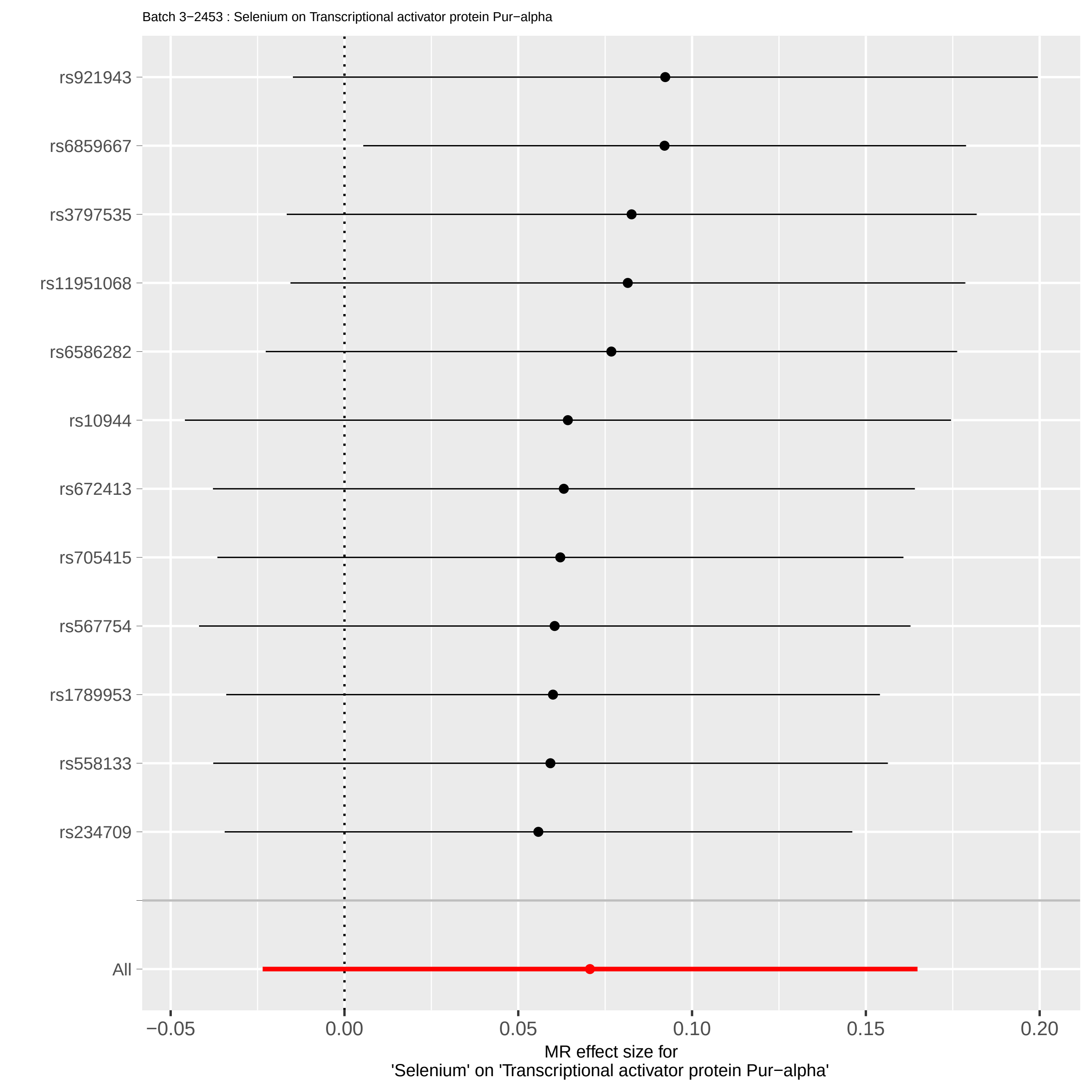

Batch 3−2453 : Selenium on Transcriptional activator protein Pur−alpha
rs921943
rs6859667
rs3797535
rs11951068
rs6586282
rs10944
rs672413
rs705415
rs567754
rs1789953
rs558133
rs234709
All
−0.05
0.00
0.05
0.10
0.15
0.20
MR effect size for
'Selenium' on 'Transcriptional activator protein Pur−alpha'

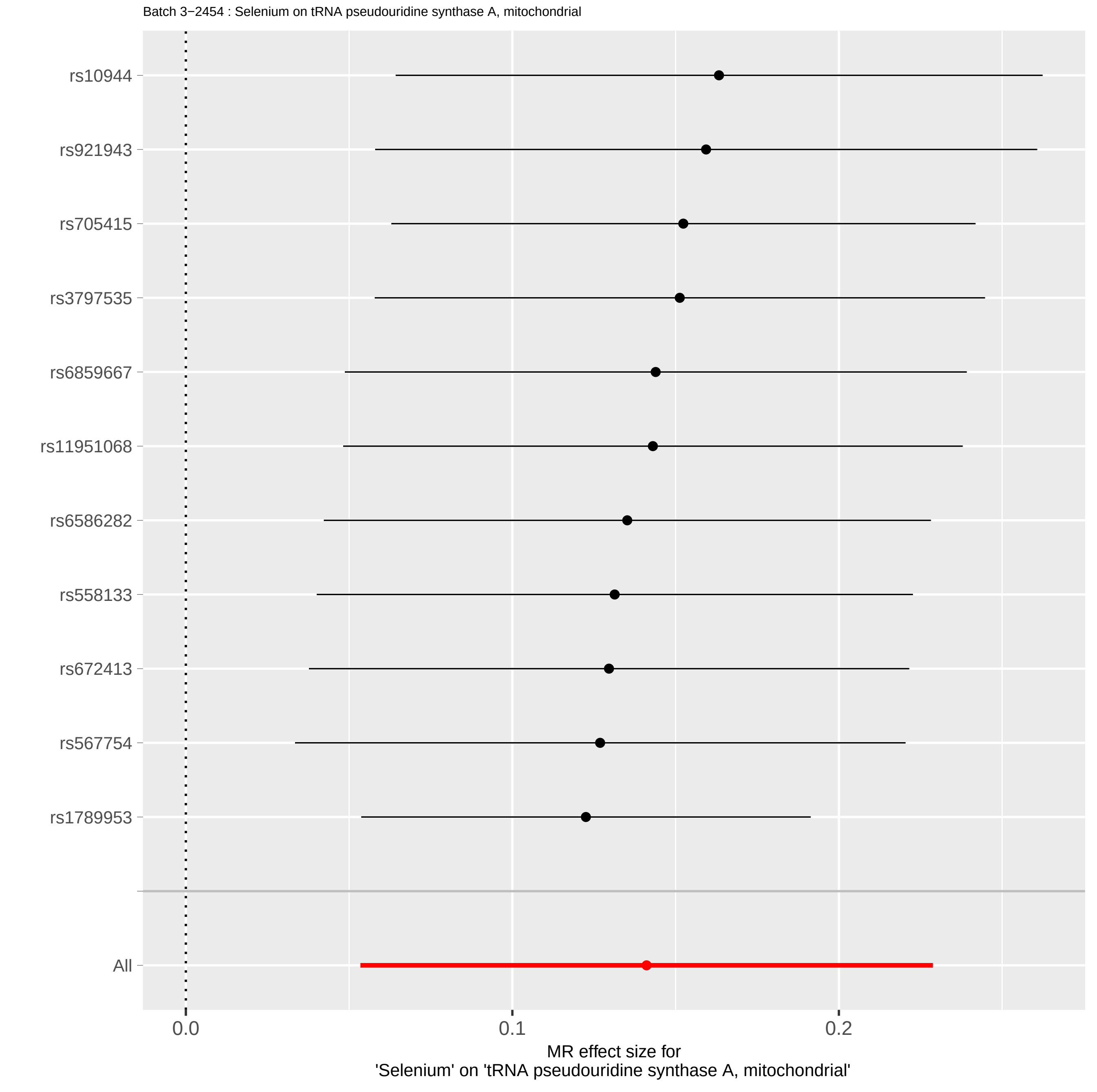

Batch 3−2454 : Selenium on tRNA pseudouridine synthase A, mitochondrial
rs10944
rs921943
rs705415
rs3797535
rs6859667
rs11951068
rs6586282
rs558133
rs672413
rs567754
rs1789953
All
0.0
0.1
0.2
MR effect size for
'Selenium' on 'tRNA pseudouridine synthase A, mitochondrial'

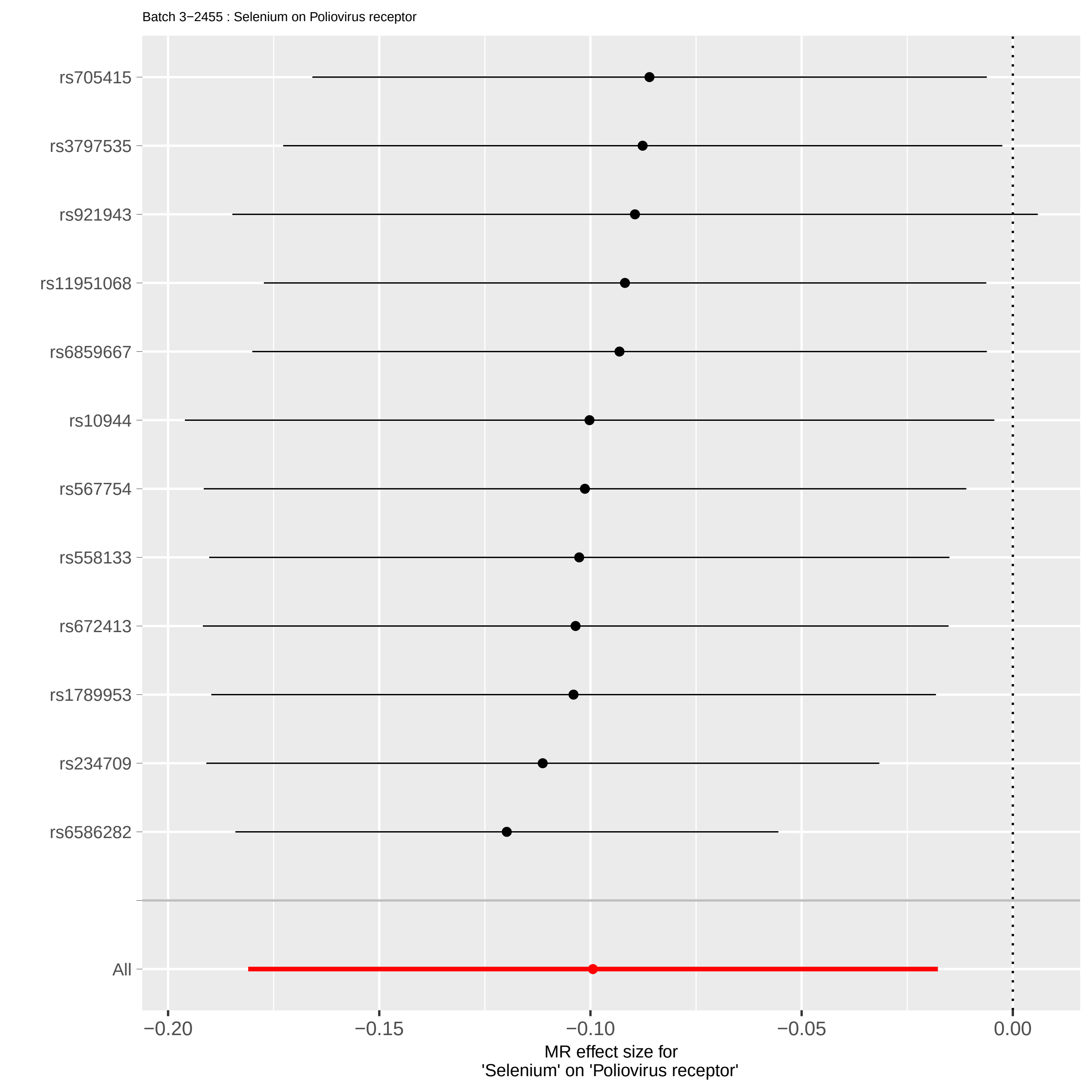

Batch 3−2455 : Selenium on Poliovirus receptor
rs705415
rs3797535
rs921943
rs11951068
rs6859667
rs10944
rs567754
rs558133
rs672413
rs1789953
rs234709
rs6586282
All
−0.20
−0.15
−0.10
−0.05
0.00
MR effect size for
'Selenium' on 'Poliovirus receptor'

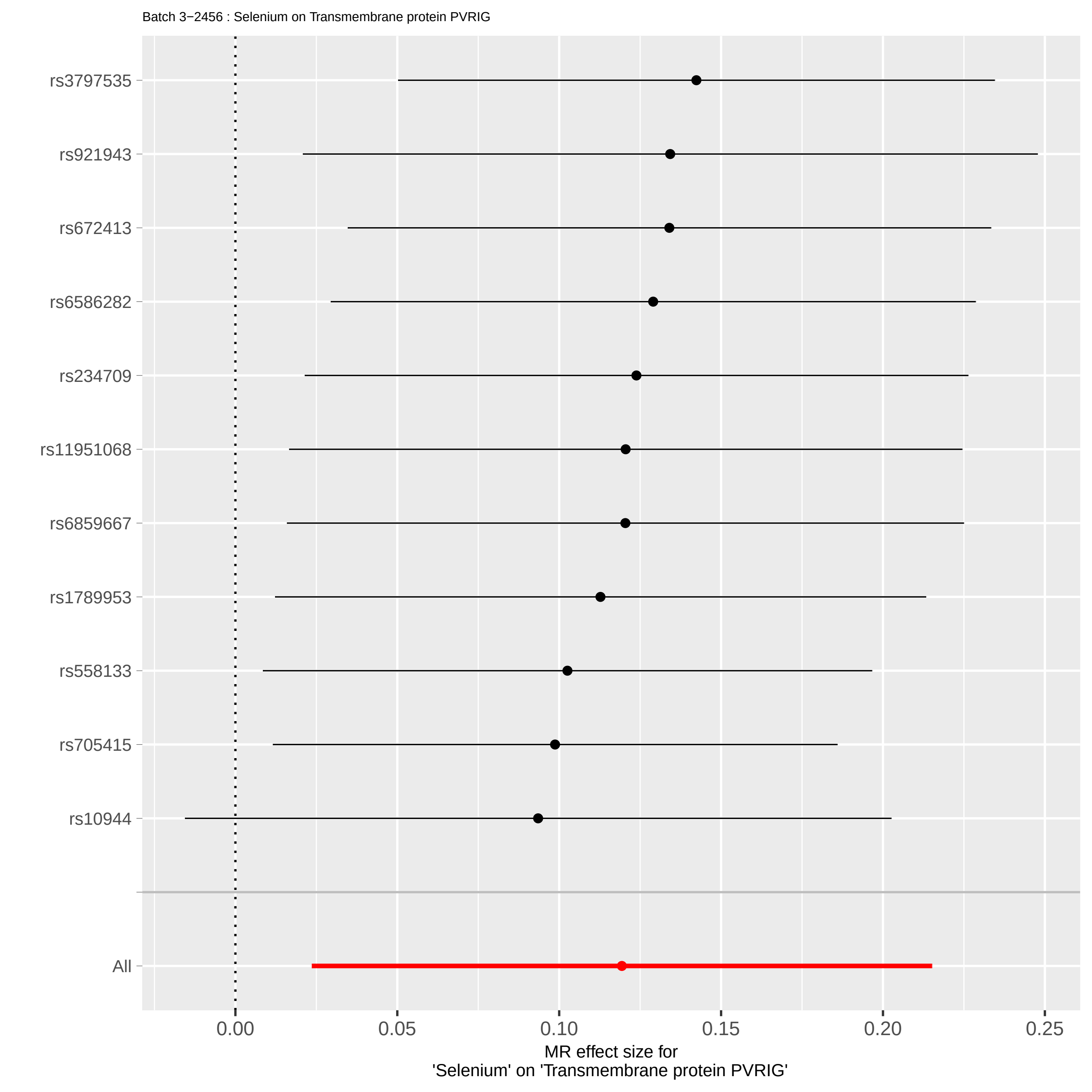

Batch 3−2456 : Selenium on Transmembrane protein PVRIG
rs3797535
rs921943
rs672413
rs6586282
rs234709
rs11951068
rs6859667
rs1789953
rs558133
rs705415
rs10944
All
0.00
0.05
0.10
0.15
0.20
0.25
MR effect size for
'Selenium' on 'Transmembrane protein PVRIG'

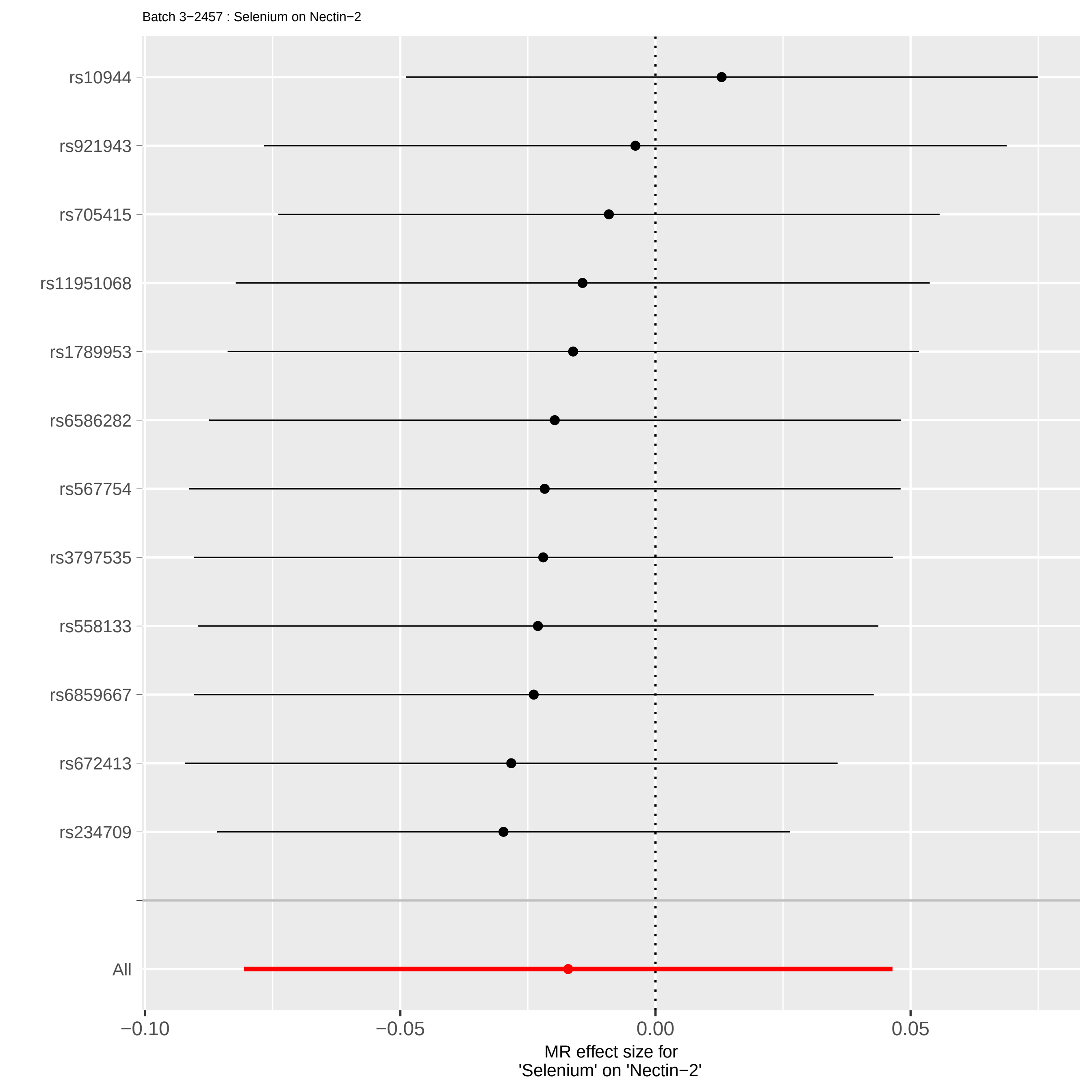

Batch 3−2457 : Selenium on Nectin−2
rs10944
rs921943
rs705415
rs11951068
rs1789953
rs6586282
rs567754
rs3797535
rs558133
rs6859667
rs672413
rs234709
All
−0.10
−0.05
0.00
0.05
MR effect size for
'Selenium' on 'Nectin−2'

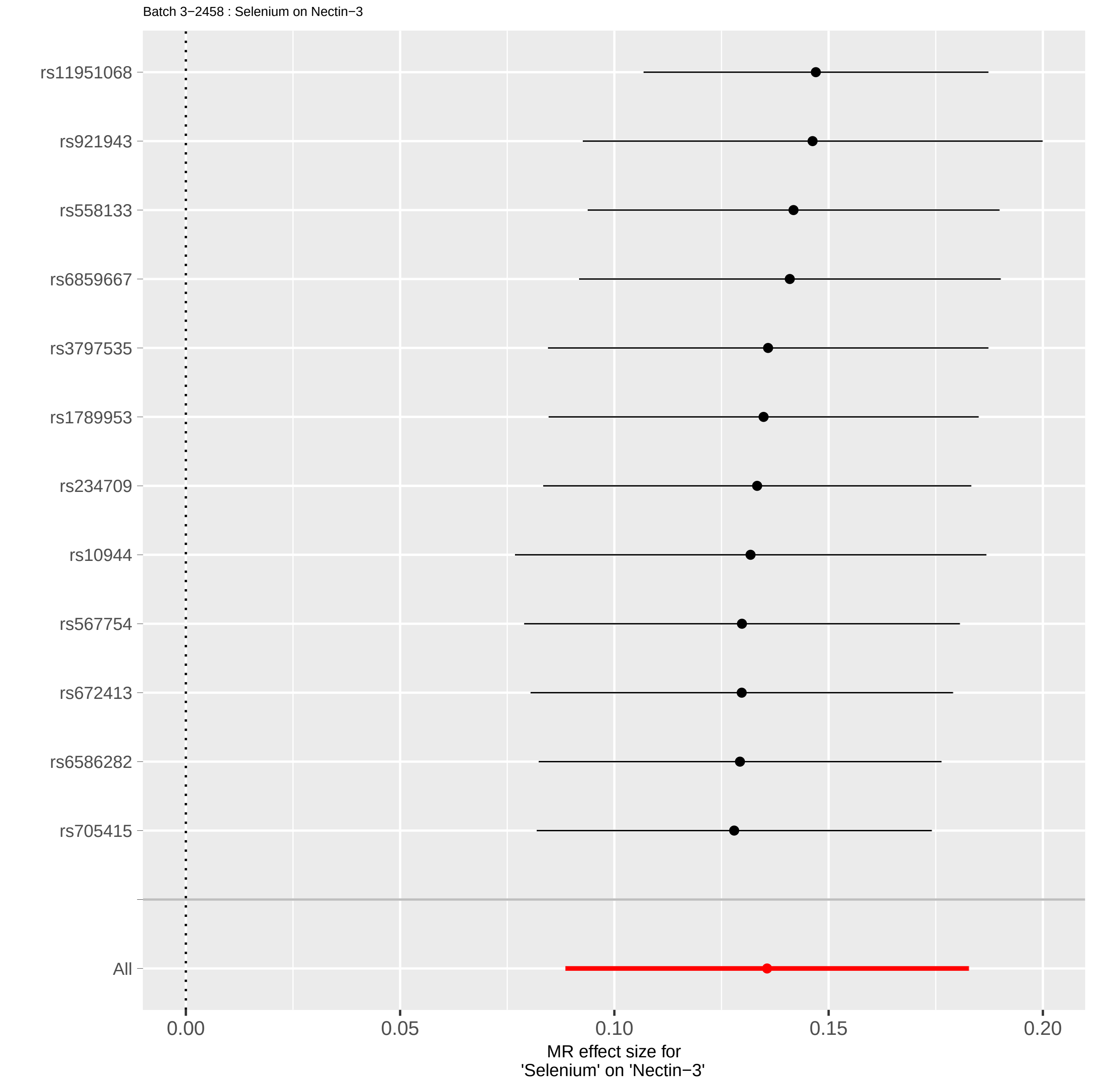
Batch 3−2458 : Selenium on Nectin−3
rs11951068
rs921943
rs558133
rs6859667
rs3797535
rs1789953
rs234709
rs10944
rs567754
rs672413
rs6586282
rs705415
All
0.00
0.05
0.10
0.15
0.20
MR effect size for
'Selenium' on 'Nectin−3'

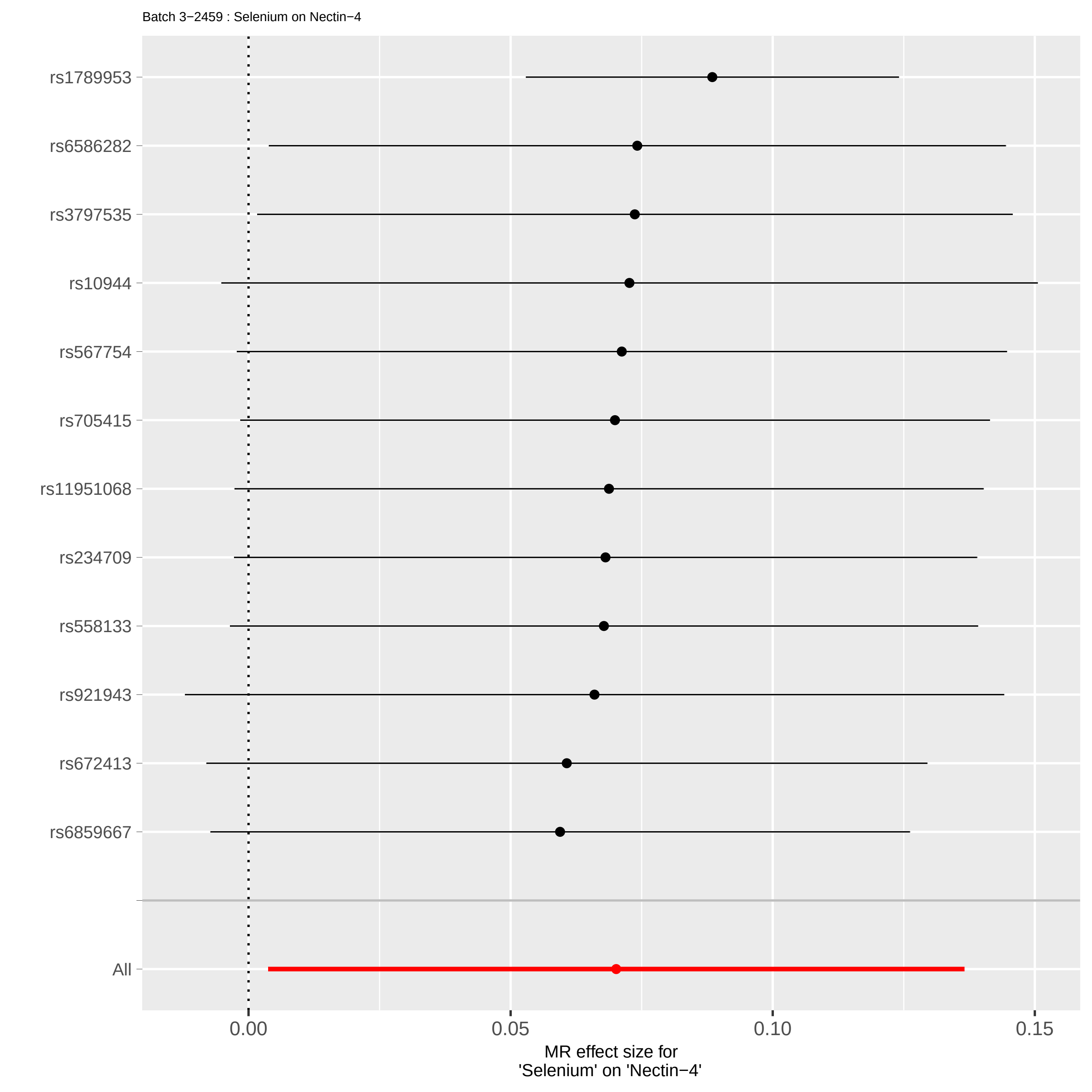

Batch 3−2459 : Selenium on Nectin−4
rs1789953
rs6586282
rs3797535
rs10944
rs567754
rs705415
rs11951068
rs234709
rs558133
rs921943
rs672413
rs6859667
All
0.00
0.05
0.10
0.15
MR effect size for
'Selenium' on 'Nectin−4'

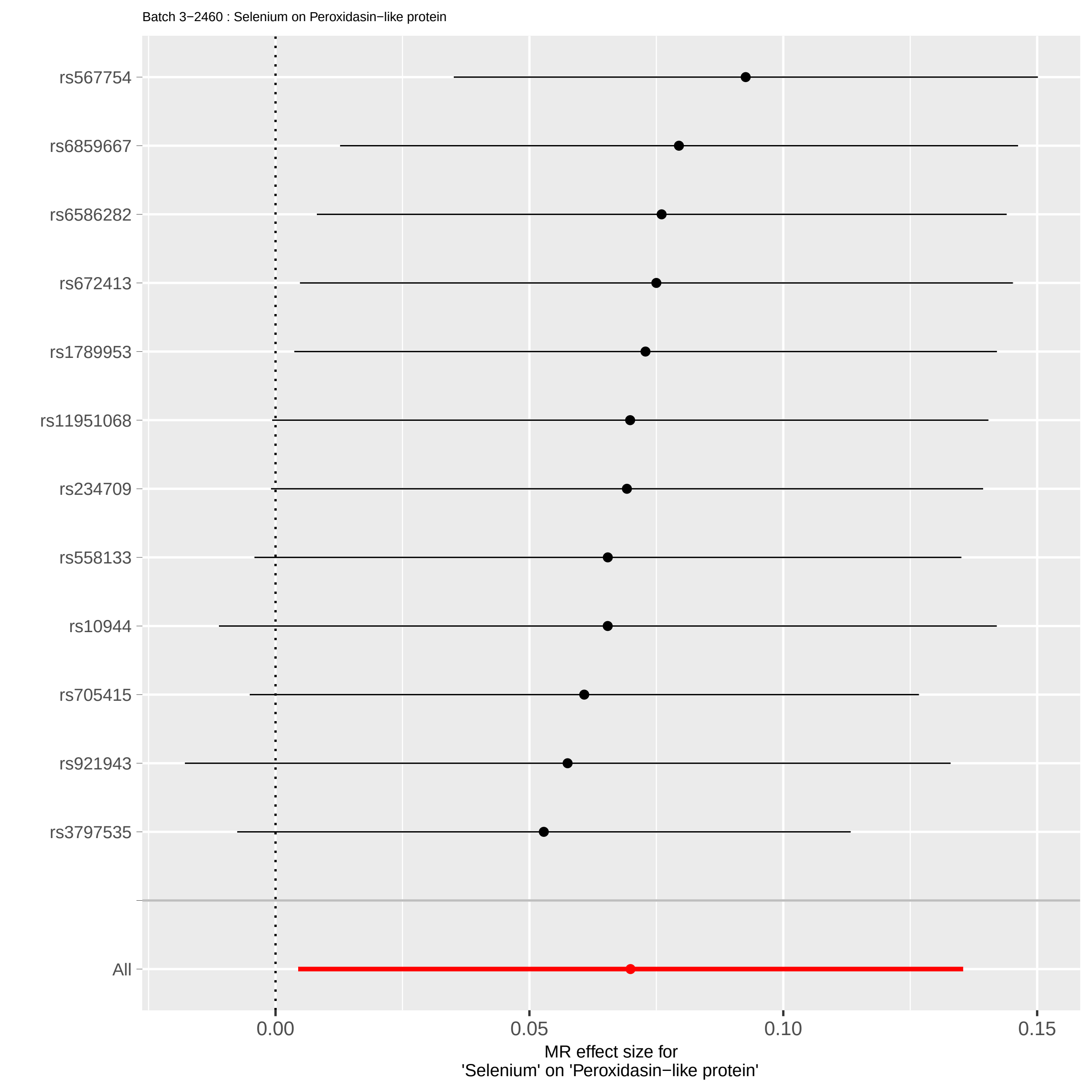

Batch 3−2460 : Selenium on Peroxidasin−like protein
rs567754
rs6859667
rs6586282
rs672413
rs1789953
rs11951068
rs234709
rs558133
rs10944
rs705415
rs921943
rs3797535
All
0.00
0.05
0.10
0.15
MR effect size for
'Selenium' on 'Peroxidasin−like protein'

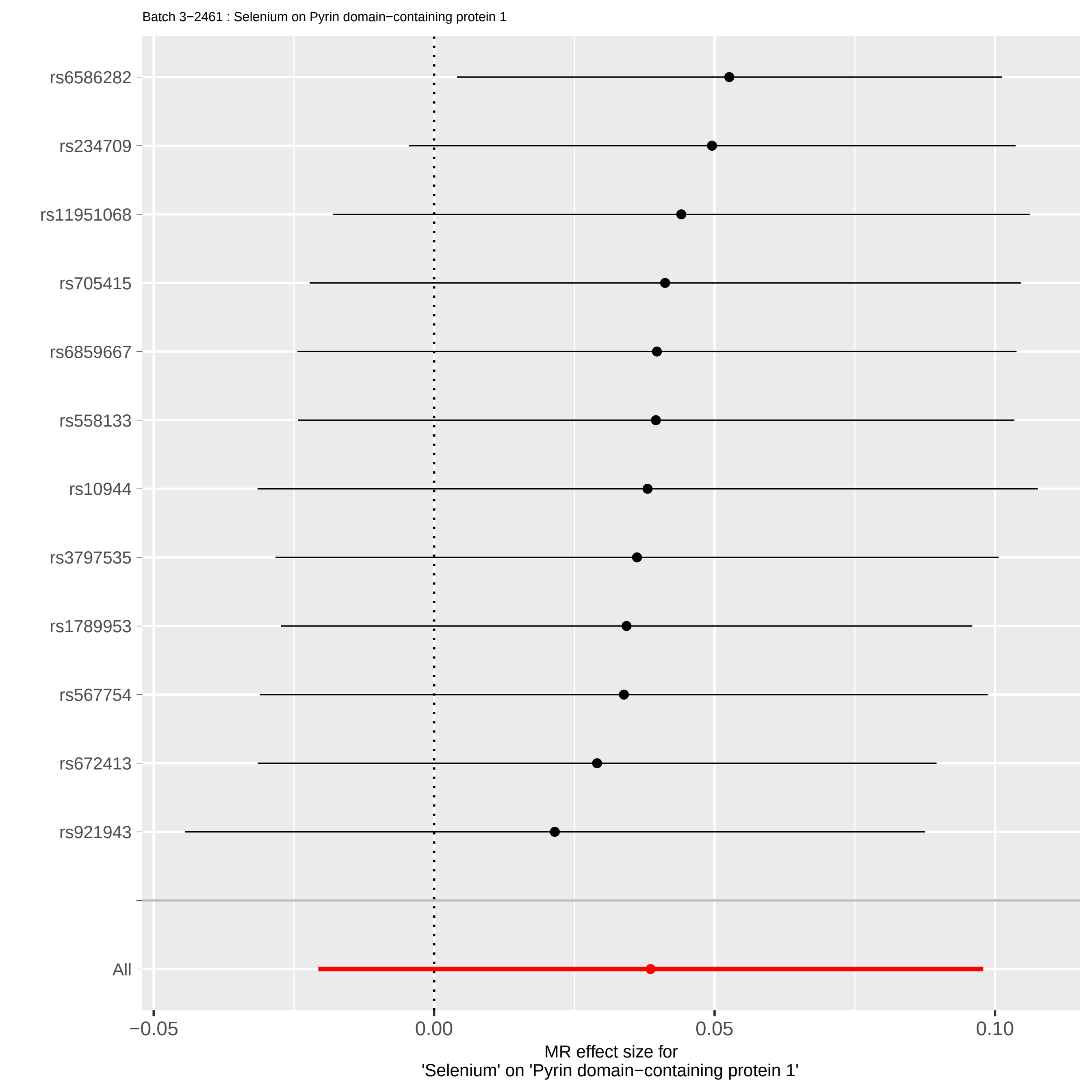
Batch 3−2461 : Selenium on Pyrin domain−containing protein 1
rs6586282
rs234709
rs11951068
rs705415
rs6859667
rs558133
rs10944
rs3797535
rs1789953
rs567754
rs672413
rs921943
All
−0.05
0.00
0.05
0.10
MR effect size for
'Selenium' on 'Pyrin domain−containing protein 1'

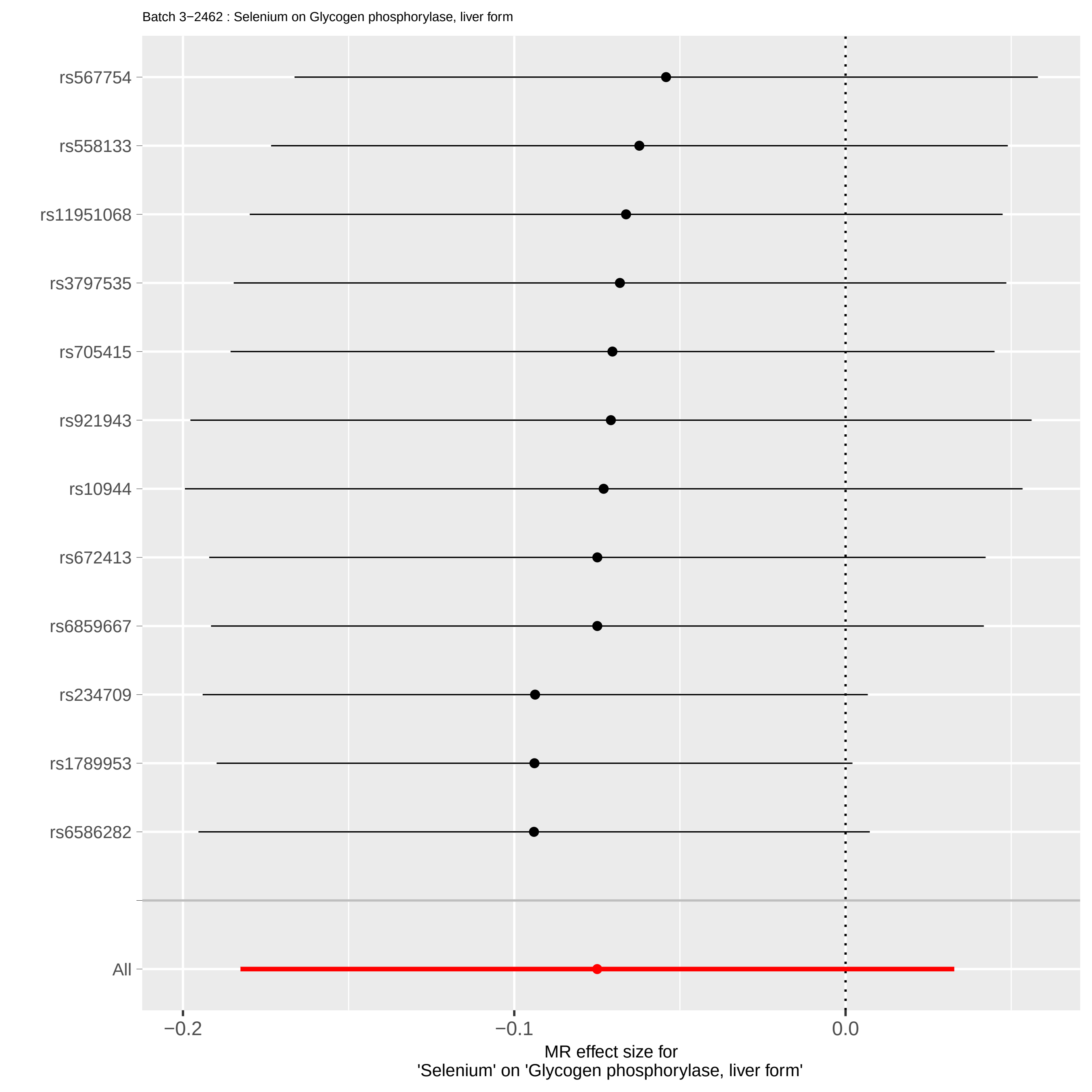
Batch 3−2462 : Selenium on Glycogen phosphorylase, liver form
rs567754
rs558133
rs11951068
rs3797535
rs705415
rs921943
rs10944
rs672413
rs6859667
rs234709
rs1789953
rs6586282
All
−0.2
−0.1
0.0
MR effect size for
'Selenium' on 'Glycogen phosphorylase, liver form'

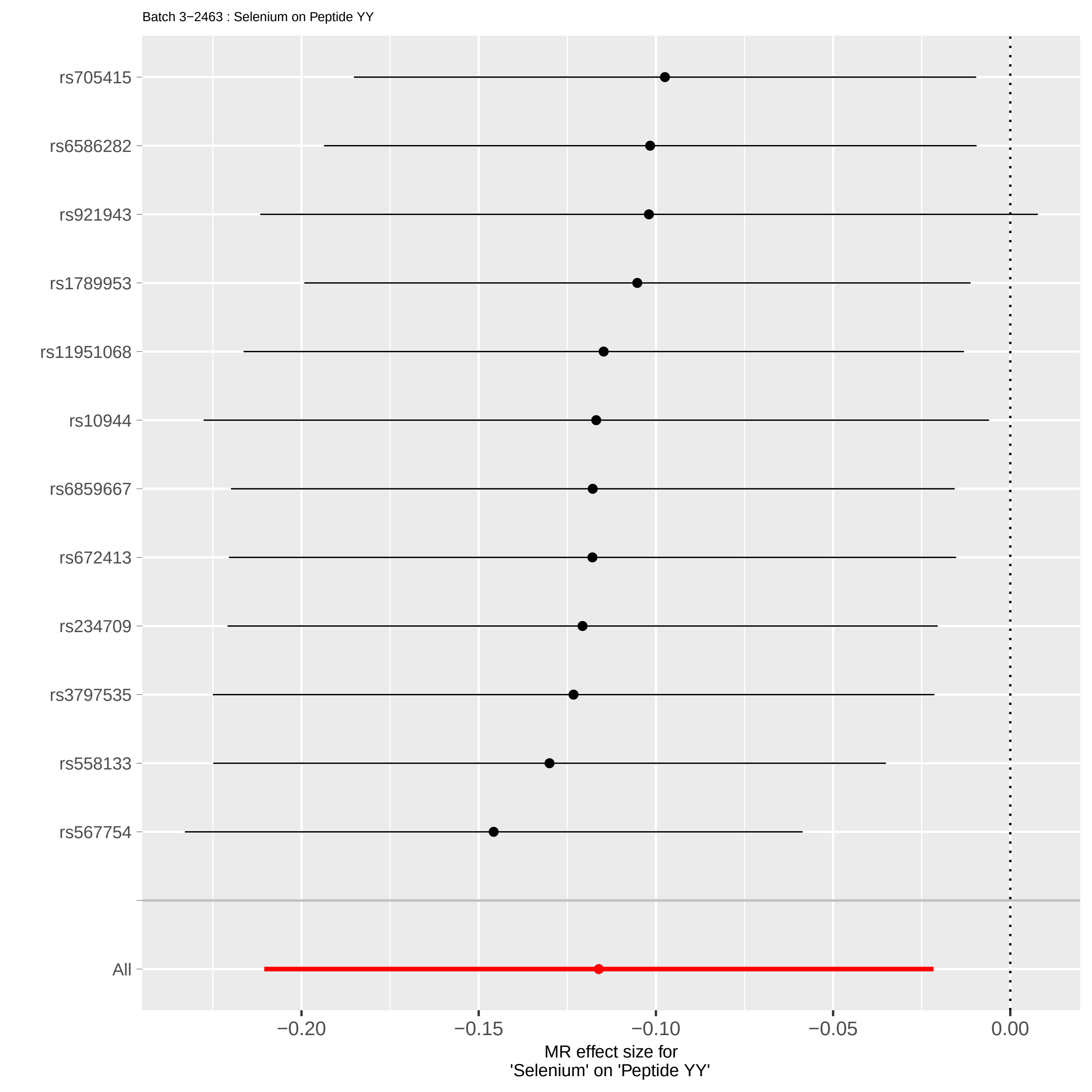

Batch 3−2463 : Selenium on Peptide YY
rs705415
rs6586282
rs921943
rs1789953
rs11951068
rs10944
rs6859667
rs672413
rs234709
rs3797535
rs558133
rs567754
All
−0.20
−0.15
−0.10
−0.05
0.00
MR effect size for
'Selenium' on 'Peptide YY'

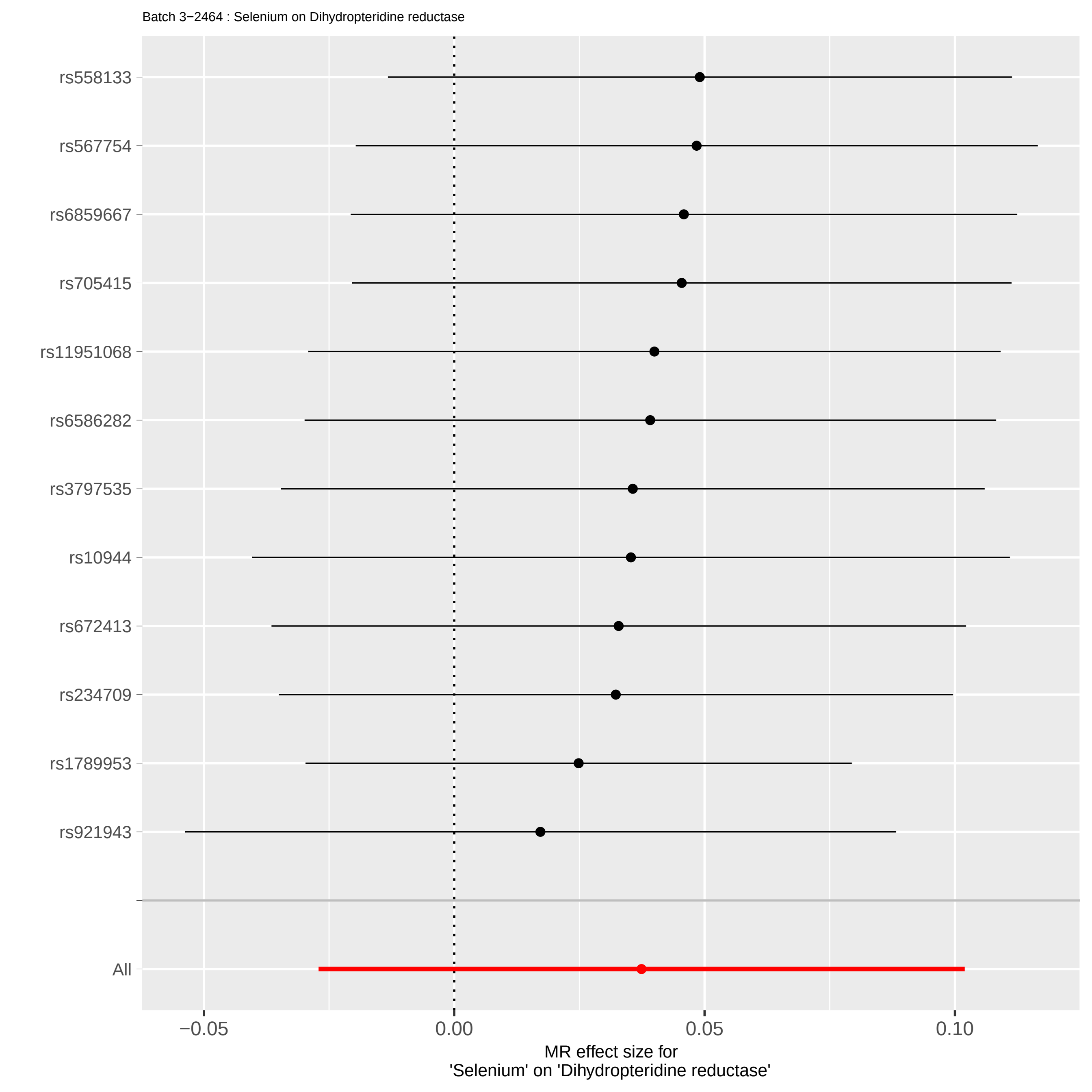

Batch 3−2464 : Selenium on Dihydropteridine reductase
rs558133
rs567754
rs6859667
rs705415
rs11951068
rs6586282
rs3797535
rs10944
rs672413
rs234709
rs1789953
rs921943
All
−0.05
0.00
0.05
0.10
MR effect size for
'Selenium' on 'Dihydropteridine reductase'

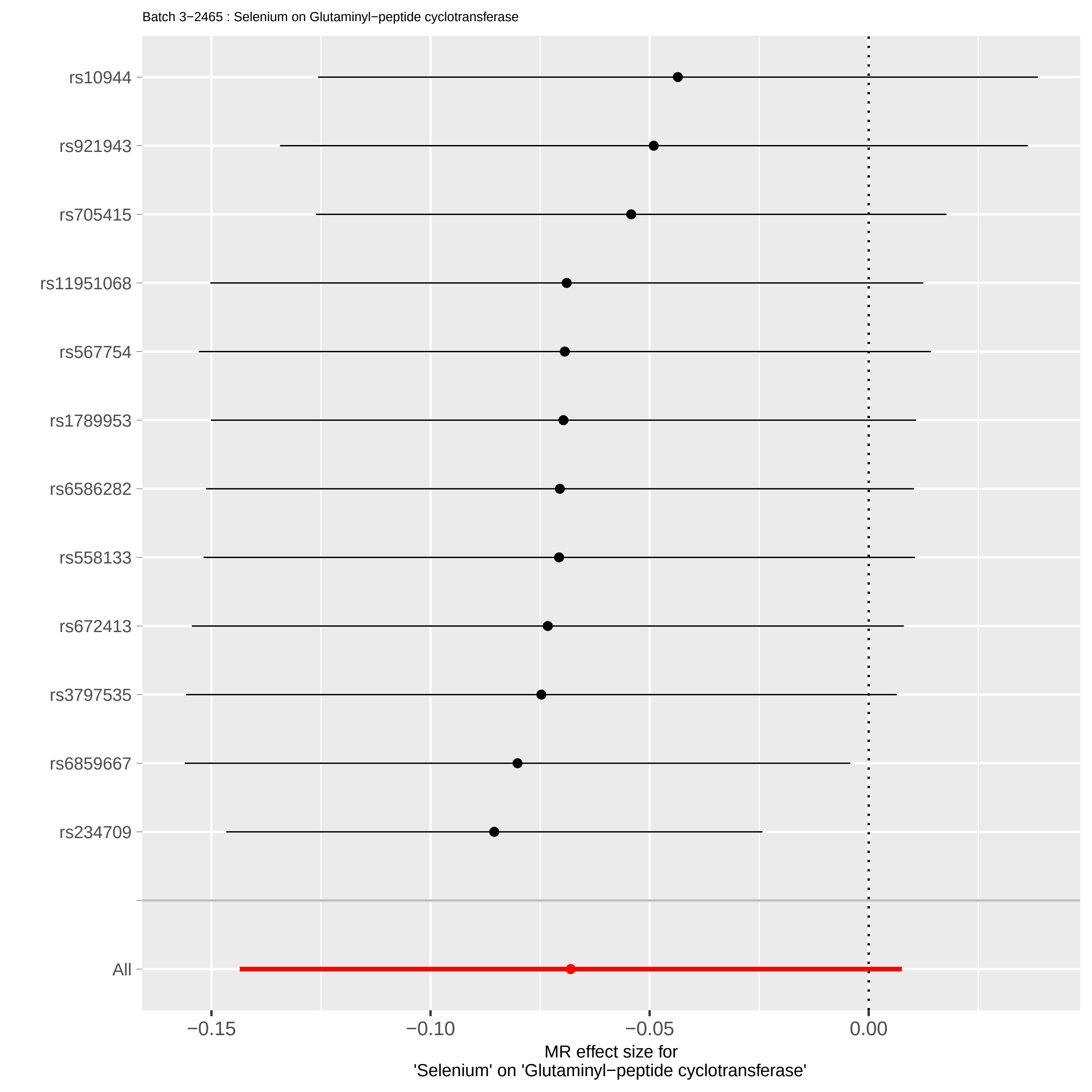
Batch 3−2465 : Selenium on Glutaminyl−peptide cyclotransferase
rs10944
rs921943
rs705415
rs11951068
rs567754
rs1789953
rs6586282
rs558133
rs672413
rs3797535
rs6859667
rs234709
All
−0.15
−0.10
−0.05
0.00
MR effect size for
'Selenium' on 'Glutaminyl−peptide cyclotransferase'

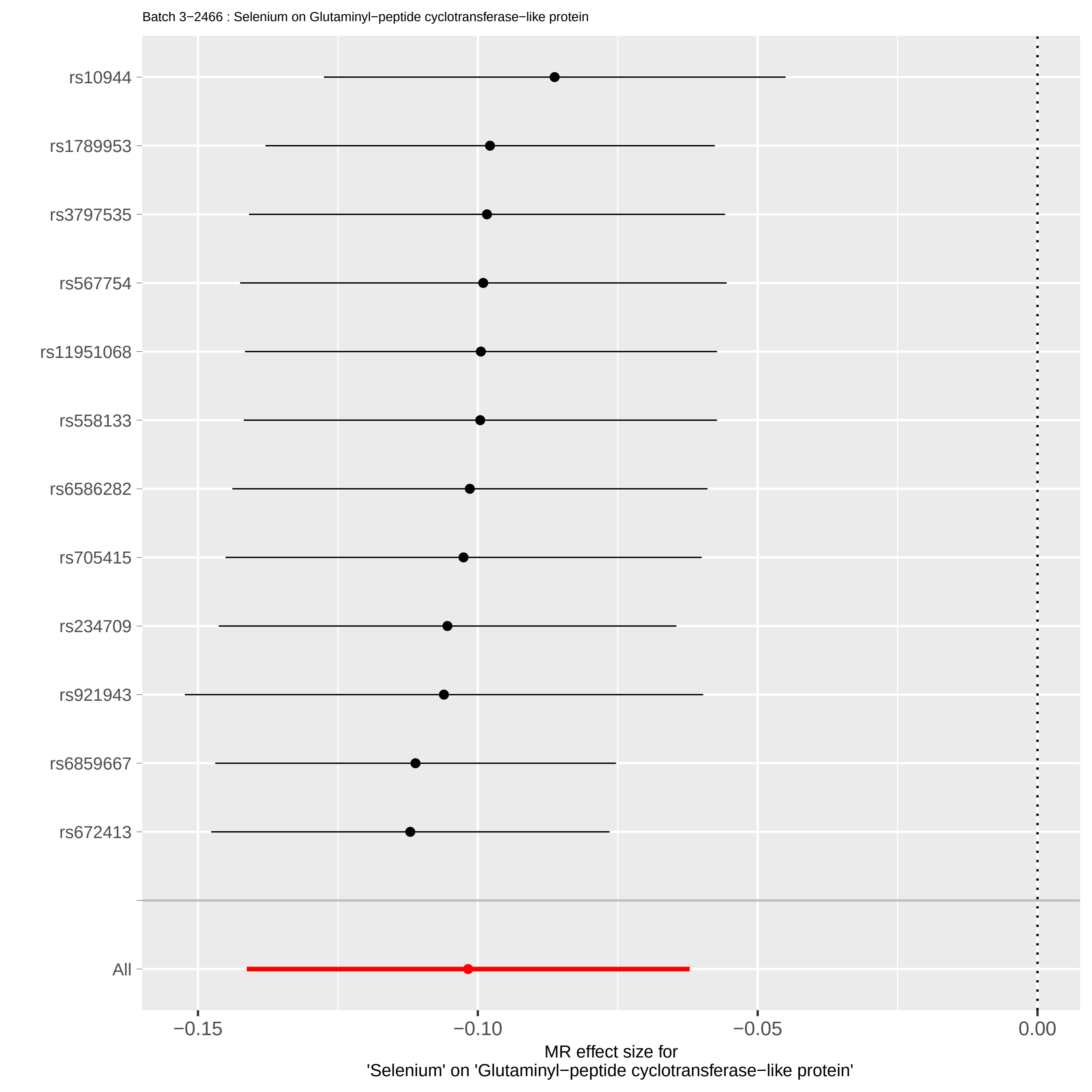

Batch 3−2466 : Selenium on Glutaminyl−peptide cyclotransferase−like protein
rs10944
rs1789953
rs3797535
rs567754
rs11951068
rs558133
rs6586282
rs705415
rs234709
rs921943
rs6859667
rs672413
All
−0.15
−0.10
−0.05
0.00
MR effect size for
'Selenium' on 'Glutaminyl−peptide cyclotransferase−like protein'

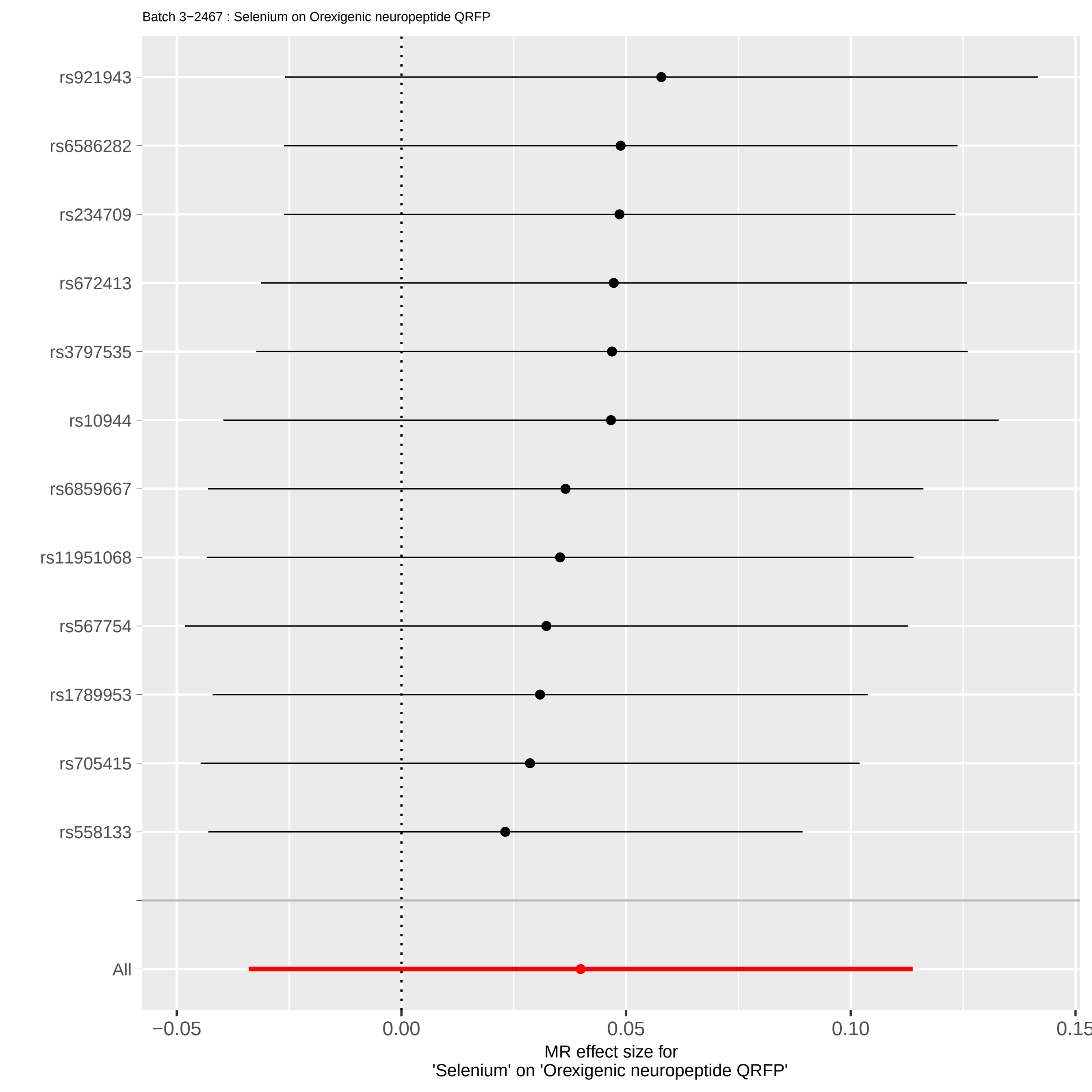
Batch 3−2467 : Selenium on Orexigenic neuropeptide QRFP
rs921943
rs6586282
rs234709
rs672413
rs3797535
rs10944
rs6859667
rs11951068
rs567754
rs1789953
rs705415
rs558133
All
−0.05
0.00
0.05
0.10
0.15
MR effect size for
'Selenium' on 'Orexigenic neuropeptide QRFP'

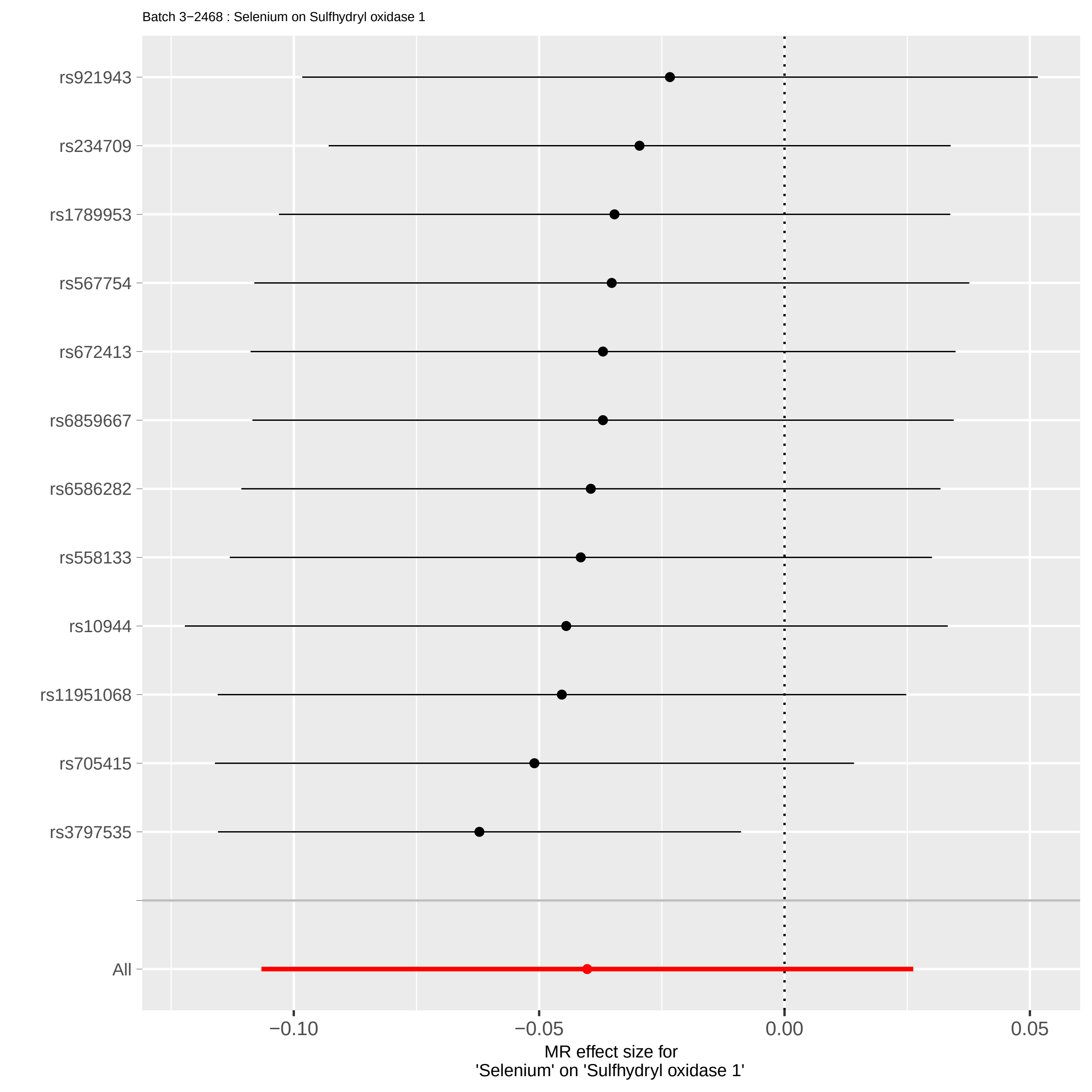

Batch 3−2468 : Selenium on Sulfhydryl oxidase 1
rs921943
rs234709
rs1789953
rs567754
rs672413
rs6859667
rs6586282
rs558133
rs10944
rs11951068
rs705415
rs3797535
All
−0.10
−0.05
0.00
0.05
MR effect size for
'Selenium' on 'Sulfhydryl oxidase 1'

Batch 3−2469 : Selenium on Sulfhydryl oxidase 1

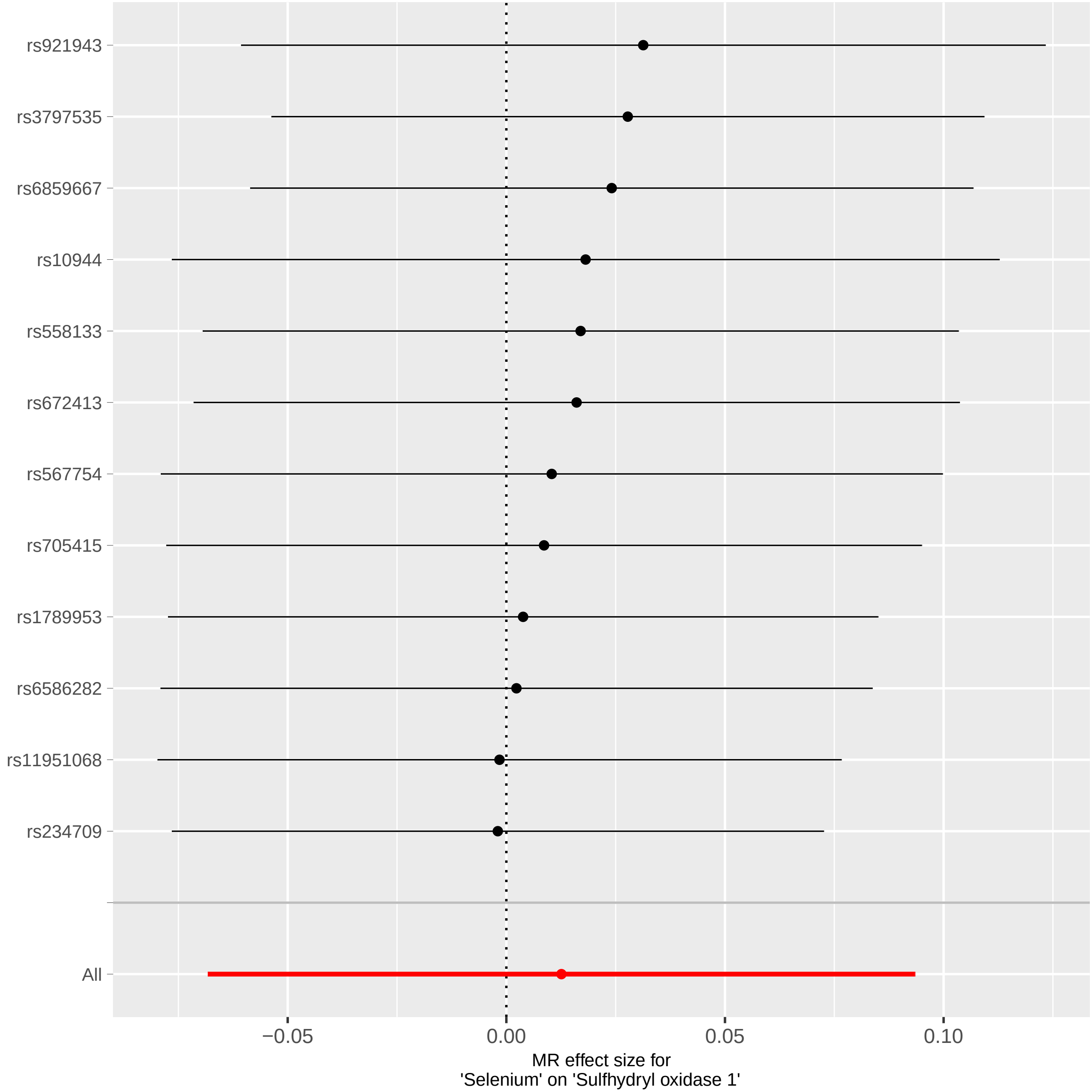

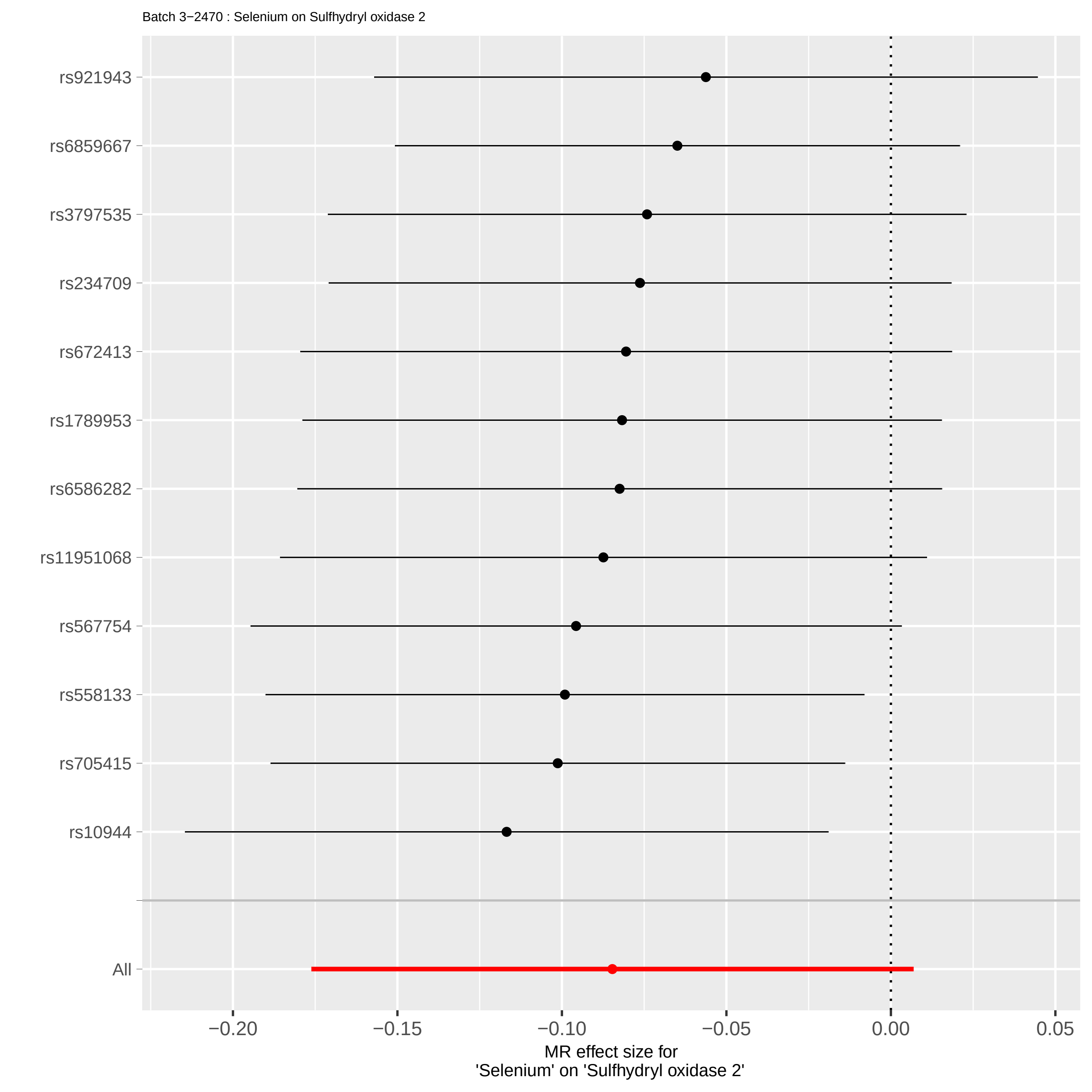
Batch 3−2470 : Selenium on Sulfhydryl oxidase 2
rs921943
rs6859667
rs3797535
rs234709
rs672413
rs1789953
rs6586282
rs11951068
rs567754
rs558133
rs705415
rs10944
All
−0.20
−0.15
−0.10
−0.05
0.00
0.05
MR effect size for
'Selenium' on 'Sulfhydryl oxidase 2'

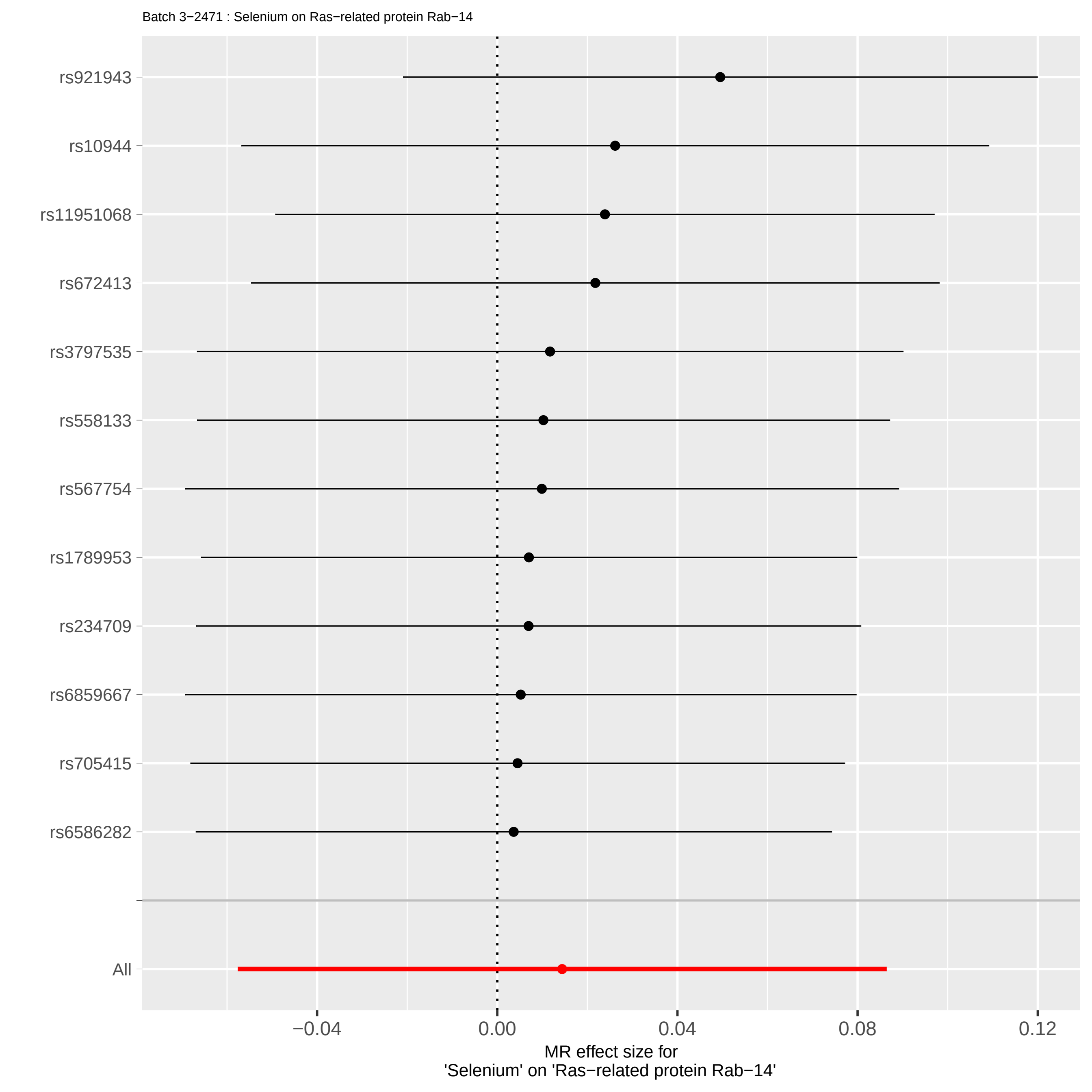

Batch 3−2471 : Selenium on Ras−related protein Rab−14
rs921943
rs10944
rs11951068
rs672413
rs3797535
rs558133
rs567754
rs1789953
rs234709
rs6859667
rs705415
rs6586282
All
−0.04
0.00
0.04
0.08
0.12
MR effect size for
'Selenium' on 'Ras−related protein Rab−14'

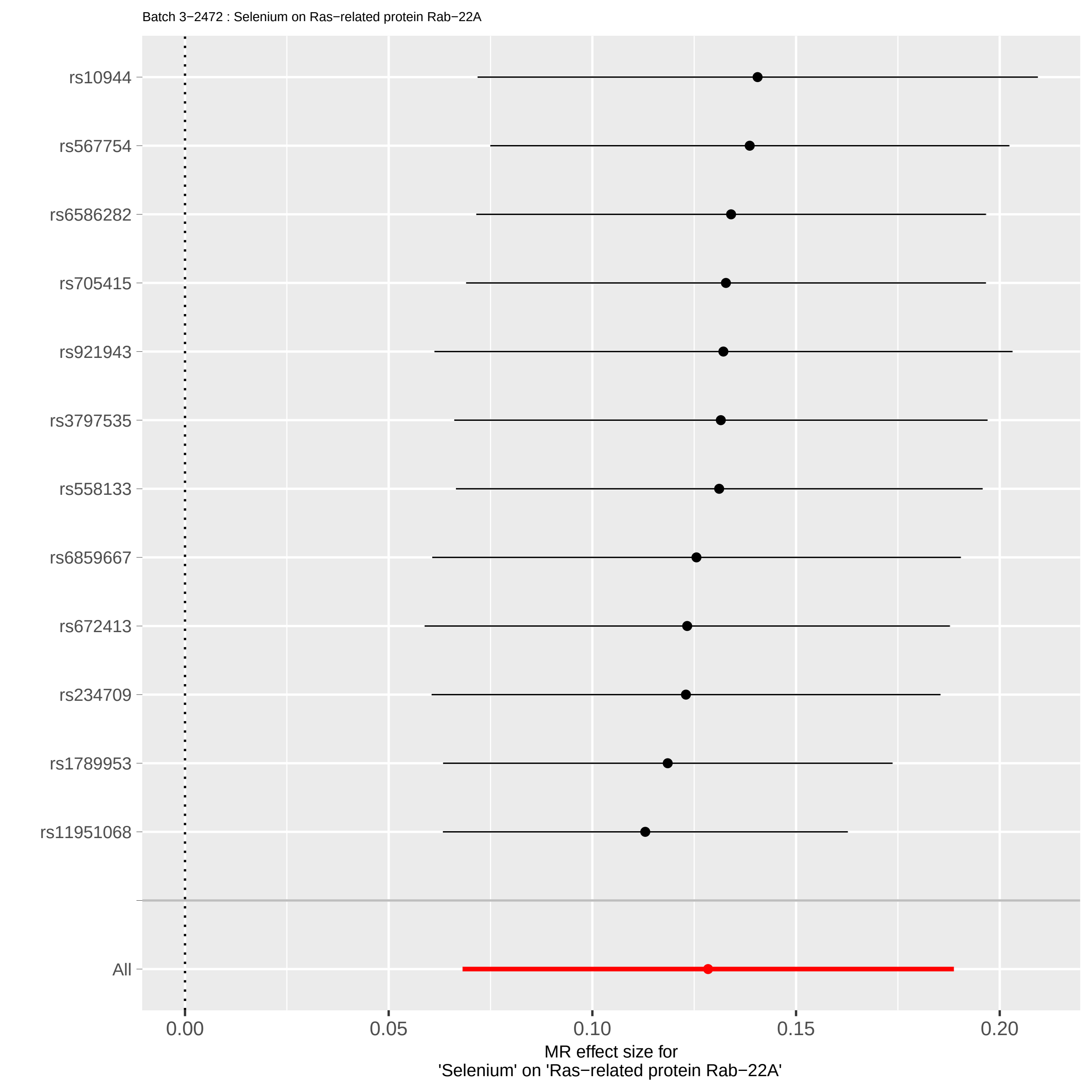
Batch 3−2472 : Selenium on Ras−related protein Rab−22A
rs10944
rs567754
rs6586282
rs705415
rs921943
rs3797535
rs558133
rs6859667
rs672413
rs234709
rs1789953
rs11951068
All
0.00
0.05
0.10
0.15
0.20
MR effect size for
'Selenium' on 'Ras−related protein Rab−22A'

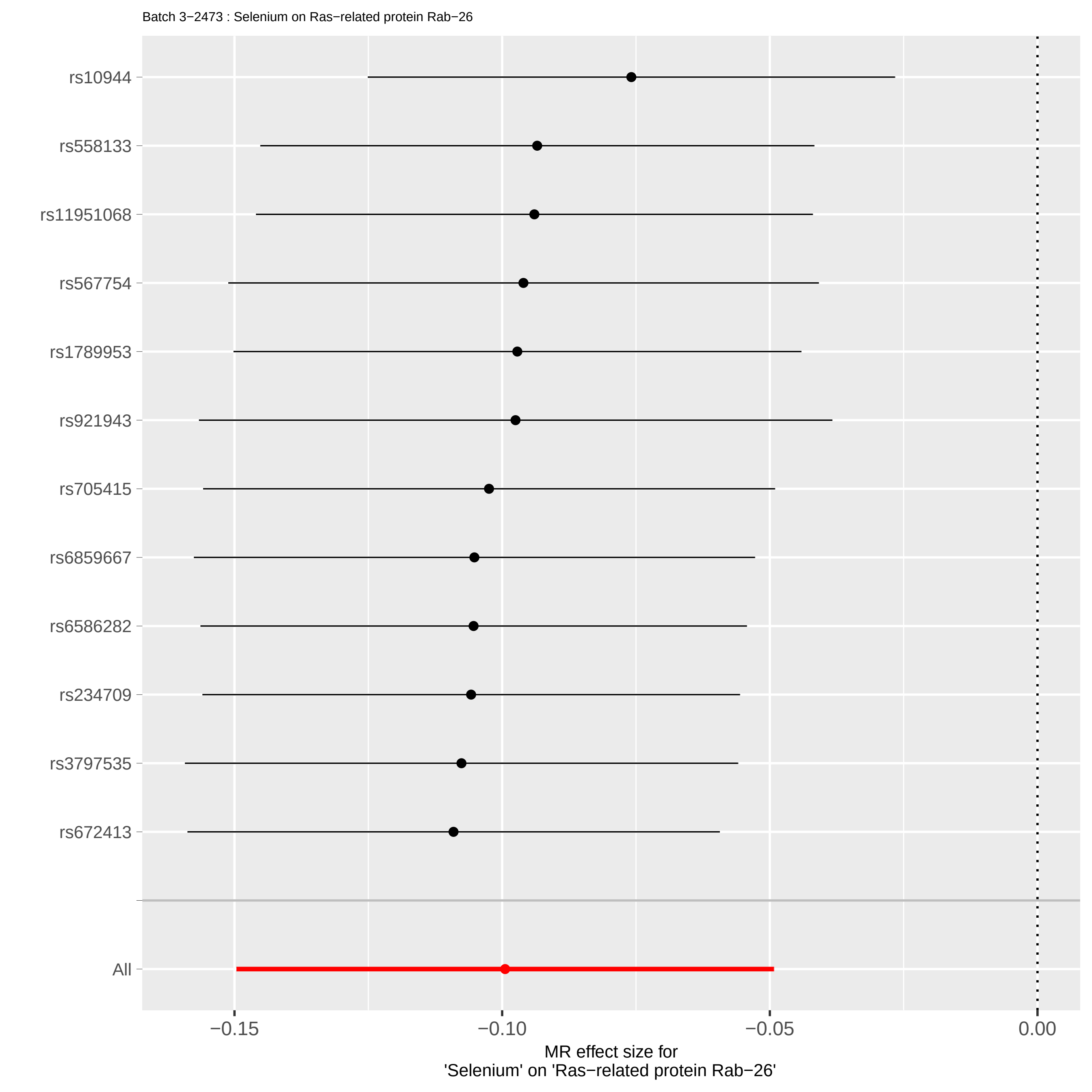

Batch 3−2473 : Selenium on Ras−related protein Rab−26
rs10944
rs558133
rs11951068
rs567754
rs1789953
rs921943
rs705415
rs6859667
rs6586282
rs234709
rs3797535
rs672413
All
−0.15
−0.10
−0.05
0.00
MR effect size for
'Selenium' on 'Ras−related protein Rab−26'

Batch 3−2474 : Selenium on Ras−related protein Rab−27A

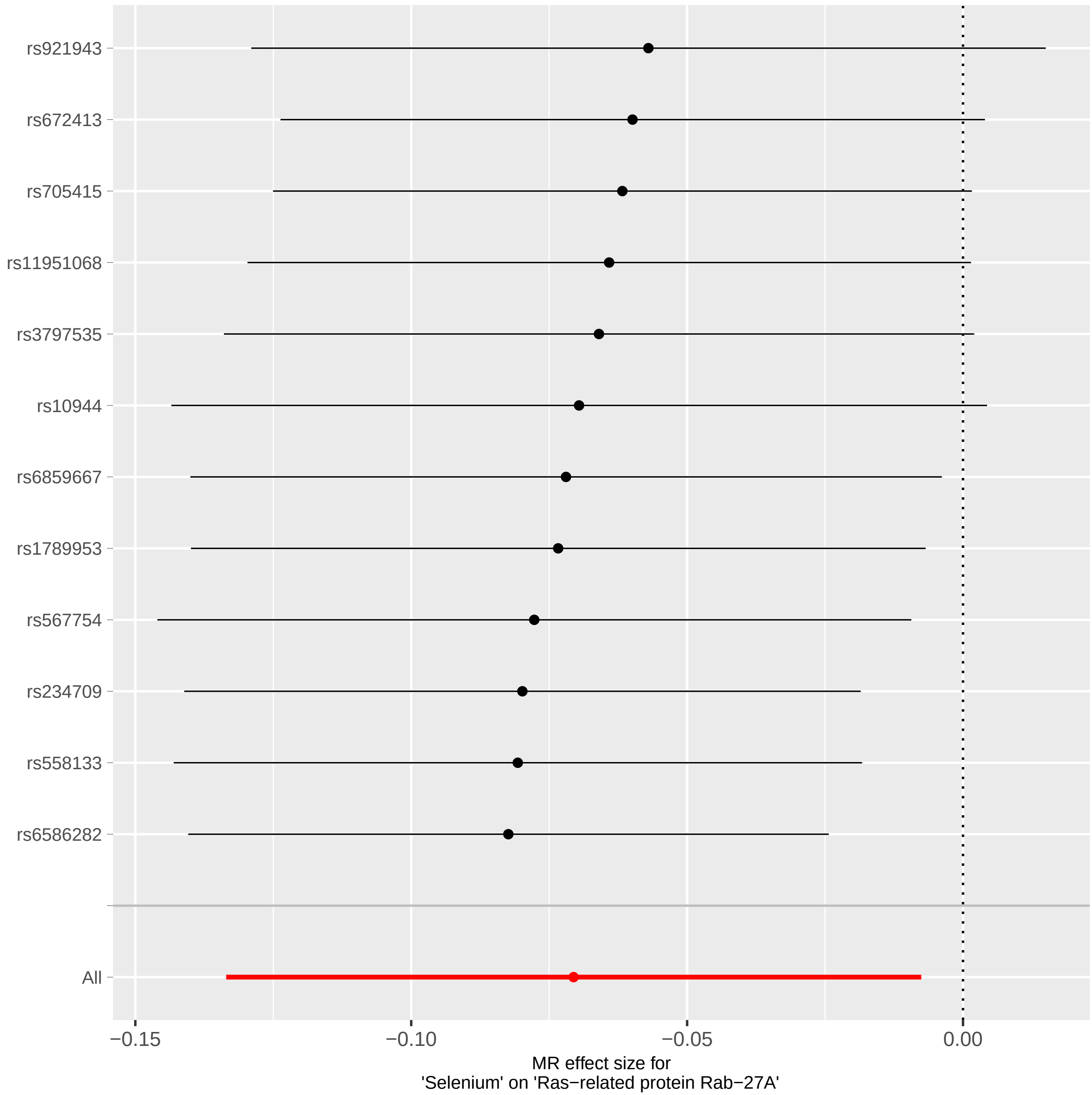

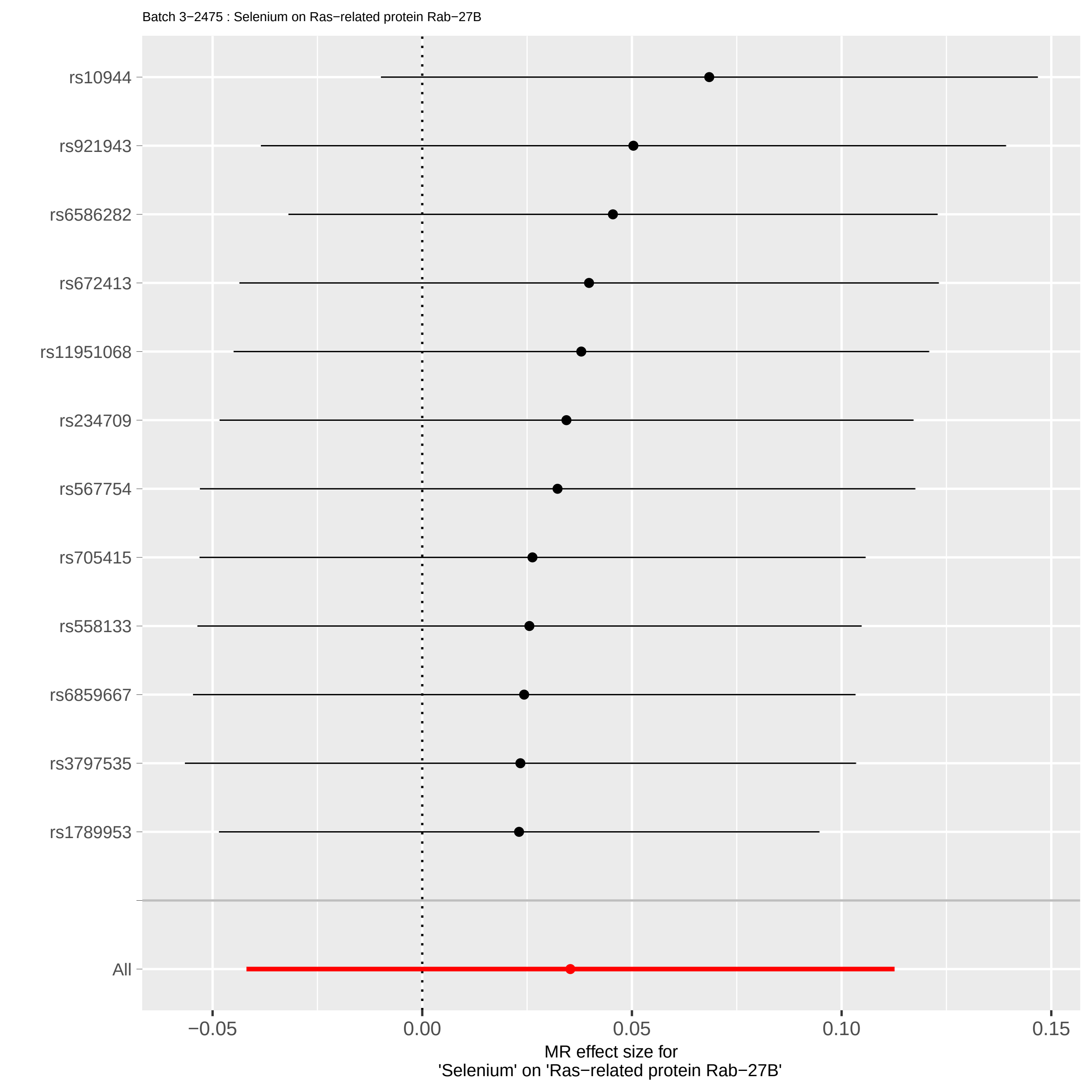

Batch 3−2475 : Selenium on Ras−related protein Rab−27B
rs10944
rs921943
rs6586282
rs672413
rs11951068
rs234709
rs567754
rs705415
rs558133
rs6859667
rs3797535
rs1789953
All
−0.05
0.00
0.05
0.10
0.15
MR effect size for
'Selenium' on 'Ras−related protein Rab−27B'

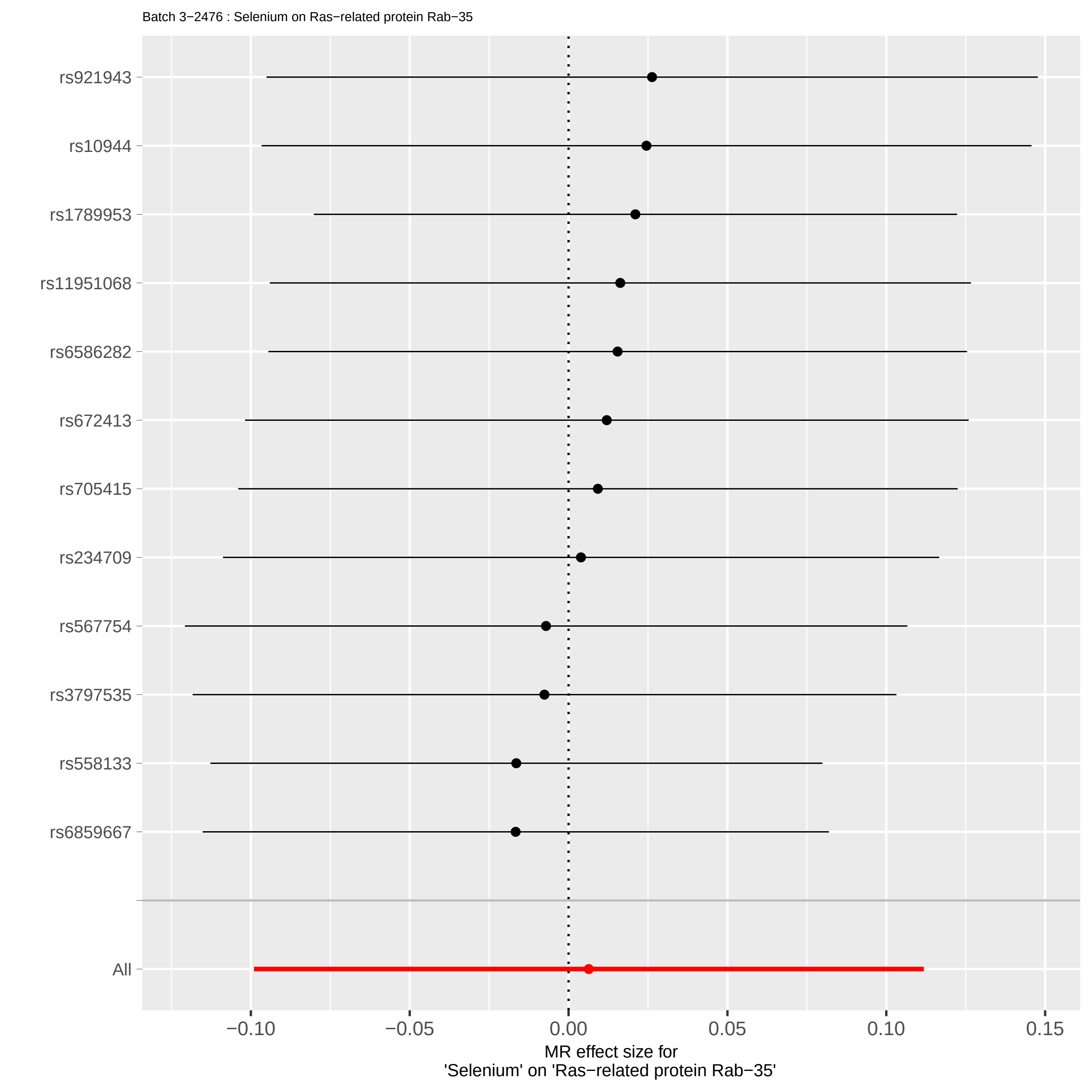

Batch 3−2476 : Selenium on Ras−related protein Rab−35
rs921943
rs10944
rs1789953
rs11951068
rs6586282
rs672413
rs705415
rs234709
rs567754
rs3797535
rs558133
rs6859667
All
−0.10
−0.05
0.00
0.05
0.10
0.15
MR effect size for
'Selenium' on 'Ras−related protein Rab−35'

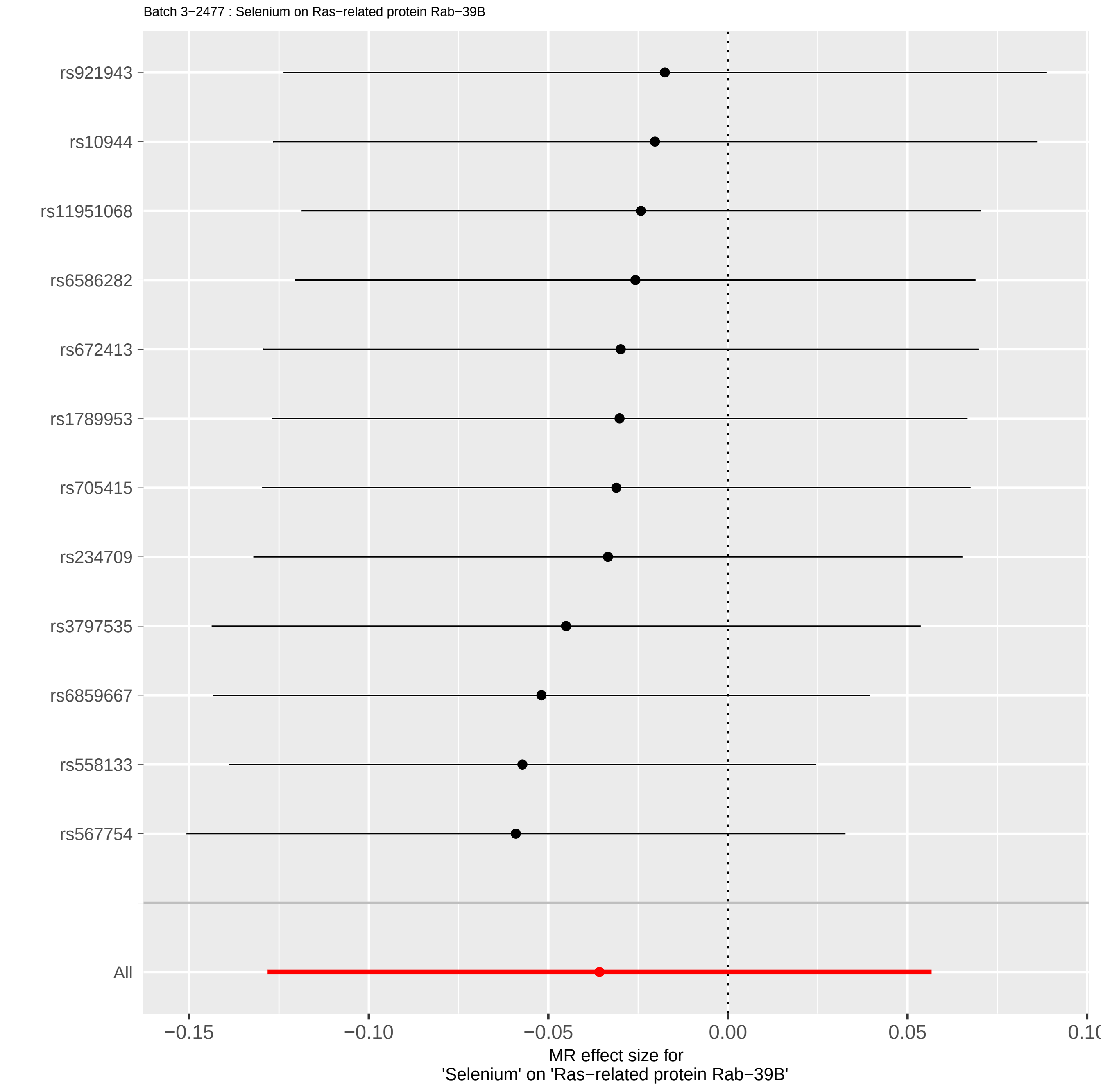

Batch 3−2477 : Selenium on Ras−related protein Rab−39B
rs921943
rs10944
rs11951068
rs6586282
rs672413
rs1789953
rs705415
rs234709
rs3797535
rs6859667
rs558133
rs567754
All
−0.15
−0.10
−0.05
0.00
0.05
0.10
MR effect size for
'Selenium' on 'Ras−related protein Rab−39B'

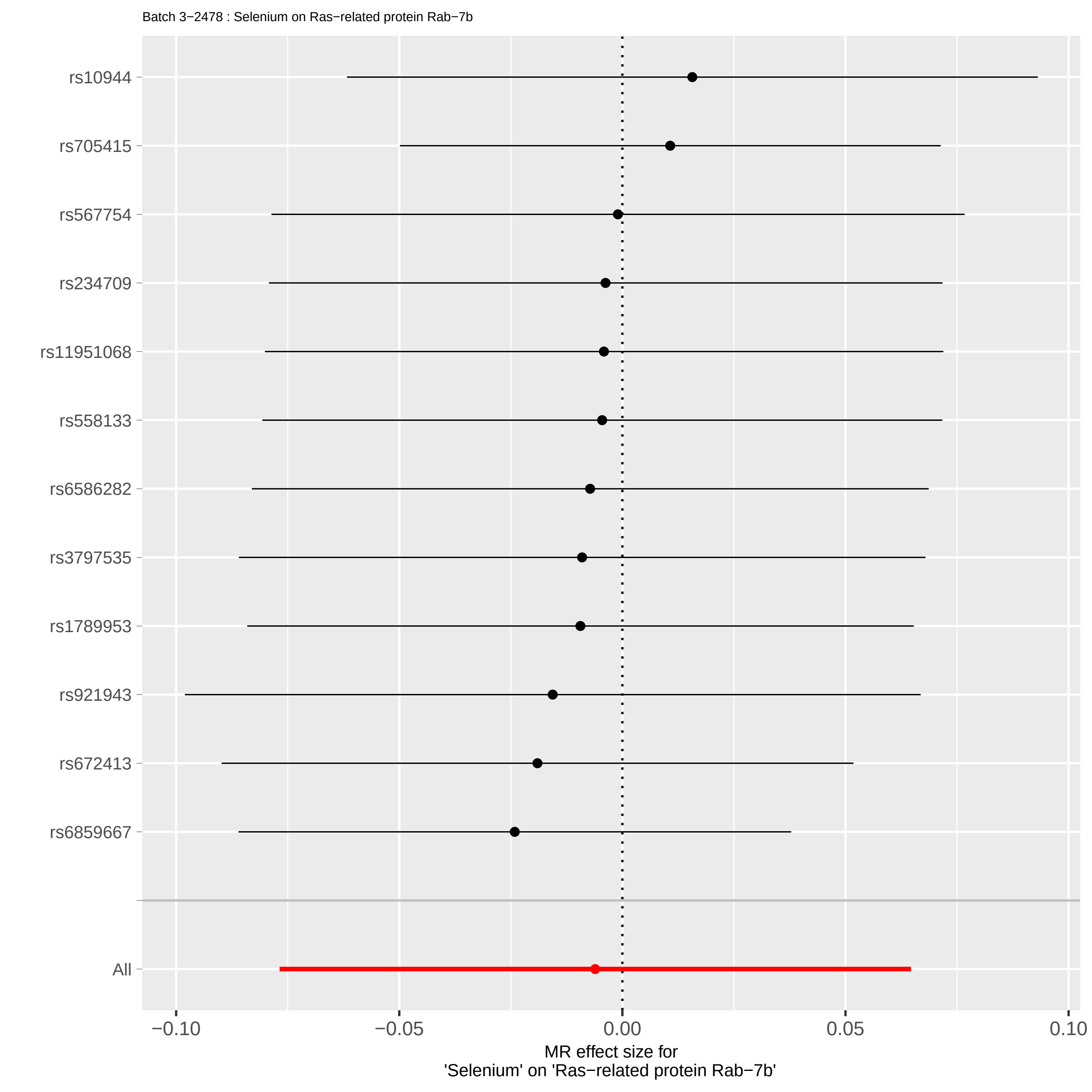

Batch 3−2478 : Selenium on Ras−related protein Rab−7b
rs10944
rs705415
rs567754
rs234709
rs11951068
rs558133
rs6586282
rs3797535
rs1789953
rs921943
rs672413
rs6859667
All
−0.10
−0.05
0.00
0.05
0.10
MR effect size for
'Selenium' on 'Ras−related protein Rab−7b'

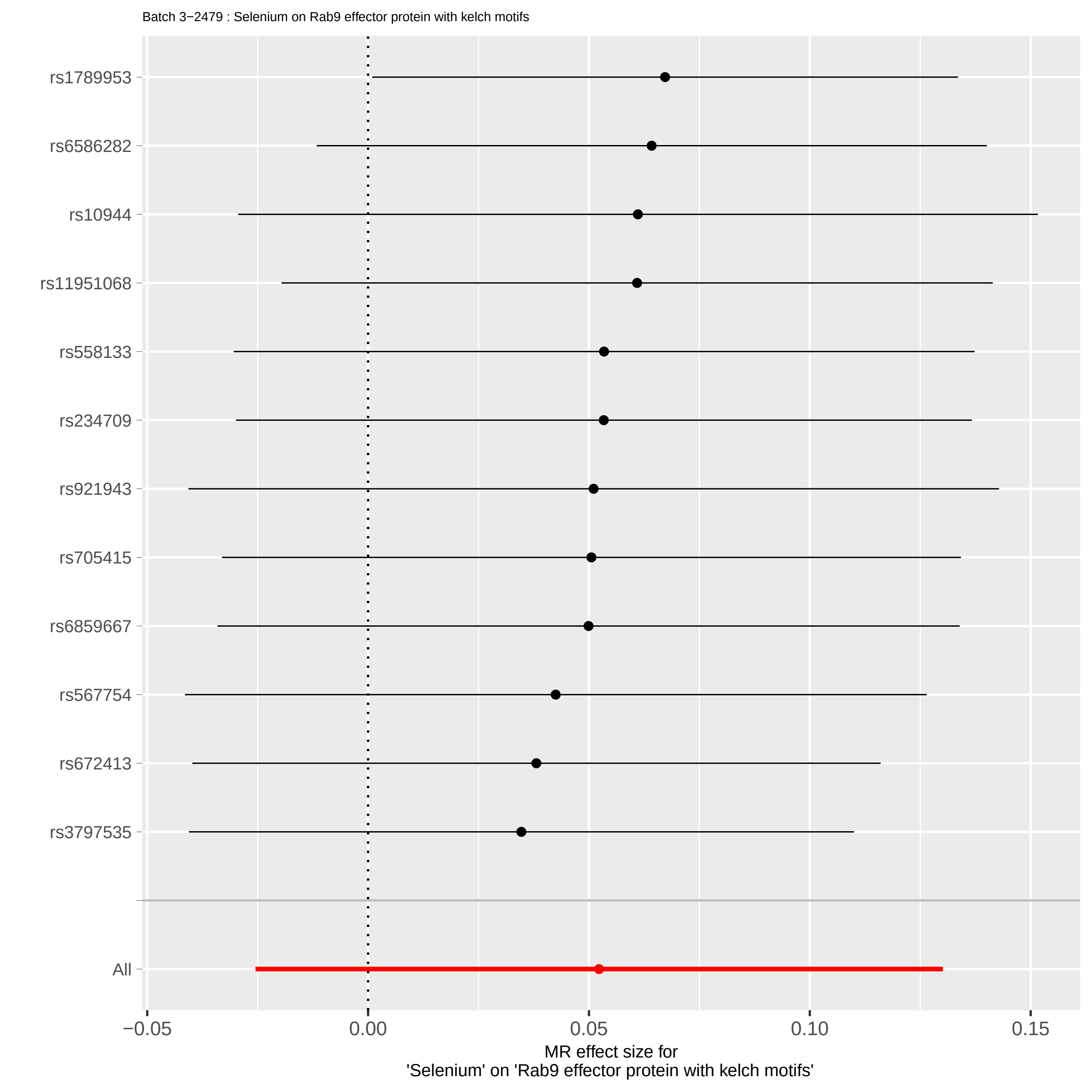

Batch 3−2479 : Selenium on Rab9 effector protein with kelch motifs
rs1789953
rs6586282
rs10944
rs11951068
rs558133
rs234709
rs921943
rs705415
rs6859667
rs567754
rs672413
rs3797535
All
−0.05
0.00
0.05
0.10
0.15
MR effect size for
'Selenium' on 'Rab9 effector protein with kelch motifs'

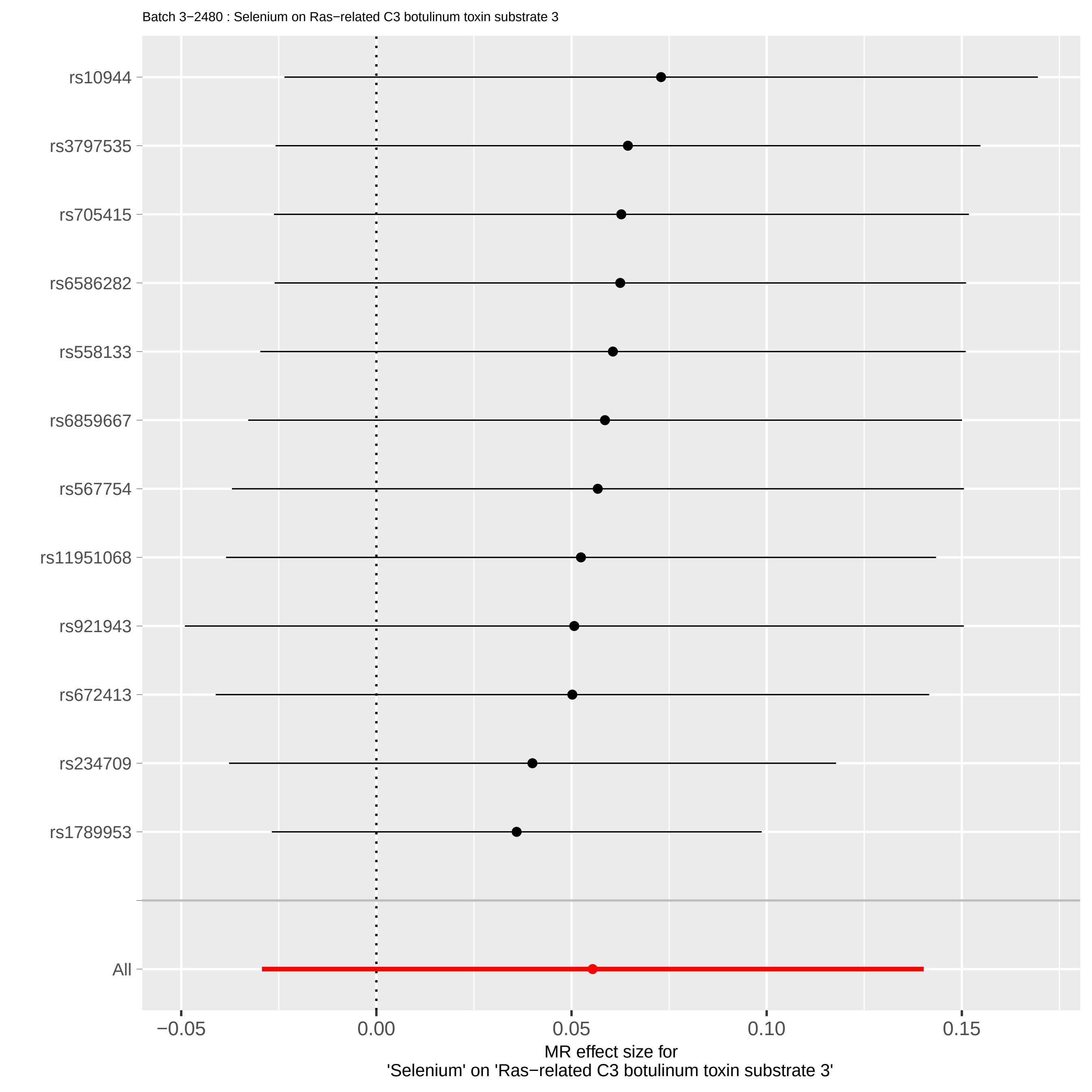
Batch 3−2480 : Selenium on Ras−related C3 botulinum toxin substrate 3
rs10944
rs3797535
rs705415
rs6586282
rs558133
rs6859667
rs567754
rs11951068
rs921943
rs672413
rs234709
rs1789953
All
−0.05
0.00
0.05
0.10
0.15
MR effect size for
'Selenium' on 'Ras−related C3 botulinum toxin substrate 3'

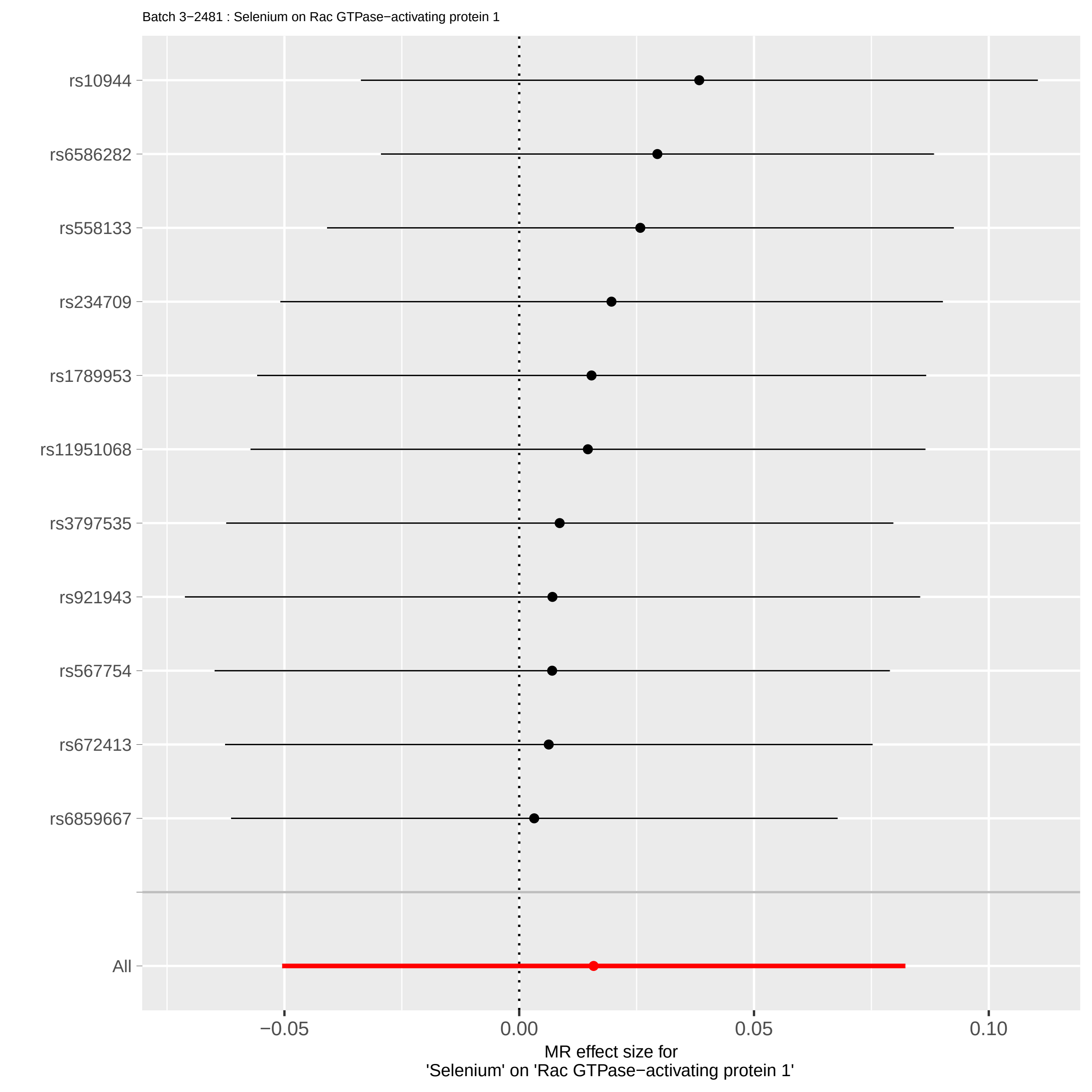

Batch 3−2481 : Selenium on Rac GTPase−activating protein 1
rs10944
rs6586282
rs558133
rs234709
rs1789953
rs11951068
rs3797535
rs921943
rs567754
rs672413
rs6859667
All
−0.05
0.00
0.05
0.10
MR effect size for
'Selenium' on 'Rac GTPase−activating protein 1'

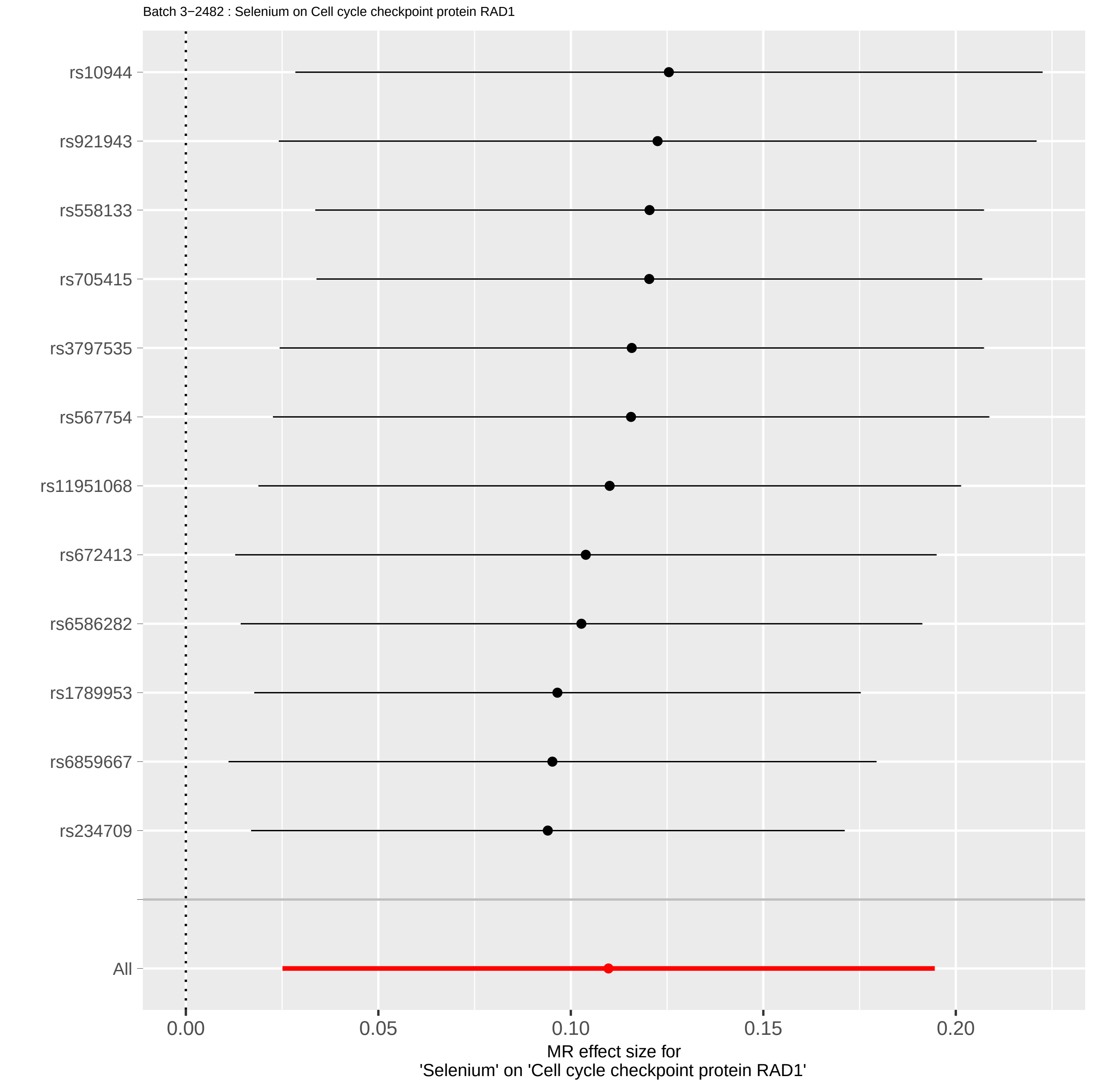

Batch 3−2482 : Selenium on Cell cycle checkpoint protein RAD1
rs10944
rs921943
rs558133
rs705415
rs3797535
rs567754
rs11951068
rs672413
rs6586282
rs1789953
rs6859667
rs234709
All
0.00
0.05
0.10
0.15
0.20
MR effect size for
'Selenium' on 'Cell cycle checkpoint protein RAD1'

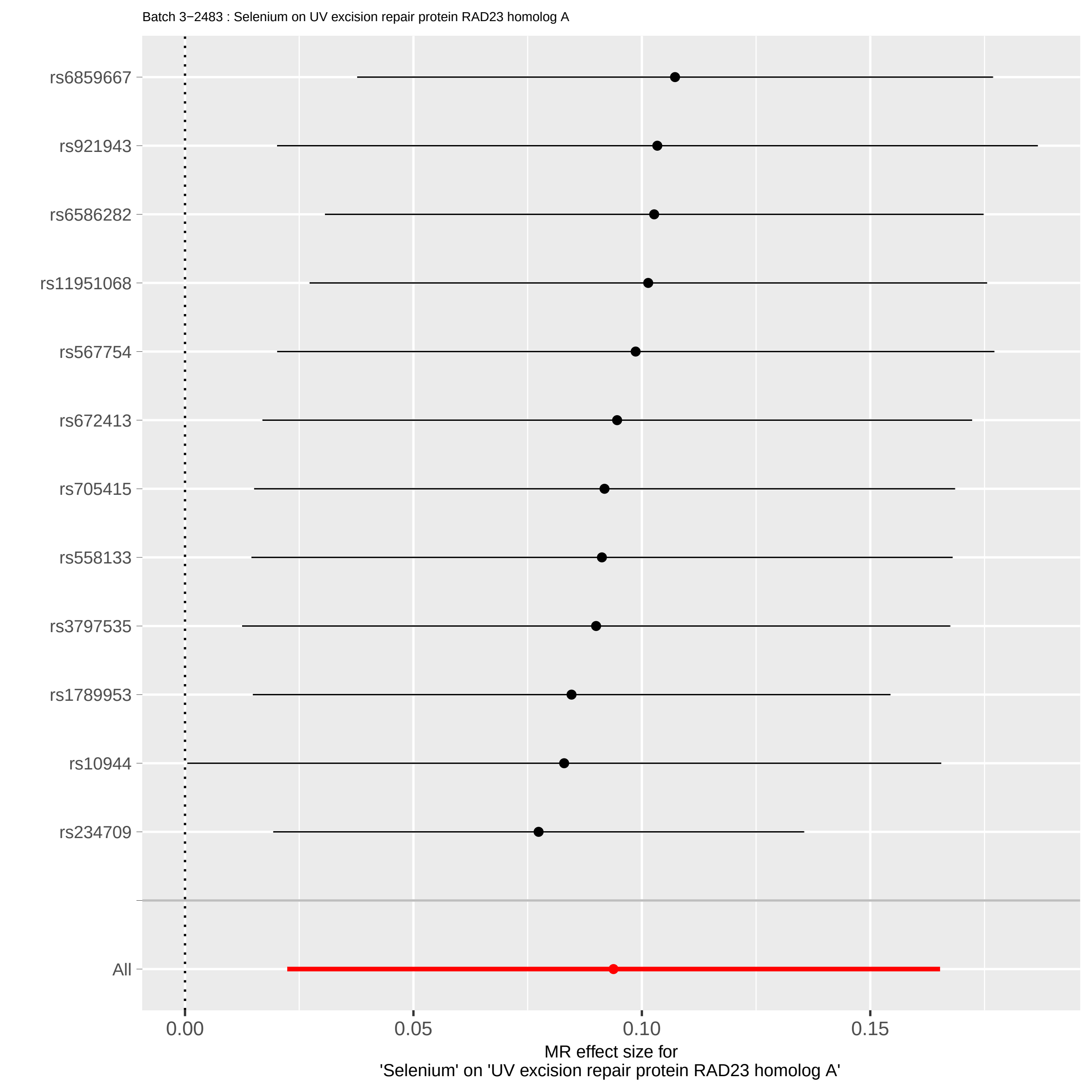

Batch 3−2483 : Selenium on UV excision repair protein RAD23 homolog A
rs6859667
rs921943
rs6586282
rs11951068
rs567754
rs672413
rs705415
rs558133
rs3797535
rs1789953
rs10944
rs234709
All
0.00
0.05
0.10
0.15
MR effect size for
'Selenium' on 'UV excision repair protein RAD23 homolog A'

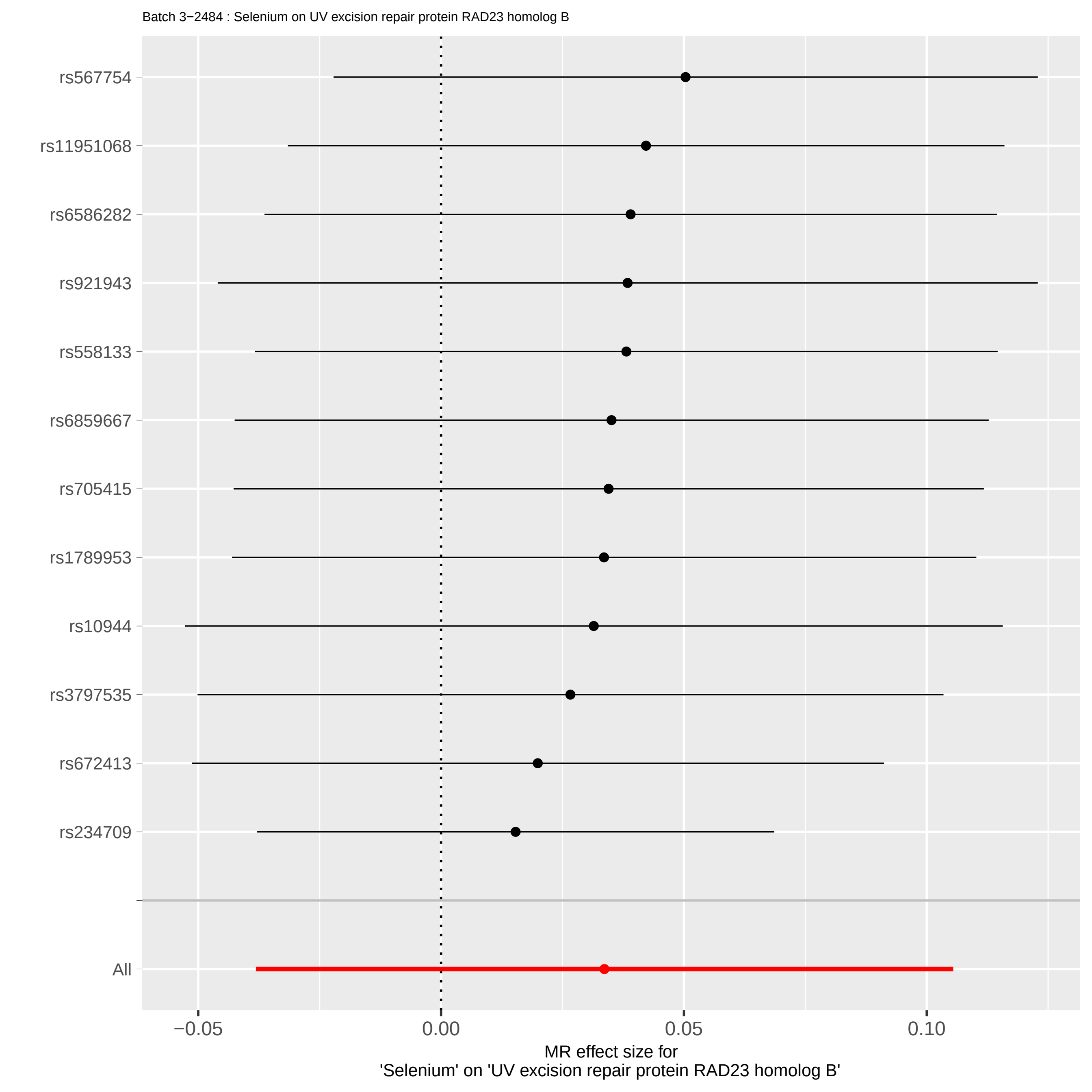

Batch 3−2484 : Selenium on UV excision repair protein RAD23 homolog B
rs567754
rs11951068
rs6586282
rs921943
rs558133
rs6859667
rs705415
rs1789953
rs10944
rs3797535
rs672413
rs234709
All
−0.05
0.00
0.05
0.10
MR effect size for
'Selenium' on 'UV excision repair protein RAD23 homolog B'

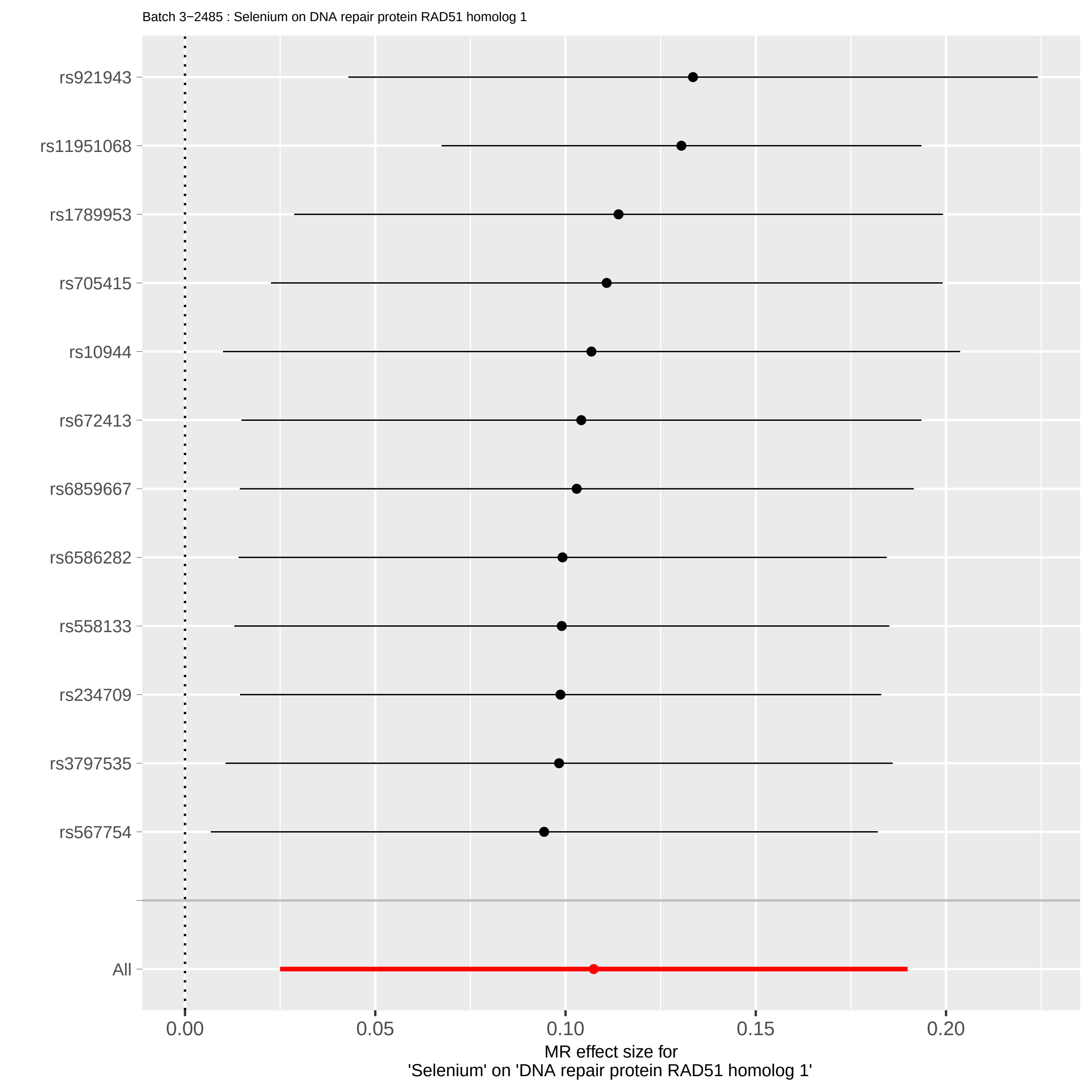
Batch 3−2485 : Selenium on DNA repair protein RAD51 homolog 1
rs921943
rs11951068
rs1789953
rs705415
rs10944
rs672413
rs6859667
rs6586282
rs558133
rs234709
rs3797535
rs567754
All
0.00
0.05
0.10
0.15
0.20
MR effect size for
'Selenium' on 'DNA repair protein RAD51 homolog 1'

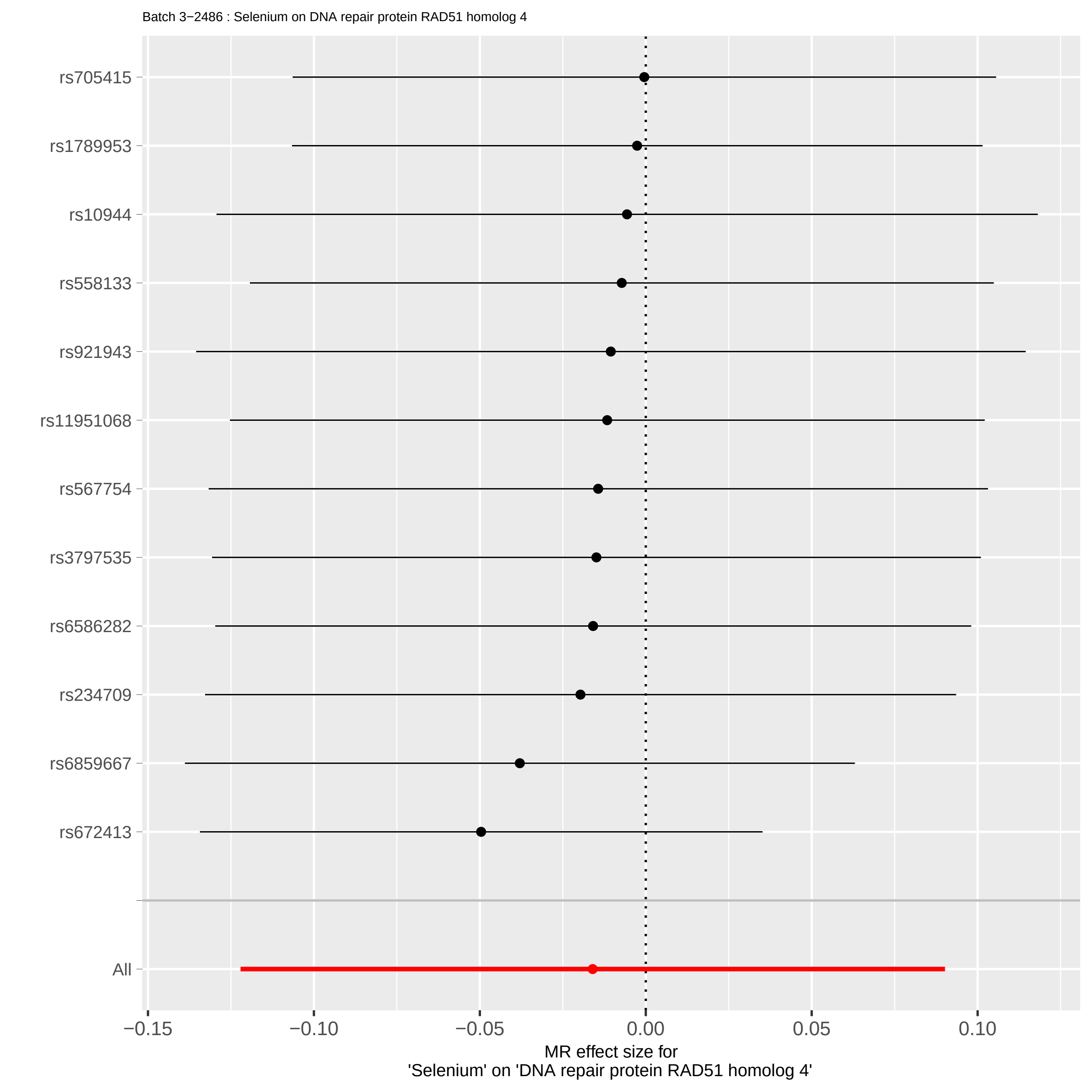
Batch 3−2486 : Selenium on DNA repair protein RAD51 homolog 4
rs705415
rs1789953
rs10944
rs558133
rs921943
rs11951068
rs567754
rs3797535
rs6586282
rs234709
rs6859667
rs672413
All
−0.15
−0.10
−0.05
0.00
0.05
0.10
MR effect size for
'Selenium' on 'DNA repair protein RAD51 homolog 4'

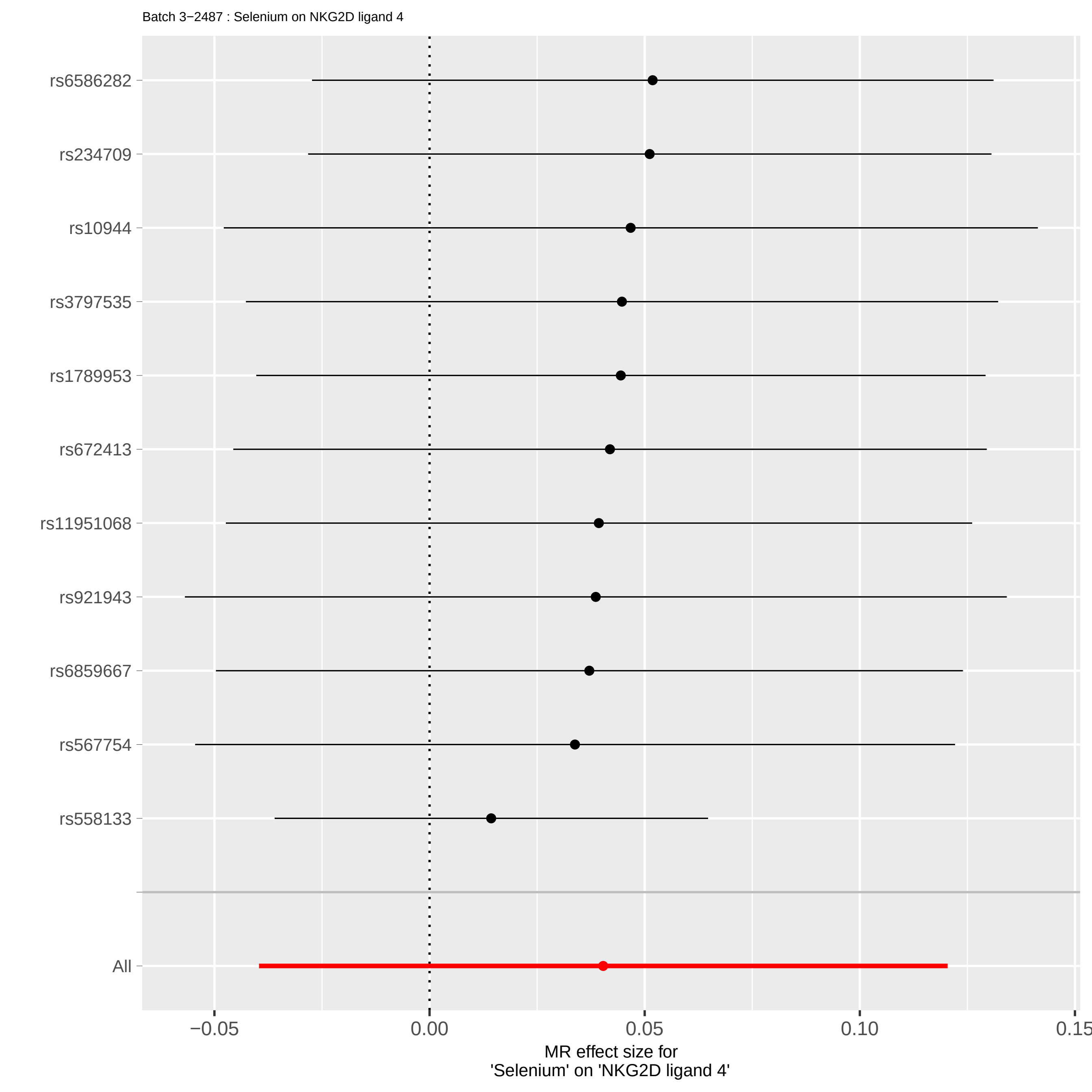

Batch 3−2487 : Selenium on NKG2D ligand 4
rs6586282
rs234709
rs10944
rs3797535
rs1789953
rs672413
rs11951068
rs921943
rs6859667
rs567754
rs558133
All
−0.05
0.00
0.05
0.10
0.15
MR effect size for
'Selenium' on 'NKG2D ligand 4'

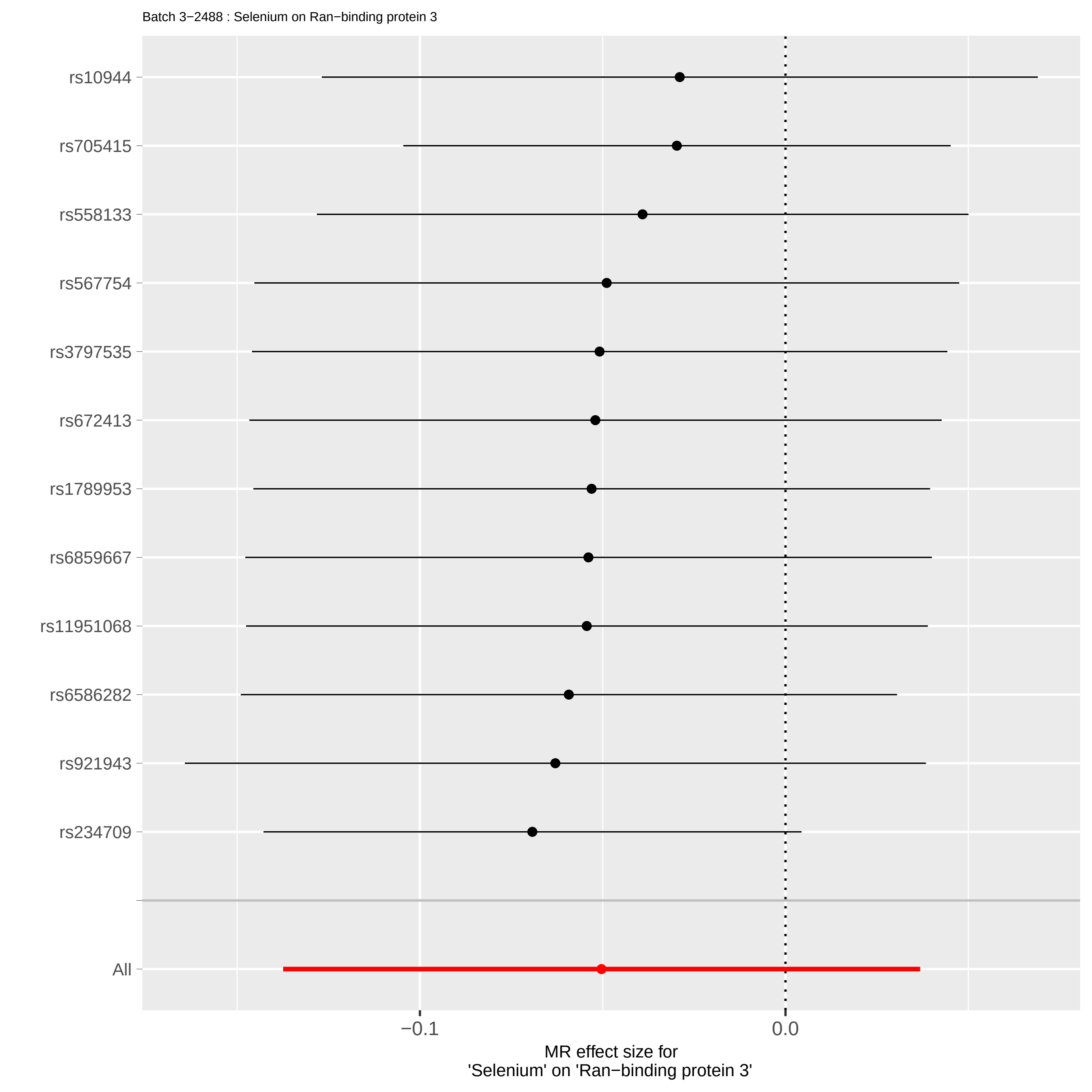

Batch 3−2488 : Selenium on Ran−binding protein 3
rs10944
rs705415
rs558133
rs567754
rs3797535
rs672413
rs1789953
rs6859667
rs11951068
rs6586282
rs921943
rs234709
All
−0.1
0.0
MR effect size for
'Selenium' on 'Ran−binding protein 3'

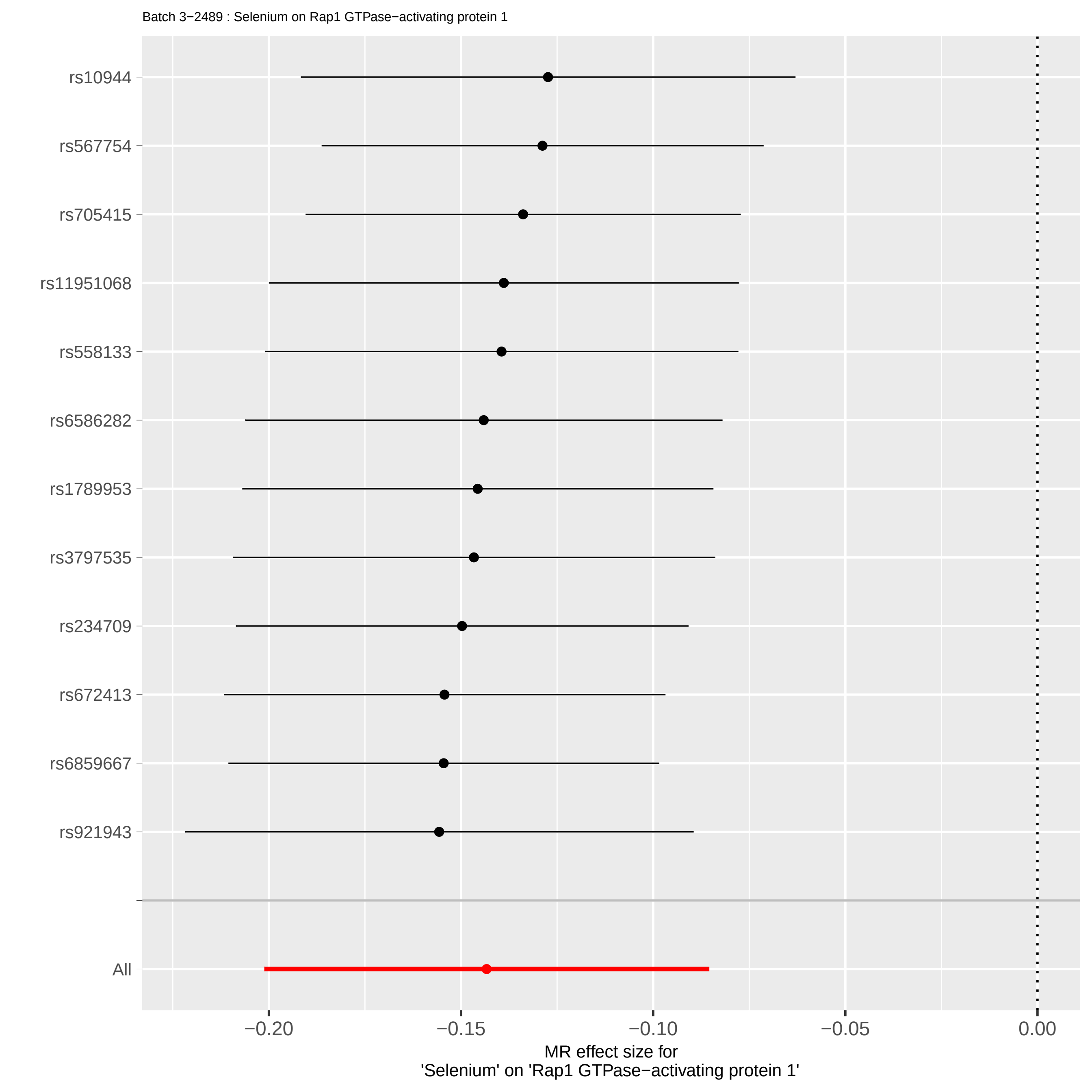

Batch 3−2489 : Selenium on Rap1 GTPase−activating protein 1
rs10944
rs567754
rs705415
rs11951068
rs558133
rs6586282
rs1789953
rs3797535
rs234709
rs672413
rs6859667
rs921943
All
−0.20
−0.15
−0.10
−0.05
0.00
MR effect size for
'Selenium' on 'Rap1 GTPase−activating protein 1'

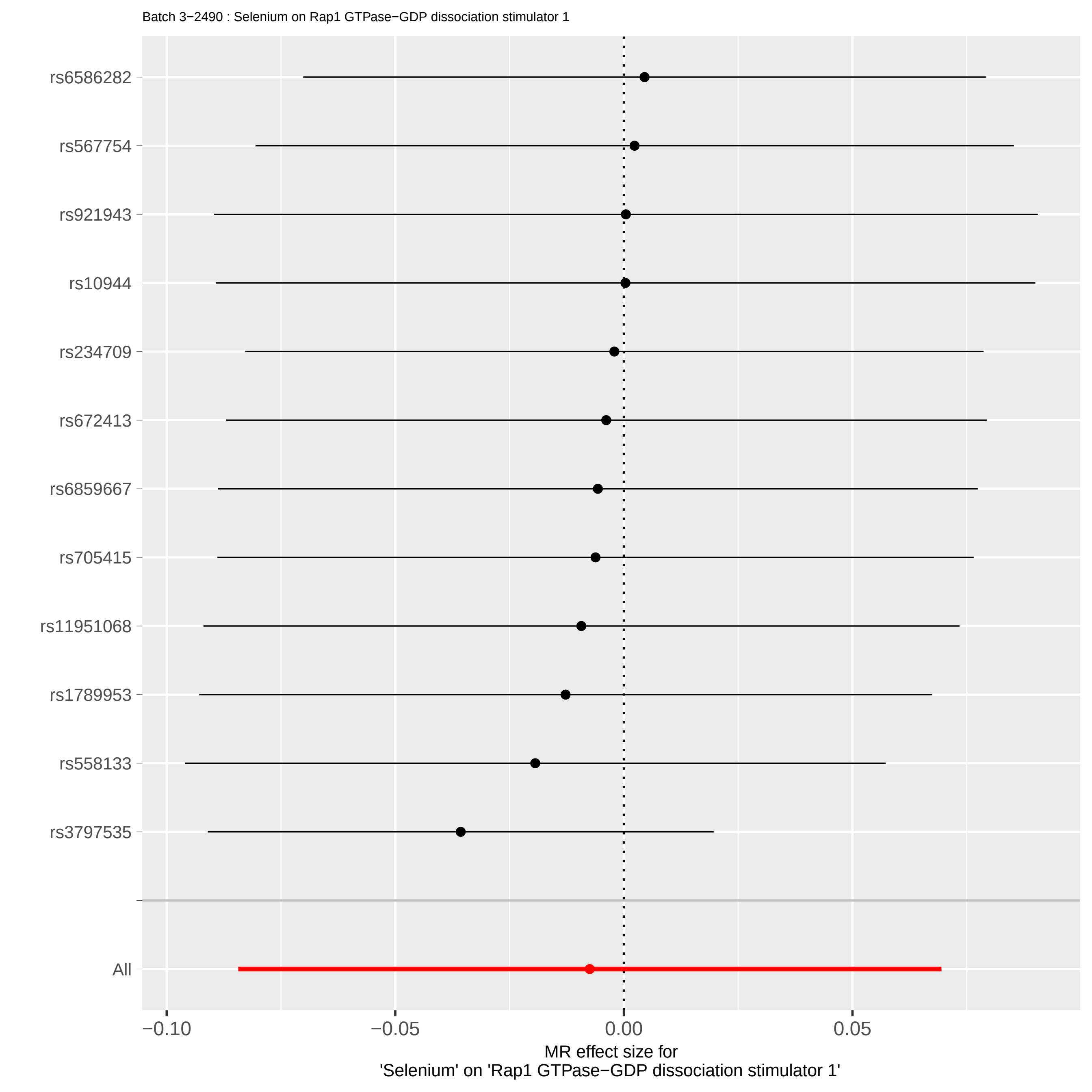

Batch 3−2490 : Selenium on Rap1 GTPase−GDP dissociation stimulator 1
rs6586282
rs567754
rs921943
rs10944
rs234709
rs672413
rs6859667
rs705415
rs11951068
rs1789953
rs558133
rs3797535
All
−0.10
−0.05
0.00
0.05
MR effect size for
'Selenium' on 'Rap1 GTPase−GDP dissociation stimulator 1'

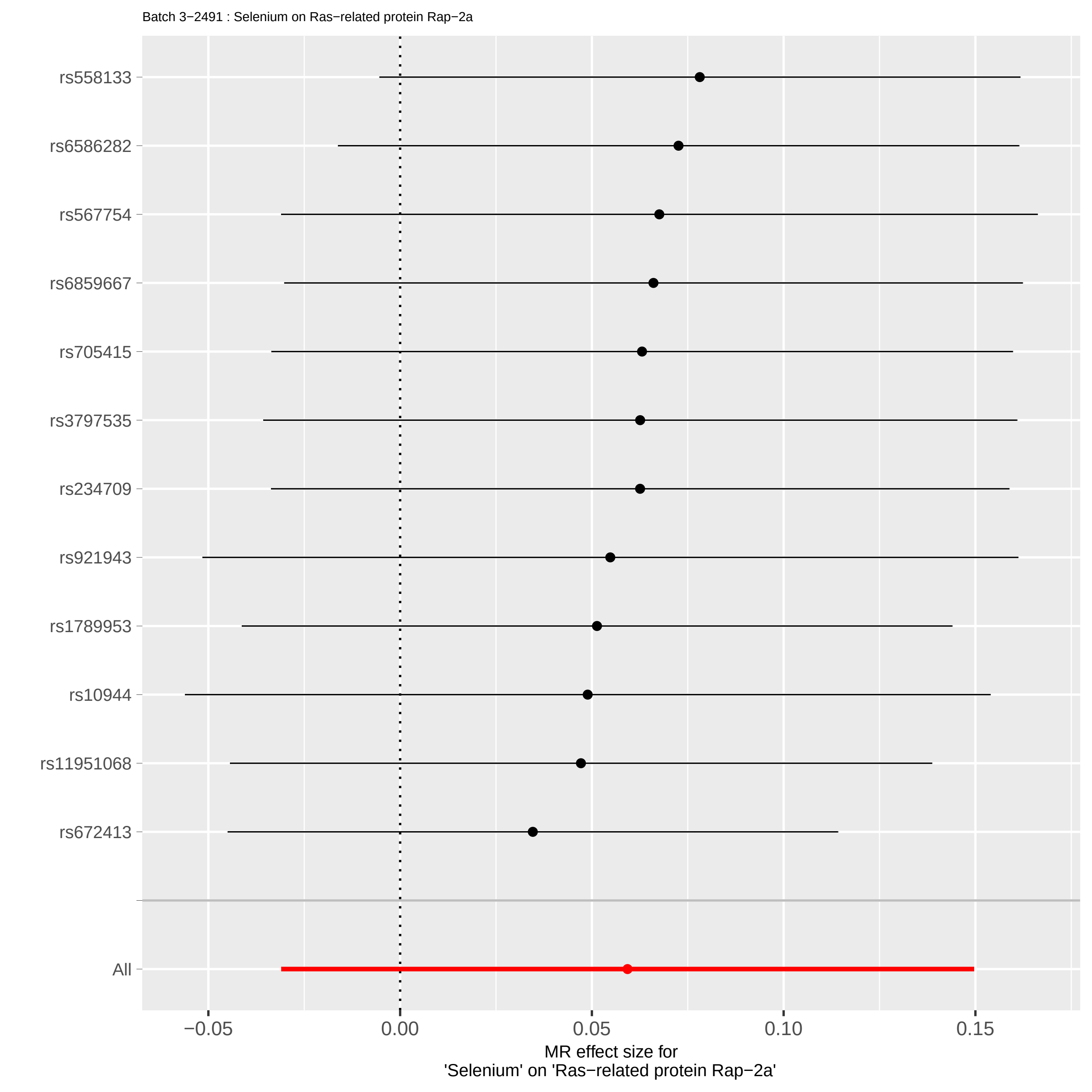
Batch 3−2491 : Selenium on Ras−related protein Rap−2a
rs558133
rs6586282
rs567754
rs6859667
rs705415
rs3797535
rs234709
rs921943
rs1789953
rs10944
rs11951068
rs672413
All
−0.05
0.00
0.05
0.10
0.15
MR effect size for
'Selenium' on 'Ras−related protein Rap−2a'

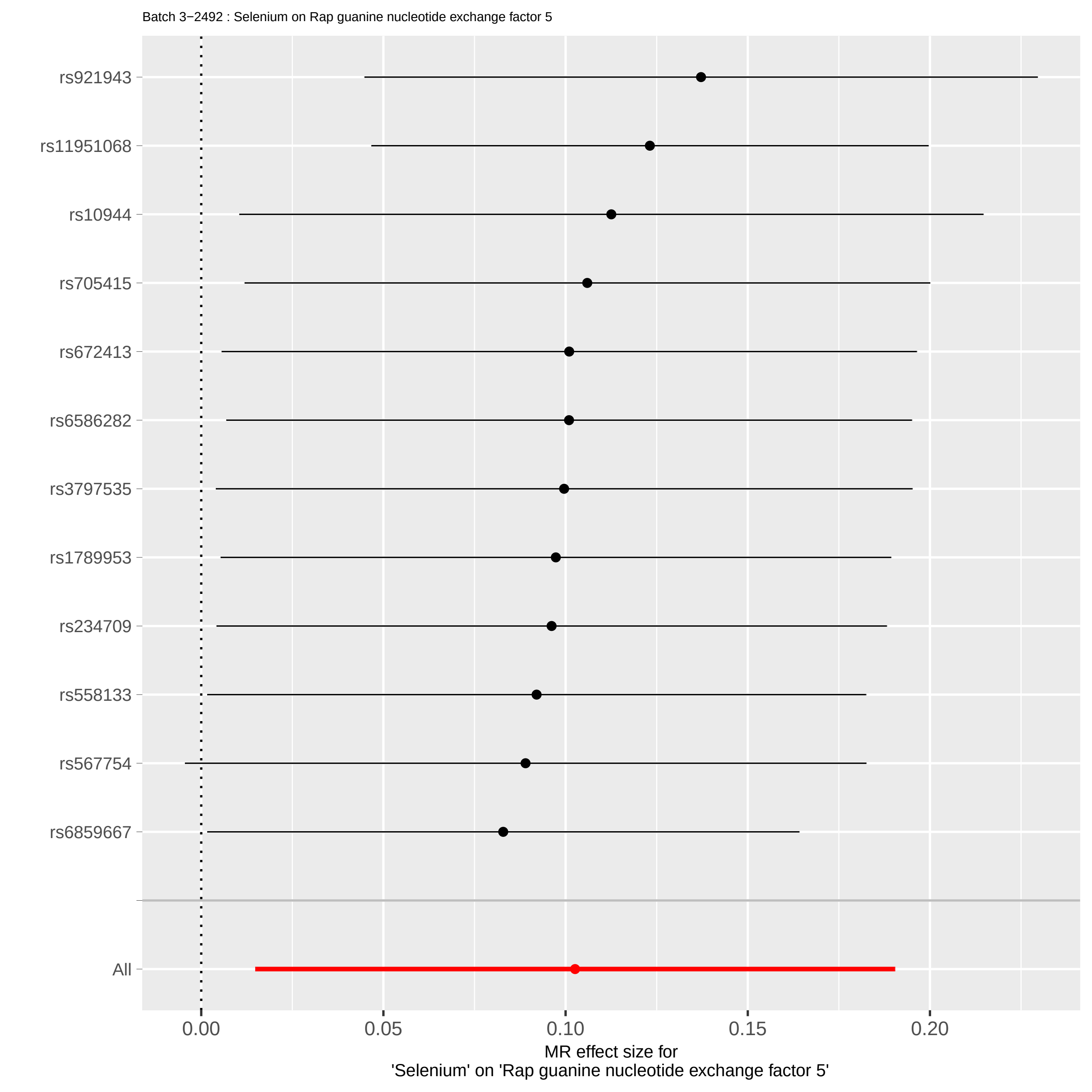

Batch 3−2492 : Selenium on Rap guanine nucleotide exchange factor 5
rs921943
rs11951068
rs10944
rs705415
rs672413
rs6586282
rs3797535
rs1789953
rs234709
rs558133
rs567754
rs6859667
All
0.00
0.05
0.10
0.15
0.20
MR effect size for
'Selenium' on 'Rap guanine nucleotide exchange factor 5'

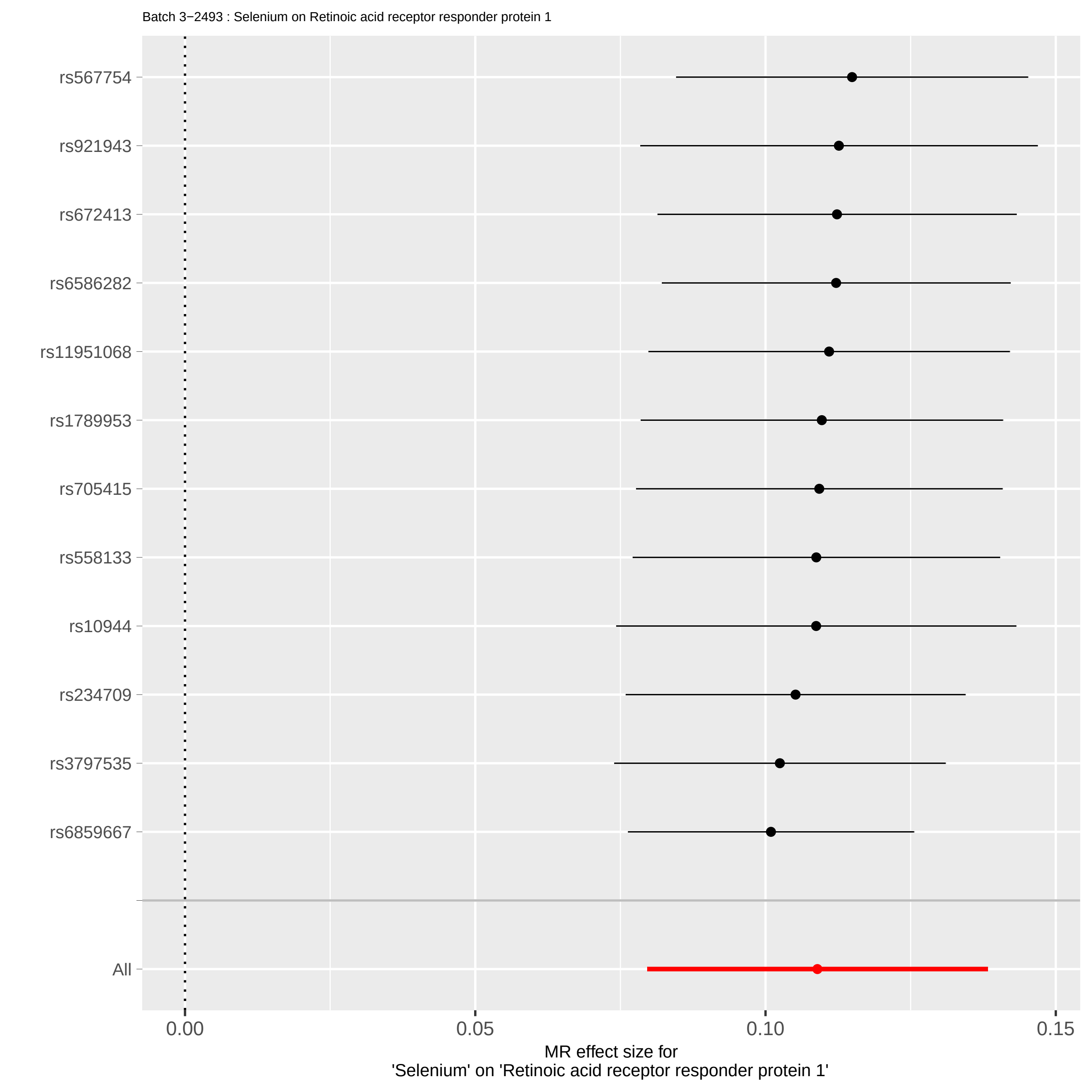

Batch 3−2493 : Selenium on Retinoic acid receptor responder protein 1
rs567754
rs921943
rs672413
rs6586282
rs11951068
rs1789953
rs705415
rs558133
rs10944
rs234709
rs3797535
rs6859667
All
0.00
0.05
0.10
0.15
MR effect size for
'Selenium' on 'Retinoic acid receptor responder protein 1'

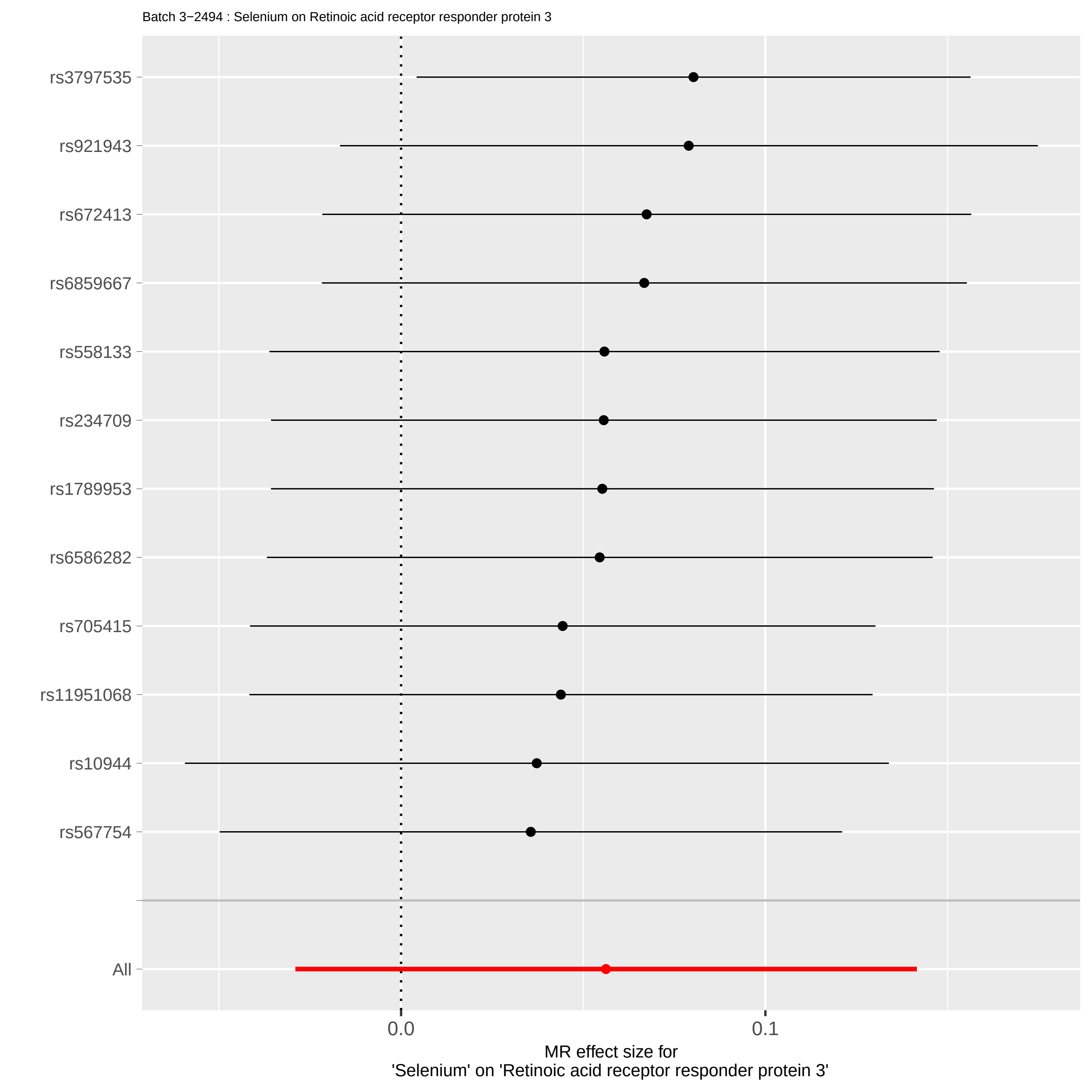

Batch 3−2494 : Selenium on Retinoic acid receptor responder protein 3
rs3797535
rs921943
rs672413
rs6859667
rs558133
rs234709
rs1789953
rs6586282
rs705415
rs11951068
rs10944
rs567754
All
0.0
0.1
MR effect size for
'Selenium' on 'Retinoic acid receptor responder protein 3'

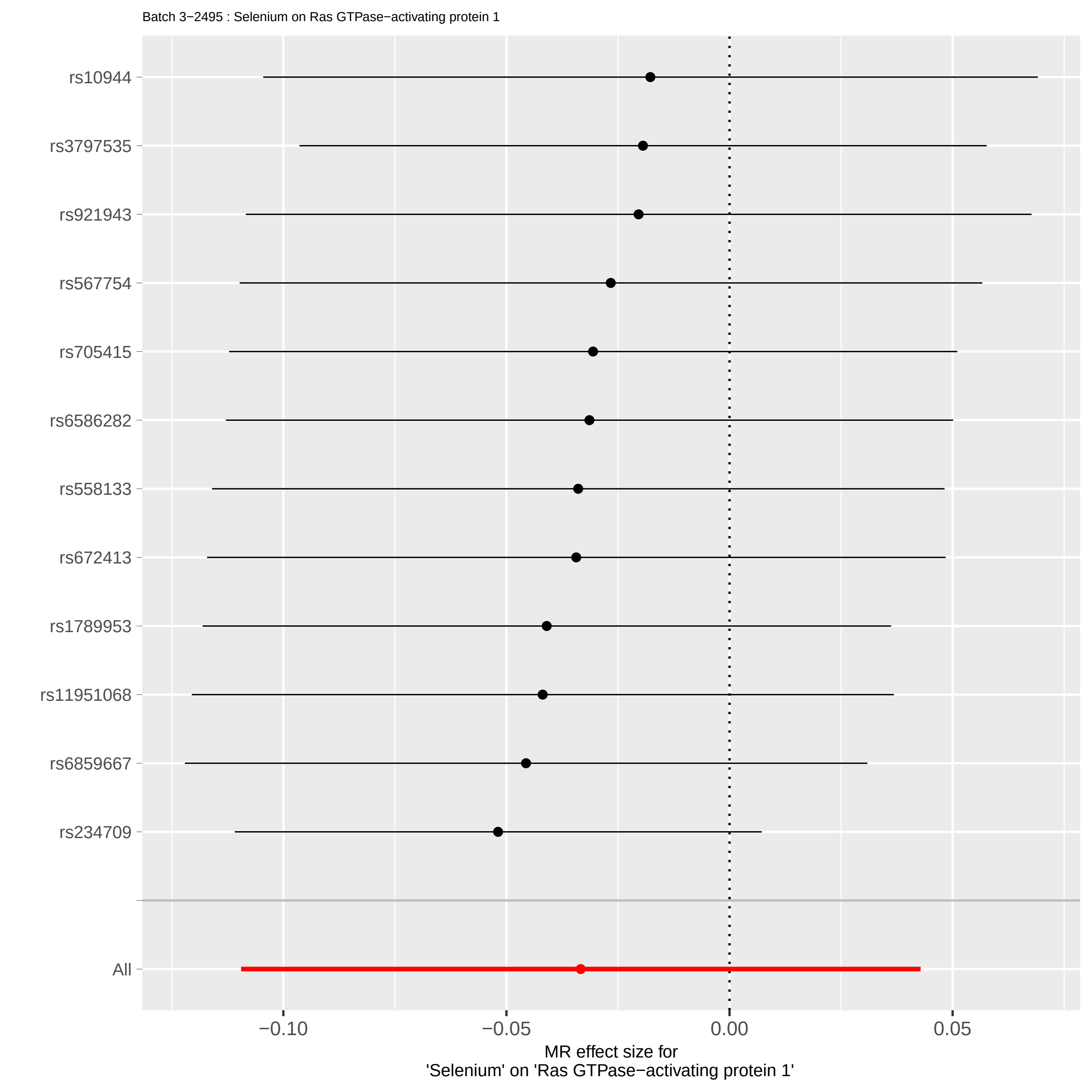

Batch 3−2495 : Selenium on Ras GTPase−activating protein 1
rs10944
rs3797535
rs921943
rs567754
rs705415
rs6586282
rs558133
rs672413
rs1789953
rs11951068
rs6859667
rs234709
All
−0.10
−0.05
0.00
0.05
MR effect size for
'Selenium' on 'Ras GTPase−activating protein 1'

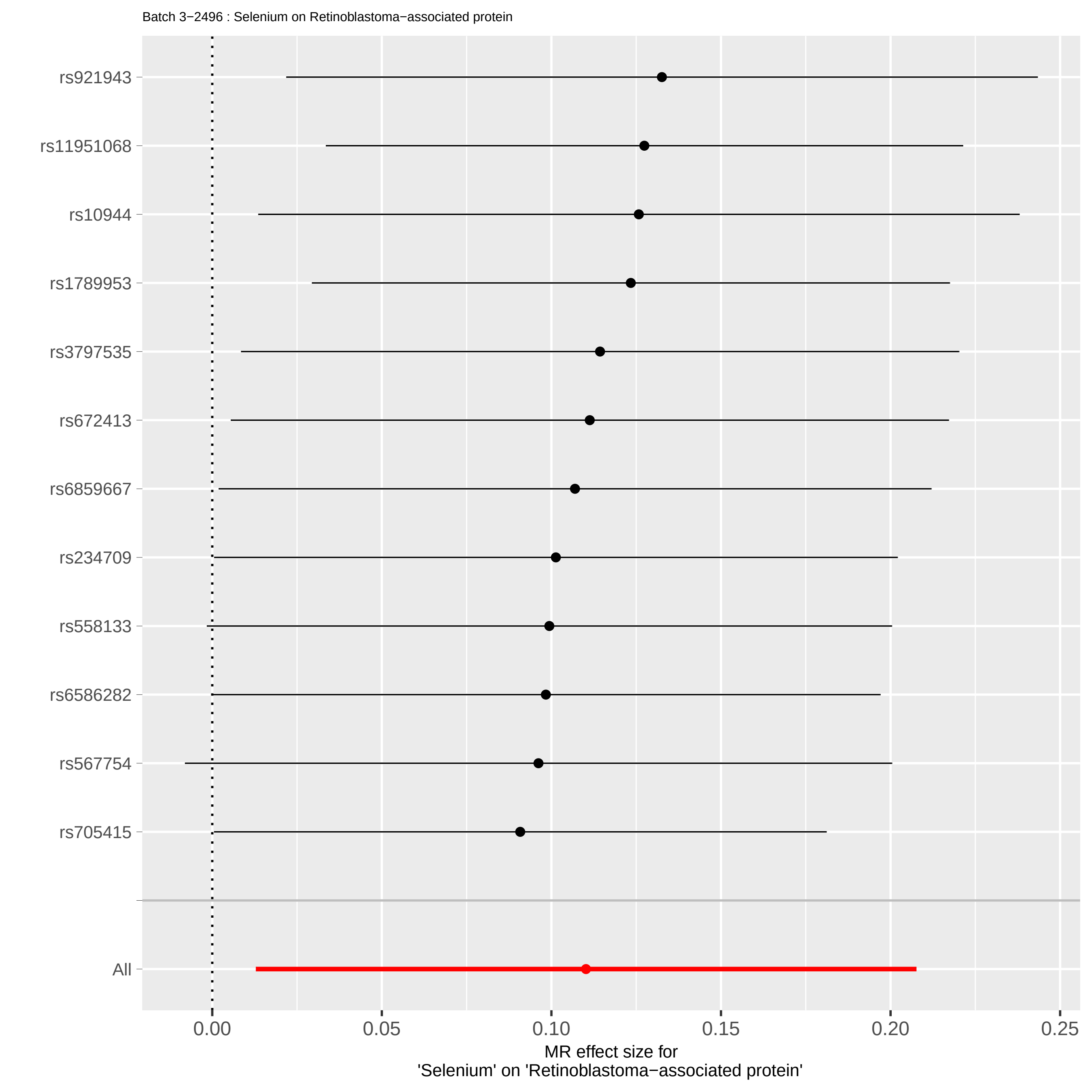

Batch 3−2496 : Selenium on Retinoblastoma−associated protein
rs921943
rs11951068
rs10944
rs1789953
rs3797535
rs672413
rs6859667
rs234709
rs558133
rs6586282
rs567754
rs705415
All
0.00
0.05
0.10
0.15
0.20
0.25
MR effect size for
'Selenium' on 'Retinoblastoma−associated protein'

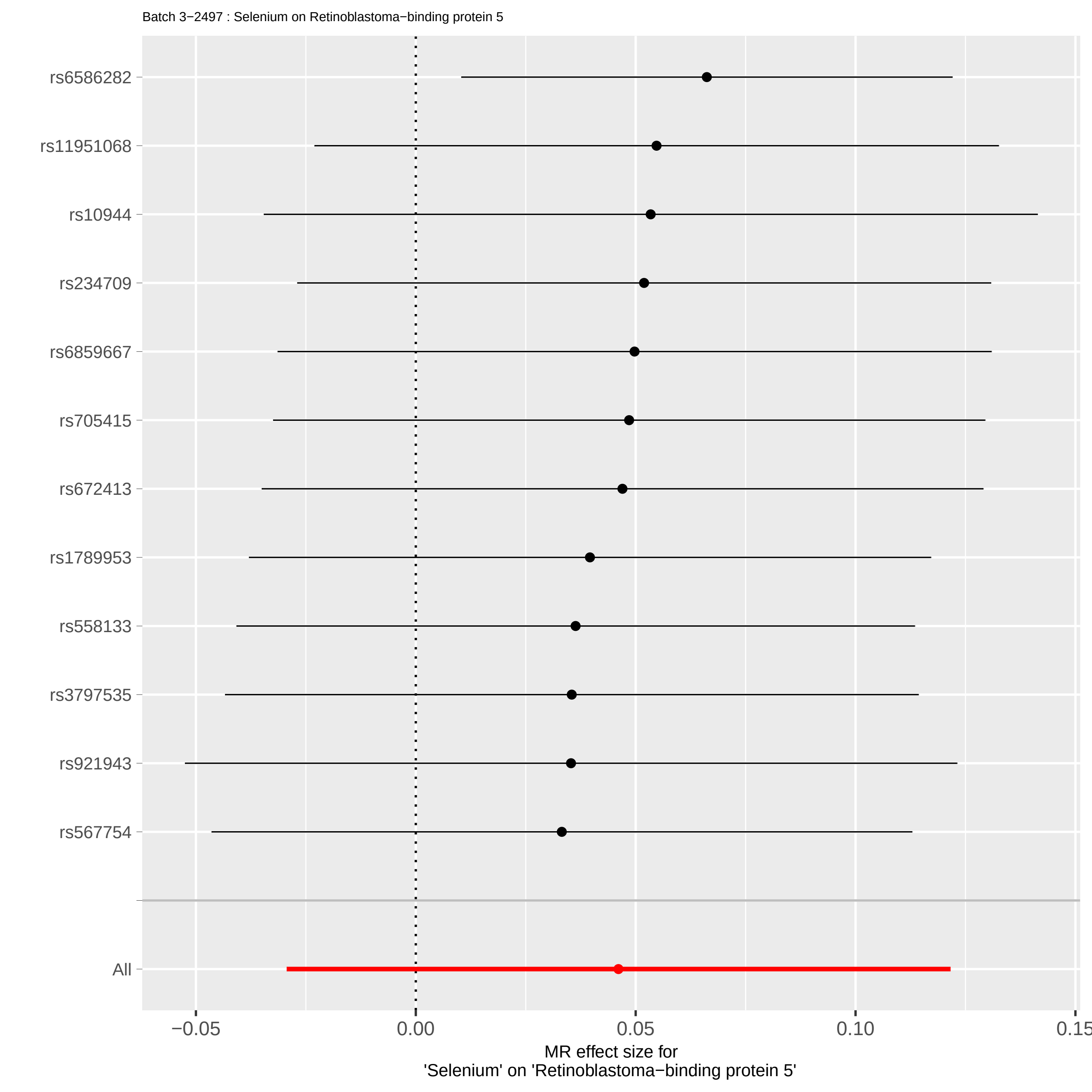
Batch 3−2497 : Selenium on Retinoblastoma−binding protein 5
rs6586282
rs11951068
rs10944
rs234709
rs6859667
rs705415
rs672413
rs1789953
rs558133
rs3797535
rs921943
rs567754
All
−0.05
0.00
0.05
0.10
0.15
MR effect size for
'Selenium' on 'Retinoblastoma−binding protein 5'

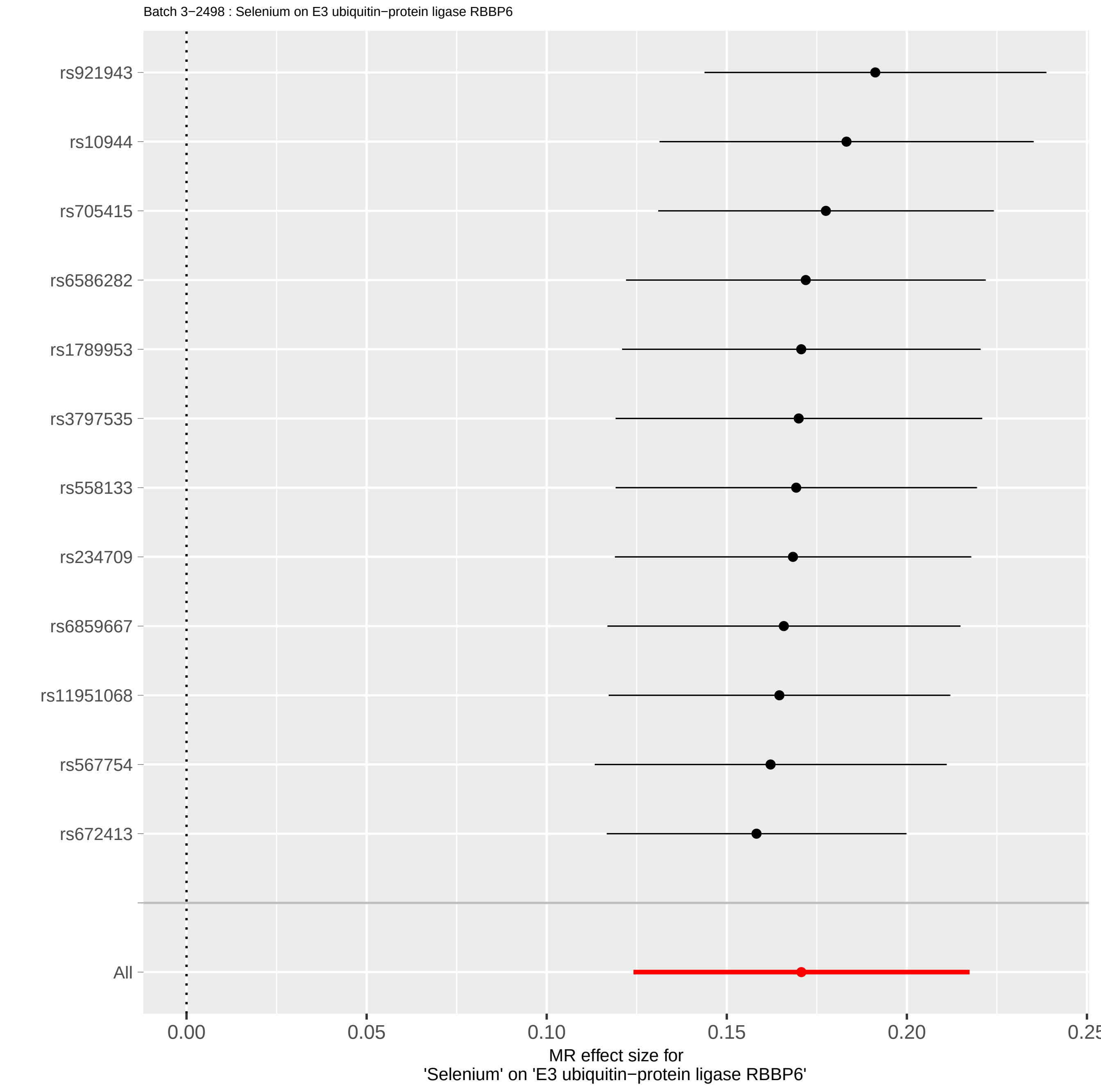

Batch 3−2498 : Selenium on E3 ubiquitin−protein ligase RBBP6
rs921943
rs10944
rs705415
rs6586282
rs1789953
rs3797535
rs558133
rs234709
rs6859667
rs11951068
rs567754
rs672413
All
0.00
0.05
0.10
0.15
0.20
0.25
MR effect size for
'Selenium' on 'E3 ubiquitin−protein ligase RBBP6'

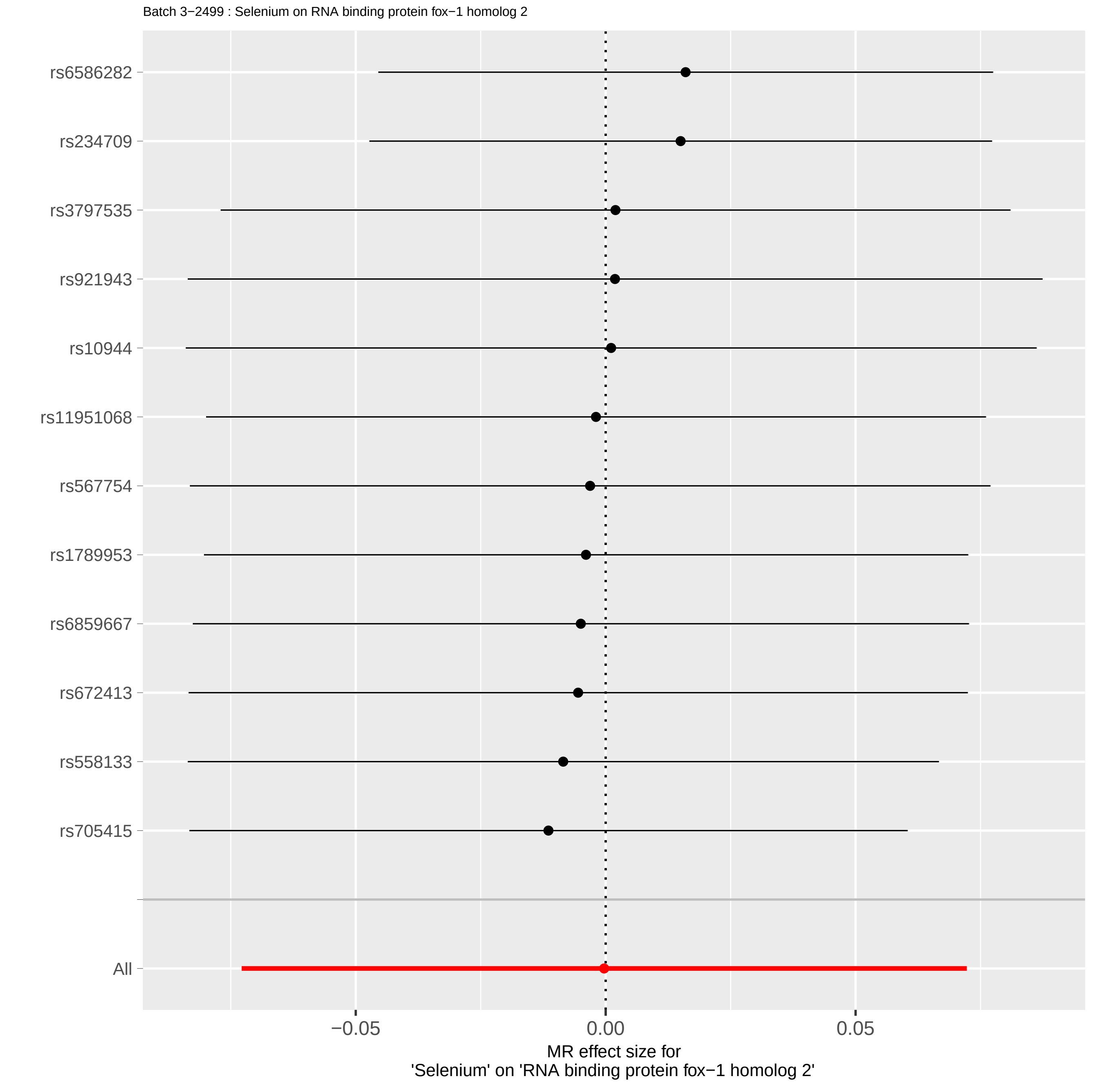

Batch 3−2499 : Selenium on RNA binding protein fox−1 homolog 2
rs6586282
rs234709
rs3797535
rs921943
rs10944
rs11951068
rs567754
rs1789953
rs6859667
rs672413
rs558133
rs705415
All
−0.05
0.00
0.05
MR effect size for
'Selenium' on 'RNA binding protein fox−1 homolog 2'

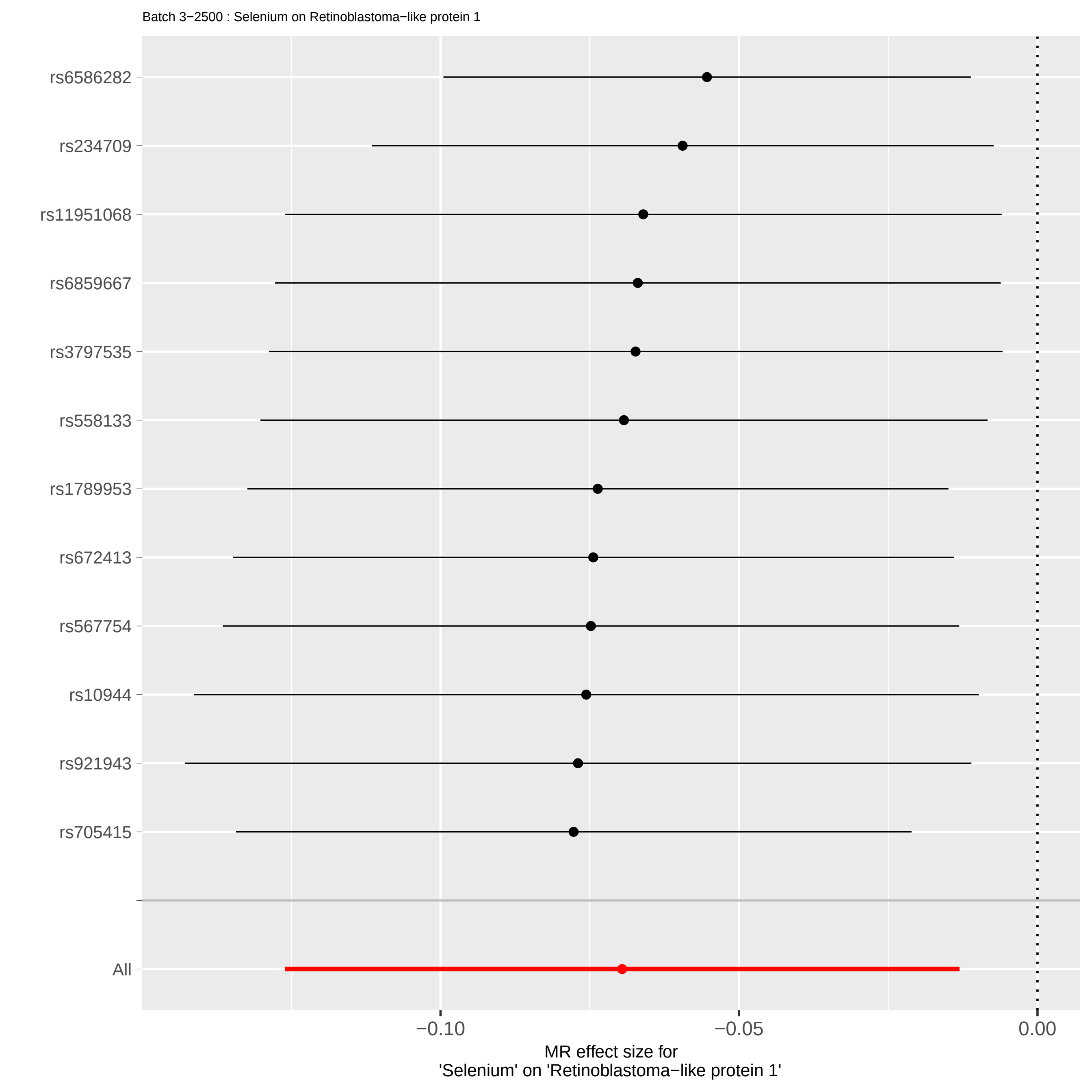

Batch 3−2500 : Selenium on Retinoblastoma−like protein 1
rs6586282
rs234709
rs11951068
rs6859667
rs3797535
rs558133
rs1789953
rs672413
rs567754
rs10944
rs921943
rs705415
All
−0.10
−0.05
0.00
MR effect size for
'Selenium' on 'Retinoblastoma−like protein 1'

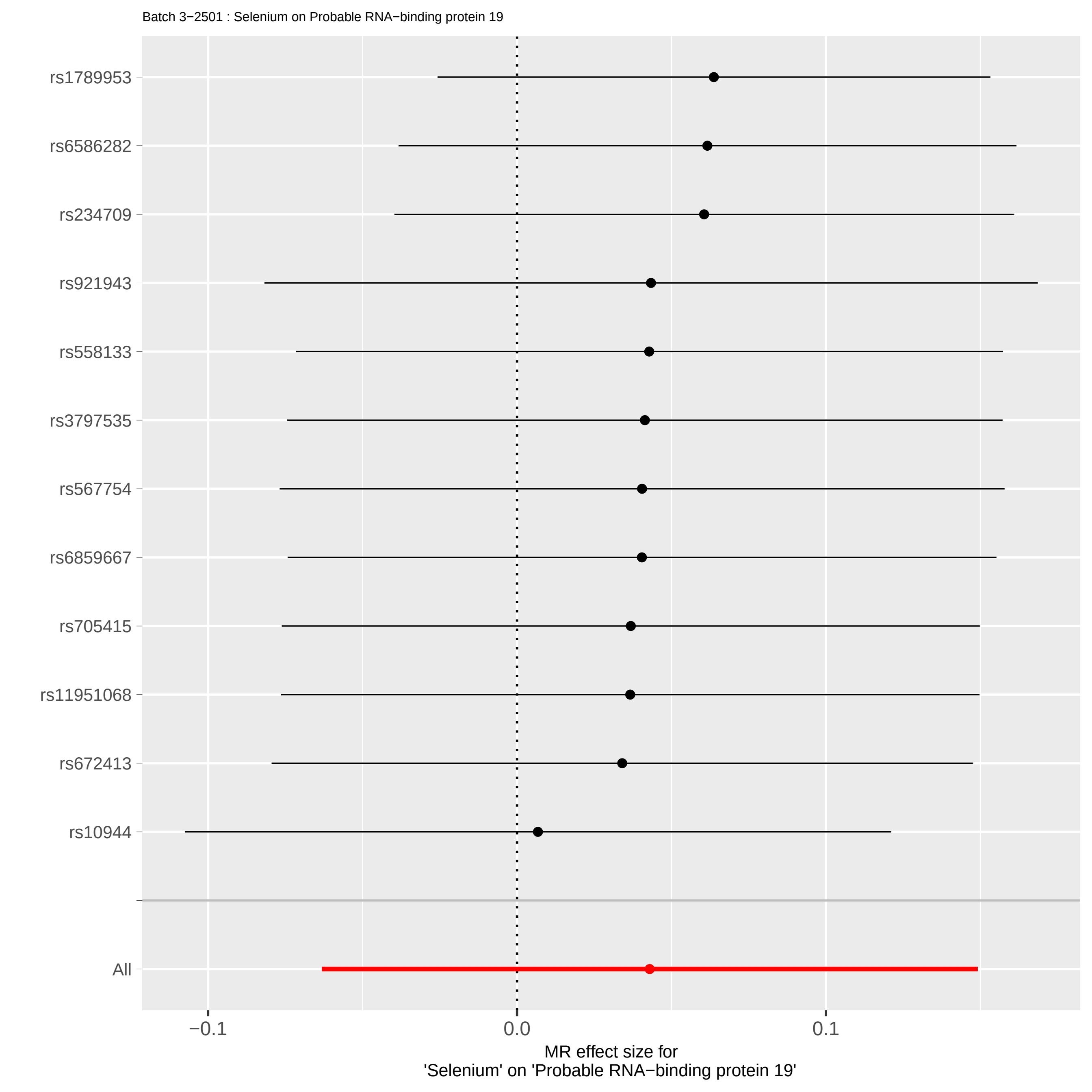

Batch 3−2501 : Selenium on Probable RNA−binding protein 19
rs1789953
rs6586282
rs234709
rs921943
rs558133
rs3797535
rs567754
rs6859667
rs705415
rs11951068
rs672413
rs10944
All
−0.1
0.0
0.1
MR effect size for
'Selenium' on 'Probable RNA−binding protein 19'

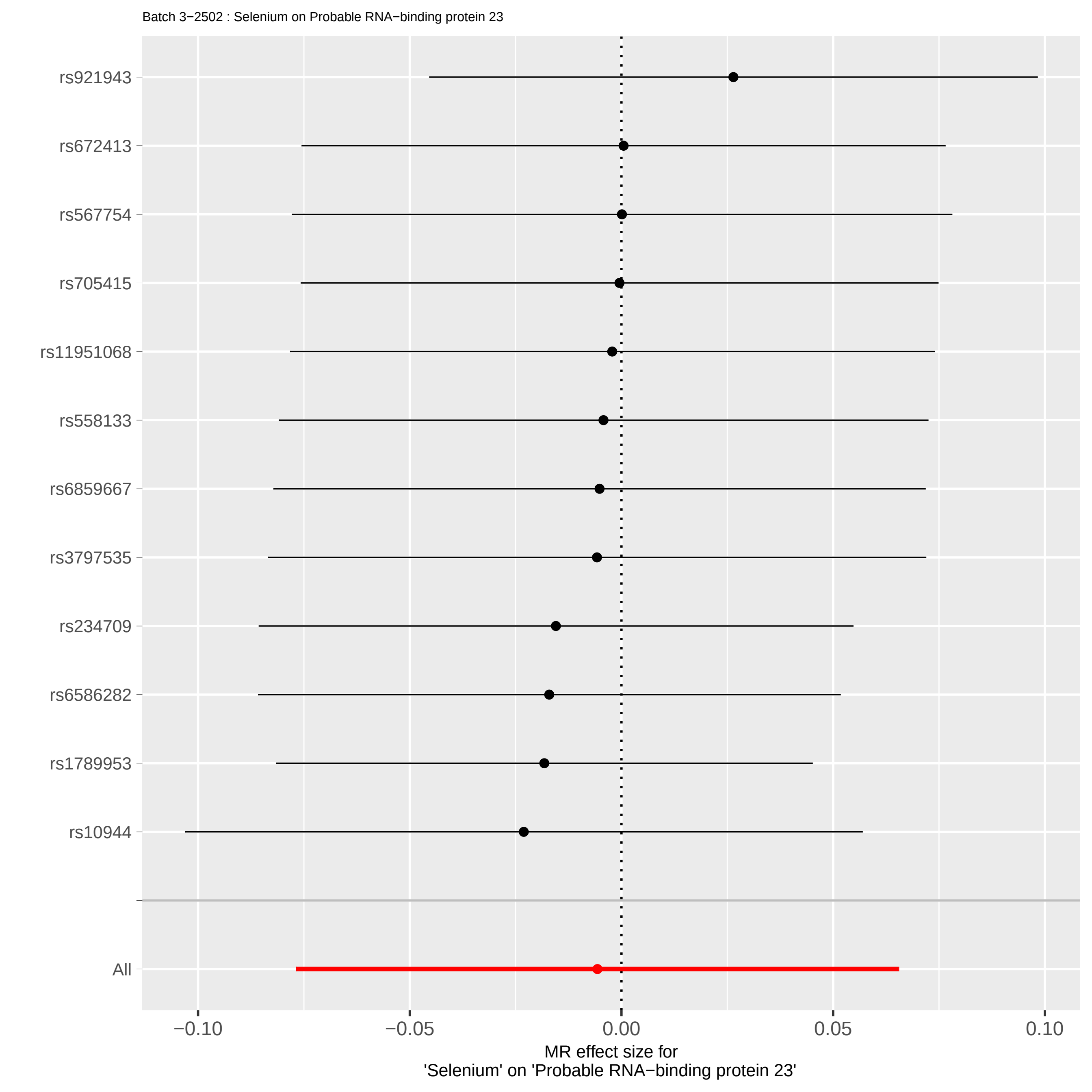

Batch 3−2502 : Selenium on Probable RNA−binding protein 23
rs921943
rs672413
rs567754
rs705415
rs11951068
rs558133
rs6859667
rs3797535
rs234709
rs6586282
rs1789953
rs10944
All
−0.10
−0.05
0.00
0.05
0.10
MR effect size for
'Selenium' on 'Probable RNA−binding protein 23'

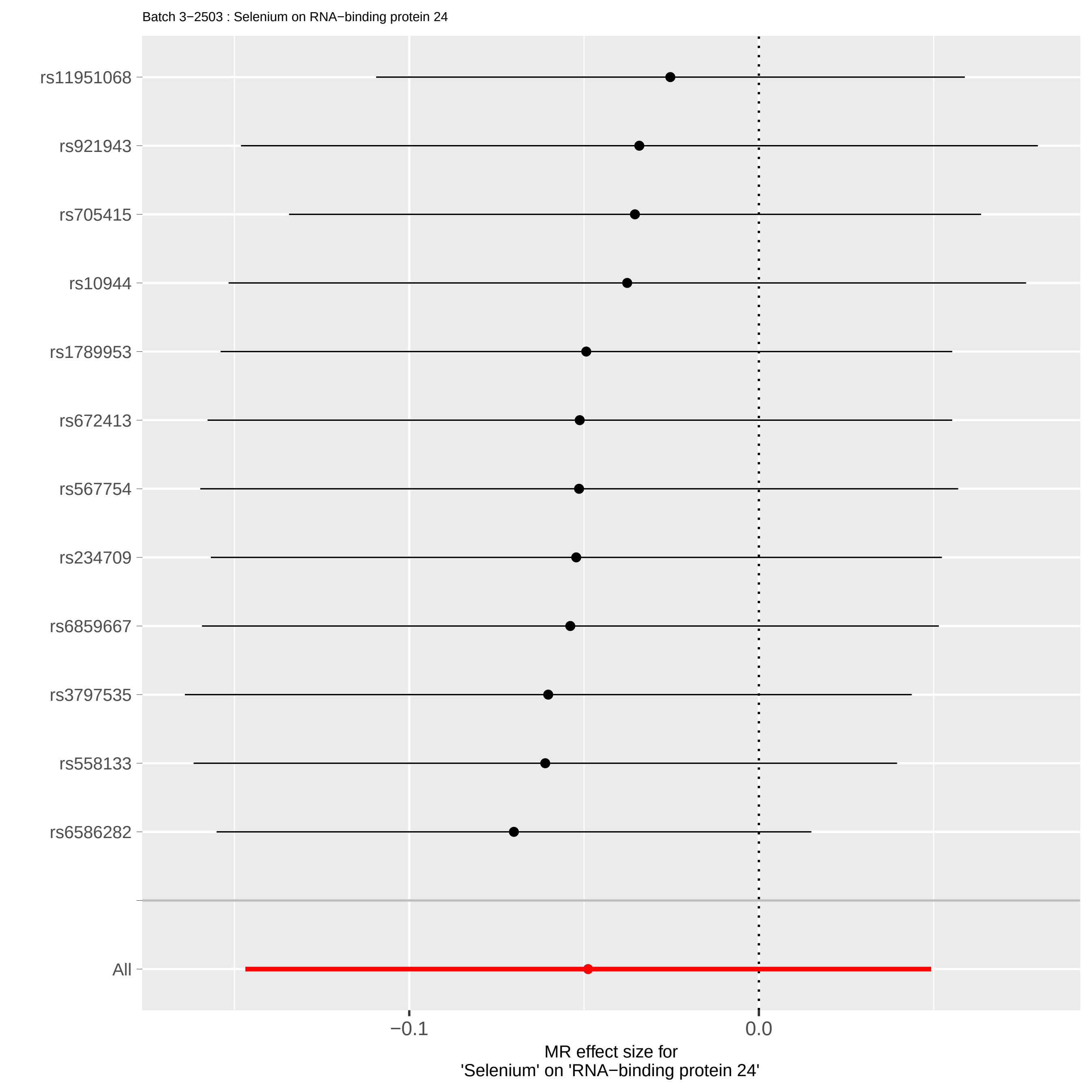
Batch 3−2503 : Selenium on RNA−binding protein 24
rs11951068
rs921943
rs705415
rs10944
rs1789953
rs672413
rs567754
rs234709
rs6859667
rs3797535
rs558133
rs6586282
All
−0.1
0.0
MR effect size for
'Selenium' on 'RNA−binding protein 24'

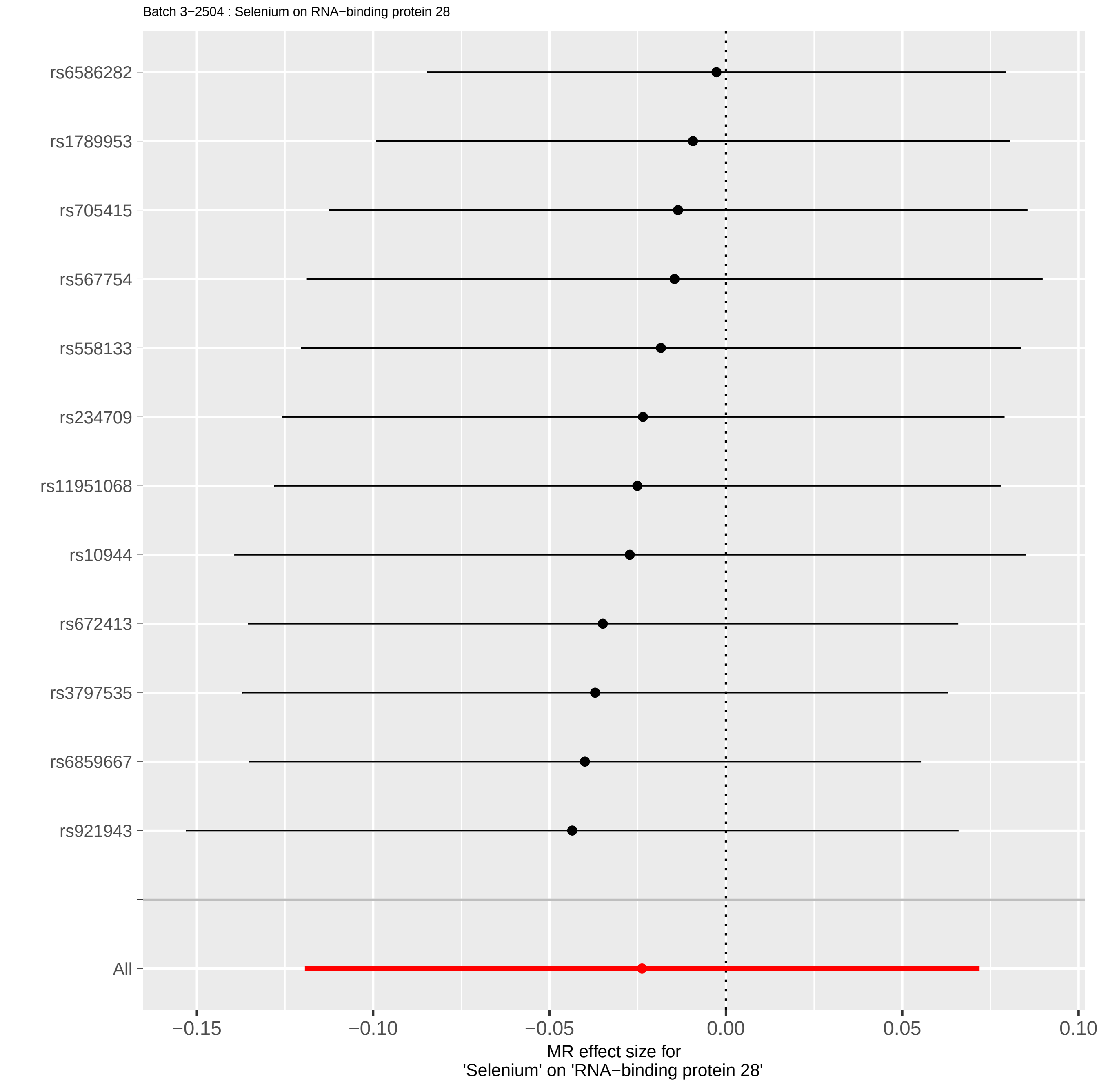

Batch 3−2504 : Selenium on RNA−binding protein 28
rs6586282
rs1789953
rs705415
rs567754
rs558133
rs234709
rs11951068
rs10944
rs672413
rs3797535
rs6859667
rs921943
All
−0.15
−0.10
−0.05
0.00
0.05
0.10
MR effect size for
'Selenium' on 'RNA−binding protein 28'

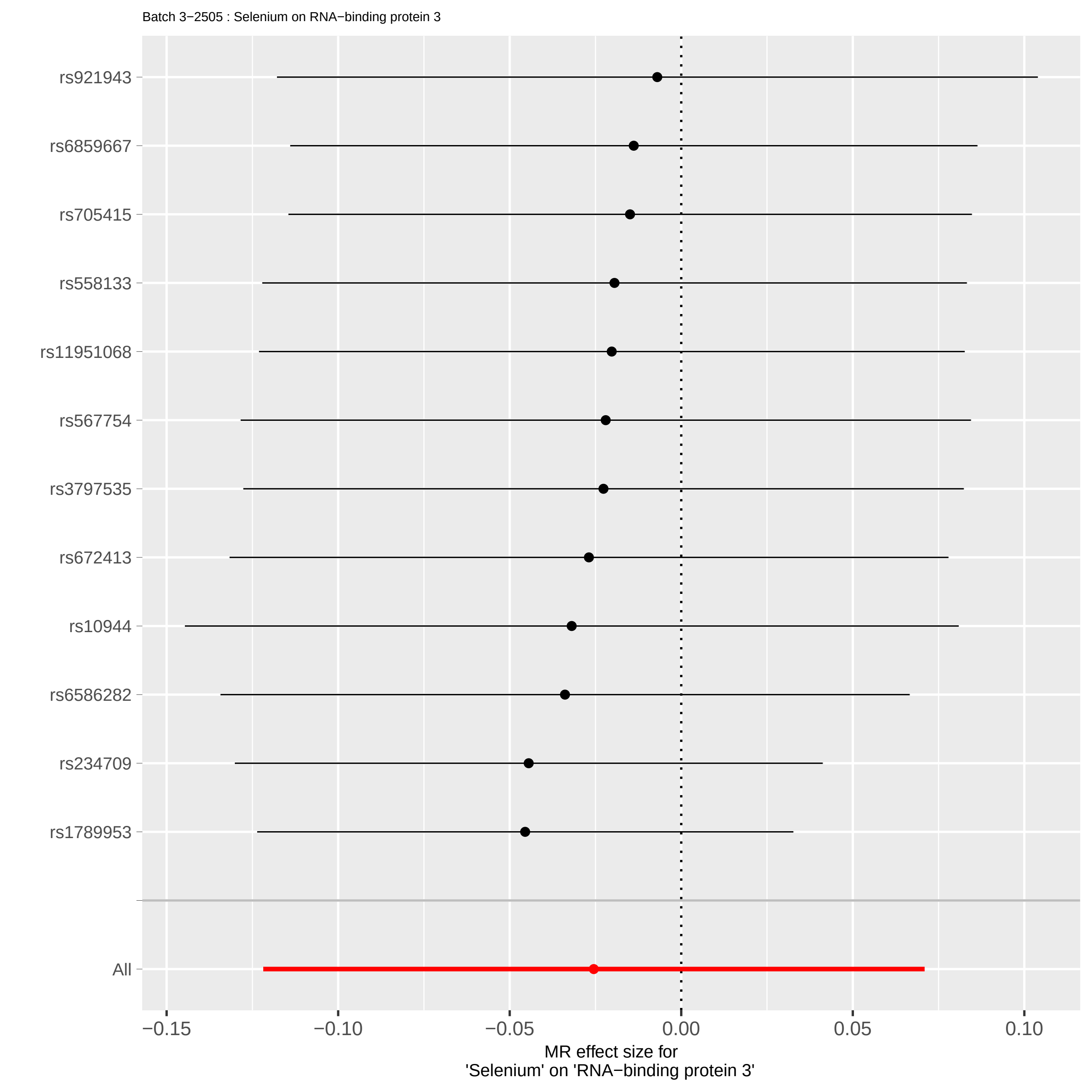

Batch 3−2505 : Selenium on RNA−binding protein 3
rs921943
rs6859667
rs705415
rs558133
rs11951068
rs567754
rs3797535
rs672413
rs10944
rs6586282
rs234709
rs1789953
All
−0.15
−0.10
−0.05
0.00
0.05
0.10
MR effect size for
'Selenium' on 'RNA−binding protein 3'

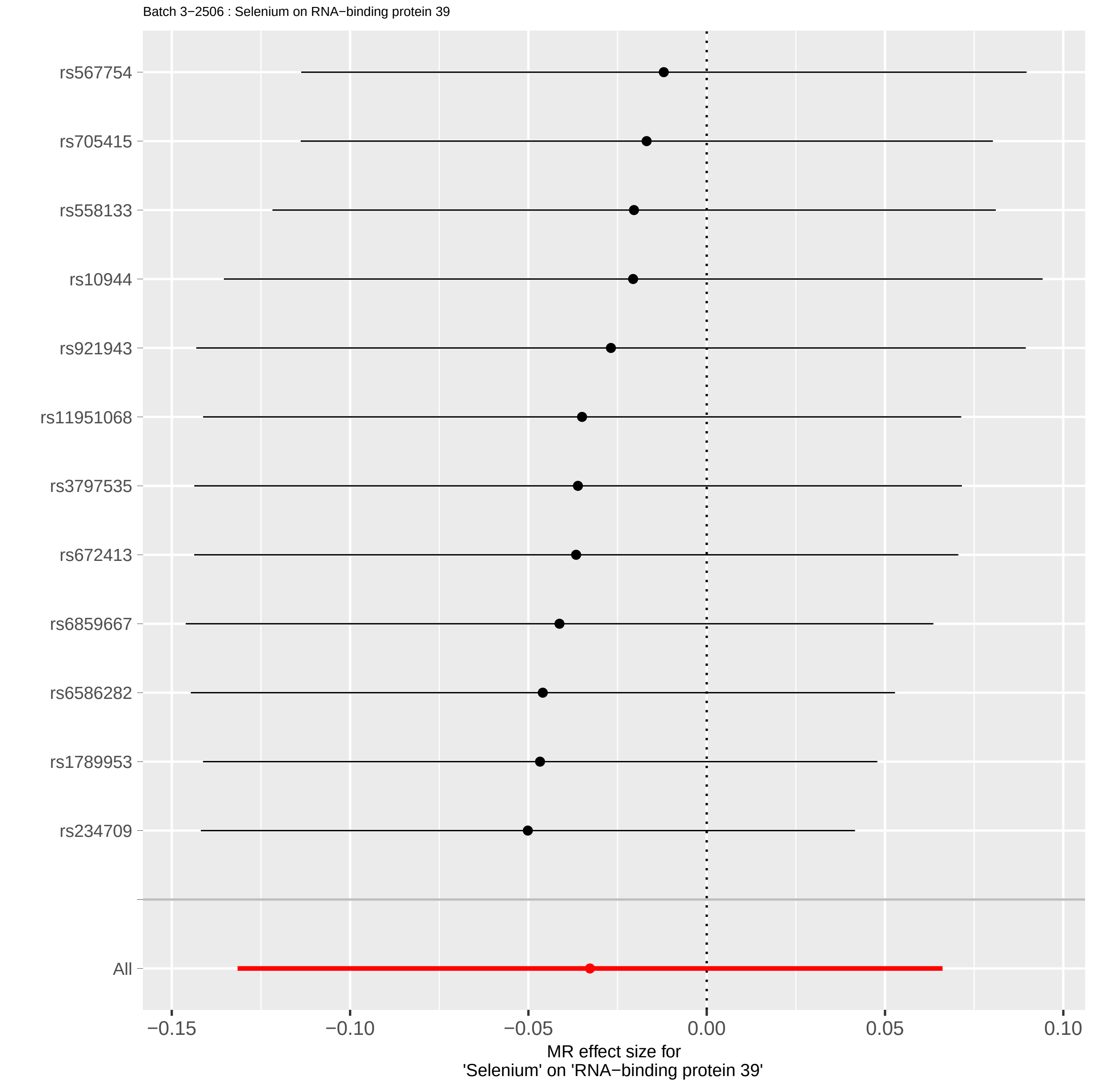

Batch 3−2506 : Selenium on RNA−binding protein 39
rs567754
rs705415
rs558133
rs10944
rs921943
rs11951068
rs3797535
rs672413
rs6859667
rs6586282
rs1789953
rs234709
All
−0.15
−0.10
−0.05
0.00
0.05
0.10
MR effect size for
'Selenium' on 'RNA−binding protein 39'

Batch 3−2507 : Selenium on Retinol−binding protein 4

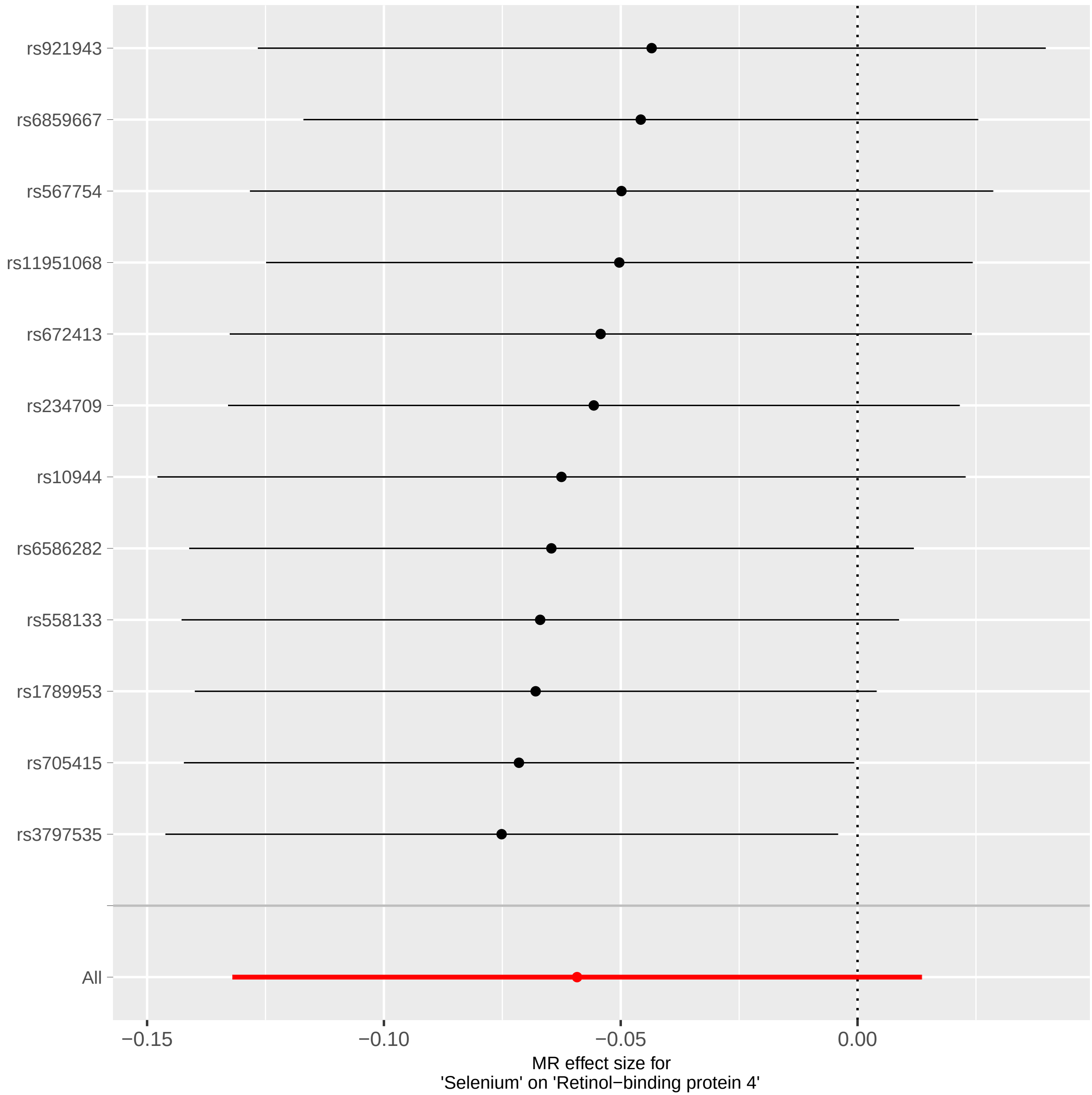

Batch 3−2508 : Selenium on Retinoid−binding protein 7

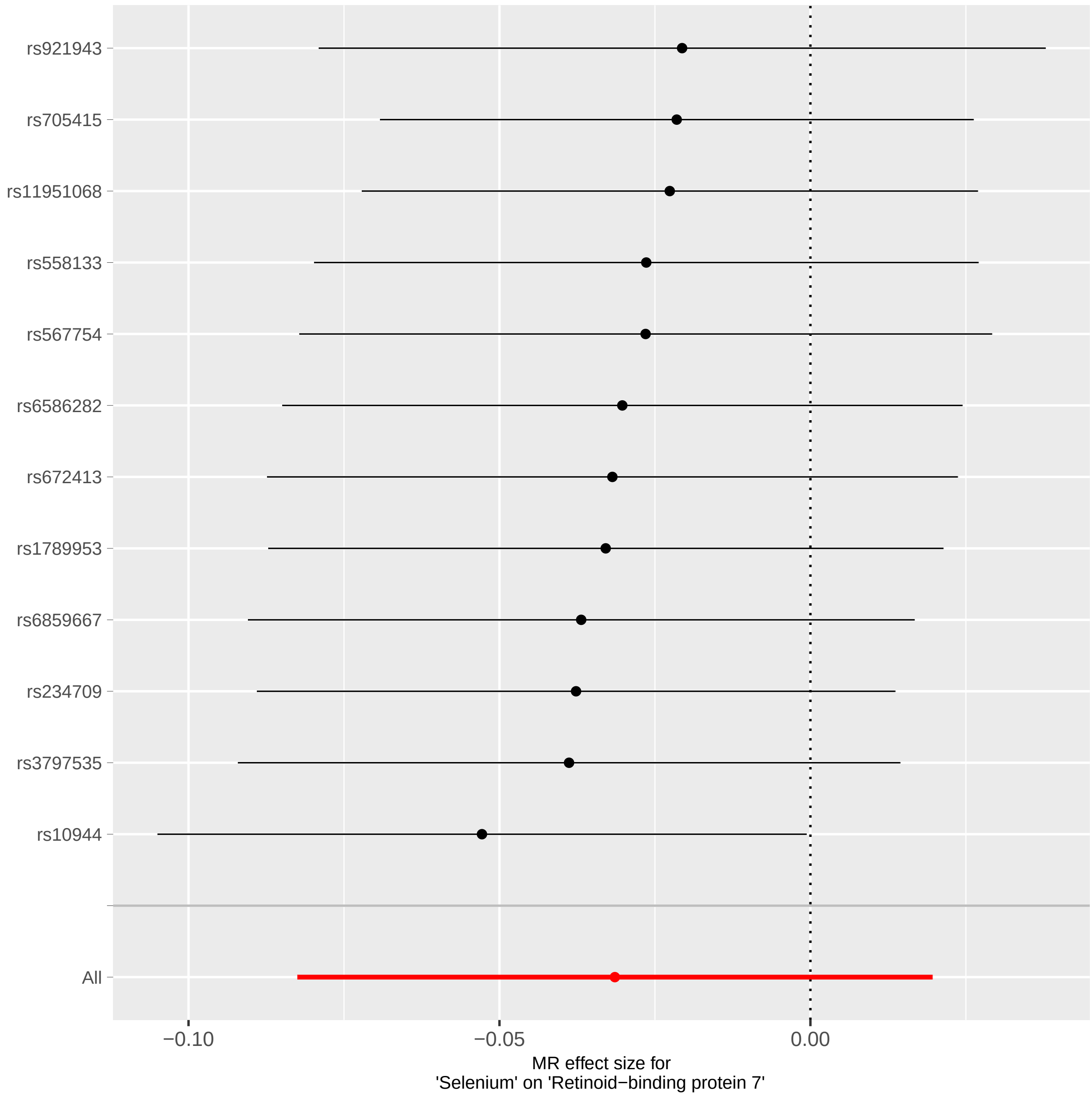

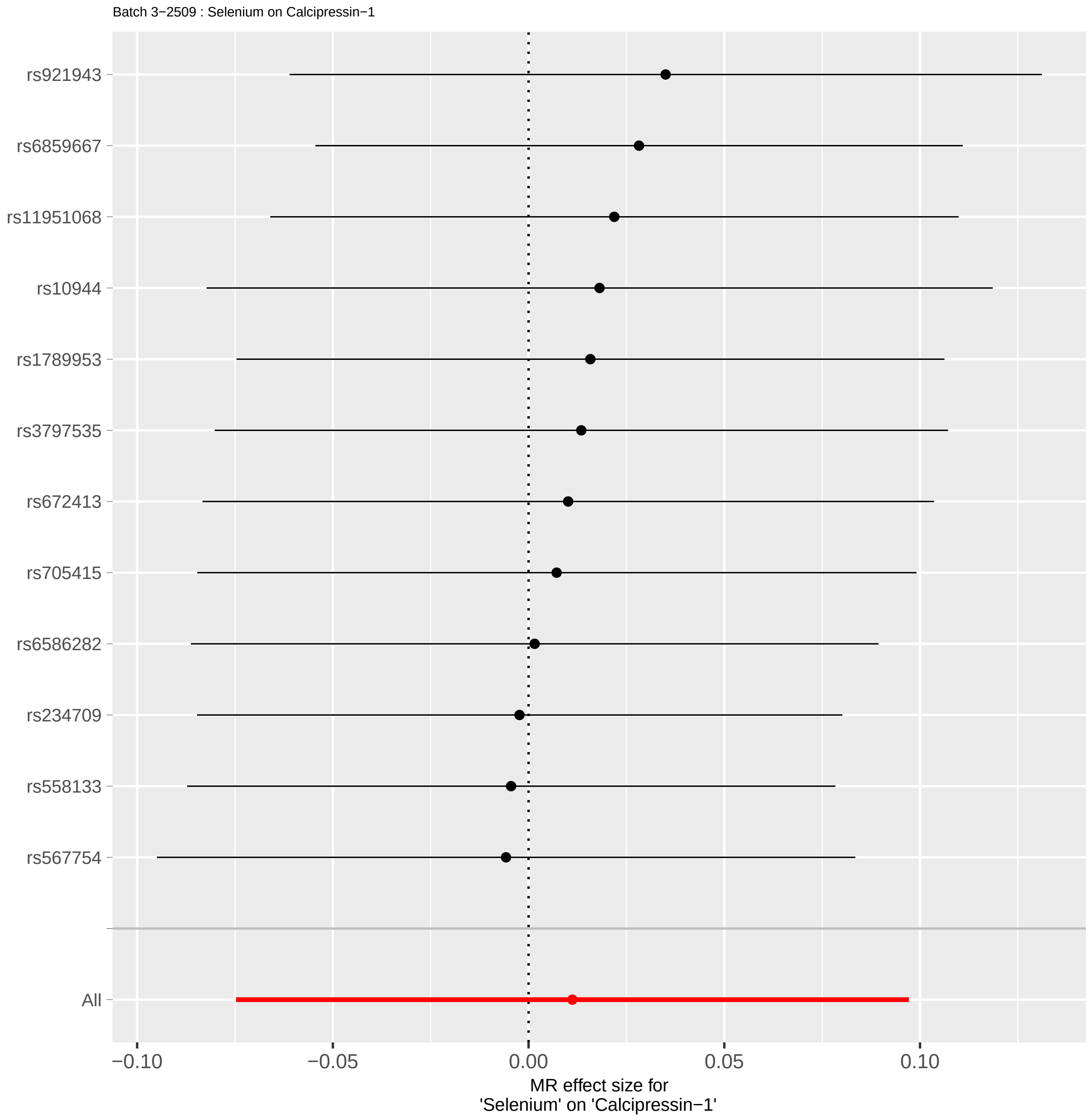

Batch 3−2509 : Selenium on Calcipressin−1
rs921943
rs6859667
rs11951068
rs10944
rs1789953
rs3797535
rs672413
rs705415
rs6586282
rs234709
rs558133
rs567754
All
−0.10
−0.05
0.00
0.05
0.10
MR effect size for
'Selenium' on 'Calcipressin−1'

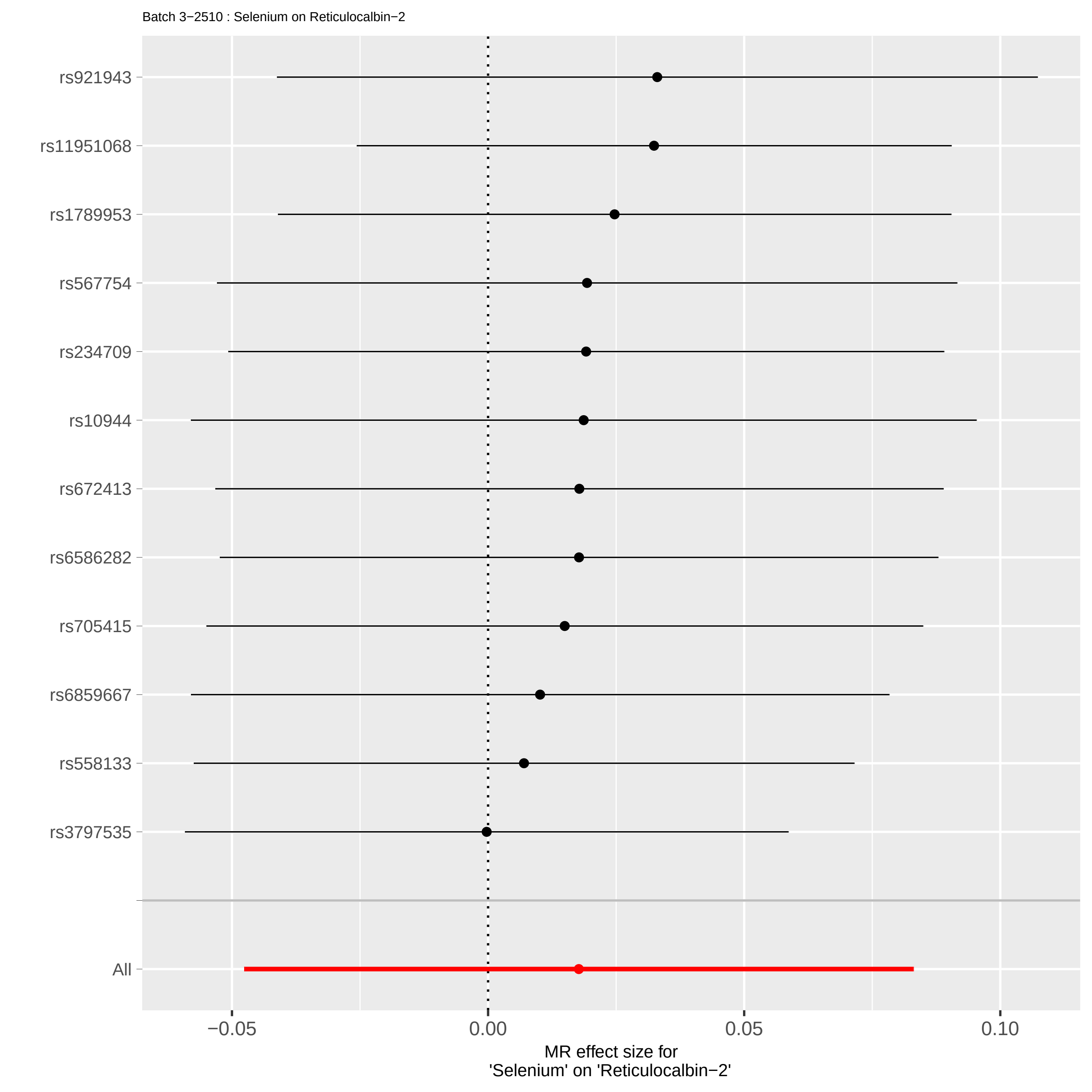
Batch 3−2510 : Selenium on Reticulocalbin−2
rs921943
rs11951068
rs1789953
rs567754
rs234709
rs10944
rs672413
rs6586282
rs705415
rs6859667
rs558133
rs3797535
All
−0.05
0.00
0.05
0.10
MR effect size for
'Selenium' on 'Reticulocalbin−2'

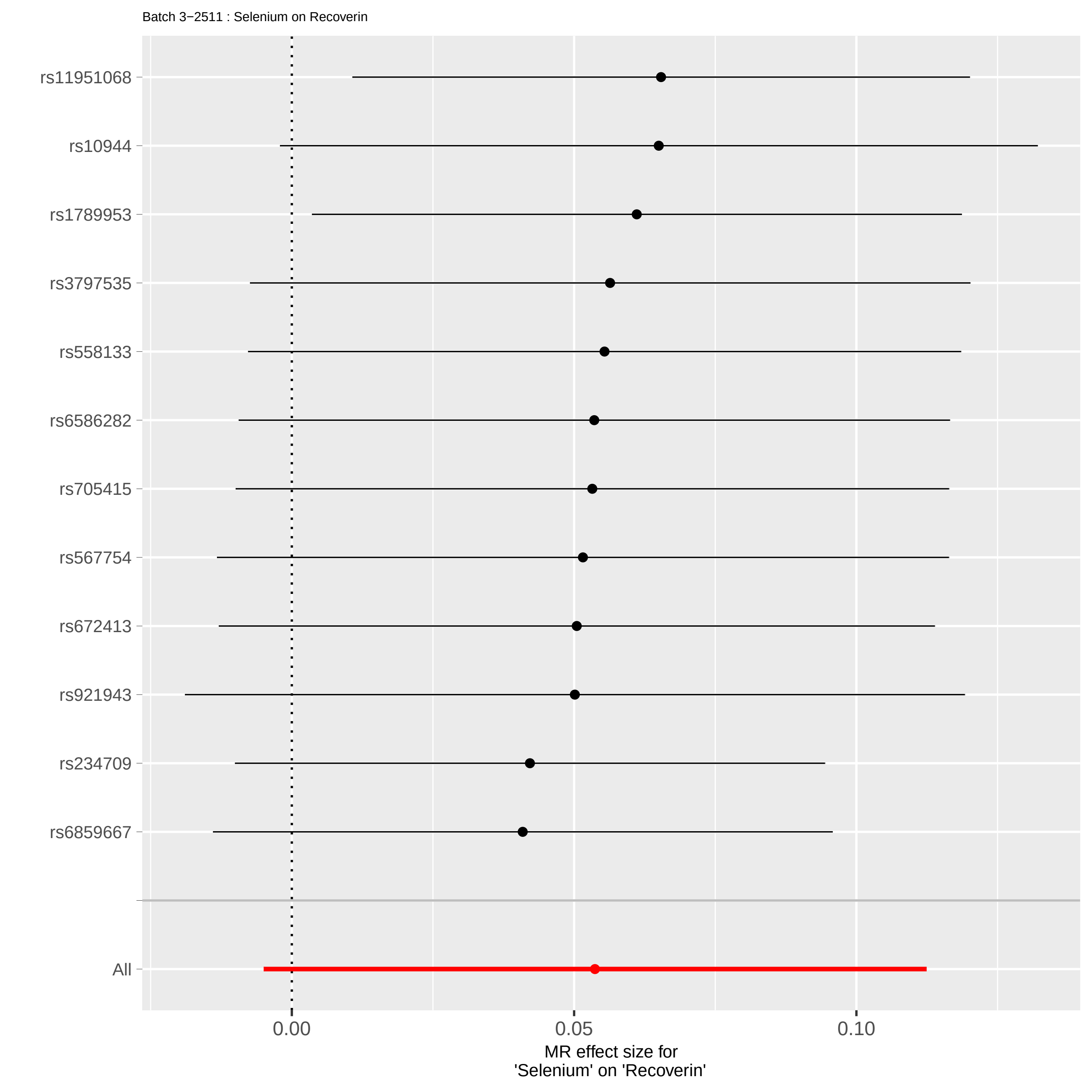
Batch 3−2511 : Selenium on Recoverin
rs11951068
rs10944
rs1789953
rs3797535
rs558133
rs6586282
rs705415
rs567754
rs672413
rs921943
rs234709
rs6859667
All
0.00
0.05
0.10
MR effect size for
'Selenium' on 'Recoverin'

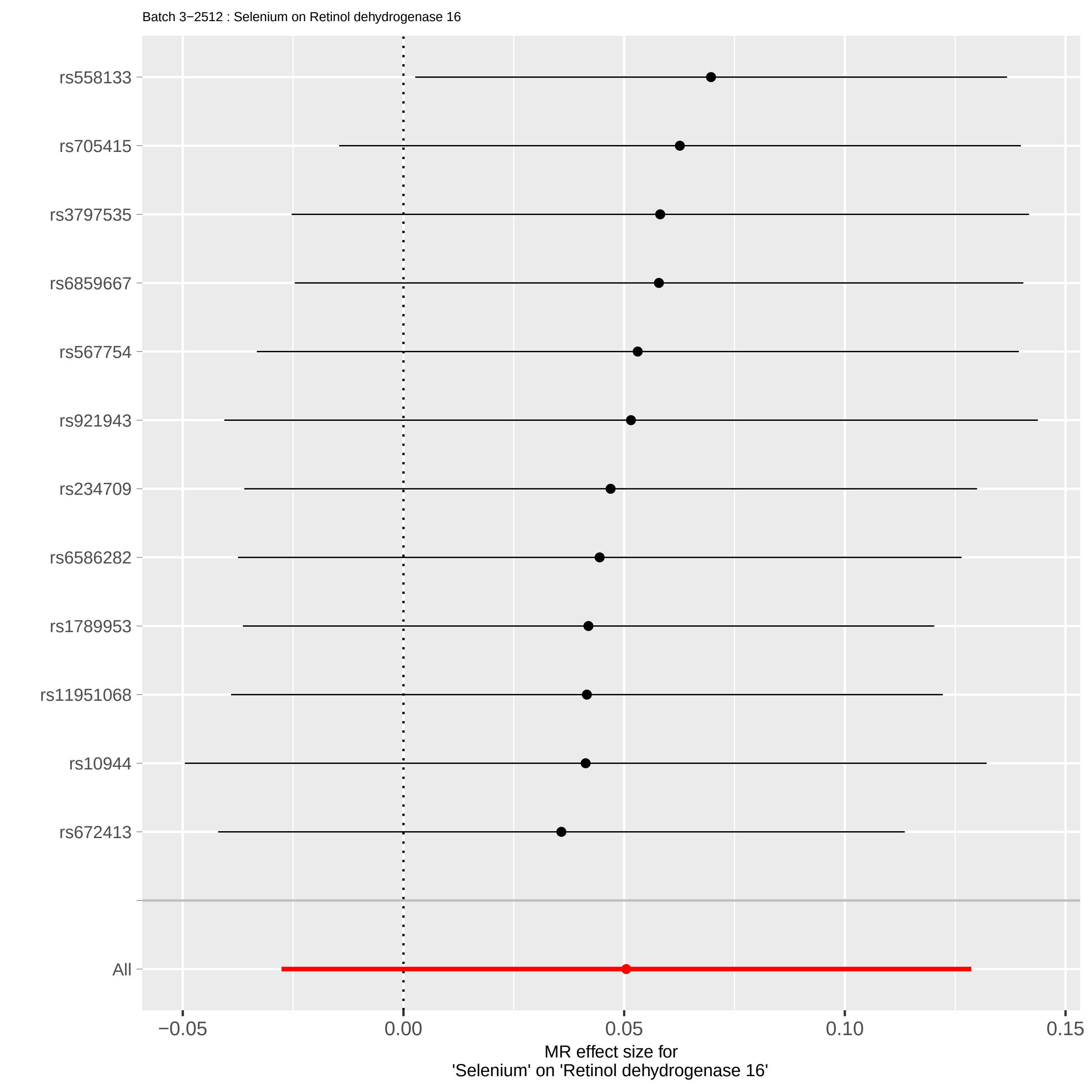

Batch 3−2512 : Selenium on Retinol dehydrogenase 16
rs558133
rs705415
rs3797535
rs6859667
rs567754
rs921943
rs234709
rs6586282
rs1789953
rs11951068
rs10944
rs672413
All
−0.05
0.00
0.05
0.10
0.15
MR effect size for
'Selenium' on 'Retinol dehydrogenase 16'

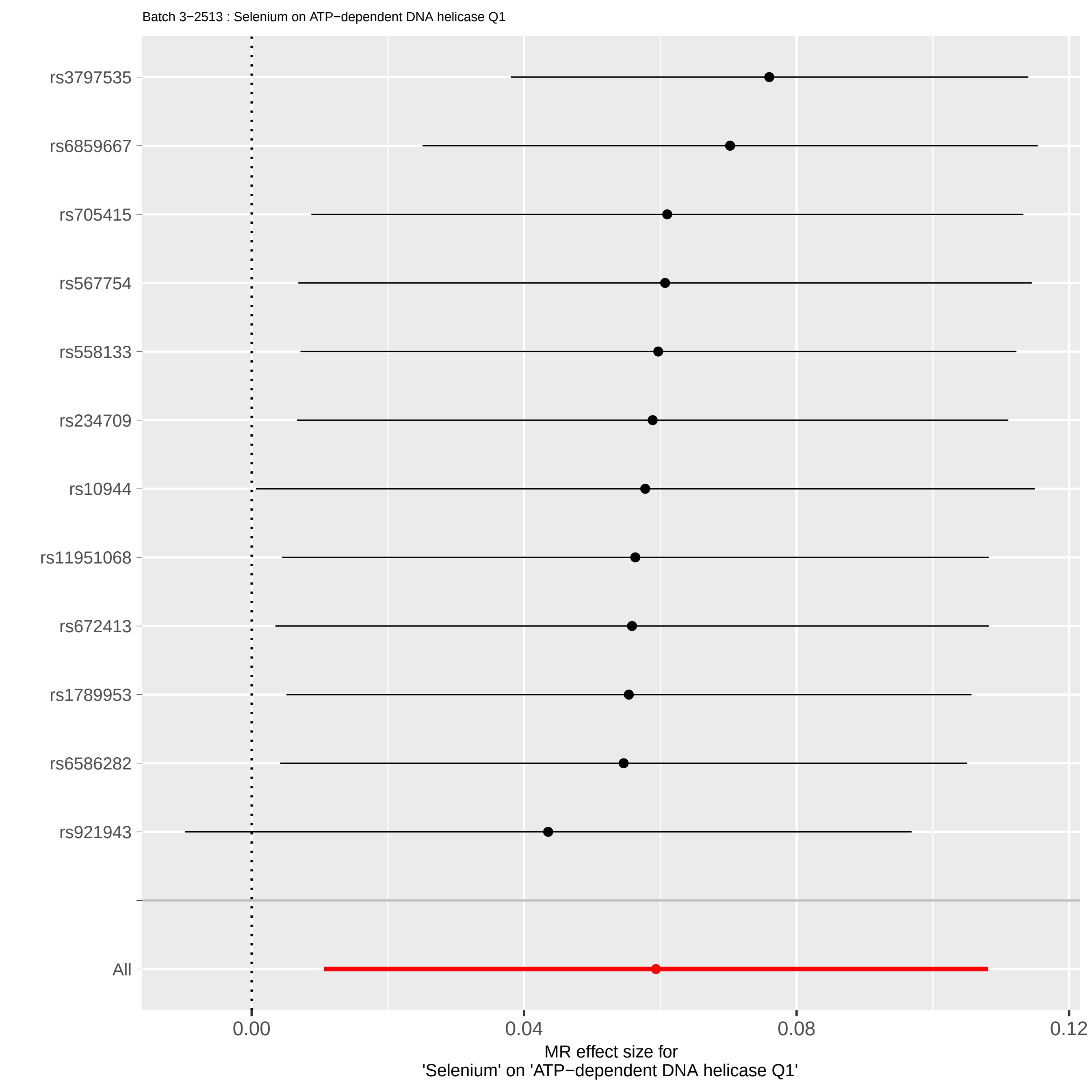
Batch 3−2513 : Selenium on ATP−dependent DNA helicase Q1
rs3797535
rs6859667
rs705415
rs567754
rs558133
rs234709
rs10944
rs11951068
rs672413
rs1789953
rs6586282
rs921943
All
0.00
0.04
0.08
0.12
MR effect size for
'Selenium' on 'ATP−dependent DNA helicase Q1'

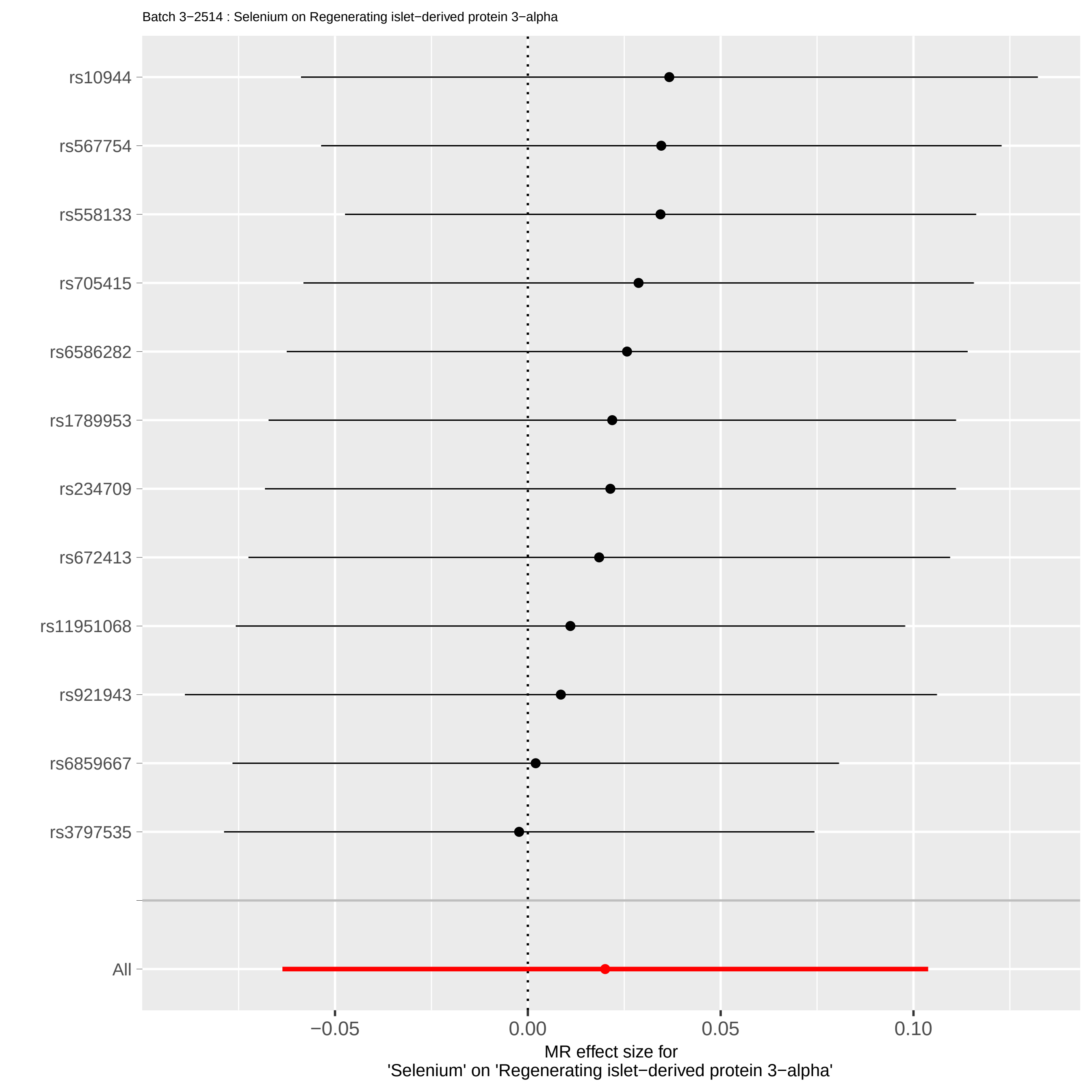

Batch 3−2514 : Selenium on Regenerating islet−derived protein 3−alpha
rs10944
rs567754
rs558133
rs705415
rs6586282
rs1789953
rs234709
rs672413
rs11951068
rs921943
rs6859667
rs3797535
All
−0.05
0.00
0.05
0.10
MR effect size for
'Selenium' on 'Regenerating islet−derived protein 3−alpha'

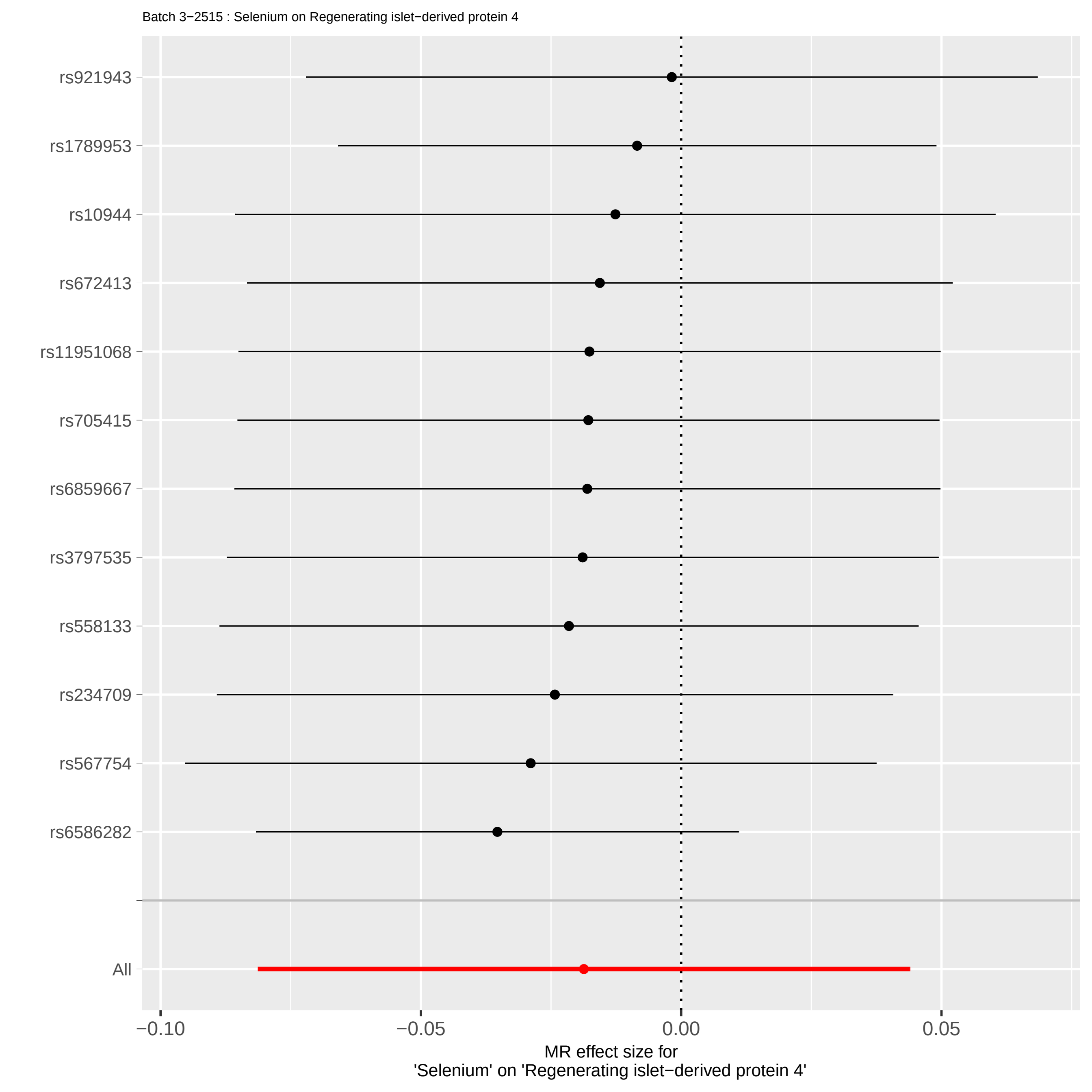

Batch 3−2515 : Selenium on Regenerating islet−derived protein 4
rs921943
rs1789953
rs10944
rs672413
rs11951068
rs705415
rs6859667
rs3797535
rs558133
rs234709
rs567754
rs6586282
All
−0.10
−0.05
0.00
0.05
MR effect size for
'Selenium' on 'Regenerating islet−derived protein 4'

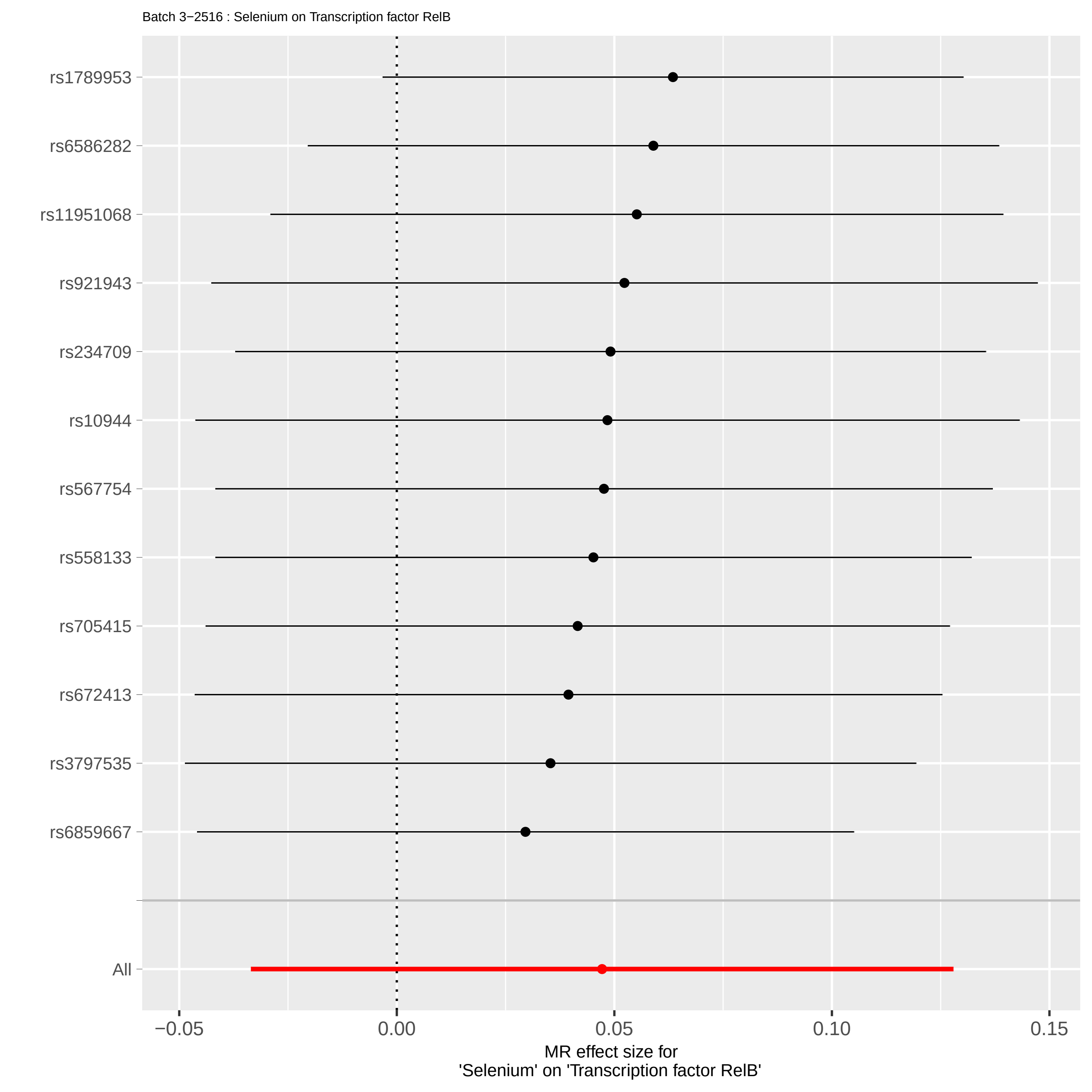
Batch 3−2516 : Selenium on Transcription factor RelB
rs1789953
rs6586282
rs11951068
rs921943
rs234709
rs10944
rs567754
rs558133
rs705415
rs672413
rs3797535
rs6859667
All
−0.05
0.00
0.05
0.10
0.15
MR effect size for
'Selenium' on 'Transcription factor RelB'

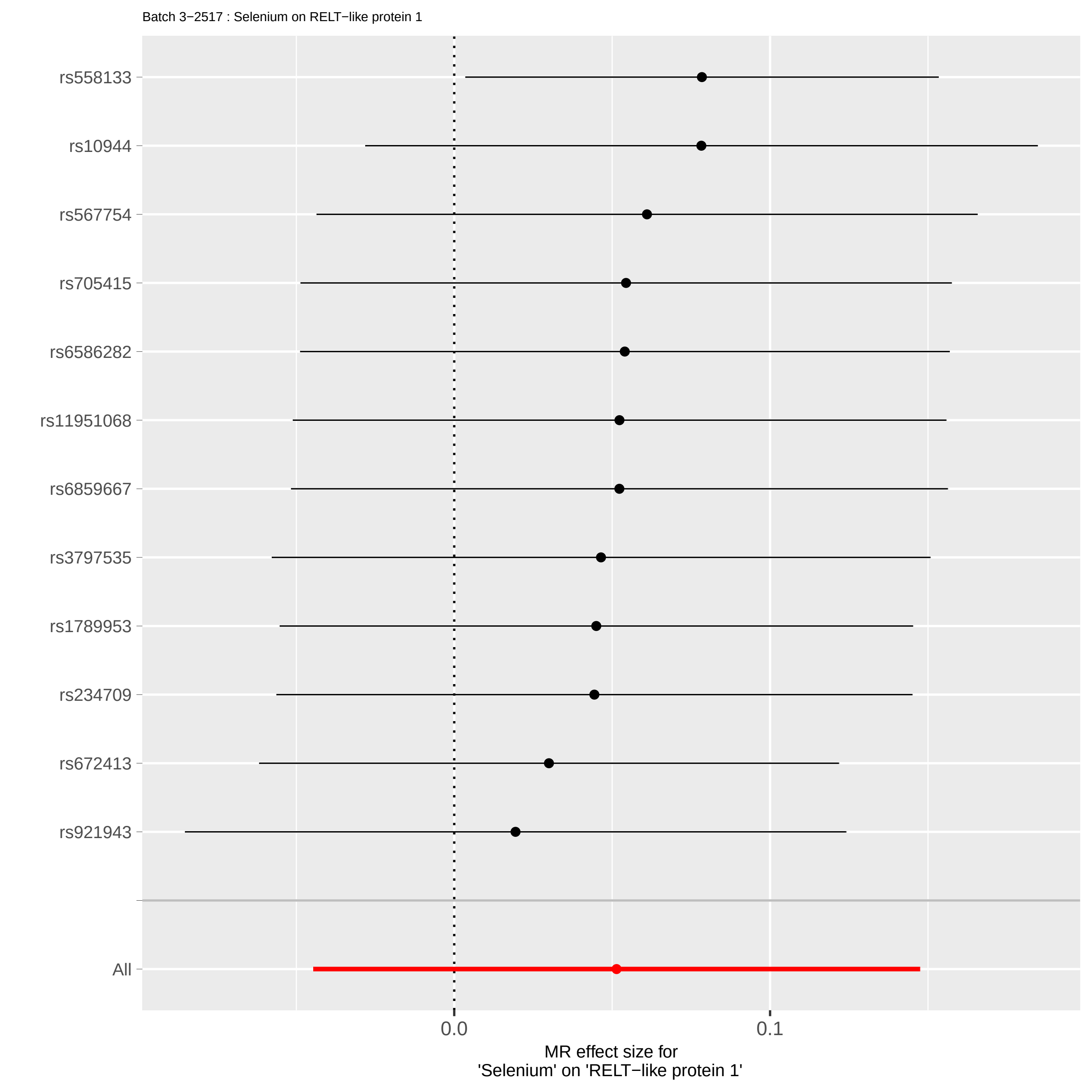

Batch 3−2517 : Selenium on RELT−like protein 1
rs558133
rs10944
rs567754
rs705415
rs6586282
rs11951068
rs6859667
rs3797535
rs1789953
rs234709
rs672413
rs921943
All
0.0
0.1
MR effect size for
'Selenium' on 'RELT−like protein 1'

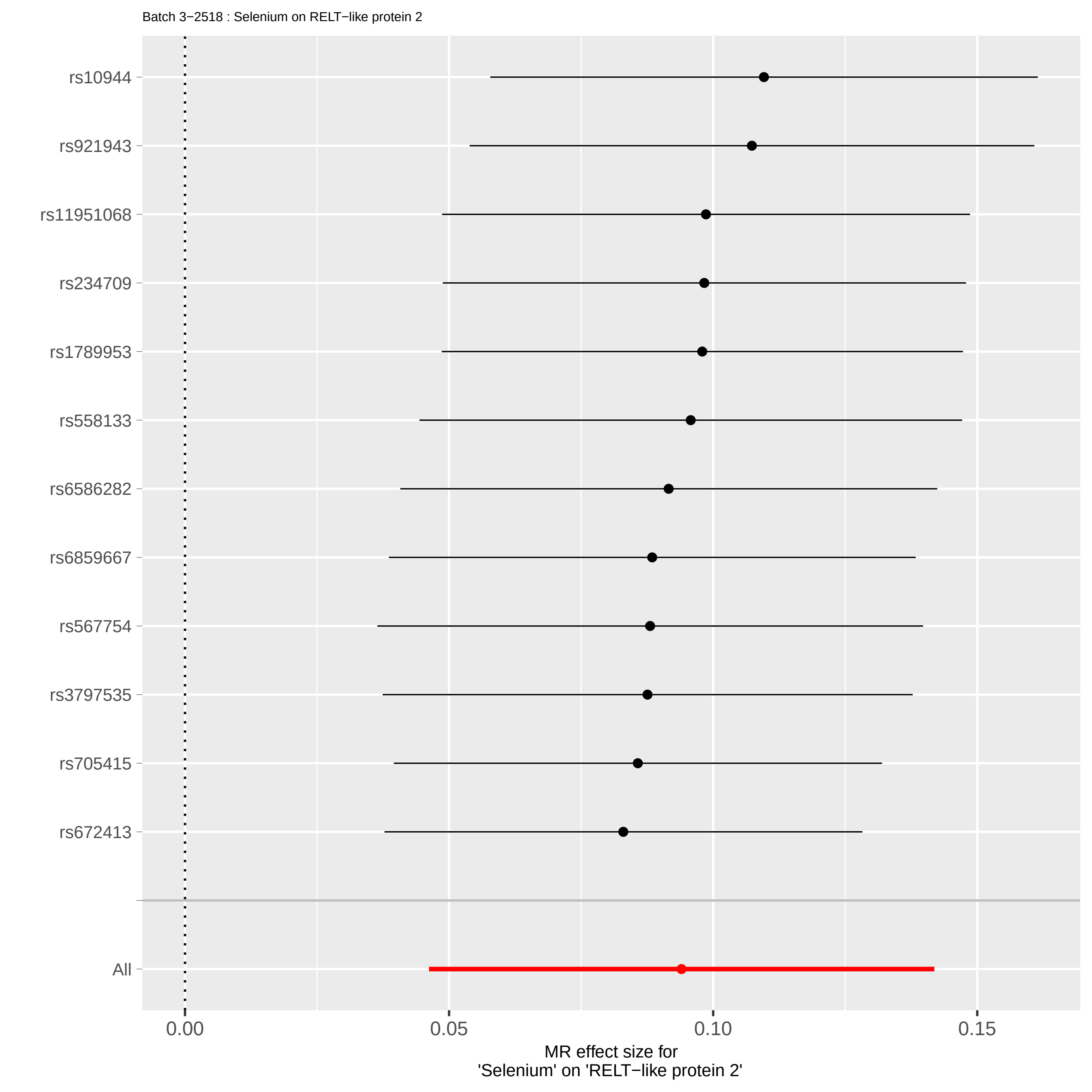

Batch 3−2518 : Selenium on RELT−like protein 2
rs10944
rs921943
rs11951068
rs234709
rs1789953
rs558133
rs6586282
rs6859667
rs567754
rs3797535
rs705415
rs672413
All
0.00
0.05
0.10
0.15
MR effect size for
'Selenium' on 'RELT−like protein 2'

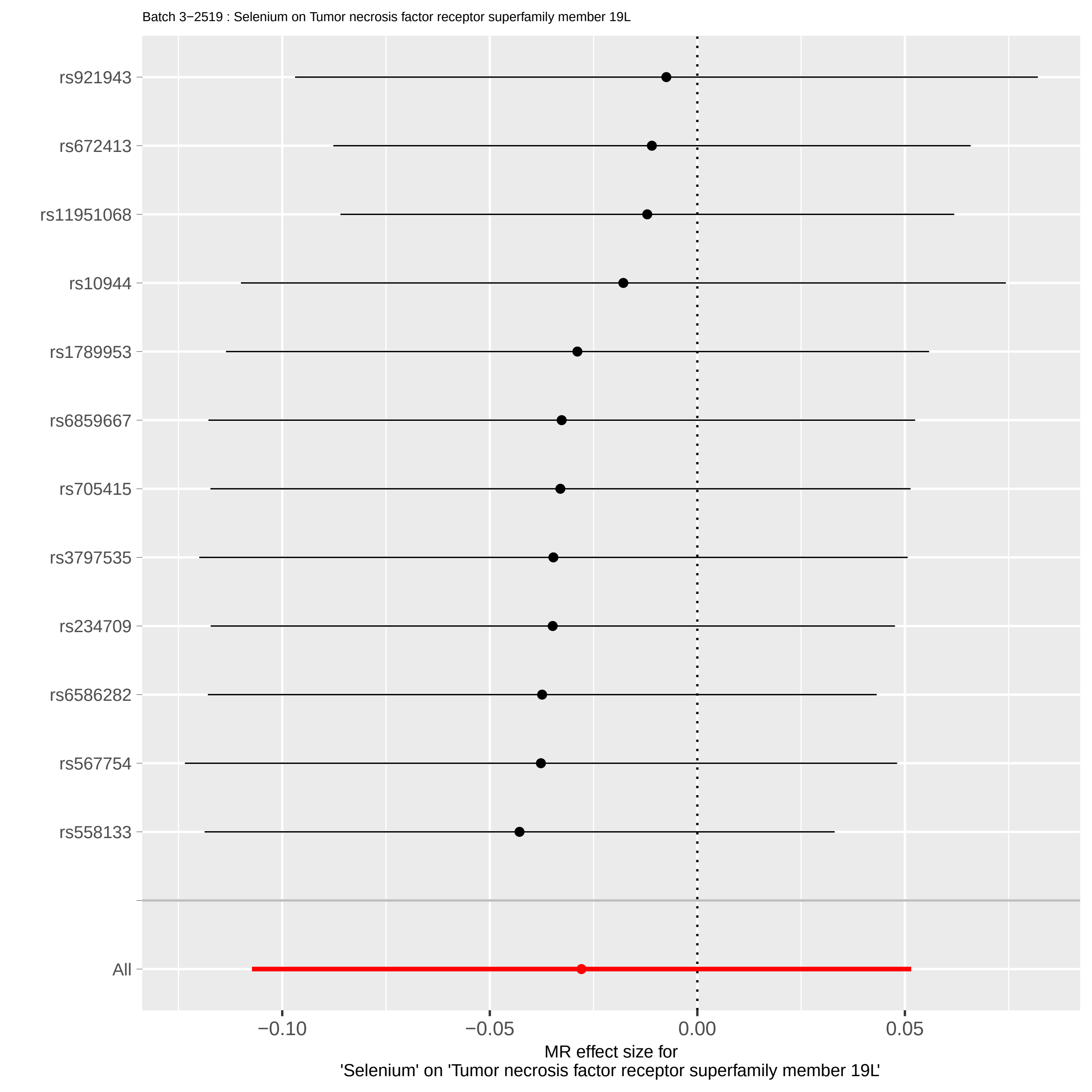

Batch 3−2519 : Selenium on Tumor necrosis factor receptor superfamily member 19L
rs921943
rs672413
rs11951068
rs10944
rs1789953
rs6859667
rs705415
rs3797535
rs234709
rs6586282
rs567754
rs558133
All
−0.10
−0.05
0.00
0.05
MR effect size for
'Selenium' on 'Tumor necrosis factor receptor superfamily member 19L'

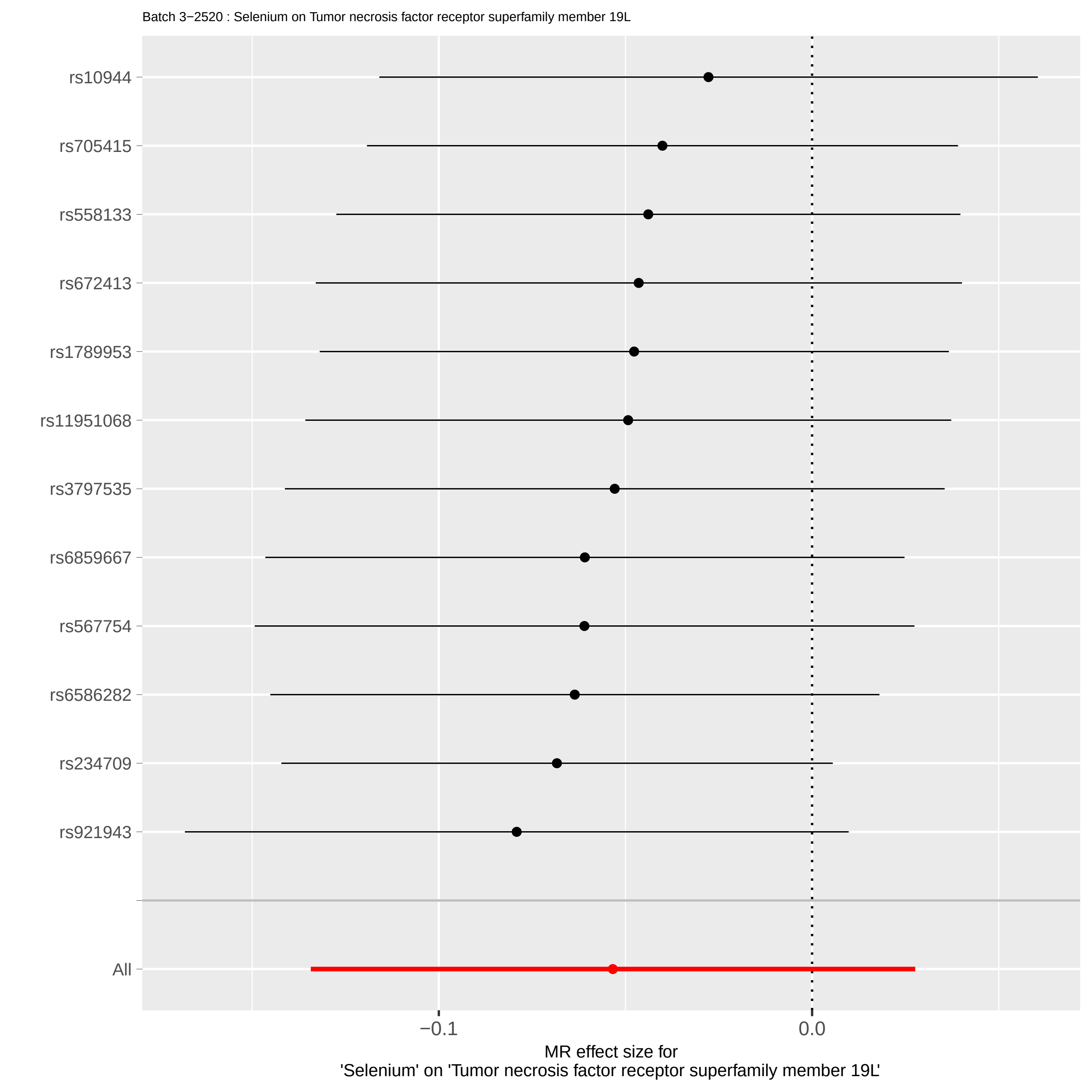

Batch 3−2520 : Selenium on Tumor necrosis factor receptor superfamily member 19L
rs10944
rs705415
rs558133
rs672413
rs1789953
rs11951068
rs3797535
rs6859667
rs567754
rs6586282
rs234709
rs921943
All
−0.1
0.0
MR effect size for
'Selenium' on 'Tumor necrosis factor receptor superfamily member 19L'

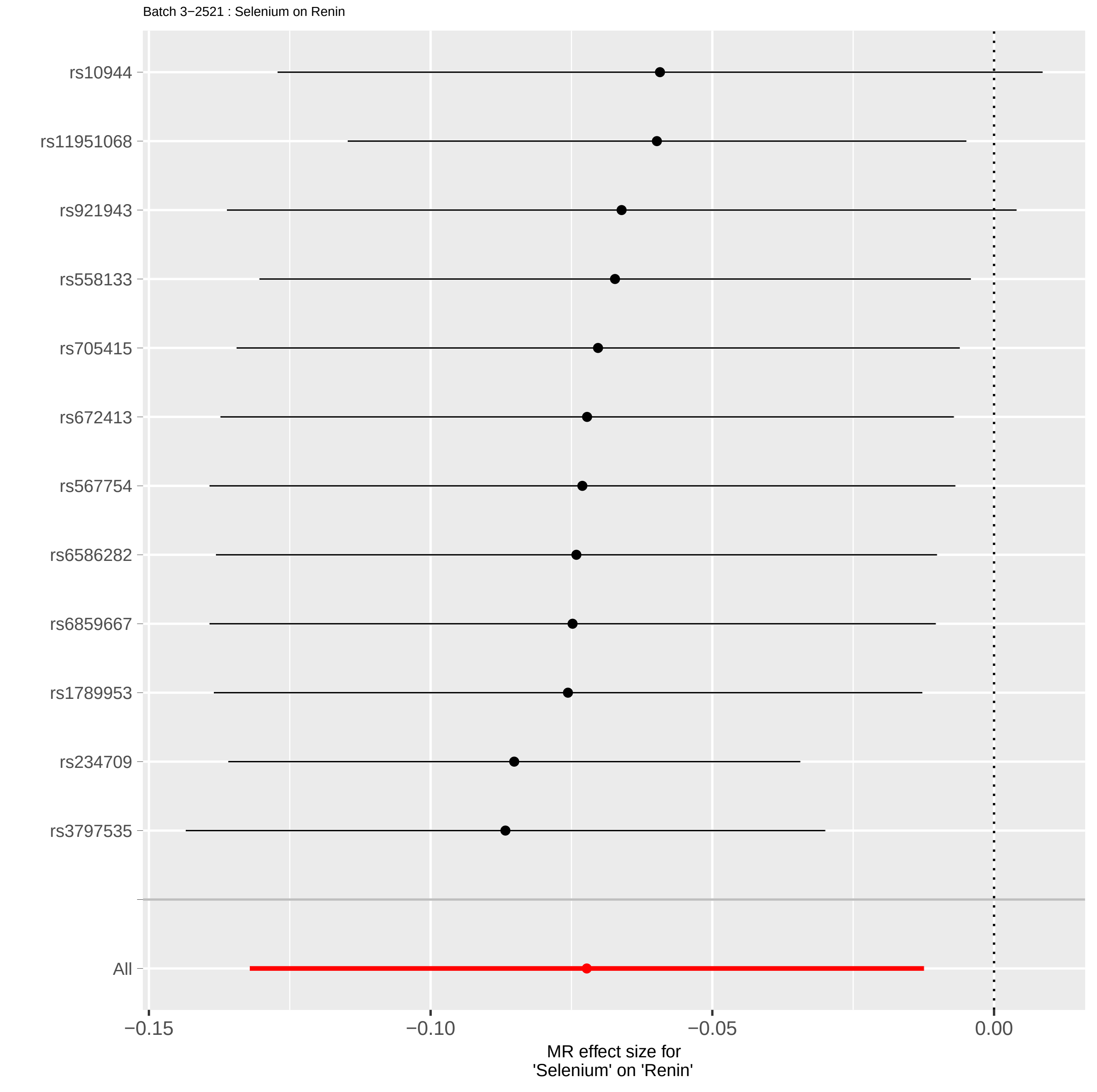

Batch 3−2521 : Selenium on Renin
rs10944
rs11951068
rs921943
rs558133
rs705415
rs672413
rs567754
rs6586282
rs6859667
rs1789953
rs234709
rs3797535
All
−0.15
−0.10
−0.05
0.00
MR effect size for
'Selenium' on 'Renin'

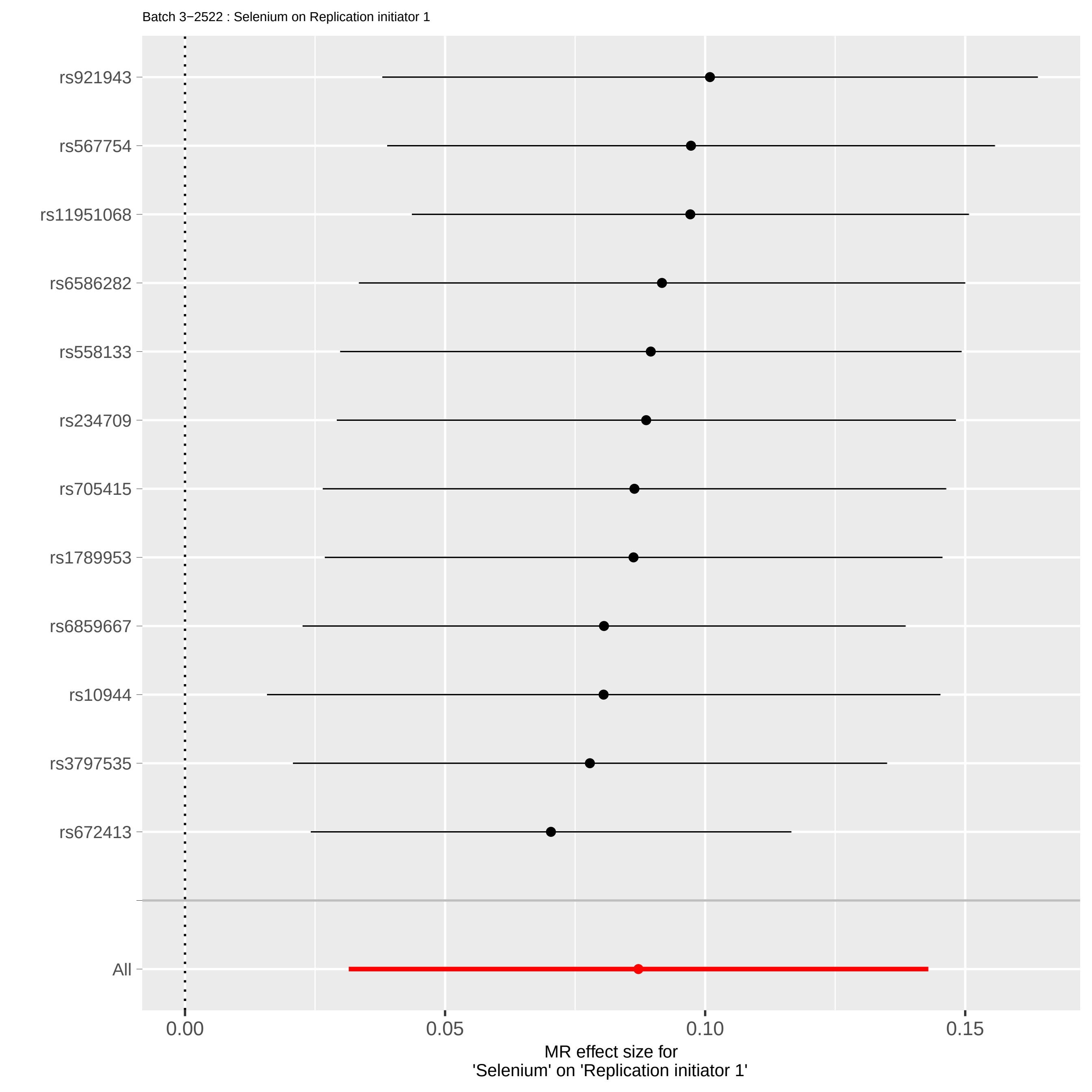

Batch 3−2522 : Selenium on Replication initiator 1
rs921943
rs567754
rs11951068
rs6586282
rs558133
rs234709
rs705415
rs1789953
rs6859667
rs10944
rs3797535
rs672413
All
0.00
0.05
0.10
0.15
MR effect size for
'Selenium' on 'Replication initiator 1'

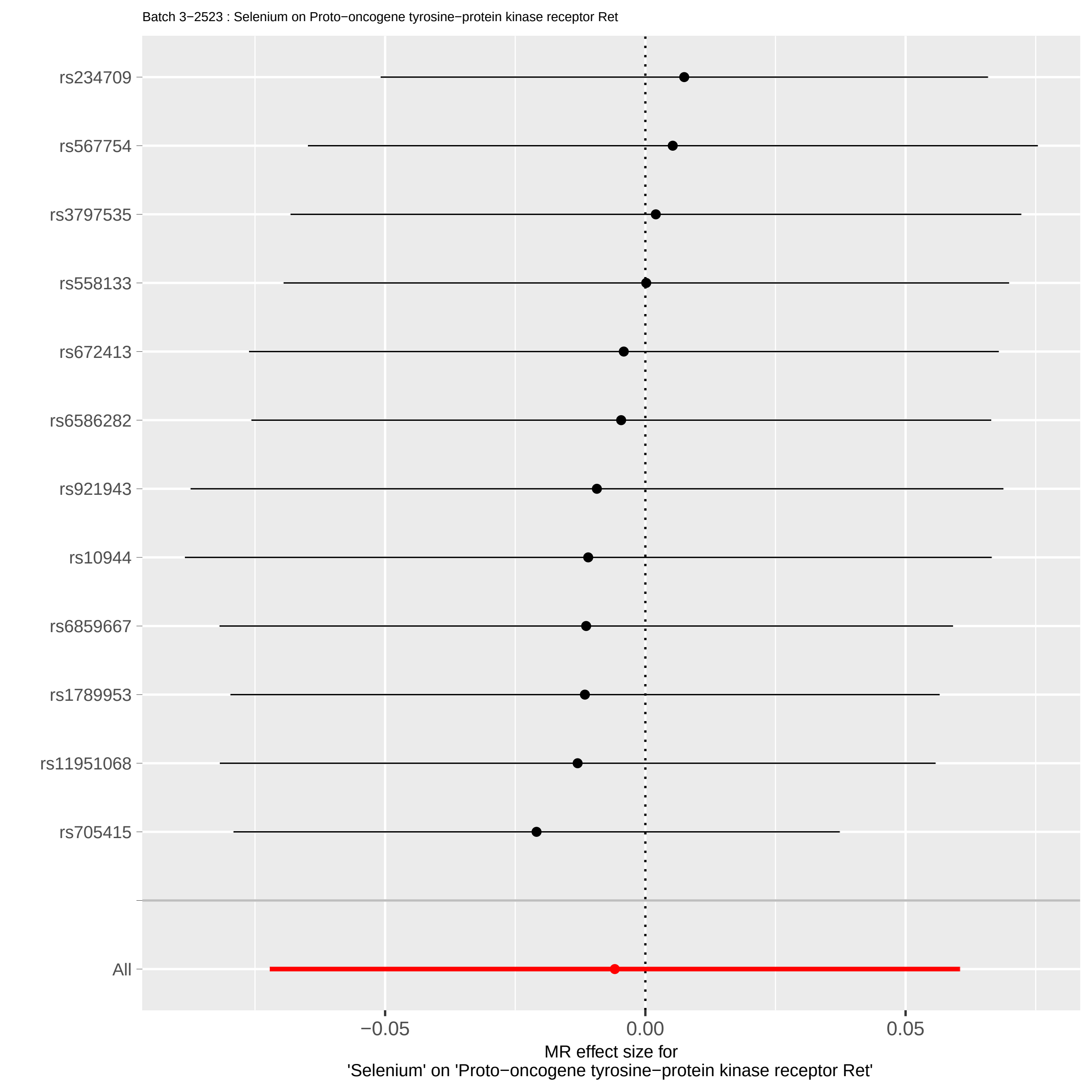

Batch 3−2523 : Selenium on Proto−oncogene tyrosine−protein kinase receptor Ret
rs234709
rs567754
rs3797535
rs558133
rs672413
rs6586282
rs921943
rs10944
rs6859667
rs1789953
rs11951068
rs705415
All
−0.05
0.00
0.05
MR effect size for
'Selenium' on 'Proto−oncogene tyrosine−protein kinase receptor Ret'

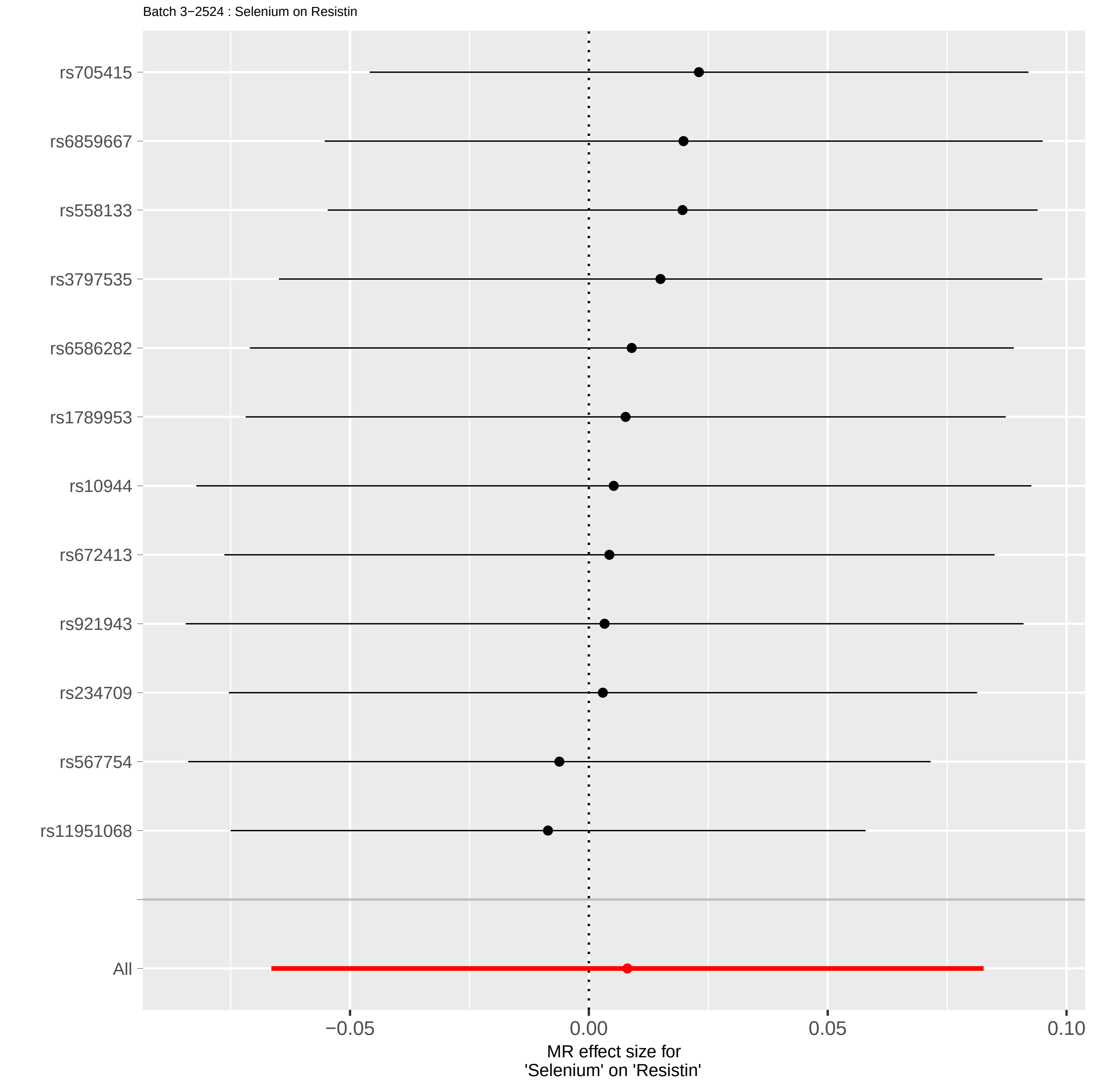

Batch 3−2524 : Selenium on Resistin
rs705415
rs6859667
rs558133
rs3797535
rs6586282
rs1789953
rs10944
rs672413
rs921943
rs234709
rs567754
rs11951068
All
−0.05
0.00
0.05
0.10
MR effect size for
'Selenium' on 'Resistin'

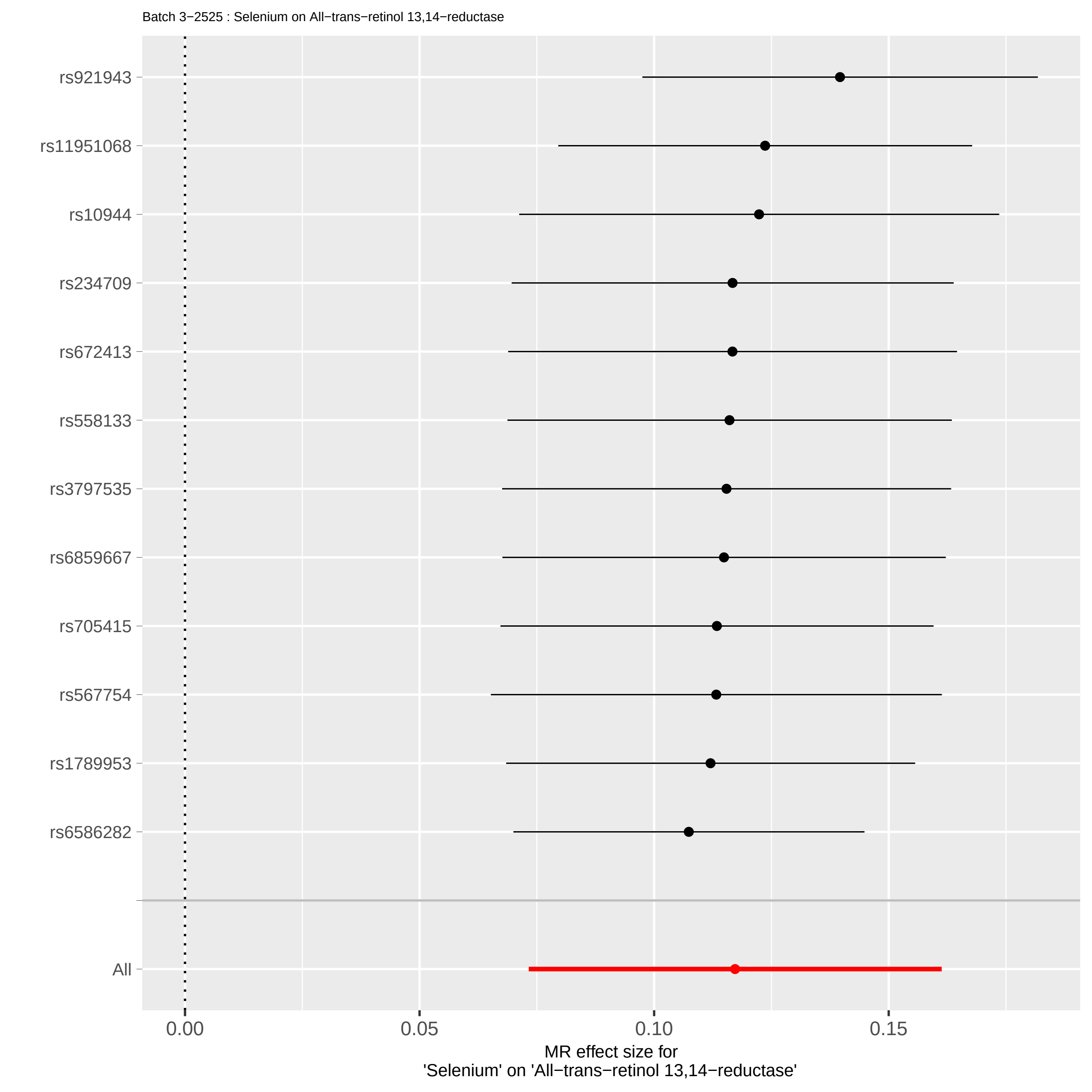

Batch 3−2525 : Selenium on All−trans−retinol 13,14−reductase
rs921943
rs11951068
rs10944
rs234709
rs672413
rs558133
rs3797535
rs6859667
rs705415
rs567754
rs1789953
rs6586282
All
0.00
0.05
0.10
0.15
MR effect size for
'Selenium' on 'All−trans−retinol 13,14−reductase'

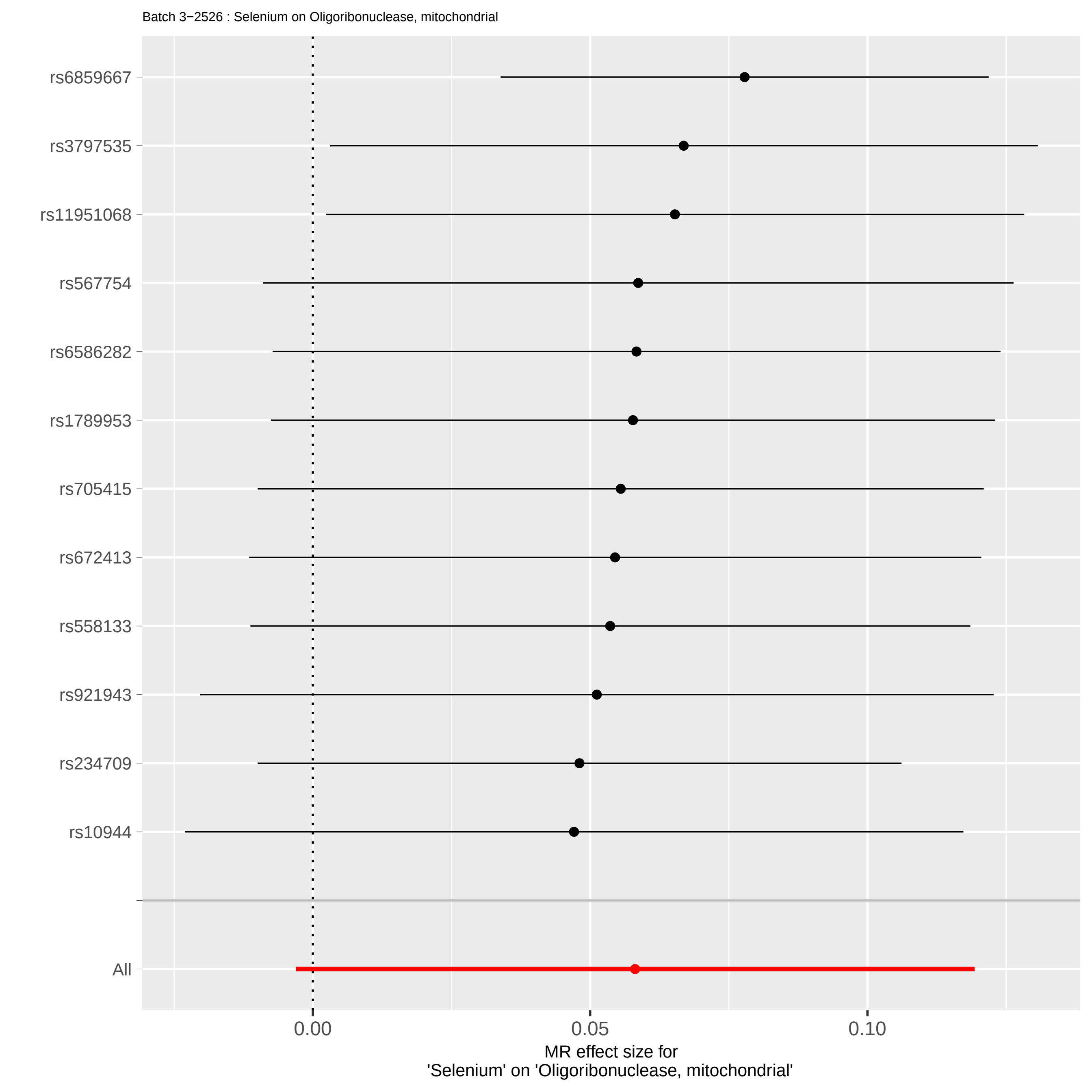
Batch 3−2526 : Selenium on Oligoribonuclease, mitochondrial
rs6859667
rs3797535
rs11951068
rs567754
rs6586282
rs1789953
rs705415
rs672413
rs558133
rs921943
rs234709
rs10944
All
0.00
0.05
0.10
MR effect size for
'Selenium' on 'Oligoribonuclease, mitochondrial'

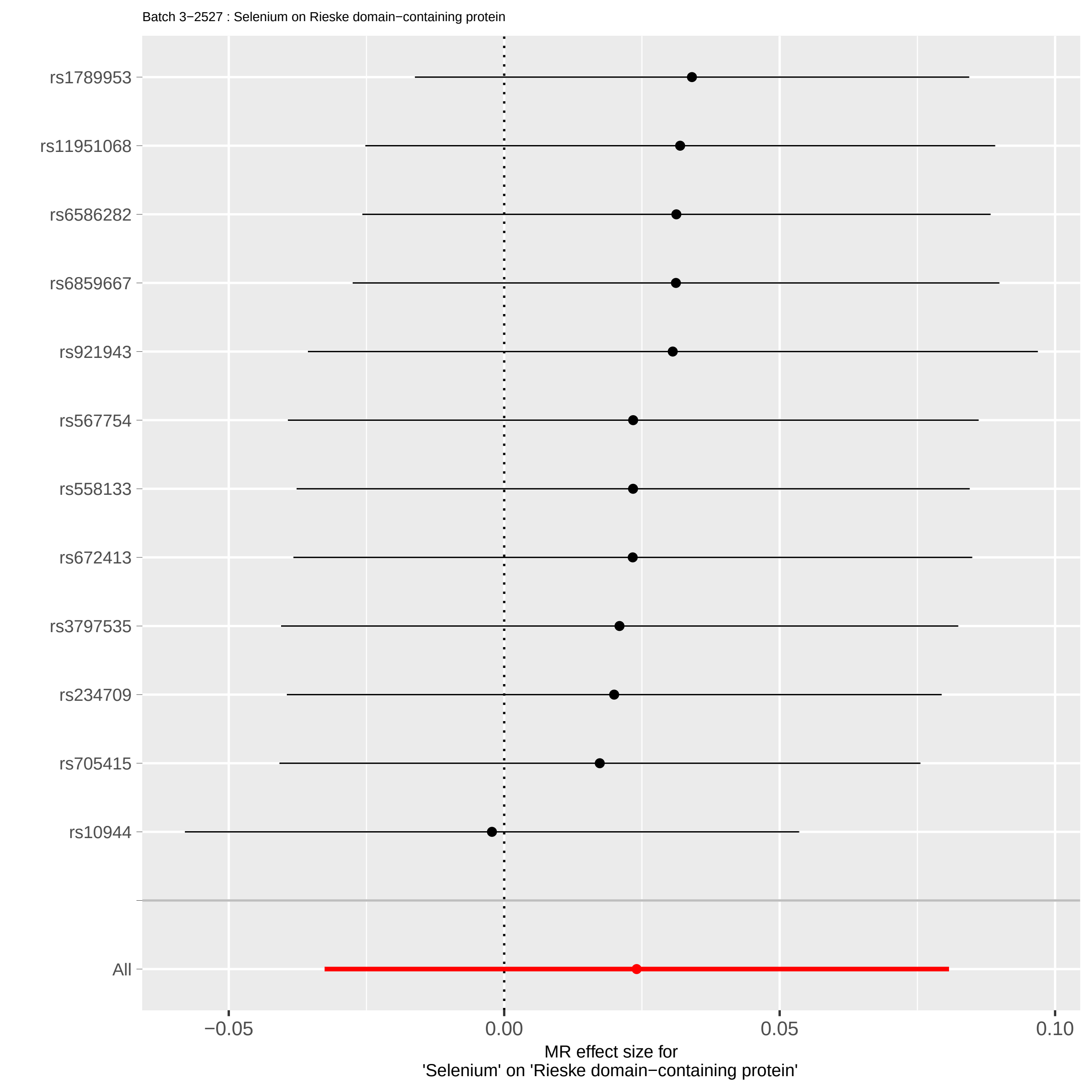

Batch 3−2527 : Selenium on Rieske domain−containing protein
rs1789953
rs11951068
rs6586282
rs6859667
rs921943
rs567754
rs558133
rs672413
rs3797535
rs234709
rs705415
rs10944
All
−0.05
0.00
0.05
0.10
MR effect size for
'Selenium' on 'Rieske domain−containing protein'

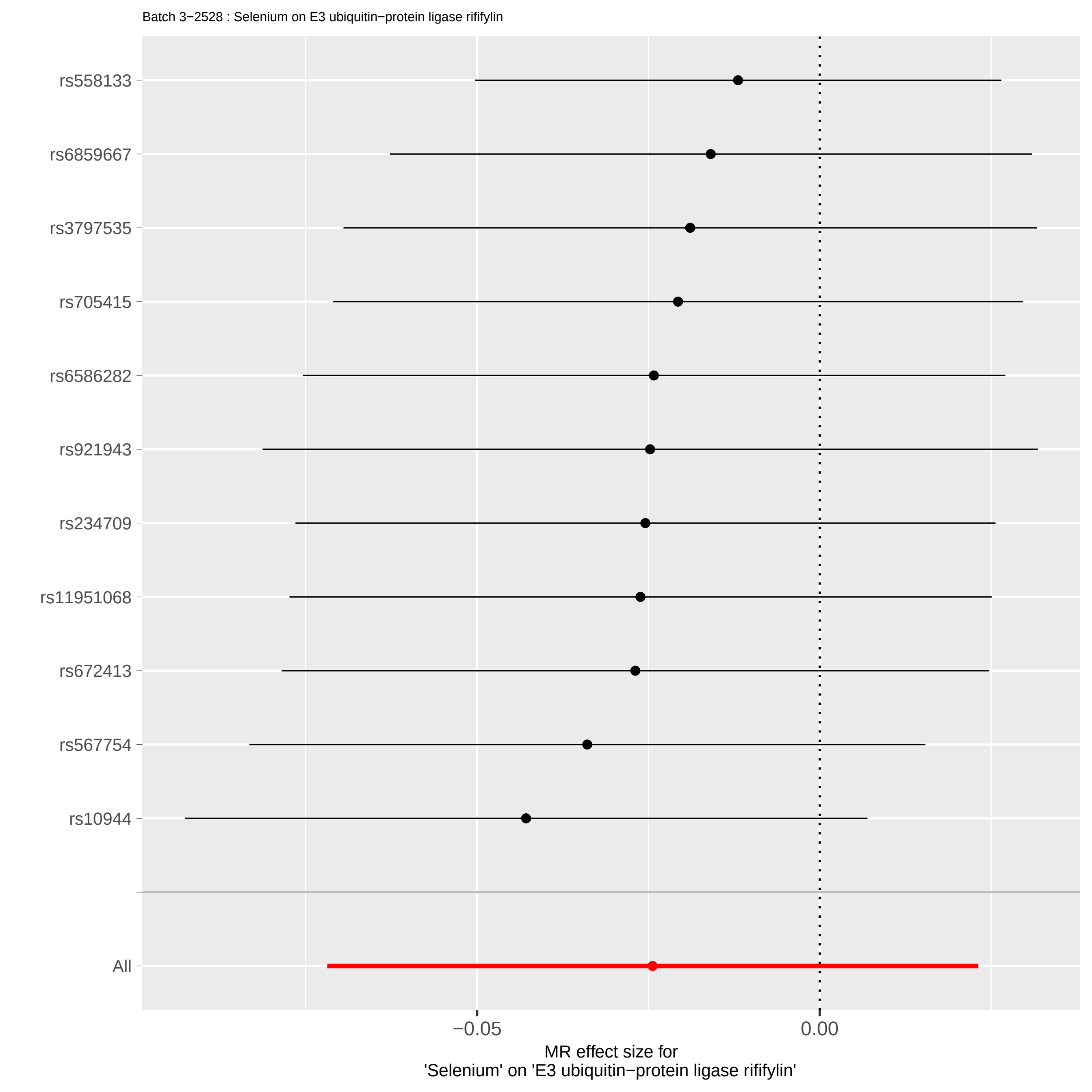
Batch 3−2528 : Selenium on E3 ubiquitin−protein ligase rififylin
rs558133
rs6859667
rs3797535
rs705415
rs6586282
rs921943
rs234709
rs11951068
rs672413
rs567754
rs10944
All
−0.05
0.00
MR effect size for
'Selenium' on 'E3 ubiquitin−protein ligase rififylin'

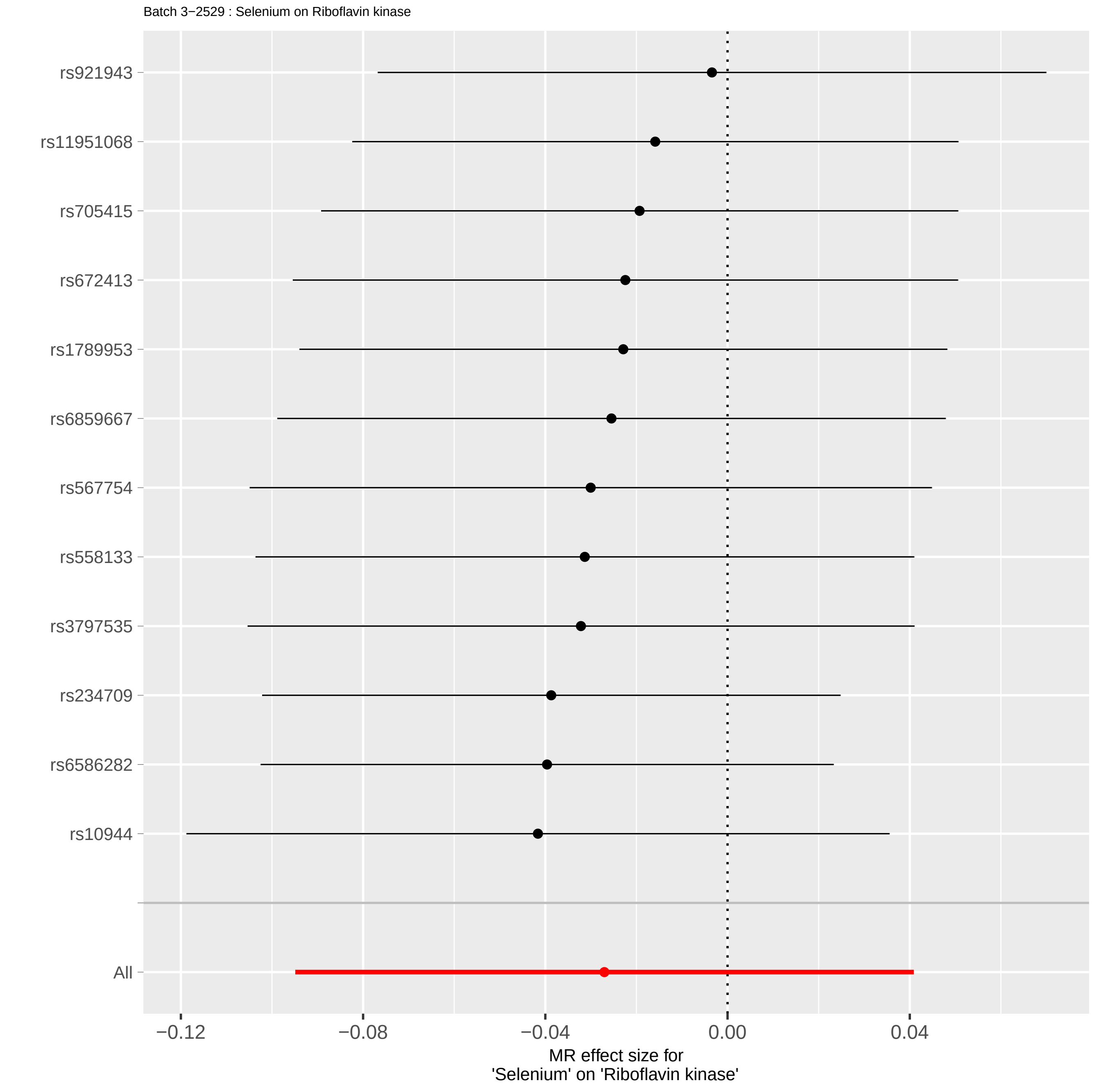

Batch 3−2529 : Selenium on Riboflavin kinase
rs921943
rs11951068
rs705415
rs672413
rs1789953
rs6859667
rs567754
rs558133
rs3797535
rs234709
rs6586282
rs10944
All
−0.12
−0.08
−0.04
0.00
0.04
MR effect size for
'Selenium' on 'Riboflavin kinase'

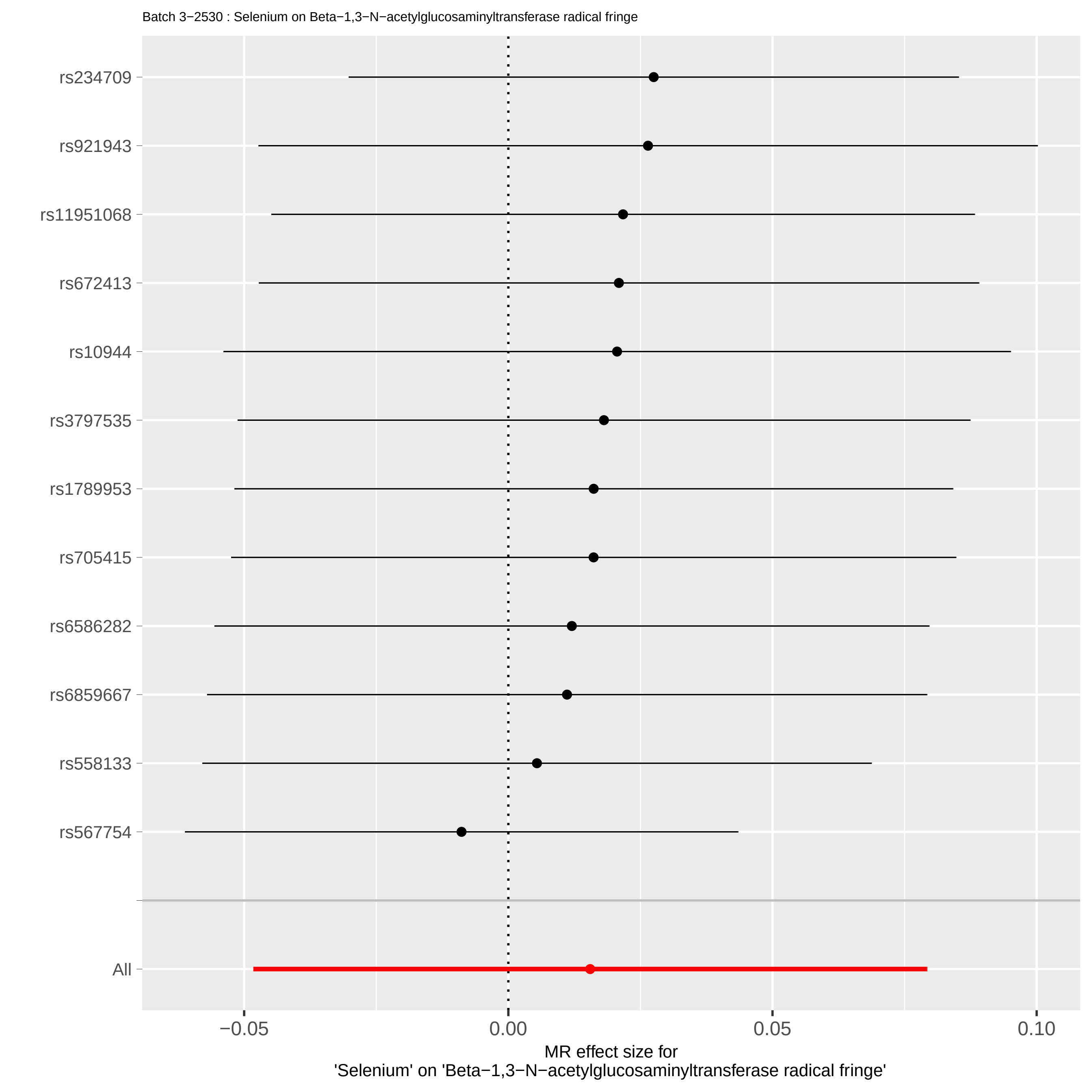

Batch 3−2530 : Selenium on Beta−1,3−N−acetylglucosaminyltransferase radical fringe
rs234709
rs921943
rs11951068
rs672413
rs10944
rs3797535
rs1789953
rs705415
rs6586282
rs6859667
rs558133
rs567754
All
−0.05
0.00
0.05
0.10
MR effect size for
'Selenium' on 'Beta−1,3−N−acetylglucosaminyltransferase radical fringe'

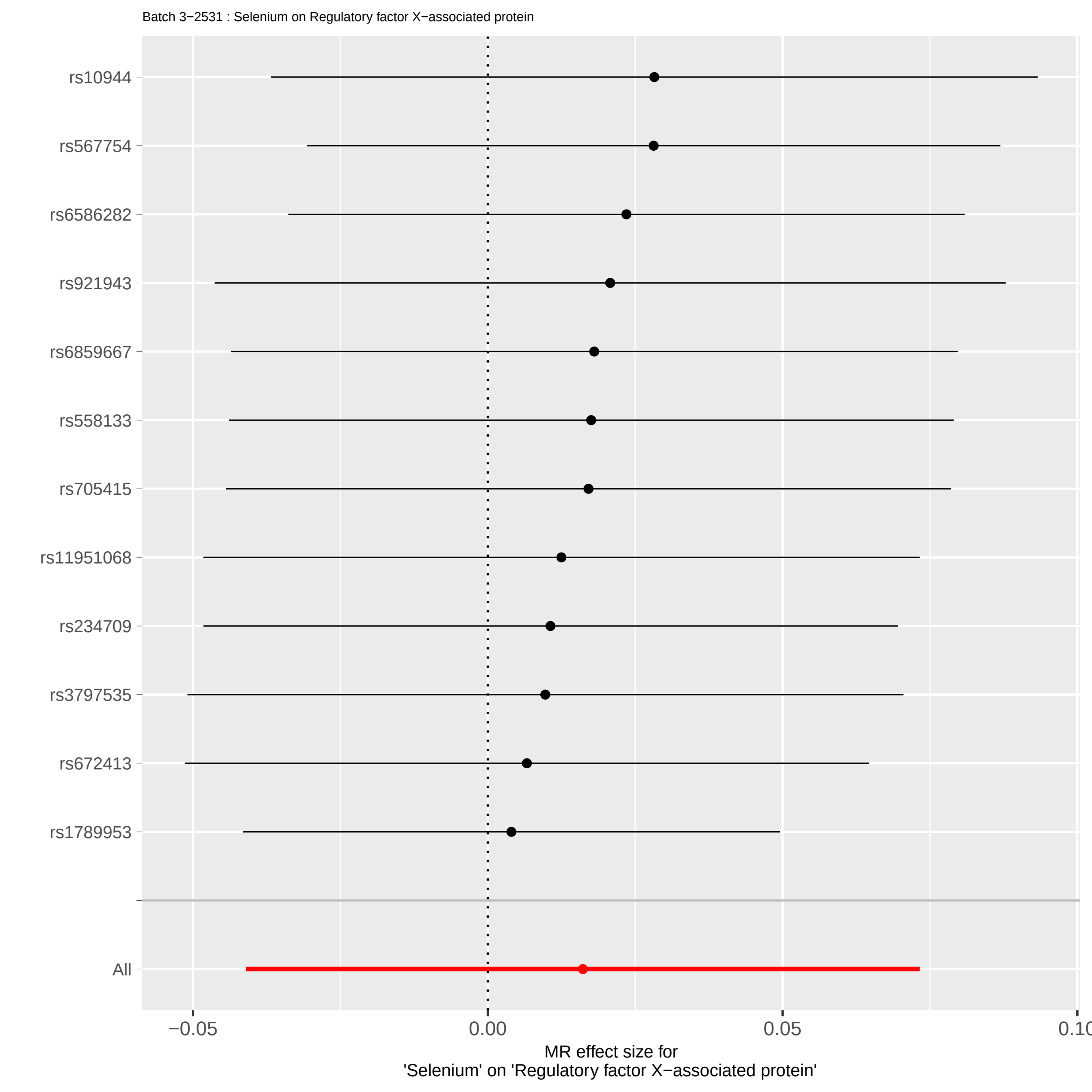

Batch 3−2531 : Selenium on Regulatory factor X−associated protein
rs10944
rs567754
rs6586282
rs921943
rs6859667
rs558133
rs705415
rs11951068
rs234709
rs3797535
rs672413
rs1789953
All
−0.05
0.00
0.05
0.10
MR effect size for
'Selenium' on 'Regulatory factor X−associated protein'

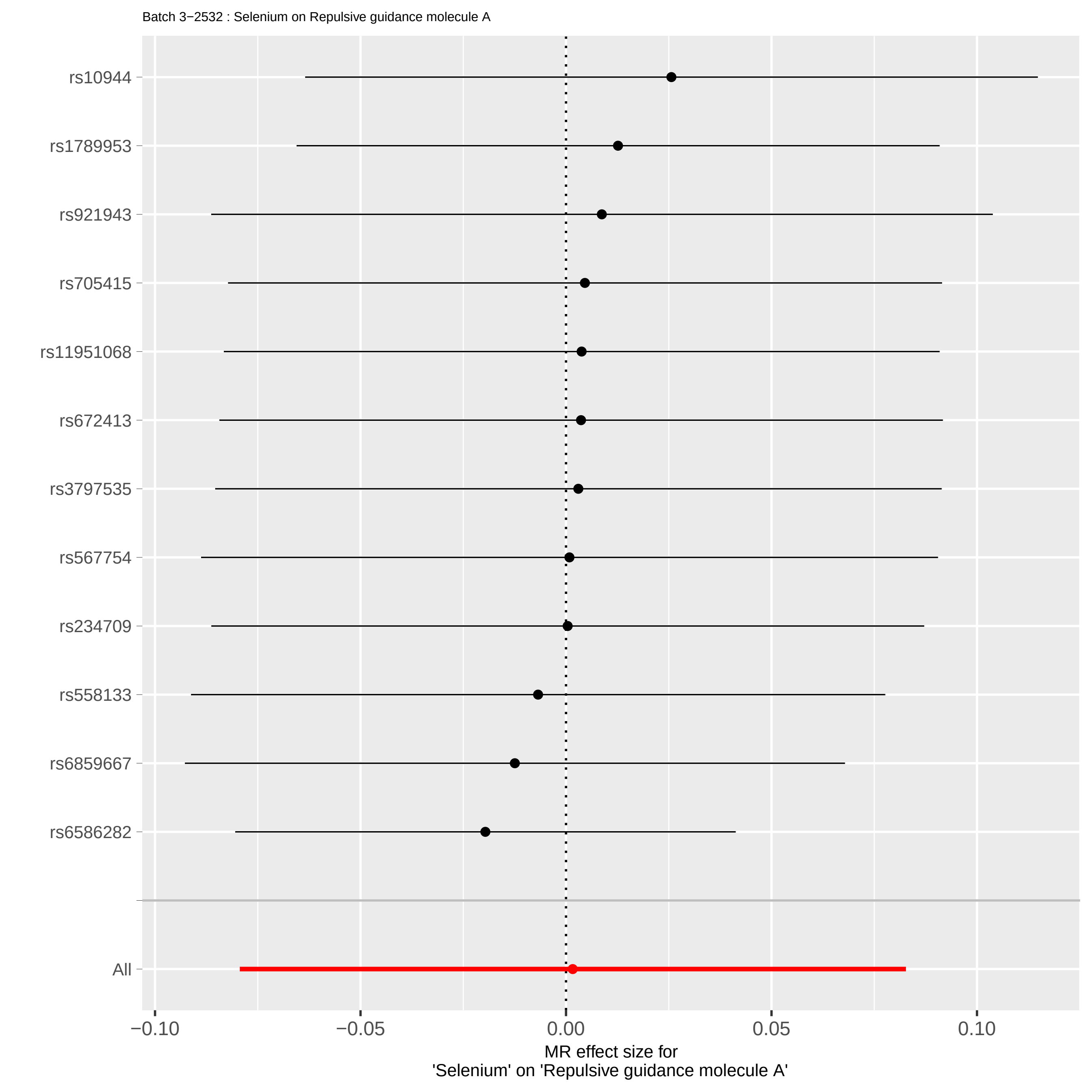
Batch 3−2532 : Selenium on Repulsive guidance molecule A
rs10944
rs1789953
rs921943
rs705415
rs11951068
rs672413
rs3797535
rs567754
rs234709
rs558133
rs6859667
rs6586282
All
−0.10
−0.05
0.00
0.05
0.10
MR effect size for
'Selenium' on 'Repulsive guidance molecule A'

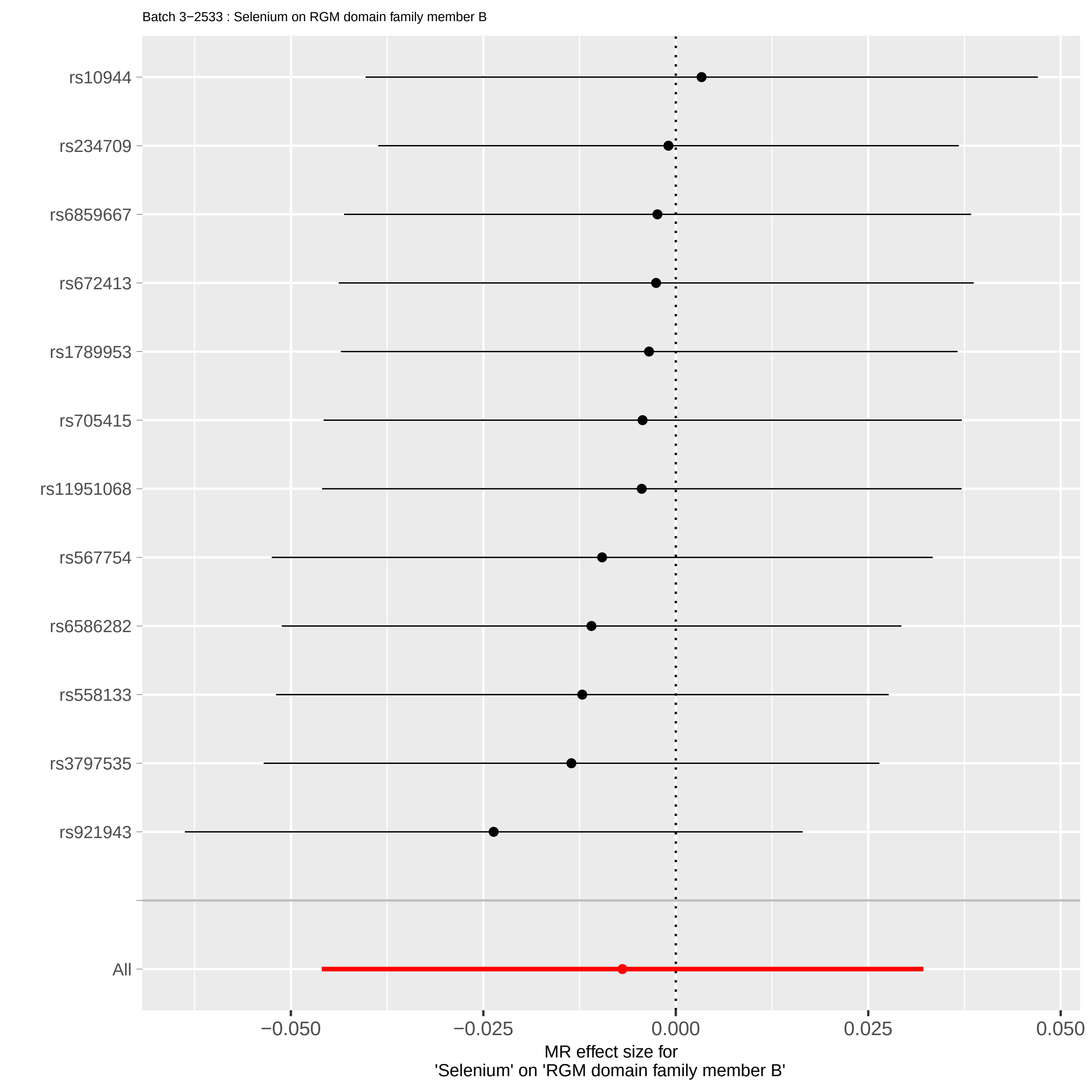

Batch 3−2533 : Selenium on RGM domain family member B
rs10944
rs234709
rs6859667
rs672413
rs1789953
rs705415
rs11951068
rs567754
rs6586282
rs558133
rs3797535
rs921943
All
−0.050
−0.025
0.000
0.025
0.050
MR effect size for
'Selenium' on 'RGM domain family member B'

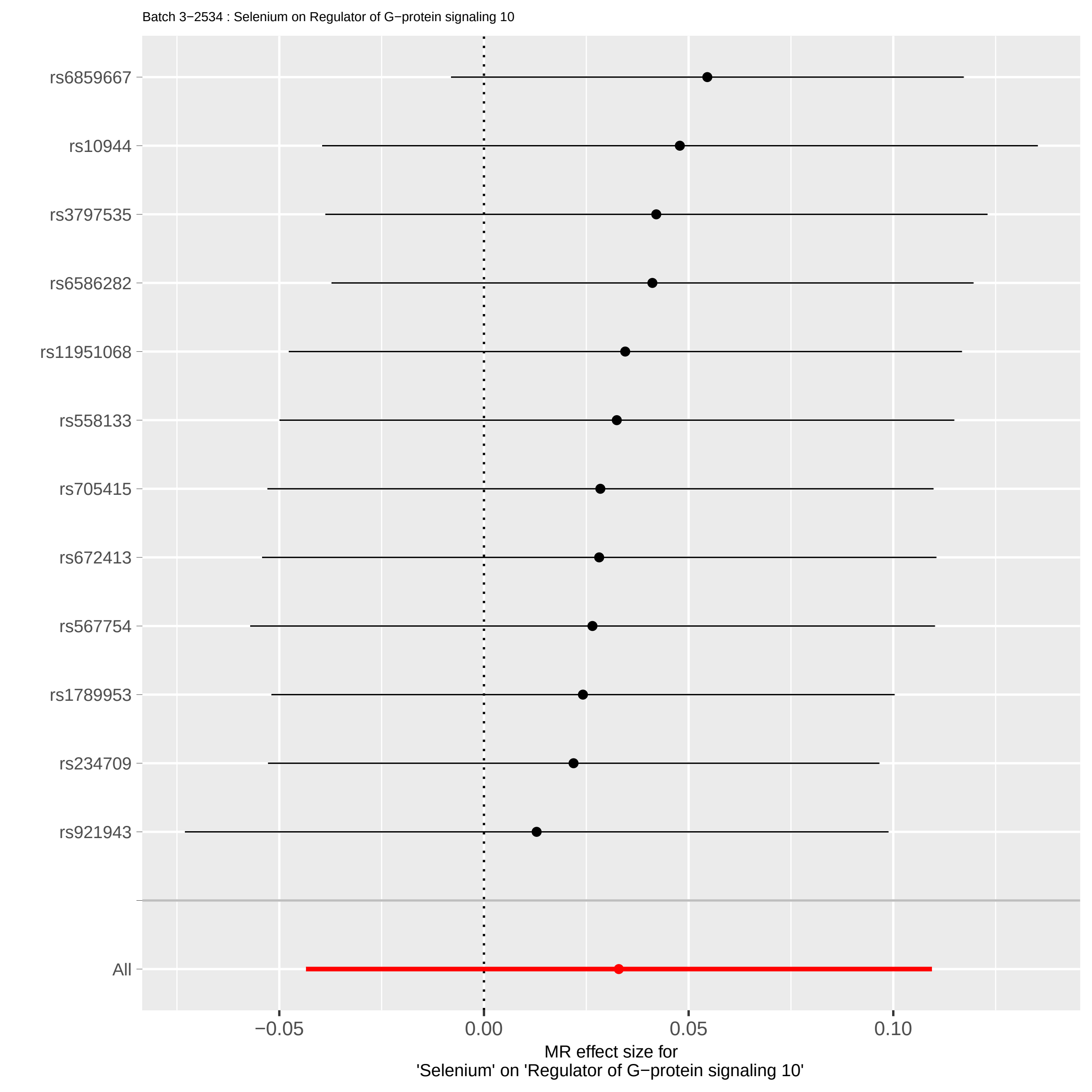

Batch 3−2534 : Selenium on Regulator of G−protein signaling 10
rs6859667
rs10944
rs3797535
rs6586282
rs11951068
rs558133
rs705415
rs672413
rs567754
rs1789953
rs234709
rs921943
All
−0.05
0.00
0.05
0.10
MR effect size for
'Selenium' on 'Regulator of G−protein signaling 10'

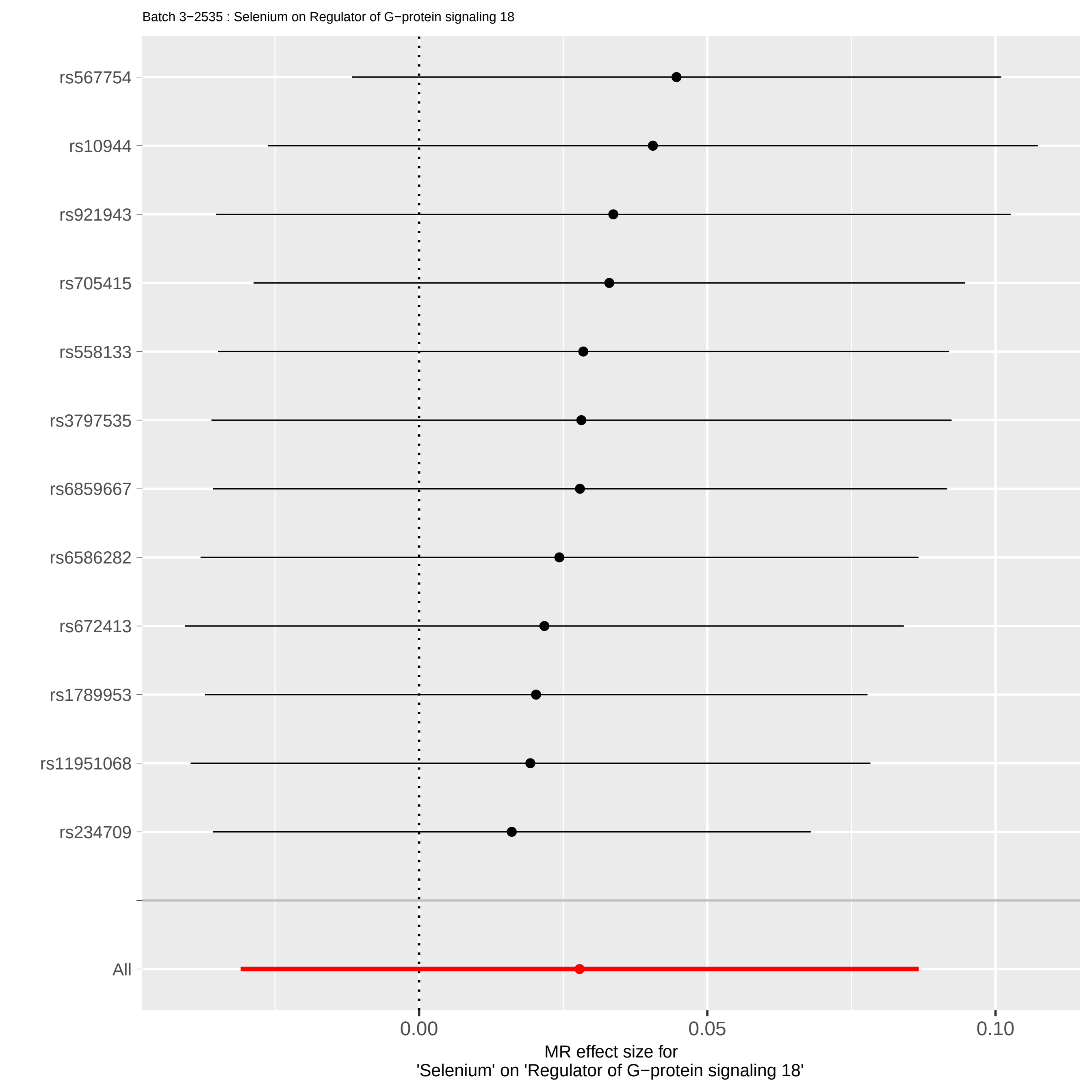
Batch 3−2535 : Selenium on Regulator of G−protein signaling 18
rs567754
rs10944
rs921943
rs705415
rs558133
rs3797535
rs6859667
rs6586282
rs672413
rs1789953
rs11951068
rs234709
All
0.00
0.05
0.10
MR effect size for
'Selenium' on 'Regulator of G−protein signaling 18'

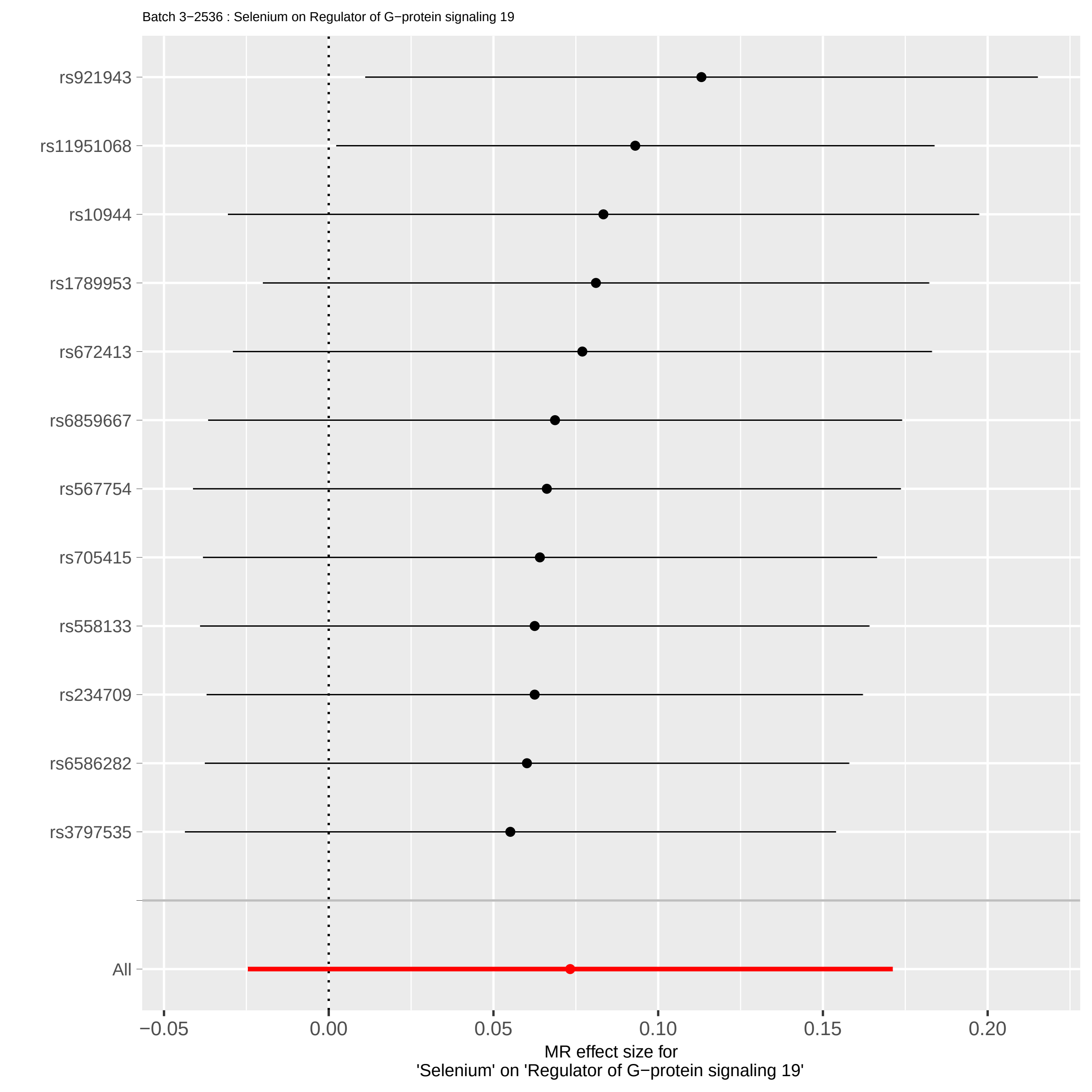

Batch 3−2536 : Selenium on Regulator of G−protein signaling 19
rs921943
rs11951068
rs10944
rs1789953
rs672413
rs6859667
rs567754
rs705415
rs558133
rs234709
rs6586282
rs3797535
All
−0.05
0.00
0.05
0.10
0.15
0.20
MR effect size for
'Selenium' on 'Regulator of G−protein signaling 19'

Batch 3−2537 : Selenium on Regulator of G−protein signaling 3

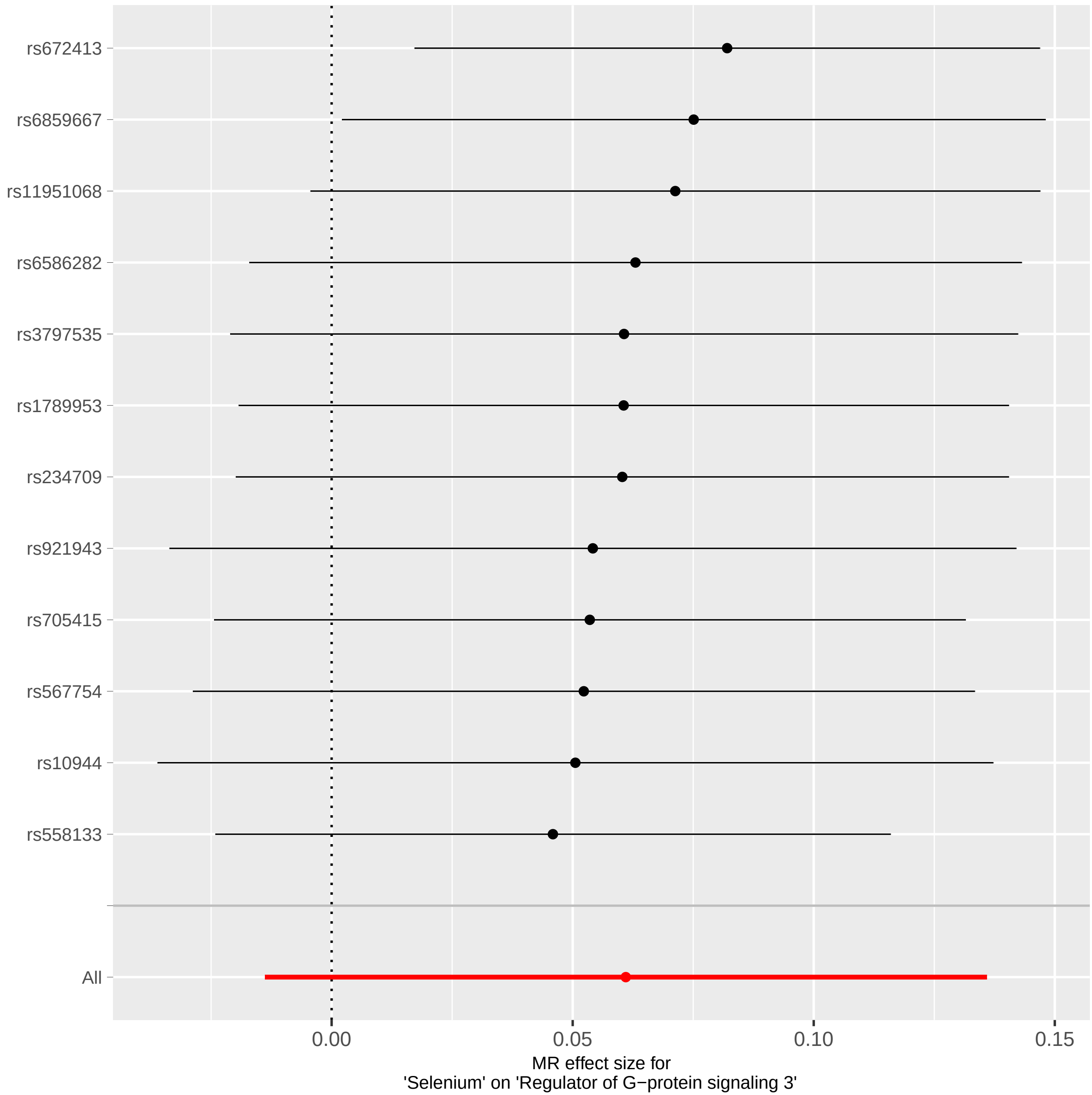

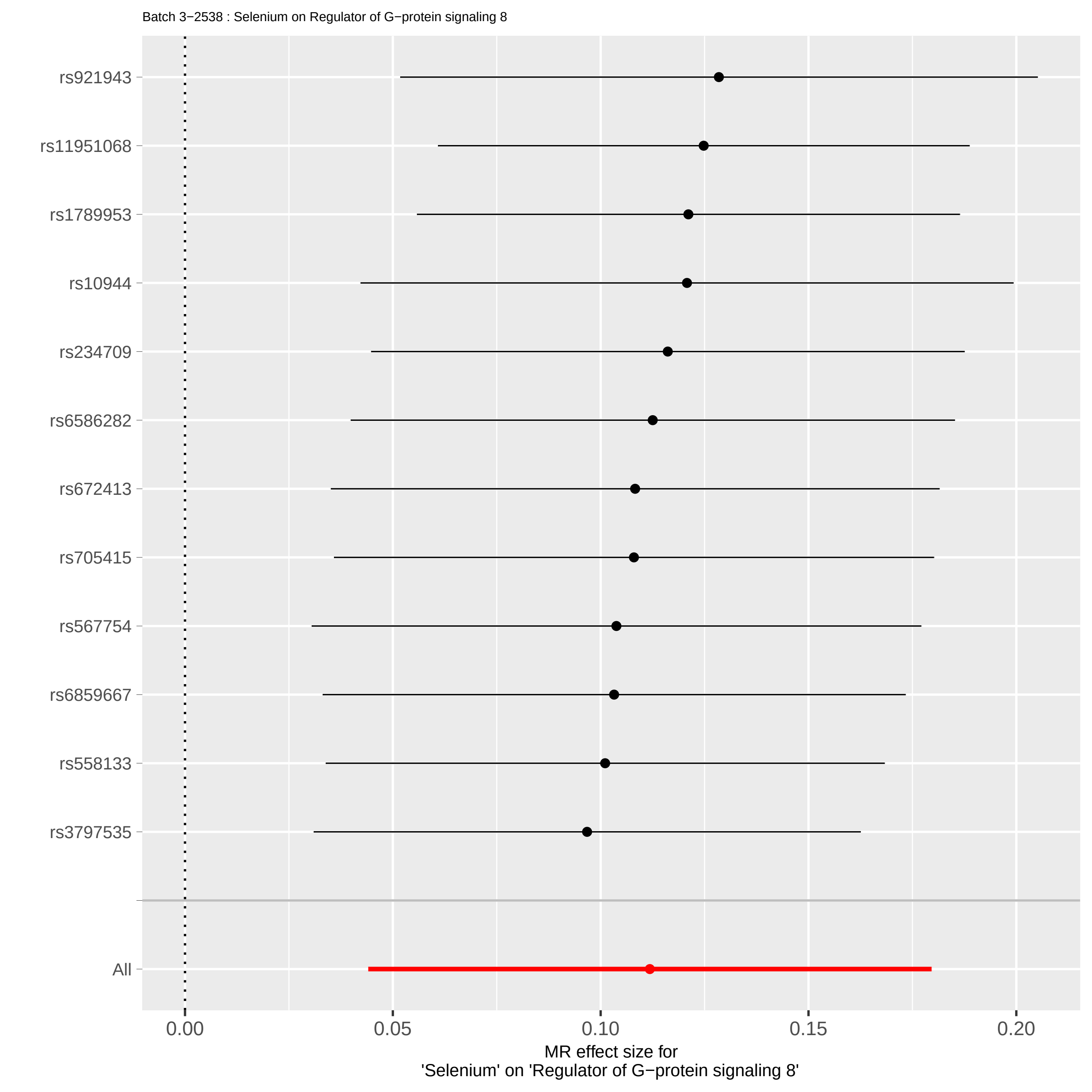

Batch 3−2538 : Selenium on Regulator of G−protein signaling 8
rs921943
rs11951068
rs1789953
rs10944
rs234709
rs6586282
rs672413
rs705415
rs567754
rs6859667
rs558133
rs3797535
All
0.00
0.05
0.10
0.15
0.20
MR effect size for
'Selenium' on 'Regulator of G−protein signaling 8'

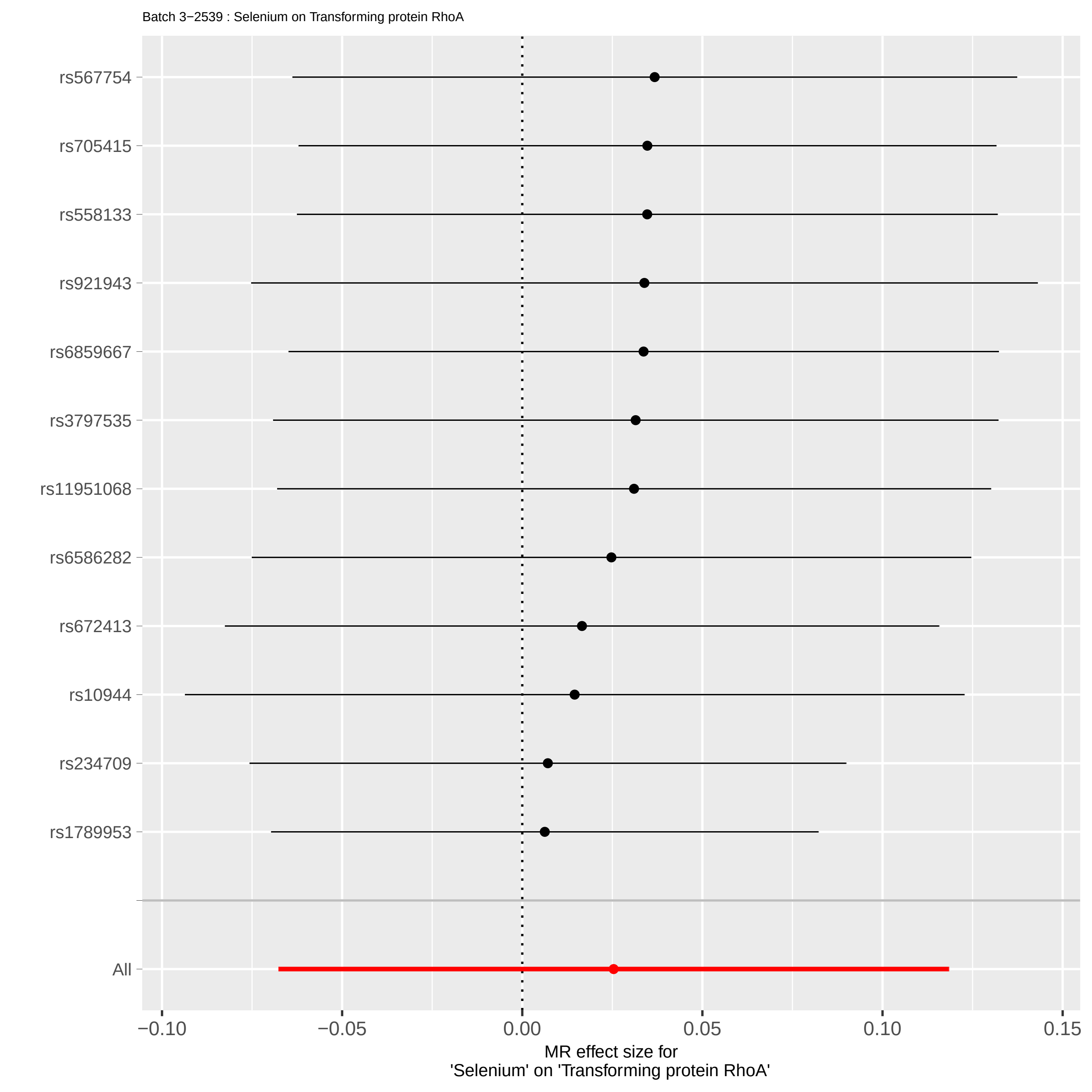
Batch 3−2539 : Selenium on Transforming protein RhoA
rs567754
rs705415
rs558133
rs921943
rs6859667
rs3797535
rs11951068
rs6586282
rs672413
rs10944
rs234709
rs1789953
All
−0.10
−0.05
0.00
0.05
0.10
0.15
MR effect size for
'Selenium' on 'Transforming protein RhoA'

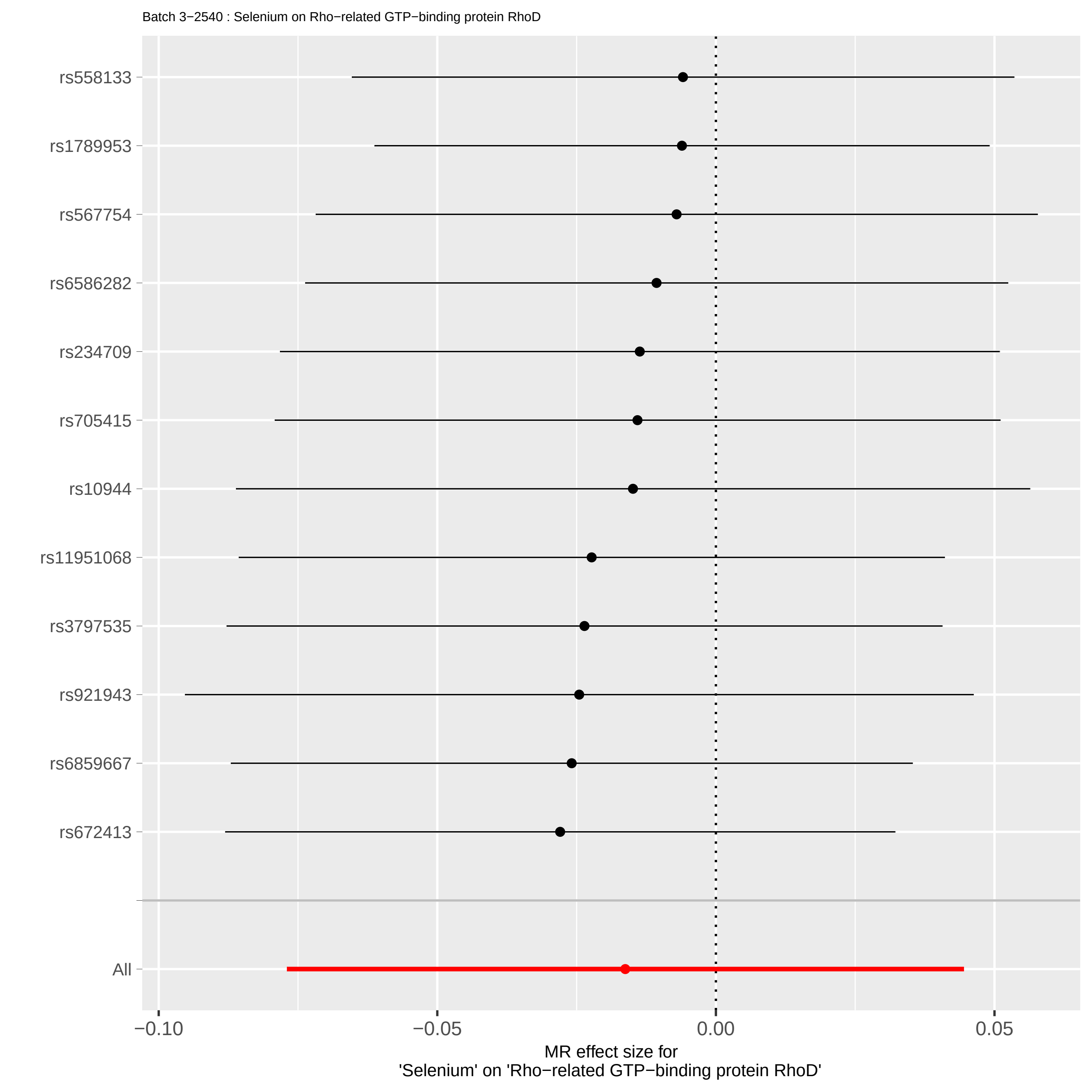

Batch 3−2540 : Selenium on Rho−related GTP−binding protein RhoD
rs558133
rs1789953
rs567754
rs6586282
rs234709
rs705415
rs10944
rs11951068
rs3797535
rs921943
rs6859667
rs672413
All
−0.10
−0.05
0.00
0.05
MR effect size for
'Selenium' on 'Rho−related GTP−binding protein RhoD'

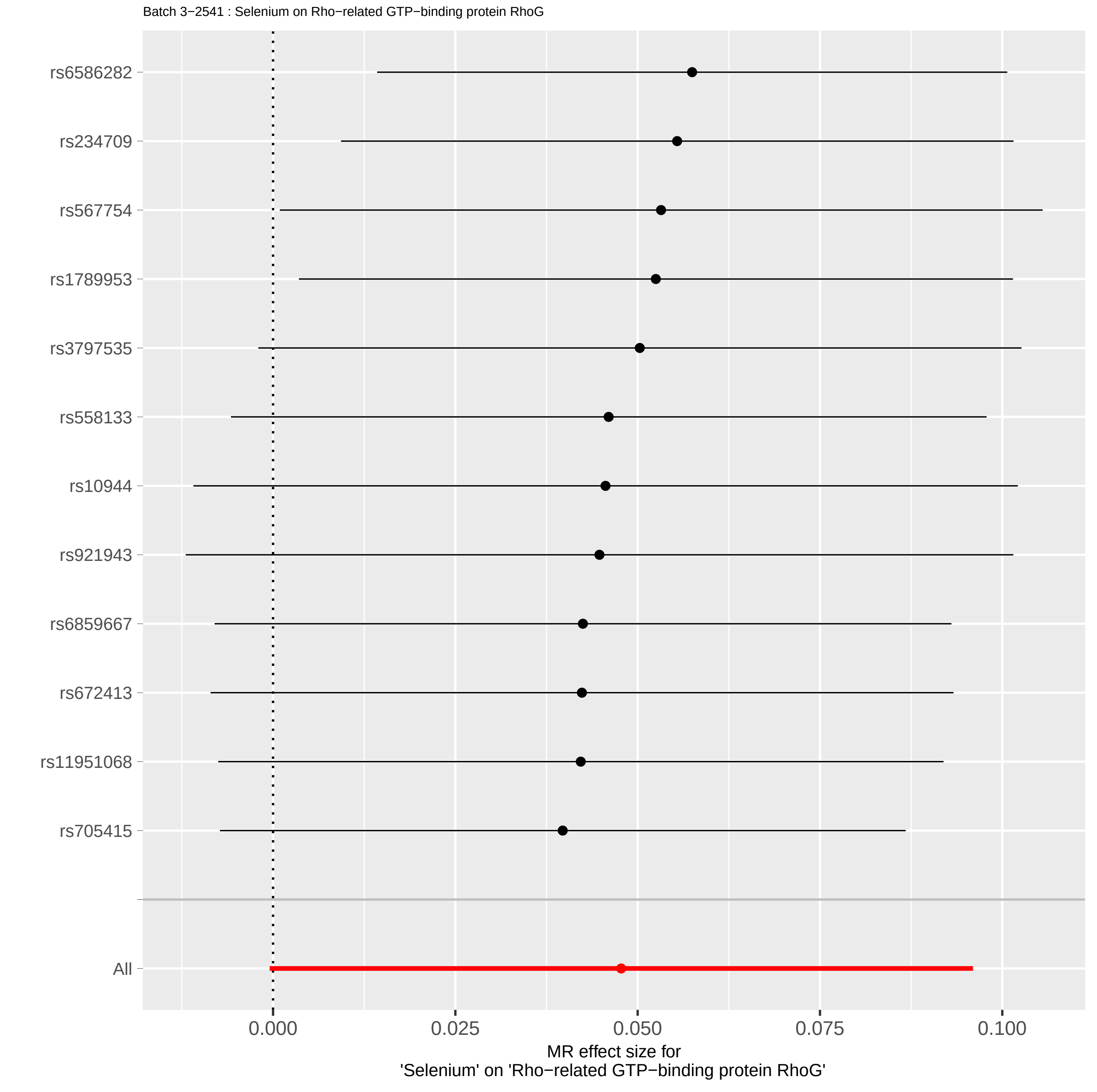

Batch 3−2541 : Selenium on Rho−related GTP−binding protein RhoG
rs6586282
rs234709
rs567754
rs1789953
rs3797535
rs558133
rs10944
rs921943
rs6859667
rs672413
rs11951068
rs705415
All
0.000
0.025
0.050
0.075
0.100
MR effect size for
'Selenium' on 'Rho−related GTP−binding protein RhoG'

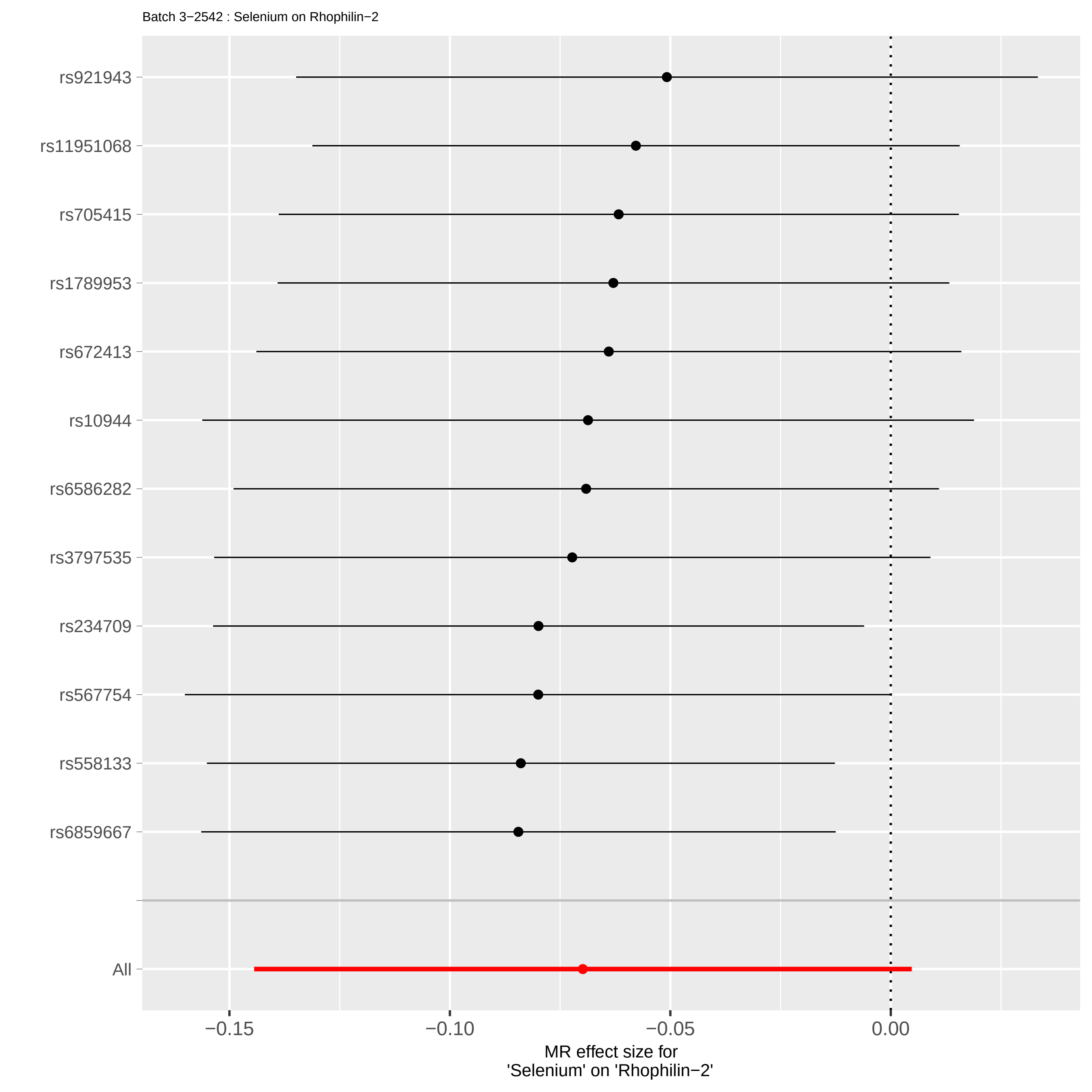
Batch 3−2542 : Selenium on Rhophilin−2
rs921943
rs11951068
rs705415
rs1789953
rs672413
rs10944
rs6586282
rs3797535
rs234709
rs567754
rs558133
rs6859667
All
−0.15
−0.10
−0.05
0.00
MR effect size for
'Selenium' on 'Rhophilin−2'

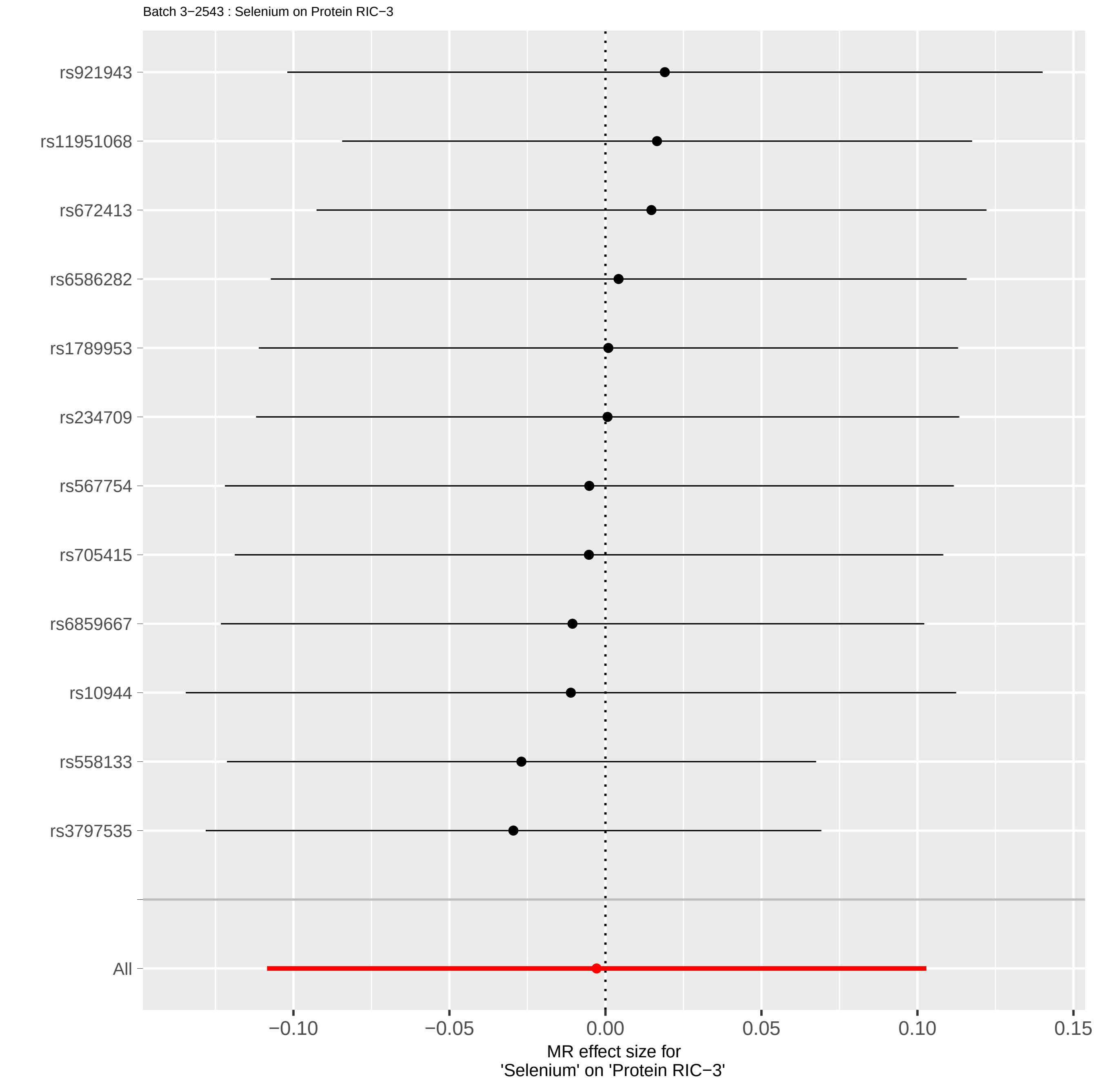

Batch 3−2543 : Selenium on Protein RIC−3
rs921943
rs11951068
rs672413
rs6586282
rs1789953
rs234709
rs567754
rs705415
rs6859667
rs10944
rs558133
rs3797535
All
−0.10
−0.05
0.00
0.05
0.10
0.15
MR effect size for
'Selenium' on 'Protein RIC−3'

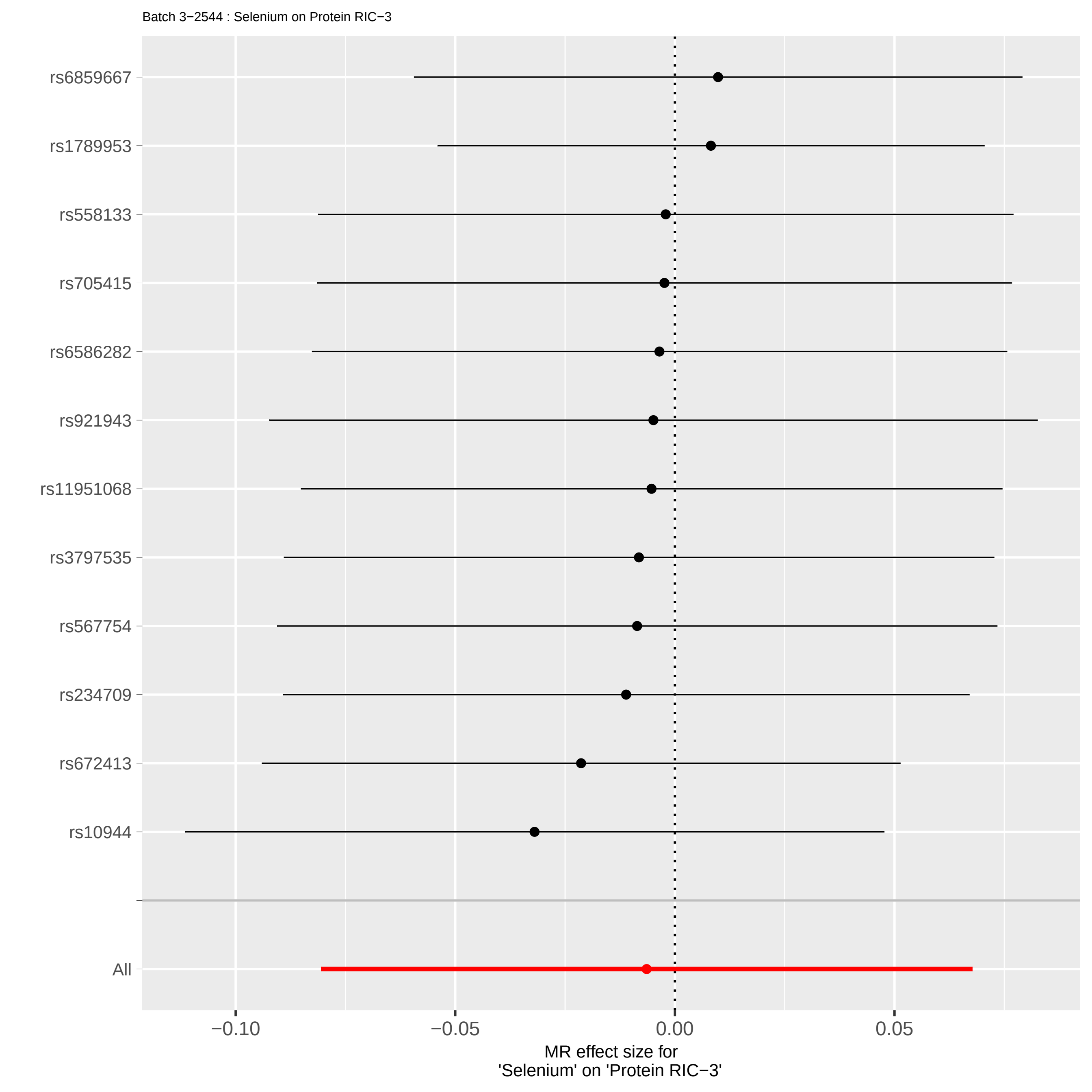

Batch 3−2544 : Selenium on Protein RIC−3
rs6859667
rs1789953
rs558133
rs705415
rs6586282
rs921943
rs11951068
rs3797535
rs567754
rs234709
rs672413
rs10944
All
−0.10
−0.05
0.00
0.05
MR effect size for
'Selenium' on 'Protein RIC−3'

Batch 3−2545 : Selenium on Receptor−interacting serine/threonine−protein kinase 2

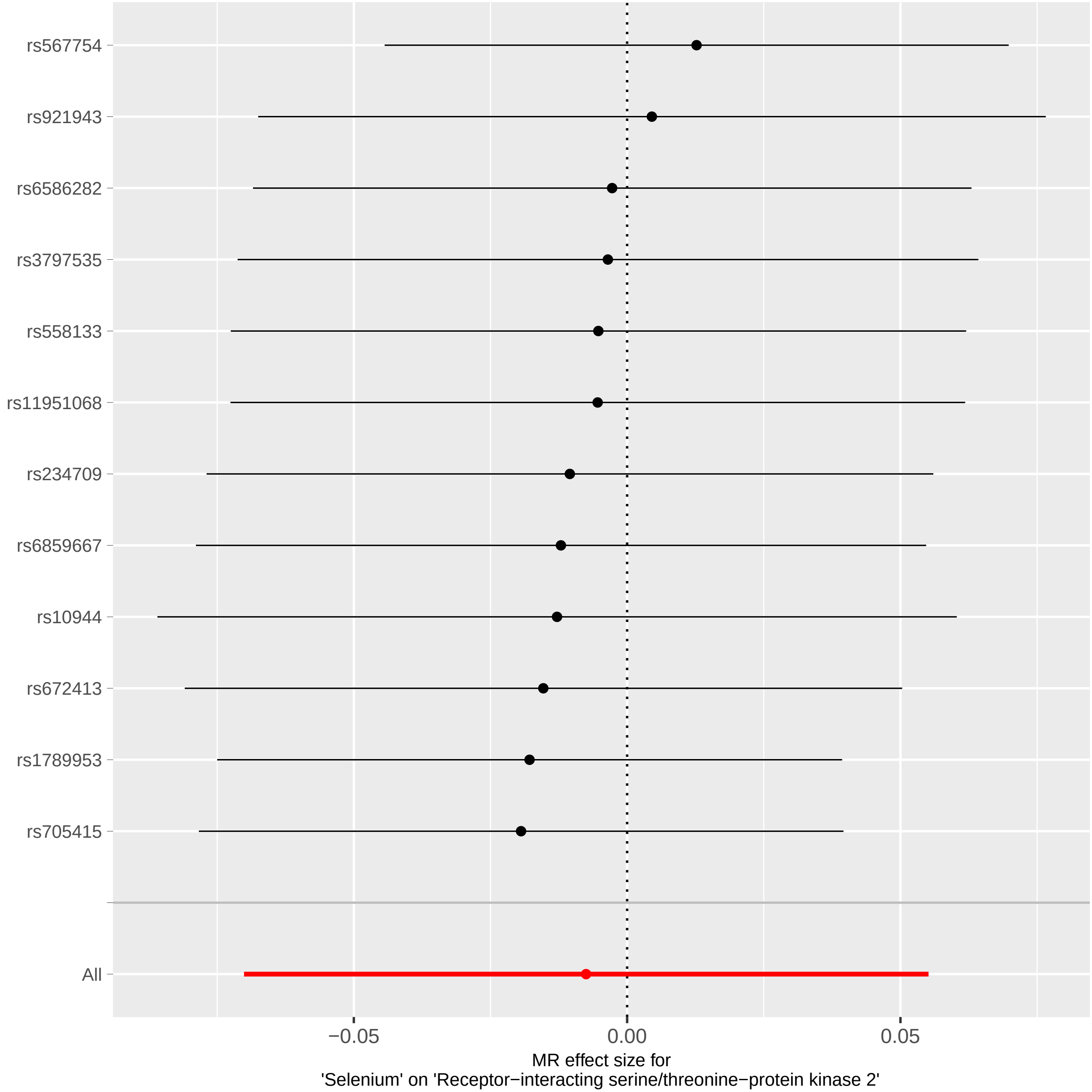

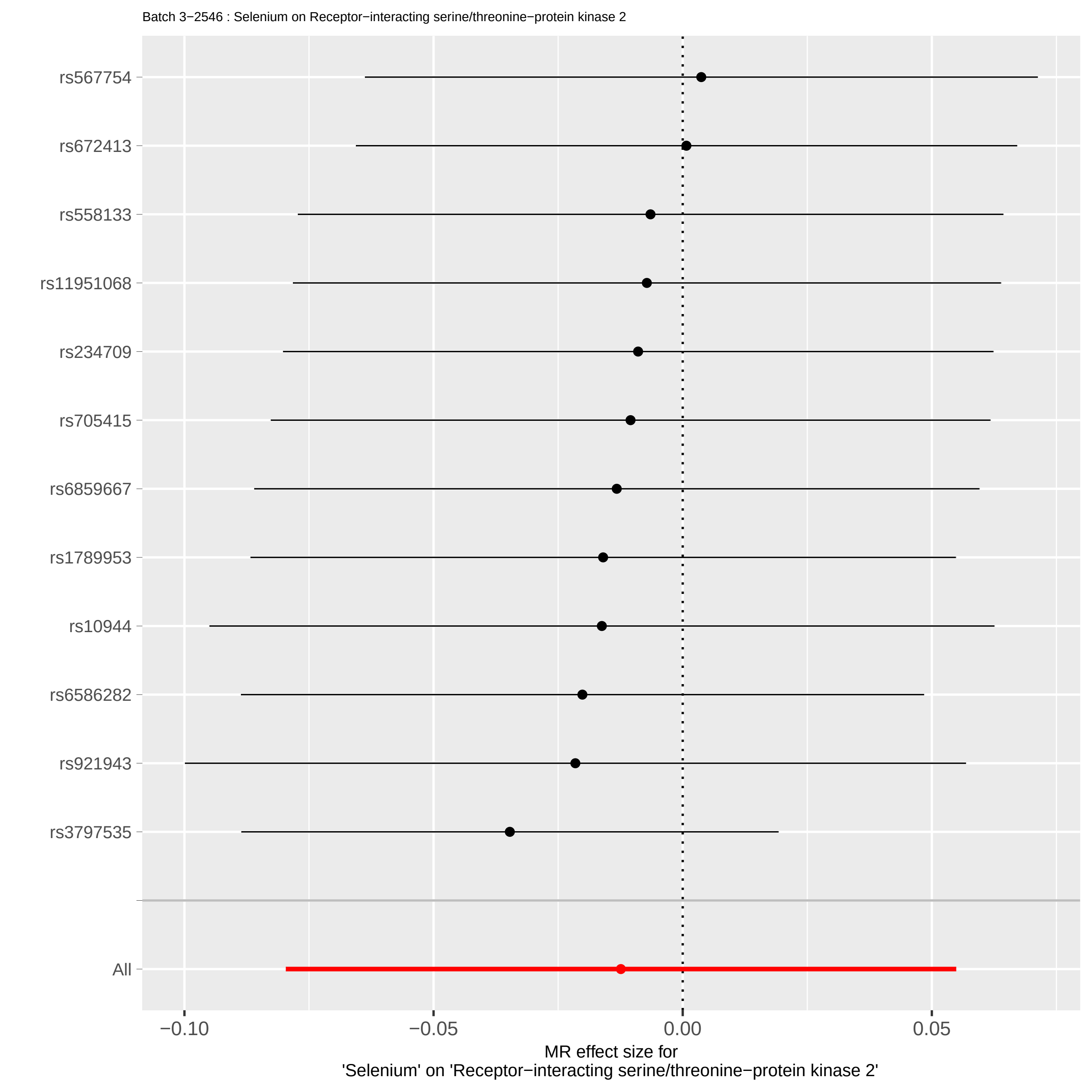

Batch 3−2546 : Selenium on Receptor−interacting serine/threonine−protein kinase 2
rs567754
rs672413
rs558133
rs11951068
rs234709
rs705415
rs6859667
rs1789953
rs10944
rs6586282
rs921943
rs3797535
All
−0.10
−0.05
0.00
0.05
MR effect size for
'Selenium' on 'Receptor−interacting serine/threonine−protein kinase 2'

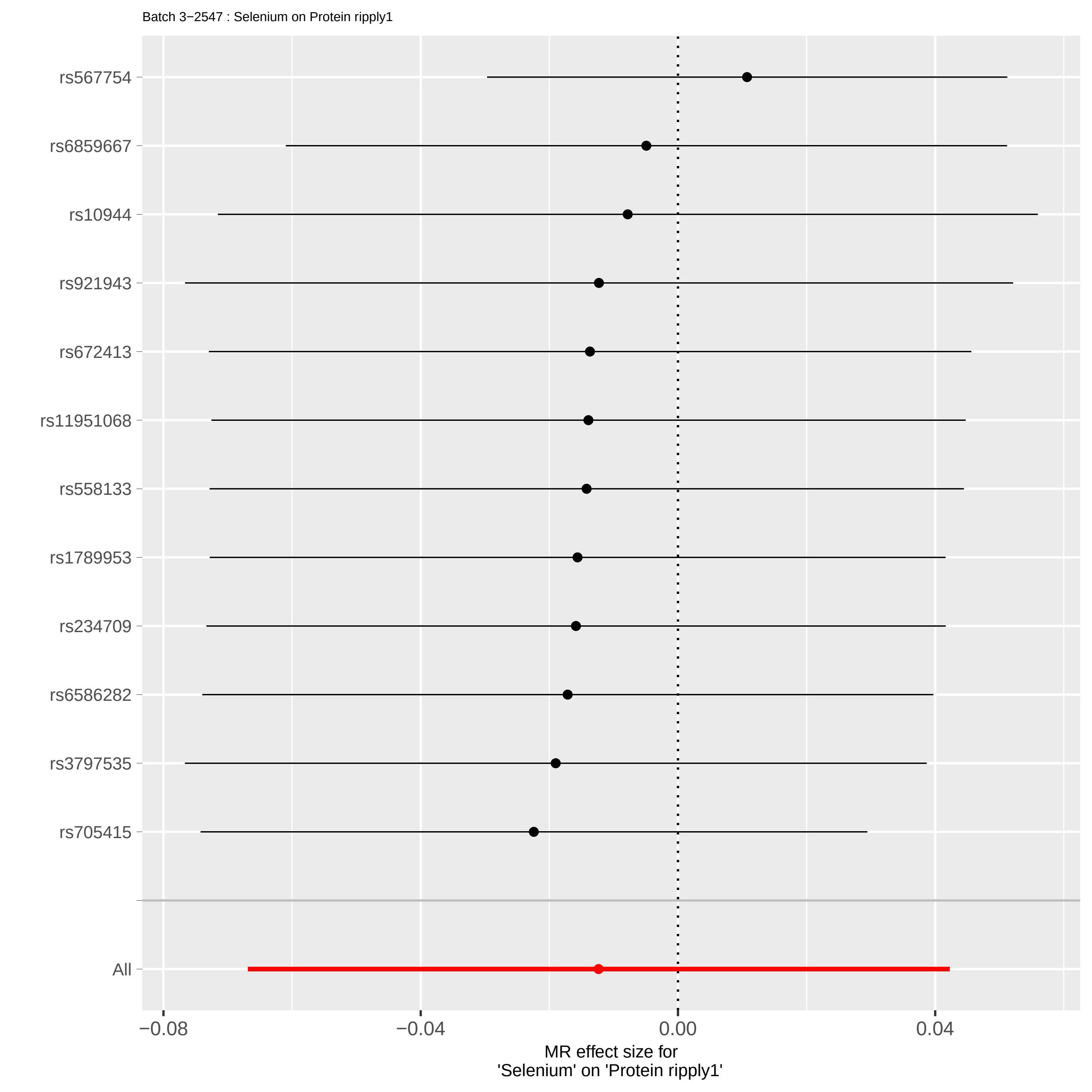
Batch 3−2547 : Selenium on Protein ripply1
rs567754
rs6859667
rs10944
rs921943
rs672413
rs11951068
rs558133
rs1789953
rs234709
rs6586282
rs3797535
rs705415
All
−0.08
−0.04
0.00
0.04
MR effect size for
'Selenium' on 'Protein ripply1'

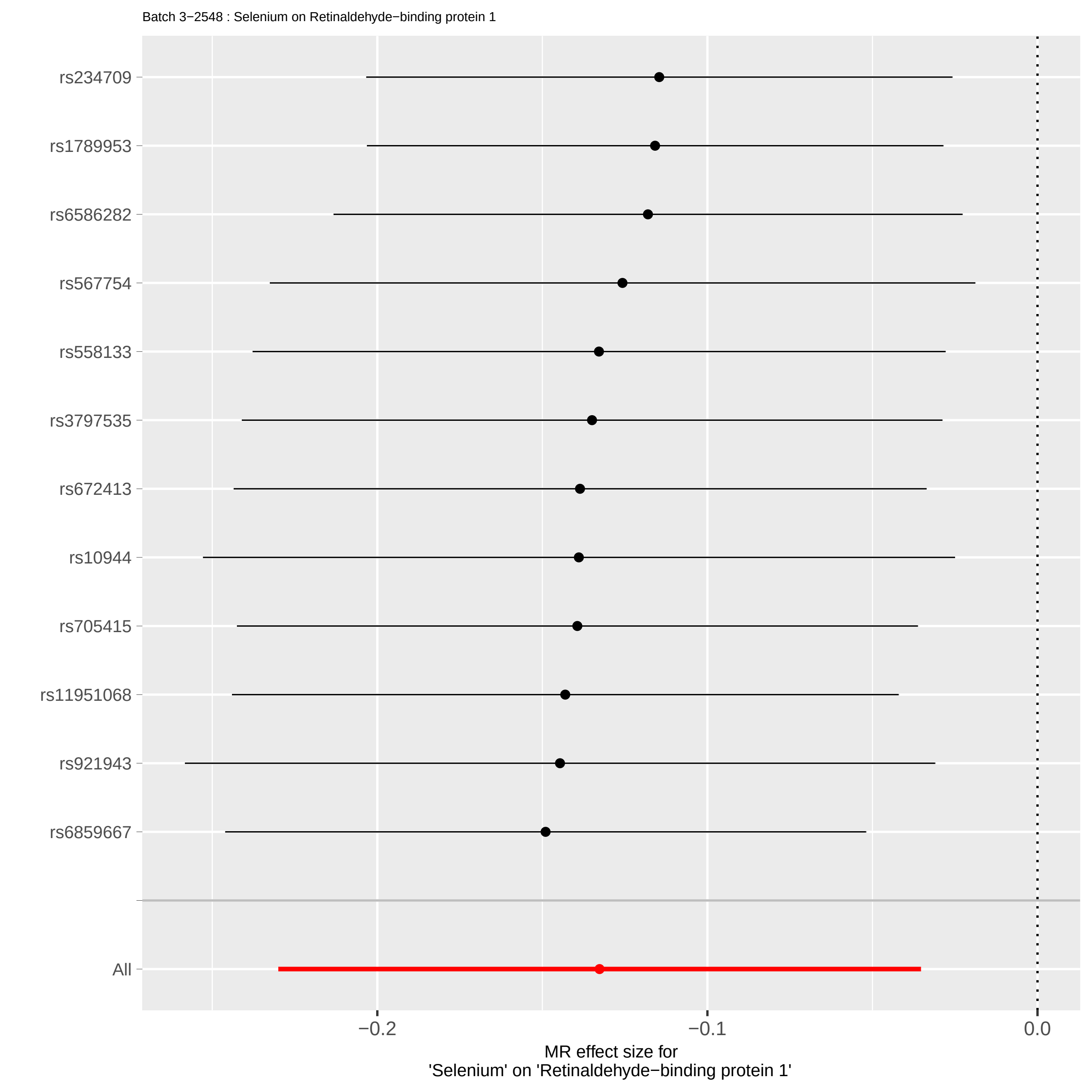
Batch 3−2548 : Selenium on Retinaldehyde−binding protein 1
rs234709
rs1789953
rs6586282
rs567754
rs558133
rs3797535
rs672413
rs10944
rs705415
rs11951068
rs921943
rs6859667
All
−0.2
−0.1
0.0
MR effect size for
'Selenium' on 'Retinaldehyde−binding protein 1'

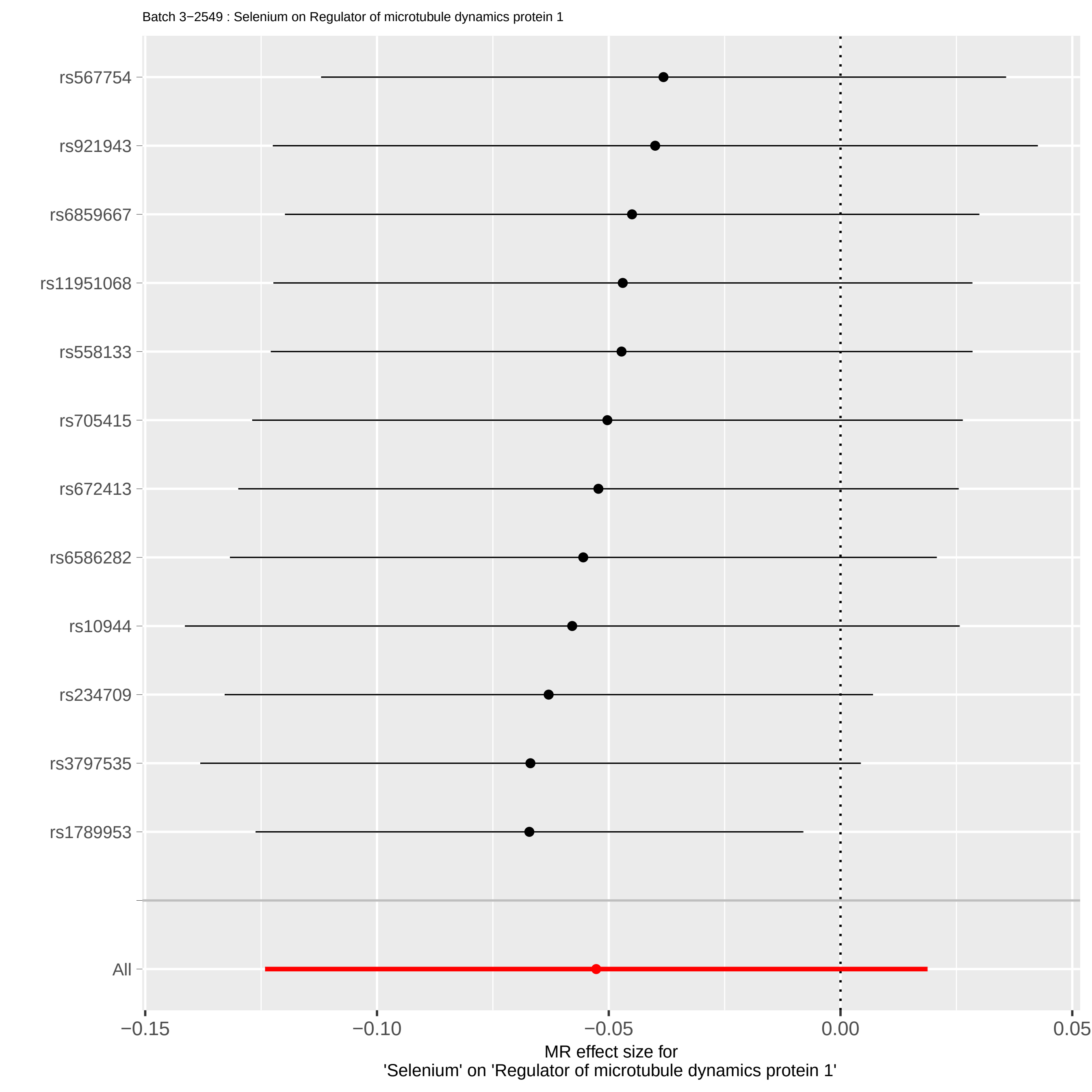

Batch 3−2549 : Selenium on Regulator of microtubule dynamics protein 1
rs567754
rs921943
rs6859667
rs11951068
rs558133
rs705415
rs672413
rs6586282
rs10944
rs234709
rs3797535
rs1789953
All
−0.15
−0.10
−0.05
0.00
0.05
MR effect size for
'Selenium' on 'Regulator of microtubule dynamics protein 1'

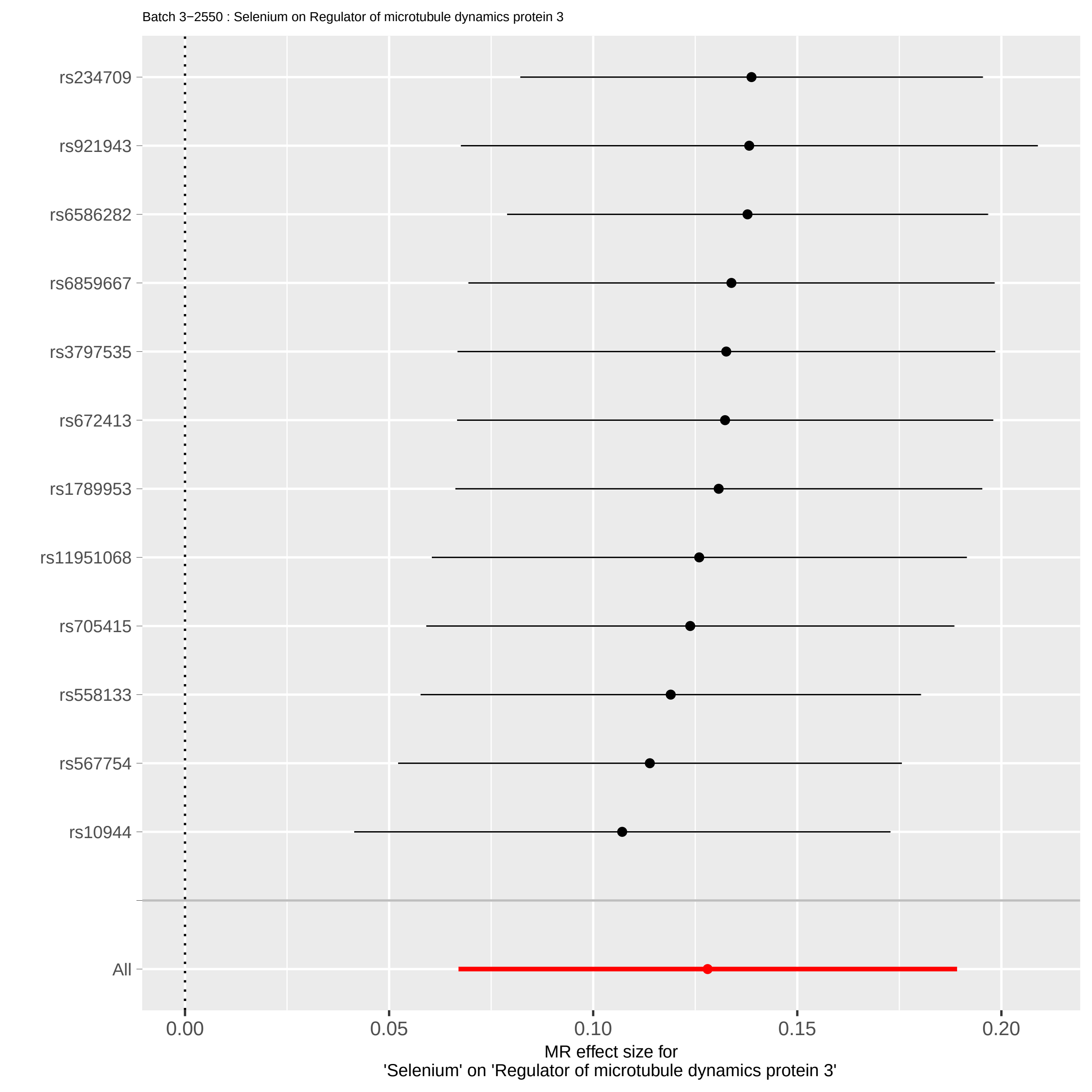

Batch 3−2550 : Selenium on Regulator of microtubule dynamics protein 3
rs234709
rs921943
rs6586282
rs6859667
rs3797535
rs672413
rs1789953
rs11951068
rs705415
rs558133
rs567754
rs10944
All
0.00
0.05
0.10
0.15
0.20
MR effect size for
'Selenium' on 'Regulator of microtubule dynamics protein 3'

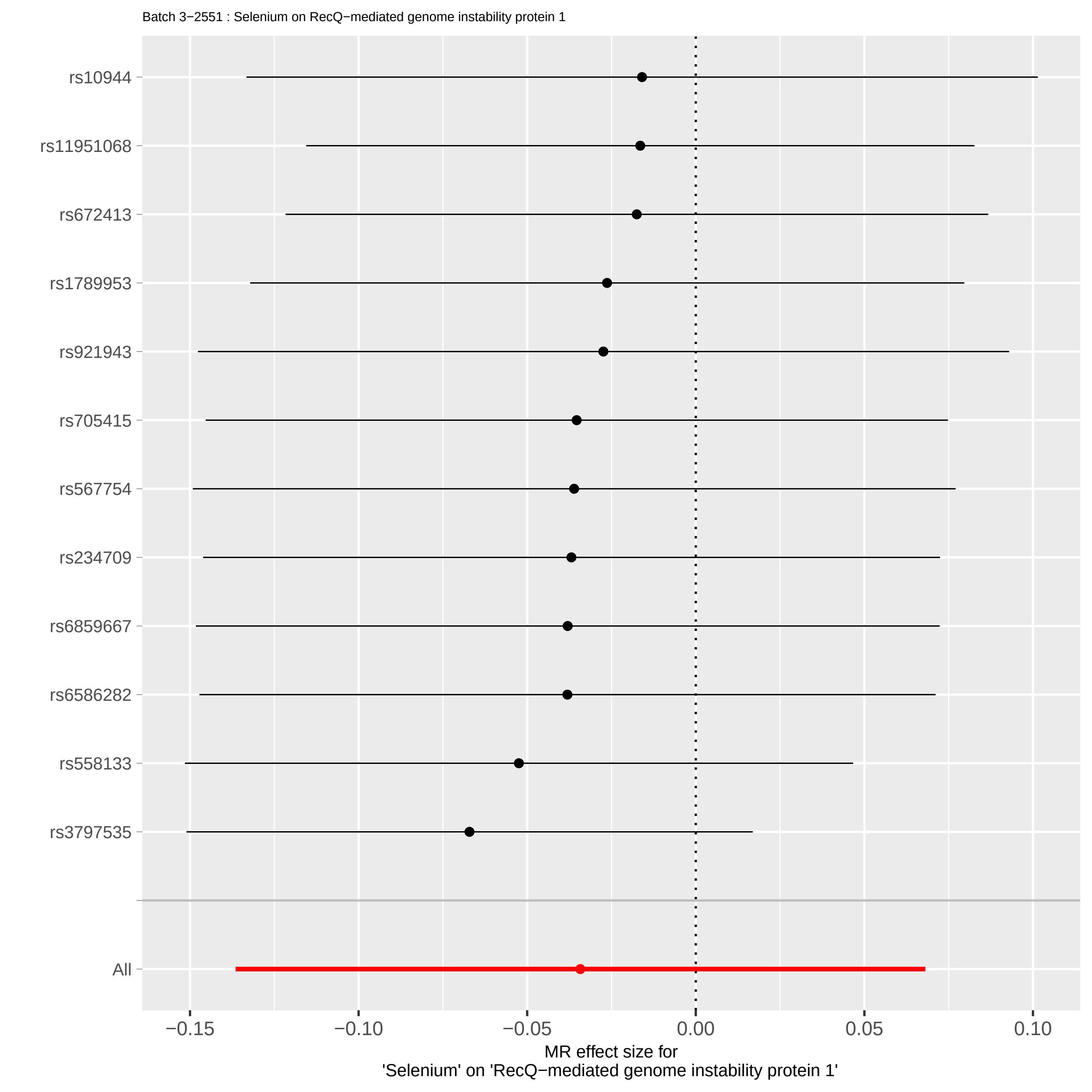
Batch 3−2551 : Selenium on RecQ−mediated genome instability protein 1
rs10944
rs11951068
rs672413
rs1789953
rs921943
rs705415
rs567754
rs234709
rs6859667
rs6586282
rs558133
rs3797535
All
−0.15
−0.10
−0.05
0.00
0.05
0.10
MR effect size for
'Selenium' on 'RecQ−mediated genome instability protein 1'

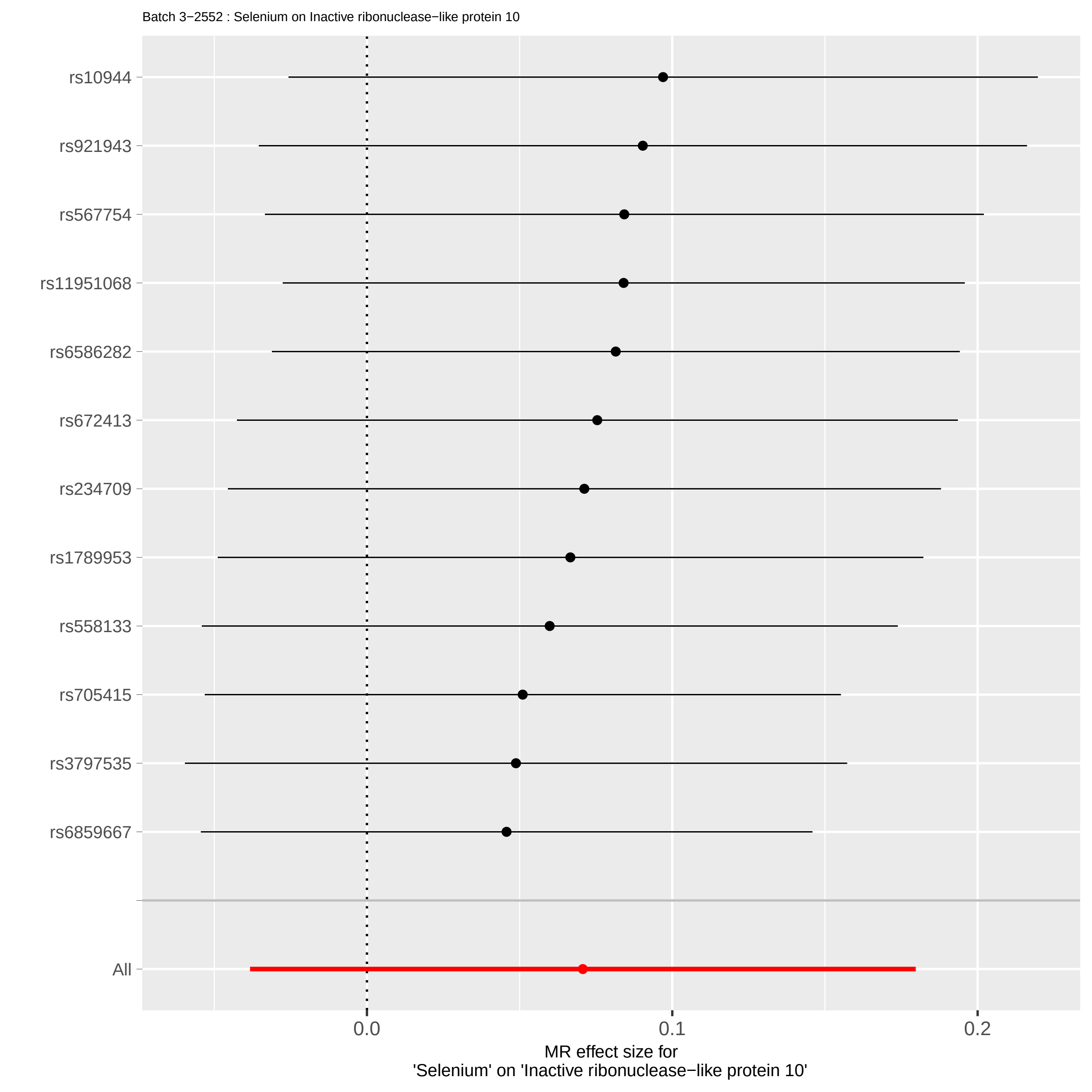

Batch 3−2552 : Selenium on Inactive ribonuclease−like protein 10
rs10944
rs921943
rs567754
rs11951068
rs6586282
rs672413
rs234709
rs1789953
rs558133
rs705415
rs3797535
rs6859667
All
0.0
0.1
0.2
MR effect size for
'Selenium' on 'Inactive ribonuclease−like protein 10'

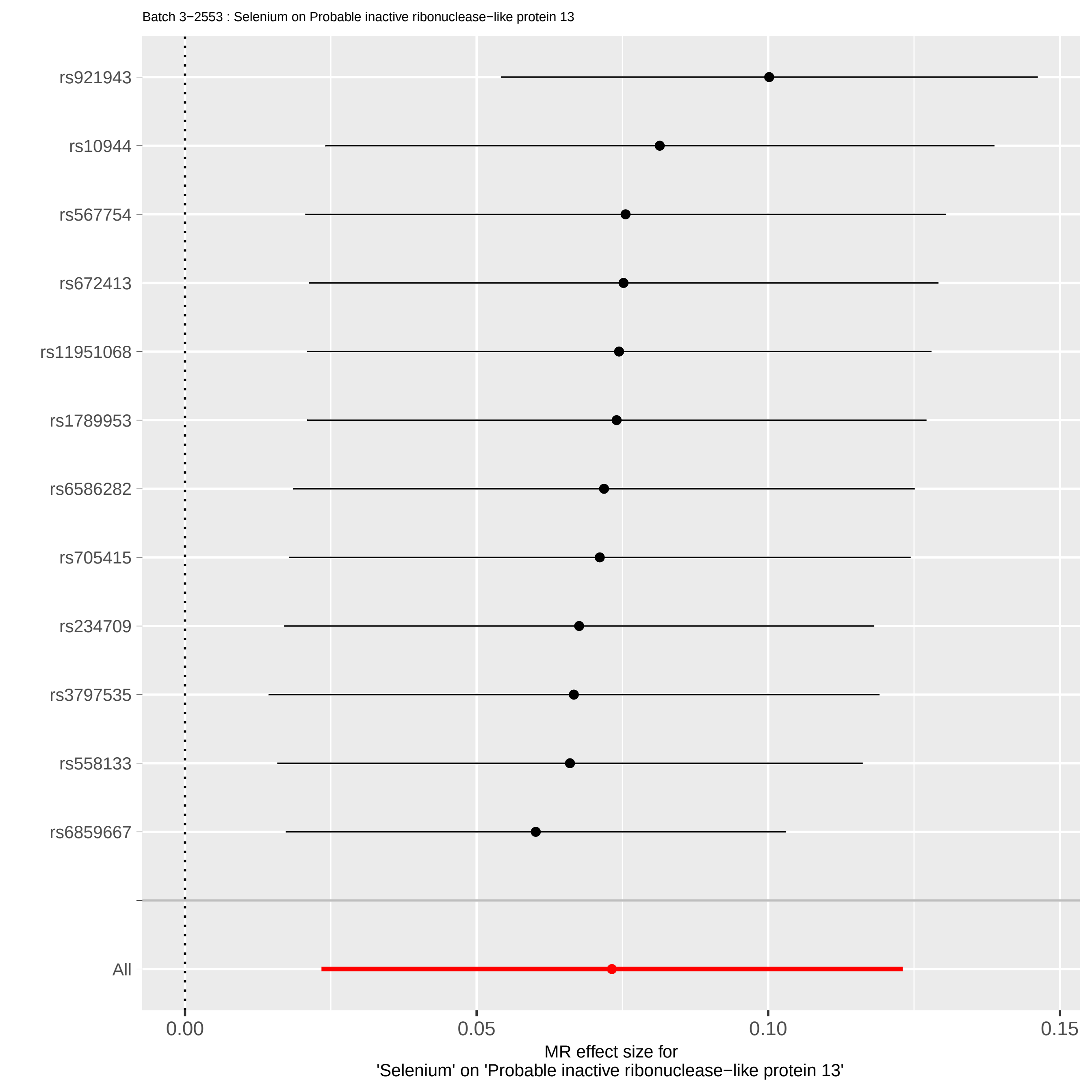

Batch 3−2553 : Selenium on Probable inactive ribonuclease−like protein 13
rs921943
rs10944
rs567754
rs672413
rs11951068
rs1789953
rs6586282
rs705415
rs234709
rs3797535
rs558133
rs6859667
All
0.00
0.05
0.10
0.15
MR effect size for
'Selenium' on 'Probable inactive ribonuclease−like protein 13'

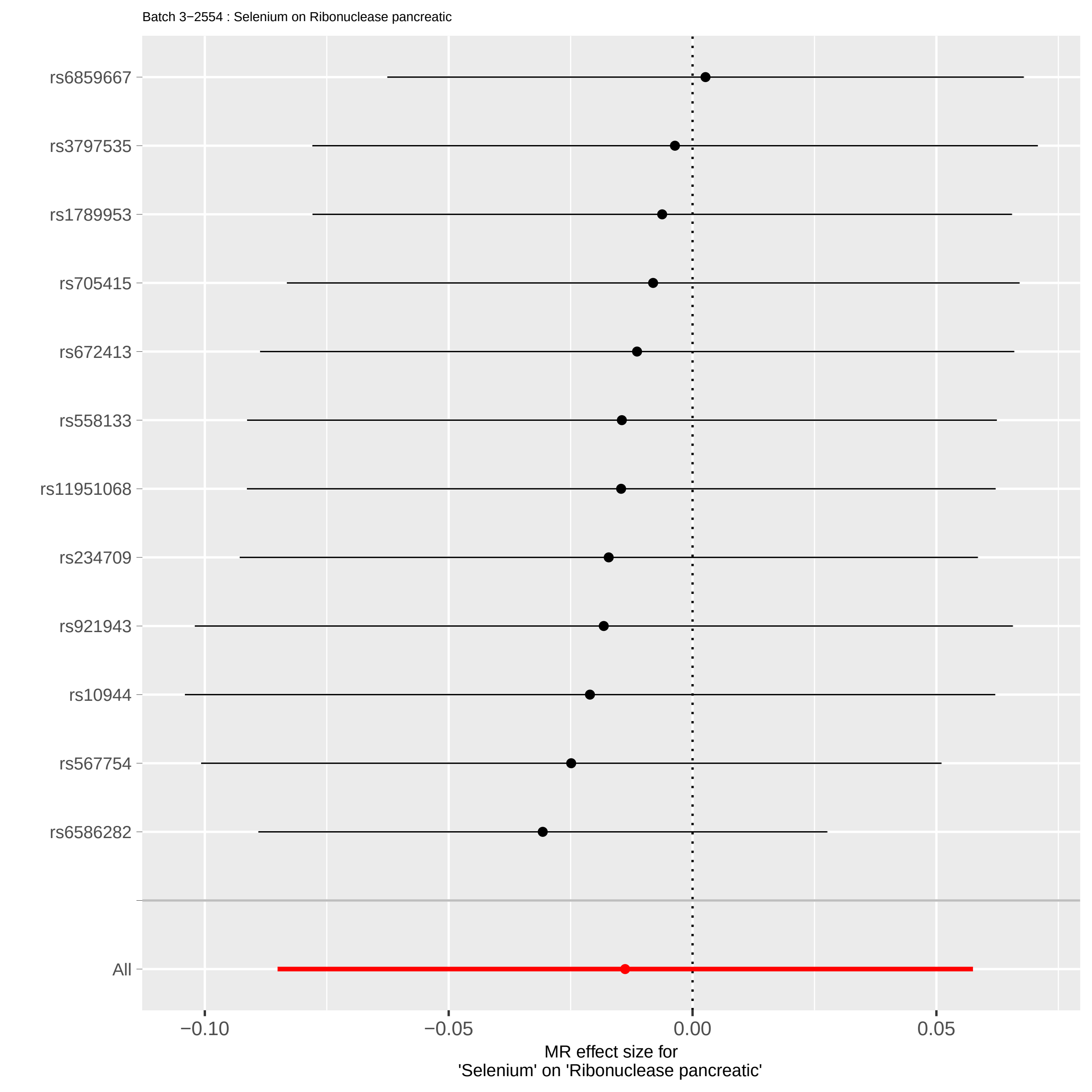
Batch 3−2554 : Selenium on Ribonuclease pancreatic
rs6859667
rs3797535
rs1789953
rs705415
rs672413
rs558133
rs11951068
rs234709
rs921943
rs10944
rs567754
rs6586282
All
−0.10
−0.05
0.00
0.05
MR effect size for
'Selenium' on 'Ribonuclease pancreatic'

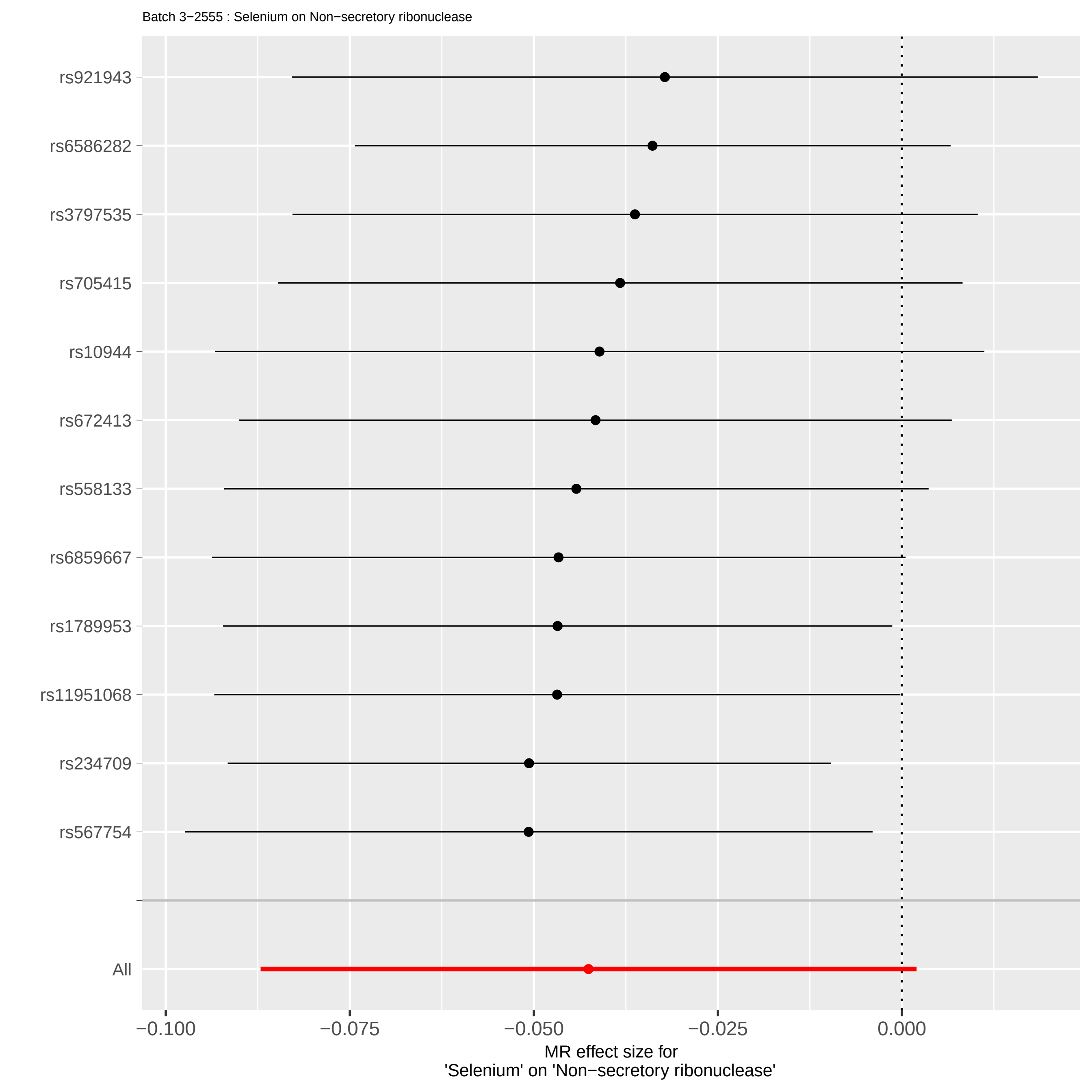

Batch 3−2555 : Selenium on Non−secretory ribonuclease
rs921943
rs6586282
rs3797535
rs705415
rs10944
rs672413
rs558133
rs6859667
rs1789953
rs11951068
rs234709
rs567754
All
−0.100
−0.075
−0.050
−0.025
0.000
MR effect size for
'Selenium' on 'Non−secretory ribonuclease'

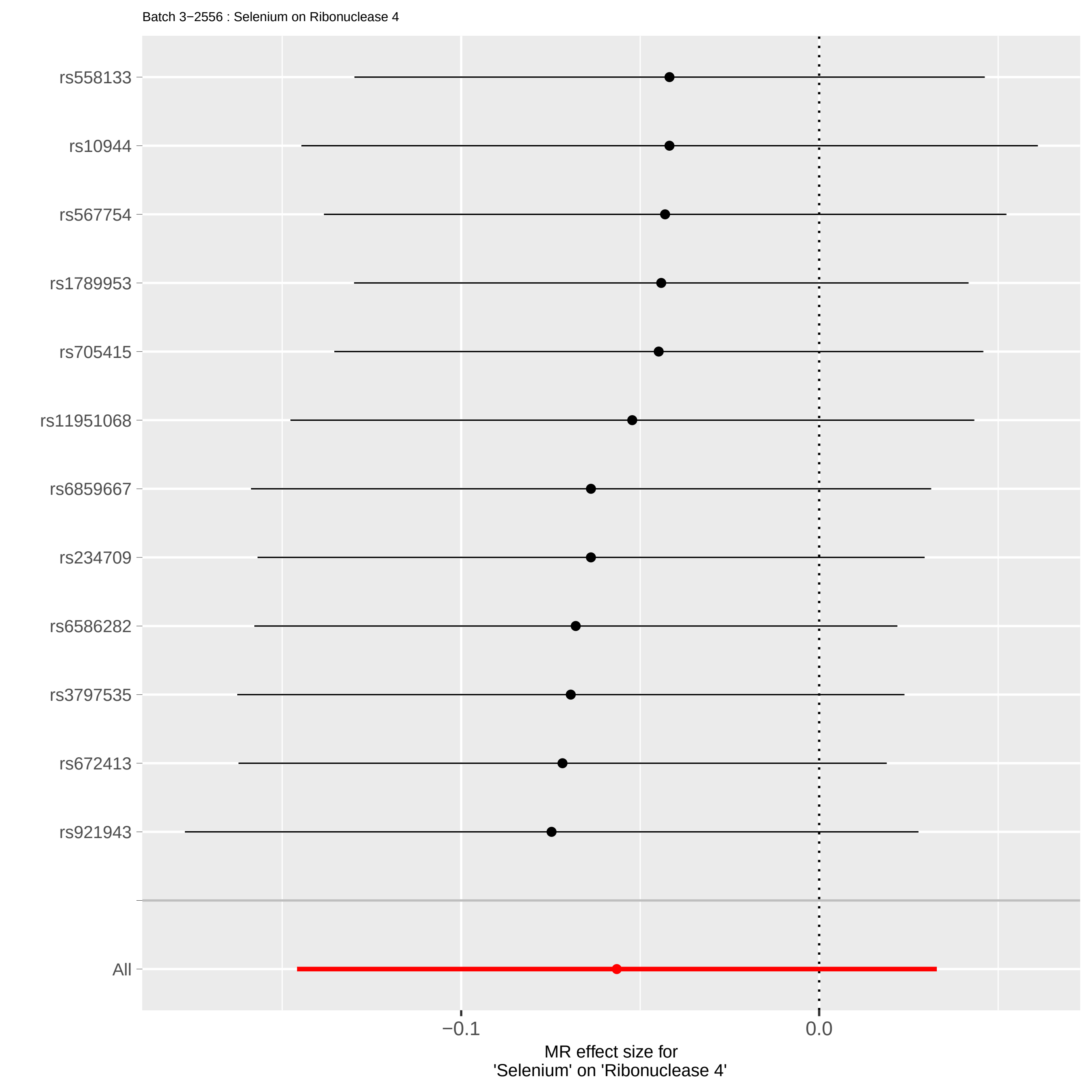
Batch 3−2556 : Selenium on Ribonuclease 4
rs558133
rs10944
rs567754
rs1789953
rs705415
rs11951068
rs6859667
rs234709
rs6586282
rs3797535
rs672413
rs921943
All
−0.1
0.0
MR effect size for
'Selenium' on 'Ribonuclease 4'

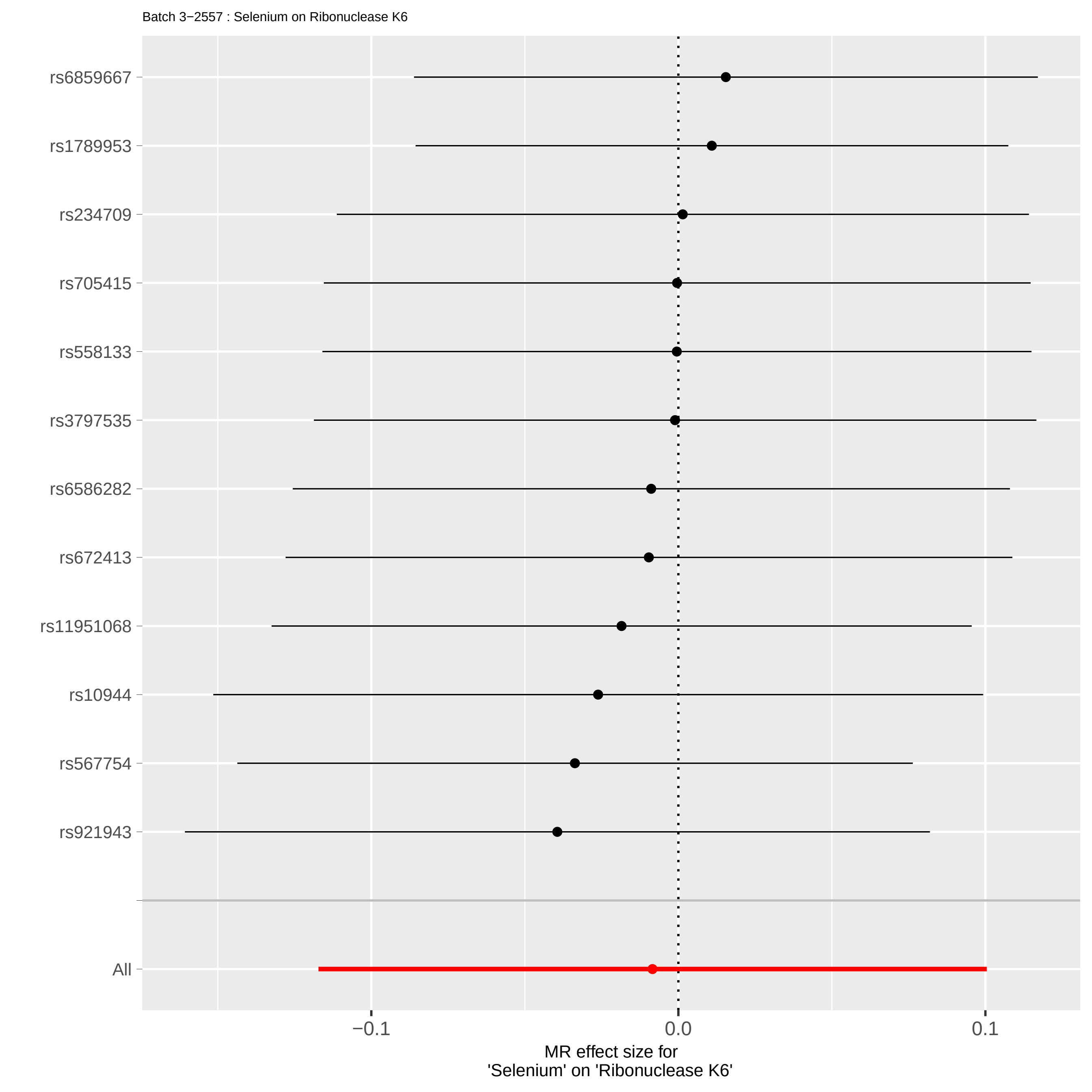

Batch 3−2557 : Selenium on Ribonuclease K6
rs6859667
rs1789953
rs234709
rs705415
rs558133
rs3797535
rs6586282
rs672413
rs11951068
rs10944
rs567754
rs921943
All
−0.1
0.0
0.1
MR effect size for
'Selenium' on 'Ribonuclease K6'

Batch 3−2558 : Selenium on Ribonuclease H1

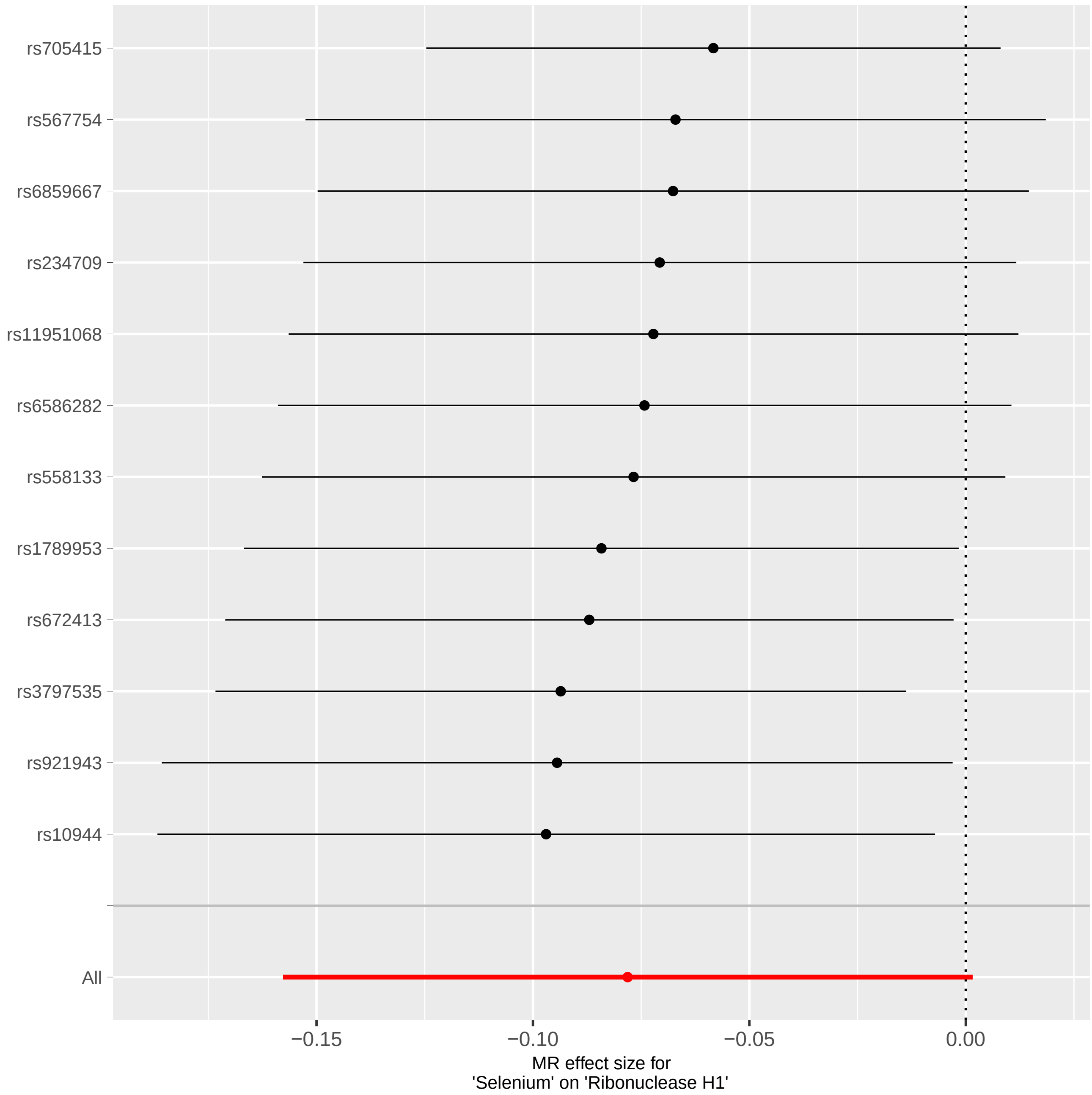

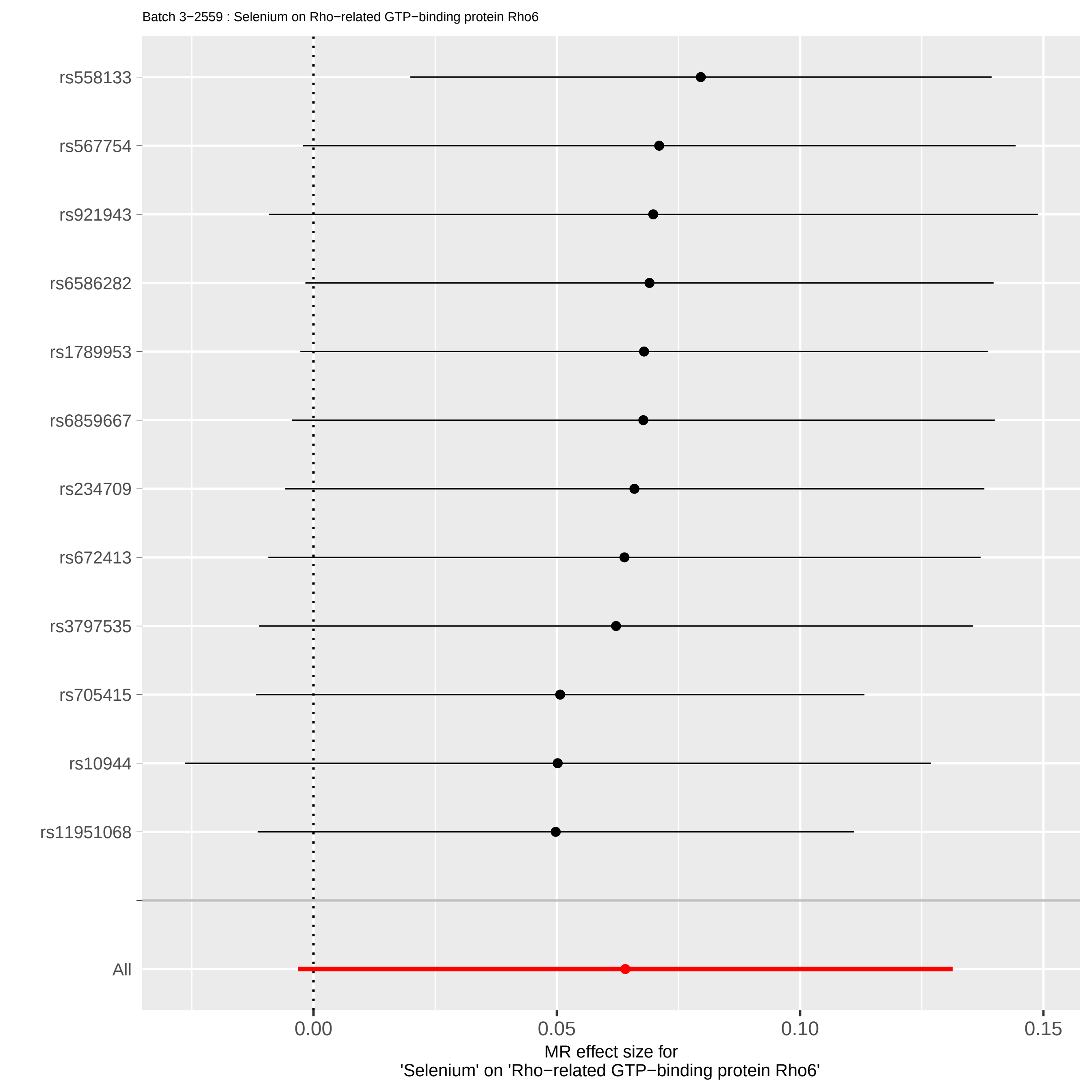

Batch 3−2559 : Selenium on Rho−related GTP−binding protein Rho6
rs558133
rs567754
rs921943
rs6586282
rs1789953
rs6859667
rs234709
rs672413
rs3797535
rs705415
rs10944
rs11951068
All
0.00
0.05
0.10
0.15
MR effect size for
'Selenium' on 'Rho−related GTP−binding protein Rho6'

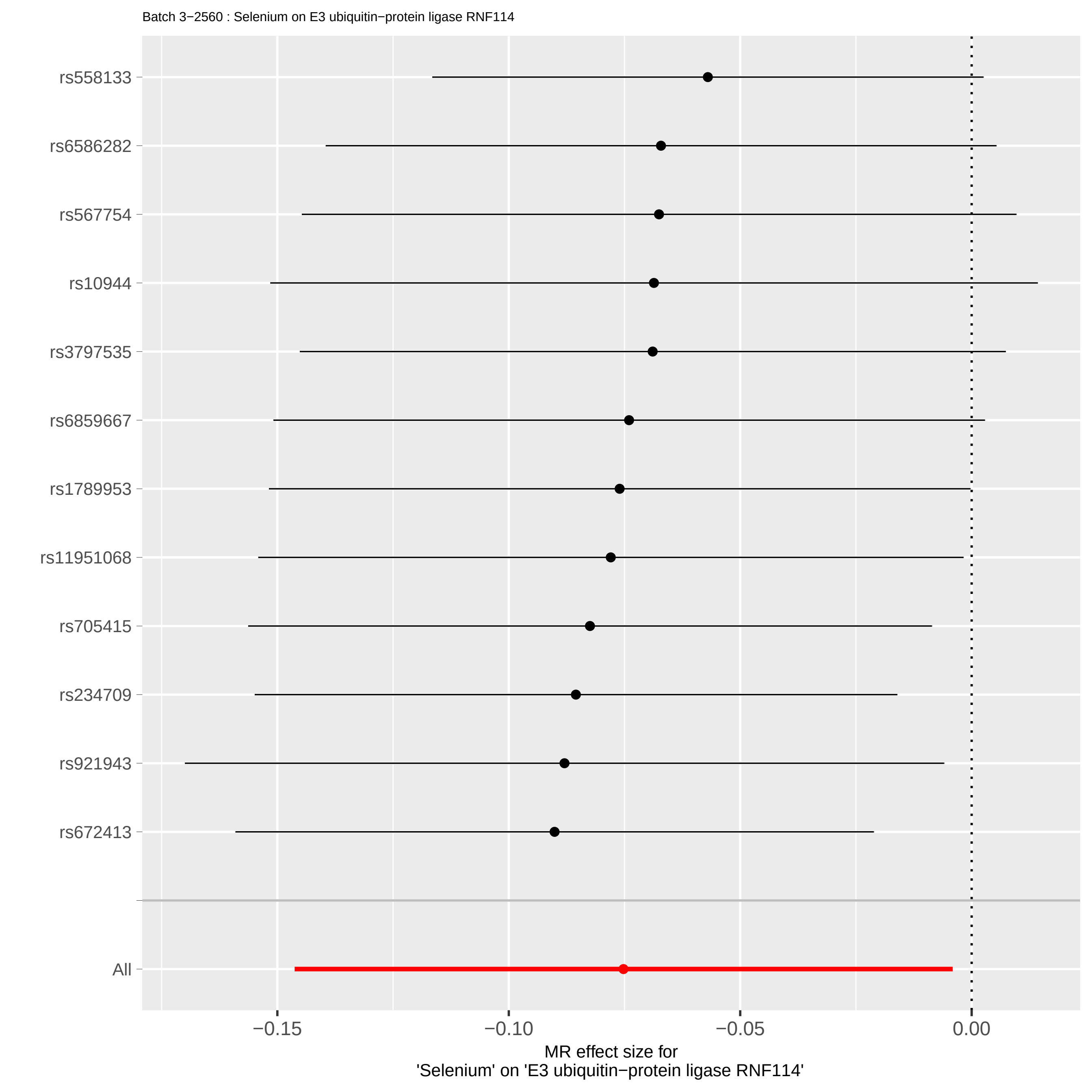

Batch 3−2560 : Selenium on E3 ubiquitin−protein ligase RNF114
rs558133
rs6586282
rs567754
rs10944
rs3797535
rs6859667
rs1789953
rs11951068
rs705415
rs234709
rs921943
rs672413
All
−0.15
−0.10
−0.05
0.00
MR effect size for
'Selenium' on 'E3 ubiquitin−protein ligase RNF114'

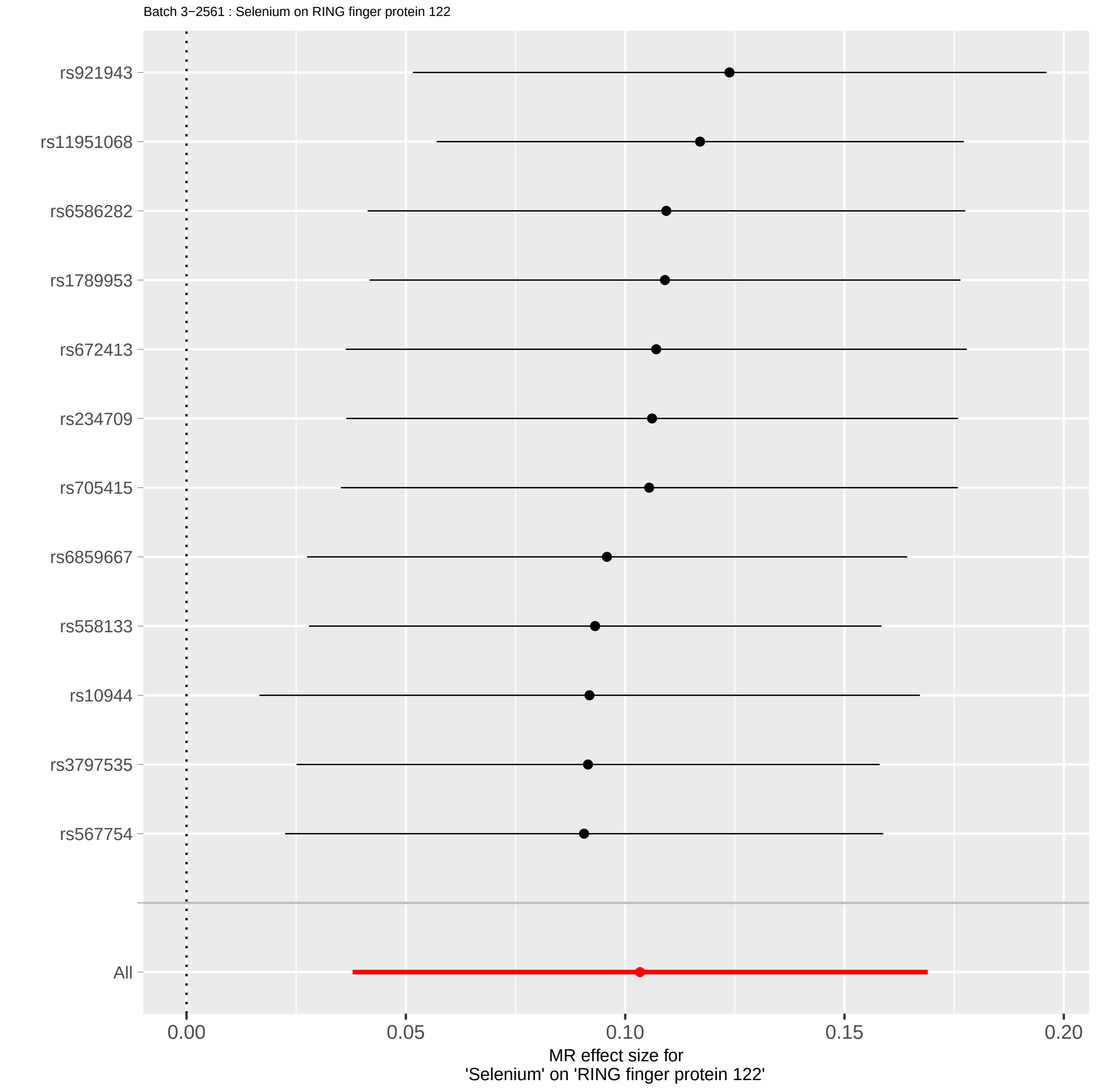
Batch 3−2561 : Selenium on RING finger protein 122
rs921943
rs11951068
rs6586282
rs1789953
rs672413
rs234709
rs705415
rs6859667
rs558133
rs10944
rs3797535
rs567754
All
0.00
0.05
0.10
0.15
0.20
MR effect size for
'Selenium' on 'RING finger protein 122'

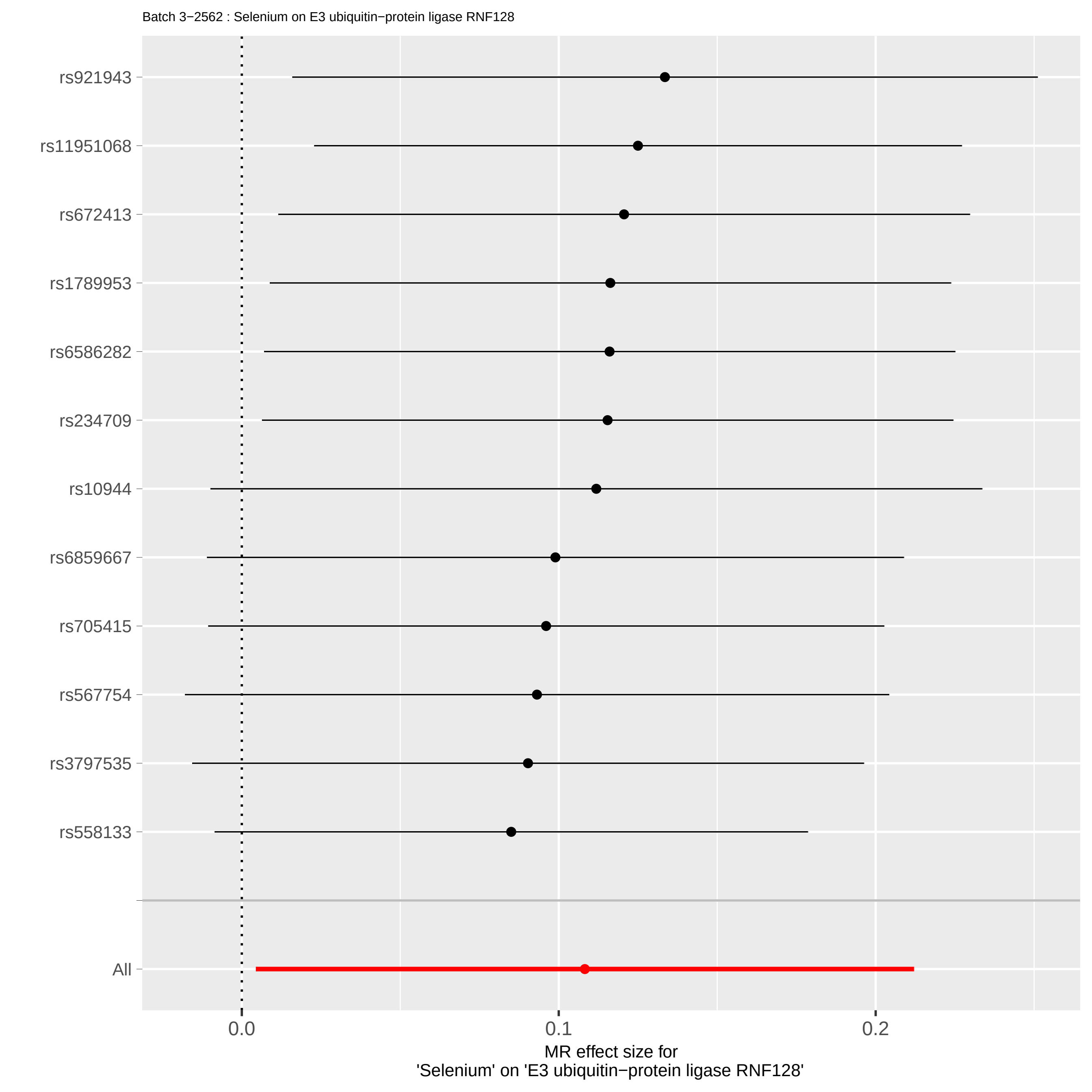

Batch 3−2562 : Selenium on E3 ubiquitin−protein ligase RNF128
rs921943
rs11951068
rs672413
rs1789953
rs6586282
rs234709
rs10944
rs6859667
rs705415
rs567754
rs3797535
rs558133
All
0.0
0.1
0.2
MR effect size for
'Selenium' on 'E3 ubiquitin−protein ligase RNF128'

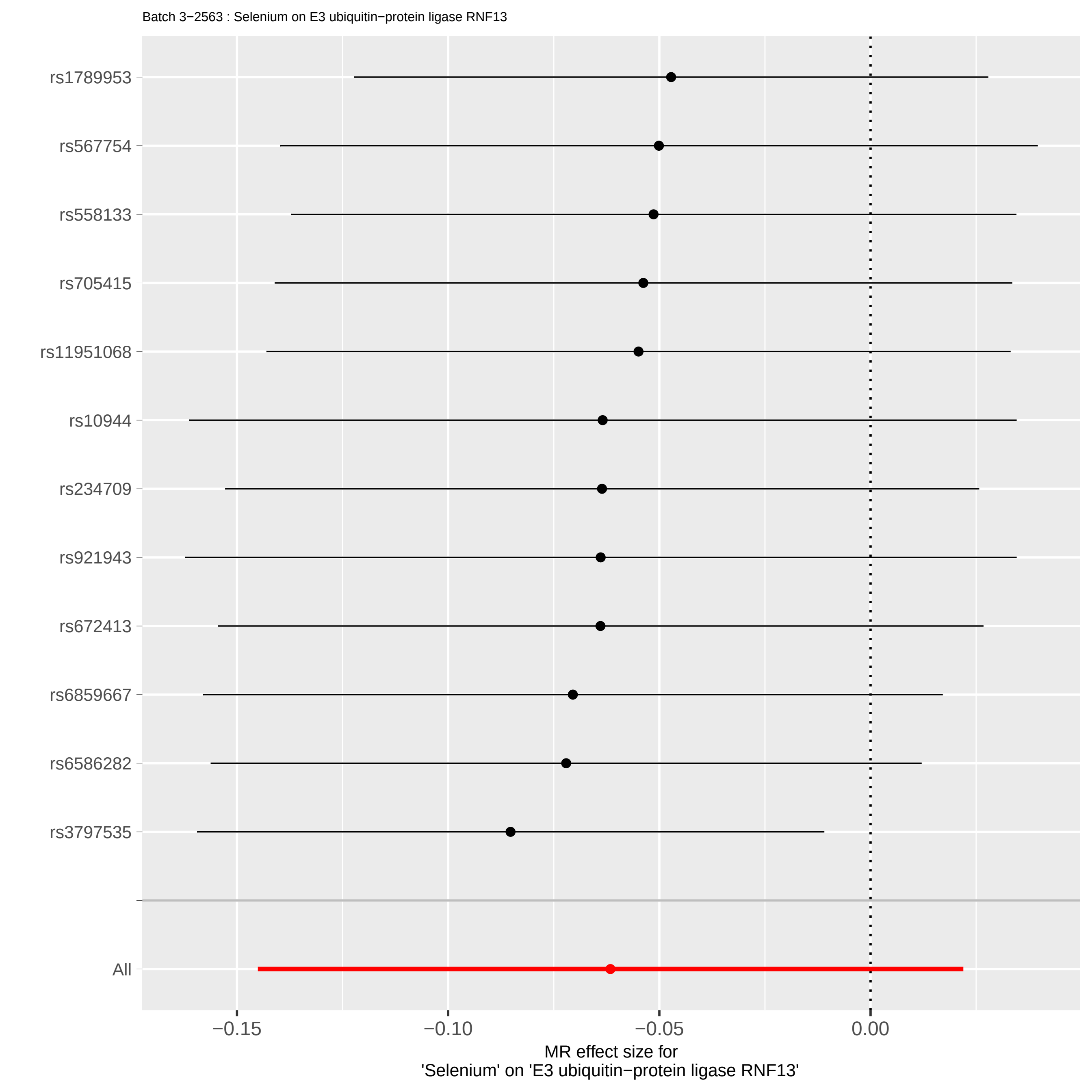
Batch 3−2563 : Selenium on E3 ubiquitin−protein ligase RNF13
rs1789953
rs567754
rs558133
rs705415
rs11951068
rs10944
rs234709
rs921943
rs672413
rs6859667
rs6586282
rs3797535
All
−0.15
−0.10
−0.05
0.00
MR effect size for
'Selenium' on 'E3 ubiquitin−protein ligase RNF13'

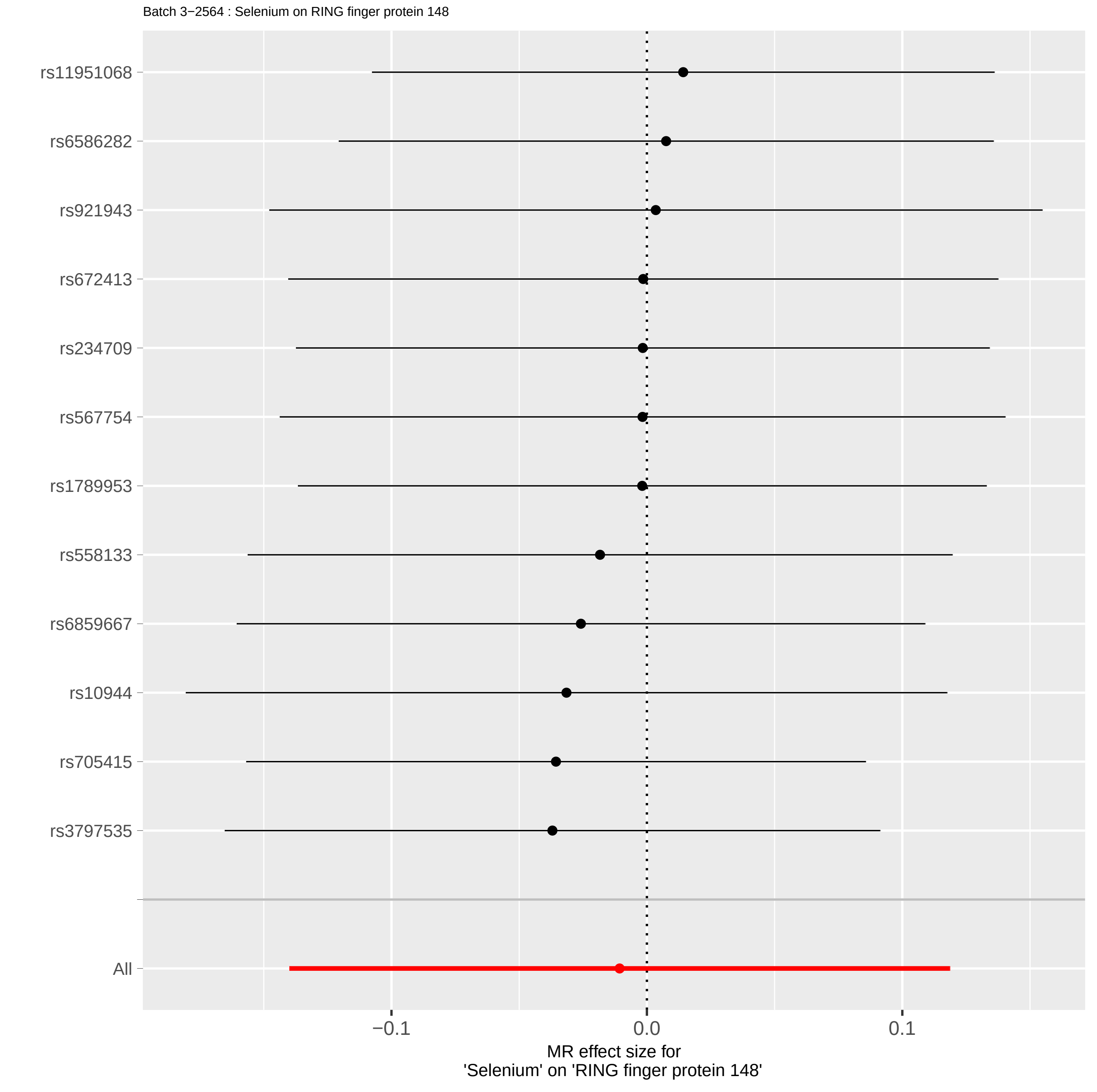

Batch 3−2564 : Selenium on RING finger protein 148
rs11951068
rs6586282
rs921943
rs672413
rs234709
rs567754
rs1789953
rs558133
rs6859667
rs10944
rs705415
rs3797535
All
−0.1
0.0
0.1
MR effect size for
'Selenium' on 'RING finger protein 148'

Batch 3−2565 : Selenium on RING finger protein 148

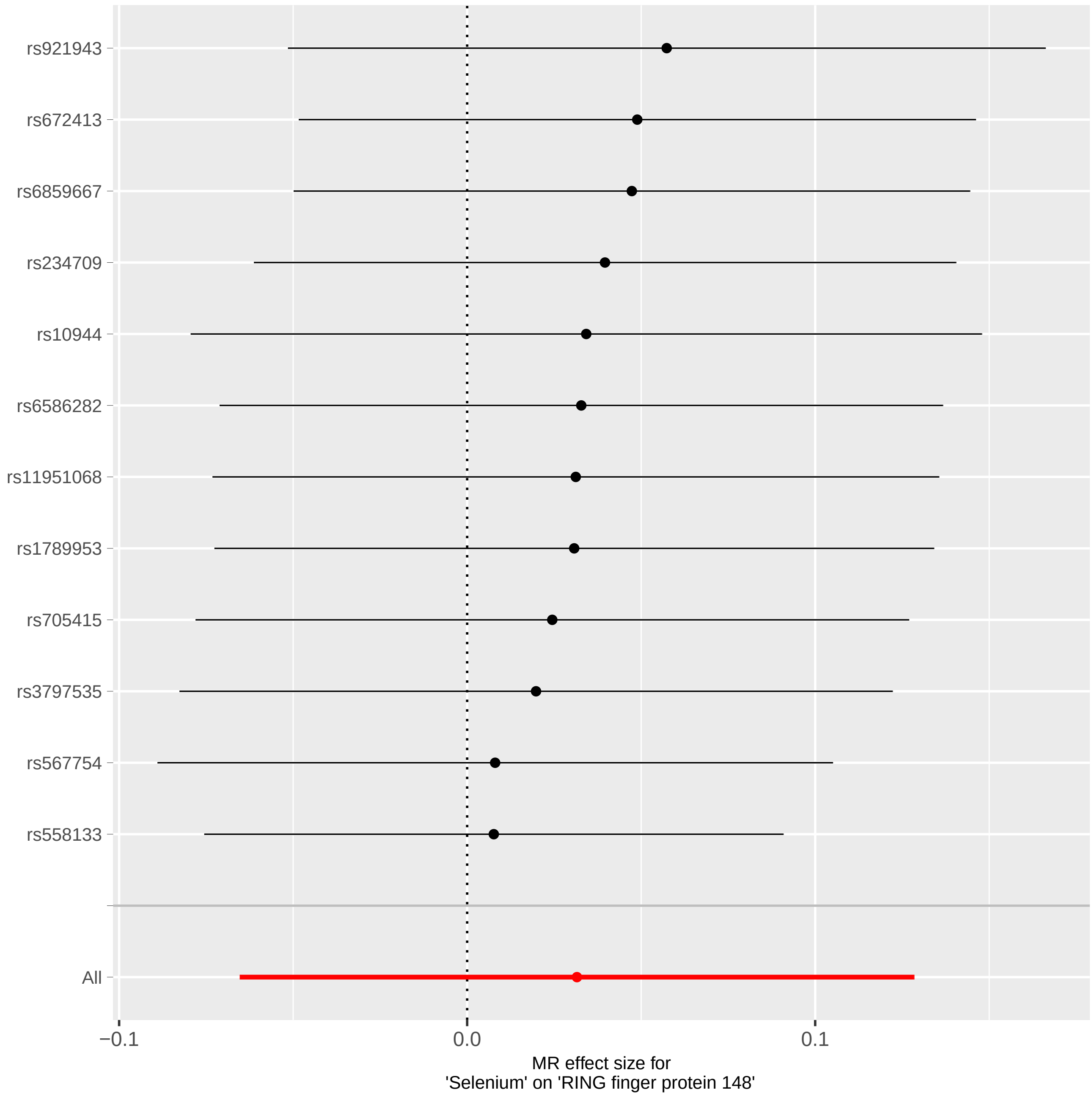

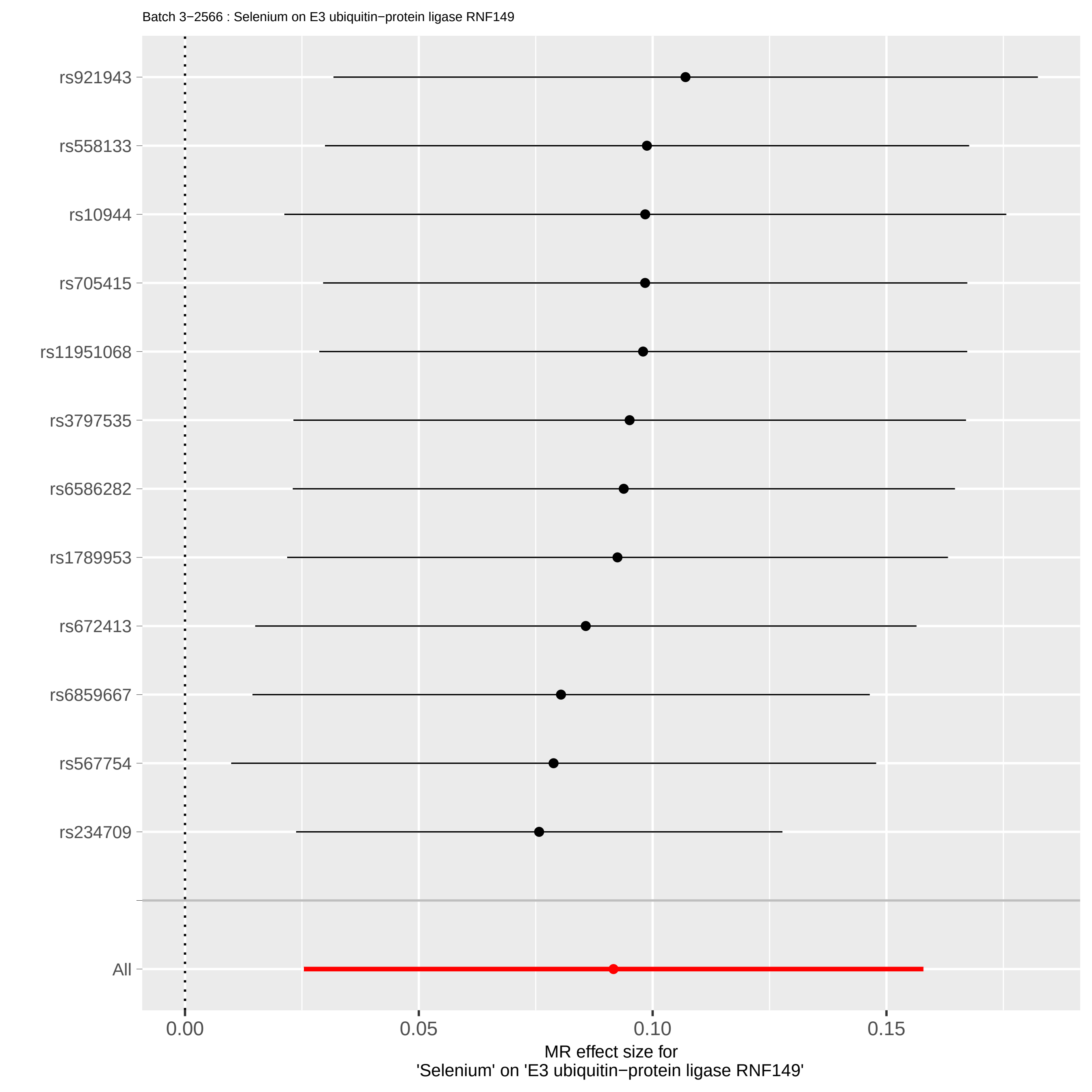
Batch 3−2566 : Selenium on E3 ubiquitin−protein ligase RNF149
rs921943
rs558133
rs10944
rs705415
rs11951068
rs3797535
rs6586282
rs1789953
rs672413
rs6859667
rs567754
rs234709
All
0.00
0.05
0.10
0.15
MR effect size for
'Selenium' on 'E3 ubiquitin−protein ligase RNF149'

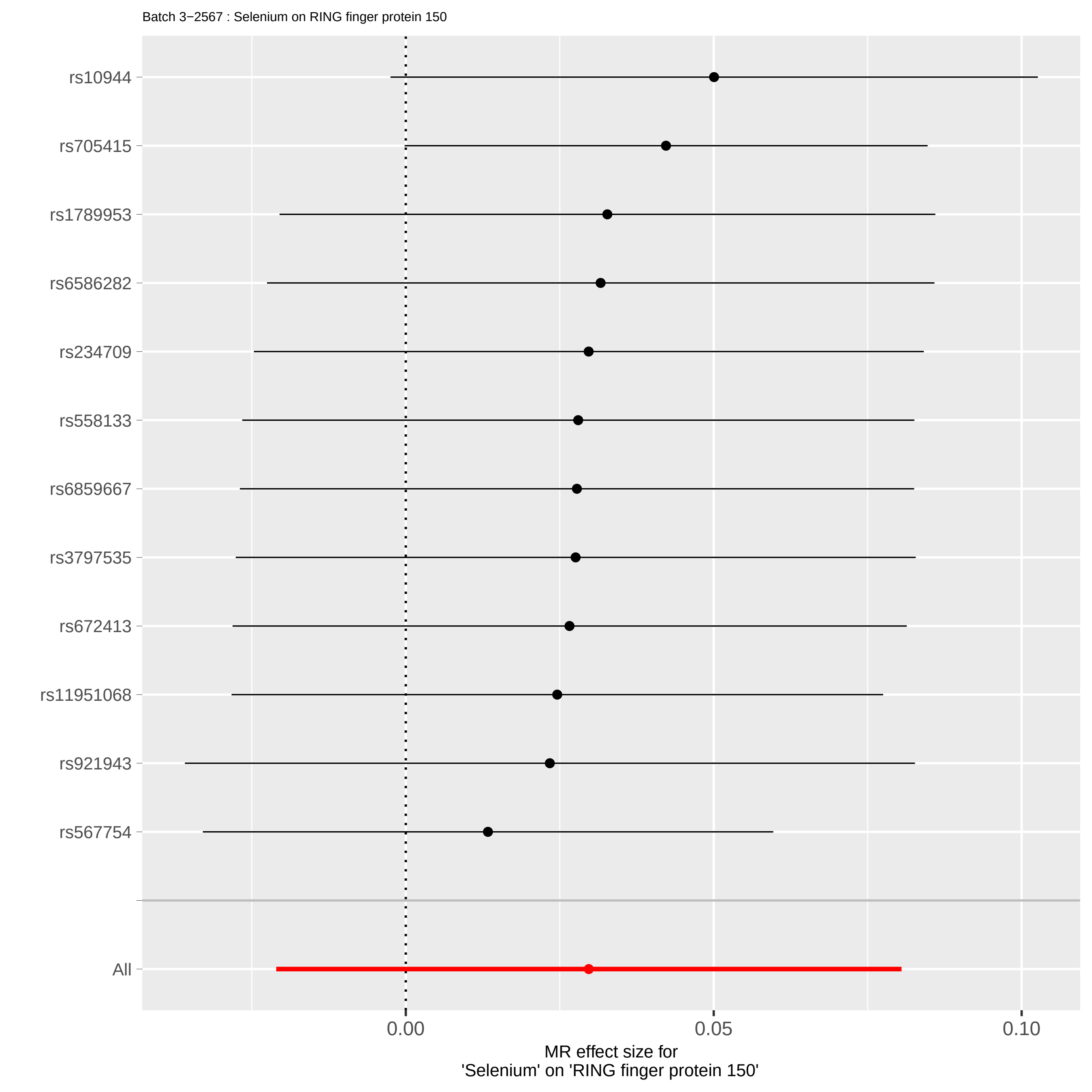

Batch 3−2567 : Selenium on RING finger protein 150
rs10944
rs705415
rs1789953
rs6586282
rs234709
rs558133
rs6859667
rs3797535
rs672413
rs11951068
rs921943
rs567754
All
0.00
0.05
0.10
MR effect size for
'Selenium' on 'RING finger protein 150'

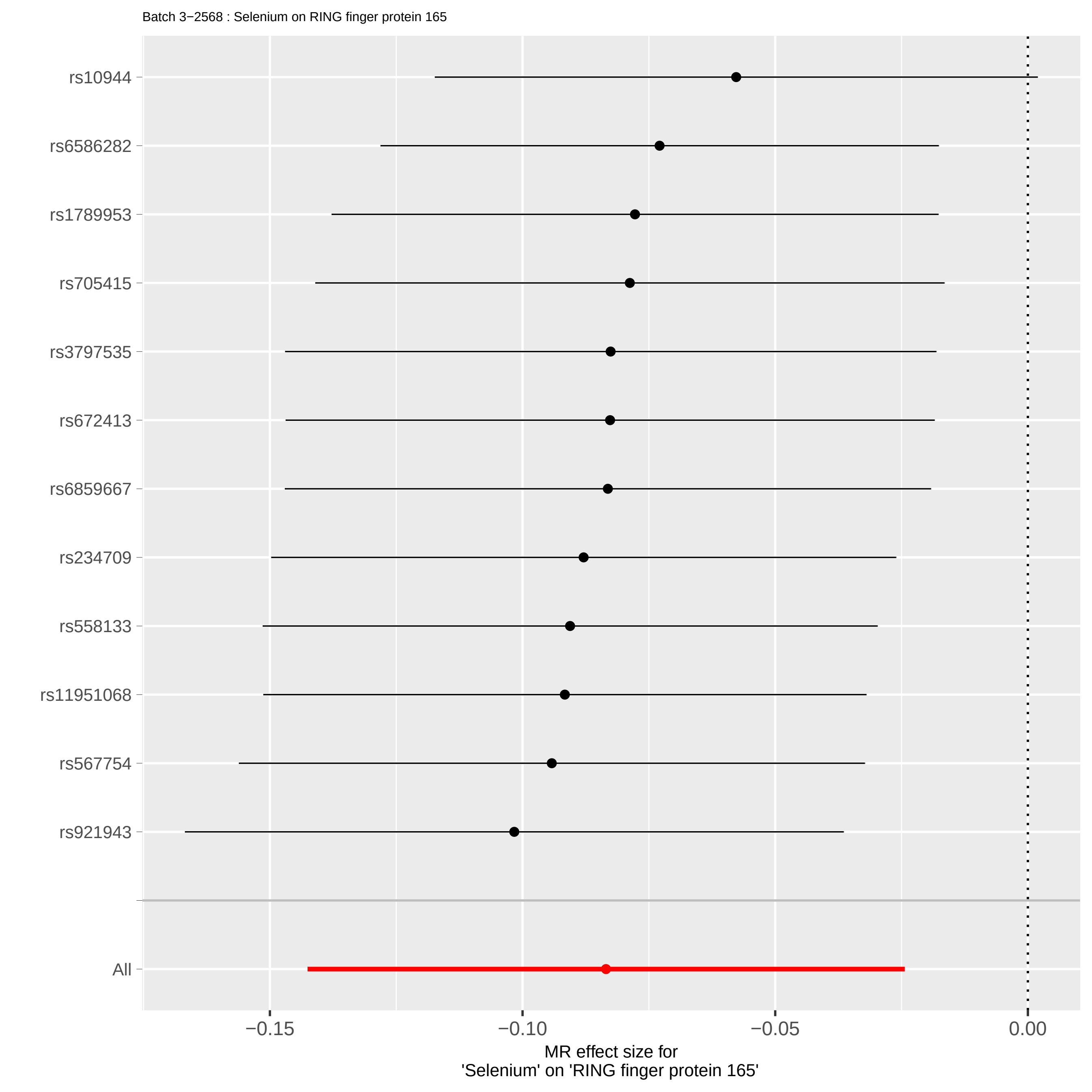

Batch 3−2568 : Selenium on RING finger protein 165
rs10944
rs6586282
rs1789953
rs705415
rs3797535
rs672413
rs6859667
rs234709
rs558133
rs11951068
rs567754
rs921943
All
−0.15
−0.10
−0.05
0.00
MR effect size for
'Selenium' on 'RING finger protein 165'

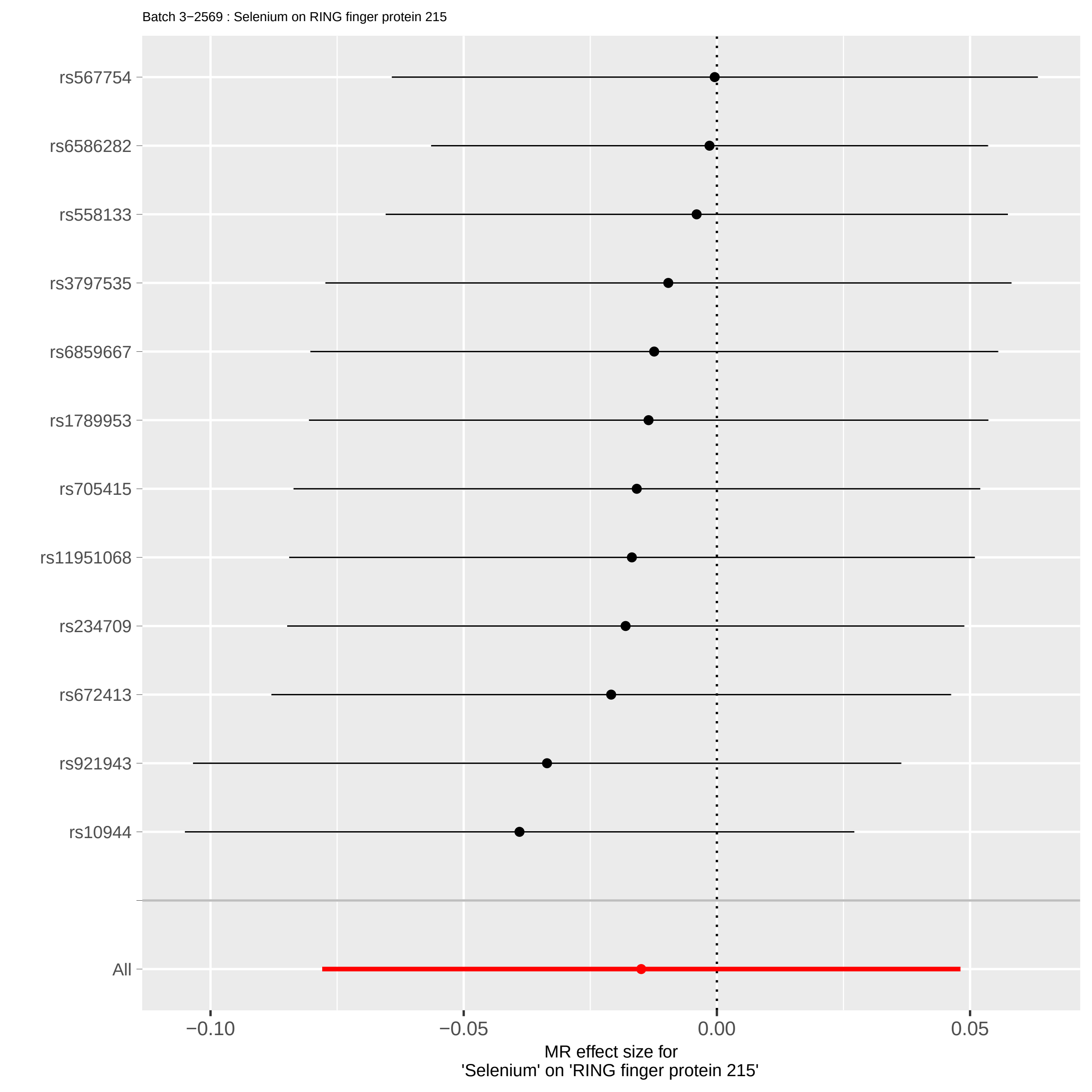
Batch 3−2569 : Selenium on RING finger protein 215
rs567754
rs6586282
rs558133
rs3797535
rs6859667
rs1789953
rs705415
rs11951068
rs234709
rs672413
rs921943
rs10944
All
−0.10
−0.05
0.00
0.05
MR effect size for
'Selenium' on 'RING finger protein 215'

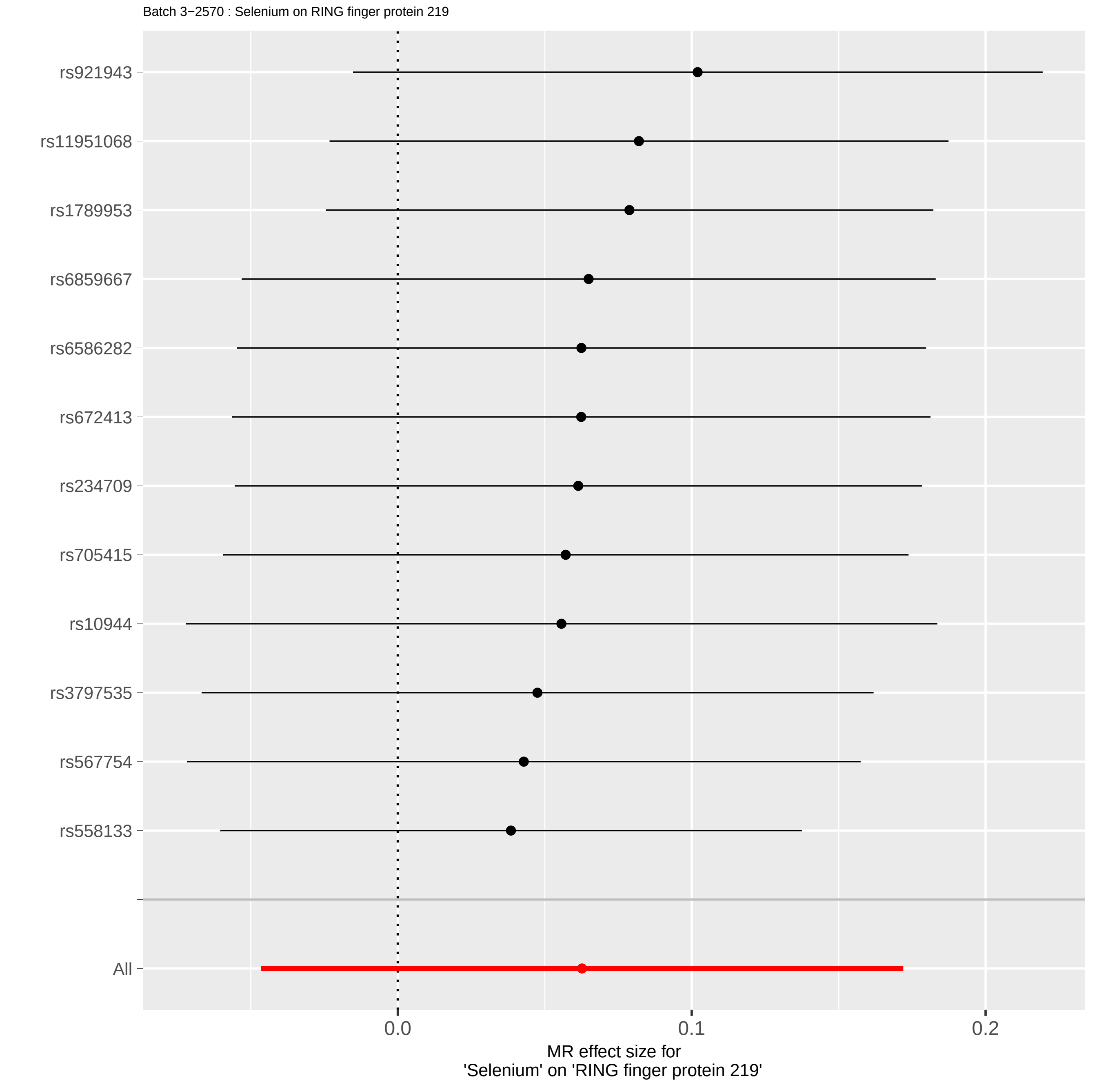

Batch 3−2570 : Selenium on RING finger protein 219
rs921943
rs11951068
rs1789953
rs6859667
rs6586282
rs672413
rs234709
rs705415
rs10944
rs3797535
rs567754
rs558133
All
0.0
0.1
0.2
MR effect size for
'Selenium' on 'RING finger protein 219'

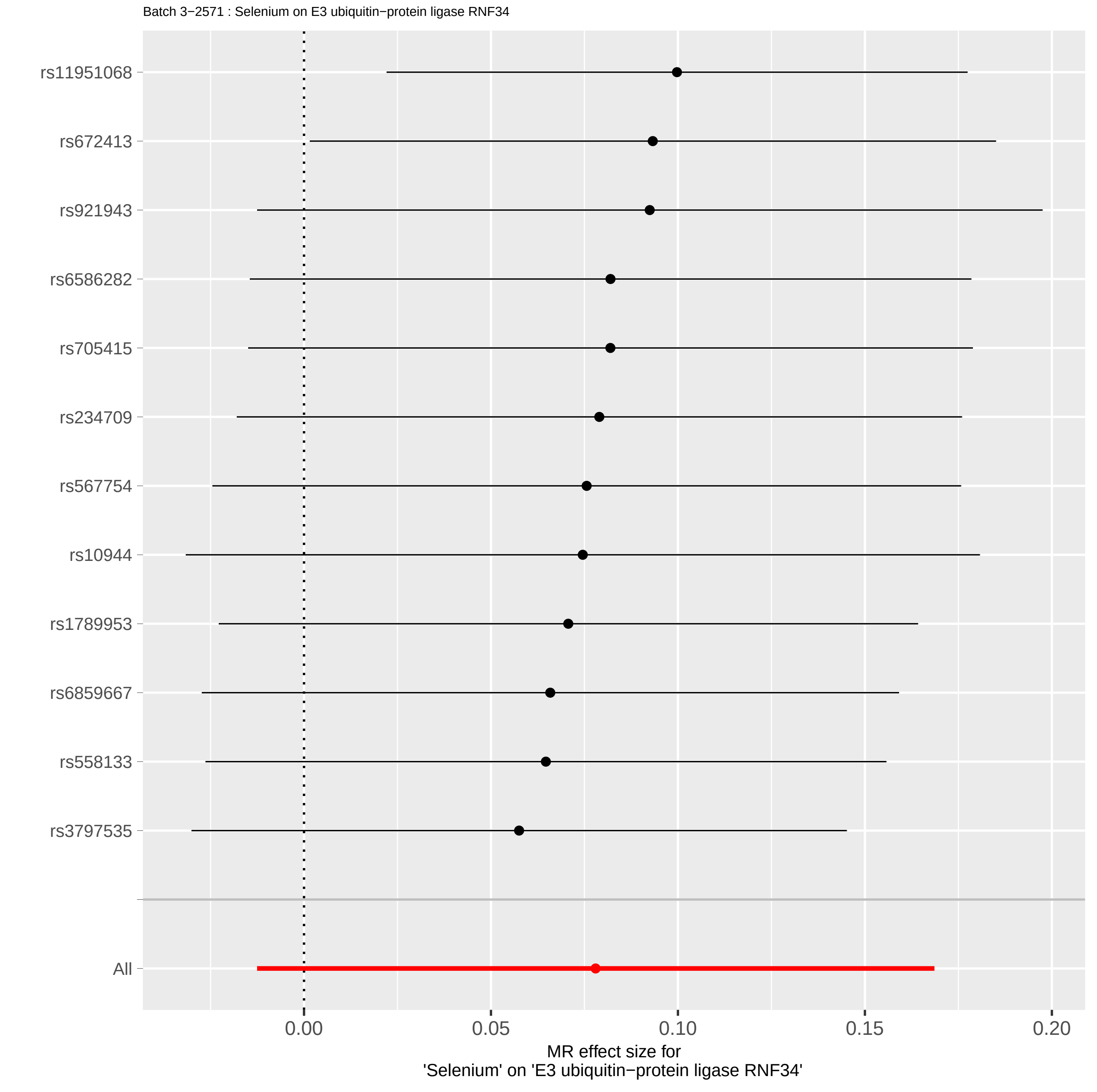

Batch 3−2571 : Selenium on E3 ubiquitin−protein ligase RNF34
rs11951068
rs672413
rs921943
rs6586282
rs705415
rs234709
rs567754
rs10944
rs1789953
rs6859667
rs558133
rs3797535
All
0.00
0.05
0.10
0.15
0.20
MR effect size for
'Selenium' on 'E3 ubiquitin−protein ligase RNF34'

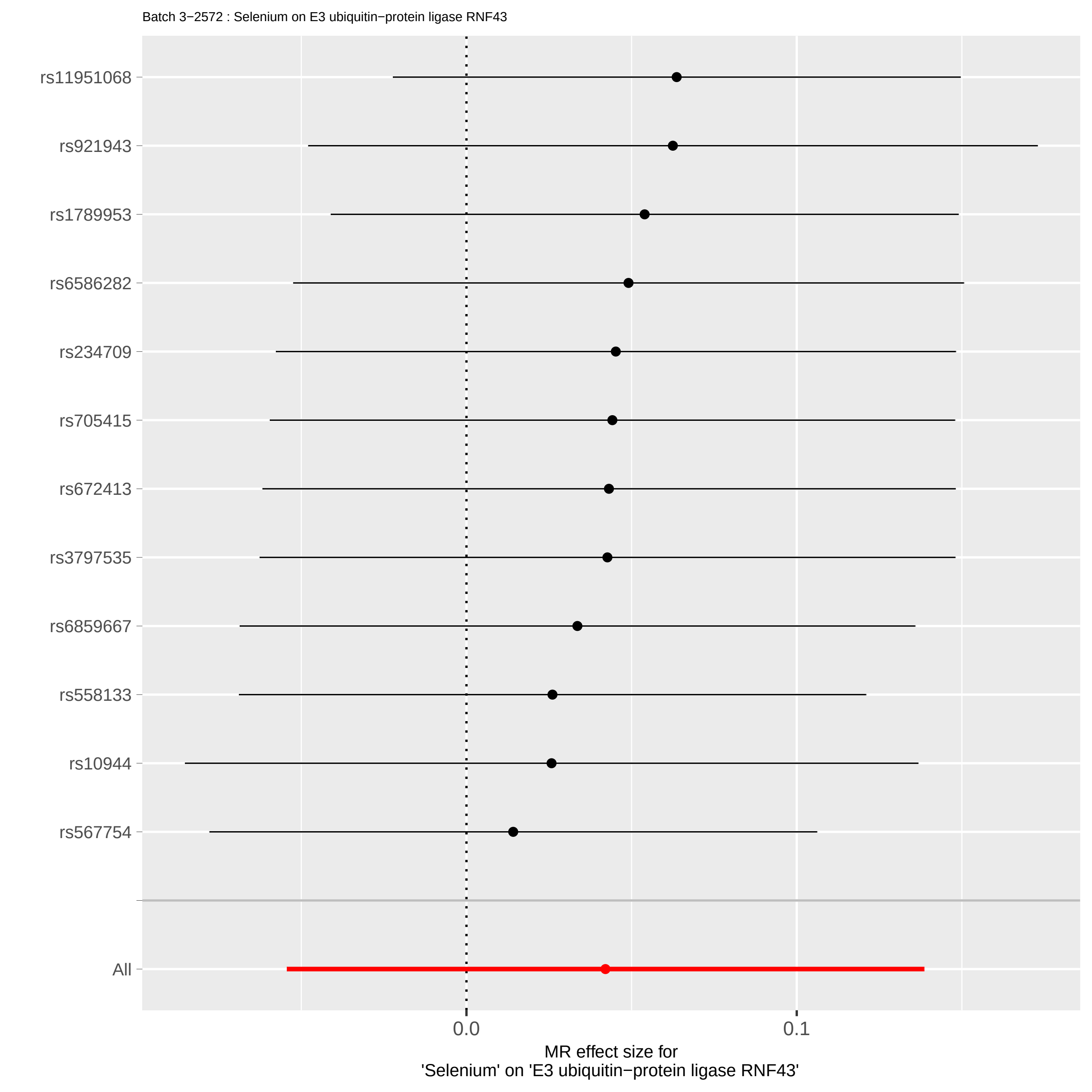

Batch 3−2572 : Selenium on E3 ubiquitin−protein ligase RNF43
rs11951068
rs921943
rs1789953
rs6586282
rs234709
rs705415
rs672413
rs3797535
rs6859667
rs558133
rs10944
rs567754
All
0.0
0.1
MR effect size for
'Selenium' on 'E3 ubiquitin−protein ligase RNF43'

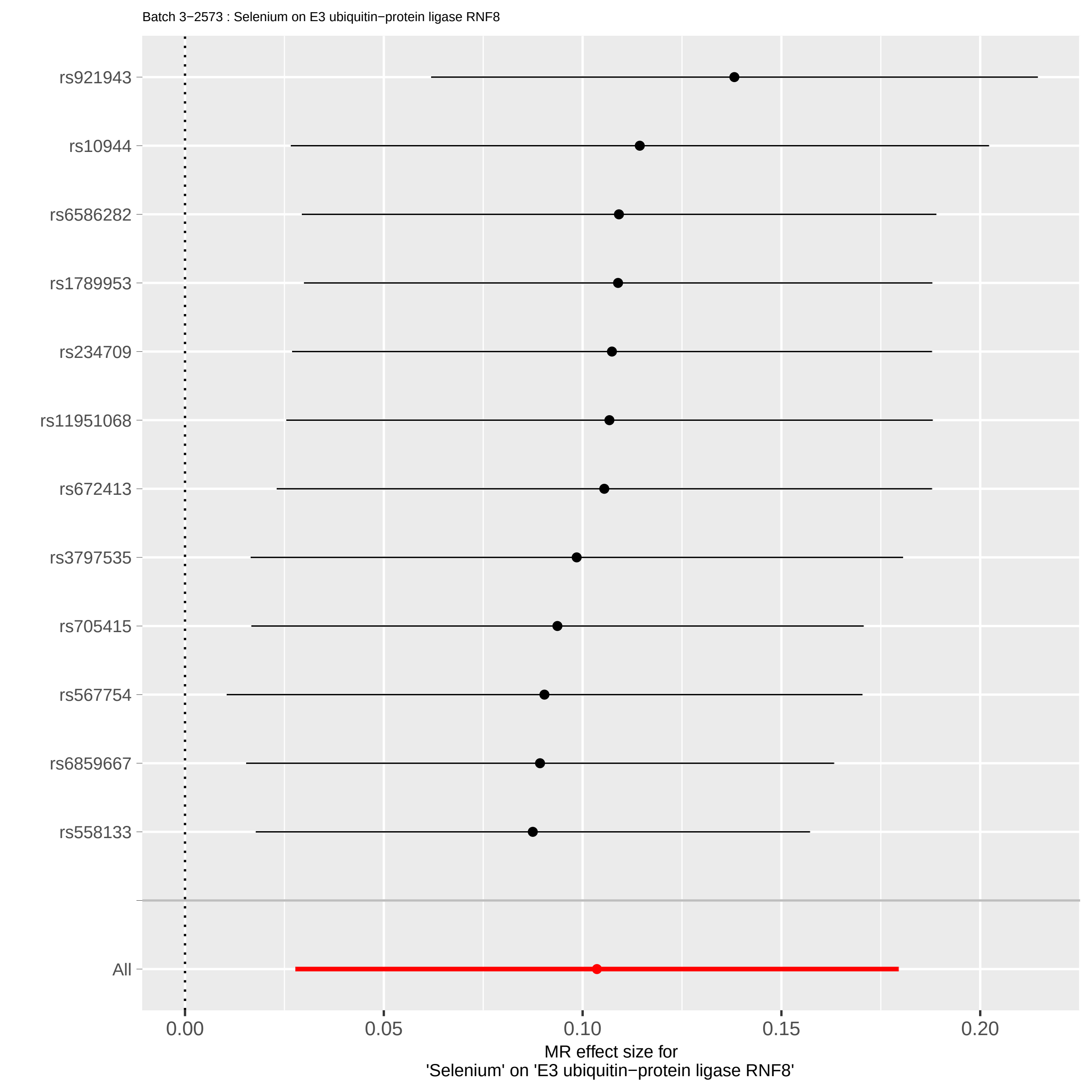

Batch 3−2573 : Selenium on E3 ubiquitin−protein ligase RNF8
rs921943
rs10944
rs6586282
rs1789953
rs234709
rs11951068
rs672413
rs3797535
rs705415
rs567754
rs6859667
rs558133
All
0.00
0.05
0.10
0.15
0.20
MR effect size for
'Selenium' on 'E3 ubiquitin−protein ligase RNF8'

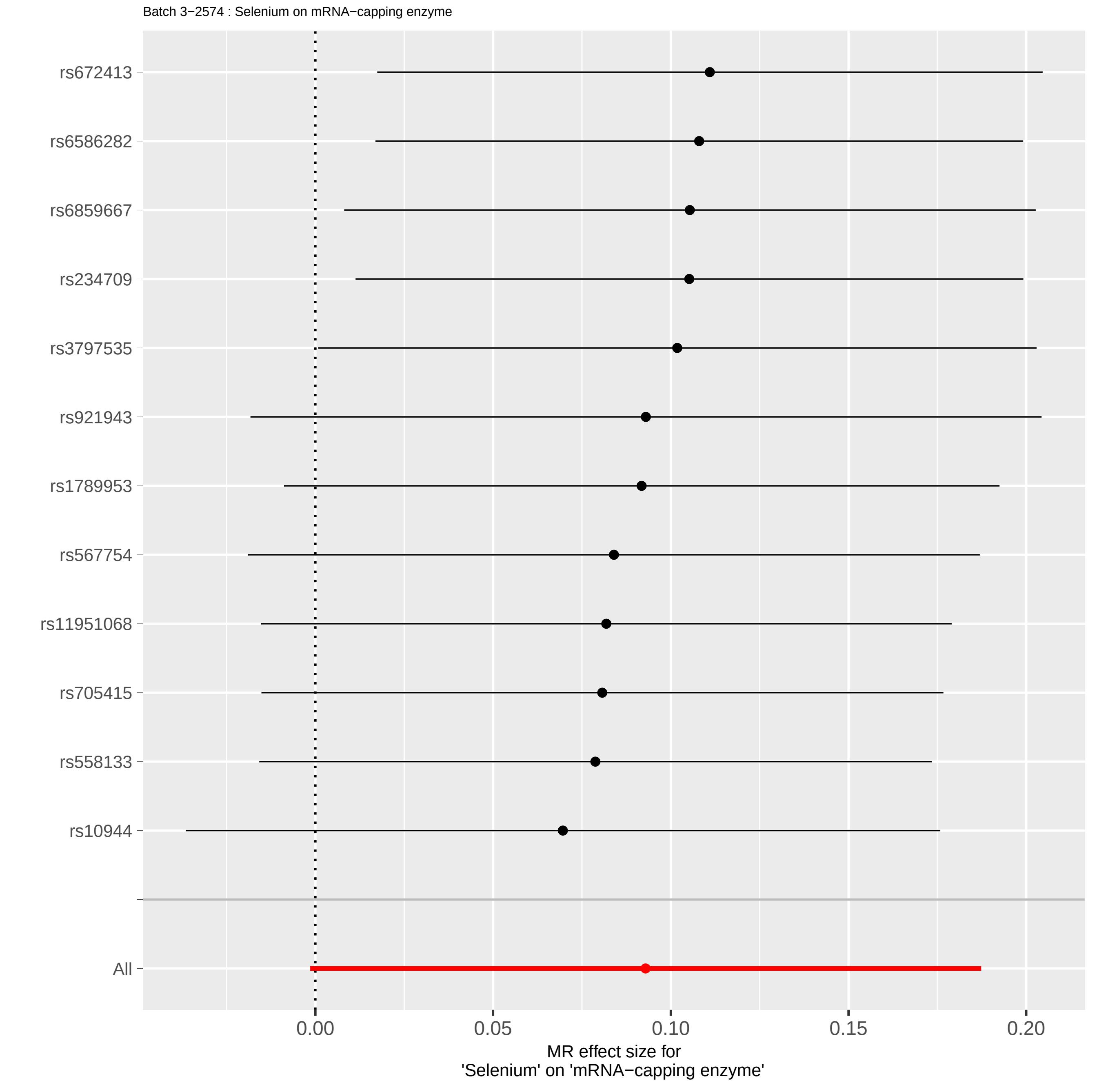

Batch 3−2574 : Selenium on mRNA−capping enzyme
rs672413
rs6586282
rs6859667
rs234709
rs3797535
rs921943
rs1789953
rs567754
rs11951068
rs705415
rs558133
rs10944
All
0.00
0.05
0.10
0.15
0.20
MR effect size for
'Selenium' on 'mRNA−capping enzyme'

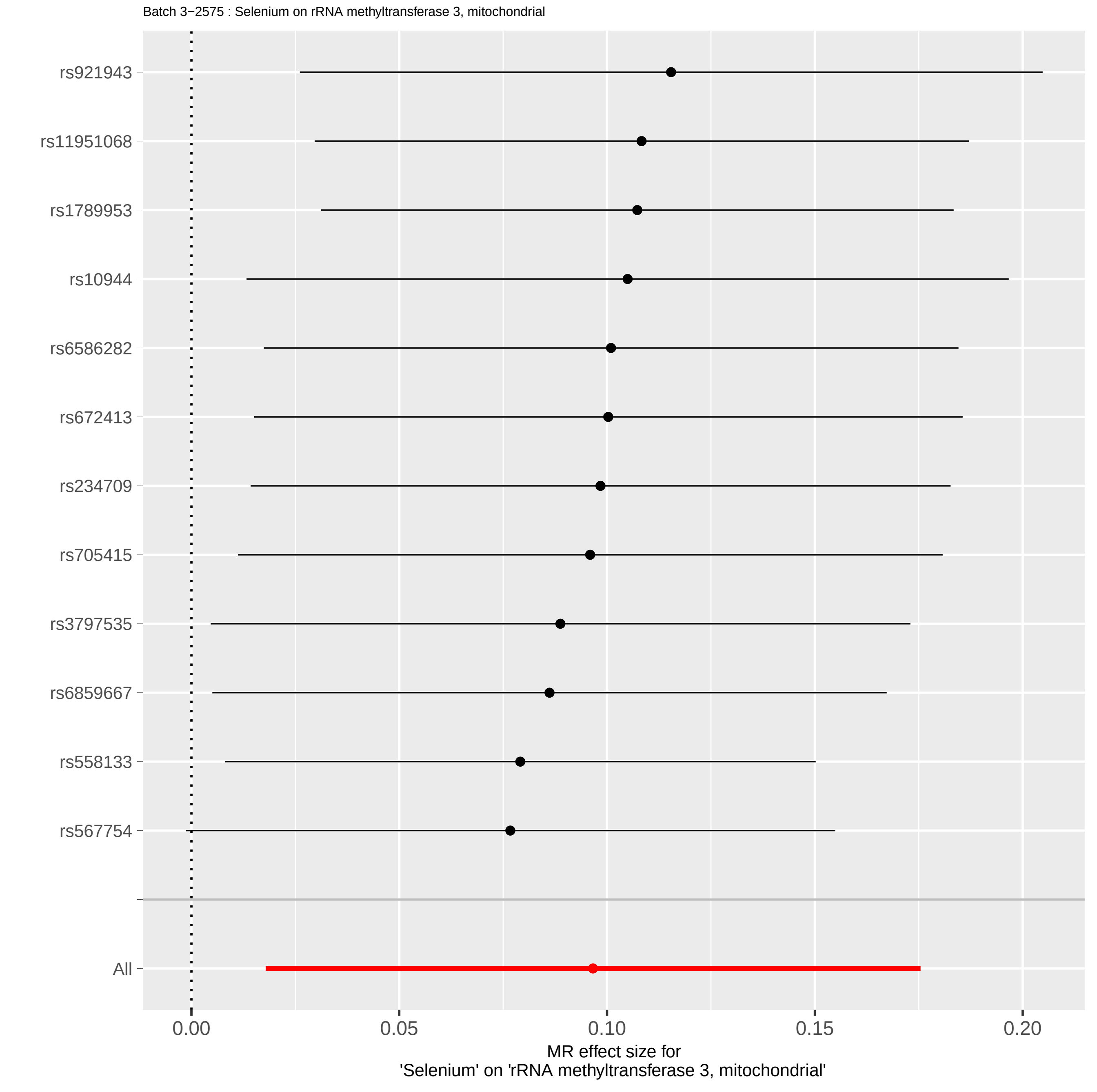

Batch 3−2575 : Selenium on rRNA methyltransferase 3, mitochondrial
rs921943
rs11951068
rs1789953
rs10944
rs6586282
rs672413
rs234709
rs705415
rs3797535
rs6859667
rs558133
rs567754
All
0.00
0.05
0.10
0.15
0.20
MR effect size for
'Selenium' on 'rRNA methyltransferase 3, mitochondrial'

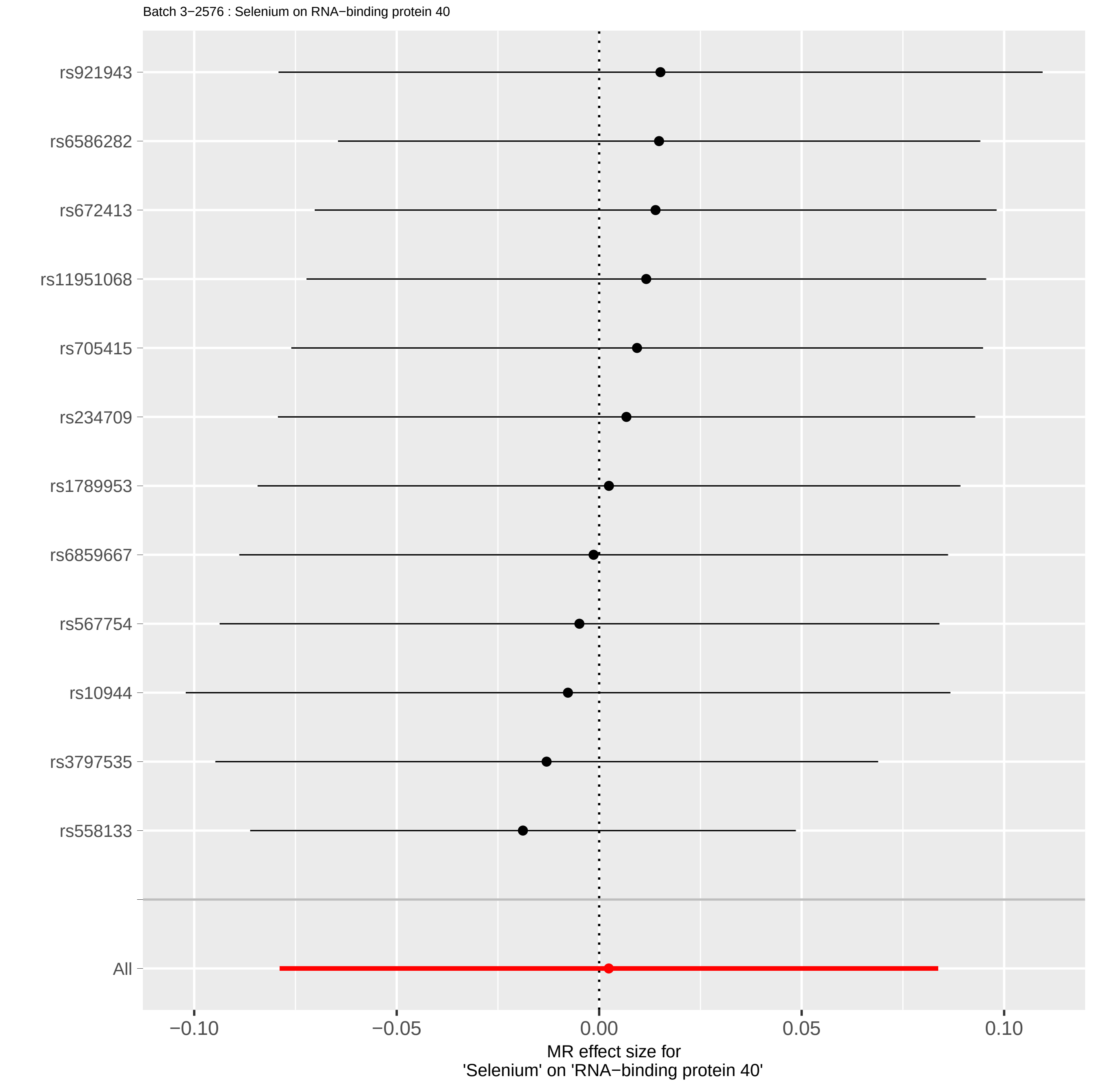

Batch 3−2576 : Selenium on RNA−binding protein 40
rs921943
rs6586282
rs672413
rs11951068
rs705415
rs234709
rs1789953
rs6859667
rs567754
rs10944
rs3797535
rs558133
All
−0.10
−0.05
0.00
0.05
0.10
MR effect size for
'Selenium' on 'RNA−binding protein 40'

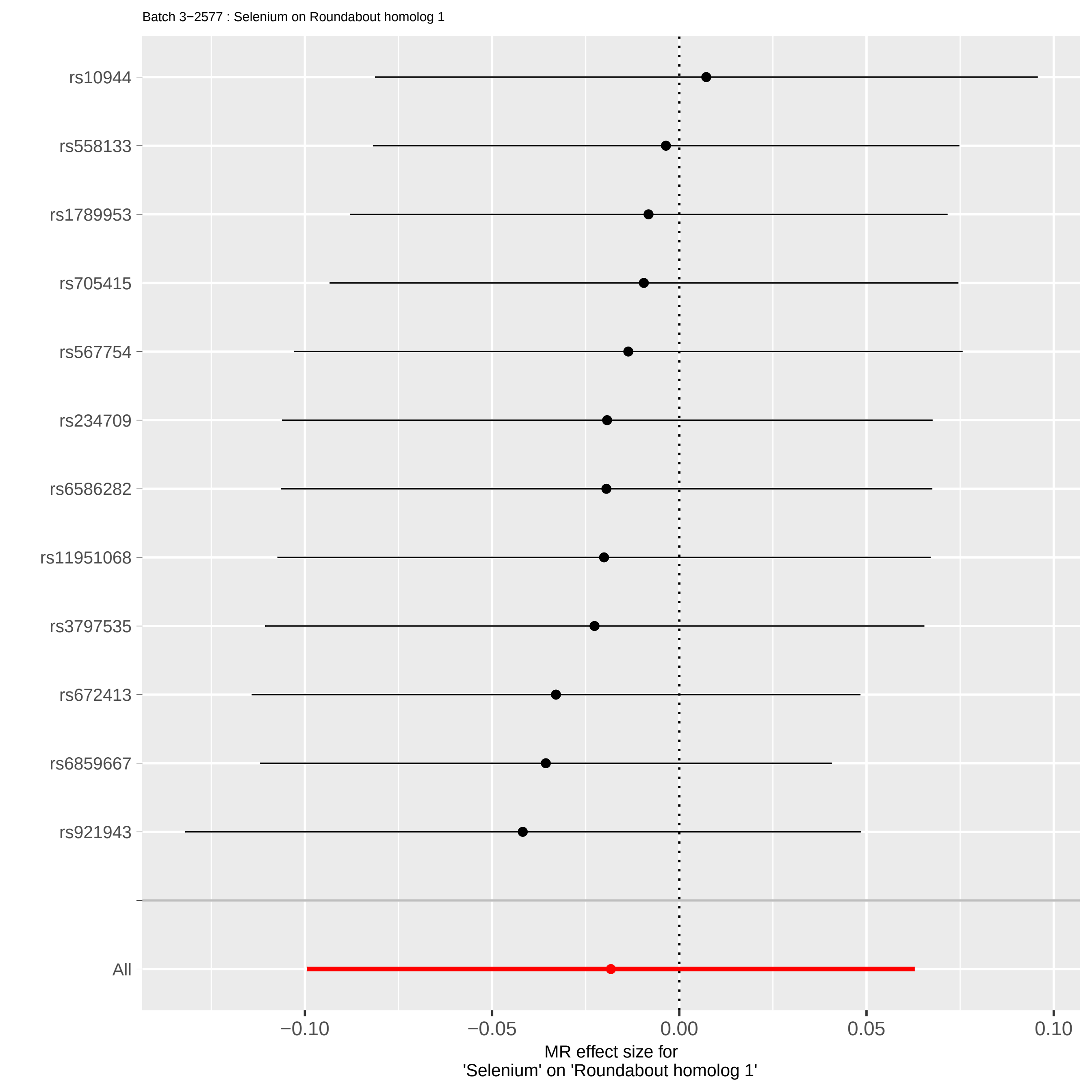

Batch 3−2577 : Selenium on Roundabout homolog 1
rs10944
rs558133
rs1789953
rs705415
rs567754
rs234709
rs6586282
rs11951068
rs3797535
rs672413
rs6859667
rs921943
All
−0.10
−0.05
0.00
0.05
0.10
MR effect size for
'Selenium' on 'Roundabout homolog 1'

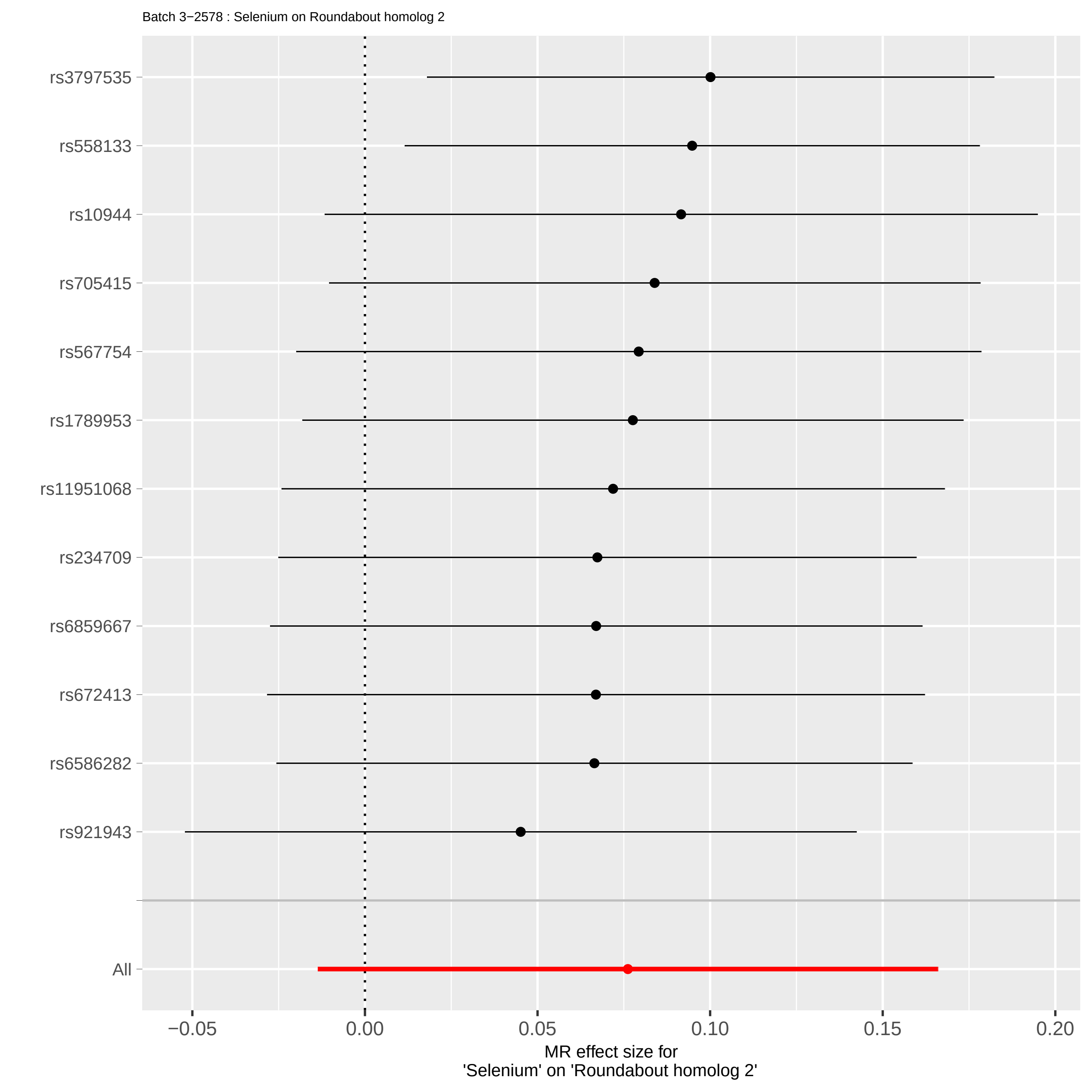
Batch 3−2578 : Selenium on Roundabout homolog 2
rs3797535
rs558133
rs10944
rs705415
rs567754
rs1789953
rs11951068
rs234709
rs6859667
rs672413
rs6586282
rs921943
All
−0.05
0.00
0.05
0.10
0.15
0.20
MR effect size for
'Selenium' on 'Roundabout homolog 2'

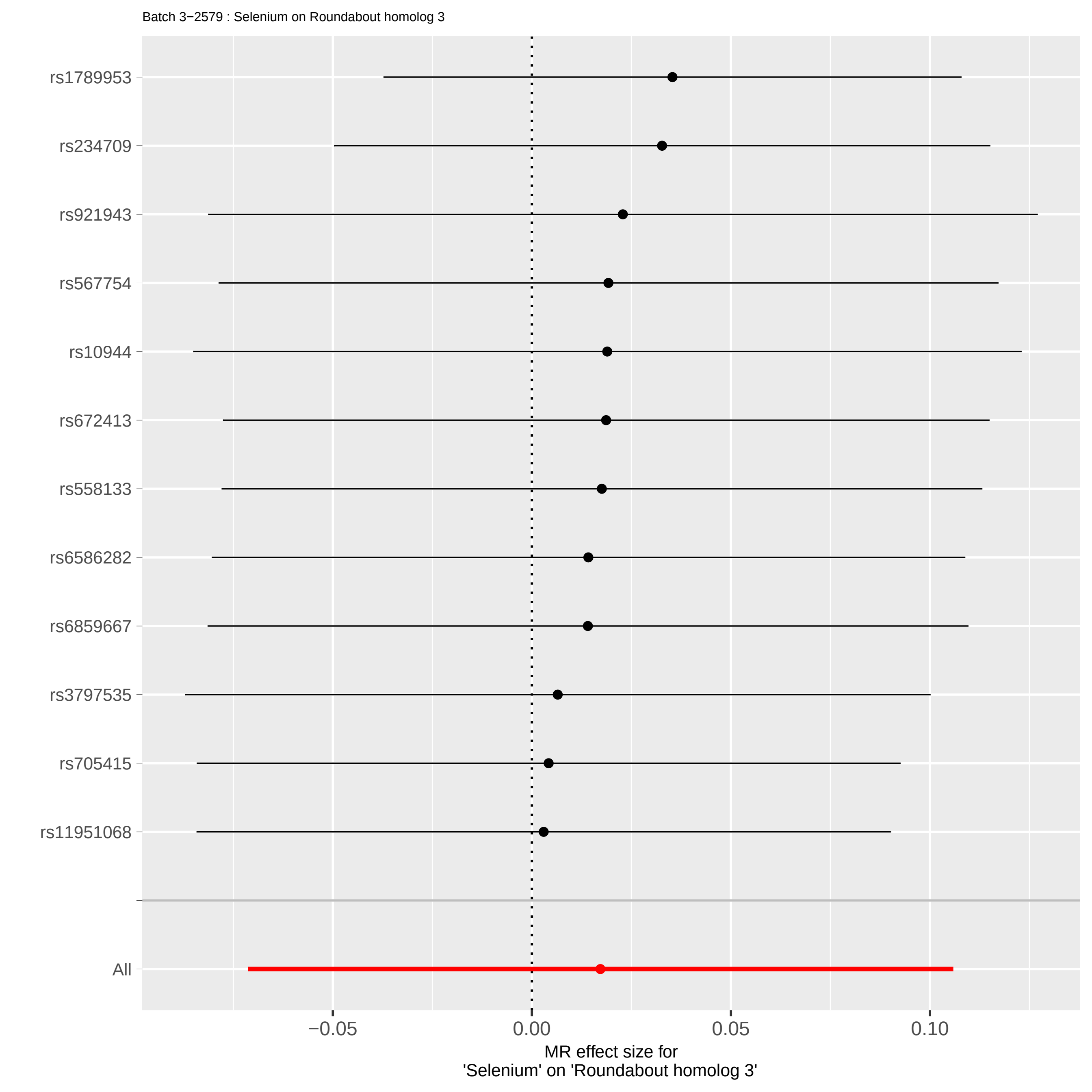

Batch 3−2579 : Selenium on Roundabout homolog 3
rs1789953
rs234709
rs921943
rs567754
rs10944
rs672413
rs558133
rs6586282
rs6859667
rs3797535
rs705415
rs11951068
All
−0.05
0.00
0.05
0.10
MR effect size for
'Selenium' on 'Roundabout homolog 3'

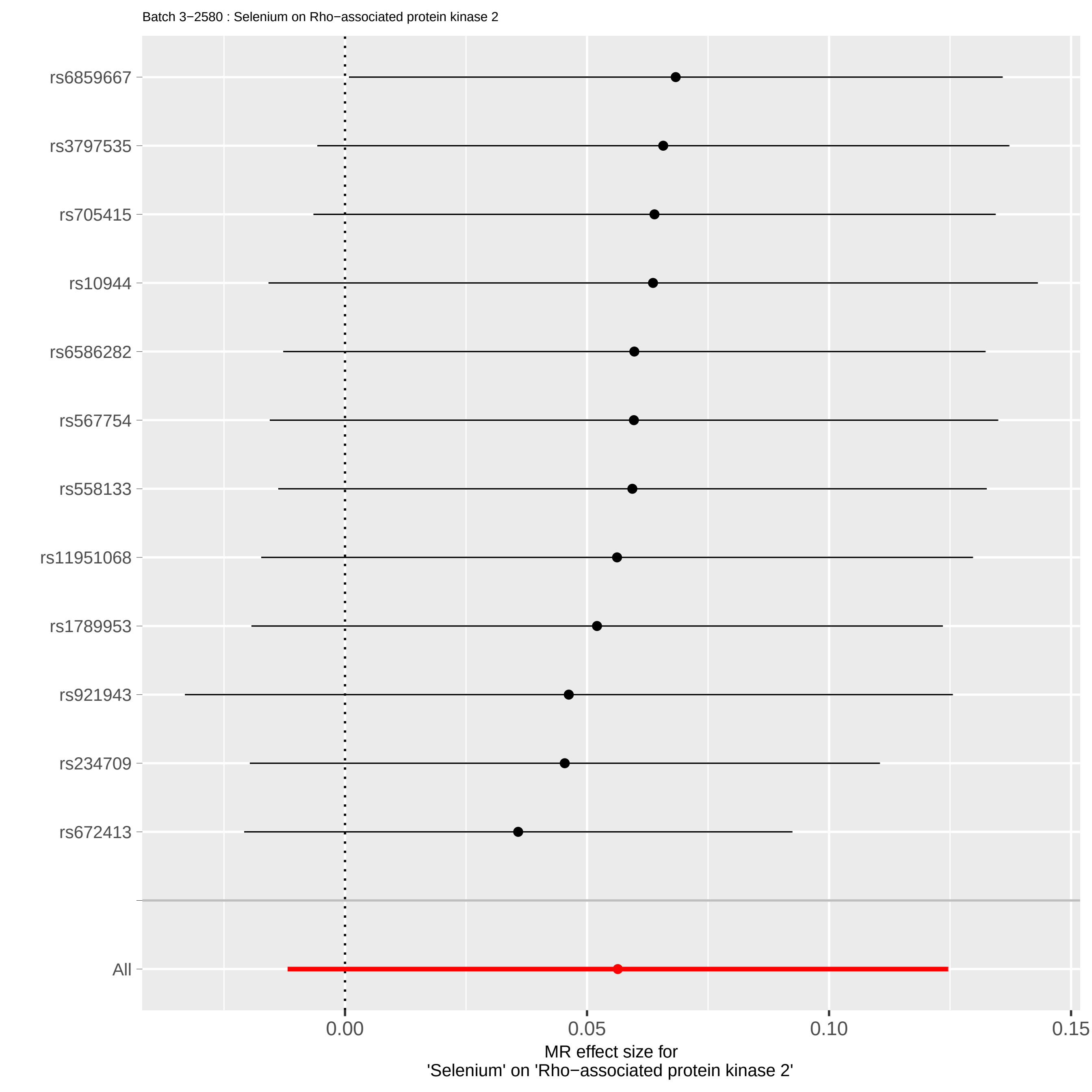

Batch 3−2580 : Selenium on Rho−associated protein kinase 2
rs6859667
rs3797535
rs705415
rs10944
rs6586282
rs567754
rs558133
rs11951068
rs1789953
rs921943
rs234709
rs672413
All
0.00
0.05
0.10
0.15
MR effect size for
'Selenium' on 'Rho−associated protein kinase 2'

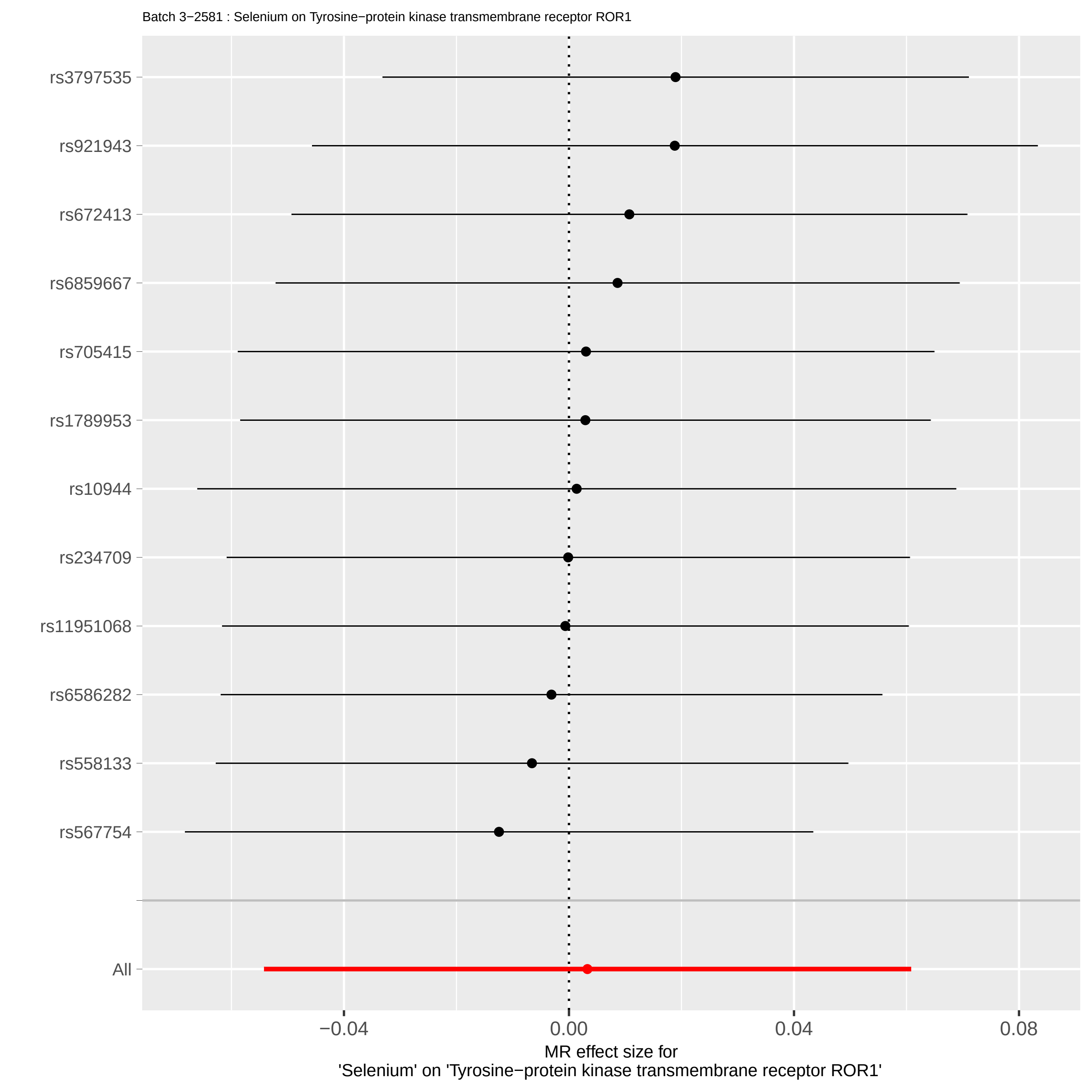

Batch 3−2581 : Selenium on Tyrosine−protein kinase transmembrane receptor ROR1
rs3797535
rs921943
rs672413
rs6859667
rs705415
rs1789953
rs10944
rs234709
rs11951068
rs6586282
rs558133
rs567754
All
−0.04
0.00
0.04
0.08
MR effect size for
'Selenium' on 'Tyrosine−protein kinase transmembrane receptor ROR1'

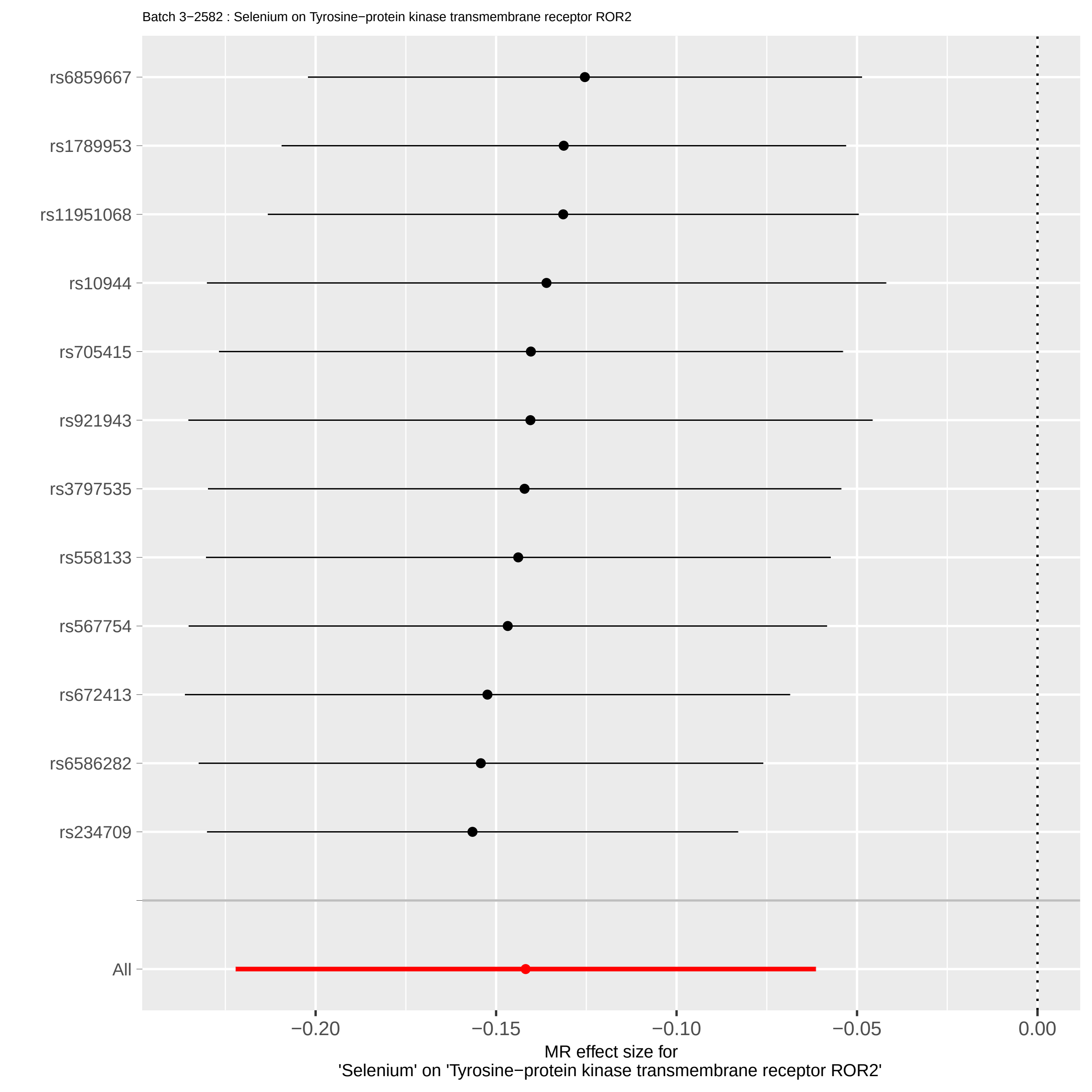

Batch 3−2582 : Selenium on Tyrosine−protein kinase transmembrane receptor ROR2
rs6859667
rs1789953
rs11951068
rs10944
rs705415
rs921943
rs3797535
rs558133
rs567754
rs672413
rs6586282
rs234709
All
−0.20
−0.15
−0.10
−0.05
0.00
MR effect size for
'Selenium' on 'Tyrosine−protein kinase transmembrane receptor ROR2'

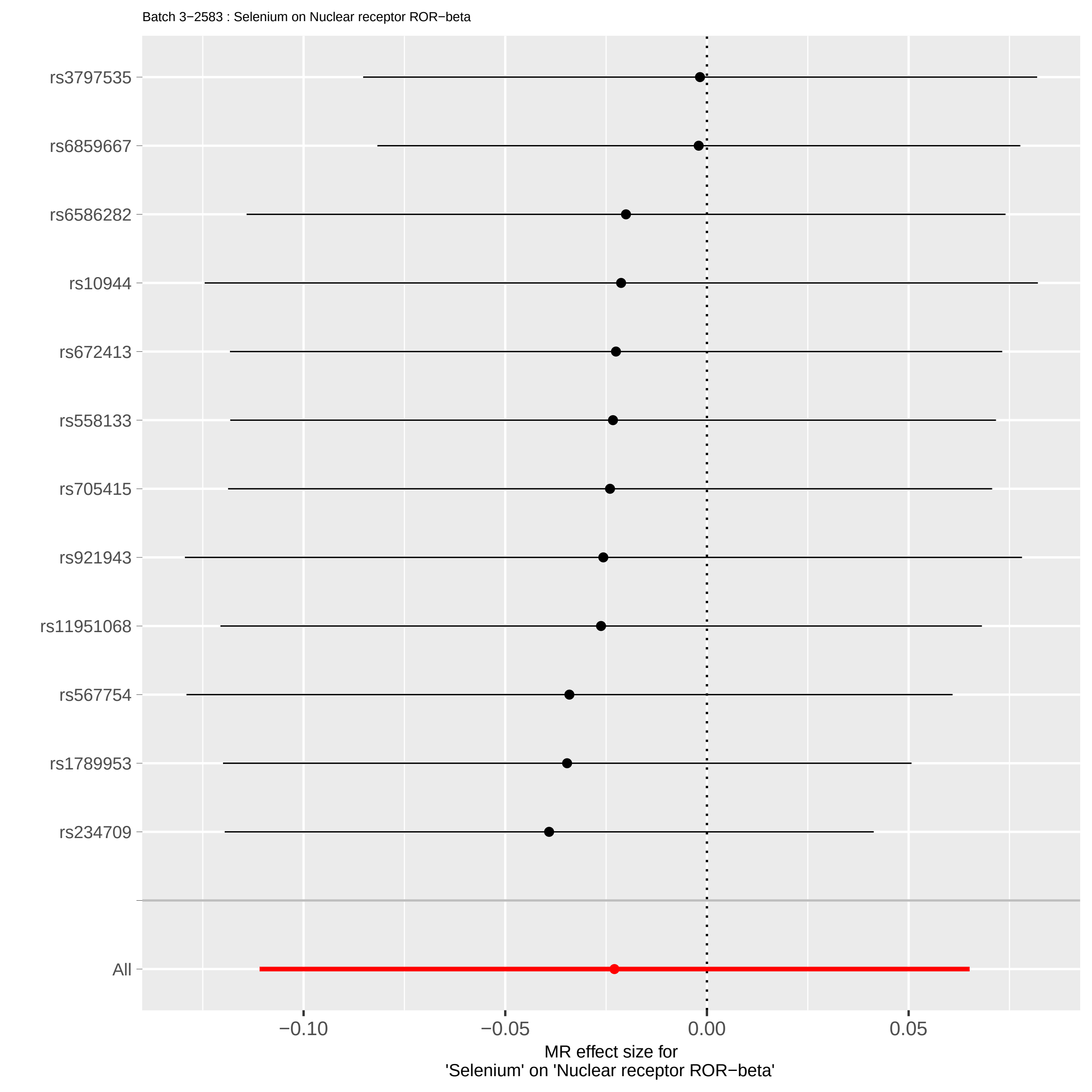
Batch 3−2583 : Selenium on Nuclear receptor ROR−beta
rs3797535
rs6859667
rs6586282
rs10944
rs672413
rs558133
rs705415
rs921943
rs11951068
rs567754
rs1789953
rs234709
All
−0.10
−0.05
0.00
0.05
MR effect size for
'Selenium' on 'Nuclear receptor ROR−beta'

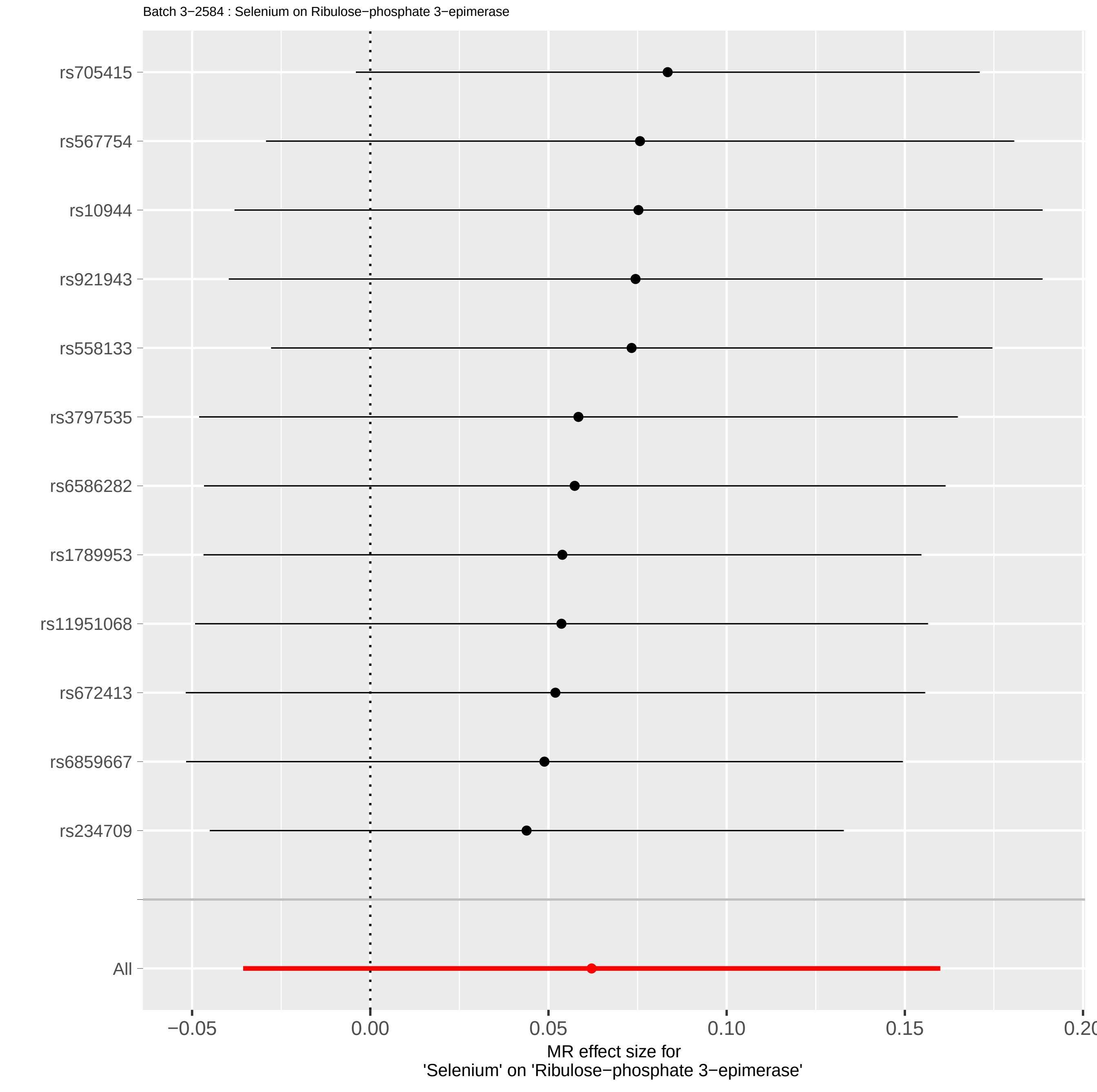
Batch 3−2584 : Selenium on Ribulose−phosphate 3−epimerase
rs705415
rs567754
rs10944
rs921943
rs558133
rs3797535
rs6586282
rs1789953
rs11951068
rs672413
rs6859667
rs234709
All
−0.05
0.00
0.05
0.10
0.15
0.20
MR effect size for
'Selenium' on 'Ribulose−phosphate 3−epimerase'

Batch 3−2585 : Selenium on Ribose−5−phosphate isomerase

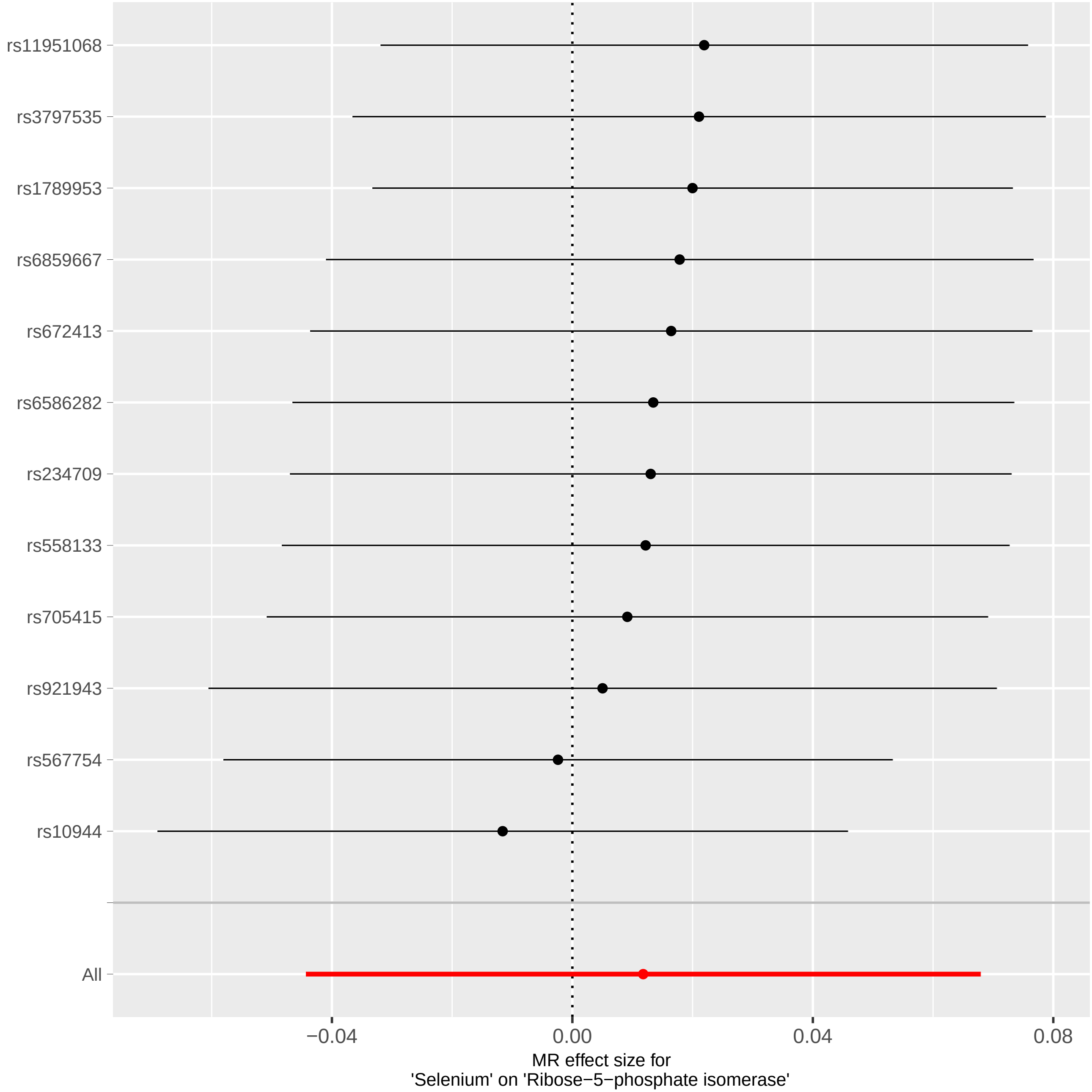

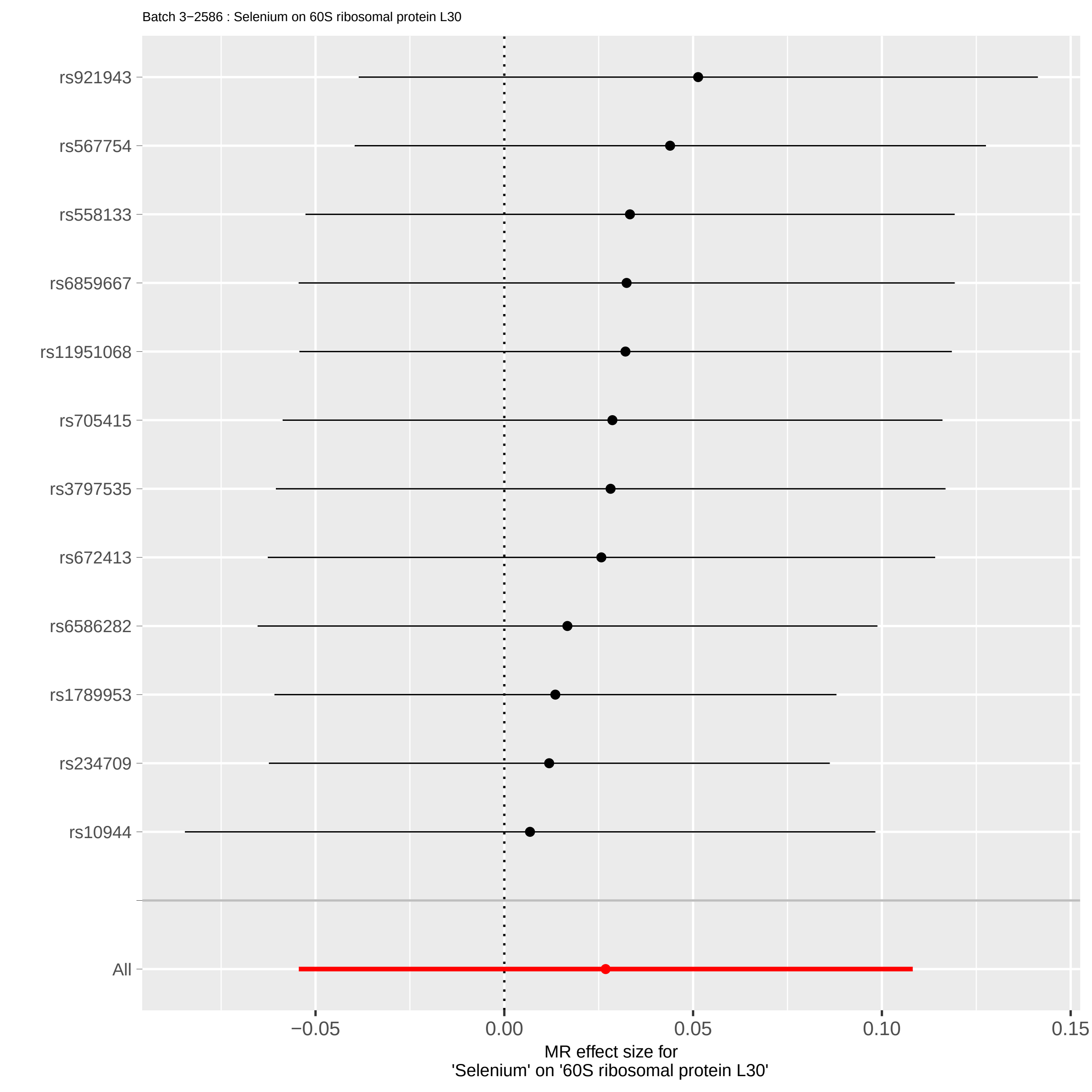
Batch 3−2586 : Selenium on 60S ribosomal protein L30
rs921943
rs567754
rs558133
rs6859667
rs11951068
rs705415
rs3797535
rs672413
rs6586282
rs1789953
rs234709
rs10944
All
−0.05
0.00
0.05
0.10
0.15
MR effect size for
'Selenium' on '60S ribosomal protein L30'

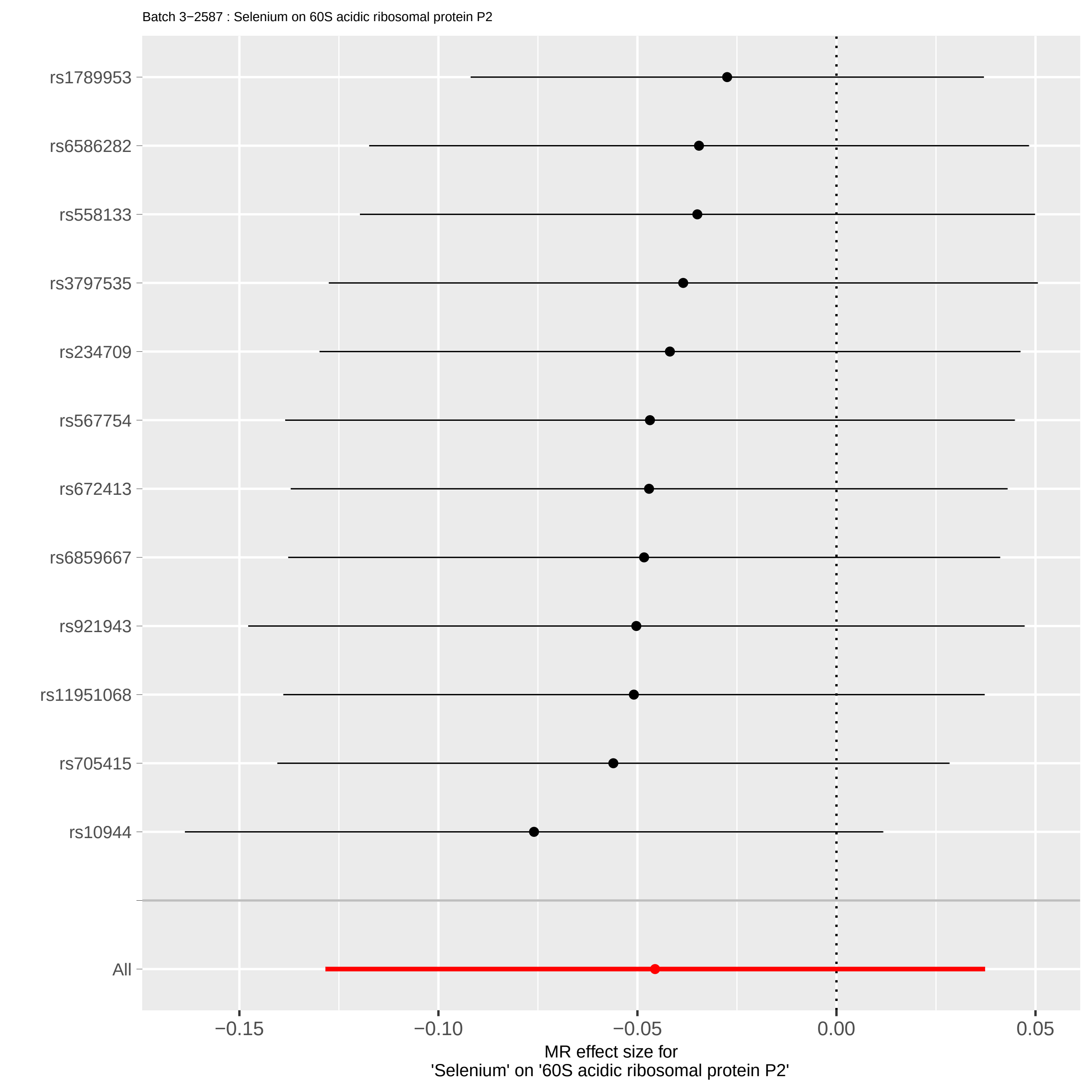

Batch 3−2587 : Selenium on 60S acidic ribosomal protein P2
rs1789953
rs6586282
rs558133
rs3797535
rs234709
rs567754
rs672413
rs6859667
rs921943
rs11951068
rs705415
rs10944
All
−0.15
−0.10
−0.05
0.00
0.05
MR effect size for
'Selenium' on '60S acidic ribosomal protein P2'

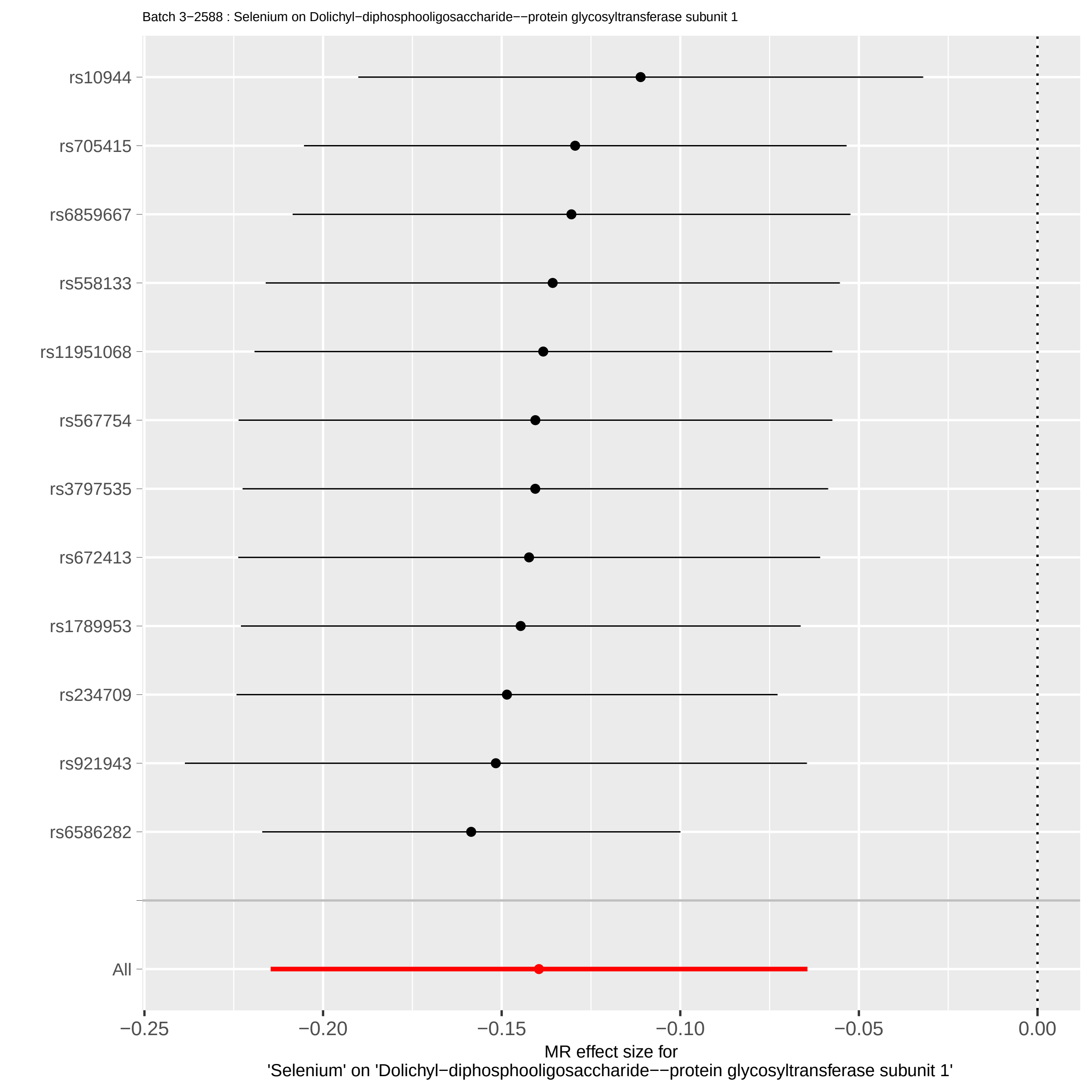

Batch 3−2588 : Selenium on Dolichyl−diphosphooligosaccharide−−protein glycosyltransferase subunit 1
rs10944
rs705415
rs6859667
rs558133
rs11951068
rs567754
rs3797535
rs672413
rs1789953
rs234709
rs921943
rs6586282
All
−0.25
−0.20
−0.15
−0.10
−0.05
0.00
MR effect size for
'Selenium' on 'Dolichyl−diphosphooligosaccharide−−protein glycosyltransferase subunit 1'

Batch 3−2589 : Selenium on Dolichyl−diphosphooligosaccharide−−protein glycosyltransferase subunit 1

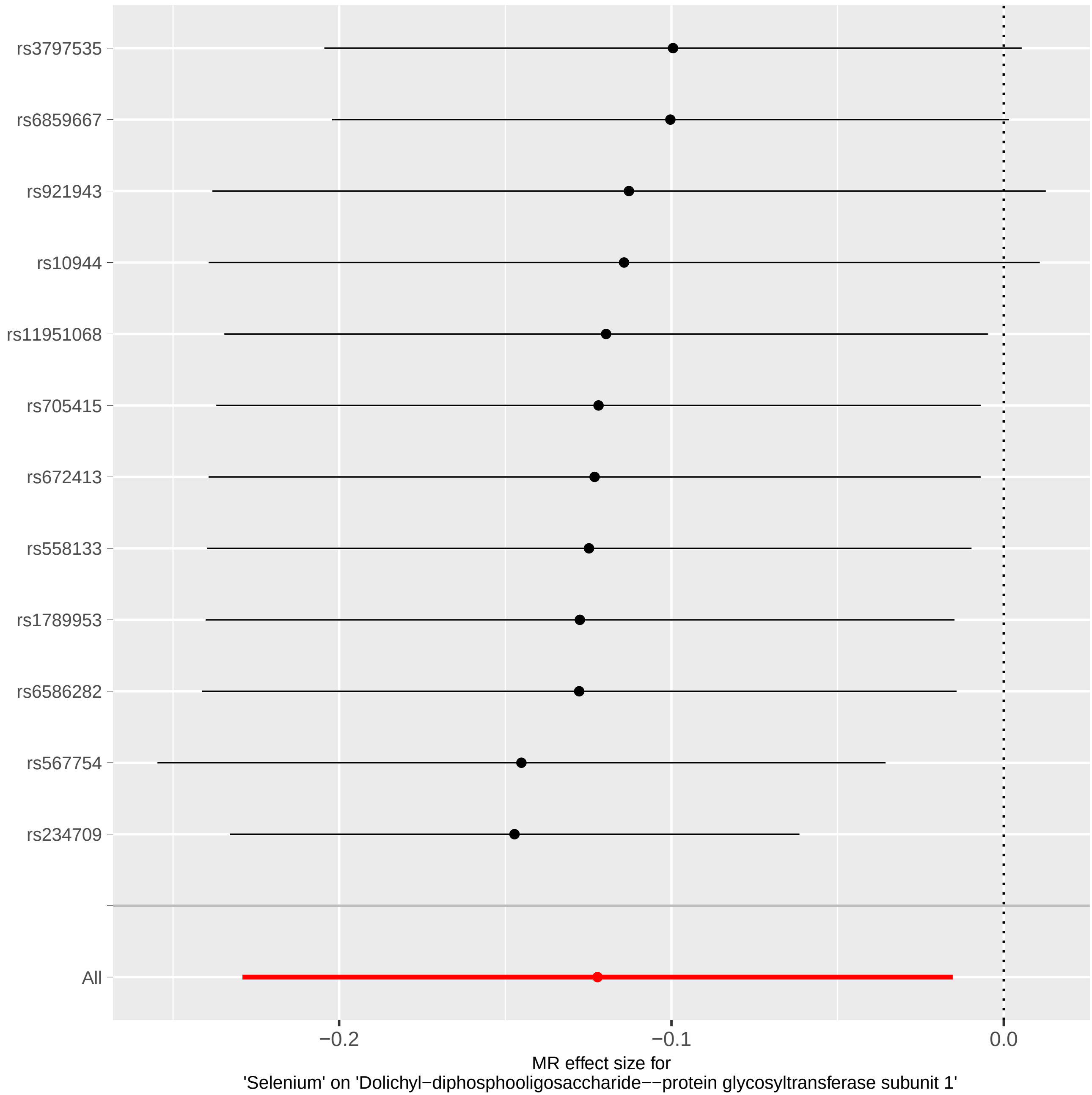

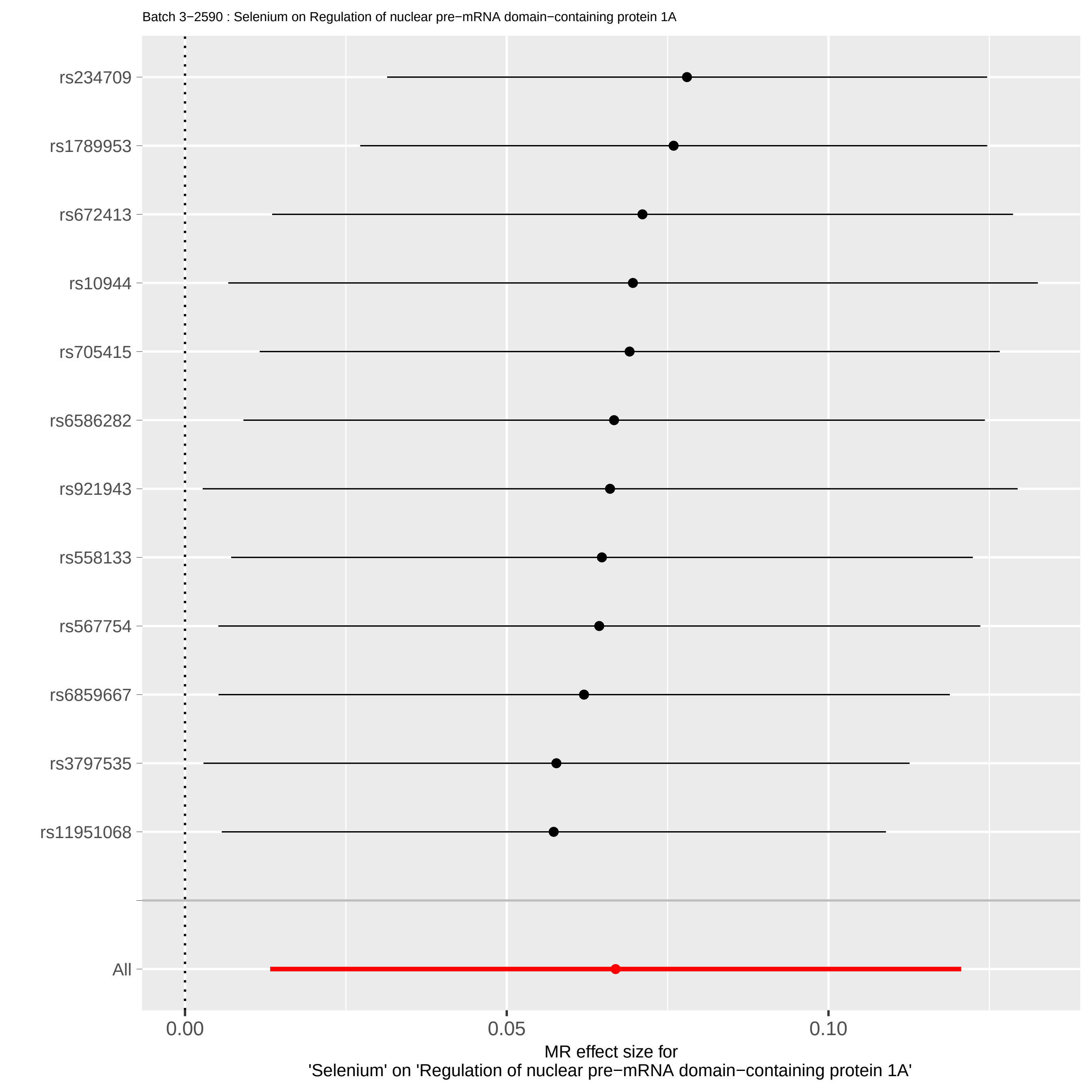

Batch 3−2590 : Selenium on Regulation of nuclear pre−mRNA domain−containing protein 1A
rs234709
rs1789953
rs672413
rs10944
rs705415
rs6586282
rs921943
rs558133
rs567754
rs6859667
rs3797535
rs11951068
All
0.00
0.05
0.10
MR effect size for
'Selenium' on 'Regulation of nuclear pre−mRNA domain−containing protein 1A'

Batch 3−2591 : Selenium on Ubiquitin+1, truncated mutation for UbB

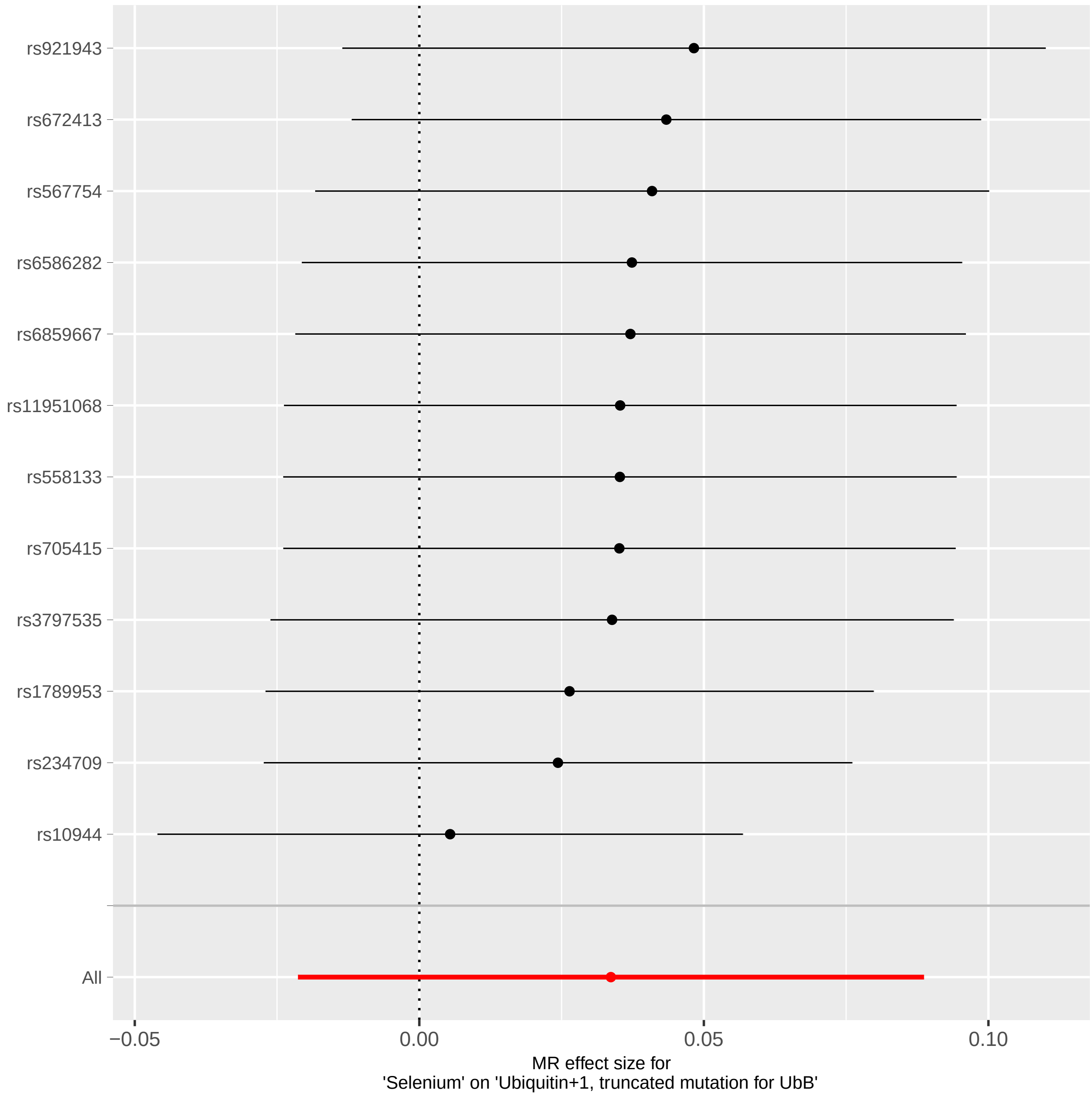

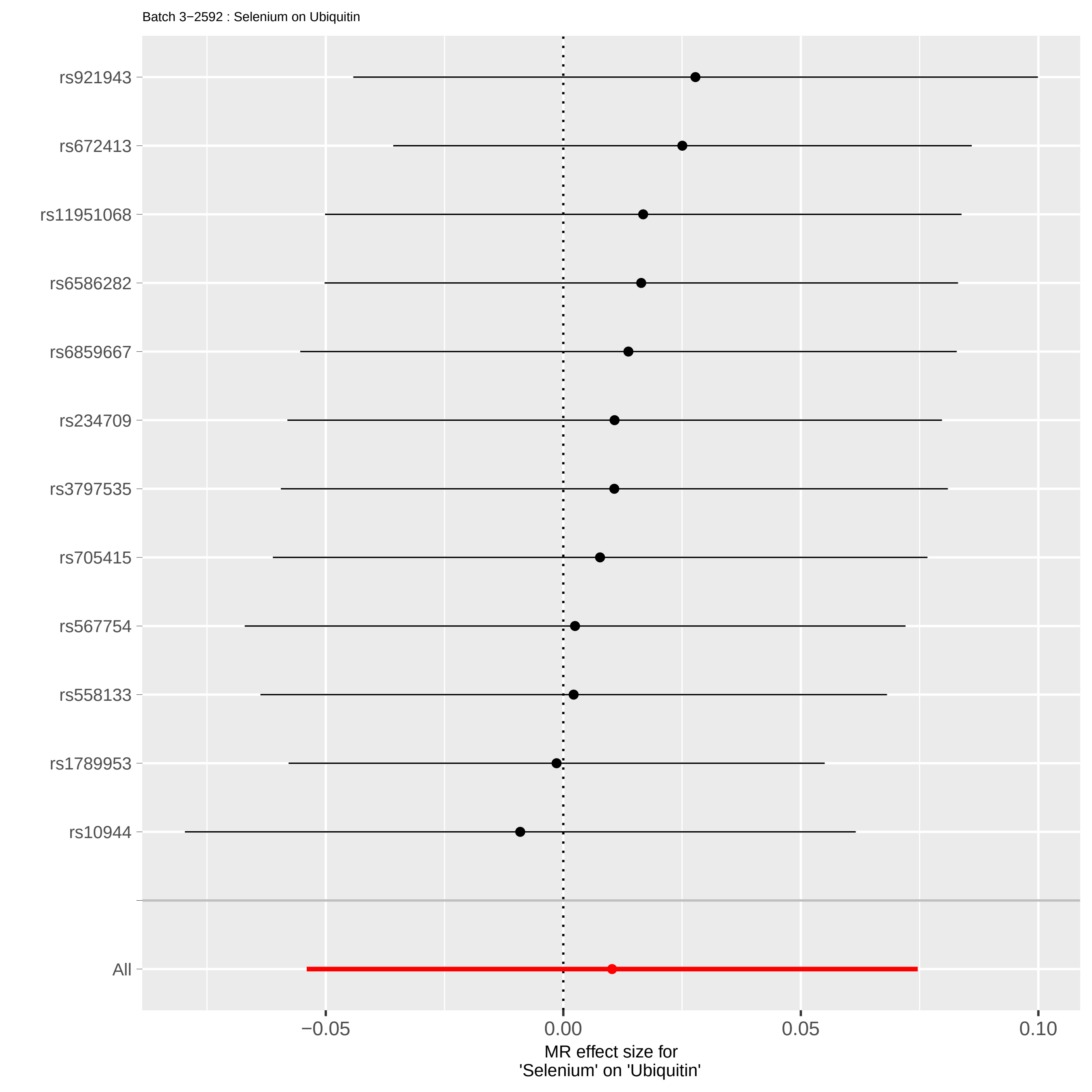
Batch 3−2592 : Selenium on Ubiquitin
rs921943
rs672413
rs11951068
rs6586282
rs6859667
rs234709
rs3797535
rs705415
rs567754
rs558133
rs1789953
rs10944
All
−0.05
0.00
0.05
0.10
MR effect size for
'Selenium' on 'Ubiquitin'

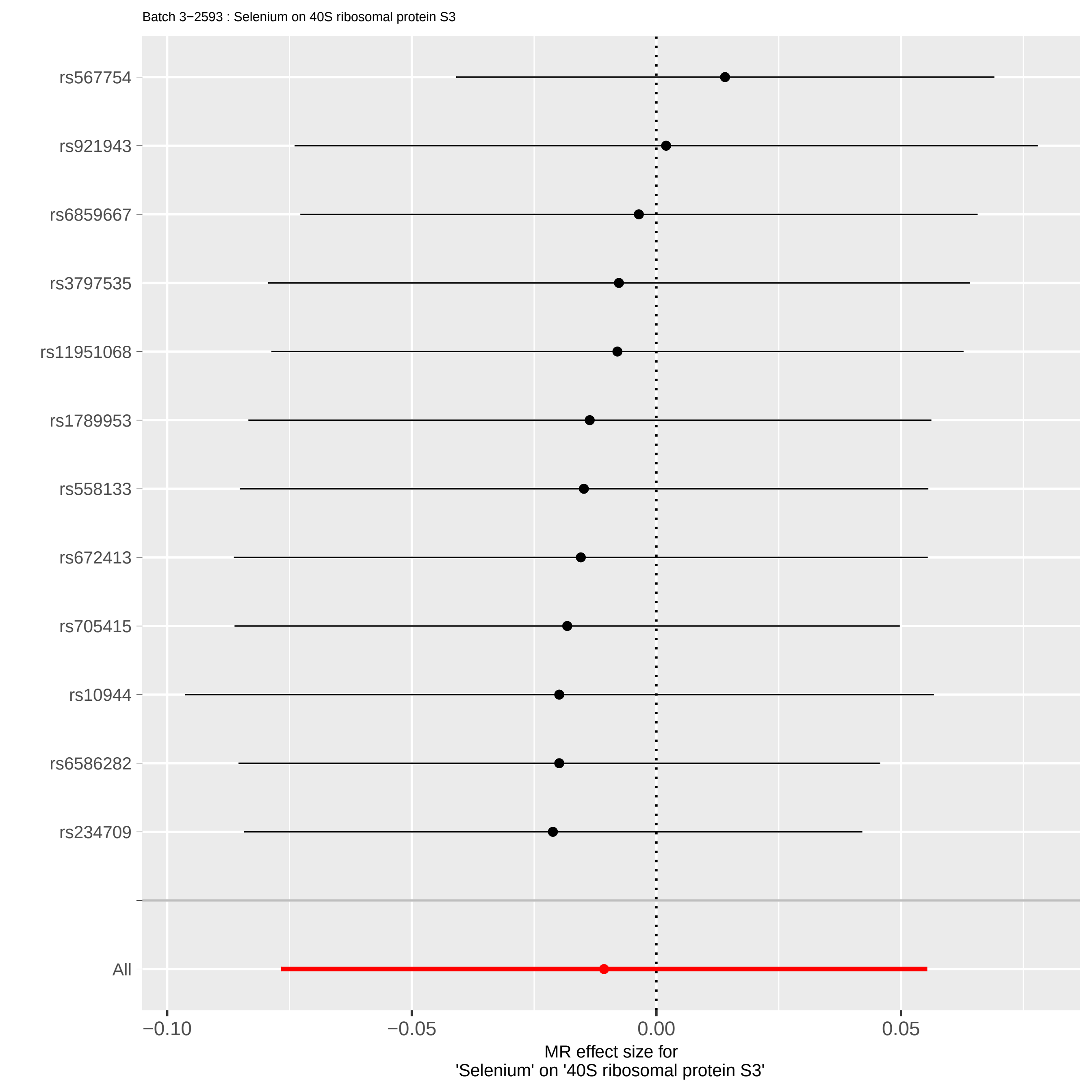
Batch 3−2593 : Selenium on 40S ribosomal protein S3
rs567754
rs921943
rs6859667
rs3797535
rs11951068
rs1789953
rs558133
rs672413
rs705415
rs10944
rs6586282
rs234709
All
−0.10
−0.05
0.00
0.05
MR effect size for
'Selenium' on '40S ribosomal protein S3'

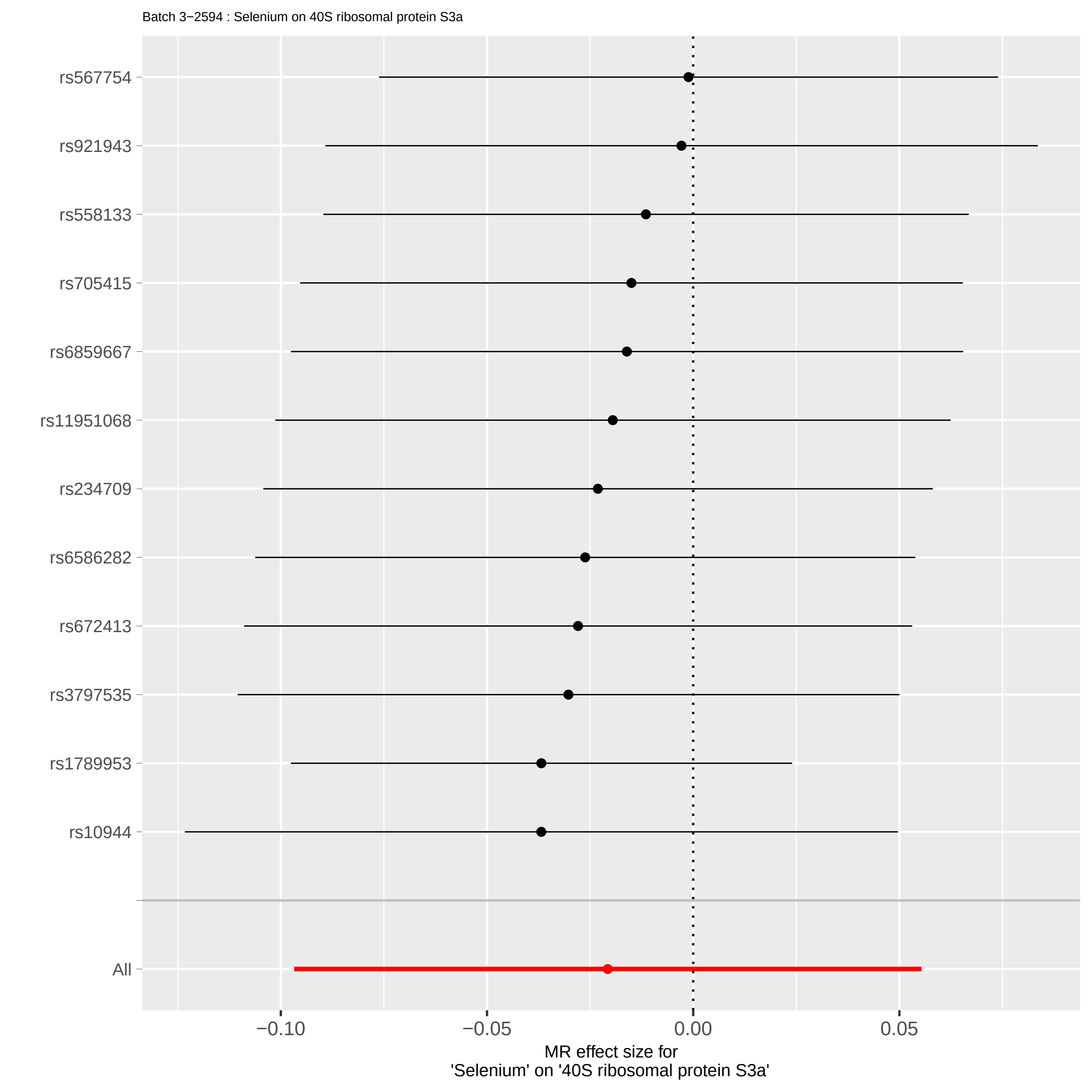
Batch 3−2594 : Selenium on 40S ribosomal protein S3a
rs567754
rs921943
rs558133
rs705415
rs6859667
rs11951068
rs234709
rs6586282
rs672413
rs3797535
rs1789953
rs10944
All
−0.10
−0.05
0.00
0.05
MR effect size for
'Selenium' on '40S ribosomal protein S3a'

Batch 3−2595 : Selenium on 40S ribosomal protein S4, X isoform

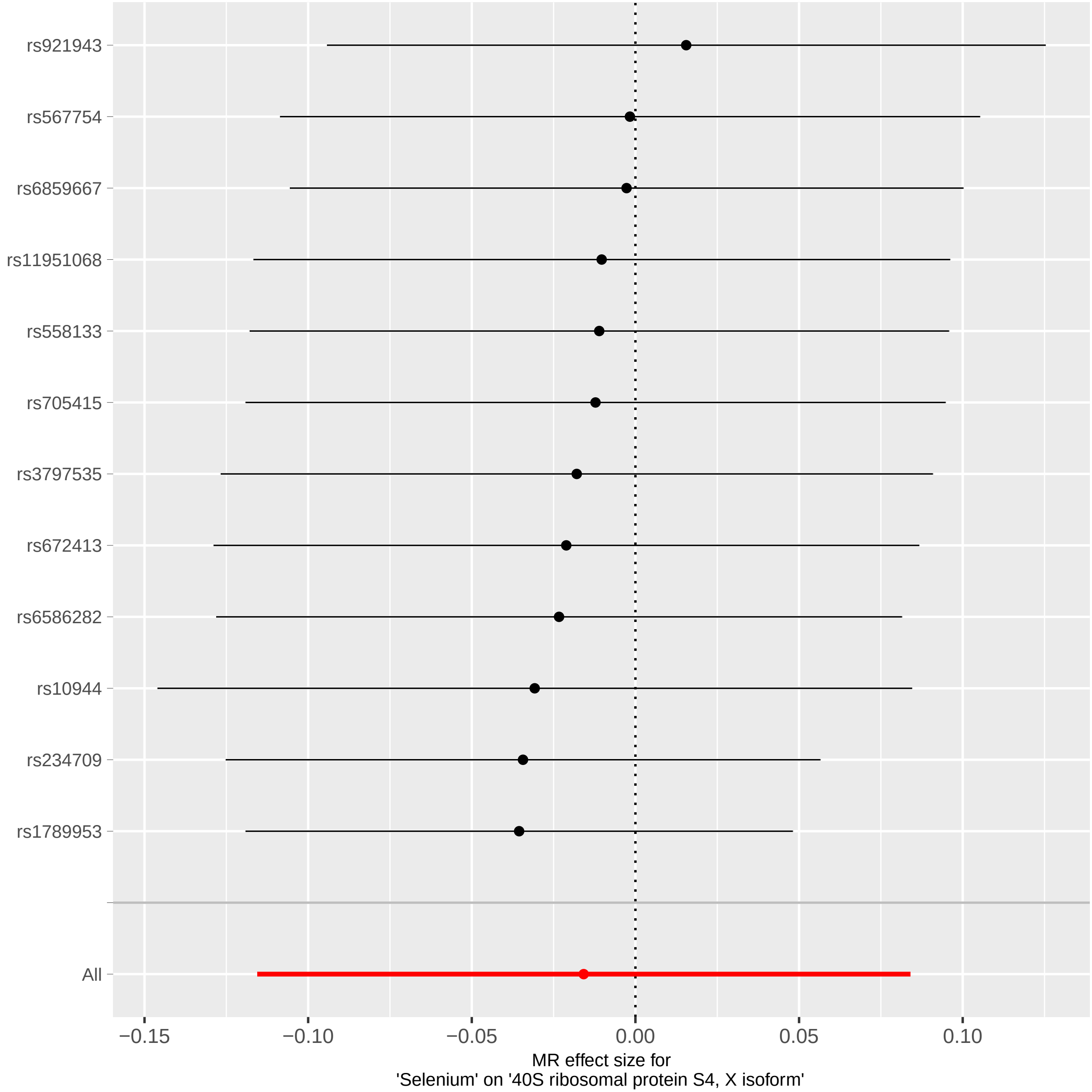

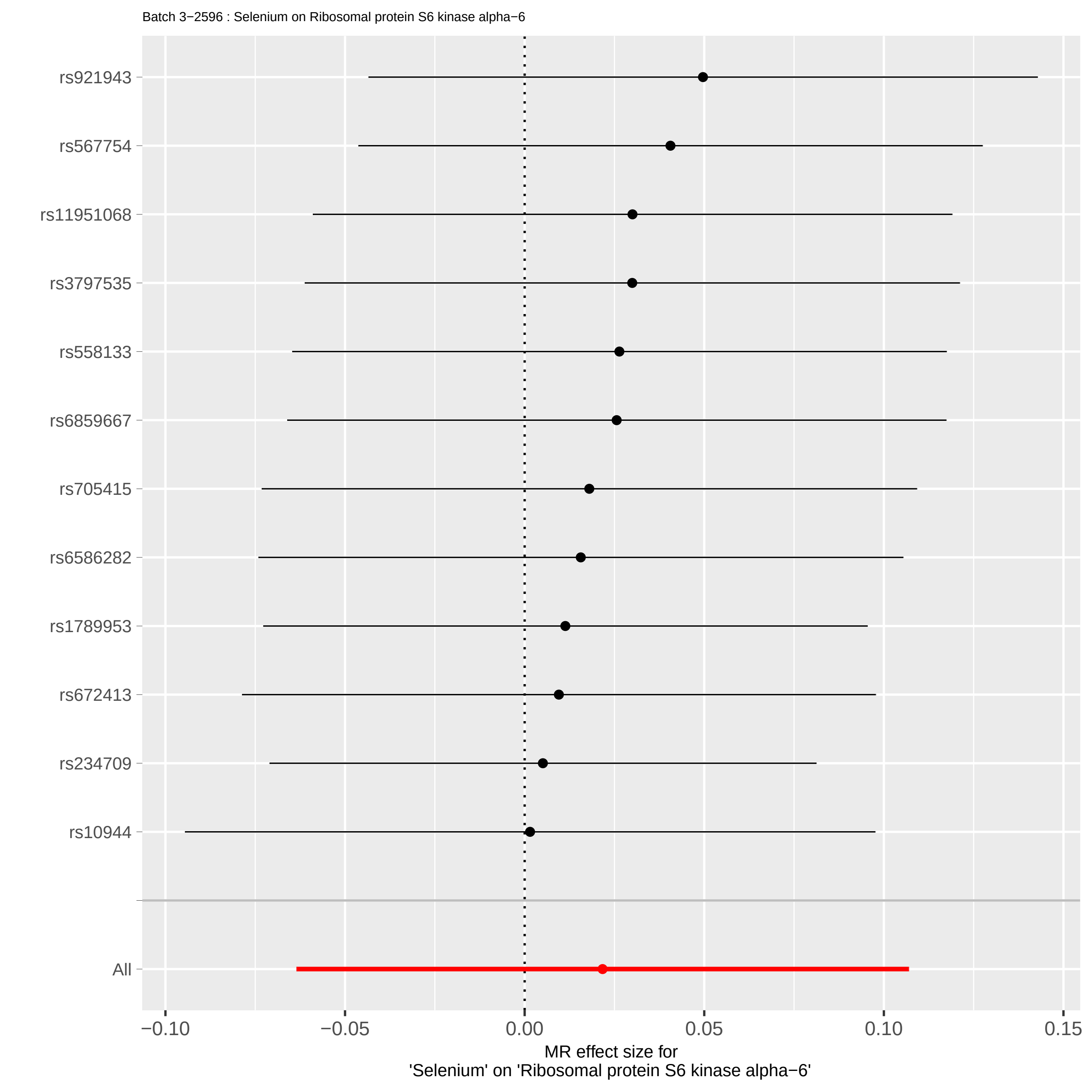

Batch 3−2596 : Selenium on Ribosomal protein S6 kinase alpha−6
rs921943
rs567754
rs11951068
rs3797535
rs558133
rs6859667
rs705415
rs6586282
rs1789953
rs672413
rs234709
rs10944
All
−0.10
−0.05
0.00
0.05
0.10
0.15
MR effect size for
'Selenium' on 'Ribosomal protein S6 kinase alpha−6'

Batch 3−2597 : Selenium on 40S ribosomal protein S7

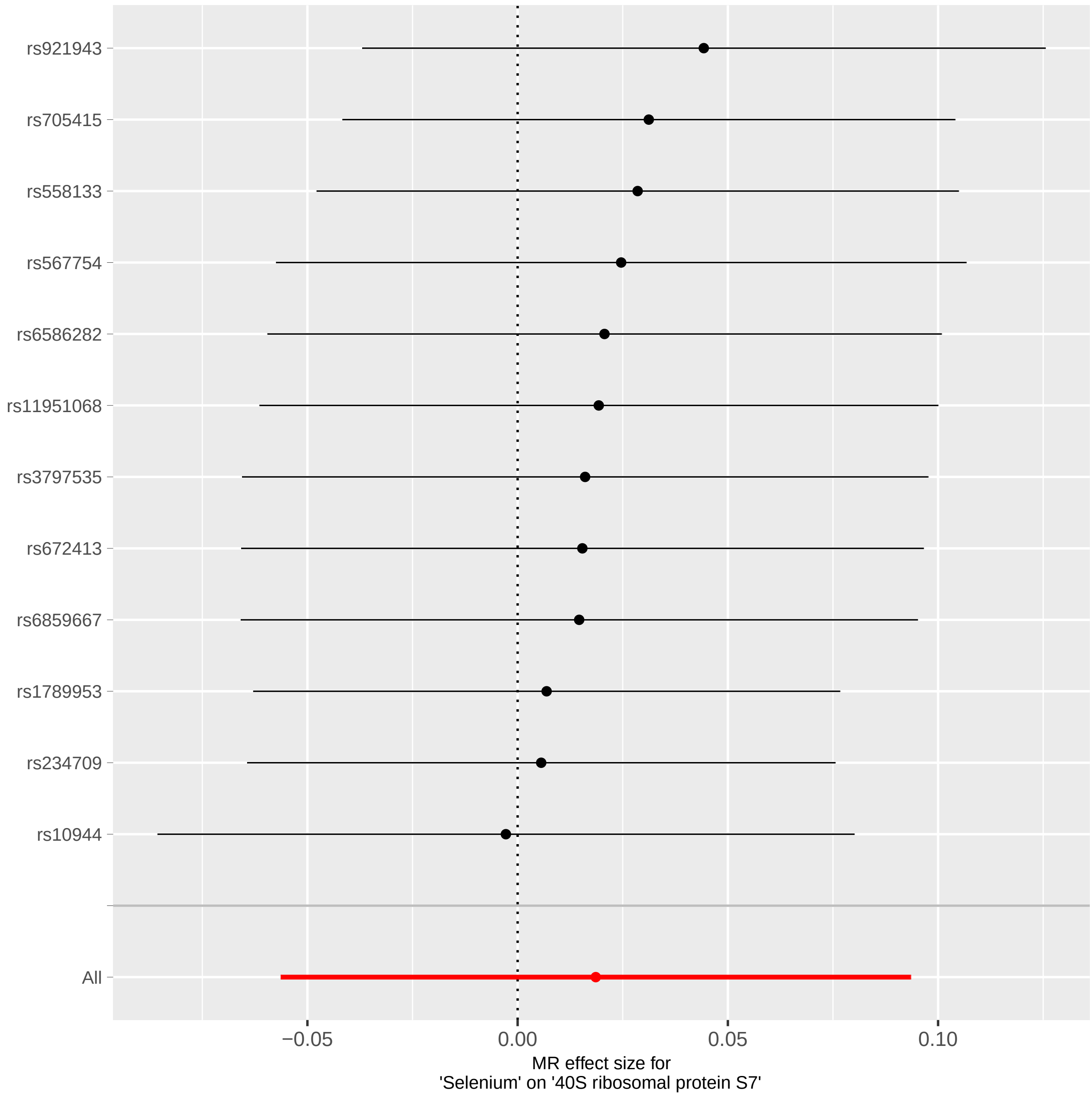

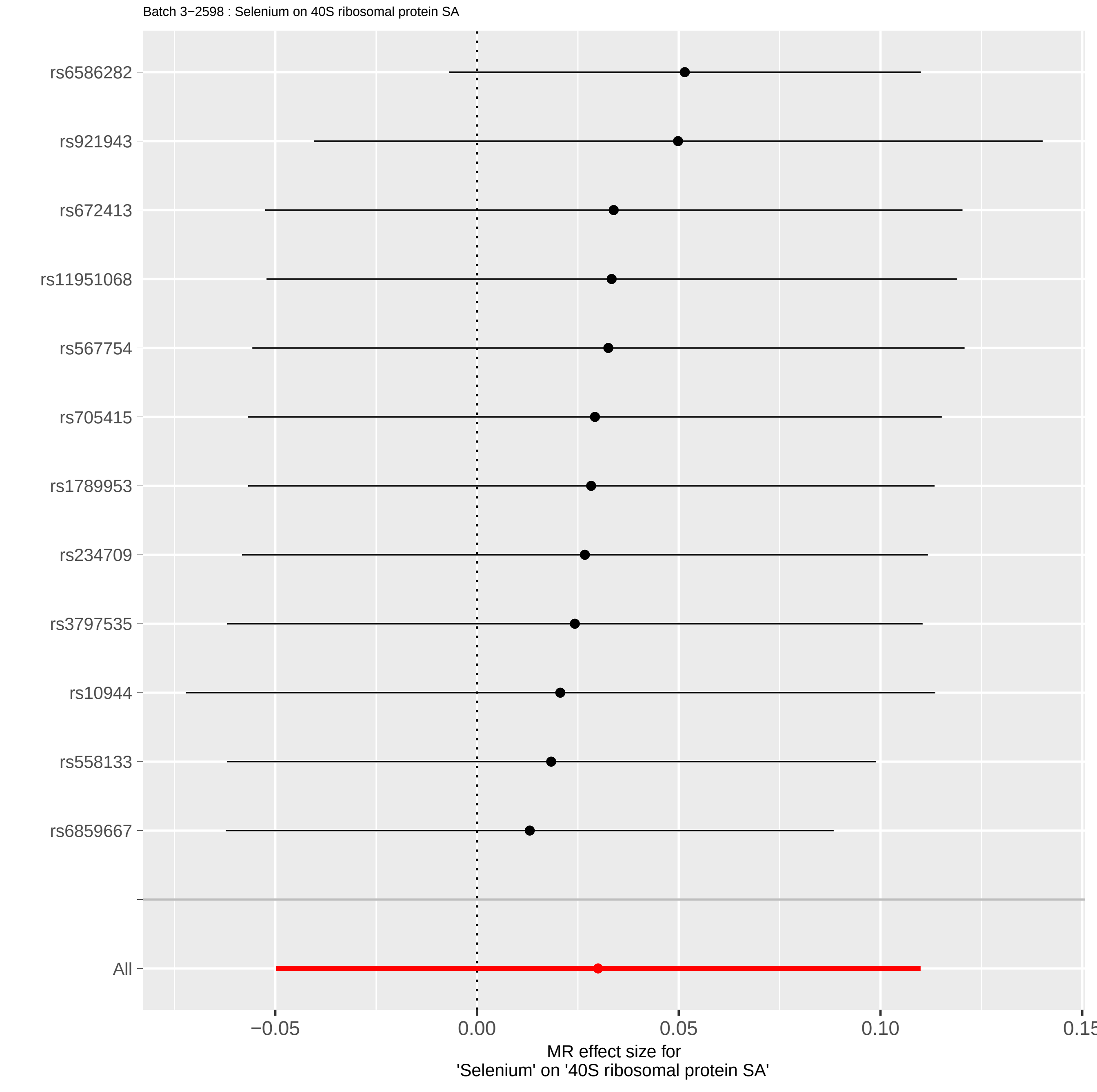

Batch 3−2598 : Selenium on 40S ribosomal protein SA
rs6586282
rs921943
rs672413
rs11951068
rs567754
rs705415
rs1789953
rs234709
rs3797535
rs10944
rs558133
rs6859667
All
−0.05
0.00
0.05
0.10
0.15
MR effect size for
'Selenium' on '40S ribosomal protein SA'

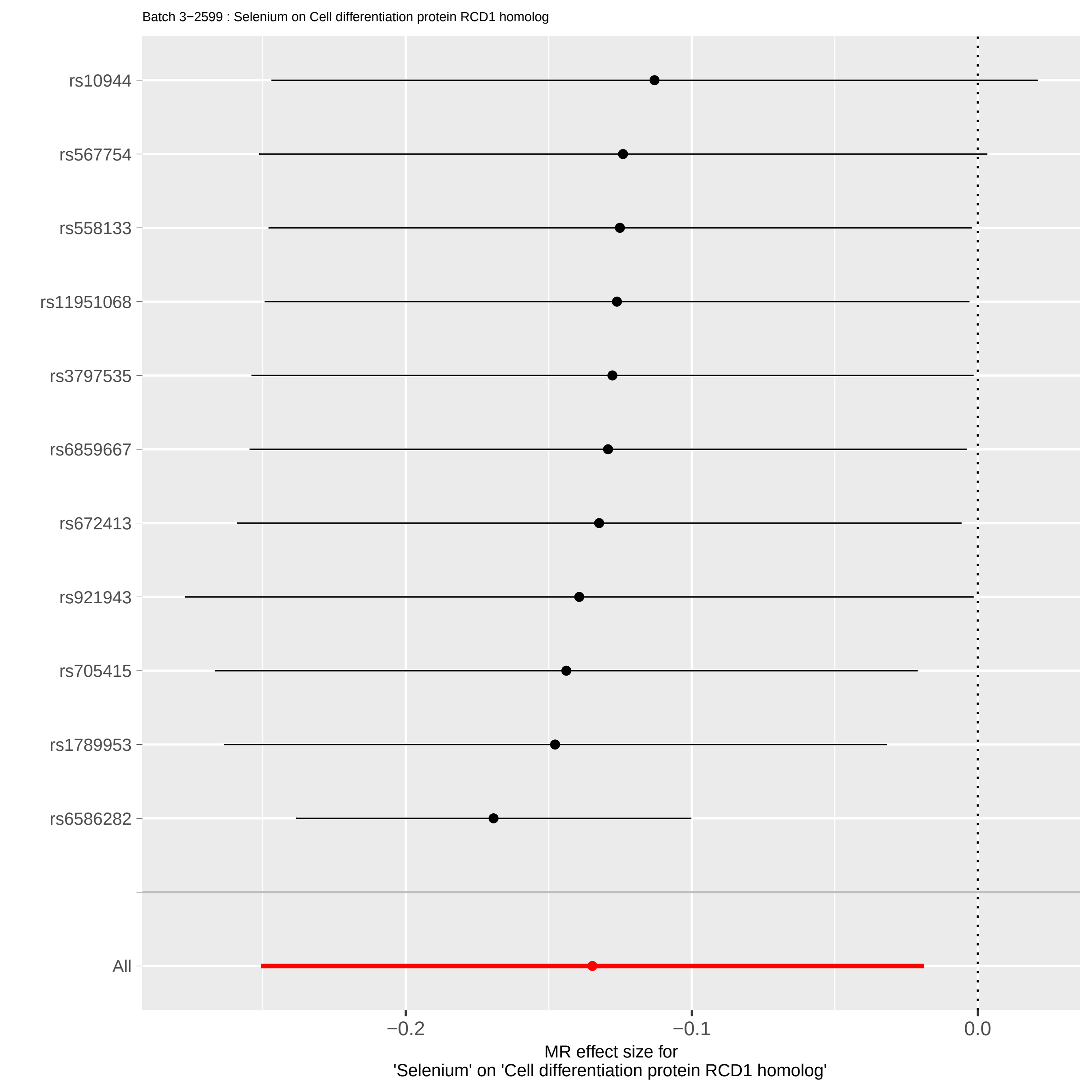

Batch 3−2599 : Selenium on Cell differentiation protein RCD1 homolog
rs10944
rs567754
rs558133
rs11951068
rs3797535
rs6859667
rs672413
rs921943
rs705415
rs1789953
rs6586282
All
−0.2
−0.1
0.0
MR effect size for
'Selenium' on 'Cell differentiation protein RCD1 homolog'

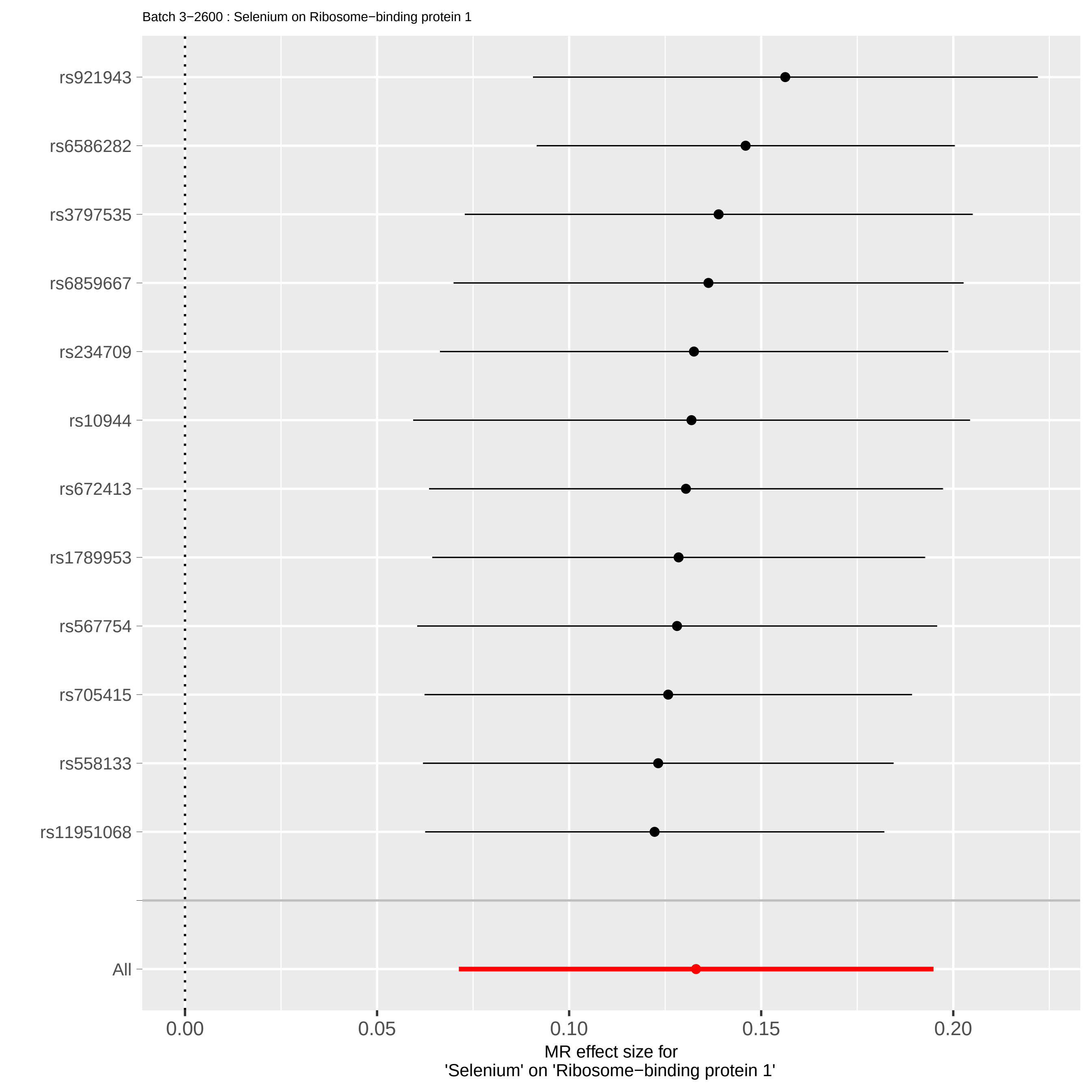

Batch 3−2600 : Selenium on Ribosome−binding protein 1
rs921943
rs6586282
rs3797535
rs6859667
rs234709
rs10944
rs672413
rs1789953
rs567754
rs705415
rs558133
rs11951068
All
0.00
0.05
0.10
0.15
0.20
MR effect size for
'Selenium' on 'Ribosome−binding protein 1'

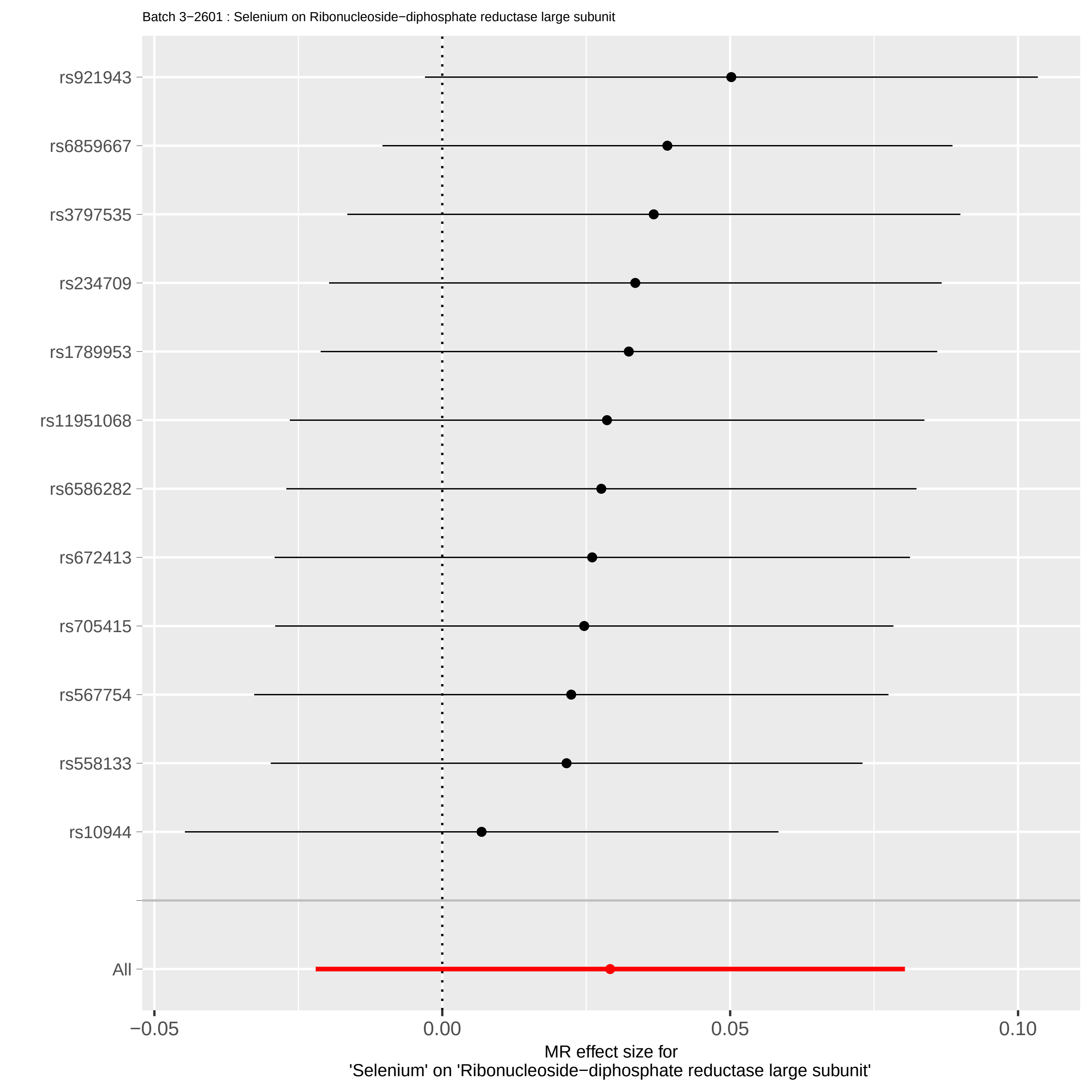

Batch 3−2601 : Selenium on Ribonucleoside−diphosphate reductase large subunit
rs921943
rs6859667
rs3797535
rs234709
rs1789953
rs11951068
rs6586282
rs672413
rs705415
rs567754
rs558133
rs10944
All
−0.05
0.00
0.05
0.10
MR effect size for
'Selenium' on 'Ribonucleoside−diphosphate reductase large subunit'

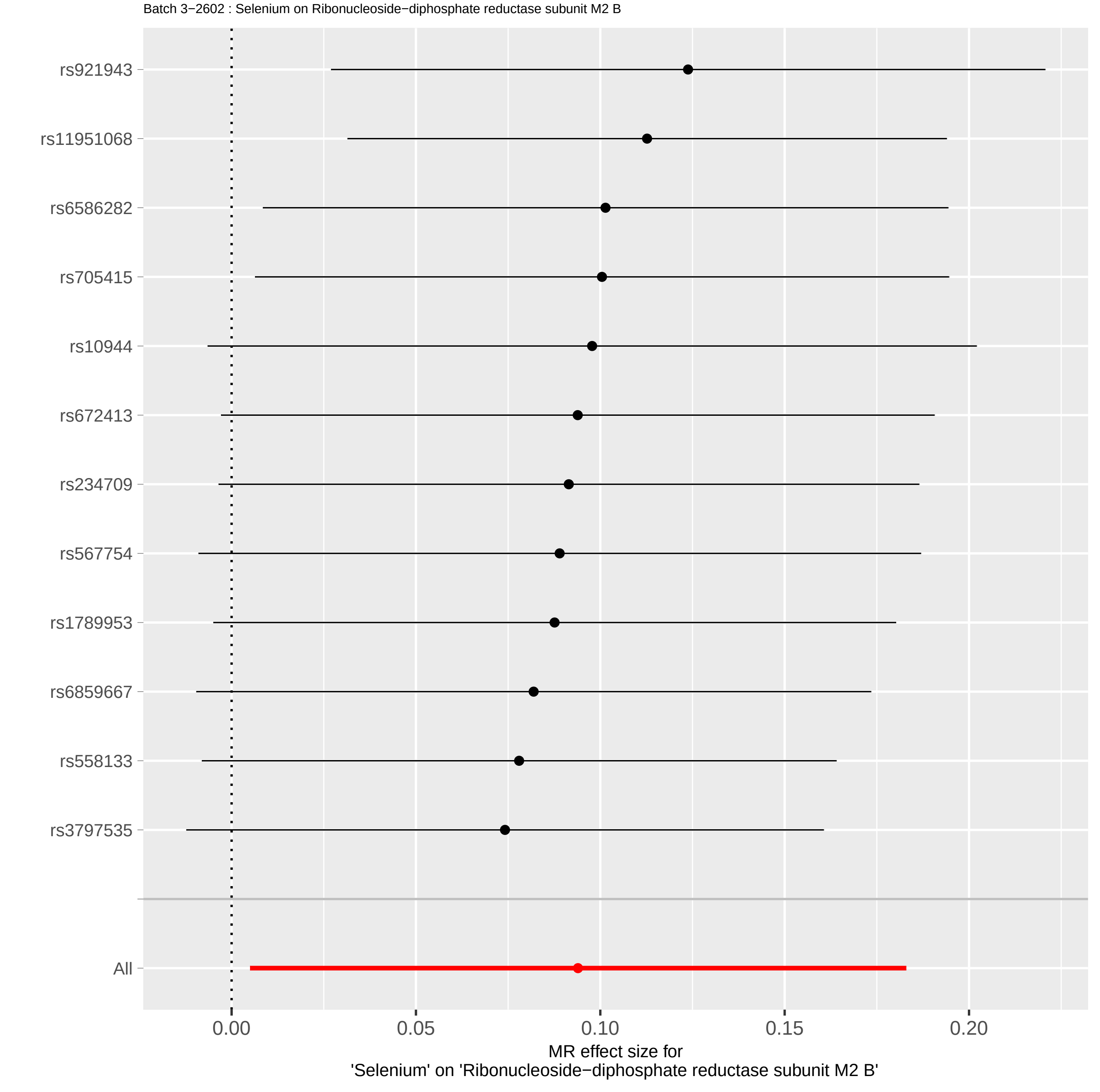

Batch 3−2602 : Selenium on Ribonucleoside−diphosphate reductase subunit M2 B
rs921943
rs11951068
rs6586282
rs705415
rs10944
rs672413
rs234709
rs567754
rs1789953
rs6859667
rs558133
rs3797535
All
0.00
0.05
0.10
0.15
0.20
MR effect size for
'Selenium' on 'Ribonucleoside−diphosphate reductase subunit M2 B'

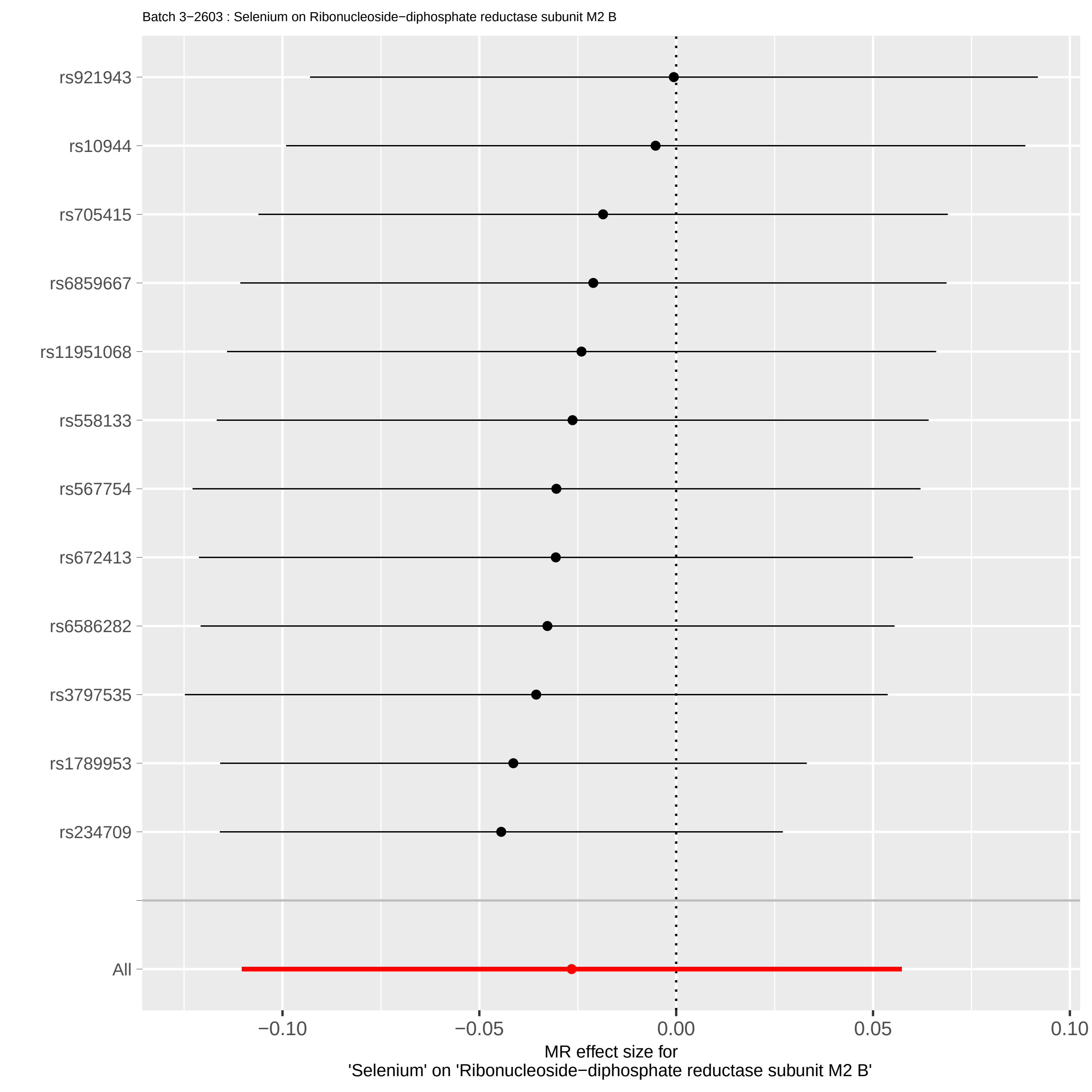

Batch 3−2603 : Selenium on Ribonucleoside−diphosphate reductase subunit M2 B
rs921943
rs10944
rs705415
rs6859667
rs11951068
rs558133
rs567754
rs672413
rs6586282
rs3797535
rs1789953
rs234709
All
−0.10
−0.05
0.00
0.05
0.10
MR effect size for
'Selenium' on 'Ribonucleoside−diphosphate reductase subunit M2 B'

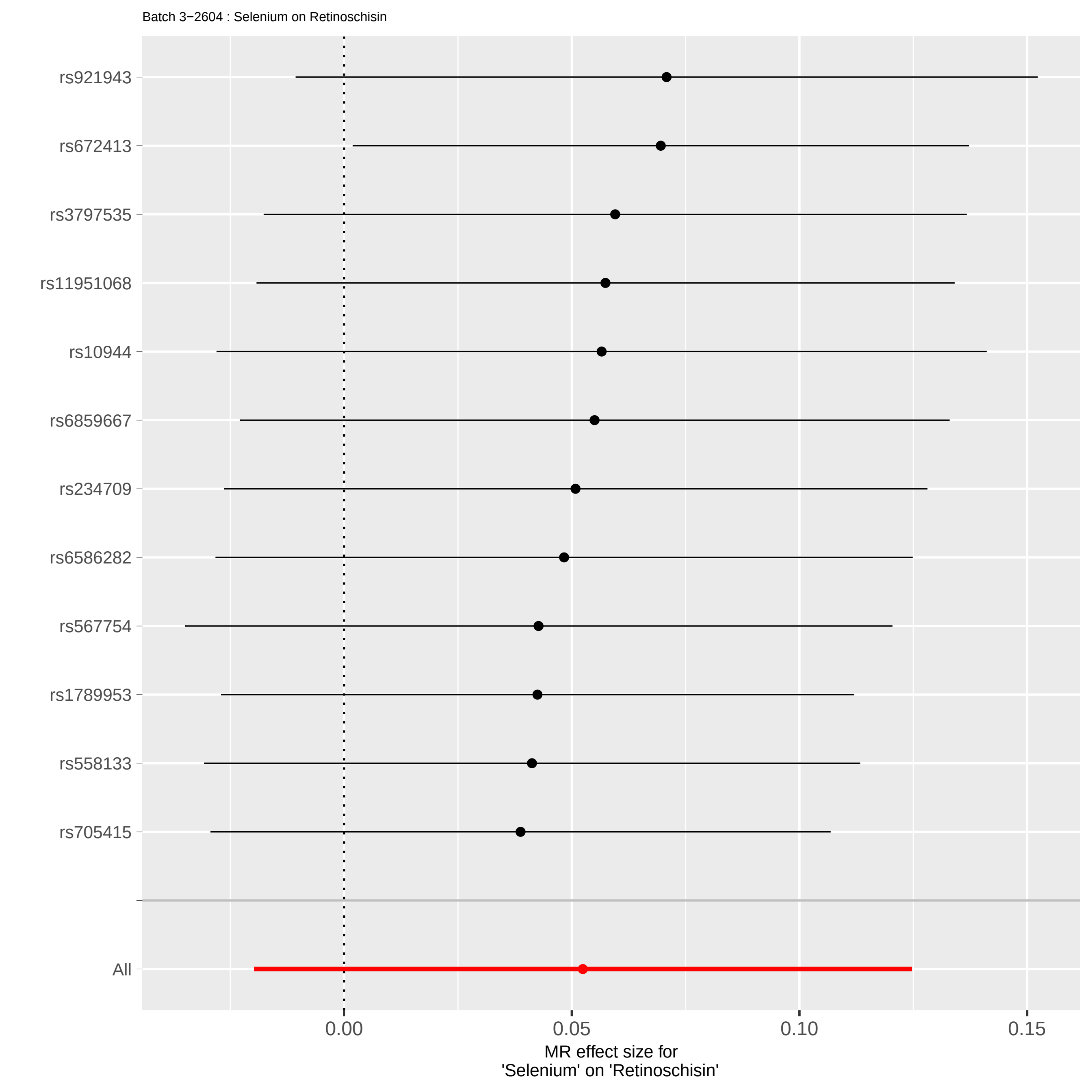
Batch 3−2604 : Selenium on Retinoschisin
rs921943
rs672413
rs3797535
rs11951068
rs10944
rs6859667
rs234709
rs6586282
rs567754
rs1789953
rs558133
rs705415
All
0.00
0.05
0.10
0.15
MR effect size for
'Selenium' on 'Retinoschisin'

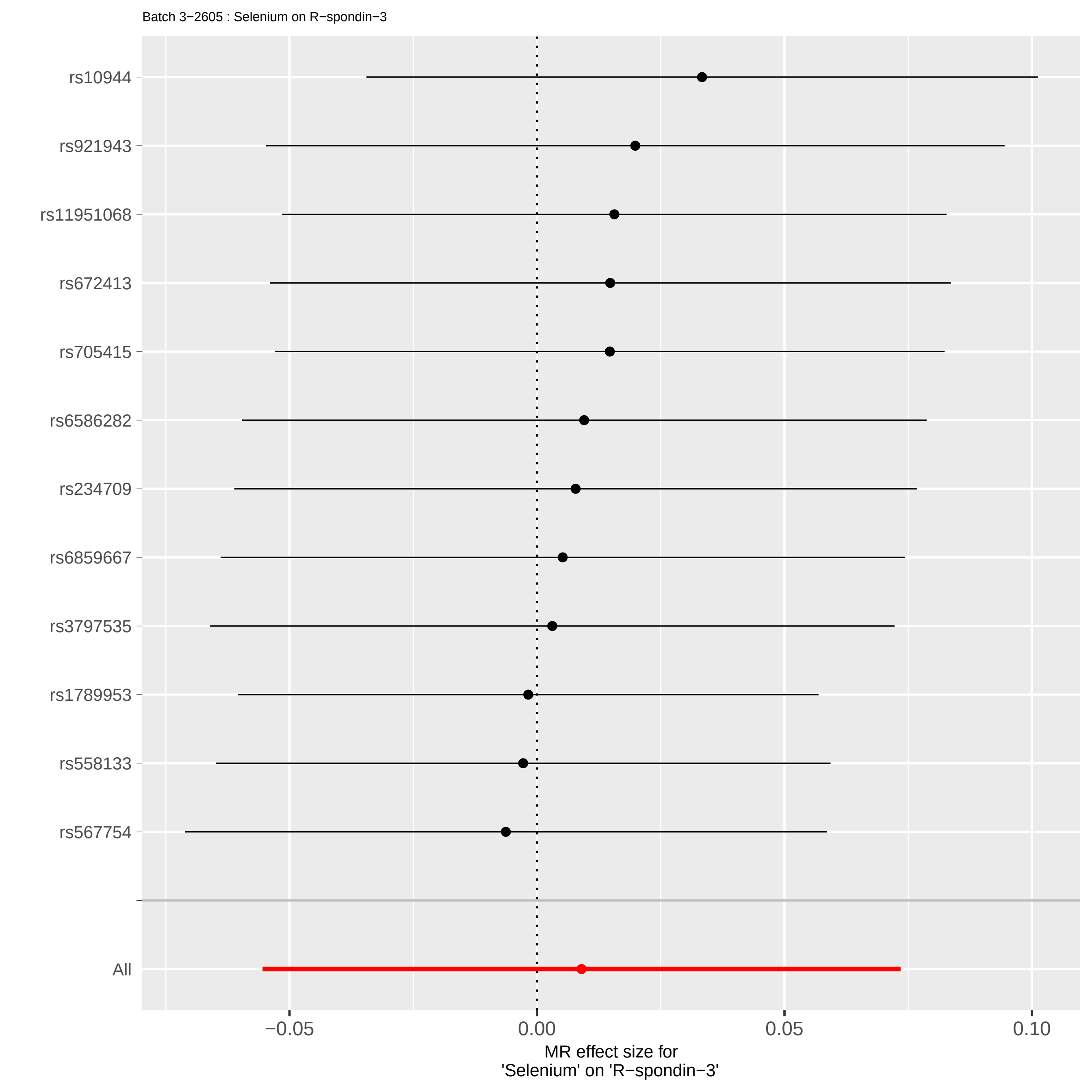

Batch 3−2605 : Selenium on R−spondin−3
rs10944
rs921943
rs11951068
rs672413
rs705415
rs6586282
rs234709
rs6859667
rs3797535
rs1789953
rs558133
rs567754
All
−0.05
0.00
0.05
0.10
MR effect size for
'Selenium' on 'R−spondin−3'

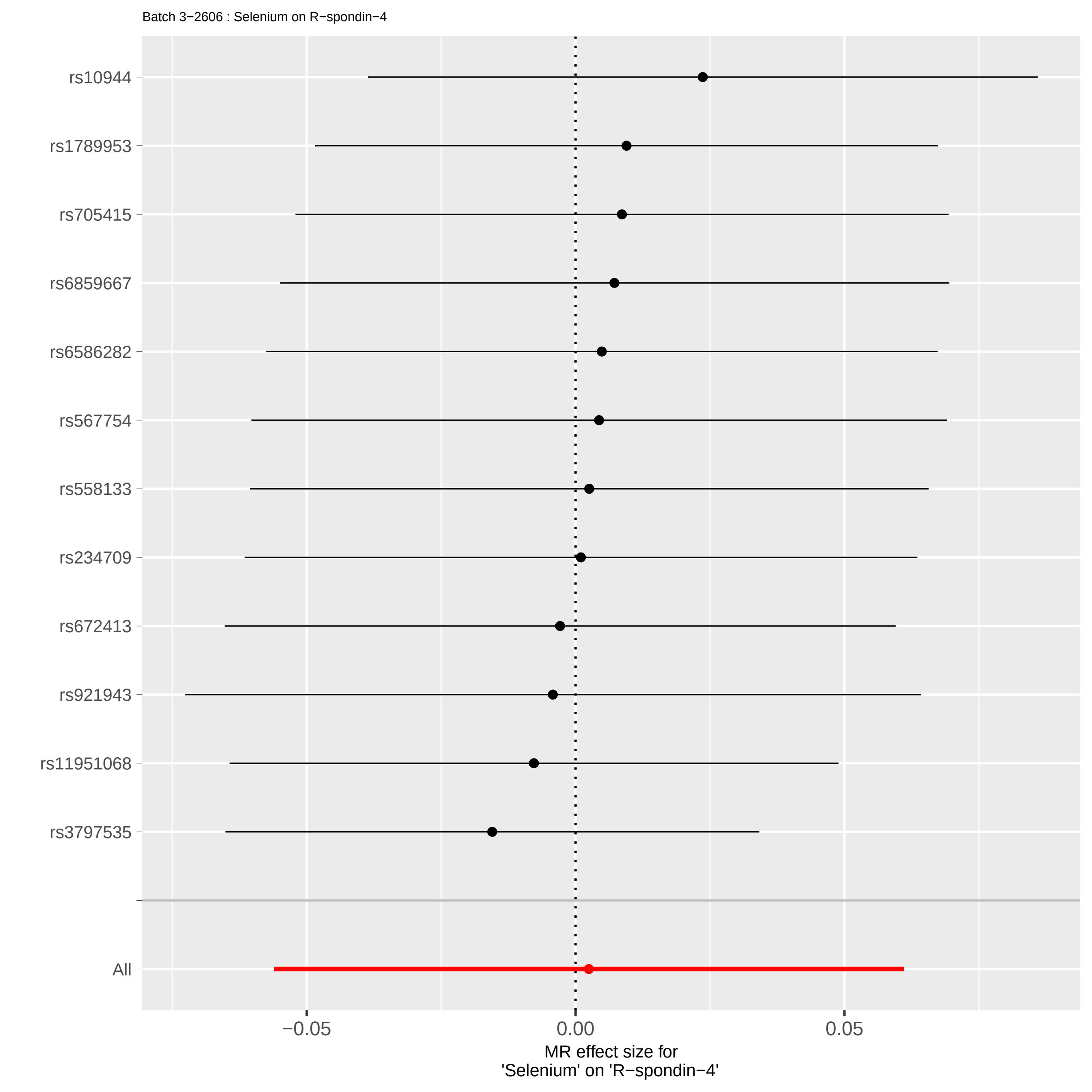

Batch 3−2606 : Selenium on R−spondin−4
rs10944
rs1789953
rs705415
rs6859667
rs6586282
rs567754
rs558133
rs234709
rs672413
rs921943
rs11951068
rs3797535
All
−0.05
0.00
0.05
MR effect size for
'Selenium' on 'R−spondin−4'

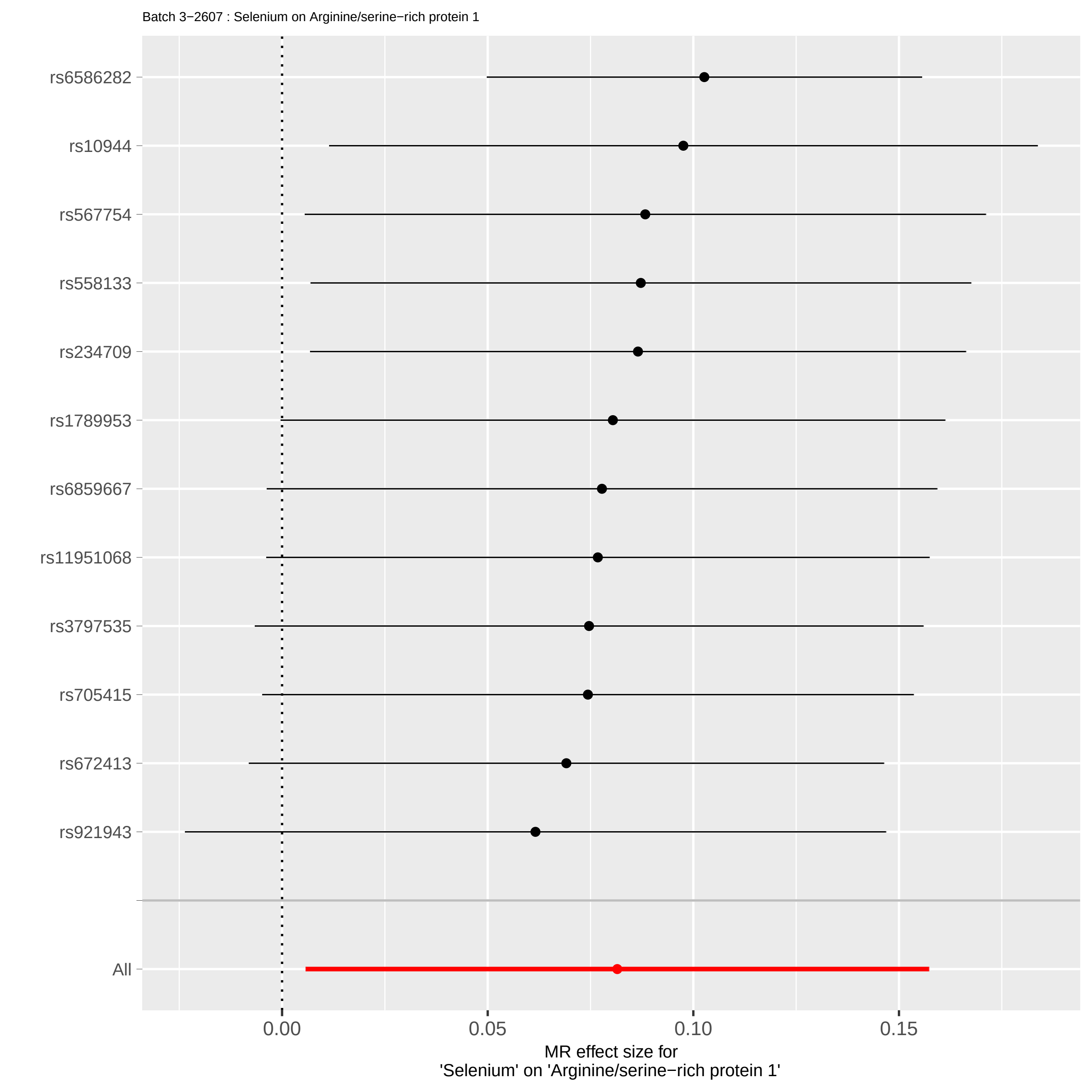
Batch 3−2607 : Selenium on Arginine/serine−rich protein 1
rs6586282
rs10944
rs567754
rs558133
rs234709
rs1789953
rs6859667
rs11951068
rs3797535
rs705415
rs672413
rs921943
All
0.00
0.05
0.10
0.15
MR effect size for
'Selenium' on 'Arginine/serine−rich protein 1'

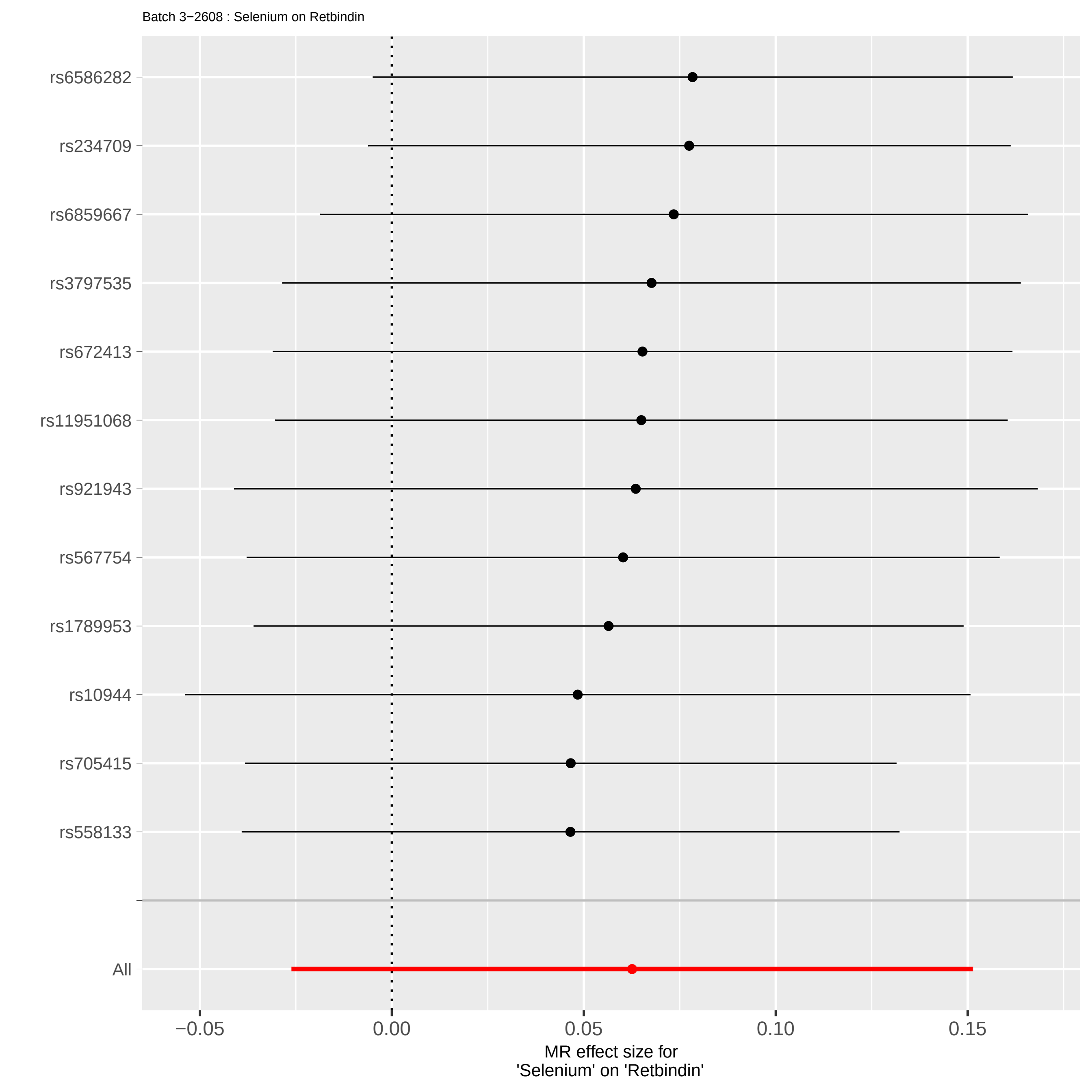

Batch 3−2608 : Selenium on Retbindin
rs6586282
rs234709
rs6859667
rs3797535
rs672413
rs11951068
rs921943
rs567754
rs1789953
rs10944
rs705415
rs558133
All
−0.05
0.00
0.05
0.10
0.15
MR effect size for
'Selenium' on 'Retbindin'

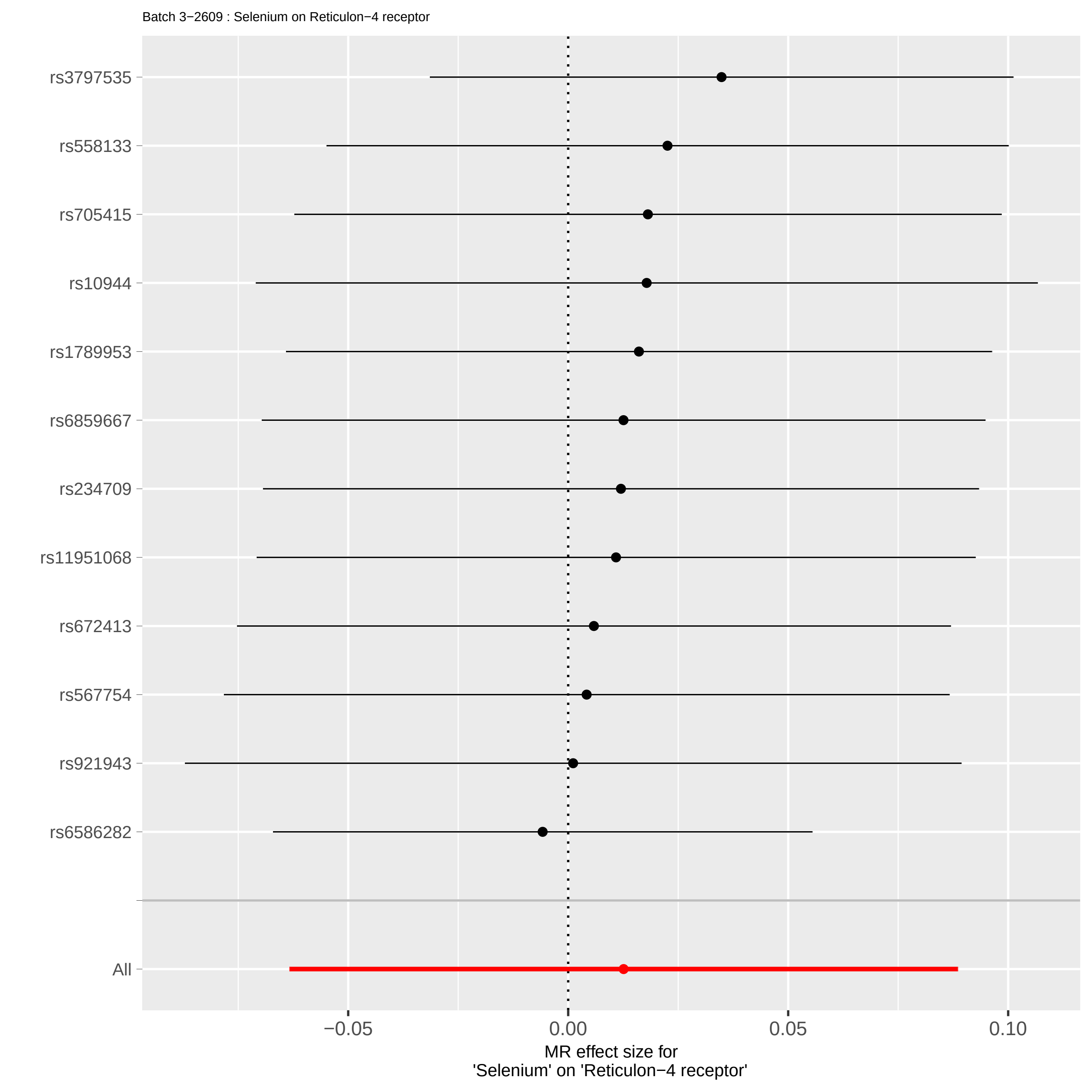

Batch 3−2609 : Selenium on Reticulon−4 receptor
rs3797535
rs558133
rs705415
rs10944
rs1789953
rs6859667
rs234709
rs11951068
rs672413
rs567754
rs921943
rs6586282
All
−0.05
0.00
0.05
0.10
MR effect size for
'Selenium' on 'Reticulon−4 receptor'

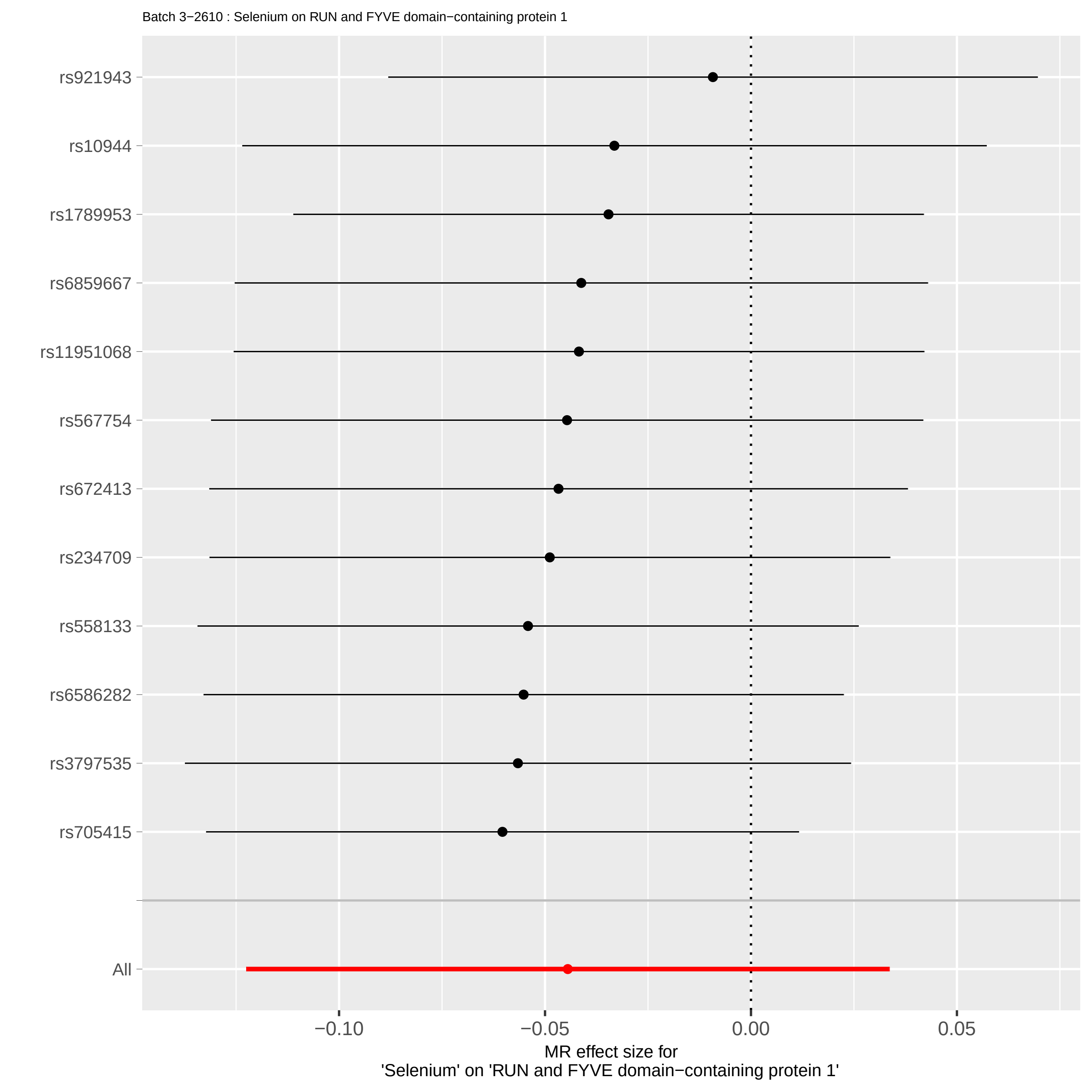

Batch 3−2610 : Selenium on RUN and FYVE domain−containing protein 1
rs921943
rs10944
rs1789953
rs6859667
rs11951068
rs567754
rs672413
rs234709
rs558133
rs6586282
rs3797535
rs705415
All
−0.10
−0.05
0.00
0.05
MR effect size for
'Selenium' on 'RUN and FYVE domain−containing protein 1'

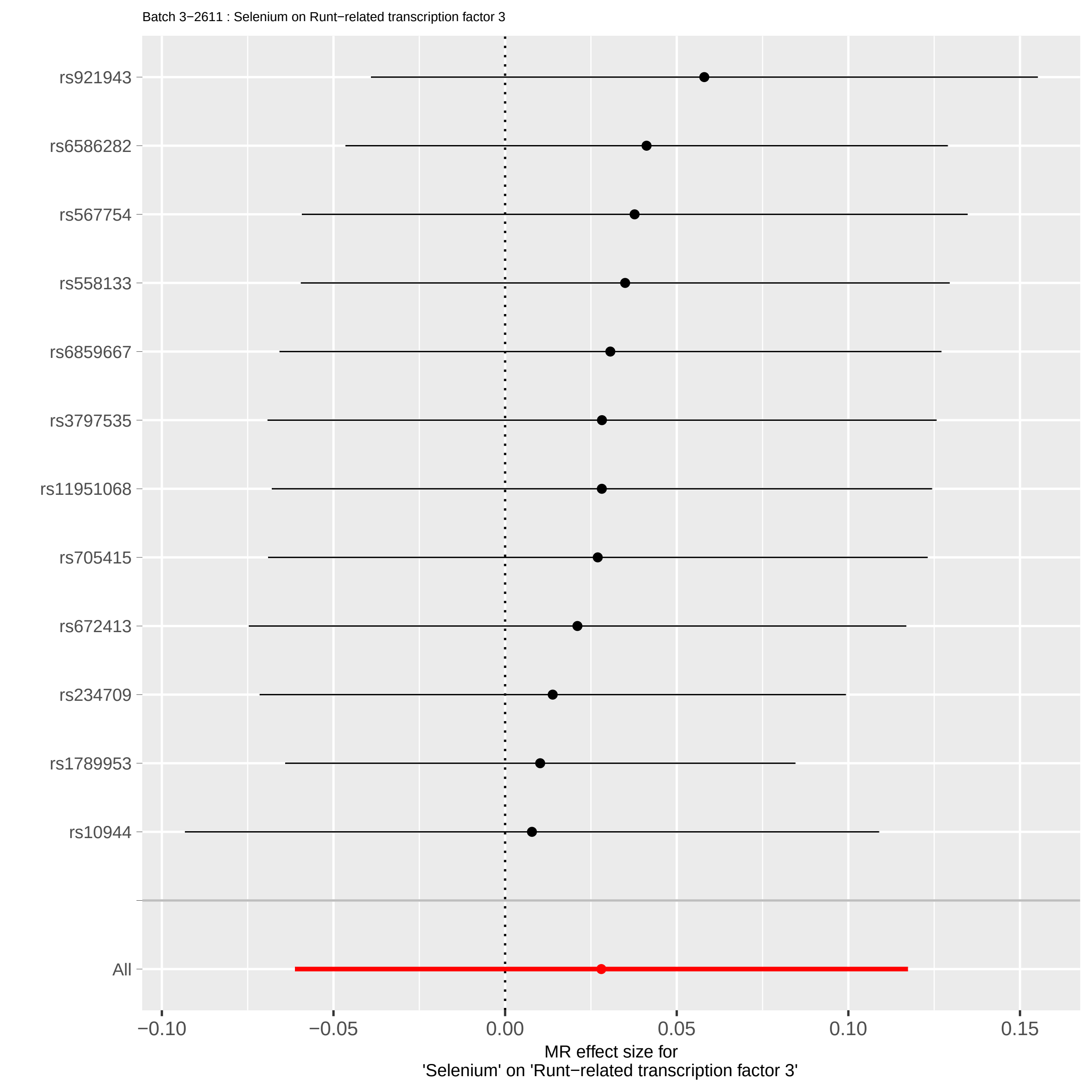

Batch 3−2611 : Selenium on Runt−related transcription factor 3
rs921943
rs6586282
rs567754
rs558133
rs6859667
rs3797535
rs11951068
rs705415
rs672413
rs234709
rs1789953
rs10944
All
−0.10
−0.05
0.00
0.05
0.10
0.15
MR effect size for
'Selenium' on 'Runt−related transcription factor 3'

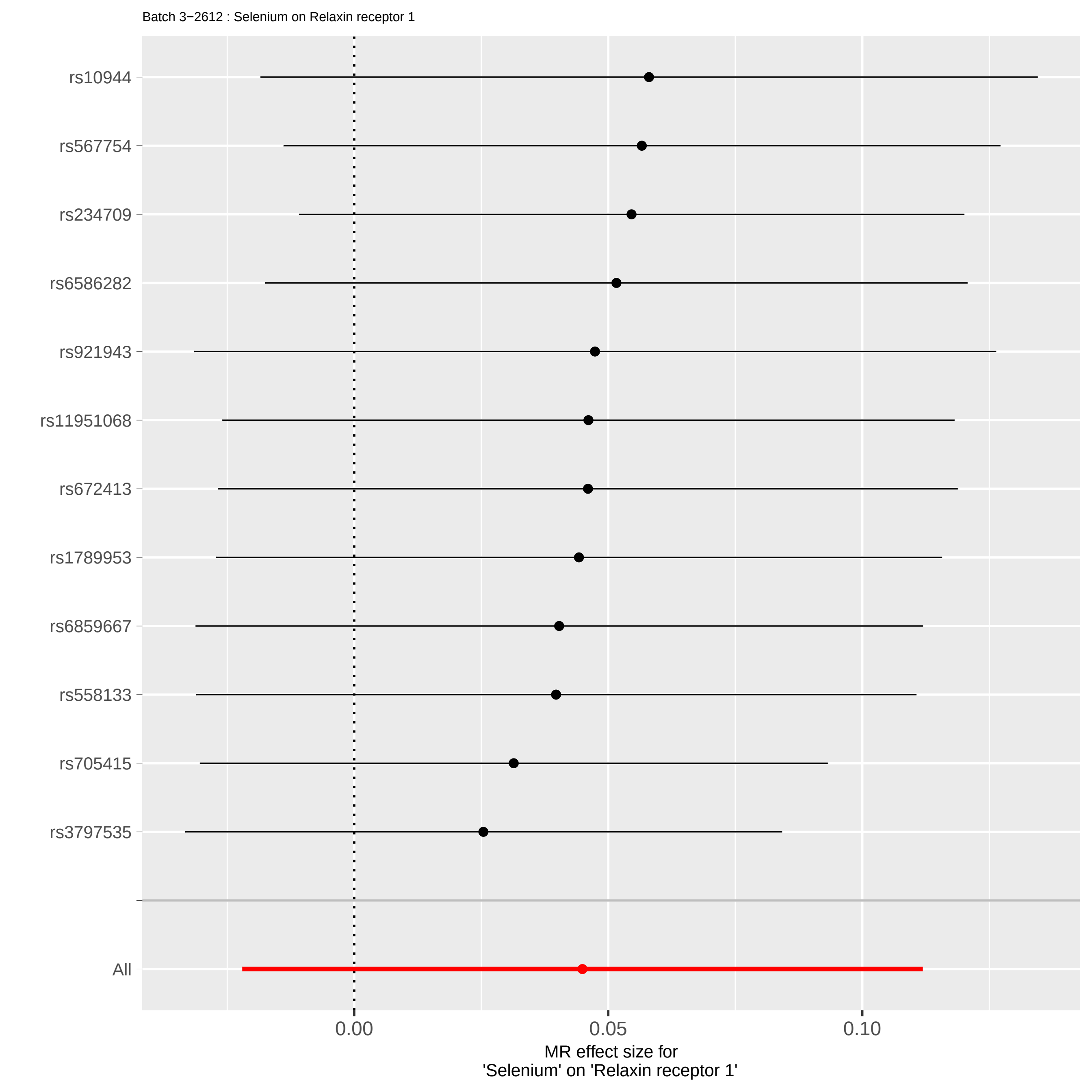

Batch 3−2612 : Selenium on Relaxin receptor 1
rs10944
rs567754
rs234709
rs6586282
rs921943
rs11951068
rs672413
rs1789953
rs6859667
rs558133
rs705415
rs3797535
All
0.00
0.05
0.10
MR effect size for
'Selenium' on 'Relaxin receptor 1'

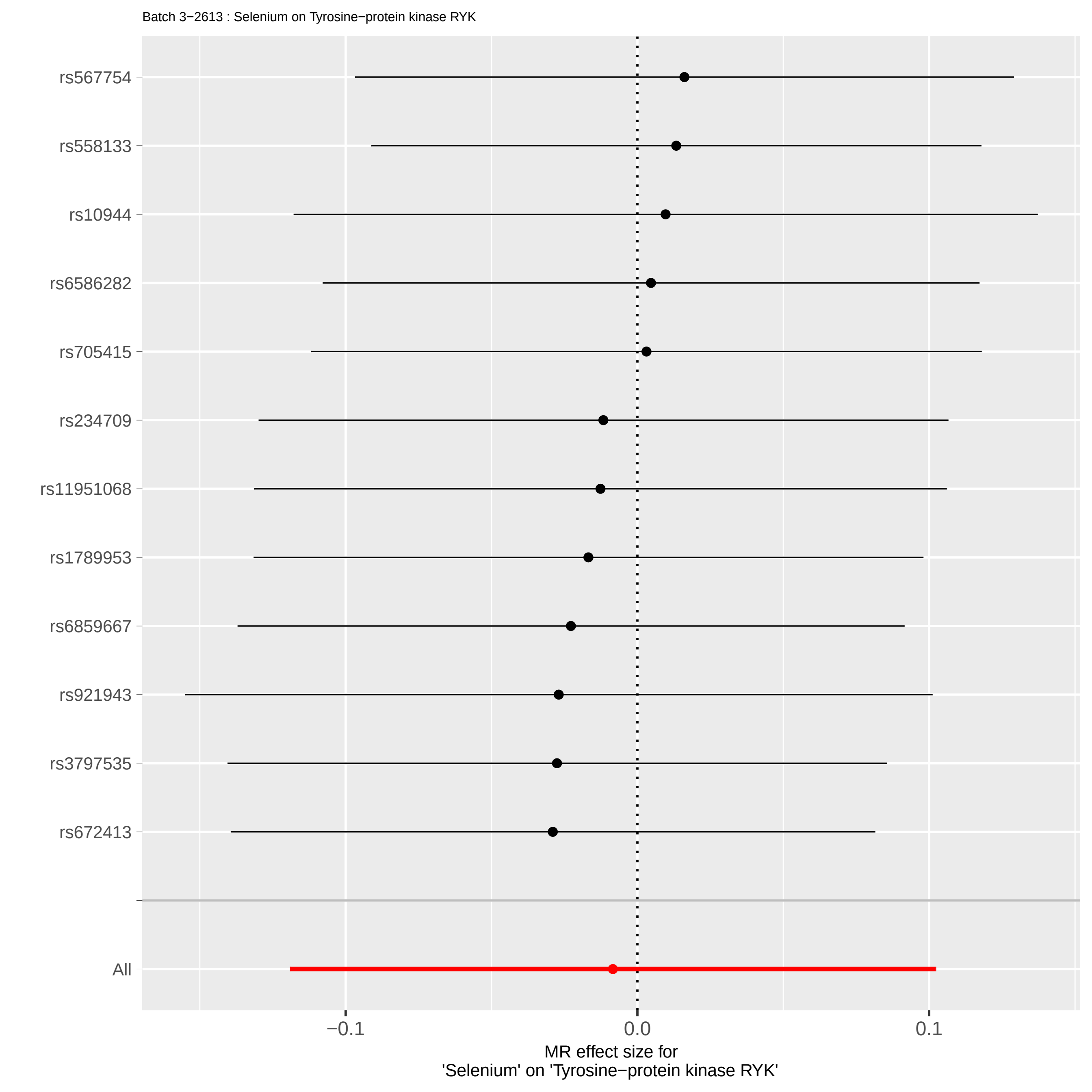

Batch 3−2613 : Selenium on Tyrosine−protein kinase RYK
rs567754
rs558133
rs10944
rs6586282
rs705415
rs234709
rs11951068
rs1789953
rs6859667
rs921943
rs3797535
rs672413
All
−0.1
0.0
0.1
MR effect size for
'Selenium' on 'Tyrosine−protein kinase RYK'

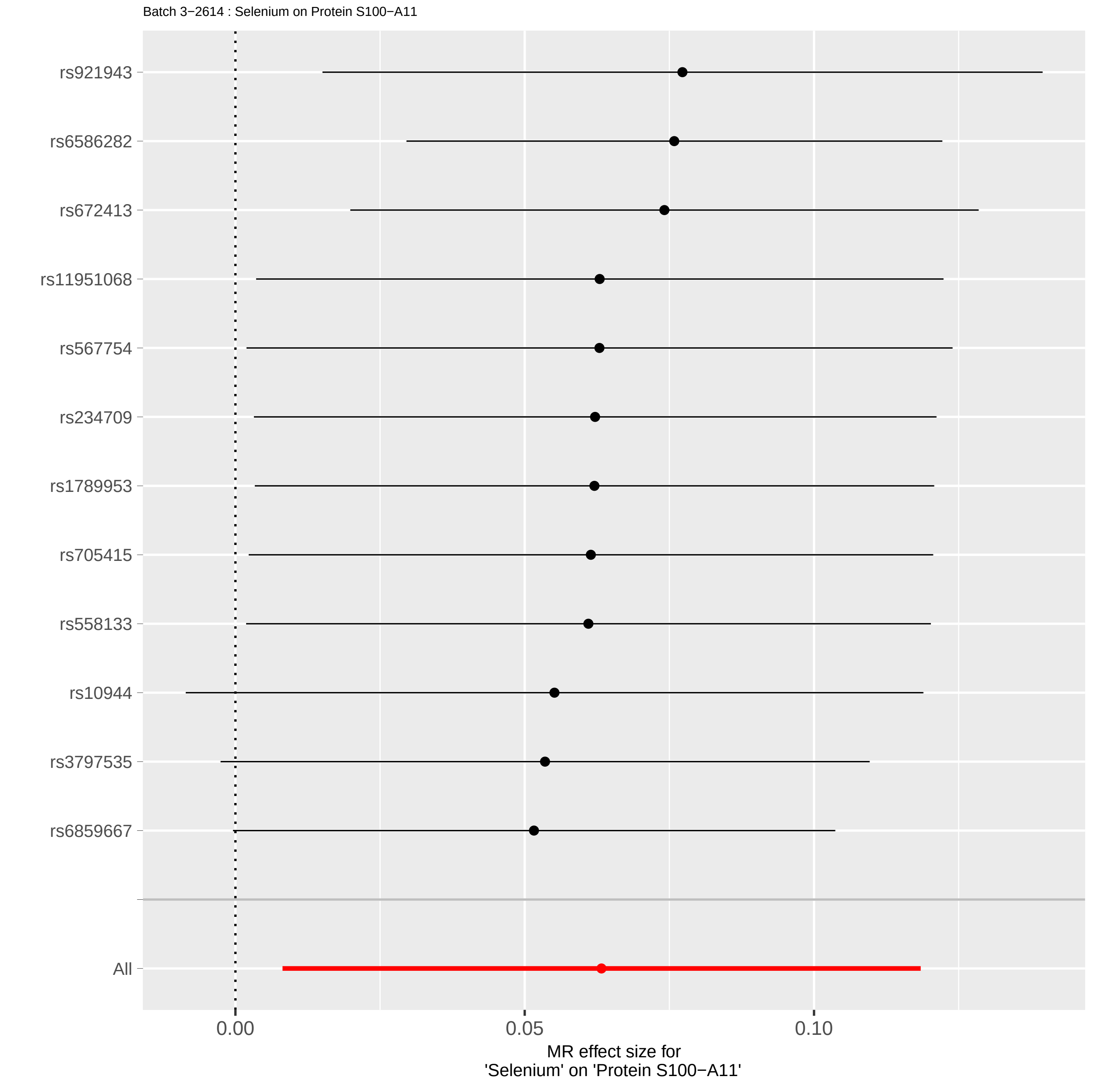

Batch 3−2614 : Selenium on Protein S100−A11
rs921943
rs6586282
rs672413
rs11951068
rs567754
rs234709
rs1789953
rs705415
rs558133
rs10944
rs3797535
rs6859667
All
0.00
0.05
0.10
MR effect size for
'Selenium' on 'Protein S100−A11'

Batch 3−2615 : Selenium on Protein S100−A12

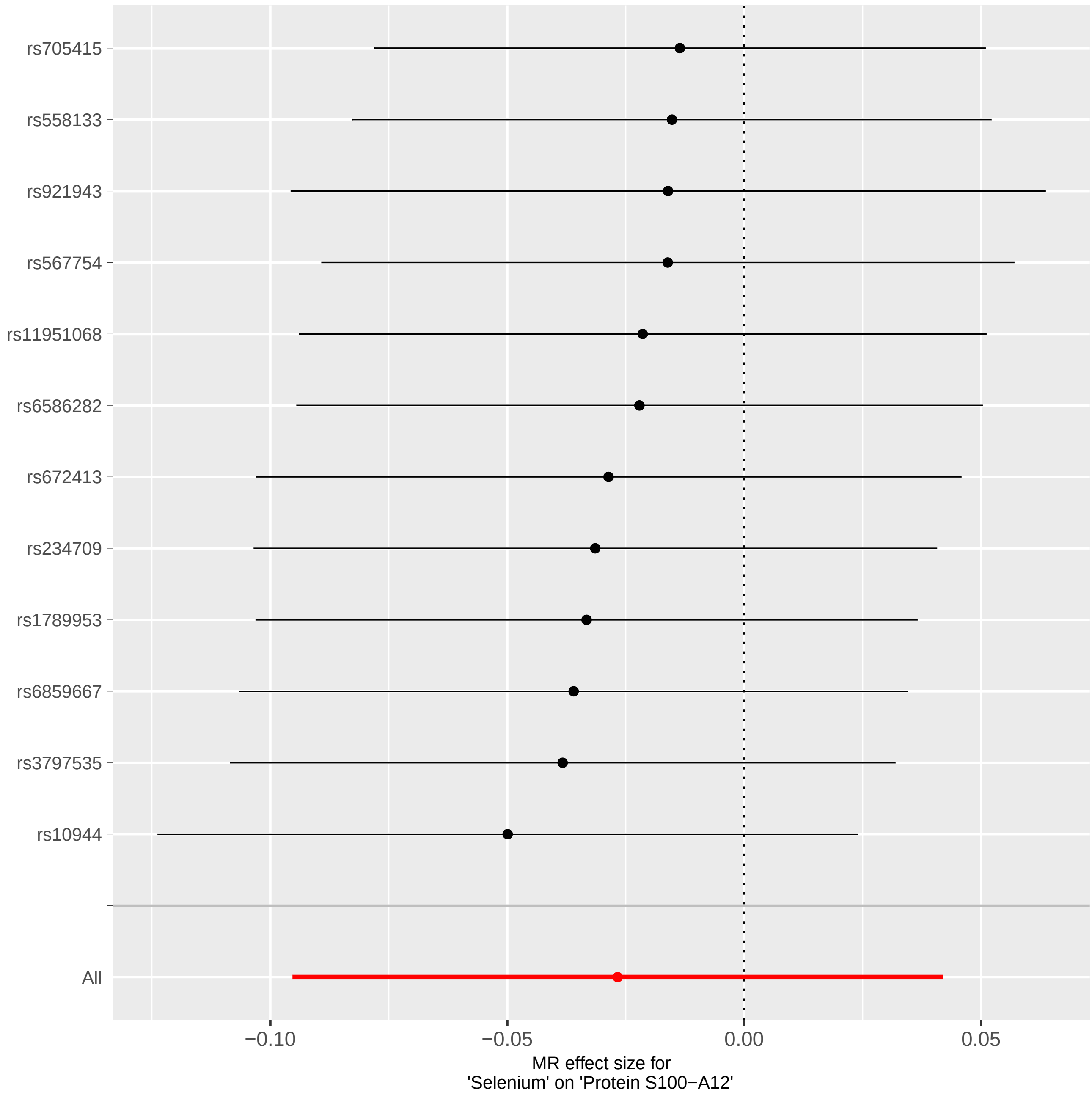

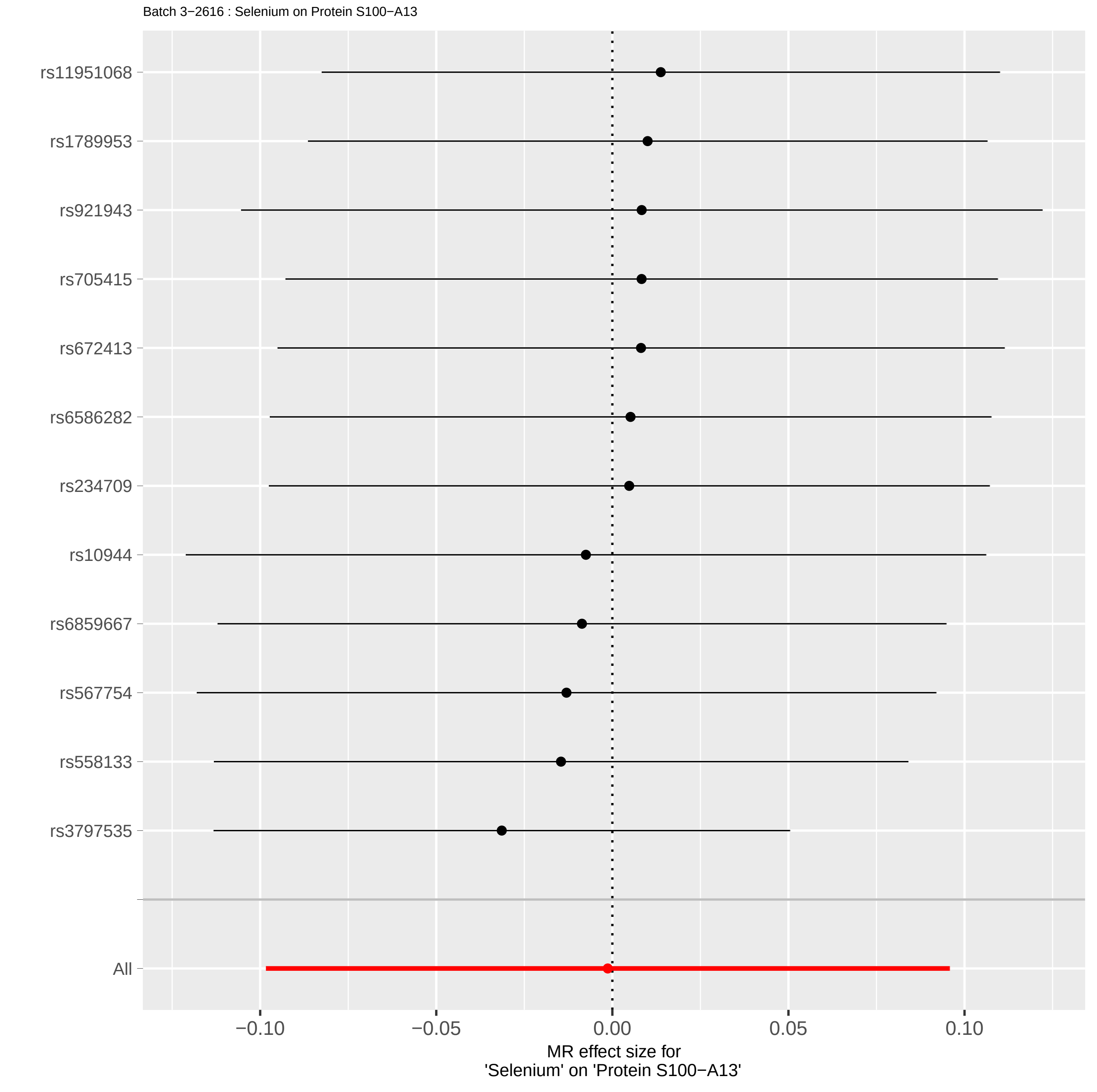
Batch 3−2616 : Selenium on Protein S100−A13
rs11951068
rs1789953
rs921943
rs705415
rs672413
rs6586282
rs234709
rs10944
rs6859667
rs567754
rs558133
rs3797535
All
−0.10
−0.05
0.00
0.05
0.10
MR effect size for
'Selenium' on 'Protein S100−A13'

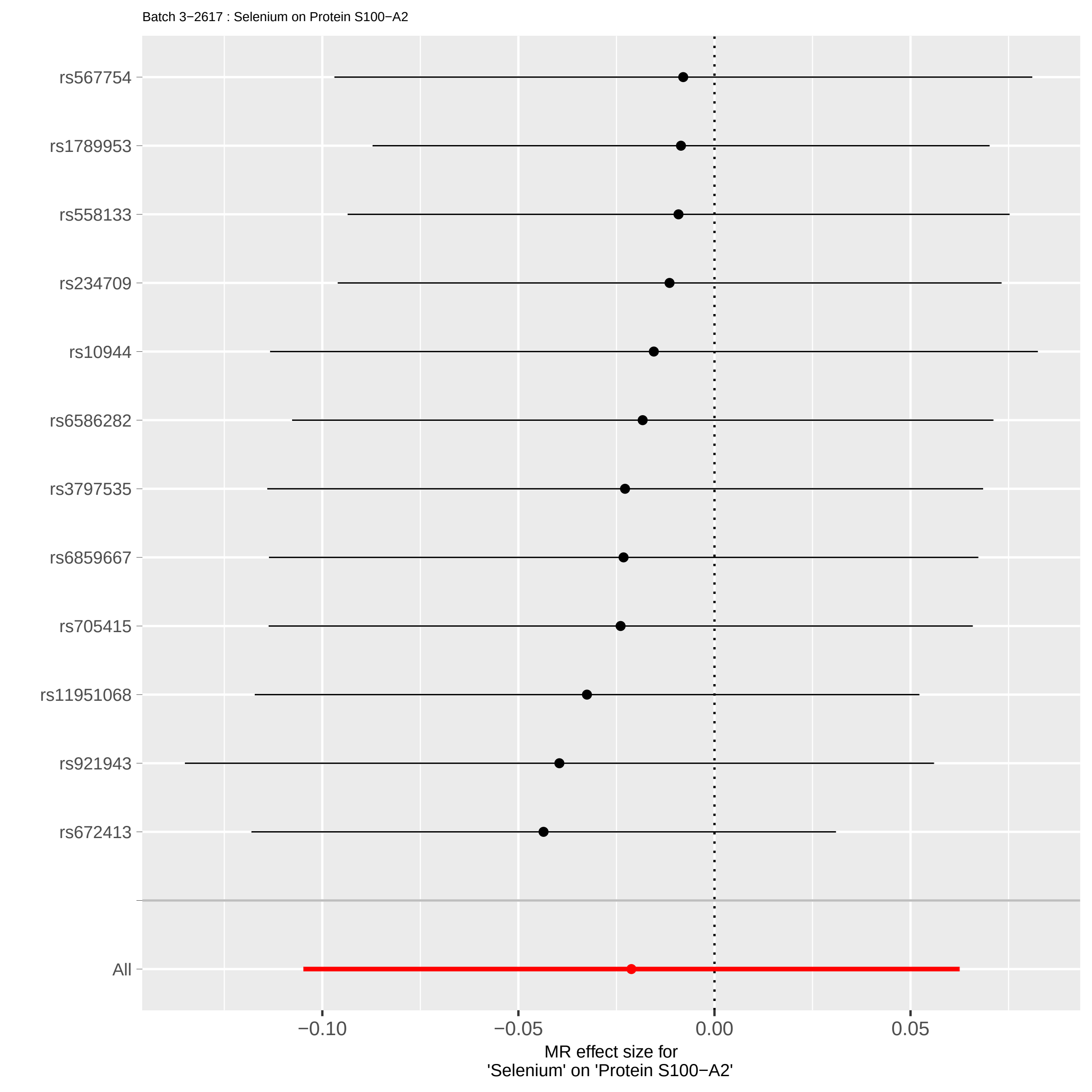

Batch 3−2617 : Selenium on Protein S100−A2
rs567754
rs1789953
rs558133
rs234709
rs10944
rs6586282
rs3797535
rs6859667
rs705415
rs11951068
rs921943
rs672413
All
−0.10
−0.05
0.00
0.05
MR effect size for
'Selenium' on 'Protein S100−A2'

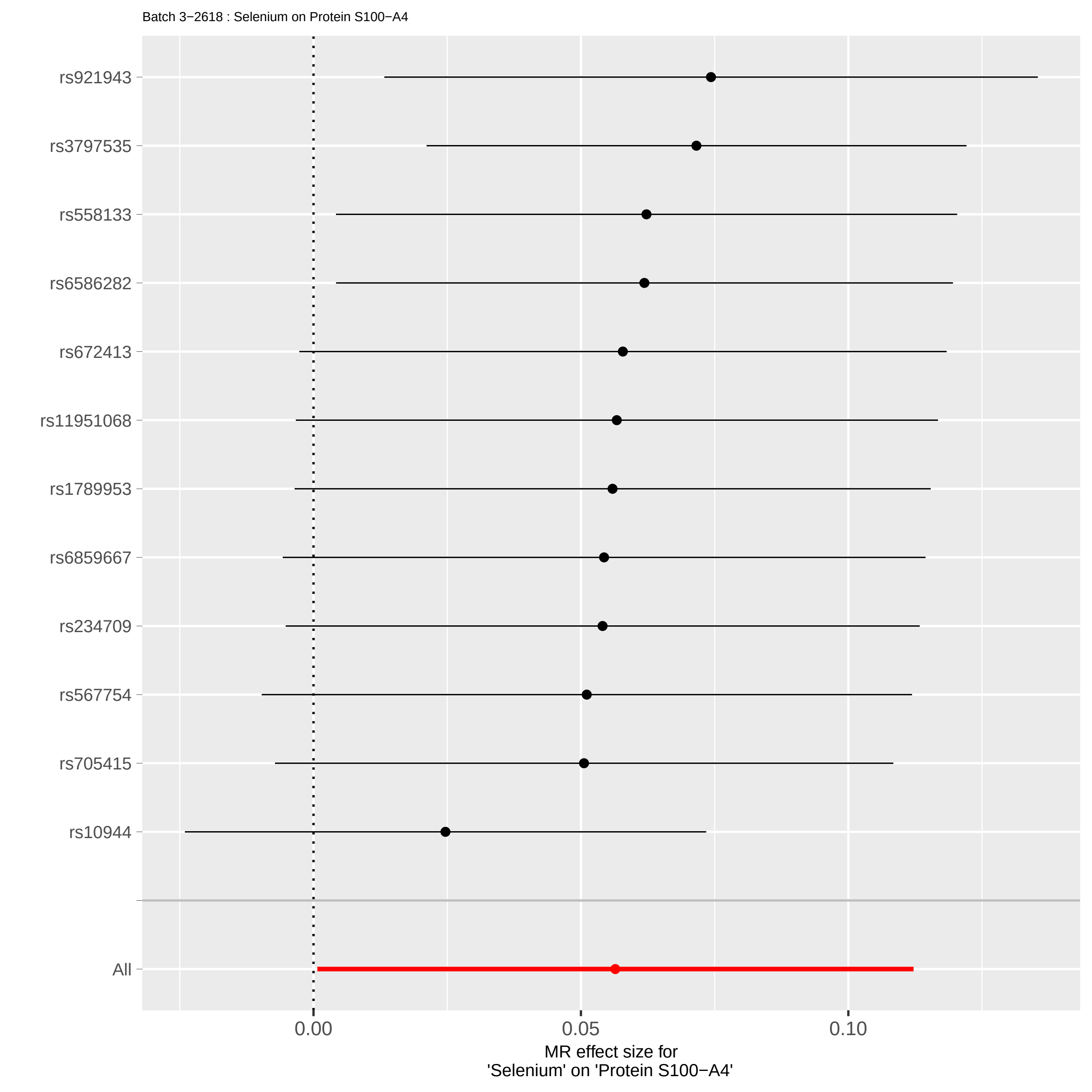
Batch 3−2618 : Selenium on Protein S100−A4
rs921943
rs3797535
rs558133
rs6586282
rs672413
rs11951068
rs1789953
rs6859667
rs234709
rs567754
rs705415
rs10944
All
0.00
0.05
0.10
MR effect size for
'Selenium' on 'Protein S100−A4'

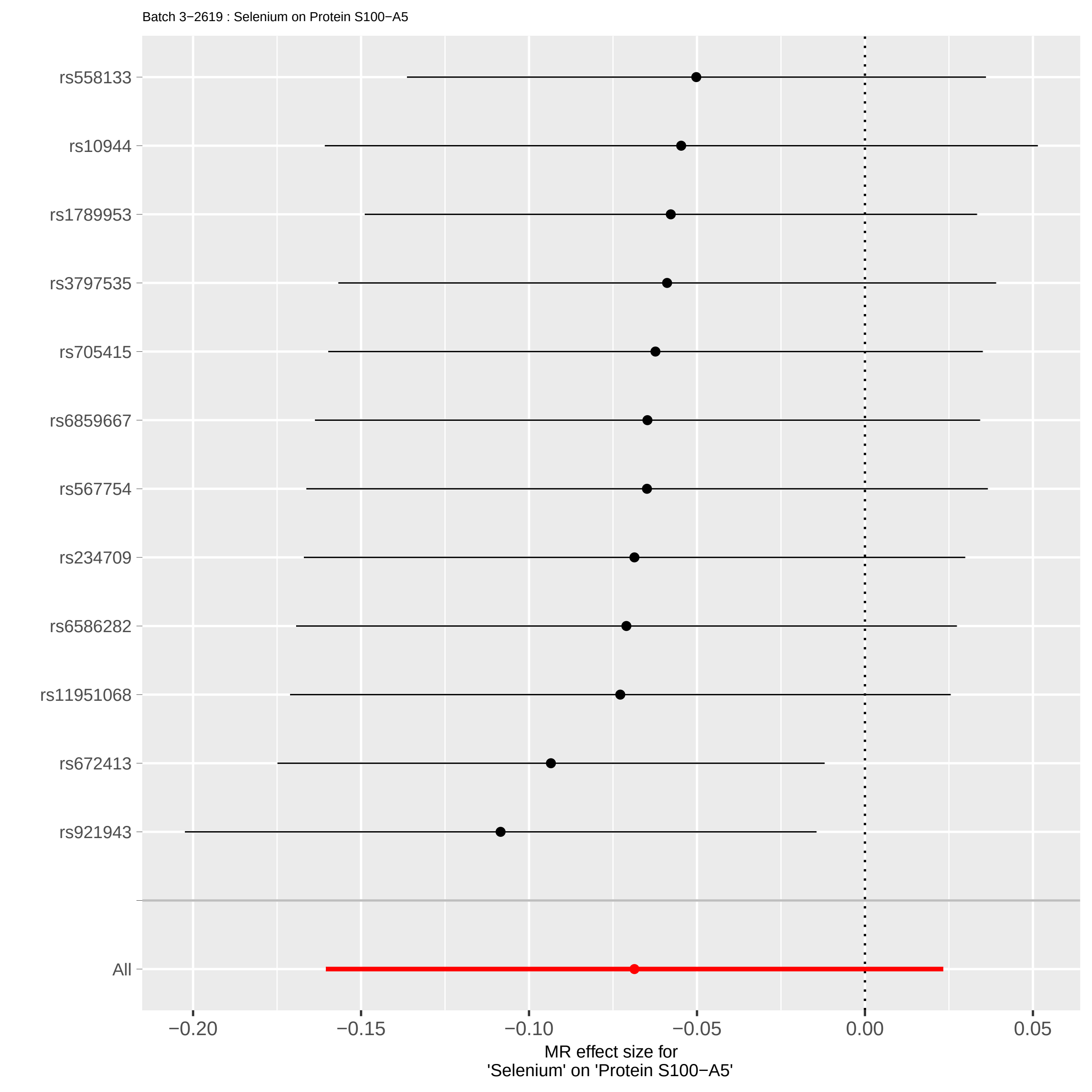

Batch 3−2619 : Selenium on Protein S100−A5
rs558133
rs10944
rs1789953
rs3797535
rs705415
rs6859667
rs567754
rs234709
rs6586282
rs11951068
rs672413
rs921943
All
−0.20
−0.15
−0.10
−0.05
0.00
0.05
MR effect size for
'Selenium' on 'Protein S100−A5'

Batch 3−2620 : Selenium on Protein S100−A6

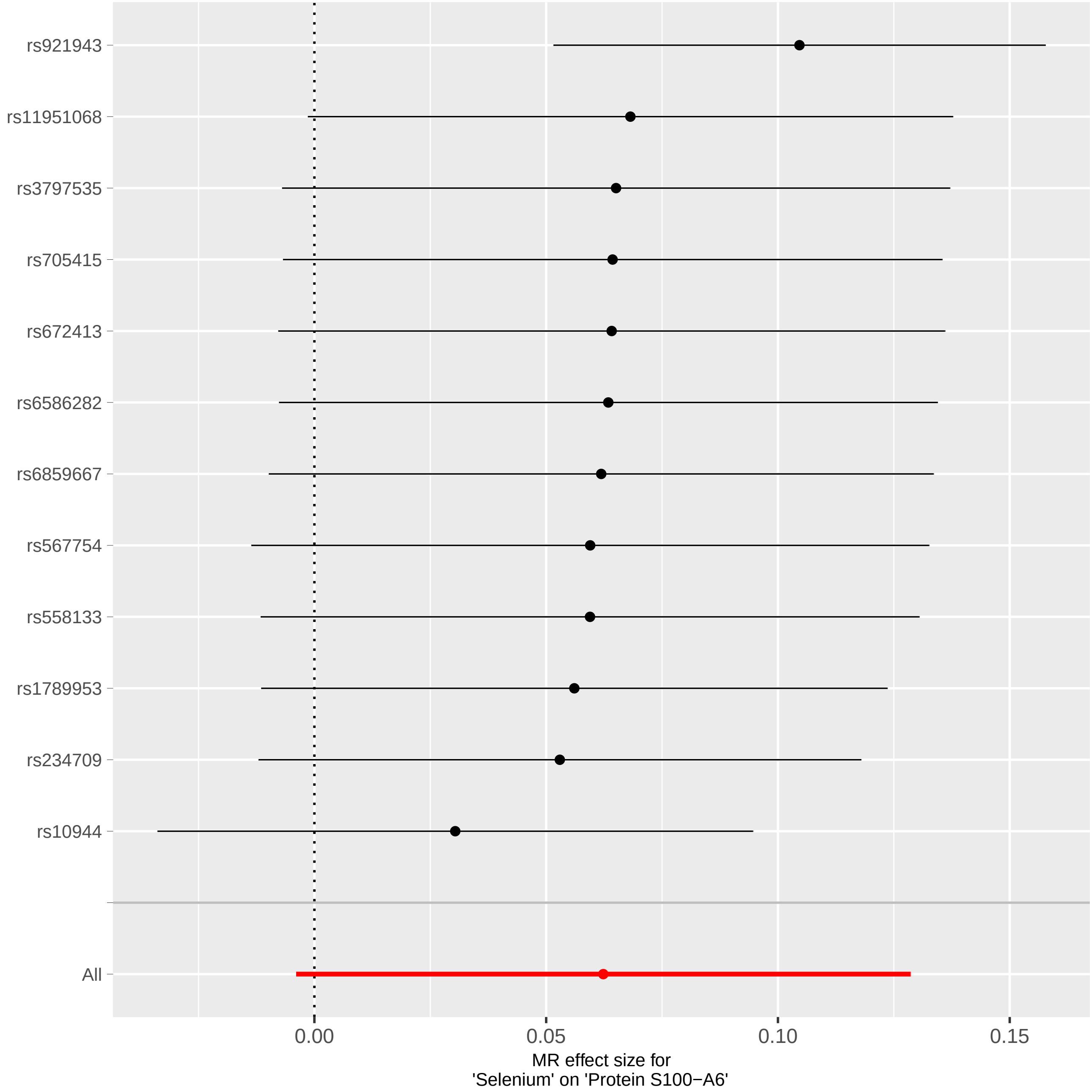

Batch 3−2621 : Selenium on Protein S100−A7

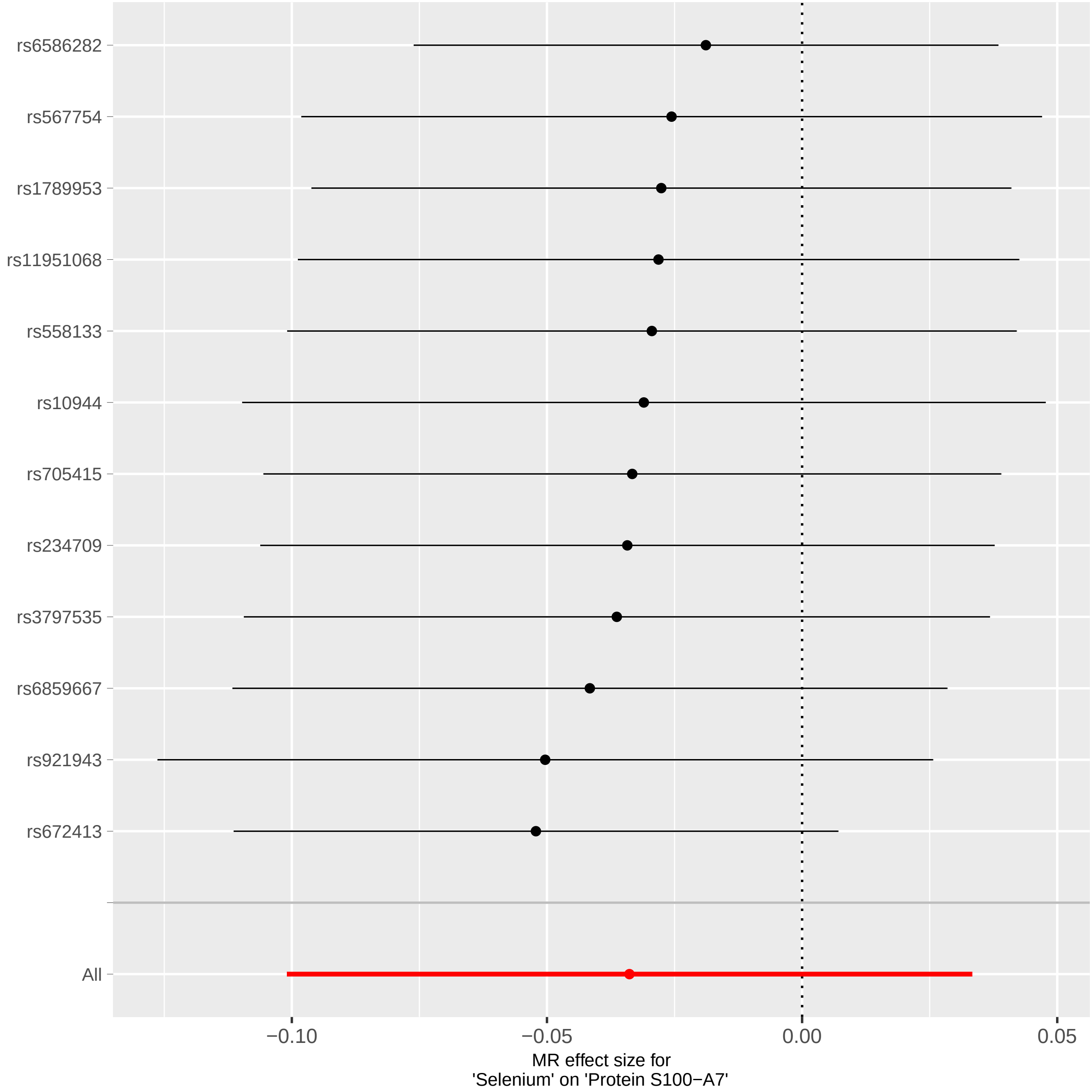

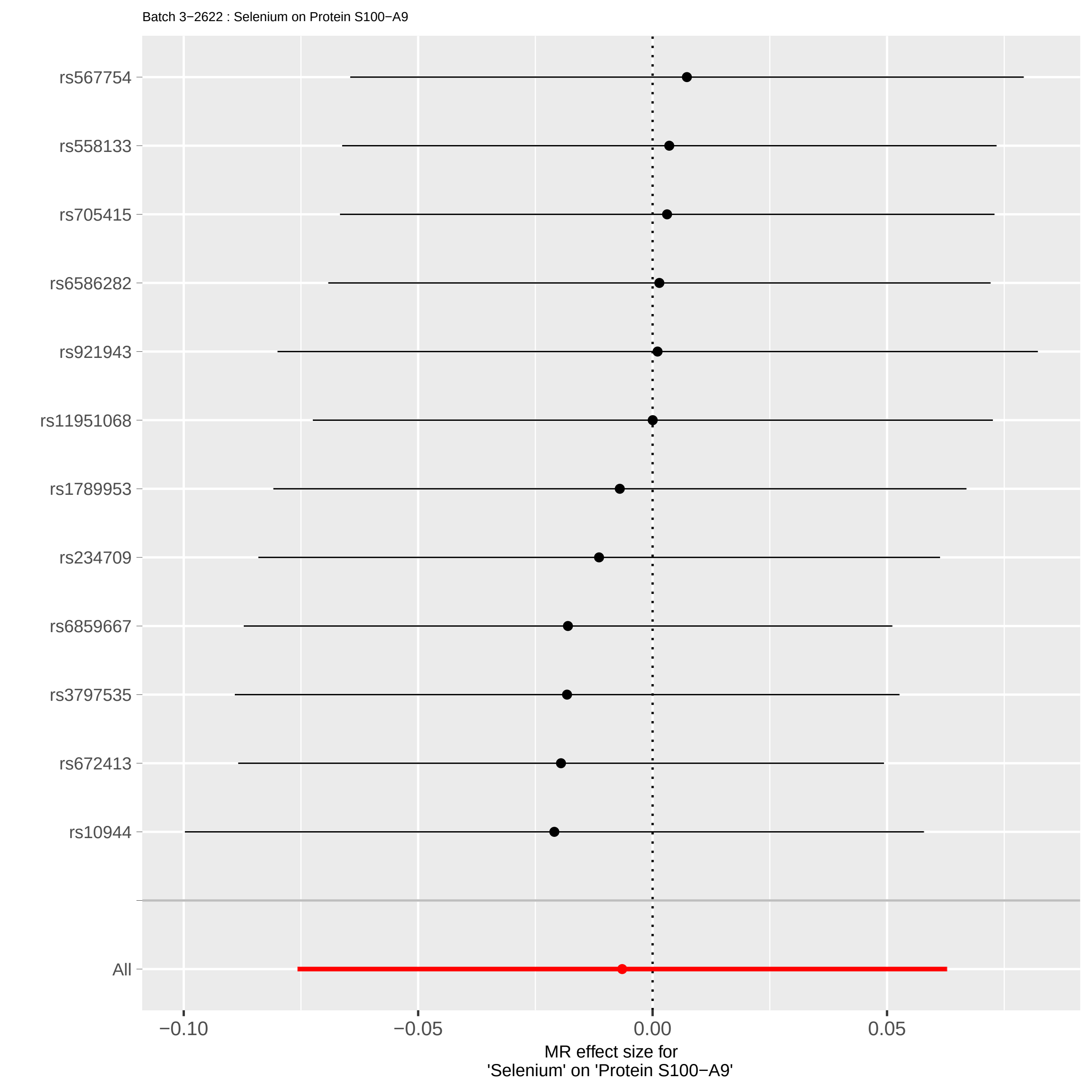

Batch 3−2622 : Selenium on Protein S100−A9
rs567754
rs558133
rs705415
rs6586282
rs921943
rs11951068
rs1789953
rs234709
rs6859667
rs3797535
rs672413
rs10944
All
−0.10
−0.05
0.00
0.05
MR effect size for
'Selenium' on 'Protein S100−A9'

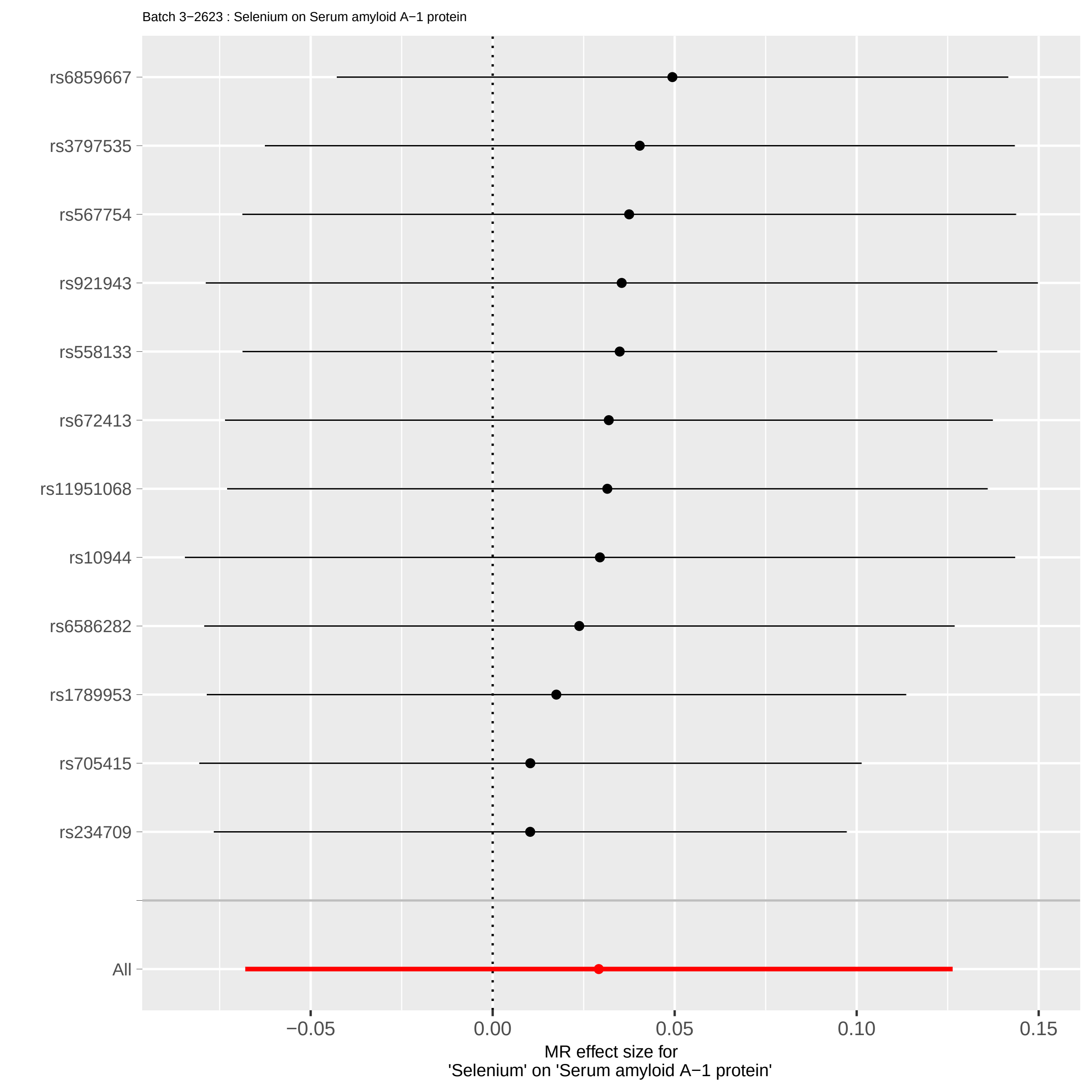

Batch 3−2623 : Selenium on Serum amyloid A−1 protein
rs6859667
rs3797535
rs567754
rs921943
rs558133
rs672413
rs11951068
rs10944
rs6586282
rs1789953
rs705415
rs234709
All
−0.05
0.00
0.05
0.10
0.15
MR effect size for
'Selenium' on 'Serum amyloid A−1 protein'

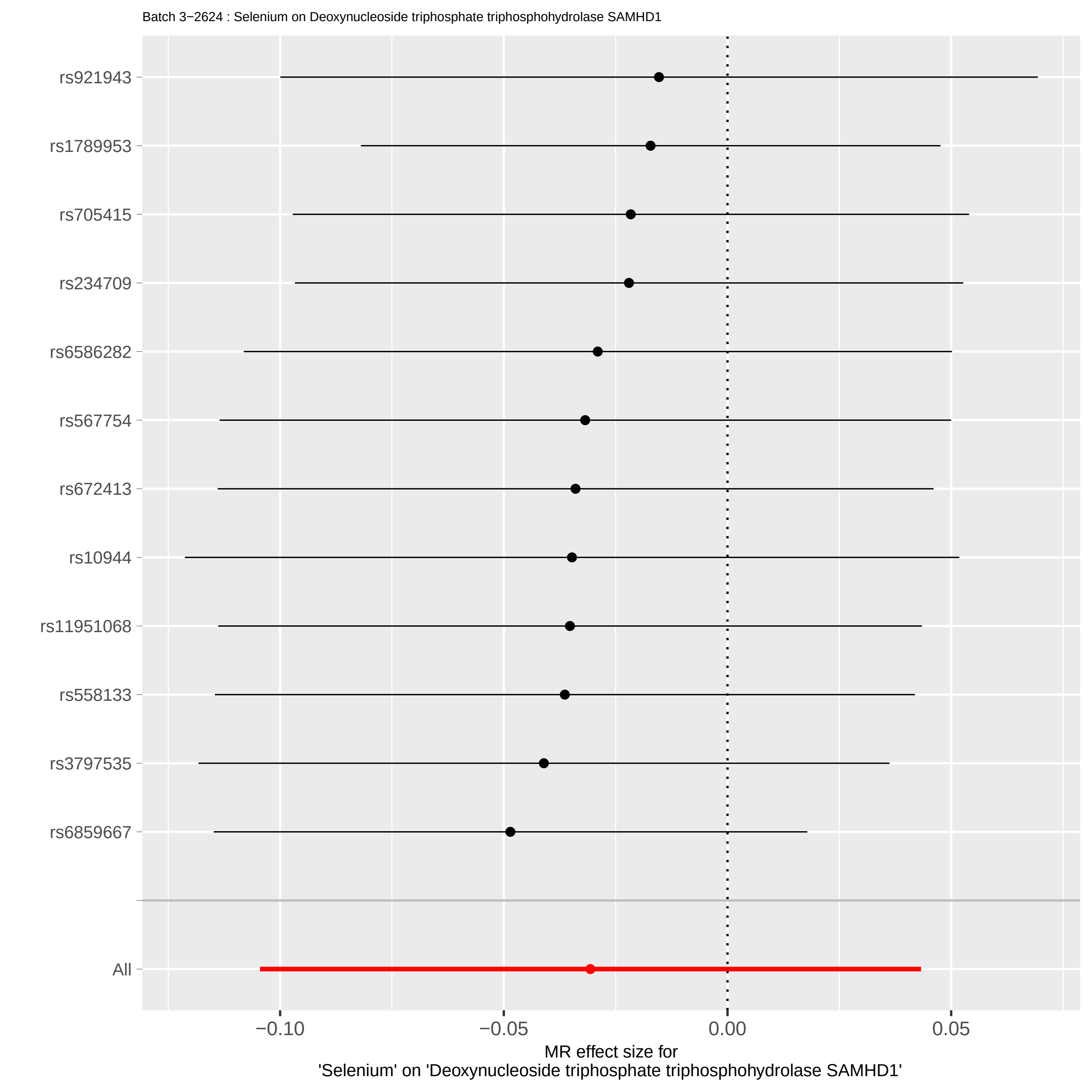

Batch 3−2624 : Selenium on Deoxynucleoside triphosphate triphosphohydrolase SAMHD1
rs921943
rs1789953
rs705415
rs234709
rs6586282
rs567754
rs672413
rs10944
rs11951068
rs558133
rs3797535
rs6859667
All
−0.10
−0.05
0.00
0.05
MR effect size for
'Selenium' on 'Deoxynucleoside triphosphate triphosphohydrolase SAMHD1'

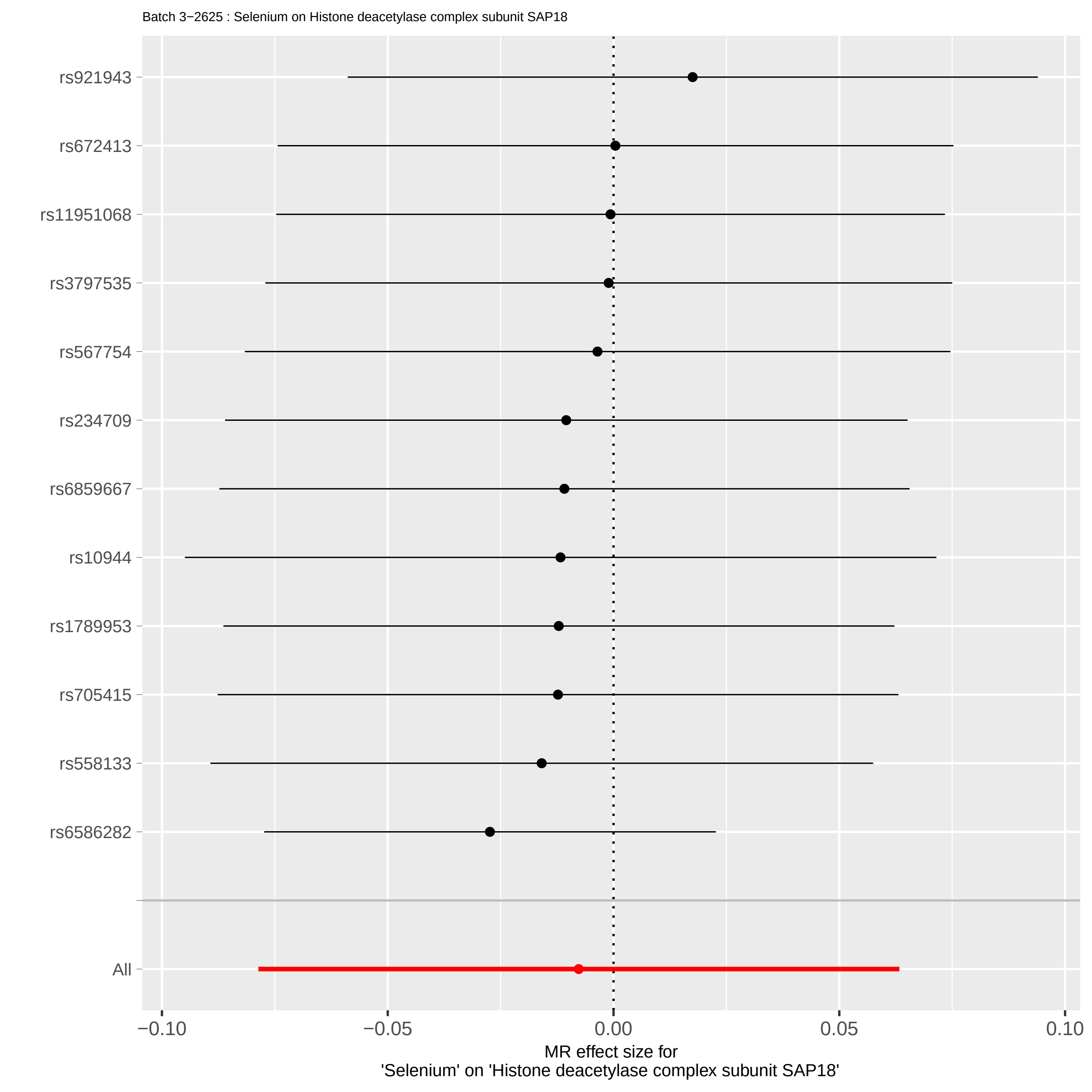
Batch 3−2625 : Selenium on Histone deacetylase complex subunit SAP18
rs921943
rs672413
rs11951068
rs3797535
rs567754
rs234709
rs6859667
rs10944
rs1789953
rs705415
rs558133
rs6586282
All
−0.10
−0.05
0.00
0.05
0.10
MR effect size for
'Selenium' on 'Histone deacetylase complex subunit SAP18'

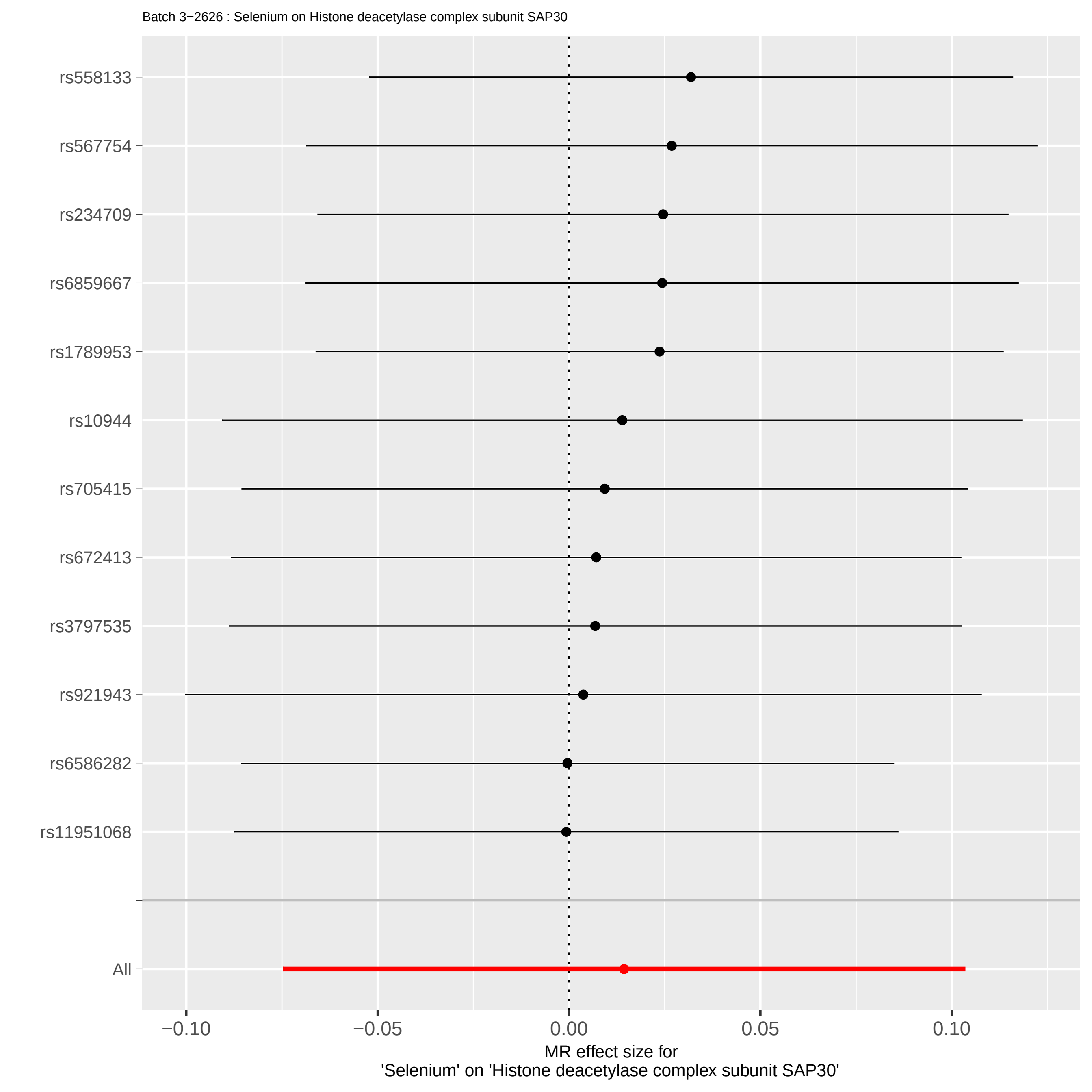
Batch 3−2626 : Selenium on Histone deacetylase complex subunit SAP30
rs558133
rs567754
rs234709
rs6859667
rs1789953
rs10944
rs705415
rs672413
rs3797535
rs921943
rs6586282
rs11951068
All
−0.10
−0.05
0.00
0.05
0.10
MR effect size for
'Selenium' on 'Histone deacetylase complex subunit SAP30'

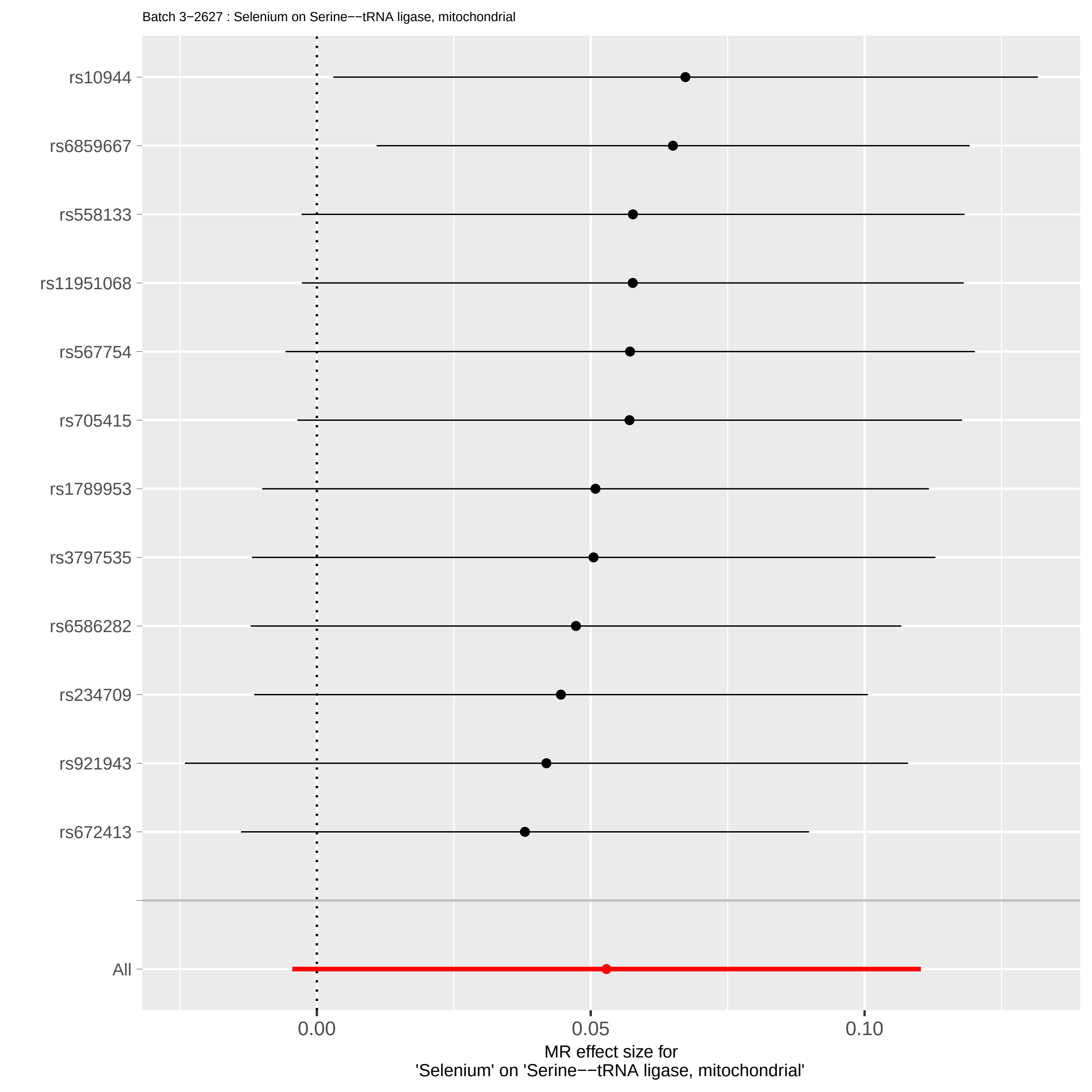

Batch 3−2627 : Selenium on Serine−−tRNA ligase, mitochondrial
rs10944
rs6859667
rs558133
rs11951068
rs567754
rs705415
rs1789953
rs3797535
rs6586282
rs234709
rs921943
rs672413
All
0.00
0.05
0.10
MR effect size for
'Selenium' on 'Serine−−tRNA ligase, mitochondrial'

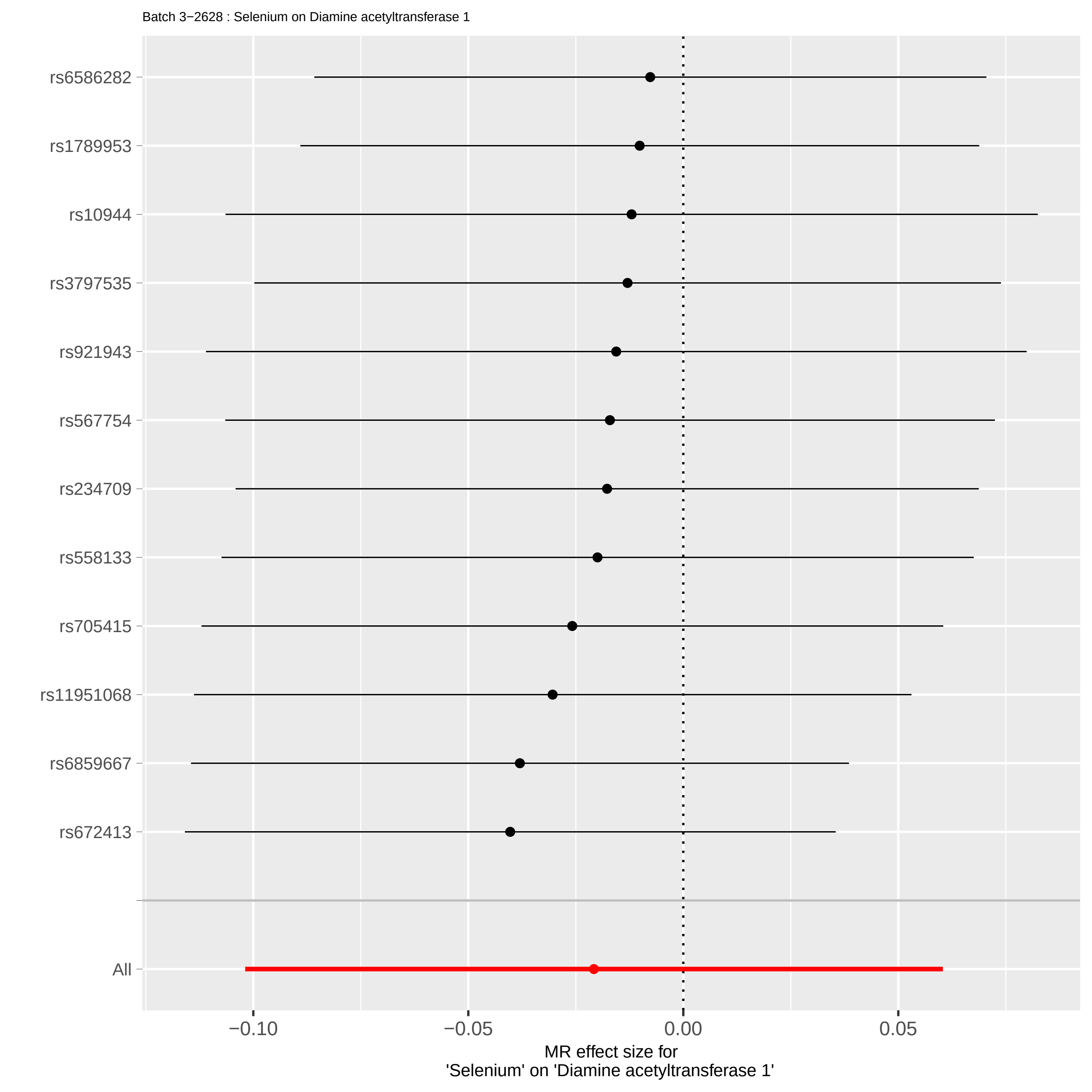

Batch 3−2628 : Selenium on Diamine acetyltransferase 1
rs6586282
rs1789953
rs10944
rs3797535
rs921943
rs567754
rs234709
rs558133
rs705415
rs11951068
rs6859667
rs672413
All
−0.10
−0.05
0.00
0.05
MR effect size for
'Selenium' on 'Diamine acetyltransferase 1'

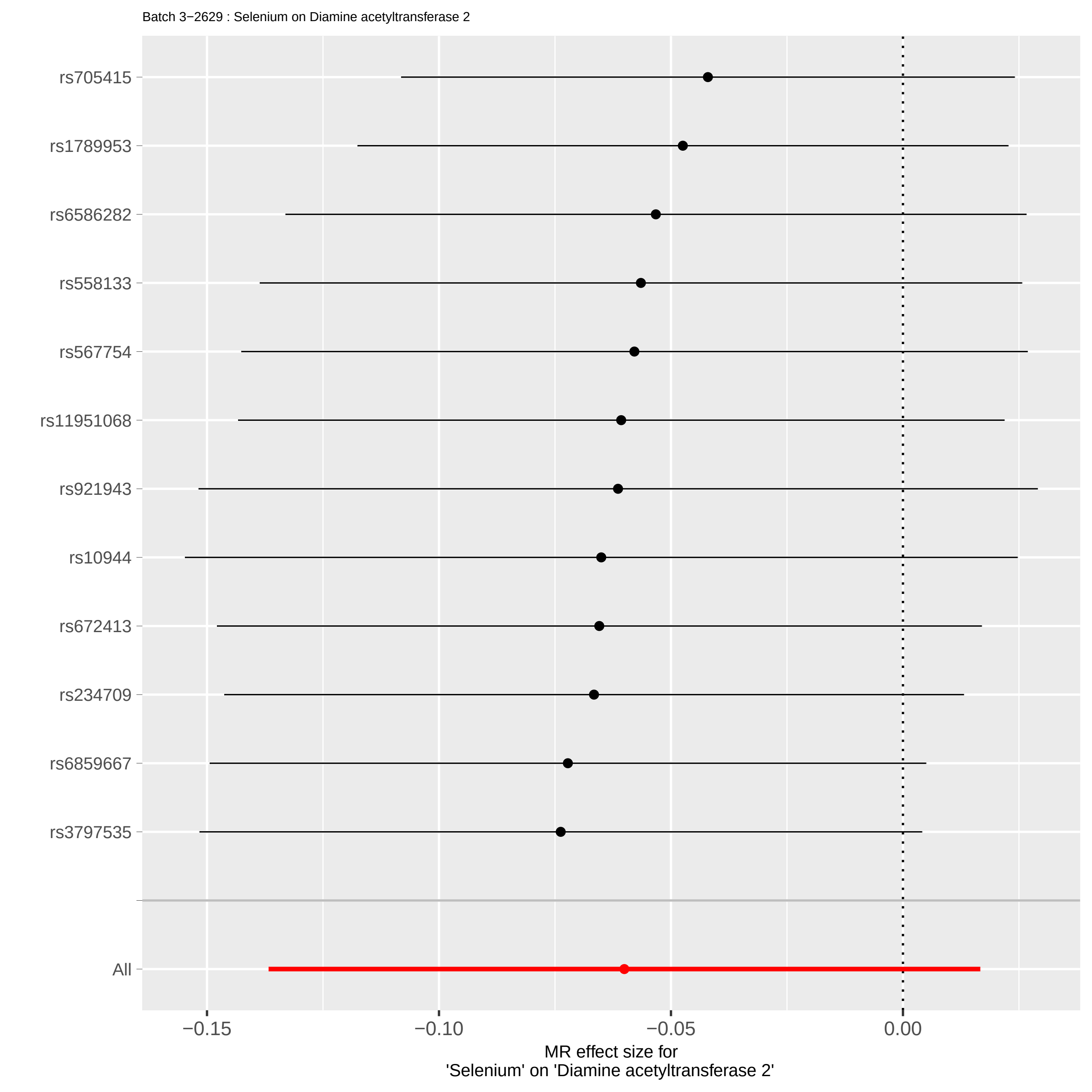

Batch 3−2629 : Selenium on Diamine acetyltransferase 2
rs705415
rs1789953
rs6586282
rs558133
rs567754
rs11951068
rs921943
rs10944
rs672413
rs234709
rs6859667
rs3797535
All
−0.15
−0.10
−0.05
0.00
MR effect size for
'Selenium' on 'Diamine acetyltransferase 2'

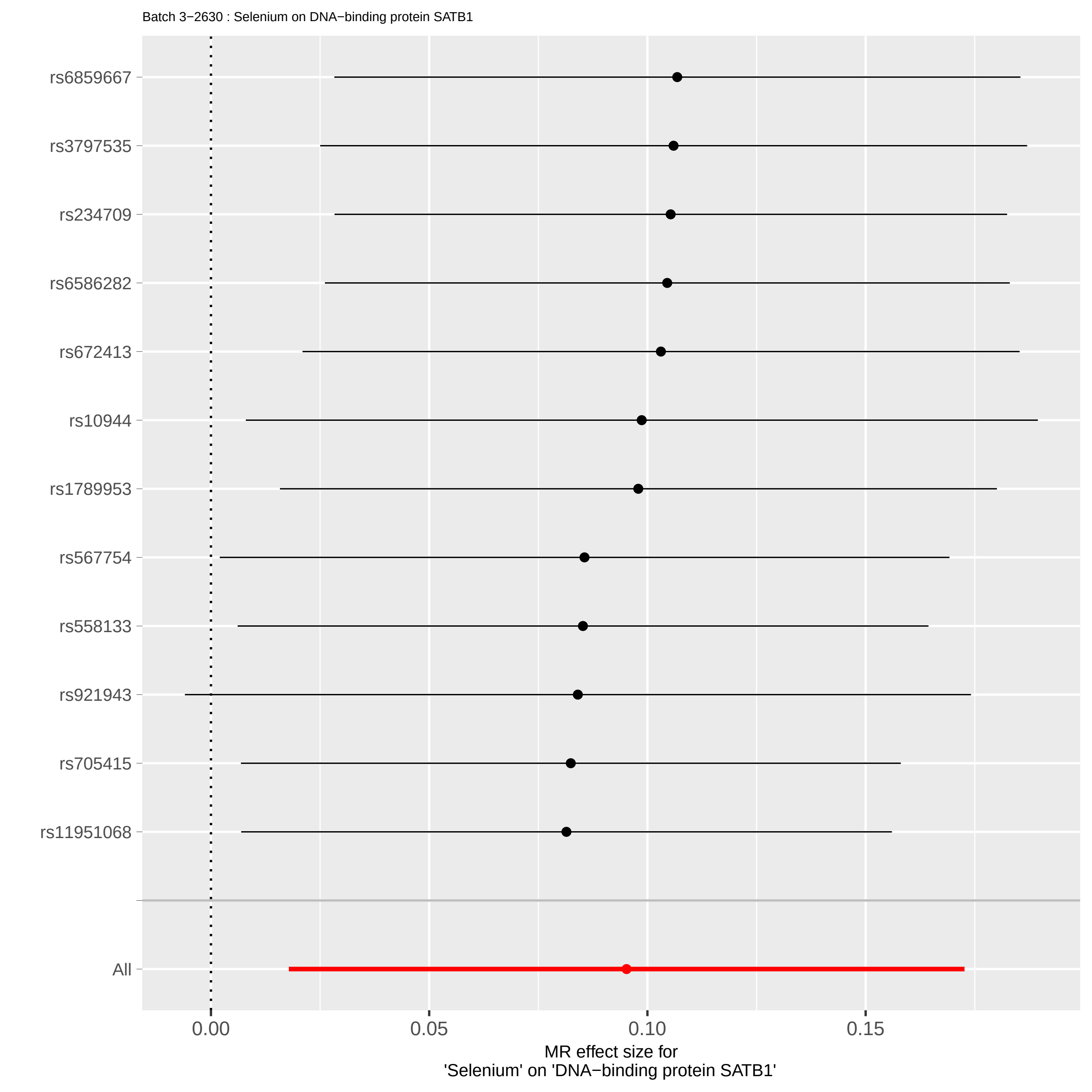

Batch 3−2630 : Selenium on DNA−binding protein SATB1
rs6859667
rs3797535
rs234709
rs6586282
rs672413
rs10944
rs1789953
rs567754
rs558133
rs921943
rs705415
rs11951068
All
0.00
0.05
0.10
0.15
MR effect size for
'Selenium' on 'DNA−binding protein SATB1'

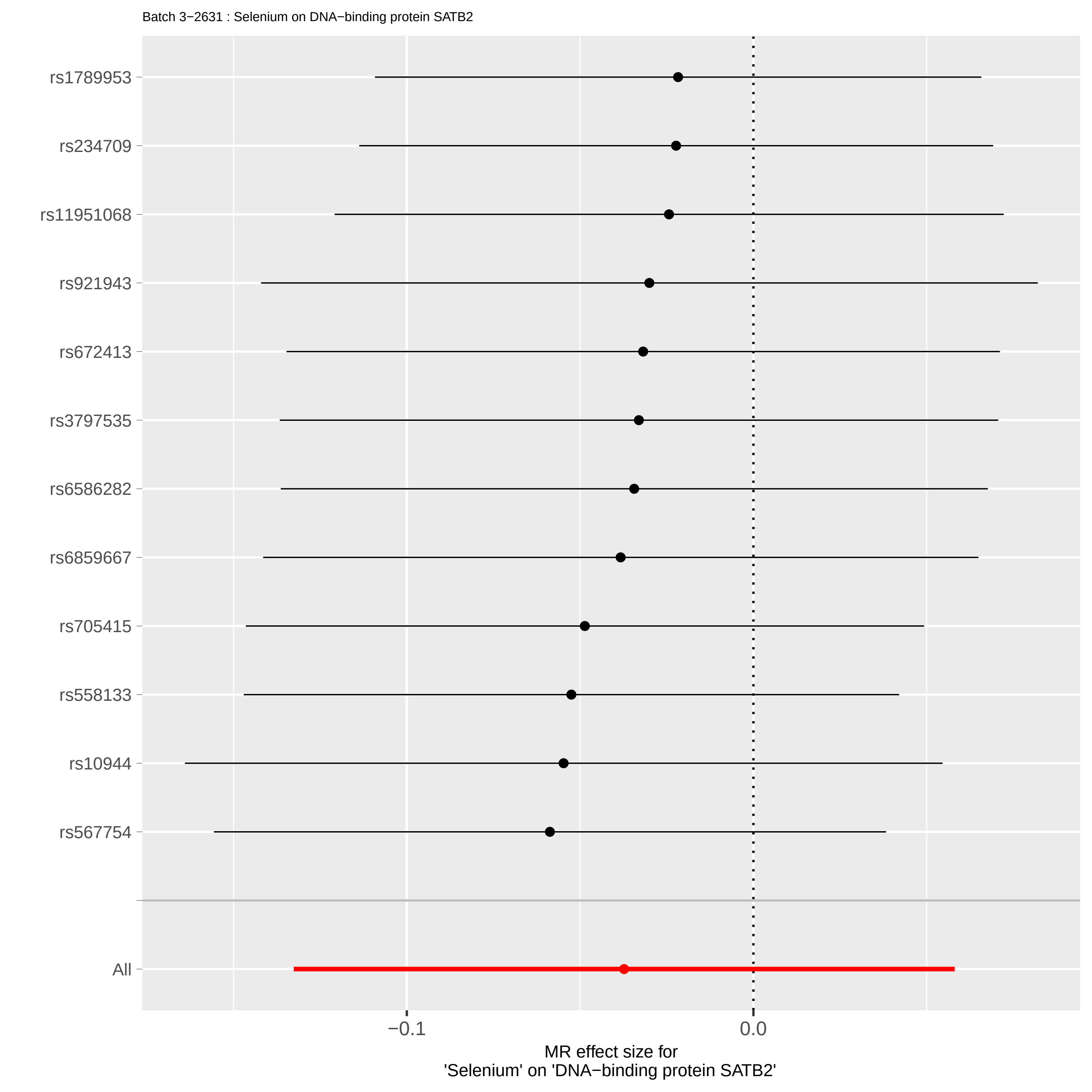

Batch 3−2631 : Selenium on DNA−binding protein SATB2
rs1789953
rs234709
rs11951068
rs921943
rs672413
rs3797535
rs6586282
rs6859667
rs705415
rs558133
rs10944
rs567754
All
−0.1
0.0
MR effect size for
'Selenium' on 'DNA−binding protein SATB2'

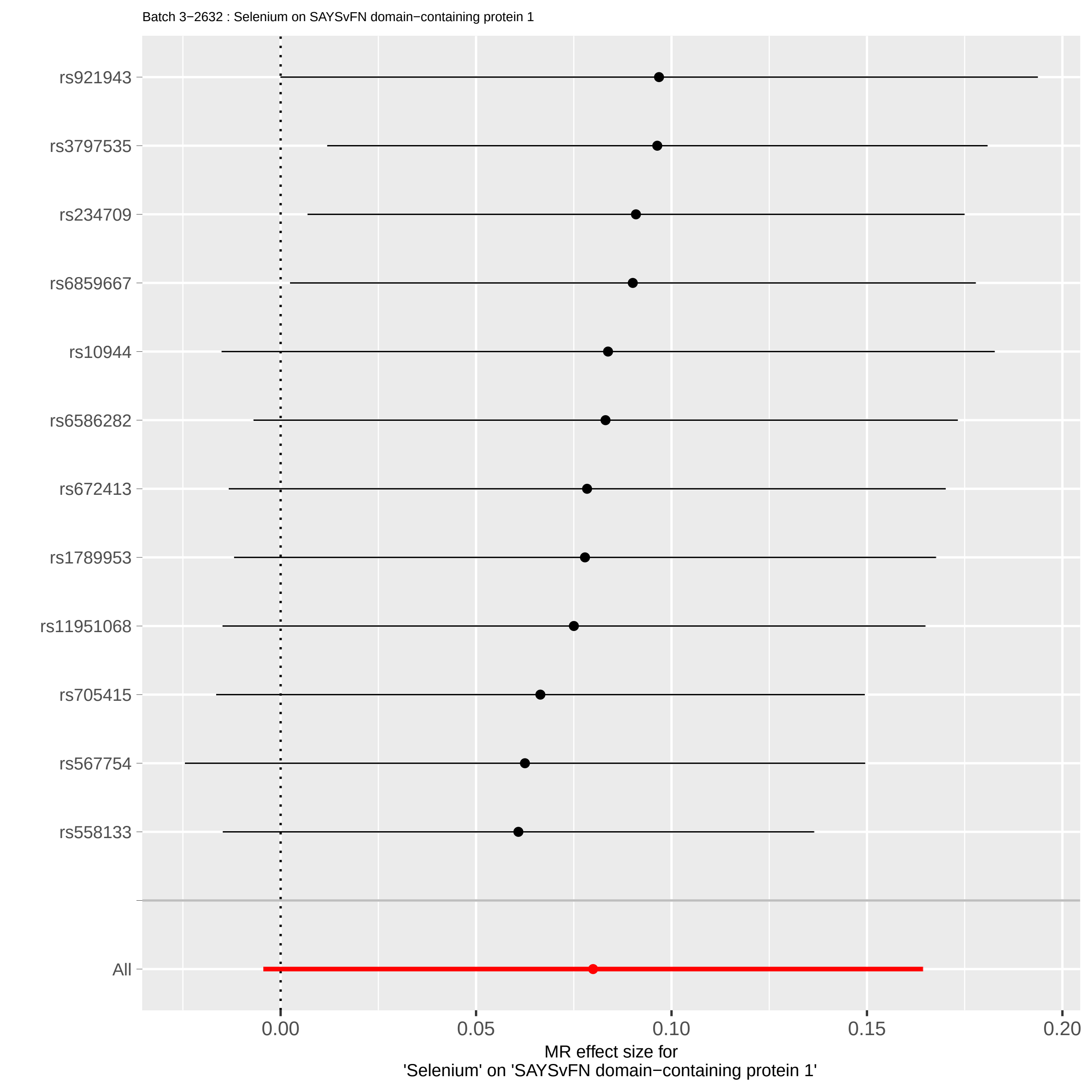

Batch 3−2632 : Selenium on SAYSvFN domain−containing protein 1
rs921943
rs3797535
rs234709
rs6859667
rs10944
rs6586282
rs672413
rs1789953
rs11951068
rs705415
rs567754
rs558133
All
0.00
0.05
0.10
0.15
0.20
MR effect size for
'Selenium' on 'SAYSvFN domain−containing protein 1'

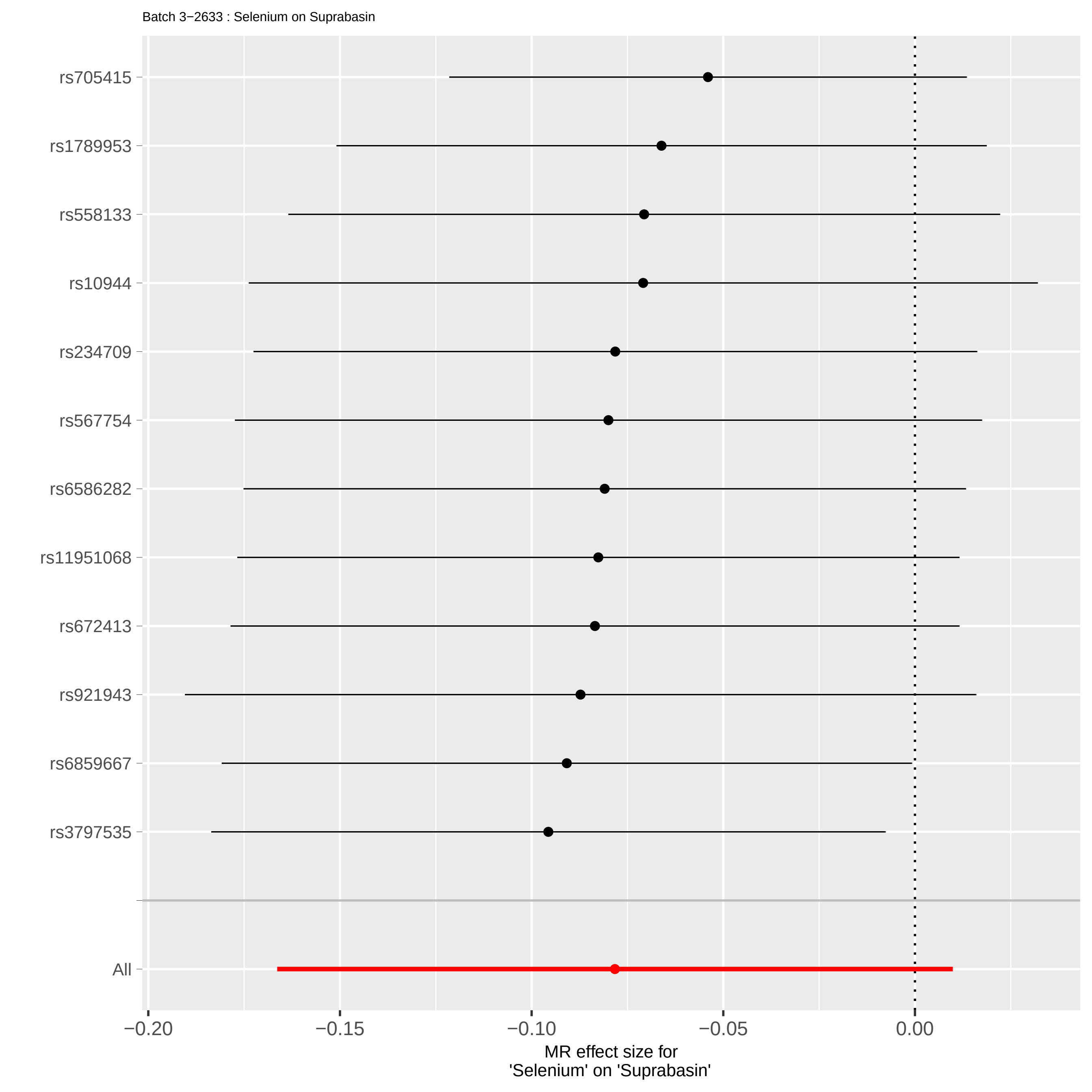

Batch 3−2633 : Selenium on Suprabasin
rs705415
rs1789953
rs558133
rs10944
rs234709
rs567754
rs6586282
rs11951068
rs672413
rs921943
rs6859667
rs3797535
All
−0.20
−0.15
−0.10
−0.05
0.00
MR effect size for
'Selenium' on 'Suprabasin'

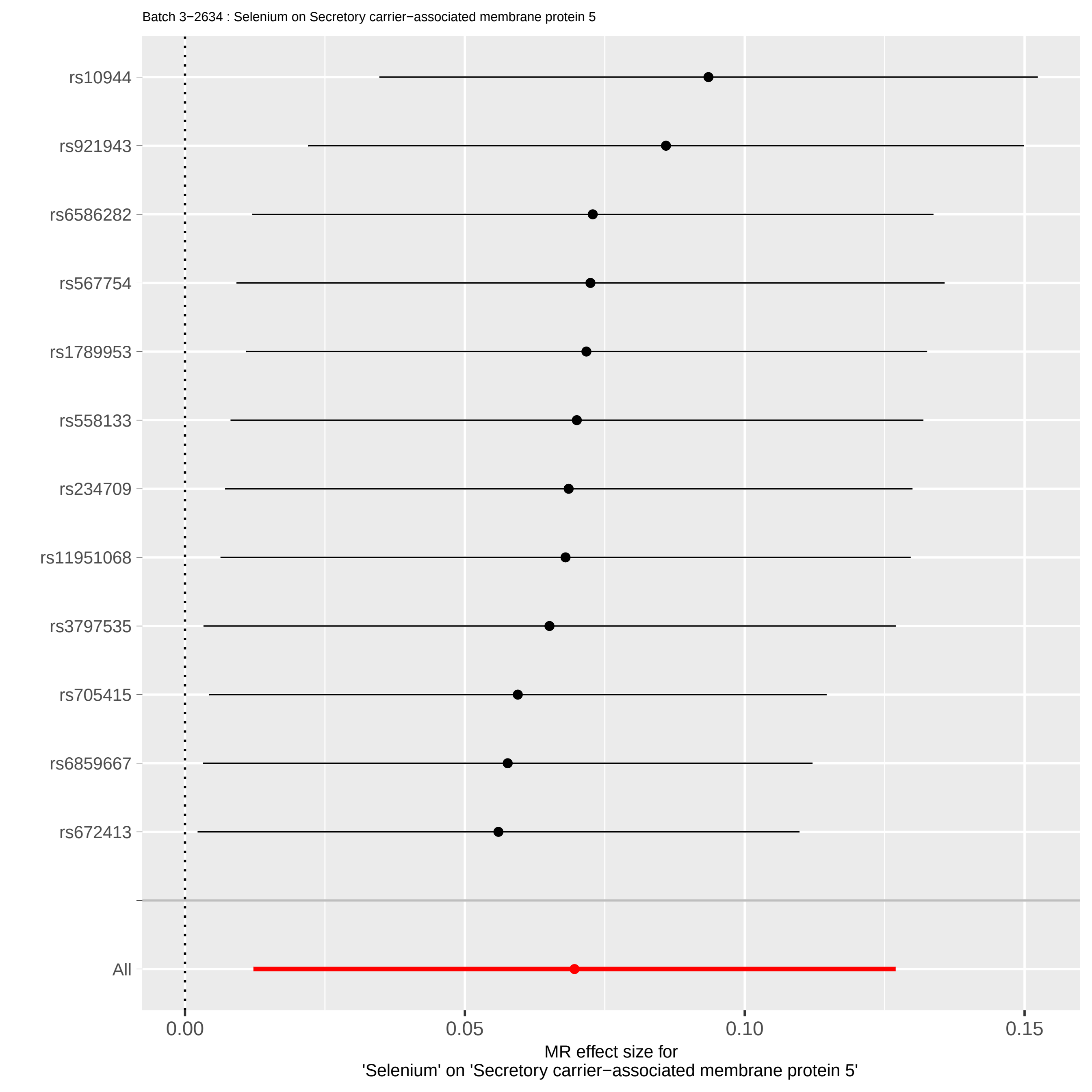

Batch 3−2634 : Selenium on Secretory carrier−associated membrane protein 5
rs10944
rs921943
rs6586282
rs567754
rs1789953
rs558133
rs234709
rs11951068
rs3797535
rs705415
rs6859667
rs672413
All
0.00
0.05
0.10
0.15
MR effect size for
'Selenium' on 'Secretory carrier−associated membrane protein 5'

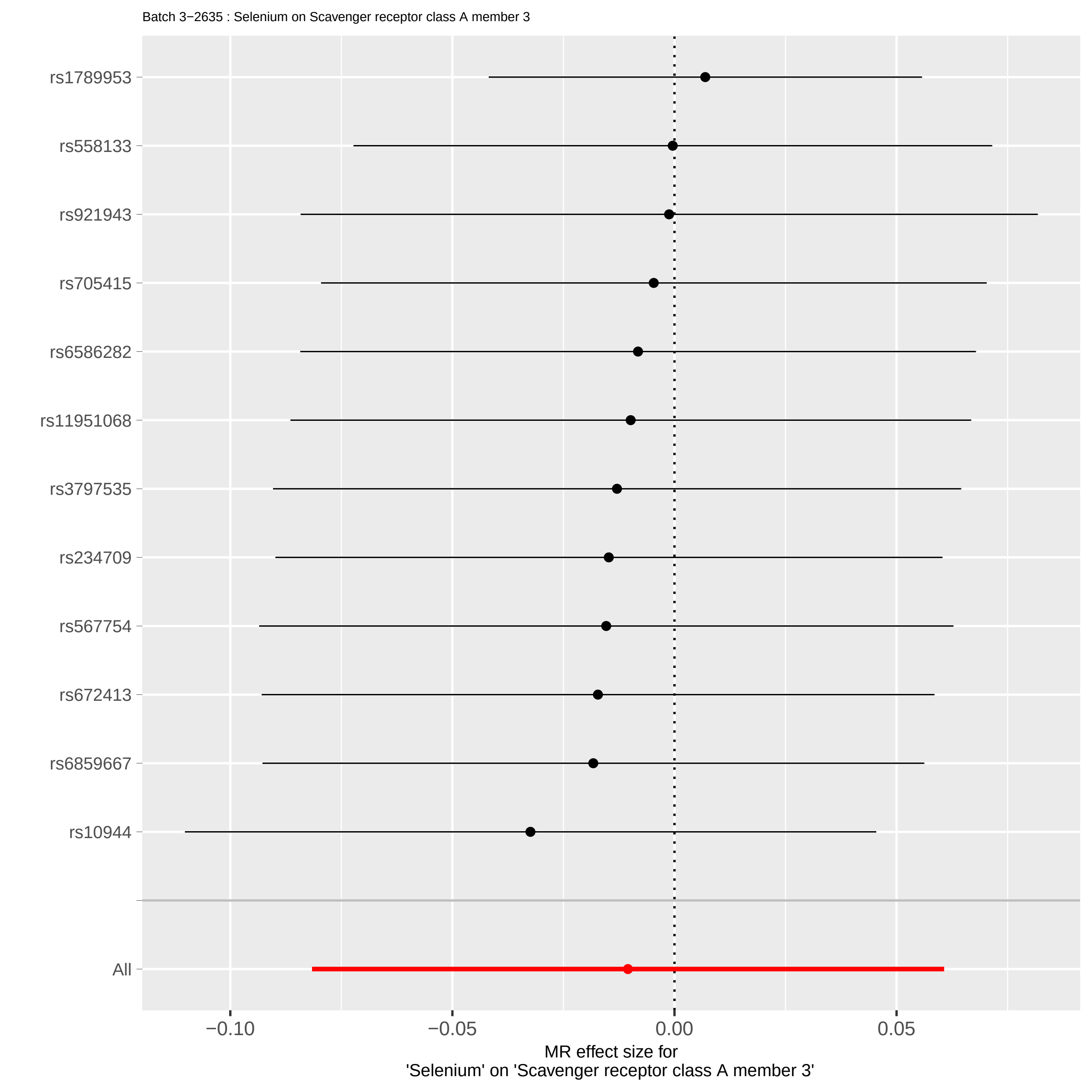

Batch 3−2635 : Selenium on Scavenger receptor class A member 3
rs1789953
rs558133
rs921943
rs705415
rs6586282
rs11951068
rs3797535
rs234709
rs567754
rs672413
rs6859667
rs10944
All
−0.10
−0.05
0.00
0.05
MR effect size for
'Selenium' on 'Scavenger receptor class A member 3'

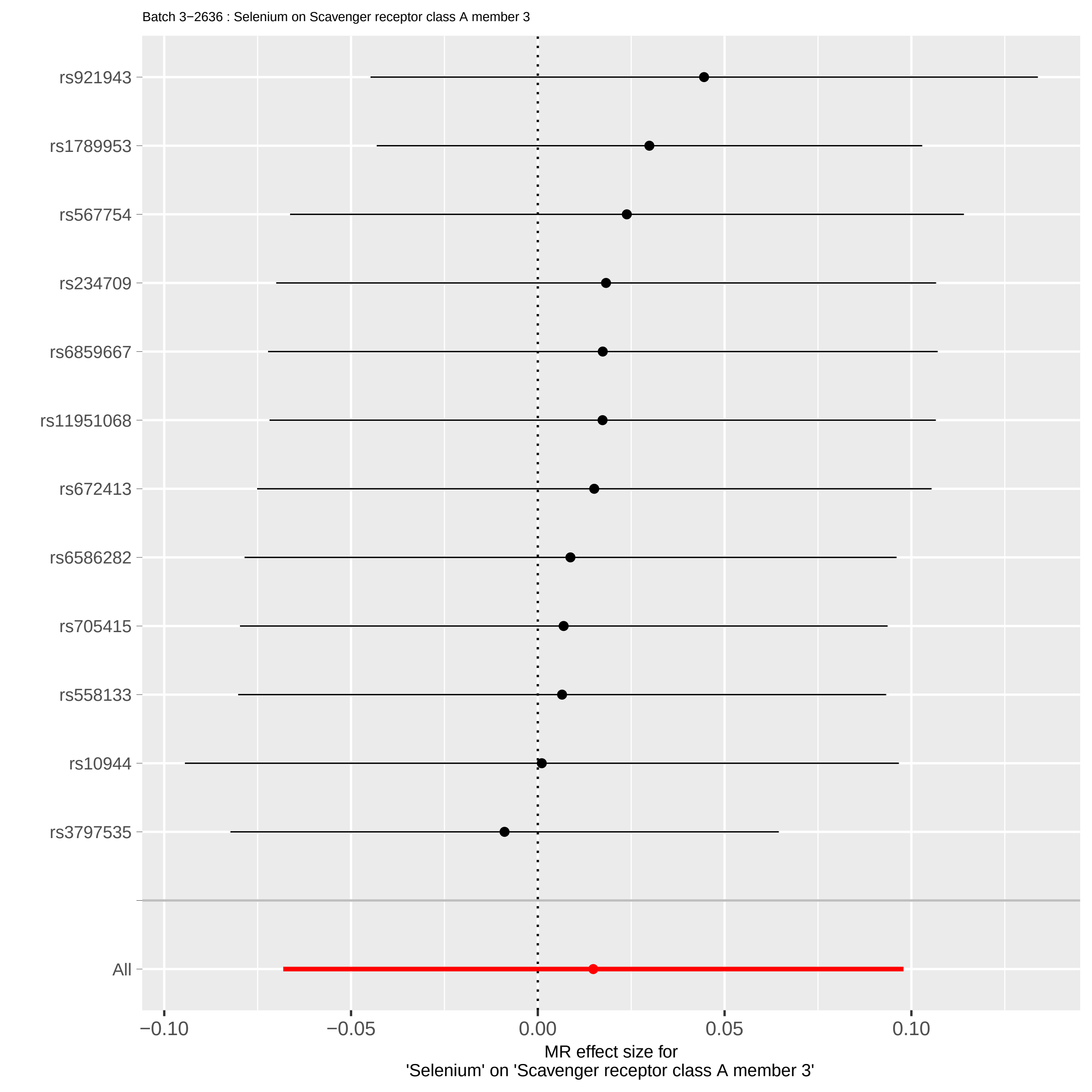

Batch 3−2636 : Selenium on Scavenger receptor class A member 3
rs921943
rs1789953
rs567754
rs234709
rs6859667
rs11951068
rs672413
rs6586282
rs705415
rs558133
rs10944
rs3797535
All
−0.10
−0.05
0.00
0.05
0.10
MR effect size for
'Selenium' on 'Scavenger receptor class A member 3'

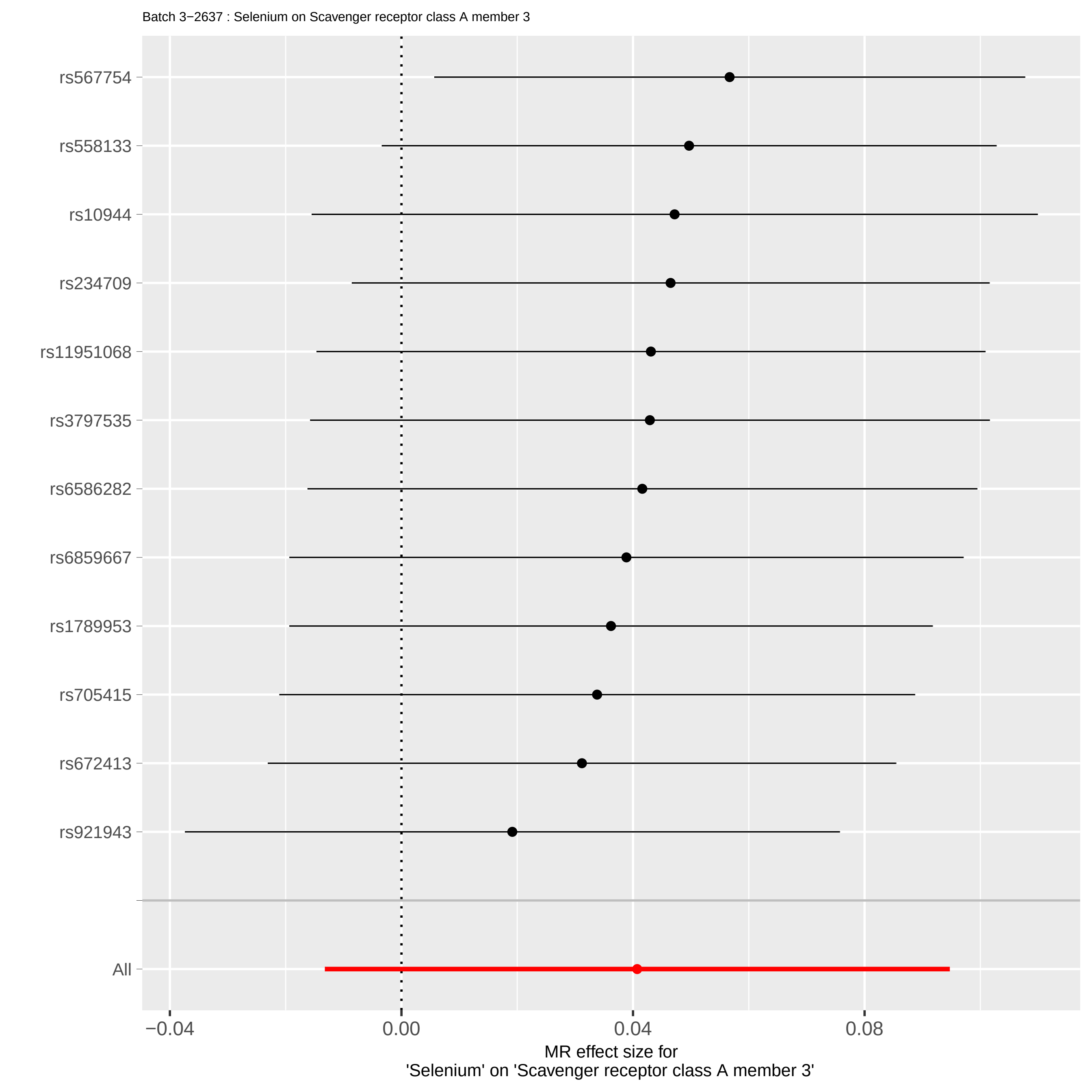
Batch 3−2637 : Selenium on Scavenger receptor class A member 3
rs567754
rs558133
rs10944
rs234709
rs11951068
rs3797535
rs6586282
rs6859667
rs1789953
rs705415
rs672413
rs921943
All
−0.04
0.00
0.04
0.08
MR effect size for
'Selenium' on 'Scavenger receptor class A member 3'

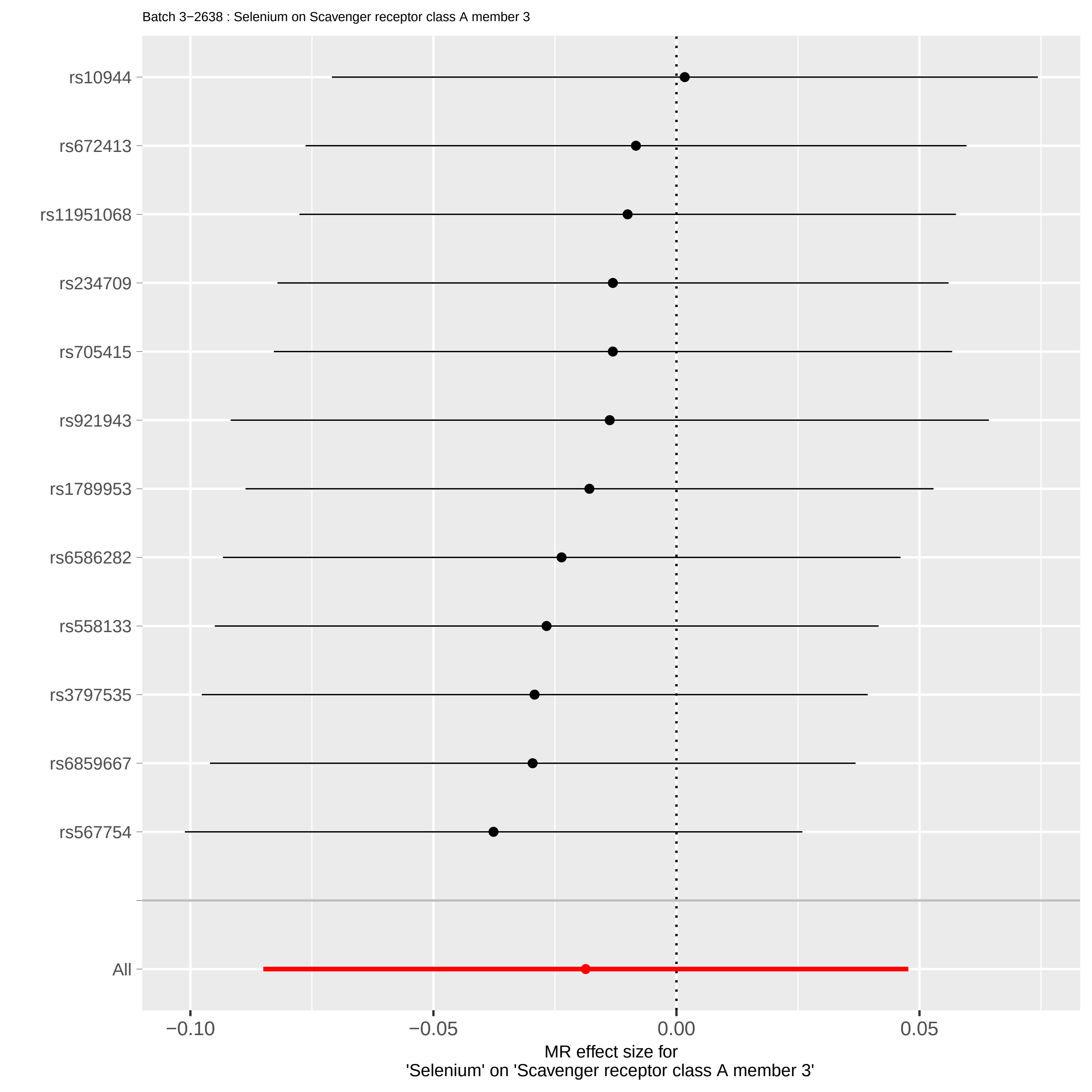

Batch 3−2638 : Selenium on Scavenger receptor class A member 3
rs10944
rs672413
rs11951068
rs234709
rs705415
rs921943
rs1789953
rs6586282
rs558133
rs3797535
rs6859667
rs567754
All
−0.10
−0.05
0.00
0.05
MR effect size for
'Selenium' on 'Scavenger receptor class A member 3'

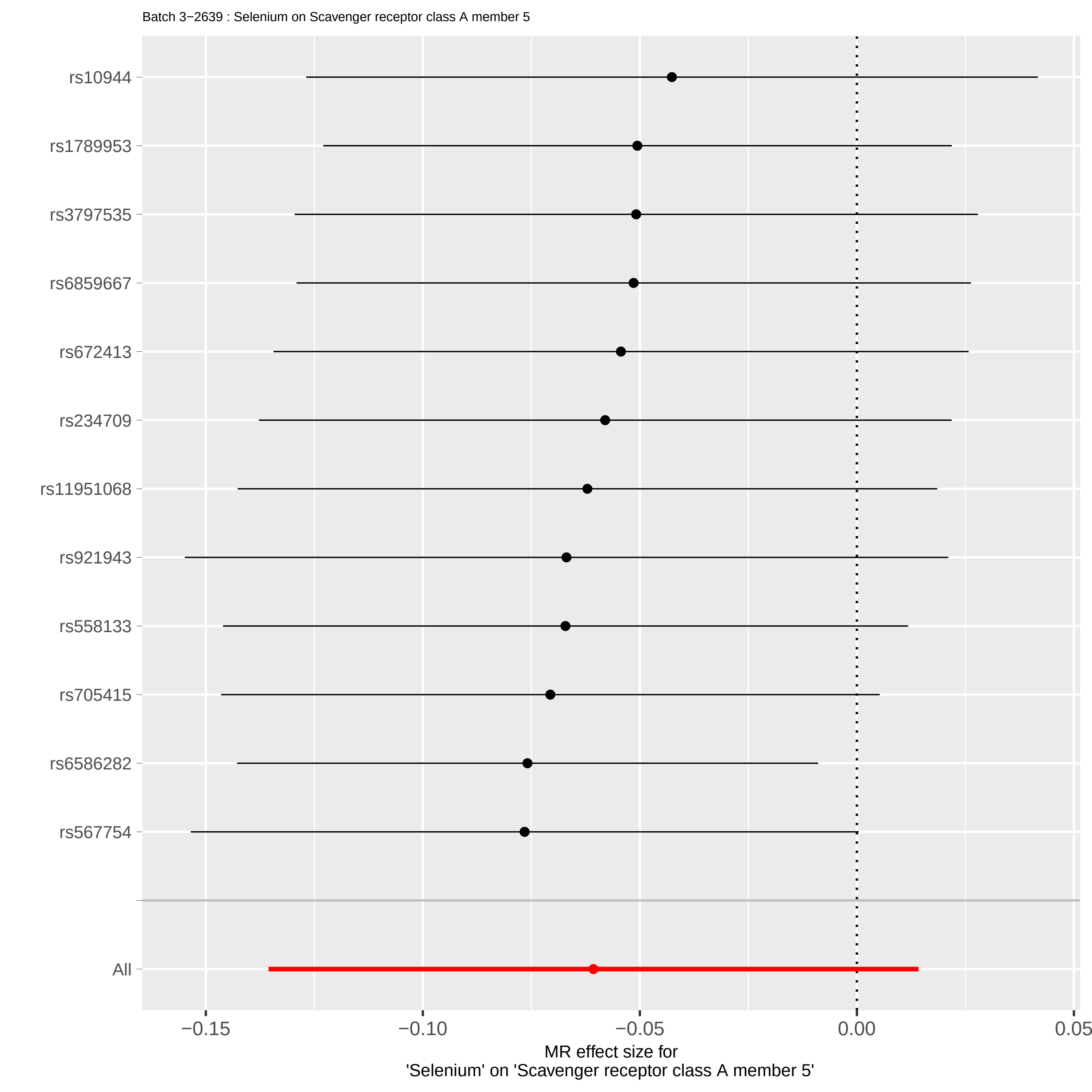

Batch 3−2639 : Selenium on Scavenger receptor class A member 5
rs10944
rs1789953
rs3797535
rs6859667
rs672413
rs234709
rs11951068
rs921943
rs558133
rs705415
rs6586282
rs567754
All
−0.15
−0.10
−0.05
0.00
0.05
MR effect size for
'Selenium' on 'Scavenger receptor class A member 5'

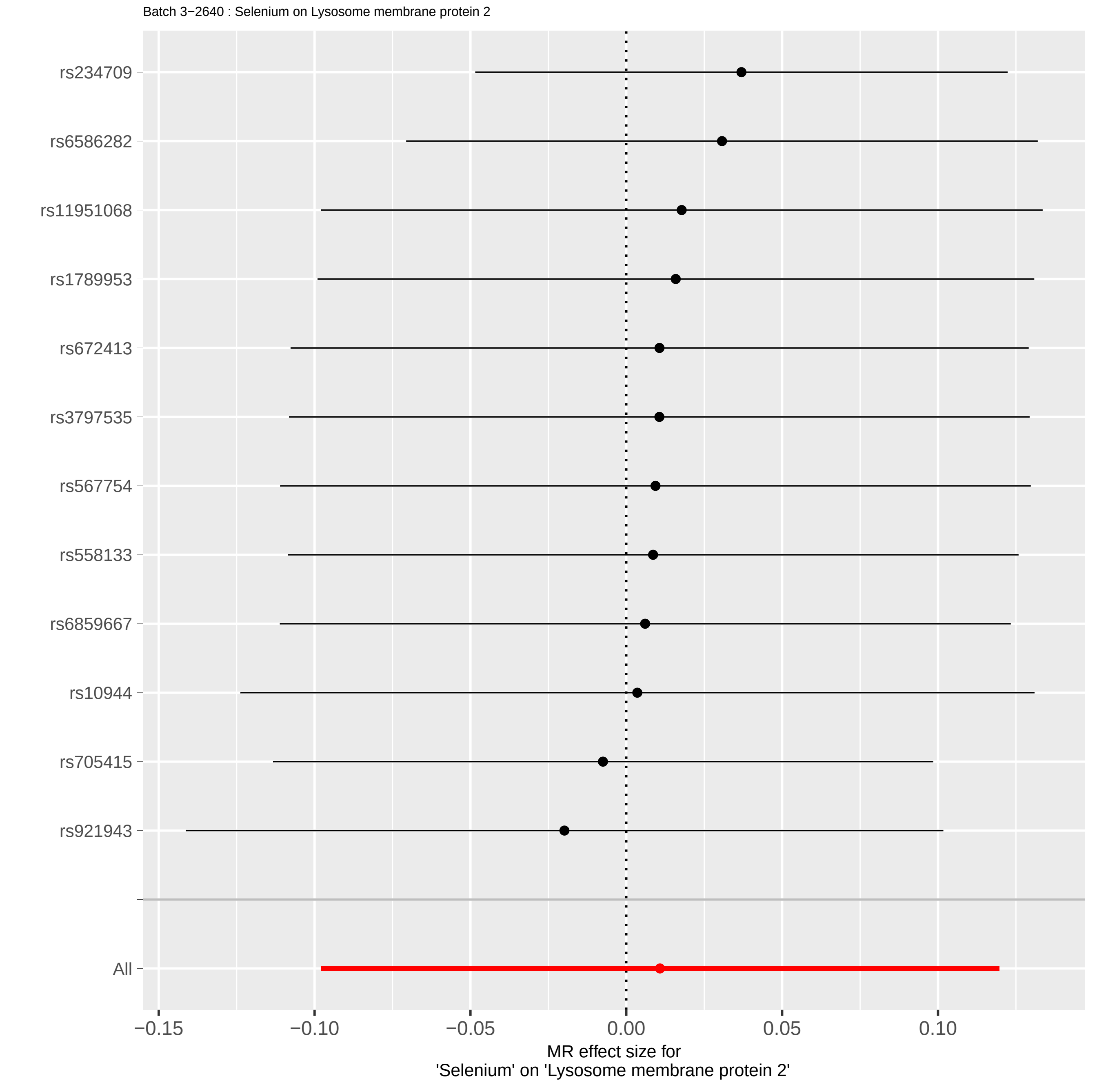

Batch 3−2640 : Selenium on Lysosome membrane protein 2
rs234709
rs6586282
rs11951068
rs1789953
rs672413
rs3797535
rs567754
rs558133
rs6859667
rs10944
rs705415
rs921943
All
−0.15
−0.10
−0.05
0.00
0.05
0.10
MR effect size for
'Selenium' on 'Lysosome membrane protein 2'

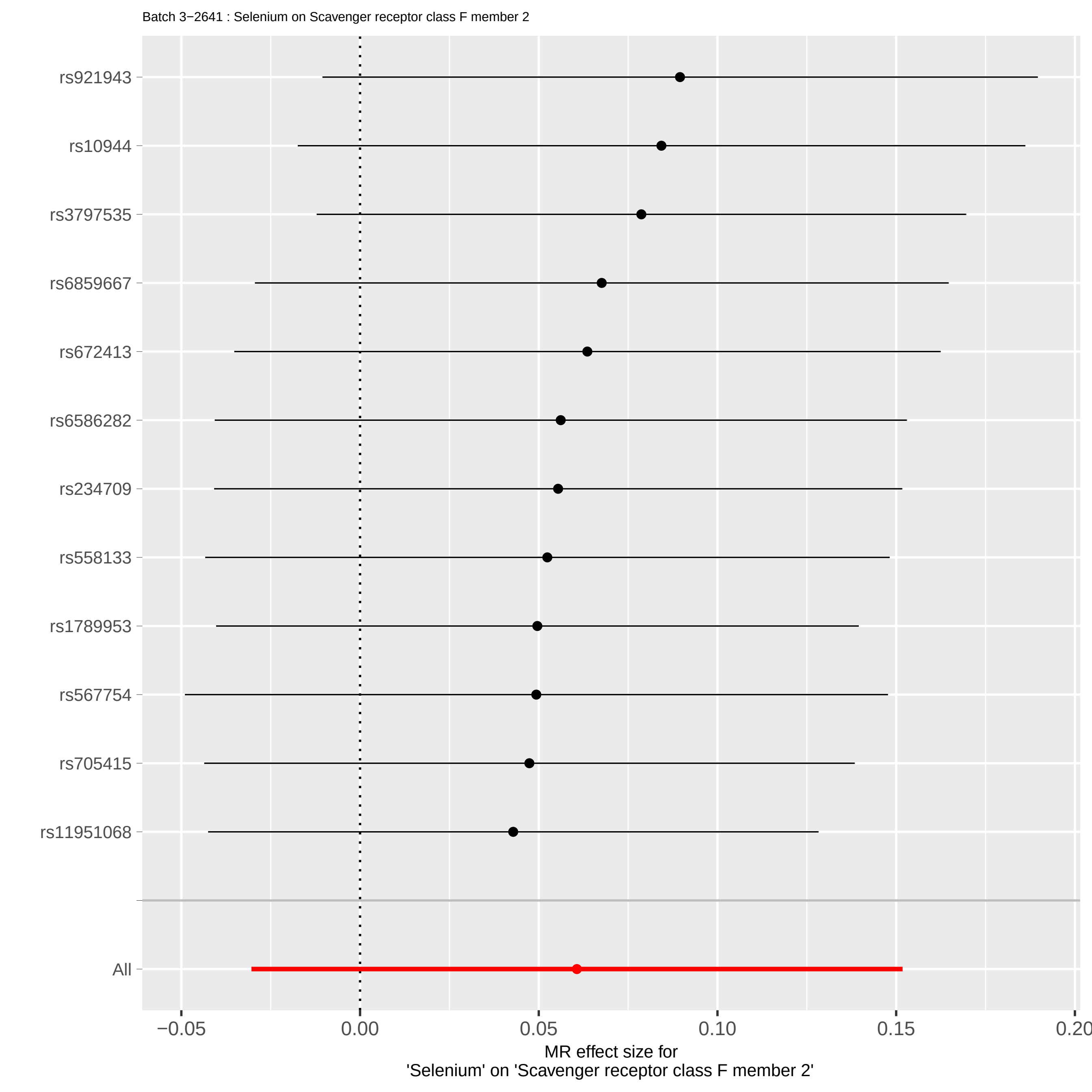
Batch 3−2641 : Selenium on Scavenger receptor class F member 2
rs921943
rs10944
rs3797535
rs6859667
rs672413
rs6586282
rs234709
rs558133
rs1789953
rs567754
rs705415
rs11951068
All
−0.05
0.00
0.05
0.10
0.15
0.20
MR effect size for
'Selenium' on 'Scavenger receptor class F member 2'

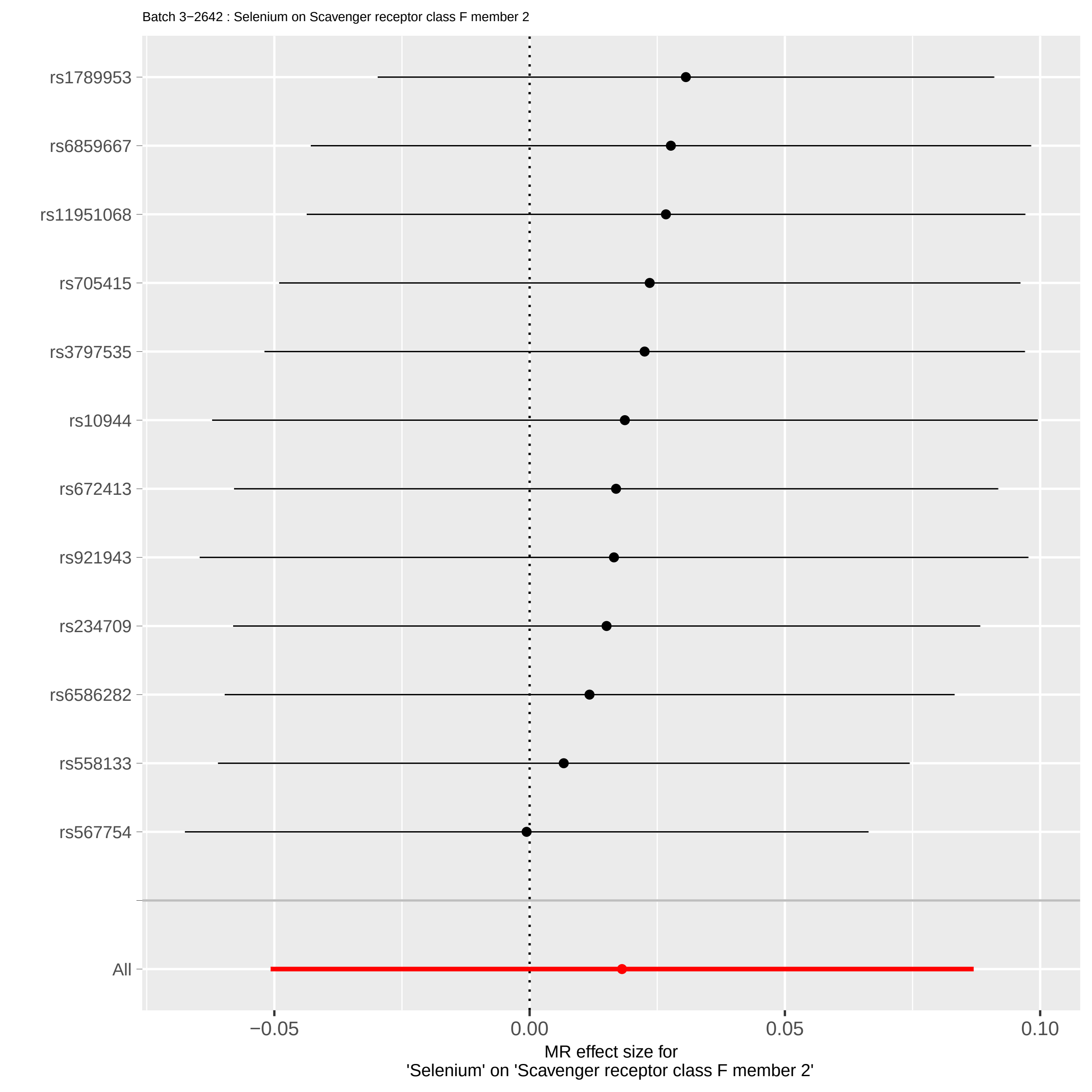

Batch 3−2642 : Selenium on Scavenger receptor class F member 2
rs1789953
rs6859667
rs11951068
rs705415
rs3797535
rs10944
rs672413
rs921943
rs234709
rs6586282
rs558133
rs567754
All
−0.05
0.00
0.05
0.10
MR effect size for
'Selenium' on 'Scavenger receptor class F member 2'

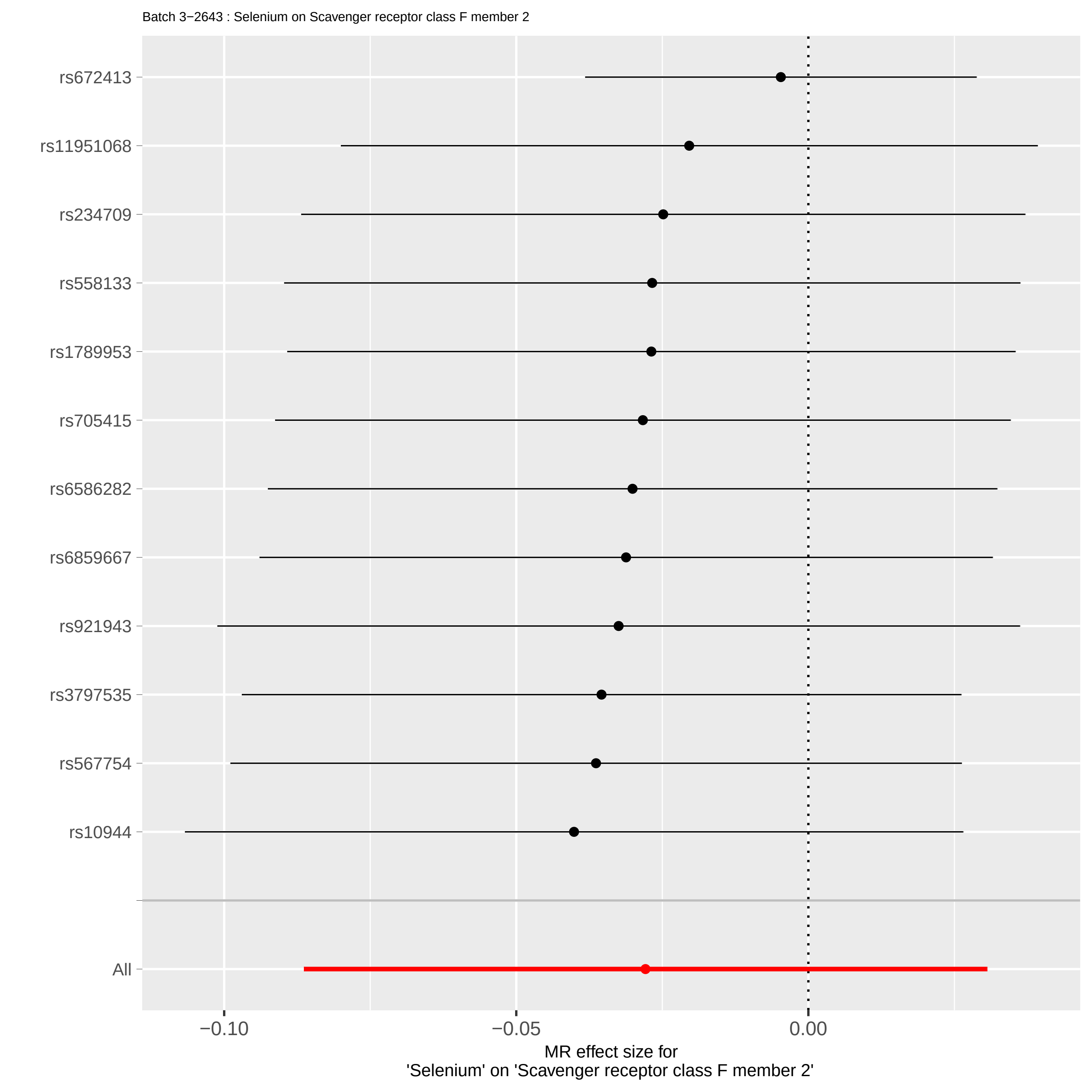
Batch 3−2643 : Selenium on Scavenger receptor class F member 2
rs672413
rs11951068
rs234709
rs558133
rs1789953
rs705415
rs6586282
rs6859667
rs921943
rs3797535
rs567754
rs10944
All
−0.10
−0.05
0.00
MR effect size for
'Selenium' on 'Scavenger receptor class F member 2'

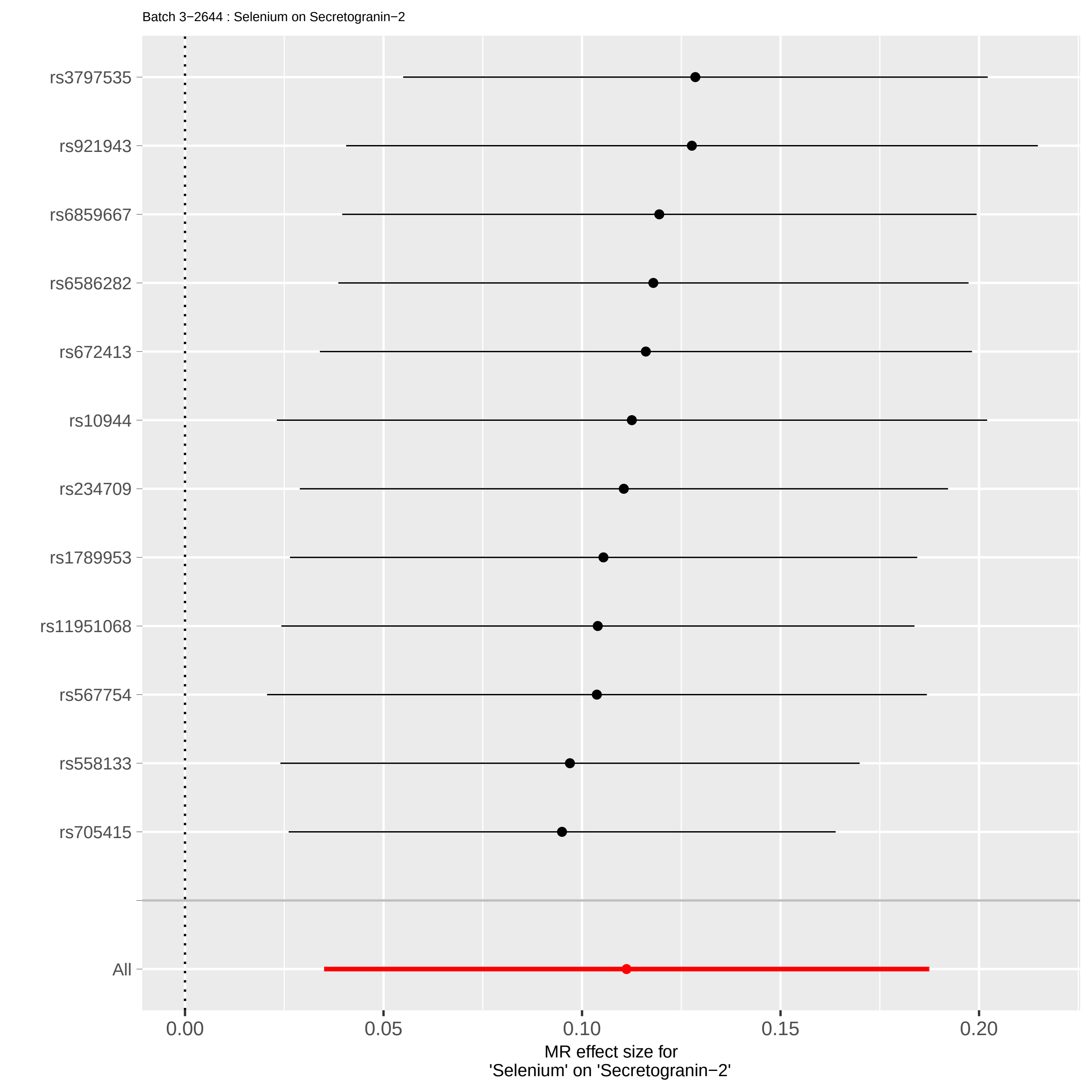

Batch 3−2644 : Selenium on Secretogranin−2
rs3797535
rs921943
rs6859667
rs6586282
rs672413
rs10944
rs234709
rs1789953
rs11951068
rs567754
rs558133
rs705415
All
0.00
0.05
0.10
0.15
0.20
MR effect size for
'Selenium' on 'Secretogranin−2'

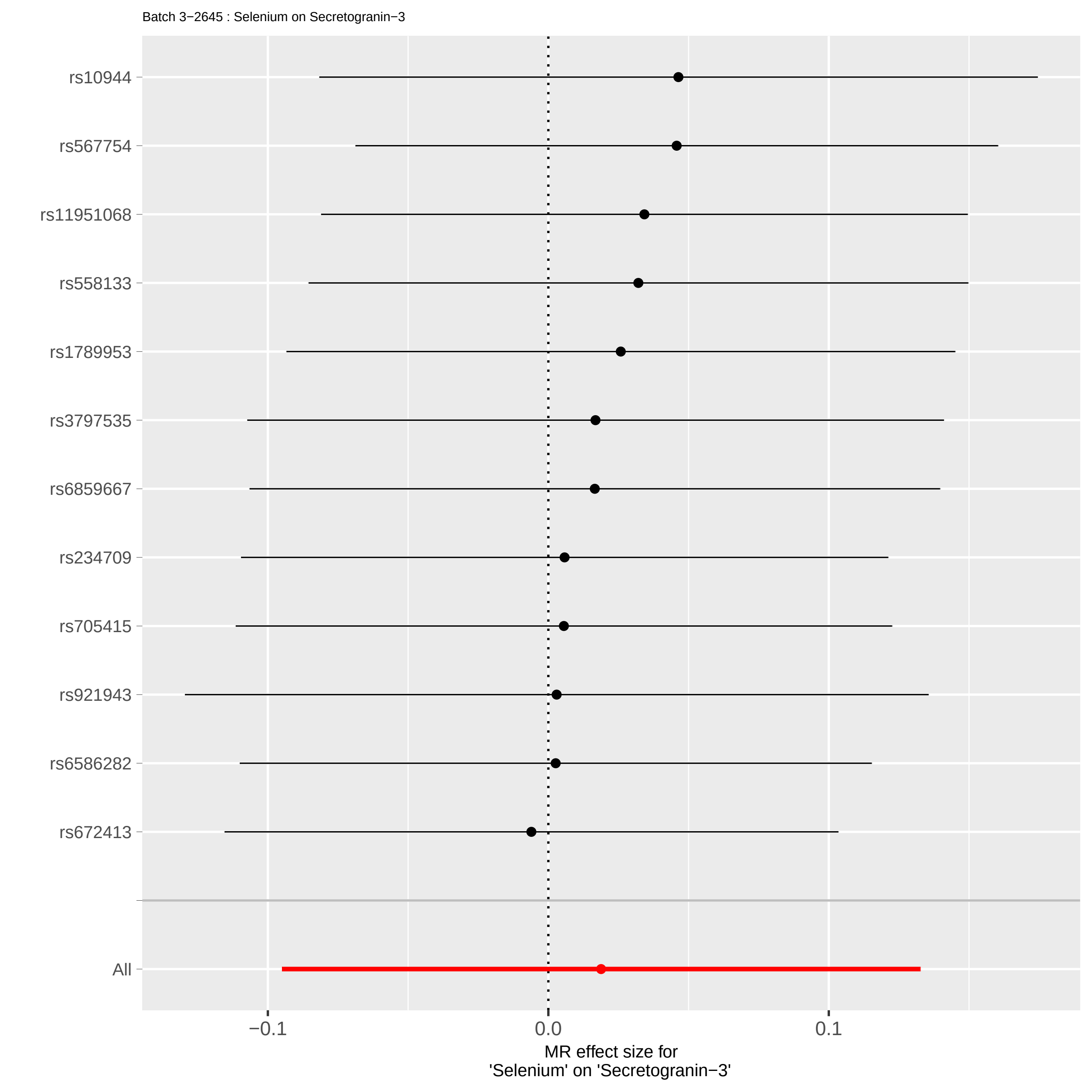

Batch 3−2645 : Selenium on Secretogranin−3
rs10944
rs567754
rs11951068
rs558133
rs1789953
rs3797535
rs6859667
rs234709
rs705415
rs921943
rs6586282
rs672413
All
−0.1
0.0
0.1
MR effect size for
'Selenium' on 'Secretogranin−3'

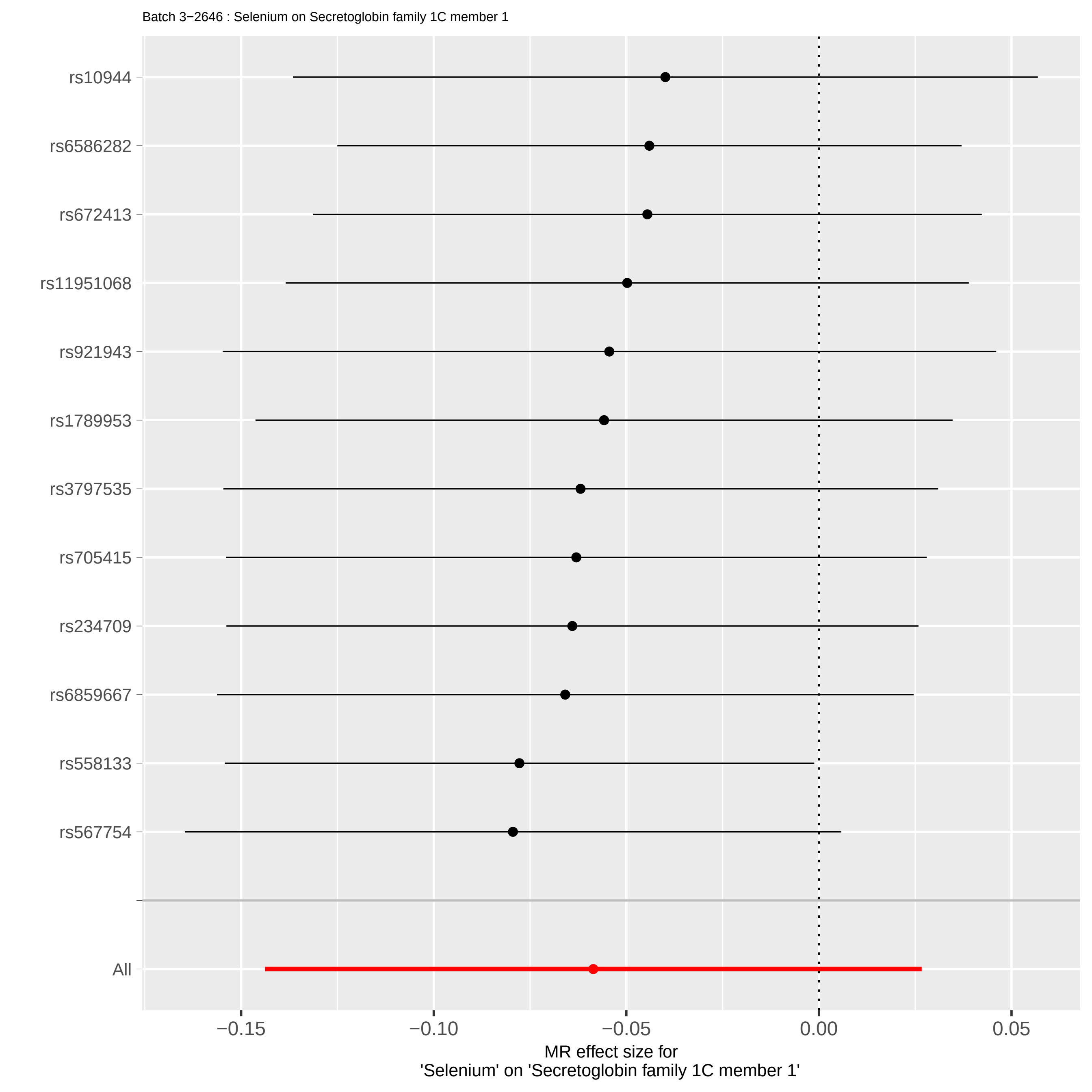
Batch 3−2646 : Selenium on Secretoglobin family 1C member 1
rs10944
rs6586282
rs672413
rs11951068
rs921943
rs1789953
rs3797535
rs705415
rs234709
rs6859667
rs558133
rs567754
All
−0.15
−0.10
−0.05
0.00
0.05
MR effect size for
'Selenium' on 'Secretoglobin family 1C member 1'

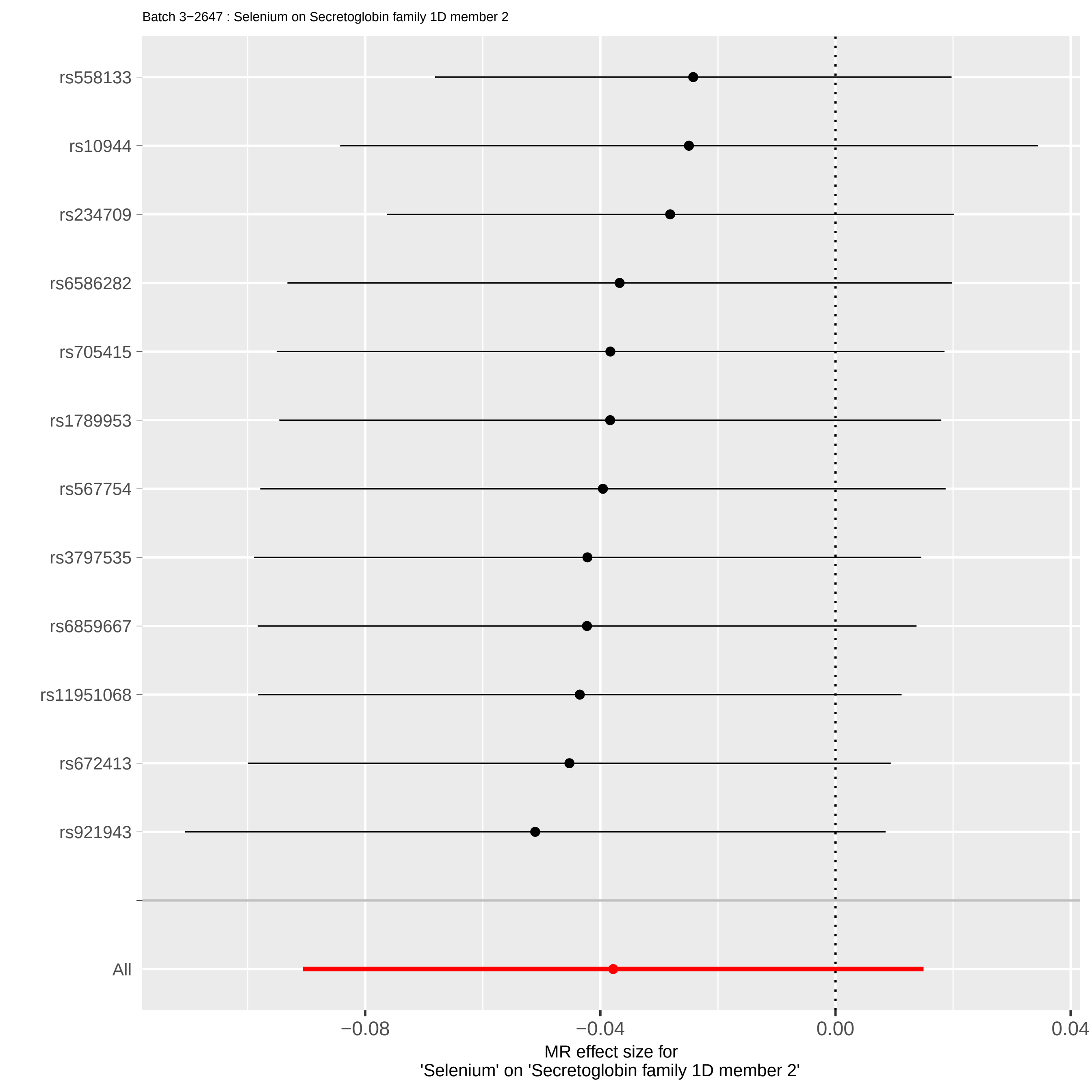

Batch 3−2647 : Selenium on Secretoglobin family 1D member 2
rs558133
rs10944
rs234709
rs6586282
rs705415
rs1789953
rs567754
rs3797535
rs6859667
rs11951068
rs672413
rs921943
All
−0.08
−0.04
0.00
0.04
MR effect size for
'Selenium' on 'Secretoglobin family 1D member 2'

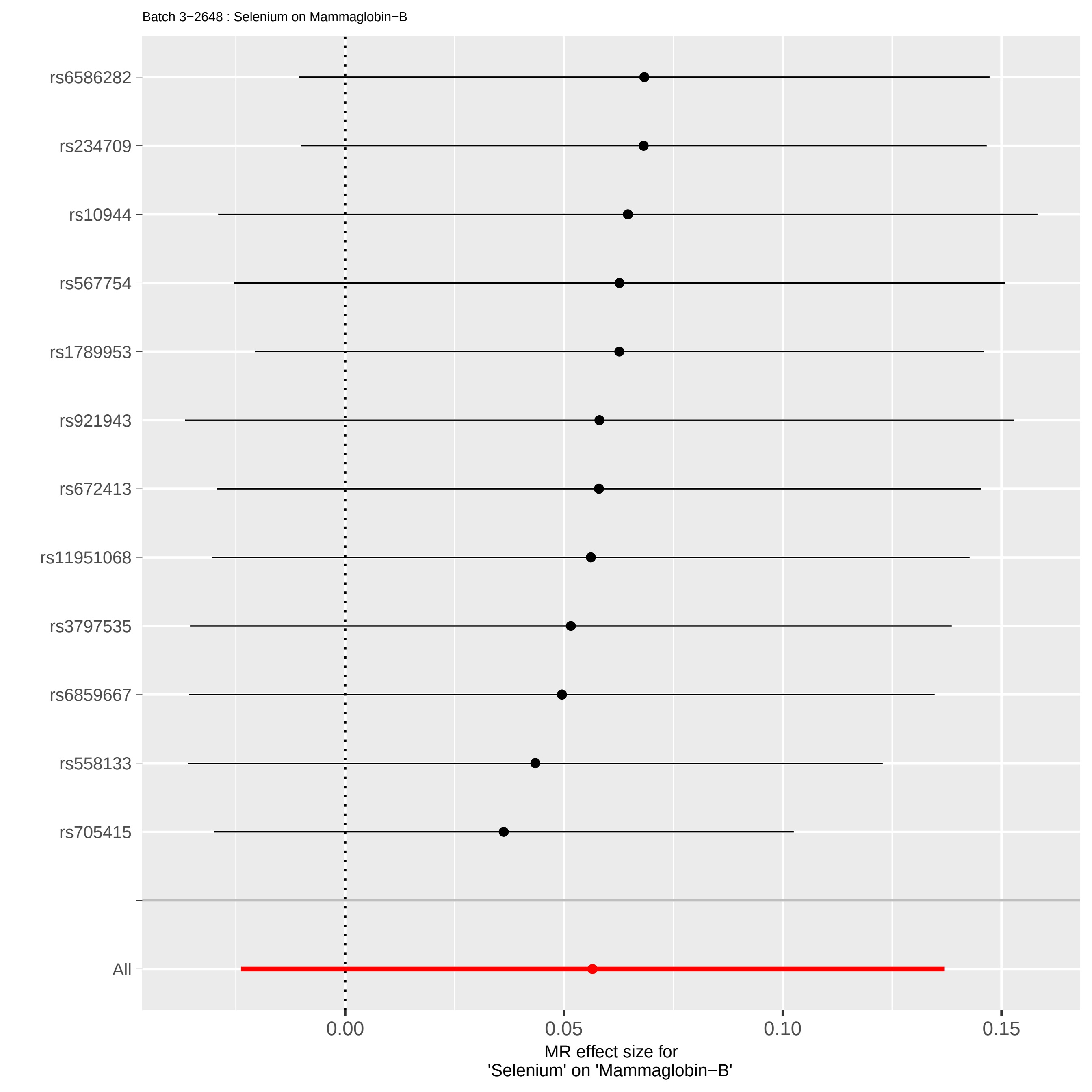

Batch 3−2648 : Selenium on Mammaglobin−B
rs6586282
rs234709
rs10944
rs567754
rs1789953
rs921943
rs672413
rs11951068
rs3797535
rs6859667
rs558133
rs705415
All
0.00
0.05
0.10
0.15
MR effect size for
'Selenium' on 'Mammaglobin−B'

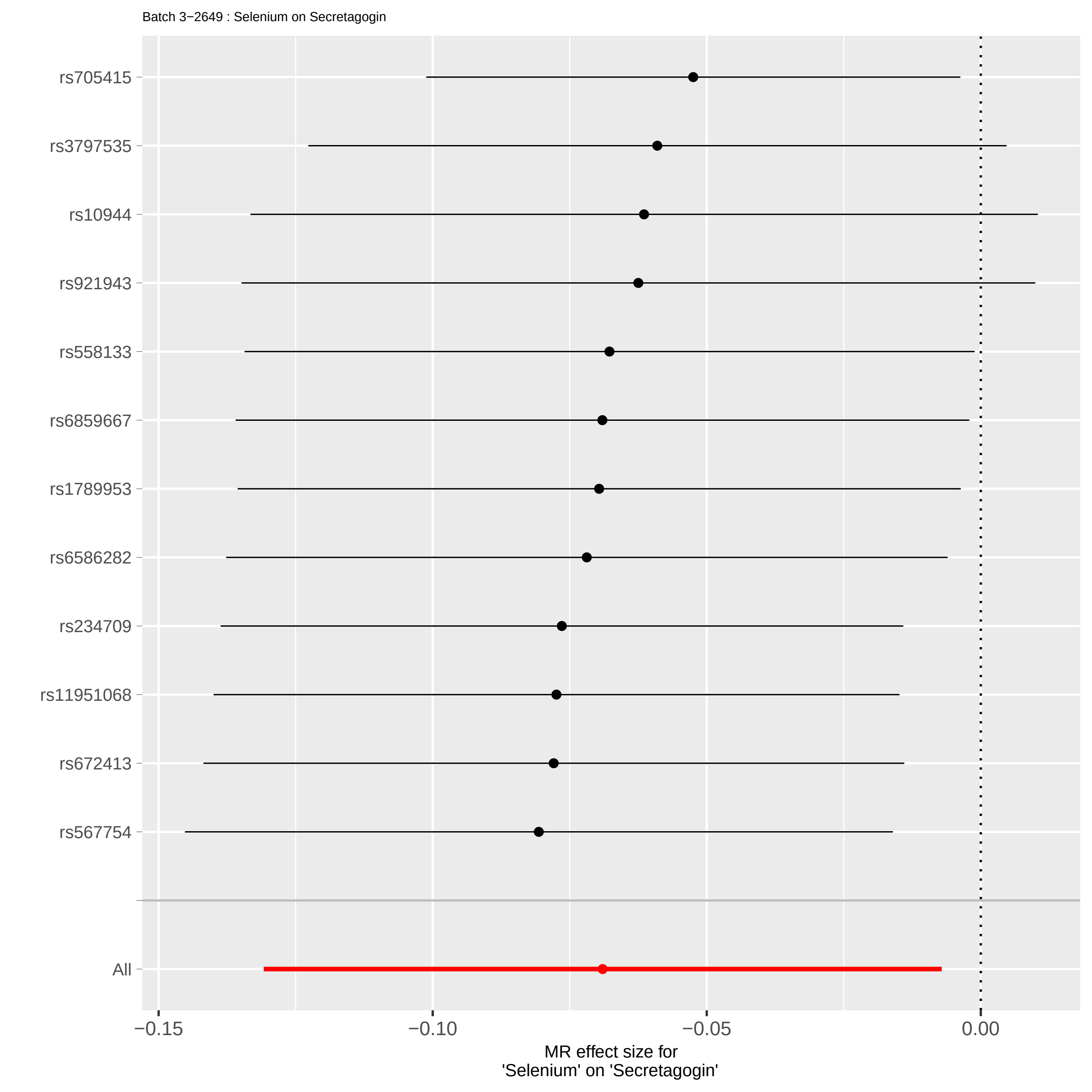

Batch 3−2649 : Selenium on Secretagogin
rs705415
rs3797535
rs10944
rs921943
rs558133
rs6859667
rs1789953
rs6586282
rs234709
rs11951068
rs672413
rs567754
All
−0.15
−0.10
−0.05
0.00
MR effect size for
'Selenium' on 'Secretagogin'

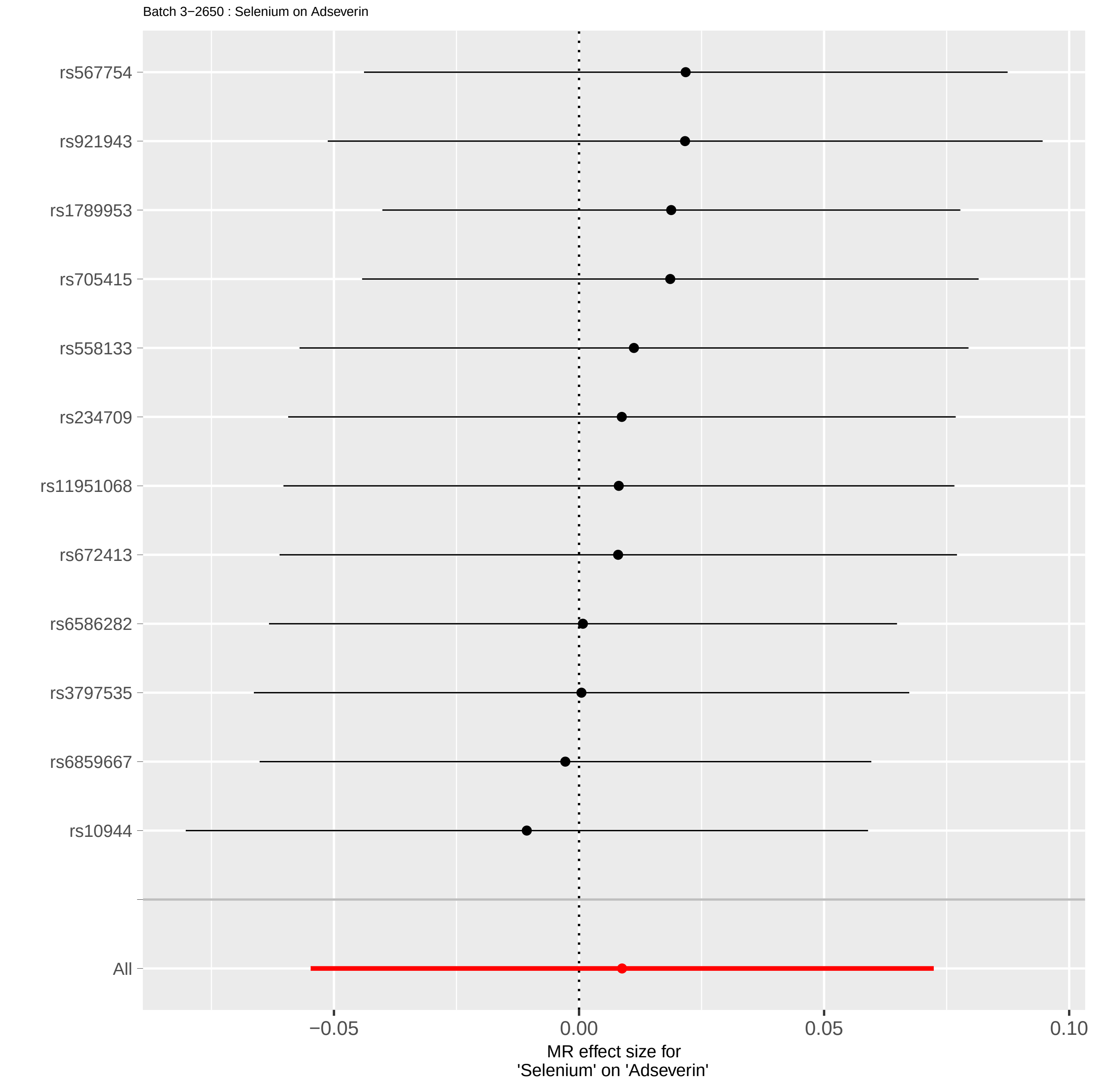

Batch 3−2650 : Selenium on Adseverin
rs567754
rs921943
rs1789953
rs705415
rs558133
rs234709
rs11951068
rs672413
rs6586282
rs3797535
rs6859667
rs10944
All
−0.05
0.00
0.05
0.10
MR effect size for
'Selenium' on 'Adseverin'

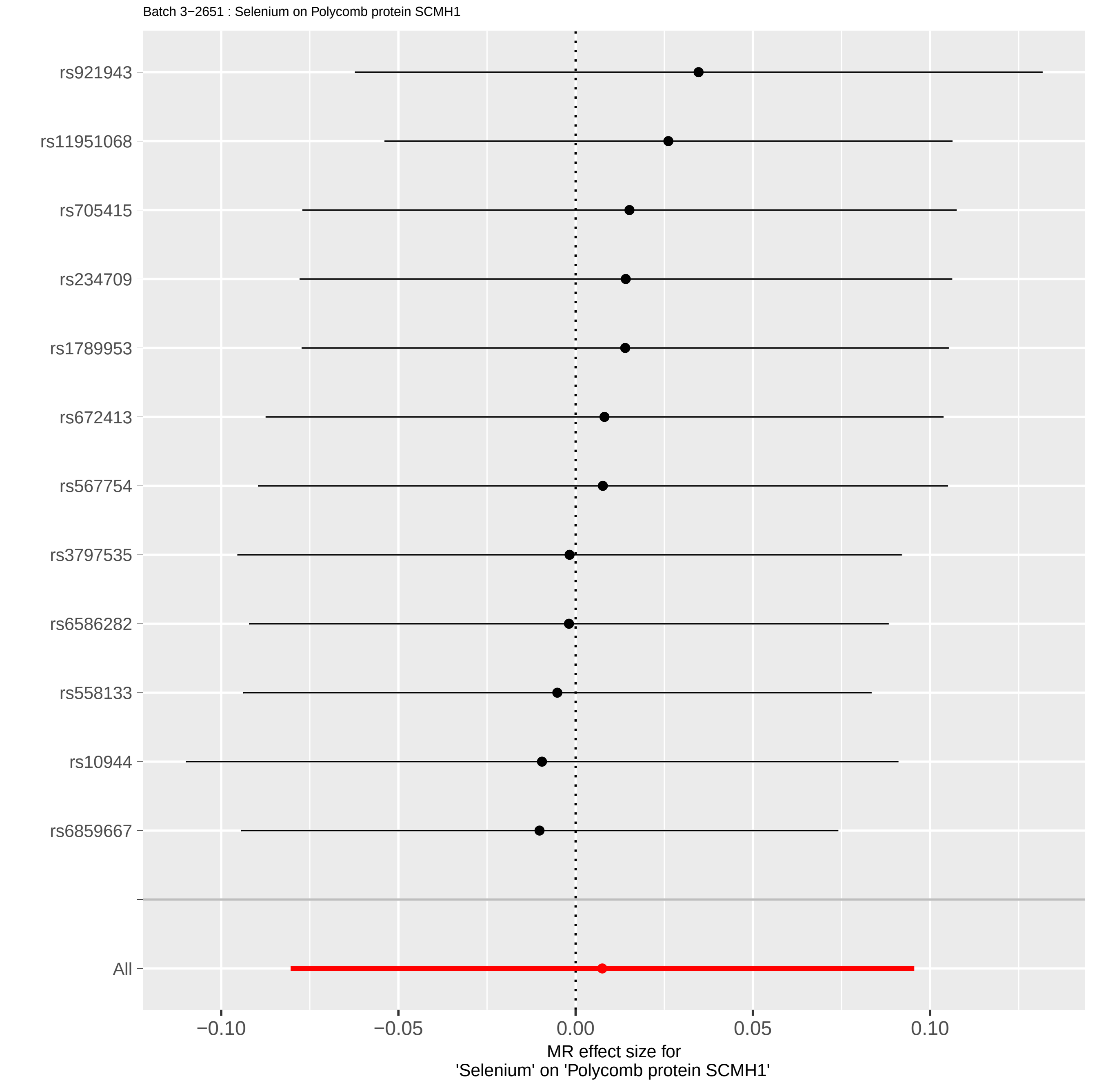

Batch 3−2651 : Selenium on Polycomb protein SCMH1
rs921943
rs11951068
rs705415
rs234709
rs1789953
rs672413
rs567754
rs3797535
rs6586282
rs558133
rs10944
rs6859667
All
−0.10
−0.05
0.00
0.05
0.10
MR effect size for
'Selenium' on 'Polycomb protein SCMH1'

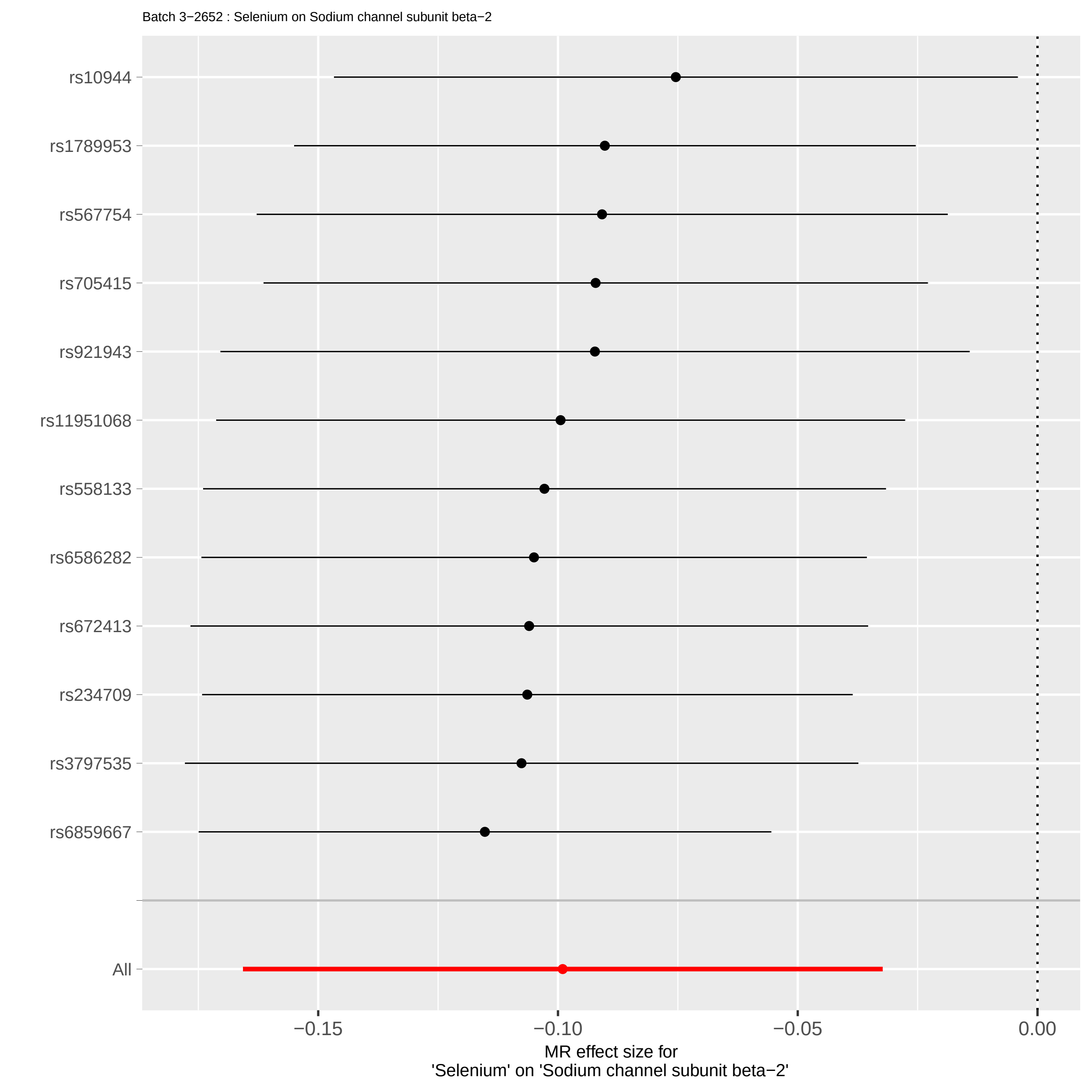

Batch 3−2652 : Selenium on Sodium channel subunit beta−2
rs10944
rs1789953
rs567754
rs705415
rs921943
rs11951068
rs558133
rs6586282
rs672413
rs234709
rs3797535
rs6859667
All
−0.15
−0.10
−0.05
0.00
MR effect size for
'Selenium' on 'Sodium channel subunit beta−2'

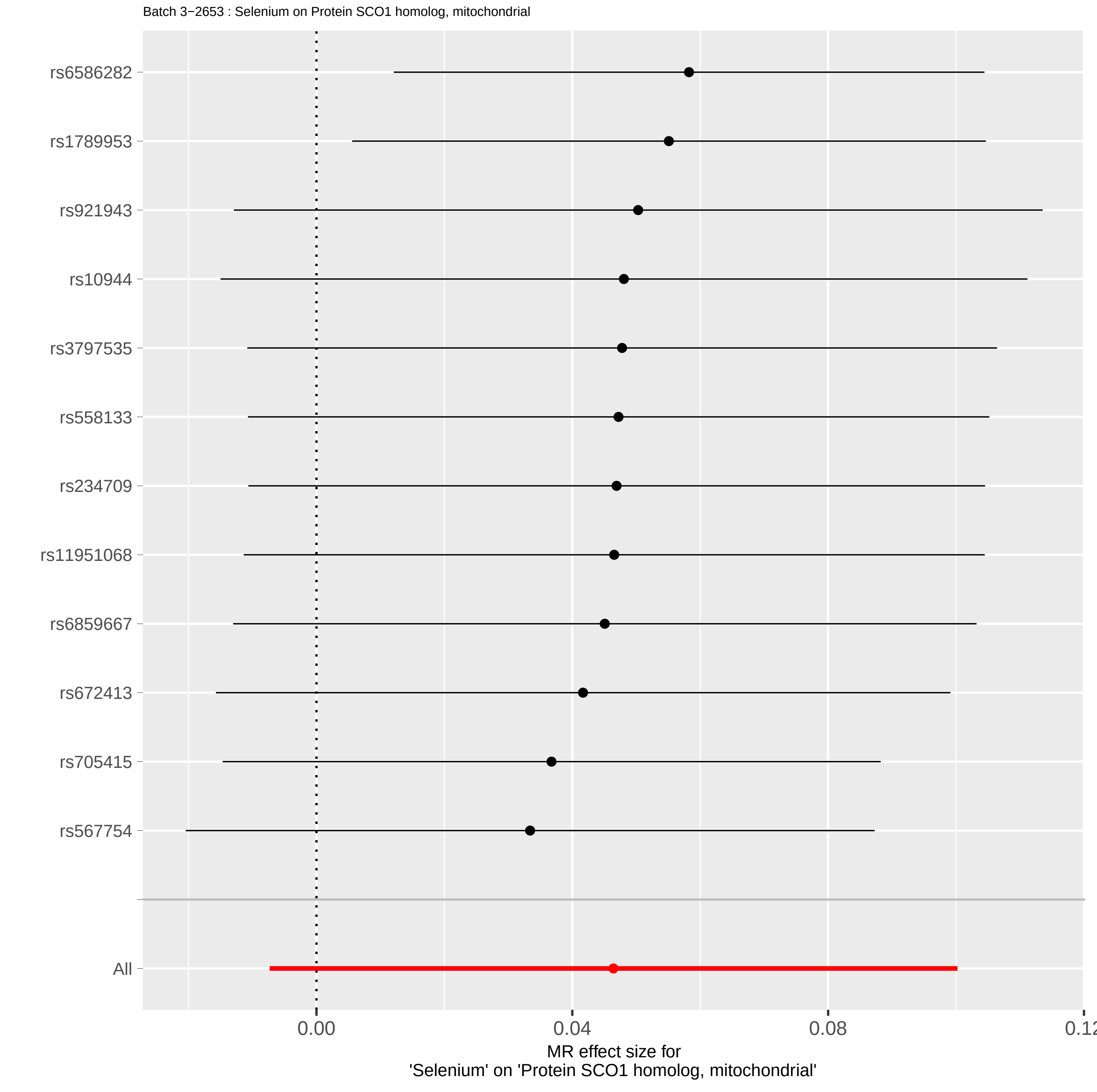

Batch 3−2653 : Selenium on Protein SCO1 homolog, mitochondrial
rs6586282
rs1789953
rs921943
rs10944
rs3797535
rs558133
rs234709
rs11951068
rs6859667
rs672413
rs705415
rs567754
All
0.00
0.04
0.08
0.12
MR effect size for
'Selenium' on 'Protein SCO1 homolog, mitochondrial'

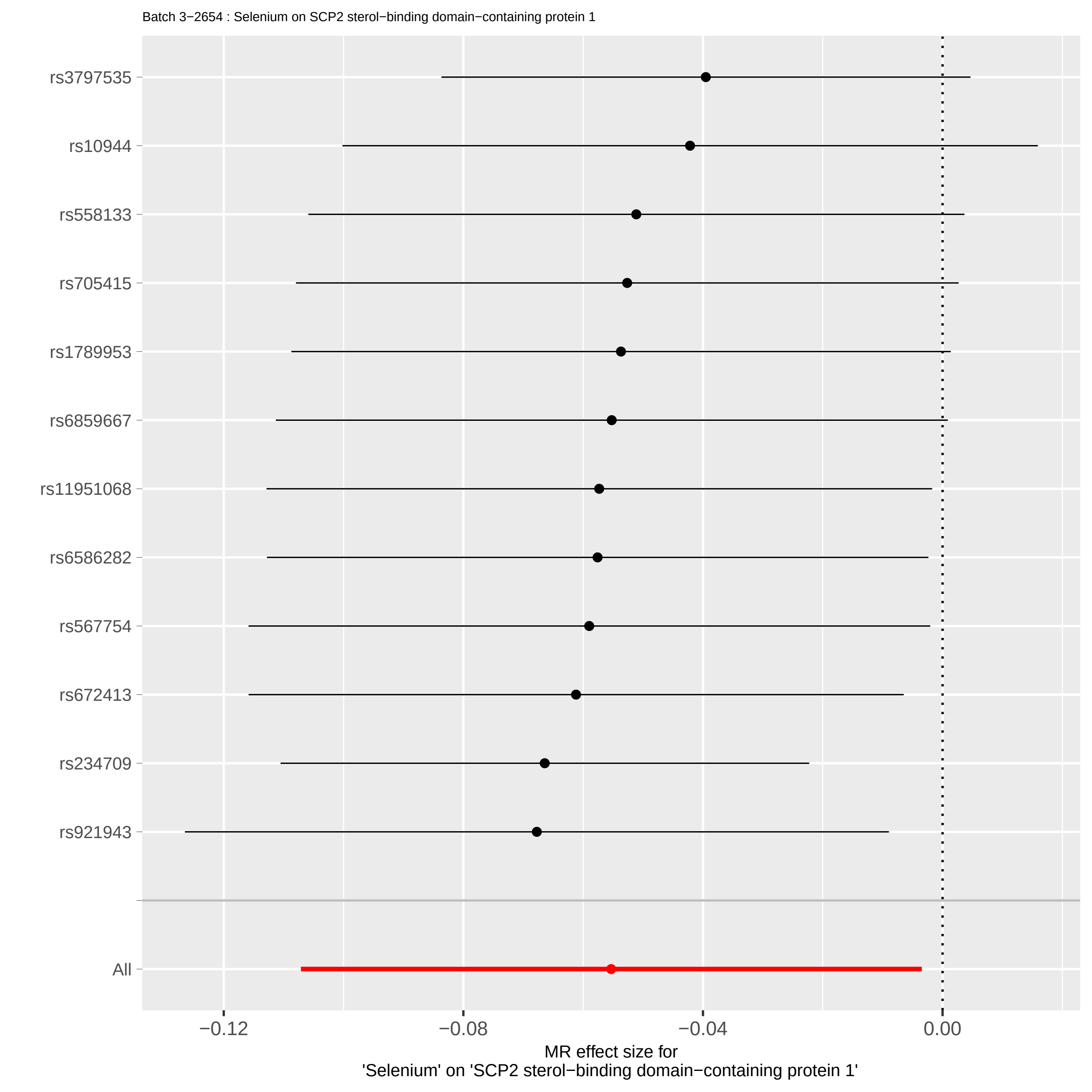

Batch 3−2654 : Selenium on SCP2 sterol−binding domain−containing protein 1
rs3797535
rs10944
rs558133
rs705415
rs1789953
rs6859667
rs11951068
rs6586282
rs567754
rs672413
rs234709
rs921943
All
−0.12
−0.08
−0.04
0.00
MR effect size for
'Selenium' on 'SCP2 sterol−binding domain−containing protein 1'

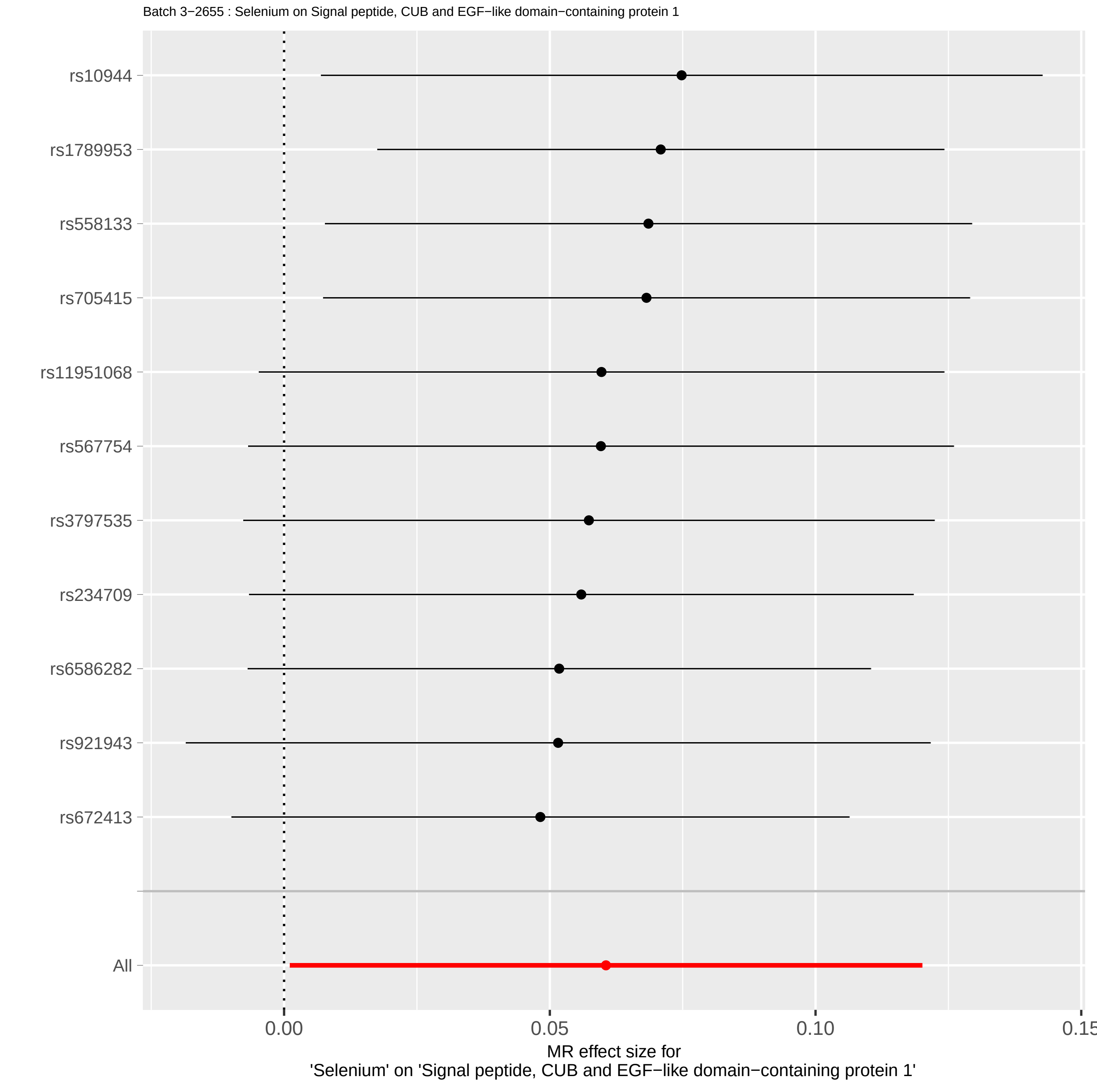

Batch 3−2655 : Selenium on Signal peptide, CUB and EGF−like domain−containing protein 1
rs10944
rs1789953
rs558133
rs705415
rs11951068
rs567754
rs3797535
rs234709
rs6586282
rs921943
rs672413
All
0.00
0.05
0.10
0.15
MR effect size for
'Selenium' on 'Signal peptide, CUB and EGF−like domain−containing protein 1'

Batch 3−2656 : Selenium on Stromal cell−derived factor 2−like protein 1

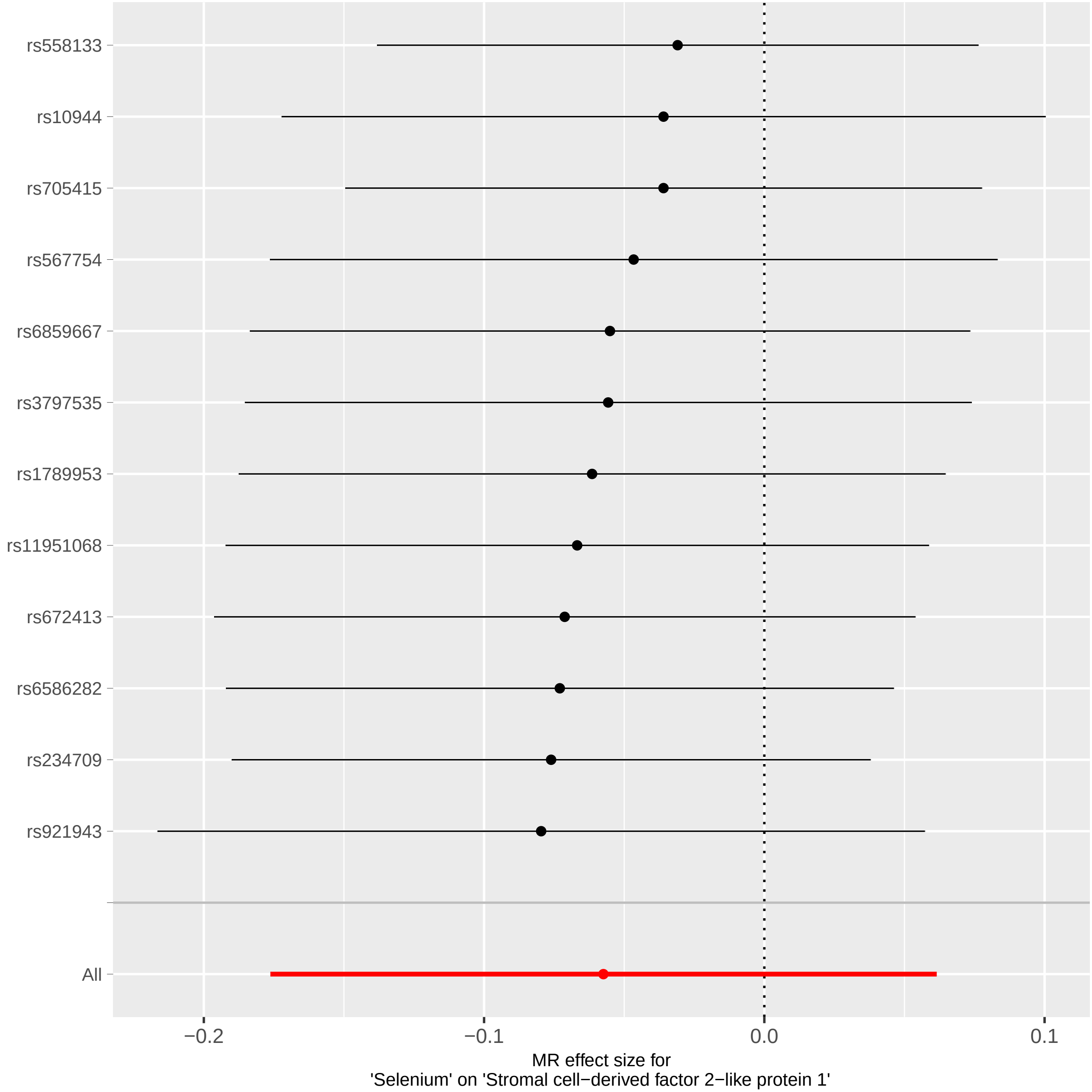

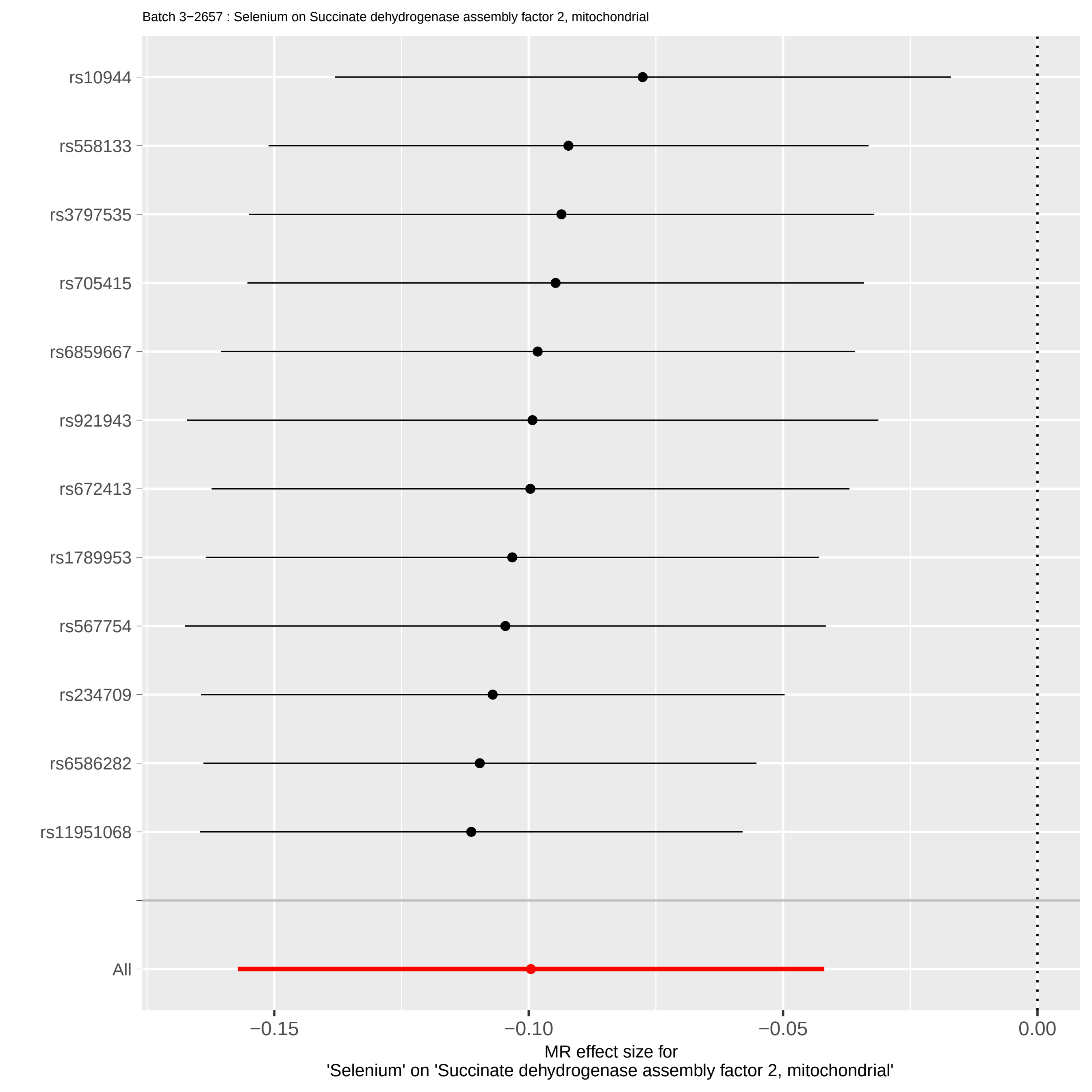

Batch 3−2657 : Selenium on Succinate dehydrogenase assembly factor 2, mitochondrial
rs10944
rs558133
rs3797535
rs705415
rs6859667
rs921943
rs672413
rs1789953
rs567754
rs234709
rs6586282
rs11951068
All
−0.15
−0.10
−0.05
0.00
MR effect size for
'Selenium' on 'Succinate dehydrogenase assembly factor 2, mitochondrial'

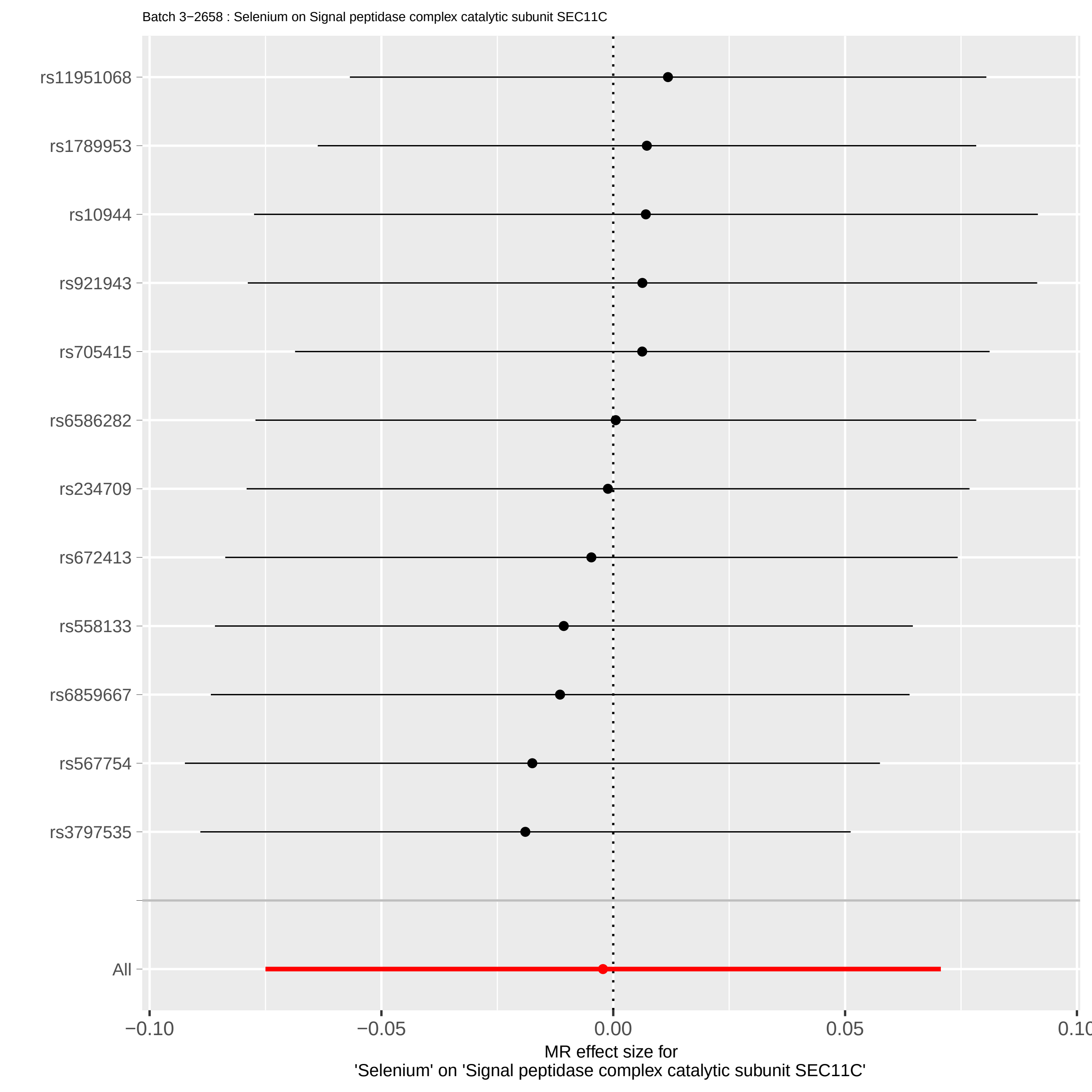

Batch 3−2658 : Selenium on Signal peptidase complex catalytic subunit SEC11C
rs11951068
rs1789953
rs10944
rs921943
rs705415
rs6586282
rs234709
rs672413
rs558133
rs6859667
rs567754
rs3797535
All
−0.10
−0.05
0.00
0.05
0.10
MR effect size for
'Selenium' on 'Signal peptidase complex catalytic subunit SEC11C'

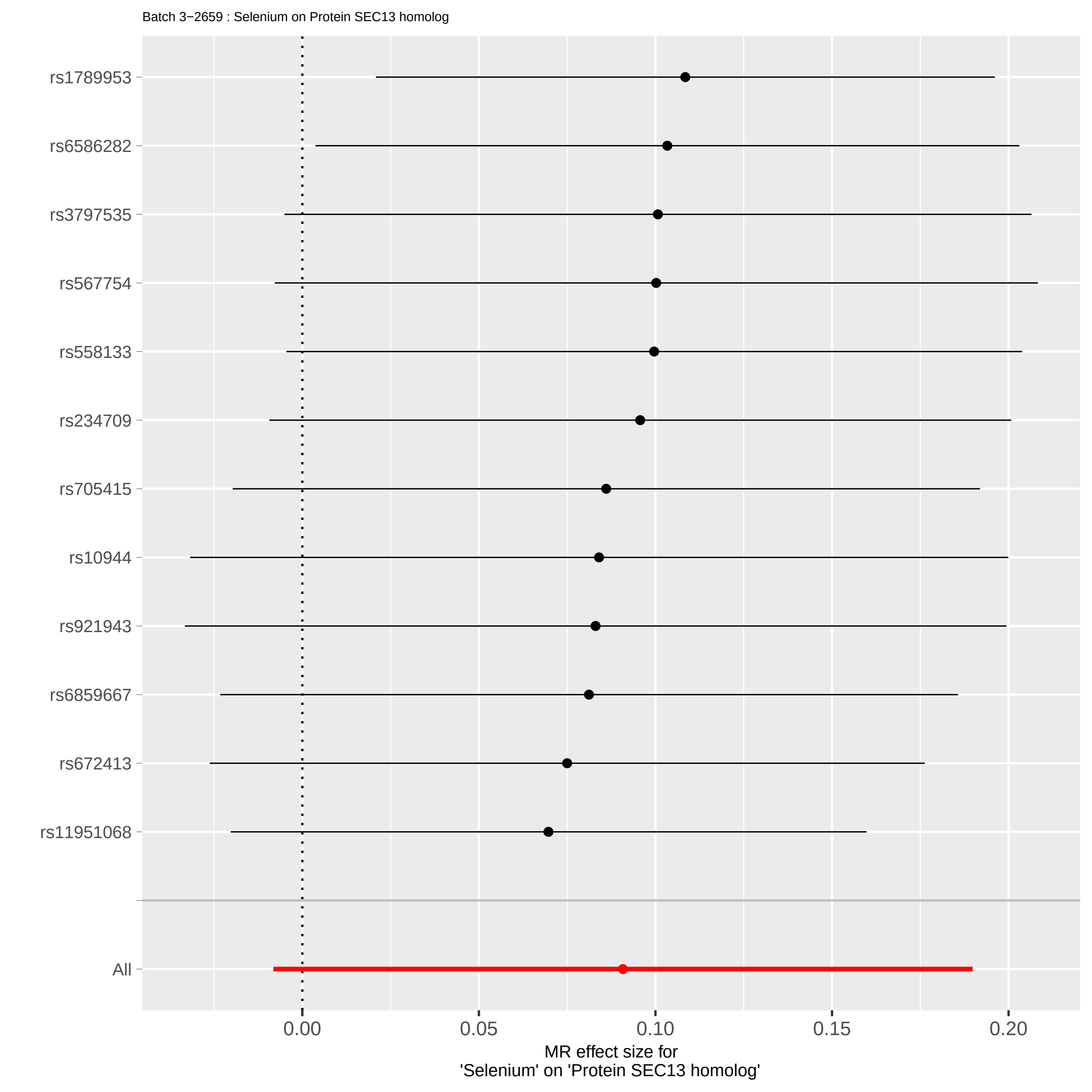

Batch 3−2659 : Selenium on Protein SEC13 homolog
rs1789953
rs6586282
rs3797535
rs567754
rs558133
rs234709
rs705415
rs10944
rs921943
rs6859667
rs672413
rs11951068
All
0.00
0.05
0.10
0.15
0.20
MR effect size for
'Selenium' on 'Protein SEC13 homolog'

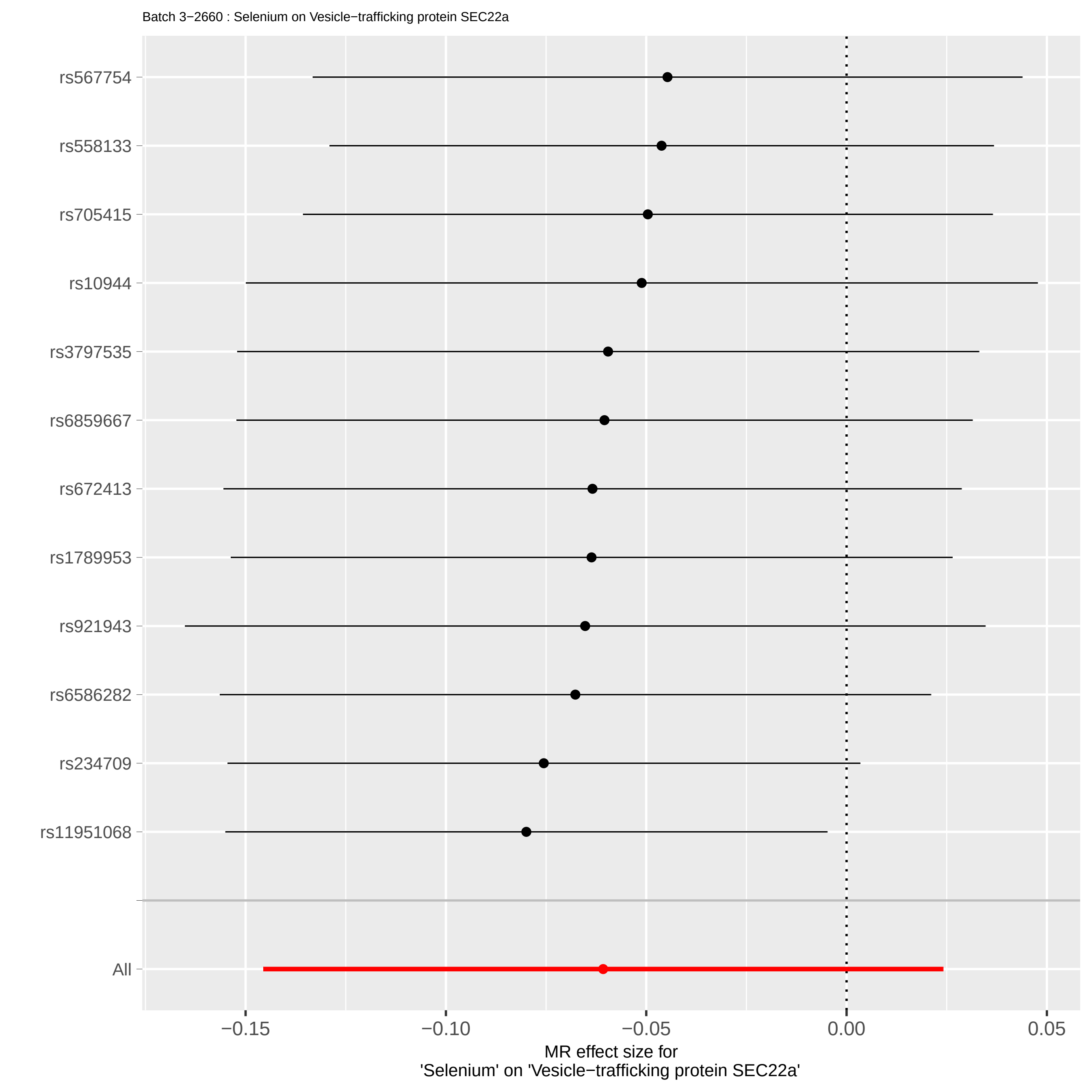

Batch 3−2660 : Selenium on Vesicle−trafficking protein SEC22a
rs567754
rs558133
rs705415
rs10944
rs3797535
rs6859667
rs672413
rs1789953
rs921943
rs6586282
rs234709
rs11951068
All
−0.15
−0.10
−0.05
0.00
0.05
MR effect size for
'Selenium' on 'Vesicle−trafficking protein SEC22a'

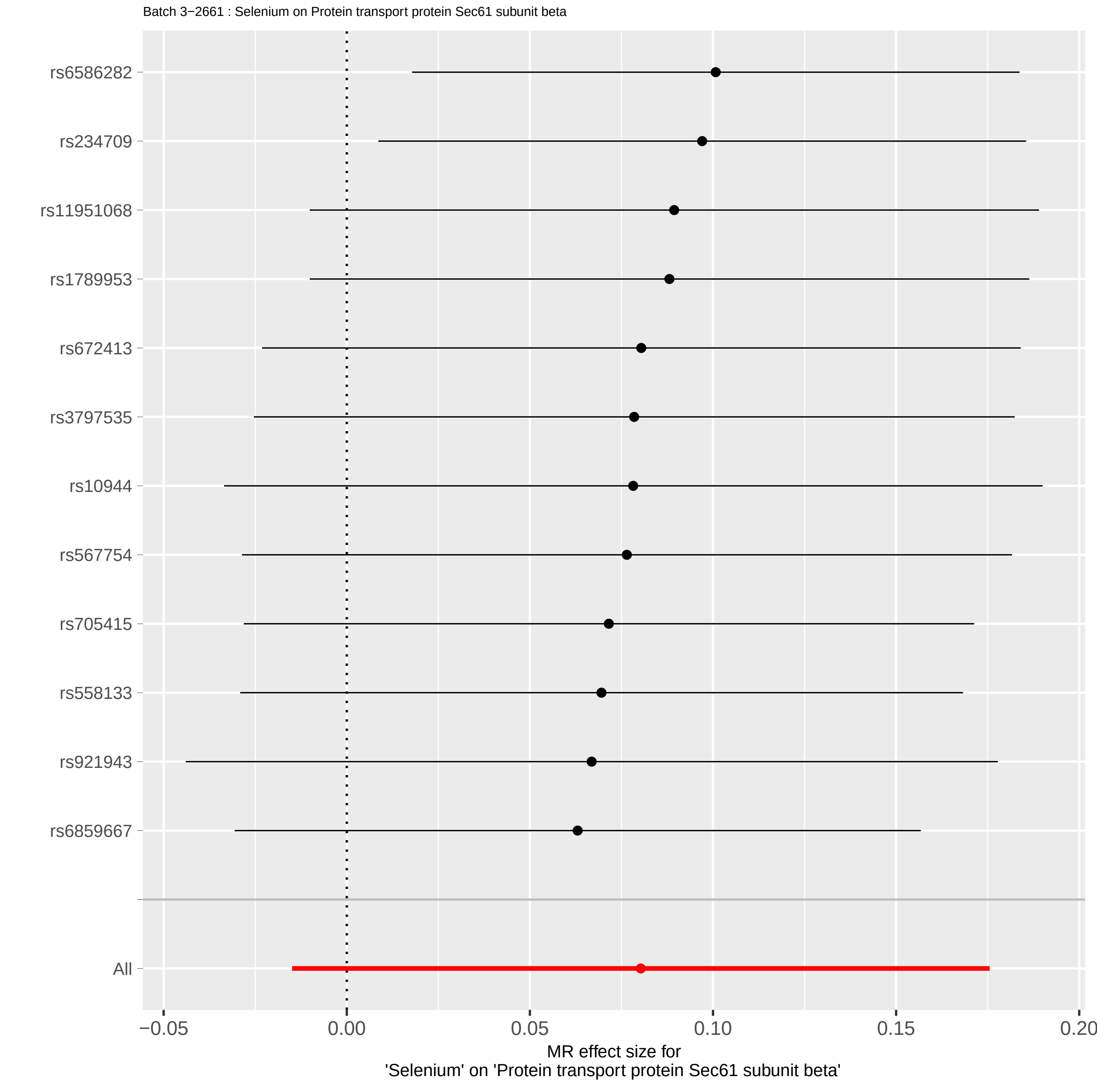

Batch 3−2661 : Selenium on Protein transport protein Sec61 subunit beta
rs6586282
rs234709
rs11951068
rs1789953
rs672413
rs3797535
rs10944
rs567754
rs705415
rs558133
rs921943
rs6859667
All
−0.05
0.00
0.05
0.10
0.15
0.20
MR effect size for
'Selenium' on 'Protein transport protein Sec61 subunit beta'

Batch 3−2662 : Selenium on Protein transport protein Sec61 subunit gamma

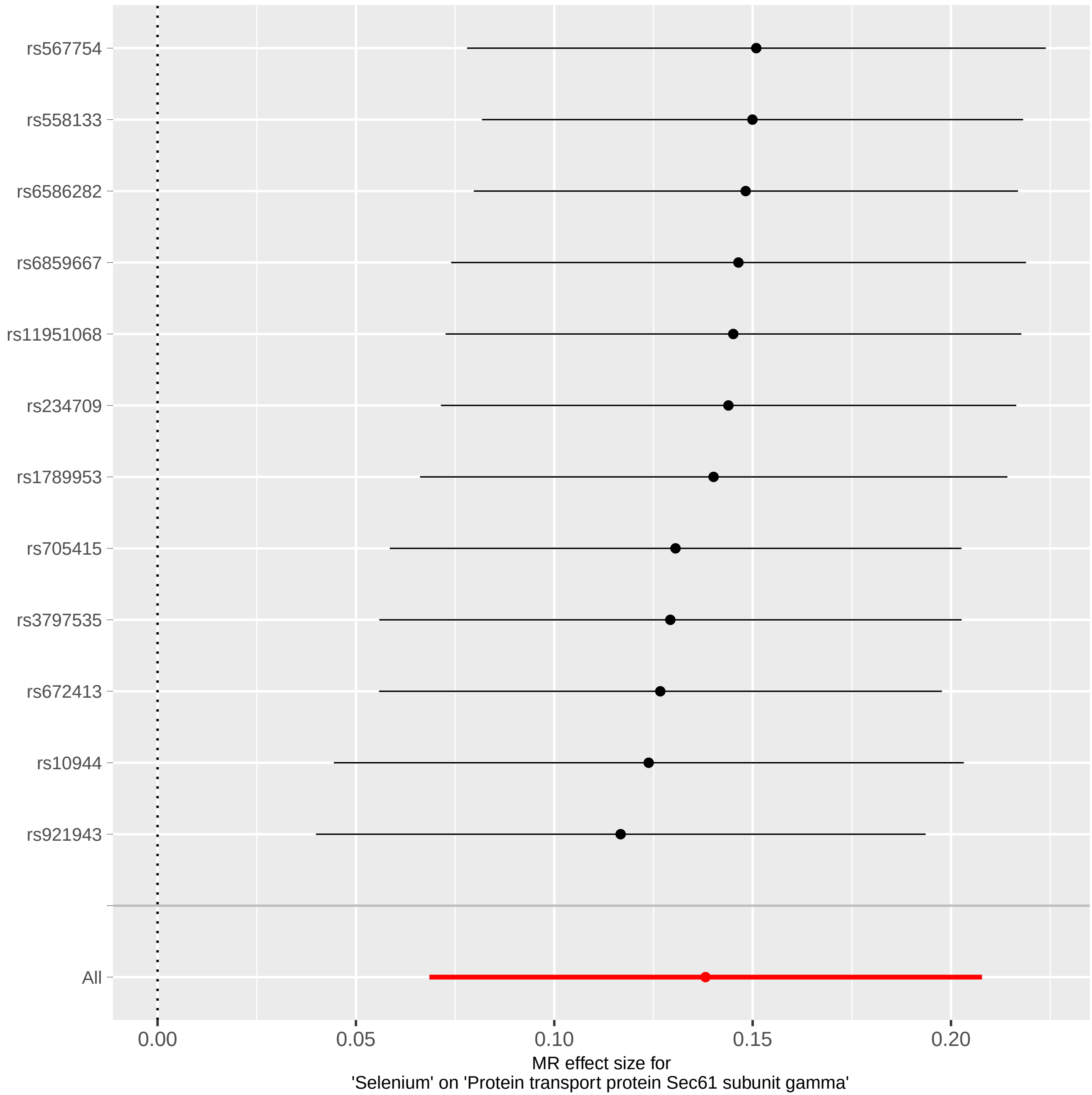

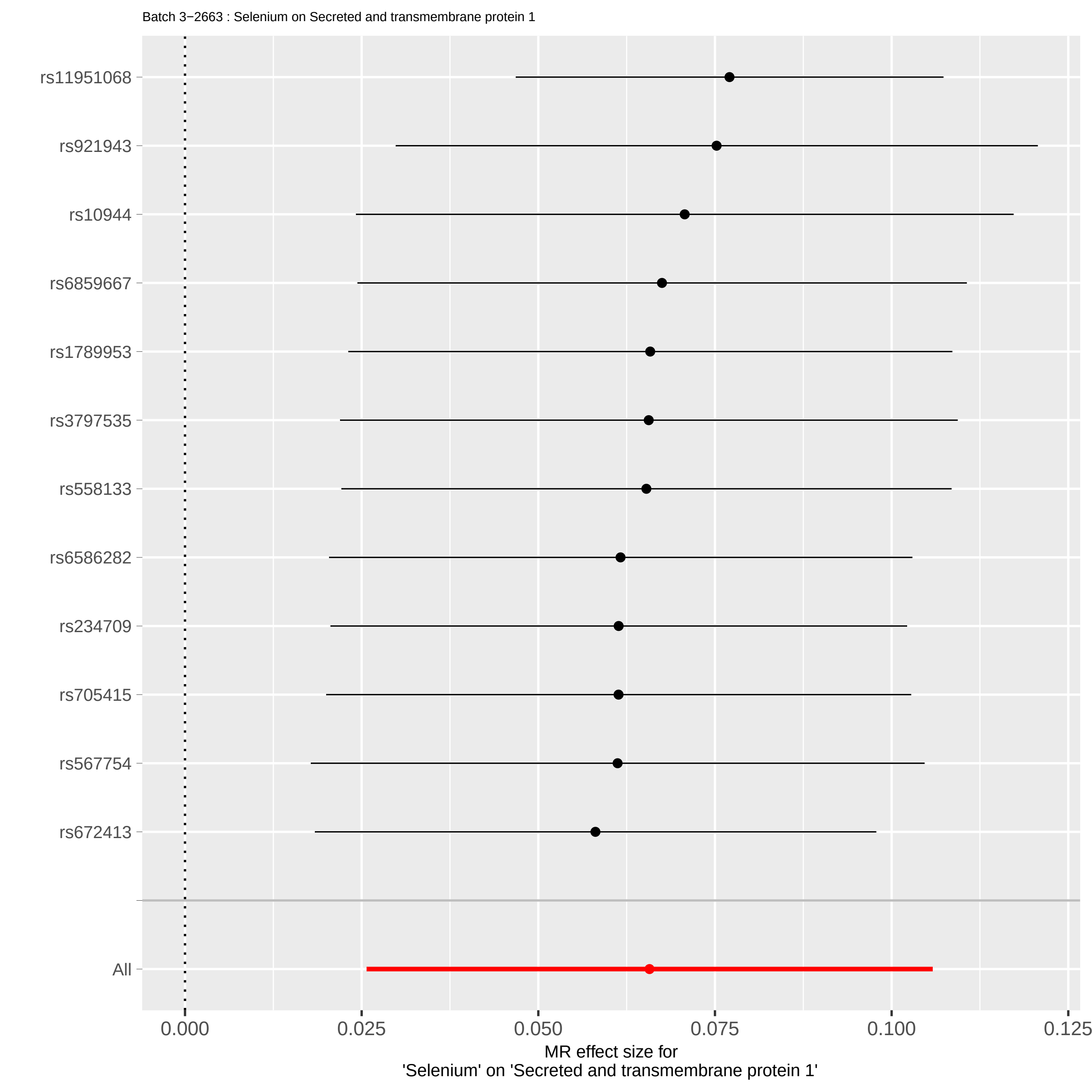

Batch 3−2663 : Selenium on Secreted and transmembrane protein 1
rs11951068
rs921943
rs10944
rs6859667
rs1789953
rs3797535
rs558133
rs6586282
rs234709
rs705415
rs567754
rs672413
All
0.000
0.025
0.050
0.075
0.100
0.125
MR effect size for
'Selenium' on 'Secreted and transmembrane protein 1'

Batch 3−2664 : Selenium on Protein sel−1 homolog 2

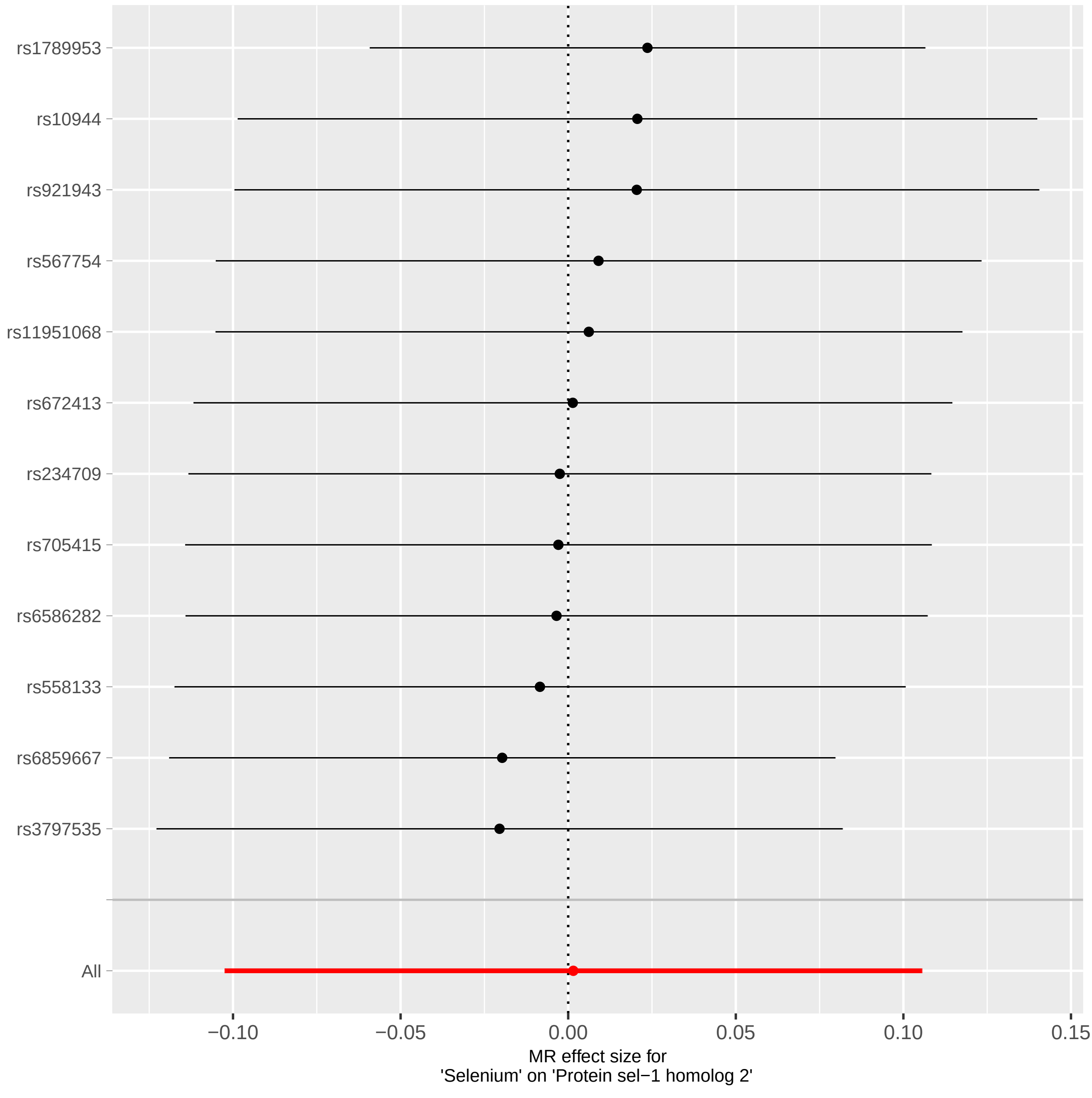

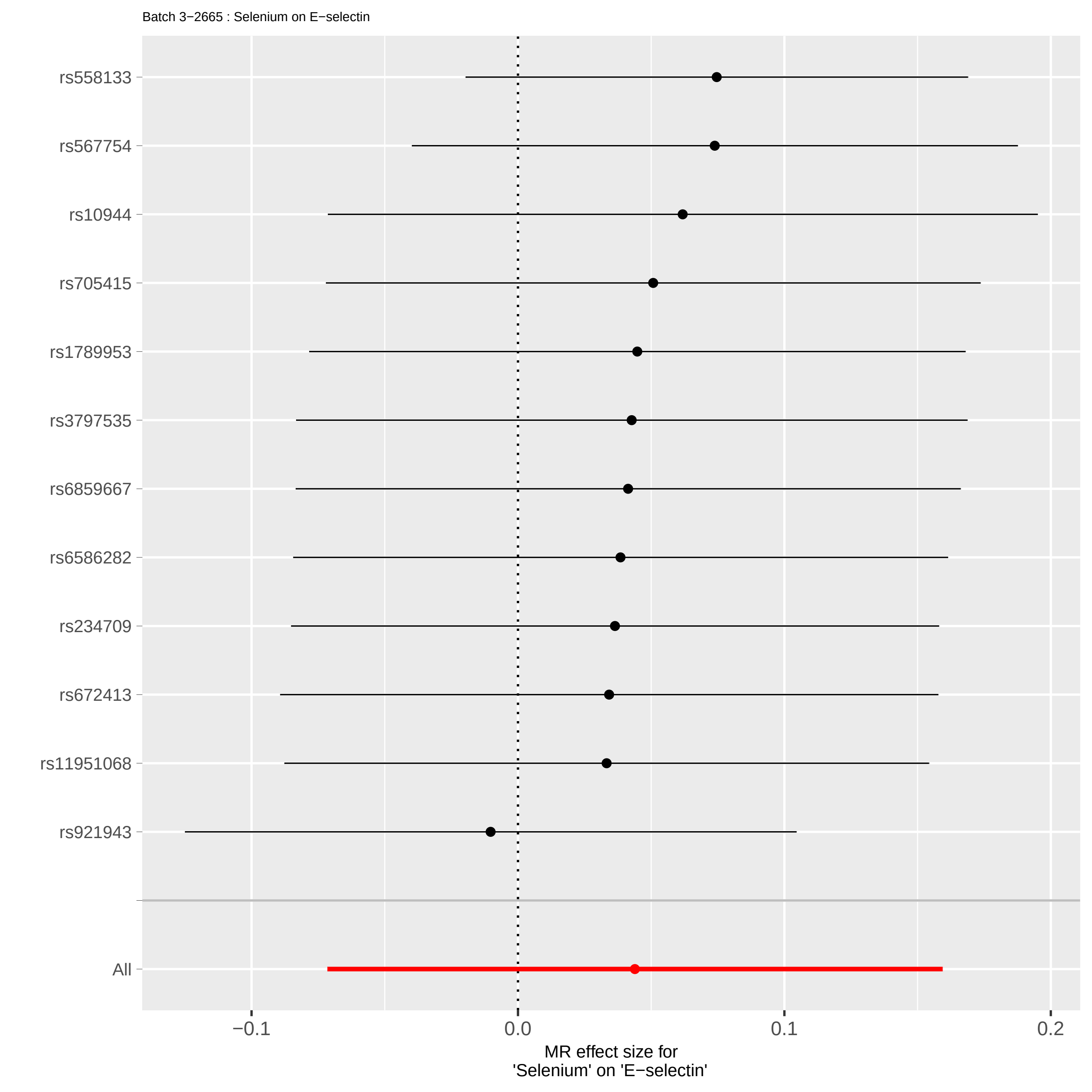

Batch 3−2665 : Selenium on E−selectin
rs558133
rs567754
rs10944
rs705415
rs1789953
rs3797535
rs6859667
rs6586282
rs234709
rs672413
rs11951068
rs921943
All
−0.1
0.0
0.1
0.2
MR effect size for
'Selenium' on 'E−selectin'

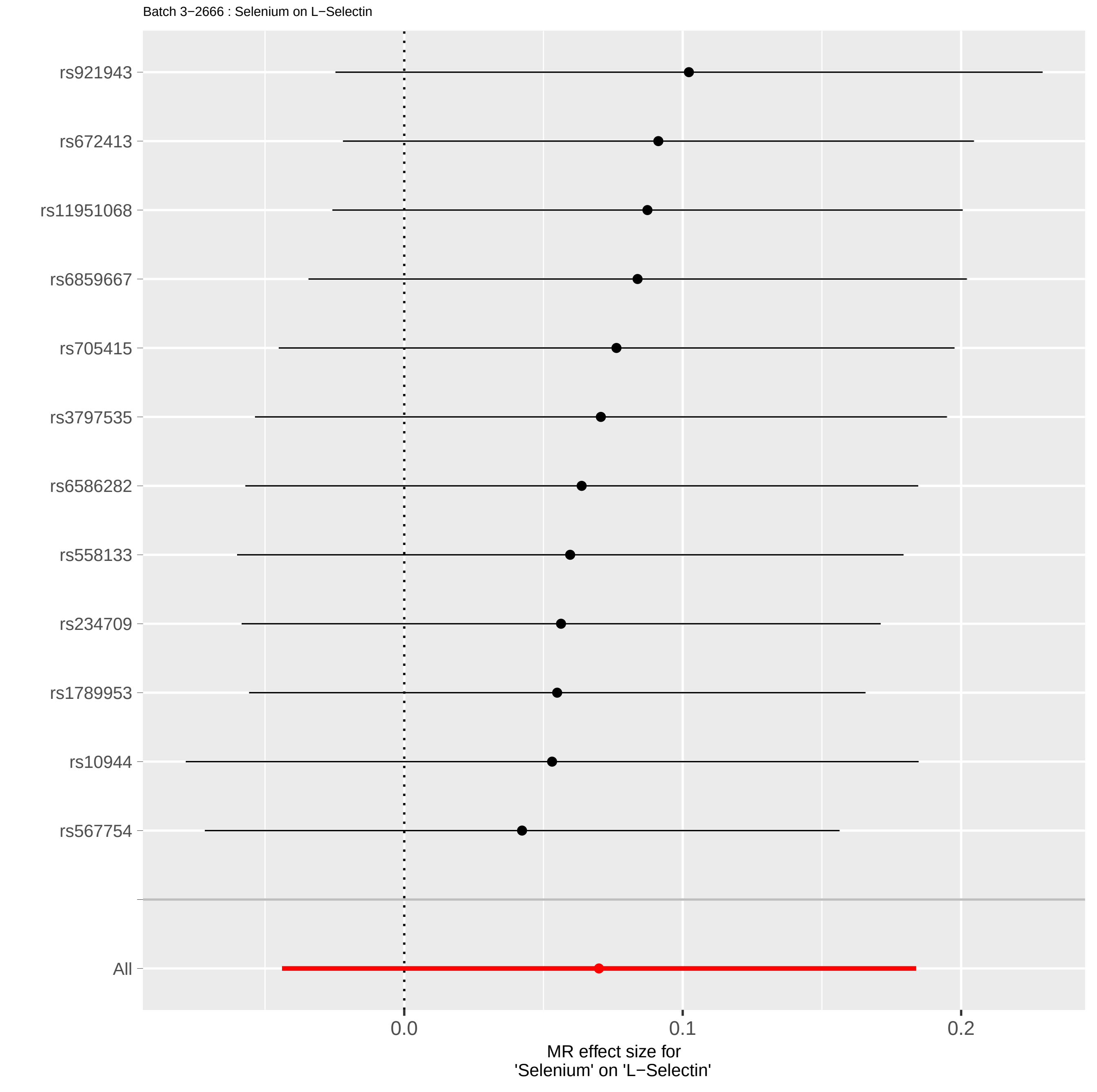

Batch 3−2666 : Selenium on L−Selectin
rs921943
rs672413
rs11951068
rs6859667
rs705415
rs3797535
rs6586282
rs558133
rs234709
rs1789953
rs10944
rs567754
All
0.0
0.1
0.2
MR effect size for
'Selenium' on 'L−Selectin'

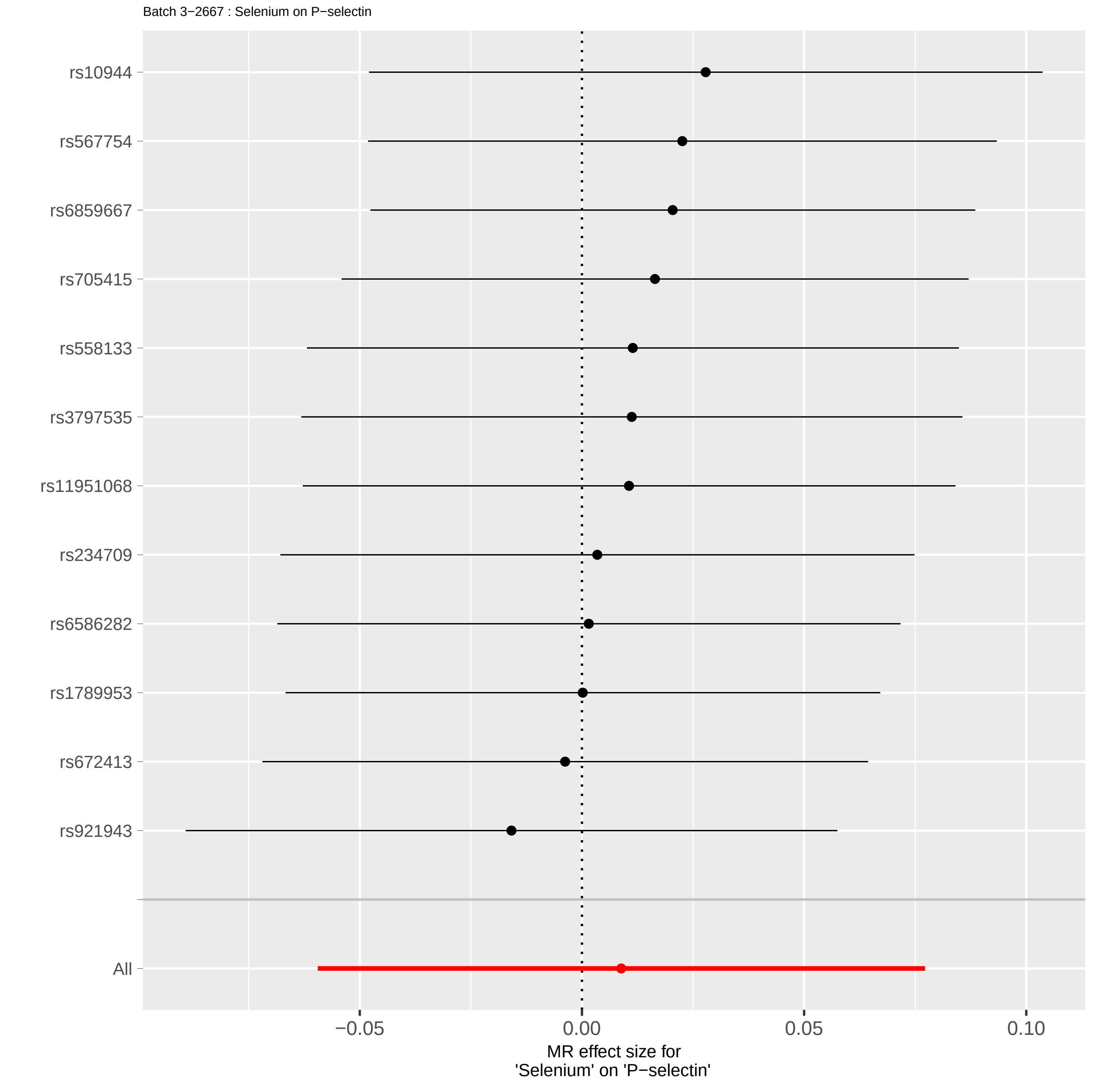

Batch 3−2667 : Selenium on P−selectin
rs10944
rs567754
rs6859667
rs705415
rs558133
rs3797535
rs11951068
rs234709
rs6586282
rs1789953
rs672413
rs921943
All
−0.05
0.00
0.05
0.10
MR effect size for
'Selenium' on 'P−selectin'

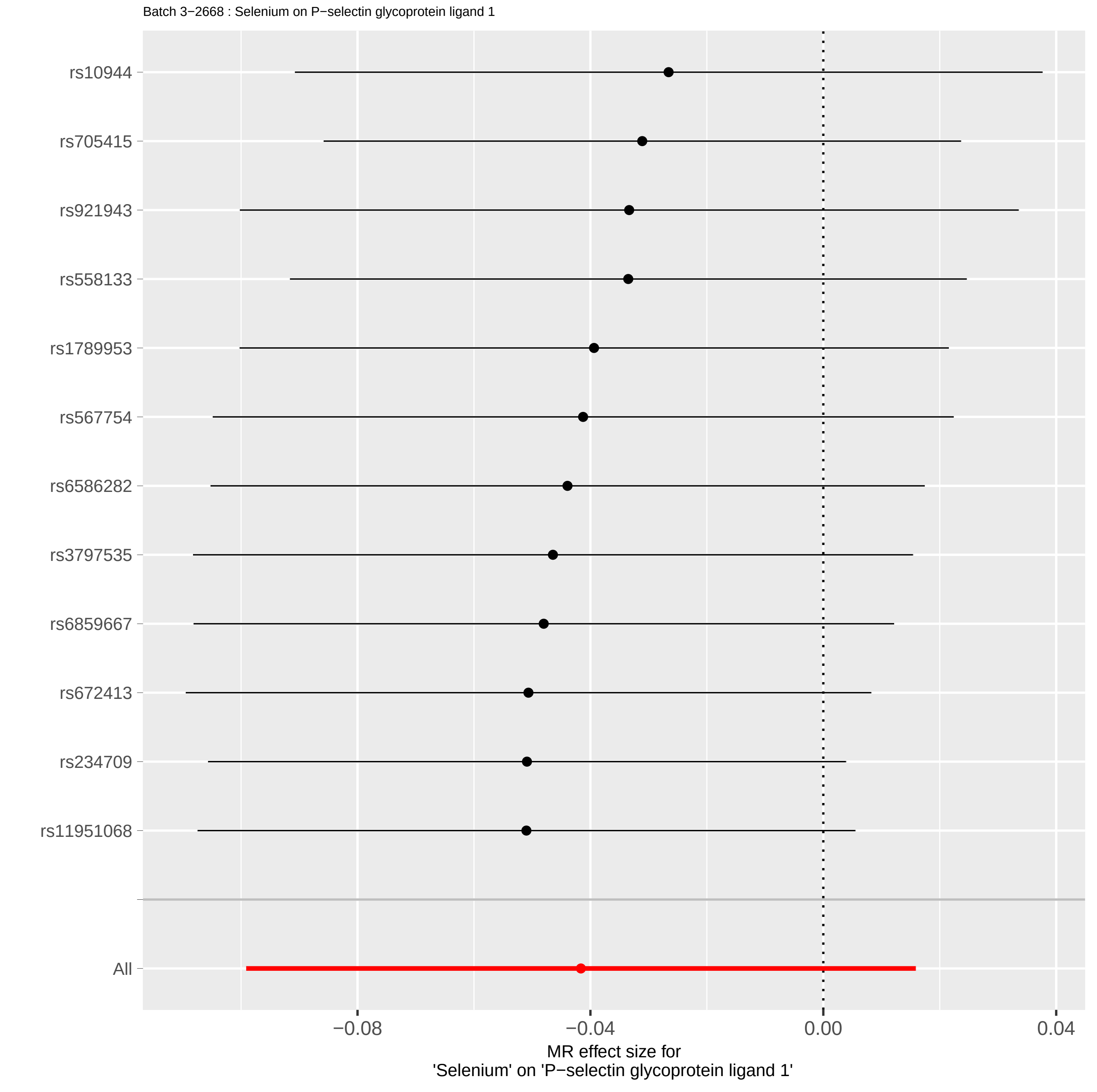

Batch 3−2668 : Selenium on P−selectin glycoprotein ligand 1
rs10944
rs705415
rs921943
rs558133
rs1789953
rs567754
rs6586282
rs3797535
rs6859667
rs672413
rs234709
rs11951068
All
−0.08
−0.04
0.00
0.04
MR effect size for
'Selenium' on 'P−selectin glycoprotein ligand 1'

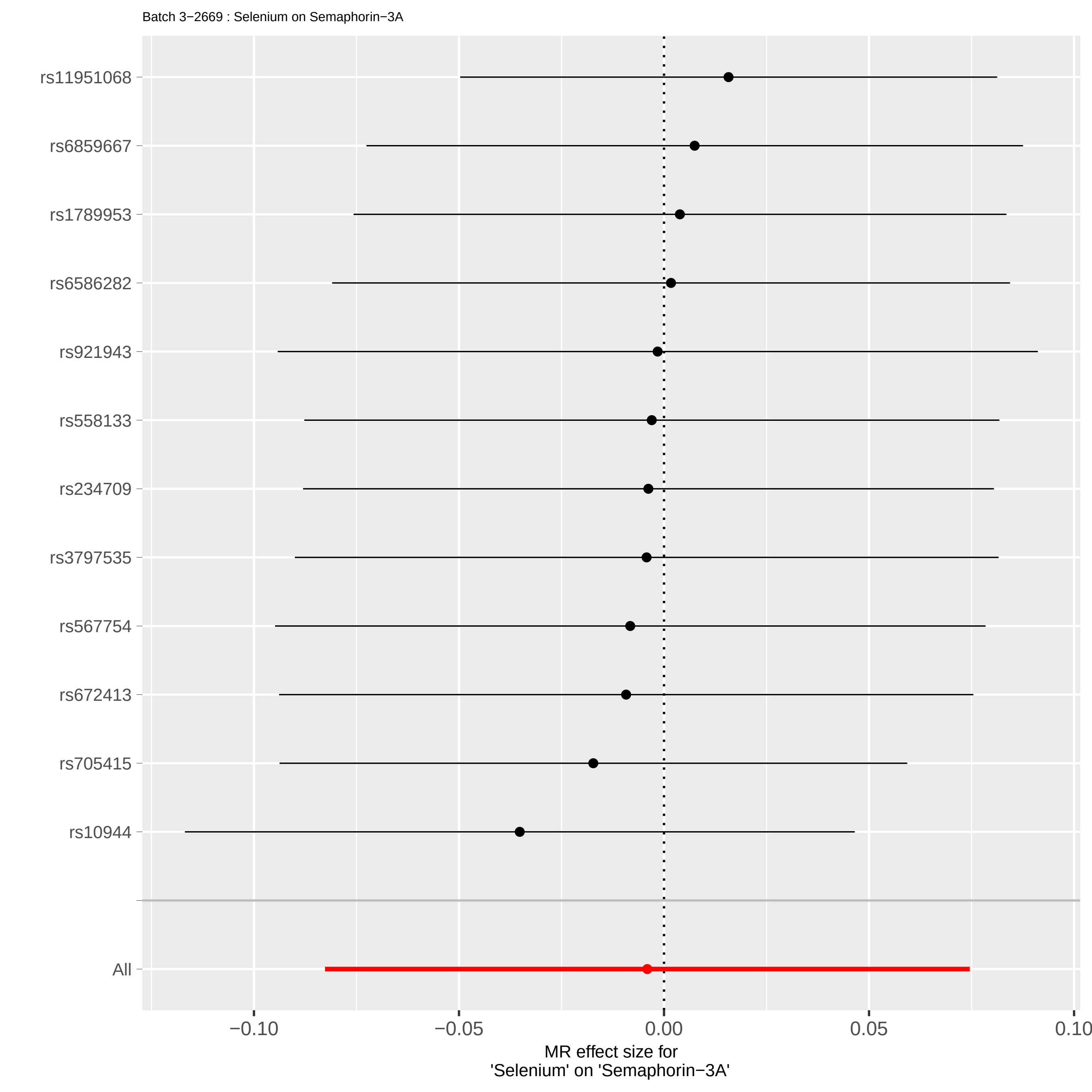

Batch 3−2669 : Selenium on Semaphorin−3A
rs11951068
rs6859667
rs1789953
rs6586282
rs921943
rs558133
rs234709
rs3797535
rs567754
rs672413
rs705415
rs10944
All
−0.10
−0.05
0.00
0.05
0.10
MR effect size for
'Selenium' on 'Semaphorin−3A'

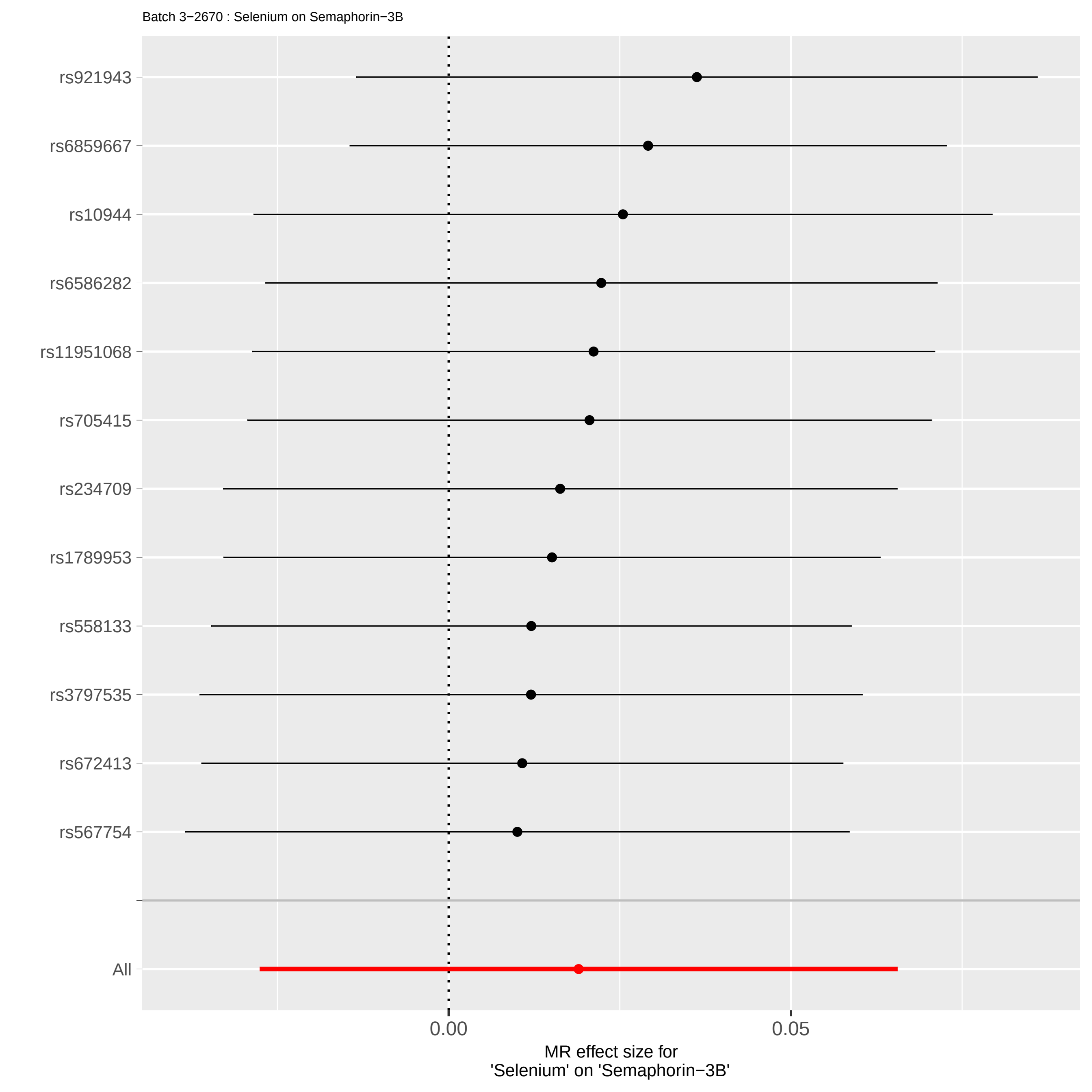
Batch 3−2670 : Selenium on Semaphorin−3B
rs921943
rs6859667
rs10944
rs6586282
rs11951068
rs705415
rs234709
rs1789953
rs558133
rs3797535
rs672413
rs567754
All
0.00
0.05
MR effect size for
'Selenium' on 'Semaphorin−3B'

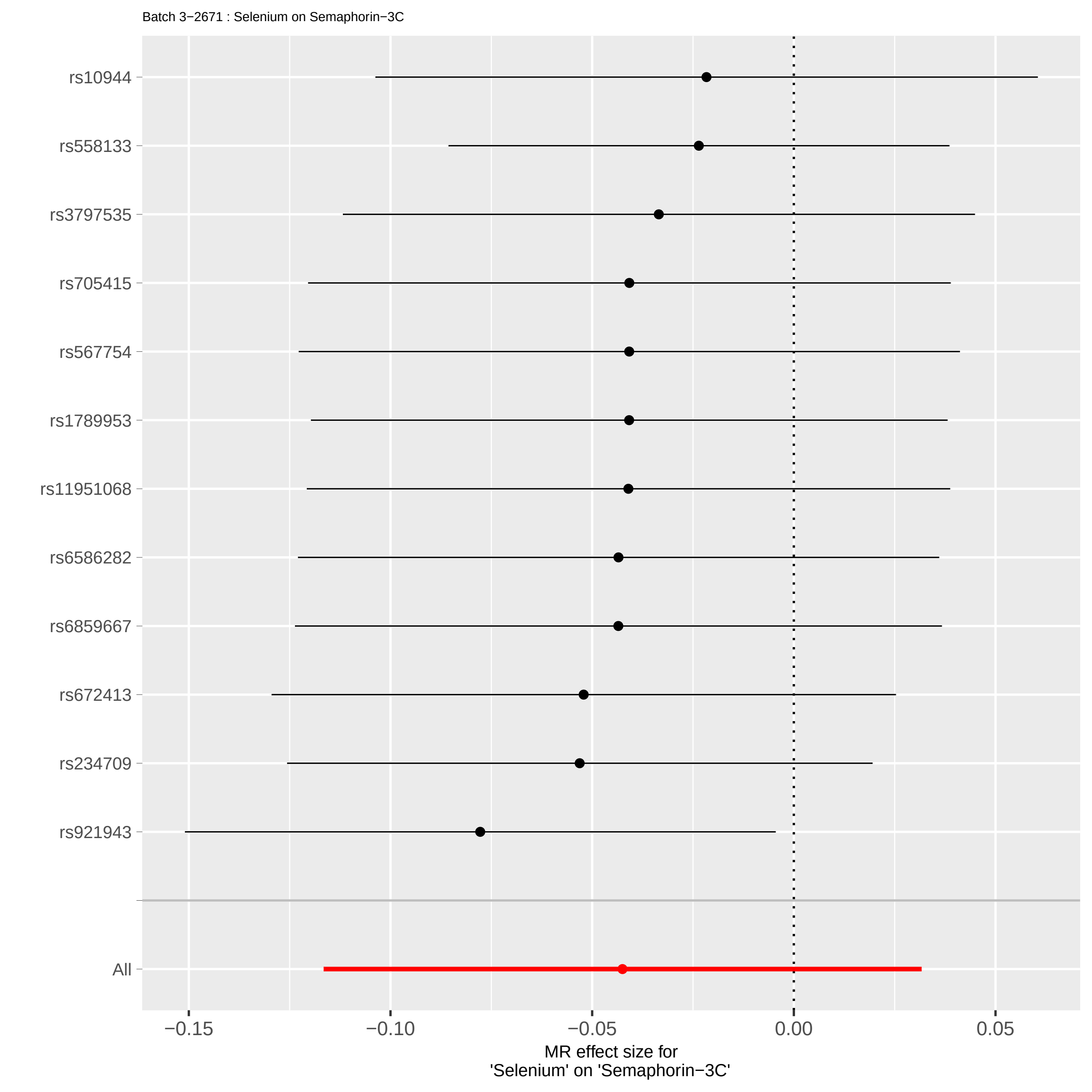

Batch 3−2671 : Selenium on Semaphorin−3C
rs10944
rs558133
rs3797535
rs705415
rs567754
rs1789953
rs11951068
rs6586282
rs6859667
rs672413
rs234709
rs921943
All
−0.15
−0.10
−0.05
0.00
0.05
MR effect size for
'Selenium' on 'Semaphorin−3C'

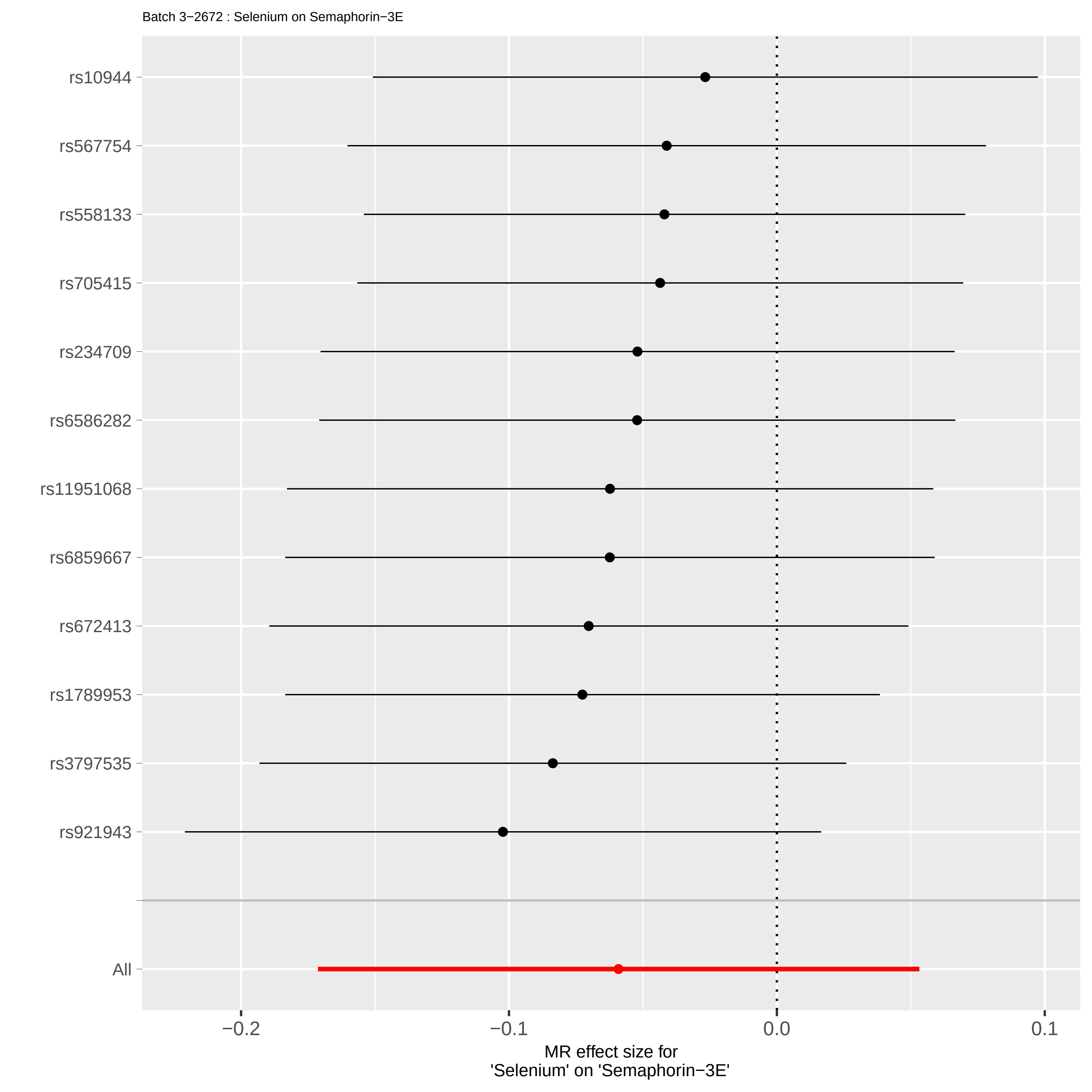
Batch 3−2672 : Selenium on Semaphorin−3E
rs10944
rs567754
rs558133
rs705415
rs234709
rs6586282
rs11951068
rs6859667
rs672413
rs1789953
rs3797535
rs921943
All
−0.2
−0.1
0.0
0.1
MR effect size for
'Selenium' on 'Semaphorin−3E'

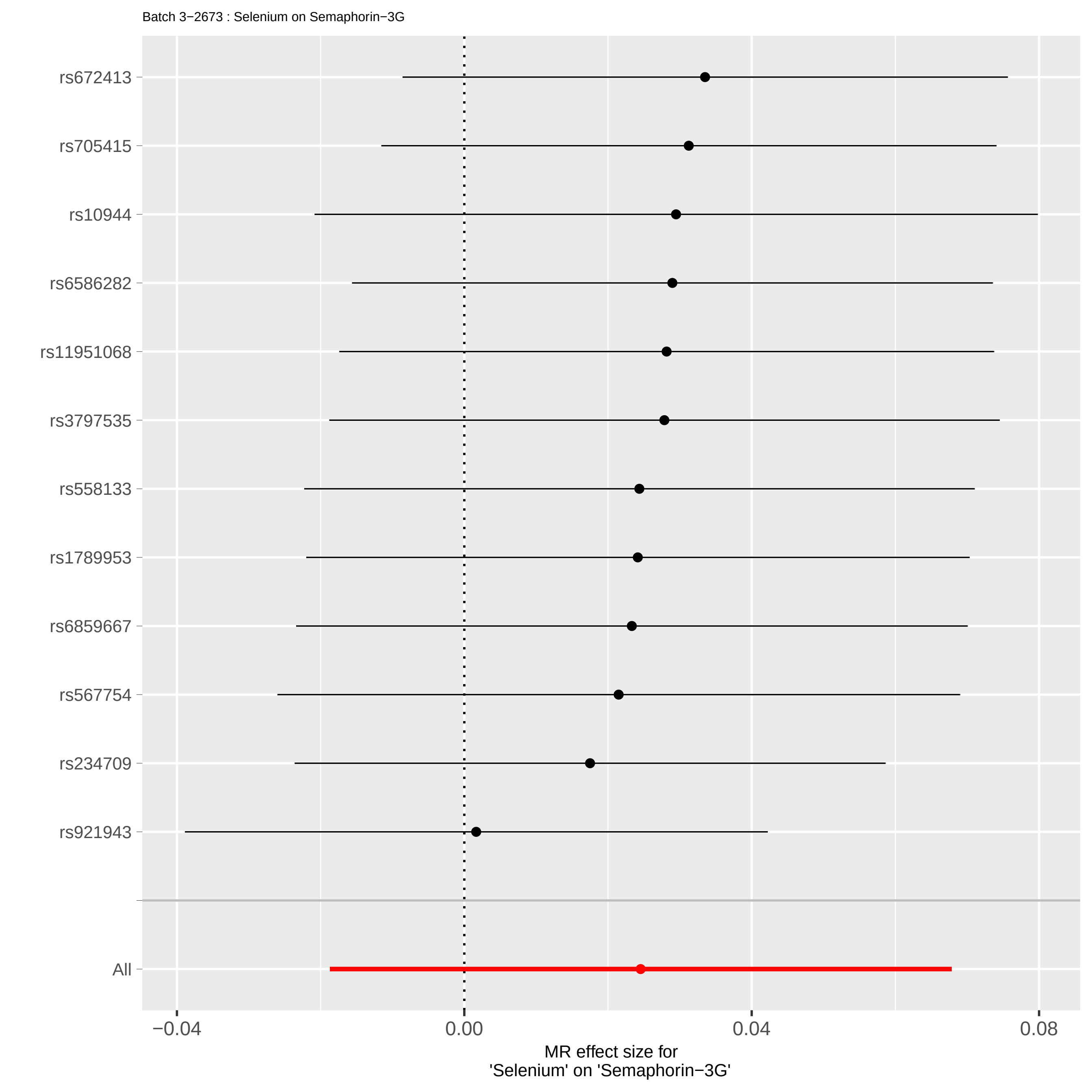

Batch 3−2673 : Selenium on Semaphorin−3G
rs672413
rs705415
rs10944
rs6586282
rs11951068
rs3797535
rs558133
rs1789953
rs6859667
rs567754
rs234709
rs921943
All
−0.04
0.00
0.04
0.08
MR effect size for
'Selenium' on 'Semaphorin−3G'

Batch 3−2674 : Selenium on Semaphorin−4C

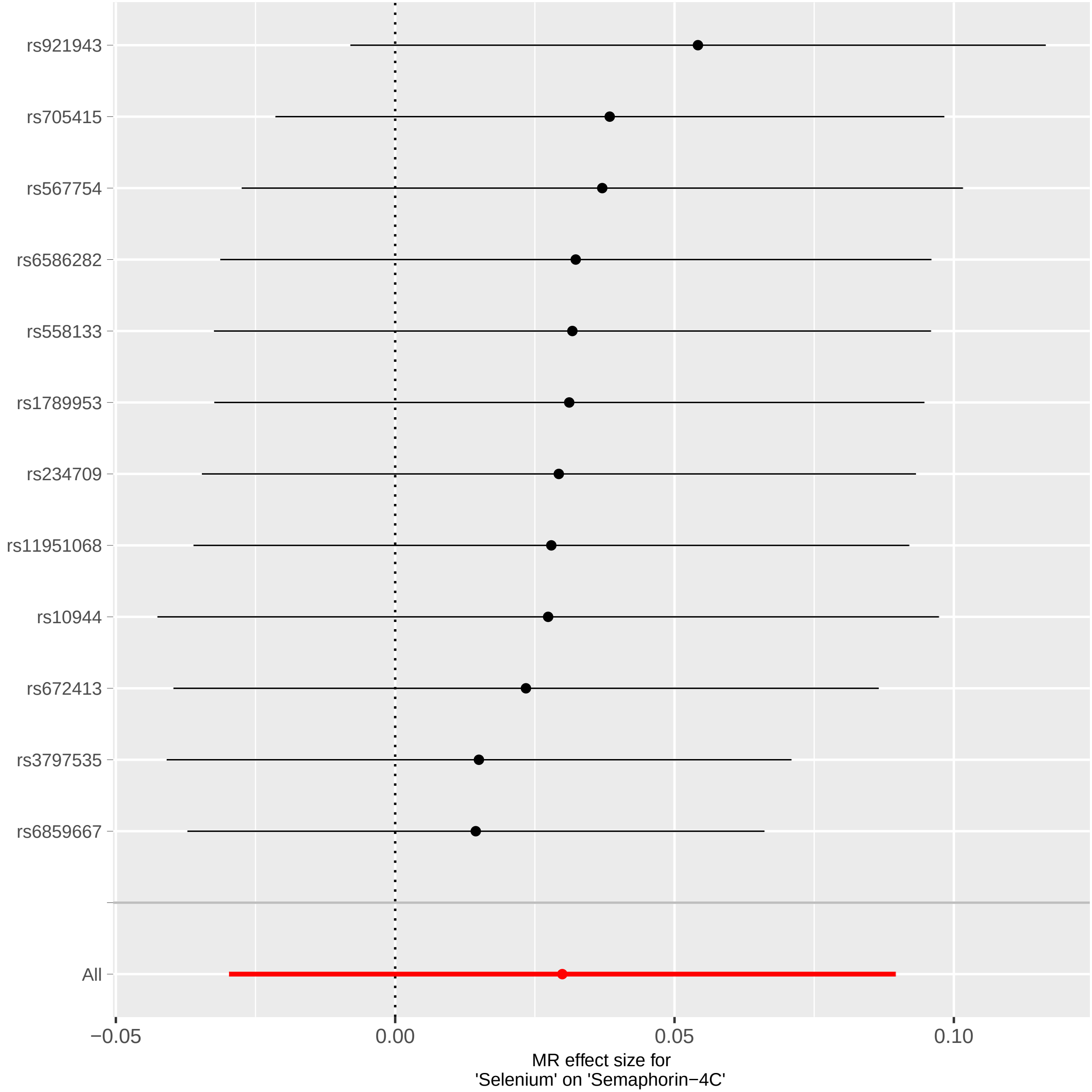

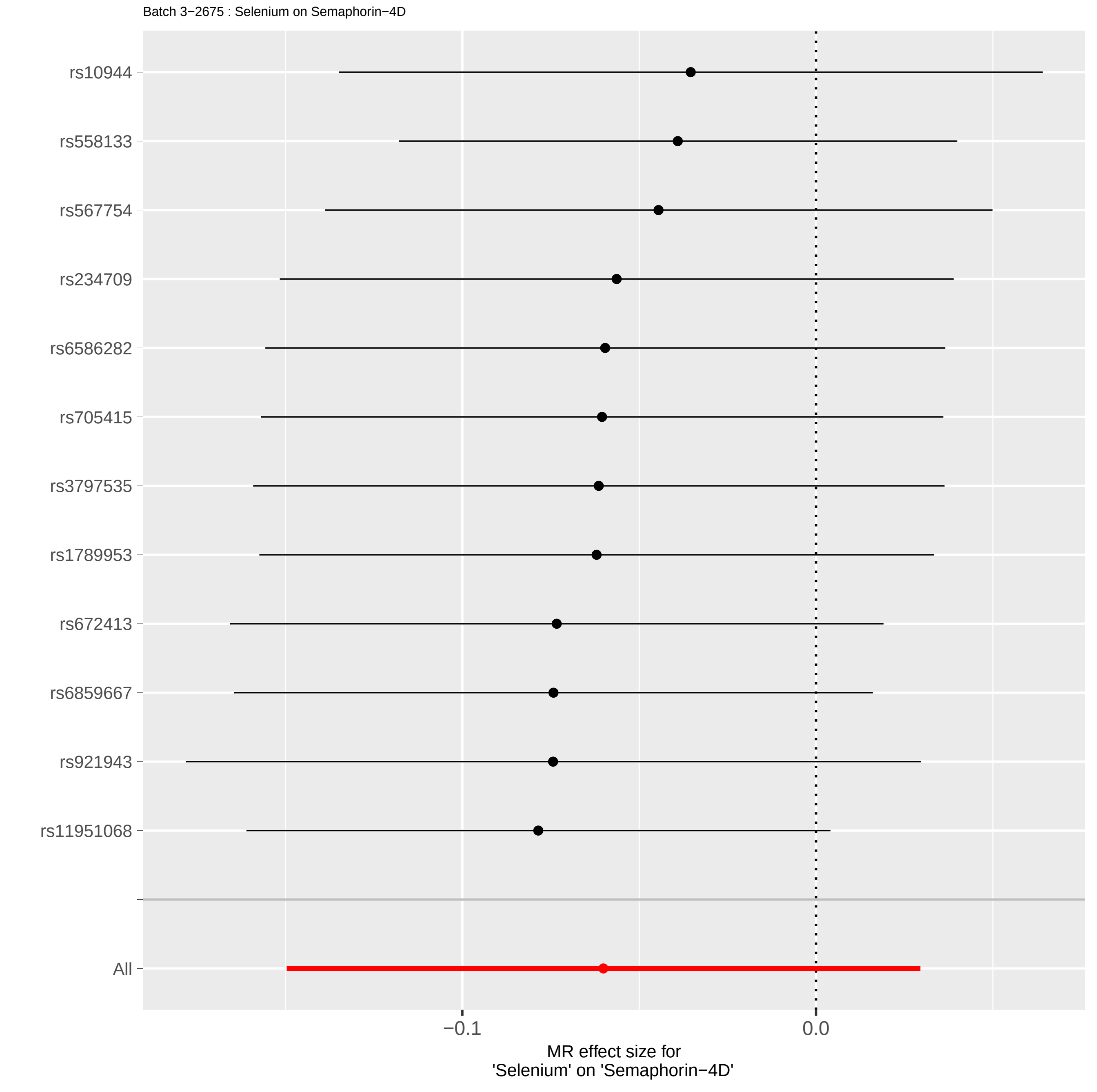

Batch 3−2675 : Selenium on Semaphorin−4D
rs10944
rs558133
rs567754
rs234709
rs6586282
rs705415
rs3797535
rs1789953
rs672413
rs6859667
rs921943
rs11951068
All
−0.1
0.0
MR effect size for
'Selenium' on 'Semaphorin−4D'

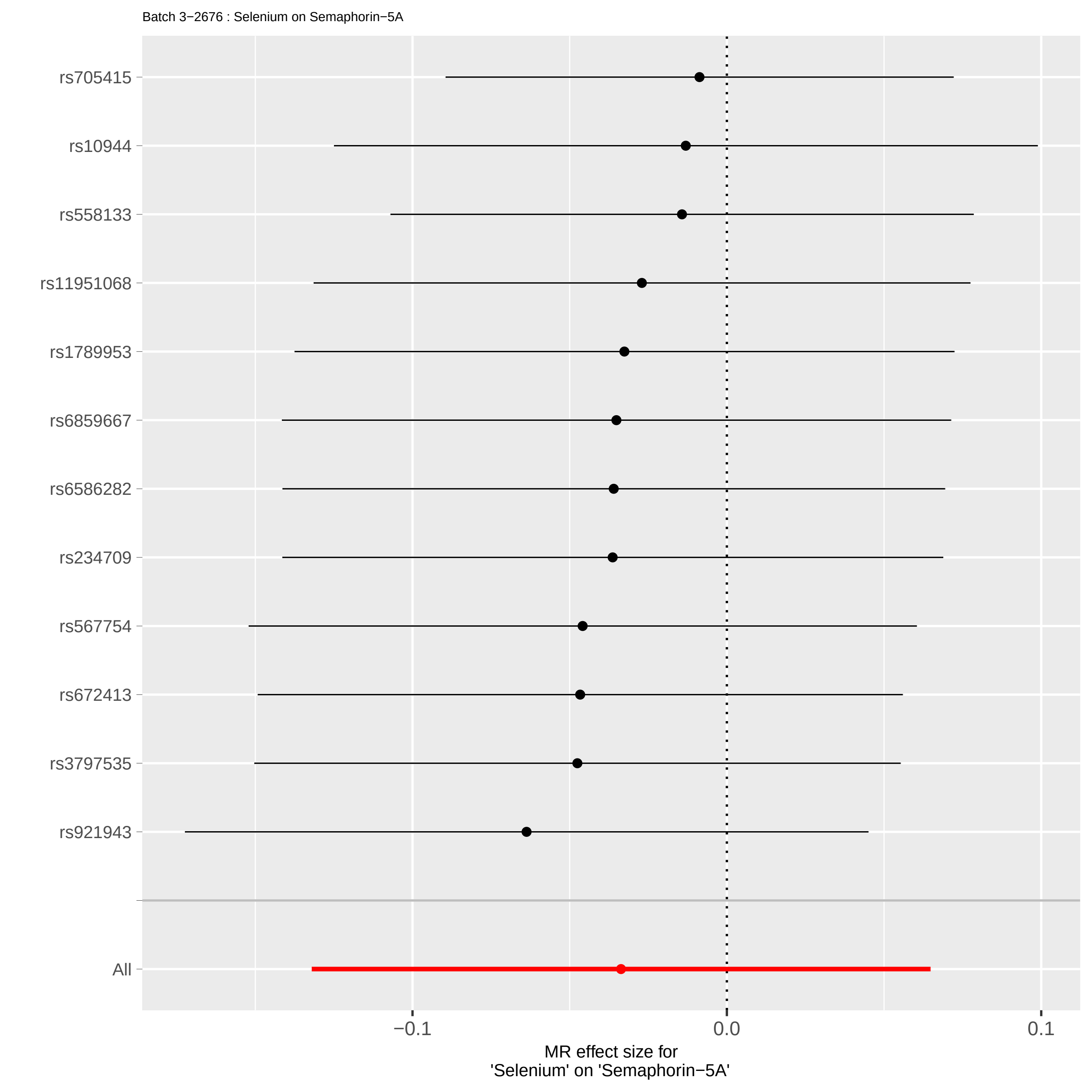

Batch 3−2676 : Selenium on Semaphorin−5A
rs705415
rs10944
rs558133
rs11951068
rs1789953
rs6859667
rs6586282
rs234709
rs567754
rs672413
rs3797535
rs921943
All
−0.1
0.0
0.1
MR effect size for
'Selenium' on 'Semaphorin−5A'

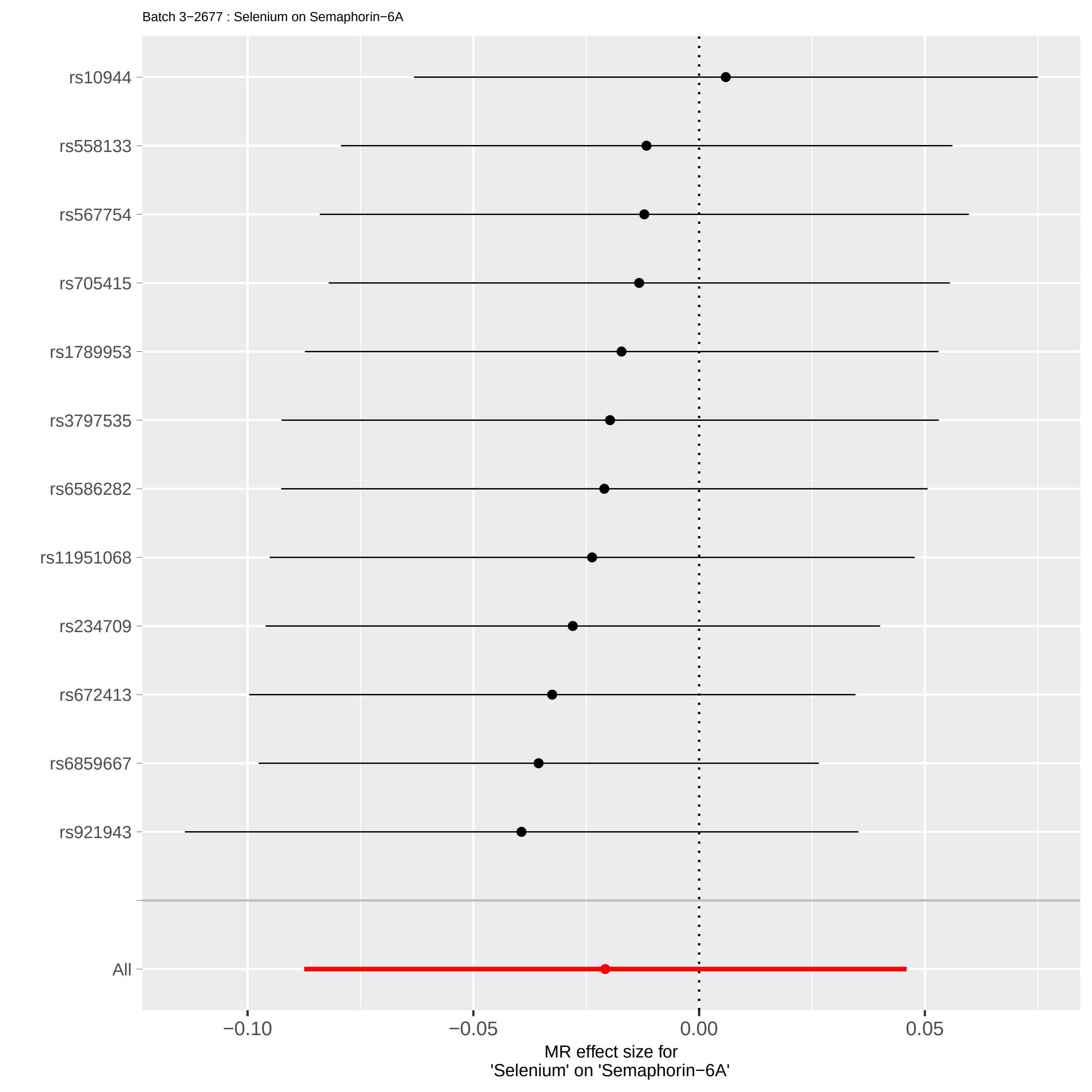

Batch 3−2677 : Selenium on Semaphorin−6A
rs10944
rs558133
rs567754
rs705415
rs1789953
rs3797535
rs6586282
rs11951068
rs234709
rs672413
rs6859667
rs921943
All
−0.10
−0.05
0.00
0.05
MR effect size for
'Selenium' on 'Semaphorin−6A'

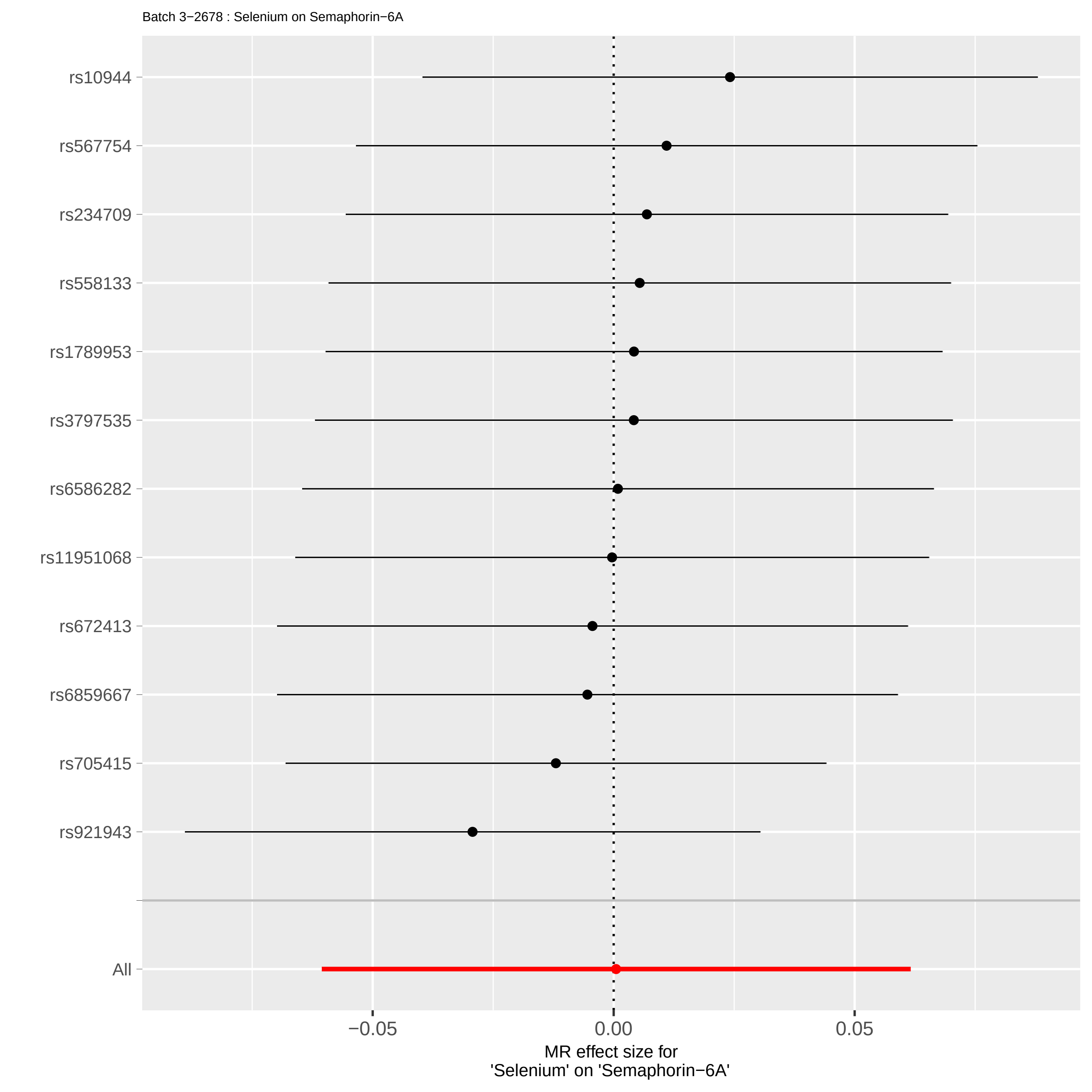

Batch 3−2678 : Selenium on Semaphorin−6A
rs10944
rs567754
rs234709
rs558133
rs1789953
rs3797535
rs6586282
rs11951068
rs672413
rs6859667
rs705415
rs921943
All
−0.05
0.00
0.05
MR effect size for
'Selenium' on 'Semaphorin−6A'

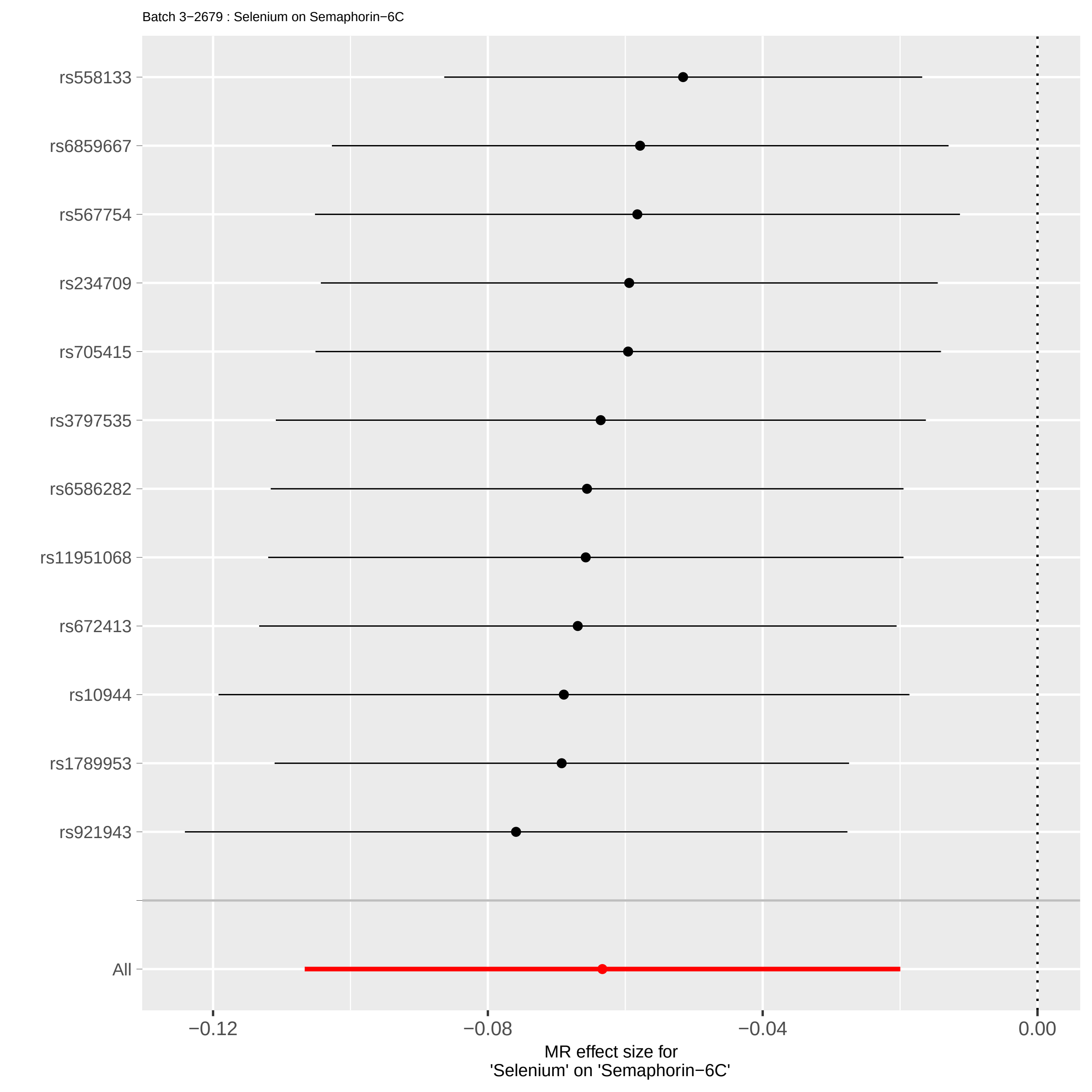

Batch 3−2679 : Selenium on Semaphorin−6C
rs558133
rs6859667
rs567754
rs234709
rs705415
rs3797535
rs6586282
rs11951068
rs672413
rs10944
rs1789953
rs921943
All
−0.12
−0.08
−0.04
0.00
MR effect size for
'Selenium' on 'Semaphorin−6C'

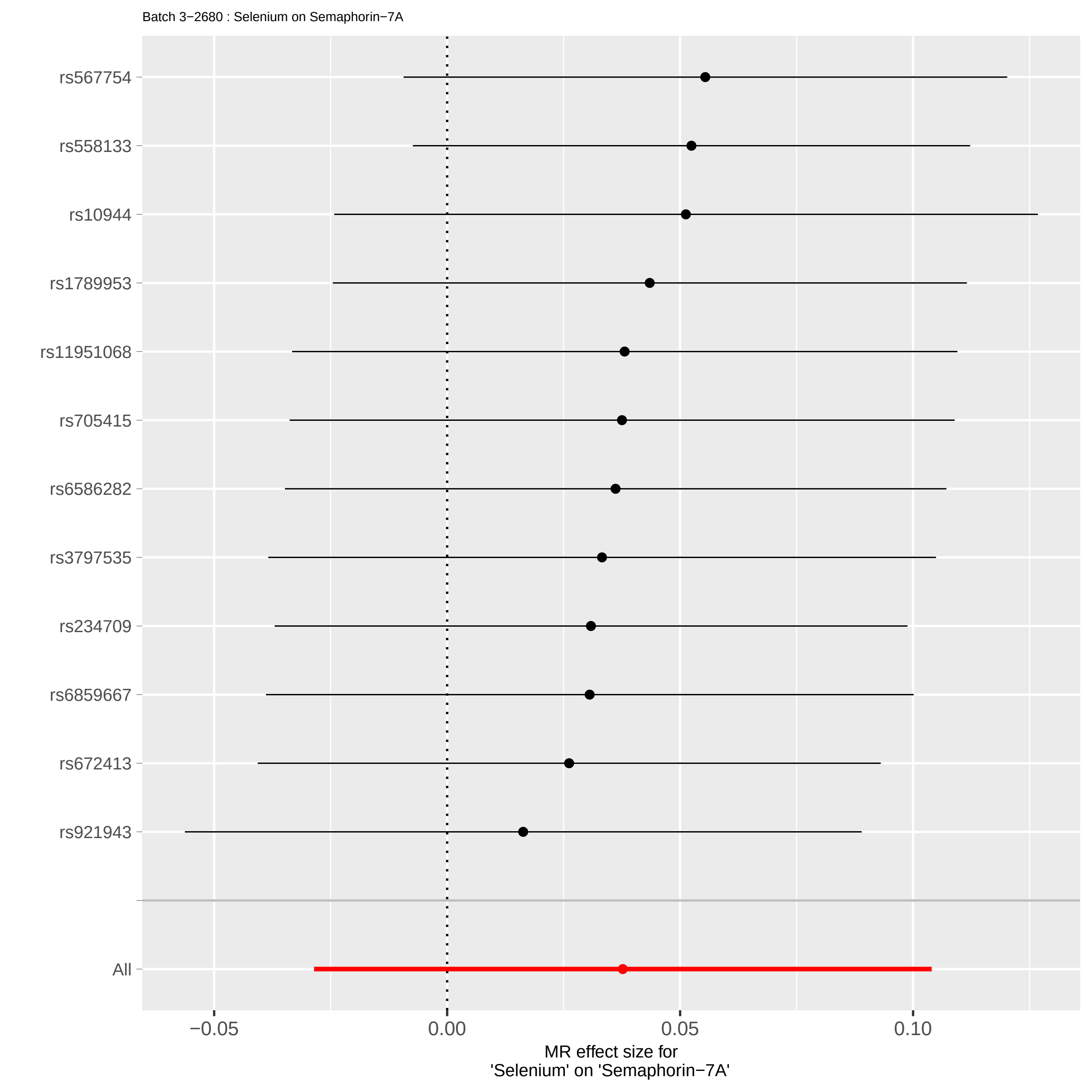

Batch 3−2680 : Selenium on Semaphorin−7A
rs567754
rs558133
rs10944
rs1789953
rs11951068
rs705415
rs6586282
rs3797535
rs234709
rs6859667
rs672413
rs921943
All
−0.05
0.00
0.05
0.10
MR effect size for
'Selenium' on 'Semaphorin−7A'

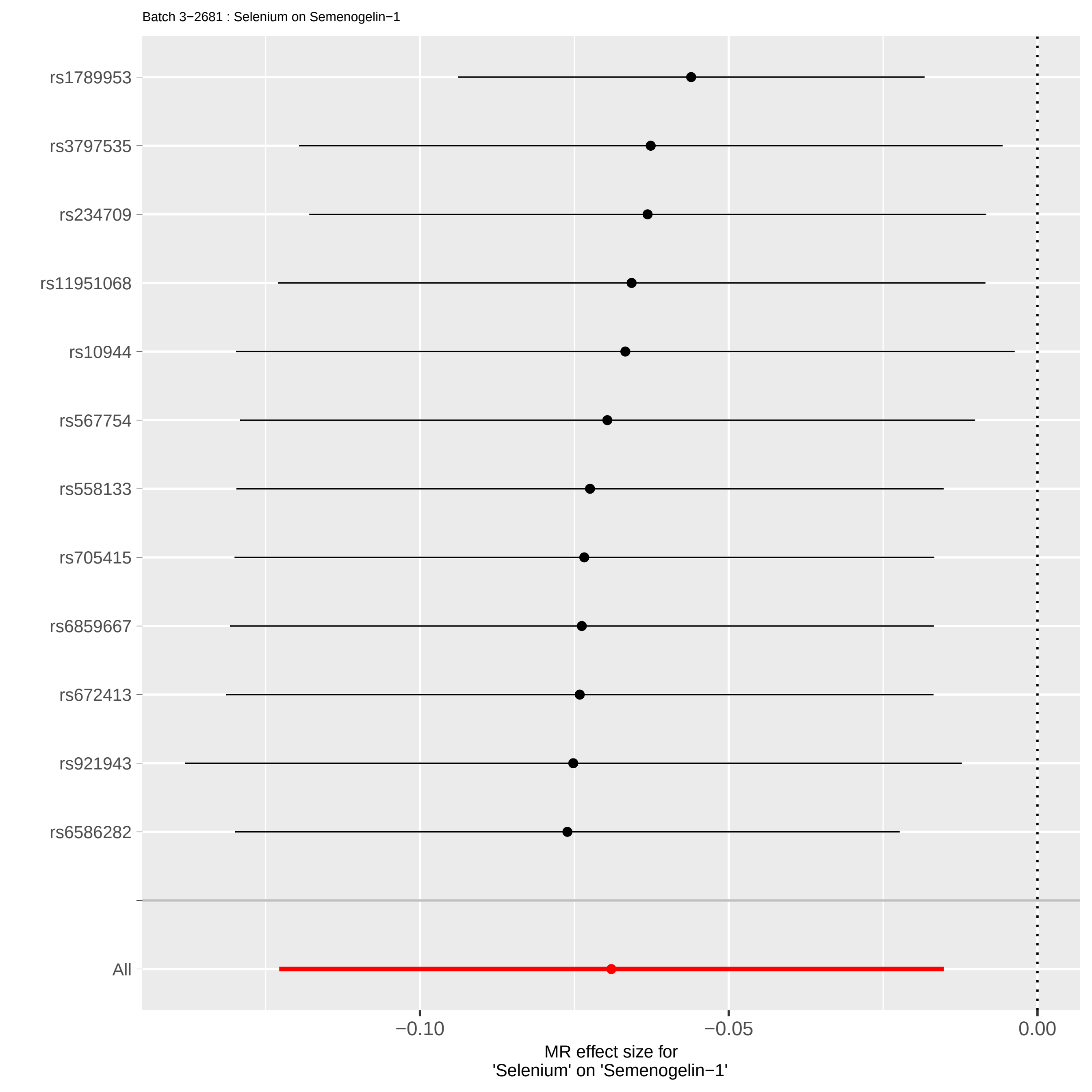

Batch 3−2681 : Selenium on Semenogelin−1
rs1789953
rs3797535
rs234709
rs11951068
rs10944
rs567754
rs558133
rs705415
rs6859667
rs672413
rs921943
rs6586282
All
−0.10
−0.05
0.00
MR effect size for
'Selenium' on 'Semenogelin−1'

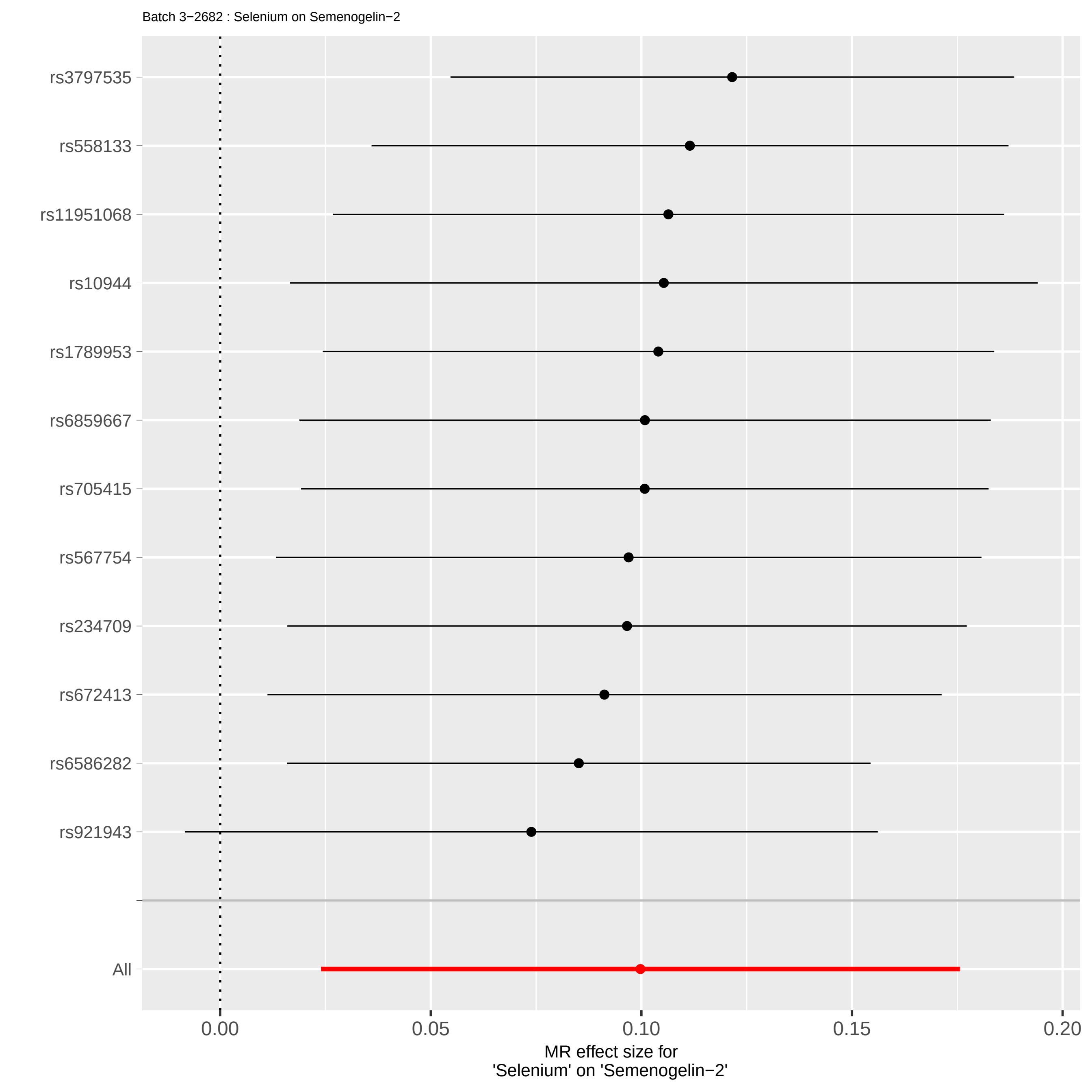
Batch 3−2682 : Selenium on Semenogelin−2
rs3797535
rs558133
rs11951068
rs10944
rs1789953
rs6859667
rs705415
rs567754
rs234709
rs672413
rs6586282
rs921943
All
0.00
0.05
0.10
0.15
0.20
MR effect size for
'Selenium' on 'Semenogelin−2'

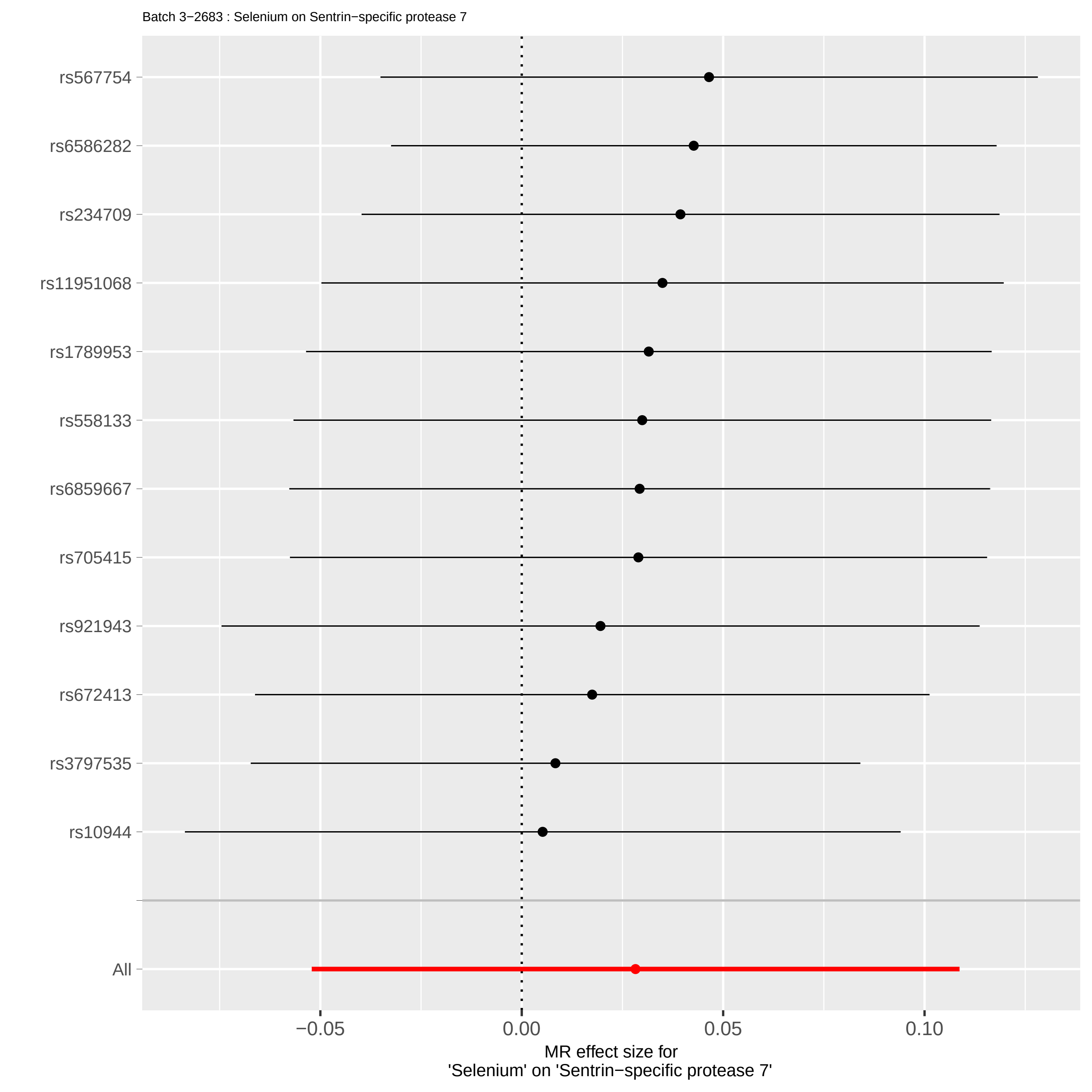

Batch 3−2683 : Selenium on Sentrin−specific protease 7
rs567754
rs6586282
rs234709
rs11951068
rs1789953
rs558133
rs6859667
rs705415
rs921943
rs672413
rs3797535
rs10944
All
−0.05
0.00
0.05
0.10
MR effect size for
'Selenium' on 'Sentrin−specific protease 7'

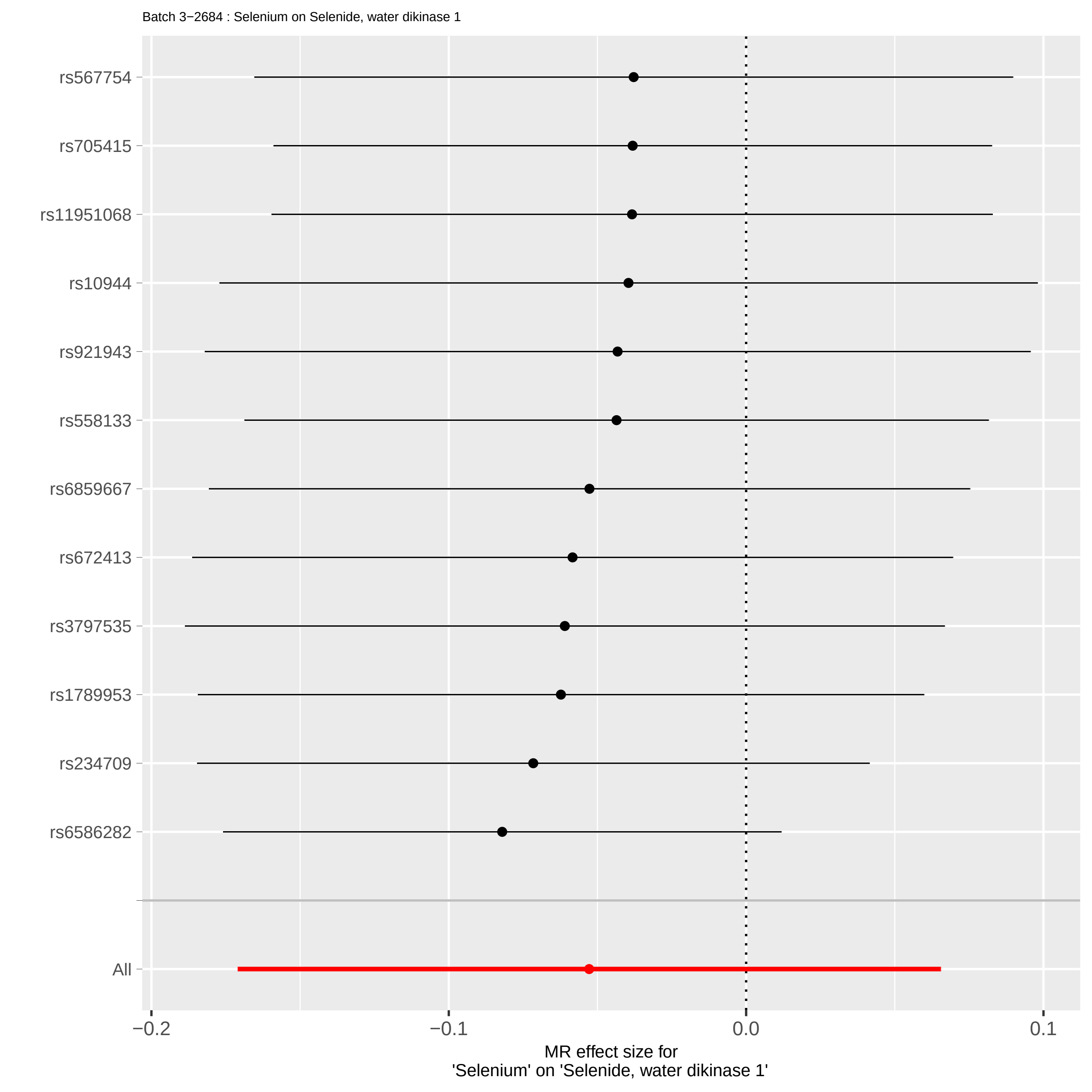

Batch 3−2684 : Selenium on Selenide, water dikinase 1
rs567754
rs705415
rs11951068
rs10944
rs921943
rs558133
rs6859667
rs672413
rs3797535
rs1789953
rs234709
rs6586282
All
−0.2
−0.1
0.0
0.1
MR effect size for
'Selenium' on 'Selenide, water dikinase 1'

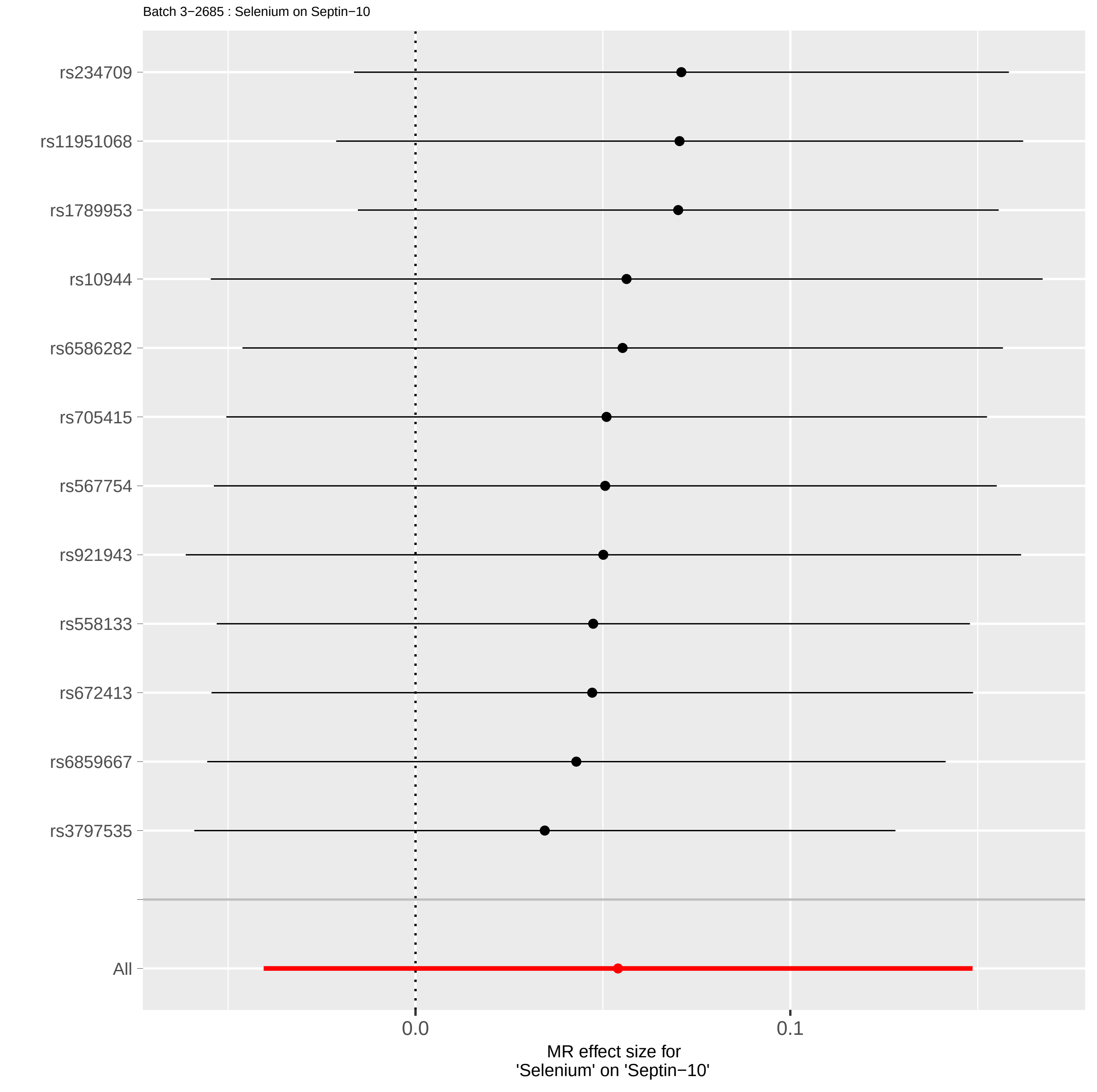

Batch 3−2685 : Selenium on Septin−10
rs234709
rs11951068
rs1789953
rs10944
rs6586282
rs705415
rs567754
rs921943
rs558133
rs672413
rs6859667
rs3797535
All
0.0
0.1
MR effect size for
'Selenium' on 'Septin−10'

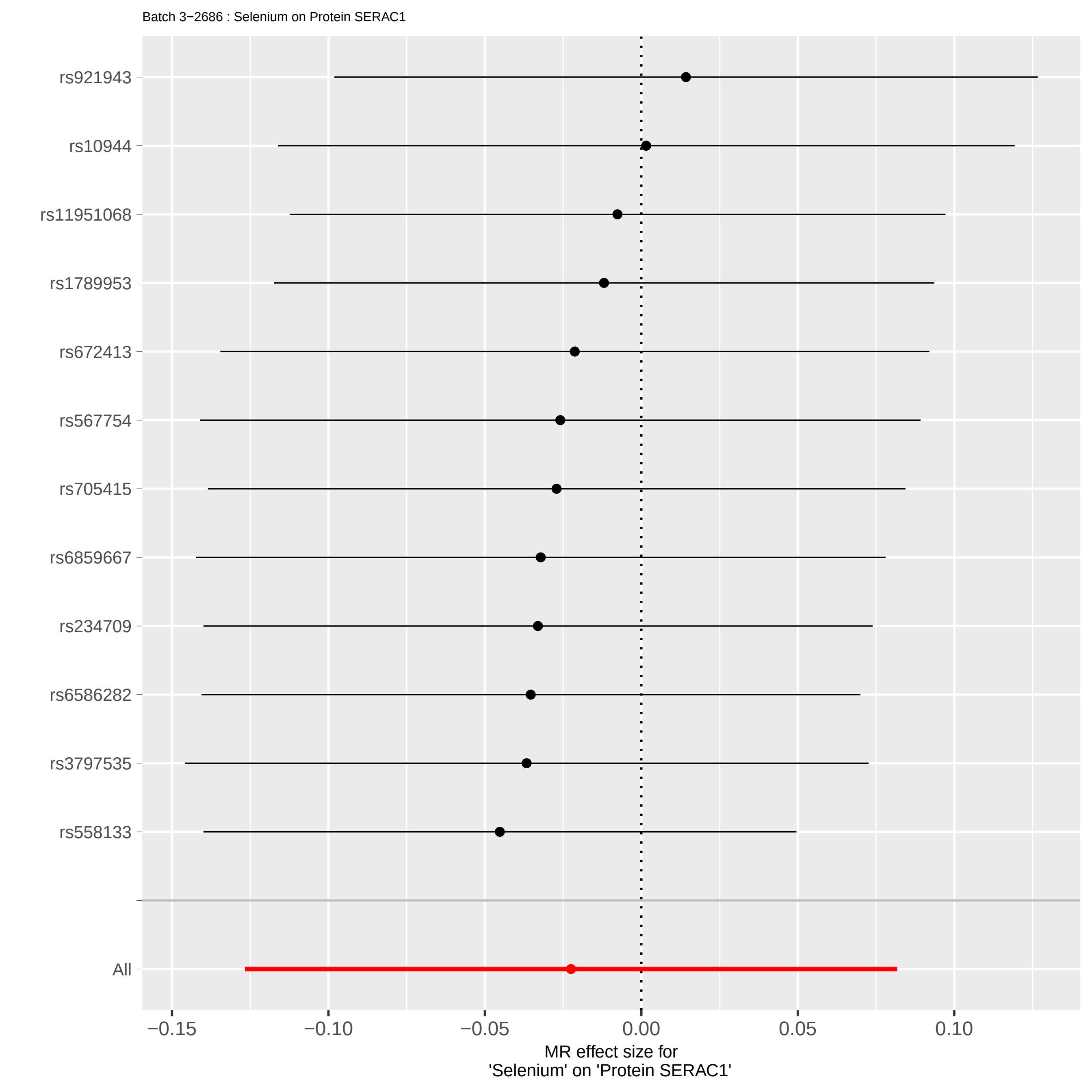

Batch 3−2686 : Selenium on Protein SERAC1
rs921943
rs10944
rs11951068
rs1789953
rs672413
rs567754
rs705415
rs6859667
rs234709
rs6586282
rs3797535
rs558133
All
−0.15
−0.10
−0.05
0.00
0.05
0.10
MR effect size for
'Selenium' on 'Protein SERAC1'

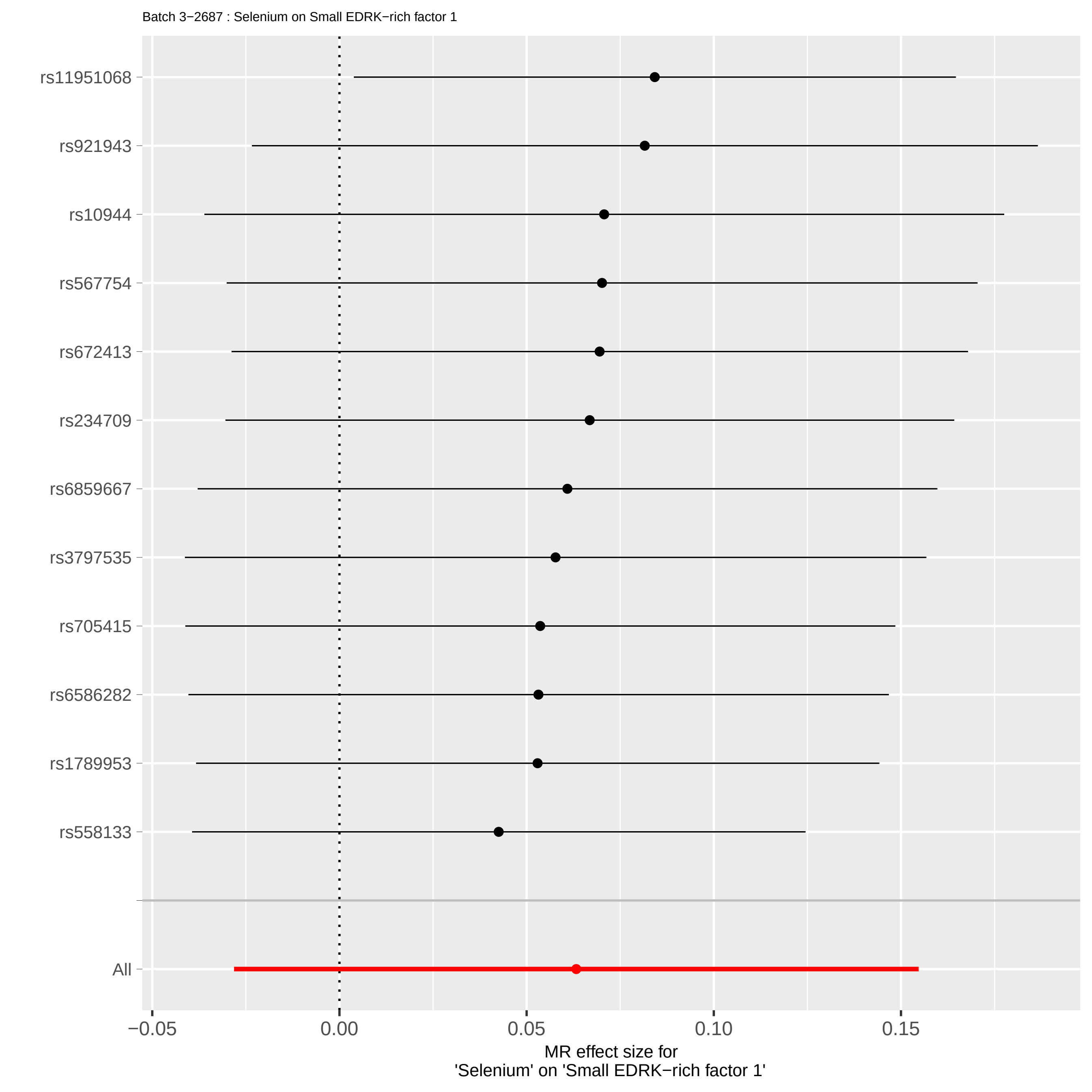

Batch 3−2687 : Selenium on Small EDRK−rich factor 1
rs11951068
rs921943
rs10944
rs567754
rs672413
rs234709
rs6859667
rs3797535
rs705415
rs6586282
rs1789953
rs558133
All
−0.05
0.00
0.05
0.10
0.15
MR effect size for
'Selenium' on 'Small EDRK−rich factor 1'

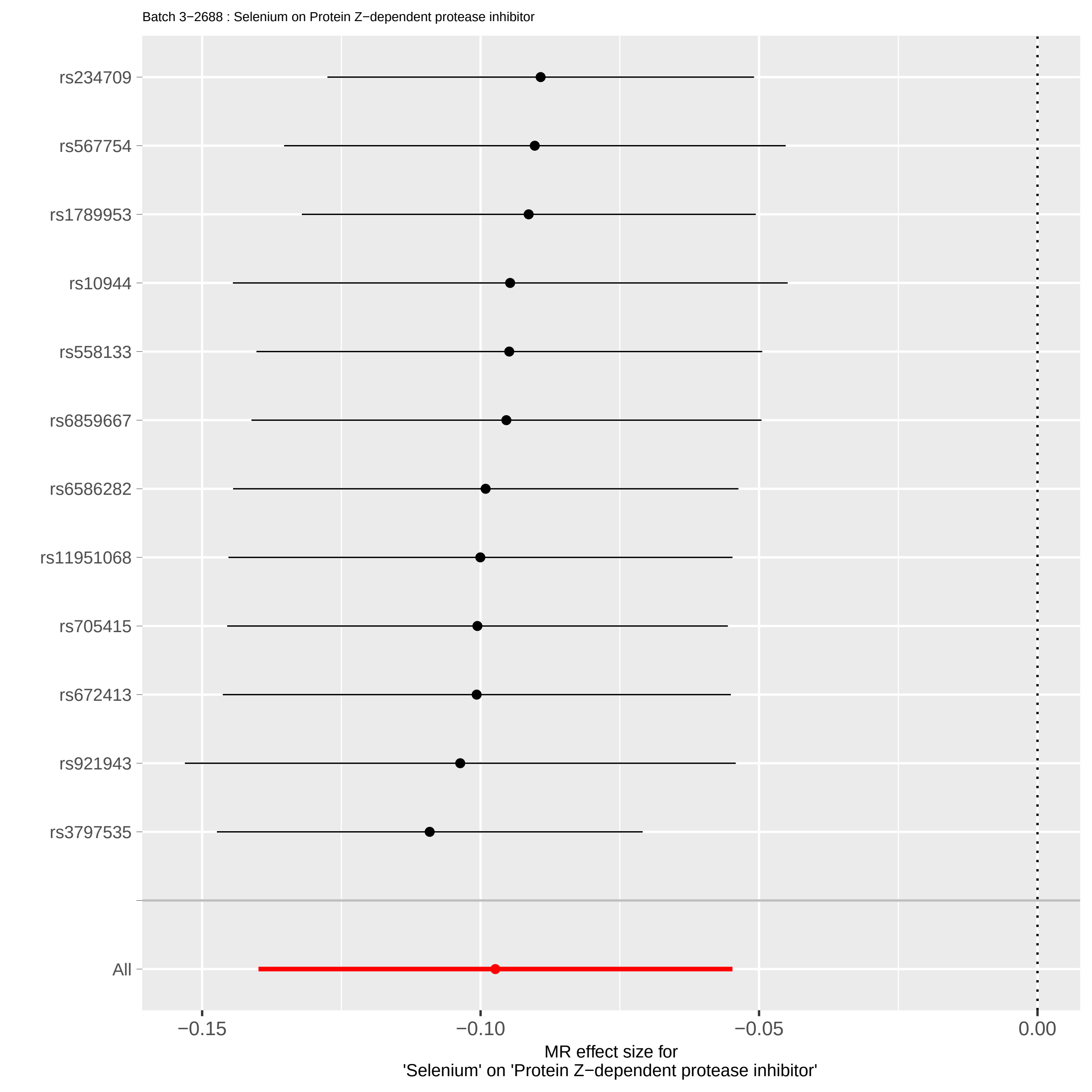

Batch 3−2688 : Selenium on Protein Z−dependent protease inhibitor
rs234709
rs567754
rs1789953
rs10944
rs558133
rs6859667
rs6586282
rs11951068
rs705415
rs672413
rs921943
rs3797535
All
−0.15
−0.10
−0.05
0.00
MR effect size for
'Selenium' on 'Protein Z−dependent protease inhibitor'

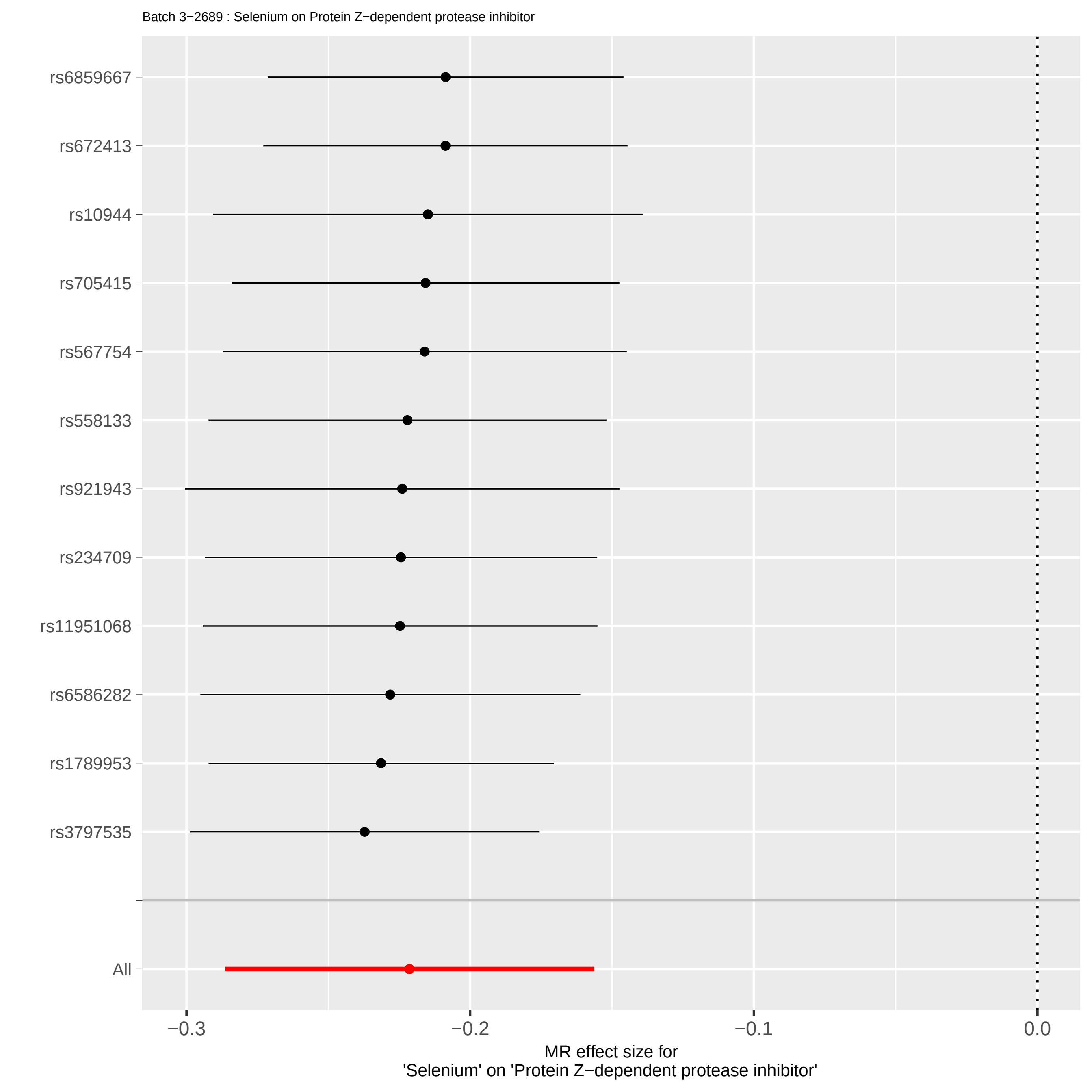
Batch 3−2689 : Selenium on Protein Z−dependent protease inhibitor
rs6859667
rs672413
rs10944
rs705415
rs567754
rs558133
rs921943
rs234709
rs11951068
rs6586282
rs1789953
rs3797535
All
−0.3
−0.2
−0.1
0.0
MR effect size for
'Selenium' on 'Protein Z−dependent protease inhibitor'

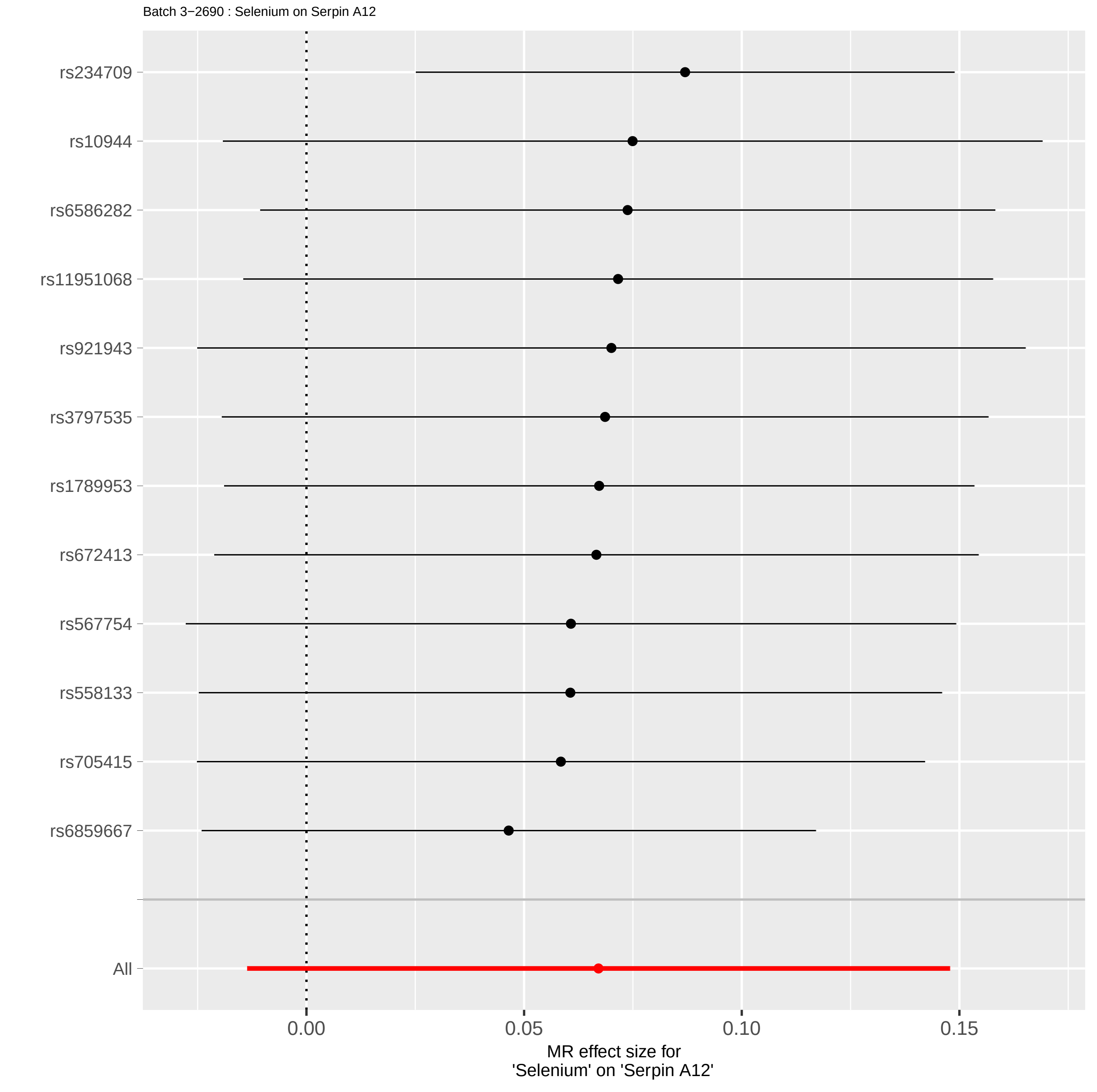
Batch 3−2690 : Selenium on Serpin A12
rs234709
rs10944
rs6586282
rs11951068
rs921943
rs3797535
rs1789953
rs672413
rs567754
rs558133
rs705415
rs6859667
All
0.00
0.05
0.10
0.15
MR effect size for
'Selenium' on 'Serpin A12'

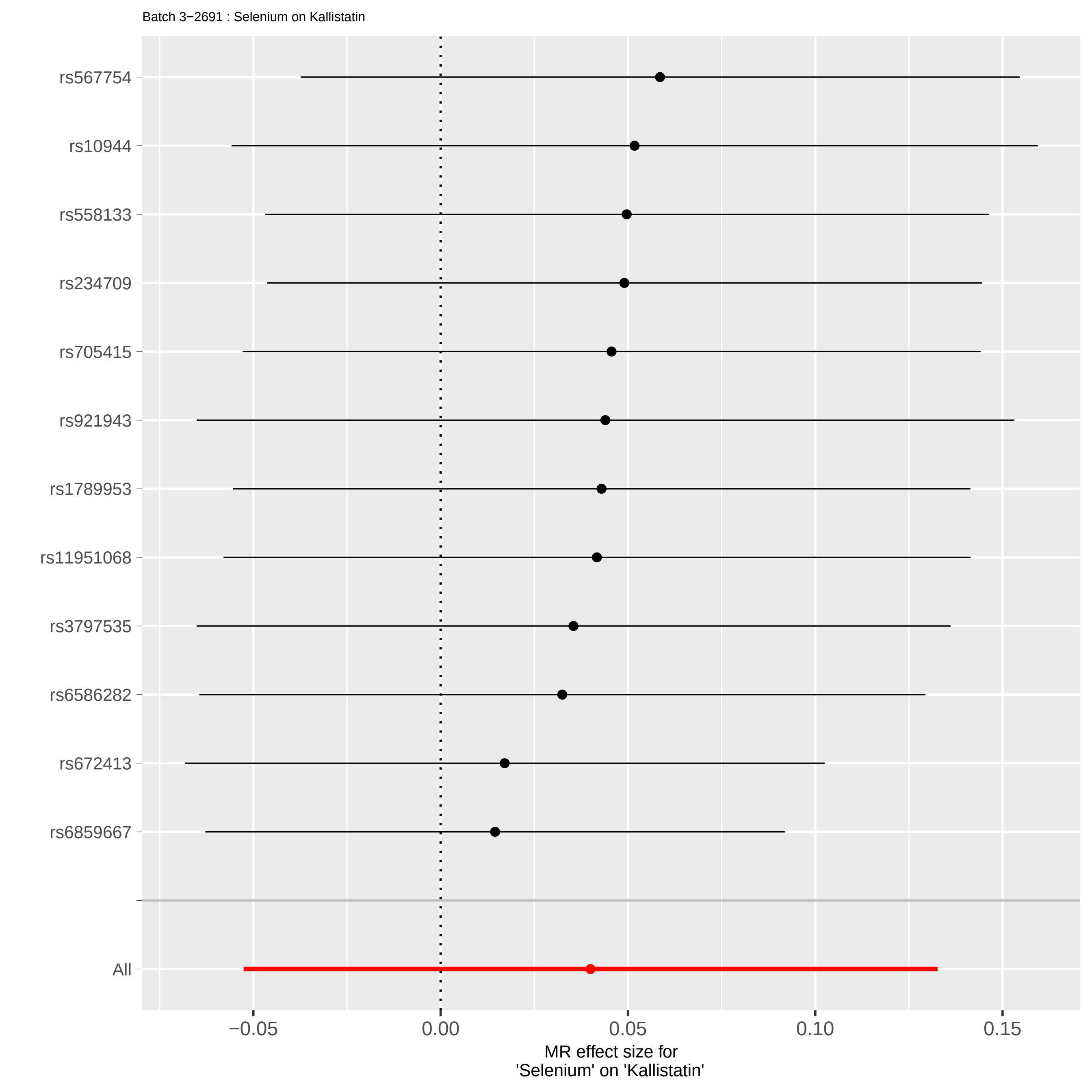

Batch 3−2691 : Selenium on Kallistatin
rs567754
rs10944
rs558133
rs234709
rs705415
rs921943
rs1789953
rs11951068
rs3797535
rs6586282
rs672413
rs6859667
All
−0.05
0.00
0.05
0.10
0.15
MR effect size for
'Selenium' on 'Kallistatin'

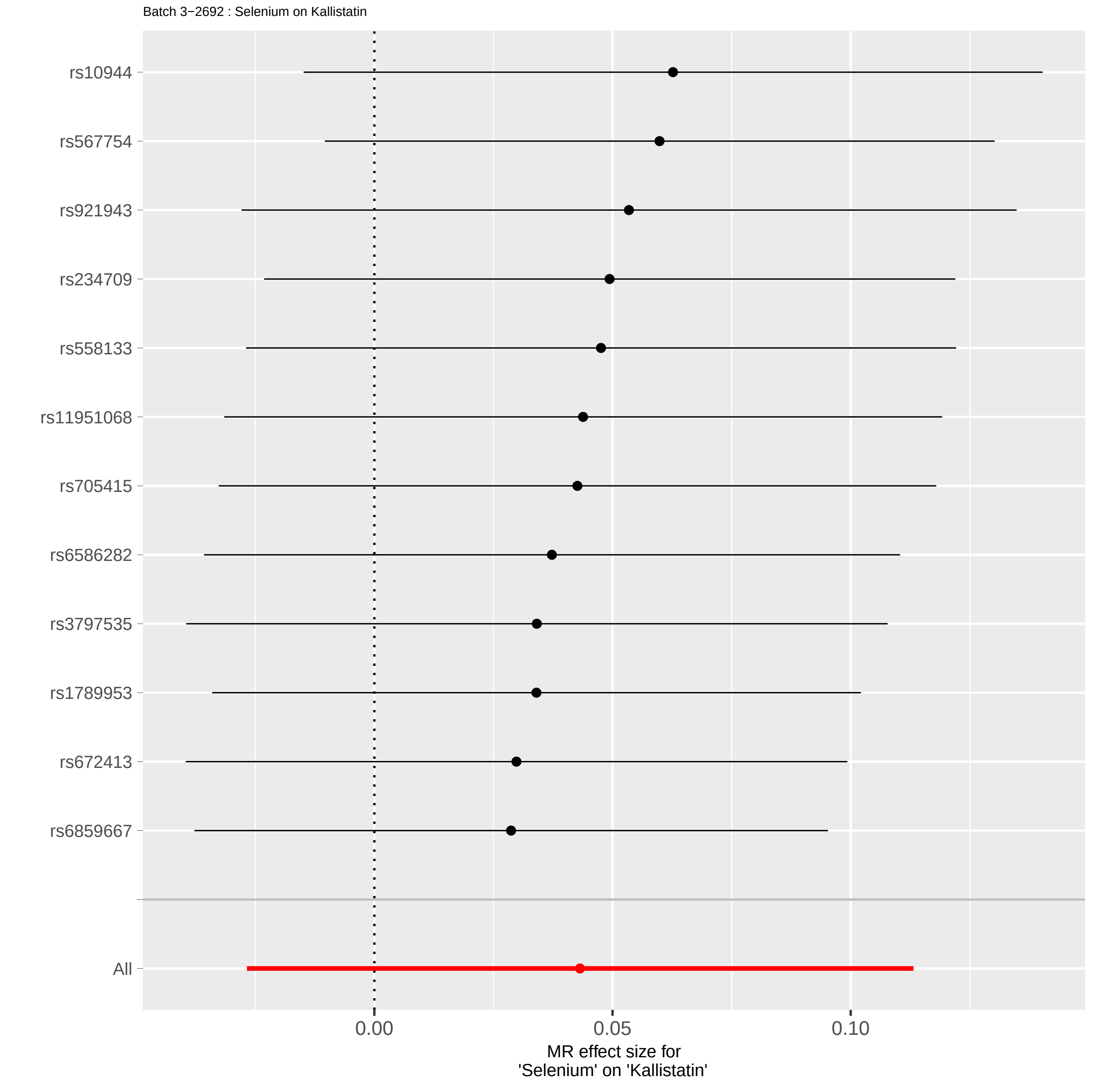
Batch 3−2692 : Selenium on Kallistatin
rs10944
rs567754
rs921943
rs234709
rs558133
rs11951068
rs705415
rs6586282
rs3797535
rs1789953
rs672413
rs6859667
All
0.00
0.05
0.10
MR effect size for
'Selenium' on 'Kallistatin'

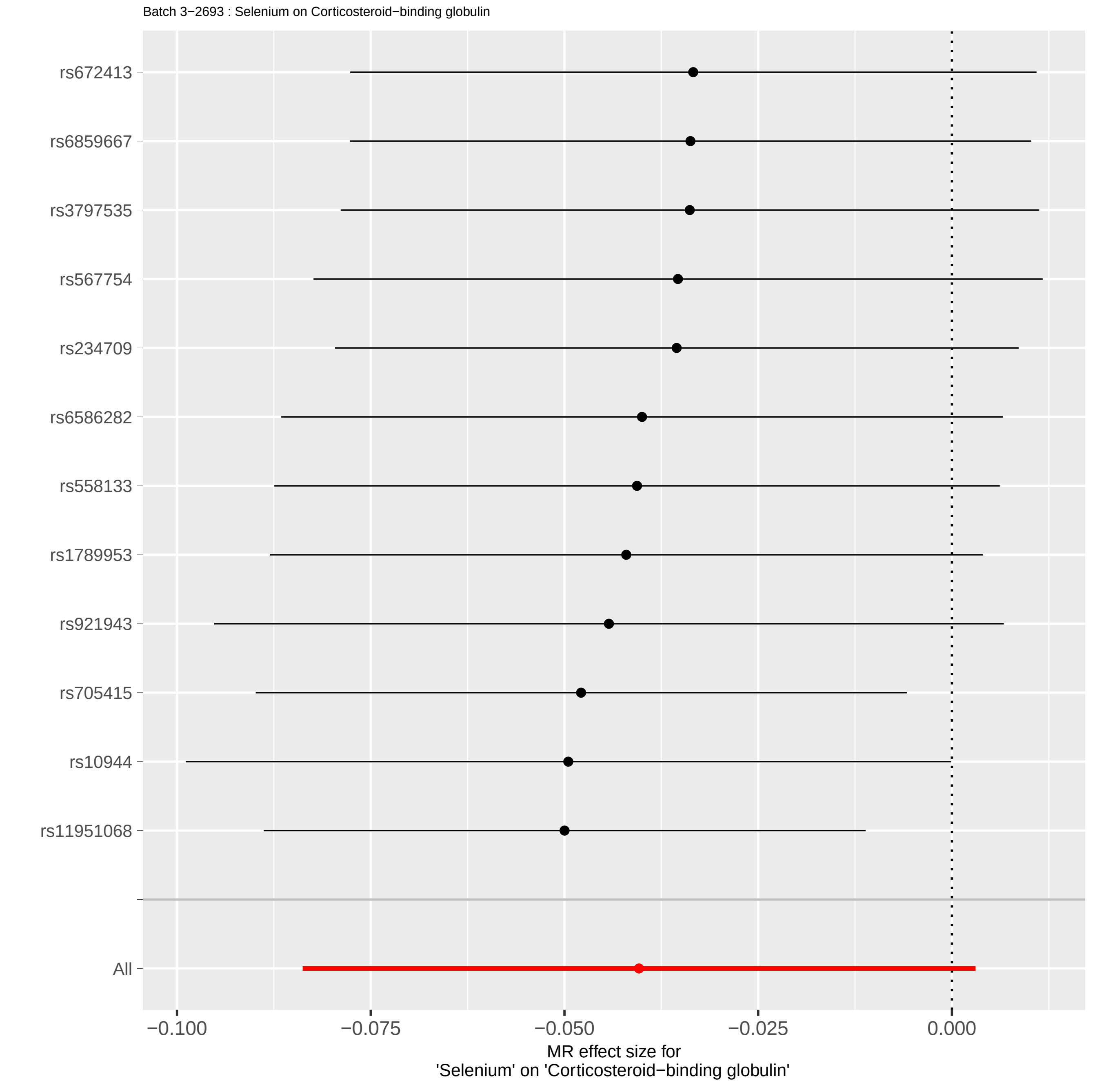

Batch 3−2693 : Selenium on Corticosteroid−binding globulin
rs672413
rs6859667
rs3797535
rs567754
rs234709
rs6586282
rs558133
rs1789953
rs921943
rs705415
rs10944
rs11951068
All
−0.100
−0.075
−0.050
−0.025
0.000
MR effect size for
'Selenium' on 'Corticosteroid−binding globulin'

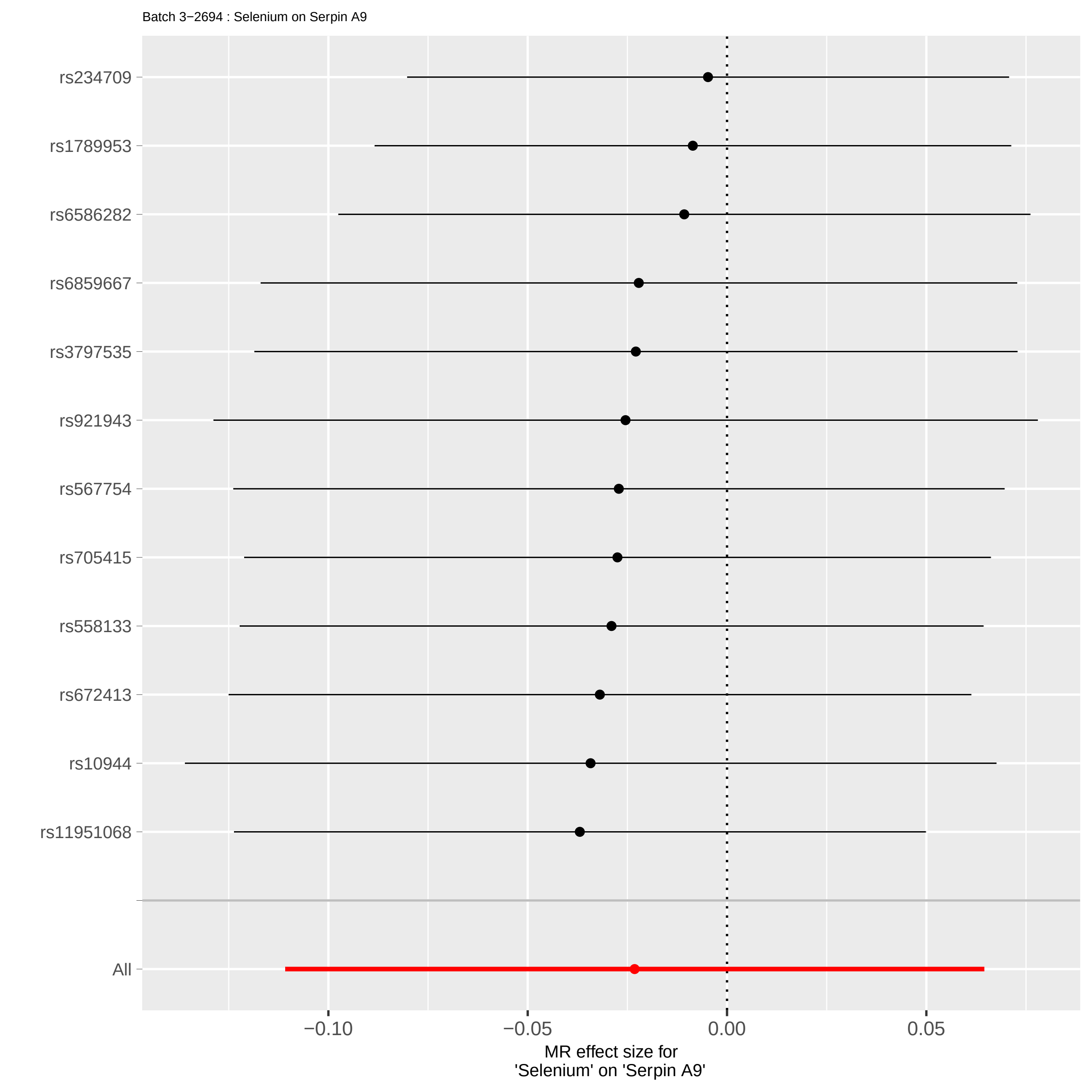

Batch 3−2694 : Selenium on Serpin A9
rs234709
rs1789953
rs6586282
rs6859667
rs3797535
rs921943
rs567754
rs705415
rs558133
rs672413
rs10944
rs11951068
All
−0.10
−0.05
0.00
0.05
MR effect size for
'Selenium' on 'Serpin A9'

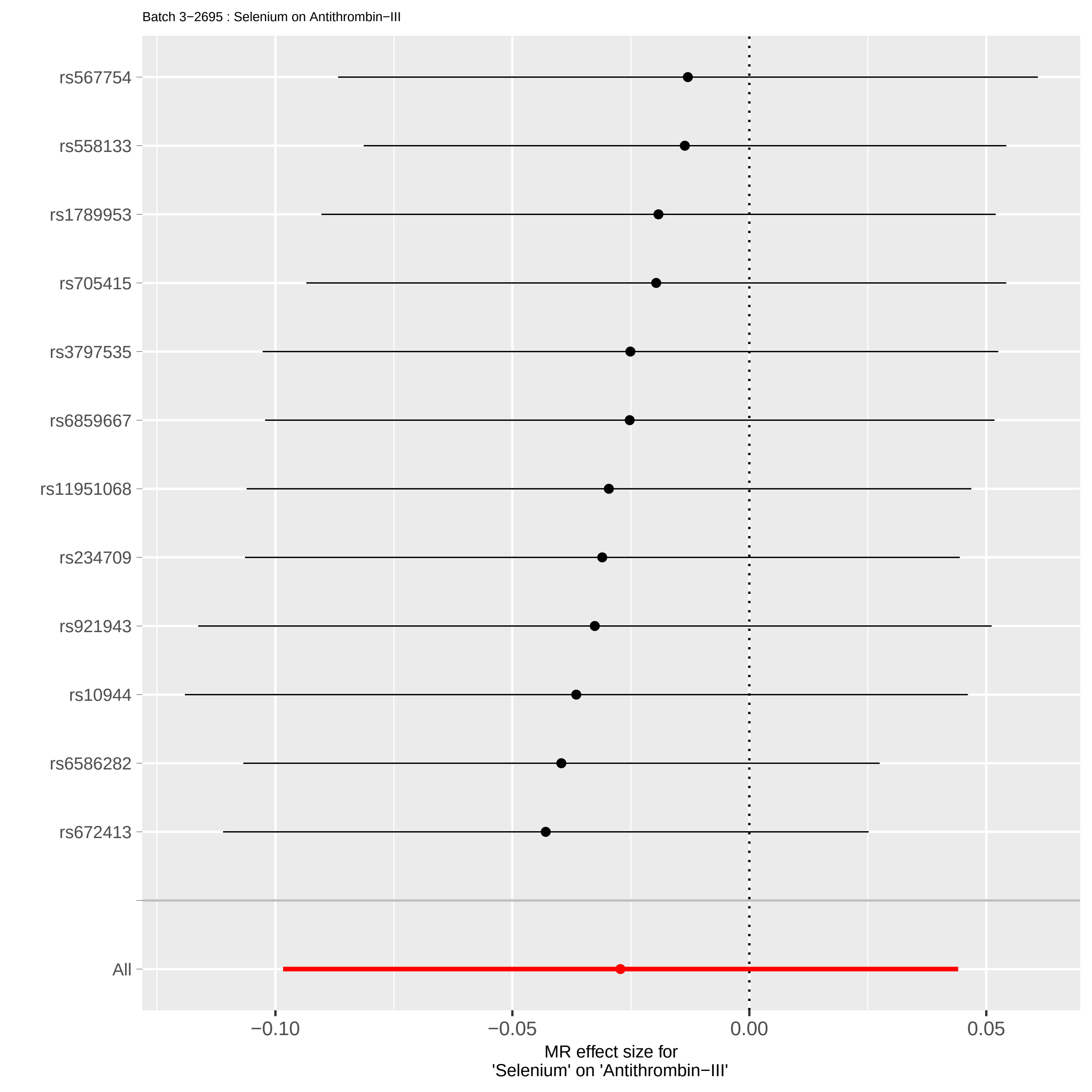

Batch 3−2695 : Selenium on Antithrombin−III
rs567754
rs558133
rs1789953
rs705415
rs3797535
rs6859667
rs11951068
rs234709
rs921943
rs10944
rs6586282
rs672413
All
−0.10
−0.05
0.00
0.05
MR effect size for
'Selenium' on 'Antithrombin−III'

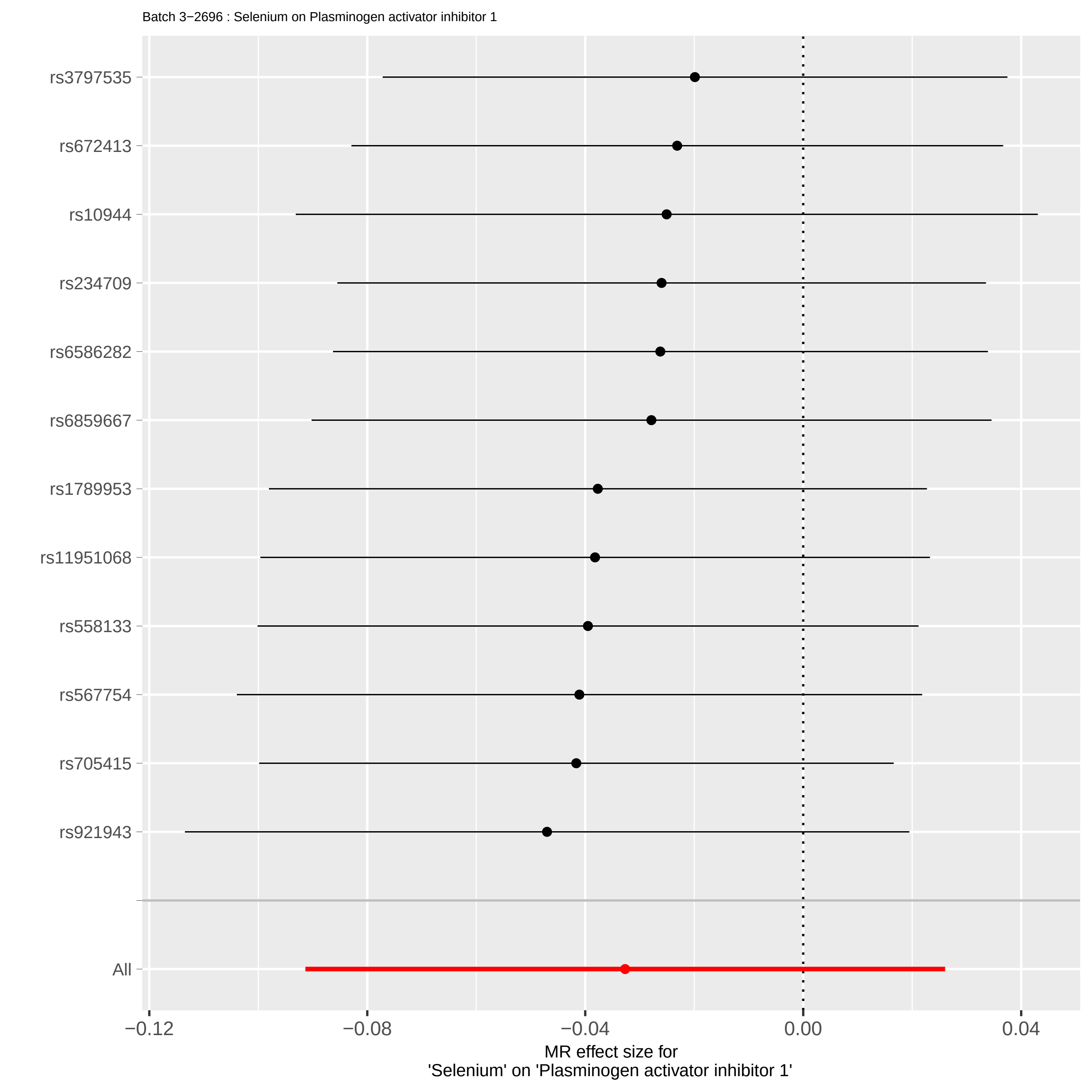

Batch 3−2696 : Selenium on Plasminogen activator inhibitor 1
rs3797535
rs672413
rs10944
rs234709
rs6586282
rs6859667
rs1789953
rs11951068
rs558133
rs567754
rs705415
rs921943
All
−0.12
−0.08
−0.04
0.00
0.04
MR effect size for
'Selenium' on 'Plasminogen activator inhibitor 1'

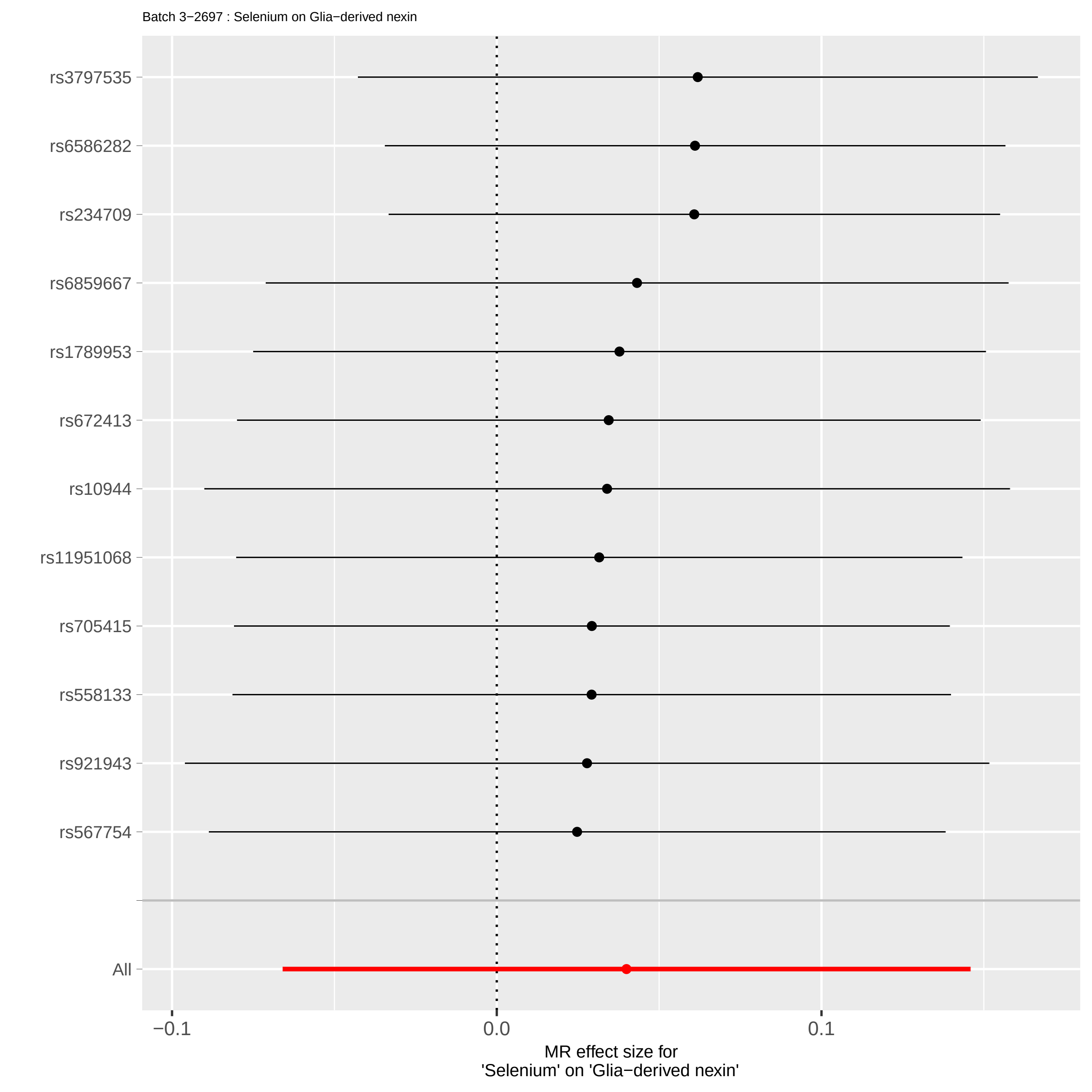

Batch 3−2697 : Selenium on Glia−derived nexin
rs3797535
rs6586282
rs234709
rs6859667
rs1789953
rs672413
rs10944
rs11951068
rs705415
rs558133
rs921943
rs567754
All
−0.1
0.0
0.1
MR effect size for
'Selenium' on 'Glia−derived nexin'

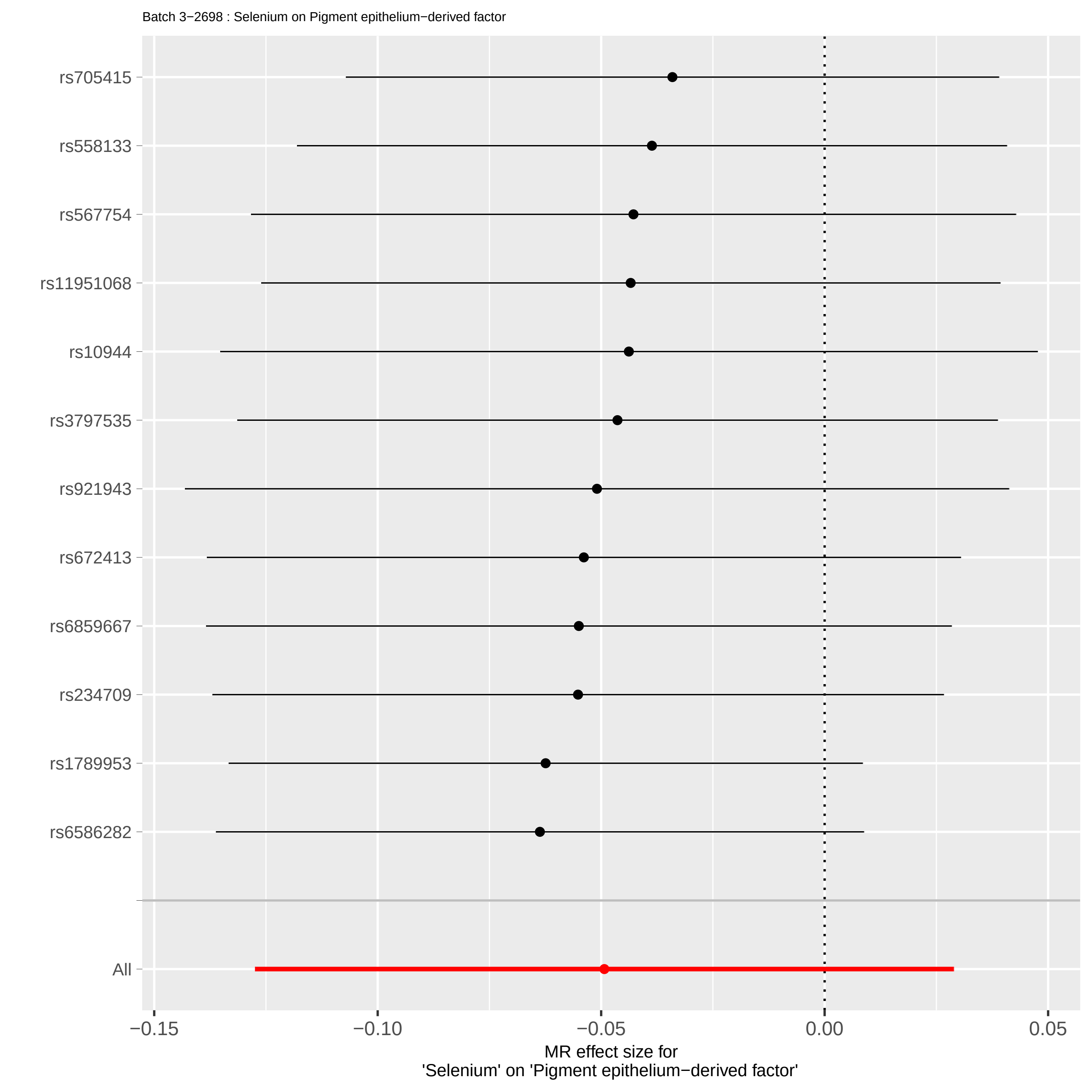

Batch 3−2698 : Selenium on Pigment epithelium−derived factor
rs705415
rs558133
rs567754
rs11951068
rs10944
rs3797535
rs921943
rs672413
rs6859667
rs234709
rs1789953
rs6586282
All
−0.15
−0.10
−0.05
0.00
0.05
MR effect size for
'Selenium' on 'Pigment epithelium−derived factor'

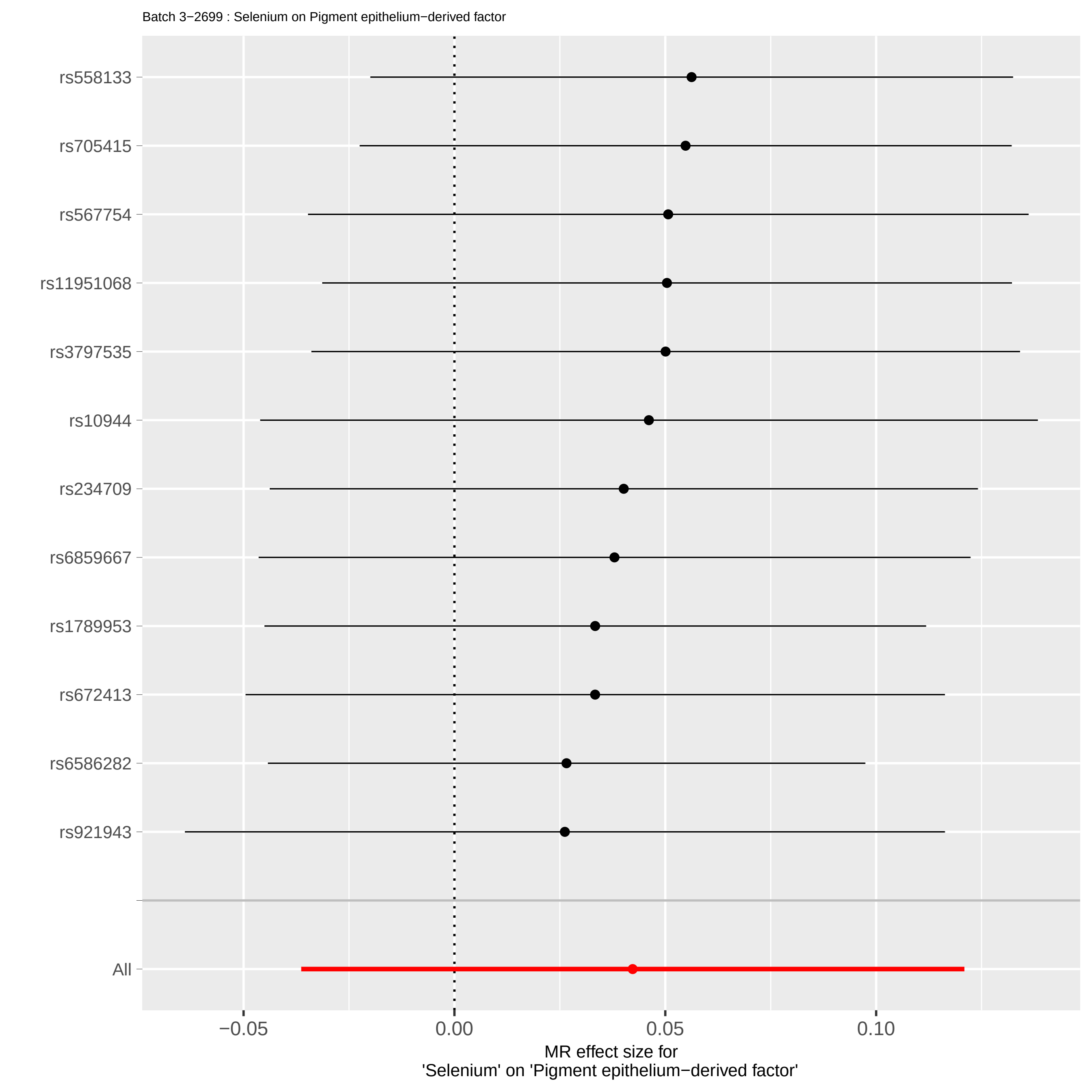

Batch 3−2699 : Selenium on Pigment epithelium−derived factor
rs558133
rs705415
rs567754
rs11951068
rs3797535
rs10944
rs234709
rs6859667
rs1789953
rs672413
rs6586282
rs921943
All
−0.05
0.00
0.05
0.10
MR effect size for
'Selenium' on 'Pigment epithelium−derived factor'

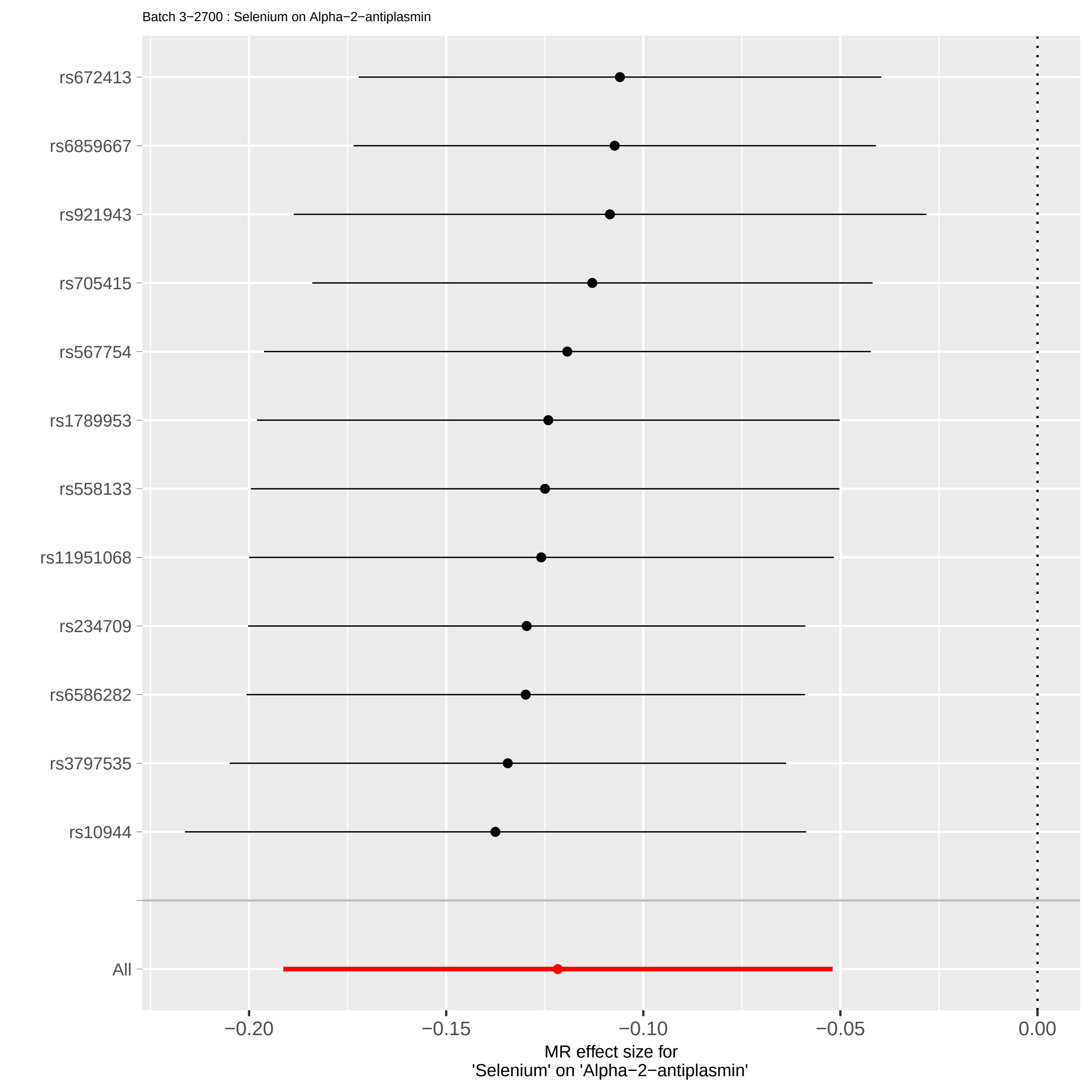
Batch 3−2700 : Selenium on Alpha−2−antiplasmin
rs672413
rs6859667
rs921943
rs705415
rs567754
rs1789953
rs558133
rs11951068
rs234709
rs6586282
rs3797535
rs10944
All
−0.20
−0.15
−0.10
−0.05
0.00
MR effect size for
'Selenium' on 'Alpha−2−antiplasmin'

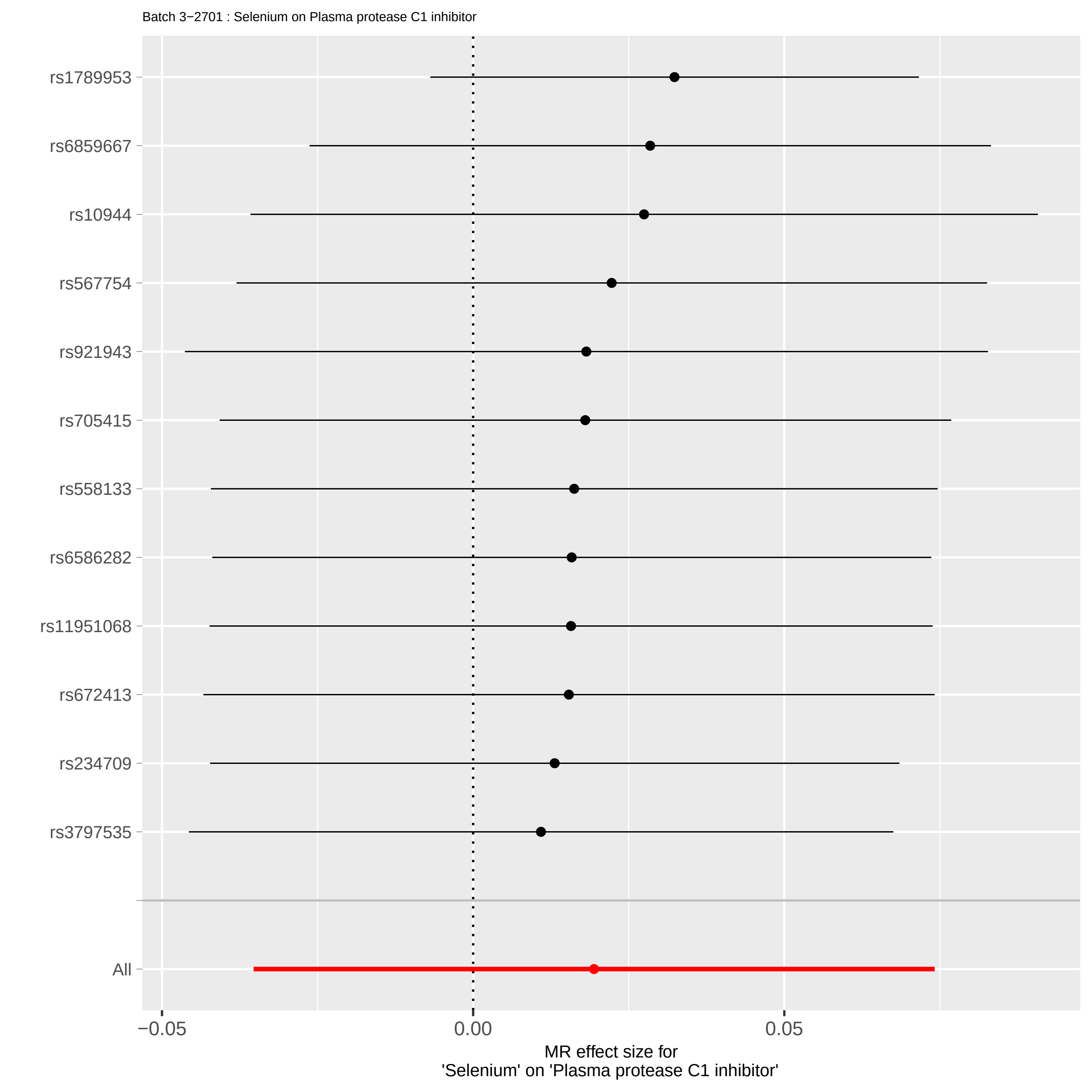

Batch 3−2701 : Selenium on Plasma protease C1 inhibitor
rs1789953
rs6859667
rs10944
rs567754
rs921943
rs705415
rs558133
rs6586282
rs11951068
rs672413
rs234709
rs3797535
All
−0.05
0.00
0.05
MR effect size for
'Selenium' on 'Plasma protease C1 inhibitor'

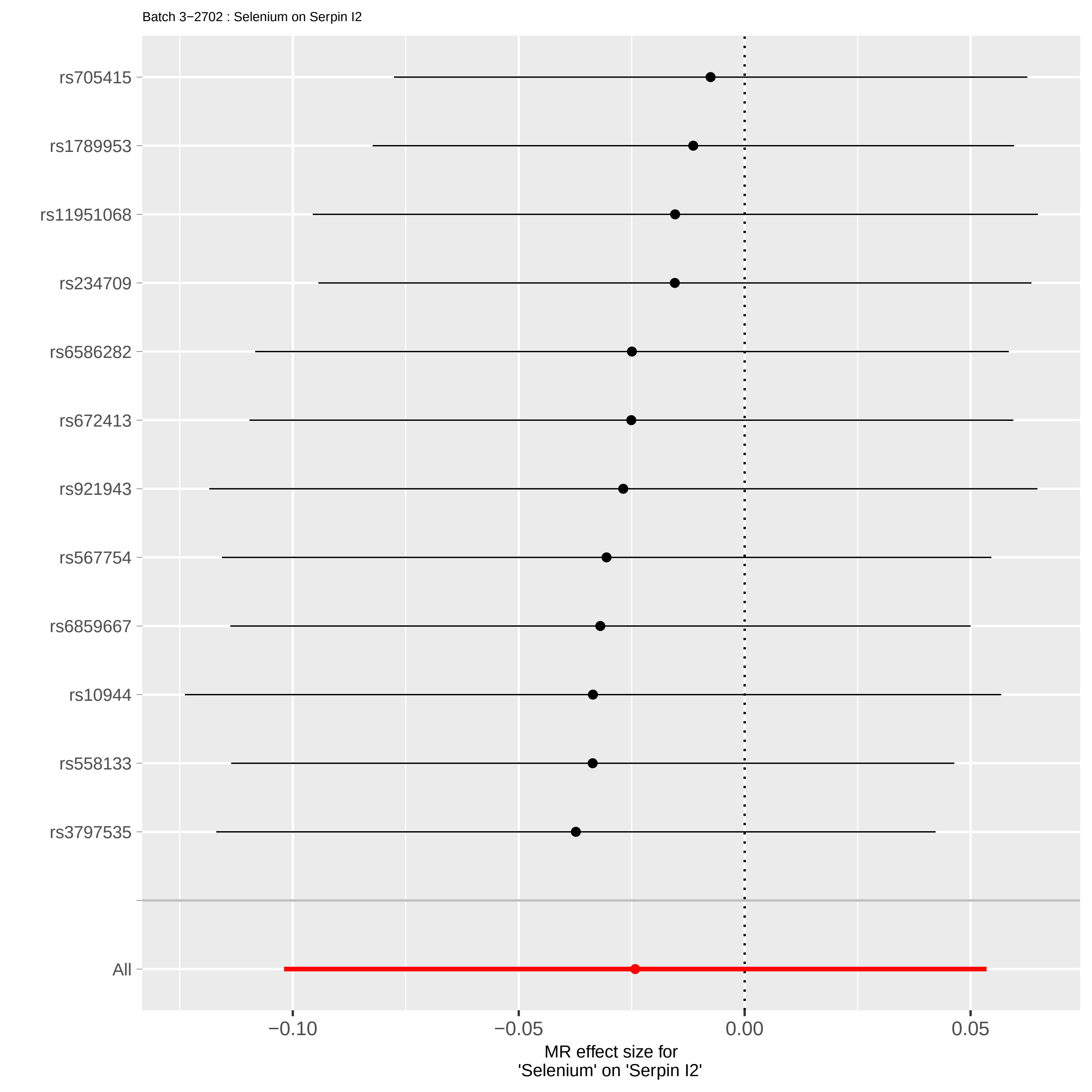

Batch 3−2702 : Selenium on Serpin I2
rs705415
rs1789953
rs11951068
rs234709
rs6586282
rs672413
rs921943
rs567754
rs6859667
rs10944
rs558133
rs3797535
All
−0.10
−0.05
0.00
0.05
MR effect size for
'Selenium' on 'Serpin I2'

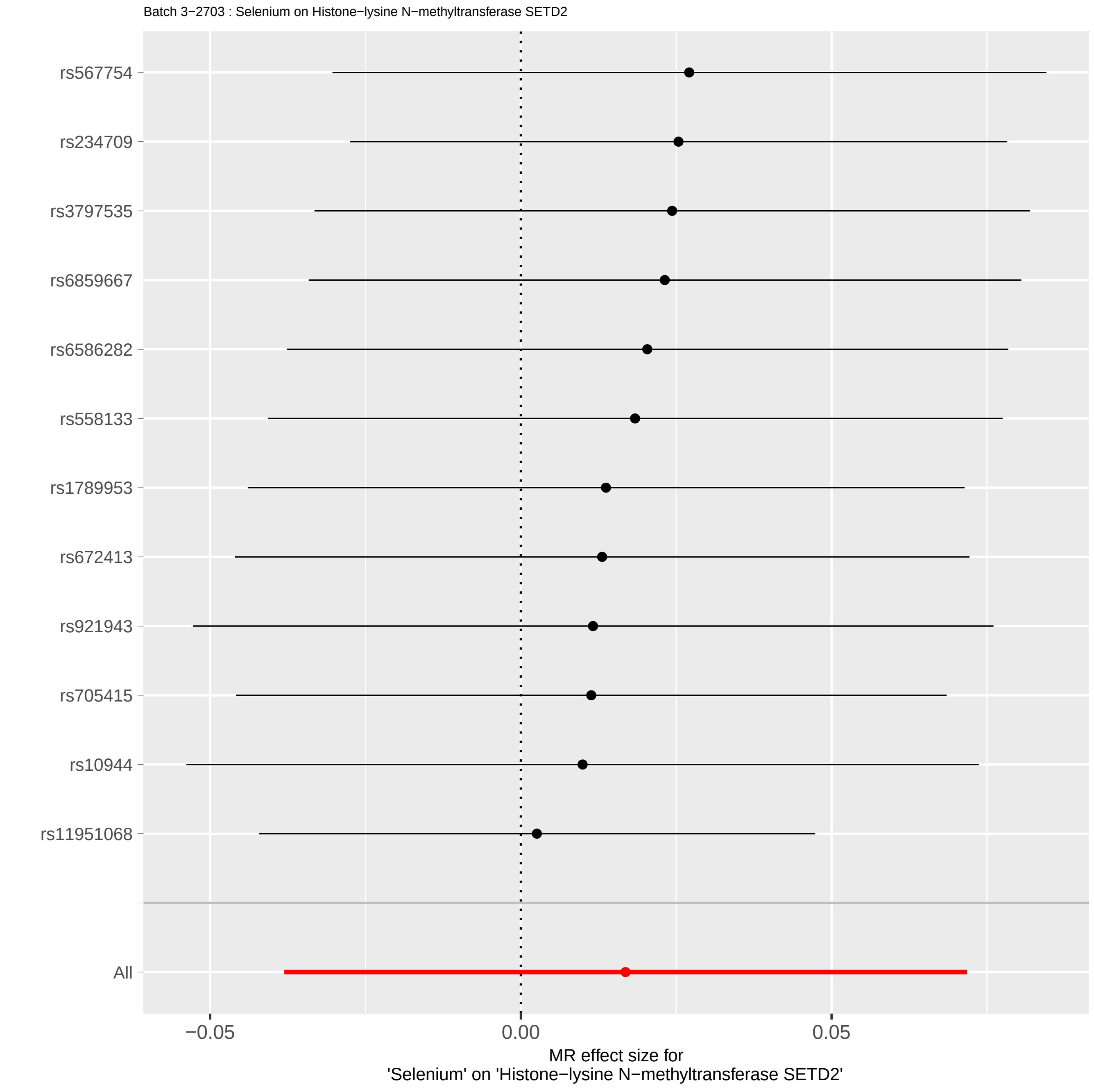

Batch 3−2703 : Selenium on Histone−lysine N−methyltransferase SETD2
rs567754
rs234709
rs3797535
rs6859667
rs6586282
rs558133
rs1789953
rs672413
rs921943
rs705415
rs10944
rs11951068
All
−0.05
0.00
0.05
MR effect size for
'Selenium' on 'Histone−lysine N−methyltransferase SETD2'

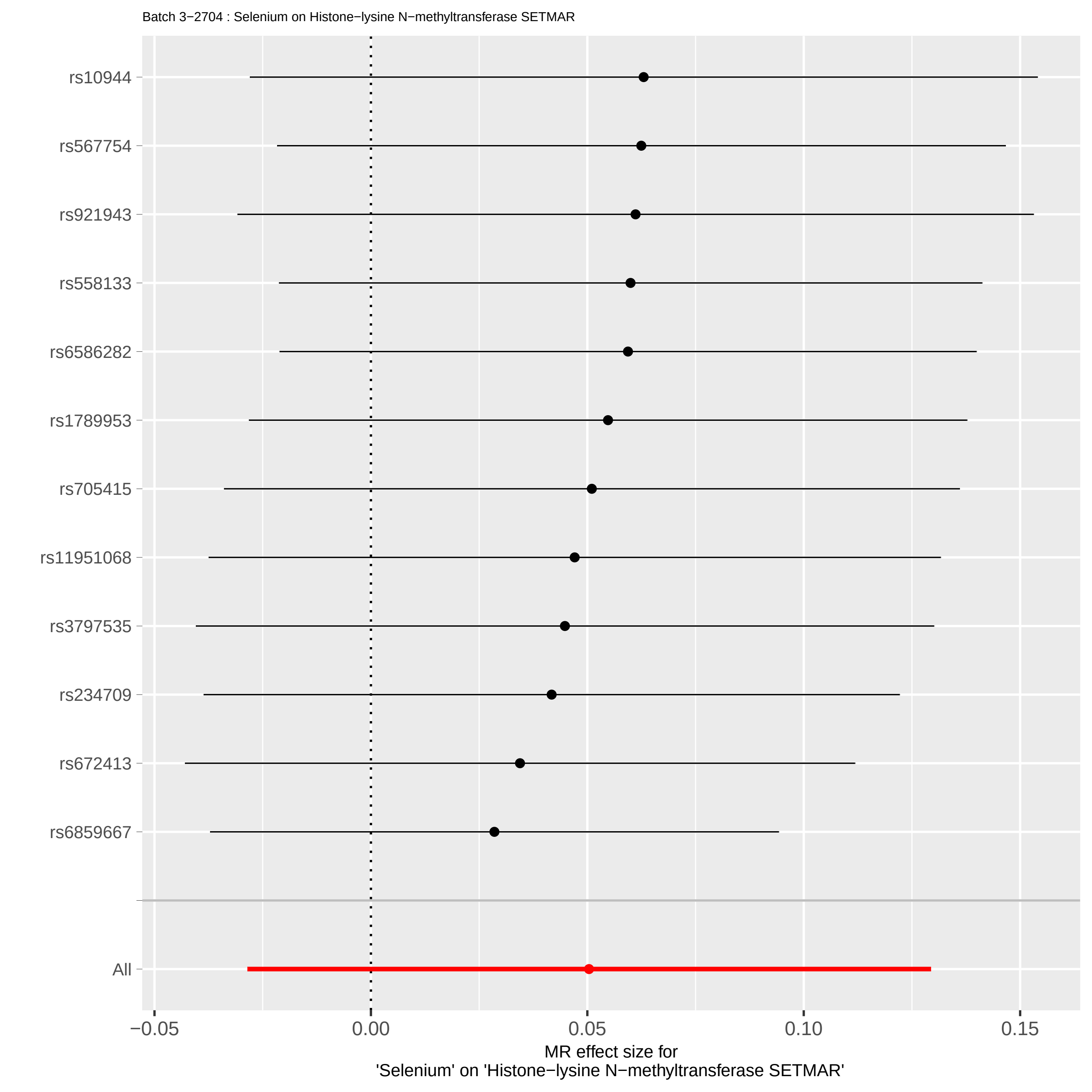

Batch 3−2704 : Selenium on Histone−lysine N−methyltransferase SETMAR
rs10944
rs567754
rs921943
rs558133
rs6586282
rs1789953
rs705415
rs11951068
rs3797535
rs234709
rs672413
rs6859667
All
−0.05
0.00
0.05
0.10
0.15
MR effect size for
'Selenium' on 'Histone−lysine N−methyltransferase SETMAR'

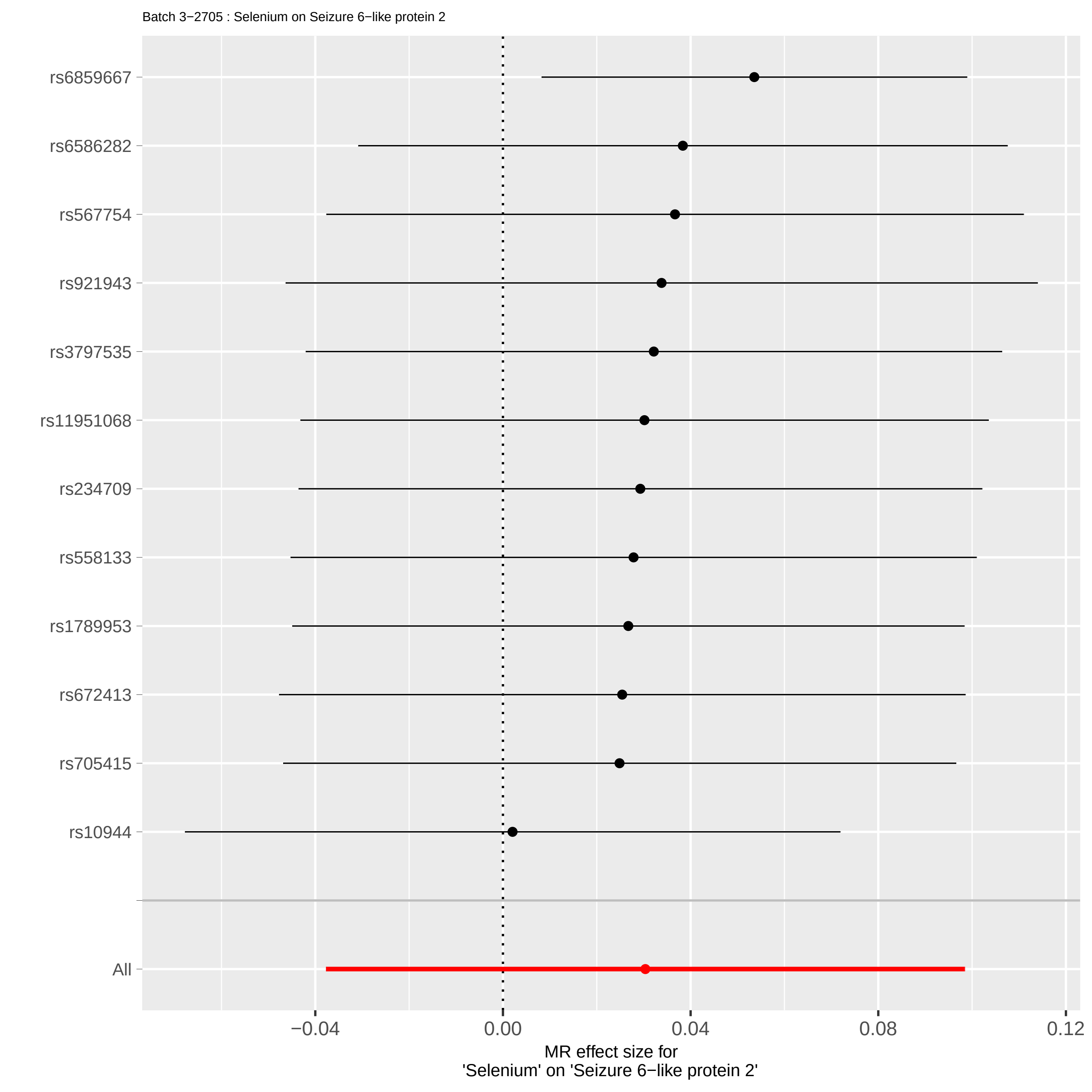

Batch 3−2705 : Selenium on Seizure 6−like protein 2
rs6859667
rs6586282
rs567754
rs921943
rs3797535
rs11951068
rs234709
rs558133
rs1789953
rs672413
rs705415
rs10944
All
−0.04
0.00
0.04
0.08
0.12
MR effect size for
'Selenium' on 'Seizure 6−like protein 2'

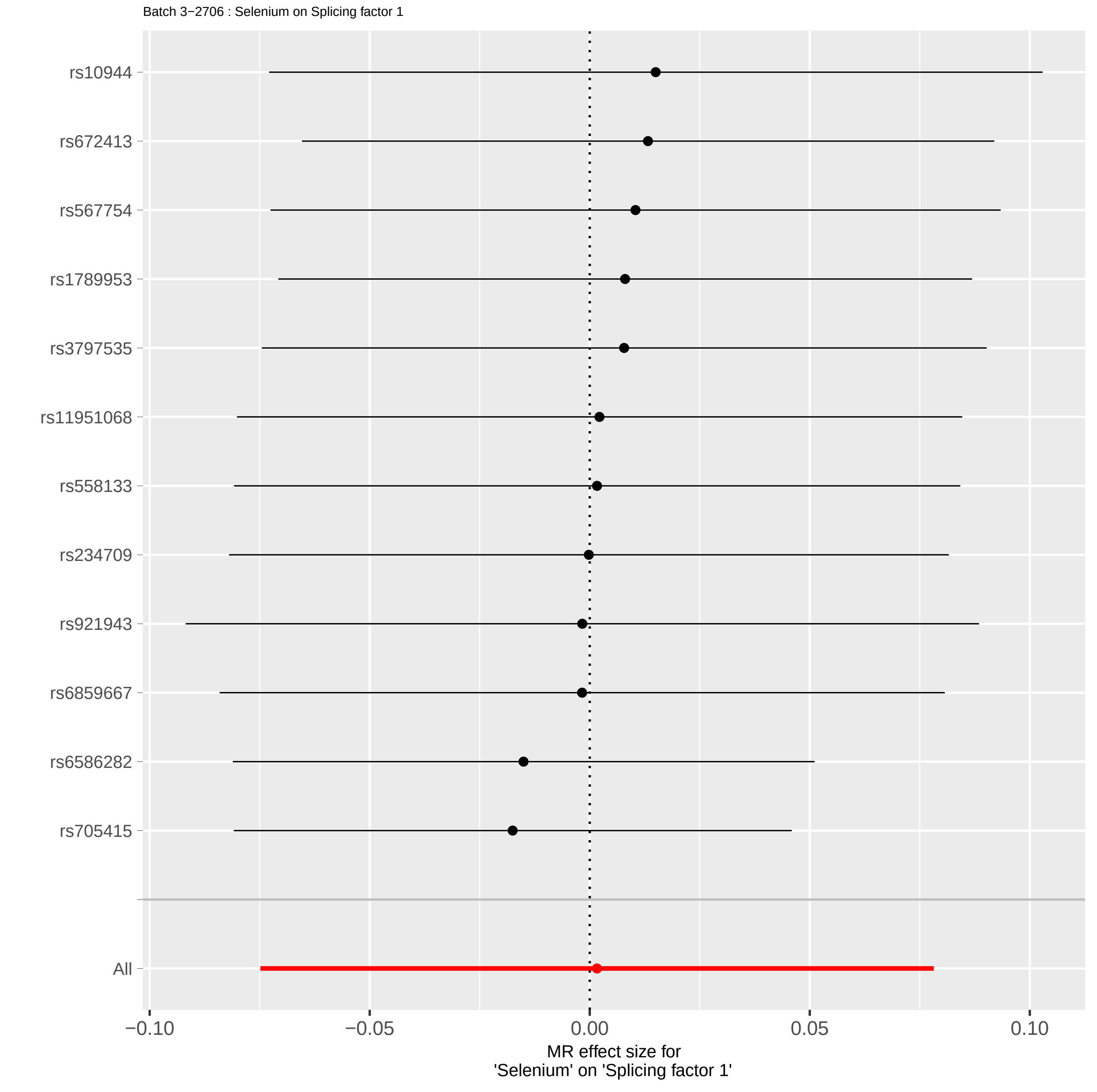
Batch 3−2706 : Selenium on Splicing factor 1
rs10944
rs672413
rs567754
rs1789953
rs3797535
rs11951068
rs558133
rs234709
rs921943
rs6859667
rs6586282
rs705415
All
−0.10
−0.05
0.00
0.05
0.10
MR effect size for
'Selenium' on 'Splicing factor 1'

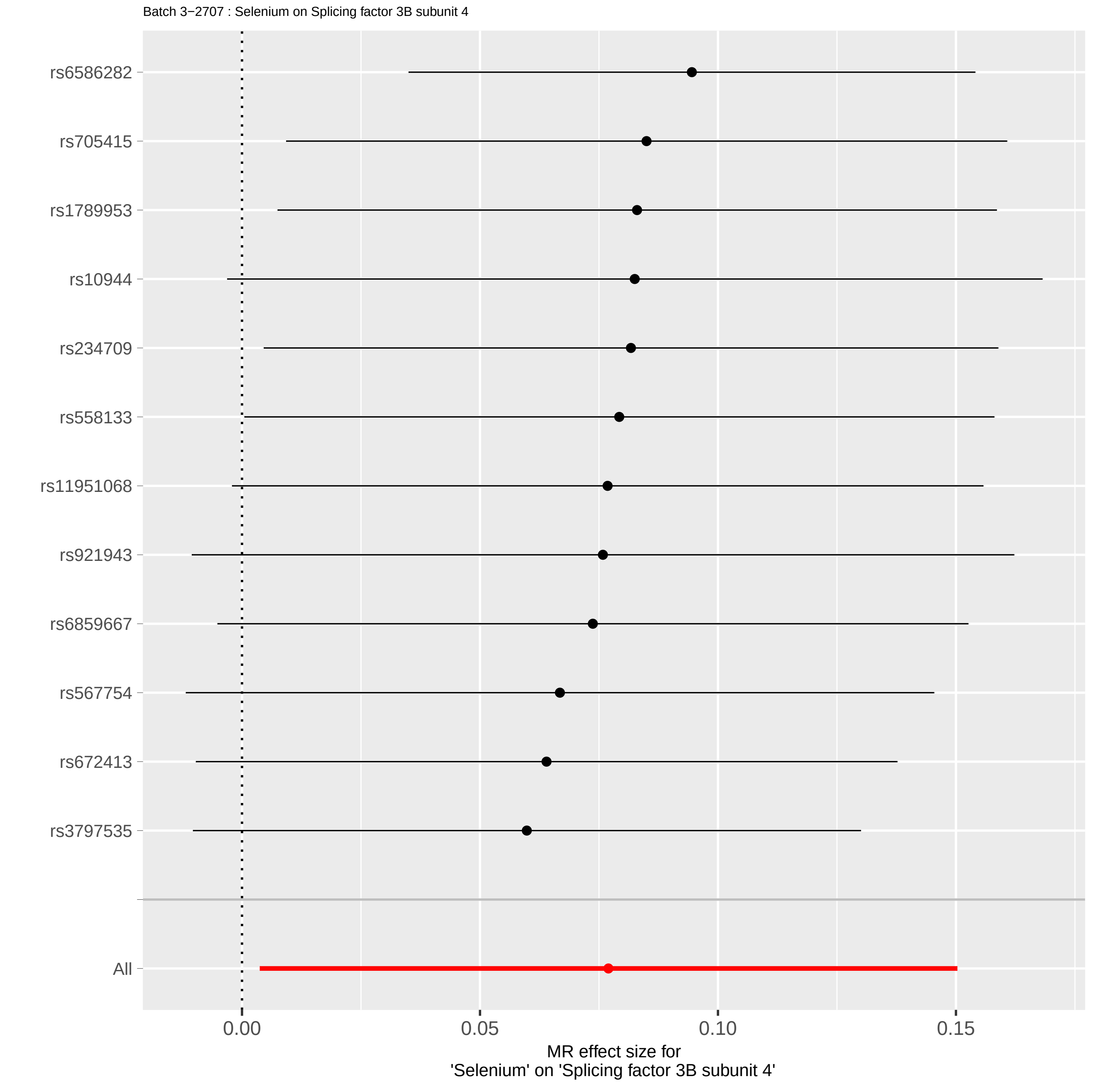

Batch 3−2707 : Selenium on Splicing factor 3B subunit 4
rs6586282
rs705415
rs1789953
rs10944
rs234709
rs558133
rs11951068
rs921943
rs6859667
rs567754
rs672413
rs3797535
All
0.00
0.05
0.10
0.15
MR effect size for
'Selenium' on 'Splicing factor 3B subunit 4'

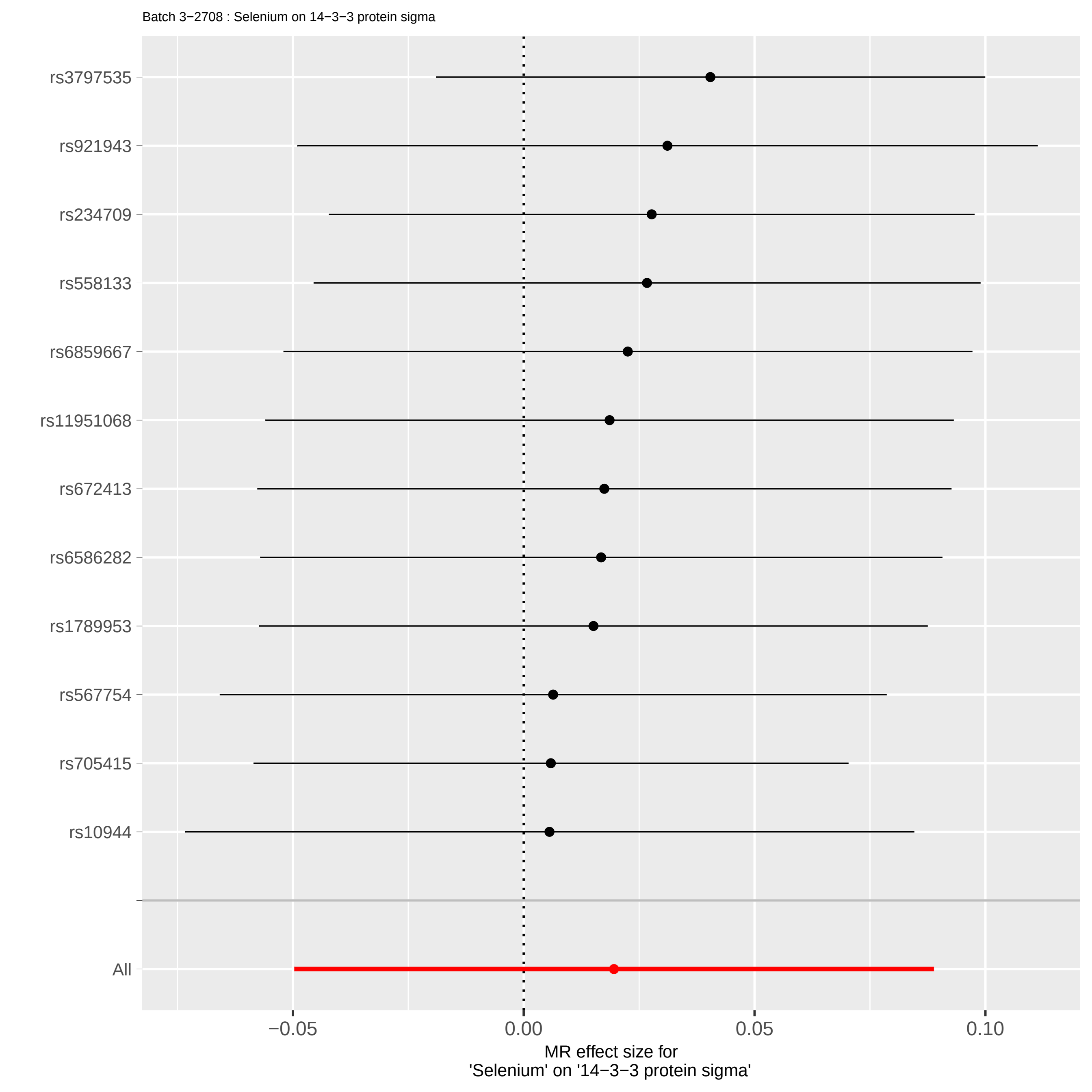

Batch 3−2708 : Selenium on 14−3−3 protein sigma
rs3797535
rs921943
rs234709
rs558133
rs6859667
rs11951068
rs672413
rs6586282
rs1789953
rs567754
rs705415
rs10944
All
−0.05
0.00
0.05
0.10
MR effect size for
'Selenium' on '14−3−3 protein sigma'

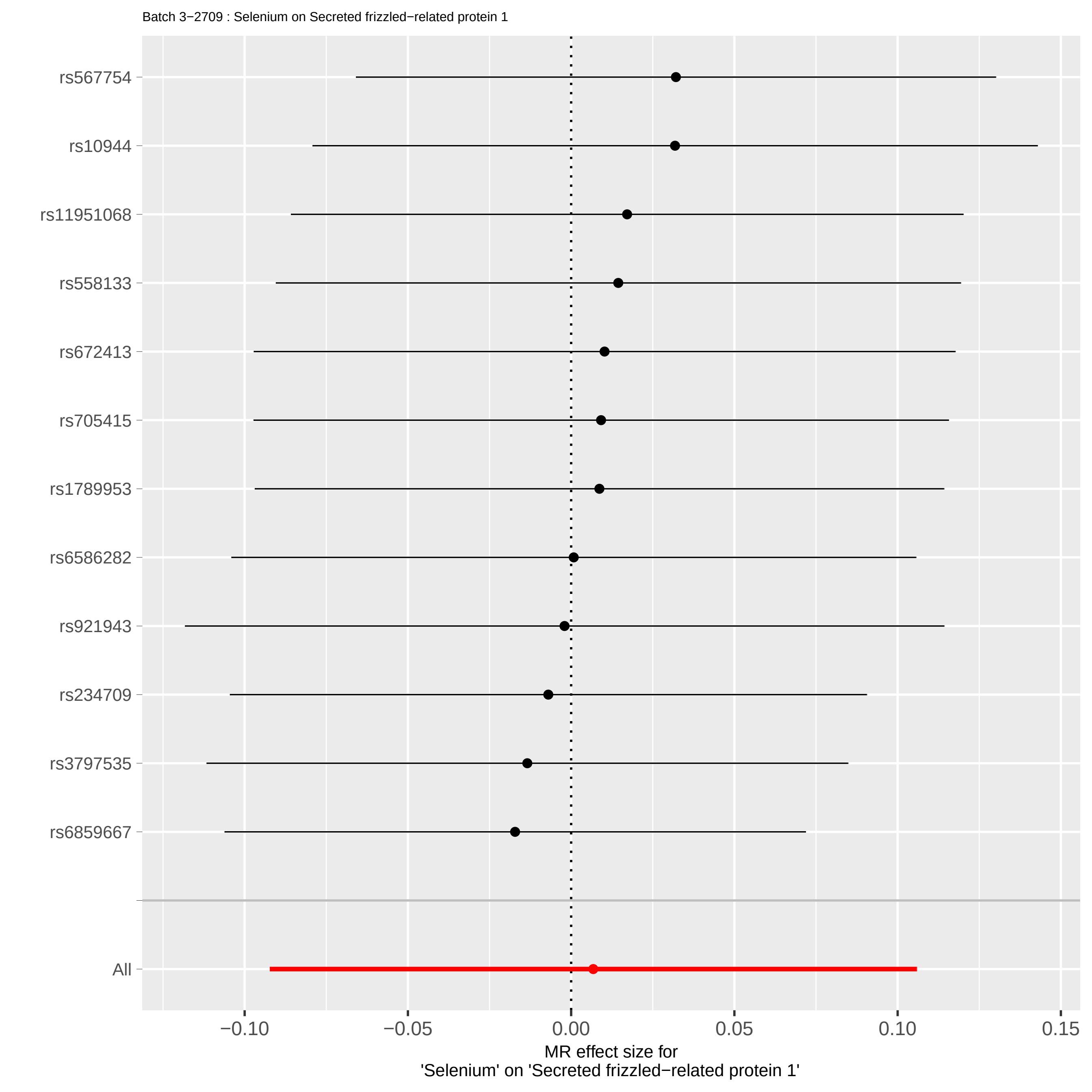

Batch 3−2709 : Selenium on Secreted frizzled−related protein 1
rs567754
rs10944
rs11951068
rs558133
rs672413
rs705415
rs1789953
rs6586282
rs921943
rs234709
rs3797535
rs6859667
All
−0.10
−0.05
0.00
0.05
0.10
0.15
MR effect size for
'Selenium' on 'Secreted frizzled−related protein 1'

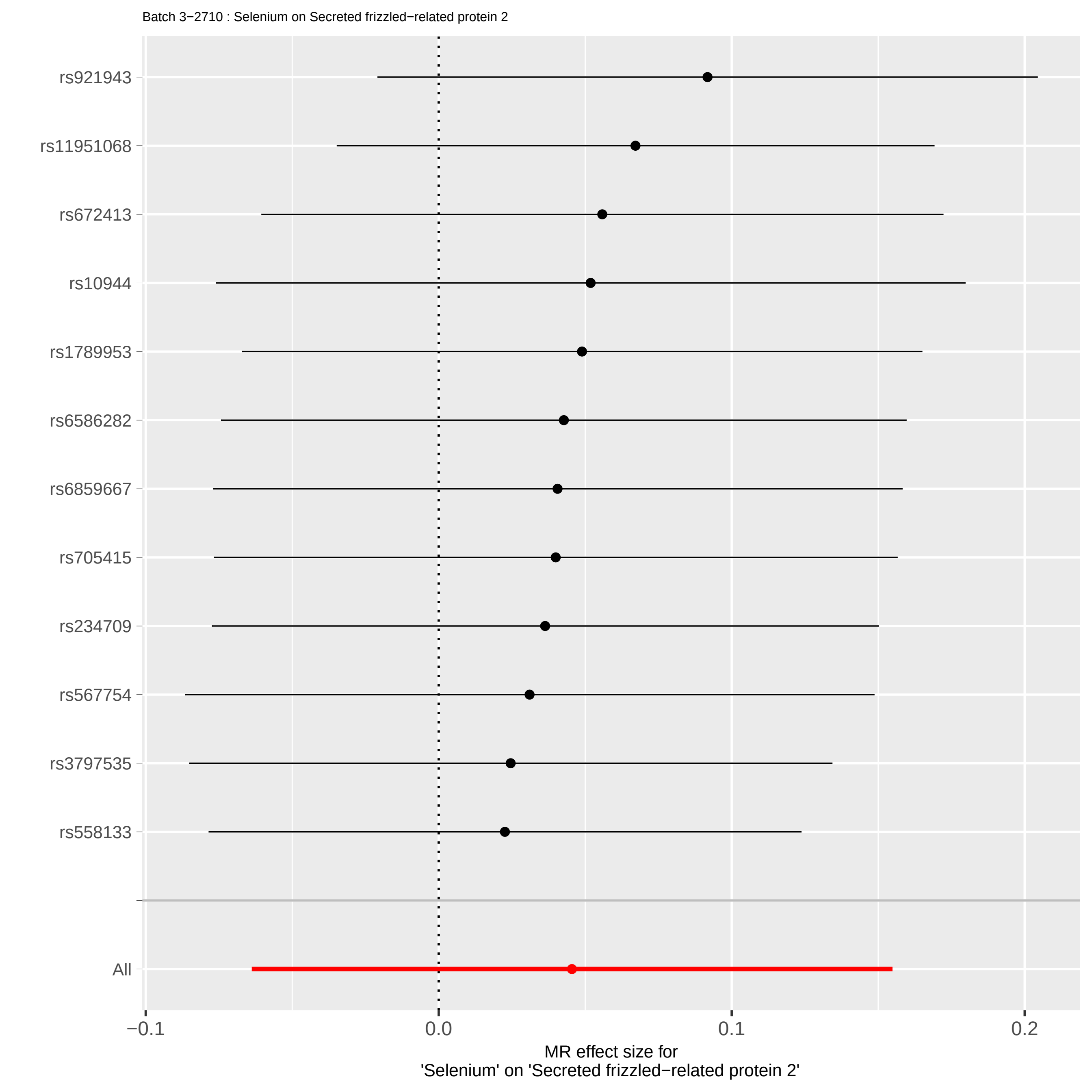

Batch 3−2710 : Selenium on Secreted frizzled−related protein 2
rs921943
rs11951068
rs672413
rs10944
rs1789953
rs6586282
rs6859667
rs705415
rs234709
rs567754
rs3797535
rs558133
All
−0.1
0.0
0.1
0.2
MR effect size for
'Selenium' on 'Secreted frizzled−related protein 2'

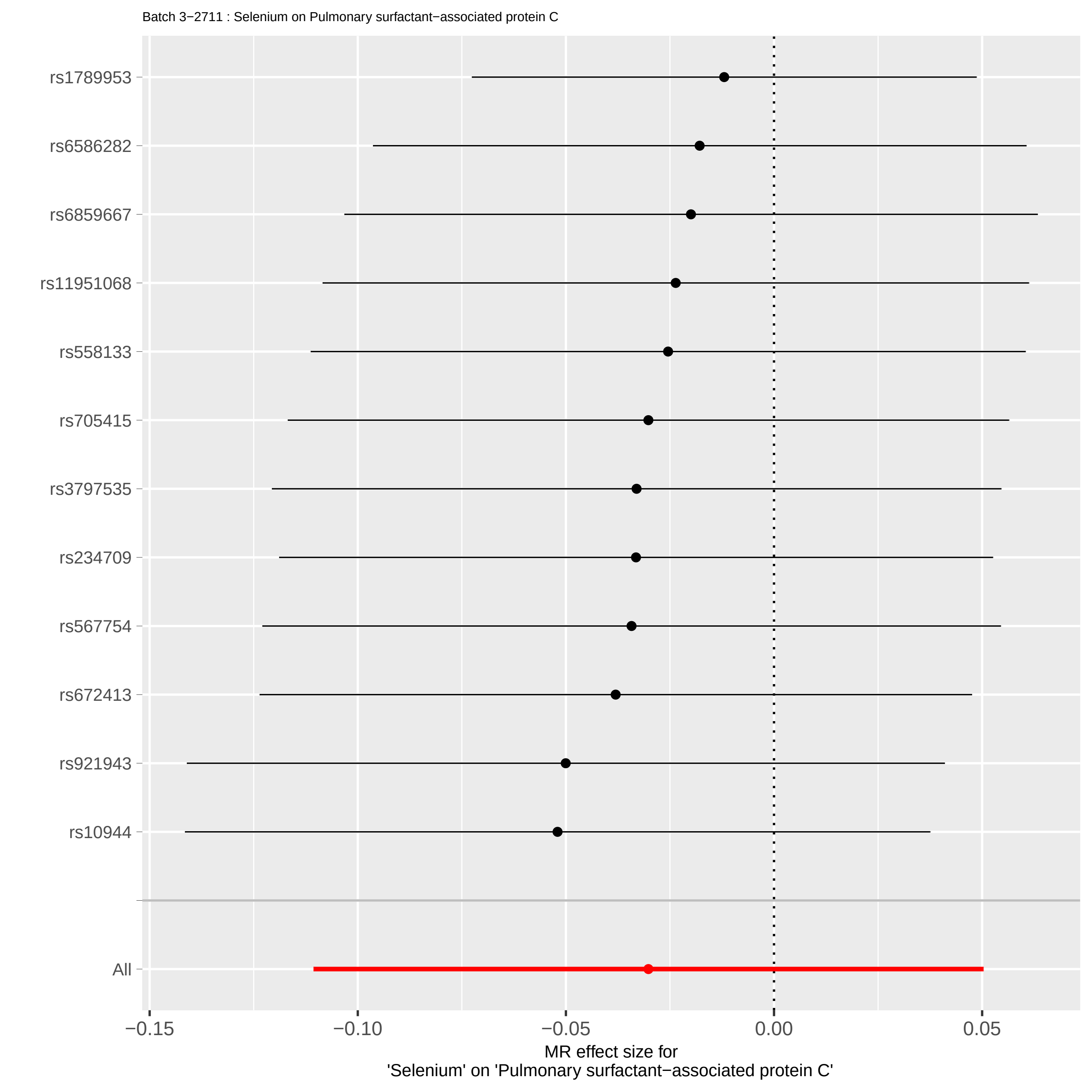
Batch 3−2711 : Selenium on Pulmonary surfactant−associated protein C
rs1789953
rs6586282
rs6859667
rs11951068
rs558133
rs705415
rs3797535
rs234709
rs567754
rs672413
rs921943
rs10944
All
−0.15
−0.10
−0.05
0.00
0.05
MR effect size for
'Selenium' on 'Pulmonary surfactant−associated protein C'

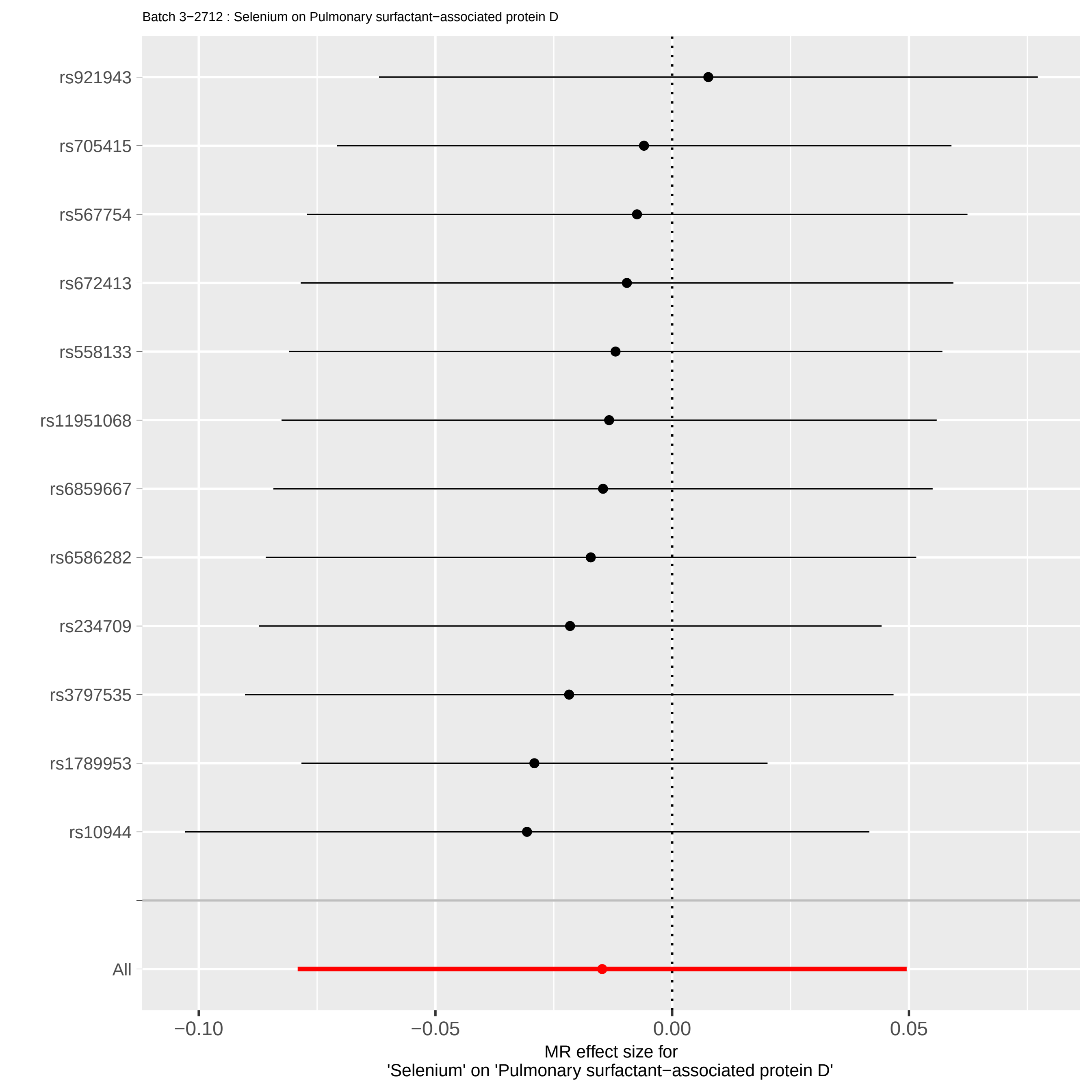

Batch 3−2712 : Selenium on Pulmonary surfactant−associated protein D
rs921943
rs705415
rs567754
rs672413
rs558133
rs11951068
rs6859667
rs6586282
rs234709
rs3797535
rs1789953
rs10944
All
−0.10
−0.05
0.00
0.05
MR effect size for
'Selenium' on 'Pulmonary surfactant−associated protein D'

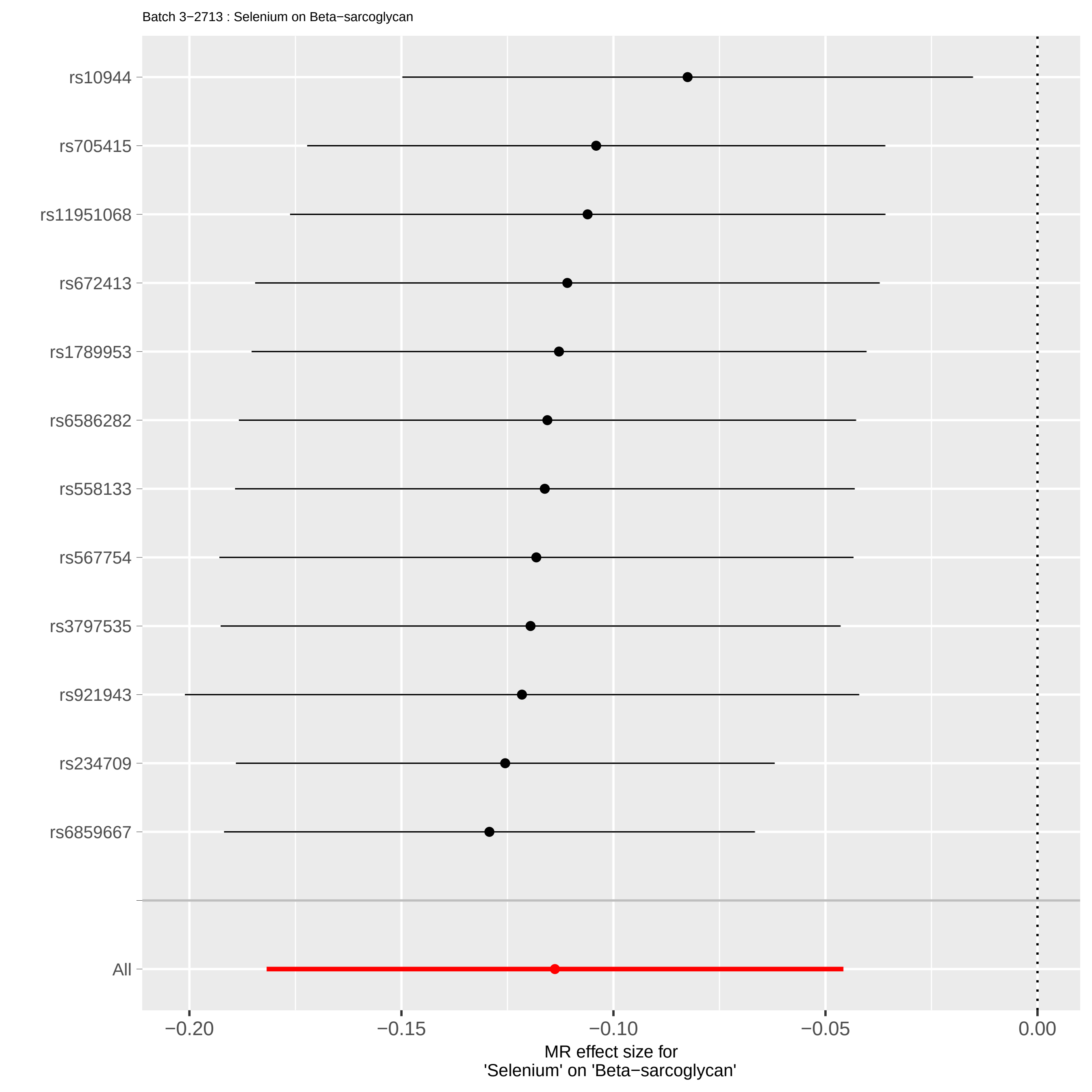
Batch 3−2713 : Selenium on Beta−sarcoglycan
rs10944
rs705415
rs11951068
rs672413
rs1789953
rs6586282
rs558133
rs567754
rs3797535
rs921943
rs234709
rs6859667
All
−0.20
−0.15
−0.10
−0.05
0.00
MR effect size for
'Selenium' on 'Beta−sarcoglycan'

Batch 3−2714 : Selenium on Small glutamine−rich tetratricopeptide repeat−containing protein alpha

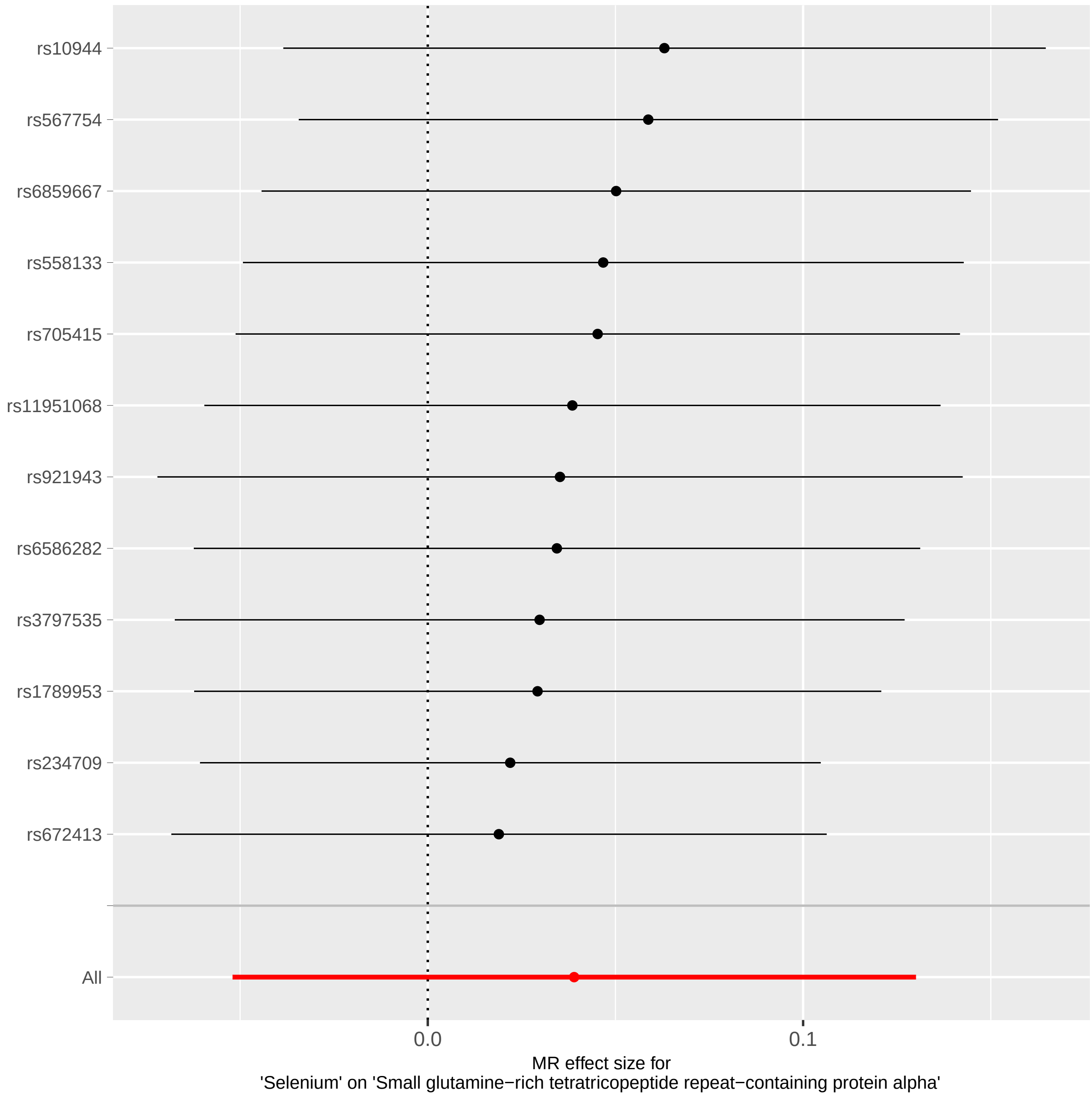

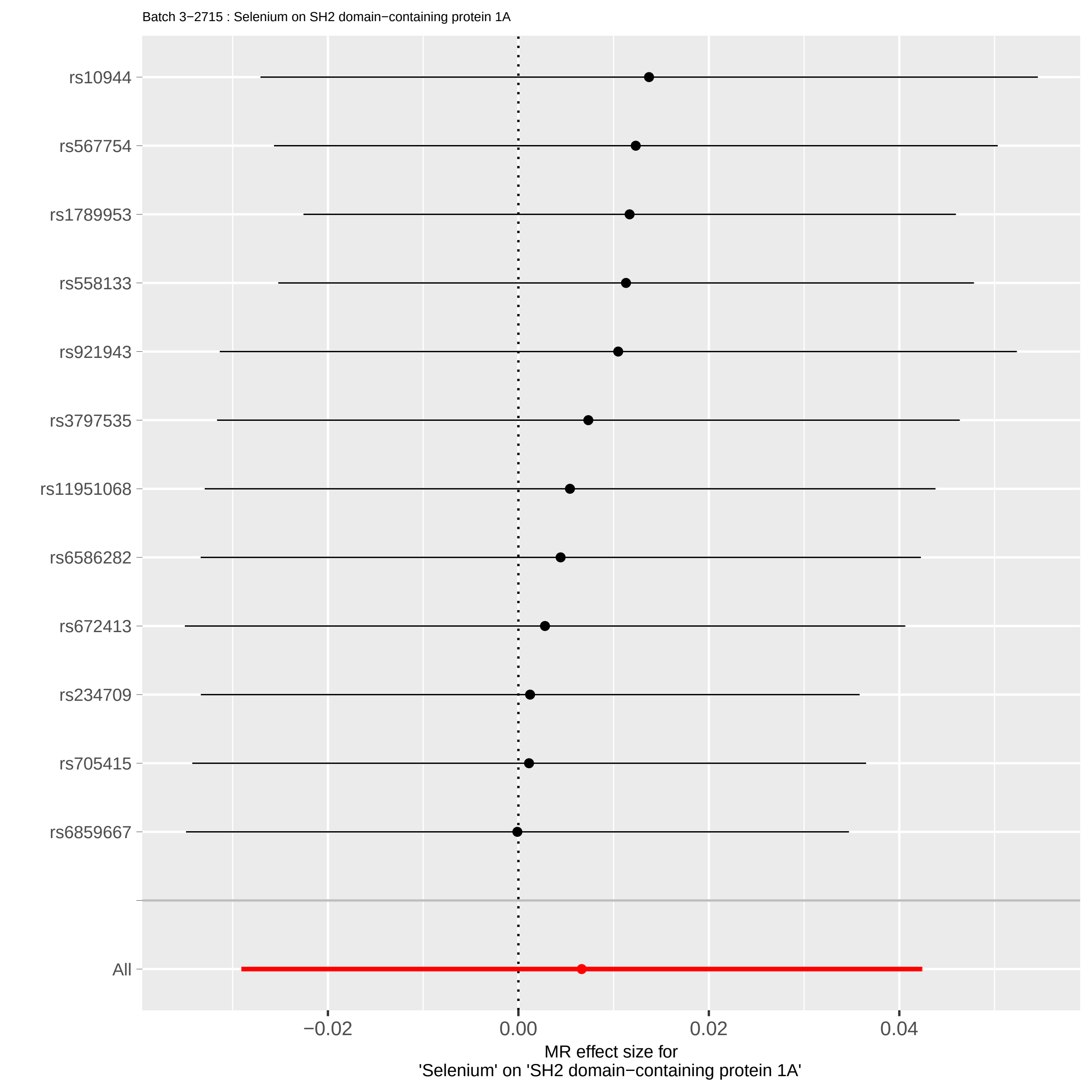

Batch 3−2715 : Selenium on SH2 domain−containing protein 1A
rs10944
rs567754
rs1789953
rs558133
rs921943
rs3797535
rs11951068
rs6586282
rs672413
rs234709
rs705415
rs6859667
All
−0.02
0.00
0.02
0.04
MR effect size for
'Selenium' on 'SH2 domain−containing protein 1A'

Batch 3−2716 : Selenium on SH3 domain−binding protein 2

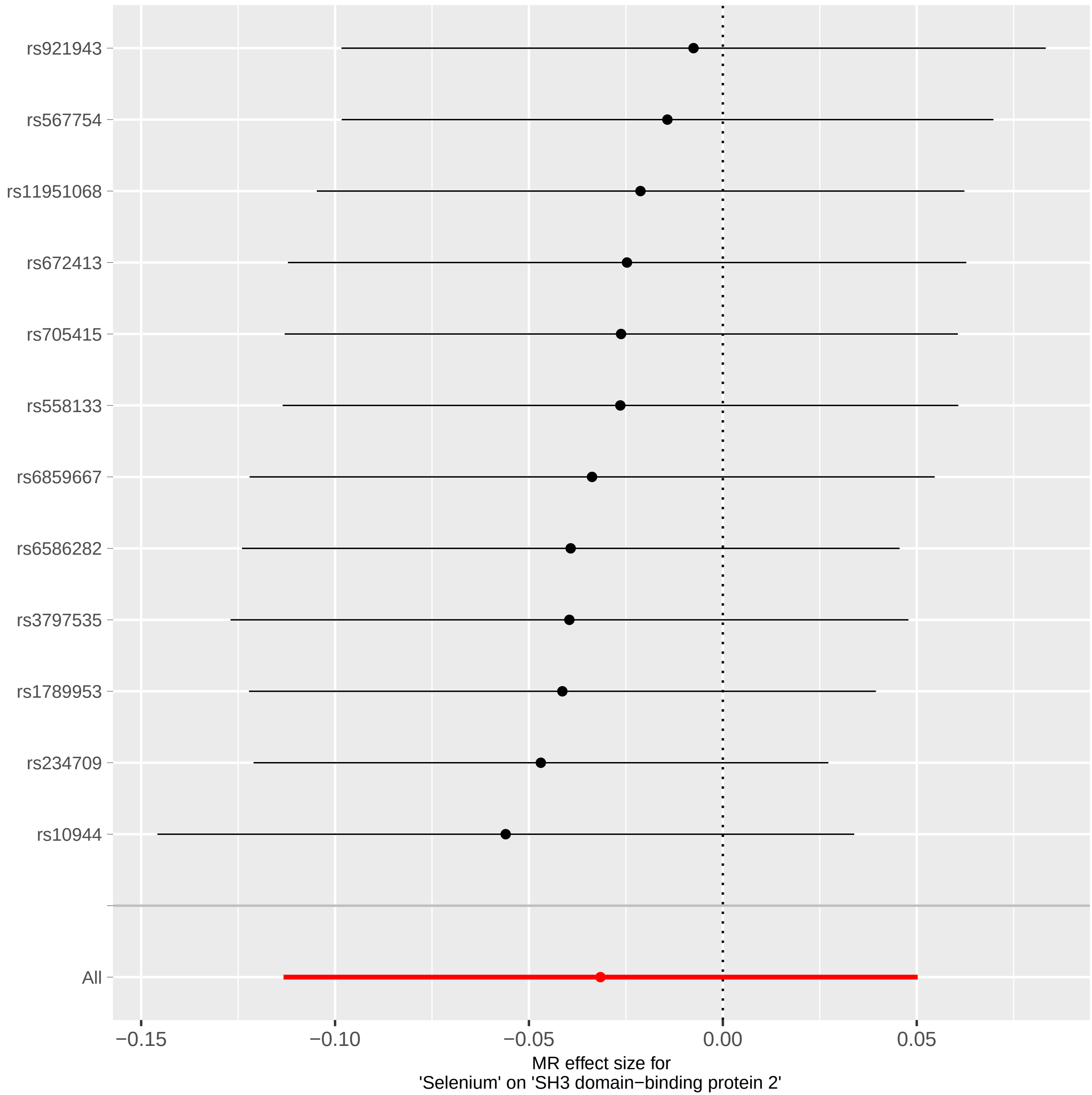

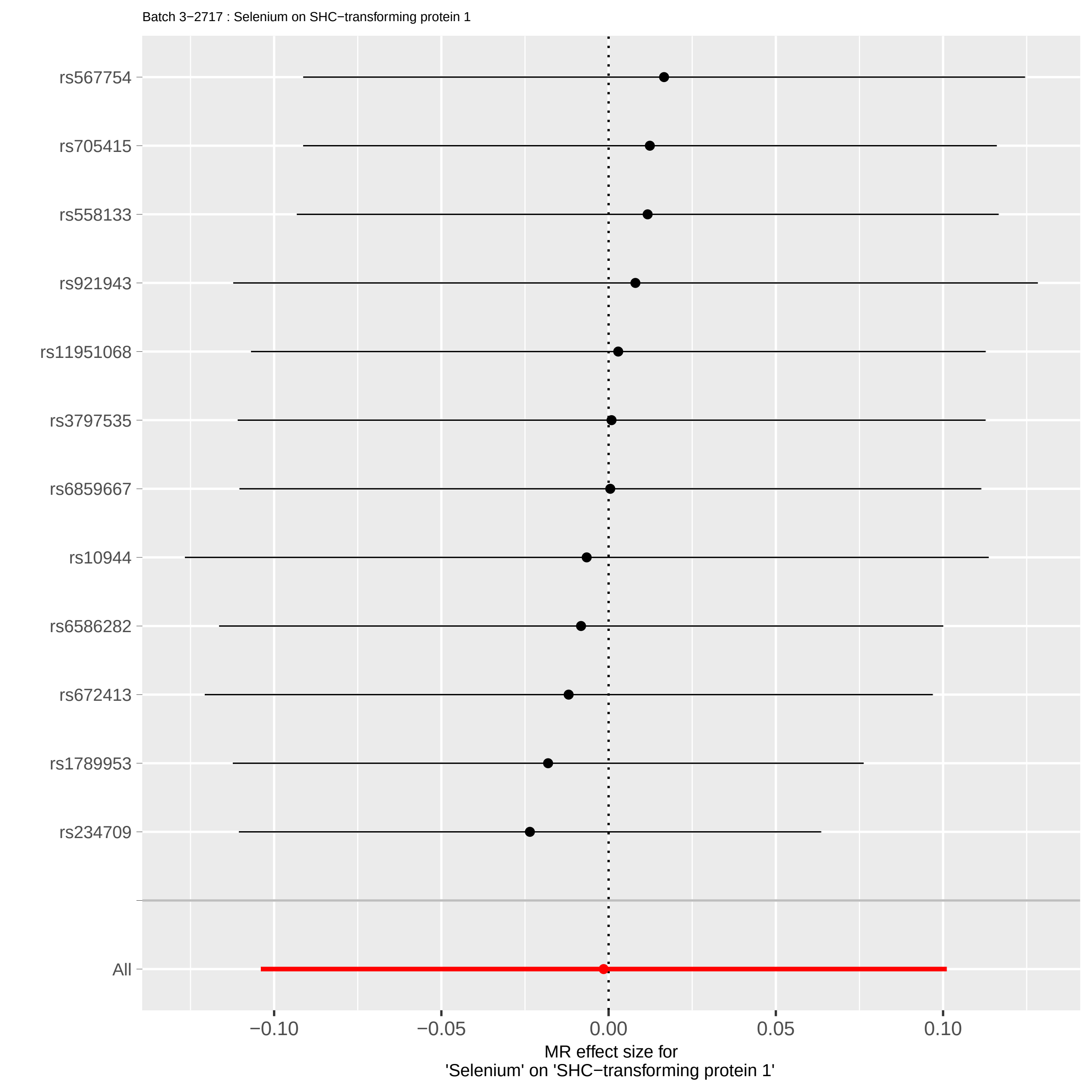

Batch 3−2717 : Selenium on SHC−transforming protein 1
rs567754
rs705415
rs558133
rs921943
rs11951068
rs3797535
rs6859667
rs10944
rs6586282
rs672413
rs1789953
rs234709
All
−0.10
−0.05
0.00
0.05
0.10
MR effect size for
'Selenium' on 'SHC−transforming protein 1'

Batch 3−2718 : Selenium on SHC−transforming protein 2

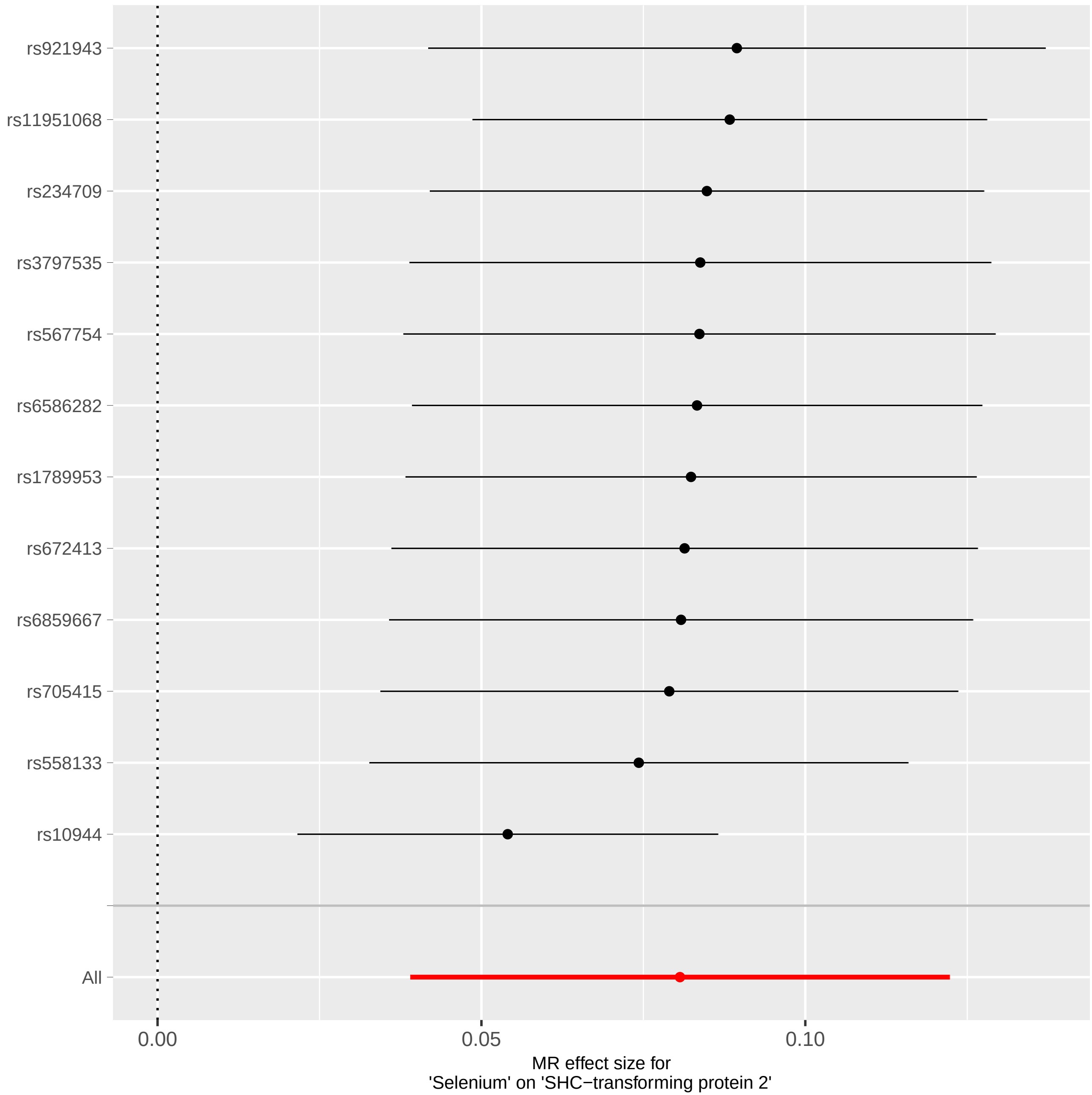

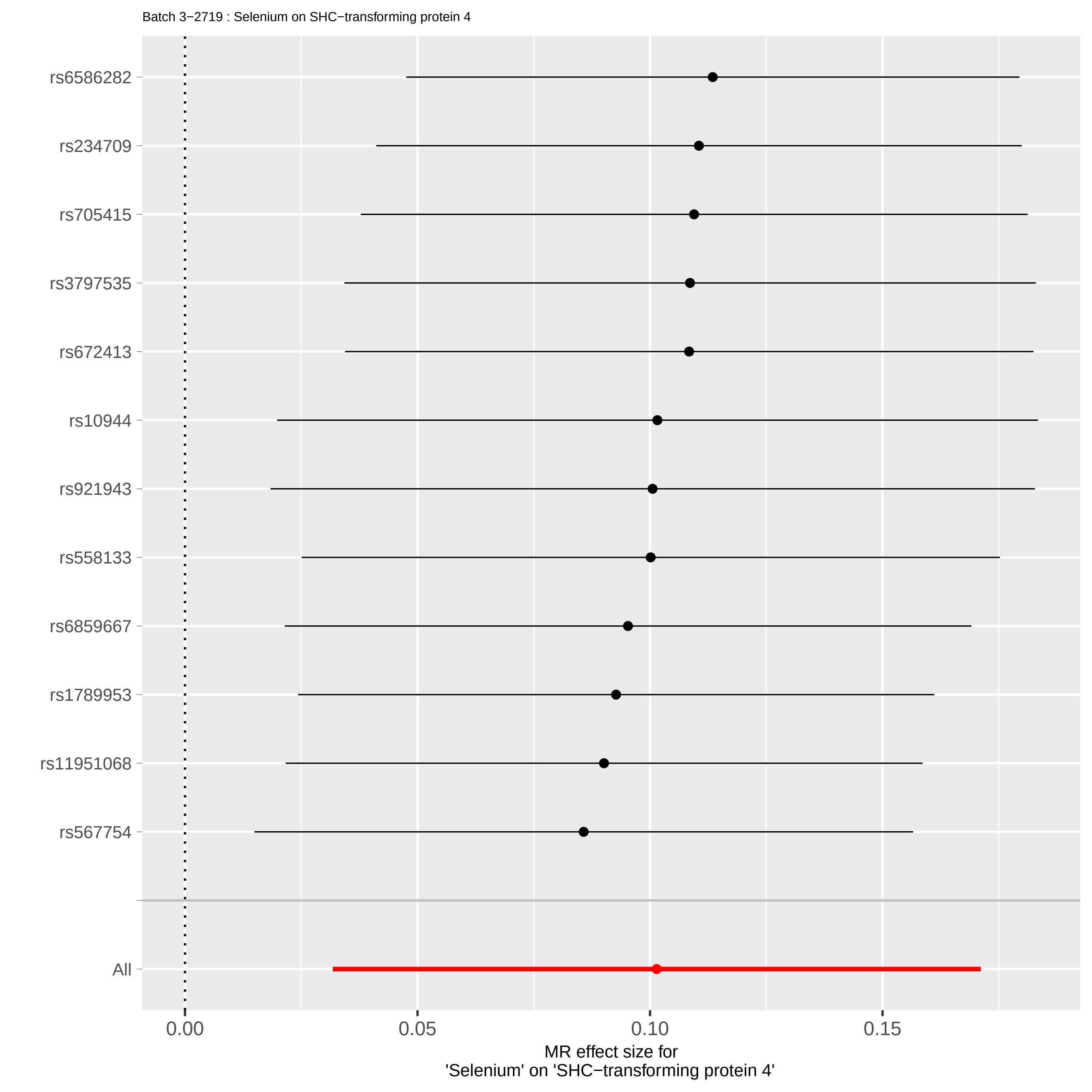

Batch 3−2719 : Selenium on SHC−transforming protein 4
rs6586282
rs234709
rs705415
rs3797535
rs672413
rs10944
rs921943
rs558133
rs6859667
rs1789953
rs11951068
rs567754
All
0.00
0.05
0.10
0.15
MR effect size for
'Selenium' on 'SHC−transforming protein 4'

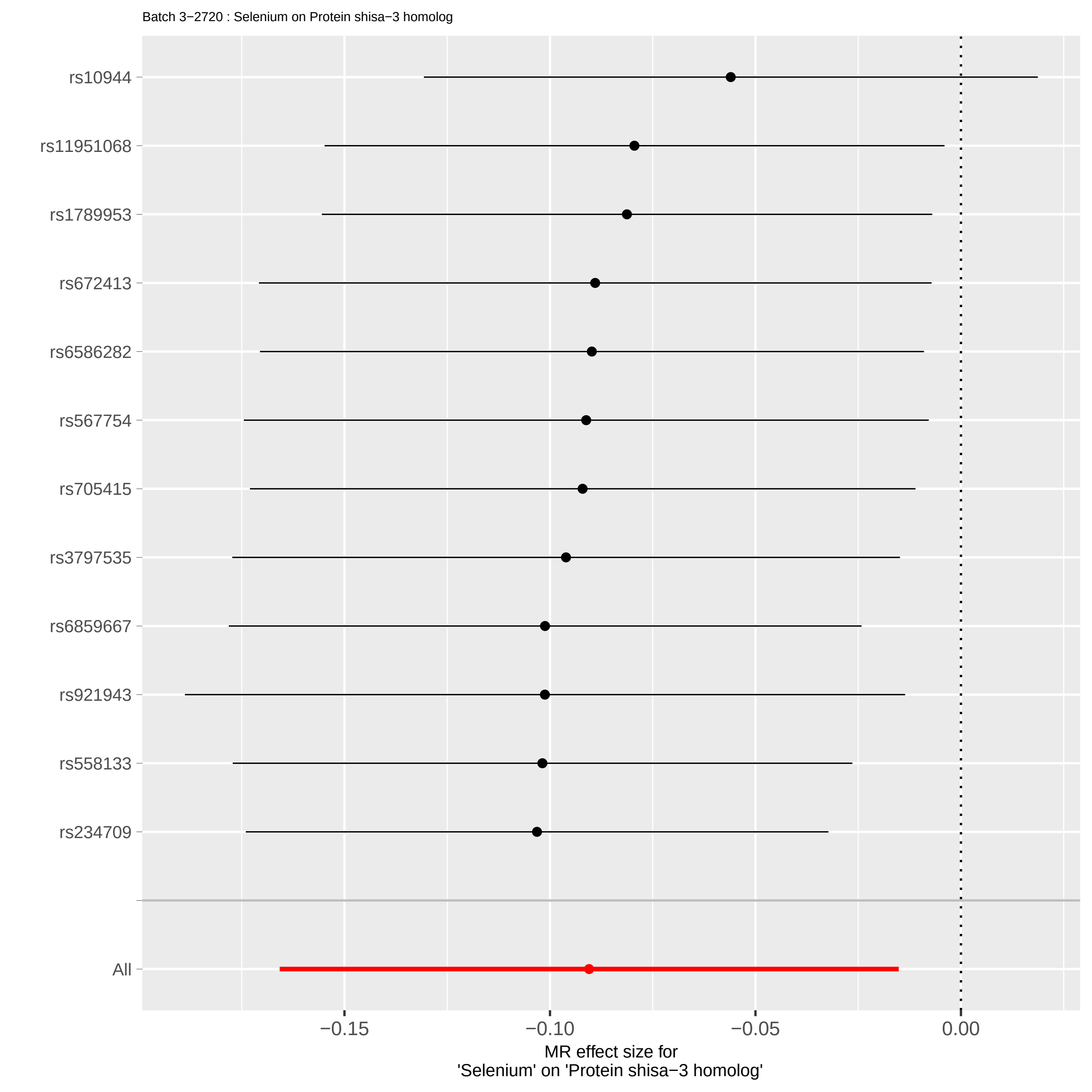
Batch 3−2720 : Selenium on Protein shisa−3 homolog
rs10944
rs11951068
rs1789953
rs672413
rs6586282
rs567754
rs705415
rs3797535
rs6859667
rs921943
rs558133
rs234709
All
−0.15
−0.10
−0.05
0.00
MR effect size for
'Selenium' on 'Protein shisa−3 homolog'

Batch 3−2721 : Selenium on Sialate O−acetylesterase

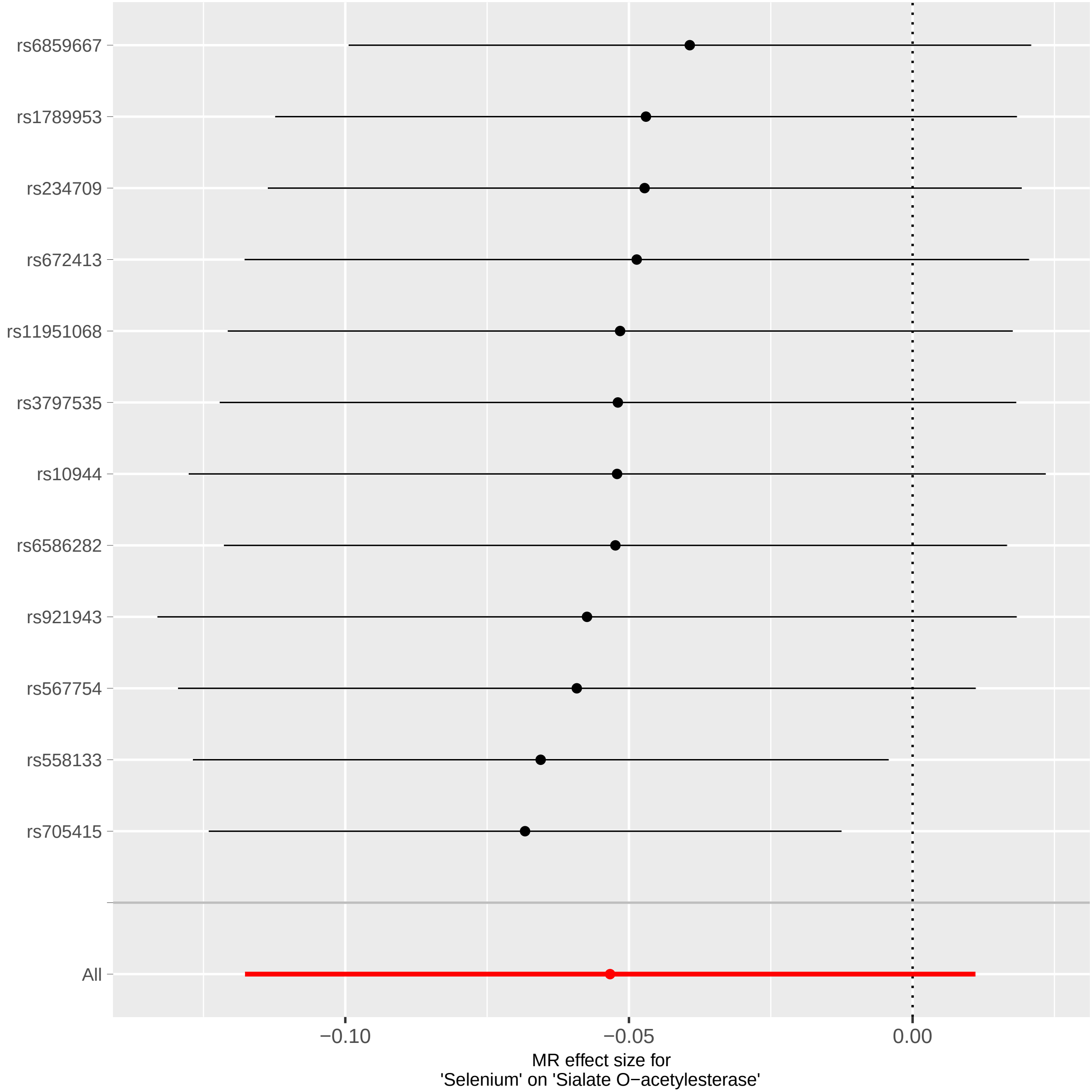

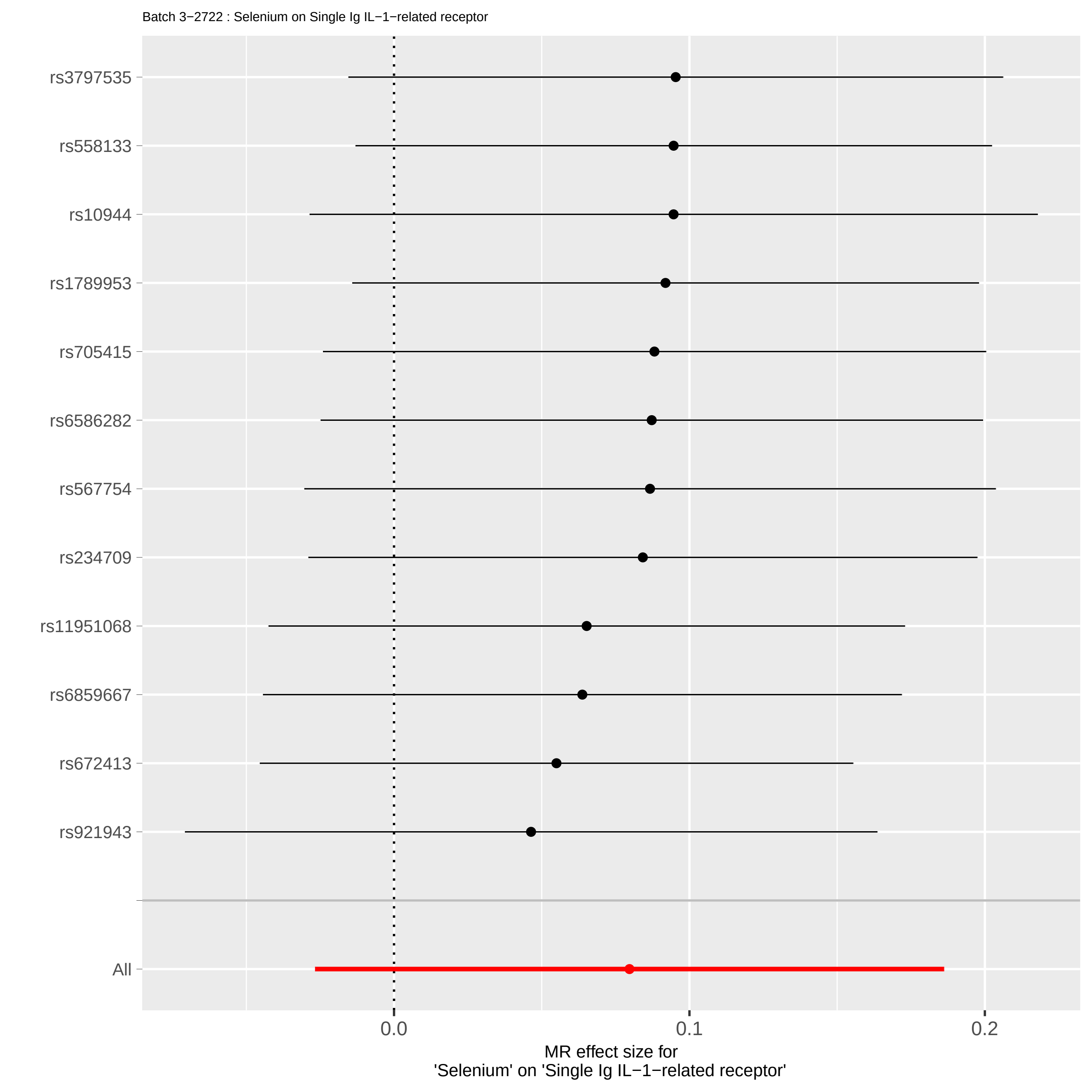

Batch 3−2722 : Selenium on Single Ig IL−1−related receptor
rs3797535
rs558133
rs10944
rs1789953
rs705415
rs6586282
rs567754
rs234709
rs11951068
rs6859667
rs672413
rs921943
All
0.0
0.1
0.2
MR effect size for
'Selenium' on 'Single Ig IL−1−related receptor'

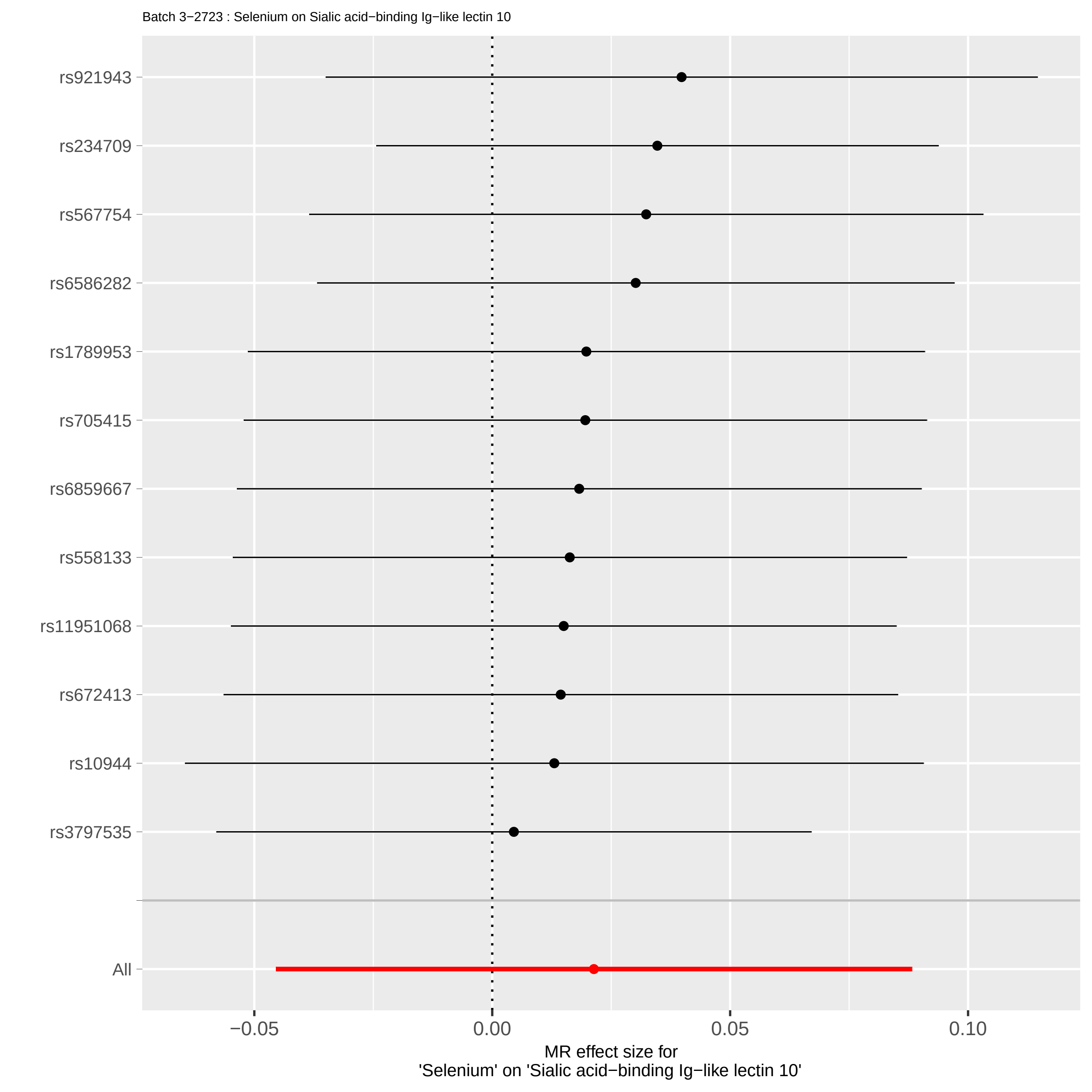

Batch 3−2723 : Selenium on Sialic acid−binding Ig−like lectin 10
rs921943
rs234709
rs567754
rs6586282
rs1789953
rs705415
rs6859667
rs558133
rs11951068
rs672413
rs10944
rs3797535
All
−0.05
0.00
0.05
0.10
MR effect size for
'Selenium' on 'Sialic acid−binding Ig−like lectin 10'

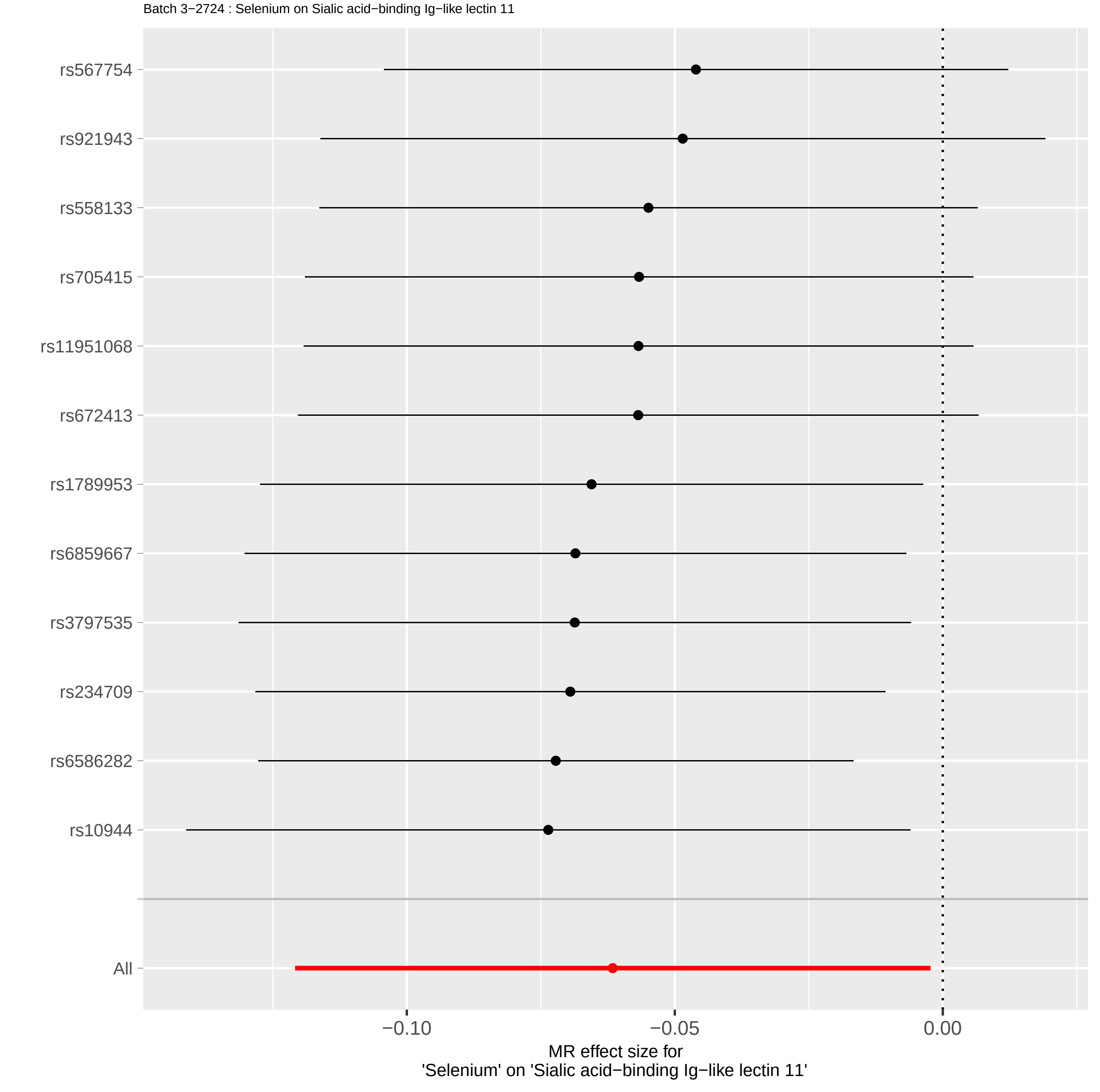

Batch 3−2724 : Selenium on Sialic acid−binding Ig−like lectin 11
rs567754
rs921943
rs558133
rs705415
rs11951068
rs672413
rs1789953
rs6859667
rs3797535
rs234709
rs6586282
rs10944
All
−0.10
−0.05
0.00
MR effect size for
'Selenium' on 'Sialic acid−binding Ig−like lectin 11'

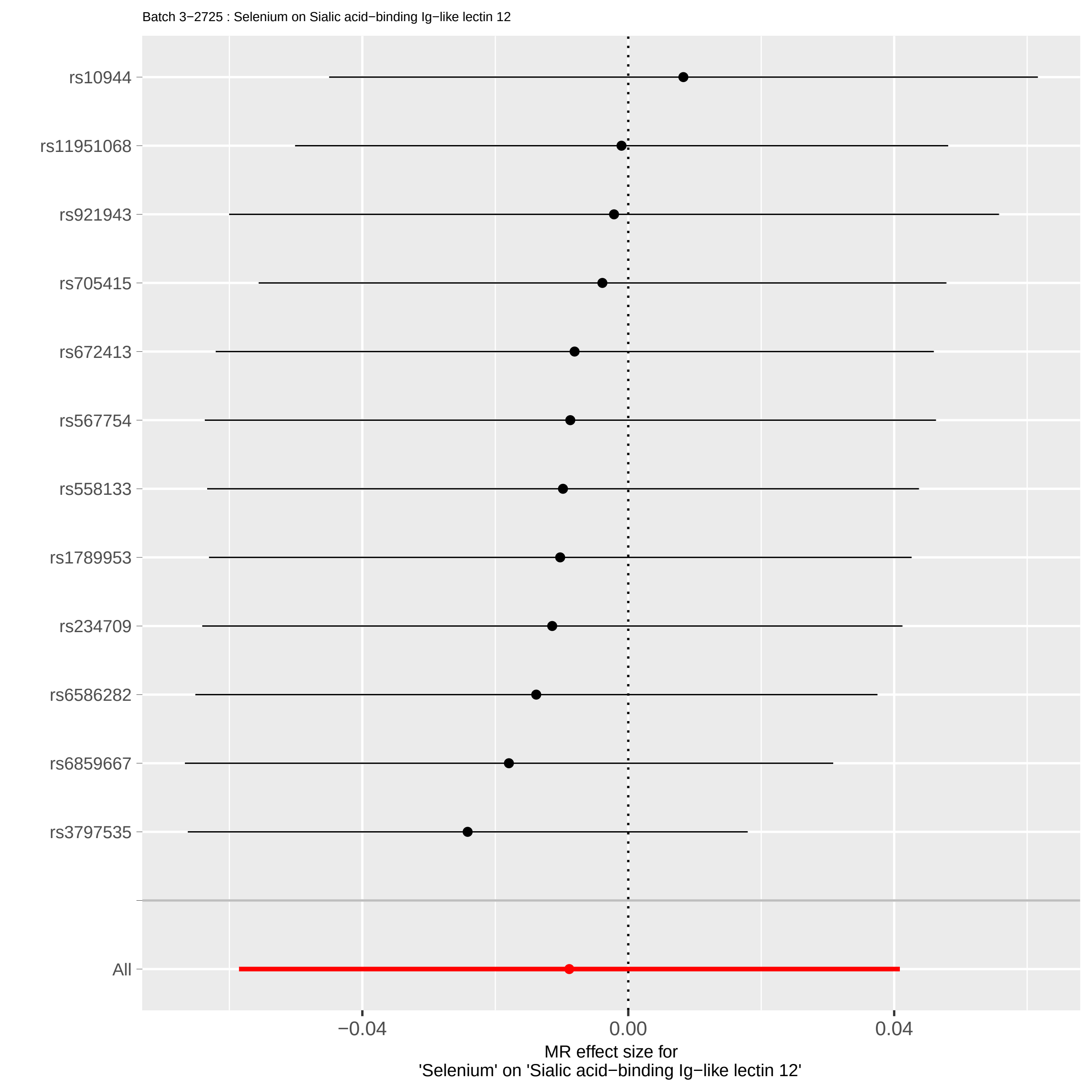
Batch 3−2725 : Selenium on Sialic acid−binding Ig−like lectin 12
rs10944
rs11951068
rs921943
rs705415
rs672413
rs567754
rs558133
rs1789953
rs234709
rs6586282
rs6859667
rs3797535
All
−0.04
0.00
0.04
MR effect size for
'Selenium' on 'Sialic acid−binding Ig−like lectin 12'

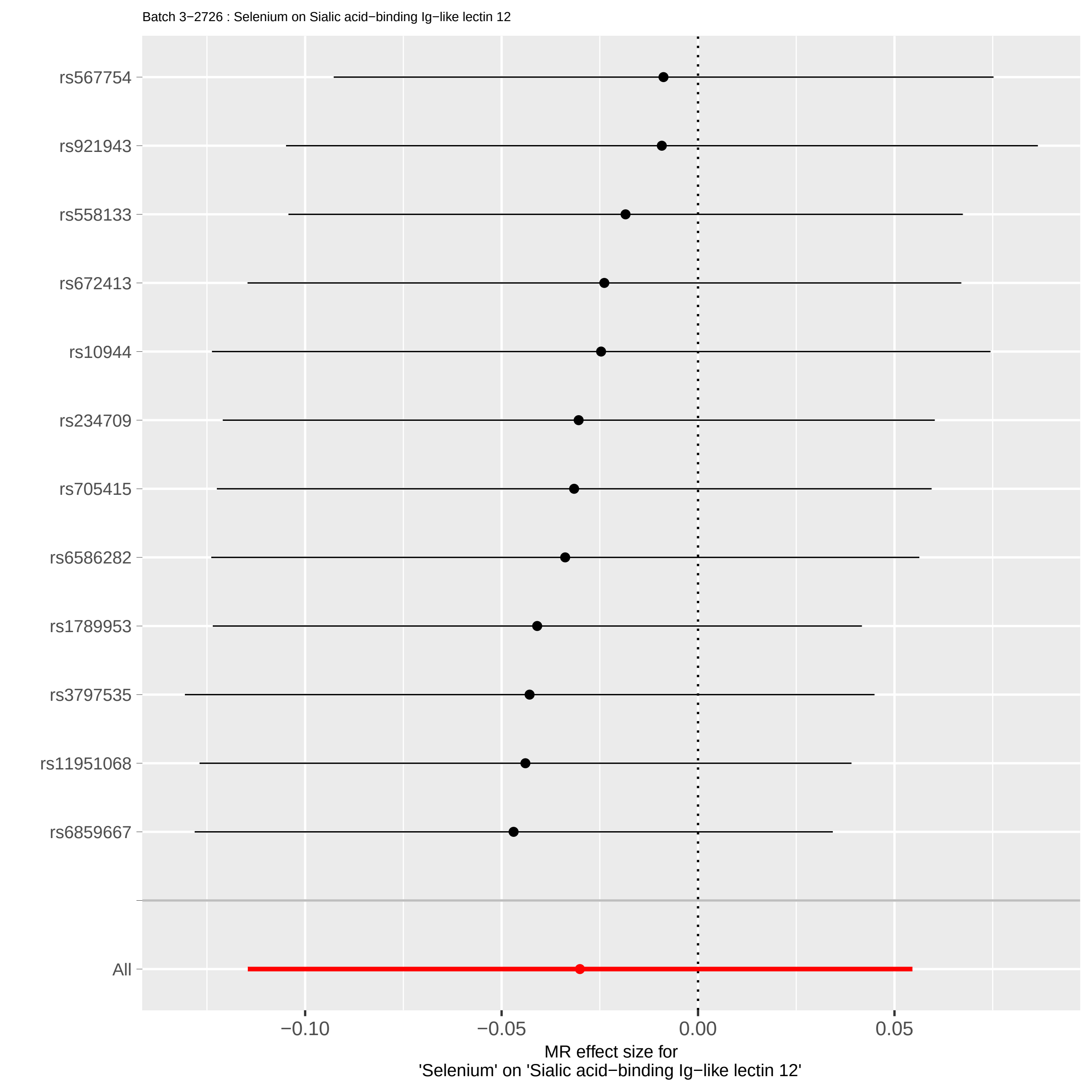
Batch 3−2726 : Selenium on Sialic acid−binding Ig−like lectin 12
rs567754
rs921943
rs558133
rs672413
rs10944
rs234709
rs705415
rs6586282
rs1789953
rs3797535
rs11951068
rs6859667
All
−0.10
−0.05
0.00
0.05
MR effect size for
'Selenium' on 'Sialic acid−binding Ig−like lectin 12'

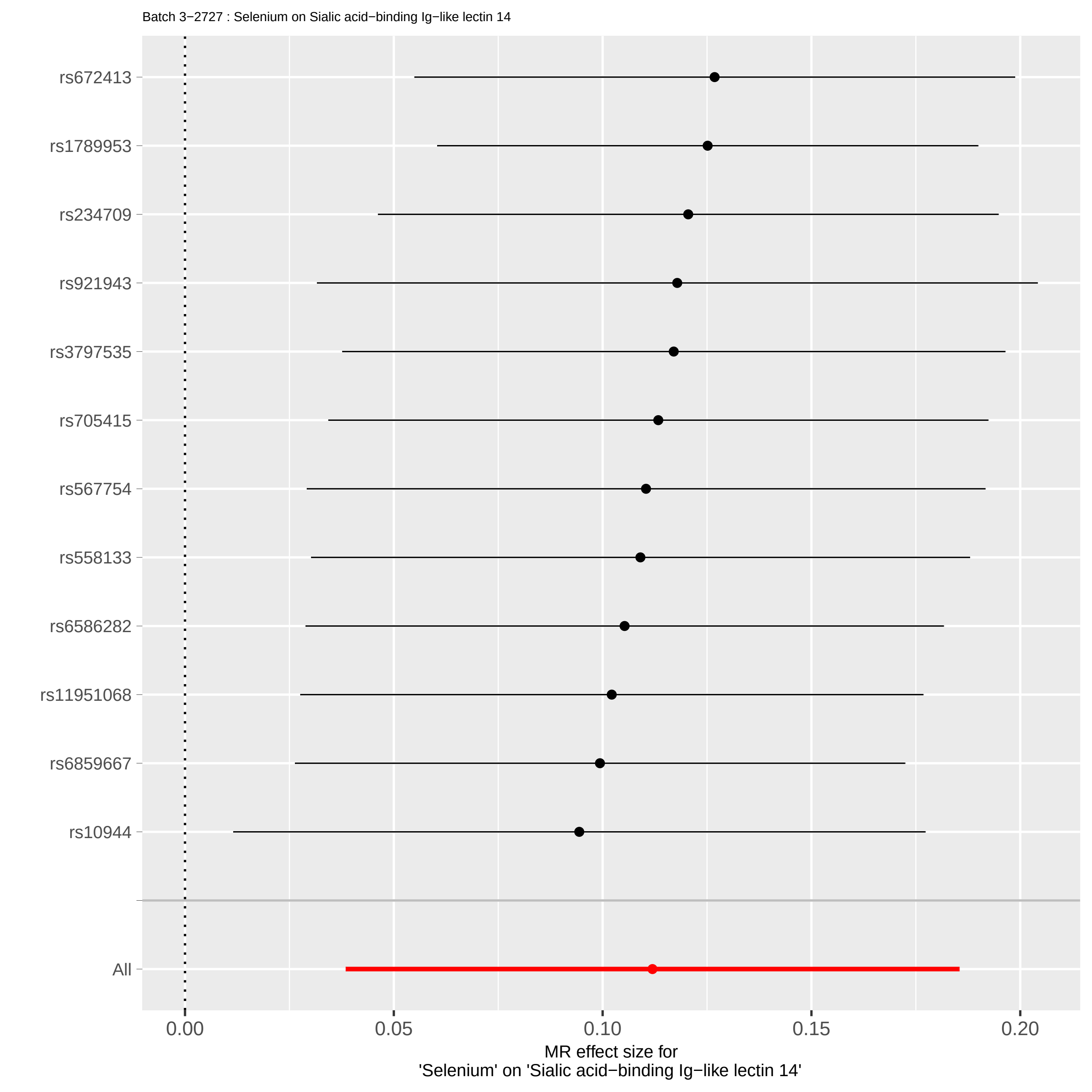

Batch 3−2727 : Selenium on Sialic acid−binding Ig−like lectin 14
rs672413
rs1789953
rs234709
rs921943
rs3797535
rs705415
rs567754
rs558133
rs6586282
rs11951068
rs6859667
rs10944
All
0.00
0.05
0.10
0.15
0.20
MR effect size for
'Selenium' on 'Sialic acid−binding Ig−like lectin 14'

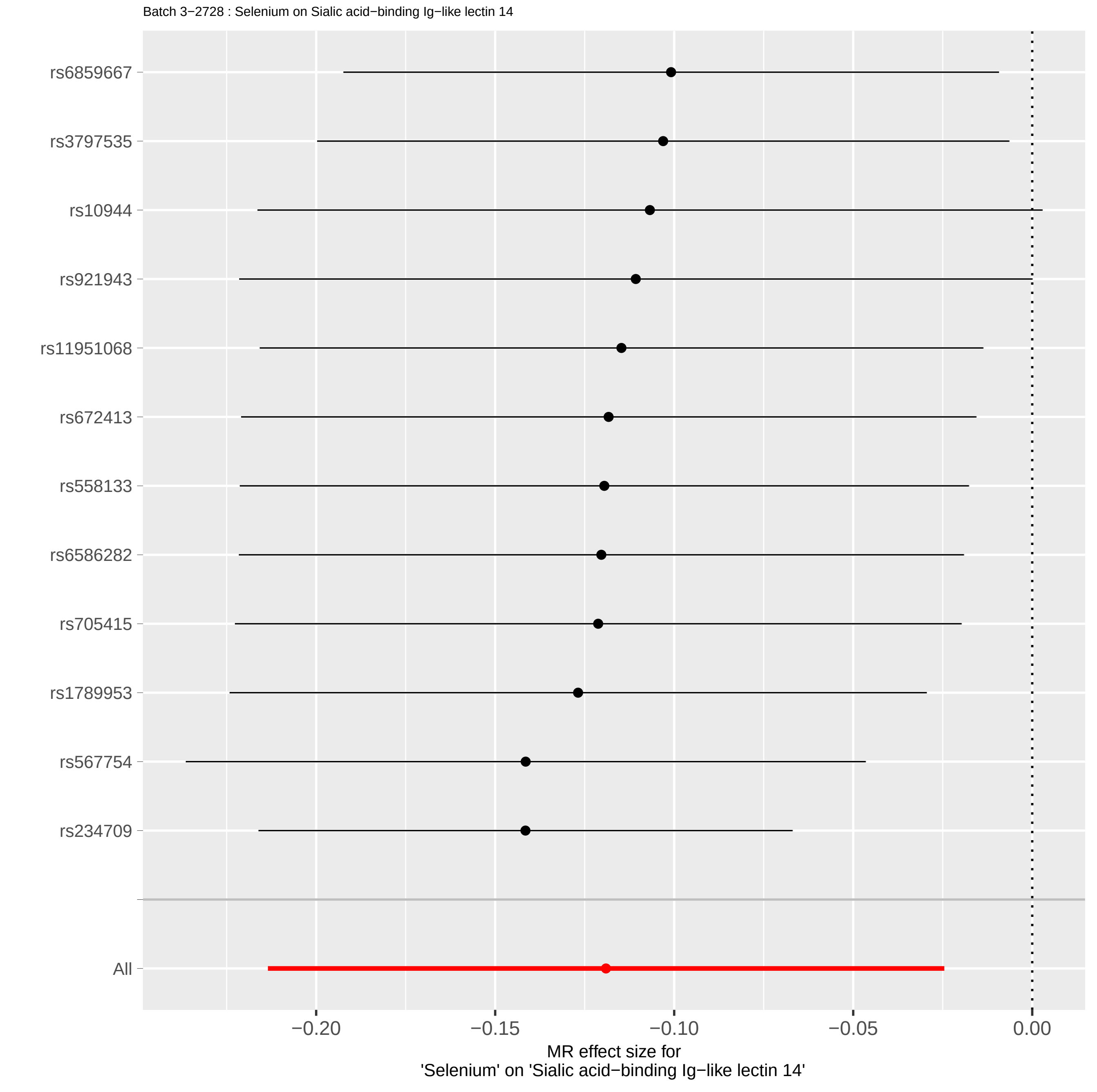

Batch 3−2728 : Selenium on Sialic acid−binding Ig−like lectin 14
rs6859667
rs3797535
rs10944
rs921943
rs11951068
rs672413
rs558133
rs6586282
rs705415
rs1789953
rs567754
rs234709
All
−0.20
−0.15
−0.10
−0.05
0.00
MR effect size for
'Selenium' on 'Sialic acid−binding Ig−like lectin 14'

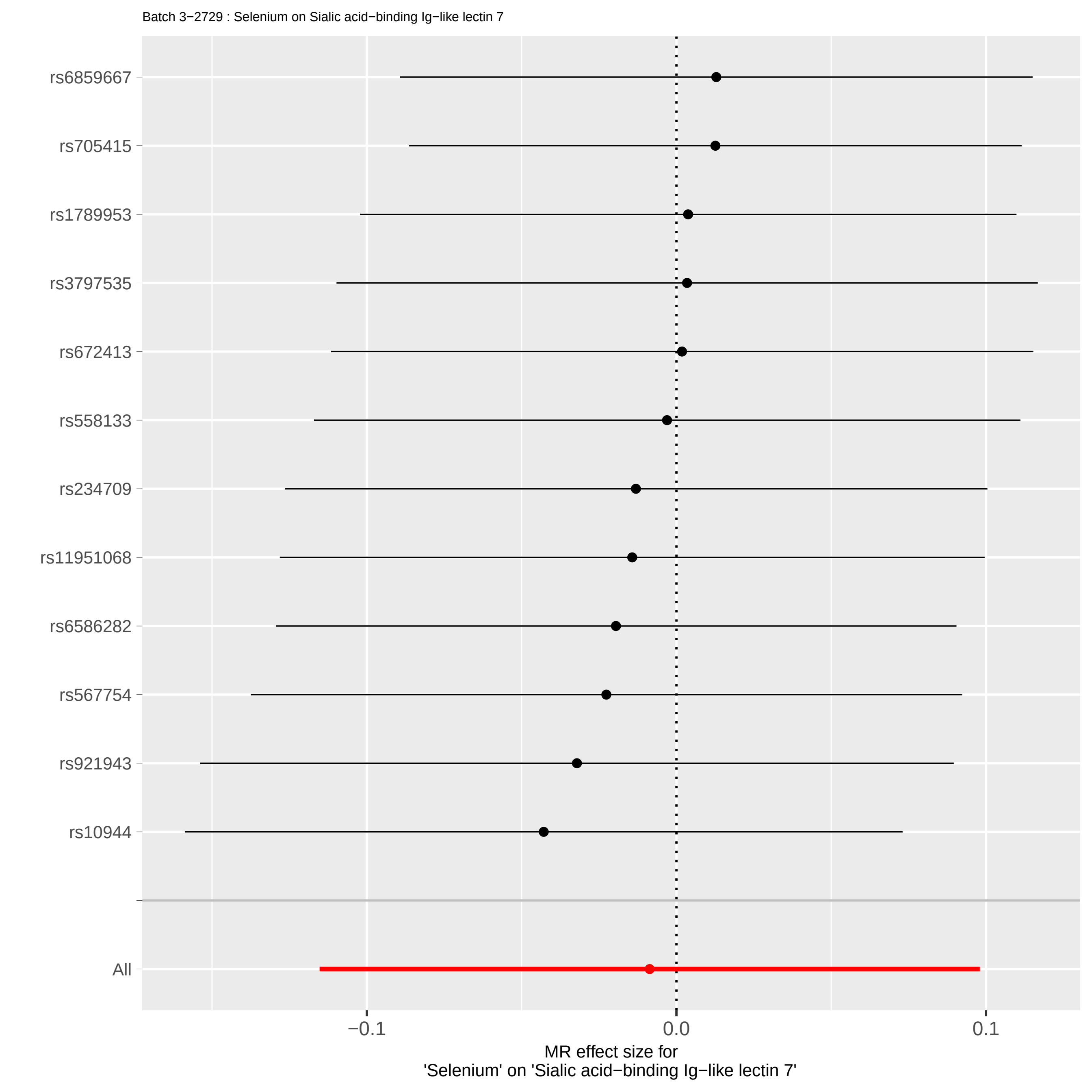

Batch 3−2729 : Selenium on Sialic acid−binding Ig−like lectin 7
rs6859667
rs705415
rs1789953
rs3797535
rs672413
rs558133
rs234709
rs11951068
rs6586282
rs567754
rs921943
rs10944
All
−0.1
0.0
0.1
MR effect size for
'Selenium' on 'Sialic acid−binding Ig−like lectin 7'

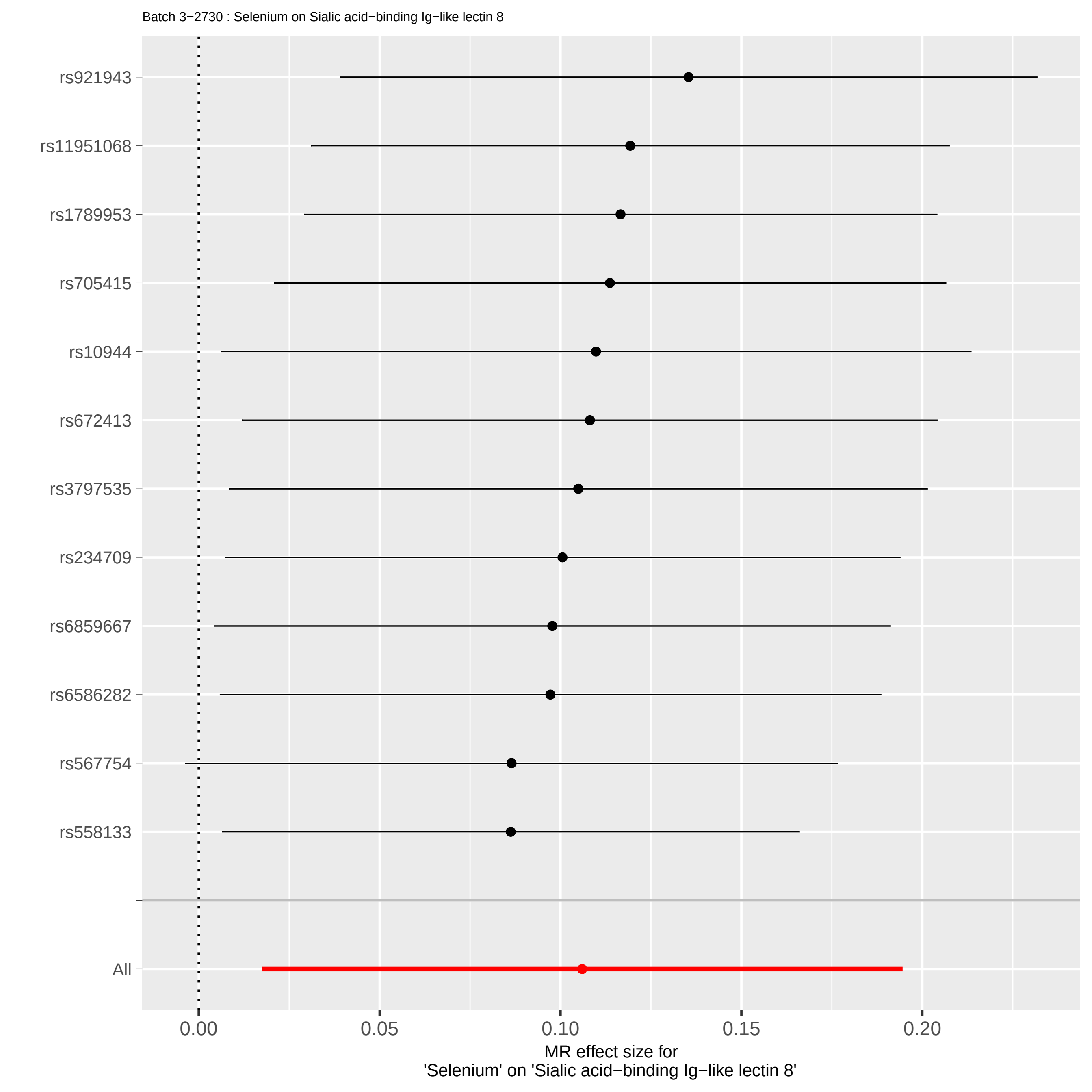
Batch 3−2730 : Selenium on Sialic acid−binding Ig−like lectin 8
rs921943
rs11951068
rs1789953
rs705415
rs10944
rs672413
rs3797535
rs234709
rs6859667
rs6586282
rs567754
rs558133
All
0.00
0.05
0.10
0.15
0.20
MR effect size for
'Selenium' on 'Sialic acid−binding Ig−like lectin 8'

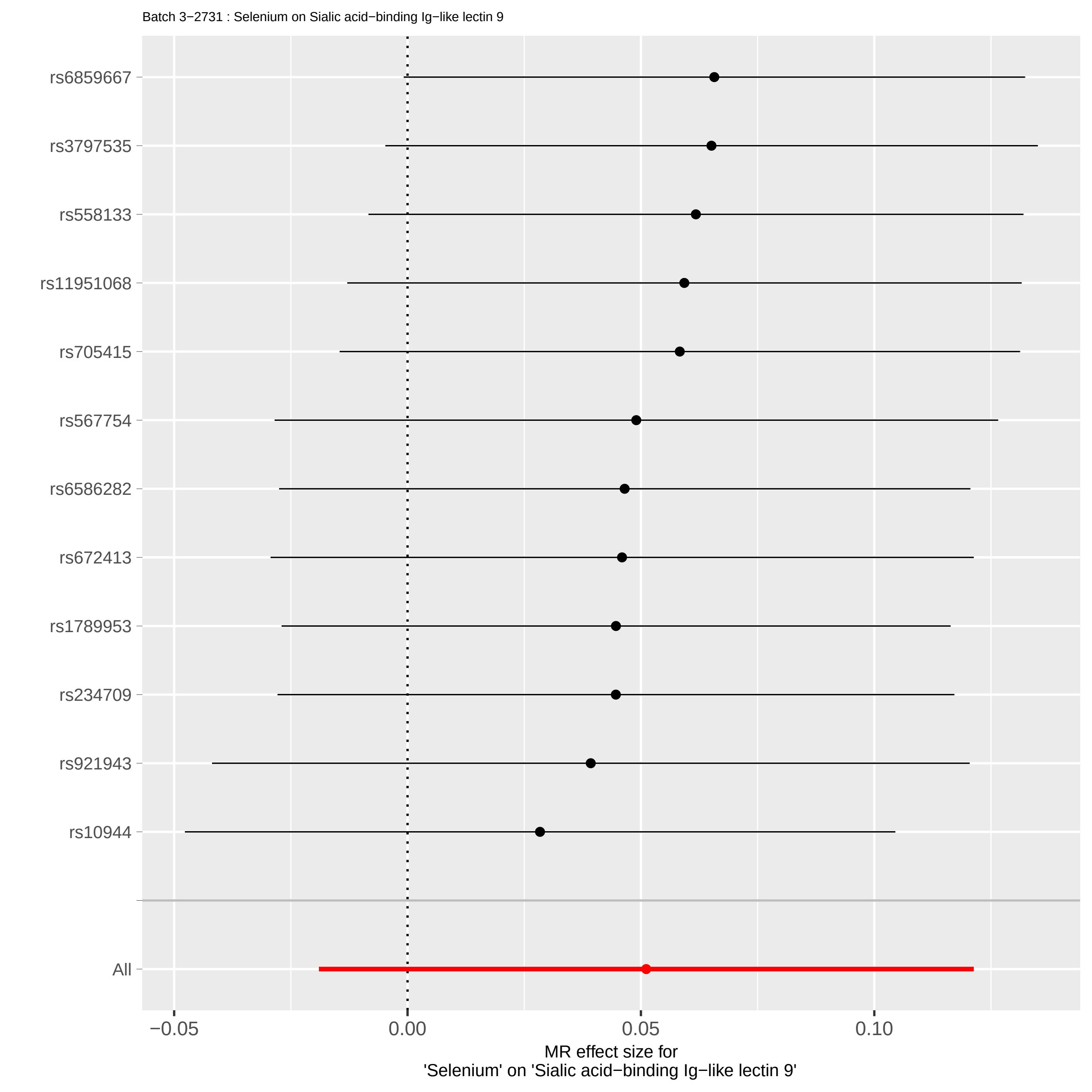

Batch 3−2731 : Selenium on Sialic acid−binding Ig−like lectin 9
rs6859667
rs3797535
rs558133
rs11951068
rs705415
rs567754
rs6586282
rs672413
rs1789953
rs234709
rs921943
rs10944
All
−0.05
0.00
0.05
0.10
MR effect size for
'Selenium' on 'Sialic acid−binding Ig−like lectin 9'

Batch 3−2732 : Selenium on Tyrosine−protein phosphatase non−receptor type substrate 1

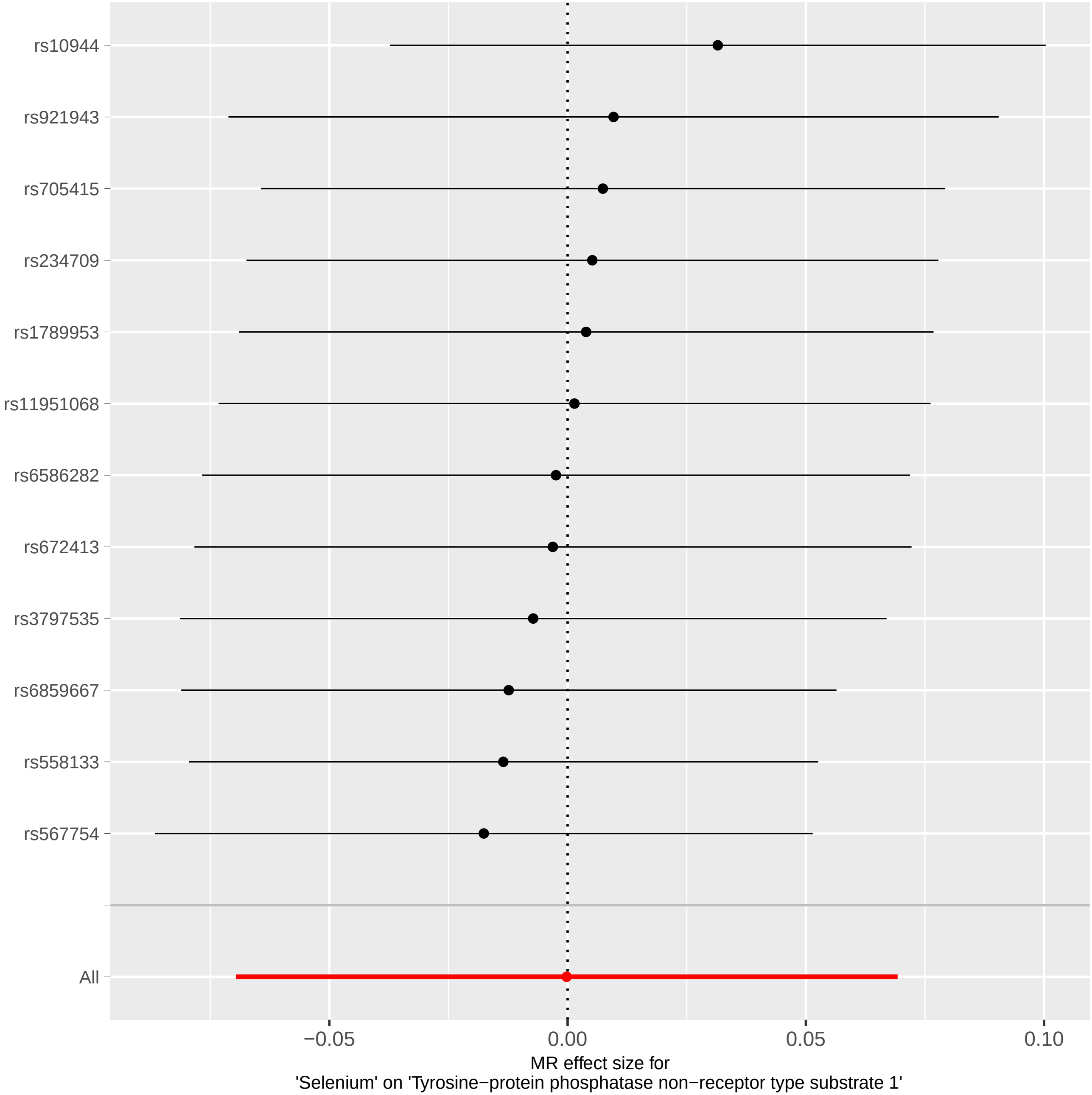

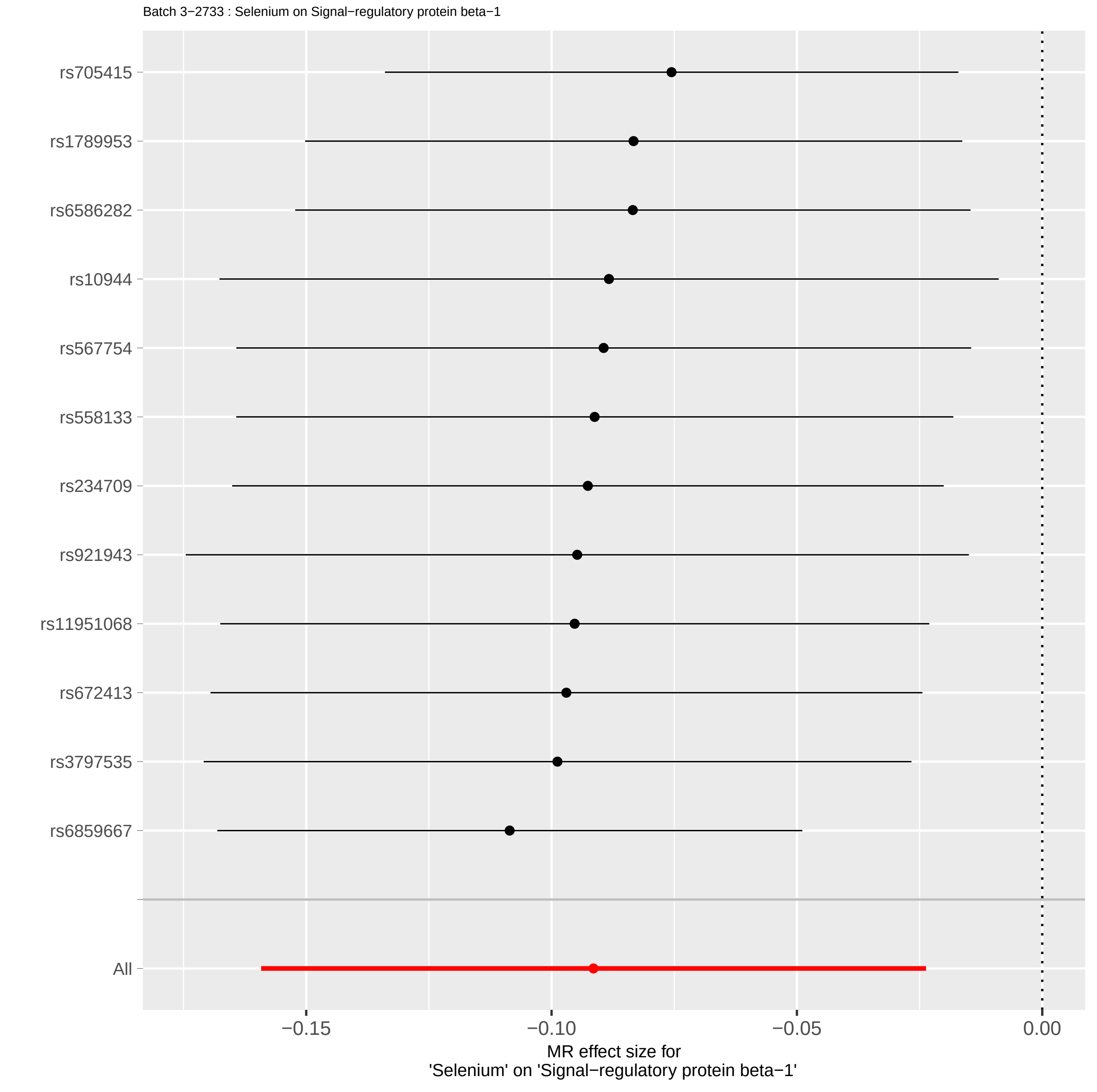

Batch 3−2733 : Selenium on Signal−regulatory protein beta−1
rs705415
rs1789953
rs6586282
rs10944
rs567754
rs558133
rs234709
rs921943
rs11951068
rs672413
rs3797535
rs6859667
All
−0.15
−0.10
−0.05
0.00
MR effect size for
'Selenium' on 'Signal−regulatory protein beta−1'

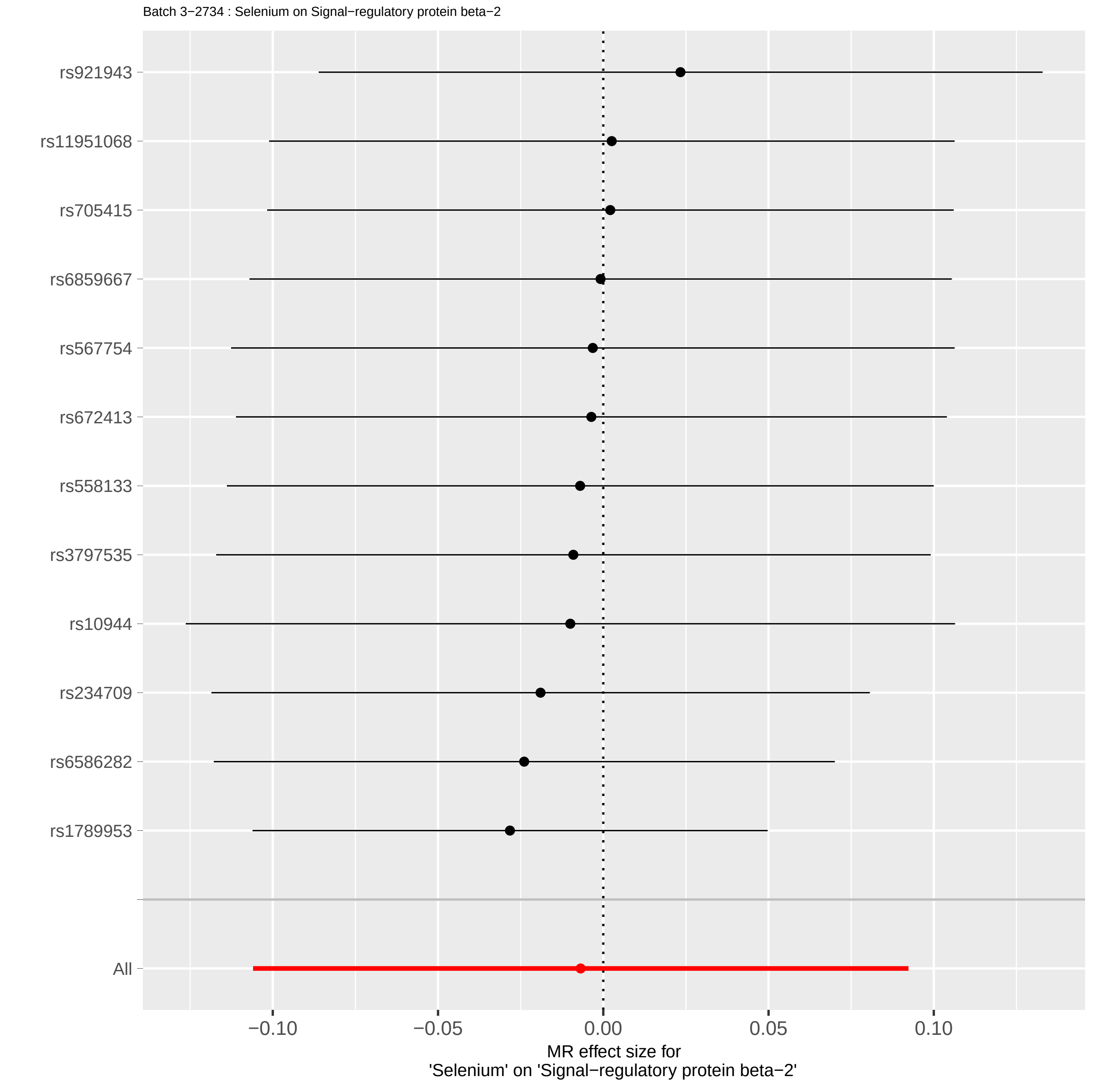

Batch 3−2734 : Selenium on Signal−regulatory protein beta−2
rs921943
rs11951068
rs705415
rs6859667
rs567754
rs672413
rs558133
rs3797535
rs10944
rs234709
rs6586282
rs1789953
All
−0.10
−0.05
0.00
0.05
0.10
MR effect size for
'Selenium' on 'Signal−regulatory protein beta−2'

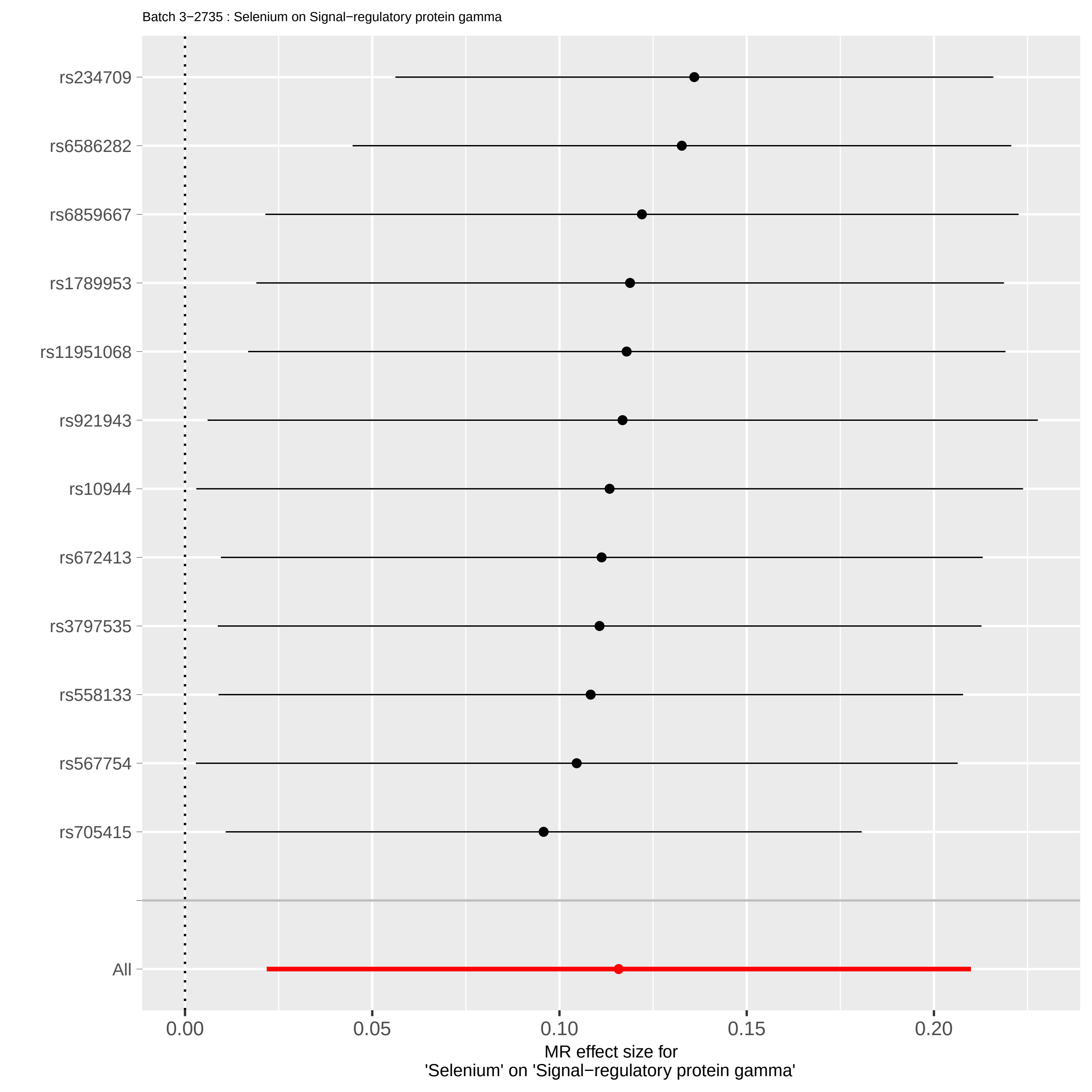
Batch 3−2735 : Selenium on Signal−regulatory protein gamma
rs234709
rs6586282
rs6859667
rs1789953
rs11951068
rs921943
rs10944
rs672413
rs3797535
rs558133
rs567754
rs705415
All
0.00
0.05
0.10
0.15
0.20
MR effect size for
'Selenium' on 'Signal−regulatory protein gamma'

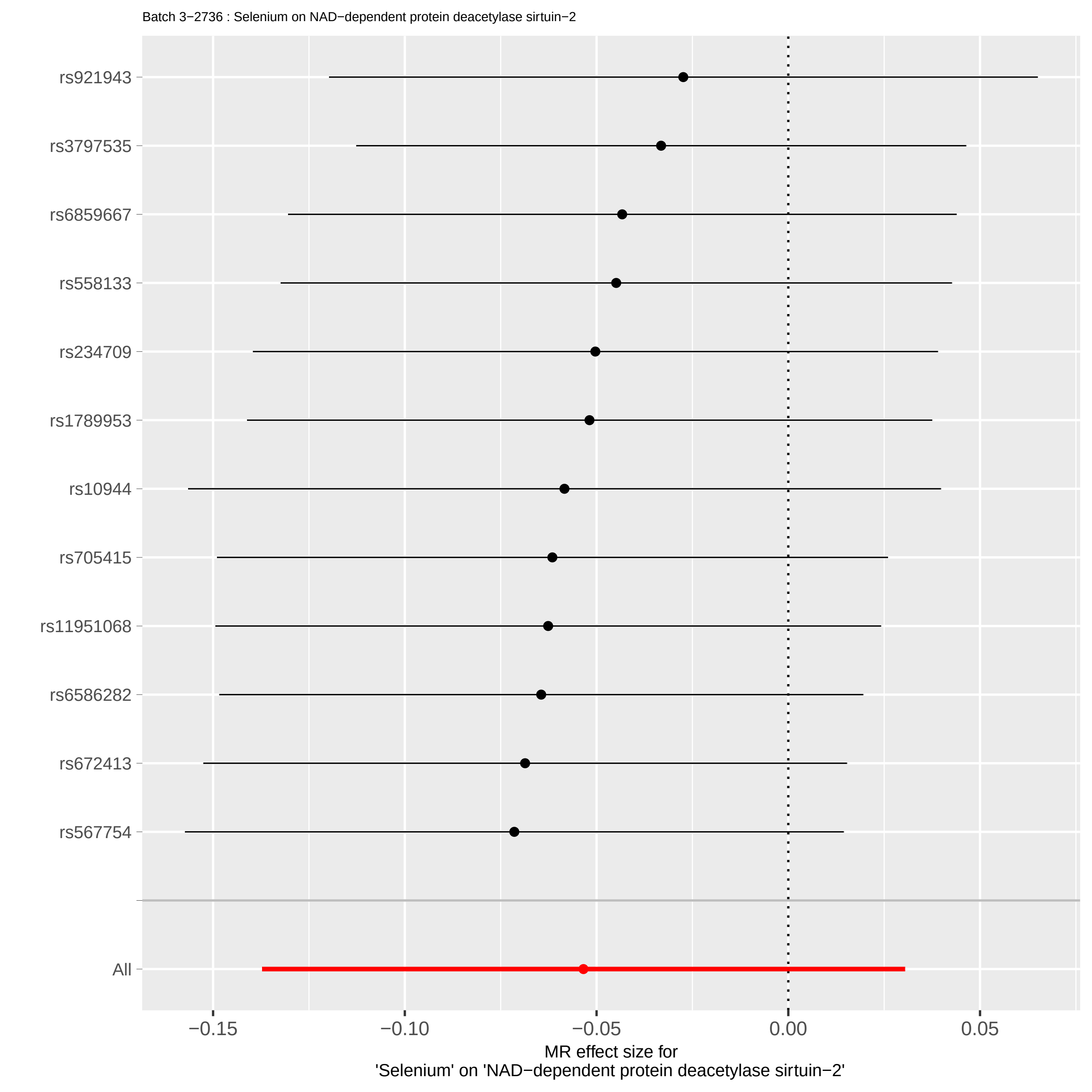

Batch 3−2736 : Selenium on NAD−dependent protein deacetylase sirtuin−2
rs921943
rs3797535
rs6859667
rs558133
rs234709
rs1789953
rs10944
rs705415
rs11951068
rs6586282
rs672413
rs567754
All
−0.15
−0.10
−0.05
0.00
0.05
MR effect size for
'Selenium' on 'NAD−dependent protein deacetylase sirtuin−2'

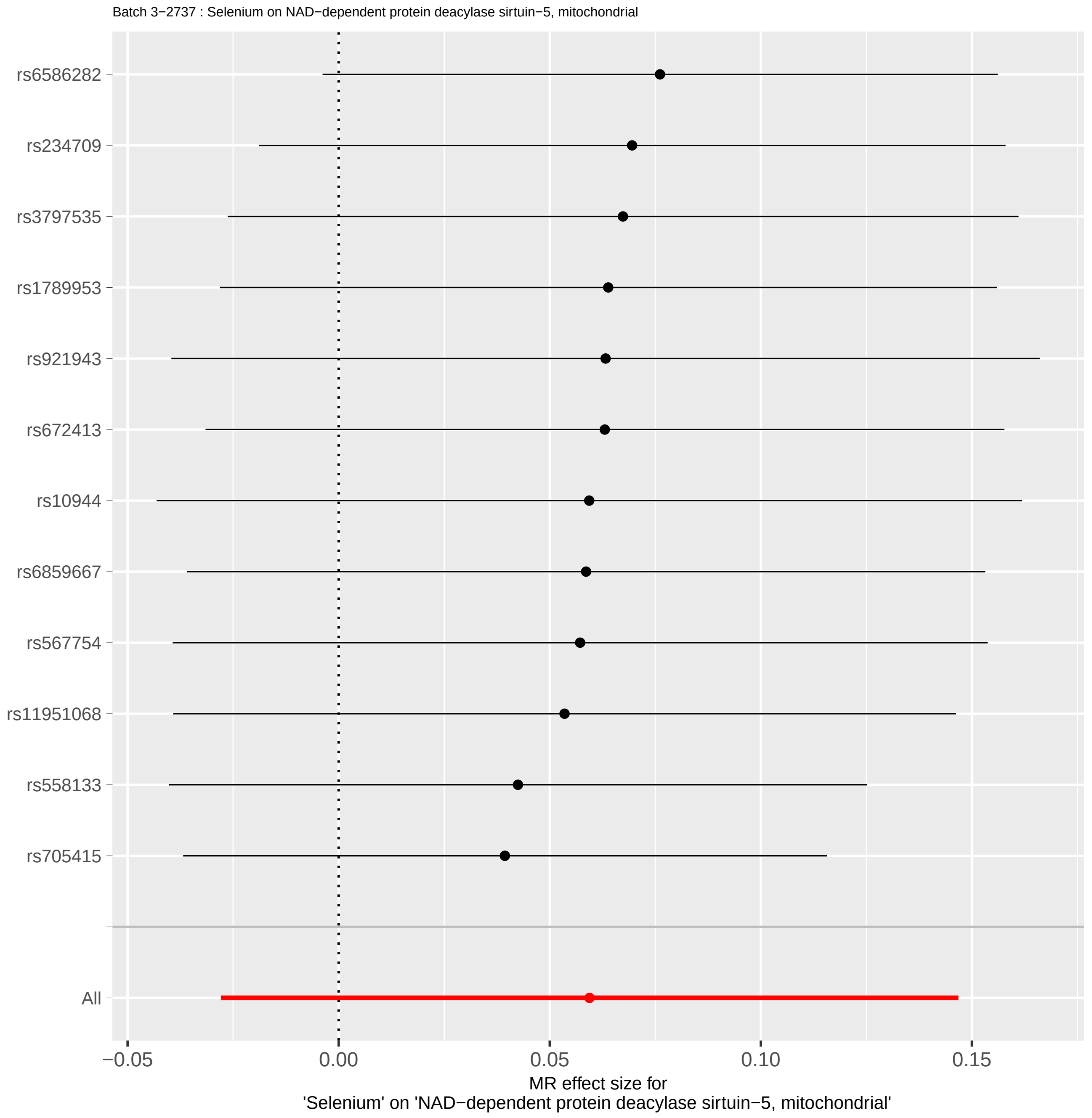

Batch 3−2737 : Selenium on NAD−dependent protein deacylase sirtuin−5, mitochondrial
rs6586282
rs234709
rs3797535
rs1789953
rs921943
rs672413
rs10944
rs6859667
rs567754
rs11951068
rs558133
rs705415
All
−0.05
0.00
0.05
0.10
0.15
MR effect size for
'Selenium' on 'NAD−dependent protein deacylase sirtuin−5, mitochondrial'

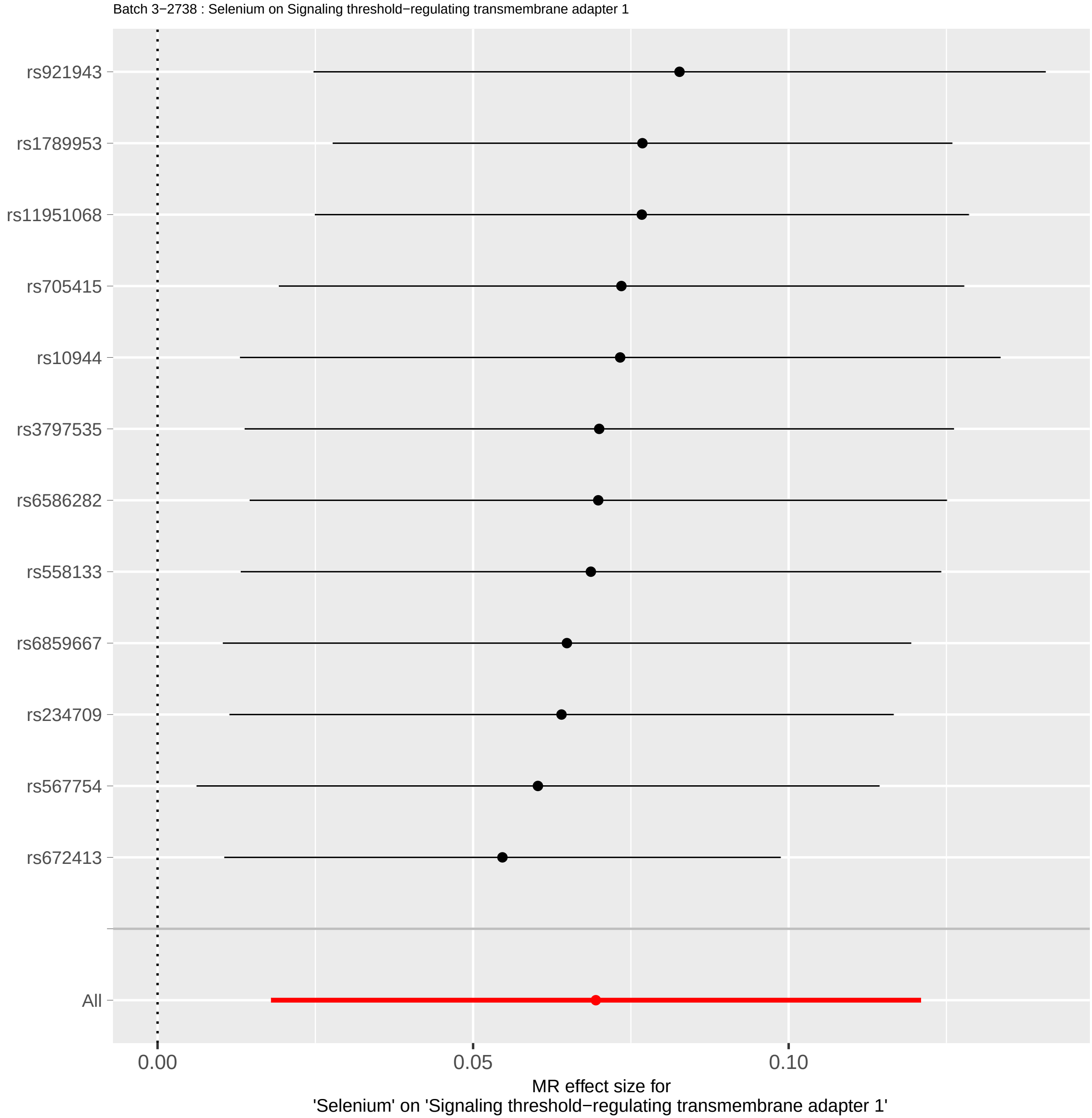

Batch 3−2738 : Selenium on Signaling threshold−regulating transmembrane adapter 1
rs921943
rs1789953
rs11951068
rs705415
rs10944
rs3797535
rs6586282
rs558133
rs6859667
rs234709
rs567754
rs672413
All
0.00
0.05
0.10
MR effect size for
'Selenium' on 'Signaling threshold−regulating transmembrane adapter 1'

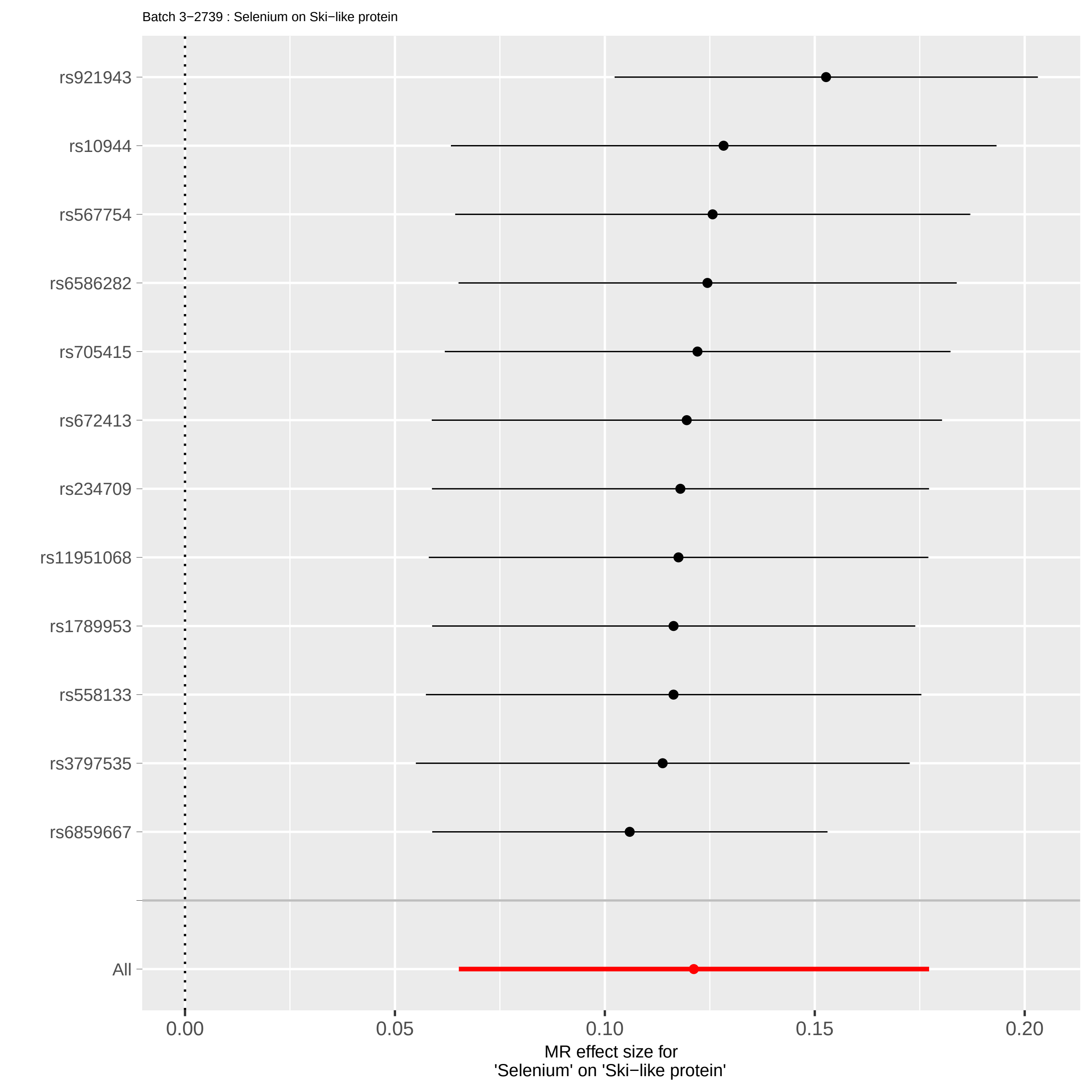

Batch 3−2739 : Selenium on Ski−like protein
rs921943
rs10944
rs567754
rs6586282
rs705415
rs672413
rs234709
rs11951068
rs1789953
rs558133
rs3797535
rs6859667
All
0.00
0.05
0.10
0.15
0.20
MR effect size for
'Selenium' on 'Ski−like protein'

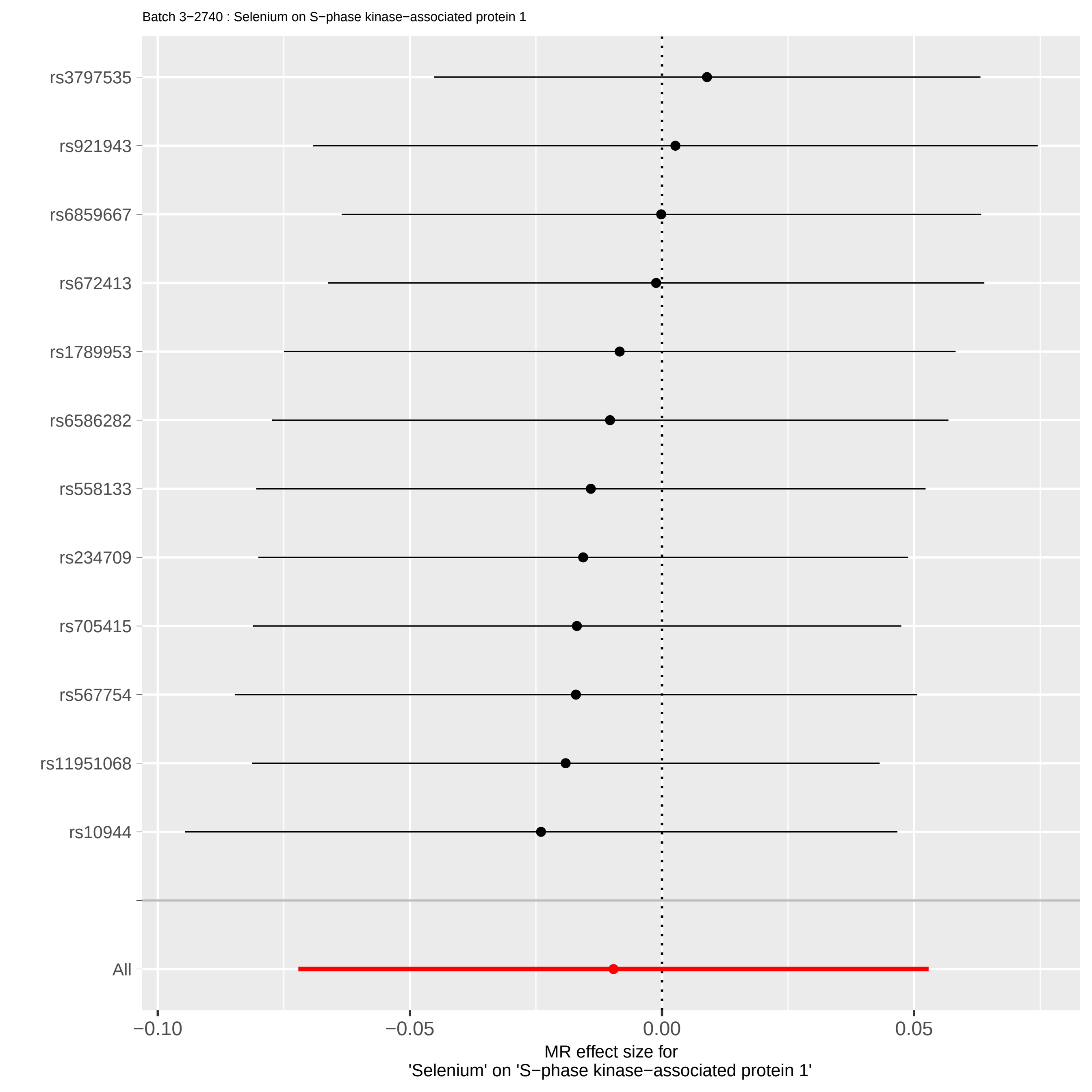

Batch 3−2740 : Selenium on S−phase kinase−associated protein 1
rs3797535
rs921943
rs6859667
rs672413
rs1789953
rs6586282
rs558133
rs234709
rs705415
rs567754
rs11951068
rs10944
All
−0.10
−0.05
0.00
0.05
MR effect size for
'Selenium' on 'S−phase kinase−associated protein 1'

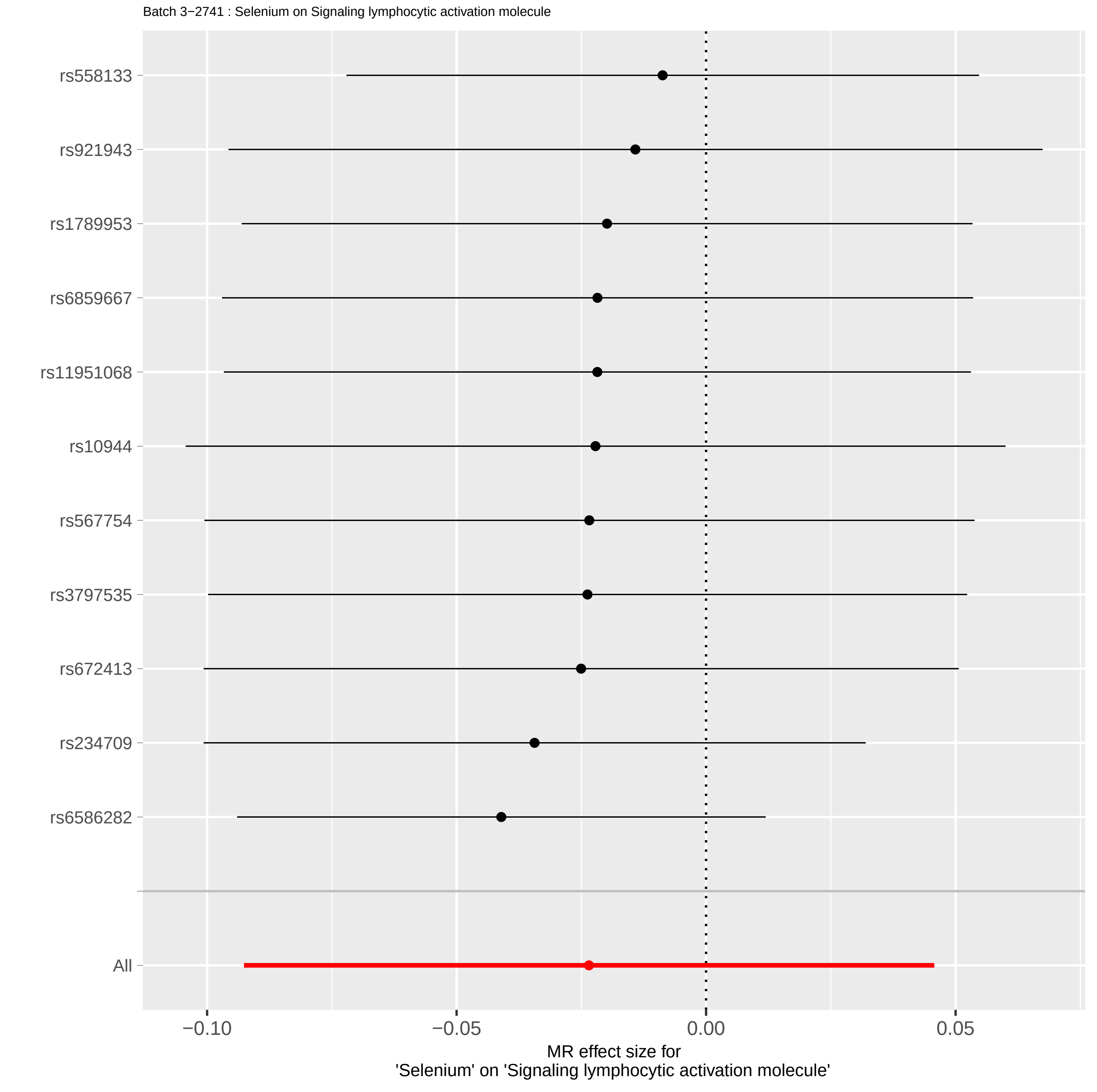

Batch 3−2741 : Selenium on Signaling lymphocytic activation molecule
rs558133
rs921943
rs1789953
rs6859667
rs11951068
rs10944
rs567754
rs3797535
rs672413
rs234709
rs6586282
All
−0.10
−0.05
0.00
0.05
MR effect size for
'Selenium' on 'Signaling lymphocytic activation molecule'

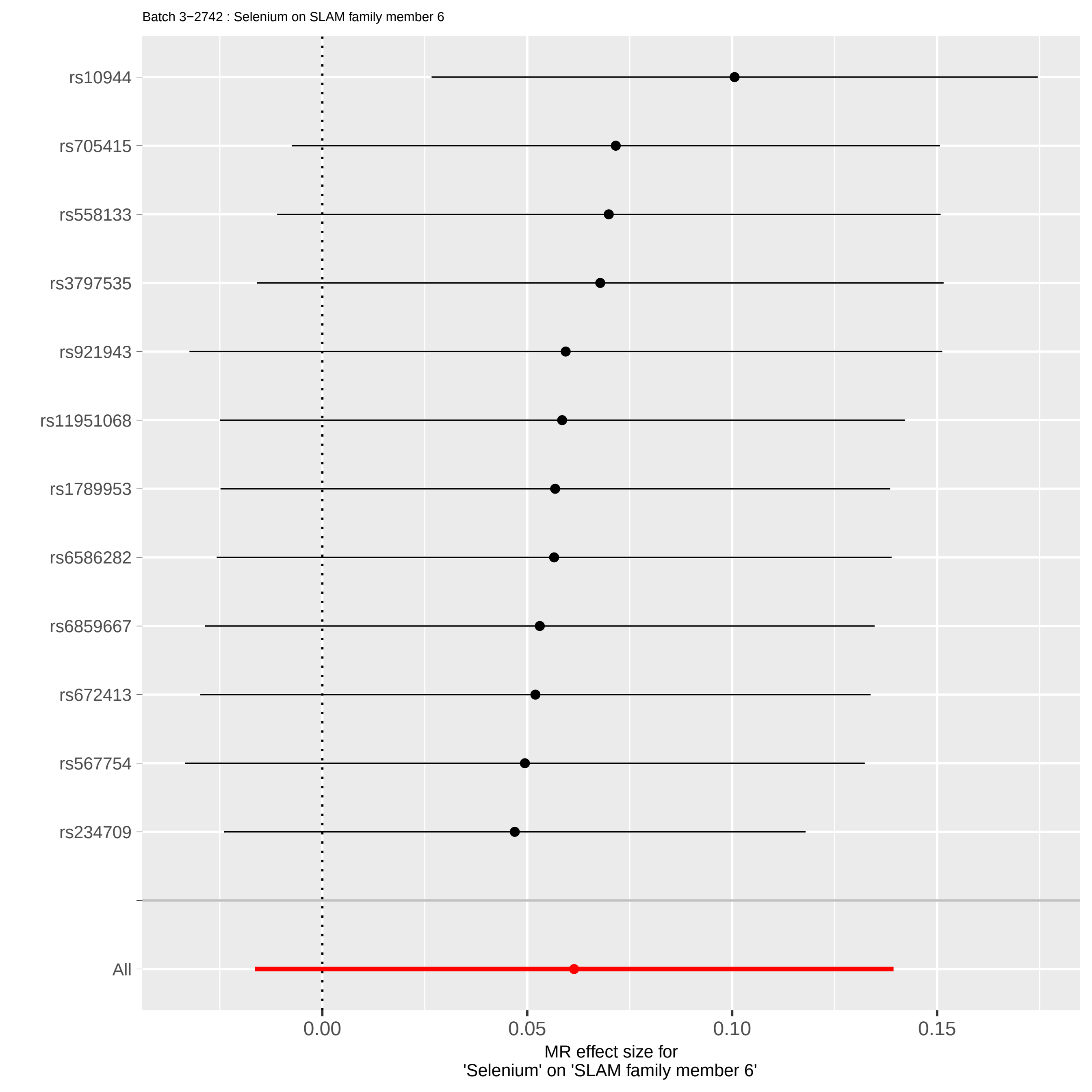
Batch 3−2742 : Selenium on SLAM family member 6
rs10944
rs705415
rs558133
rs3797535
rs921943
rs11951068
rs1789953
rs6586282
rs6859667
rs672413
rs567754
rs234709
All
0.00
0.05
0.10
0.15
MR effect size for
'Selenium' on 'SLAM family member 6'

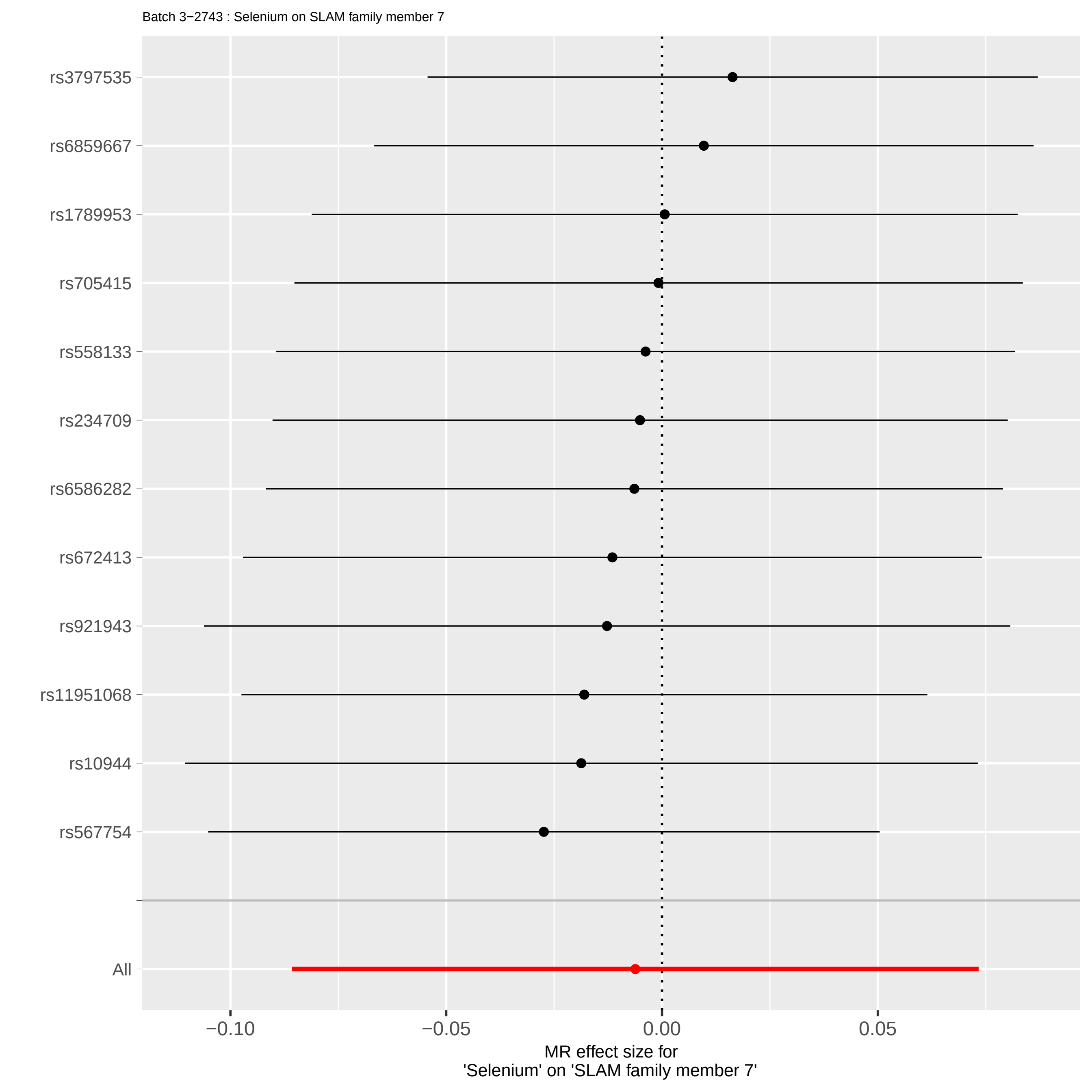

Batch 3−2743 : Selenium on SLAM family member 7
rs3797535
rs6859667
rs1789953
rs705415
rs558133
rs234709
rs6586282
rs672413
rs921943
rs11951068
rs10944
rs567754
All
−0.10
−0.05
0.00
0.05
MR effect size for
'Selenium' on 'SLAM family member 7'

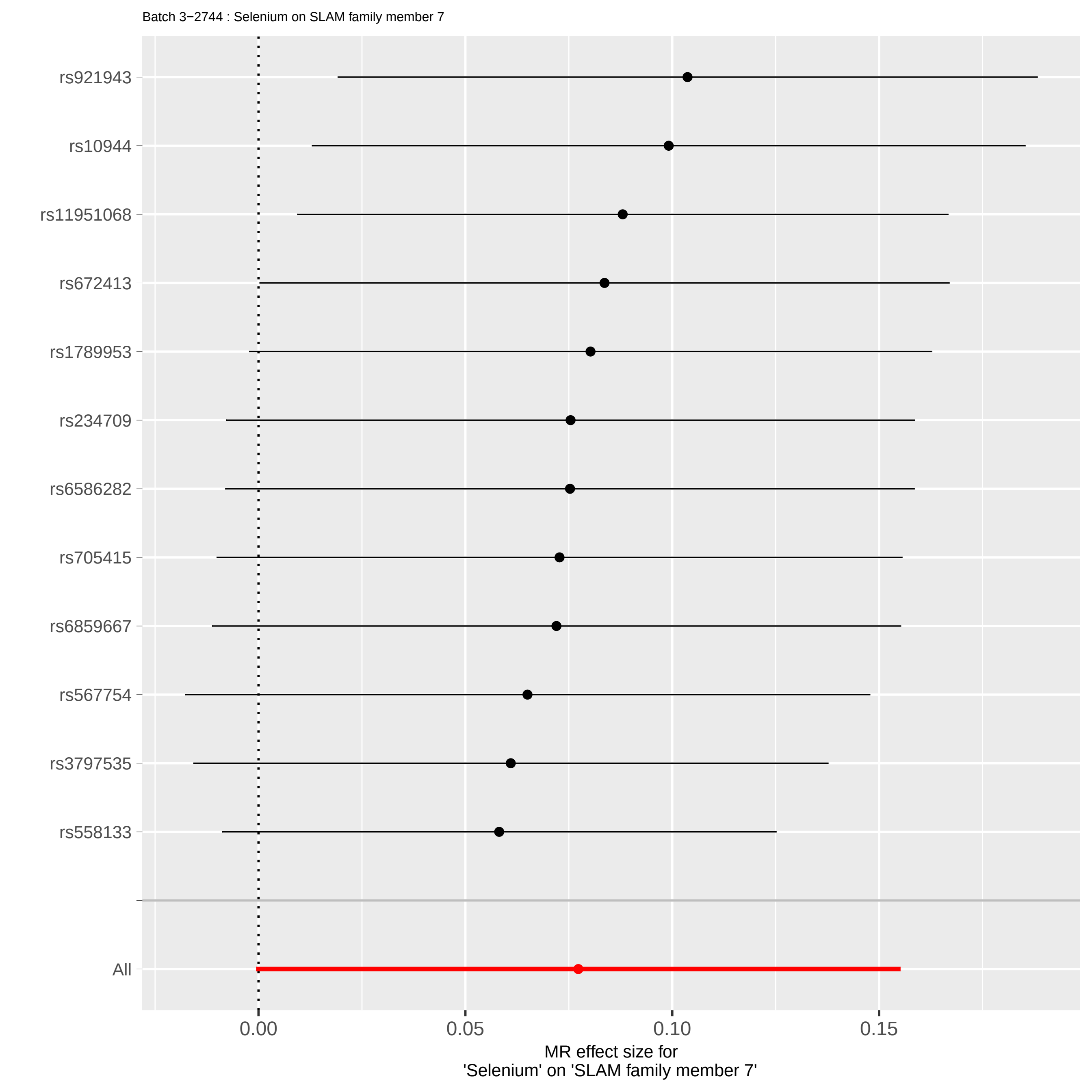

Batch 3−2744 : Selenium on SLAM family member 7
rs921943
rs10944
rs11951068
rs672413
rs1789953
rs234709
rs6586282
rs705415
rs6859667
rs567754
rs3797535
rs558133
All
0.00
0.05
0.10
0.15
MR effect size for
'Selenium' on 'SLAM family member 7'

Batch 3−2745 : Selenium on SLAM family member 8

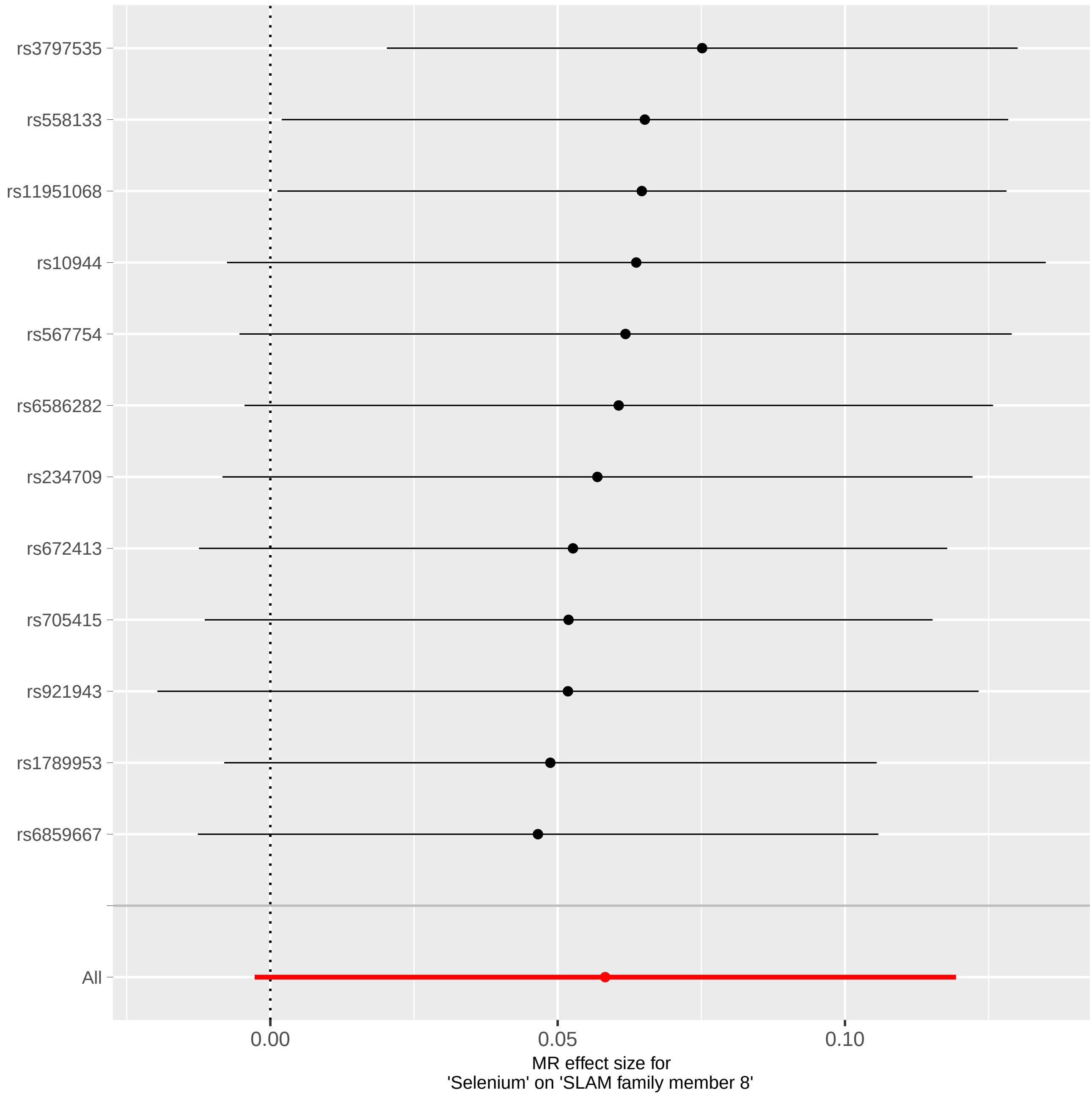

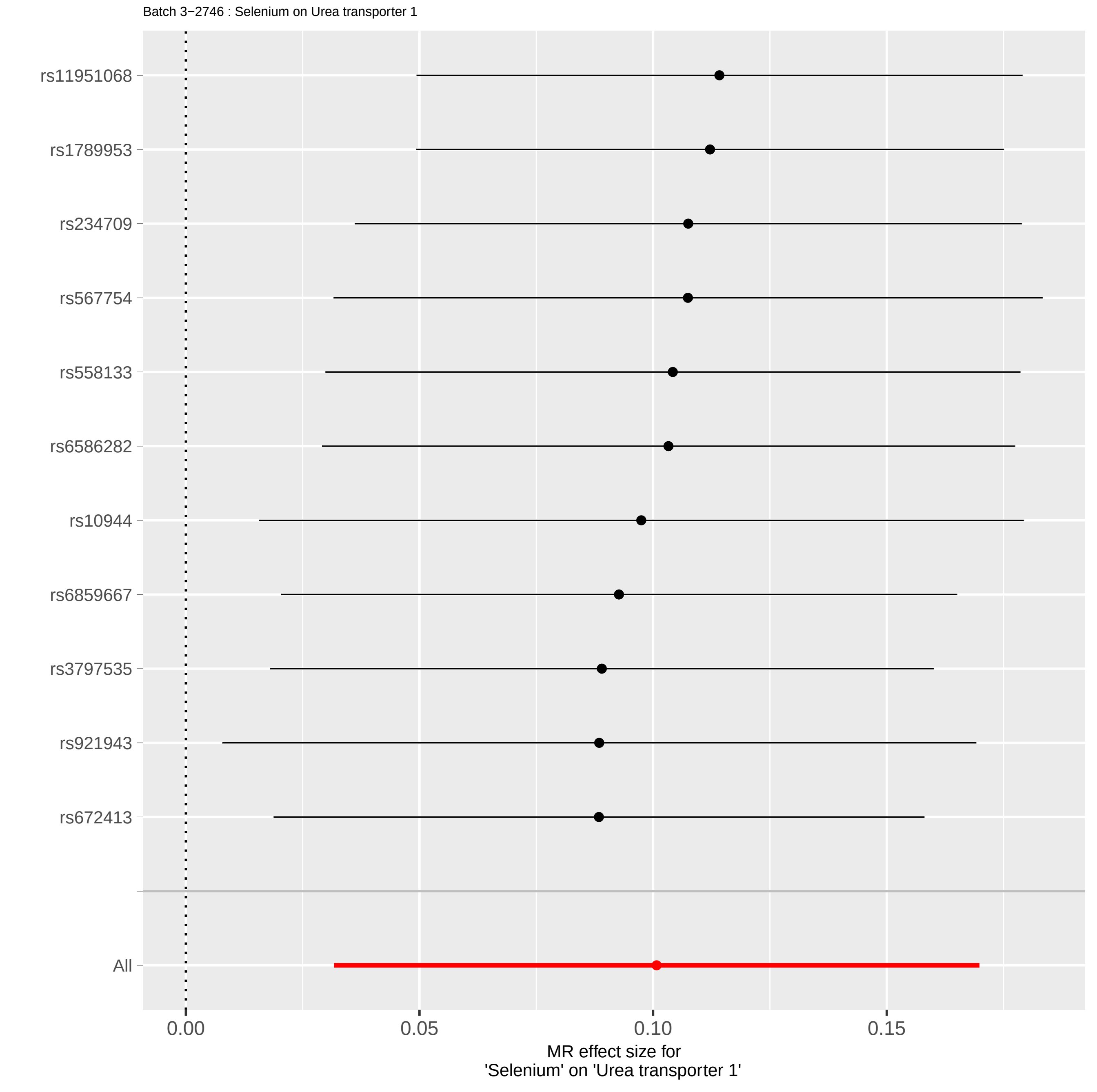

Batch 3−2746 : Selenium on Urea transporter 1
rs11951068
rs1789953
rs234709
rs567754
rs558133
rs6586282
rs10944
rs6859667
rs3797535
rs921943
rs672413
All
0.00
0.05
0.10
0.15
MR effect size for
'Selenium' on 'Urea transporter 1'

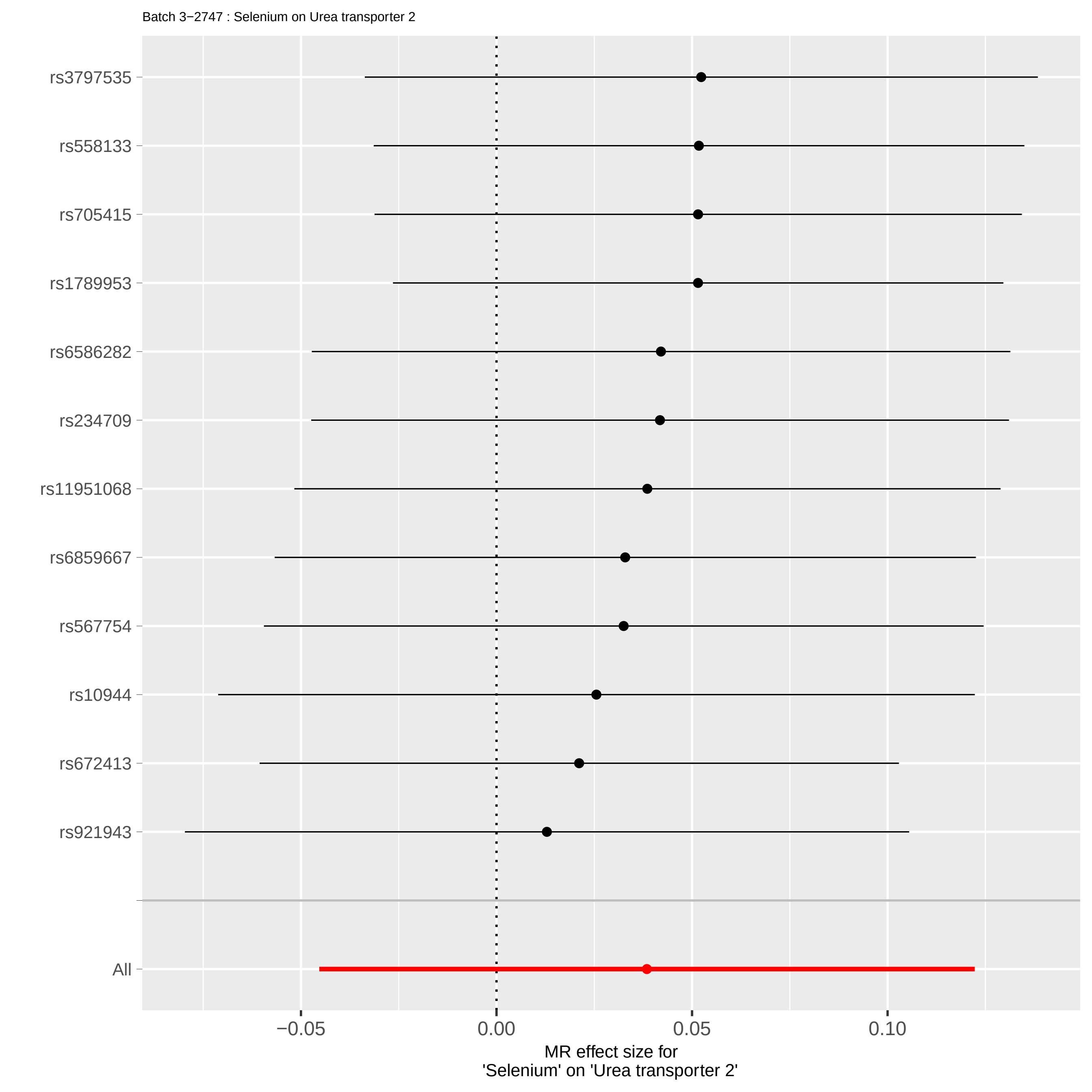

Batch 3−2747 : Selenium on Urea transporter 2
rs3797535
rs558133
rs705415
rs1789953
rs6586282
rs234709
rs11951068
rs6859667
rs567754
rs10944
rs672413
rs921943
All
−0.05
0.00
0.05
0.10
MR effect size for
'Selenium' on 'Urea transporter 2'

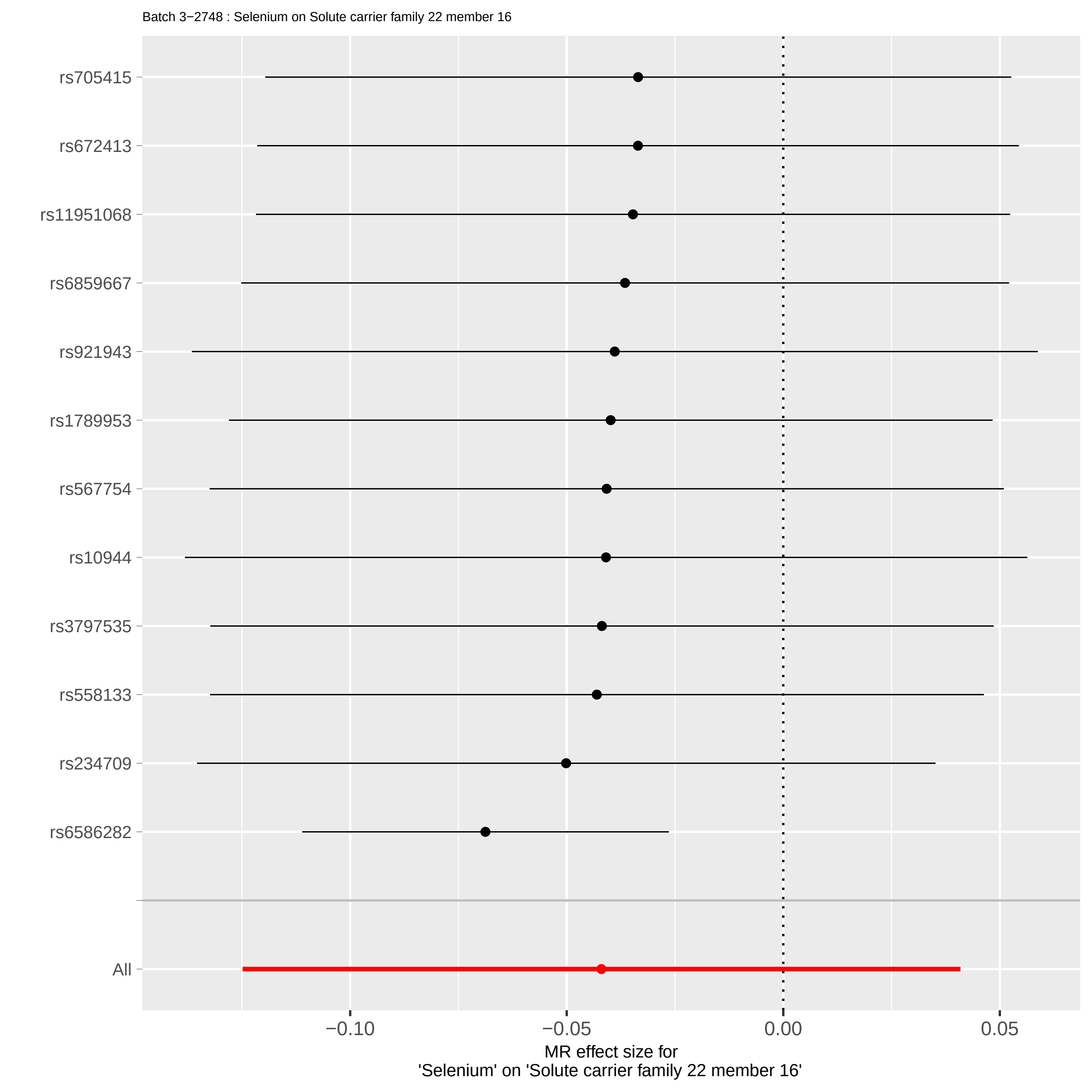

Batch 3−2748 : Selenium on Solute carrier family 22 member 16
rs705415
rs672413
rs11951068
rs6859667
rs921943
rs1789953
rs567754
rs10944
rs3797535
rs558133
rs234709
rs6586282
All
−0.10
−0.05
0.00
0.05
MR effect size for
'Selenium' on 'Solute carrier family 22 member 16'

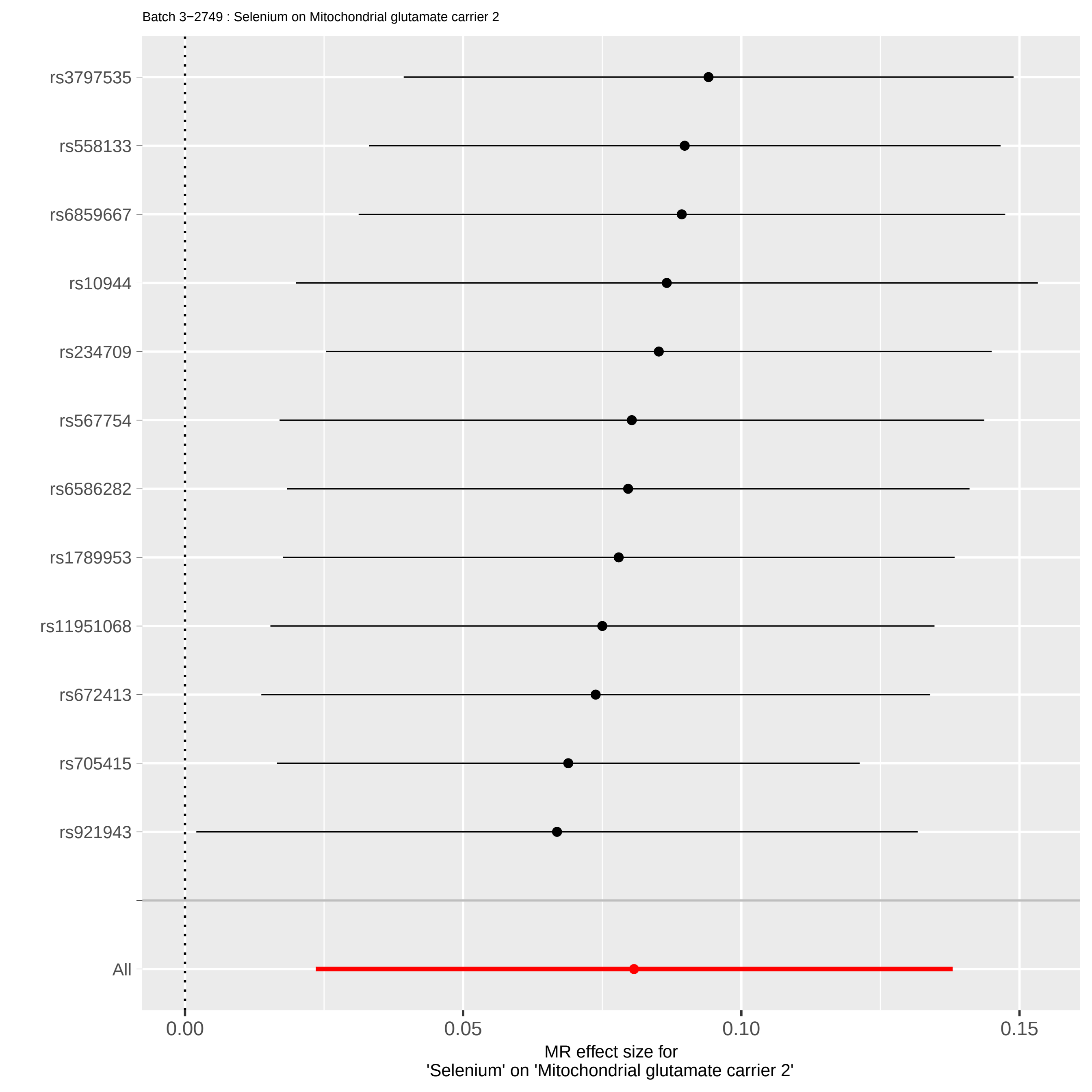

Batch 3−2749 : Selenium on Mitochondrial glutamate carrier 2
rs3797535
rs558133
rs6859667
rs10944
rs234709
rs567754
rs6586282
rs1789953
rs11951068
rs672413
rs705415
rs921943
All
0.00
0.05
0.10
0.15
MR effect size for
'Selenium' on 'Mitochondrial glutamate carrier 2'

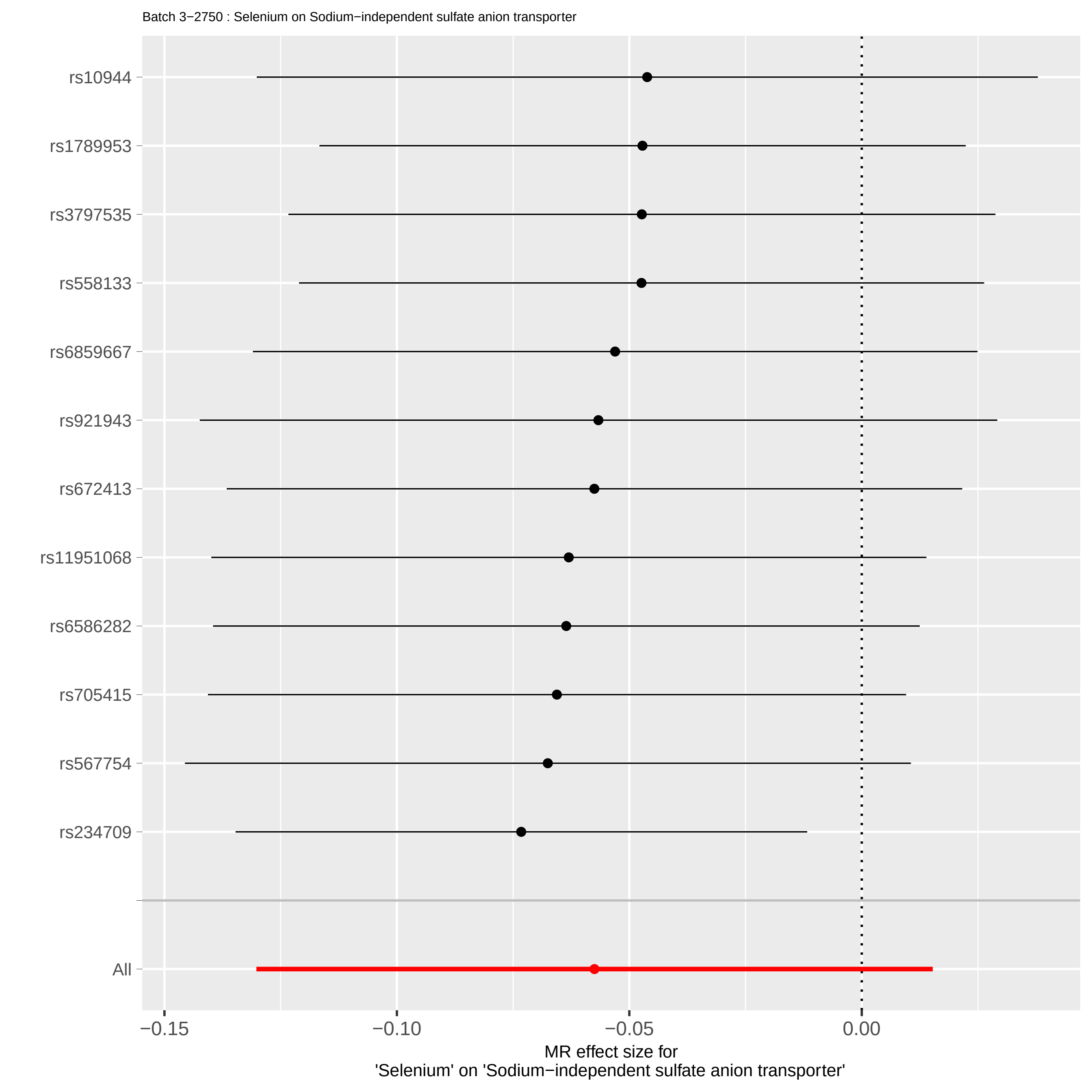

Batch 3−2750 : Selenium on Sodium−independent sulfate anion transporter
rs10944
rs1789953
rs3797535
rs558133
rs6859667
rs921943
rs672413
rs11951068
rs6586282
rs705415
rs567754
rs234709
All
−0.15
−0.10
−0.05
0.00
MR effect size for
'Selenium' on 'Sodium−independent sulfate anion transporter'

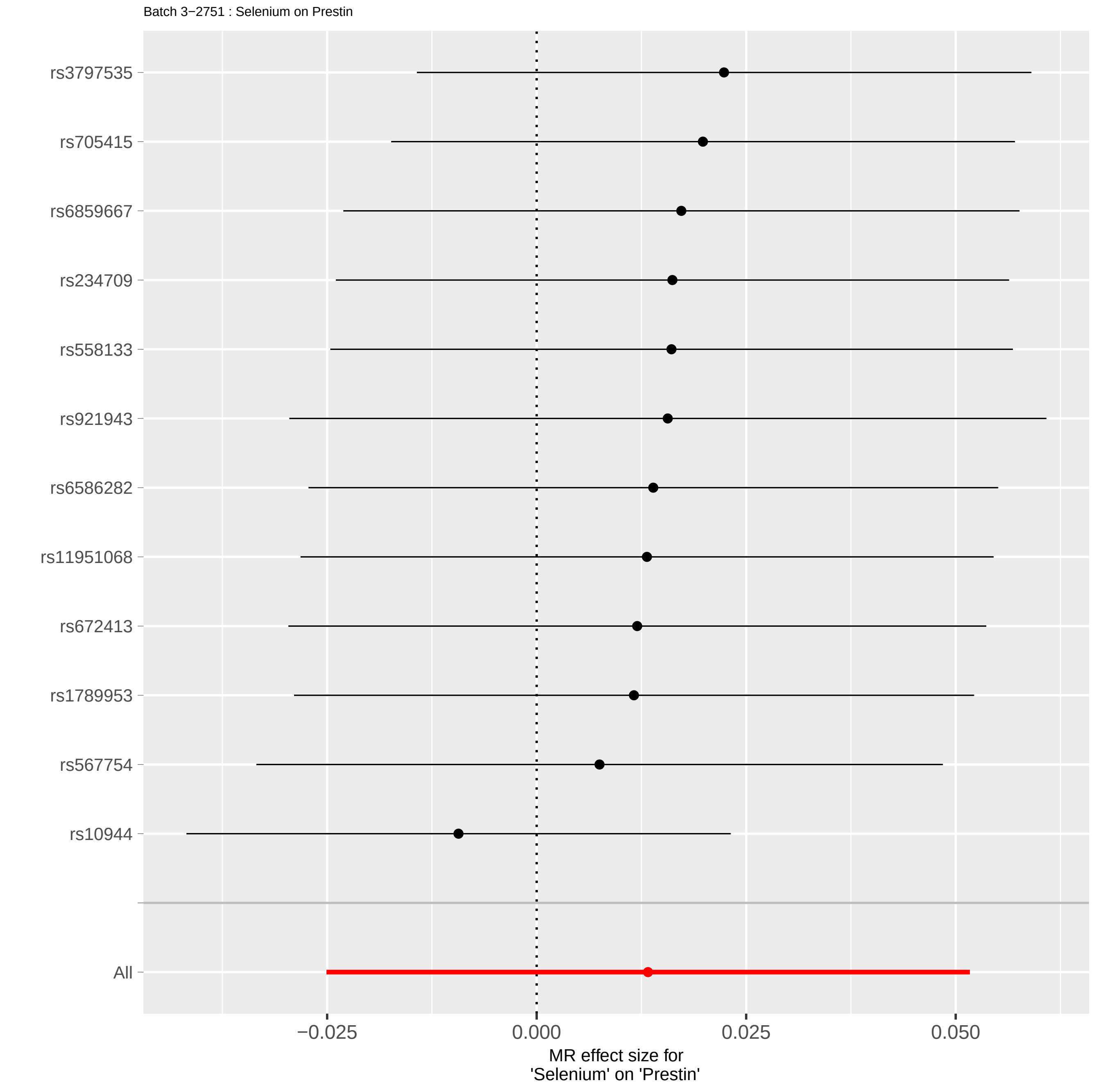

Batch 3−2751 : Selenium on Prestin
rs3797535
rs705415
rs6859667
rs234709
rs558133
rs921943
rs6586282
rs11951068
rs672413
rs1789953
rs567754
rs10944
All
−0.025
0.000
0.025
0.050
MR effect size for
'Selenium' on 'Prestin'

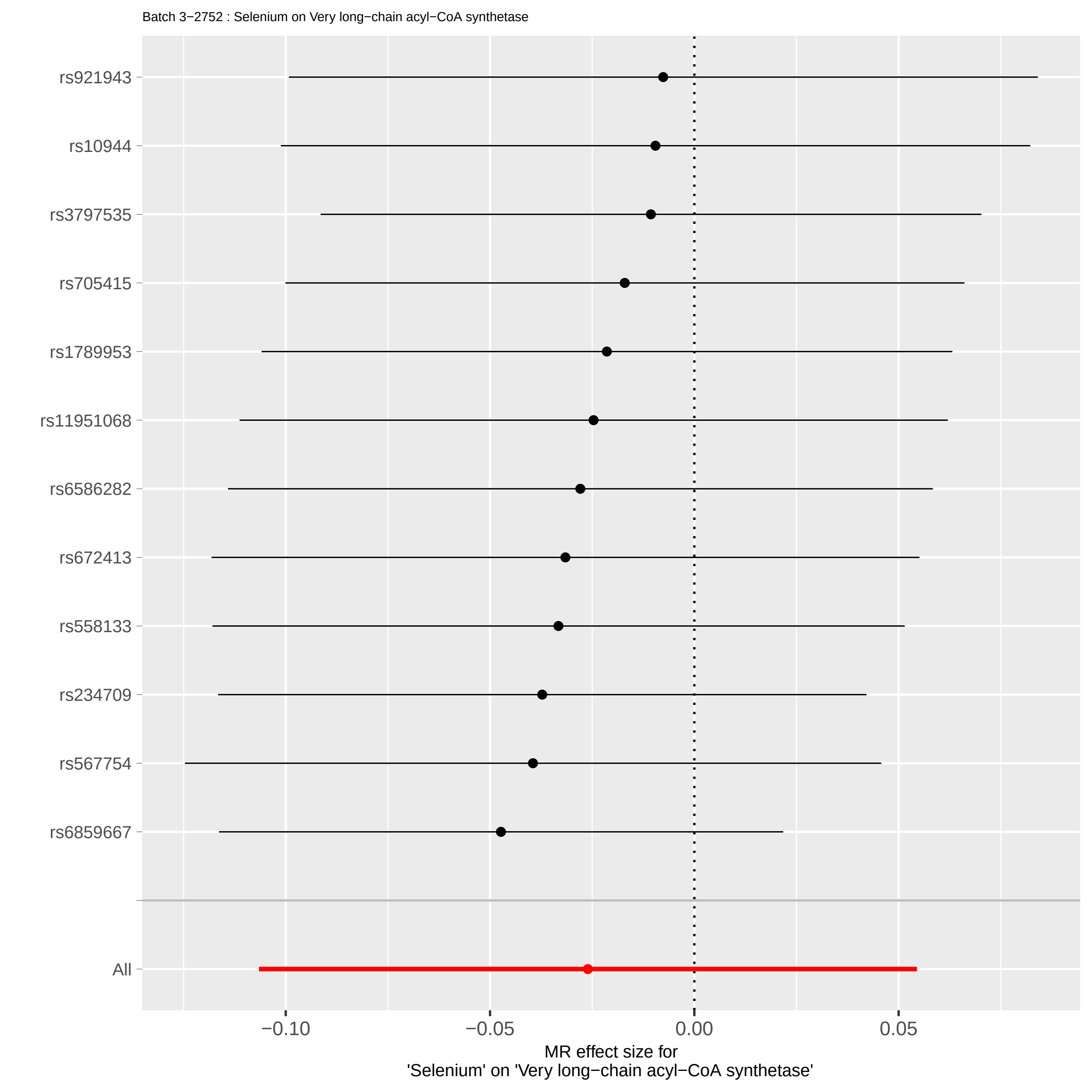
Batch 3−2752 : Selenium on Very long−chain acyl−CoA synthetase
rs921943
rs10944
rs3797535
rs705415
rs1789953
rs11951068
rs6586282
rs672413
rs558133
rs234709
rs567754
rs6859667
All
−0.10
−0.05
0.00
0.05
MR effect size for
'Selenium' on 'Very long−chain acyl−CoA synthetase'

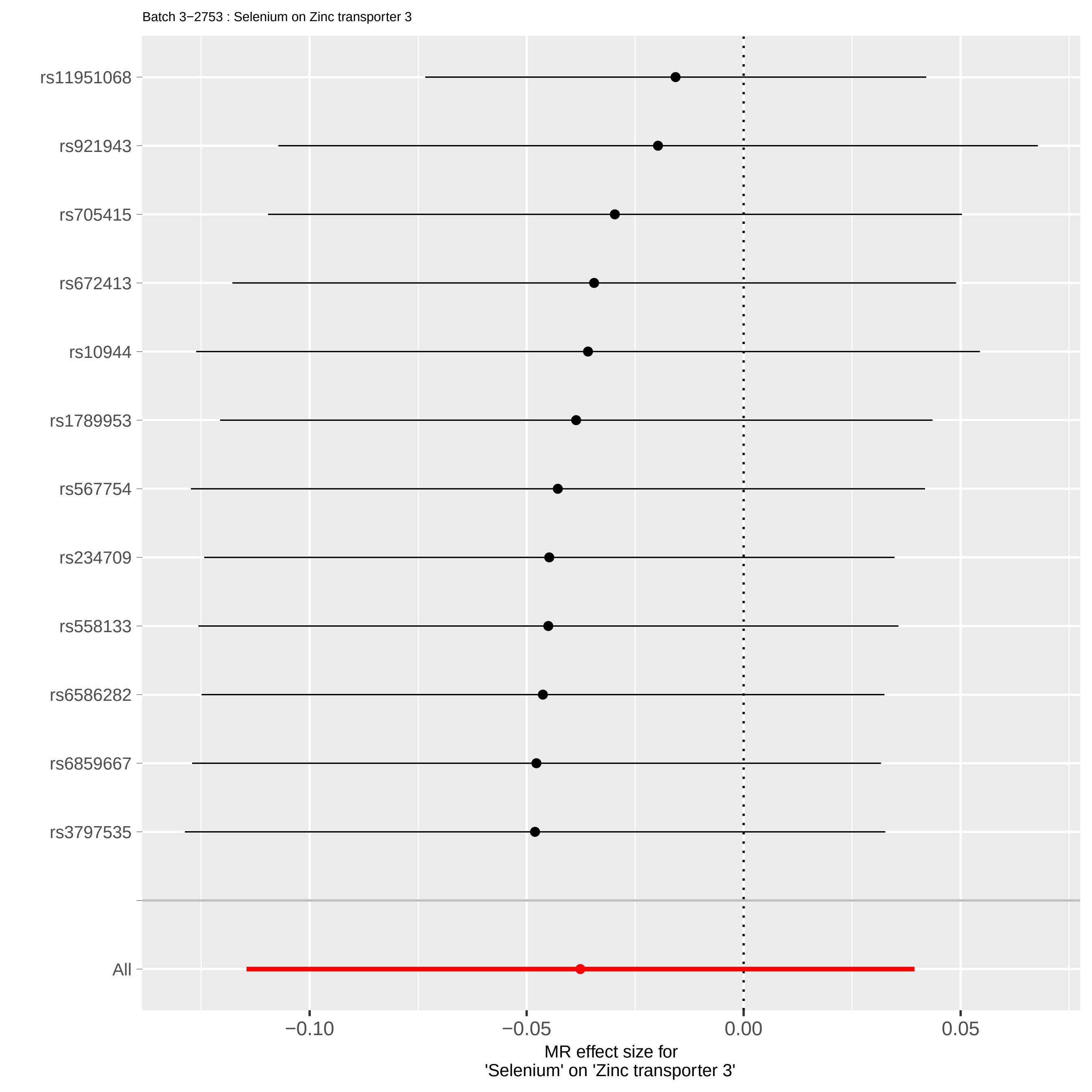

Batch 3−2753 : Selenium on Zinc transporter 3
rs11951068
rs921943
rs705415
rs672413
rs10944
rs1789953
rs567754
rs234709
rs558133
rs6586282
rs6859667
rs3797535
All
−0.10
−0.05
0.00
0.05
MR effect size for
'Selenium' on 'Zinc transporter 3'

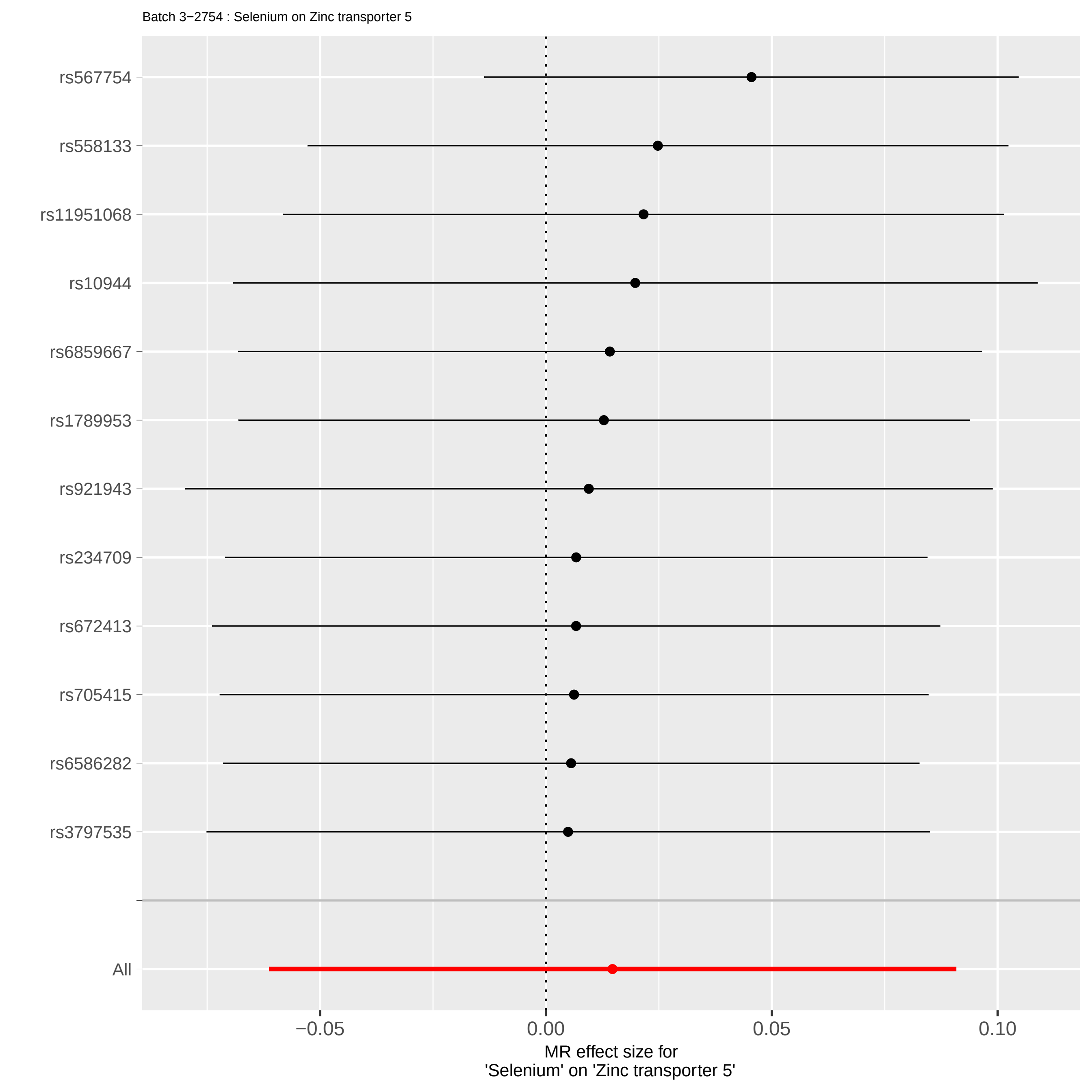

Batch 3−2754 : Selenium on Zinc transporter 5
rs567754
rs558133
rs11951068
rs10944
rs6859667
rs1789953
rs921943
rs234709
rs672413
rs705415
rs6586282
rs3797535
All
−0.05
0.00
0.05
0.10
MR effect size for
'Selenium' on 'Zinc transporter 5'

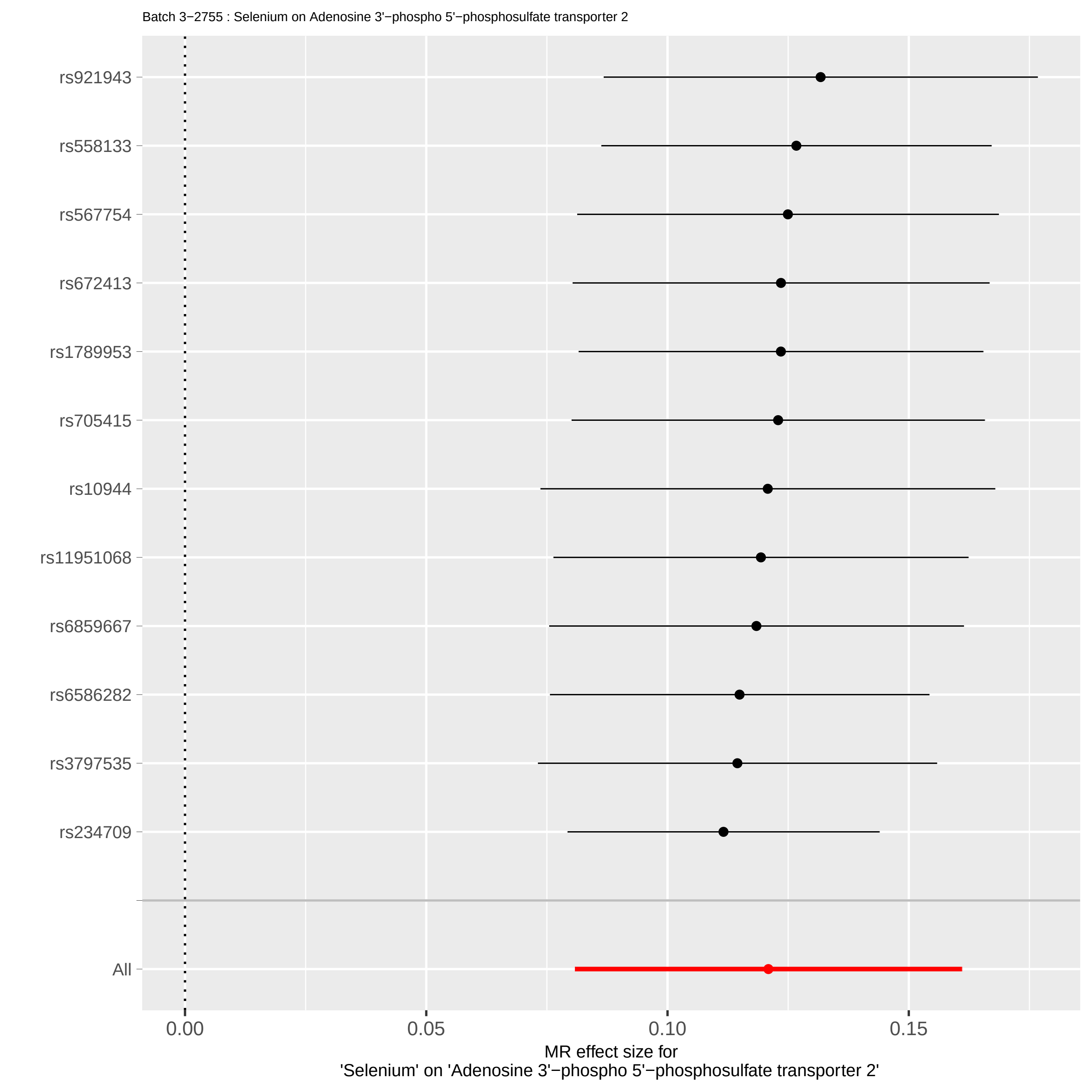

Batch 3−2755 : Selenium on Adenosine 3'−phospho 5'−phosphosulfate transporter 2
rs921943
rs558133
rs567754
rs672413
rs1789953
rs705415
rs10944
rs11951068
rs6859667
rs6586282
rs3797535
rs234709
All
0.00
0.05
0.10
0.15
MR effect size for
'Selenium' on 'Adenosine 3'−phospho 5'−phosphosulfate transporter 2'

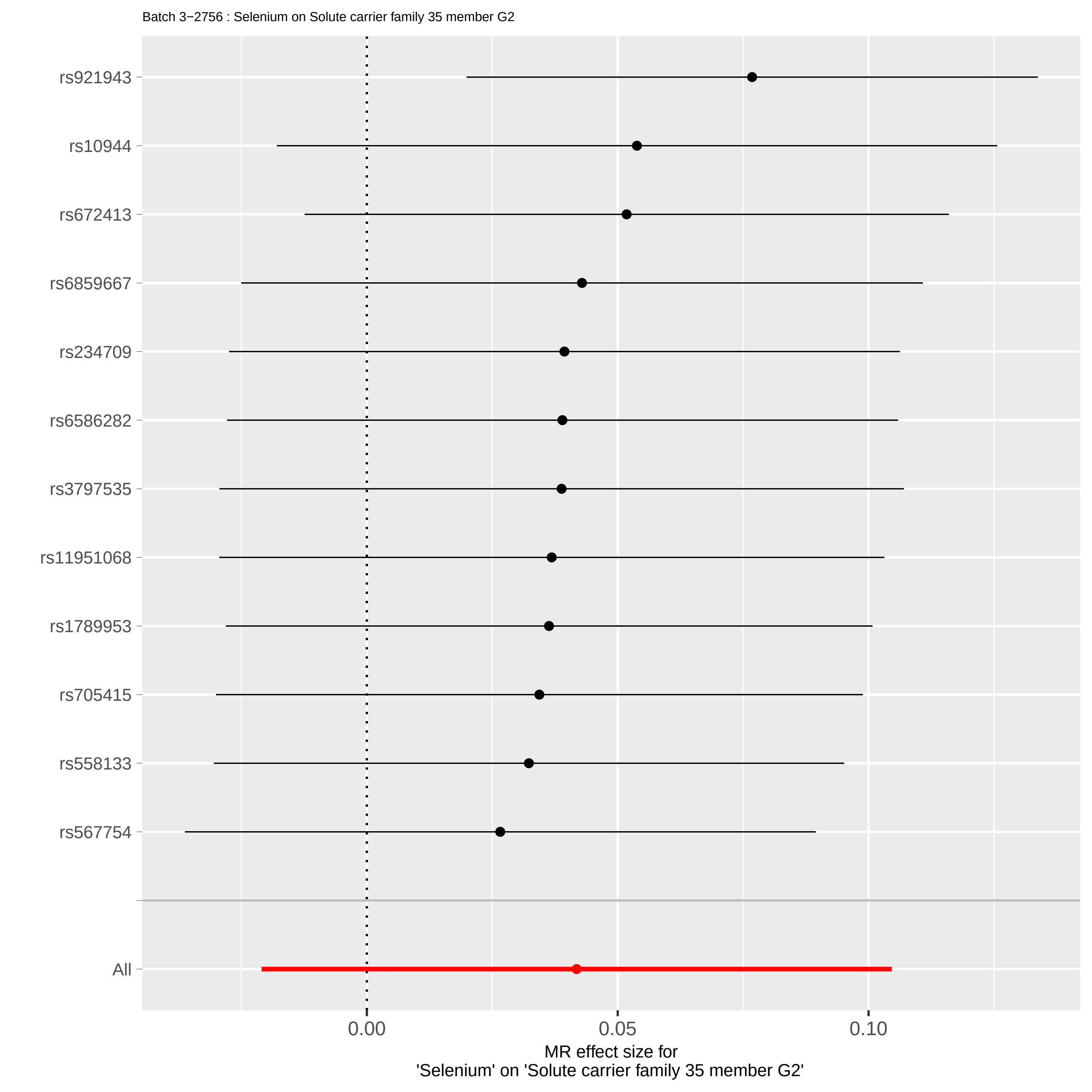

Batch 3−2756 : Selenium on Solute carrier family 35 member G2
rs921943
rs10944
rs672413
rs6859667
rs234709
rs6586282
rs3797535
rs11951068
rs1789953
rs705415
rs558133
rs567754
All
0.00
0.05
0.10
MR effect size for
'Selenium' on 'Solute carrier family 35 member G2'

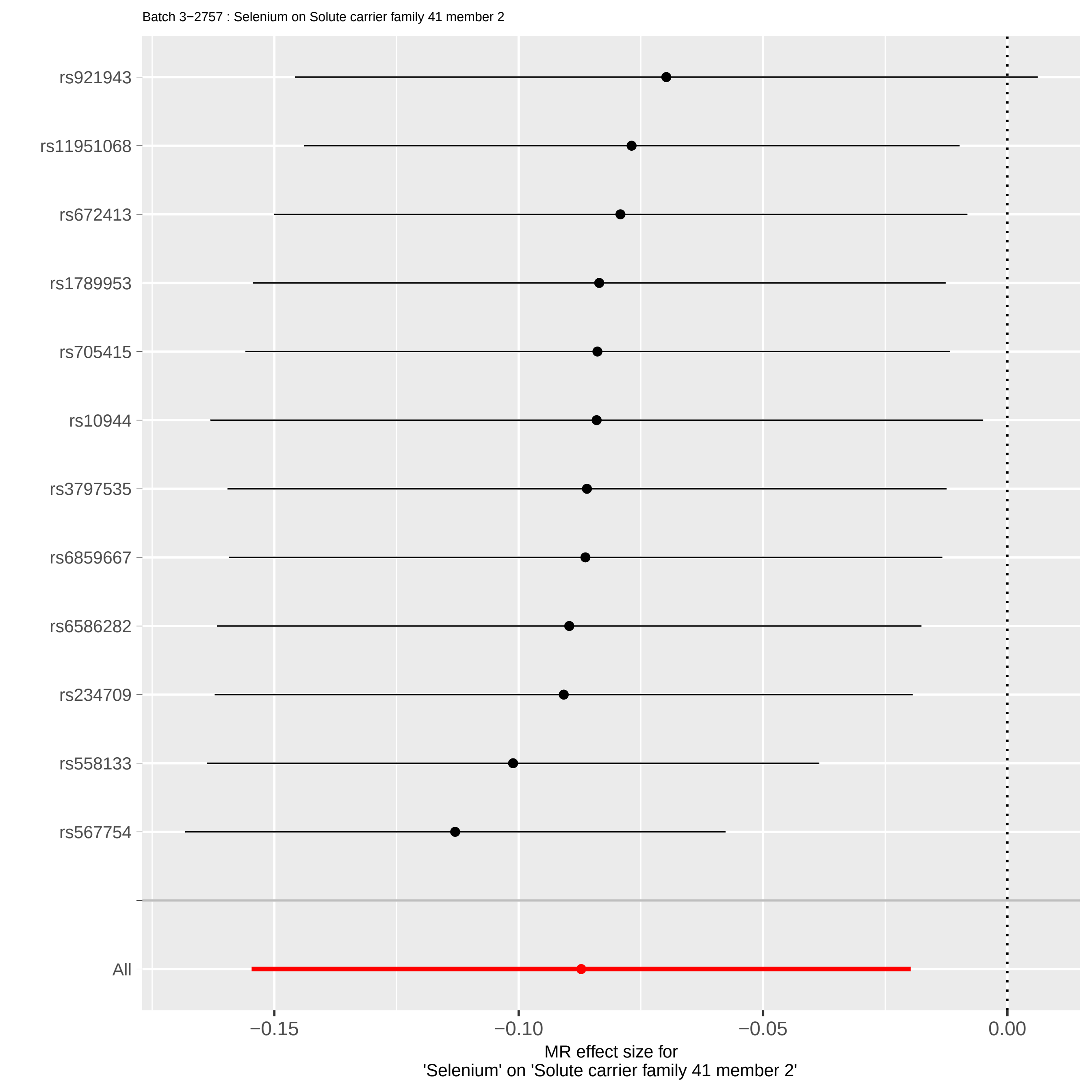

Batch 3−2757 : Selenium on Solute carrier family 41 member 2
rs921943
rs11951068
rs672413
rs1789953
rs705415
rs10944
rs3797535
rs6859667
rs6586282
rs234709
rs558133
rs567754
All
−0.15
−0.10
−0.05
0.00
MR effect size for
'Selenium' on 'Solute carrier family 41 member 2'

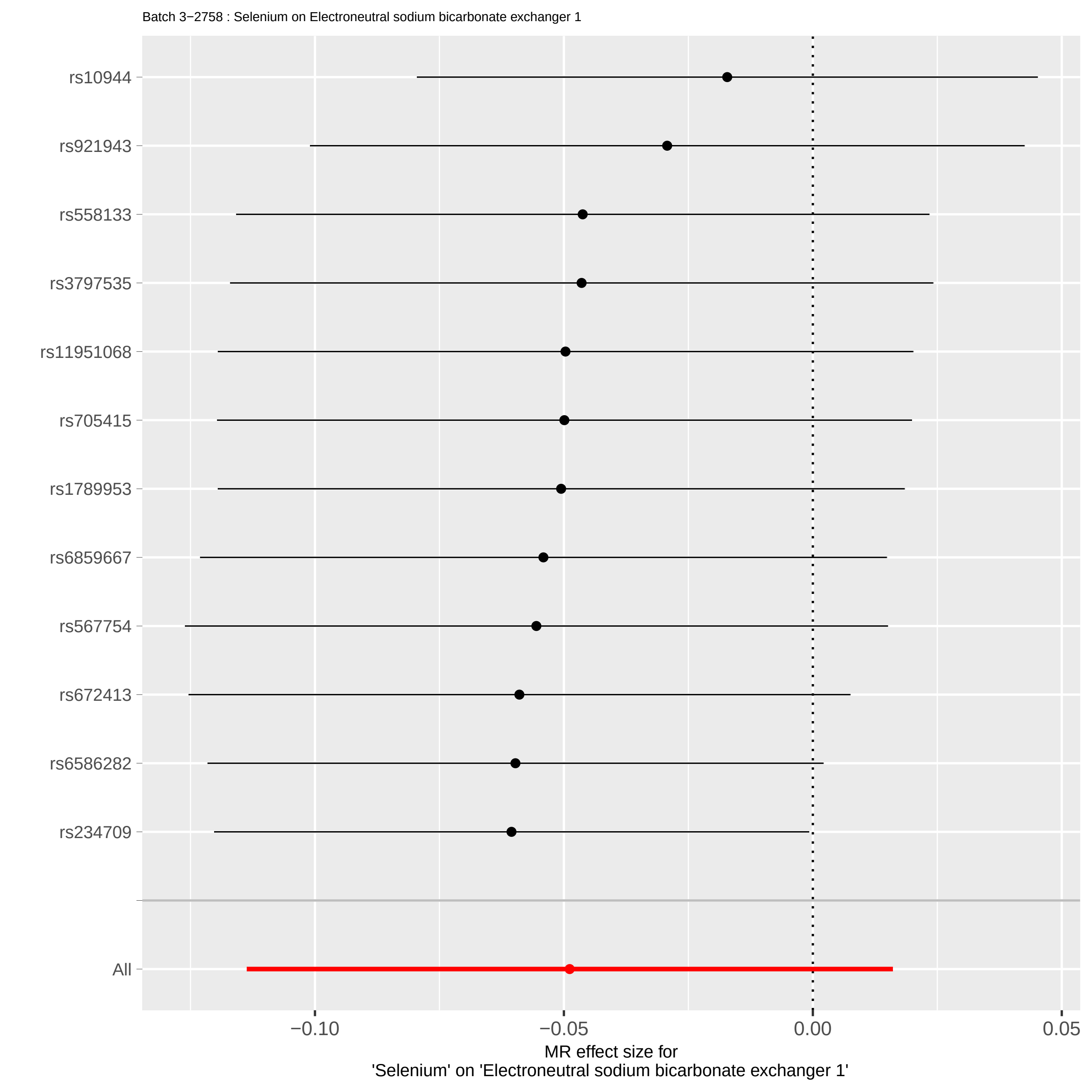
Batch 3−2758 : Selenium on Electroneutral sodium bicarbonate exchanger 1
rs10944
rs921943
rs558133
rs3797535
rs11951068
rs705415
rs1789953
rs6859667
rs567754
rs672413
rs6586282
rs234709
All
−0.10
−0.05
0.00
0.05
MR effect size for
'Selenium' on 'Electroneutral sodium bicarbonate exchanger 1'

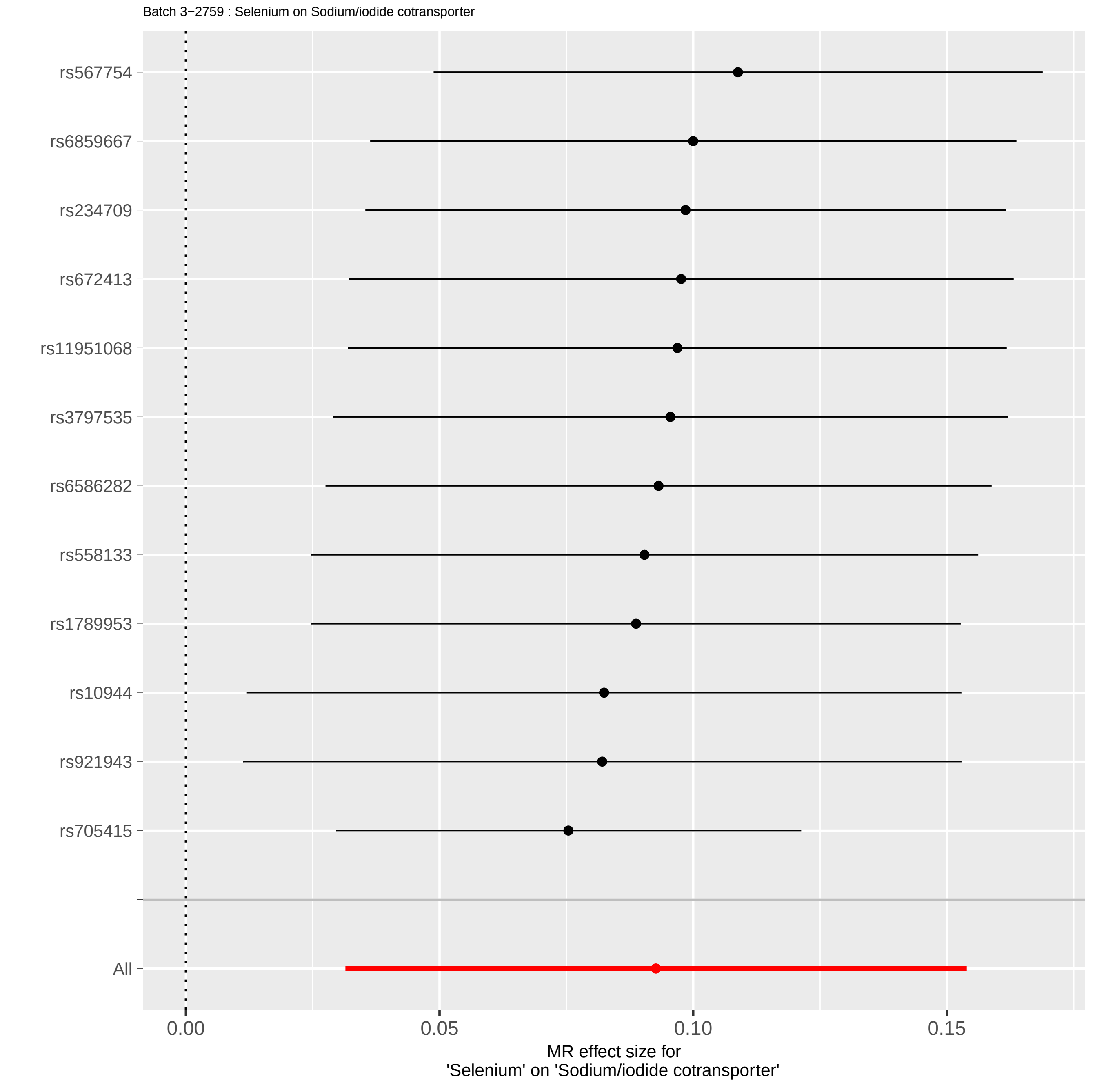

Batch 3−2759 : Selenium on Sodium/iodide cotransporter
rs567754
rs6859667
rs234709
rs672413
rs11951068
rs3797535
rs6586282
rs558133
rs1789953
rs10944
rs921943
rs705415
All
0.00
0.05
0.10
0.15
MR effect size for
'Selenium' on 'Sodium/iodide cotransporter'

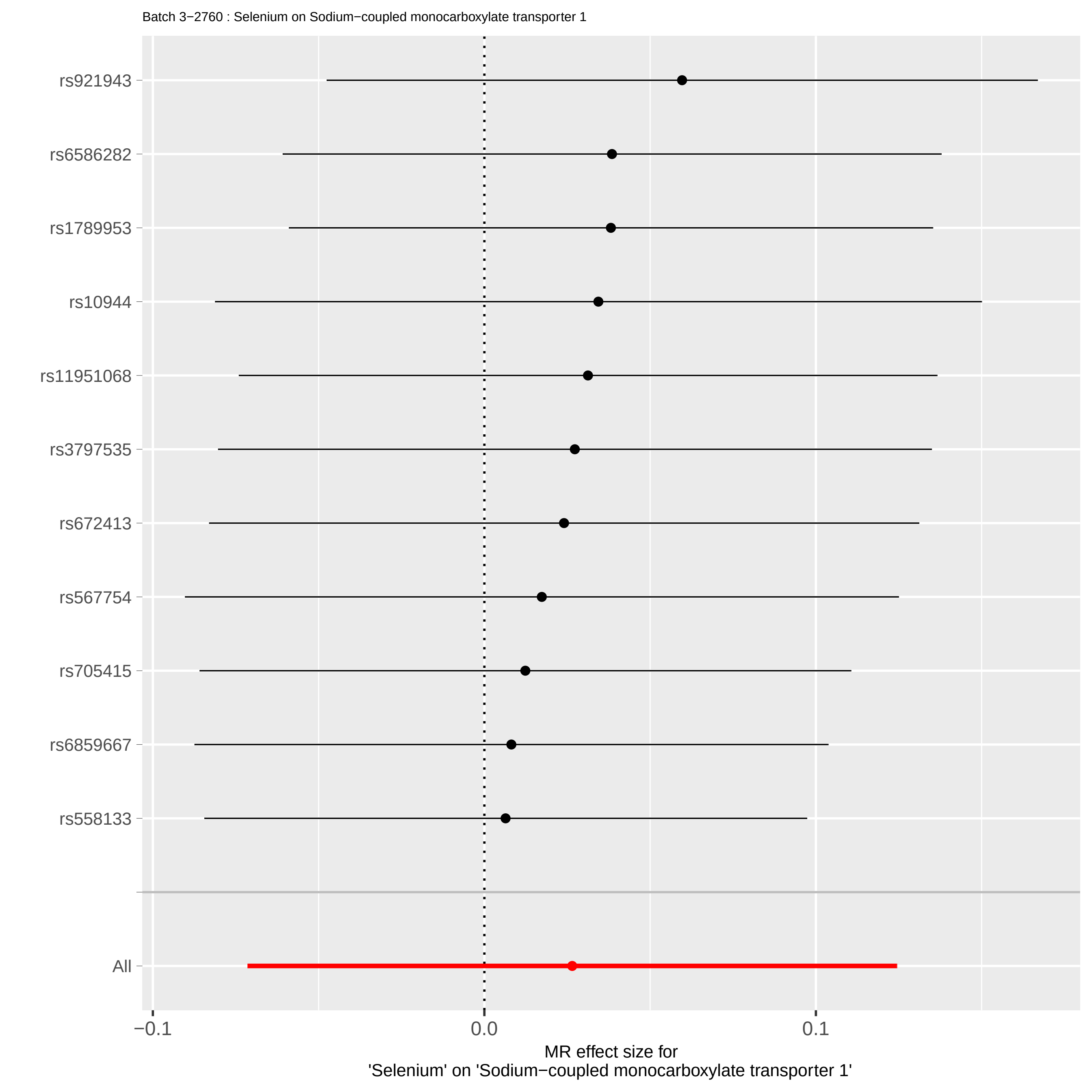

Batch 3−2760 : Selenium on Sodium−coupled monocarboxylate transporter 1
rs921943
rs6586282
rs1789953
rs10944
rs11951068
rs3797535
rs672413
rs567754
rs705415
rs6859667
rs558133
All
−0.1
0.0
0.1
MR effect size for
'Selenium' on 'Sodium−coupled monocarboxylate transporter 1'

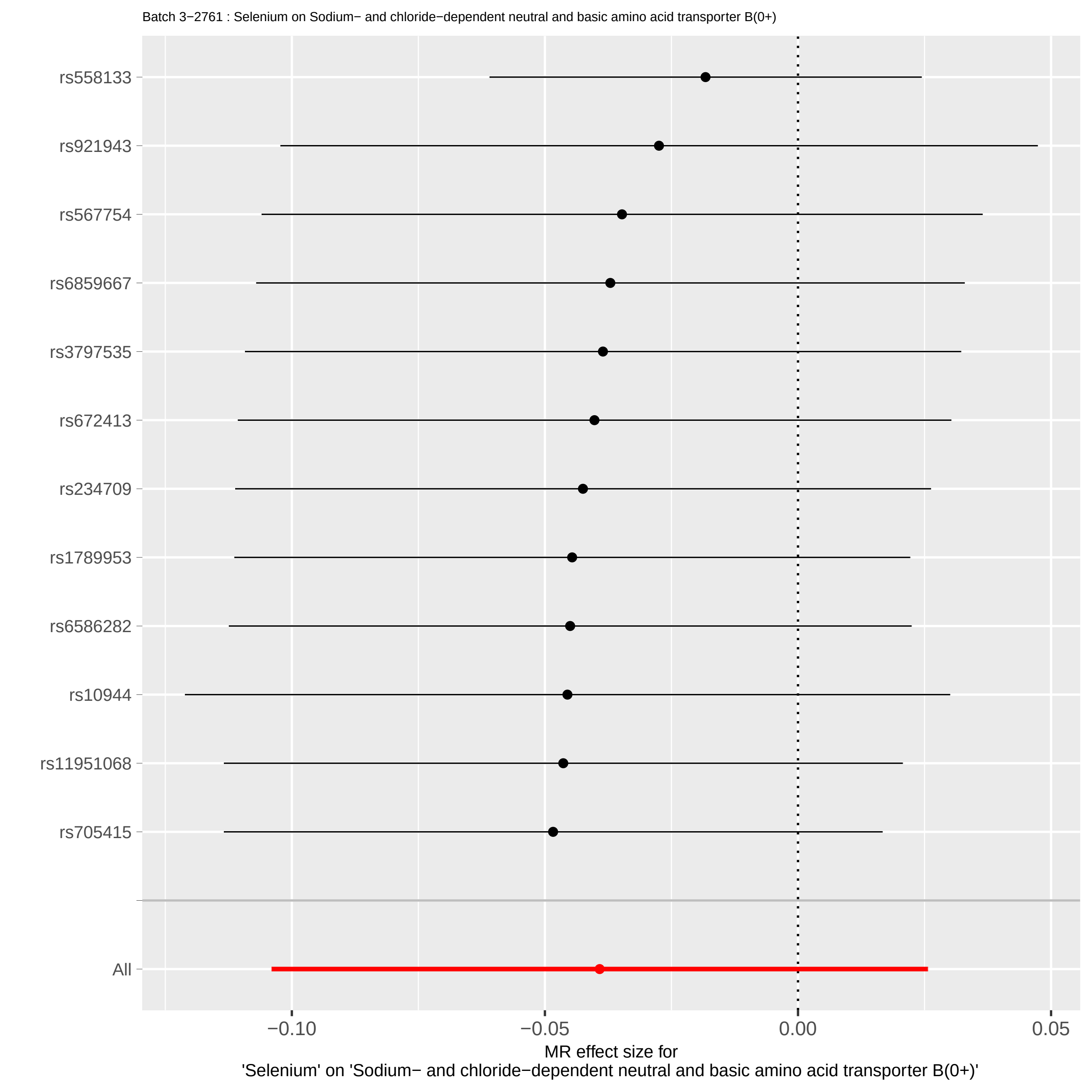

Batch 3−2761 : Selenium on Sodium− and chloride−dependent neutral and basic amino acid transporter B(0+)
rs558133
rs921943
rs567754
rs6859667
rs3797535
rs672413
rs234709
rs1789953
rs6586282
rs10944
rs11951068
rs705415
All
−0.10
−0.05
0.00
0.05
MR effect size for
'Selenium' on 'Sodium− and chloride−dependent neutral and basic amino acid transporter B(0+)'

Batch 3−2762 : Selenium on Orphan sodium− and chloride−dependent neurotransmitter transporter NTT5

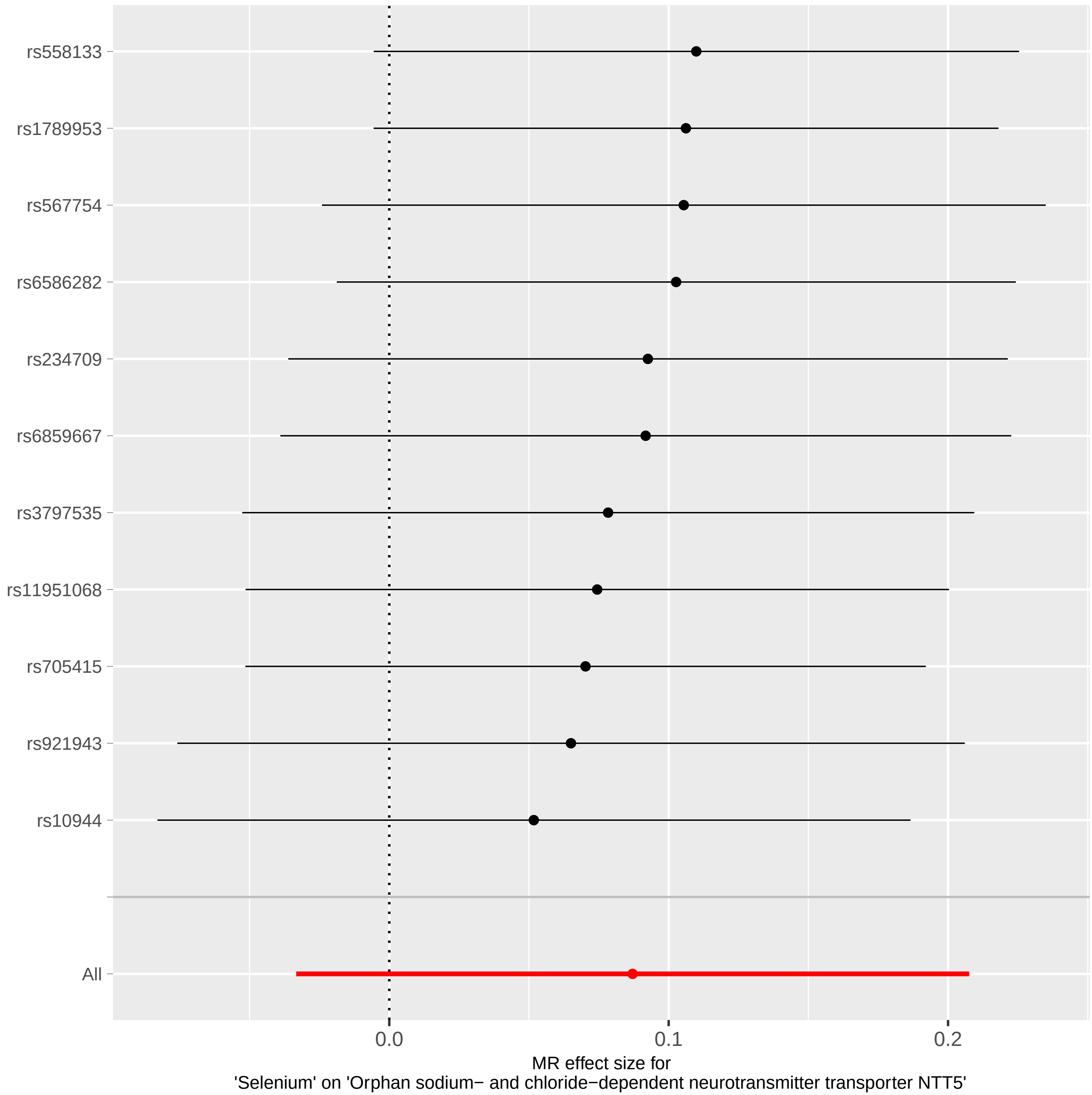

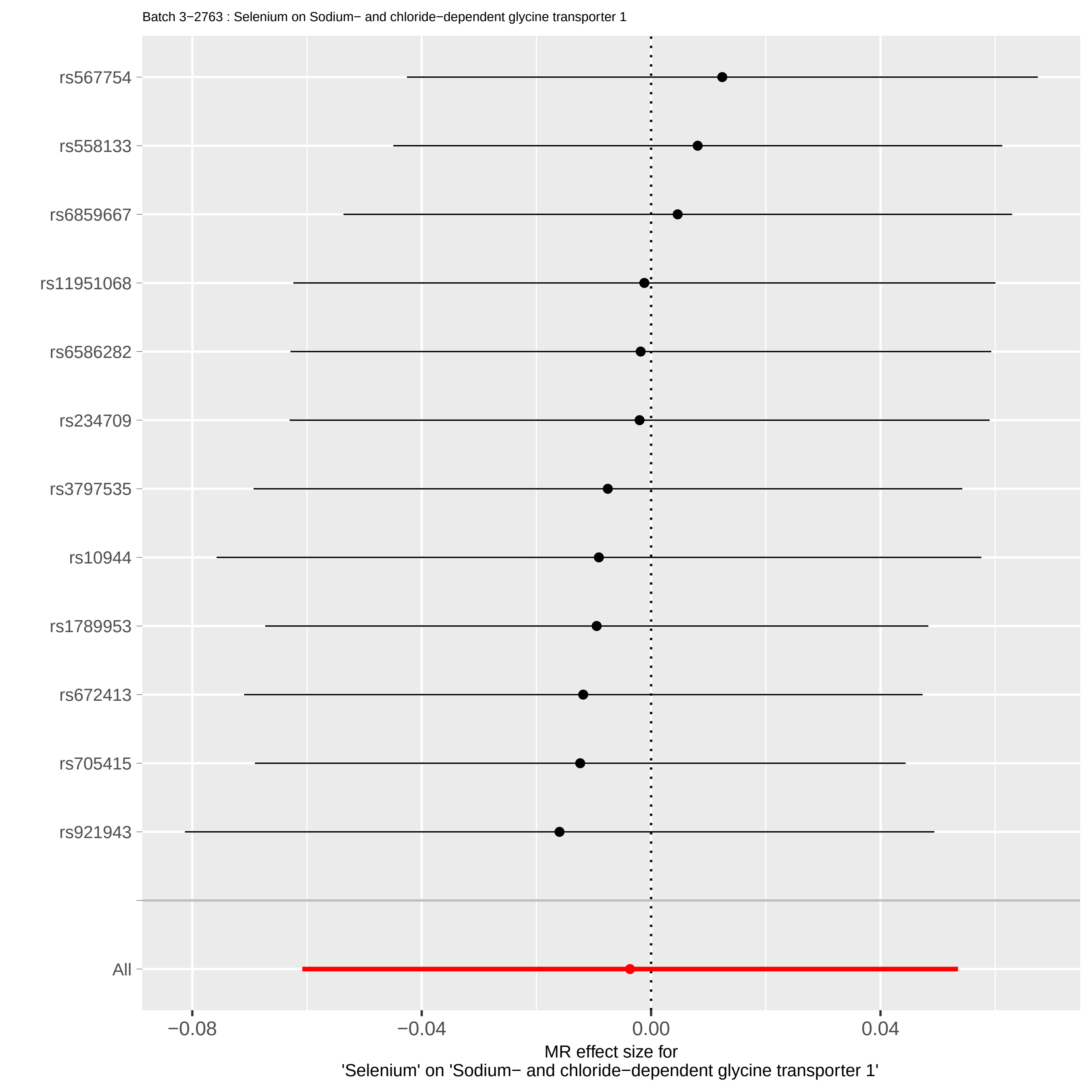
Batch 3−2763 : Selenium on Sodium− and chloride−dependent glycine transporter 1
rs567754
rs558133
rs6859667
rs11951068
rs6586282
rs234709
rs3797535
rs10944
rs1789953
rs672413
rs705415
rs921943
All
−0.08
−0.04
0.00
0.04
MR effect size for
'Selenium' on 'Sodium− and chloride−dependent glycine transporter 1'

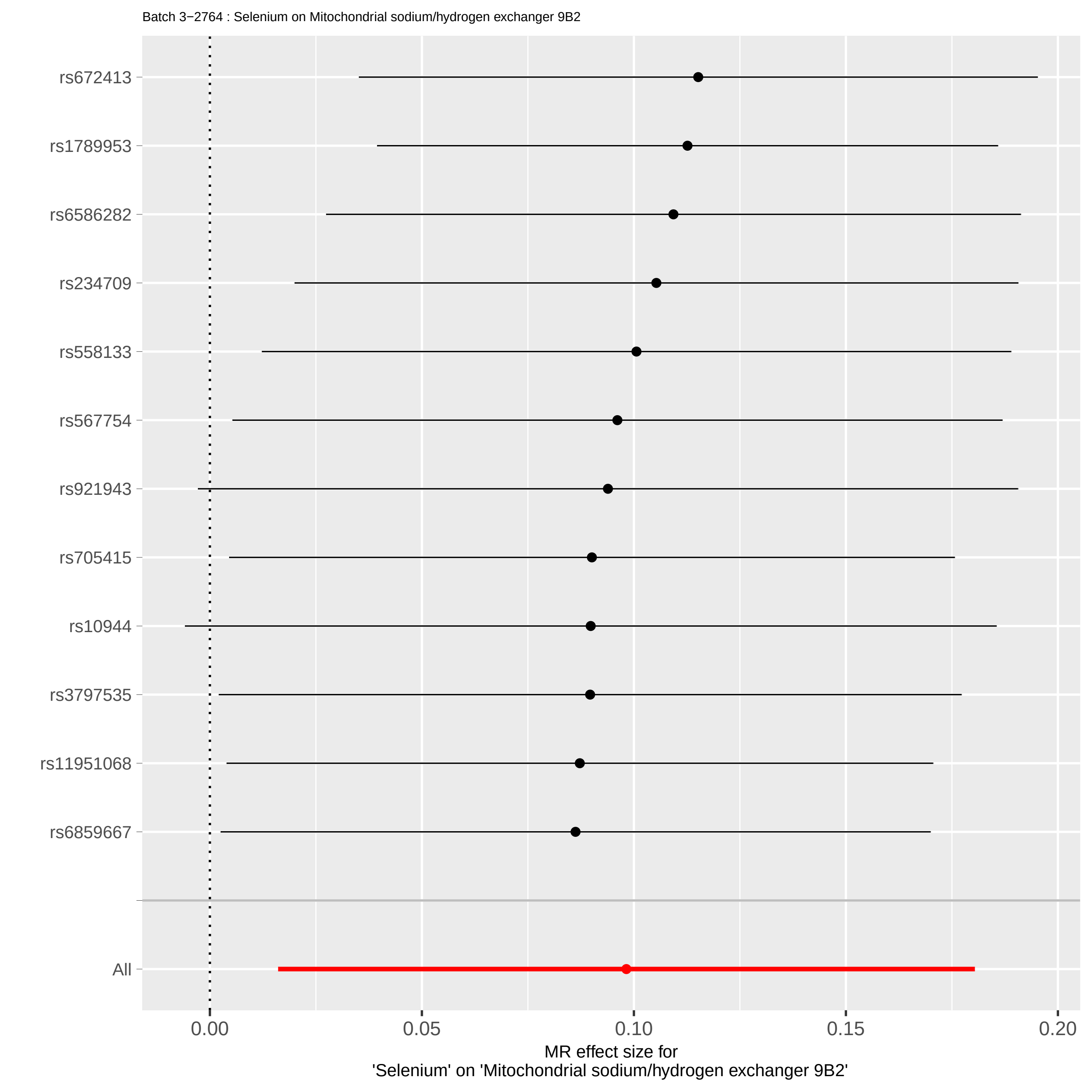

Batch 3−2764 : Selenium on Mitochondrial sodium/hydrogen exchanger 9B2
rs672413
rs1789953
rs6586282
rs234709
rs558133
rs567754
rs921943
rs705415
rs10944
rs3797535
rs11951068
rs6859667
All
0.00
0.05
0.10
0.15
0.20
MR effect size for
'Selenium' on 'Mitochondrial sodium/hydrogen exchanger 9B2'

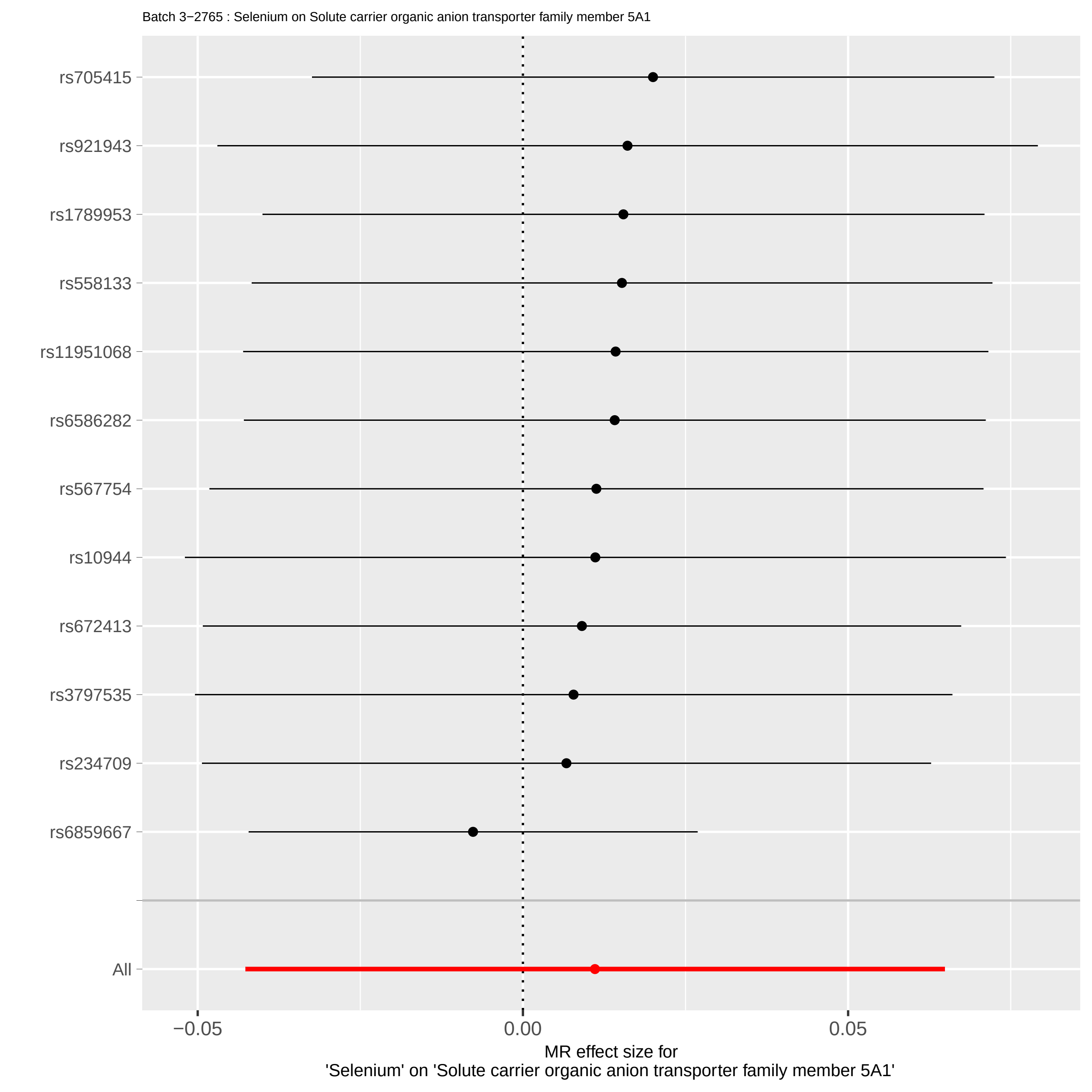

Batch 3−2765 : Selenium on Solute carrier organic anion transporter family member 5A1
rs705415
rs921943
rs1789953
rs558133
rs11951068
rs6586282
rs567754
rs10944
rs672413
rs3797535
rs234709
rs6859667
All
−0.05
0.00
0.05
MR effect size for
'Selenium' on 'Solute carrier organic anion transporter family member 5A1'

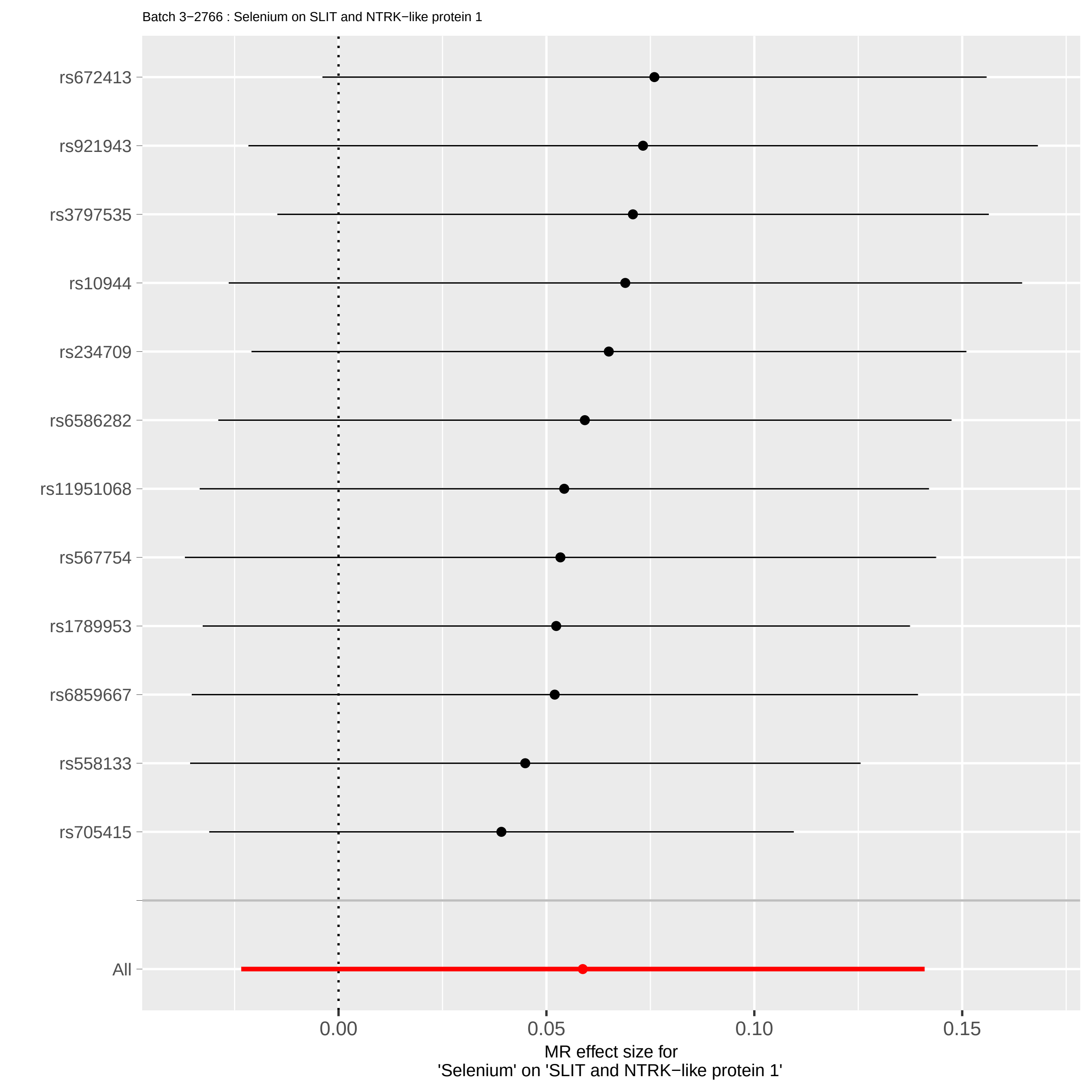

Batch 3−2766 : Selenium on SLIT and NTRK−like protein 1
rs672413
rs921943
rs3797535
rs10944
rs234709
rs6586282
rs11951068
rs567754
rs1789953
rs6859667
rs558133
rs705415
All
0.00
0.05
0.10
0.15
MR effect size for
'Selenium' on 'SLIT and NTRK−like protein 1'

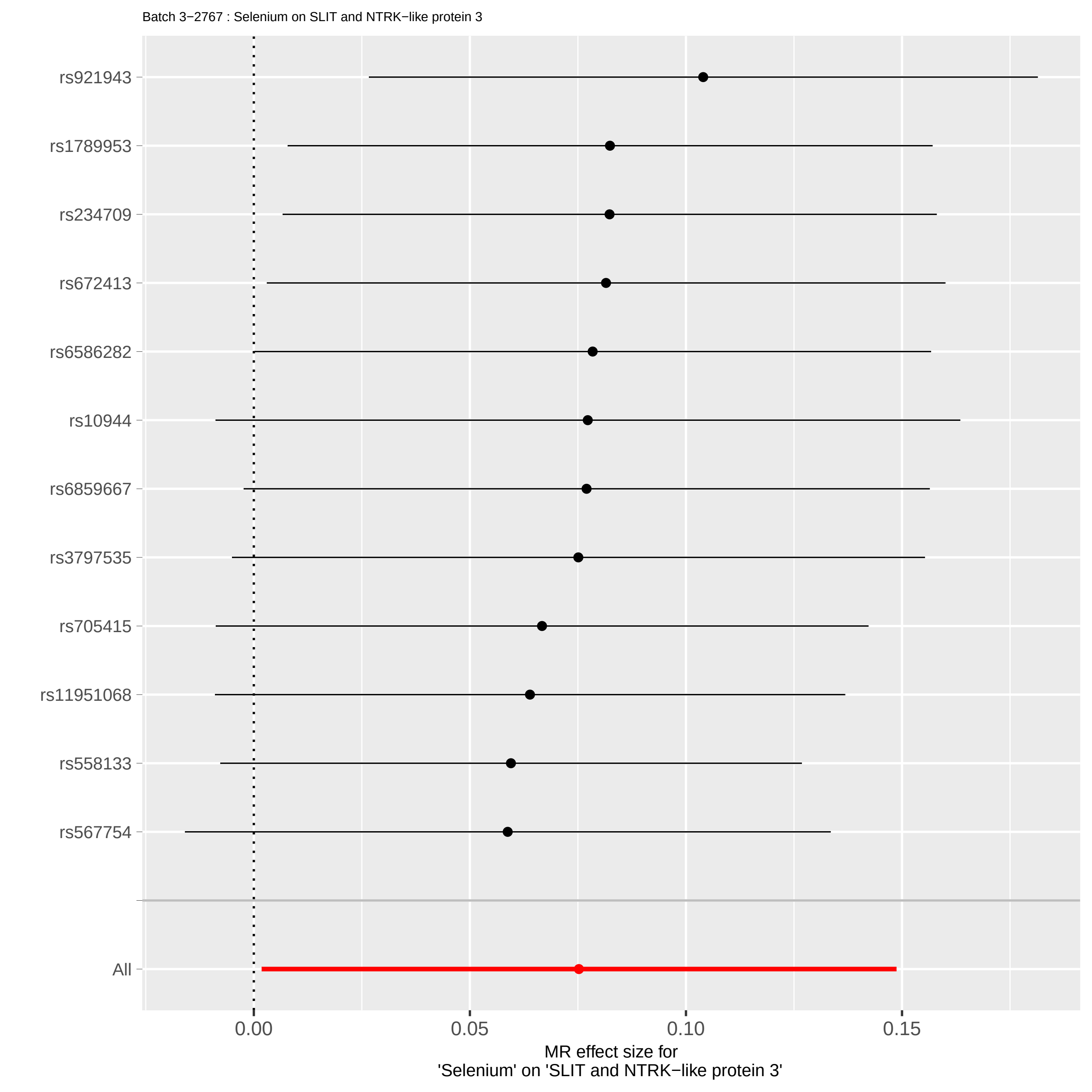

Batch 3−2767 : Selenium on SLIT and NTRK−like protein 3
rs921943
rs1789953
rs234709
rs672413
rs6586282
rs10944
rs6859667
rs3797535
rs705415
rs11951068
rs558133
rs567754
All
0.00
0.05
0.10
0.15
MR effect size for
'Selenium' on 'SLIT and NTRK−like protein 3'

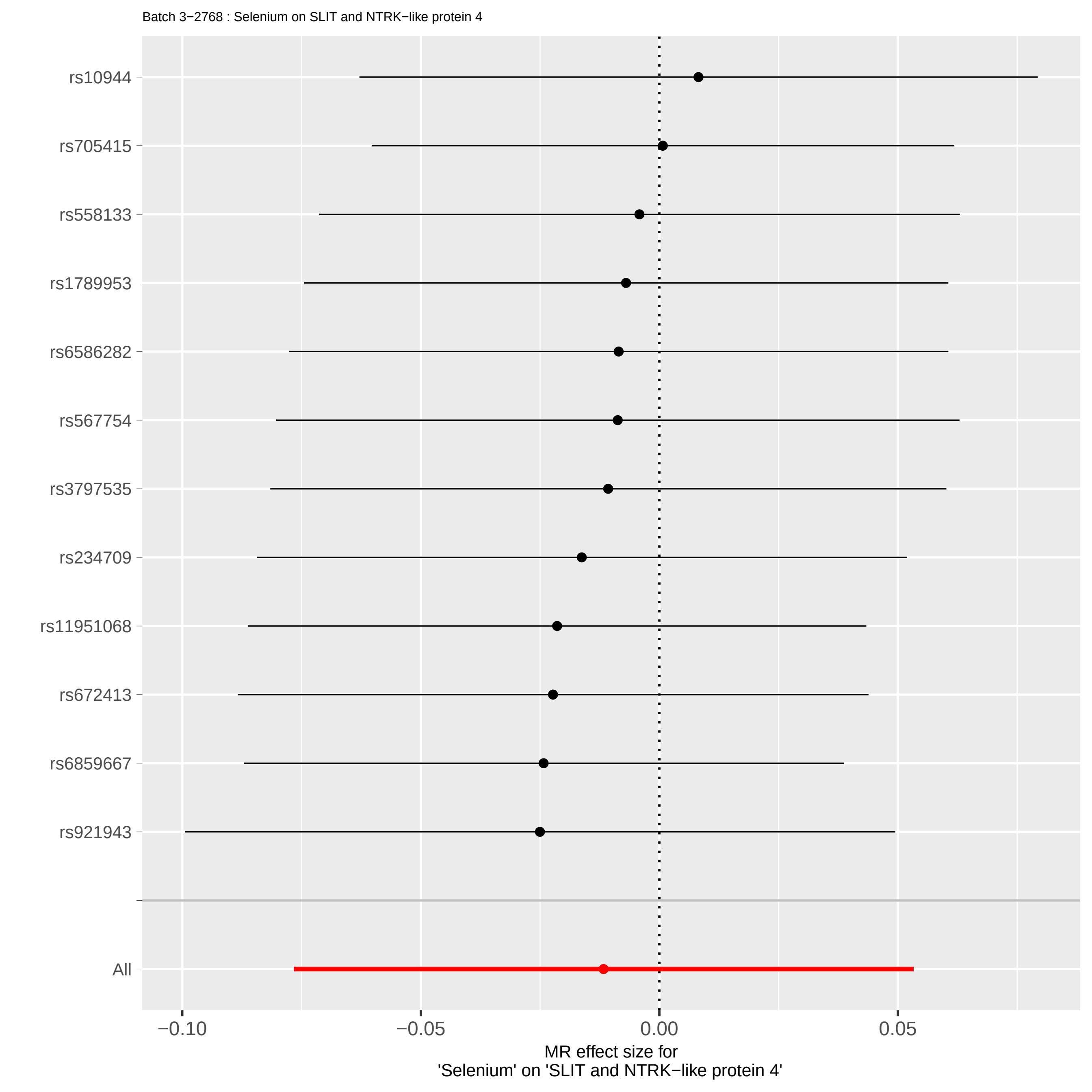
Batch 3−2768 : Selenium on SLIT and NTRK−like protein 4
rs10944
rs705415
rs558133
rs1789953
rs6586282
rs567754
rs3797535
rs234709
rs11951068
rs672413
rs6859667
rs921943
All
−0.10
−0.05
0.00
0.05
MR effect size for
'Selenium' on 'SLIT and NTRK−like protein 4'

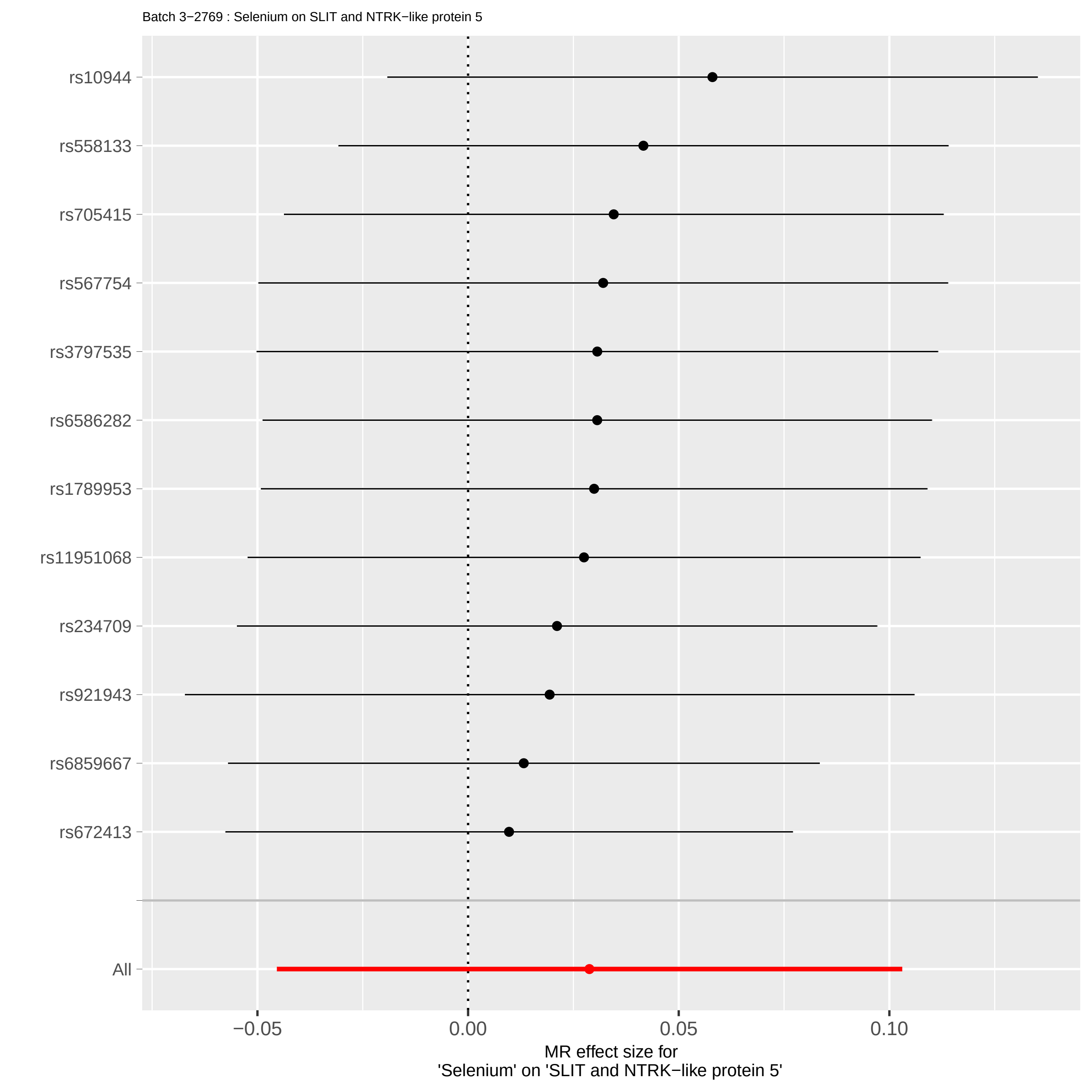

Batch 3−2769 : Selenium on SLIT and NTRK−like protein 5
rs10944
rs558133
rs705415
rs567754
rs3797535
rs6586282
rs1789953
rs11951068
rs234709
rs921943
rs6859667
rs672413
All
−0.05
0.00
0.05
0.10
MR effect size for
'Selenium' on 'SLIT and NTRK−like protein 5'

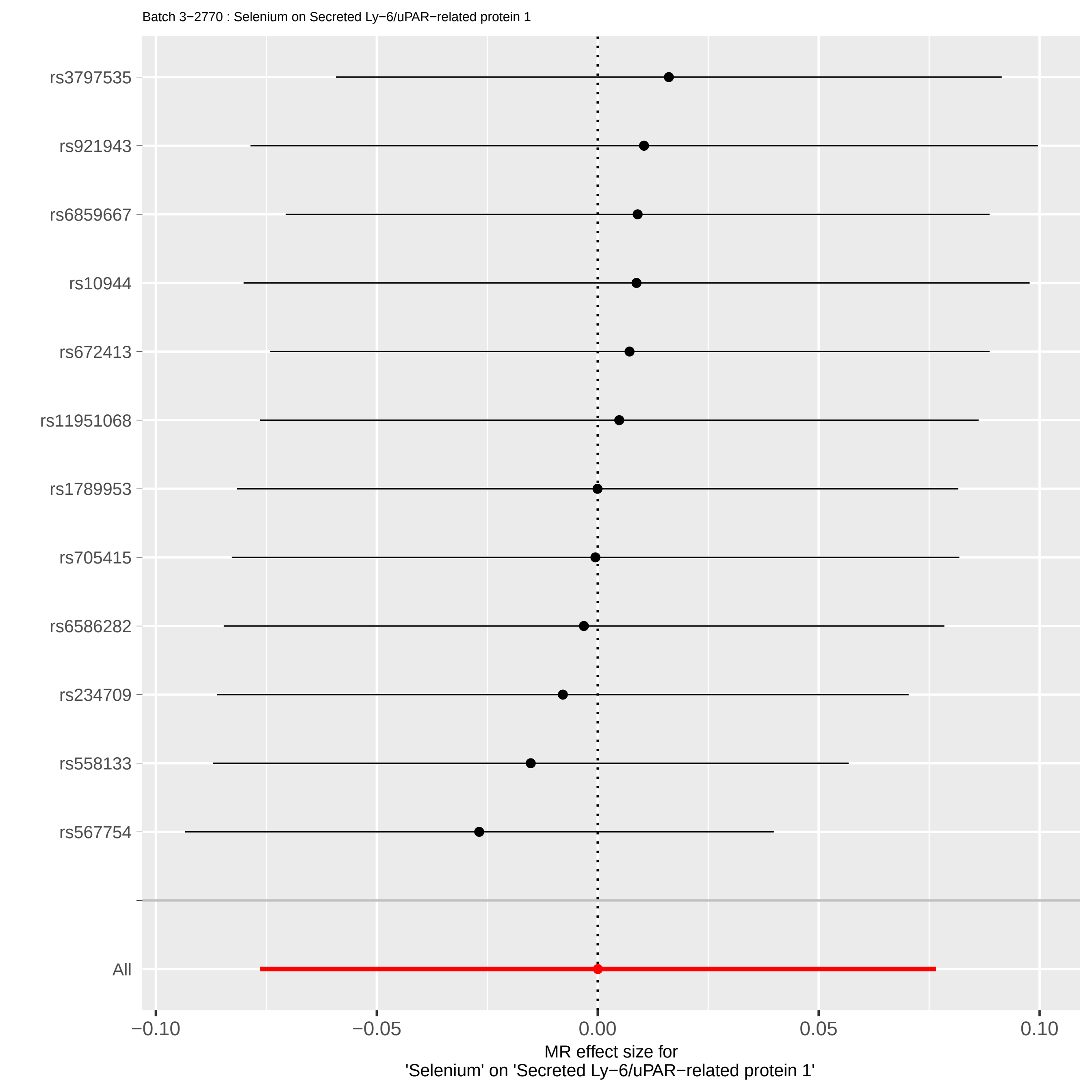

Batch 3−2770 : Selenium on Secreted Ly−6/uPAR−related protein 1
rs3797535
rs921943
rs6859667
rs10944
rs672413
rs11951068
rs1789953
rs705415
rs6586282
rs234709
rs558133
rs567754
All
−0.10
−0.05
0.00
0.05
0.10
MR effect size for
'Selenium' on 'Secreted Ly−6/uPAR−related protein 1'

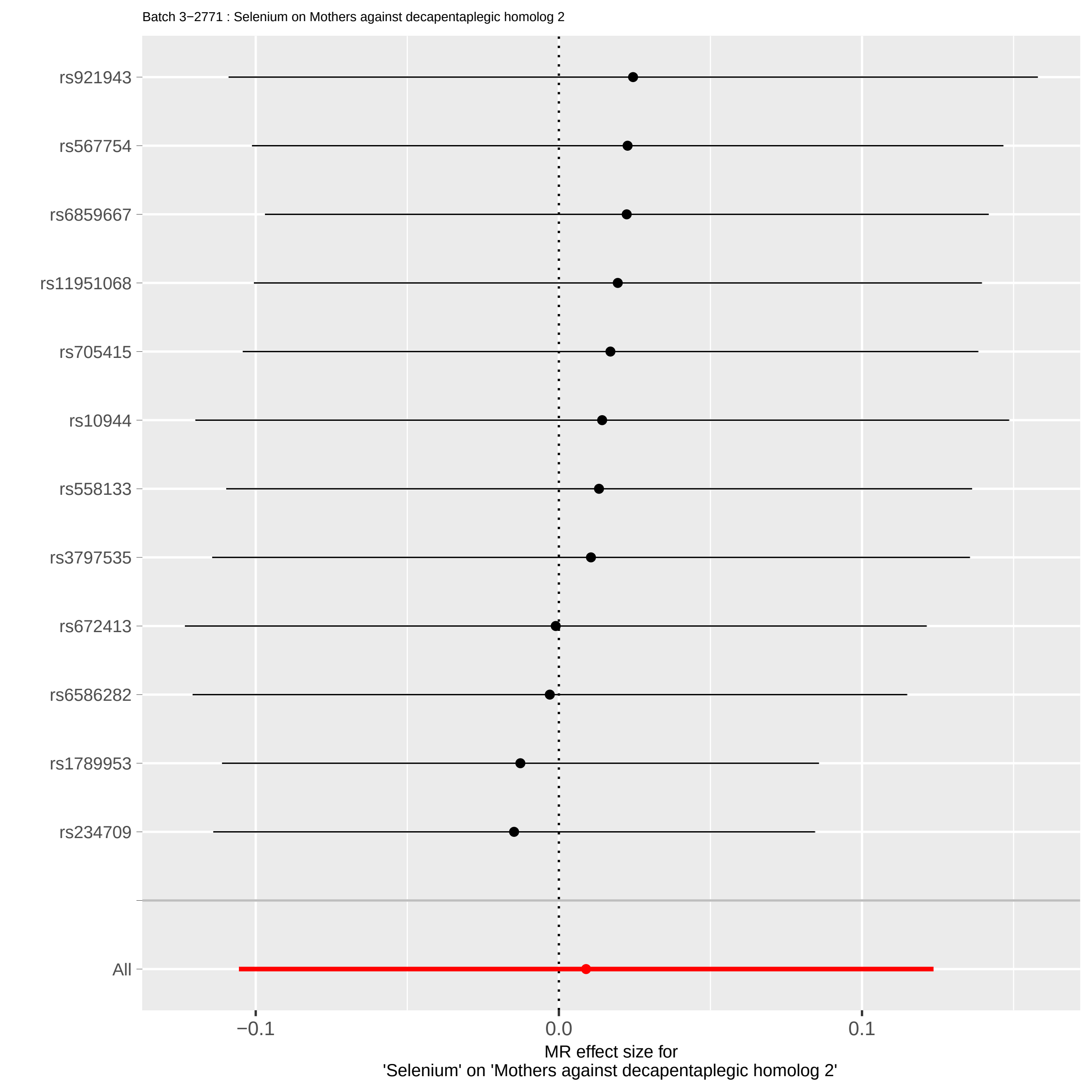

Batch 3−2771 : Selenium on Mothers against decapentaplegic homolog 2
rs921943
rs567754
rs6859667
rs11951068
rs705415
rs10944
rs558133
rs3797535
rs672413
rs6586282
rs1789953
rs234709
All
−0.1
0.0
0.1
MR effect size for
'Selenium' on 'Mothers against decapentaplegic homolog 2'

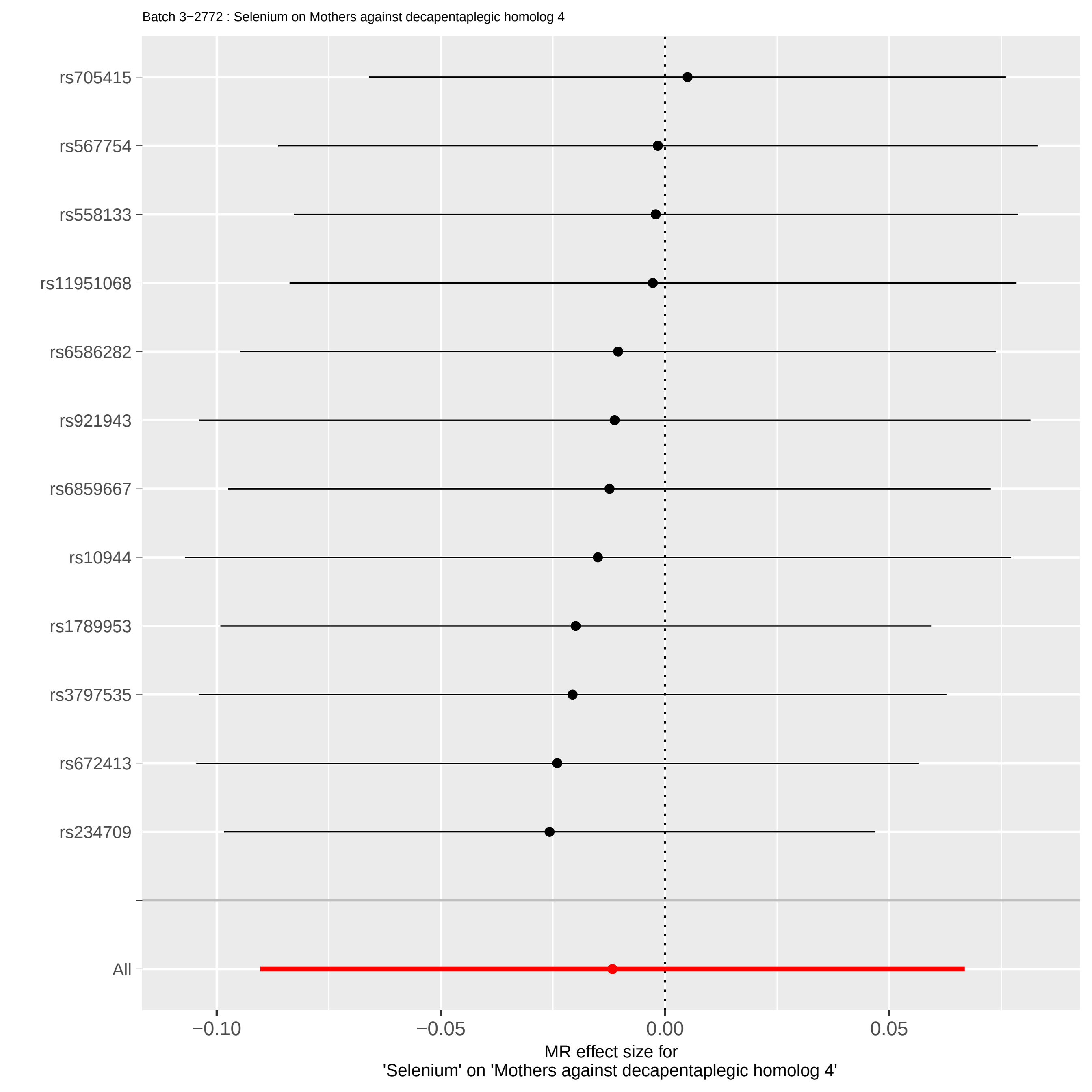
Batch 3−2772 : Selenium on Mothers against decapentaplegic homolog 4
rs705415
rs567754
rs558133
rs11951068
rs6586282
rs921943
rs6859667
rs10944
rs1789953
rs3797535
rs672413
rs234709
All
−0.10
−0.05
0.00
0.05
MR effect size for
'Selenium' on 'Mothers against decapentaplegic homolog 4'

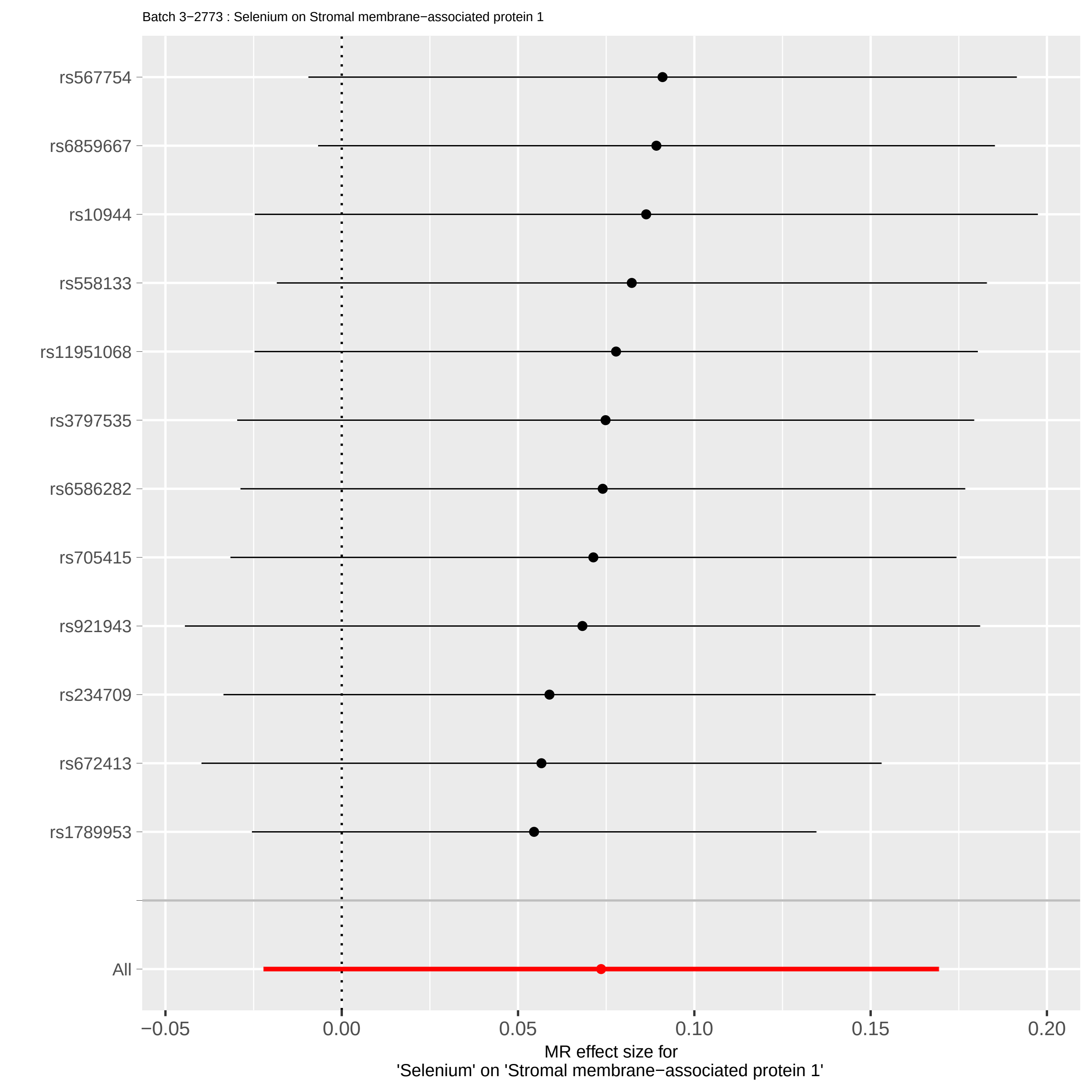

Batch 3−2773 : Selenium on Stromal membrane−associated protein 1
rs567754
rs6859667
rs10944
rs558133
rs11951068
rs3797535
rs6586282
rs705415
rs921943
rs234709
rs672413
rs1789953
All
−0.05
0.00
0.05
0.10
0.15
0.20
MR effect size for
'Selenium' on 'Stromal membrane−associated protein 1'

Batch 3−2774 : Selenium on Structural maintenance of chromosomes protein 3

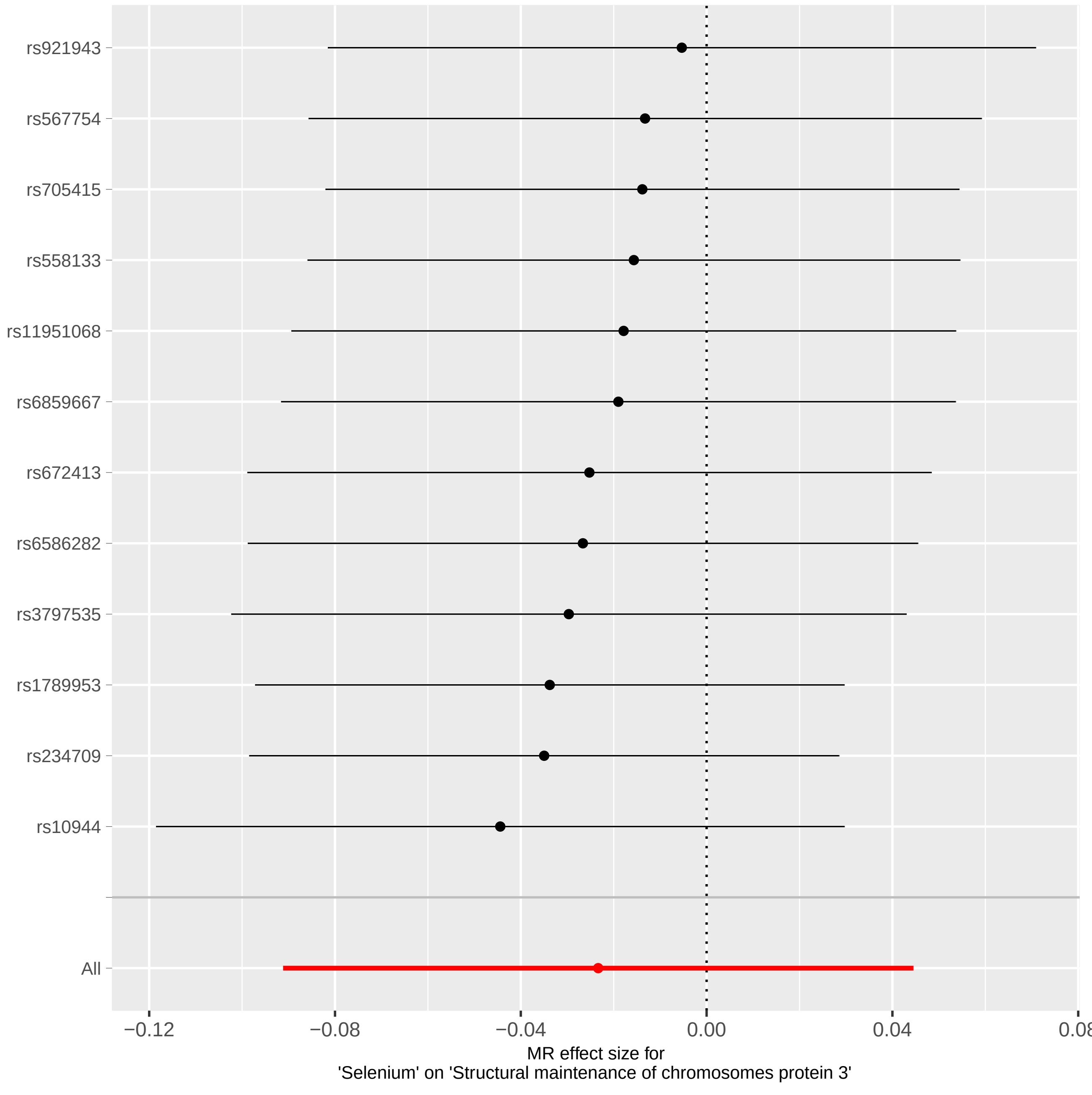

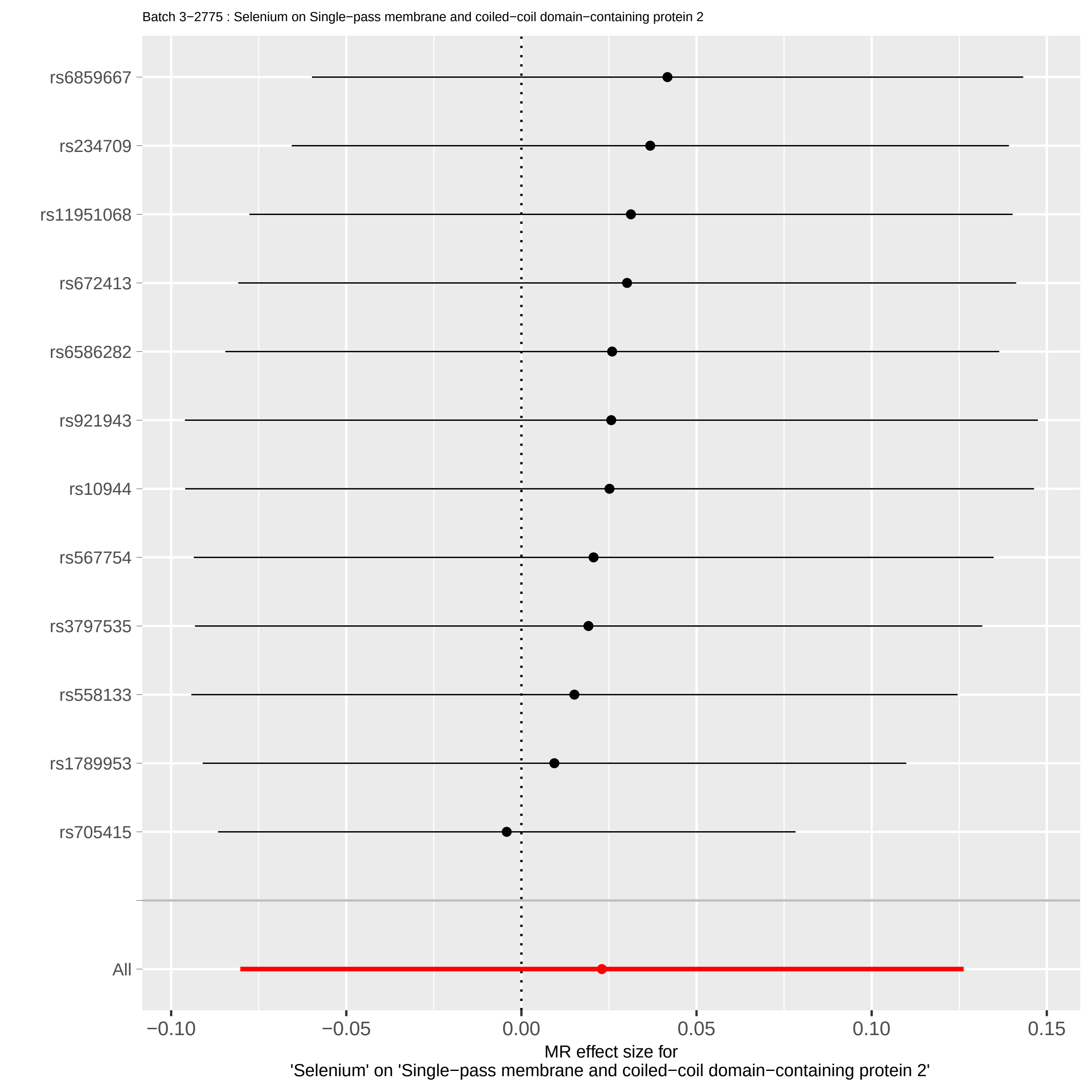
Batch 3−2775 : Selenium on Single−pass membrane and coiled−coil domain−containing protein 2
rs6859667
rs234709
rs11951068
rs672413
rs6586282
rs921943
rs10944
rs567754
rs3797535
rs558133
rs1789953
rs705415
All
−0.10
−0.05
0.00
0.05
0.10
0.15
MR effect size for
'Selenium' on 'Single−pass membrane and coiled−coil domain−containing protein 2'

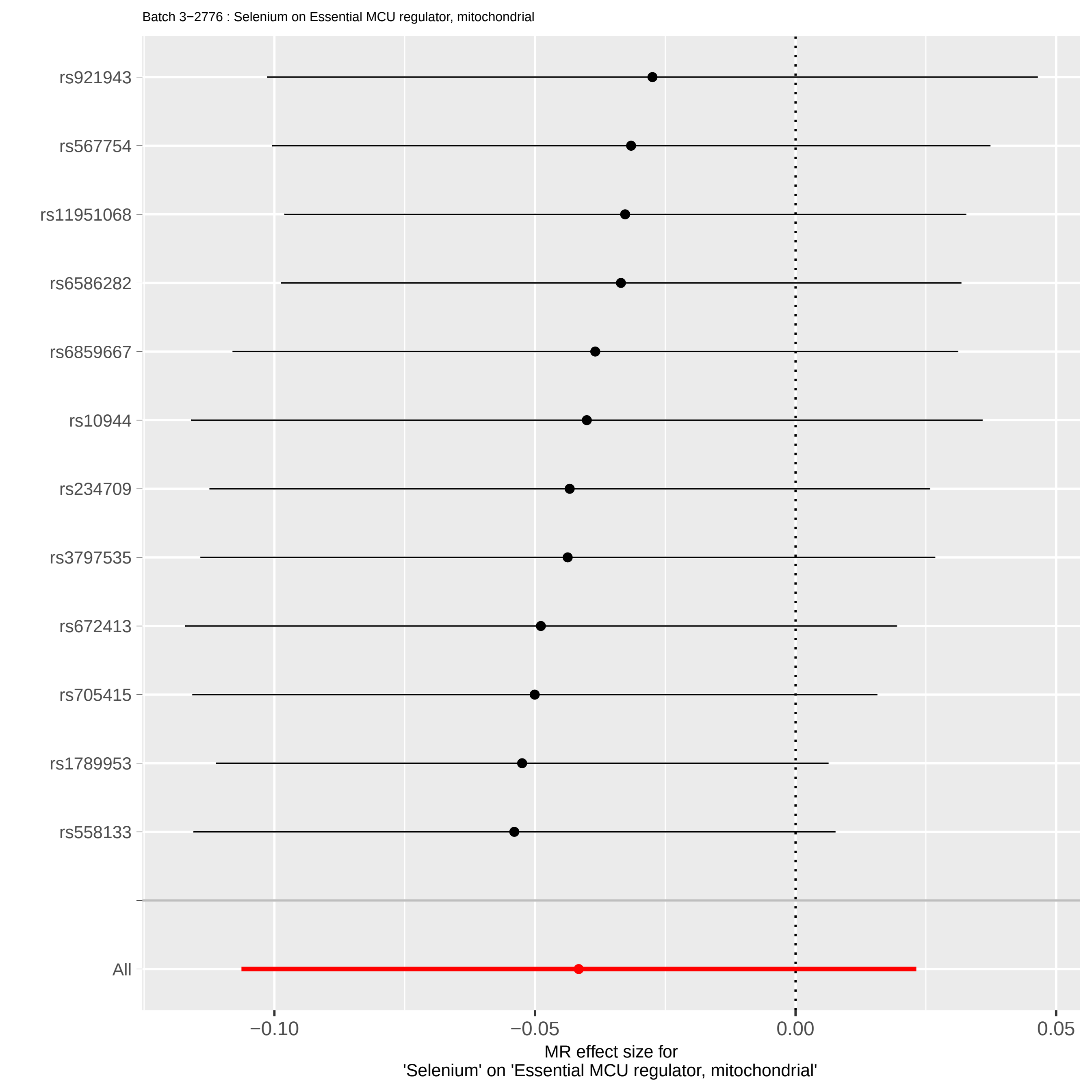

Batch 3−2776 : Selenium on Essential MCU regulator, mitochondrial
rs921943
rs567754
rs11951068
rs6586282
rs6859667
rs10944
rs234709
rs3797535
rs672413
rs705415
rs1789953
rs558133
All
−0.10
−0.05
0.00
0.05
MR effect size for
'Selenium' on 'Essential MCU regulator, mitochondrial'

Batch 3−2777 : Selenium on Small integral membrane protein 10

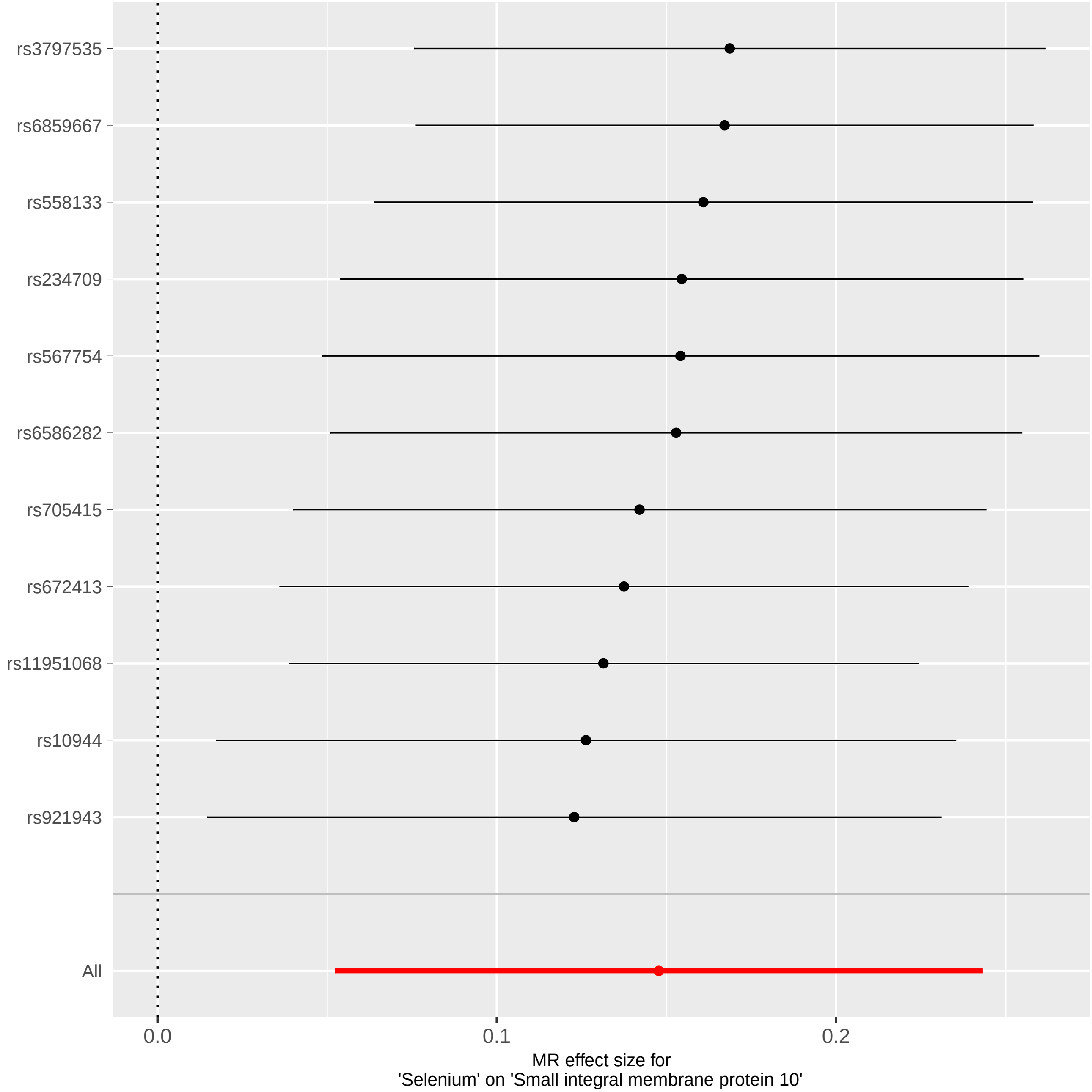

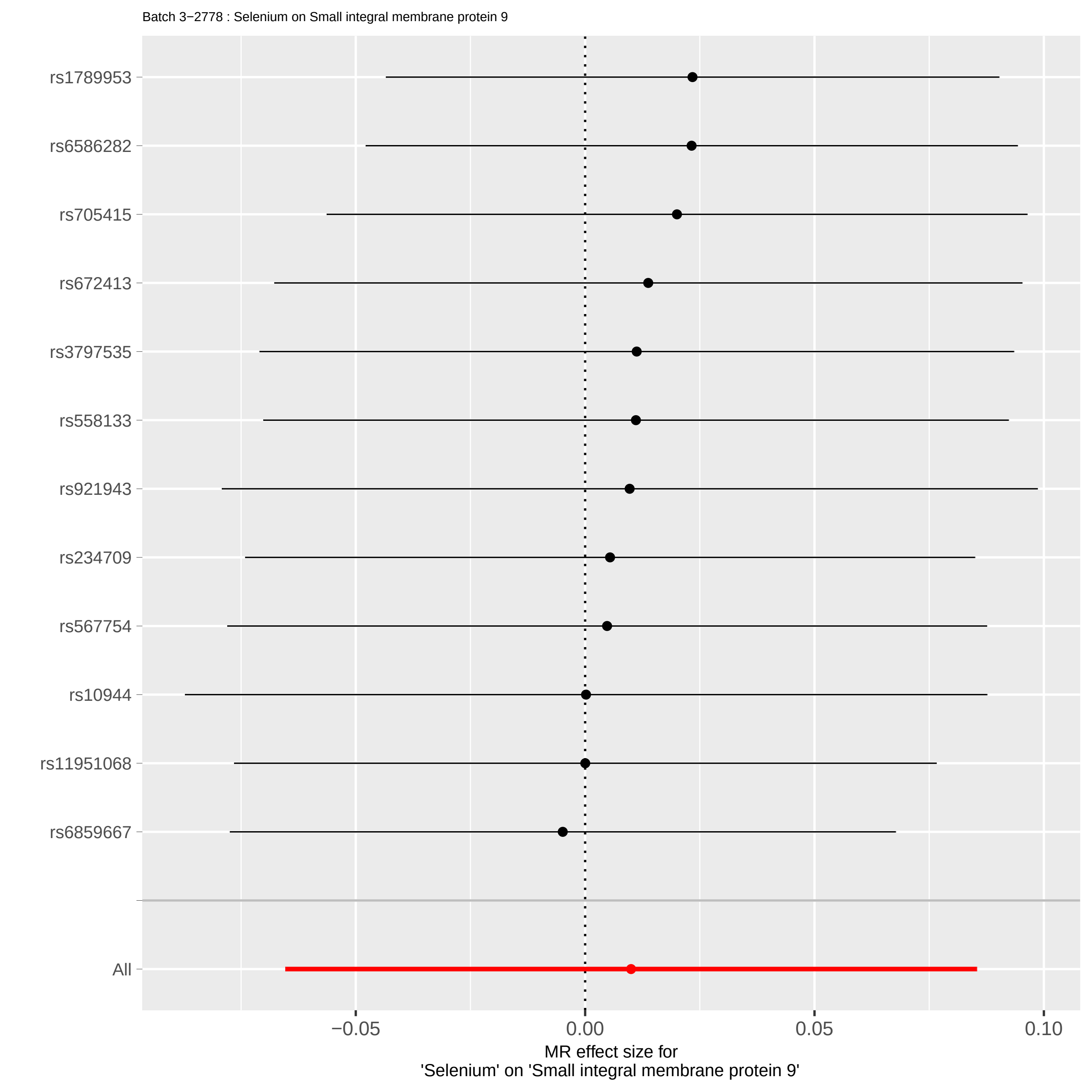

Batch 3−2778 : Selenium on Small integral membrane protein 9
rs1789953
rs6586282
rs705415
rs672413
rs3797535
rs558133
rs921943
rs234709
rs567754
rs10944
rs11951068
rs6859667
All
−0.05
0.00
0.05
0.10
MR effect size for
'Selenium' on 'Small integral membrane protein 9'

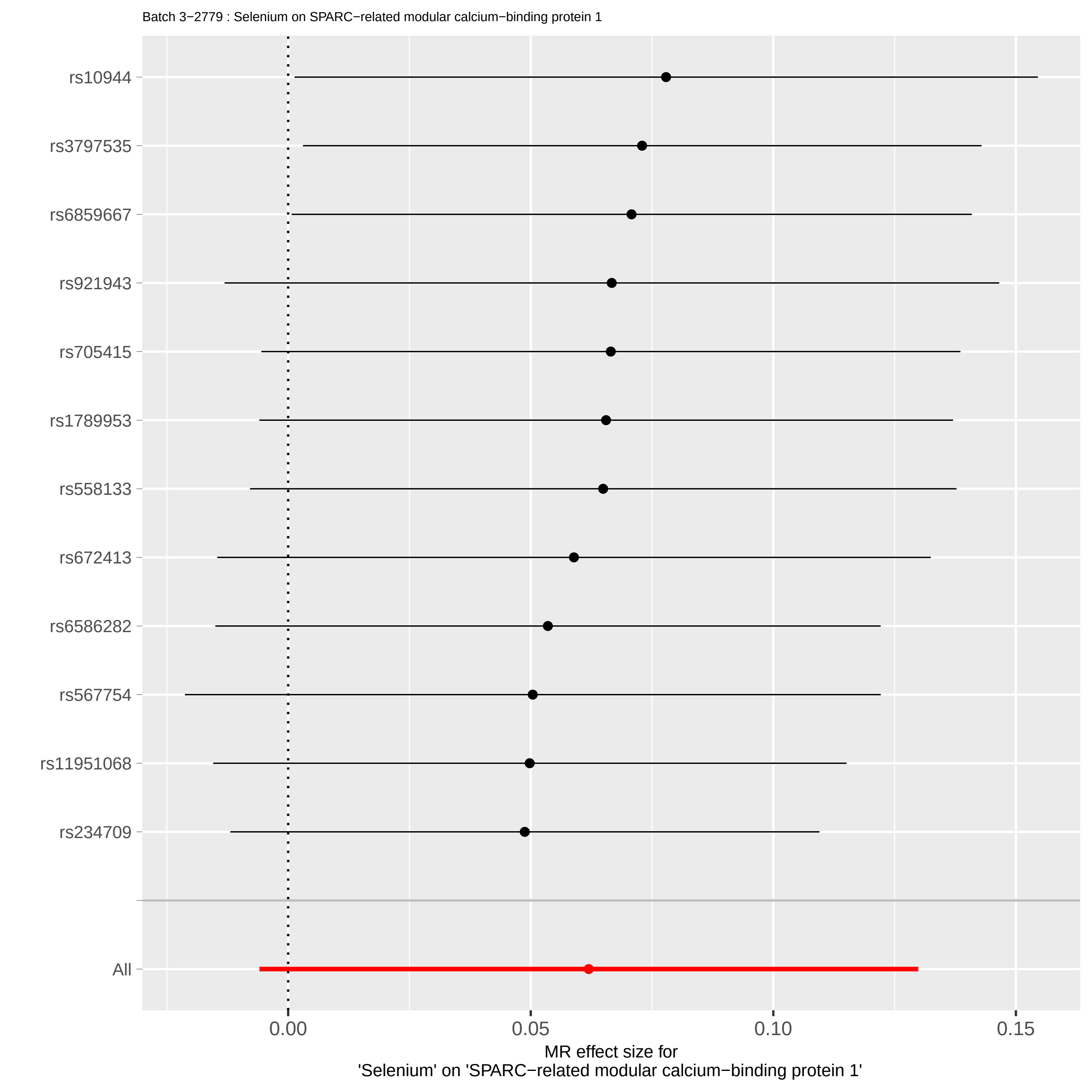

Batch 3−2779 : Selenium on SPARC−related modular calcium−binding protein 1
rs10944
rs3797535
rs6859667
rs921943
rs705415
rs1789953
rs558133
rs672413
rs6586282
rs567754
rs11951068
rs234709
All
0.00
0.05
0.10
0.15
MR effect size for
'Selenium' on 'SPARC−related modular calcium−binding protein 1'

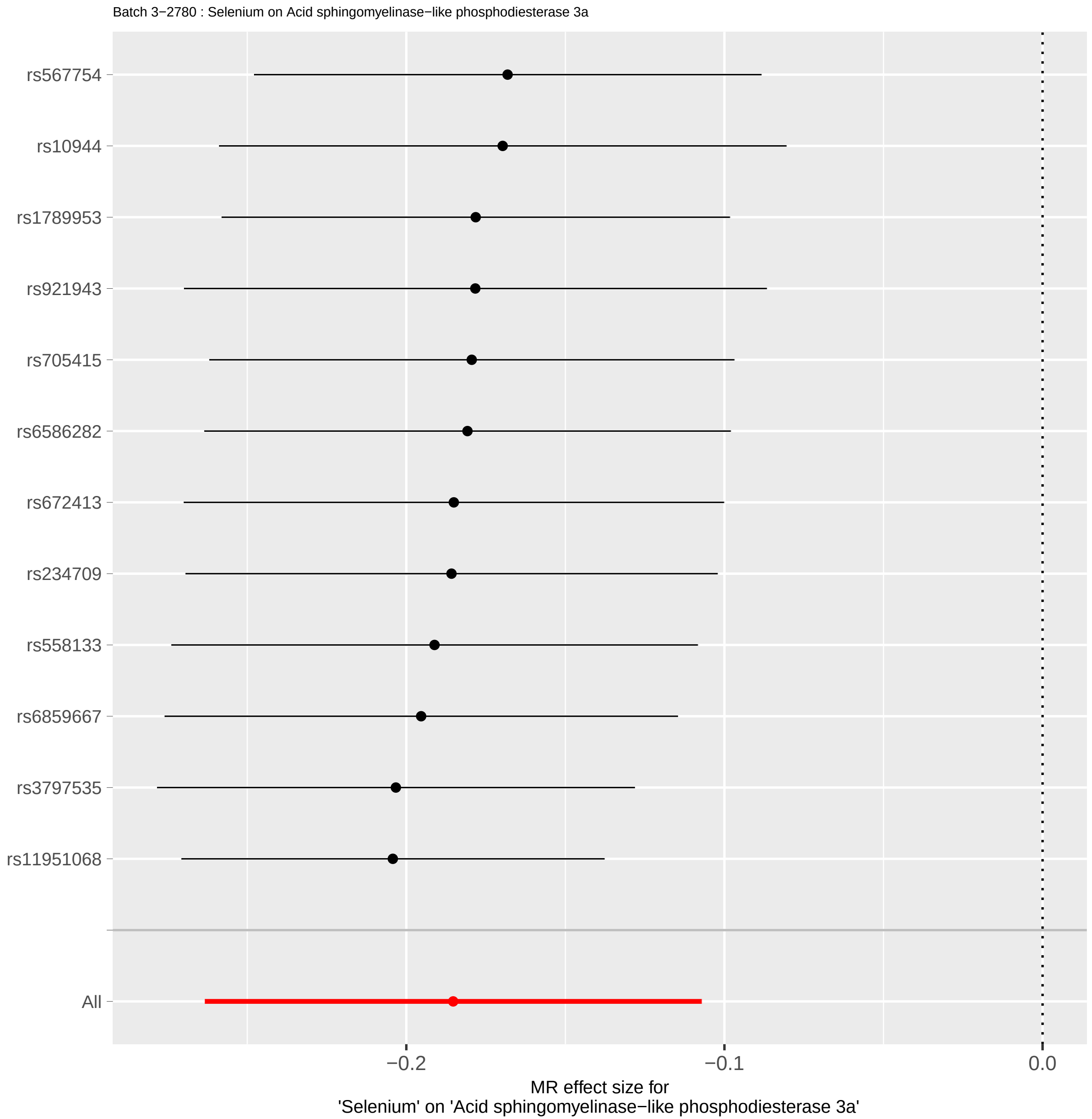

Batch 3−2780 : Selenium on Acid sphingomyelinase−like phosphodiesterase 3a
rs567754
rs10944
rs1789953
rs921943
rs705415
rs6586282
rs672413
rs234709
rs558133
rs6859667
rs3797535
rs11951068
All
−0.2
−0.1
0.0
MR effect size for
'Selenium' on 'Acid sphingomyelinase−like phosphodiesterase 3a'

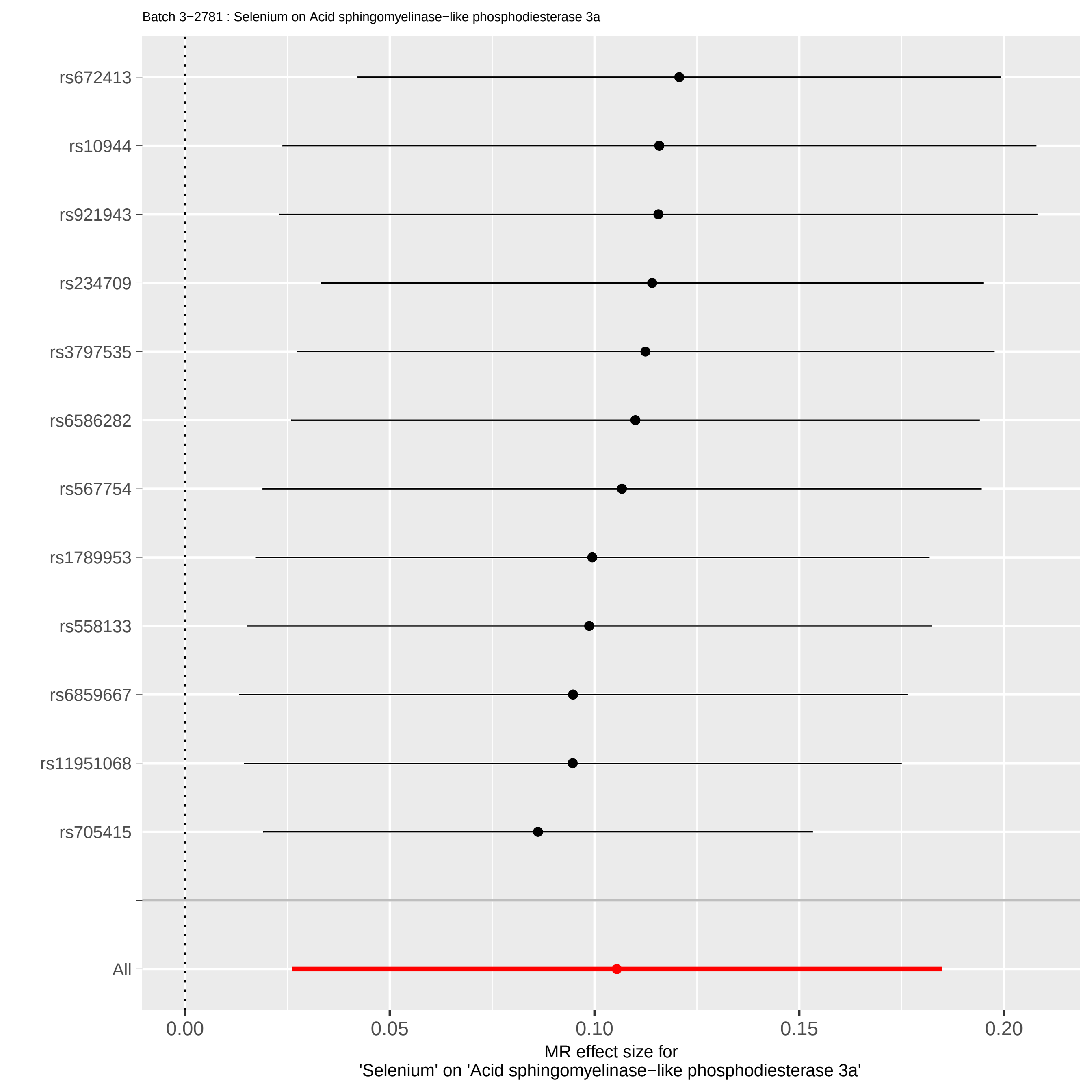

Batch 3−2781 : Selenium on Acid sphingomyelinase−like phosphodiesterase 3a
rs672413
rs10944
rs921943
rs234709
rs3797535
rs6586282
rs567754
rs1789953
rs558133
rs6859667
rs11951068
rs705415
All
0.00
0.05
0.10
0.15
0.20
MR effect size for
'Selenium' on 'Acid sphingomyelinase−like phosphodiesterase 3a'

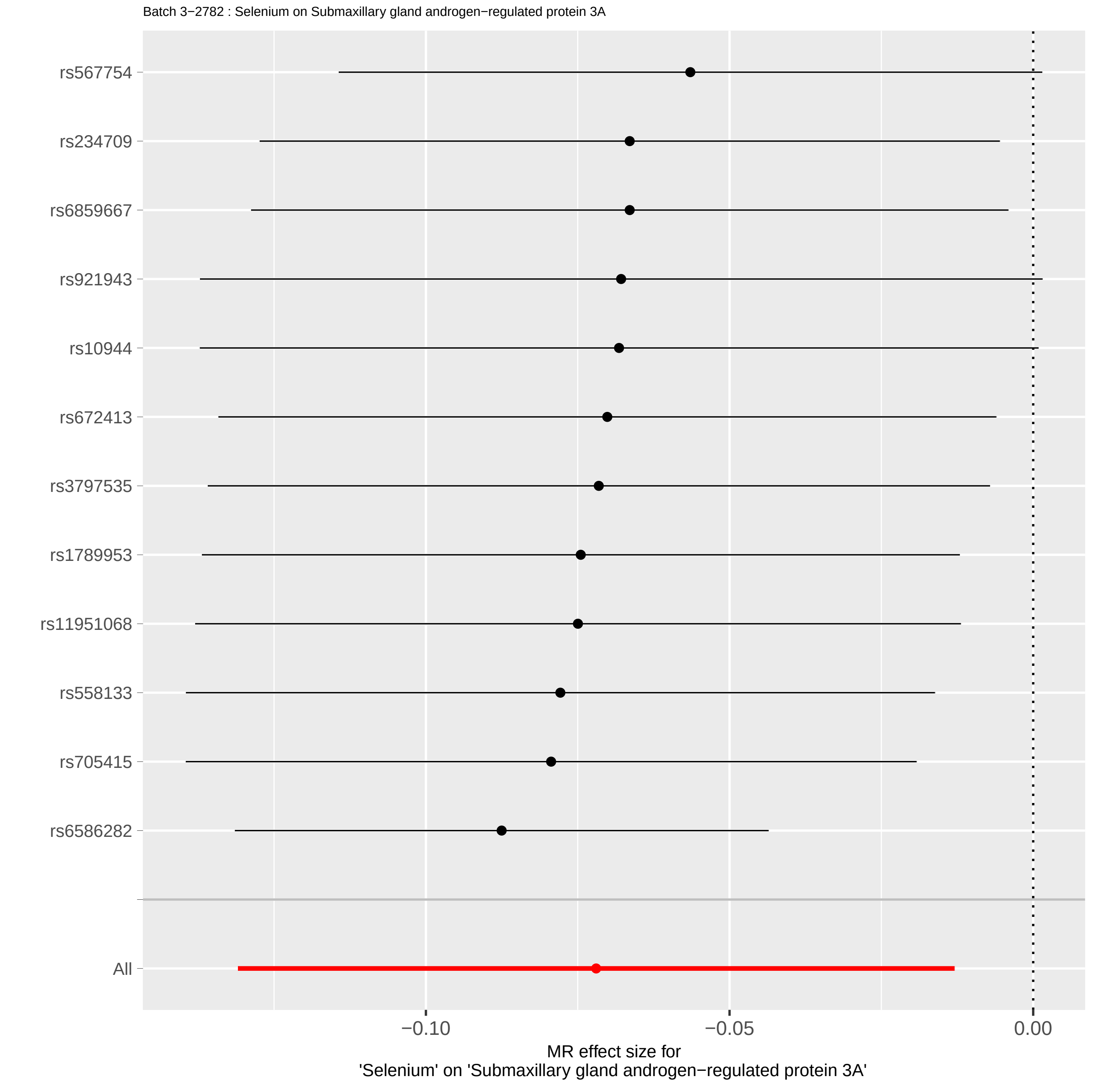

Batch 3−2782 : Selenium on Submaxillary gland androgen−regulated protein 3A
rs567754
rs234709
rs6859667
rs921943
rs10944
rs672413
rs3797535
rs1789953
rs11951068
rs558133
rs705415
rs6586282
All
−0.10
−0.05
0.00
MR effect size for
'Selenium' on 'Submaxillary gland androgen−regulated protein 3A'

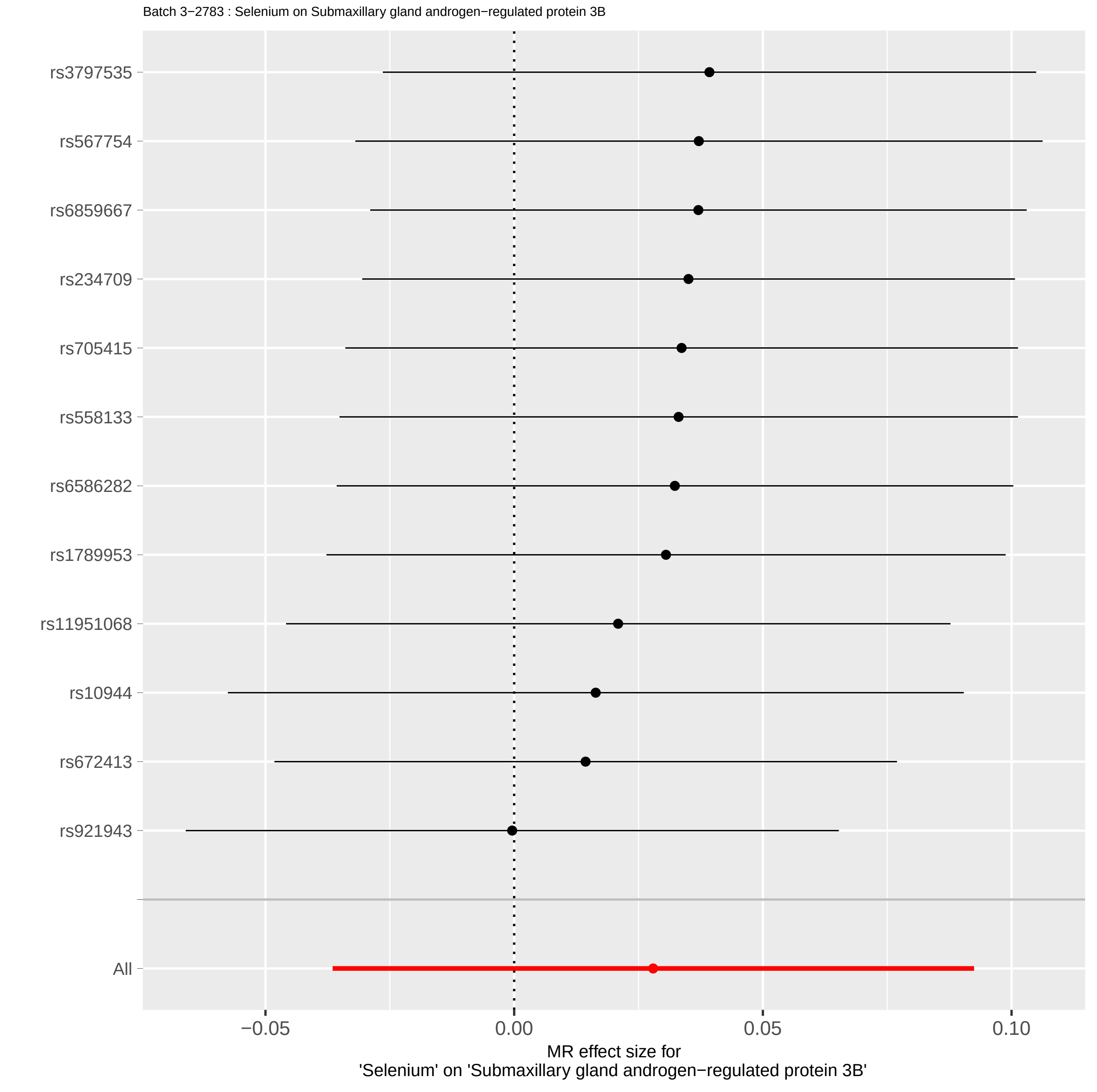

Batch 3−2783 : Selenium on Submaxillary gland androgen−regulated protein 3B
rs3797535
rs567754
rs6859667
rs234709
rs705415
rs558133
rs6586282
rs1789953
rs11951068
rs10944
rs672413
rs921943
All
−0.05
0.00
0.05
0.10
MR effect size for
'Selenium' on 'Submaxillary gland androgen−regulated protein 3B'

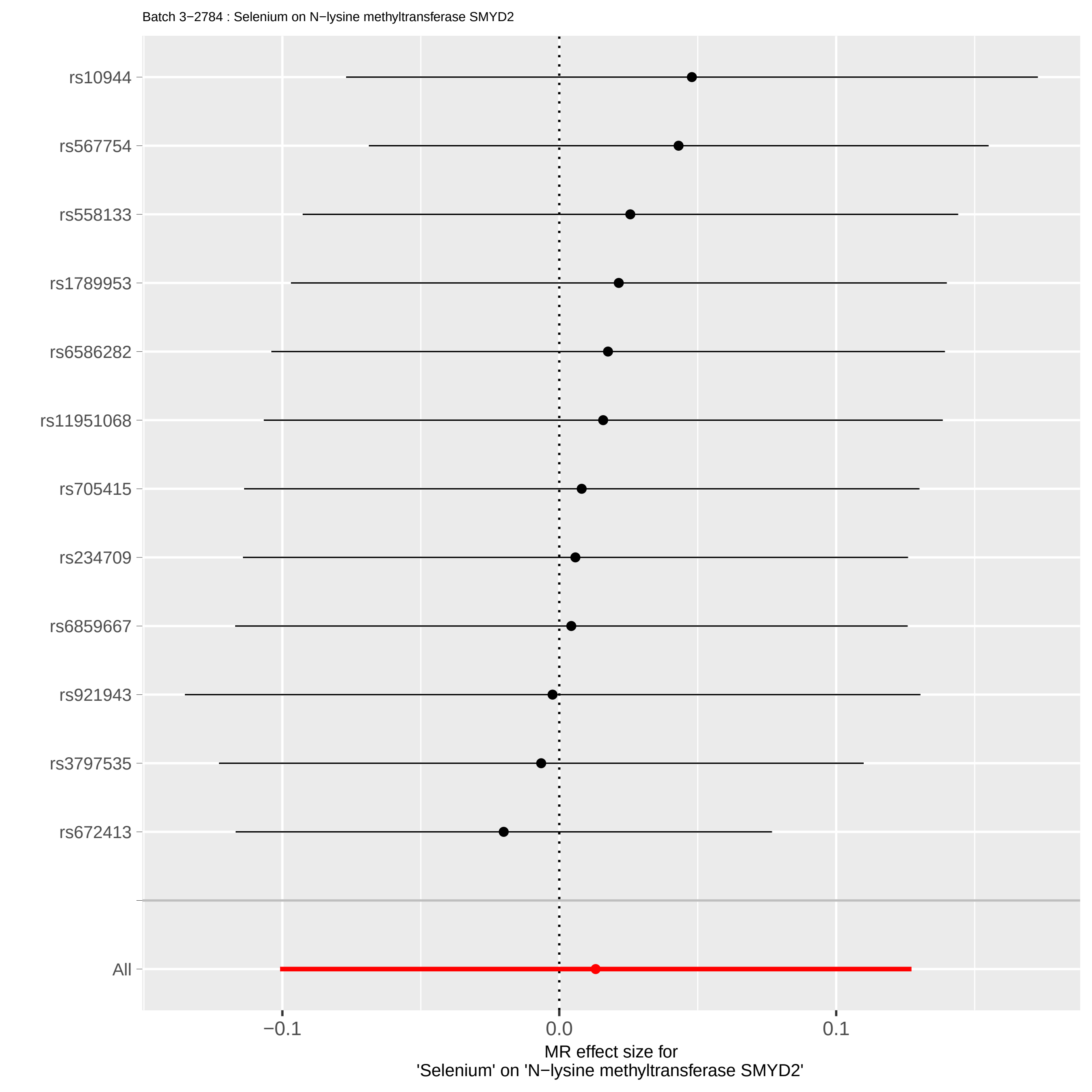

Batch 3−2784 : Selenium on N−lysine methyltransferase SMYD2
rs10944
rs567754
rs558133
rs1789953
rs6586282
rs11951068
rs705415
rs234709
rs6859667
rs921943
rs3797535
rs672413
All
−0.1
0.0
0.1
MR effect size for
'Selenium' on 'N−lysine methyltransferase SMYD2'

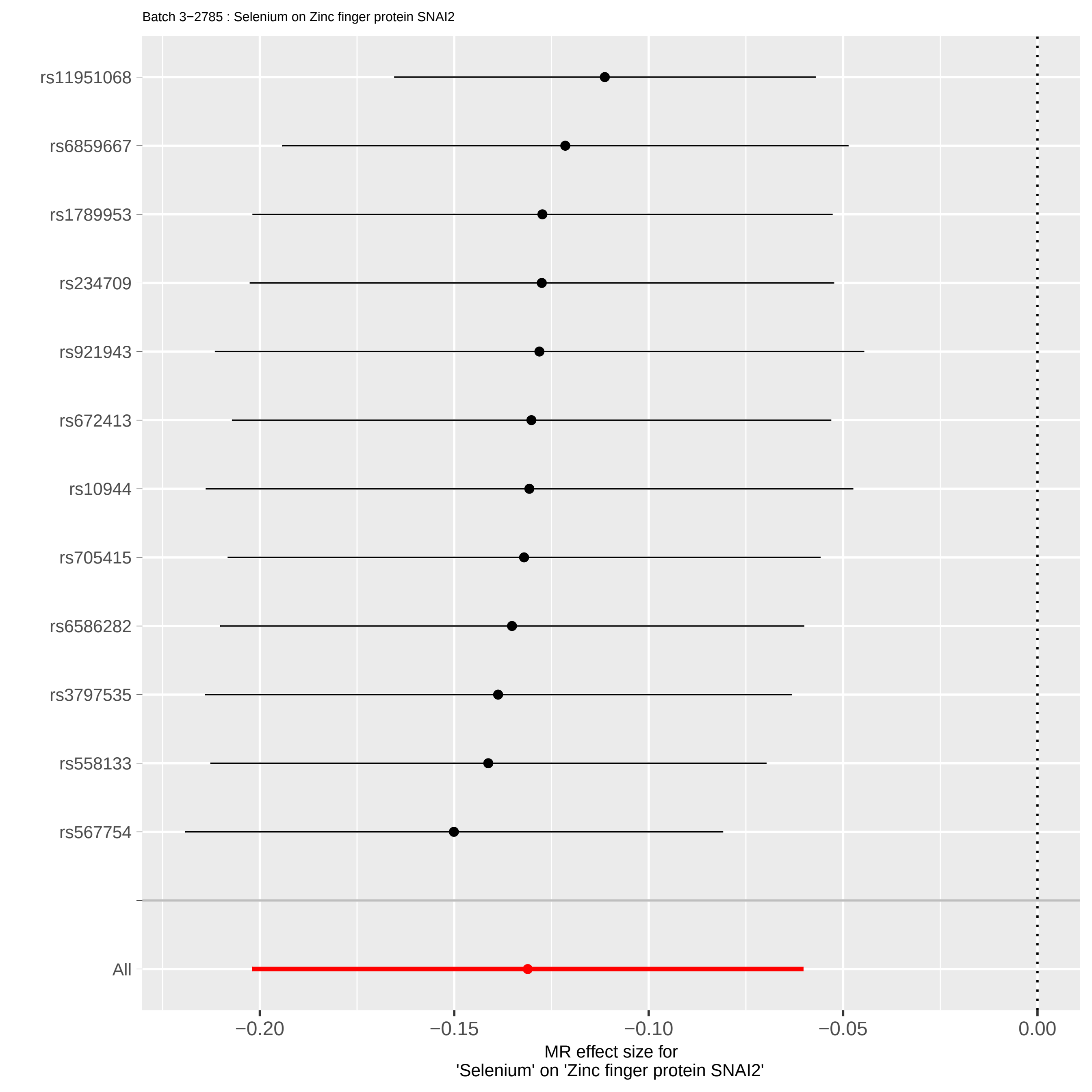

Batch 3−2785 : Selenium on Zinc finger protein SNAI2
rs11951068
rs6859667
rs1789953
rs234709
rs921943
rs672413
rs10944
rs705415
rs6586282
rs3797535
rs558133
rs567754
All
−0.20
−0.15
−0.10
−0.05
0.00
MR effect size for
'Selenium' on 'Zinc finger protein SNAI2'

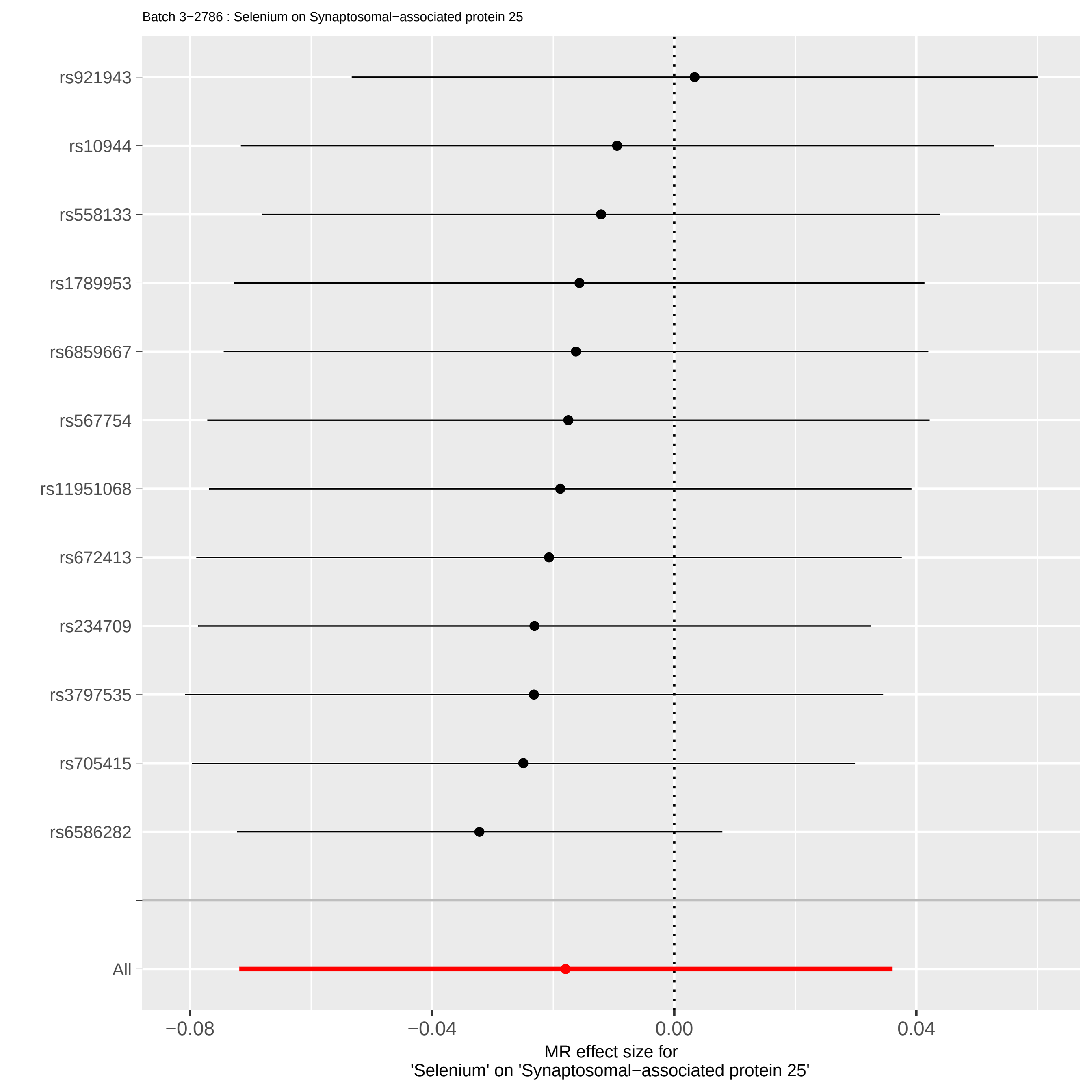

Batch 3−2786 : Selenium on Synaptosomal−associated protein 25
rs921943
rs10944
rs558133
rs1789953
rs6859667
rs567754
rs11951068
rs672413
rs234709
rs3797535
rs705415
rs6586282
All
−0.08
−0.04
0.00
0.04
MR effect size for
'Selenium' on 'Synaptosomal−associated protein 25'

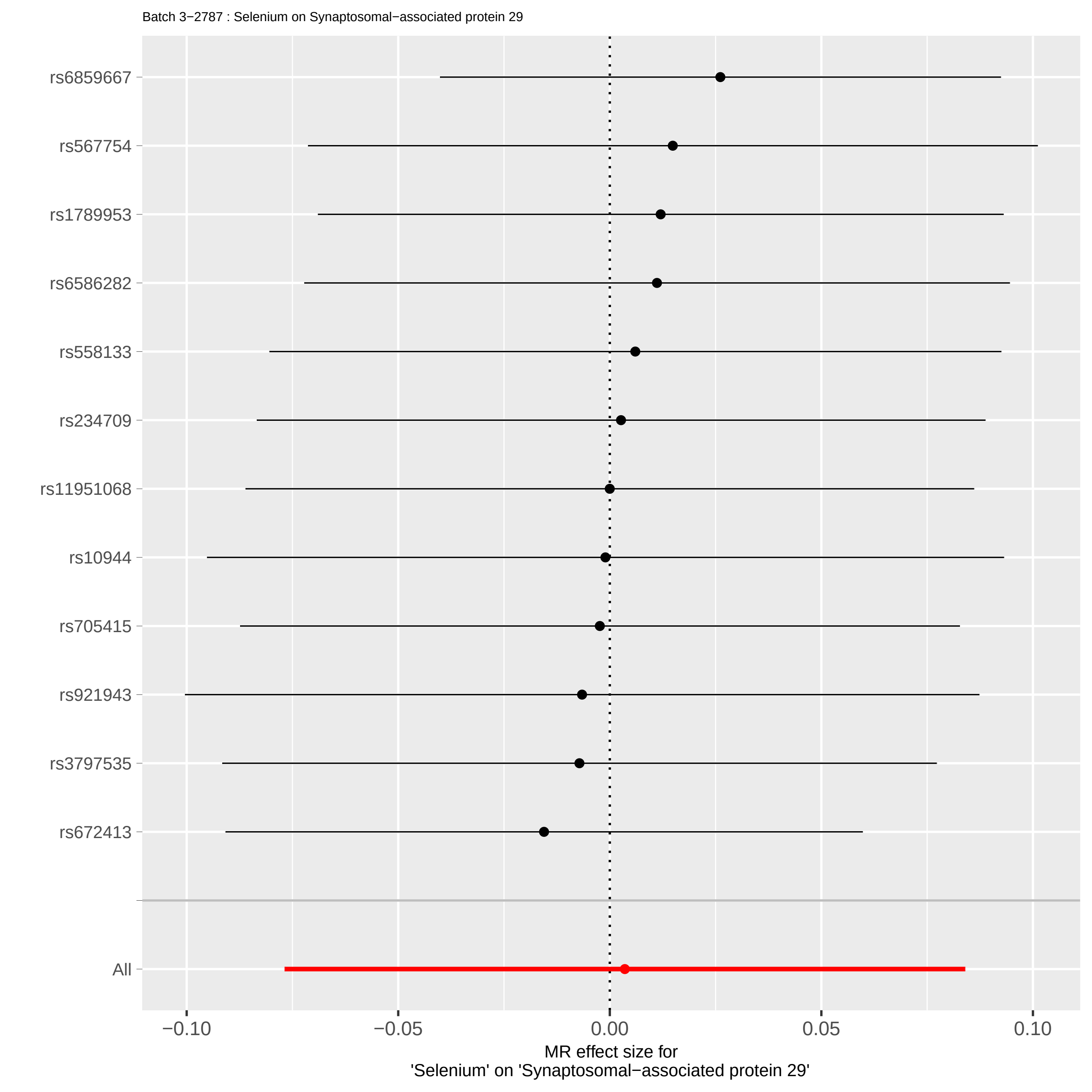

Batch 3−2787 : Selenium on Synaptosomal−associated protein 29
rs6859667
rs567754
rs1789953
rs6586282
rs558133
rs234709
rs11951068
rs10944
rs705415
rs921943
rs3797535
rs672413
All
−0.10
−0.05
0.00
0.05
0.10
MR effect size for
'Selenium' on 'Synaptosomal−associated protein 29'

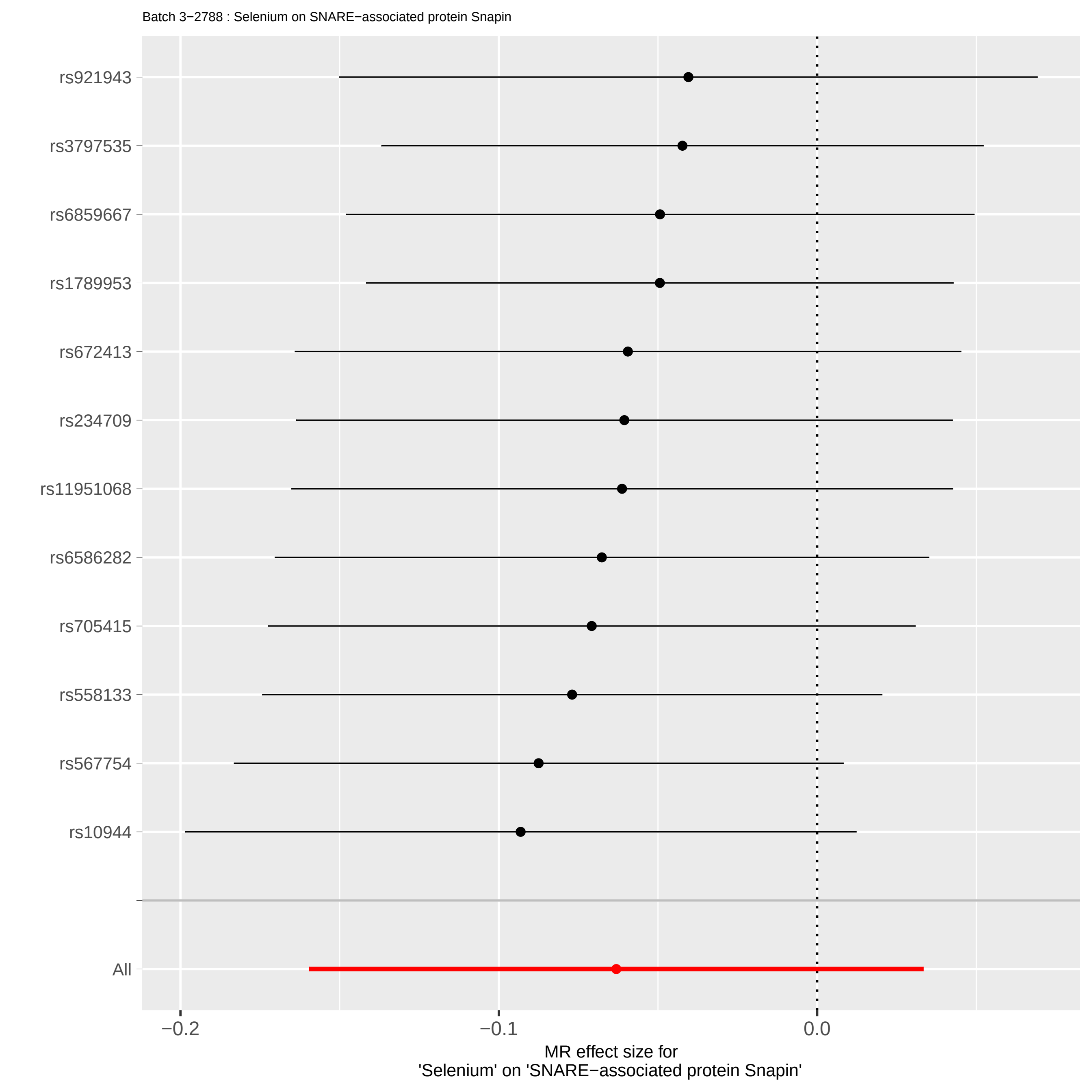

Batch 3−2788 : Selenium on SNARE−associated protein Snapin
rs921943
rs3797535
rs6859667
rs1789953
rs672413
rs234709
rs11951068
rs6586282
rs705415
rs558133
rs567754
rs10944
All
−0.2
−0.1
0.0
MR effect size for
'Selenium' on 'SNARE−associated protein Snapin'

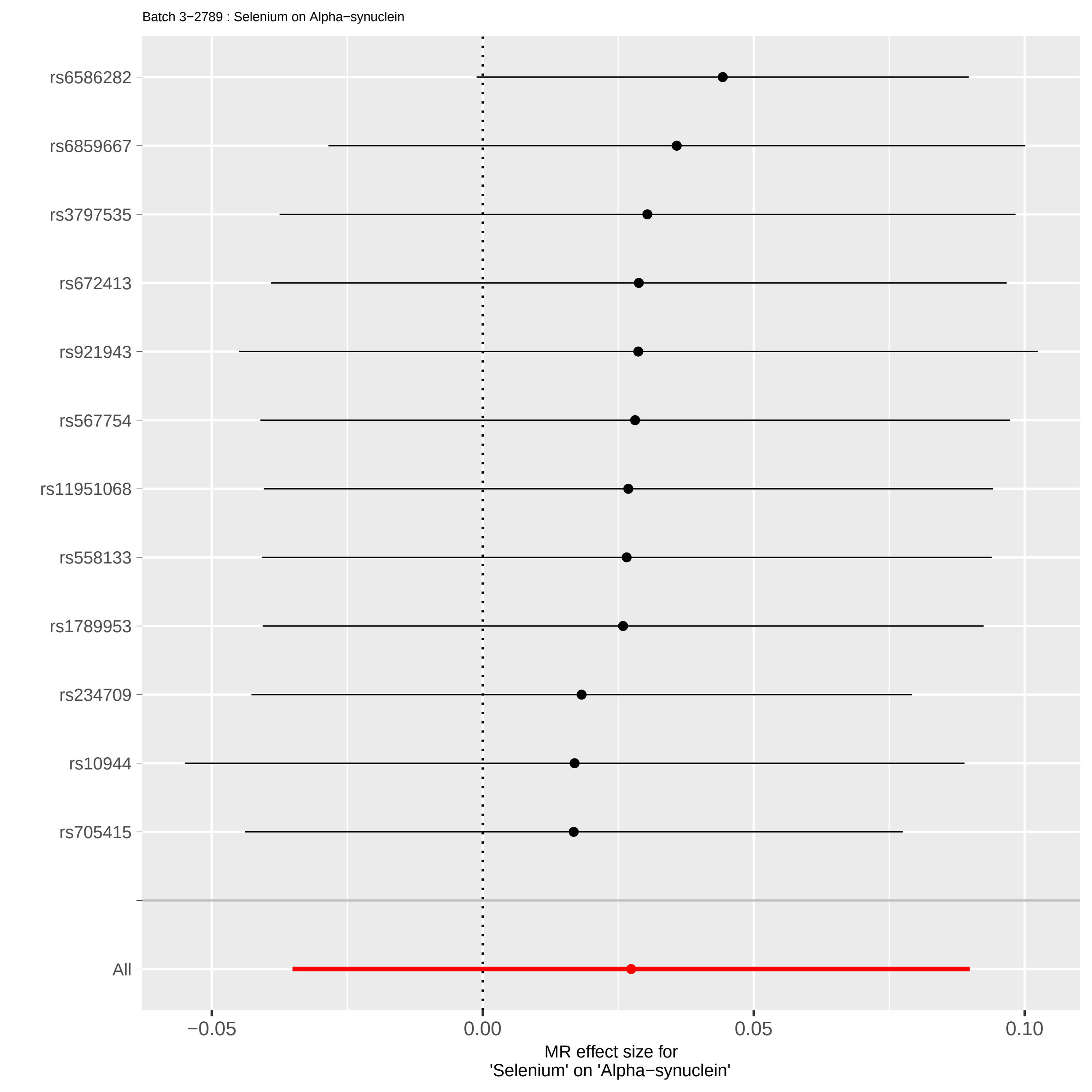

Batch 3−2789 : Selenium on Alpha−synuclein
rs6586282
rs6859667
rs3797535
rs672413
rs921943
rs567754
rs11951068
rs558133
rs1789953
rs234709
rs10944
rs705415
All
−0.05
0.00
0.05
0.10
MR effect size for
'Selenium' on 'Alpha−synuclein'

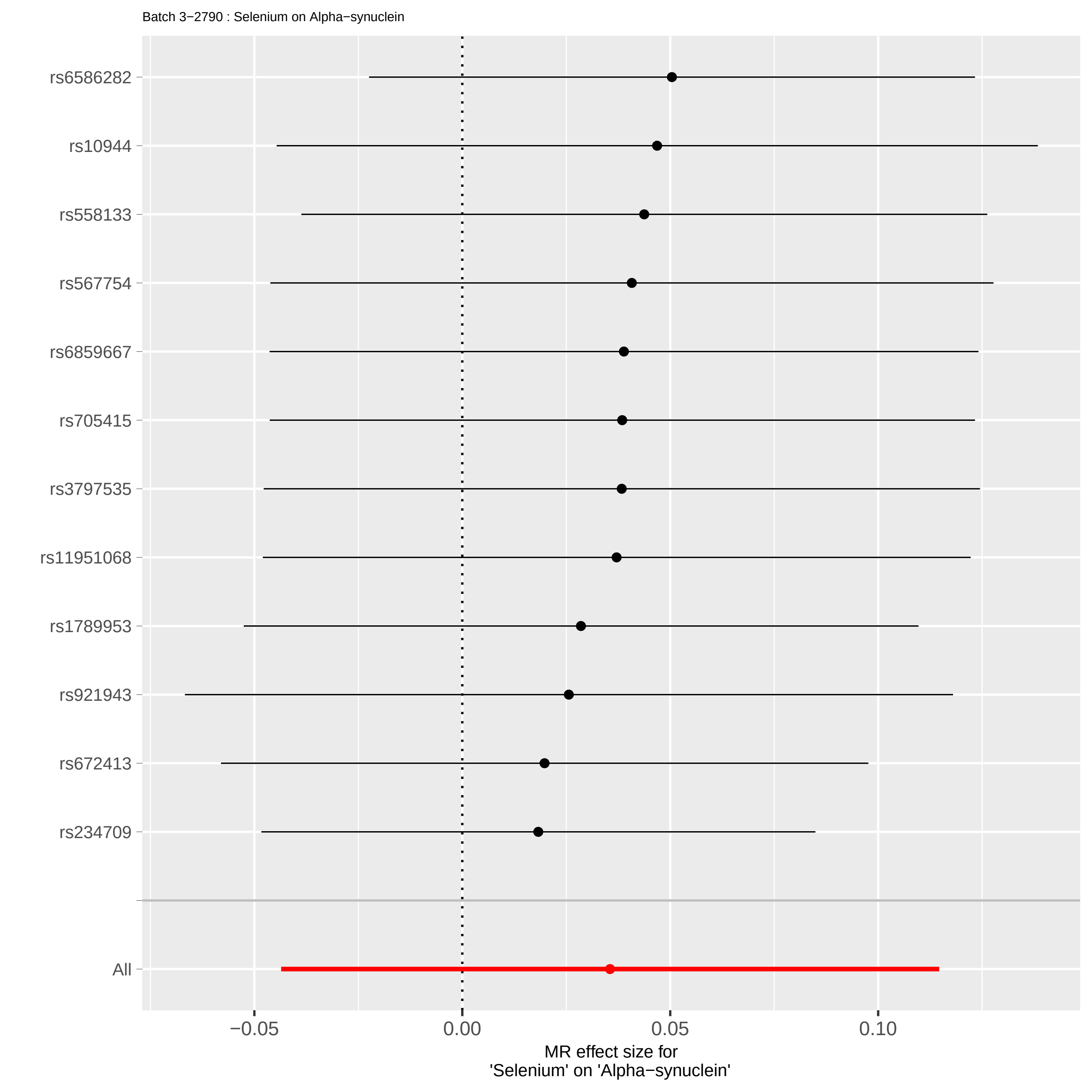

Batch 3−2790 : Selenium on Alpha−synuclein
rs6586282
rs10944
rs558133
rs567754
rs6859667
rs705415
rs3797535
rs11951068
rs1789953
rs921943
rs672413
rs234709
All
−0.05
0.00
0.05
0.10
MR effect size for
'Selenium' on 'Alpha−synuclein'

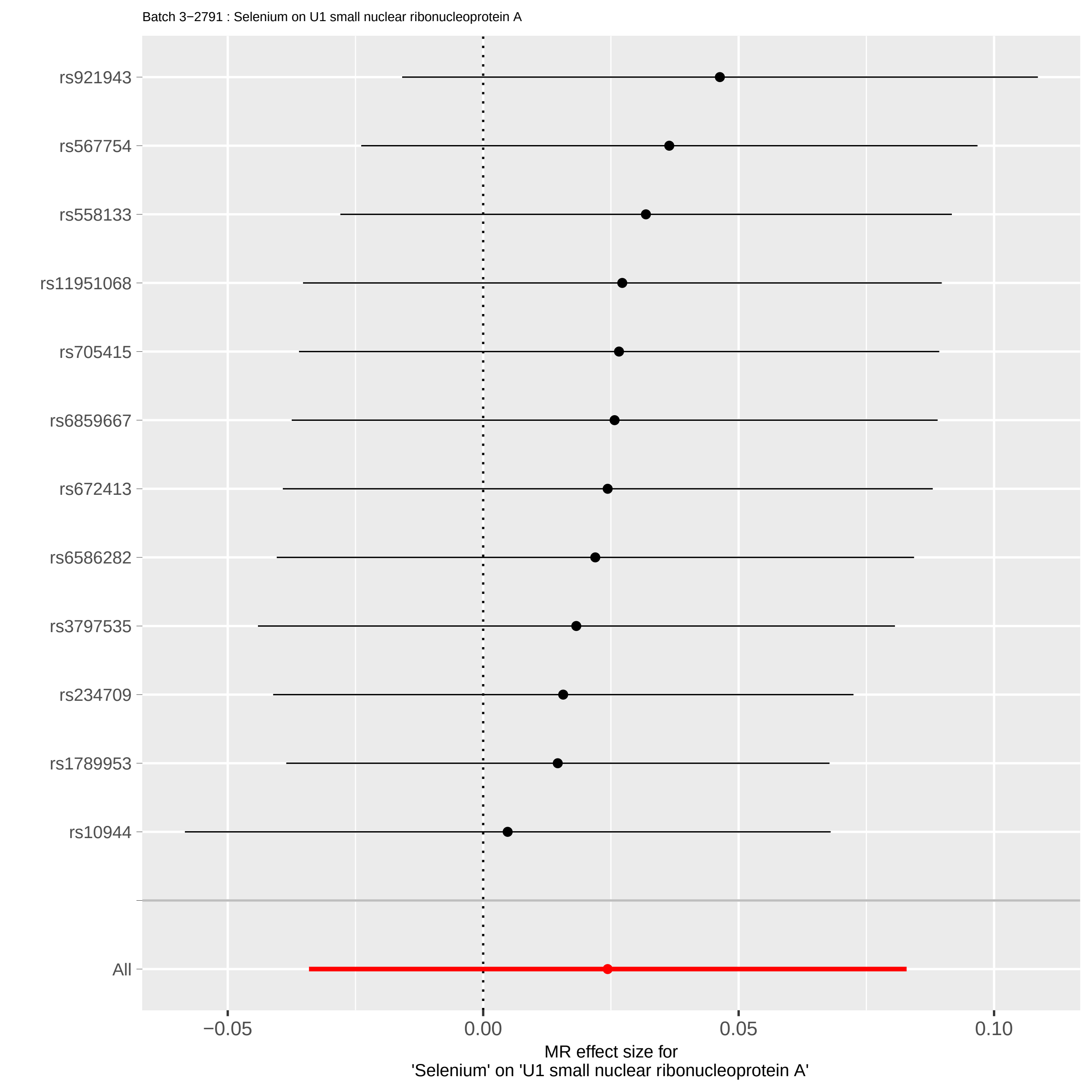

Batch 3−2791 : Selenium on U1 small nuclear ribonucleoprotein A
rs921943
rs567754
rs558133
rs11951068
rs705415
rs6859667
rs672413
rs6586282
rs3797535
rs234709
rs1789953
rs10944
All
−0.05
0.00
0.05
0.10
MR effect size for
'Selenium' on 'U1 small nuclear ribonucleoprotein A'

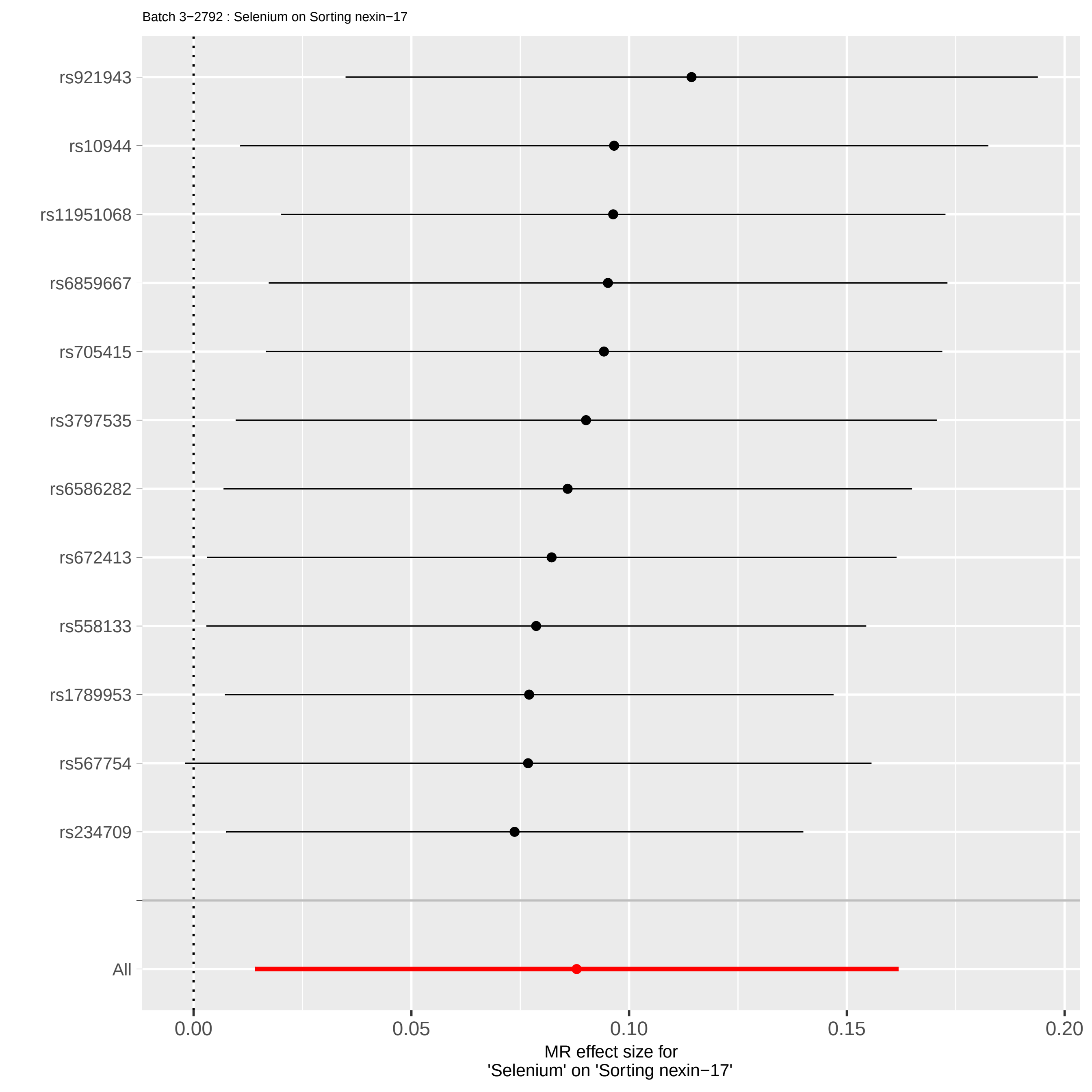

Batch 3−2792 : Selenium on Sorting nexin−17
rs921943
rs10944
rs11951068
rs6859667
rs705415
rs3797535
rs6586282
rs672413
rs558133
rs1789953
rs567754
rs234709
All
0.00
0.05
0.10
0.15
0.20
MR effect size for
'Selenium' on 'Sorting nexin−17'

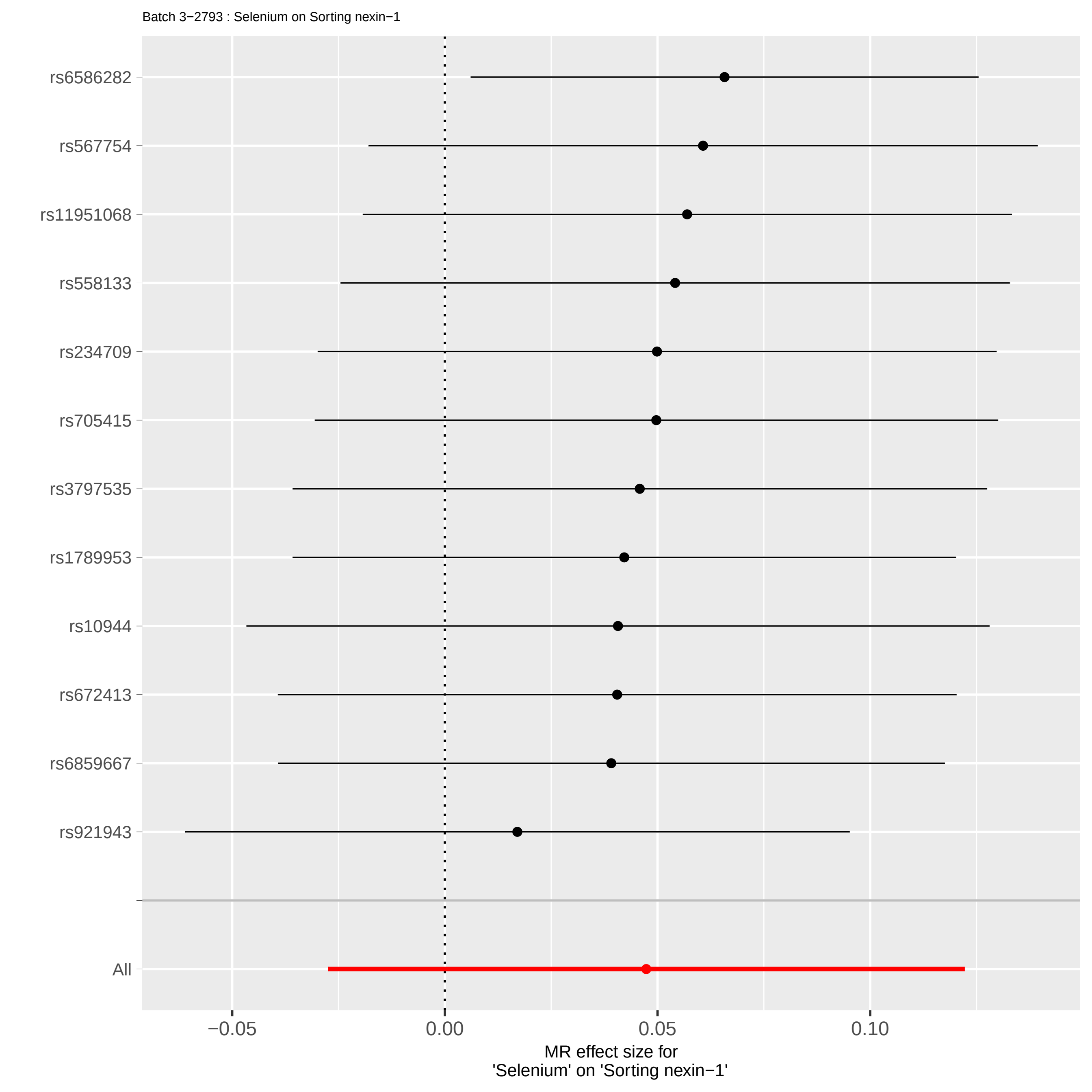

Batch 3−2793 : Selenium on Sorting nexin−1
rs6586282
rs567754
rs11951068
rs558133
rs234709
rs705415
rs3797535
rs1789953
rs10944
rs672413
rs6859667
rs921943
All
−0.05
0.00
0.05
0.10
MR effect size for
'Selenium' on 'Sorting nexin−1'

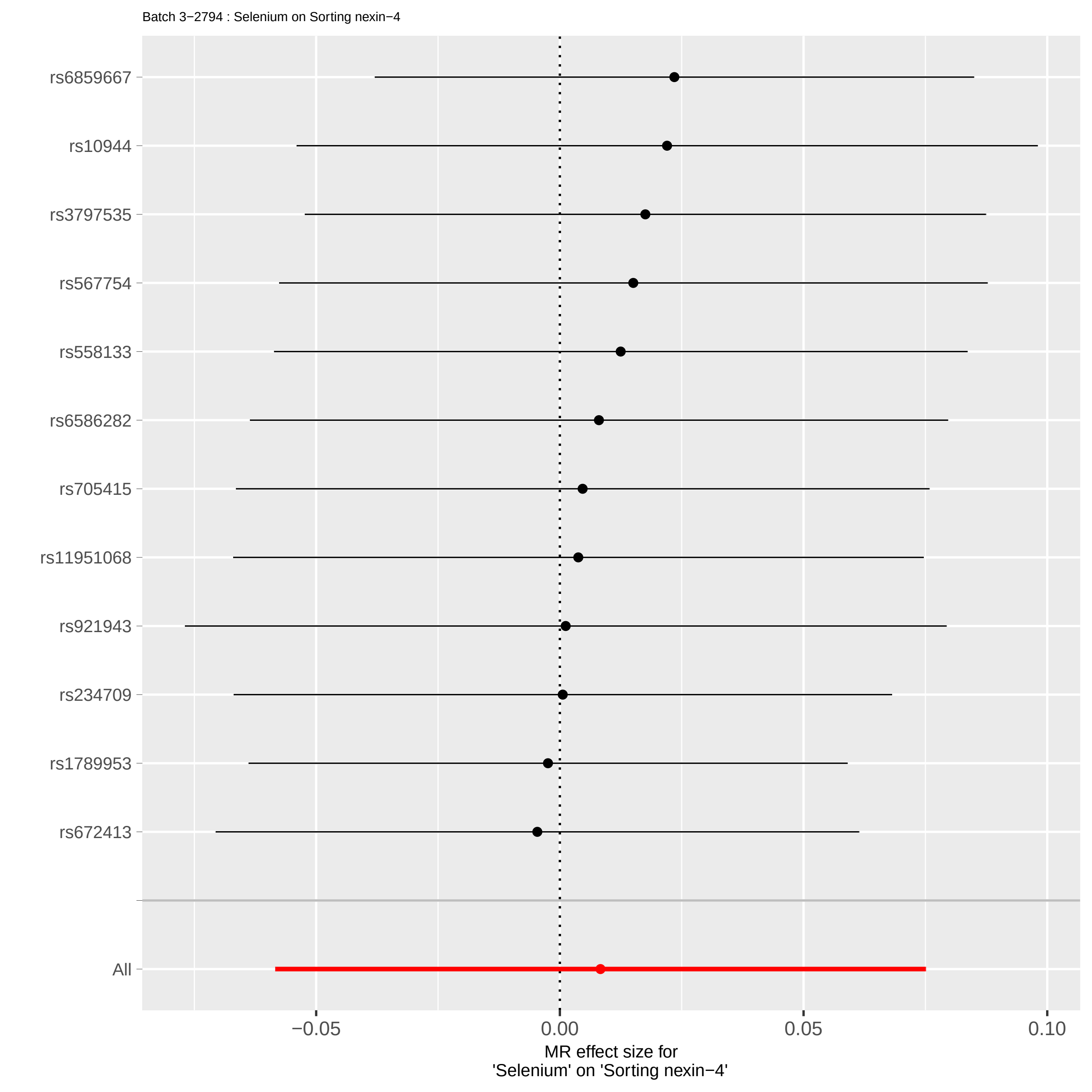
Batch 3−2794 : Selenium on Sorting nexin−4
rs6859667
rs10944
rs3797535
rs567754
rs558133
rs6586282
rs705415
rs11951068
rs921943
rs234709
rs1789953
rs672413
All
−0.05
0.00
0.05
0.10
MR effect size for
'Selenium' on 'Sorting nexin−4'

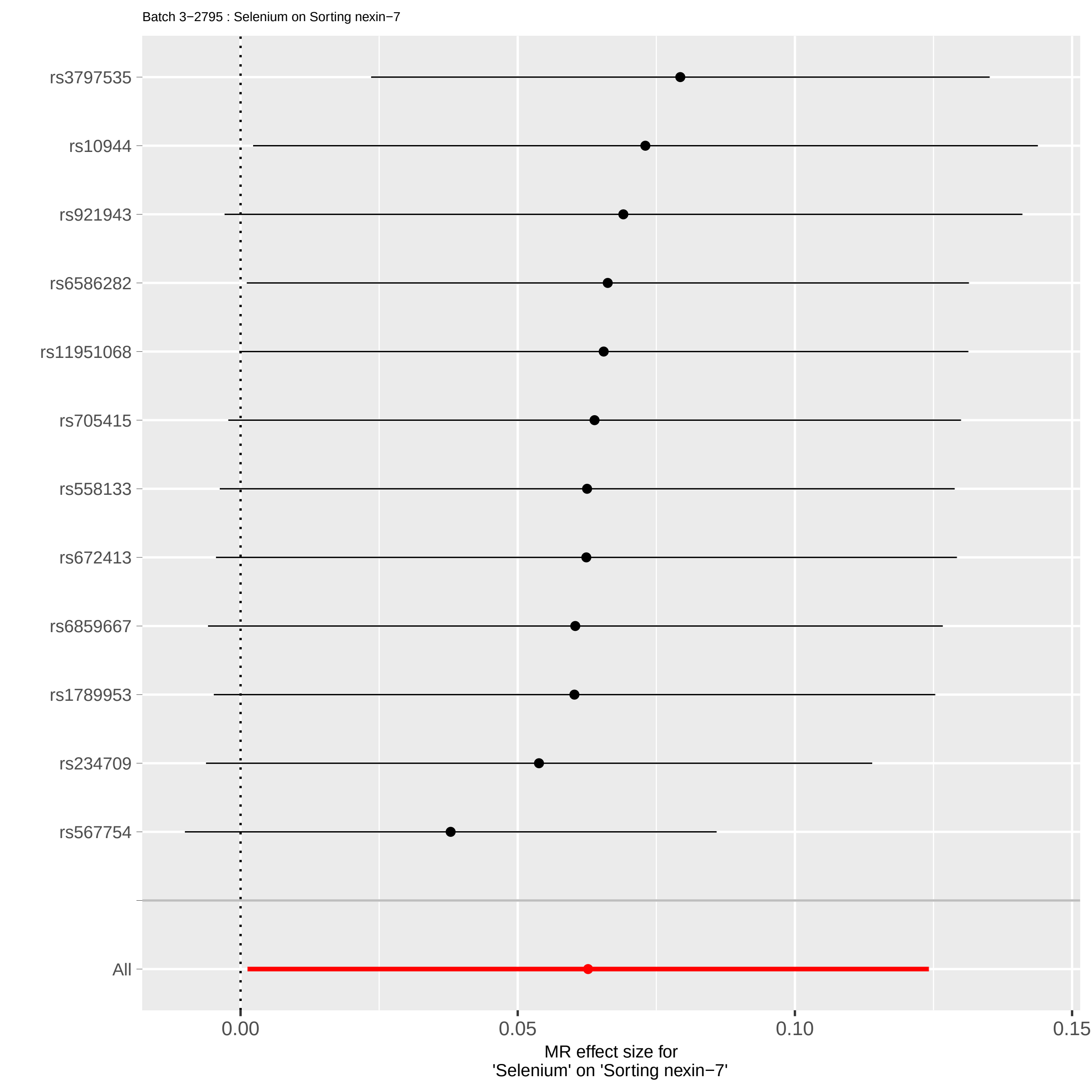

Batch 3−2795 : Selenium on Sorting nexin−7
rs3797535
rs10944
rs921943
rs6586282
rs11951068
rs705415
rs558133
rs672413
rs6859667
rs1789953
rs234709
rs567754
All
0.00
0.05
0.10
0.15
MR effect size for
'Selenium' on 'Sorting nexin−7'

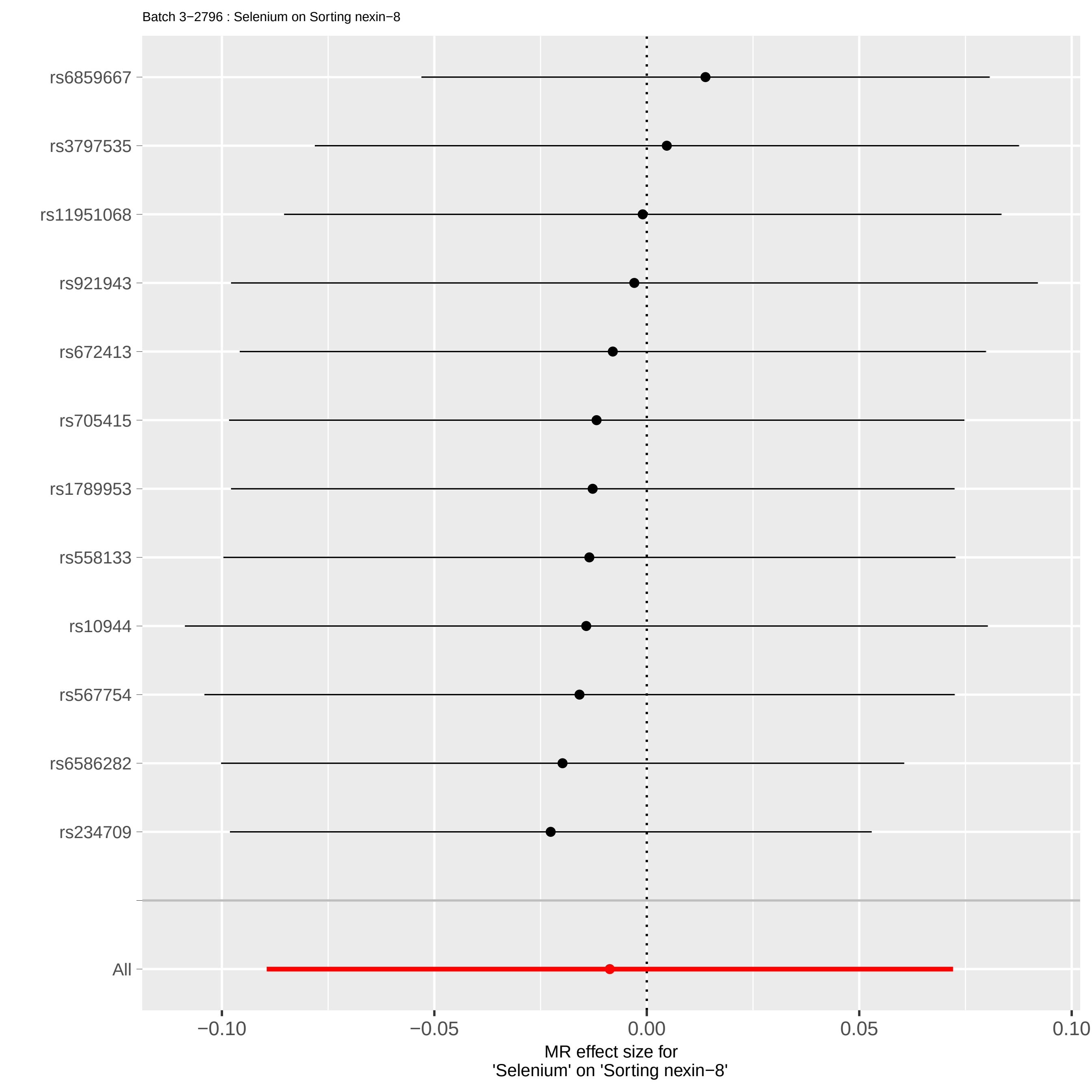
Batch 3−2796 : Selenium on Sorting nexin−8
rs6859667
rs3797535
rs11951068
rs921943
rs672413
rs705415
rs1789953
rs558133
rs10944
rs567754
rs6586282
rs234709
All
−0.10
−0.05
0.00
0.05
0.10
MR effect size for
'Selenium' on 'Sorting nexin−8'

Batch 3−2797 : Selenium on Suppressor of cytokine signaling 3

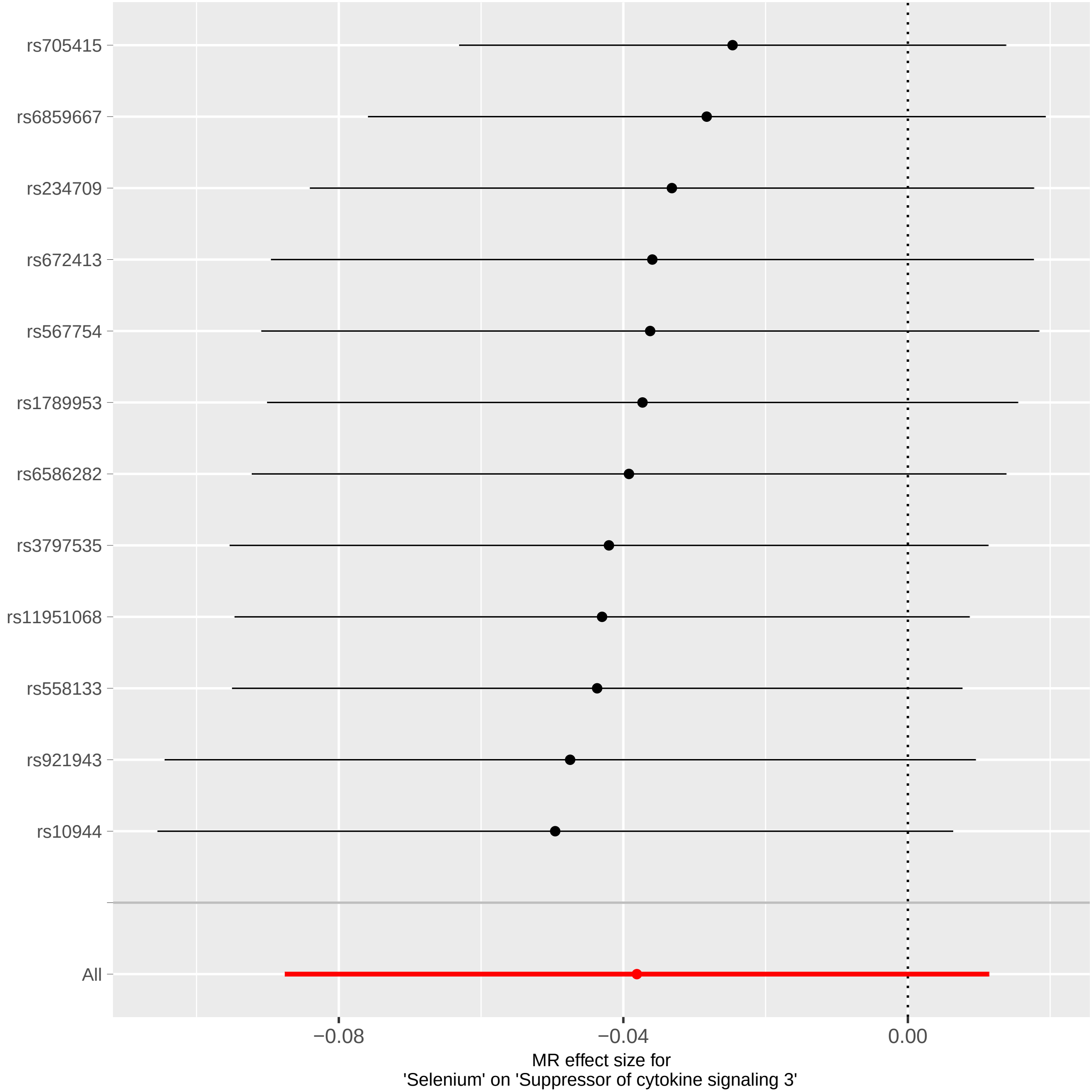

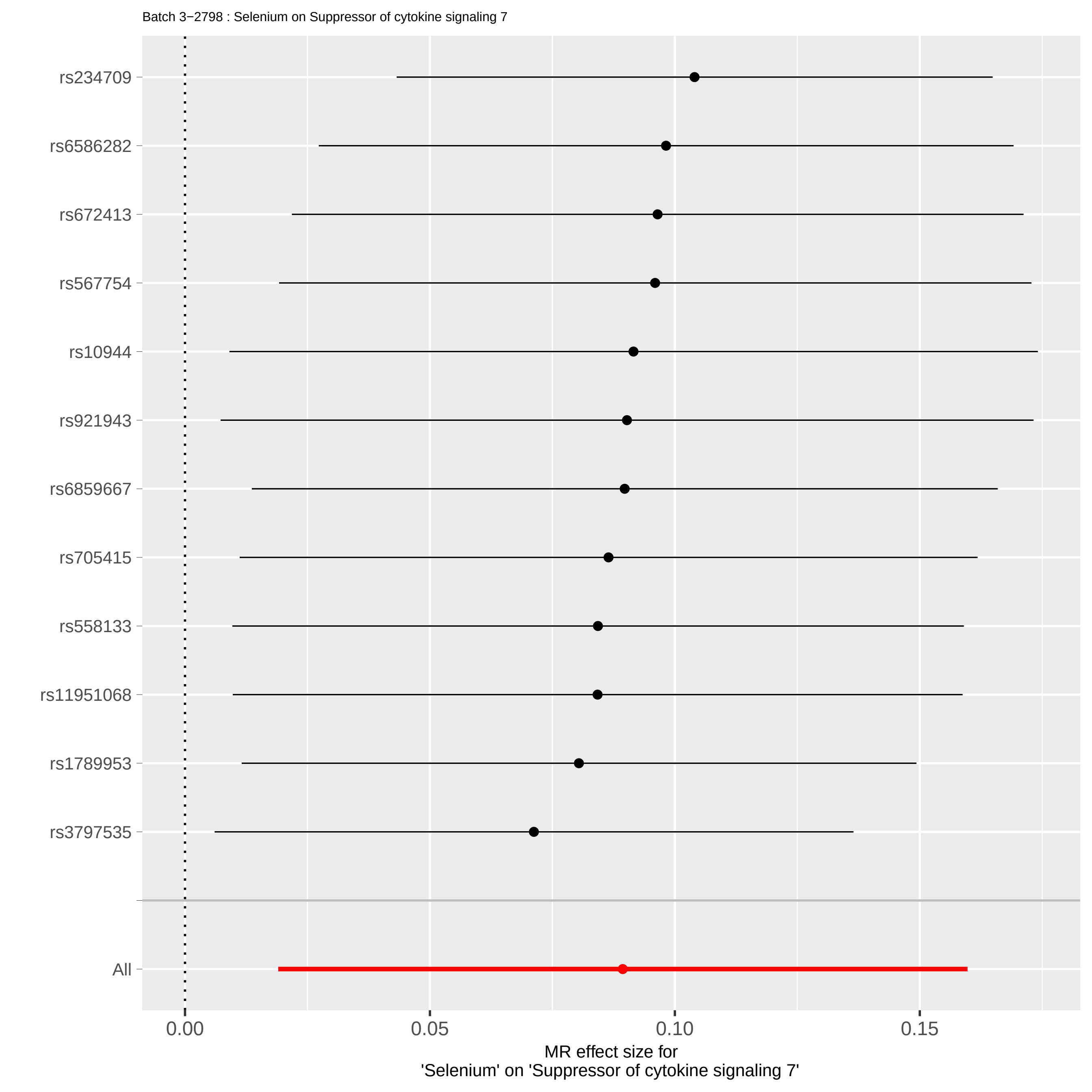

Batch 3−2798 : Selenium on Suppressor of cytokine signaling 7
rs234709
rs6586282
rs672413
rs567754
rs10944
rs921943
rs6859667
rs705415
rs558133
rs11951068
rs1789953
rs3797535
All
0.00
0.05
0.10
0.15
MR effect size for
'Selenium' on 'Suppressor of cytokine signaling 7'

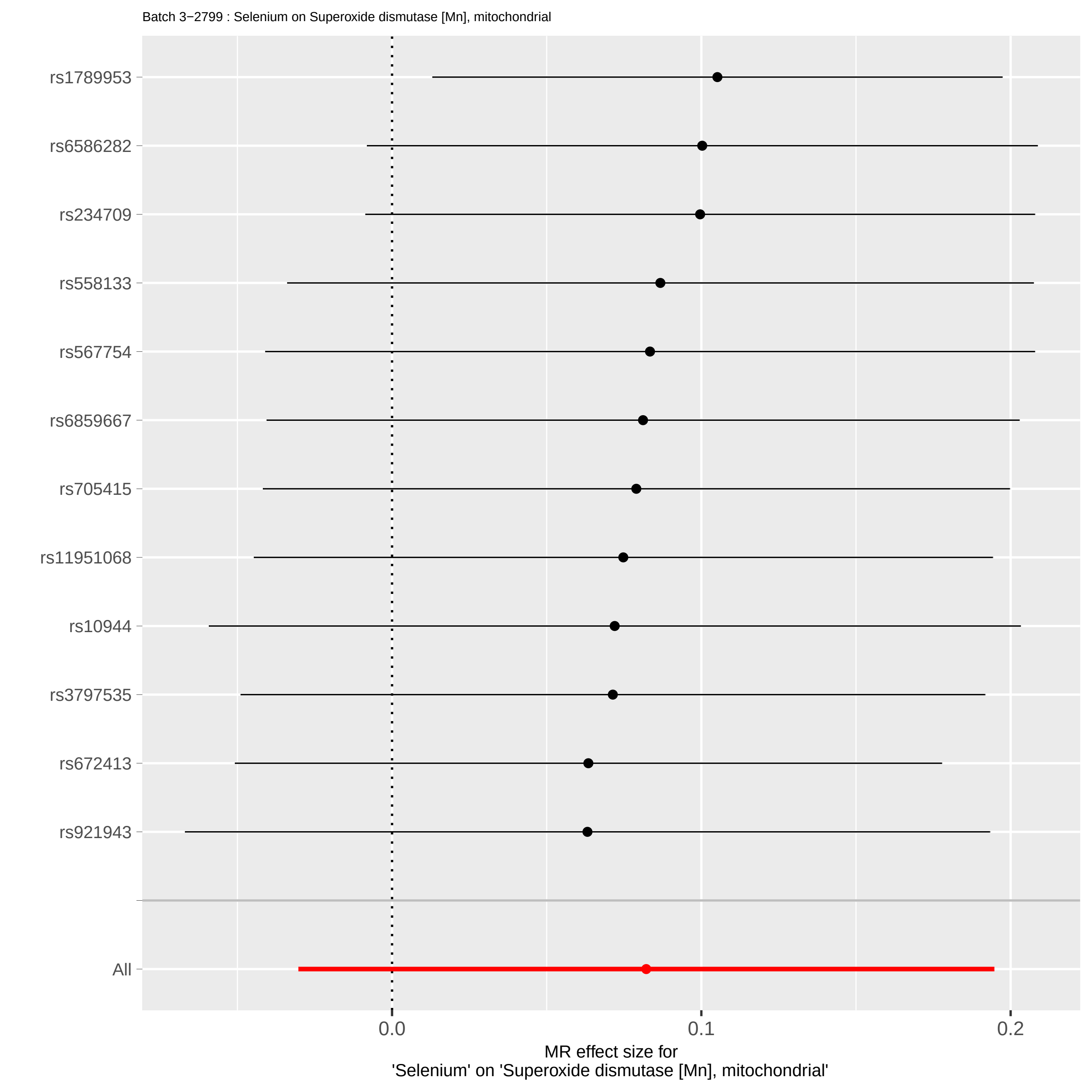
Batch 3−2799 : Selenium on Superoxide dismutase [Mn], mitochondrial
rs1789953
rs6586282
rs234709
rs558133
rs567754
rs6859667
rs705415
rs11951068
rs10944
rs3797535
rs672413
rs921943
All
0.0
0.1
0.2
MR effect size for
'Selenium' on 'Superoxide dismutase [Mn], mitochondrial'

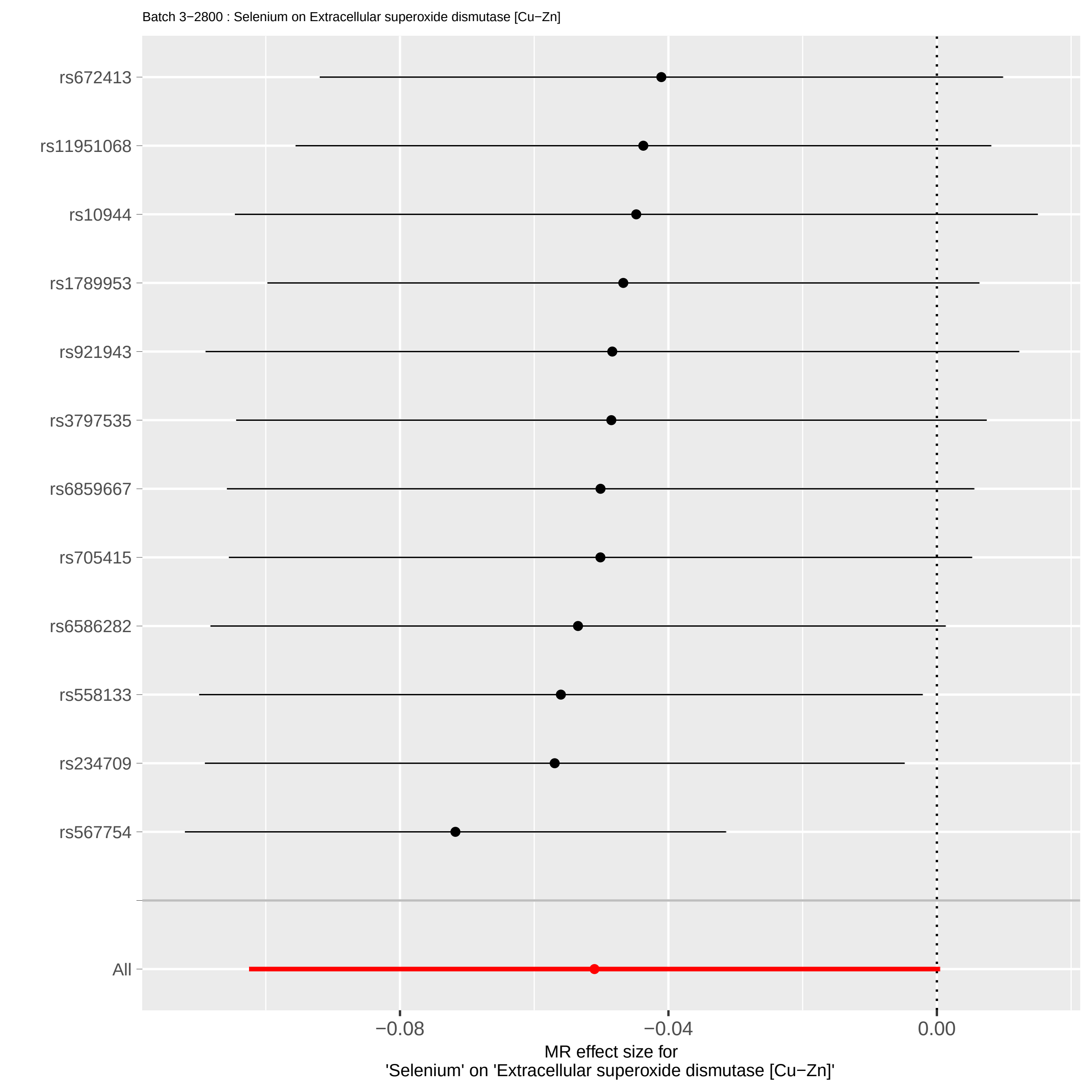
Batch 3−2800 : Selenium on Extracellular superoxide dismutase [Cu−Zn]
rs672413
rs11951068
rs10944
rs1789953
rs921943
rs3797535
rs6859667
rs705415
rs6586282
rs558133
rs234709
rs567754
All
−0.08
−0.04
0.00
MR effect size for
'Selenium' on 'Extracellular superoxide dismutase [Cu−Zn]'

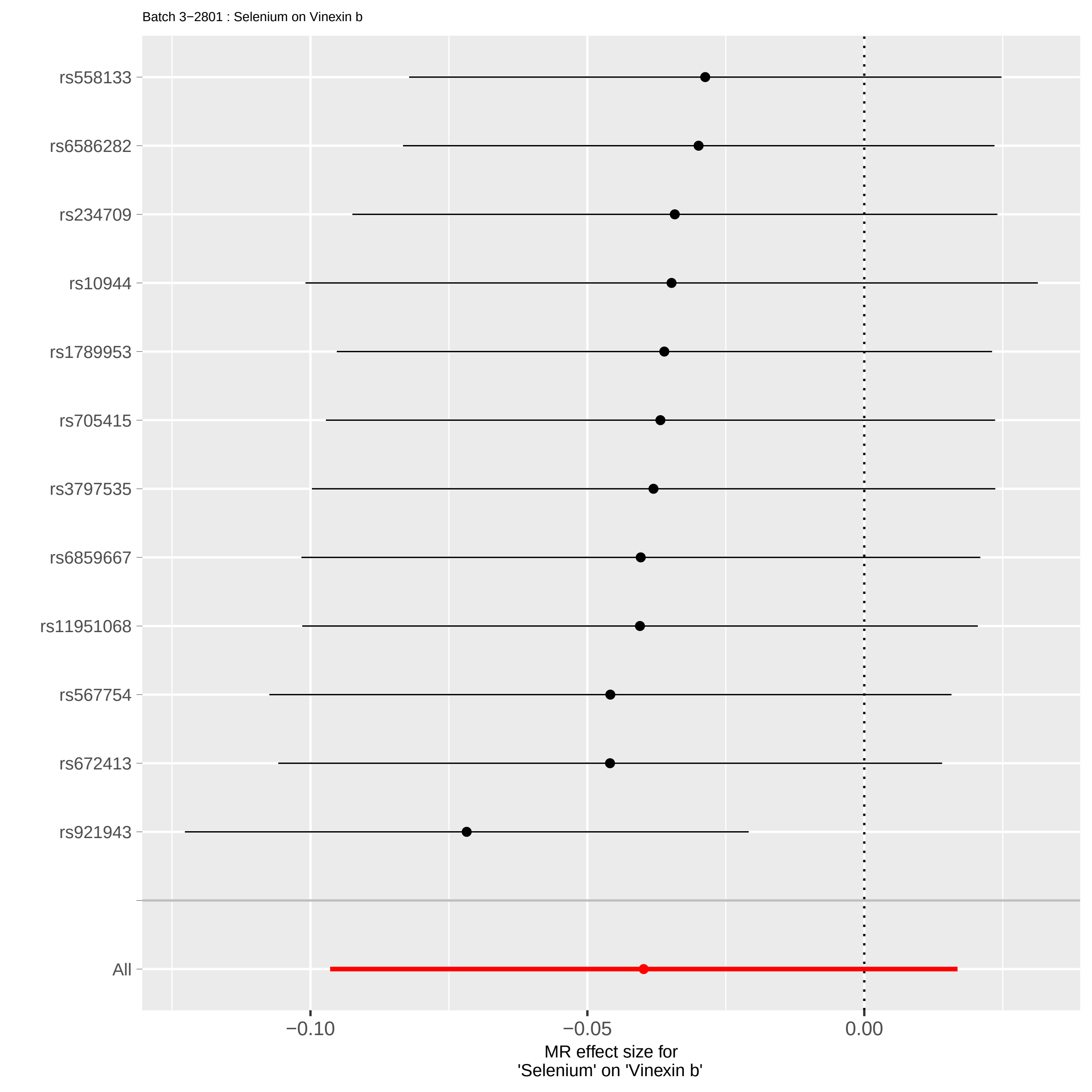

Batch 3−2801 : Selenium on Vinexin b
rs558133
rs6586282
rs234709
rs10944
rs1789953
rs705415
rs3797535
rs6859667
rs11951068
rs567754
rs672413
rs921943
All
−0.10
−0.05
0.00
MR effect size for
'Selenium' on 'Vinexin b'

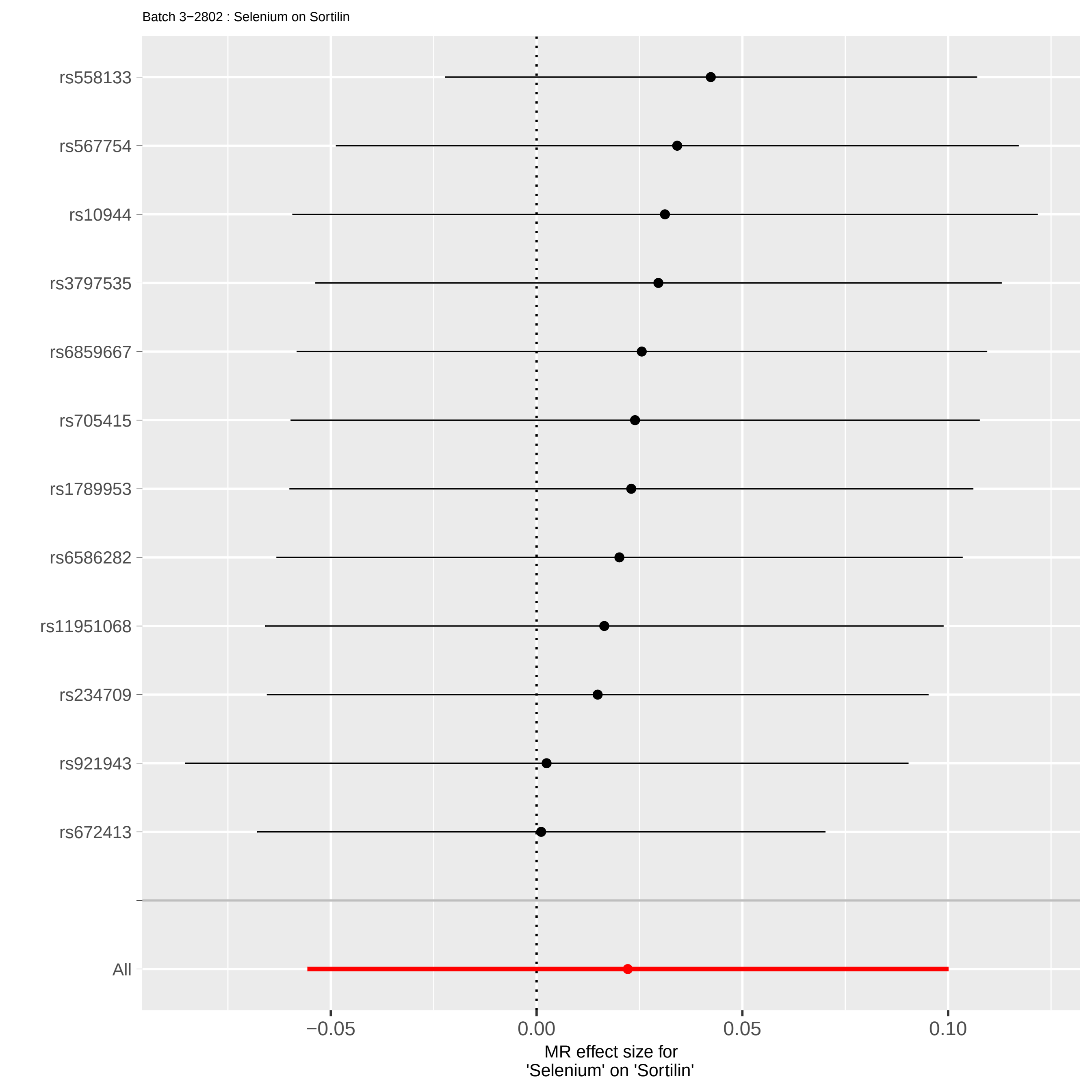

Batch 3−2802 : Selenium on Sortilin
rs558133
rs567754
rs10944
rs3797535
rs6859667
rs705415
rs1789953
rs6586282
rs11951068
rs234709
rs921943
rs672413
All
−0.05
0.00
0.05
0.10
MR effect size for
'Selenium' on 'Sortilin'

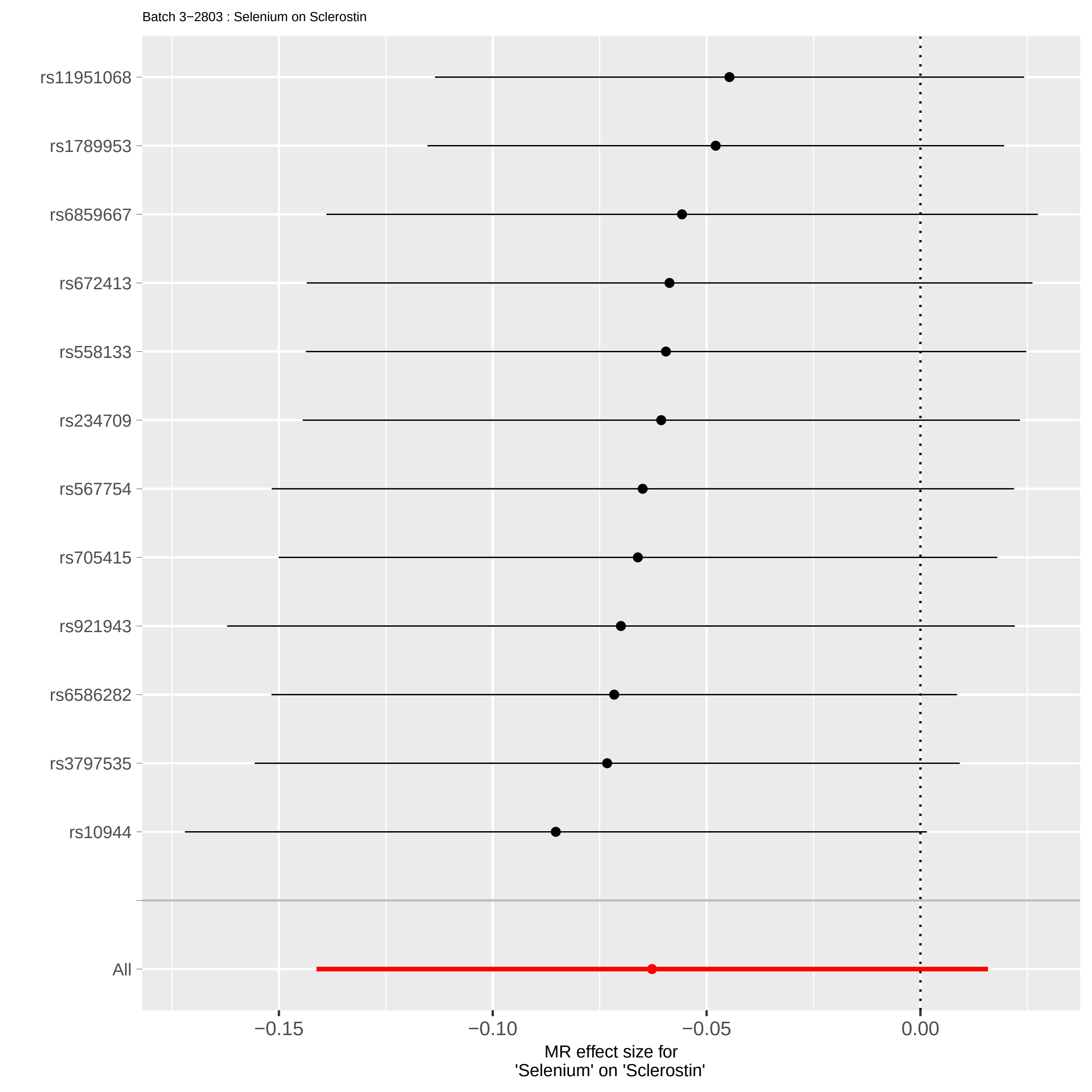

Batch 3−2803 : Selenium on Sclerostin
rs11951068
rs1789953
rs6859667
rs672413
rs558133
rs234709
rs567754
rs705415
rs921943
rs6586282
rs3797535
rs10944
All
−0.15
−0.10
−0.05
0.00
MR effect size for
'Selenium' on 'Sclerostin'

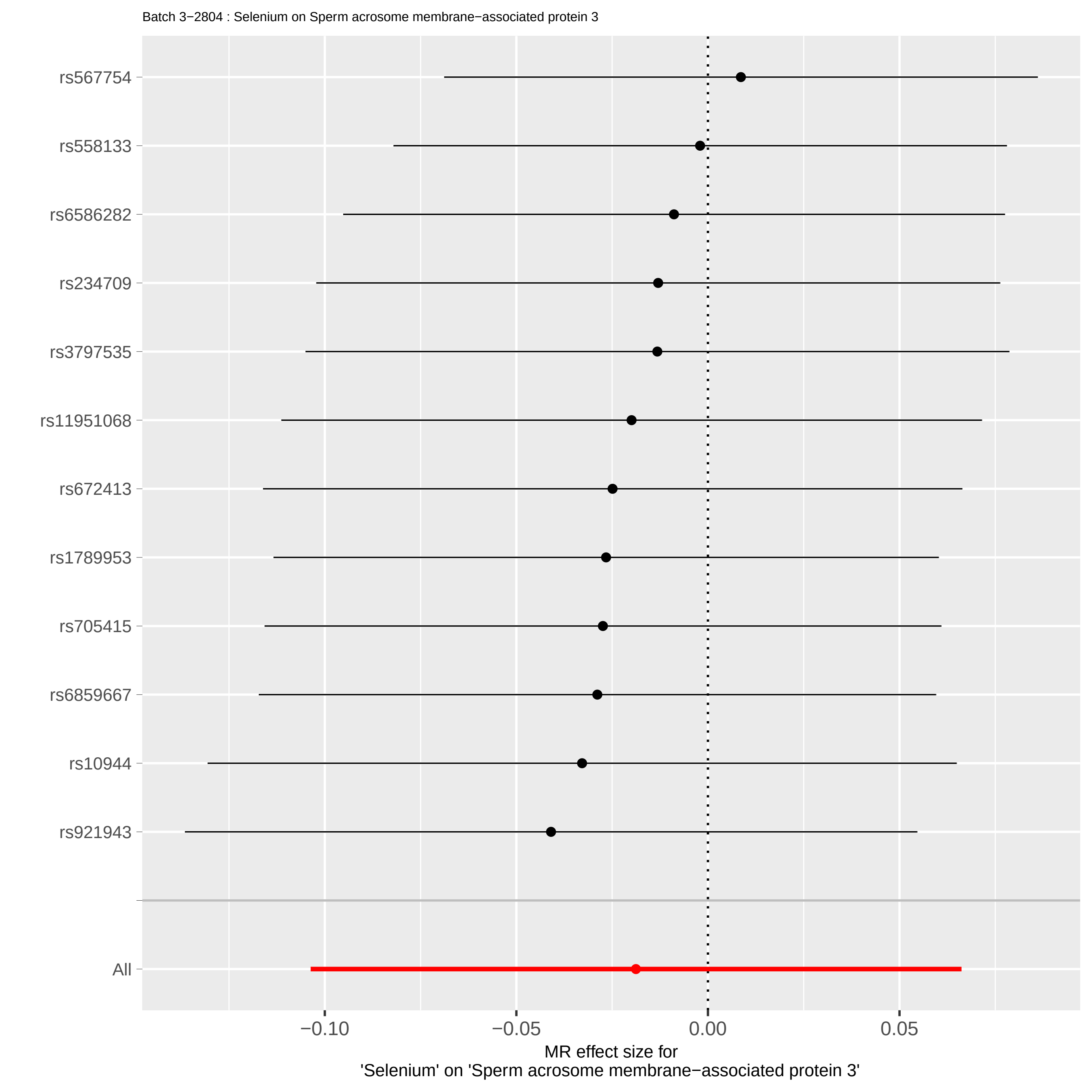
Batch 3−2804 : Selenium on Sperm acrosome membrane−associated protein 3
rs567754
rs558133
rs6586282
rs234709
rs3797535
rs11951068
rs672413
rs1789953
rs705415
rs6859667
rs10944
rs921943
All
−0.10
−0.05
0.00
0.05
MR effect size for
'Selenium' on 'Sperm acrosome membrane−associated protein 3'

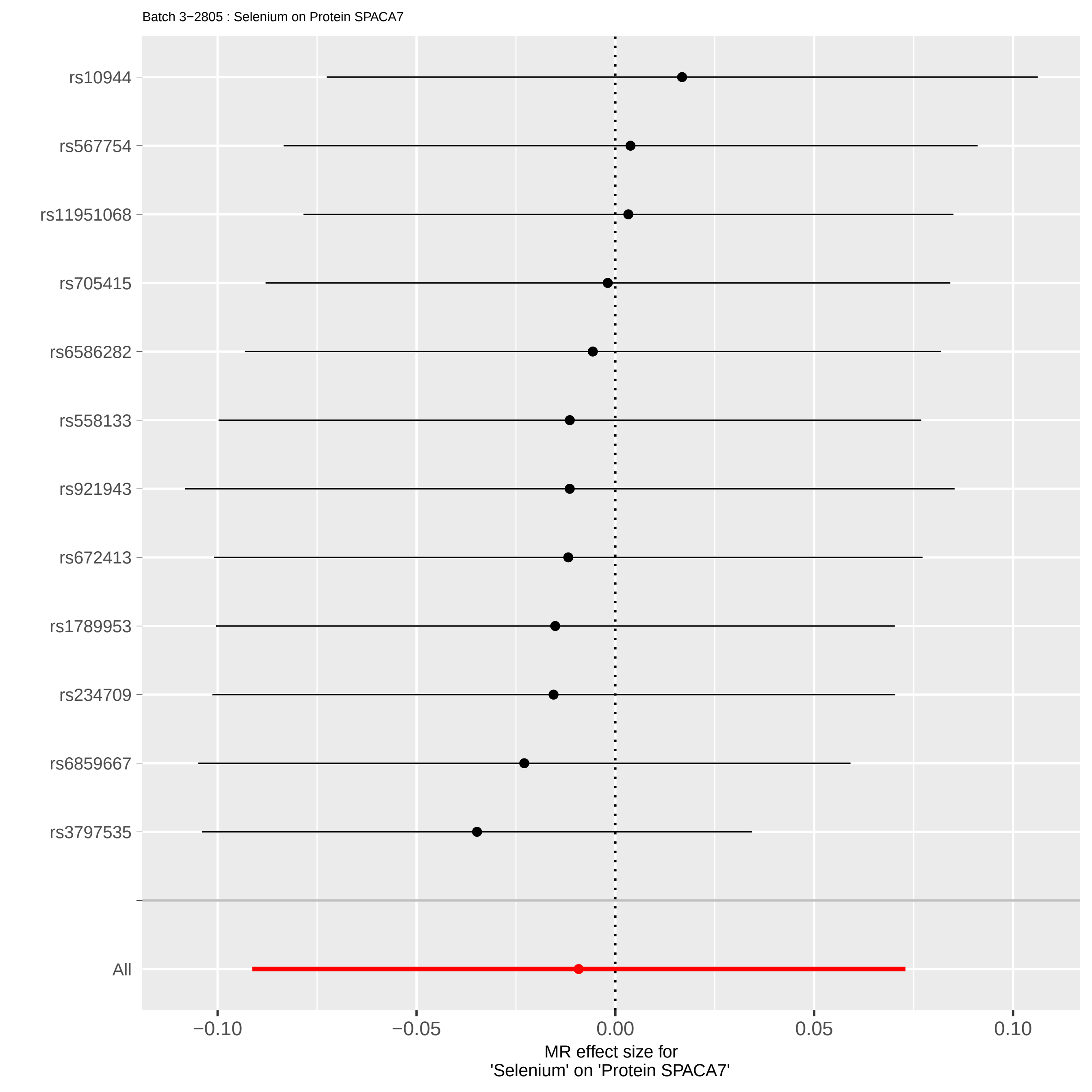

Batch 3−2805 : Selenium on Protein SPACA7
rs10944
rs567754
rs11951068
rs705415
rs6586282
rs558133
rs921943
rs672413
rs1789953
rs234709
rs6859667
rs3797535
All
−0.10
−0.05
0.00
0.05
0.10
MR effect size for
'Selenium' on 'Protein SPACA7'

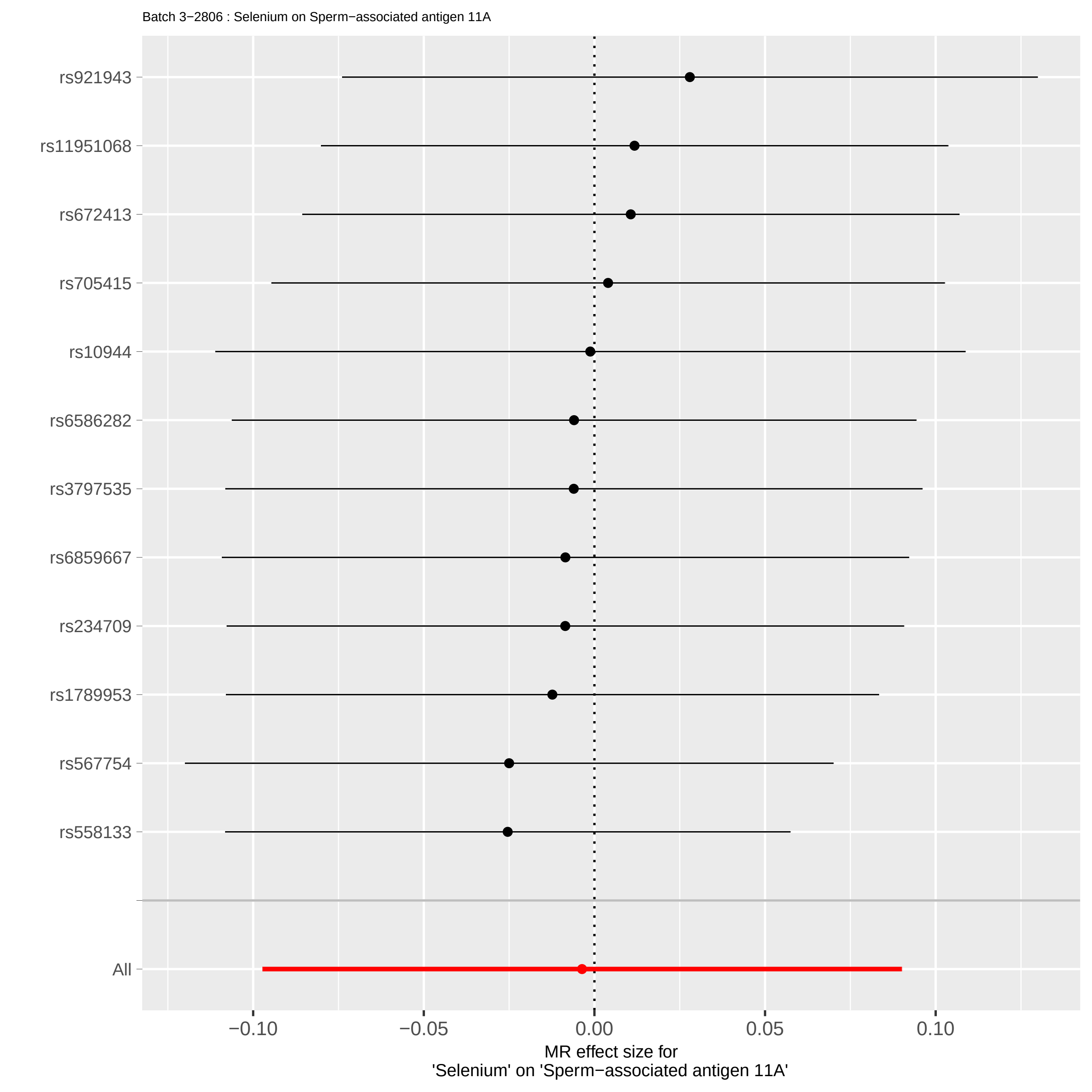
Batch 3−2806 : Selenium on Sperm−associated antigen 11A
rs921943
rs11951068
rs672413
rs705415
rs10944
rs6586282
rs3797535
rs6859667
rs234709
rs1789953
rs567754
rs558133
All
−0.10
−0.05
0.00
0.05
0.10
MR effect size for
'Selenium' on 'Sperm−associated antigen 11A'

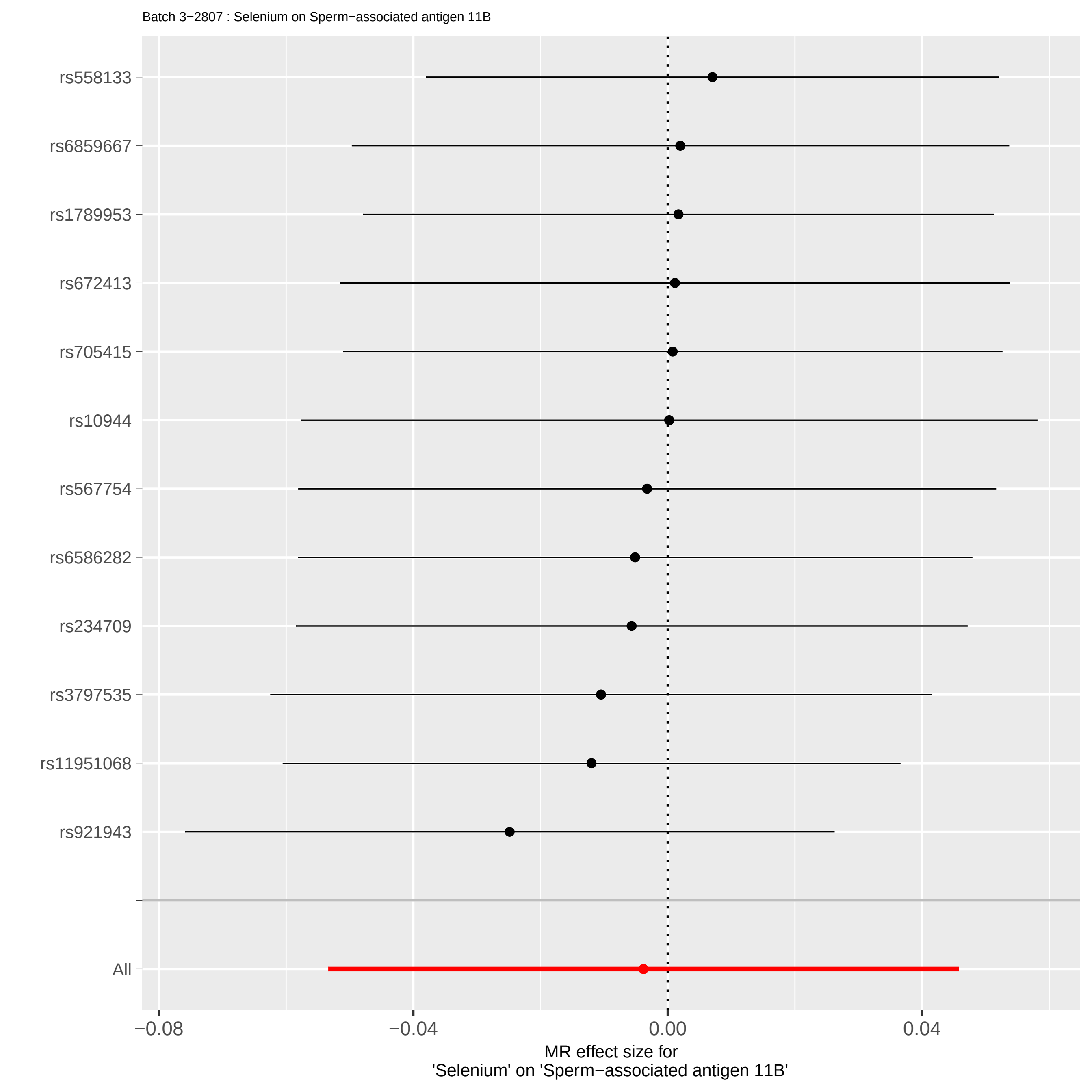

Batch 3−2807 : Selenium on Sperm−associated antigen 11B
rs558133
rs6859667
rs1789953
rs672413
rs705415
rs10944
rs567754
rs6586282
rs234709
rs3797535
rs11951068
rs921943
All
−0.08
−0.04
0.00
0.04
MR effect size for
'Selenium' on 'Sperm−associated antigen 11B'

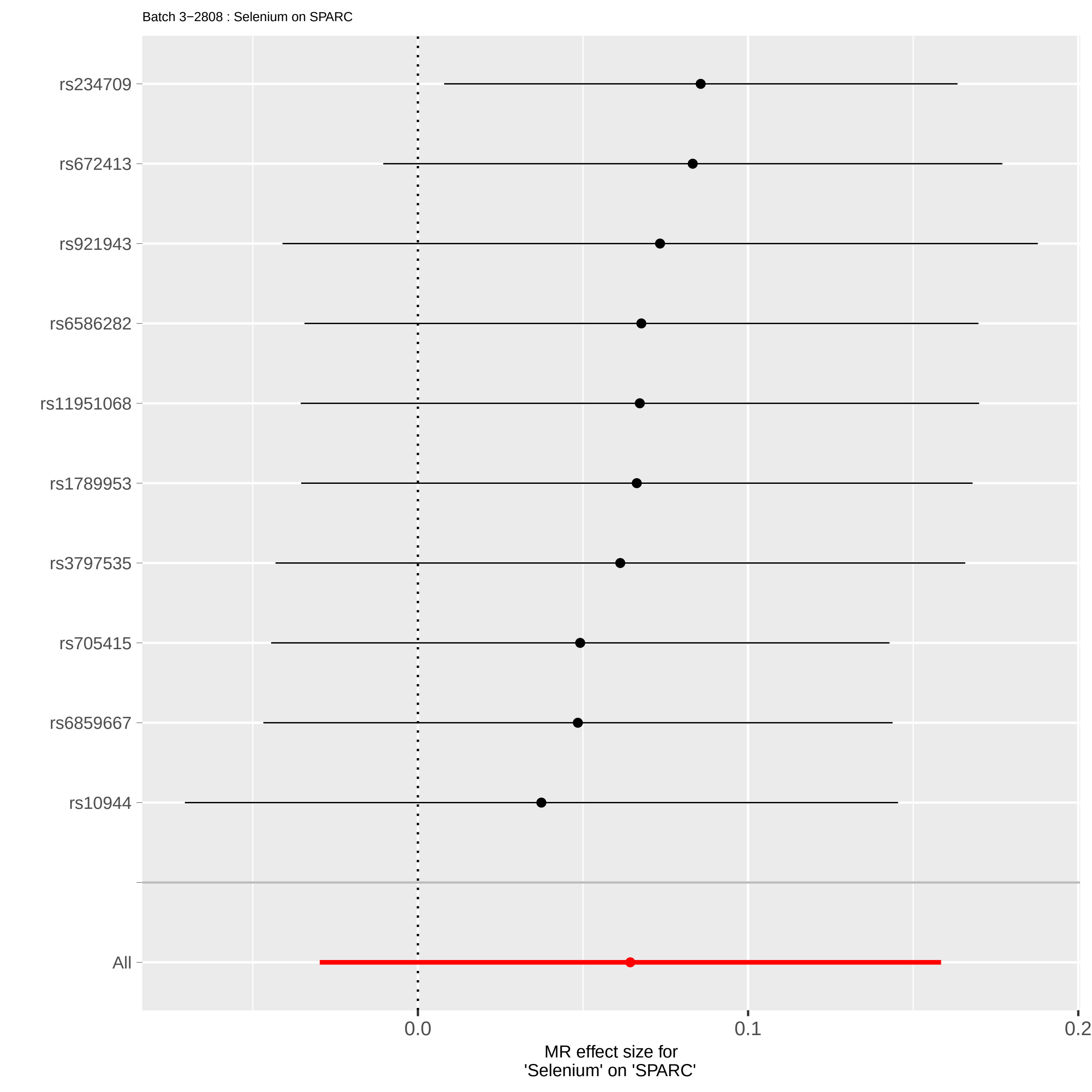

Batch 3−2808 : Selenium on SPARC
rs234709
rs672413
rs921943
rs6586282
rs11951068
rs1789953
rs3797535
rs705415
rs6859667
rs10944
All
0.0
0.1
0.2
MR effect size for
'Selenium' on 'SPARC'

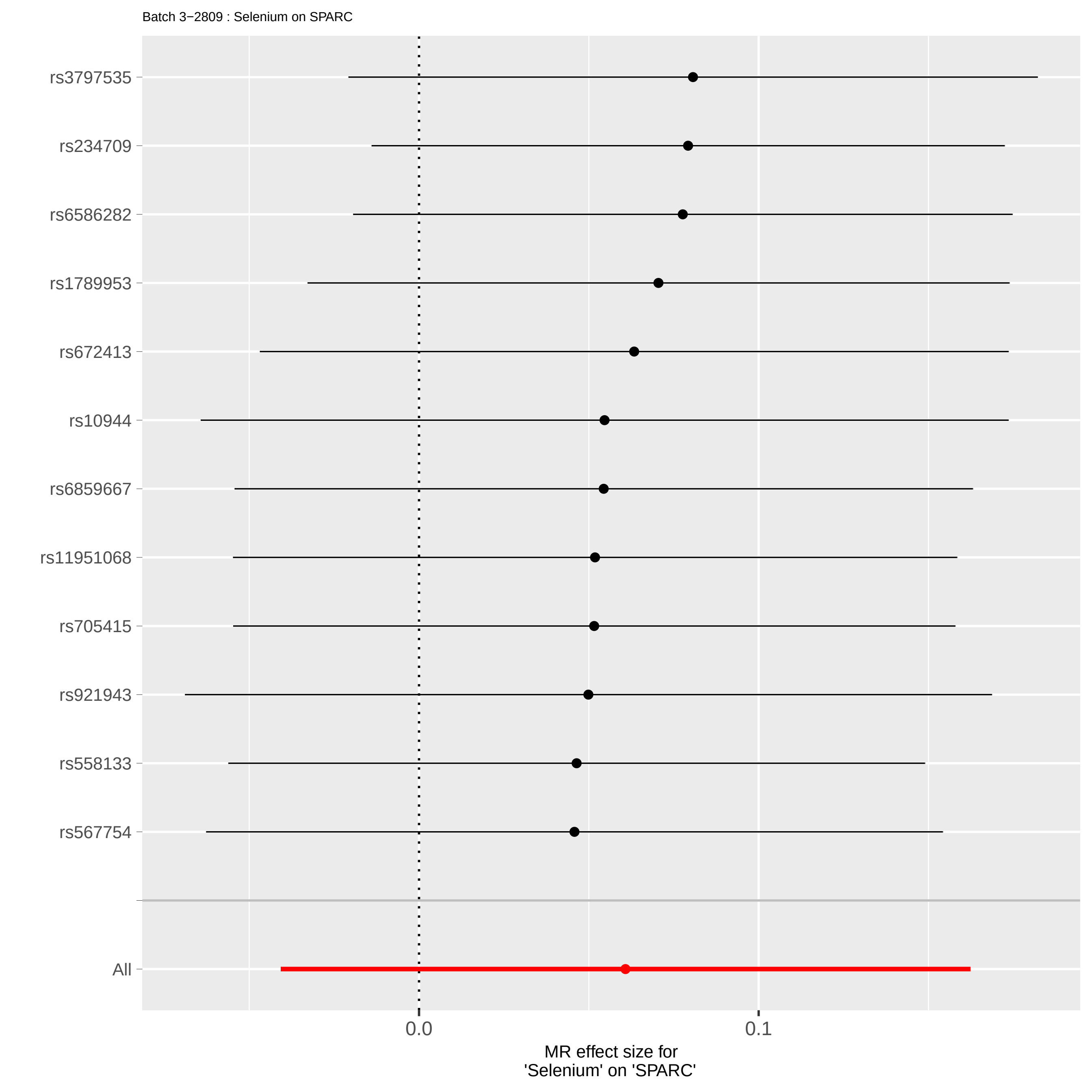

Batch 3−2809 : Selenium on SPARC
rs3797535
rs234709
rs6586282
rs1789953
rs672413
rs10944
rs6859667
rs11951068
rs705415
rs921943
rs558133
rs567754
All
0.0
0.1
MR effect size for
'Selenium' on 'SPARC'

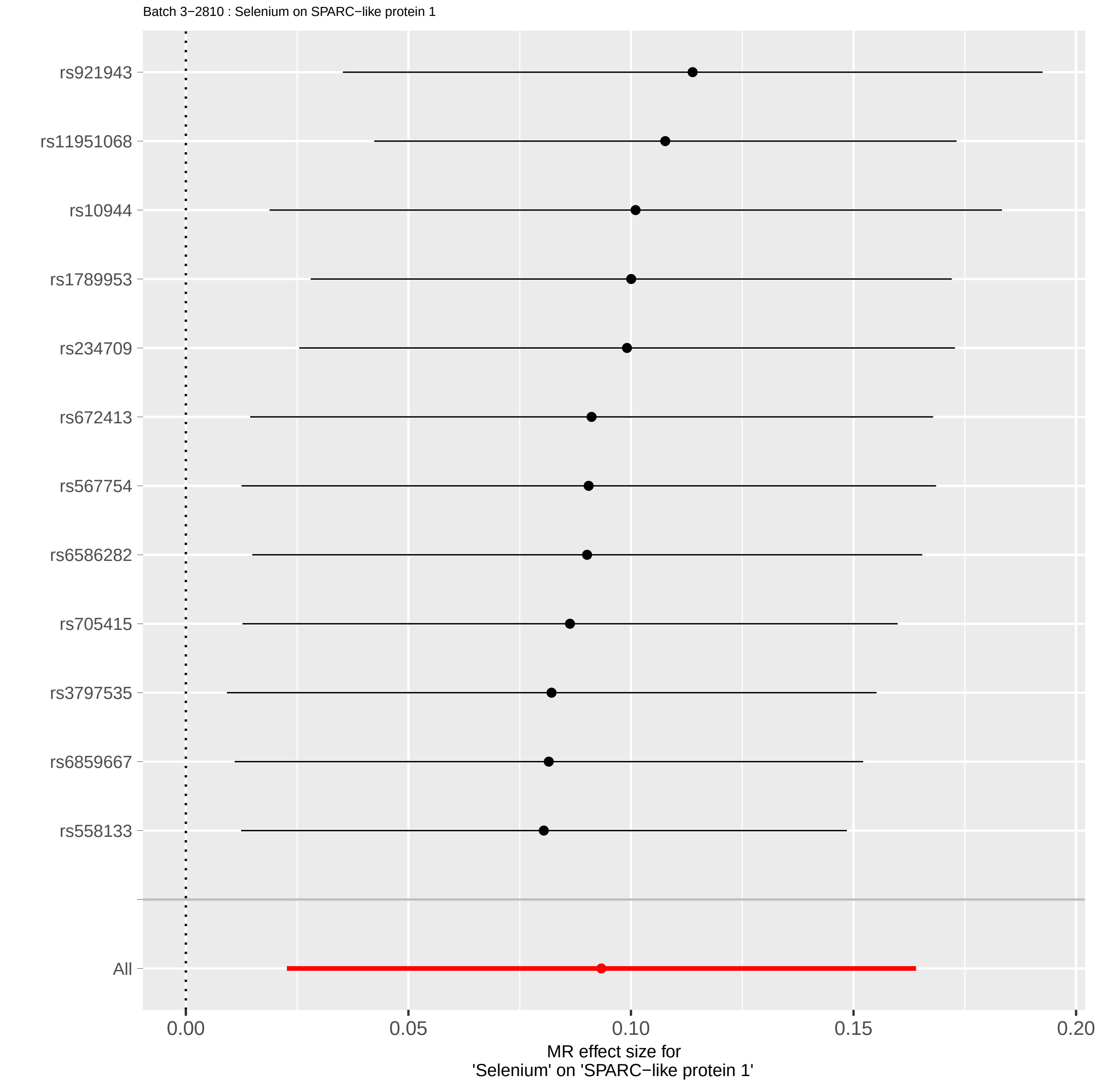

Batch 3−2810 : Selenium on SPARC−like protein 1
rs921943
rs11951068
rs10944
rs1789953
rs234709
rs672413
rs567754
rs6586282
rs705415
rs3797535
rs6859667
rs558133
All
0.00
0.05
0.10
0.15
0.20
MR effect size for
'Selenium' on 'SPARC−like protein 1'

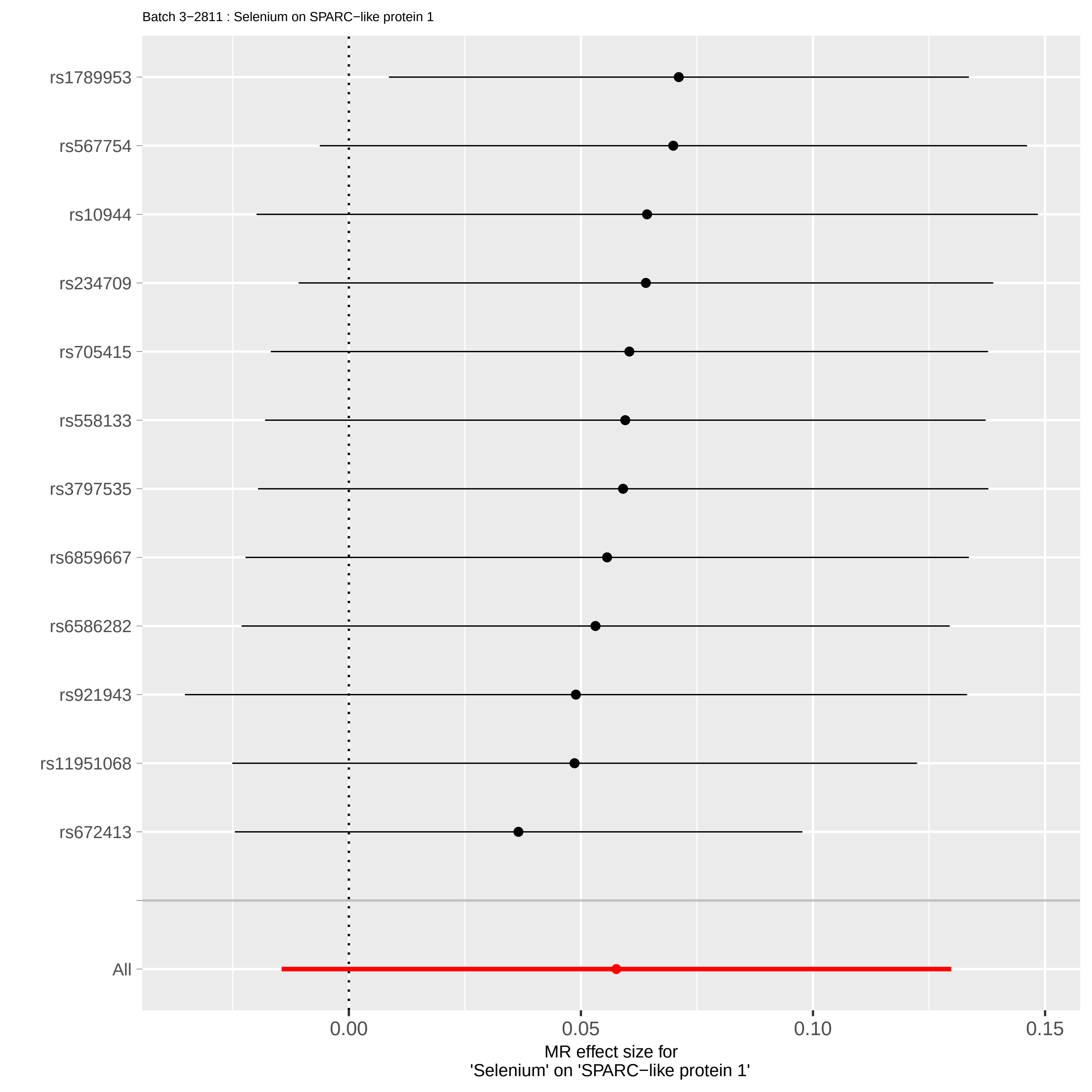

Batch 3−2811 : Selenium on SPARC−like protein 1
rs1789953
rs567754
rs10944
rs234709
rs705415
rs558133
rs3797535
rs6859667
rs6586282
rs921943
rs11951068
rs672413
All
0.00
0.05
0.10
0.15
MR effect size for
'Selenium' on 'SPARC−like protein 1'

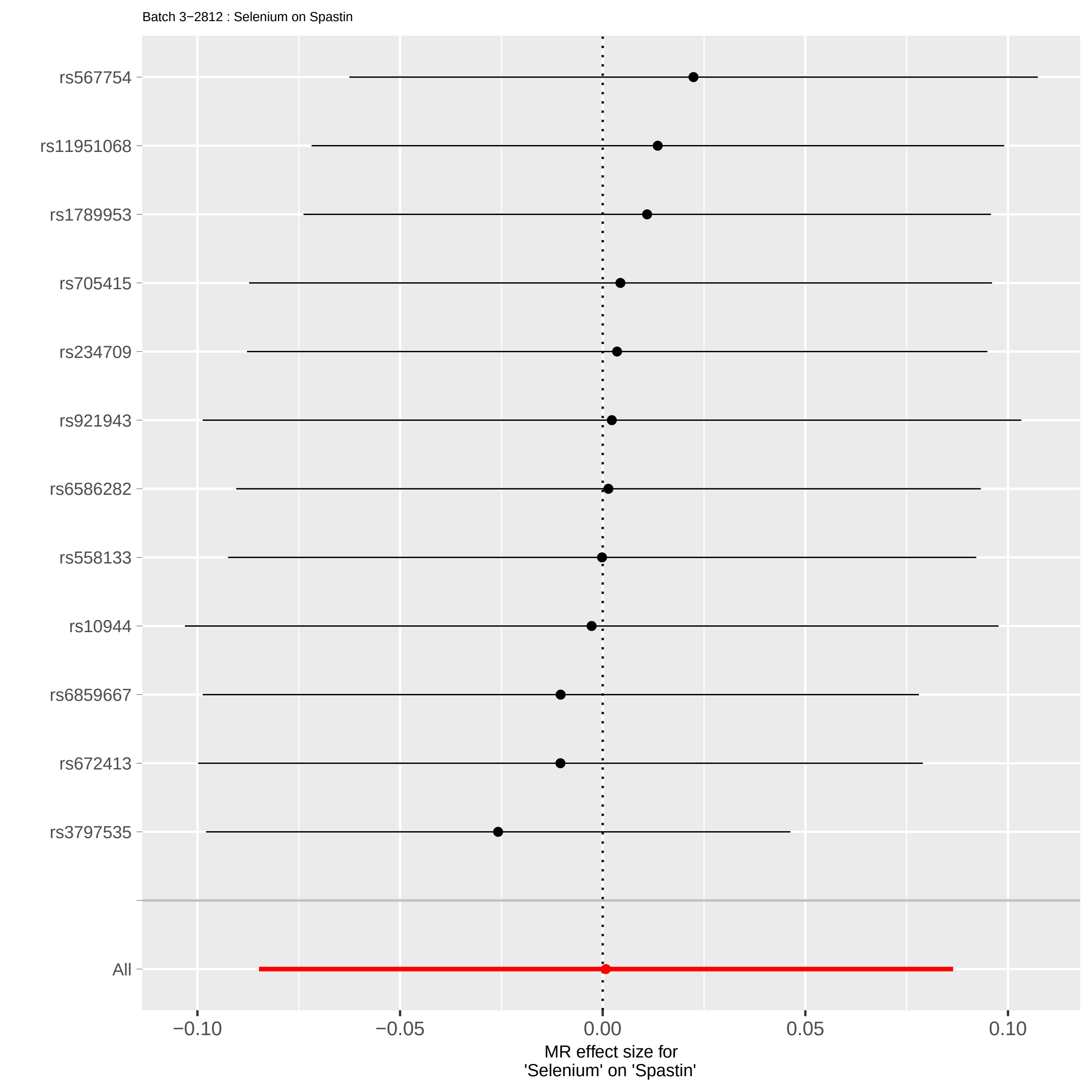

Batch 3−2812 : Selenium on Spastin
rs567754
rs11951068
rs1789953
rs705415
rs234709
rs921943
rs6586282
rs558133
rs10944
rs6859667
rs672413
rs3797535
All
−0.10
−0.05
0.00
0.05
0.10
MR effect size for
'Selenium' on 'Spastin'

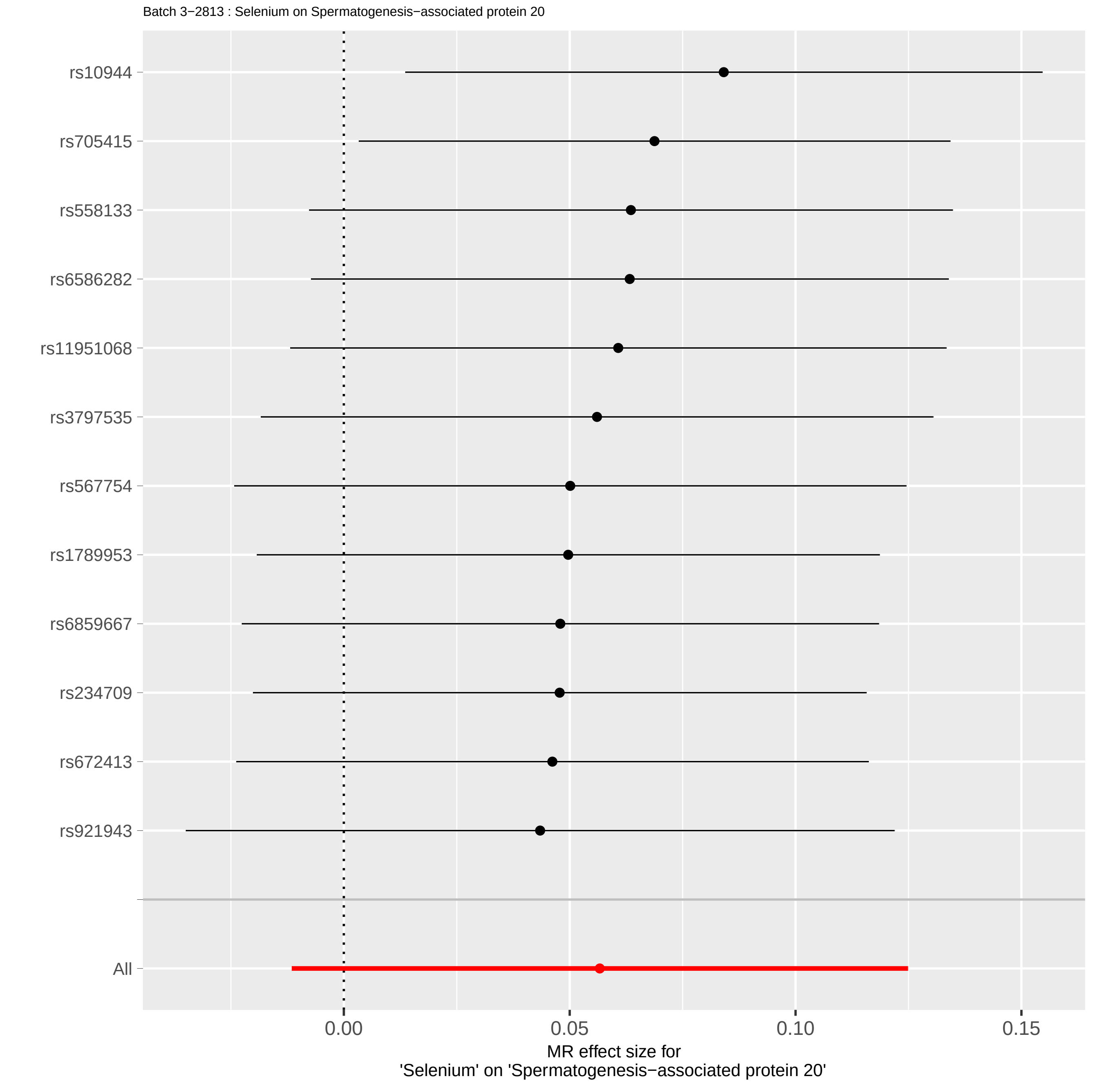
Batch 3−2813 : Selenium on Spermatogenesis−associated protein 20
rs10944
rs705415
rs558133
rs6586282
rs11951068
rs3797535
rs567754
rs1789953
rs6859667
rs234709
rs672413
rs921943
All
0.00
0.05
0.10
0.15
MR effect size for
'Selenium' on 'Spermatogenesis−associated protein 20'

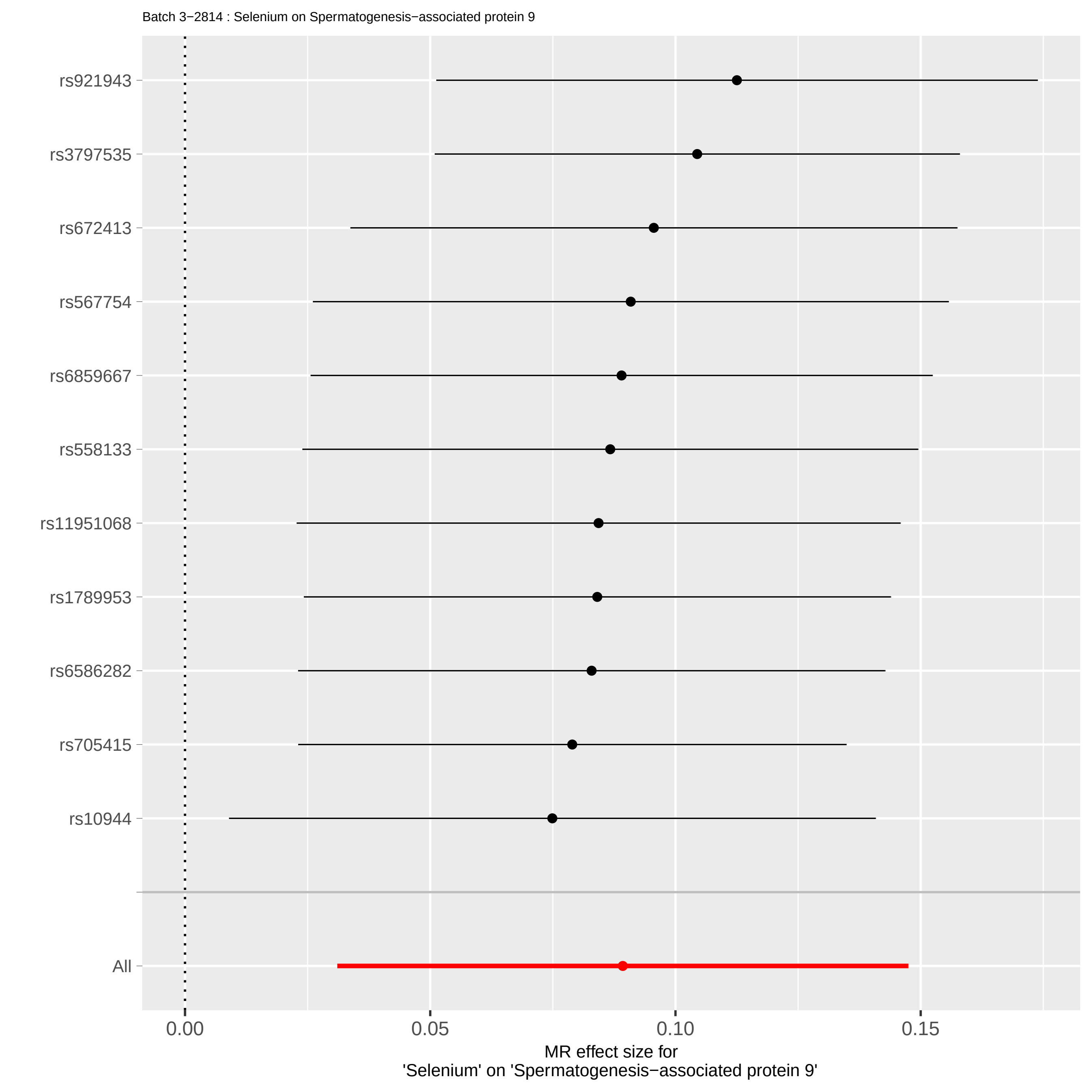

Batch 3−2814 : Selenium on Spermatogenesis−associated protein 9
rs921943
rs3797535
rs672413
rs567754
rs6859667
rs558133
rs11951068
rs1789953
rs6586282
rs705415
rs10944
All
0.00
0.05
0.10
0.15
MR effect size for
'Selenium' on 'Spermatogenesis−associated protein 9'

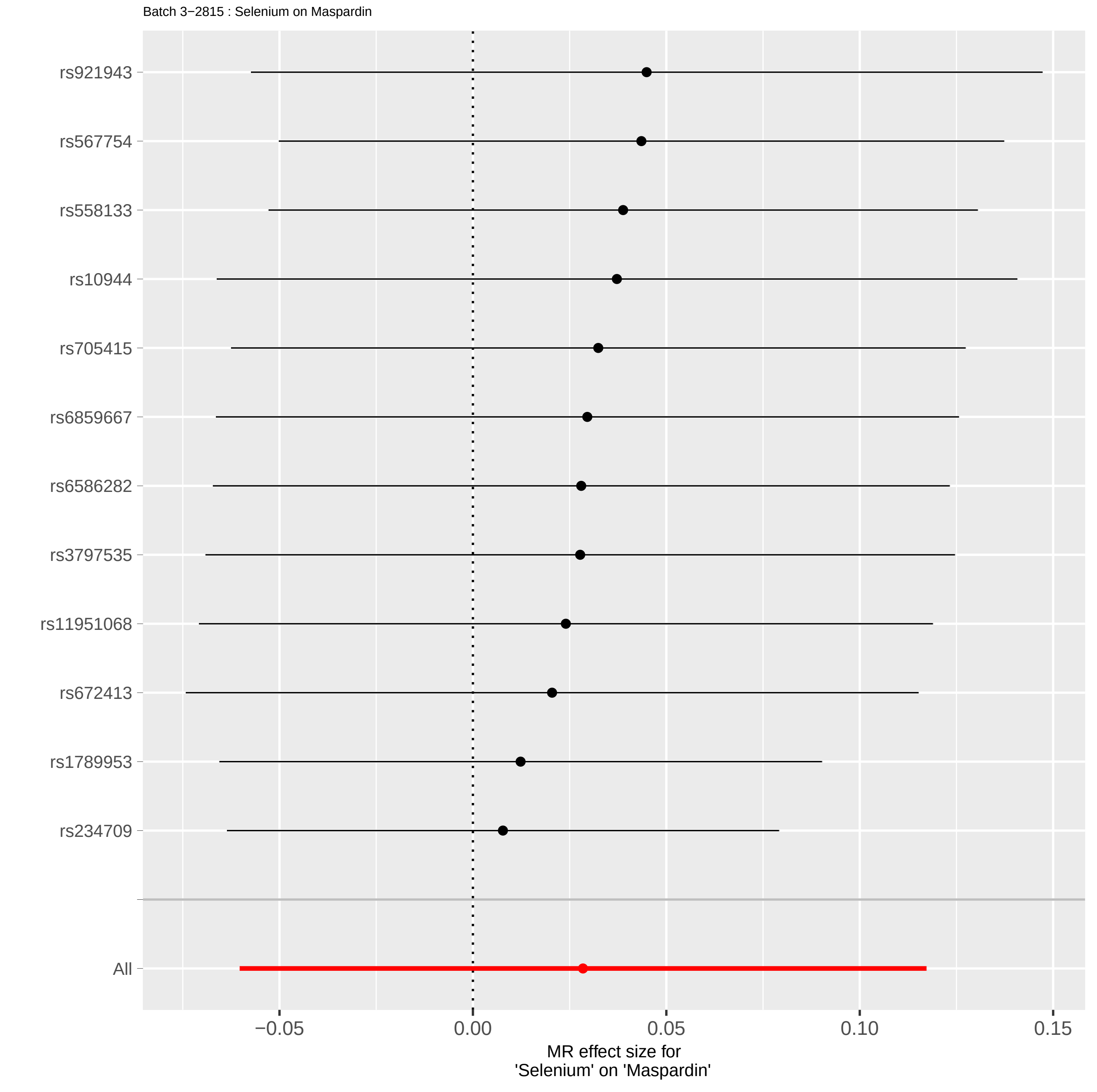

Batch 3−2815 : Selenium on Maspardin
rs921943
rs567754
rs558133
rs10944
rs705415
rs6859667
rs6586282
rs3797535
rs11951068
rs672413
rs1789953
rs234709
All
−0.05
0.00
0.05
0.10
0.15
MR effect size for
'Selenium' on 'Maspardin'

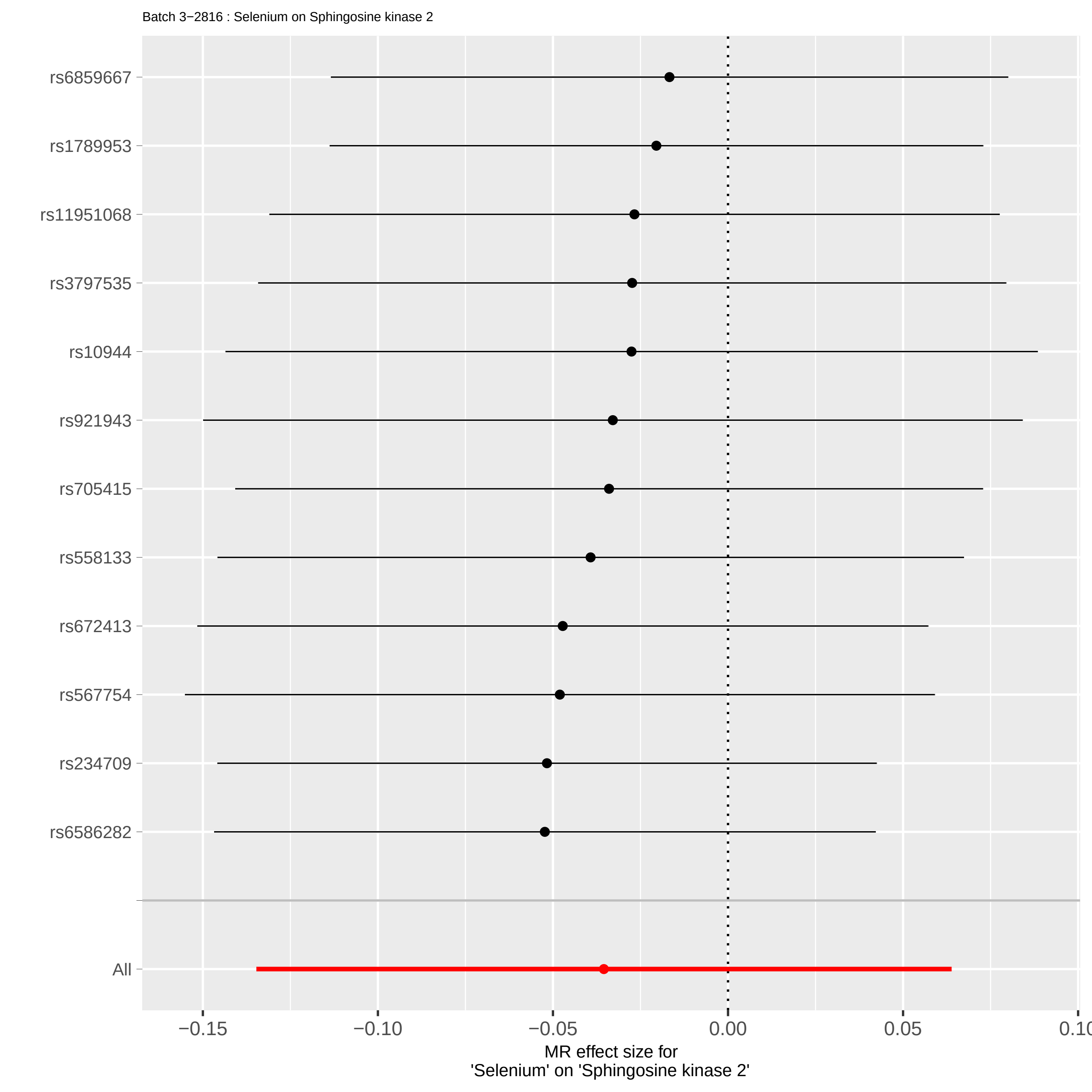

Batch 3−2816 : Selenium on Sphingosine kinase 2
rs6859667
rs1789953
rs11951068
rs3797535
rs10944
rs921943
rs705415
rs558133
rs672413
rs567754
rs234709
rs6586282
All
−0.15
−0.10
−0.05
0.00
0.05
0.10
MR effect size for
'Selenium' on 'Sphingosine kinase 2'

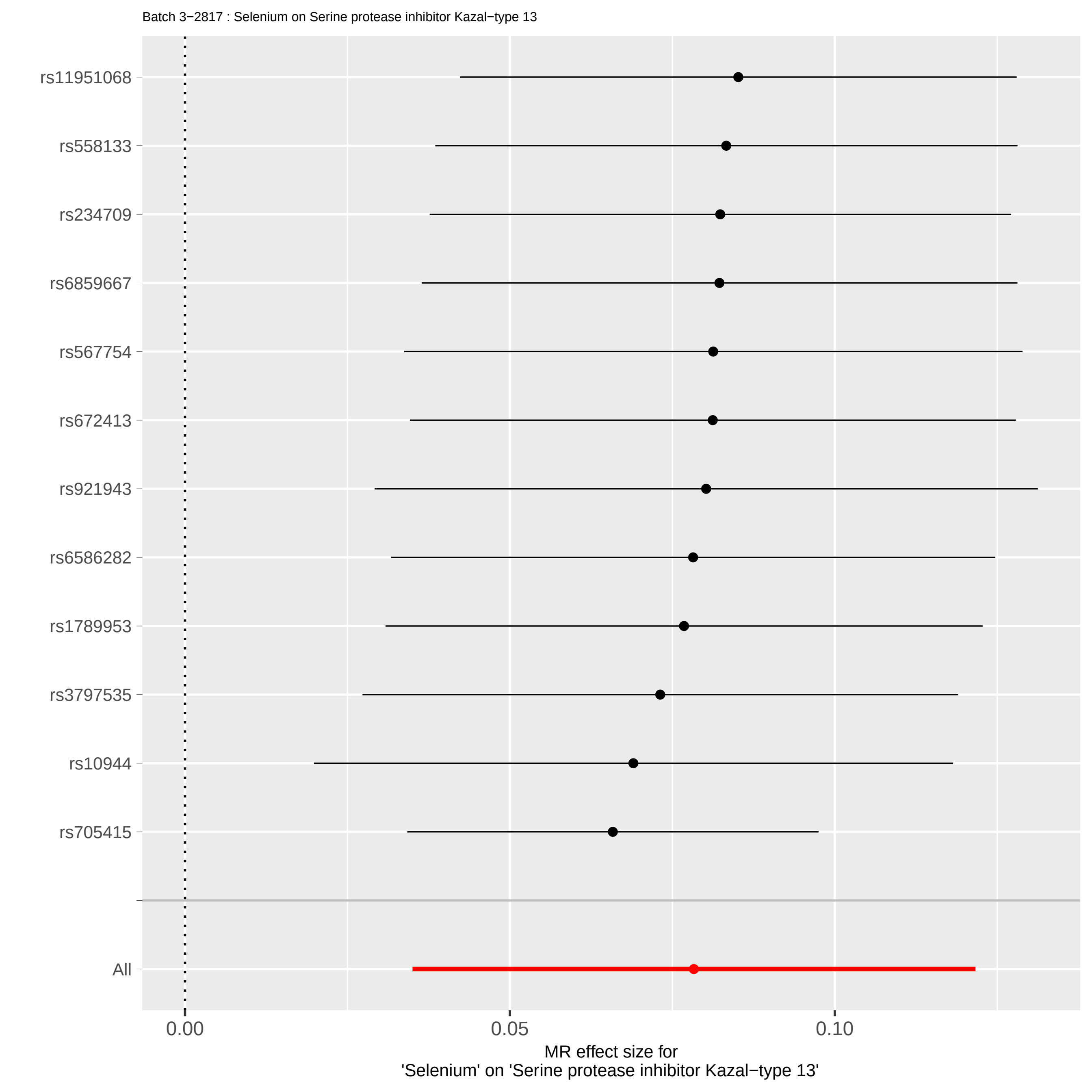

Batch 3−2817 : Selenium on Serine protease inhibitor Kazal−type 13
rs11951068
rs558133
rs234709
rs6859667
rs567754
rs672413
rs921943
rs6586282
rs1789953
rs3797535
rs10944
rs705415
All
0.00
0.05
0.10
MR effect size for
'Selenium' on 'Serine protease inhibitor Kazal−type 13'

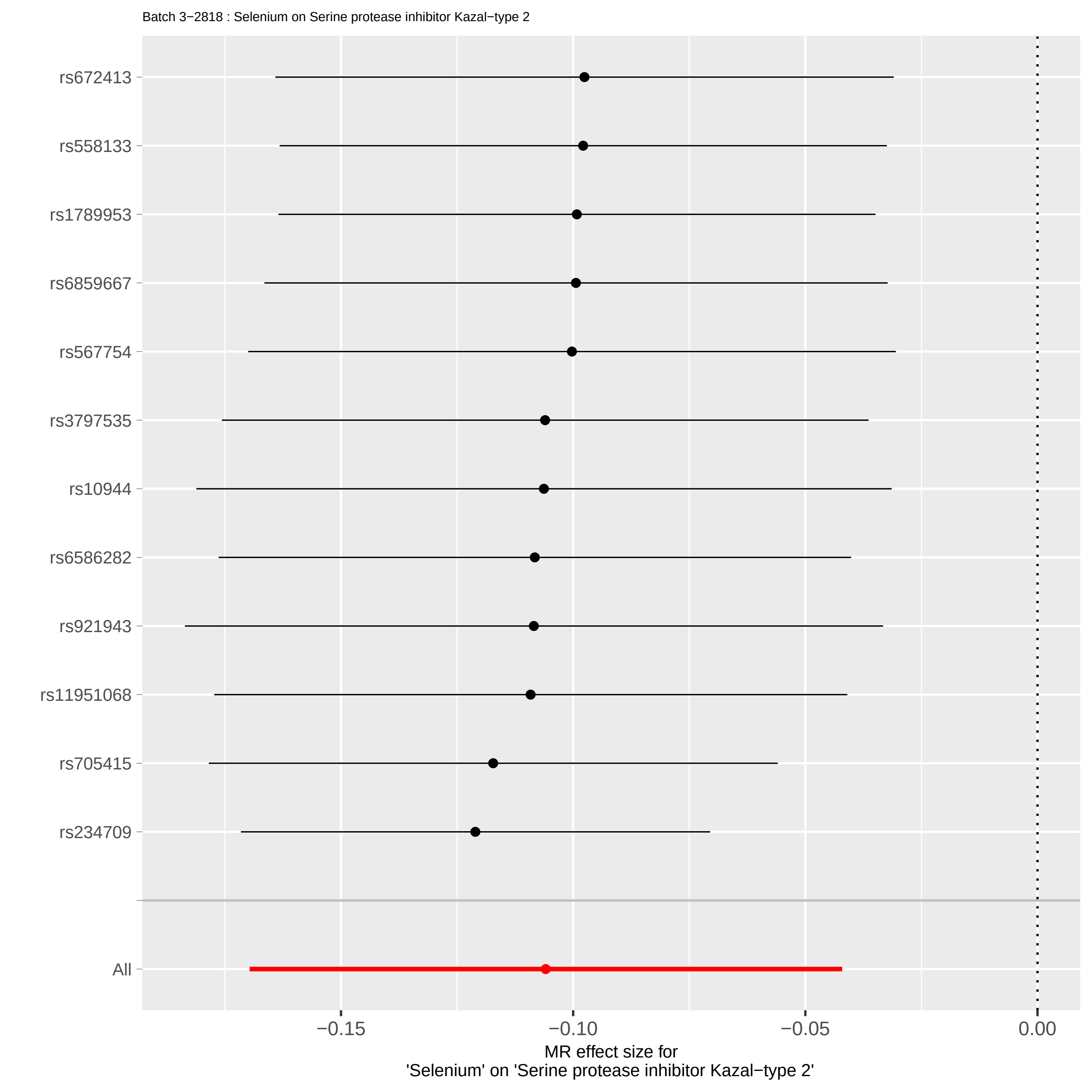

Batch 3−2818 : Selenium on Serine protease inhibitor Kazal−type 2
rs672413
rs558133
rs1789953
rs6859667
rs567754
rs3797535
rs10944
rs6586282
rs921943
rs11951068
rs705415
rs234709
All
−0.15
−0.10
−0.05
0.00
MR effect size for
'Selenium' on 'Serine protease inhibitor Kazal−type 2'

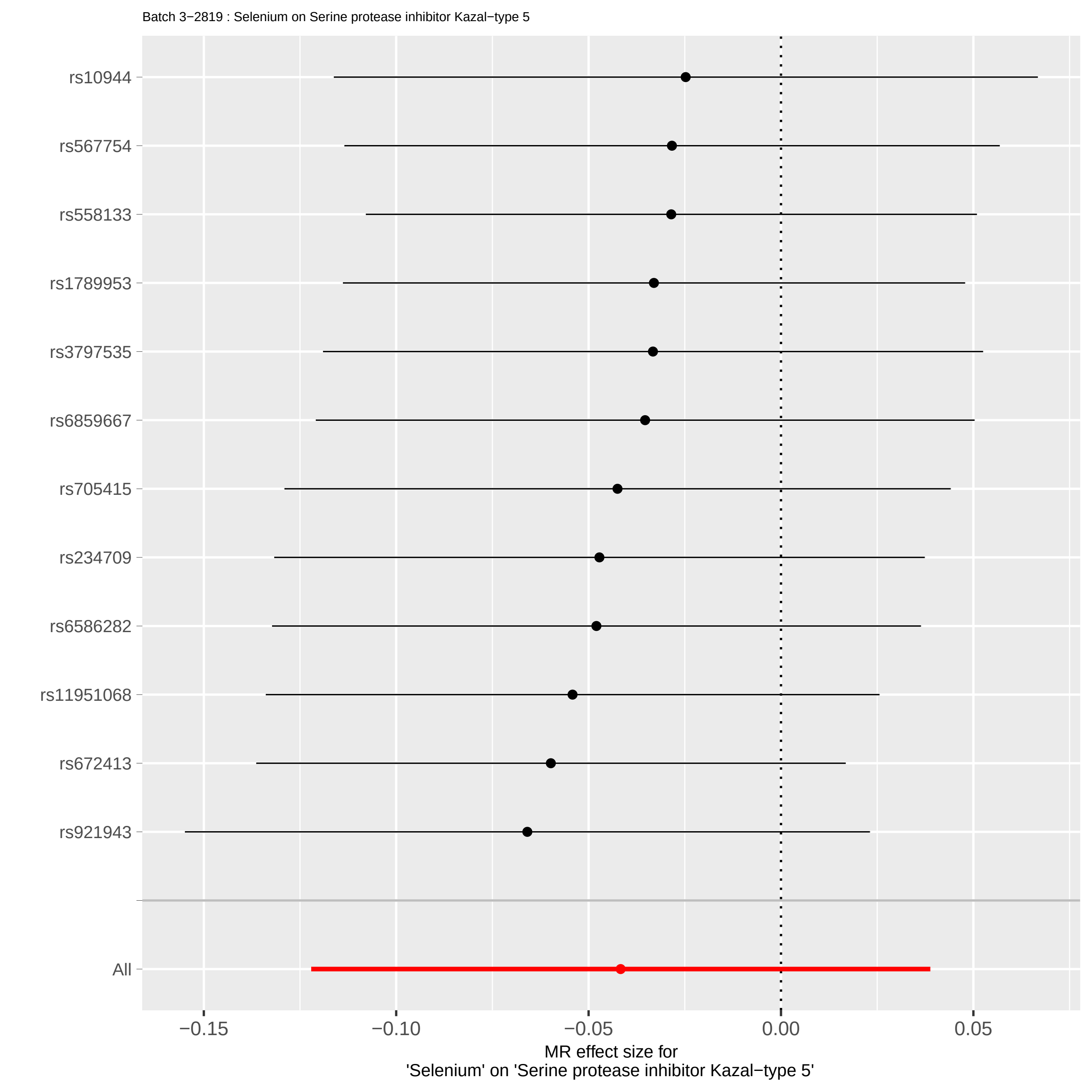

Batch 3−2819 : Selenium on Serine protease inhibitor Kazal−type 5
rs10944
rs567754
rs558133
rs1789953
rs3797535
rs6859667
rs705415
rs234709
rs6586282
rs11951068
rs672413
rs921943
All
−0.15
−0.10
−0.05
0.00
0.05
MR effect size for
'Selenium' on 'Serine protease inhibitor Kazal−type 5'

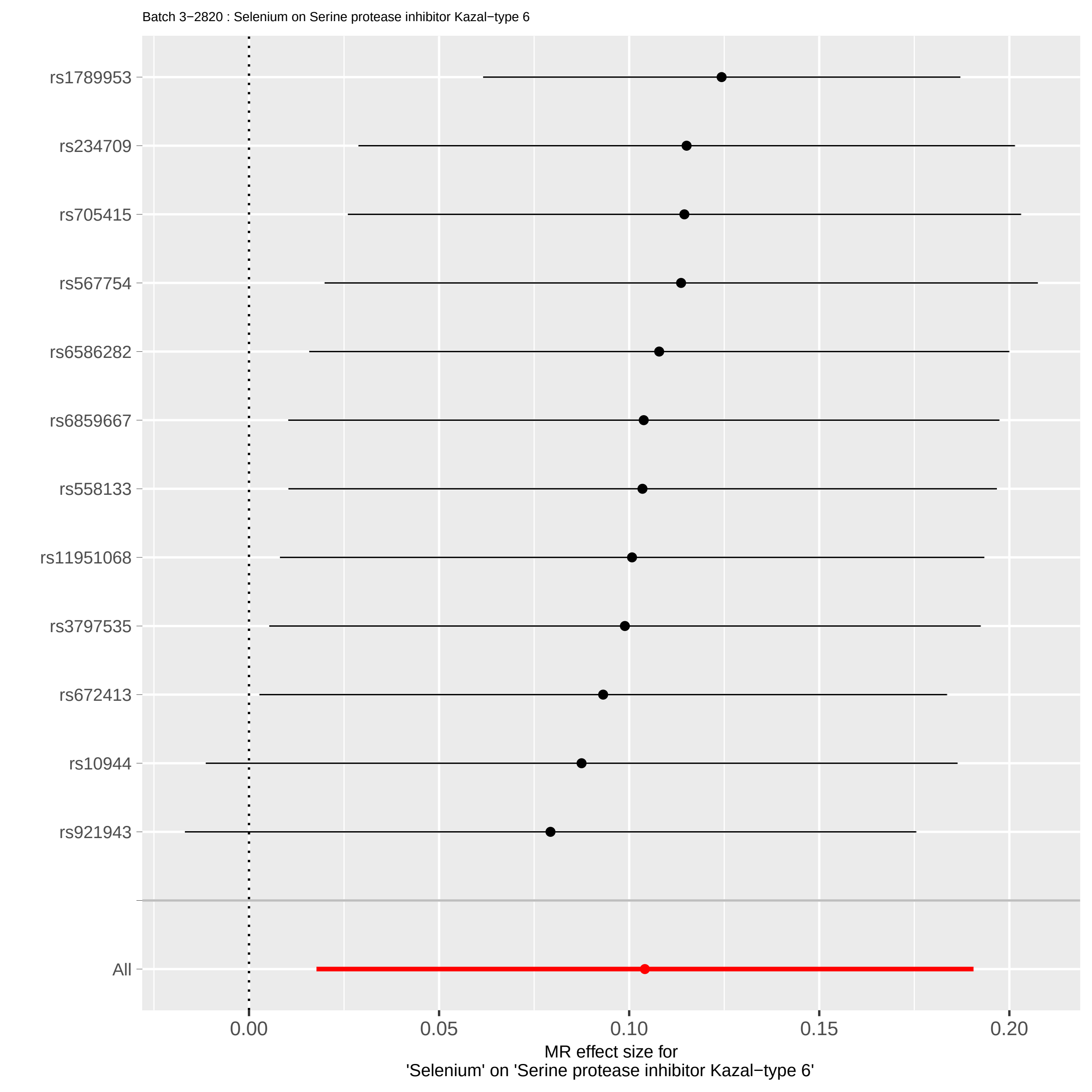
Batch 3−2820 : Selenium on Serine protease inhibitor Kazal−type 6
rs1789953
rs234709
rs705415
rs567754
rs6586282
rs6859667
rs558133
rs11951068
rs3797535
rs672413
rs10944
rs921943
All
0.00
0.05
0.10
0.15
0.20
MR effect size for
'Selenium' on 'Serine protease inhibitor Kazal−type 6'

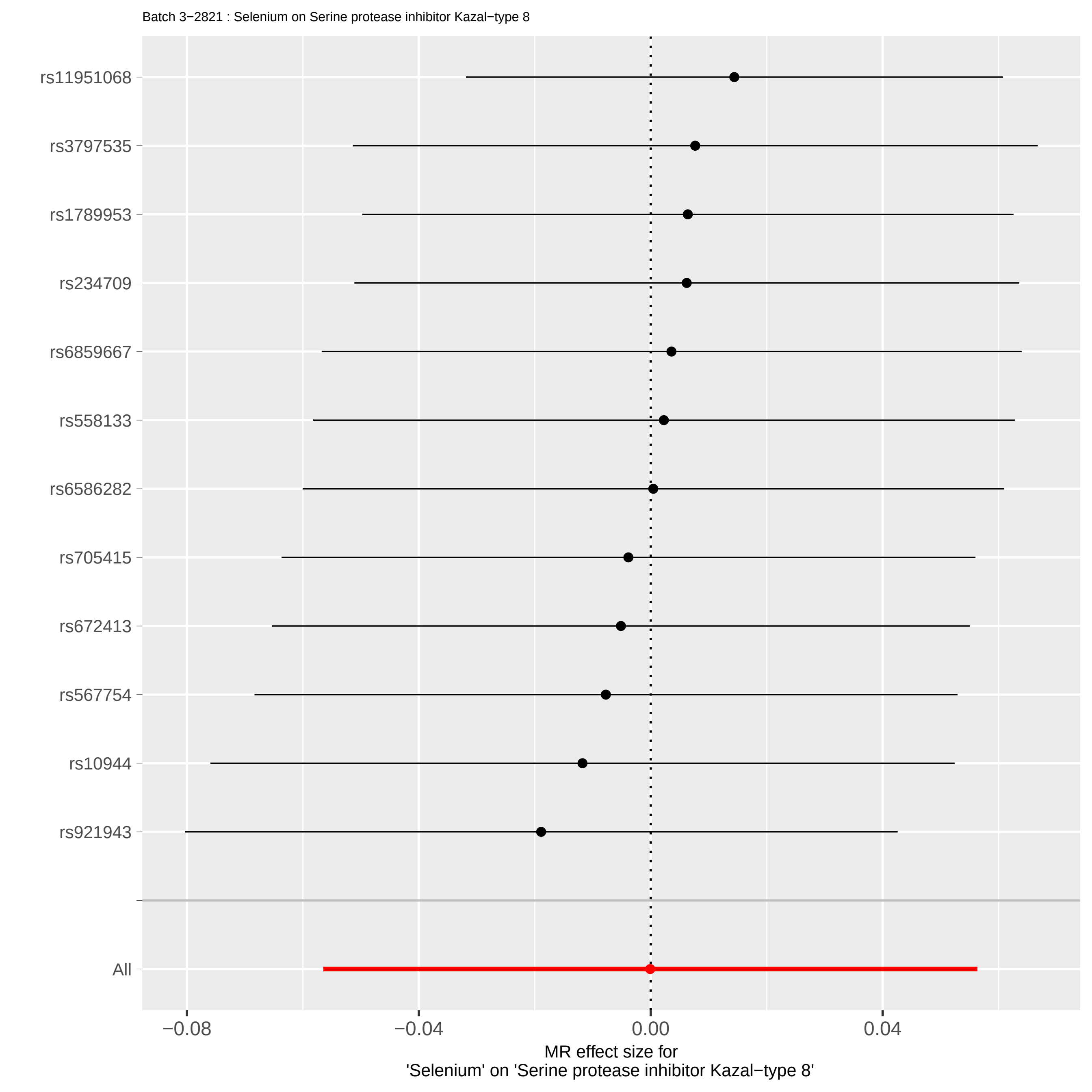

Batch 3−2821 : Selenium on Serine protease inhibitor Kazal−type 8
rs11951068
rs3797535
rs1789953
rs234709
rs6859667
rs558133
rs6586282
rs705415
rs672413
rs567754
rs10944
rs921943
All
−0.08
−0.04
0.00
0.04
MR effect size for
'Selenium' on 'Serine protease inhibitor Kazal−type 8'

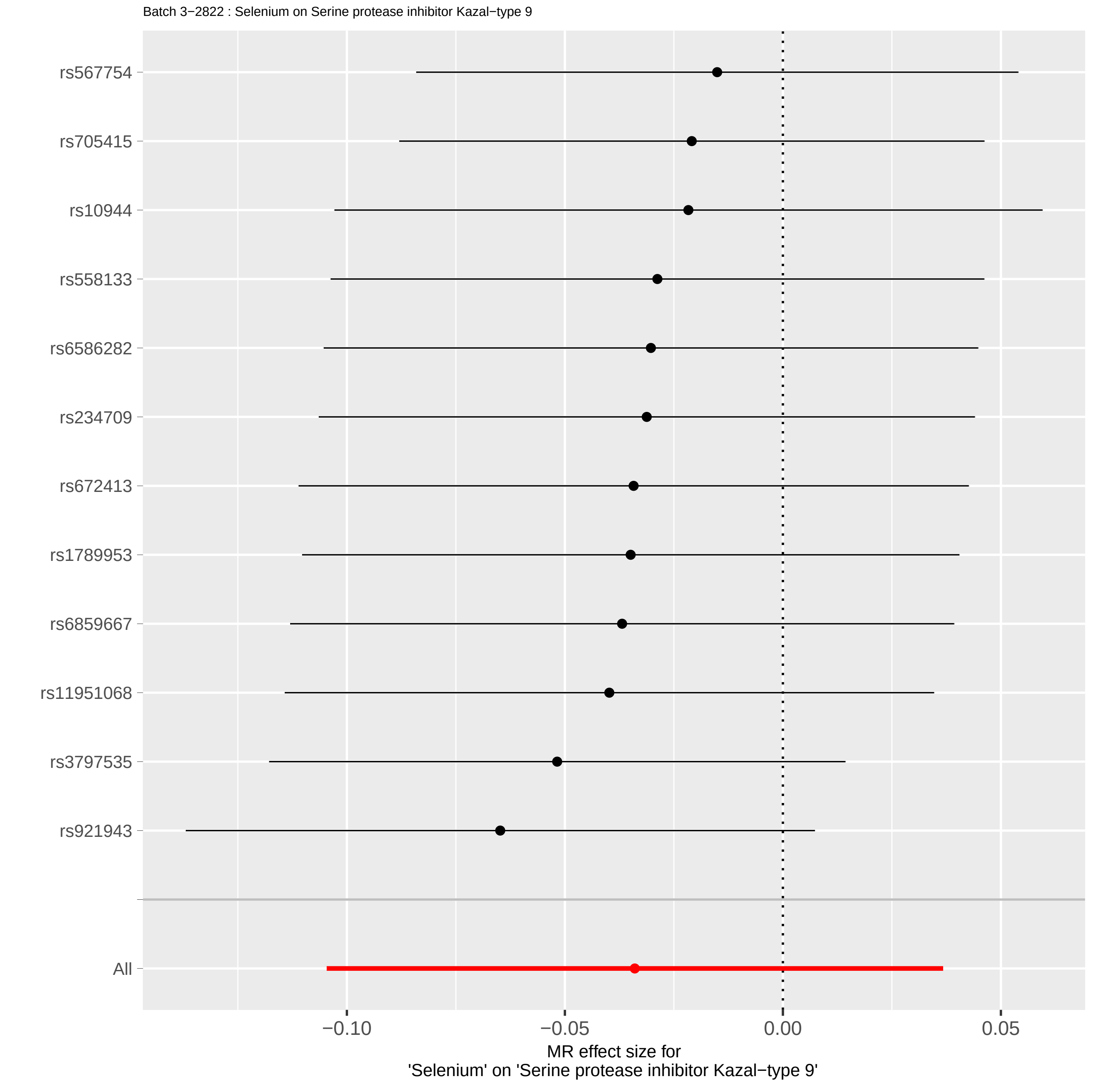
Batch 3−2822 : Selenium on Serine protease inhibitor Kazal−type 9
rs567754
rs705415
rs10944
rs558133
rs6586282
rs234709
rs672413
rs1789953
rs6859667
rs11951068
rs3797535
rs921943
All
−0.10
−0.05
0.00
0.05
MR effect size for
'Selenium' on 'Serine protease inhibitor Kazal−type 9'

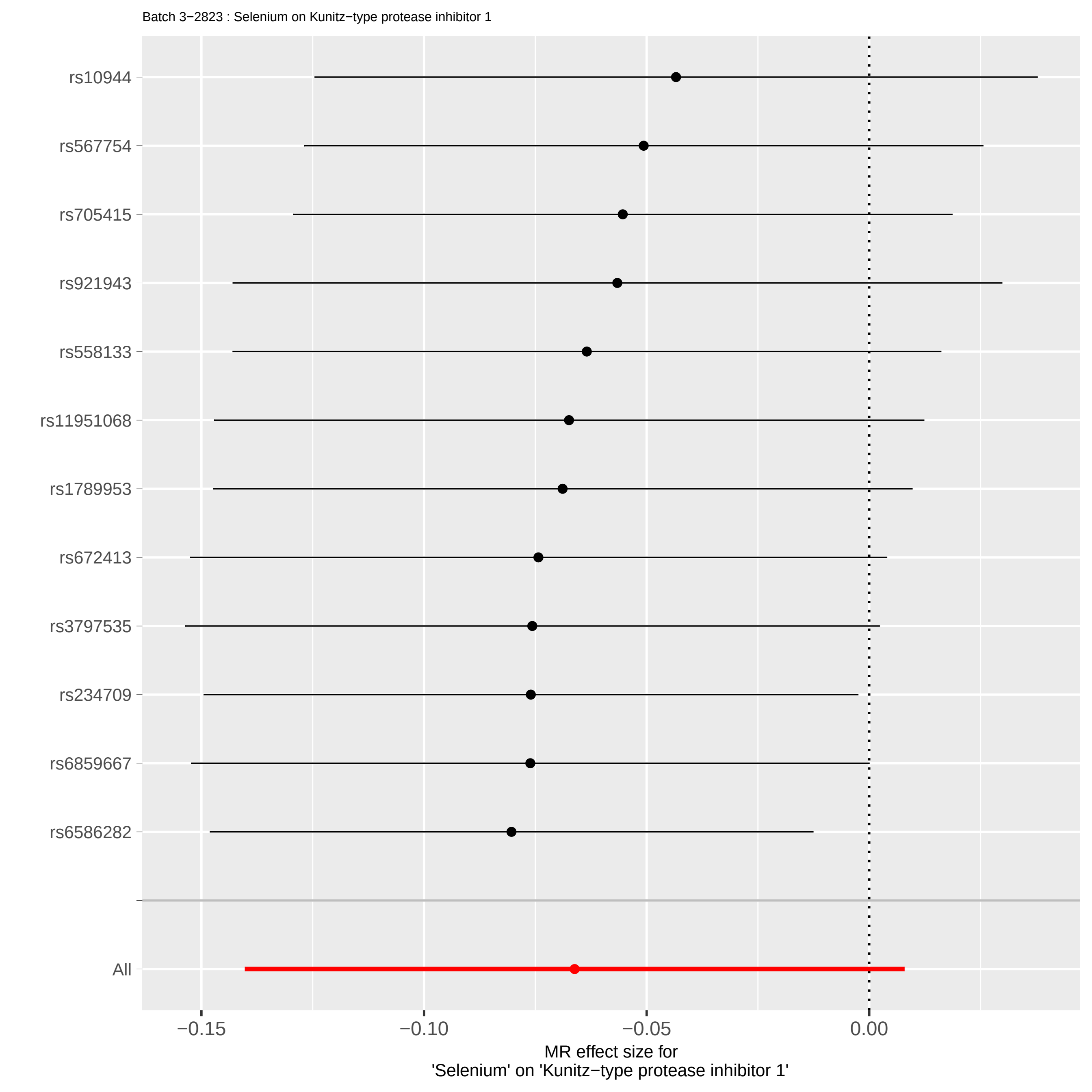

Batch 3−2823 : Selenium on Kunitz−type protease inhibitor 1
rs10944
rs567754
rs705415
rs921943
rs558133
rs11951068
rs1789953
rs672413
rs3797535
rs234709
rs6859667
rs6586282
All
−0.15
−0.10
−0.05
0.00
MR effect size for
'Selenium' on 'Kunitz−type protease inhibitor 1'

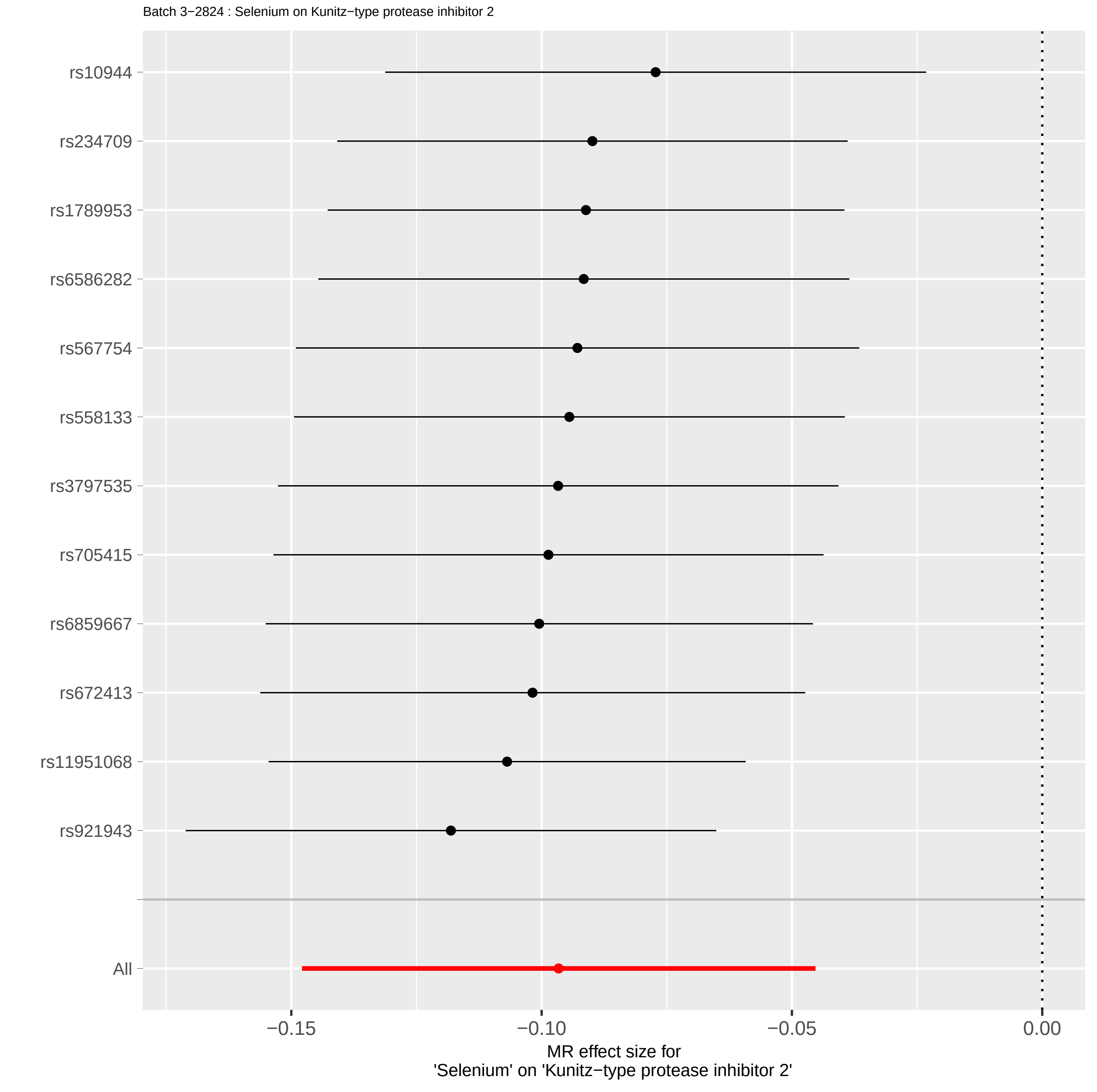

Batch 3−2824 : Selenium on Kunitz−type protease inhibitor 2
rs10944
rs234709
rs1789953
rs6586282
rs567754
rs558133
rs3797535
rs705415
rs6859667
rs672413
rs11951068
rs921943
All
−0.15
−0.10
−0.05
0.00
MR effect size for
'Selenium' on 'Kunitz−type protease inhibitor 2'

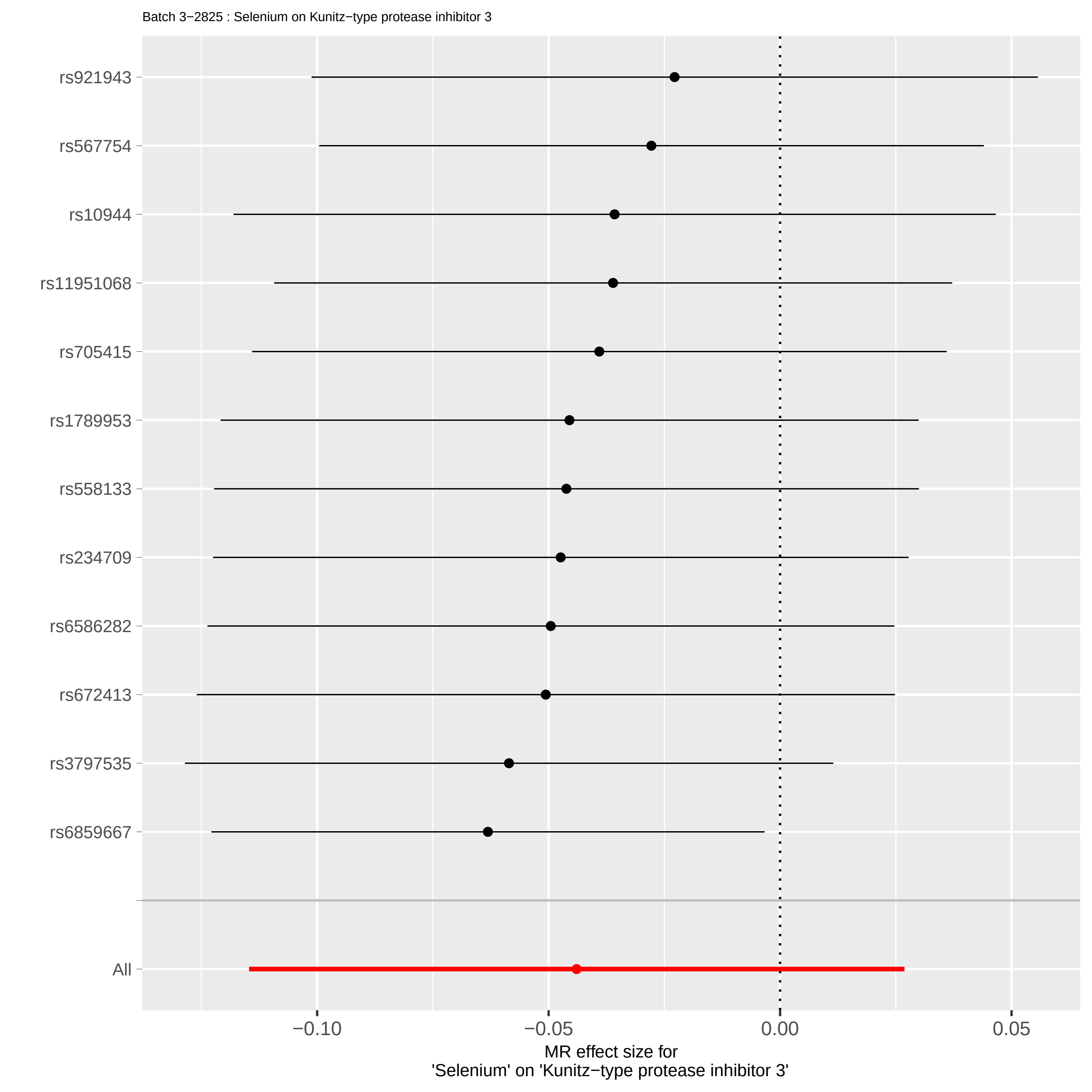

Batch 3−2825 : Selenium on Kunitz−type protease inhibitor 3
rs921943
rs567754
rs10944
rs11951068
rs705415
rs1789953
rs558133
rs234709
rs6586282
rs672413
rs3797535
rs6859667
All
−0.10
−0.05
0.00
0.05
MR effect size for
'Selenium' on 'Kunitz−type protease inhibitor 3'

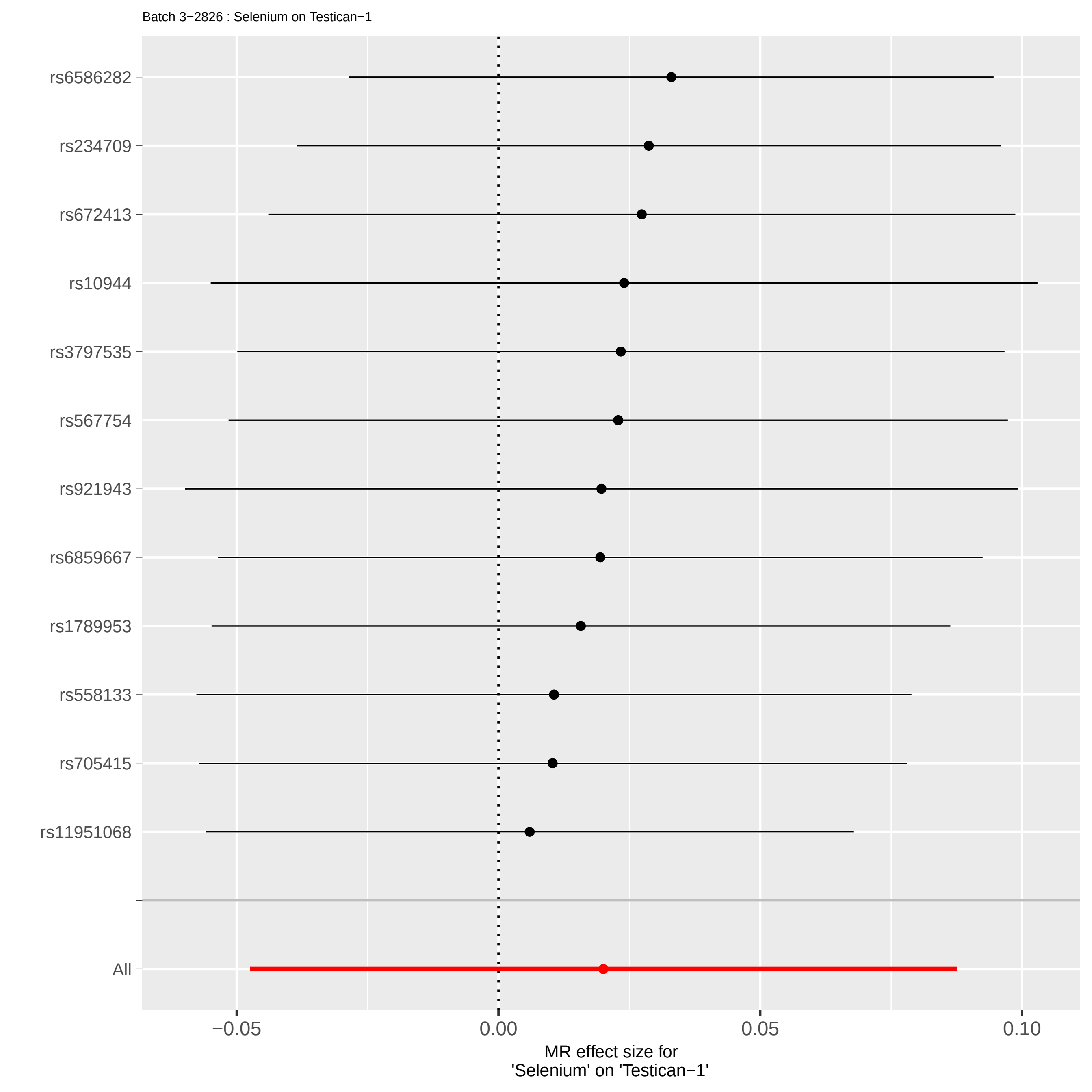

Batch 3−2826 : Selenium on Testican−1
rs6586282
rs234709
rs672413
rs10944
rs3797535
rs567754
rs921943
rs6859667
rs1789953
rs558133
rs705415
rs11951068
All
−0.05
0.00
0.05
0.10
MR effect size for
'Selenium' on 'Testican−1'

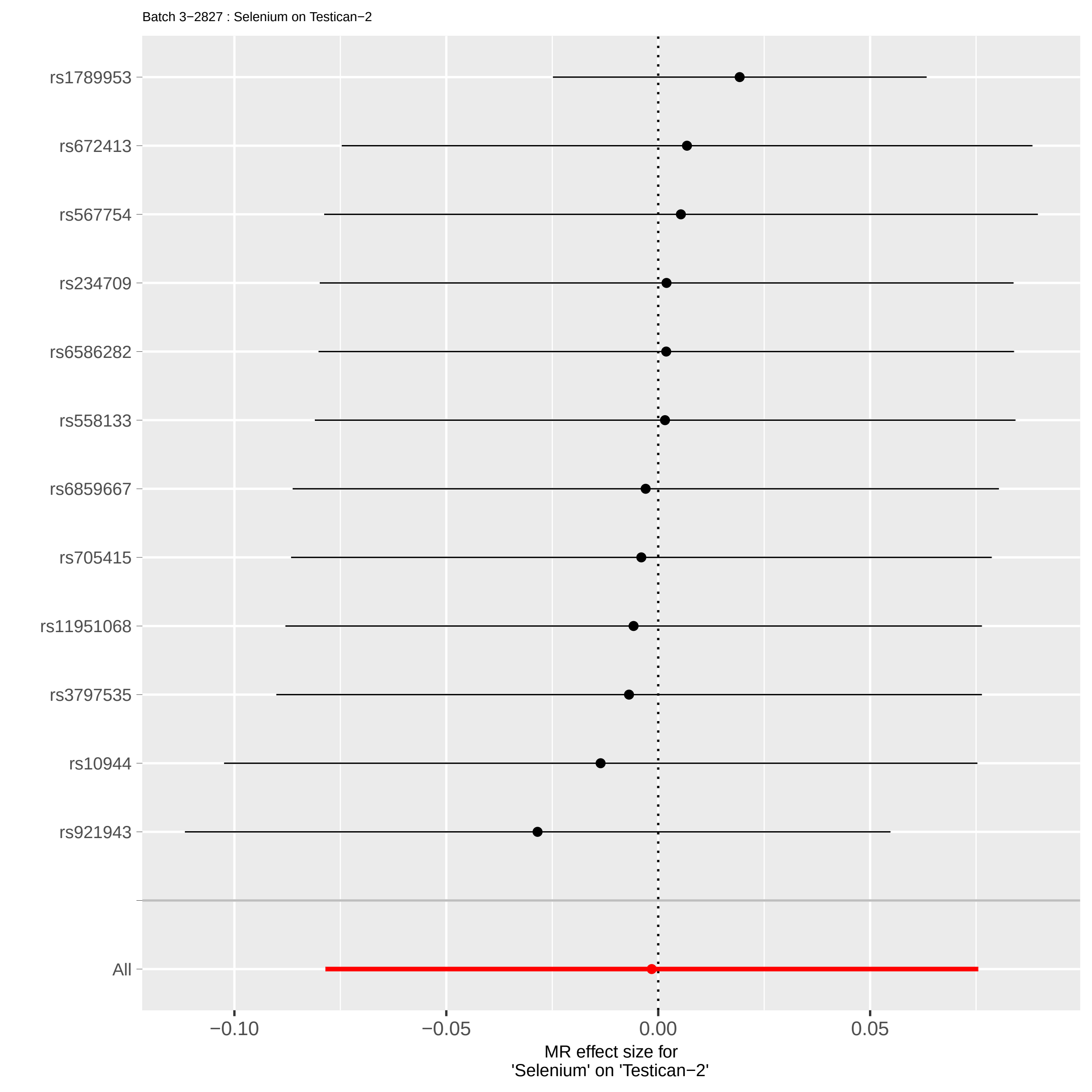

Batch 3−2827 : Selenium on Testican−2
rs1789953
rs672413
rs567754
rs234709
rs6586282
rs558133
rs6859667
rs705415
rs11951068
rs3797535
rs10944
rs921943
All
−0.10
−0.05
0.00
0.05
MR effect size for
'Selenium' on 'Testican−2'

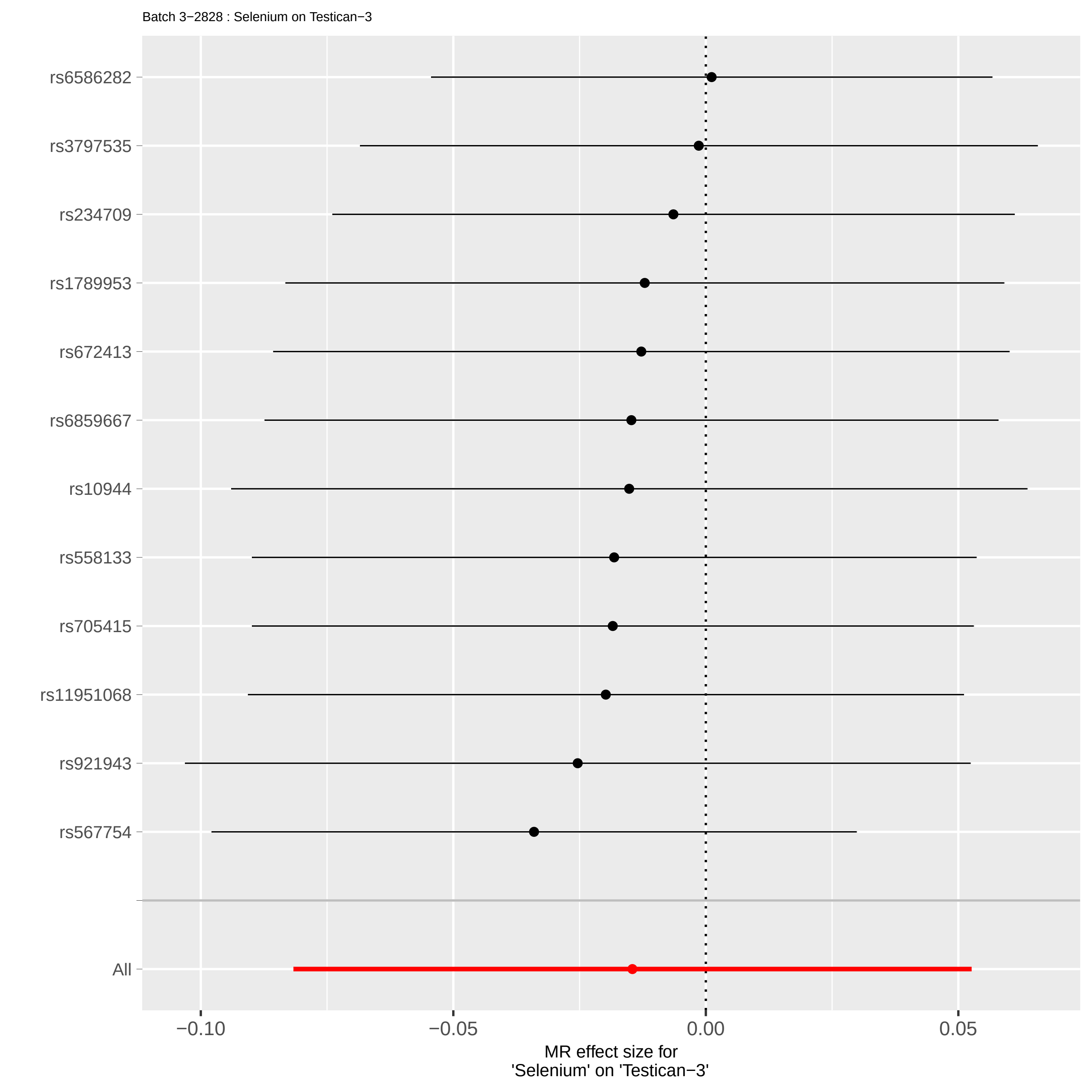
Batch 3−2828 : Selenium on Testican−3
rs6586282
rs3797535
rs234709
rs1789953
rs672413
rs6859667
rs10944
rs558133
rs705415
rs11951068
rs921943
rs567754
All
−0.10
−0.05
0.00
0.05
MR effect size for
'Selenium' on 'Testican−3'

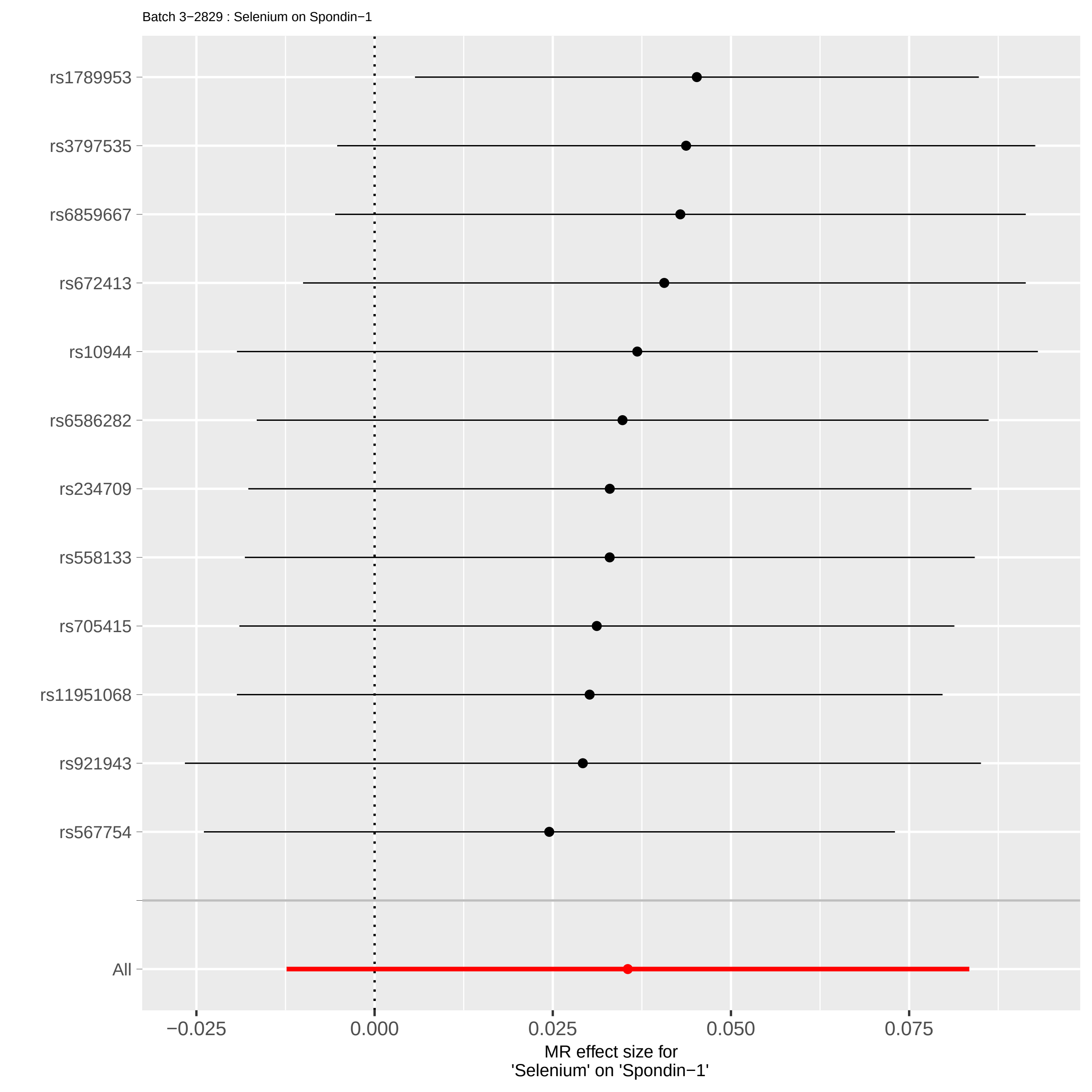
Batch 3−2829 : Selenium on Spondin−1
rs1789953
rs3797535
rs6859667
rs672413
rs10944
rs6586282
rs234709
rs558133
rs705415
rs11951068
rs921943
rs567754
All
−0.025
0.000
0.025
0.050
0.075
MR effect size for
'Selenium' on 'Spondin−1'

Batch 3−2830 : Selenium on Speckle−type POZ protein

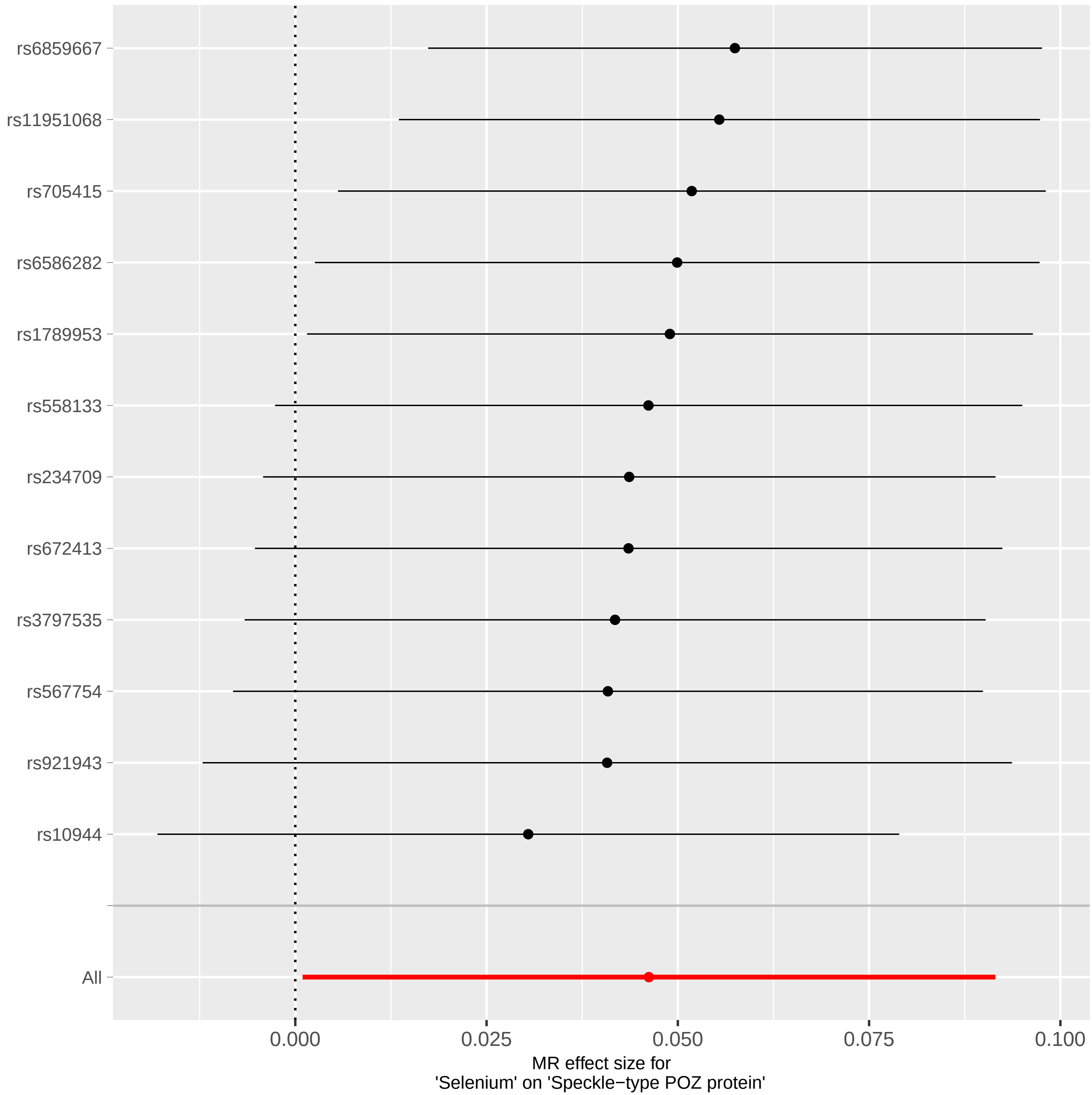

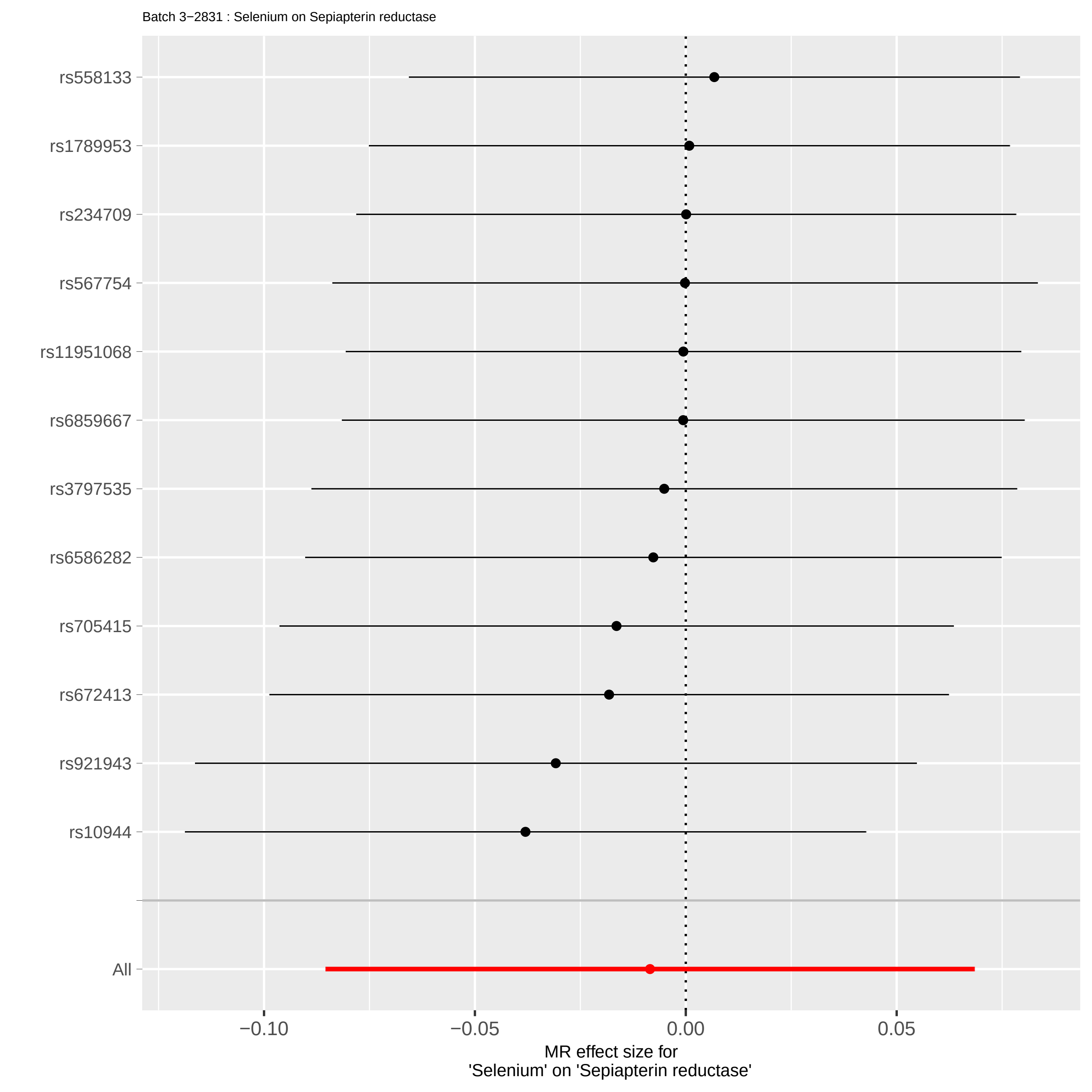

Batch 3−2831 : Selenium on Sepiapterin reductase
rs558133
rs1789953
rs234709
rs567754
rs11951068
rs6859667
rs3797535
rs6586282
rs705415
rs672413
rs921943
rs10944
All
−0.10
−0.05
0.00
0.05
MR effect size for
'Selenium' on 'Sepiapterin reductase'

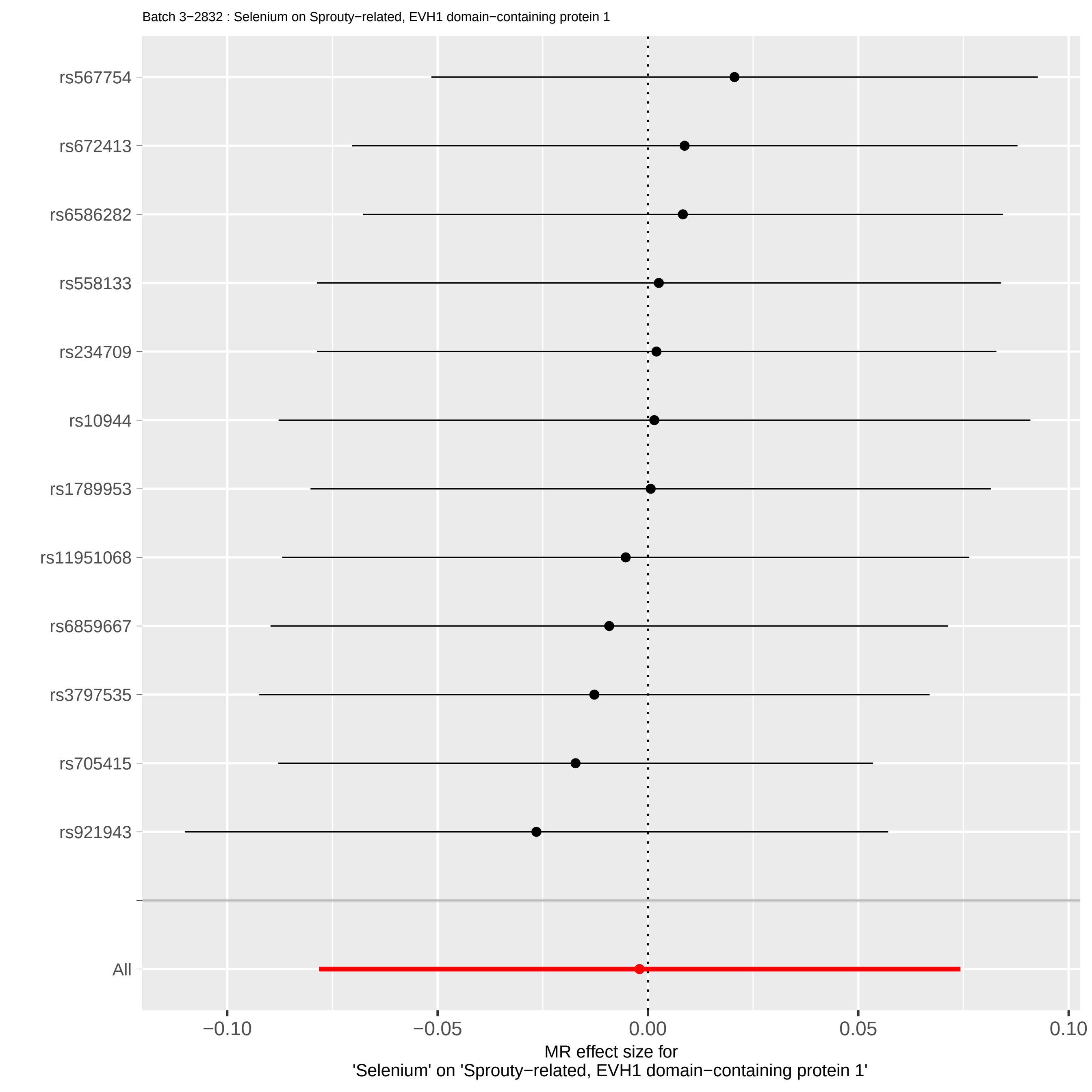

Batch 3−2832 : Selenium on Sprouty−related, EVH1 domain−containing protein 1
rs567754
rs672413
rs6586282
rs558133
rs234709
rs10944
rs1789953
rs11951068
rs6859667
rs3797535
rs705415
rs921943
All
−0.10
−0.05
0.00
0.05
0.10
MR effect size for
'Selenium' on 'Sprouty−related, EVH1 domain−containing protein 1'

Batch 3−2833 : Selenium on Protein sprouty homolog 1

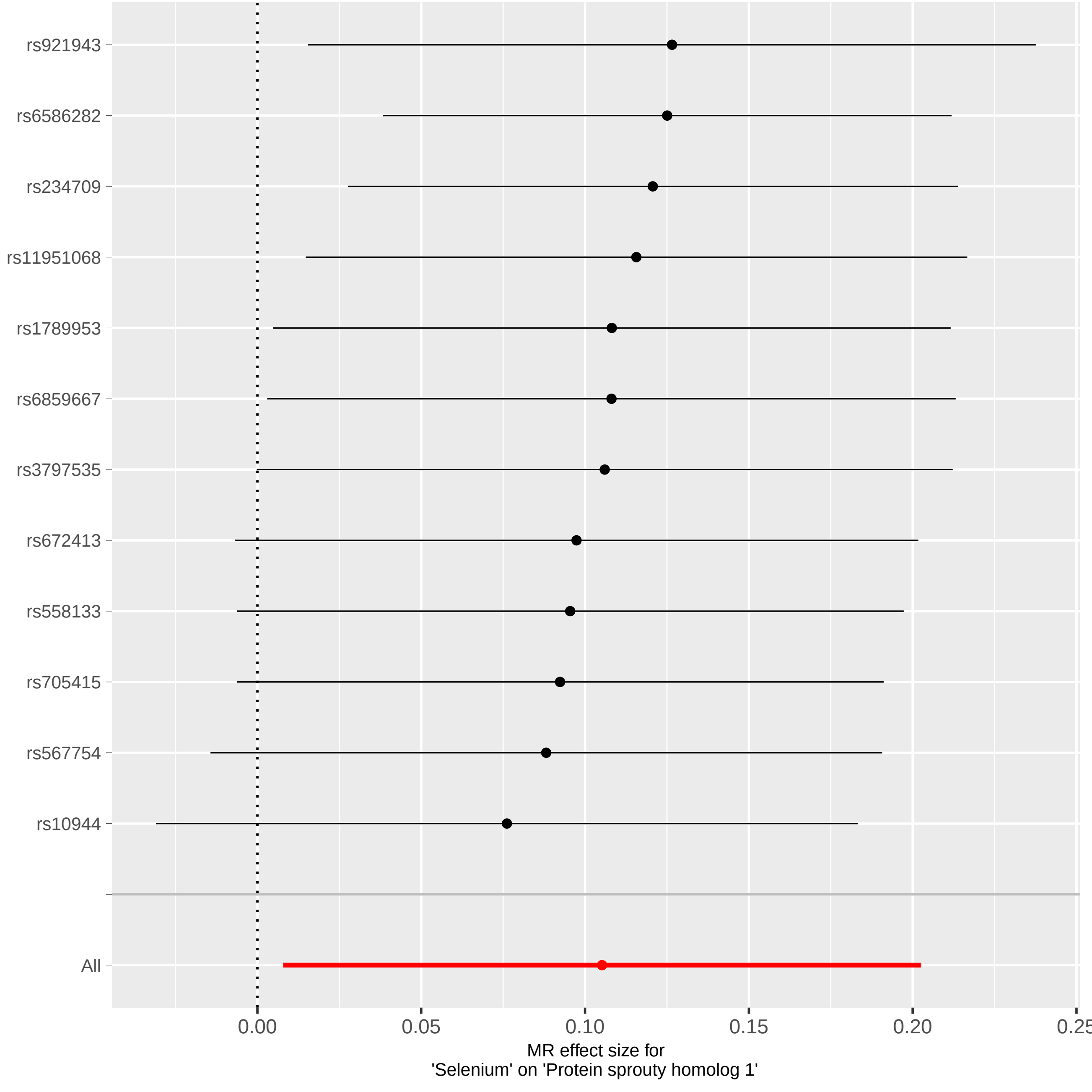

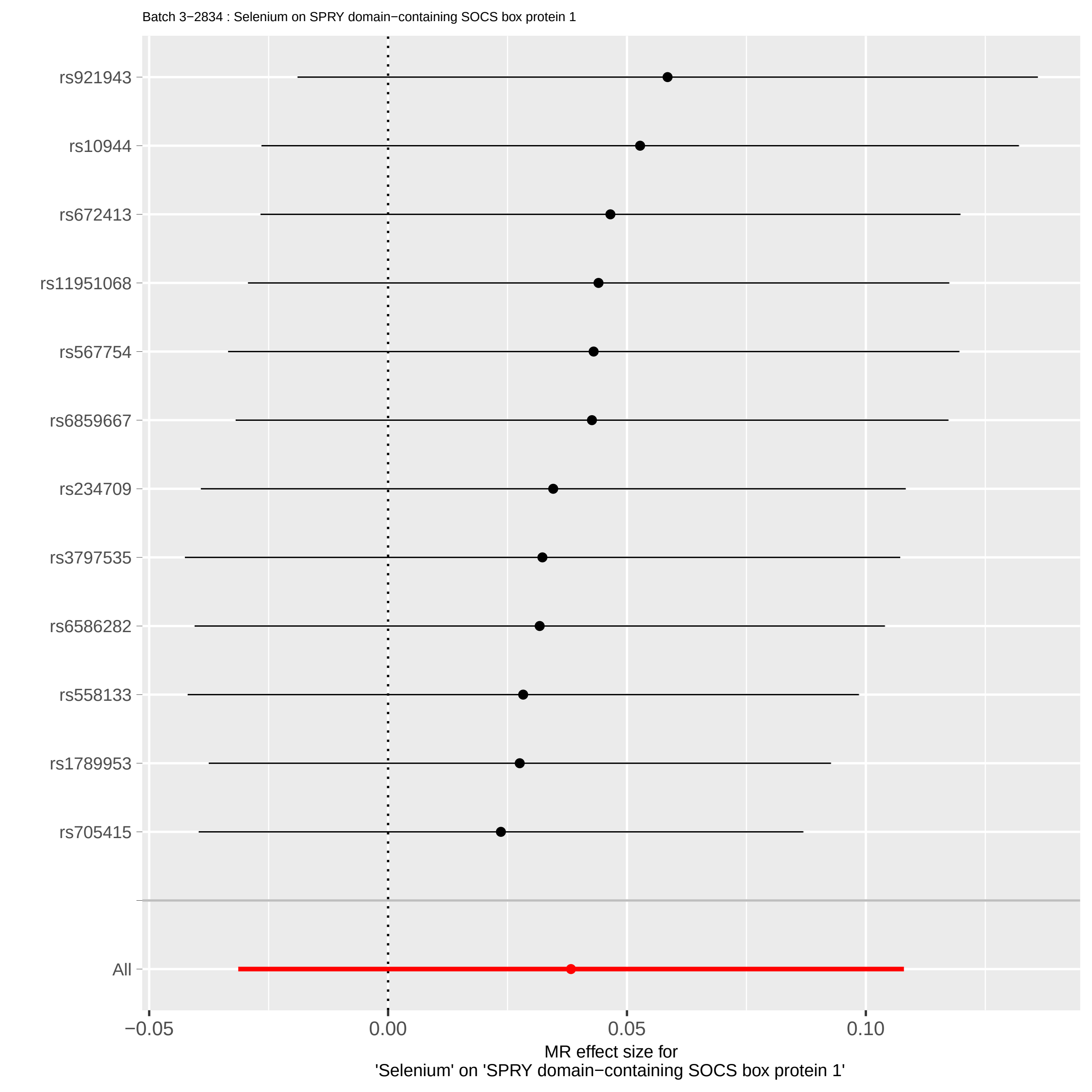

Batch 3−2834 : Selenium on SPRY domain−containing SOCS box protein 1
rs921943
rs10944
rs672413
rs11951068
rs567754
rs6859667
rs234709
rs3797535
rs6586282
rs558133
rs1789953
rs705415
All
−0.05
0.00
0.05
0.10
MR effect size for
'Selenium' on 'SPRY domain−containing SOCS box protein 1'

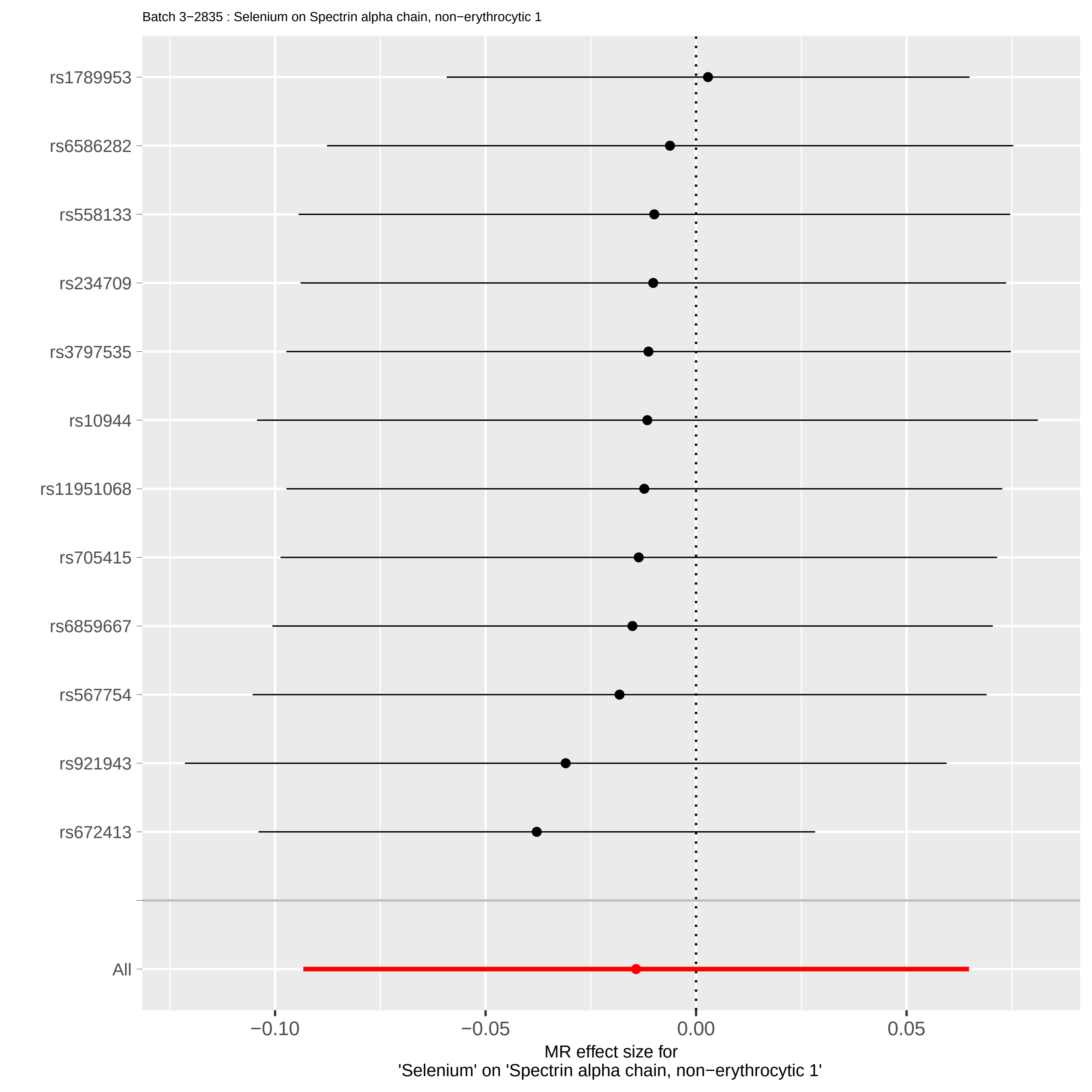
Batch 3−2835 : Selenium on Spectrin alpha chain, non−erythrocytic 1
rs1789953
rs6586282
rs558133
rs234709
rs3797535
rs10944
rs11951068
rs705415
rs6859667
rs567754
rs921943
rs672413
All
−0.10
−0.05
0.00
0.05
MR effect size for
'Selenium' on 'Spectrin alpha chain, non−erythrocytic 1'

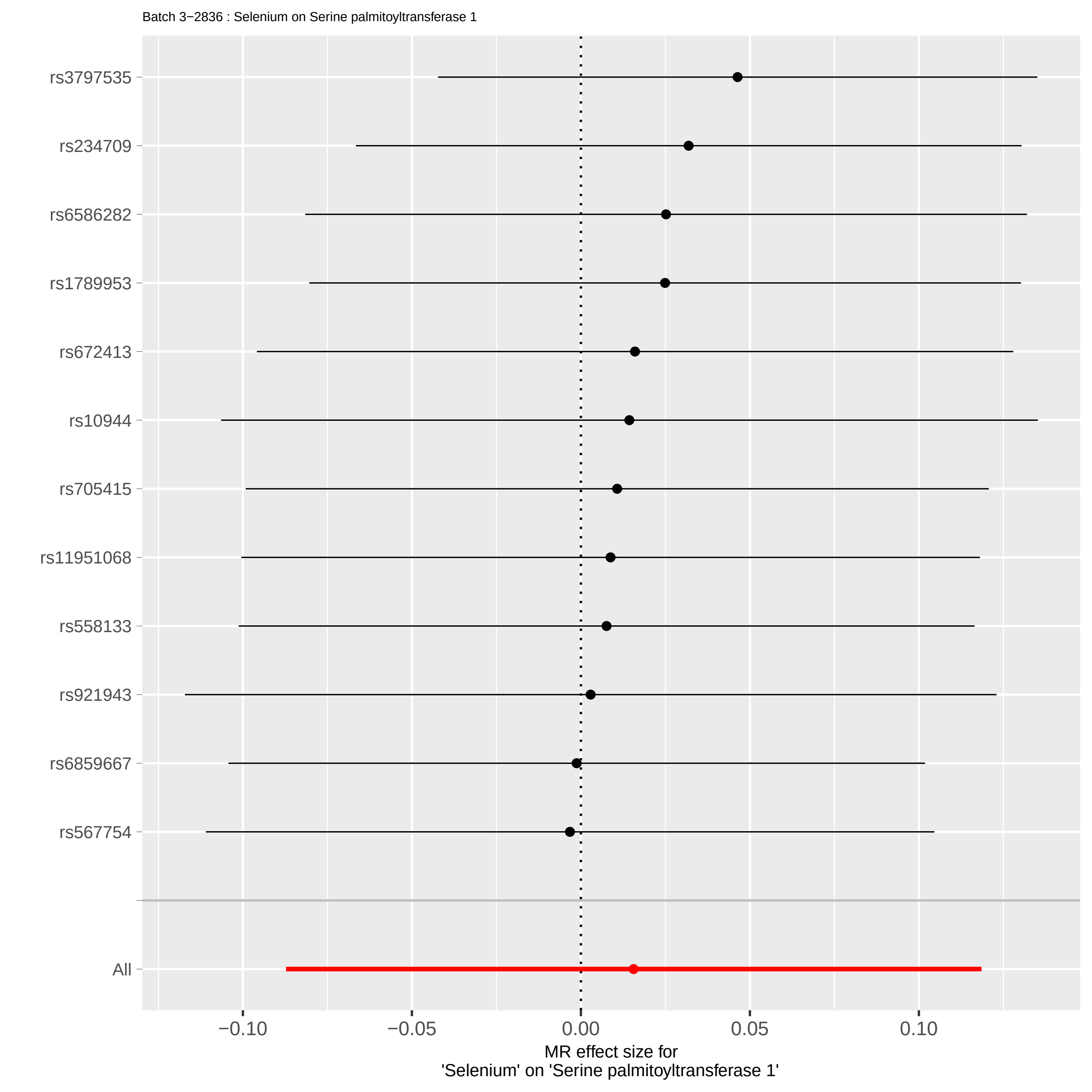

Batch 3−2836 : Selenium on Serine palmitoyltransferase 1
rs3797535
rs234709
rs6586282
rs1789953
rs672413
rs10944
rs705415
rs11951068
rs558133
rs921943
rs6859667
rs567754
All
−0.10
−0.05
0.00
0.05
0.10
MR effect size for
'Selenium' on 'Serine palmitoyltransferase 1'

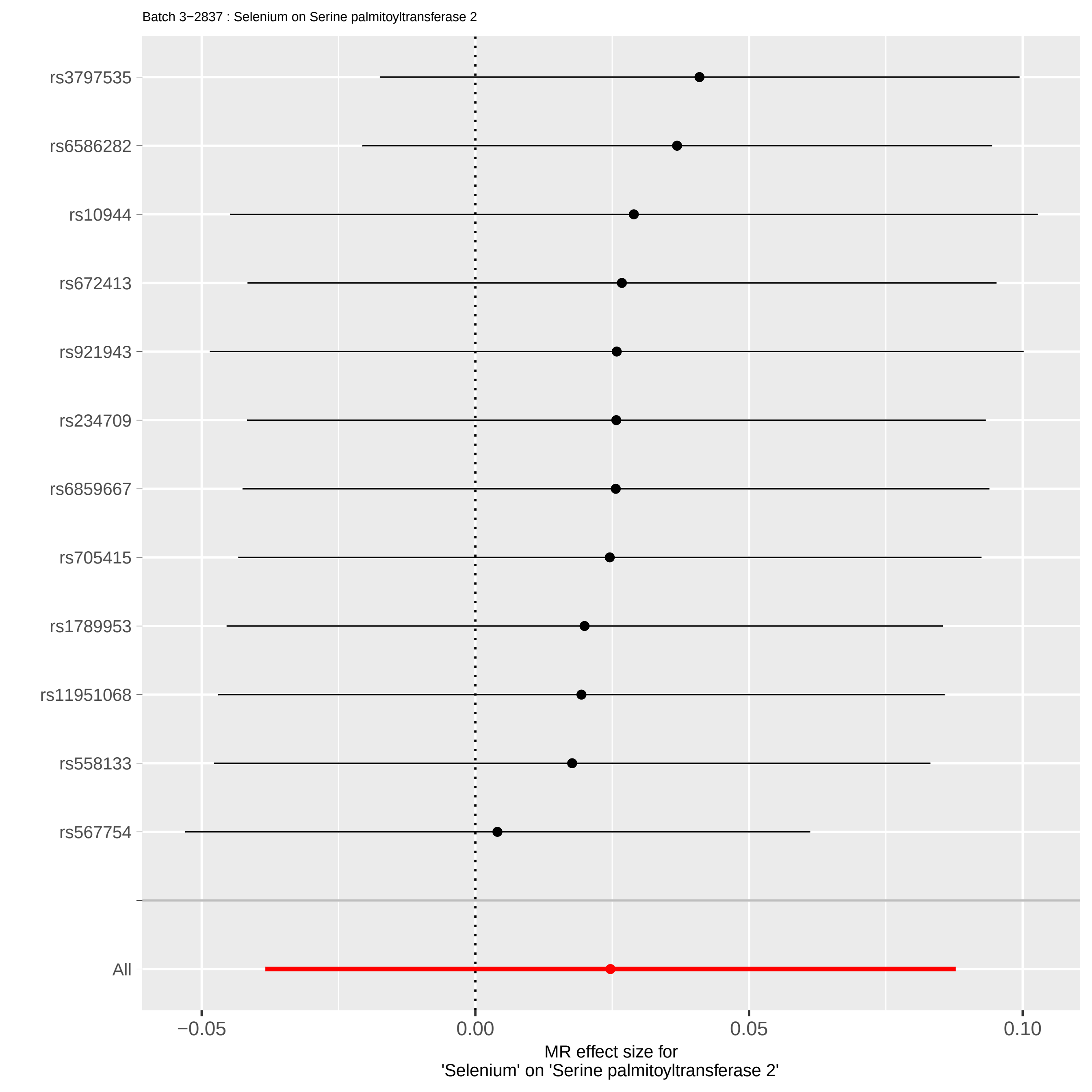

Batch 3−2837 : Selenium on Serine palmitoyltransferase 2
rs3797535
rs6586282
rs10944
rs672413
rs921943
rs234709
rs6859667
rs705415
rs1789953
rs11951068
rs558133
rs567754
All
−0.05
0.00
0.05
0.10
MR effect size for
'Selenium' on 'Serine palmitoyltransferase 2'

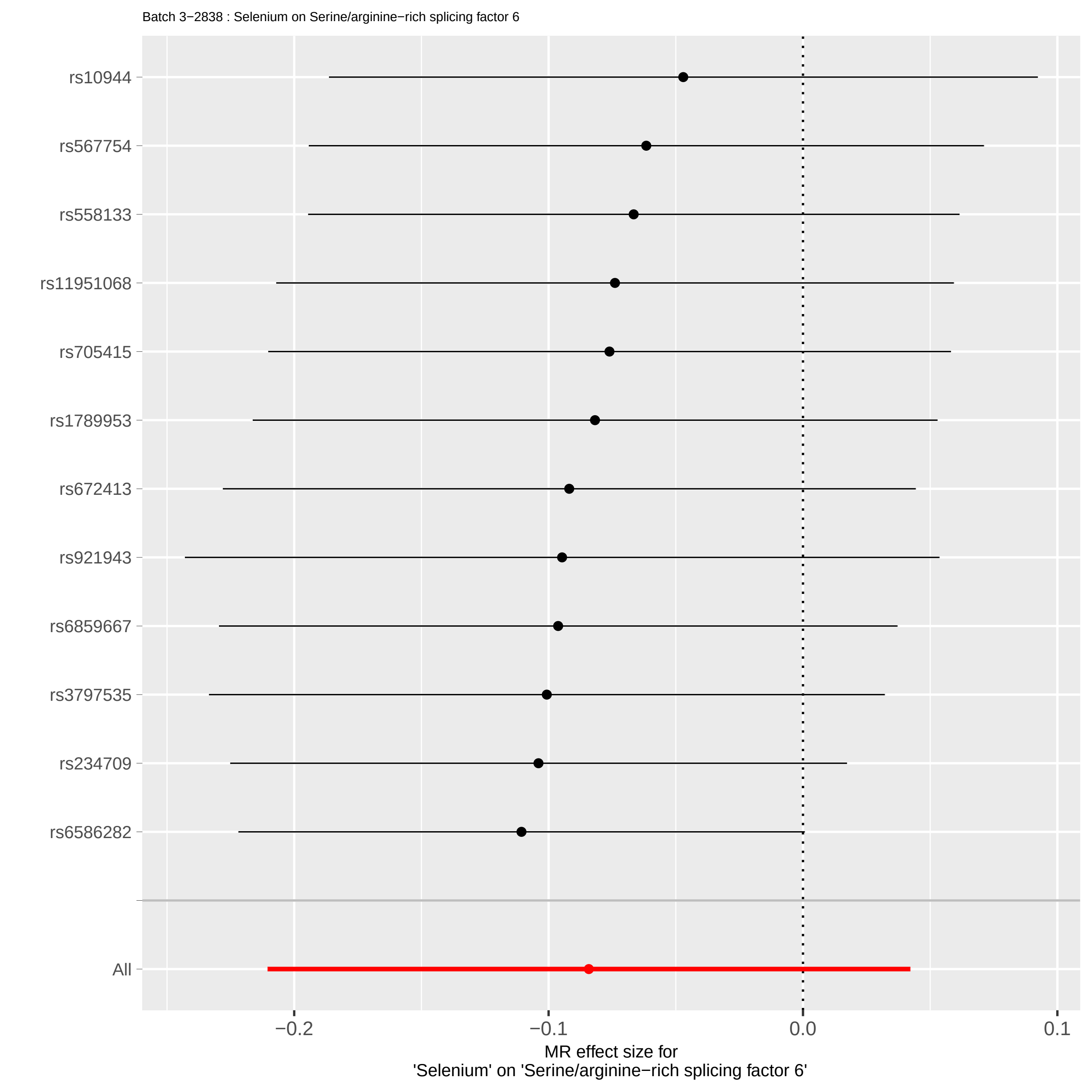
Batch 3−2838 : Selenium on Serine/arginine−rich splicing factor 6
rs10944
rs567754
rs558133
rs11951068
rs705415
rs1789953
rs672413
rs921943
rs6859667
rs3797535
rs234709
rs6586282
All
−0.2
−0.1
0.0
0.1
MR effect size for
'Selenium' on 'Serine/arginine−rich splicing factor 6'

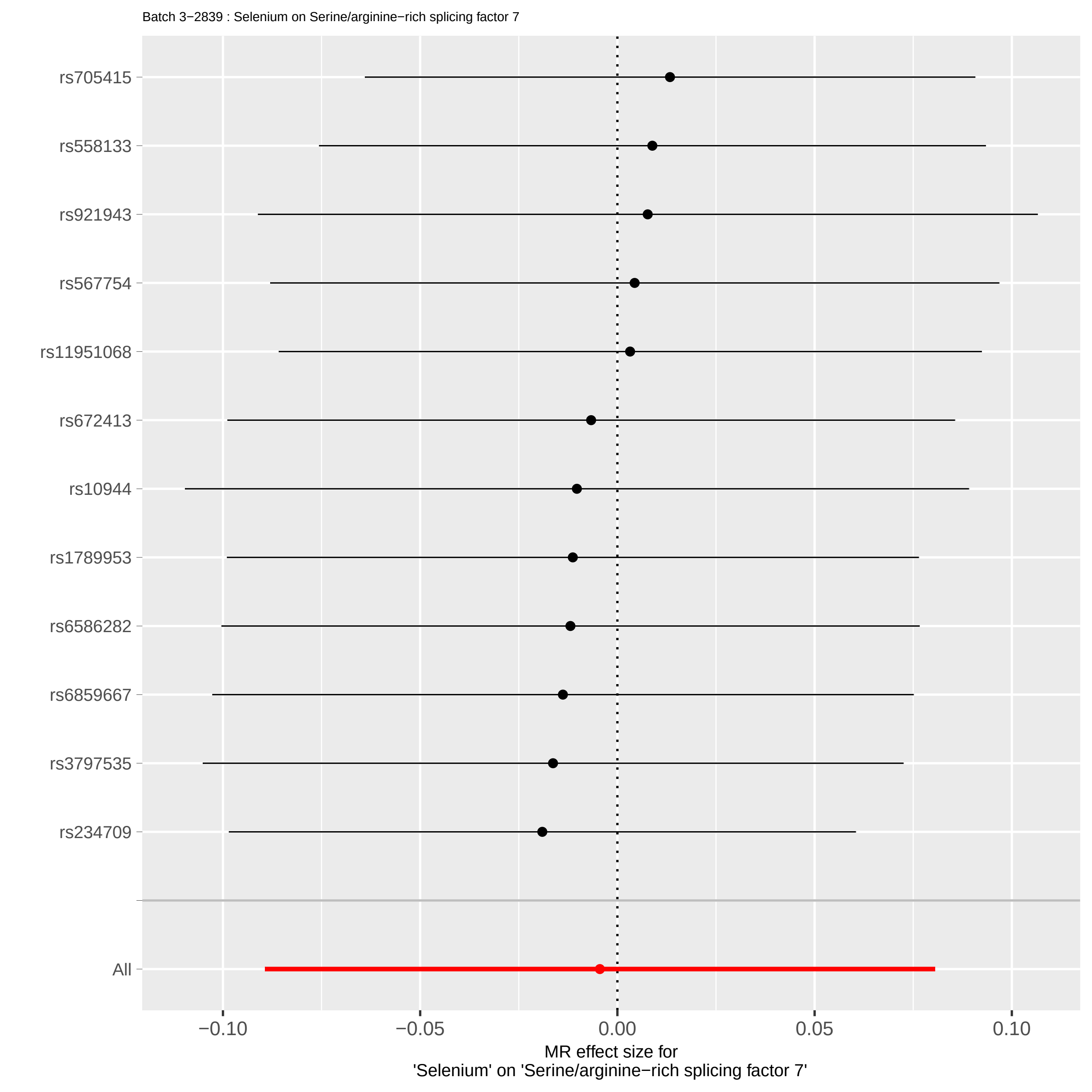

Batch 3−2839 : Selenium on Serine/arginine−rich splicing factor 7
rs705415
rs558133
rs921943
rs567754
rs11951068
rs672413
rs10944
rs1789953
rs6586282
rs6859667
rs3797535
rs234709
All
−0.10
−0.05
0.00
0.05
0.10
MR effect size for
'Selenium' on 'Serine/arginine−rich splicing factor 7'

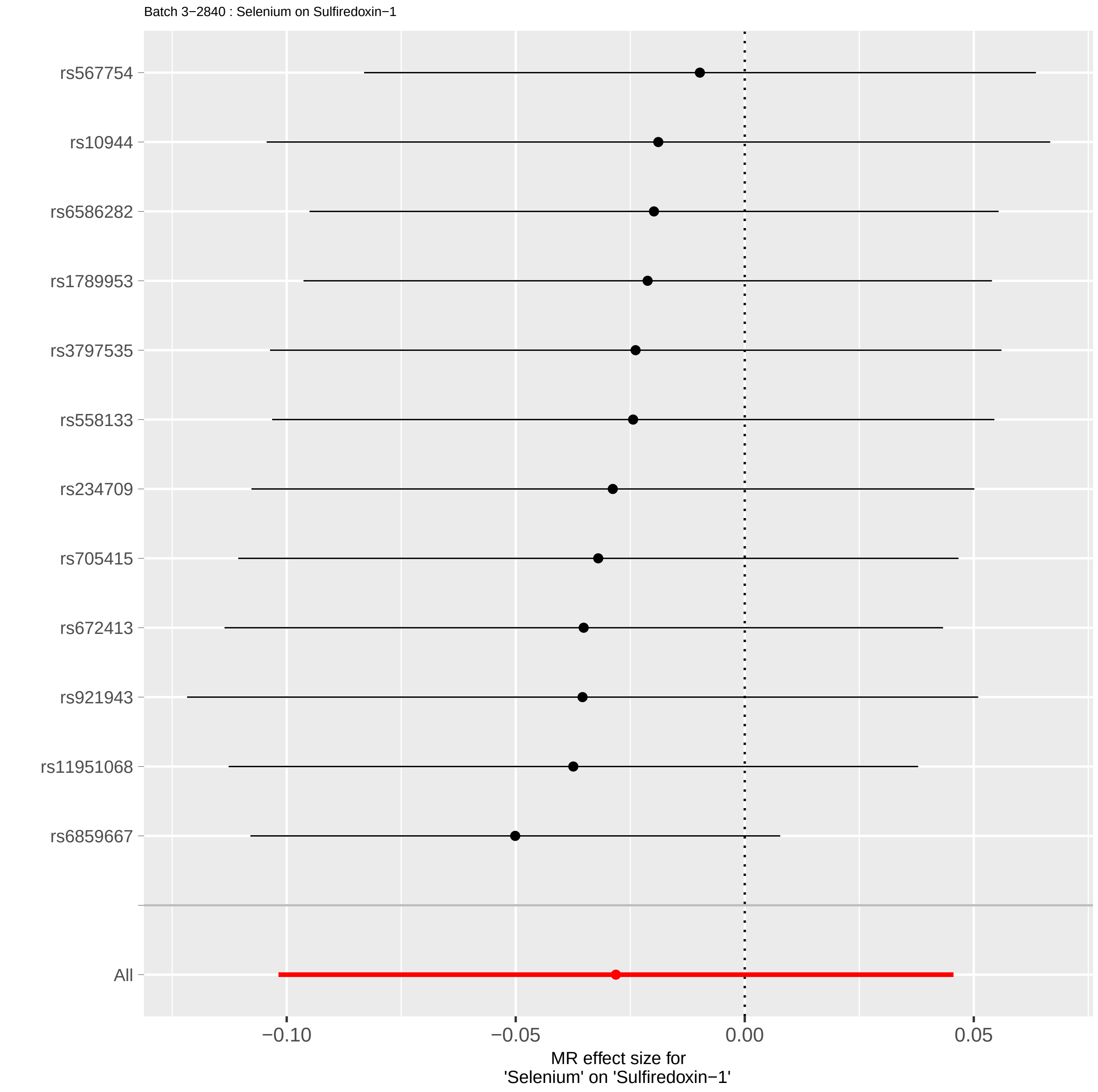

Batch 3−2840 : Selenium on Sulfiredoxin−1
rs567754
rs10944
rs6586282
rs1789953
rs3797535
rs558133
rs234709
rs705415
rs672413
rs921943
rs11951068
rs6859667
All
−0.10
−0.05
0.00
0.05
MR effect size for
'Selenium' on 'Sulfiredoxin−1'

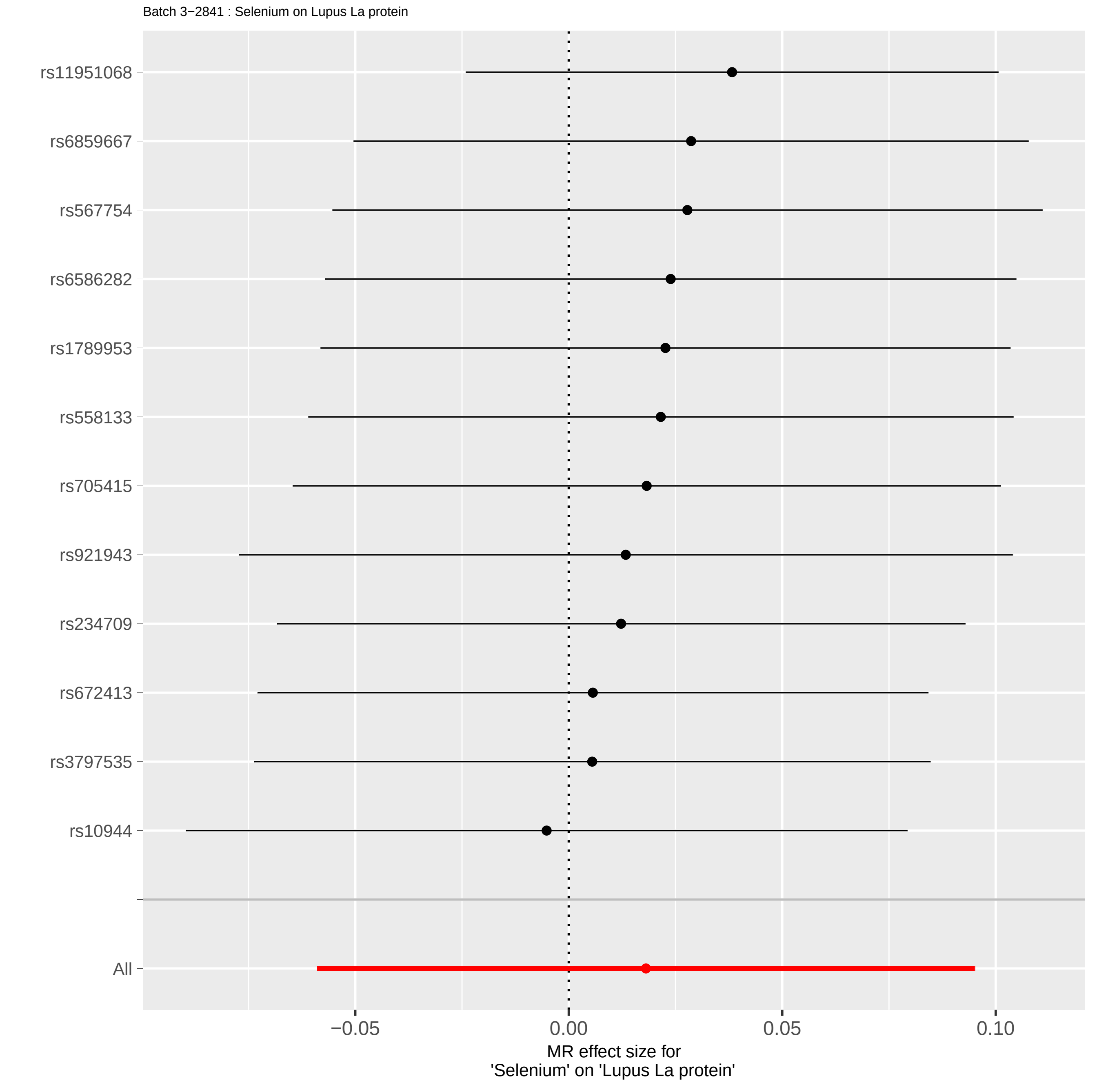
Batch 3−2841 : Selenium on Lupus La protein
rs11951068
rs6859667
rs567754
rs6586282
rs1789953
rs558133
rs705415
rs921943
rs234709
rs672413
rs3797535
rs10944
All
−0.05
0.00
0.05
0.10
MR effect size for
'Selenium' on 'Lupus La protein'

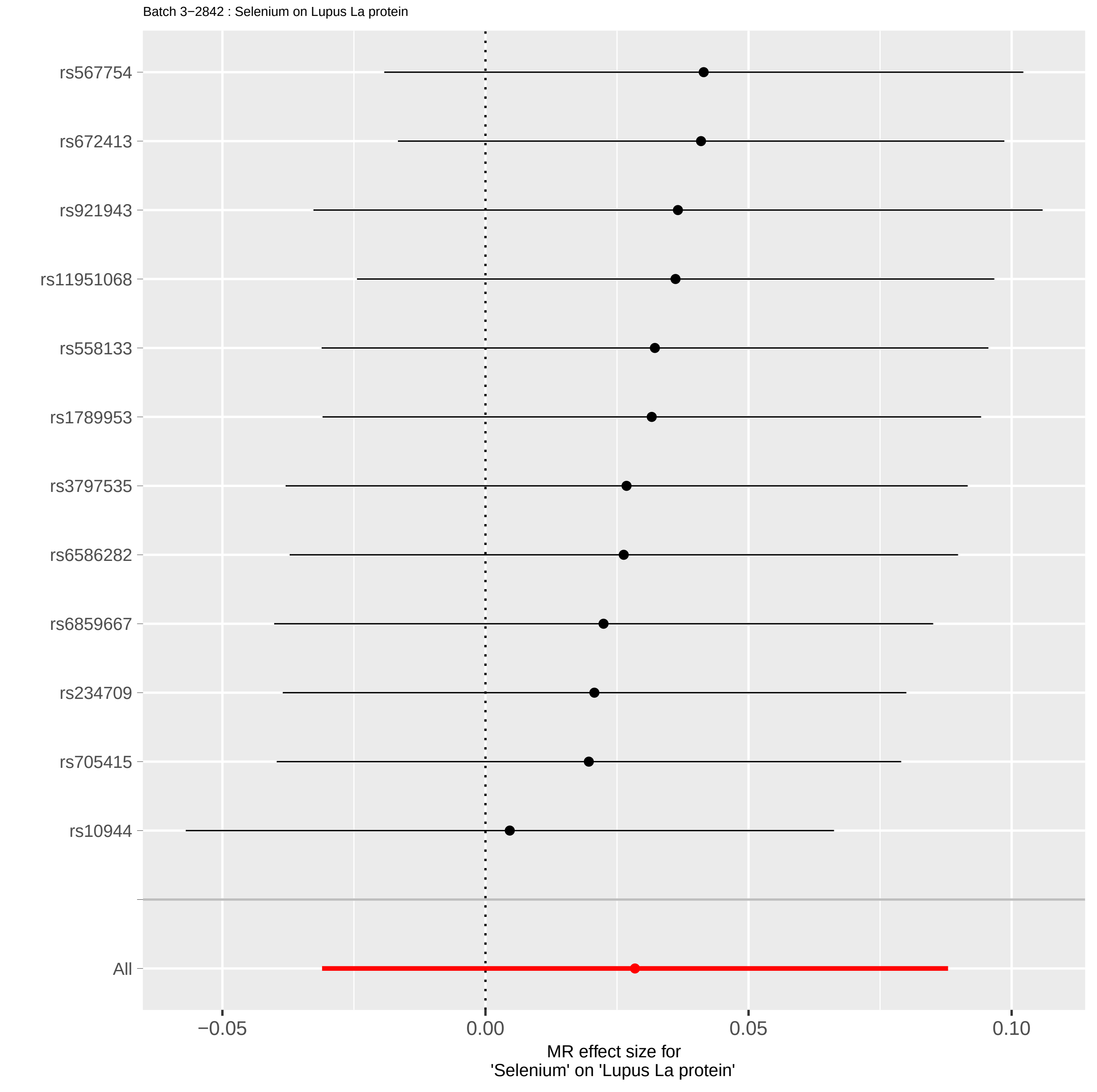

Batch 3−2842 : Selenium on Lupus La protein
rs567754
rs672413
rs921943
rs11951068
rs558133
rs1789953
rs3797535
rs6586282
rs6859667
rs234709
rs705415
rs10944
All
−0.05
0.00
0.05
0.10
MR effect size for
'Selenium' on 'Lupus La protein'

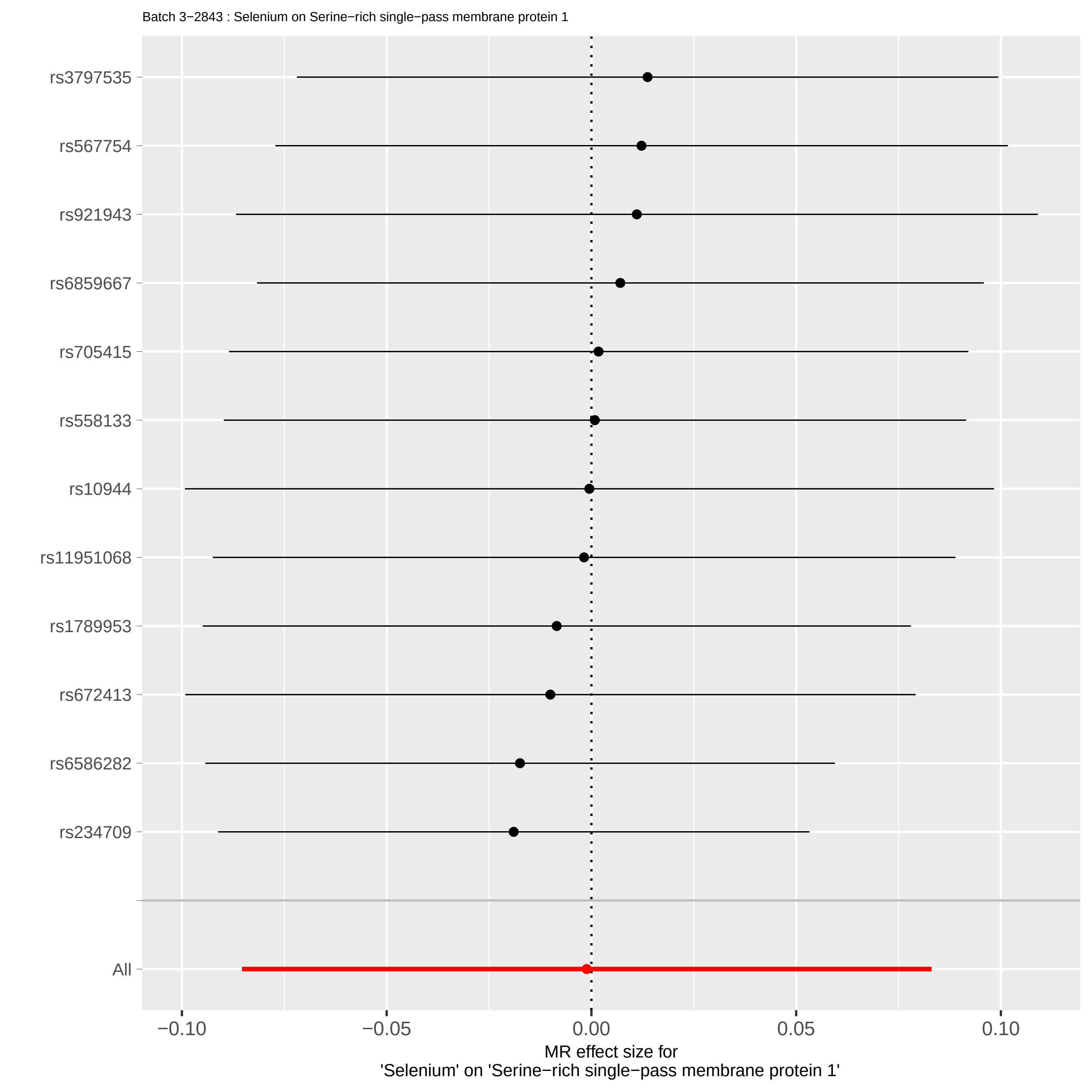
Batch 3−2843 : Selenium on Serine−rich single−pass membrane protein 1
rs3797535
rs567754
rs921943
rs6859667
rs705415
rs558133
rs10944
rs11951068
rs1789953
rs672413
rs6586282
rs234709
All
−0.10
−0.05
0.00
0.05
0.10
MR effect size for
'Selenium' on 'Serine−rich single−pass membrane protein 1'

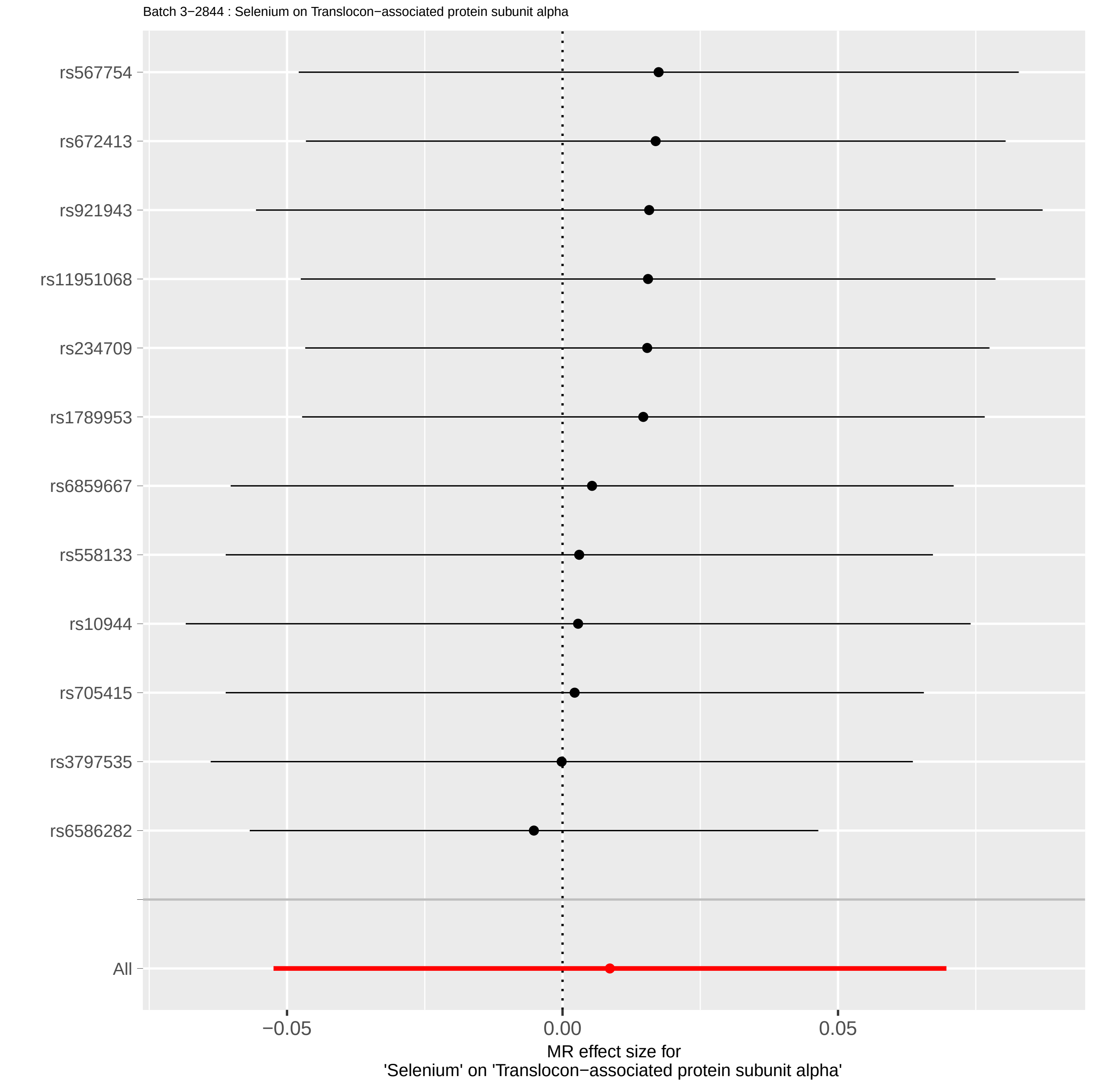

Batch 3−2844 : Selenium on Translocon−associated protein subunit alpha
rs567754
rs672413
rs921943
rs11951068
rs234709
rs1789953
rs6859667
rs558133
rs10944
rs705415
rs3797535
rs6586282
All
−0.05
0.00
0.05
MR effect size for
'Selenium' on 'Translocon−associated protein subunit alpha'

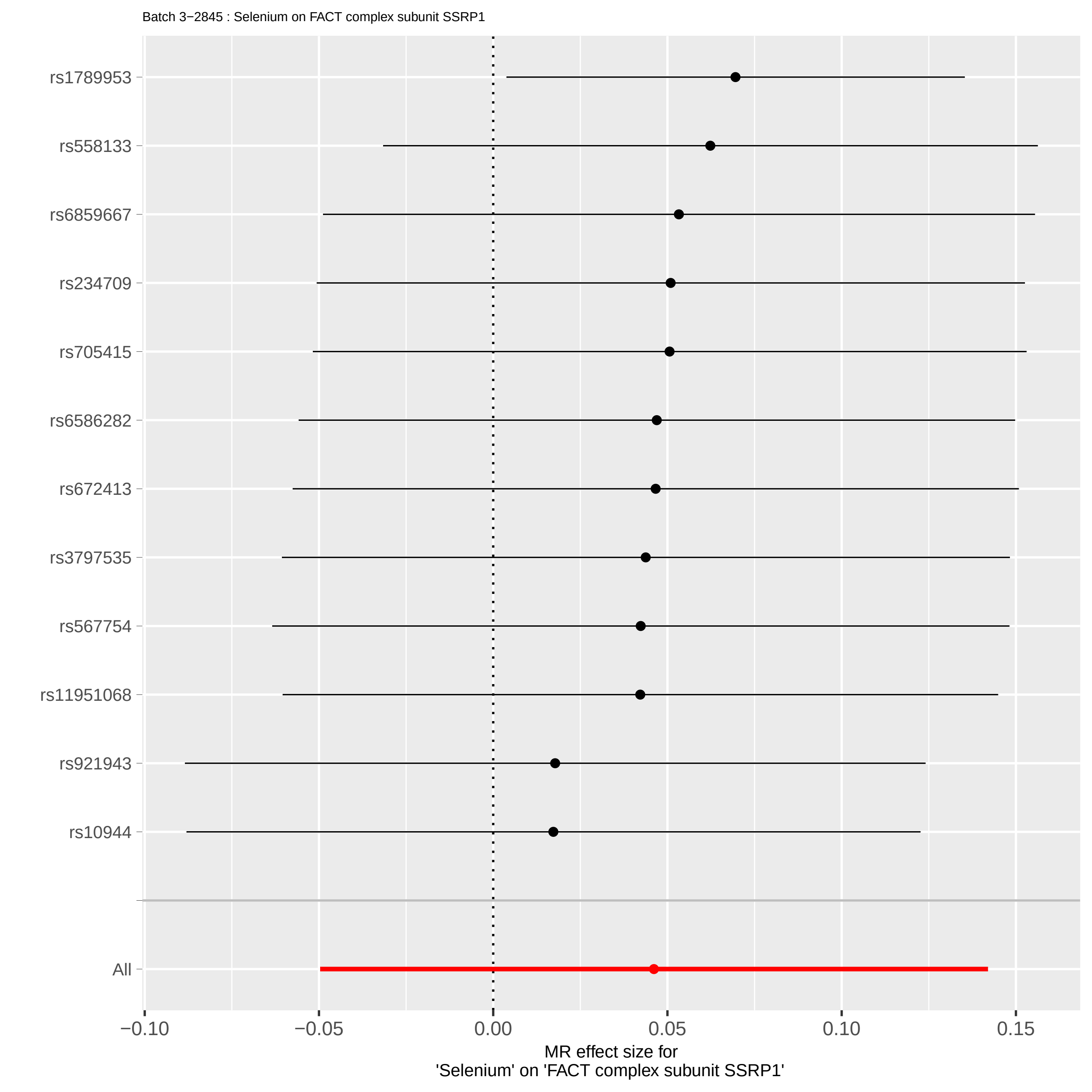

Batch 3−2845 : Selenium on FACT complex subunit SSRP1
rs1789953
rs558133
rs6859667
rs234709
rs705415
rs6586282
rs672413
rs3797535
rs567754
rs11951068
rs921943
rs10944
All
−0.10
−0.05
0.00
0.05
0.10
0.15
MR effect size for
'Selenium' on 'FACT complex subunit SSRP1'

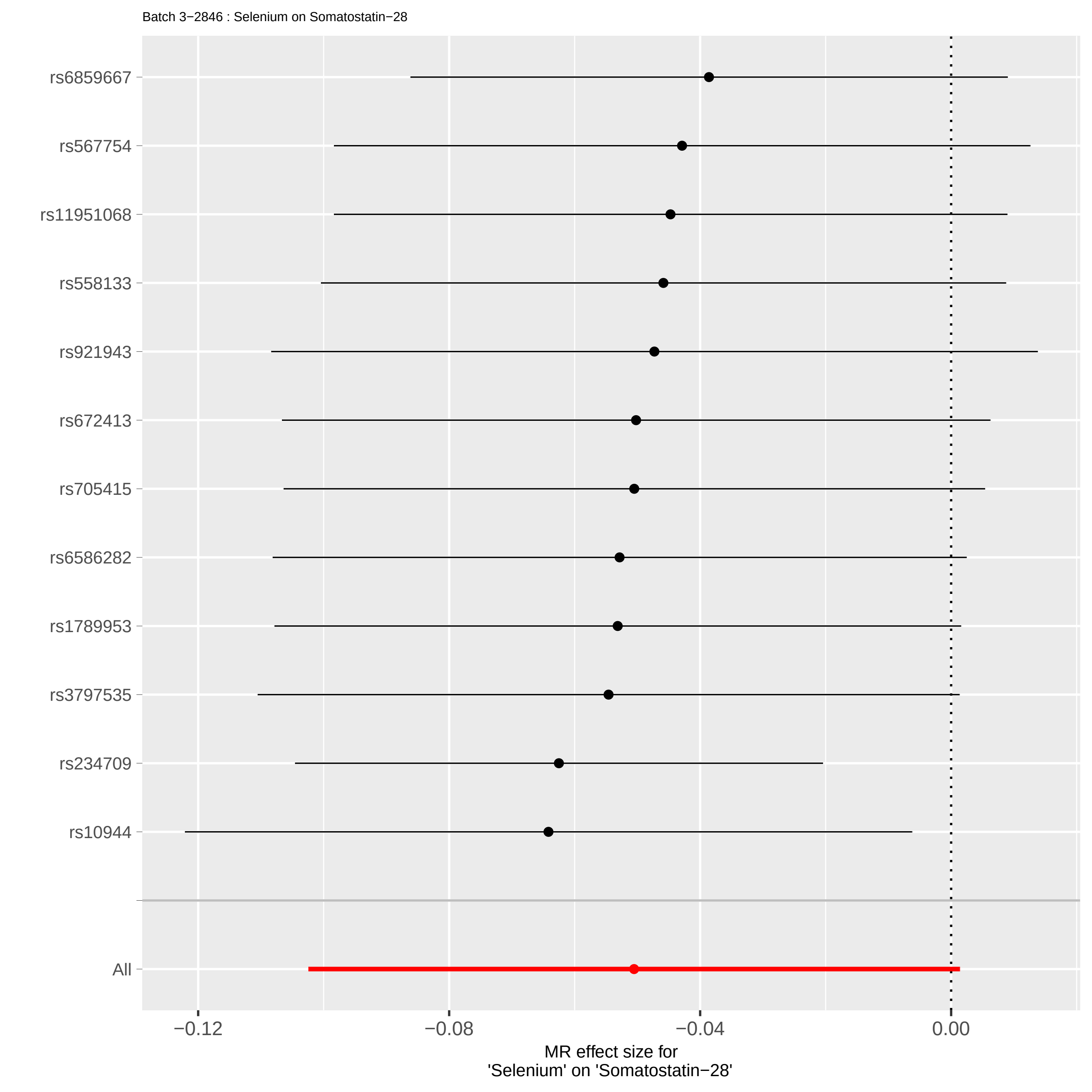

Batch 3−2846 : Selenium on Somatostatin−28
rs6859667
rs567754
rs11951068
rs558133
rs921943
rs672413
rs705415
rs6586282
rs1789953
rs3797535
rs234709
rs10944
All
−0.12
−0.08
−0.04
0.00
MR effect size for
'Selenium' on 'Somatostatin−28'

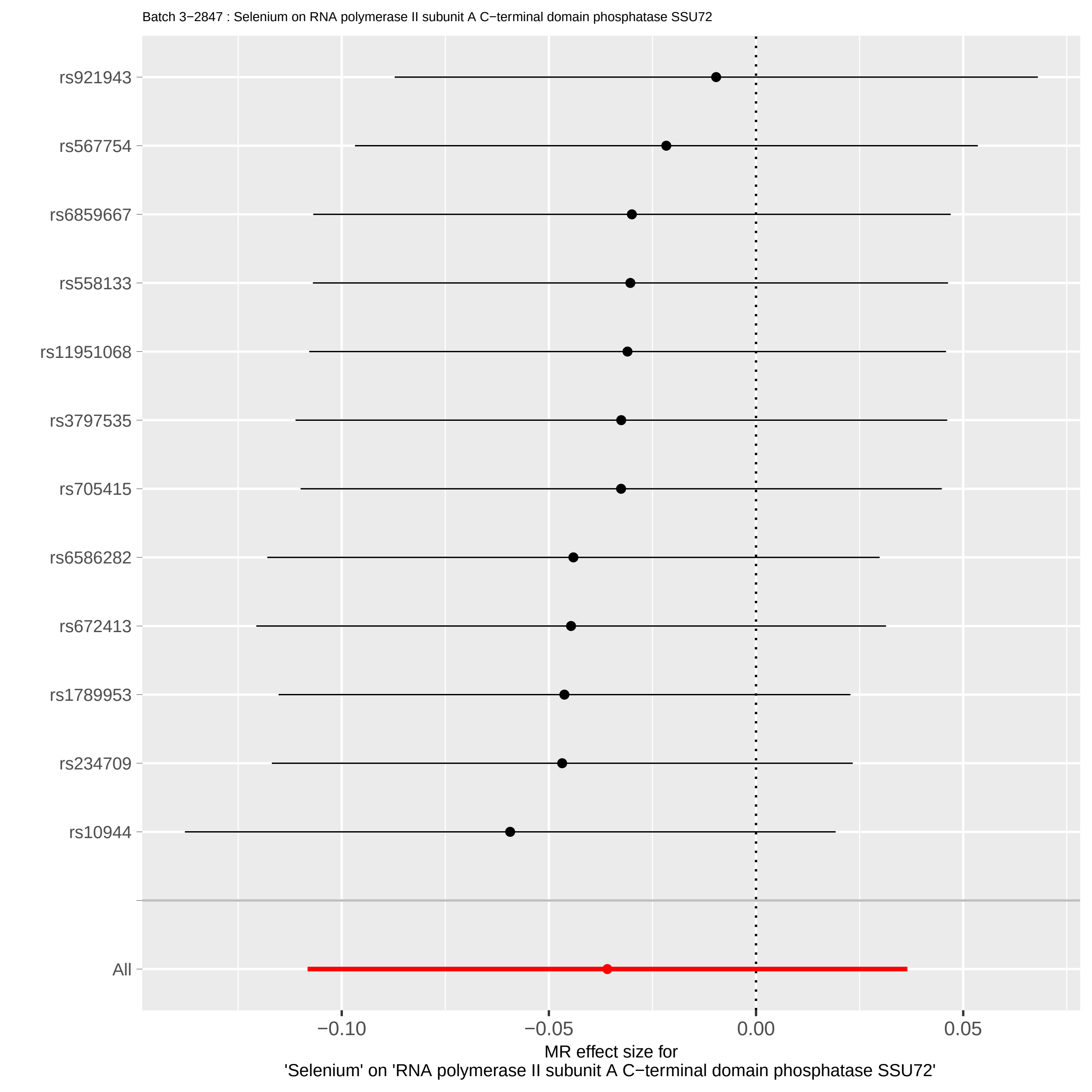

Batch 3−2847 : Selenium on RNA polymerase II subunit A C−terminal domain phosphatase SSU72
rs921943
rs567754
rs6859667
rs558133
rs11951068
rs3797535
rs705415
rs6586282
rs672413
rs1789953
rs234709
rs10944
All
−0.10
−0.05
0.00
0.05
MR effect size for
'Selenium' on 'RNA polymerase II subunit A C−terminal domain phosphatase SSU72'

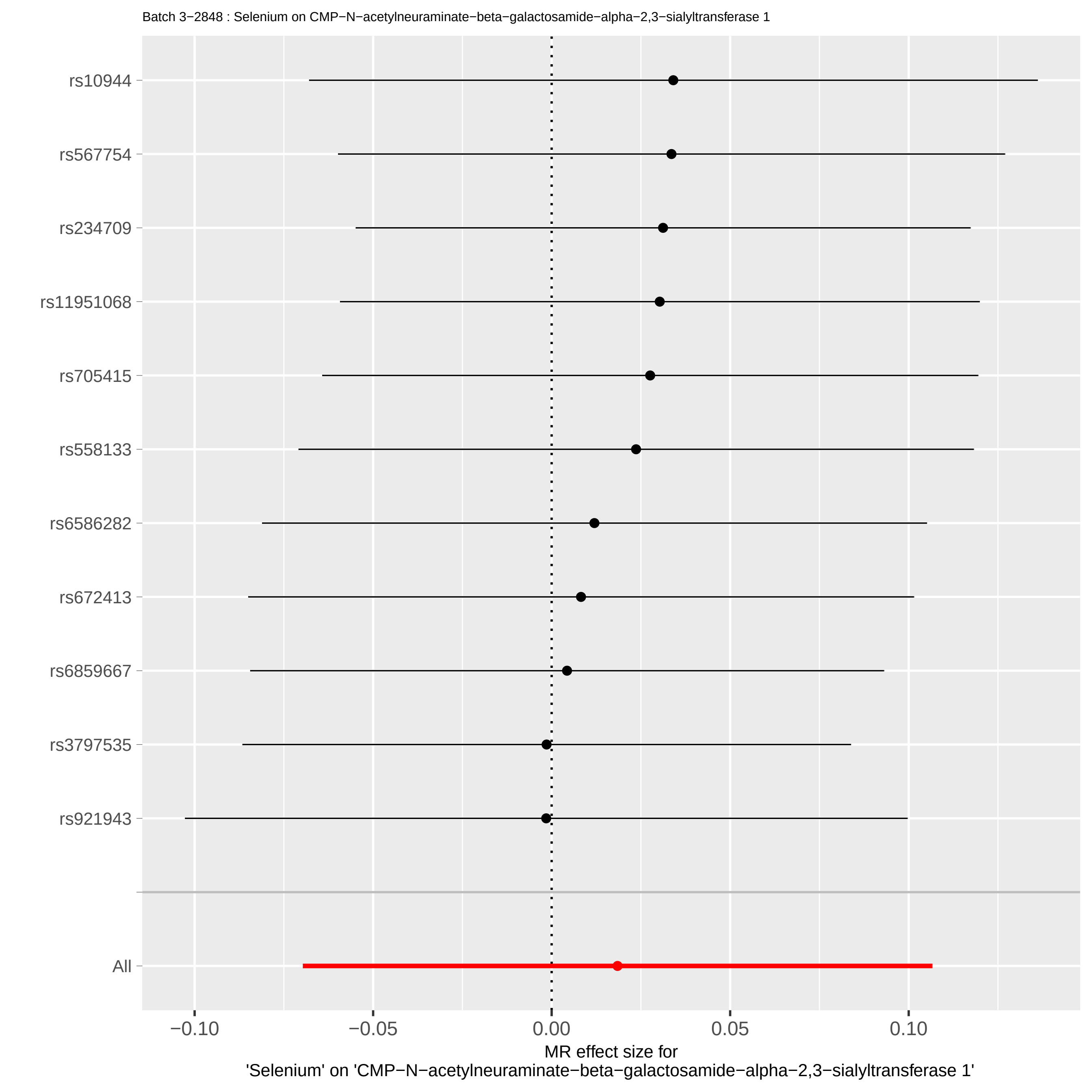
Batch 3−2848 : Selenium on CMP−N−acetylneuraminate−beta−galactosamide−alpha−2,3−sialyltransferase 1
rs10944
rs567754
rs234709
rs11951068
rs705415
rs558133
rs6586282
rs672413
rs6859667
rs3797535
rs921943
All
−0.10
−0.05
0.00
0.05
0.10
MR effect size for
'Selenium' on 'CMP−N−acetylneuraminate−beta−galactosamide−alpha−2,3−sialyltransferase 1'

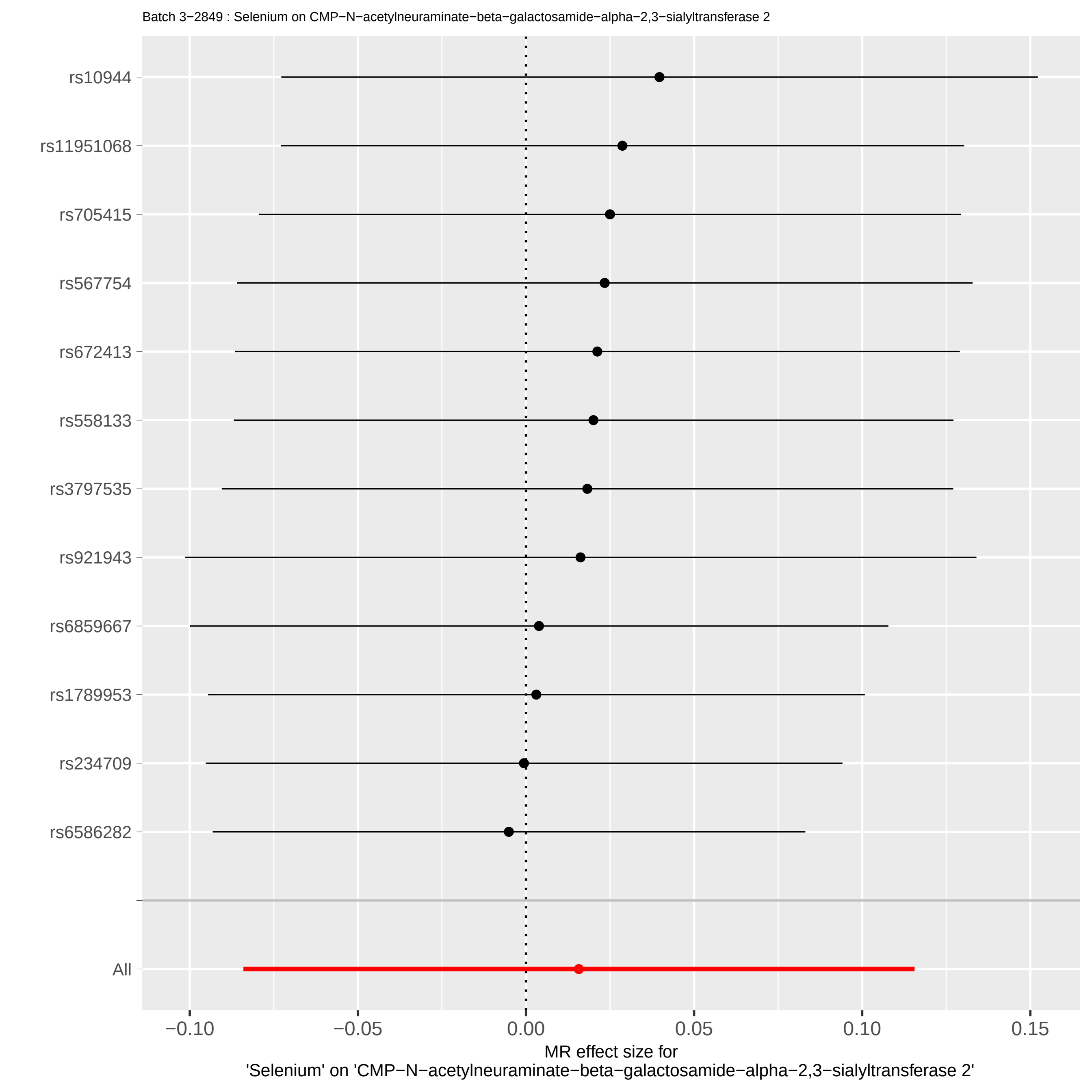

Batch 3−2849 : Selenium on CMP−N−acetylneuraminate−beta−galactosamide−alpha−2,3−sialyltransferase 2
rs10944
rs11951068
rs705415
rs567754
rs672413
rs558133
rs3797535
rs921943
rs6859667
rs1789953
rs234709
rs6586282
All
−0.10
−0.05
0.00
0.05
0.10
0.15
MR effect size for
'Selenium' on 'CMP−N−acetylneuraminate−beta−galactosamide−alpha−2,3−sialyltransferase 2'

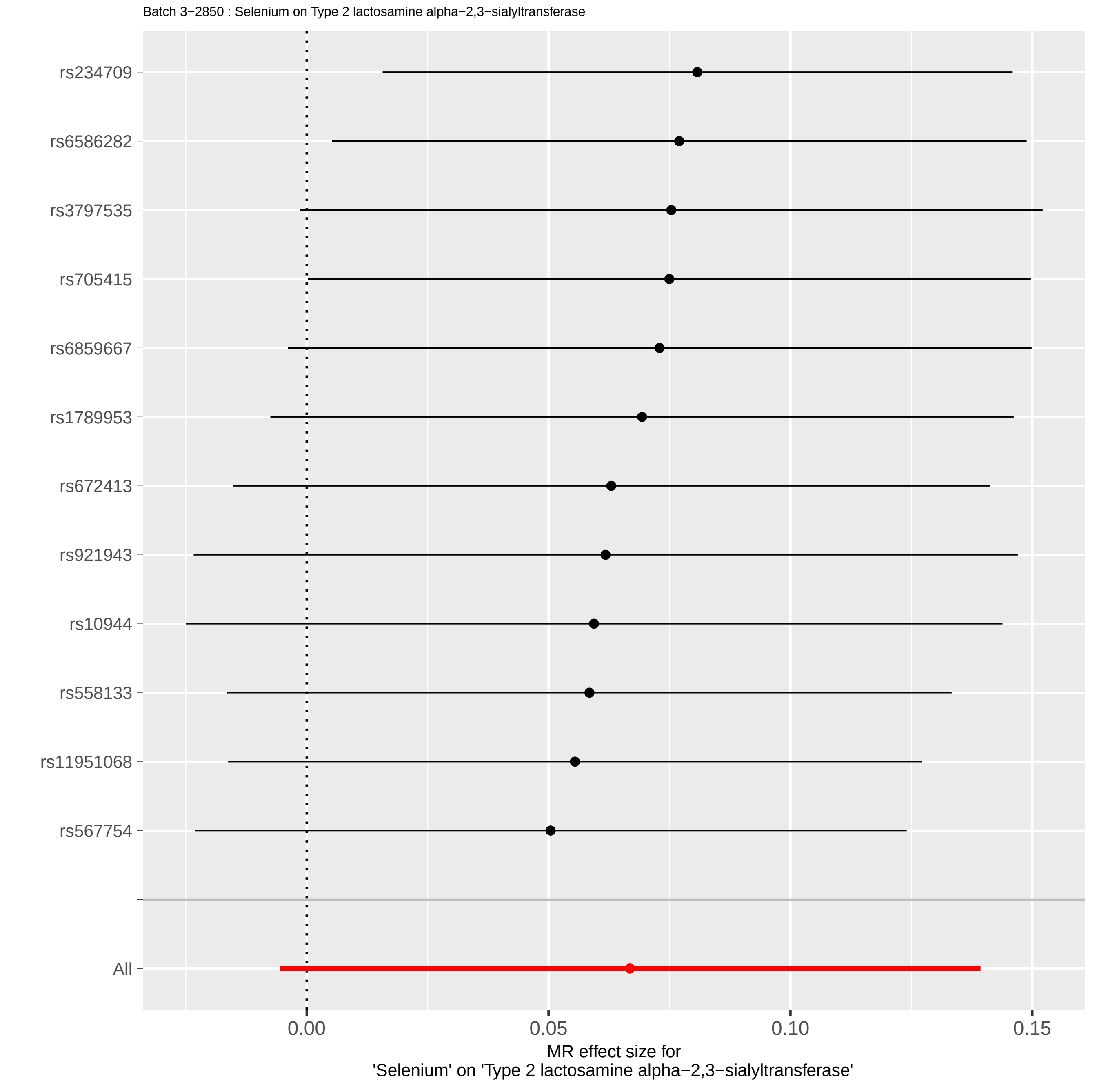

Batch 3−2850 : Selenium on Type 2 lactosamine alpha−2,3−sialyltransferase
rs234709
rs6586282
rs3797535
rs705415
rs6859667
rs1789953
rs672413
rs921943
rs10944
rs558133
rs11951068
rs567754
All
0.00
0.05
0.10
0.15
MR effect size for
'Selenium' on 'Type 2 lactosamine alpha−2,3−sialyltransferase'

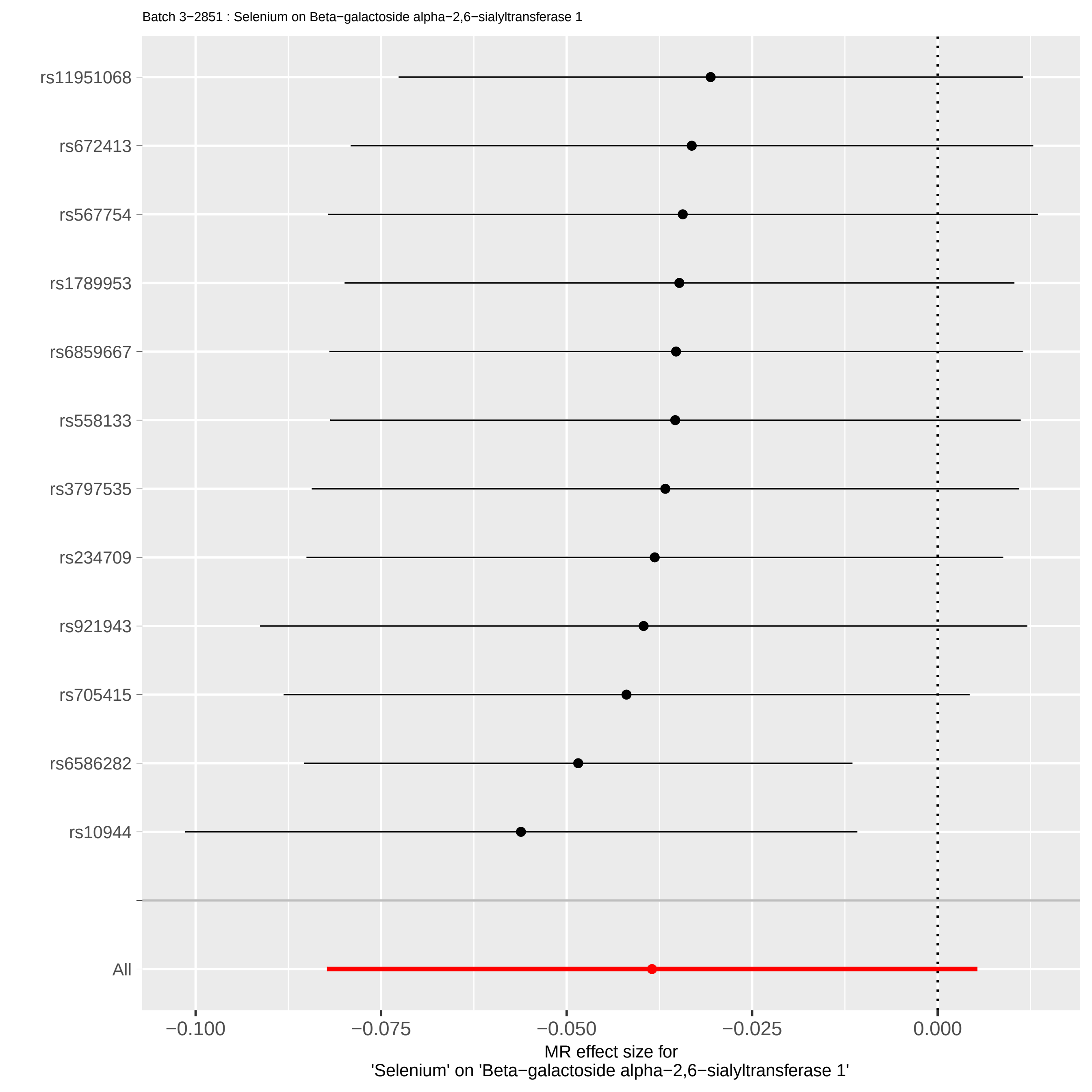

Batch 3−2851 : Selenium on Beta−galactoside alpha−2,6−sialyltransferase 1
rs11951068
rs672413
rs567754
rs1789953
rs6859667
rs558133
rs3797535
rs234709
rs921943
rs705415
rs6586282
rs10944
All
−0.100
−0.075
−0.050
−0.025
0.000
MR effect size for
'Selenium' on 'Beta−galactoside alpha−2,6−sialyltransferase 1'

Batch 3−2852 : Selenium on Alpha−N−acetylgalactosaminide alpha−2,6−sialyltransferase 1

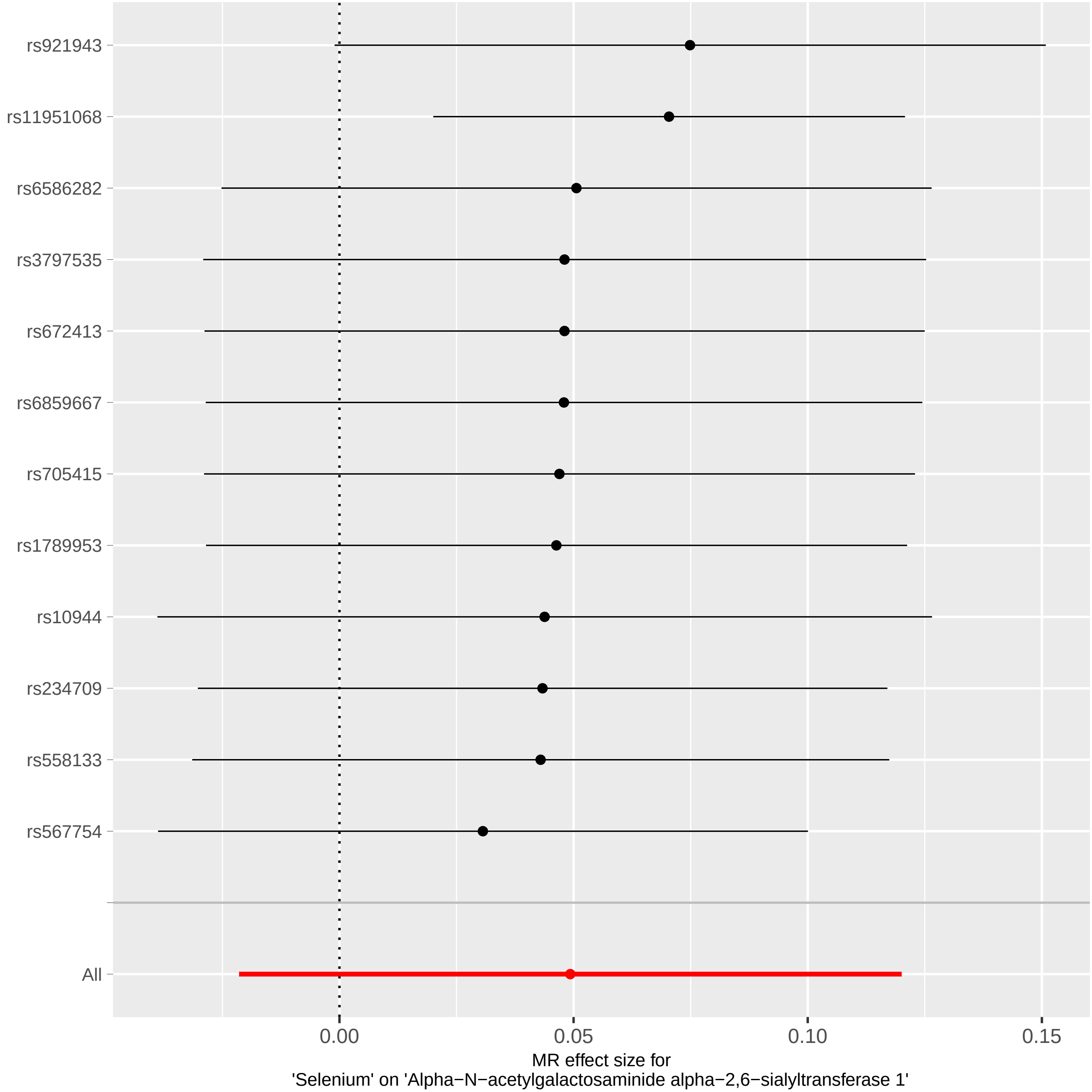

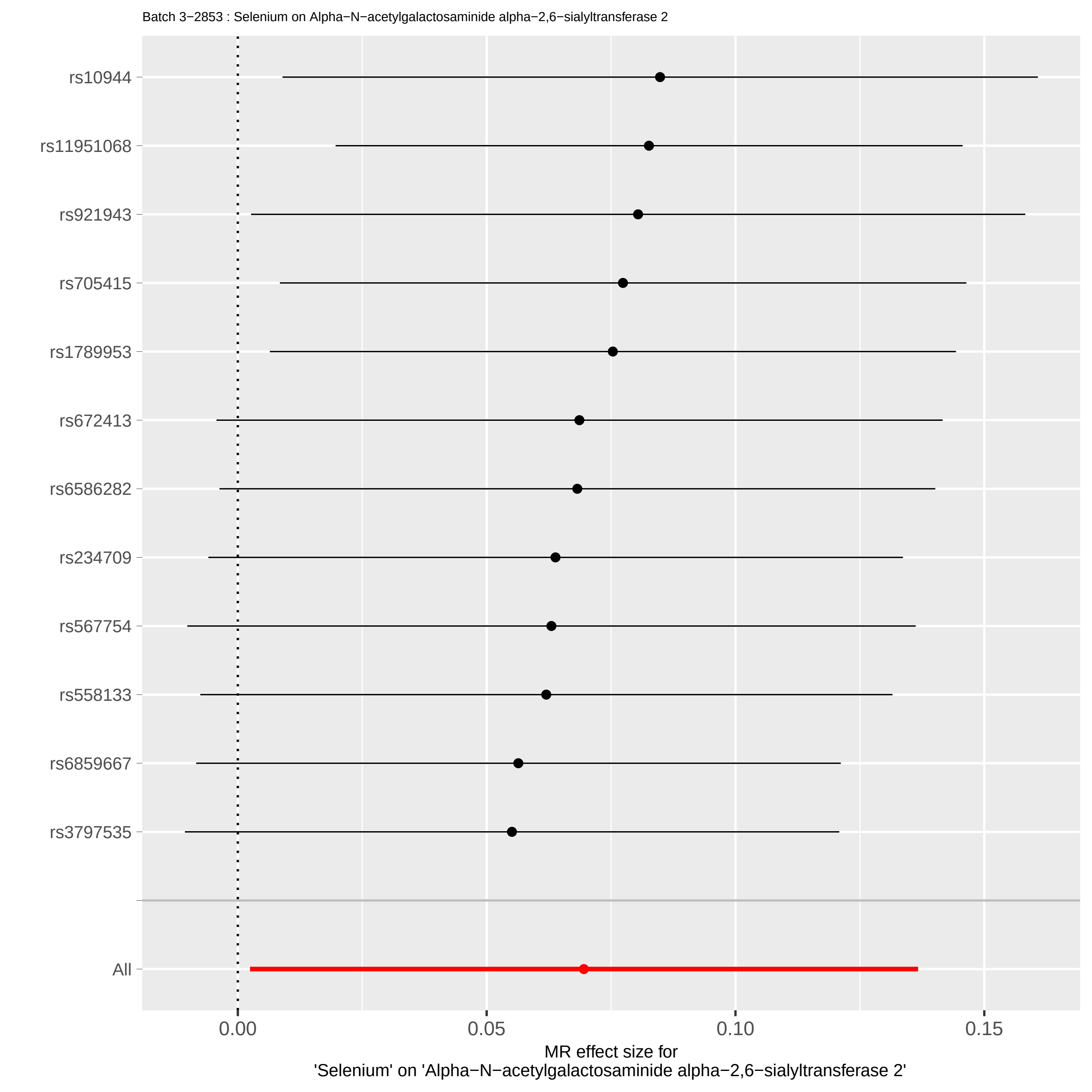

Batch 3−2853 : Selenium on Alpha−N−acetylgalactosaminide alpha−2,6−sialyltransferase 2
rs10944
rs11951068
rs921943
rs705415
rs1789953
rs672413
rs6586282
rs234709
rs567754
rs558133
rs6859667
rs3797535
All
0.00
0.05
0.10
0.15
MR effect size for
'Selenium' on 'Alpha−N−acetylgalactosaminide alpha−2,6−sialyltransferase 2'

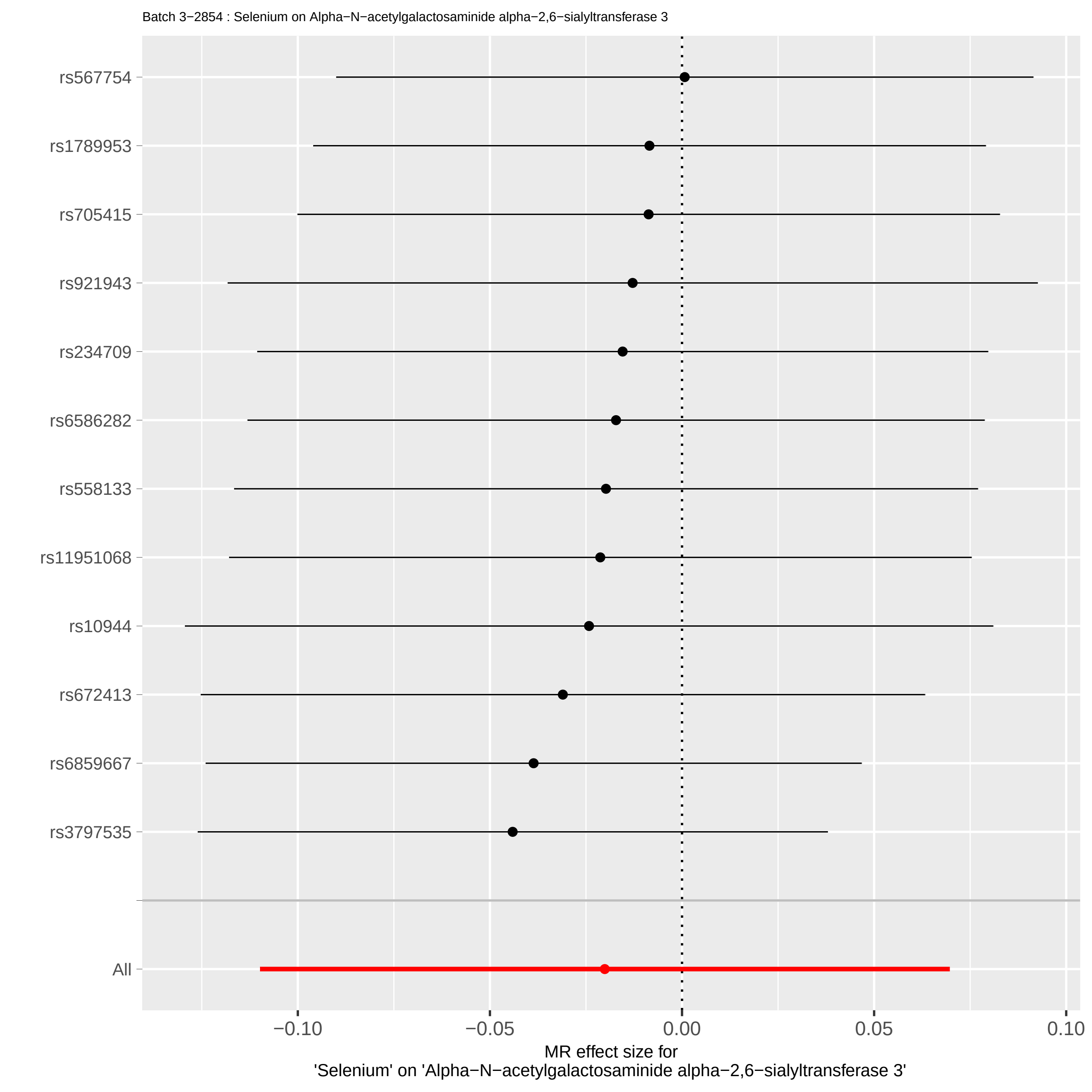

Batch 3−2854 : Selenium on Alpha−N−acetylgalactosaminide alpha−2,6−sialyltransferase 3
rs567754
rs1789953
rs705415
rs921943
rs234709
rs6586282
rs558133
rs11951068
rs10944
rs672413
rs6859667
rs3797535
All
−0.10
−0.05
0.00
0.05
0.10
MR effect size for
'Selenium' on 'Alpha−N−acetylgalactosaminide alpha−2,6−sialyltransferase 3'

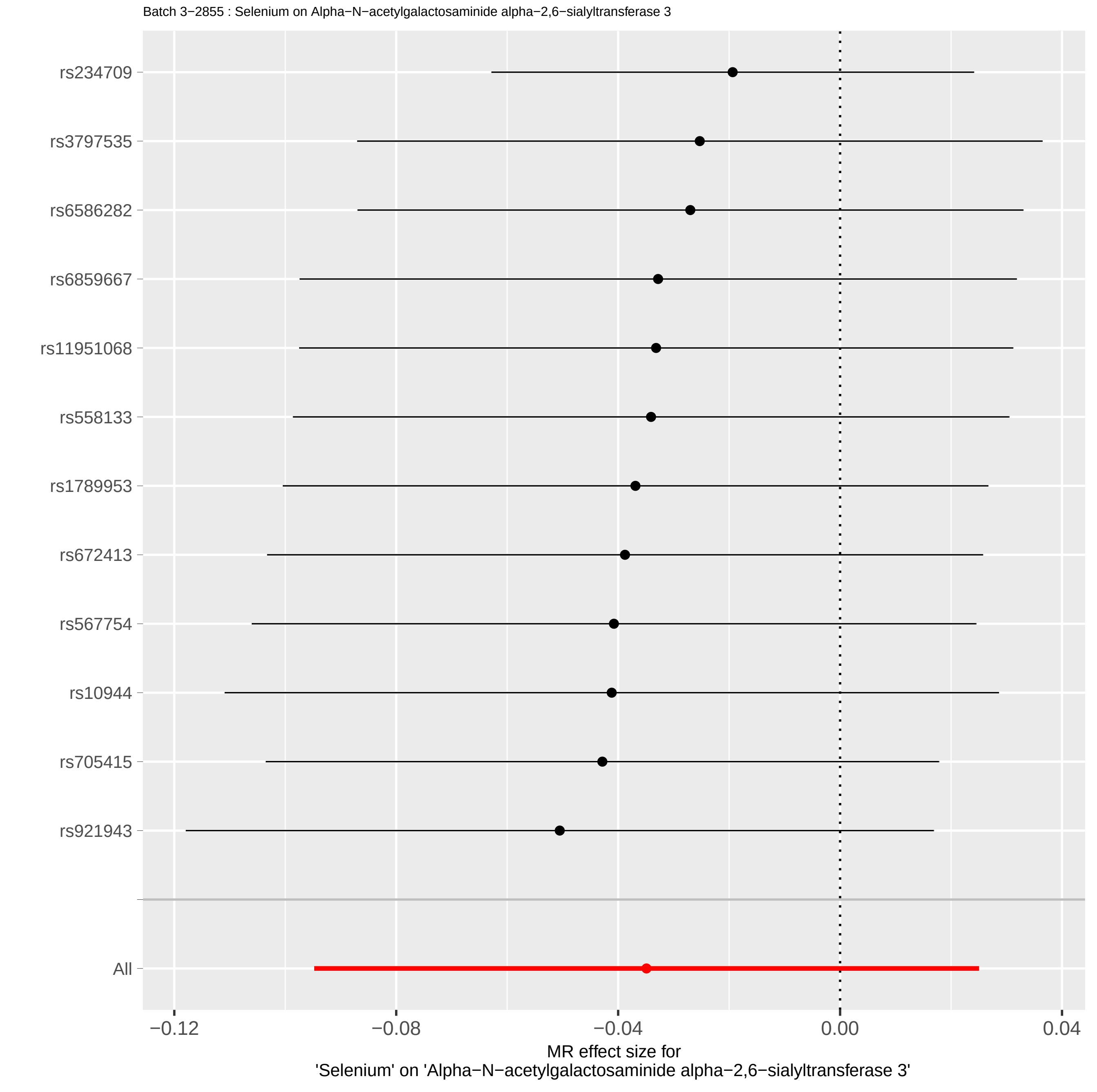

Batch 3−2855 : Selenium on Alpha−N−acetylgalactosaminide alpha−2,6−sialyltransferase 3
rs234709
rs3797535
rs6586282
rs6859667
rs11951068
rs558133
rs1789953
rs672413
rs567754
rs10944
rs705415
rs921943
All
−0.12
−0.08
−0.04
0.00
0.04
MR effect size for
'Selenium' on 'Alpha−N−acetylgalactosaminide alpha−2,6−sialyltransferase 3'

Batch 3−2856 : Selenium on Alpha−N−acetylgalactosaminide alpha−2,6−sialyltransferase 5

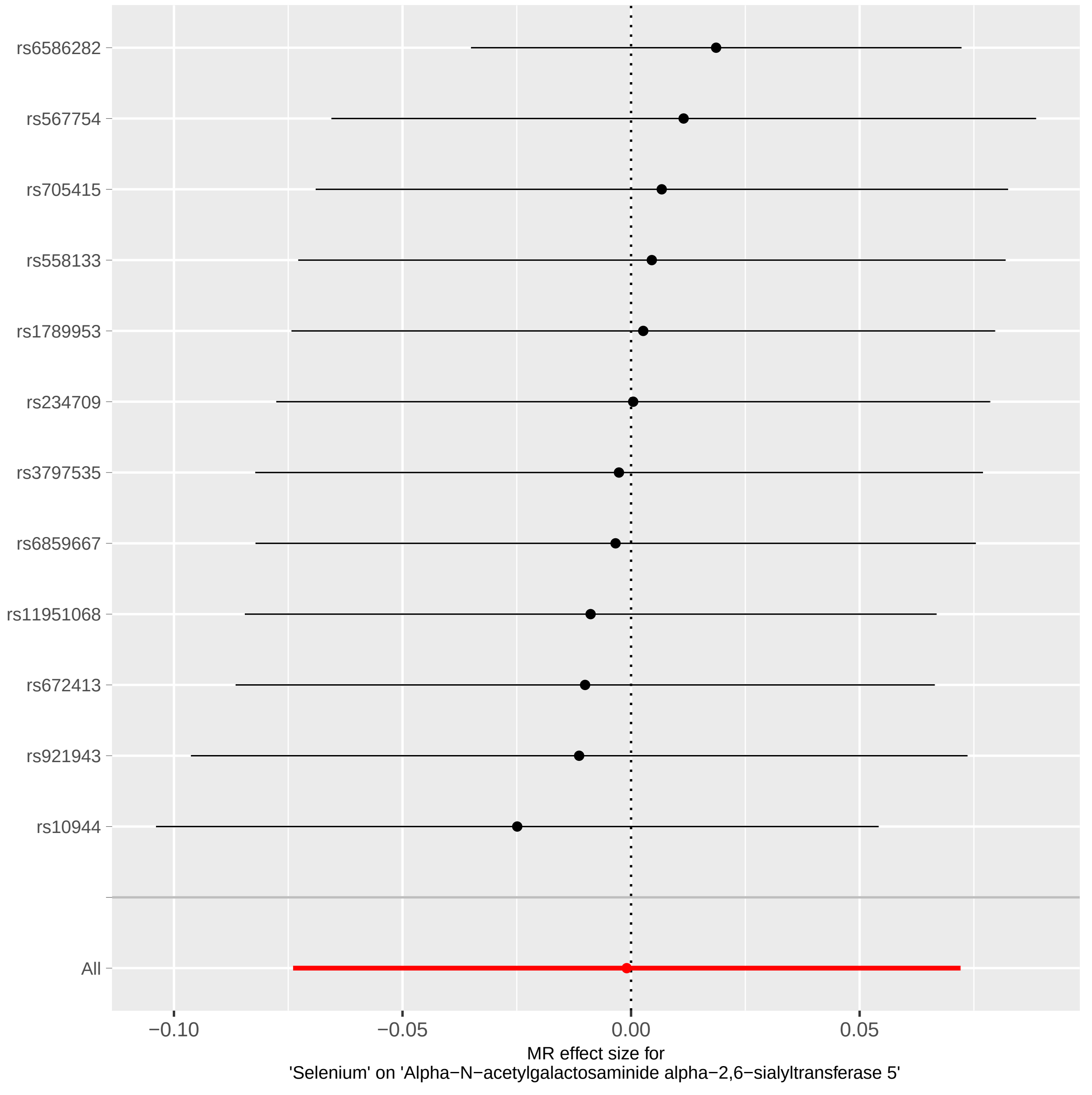

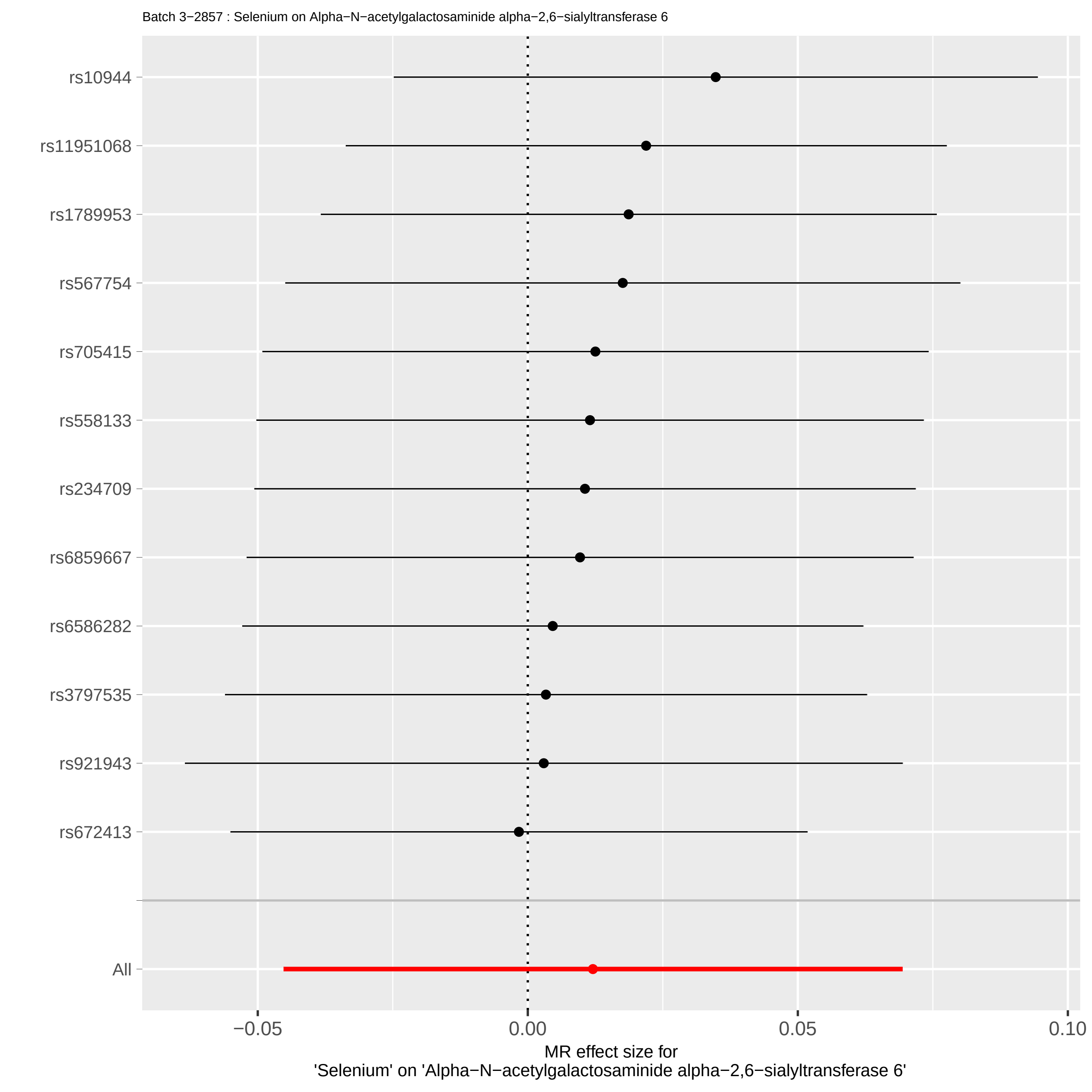

Batch 3−2857 : Selenium on Alpha−N−acetylgalactosaminide alpha−2,6−sialyltransferase 6
rs10944
rs11951068
rs1789953
rs567754
rs705415
rs558133
rs234709
rs6859667
rs6586282
rs3797535
rs921943
rs672413
All
−0.05
0.00
0.05
0.10
MR effect size for
'Selenium' on 'Alpha−N−acetylgalactosaminide alpha−2,6−sialyltransferase 6'

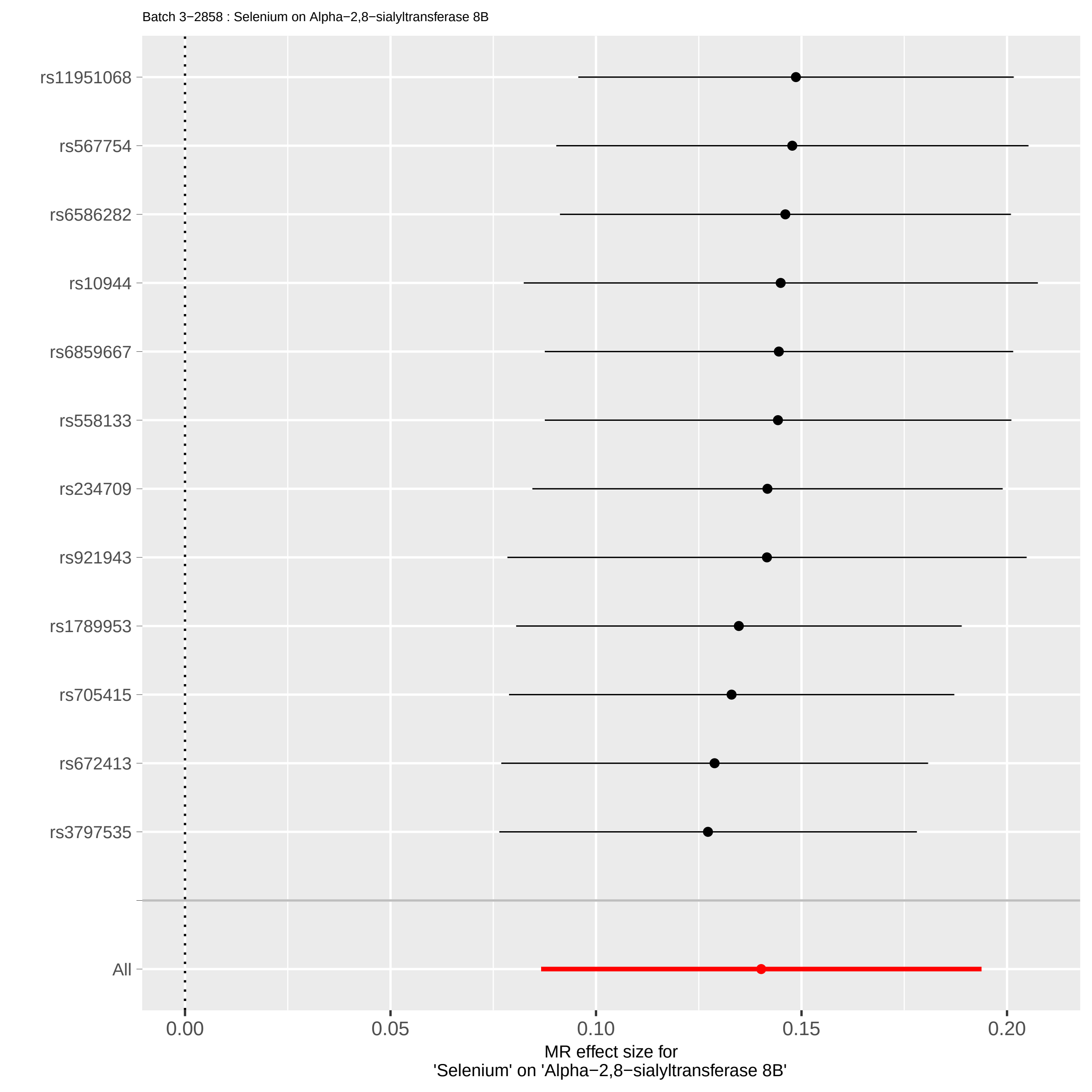

Batch 3−2858 : Selenium on Alpha−2,8−sialyltransferase 8B
rs11951068
rs567754
rs6586282
rs10944
rs6859667
rs558133
rs234709
rs921943
rs1789953
rs705415
rs672413
rs3797535
All
0.00
0.05
0.10
0.15
0.20
MR effect size for
'Selenium' on 'Alpha−2,8−sialyltransferase 8B'

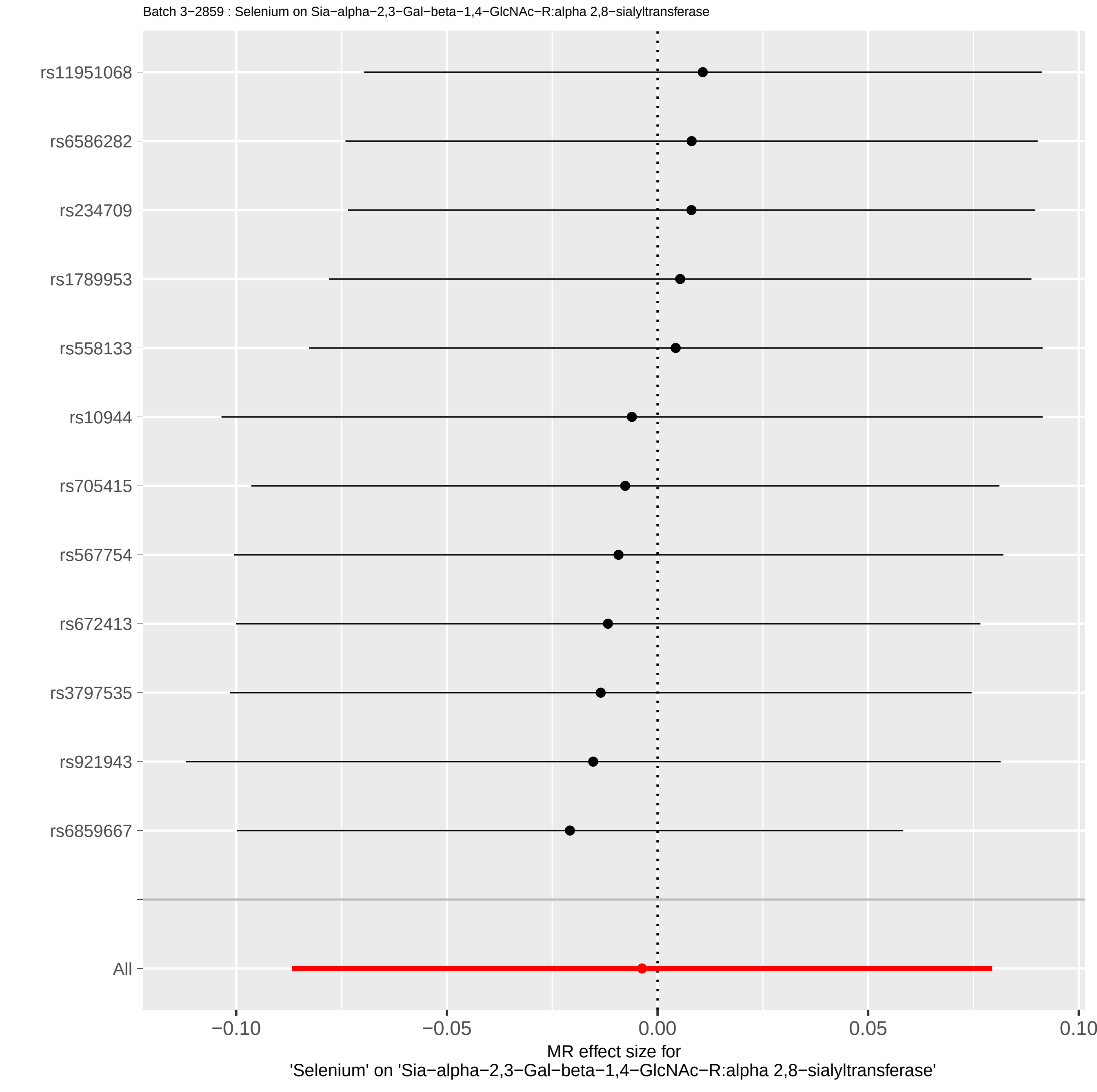
Batch 3−2859 : Selenium on Sia−alpha−2,3−Gal−beta−1,4−GlcNAc−R:alpha 2,8−sialyltransferase
rs11951068
rs6586282
rs234709
rs1789953
rs558133
rs10944
rs705415
rs567754
rs672413
rs3797535
rs921943
rs6859667
All
−0.10
−0.05
0.00
0.05
0.10
MR effect size for
'Selenium' on 'Sia−alpha−2,3−Gal−beta−1,4−GlcNAc−R:alpha 2,8−sialyltransferase'

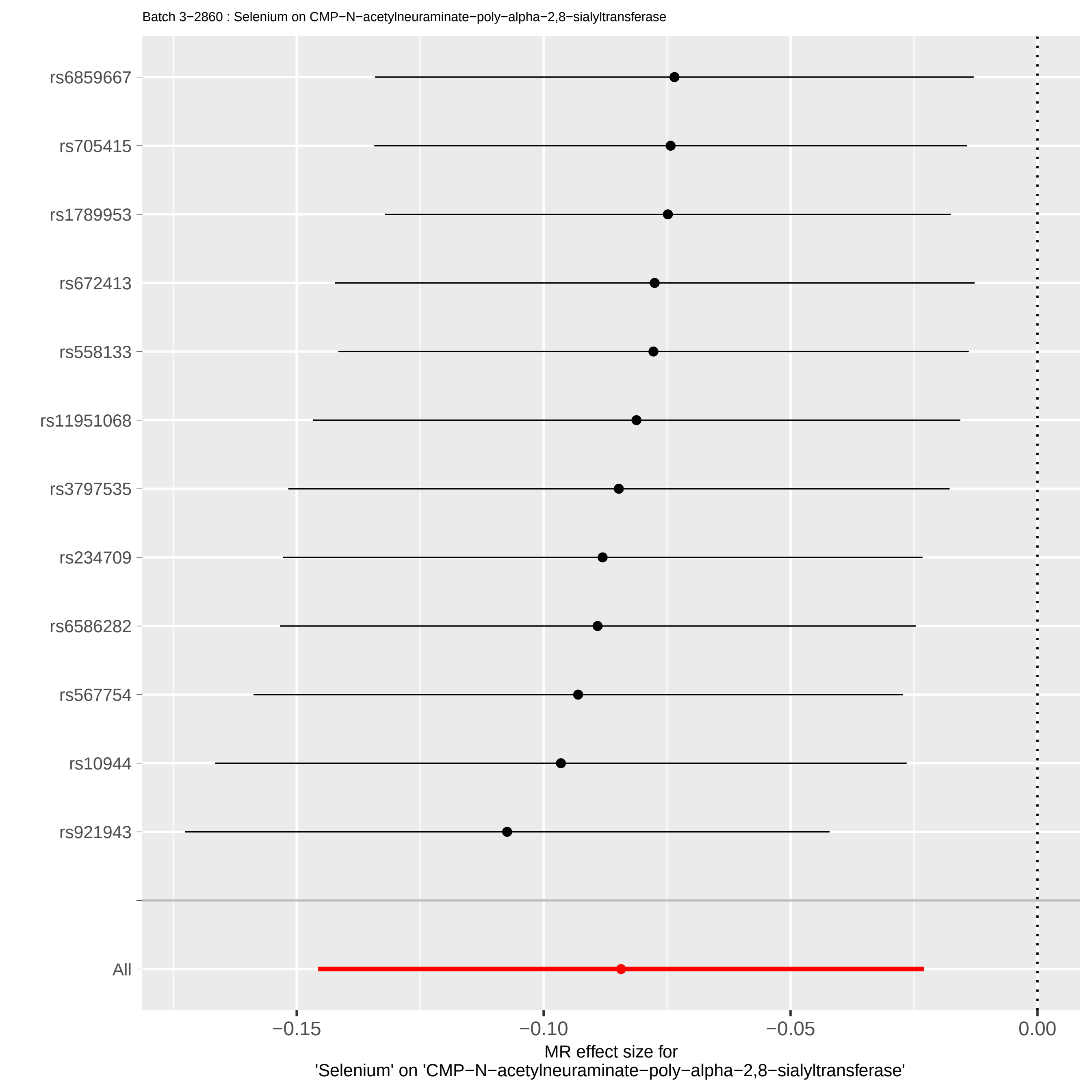
Batch 3−2860 : Selenium on CMP−N−acetylneuraminate−poly−alpha−2,8−sialyltransferase
rs6859667
rs705415
rs1789953
rs672413
rs558133
rs11951068
rs3797535
rs234709
rs6586282
rs567754
rs10944
rs921943
All
−0.15
−0.10
−0.05
0.00
MR effect size for
'Selenium' on 'CMP−N−acetylneuraminate−poly−alpha−2,8−sialyltransferase'

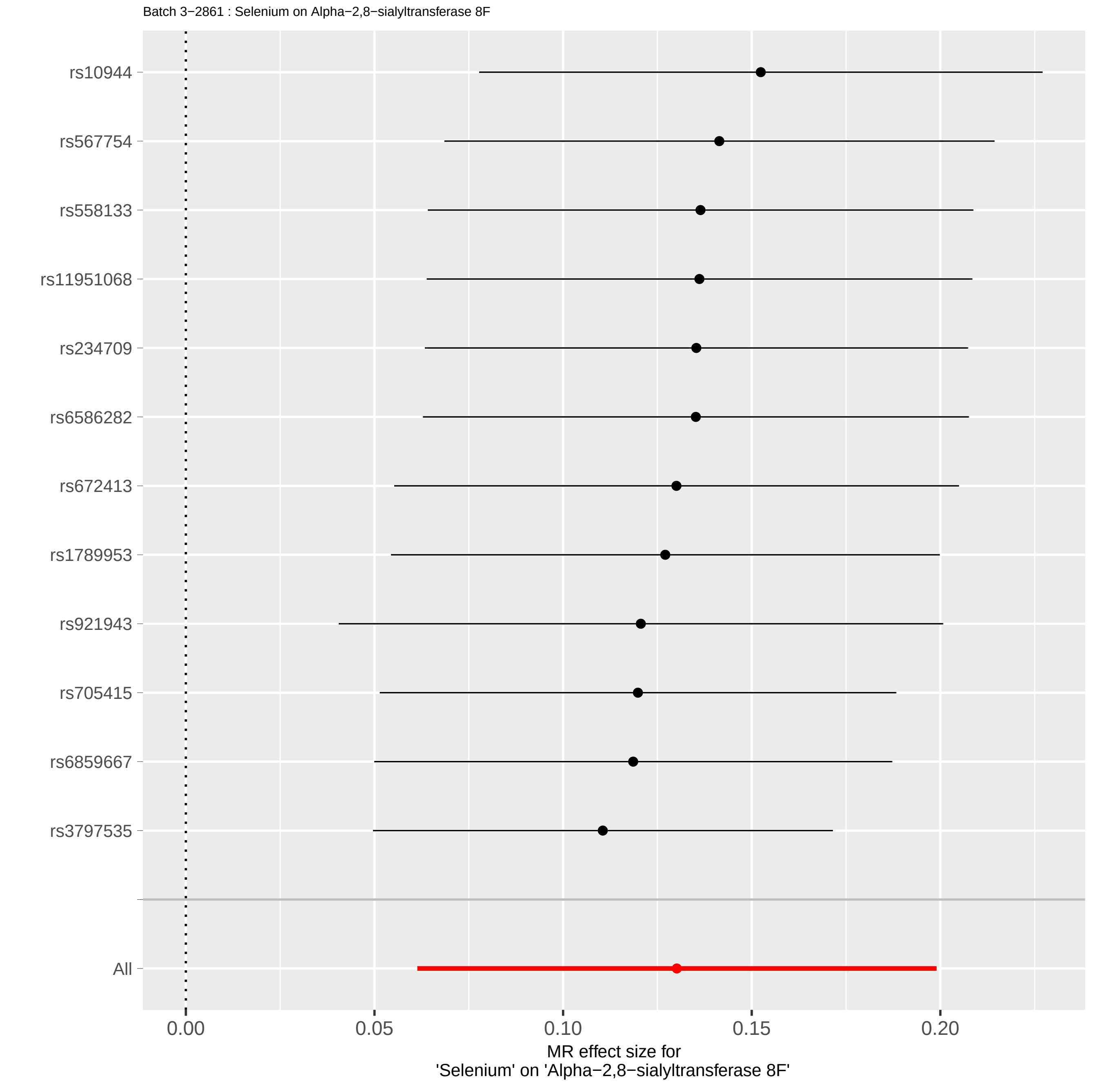

Batch 3−2861 : Selenium on Alpha−2,8−sialyltransferase 8F
rs10944
rs567754
rs558133
rs11951068
rs234709
rs6586282
rs672413
rs1789953
rs921943
rs705415
rs6859667
rs3797535
All
0.00
0.05
0.10
0.15
0.20
MR effect size for
'Selenium' on 'Alpha−2,8−sialyltransferase 8F'

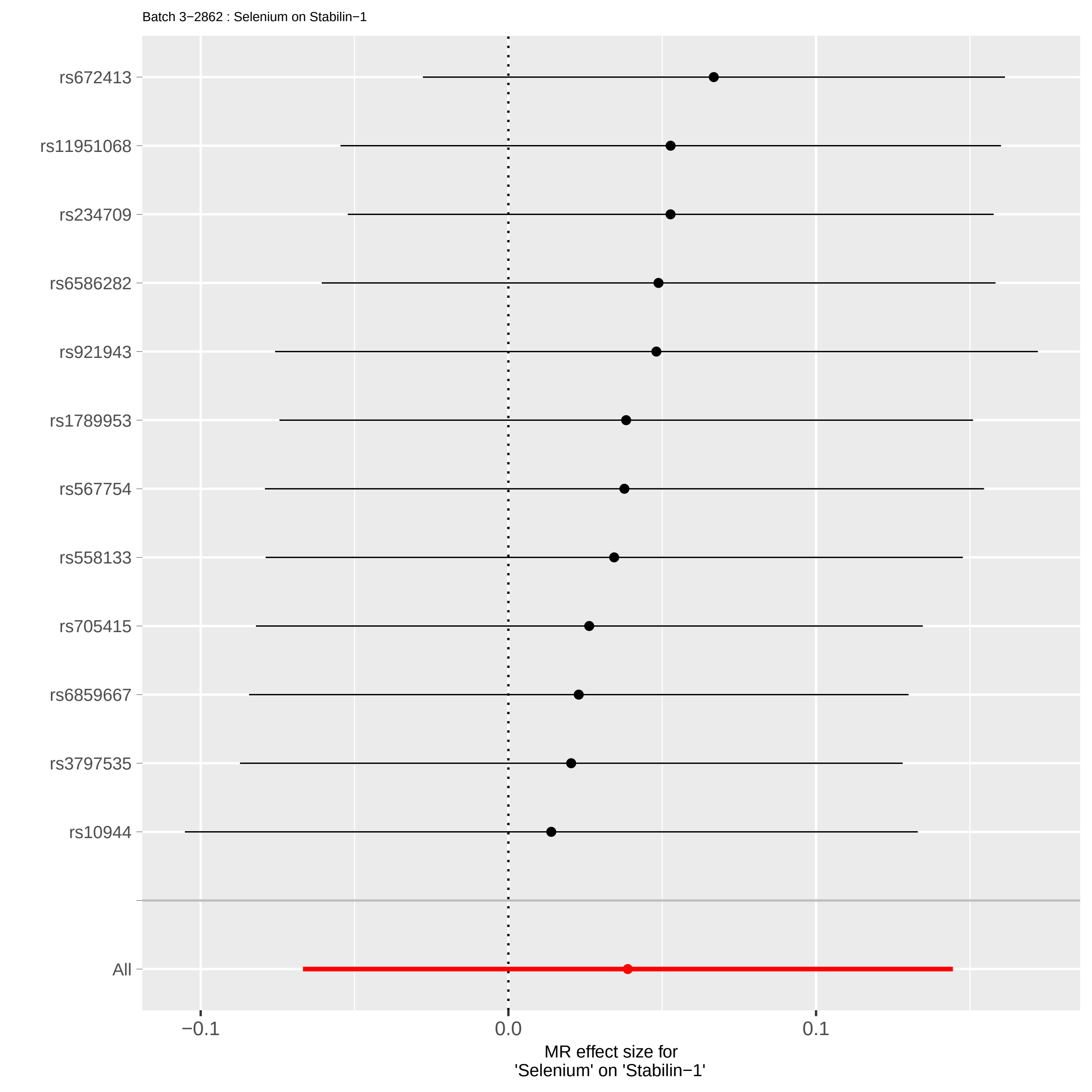

Batch 3−2862 : Selenium on Stabilin−1
rs672413
rs11951068
rs234709
rs6586282
rs921943
rs1789953
rs567754
rs558133
rs705415
rs6859667
rs3797535
rs10944
All
−0.1
0.0
0.1
MR effect size for
'Selenium' on 'Stabilin−1'

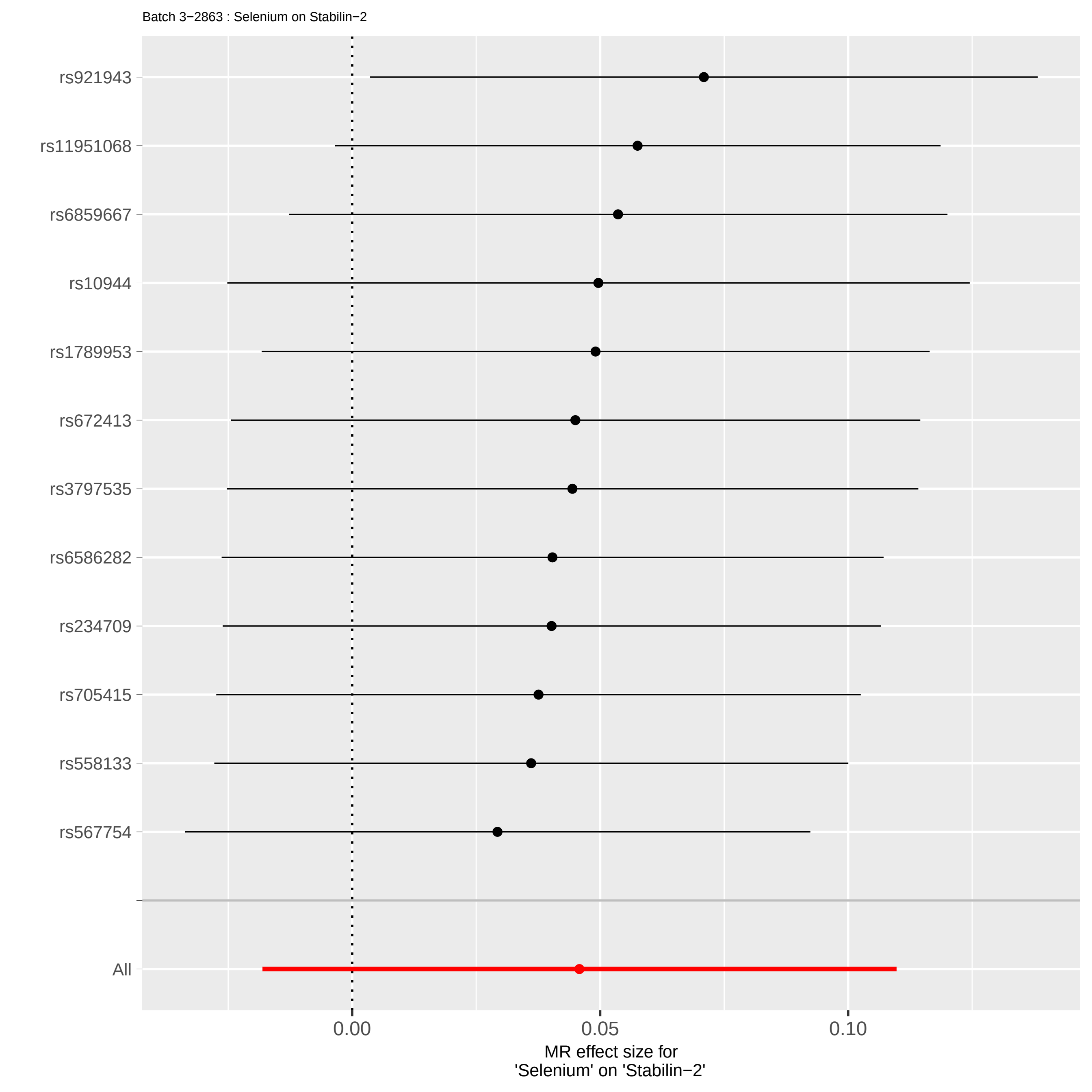

Batch 3−2863 : Selenium on Stabilin−2
rs921943
rs11951068
rs6859667
rs10944
rs1789953
rs672413
rs3797535
rs6586282
rs234709
rs705415
rs558133
rs567754
All
0.00
0.05
0.10
MR effect size for
'Selenium' on 'Stabilin−2'

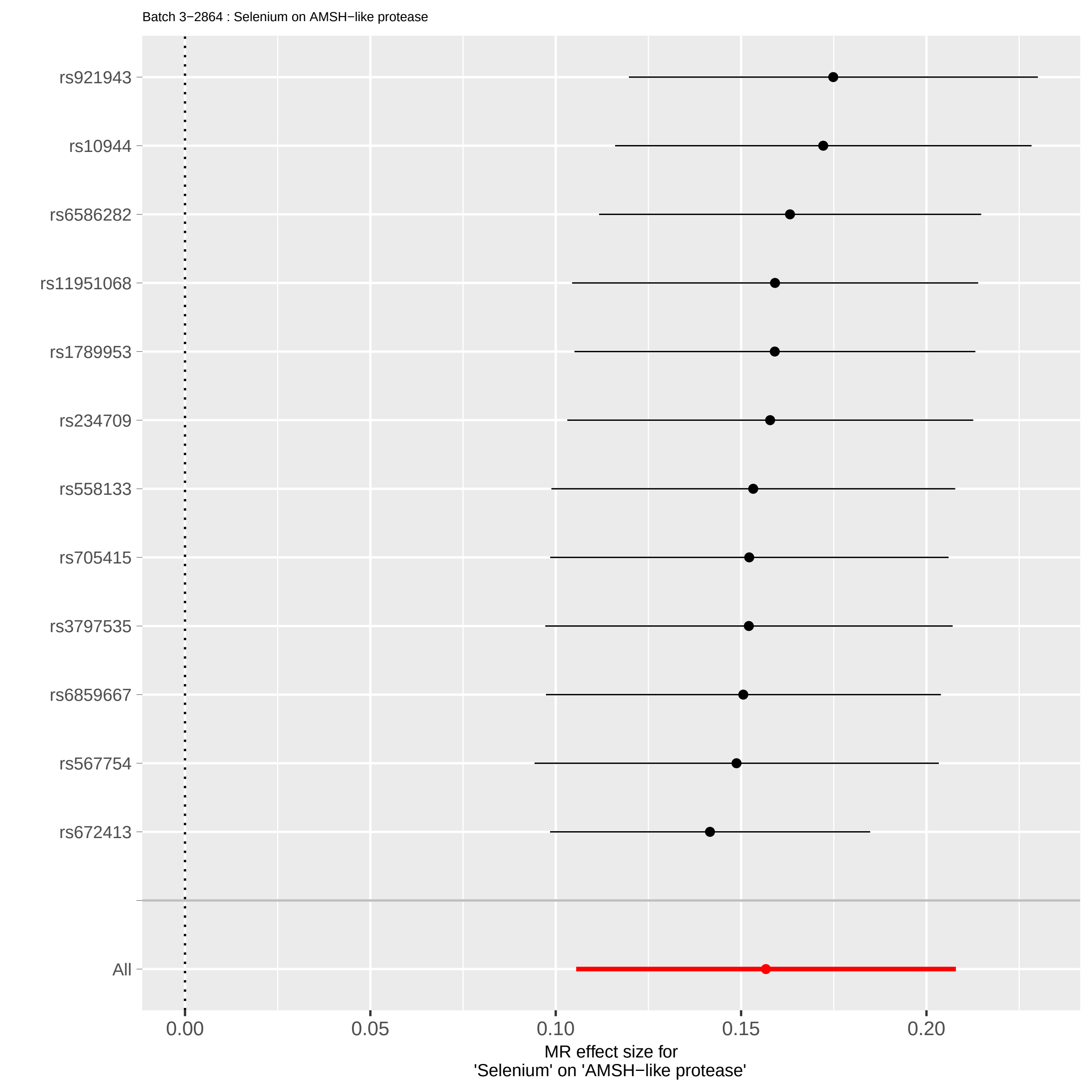

Batch 3−2864 : Selenium on AMSH−like protease
rs921943
rs10944
rs6586282
rs11951068
rs1789953
rs234709
rs558133
rs705415
rs3797535
rs6859667
rs567754
rs672413
All
0.00
0.05
0.10
0.15
0.20
MR effect size for
'Selenium' on 'AMSH−like protease'

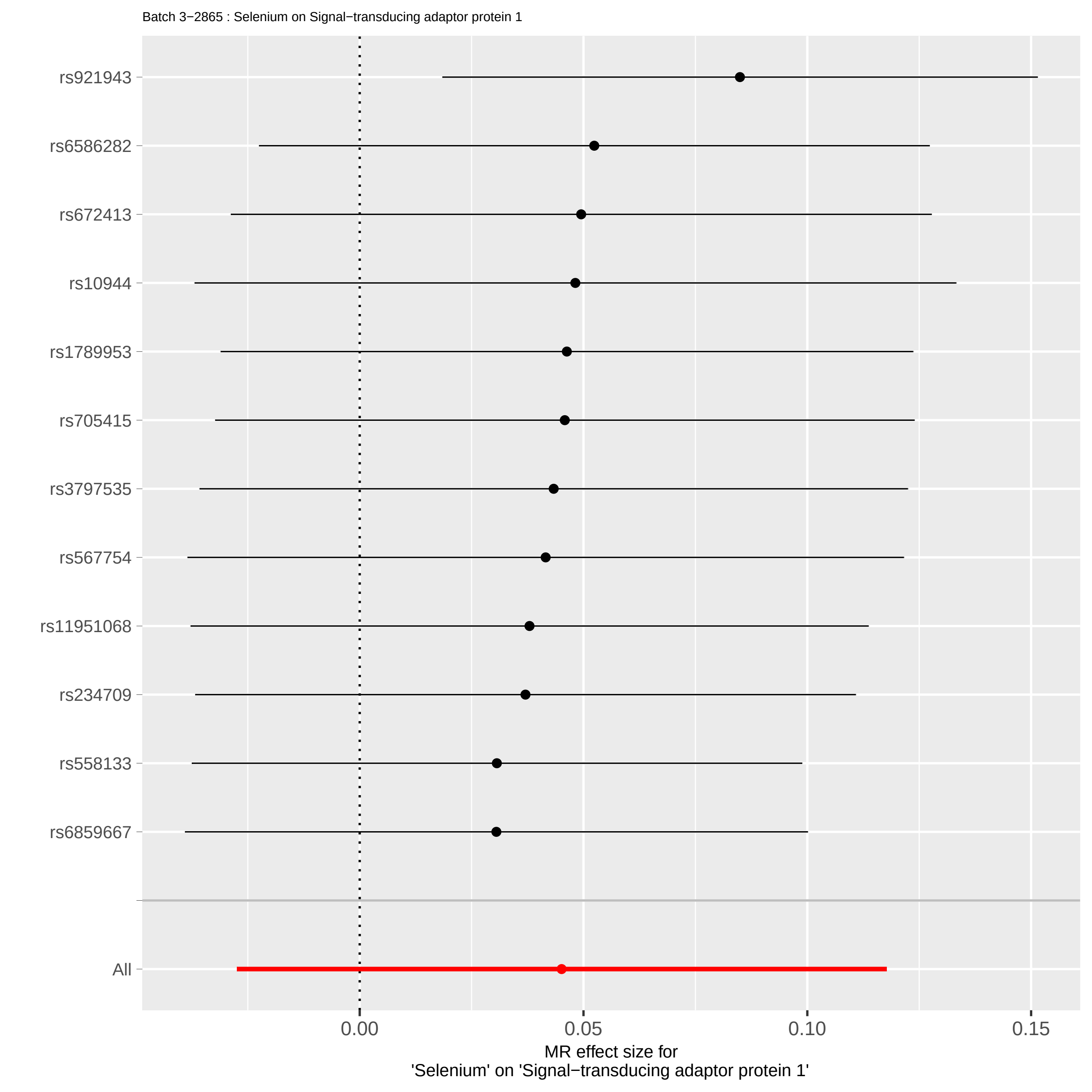

Batch 3−2865 : Selenium on Signal−transducing adaptor protein 1
rs921943
rs6586282
rs672413
rs10944
rs1789953
rs705415
rs3797535
rs567754
rs11951068
rs234709
rs558133
rs6859667
All
0.00
0.05
0.10
0.15
MR effect size for
'Selenium' on 'Signal−transducing adaptor protein 1'

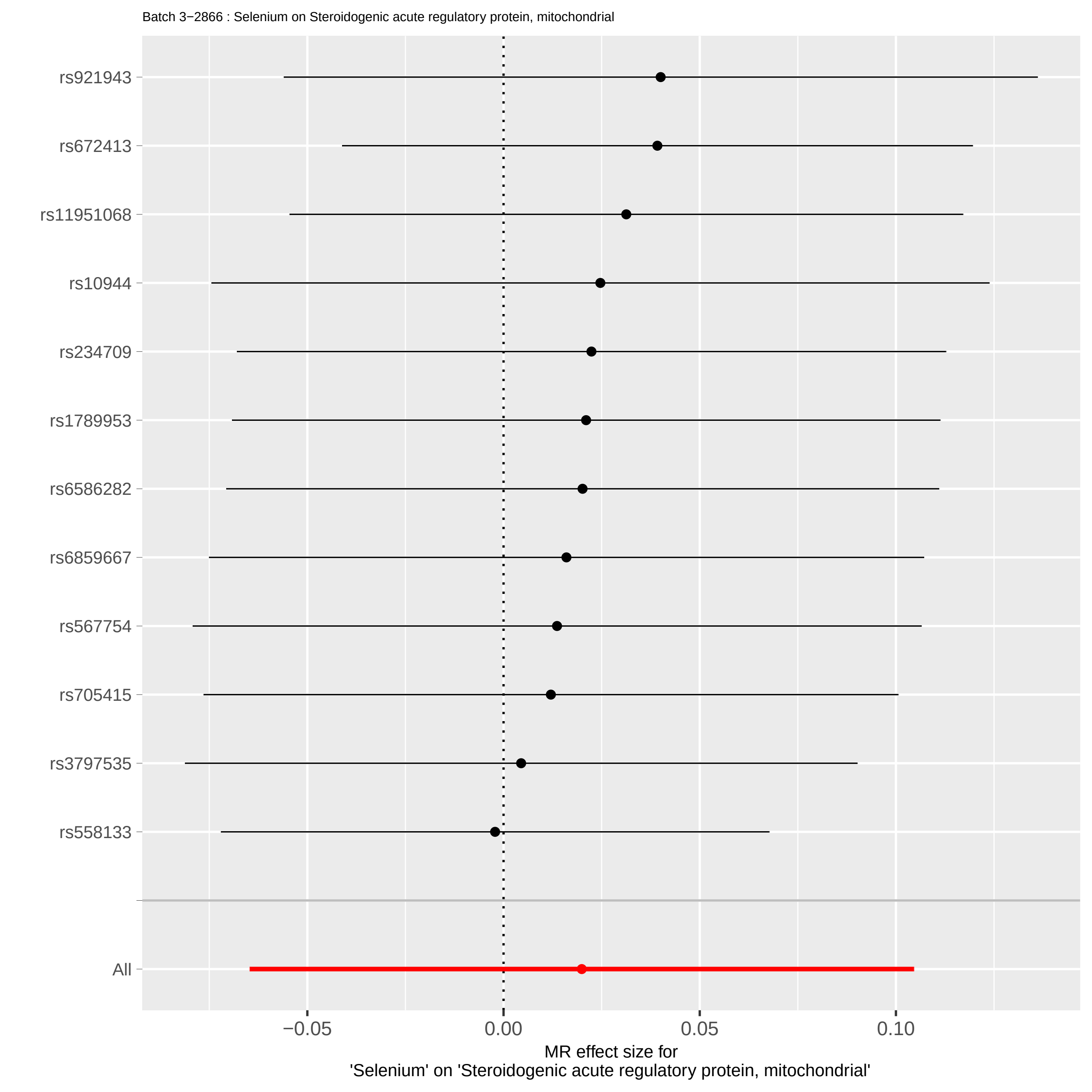

Batch 3−2866 : Selenium on Steroidogenic acute regulatory protein, mitochondrial
rs921943
rs672413
rs11951068
rs10944
rs234709
rs1789953
rs6586282
rs6859667
rs567754
rs705415
rs3797535
rs558133
All
−0.05
0.00
0.05
0.10
MR effect size for
'Selenium' on 'Steroidogenic acute regulatory protein, mitochondrial'

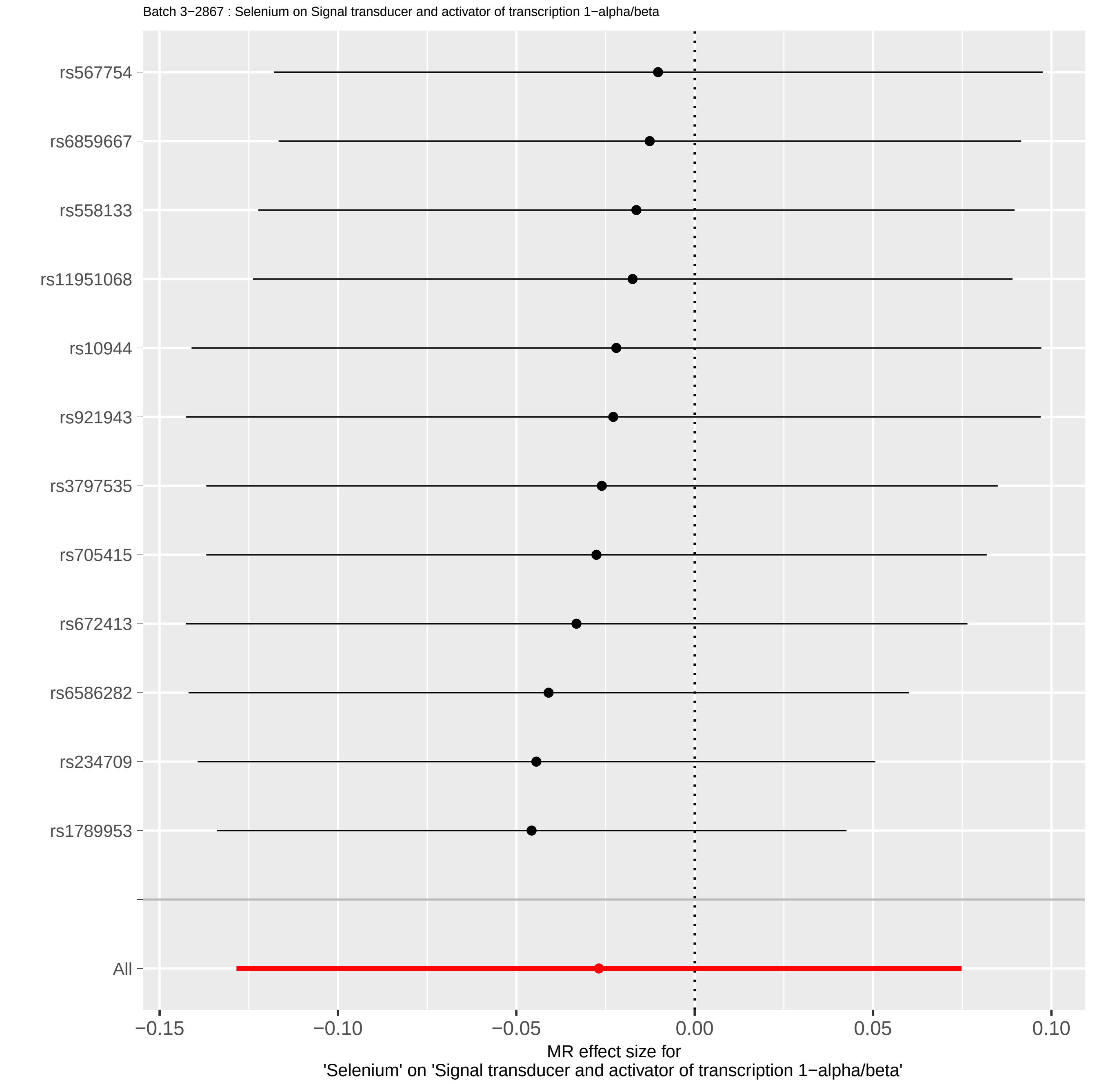

Batch 3−2867 : Selenium on Signal transducer and activator of transcription 1−alpha/beta
rs567754
rs6859667
rs558133
rs11951068
rs10944
rs921943
rs3797535
rs705415
rs672413
rs6586282
rs234709
rs1789953
All
−0.15
−0.10
−0.05
0.00
0.05
0.10
MR effect size for
'Selenium' on 'Signal transducer and activator of transcription 1−alpha/beta'

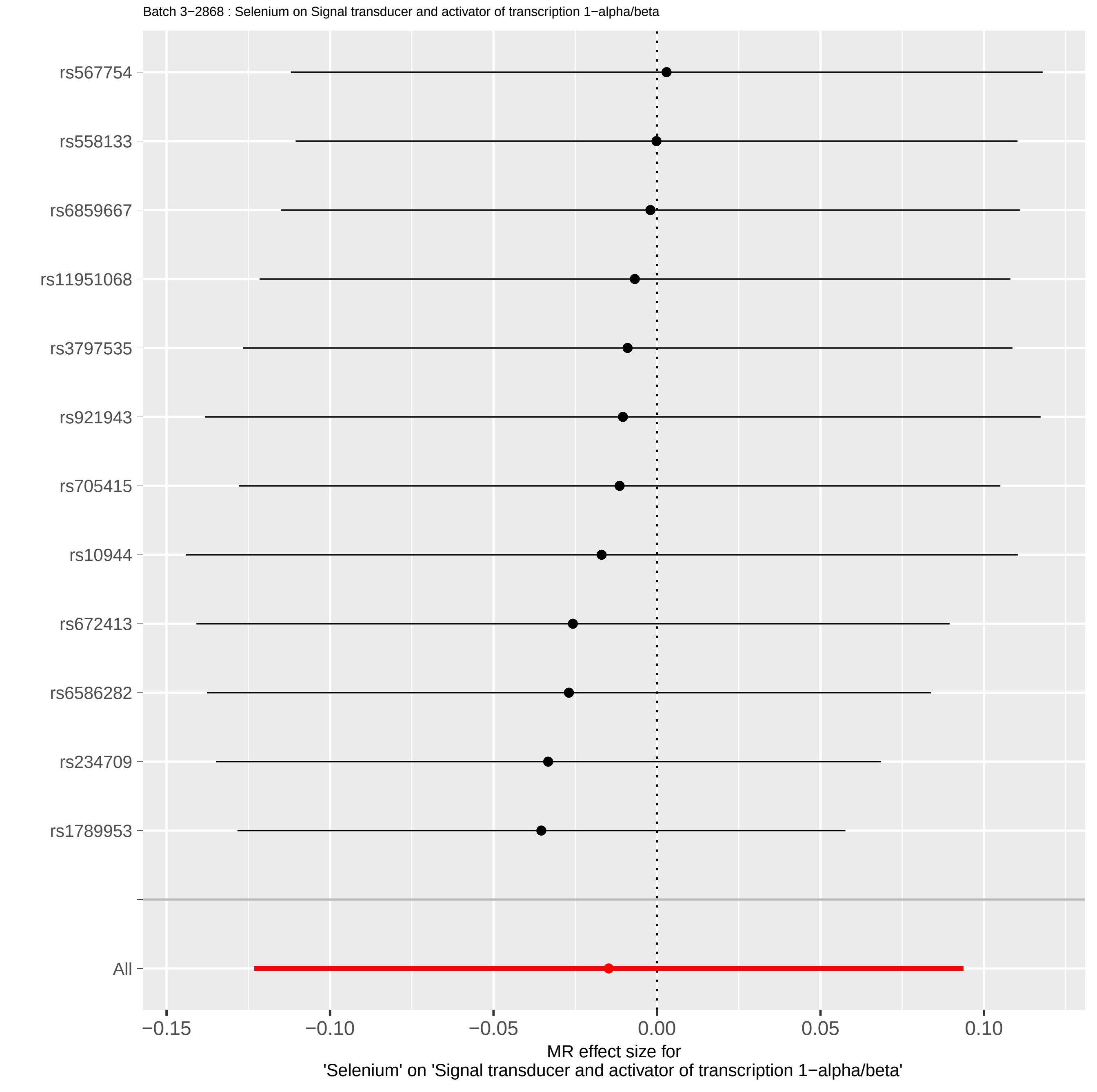

Batch 3−2868 : Selenium on Signal transducer and activator of transcription 1−alpha/beta
rs567754
rs558133
rs6859667
rs11951068
rs3797535
rs921943
rs705415
rs10944
rs672413
rs6586282
rs234709
rs1789953
All
−0.15
−0.10
−0.05
0.00
0.05
0.10
MR effect size for
'Selenium' on 'Signal transducer and activator of transcription 1−alpha/beta'

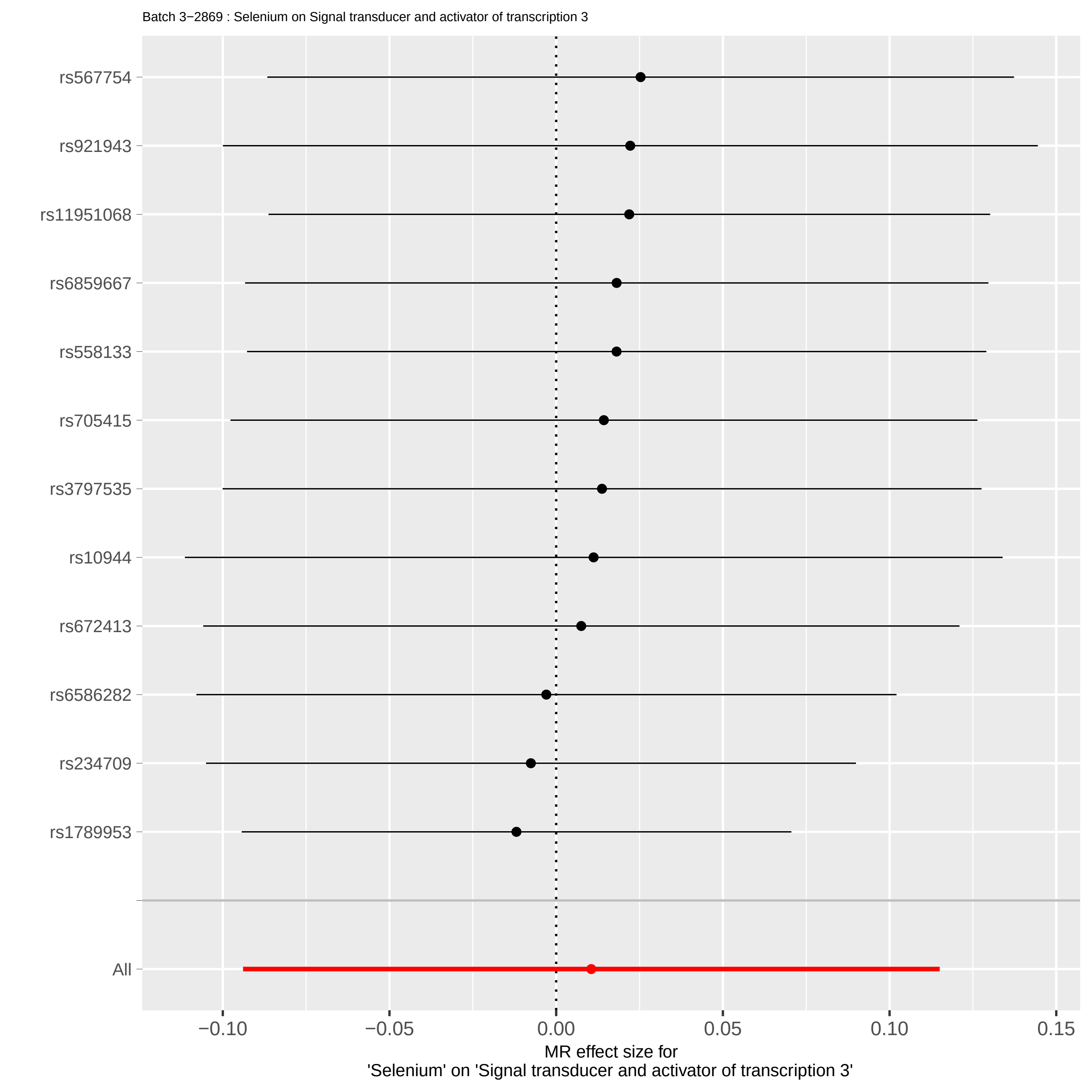

Batch 3−2869 : Selenium on Signal transducer and activator of transcription 3
rs567754
rs921943
rs11951068
rs6859667
rs558133
rs705415
rs3797535
rs10944
rs672413
rs6586282
rs234709
rs1789953
All
−0.10
−0.05
0.00
0.05
0.10
0.15
MR effect size for
'Selenium' on 'Signal transducer and activator of transcription 3'

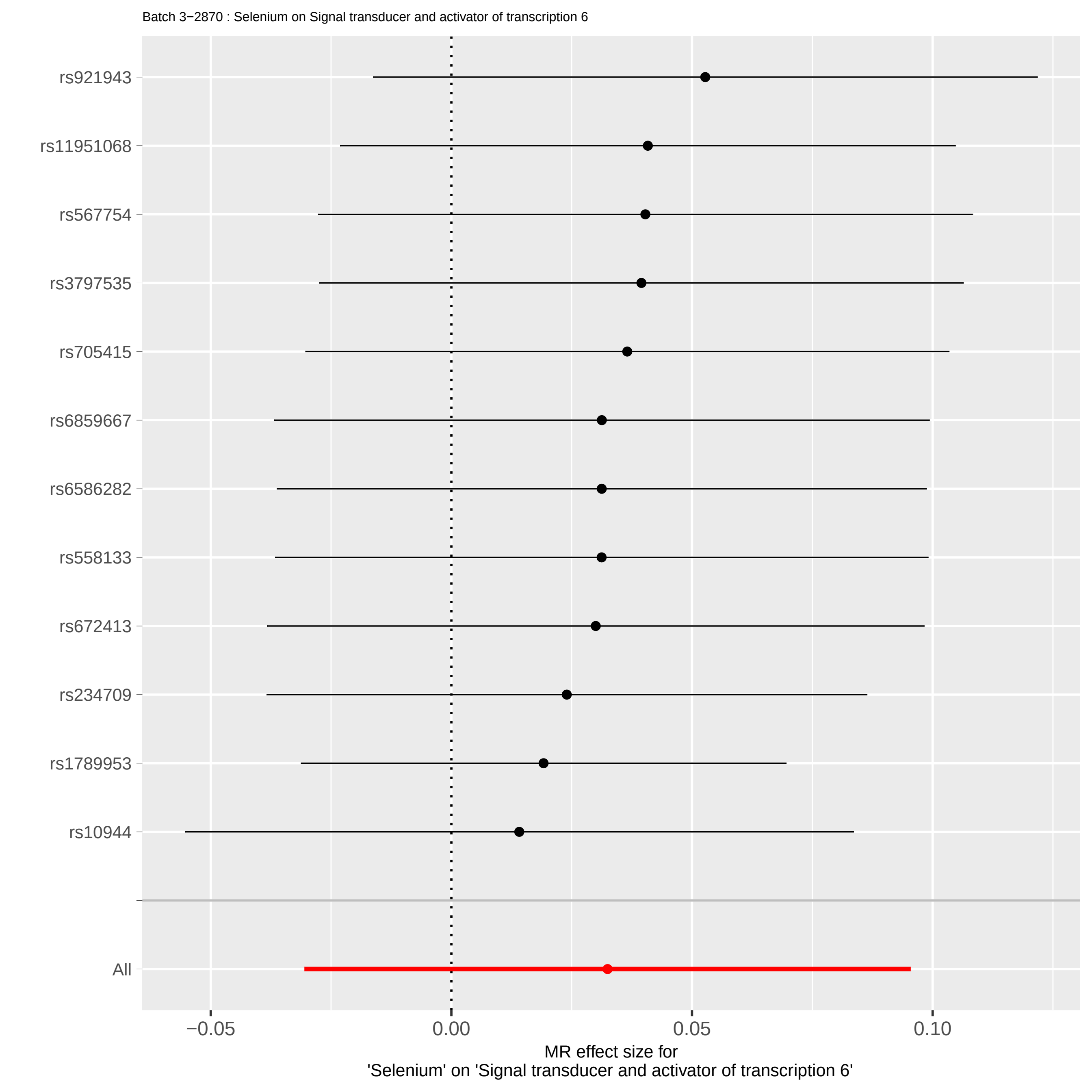

Batch 3−2870 : Selenium on Signal transducer and activator of transcription 6
rs921943
rs11951068
rs567754
rs3797535
rs705415
rs6859667
rs6586282
rs558133
rs672413
rs234709
rs1789953
rs10944
All
−0.05
0.00
0.05
0.10
MR effect size for
'Selenium' on 'Signal transducer and activator of transcription 6'

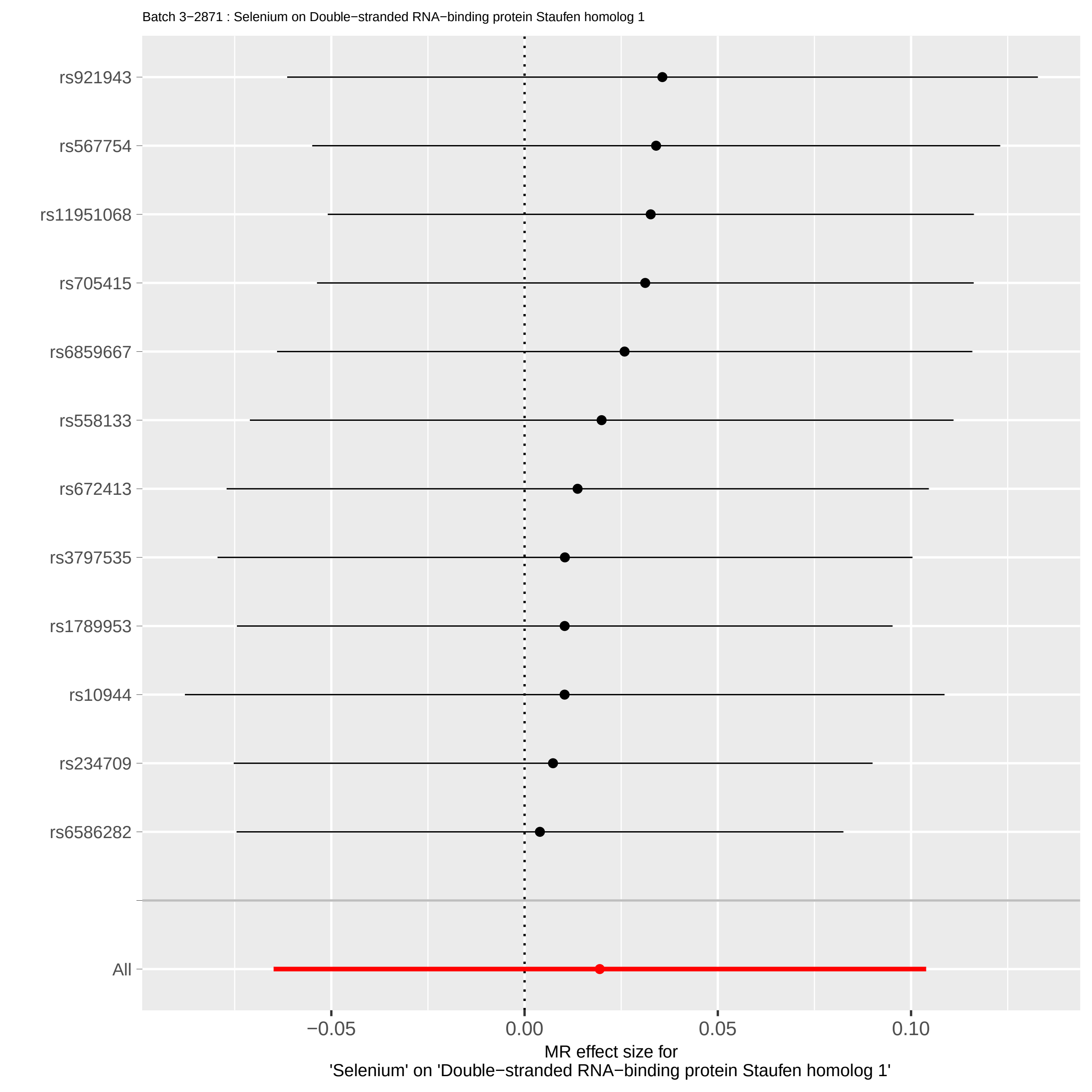

Batch 3−2871 : Selenium on Double−stranded RNA−binding protein Staufen homolog 1
rs921943
rs567754
rs11951068
rs705415
rs6859667
rs558133
rs672413
rs3797535
rs1789953
rs10944
rs234709
rs6586282
All
−0.05
0.00
0.05
0.10
MR effect size for
'Selenium' on 'Double−stranded RNA−binding protein Staufen homolog 1'

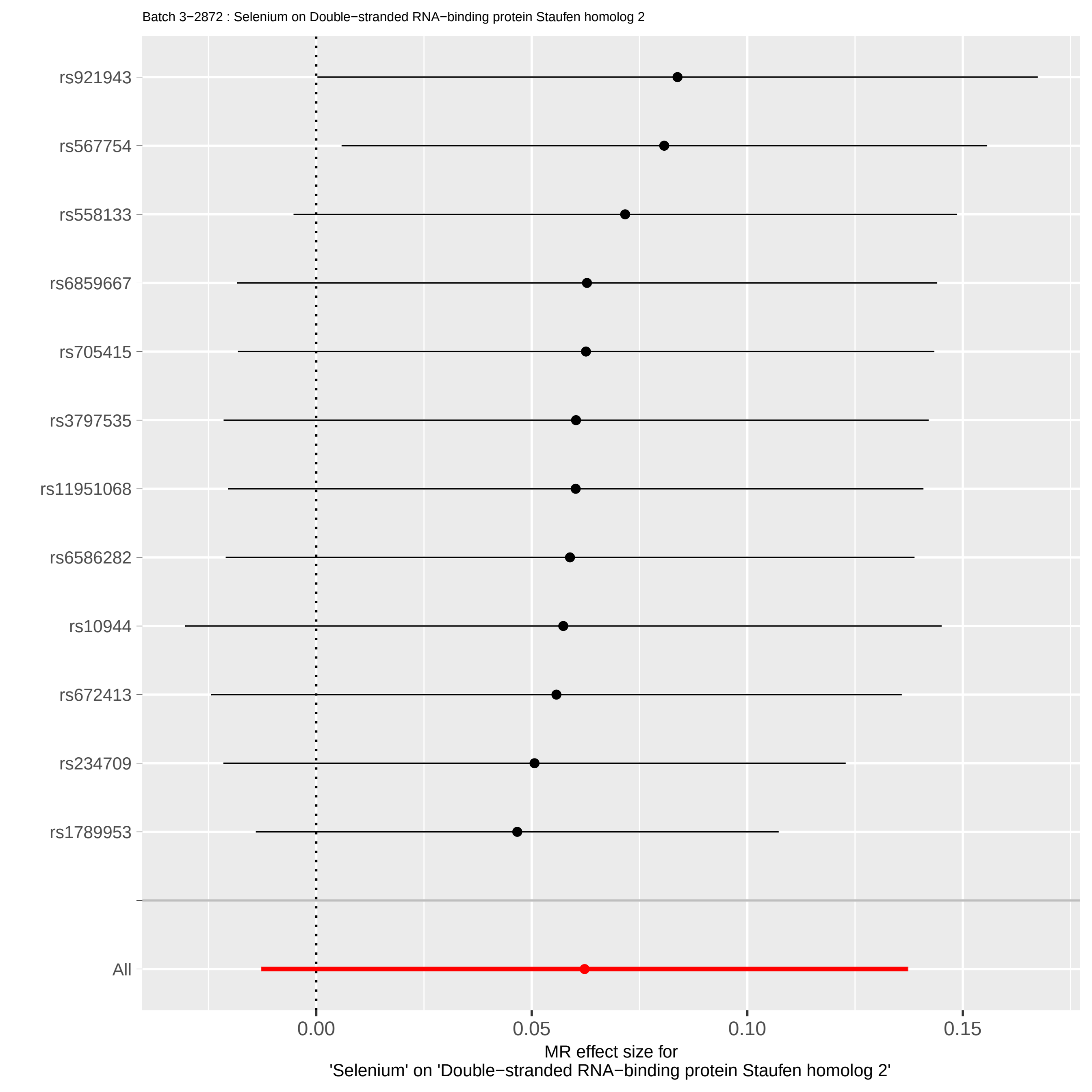

Batch 3−2872 : Selenium on Double−stranded RNA−binding protein Staufen homolog 2
rs921943
rs567754
rs558133
rs6859667
rs705415
rs3797535
rs11951068
rs6586282
rs10944
rs672413
rs234709
rs1789953
All
0.00
0.05
0.10
0.15
MR effect size for
'Selenium' on 'Double−stranded RNA−binding protein Staufen homolog 2'

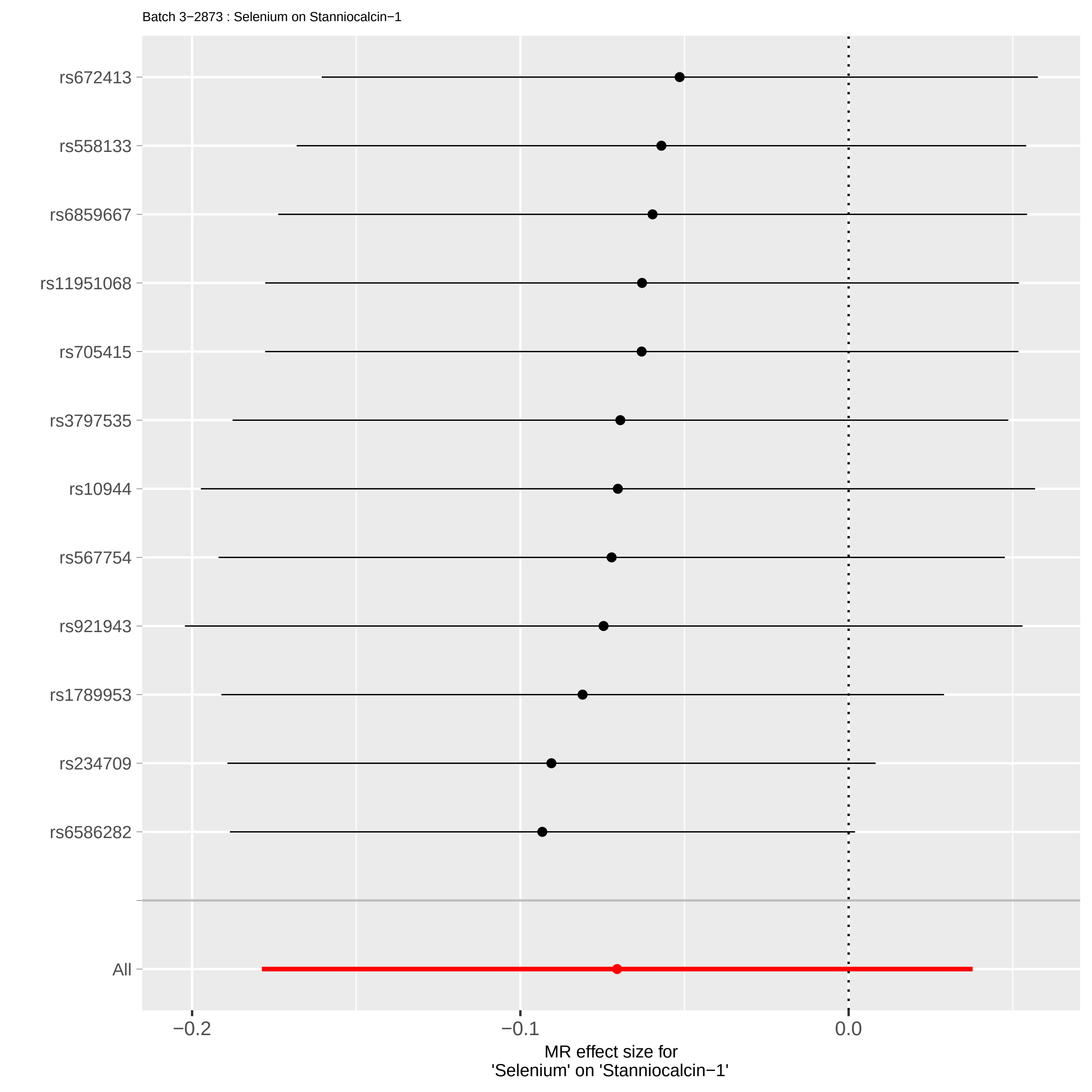

Batch 3−2873 : Selenium on Stanniocalcin−1
rs672413
rs558133
rs6859667
rs11951068
rs705415
rs3797535
rs10944
rs567754
rs921943
rs1789953
rs234709
rs6586282
All
−0.2
−0.1
0.0
MR effect size for
'Selenium' on 'Stanniocalcin−1'

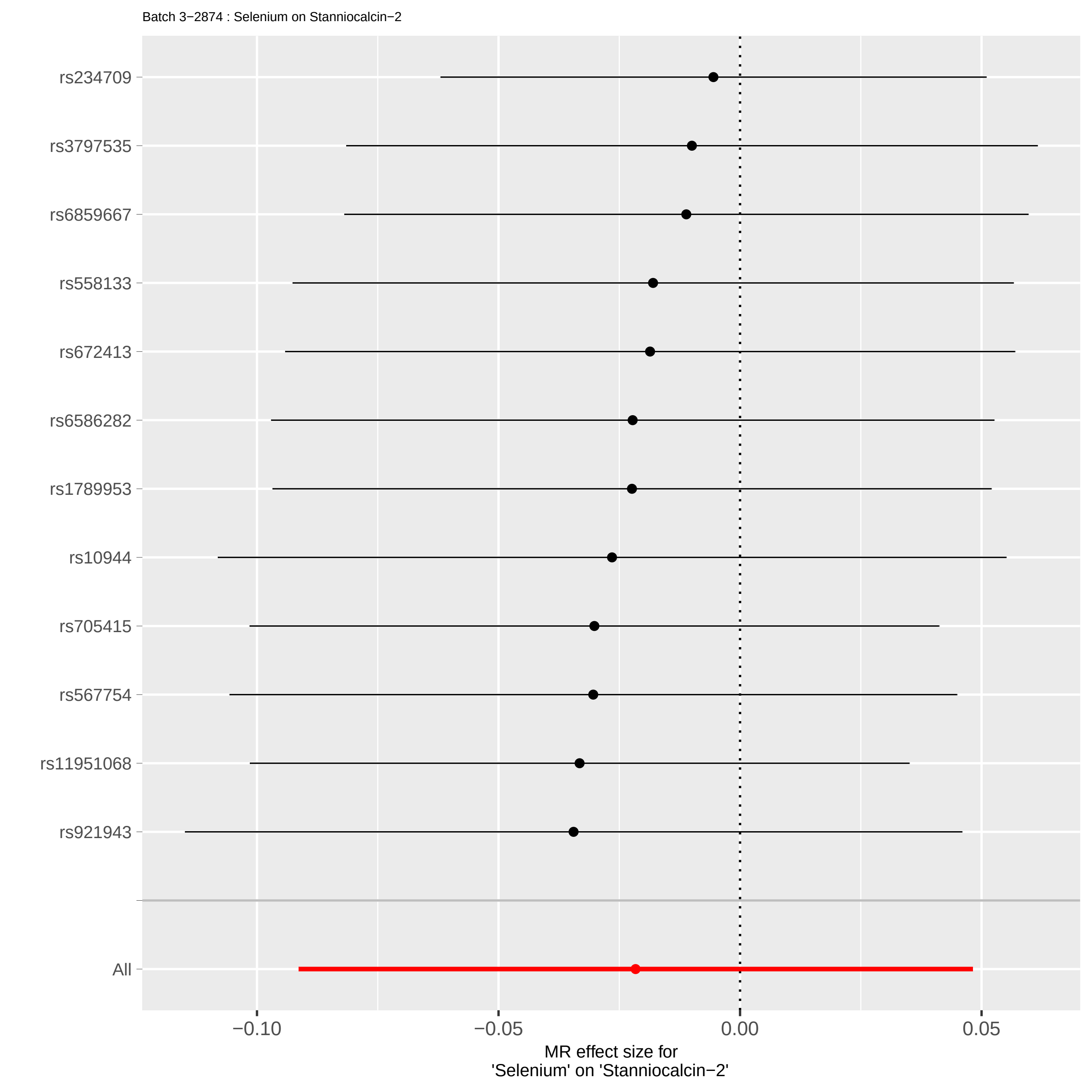

Batch 3−2874 : Selenium on Stanniocalcin−2
rs234709
rs3797535
rs6859667
rs558133
rs672413
rs6586282
rs1789953
rs10944
rs705415
rs567754
rs11951068
rs921943
All
−0.10
−0.05
0.00
0.05
MR effect size for
'Selenium' on 'Stanniocalcin−2'

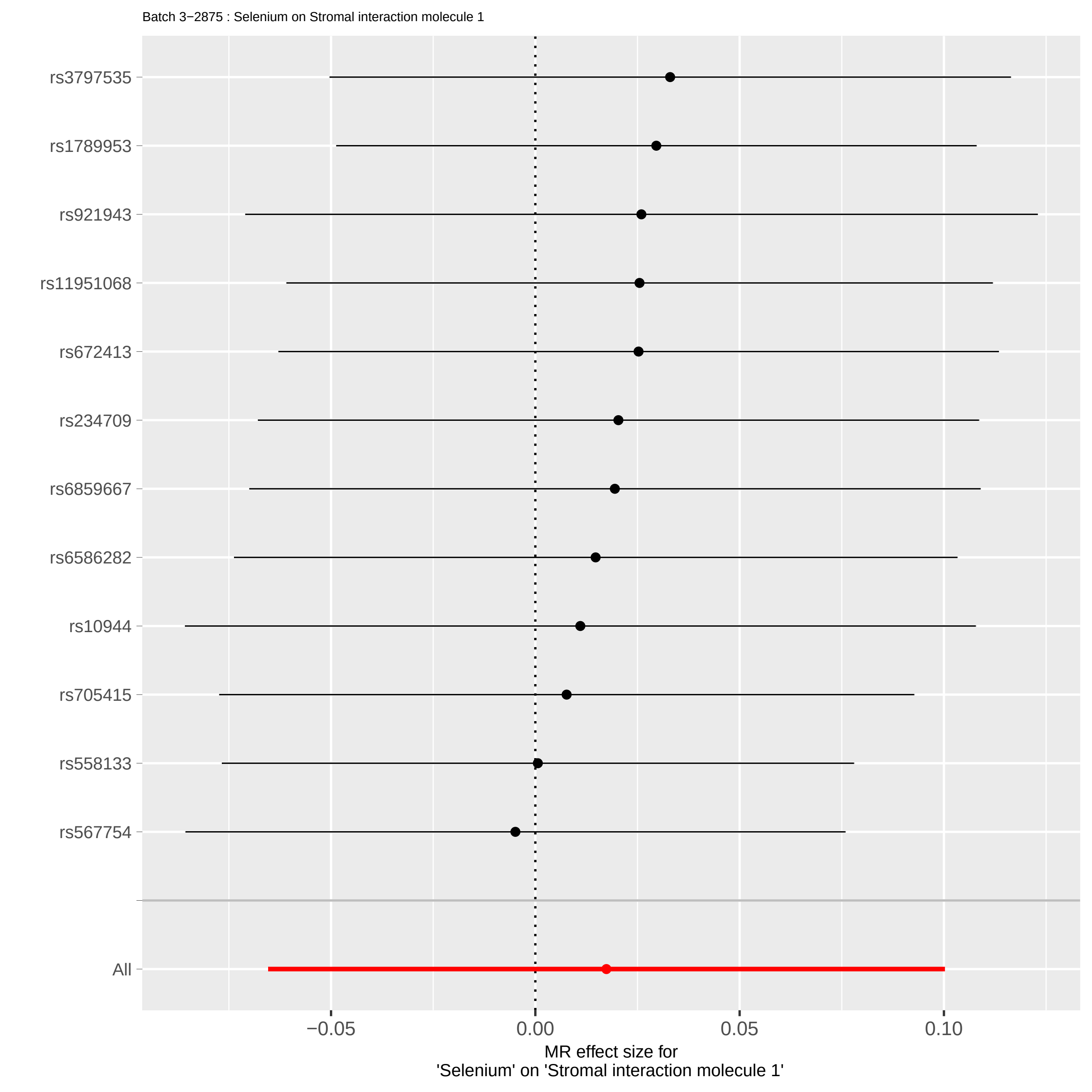

Batch 3−2875 : Selenium on Stromal interaction molecule 1
rs3797535
rs1789953
rs921943
rs11951068
rs672413
rs234709
rs6859667
rs6586282
rs10944
rs705415
rs558133
rs567754
All
−0.05
0.00
0.05
0.10
MR effect size for
'Selenium' on 'Stromal interaction molecule 1'

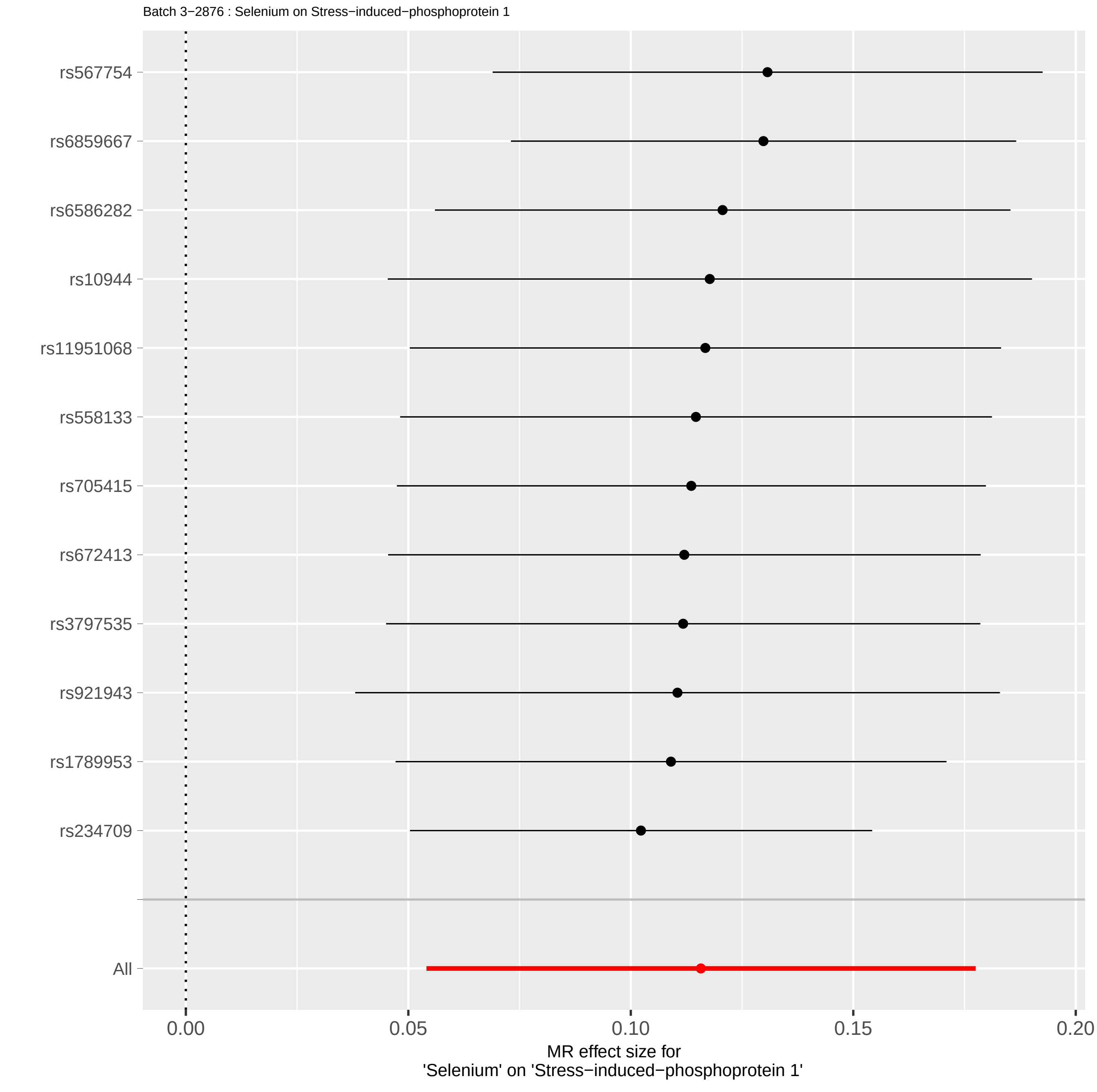

Batch 3−2876 : Selenium on Stress−induced−phosphoprotein 1
rs567754
rs6859667
rs6586282
rs10944
rs11951068
rs558133
rs705415
rs672413
rs3797535
rs921943
rs1789953
rs234709
All
0.00
0.05
0.10
0.15
0.20
MR effect size for
'Selenium' on 'Stress−induced−phosphoprotein 1'

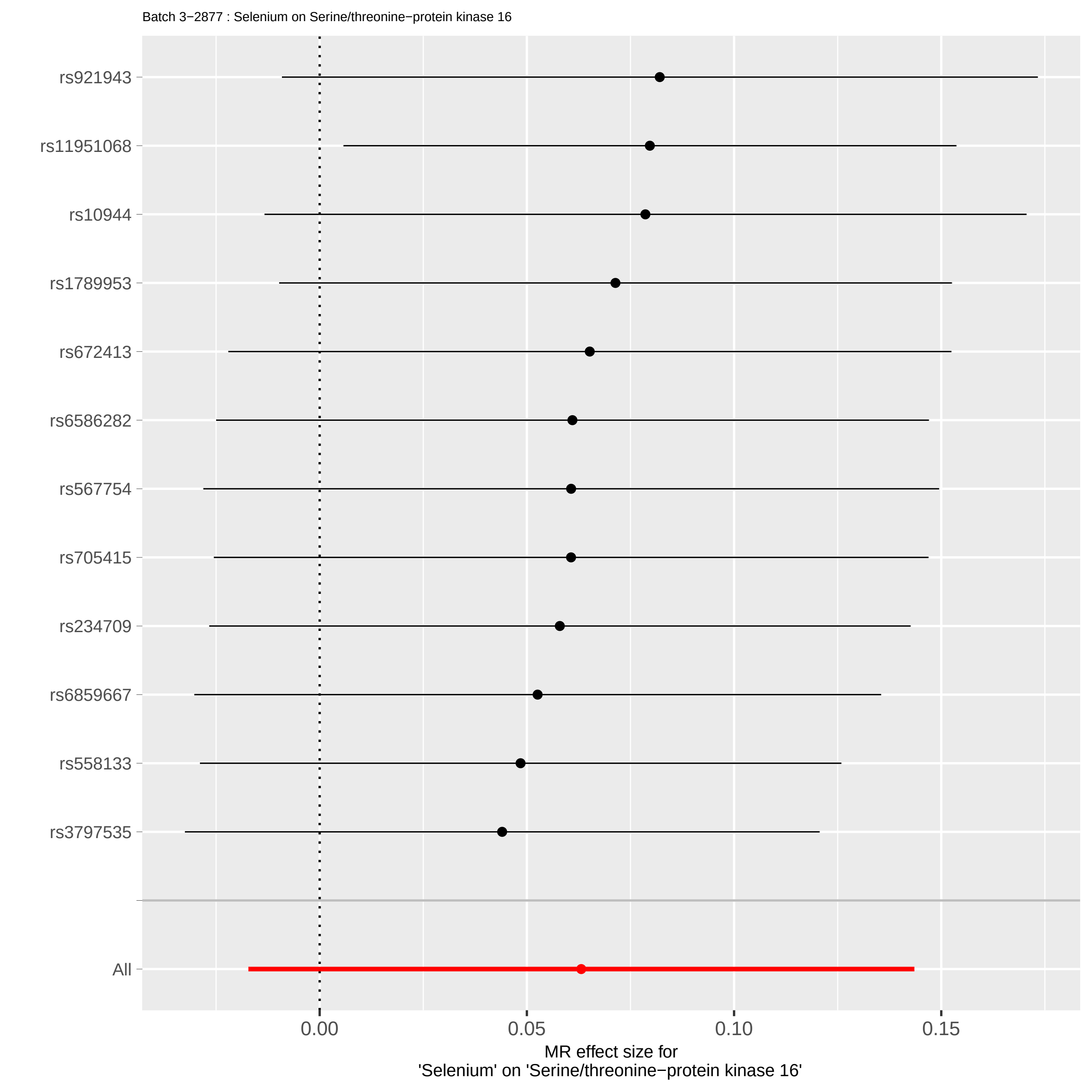

Batch 3−2877 : Selenium on Serine/threonine−protein kinase 16
rs921943
rs11951068
rs10944
rs1789953
rs672413
rs6586282
rs567754
rs705415
rs234709
rs6859667
rs558133
rs3797535
All
0.00
0.05
0.10
0.15
MR effect size for
'Selenium' on 'Serine/threonine−protein kinase 16'

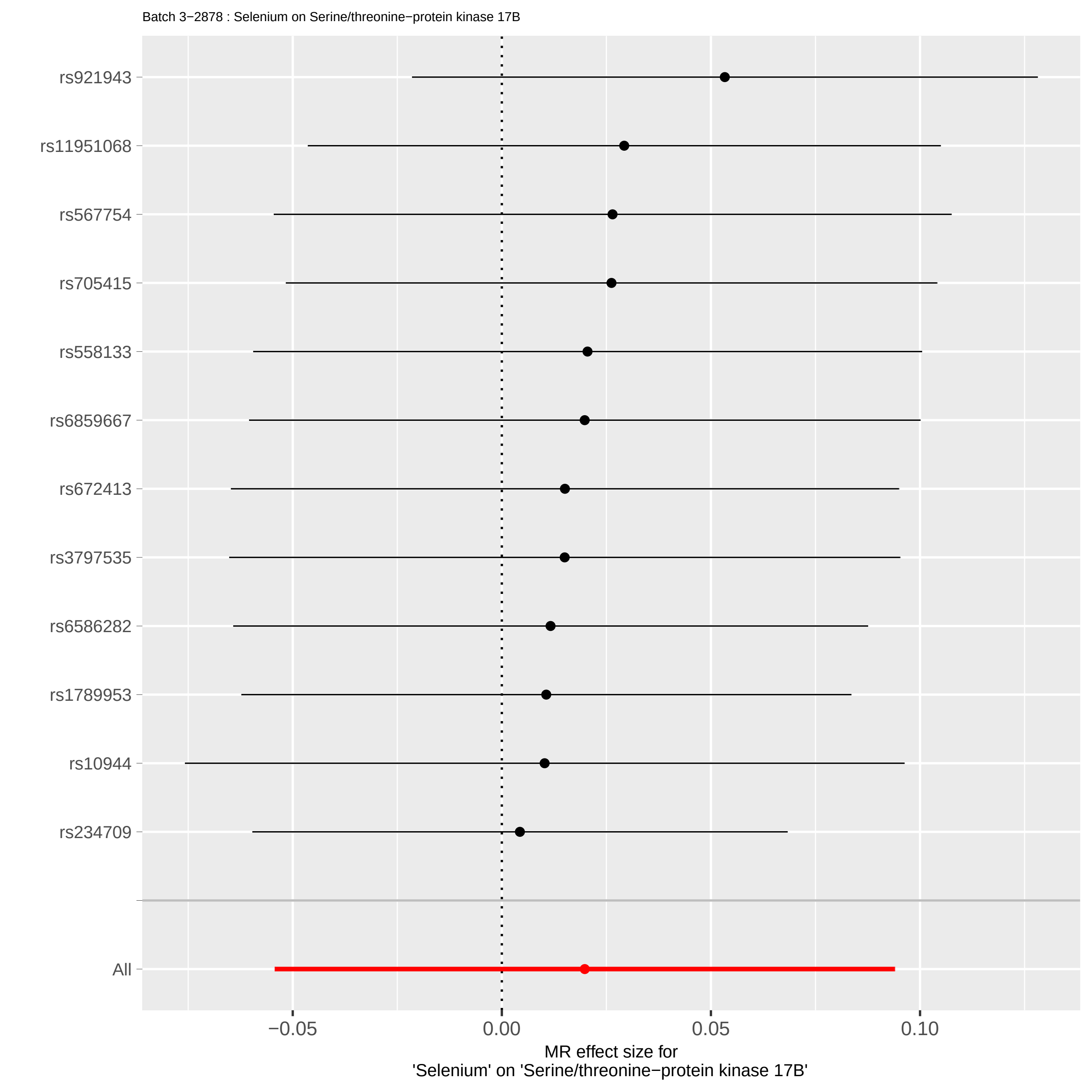

Batch 3−2878 : Selenium on Serine/threonine−protein kinase 17B
rs921943
rs11951068
rs567754
rs705415
rs558133
rs6859667
rs672413
rs3797535
rs6586282
rs1789953
rs10944
rs234709
All
−0.05
0.00
0.05
0.10
MR effect size for
'Selenium' on 'Serine/threonine−protein kinase 17B'

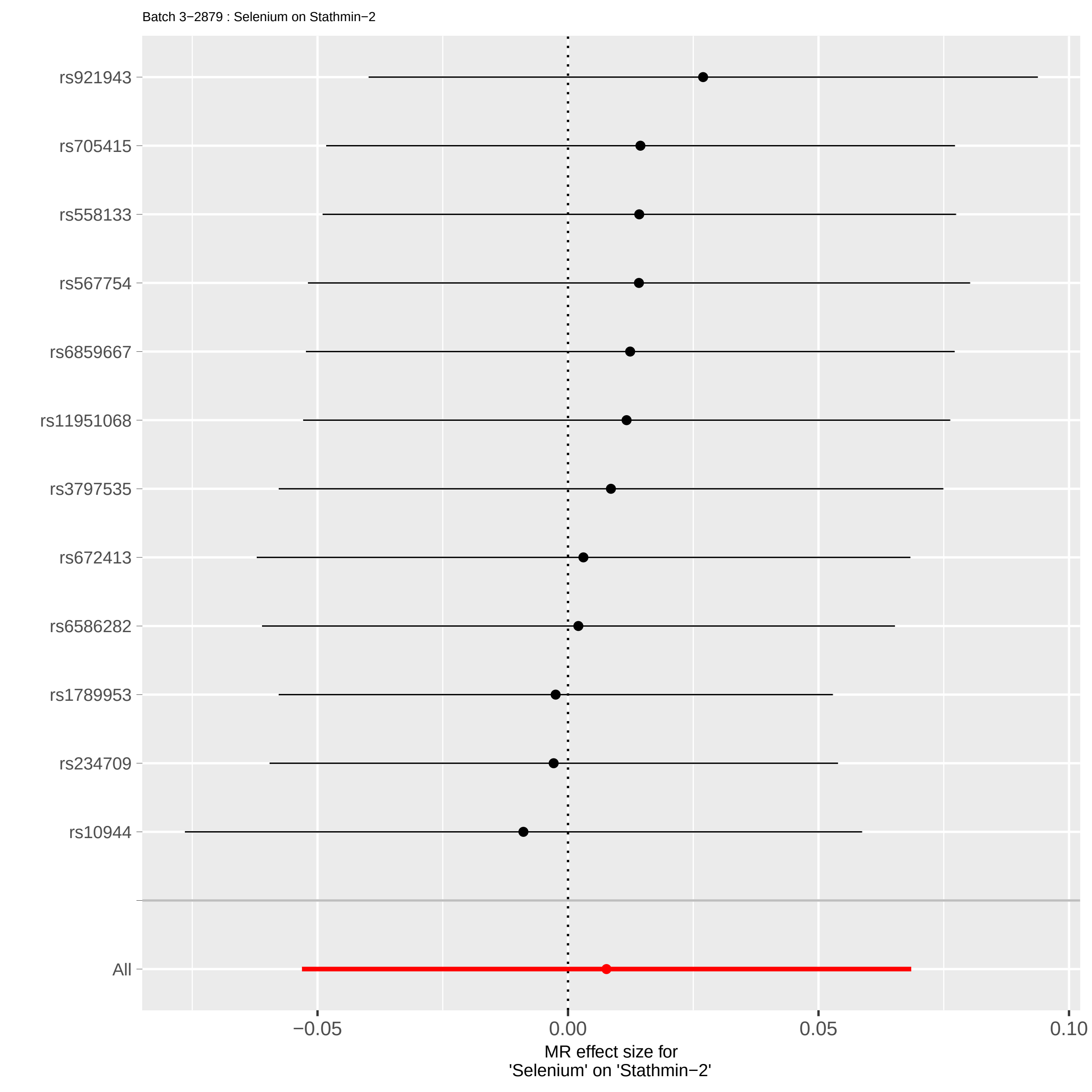

Batch 3−2879 : Selenium on Stathmin−2
rs921943
rs705415
rs558133
rs567754
rs6859667
rs11951068
rs3797535
rs672413
rs6586282
rs1789953
rs234709
rs10944
All
−0.05
0.00
0.05
0.10
MR effect size for
'Selenium' on 'Stathmin−2'

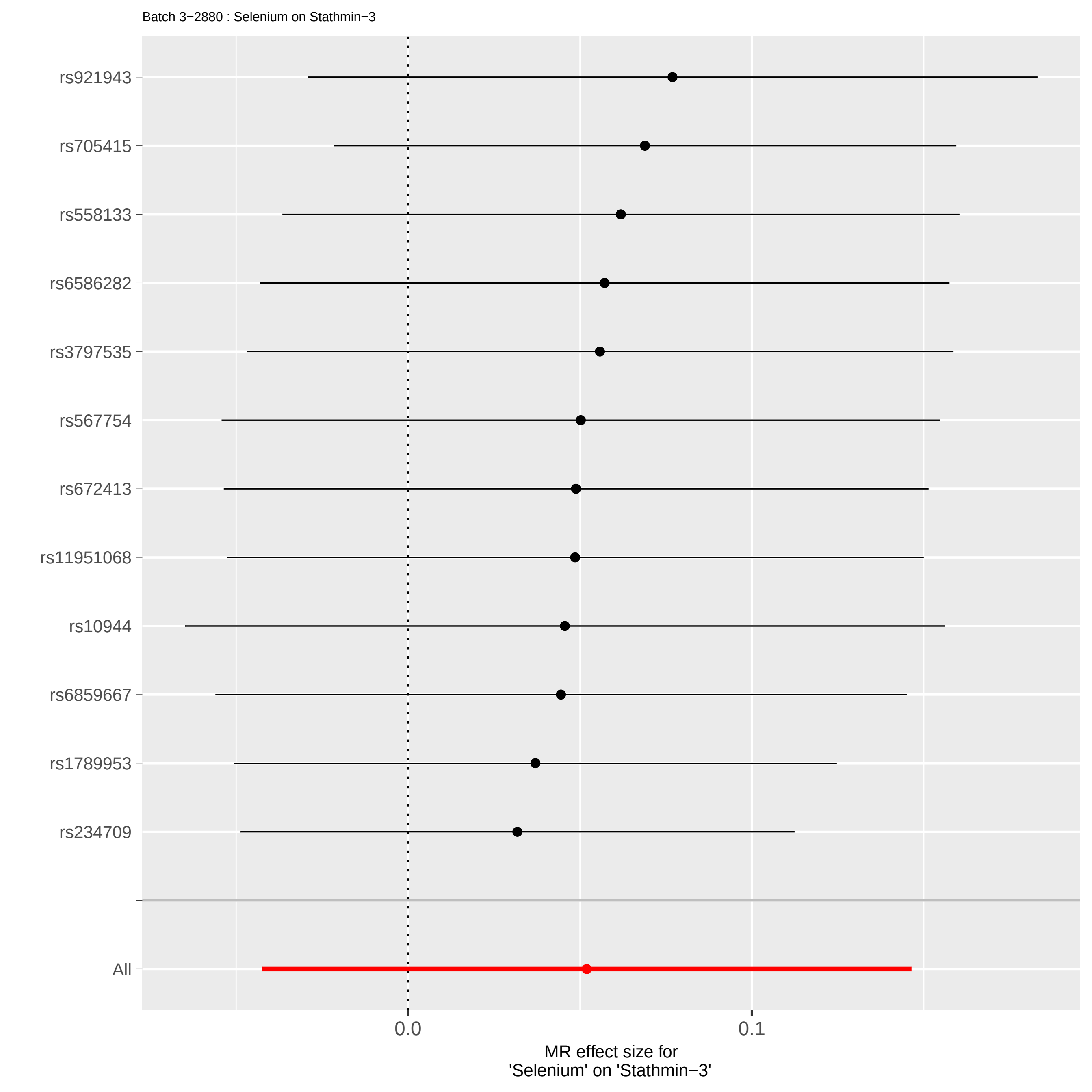

Batch 3−2880 : Selenium on Stathmin−3
rs921943
rs705415
rs558133
rs6586282
rs3797535
rs567754
rs672413
rs11951068
rs10944
rs6859667
rs1789953
rs234709
All
0.0
0.1
MR effect size for
'Selenium' on 'Stathmin−3'

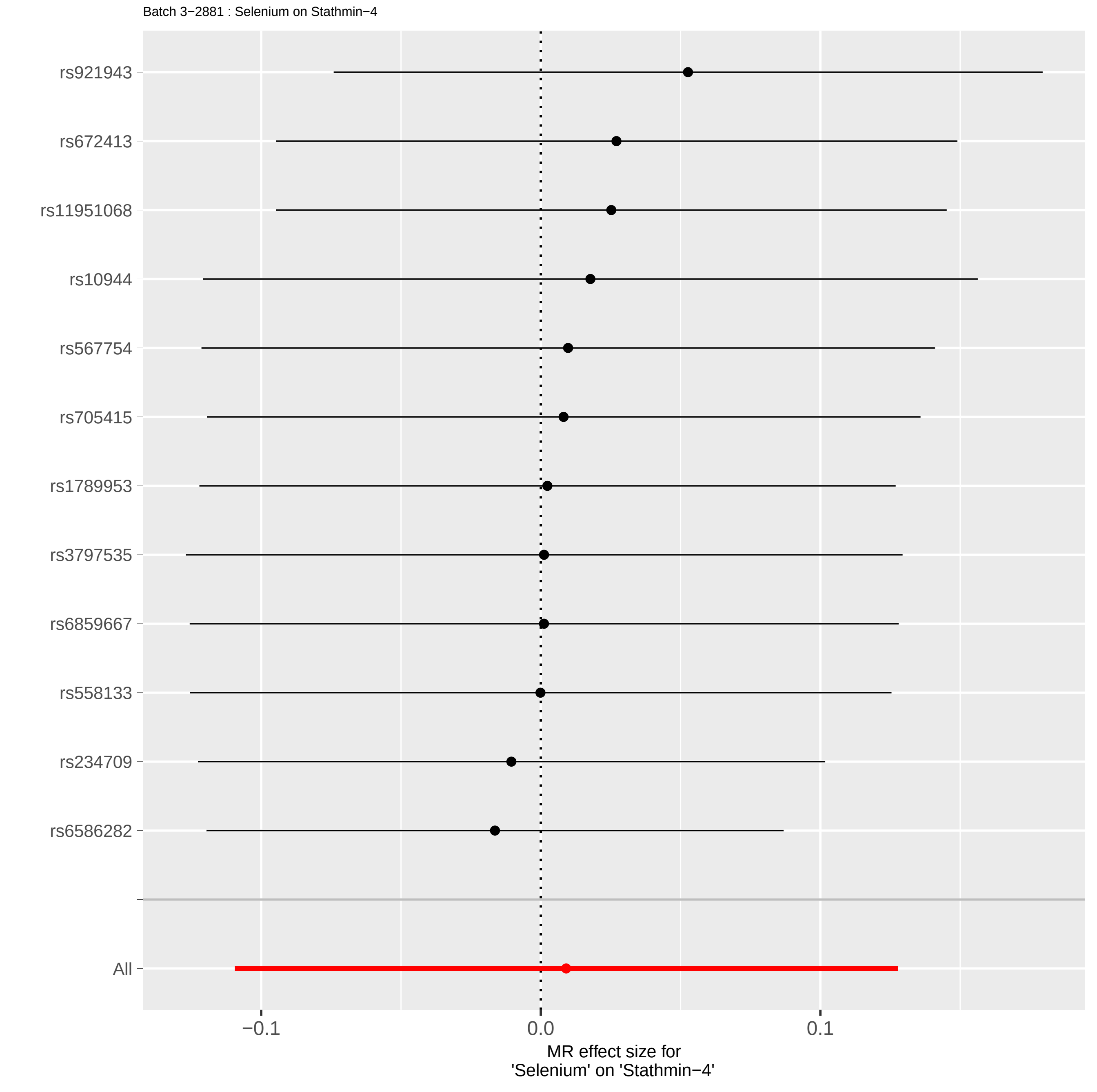
Batch 3−2881 : Selenium on Stathmin−4
rs921943
rs672413
rs11951068
rs10944
rs567754
rs705415
rs1789953
rs3797535
rs6859667
rs558133
rs234709
rs6586282
All
−0.1
0.0
0.1
MR effect size for
'Selenium' on 'Stathmin−4'

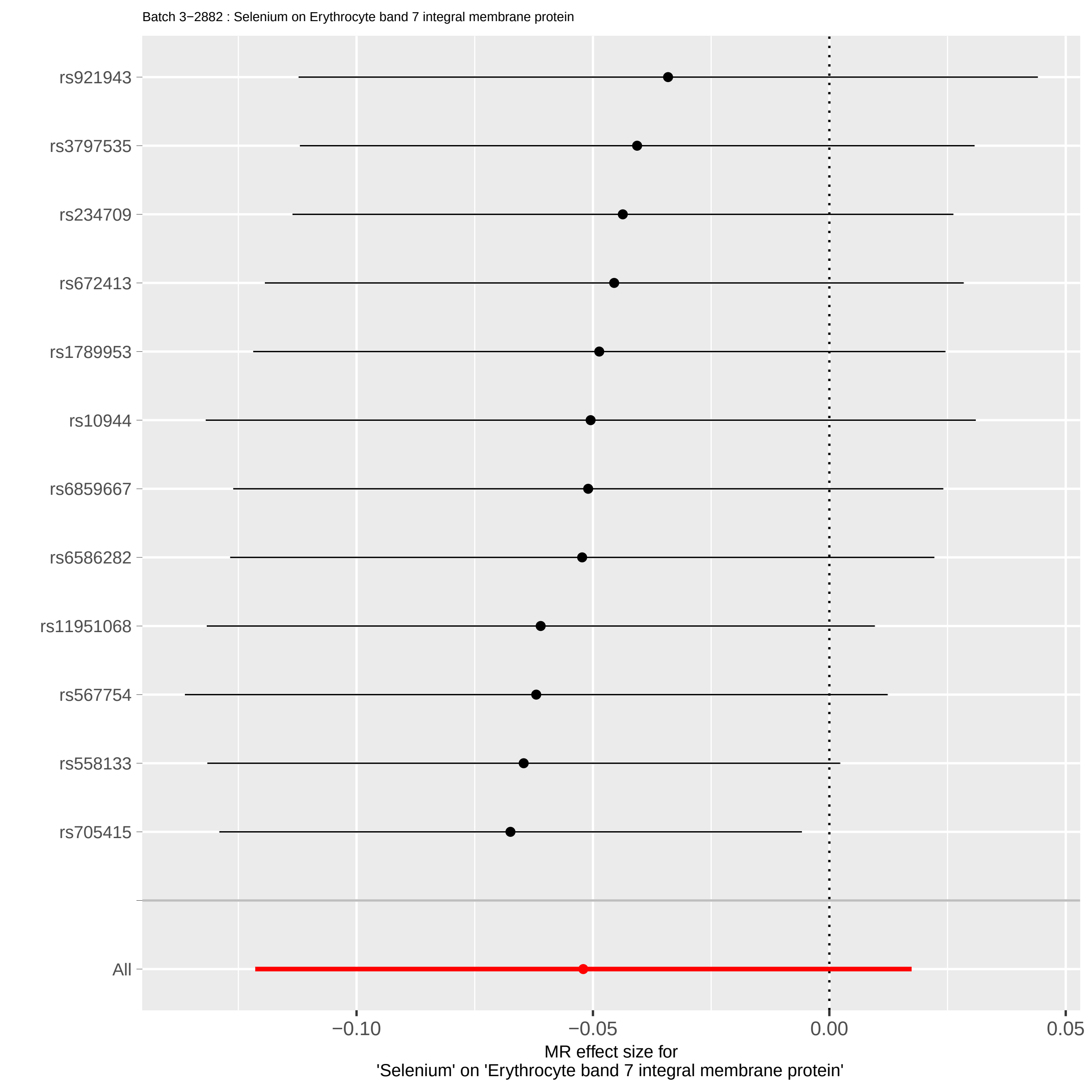

Batch 3−2882 : Selenium on Erythrocyte band 7 integral membrane protein
rs921943
rs3797535
rs234709
rs672413
rs1789953
rs10944
rs6859667
rs6586282
rs11951068
rs567754
rs558133
rs705415
All
−0.10
−0.05
0.00
0.05
MR effect size for
'Selenium' on 'Erythrocyte band 7 integral membrane protein'

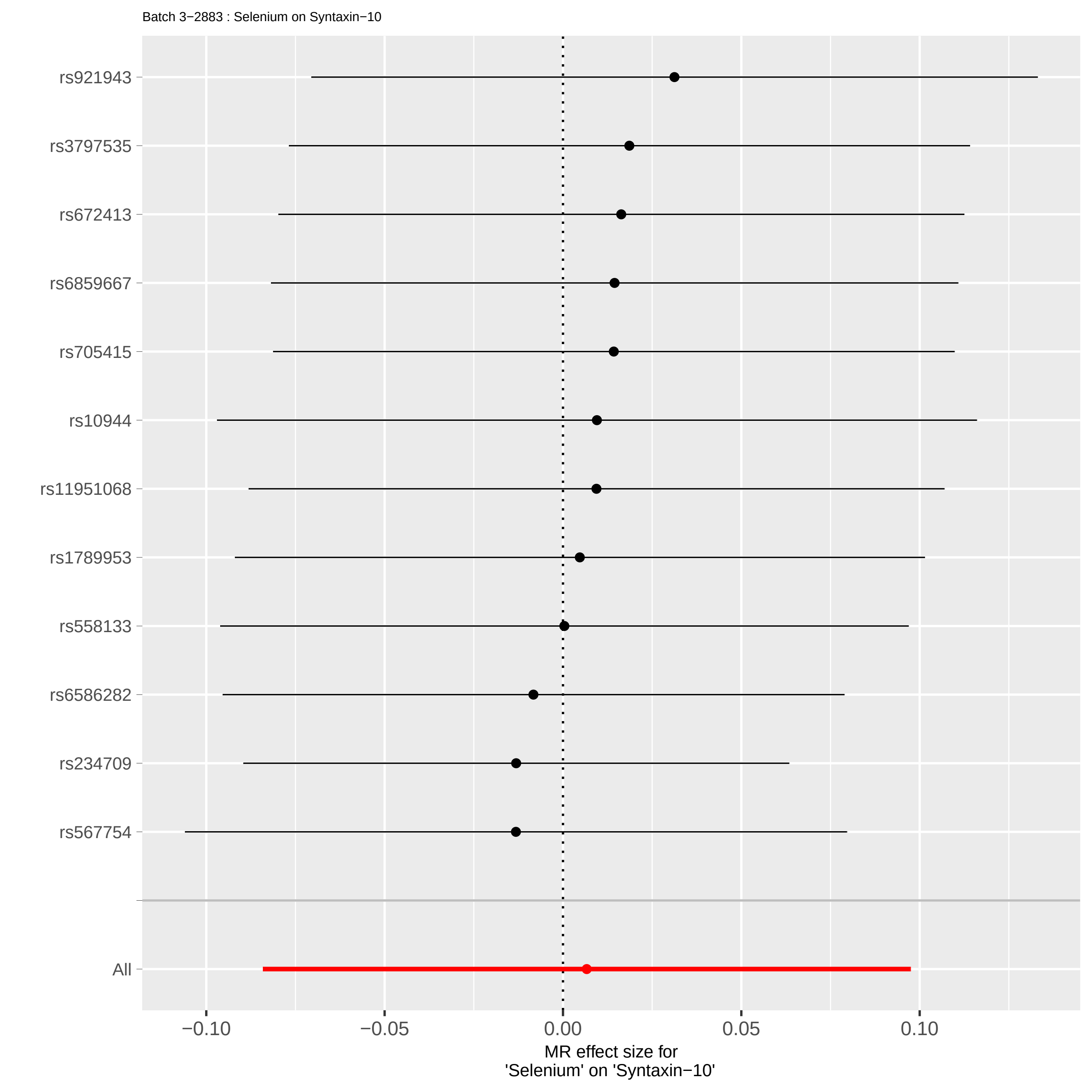

Batch 3−2883 : Selenium on Syntaxin−10
rs921943
rs3797535
rs672413
rs6859667
rs705415
rs10944
rs11951068
rs1789953
rs558133
rs6586282
rs234709
rs567754
All
−0.10
−0.05
0.00
0.05
0.10
MR effect size for
'Selenium' on 'Syntaxin−10'

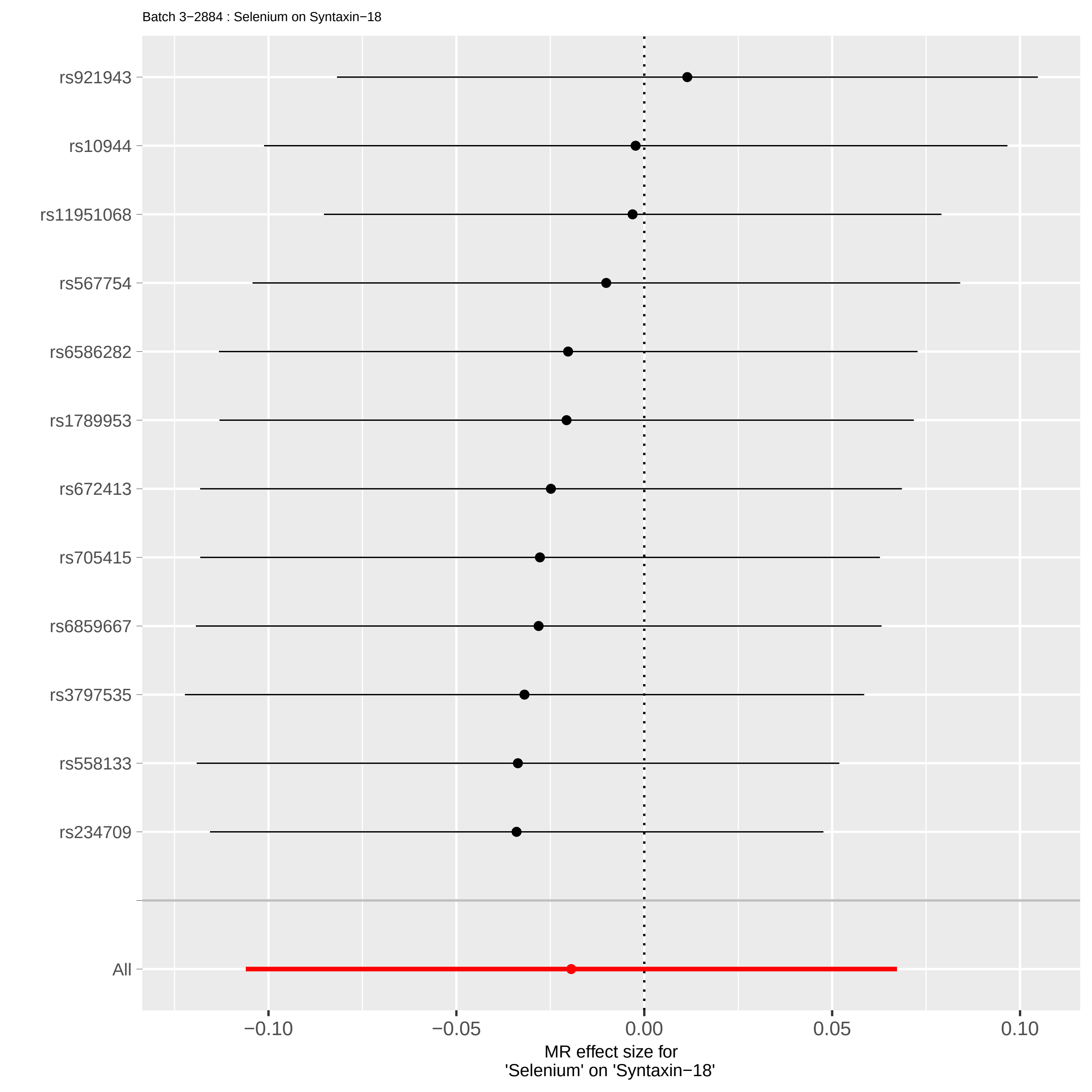
Batch 3−2884 : Selenium on Syntaxin−18
rs921943
rs10944
rs11951068
rs567754
rs6586282
rs1789953
rs672413
rs705415
rs6859667
rs3797535
rs558133
rs234709
All
−0.10
−0.05
0.00
0.05
0.10
MR effect size for
'Selenium' on 'Syntaxin−18'

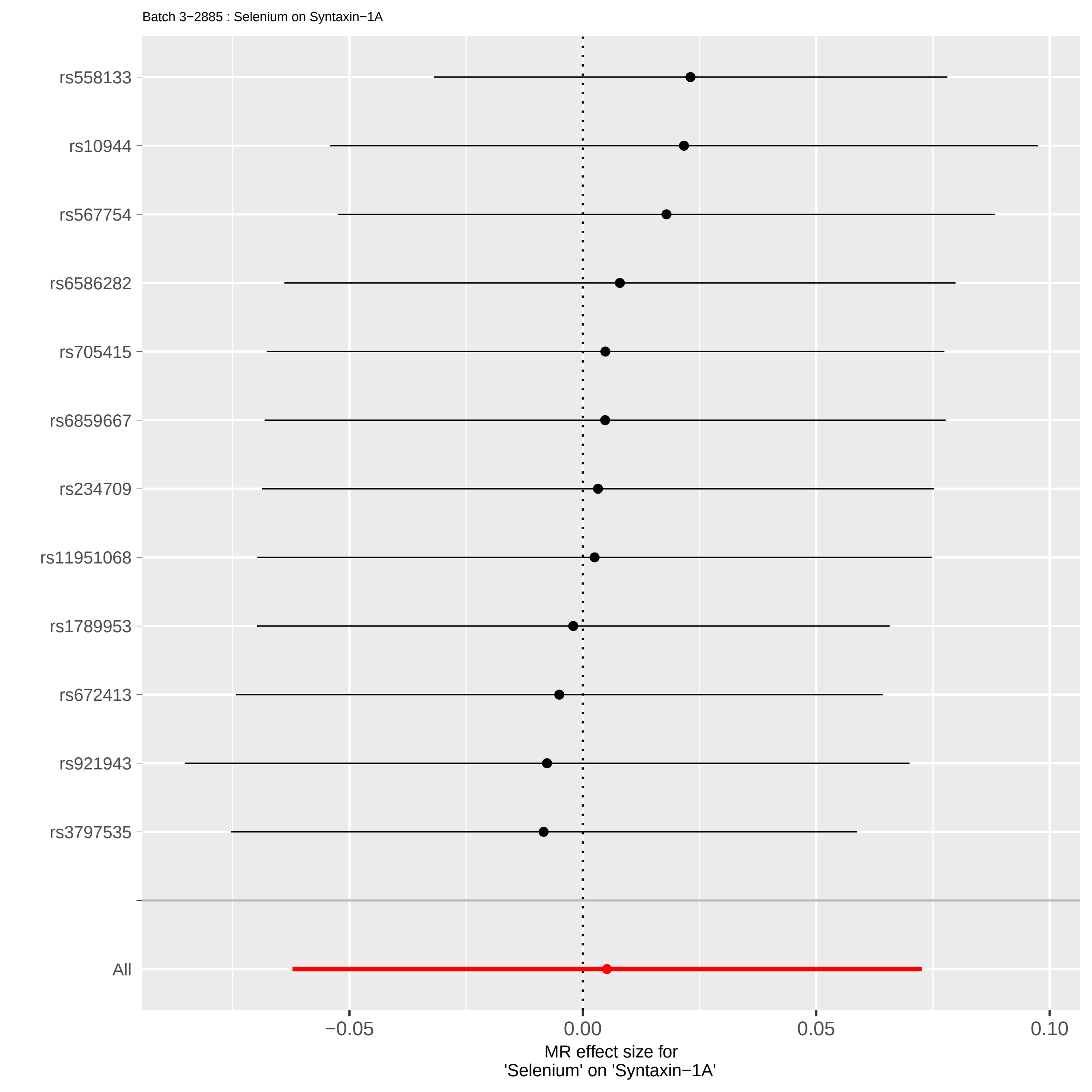
Batch 3−2885 : Selenium on Syntaxin−1A
rs558133
rs10944
rs567754
rs6586282
rs705415
rs6859667
rs234709
rs11951068
rs1789953
rs672413
rs921943
rs3797535
All
−0.05
0.00
0.05
0.10
MR effect size for
'Selenium' on 'Syntaxin−1A'

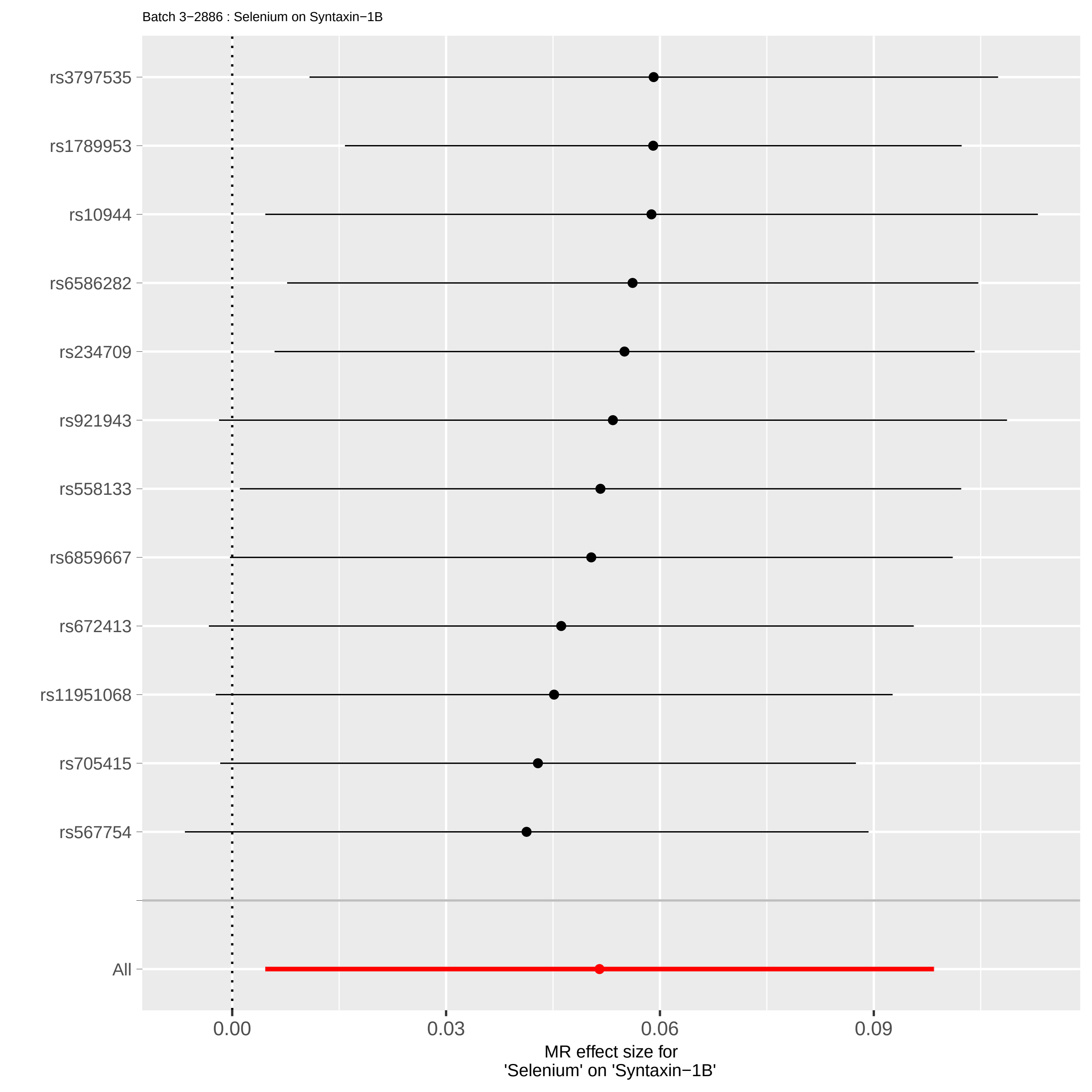

Batch 3−2886 : Selenium on Syntaxin−1B
rs3797535
rs1789953
rs10944
rs6586282
rs234709
rs921943
rs558133
rs6859667
rs672413
rs11951068
rs705415
rs567754
All
0.00
0.03
0.06
0.09
MR effect size for
'Selenium' on 'Syntaxin−1B'

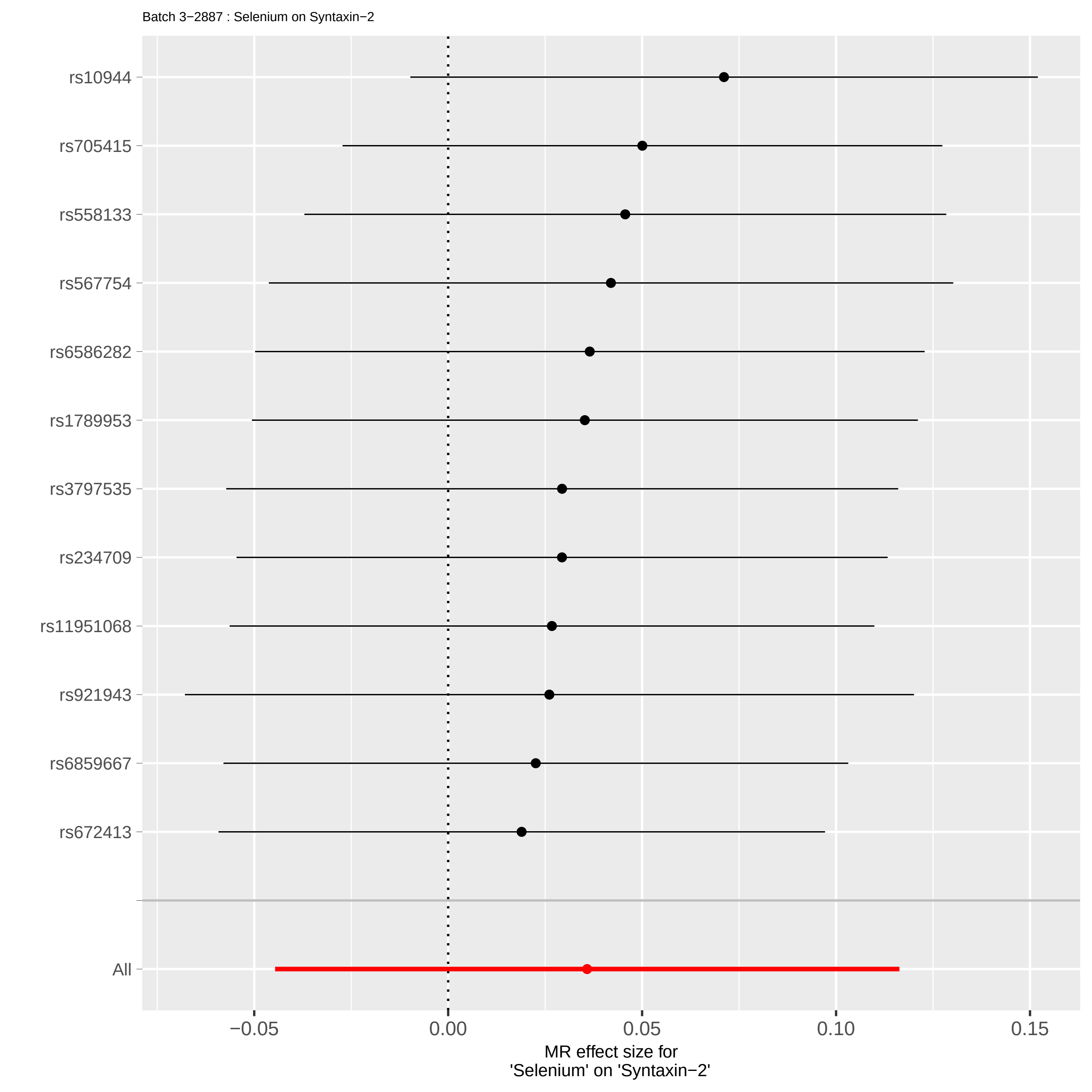

Batch 3−2887 : Selenium on Syntaxin−2
rs10944
rs705415
rs558133
rs567754
rs6586282
rs1789953
rs3797535
rs234709
rs11951068
rs921943
rs6859667
rs672413
All
−0.05
0.00
0.05
0.10
0.15
MR effect size for
'Selenium' on 'Syntaxin−2'

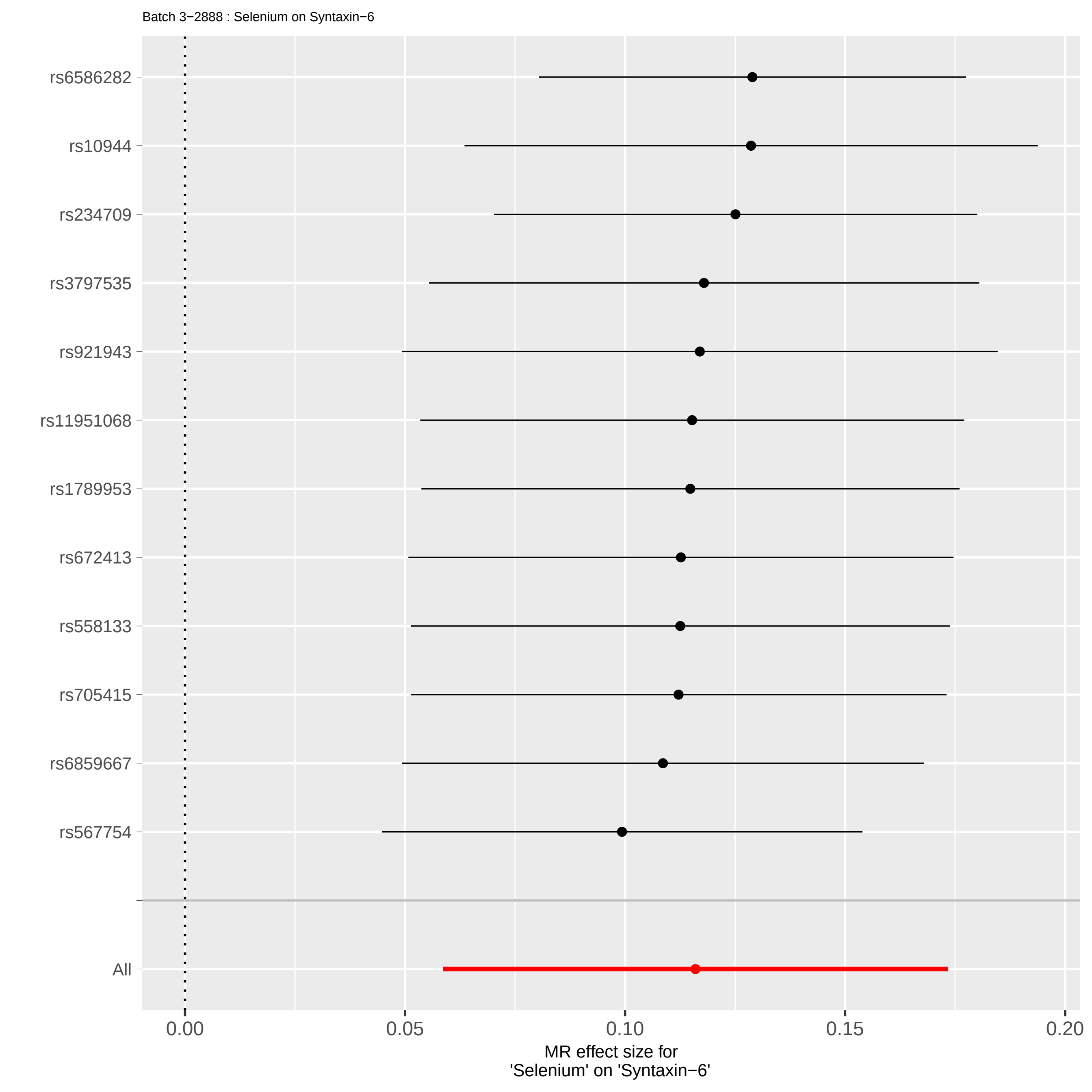
Batch 3−2888 : Selenium on Syntaxin−6
rs6586282
rs10944
rs234709
rs3797535
rs921943
rs11951068
rs1789953
rs672413
rs558133
rs705415
rs6859667
rs567754
All
0.00
0.05
0.10
0.15
0.20
MR effect size for
'Selenium' on 'Syntaxin−6'

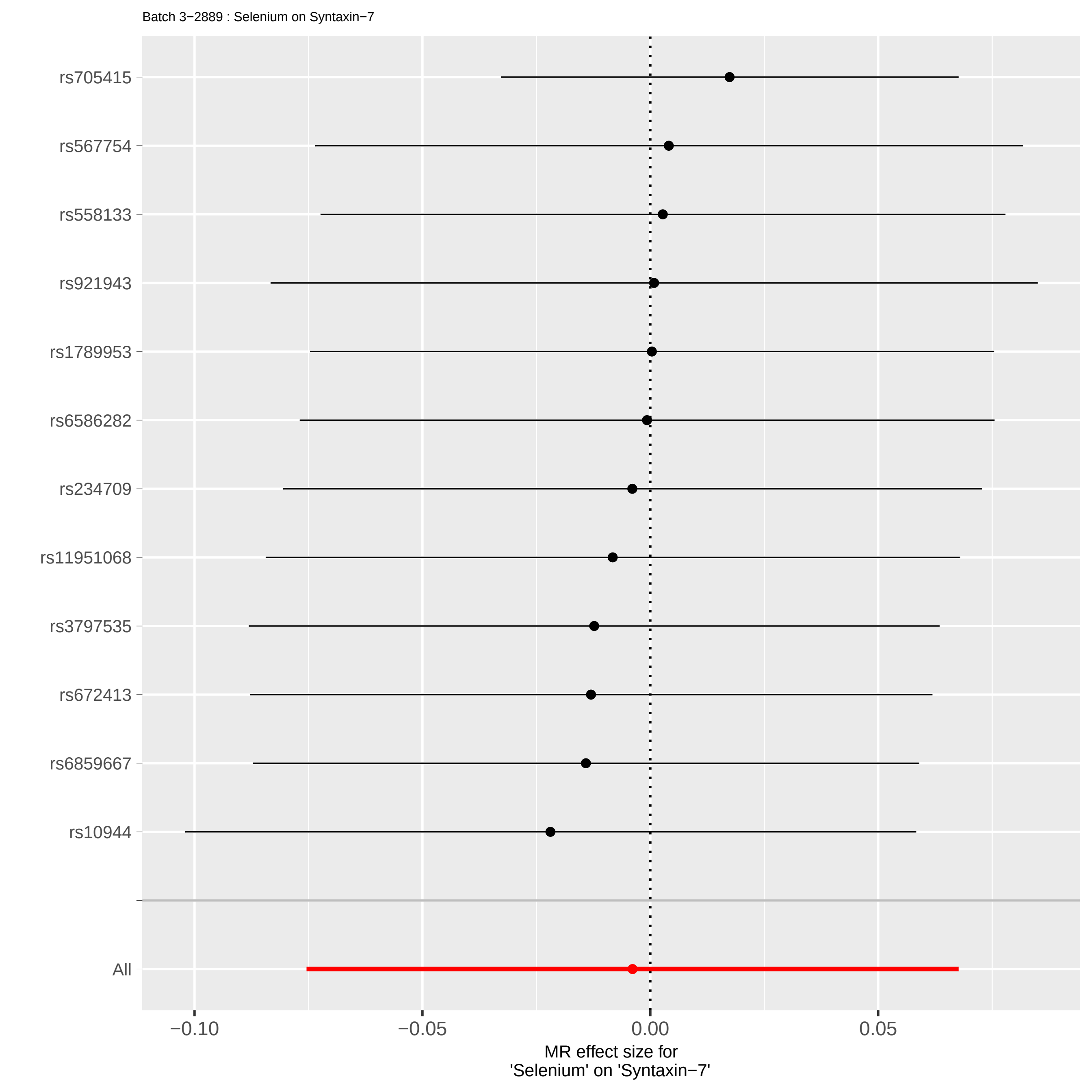

Batch 3−2889 : Selenium on Syntaxin−7
rs705415
rs567754
rs558133
rs921943
rs1789953
rs6586282
rs234709
rs11951068
rs3797535
rs672413
rs6859667
rs10944
All
−0.10
−0.05
0.00
0.05
MR effect size for
'Selenium' on 'Syntaxin−7'

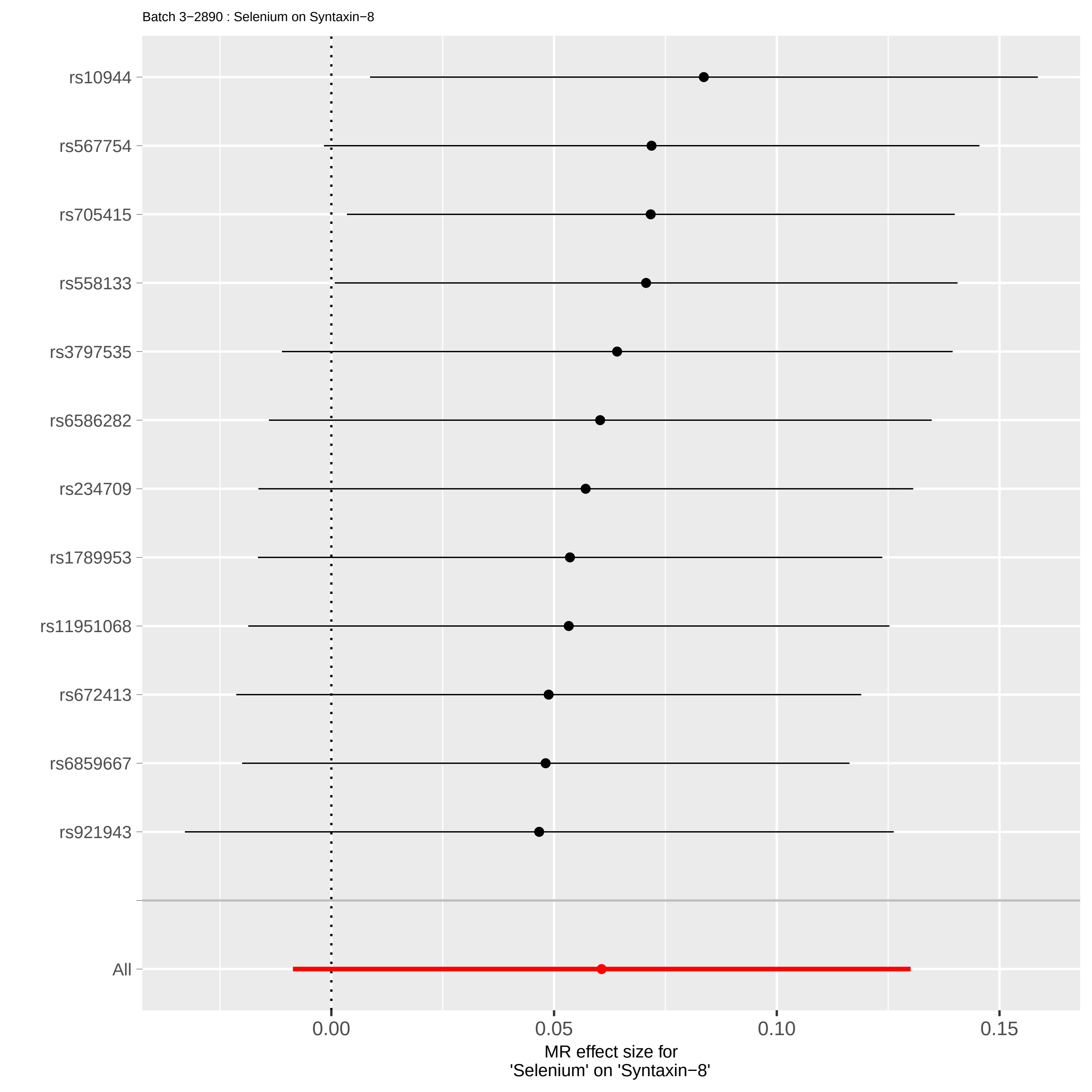

Batch 3−2890 : Selenium on Syntaxin−8
rs10944
rs567754
rs705415
rs558133
rs3797535
rs6586282
rs234709
rs1789953
rs11951068
rs672413
rs6859667
rs921943
All
0.00
0.05
0.10
0.15
MR effect size for
'Selenium' on 'Syntaxin−8'

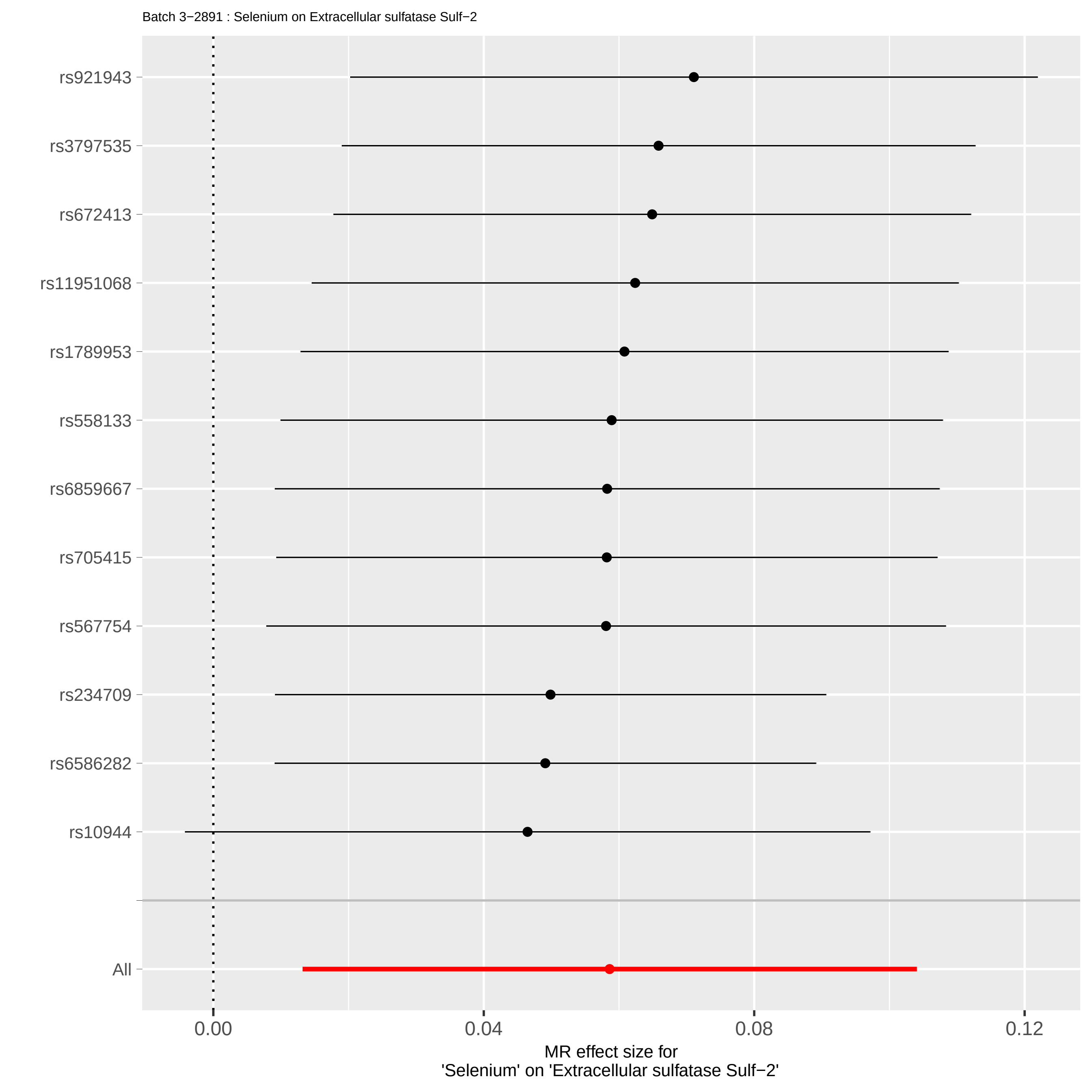
Batch 3−2891 : Selenium on Extracellular sulfatase Sulf−2
rs921943
rs3797535
rs672413
rs11951068
rs1789953
rs558133
rs6859667
rs705415
rs567754
rs234709
rs6586282
rs10944
All
0.00
0.04
0.08
0.12
MR effect size for
'Selenium' on 'Extracellular sulfatase Sulf−2'

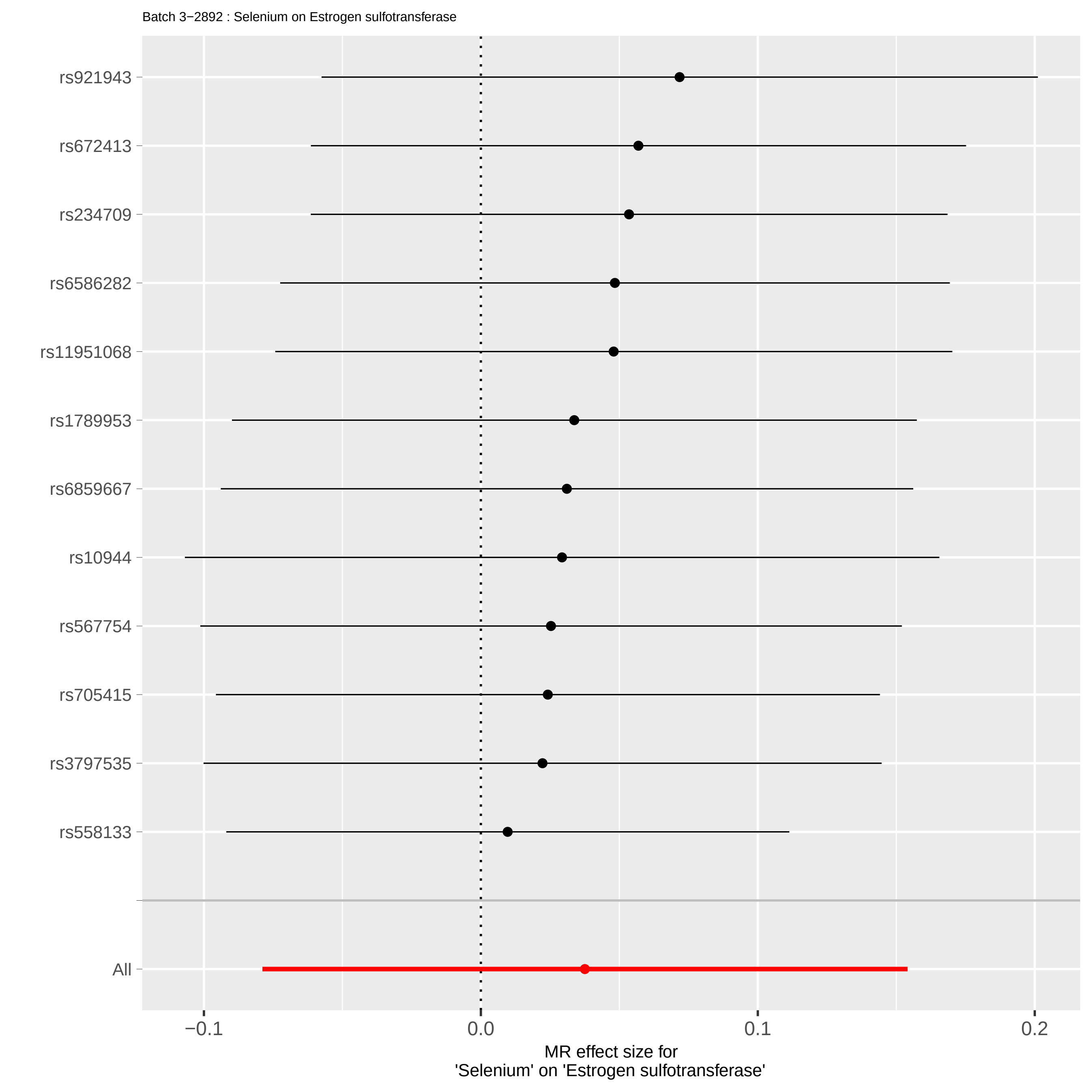

Batch 3−2892 : Selenium on Estrogen sulfotransferase
rs921943
rs672413
rs234709
rs6586282
rs11951068
rs1789953
rs6859667
rs10944
rs567754
rs705415
rs3797535
rs558133
All
−0.1
0.0
0.1
0.2
MR effect size for
'Selenium' on 'Estrogen sulfotransferase'

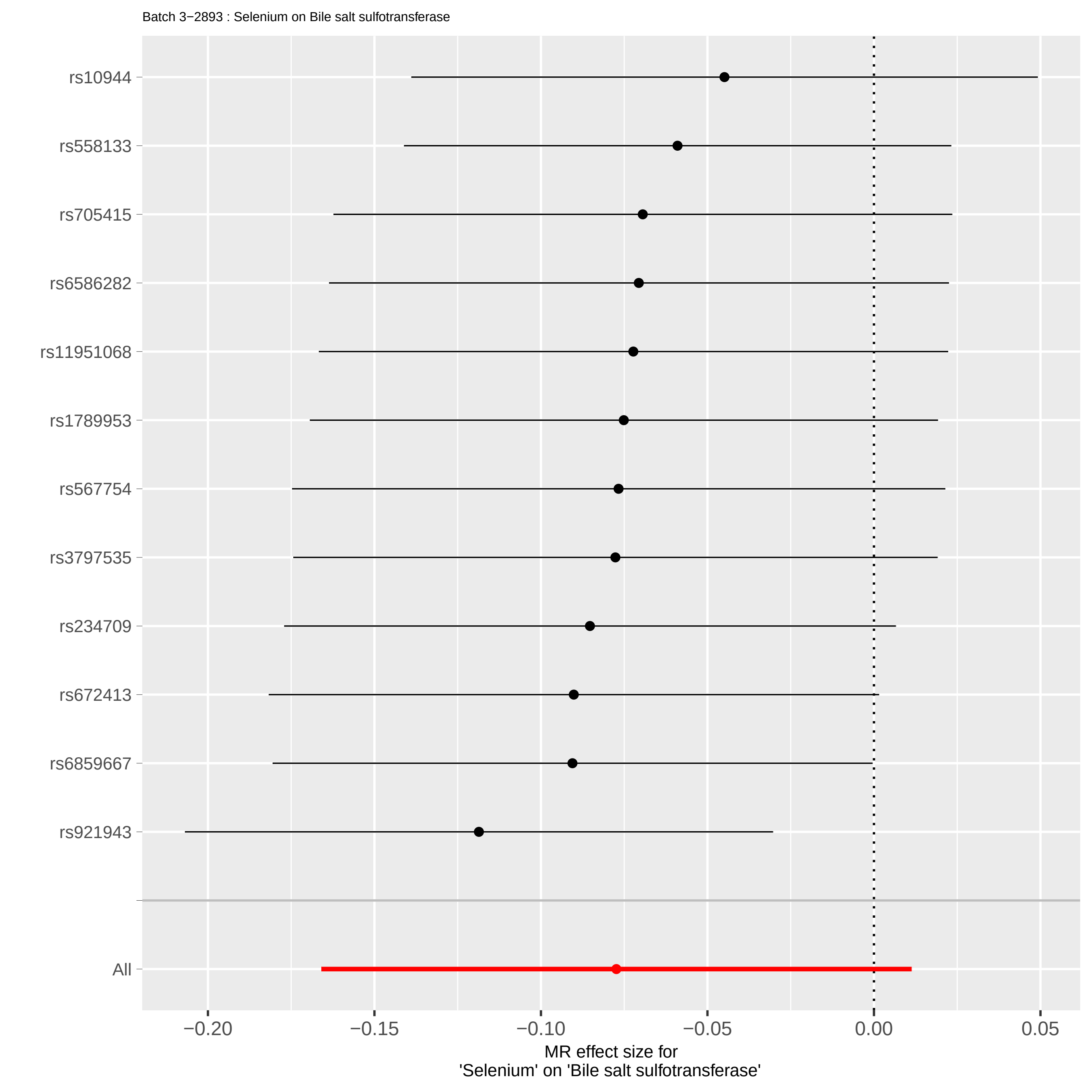
Batch 3−2893 : Selenium on Bile salt sulfotransferase
rs10944
rs558133
rs705415
rs6586282
rs11951068
rs1789953
rs567754
rs3797535
rs234709
rs672413
rs6859667
rs921943
All
−0.20
−0.15
−0.10
−0.05
0.00
0.05
MR effect size for
'Selenium' on 'Bile salt sulfotransferase'

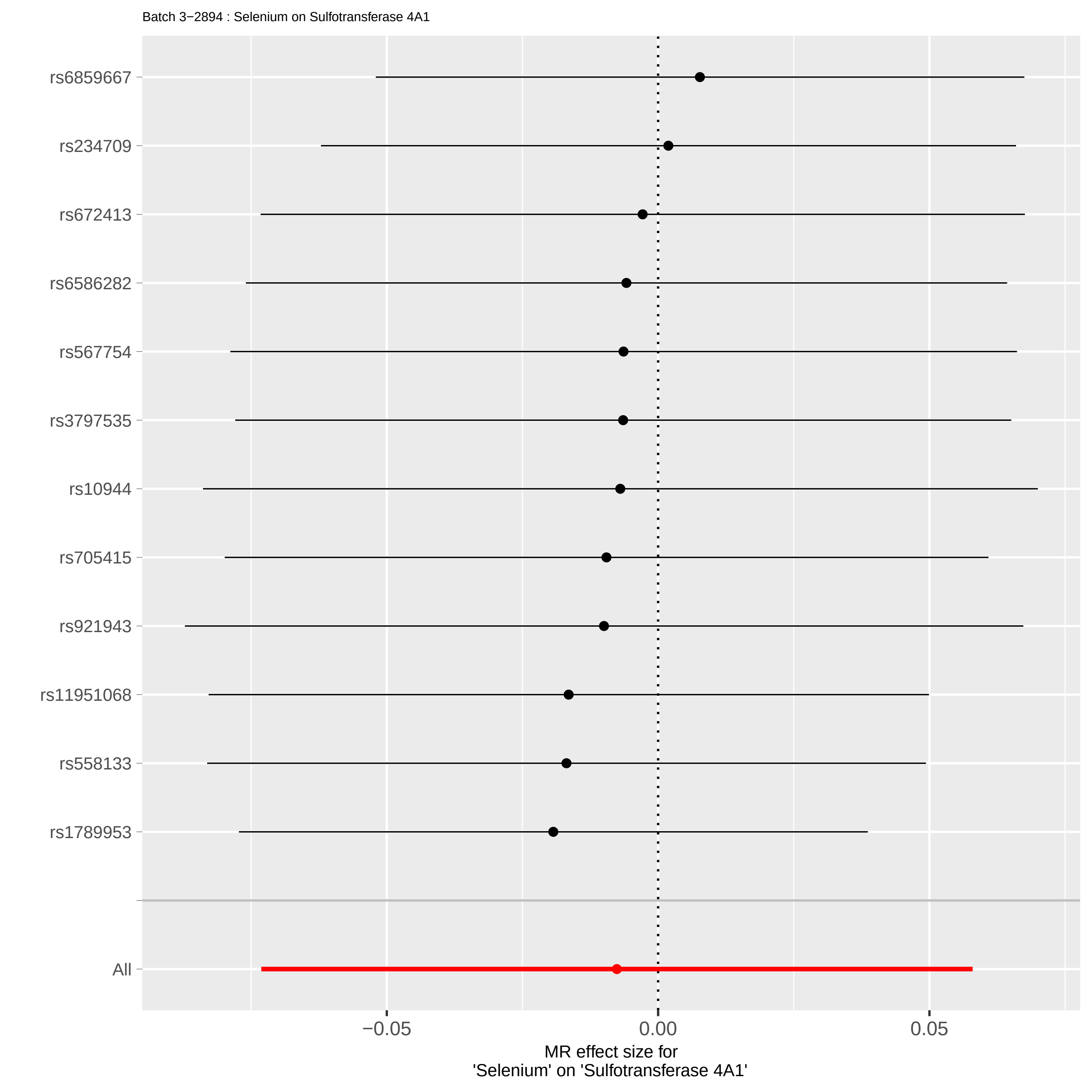

Batch 3−2894 : Selenium on Sulfotransferase 4A1
rs6859667
rs234709
rs672413
rs6586282
rs567754
rs3797535
rs10944
rs705415
rs921943
rs11951068
rs558133
rs1789953
All
−0.05
0.00
0.05
MR effect size for
'Selenium' on 'Sulfotransferase 4A1'

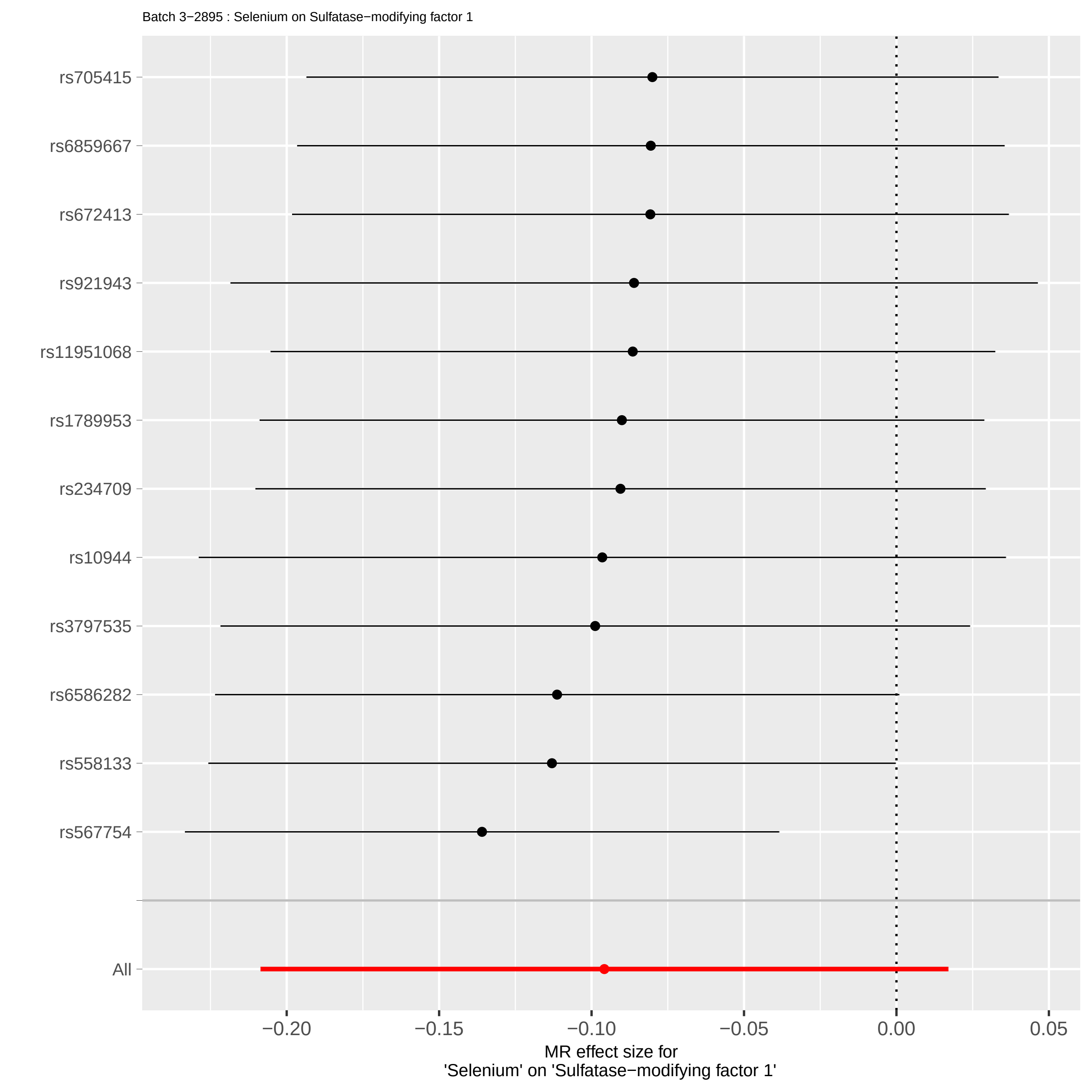

Batch 3−2895 : Selenium on Sulfatase−modifying factor 1
rs705415
rs6859667
rs672413
rs921943
rs11951068
rs1789953
rs234709
rs10944
rs3797535
rs6586282
rs558133
rs567754
All
−0.20
−0.15
−0.10
−0.05
0.00
0.05
MR effect size for
'Selenium' on 'Sulfatase−modifying factor 1'

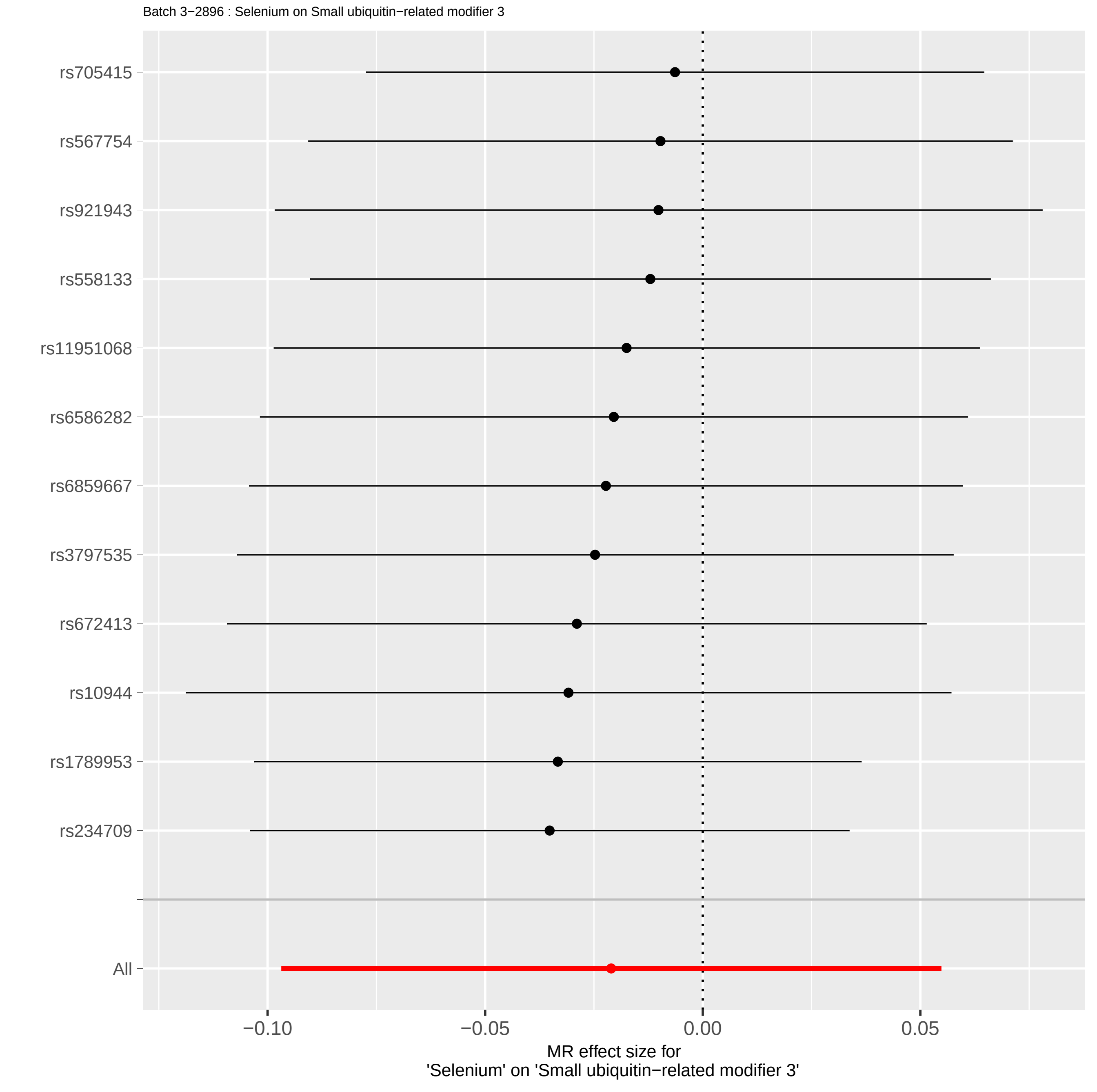

Batch 3−2896 : Selenium on Small ubiquitin−related modifier 3
rs705415
rs567754
rs921943
rs558133
rs11951068
rs6586282
rs6859667
rs3797535
rs672413
rs10944
rs1789953
rs234709
All
−0.10
−0.05
0.00
0.05
MR effect size for
'Selenium' on 'Small ubiquitin−related modifier 3'

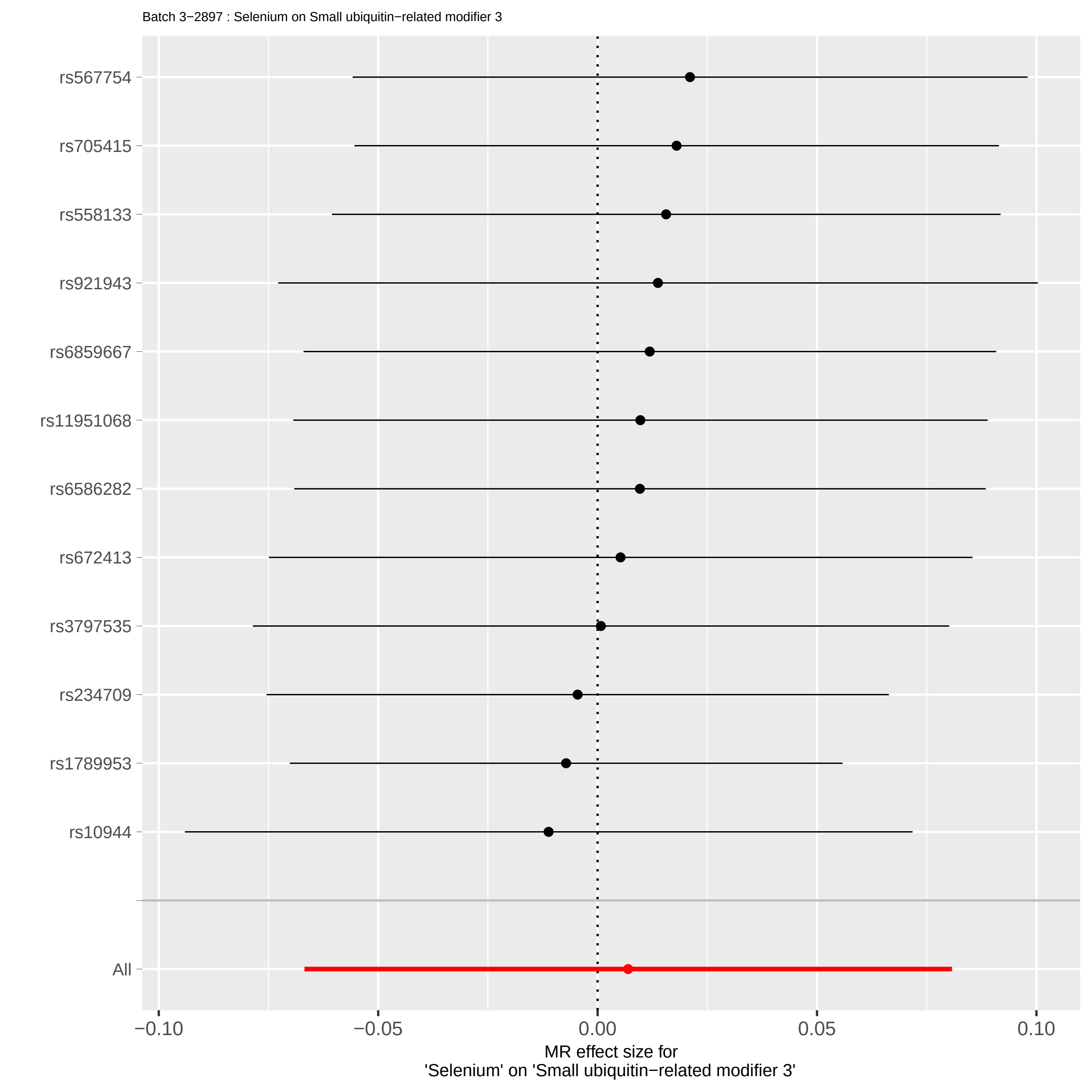

Batch 3−2897 : Selenium on Small ubiquitin−related modifier 3
rs567754
rs705415
rs558133
rs921943
rs6859667
rs11951068
rs6586282
rs672413
rs3797535
rs234709
rs1789953
rs10944
All
−0.10
−0.05
0.00
0.05
0.10
MR effect size for
'Selenium' on 'Small ubiquitin−related modifier 3'

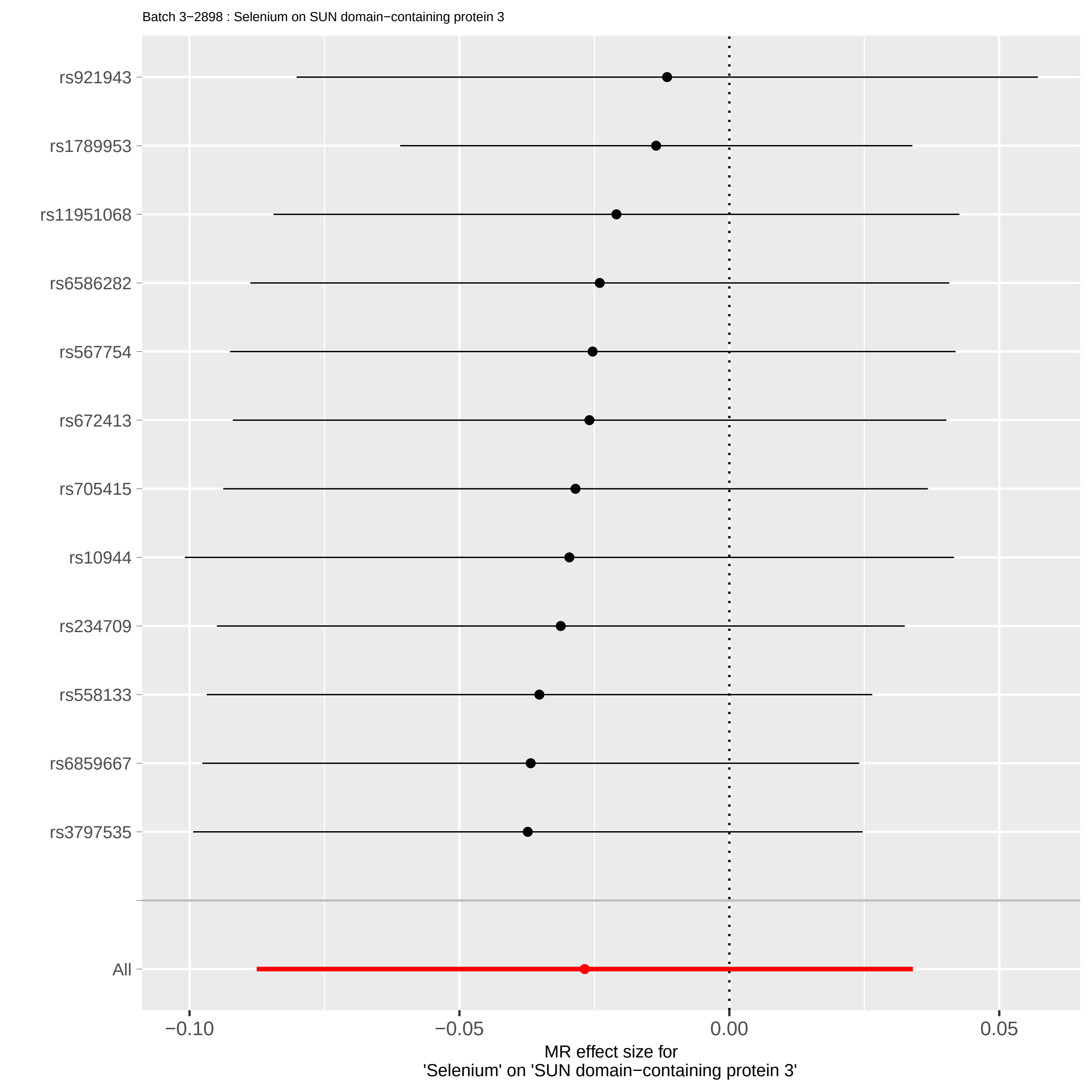

Batch 3−2898 : Selenium on SUN domain−containing protein 3
rs921943
rs1789953
rs11951068
rs6586282
rs567754
rs672413
rs705415
rs10944
rs234709
rs558133
rs6859667
rs3797535
All
−0.10
−0.05
0.00
0.05
MR effect size for
'Selenium' on 'SUN domain−containing protein 3'

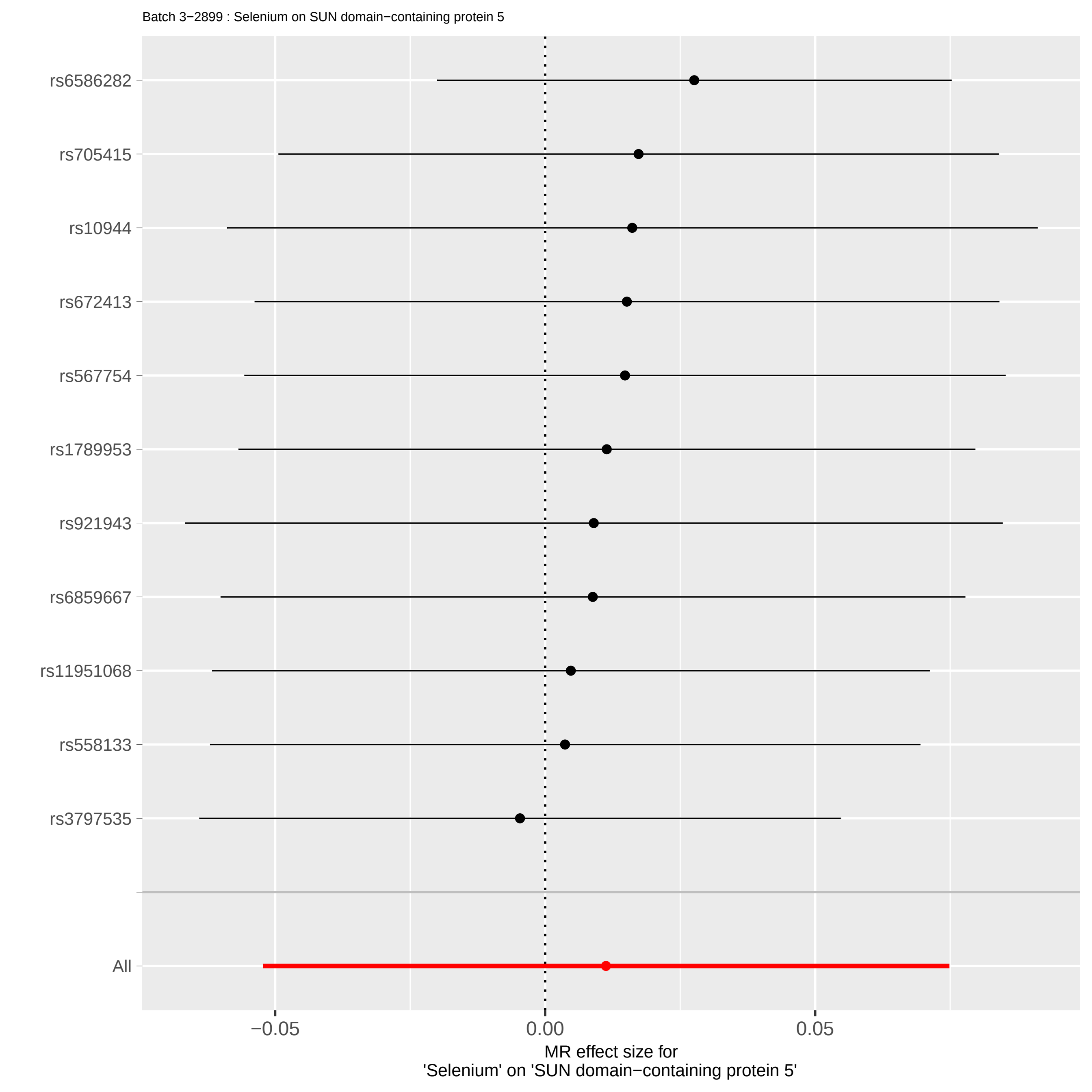
Batch 3−2899 : Selenium on SUN domain−containing protein 5
rs6586282
rs705415
rs10944
rs672413
rs567754
rs1789953
rs921943
rs6859667
rs11951068
rs558133
rs3797535
All
−0.05
0.00
0.05
MR effect size for
'Selenium' on 'SUN domain−containing protein 5'

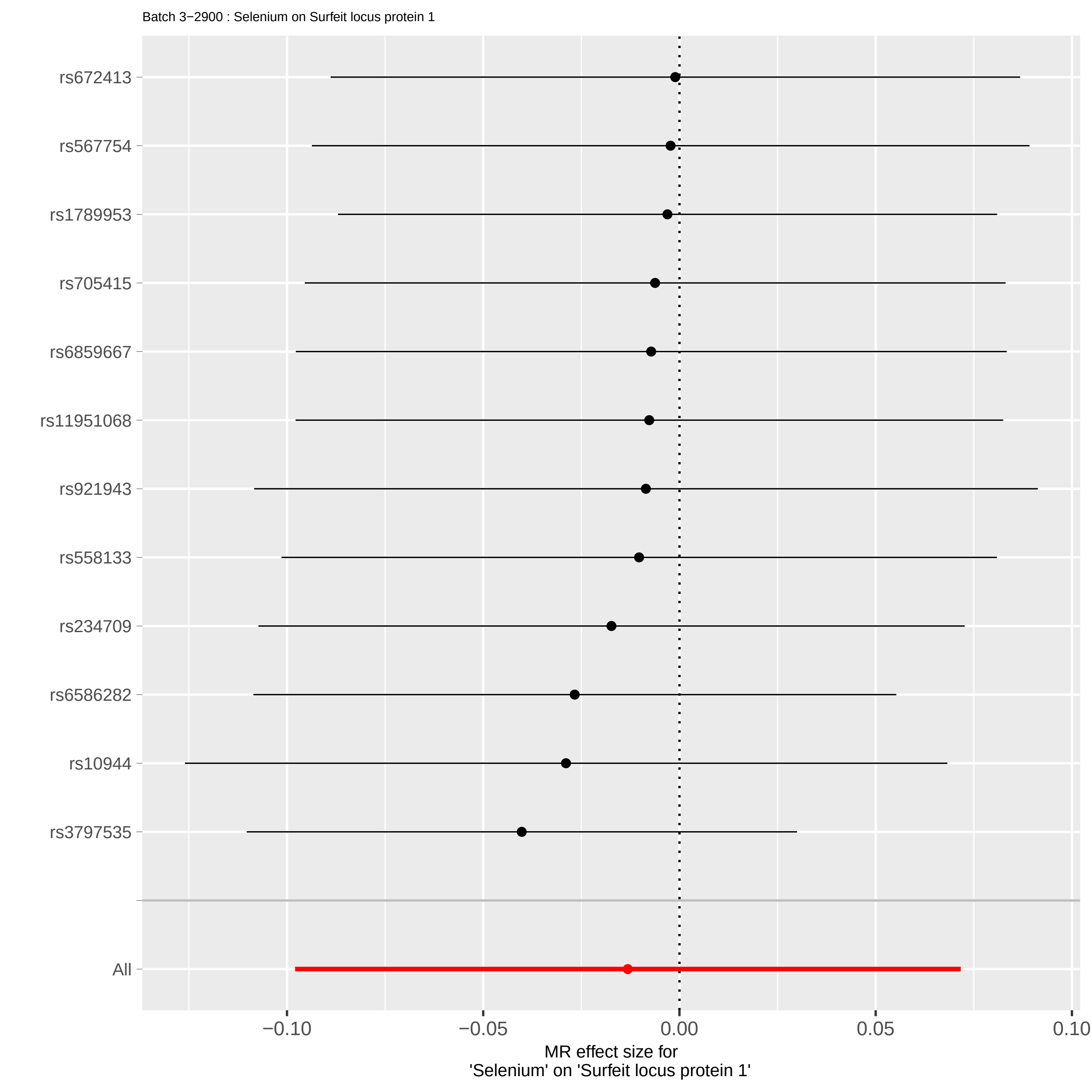
Batch 3−2900 : Selenium on Surfeit locus protein 1
rs672413
rs567754
rs1789953
rs705415
rs6859667
rs11951068
rs921943
rs558133
rs234709
rs6586282
rs10944
rs3797535
All
−0.10
−0.05
0.00
0.05
0.10
MR effect size for
'Selenium' on 'Surfeit locus protein 1'

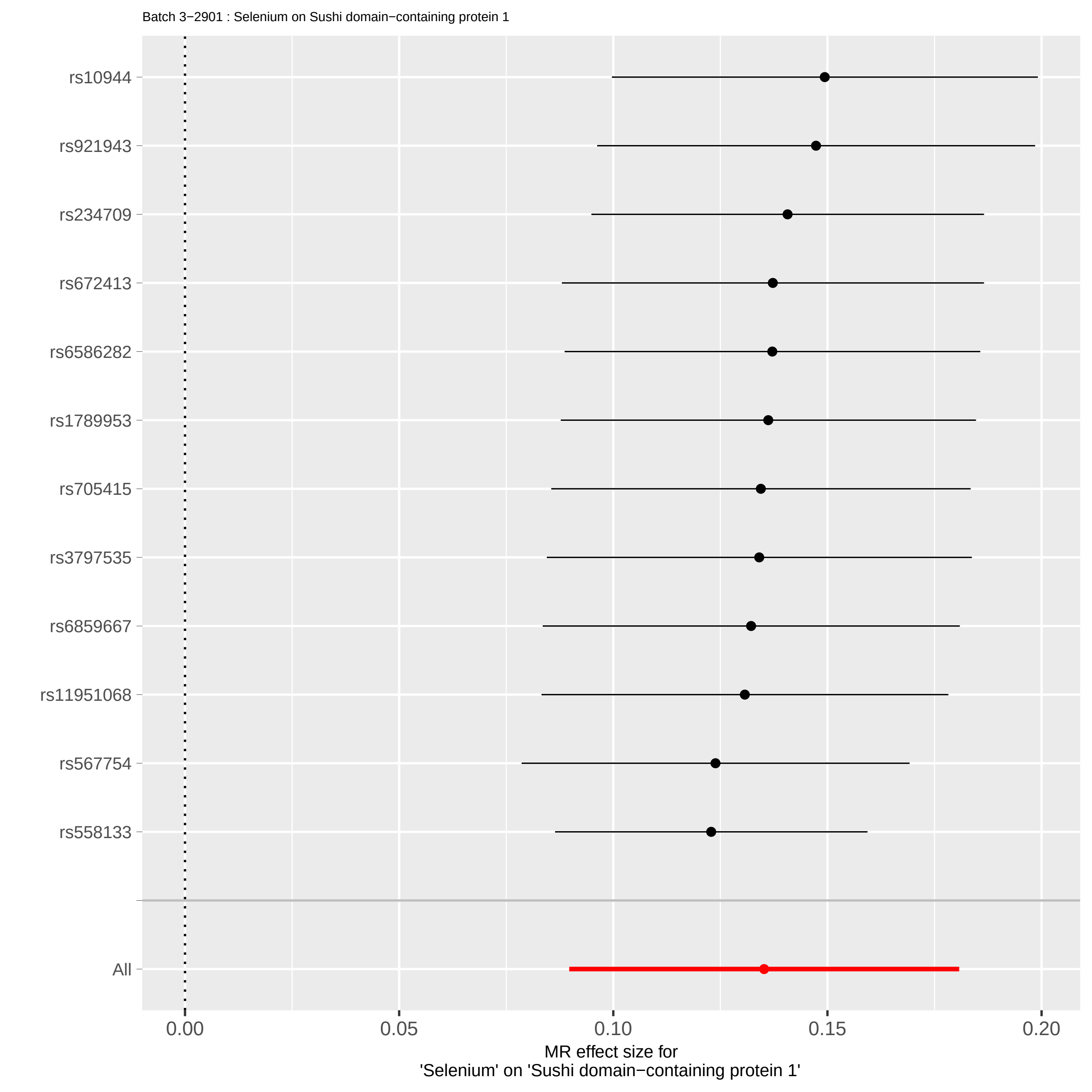

Batch 3−2901 : Selenium on Sushi domain−containing protein 1
rs10944
rs921943
rs234709
rs672413
rs6586282
rs1789953
rs705415
rs3797535
rs6859667
rs11951068
rs567754
rs558133
All
0.00
0.05
0.10
0.15
0.20
MR effect size for
'Selenium' on 'Sushi domain−containing protein 1'

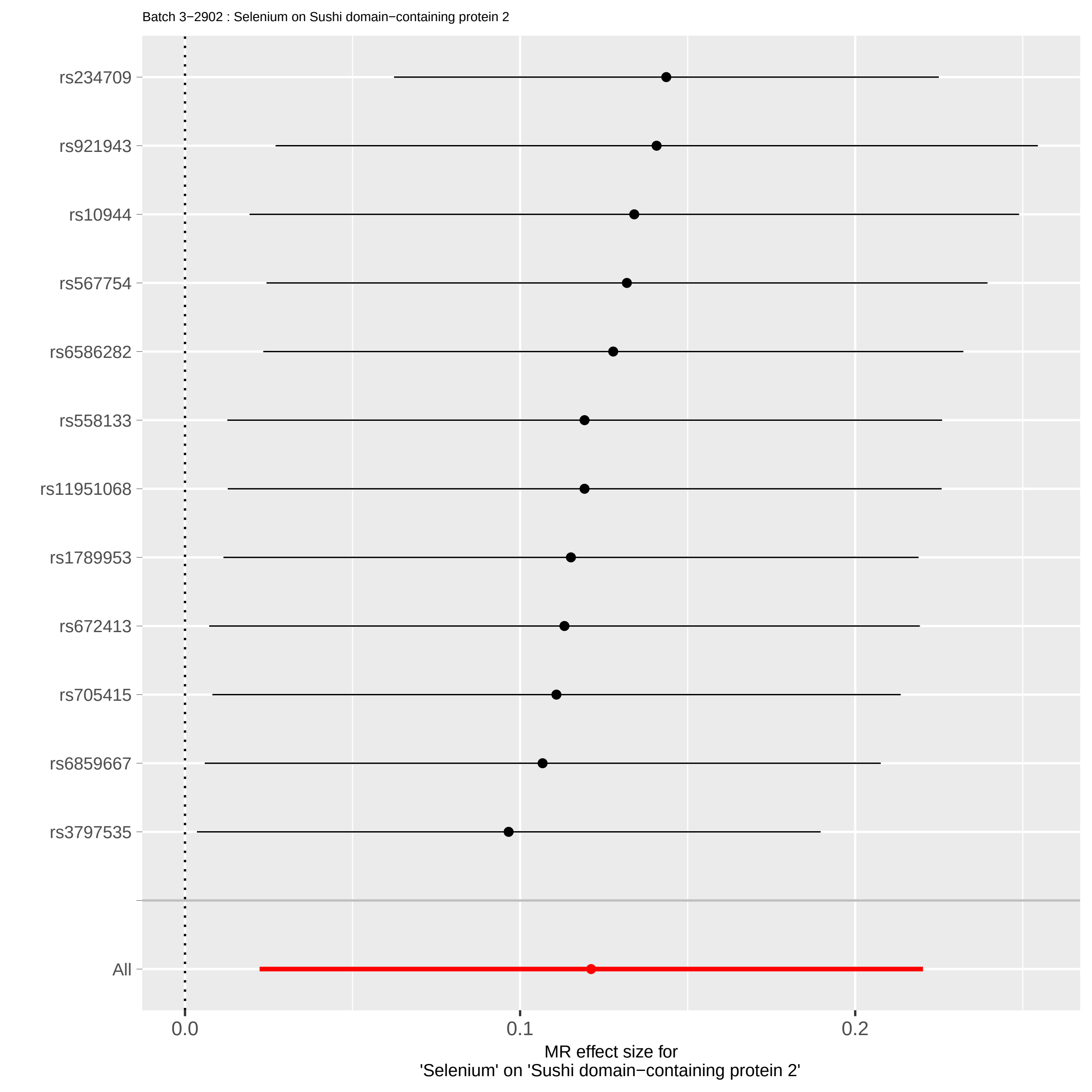

Batch 3−2902 : Selenium on Sushi domain−containing protein 2
rs234709
rs921943
rs10944
rs567754
rs6586282
rs558133
rs11951068
rs1789953
rs672413
rs705415
rs6859667
rs3797535
All
0.0
0.1
0.2
MR effect size for
'Selenium' on 'Sushi domain−containing protein 2'

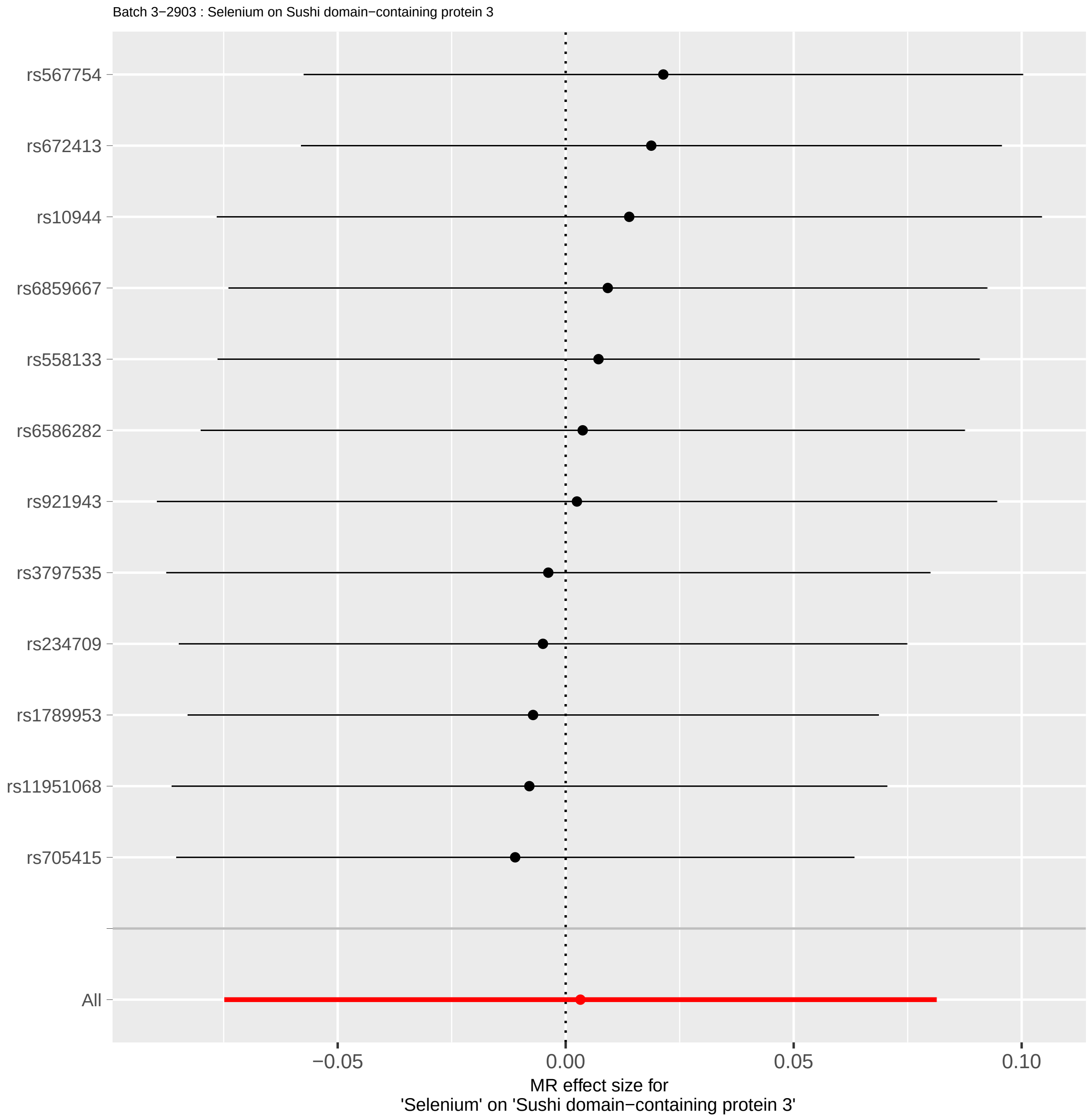
Batch 3−2903 : Selenium on Sushi domain−containing protein 3
rs567754
rs672413
rs10944
rs6859667
rs558133
rs6586282
rs921943
rs3797535
rs234709
rs1789953
rs11951068
rs705415
All
−0.05
0.00
0.05
0.10
MR effect size for
'Selenium' on 'Sushi domain−containing protein 3'

Batch 3−2904 : Selenium on Histone−lysine N−methyltransferase SUV420H2

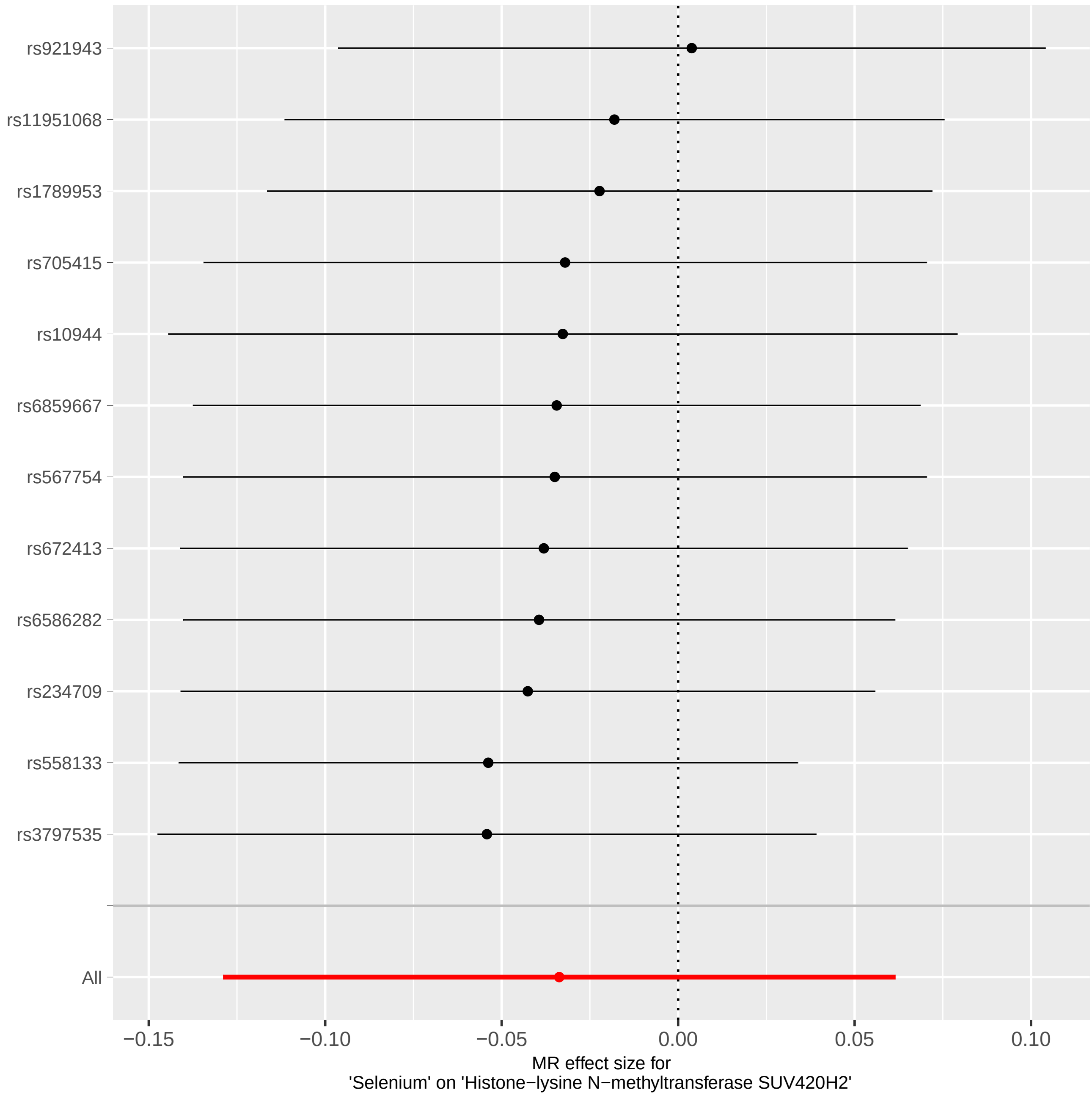

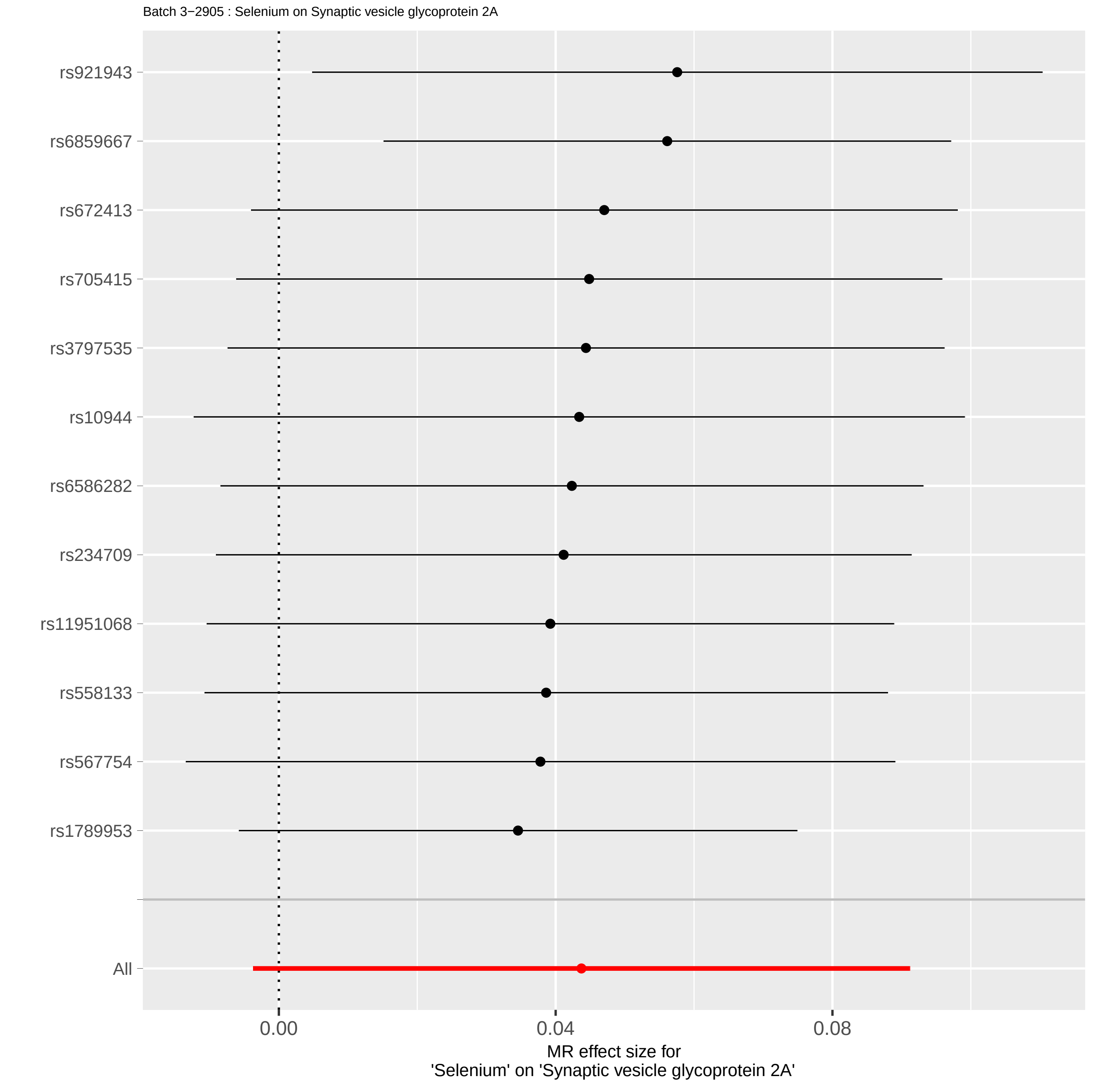
Batch 3−2905 : Selenium on Synaptic vesicle glycoprotein 2A
rs921943
rs6859667
rs672413
rs705415
rs3797535
rs10944
rs6586282
rs234709
rs11951068
rs558133
rs567754
rs1789953
All
0.00
0.04
0.08
MR effect size for
'Selenium' on 'Synaptic vesicle glycoprotein 2A'

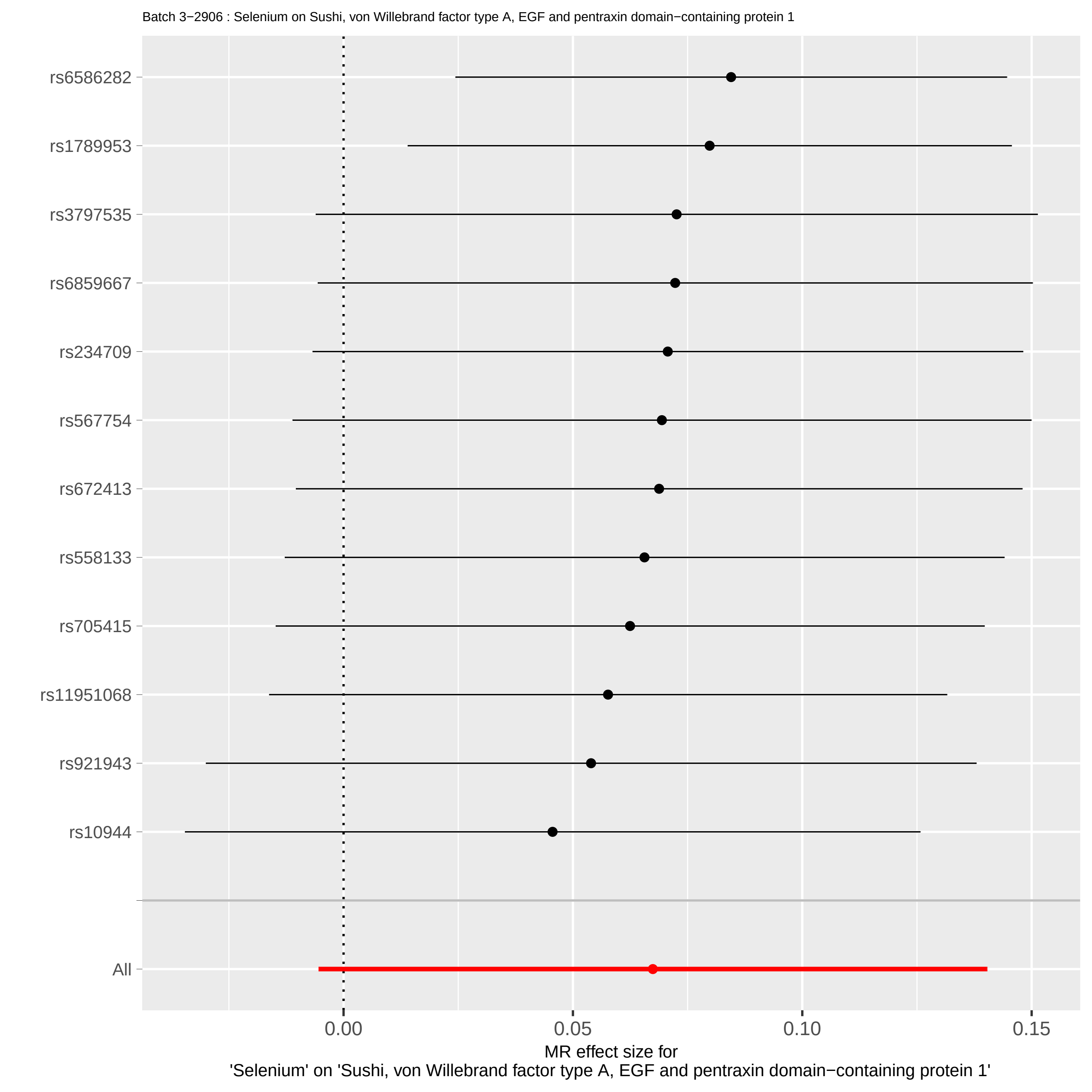
Batch 3−2906 : Selenium on Sushi, von Willebrand factor type A, EGF and pentraxin domain−containing protein 1
rs6586282
rs1789953
rs3797535
rs6859667
rs234709
rs567754
rs672413
rs558133
rs705415
rs11951068
rs921943
rs10944
All
0.00
0.05
0.10
0.15
MR effect size for
'Selenium' on 'Sushi, von Willebrand factor type A, EGF and pentraxin domain−containing protein 1'

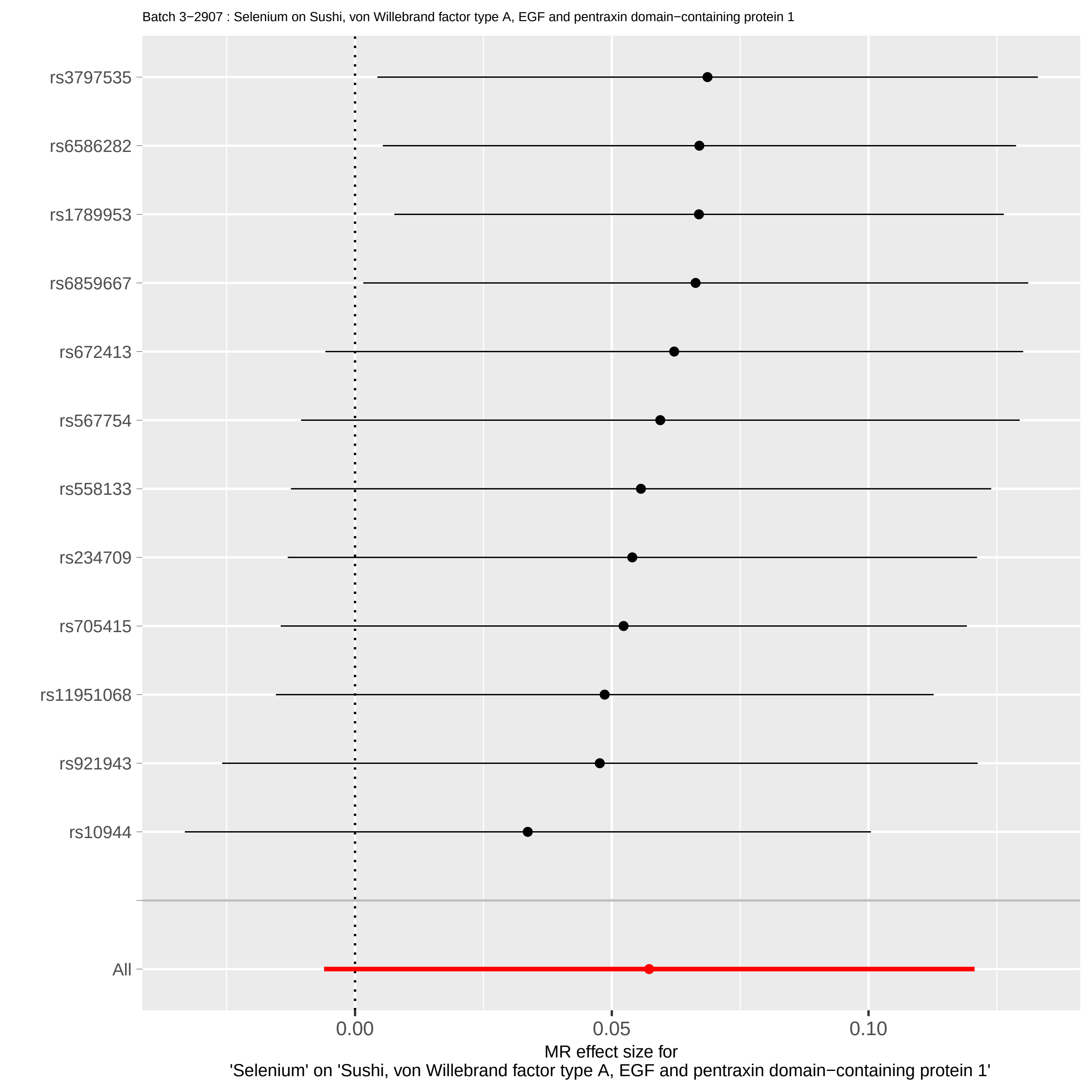
Batch 3−2907 : Selenium on Sushi, von Willebrand factor type A, EGF and pentraxin domain−containing protein 1
rs3797535
rs6586282
rs1789953
rs6859667
rs672413
rs567754
rs558133
rs234709
rs705415
rs11951068
rs921943
rs10944
All
0.00
0.05
0.10
MR effect size for
'Selenium' on 'Sushi, von Willebrand factor type A, EGF and pentraxin domain−containing protein 1'

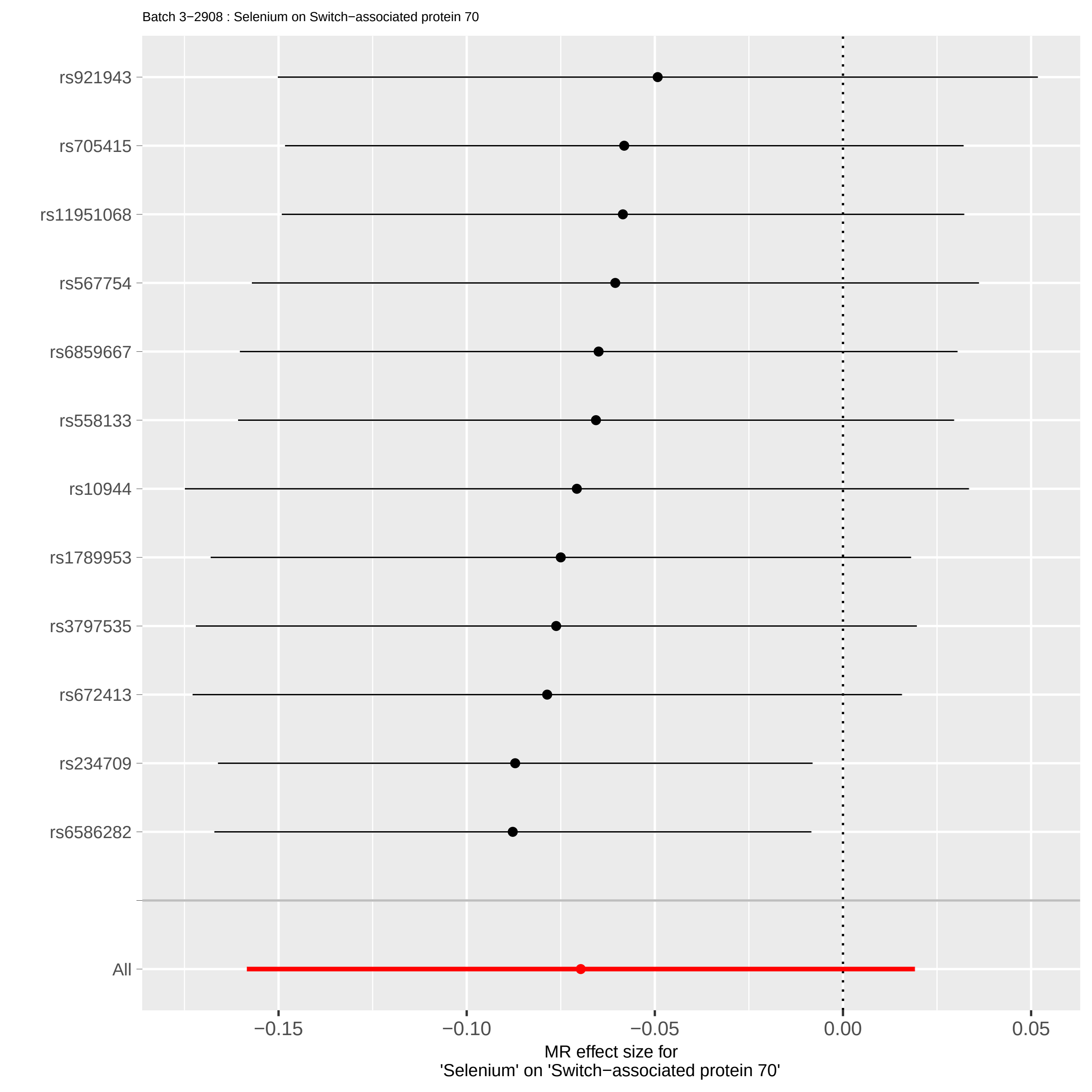

Batch 3−2908 : Selenium on Switch−associated protein 70
rs921943
rs705415
rs11951068
rs567754
rs6859667
rs558133
rs10944
rs1789953
rs3797535
rs672413
rs234709
rs6586282
All
−0.15
−0.10
−0.05
0.00
0.05
MR effect size for
'Selenium' on 'Switch−associated protein 70'

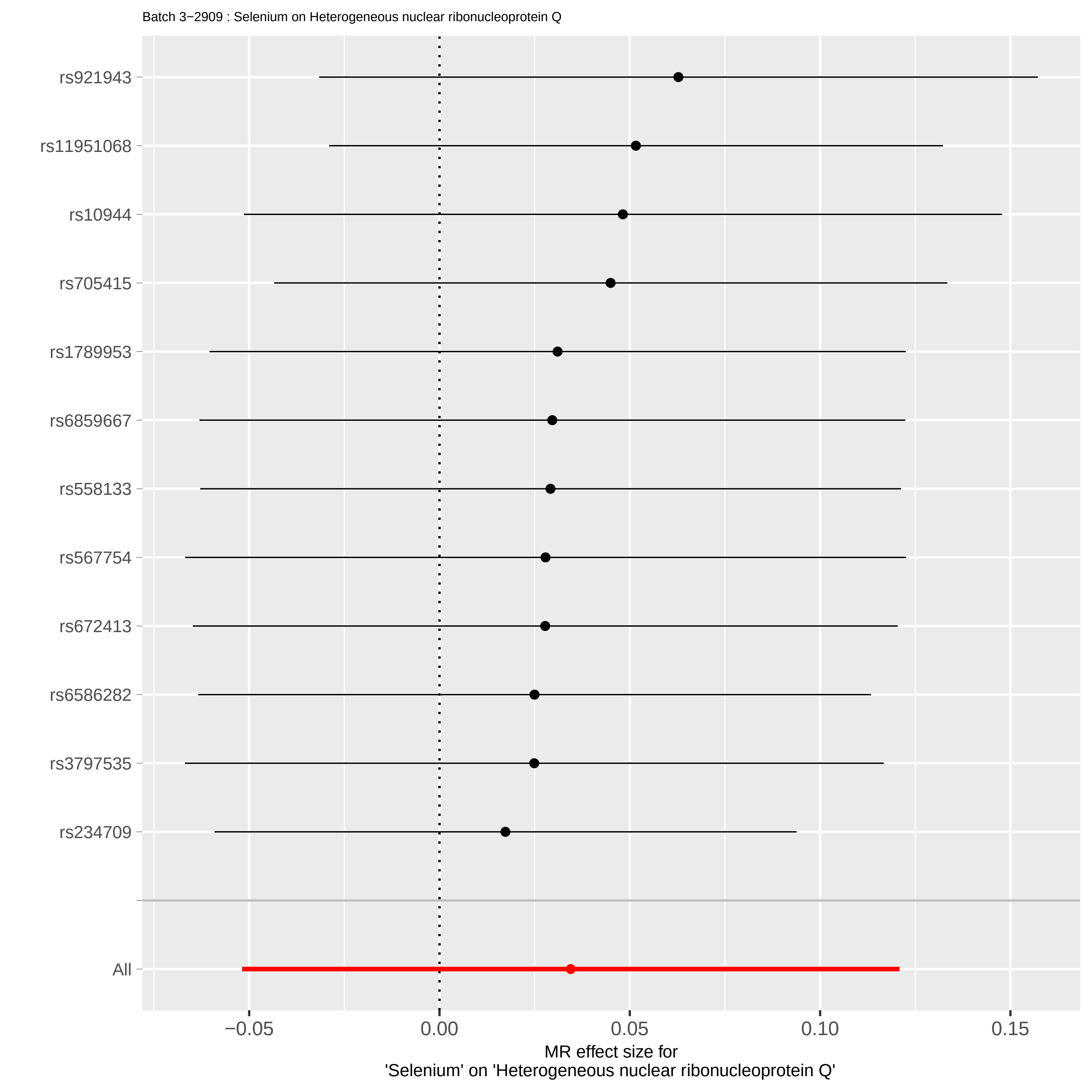

Batch 3−2909 : Selenium on Heterogeneous nuclear ribonucleoprotein Q
rs921943
rs11951068
rs10944
rs705415
rs1789953
rs6859667
rs558133
rs567754
rs672413
rs6586282
rs3797535
rs234709
All
−0.05
0.00
0.05
0.10
0.15
MR effect size for
'Selenium' on 'Heterogeneous nuclear ribonucleoprotein Q'

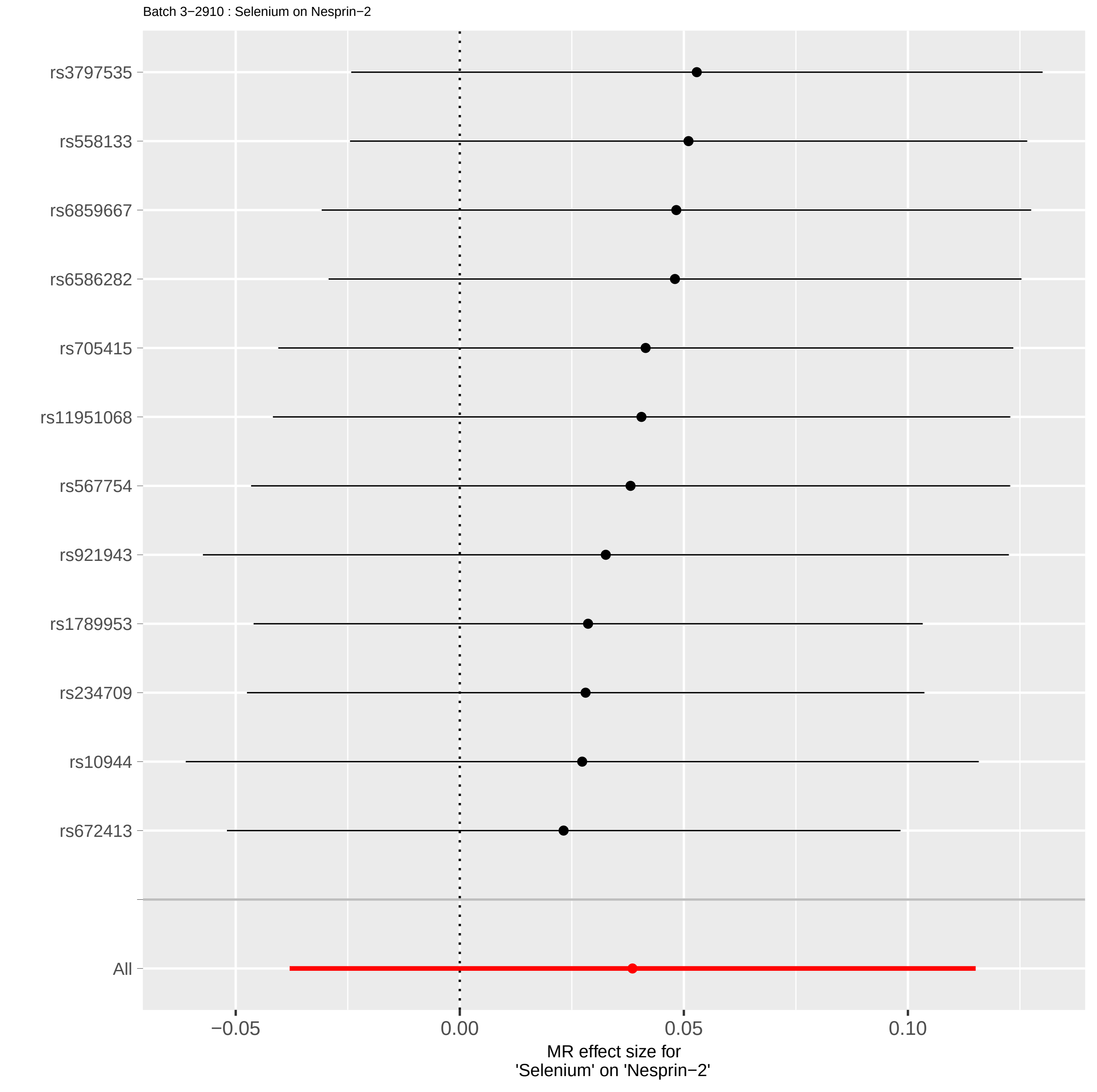

Batch 3−2910 : Selenium on Nesprin−2
rs3797535
rs558133
rs6859667
rs6586282
rs705415
rs11951068
rs567754
rs921943
rs1789953
rs234709
rs10944
rs672413
All
−0.05
0.00
0.05
0.10
MR effect size for
'Selenium' on 'Nesprin−2'

Batch 3−2911 : Selenium on Synaptotagmin−11

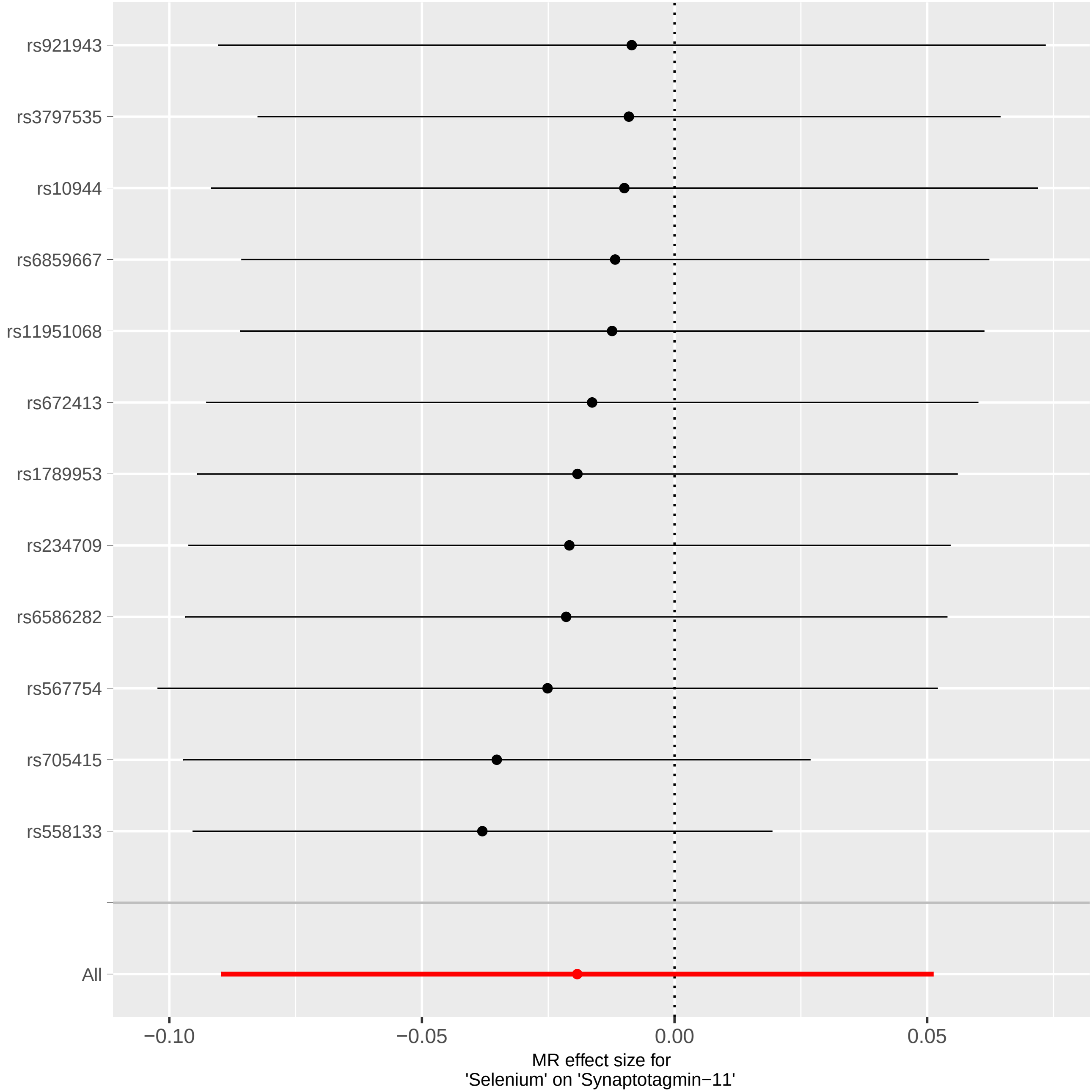

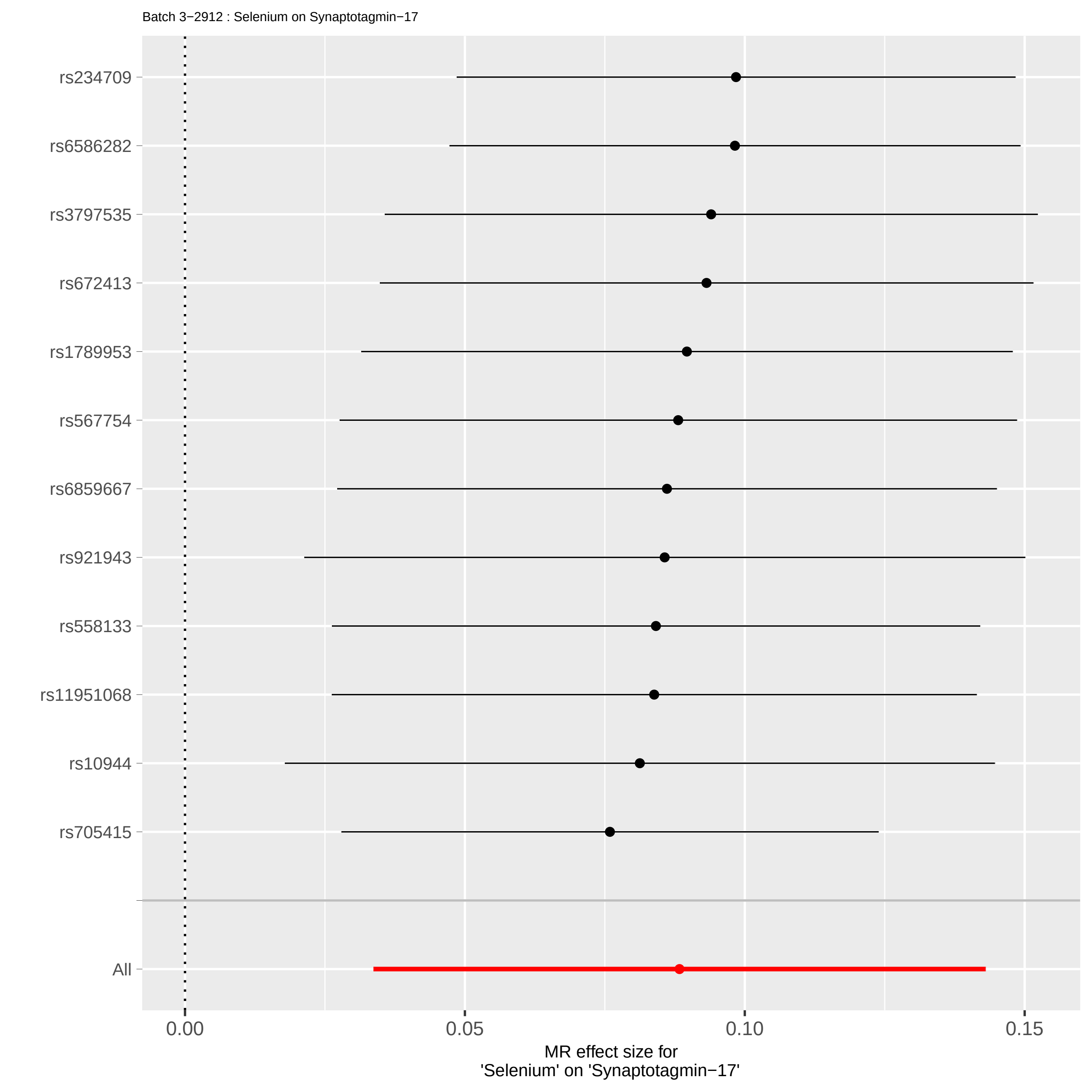

Batch 3−2912 : Selenium on Synaptotagmin−17
rs234709
rs6586282
rs3797535
rs672413
rs1789953
rs567754
rs6859667
rs921943
rs558133
rs11951068
rs10944
rs705415
All
0.00
0.05
0.10
0.15
MR effect size for
'Selenium' on 'Synaptotagmin−17'

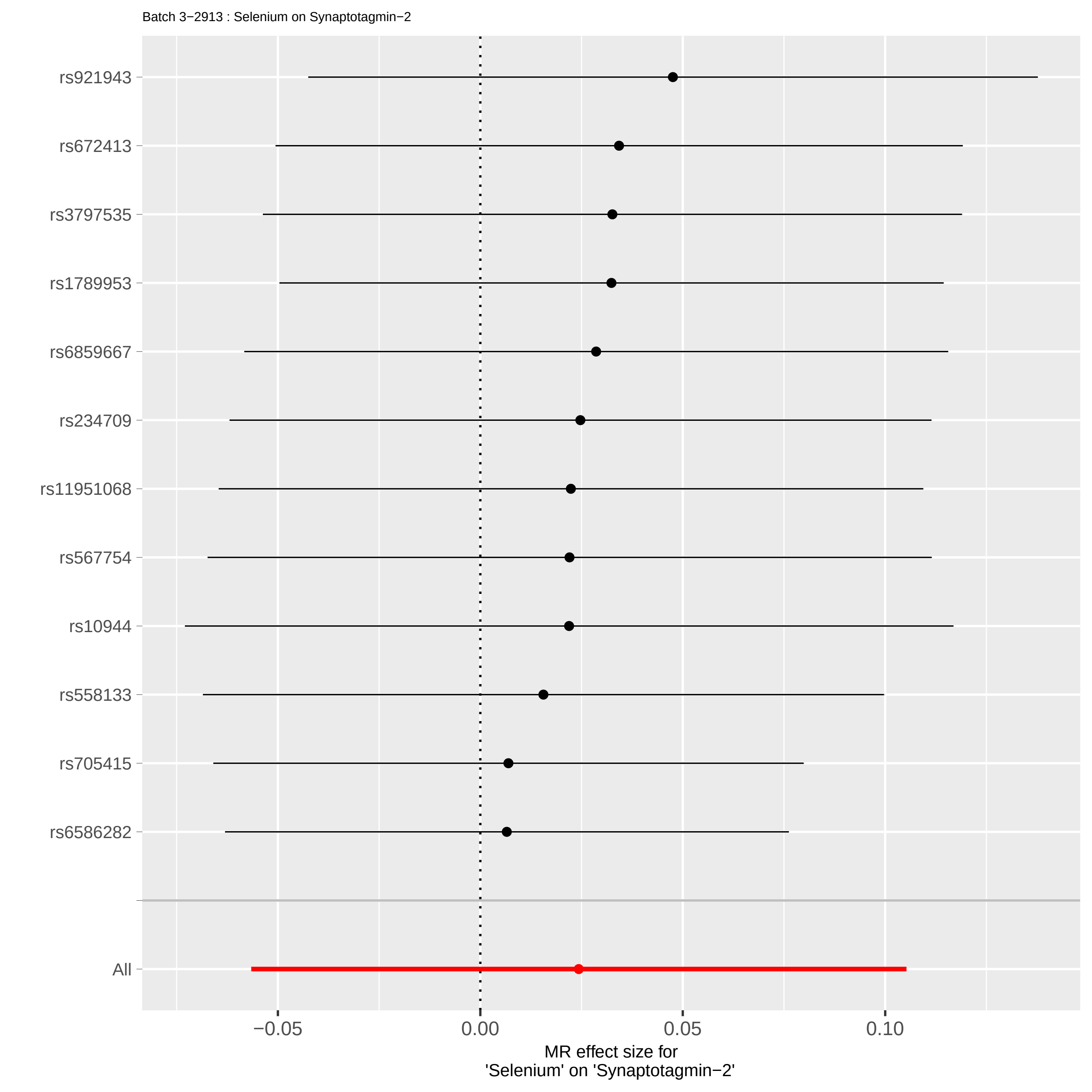

Batch 3−2913 : Selenium on Synaptotagmin−2
rs921943
rs672413
rs3797535
rs1789953
rs6859667
rs234709
rs11951068
rs567754
rs10944
rs558133
rs705415
rs6586282
All
−0.05
0.00
0.05
0.10
MR effect size for
'Selenium' on 'Synaptotagmin−2'

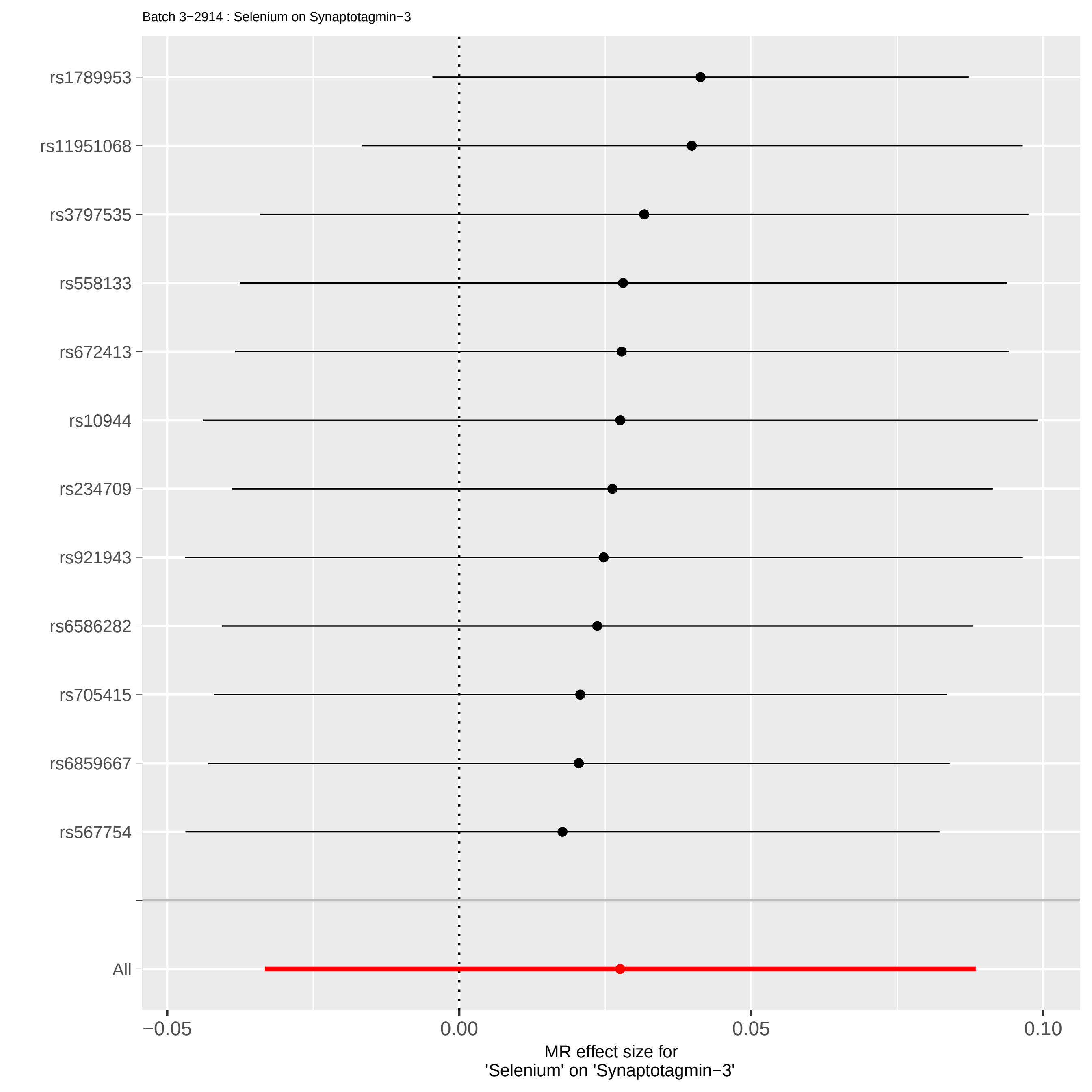

Batch 3−2914 : Selenium on Synaptotagmin−3
rs1789953
rs11951068
rs3797535
rs558133
rs672413
rs10944
rs234709
rs921943
rs6586282
rs705415
rs6859667
rs567754
All
−0.05
0.00
0.05
0.10
MR effect size for
'Selenium' on 'Synaptotagmin−3'

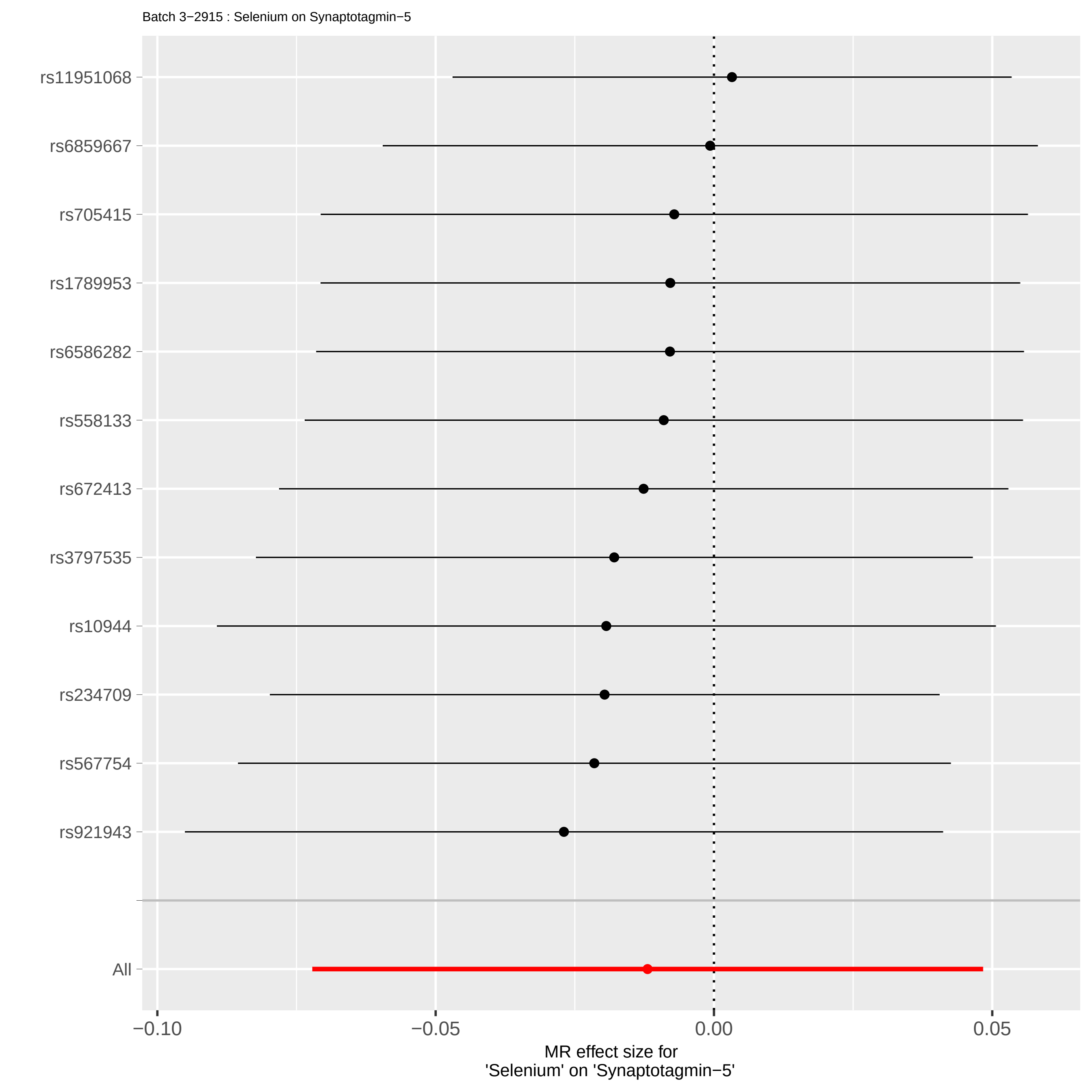
Batch 3−2915 : Selenium on Synaptotagmin−5
rs11951068
rs6859667
rs705415
rs1789953
rs6586282
rs558133
rs672413
rs3797535
rs10944
rs234709
rs567754
rs921943
All
−0.10
−0.05
0.00
0.05
MR effect size for
'Selenium' on 'Synaptotagmin−5'

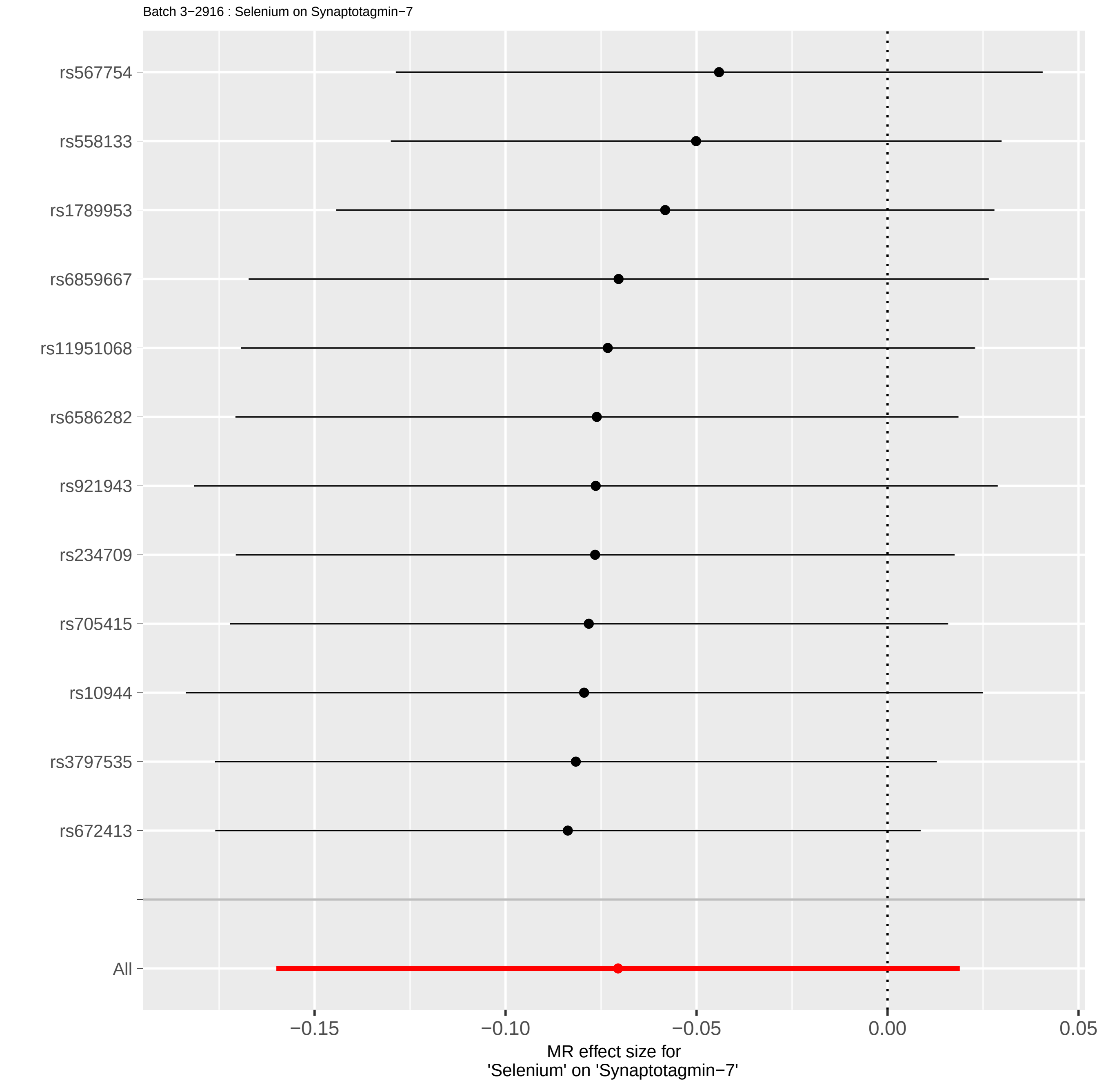

Batch 3−2916 : Selenium on Synaptotagmin−7
rs567754
rs558133
rs1789953
rs6859667
rs11951068
rs6586282
rs921943
rs234709
rs705415
rs10944
rs3797535
rs672413
All
−0.15
−0.10
−0.05
0.00
0.05
MR effect size for
'Selenium' on 'Synaptotagmin−7'

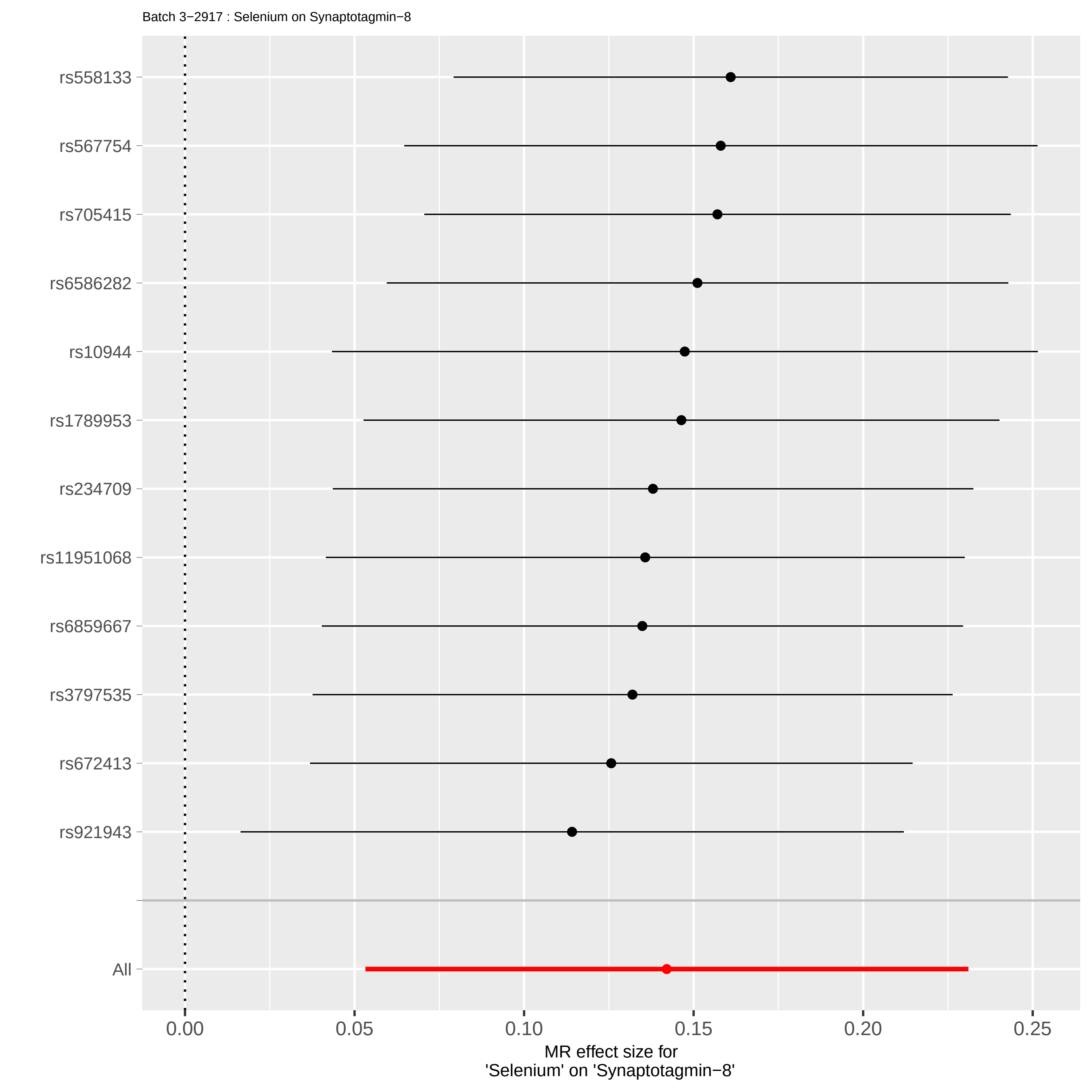

Batch 3−2917 : Selenium on Synaptotagmin−8
rs558133
rs567754
rs705415
rs6586282
rs10944
rs1789953
rs234709
rs11951068
rs6859667
rs3797535
rs672413
rs921943
All
0.00
0.05
0.10
0.15
0.20
0.25
MR effect size for
'Selenium' on 'Synaptotagmin−8'

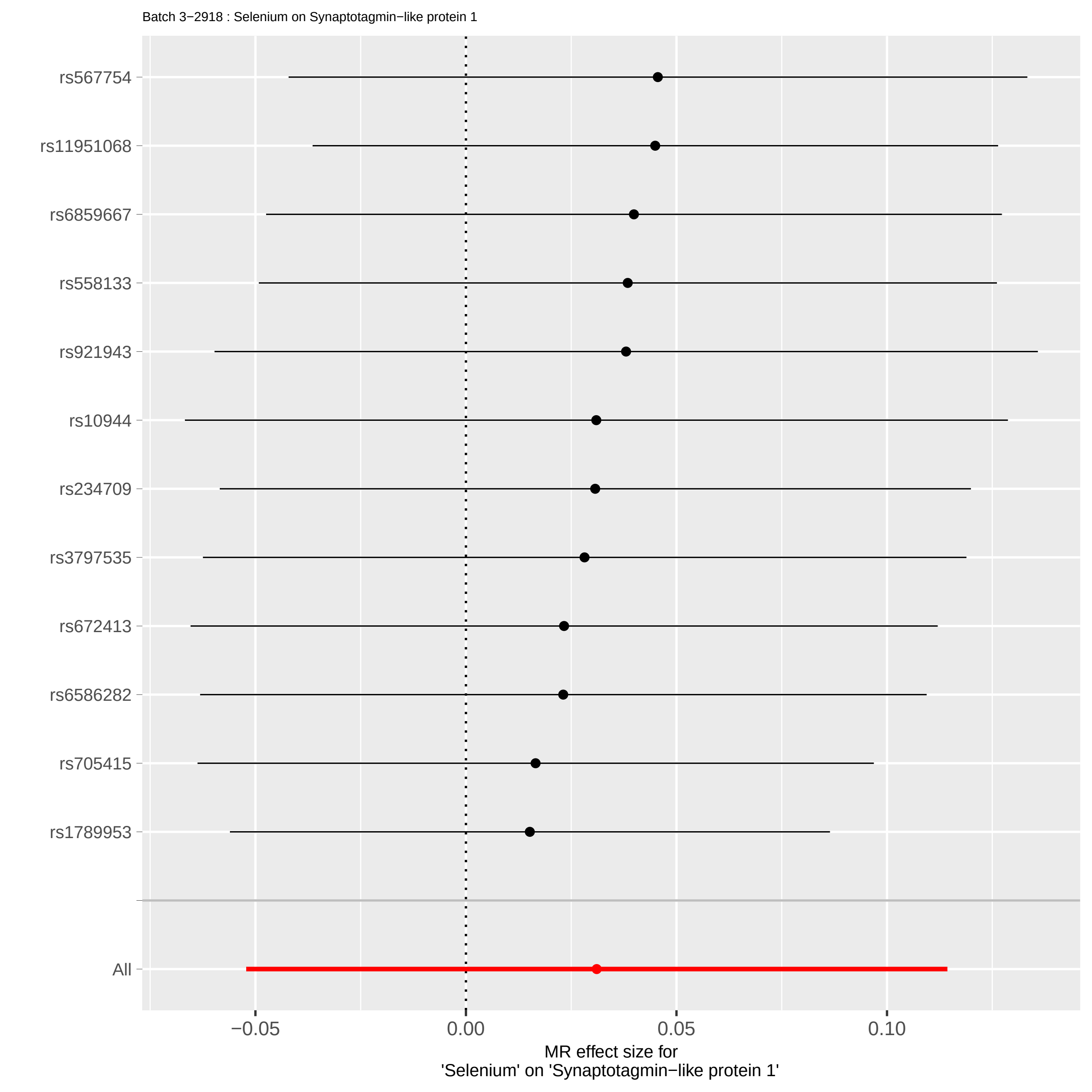
Batch 3−2918 : Selenium on Synaptotagmin−like protein 1
rs567754
rs11951068
rs6859667
rs558133
rs921943
rs10944
rs234709
rs3797535
rs672413
rs6586282
rs705415
rs1789953
All
−0.05
0.00
0.05
0.10
MR effect size for
'Selenium' on 'Synaptotagmin−like protein 1'

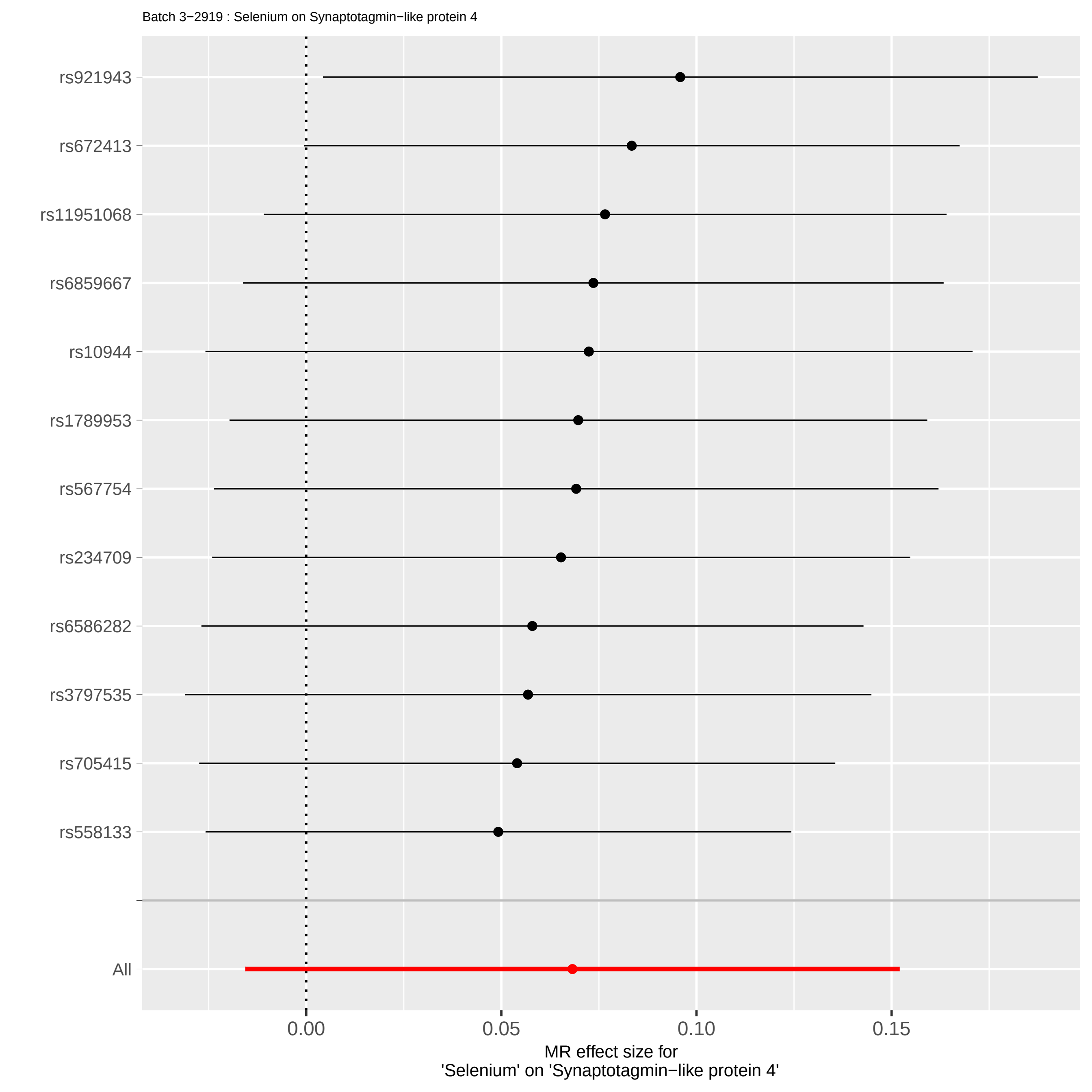

Batch 3−2919 : Selenium on Synaptotagmin−like protein 4
rs921943
rs672413
rs11951068
rs6859667
rs10944
rs1789953
rs567754
rs234709
rs6586282
rs3797535
rs705415
rs558133
All
0.00
0.05
0.10
0.15
MR effect size for
'Selenium' on 'Synaptotagmin−like protein 4'

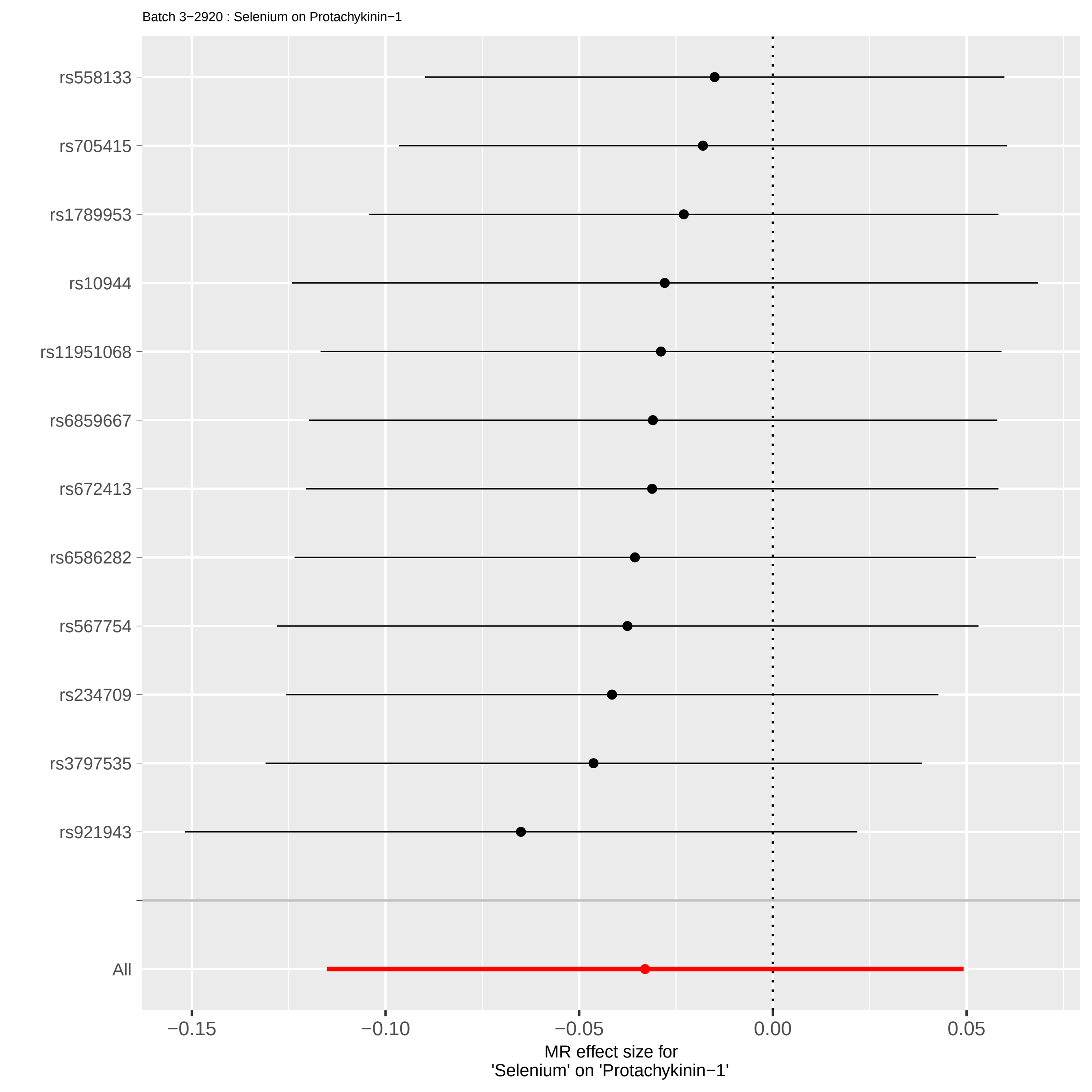

Batch 3−2920 : Selenium on Protachykinin−1
rs558133
rs705415
rs1789953
rs10944
rs11951068
rs6859667
rs672413
rs6586282
rs567754
rs234709
rs3797535
rs921943
All
−0.15
−0.10
−0.05
0.00
0.05
MR effect size for
'Selenium' on 'Protachykinin−1'

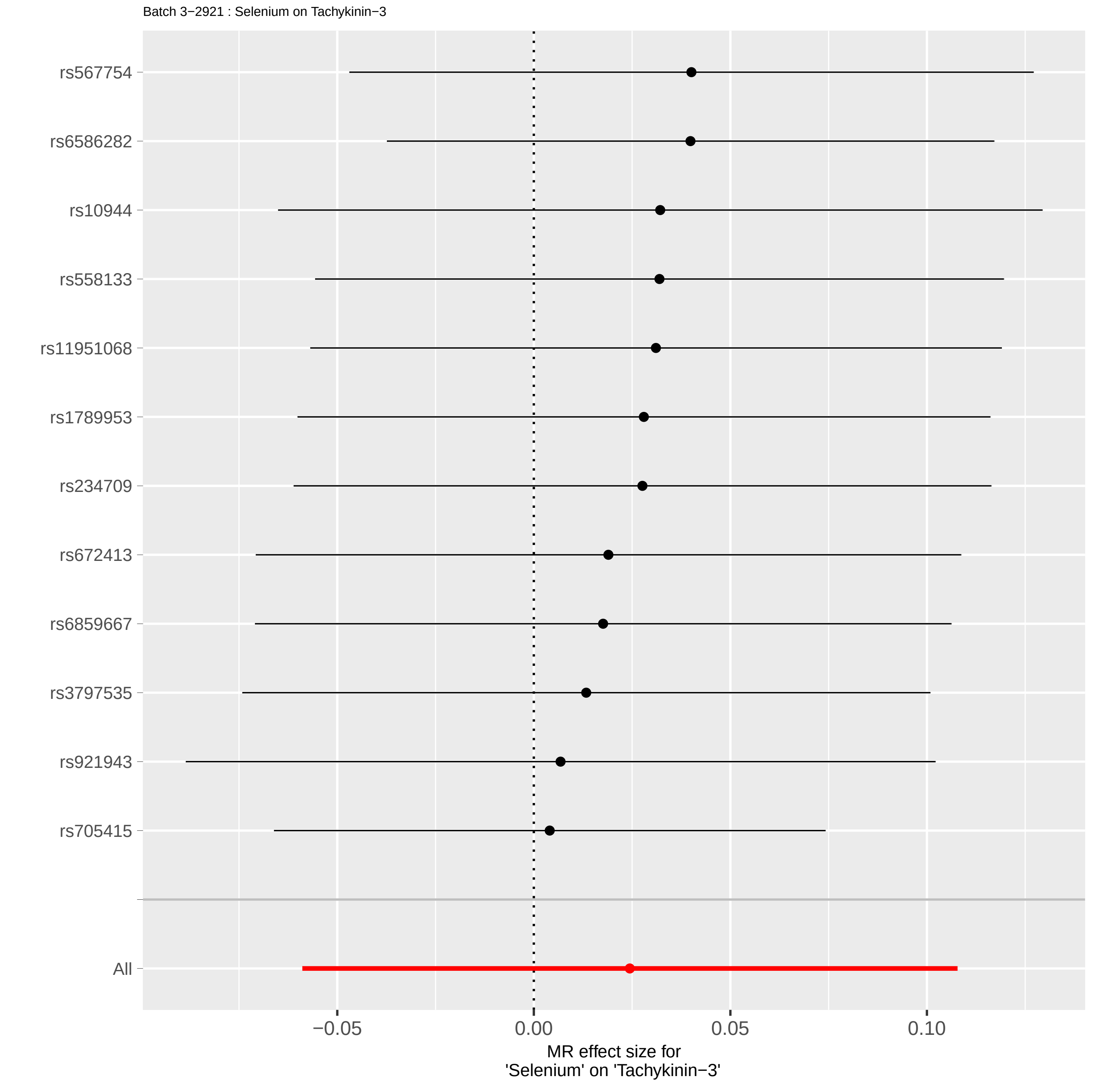

Batch 3−2921 : Selenium on Tachykinin−3
rs567754
rs6586282
rs10944
rs558133
rs11951068
rs1789953
rs234709
rs672413
rs6859667
rs3797535
rs921943
rs705415
All
−0.05
0.00
0.05
0.10
MR effect size for
'Selenium' on 'Tachykinin−3'

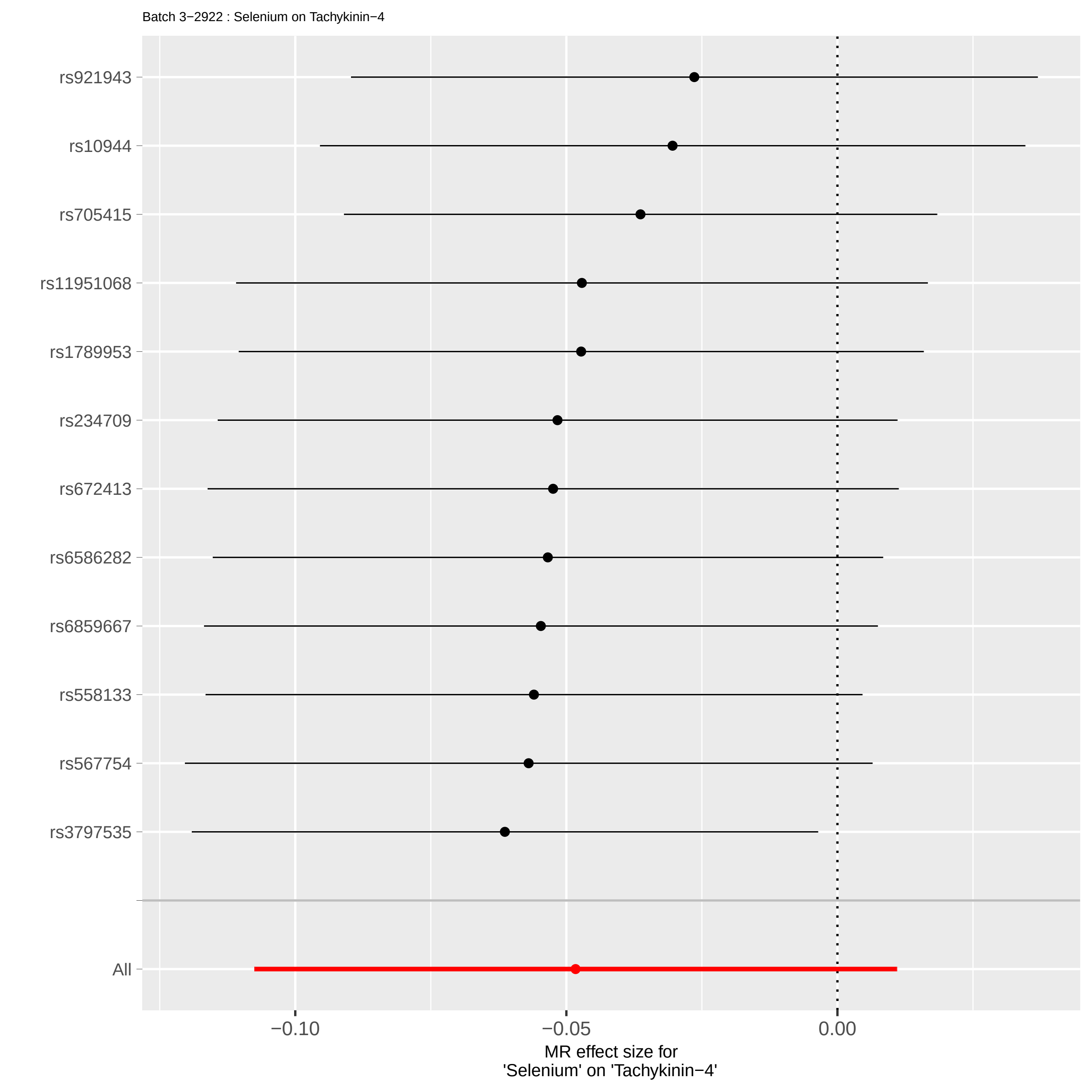

Batch 3−2922 : Selenium on Tachykinin−4
rs921943
rs10944
rs705415
rs11951068
rs1789953
rs234709
rs672413
rs6586282
rs6859667
rs558133
rs567754
rs3797535
All
−0.10
−0.05
0.00
MR effect size for
'Selenium' on 'Tachykinin−4'

Batch 3−2923 : Selenium on Tumor−associated calcium signal transducer 2

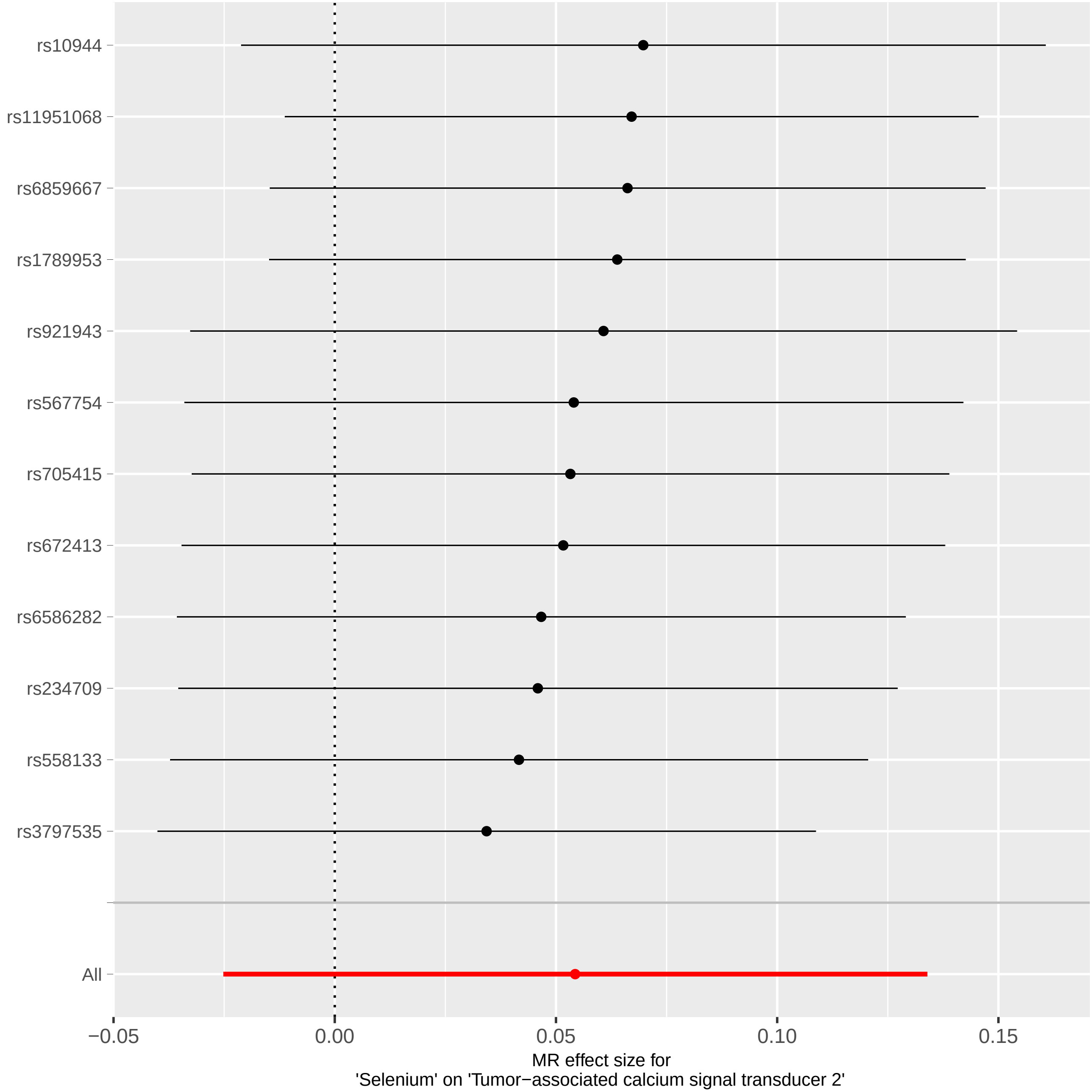

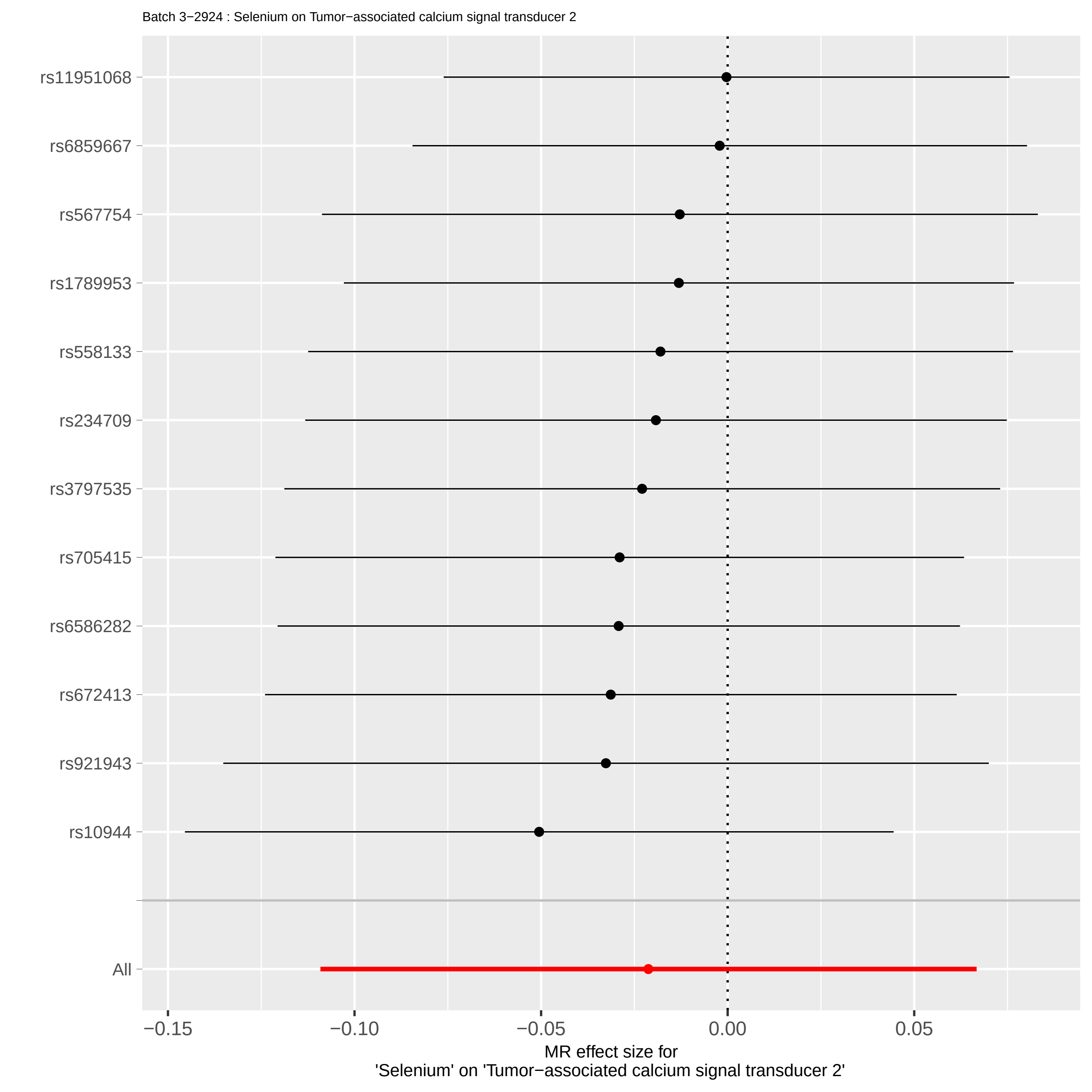

Batch 3−2924 : Selenium on Tumor−associated calcium signal transducer 2
rs11951068
rs6859667
rs567754
rs1789953
rs558133
rs234709
rs3797535
rs705415
rs6586282
rs672413
rs921943
rs10944
All
−0.15
−0.10
−0.05
0.00
0.05
MR effect size for
'Selenium' on 'Tumor−associated calcium signal transducer 2'

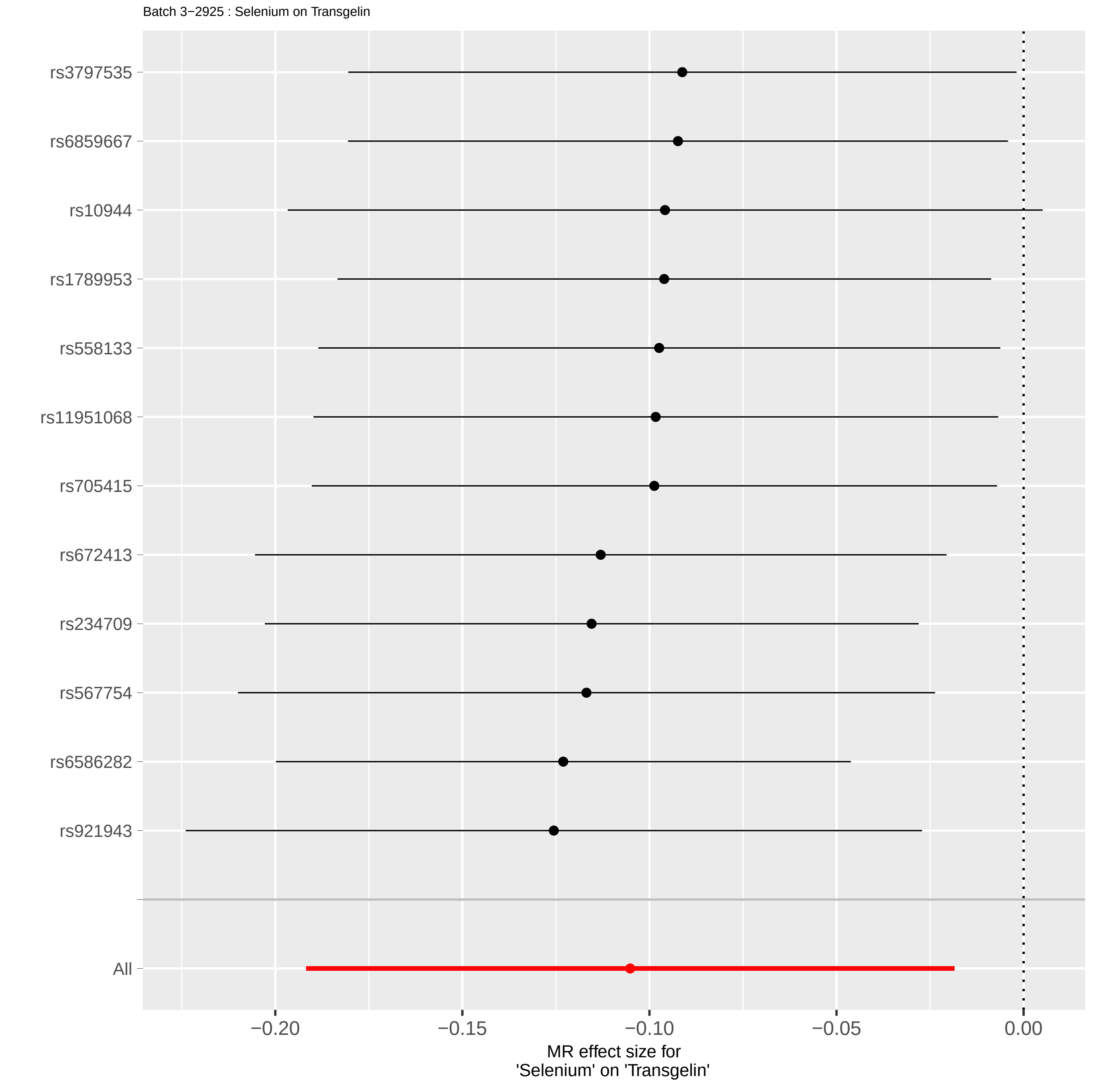

Batch 3−2925 : Selenium on Transgelin
rs3797535
rs6859667
rs10944
rs1789953
rs558133
rs11951068
rs705415
rs672413
rs234709
rs567754
rs6586282
rs921943
All
−0.20
−0.15
−0.10
−0.05
0.00
MR effect size for
'Selenium' on 'Transgelin'

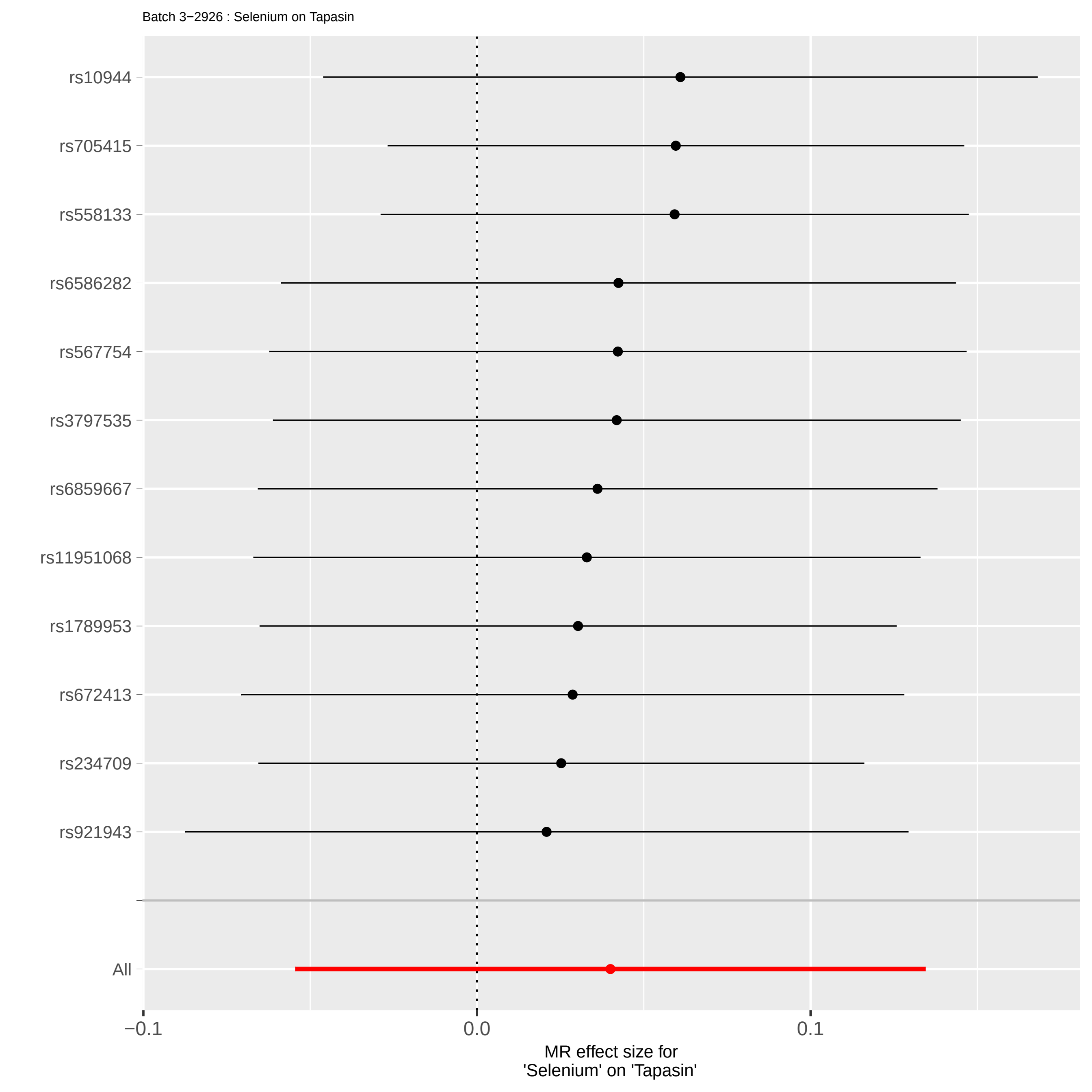
Batch 3−2926 : Selenium on Tapasin
rs10944
rs705415
rs558133
rs6586282
rs567754
rs3797535
rs6859667
rs11951068
rs1789953
rs672413
rs234709
rs921943
All
−0.1
0.0
0.1
MR effect size for
'Selenium' on 'Tapasin'

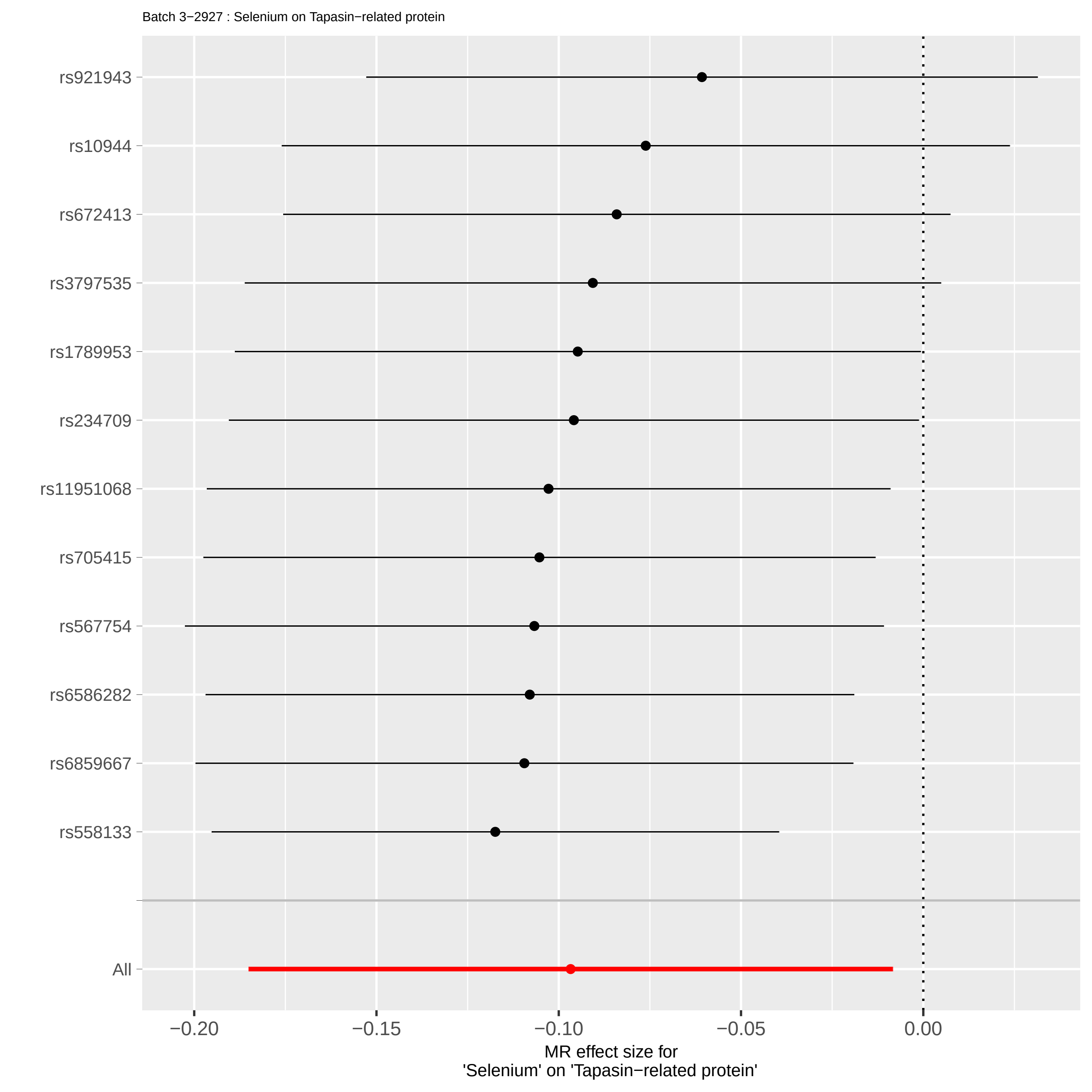

Batch 3−2927 : Selenium on Tapasin−related protein
rs921943
rs10944
rs672413
rs3797535
rs1789953
rs234709
rs11951068
rs705415
rs567754
rs6586282
rs6859667
rs558133
All
−0.20
−0.15
−0.10
−0.05
0.00
MR effect size for
'Selenium' on 'Tapasin−related protein'

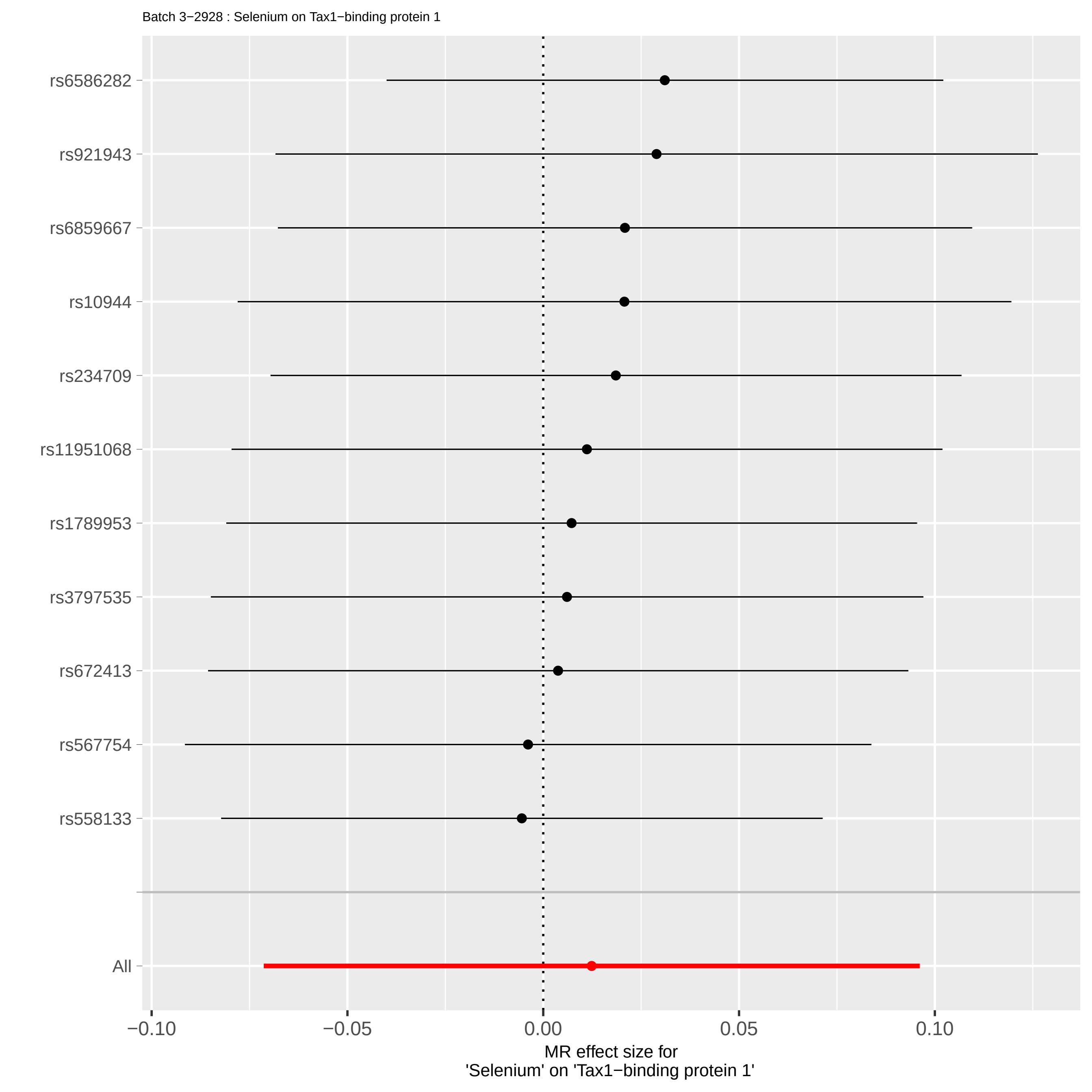

Batch 3−2928 : Selenium on Tax1−binding protein 1
rs6586282
rs921943
rs6859667
rs10944
rs234709
rs11951068
rs1789953
rs3797535
rs672413
rs567754
rs558133
All
−0.10
−0.05
0.00
0.05
0.10
MR effect size for
'Selenium' on 'Tax1−binding protein 1'

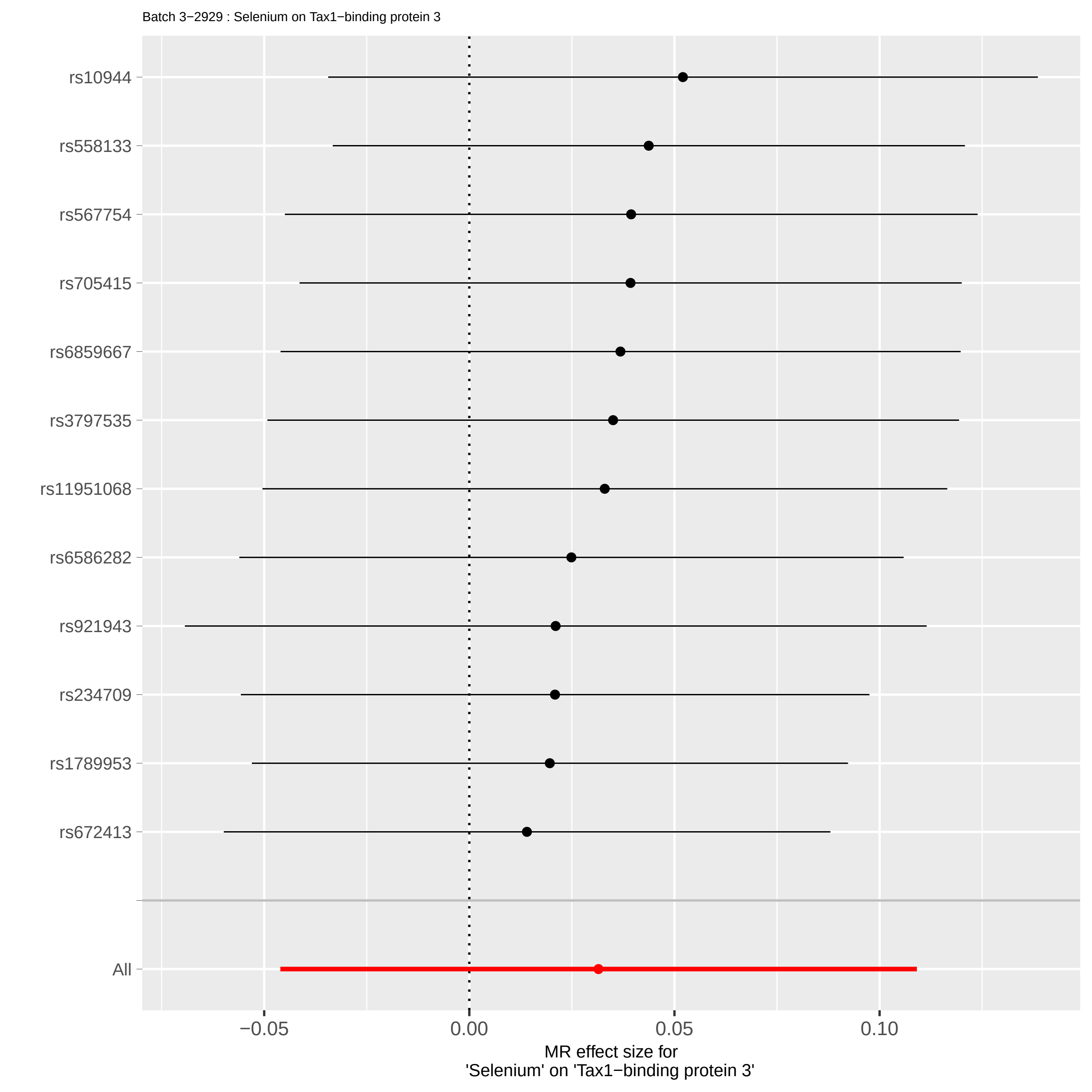

Batch 3−2929 : Selenium on Tax1−binding protein 3
rs10944
rs558133
rs567754
rs705415
rs6859667
rs3797535
rs11951068
rs6586282
rs921943
rs234709
rs1789953
rs672413
All
−0.05
0.00
0.05
0.10
MR effect size for
'Selenium' on 'Tax1−binding protein 3'

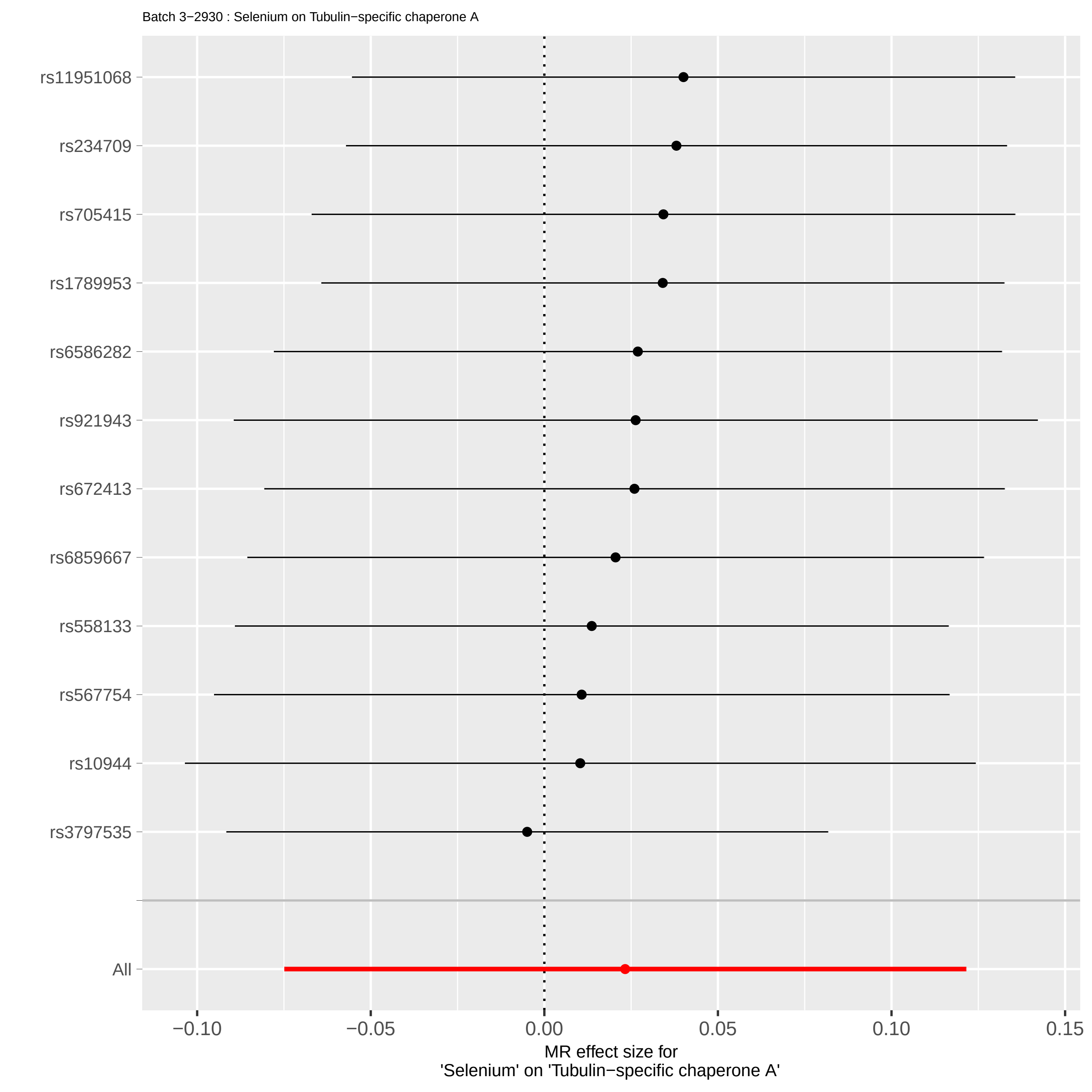

Batch 3−2930 : Selenium on Tubulin−specific chaperone A
rs11951068
rs234709
rs705415
rs1789953
rs6586282
rs921943
rs672413
rs6859667
rs558133
rs567754
rs10944
rs3797535
All
−0.10
−0.05
0.00
0.05
0.10
0.15
MR effect size for
'Selenium' on 'Tubulin−specific chaperone A'

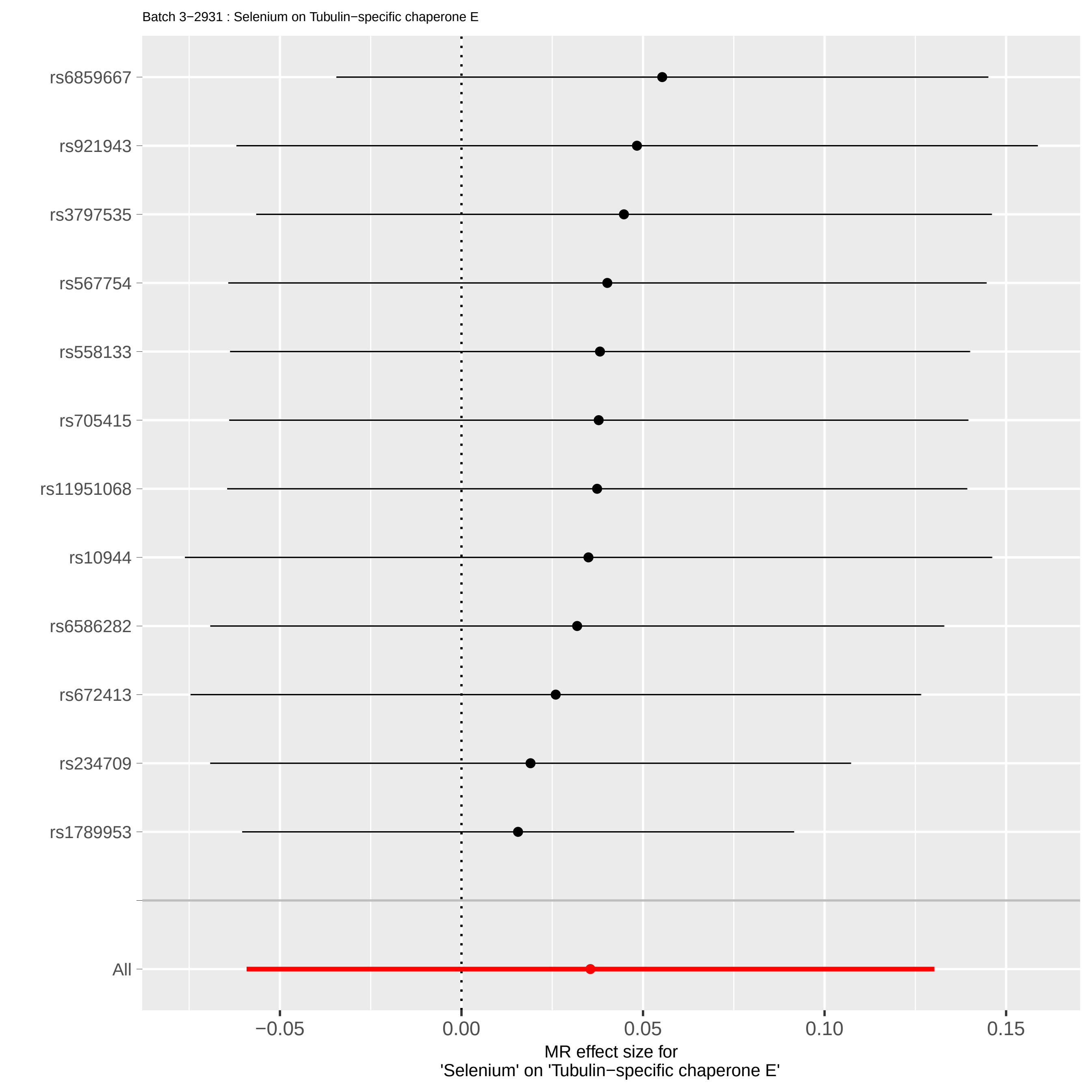

Batch 3−2931 : Selenium on Tubulin−specific chaperone E
rs6859667
rs921943
rs3797535
rs567754
rs558133
rs705415
rs11951068
rs10944
rs6586282
rs672413
rs234709
rs1789953
All
−0.05
0.00
0.05
0.10
0.15
MR effect size for
'Selenium' on 'Tubulin−specific chaperone E'

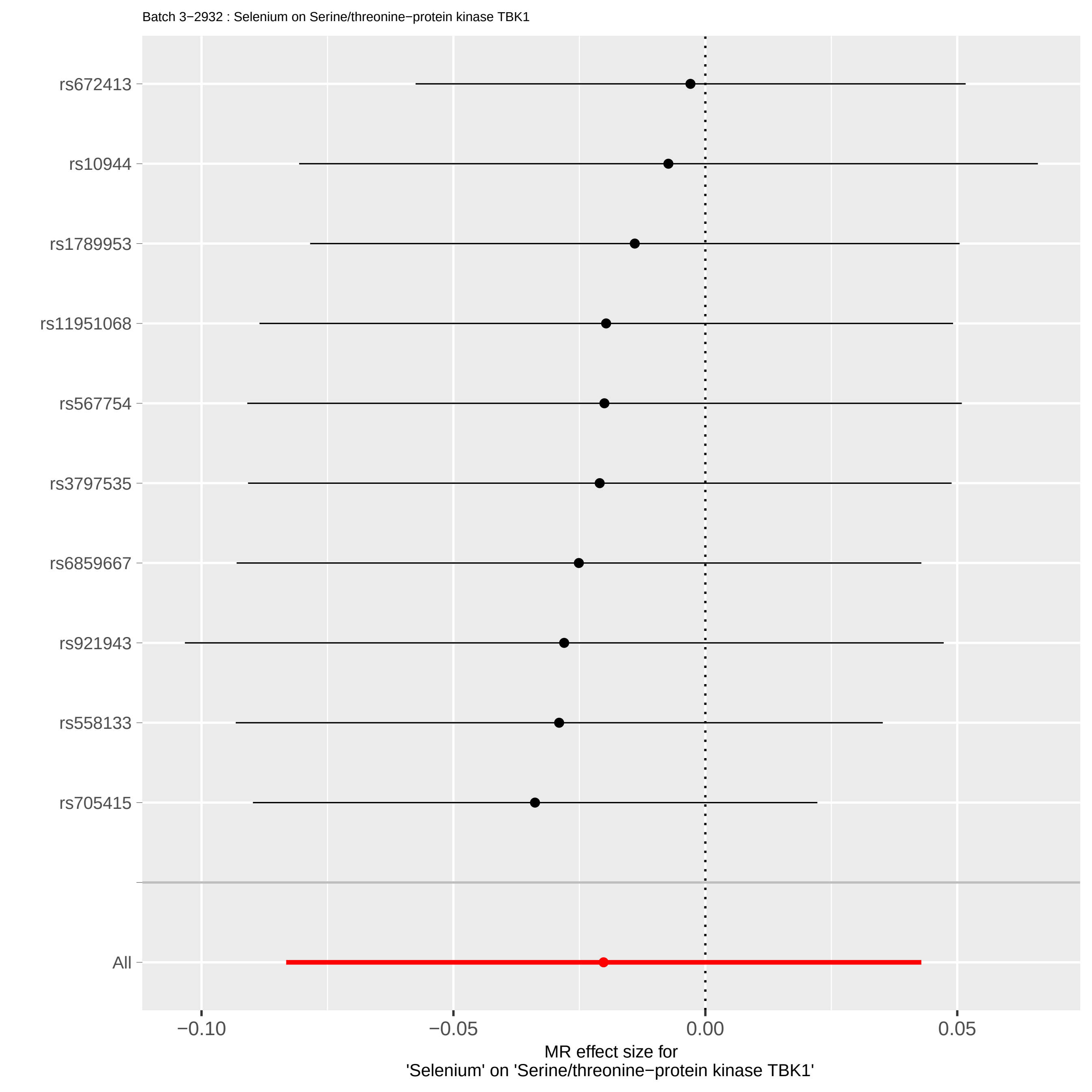

Batch 3−2932 : Selenium on Serine/threonine−protein kinase TBK1
rs672413
rs10944
rs1789953
rs11951068
rs567754
rs3797535
rs6859667
rs921943
rs558133
rs705415
All
−0.10
−0.05
0.00
0.05
MR effect size for
'Selenium' on 'Serine/threonine−protein kinase TBK1'

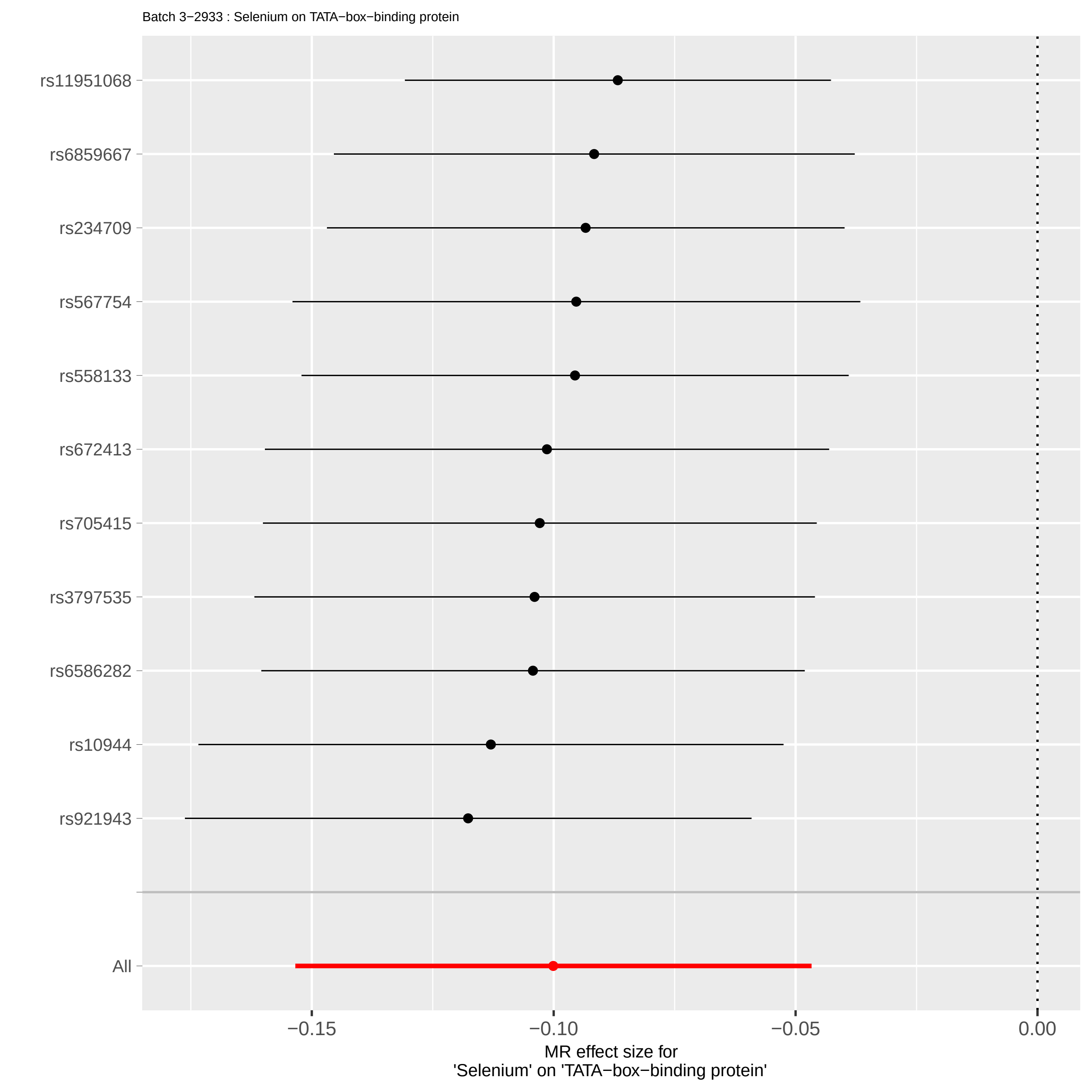

Batch 3−2933 : Selenium on TATA−box−binding protein
rs11951068
rs6859667
rs234709
rs567754
rs558133
rs672413
rs705415
rs3797535
rs6586282
rs10944
rs921943
All
−0.15
−0.10
−0.05
0.00
MR effect size for
'Selenium' on 'TATA−box−binding protein'

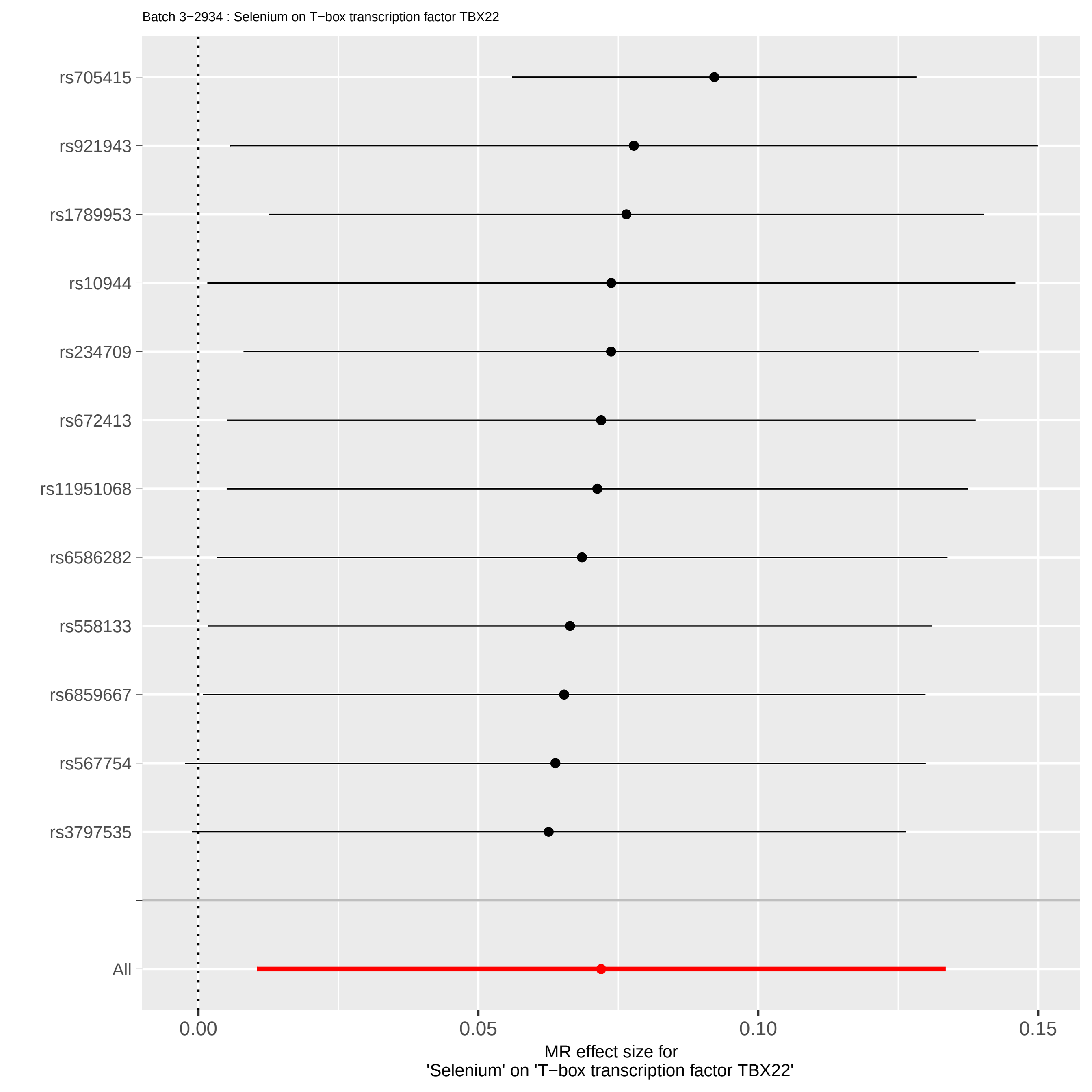

Batch 3−2934 : Selenium on T−box transcription factor TBX22
rs705415
rs921943
rs1789953
rs10944
rs234709
rs672413
rs11951068
rs6586282
rs558133
rs6859667
rs567754
rs3797535
All
0.00
0.05
0.10
0.15
MR effect size for
'Selenium' on 'T−box transcription factor TBX22'

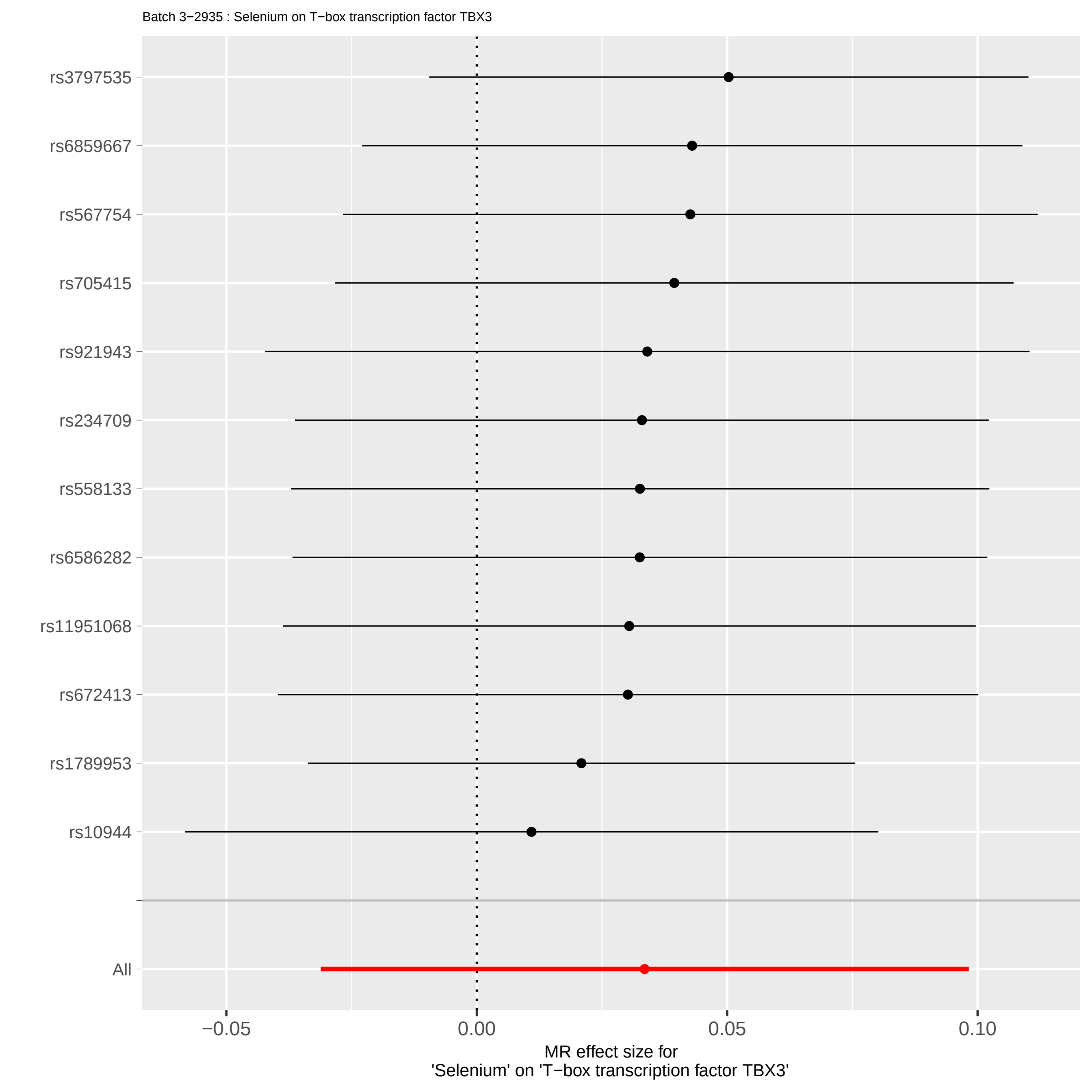

Batch 3−2935 : Selenium on T−box transcription factor TBX3
rs3797535
rs6859667
rs567754
rs705415
rs921943
rs234709
rs558133
rs6586282
rs11951068
rs672413
rs1789953
rs10944
All
−0.05
0.00
0.05
0.10
MR effect size for
'Selenium' on 'T−box transcription factor TBX3'

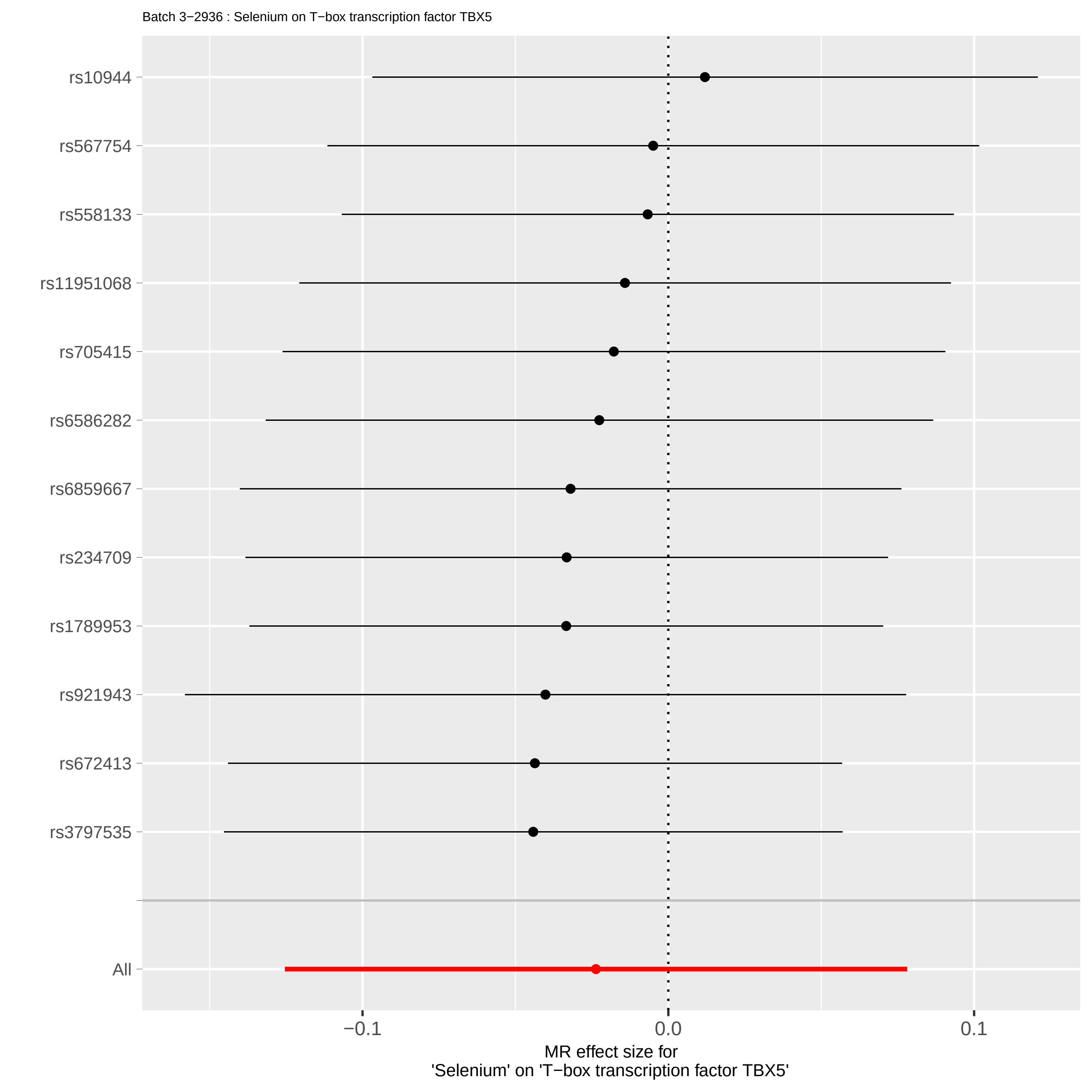
Batch 3−2936 : Selenium on T−box transcription factor TBX5
rs10944
rs567754
rs558133
rs11951068
rs705415
rs6586282
rs6859667
rs234709
rs1789953
rs921943
rs672413
rs3797535
All
−0.1
0.0
0.1
MR effect size for
'Selenium' on 'T−box transcription factor TBX5'

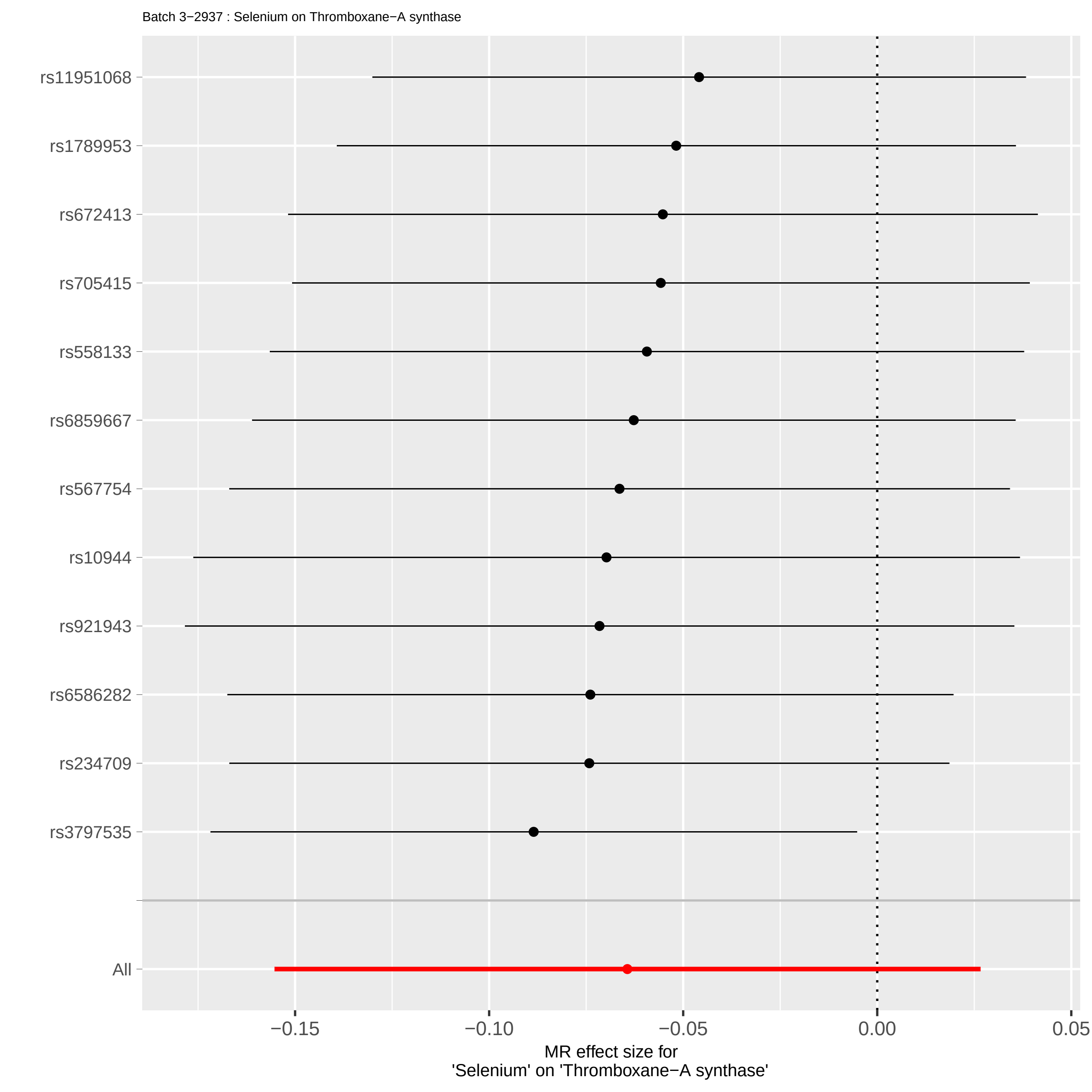
Batch 3−2937 : Selenium on Thromboxane−A synthase
rs11951068
rs1789953
rs672413
rs705415
rs558133
rs6859667
rs567754
rs10944
rs921943
rs6586282
rs234709
rs3797535
All
−0.15
−0.10
−0.05
0.00
0.05
MR effect size for
'Selenium' on 'Thromboxane−A synthase'

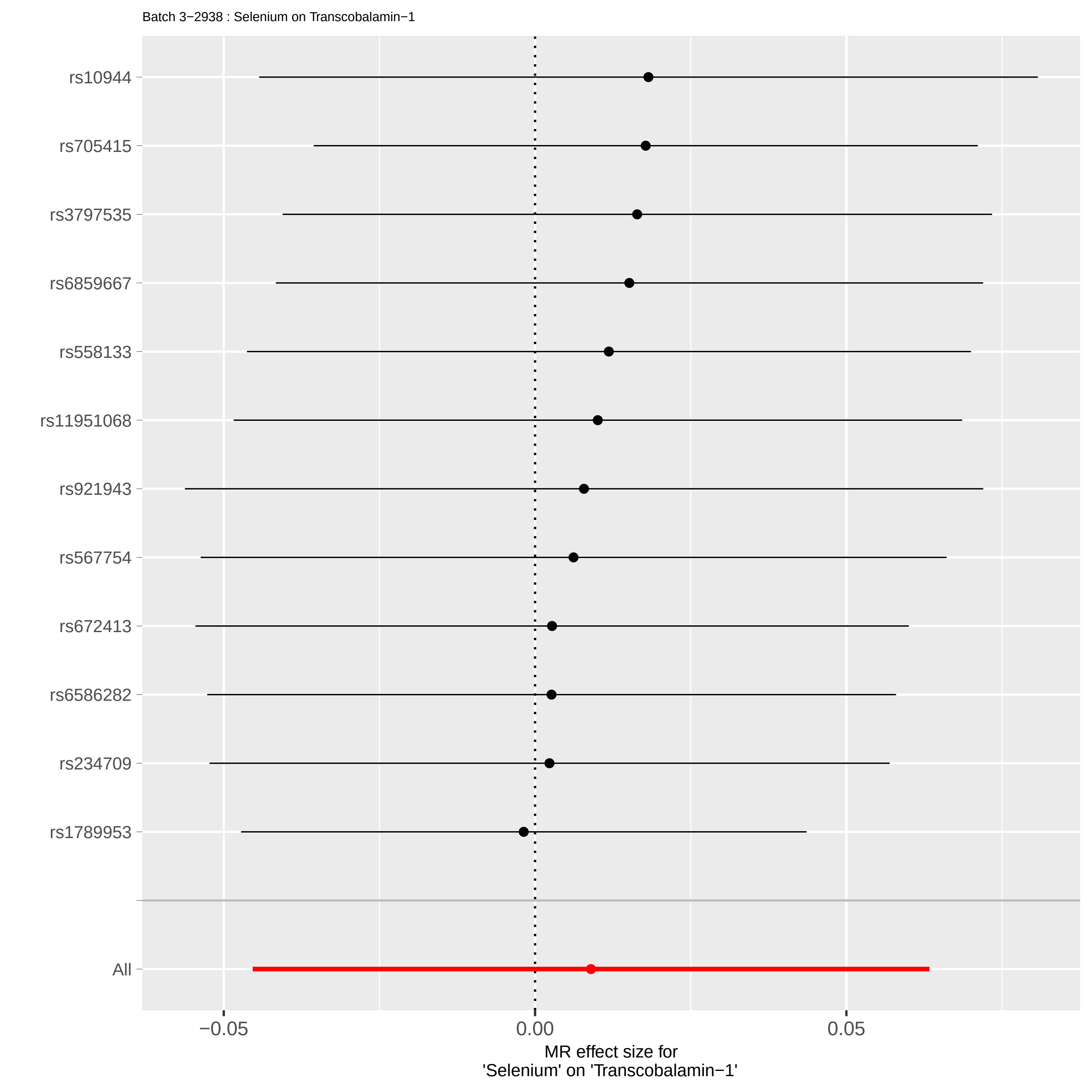
Batch 3−2938 : Selenium on Transcobalamin−1
rs10944
rs705415
rs3797535
rs6859667
rs558133
rs11951068
rs921943
rs567754
rs672413
rs6586282
rs234709
rs1789953
All
−0.05
0.00
0.05
MR effect size for
'Selenium' on 'Transcobalamin−1'

Batch 3−2939 : Selenium on Holo−Transcobalamin−2

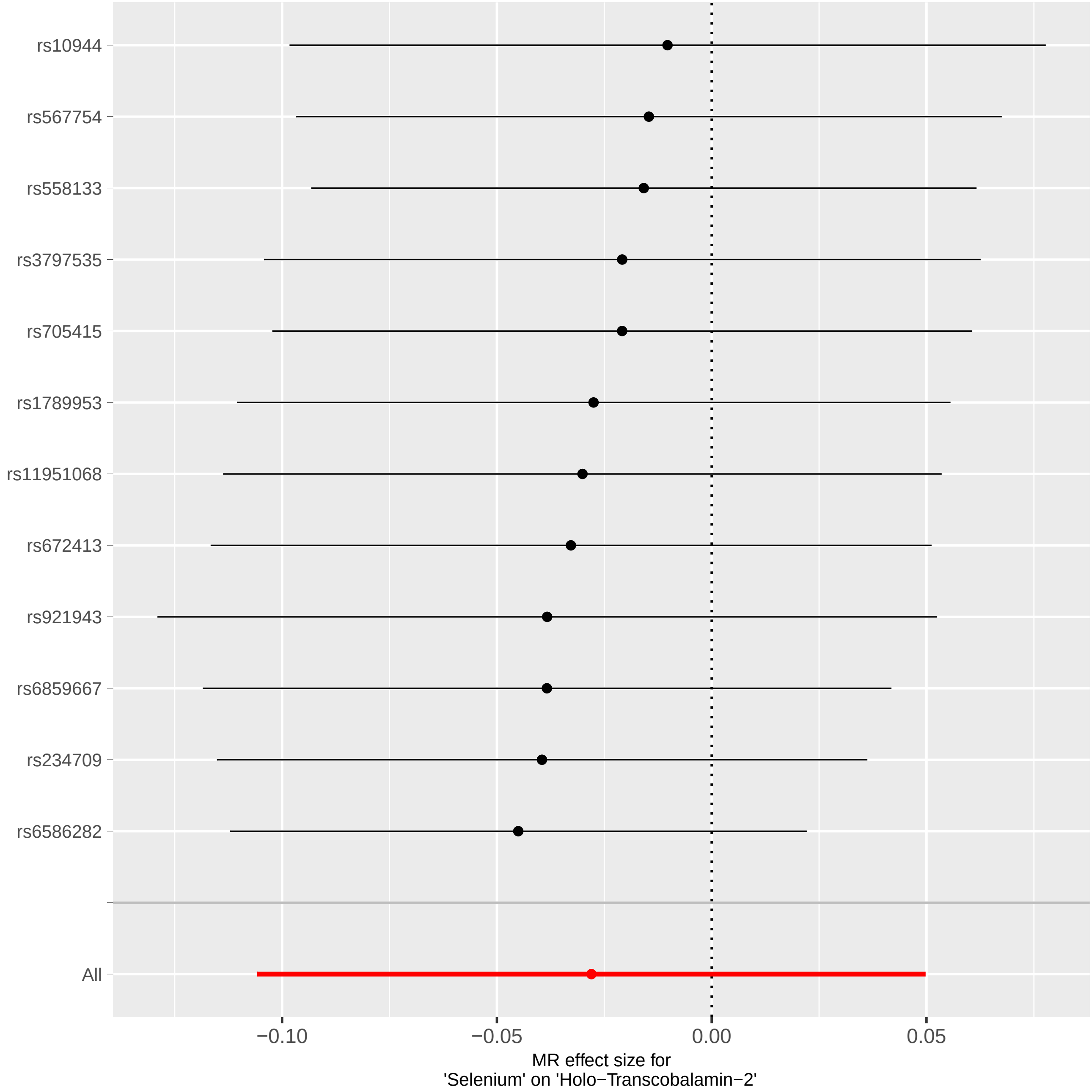

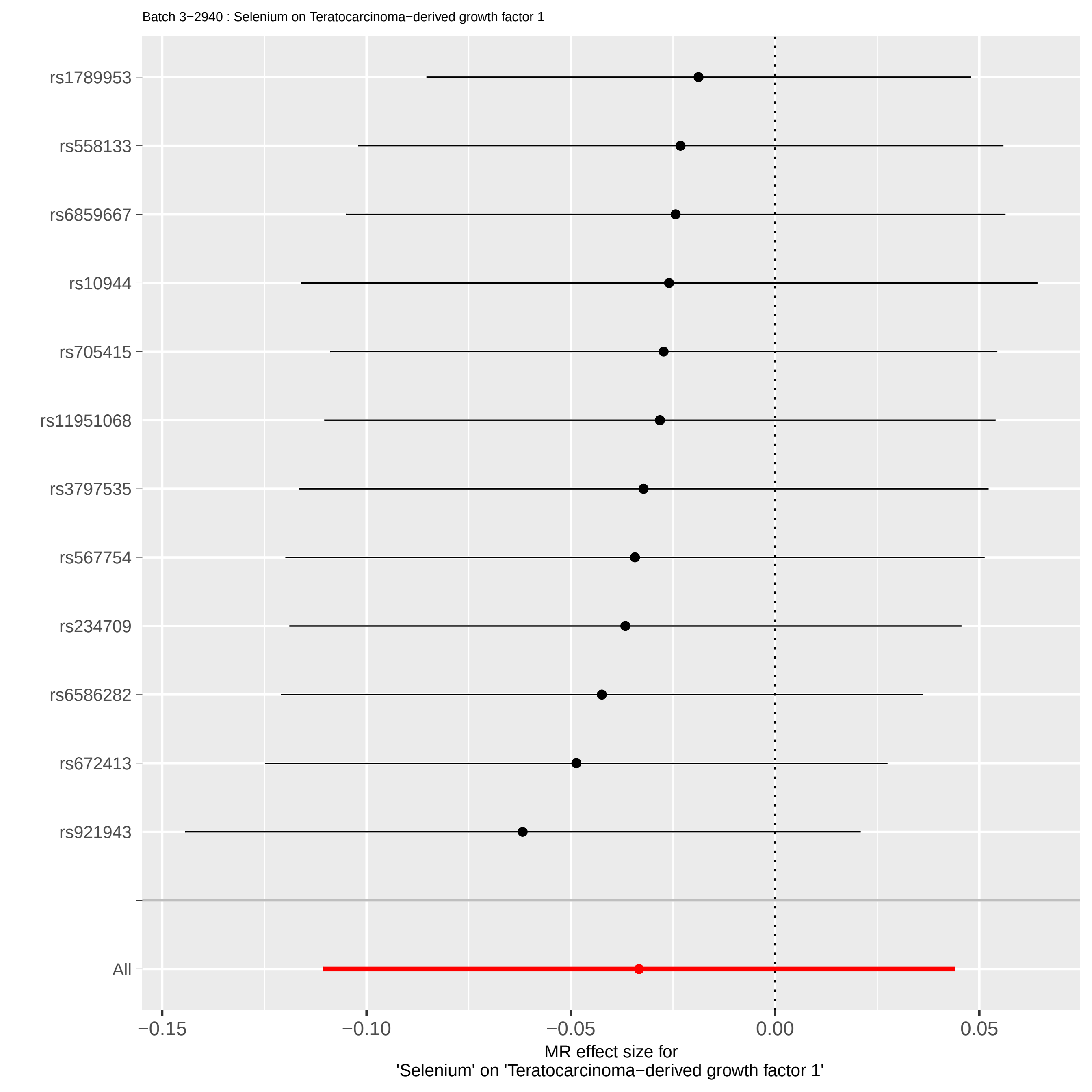
Batch 3−2940 : Selenium on Teratocarcinoma−derived growth factor 1
rs1789953
rs558133
rs6859667
rs10944
rs705415
rs11951068
rs3797535
rs567754
rs234709
rs6586282
rs672413
rs921943
All
−0.15
−0.10
−0.05
0.00
0.05
MR effect size for
'Selenium' on 'Teratocarcinoma−derived growth factor 1'

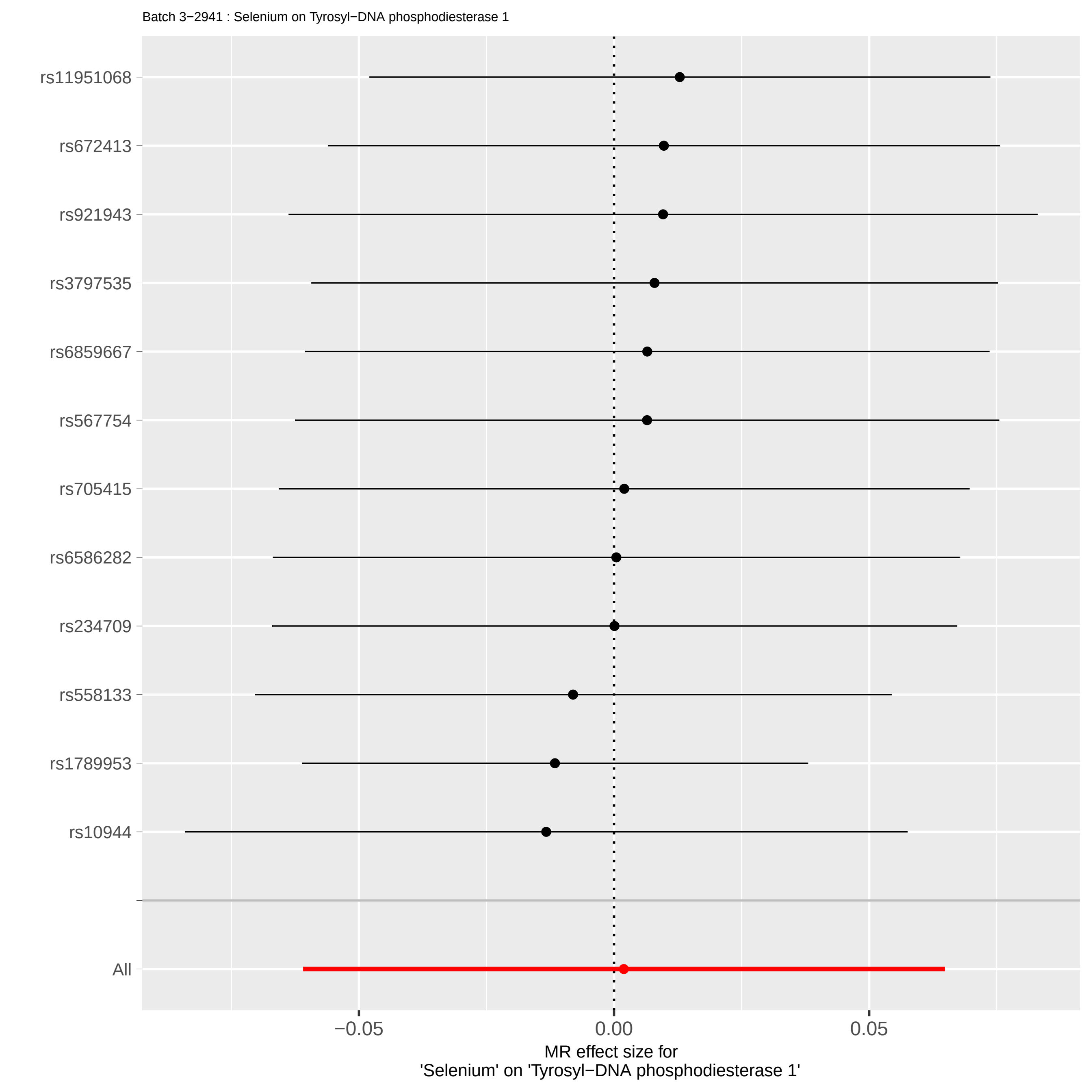
Batch 3−2941 : Selenium on Tyrosyl−DNA phosphodiesterase 1
rs11951068
rs672413
rs921943
rs3797535
rs6859667
rs567754
rs705415
rs6586282
rs234709
rs558133
rs1789953
rs10944
All
−0.05
0.00
0.05
MR effect size for
'Selenium' on 'Tyrosyl−DNA phosphodiesterase 1'

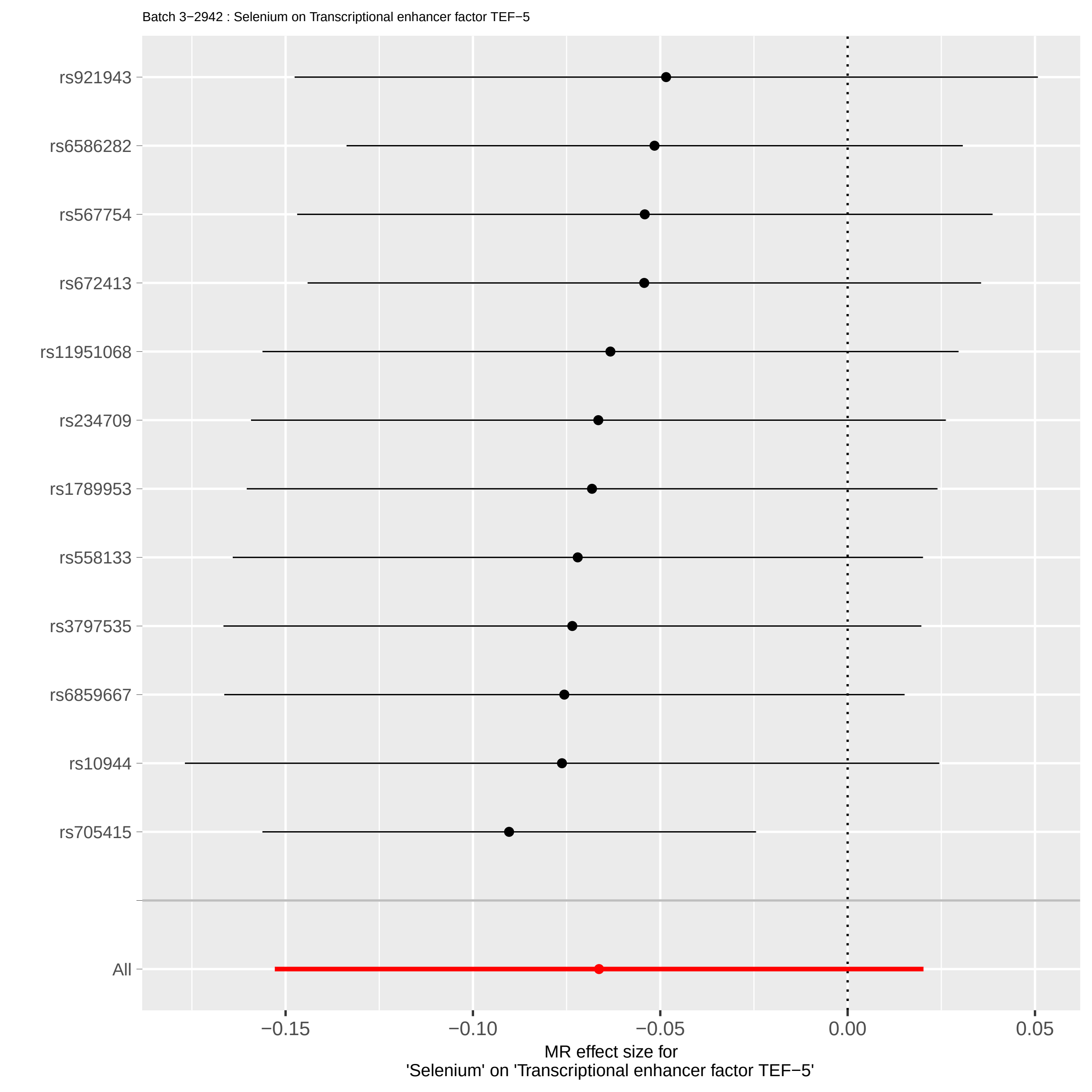

Batch 3−2942 : Selenium on Transcriptional enhancer factor TEF−5
rs921943
rs6586282
rs567754
rs672413
rs11951068
rs234709
rs1789953
rs558133
rs3797535
rs6859667
rs10944
rs705415
All
−0.15
−0.10
−0.05
0.00
0.05
MR effect size for
'Selenium' on 'Transcriptional enhancer factor TEF−5'

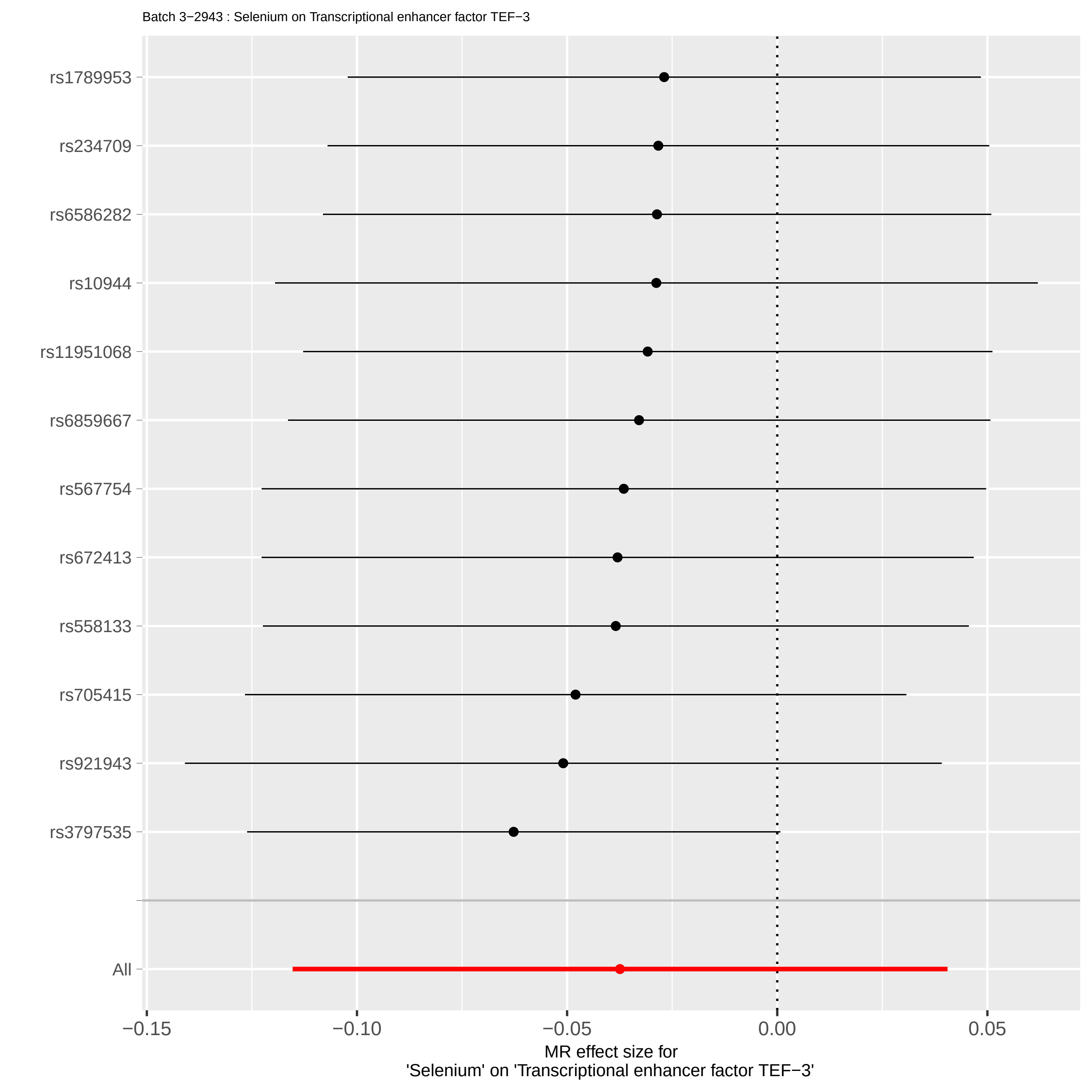

Batch 3−2943 : Selenium on Transcriptional enhancer factor TEF−3
rs1789953
rs234709
rs6586282
rs10944
rs11951068
rs6859667
rs567754
rs672413
rs558133
rs705415
rs921943
rs3797535
All
−0.15
−0.10
−0.05
0.00
0.05
MR effect size for
'Selenium' on 'Transcriptional enhancer factor TEF−3'

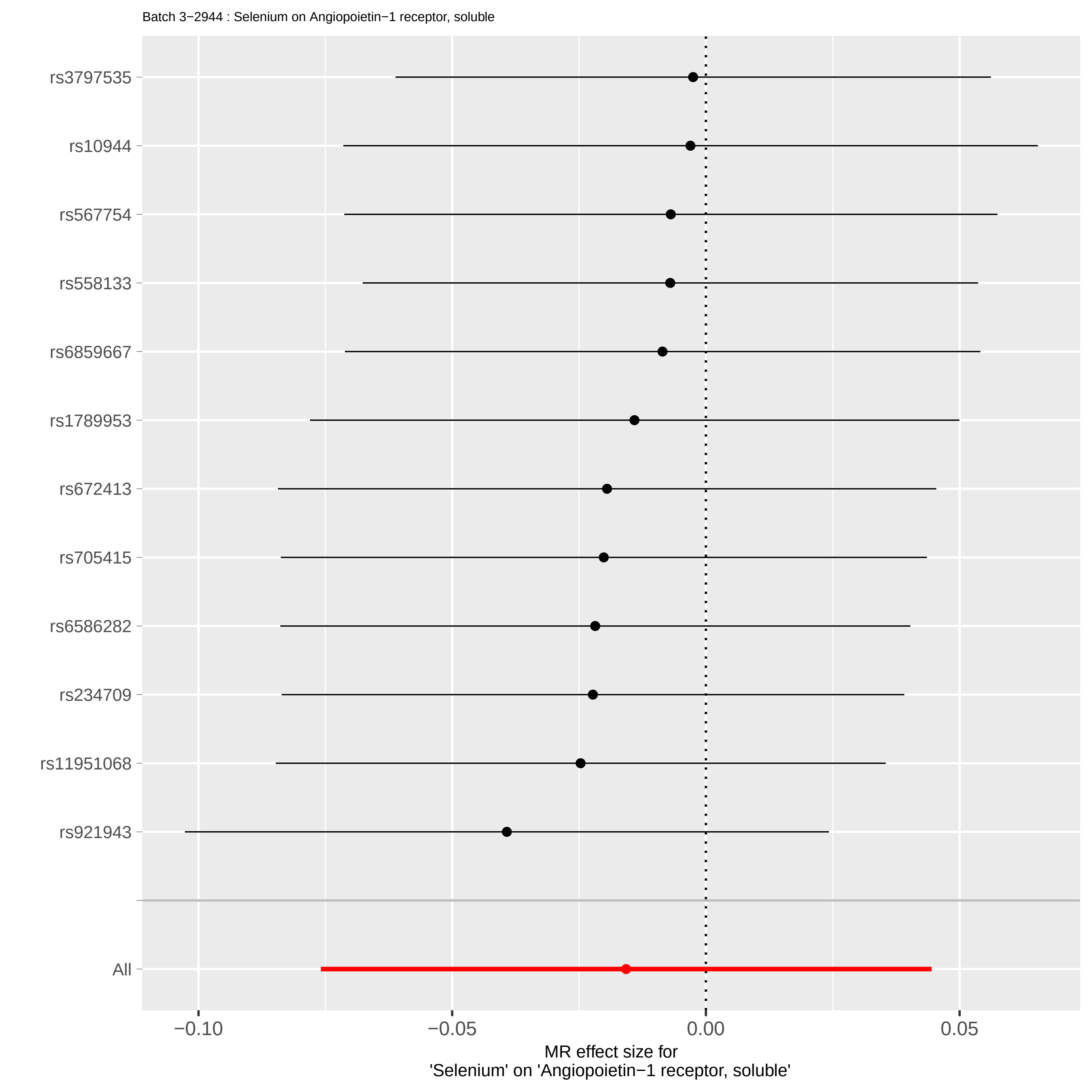

Batch 3−2944 : Selenium on Angiopoietin−1 receptor, soluble
rs3797535
rs10944
rs567754
rs558133
rs6859667
rs1789953
rs672413
rs705415
rs6586282
rs234709
rs11951068
rs921943
All
−0.10
−0.05
0.00
0.05
MR effect size for
'Selenium' on 'Angiopoietin−1 receptor, soluble'

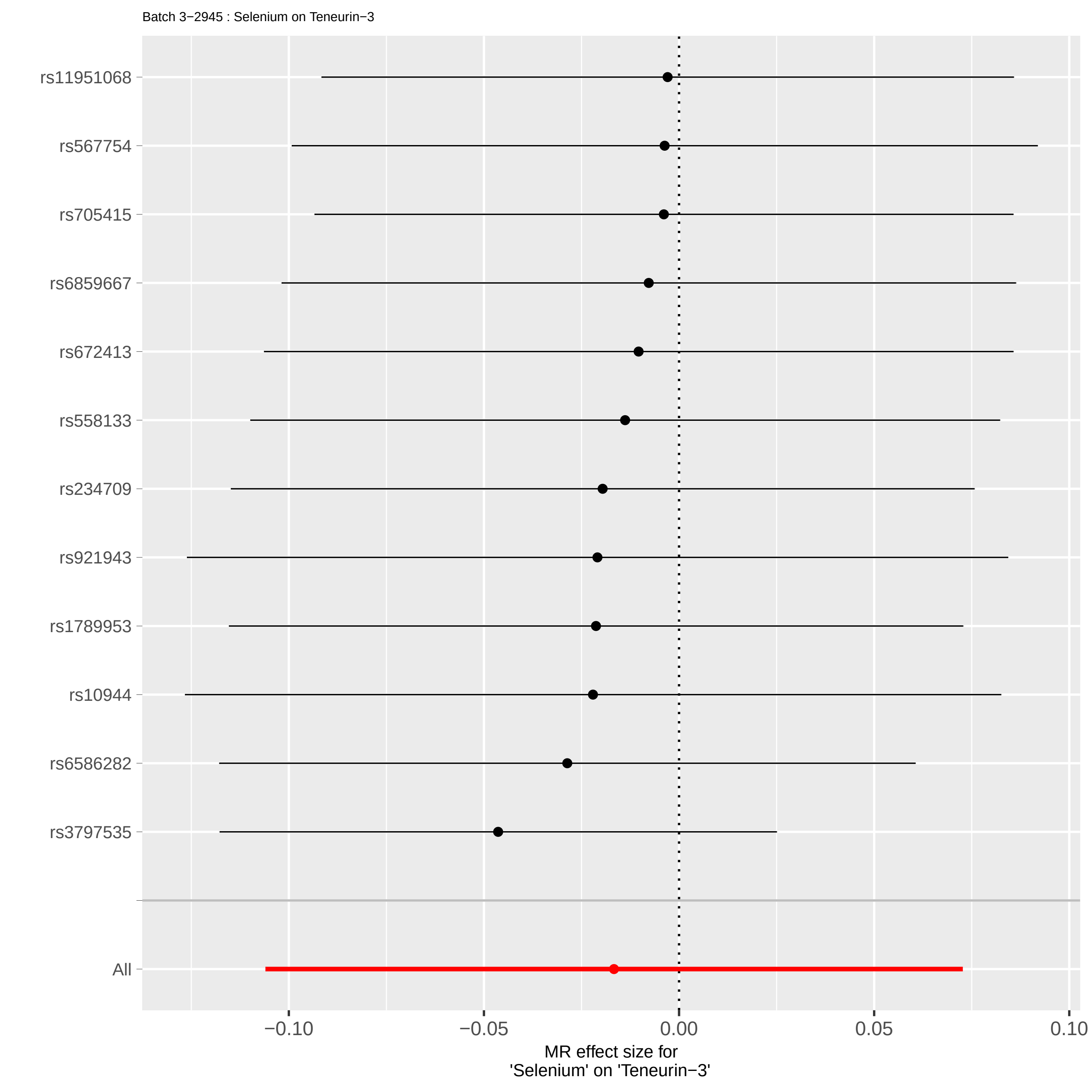
Batch 3−2945 : Selenium on Teneurin−3
rs11951068
rs567754
rs705415
rs6859667
rs672413
rs558133
rs234709
rs921943
rs1789953
rs10944
rs6586282
rs3797535
All
−0.10
−0.05
0.00
0.05
0.10
MR effect size for
'Selenium' on 'Teneurin−3'

Batch 3−2946 : Selenium on Teneurin−4

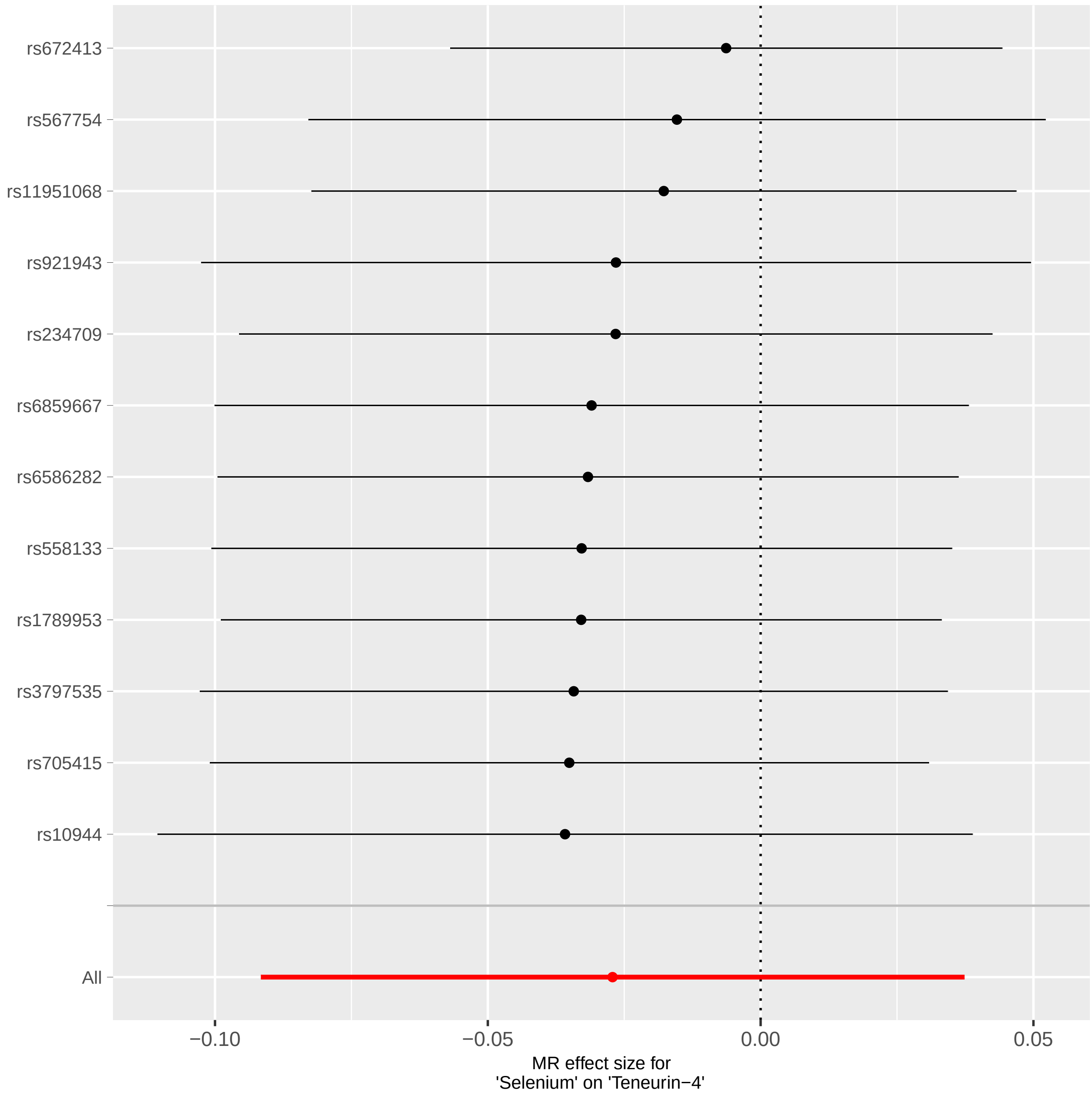

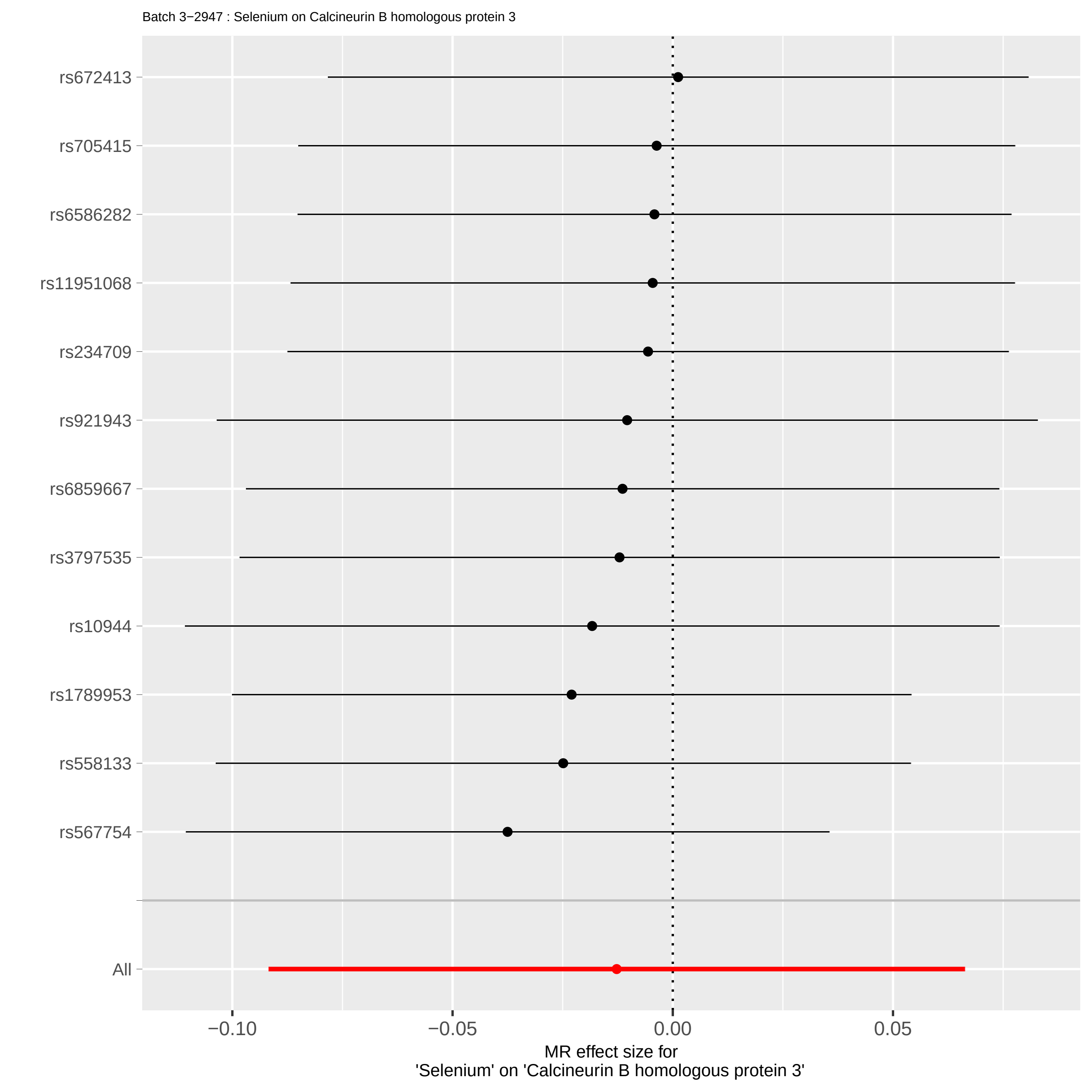

Batch 3−2947 : Selenium on Calcineurin B homologous protein 3
rs672413
rs705415
rs6586282
rs11951068
rs234709
rs921943
rs6859667
rs3797535
rs10944
rs1789953
rs558133
rs567754
All
−0.10
−0.05
0.00
0.05
MR effect size for
'Selenium' on 'Calcineurin B homologous protein 3'

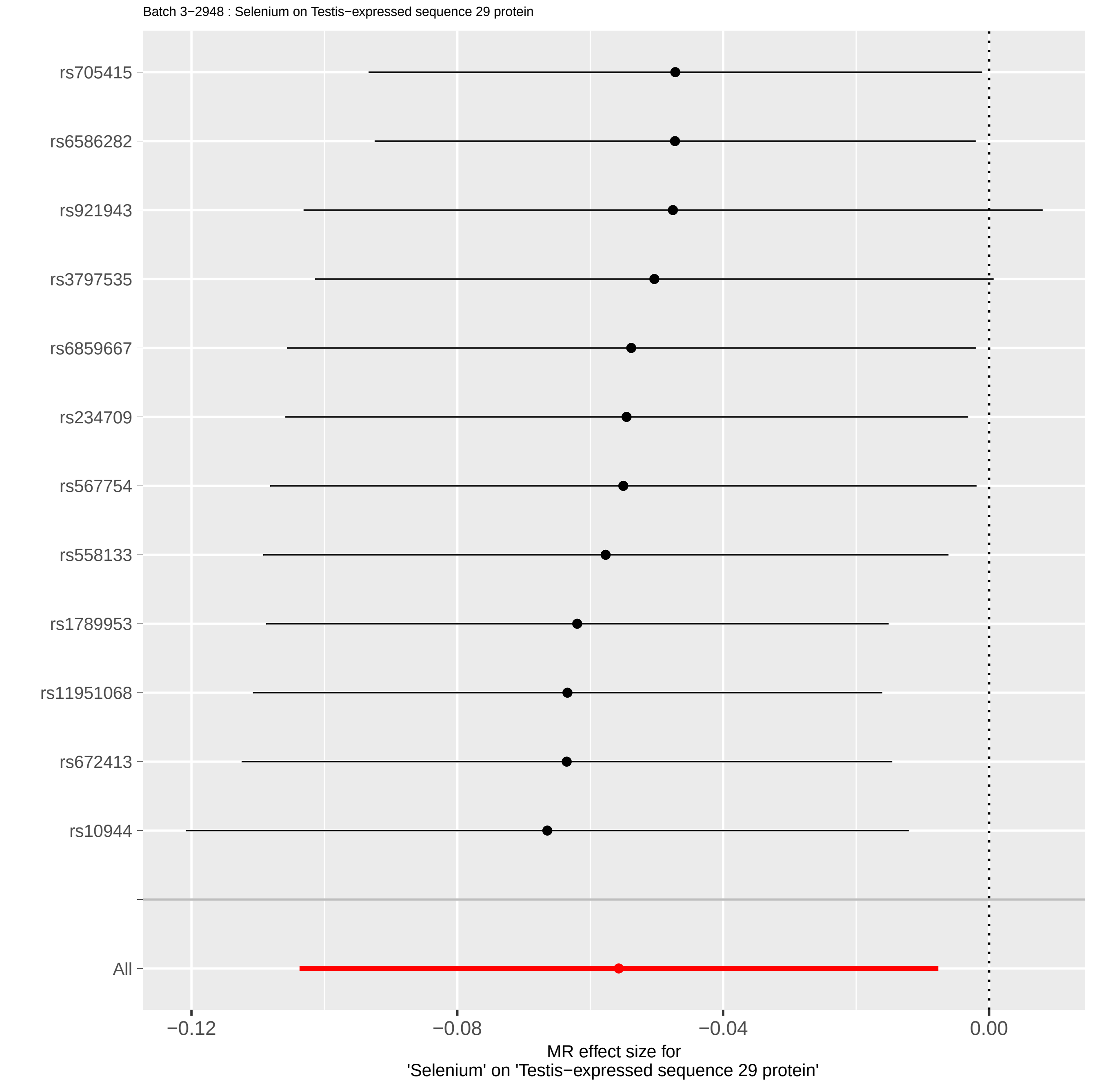
Batch 3−2948 : Selenium on Testis−expressed sequence 29 protein
rs705415
rs6586282
rs921943
rs3797535
rs6859667
rs234709
rs567754
rs558133
rs1789953
rs11951068
rs672413
rs10944
All
−0.12
−0.08
−0.04
0.00
MR effect size for
'Selenium' on 'Testis−expressed sequence 29 protein'

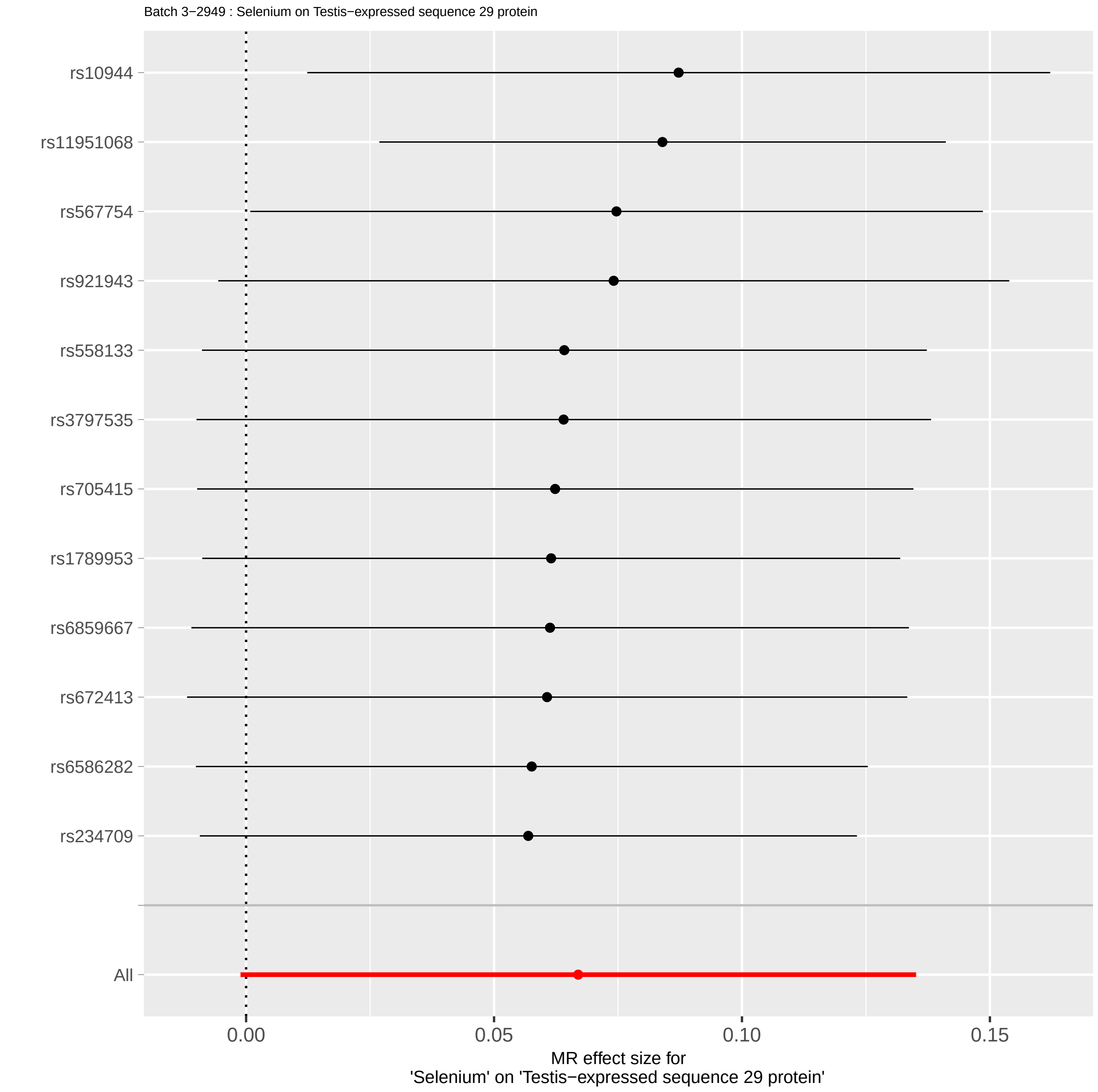

Batch 3−2949 : Selenium on Testis−expressed sequence 29 protein
rs10944
rs11951068
rs567754
rs921943
rs558133
rs3797535
rs705415
rs1789953
rs6859667
rs672413
rs6586282
rs234709
All
0.00
0.05
0.10
0.15
MR effect size for
'Selenium' on 'Testis−expressed sequence 29 protein'

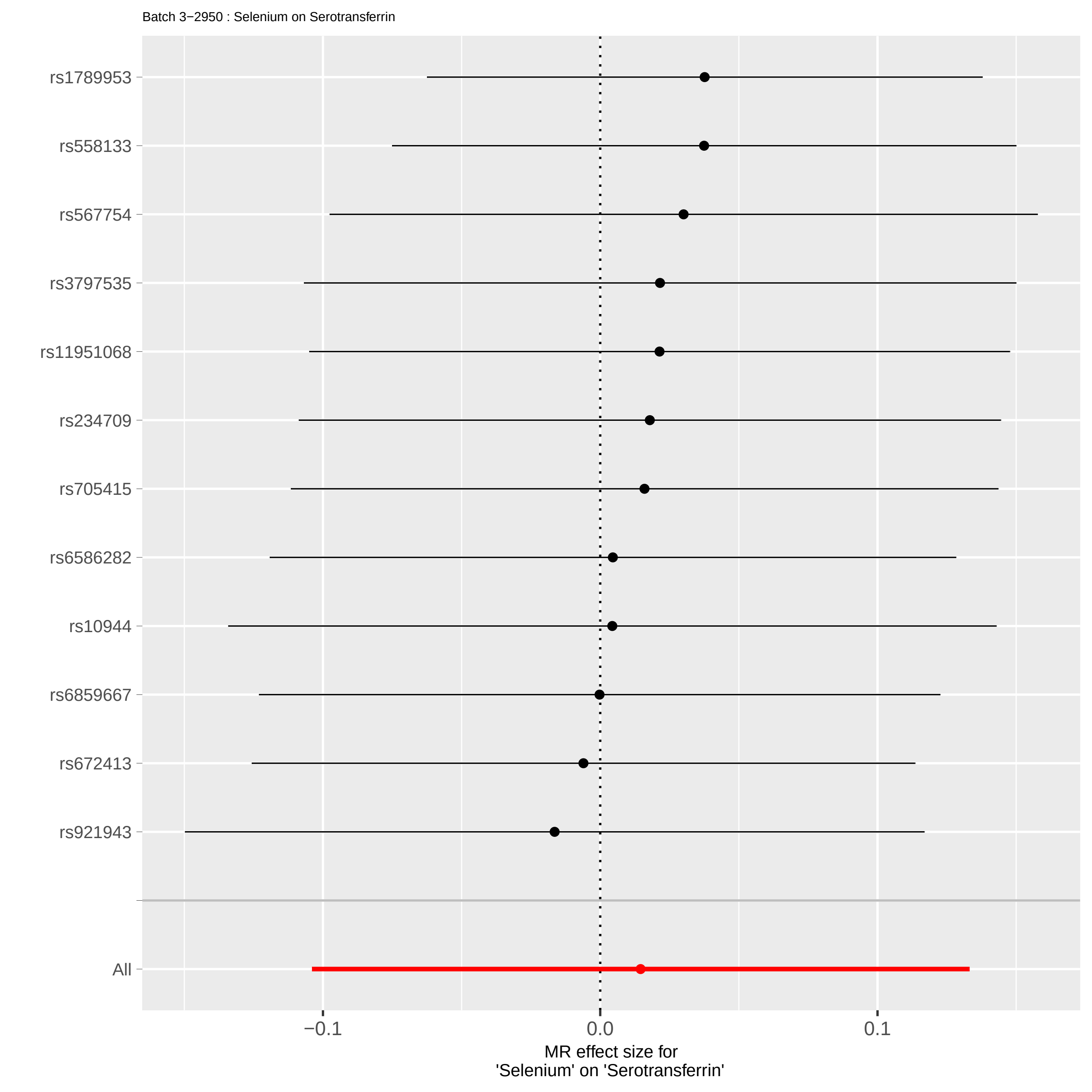
Batch 3−2950 : Selenium on Serotransferrin
rs1789953
rs558133
rs567754
rs3797535
rs11951068
rs234709
rs705415
rs6586282
rs10944
rs6859667
rs672413
rs921943
All
−0.1
0.0
0.1
MR effect size for
'Selenium' on 'Serotransferrin'

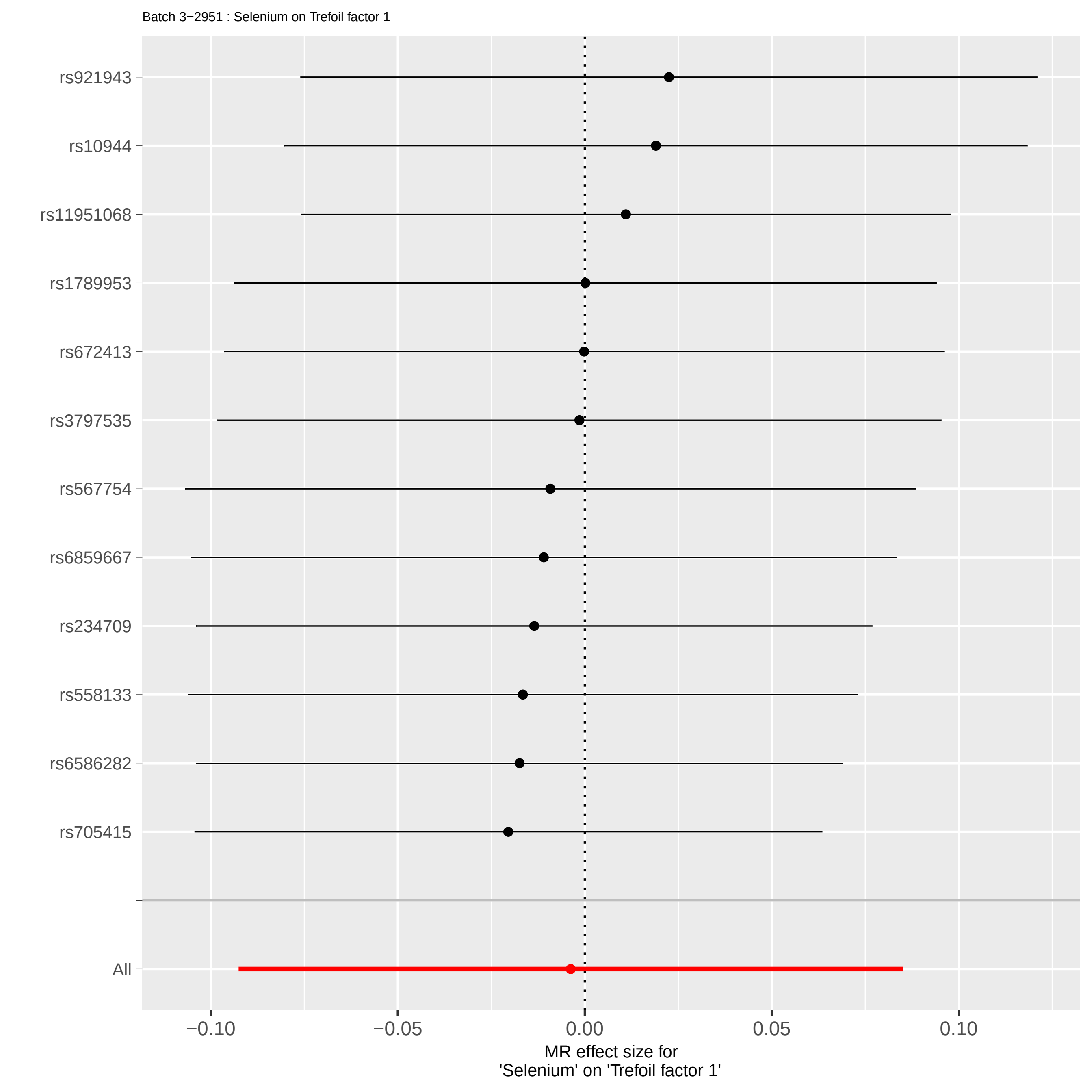

Batch 3−2951 : Selenium on Trefoil factor 1
rs921943
rs10944
rs11951068
rs1789953
rs672413
rs3797535
rs567754
rs6859667
rs234709
rs558133
rs6586282
rs705415
All
−0.10
−0.05
0.00
0.05
0.10
MR effect size for
'Selenium' on 'Trefoil factor 1'

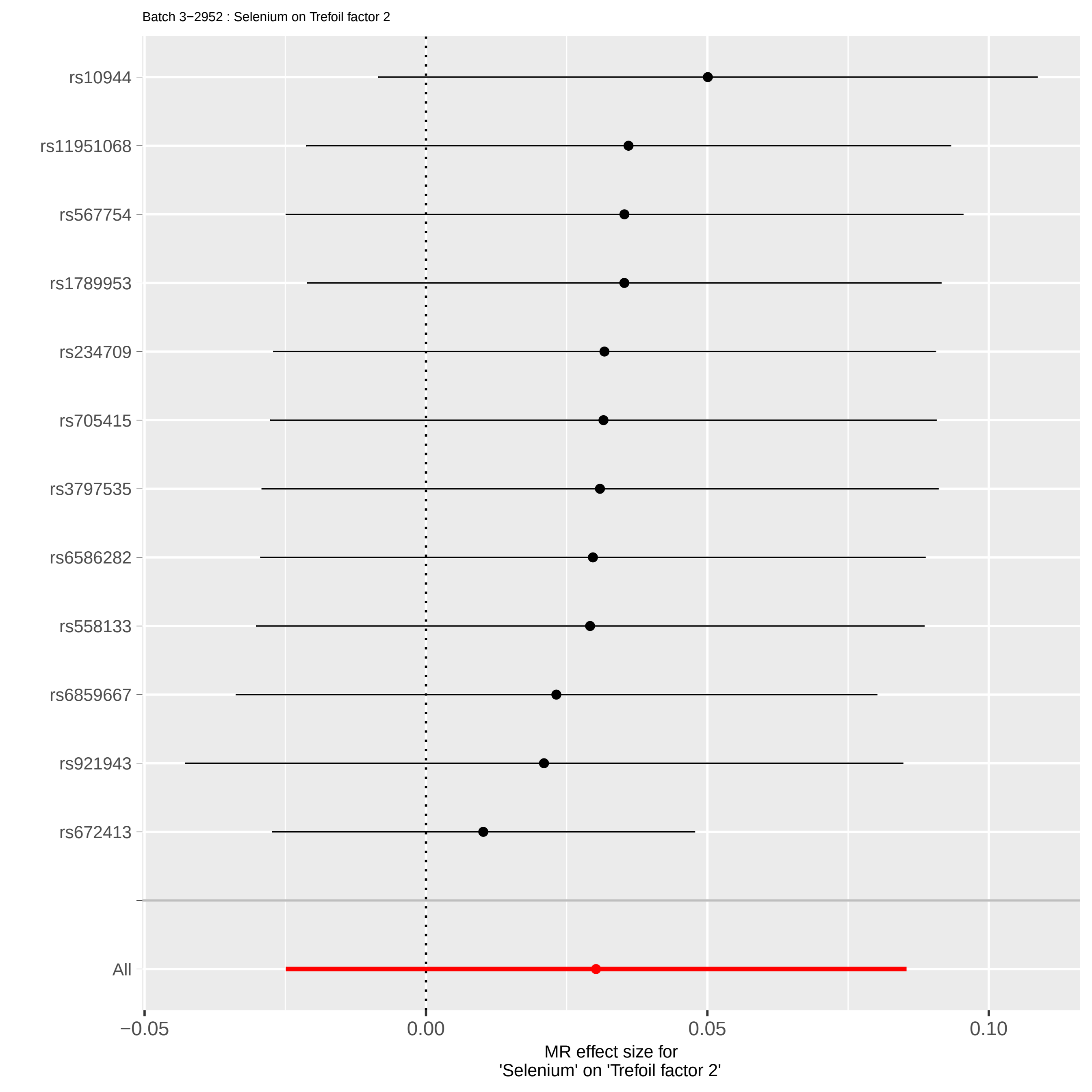

Batch 3−2952 : Selenium on Trefoil factor 2
rs10944
rs11951068
rs567754
rs1789953
rs234709
rs705415
rs3797535
rs6586282
rs558133
rs6859667
rs921943
rs672413
All
−0.05
0.00
0.05
0.10
MR effect size for
'Selenium' on 'Trefoil factor 2'

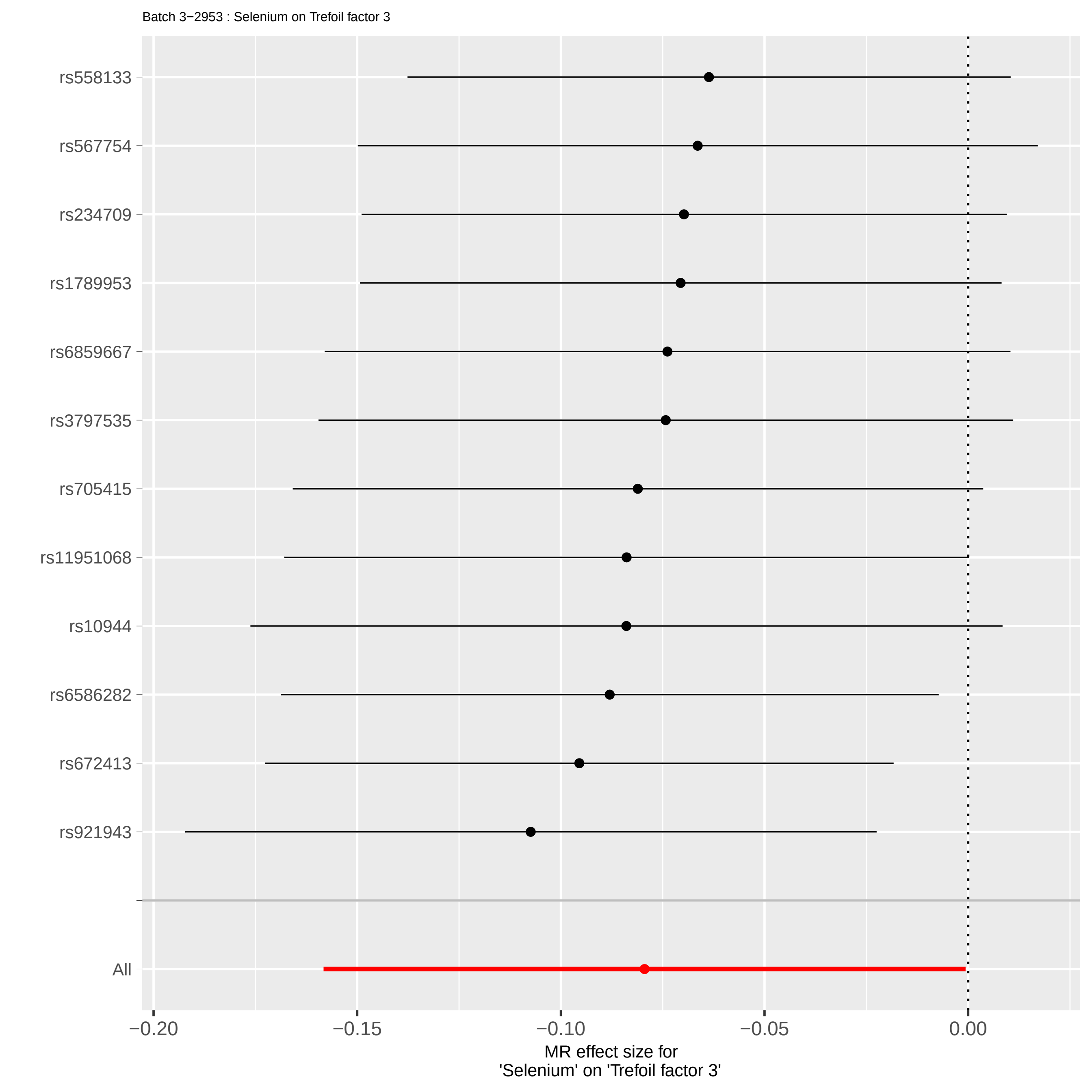

Batch 3−2953 : Selenium on Trefoil factor 3
rs558133
rs567754
rs234709
rs1789953
rs6859667
rs3797535
rs705415
rs11951068
rs10944
rs6586282
rs672413
rs921943
All
−0.20
−0.15
−0.10
−0.05
0.00
MR effect size for
'Selenium' on 'Trefoil factor 3'

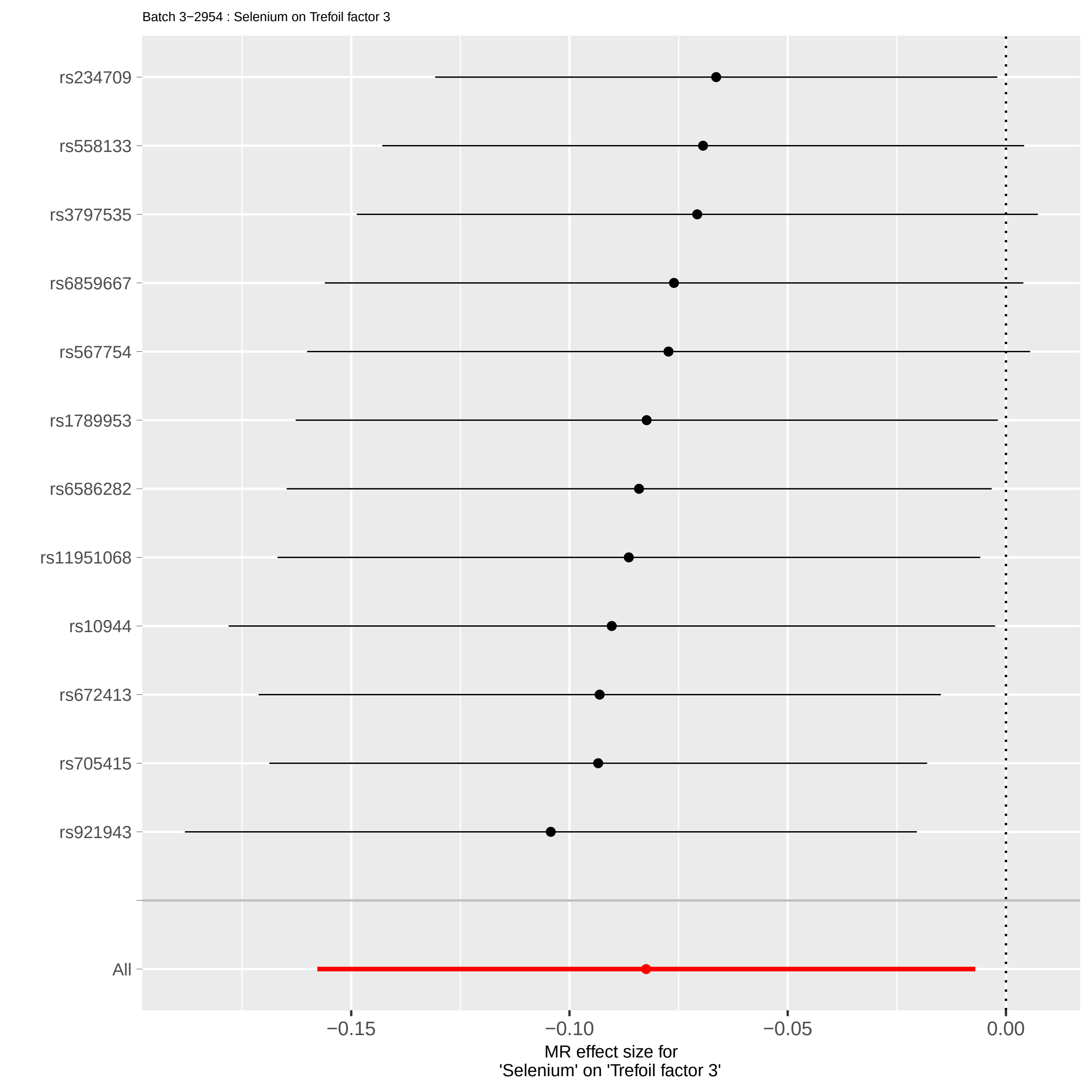

Batch 3−2954 : Selenium on Trefoil factor 3
rs234709
rs558133
rs3797535
rs6859667
rs567754
rs1789953
rs6586282
rs11951068
rs10944
rs672413
rs705415
rs921943
All
−0.15
−0.10
−0.05
0.00
MR effect size for
'Selenium' on 'Trefoil factor 3'

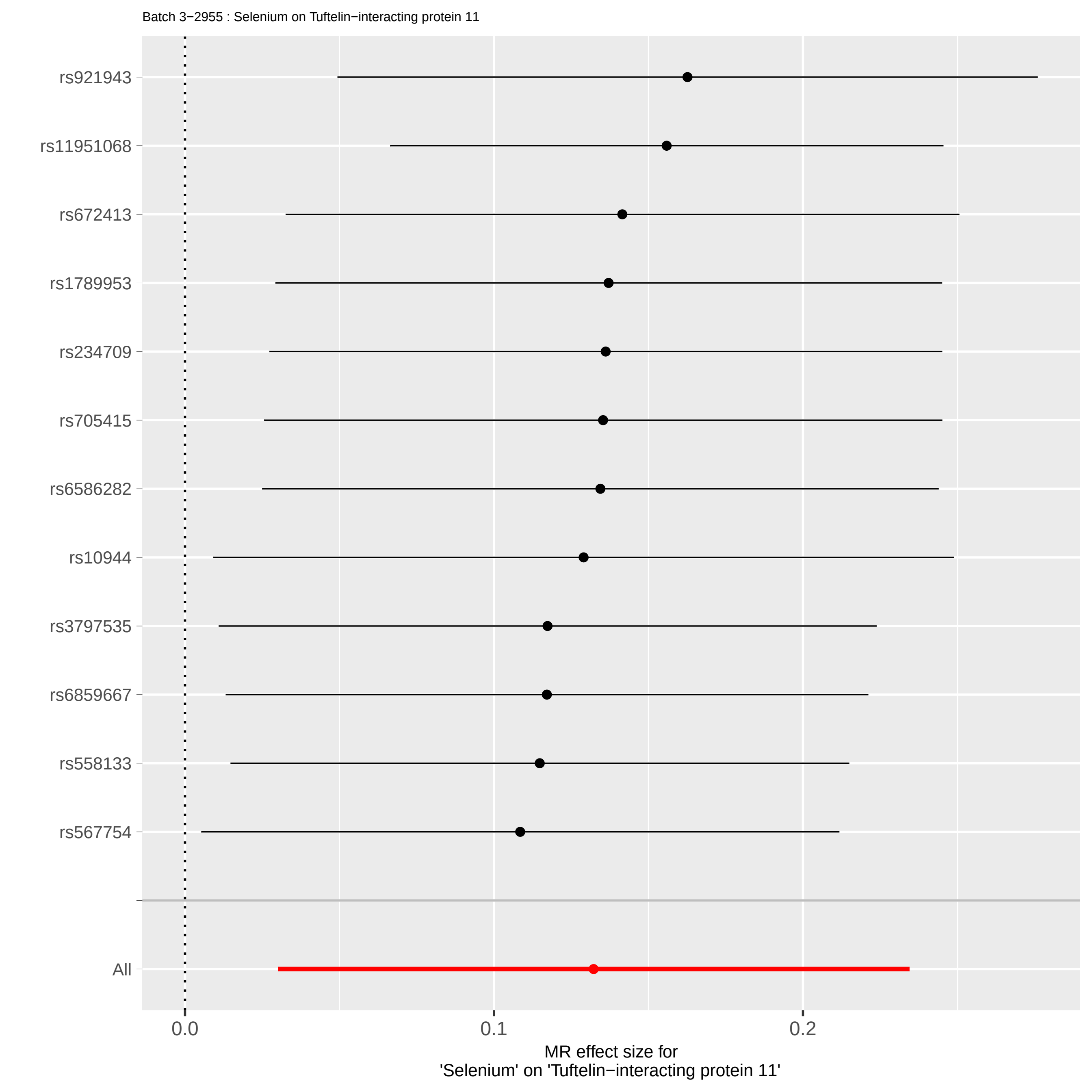
Batch 3−2955 : Selenium on Tuftelin−interacting protein 11
rs921943
rs11951068
rs672413
rs1789953
rs234709
rs705415
rs6586282
rs10944
rs3797535
rs6859667
rs558133
rs567754
All
0.0
0.1
0.2
MR effect size for
'Selenium' on 'Tuftelin−interacting protein 11'

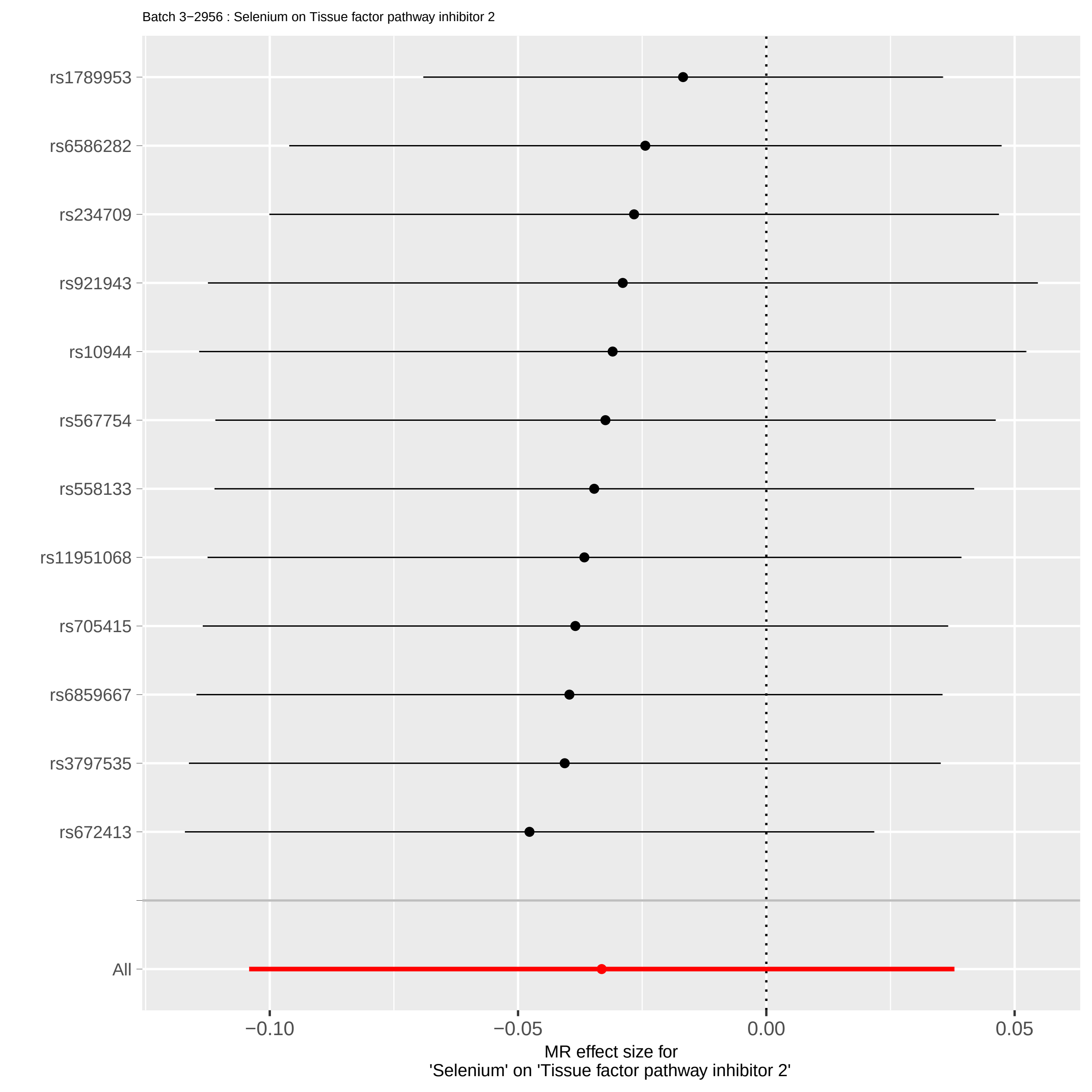

Batch 3−2956 : Selenium on Tissue factor pathway inhibitor 2
rs1789953
rs6586282
rs234709
rs921943
rs10944
rs567754
rs558133
rs11951068
rs705415
rs6859667
rs3797535
rs672413
All
−0.10
−0.05
0.00
0.05
MR effect size for
'Selenium' on 'Tissue factor pathway inhibitor 2'

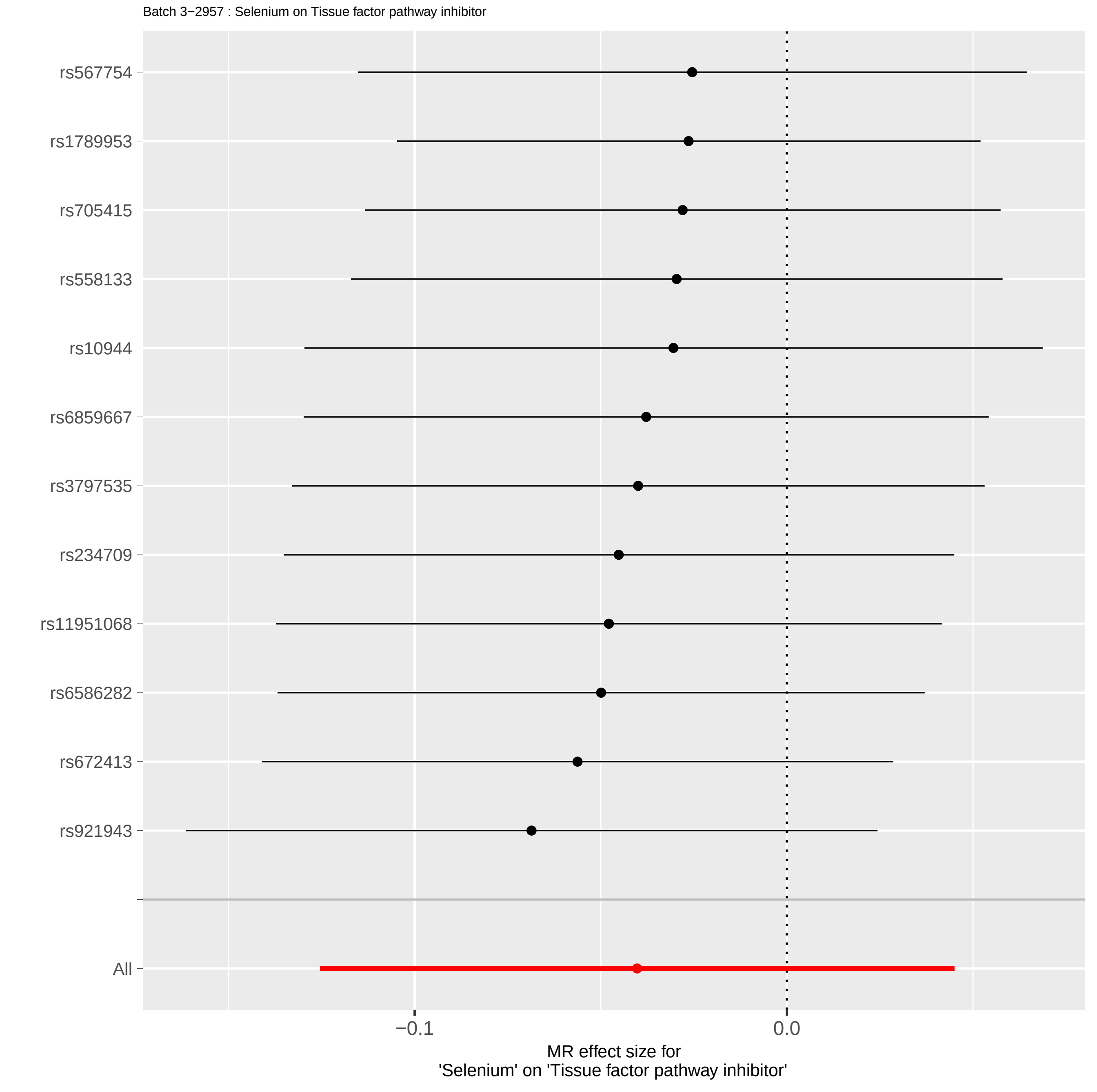

Batch 3−2957 : Selenium on Tissue factor pathway inhibitor
rs567754
rs1789953
rs705415
rs558133
rs10944
rs6859667
rs3797535
rs234709
rs11951068
rs6586282
rs672413
rs921943
All
−0.1
0.0
MR effect size for
'Selenium' on 'Tissue factor pathway inhibitor'

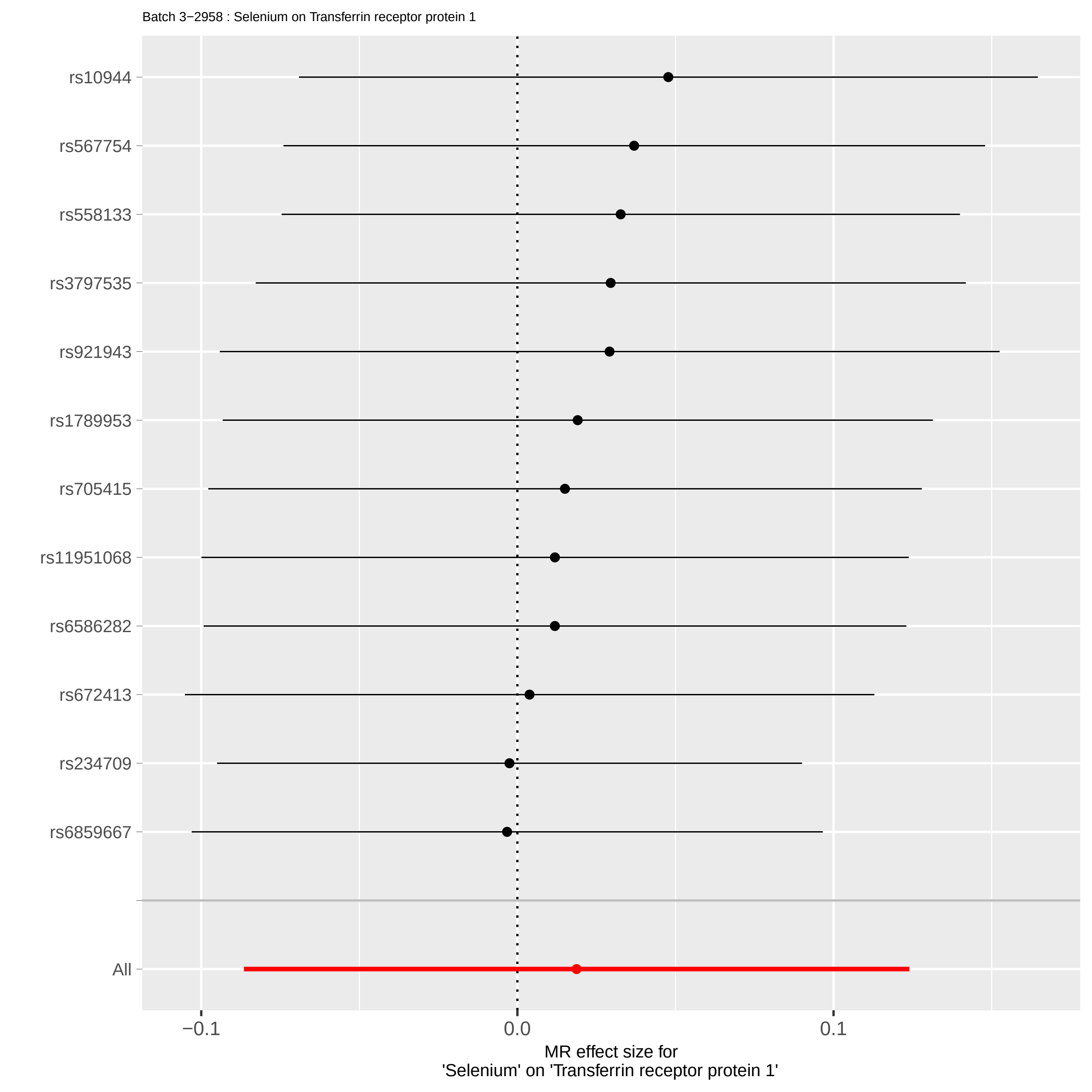

Batch 3−2958 : Selenium on Transferrin receptor protein 1
rs10944
rs567754
rs558133
rs3797535
rs921943
rs1789953
rs705415
rs11951068
rs6586282
rs672413
rs234709
rs6859667
All
−0.1
0.0
0.1
MR effect size for
'Selenium' on 'Transferrin receptor protein 1'

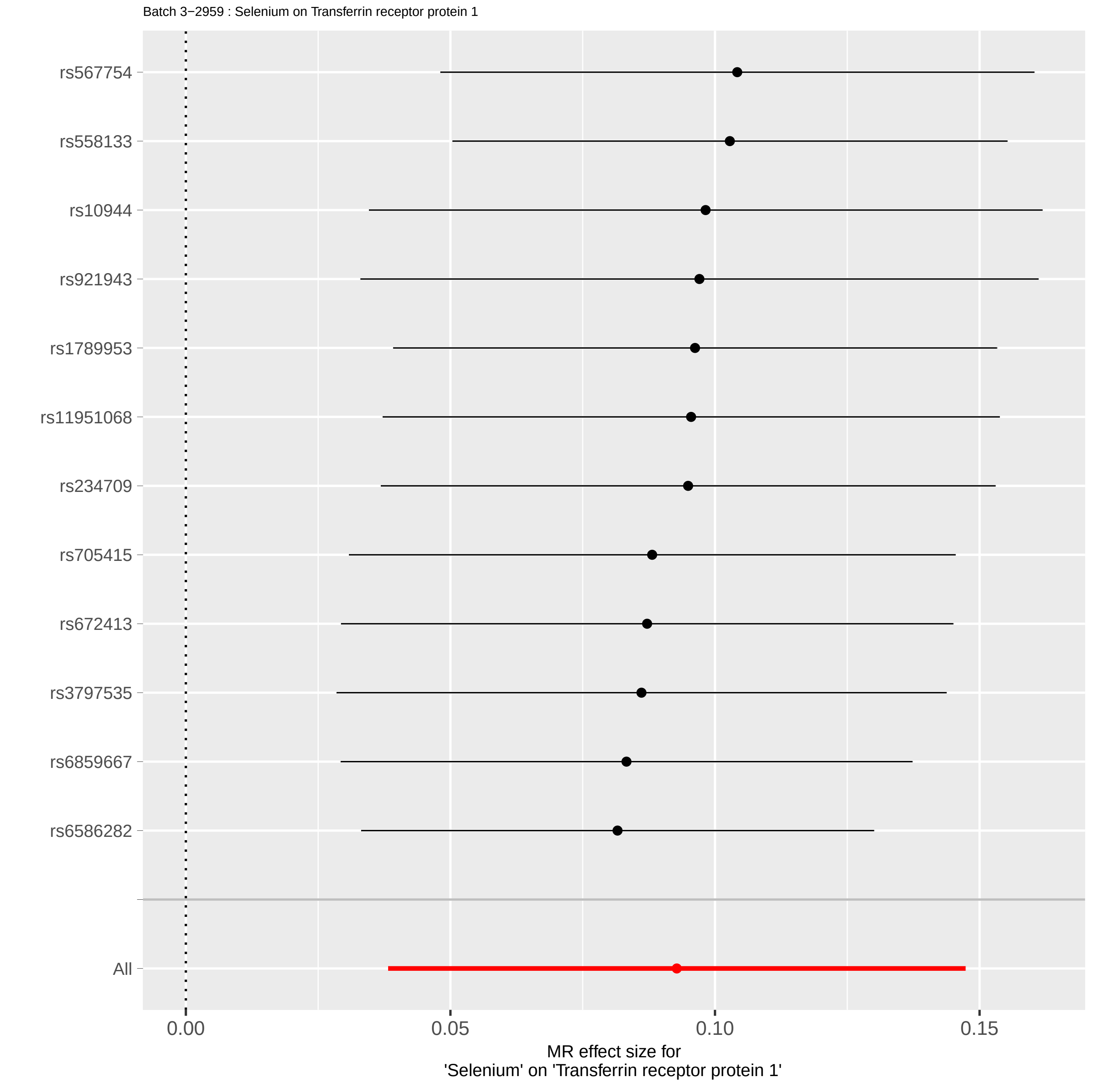

Batch 3−2959 : Selenium on Transferrin receptor protein 1
rs567754
rs558133
rs10944
rs921943
rs1789953
rs11951068
rs234709
rs705415
rs672413
rs3797535
rs6859667
rs6586282
All
0.00
0.05
0.10
0.15
MR effect size for
'Selenium' on 'Transferrin receptor protein 1'

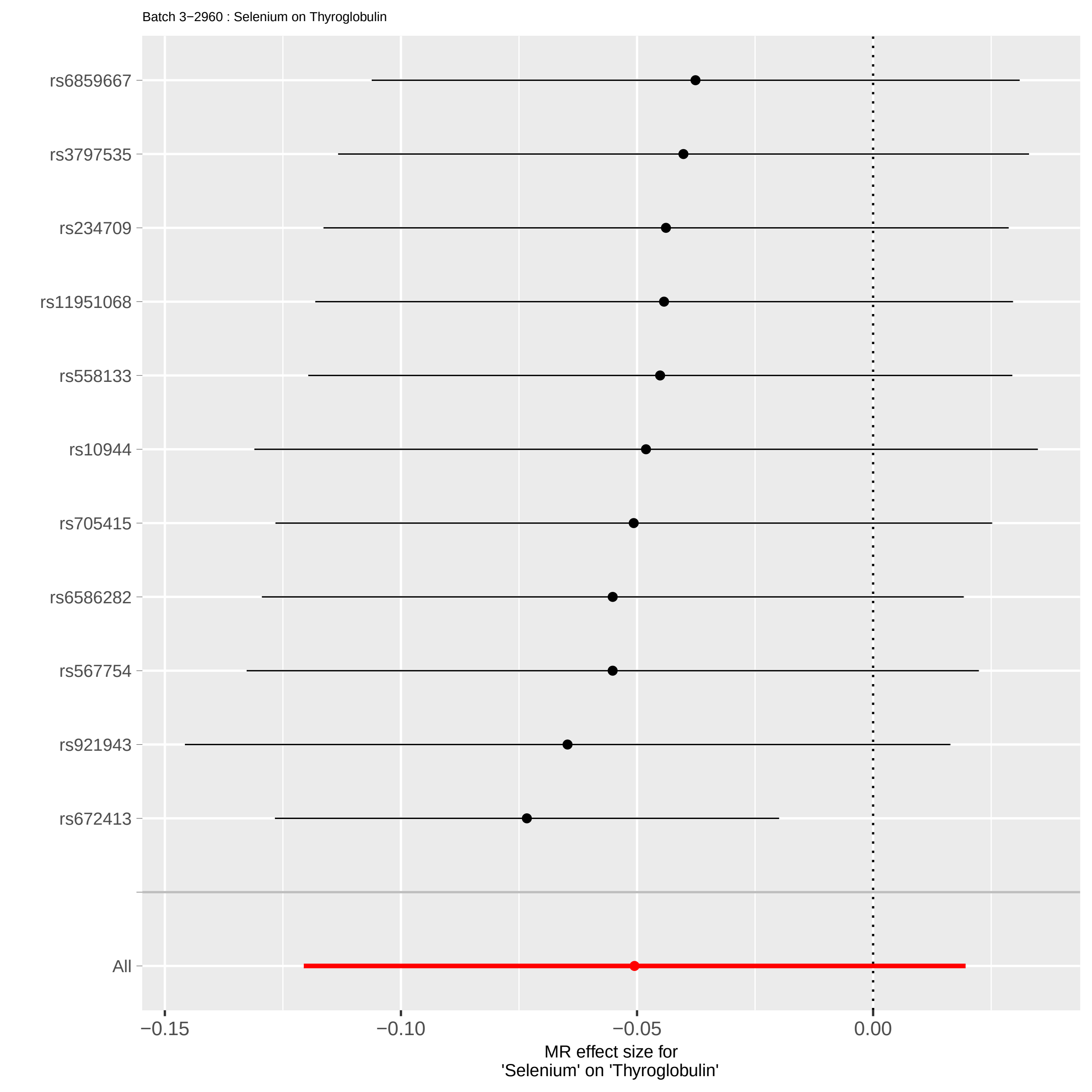
Batch 3−2960 : Selenium on Thyroglobulin
rs6859667
rs3797535
rs234709
rs11951068
rs558133
rs10944
rs705415
rs6586282
rs567754
rs921943
rs672413
All
−0.15
−0.10
−0.05
0.00
MR effect size for
'Selenium' on 'Thyroglobulin'

Batch 3−2961 : Selenium on Transforming growth factor alpha

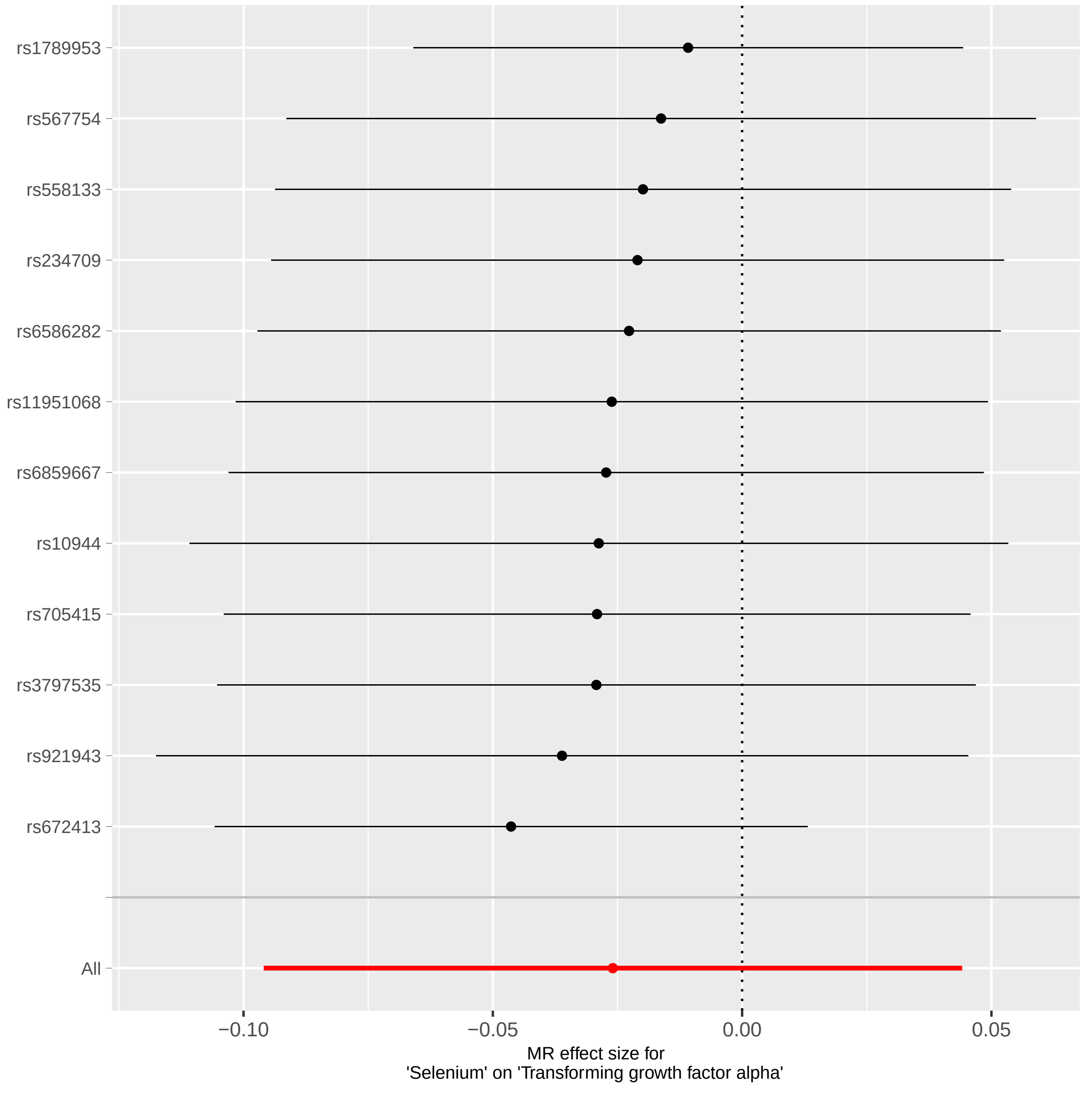

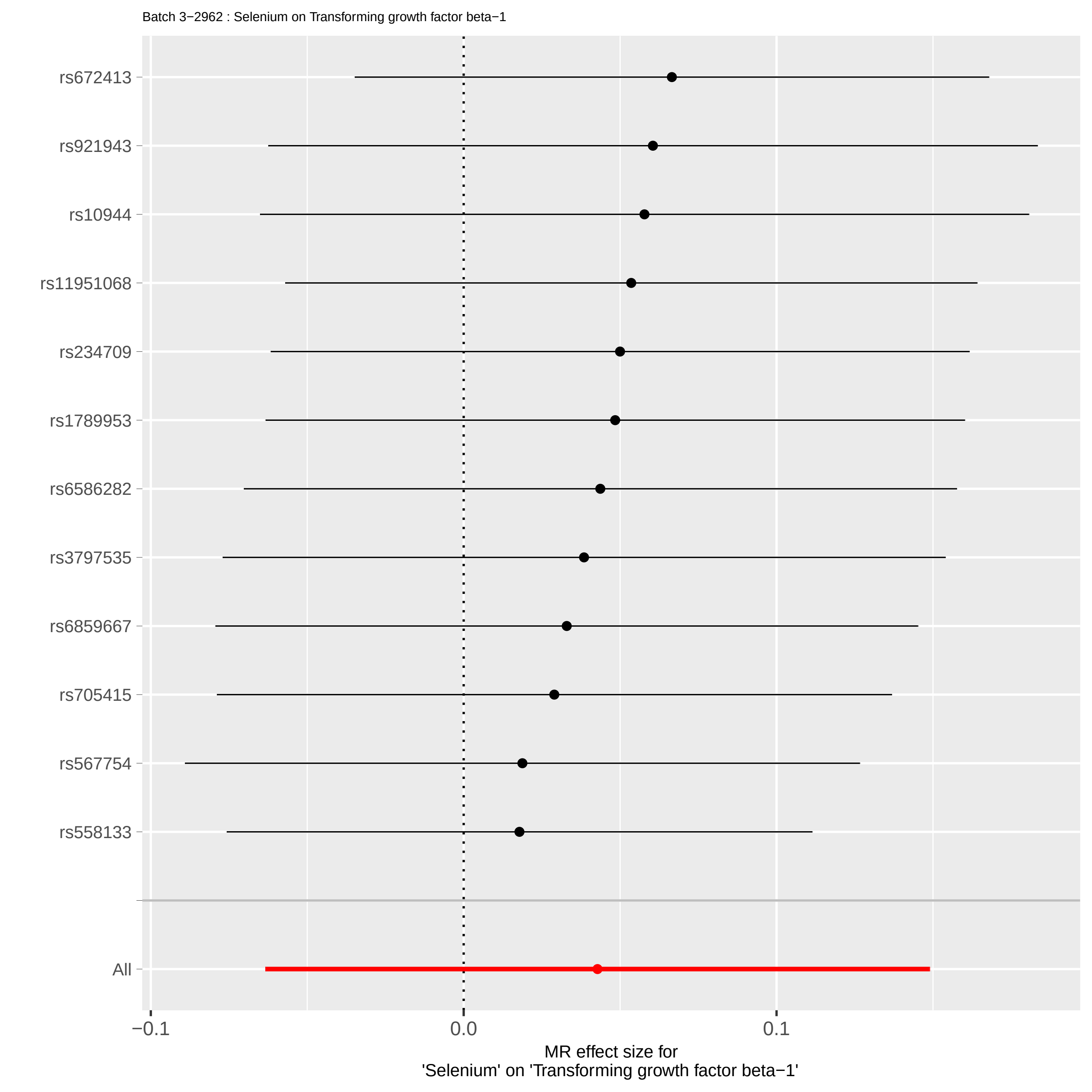

Batch 3−2962 : Selenium on Transforming growth factor beta−1
rs672413
rs921943
rs10944
rs11951068
rs234709
rs1789953
rs6586282
rs3797535
rs6859667
rs705415
rs567754
rs558133
All
−0.1
0.0
0.1
MR effect size for
'Selenium' on 'Transforming growth factor beta−1'

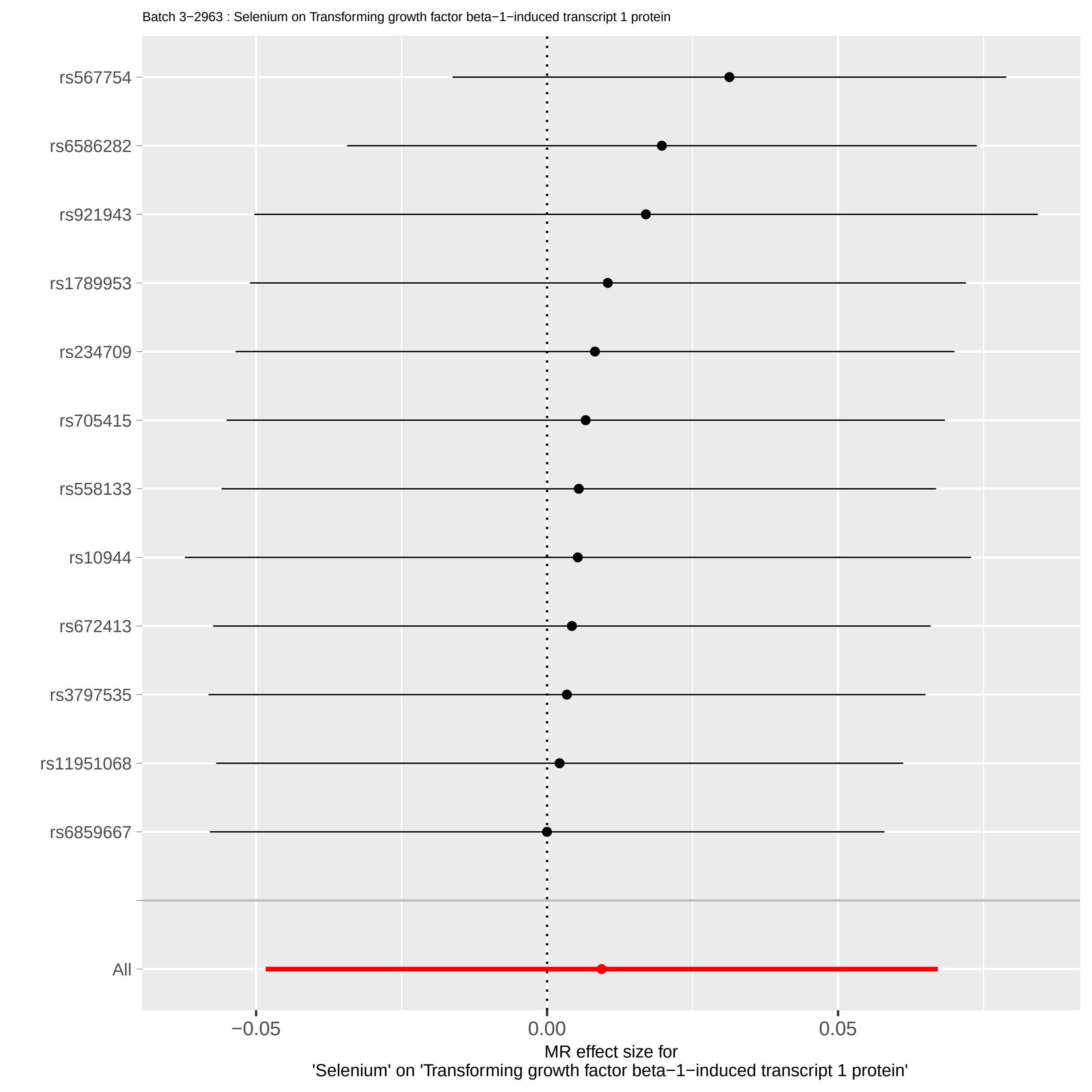
Batch 3−2963 : Selenium on Transforming growth factor beta−1−induced transcript 1 protein
rs567754
rs6586282
rs921943
rs1789953
rs234709
rs705415
rs558133
rs10944
rs672413
rs3797535
rs11951068
rs6859667
All
−0.05
0.00
0.05
MR effect size for
'Selenium' on 'Transforming growth factor beta−1−induced transcript 1 protein'

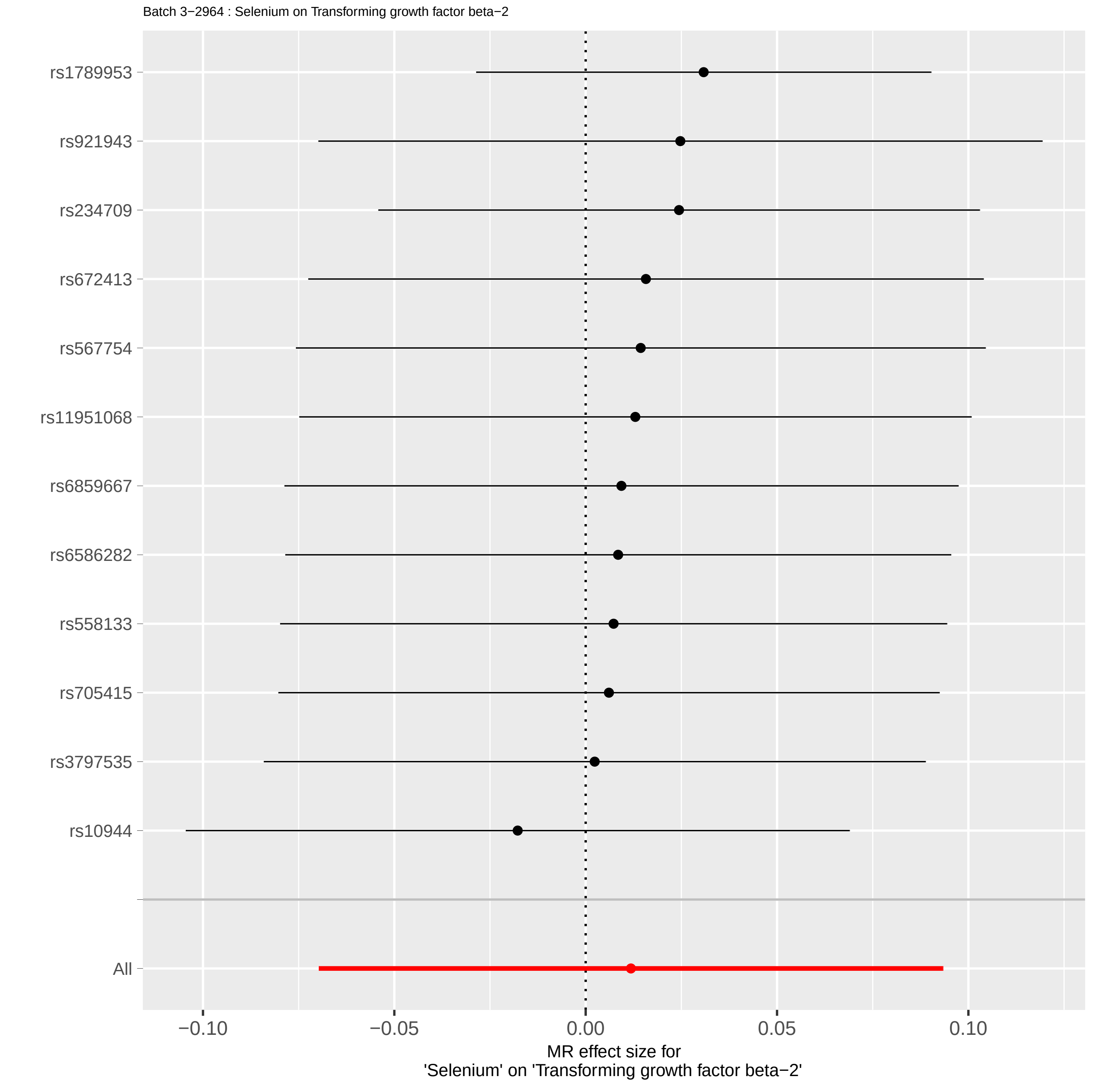

Batch 3−2964 : Selenium on Transforming growth factor beta−2
rs1789953
rs921943
rs234709
rs672413
rs567754
rs11951068
rs6859667
rs6586282
rs558133
rs705415
rs3797535
rs10944
All
−0.10
−0.05
0.00
0.05
0.10
MR effect size for
'Selenium' on 'Transforming growth factor beta−2'

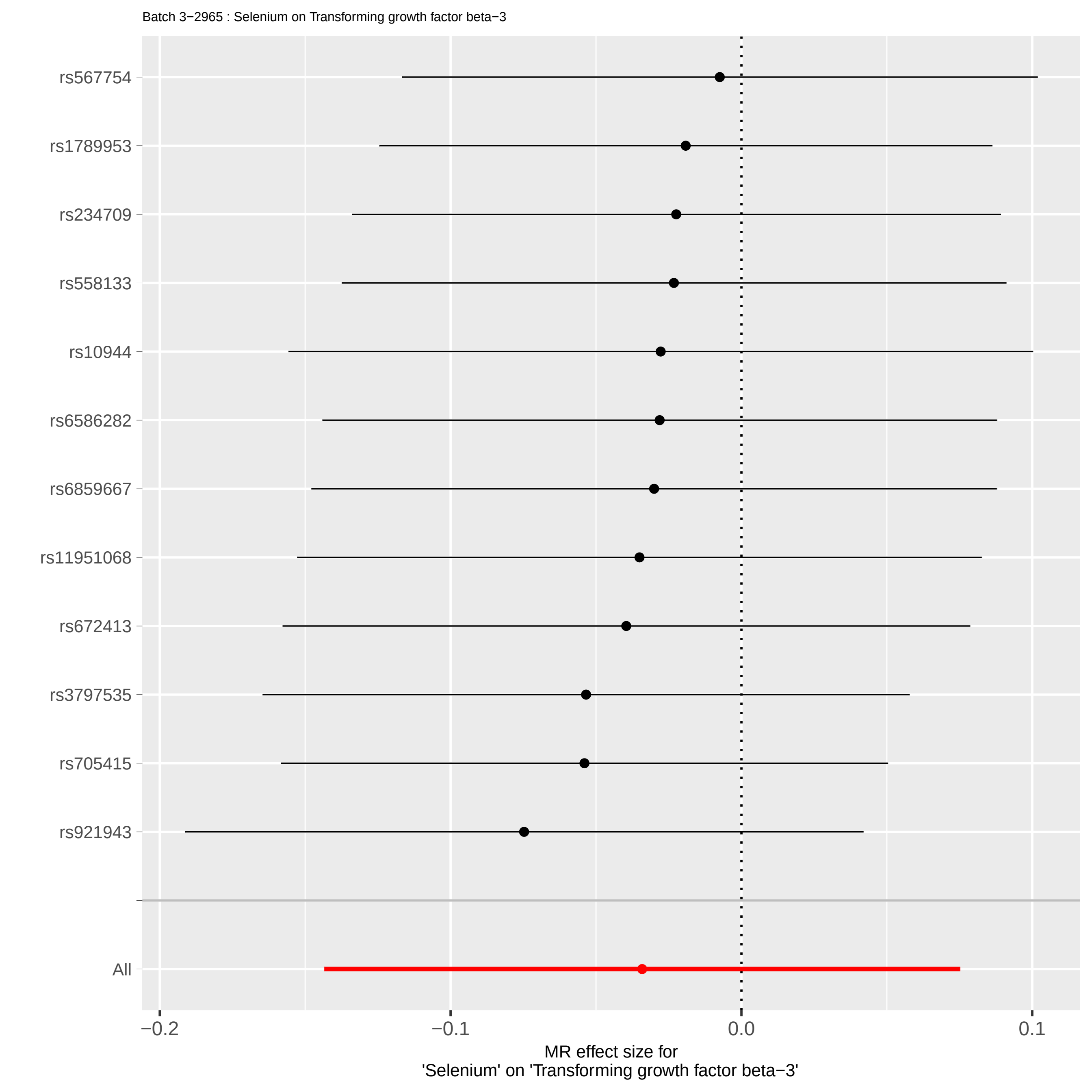

Batch 3−2965 : Selenium on Transforming growth factor beta−3
rs567754
rs1789953
rs234709
rs558133
rs10944
rs6586282
rs6859667
rs11951068
rs672413
rs3797535
rs705415
rs921943
All
−0.2
−0.1
0.0
0.1
MR effect size for
'Selenium' on 'Transforming growth factor beta−3'

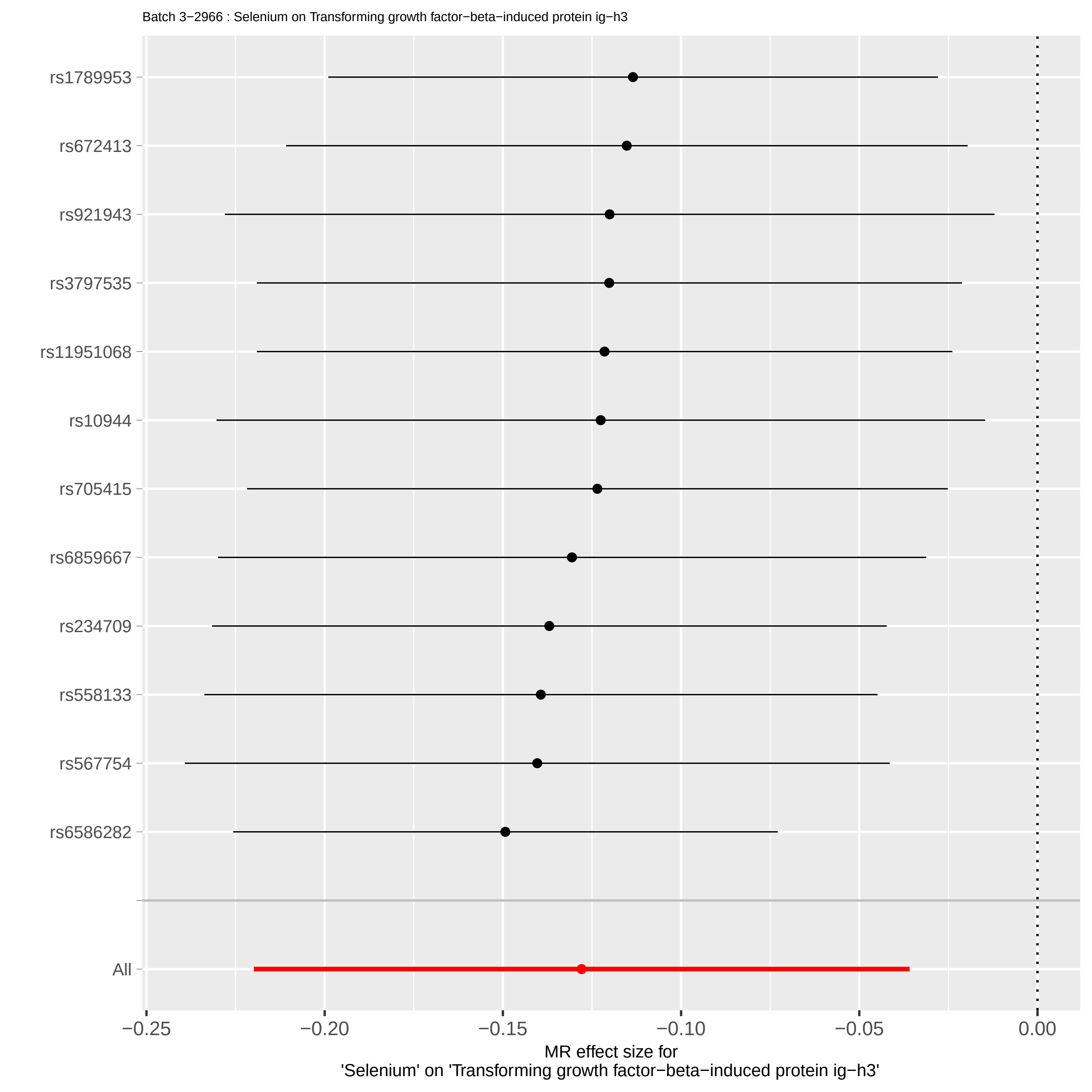

Batch 3−2966 : Selenium on Transforming growth factor−beta−induced protein ig−h3
rs1789953
rs672413
rs921943
rs3797535
rs11951068
rs10944
rs705415
rs6859667
rs234709
rs558133
rs567754
rs6586282
All
−0.25
−0.20
−0.15
−0.10
−0.05
0.00
MR effect size for
'Selenium' on 'Transforming growth factor−beta−induced protein ig−h3'

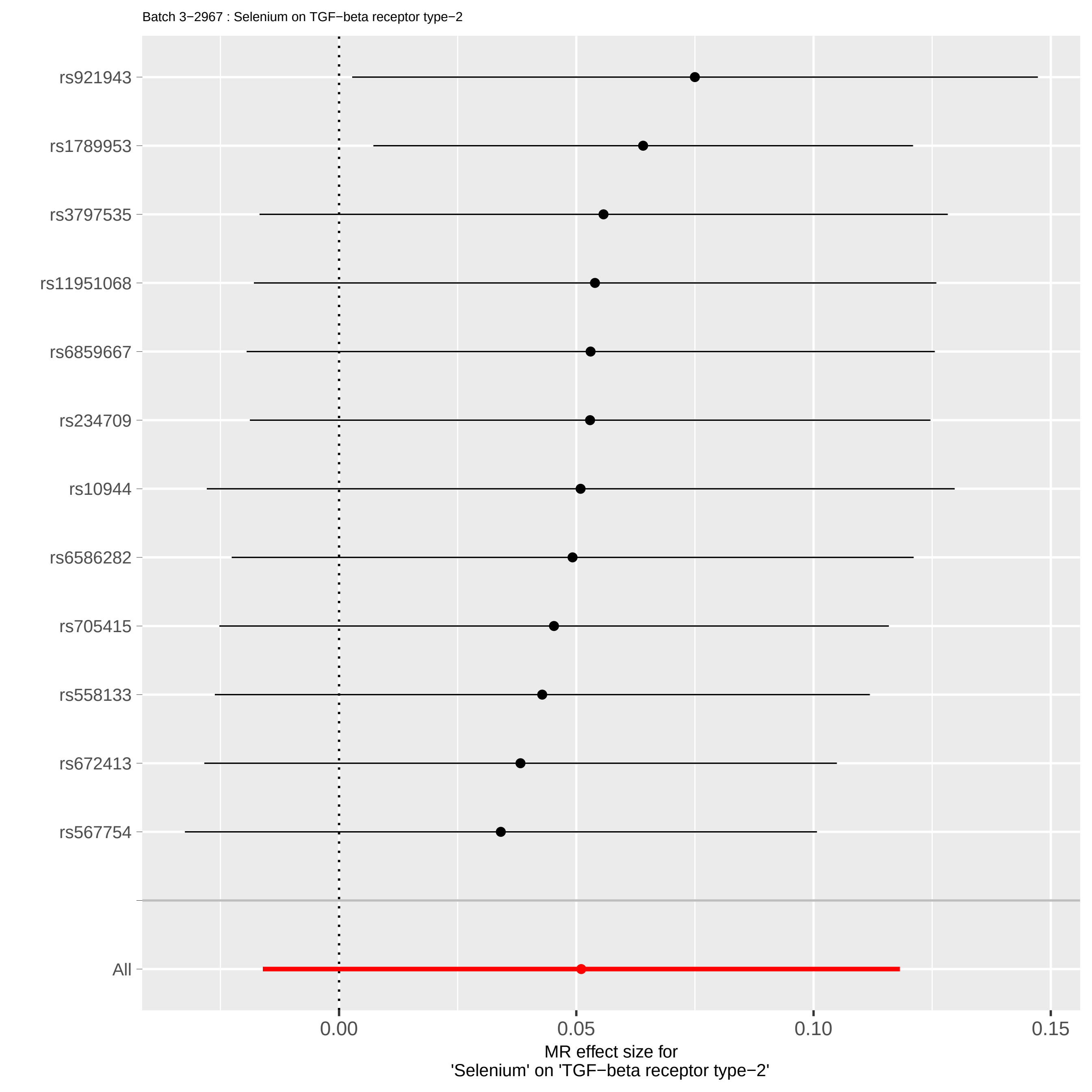

Batch 3−2967 : Selenium on TGF−beta receptor type−2
rs921943
rs1789953
rs3797535
rs11951068
rs6859667
rs234709
rs10944
rs6586282
rs705415
rs558133
rs672413
rs567754
All
0.00
0.05
0.10
0.15
MR effect size for
'Selenium' on 'TGF−beta receptor type−2'

Batch 3−2968 : Selenium on Transforming growth factor beta receptor type 3

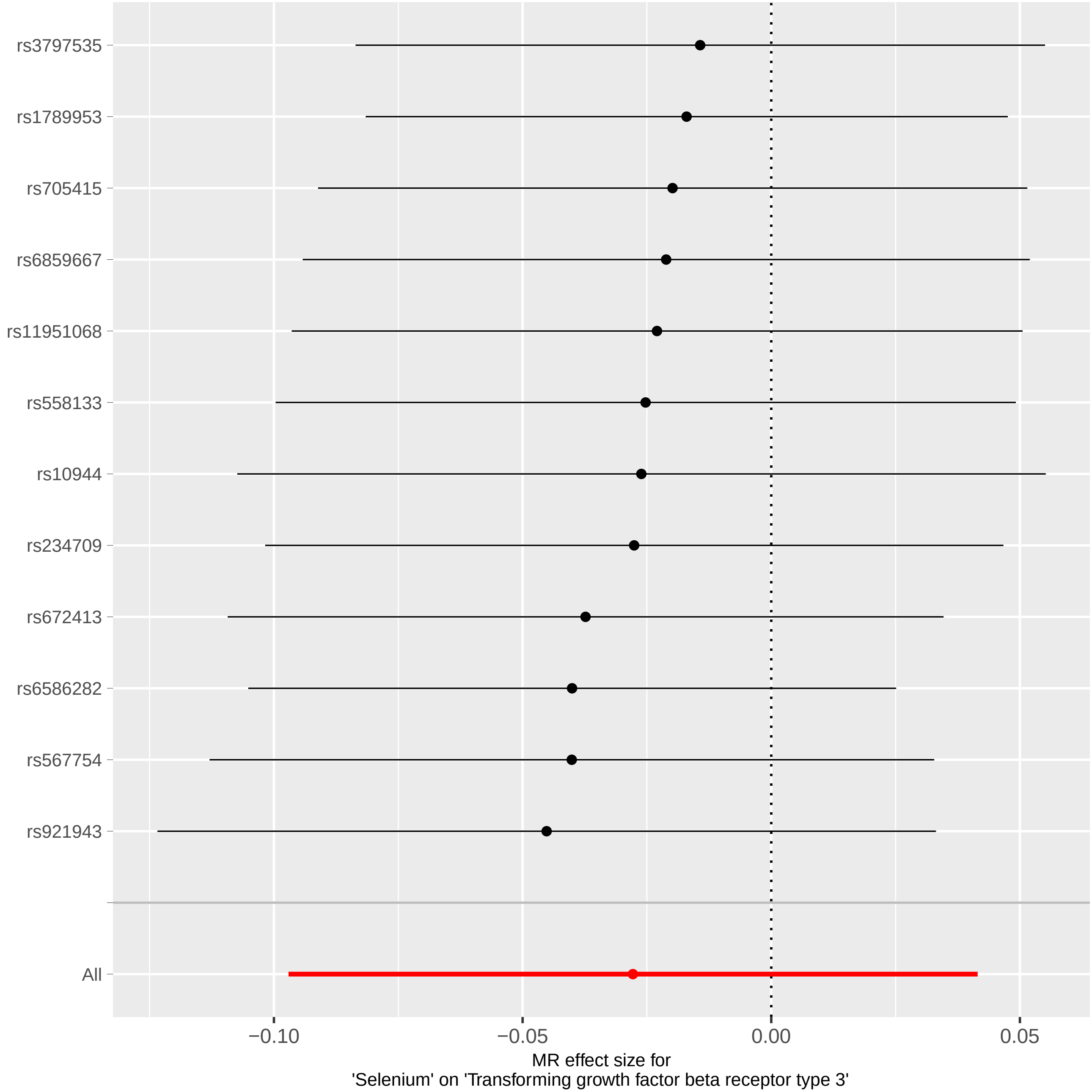

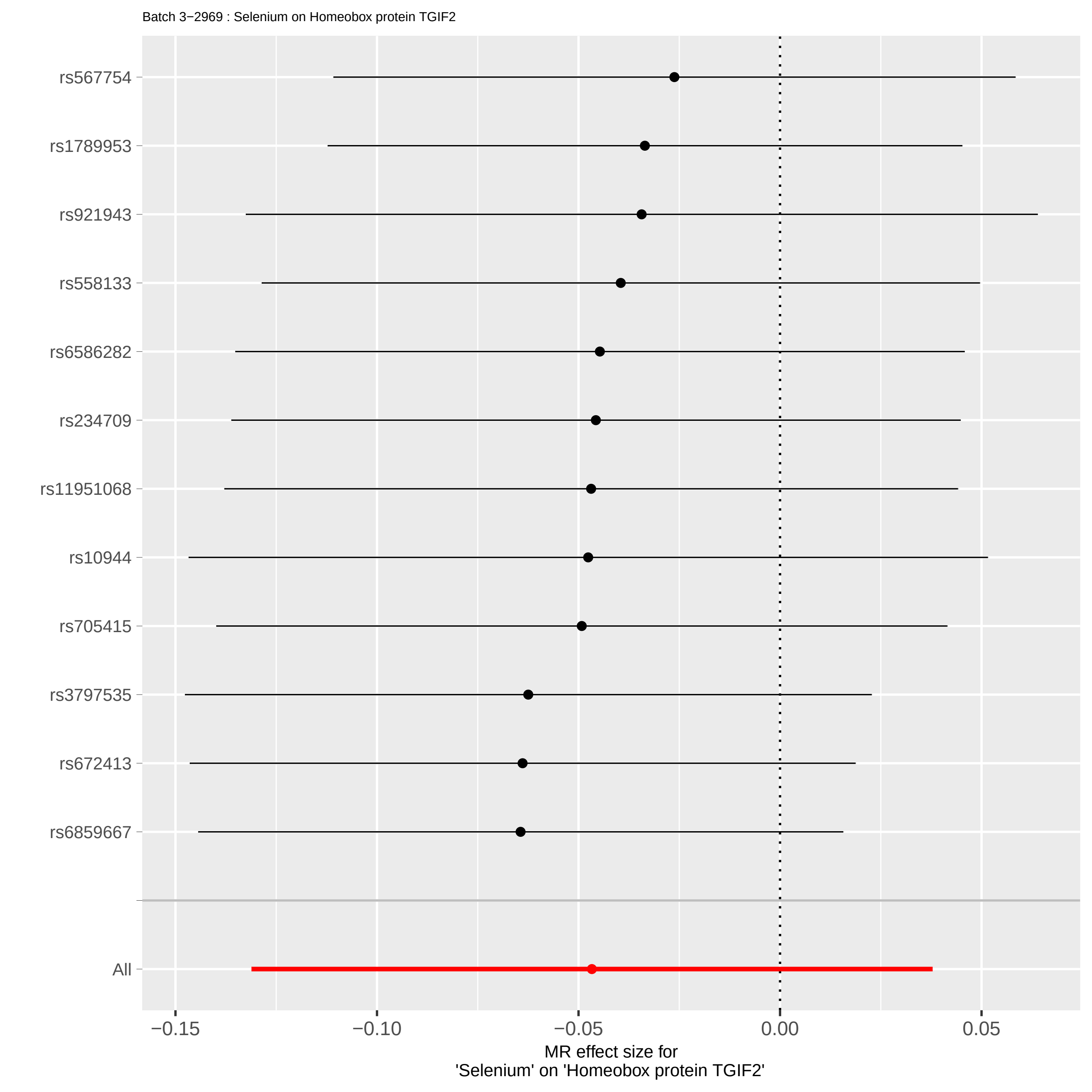

Batch 3−2969 : Selenium on Homeobox protein TGIF2
rs567754
rs1789953
rs921943
rs558133
rs6586282
rs234709
rs11951068
rs10944
rs705415
rs3797535
rs672413
rs6859667
All
−0.15
−0.10
−0.05
0.00
0.05
MR effect size for
'Selenium' on 'Homeobox protein TGIF2'

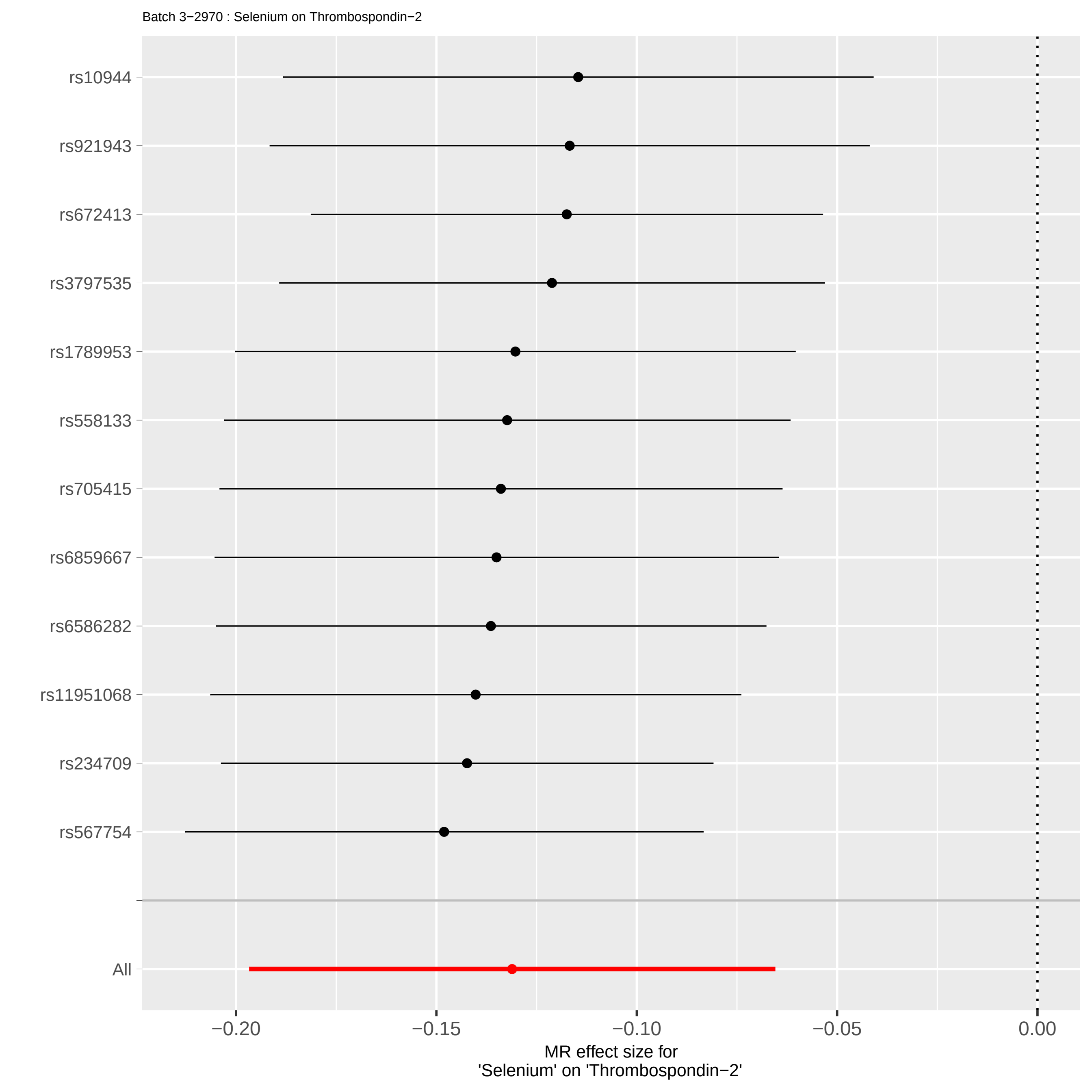

Batch 3−2970 : Selenium on Thrombospondin−2
rs10944
rs921943
rs672413
rs3797535
rs1789953
rs558133
rs705415
rs6859667
rs6586282
rs11951068
rs234709
rs567754
All
−0.20
−0.15
−0.10
−0.05
0.00
MR effect size for
'Selenium' on 'Thrombospondin−2'

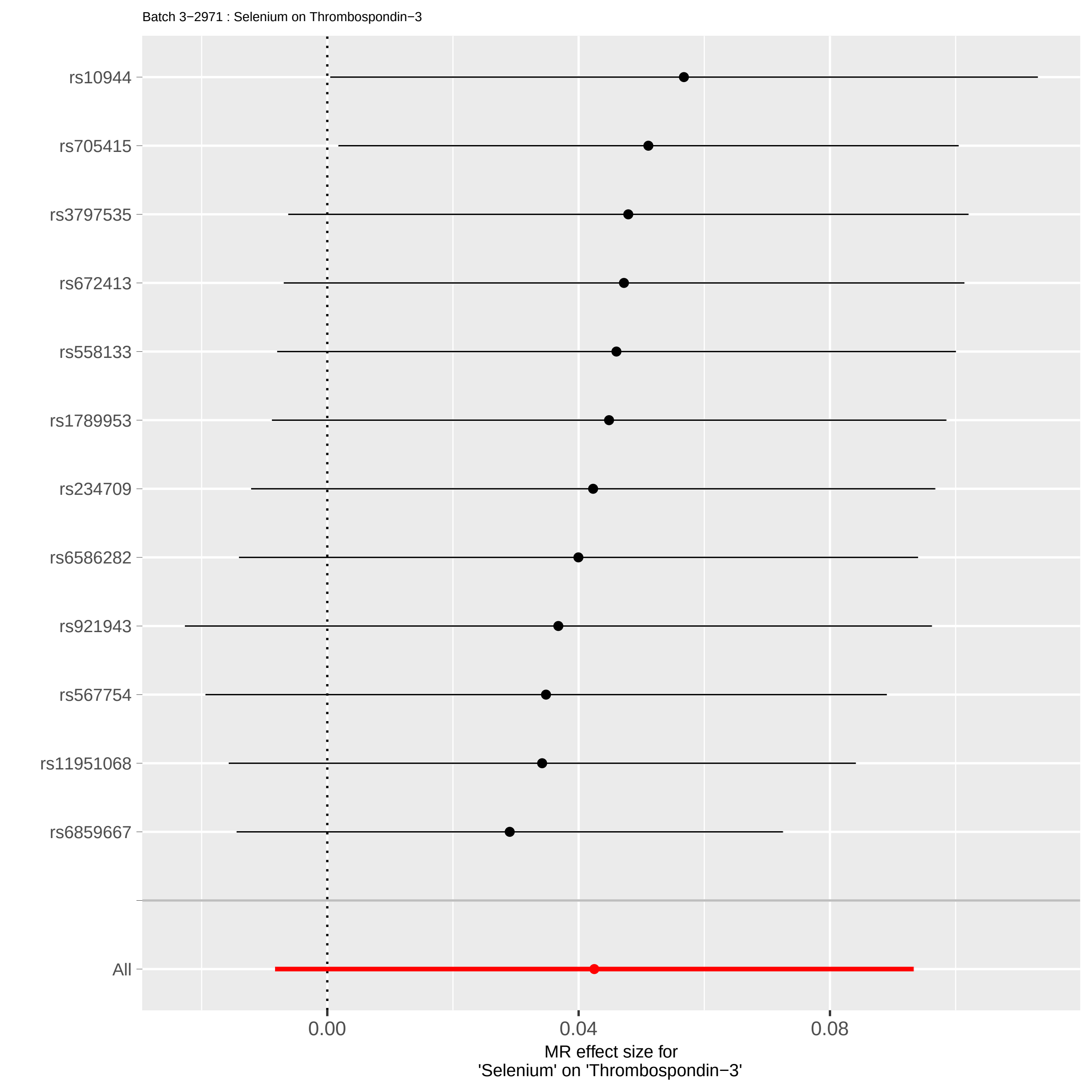

Batch 3−2971 : Selenium on Thrombospondin−3
rs10944
rs705415
rs3797535
rs672413
rs558133
rs1789953
rs234709
rs6586282
rs921943
rs567754
rs11951068
rs6859667
All
0.00
0.04
0.08
MR effect size for
'Selenium' on 'Thrombospondin−3'

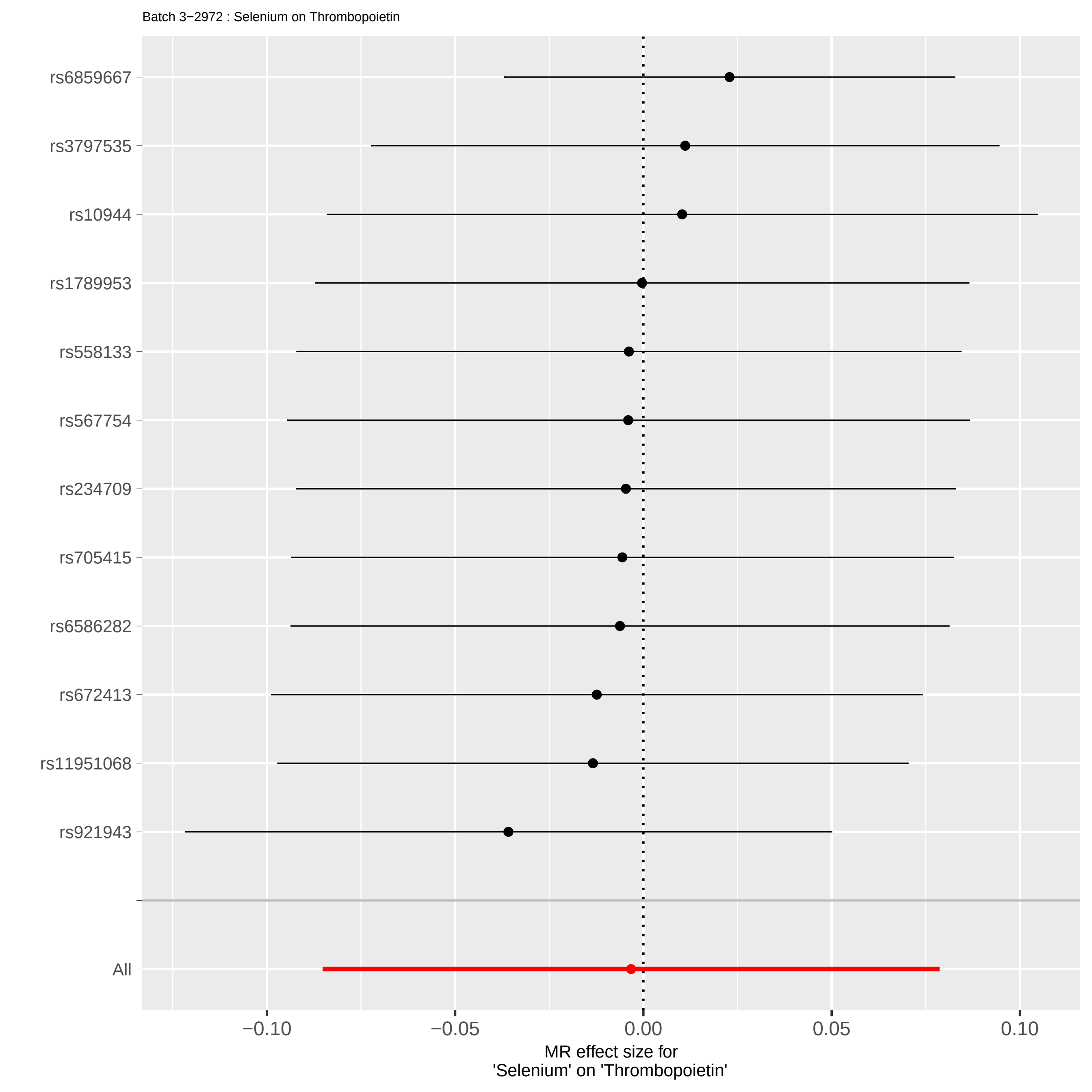

Batch 3−2972 : Selenium on Thrombopoietin
rs6859667
rs3797535
rs10944
rs1789953
rs558133
rs567754
rs234709
rs705415
rs6586282
rs672413
rs11951068
rs921943
All
−0.10
−0.05
0.00
0.05
0.10
MR effect size for
'Selenium' on 'Thrombopoietin'

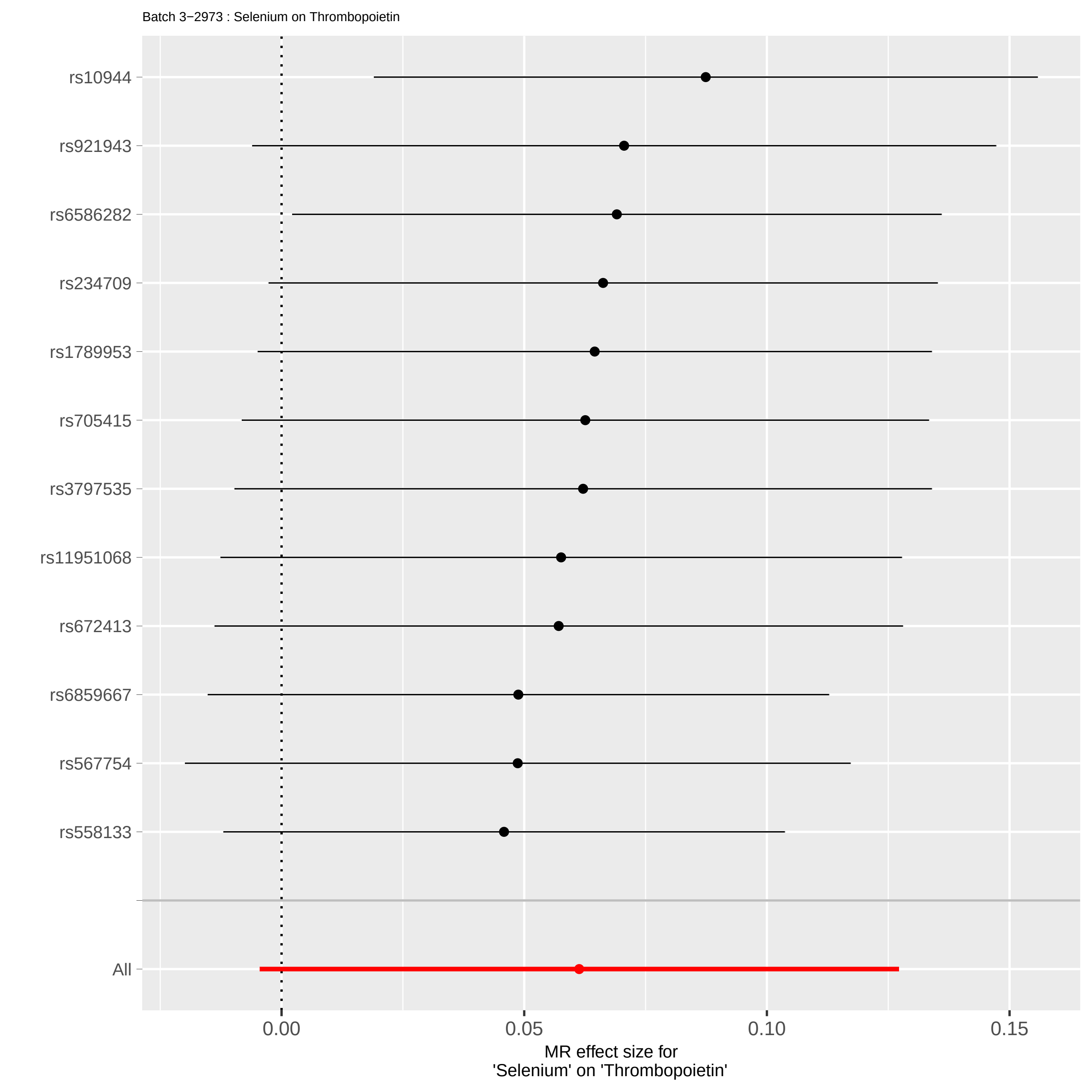

Batch 3−2973 : Selenium on Thrombopoietin
rs10944
rs921943
rs6586282
rs234709
rs1789953
rs705415
rs3797535
rs11951068
rs672413
rs6859667
rs567754
rs558133
All
0.00
0.05
0.10
0.15
MR effect size for
'Selenium' on 'Thrombopoietin'

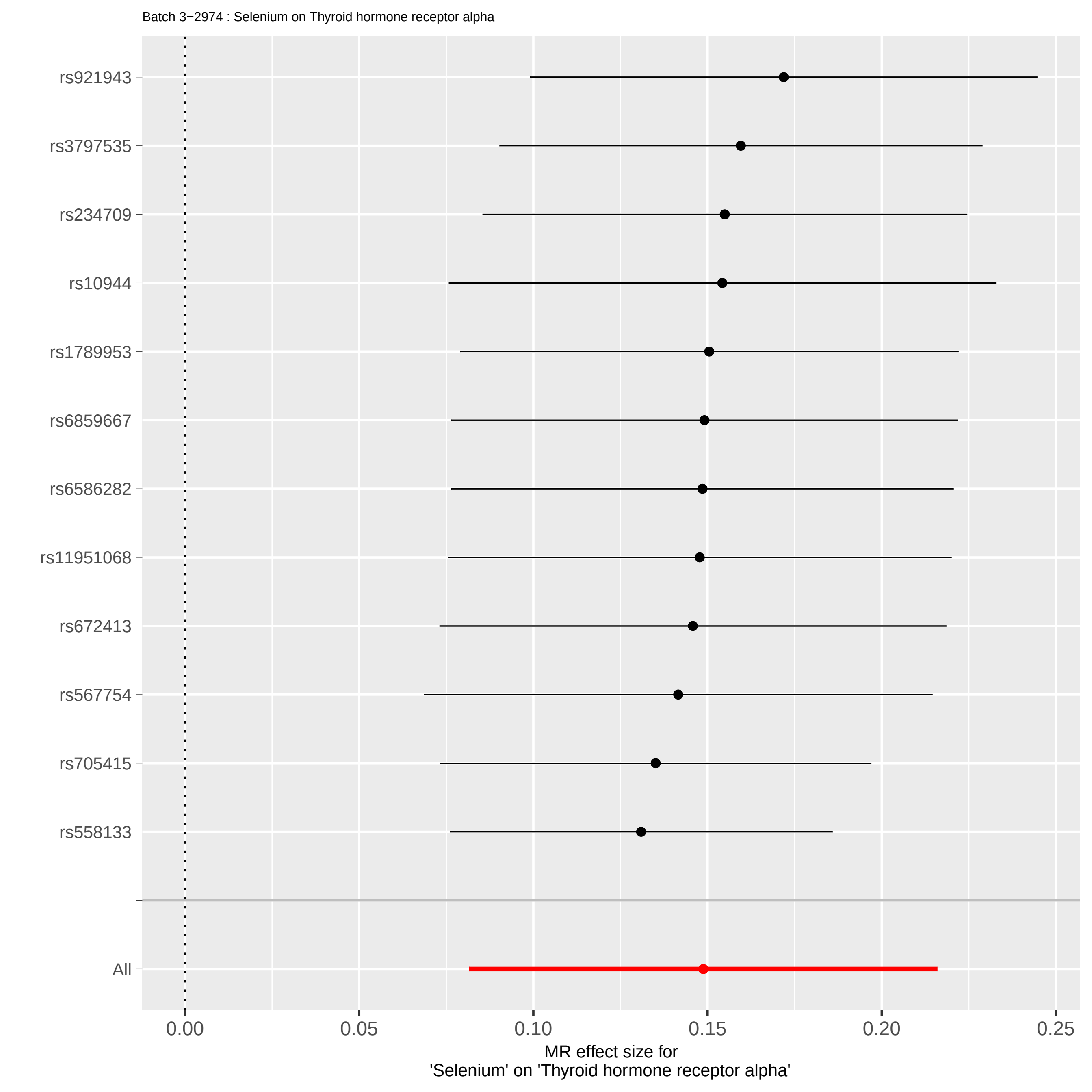

Batch 3−2974 : Selenium on Thyroid hormone receptor alpha
rs921943
rs3797535
rs234709
rs10944
rs1789953
rs6859667
rs6586282
rs11951068
rs672413
rs567754
rs705415
rs558133
All
0.00
0.05
0.10
0.15
0.20
0.25
MR effect size for
'Selenium' on 'Thyroid hormone receptor alpha'

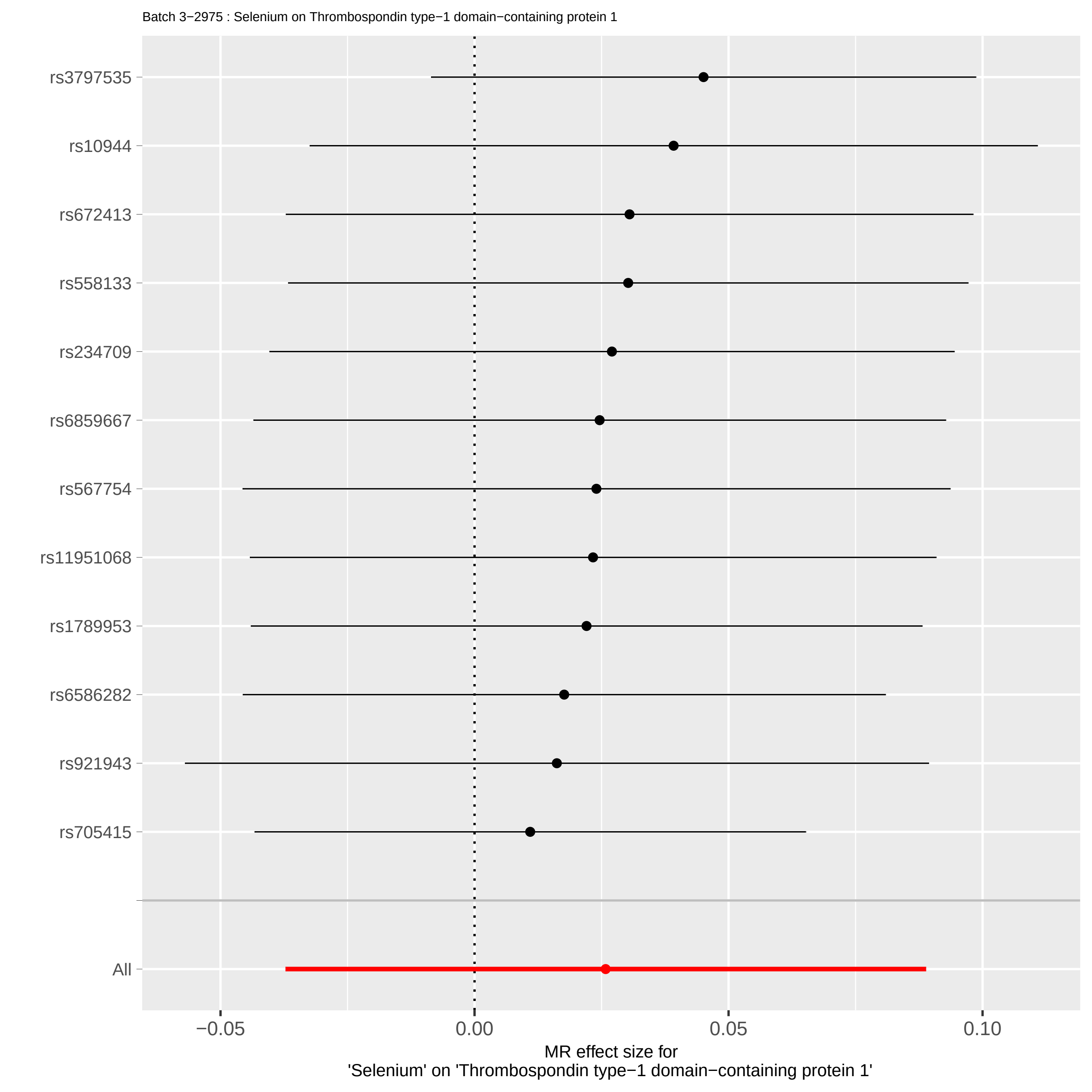
Batch 3−2975 : Selenium on Thrombospondin type−1 domain−containing protein 1
rs3797535
rs10944
rs672413
rs558133
rs234709
rs6859667
rs567754
rs11951068
rs1789953
rs6586282
rs921943
rs705415
All
−0.05
0.00
0.05
0.10
MR effect size for
'Selenium' on 'Thrombospondin type−1 domain−containing protein 1'

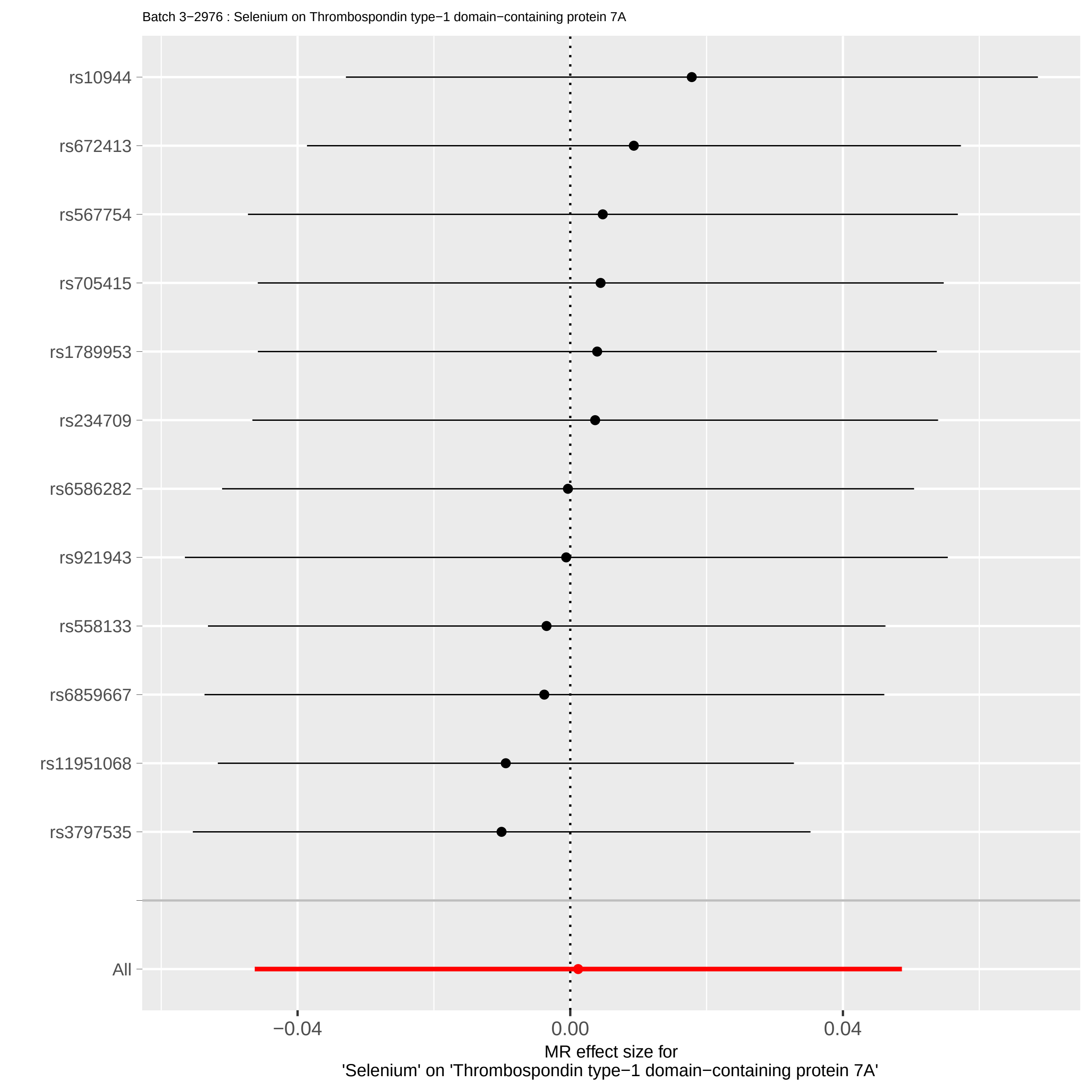

Batch 3−2976 : Selenium on Thrombospondin type−1 domain−containing protein 7A
rs10944
rs672413
rs567754
rs705415
rs1789953
rs234709
rs6586282
rs921943
rs558133
rs6859667
rs11951068
rs3797535
All
−0.04
0.00
0.04
MR effect size for
'Selenium' on 'Thrombospondin type−1 domain−containing protein 7A'

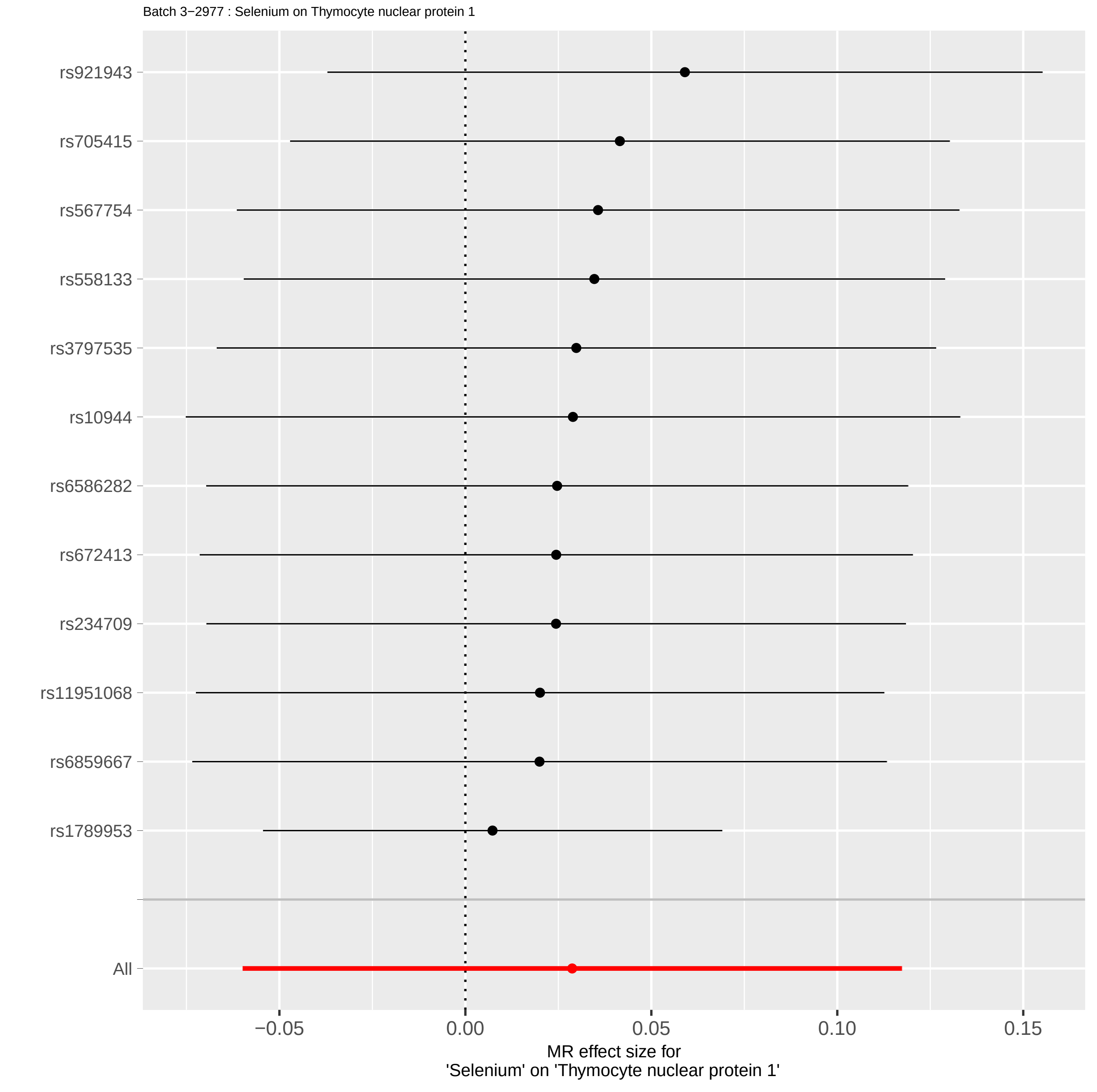
Batch 3−2977 : Selenium on Thymocyte nuclear protein 1
rs921943
rs705415
rs567754
rs558133
rs3797535
rs10944
rs6586282
rs672413
rs234709
rs11951068
rs6859667
rs1789953
All
−0.05
0.00
0.05
0.10
0.15
MR effect size for
'Selenium' on 'Thymocyte nuclear protein 1'

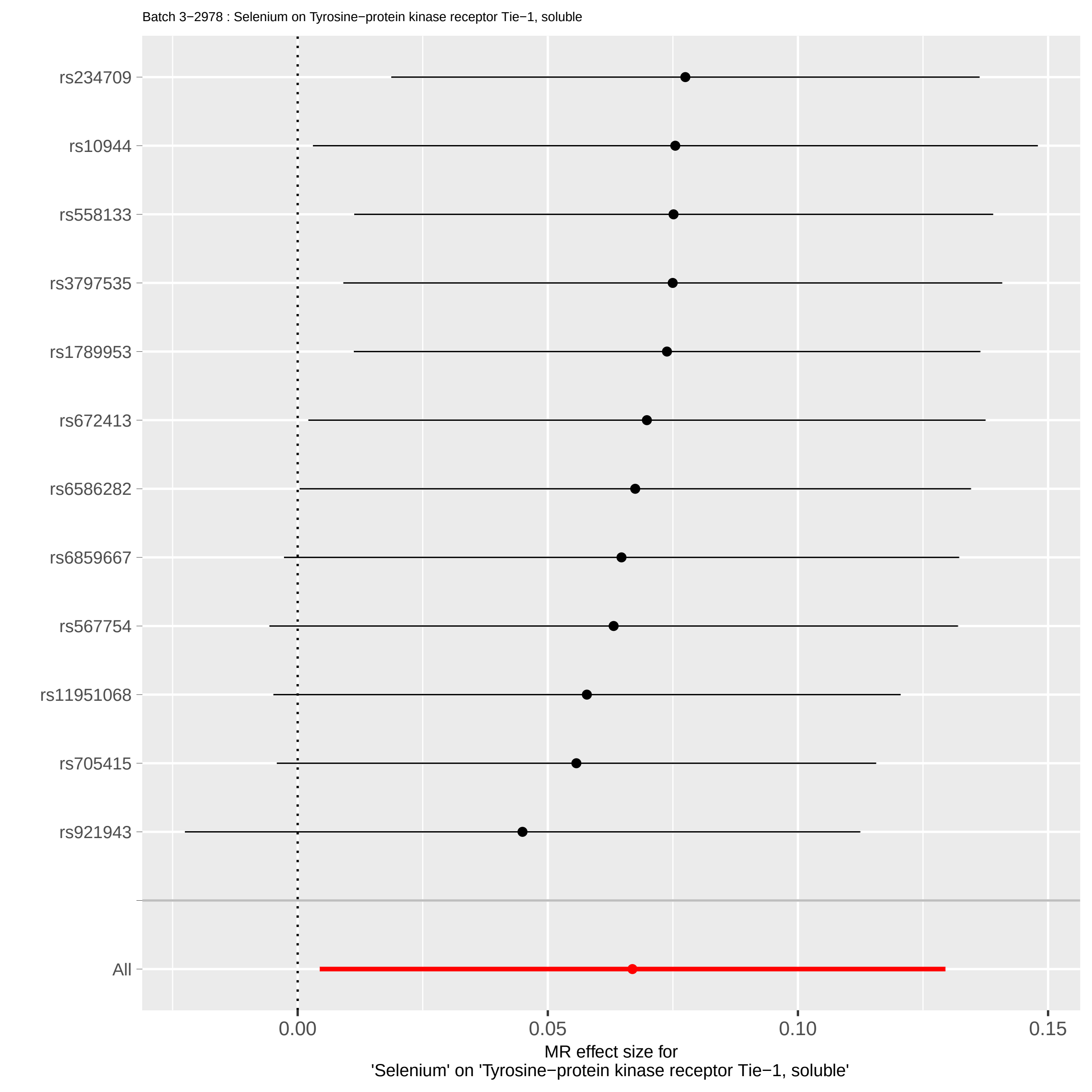

Batch 3−2978 : Selenium on Tyrosine−protein kinase receptor Tie−1, soluble
rs234709
rs10944
rs558133
rs3797535
rs1789953
rs672413
rs6586282
rs6859667
rs567754
rs11951068
rs705415
rs921943
All
0.00
0.05
0.10
0.15
MR effect size for
'Selenium' on 'Tyrosine−protein kinase receptor Tie−1, soluble'

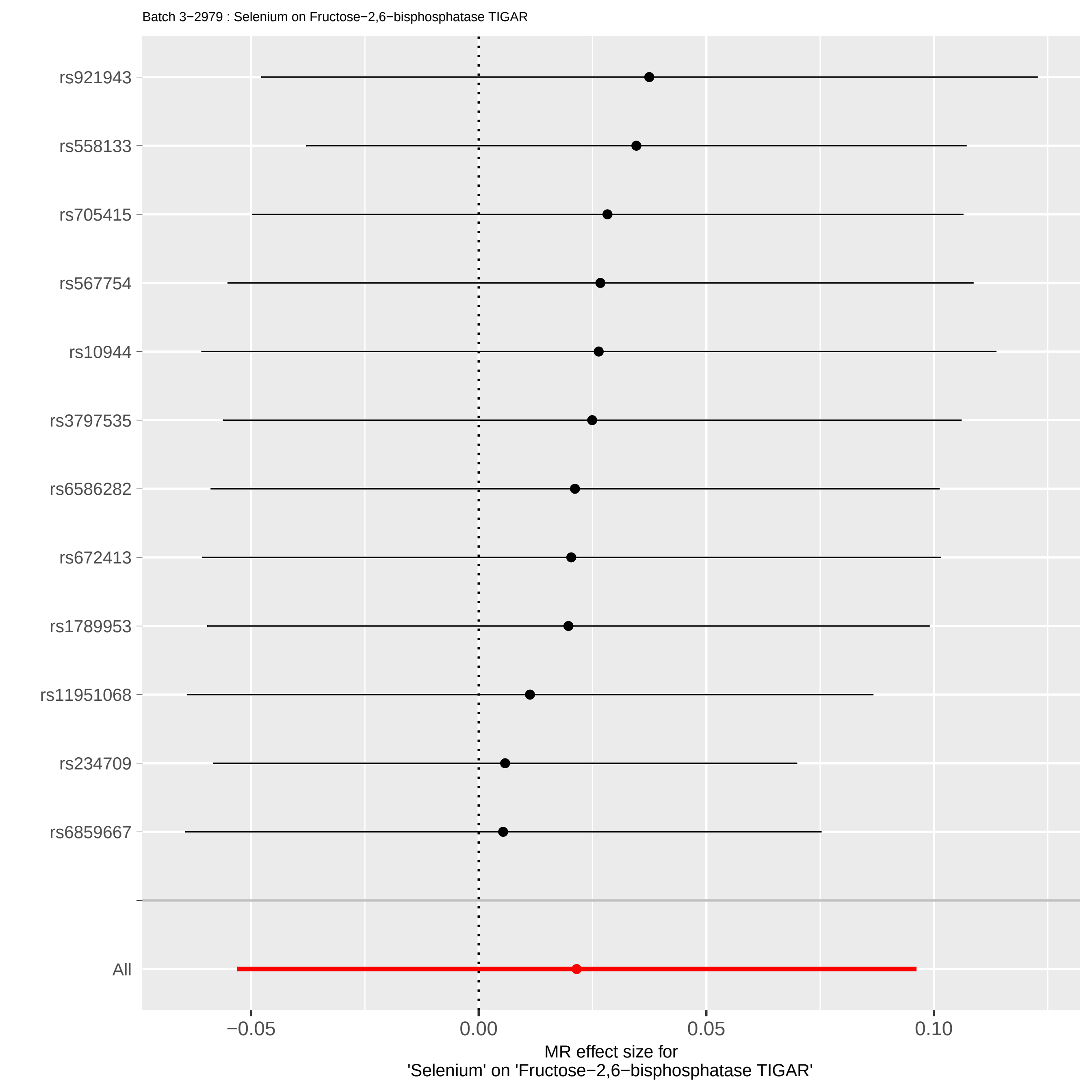
Batch 3−2979 : Selenium on Fructose−2,6−bisphosphatase TIGAR
rs921943
rs558133
rs705415
rs567754
rs10944
rs3797535
rs6586282
rs672413
rs1789953
rs11951068
rs234709
rs6859667
All
−0.05
0.00
0.05
0.10
MR effect size for
'Selenium' on 'Fructose−2,6−bisphosphatase TIGAR'

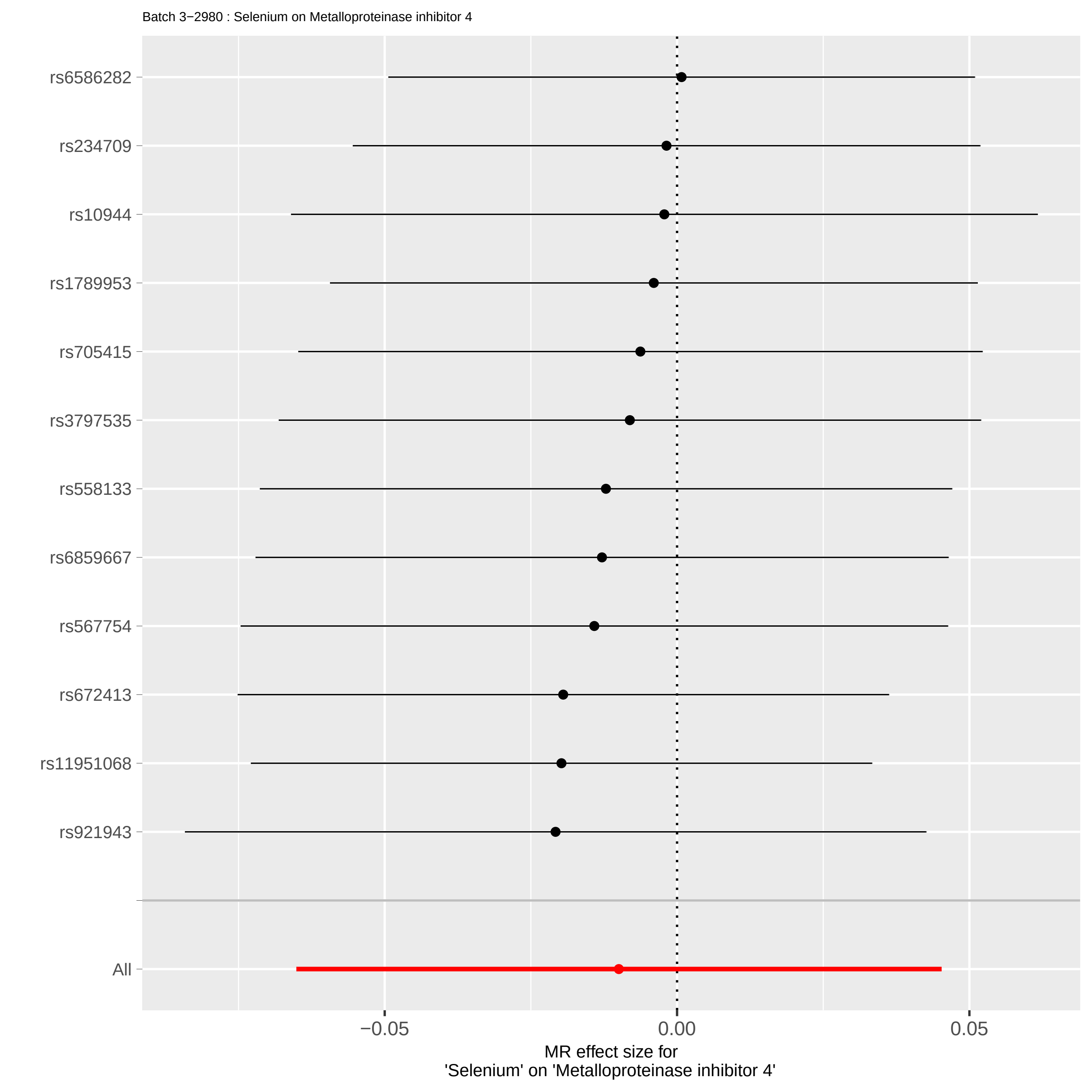
Batch 3−2980 : Selenium on Metalloproteinase inhibitor 4
rs6586282
rs234709
rs10944
rs1789953
rs705415
rs3797535
rs558133
rs6859667
rs567754
rs672413
rs11951068
rs921943
All
−0.05
0.00
0.05
MR effect size for
'Selenium' on 'Metalloproteinase inhibitor 4'

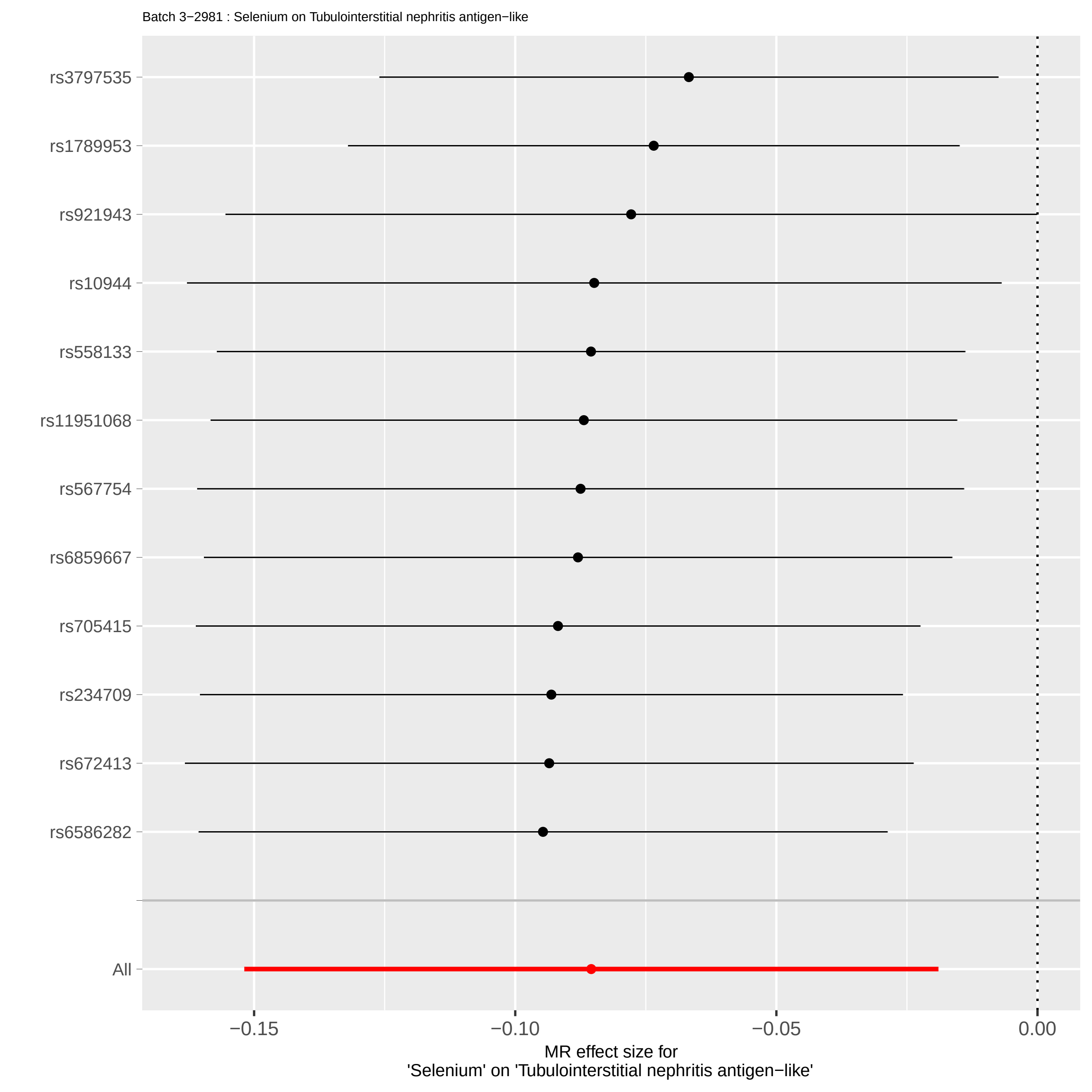

Batch 3−2981 : Selenium on Tubulointerstitial nephritis antigen−like
rs3797535
rs1789953
rs921943
rs10944
rs558133
rs11951068
rs567754
rs6859667
rs705415
rs234709
rs672413
rs6586282
All
−0.15
−0.10
−0.05
0.00
MR effect size for
'Selenium' on 'Tubulointerstitial nephritis antigen−like'

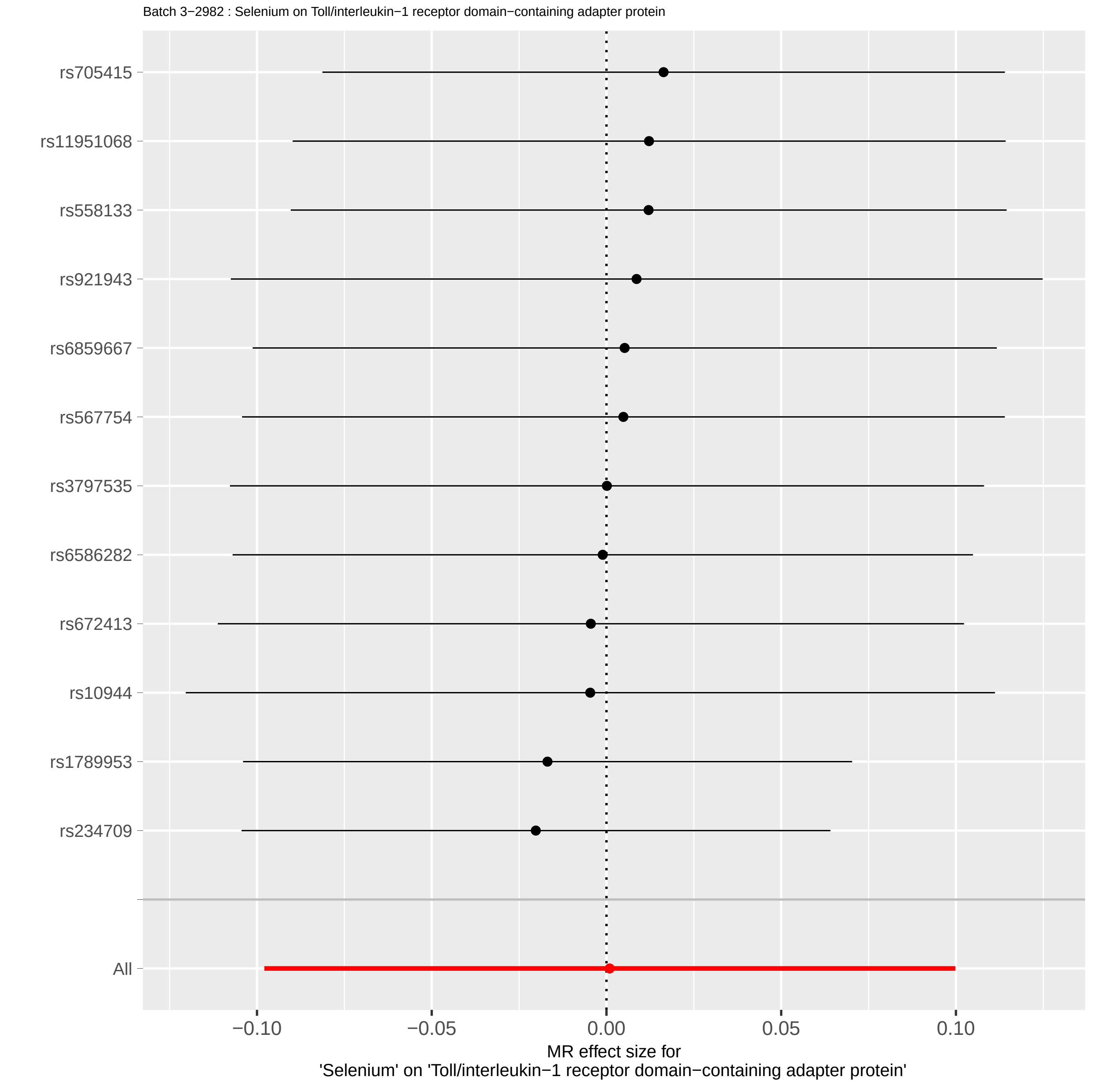

Batch 3−2982 : Selenium on Toll/interleukin−1 receptor domain−containing adapter protein
rs705415
rs11951068
rs558133
rs921943
rs6859667
rs567754
rs3797535
rs6586282
rs672413
rs10944
rs1789953
rs234709
All
−0.10
−0.05
0.00
0.05
0.10
MR effect size for
'Selenium' on 'Toll/interleukin−1 receptor domain−containing adapter protein'

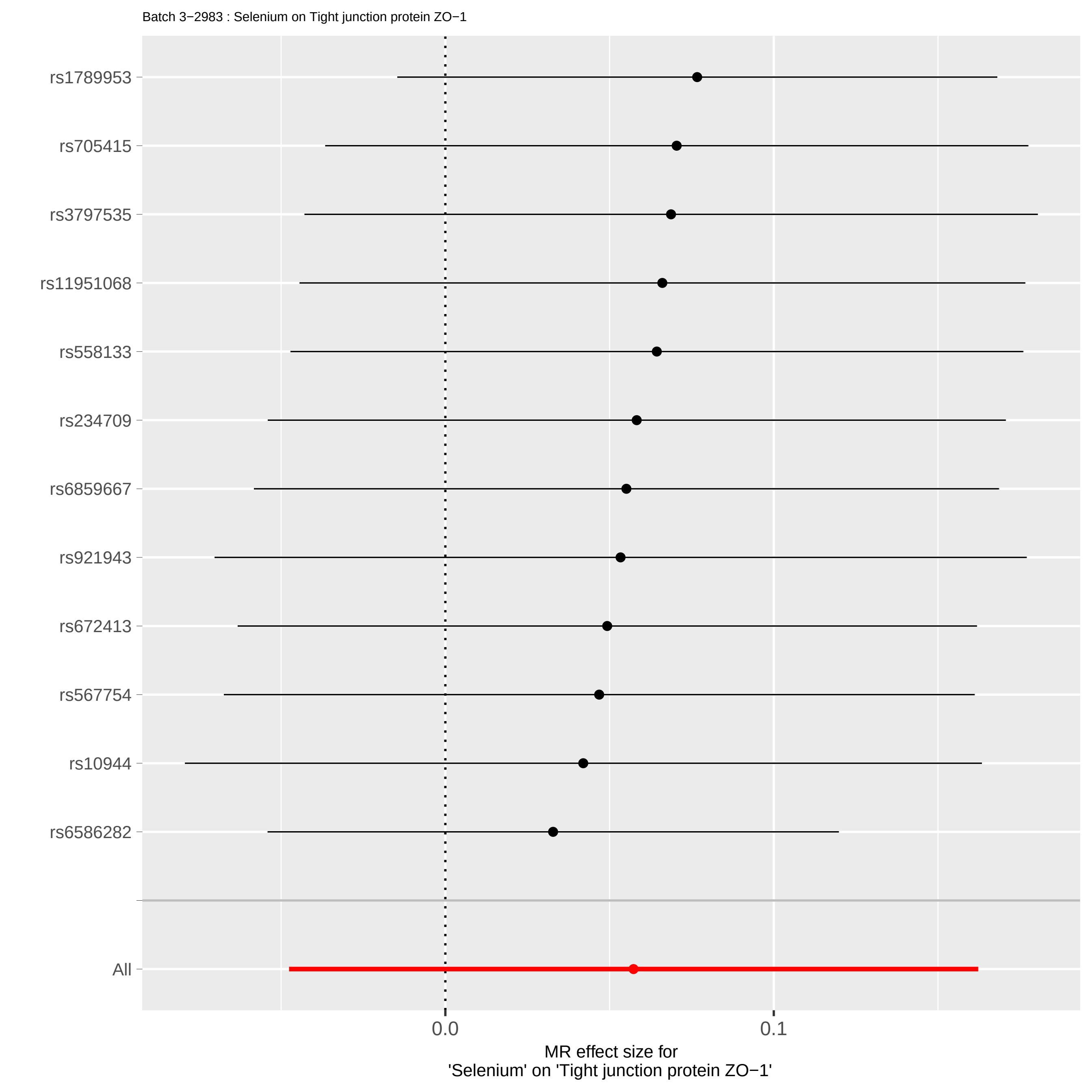

Batch 3−2983 : Selenium on Tight junction protein ZO−1
rs1789953
rs705415
rs3797535
rs11951068
rs558133
rs234709
rs6859667
rs921943
rs672413
rs567754
rs10944
rs6586282
All
0.0
0.1
MR effect size for
'Selenium' on 'Tight junction protein ZO−1'

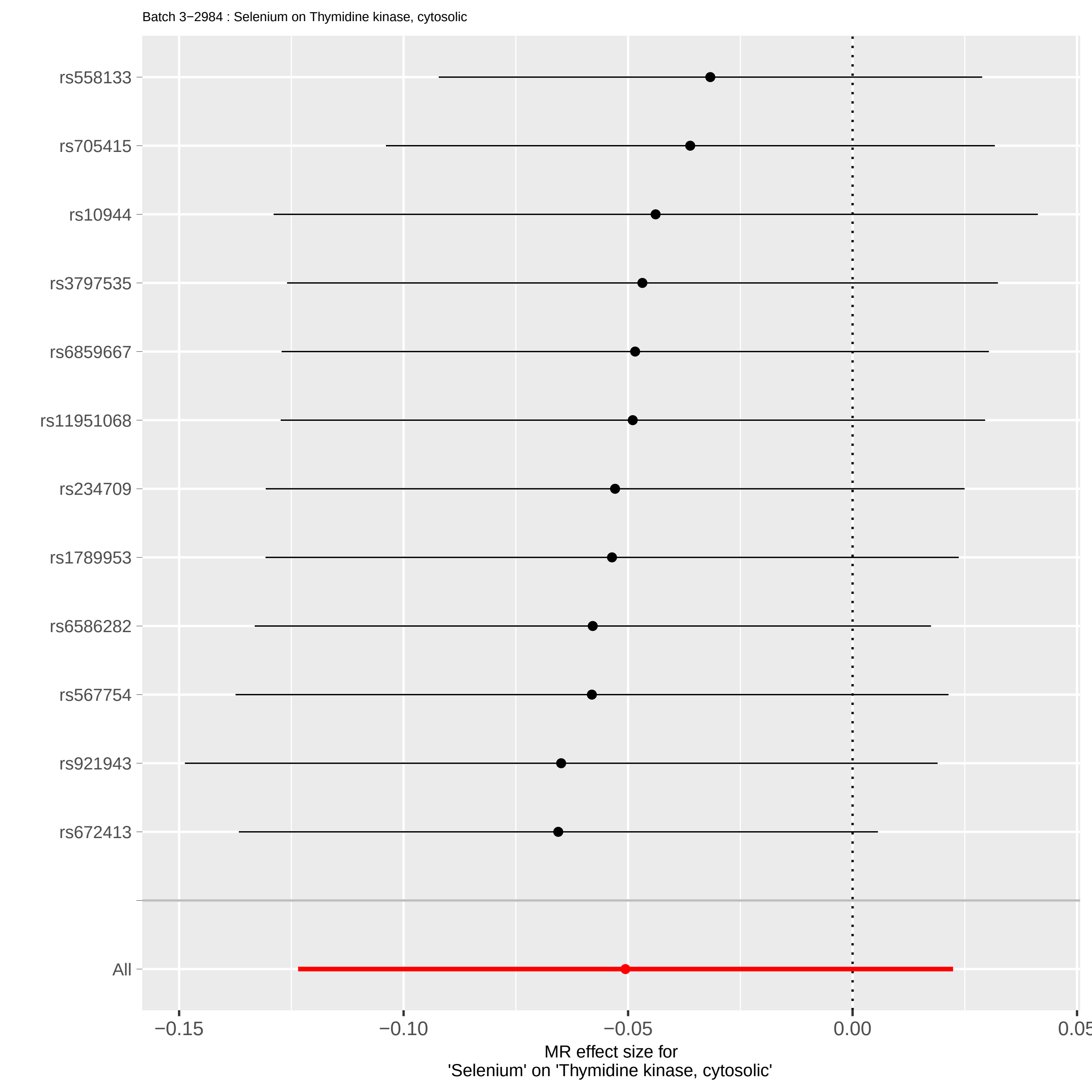

Batch 3−2984 : Selenium on Thymidine kinase, cytosolic
rs558133
rs705415
rs10944
rs3797535
rs6859667
rs11951068
rs234709
rs1789953
rs6586282
rs567754
rs921943
rs672413
All
−0.15
−0.10
−0.05
0.00
0.05
MR effect size for
'Selenium' on 'Thymidine kinase, cytosolic'

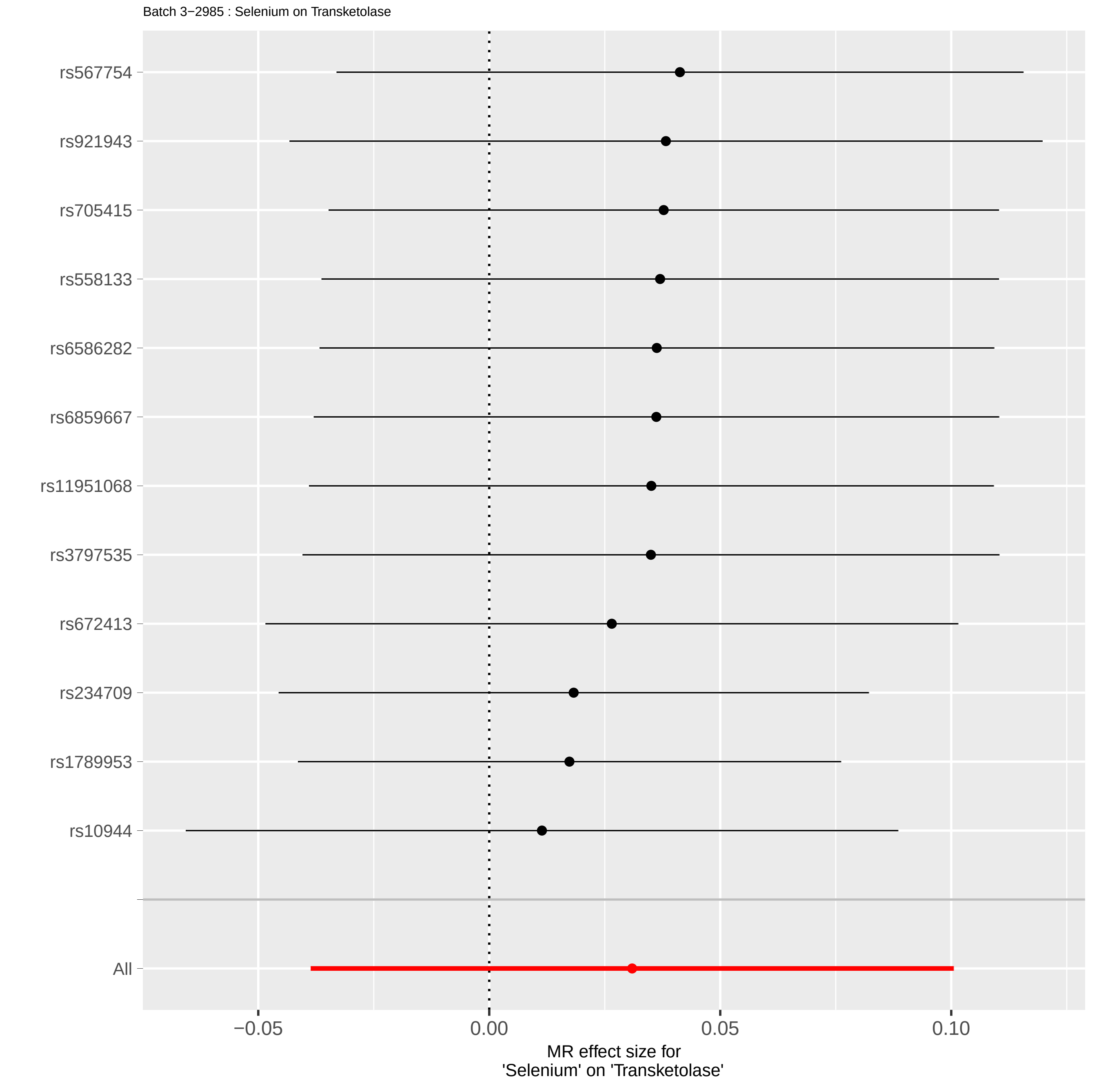
Batch 3−2985 : Selenium on Transketolase
rs567754
rs921943
rs705415
rs558133
rs6586282
rs6859667
rs11951068
rs3797535
rs672413
rs234709
rs1789953
rs10944
All
−0.05
0.00
0.05
0.10
MR effect size for
'Selenium' on 'Transketolase'

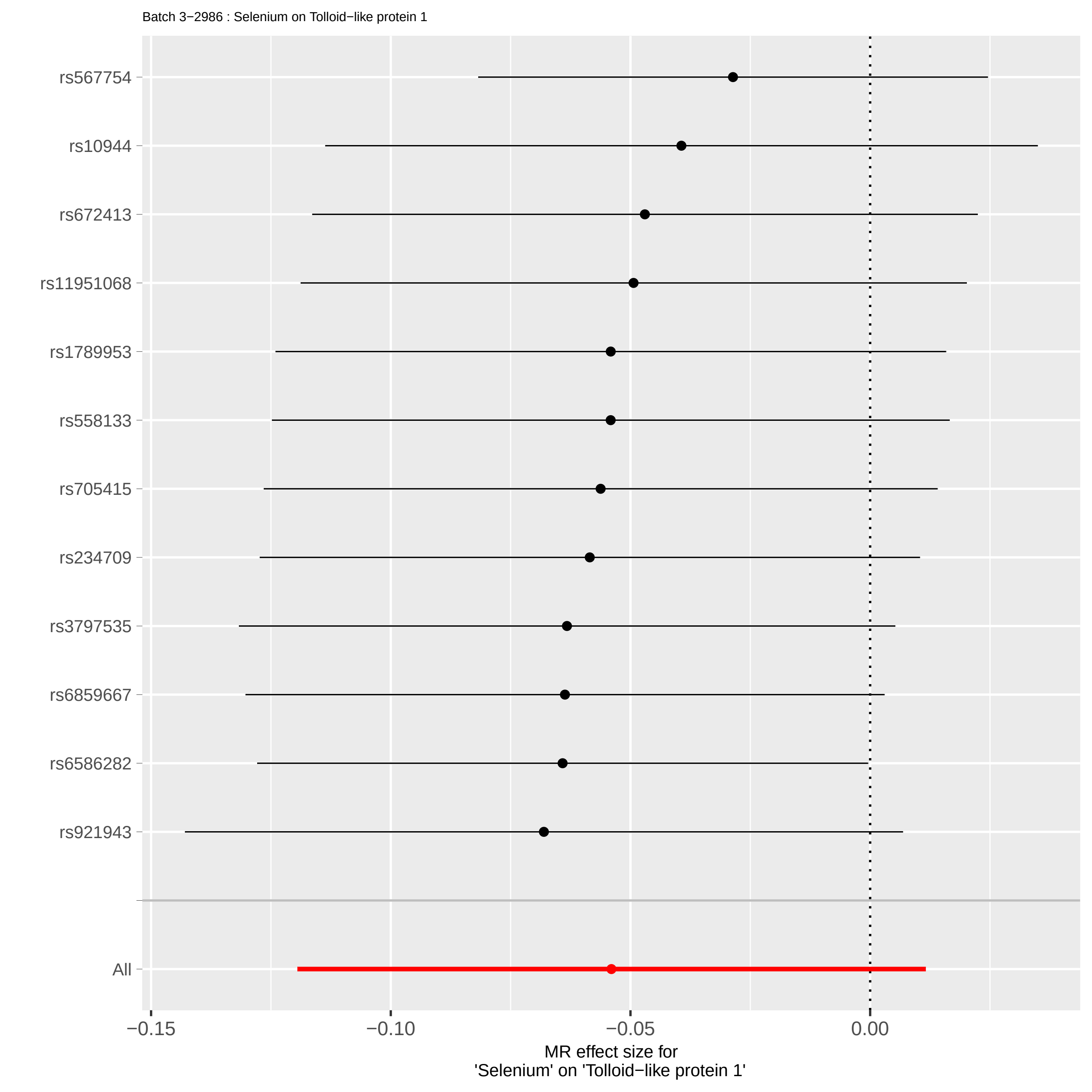

Batch 3−2986 : Selenium on Tolloid−like protein 1
rs567754
rs10944
rs672413
rs11951068
rs1789953
rs558133
rs705415
rs234709
rs3797535
rs6859667
rs6586282
rs921943
All
−0.15
−0.10
−0.05
0.00
MR effect size for
'Selenium' on 'Tolloid−like protein 1'

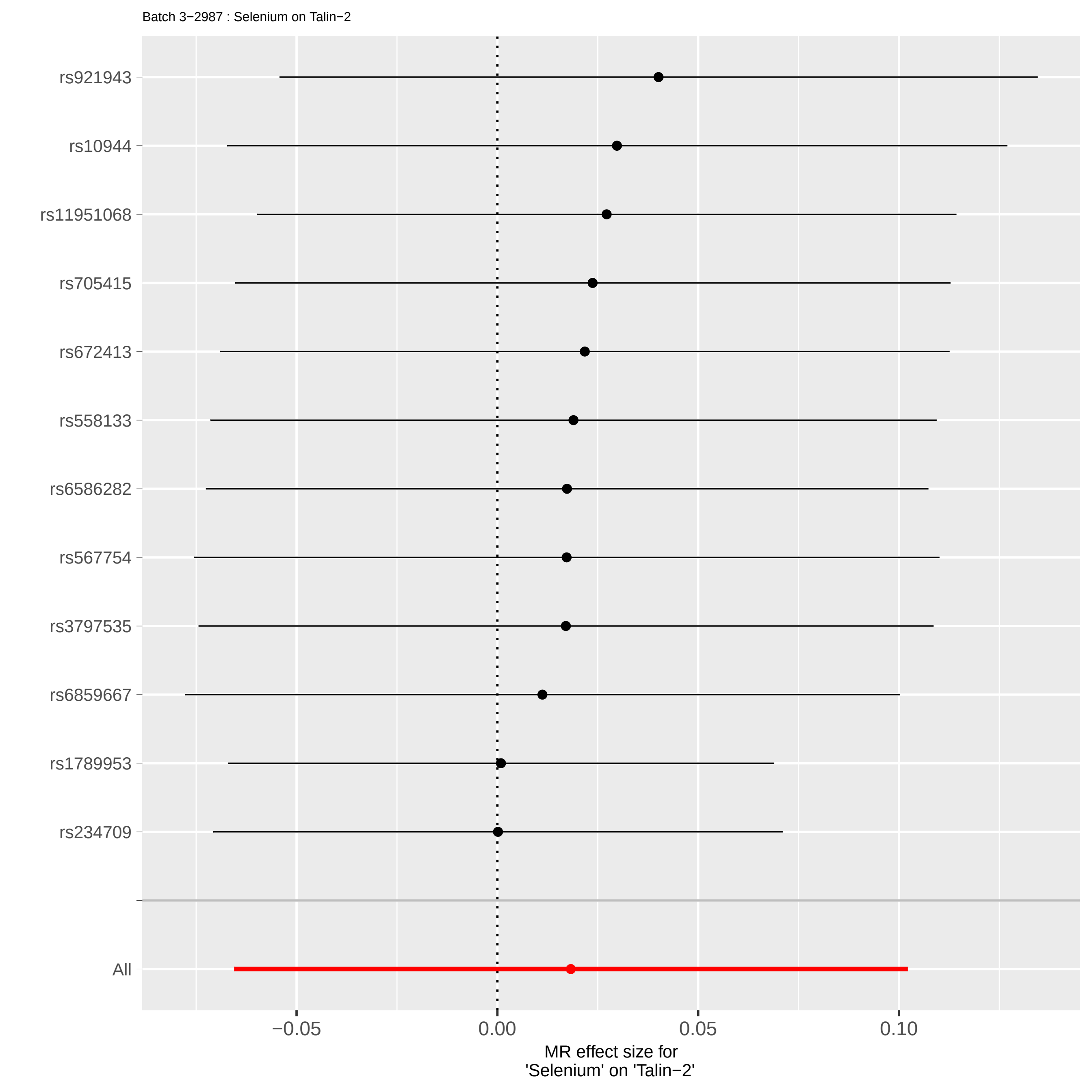
Batch 3−2987 : Selenium on Talin−2
rs921943
rs10944
rs11951068
rs705415
rs672413
rs558133
rs6586282
rs567754
rs3797535
rs6859667
rs1789953
rs234709
All
−0.05
0.00
0.05
0.10
MR effect size for
'Selenium' on 'Talin−2'

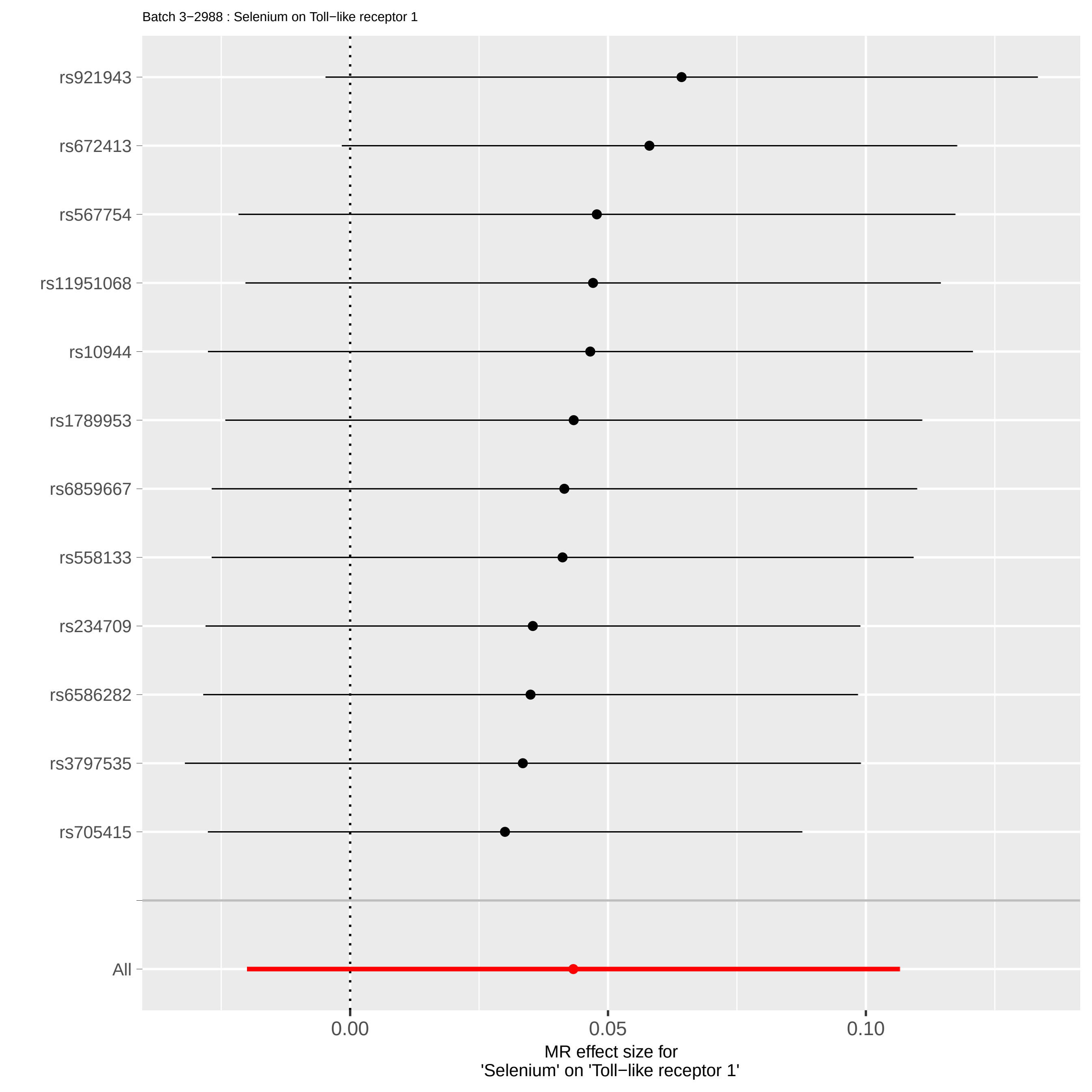

Batch 3−2988 : Selenium on Toll−like receptor 1
rs921943
rs672413
rs567754
rs11951068
rs10944
rs1789953
rs6859667
rs558133
rs234709
rs6586282
rs3797535
rs705415
All
0.00
0.05
0.10
MR effect size for
'Selenium' on 'Toll−like receptor 1'

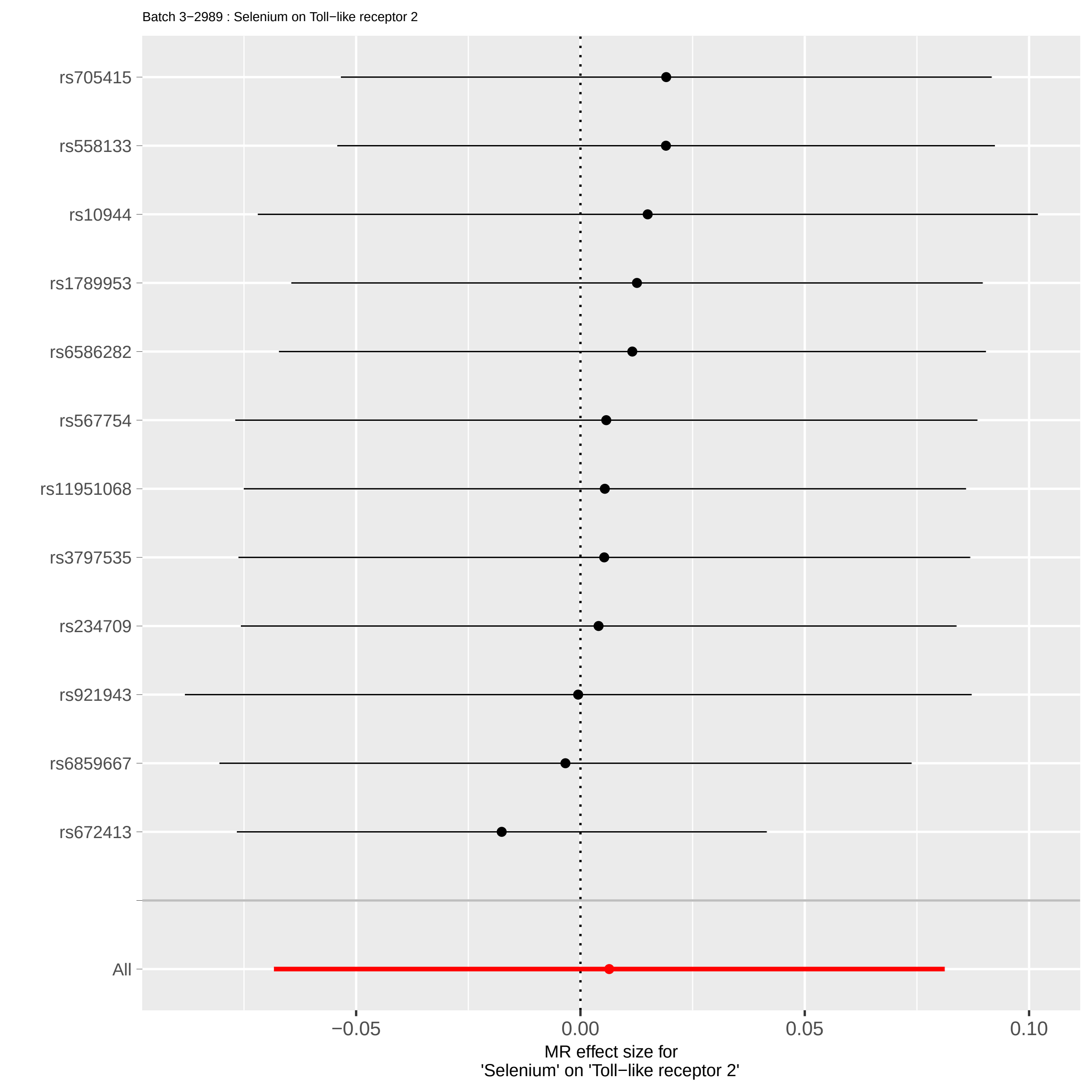

Batch 3−2989 : Selenium on Toll−like receptor 2
rs705415
rs558133
rs10944
rs1789953
rs6586282
rs567754
rs11951068
rs3797535
rs234709
rs921943
rs6859667
rs672413
All
−0.05
0.00
0.05
0.10
MR effect size for
'Selenium' on 'Toll−like receptor 2'

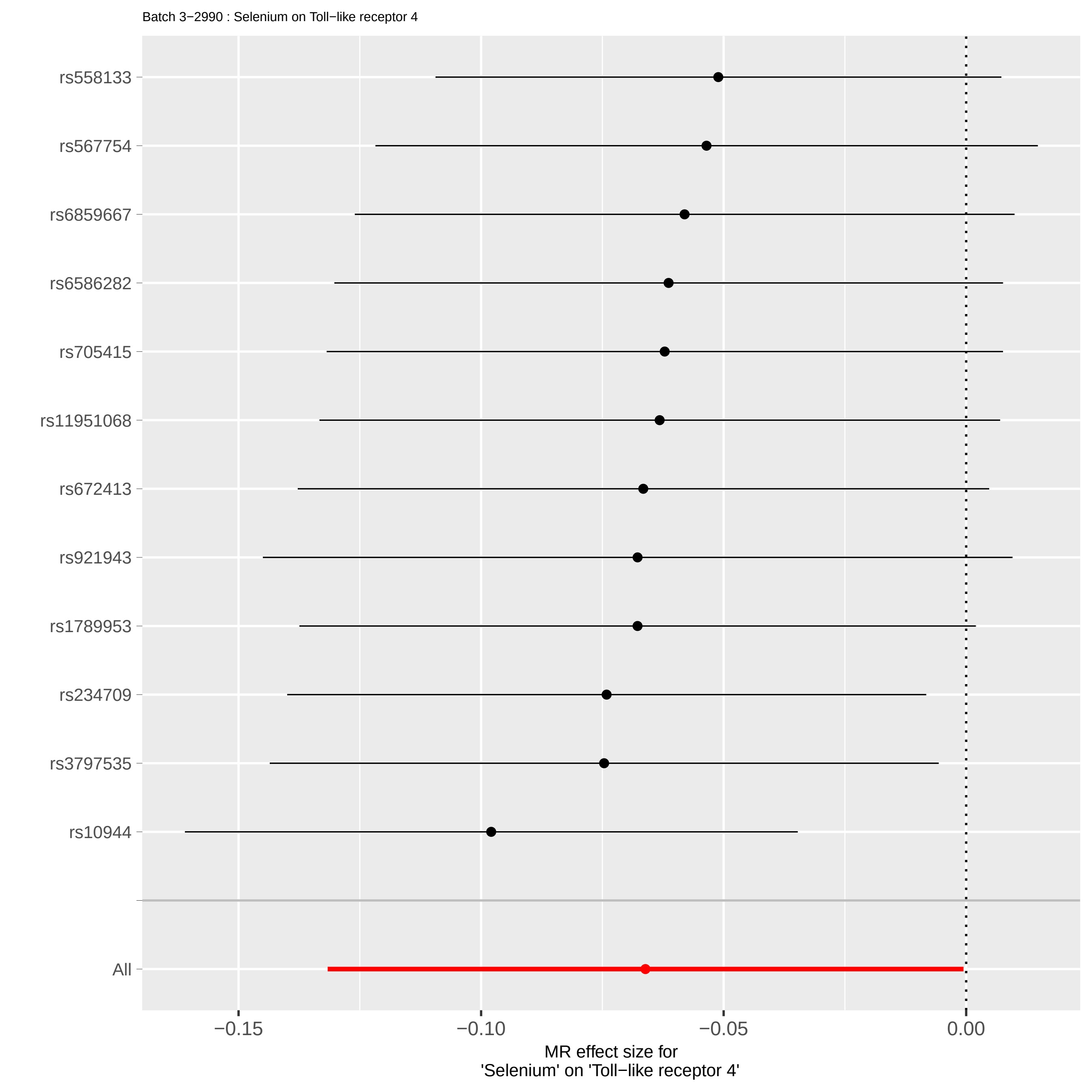

Batch 3−2990 : Selenium on Toll−like receptor 4
rs558133
rs567754
rs6859667
rs6586282
rs705415
rs11951068
rs672413
rs921943
rs1789953
rs234709
rs3797535
rs10944
All
−0.15
−0.10
−0.05
0.00
MR effect size for
'Selenium' on 'Toll−like receptor 4'

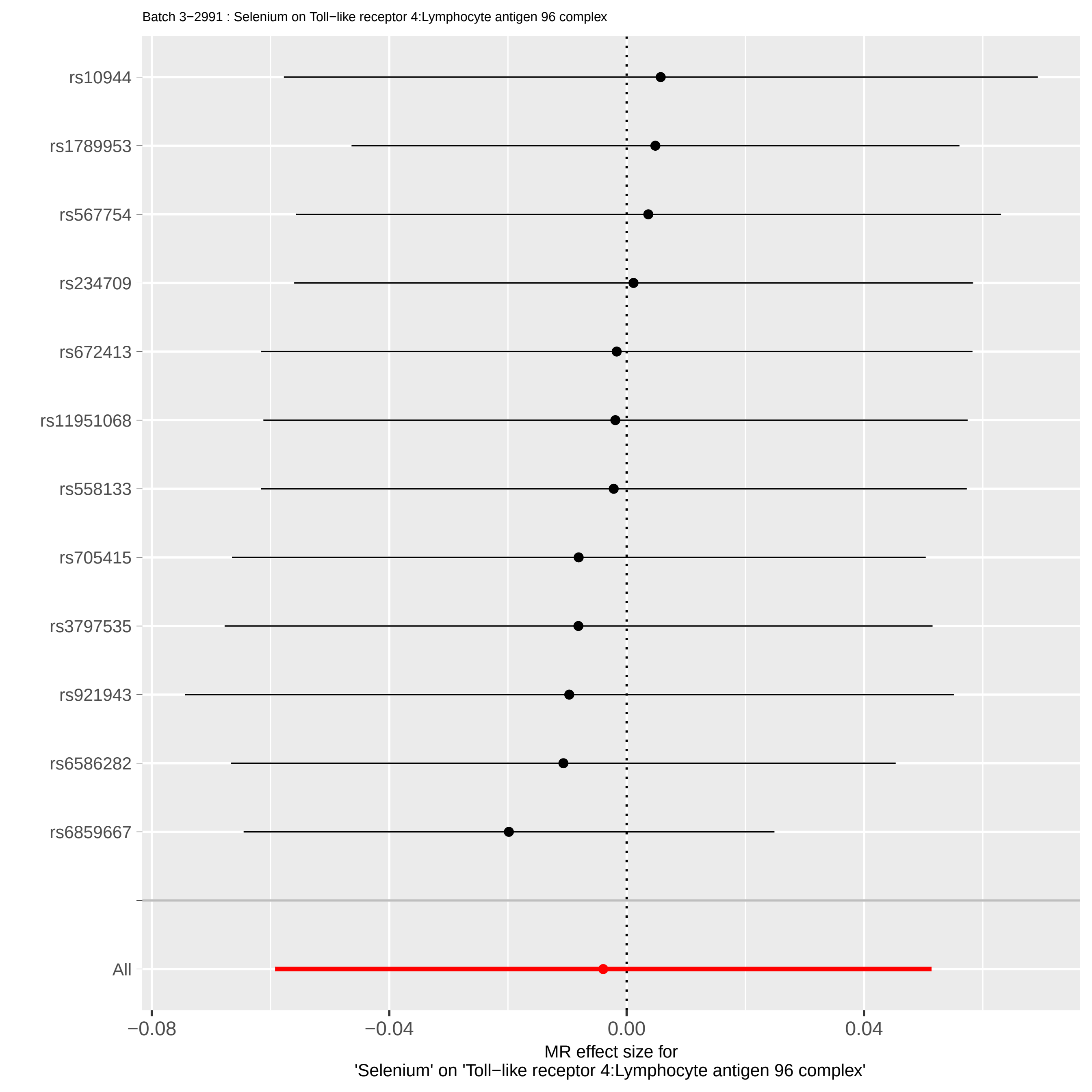

Batch 3−2991 : Selenium on Toll−like receptor 4:Lymphocyte antigen 96 complex
rs10944
rs1789953
rs567754
rs234709
rs672413
rs11951068
rs558133
rs705415
rs3797535
rs921943
rs6586282
rs6859667
All
−0.08
−0.04
0.00
0.04
MR effect size for
'Selenium' on 'Toll−like receptor 4:Lymphocyte antigen 96 complex'

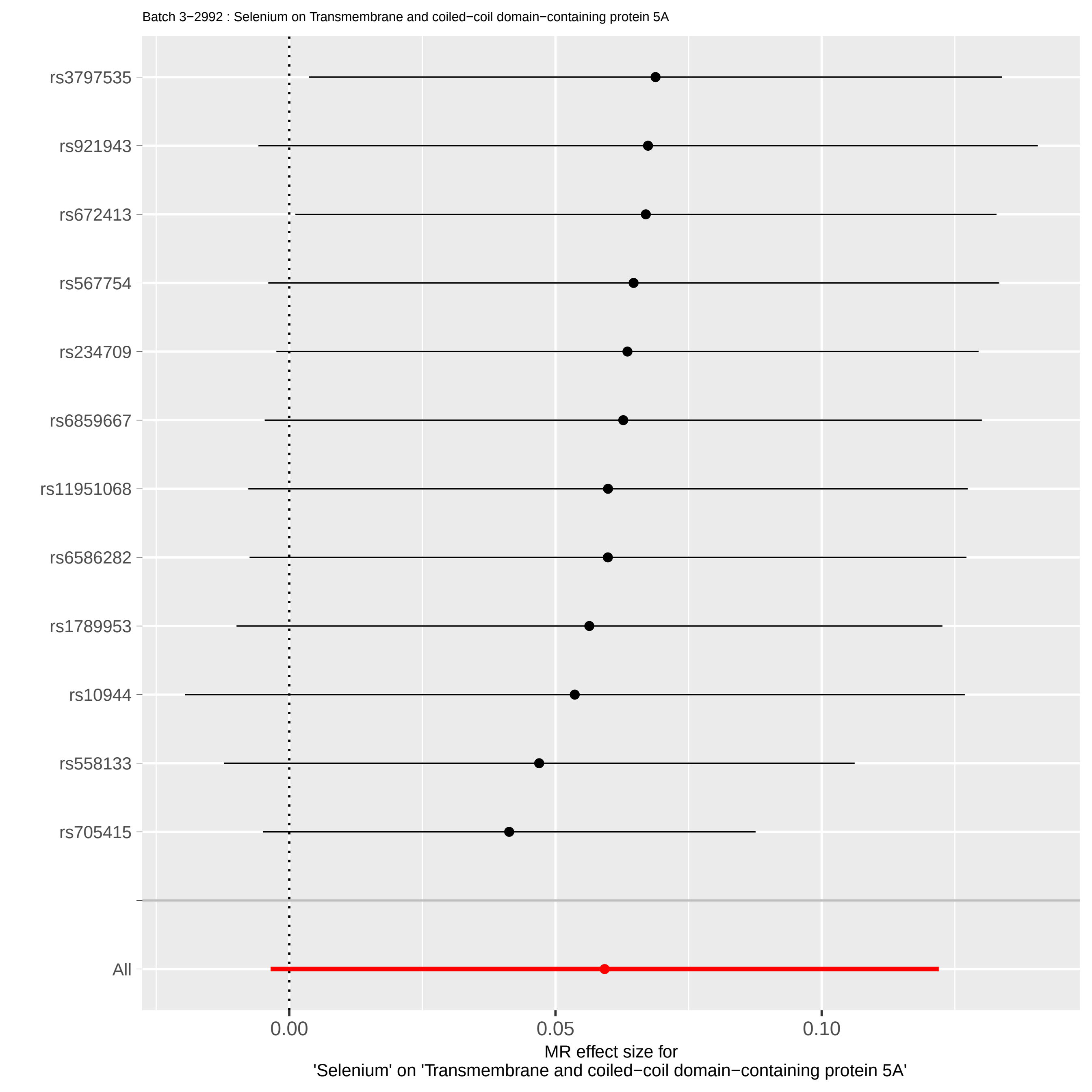

Batch 3−2992 : Selenium on Transmembrane and coiled−coil domain−containing protein 5A
rs3797535
rs921943
rs672413
rs567754
rs234709
rs6859667
rs11951068
rs6586282
rs1789953
rs10944
rs558133
rs705415
All
0.00
0.05
0.10
MR effect size for
'Selenium' on 'Transmembrane and coiled−coil domain−containing protein 5A'

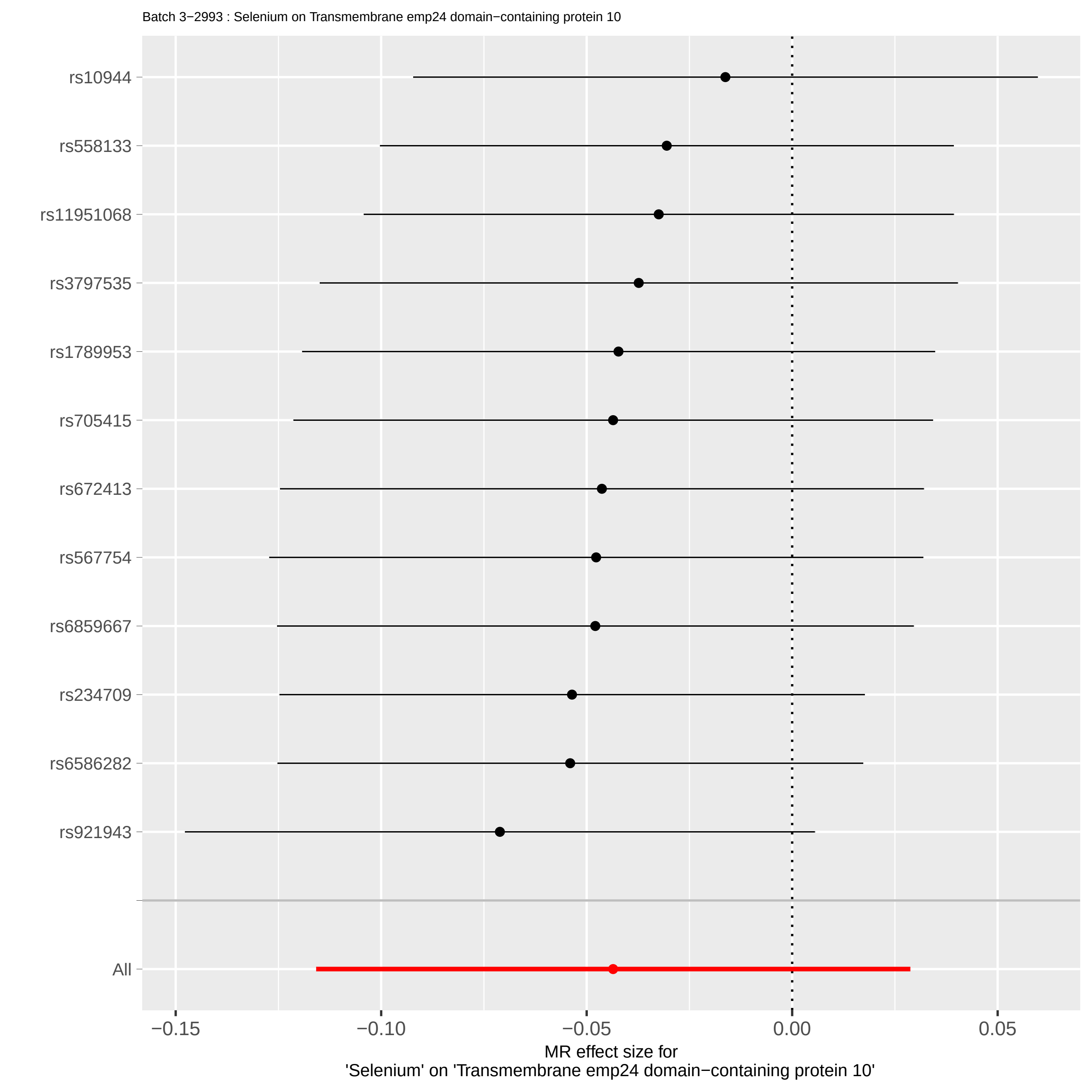

Batch 3−2993 : Selenium on Transmembrane emp24 domain−containing protein 10
rs10944
rs558133
rs11951068
rs3797535
rs1789953
rs705415
rs672413
rs567754
rs6859667
rs234709
rs6586282
rs921943
All
−0.15
−0.10
−0.05
0.00
0.05
MR effect size for
'Selenium' on 'Transmembrane emp24 domain−containing protein 10'

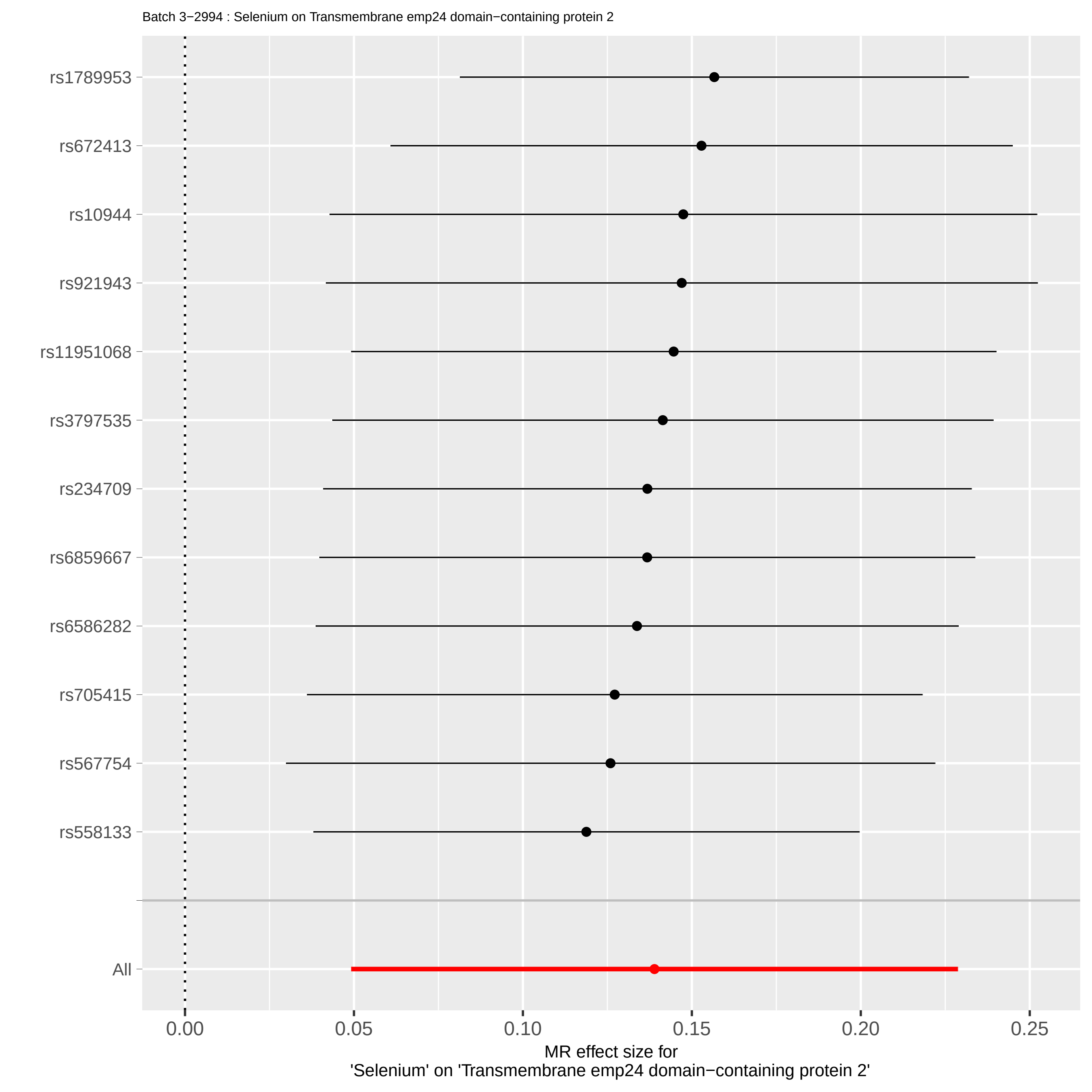

Batch 3−2994 : Selenium on Transmembrane emp24 domain−containing protein 2
rs1789953
rs672413
rs10944
rs921943
rs11951068
rs3797535
rs234709
rs6859667
rs6586282
rs705415
rs567754
rs558133
All
0.00
0.05
0.10
0.15
0.20
0.25
MR effect size for
'Selenium' on 'Transmembrane emp24 domain−containing protein 2'

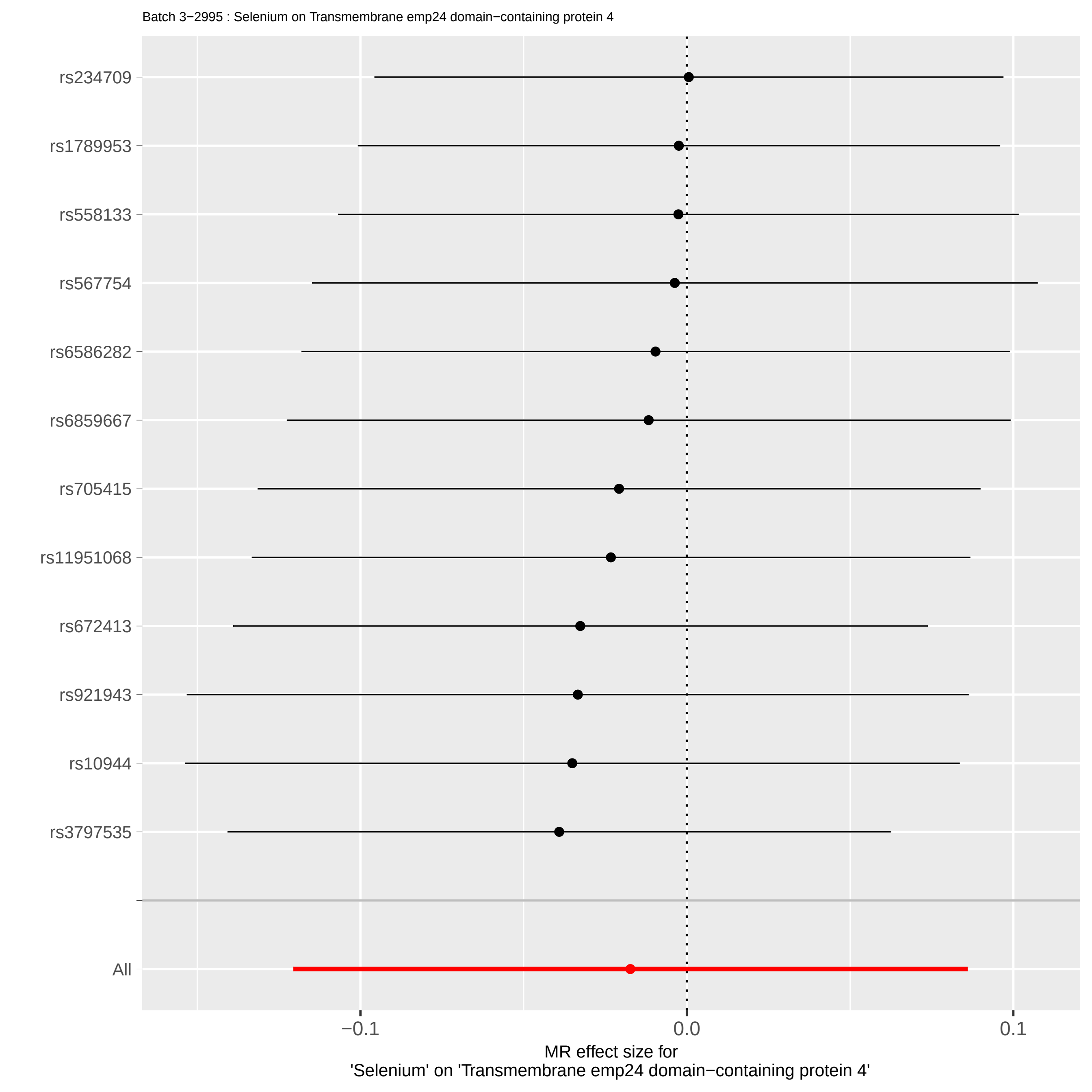

Batch 3−2995 : Selenium on Transmembrane emp24 domain−containing protein 4
rs234709
rs1789953
rs558133
rs567754
rs6586282
rs6859667
rs705415
rs11951068
rs672413
rs921943
rs10944
rs3797535
All
−0.1
0.0
0.1
MR effect size for
'Selenium' on 'Transmembrane emp24 domain−containing protein 4'

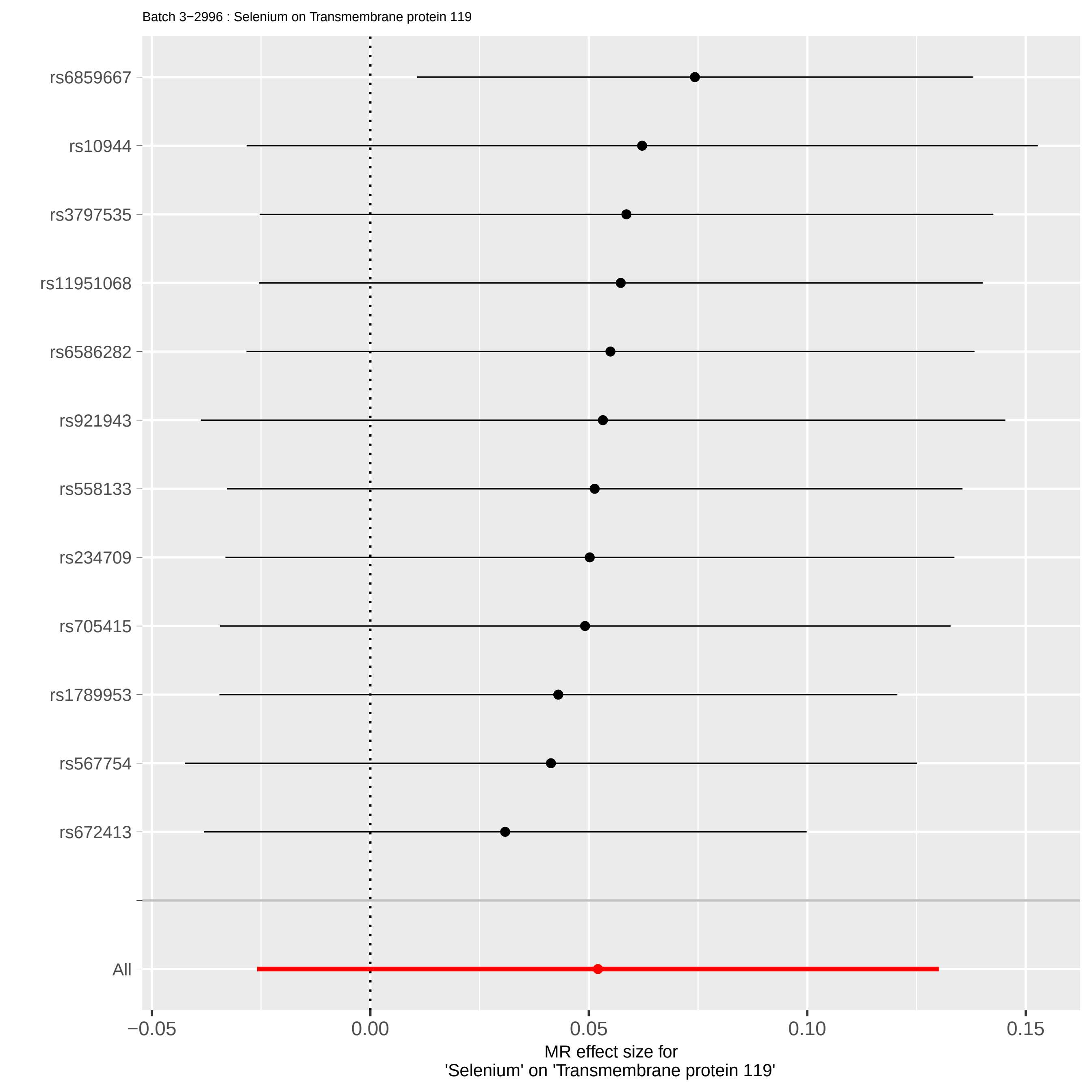

Batch 3−2996 : Selenium on Transmembrane protein 119
rs6859667
rs10944
rs3797535
rs11951068
rs6586282
rs921943
rs558133
rs234709
rs705415
rs1789953
rs567754
rs672413
All
−0.05
0.00
0.05
0.10
0.15
MR effect size for
'Selenium' on 'Transmembrane protein 119'

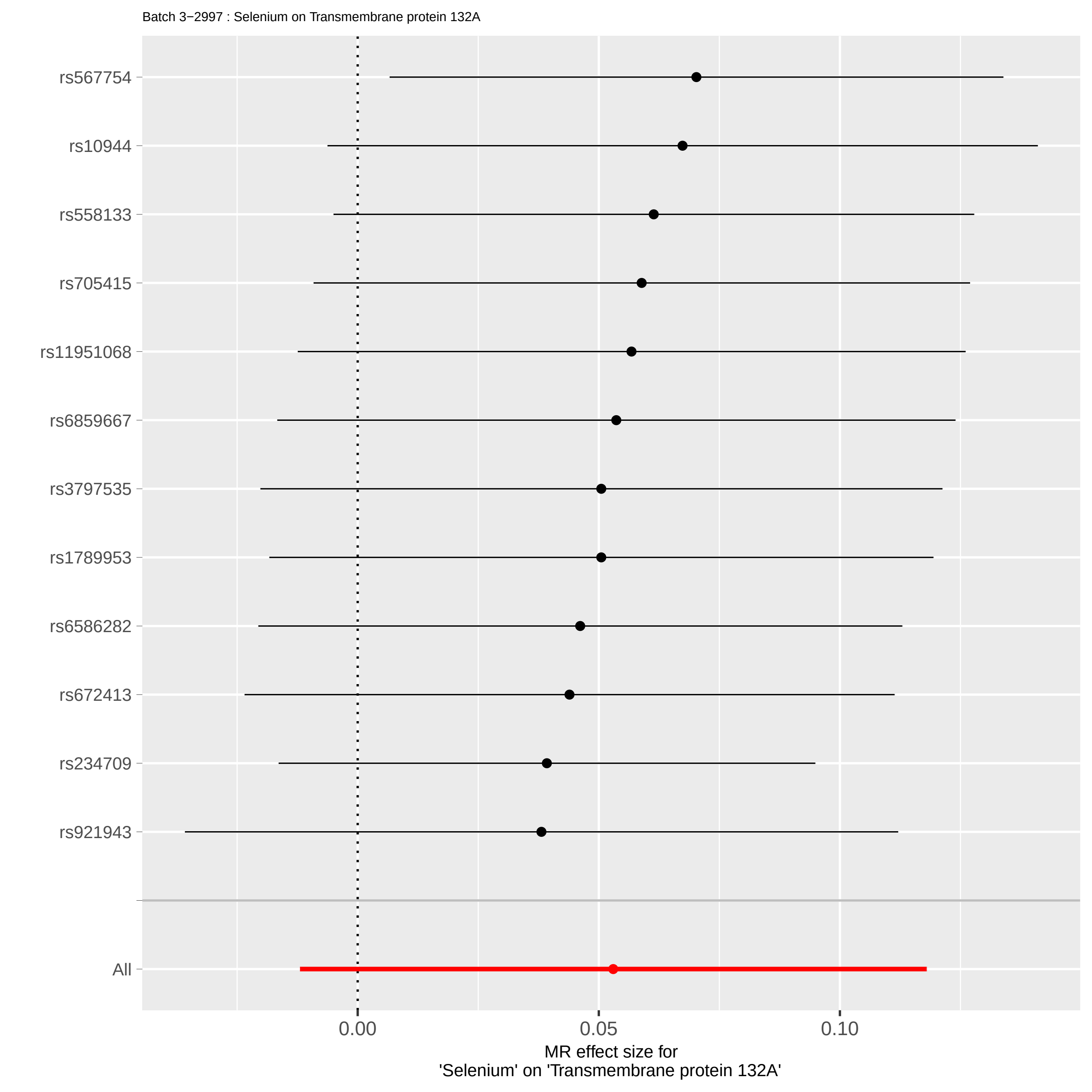

Batch 3−2997 : Selenium on Transmembrane protein 132A
rs567754
rs10944
rs558133
rs705415
rs11951068
rs6859667
rs3797535
rs1789953
rs6586282
rs672413
rs234709
rs921943
All
0.00
0.05
0.10
MR effect size for
'Selenium' on 'Transmembrane protein 132A'

Batch 3−2998 : Selenium on Transmembrane protein 132B

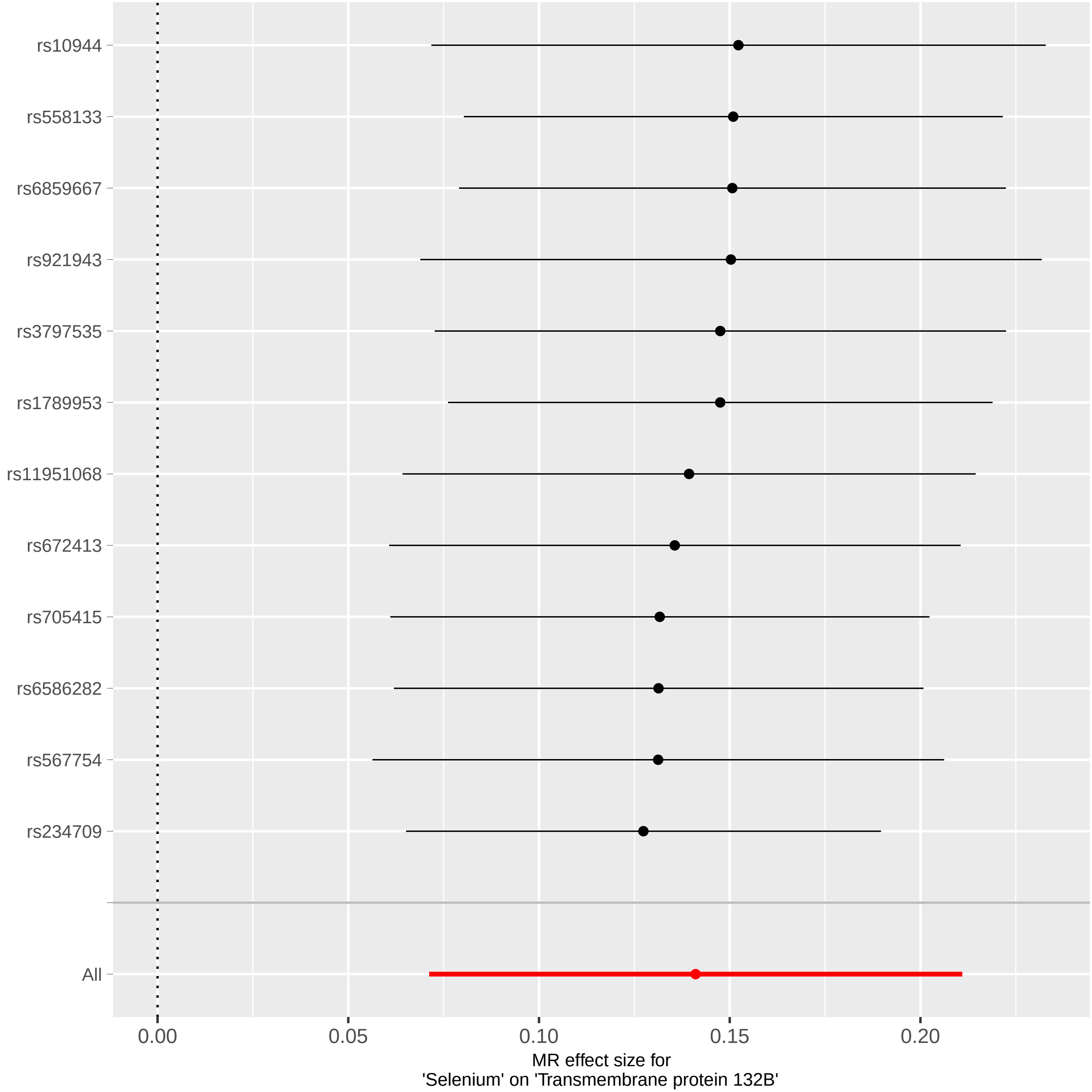

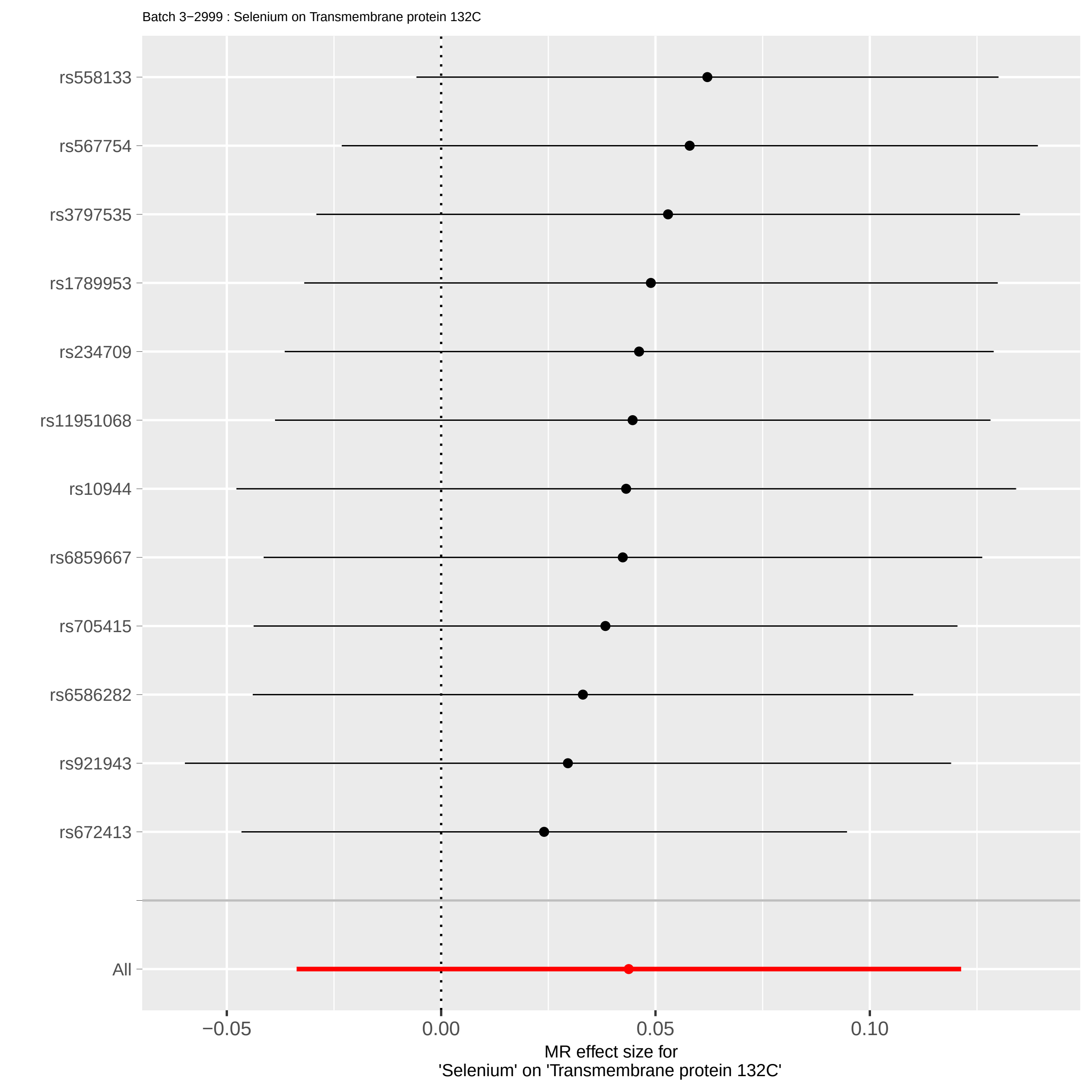

Batch 3−2999 : Selenium on Transmembrane protein 132C
rs558133
rs567754
rs3797535
rs1789953
rs234709
rs11951068
rs10944
rs6859667
rs705415
rs6586282
rs921943
rs672413
All
−0.05
0.00
0.05
0.10
MR effect size for
'Selenium' on 'Transmembrane protein 132C'

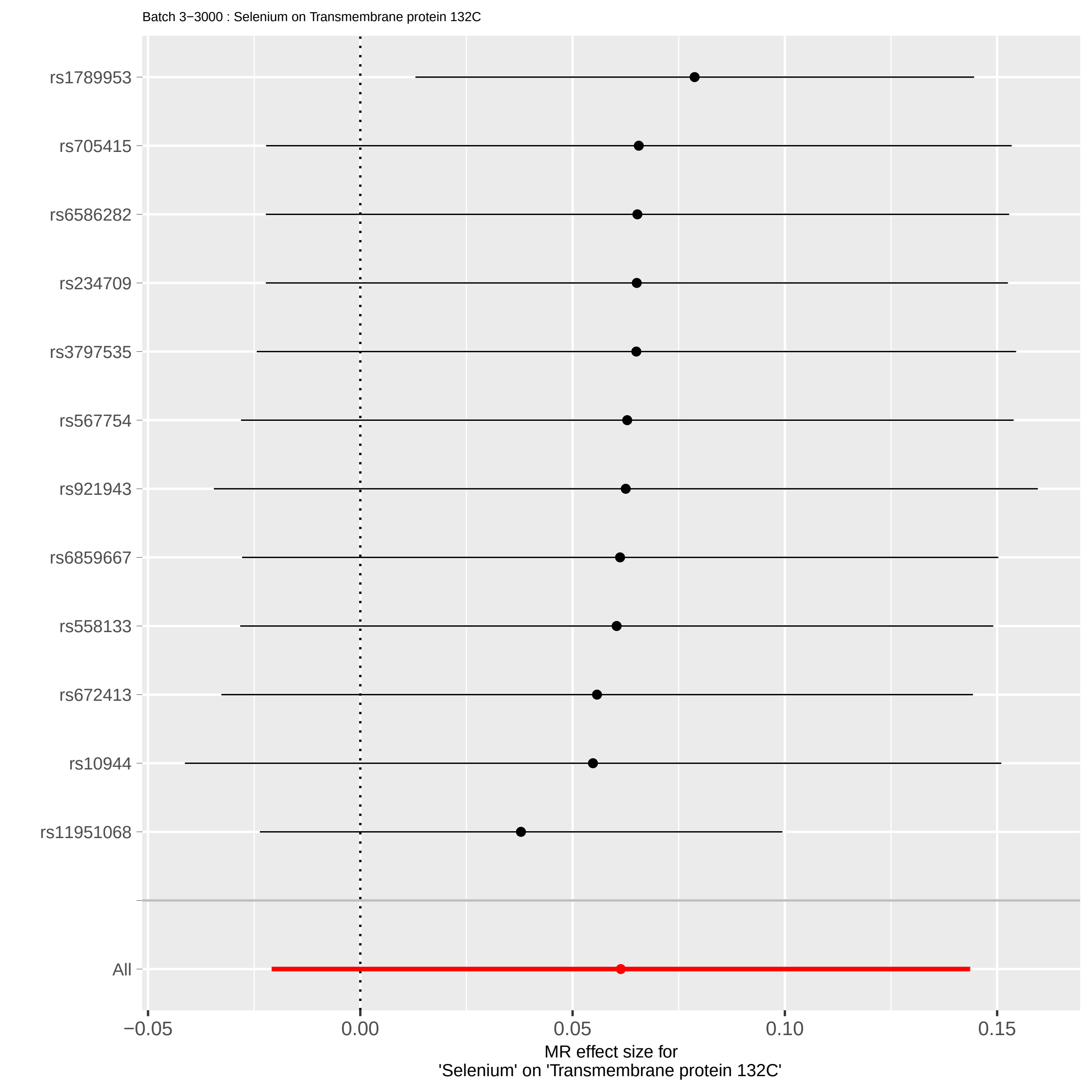

Batch 3−3000 : Selenium on Transmembrane protein 132C
rs1789953
rs705415
rs6586282
rs234709
rs3797535
rs567754
rs921943
rs6859667
rs558133
rs672413
rs10944
rs11951068
All
−0.05
0.00
0.05
0.10
0.15
MR effect size for
'Selenium' on 'Transmembrane protein 132C'

Batch 3−3001 : Selenium on Transmembrane protein 132D

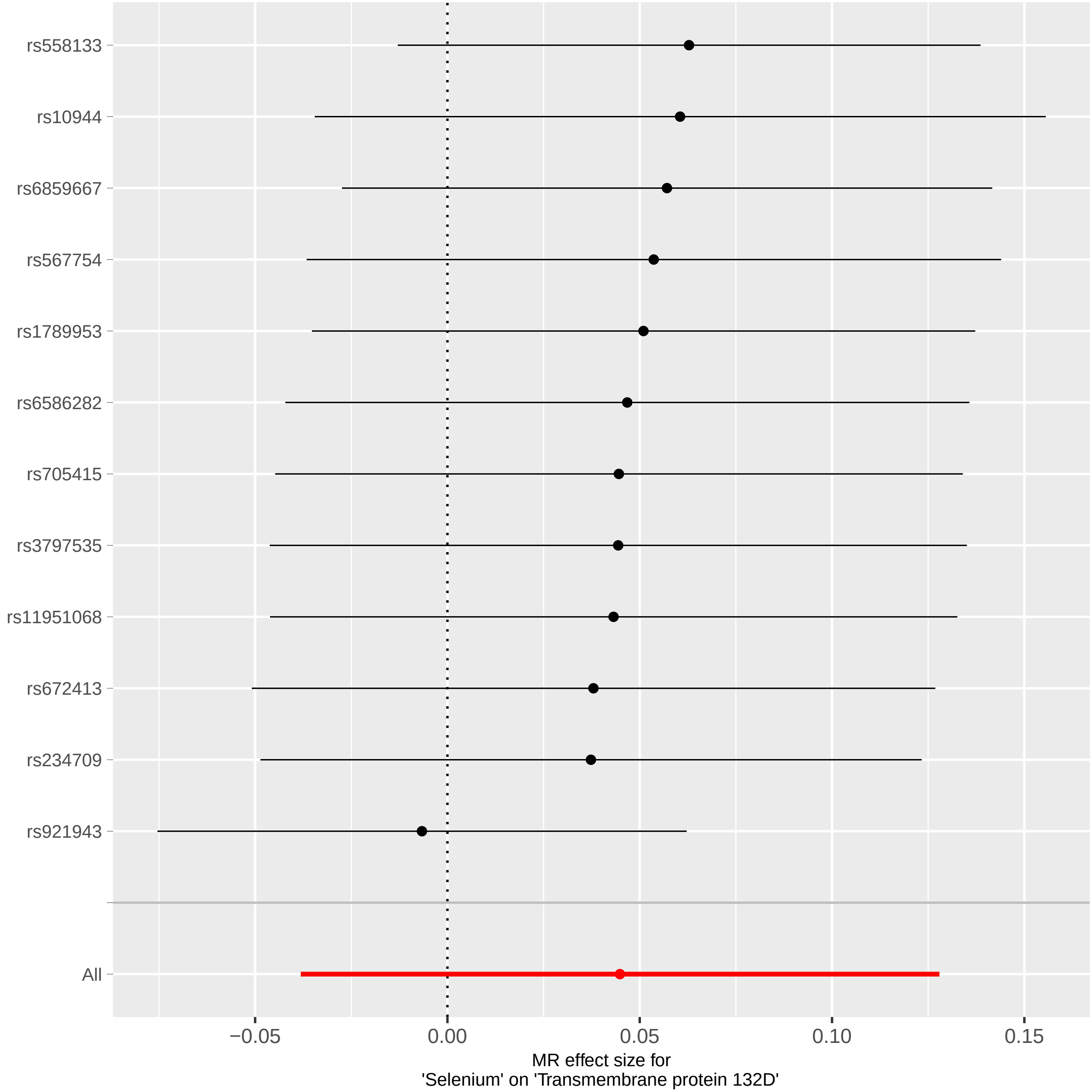

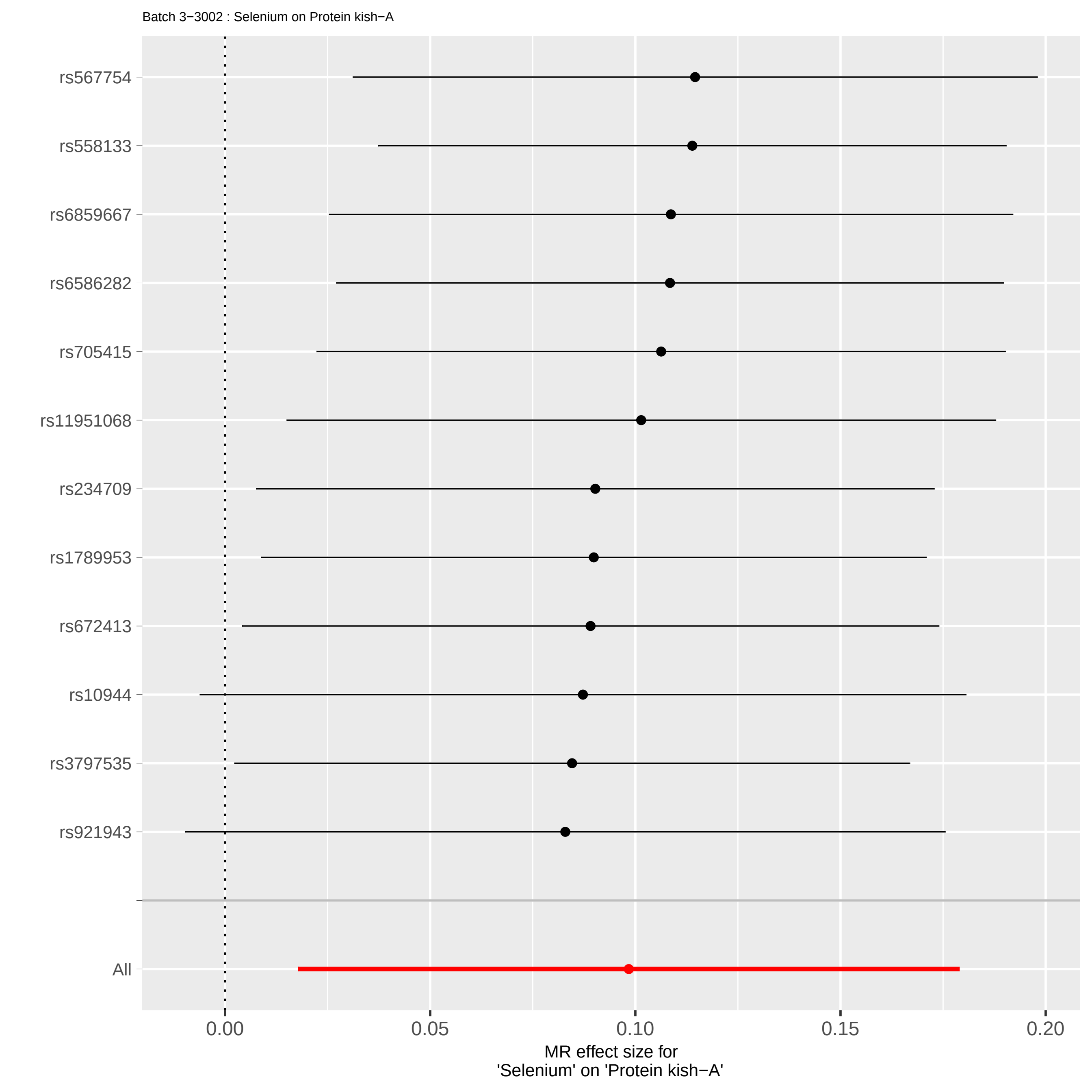
Batch 3−3002 : Selenium on Protein kish−A
rs567754
rs558133
rs6859667
rs6586282
rs705415
rs11951068
rs234709
rs1789953
rs672413
rs10944
rs3797535
rs921943
All
0.00
0.05
0.10
0.15
0.20
MR effect size for
'Selenium' on 'Protein kish−A'

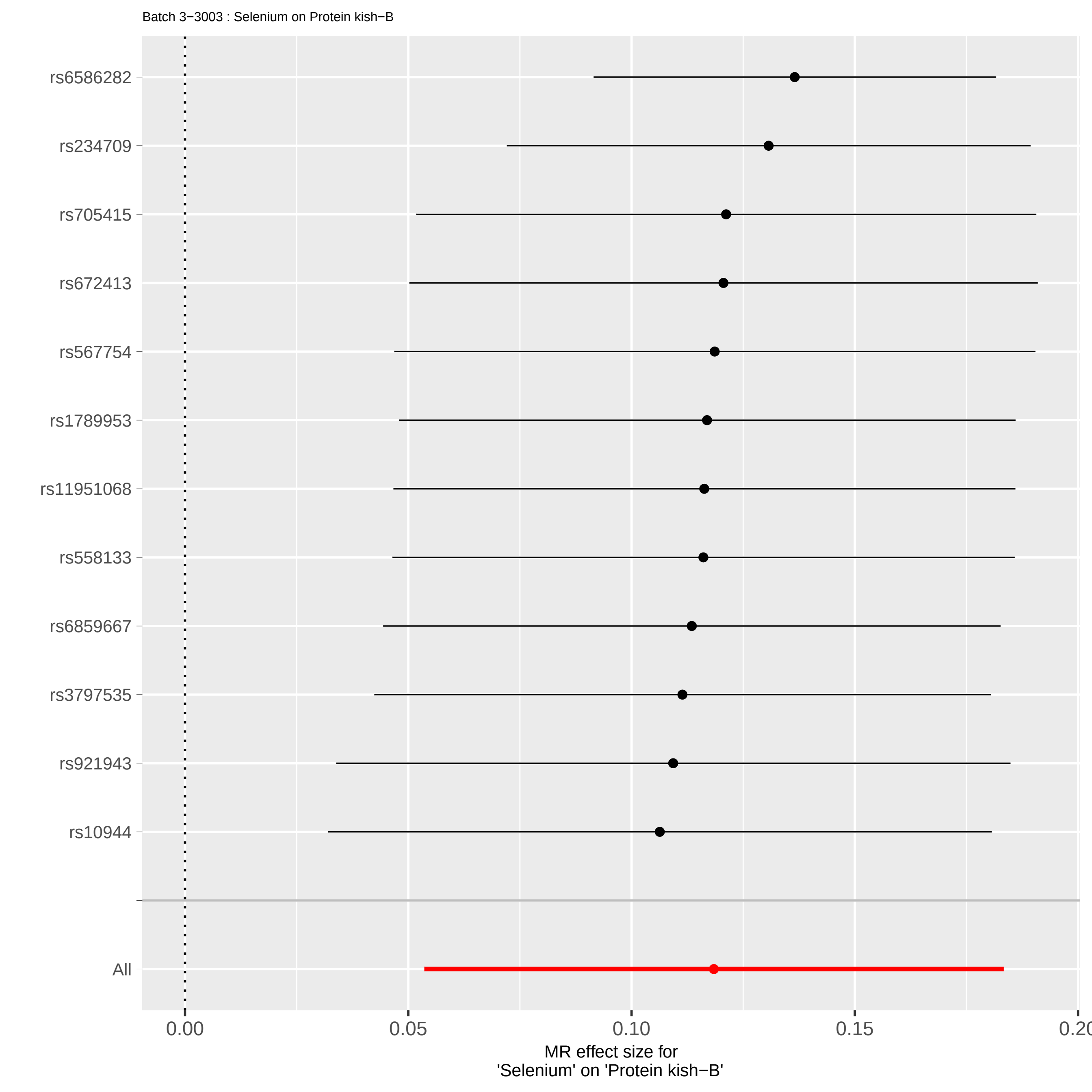

Batch 3−3003 : Selenium on Protein kish−B
rs6586282
rs234709
rs705415
rs672413
rs567754
rs1789953
rs11951068
rs558133
rs6859667
rs3797535
rs921943
rs10944
All
0.00
0.05
0.10
0.15
0.20
MR effect size for
'Selenium' on 'Protein kish−B'

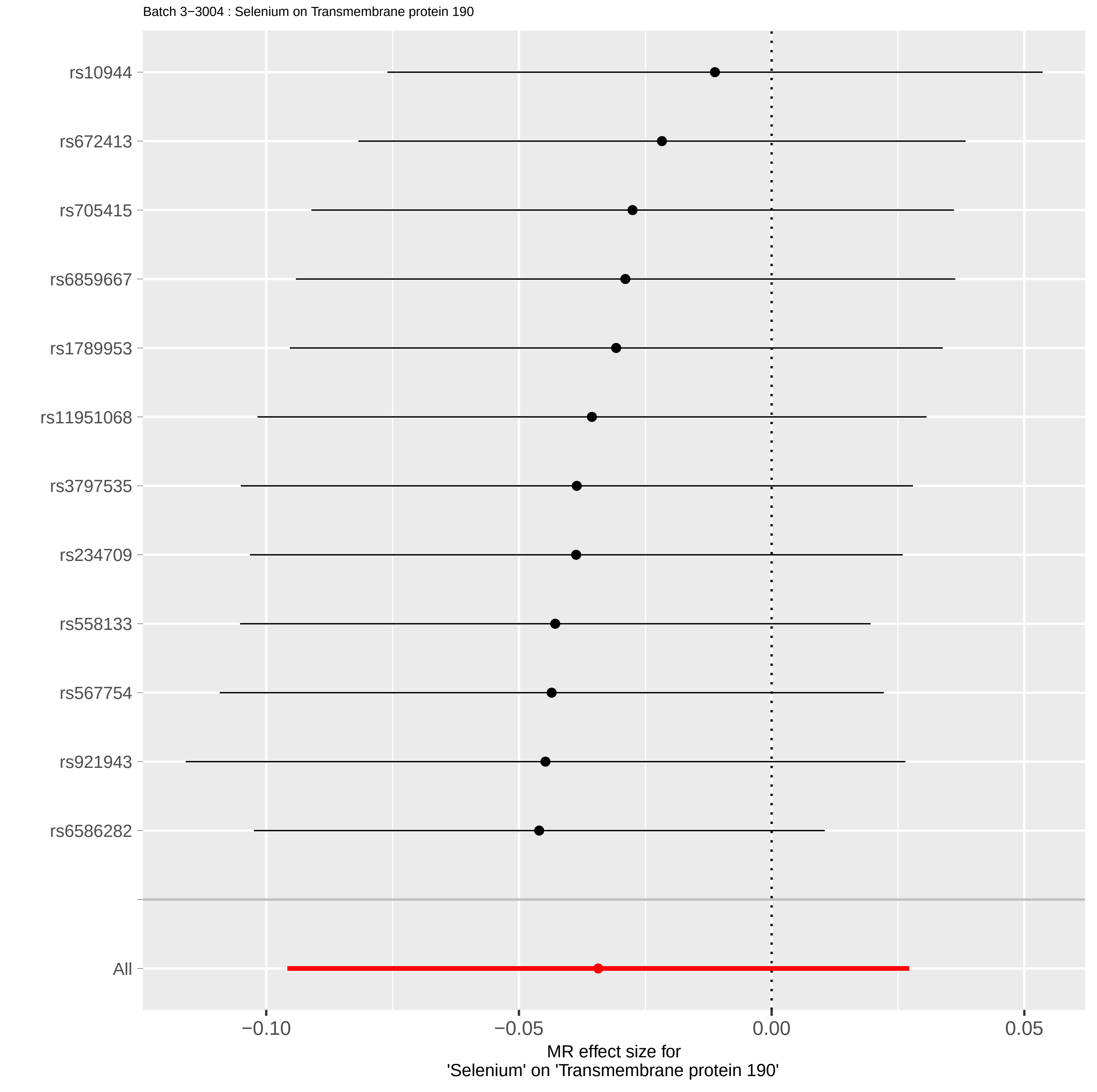

Batch 3−3004 : Selenium on Transmembrane protein 190
rs10944
rs672413
rs705415
rs6859667
rs1789953
rs11951068
rs3797535
rs234709
rs558133
rs567754
rs921943
rs6586282
All
−0.10
−0.05
0.00
0.05
MR effect size for
'Selenium' on 'Transmembrane protein 190'

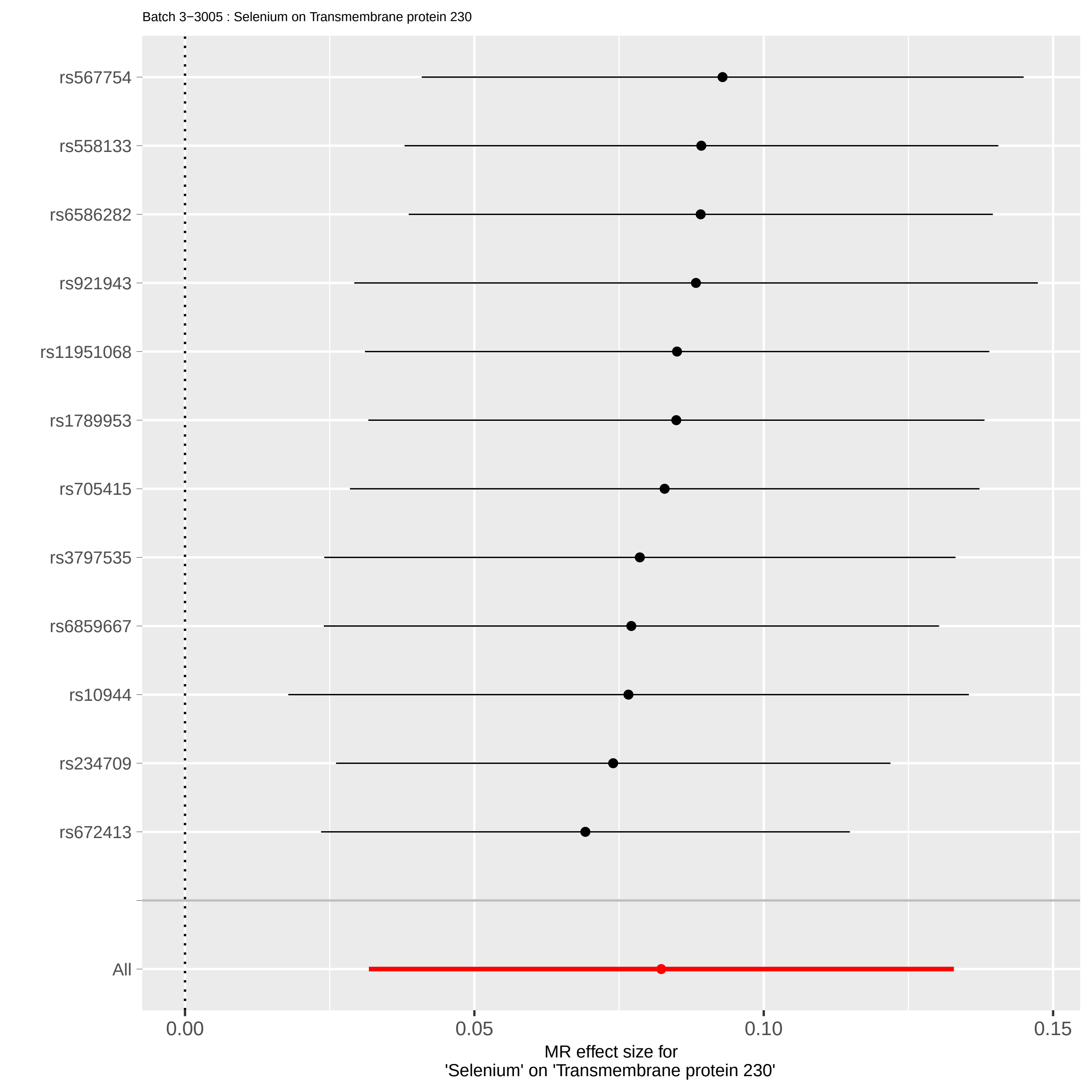

Batch 3−3005 : Selenium on Transmembrane protein 230
rs567754
rs558133
rs6586282
rs921943
rs11951068
rs1789953
rs705415
rs3797535
rs6859667
rs10944
rs234709
rs672413
All
0.00
0.05
0.10
0.15
MR effect size for
'Selenium' on 'Transmembrane protein 230'

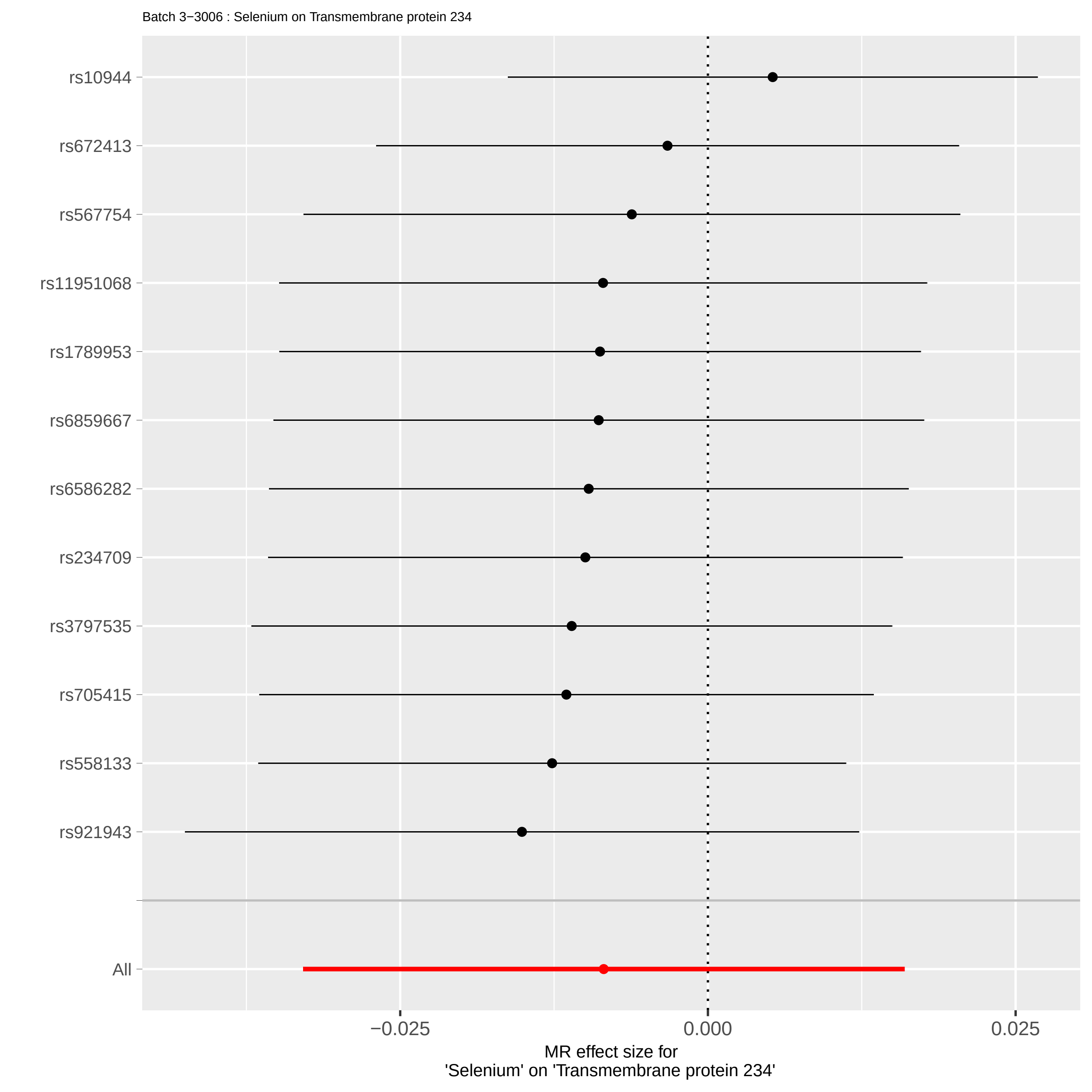

Batch 3−3006 : Selenium on Transmembrane protein 234
rs10944
rs672413
rs567754
rs11951068
rs1789953
rs6859667
rs6586282
rs234709
rs3797535
rs705415
rs558133
rs921943
All
−0.025
0.000
0.025
MR effect size for
'Selenium' on 'Transmembrane protein 234'

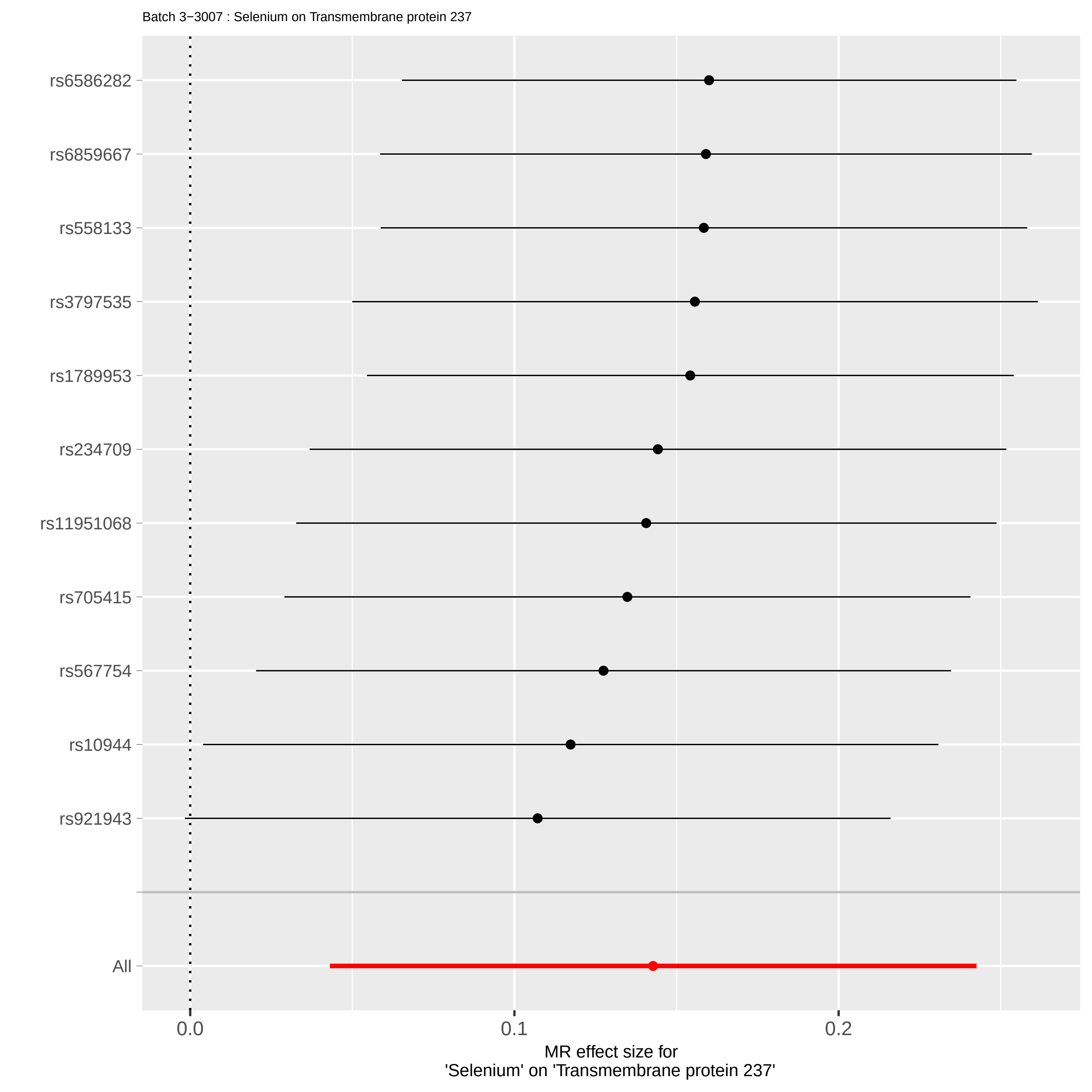
Batch 3−3007 : Selenium on Transmembrane protein 237
rs6586282
rs6859667
rs558133
rs3797535
rs1789953
rs234709
rs11951068
rs705415
rs567754
rs10944
rs921943
All
0.0
0.1
0.2
MR effect size for
'Selenium' on 'Transmembrane protein 237'

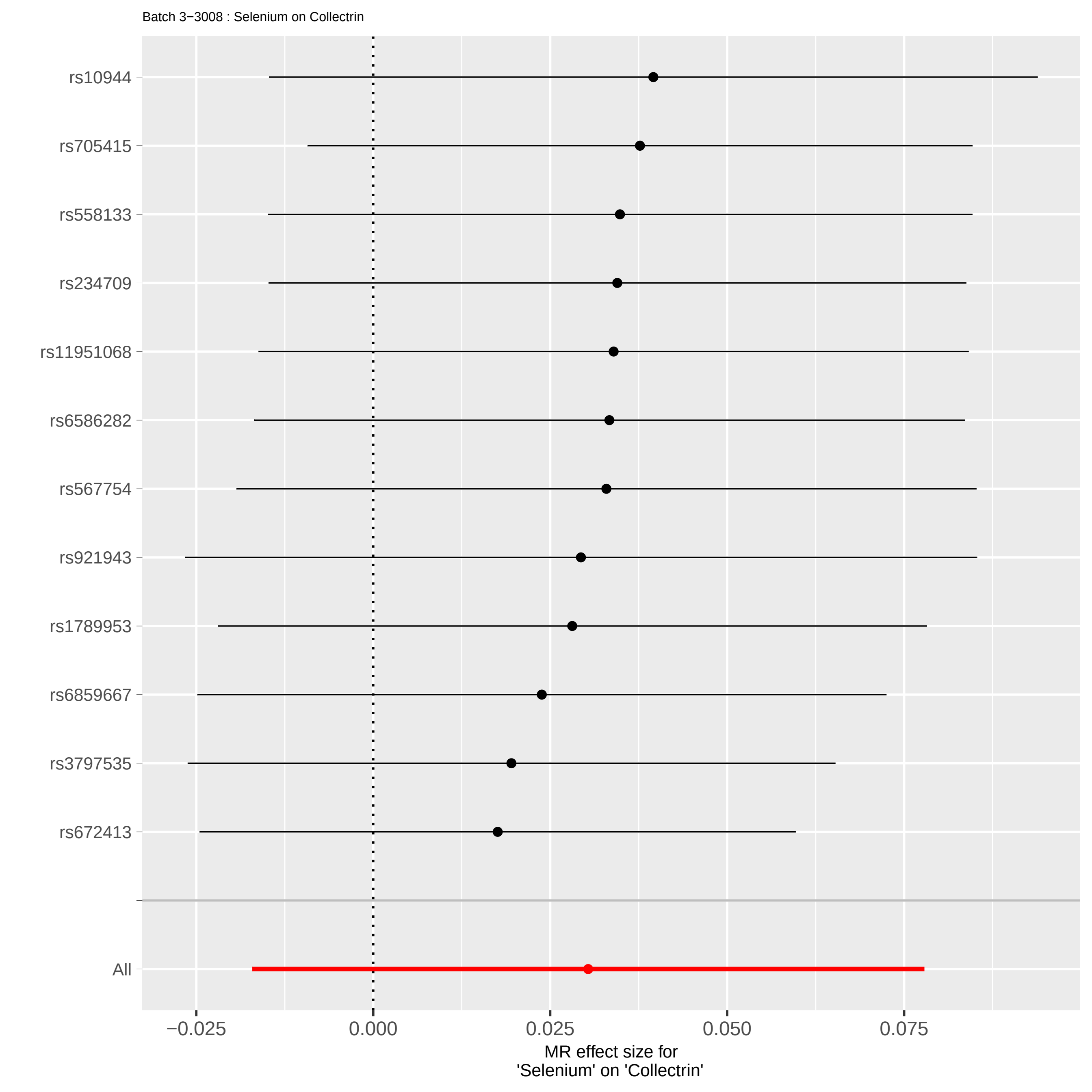

Batch 3−3008 : Selenium on Collectrin
rs10944
rs705415
rs558133
rs234709
rs11951068
rs6586282
rs567754
rs921943
rs1789953
rs6859667
rs3797535
rs672413
All
−0.025
0.000
0.025
0.050
0.075
MR effect size for
'Selenium' on 'Collectrin'

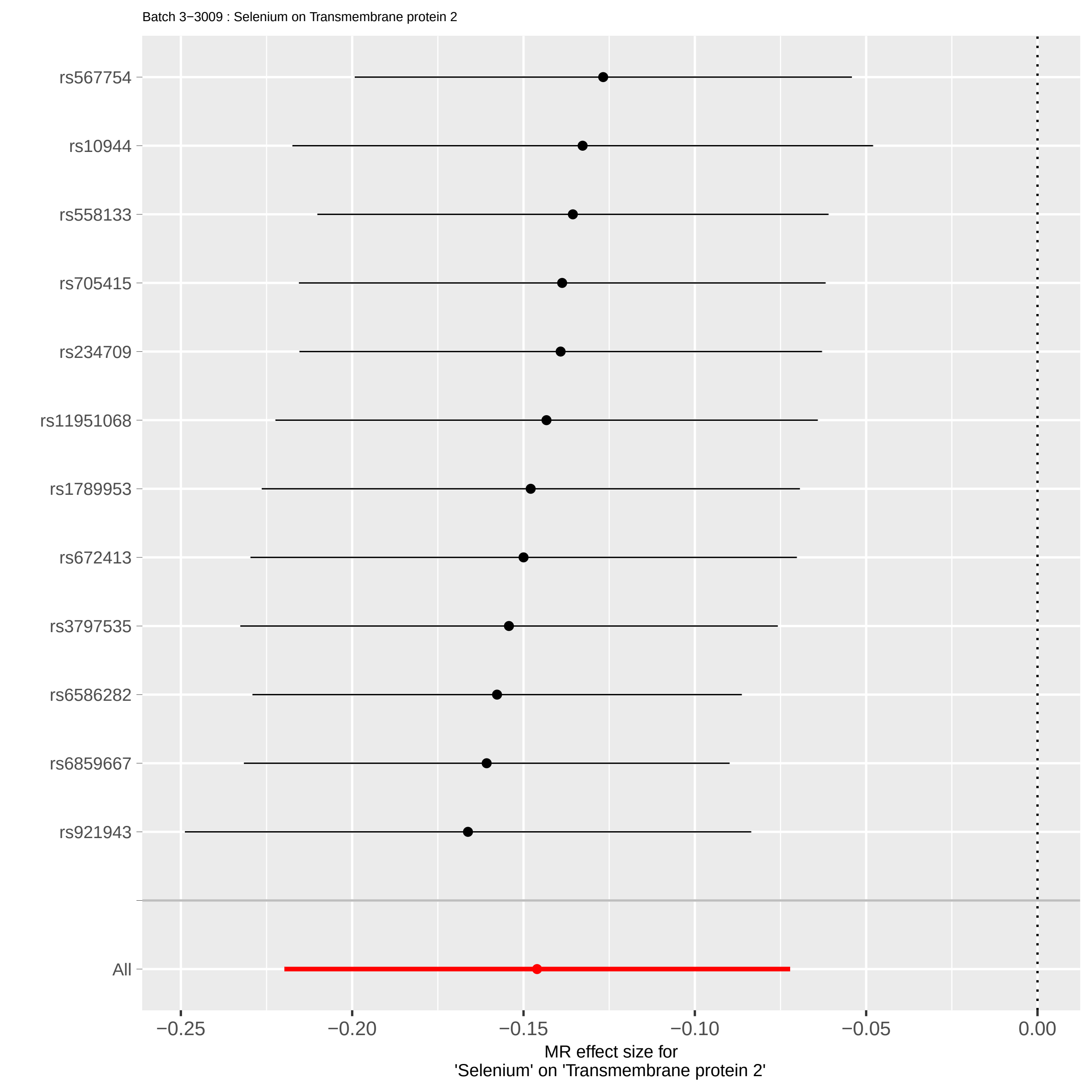

Batch 3−3009 : Selenium on Transmembrane protein 2
rs567754
rs10944
rs558133
rs705415
rs234709
rs11951068
rs1789953
rs672413
rs3797535
rs6586282
rs6859667
rs921943
All
−0.25
−0.20
−0.15
−0.10
−0.05
0.00
MR effect size for
'Selenium' on 'Transmembrane protein 2'

Batch 3−3010 : Selenium on Transmembrane protein 40

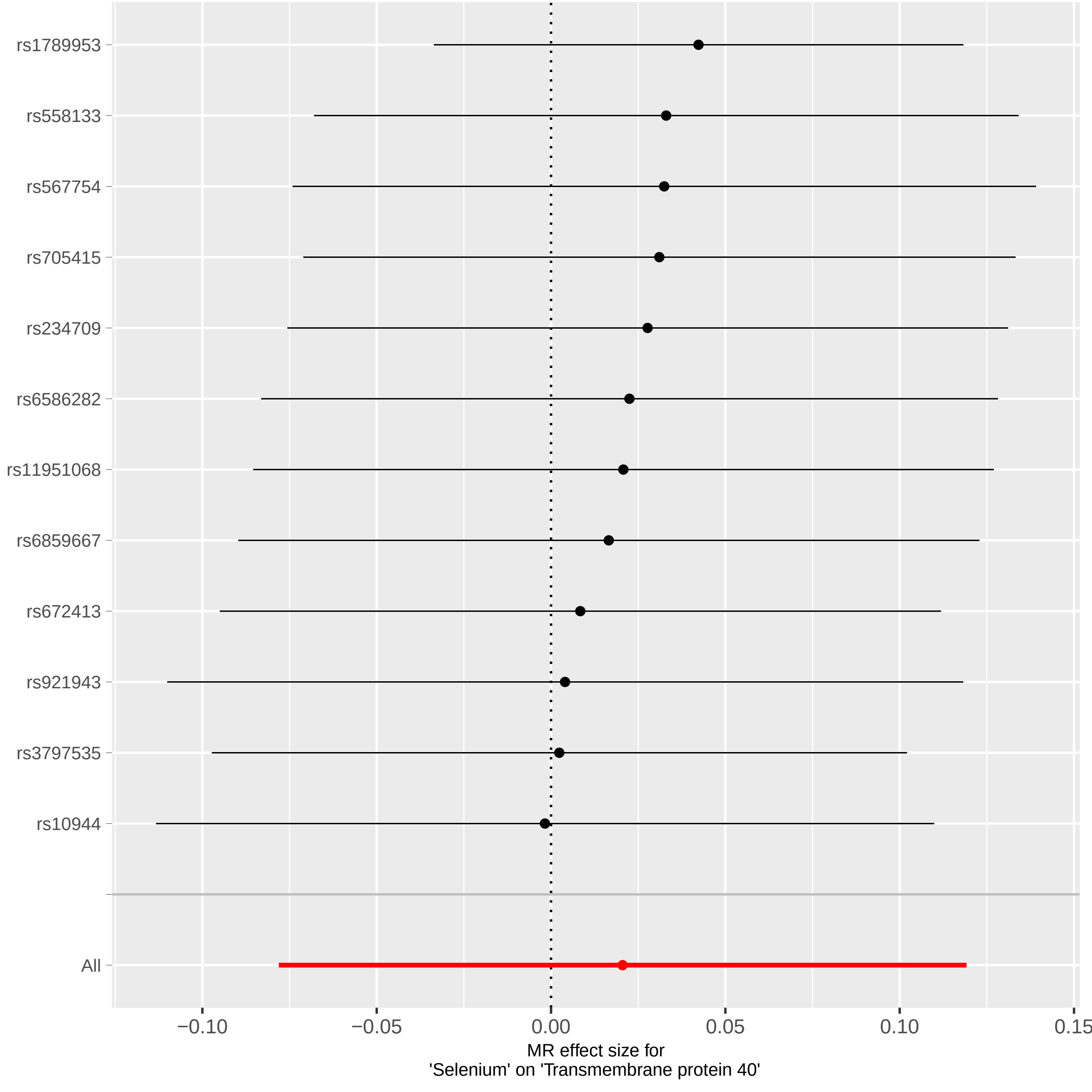

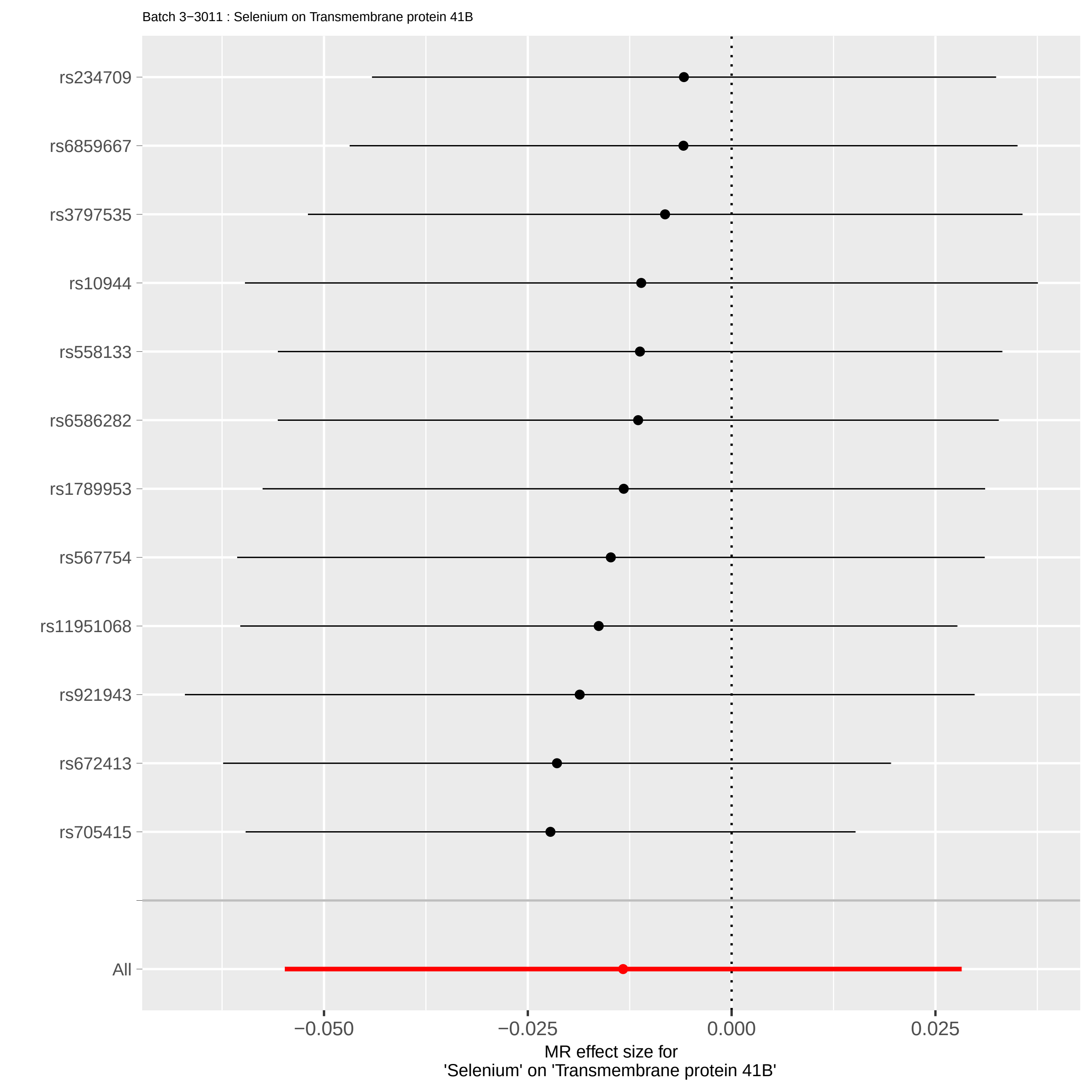

Batch 3−3011 : Selenium on Transmembrane protein 41B
rs234709
rs6859667
rs3797535
rs10944
rs558133
rs6586282
rs1789953
rs567754
rs11951068
rs921943
rs672413
rs705415
All
−0.050
−0.025
0.000
0.025
MR effect size for
'Selenium' on 'Transmembrane protein 41B'

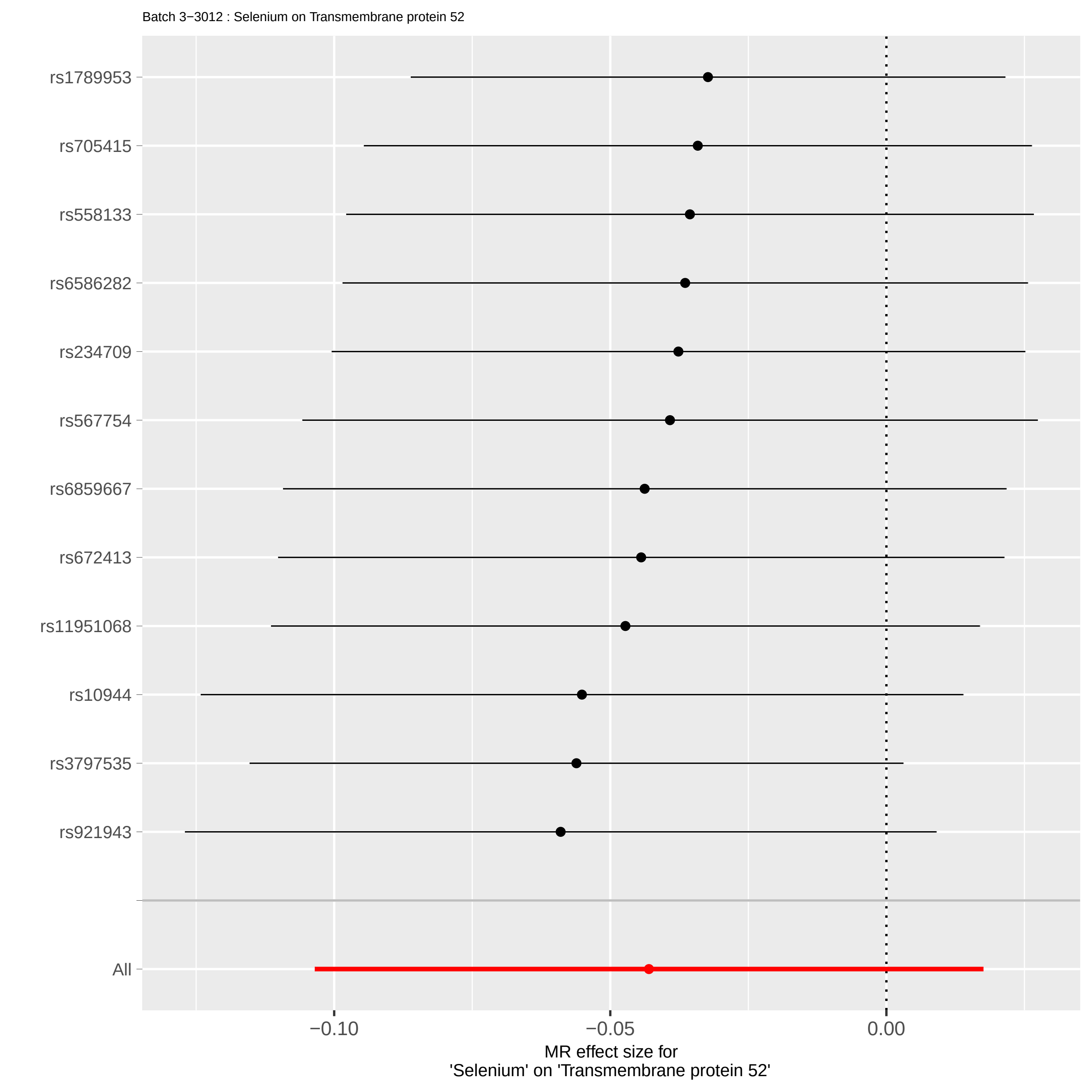

Batch 3−3012 : Selenium on Transmembrane protein 52
rs1789953
rs705415
rs558133
rs6586282
rs234709
rs567754
rs6859667
rs672413
rs11951068
rs10944
rs3797535
rs921943
All
−0.10
−0.05
0.00
MR effect size for
'Selenium' on 'Transmembrane protein 52'

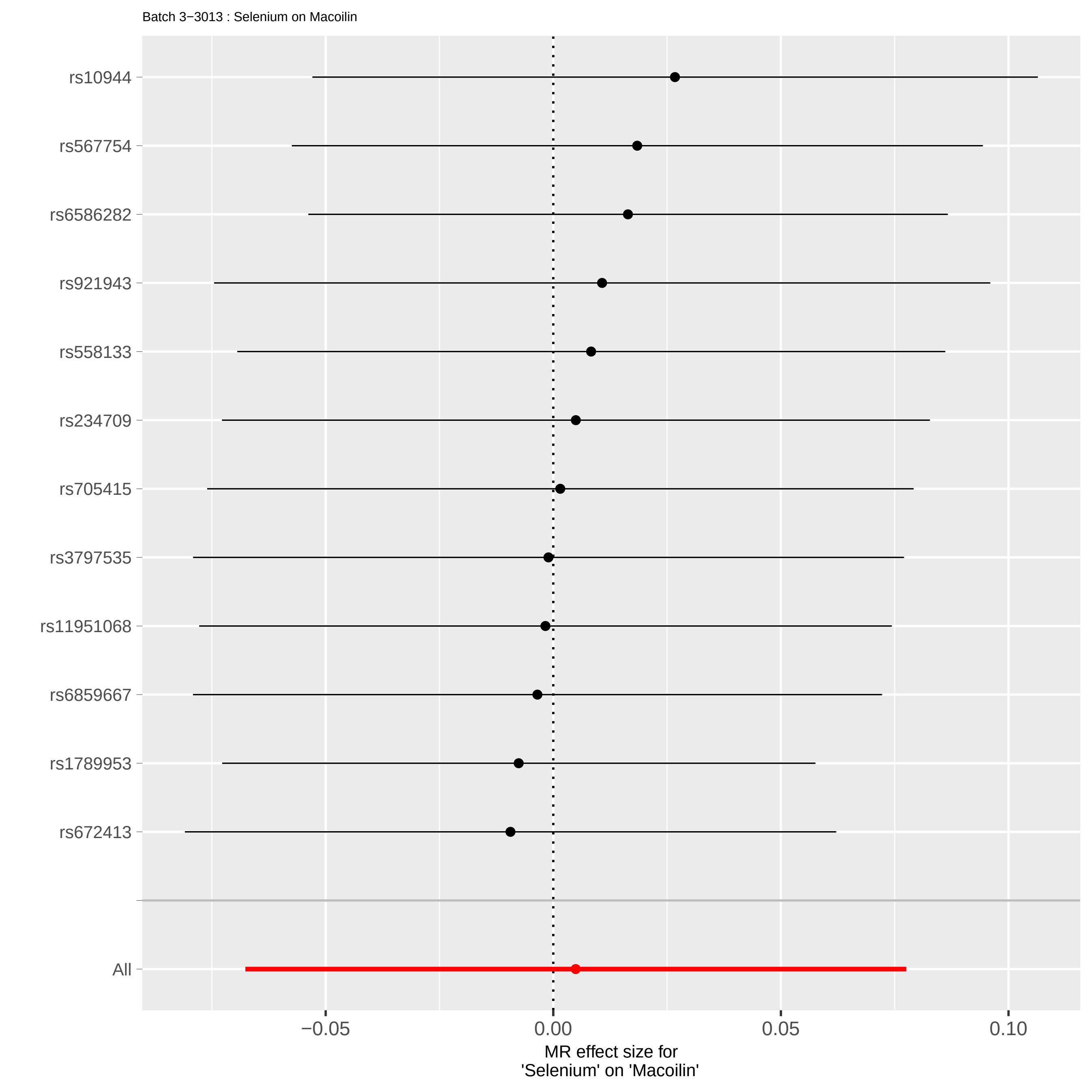

Batch 3−3013 : Selenium on Macoilin
rs10944
rs567754
rs6586282
rs921943
rs558133
rs234709
rs705415
rs3797535
rs11951068
rs6859667
rs1789953
rs672413
All
−0.05
0.00
0.05
0.10
MR effect size for
'Selenium' on 'Macoilin'

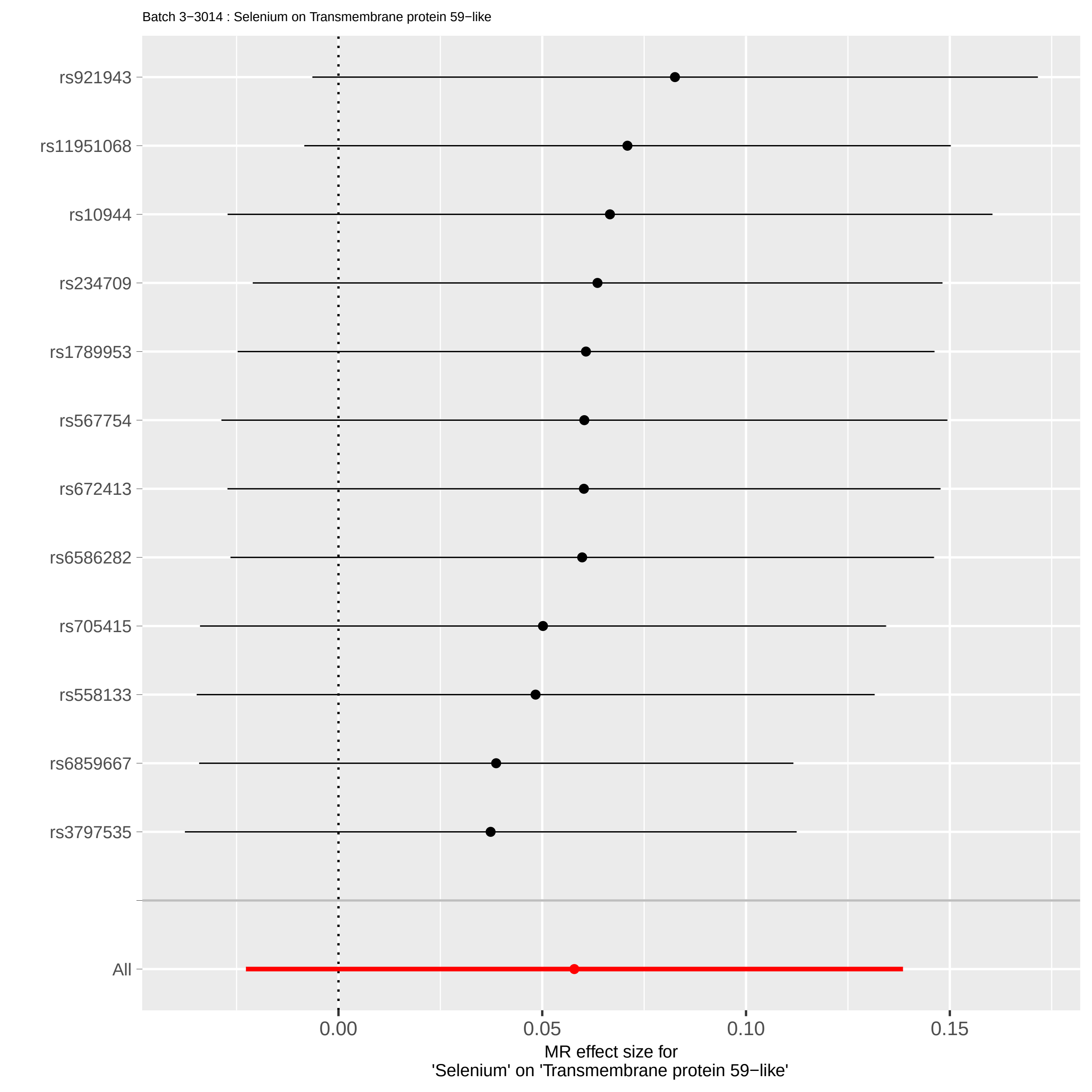

Batch 3−3014 : Selenium on Transmembrane protein 59−like
rs921943
rs11951068
rs10944
rs234709
rs1789953
rs567754
rs672413
rs6586282
rs705415
rs558133
rs6859667
rs3797535
All
0.00
0.05
0.10
0.15
MR effect size for
'Selenium' on 'Transmembrane protein 59−like'

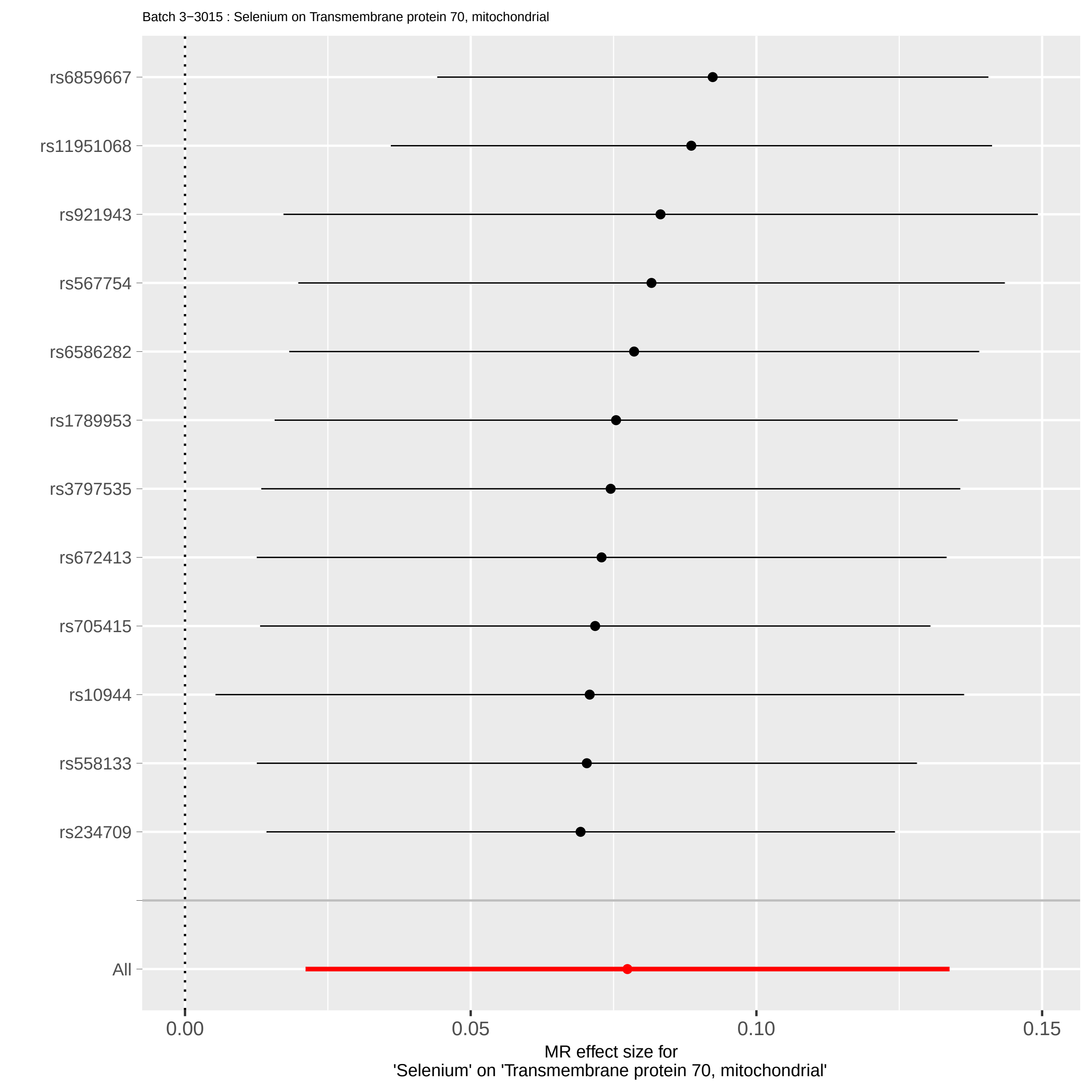

Batch 3−3015 : Selenium on Transmembrane protein 70, mitochondrial
rs6859667
rs11951068
rs921943
rs567754
rs6586282
rs1789953
rs3797535
rs672413
rs705415
rs10944
rs558133
rs234709
All
0.00
0.05
0.10
0.15
MR effect size for
'Selenium' on 'Transmembrane protein 70, mitochondrial'

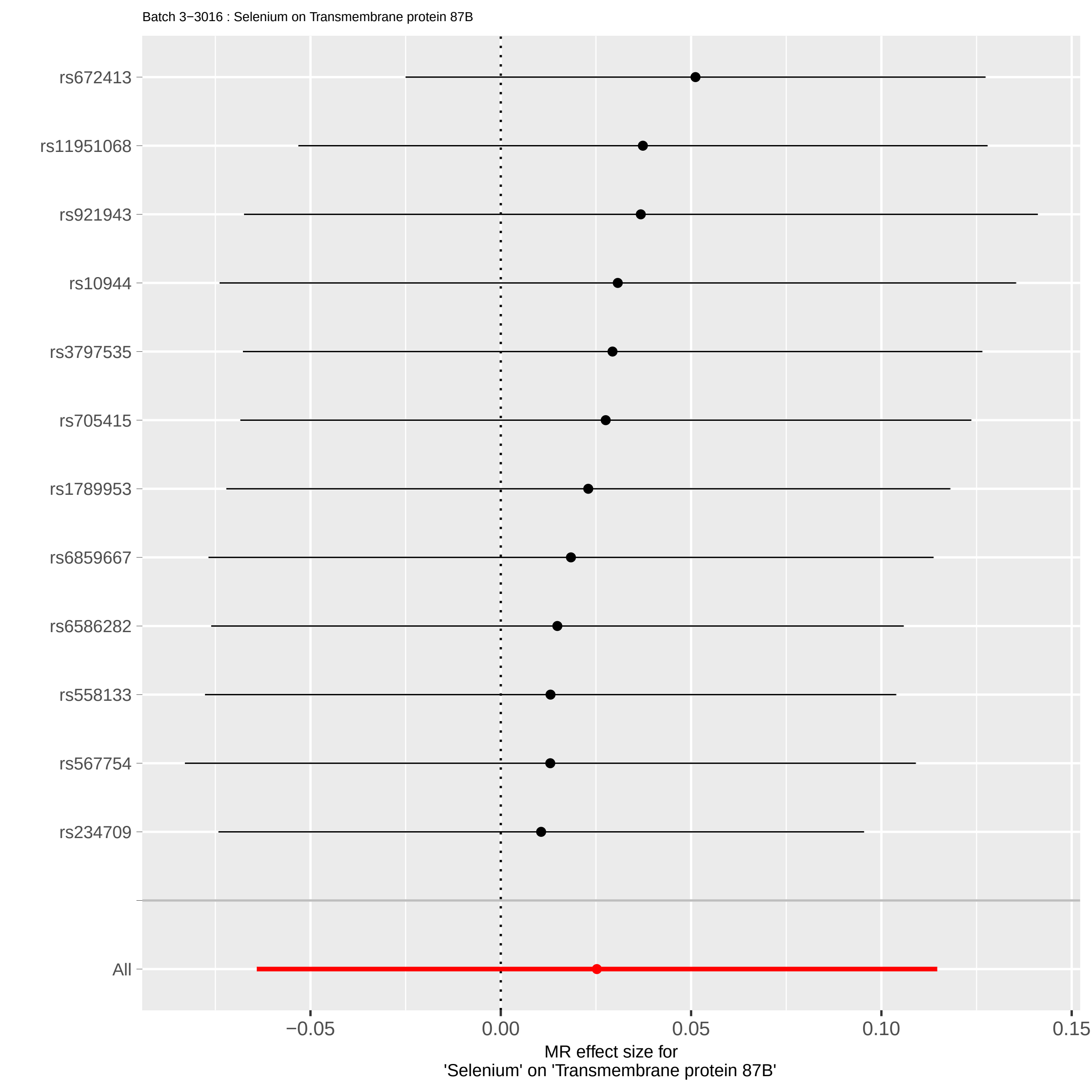

Batch 3−3016 : Selenium on Transmembrane protein 87B
rs672413
rs11951068
rs921943
rs10944
rs3797535
rs705415
rs1789953
rs6859667
rs6586282
rs558133
rs567754
rs234709
All
−0.05
0.00
0.05
0.10
0.15
MR effect size for
'Selenium' on 'Transmembrane protein 87B'

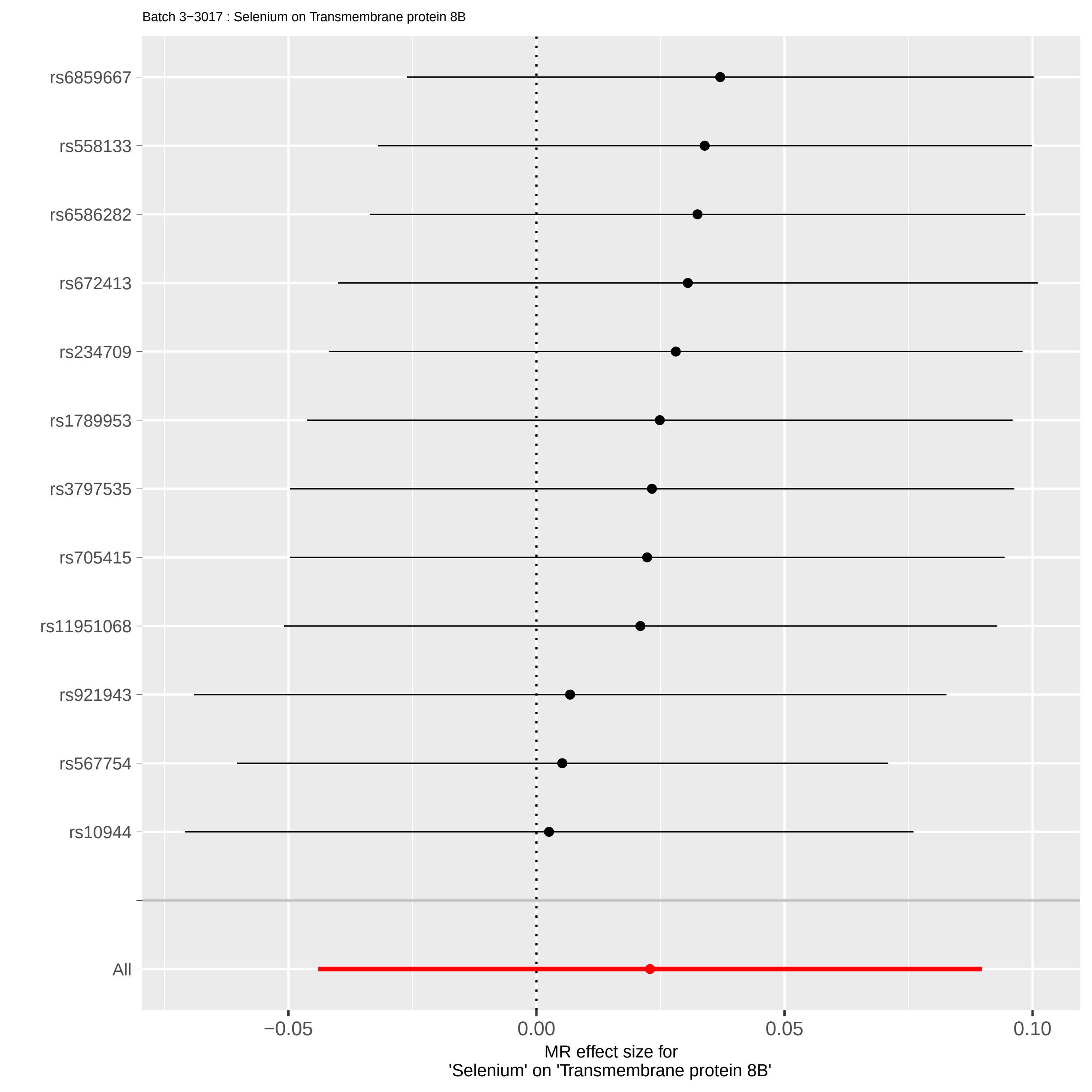
Batch 3−3017 : Selenium on Transmembrane protein 8B
rs6859667
rs558133
rs6586282
rs672413
rs234709
rs1789953
rs3797535
rs705415
rs11951068
rs921943
rs567754
rs10944
All
−0.05
0.00
0.05
0.10
MR effect size for
'Selenium' on 'Transmembrane protein 8B'

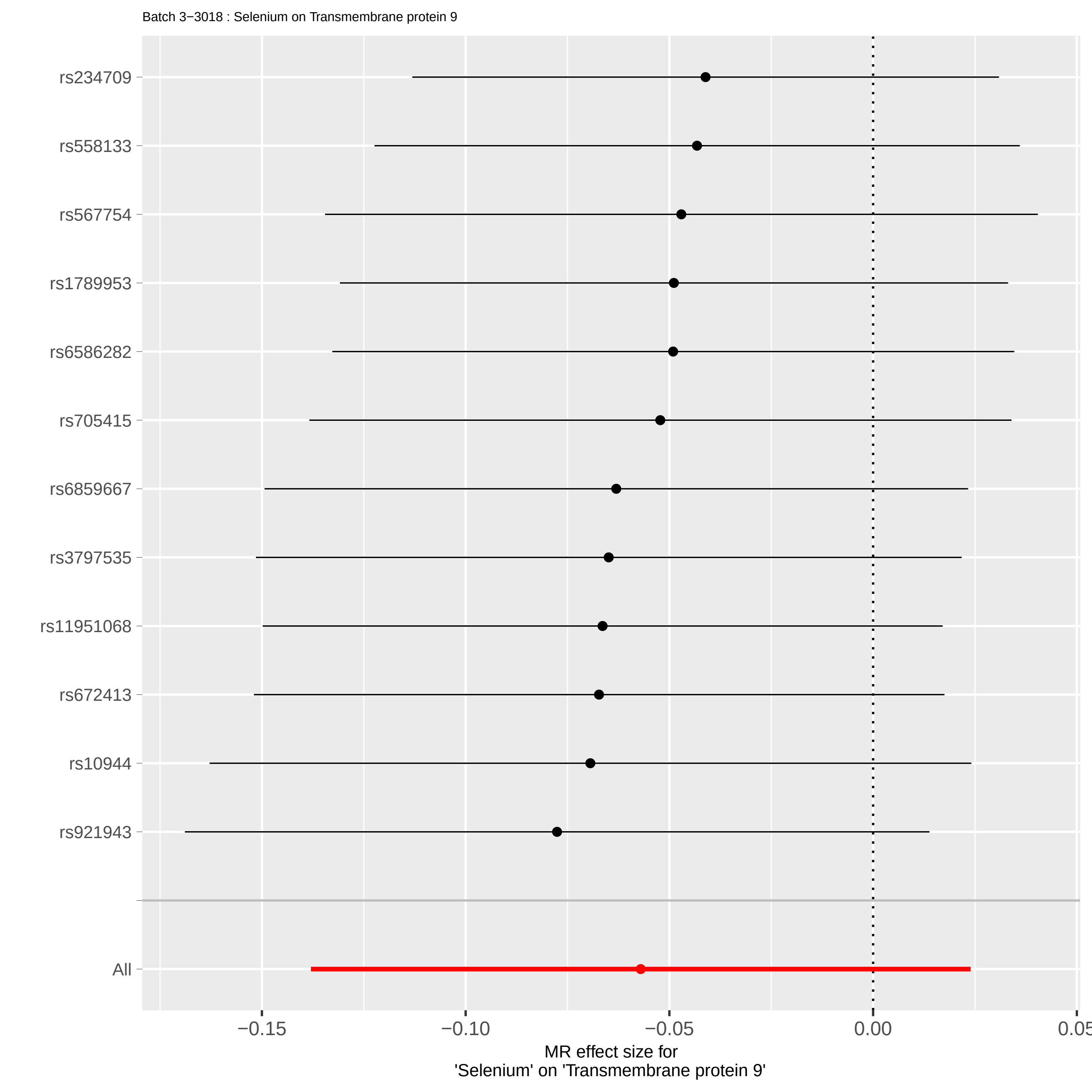
Batch 3−3018 : Selenium on Transmembrane protein 9
rs234709
rs558133
rs567754
rs1789953
rs6586282
rs705415
rs6859667
rs3797535
rs11951068
rs672413
rs10944
rs921943
All
−0.15
−0.10
−0.05
0.00
0.05
MR effect size for
'Selenium' on 'Transmembrane protein 9'

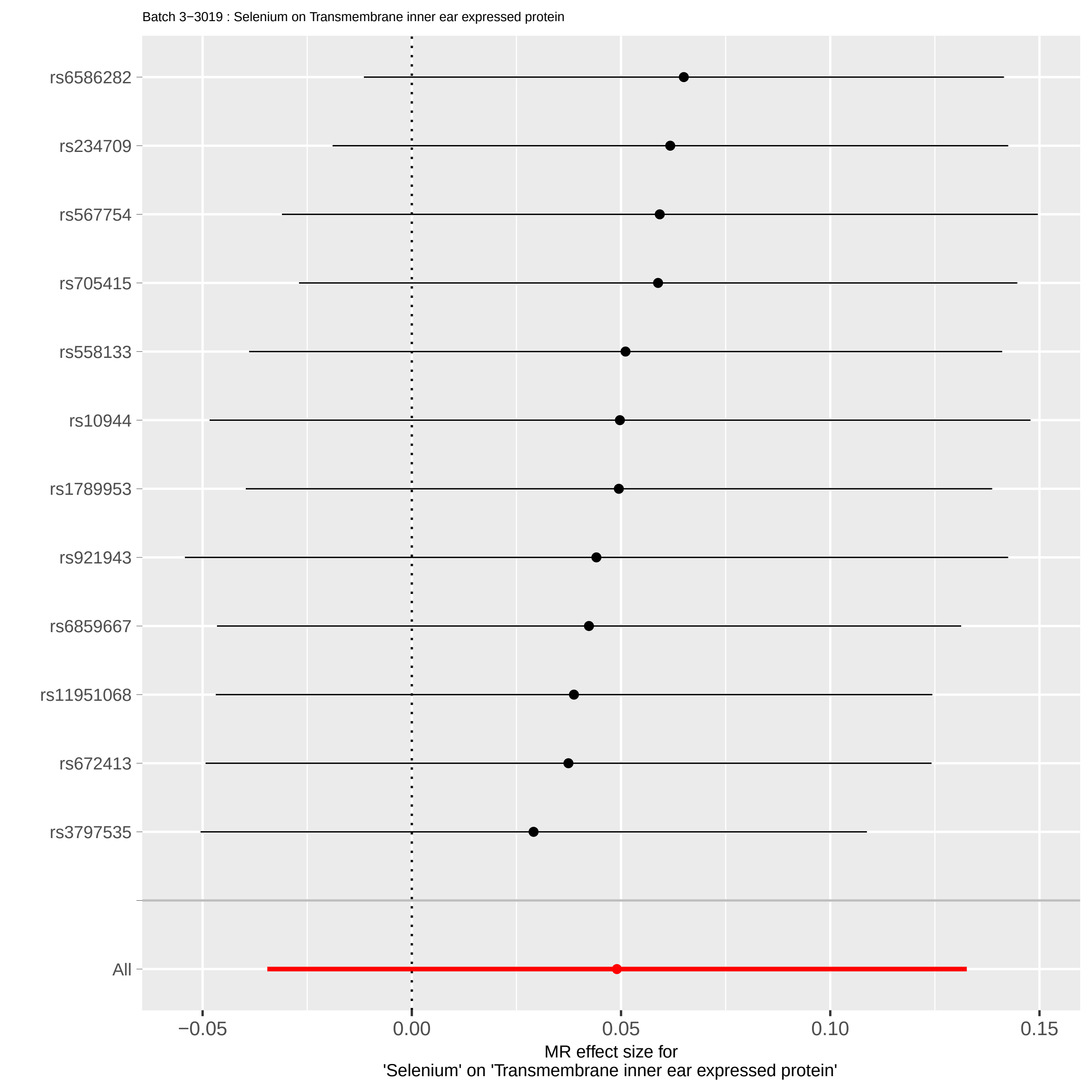

Batch 3−3019 : Selenium on Transmembrane inner ear expressed protein
rs6586282
rs234709
rs567754
rs705415
rs558133
rs10944
rs1789953
rs921943
rs6859667
rs11951068
rs672413
rs3797535
All
−0.05
0.00
0.05
0.10
0.15
MR effect size for
'Selenium' on 'Transmembrane inner ear expressed protein'

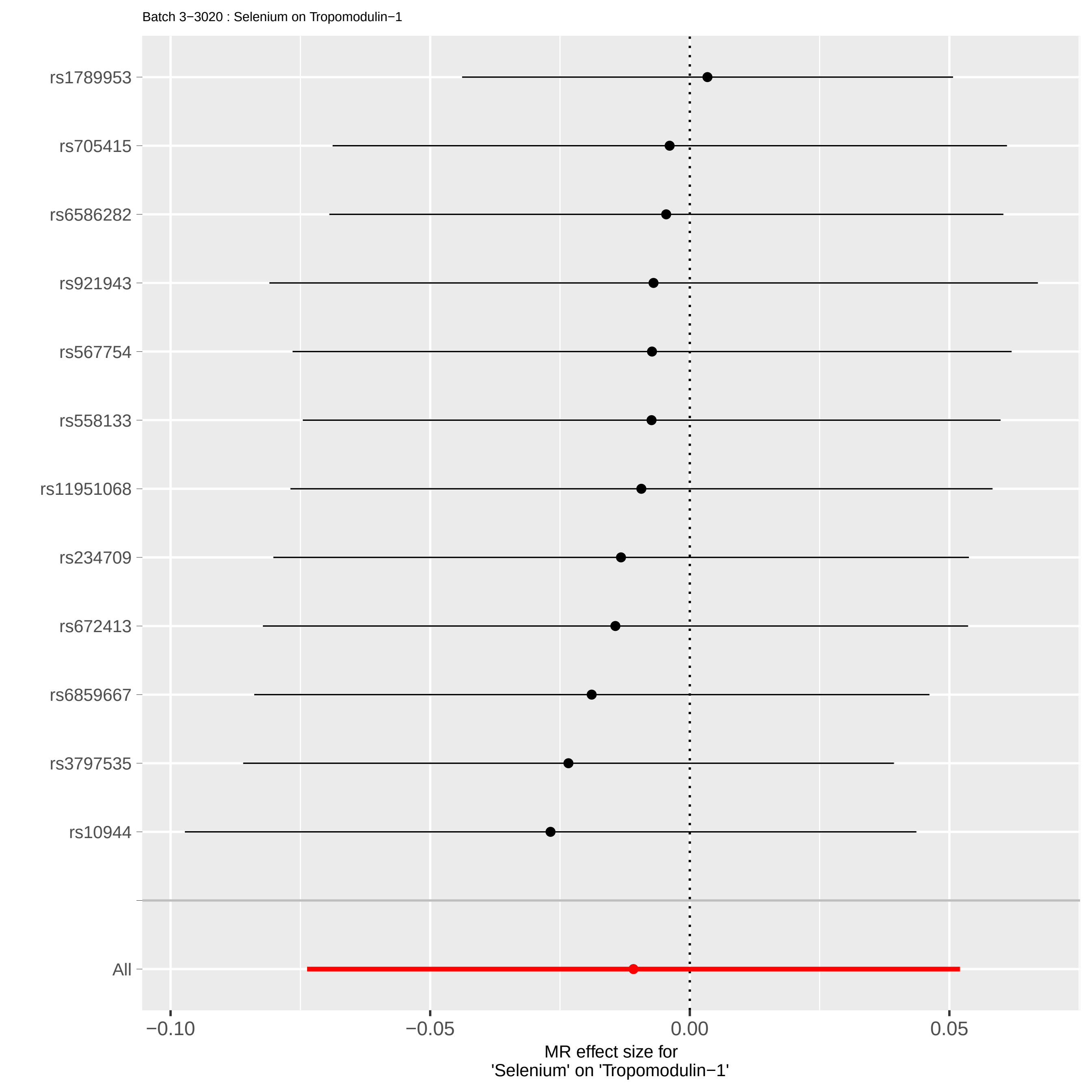
Batch 3−3020 : Selenium on Tropomodulin−1
rs1789953
rs705415
rs6586282
rs921943
rs567754
rs558133
rs11951068
rs234709
rs672413
rs6859667
rs3797535
rs10944
All
−0.10
−0.05
0.00
0.05
MR effect size for
'Selenium' on 'Tropomodulin−1'

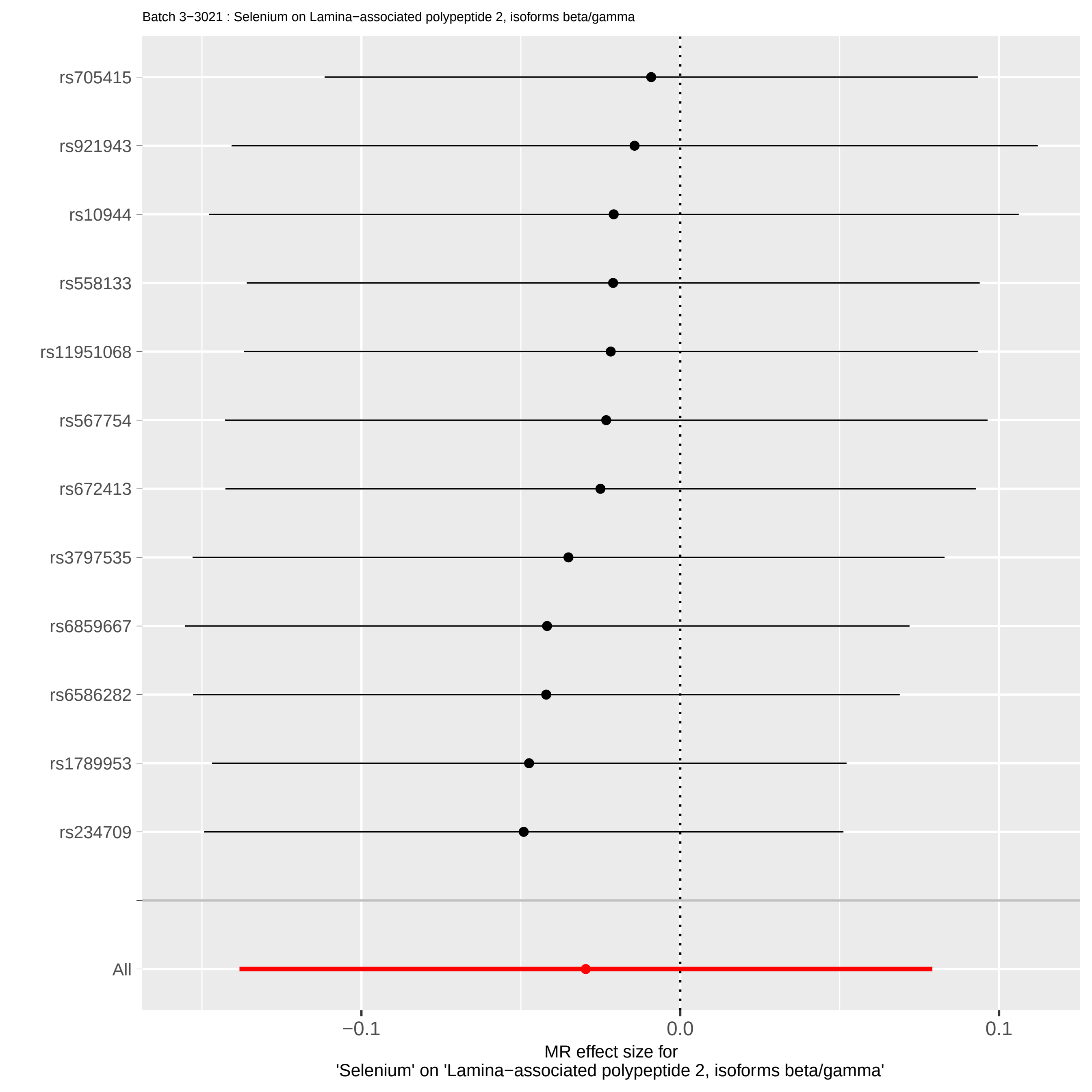

Batch 3−3021 : Selenium on Lamina−associated polypeptide 2, isoforms beta/gamma
rs705415
rs921943
rs10944
rs558133
rs11951068
rs567754
rs672413
rs3797535
rs6859667
rs6586282
rs1789953
rs234709
All
−0.1
0.0
0.1
MR effect size for
'Selenium' on 'Lamina−associated polypeptide 2, isoforms beta/gamma'

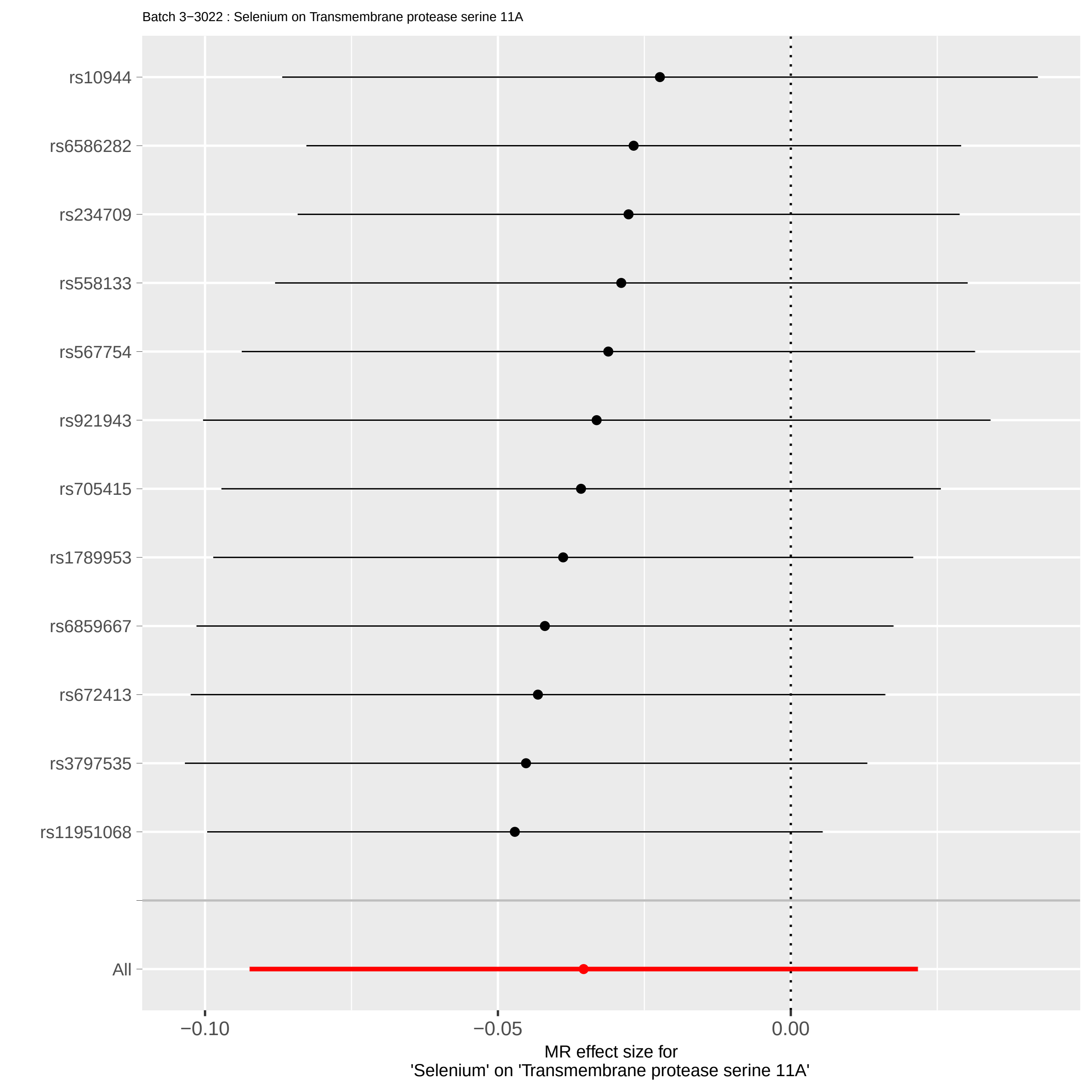

Batch 3−3022 : Selenium on Transmembrane protease serine 11A
rs10944
rs6586282
rs234709
rs558133
rs567754
rs921943
rs705415
rs1789953
rs6859667
rs672413
rs3797535
rs11951068
All
−0.10
−0.05
0.00
MR effect size for
'Selenium' on 'Transmembrane protease serine 11A'

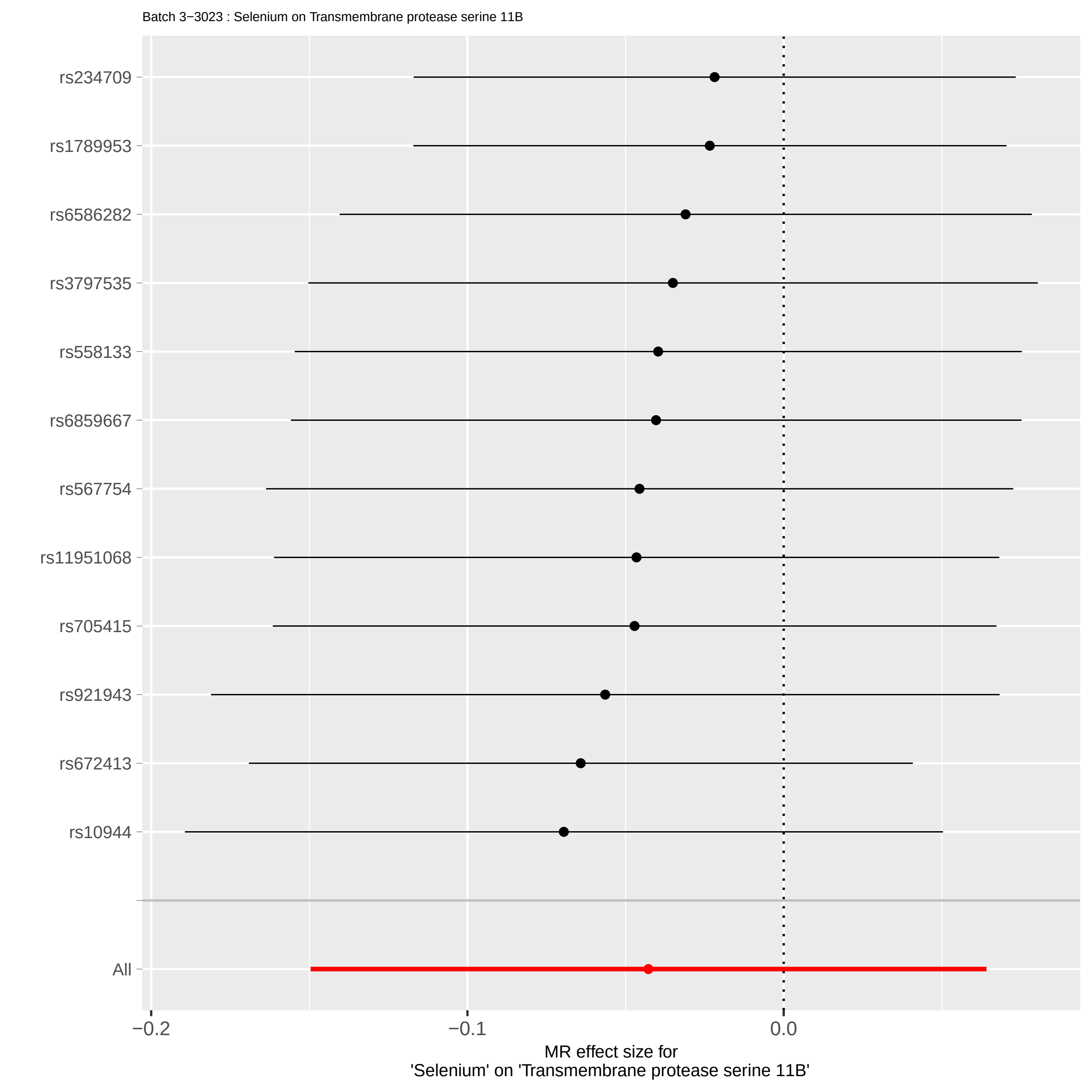

Batch 3−3023 : Selenium on Transmembrane protease serine 11B
rs234709
rs1789953
rs6586282
rs3797535
rs558133
rs6859667
rs567754
rs11951068
rs705415
rs921943
rs672413
rs10944
All
−0.2
−0.1
0.0
MR effect size for
'Selenium' on 'Transmembrane protease serine 11B'

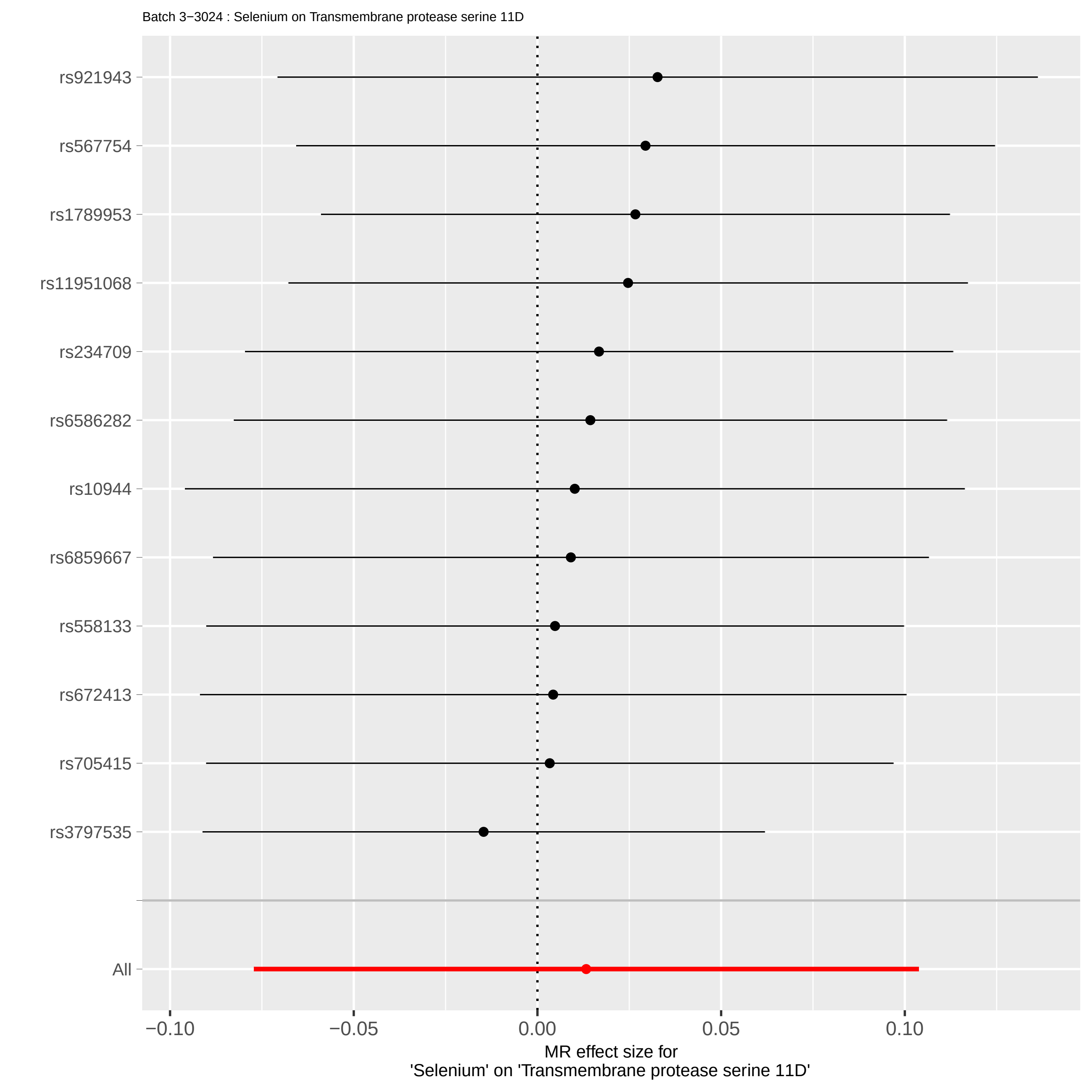
Batch 3−3024 : Selenium on Transmembrane protease serine 11D
rs921943
rs567754
rs1789953
rs11951068
rs234709
rs6586282
rs10944
rs6859667
rs558133
rs672413
rs705415
rs3797535
All
−0.10
−0.05
0.00
0.05
0.10
MR effect size for
'Selenium' on 'Transmembrane protease serine 11D'

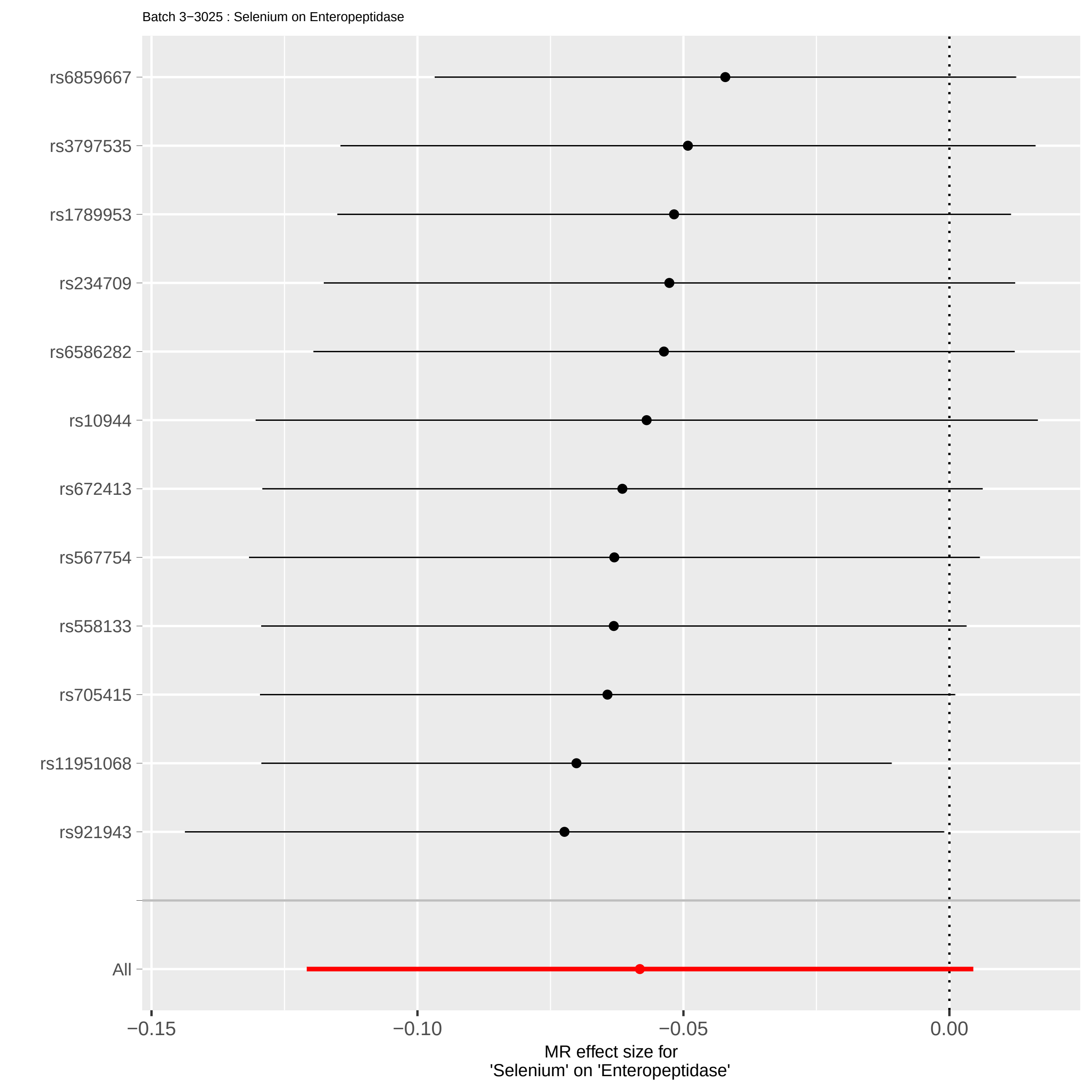

Batch 3−3025 : Selenium on Enteropeptidase
rs6859667
rs3797535
rs1789953
rs234709
rs6586282
rs10944
rs672413
rs567754
rs558133
rs705415
rs11951068
rs921943
All
−0.15
−0.10
−0.05
0.00
MR effect size for
'Selenium' on 'Enteropeptidase'

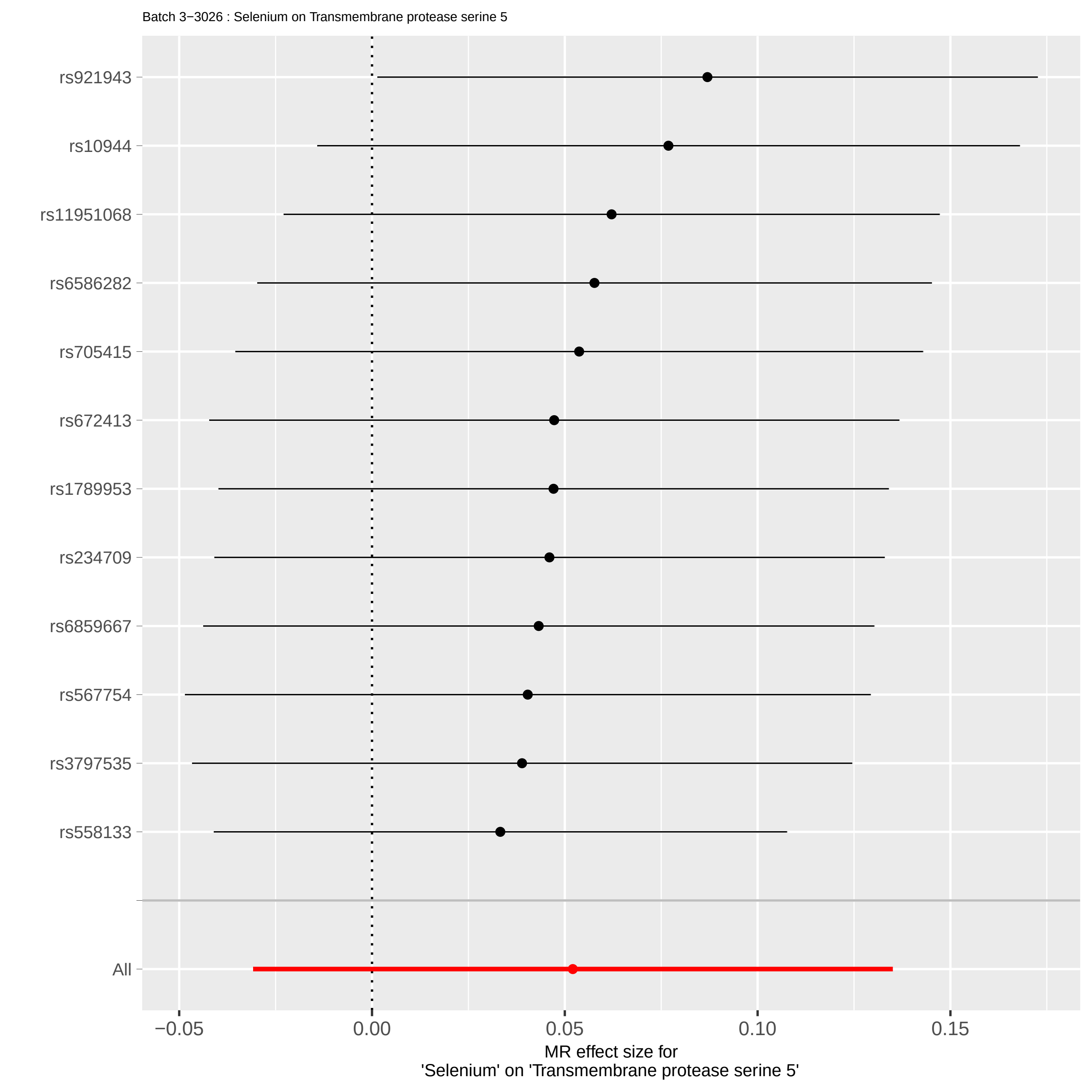

Batch 3−3026 : Selenium on Transmembrane protease serine 5
rs921943
rs10944
rs11951068
rs6586282
rs705415
rs672413
rs1789953
rs234709
rs6859667
rs567754
rs3797535
rs558133
All
−0.05
0.00
0.05
0.10
0.15
MR effect size for
'Selenium' on 'Transmembrane protease serine 5'

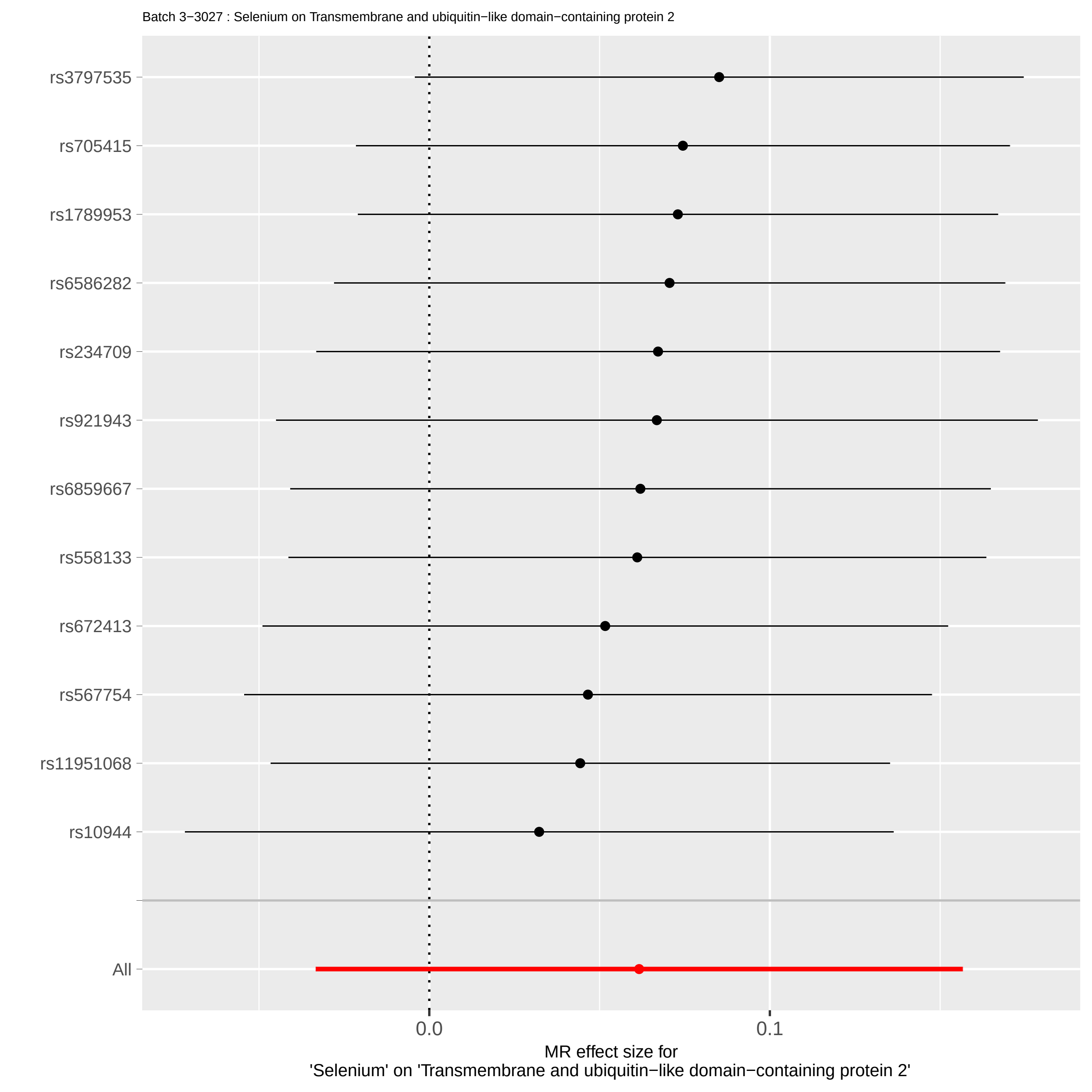
Batch 3−3027 : Selenium on Transmembrane and ubiquitin−like domain−containing protein 2
rs3797535
rs705415
rs1789953
rs6586282
rs234709
rs921943
rs6859667
rs558133
rs672413
rs567754
rs11951068
rs10944
All
0.0
0.1
MR effect size for
'Selenium' on 'Transmembrane and ubiquitin−like domain−containing protein 2'

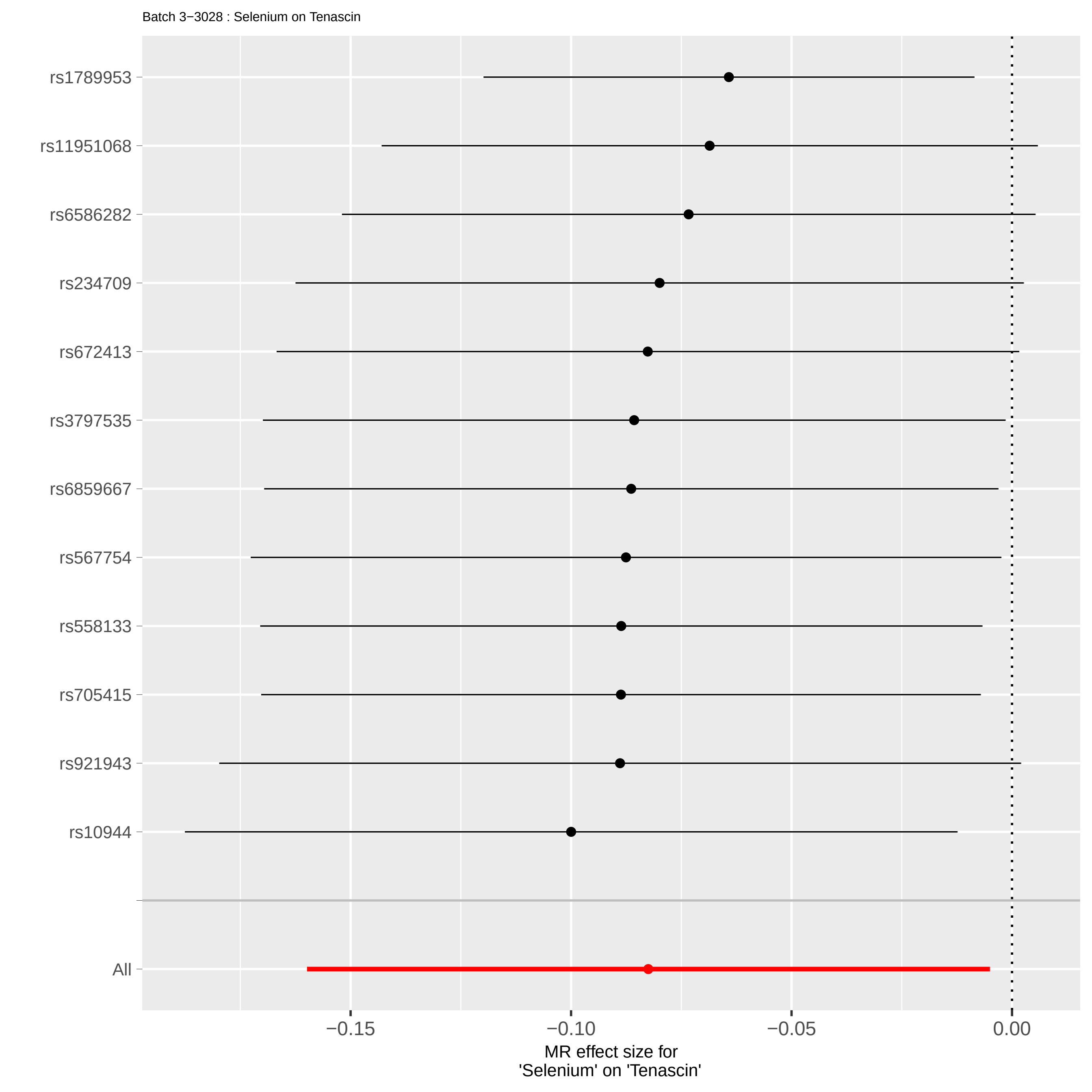
Batch 3−3028 : Selenium on Tenascin
rs1789953
rs11951068
rs6586282
rs234709
rs672413
rs3797535
rs6859667
rs567754
rs558133
rs705415
rs921943
rs10944
All
−0.15
−0.10
−0.05
0.00
MR effect size for
'Selenium' on 'Tenascin'

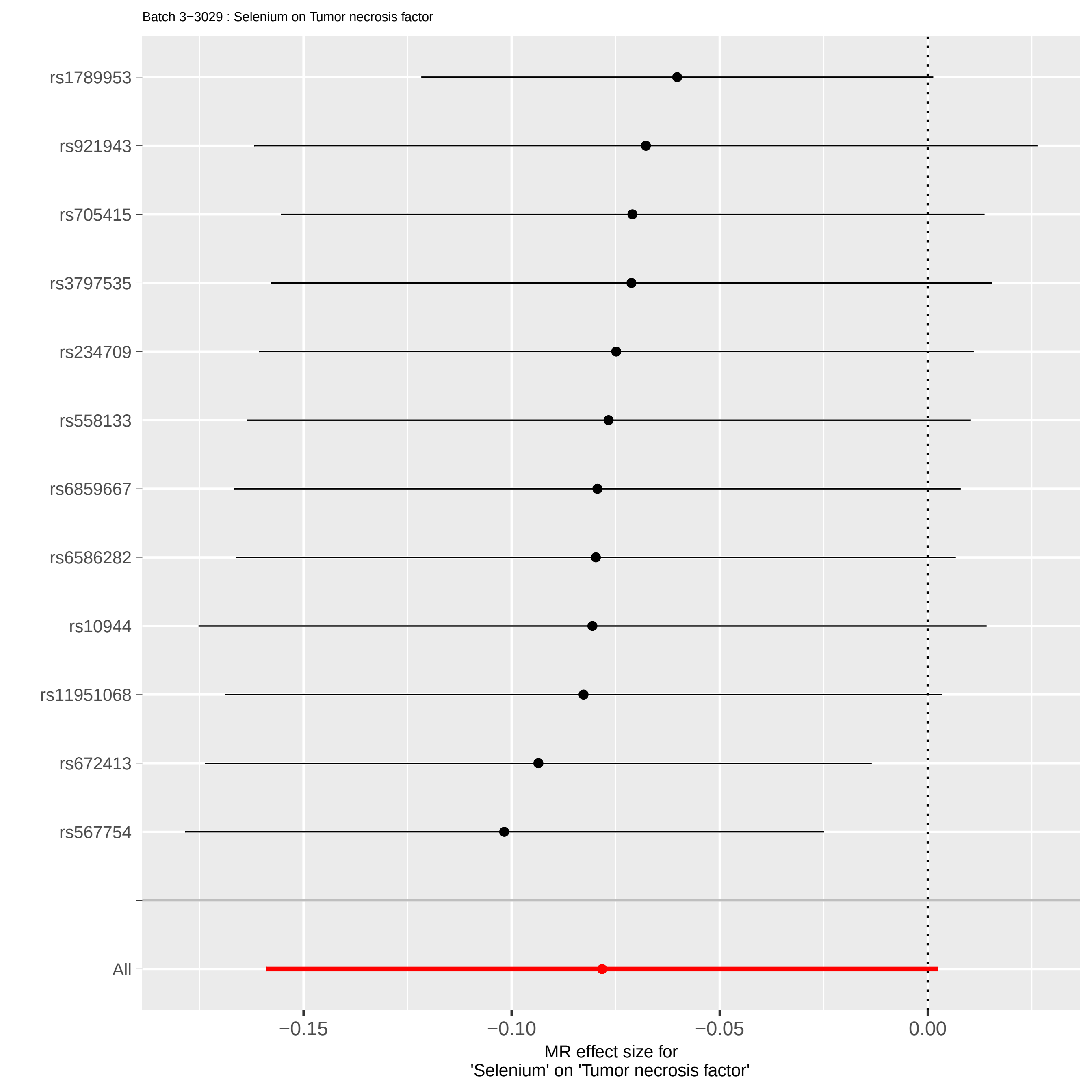

Batch 3−3029 : Selenium on Tumor necrosis factor
rs1789953
rs921943
rs705415
rs3797535
rs234709
rs558133
rs6859667
rs6586282
rs10944
rs11951068
rs672413
rs567754
All
−0.15
−0.10
−0.05
0.00
MR effect size for
'Selenium' on 'Tumor necrosis factor'

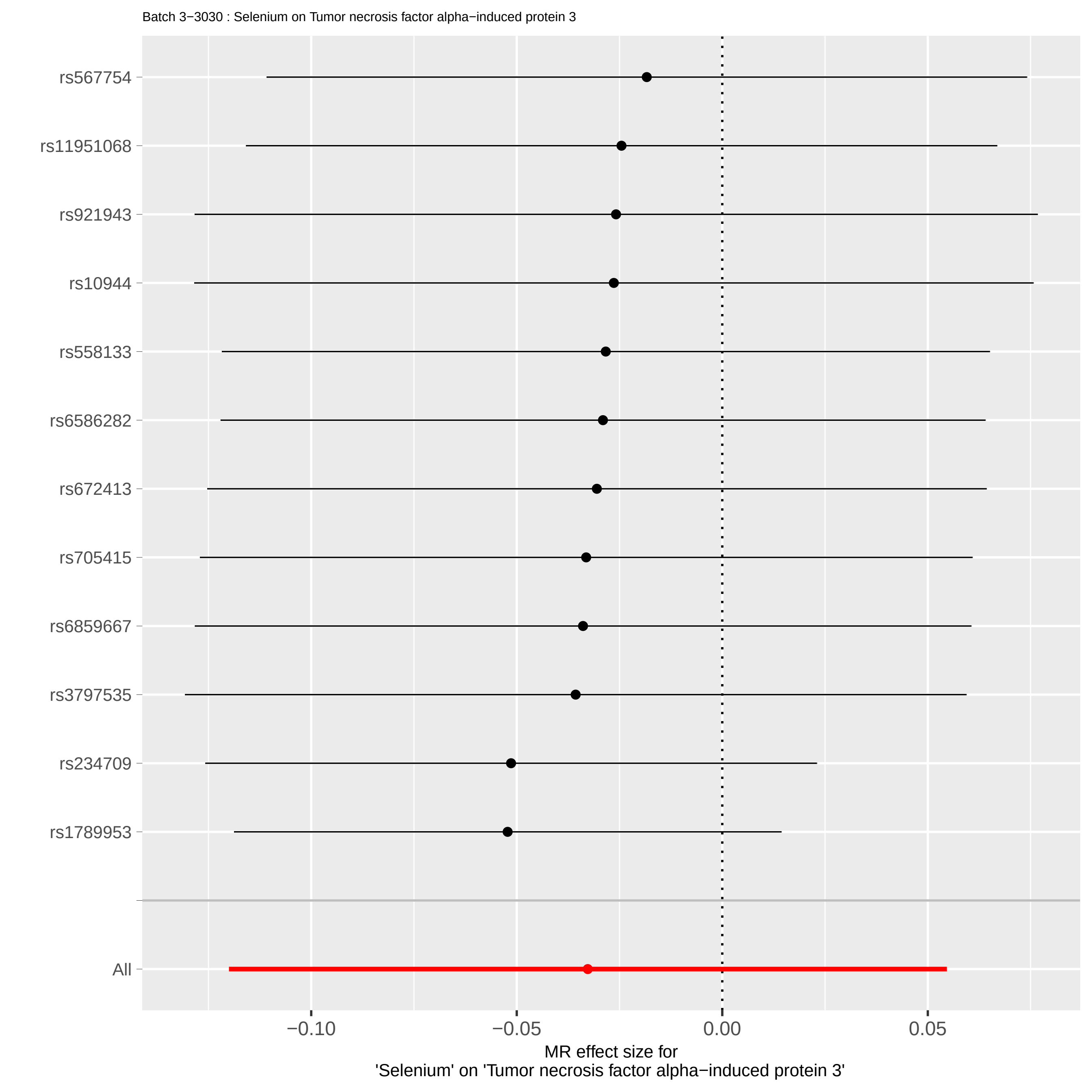
Batch 3−3030 : Selenium on Tumor necrosis factor alpha−induced protein 3
rs567754
rs11951068
rs921943
rs10944
rs558133
rs6586282
rs672413
rs705415
rs6859667
rs3797535
rs234709
rs1789953
All
−0.10
−0.05
0.00
0.05
MR effect size for
'Selenium' on 'Tumor necrosis factor alpha−induced protein 3'

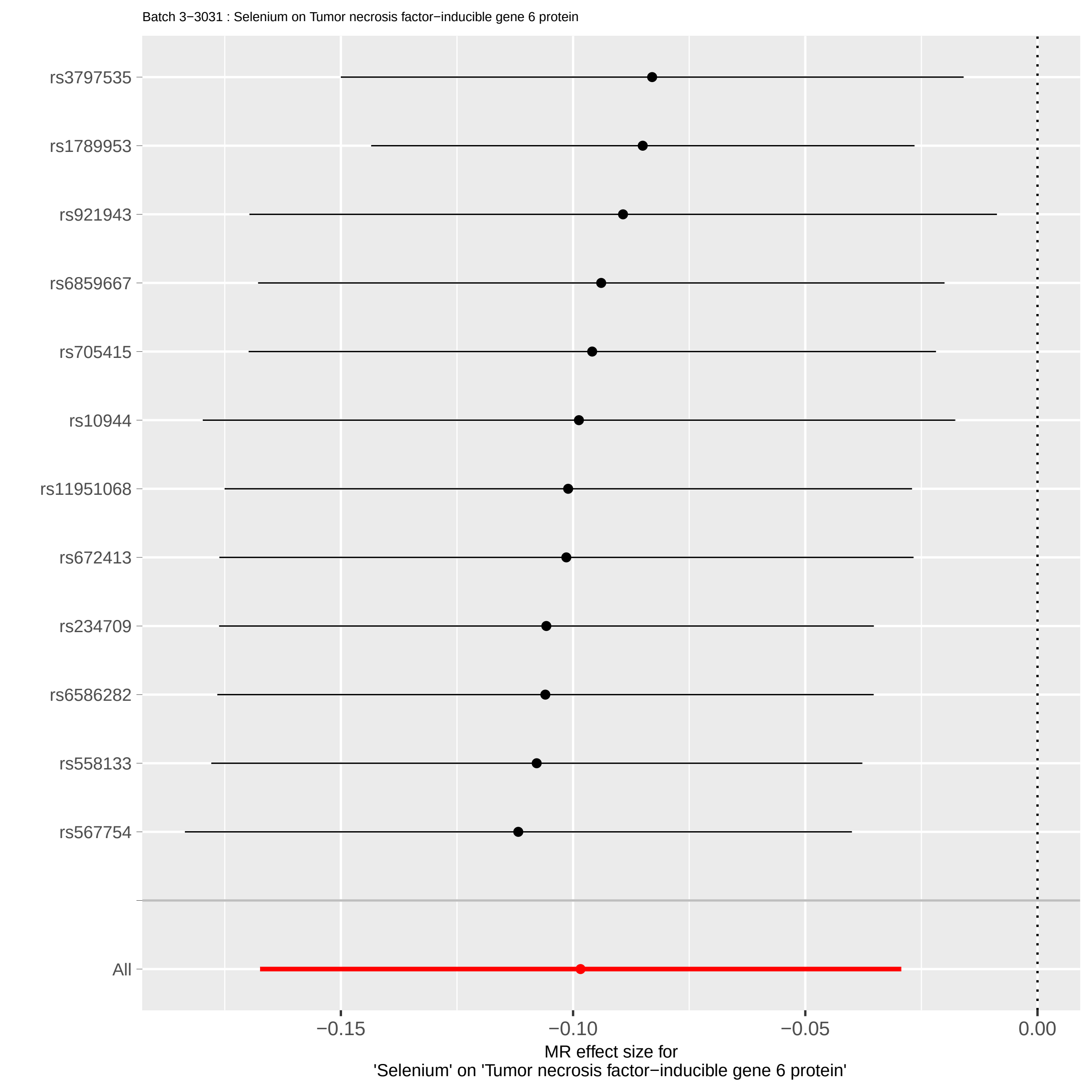

Batch 3−3031 : Selenium on Tumor necrosis factor−inducible gene 6 protein
rs3797535
rs1789953
rs921943
rs6859667
rs705415
rs10944
rs11951068
rs672413
rs234709
rs6586282
rs558133
rs567754
All
−0.15
−0.10
−0.05
0.00
MR effect size for
'Selenium' on 'Tumor necrosis factor−inducible gene 6 protein'

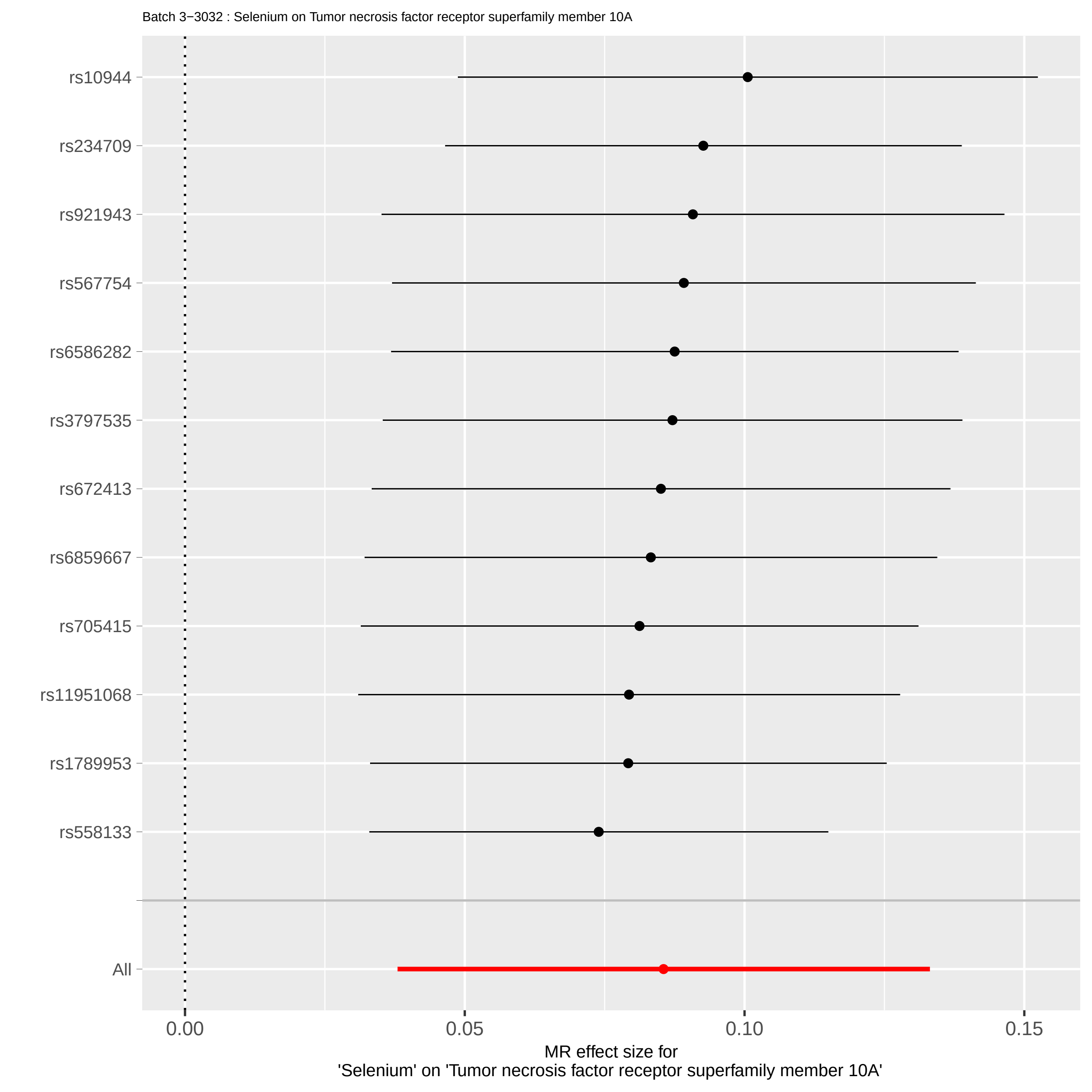
Batch 3−3032 : Selenium on Tumor necrosis factor receptor superfamily member 10A
rs10944
rs234709
rs921943
rs567754
rs6586282
rs3797535
rs672413
rs6859667
rs705415
rs11951068
rs1789953
rs558133
All
0.00
0.05
0.10
0.15
MR effect size for
'Selenium' on 'Tumor necrosis factor receptor superfamily member 10A'

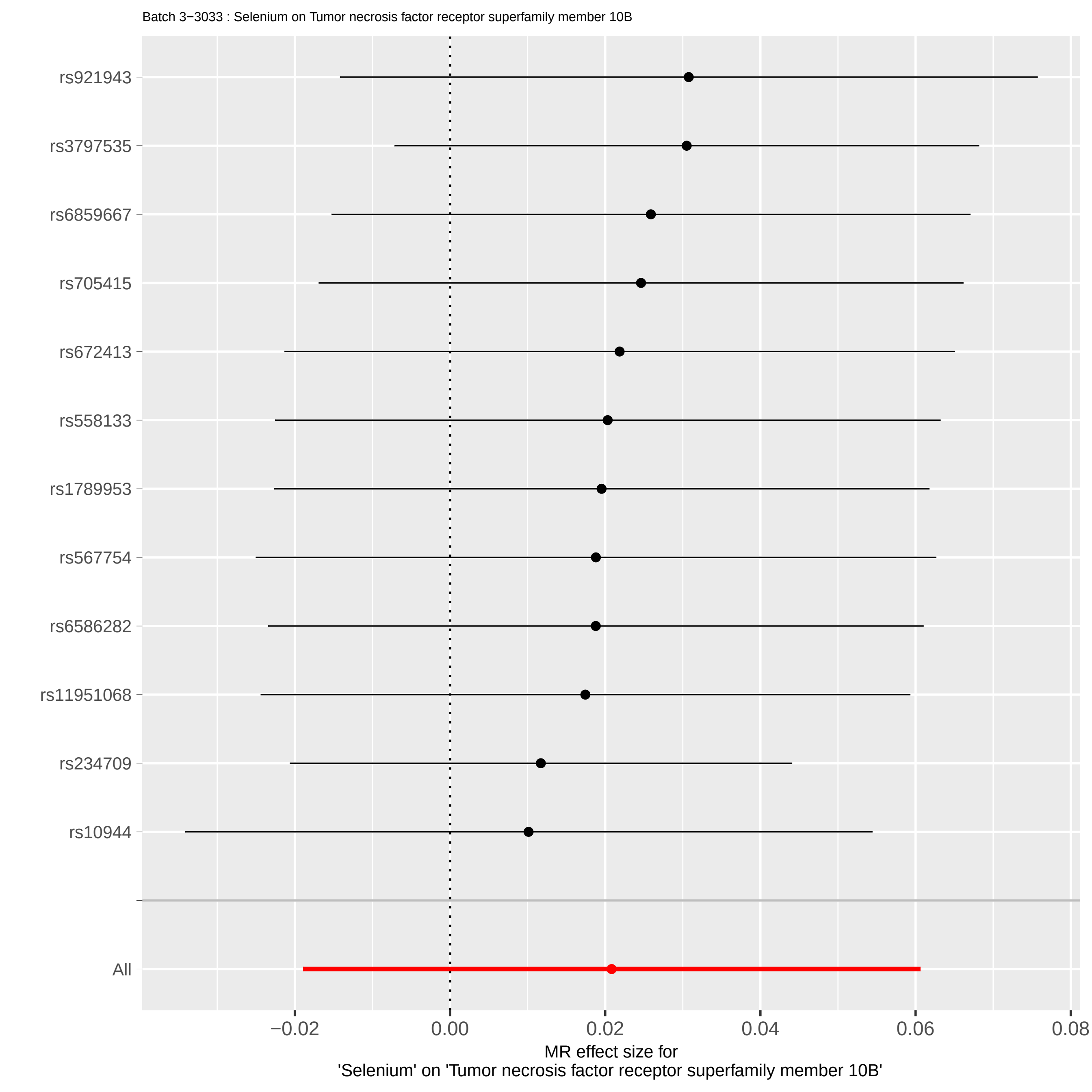

Batch 3−3033 : Selenium on Tumor necrosis factor receptor superfamily member 10B
rs921943
rs3797535
rs6859667
rs705415
rs672413
rs558133
rs1789953
rs567754
rs6586282
rs11951068
rs234709
rs10944
All
−0.02
0.00
0.02
0.04
0.06
0.08
MR effect size for
'Selenium' on 'Tumor necrosis factor receptor superfamily member 10B'

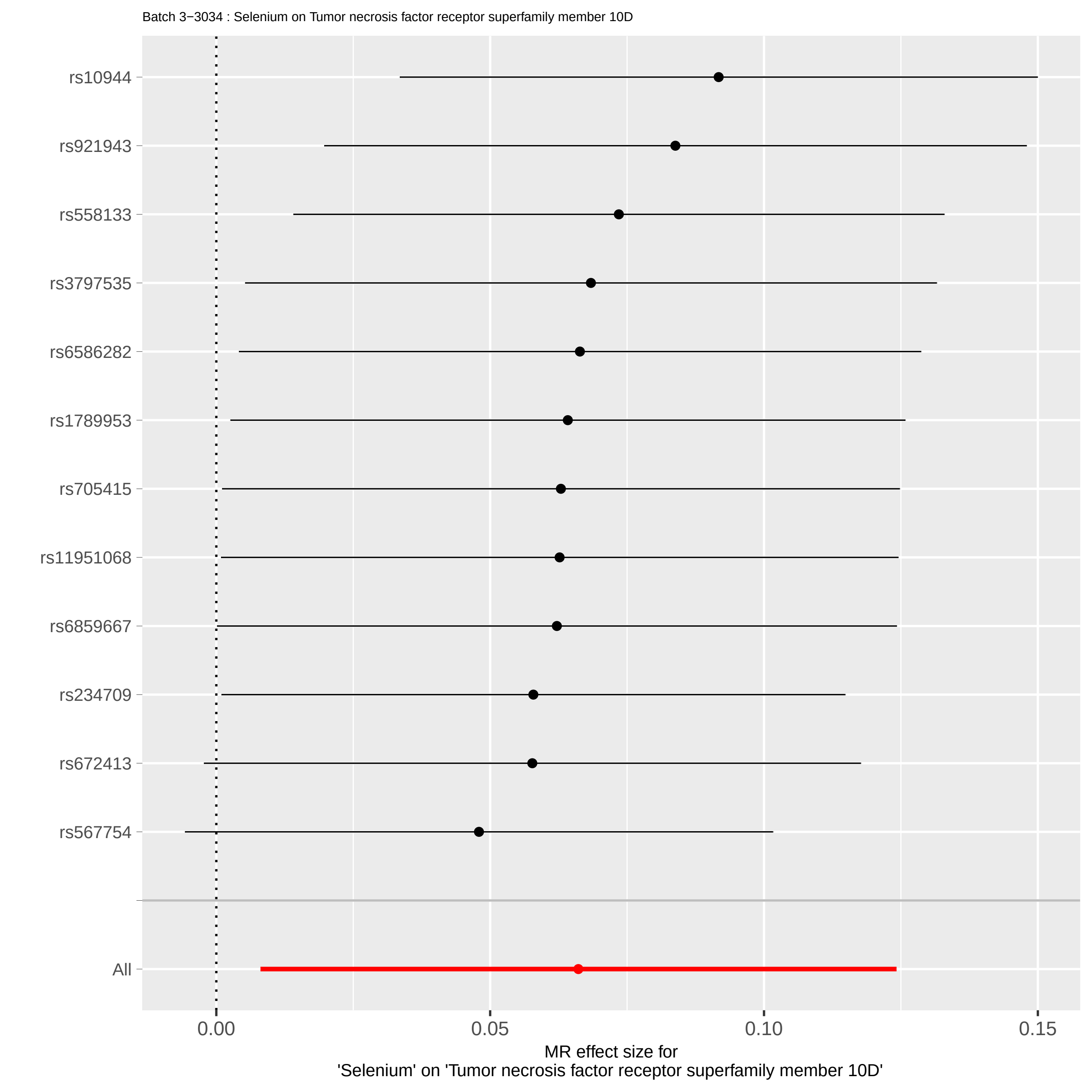

Batch 3−3034 : Selenium on Tumor necrosis factor receptor superfamily member 10D
rs10944
rs921943
rs558133
rs3797535
rs6586282
rs1789953
rs705415
rs11951068
rs6859667
rs234709
rs672413
rs567754
All
0.00
0.05
0.10
0.15
MR effect size for
'Selenium' on 'Tumor necrosis factor receptor superfamily member 10D'

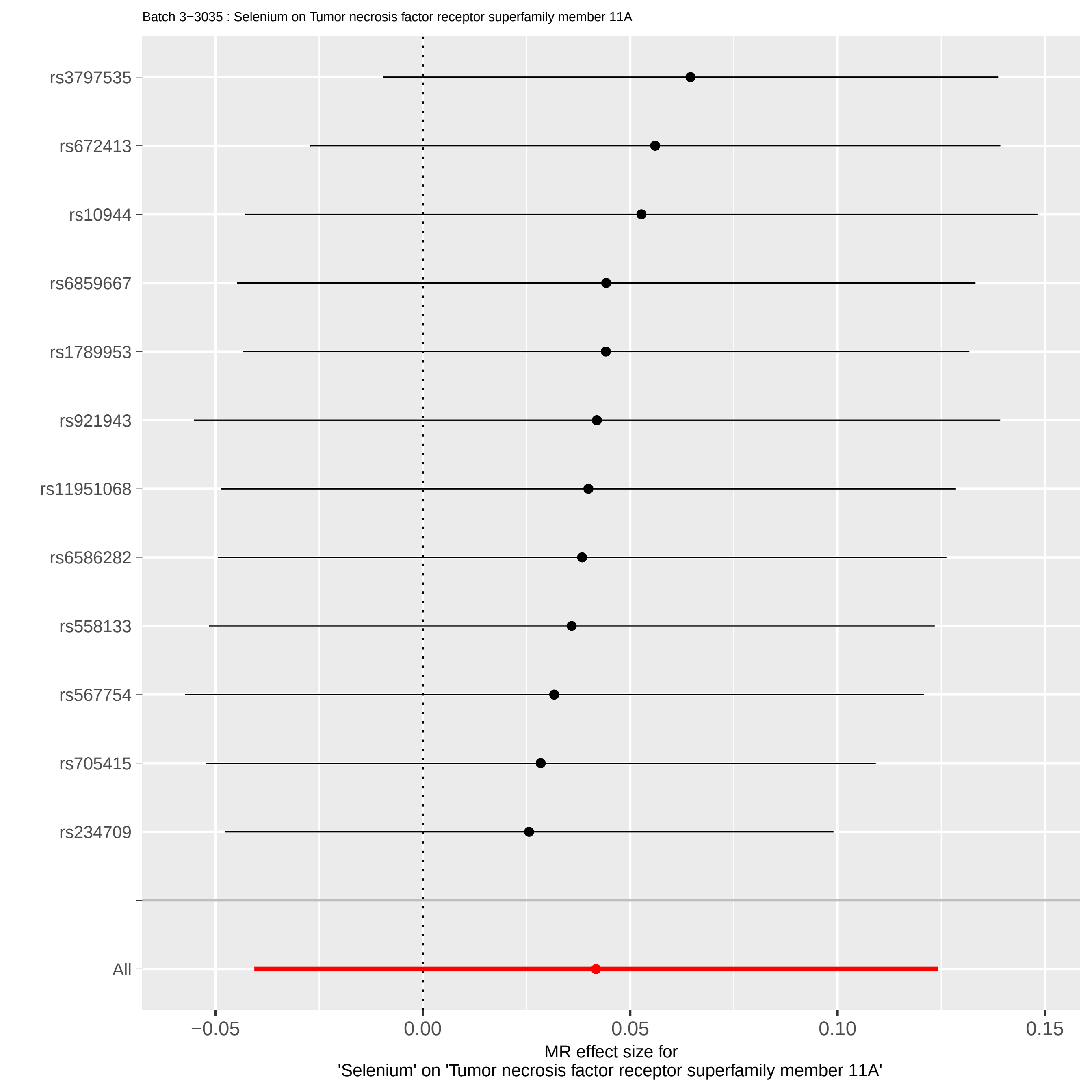
Batch 3−3035 : Selenium on Tumor necrosis factor receptor superfamily member 11A
rs3797535
rs672413
rs10944
rs6859667
rs1789953
rs921943
rs11951068
rs6586282
rs558133
rs567754
rs705415
rs234709
All
−0.05
0.00
0.05
0.10
0.15
MR effect size for
'Selenium' on 'Tumor necrosis factor receptor superfamily member 11A'

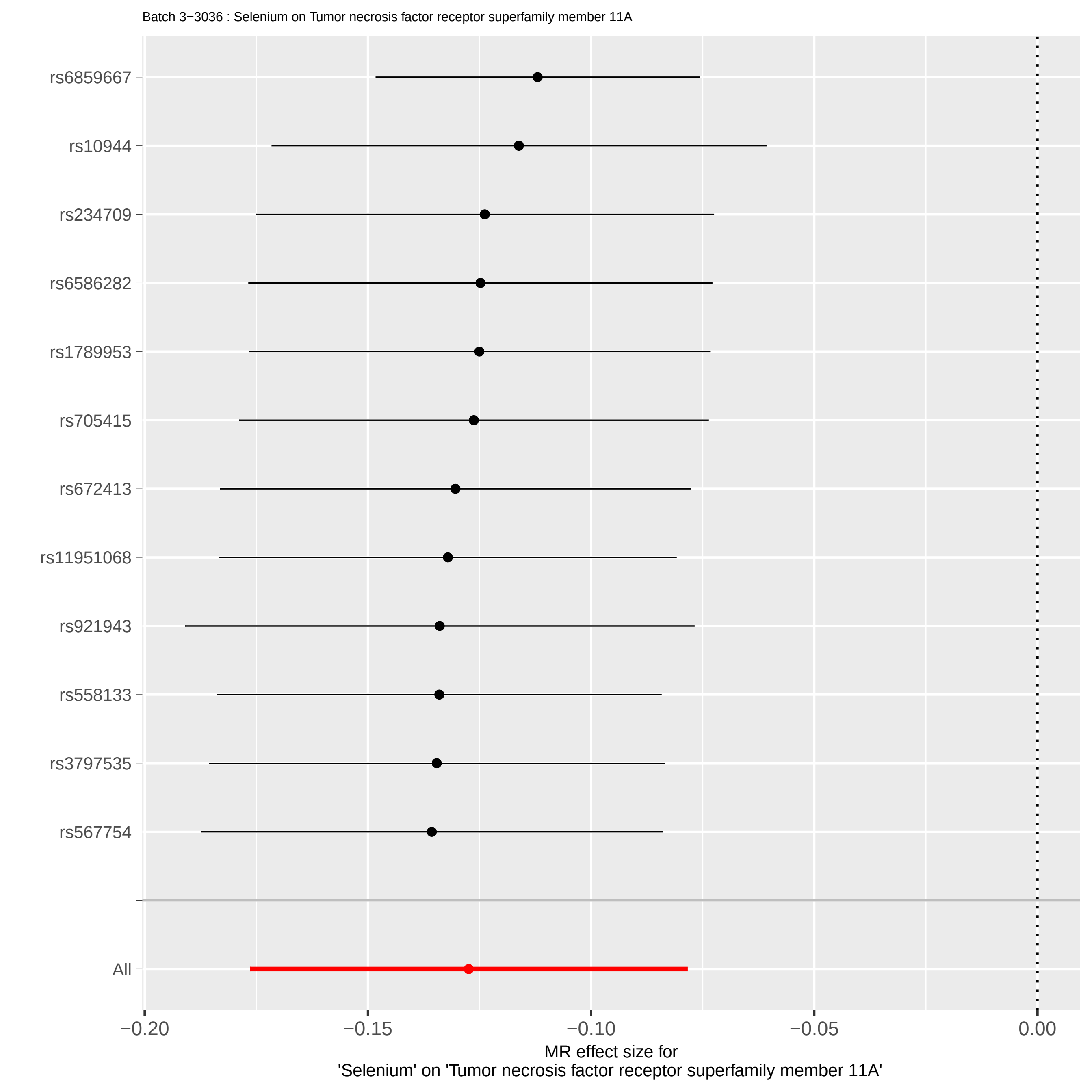
Batch 3−3036 : Selenium on Tumor necrosis factor receptor superfamily member 11A
rs6859667
rs10944
rs234709
rs6586282
rs1789953
rs705415
rs672413
rs11951068
rs921943
rs558133
rs3797535
rs567754
All
−0.20
−0.15
−0.10
−0.05
0.00
MR effect size for
'Selenium' on 'Tumor necrosis factor receptor superfamily member 11A'

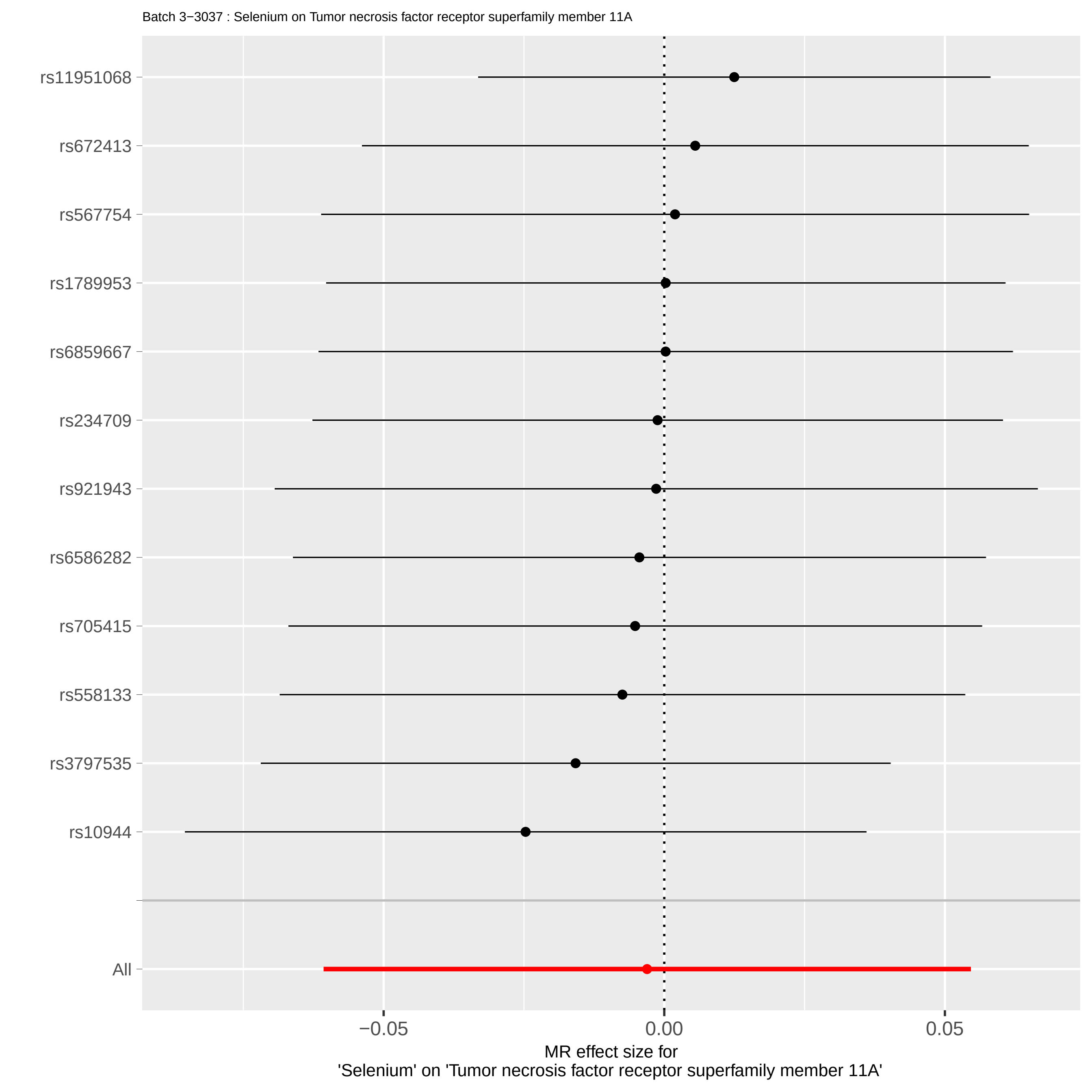

Batch 3−3037 : Selenium on Tumor necrosis factor receptor superfamily member 11A
rs11951068
rs672413
rs567754
rs1789953
rs6859667
rs234709
rs921943
rs6586282
rs705415
rs558133
rs3797535
rs10944
All
−0.05
0.00
0.05
MR effect size for
'Selenium' on 'Tumor necrosis factor receptor superfamily member 11A'

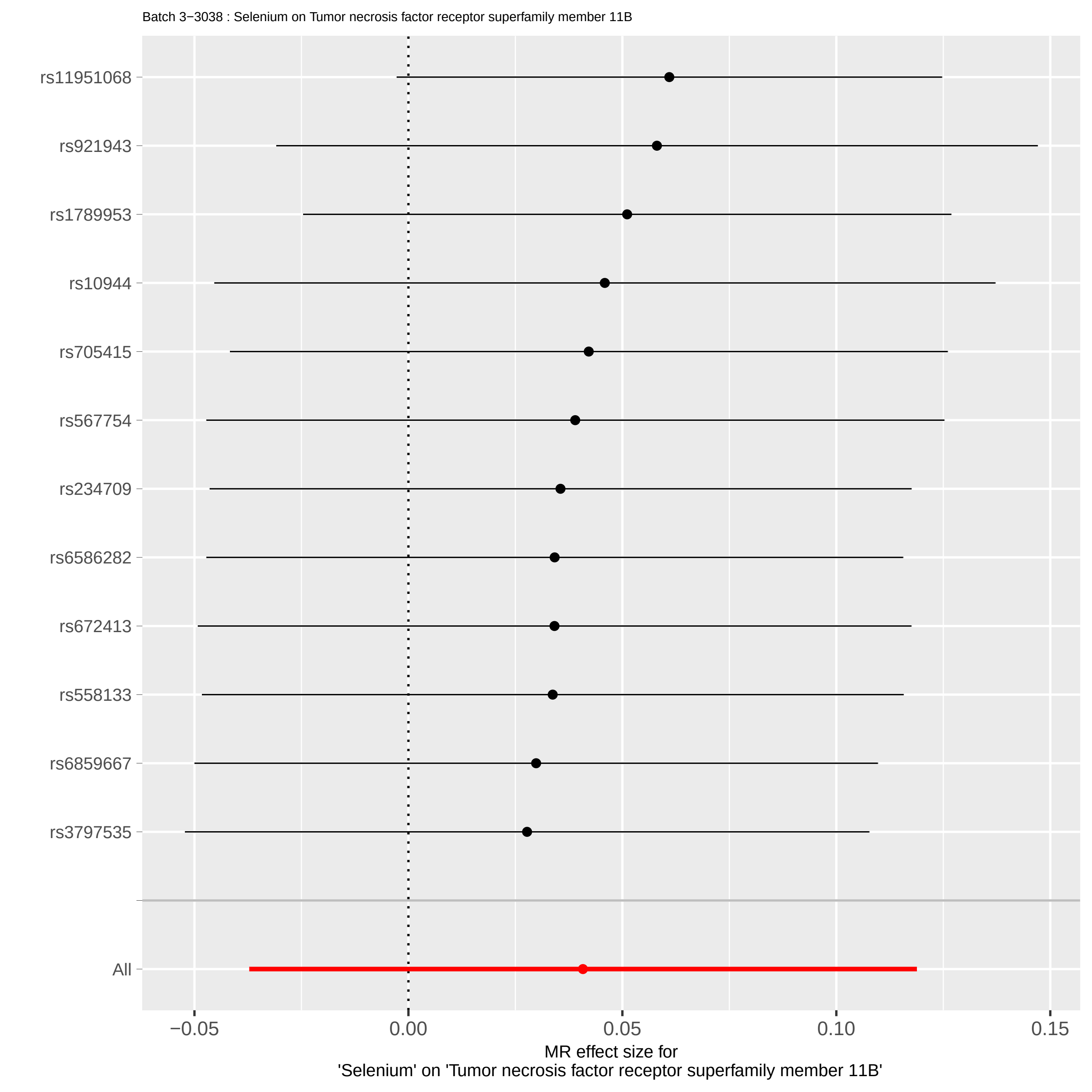
Batch 3−3038 : Selenium on Tumor necrosis factor receptor superfamily member 11B
rs11951068
rs921943
rs1789953
rs10944
rs705415
rs567754
rs234709
rs6586282
rs672413
rs558133
rs6859667
rs3797535
All
−0.05
0.00
0.05
0.10
0.15
MR effect size for
'Selenium' on 'Tumor necrosis factor receptor superfamily member 11B'

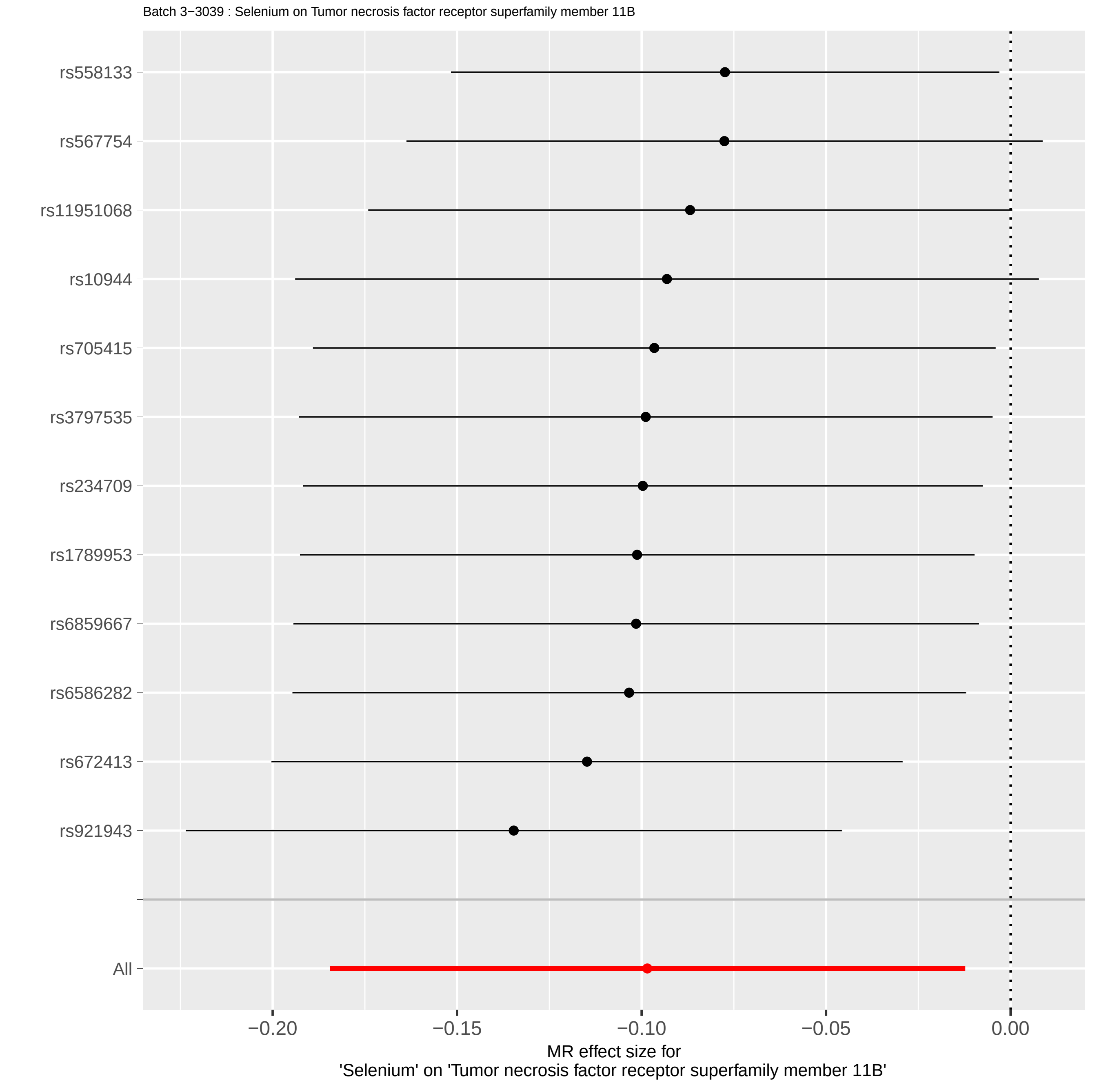

Batch 3−3039 : Selenium on Tumor necrosis factor receptor superfamily member 11B
rs558133
rs567754
rs11951068
rs10944
rs705415
rs3797535
rs234709
rs1789953
rs6859667
rs6586282
rs672413
rs921943
All
−0.20
−0.15
−0.10
−0.05
0.00
MR effect size for
'Selenium' on 'Tumor necrosis factor receptor superfamily member 11B'

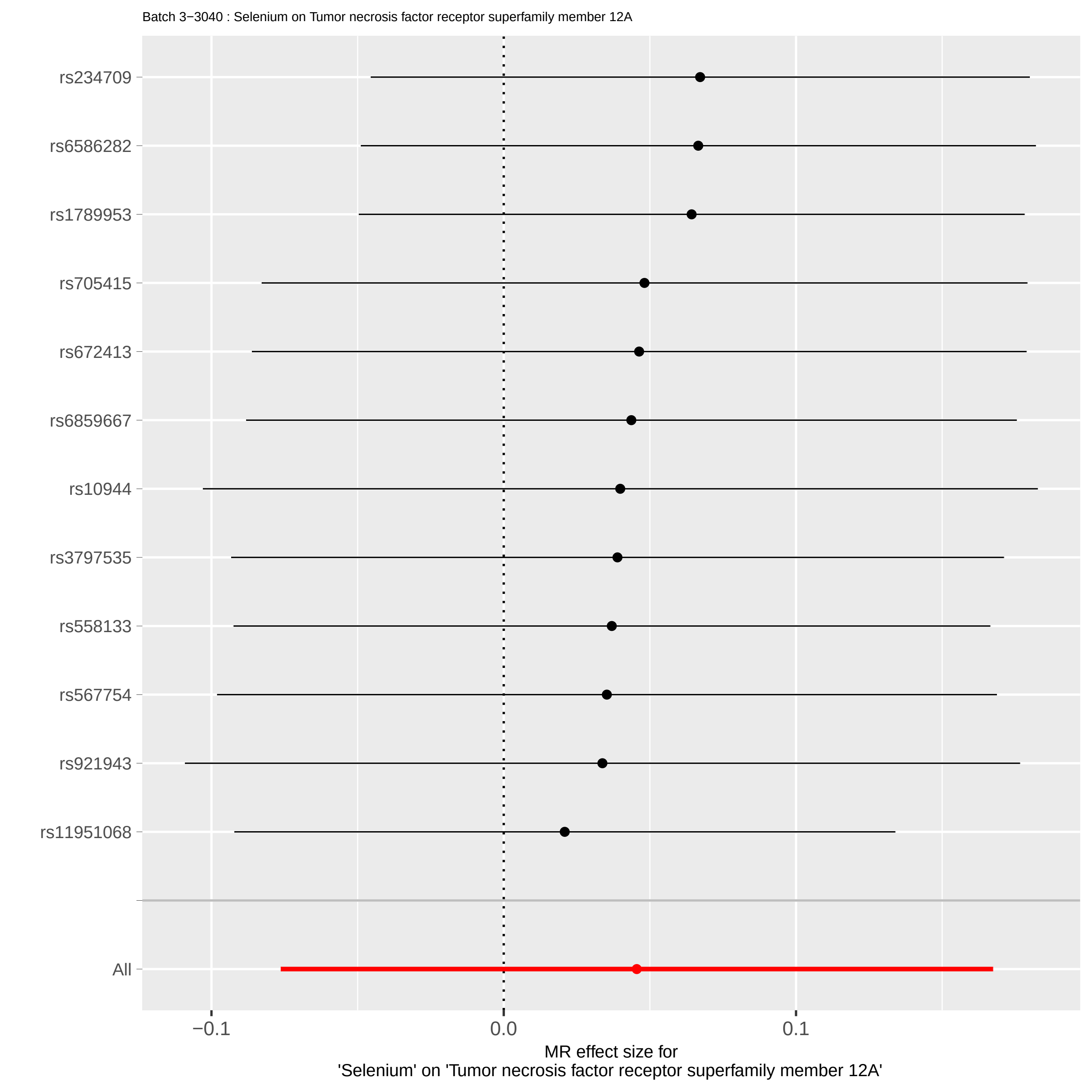

Batch 3−3040 : Selenium on Tumor necrosis factor receptor superfamily member 12A
rs234709
rs6586282
rs1789953
rs705415
rs672413
rs6859667
rs10944
rs3797535
rs558133
rs567754
rs921943
rs11951068
All
−0.1
0.0
0.1
MR effect size for
'Selenium' on 'Tumor necrosis factor receptor superfamily member 12A'

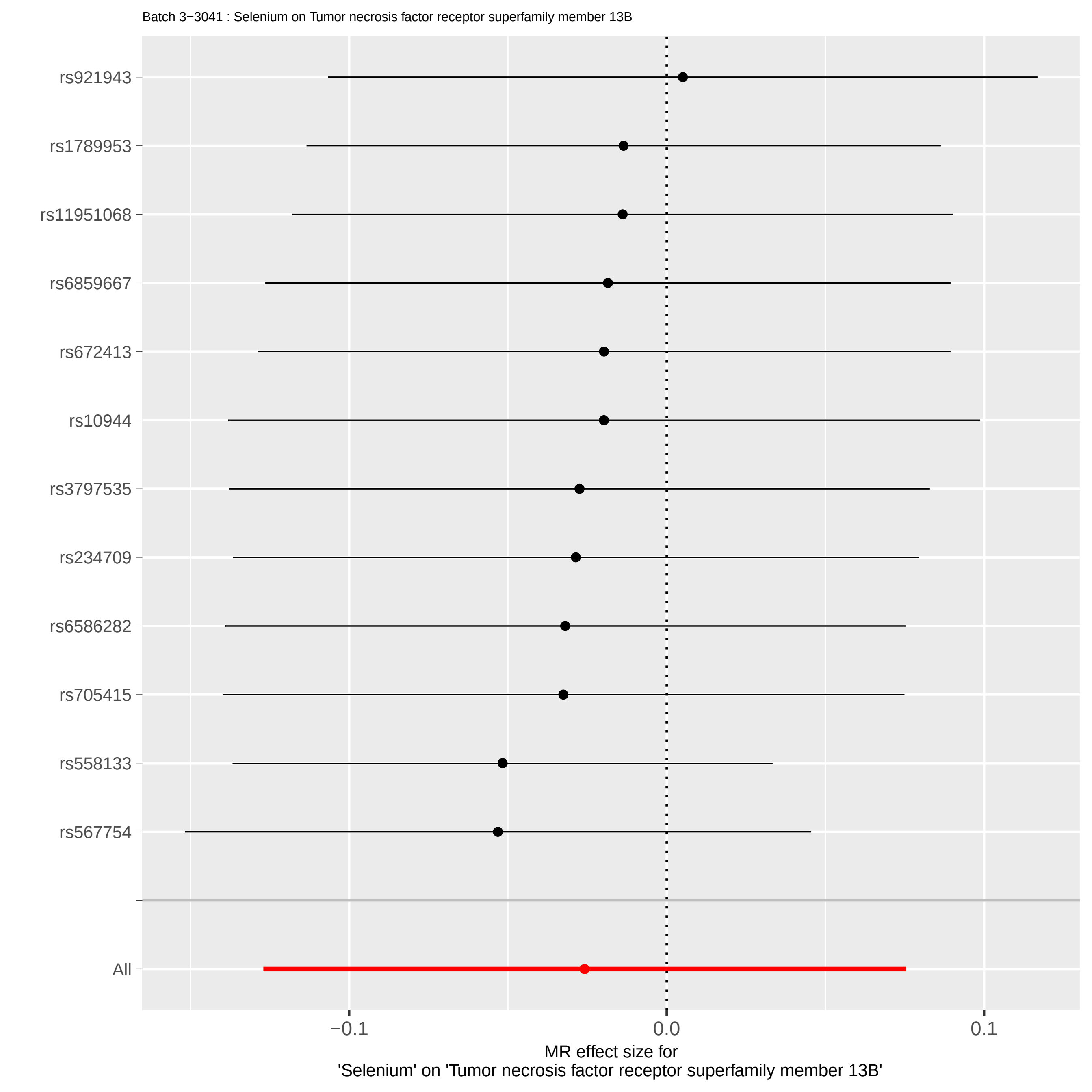

Batch 3−3041 : Selenium on Tumor necrosis factor receptor superfamily member 13B
rs921943
rs1789953
rs11951068
rs6859667
rs672413
rs10944
rs3797535
rs234709
rs6586282
rs705415
rs558133
rs567754
All
−0.1
0.0
0.1
MR effect size for
'Selenium' on 'Tumor necrosis factor receptor superfamily member 13B'

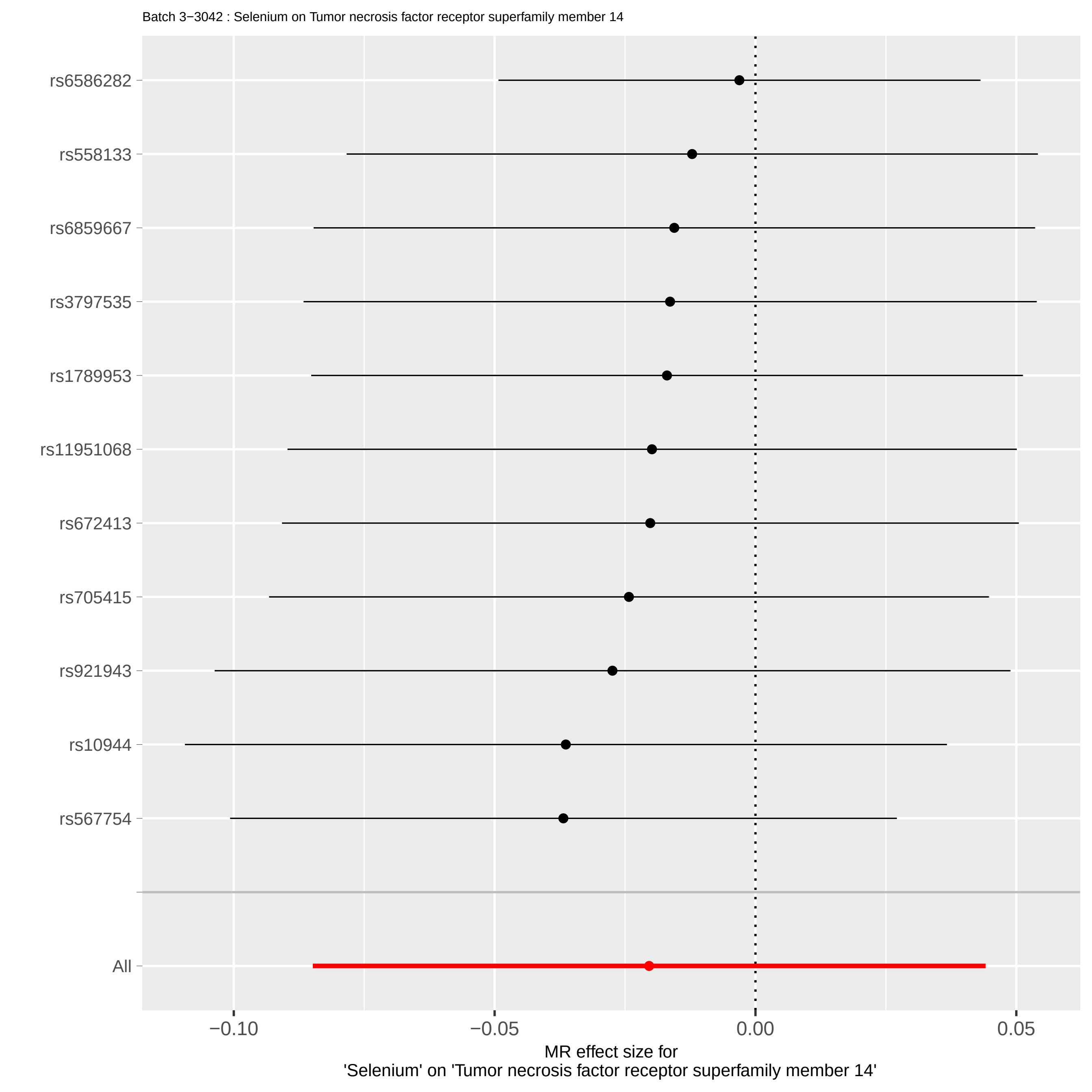

Batch 3−3042 : Selenium on Tumor necrosis factor receptor superfamily member 14
rs6586282
rs558133
rs6859667
rs3797535
rs1789953
rs11951068
rs672413
rs705415
rs921943
rs10944
rs567754
All
−0.10
−0.05
0.00
0.05
MR effect size for
'Selenium' on 'Tumor necrosis factor receptor superfamily member 14'

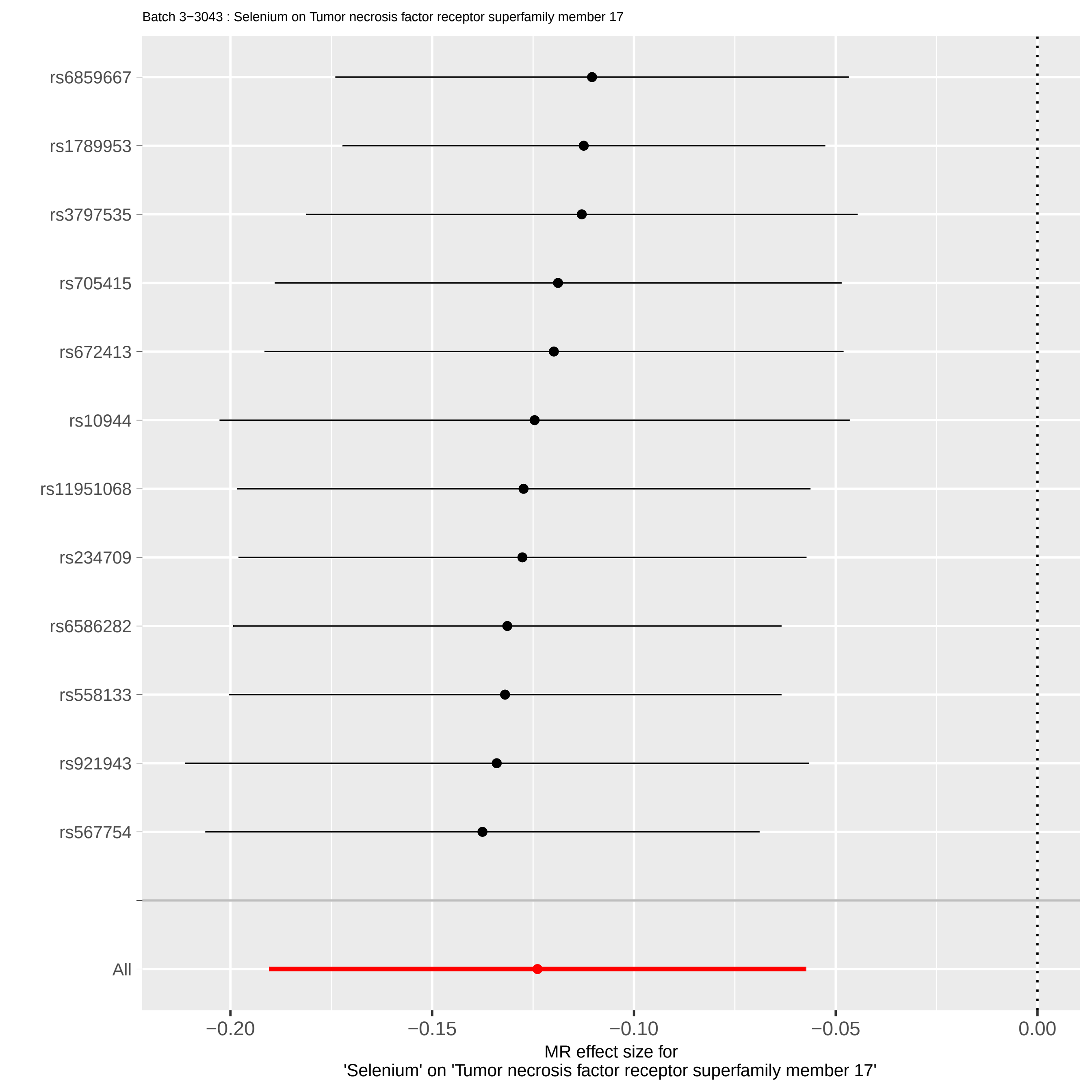

Batch 3−3043 : Selenium on Tumor necrosis factor receptor superfamily member 17
rs6859667
rs1789953
rs3797535
rs705415
rs672413
rs10944
rs11951068
rs234709
rs6586282
rs558133
rs921943
rs567754
All
−0.20
−0.15
−0.10
−0.05
0.00
MR effect size for
'Selenium' on 'Tumor necrosis factor receptor superfamily member 17'

Batch 3−3044 : Selenium on Tumor necrosis factor receptor superfamily member 18

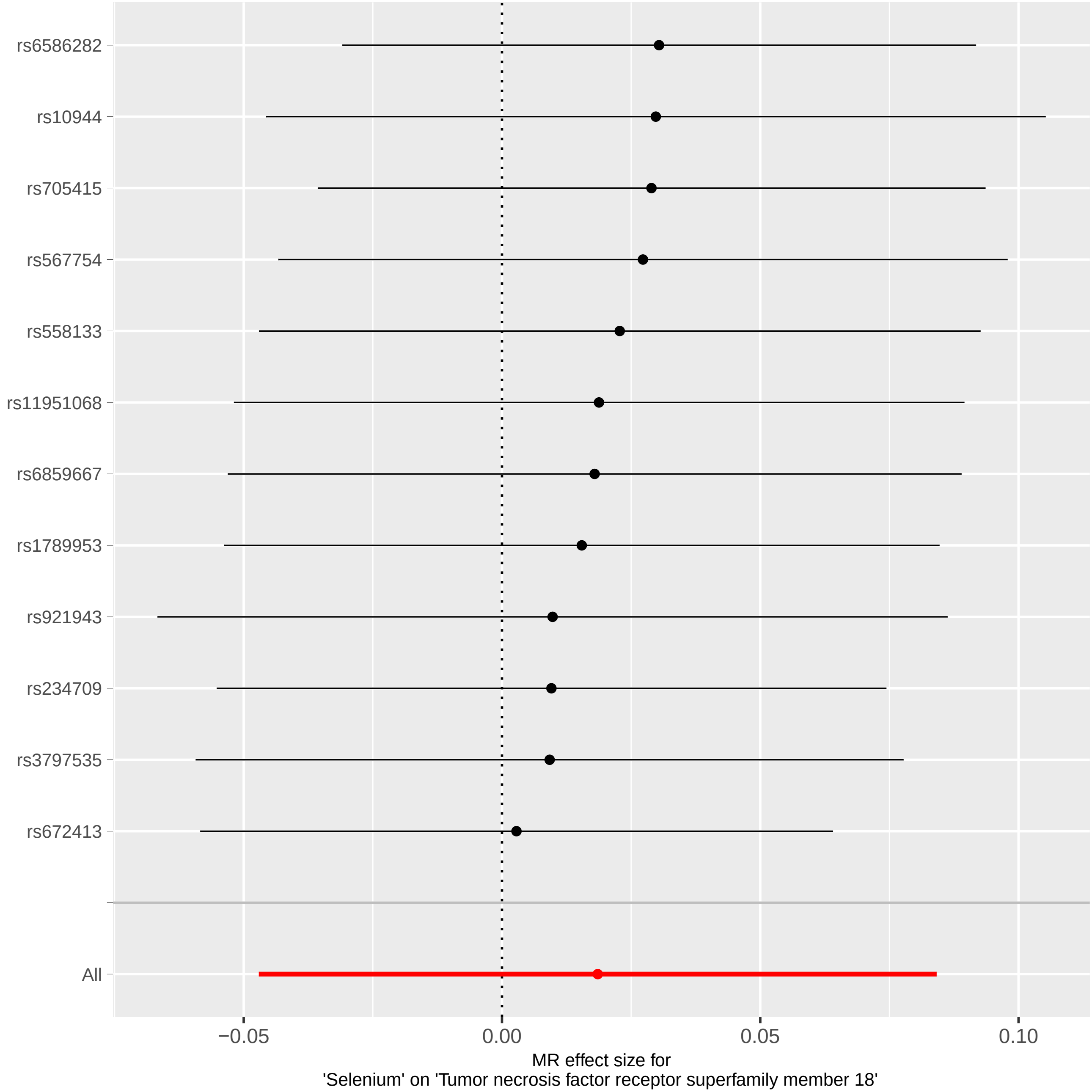

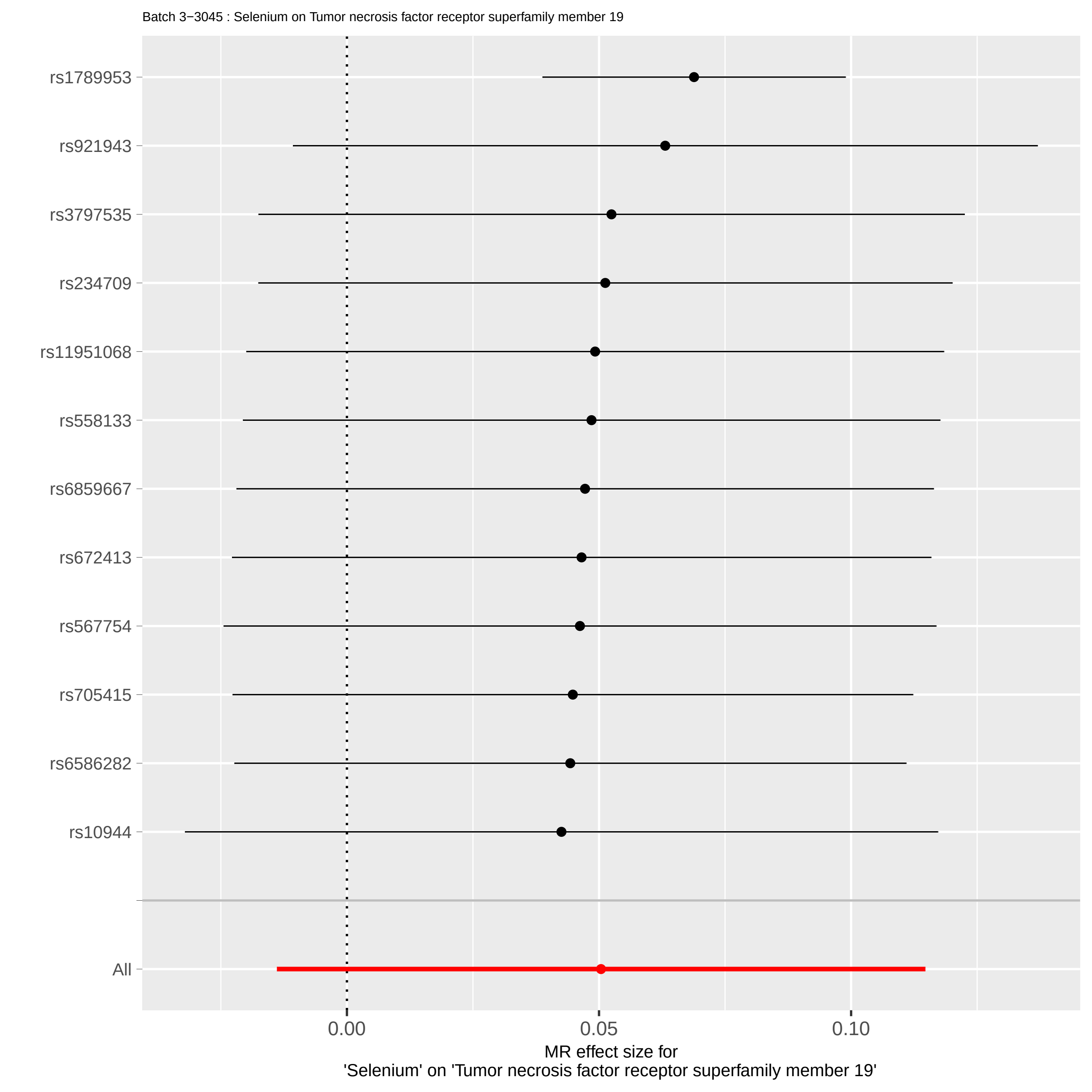
Batch 3−3045 : Selenium on Tumor necrosis factor receptor superfamily member 19
rs1789953
rs921943
rs3797535
rs234709
rs11951068
rs558133
rs6859667
rs672413
rs567754
rs705415
rs6586282
rs10944
All
0.00
0.05
0.10
MR effect size for
'Selenium' on 'Tumor necrosis factor receptor superfamily member 19'

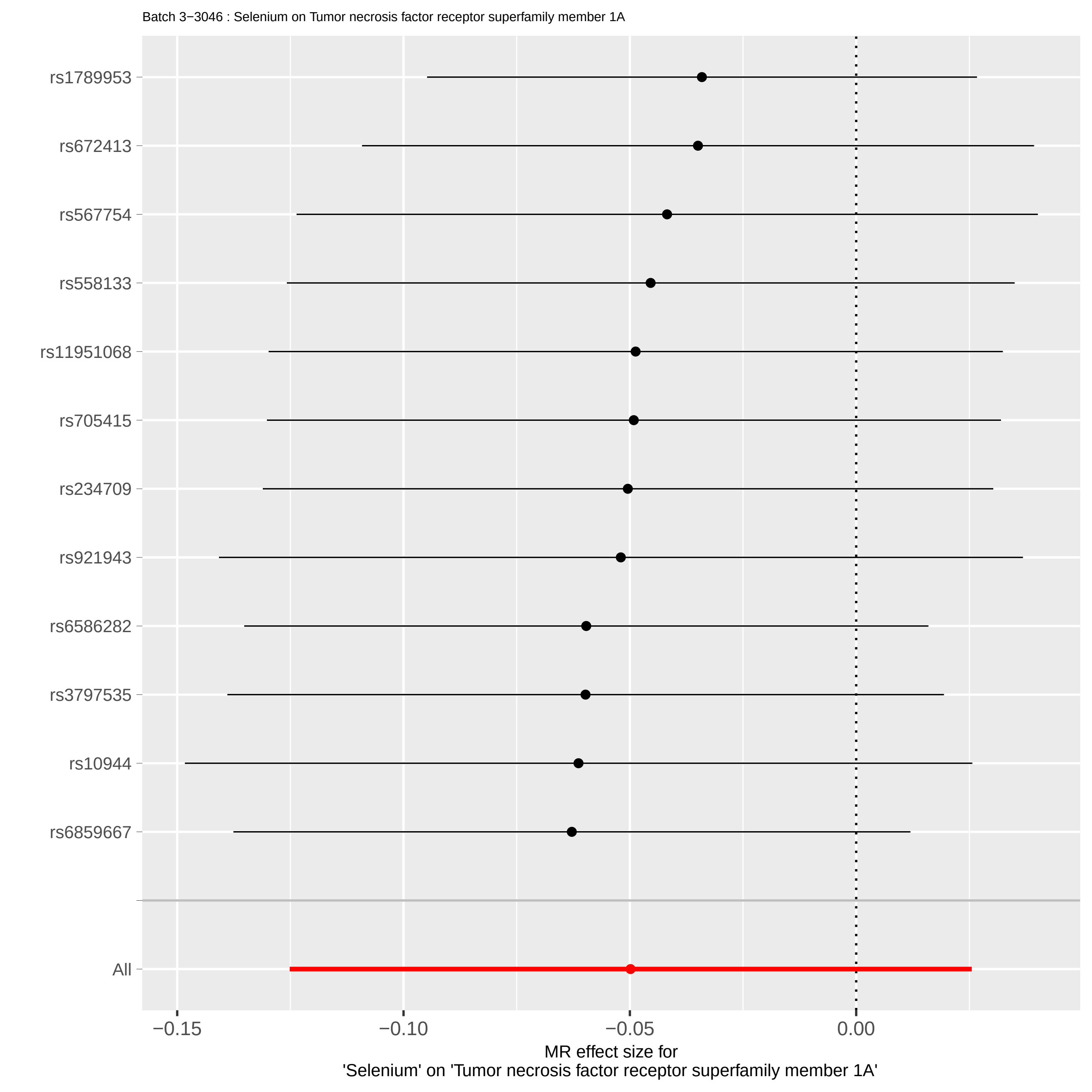

Batch 3−3046 : Selenium on Tumor necrosis factor receptor superfamily member 1A
rs1789953
rs672413
rs567754
rs558133
rs11951068
rs705415
rs234709
rs921943
rs6586282
rs3797535
rs10944
rs6859667
All
−0.15
−0.10
−0.05
0.00
MR effect size for
'Selenium' on 'Tumor necrosis factor receptor superfamily member 1A'

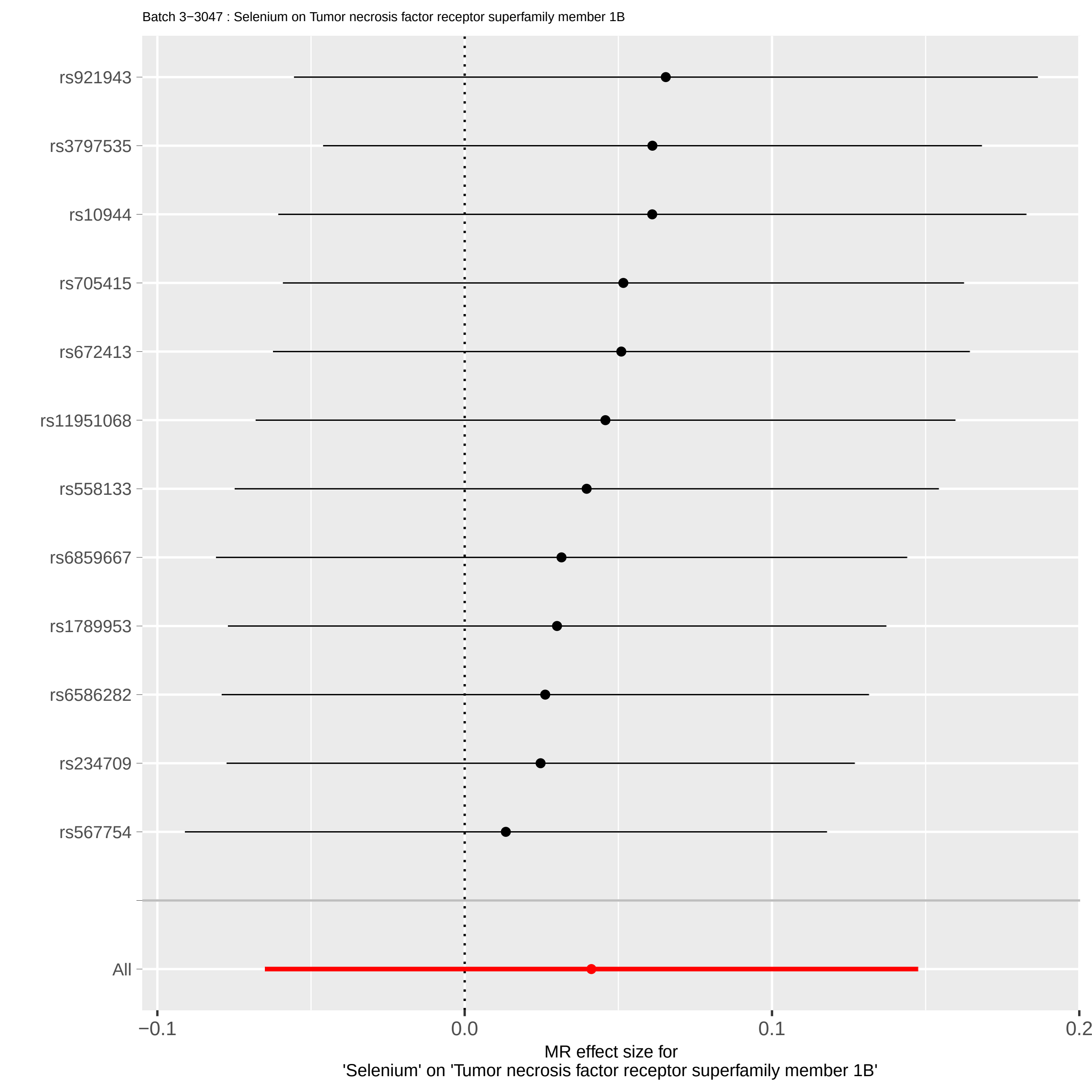

Batch 3−3047 : Selenium on Tumor necrosis factor receptor superfamily member 1B
rs921943
rs3797535
rs10944
rs705415
rs672413
rs11951068
rs558133
rs6859667
rs1789953
rs6586282
rs234709
rs567754
All
−0.1
0.0
0.1
0.2
MR effect size for
'Selenium' on 'Tumor necrosis factor receptor superfamily member 1B'

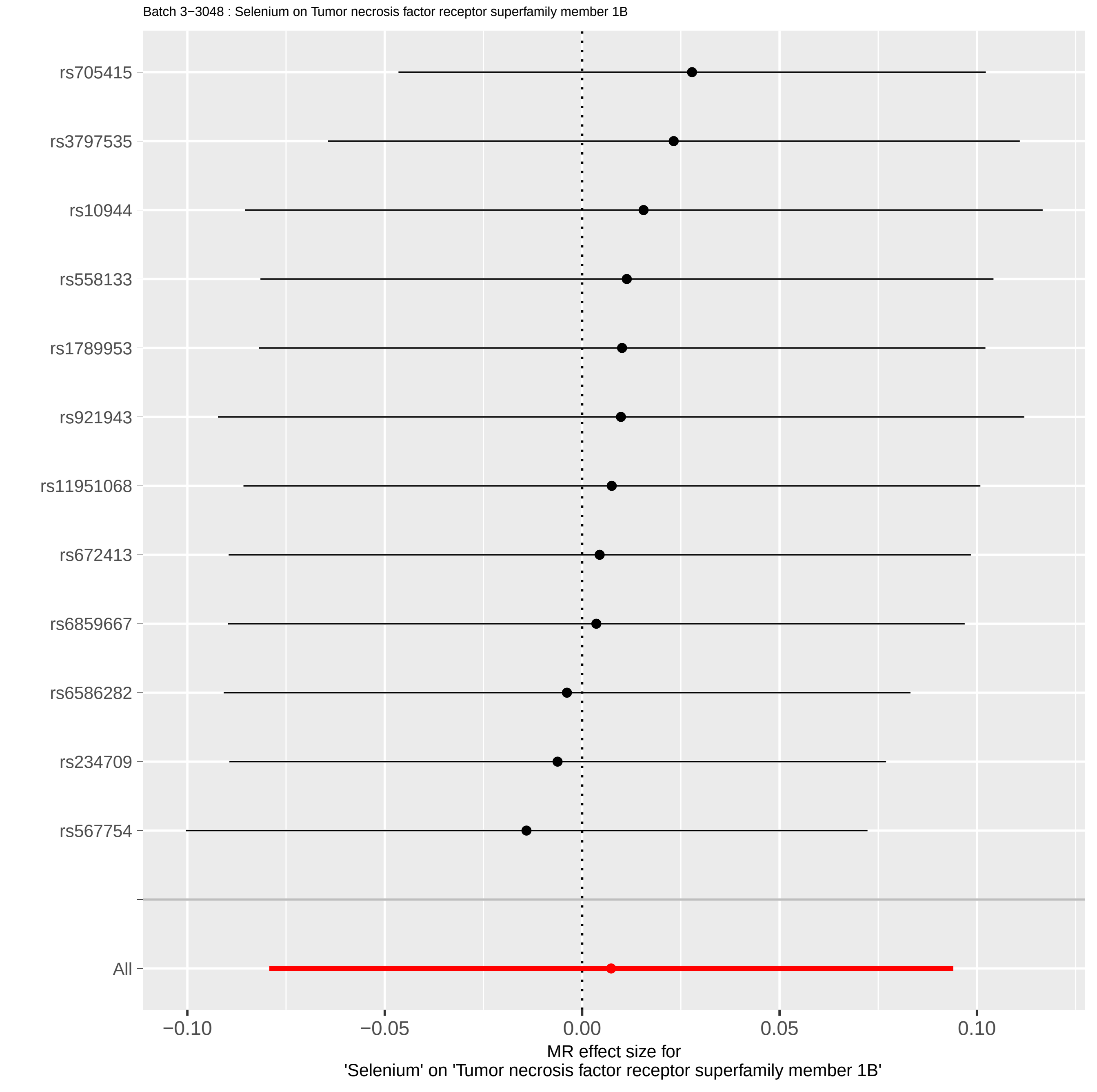

Batch 3−3048 : Selenium on Tumor necrosis factor receptor superfamily member 1B
rs705415
rs3797535
rs10944
rs558133
rs1789953
rs921943
rs11951068
rs672413
rs6859667
rs6586282
rs234709
rs567754
All
−0.10
−0.05
0.00
0.05
0.10
MR effect size for
'Selenium' on 'Tumor necrosis factor receptor superfamily member 1B'

Batch 3−3049 : Selenium on Tumor necrosis factor receptor superfamily member 21

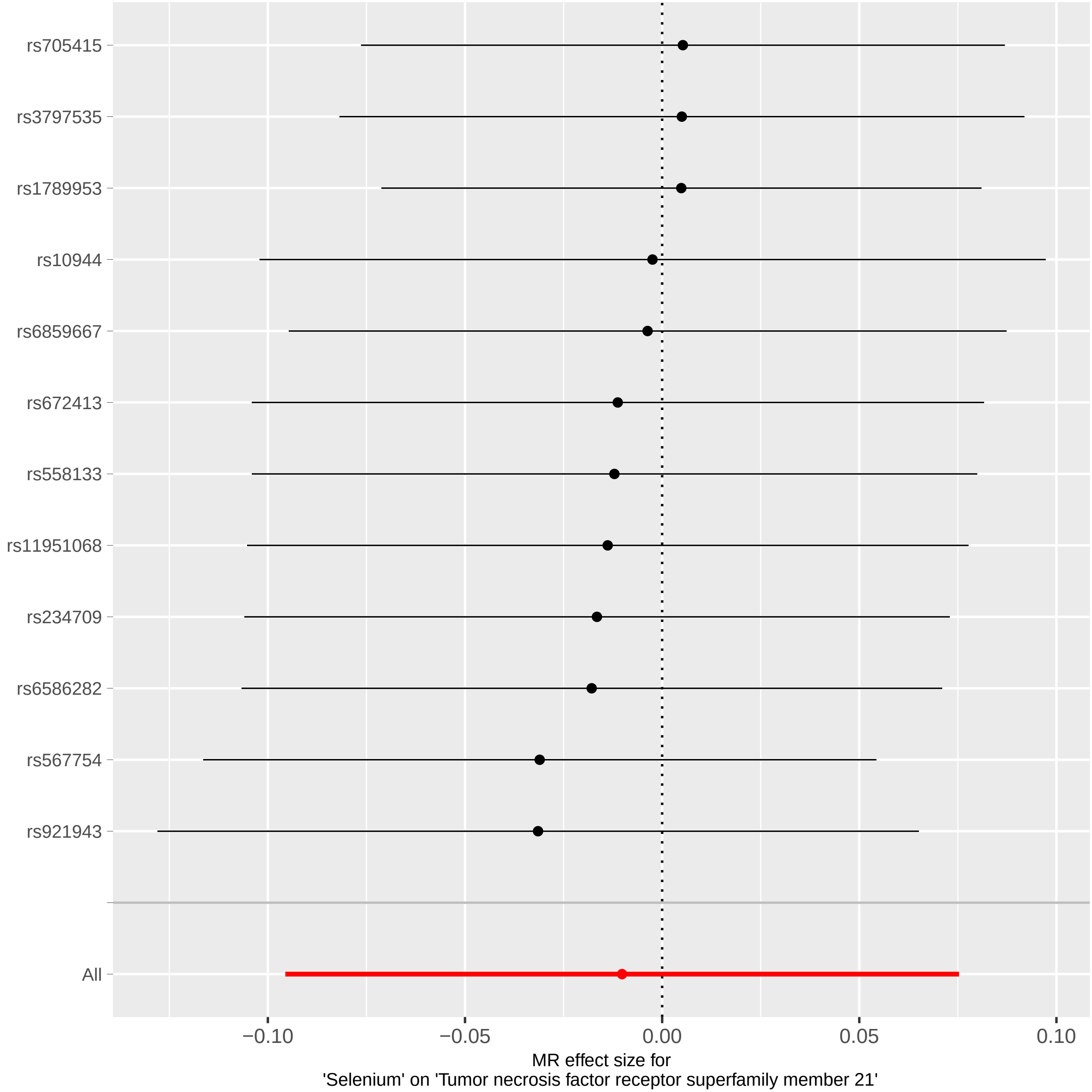

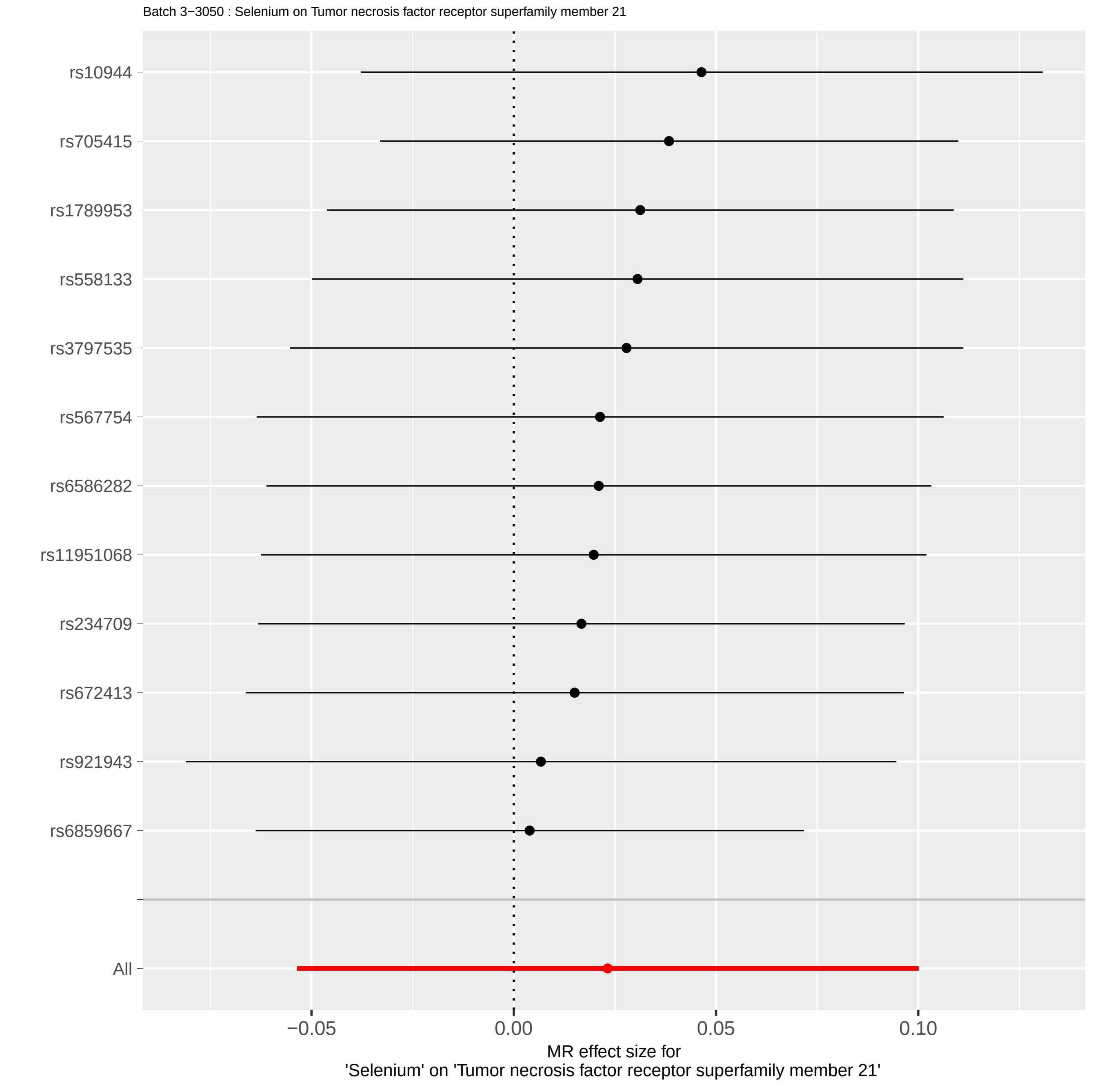

Batch 3−3050 : Selenium on Tumor necrosis factor receptor superfamily member 21
rs10944
rs705415
rs1789953
rs558133
rs3797535
rs567754
rs6586282
rs11951068
rs234709
rs672413
rs921943
rs6859667
All
−0.05
0.00
0.05
0.10
MR effect size for
'Selenium' on 'Tumor necrosis factor receptor superfamily member 21'

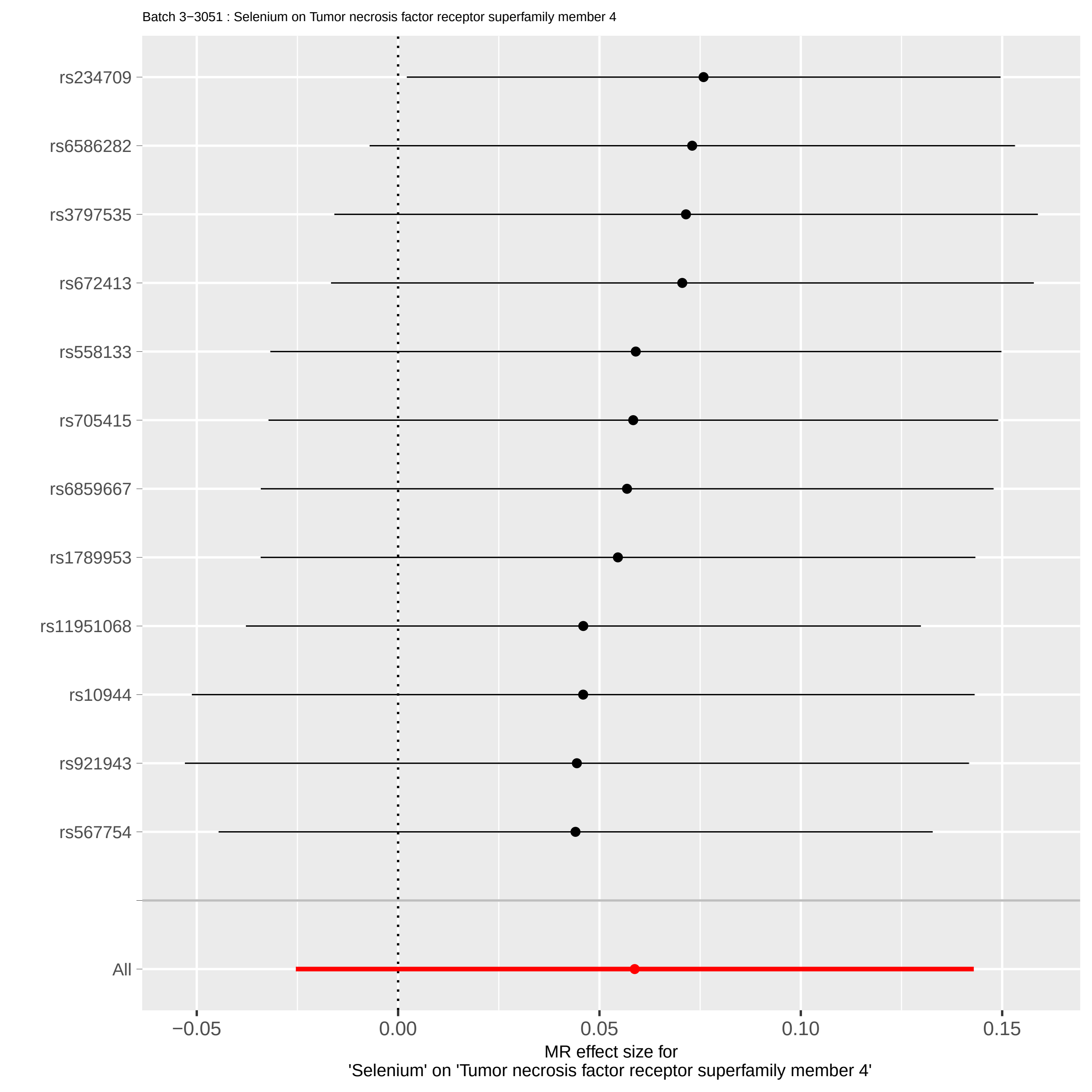

Batch 3−3051 : Selenium on Tumor necrosis factor receptor superfamily member 4
rs234709
rs6586282
rs3797535
rs672413
rs558133
rs705415
rs6859667
rs1789953
rs11951068
rs10944
rs921943
rs567754
All
−0.05
0.00
0.05
0.10
0.15
MR effect size for
'Selenium' on 'Tumor necrosis factor receptor superfamily member 4'

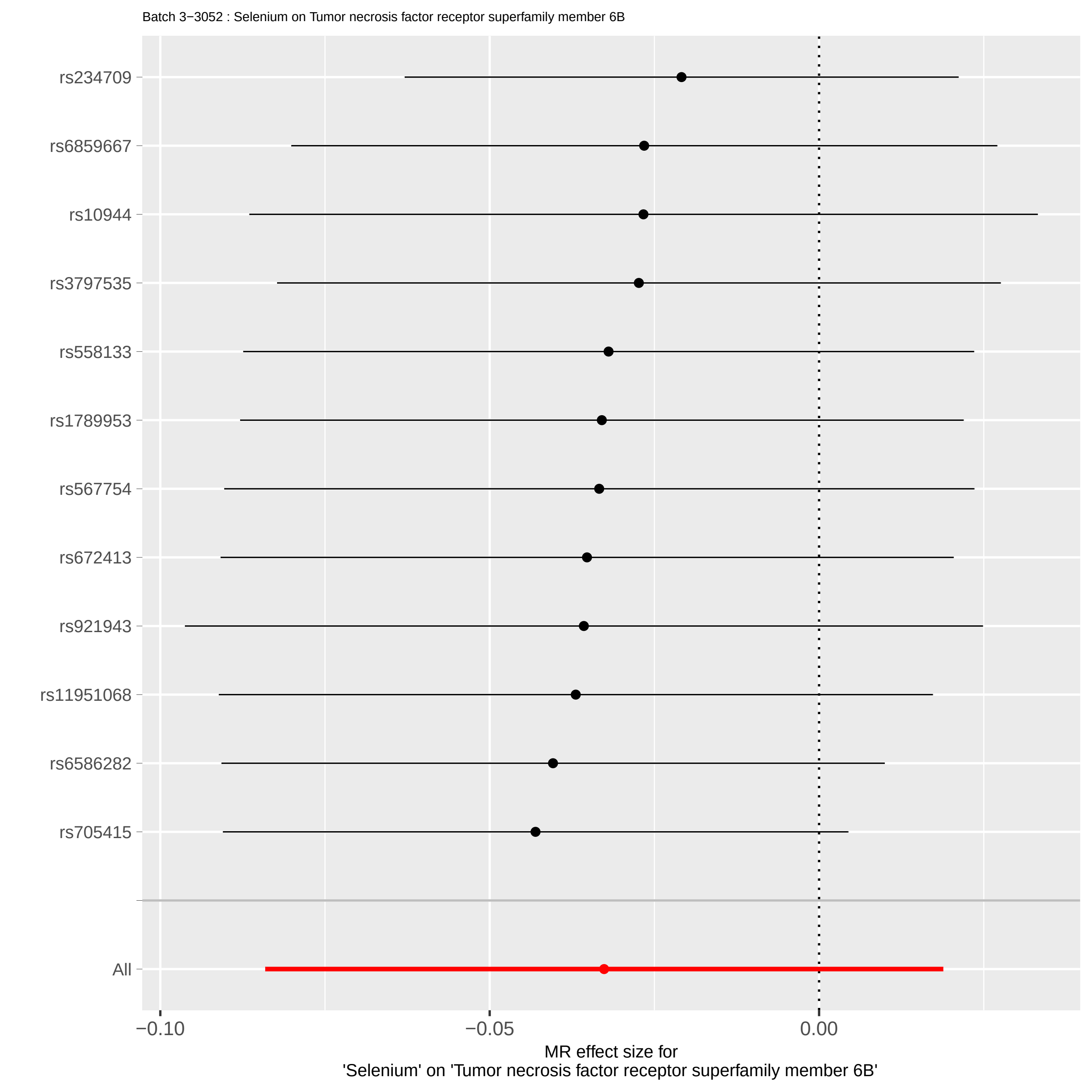
Batch 3−3052 : Selenium on Tumor necrosis factor receptor superfamily member 6B
rs234709
rs6859667
rs10944
rs3797535
rs558133
rs1789953
rs567754
rs672413
rs921943
rs11951068
rs6586282
rs705415
All
−0.10
−0.05
0.00
MR effect size for
'Selenium' on 'Tumor necrosis factor receptor superfamily member 6B'

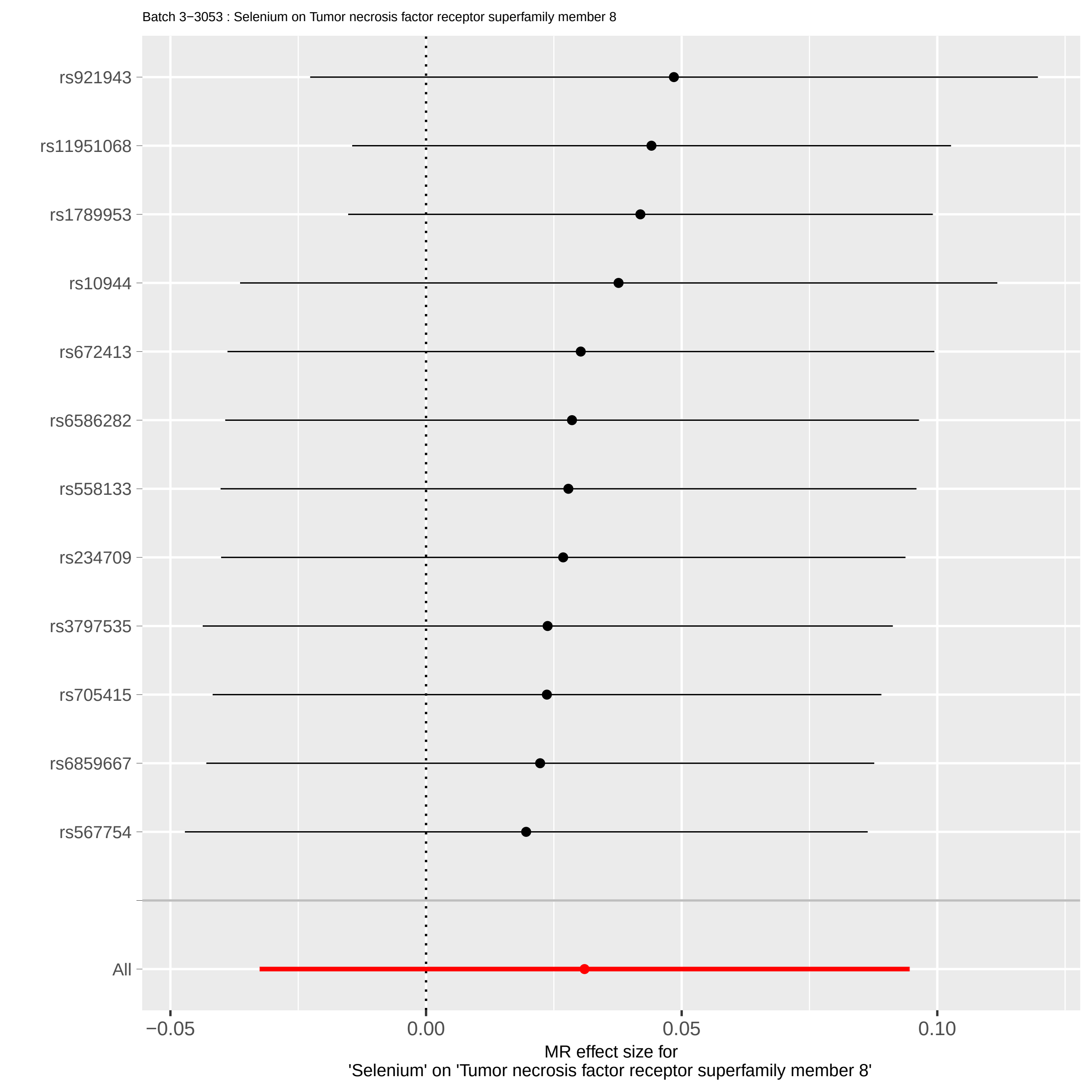

Batch 3−3053 : Selenium on Tumor necrosis factor receptor superfamily member 8
rs921943
rs11951068
rs1789953
rs10944
rs672413
rs6586282
rs558133
rs234709
rs3797535
rs705415
rs6859667
rs567754
All
−0.05
0.00
0.05
0.10
MR effect size for
'Selenium' on 'Tumor necrosis factor receptor superfamily member 8'

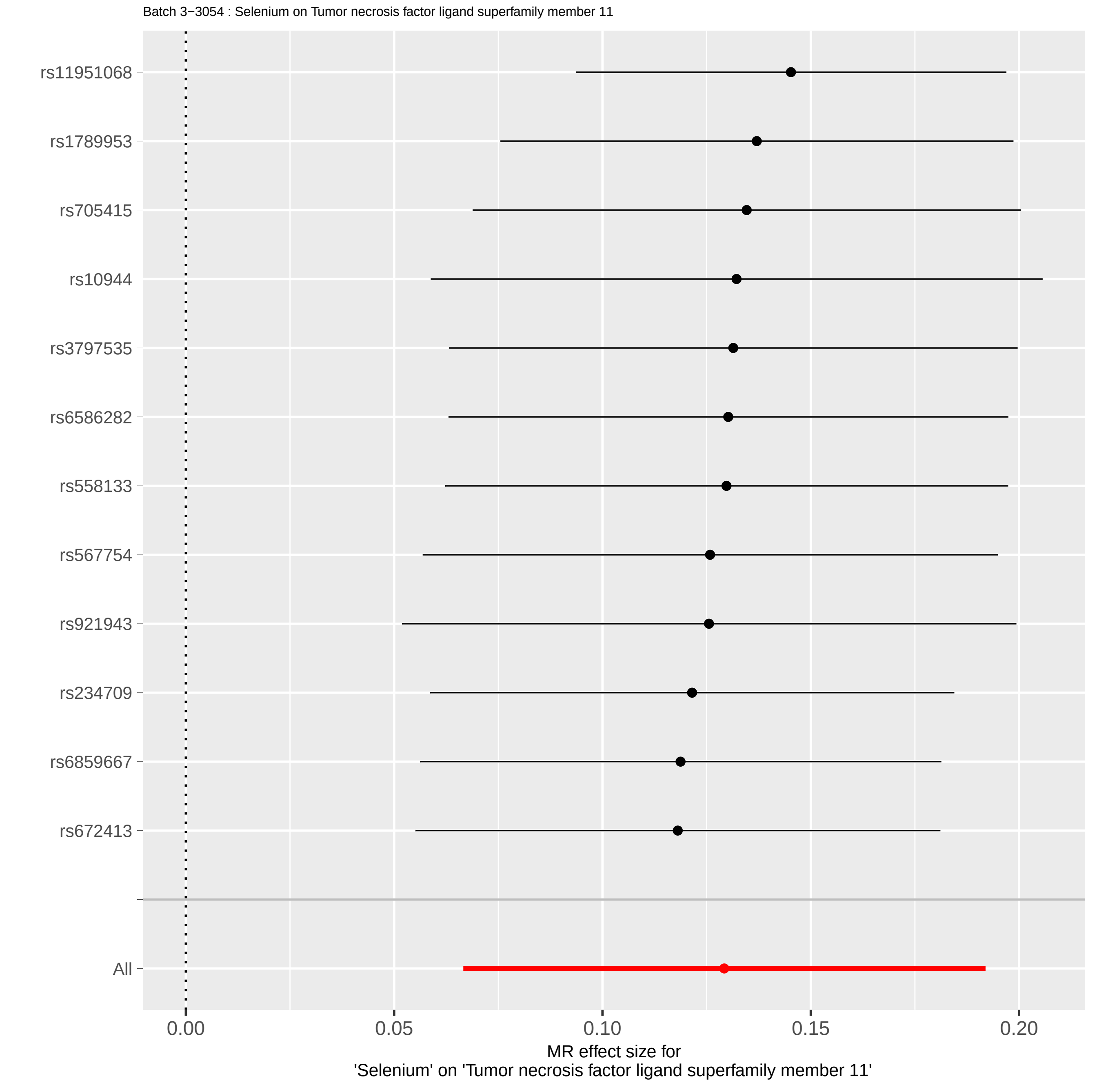

Batch 3−3054 : Selenium on Tumor necrosis factor ligand superfamily member 11
rs11951068
rs1789953
rs705415
rs10944
rs3797535
rs6586282
rs558133
rs567754
rs921943
rs234709
rs6859667
rs672413
All
0.00
0.05
0.10
0.15
0.20
MR effect size for
'Selenium' on 'Tumor necrosis factor ligand superfamily member 11'

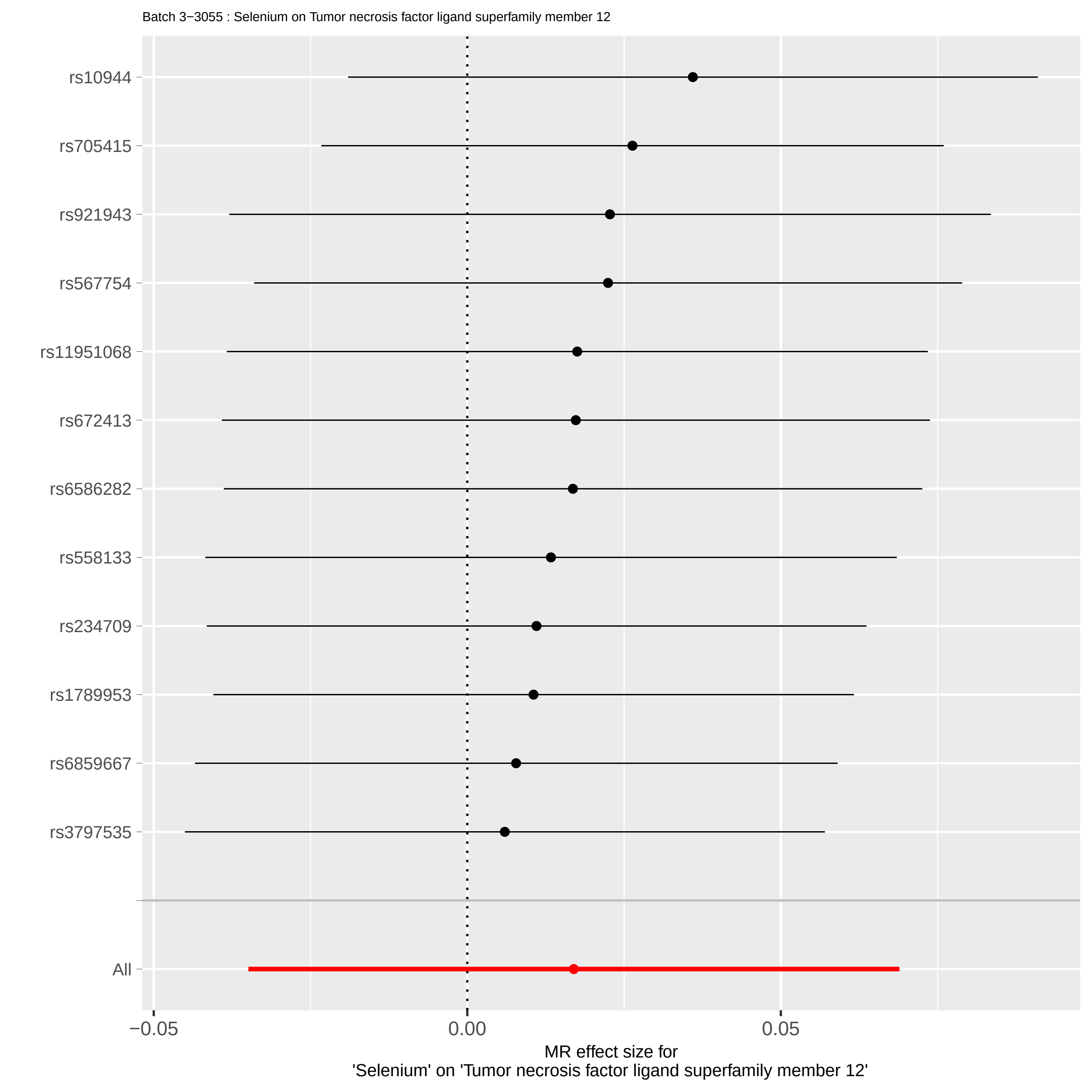

Batch 3−3055 : Selenium on Tumor necrosis factor ligand superfamily member 12
rs10944
rs705415
rs921943
rs567754
rs11951068
rs672413
rs6586282
rs558133
rs234709
rs1789953
rs6859667
rs3797535
All
−0.05
0.00
0.05
MR effect size for
'Selenium' on 'Tumor necrosis factor ligand superfamily member 12'

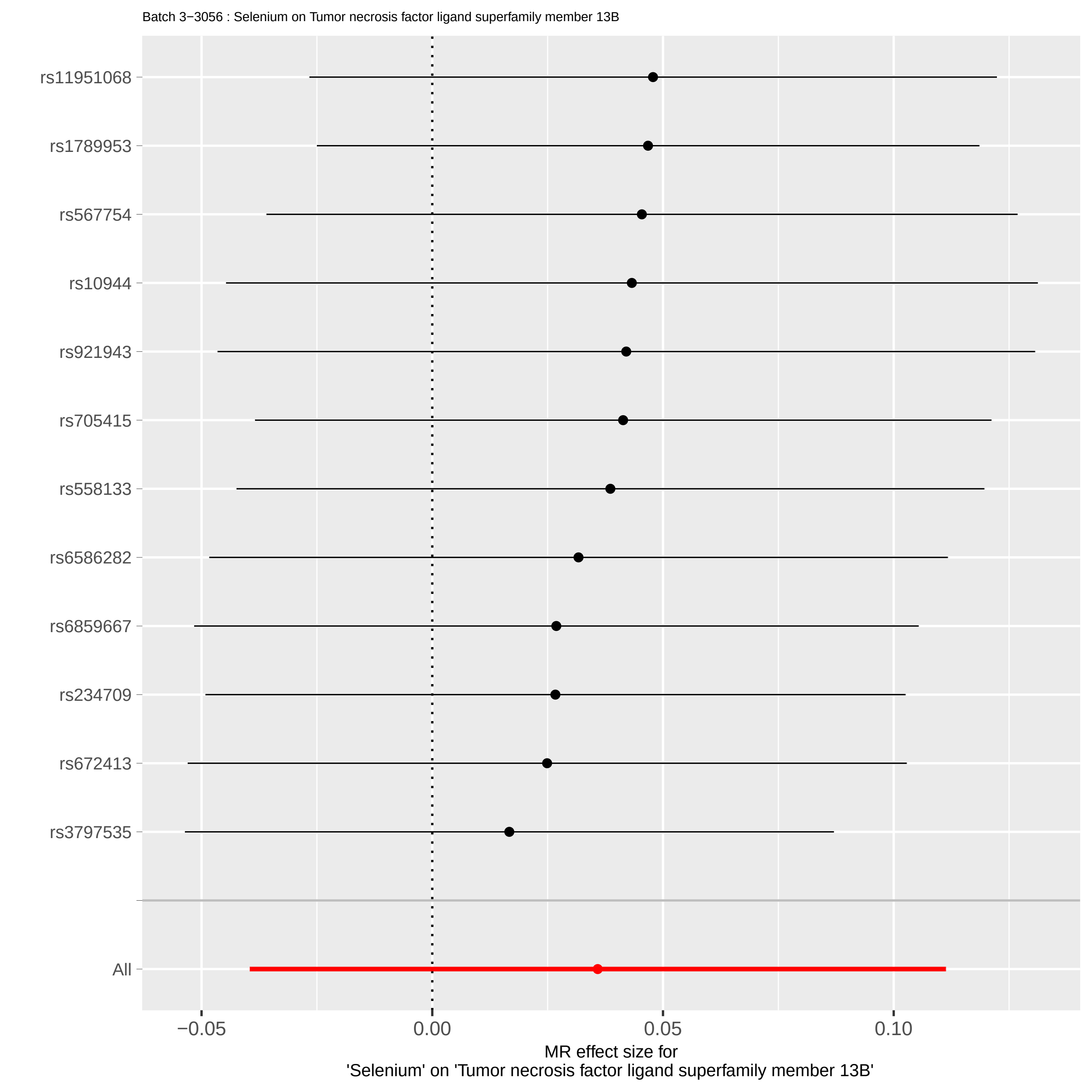

Batch 3−3056 : Selenium on Tumor necrosis factor ligand superfamily member 13B
rs11951068
rs1789953
rs567754
rs10944
rs921943
rs705415
rs558133
rs6586282
rs6859667
rs234709
rs672413
rs3797535
All
−0.05
0.00
0.05
0.10
MR effect size for
'Selenium' on 'Tumor necrosis factor ligand superfamily member 13B'

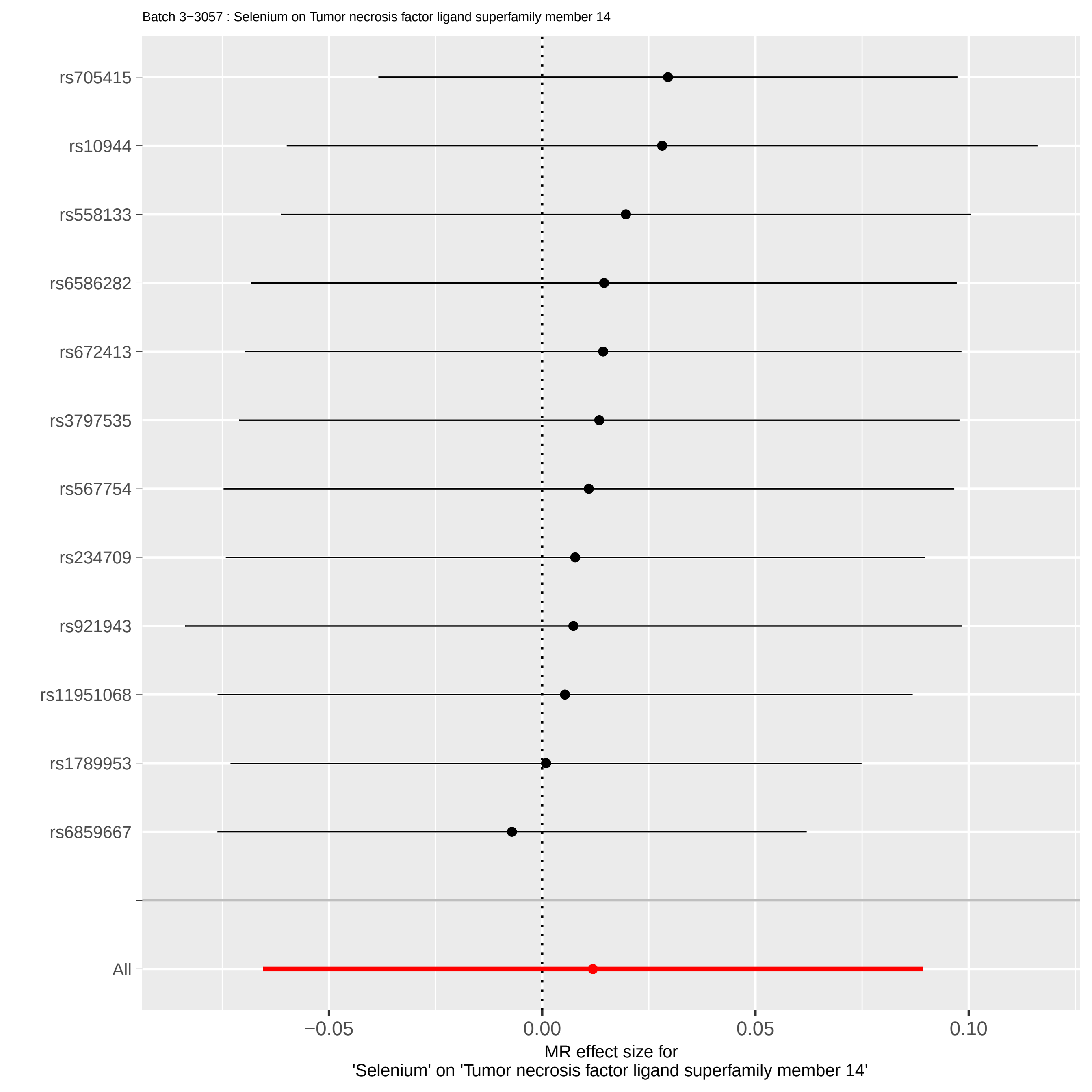
Batch 3−3057 : Selenium on Tumor necrosis factor ligand superfamily member 14
rs705415
rs10944
rs558133
rs6586282
rs672413
rs3797535
rs567754
rs234709
rs921943
rs11951068
rs1789953
rs6859667
All
−0.05
0.00
0.05
0.10
MR effect size for
'Selenium' on 'Tumor necrosis factor ligand superfamily member 14'

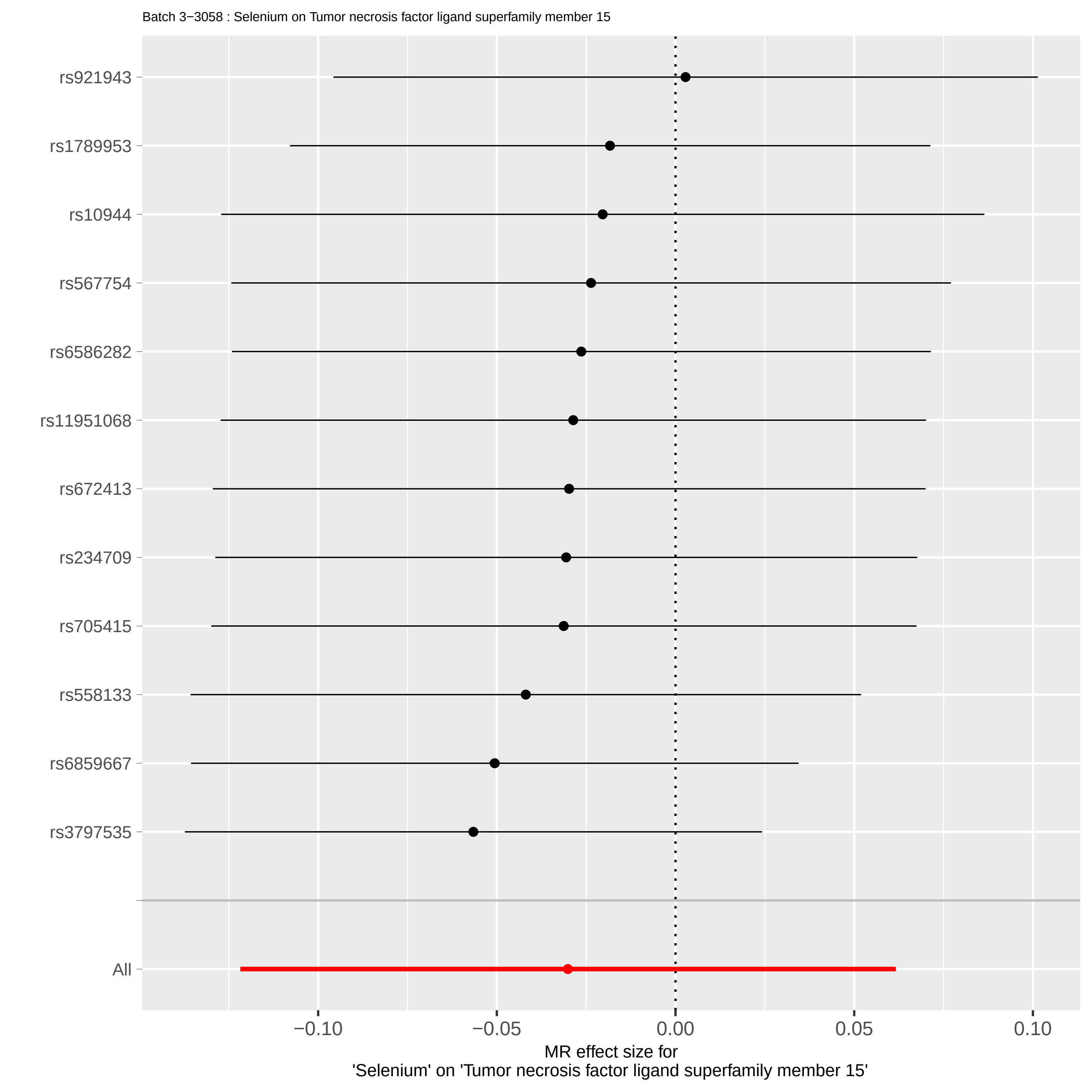

Batch 3−3058 : Selenium on Tumor necrosis factor ligand superfamily member 15
rs921943
rs1789953
rs10944
rs567754
rs6586282
rs11951068
rs672413
rs234709
rs705415
rs558133
rs6859667
rs3797535
All
−0.10
−0.05
0.00
0.05
0.10
MR effect size for
'Selenium' on 'Tumor necrosis factor ligand superfamily member 15'

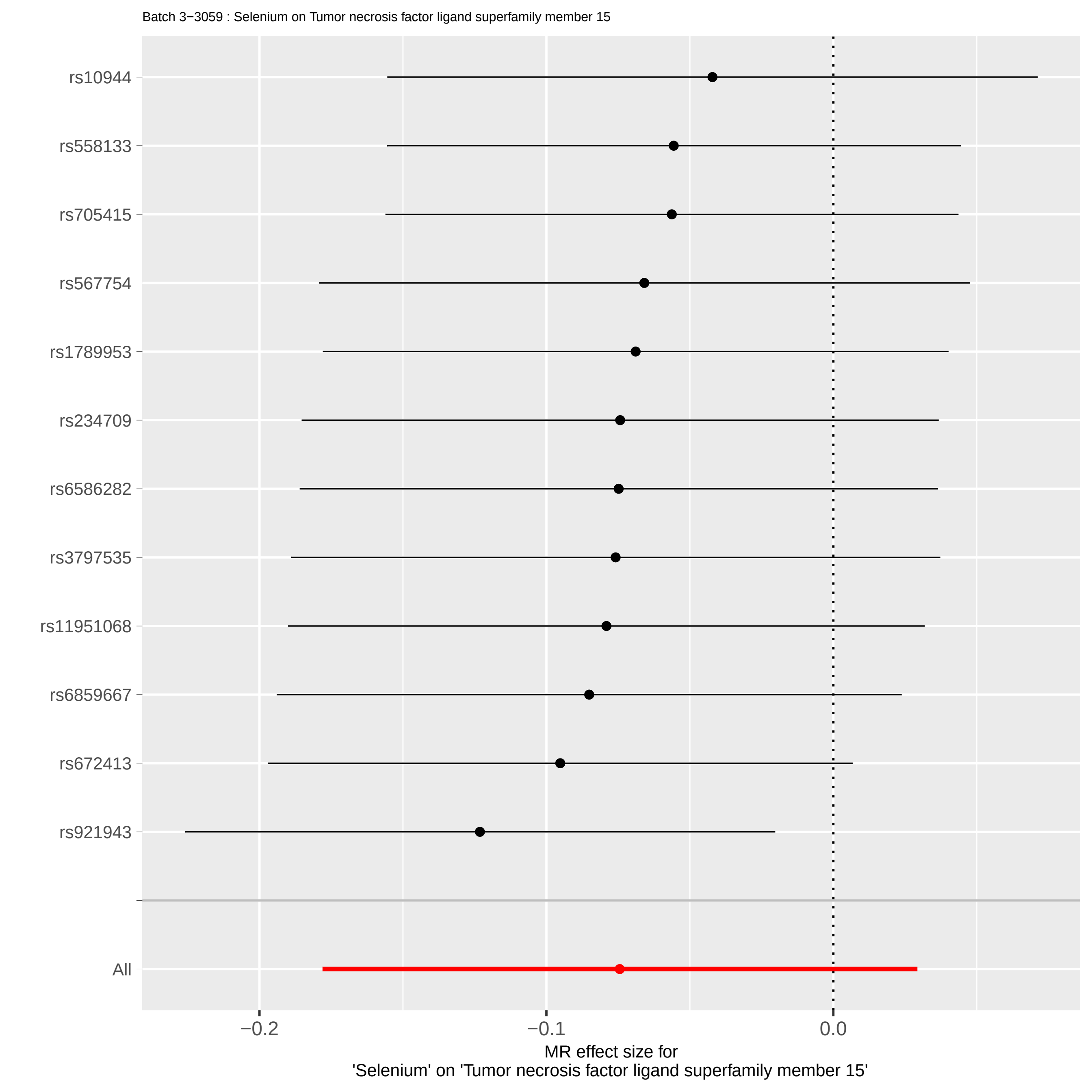

Batch 3−3059 : Selenium on Tumor necrosis factor ligand superfamily member 15
rs10944
rs558133
rs705415
rs567754
rs1789953
rs234709
rs6586282
rs3797535
rs11951068
rs6859667
rs672413
rs921943
All
−0.2
−0.1
0.0
MR effect size for
'Selenium' on 'Tumor necrosis factor ligand superfamily member 15'

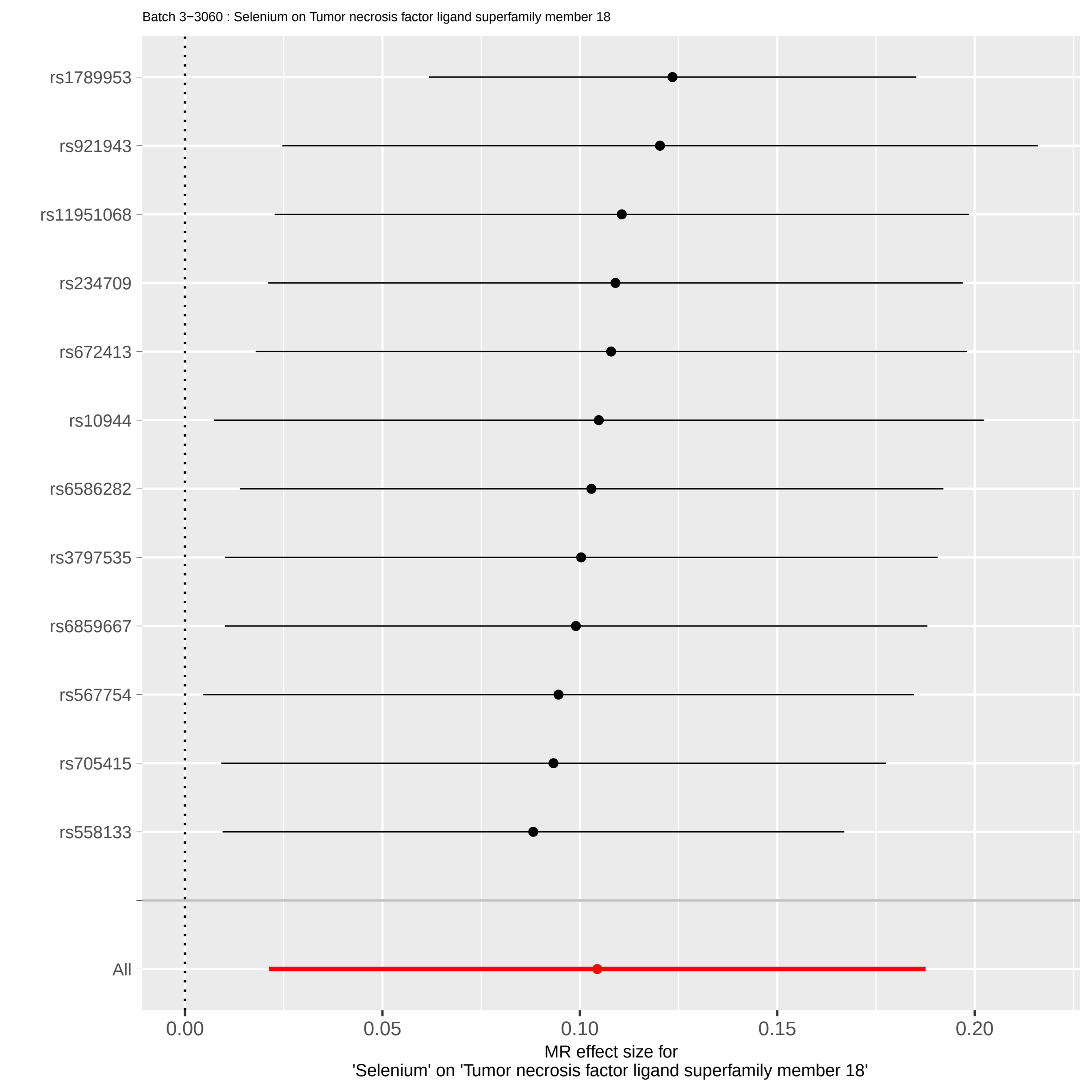

Batch 3−3060 : Selenium on Tumor necrosis factor ligand superfamily member 18
rs1789953
rs921943
rs11951068
rs234709
rs672413
rs10944
rs6586282
rs3797535
rs6859667
rs567754
rs705415
rs558133
All
0.00
0.05
0.10
0.15
0.20
MR effect size for
'Selenium' on 'Tumor necrosis factor ligand superfamily member 18'

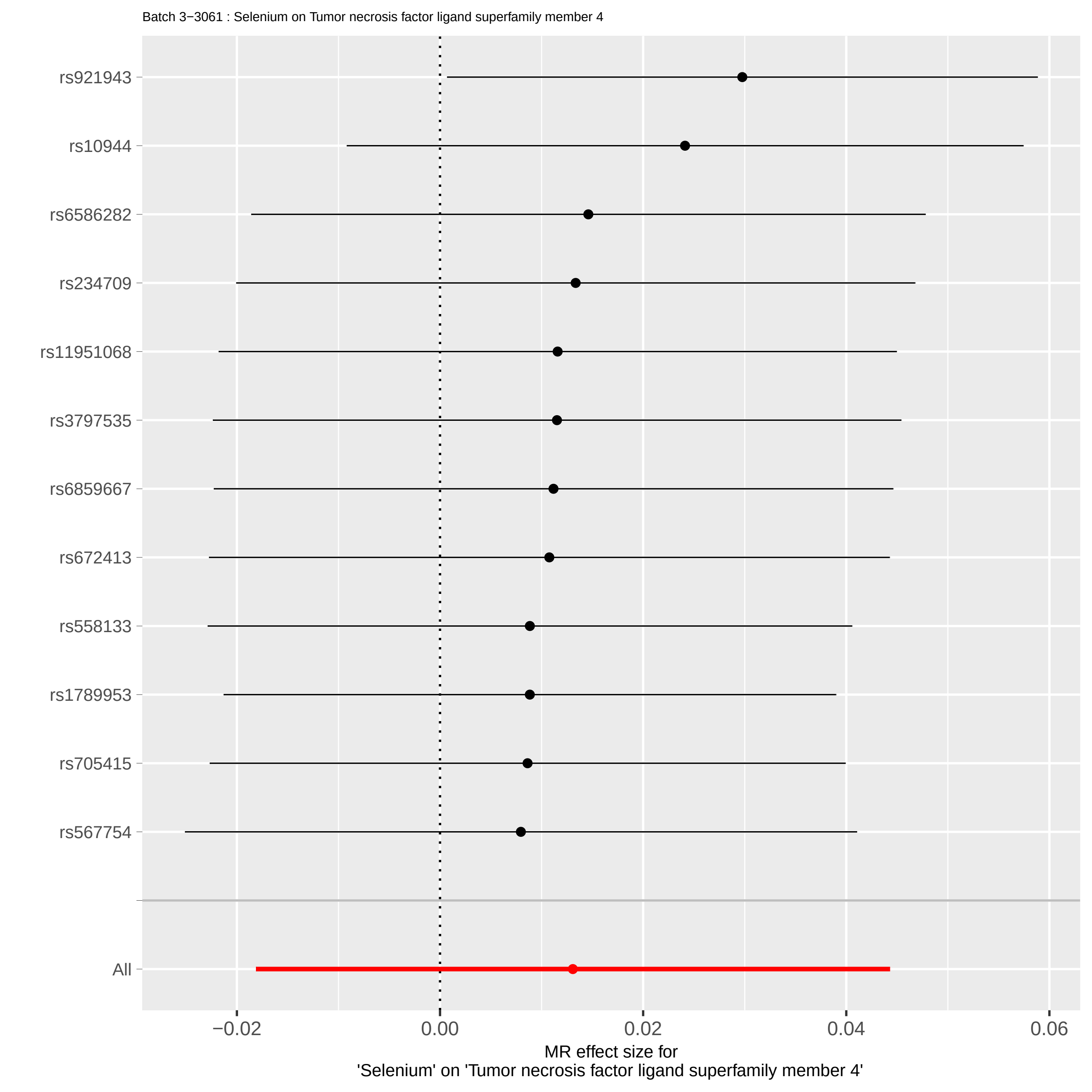
Batch 3−3061 : Selenium on Tumor necrosis factor ligand superfamily member 4
rs921943
rs10944
rs6586282
rs234709
rs11951068
rs3797535
rs6859667
rs672413
rs558133
rs1789953
rs705415
rs567754
All
−0.02
0.00
0.02
0.04
0.06
MR effect size for
'Selenium' on 'Tumor necrosis factor ligand superfamily member 4'

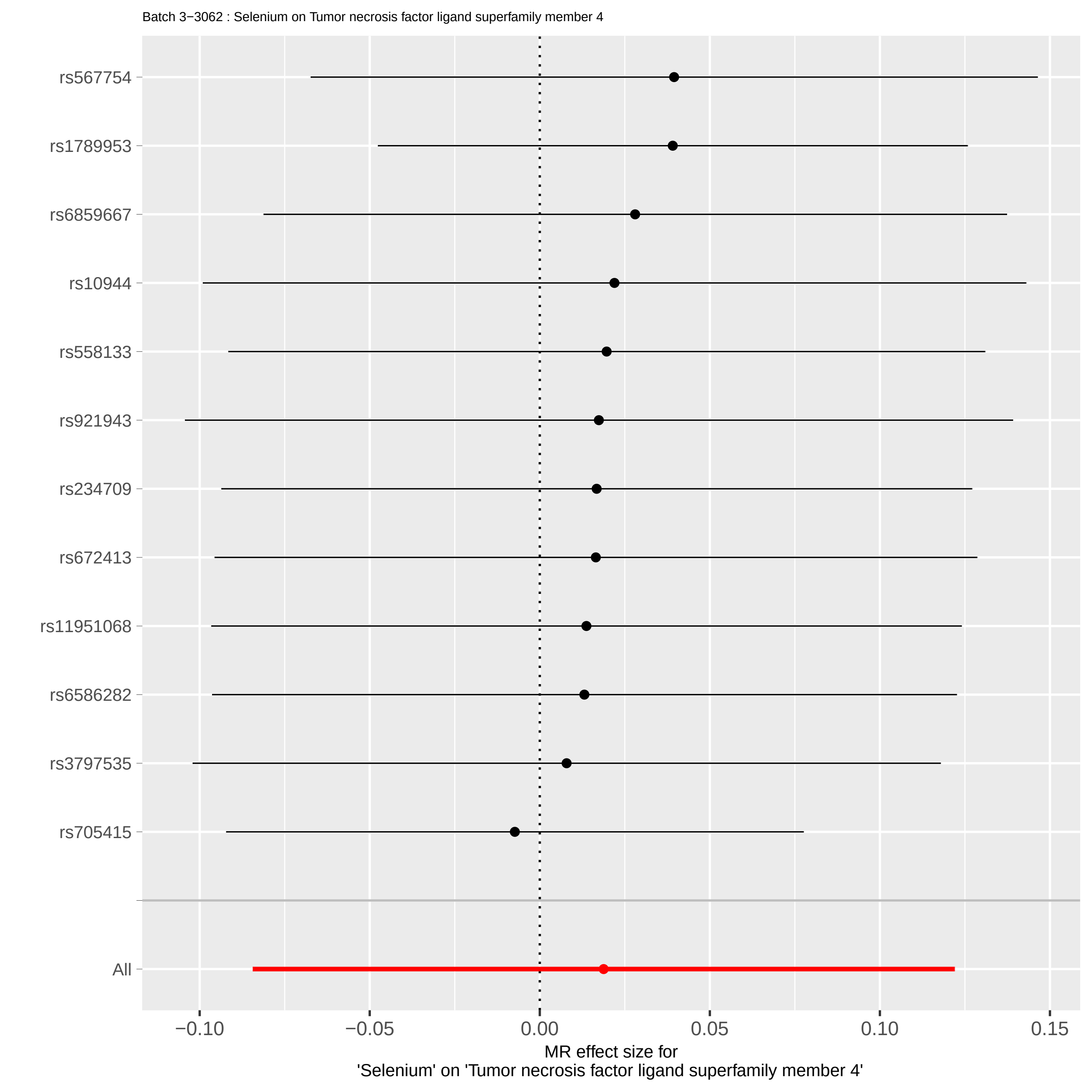
Batch 3−3062 : Selenium on Tumor necrosis factor ligand superfamily member 4
rs567754
rs1789953
rs6859667
rs10944
rs558133
rs921943
rs234709
rs672413
rs11951068
rs6586282
rs3797535
rs705415
All
−0.10
−0.05
0.00
0.05
0.10
0.15
MR effect size for
'Selenium' on 'Tumor necrosis factor ligand superfamily member 4'

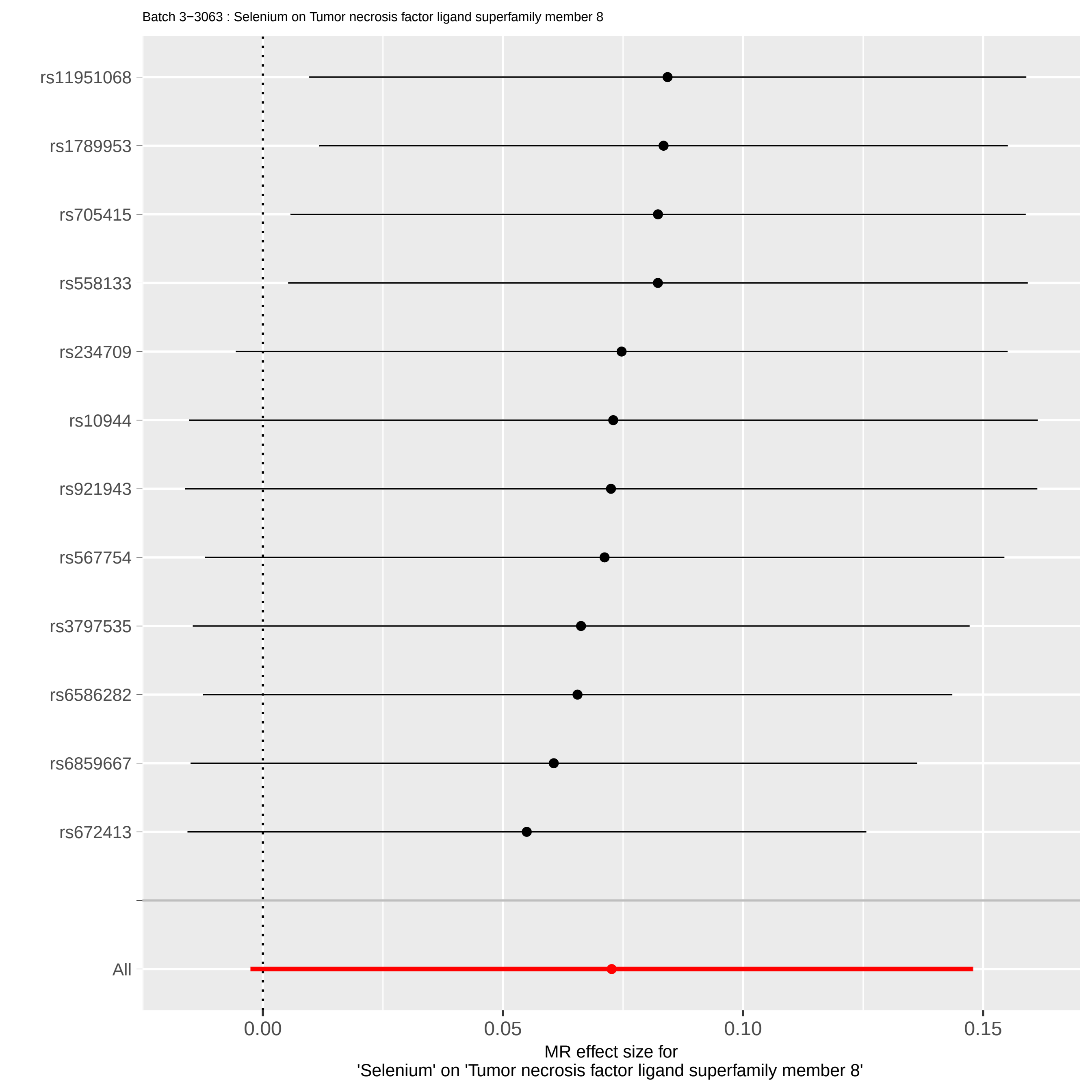

Batch 3−3063 : Selenium on Tumor necrosis factor ligand superfamily member 8
rs11951068
rs1789953
rs705415
rs558133
rs234709
rs10944
rs921943
rs567754
rs3797535
rs6586282
rs6859667
rs672413
All
0.00
0.05
0.10
0.15
MR effect size for
'Selenium' on 'Tumor necrosis factor ligand superfamily member 8'

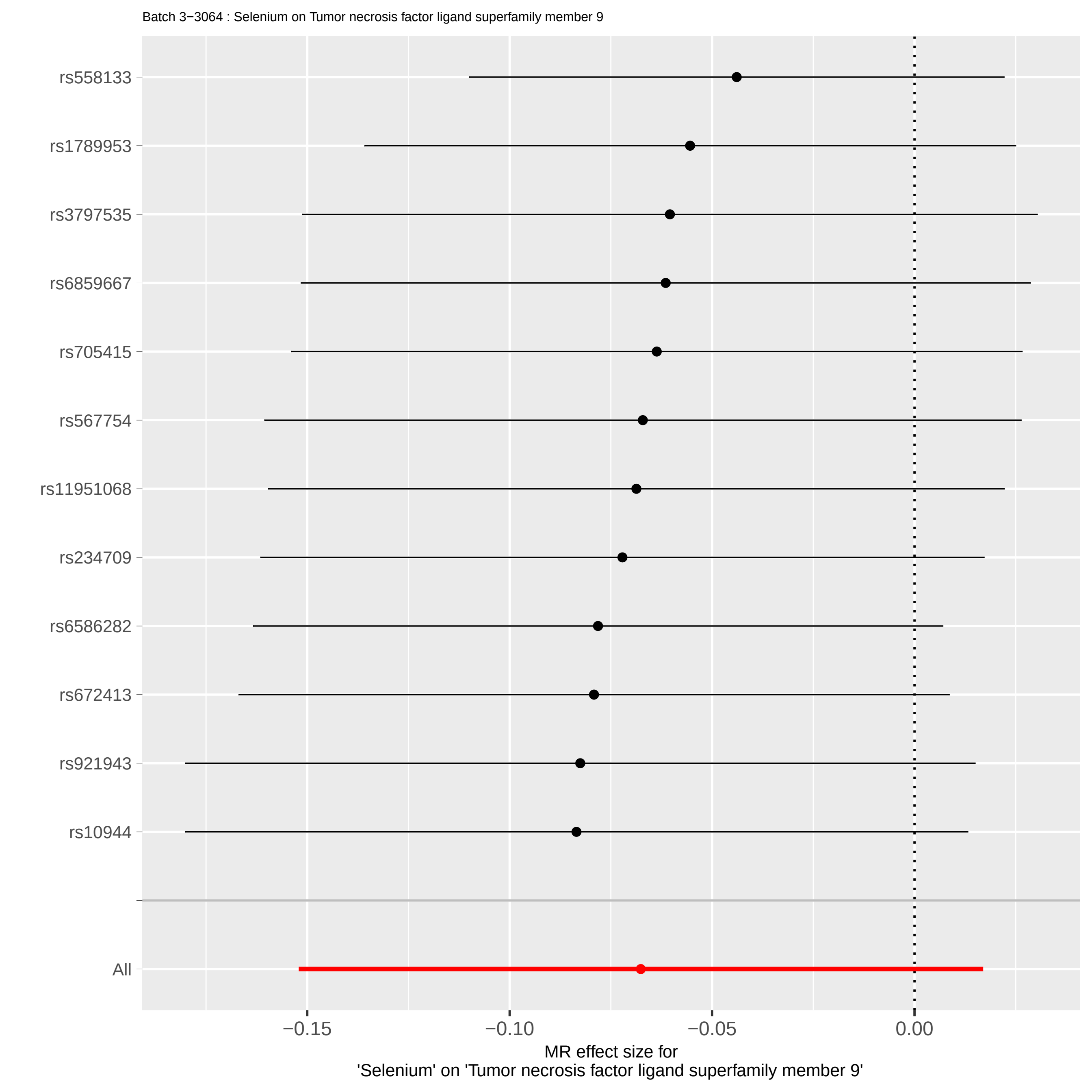

Batch 3−3064 : Selenium on Tumor necrosis factor ligand superfamily member 9
rs558133
rs1789953
rs3797535
rs6859667
rs705415
rs567754
rs11951068
rs234709
rs6586282
rs672413
rs921943
rs10944
All
−0.15
−0.10
−0.05
0.00
MR effect size for
'Selenium' on 'Tumor necrosis factor ligand superfamily member 9'

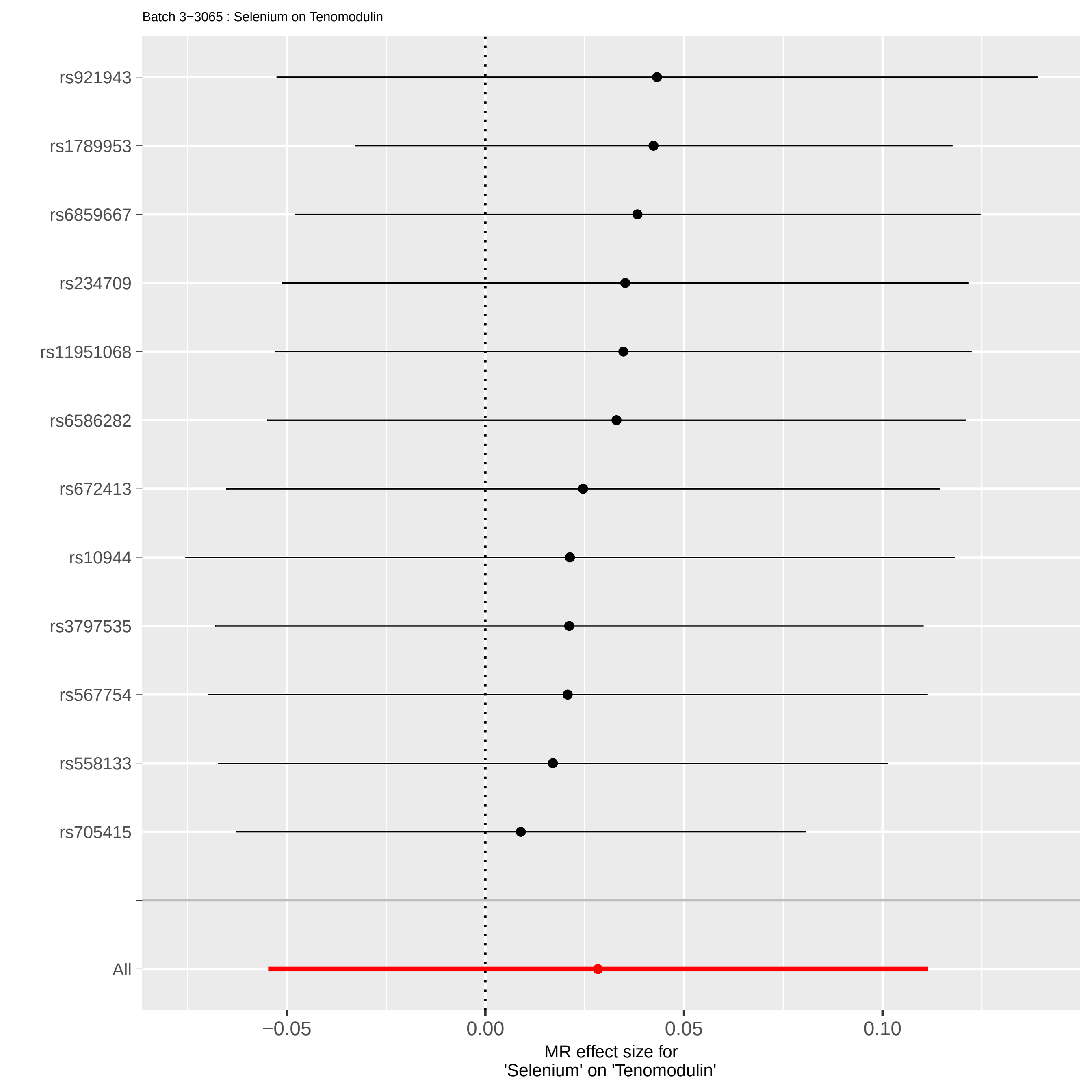

Batch 3−3065 : Selenium on Tenomodulin
rs921943
rs1789953
rs6859667
rs234709
rs11951068
rs6586282
rs672413
rs10944
rs3797535
rs567754
rs558133
rs705415
All
−0.05
0.00
0.05
0.10
MR effect size for
'Selenium' on 'Tenomodulin'

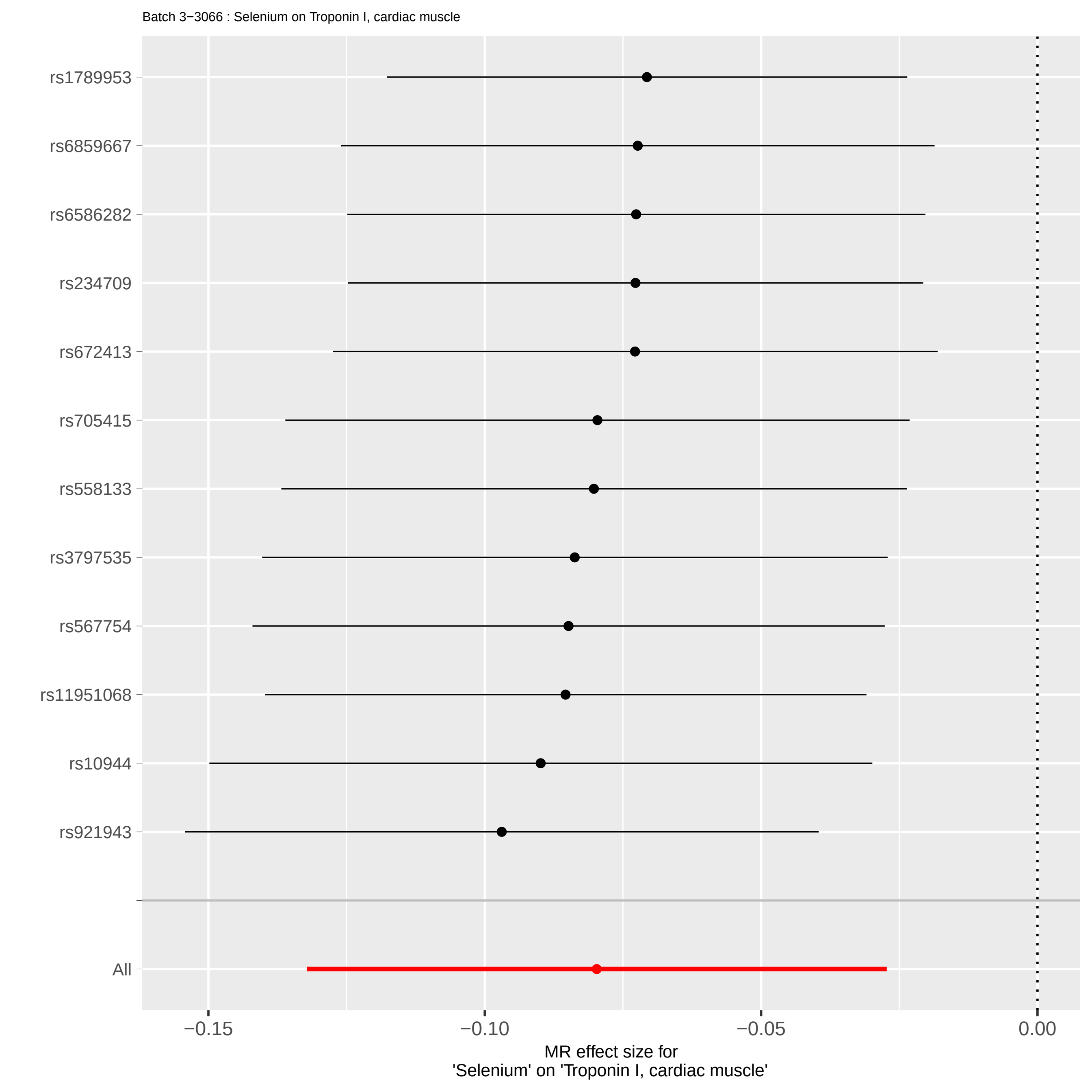

Batch 3−3066 : Selenium on Troponin I, cardiac muscle
rs1789953
rs6859667
rs6586282
rs234709
rs672413
rs705415
rs558133
rs3797535
rs567754
rs11951068
rs10944
rs921943
All
−0.15
−0.10
−0.05
0.00
MR effect size for
'Selenium' on 'Troponin I, cardiac muscle'

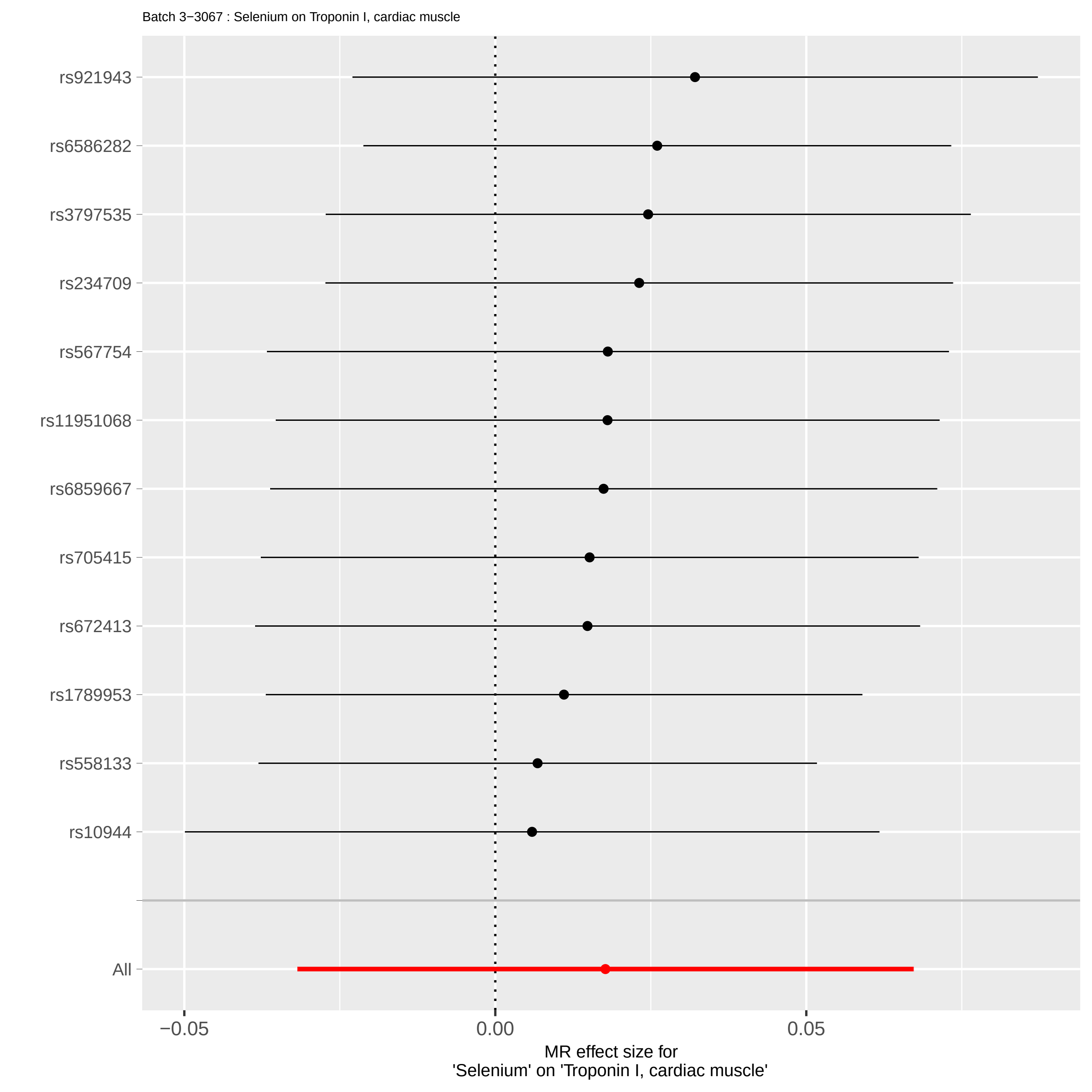
Batch 3−3067 : Selenium on Troponin I, cardiac muscle
rs921943
rs6586282
rs3797535
rs234709
rs567754
rs11951068
rs6859667
rs705415
rs672413
rs1789953
rs558133
rs10944
All
−0.05
0.00
0.05
MR effect size for
'Selenium' on 'Troponin I, cardiac muscle'

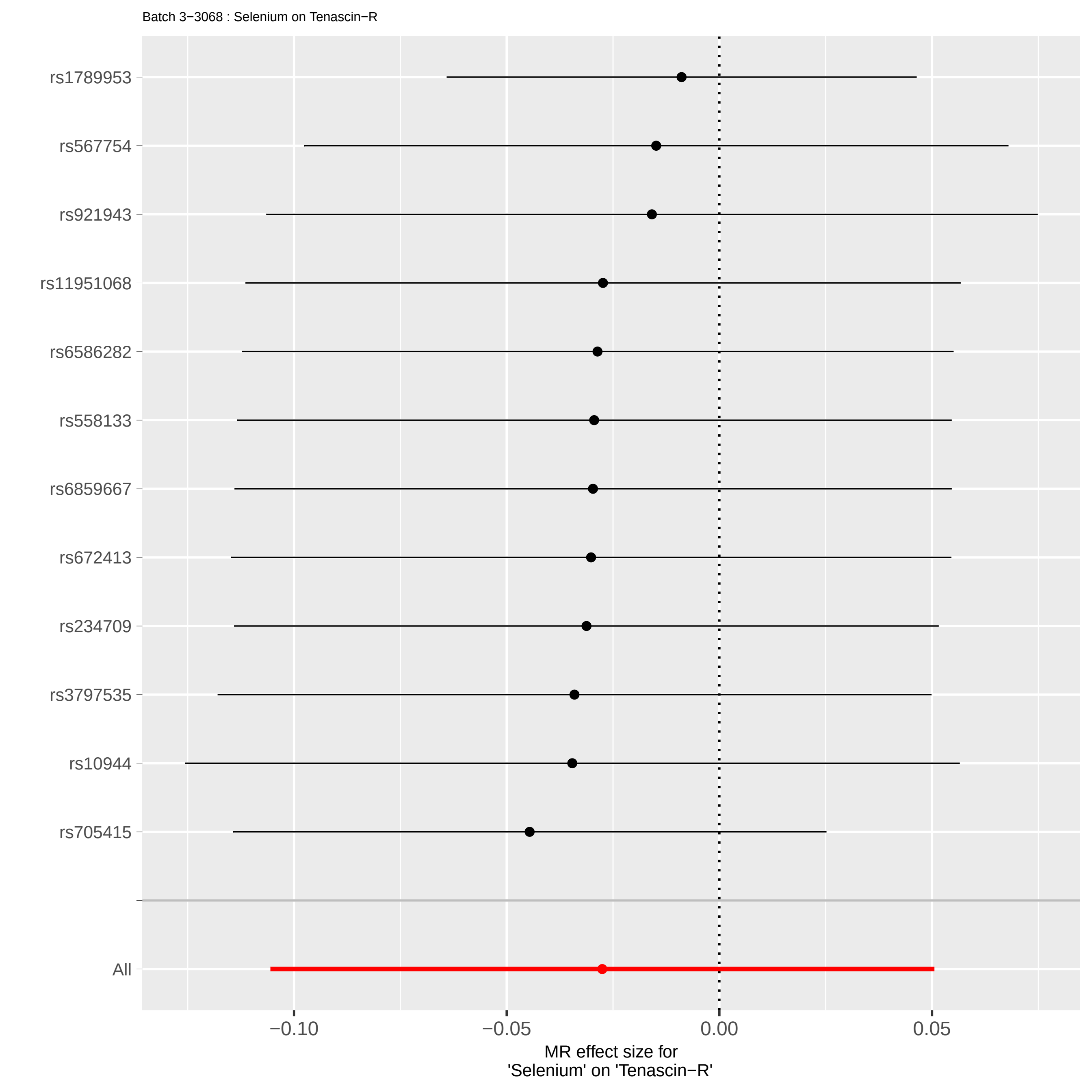

Batch 3−3068 : Selenium on Tenascin−R
rs1789953
rs567754
rs921943
rs11951068
rs6586282
rs558133
rs6859667
rs672413
rs234709
rs3797535
rs10944
rs705415
All
−0.10
−0.05
0.00
0.05
MR effect size for
'Selenium' on 'Tenascin−R'

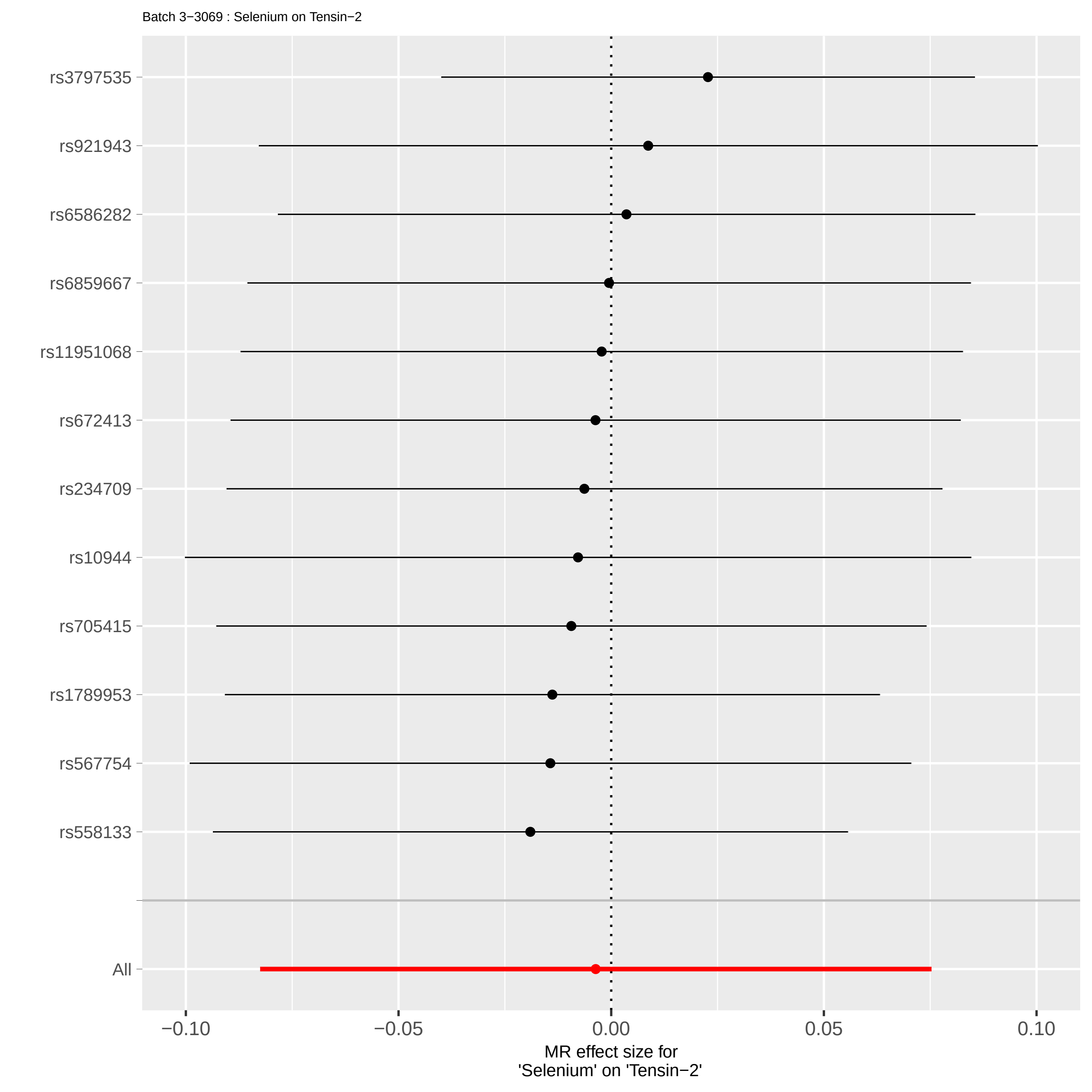
Batch 3−3069 : Selenium on Tensin−2
rs3797535
rs921943
rs6586282
rs6859667
rs11951068
rs672413
rs234709
rs10944
rs705415
rs1789953
rs567754
rs558133
All
−0.10
−0.05
0.00
0.05
0.10
MR effect size for
'Selenium' on 'Tensin−2'

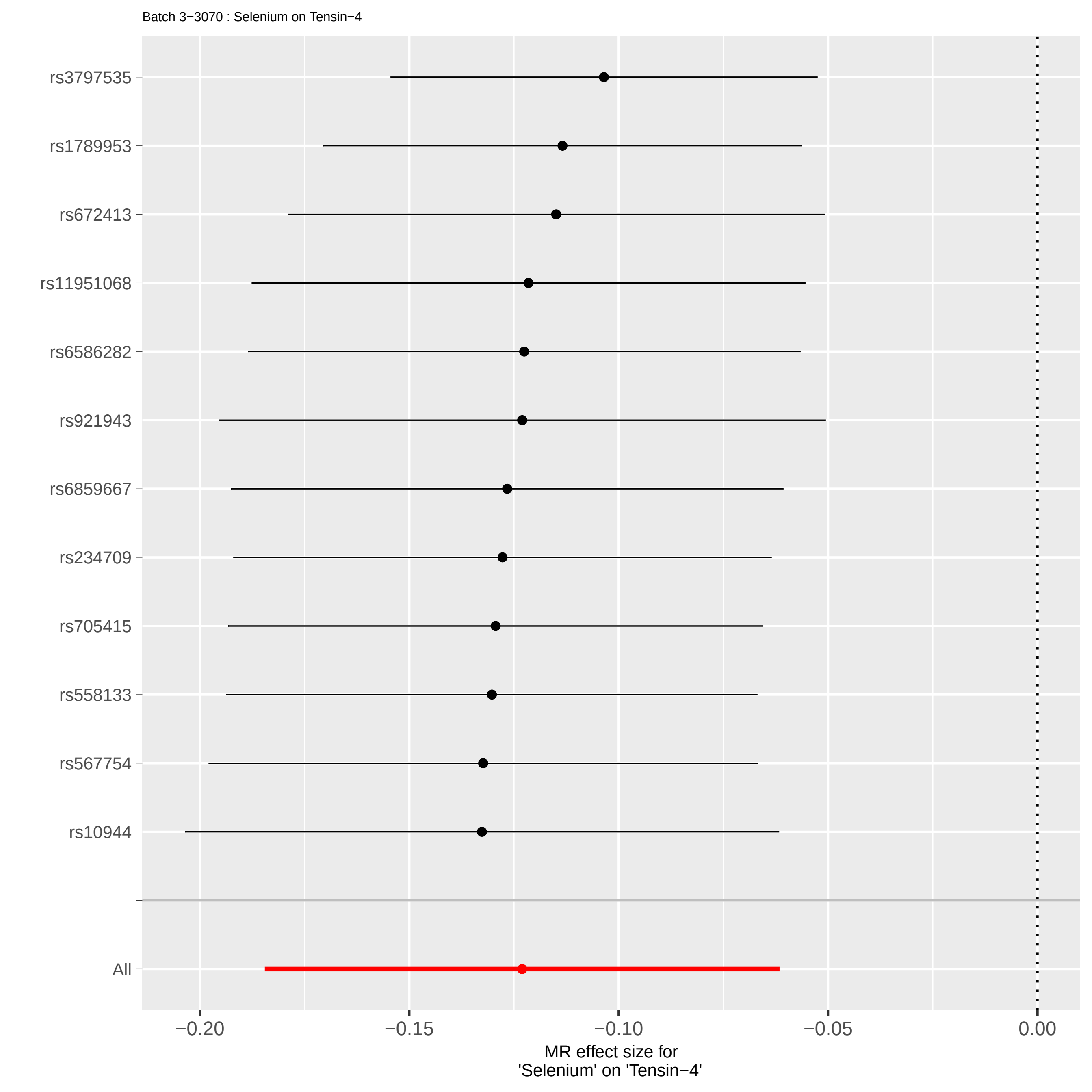

Batch 3−3070 : Selenium on Tensin−4
rs3797535
rs1789953
rs672413
rs11951068
rs6586282
rs921943
rs6859667
rs234709
rs705415
rs558133
rs567754
rs10944
All
−0.20
−0.15
−0.10
−0.05
0.00
MR effect size for
'Selenium' on 'Tensin−4'

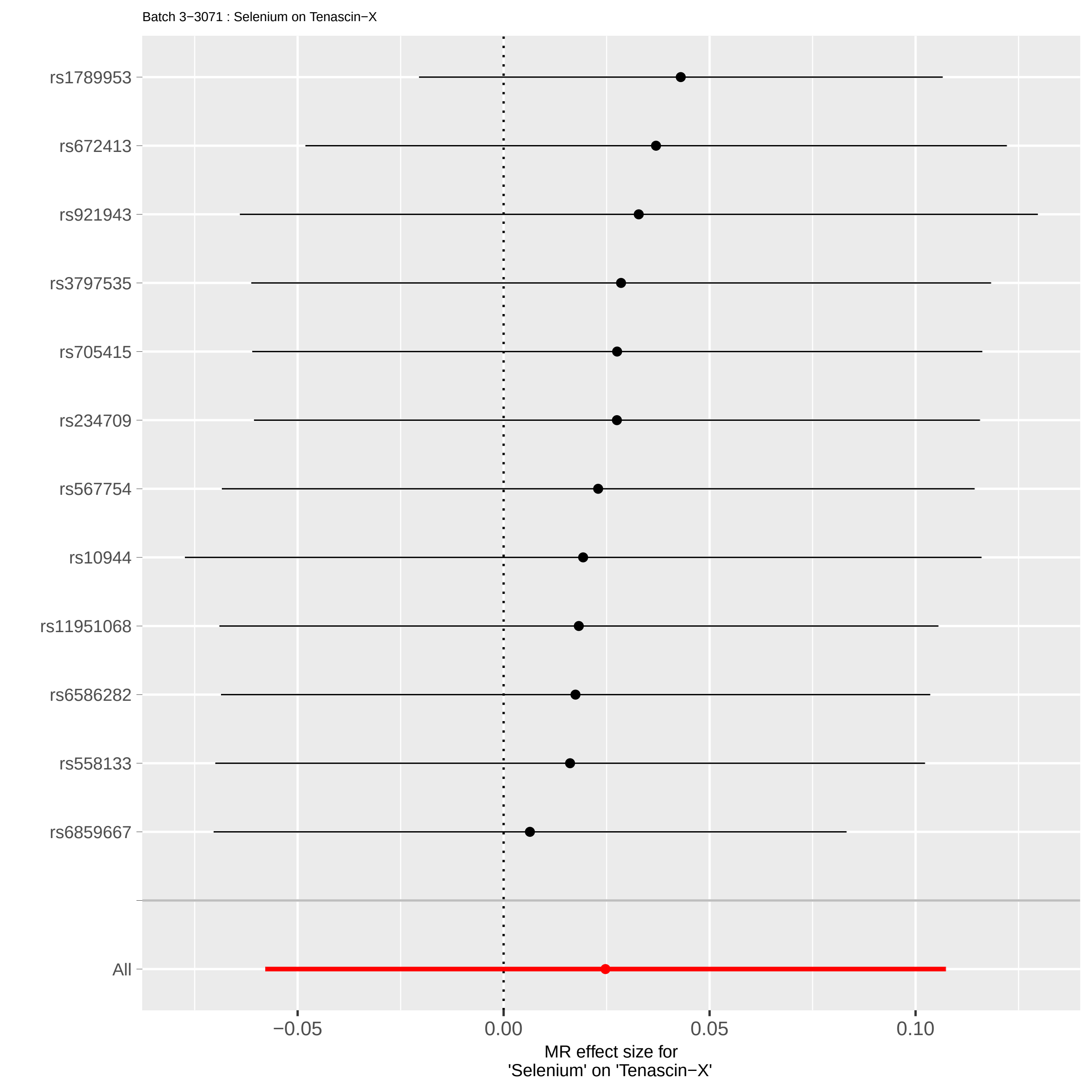

Batch 3−3071 : Selenium on Tenascin−X
rs1789953
rs672413
rs921943
rs3797535
rs705415
rs234709
rs567754
rs10944
rs11951068
rs6586282
rs558133
rs6859667
All
−0.05
0.00
0.05
0.10
MR effect size for
'Selenium' on 'Tenascin−X'

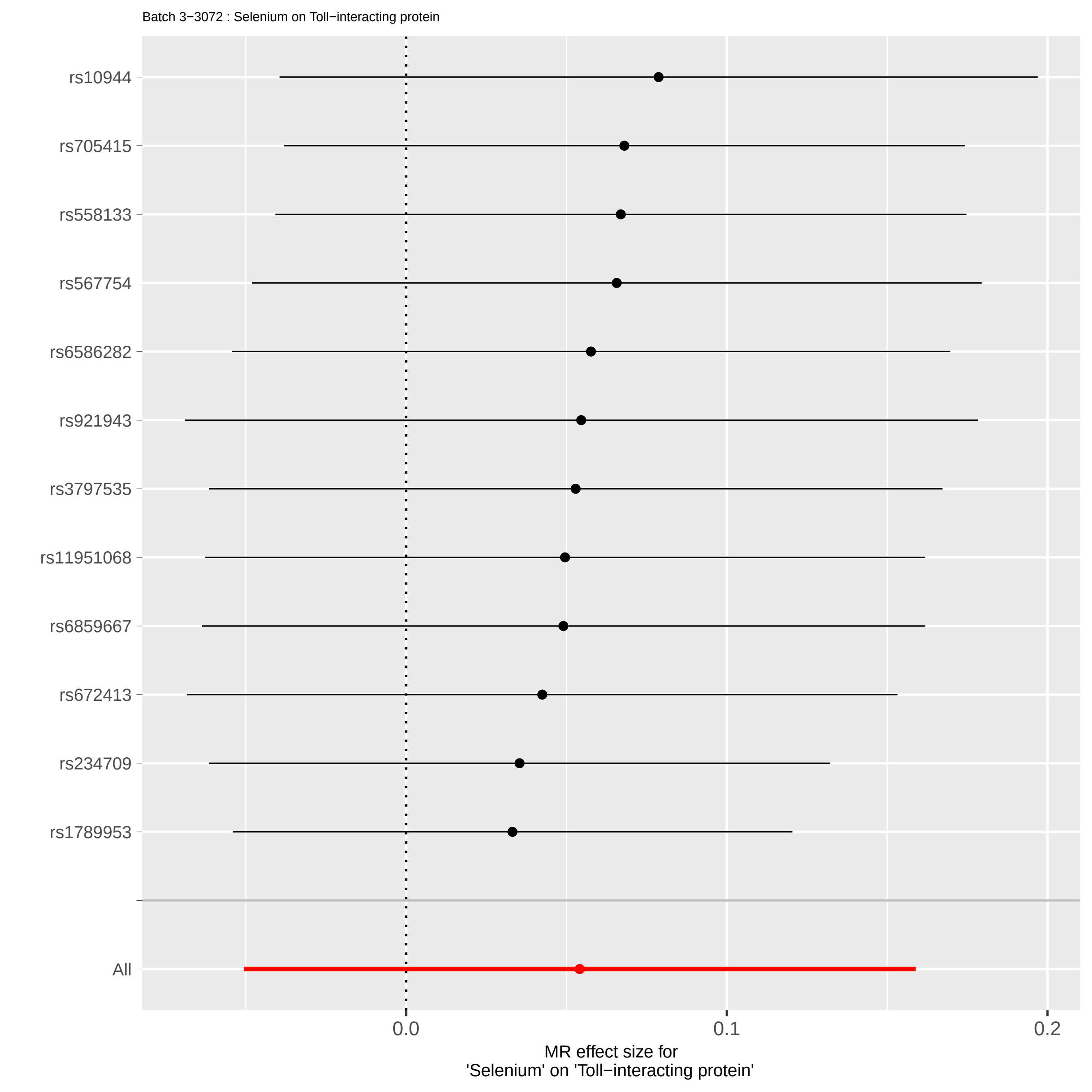
Batch 3−3072 : Selenium on Toll−interacting protein
rs10944
rs705415
rs558133
rs567754
rs6586282
rs921943
rs3797535
rs11951068
rs6859667
rs672413
rs234709
rs1789953
All
0.0
0.1
0.2
MR effect size for
'Selenium' on 'Toll−interacting protein'

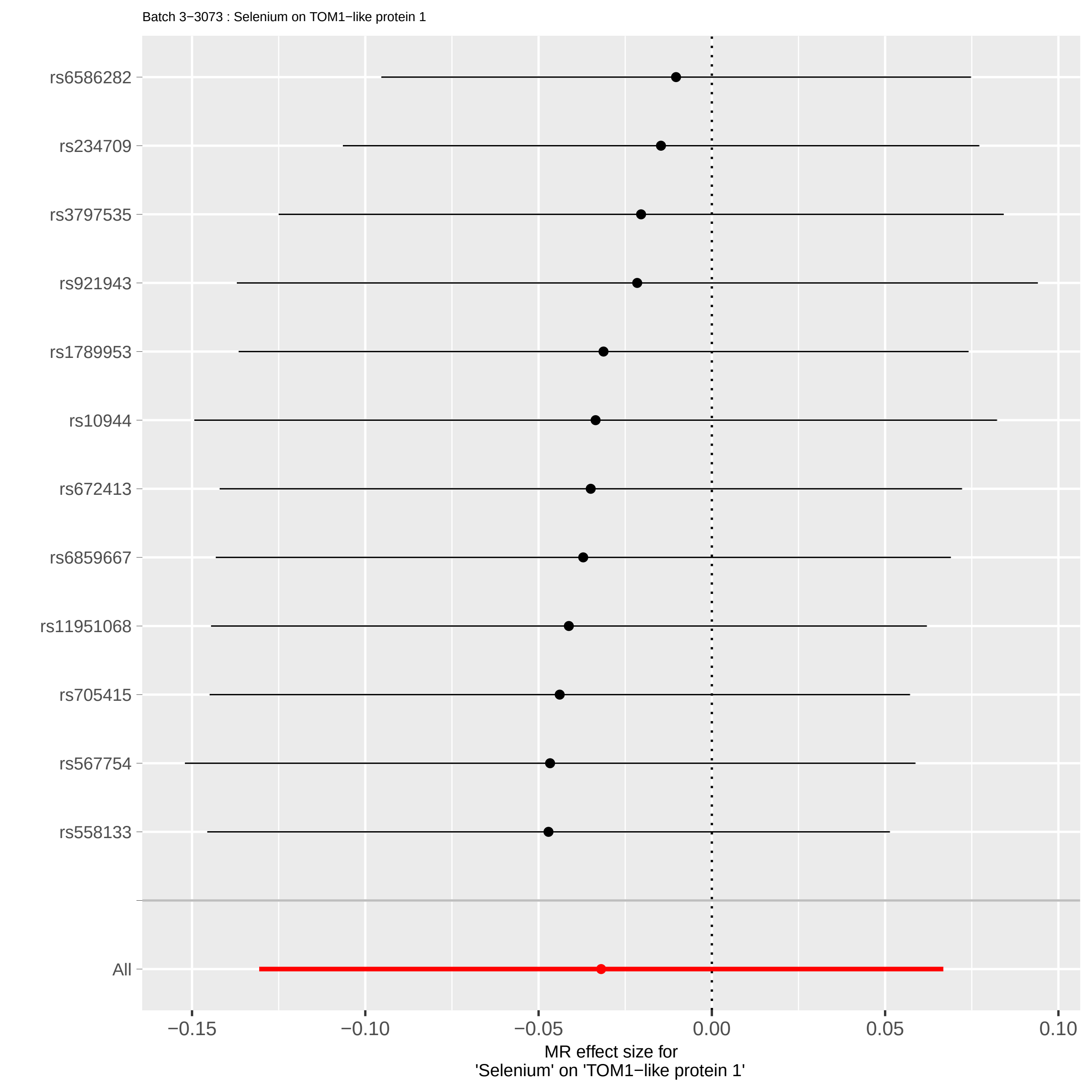

Batch 3−3073 : Selenium on TOM1−like protein 1
rs6586282
rs234709
rs3797535
rs921943
rs1789953
rs10944
rs672413
rs6859667
rs11951068
rs705415
rs567754
rs558133
All
−0.15
−0.10
−0.05
0.00
0.05
0.10
MR effect size for
'Selenium' on 'TOM1−like protein 1'

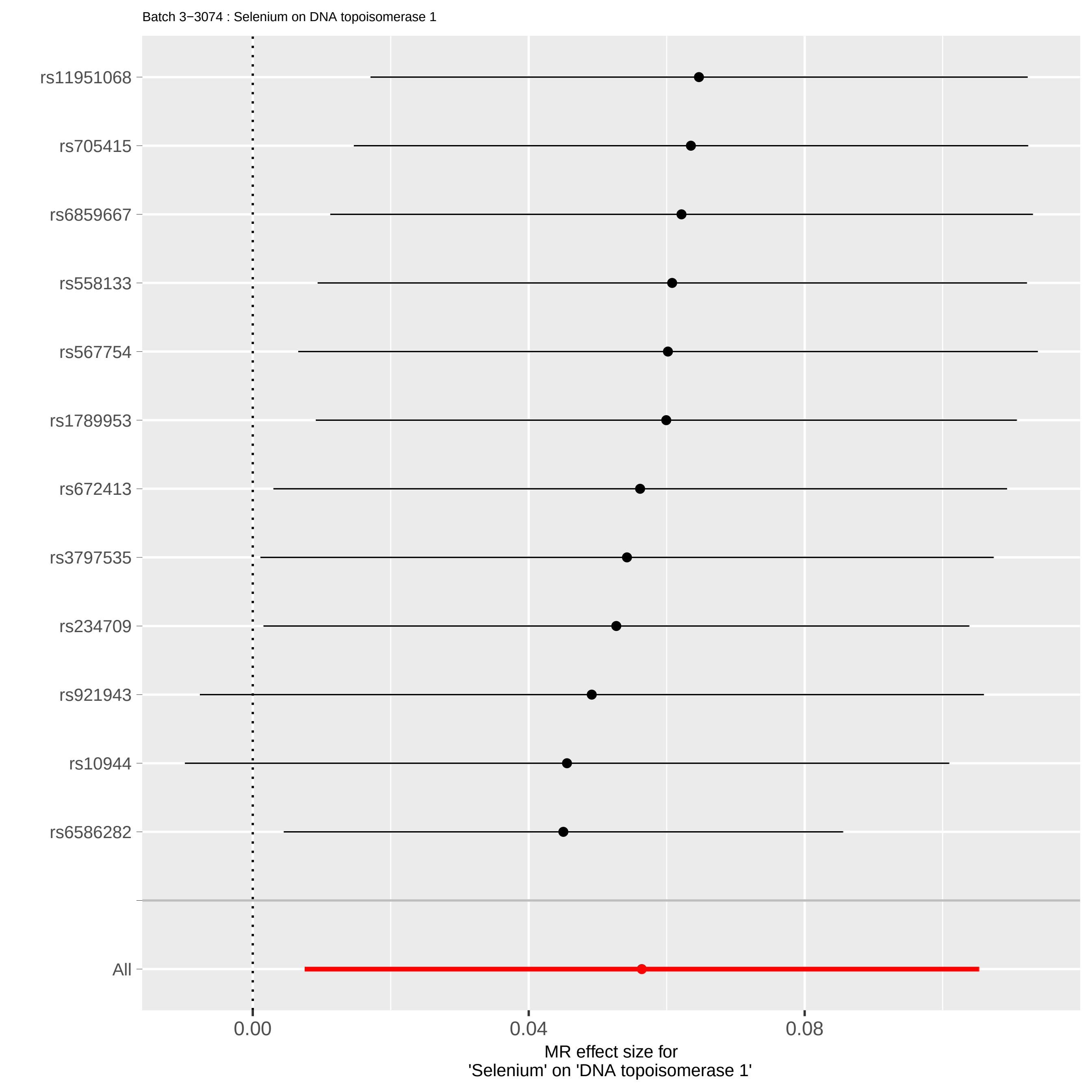
Batch 3−3074 : Selenium on DNA topoisomerase 1
rs11951068
rs705415
rs6859667
rs558133
rs567754
rs1789953
rs672413
rs3797535
rs234709
rs921943
rs10944
rs6586282
All
0.00
0.04
0.08
MR effect size for
'Selenium' on 'DNA topoisomerase 1'

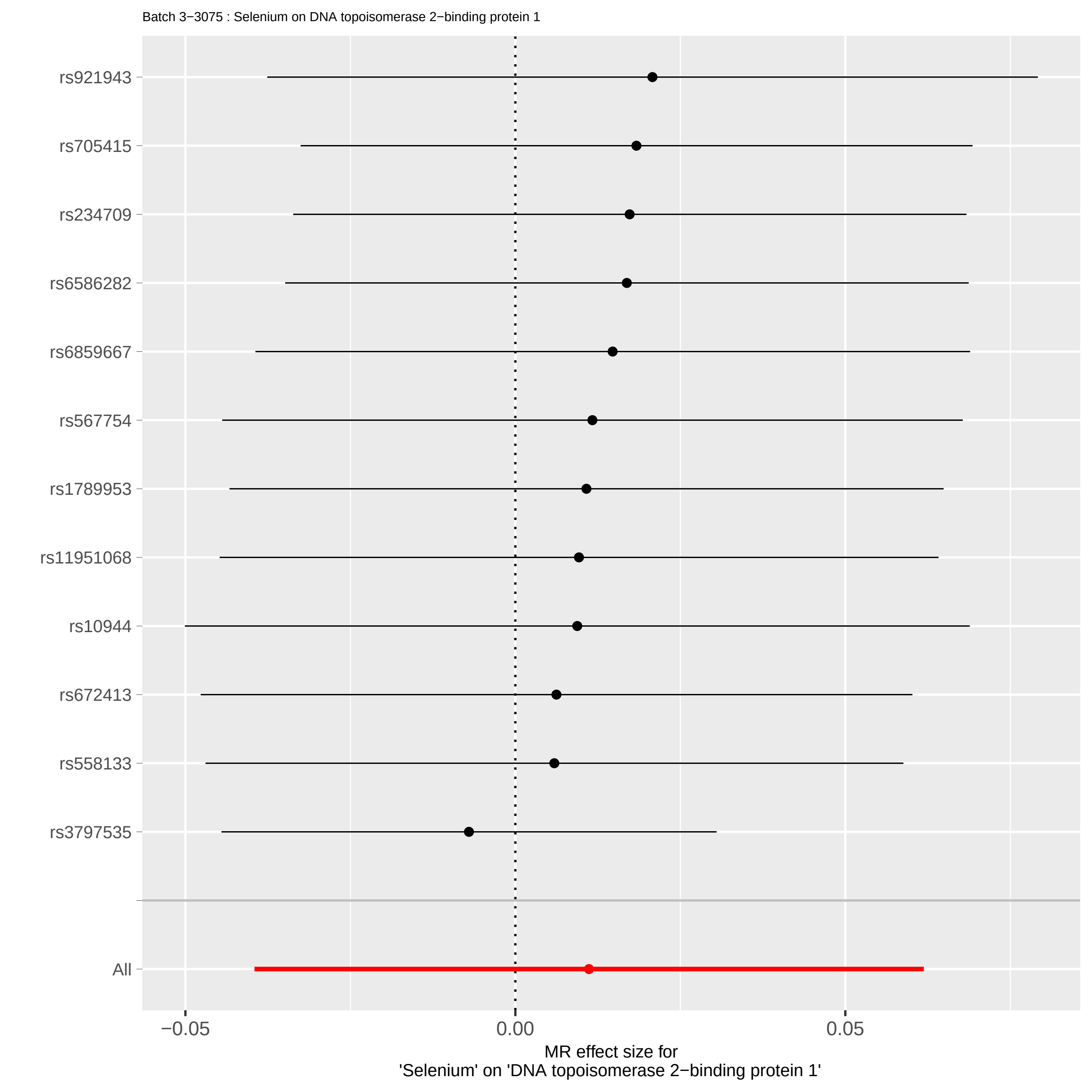

Batch 3−3075 : Selenium on DNA topoisomerase 2−binding protein 1
rs921943
rs705415
rs234709
rs6586282
rs6859667
rs567754
rs1789953
rs11951068
rs10944
rs672413
rs558133
rs3797535
All
−0.05
0.00
0.05
MR effect size for
'Selenium' on 'DNA topoisomerase 2−binding protein 1'

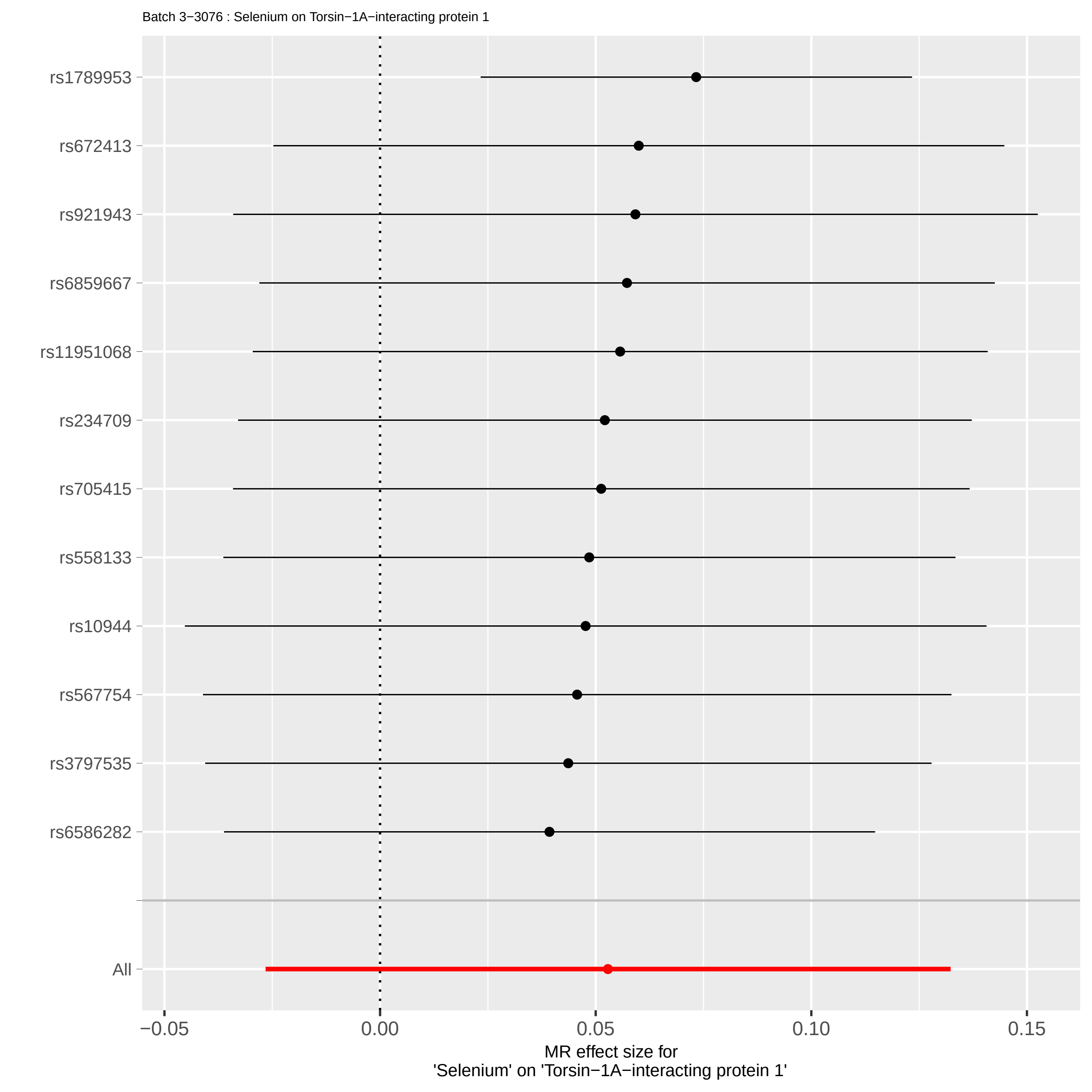

Batch 3−3076 : Selenium on Torsin−1A−interacting protein 1
rs1789953
rs672413
rs921943
rs6859667
rs11951068
rs234709
rs705415
rs558133
rs10944
rs567754
rs3797535
rs6586282
All
−0.05
0.00
0.05
0.10
0.15
MR effect size for
'Selenium' on 'Torsin−1A−interacting protein 1'

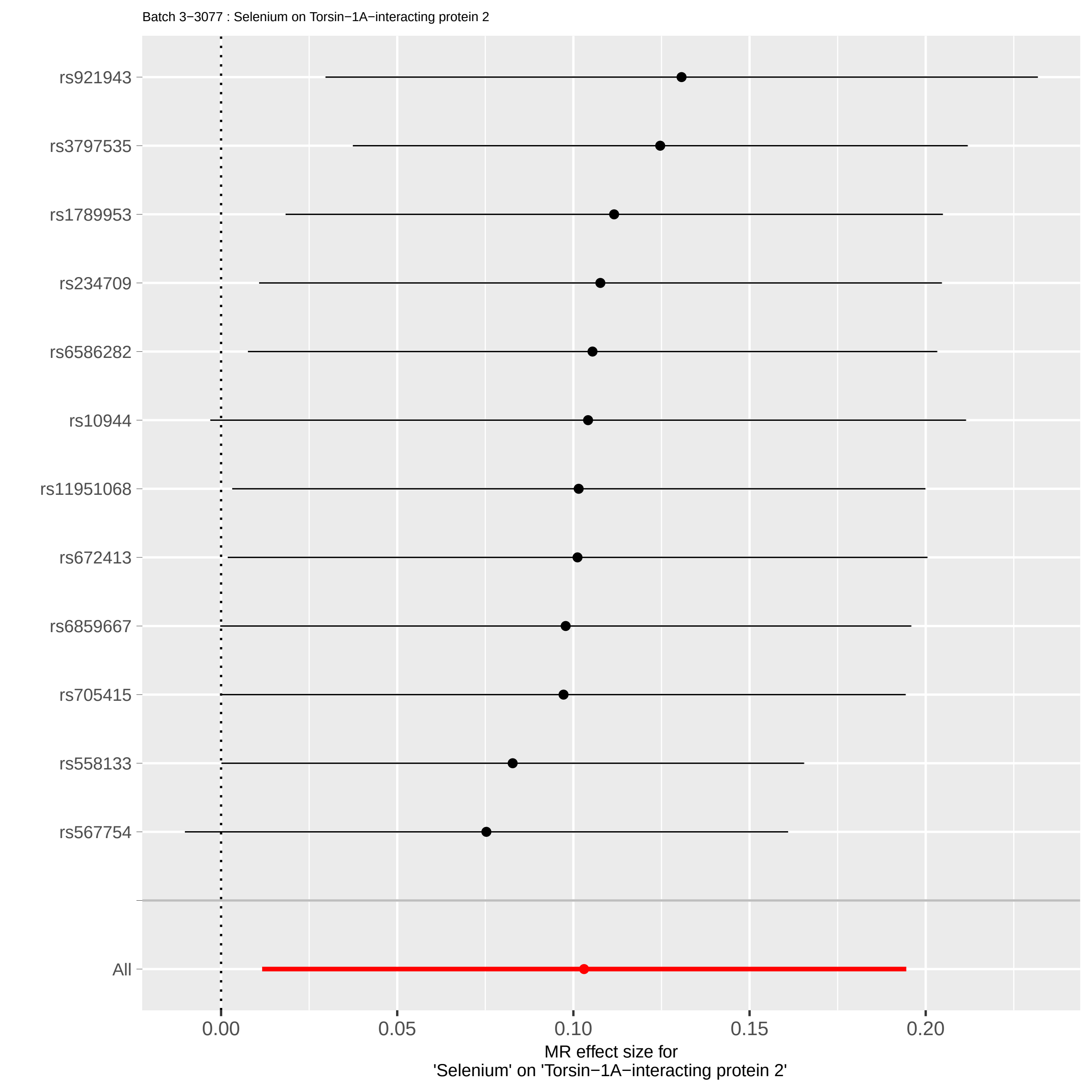

Batch 3−3077 : Selenium on Torsin−1A−interacting protein 2
rs921943
rs3797535
rs1789953
rs234709
rs6586282
rs10944
rs11951068
rs672413
rs6859667
rs705415
rs558133
rs567754
All
0.00
0.05
0.10
0.15
0.20
MR effect size for
'Selenium' on 'Torsin−1A−interacting protein 2'

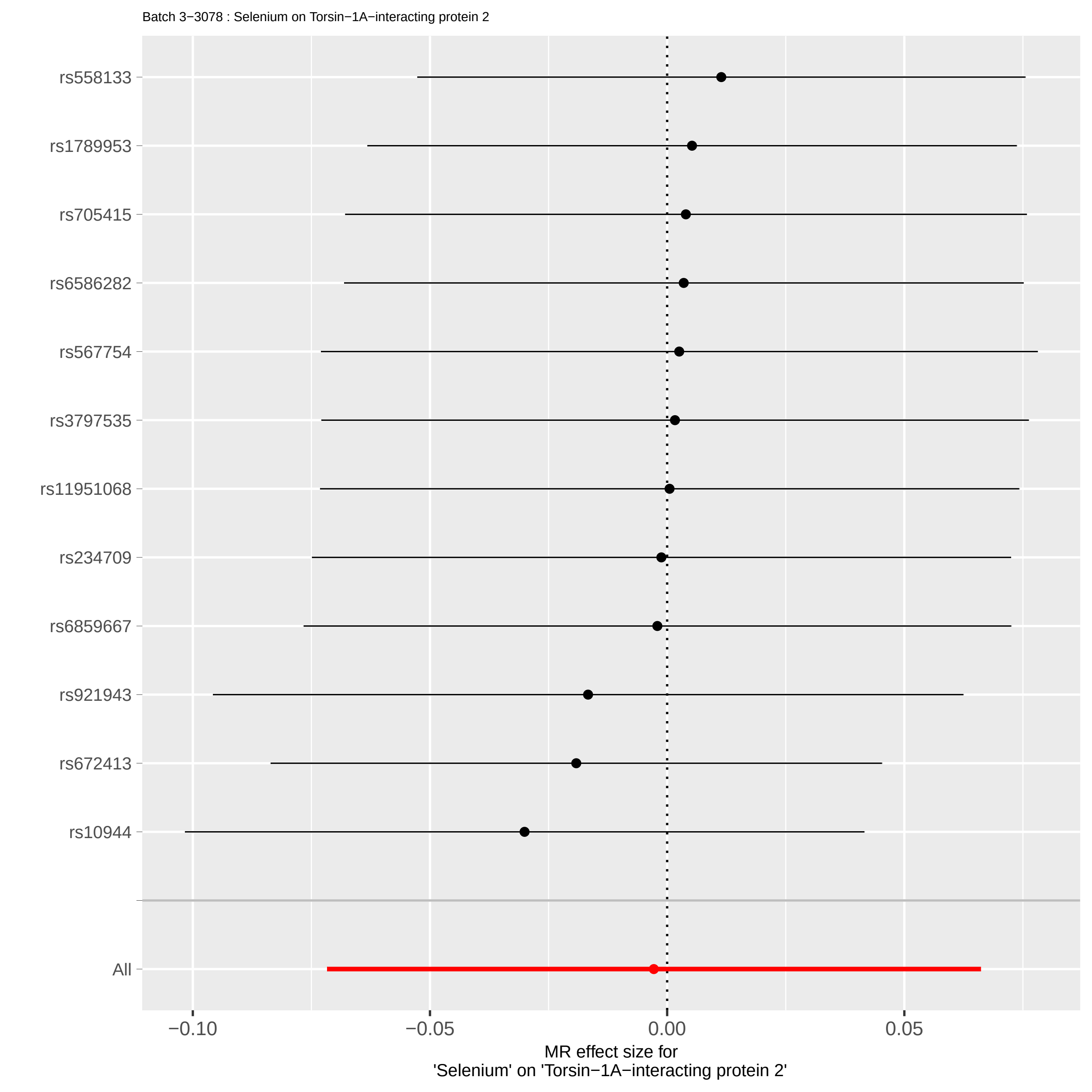

Batch 3−3078 : Selenium on Torsin−1A−interacting protein 2
rs558133
rs1789953
rs705415
rs6586282
rs567754
rs3797535
rs11951068
rs234709
rs6859667
rs921943
rs672413
rs10944
All
−0.10
−0.05
0.00
0.05
MR effect size for
'Selenium' on 'Torsin−1A−interacting protein 2'

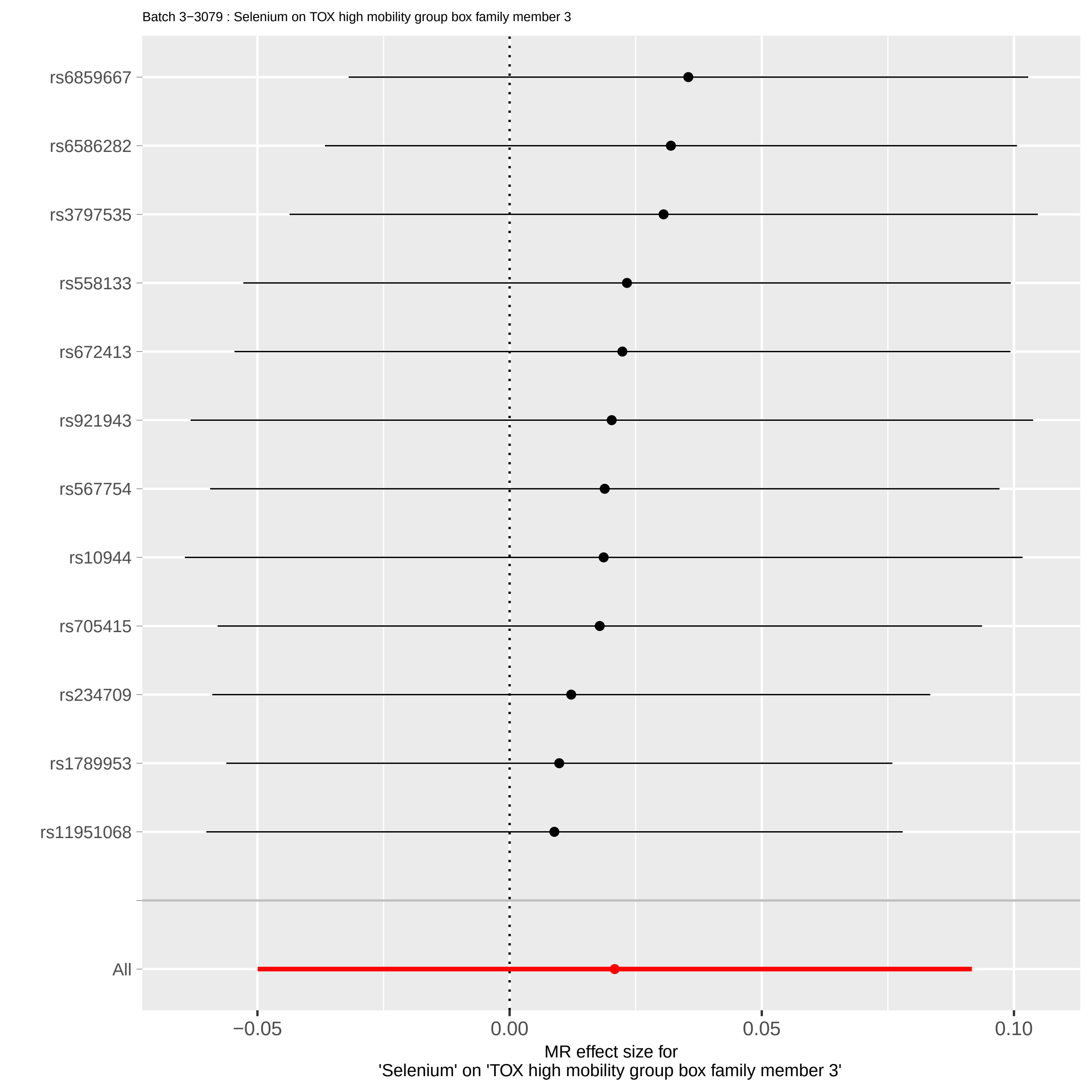

Batch 3−3079 : Selenium on TOX high mobility group box family member 3
rs6859667
rs6586282
rs3797535
rs558133
rs672413
rs921943
rs567754
rs10944
rs705415
rs234709
rs1789953
rs11951068
All
−0.05
0.00
0.05
0.10
MR effect size for
'Selenium' on 'TOX high mobility group box family member 3'

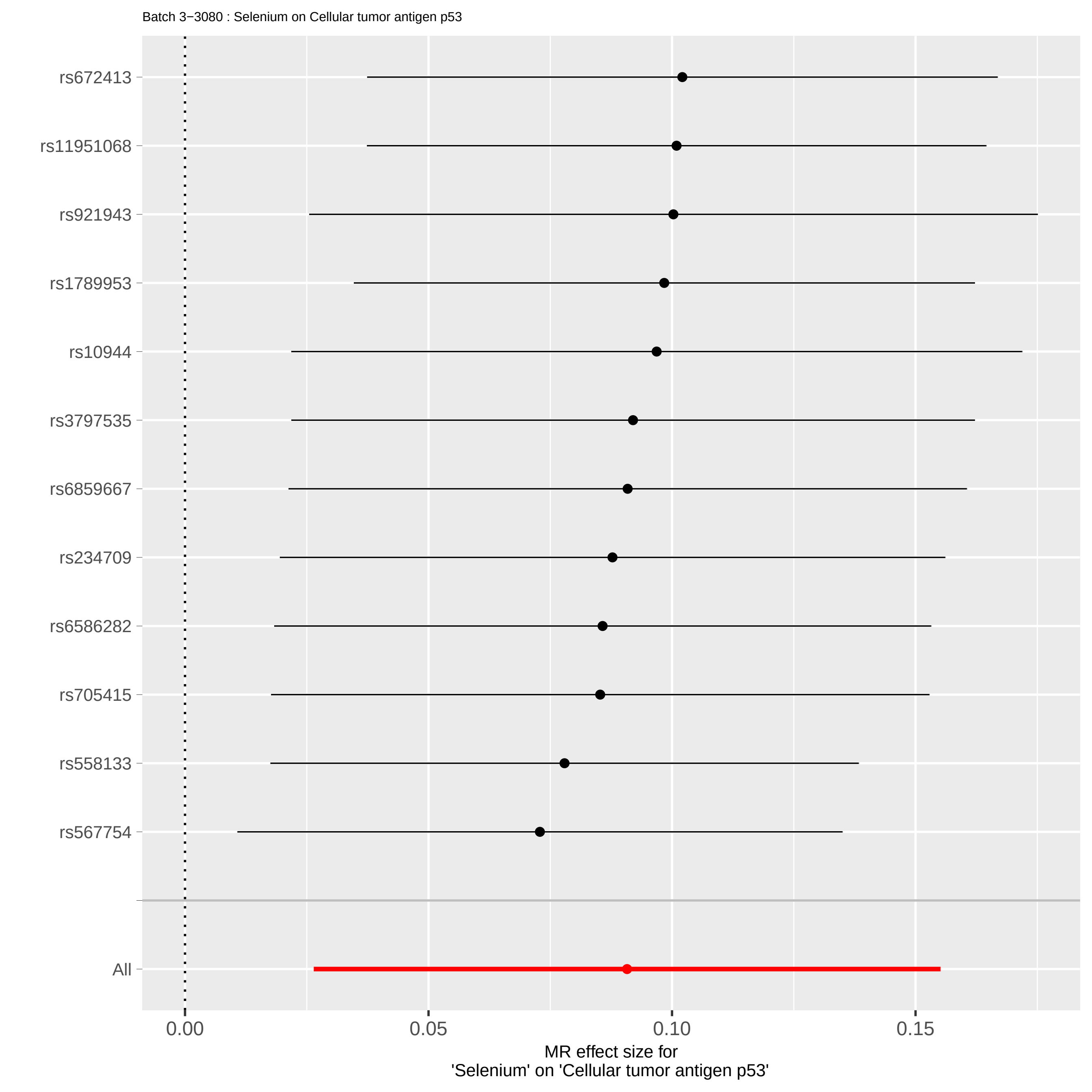

Batch 3−3080 : Selenium on Cellular tumor antigen p53
rs672413
rs11951068
rs921943
rs1789953
rs10944
rs3797535
rs6859667
rs234709
rs6586282
rs705415
rs558133
rs567754
All
0.00
0.05
0.10
0.15
MR effect size for
'Selenium' on 'Cellular tumor antigen p53'

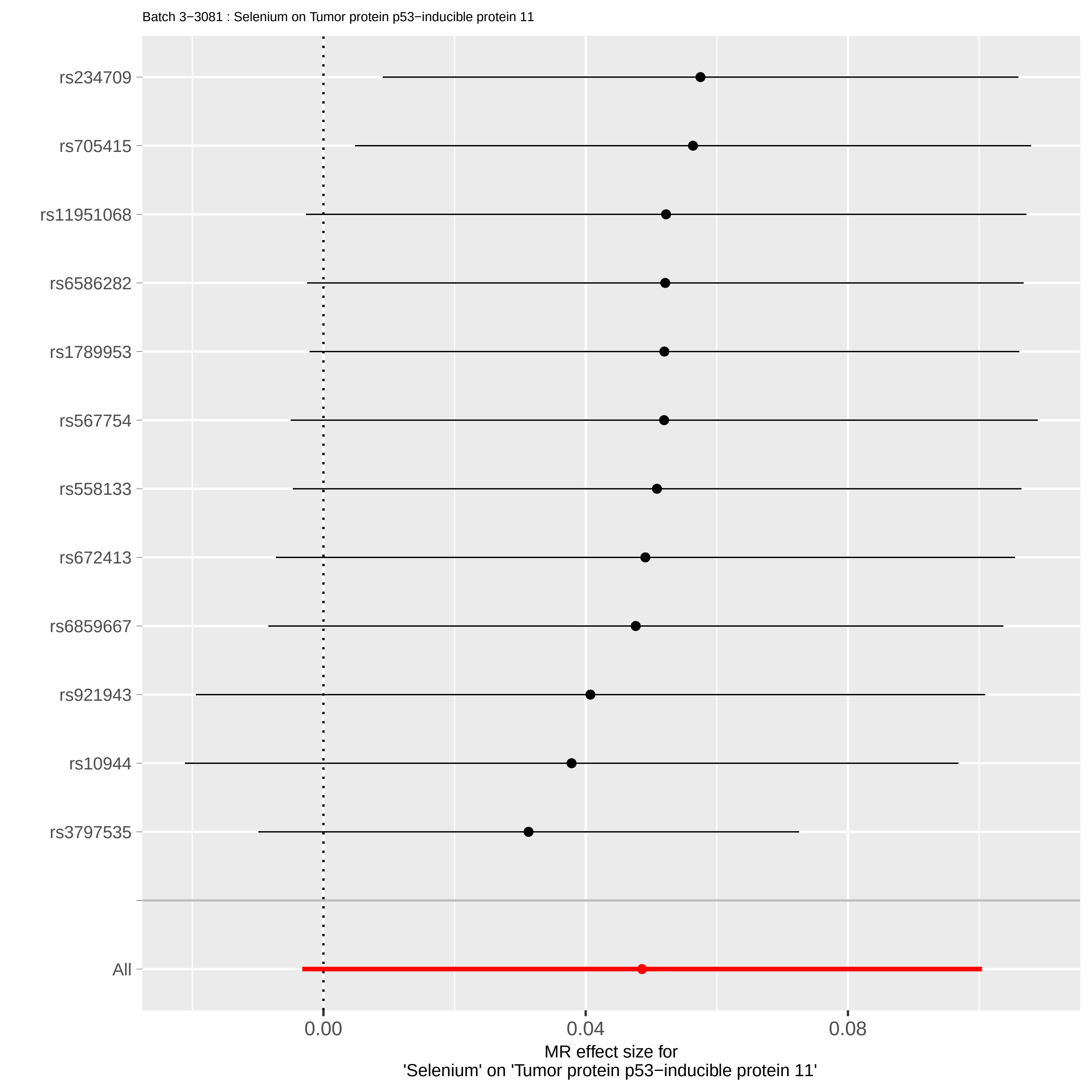

Batch 3−3081 : Selenium on Tumor protein p53−inducible protein 11
rs234709
rs705415
rs11951068
rs6586282
rs1789953
rs567754
rs558133
rs672413
rs6859667
rs921943
rs10944
rs3797535
All
0.00
0.04
0.08
MR effect size for
'Selenium' on 'Tumor protein p53−inducible protein 11'

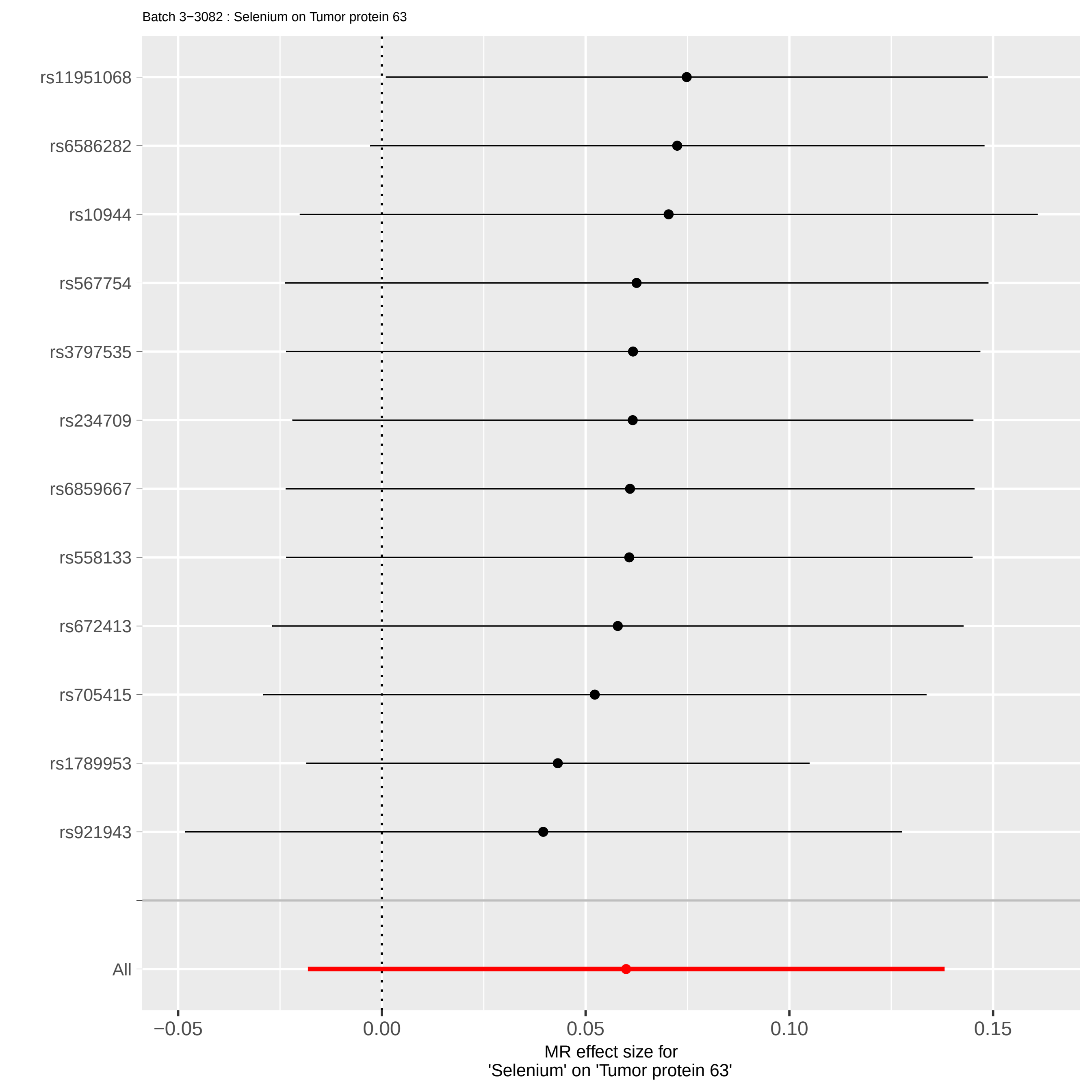

Batch 3−3082 : Selenium on Tumor protein 63
rs11951068
rs6586282
rs10944
rs567754
rs3797535
rs234709
rs6859667
rs558133
rs672413
rs705415
rs1789953
rs921943
All
−0.05
0.00
0.05
0.10
0.15
MR effect size for
'Selenium' on 'Tumor protein 63'

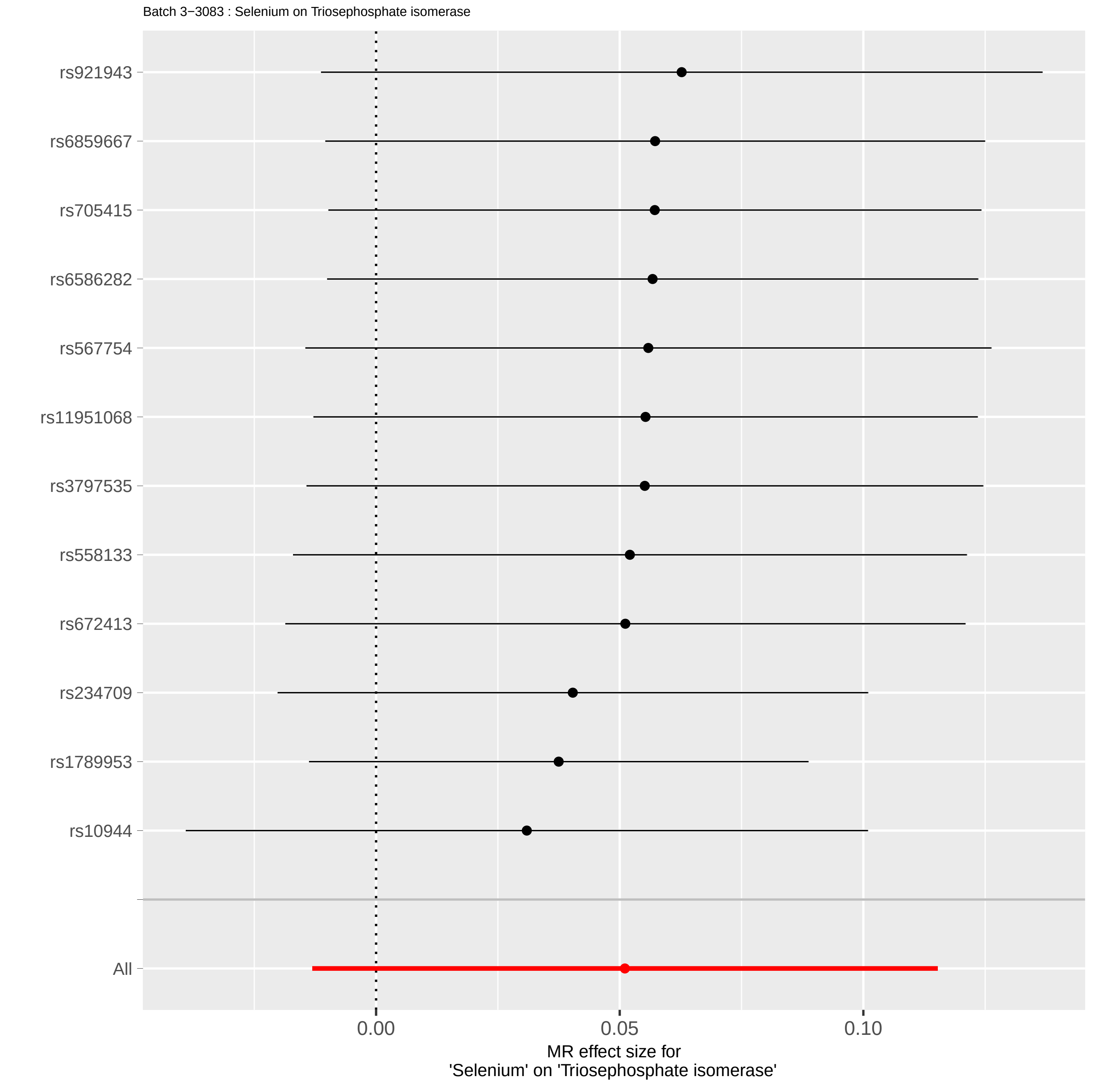

Batch 3−3083 : Selenium on Triosephosphate isomerase
rs921943
rs6859667
rs705415
rs6586282
rs567754
rs11951068
rs3797535
rs558133
rs672413
rs234709
rs1789953
rs10944
All
0.00
0.05
0.10
MR effect size for
'Selenium' on 'Triosephosphate isomerase'

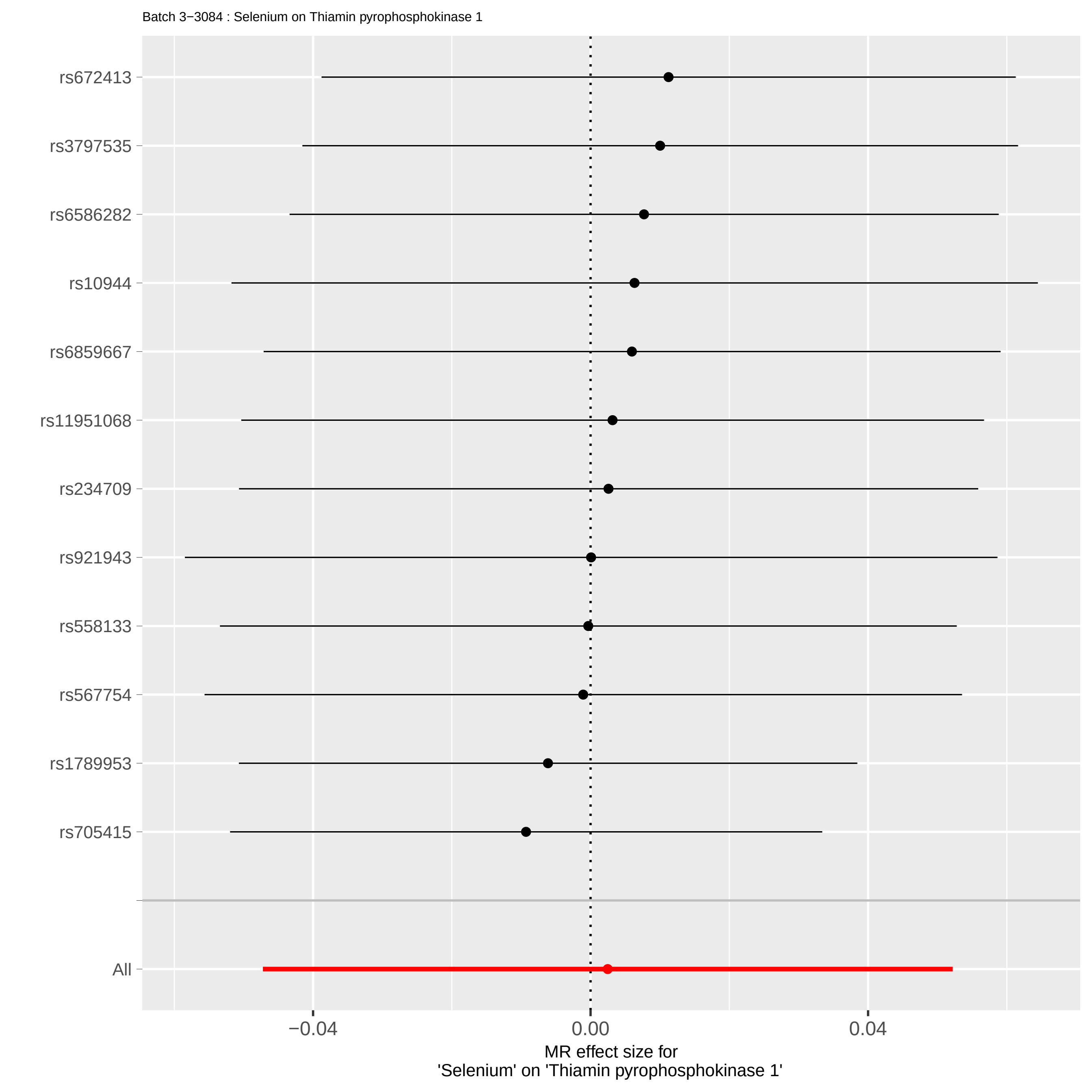

Batch 3−3084 : Selenium on Thiamin pyrophosphokinase 1
rs672413
rs3797535
rs6586282
rs10944
rs6859667
rs11951068
rs234709
rs921943
rs558133
rs567754
rs1789953
rs705415
All
−0.04
0.00
0.04
MR effect size for
'Selenium' on 'Thiamin pyrophosphokinase 1'

Batch 3−3085 : Selenium on Tropomyosin alpha−1 chain

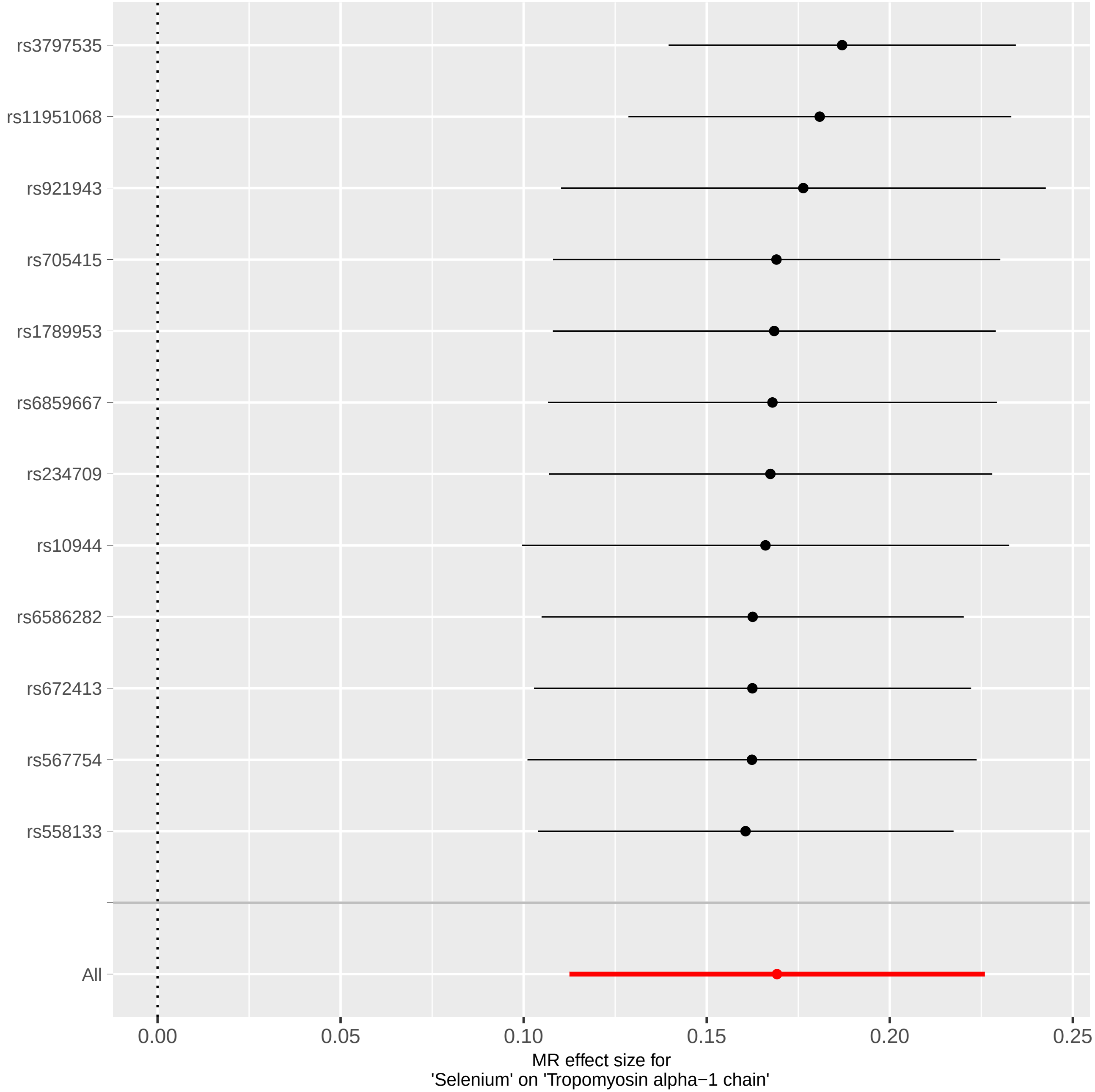

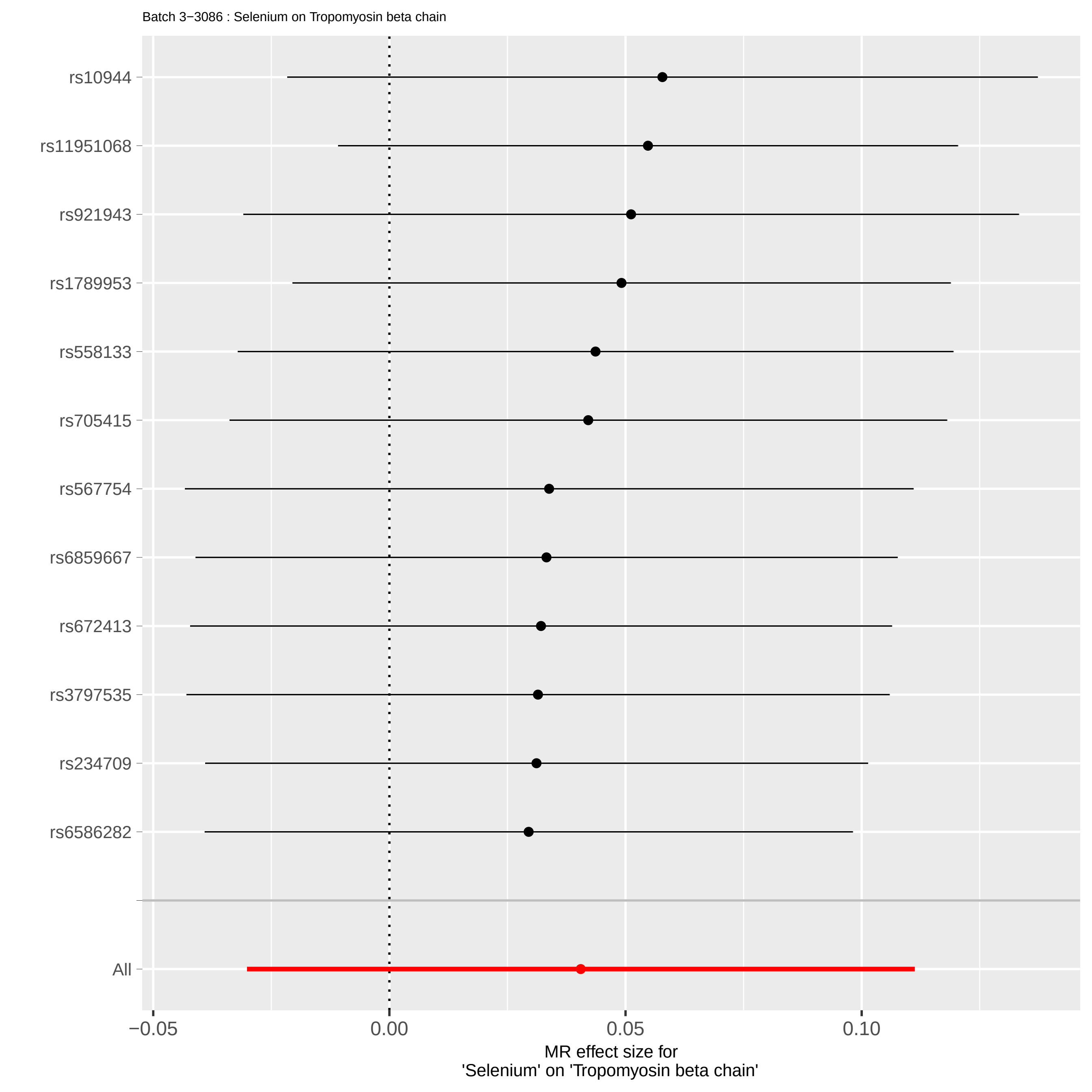

Batch 3−3086 : Selenium on Tropomyosin beta chain
rs10944
rs11951068
rs921943
rs1789953
rs558133
rs705415
rs567754
rs6859667
rs672413
rs3797535
rs234709
rs6586282
All
−0.05
0.00
0.05
0.10
MR effect size for
'Selenium' on 'Tropomyosin beta chain'

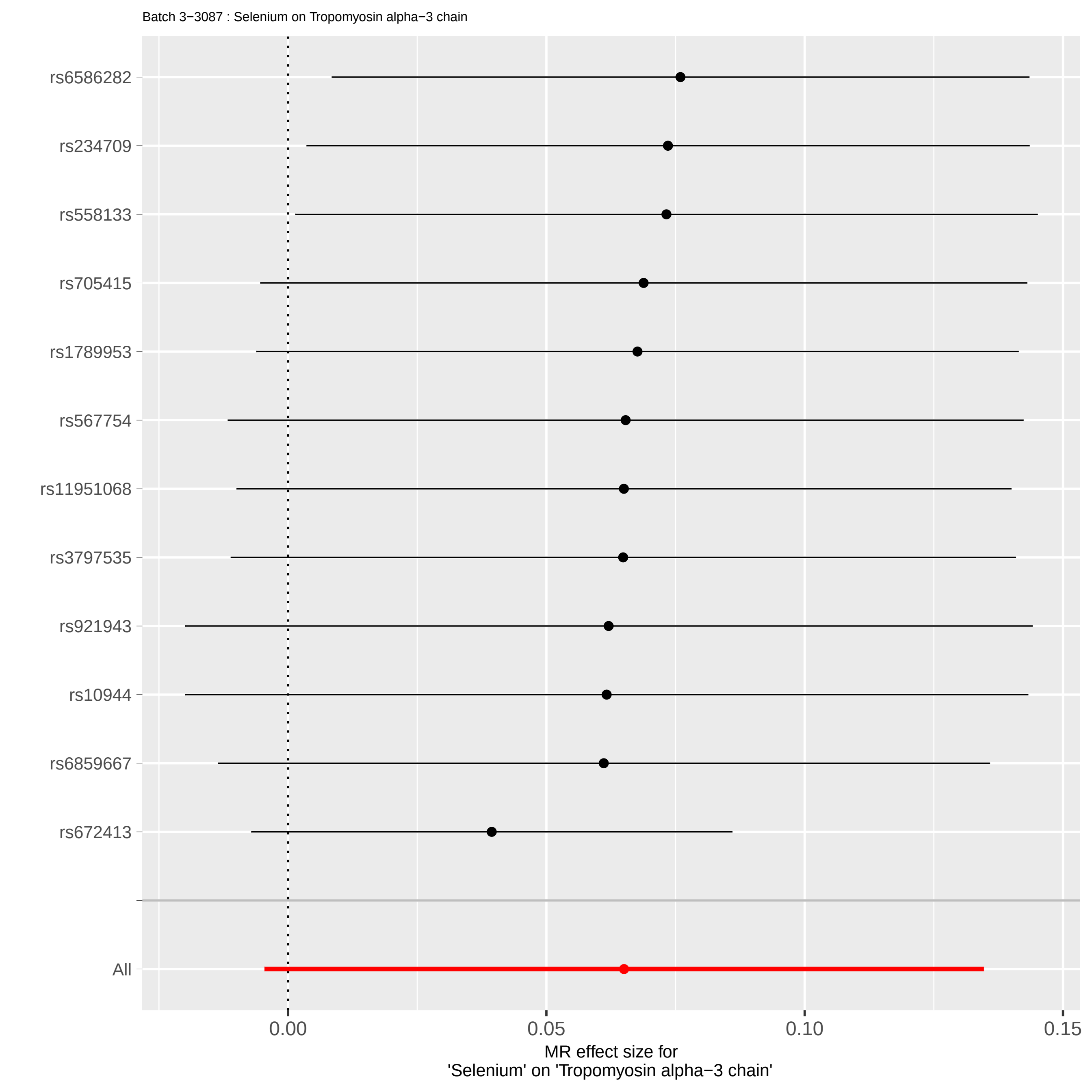

Batch 3−3087 : Selenium on Tropomyosin alpha−3 chain
rs6586282
rs234709
rs558133
rs705415
rs1789953
rs567754
rs11951068
rs3797535
rs921943
rs10944
rs6859667
rs672413
All
0.00
0.05
0.10
0.15
MR effect size for
'Selenium' on 'Tropomyosin alpha−3 chain'

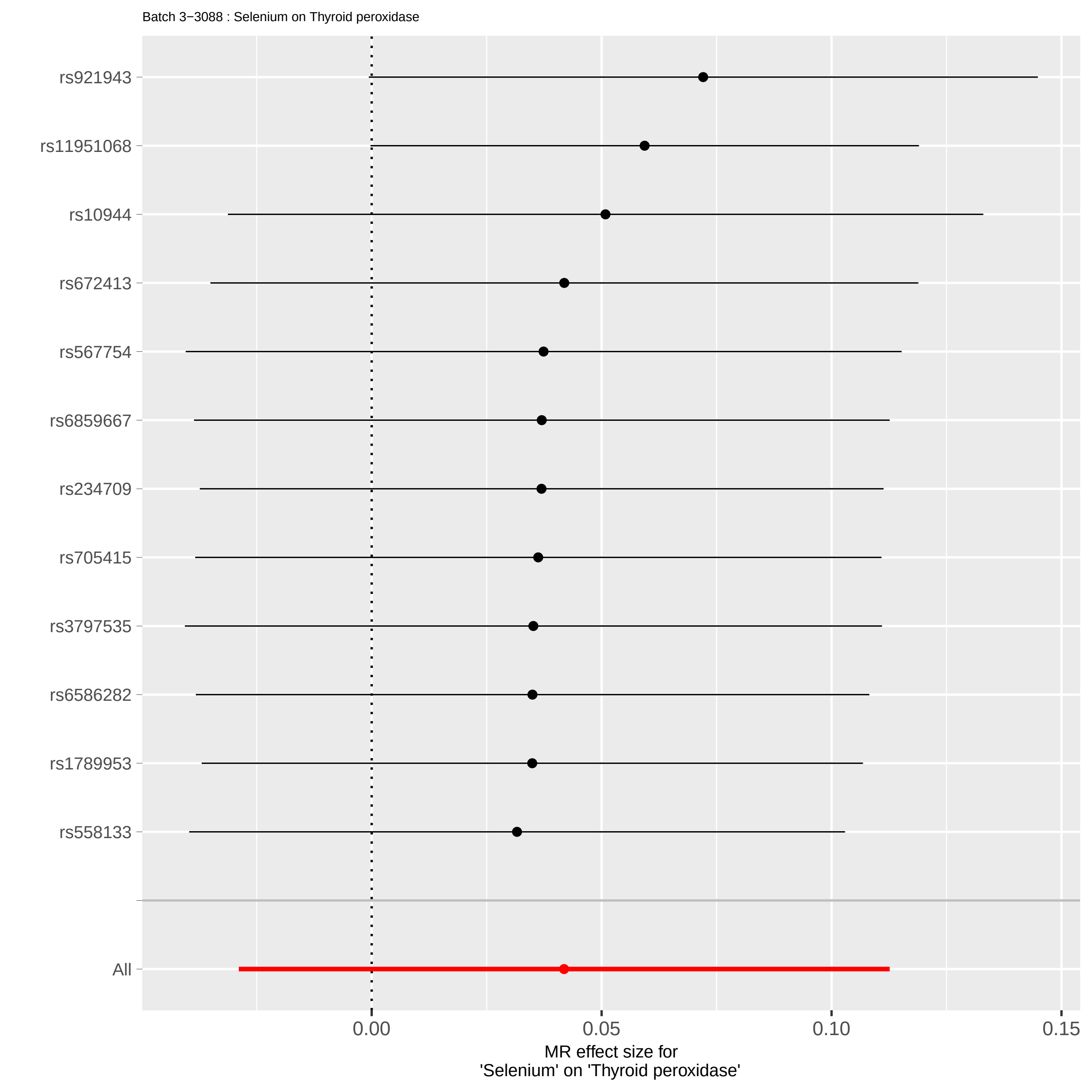
Batch 3−3088 : Selenium on Thyroid peroxidase
rs921943
rs11951068
rs10944
rs672413
rs567754
rs6859667
rs234709
rs705415
rs3797535
rs6586282
rs1789953
rs558133
All
0.00
0.05
0.10
0.15
MR effect size for
'Selenium' on 'Thyroid peroxidase'

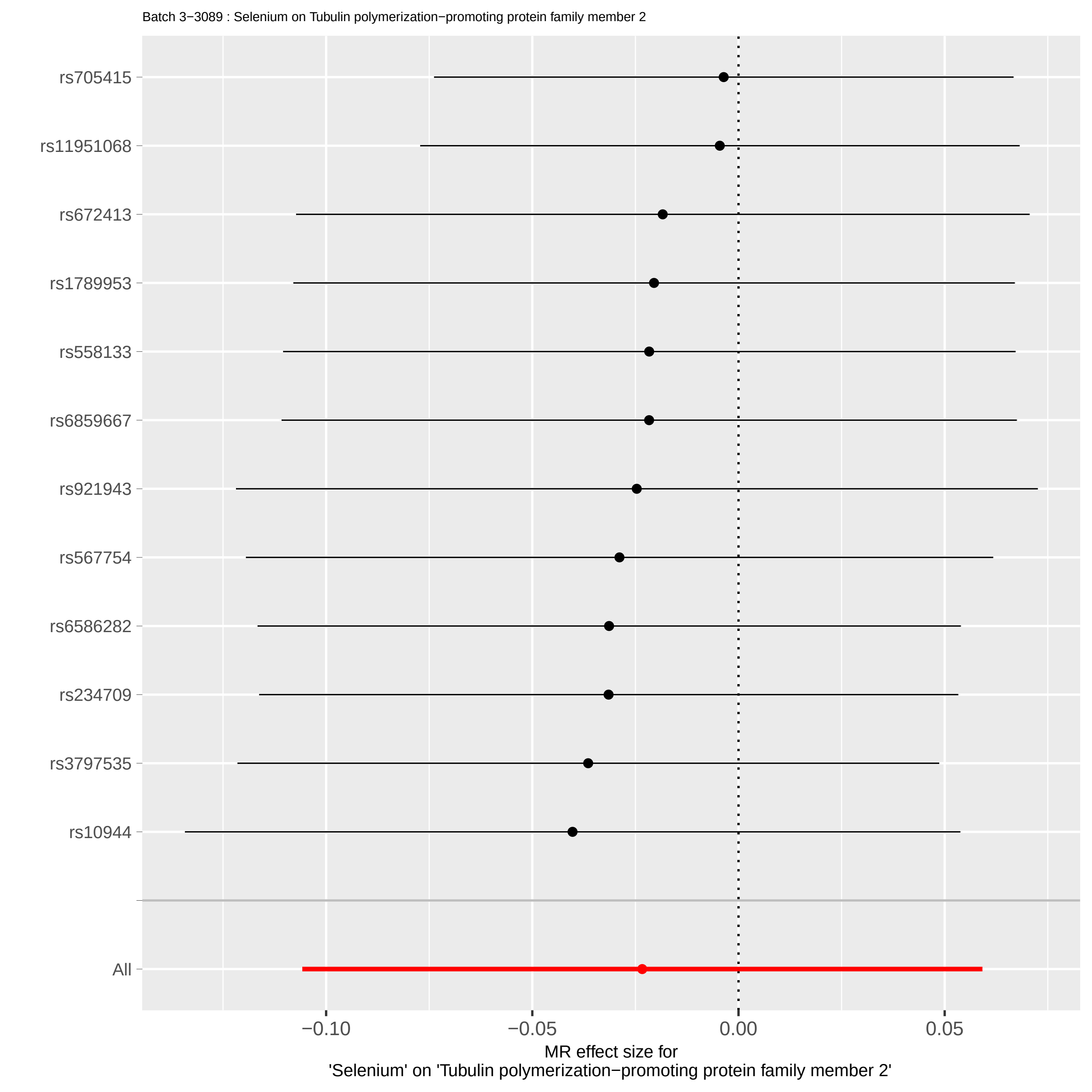

Batch 3−3089 : Selenium on Tubulin polymerization−promoting protein family member 2
rs705415
rs11951068
rs672413
rs1789953
rs558133
rs6859667
rs921943
rs567754
rs6586282
rs234709
rs3797535
rs10944
All
−0.10
−0.05
0.00
0.05
MR effect size for
'Selenium' on 'Tubulin polymerization−promoting protein family member 2'

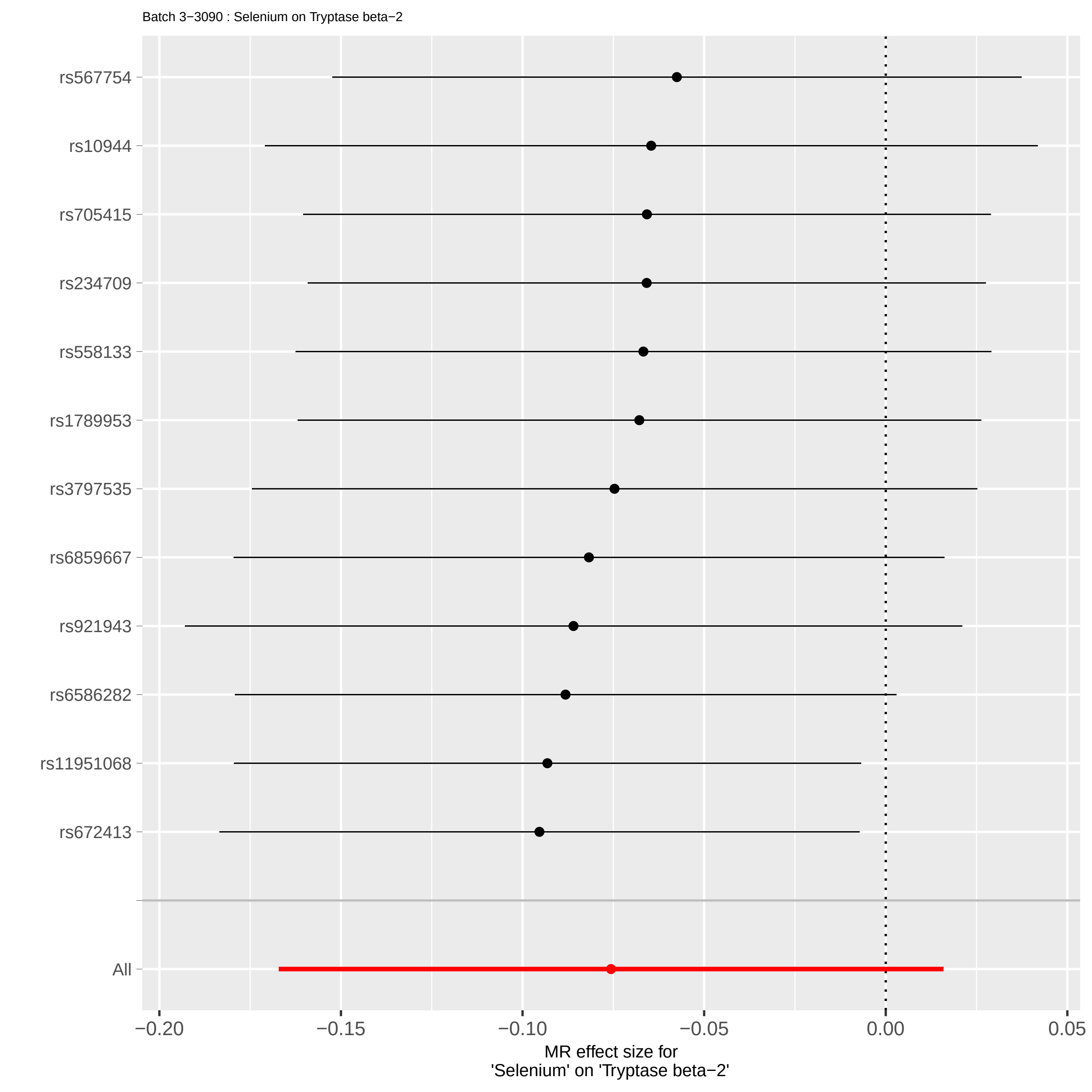
Batch 3−3090 : Selenium on Tryptase beta−2
rs567754
rs10944
rs705415
rs234709
rs558133
rs1789953
rs3797535
rs6859667
rs921943
rs6586282
rs11951068
rs672413
All
−0.20
−0.15
−0.10
−0.05
0.00
0.05
MR effect size for
'Selenium' on 'Tryptase beta−2'

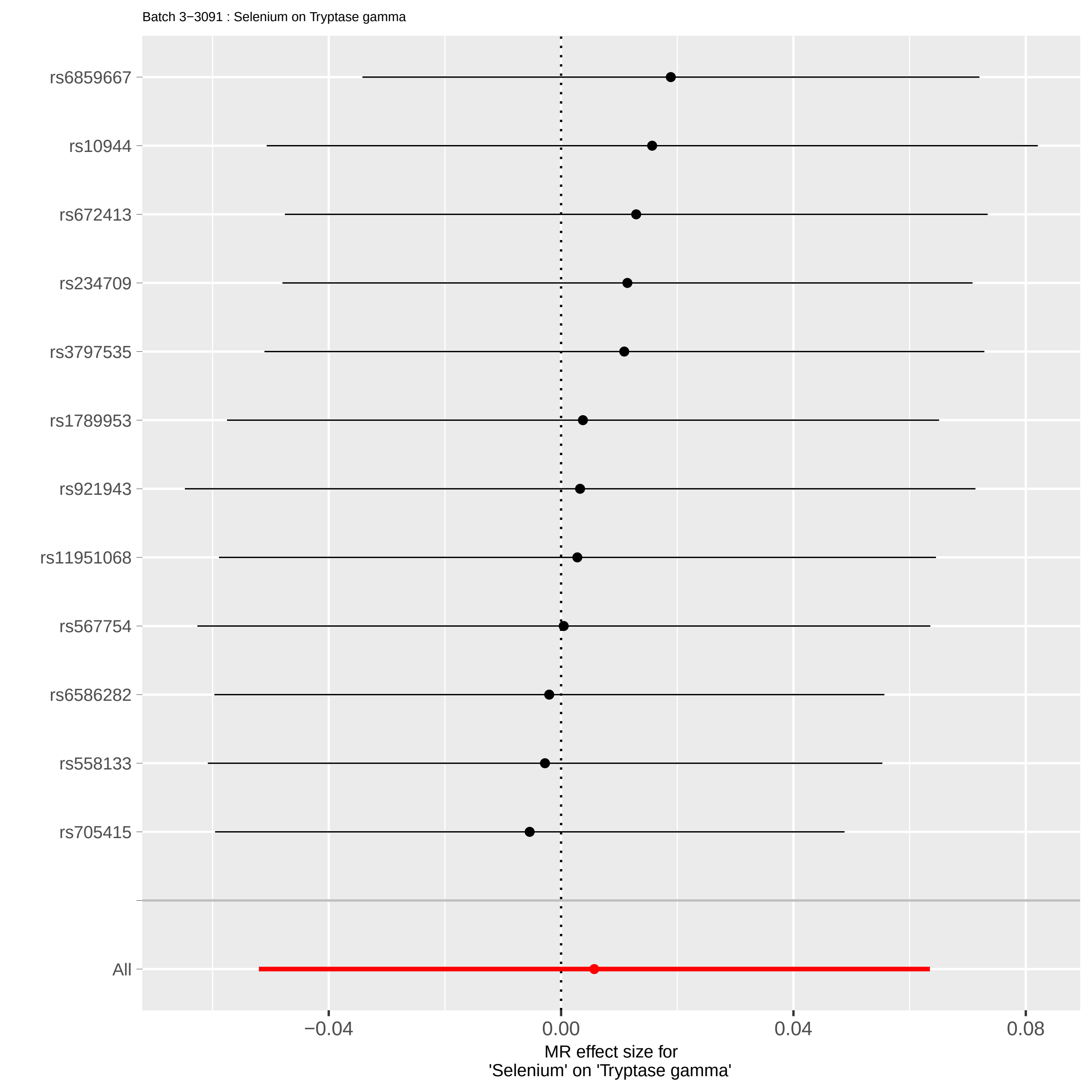

Batch 3−3091 : Selenium on Tryptase gamma
rs6859667
rs10944
rs672413
rs234709
rs3797535
rs1789953
rs921943
rs11951068
rs567754
rs6586282
rs558133
rs705415
All
−0.04
0.00
0.04
0.08
MR effect size for
'Selenium' on 'Tryptase gamma'

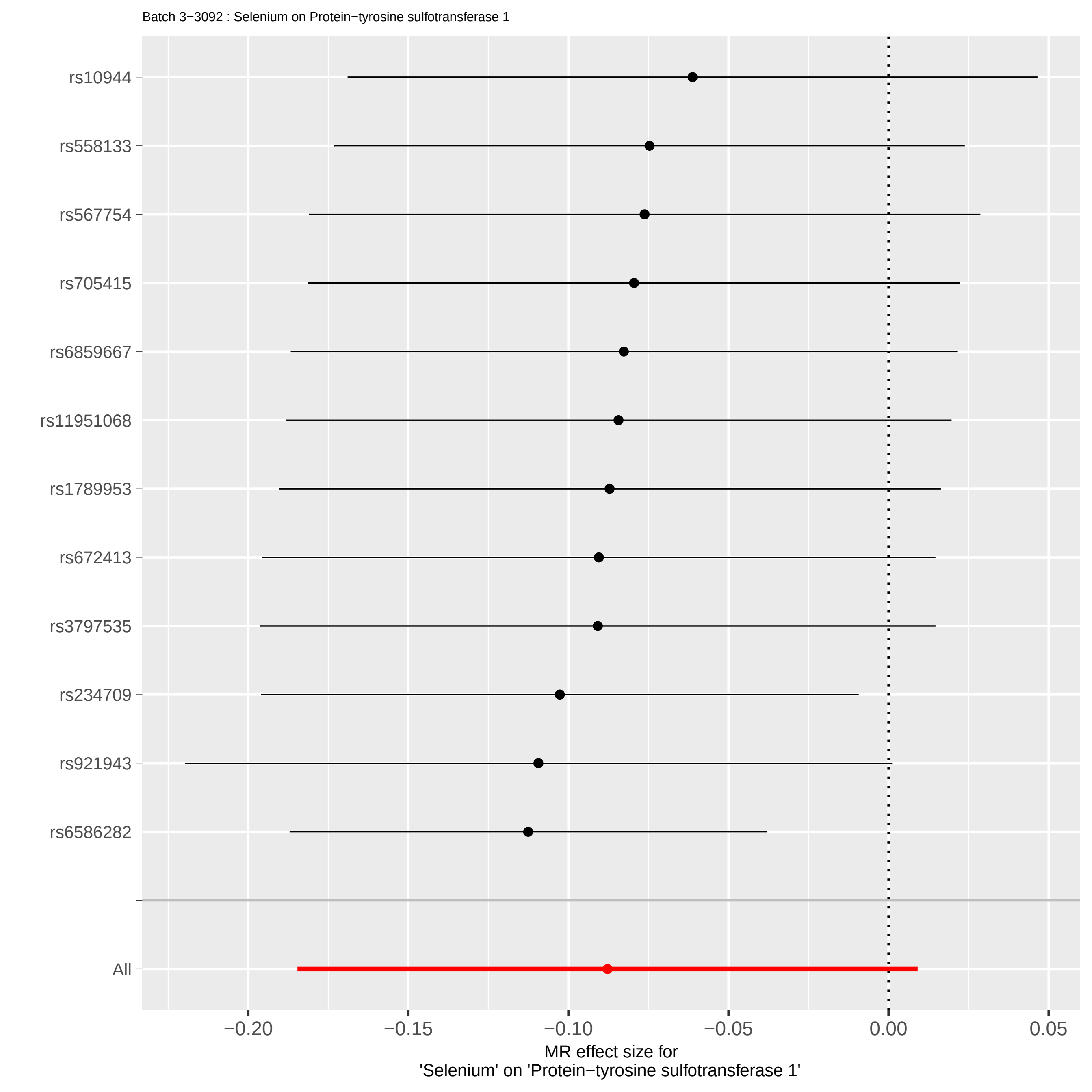

Batch 3−3092 : Selenium on Protein−tyrosine sulfotransferase 1
rs10944
rs558133
rs567754
rs705415
rs6859667
rs11951068
rs1789953
rs672413
rs3797535
rs234709
rs921943
rs6586282
All
−0.20
−0.15
−0.10
−0.05
0.00
0.05
MR effect size for
'Selenium' on 'Protein−tyrosine sulfotransferase 1'

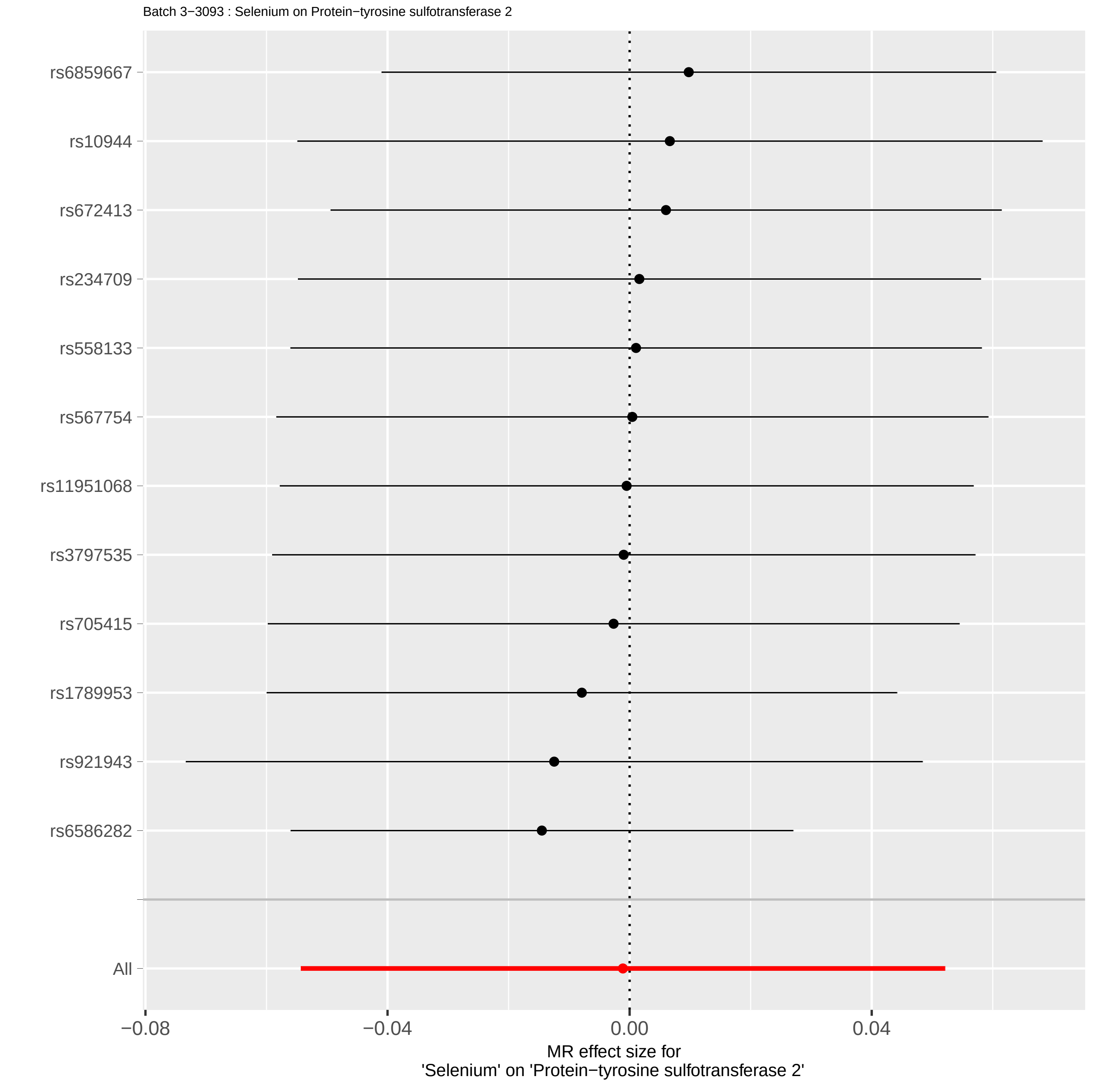

Batch 3−3093 : Selenium on Protein−tyrosine sulfotransferase 2
rs6859667
rs10944
rs672413
rs234709
rs558133
rs567754
rs11951068
rs3797535
rs705415
rs1789953
rs921943
rs6586282
All
−0.08
−0.04
0.00
0.04
MR effect size for
'Selenium' on 'Protein−tyrosine sulfotransferase 2'

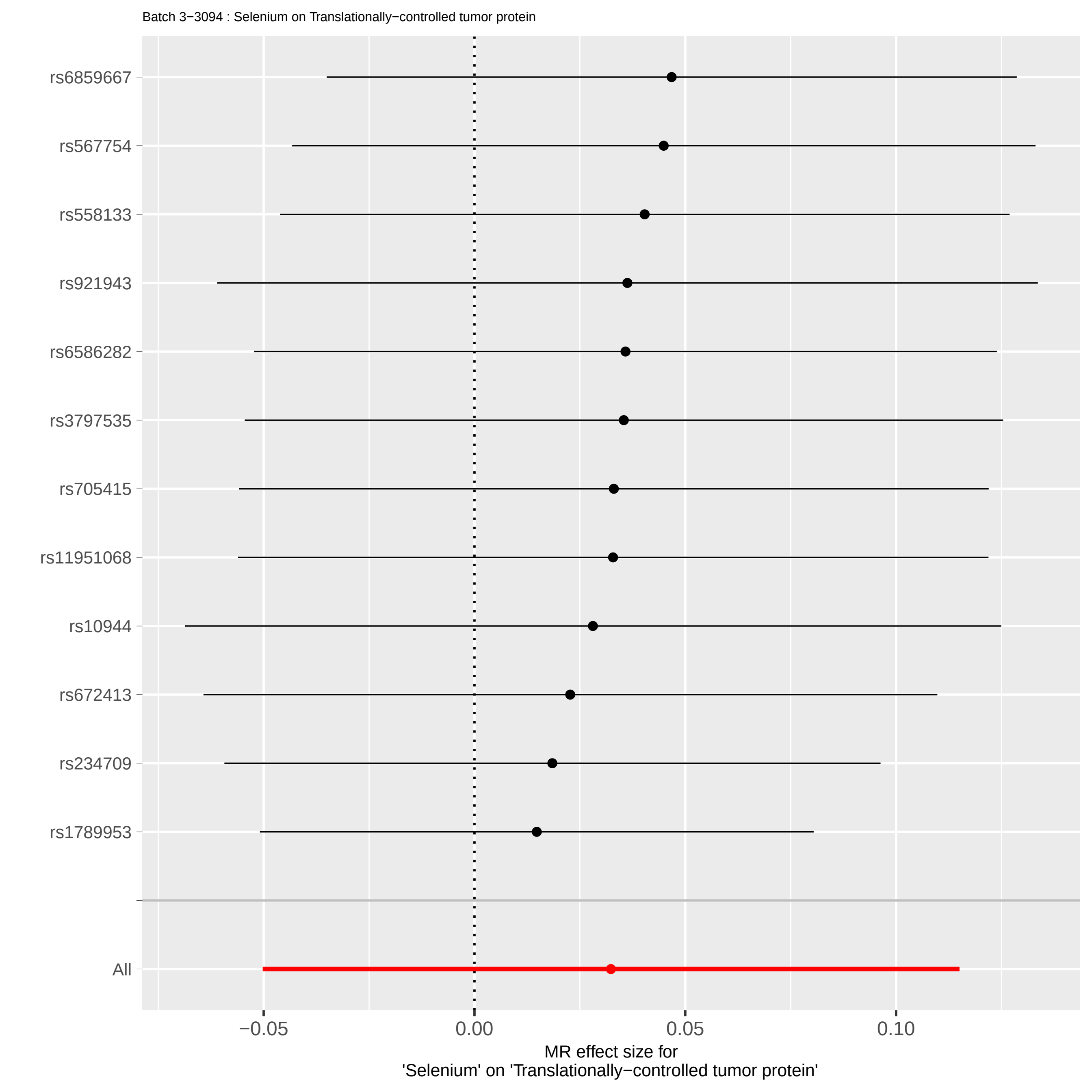

Batch 3−3094 : Selenium on Translationally−controlled tumor protein
rs6859667
rs567754
rs558133
rs921943
rs6586282
rs3797535
rs705415
rs11951068
rs10944
rs672413
rs234709
rs1789953
All
−0.05
0.00
0.05
0.10
MR effect size for
'Selenium' on 'Translationally−controlled tumor protein'

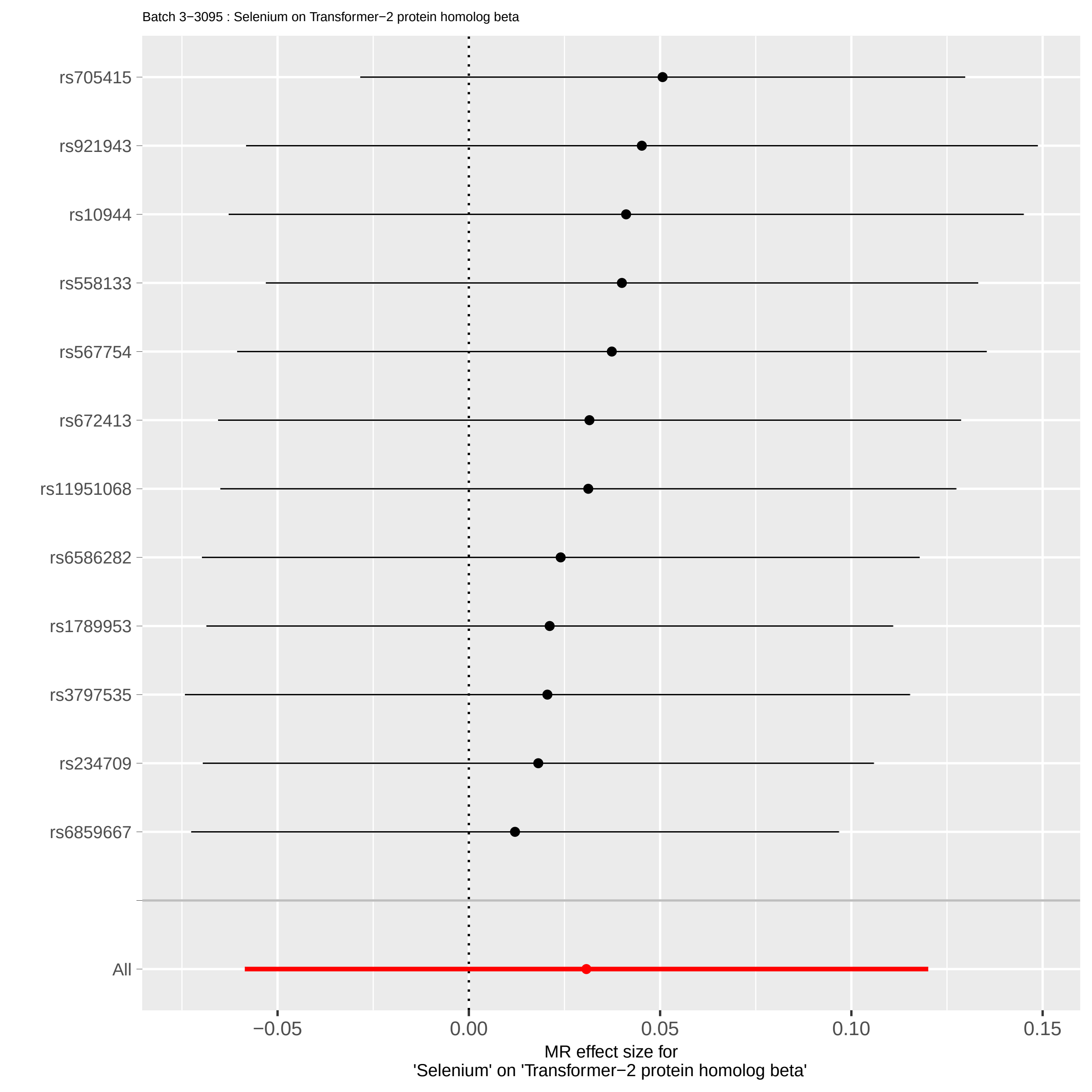

Batch 3−3095 : Selenium on Transformer−2 protein homolog beta
rs705415
rs921943
rs10944
rs558133
rs567754
rs672413
rs11951068
rs6586282
rs1789953
rs3797535
rs234709
rs6859667
All
−0.05
0.00
0.05
0.10
0.15
MR effect size for
'Selenium' on 'Transformer−2 protein homolog beta'

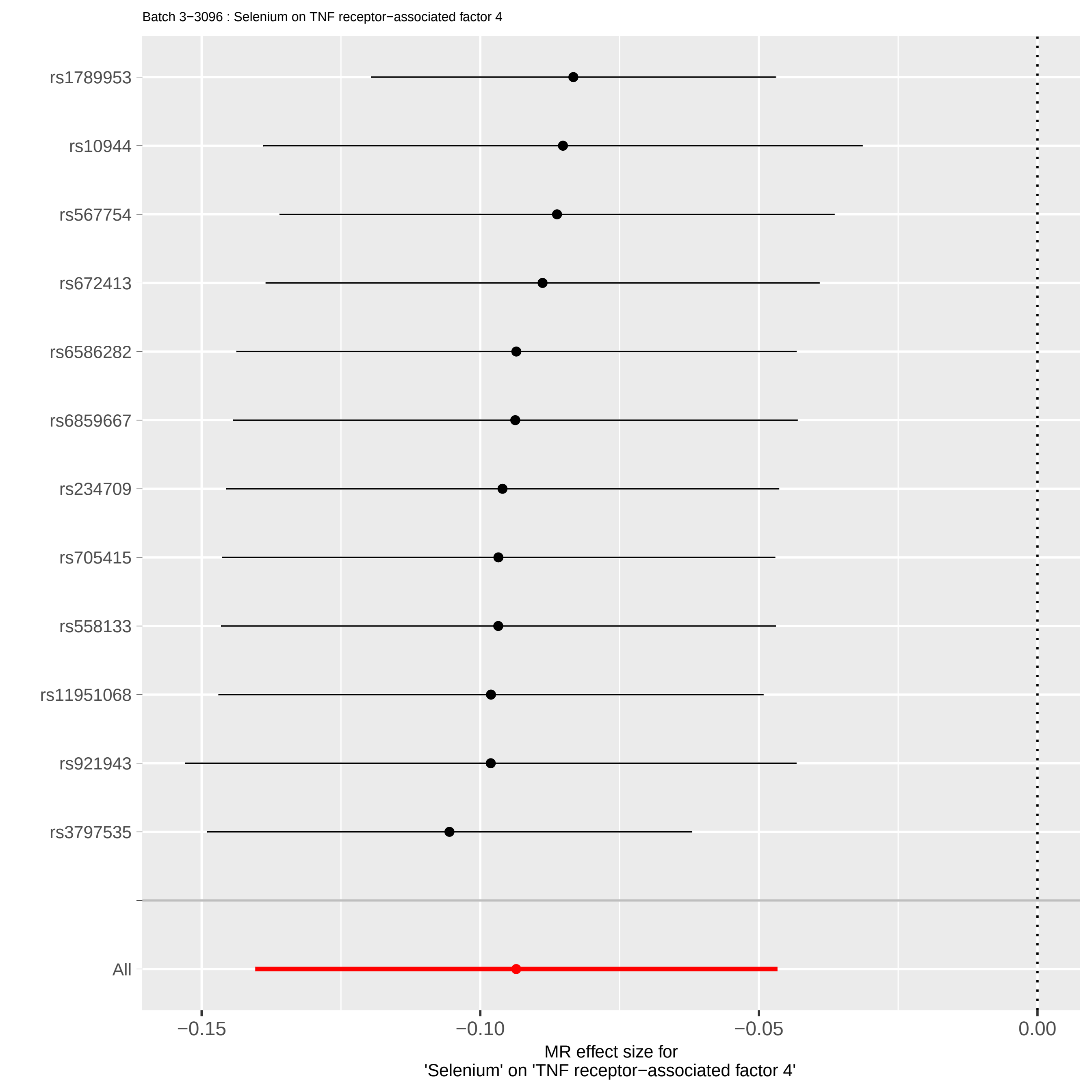

Batch 3−3096 : Selenium on TNF receptor−associated factor 4
rs1789953
rs10944
rs567754
rs672413
rs6586282
rs6859667
rs234709
rs705415
rs558133
rs11951068
rs921943
rs3797535
All
−0.15
−0.10
−0.05
0.00
MR effect size for
'Selenium' on 'TNF receptor−associated factor 4'

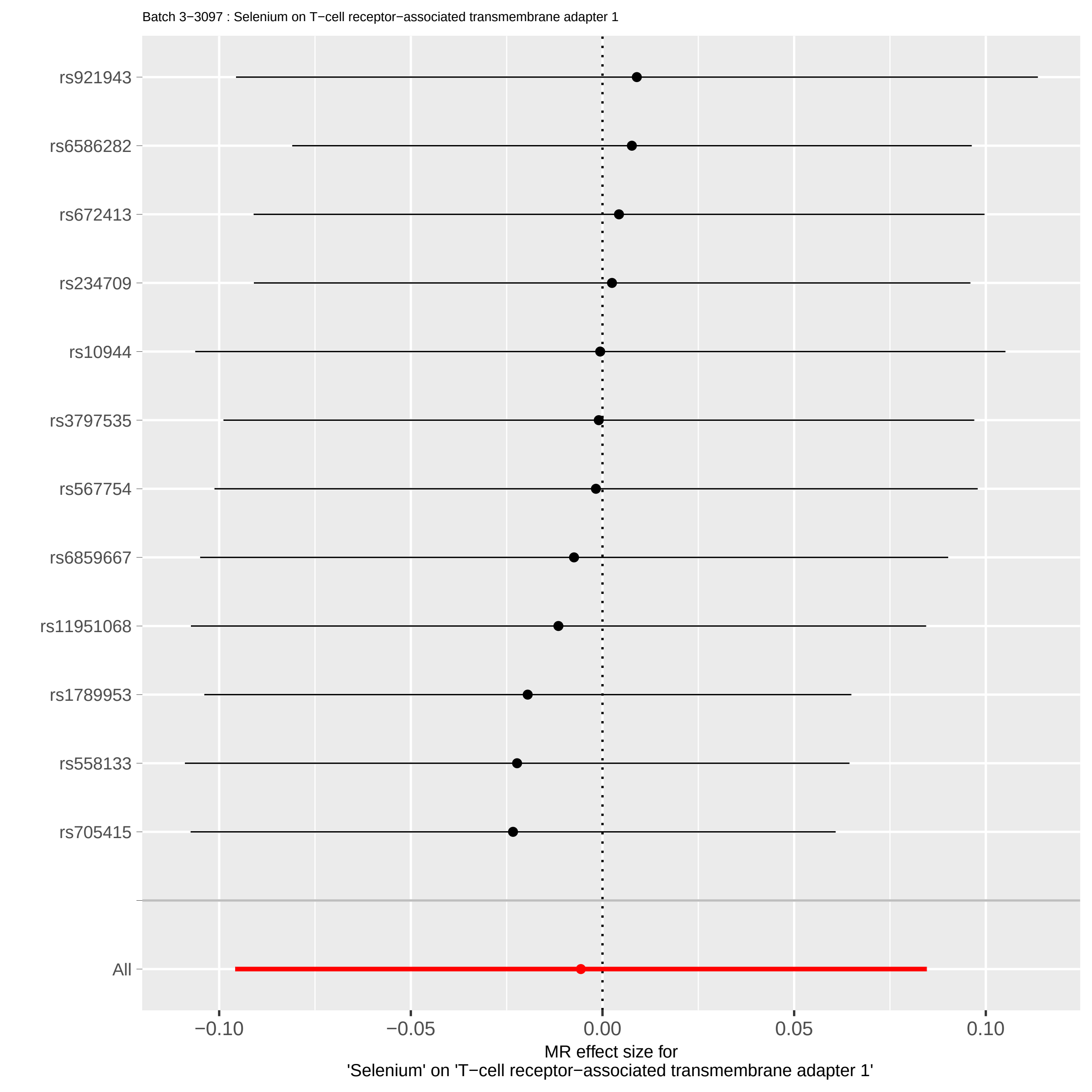

Batch 3−3097 : Selenium on T−cell receptor−associated transmembrane adapter 1
rs921943
rs6586282
rs672413
rs234709
rs10944
rs3797535
rs567754
rs6859667
rs11951068
rs1789953
rs558133
rs705415
All
−0.10
−0.05
0.00
0.05
0.10
MR effect size for
'Selenium' on 'T−cell receptor−associated transmembrane adapter 1'

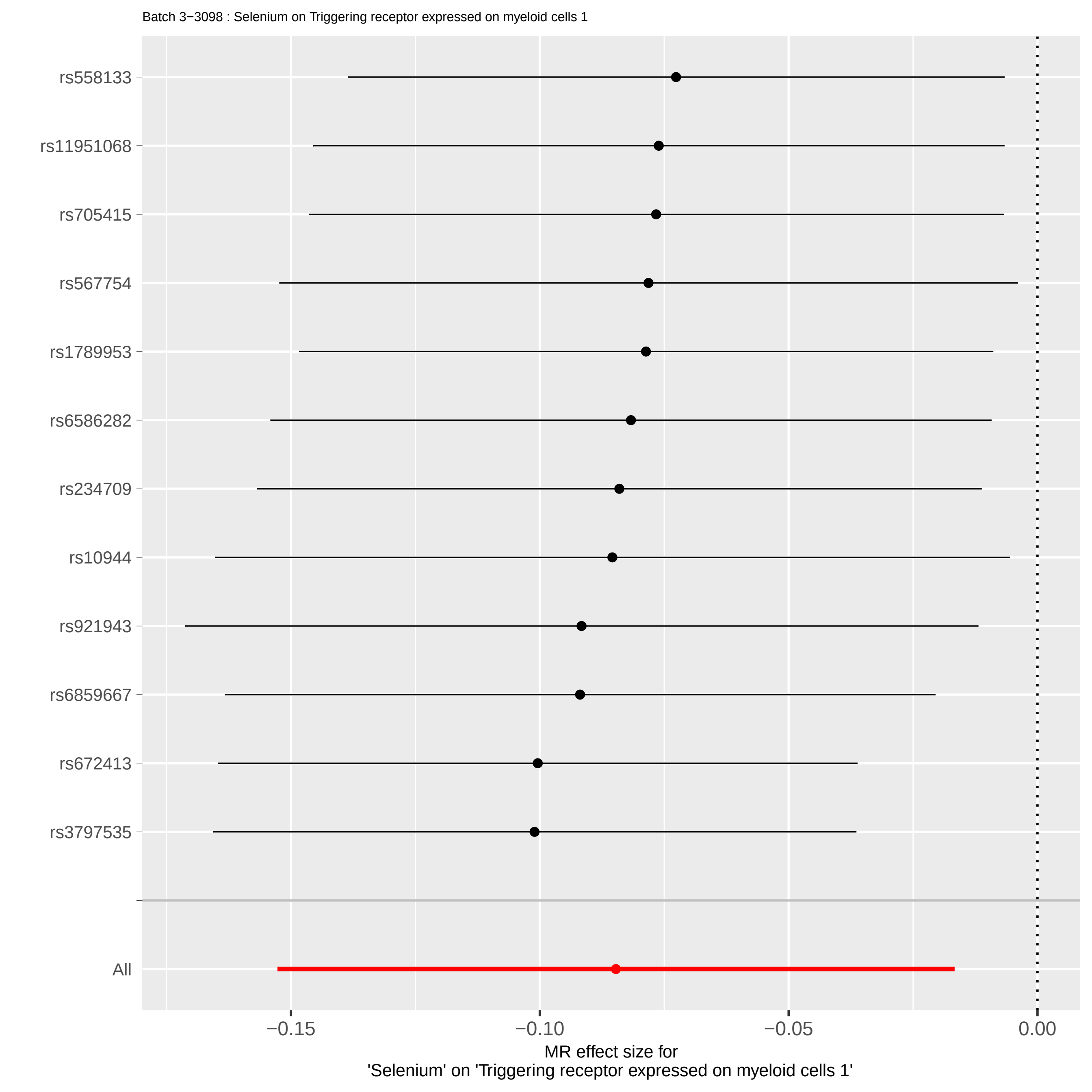

Batch 3−3098 : Selenium on Triggering receptor expressed on myeloid cells 1
rs558133
rs11951068
rs705415
rs567754
rs1789953
rs6586282
rs234709
rs10944
rs921943
rs6859667
rs672413
rs3797535
All
−0.15
−0.10
−0.05
0.00
MR effect size for
'Selenium' on 'Triggering receptor expressed on myeloid cells 1'

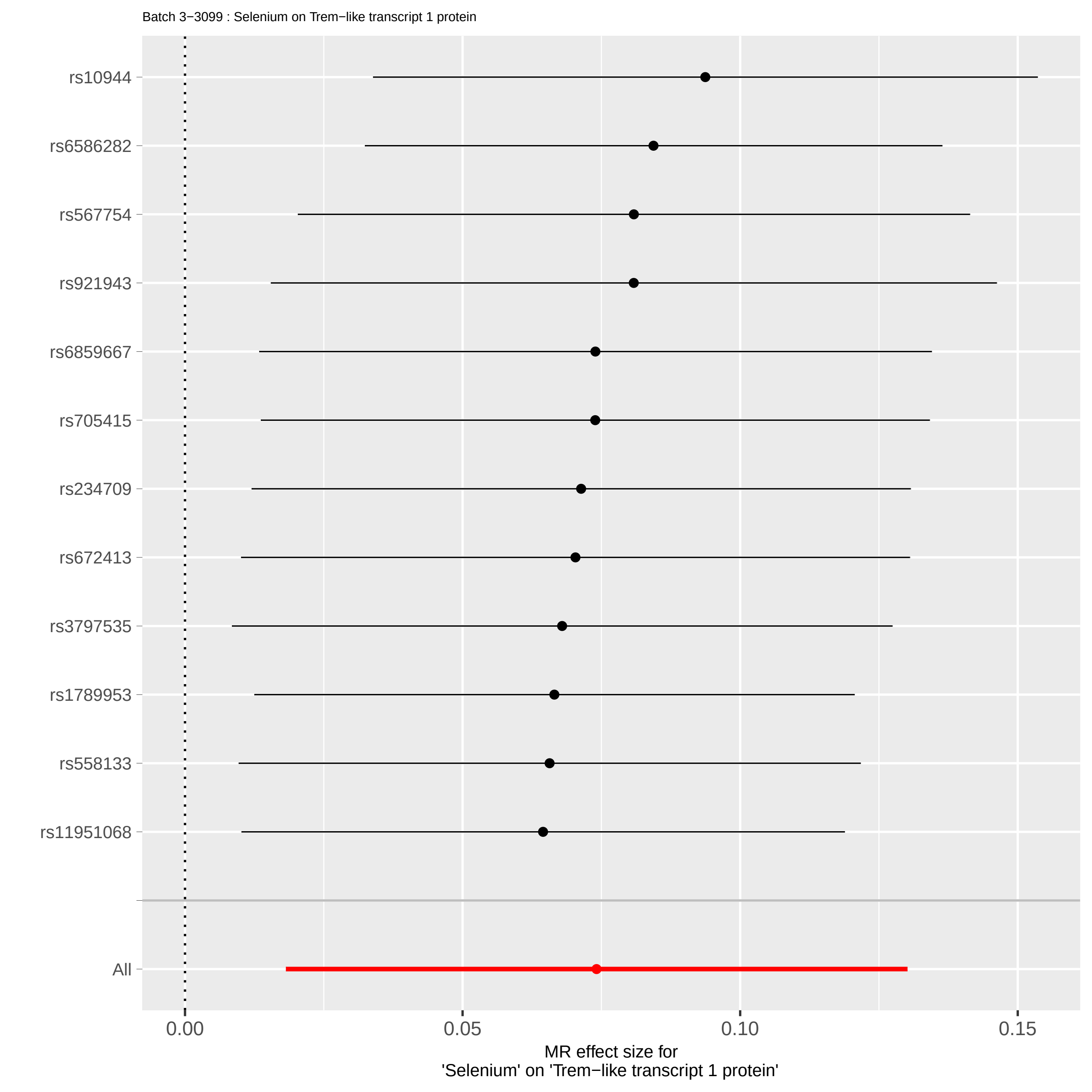

Batch 3−3099 : Selenium on Trem−like transcript 1 protein
rs10944
rs6586282
rs567754
rs921943
rs6859667
rs705415
rs234709
rs672413
rs3797535
rs1789953
rs558133
rs11951068
All
0.00
0.05
0.10
0.15
MR effect size for
'Selenium' on 'Trem−like transcript 1 protein'

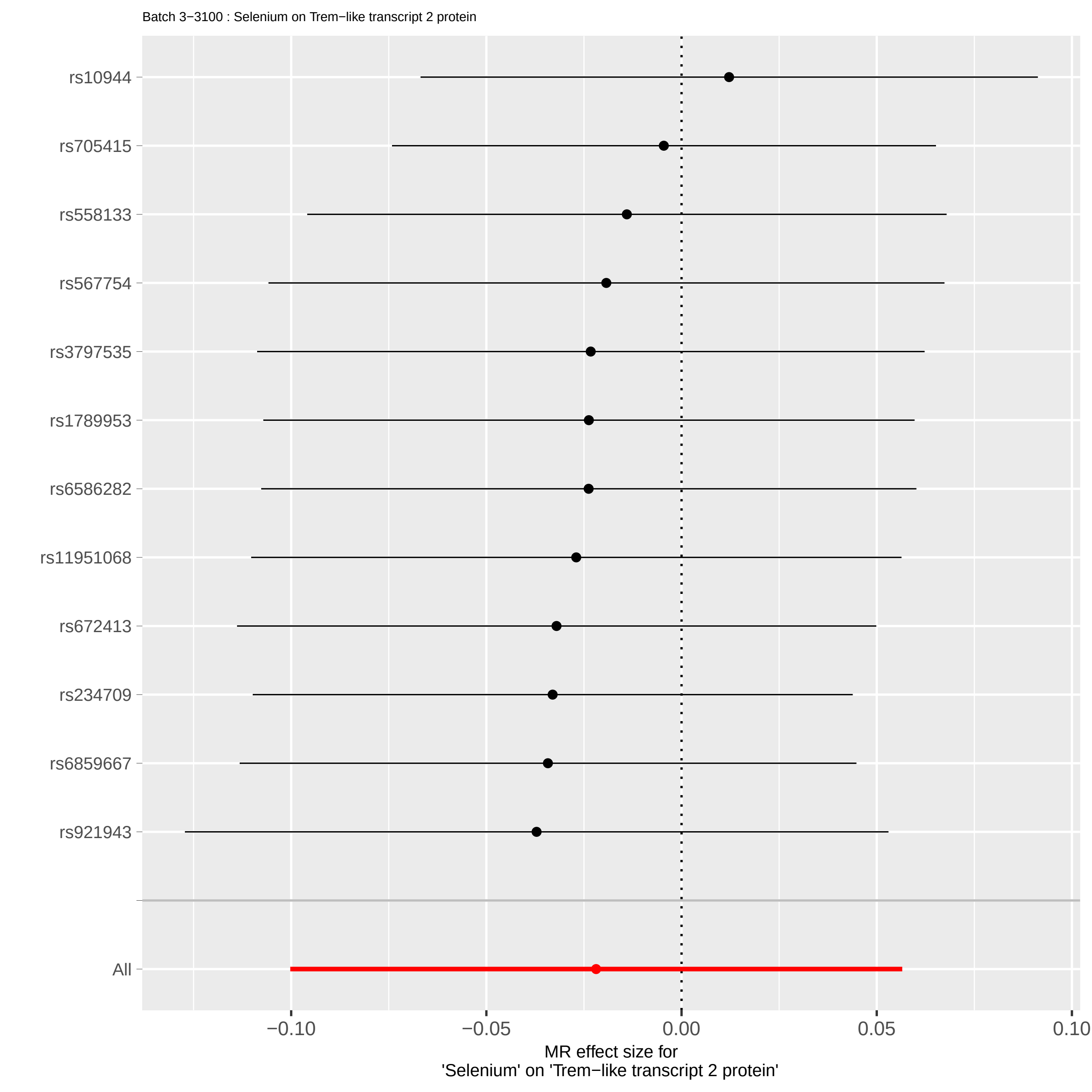

Batch 3−3100 : Selenium on Trem−like transcript 2 protein
rs10944
rs705415
rs558133
rs567754
rs3797535
rs1789953
rs6586282
rs11951068
rs672413
rs234709
rs6859667
rs921943
All
−0.10
−0.05
0.00
0.05
0.10
MR effect size for
'Selenium' on 'Trem−like transcript 2 protein'

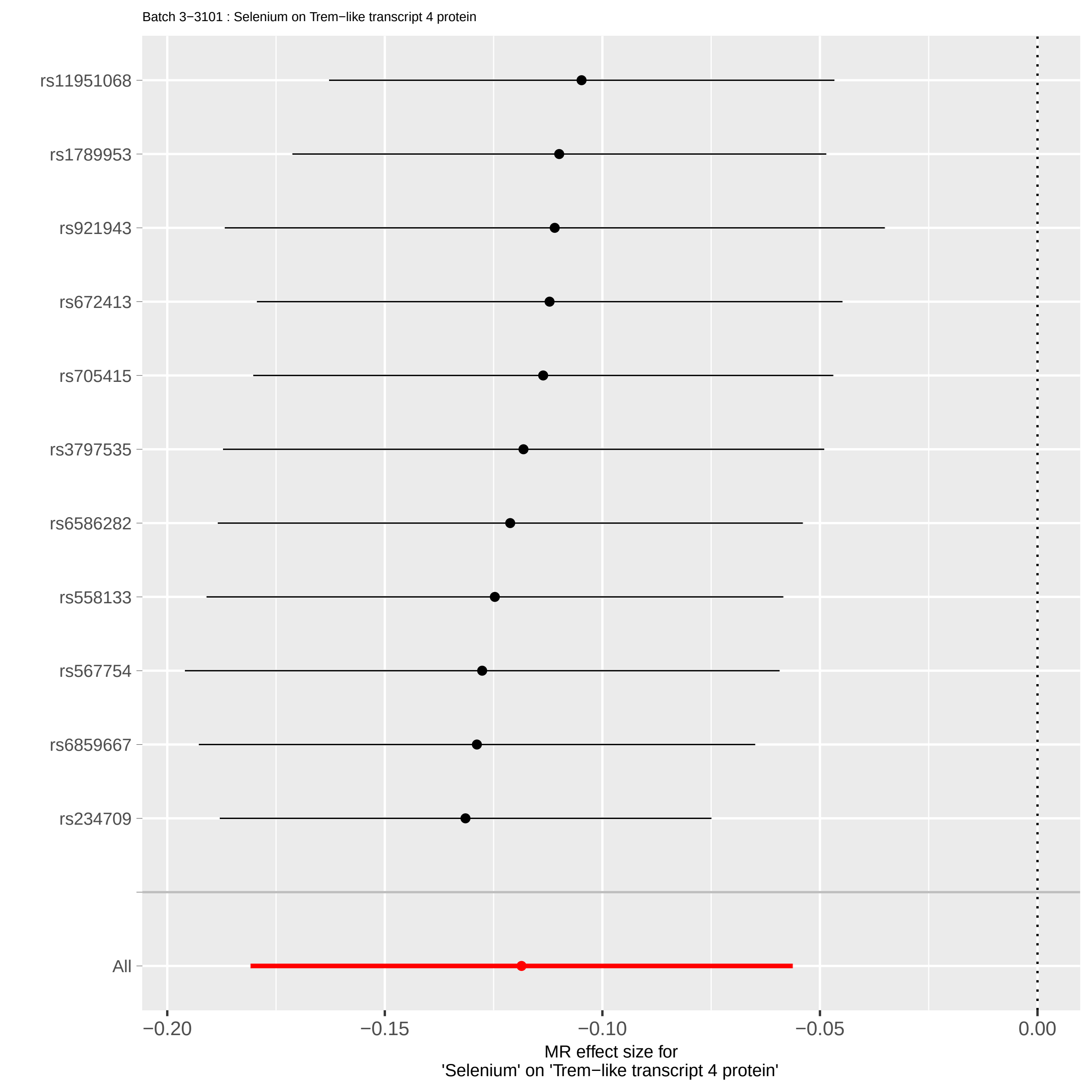

Batch 3−3101 : Selenium on Trem−like transcript 4 protein
rs11951068
rs1789953
rs921943
rs672413
rs705415
rs3797535
rs6586282
rs558133
rs567754
rs6859667
rs234709
All
−0.20
−0.15
−0.10
−0.05
0.00
MR effect size for
'Selenium' on 'Trem−like transcript 4 protein'

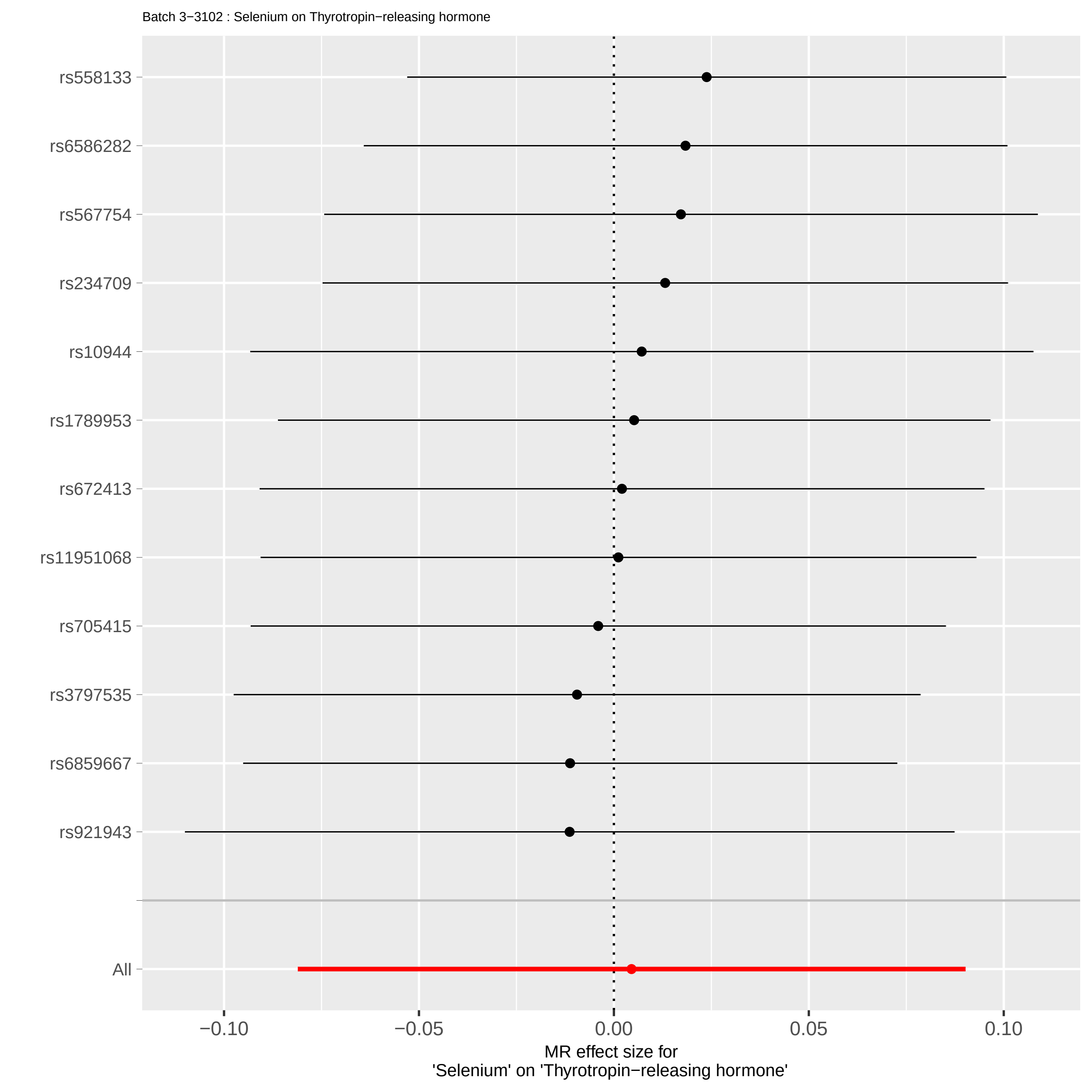

Batch 3−3102 : Selenium on Thyrotropin−releasing hormone
rs558133
rs6586282
rs567754
rs234709
rs10944
rs1789953
rs672413
rs11951068
rs705415
rs3797535
rs6859667
rs921943
All
−0.10
−0.05
0.00
0.05
0.10
MR effect size for
'Selenium' on 'Thyrotropin−releasing hormone'

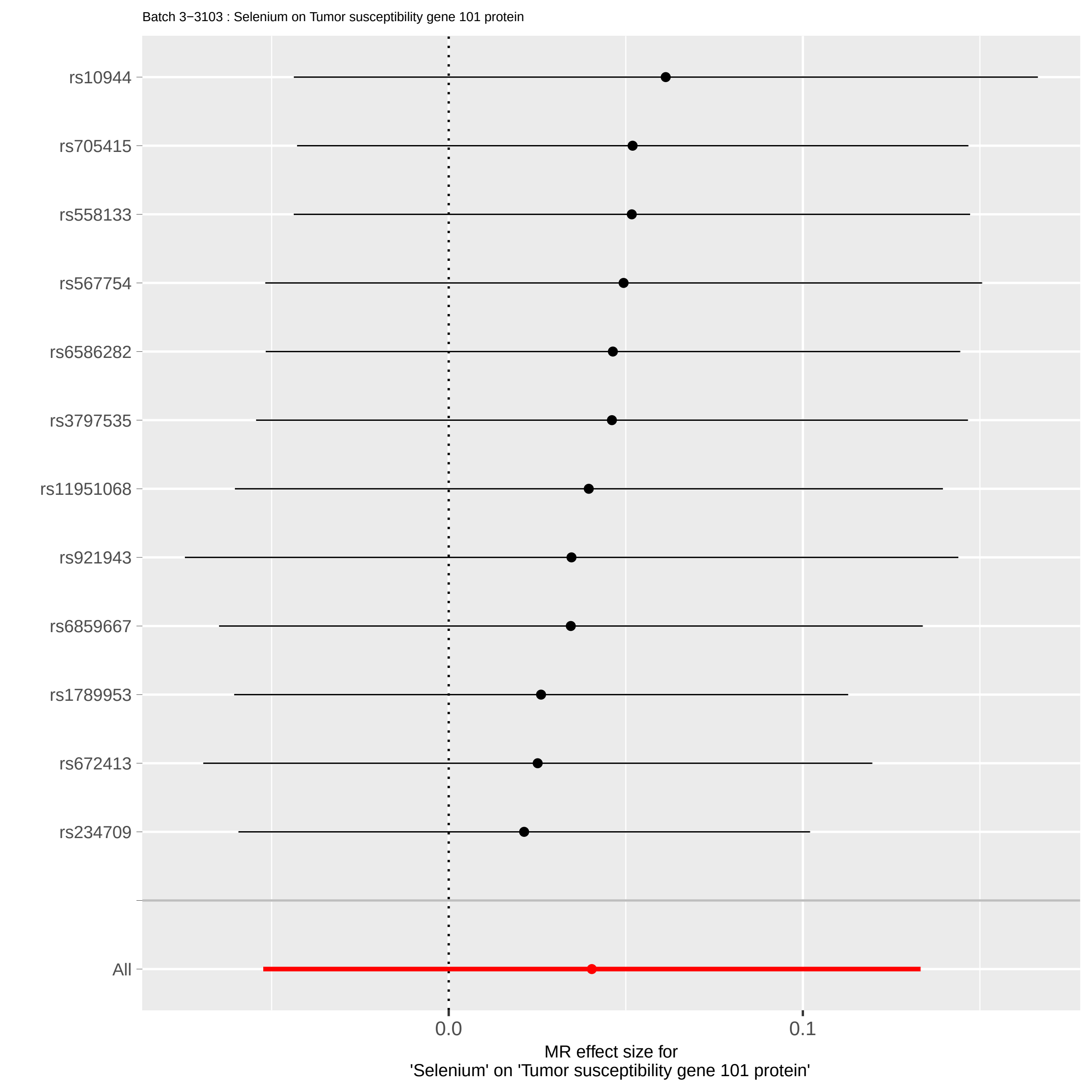

Batch 3−3103 : Selenium on Tumor susceptibility gene 101 protein
rs10944
rs705415
rs558133
rs567754
rs6586282
rs3797535
rs11951068
rs921943
rs6859667
rs1789953
rs672413
rs234709
All
0.0
0.1
MR effect size for
'Selenium' on 'Tumor susceptibility gene 101 protein'

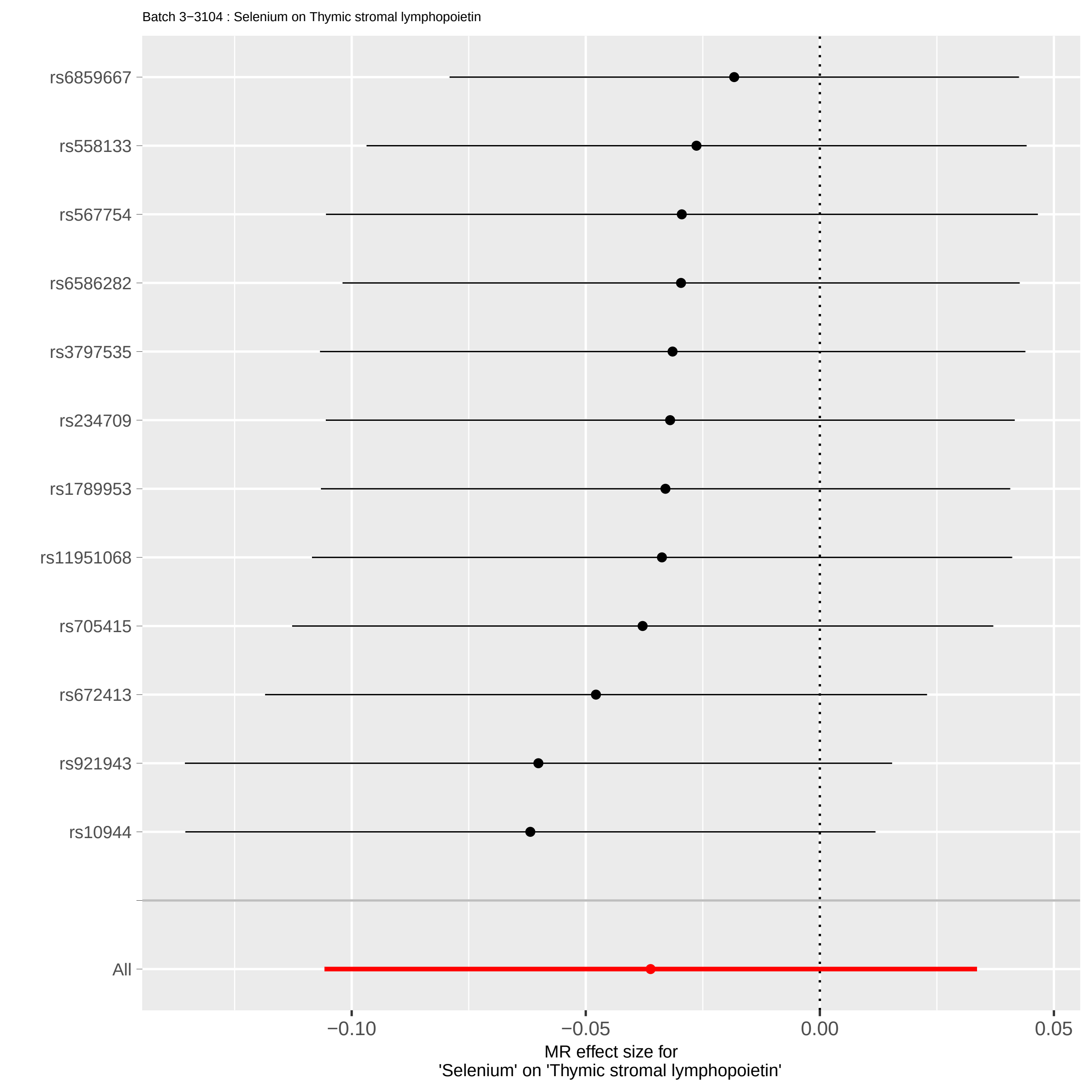

Batch 3−3104 : Selenium on Thymic stromal lymphopoietin
rs6859667
rs558133
rs567754
rs6586282
rs3797535
rs234709
rs1789953
rs11951068
rs705415
rs672413
rs921943
rs10944
All
−0.10
−0.05
0.00
0.05
MR effect size for
'Selenium' on 'Thymic stromal lymphopoietin'

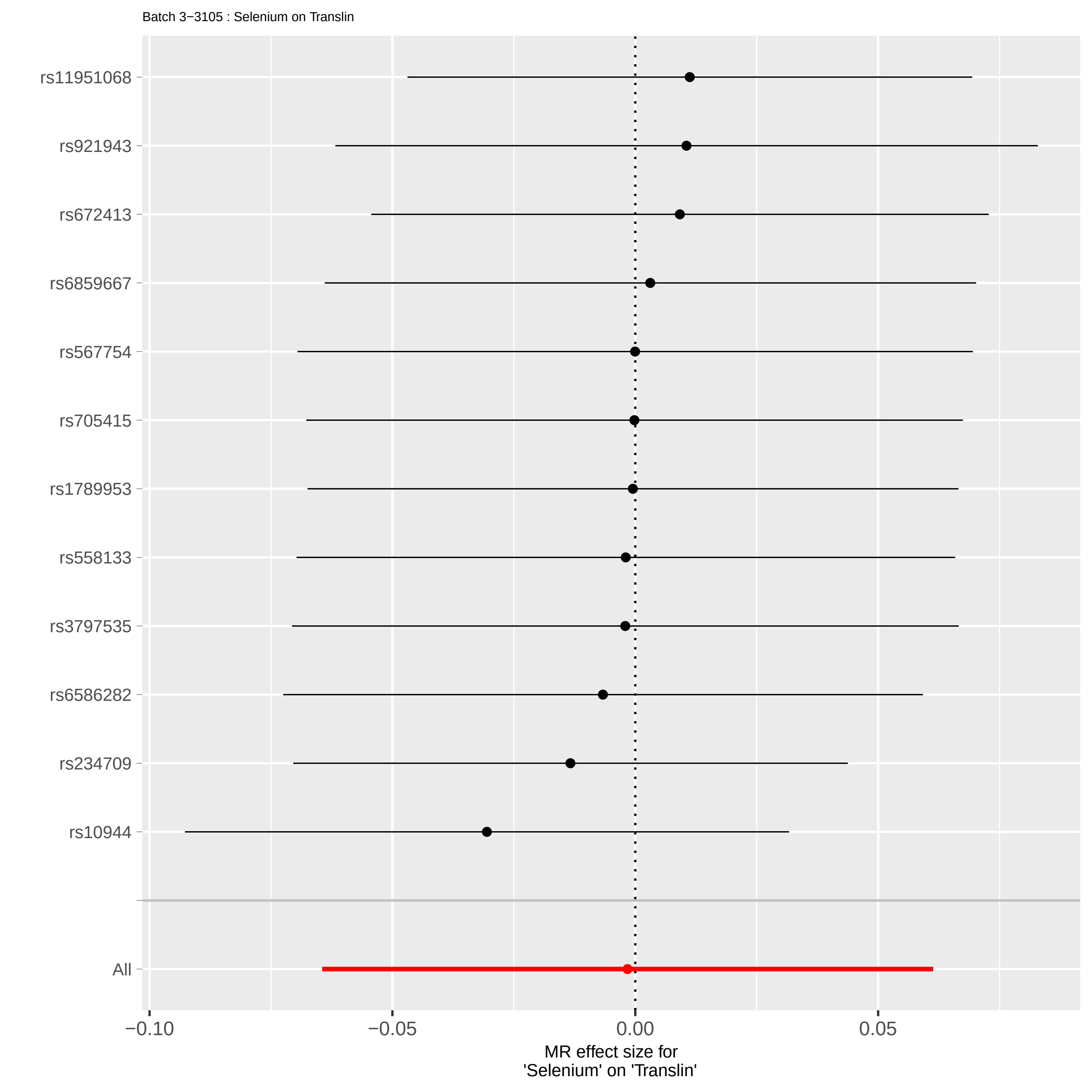

Batch 3−3105 : Selenium on Translin
rs11951068
rs921943
rs672413
rs6859667
rs567754
rs705415
rs1789953
rs558133
rs3797535
rs6586282
rs234709
rs10944
All
−0.10
−0.05
0.00
0.05
MR effect size for
'Selenium' on 'Translin'

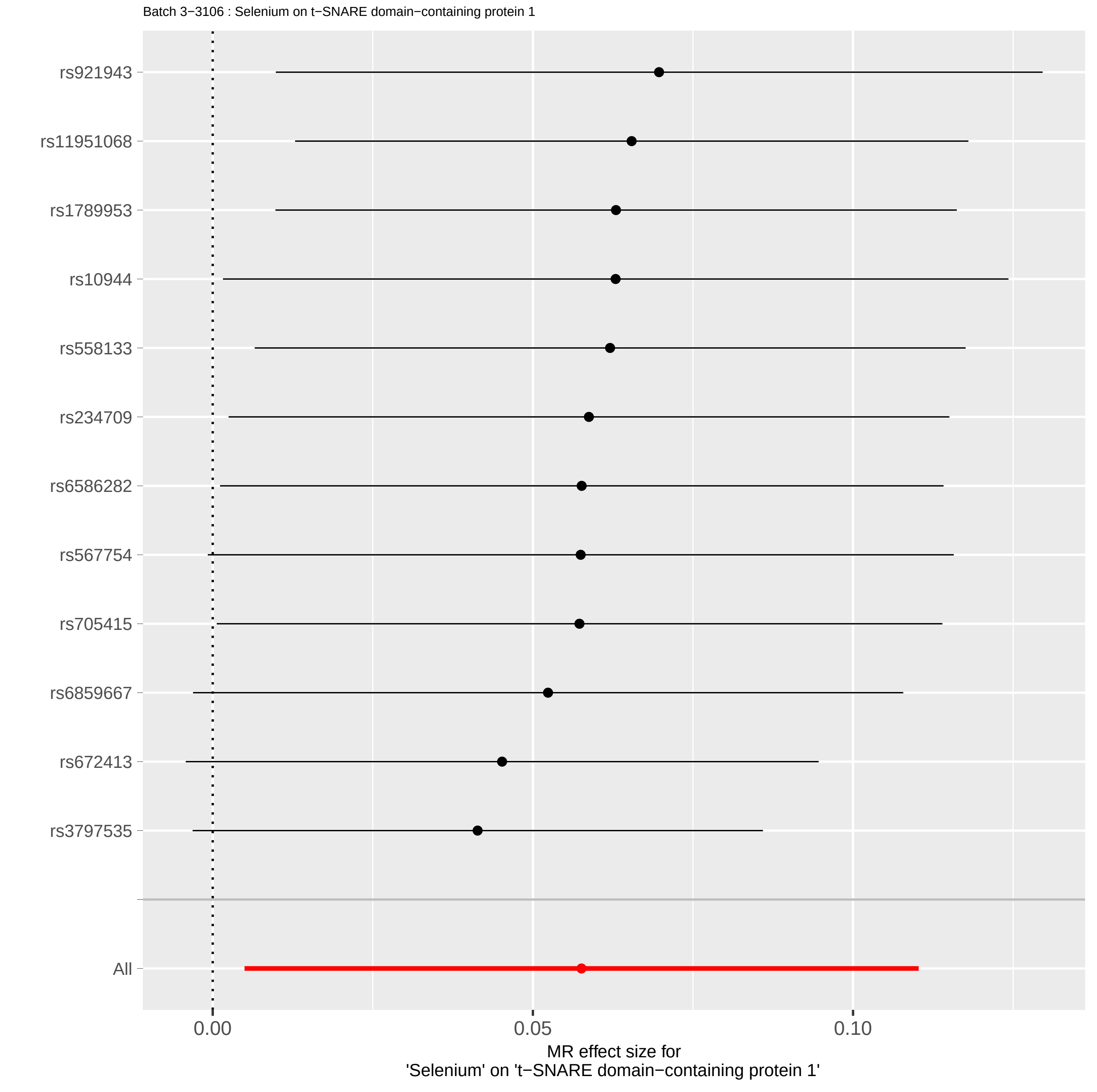
Batch 3−3106 : Selenium on t−SNARE domain−containing protein 1
rs921943
rs11951068
rs1789953
rs10944
rs558133
rs234709
rs6586282
rs567754
rs705415
rs6859667
rs672413
rs3797535
All
0.00
0.05
0.10
MR effect size for
'Selenium' on 't−SNARE domain−containing protein 1'

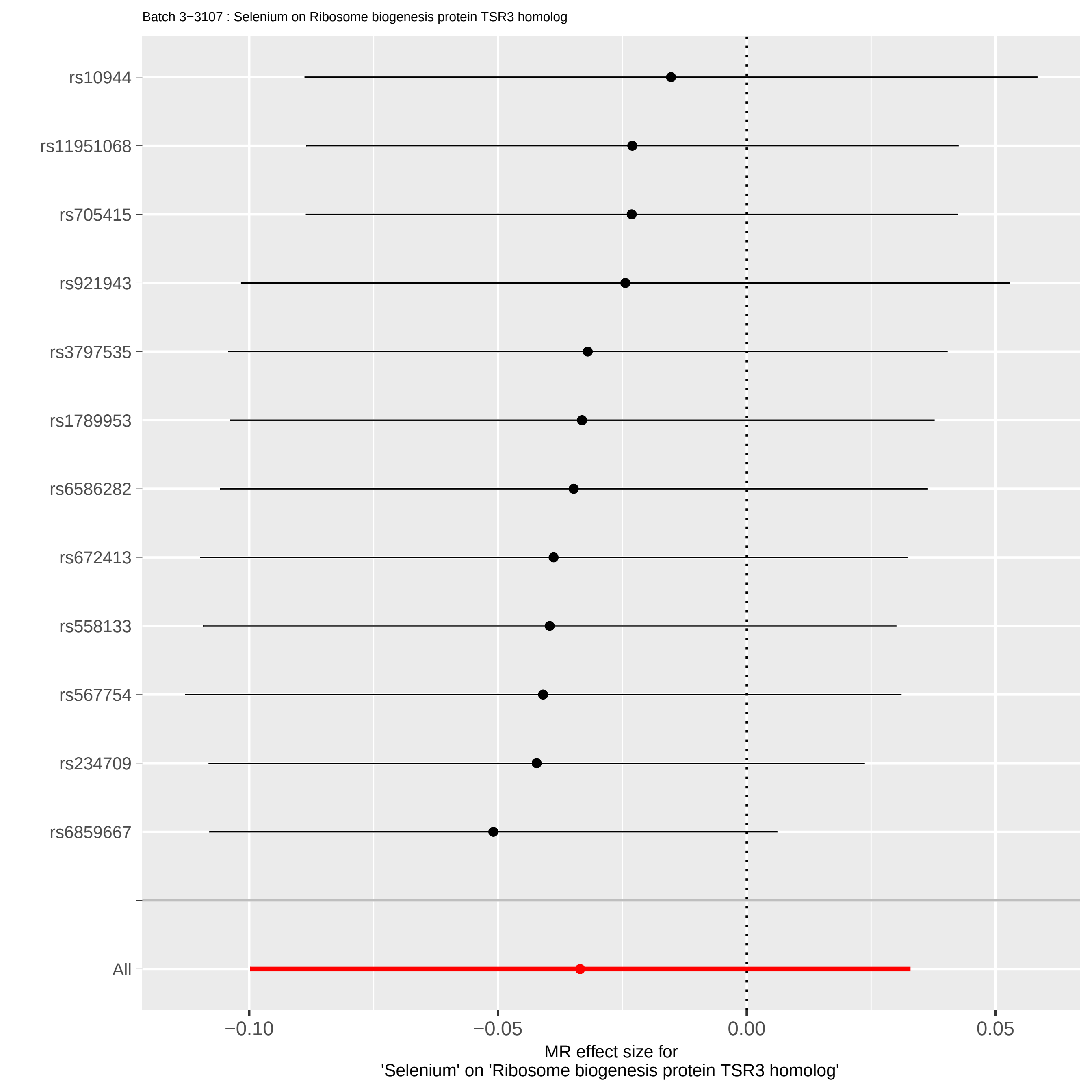

Batch 3−3107 : Selenium on Ribosome biogenesis protein TSR3 homolog
rs10944
rs11951068
rs705415
rs921943
rs3797535
rs1789953
rs6586282
rs672413
rs558133
rs567754
rs234709
rs6859667
All
−0.10
−0.05
0.00
0.05
MR effect size for
'Selenium' on 'Ribosome biogenesis protein TSR3 homolog'

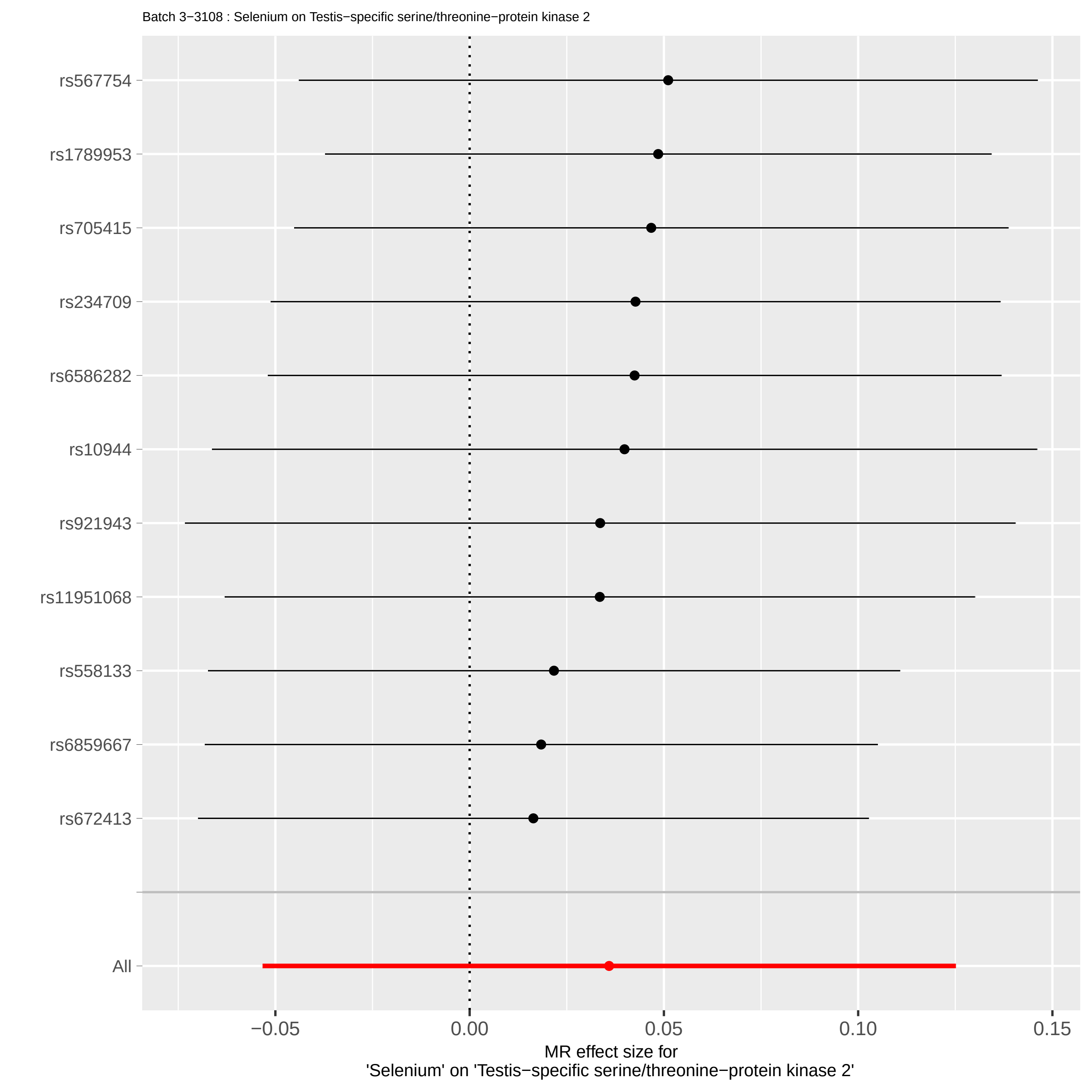

Batch 3−3108 : Selenium on Testis−specific serine/threonine−protein kinase 2
rs567754
rs1789953
rs705415
rs234709
rs6586282
rs10944
rs921943
rs11951068
rs558133
rs6859667
rs672413
All
−0.05
0.00
0.05
0.10
0.15
MR effect size for
'Selenium' on 'Testis−specific serine/threonine−protein kinase 2'

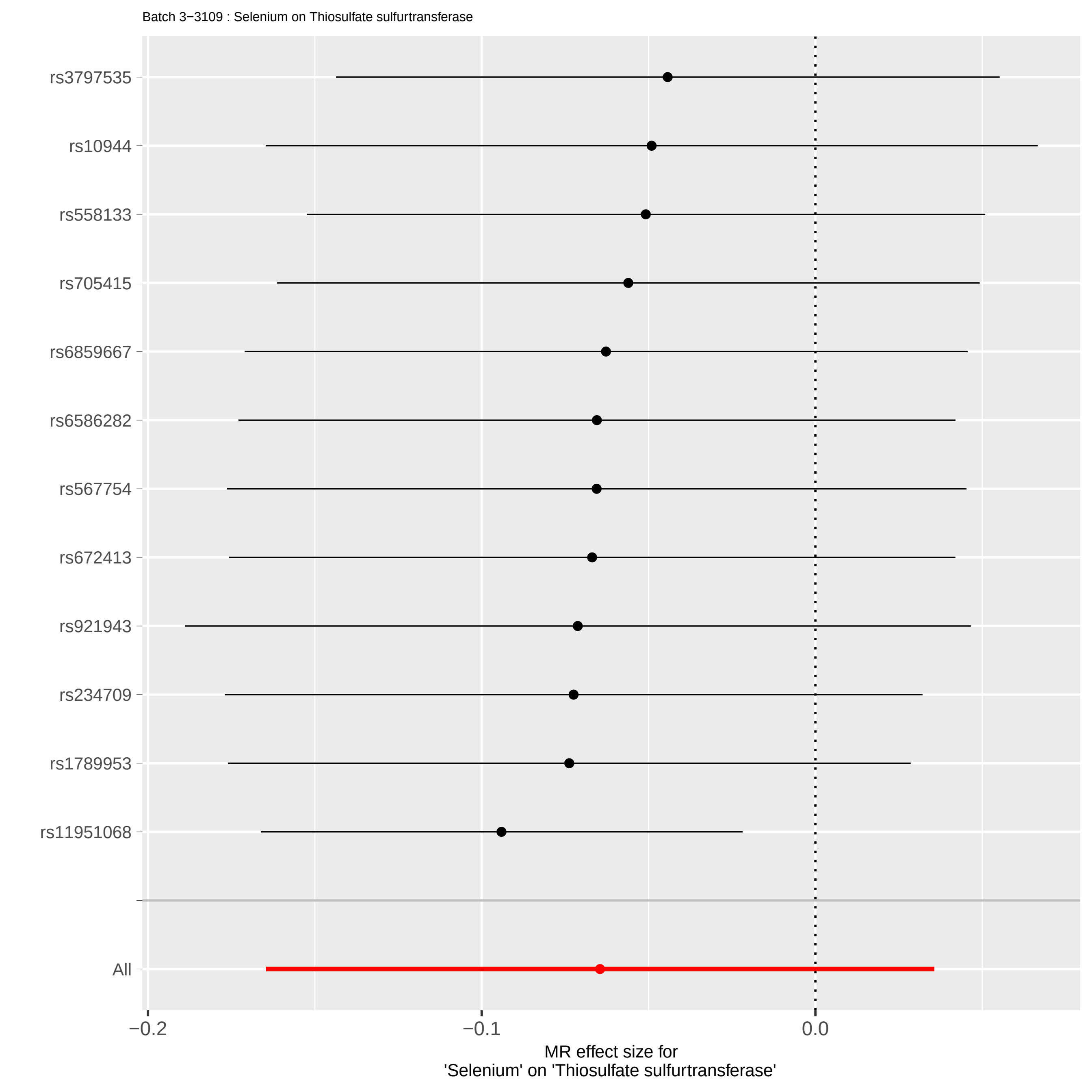

Batch 3−3109 : Selenium on Thiosulfate sulfurtransferase
rs3797535
rs10944
rs558133
rs705415
rs6859667
rs6586282
rs567754
rs672413
rs921943
rs234709
rs1789953
rs11951068
All
−0.2
−0.1
0.0
MR effect size for
'Selenium' on 'Thiosulfate sulfurtransferase'

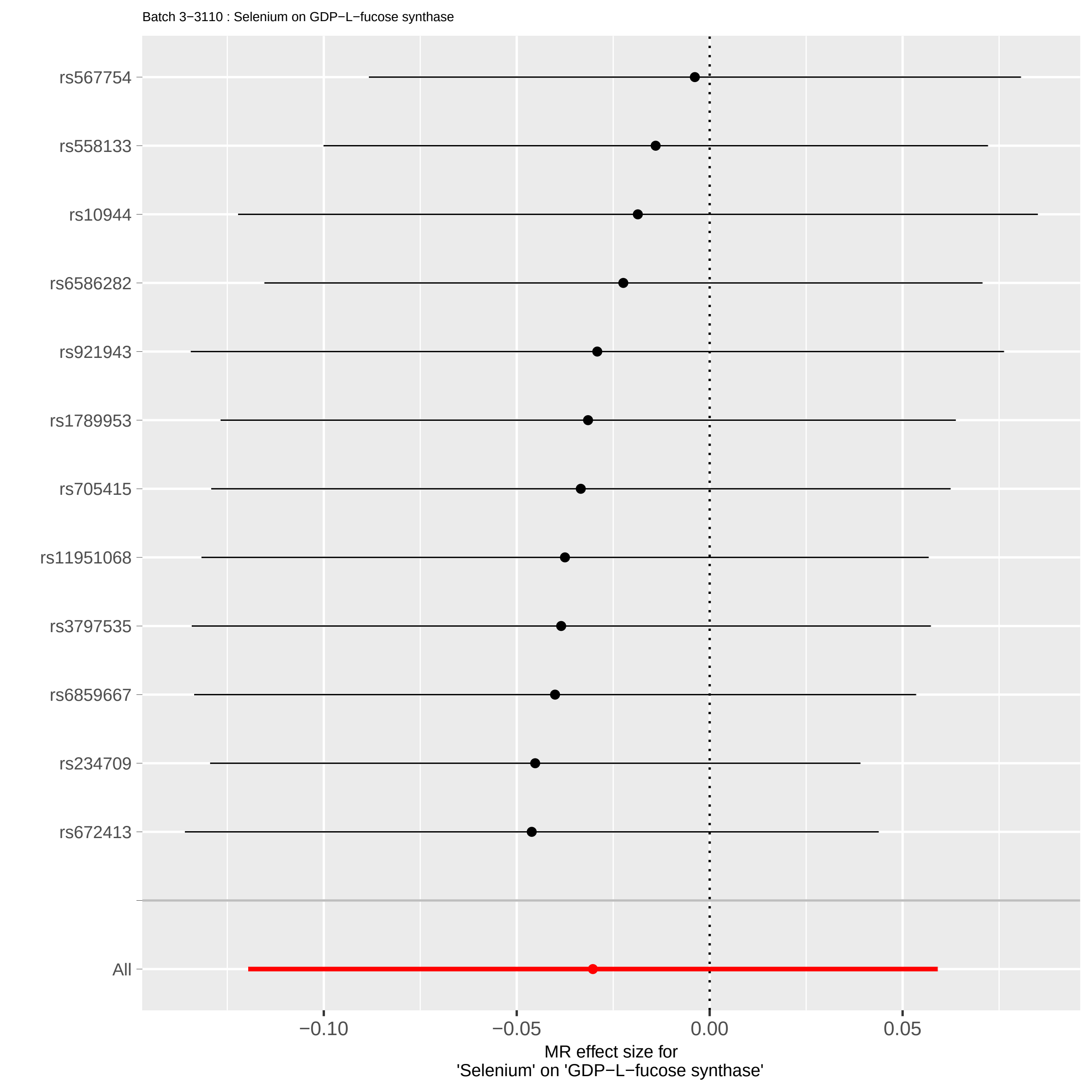

Batch 3−3110 : Selenium on GDP−L−fucose synthase
rs567754
rs558133
rs10944
rs6586282
rs921943
rs1789953
rs705415
rs11951068
rs3797535
rs6859667
rs234709
rs672413
All
−0.10
−0.05
0.00
0.05
MR effect size for
'Selenium' on 'GDP−L−fucose synthase'

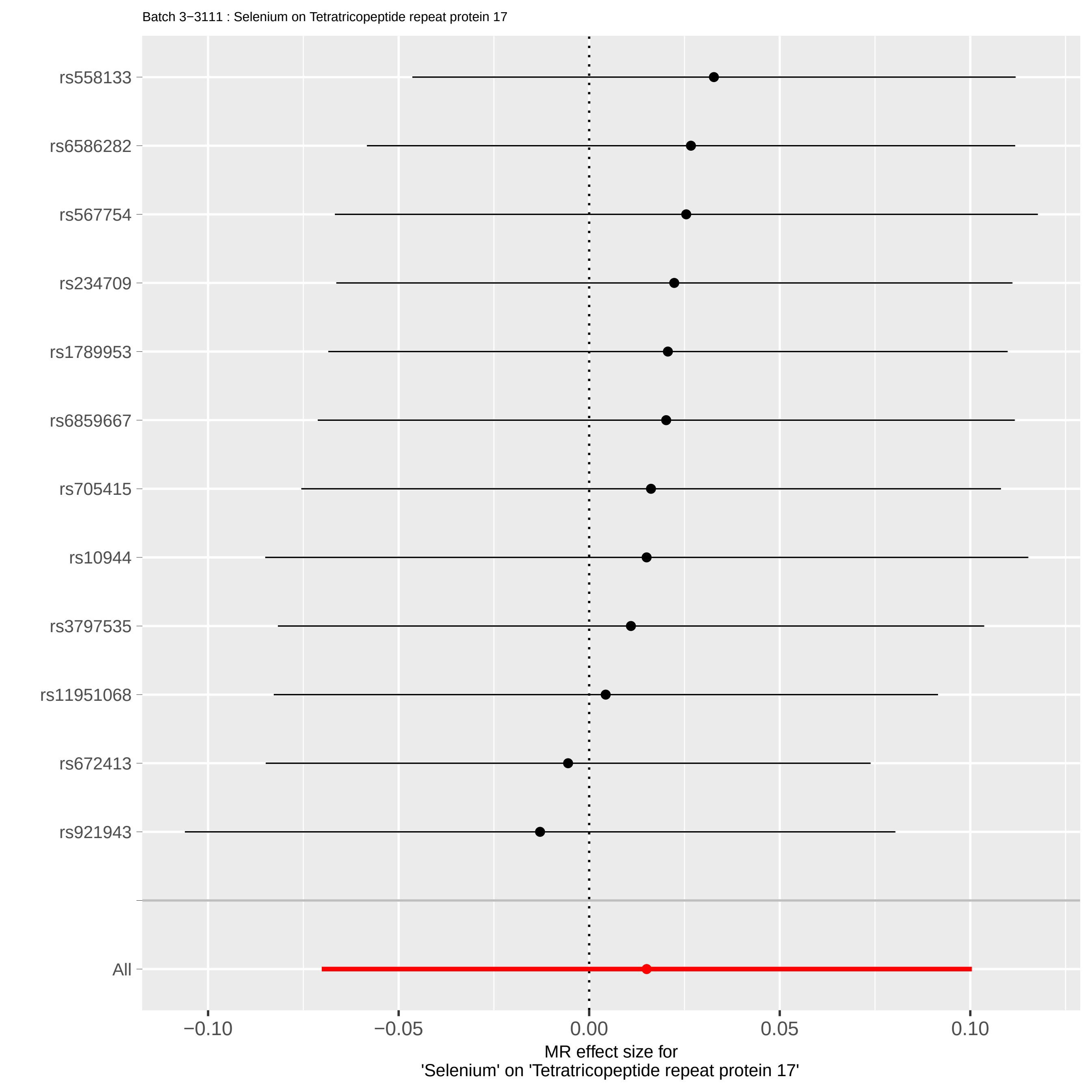

Batch 3−3111 : Selenium on Tetratricopeptide repeat protein 17
rs558133
rs6586282
rs567754
rs234709
rs1789953
rs6859667
rs705415
rs10944
rs3797535
rs11951068
rs672413
rs921943
All
−0.10
−0.05
0.00
0.05
0.10
MR effect size for
'Selenium' on 'Tetratricopeptide repeat protein 17'

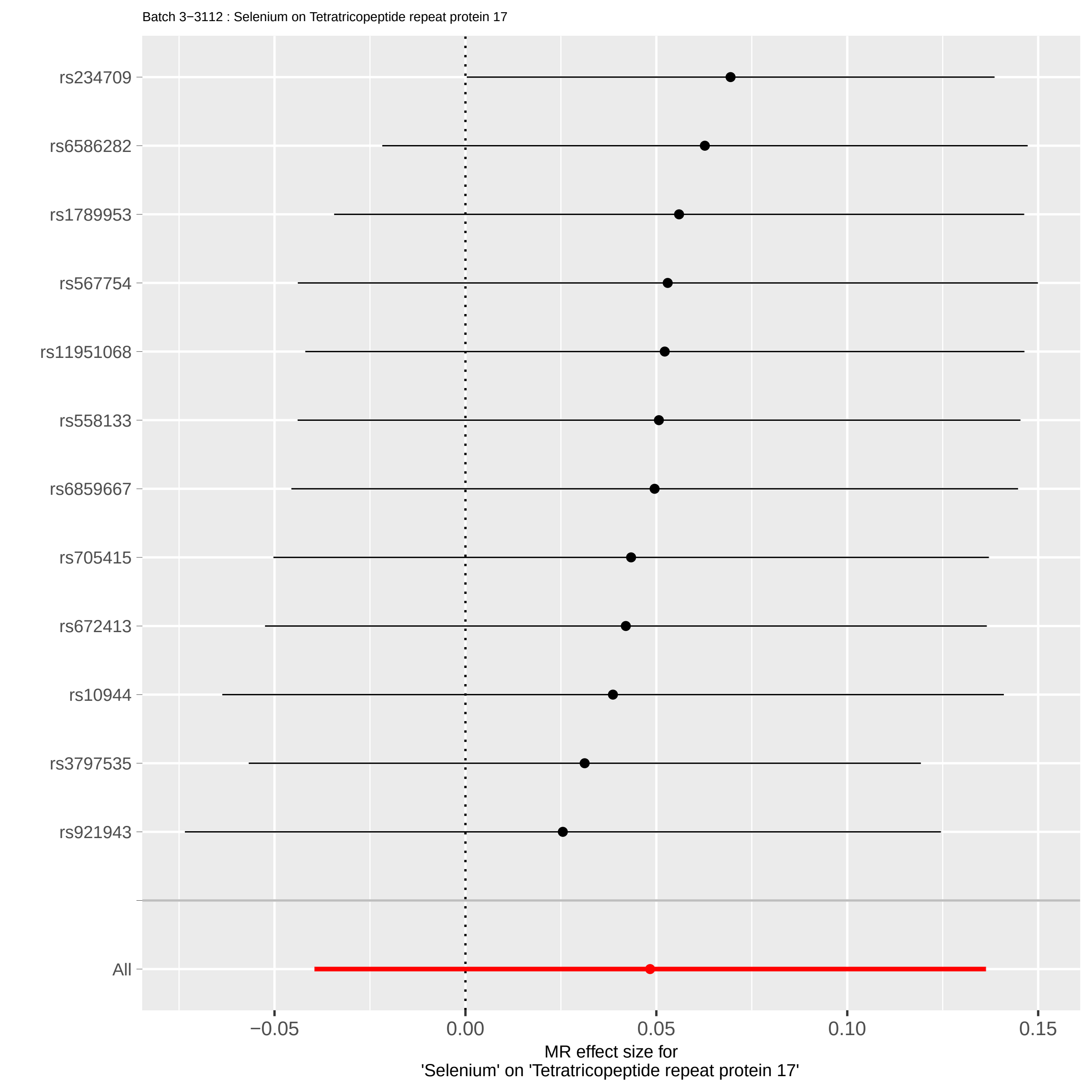

Batch 3−3112 : Selenium on Tetratricopeptide repeat protein 17
rs234709
rs6586282
rs1789953
rs567754
rs11951068
rs558133
rs6859667
rs705415
rs672413
rs10944
rs3797535
rs921943
All
−0.05
0.00
0.05
0.10
0.15
MR effect size for
'Selenium' on 'Tetratricopeptide repeat protein 17'

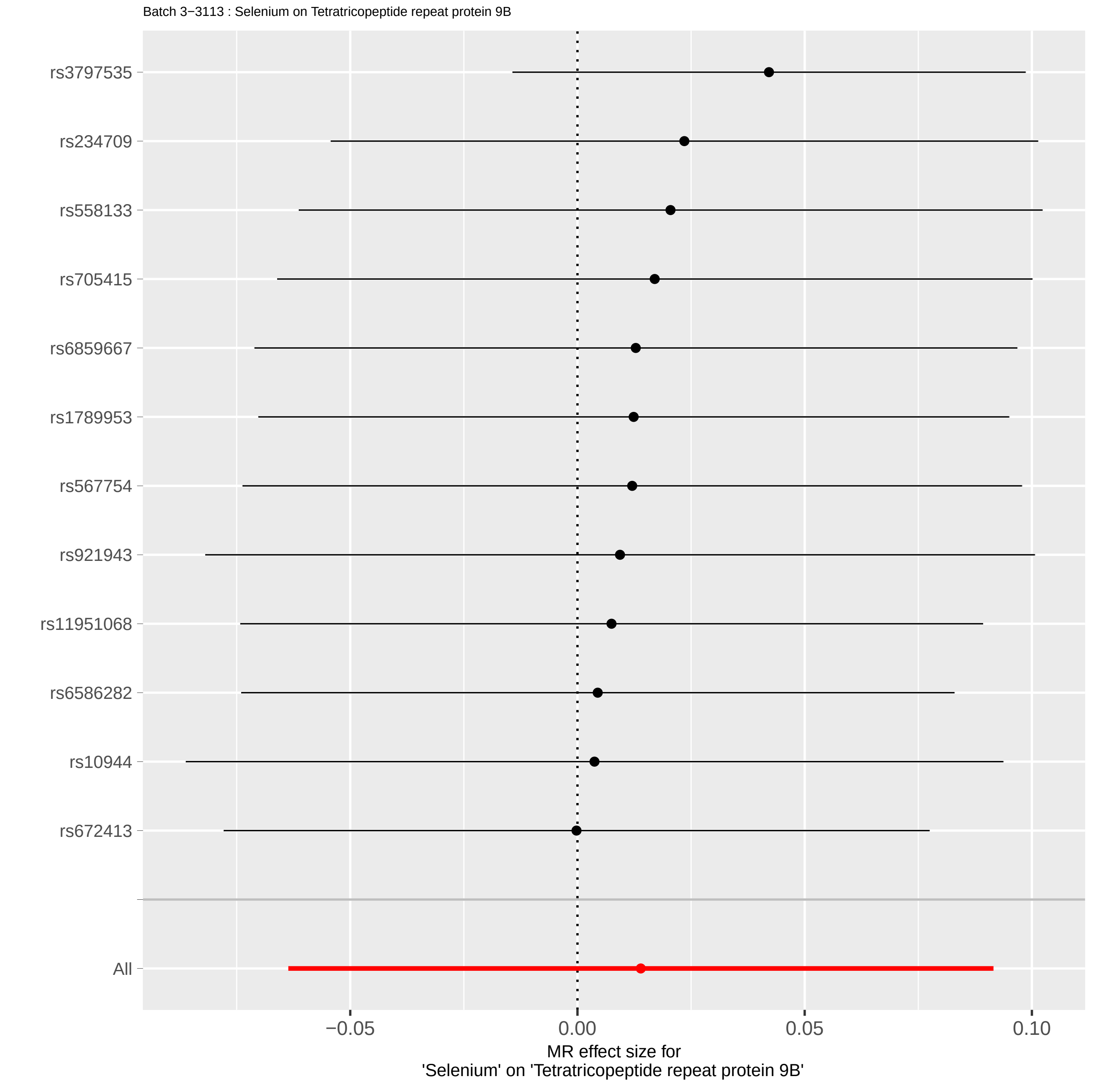

Batch 3−3113 : Selenium on Tetratricopeptide repeat protein 9B
rs3797535
rs234709
rs558133
rs705415
rs6859667
rs1789953
rs567754
rs921943
rs11951068
rs6586282
rs10944
rs672413
All
−0.05
0.00
0.05
0.10
MR effect size for
'Selenium' on 'Tetratricopeptide repeat protein 9B'

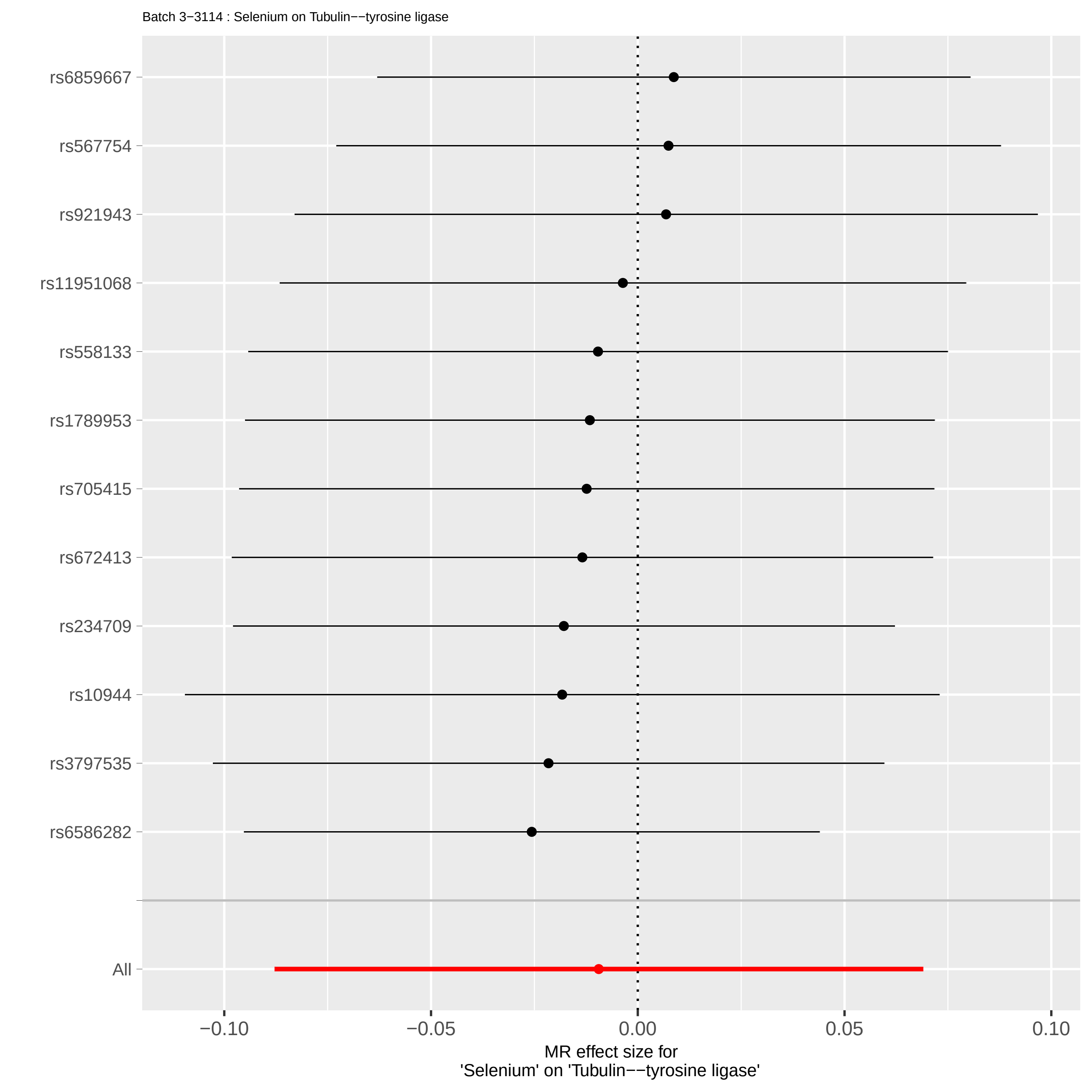

Batch 3−3114 : Selenium on Tubulin−−tyrosine ligase
rs6859667
rs567754
rs921943
rs11951068
rs558133
rs1789953
rs705415
rs672413
rs234709
rs10944
rs3797535
rs6586282
All
−0.10
−0.05
0.00
0.05
0.10
MR effect size for
'Selenium' on 'Tubulin−−tyrosine ligase'

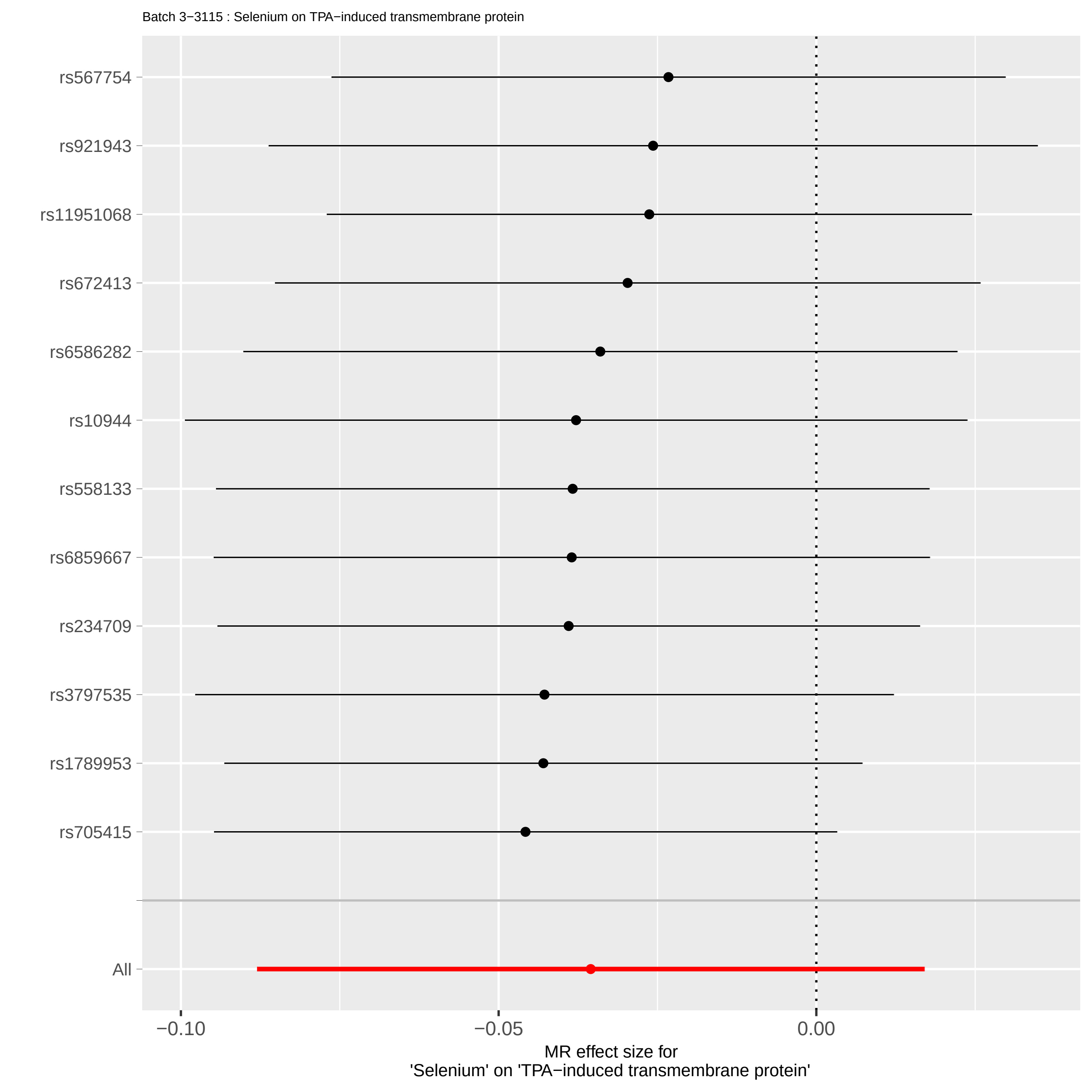

Batch 3−3115 : Selenium on TPA−induced transmembrane protein
rs567754
rs921943
rs11951068
rs672413
rs6586282
rs10944
rs558133
rs6859667
rs234709
rs3797535
rs1789953
rs705415
All
−0.10
−0.05
0.00
MR effect size for
'Selenium' on 'TPA−induced transmembrane protein'

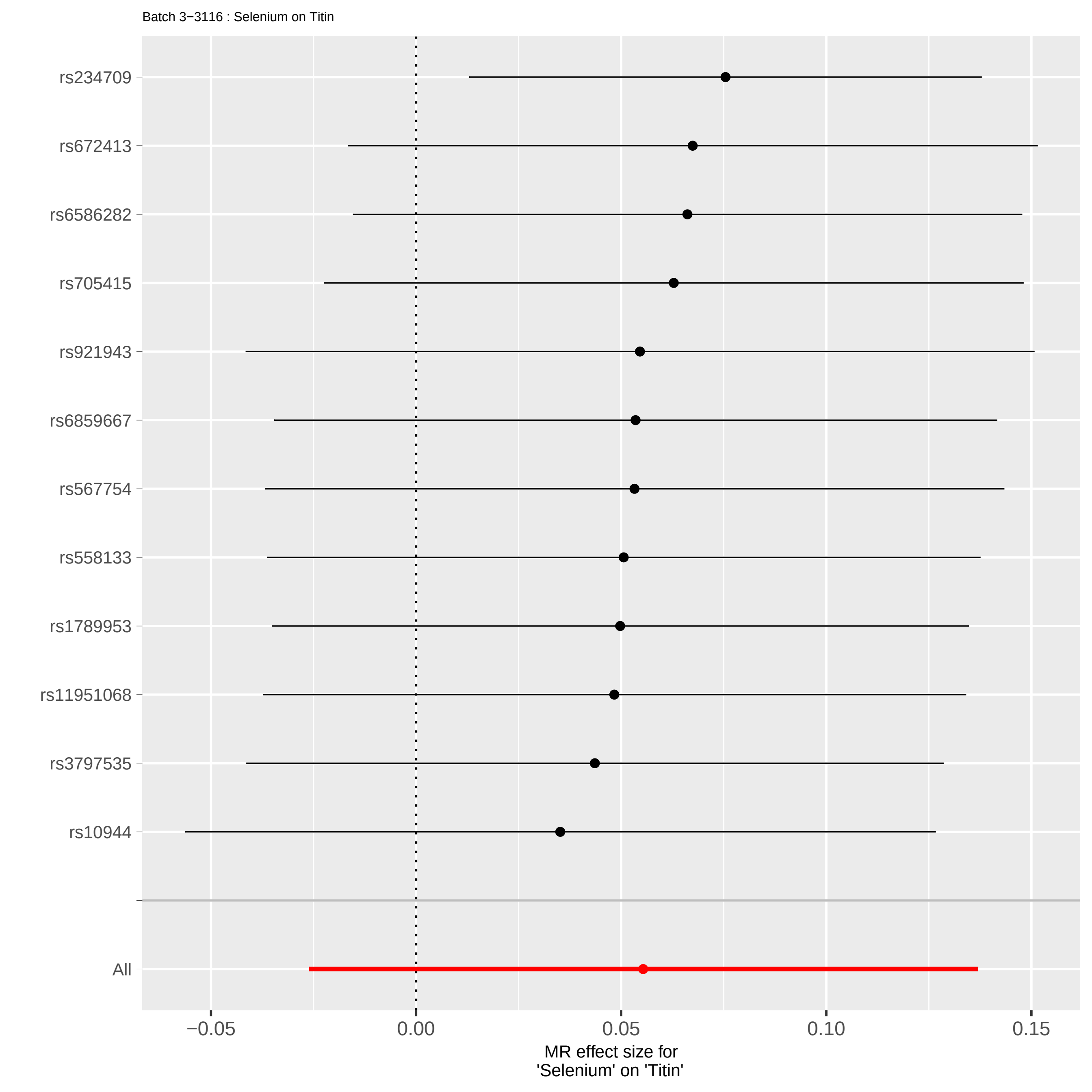

Batch 3−3116 : Selenium on Titin
rs234709
rs672413
rs6586282
rs705415
rs921943
rs6859667
rs567754
rs558133
rs1789953
rs11951068
rs3797535
rs10944
All
−0.05
0.00
0.05
0.10
0.15
MR effect size for
'Selenium' on 'Titin'

Batch 3−3117 : Selenium on Tuftelin

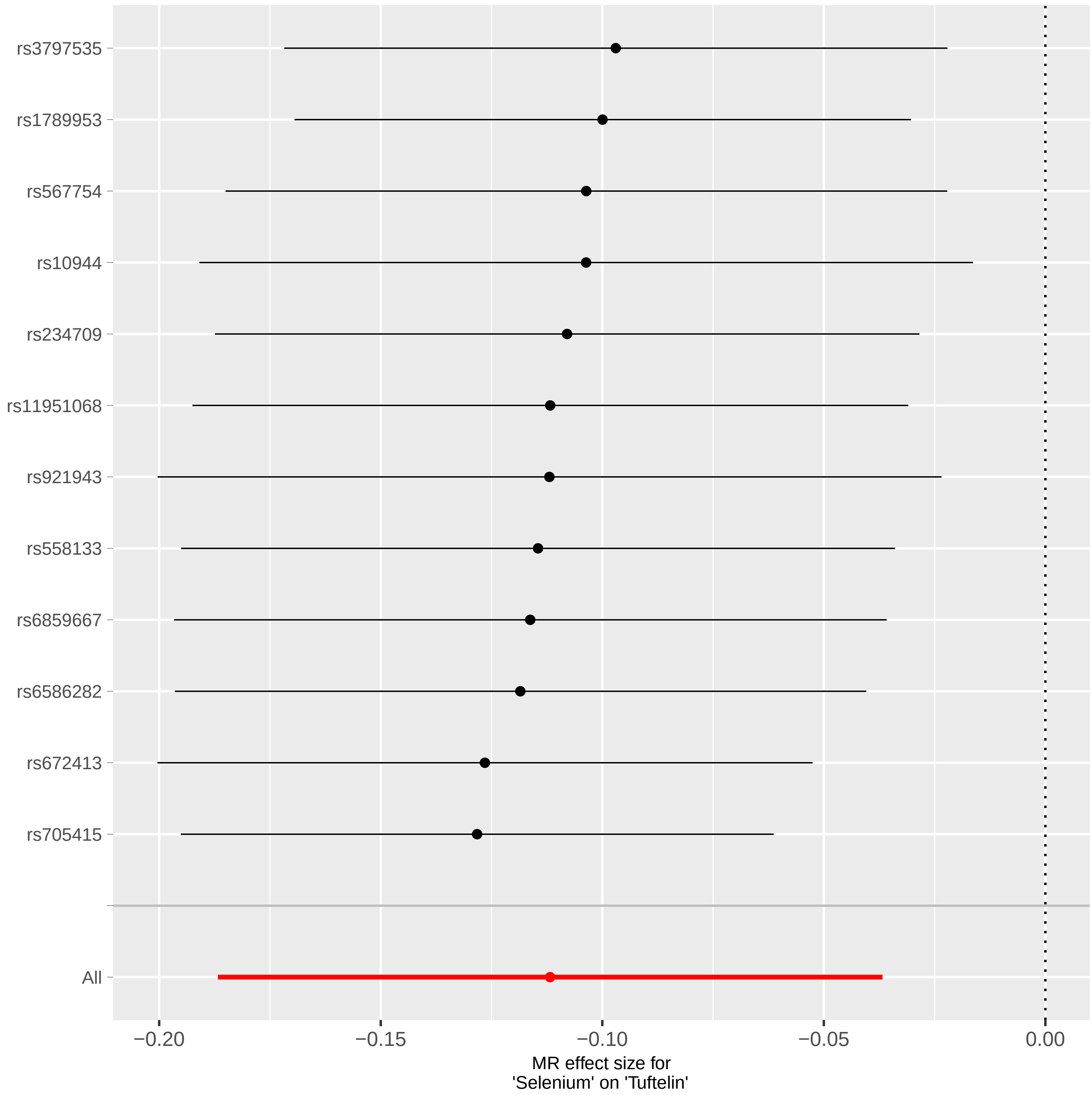

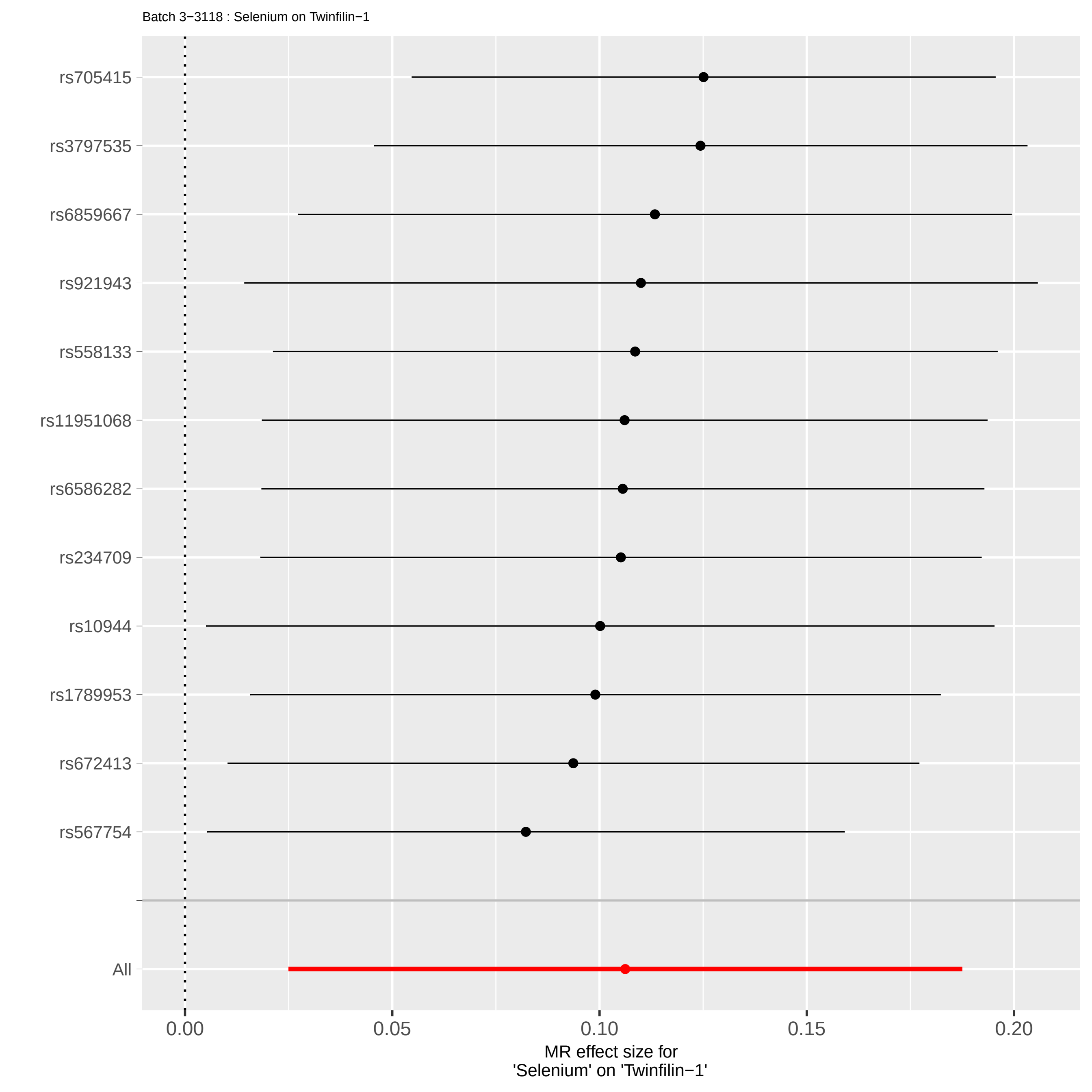

Batch 3−3118 : Selenium on Twinfilin−1
rs705415
rs3797535
rs6859667
rs921943
rs558133
rs11951068
rs6586282
rs234709
rs10944
rs1789953
rs672413
rs567754
All
0.00
0.05
0.10
0.15
0.20
MR effect size for
'Selenium' on 'Twinfilin−1'

Batch 3−3119 : Selenium on Twisted gastrulation protein homolog 1

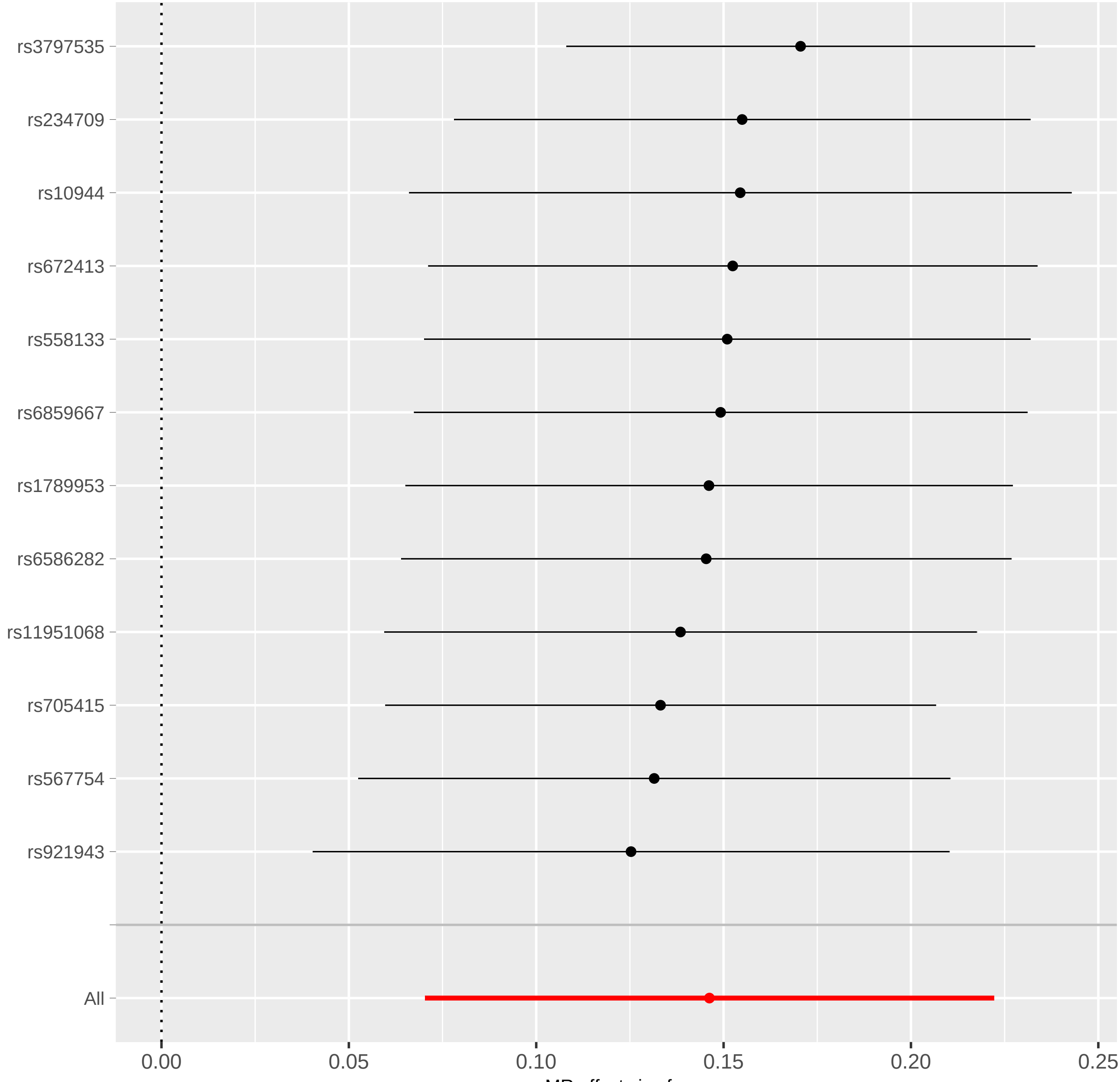

MR effect size for
'Selenium' on 'Twisted gastrulation protein homolog 1'

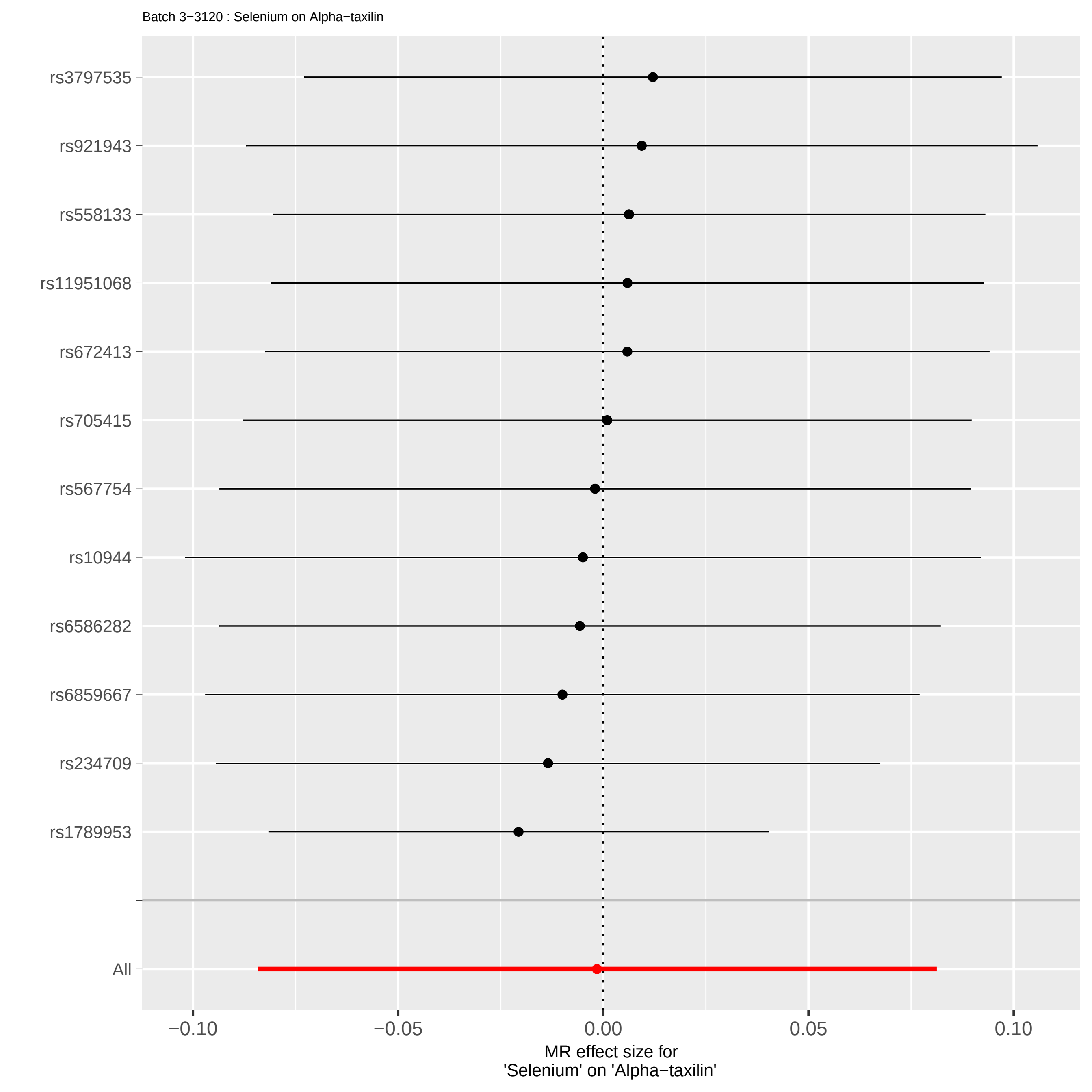
Batch 3−3120 : Selenium on Alpha−taxilin
rs3797535
rs921943
rs558133
rs11951068
rs672413
rs705415
rs567754
rs10944
rs6586282
rs6859667
rs234709
rs1789953
All
−0.10
−0.05
0.00
0.05
0.10
MR effect size for
'Selenium' on 'Alpha−taxilin'

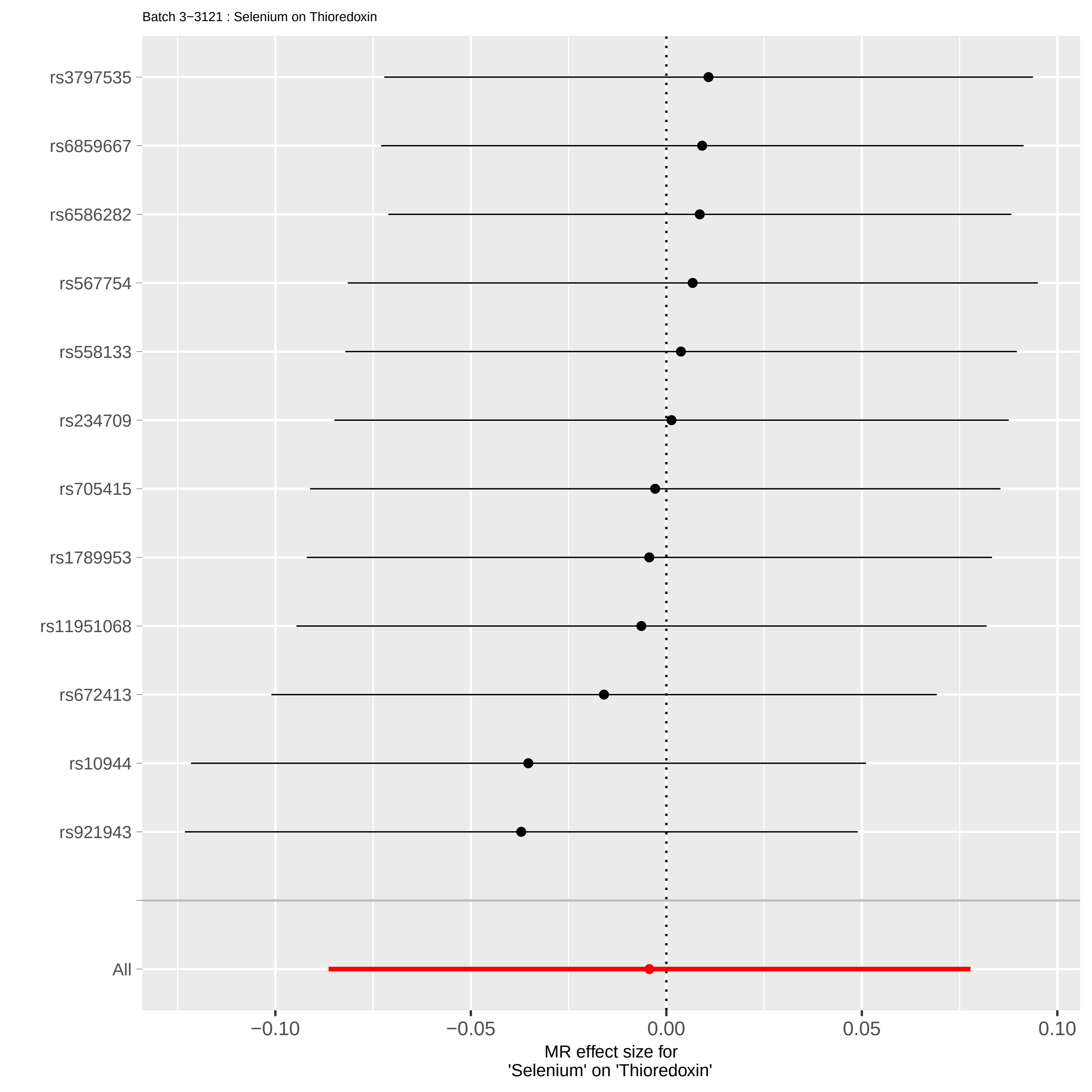

Batch 3−3121 : Selenium on Thioredoxin
rs3797535
rs6859667
rs6586282
rs567754
rs558133
rs234709
rs705415
rs1789953
rs11951068
rs672413
rs10944
rs921943
All
−0.10
−0.05
0.00
0.05
0.10
MR effect size for
'Selenium' on 'Thioredoxin'

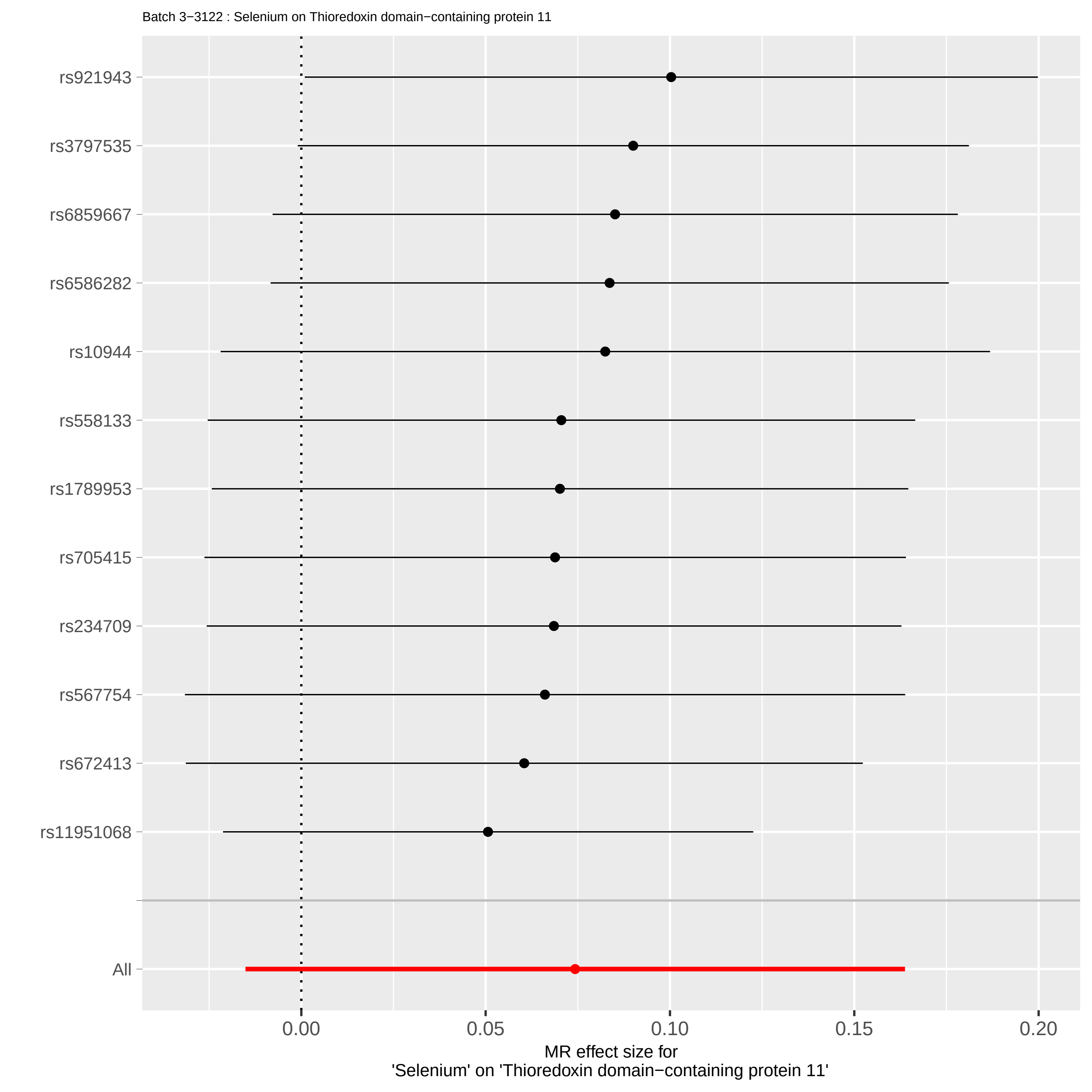
Batch 3−3122 : Selenium on Thioredoxin domain−containing protein 11
rs921943
rs3797535
rs6859667
rs6586282
rs10944
rs558133
rs1789953
rs705415
rs234709
rs567754
rs672413
rs11951068
All
0.00
0.05
0.10
0.15
0.20
MR effect size for
'Selenium' on 'Thioredoxin domain−containing protein 11'

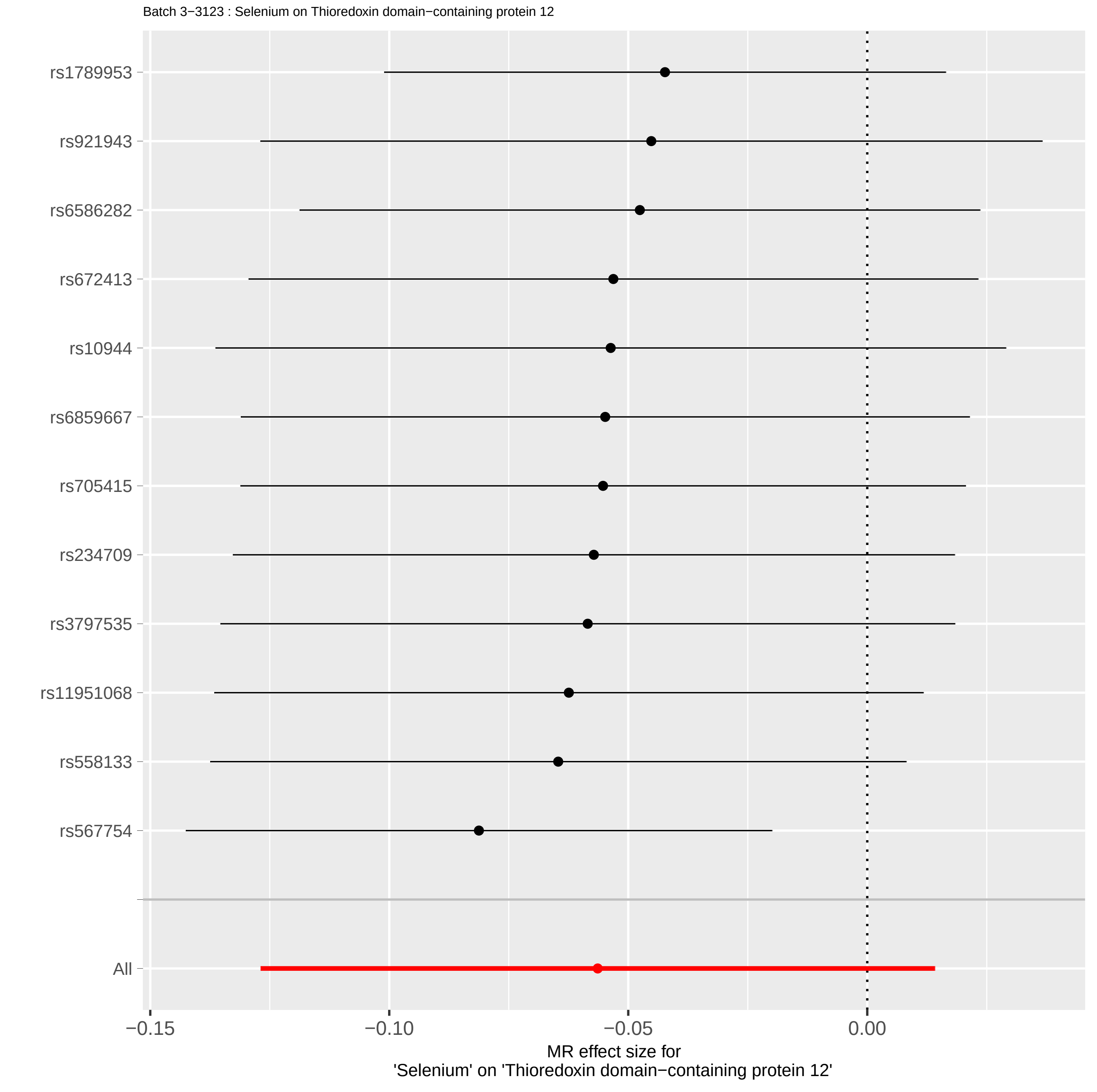

Batch 3−3123 : Selenium on Thioredoxin domain−containing protein 12
rs1789953
rs921943
rs6586282
rs672413
rs10944
rs6859667
rs705415
rs234709
rs3797535
rs11951068
rs558133
rs567754
All
−0.15
−0.10
−0.05
0.00
MR effect size for
'Selenium' on 'Thioredoxin domain−containing protein 12'

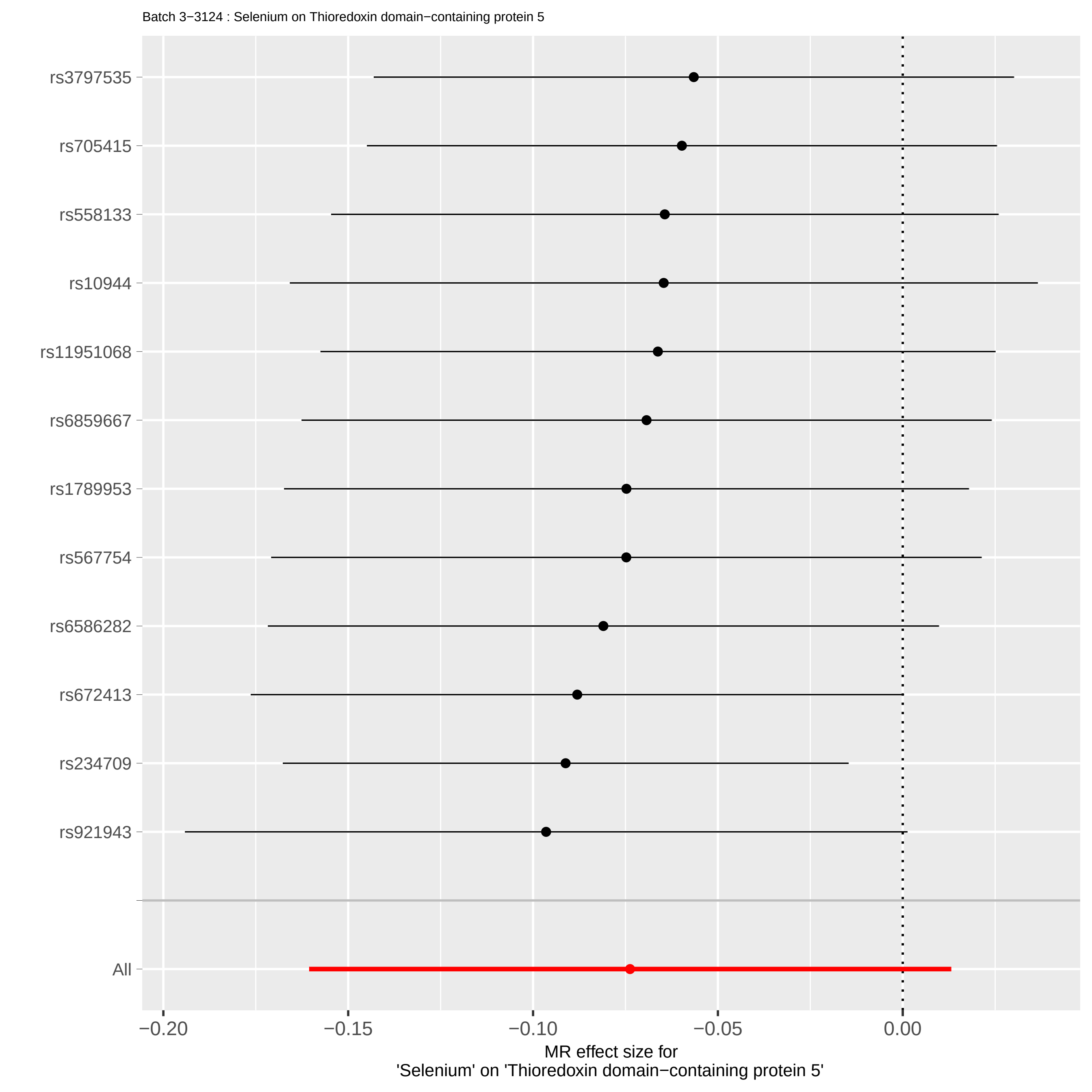

Batch 3−3124 : Selenium on Thioredoxin domain−containing protein 5
rs3797535
rs705415
rs558133
rs10944
rs11951068
rs6859667
rs1789953
rs567754
rs6586282
rs672413
rs234709
rs921943
All
−0.20
−0.15
−0.10
−0.05
0.00
MR effect size for
'Selenium' on 'Thioredoxin domain−containing protein 5'

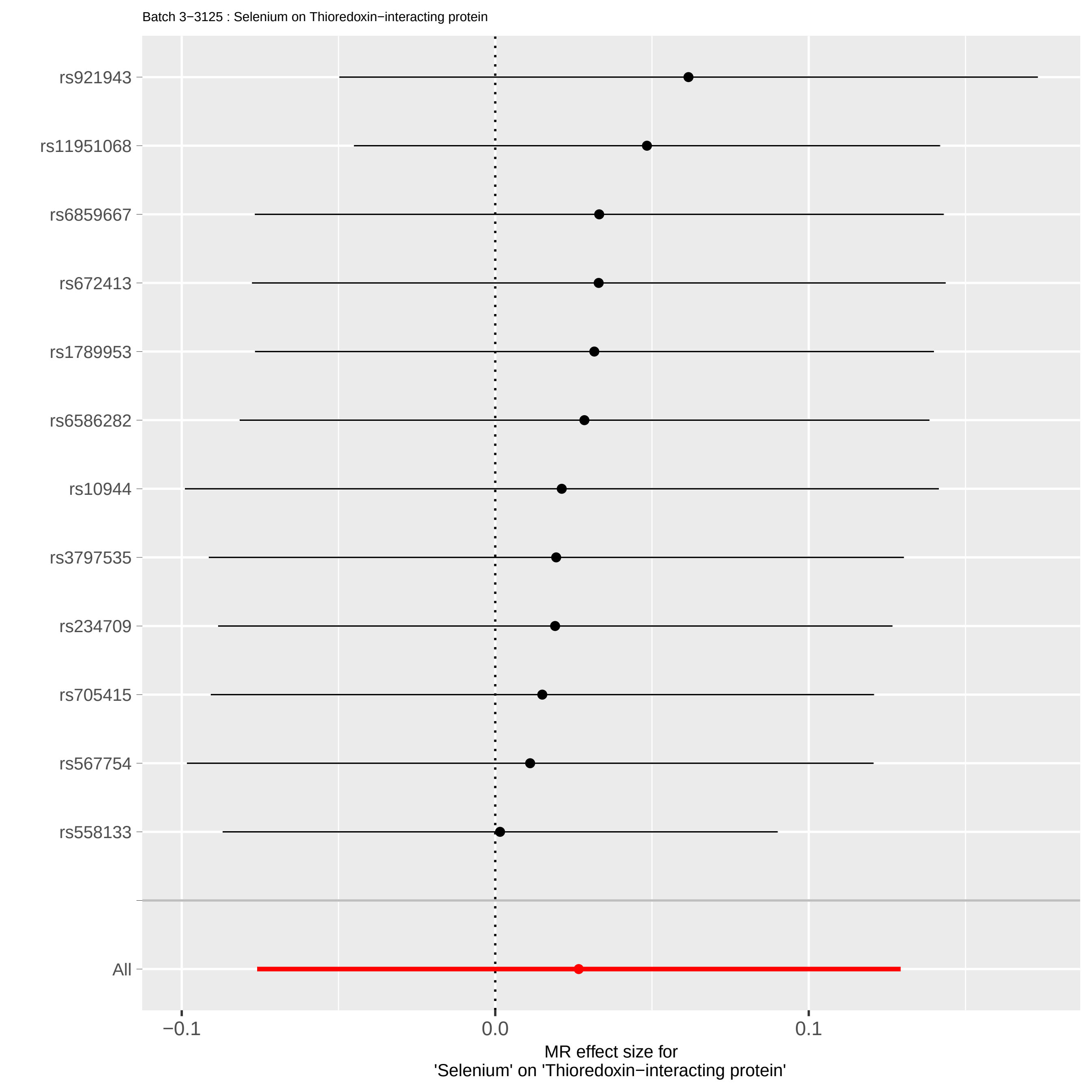

Batch 3−3125 : Selenium on Thioredoxin−interacting protein
rs921943
rs11951068
rs6859667
rs672413
rs1789953
rs6586282
rs10944
rs3797535
rs234709
rs705415
rs567754
rs558133
All
−0.1
0.0
0.1
MR effect size for
'Selenium' on 'Thioredoxin−interacting protein'

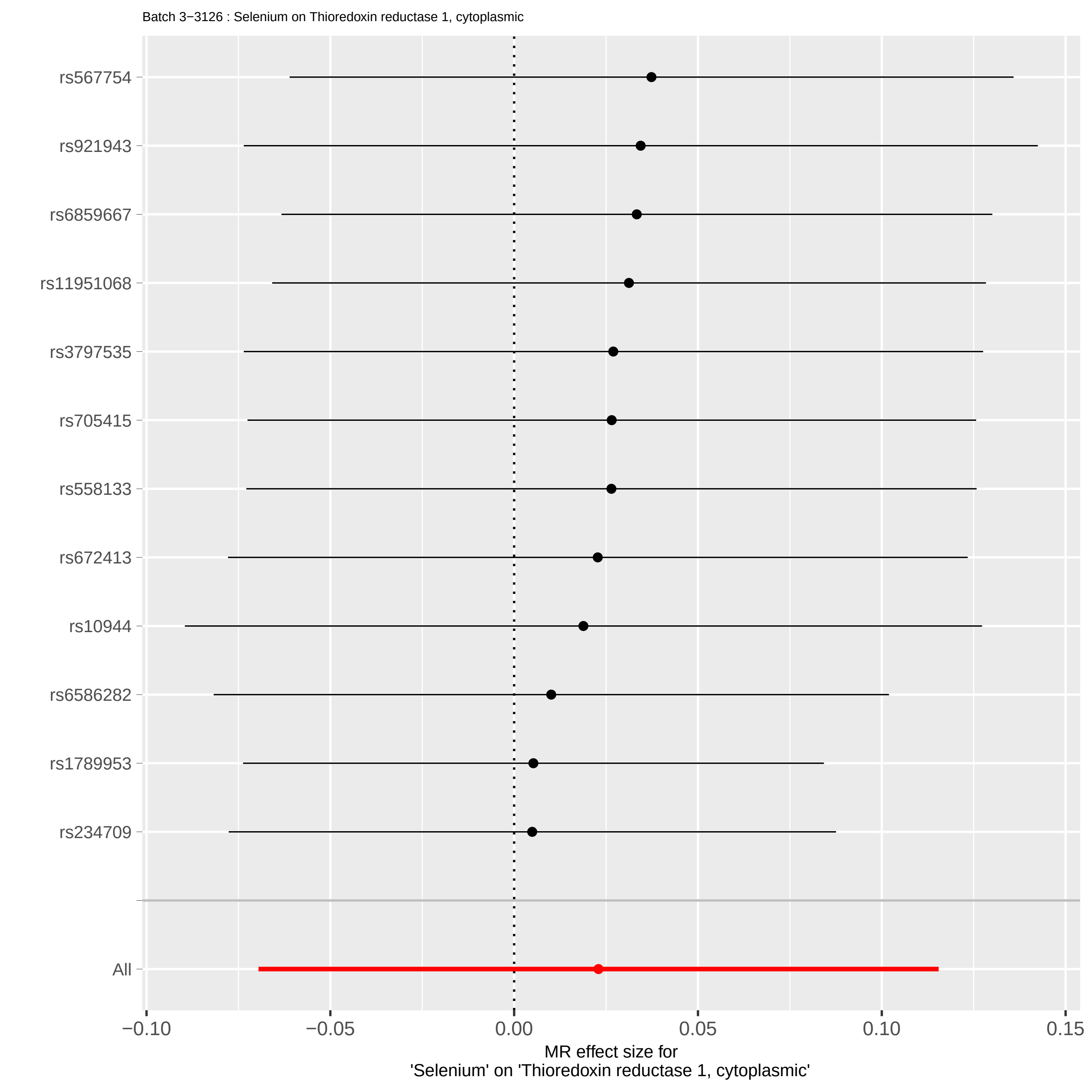

Batch 3−3126 : Selenium on Thioredoxin reductase 1, cytoplasmic
rs567754
rs921943
rs6859667
rs11951068
rs3797535
rs705415
rs558133
rs672413
rs10944
rs6586282
rs1789953
rs234709
All
−0.10
−0.05
0.00
0.05
0.10
0.15
MR effect size for
'Selenium' on 'Thioredoxin reductase 1, cytoplasmic'

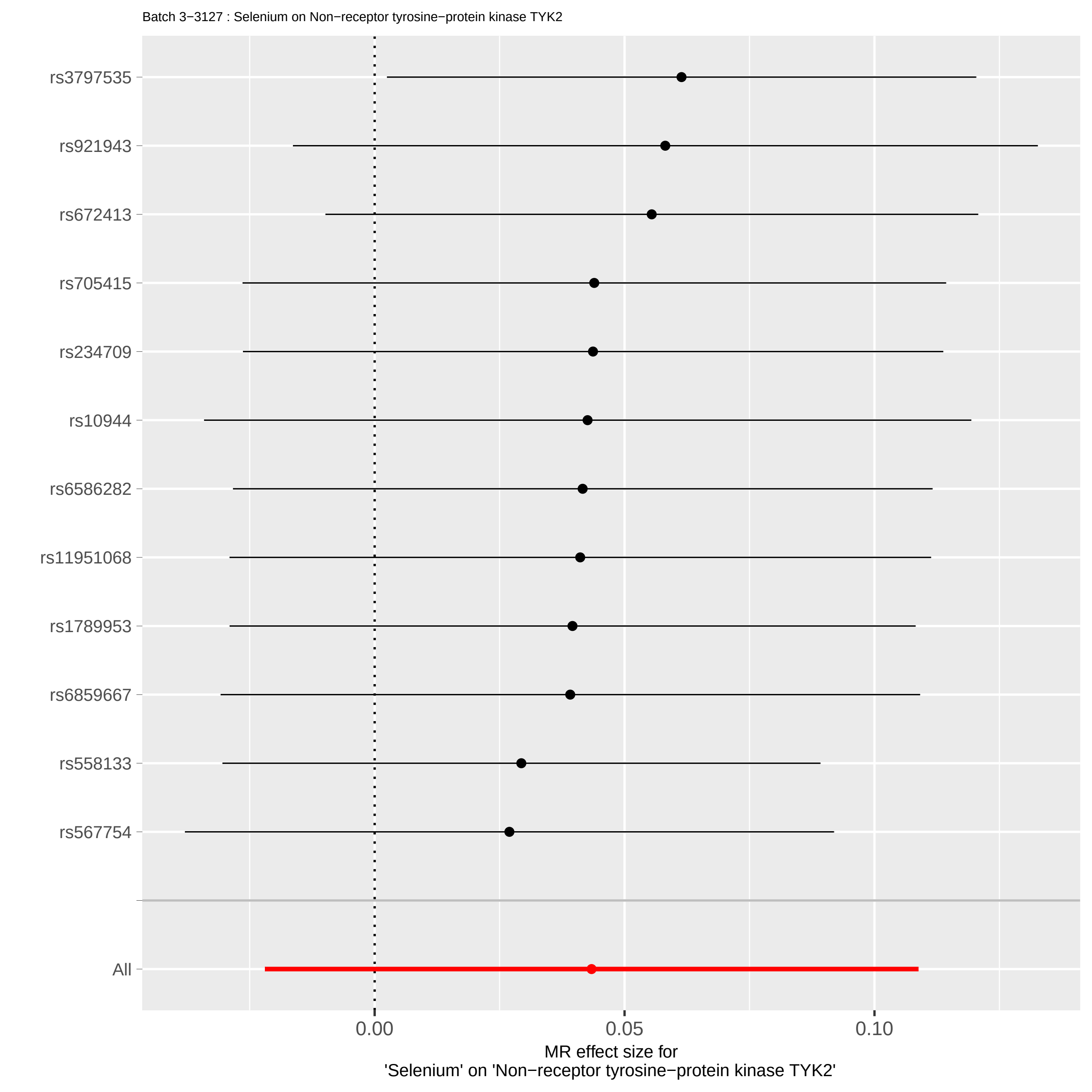
Batch 3−3127 : Selenium on Non−receptor tyrosine−protein kinase TYK2
rs3797535
rs921943
rs672413
rs705415
rs234709
rs10944
rs6586282
rs11951068
rs1789953
rs6859667
rs558133
rs567754
All
0.00
0.05
0.10
MR effect size for
'Selenium' on 'Non−receptor tyrosine−protein kinase TYK2'

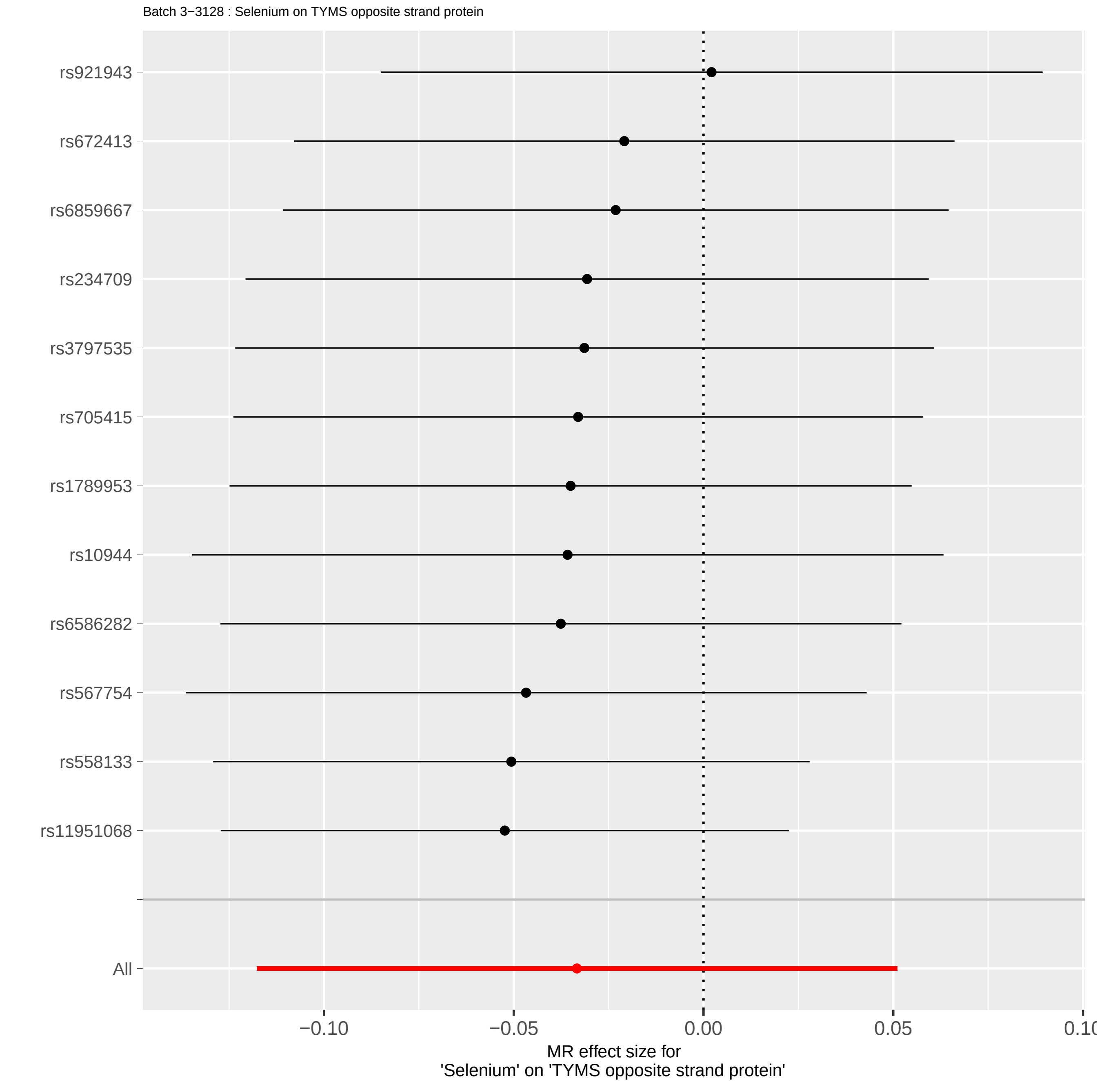

Batch 3−3128 : Selenium on TYMS opposite strand protein
rs921943
rs672413
rs6859667
rs234709
rs3797535
rs705415
rs1789953
rs10944
rs6586282
rs567754
rs558133
rs11951068
All
−0.10
−0.05
0.00
0.05
0.10
MR effect size for
'Selenium' on 'TYMS opposite strand protein'

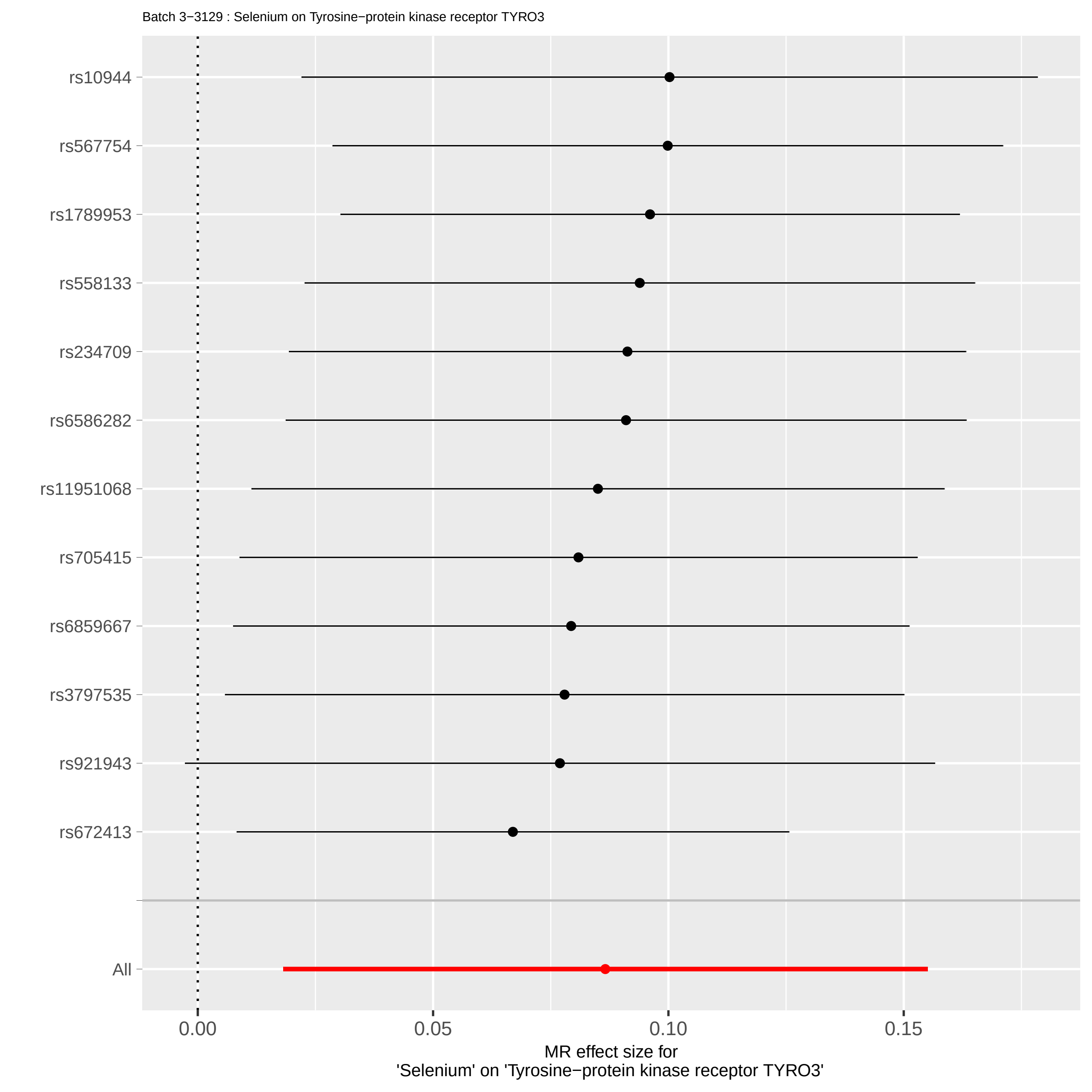

Batch 3−3129 : Selenium on Tyrosine−protein kinase receptor TYRO3
rs10944
rs567754
rs1789953
rs558133
rs234709
rs6586282
rs11951068
rs705415
rs6859667
rs3797535
rs921943
rs672413
All
0.00
0.05
0.10
0.15
MR effect size for
'Selenium' on 'Tyrosine−protein kinase receptor TYRO3'

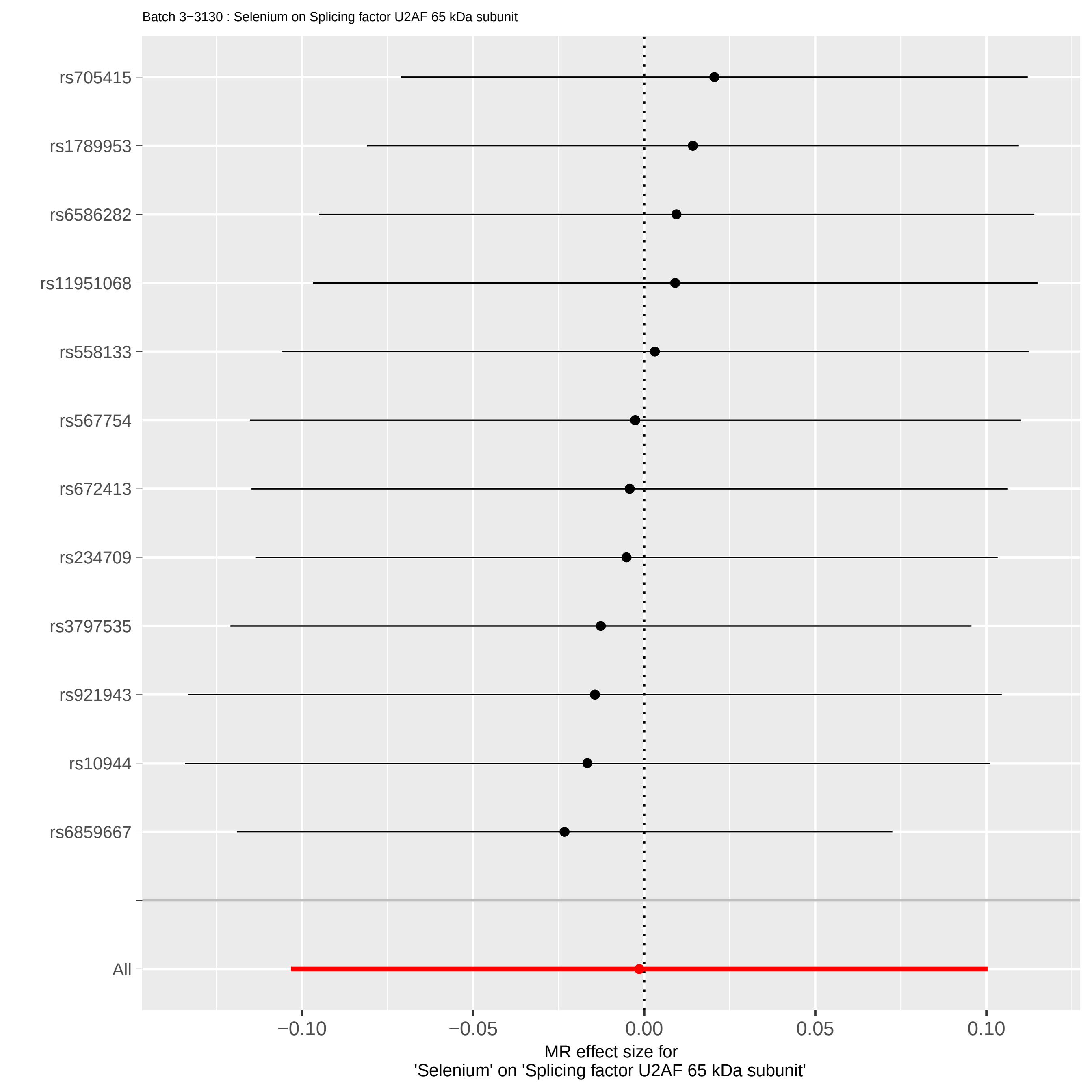

Batch 3−3130 : Selenium on Splicing factor U2AF 65 kDa subunit
rs705415
rs1789953
rs6586282
rs11951068
rs558133
rs567754
rs672413
rs234709
rs3797535
rs921943
rs10944
rs6859667
All
−0.10
−0.05
0.00
0.05
0.10
MR effect size for
'Selenium' on 'Splicing factor U2AF 65 kDa subunit'

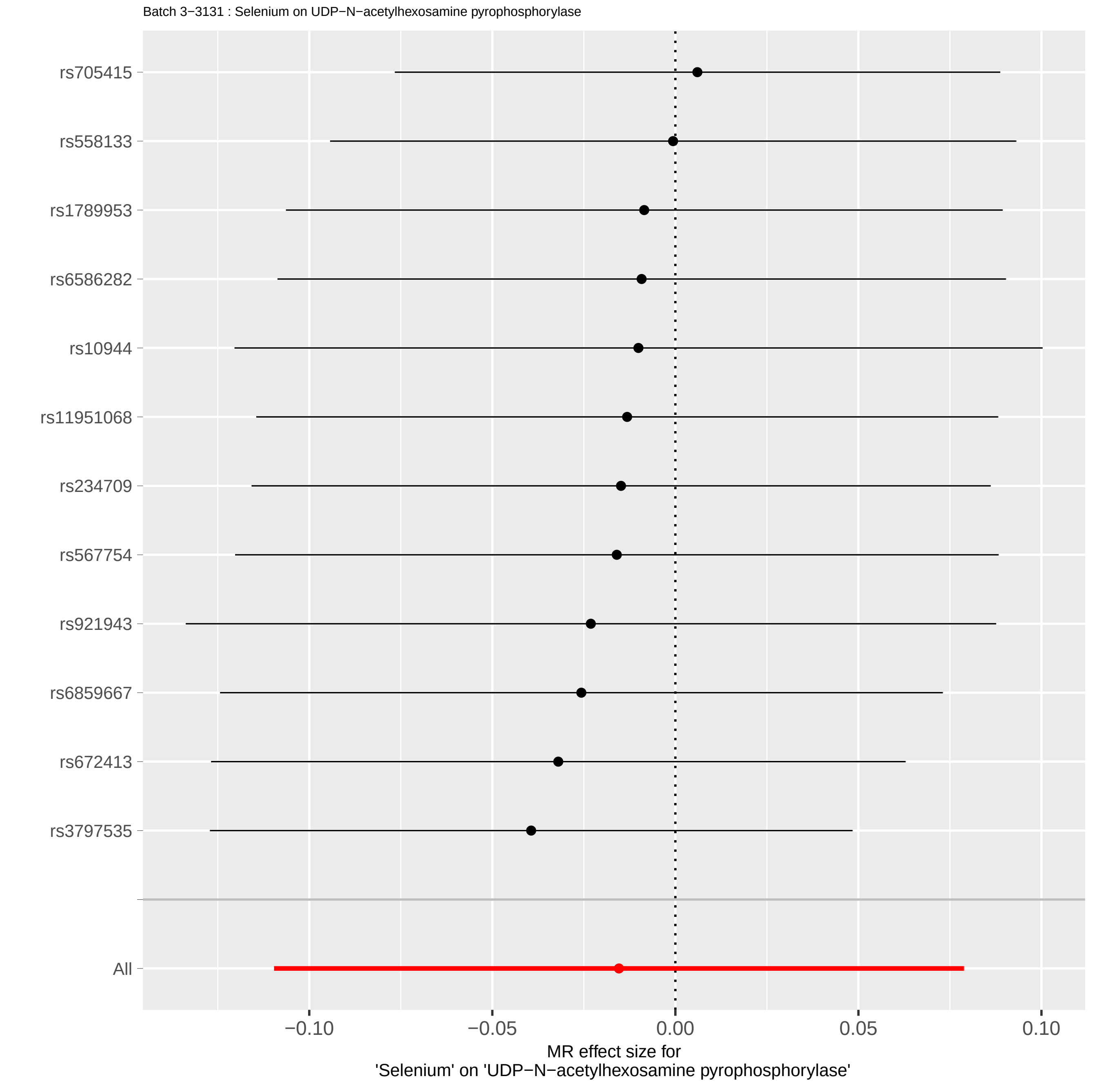

Batch 3−3131 : Selenium on UDP−N−acetylhexosamine pyrophosphorylase
rs705415
rs558133
rs1789953
rs6586282
rs10944
rs11951068
rs234709
rs567754
rs921943
rs6859667
rs672413
rs3797535
All
−0.10
−0.05
0.00
0.05
0.10
MR effect size for
'Selenium' on 'UDP−N−acetylhexosamine pyrophosphorylase'

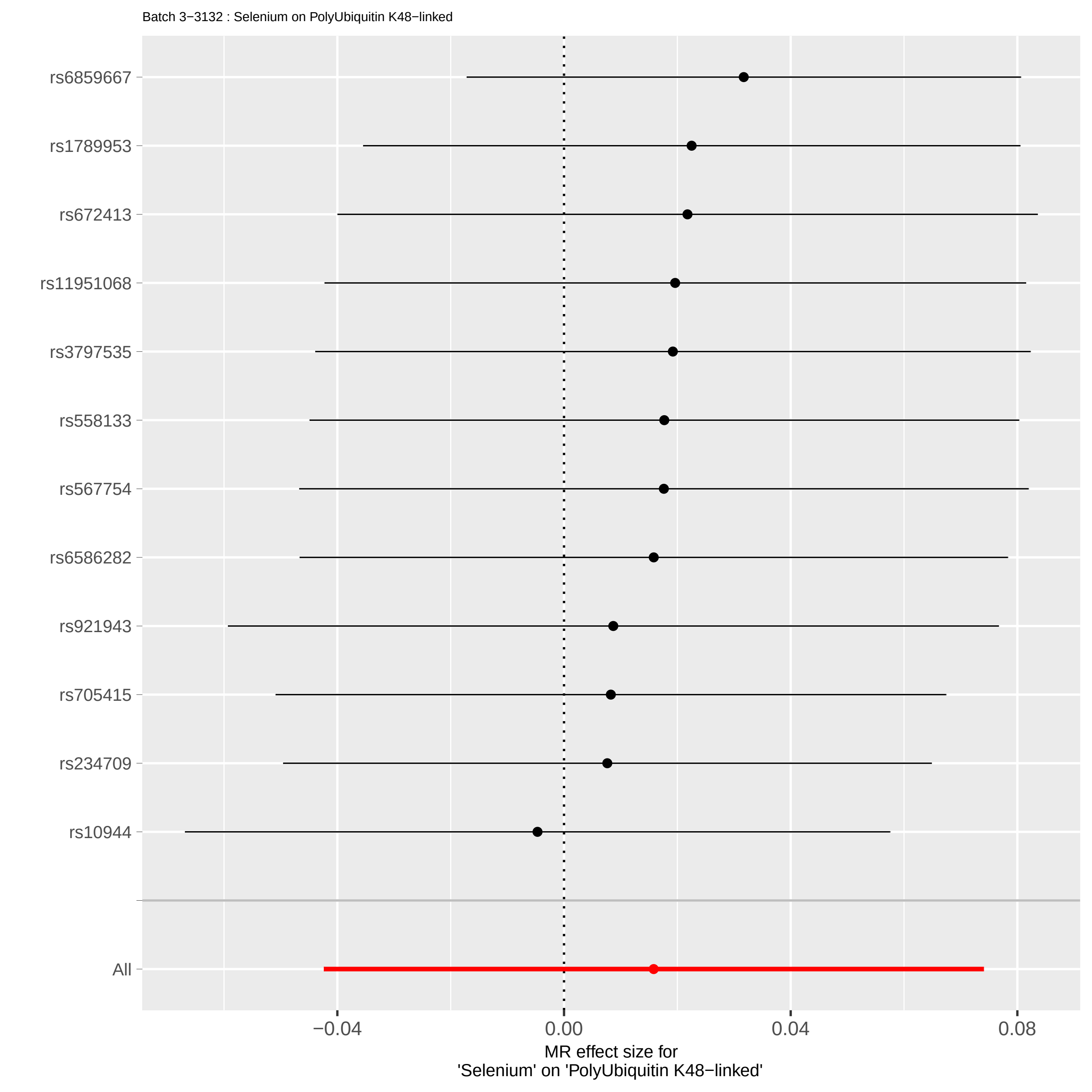
Batch 3−3132 : Selenium on PolyUbiquitin K48−linked
rs6859667
rs1789953
rs672413
rs11951068
rs3797535
rs558133
rs567754
rs6586282
rs921943
rs705415
rs234709
rs10944
All
−0.04
0.00
0.04
0.08
MR effect size for
'Selenium' on 'PolyUbiquitin K48−linked'

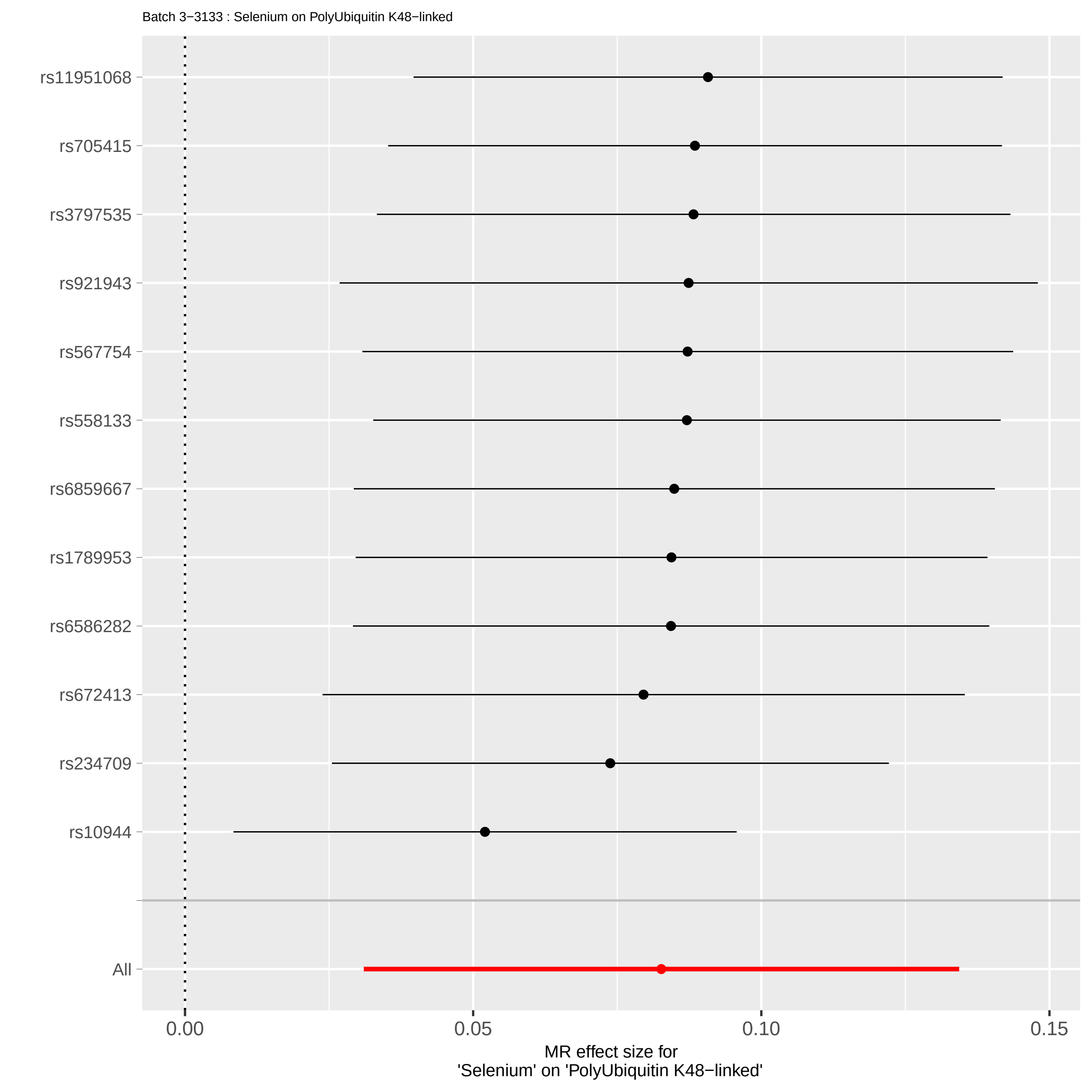
Batch 3−3133 : Selenium on PolyUbiquitin K48−linked
rs11951068
rs705415
rs3797535
rs921943
rs567754
rs558133
rs6859667
rs1789953
rs6586282
rs672413
rs234709
rs10944
All
0.00
0.05
0.10
0.15
MR effect size for
'Selenium' on 'PolyUbiquitin K48−linked'

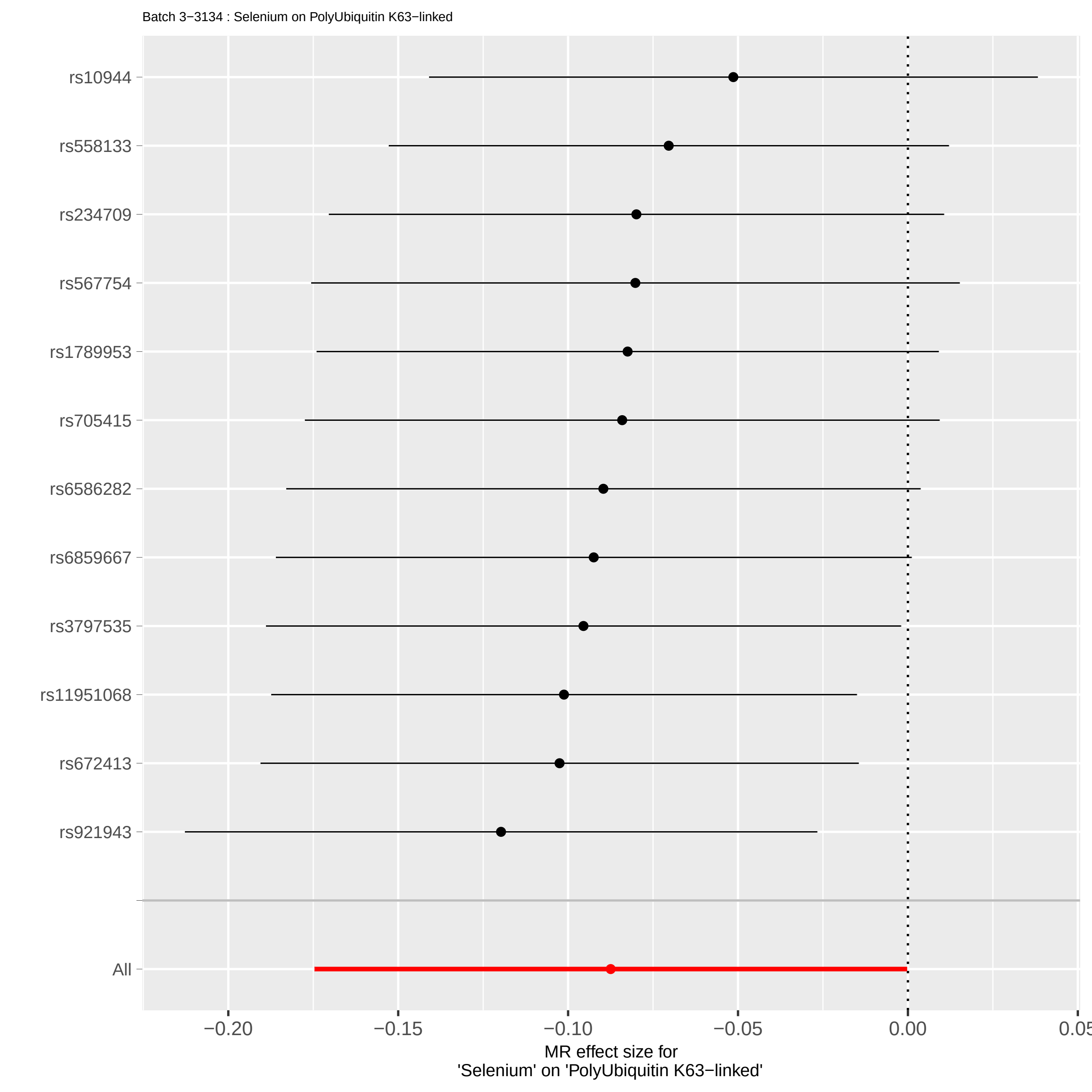

Batch 3−3134 : Selenium on PolyUbiquitin K63−linked
rs10944
rs558133
rs234709
rs567754
rs1789953
rs705415
rs6586282
rs6859667
rs3797535
rs11951068
rs672413
rs921943
All
−0.20
−0.15
−0.10
−0.05
0.00
0.05
MR effect size for
'Selenium' on 'PolyUbiquitin K63−linked'

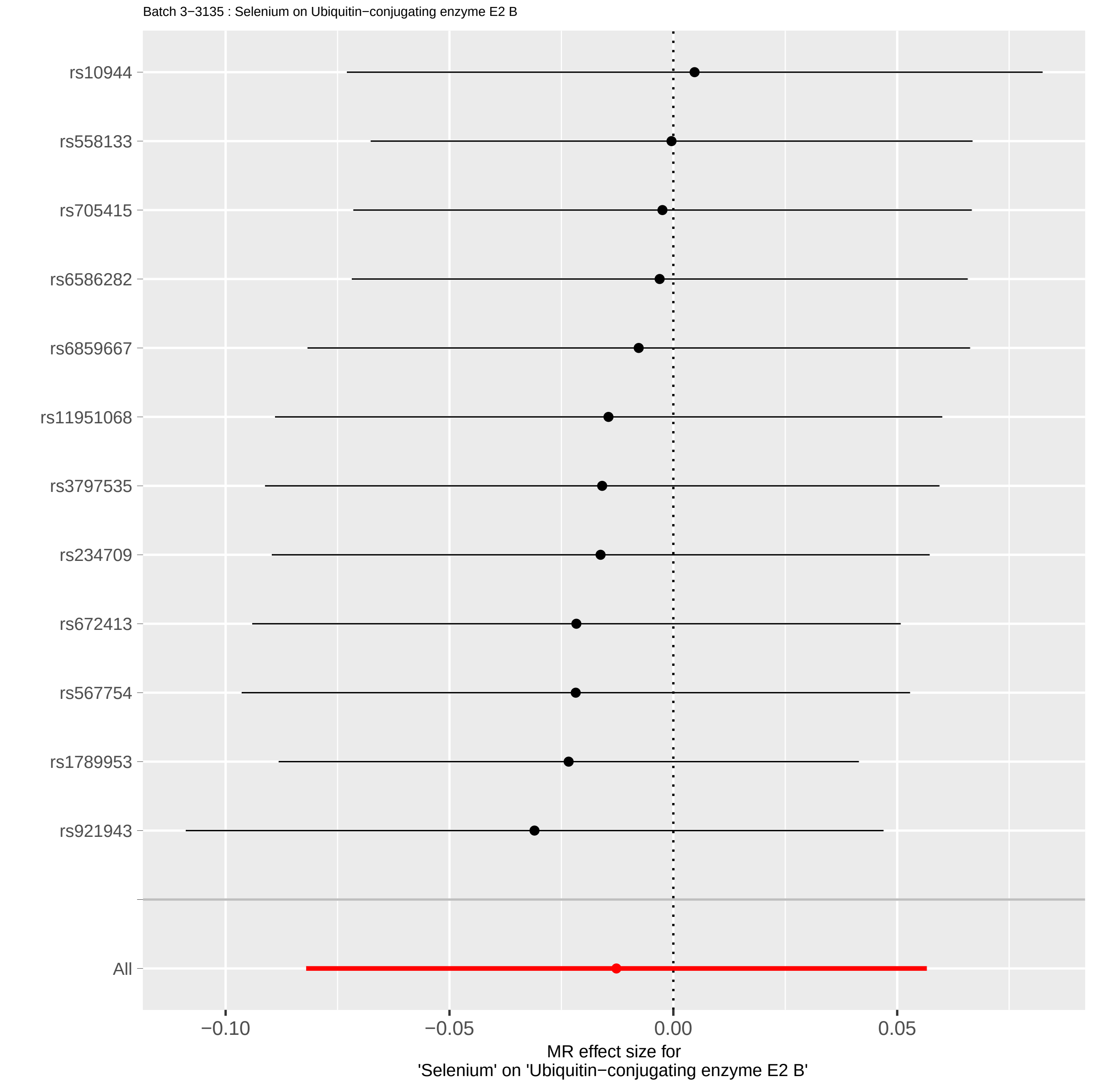

Batch 3−3135 : Selenium on Ubiquitin−conjugating enzyme E2 B
rs10944
rs558133
rs705415
rs6586282
rs6859667
rs11951068
rs3797535
rs234709
rs672413
rs567754
rs1789953
rs921943
All
−0.10
−0.05
0.00
0.05
MR effect size for
'Selenium' on 'Ubiquitin−conjugating enzyme E2 B'

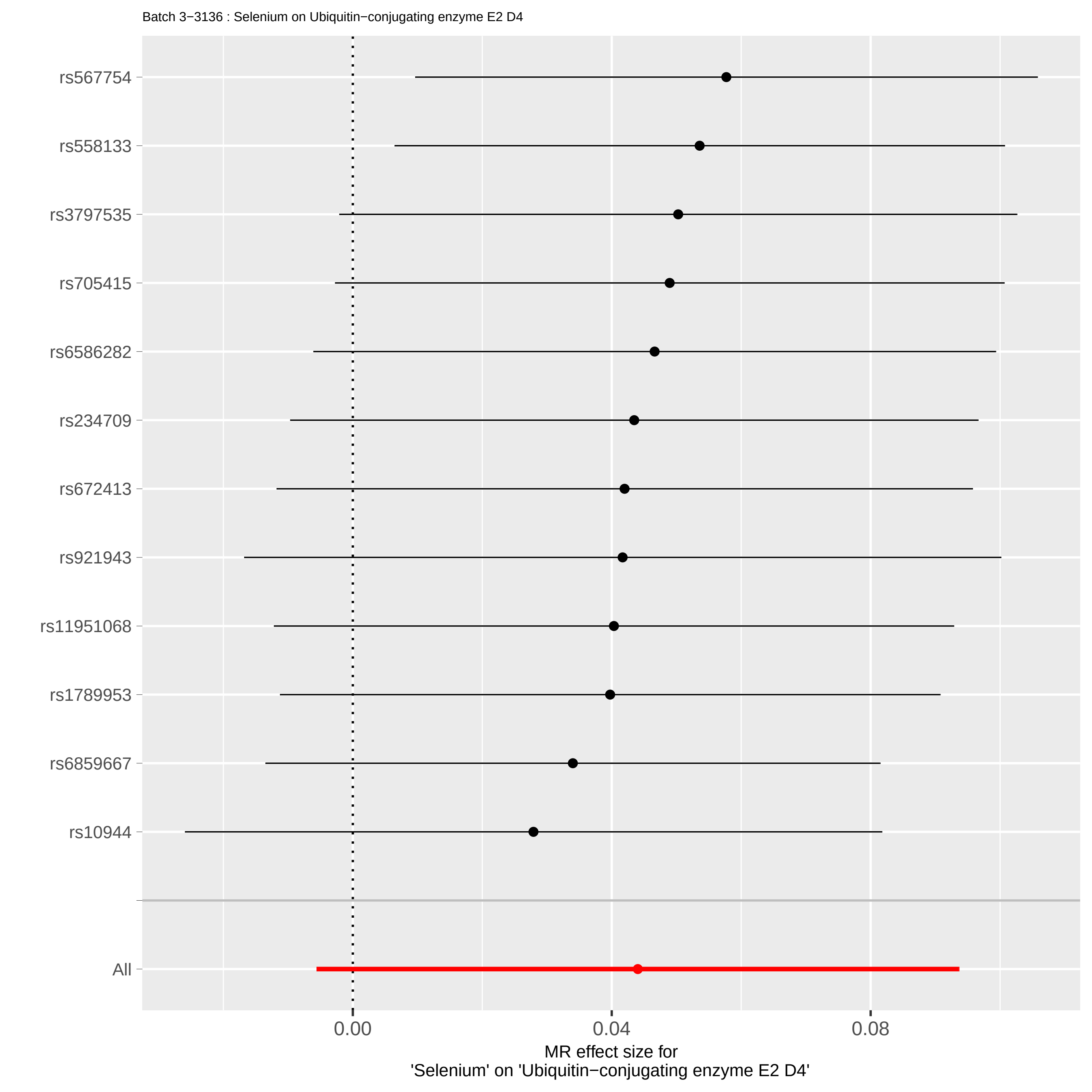

Batch 3−3136 : Selenium on Ubiquitin−conjugating enzyme E2 D4
rs567754
rs558133
rs3797535
rs705415
rs6586282
rs234709
rs672413
rs921943
rs11951068
rs1789953
rs6859667
rs10944
All
0.00
0.04
0.08
MR effect size for
'Selenium' on 'Ubiquitin−conjugating enzyme E2 D4'

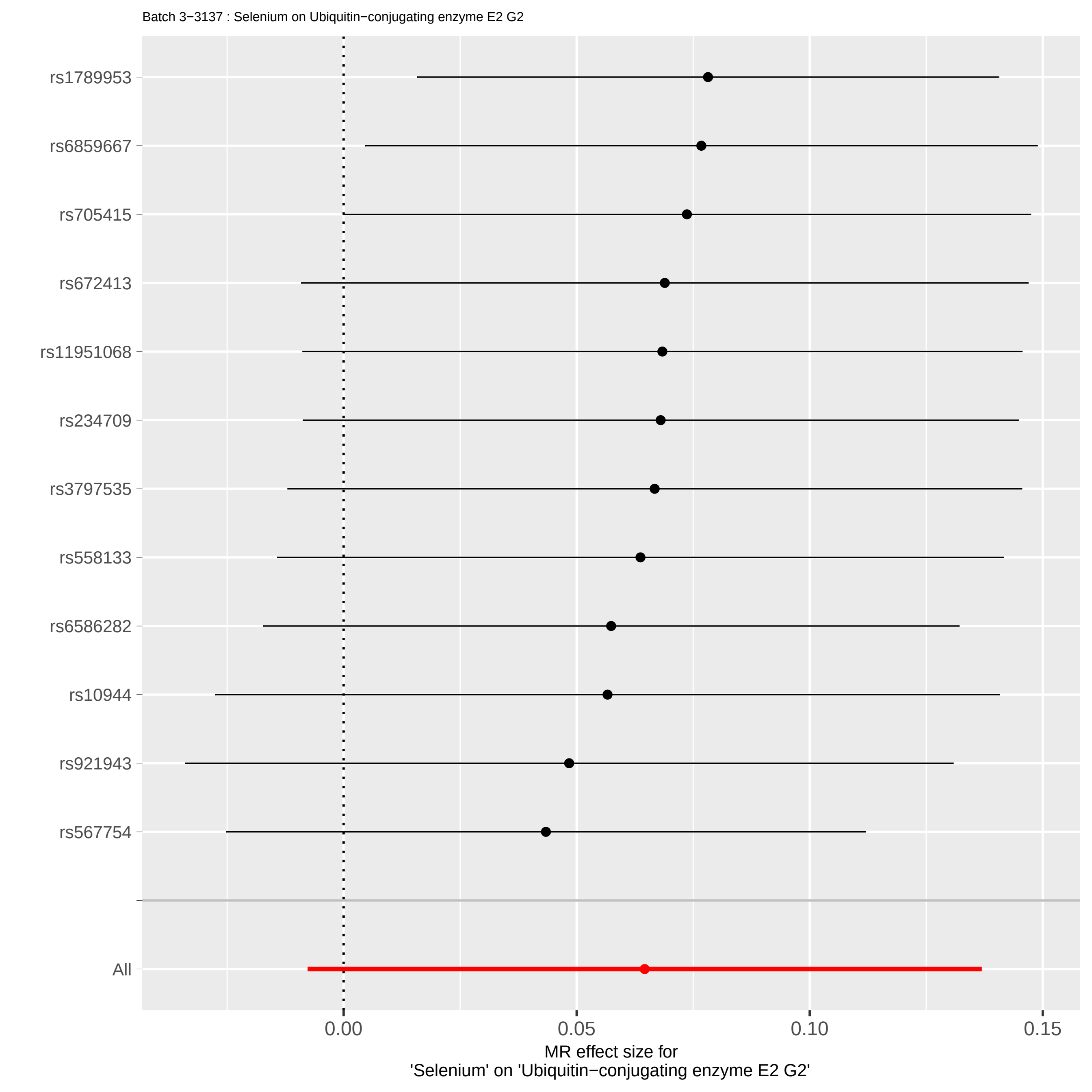

Batch 3−3137 : Selenium on Ubiquitin−conjugating enzyme E2 G2
rs1789953
rs6859667
rs705415
rs672413
rs11951068
rs234709
rs3797535
rs558133
rs6586282
rs10944
rs921943
rs567754
All
0.00
0.05
0.10
0.15
MR effect size for
'Selenium' on 'Ubiquitin−conjugating enzyme E2 G2'

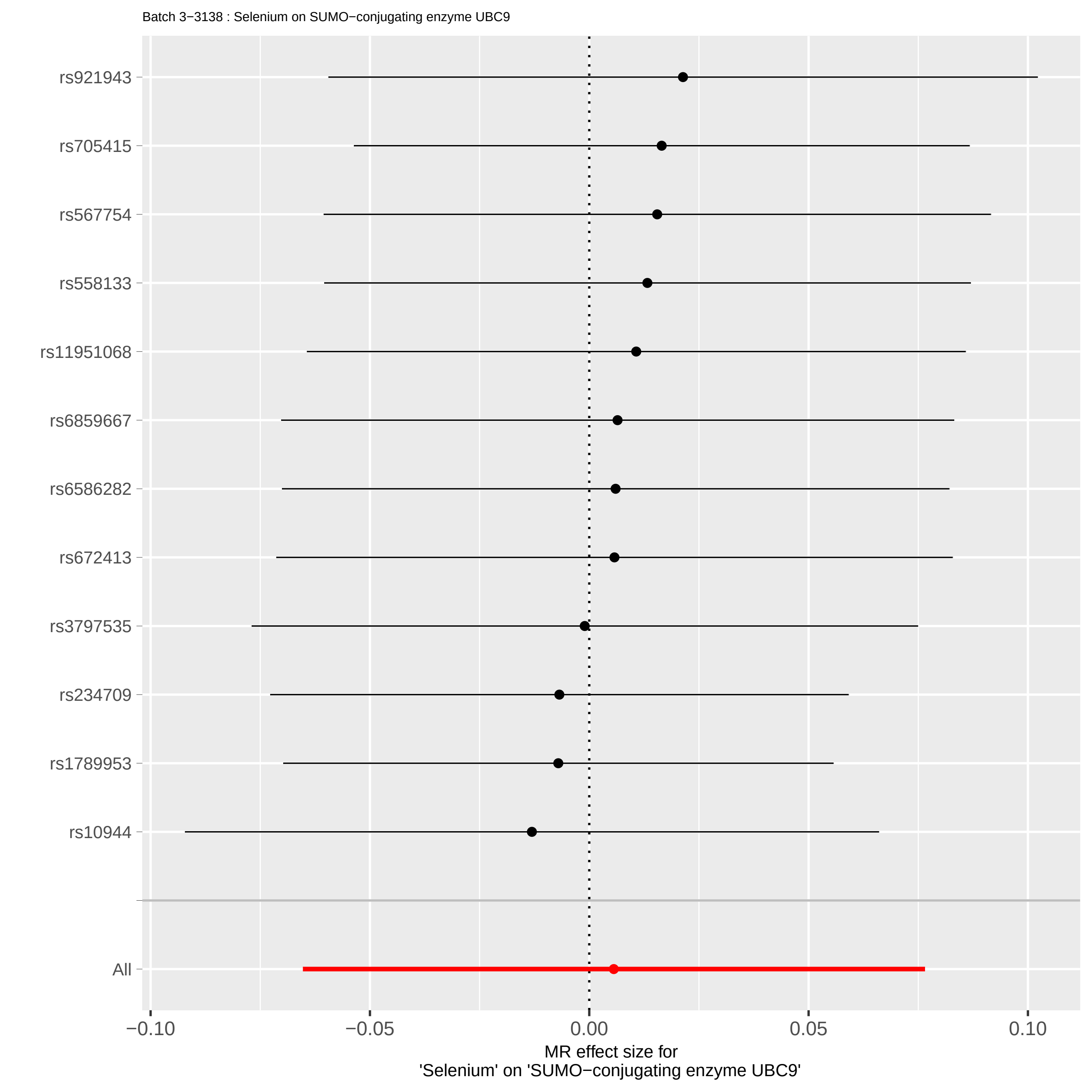

Batch 3−3138 : Selenium on SUMO−conjugating enzyme UBC9
rs921943
rs705415
rs567754
rs558133
rs11951068
rs6859667
rs6586282
rs672413
rs3797535
rs234709
rs1789953
rs10944
All
−0.10
−0.05
0.00
0.05
0.10
MR effect size for
'Selenium' on 'SUMO−conjugating enzyme UBC9'

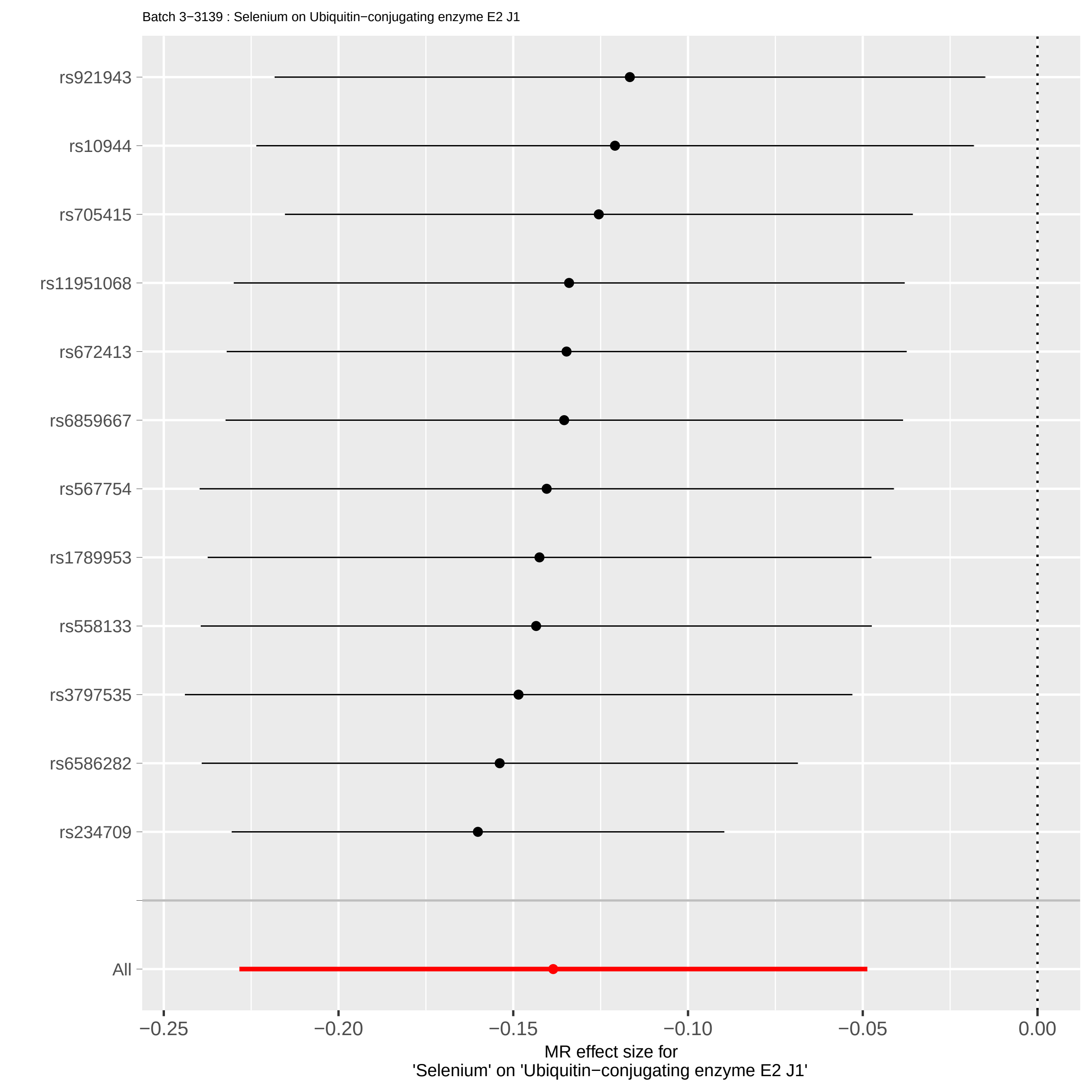
Batch 3−3139 : Selenium on Ubiquitin−conjugating enzyme E2 J1
rs921943
rs10944
rs705415
rs11951068
rs672413
rs6859667
rs567754
rs1789953
rs558133
rs3797535
rs6586282
rs234709
All
−0.25
−0.20
−0.15
−0.10
−0.05
0.00
MR effect size for
'Selenium' on 'Ubiquitin−conjugating enzyme E2 J1'

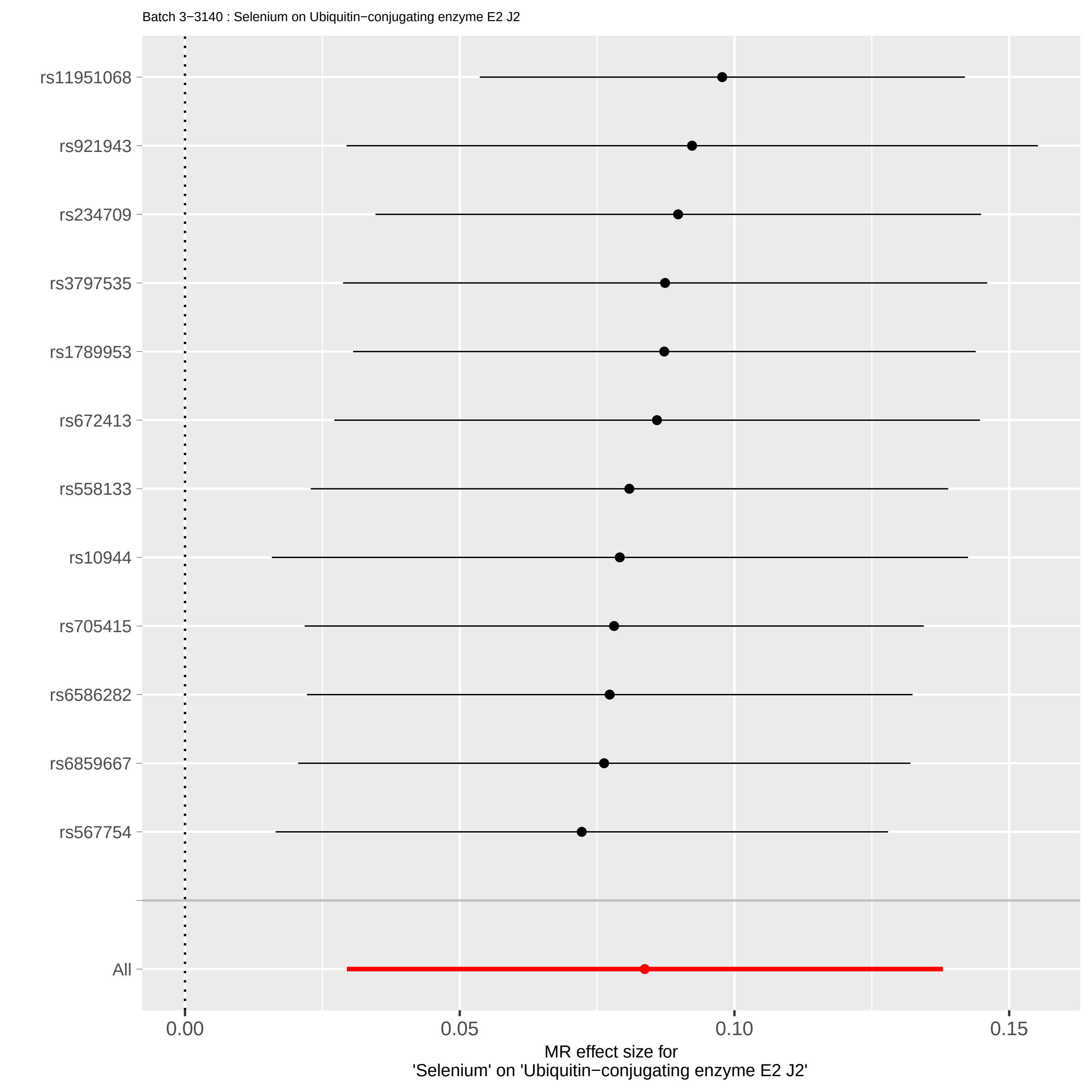

Batch 3−3140 : Selenium on Ubiquitin−conjugating enzyme E2 J2
rs11951068
rs921943
rs234709
rs3797535
rs1789953
rs672413
rs558133
rs10944
rs705415
rs6586282
rs6859667
rs567754
All
0.00
0.05
0.10
0.15
MR effect size for
'Selenium' on 'Ubiquitin−conjugating enzyme E2 J2'

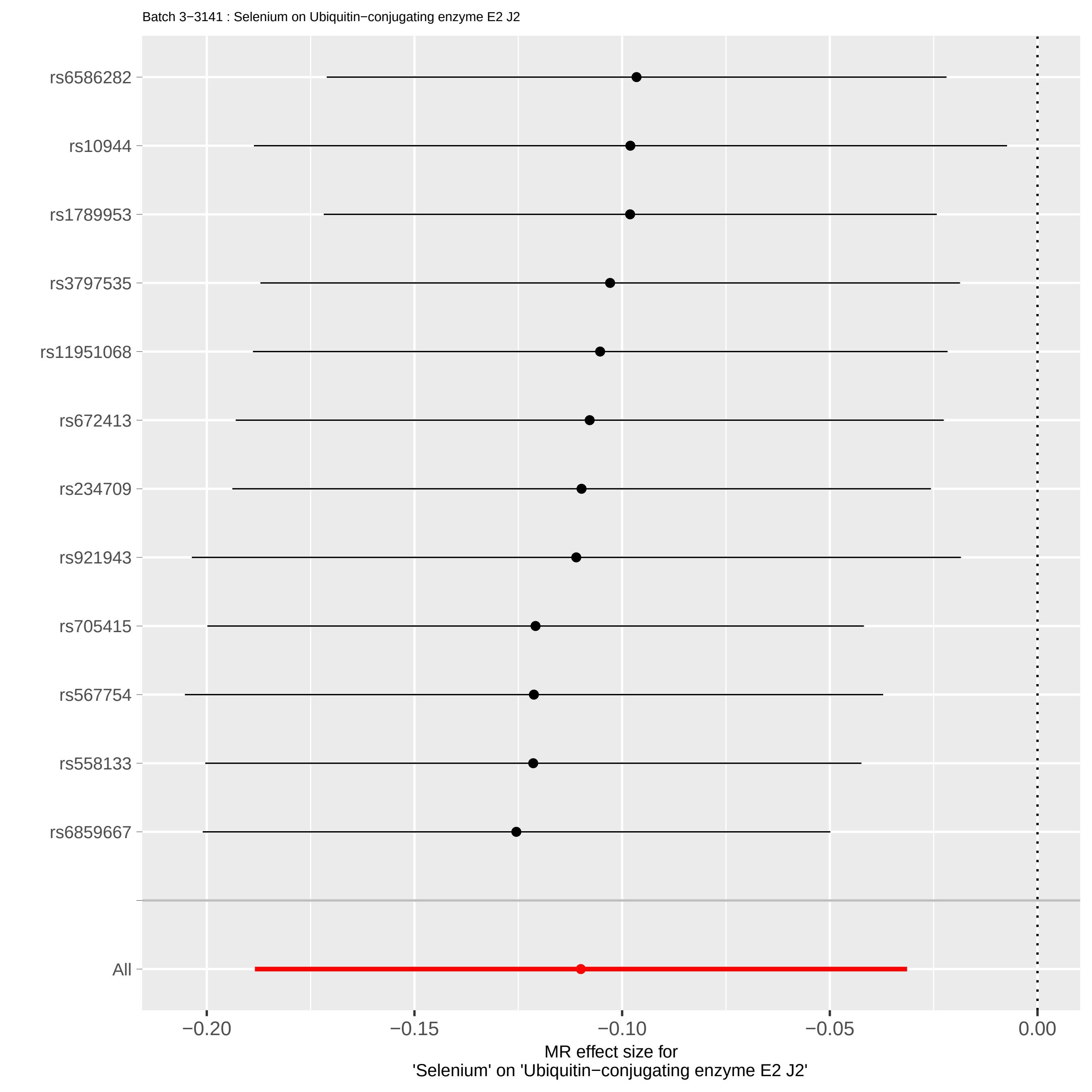

Batch 3−3141 : Selenium on Ubiquitin−conjugating enzyme E2 J2
rs6586282
rs10944
rs1789953
rs3797535
rs11951068
rs672413
rs234709
rs921943
rs705415
rs567754
rs558133
rs6859667
All
−0.20
−0.15
−0.10
−0.05
0.00
MR effect size for
'Selenium' on 'Ubiquitin−conjugating enzyme E2 J2'

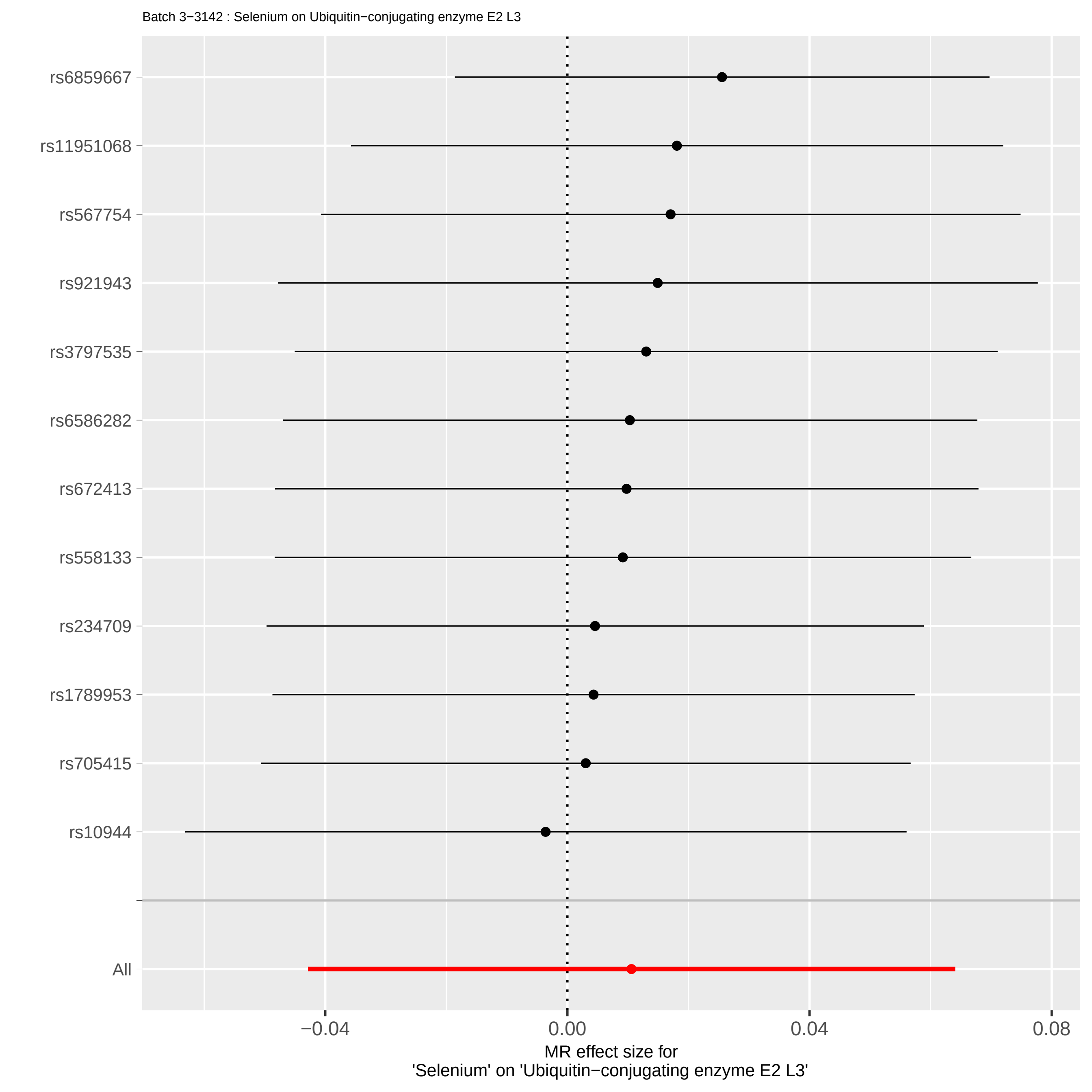

Batch 3−3142 : Selenium on Ubiquitin−conjugating enzyme E2 L3
rs6859667
rs11951068
rs567754
rs921943
rs3797535
rs6586282
rs672413
rs558133
rs234709
rs1789953
rs705415
rs10944
All
−0.04
0.00
0.04
0.08
MR effect size for
'Selenium' on 'Ubiquitin−conjugating enzyme E2 L3'

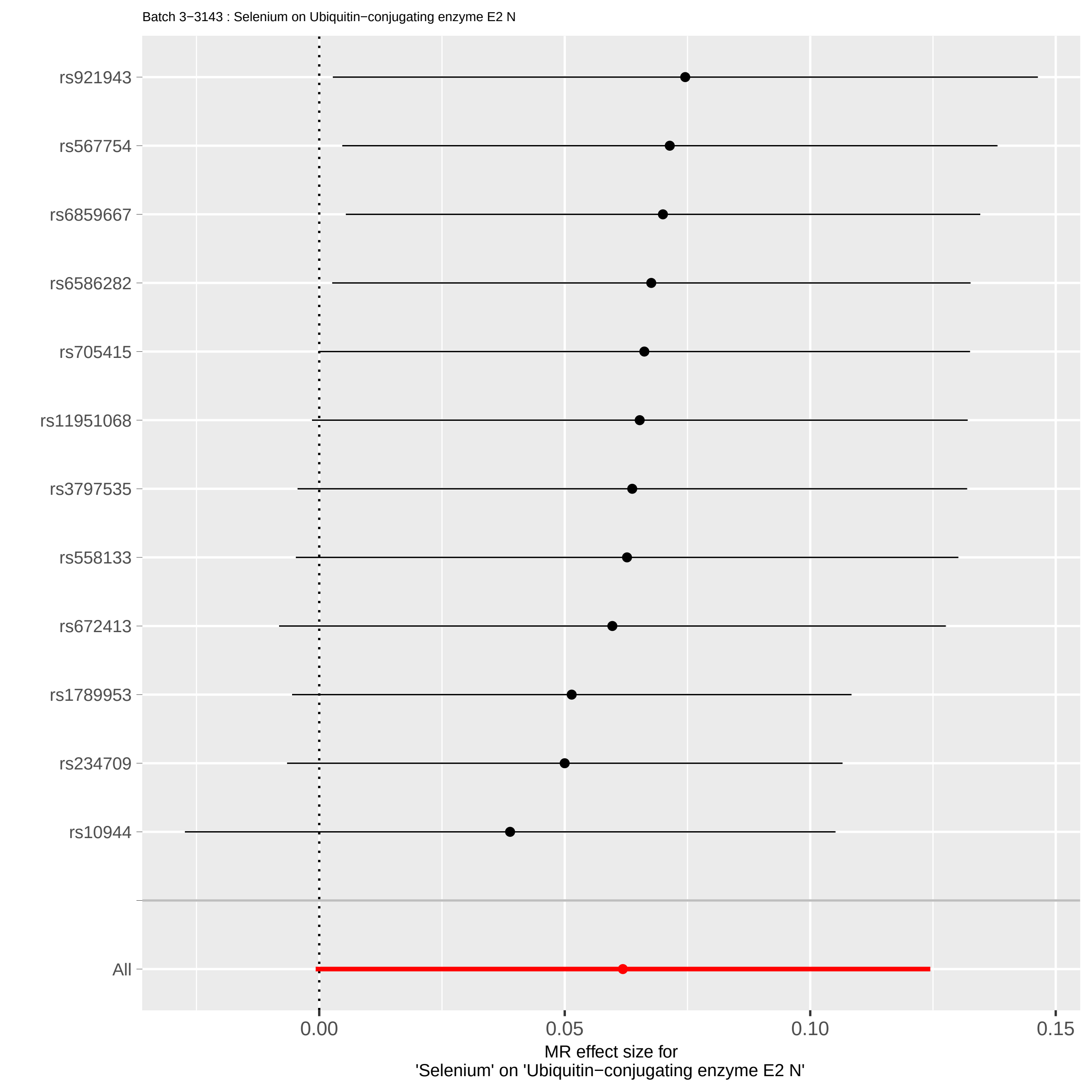

Batch 3−3143 : Selenium on Ubiquitin−conjugating enzyme E2 N
rs921943
rs567754
rs6859667
rs6586282
rs705415
rs11951068
rs3797535
rs558133
rs672413
rs1789953
rs234709
rs10944
All
0.00
0.05
0.10
0.15
MR effect size for
'Selenium' on 'Ubiquitin−conjugating enzyme E2 N'

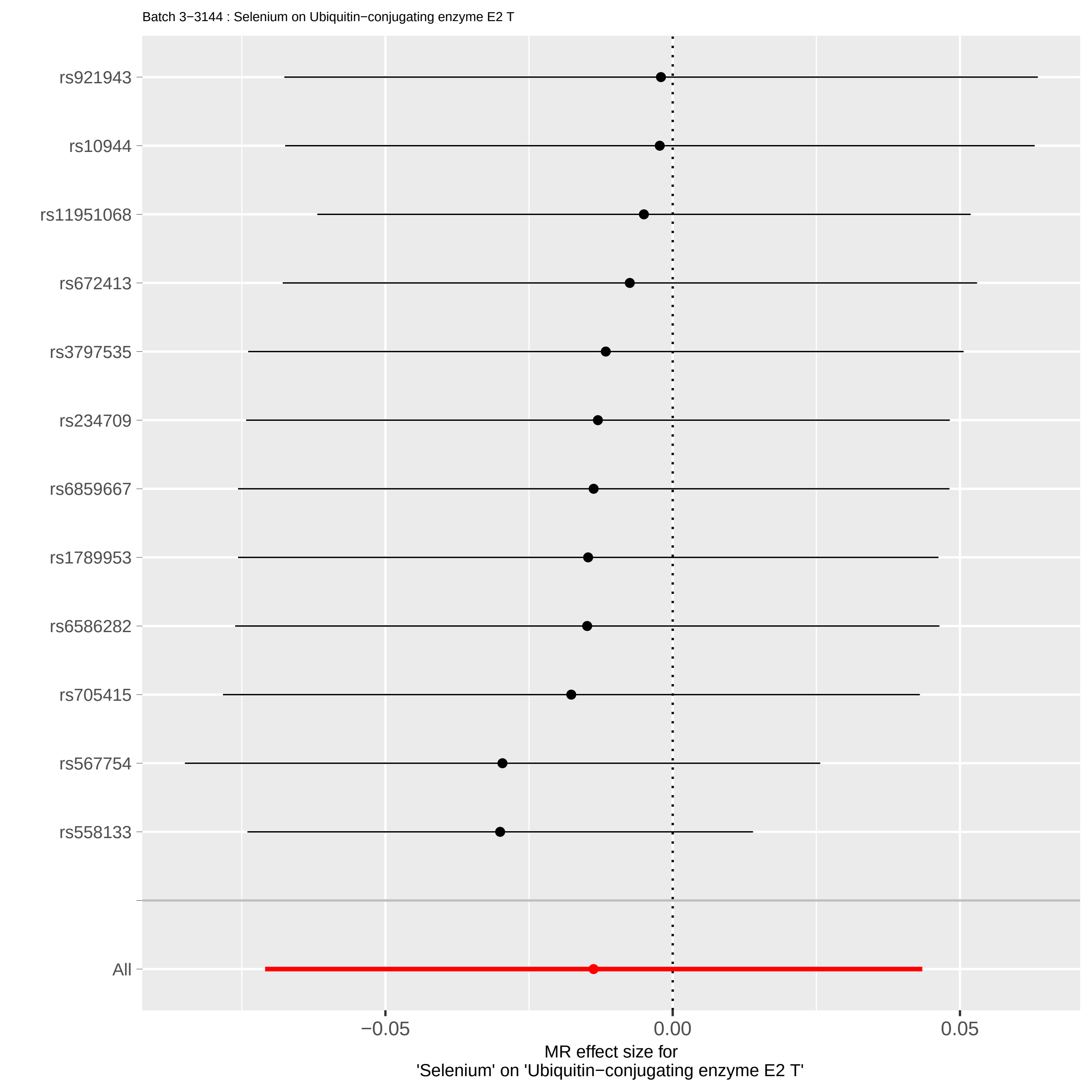

Batch 3−3144 : Selenium on Ubiquitin−conjugating enzyme E2 T
rs921943
rs10944
rs11951068
rs672413
rs3797535
rs234709
rs6859667
rs1789953
rs6586282
rs705415
rs567754
rs558133
All
−0.05
0.00
0.05
MR effect size for
'Selenium' on 'Ubiquitin−conjugating enzyme E2 T'

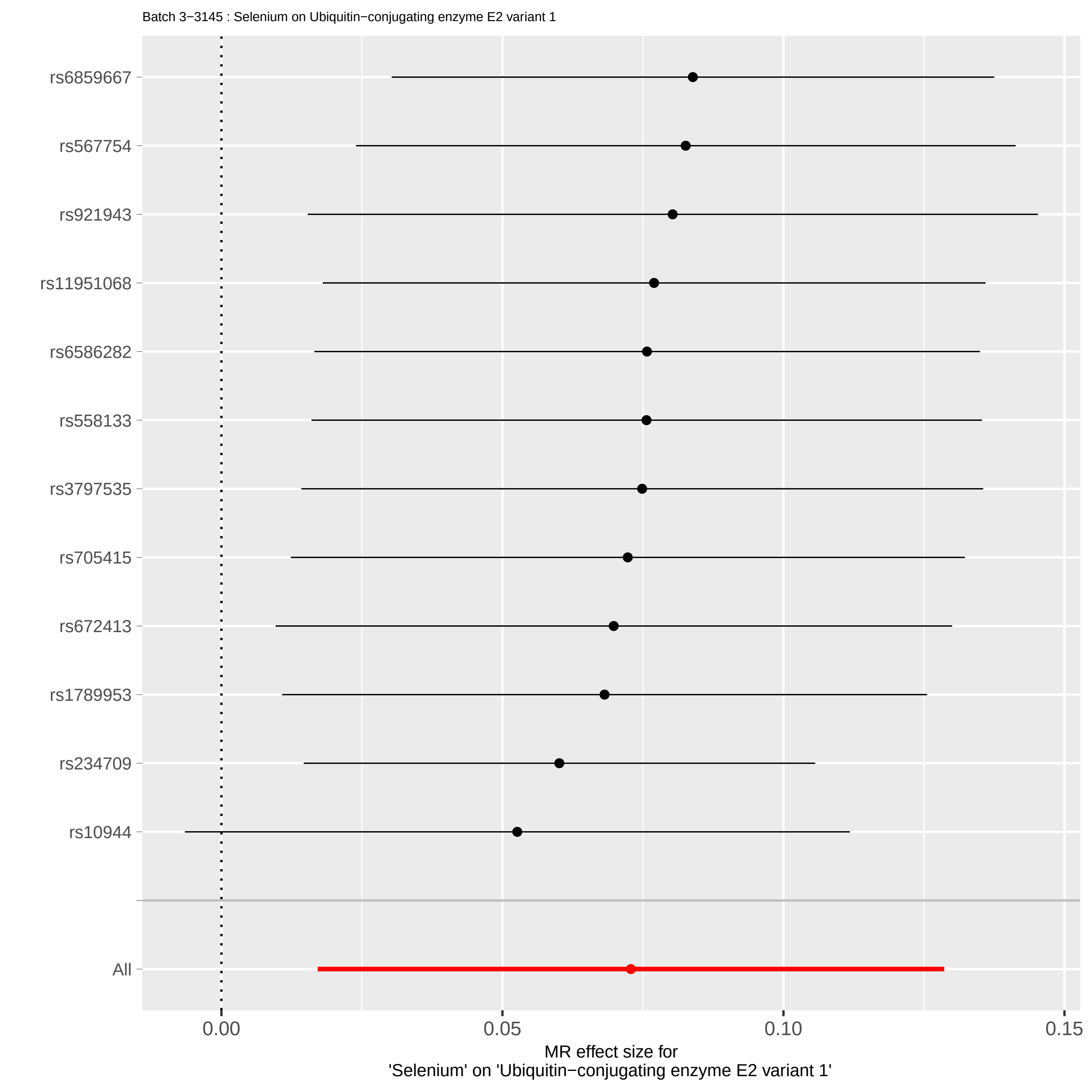
Batch 3−3145 : Selenium on Ubiquitin−conjugating enzyme E2 variant 1
rs6859667
rs567754
rs921943
rs11951068
rs6586282
rs558133
rs3797535
rs705415
rs672413
rs1789953
rs234709
rs10944
All
0.00
0.05
0.10
0.15
MR effect size for
'Selenium' on 'Ubiquitin−conjugating enzyme E2 variant 1'

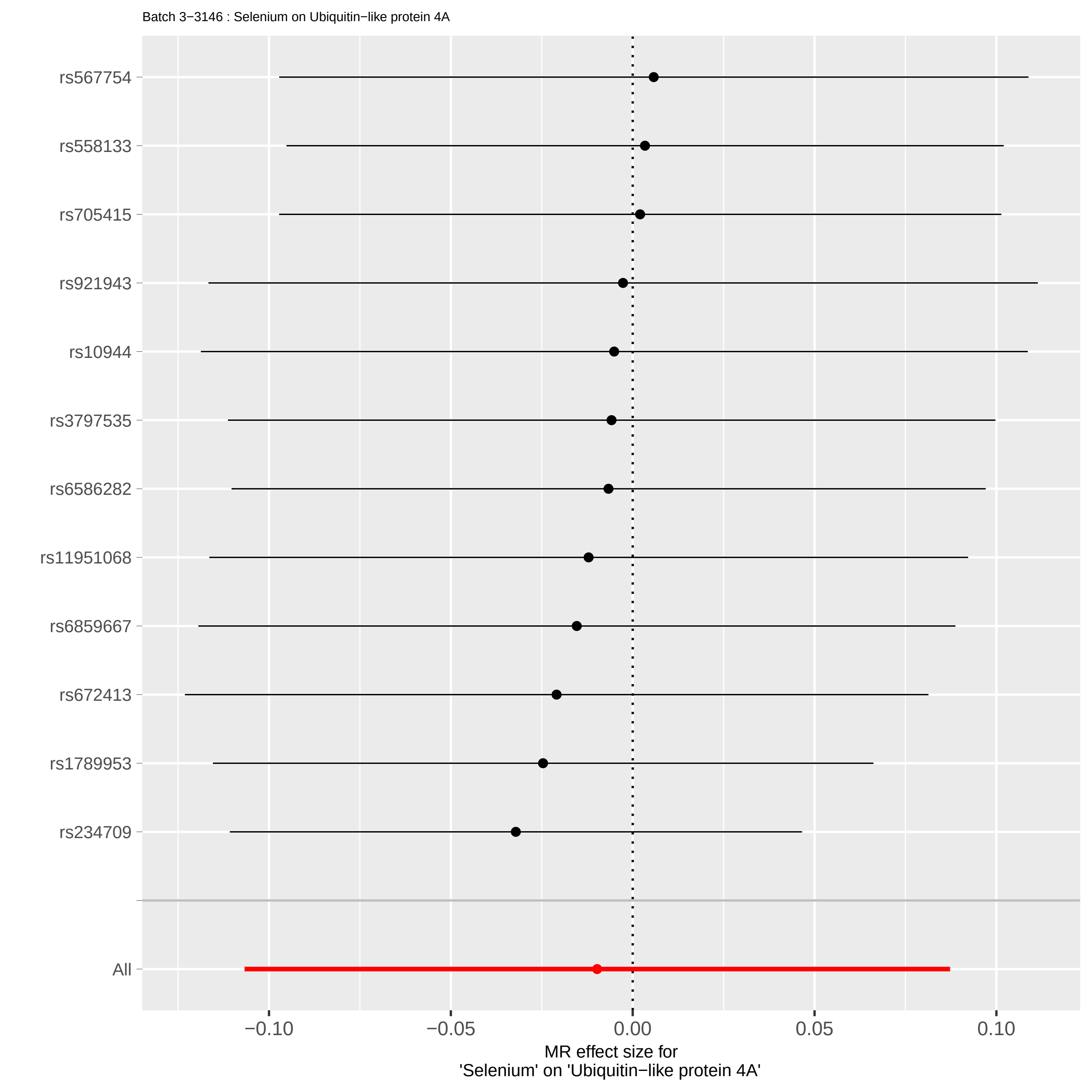

Batch 3−3146 : Selenium on Ubiquitin−like protein 4A
rs567754
rs558133
rs705415
rs921943
rs10944
rs3797535
rs6586282
rs11951068
rs6859667
rs672413
rs1789953
rs234709
All
−0.10
−0.05
0.00
0.05
0.10
MR effect size for
'Selenium' on 'Ubiquitin−like protein 4A'

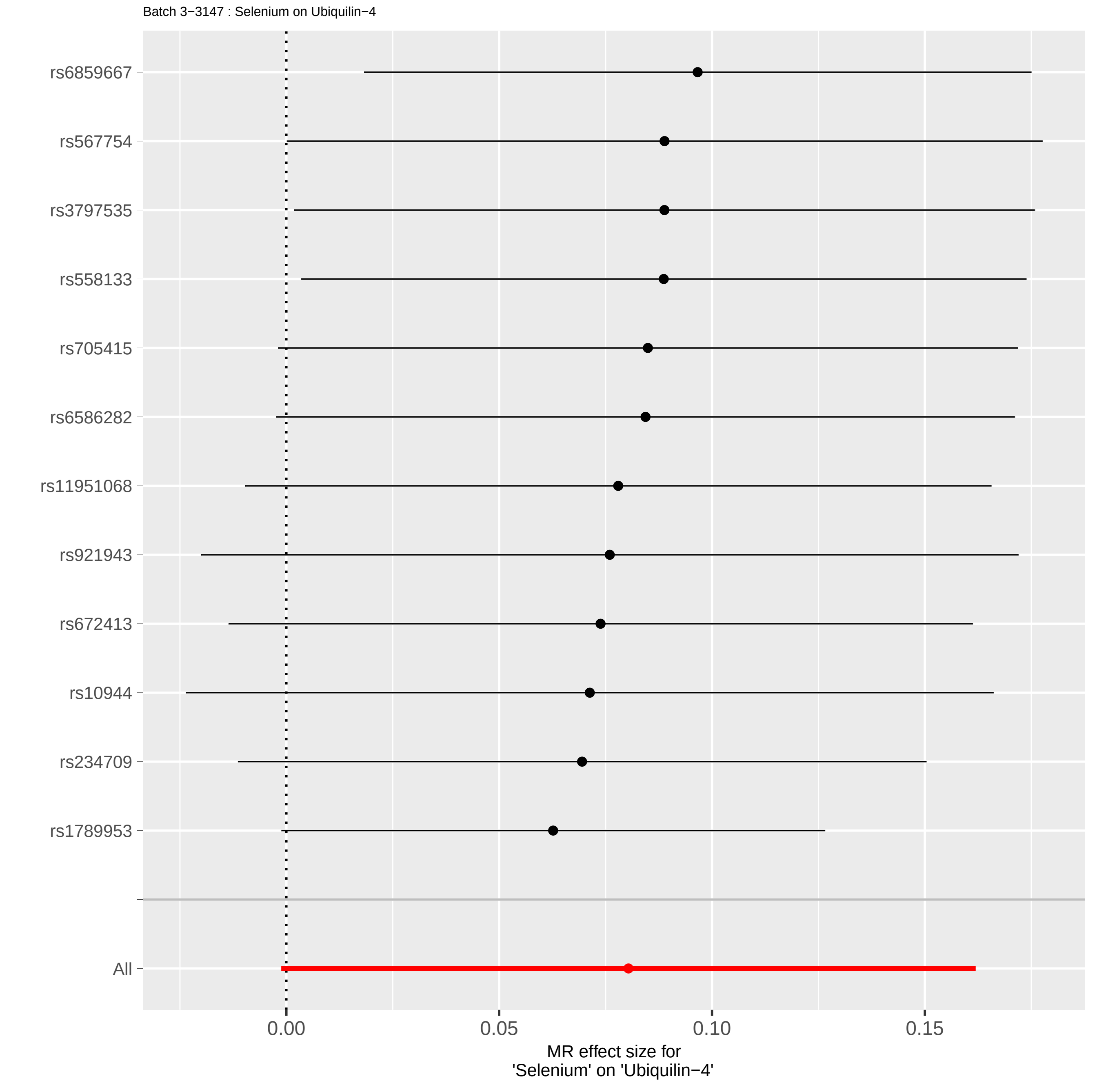
Batch 3−3147 : Selenium on Ubiquilin−4
rs6859667
rs567754
rs3797535
rs558133
rs705415
rs6586282
rs11951068
rs921943
rs672413
rs10944
rs234709
rs1789953
All
0.00
0.05
0.10
0.15
MR effect size for
'Selenium' on 'Ubiquilin−4'

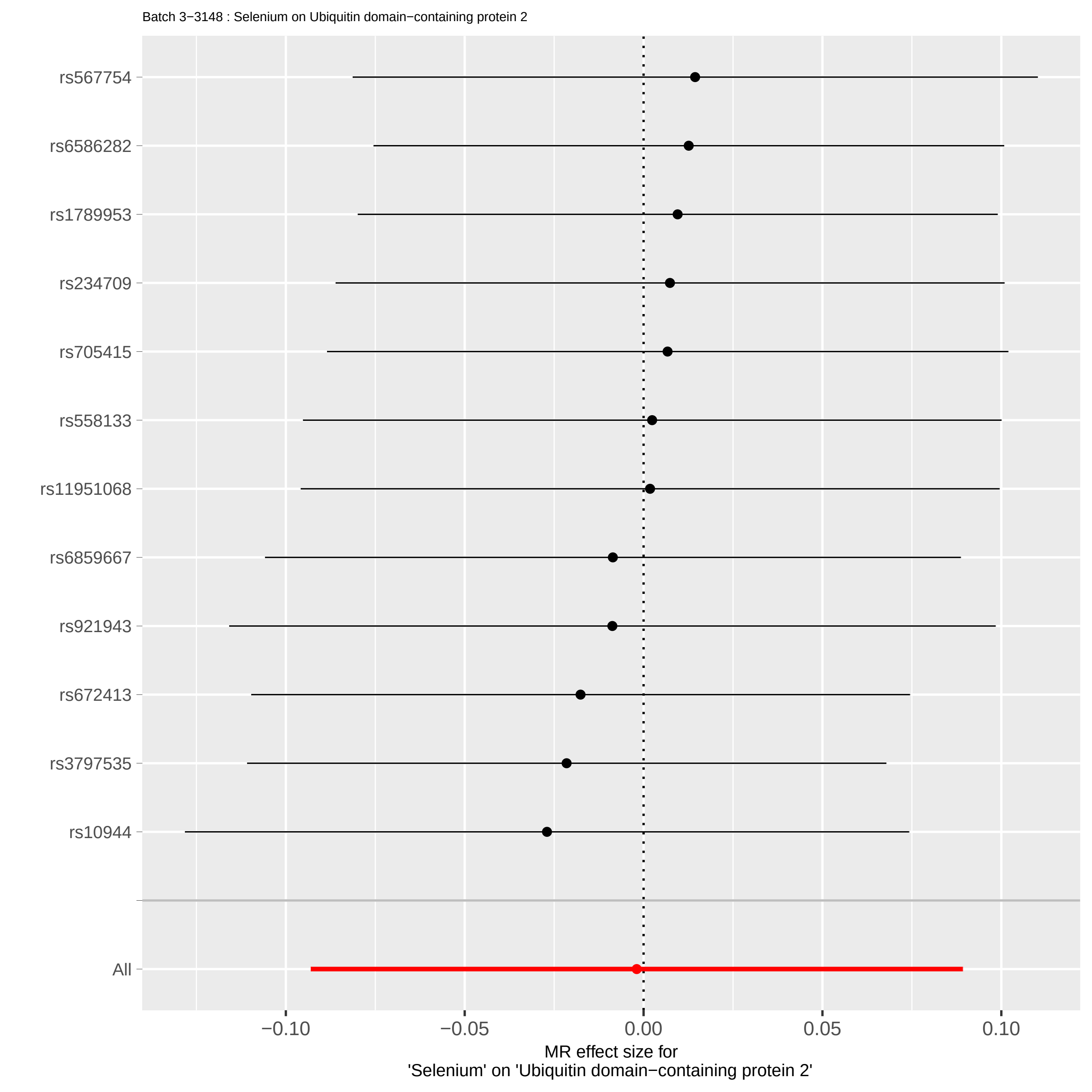

Batch 3−3148 : Selenium on Ubiquitin domain−containing protein 2
rs567754
rs6586282
rs1789953
rs234709
rs705415
rs558133
rs11951068
rs6859667
rs921943
rs672413
rs3797535
rs10944
All
−0.10
−0.05
0.00
0.05
0.10
MR effect size for
'Selenium' on 'Ubiquitin domain−containing protein 2'

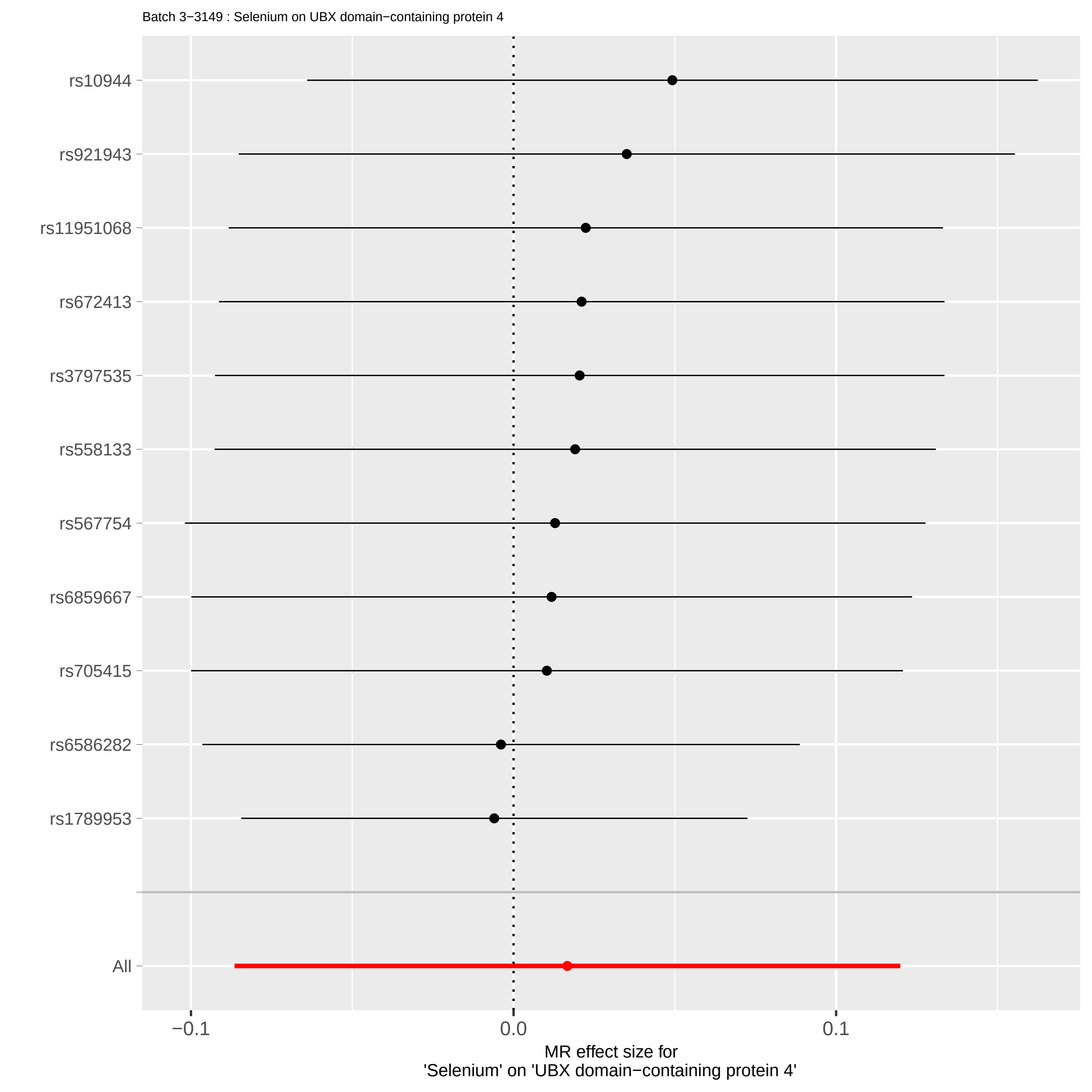
Batch 3−3149 : Selenium on UBX domain−containing protein 4
rs10944
rs921943
rs11951068
rs672413
rs3797535
rs558133
rs567754
rs6859667
rs705415
rs6586282
rs1789953
All
−0.1
0.0
0.1
MR effect size for
'Selenium' on 'UBX domain−containing protein 4'

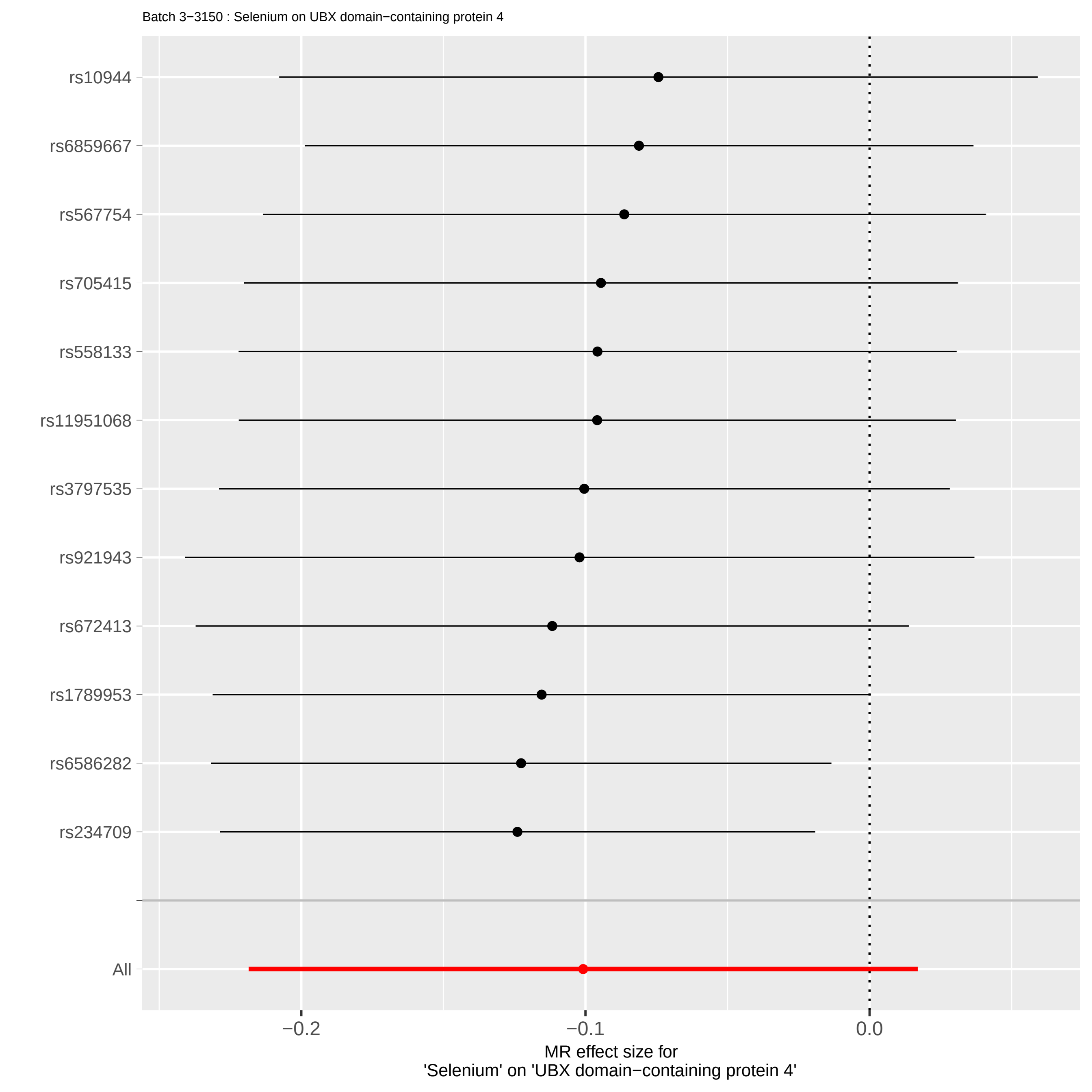

Batch 3−3150 : Selenium on UBX domain−containing protein 4
rs10944
rs6859667
rs567754
rs705415
rs558133
rs11951068
rs3797535
rs921943
rs672413
rs1789953
rs6586282
rs234709
All
−0.2
−0.1
0.0
MR effect size for
'Selenium' on 'UBX domain−containing protein 4'

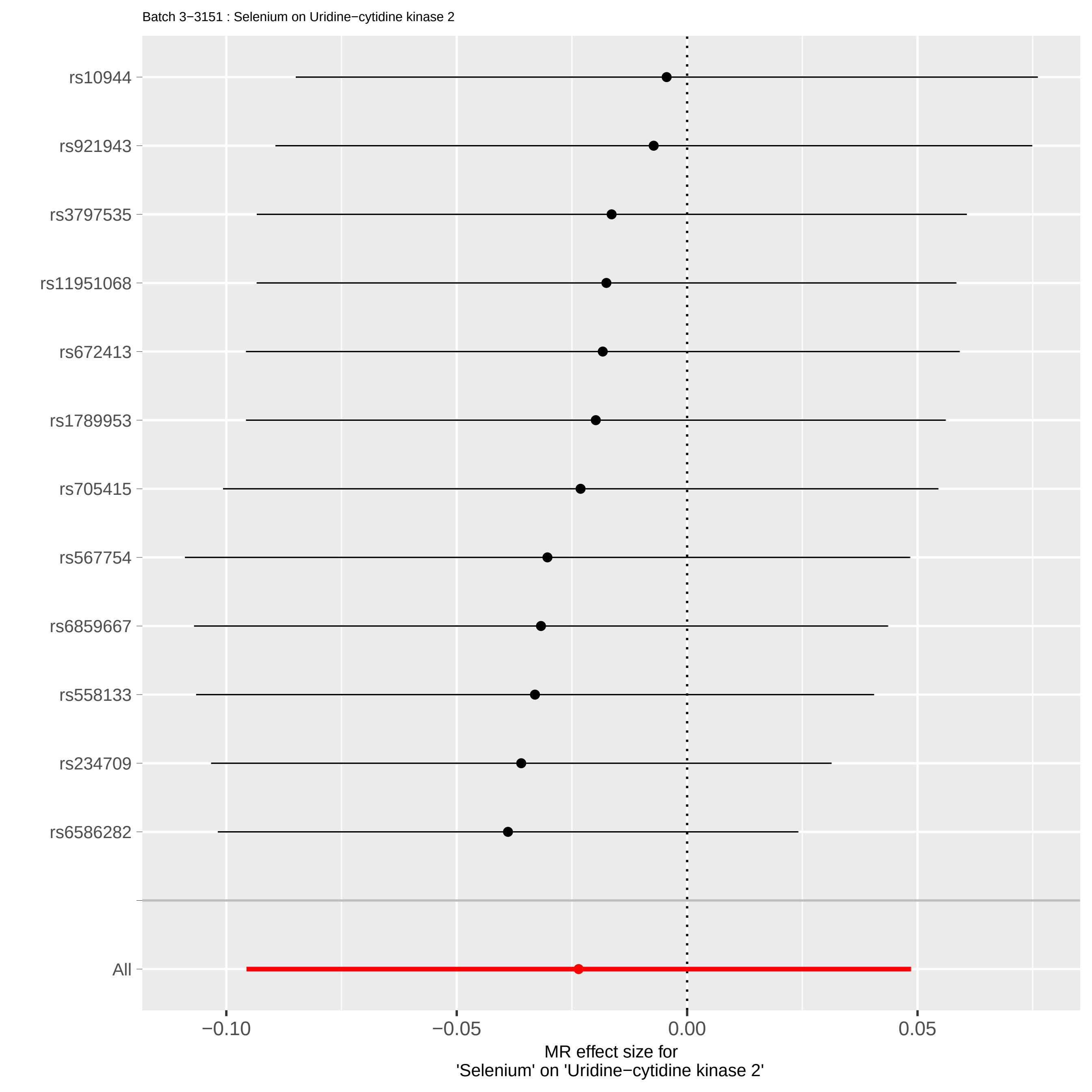
Batch 3−3151 : Selenium on Uridine−cytidine kinase 2
rs10944
rs921943
rs3797535
rs11951068
rs672413
rs1789953
rs705415
rs567754
rs6859667
rs558133
rs234709
rs6586282
All
−0.10
−0.05
0.00
0.05
MR effect size for
'Selenium' on 'Uridine−cytidine kinase 2'

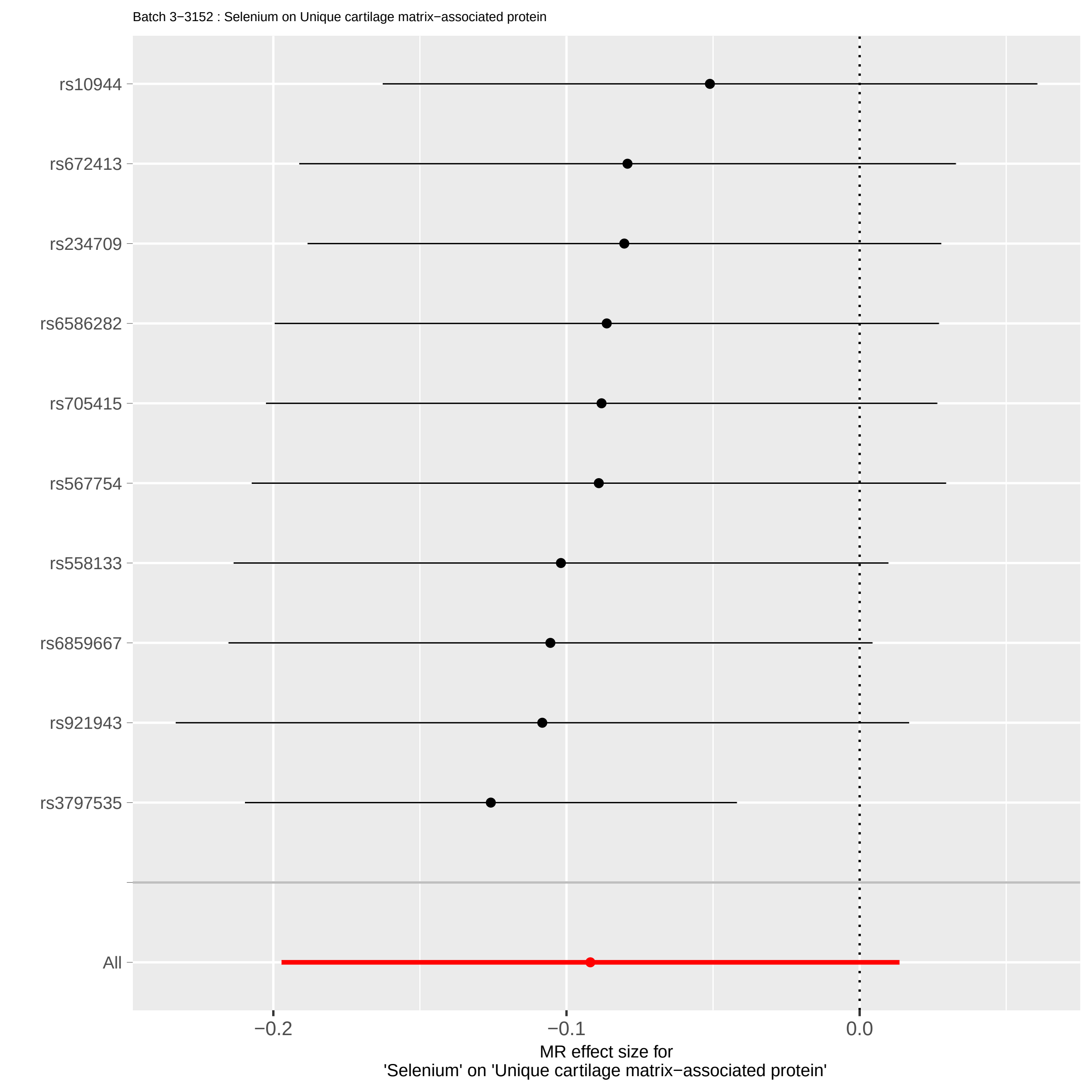

Batch 3−3152 : Selenium on Unique cartilage matrix−associated protein
rs10944
rs672413
rs234709
rs6586282
rs705415
rs567754
rs558133
rs6859667
rs921943
rs3797535
All
−0.2
−0.1
0.0
MR effect size for
'Selenium' on 'Unique cartilage matrix−associated protein'

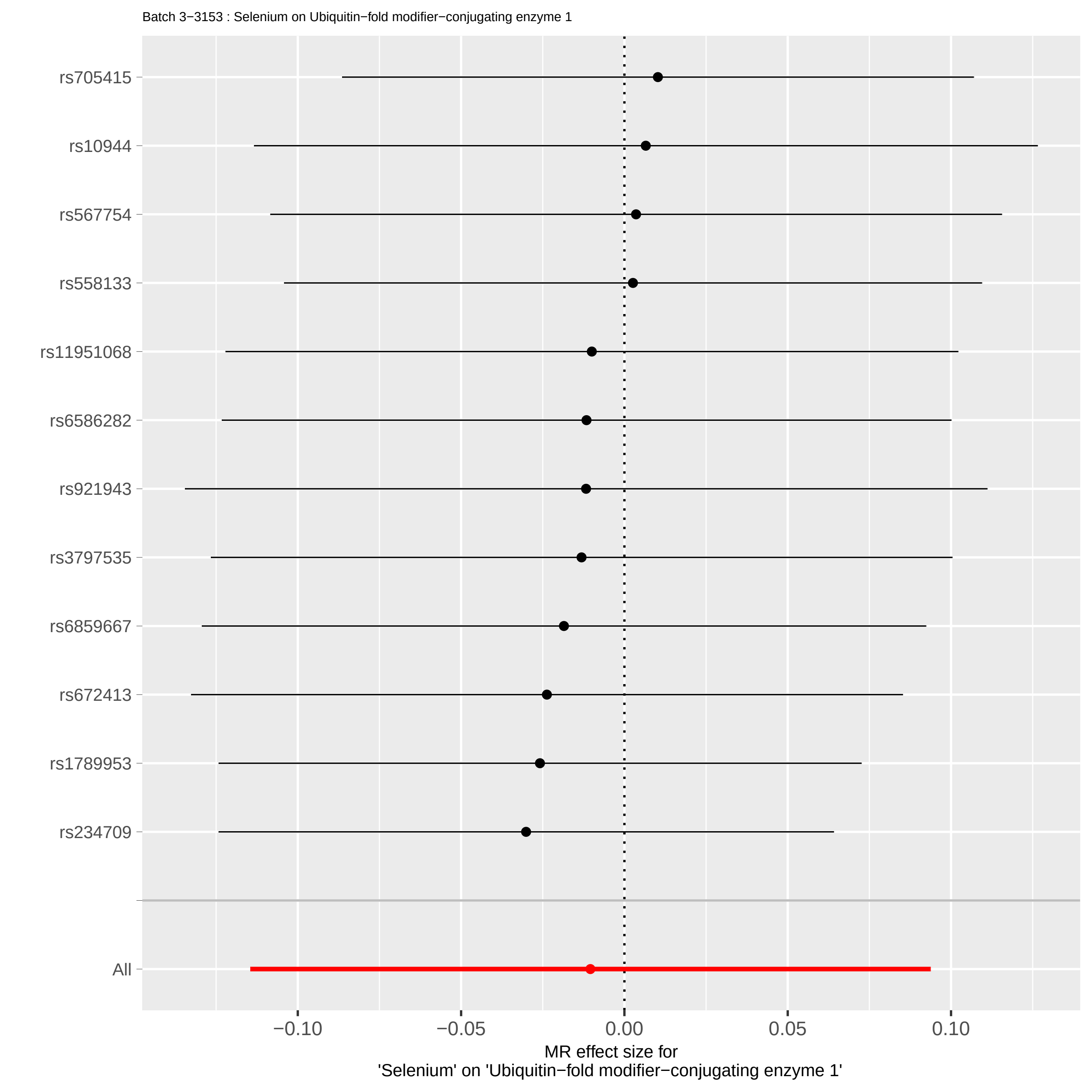

Batch 3−3153 : Selenium on Ubiquitin−fold modifier−conjugating enzyme 1
rs705415
rs10944
rs567754
rs558133
rs11951068
rs6586282
rs921943
rs3797535
rs6859667
rs672413
rs1789953
rs234709
All
−0.10
−0.05
0.00
0.05
0.10
MR effect size for
'Selenium' on 'Ubiquitin−fold modifier−conjugating enzyme 1'

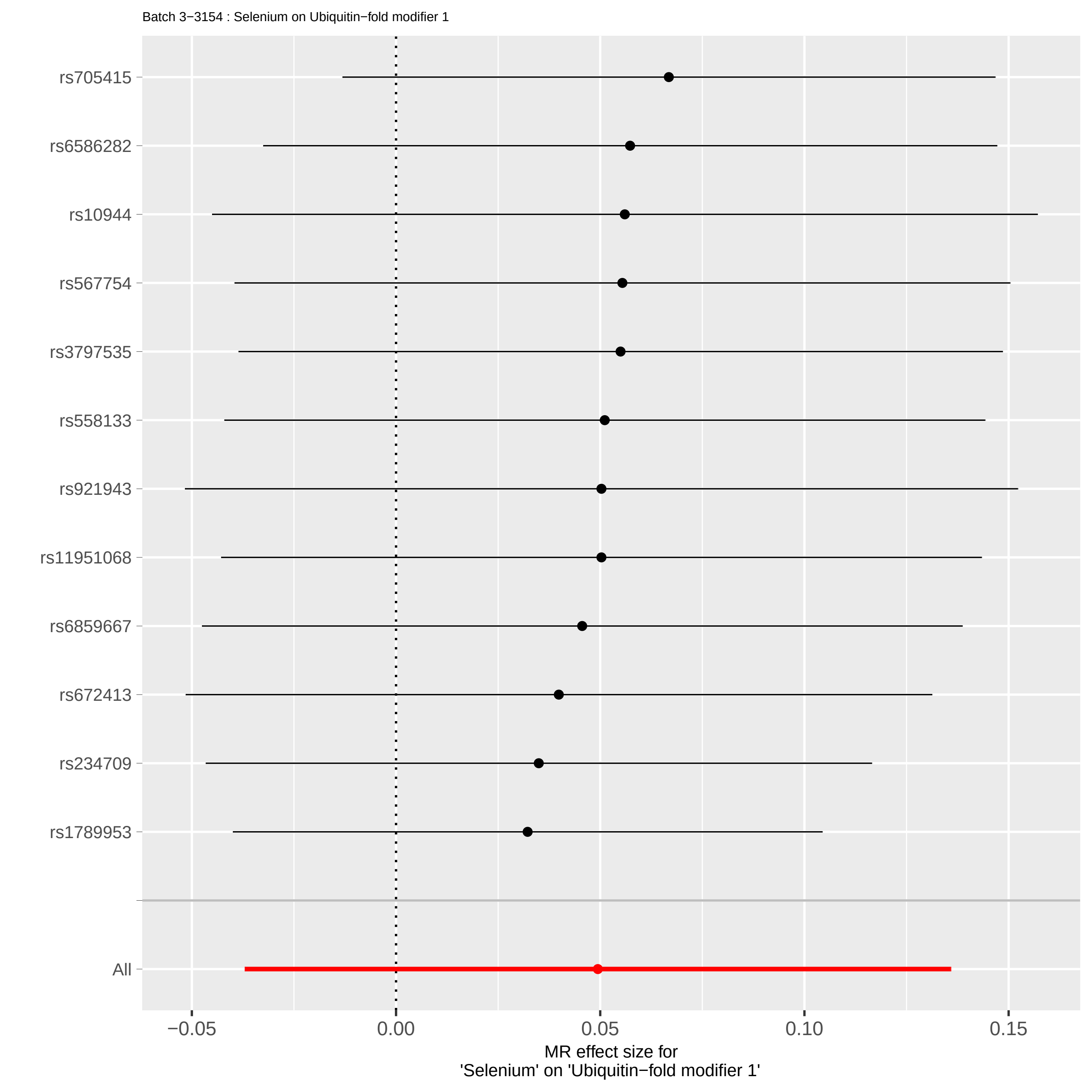

Batch 3−3154 : Selenium on Ubiquitin−fold modifier 1
rs705415
rs6586282
rs10944
rs567754
rs3797535
rs558133
rs921943
rs11951068
rs6859667
rs672413
rs234709
rs1789953
All
−0.05
0.00
0.05
0.10
0.15
MR effect size for
'Selenium' on 'Ubiquitin−fold modifier 1'

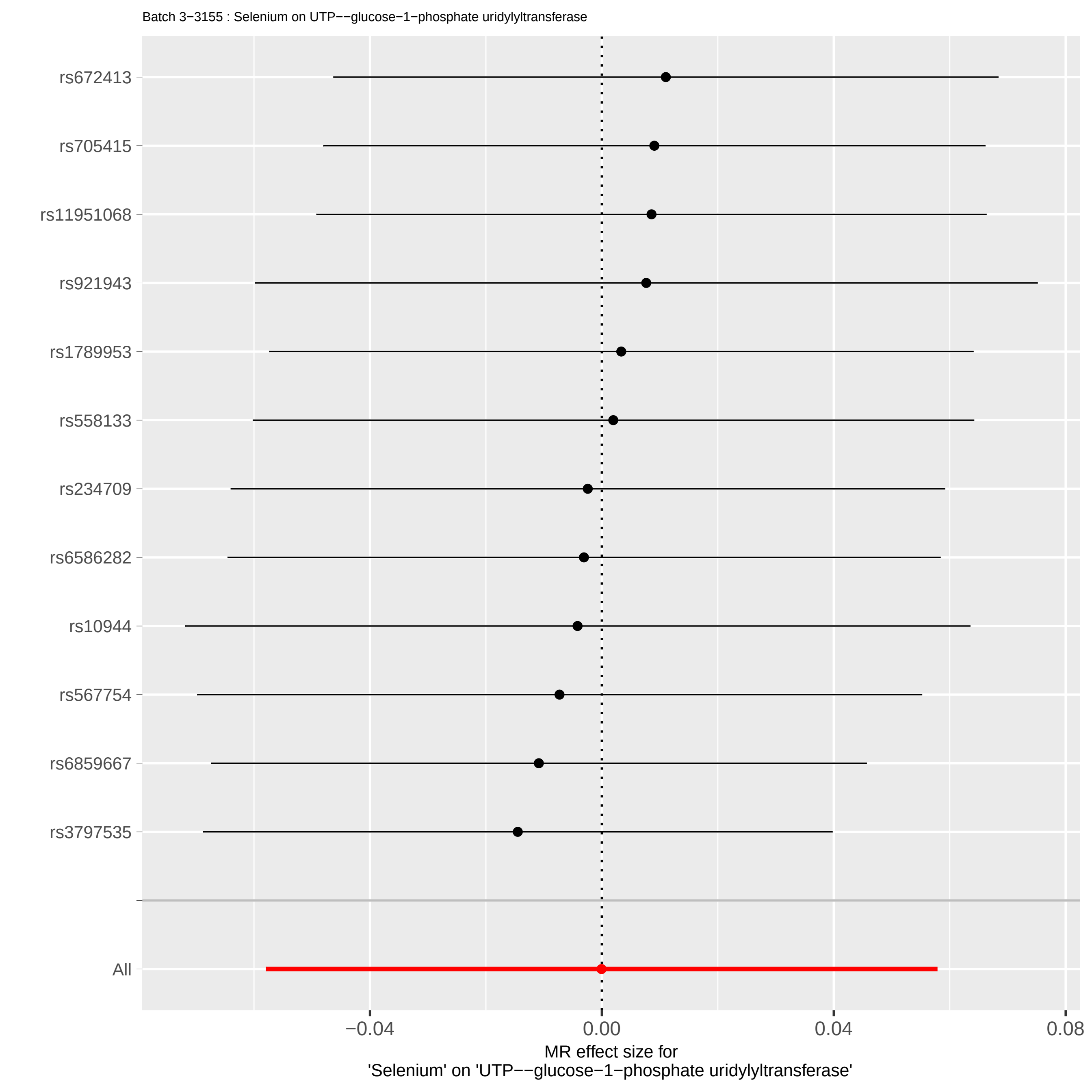

Batch 3−3155 : Selenium on UTP−−glucose−1−phosphate uridylyltransferase
rs672413
rs705415
rs11951068
rs921943
rs1789953
rs558133
rs234709
rs6586282
rs10944
rs567754
rs6859667
rs3797535
All
−0.04
0.00
0.04
0.08
MR effect size for
'Selenium' on 'UTP−−glucose−1−phosphate uridylyltransferase'

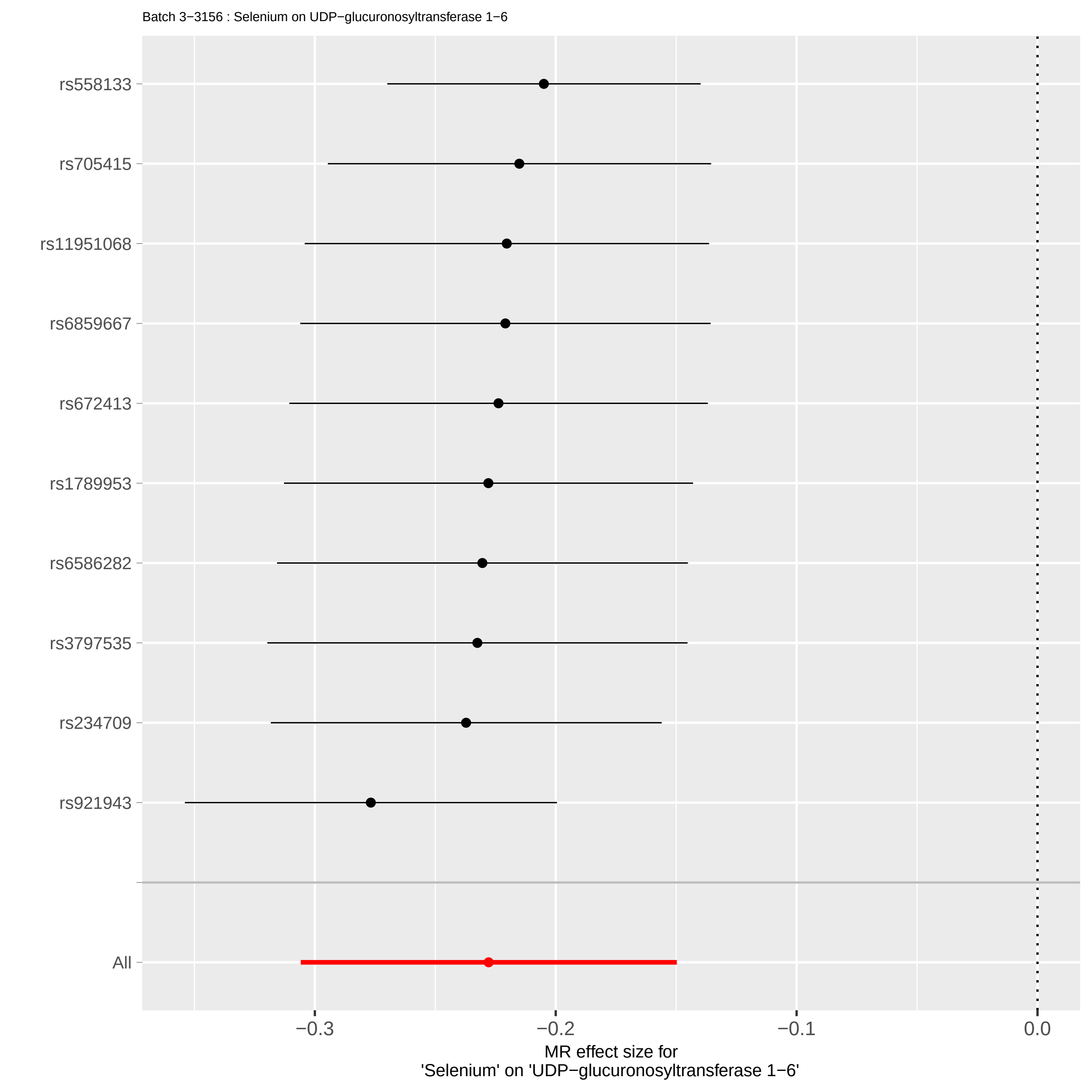

Batch 3−3156 : Selenium on UDP−glucuronosyltransferase 1−6
rs558133
rs705415
rs11951068
rs6859667
rs672413
rs1789953
rs6586282
rs3797535
rs234709
rs921943
All
−0.3
−0.2
−0.1
0.0
MR effect size for
'Selenium' on 'UDP−glucuronosyltransferase 1−6'

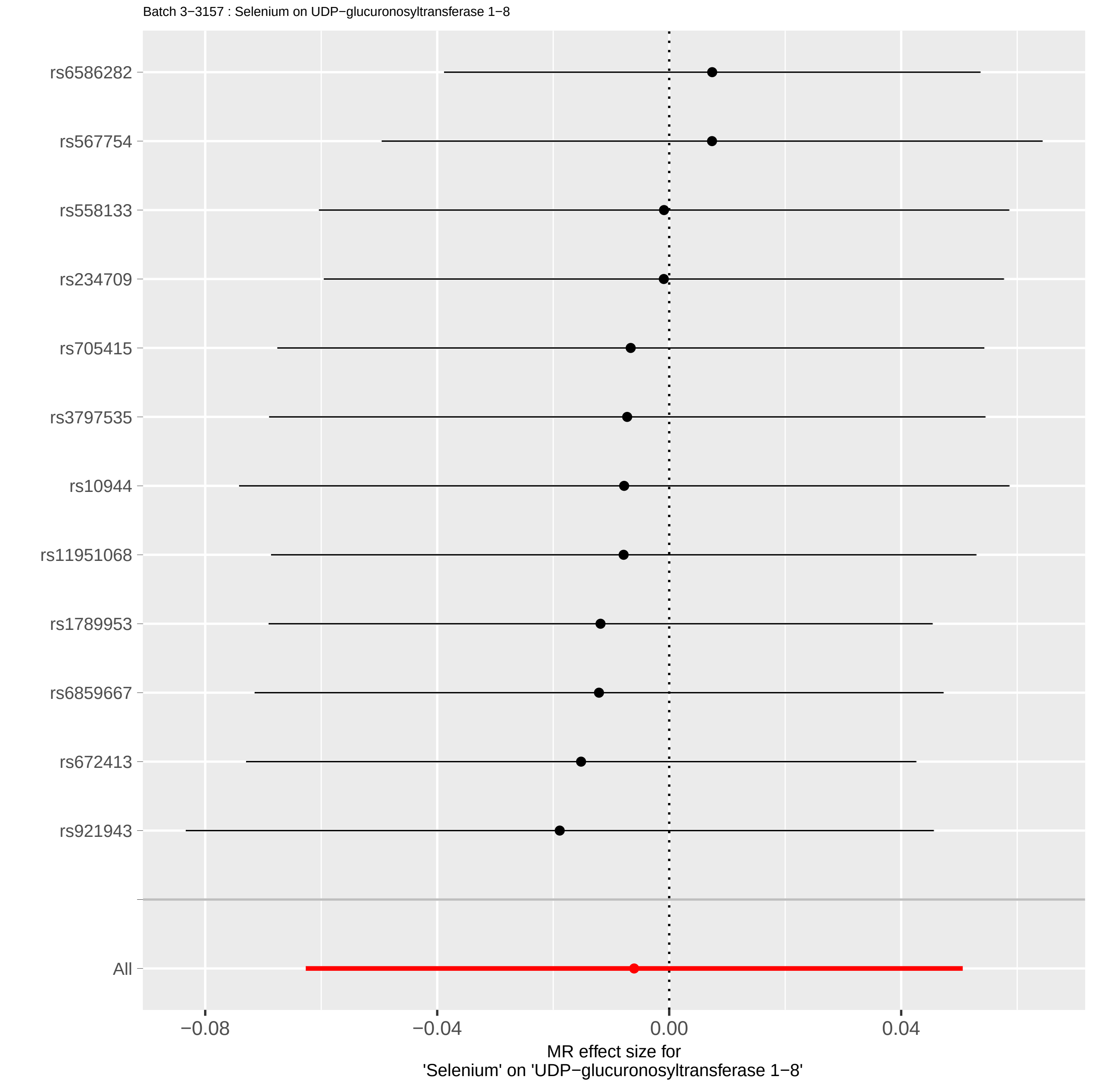

Batch 3−3157 : Selenium on UDP−glucuronosyltransferase 1−8
rs6586282
rs567754
rs558133
rs234709
rs705415
rs3797535
rs10944
rs11951068
rs1789953
rs6859667
rs672413
rs921943
All
−0.08
−0.04
0.00
0.04
MR effect size for
'Selenium' on 'UDP−glucuronosyltransferase 1−8'

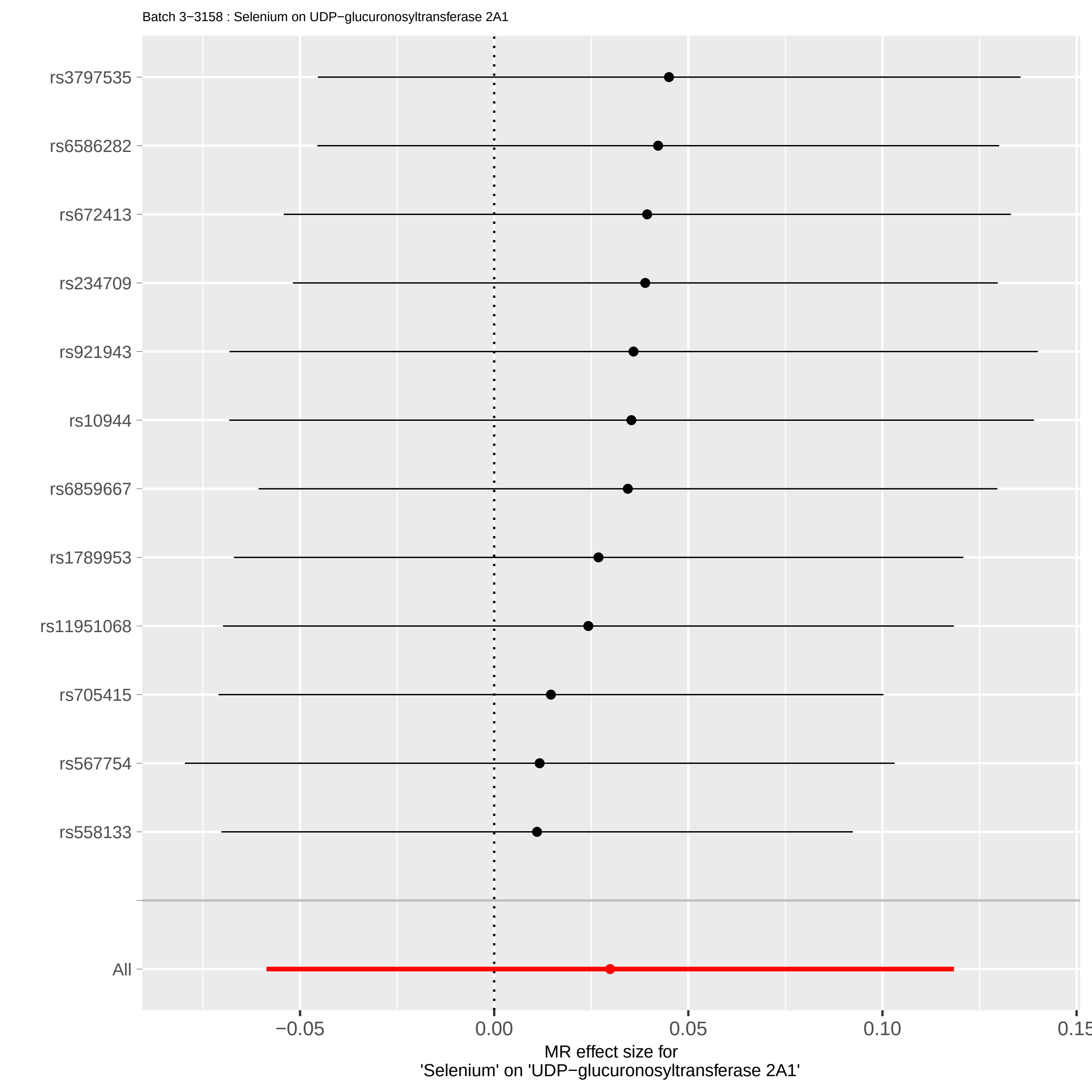

Batch 3−3158 : Selenium on UDP−glucuronosyltransferase 2A1
rs3797535
rs6586282
rs672413
rs234709
rs921943
rs10944
rs6859667
rs1789953
rs11951068
rs705415
rs567754
rs558133
All
−0.05
0.00
0.05
0.10
0.15
MR effect size for
'Selenium' on 'UDP−glucuronosyltransferase 2A1'

Batch 3−3159 : Selenium on NKG2D ligand 1

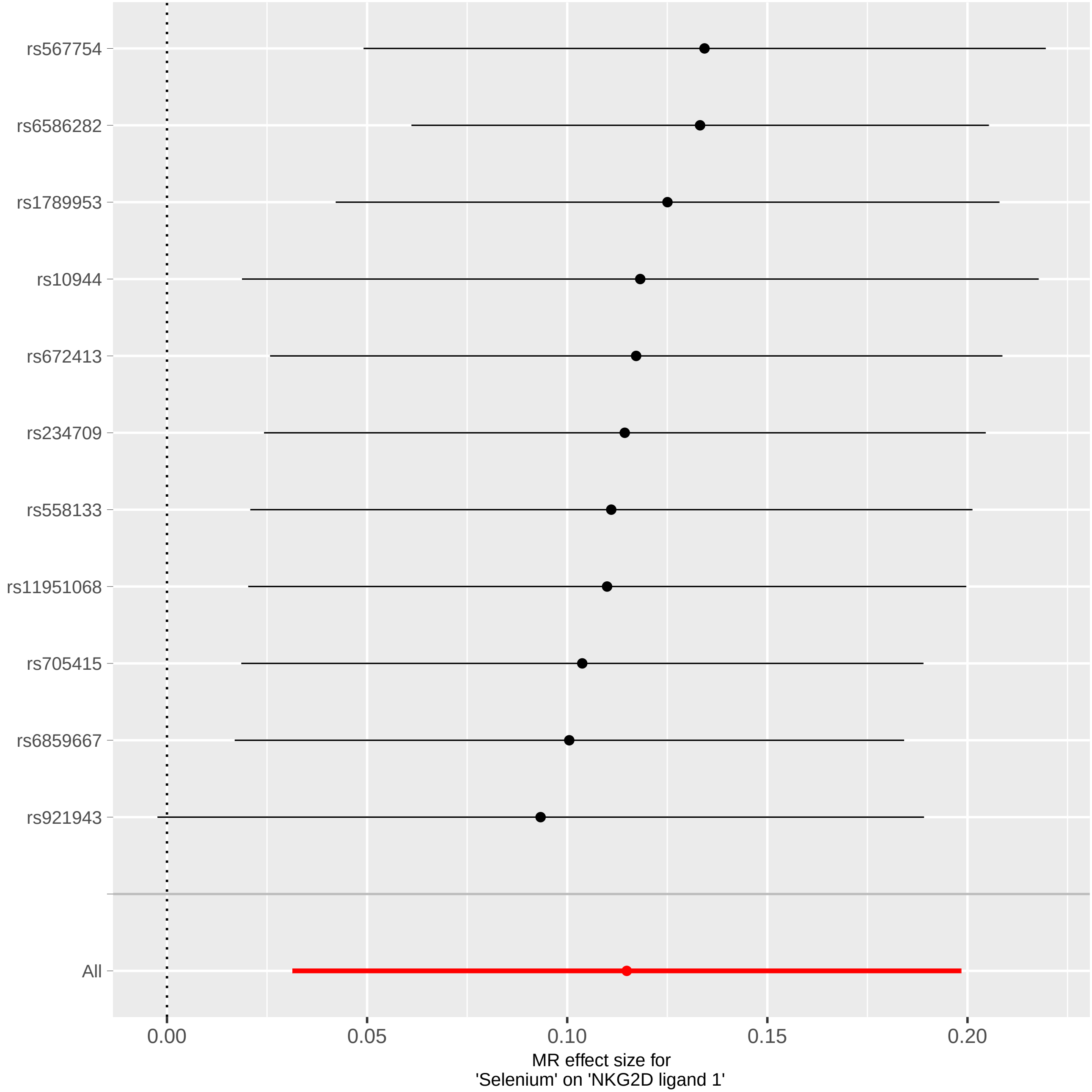

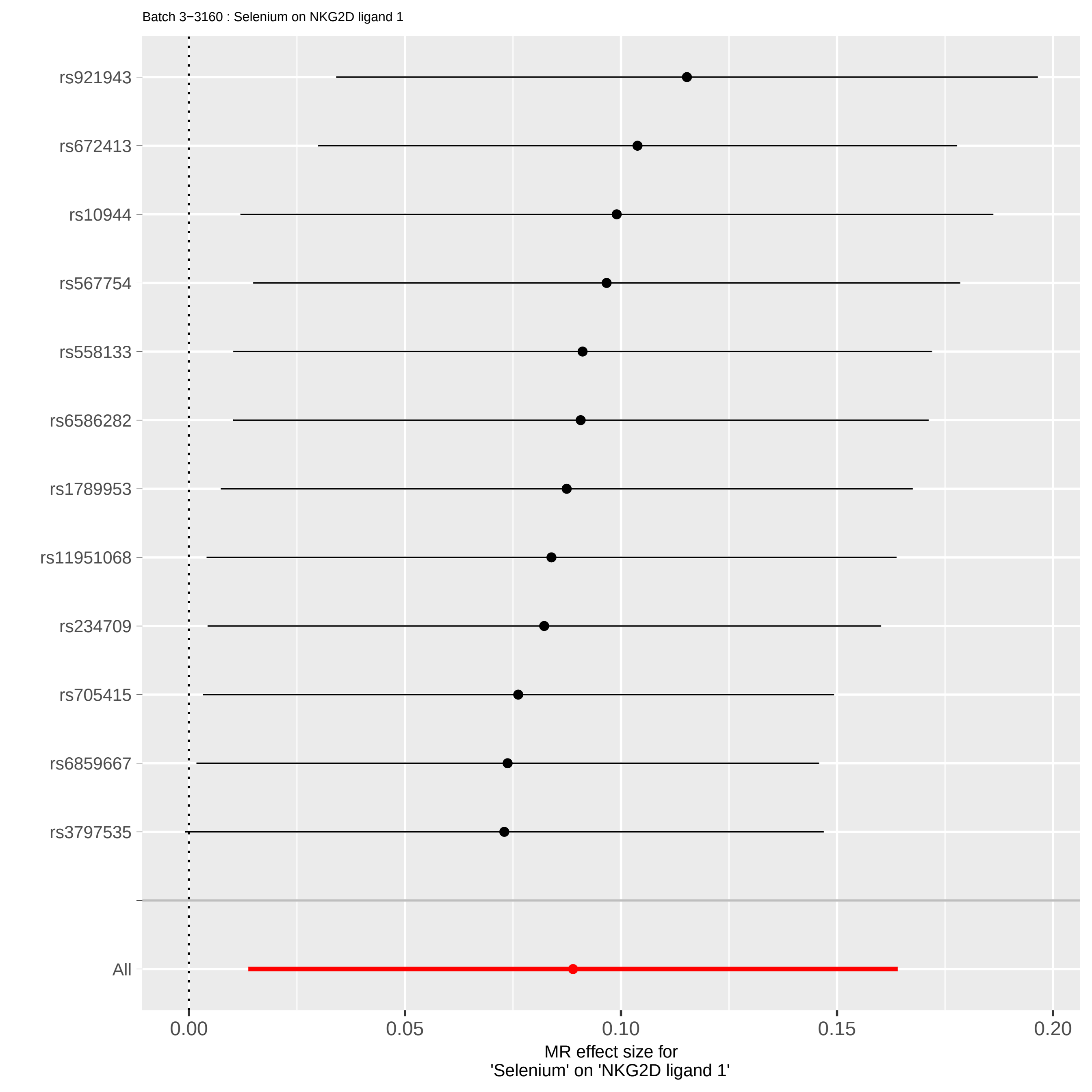

Batch 3−3160 : Selenium on NKG2D ligand 1
rs921943
rs672413
rs10944
rs567754
rs558133
rs6586282
rs1789953
rs11951068
rs234709
rs705415
rs6859667
rs3797535
All
0.00
0.05
0.10
0.15
0.20
MR effect size for
'Selenium' on 'NKG2D ligand 1'

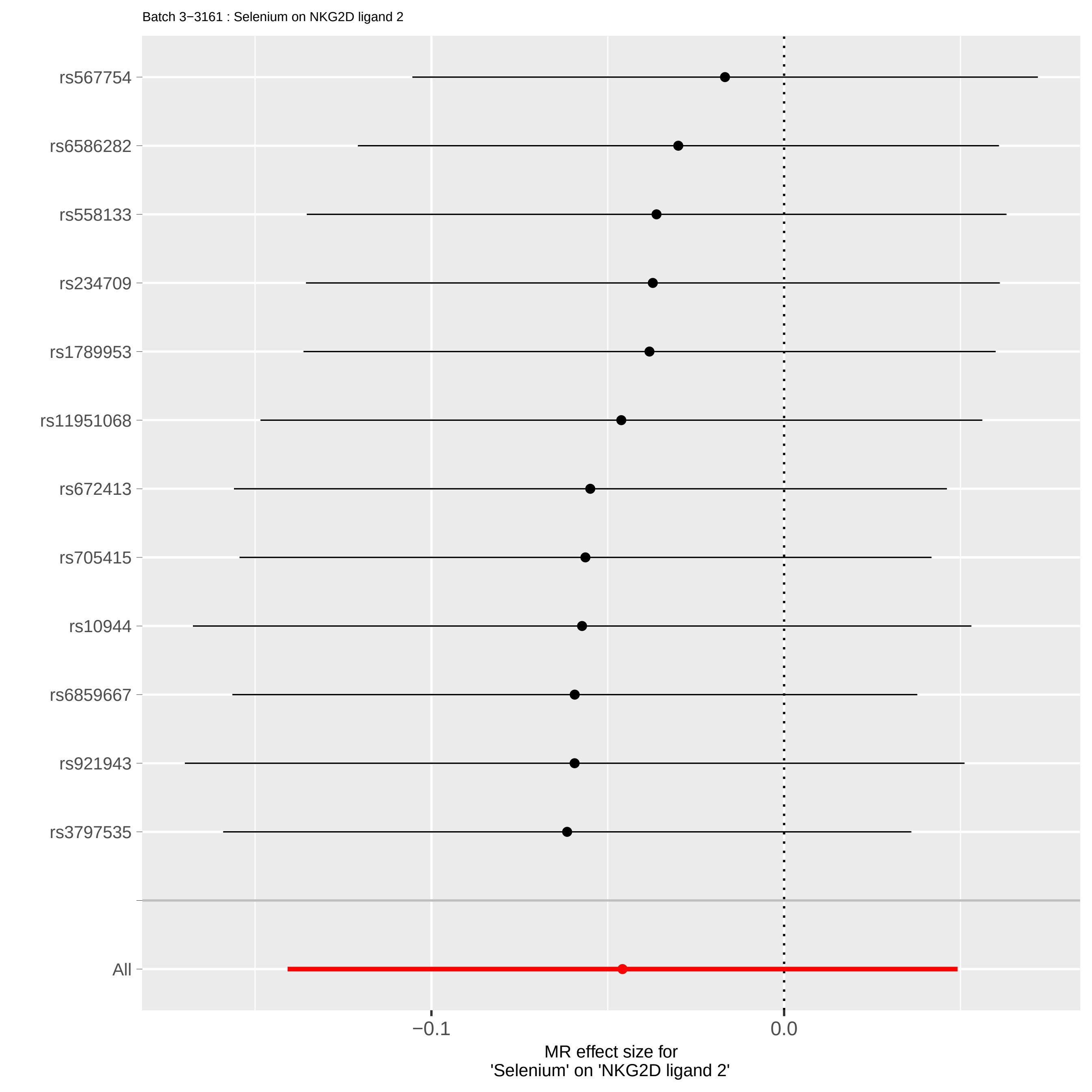

Batch 3−3161 : Selenium on NKG2D ligand 2
rs567754
rs6586282
rs558133
rs234709
rs1789953
rs11951068
rs672413
rs705415
rs10944
rs6859667
rs921943
rs3797535
All
−0.1
0.0
MR effect size for
'Selenium' on 'NKG2D ligand 2'

Batch 3−3162 : Selenium on NKG2D ligand 3

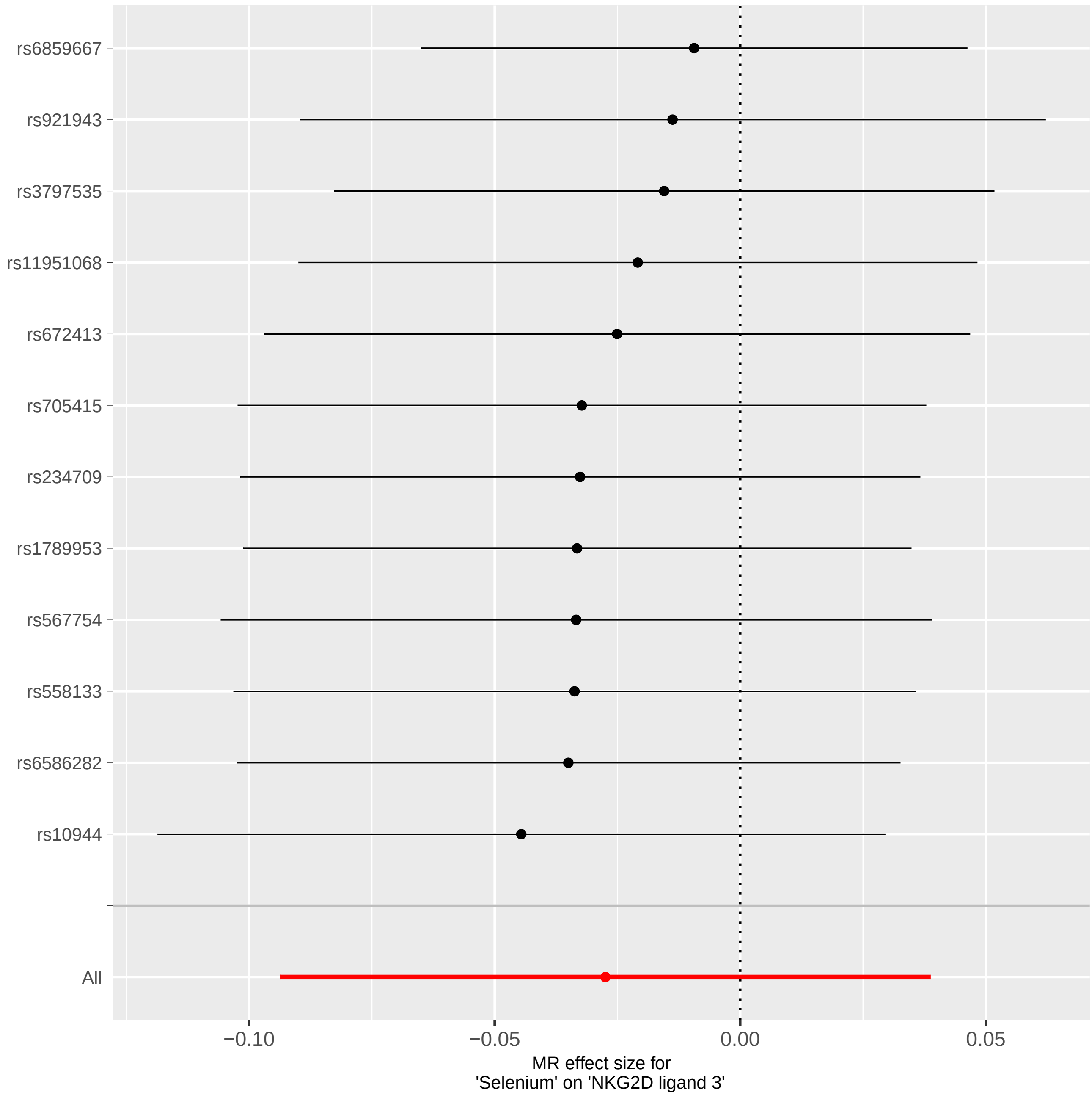

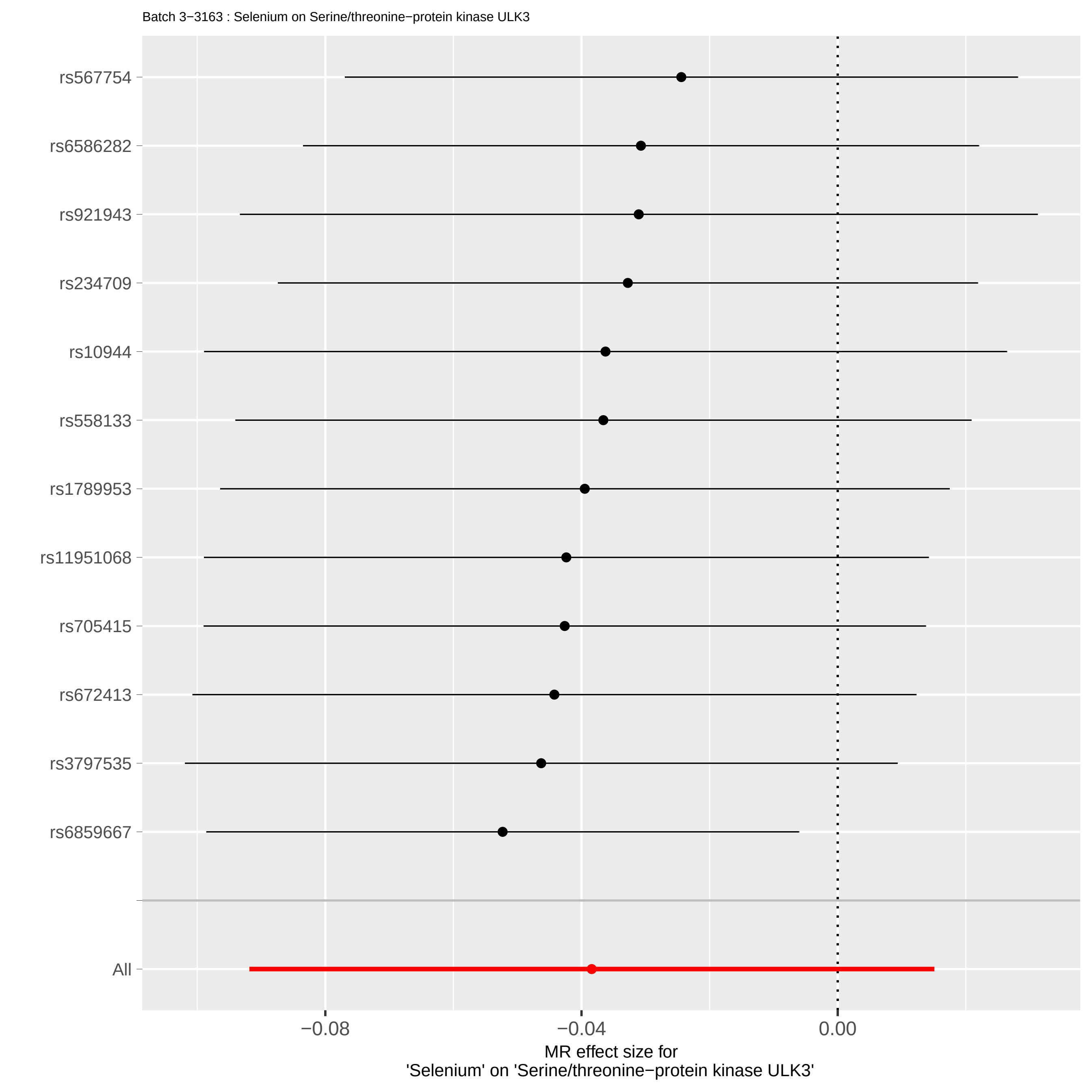

Batch 3−3163 : Selenium on Serine/threonine−protein kinase ULK3
rs567754
rs6586282
rs921943
rs234709
rs10944
rs558133
rs1789953
rs11951068
rs705415
rs672413
rs3797535
rs6859667
All
−0.08
−0.04
0.00
MR effect size for
'Selenium' on 'Serine/threonine−protein kinase ULK3'

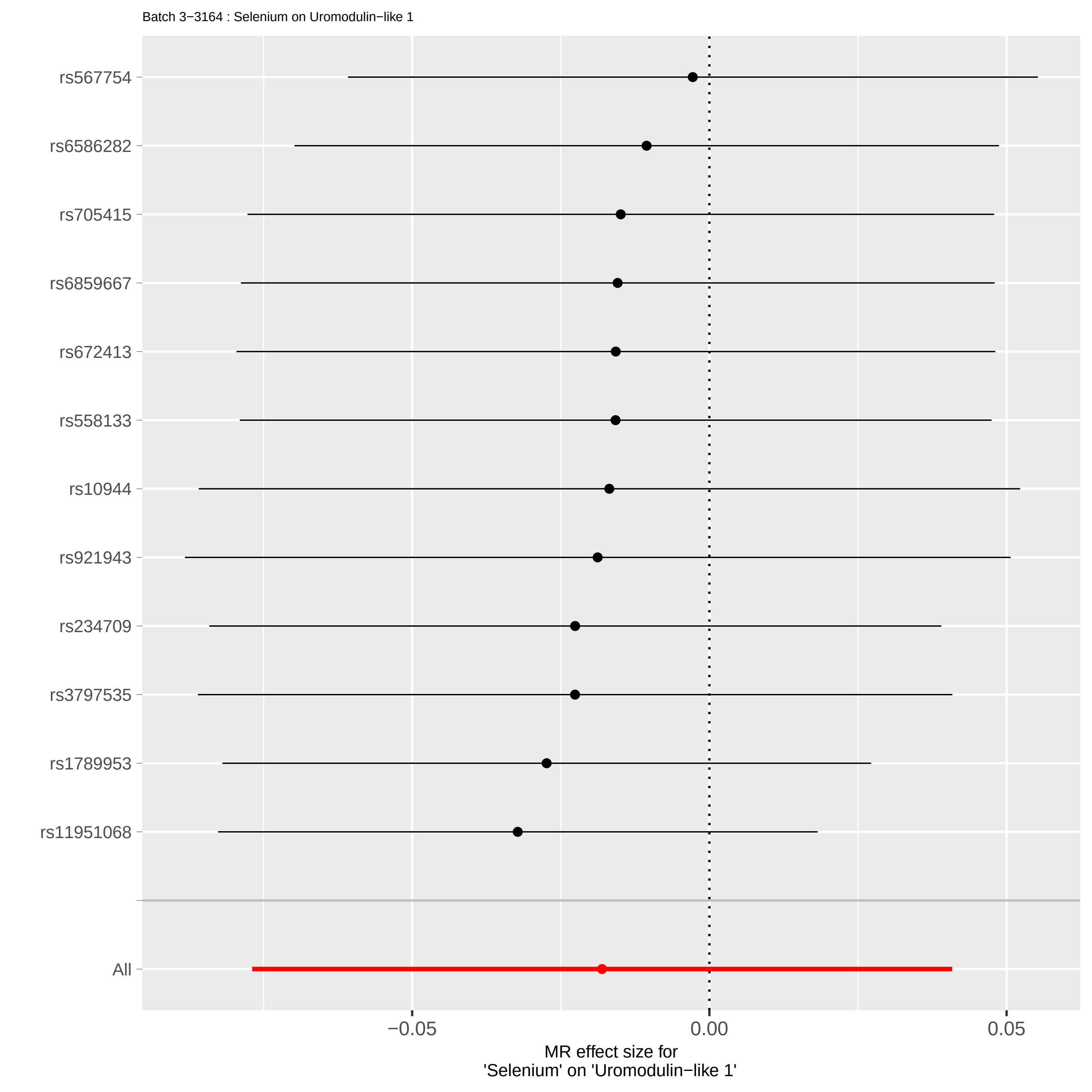

Batch 3−3164 : Selenium on Uromodulin−like 1
rs567754
rs6586282
rs705415
rs6859667
rs672413
rs558133
rs10944
rs921943
rs234709
rs3797535
rs1789953
rs11951068
All
−0.05
0.00
0.05
MR effect size for
'Selenium' on 'Uromodulin−like 1'

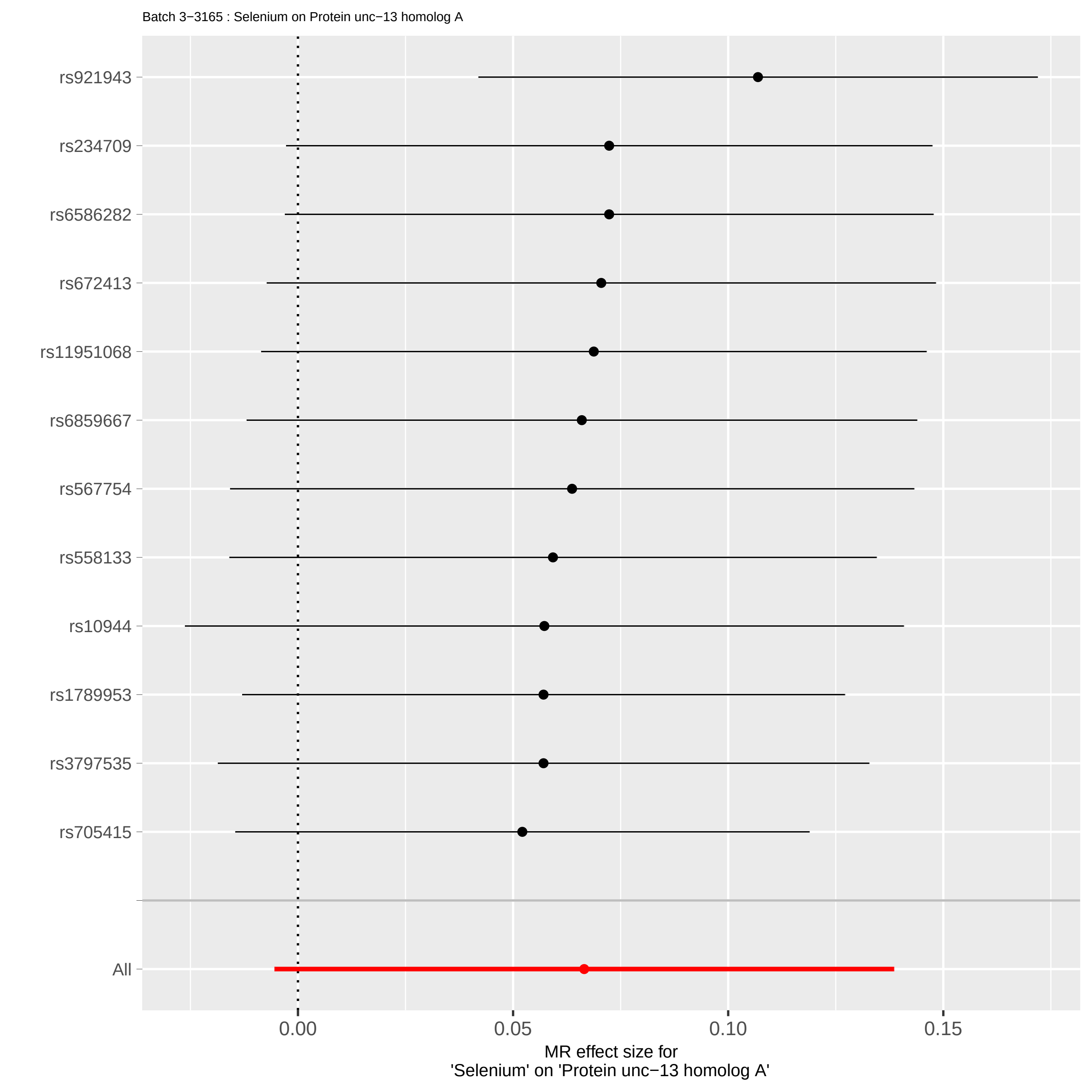
Batch 3−3165 : Selenium on Protein unc−13 homolog A
rs921943
rs234709
rs6586282
rs672413
rs11951068
rs6859667
rs567754
rs558133
rs10944
rs1789953
rs3797535
rs705415
All
0.00
0.05
0.10
0.15
MR effect size for
'Selenium' on 'Protein unc−13 homolog A'

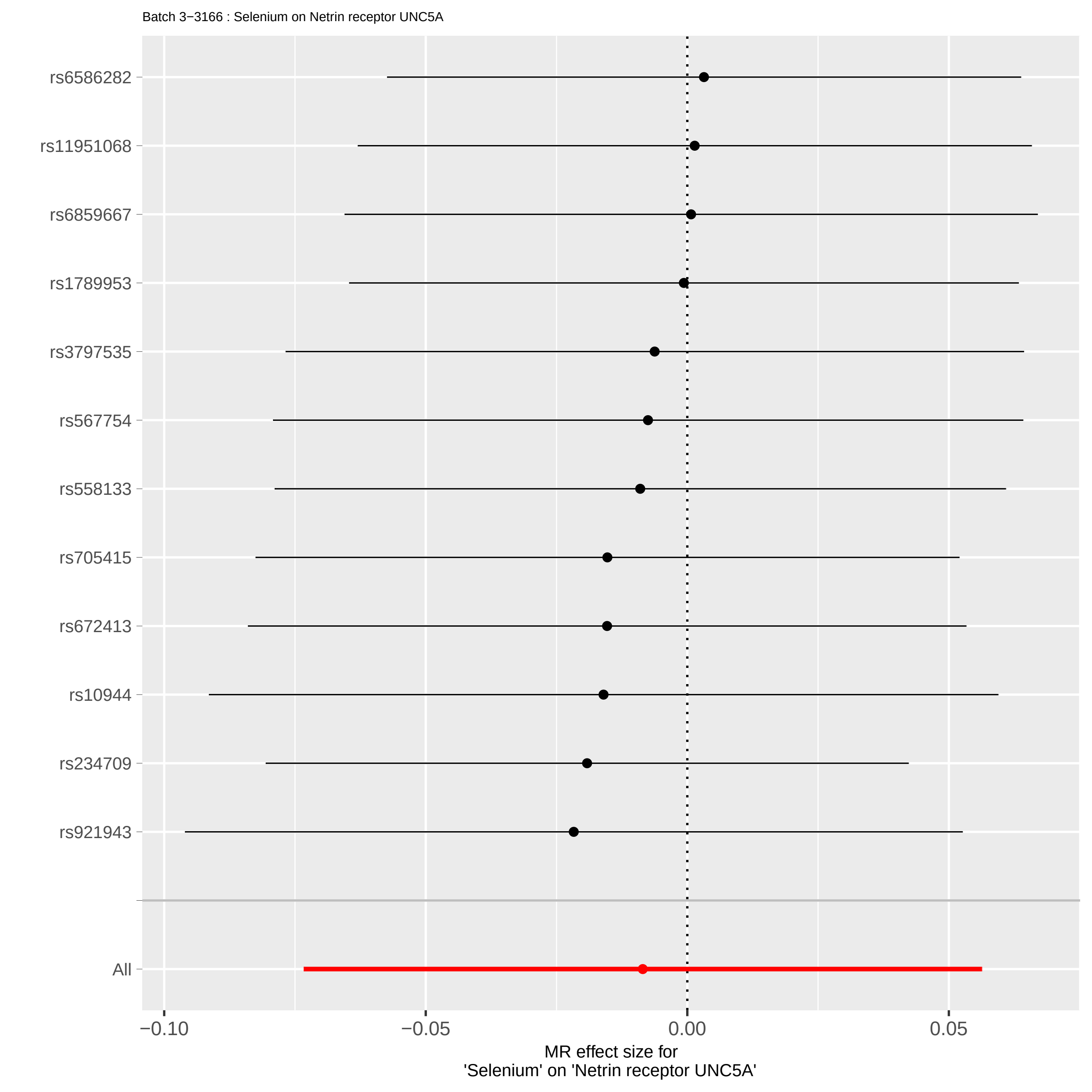

Batch 3−3166 : Selenium on Netrin receptor UNC5A
rs6586282
rs11951068
rs6859667
rs1789953
rs3797535
rs567754
rs558133
rs705415
rs672413
rs10944
rs234709
rs921943
All
−0.10
−0.05
0.00
0.05
MR effect size for
'Selenium' on 'Netrin receptor UNC5A'

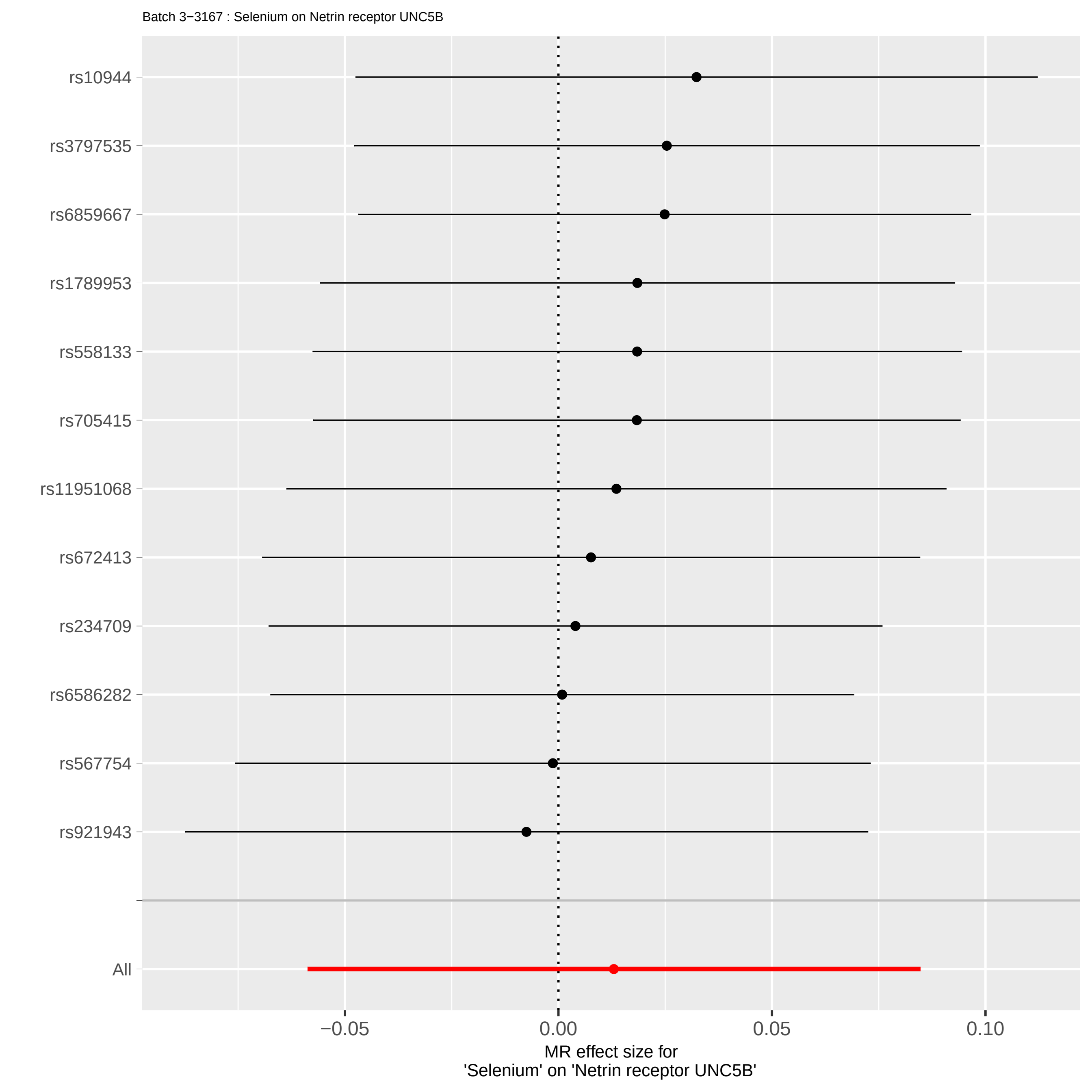
Batch 3−3167 : Selenium on Netrin receptor UNC5B
rs10944
rs3797535
rs6859667
rs1789953
rs558133
rs705415
rs11951068
rs672413
rs234709
rs6586282
rs567754
rs921943
All
−0.05
0.00
0.05
0.10
MR effect size for
'Selenium' on 'Netrin receptor UNC5B'

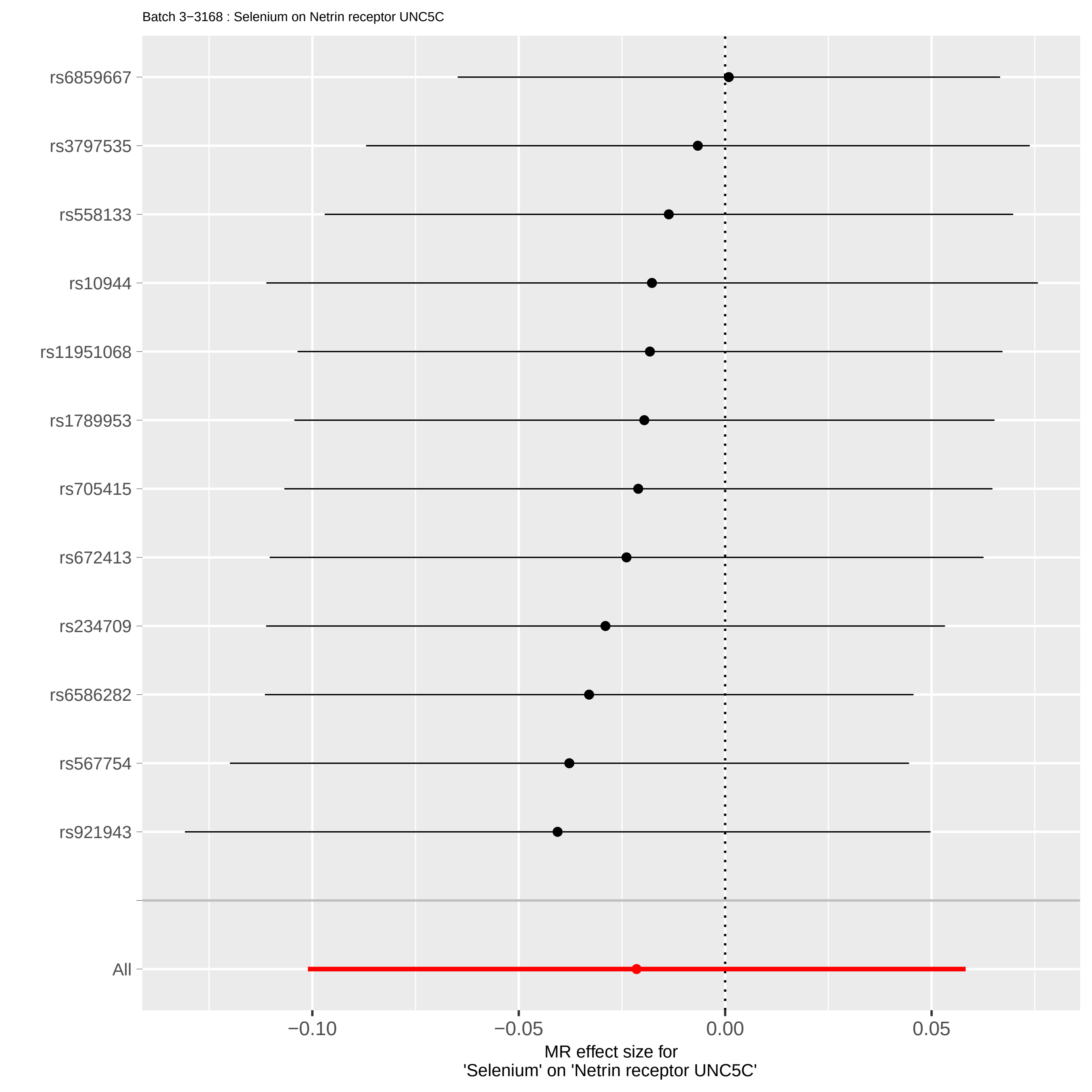
Batch 3−3168 : Selenium on Netrin receptor UNC5C
rs6859667
rs3797535
rs558133
rs10944
rs11951068
rs1789953
rs705415
rs672413
rs234709
rs6586282
rs567754
rs921943
All
−0.10
−0.05
0.00
0.05
MR effect size for
'Selenium' on 'Netrin receptor UNC5C'

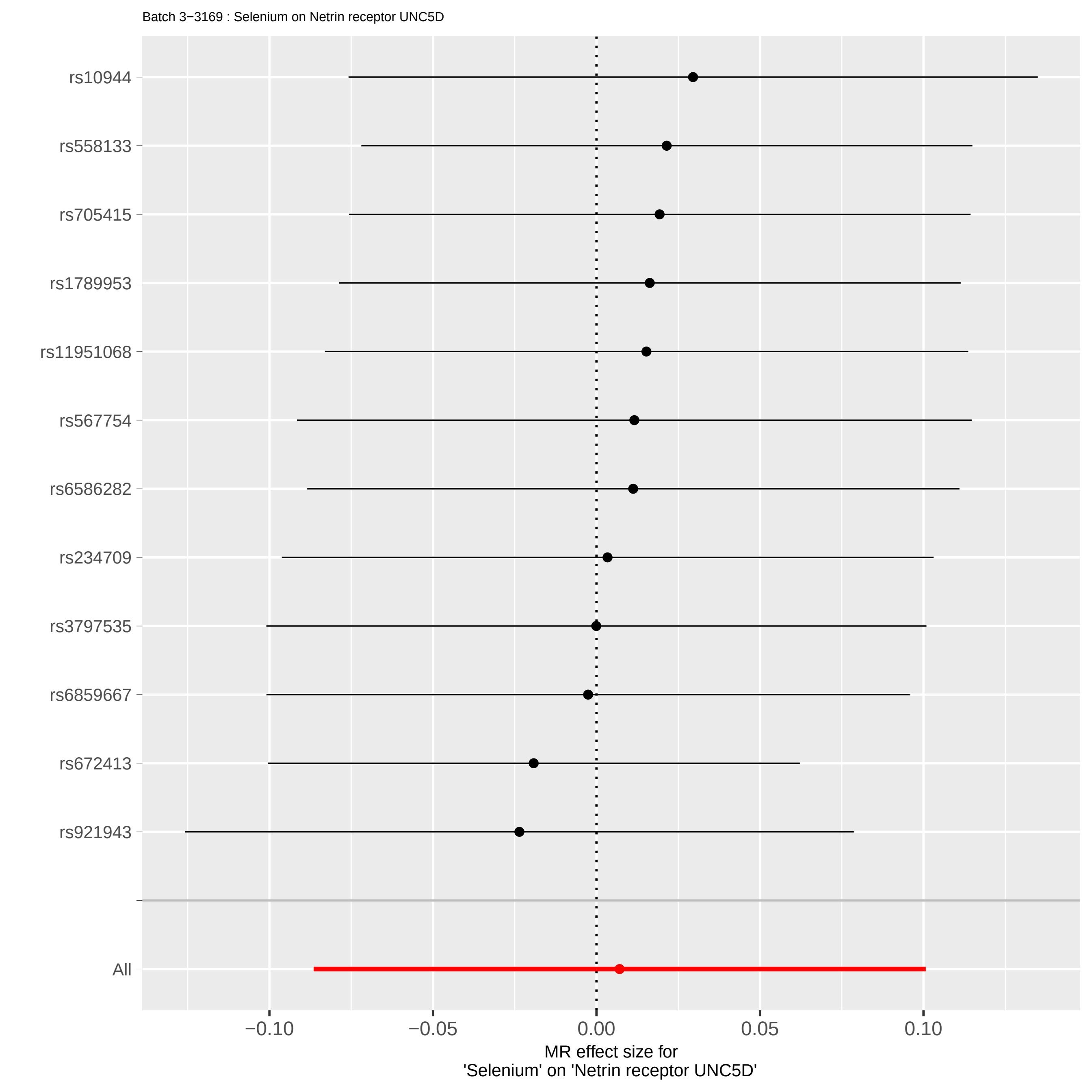

Batch 3−3169 : Selenium on Netrin receptor UNC5D
rs10944
rs558133
rs705415
rs1789953
rs11951068
rs567754
rs6586282
rs234709
rs3797535
rs6859667
rs672413
rs921943
All
−0.10
−0.05
0.00
0.05
0.10
MR effect size for
'Selenium' on 'Netrin receptor UNC5D'

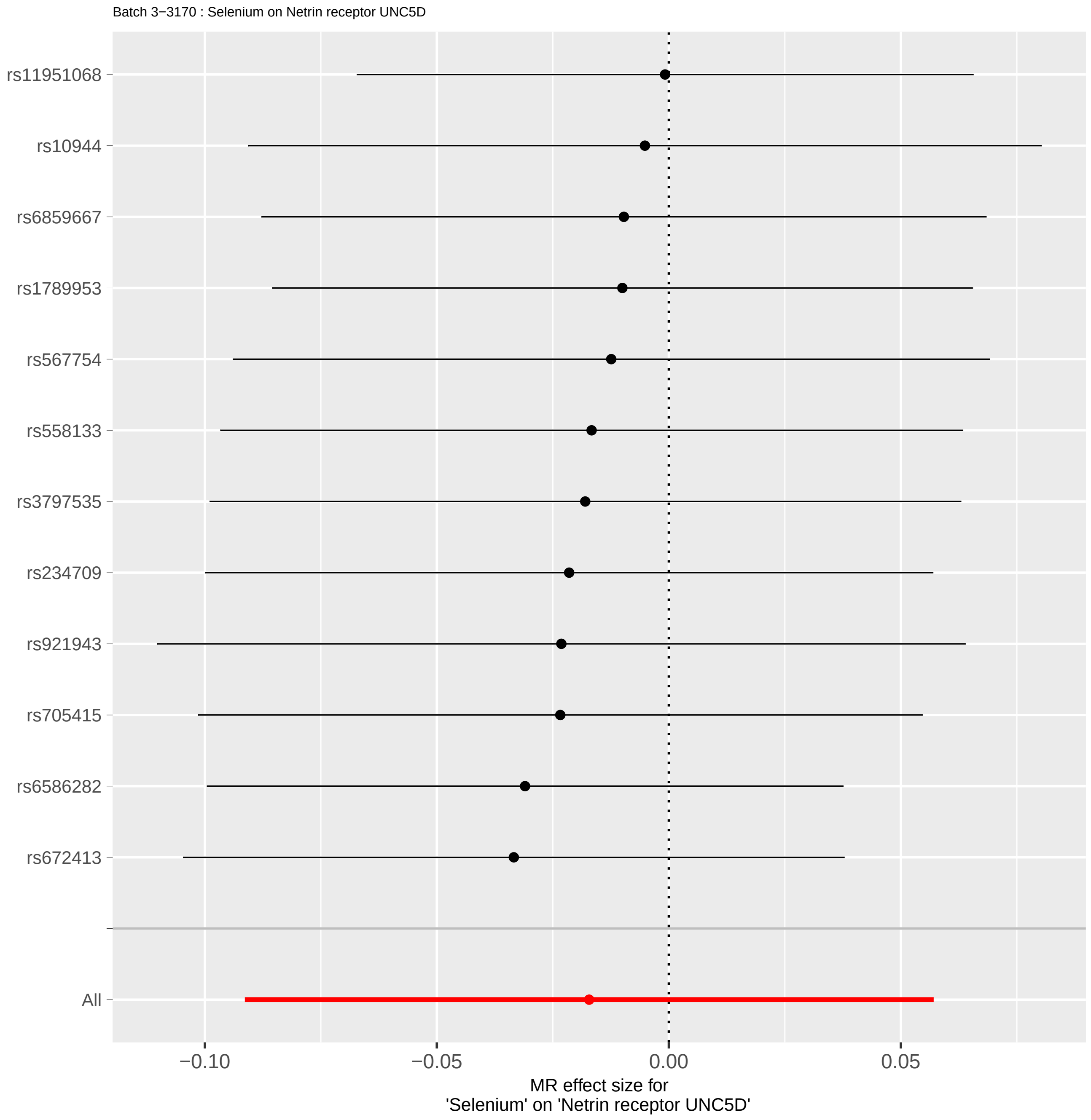
Batch 3−3170 : Selenium on Netrin receptor UNC5D
rs11951068
rs10944
rs6859667
rs1789953
rs567754
rs558133
rs3797535
rs234709
rs921943
rs705415
rs6586282
rs672413
All
−0.10
−0.05
0.00
0.05
MR effect size for
'Selenium' on 'Netrin receptor UNC5D'

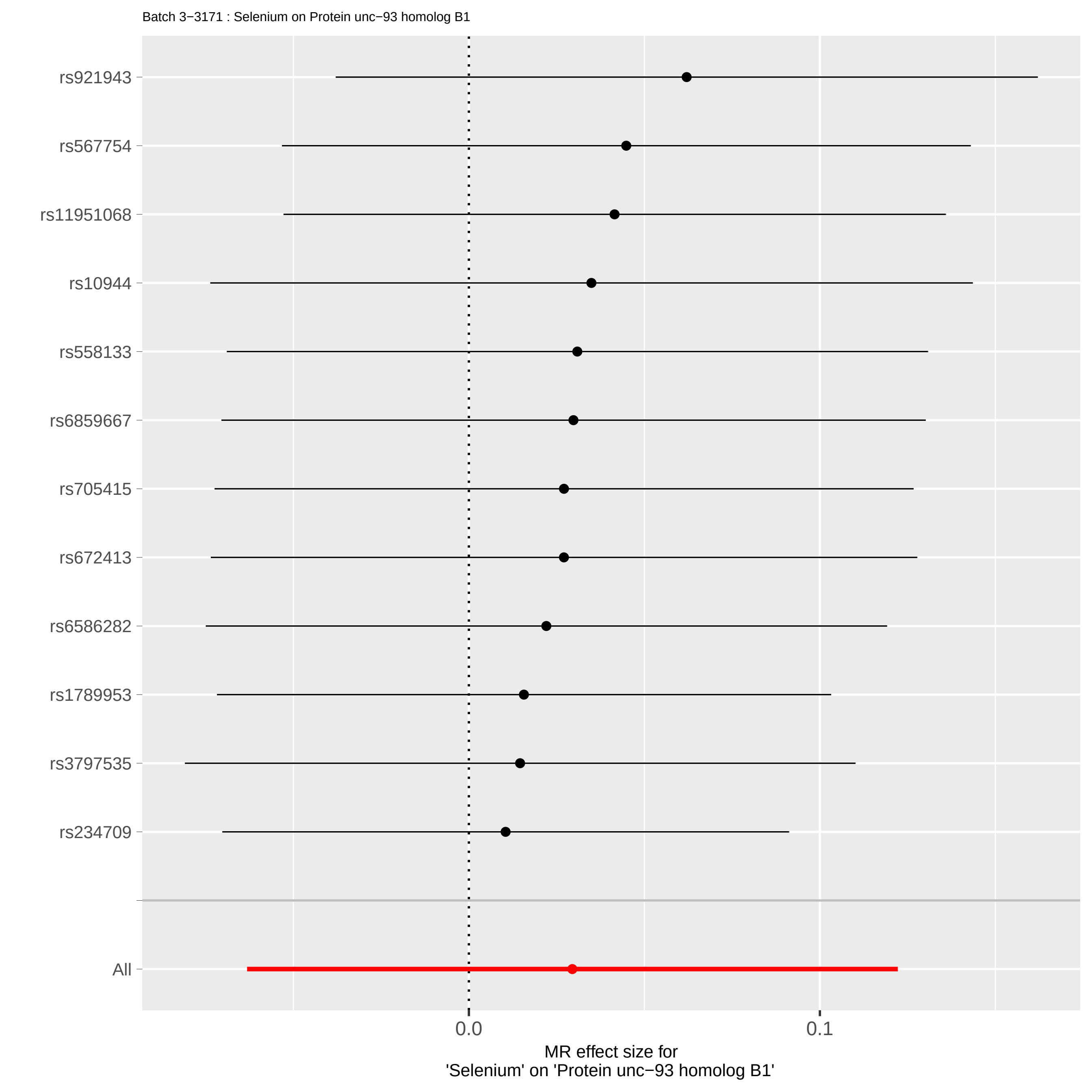

Batch 3−3171 : Selenium on Protein unc−93 homolog B1
rs921943
rs567754
rs11951068
rs10944
rs558133
rs6859667
rs705415
rs672413
rs6586282
rs1789953
rs3797535
rs234709
All
0.0
0.1
MR effect size for
'Selenium' on 'Protein unc−93 homolog B1'

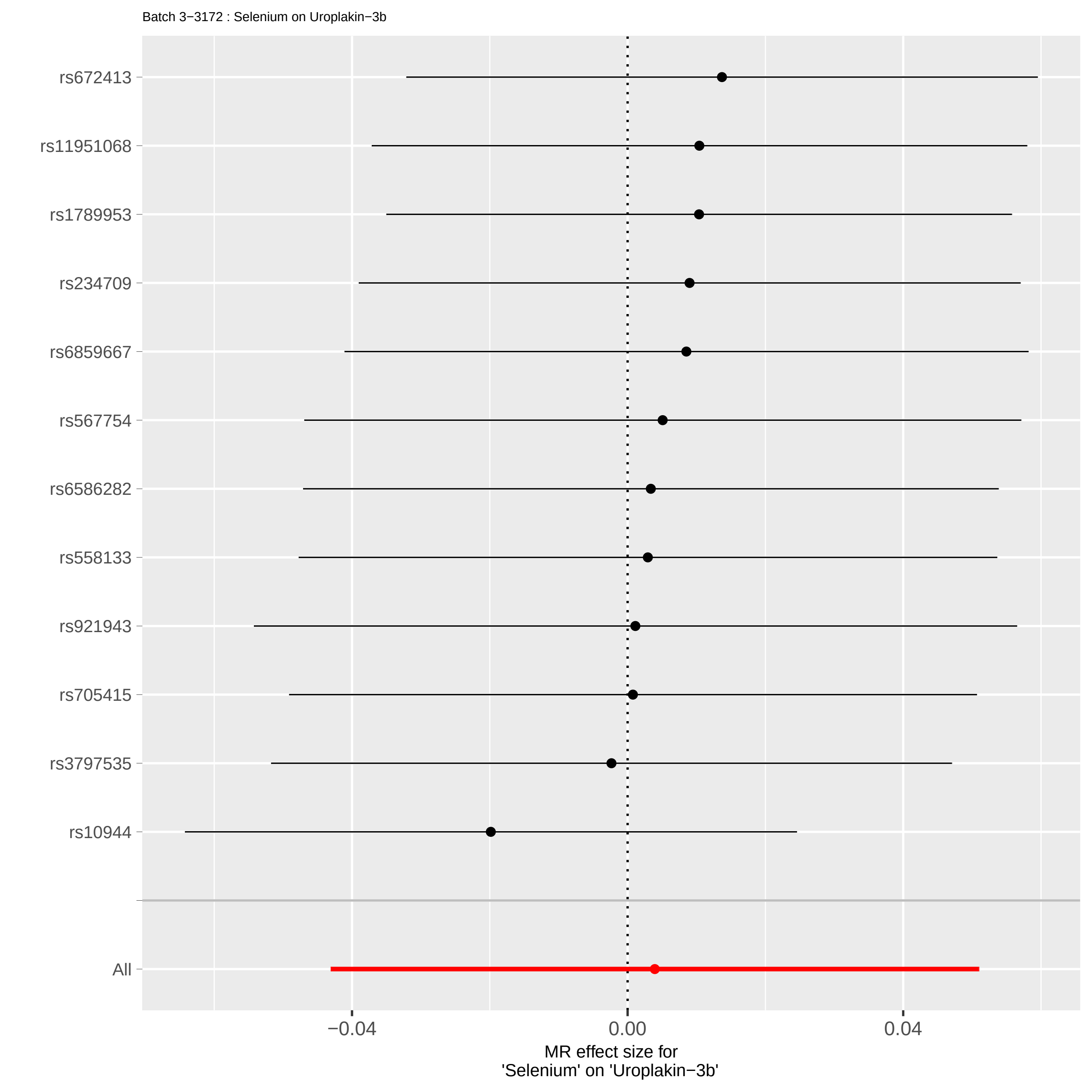
Batch 3−3172 : Selenium on Uroplakin−3b
rs672413
rs11951068
rs1789953
rs234709
rs6859667
rs567754
rs6586282
rs558133
rs921943
rs705415
rs3797535
rs10944
All
−0.04
0.00
0.04
MR effect size for
'Selenium' on 'Uroplakin−3b'

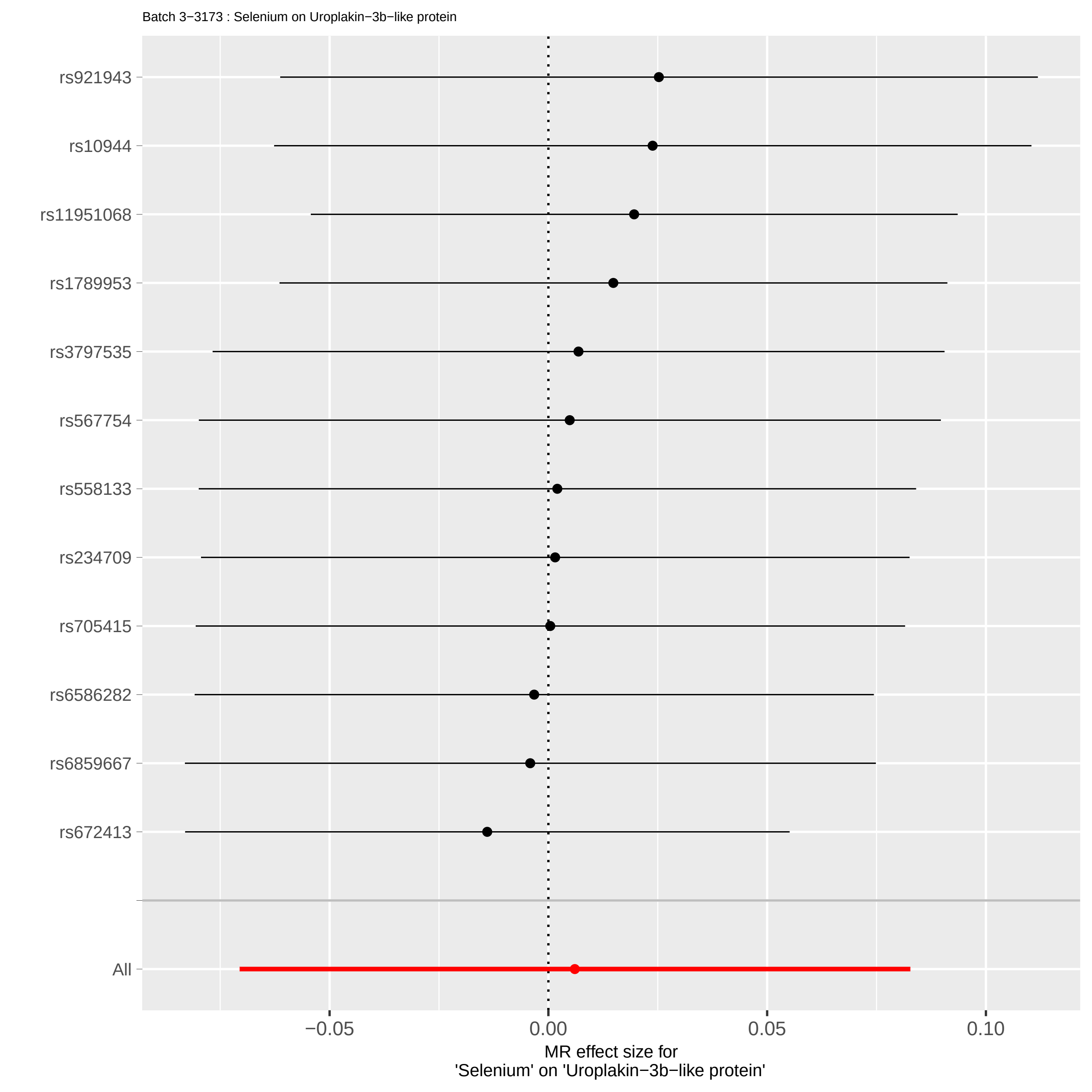
Batch 3−3173 : Selenium on Uroplakin−3b−like protein
rs921943
rs10944
rs11951068
rs1789953
rs3797535
rs567754
rs558133
rs234709
rs705415
rs6586282
rs6859667
rs672413
All
−0.05
0.00
0.05
0.10
MR effect size for
'Selenium' on 'Uroplakin−3b−like protein'

Batch 3−3174 : Selenium on Ubiquinol−cytochrome−c reductase complex assembly factor 3

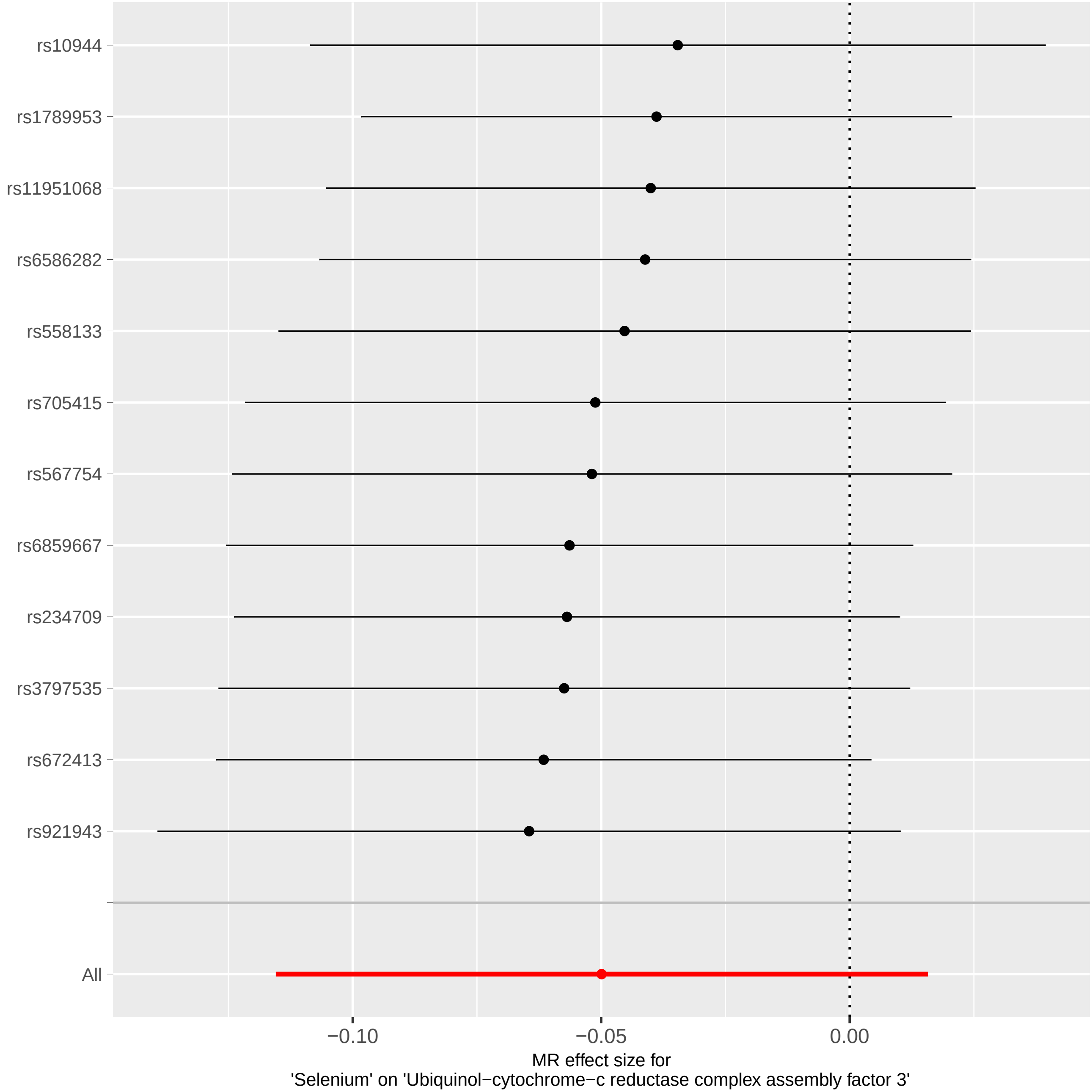

Batch 3−3175 : Selenium on Cytochrome b−c1 complex subunit 7

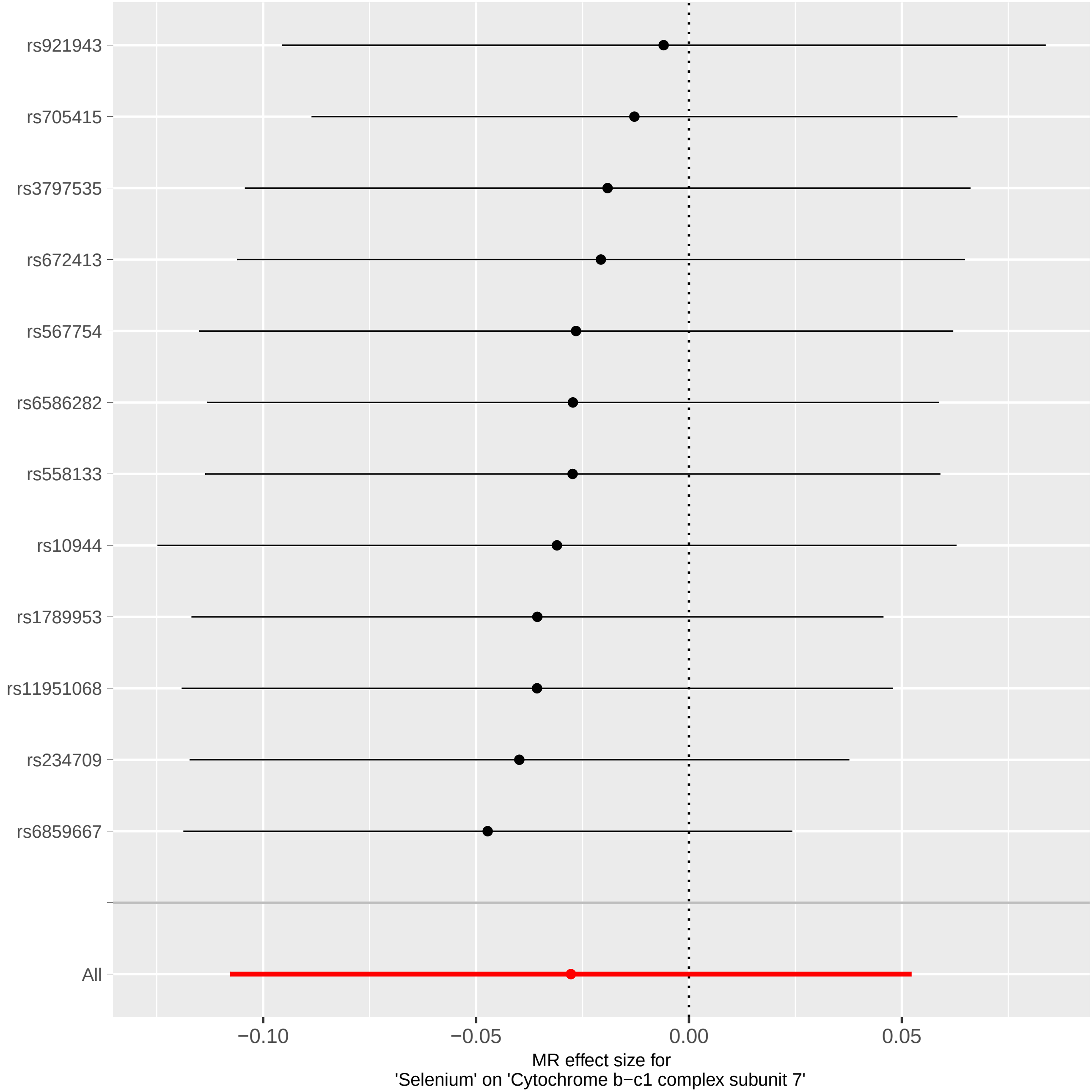

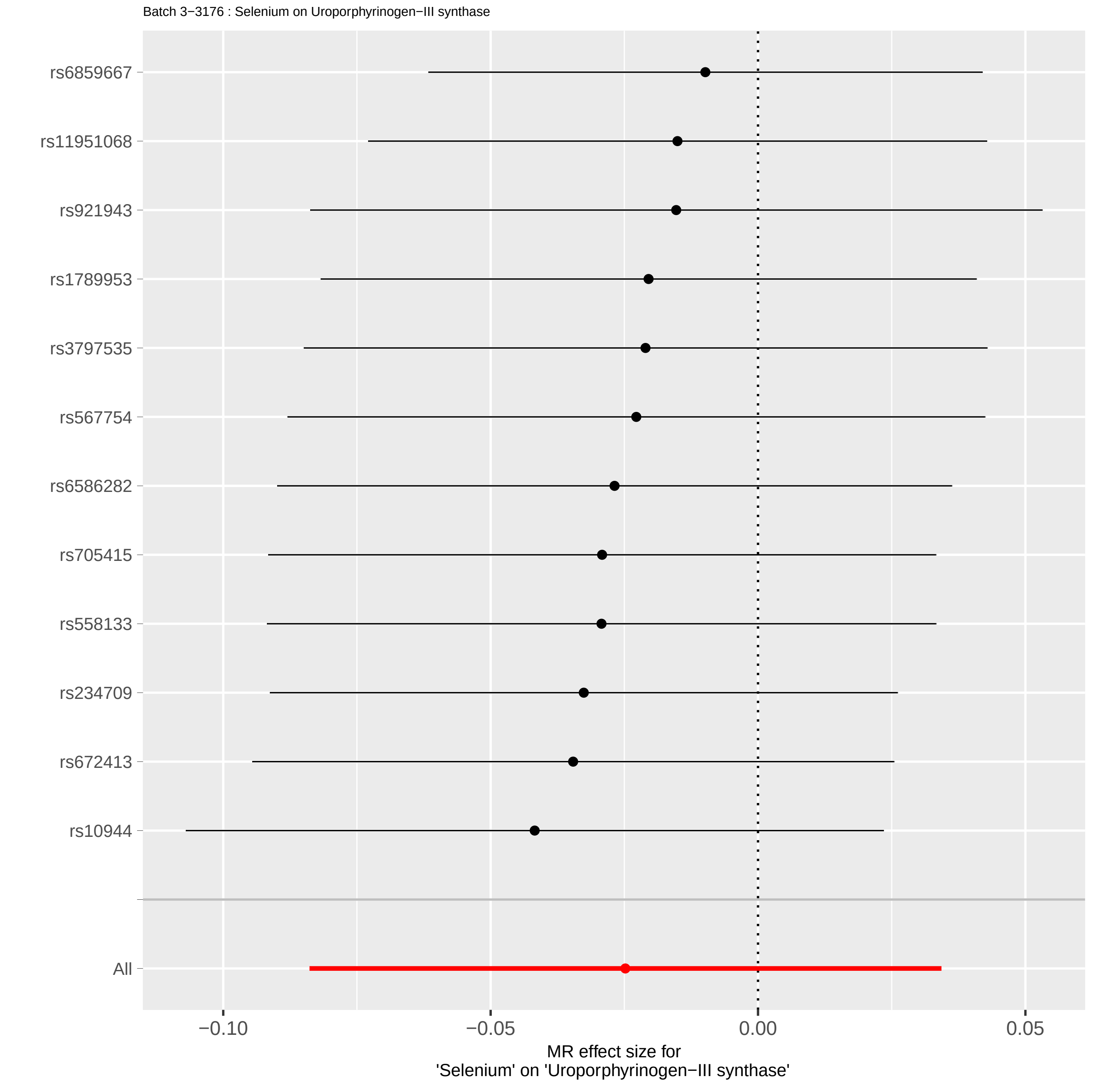

Batch 3−3176 : Selenium on Uroporphyrinogen−III synthase
rs6859667
rs11951068
rs921943
rs1789953
rs3797535
rs567754
rs6586282
rs705415
rs558133
rs234709
rs672413
rs10944
All
−0.10
−0.05
0.00
0.05
MR effect size for
'Selenium' on 'Uroporphyrinogen−III synthase'

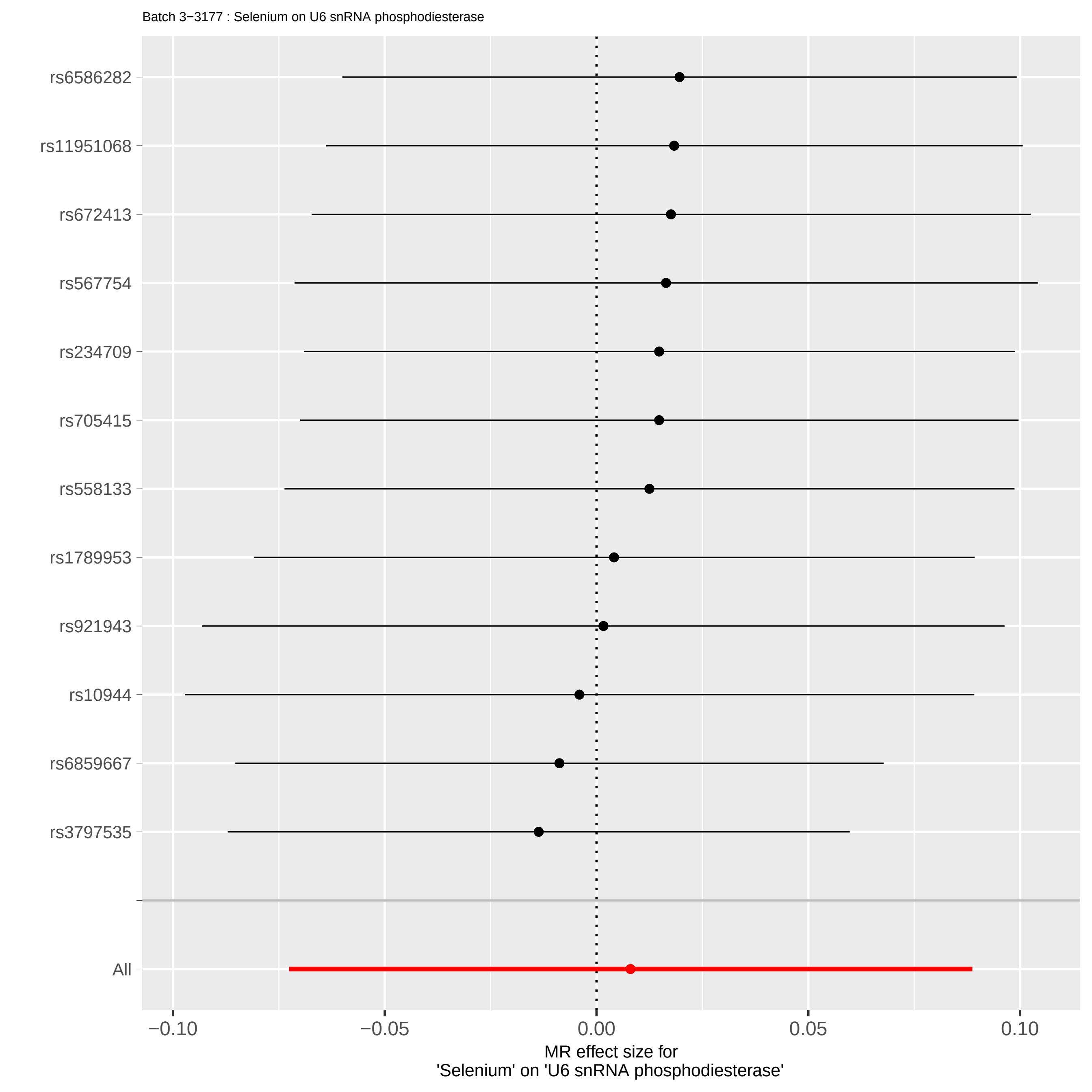

Batch 3−3177 : Selenium on U6 snRNA phosphodiesterase
rs6586282
rs11951068
rs672413
rs567754
rs234709
rs705415
rs558133
rs1789953
rs921943
rs10944
rs6859667
rs3797535
All
−0.10
−0.05
0.00
0.05
0.10
MR effect size for
'Selenium' on 'U6 snRNA phosphodiesterase'

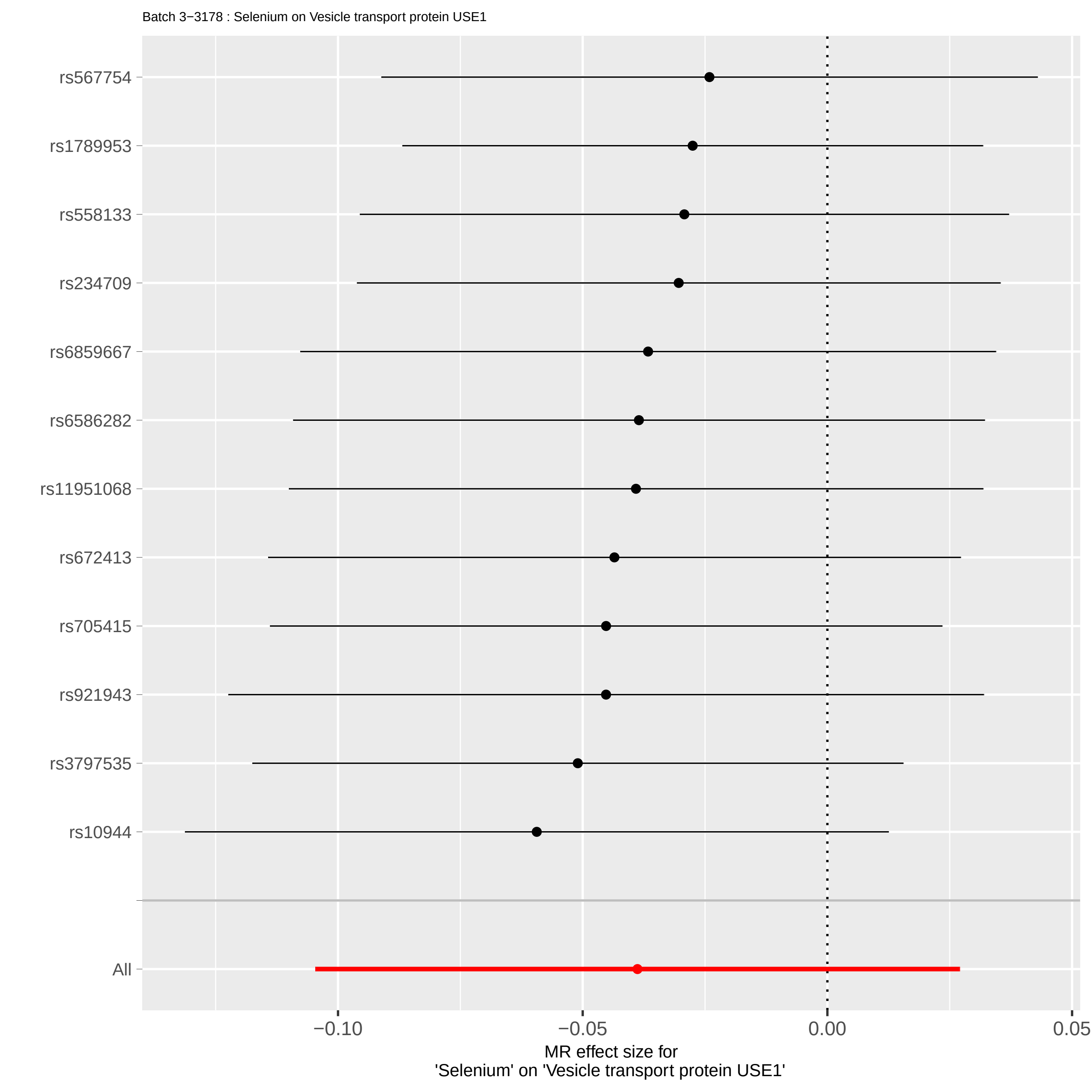
Batch 3−3178 : Selenium on Vesicle transport protein USE1
rs567754
rs1789953
rs558133
rs234709
rs6859667
rs6586282
rs11951068
rs672413
rs705415
rs921943
rs3797535
rs10944
All
−0.10
−0.05
0.00
0.05
MR effect size for
'Selenium' on 'Vesicle transport protein USE1'

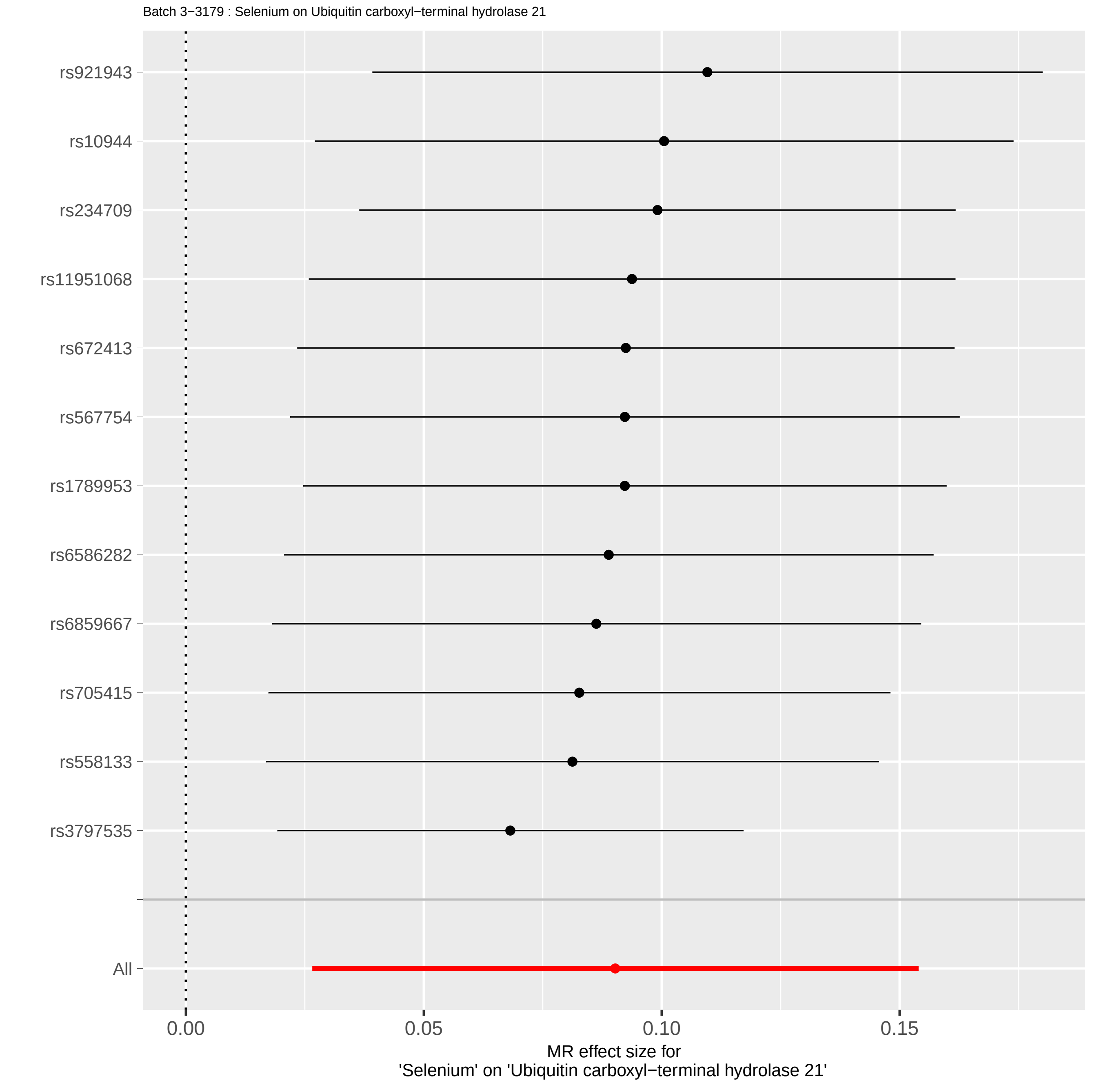

Batch 3−3179 : Selenium on Ubiquitin carboxyl−terminal hydrolase 21
rs921943
rs10944
rs234709
rs11951068
rs672413
rs567754
rs1789953
rs6586282
rs6859667
rs705415
rs558133
rs3797535
All
0.00
0.05
0.10
0.15
MR effect size for
'Selenium' on 'Ubiquitin carboxyl−terminal hydrolase 21'

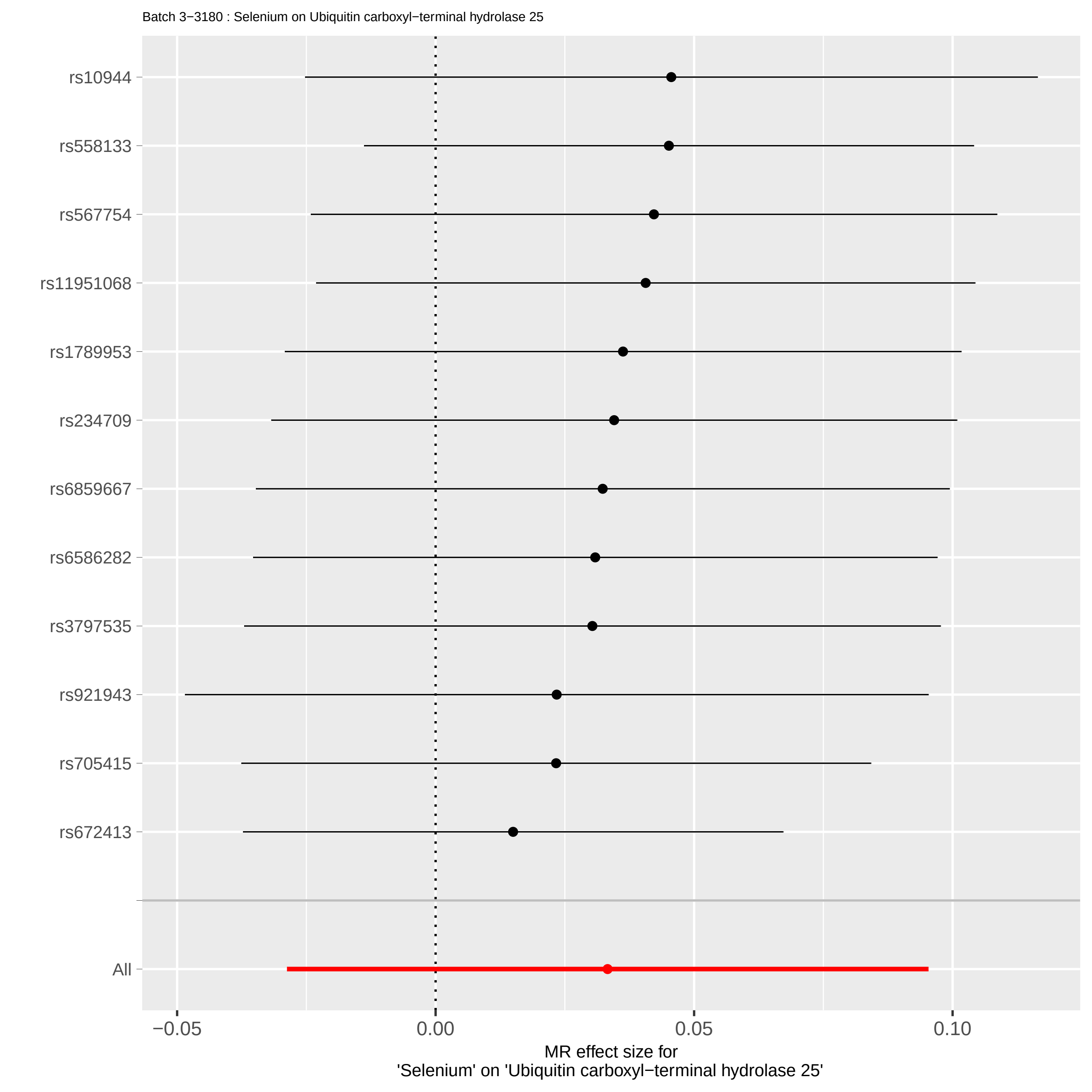

Batch 3−3180 : Selenium on Ubiquitin carboxyl−terminal hydrolase 25
rs10944
rs558133
rs567754
rs11951068
rs1789953
rs234709
rs6859667
rs6586282
rs3797535
rs921943
rs705415
rs672413
All
−0.05
0.00
0.05
0.10
MR effect size for
'Selenium' on 'Ubiquitin carboxyl−terminal hydrolase 25'

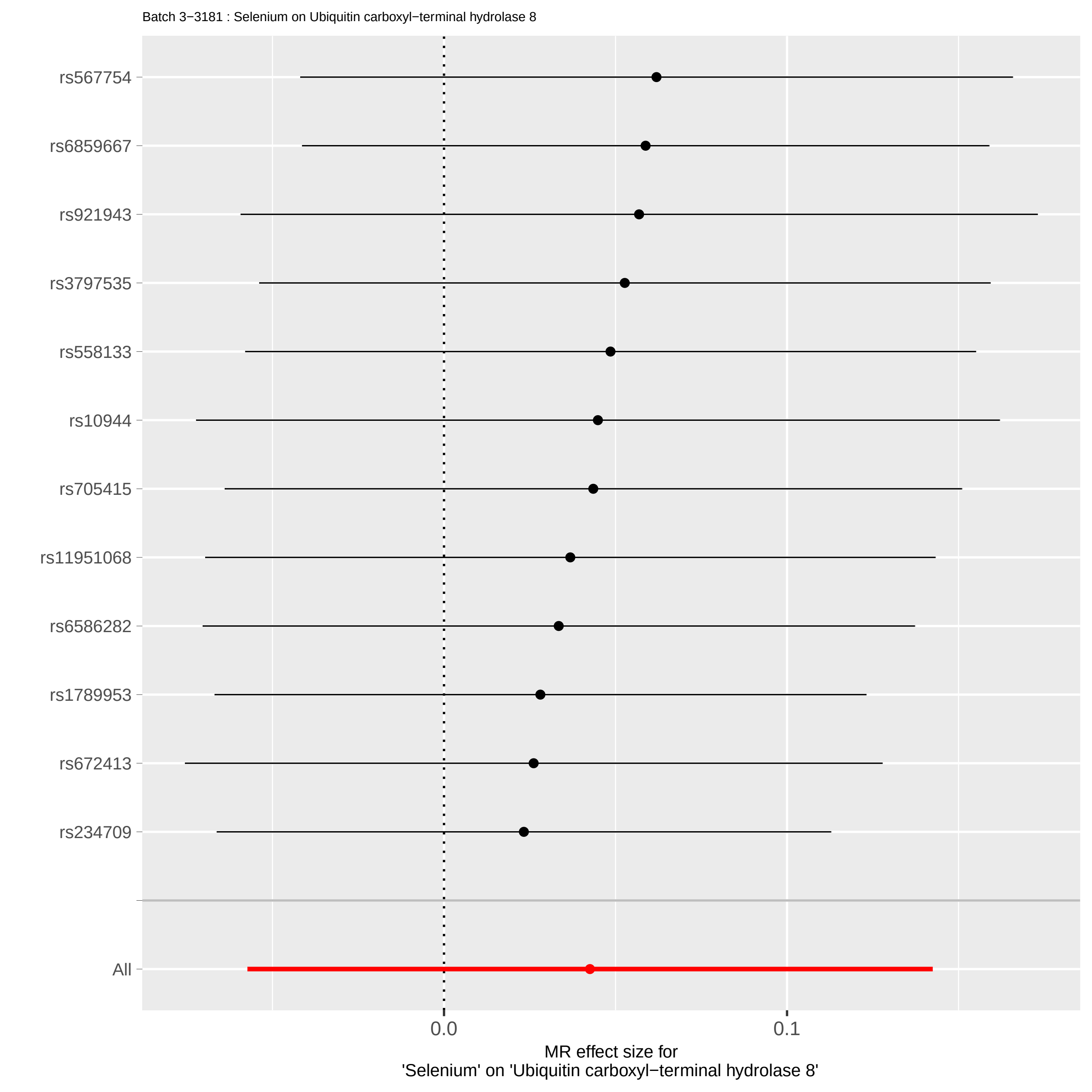

Batch 3−3181 : Selenium on Ubiquitin carboxyl−terminal hydrolase 8
rs567754
rs6859667
rs921943
rs3797535
rs558133
rs10944
rs705415
rs11951068
rs6586282
rs1789953
rs672413
rs234709
All
0.0
0.1
MR effect size for
'Selenium' on 'Ubiquitin carboxyl−terminal hydrolase 8'

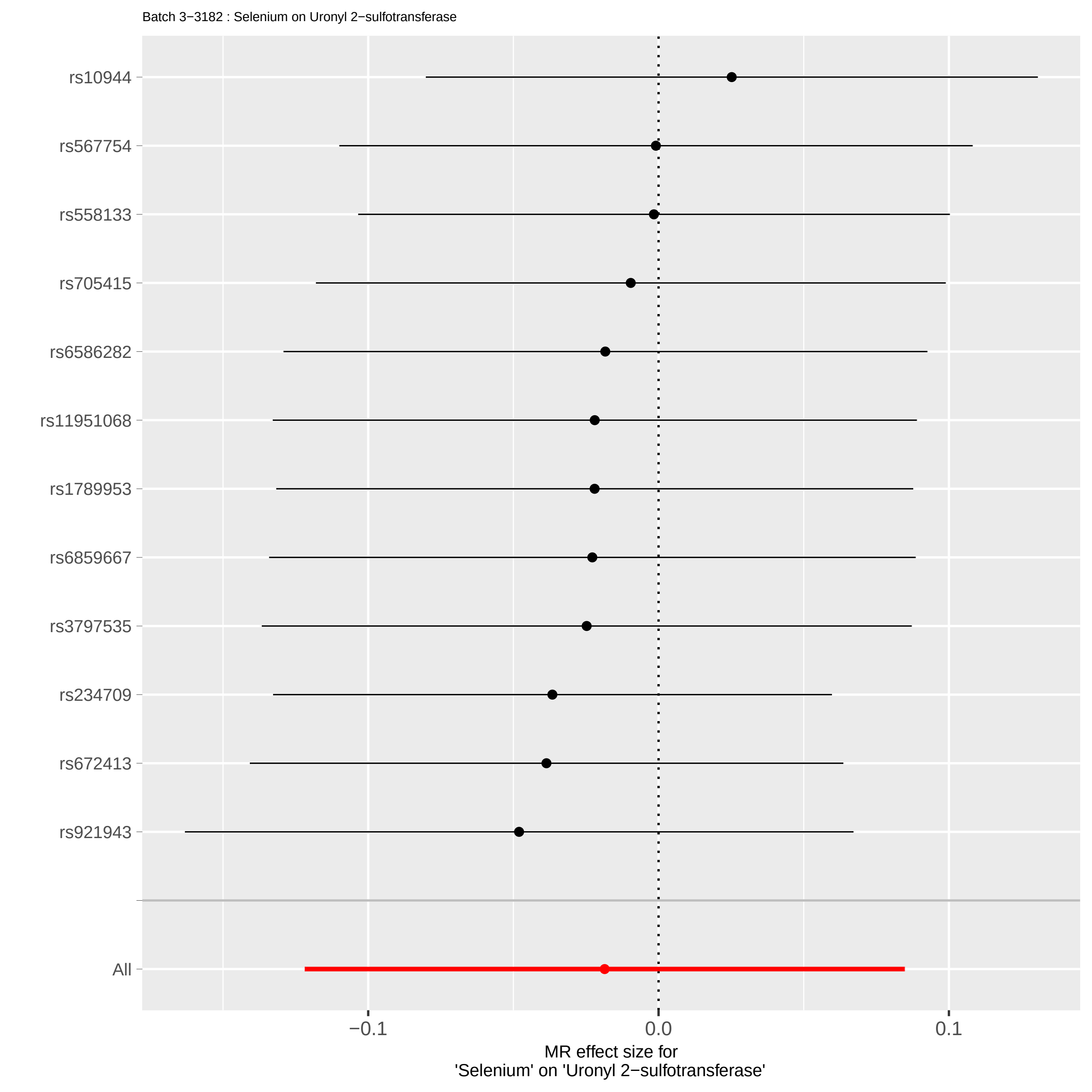

Batch 3−3182 : Selenium on Uronyl 2−sulfotransferase
rs10944
rs567754
rs558133
rs705415
rs6586282
rs11951068
rs1789953
rs6859667
rs3797535
rs234709
rs672413
rs921943
All
−0.1
0.0
0.1
MR effect size for
'Selenium' on 'Uronyl 2−sulfotransferase'

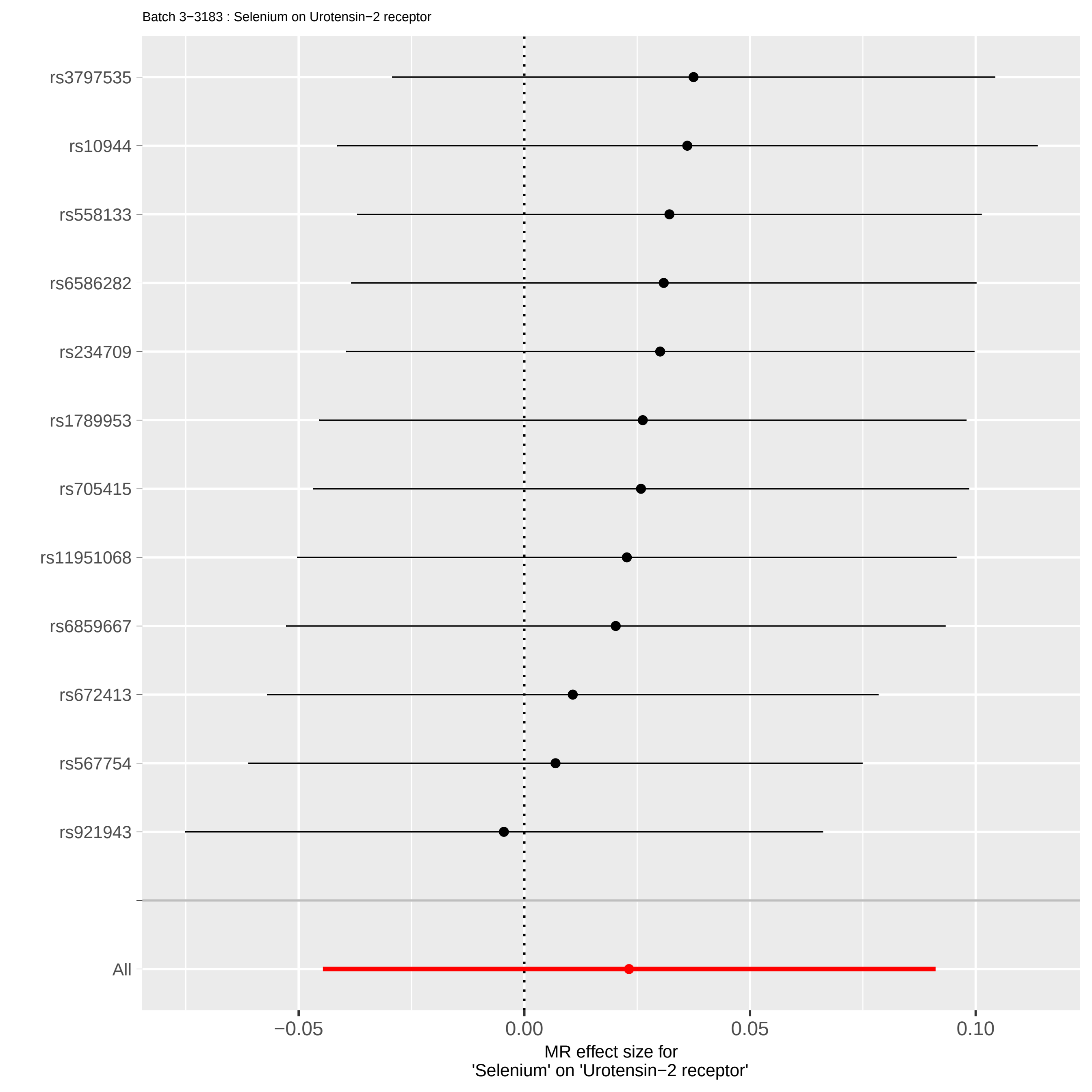
Batch 3−3183 : Selenium on Urotensin−2 receptor
rs3797535
rs10944
rs558133
rs6586282
rs234709
rs1789953
rs705415
rs11951068
rs6859667
rs672413
rs567754
rs921943
All
−0.05
0.00
0.05
0.10
MR effect size for
'Selenium' on 'Urotensin−2 receptor'

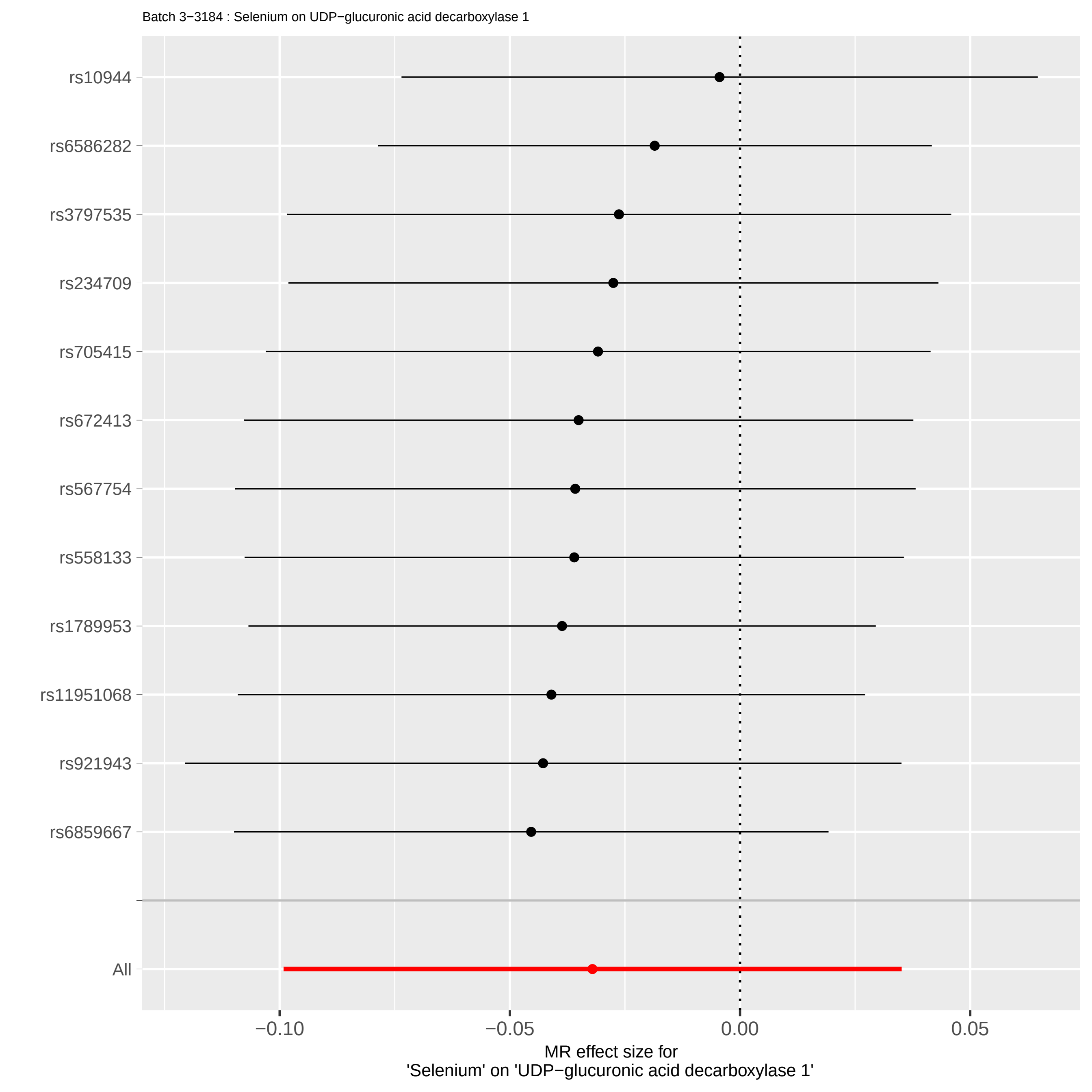

Batch 3−3184 : Selenium on UDP−glucuronic acid decarboxylase 1
rs10944
rs6586282
rs3797535
rs234709
rs705415
rs672413
rs567754
rs558133
rs1789953
rs11951068
rs921943
rs6859667
All
−0.10
−0.05
0.00
0.05
MR effect size for
'Selenium' on 'UDP−glucuronic acid decarboxylase 1'

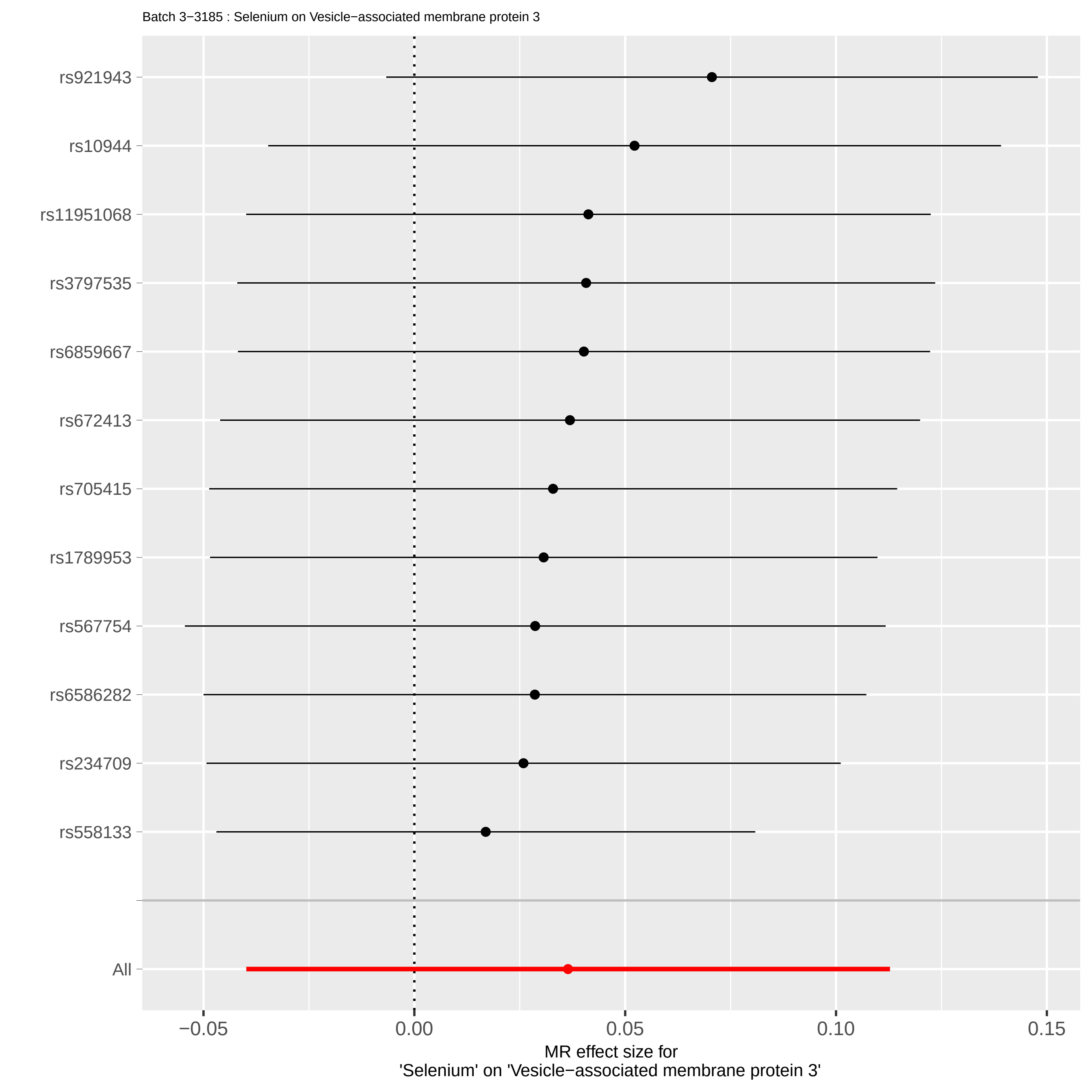

Batch 3−3185 : Selenium on Vesicle−associated membrane protein 3
rs921943
rs10944
rs11951068
rs3797535
rs6859667
rs672413
rs705415
rs1789953
rs567754
rs6586282
rs234709
rs558133
All
−0.05
0.00
0.05
0.10
0.15
MR effect size for
'Selenium' on 'Vesicle−associated membrane protein 3'

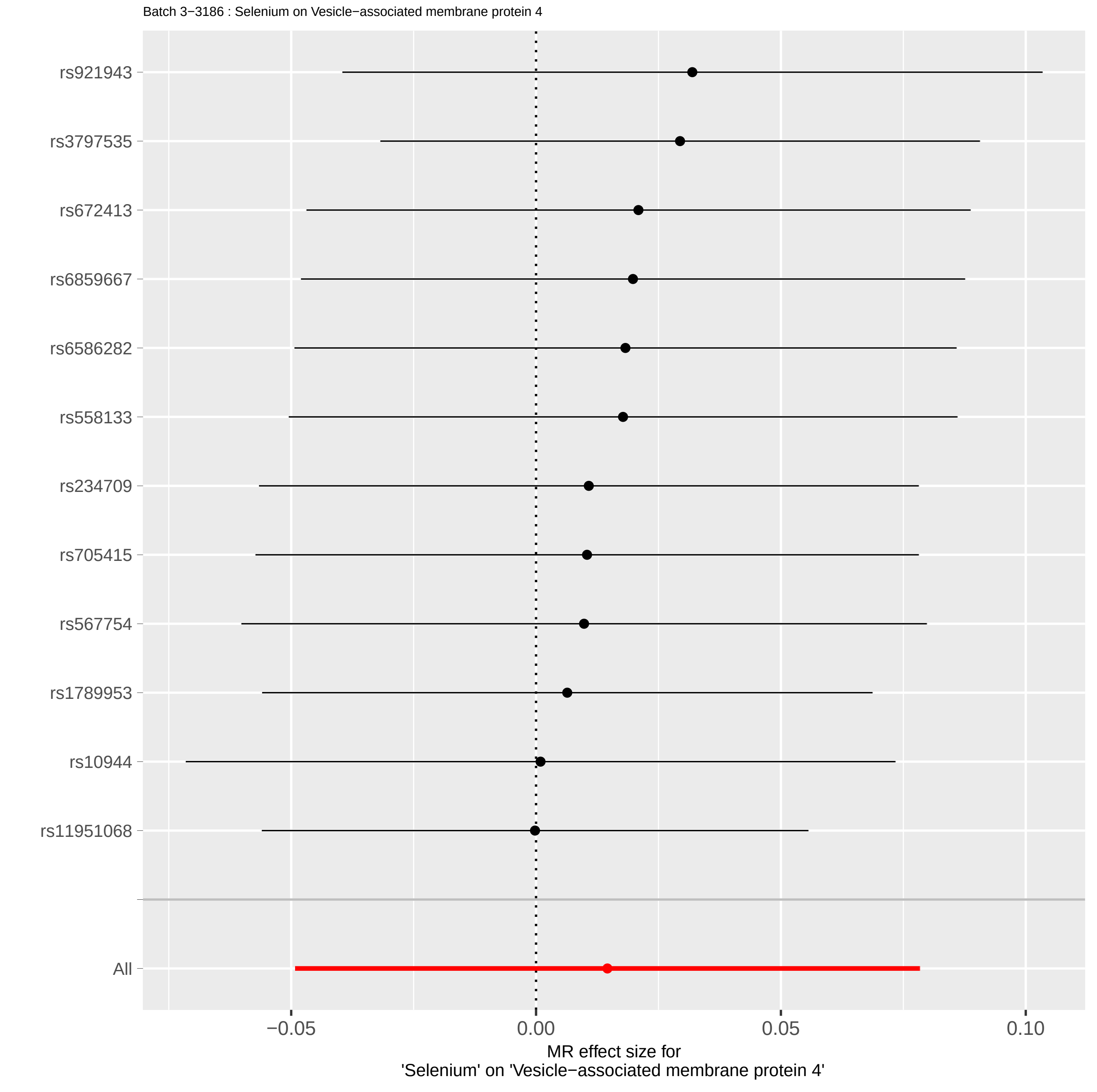
Batch 3−3186 : Selenium on Vesicle−associated membrane protein 4
rs921943
rs3797535
rs672413
rs6859667
rs6586282
rs558133
rs234709
rs705415
rs567754
rs1789953
rs10944
rs11951068
All
−0.05
0.00
0.05
0.10
MR effect size for
'Selenium' on 'Vesicle−associated membrane protein 4'

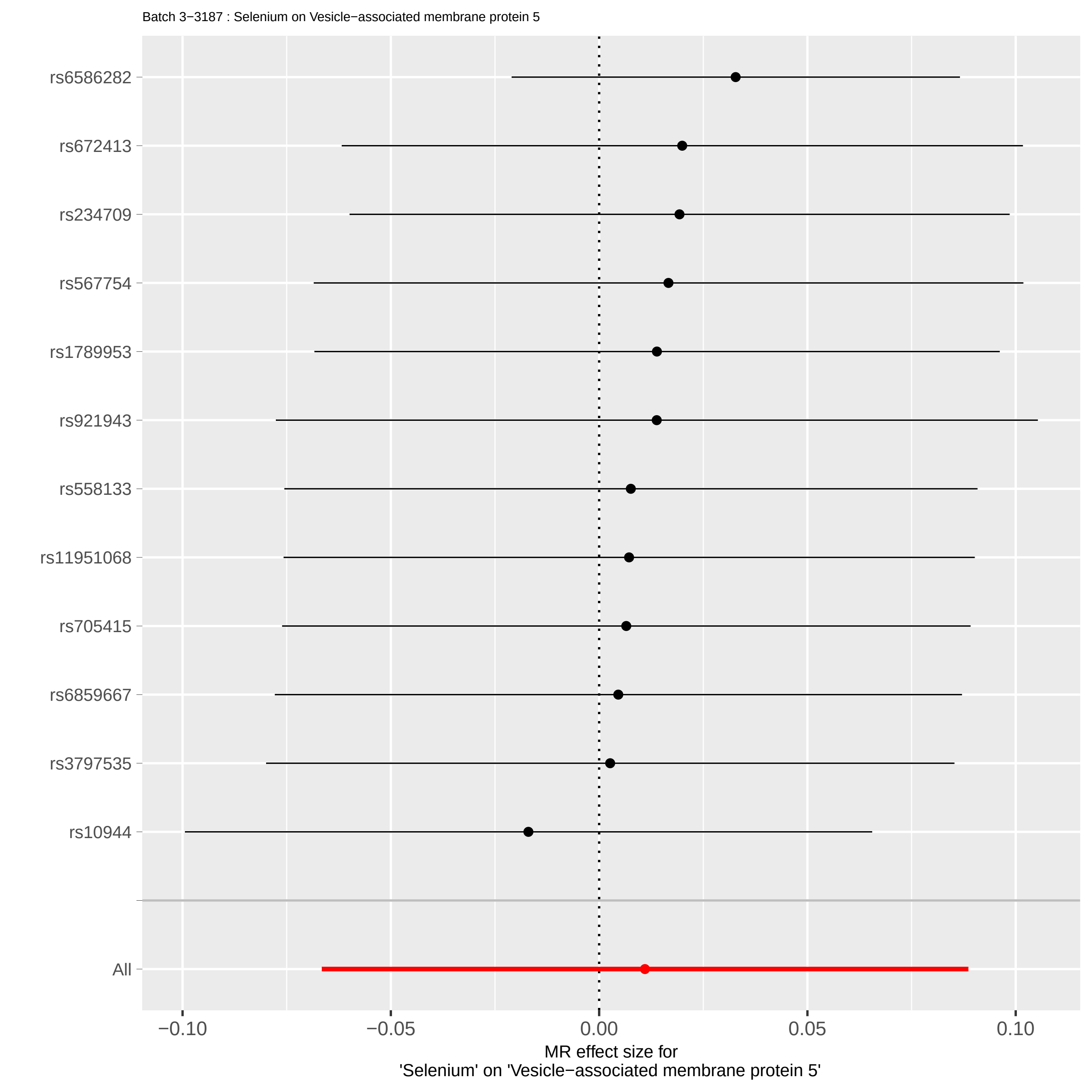
Batch 3−3187 : Selenium on Vesicle−associated membrane protein 5
rs6586282
rs672413
rs234709
rs567754
rs1789953
rs921943
rs558133
rs11951068
rs705415
rs6859667
rs3797535
rs10944
All
−0.10
−0.05
0.00
0.05
0.10
MR effect size for
'Selenium' on 'Vesicle−associated membrane protein 5'

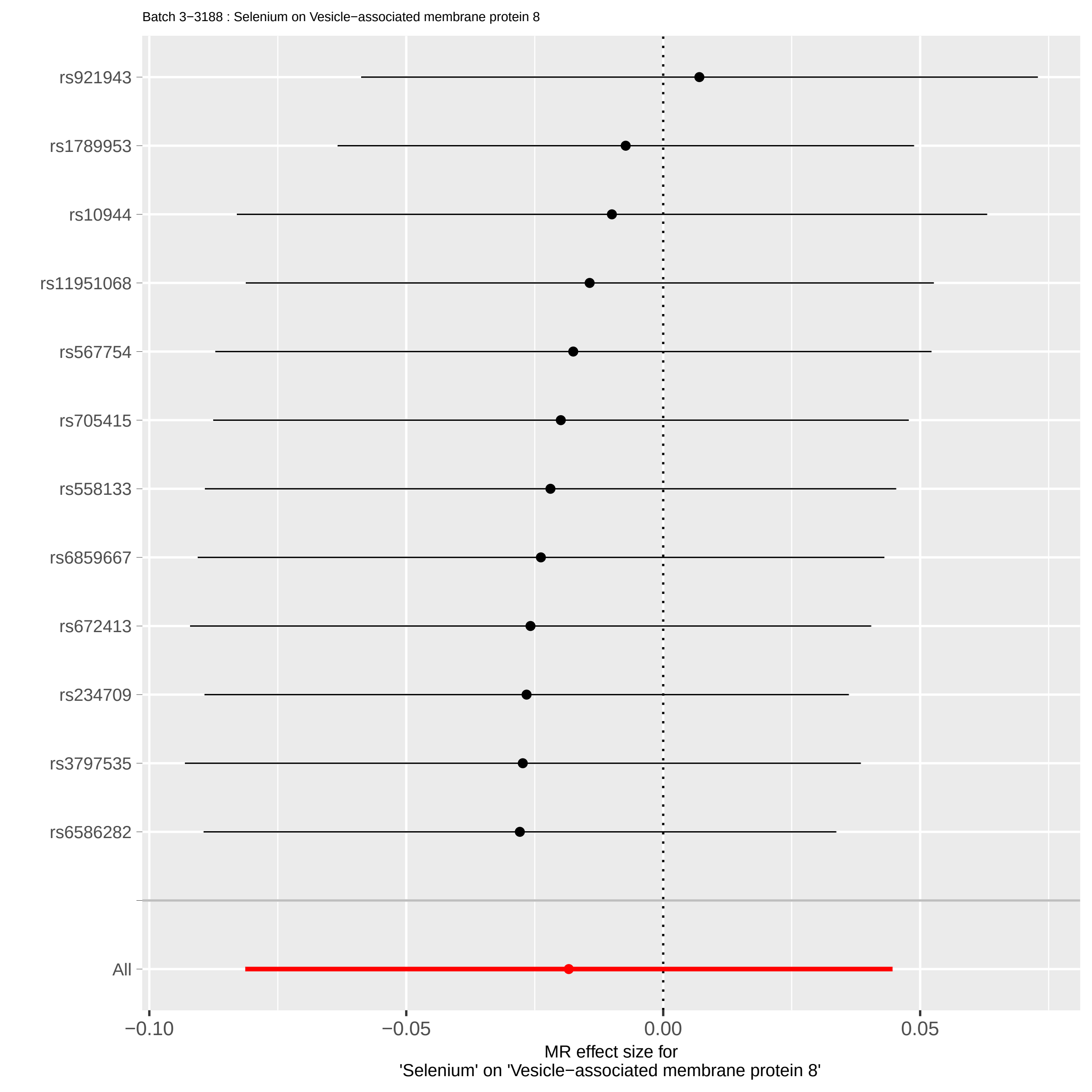

Batch 3−3188 : Selenium on Vesicle−associated membrane protein 8
rs921943
rs1789953
rs10944
rs11951068
rs567754
rs705415
rs558133
rs6859667
rs672413
rs234709
rs3797535
rs6586282
All
−0.10
−0.05
0.00
0.05
MR effect size for
'Selenium' on 'Vesicle−associated membrane protein 8'

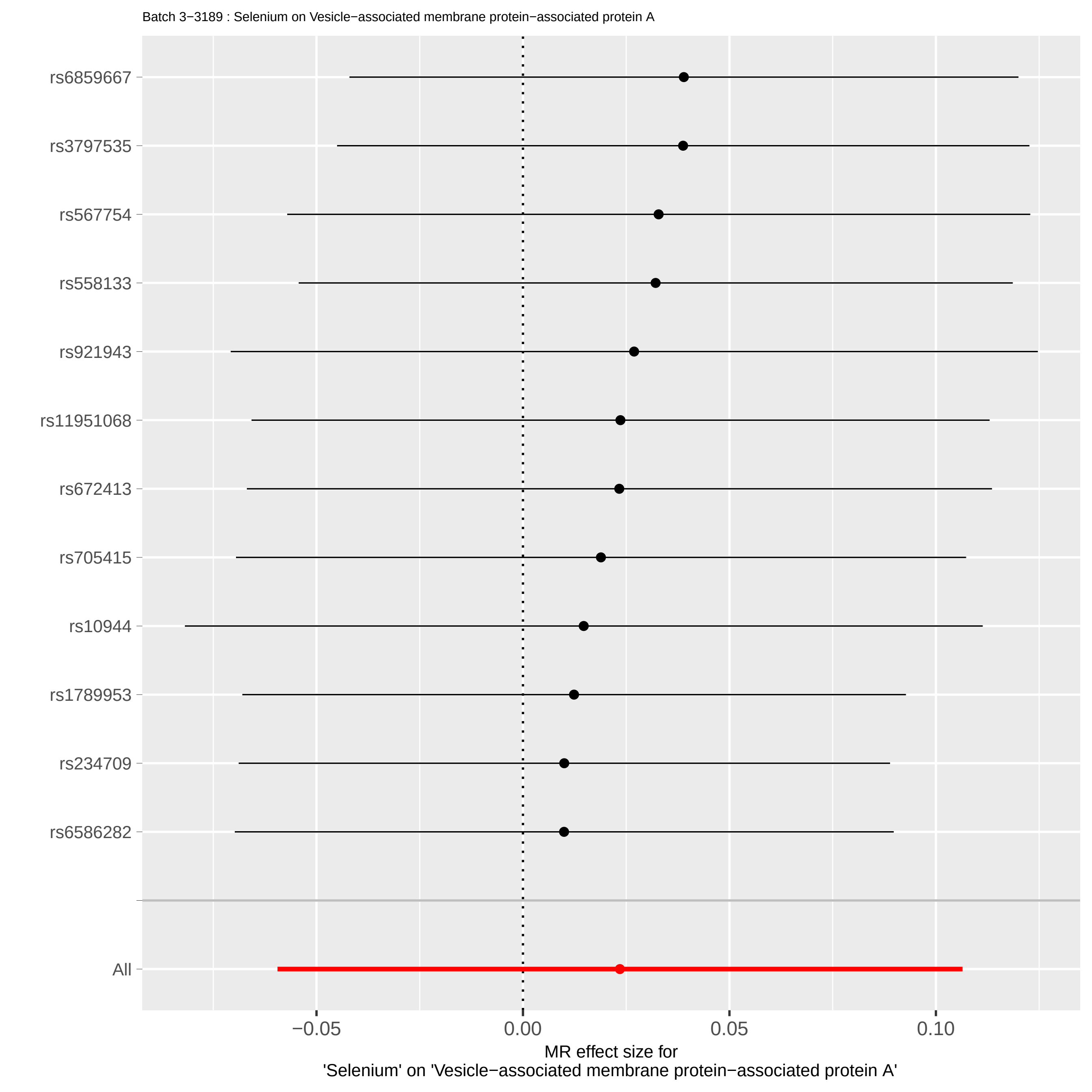

Batch 3−3189 : Selenium on Vesicle−associated membrane protein−associated protein A
rs6859667
rs3797535
rs567754
rs558133
rs921943
rs11951068
rs672413
rs705415
rs10944
rs1789953
rs234709
rs6586282
All
−0.05
0.00
0.05
0.10
MR effect size for
'Selenium' on 'Vesicle−associated membrane protein−associated protein A'

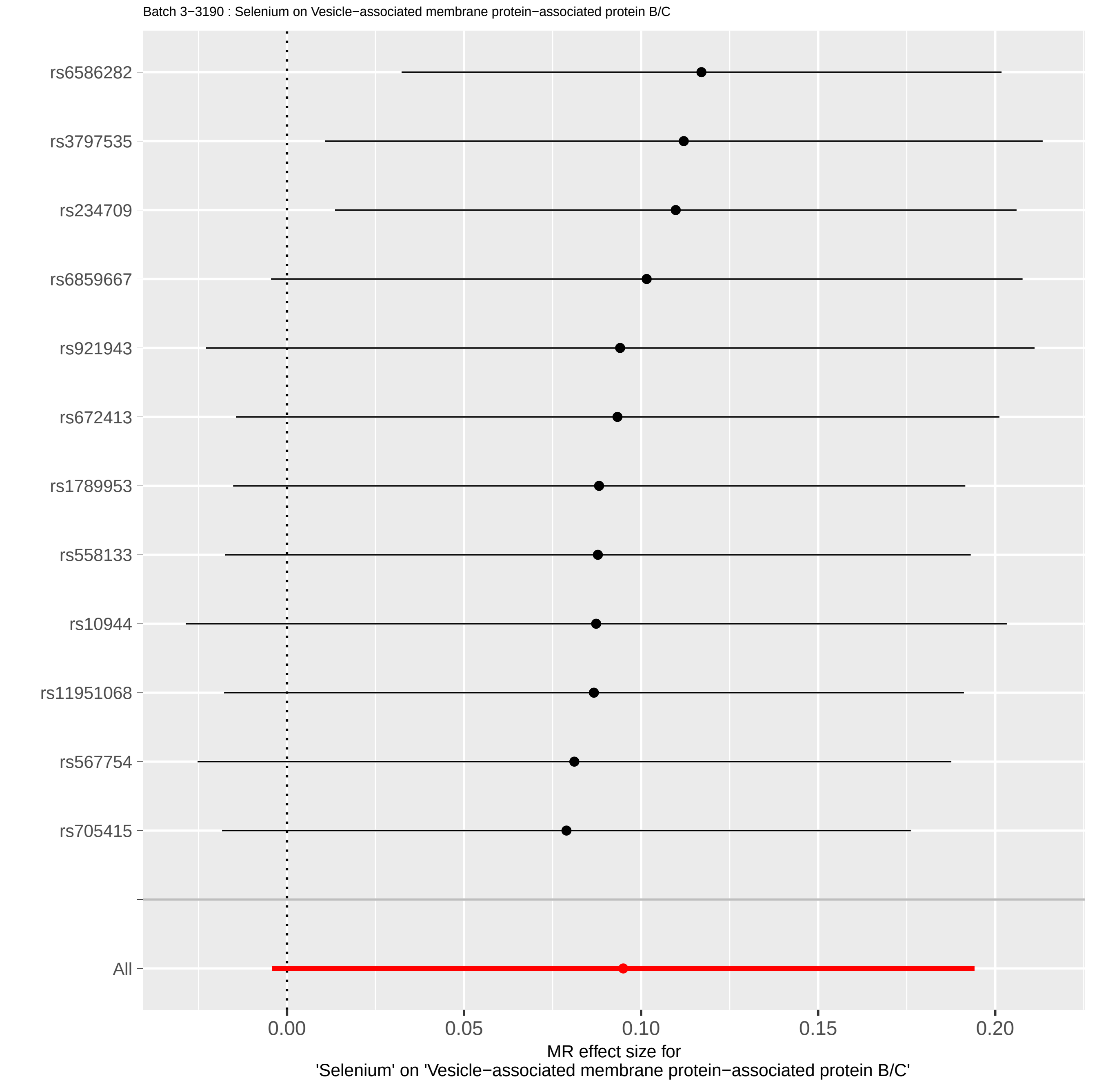

Batch 3−3190 : Selenium on Vesicle−associated membrane protein−associated protein B/C
rs6586282
rs3797535
rs234709
rs6859667
rs921943
rs672413
rs1789953
rs558133
rs10944
rs11951068
rs567754
rs705415
All
0.00
0.05
0.10
0.15
0.20
MR effect size for
'Selenium' on 'Vesicle−associated membrane protein−associated protein B/C'

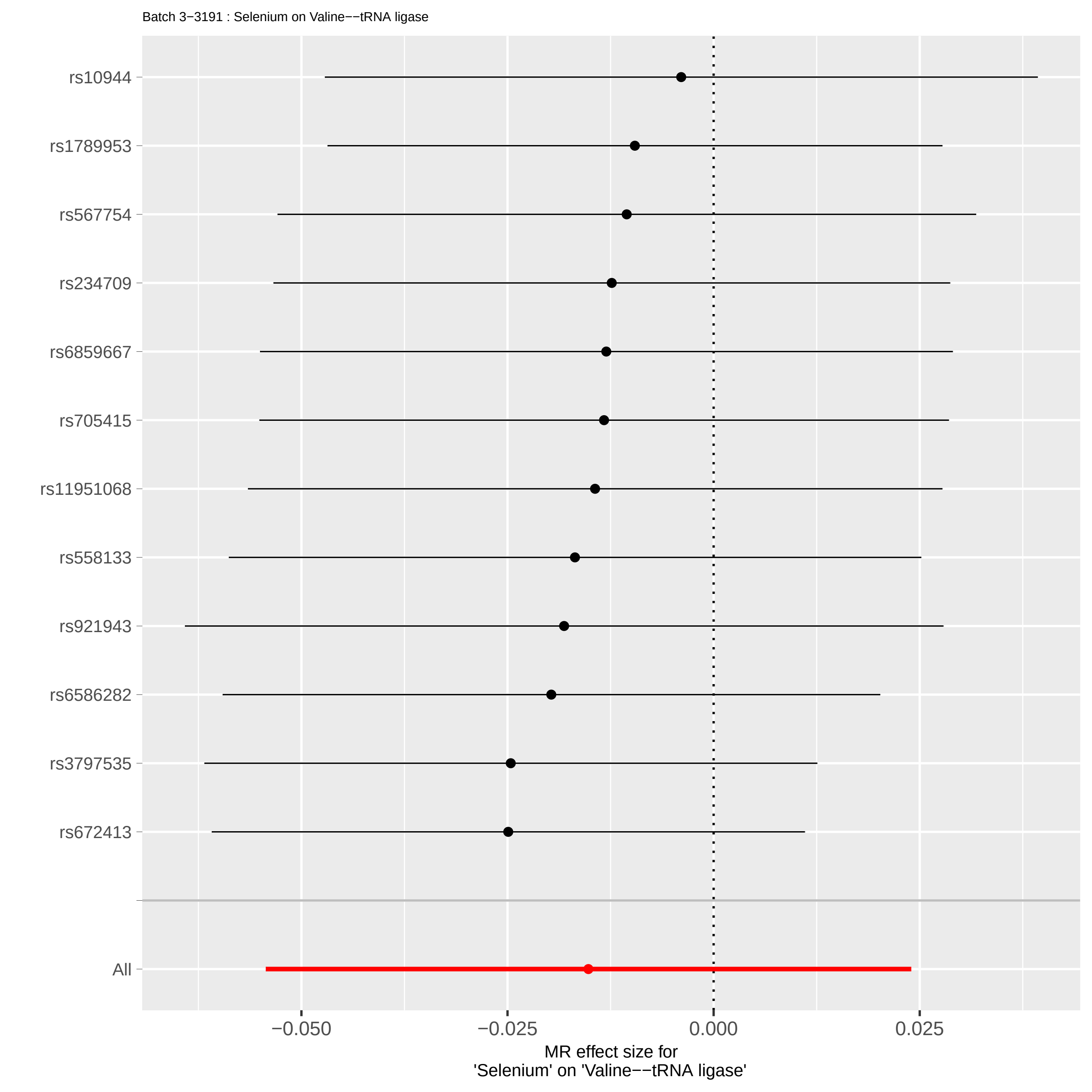

Batch 3−3191 : Selenium on Valine−−tRNA ligase
rs10944
rs1789953
rs567754
rs234709
rs6859667
rs705415
rs11951068
rs558133
rs921943
rs6586282
rs3797535
rs672413
All
−0.050
−0.025
0.000
0.025
MR effect size for
'Selenium' on 'Valine−−tRNA ligase'

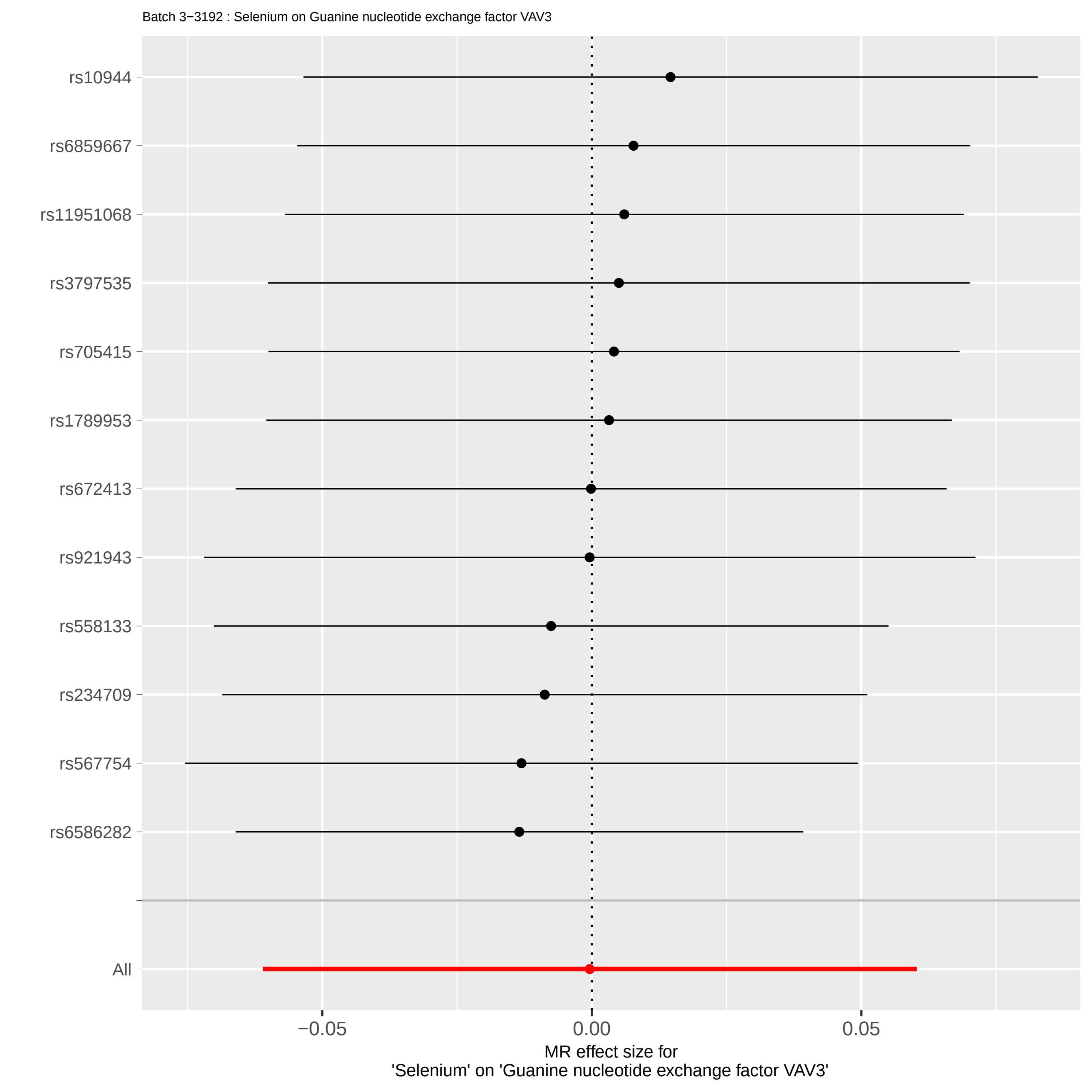
Batch 3−3192 : Selenium on Guanine nucleotide exchange factor VAV3
rs10944
rs6859667
rs11951068
rs3797535
rs705415
rs1789953
rs672413
rs921943
rs558133
rs234709
rs567754
rs6586282
All
−0.05
0.00
0.05
MR effect size for
'Selenium' on 'Guanine nucleotide exchange factor VAV3'

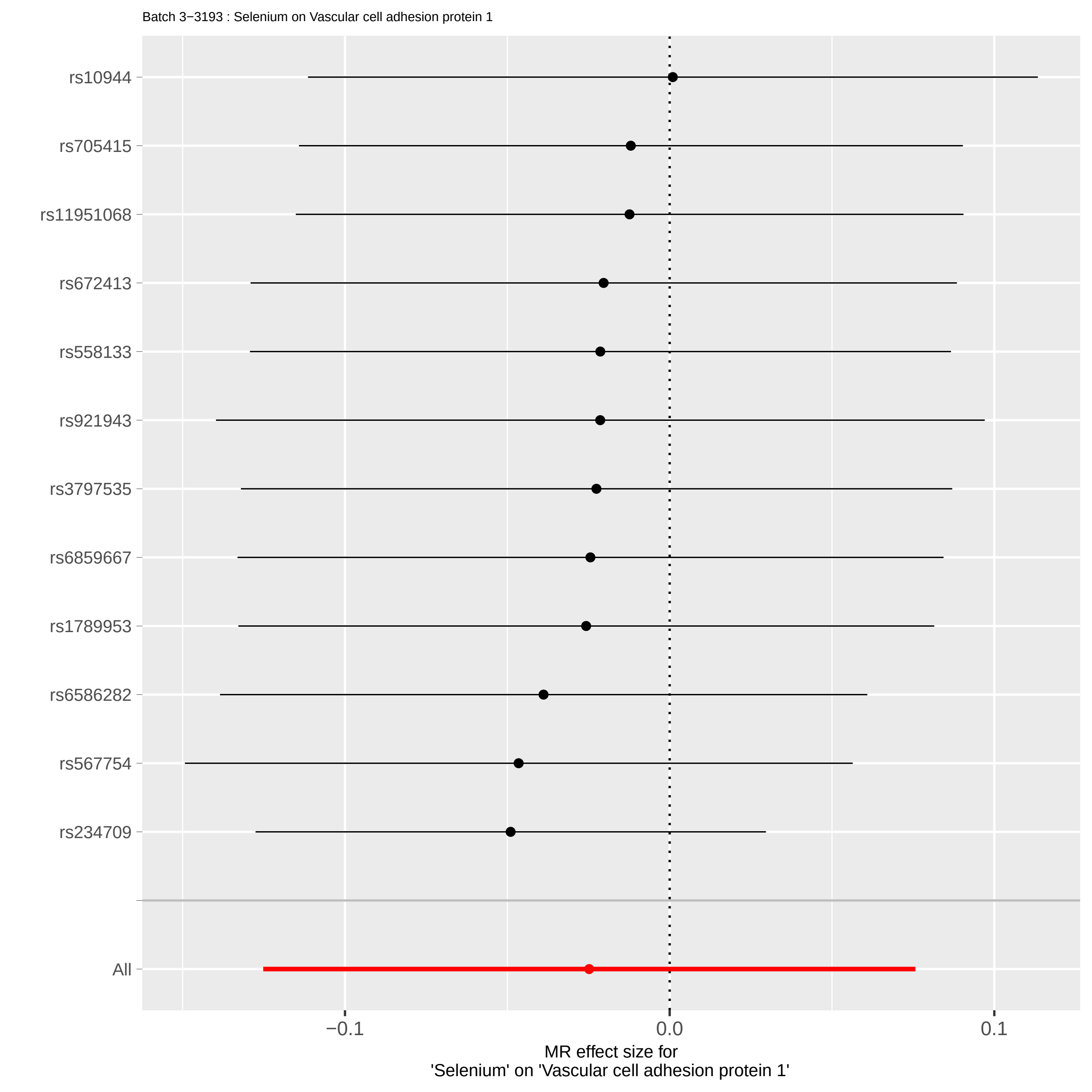

Batch 3−3193 : Selenium on Vascular cell adhesion protein 1
rs10944
rs705415
rs11951068
rs672413
rs558133
rs921943
rs3797535
rs6859667
rs1789953
rs6586282
rs567754
rs234709
All
−0.1
0.0
0.1
MR effect size for
'Selenium' on 'Vascular cell adhesion protein 1'

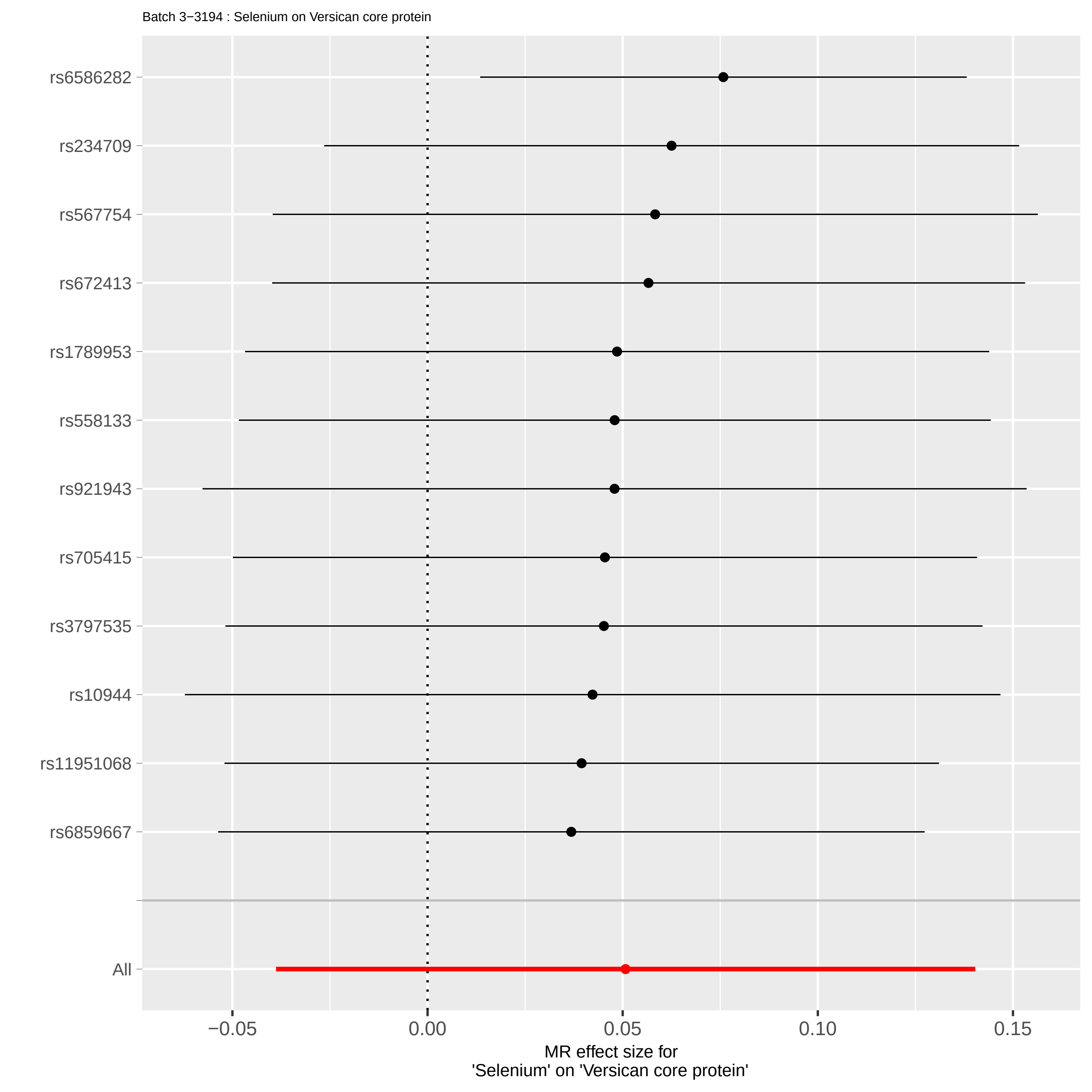
Batch 3−3194 : Selenium on Versican core protein
rs6586282
rs234709
rs567754
rs672413
rs1789953
rs558133
rs921943
rs705415
rs3797535
rs10944
rs11951068
rs6859667
All
−0.05
0.00
0.05
0.10
0.15
MR effect size for
'Selenium' on 'Versican core protein'

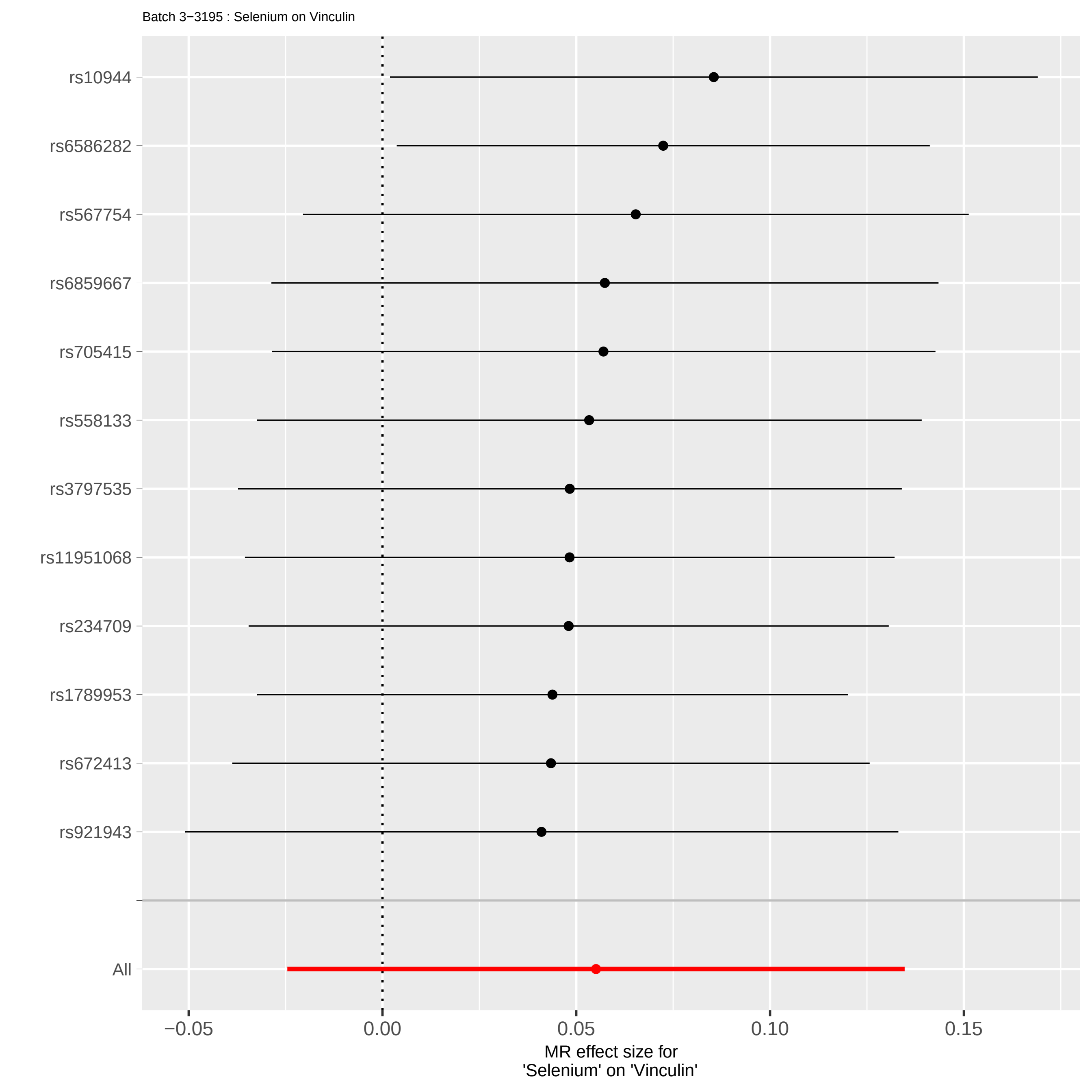

Batch 3−3195 : Selenium on Vinculin
rs10944
rs6586282
rs567754
rs6859667
rs705415
rs558133
rs3797535
rs11951068
rs234709
rs1789953
rs672413
rs921943
All
−0.05
0.00
0.05
0.10
0.15
MR effect size for
'Selenium' on 'Vinculin'

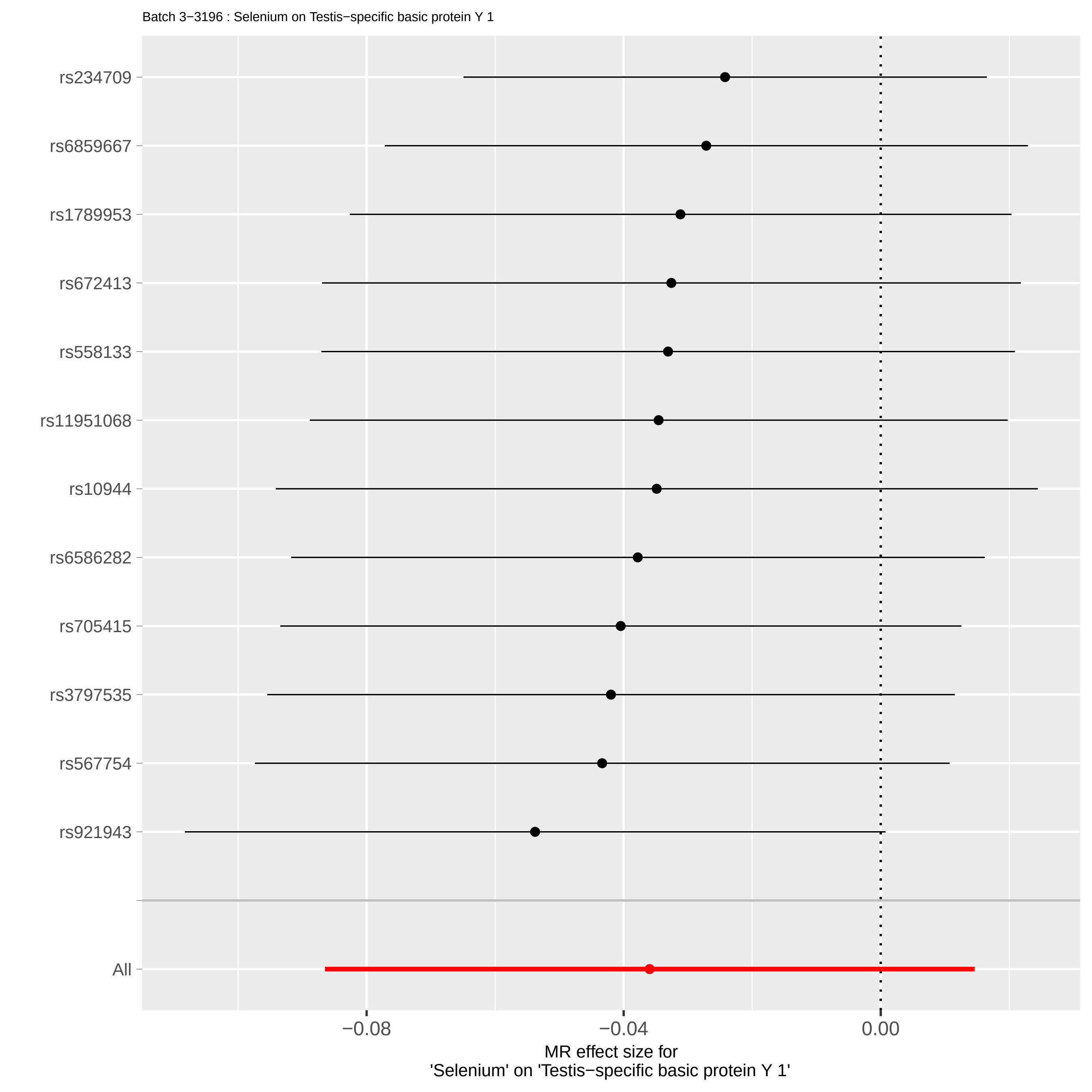

Batch 3−3196 : Selenium on Testis−specific basic protein Y 1
rs234709
rs6859667
rs1789953
rs672413
rs558133
rs11951068
rs10944
rs6586282
rs705415
rs3797535
rs567754
rs921943
All
−0.08
−0.04
0.00
MR effect size for
'Selenium' on 'Testis−specific basic protein Y 1'

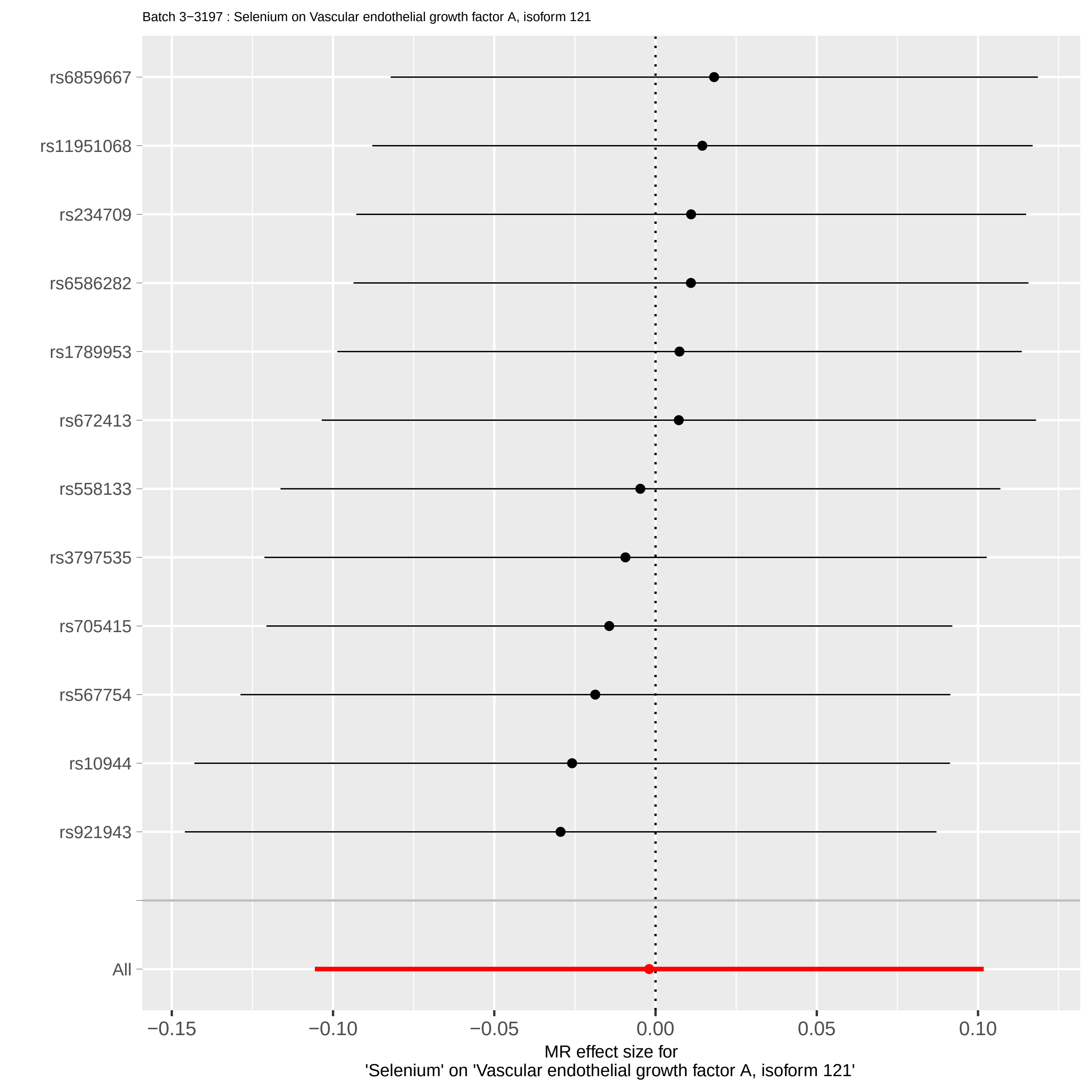

Batch 3−3197 : Selenium on Vascular endothelial growth factor A, isoform 121
rs6859667
rs11951068
rs234709
rs6586282
rs1789953
rs672413
rs558133
rs3797535
rs705415
rs567754
rs10944
rs921943
All
−0.15
−0.10
−0.05
0.00
0.05
0.10
MR effect size for
'Selenium' on 'Vascular endothelial growth factor A, isoform 121'

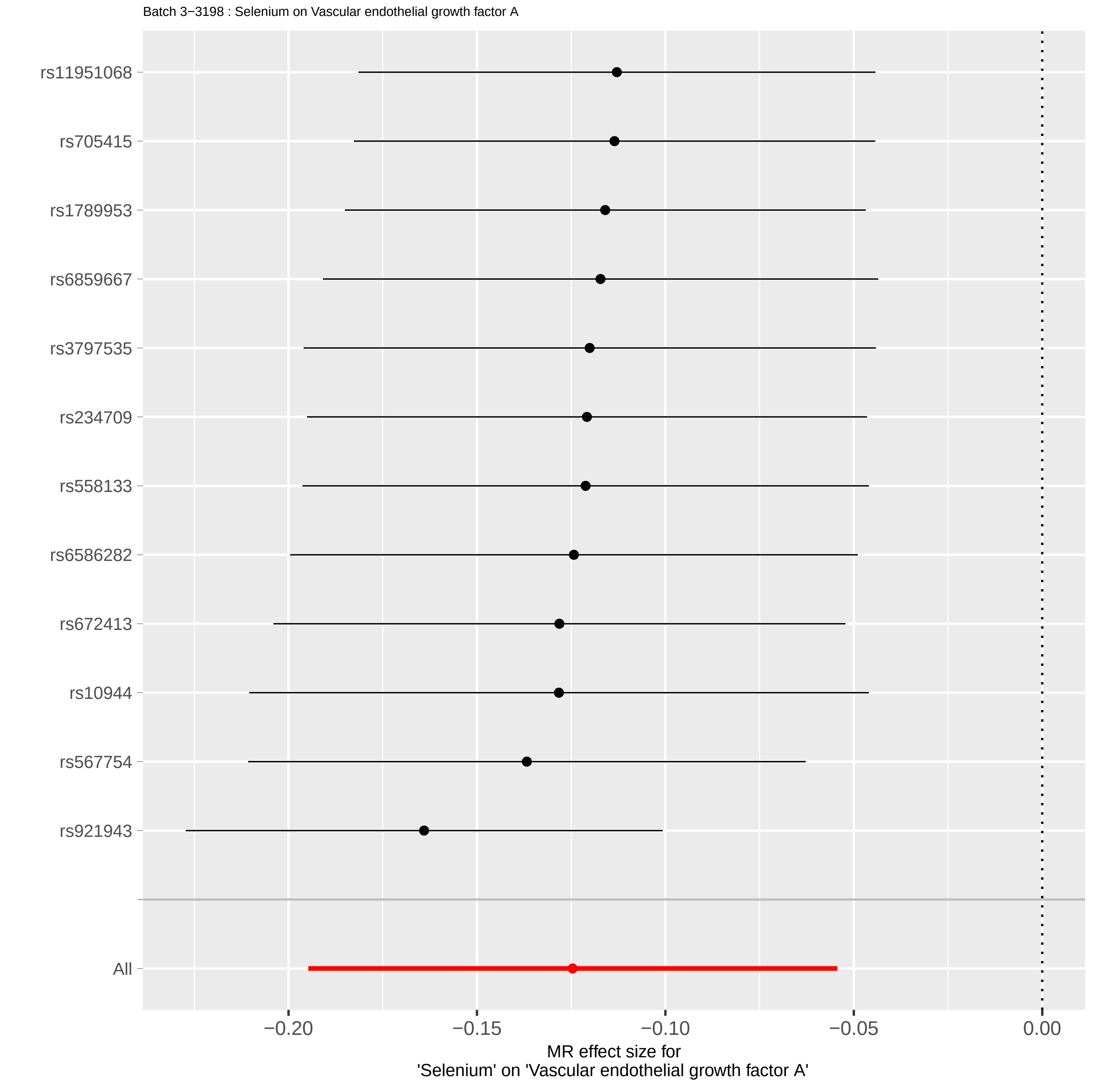
Batch 3−3198 : Selenium on Vascular endothelial growth factor A
rs11951068
rs705415
rs1789953
rs6859667
rs3797535
rs234709
rs558133
rs6586282
rs672413
rs10944
rs567754
rs921943
All
−0.20
−0.15
−0.10
−0.05
0.00
MR effect size for
'Selenium' on 'Vascular endothelial growth factor A'

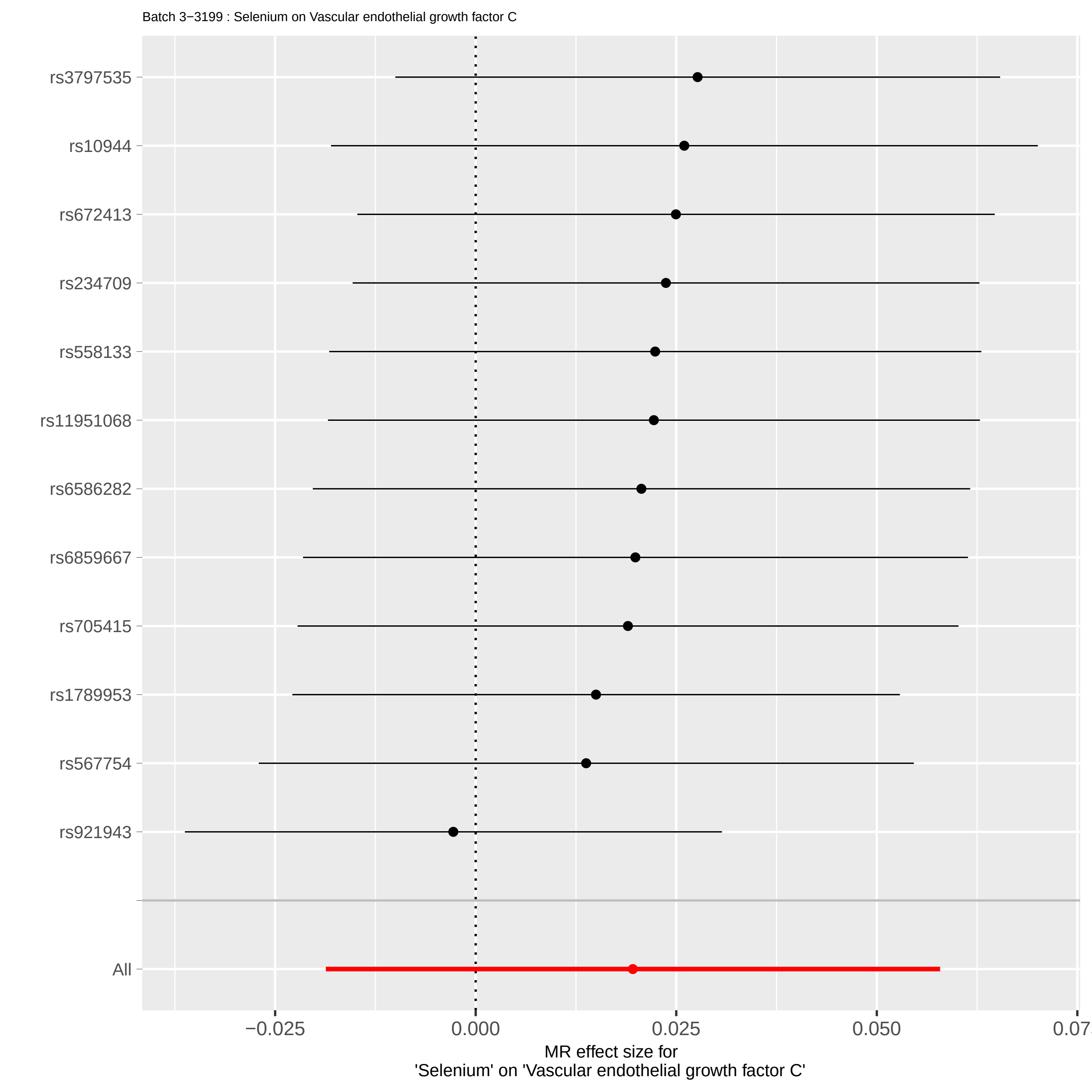

Batch 3−3199 : Selenium on Vascular endothelial growth factor C
rs3797535
rs10944
rs672413
rs234709
rs558133
rs11951068
rs6586282
rs6859667
rs705415
rs1789953
rs567754
rs921943
All
−0.025
0.000
0.025
0.050
MR effect size for
'Selenium' on 'Vascular endothelial growth factor C'

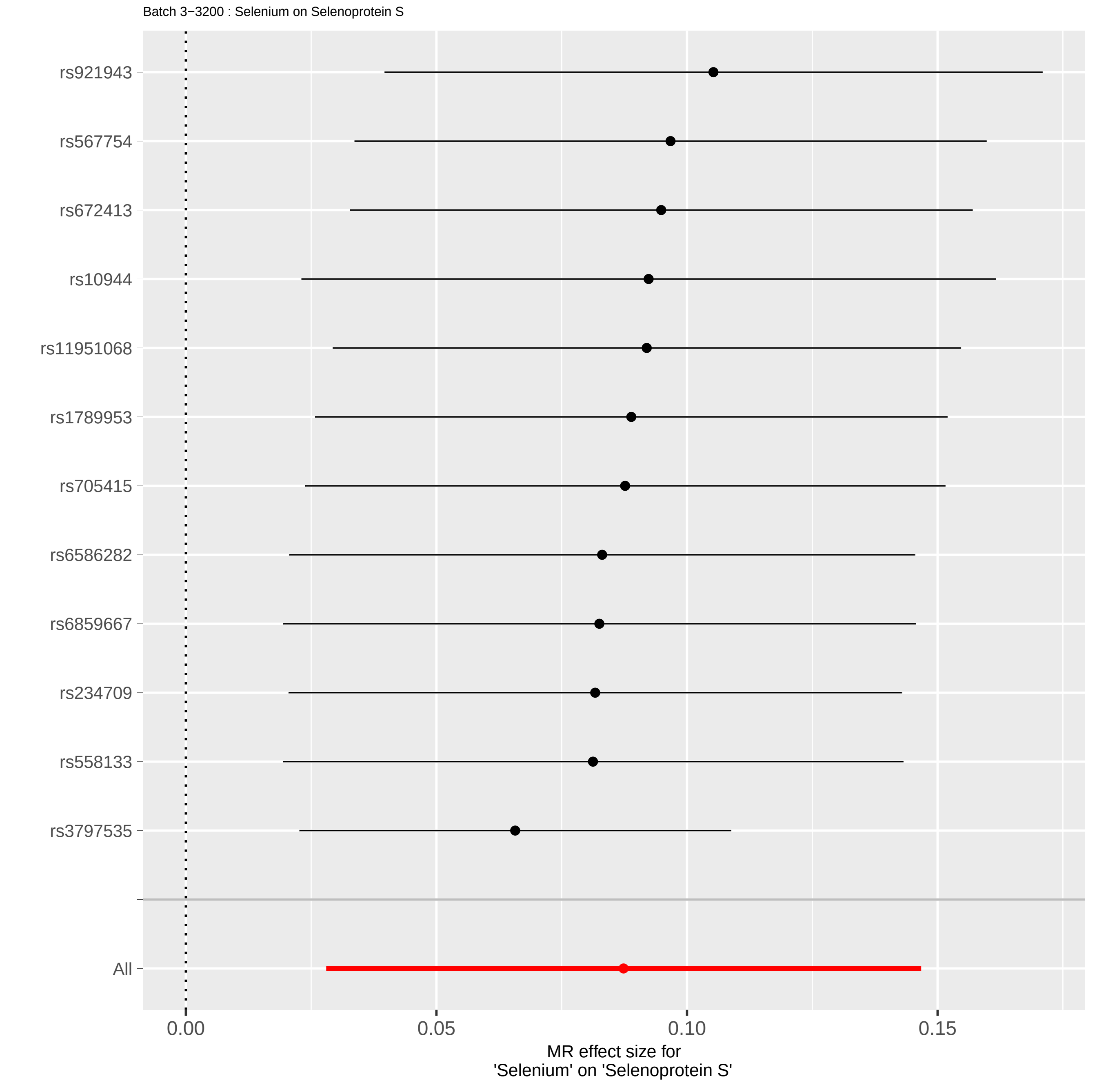

Batch 3−3200 : Selenium on Selenoprotein S
rs921943
rs567754
rs672413
rs10944
rs11951068
rs1789953
rs705415
rs6586282
rs6859667
rs234709
rs558133
rs3797535
All
0.00
0.05
0.10
0.15
MR effect size for
'Selenium' on 'Selenoprotein S'

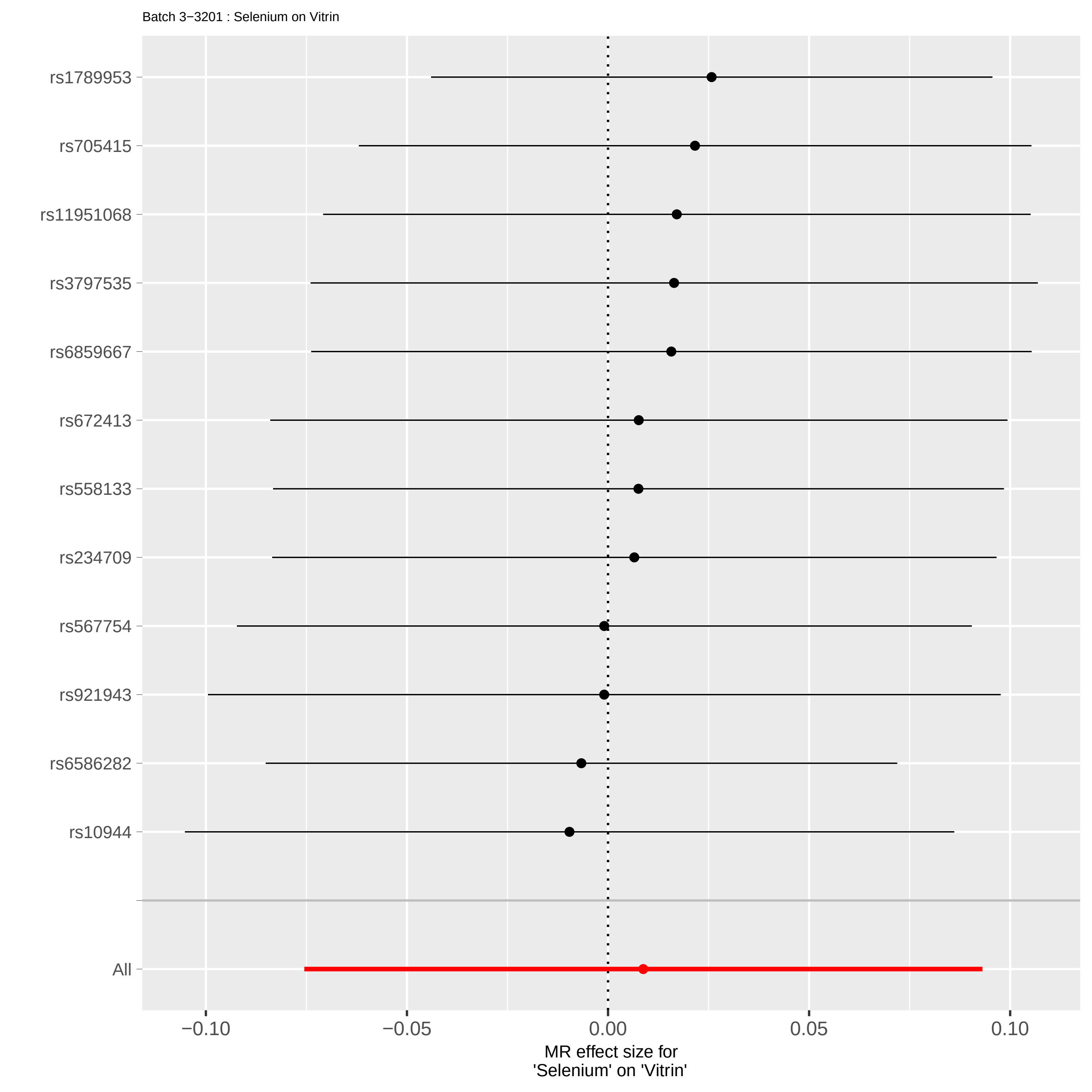

Batch 3−3201 : Selenium on Vitrin
rs1789953
rs705415
rs11951068
rs3797535
rs6859667
rs672413
rs558133
rs234709
rs567754
rs921943
rs6586282
rs10944
All
−0.10
−0.05
0.00
0.05
0.10
MR effect size for
'Selenium' on 'Vitrin'

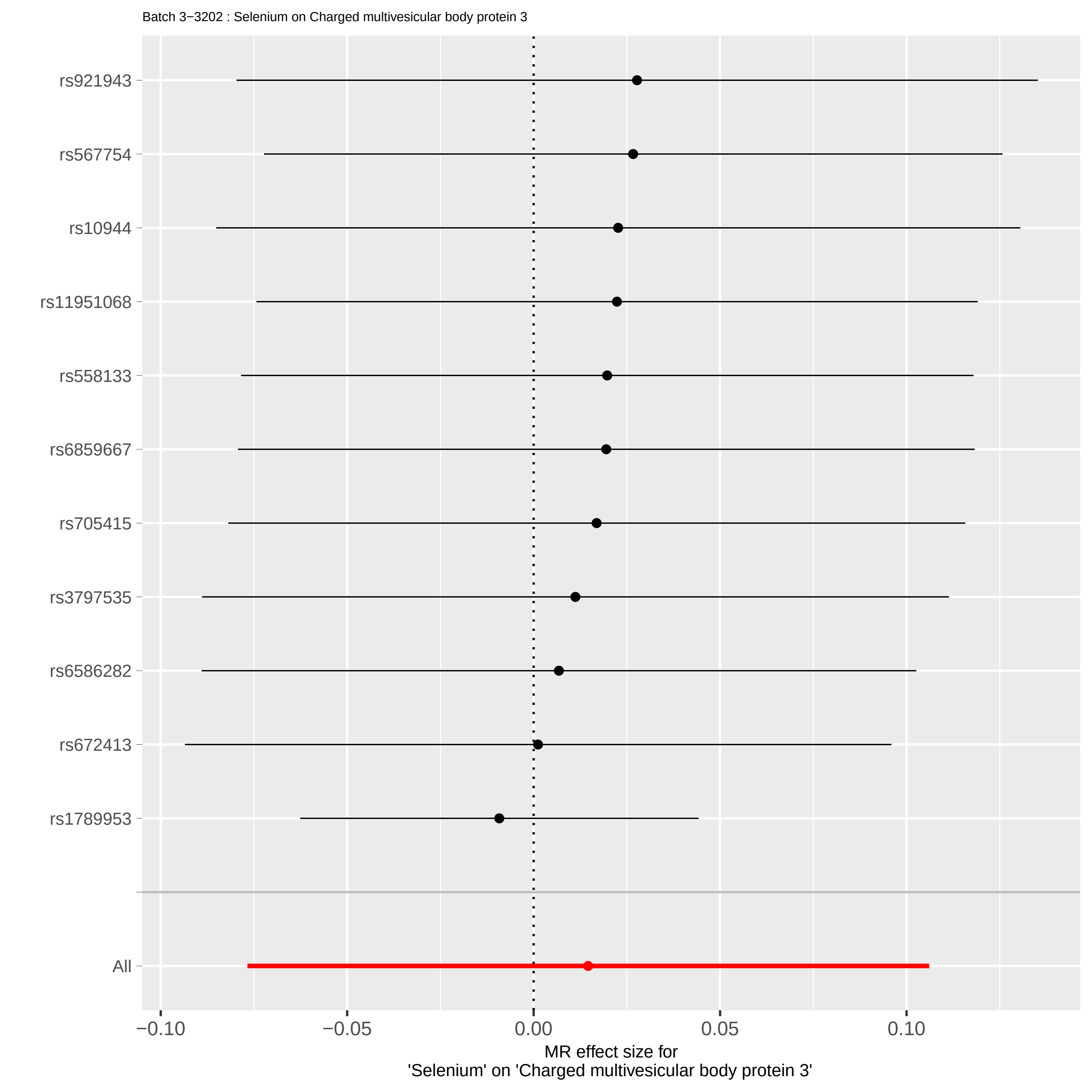

Batch 3−3202 : Selenium on Charged multivesicular body protein 3
rs921943
rs567754
rs10944
rs11951068
rs558133
rs6859667
rs705415
rs3797535
rs6586282
rs672413
rs1789953
All
−0.10
−0.05
0.00
0.05
0.10
MR effect size for
'Selenium' on 'Charged multivesicular body protein 3'

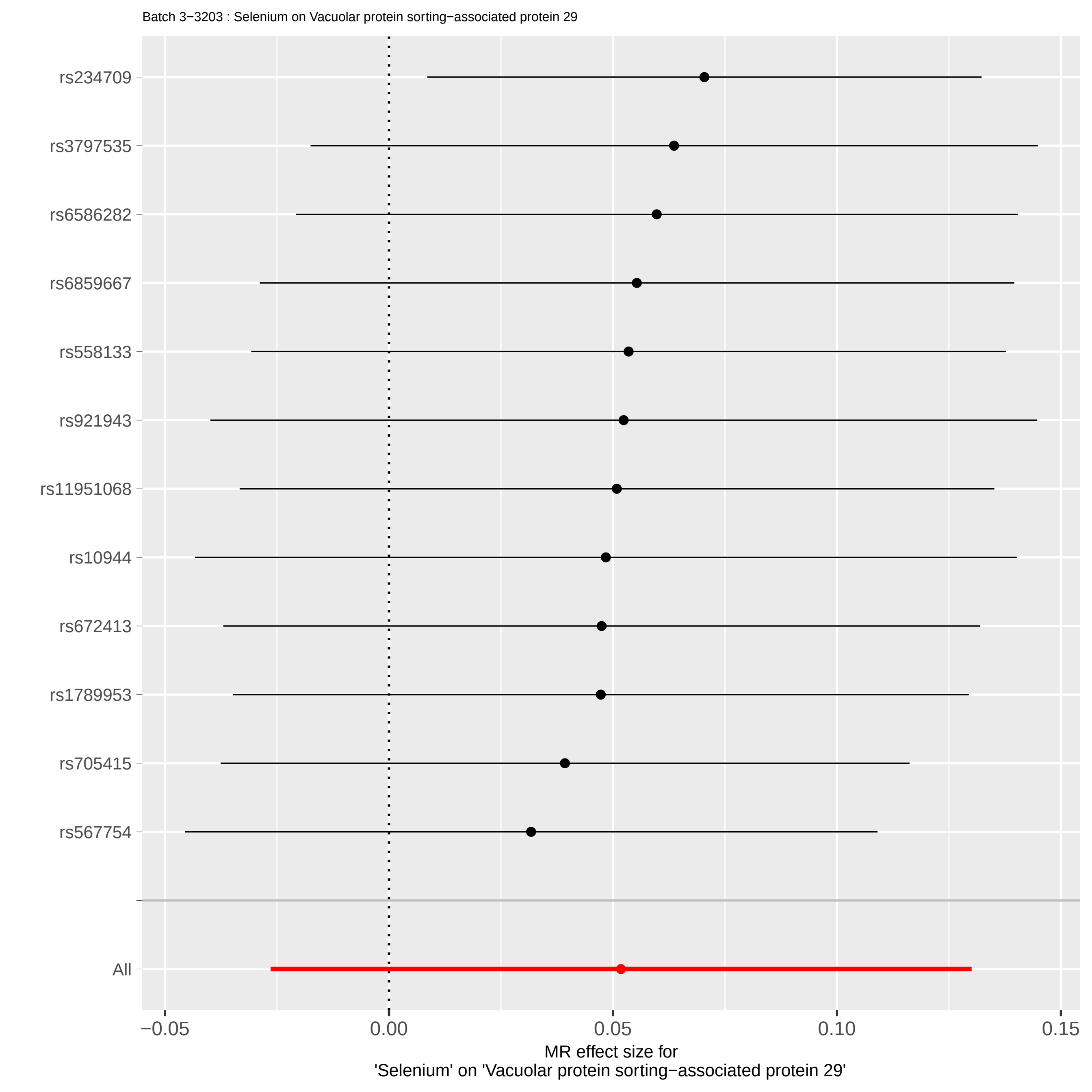

Batch 3−3203 : Selenium on Vacuolar protein sorting−associated protein 29
rs234709
rs3797535
rs6586282
rs6859667
rs558133
rs921943
rs11951068
rs10944
rs672413
rs1789953
rs705415
rs567754
All
−0.05
0.00
0.05
0.10
0.15
MR effect size for
'Selenium' on 'Vacuolar protein sorting−associated protein 29'

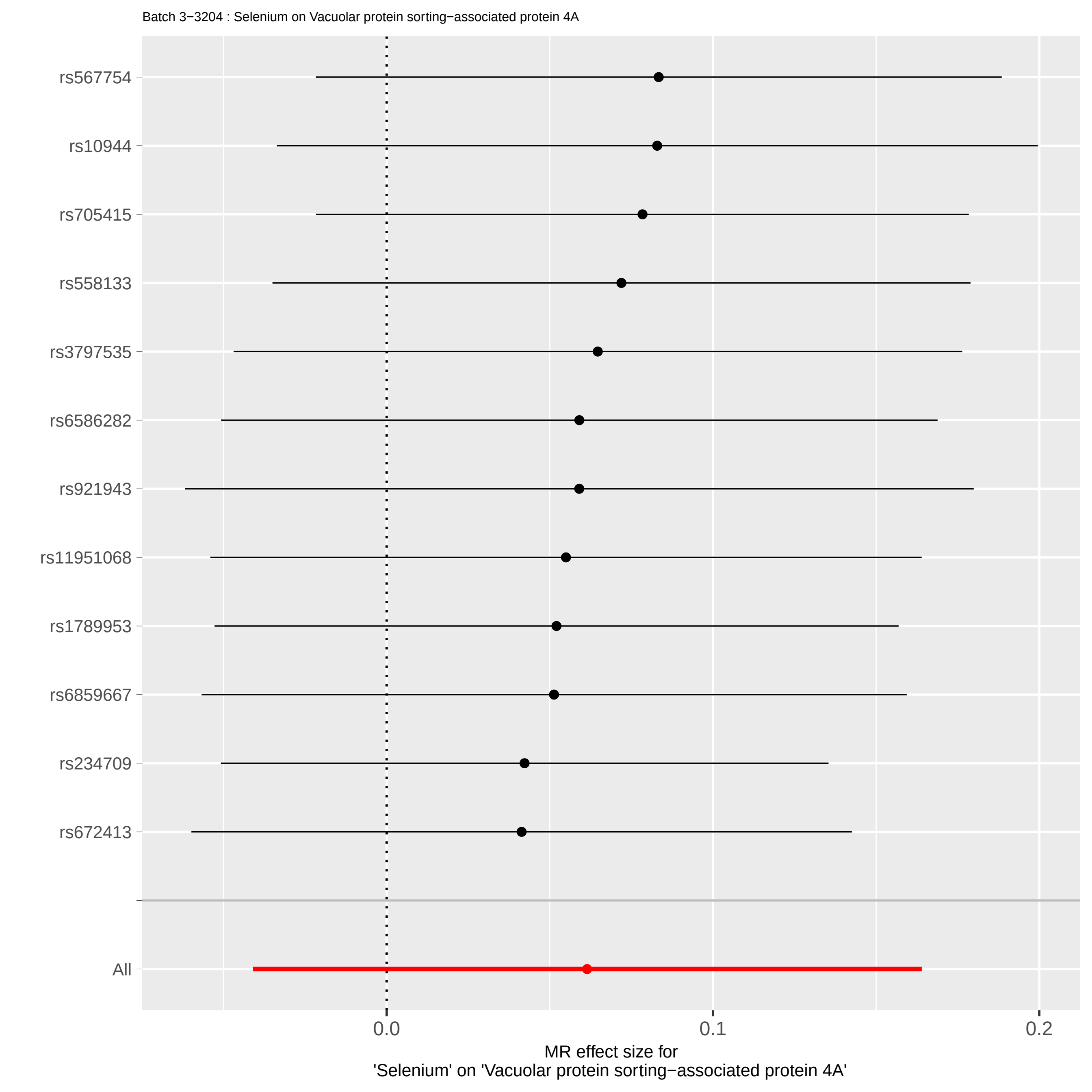

Batch 3−3204 : Selenium on Vacuolar protein sorting−associated protein 4A
rs567754
rs10944
rs705415
rs558133
rs3797535
rs6586282
rs921943
rs11951068
rs1789953
rs6859667
rs234709
rs672413
All
0.0
0.1
0.2
MR effect size for
'Selenium' on 'Vacuolar protein sorting−associated protein 4A'

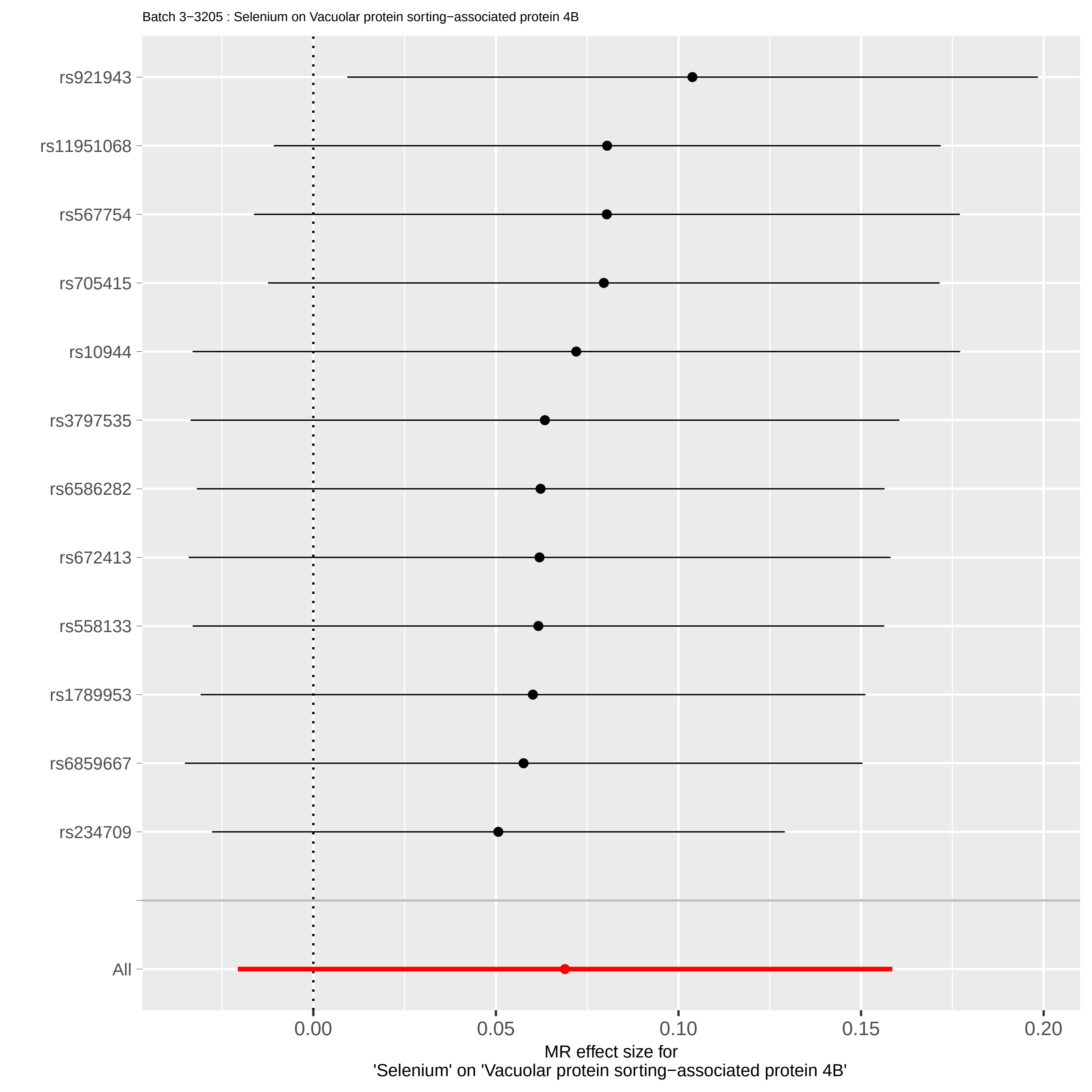

Batch 3−3205 : Selenium on Vacuolar protein sorting−associated protein 4B
rs921943
rs11951068
rs567754
rs705415
rs10944
rs3797535
rs6586282
rs672413
rs558133
rs1789953
rs6859667
rs234709
All
0.00
0.05
0.10
0.15
0.20
MR effect size for
'Selenium' on 'Vacuolar protein sorting−associated protein 4B'

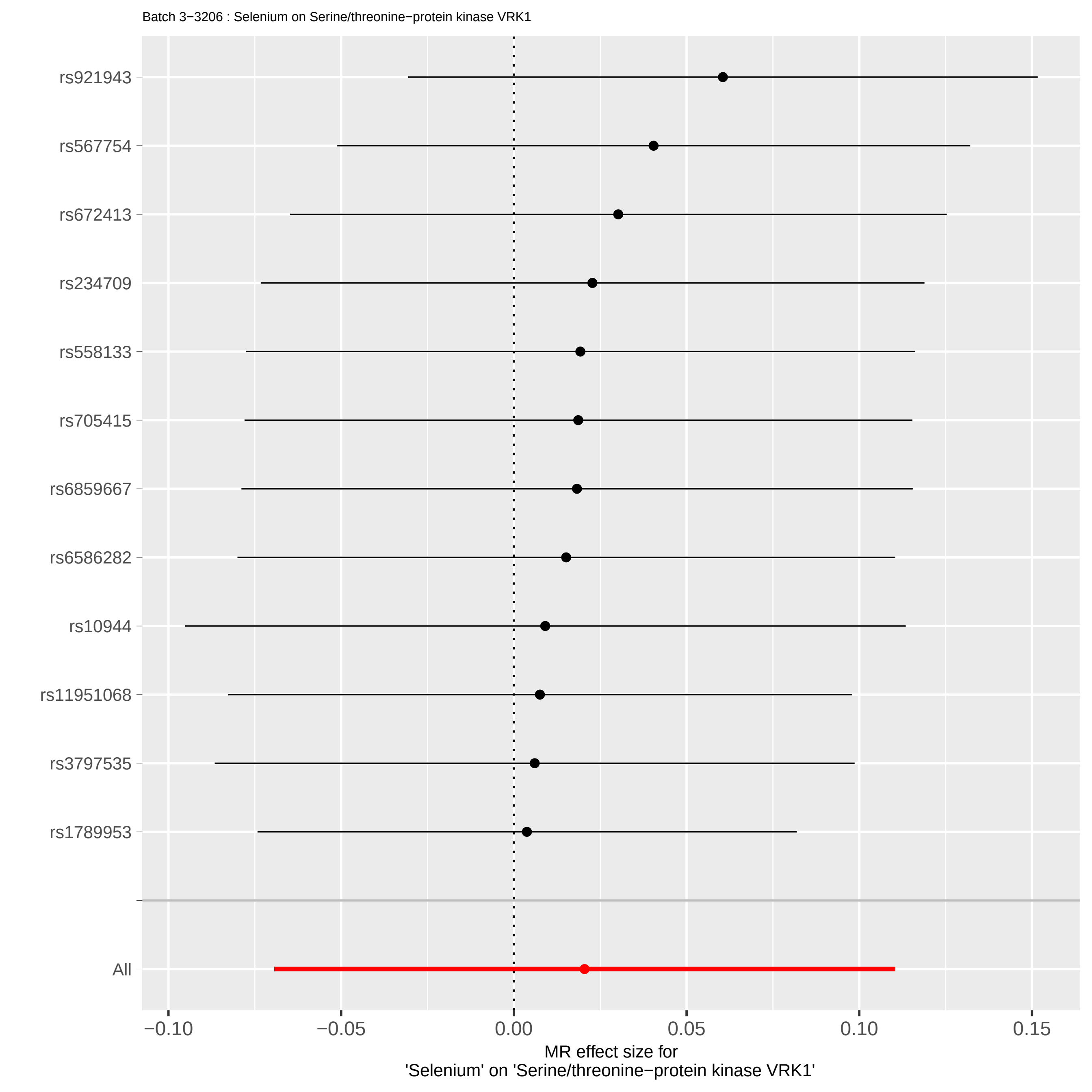

Batch 3−3206 : Selenium on Serine/threonine−protein kinase VRK1
rs921943
rs567754
rs672413
rs234709
rs558133
rs705415
rs6859667
rs6586282
rs10944
rs11951068
rs3797535
rs1789953
All
−0.10
−0.05
0.00
0.05
0.10
0.15
MR effect size for
'Selenium' on 'Serine/threonine−protein kinase VRK1'

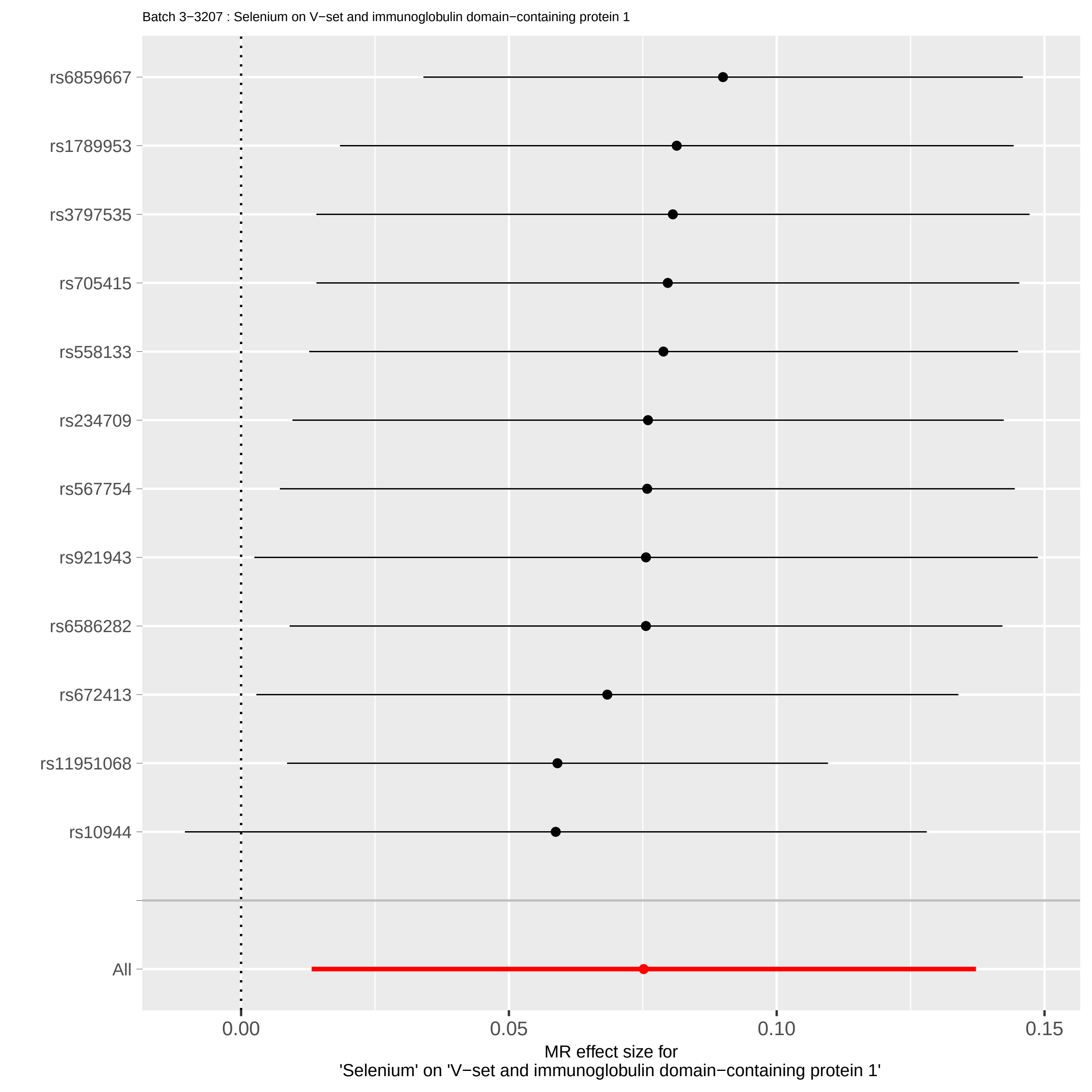

Batch 3−3207 : Selenium on V−set and immunoglobulin domain−containing protein 1
rs6859667
rs1789953
rs3797535
rs705415
rs558133
rs234709
rs567754
rs921943
rs6586282
rs672413
rs11951068
rs10944
All
0.00
0.05
0.10
0.15
MR effect size for
'Selenium' on 'V−set and immunoglobulin domain−containing protein 1'

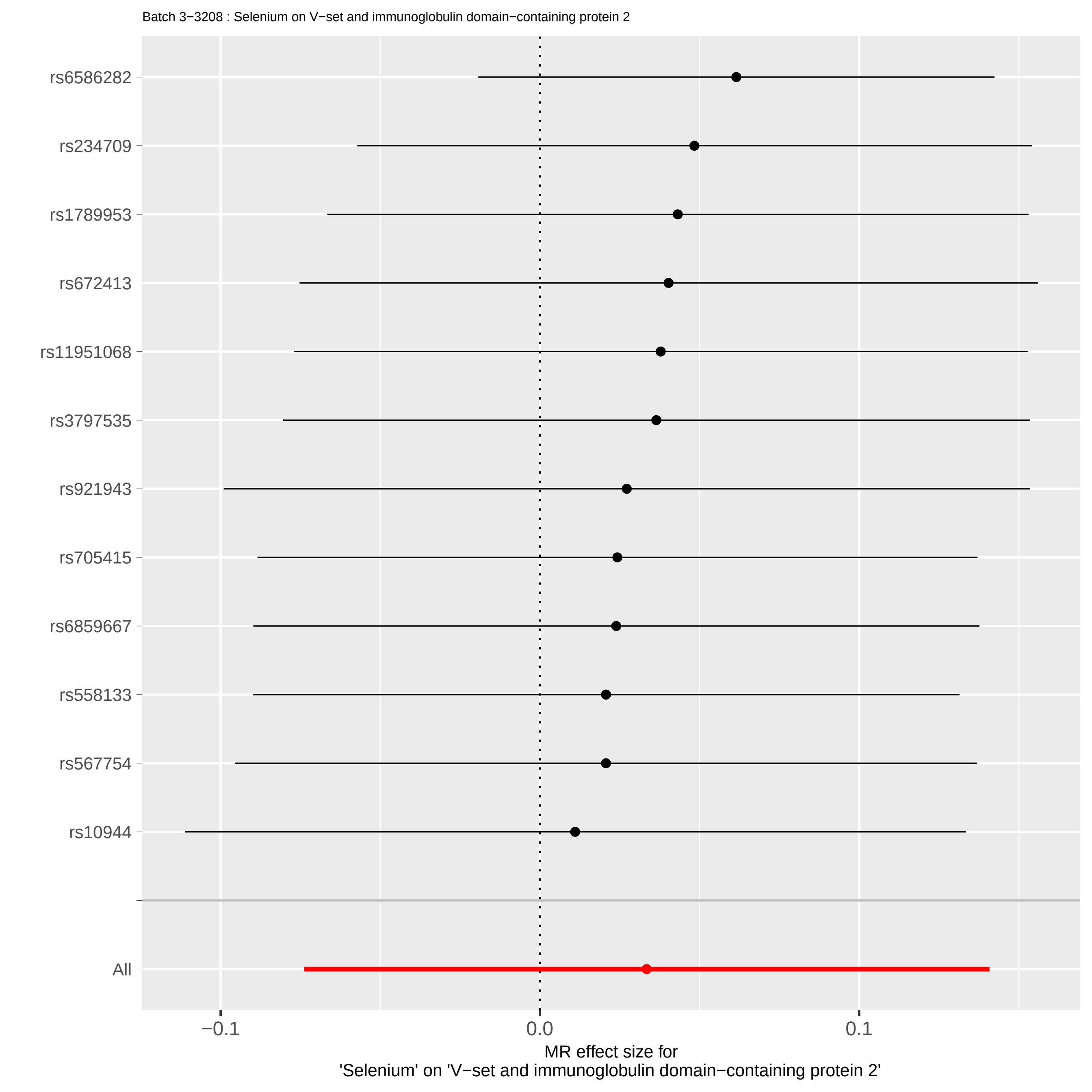

Batch 3−3208 : Selenium on V−set and immunoglobulin domain−containing protein 2
rs6586282
rs234709
rs1789953
rs672413
rs11951068
rs3797535
rs921943
rs705415
rs6859667
rs558133
rs567754
rs10944
All
−0.1
0.0
0.1
MR effect size for
'Selenium' on 'V−set and immunoglobulin domain−containing protein 2'

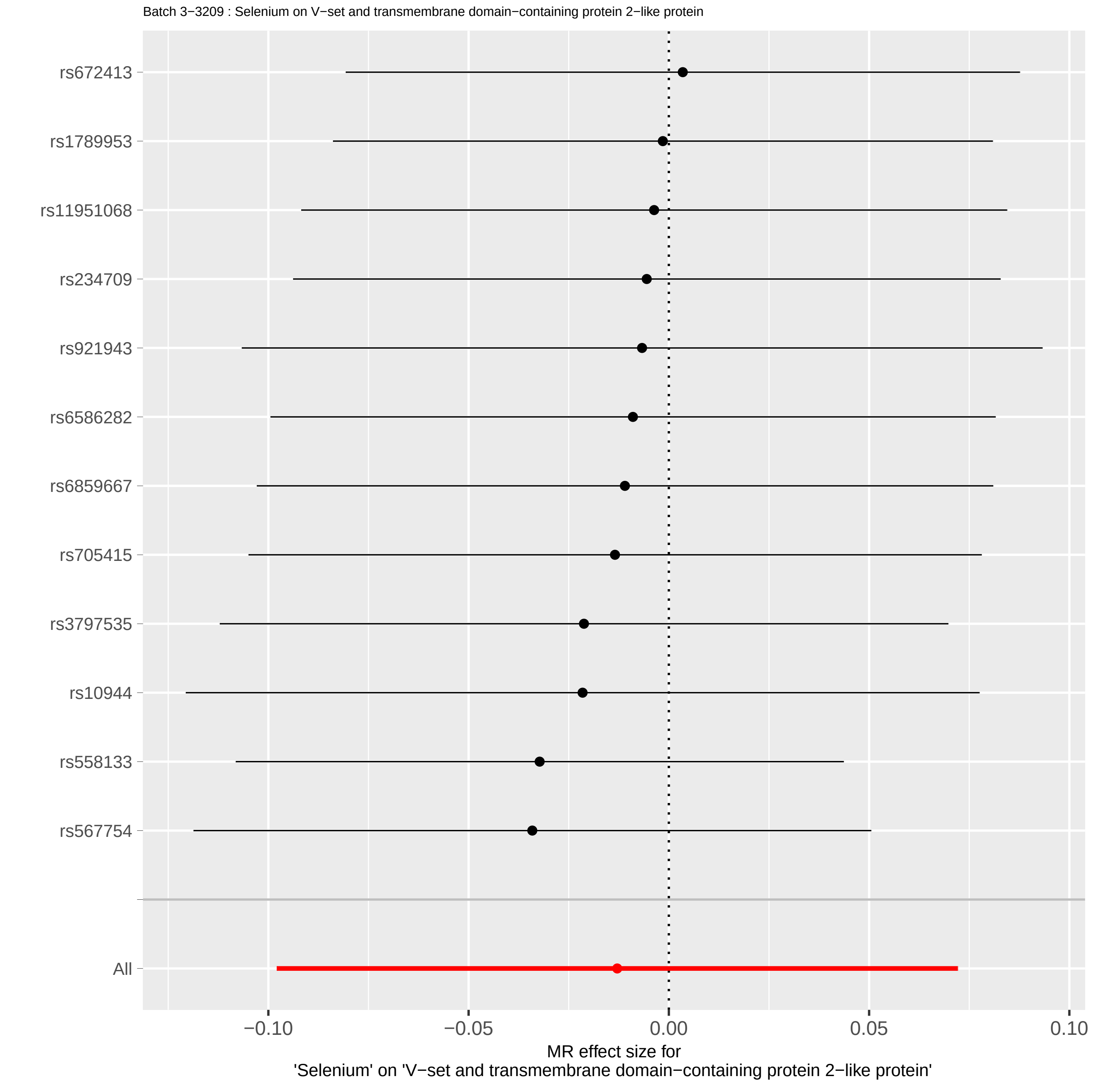
Batch 3−3209 : Selenium on V−set and transmembrane domain−containing protein 2−like protein
rs672413
rs1789953
rs11951068
rs234709
rs921943
rs6586282
rs6859667
rs705415
rs3797535
rs10944
rs558133
rs567754
All
−0.10
−0.05
0.00
0.05
0.10
MR effect size for
'Selenium' on 'V−set and transmembrane domain−containing protein 2−like protein'

Batch 3−3210 : Selenium on V−set and transmembrane domain−containing protein 4

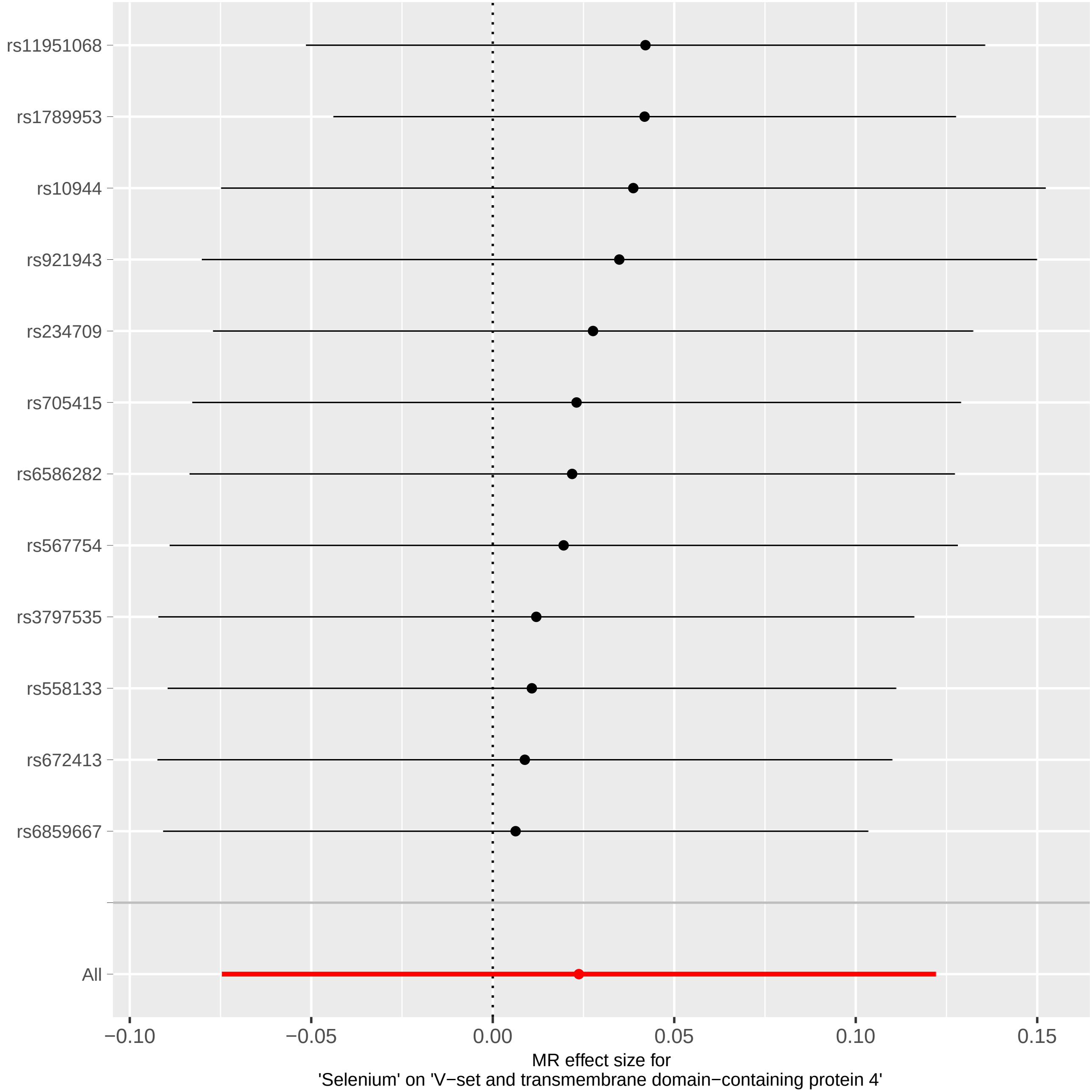

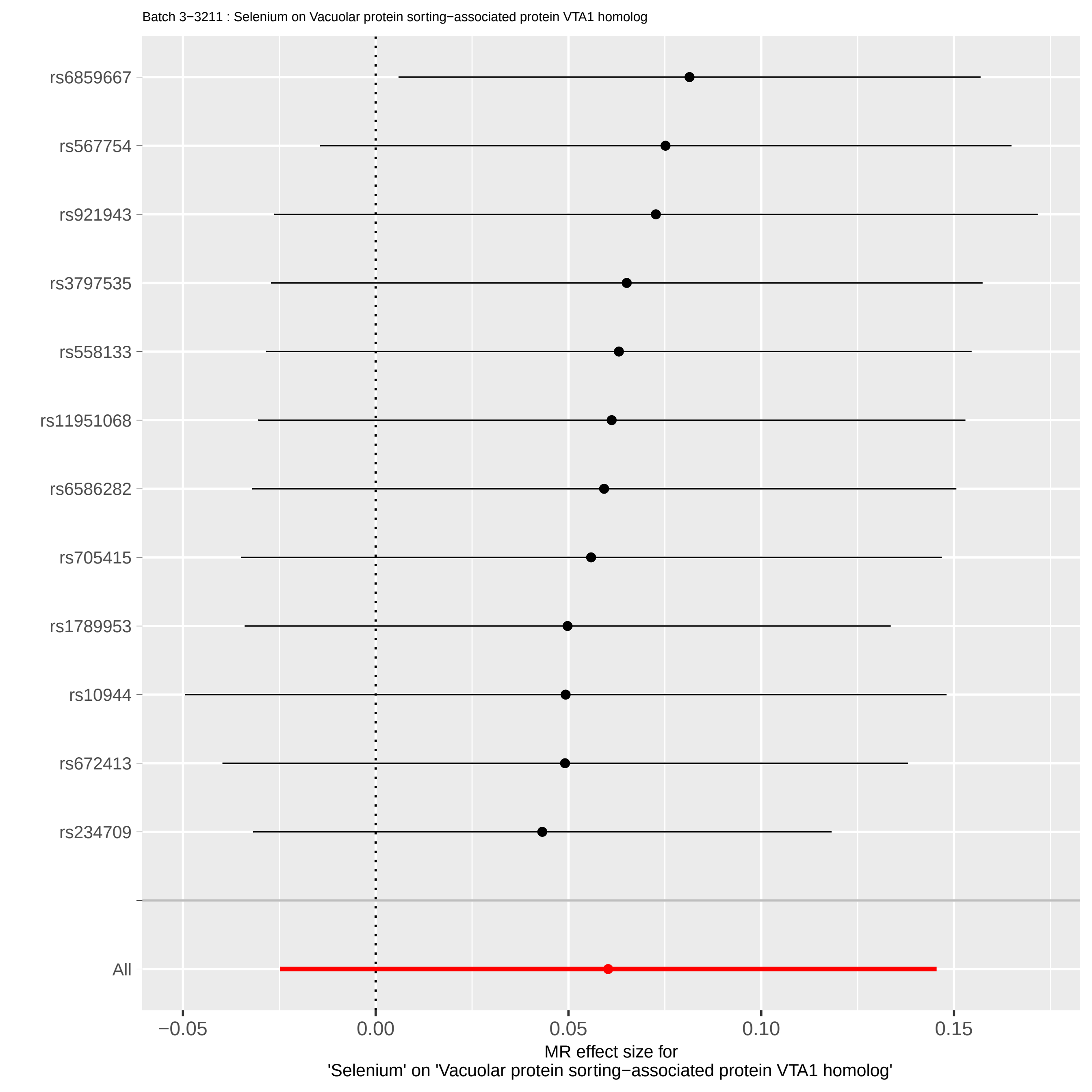

Batch 3−3211 : Selenium on Vacuolar protein sorting−associated protein VTA1 homolog
rs6859667
rs567754
rs921943
rs3797535
rs558133
rs11951068
rs6586282
rs705415
rs1789953
rs10944
rs672413
rs234709
All
−0.05
0.00
0.05
0.10
0.15
MR effect size for
'Selenium' on 'Vacuolar protein sorting−associated protein VTA1 homolog'

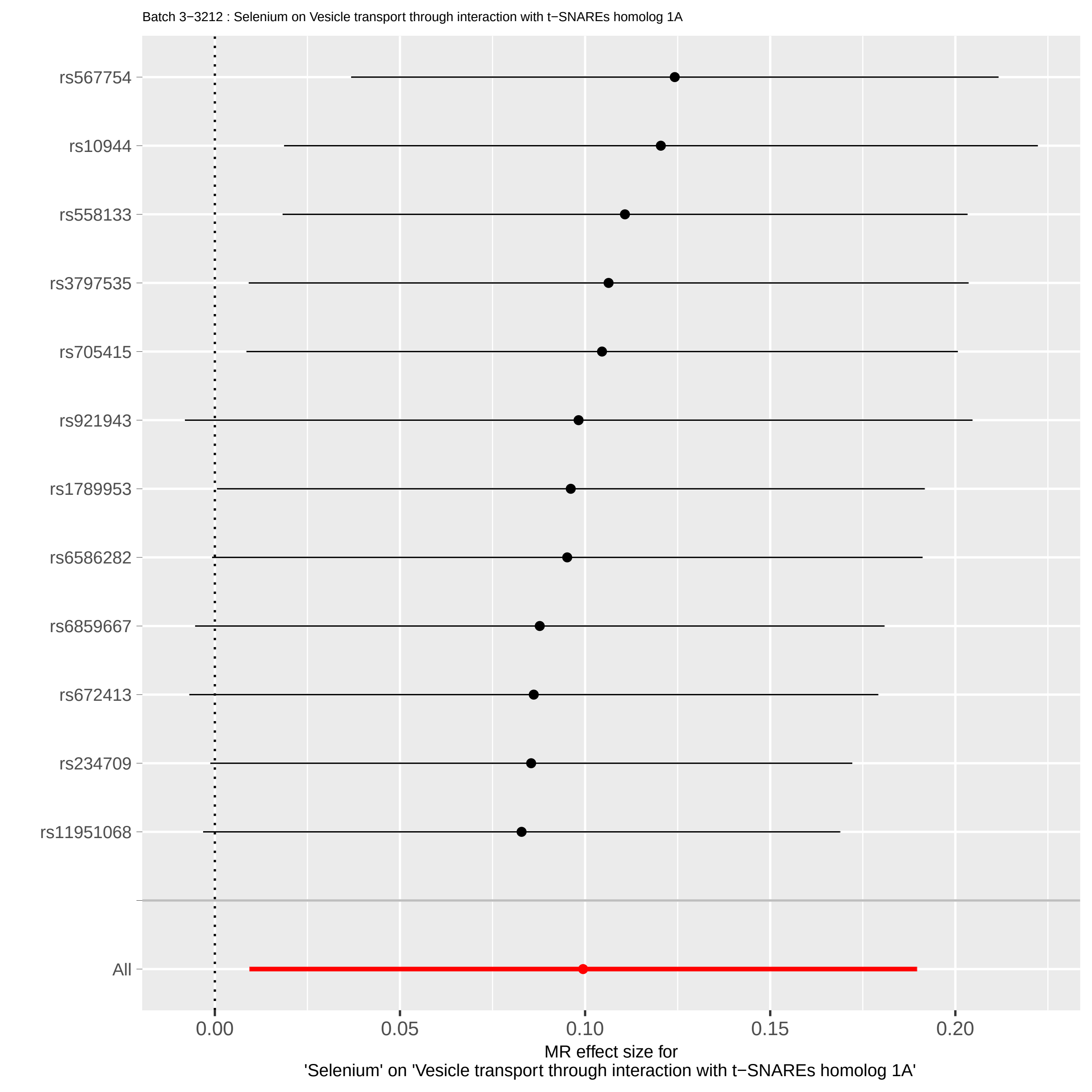

Batch 3−3212 : Selenium on Vesicle transport through interaction with t−SNAREs homolog 1A
rs567754
rs10944
rs558133
rs3797535
rs705415
rs921943
rs1789953
rs6586282
rs6859667
rs672413
rs234709
rs11951068
All
0.00
0.05
0.10
0.15
0.20
MR effect size for
'Selenium' on 'Vesicle transport through interaction with t−SNAREs homolog 1A'

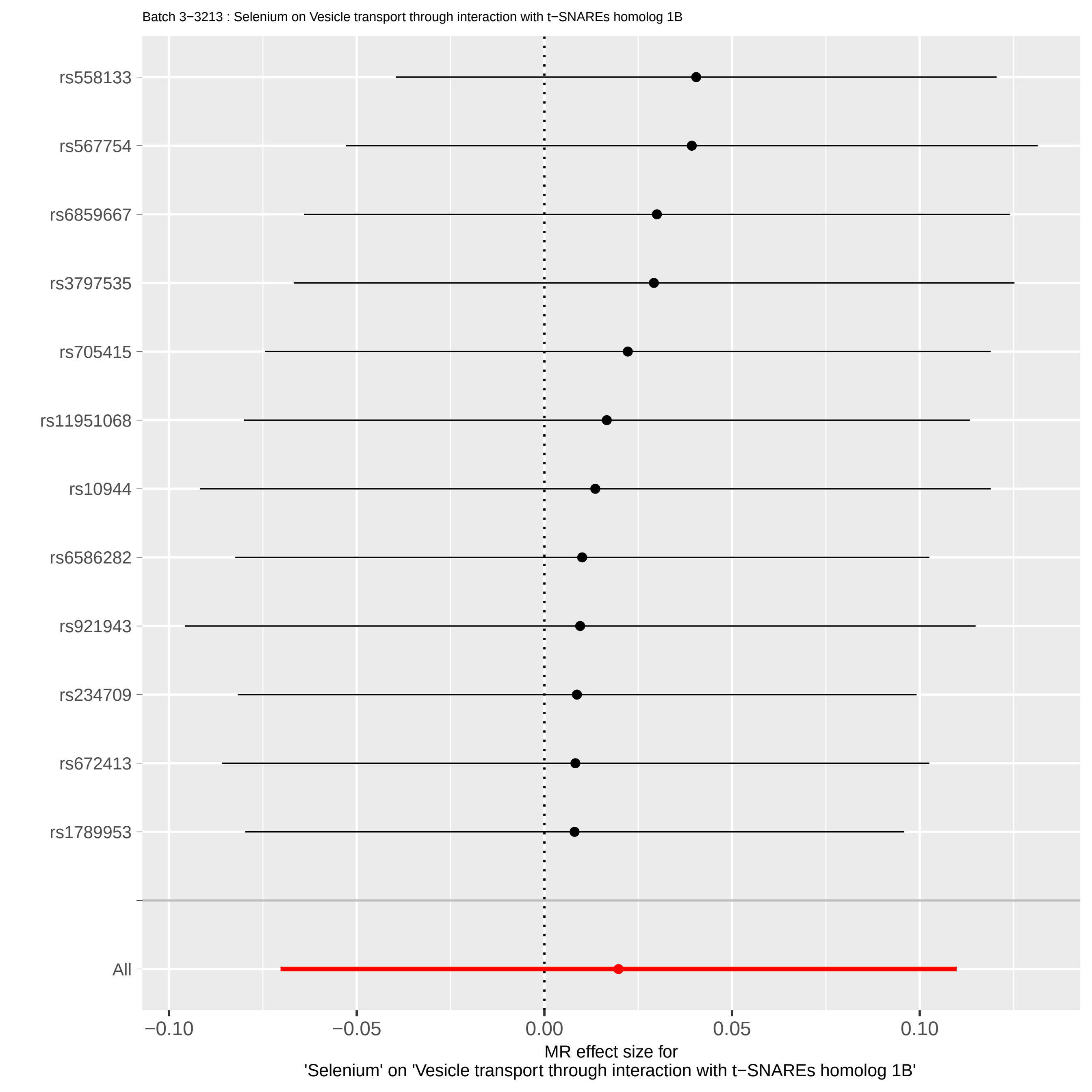

Batch 3−3213 : Selenium on Vesicle transport through interaction with t−SNAREs homolog 1B
rs558133
rs567754
rs6859667
rs3797535
rs705415
rs11951068
rs10944
rs6586282
rs921943
rs234709
rs672413
rs1789953
All
−0.10
−0.05
0.00
0.05
0.10
MR effect size for
'Selenium' on 'Vesicle transport through interaction with t−SNAREs homolog 1B'

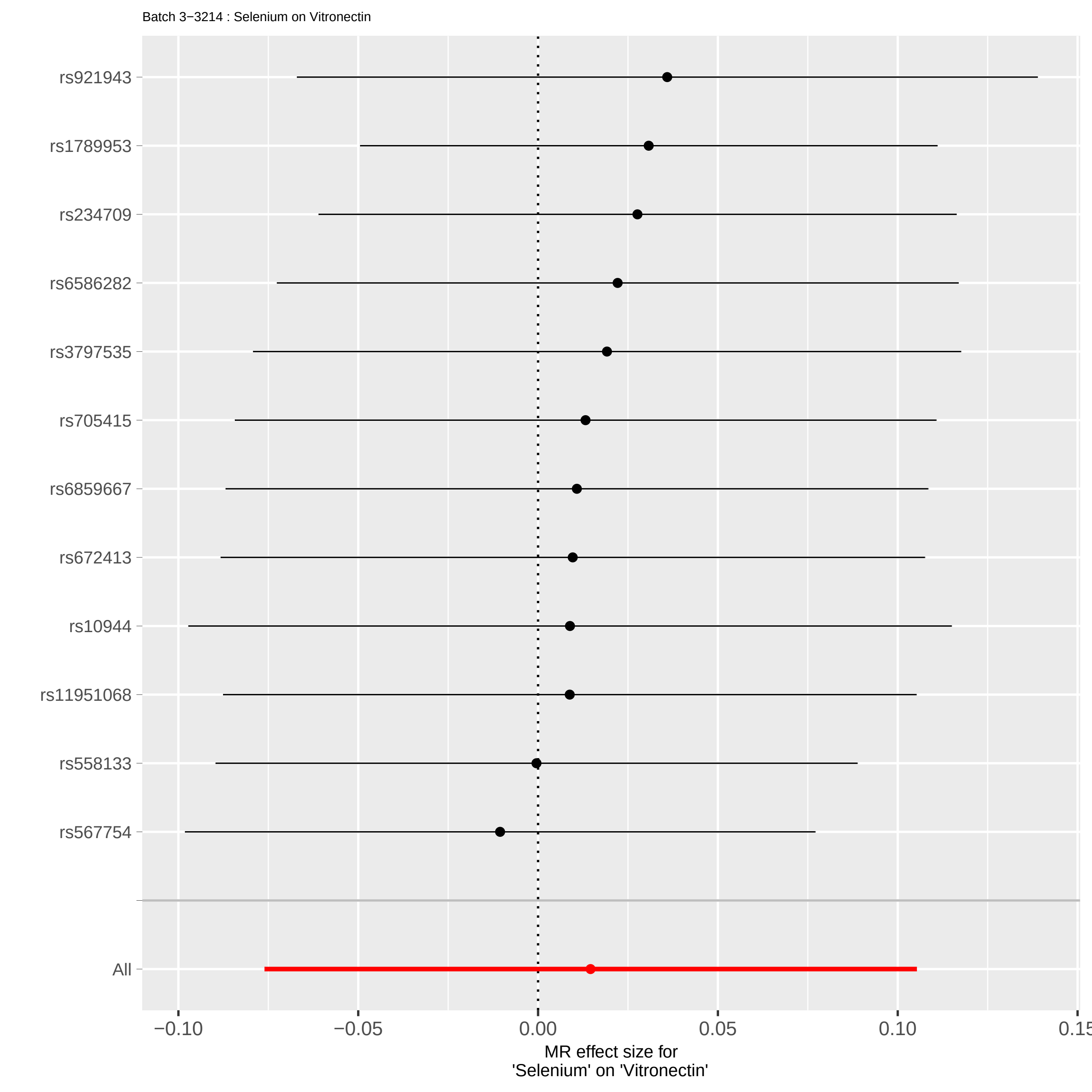

Batch 3−3214 : Selenium on Vitronectin
rs921943
rs1789953
rs234709
rs6586282
rs3797535
rs705415
rs6859667
rs672413
rs10944
rs11951068
rs558133
rs567754
All
−0.10
−0.05
0.00
0.05
0.10
0.15
MR effect size for
'Selenium' on 'Vitronectin'

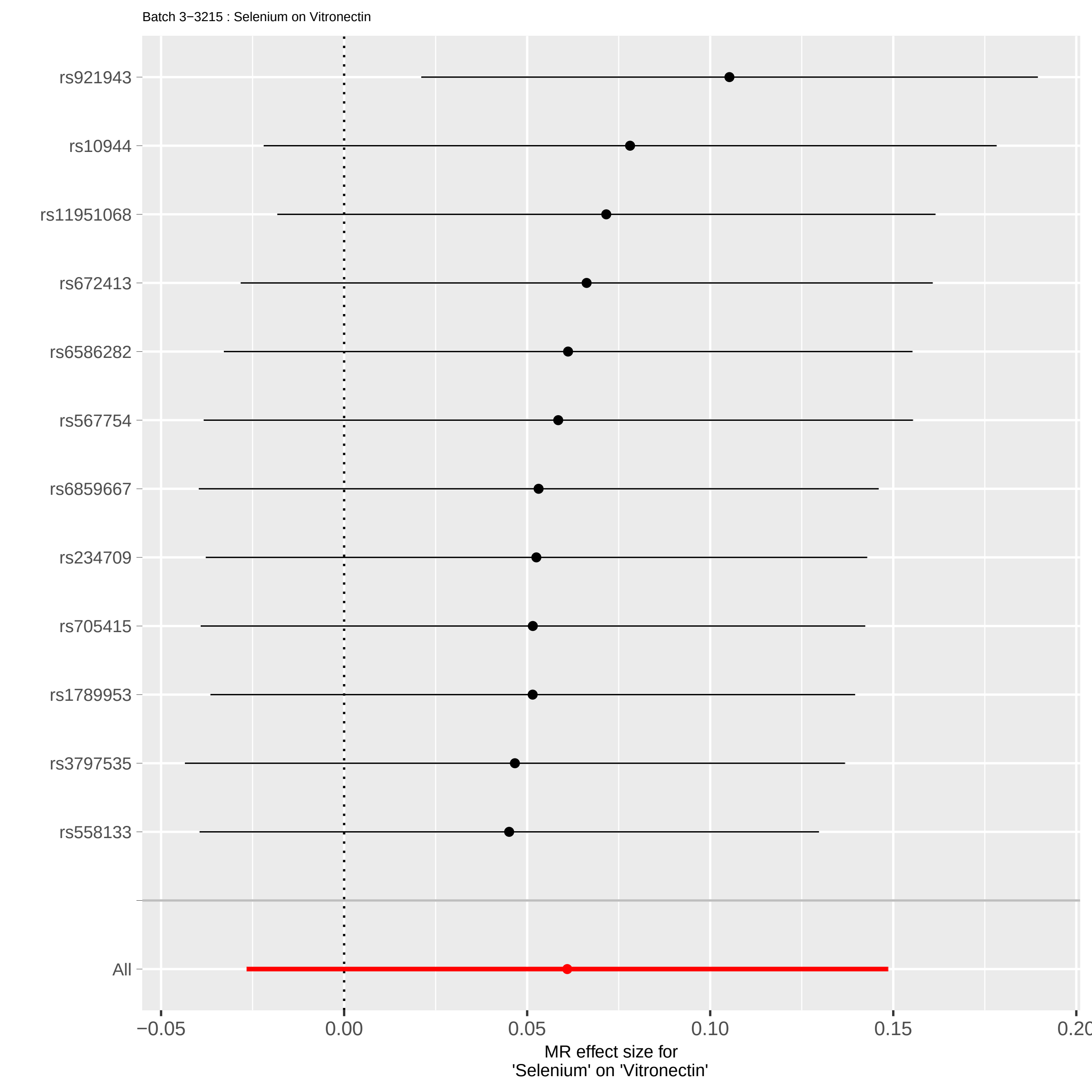

Batch 3−3215 : Selenium on Vitronectin
rs921943
rs10944
rs11951068
rs672413
rs6586282
rs567754
rs6859667
rs234709
rs705415
rs1789953
rs3797535
rs558133
All
−0.05
0.00
0.05
0.10
0.15
0.20
MR effect size for
'Selenium' on 'Vitronectin'

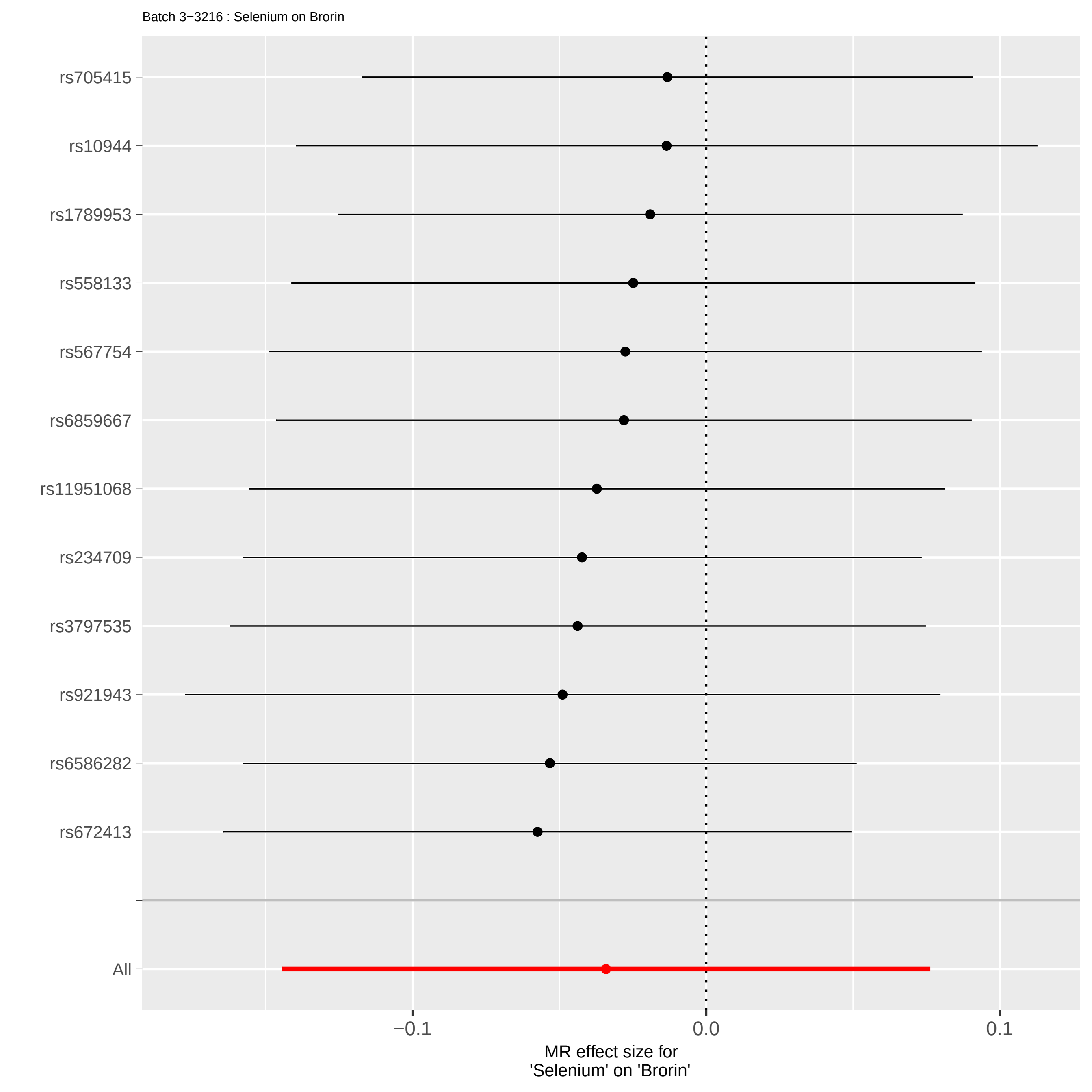

Batch 3−3216 : Selenium on Brorin
rs705415
rs10944
rs1789953
rs558133
rs567754
rs6859667
rs11951068
rs234709
rs3797535
rs921943
rs6586282
rs672413
All
−0.1
0.0
0.1
MR effect size for
'Selenium' on 'Brorin'

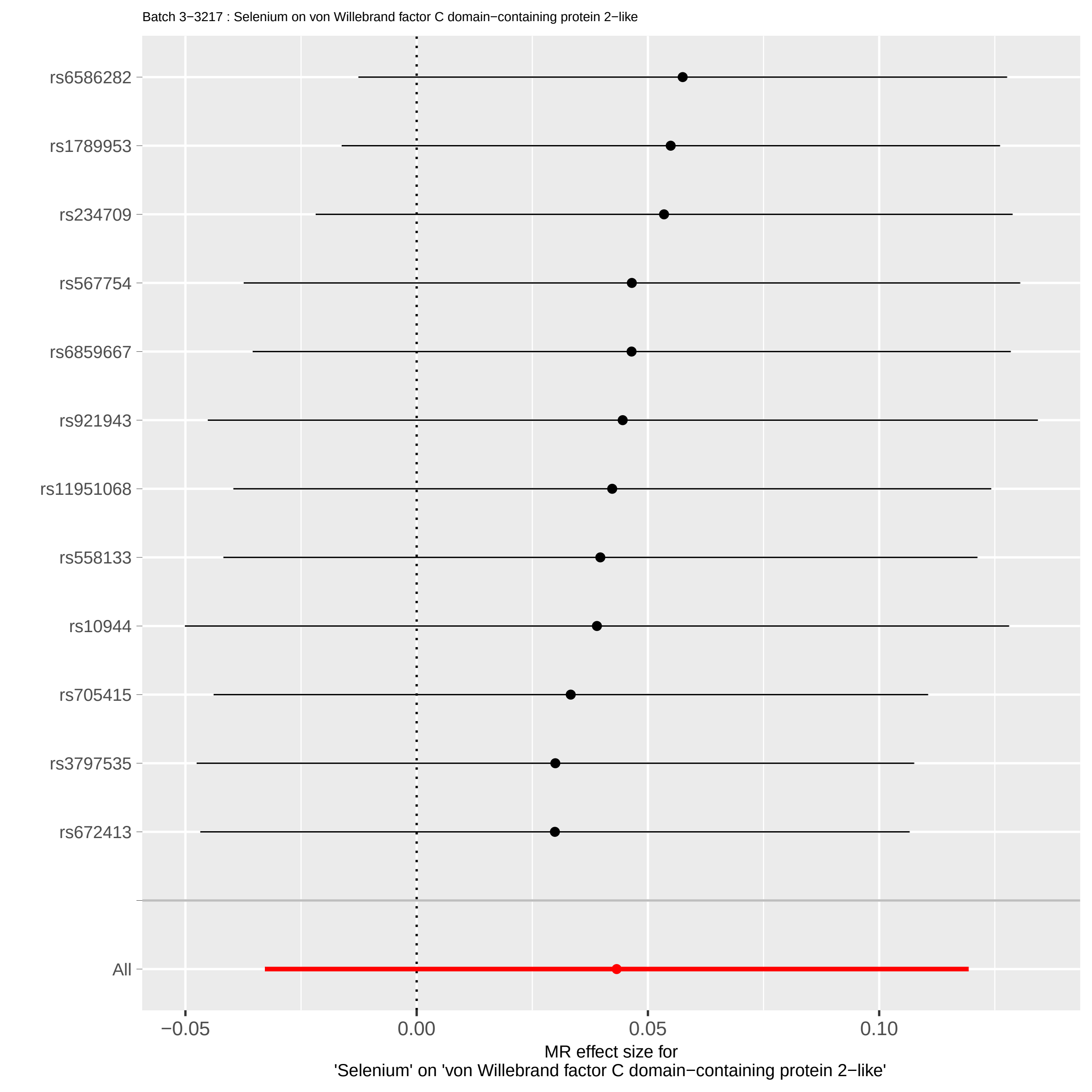

Batch 3−3217 : Selenium on von Willebrand factor C domain−containing protein 2−like
rs6586282
rs1789953
rs234709
rs567754
rs6859667
rs921943
rs11951068
rs558133
rs10944
rs705415
rs3797535
rs672413
All
−0.05
0.00
0.05
0.10
MR effect size for
'Selenium' on 'von Willebrand factor C domain−containing protein 2−like'

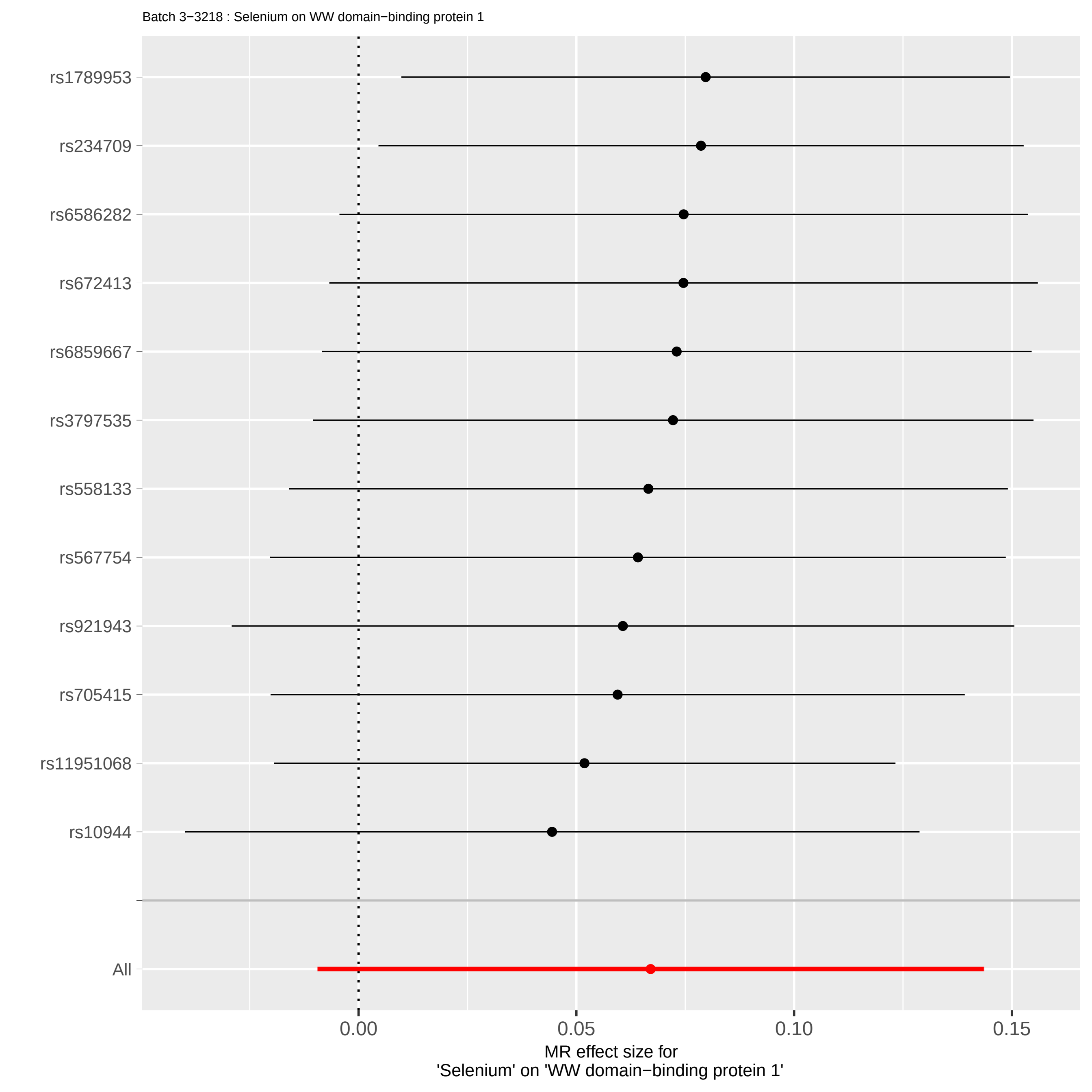

Batch 3−3218 : Selenium on WW domain−binding protein 1
rs1789953
rs234709
rs6586282
rs672413
rs6859667
rs3797535
rs558133
rs567754
rs921943
rs705415
rs11951068
rs10944
All
0.00
0.05
0.10
0.15
MR effect size for
'Selenium' on 'WW domain−binding protein 1'

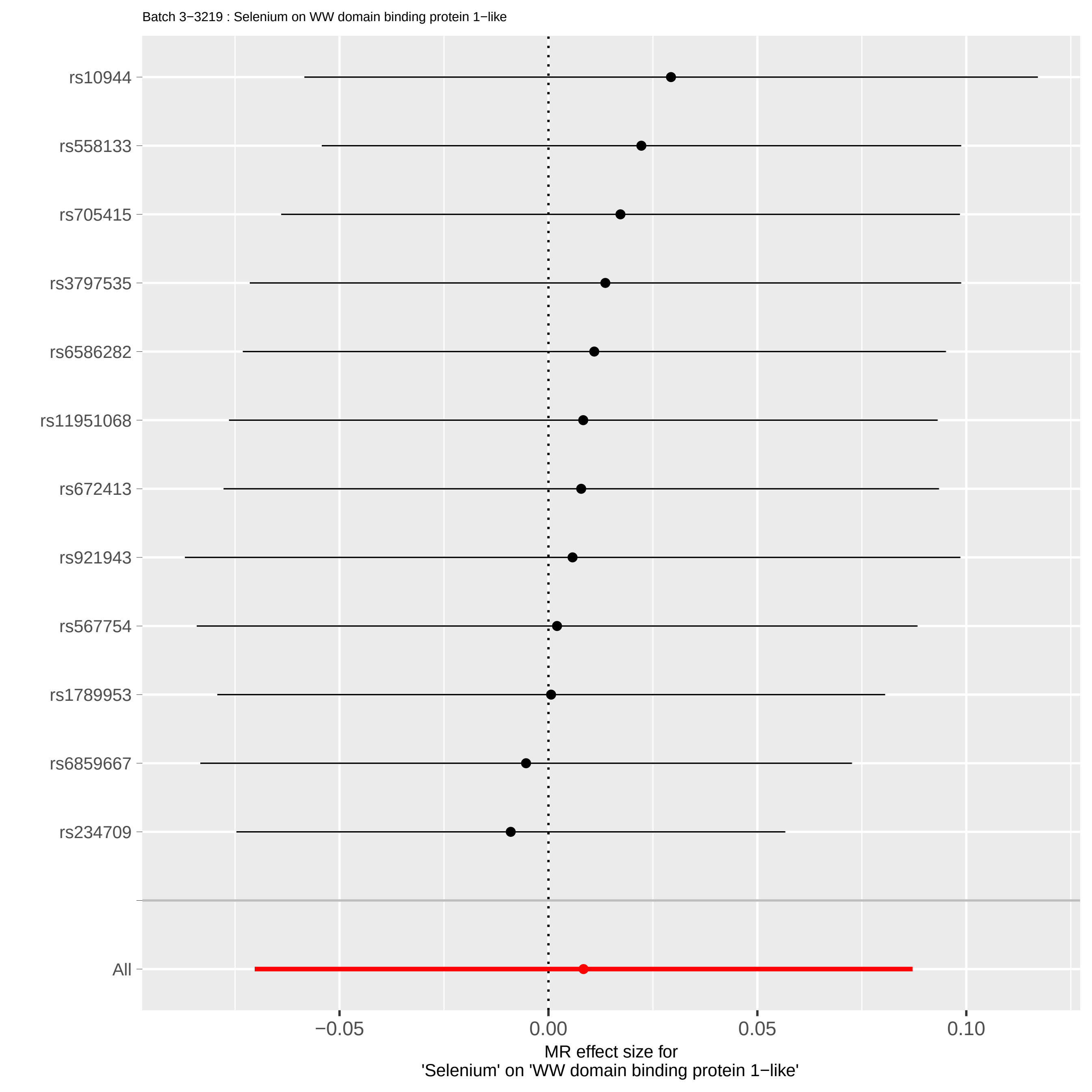

Batch 3−3219 : Selenium on WW domain binding protein 1−like
rs10944
rs558133
rs705415
rs3797535
rs6586282
rs11951068
rs672413
rs921943
rs567754
rs1789953
rs6859667
rs234709
All
−0.05
0.00
0.05
0.10
MR effect size for
'Selenium' on 'WW domain binding protein 1−like'

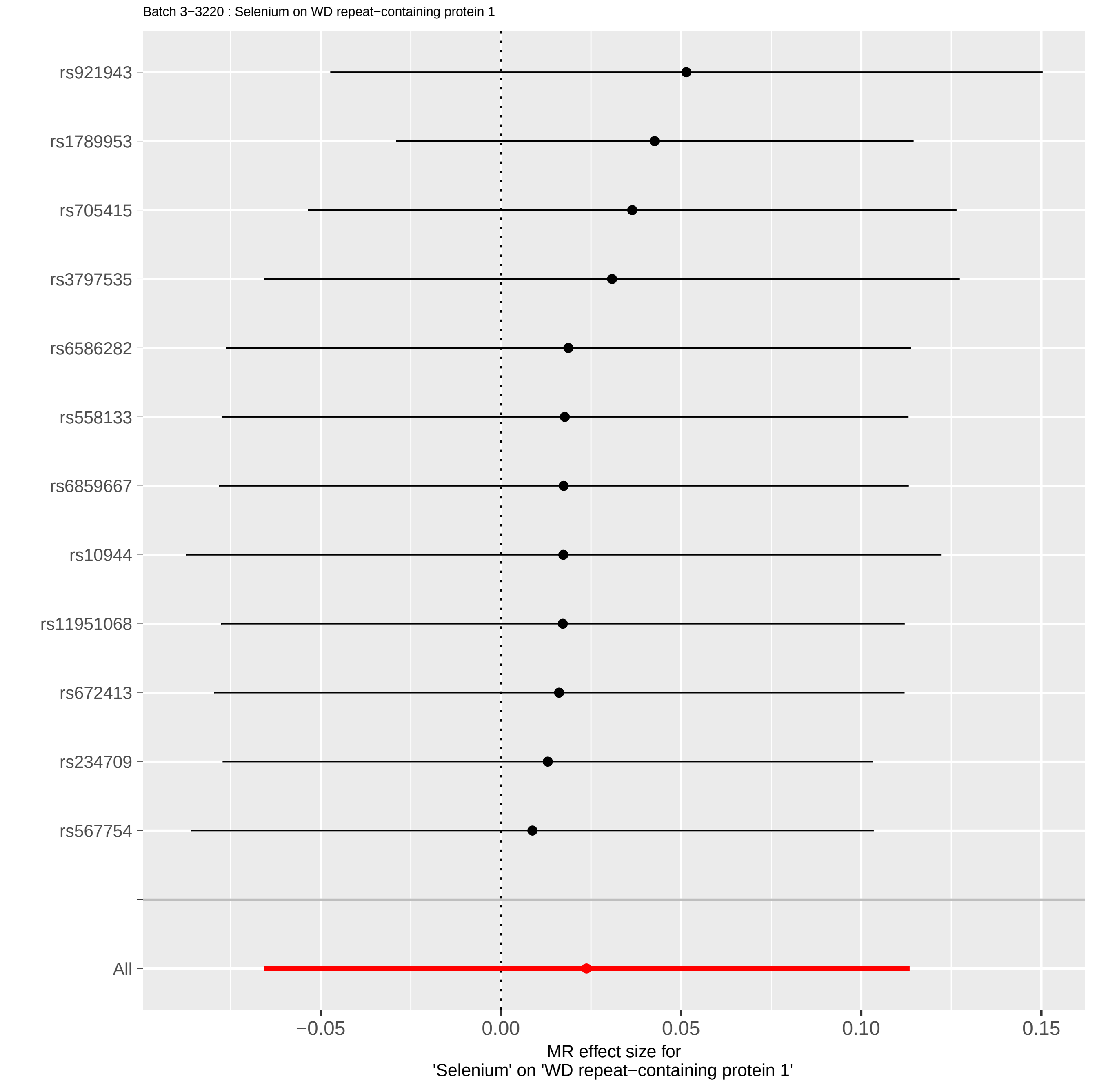

Batch 3−3220 : Selenium on WD repeat−containing protein 1
rs921943
rs1789953
rs705415
rs3797535
rs6586282
rs558133
rs6859667
rs10944
rs11951068
rs672413
rs234709
rs567754
All
−0.05
0.00
0.05
0.10
0.15
MR effect size for
'Selenium' on 'WD repeat−containing protein 1'

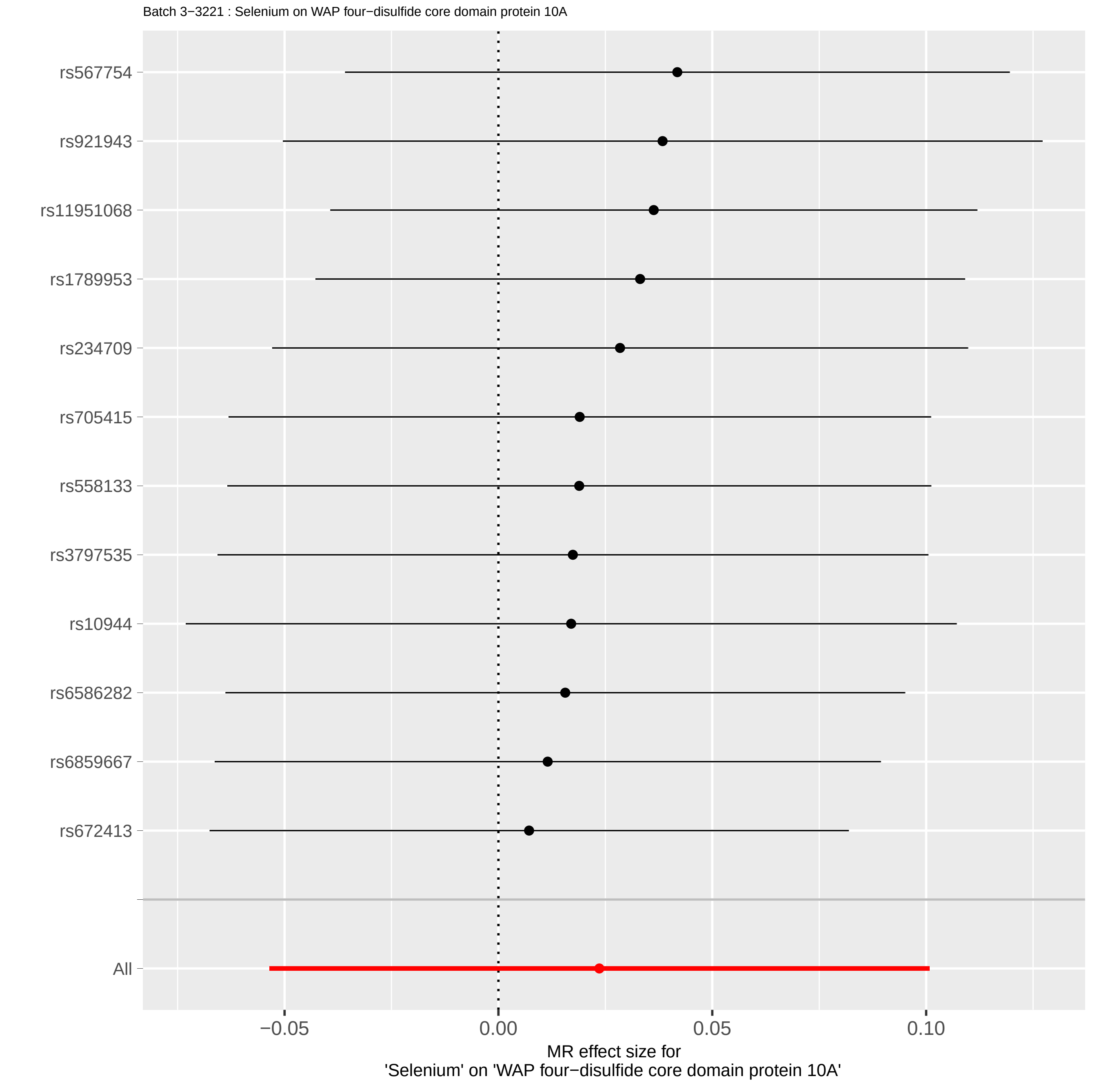

Batch 3−3221 : Selenium on WAP four−disulfide core domain protein 10A
rs567754
rs921943
rs11951068
rs1789953
rs234709
rs705415
rs558133
rs3797535
rs10944
rs6586282
rs6859667
rs672413
All
−0.05
0.00
0.05
0.10
MR effect size for
'Selenium' on 'WAP four−disulfide core domain protein 10A'

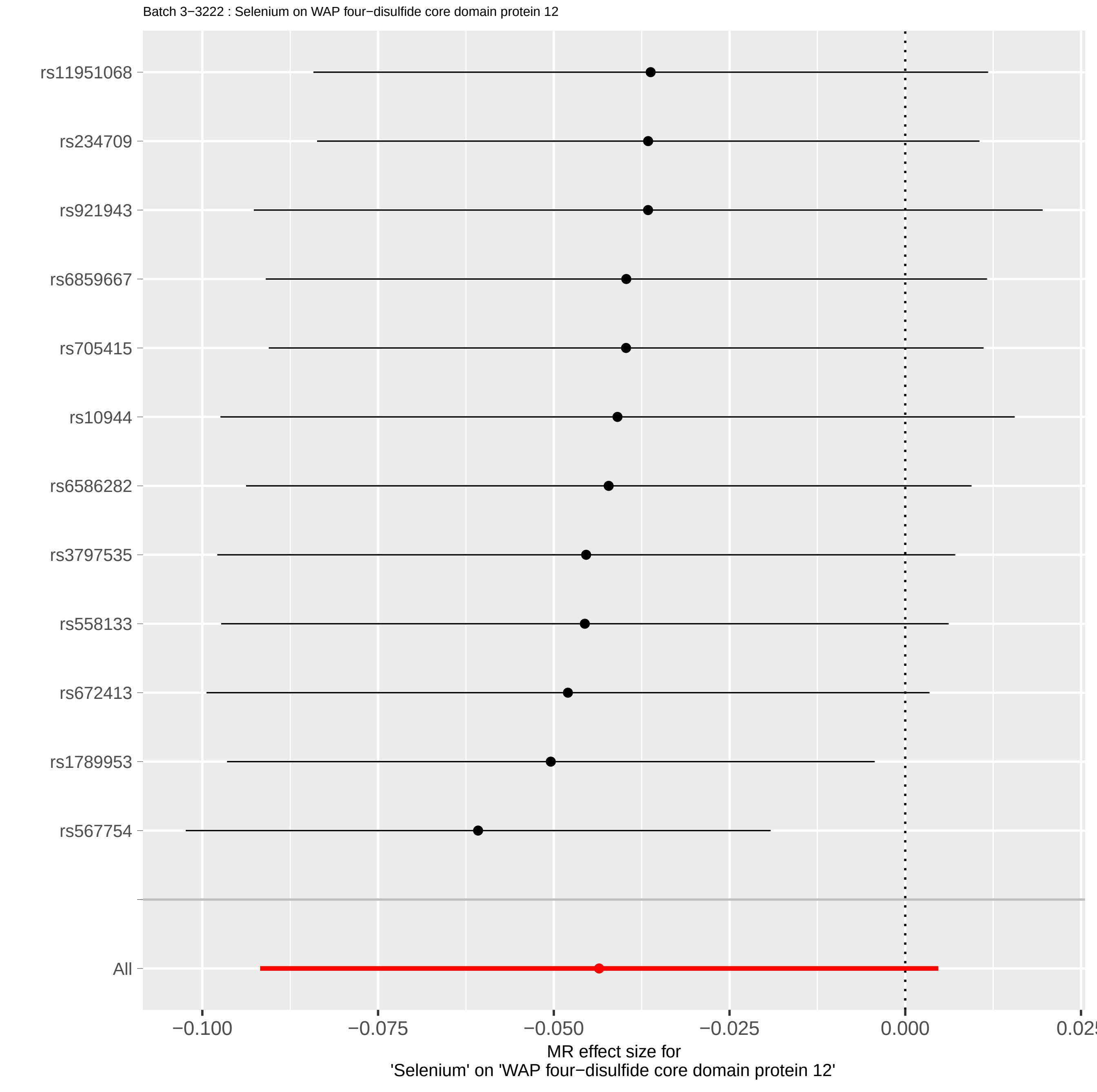

Batch 3−3222 : Selenium on WAP four−disulfide core domain protein 12
rs11951068
rs234709
rs921943
rs6859667
rs705415
rs10944
rs6586282
rs3797535
rs558133
rs672413
rs1789953
rs567754
All
−0.100
−0.075
−0.050
−0.025
0.000
MR effect size for
'Selenium' on 'WAP four−disulfide core domain protein 12'

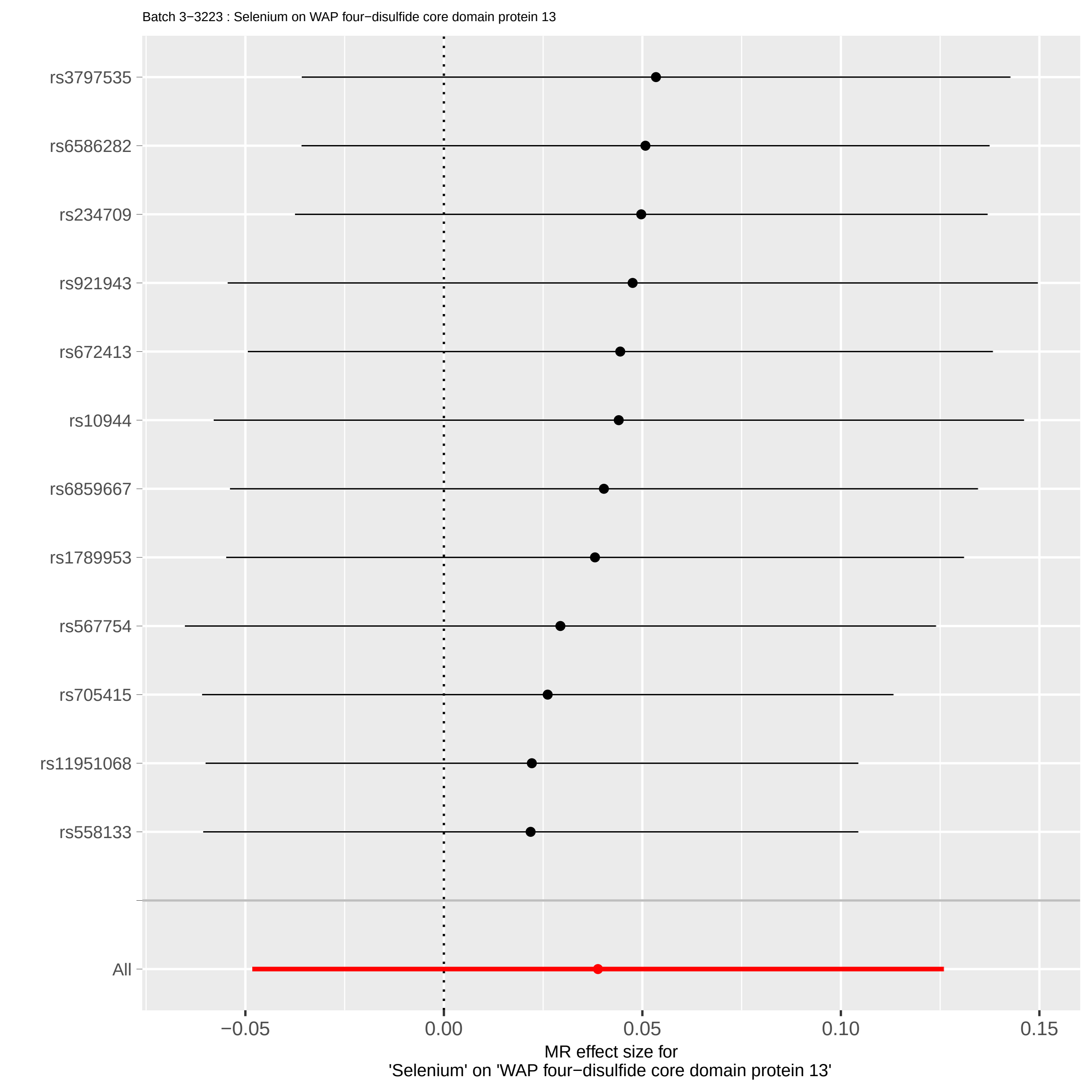
Batch 3−3223 : Selenium on WAP four−disulfide core domain protein 13
rs3797535
rs6586282
rs234709
rs921943
rs672413
rs10944
rs6859667
rs1789953
rs567754
rs705415
rs11951068
rs558133
All
−0.05
0.00
0.05
0.10
0.15
MR effect size for
'Selenium' on 'WAP four−disulfide core domain protein 13'

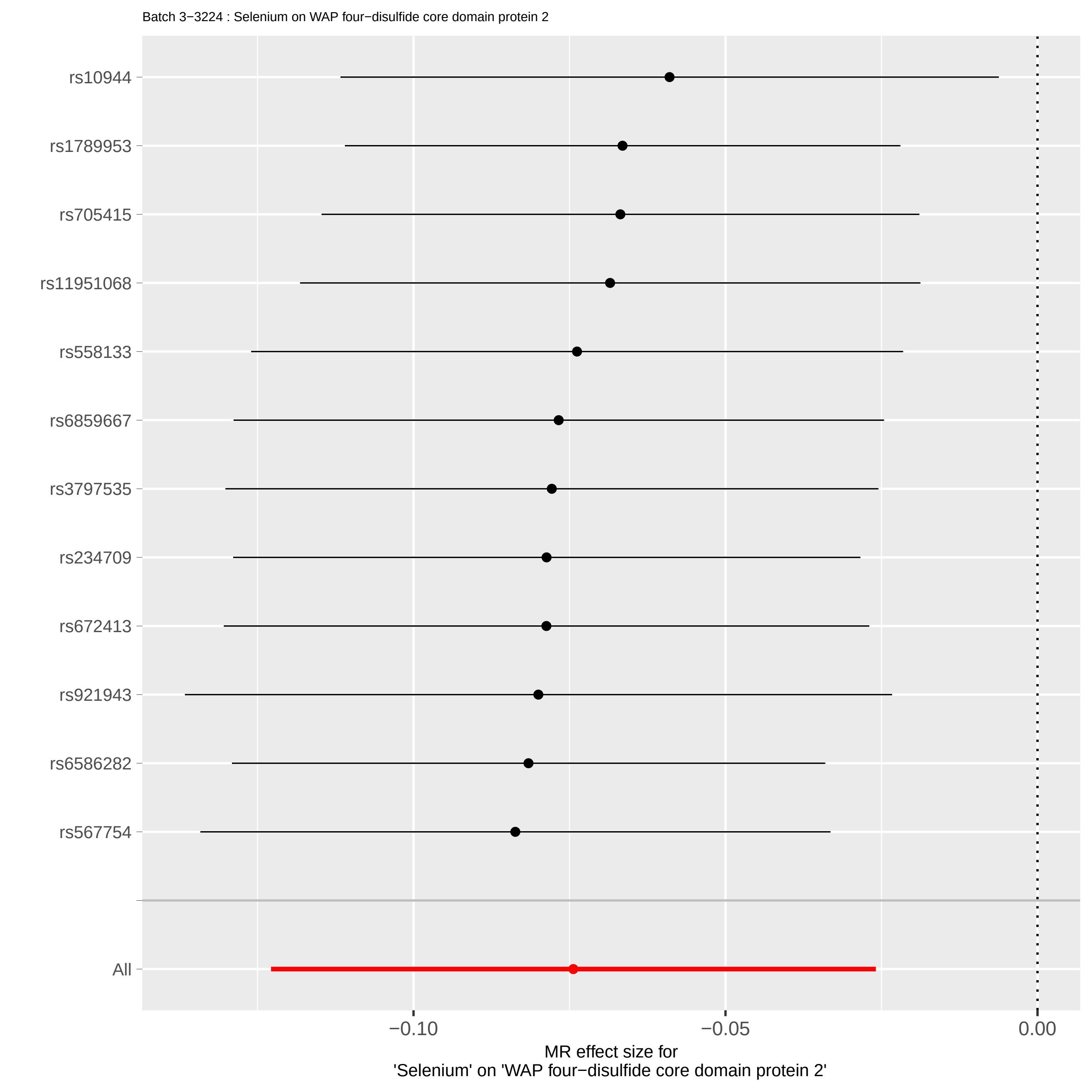

Batch 3−3224 : Selenium on WAP four−disulfide core domain protein 2
rs10944
rs1789953
rs705415
rs11951068
rs558133
rs6859667
rs3797535
rs234709
rs672413
rs921943
rs6586282
rs567754
All
−0.10
−0.05
0.00
MR effect size for
'Selenium' on 'WAP four−disulfide core domain protein 2'

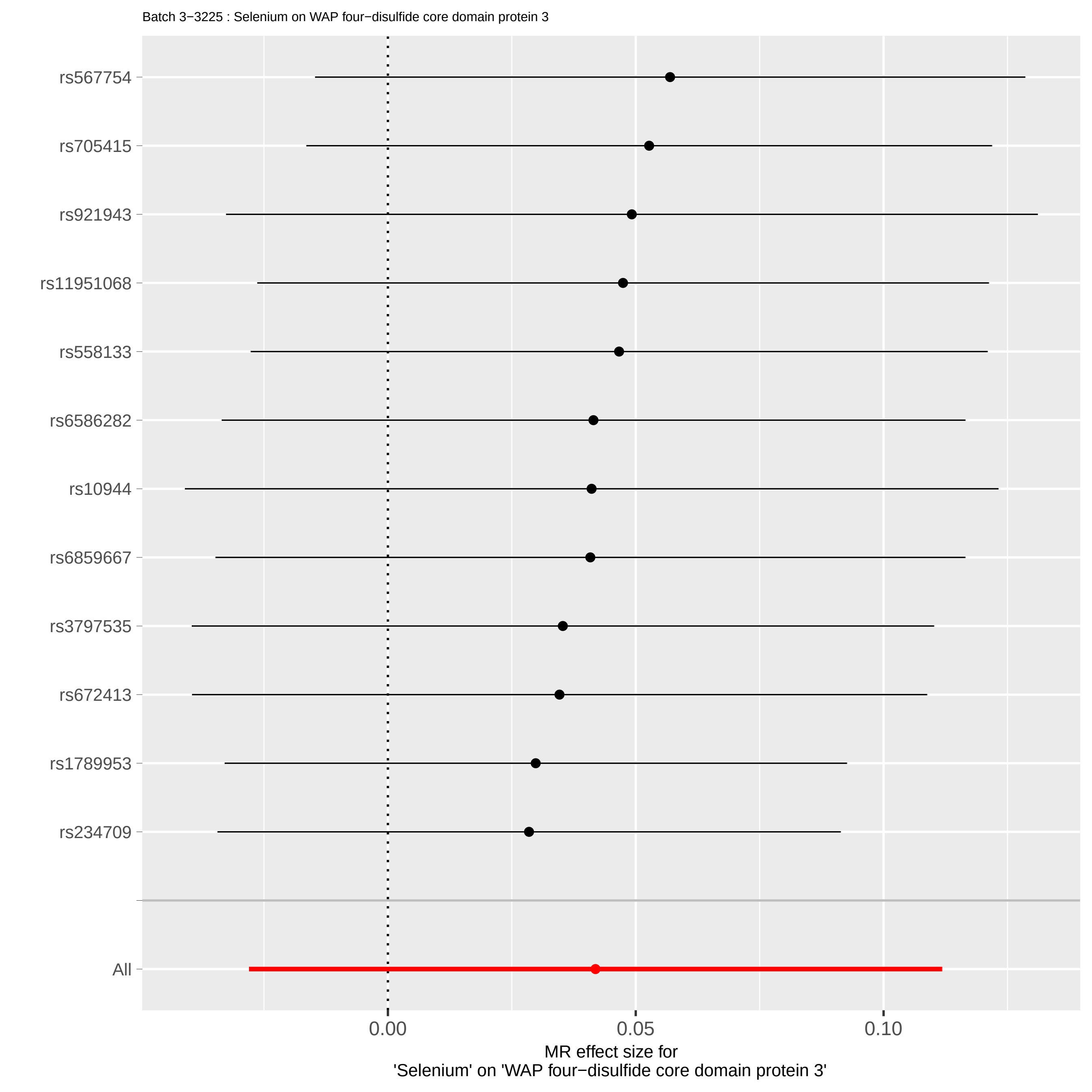

Batch 3−3225 : Selenium on WAP four−disulfide core domain protein 3
rs567754
rs705415
rs921943
rs11951068
rs558133
rs6586282
rs10944
rs6859667
rs3797535
rs672413
rs1789953
rs234709
All
0.00
0.05
0.10
MR effect size for
'Selenium' on 'WAP four−disulfide core domain protein 3'

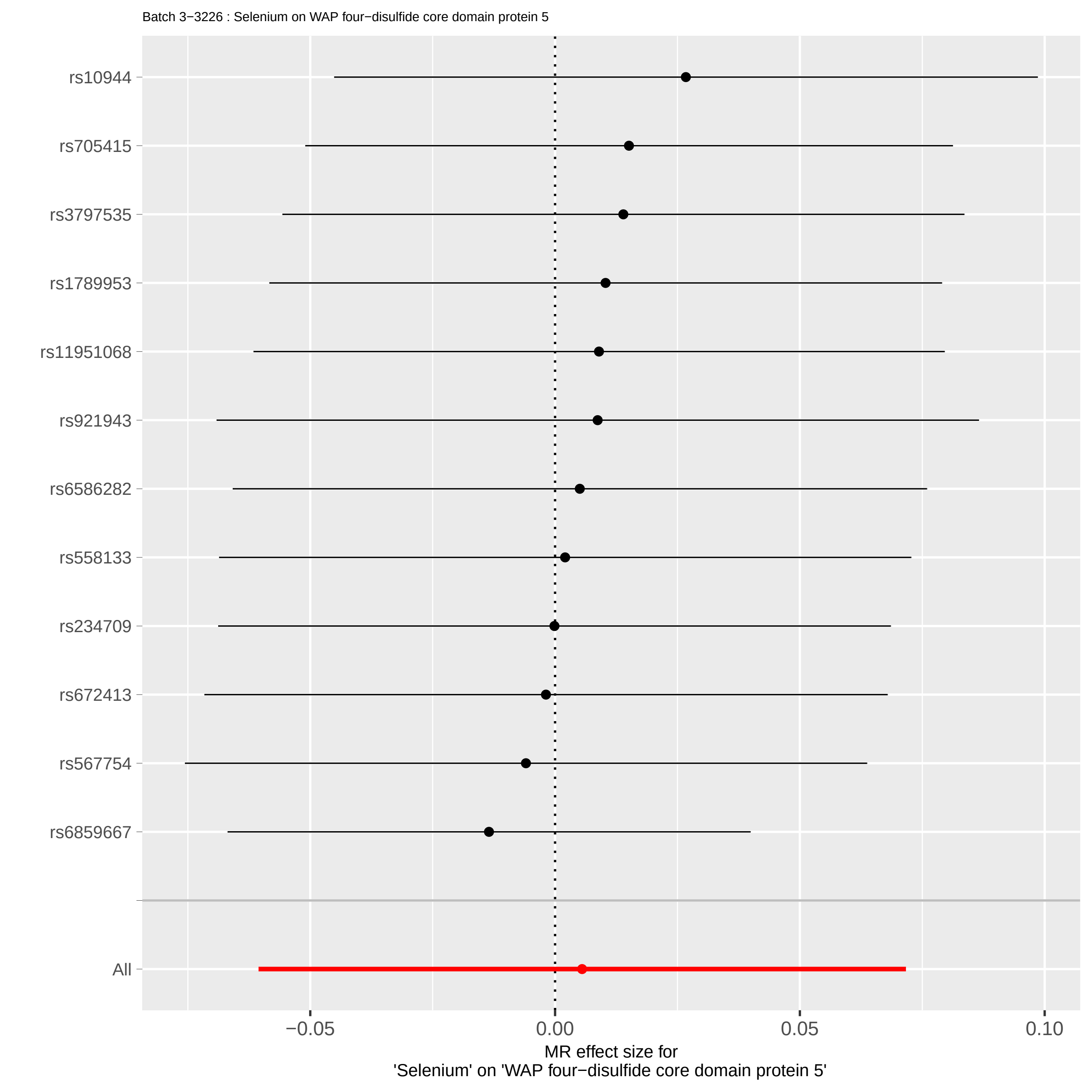

Batch 3−3226 : Selenium on WAP four−disulfide core domain protein 5
rs10944
rs705415
rs3797535
rs1789953
rs11951068
rs921943
rs6586282
rs558133
rs234709
rs672413
rs567754
rs6859667
All
−0.05
0.00
0.05
0.10
MR effect size for
'Selenium' on 'WAP four−disulfide core domain protein 5'

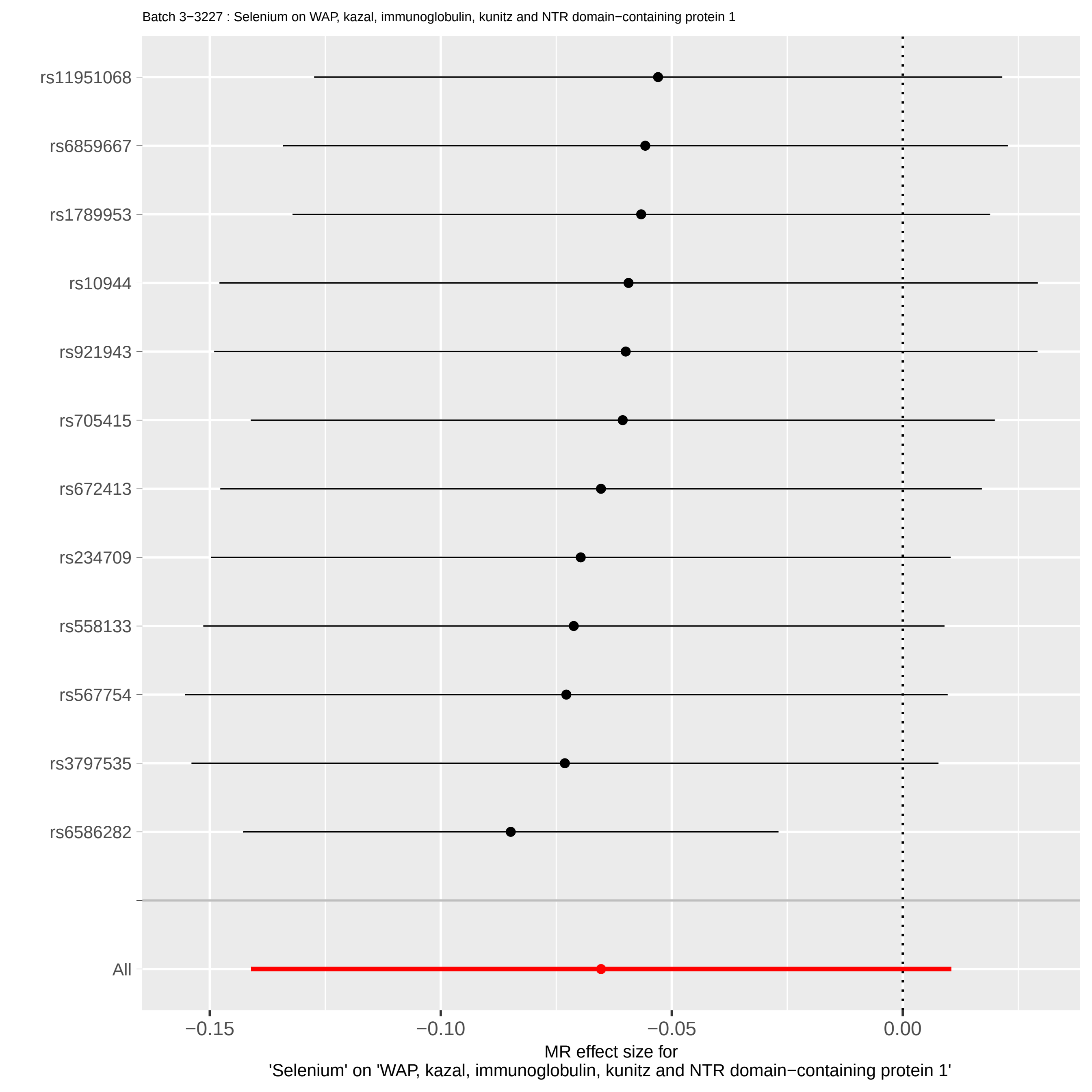
Batch 3−3227 : Selenium on WAP, kazal, immunoglobulin, kunitz and NTR domain−containing protein 1
rs11951068
rs6859667
rs1789953
rs10944
rs921943
rs705415
rs672413
rs234709
rs558133
rs567754
rs3797535
rs6586282
All
−0.15
−0.10
−0.05
0.00
MR effect size for
'Selenium' on 'WAP, kazal, immunoglobulin, kunitz and NTR domain−containing protein 1'

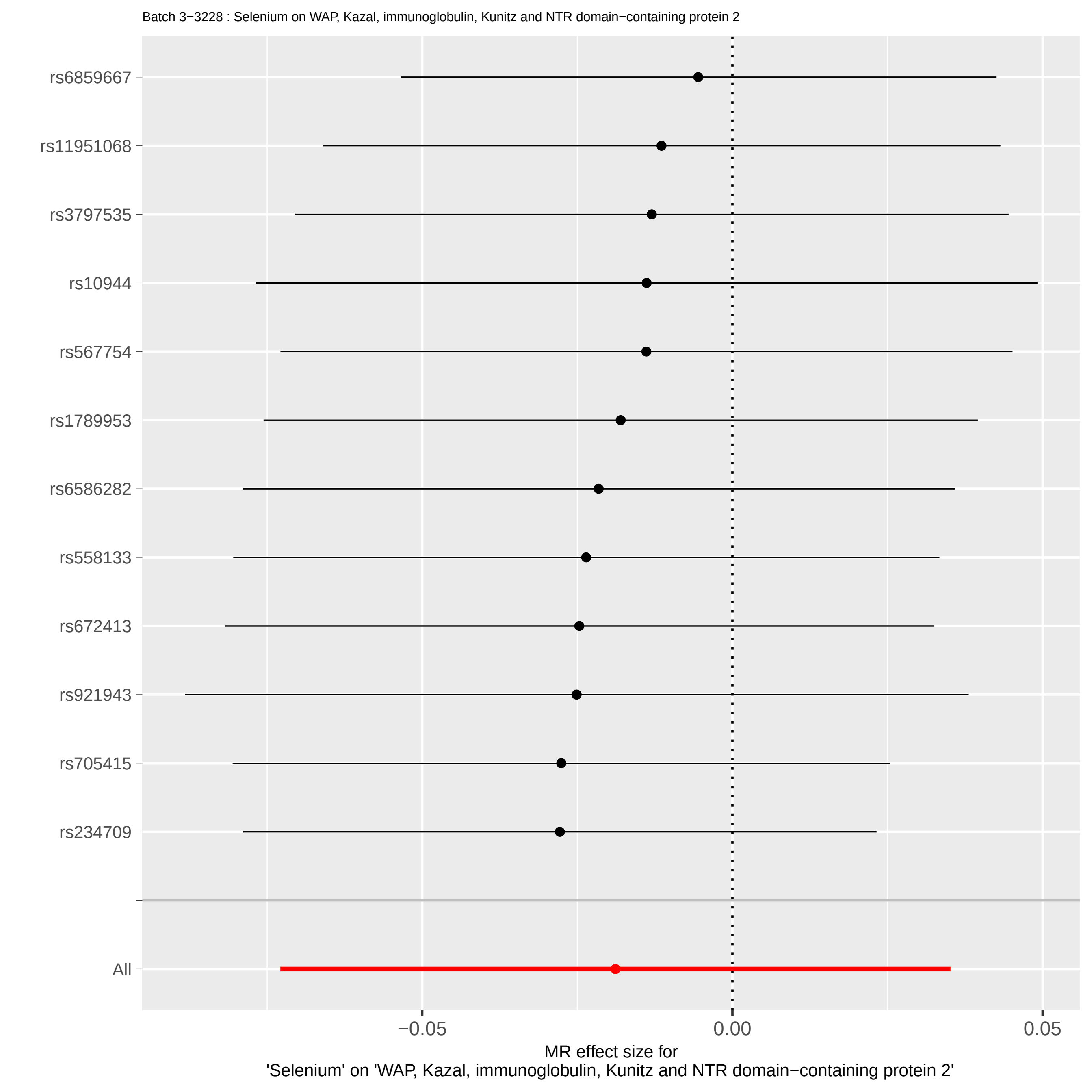
Batch 3−3228 : Selenium on WAP, Kazal, immunoglobulin, Kunitz and NTR domain−containing protein 2
rs6859667
rs11951068
rs3797535
rs10944
rs567754
rs1789953
rs6586282
rs558133
rs672413
rs921943
rs705415
rs234709
All
−0.05
0.00
0.05
MR effect size for
'Selenium' on 'WAP, Kazal, immunoglobulin, Kunitz and NTR domain−containing protein 2'

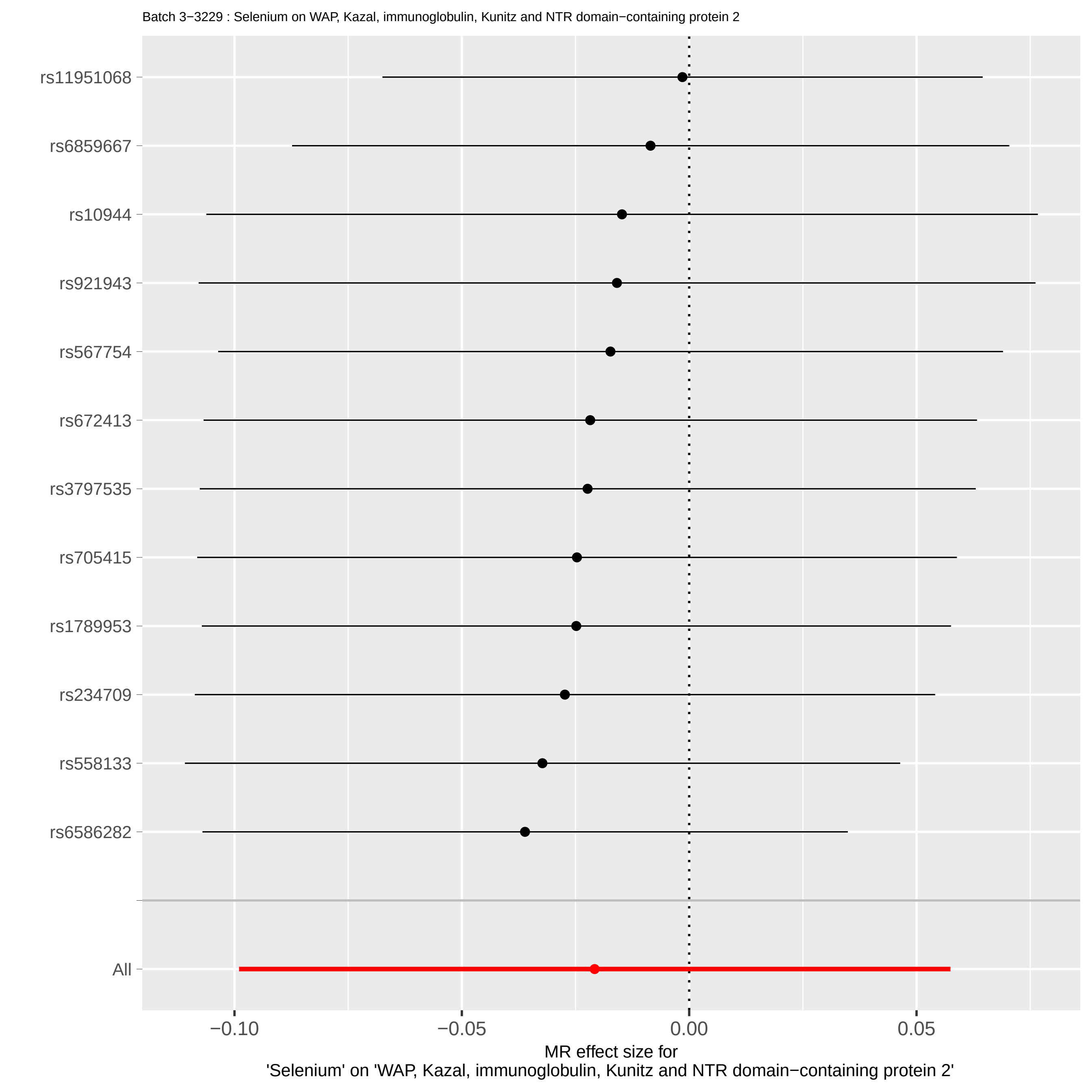

Batch 3−3229 : Selenium on WAP, Kazal, immunoglobulin, Kunitz and NTR domain−containing protein 2
rs11951068
rs6859667
rs10944
rs921943
rs567754
rs672413
rs3797535
rs705415
rs1789953
rs234709
rs558133
rs6586282
All
−0.10
−0.05
0.00
0.05
MR effect size for
'Selenium' on 'WAP, Kazal, immunoglobulin, Kunitz and NTR domain−containing protein 2'

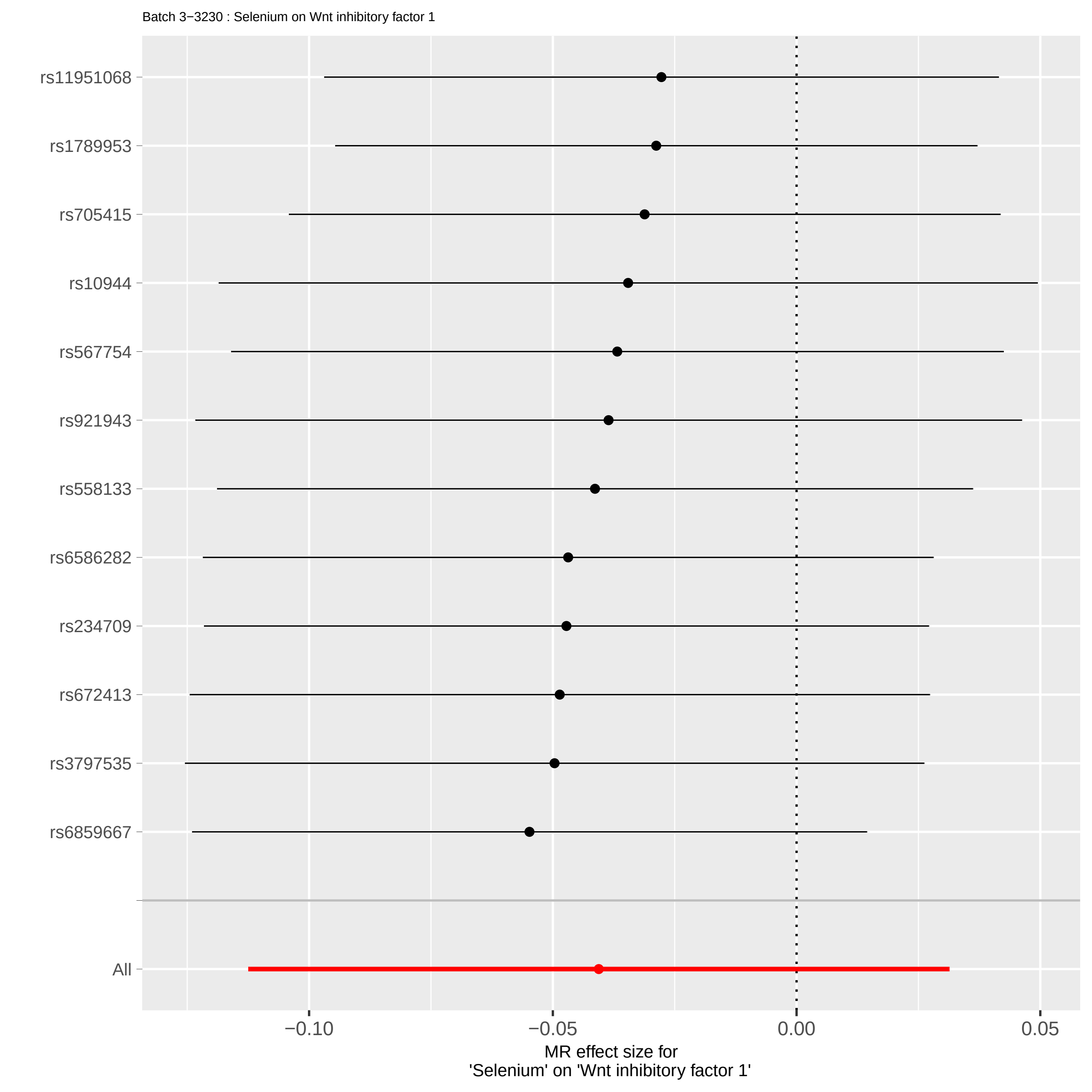

Batch 3−3230 : Selenium on Wnt inhibitory factor 1
rs11951068
rs1789953
rs705415
rs10944
rs567754
rs921943
rs558133
rs6586282
rs234709
rs672413
rs3797535
rs6859667
All
−0.10
−0.05
0.00
0.05
MR effect size for
'Selenium' on 'Wnt inhibitory factor 1'

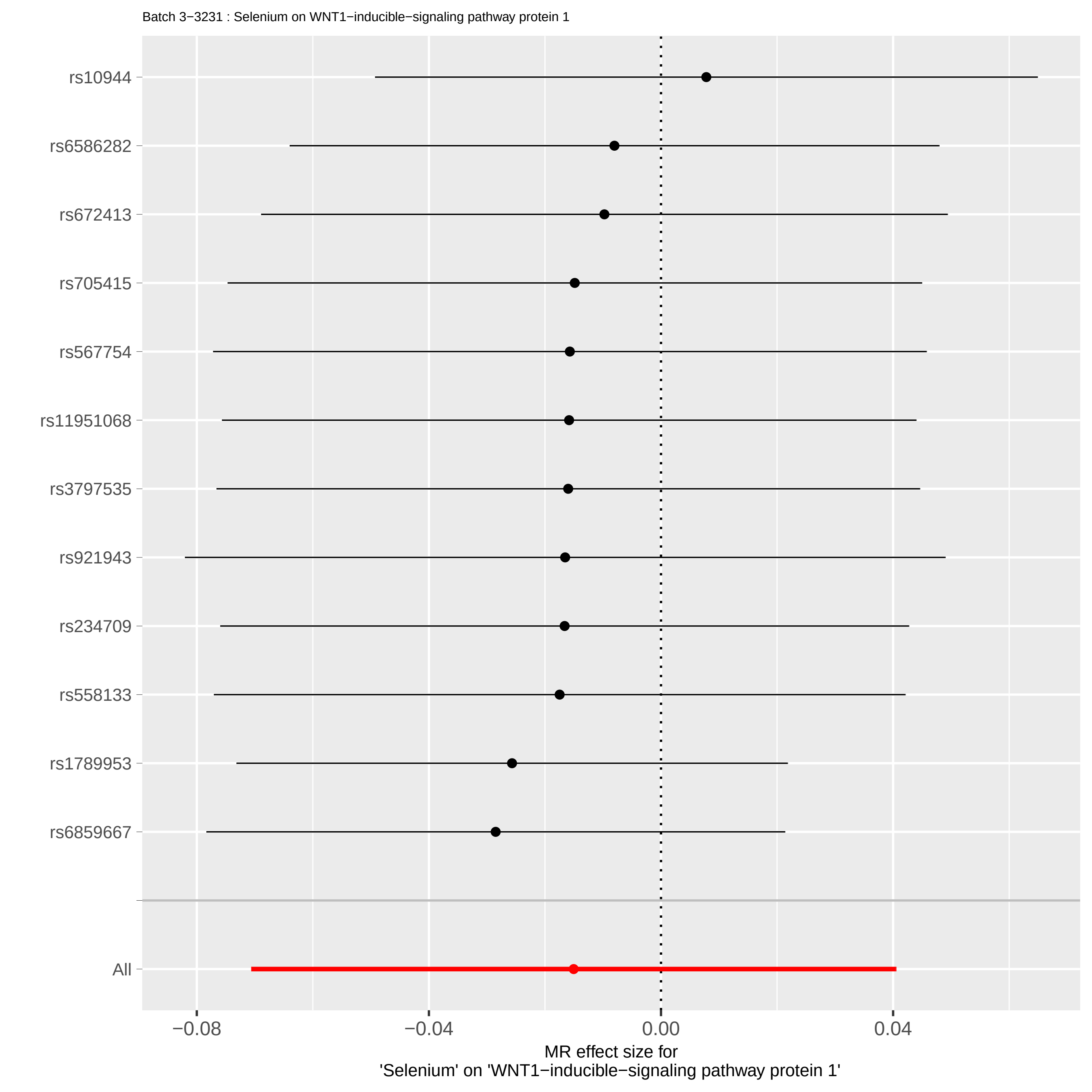

Batch 3−3231 : Selenium on WNT1−inducible−signaling pathway protein 1
rs10944
rs6586282
rs672413
rs705415
rs567754
rs11951068
rs3797535
rs921943
rs234709
rs558133
rs1789953
rs6859667
All
−0.08
−0.04
0.00
0.04
MR effect size for
'Selenium' on 'WNT1−inducible−signaling pathway protein 1'

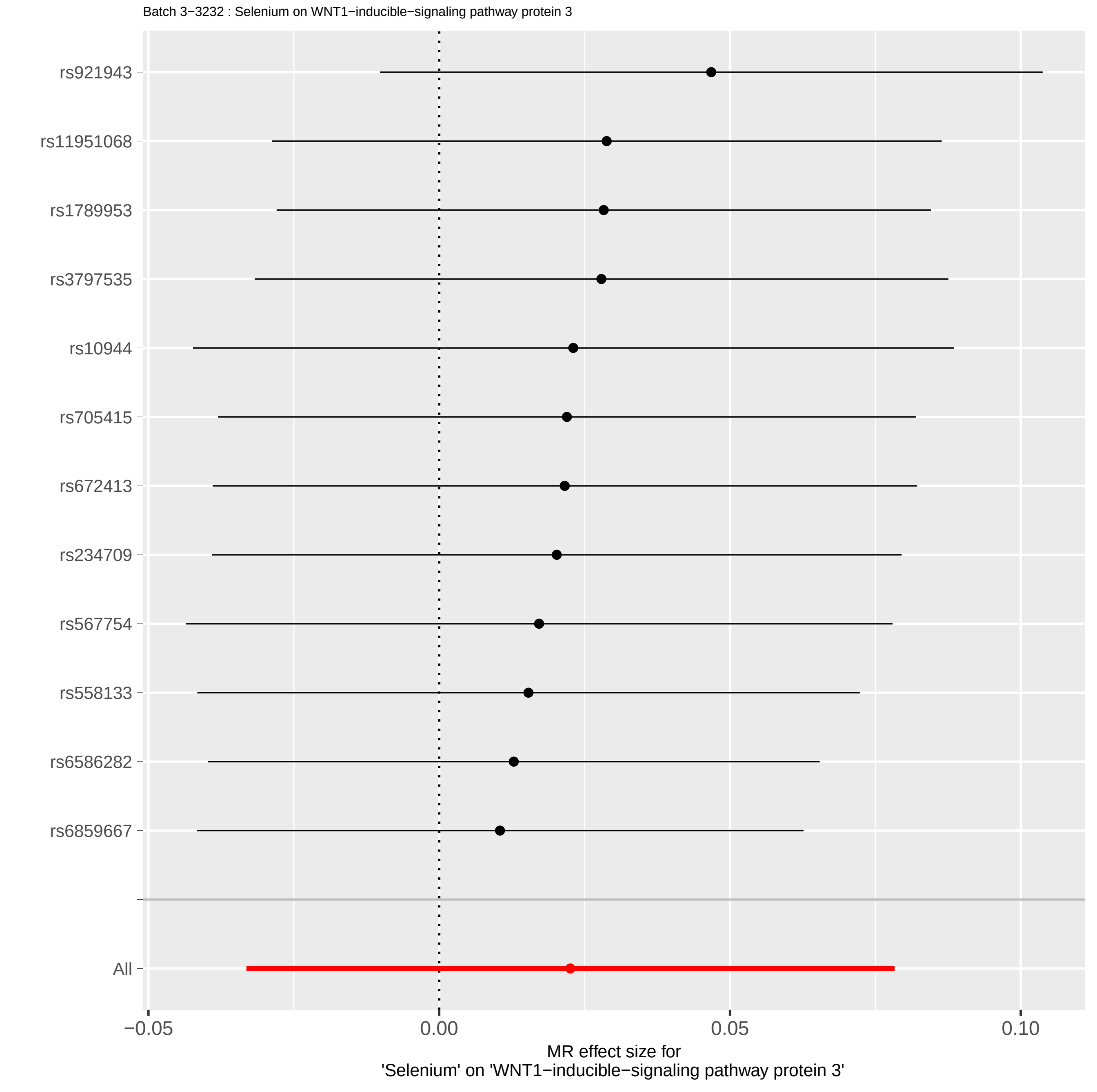

Batch 3−3232 : Selenium on WNT1−inducible−signaling pathway protein 3
rs921943
rs11951068
rs1789953
rs3797535
rs10944
rs705415
rs672413
rs234709
rs567754
rs558133
rs6586282
rs6859667
All
−0.05
0.00
0.05
0.10
MR effect size for
'Selenium' on 'WNT1−inducible−signaling pathway protein 3'

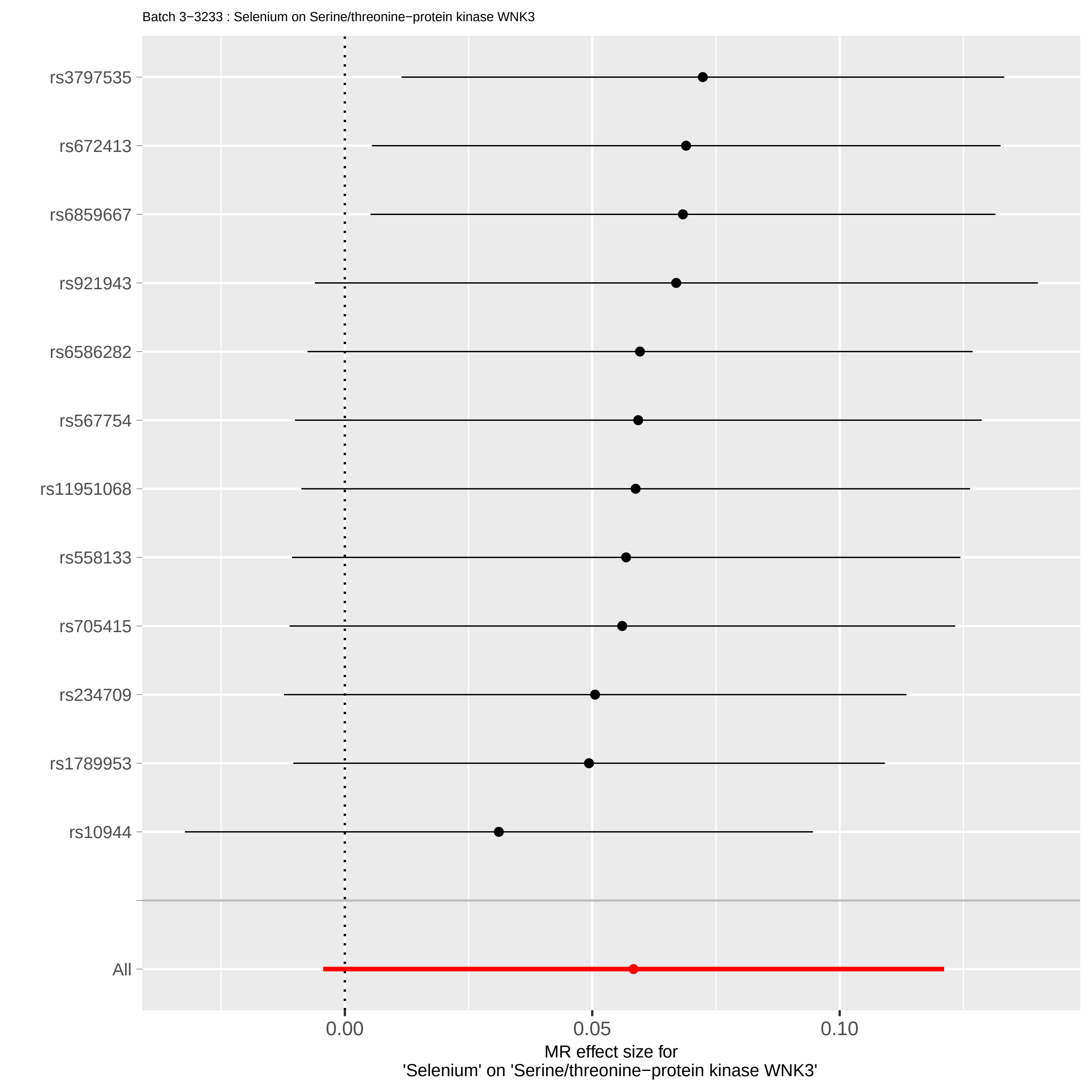

Batch 3−3233 : Selenium on Serine/threonine−protein kinase WNK3
rs3797535
rs672413
rs6859667
rs921943
rs6586282
rs567754
rs11951068
rs558133
rs705415
rs234709
rs1789953
rs10944
All
0.00
0.05
0.10
MR effect size for
'Selenium' on 'Serine/threonine−protein kinase WNK3'

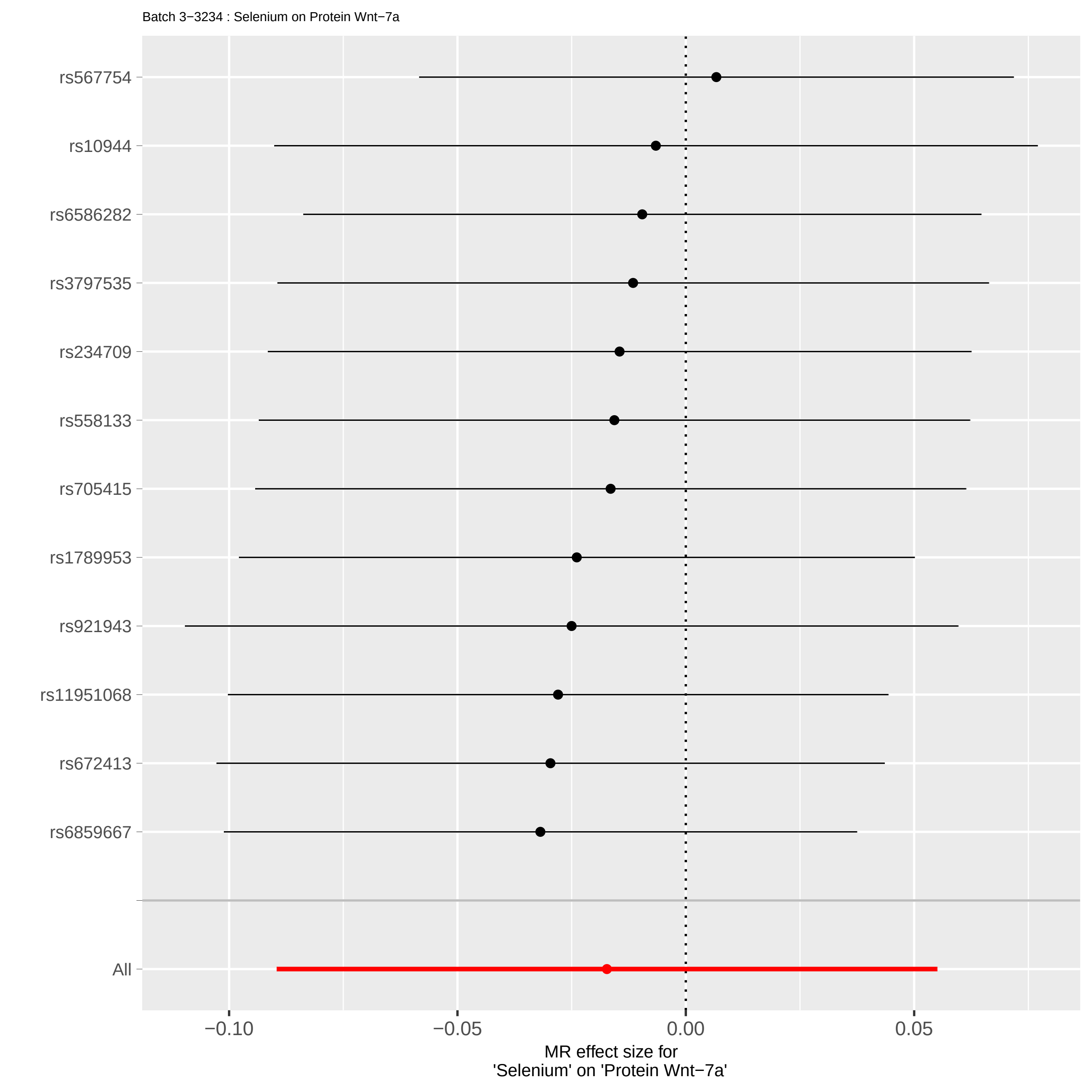

Batch 3−3234 : Selenium on Protein Wnt−7a
rs567754
rs10944
rs6586282
rs3797535
rs234709
rs558133
rs705415
rs1789953
rs921943
rs11951068
rs672413
rs6859667
All
−0.10
−0.05
0.00
0.05
MR effect size for
'Selenium' on 'Protein Wnt−7a'

Batch 3−3235 : Selenium on WSC domain−containing protein 2

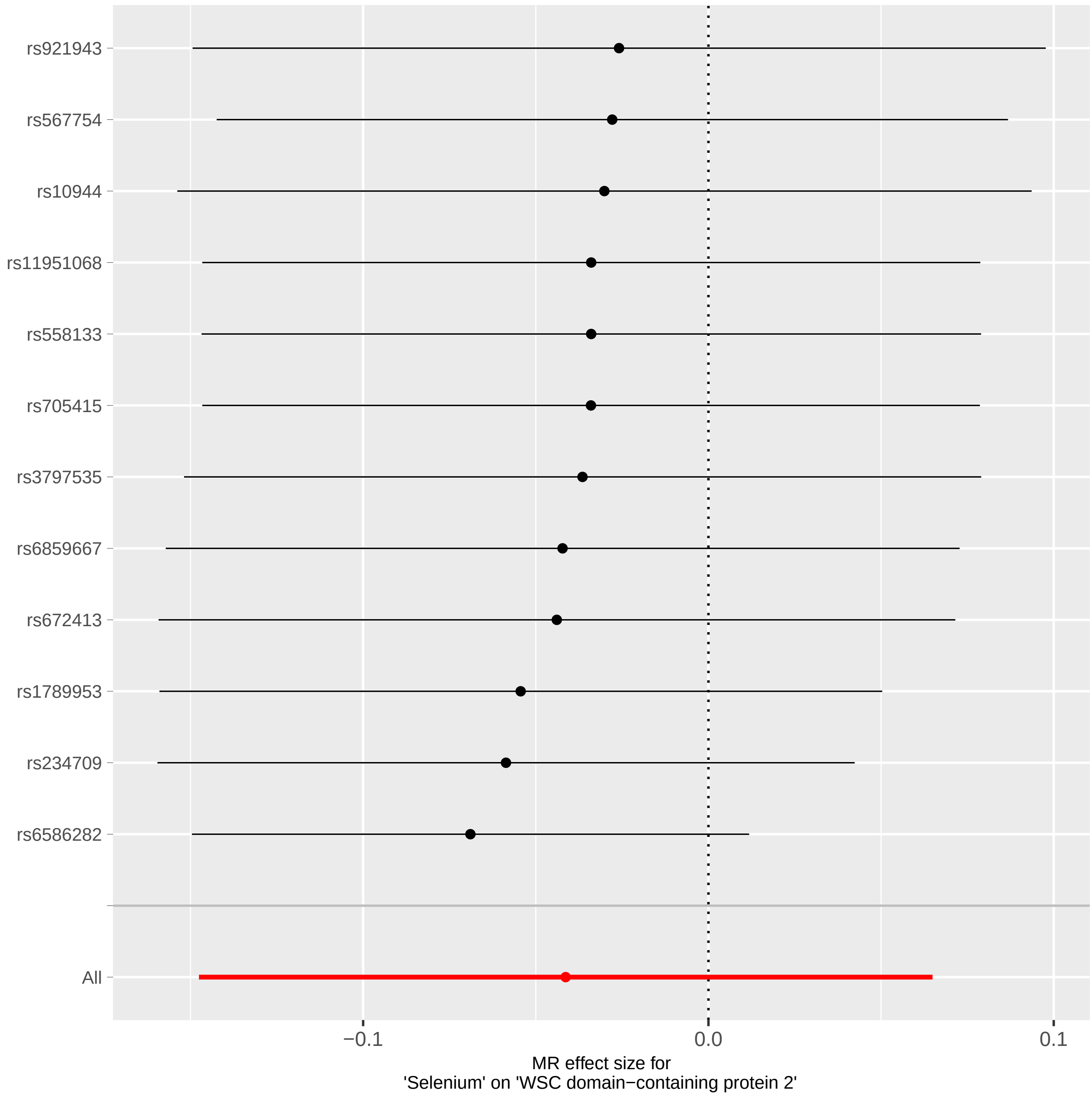

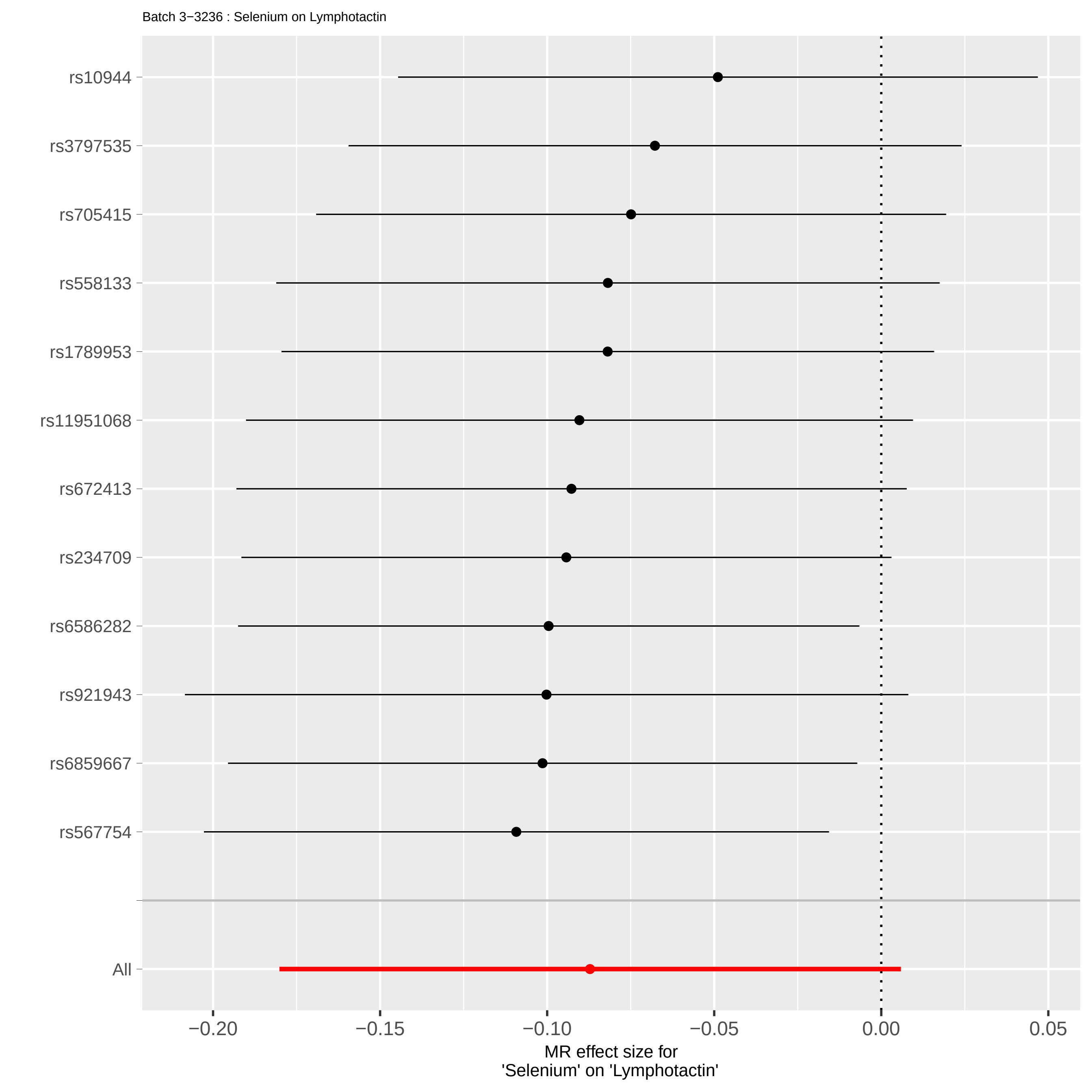

Batch 3−3236 : Selenium on Lymphotactin
rs10944
rs3797535
rs705415
rs558133
rs1789953
rs11951068
rs672413
rs234709
rs6586282
rs921943
rs6859667
rs567754
All
−0.20
−0.15
−0.10
−0.05
0.00
0.05
MR effect size for
'Selenium' on 'Lymphotactin'

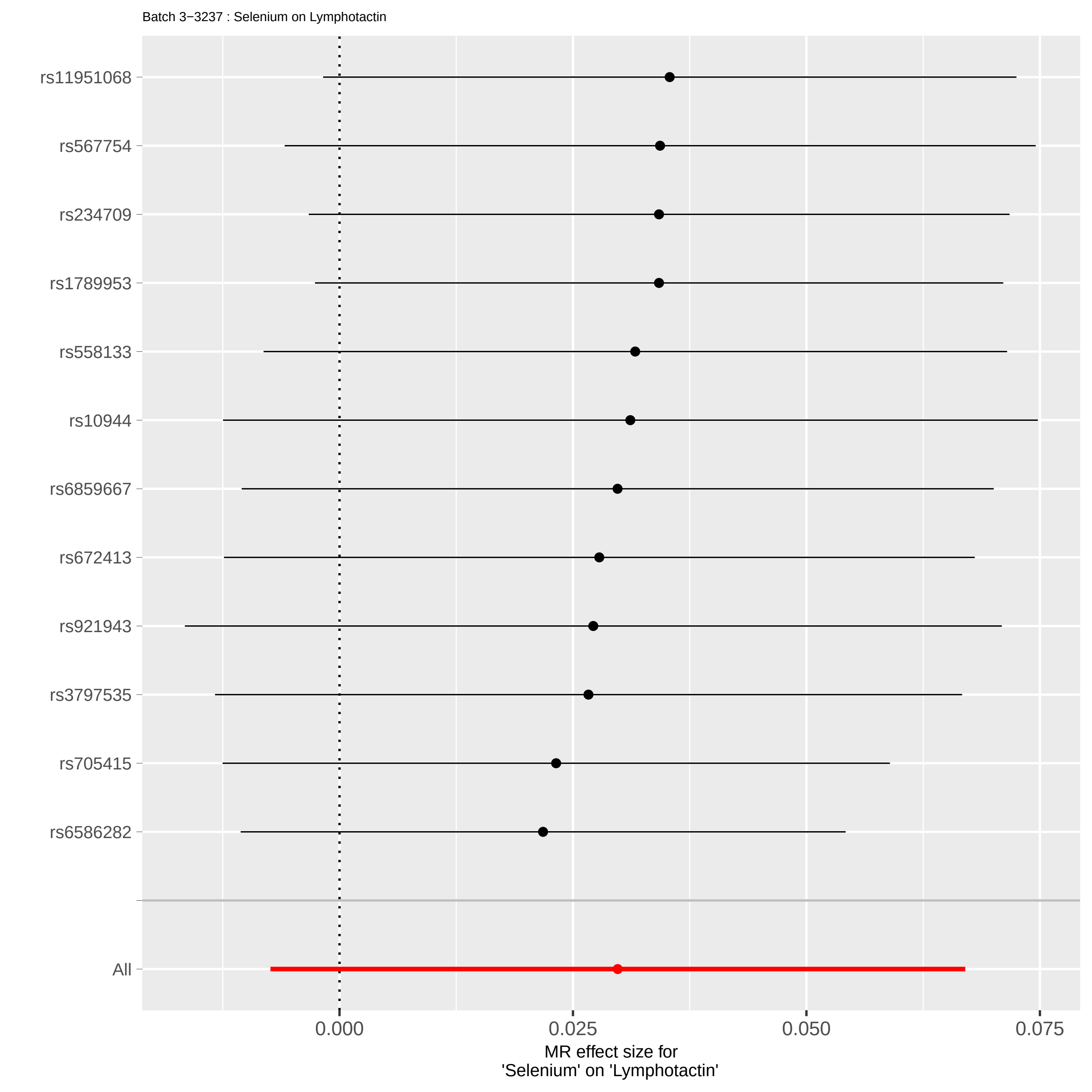

Batch 3−3237 : Selenium on Lymphotactin
rs11951068
rs567754
rs234709
rs1789953
rs558133
rs10944
rs6859667
rs672413
rs921943
rs3797535
rs705415
rs6586282
All
0.000
0.025
0.050
0.075
MR effect size for
'Selenium' on 'Lymphotactin'

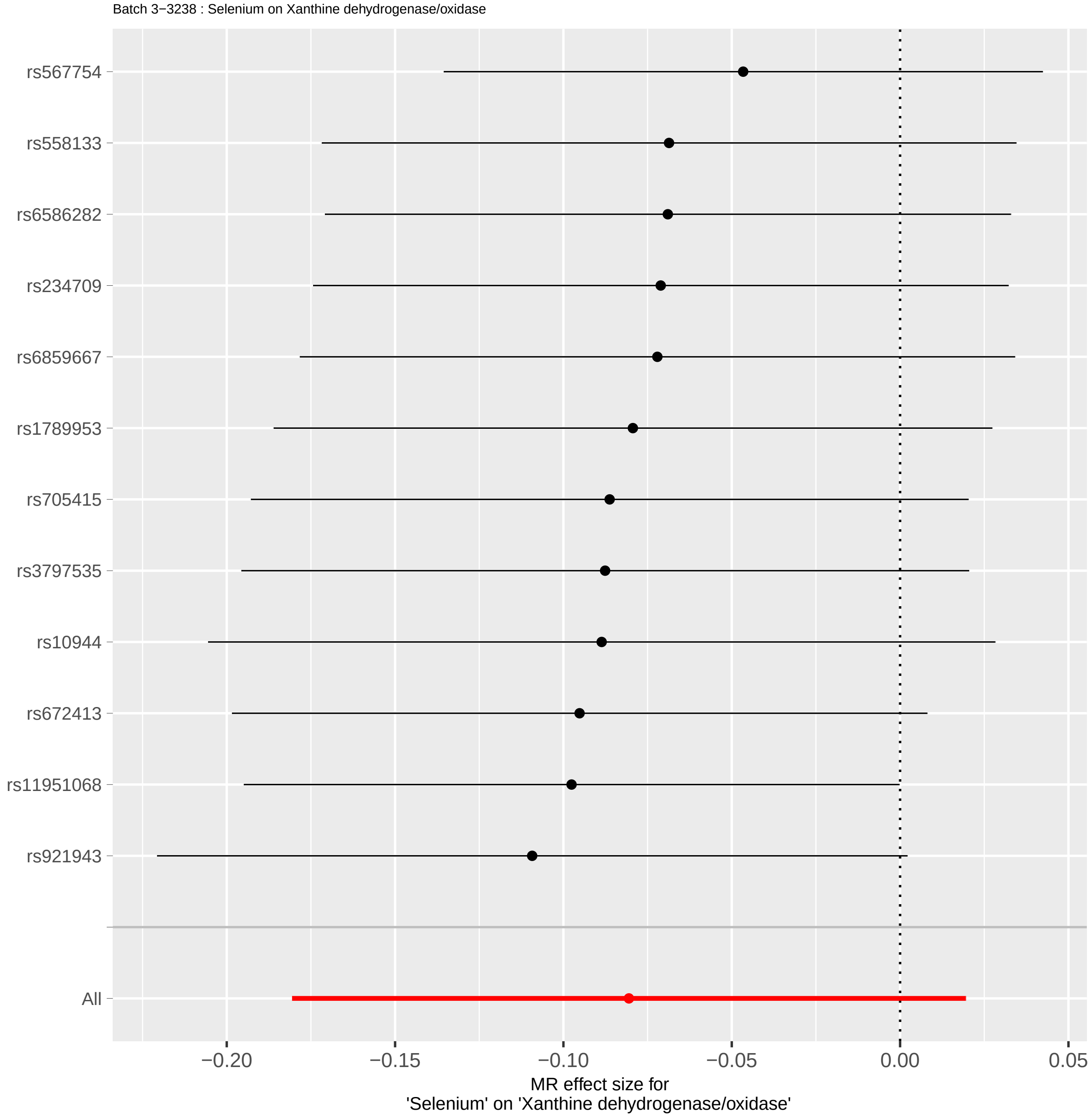
Batch 3−3238 : Selenium on Xanthine dehydrogenase/oxidase
rs567754
rs558133
rs6586282
rs234709
rs6859667
rs1789953
rs705415
rs3797535
rs10944
rs672413
rs11951068
rs921943
All
−0.20
−0.15
−0.10
−0.05
0.00
0.05
MR effect size for
'Selenium' on 'Xanthine dehydrogenase/oxidase'

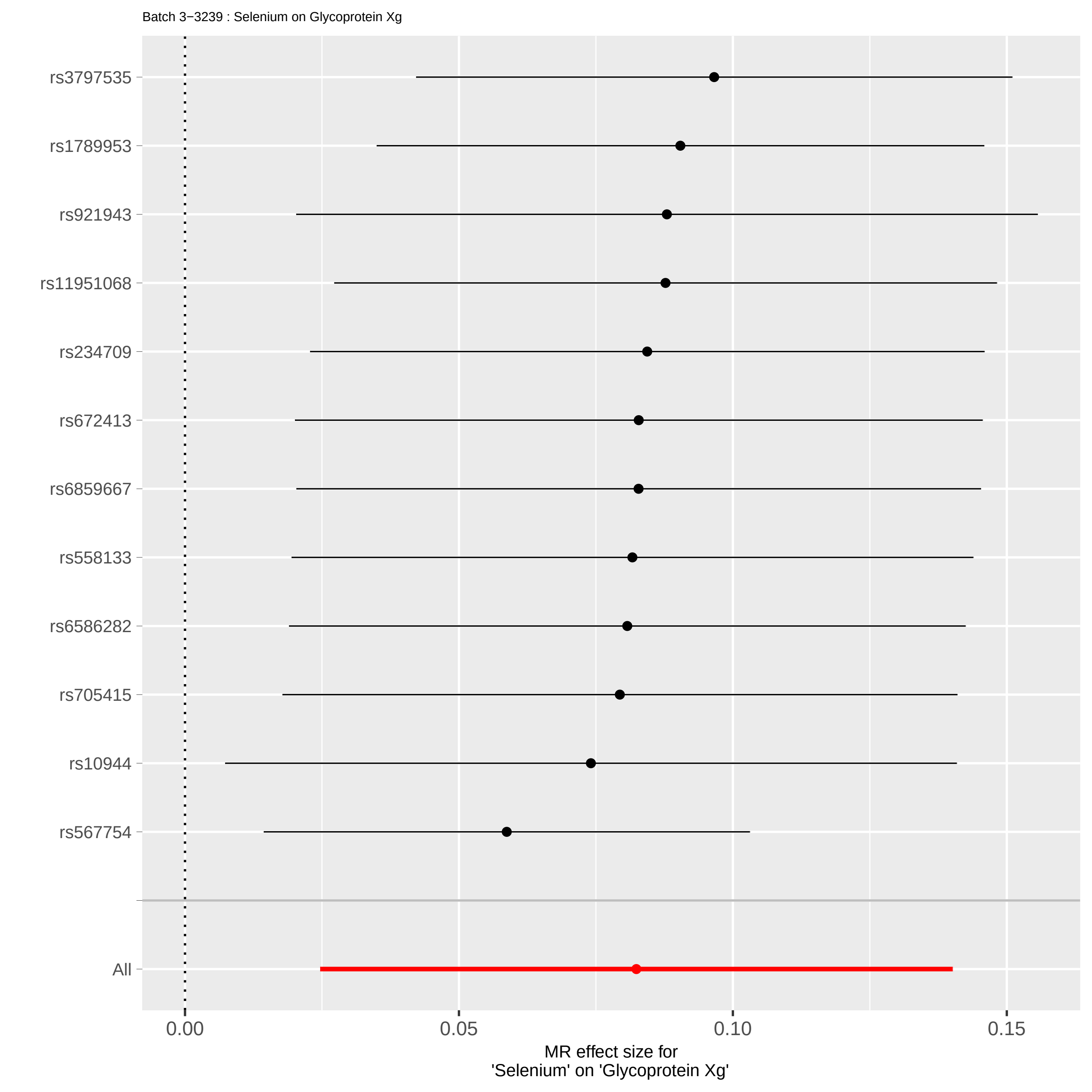

Batch 3−3239 : Selenium on Glycoprotein Xg
rs3797535
rs1789953
rs921943
rs11951068
rs234709
rs672413
rs6859667
rs558133
rs6586282
rs705415
rs10944
rs567754
All
0.00
0.05
0.10
0.15
MR effect size for
'Selenium' on 'Glycoprotein Xg'

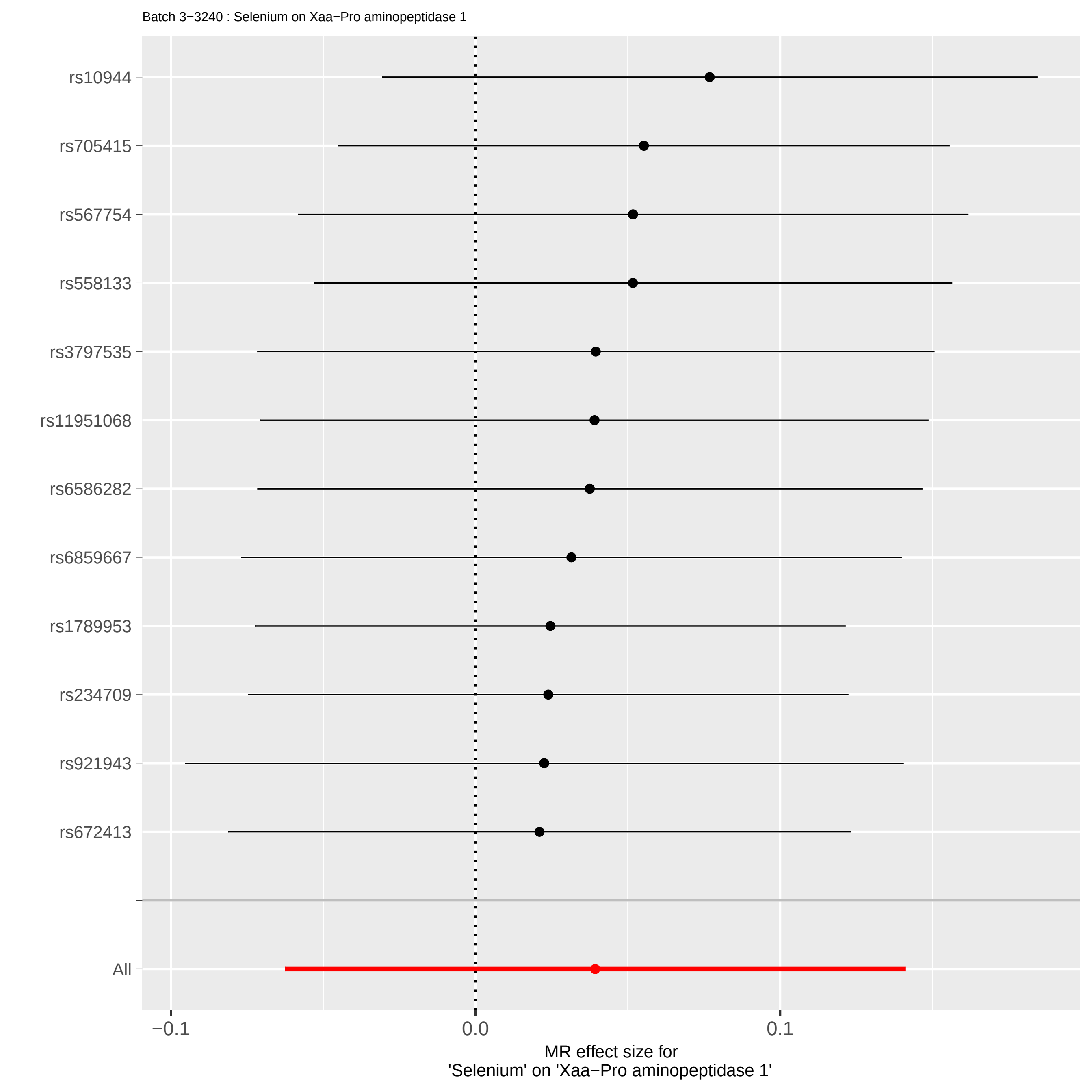

Batch 3−3240 : Selenium on Xaa−Pro aminopeptidase 1
rs10944
rs705415
rs567754
rs558133
rs3797535
rs11951068
rs6586282
rs6859667
rs1789953
rs234709
rs921943
rs672413
All
−0.1
0.0
0.1
MR effect size for
'Selenium' on 'Xaa−Pro aminopeptidase 1'

Batch 3−3241 : Selenium on DNA repair protein XRCC1

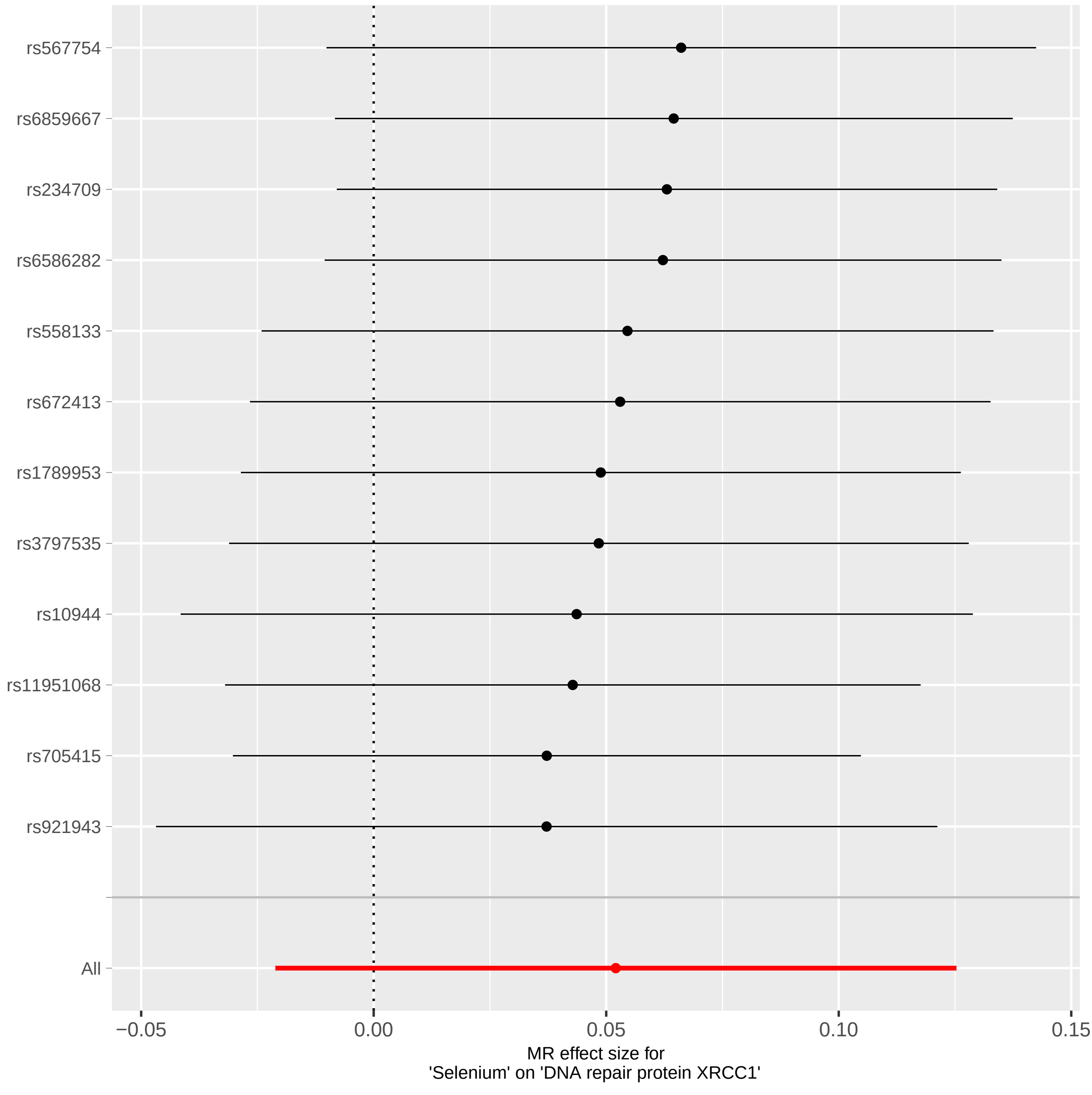

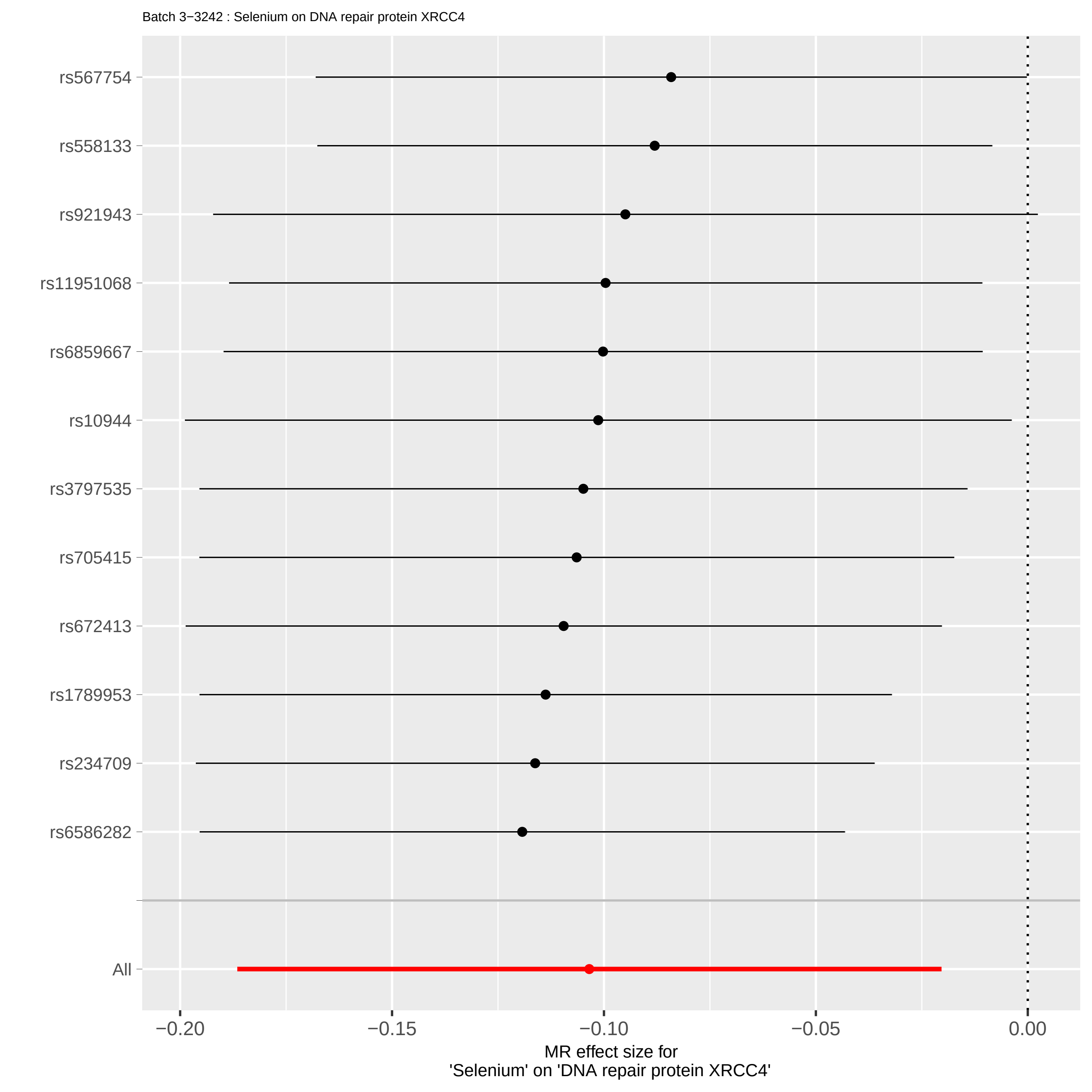

Batch 3−3242 : Selenium on DNA repair protein XRCC4
rs567754
rs558133
rs921943
rs11951068
rs6859667
rs10944
rs3797535
rs705415
rs672413
rs1789953
rs234709
rs6586282
All
−0.20
−0.15
−0.10
−0.05
0.00
MR effect size for
'Selenium' on 'DNA repair protein XRCC4'

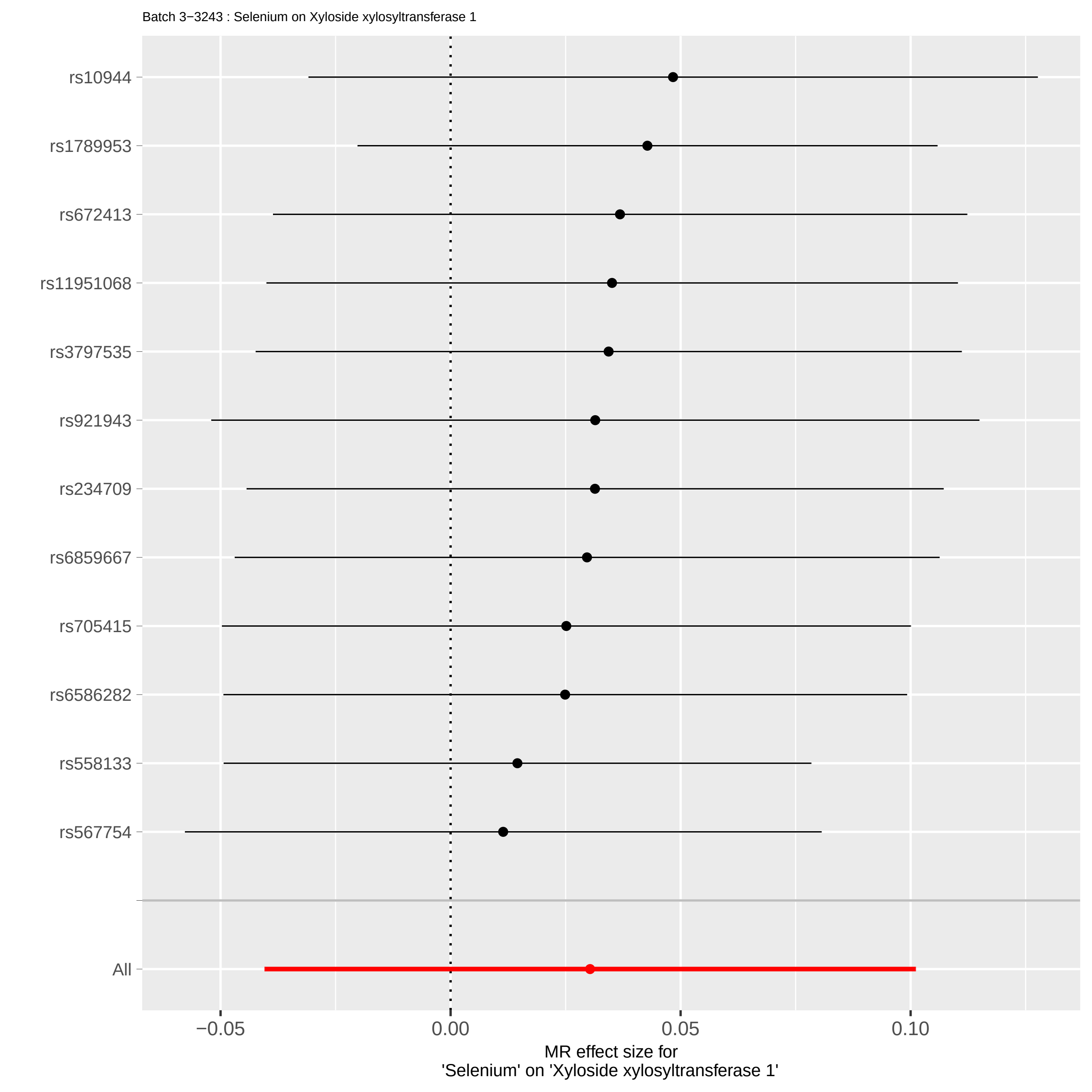

Batch 3−3243 : Selenium on Xyloside xylosyltransferase 1
rs10944
rs1789953
rs672413
rs11951068
rs3797535
rs921943
rs234709
rs6859667
rs705415
rs6586282
rs558133
rs567754
All
−0.05
0.00
0.05
0.10
MR effect size for
'Selenium' on 'Xyloside xylosyltransferase 1'

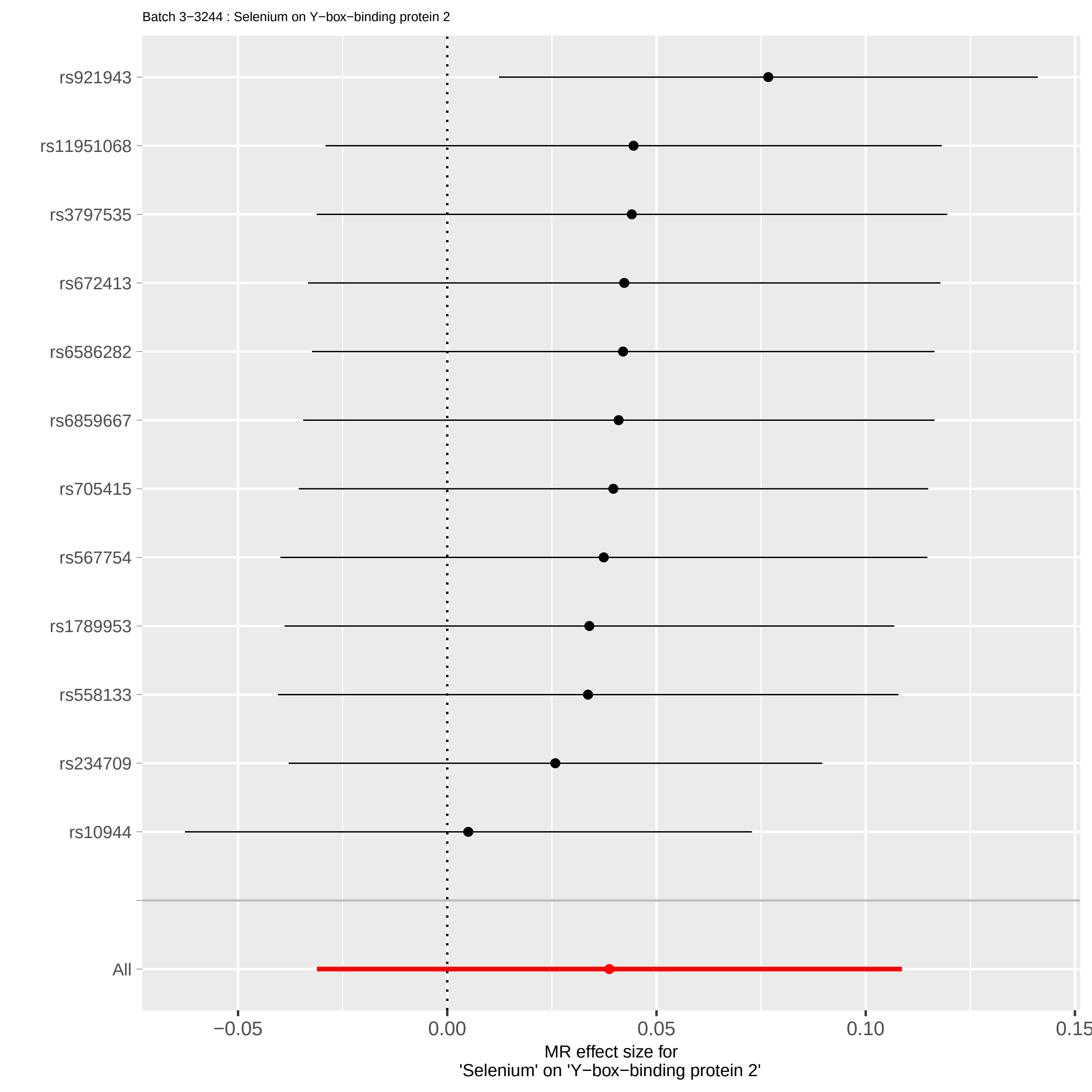

Batch 3−3244 : Selenium on Y−box−binding protein 2
rs921943
rs11951068
rs3797535
rs672413
rs6586282
rs6859667
rs705415
rs567754
rs1789953
rs558133
rs234709
rs10944
All
−0.05
0.00
0.05
0.10
0.15
MR effect size for
'Selenium' on 'Y−box−binding protein 2'

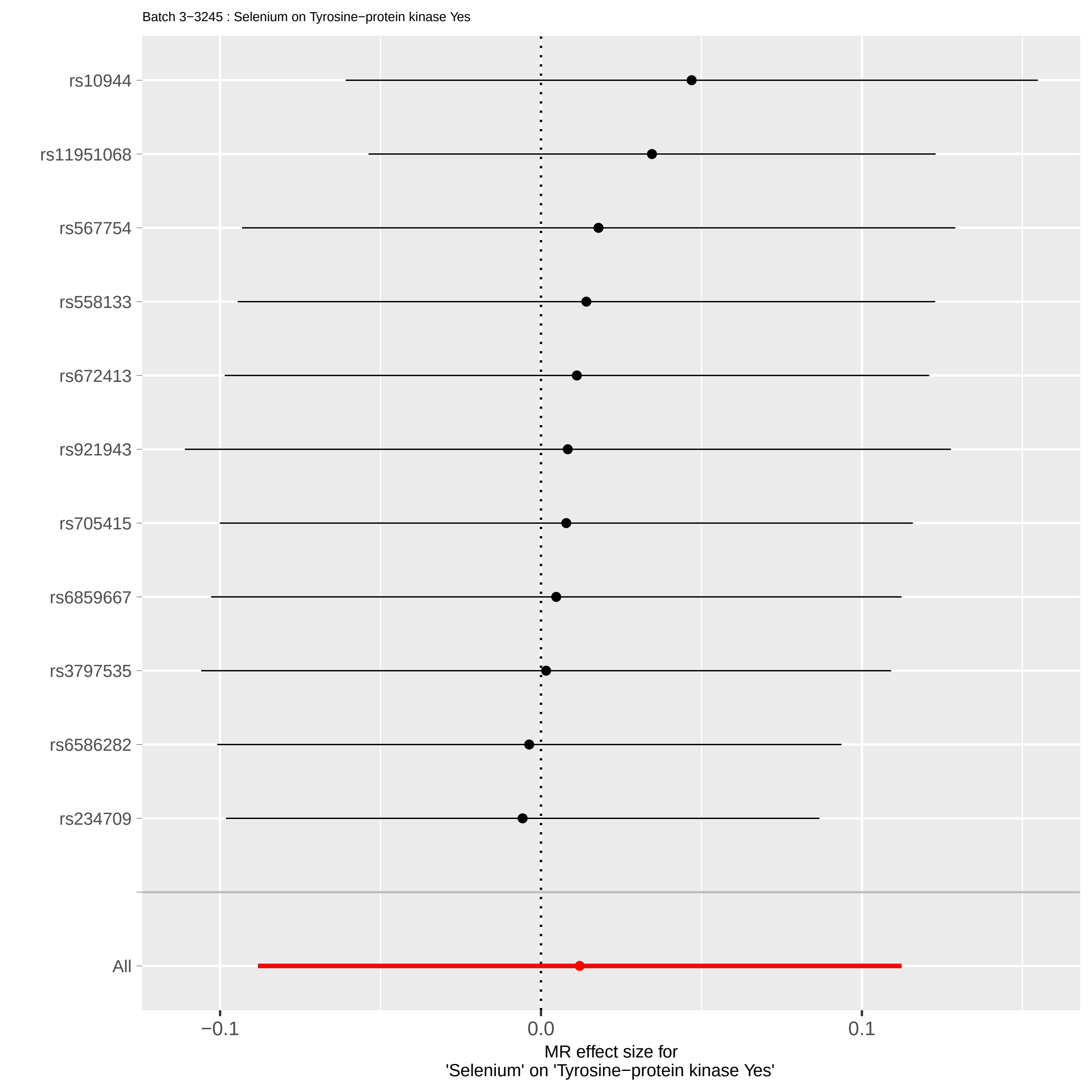

Batch 3−3245 : Selenium on Tyrosine−protein kinase Yes
rs10944
rs11951068
rs567754
rs558133
rs672413
rs921943
rs705415
rs6859667
rs3797535
rs6586282
rs234709
All
−0.1
0.0
0.1
MR effect size for
'Selenium' on 'Tyrosine−protein kinase Yes'

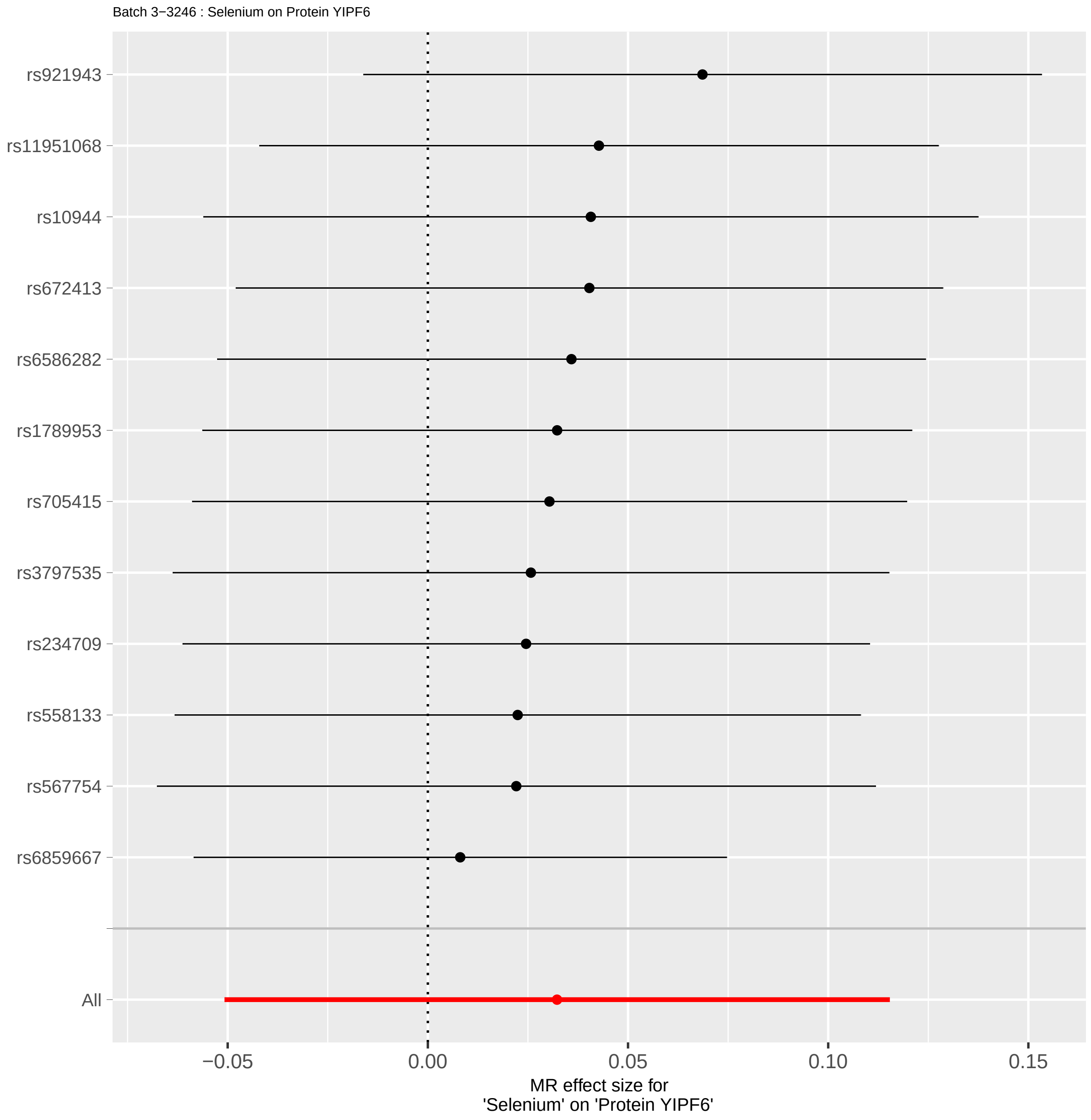

Batch 3−3246 : Selenium on Protein YIPF6
rs921943
rs11951068
rs10944
rs672413
rs6586282
rs1789953
rs705415
rs3797535
rs234709
rs558133
rs567754
rs6859667
All
−0.05
0.00
0.05
0.10
0.15
MR effect size for
'Selenium' on 'Protein YIPF6'

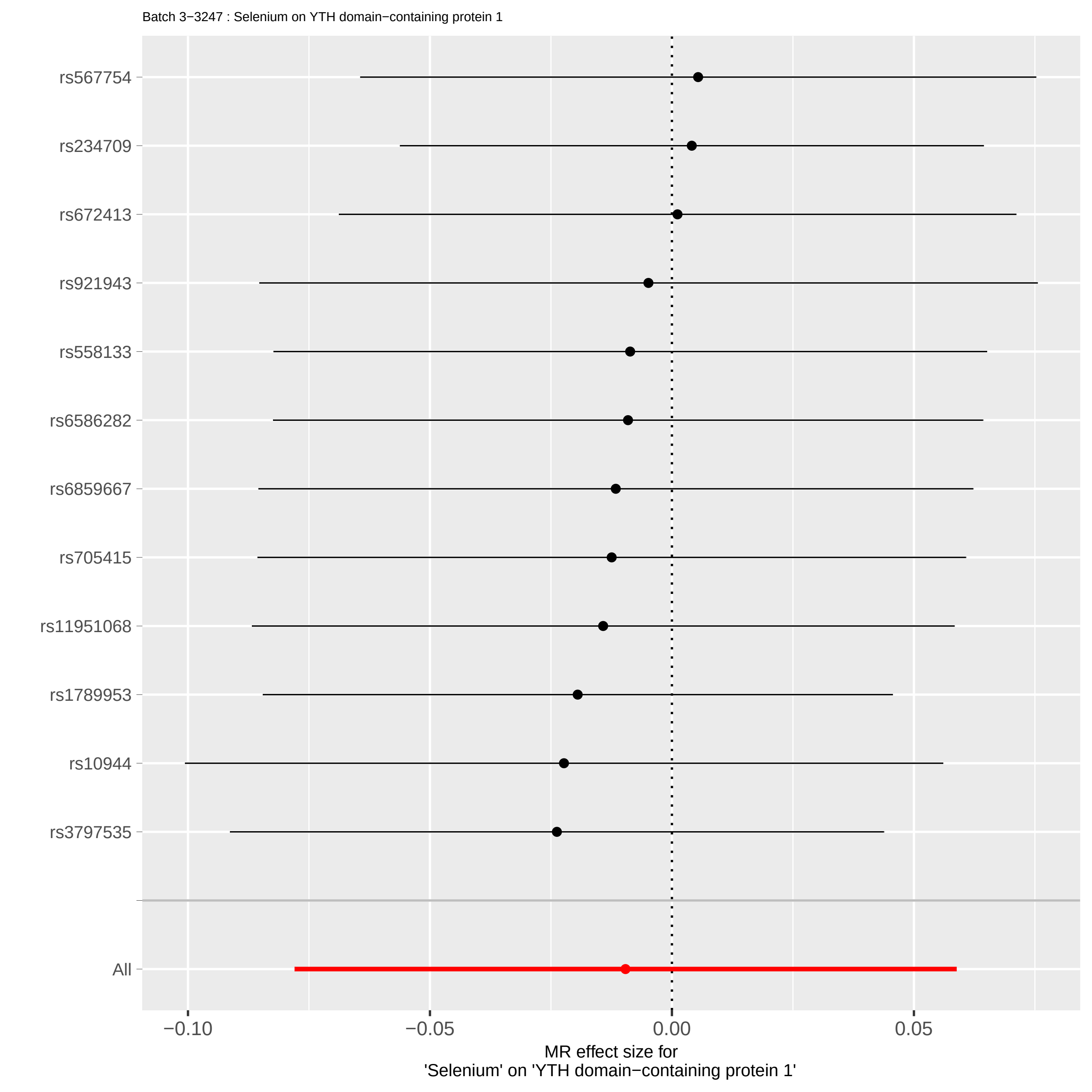

Batch 3−3247 : Selenium on YTH domain−containing protein 1
rs567754
rs234709
rs672413
rs921943
rs558133
rs6586282
rs6859667
rs705415
rs11951068
rs1789953
rs10944
rs3797535
All
−0.10
−0.05
0.00
0.05
MR effect size for
'Selenium' on 'YTH domain−containing protein 1'

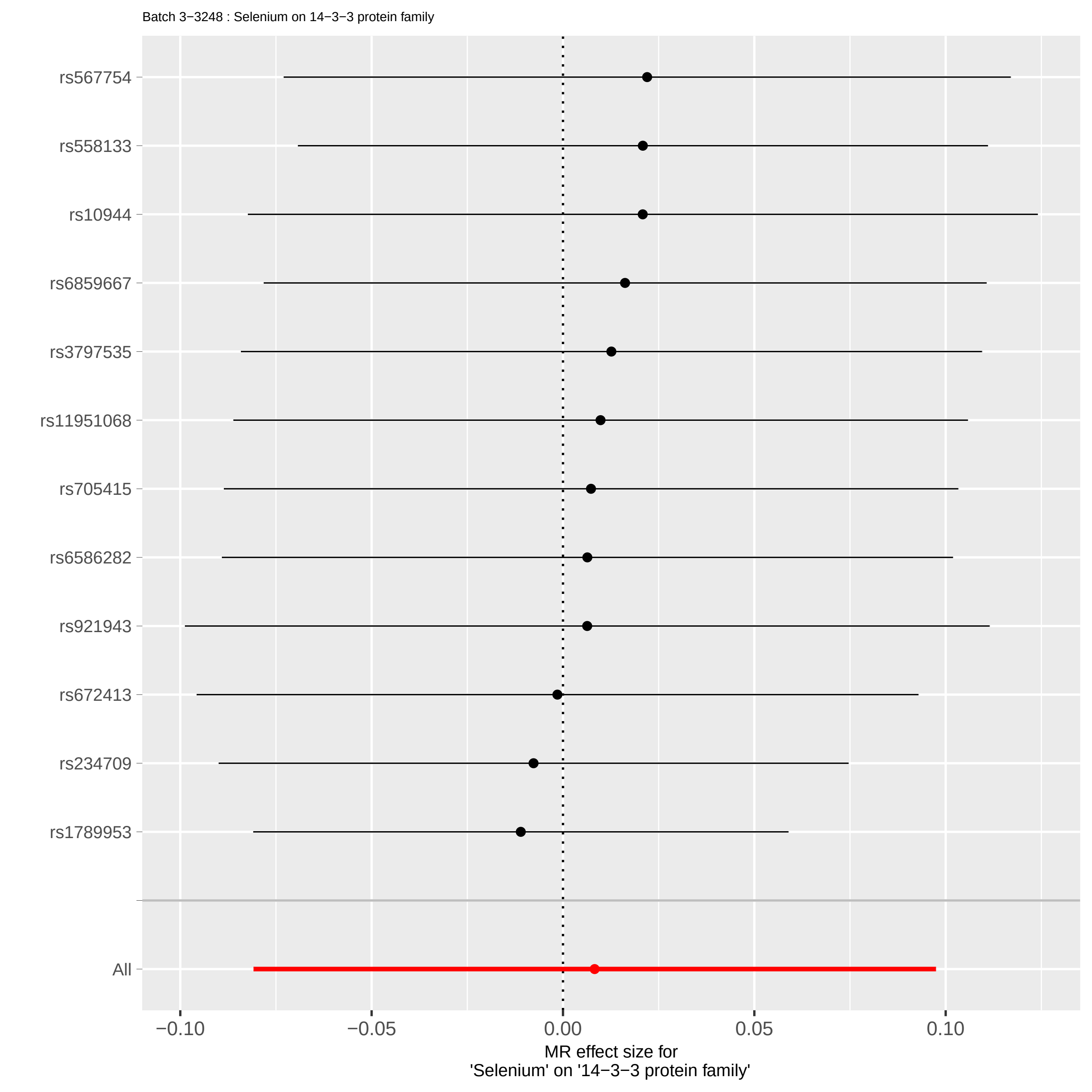
Batch 3−3248 : Selenium on 14−3−3 protein family
rs567754
rs558133
rs10944
rs6859667
rs3797535
rs11951068
rs705415
rs6586282
rs921943
rs672413
rs234709
rs1789953
All
−0.10
−0.05
0.00
0.05
0.10
MR effect size for
'Selenium' on '14−3−3 protein family'

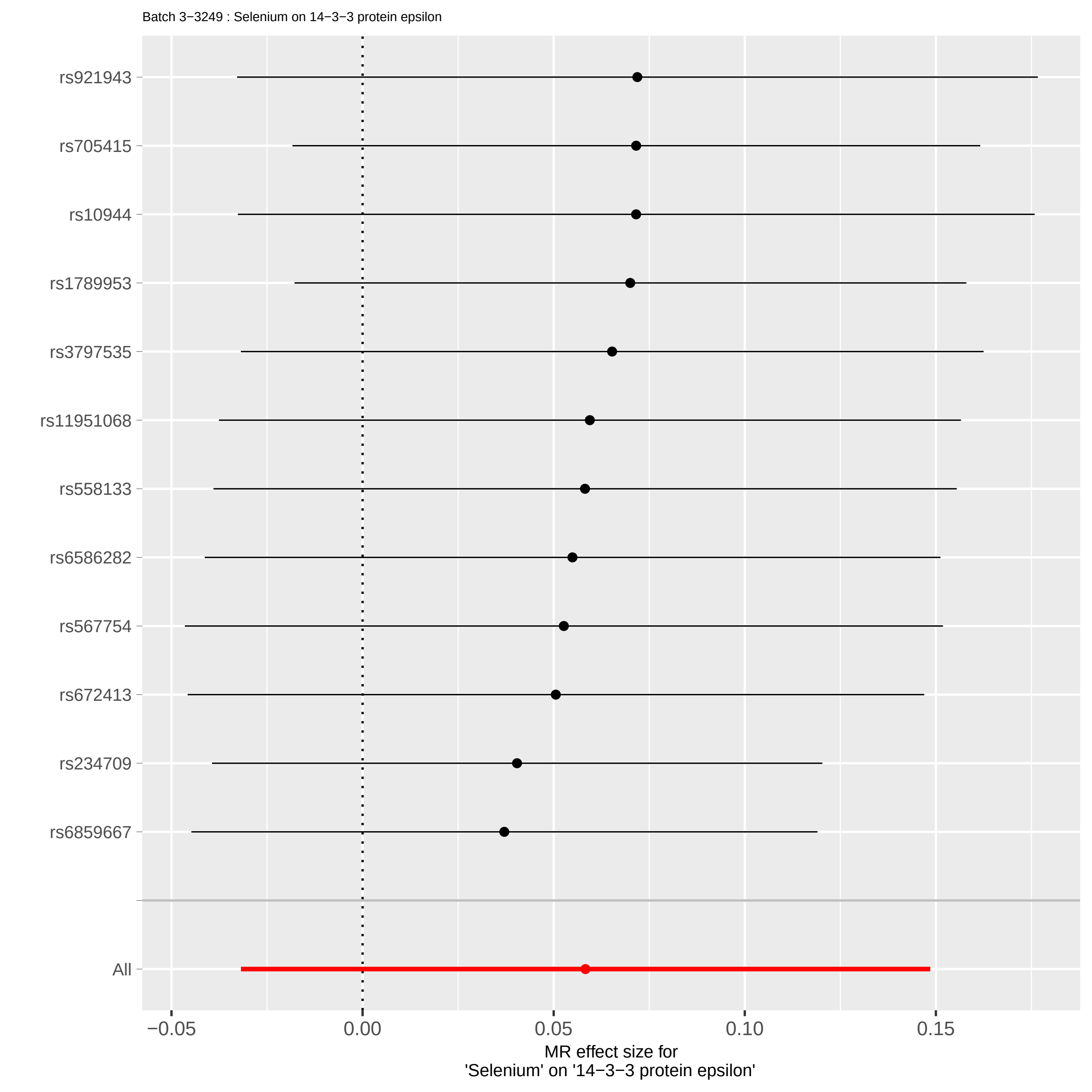
Batch 3−3249 : Selenium on 14−3−3 protein epsilon
rs921943
rs705415
rs10944
rs1789953
rs3797535
rs11951068
rs558133
rs6586282
rs567754
rs672413
rs234709
rs6859667
All
−0.05
0.00
0.05
0.10
0.15
MR effect size for
'Selenium' on '14−3−3 protein epsilon'

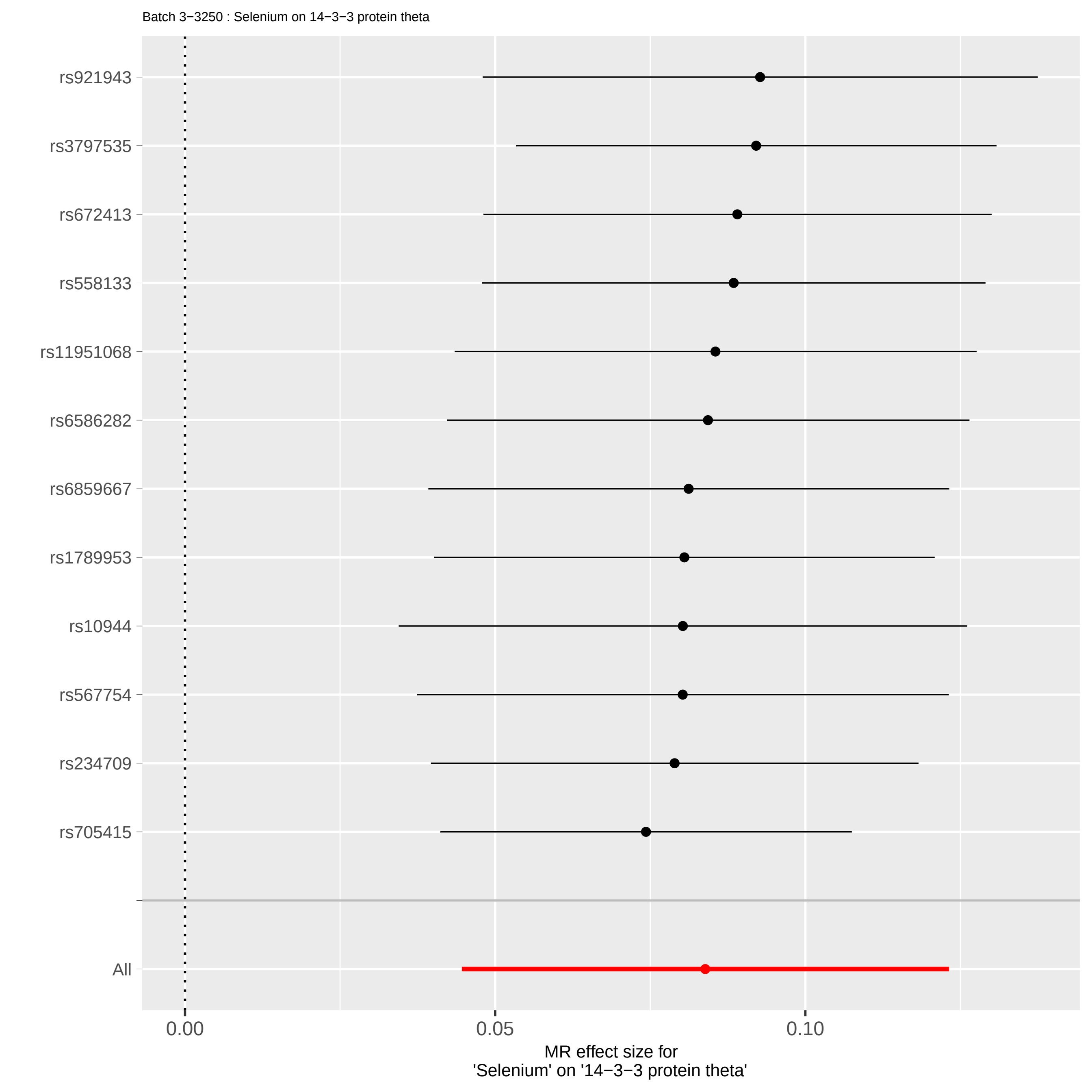
Batch 3−3250 : Selenium on 14−3−3 protein theta
rs921943
rs3797535
rs672413
rs558133
rs11951068
rs6586282
rs6859667
rs1789953
rs10944
rs567754
rs234709
rs705415
All
0.00
0.05
0.10
MR effect size for
'Selenium' on '14−3−3 protein theta'

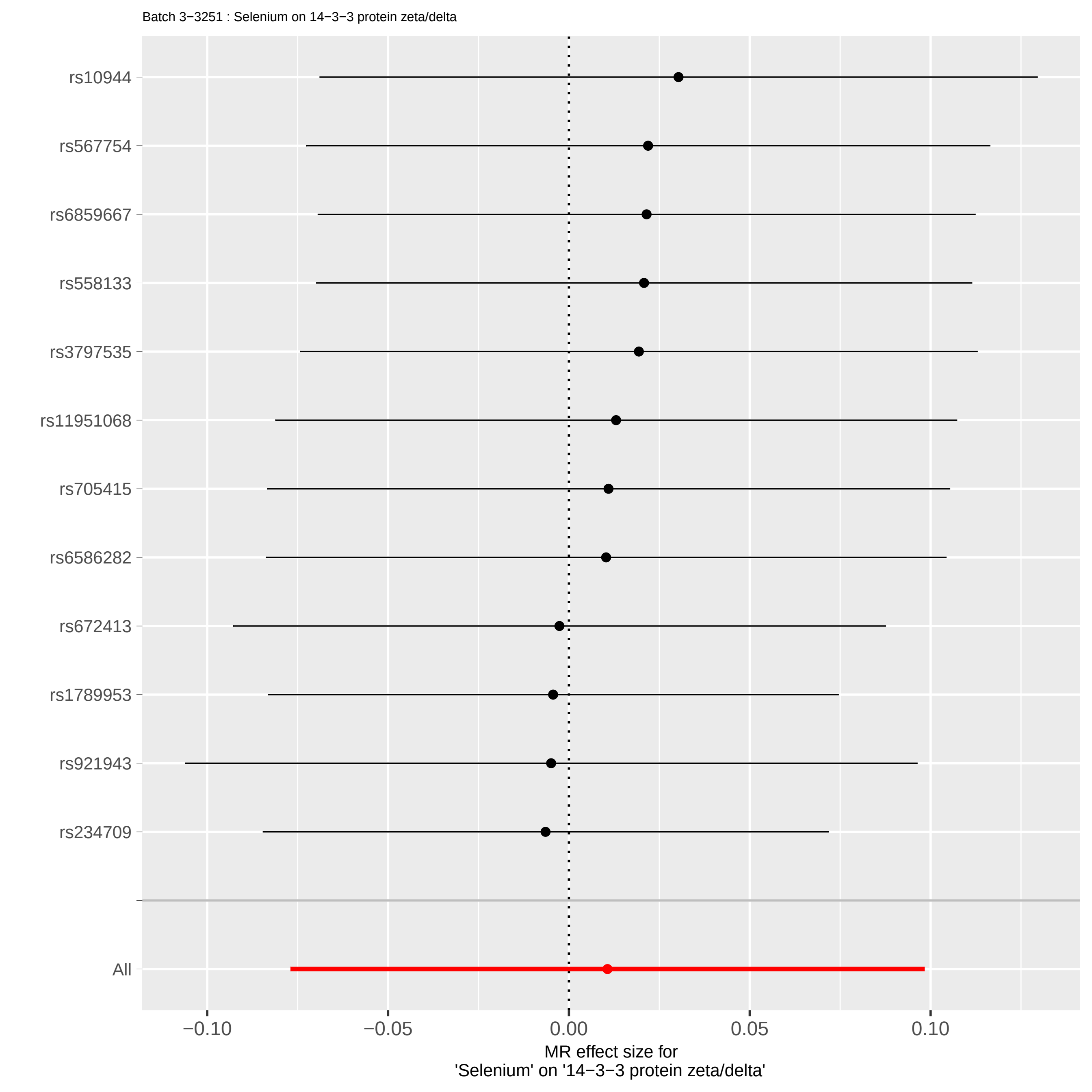
Batch 3−3251 : Selenium on 14−3−3 protein zeta/delta
rs10944
rs567754
rs6859667
rs558133
rs3797535
rs11951068
rs705415
rs6586282
rs672413
rs1789953
rs921943
rs234709
All
−0.10
−0.05
0.00
0.05
0.10
MR effect size for
'Selenium' on '14−3−3 protein zeta/delta'

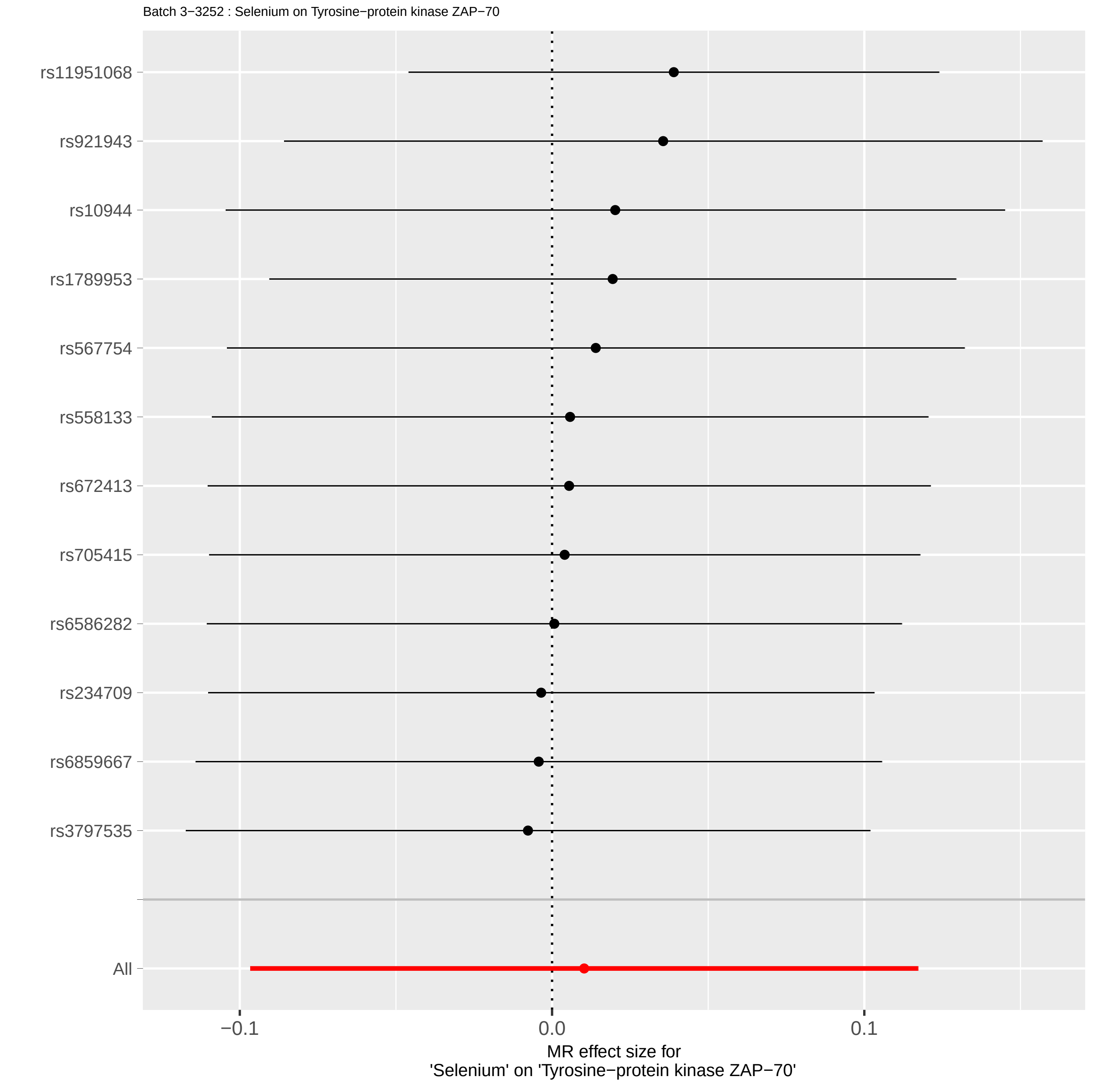

Batch 3−3252 : Selenium on Tyrosine−protein kinase ZAP−70
rs11951068
rs921943
rs10944
rs1789953
rs567754
rs558133
rs672413
rs705415
rs6586282
rs234709
rs6859667
rs3797535
All
−0.1
0.0
0.1
MR effect size for
'Selenium' on 'Tyrosine−protein kinase ZAP−70'

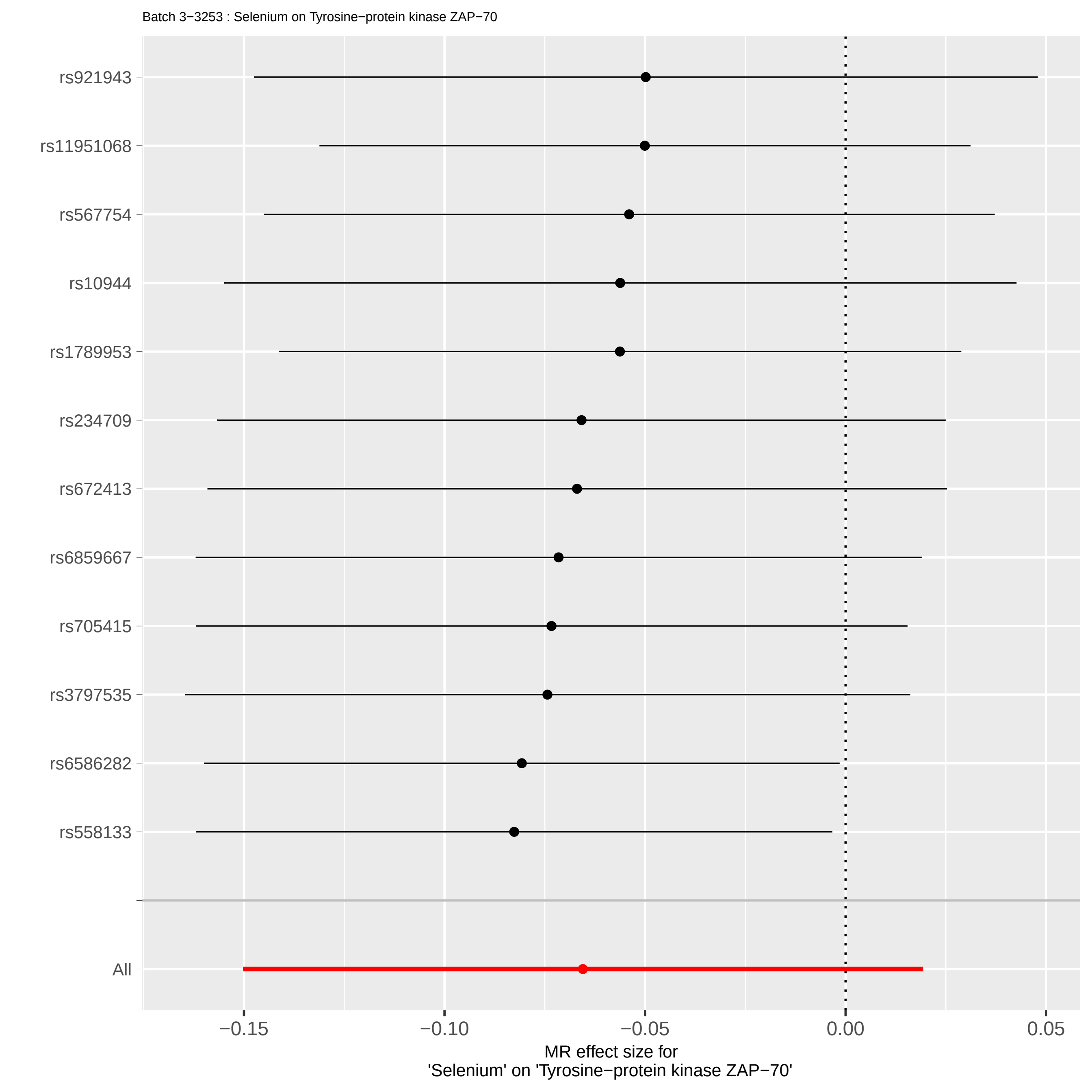

Batch 3−3253 : Selenium on Tyrosine−protein kinase ZAP−70
rs921943
rs11951068
rs567754
rs10944
rs1789953
rs234709
rs672413
rs6859667
rs705415
rs3797535
rs6586282
rs558133
All
−0.15
−0.10
−0.05
0.00
0.05
MR effect size for
'Selenium' on 'Tyrosine−protein kinase ZAP−70'

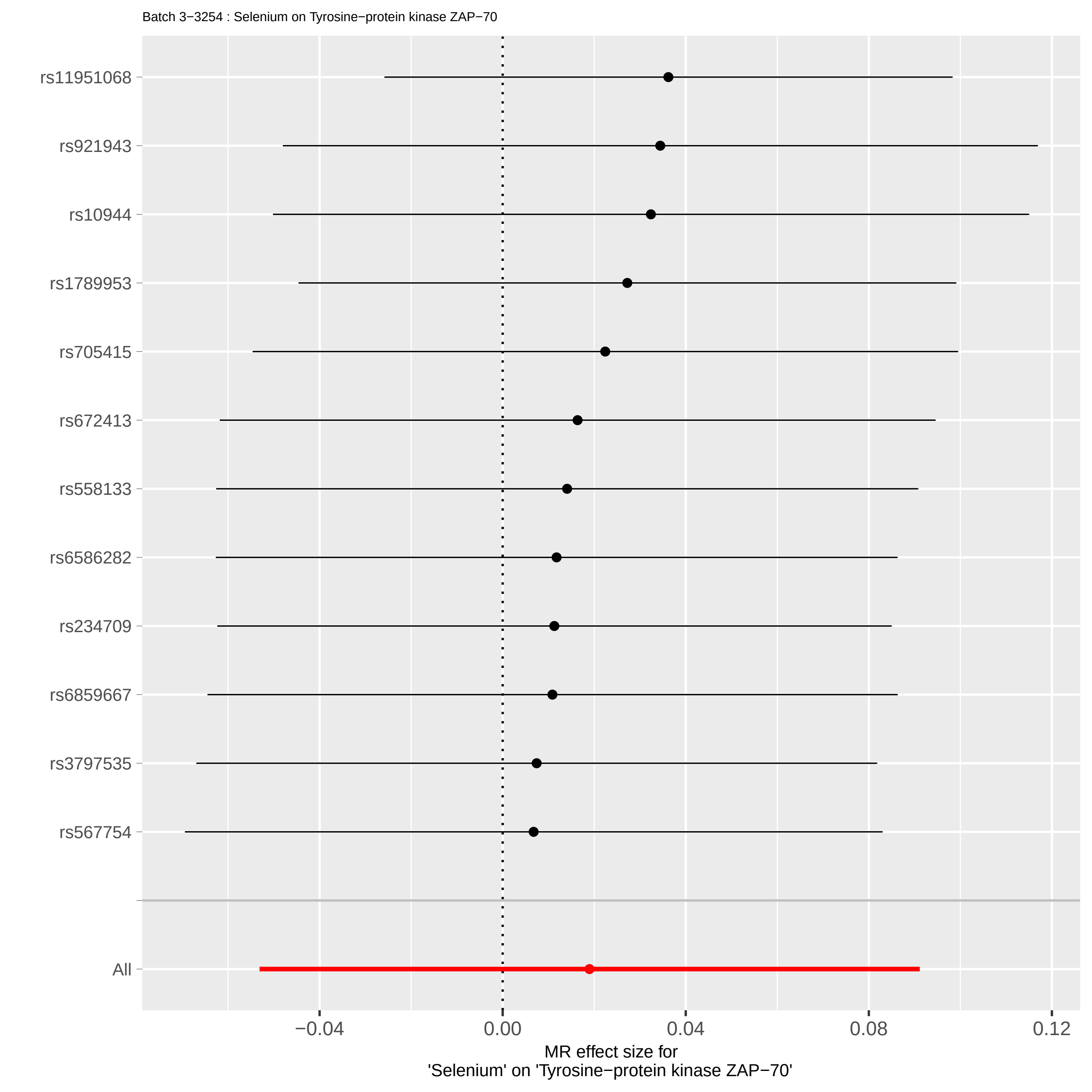

Batch 3−3254 : Selenium on Tyrosine−protein kinase ZAP−70
rs11951068
rs921943
rs10944
rs1789953
rs705415
rs672413
rs558133
rs6586282
rs234709
rs6859667
rs3797535
rs567754
All
−0.04
0.00
0.04
0.08
0.12
MR effect size for
'Selenium' on 'Tyrosine−protein kinase ZAP−70'

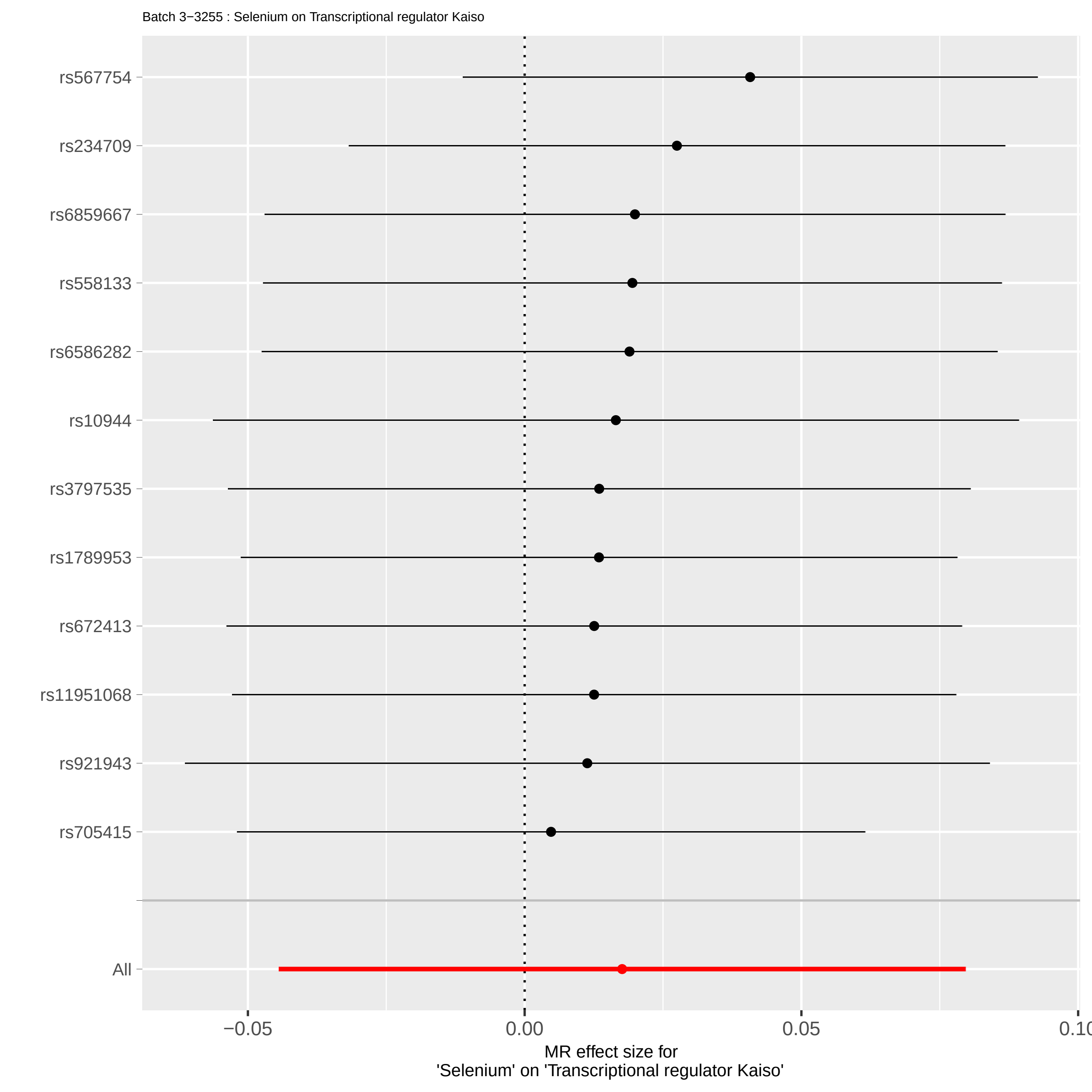

Batch 3−3255 : Selenium on Transcriptional regulator Kaiso
rs567754
rs234709
rs6859667
rs558133
rs6586282
rs10944
rs3797535
rs1789953
rs672413
rs11951068
rs921943
rs705415
All
−0.05
0.00
0.05
0.10
MR effect size for
'Selenium' on 'Transcriptional regulator Kaiso'

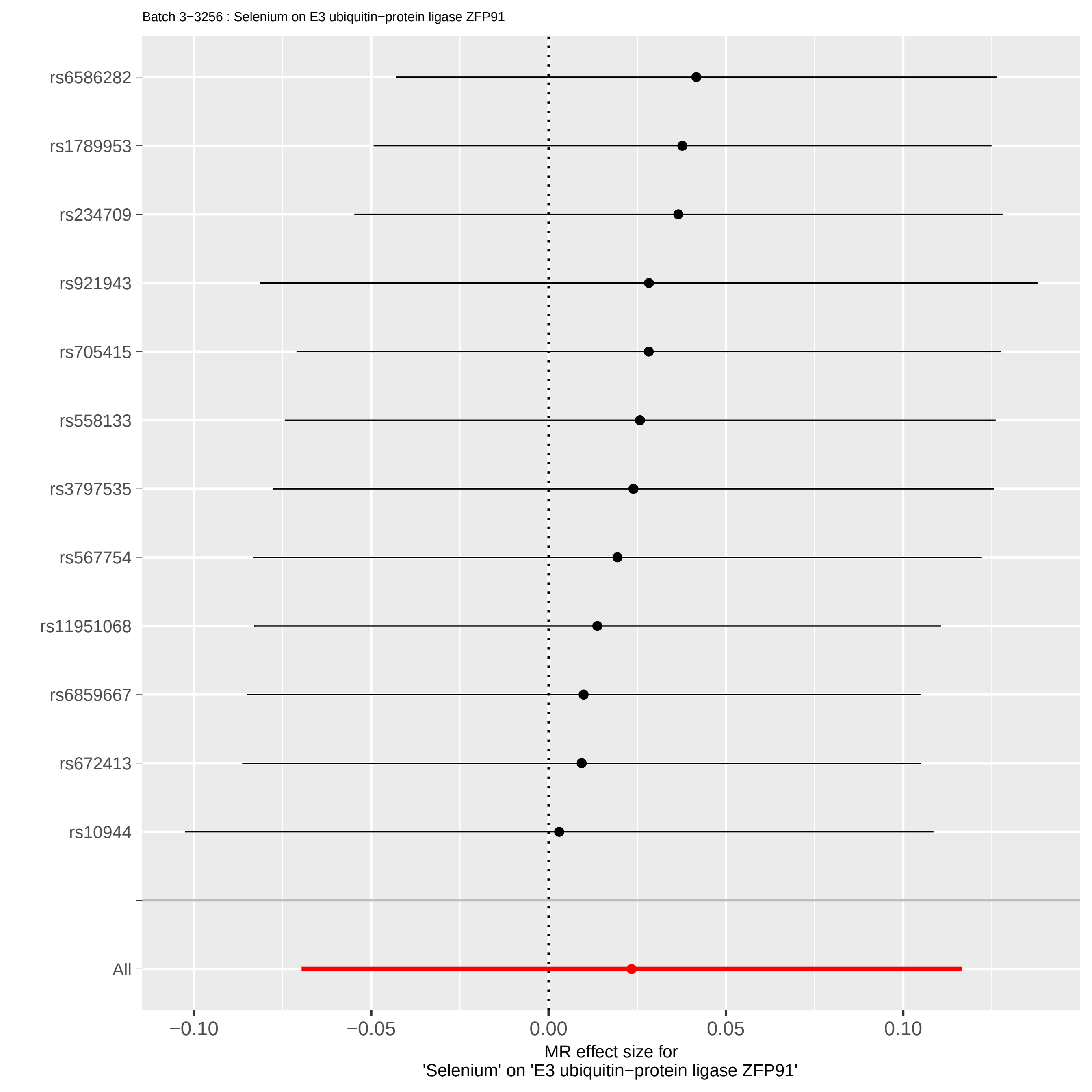
Batch 3−3256 : Selenium on E3 ubiquitin−protein ligase ZFP91
rs6586282
rs1789953
rs234709
rs921943
rs705415
rs558133
rs3797535
rs567754
rs11951068
rs6859667
rs672413
rs10944
All
−0.10
−0.05
0.00
0.05
0.10
MR effect size for
'Selenium' on 'E3 ubiquitin−protein ligase ZFP91'

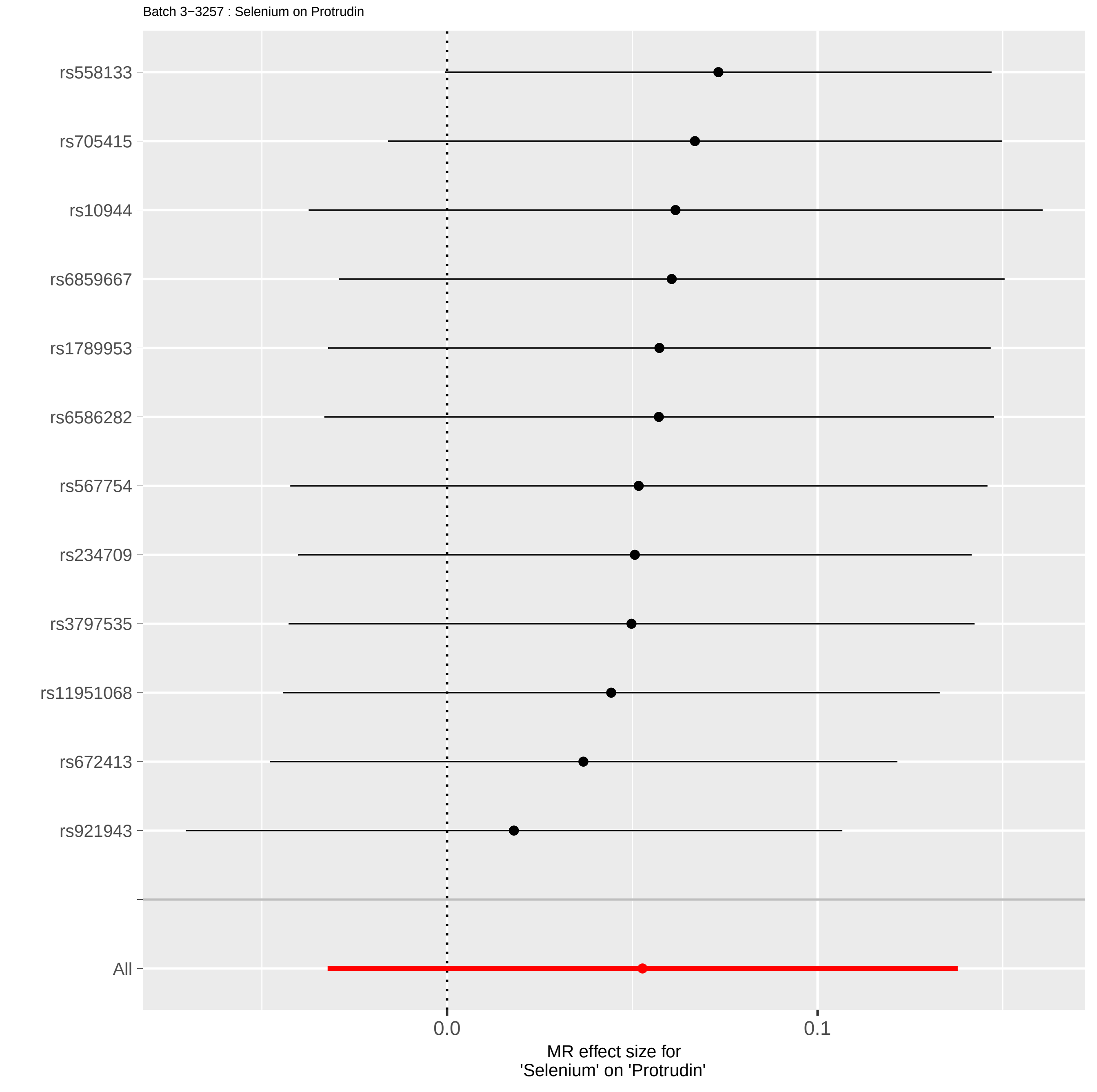

Batch 3−3257 : Selenium on Protrudin
rs558133
rs705415
rs10944
rs6859667
rs1789953
rs6586282
rs567754
rs234709
rs3797535
rs11951068
rs672413
rs921943
All
0.0
0.1
MR effect size for
'Selenium' on 'Protrudin'

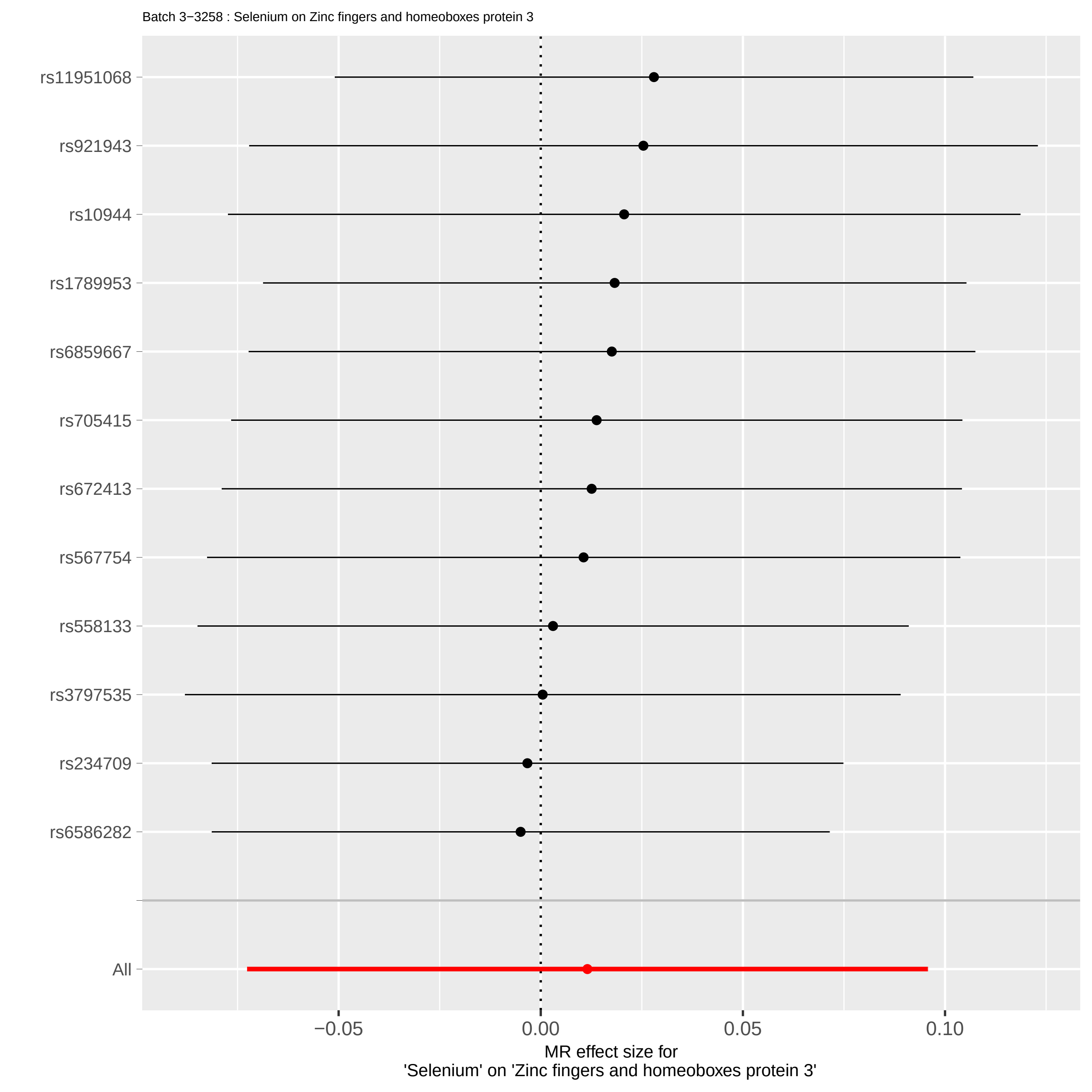

Batch 3−3258 : Selenium on Zinc fingers and homeoboxes protein 3
rs11951068
rs921943
rs10944
rs1789953
rs6859667
rs705415
rs672413
rs567754
rs558133
rs3797535
rs234709
rs6586282
All
−0.05
0.00
0.05
0.10
MR effect size for
'Selenium' on 'Zinc fingers and homeoboxes protein 3'

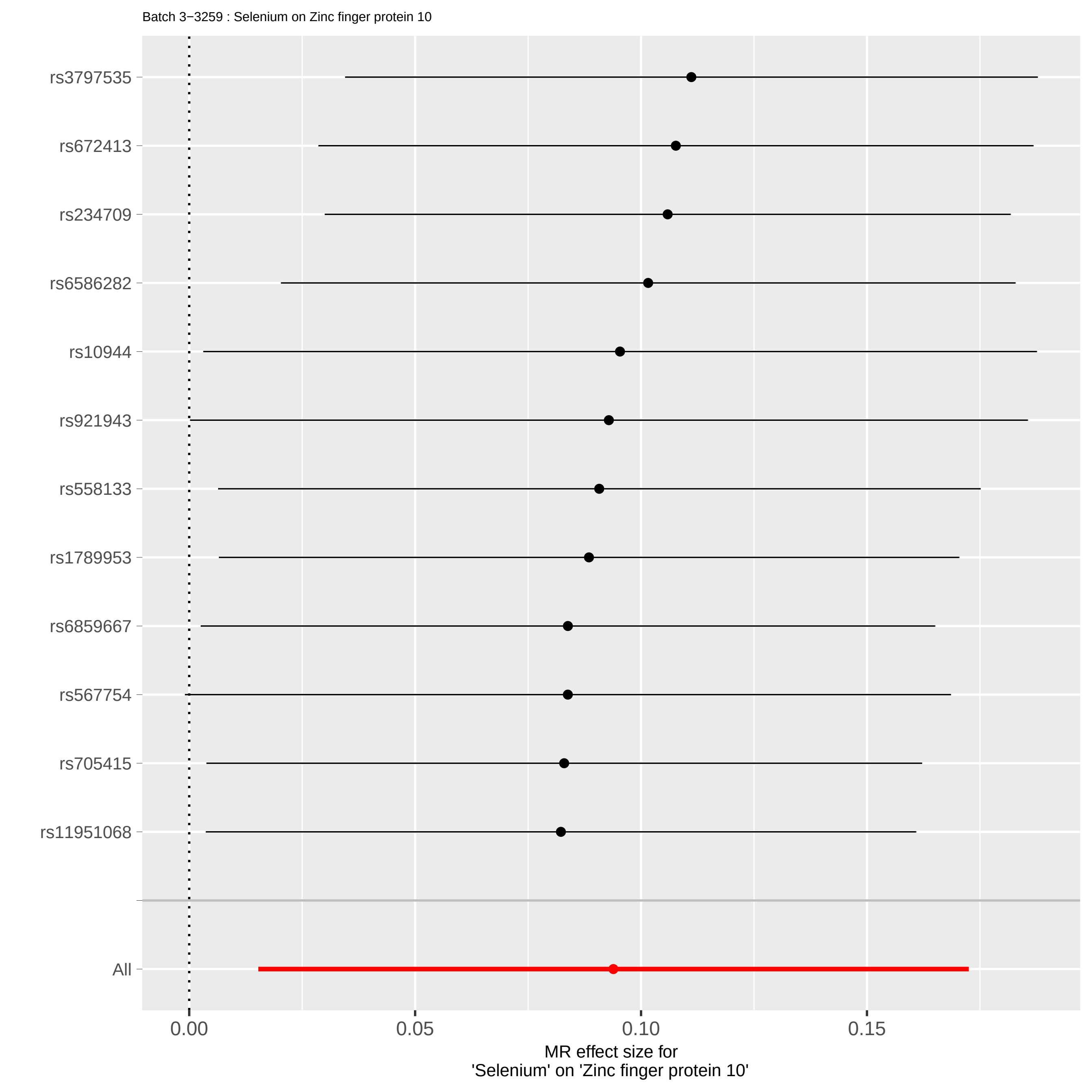
Batch 3−3259 : Selenium on Zinc finger protein 10
rs3797535
rs672413
rs234709
rs6586282
rs10944
rs921943
rs558133
rs1789953
rs6859667
rs567754
rs705415
rs11951068
All
0.00
0.05
0.10
0.15
MR effect size for
'Selenium' on 'Zinc finger protein 10'

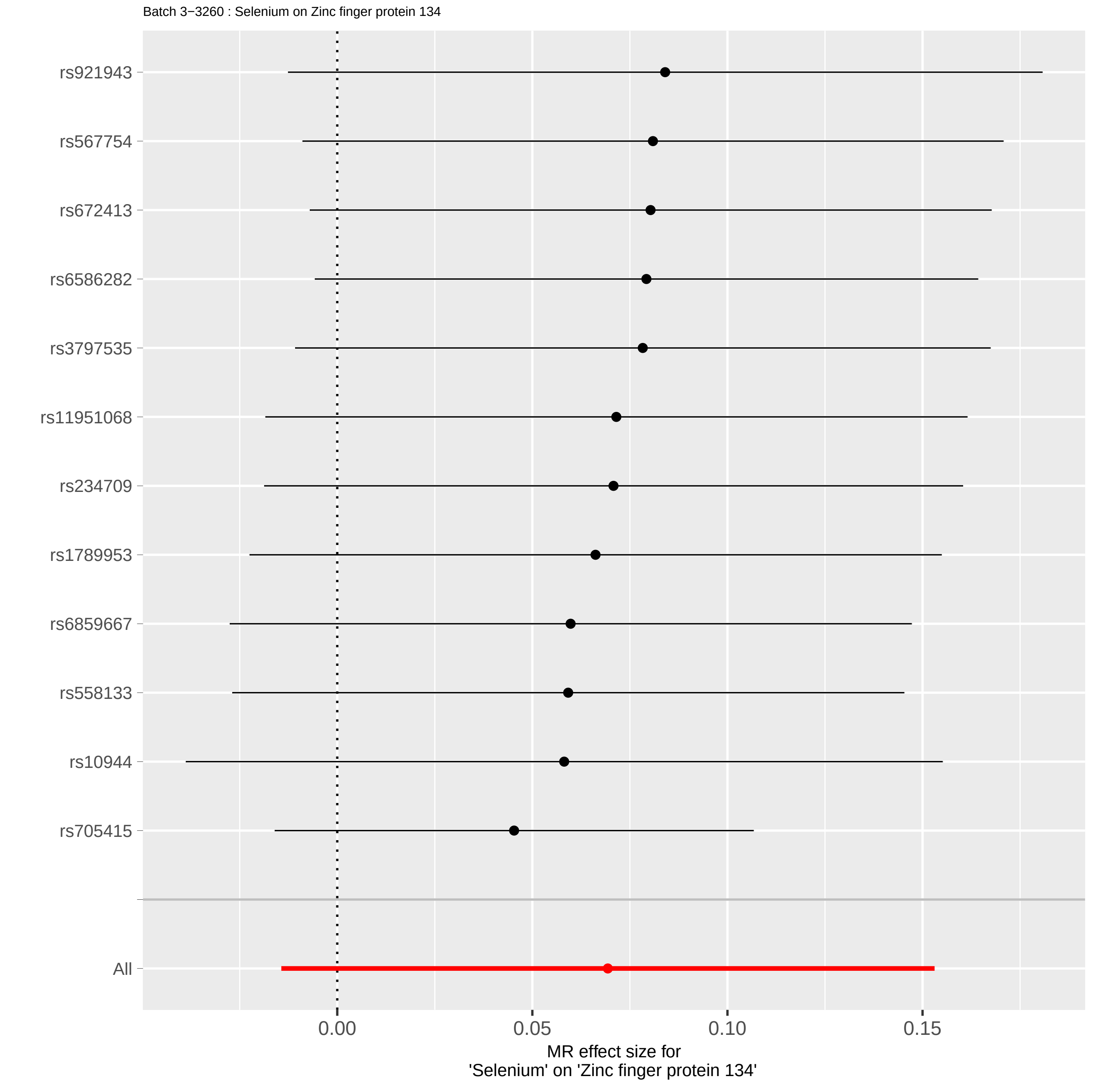

Batch 3−3260 : Selenium on Zinc finger protein 134
rs921943
rs567754
rs672413
rs6586282
rs3797535
rs11951068
rs234709
rs1789953
rs6859667
rs558133
rs10944
rs705415
All
0.00
0.05
0.10
0.15
MR effect size for
'Selenium' on 'Zinc finger protein 134'

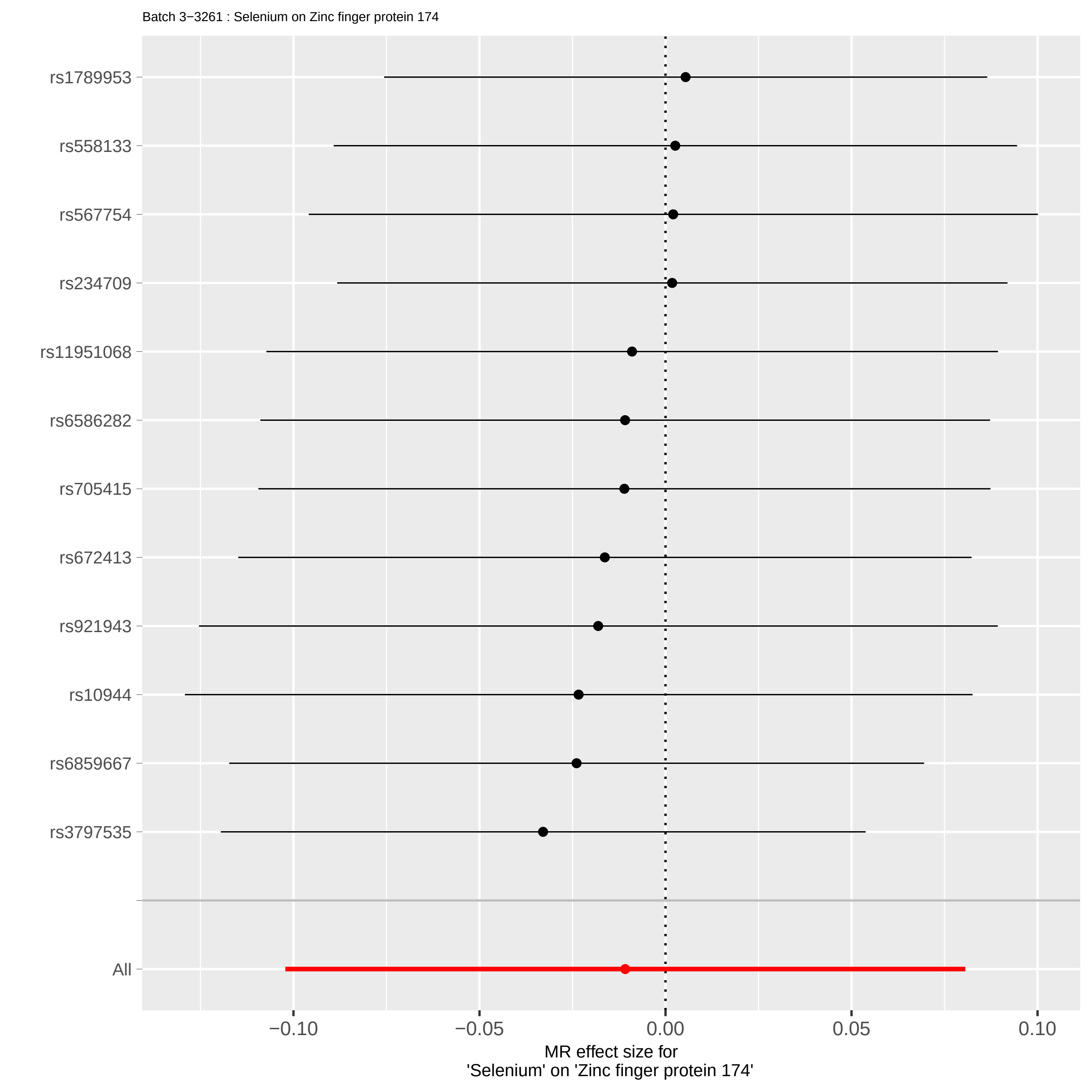

Batch 3−3261 : Selenium on Zinc finger protein 174
rs1789953
rs558133
rs567754
rs234709
rs11951068
rs6586282
rs705415
rs672413
rs921943
rs10944
rs6859667
rs3797535
All
−0.10
−0.05
0.00
0.05
0.10
MR effect size for
'Selenium' on 'Zinc finger protein 174'

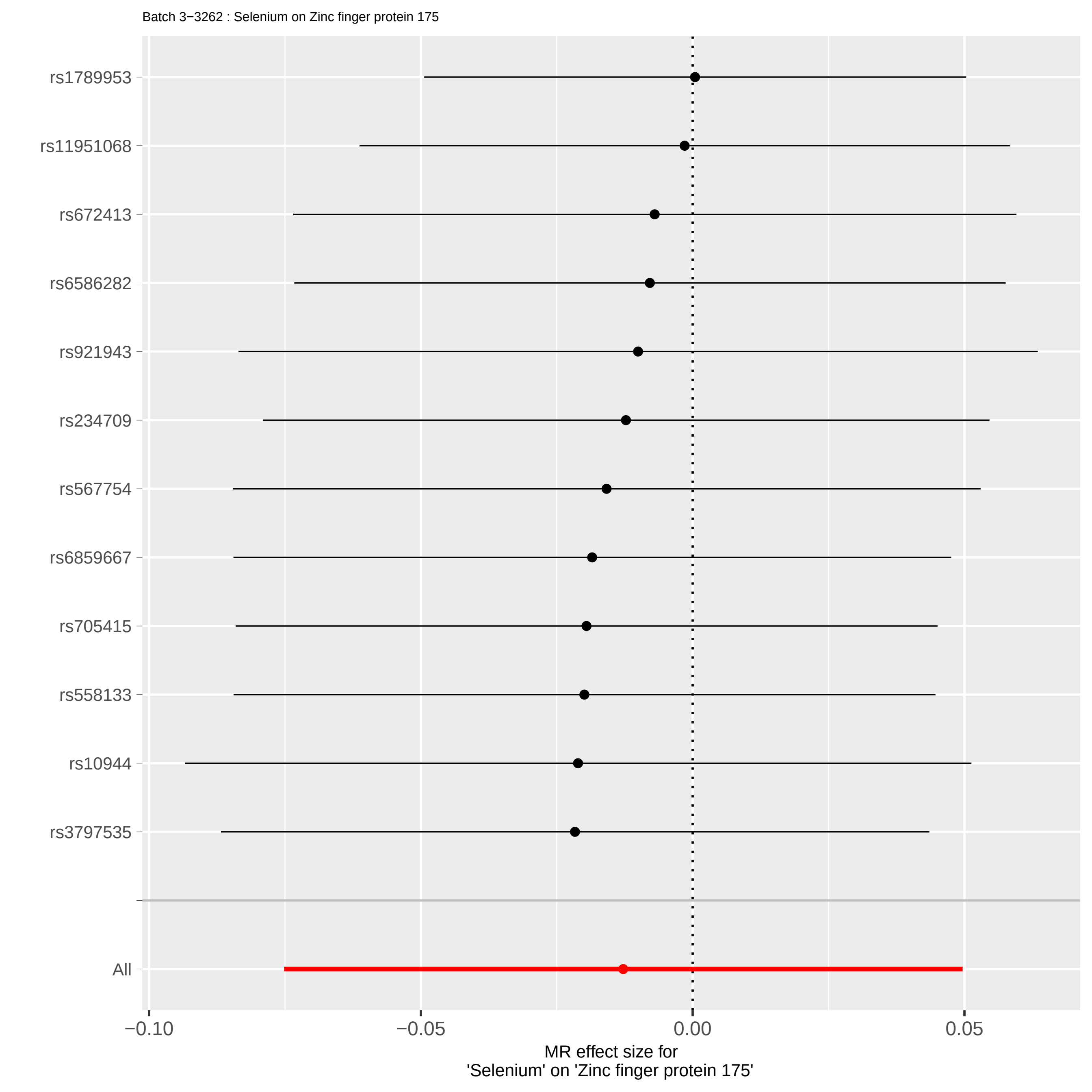
Batch 3−3262 : Selenium on Zinc finger protein 175
rs1789953
rs11951068
rs672413
rs6586282
rs921943
rs234709
rs567754
rs6859667
rs705415
rs558133
rs10944
rs3797535
All
−0.10
−0.05
0.00
0.05
MR effect size for
'Selenium' on 'Zinc finger protein 175'

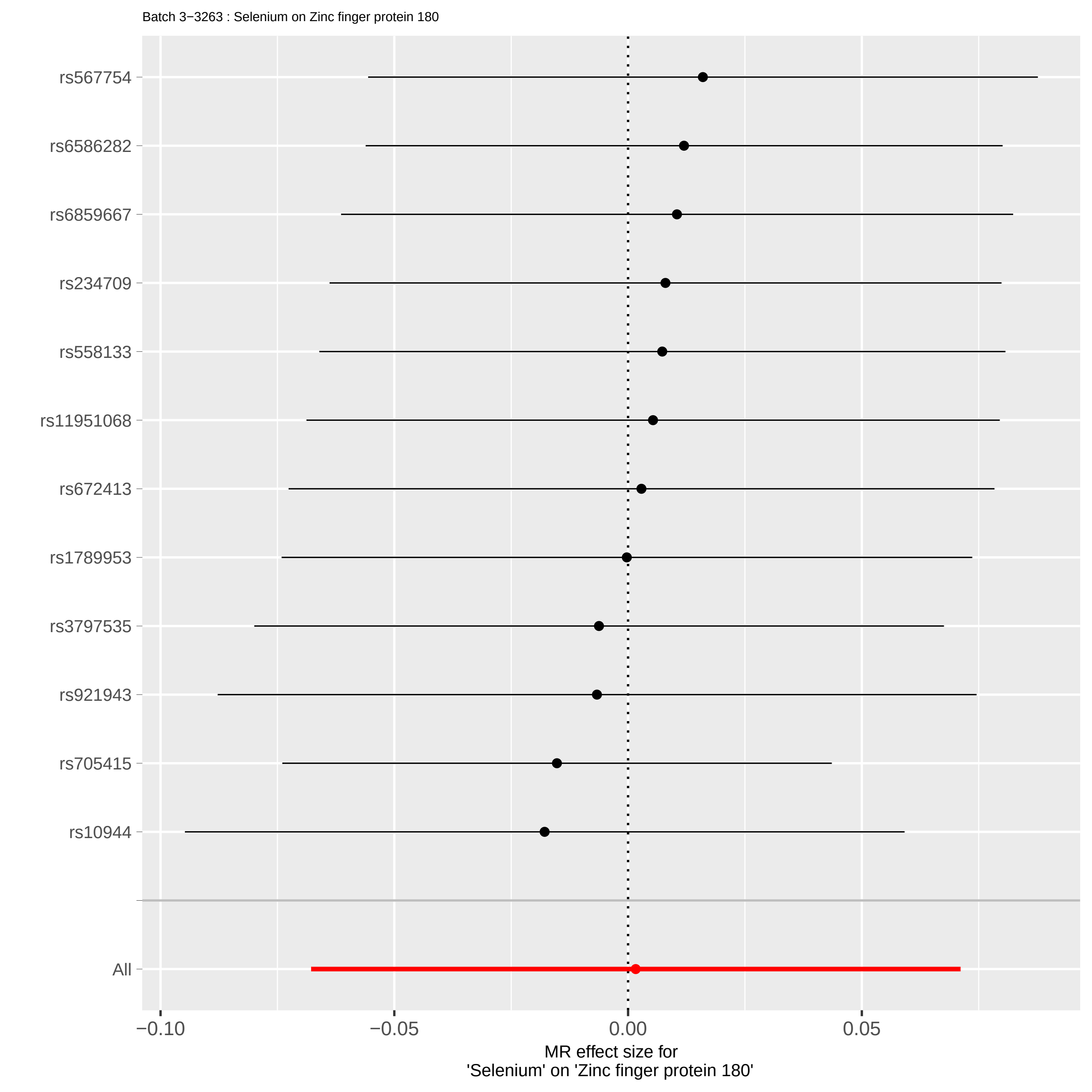
Batch 3−3263 : Selenium on Zinc finger protein 180
rs567754
rs6586282
rs6859667
rs234709
rs558133
rs11951068
rs672413
rs1789953
rs3797535
rs921943
rs705415
rs10944
All
−0.10
−0.05
0.00
0.05
MR effect size for
'Selenium' on 'Zinc finger protein 180'

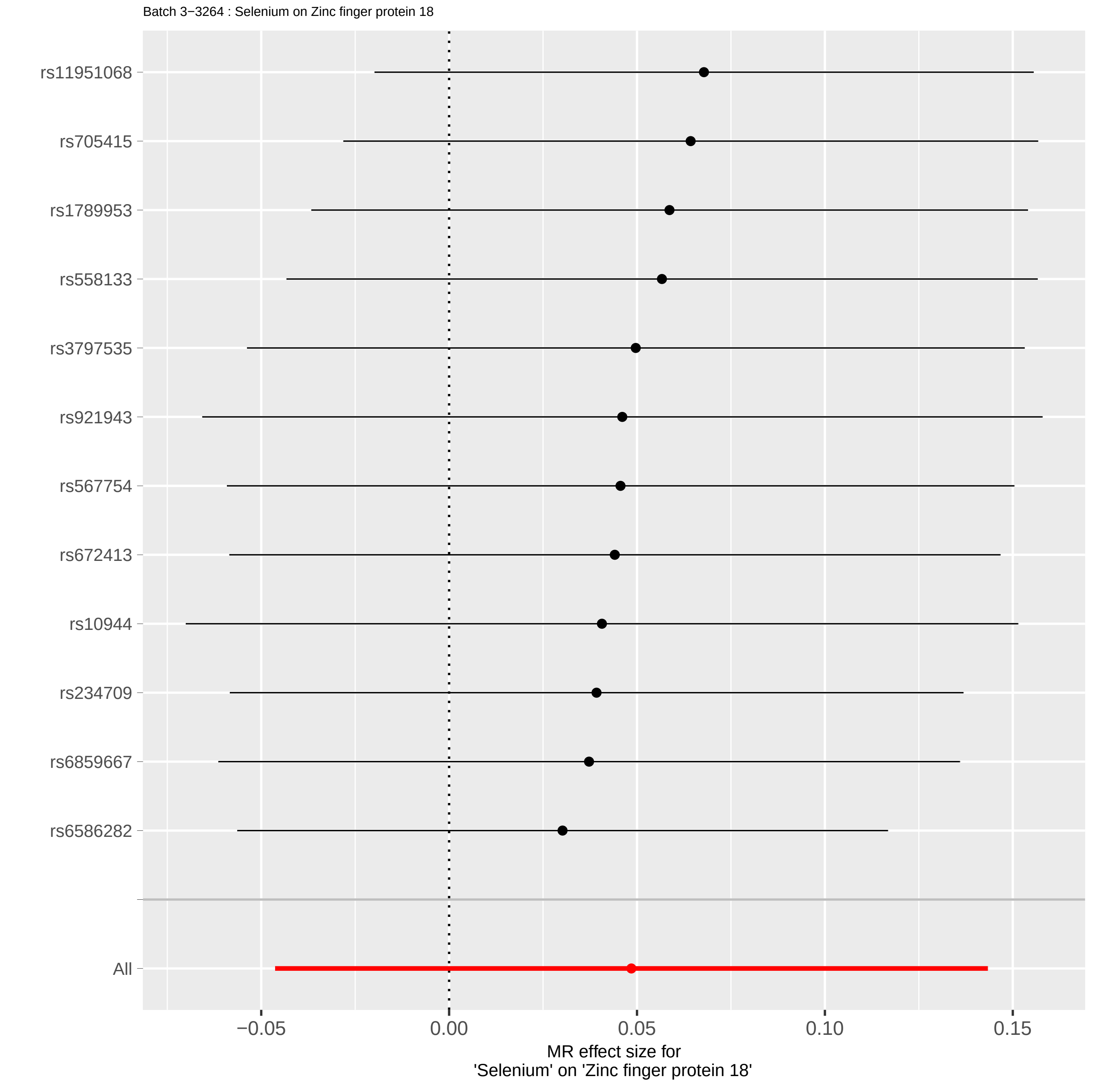

Batch 3−3264 : Selenium on Zinc finger protein 18
rs11951068
rs705415
rs1789953
rs558133
rs3797535
rs921943
rs567754
rs672413
rs10944
rs234709
rs6859667
rs6586282
All
−0.05
0.00
0.05
0.10
0.15
MR effect size for
'Selenium' on 'Zinc finger protein 18'

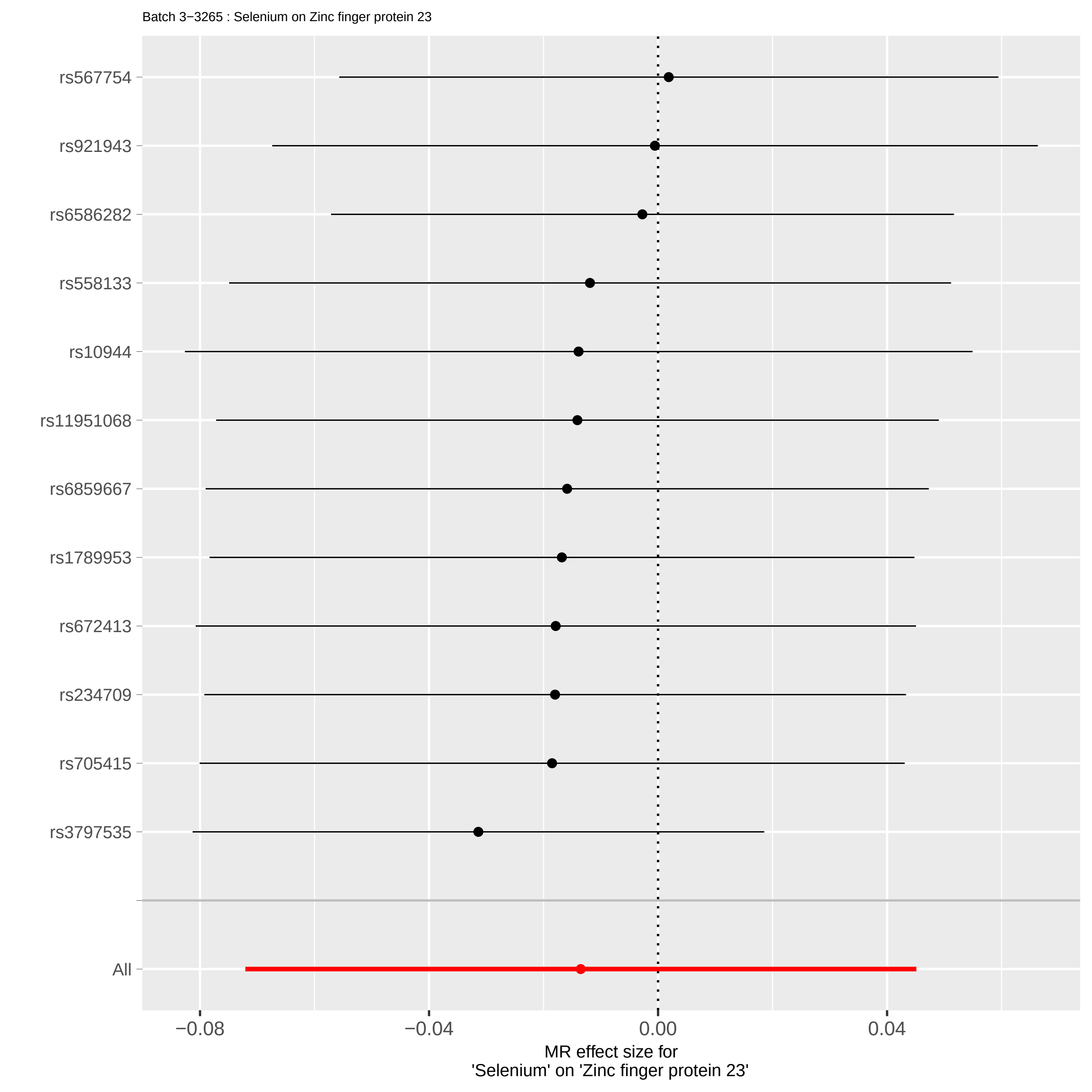
Batch 3−3265 : Selenium on Zinc finger protein 23
rs567754
rs921943
rs6586282
rs558133
rs10944
rs11951068
rs6859667
rs1789953
rs672413
rs234709
rs705415
rs3797535
All
−0.08
−0.04
0.00
0.04
MR effect size for
'Selenium' on 'Zinc finger protein 23'

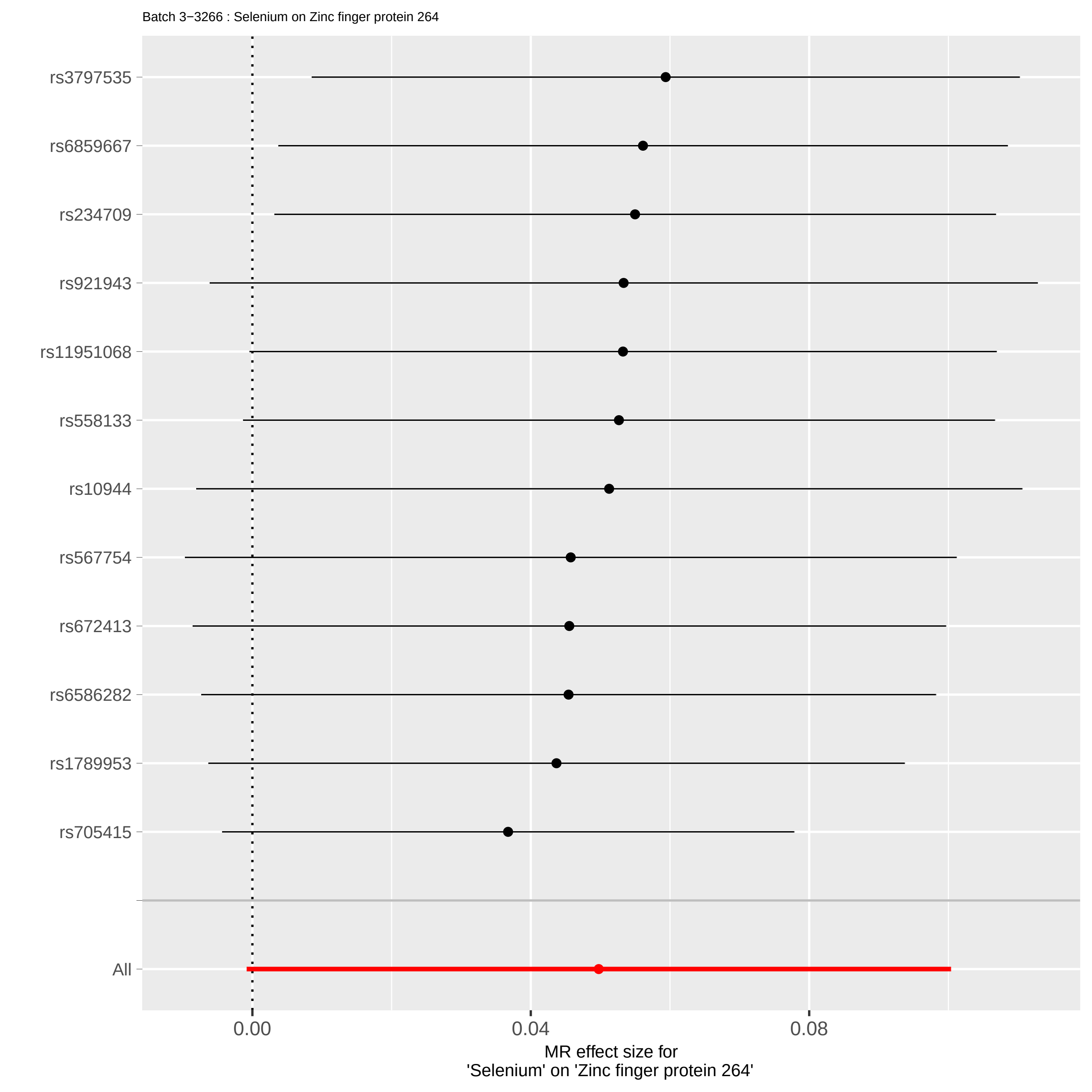

Batch 3−3266 : Selenium on Zinc finger protein 264
rs3797535
rs6859667
rs234709
rs921943
rs11951068
rs558133
rs10944
rs567754
rs672413
rs6586282
rs1789953
rs705415
All
0.00
0.04
0.08
MR effect size for
'Selenium' on 'Zinc finger protein 264'

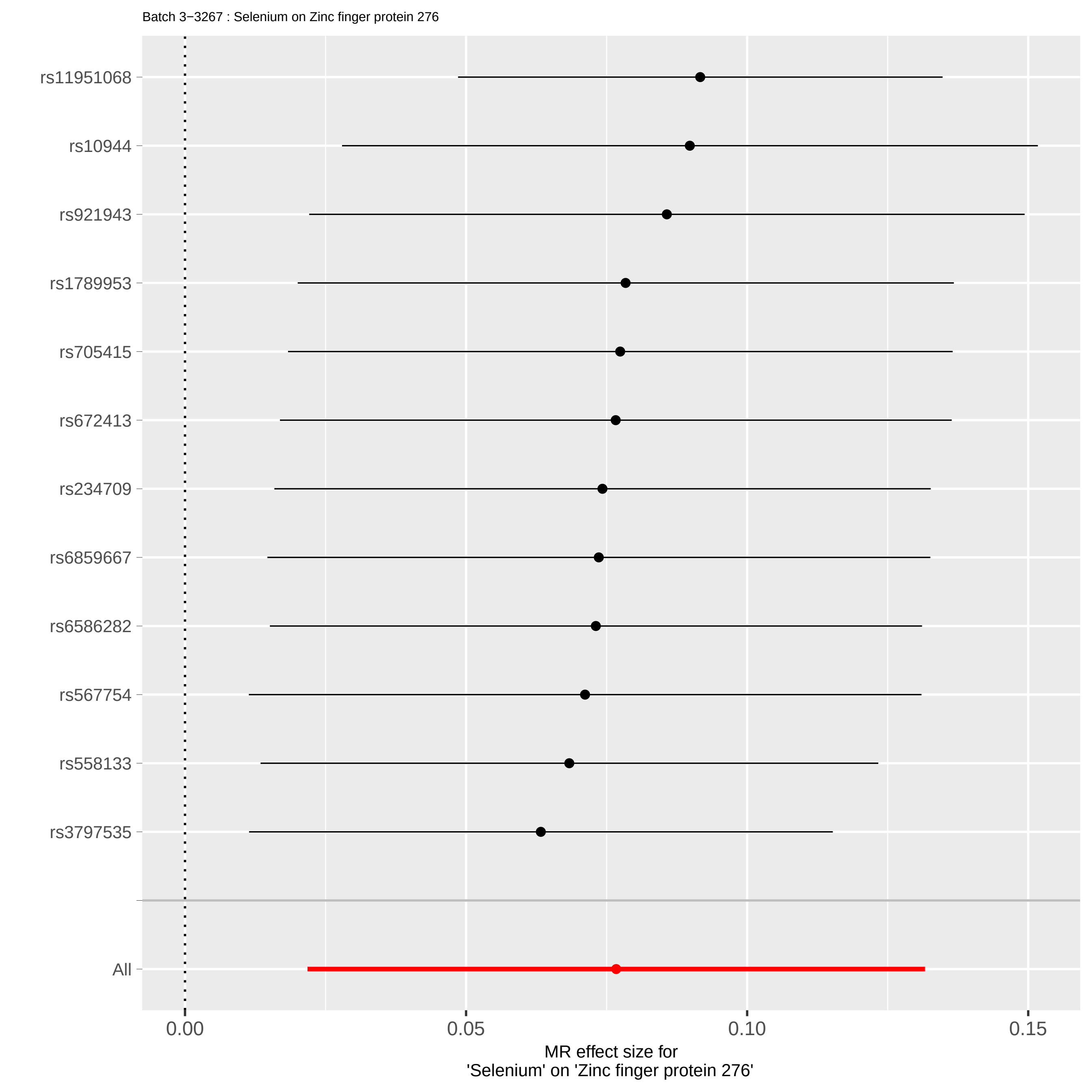
Batch 3−3267 : Selenium on Zinc finger protein 276
rs11951068
rs10944
rs921943
rs1789953
rs705415
rs672413
rs234709
rs6859667
rs6586282
rs567754
rs558133
rs3797535
All
0.00
0.05
0.10
0.15
MR effect size for
'Selenium' on 'Zinc finger protein 276'

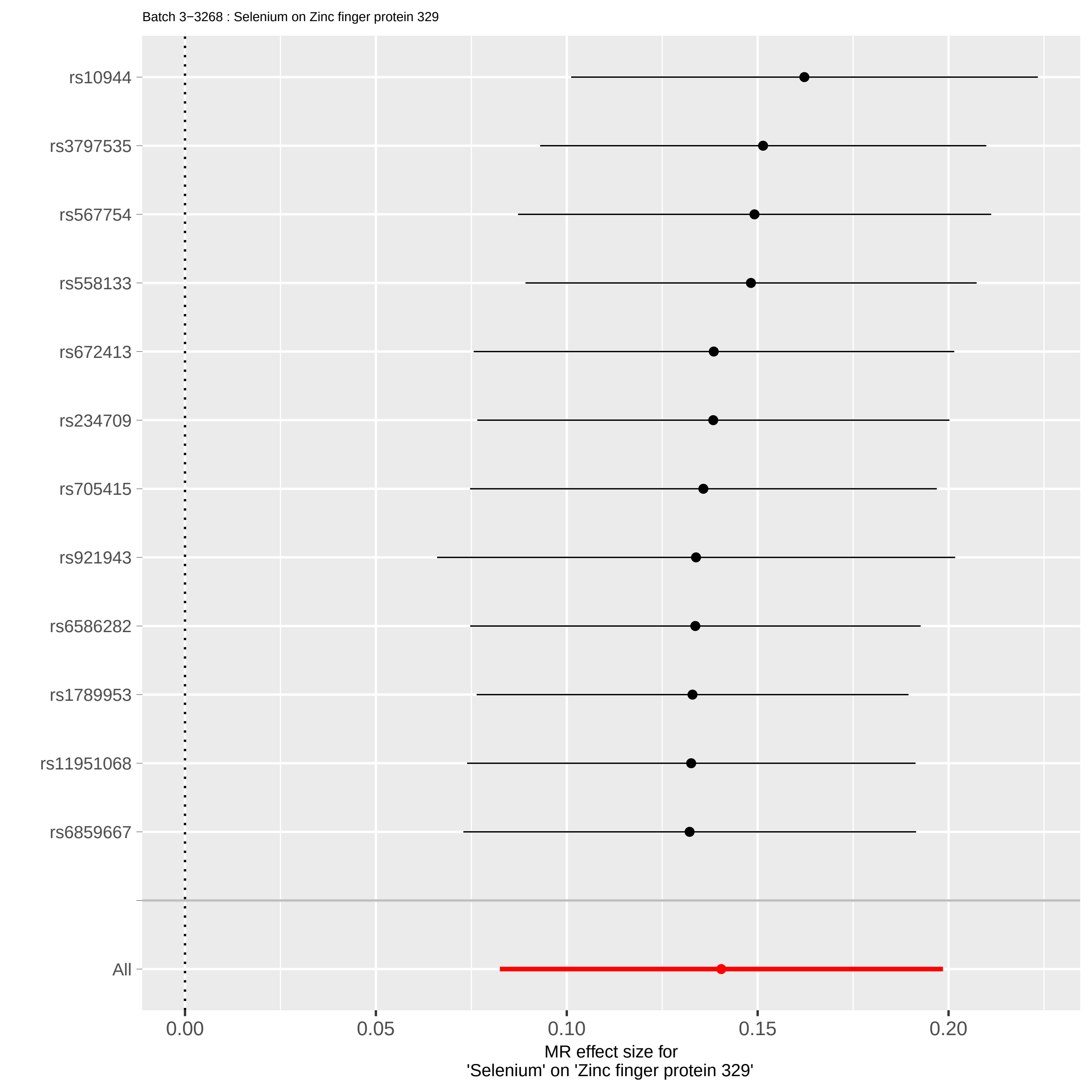

Batch 3−3268 : Selenium on Zinc finger protein 329
rs10944
rs3797535
rs567754
rs558133
rs672413
rs234709
rs705415
rs921943
rs6586282
rs1789953
rs11951068
rs6859667
All
0.00
0.05
0.10
0.15
0.20
MR effect size for
'Selenium' on 'Zinc finger protein 329'

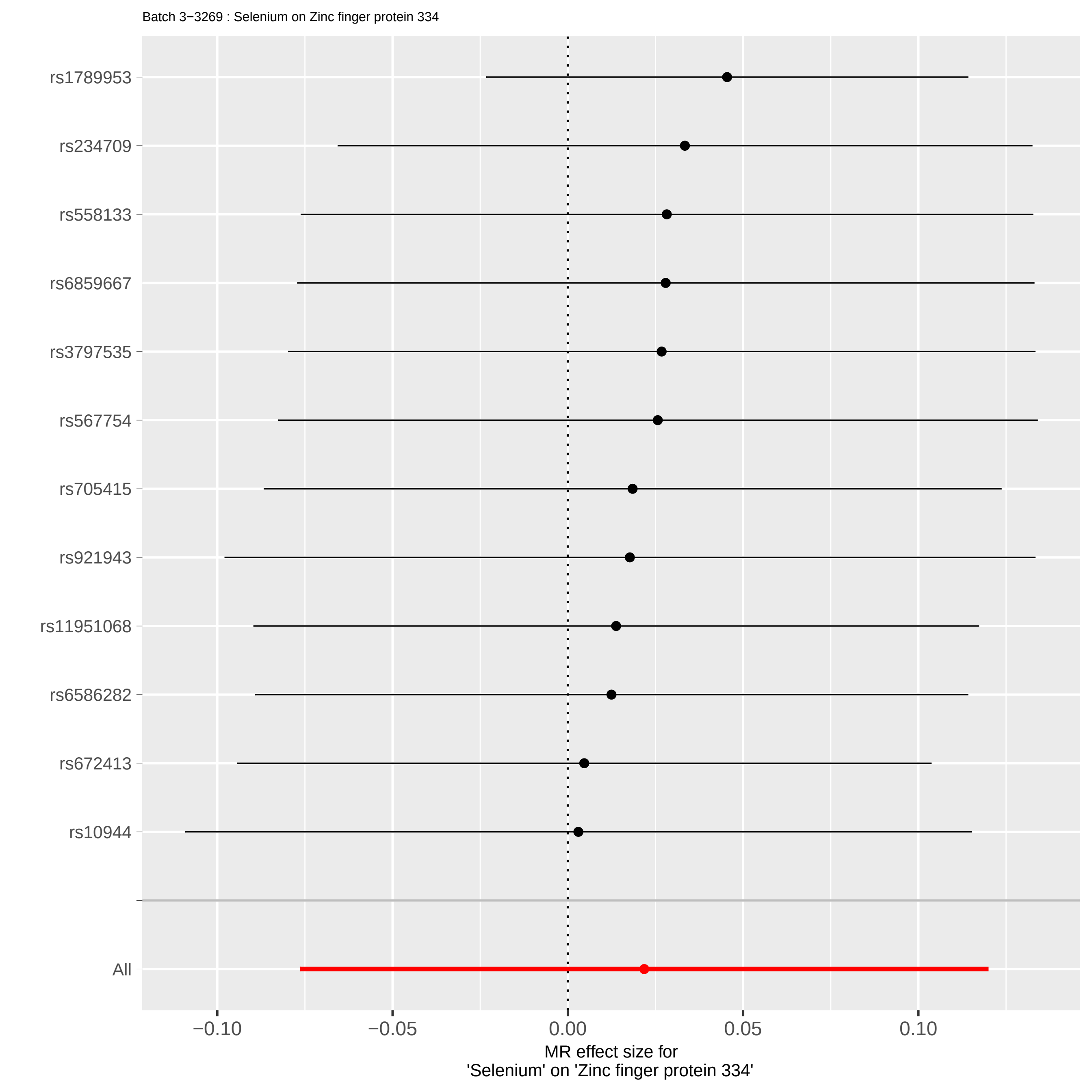

Batch 3−3269 : Selenium on Zinc finger protein 334
rs1789953
rs234709
rs558133
rs6859667
rs3797535
rs567754
rs705415
rs921943
rs11951068
rs6586282
rs672413
rs10944
All
−0.10
−0.05
0.00
0.05
0.10
MR effect size for
'Selenium' on 'Zinc finger protein 334'

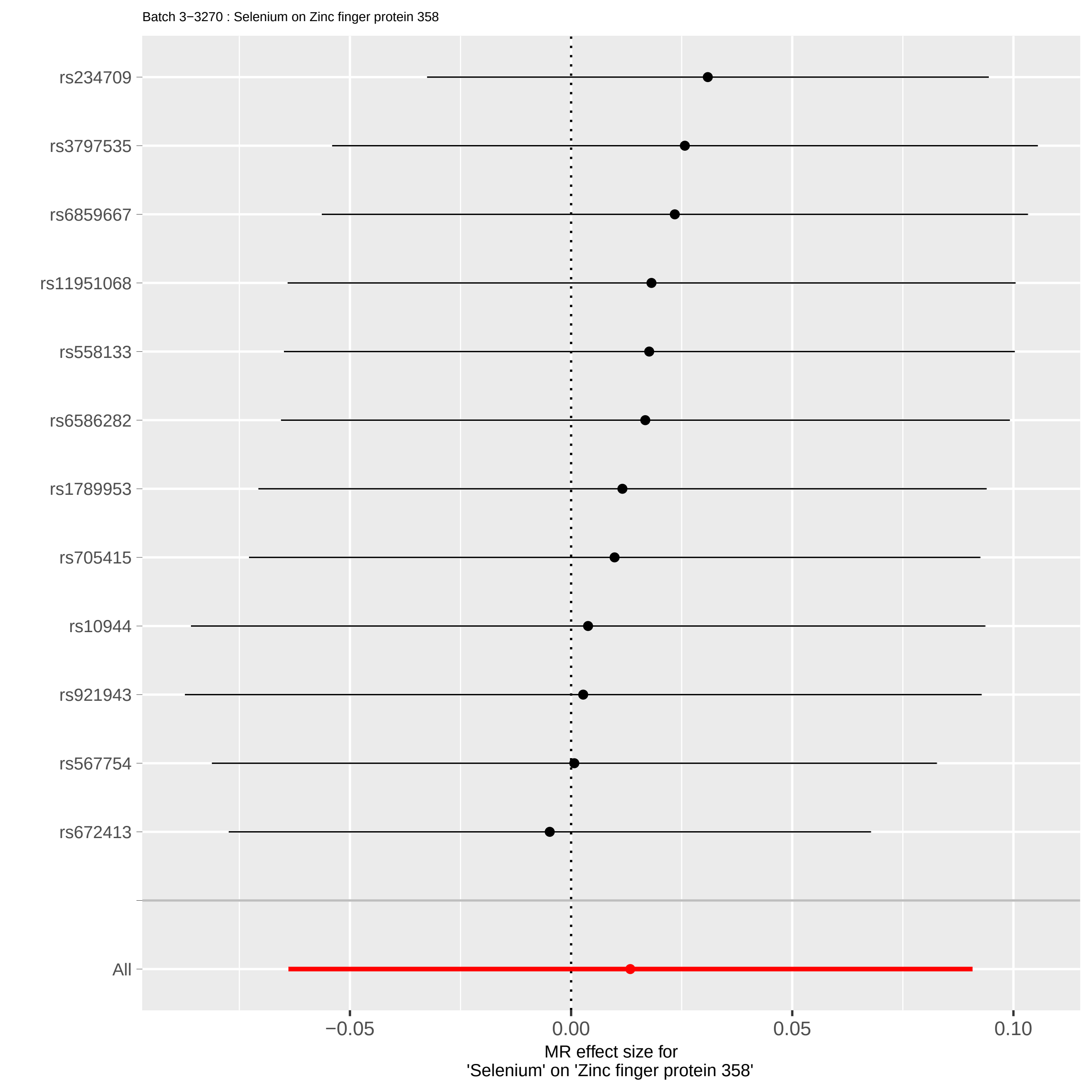

Batch 3−3270 : Selenium on Zinc finger protein 358
rs234709
rs3797535
rs6859667
rs11951068
rs558133
rs6586282
rs1789953
rs705415
rs10944
rs921943
rs567754
rs672413
All
−0.05
0.00
0.05
0.10
MR effect size for
'Selenium' on 'Zinc finger protein 358'

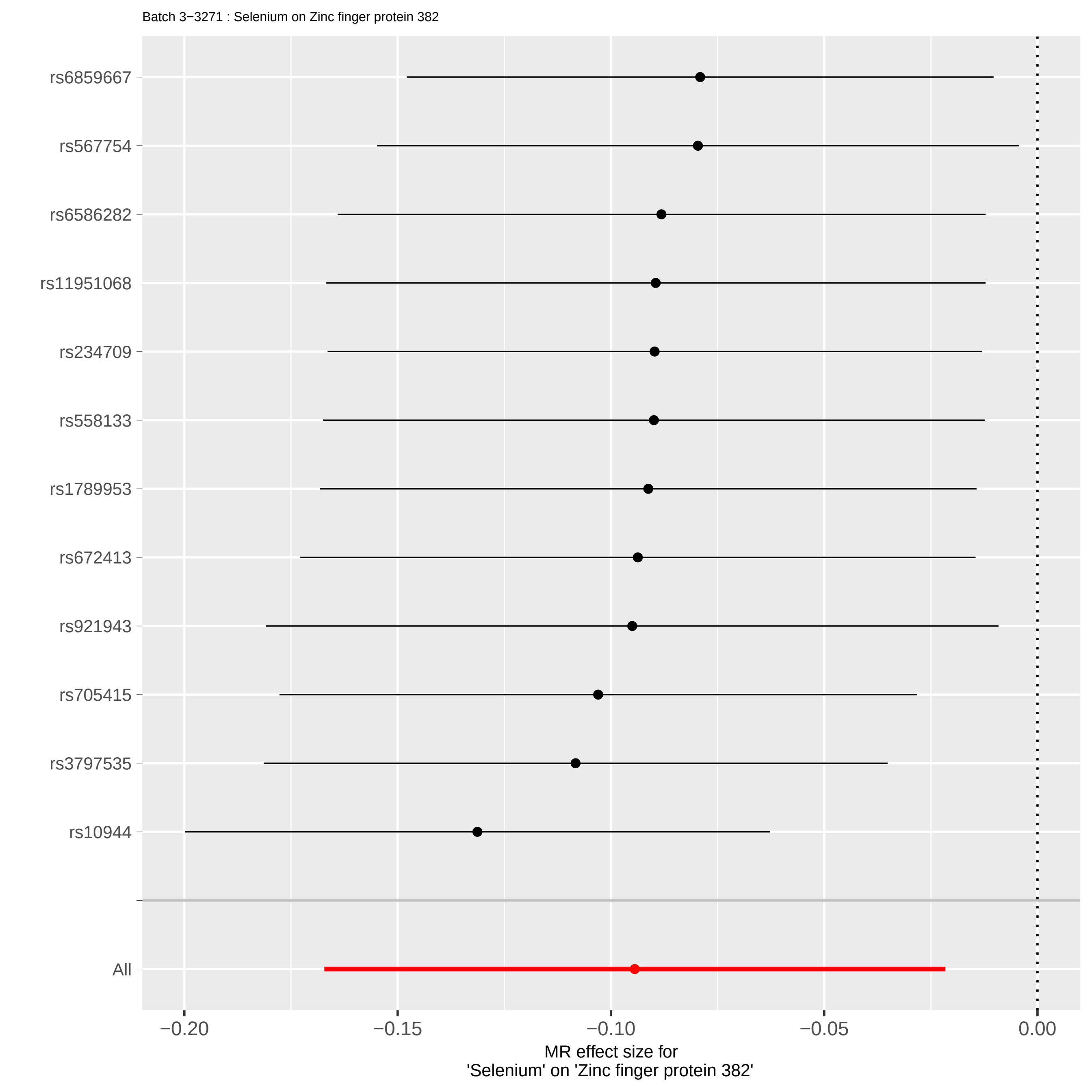
Batch 3−3271 : Selenium on Zinc finger protein 382
rs6859667
rs567754
rs6586282
rs11951068
rs234709
rs558133
rs1789953
rs672413
rs921943
rs705415
rs3797535
rs10944
All
−0.20
−0.15
−0.10
−0.05
0.00
MR effect size for
'Selenium' on 'Zinc finger protein 382'

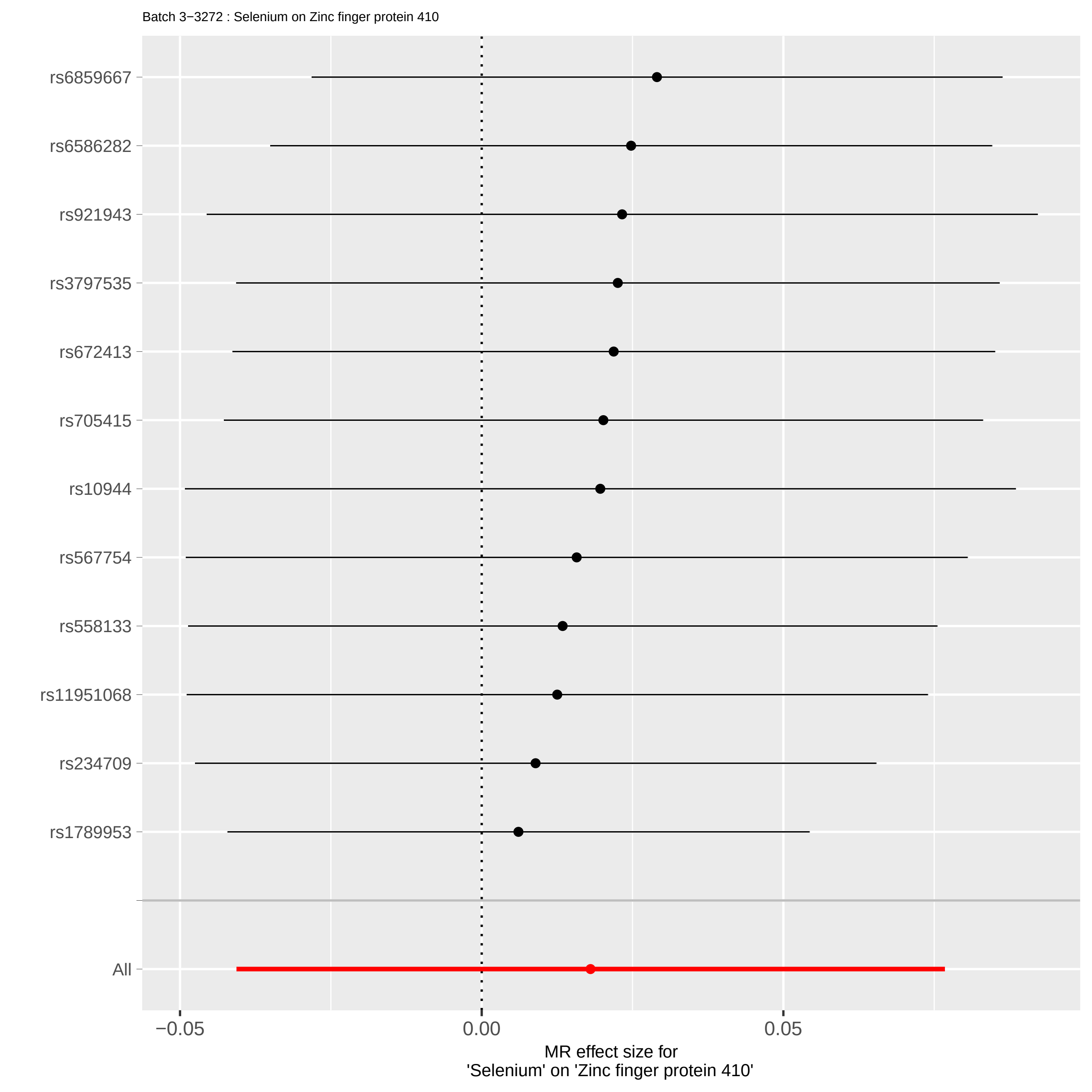

Batch 3−3272 : Selenium on Zinc finger protein 410
rs6859667
rs6586282
rs921943
rs3797535
rs672413
rs705415
rs10944
rs567754
rs558133
rs11951068
rs234709
rs1789953
All
−0.05
0.00
0.05
MR effect size for
'Selenium' on 'Zinc finger protein 410'

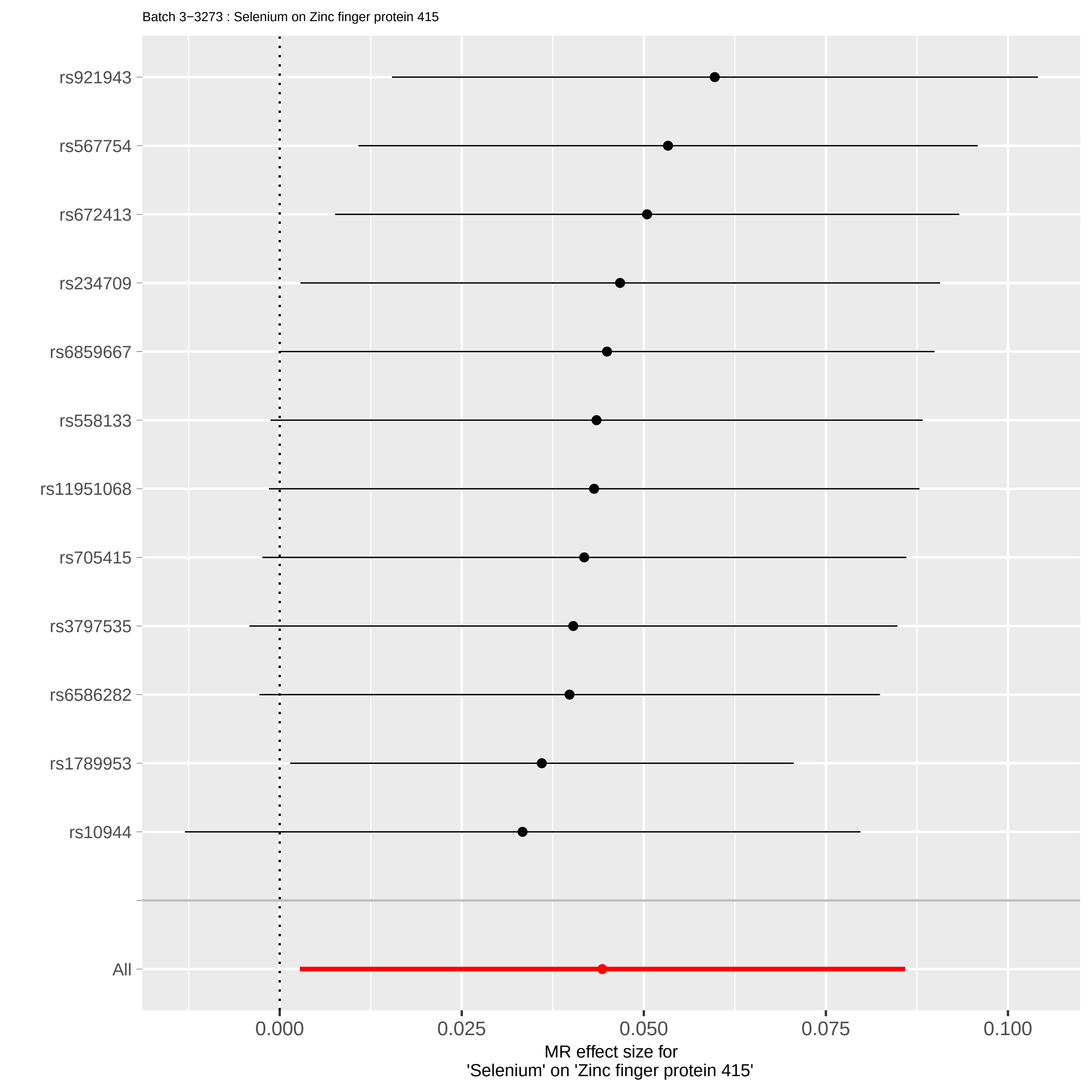

Batch 3−3273 : Selenium on Zinc finger protein 415
rs921943
rs567754
rs672413
rs234709
rs6859667
rs558133
rs11951068
rs705415
rs3797535
rs6586282
rs1789953
rs10944
All
0.000
0.025
0.050
0.075
0.100
MR effect size for
'Selenium' on 'Zinc finger protein 415'

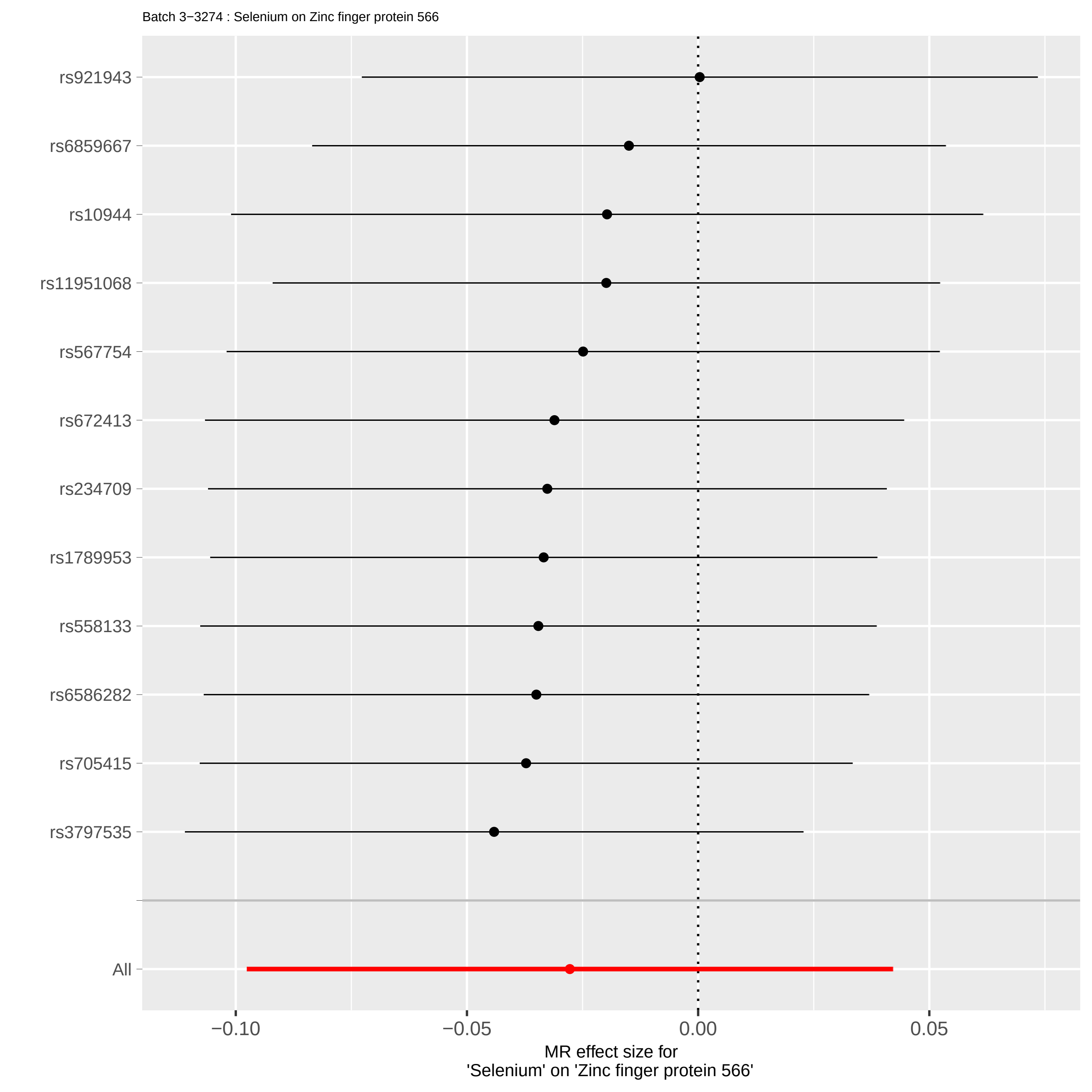

Batch 3−3274 : Selenium on Zinc finger protein 566
rs921943
rs6859667
rs10944
rs11951068
rs567754
rs672413
rs234709
rs1789953
rs558133
rs6586282
rs705415
rs3797535
All
−0.10
−0.05
0.00
0.05
MR effect size for
'Selenium' on 'Zinc finger protein 566'

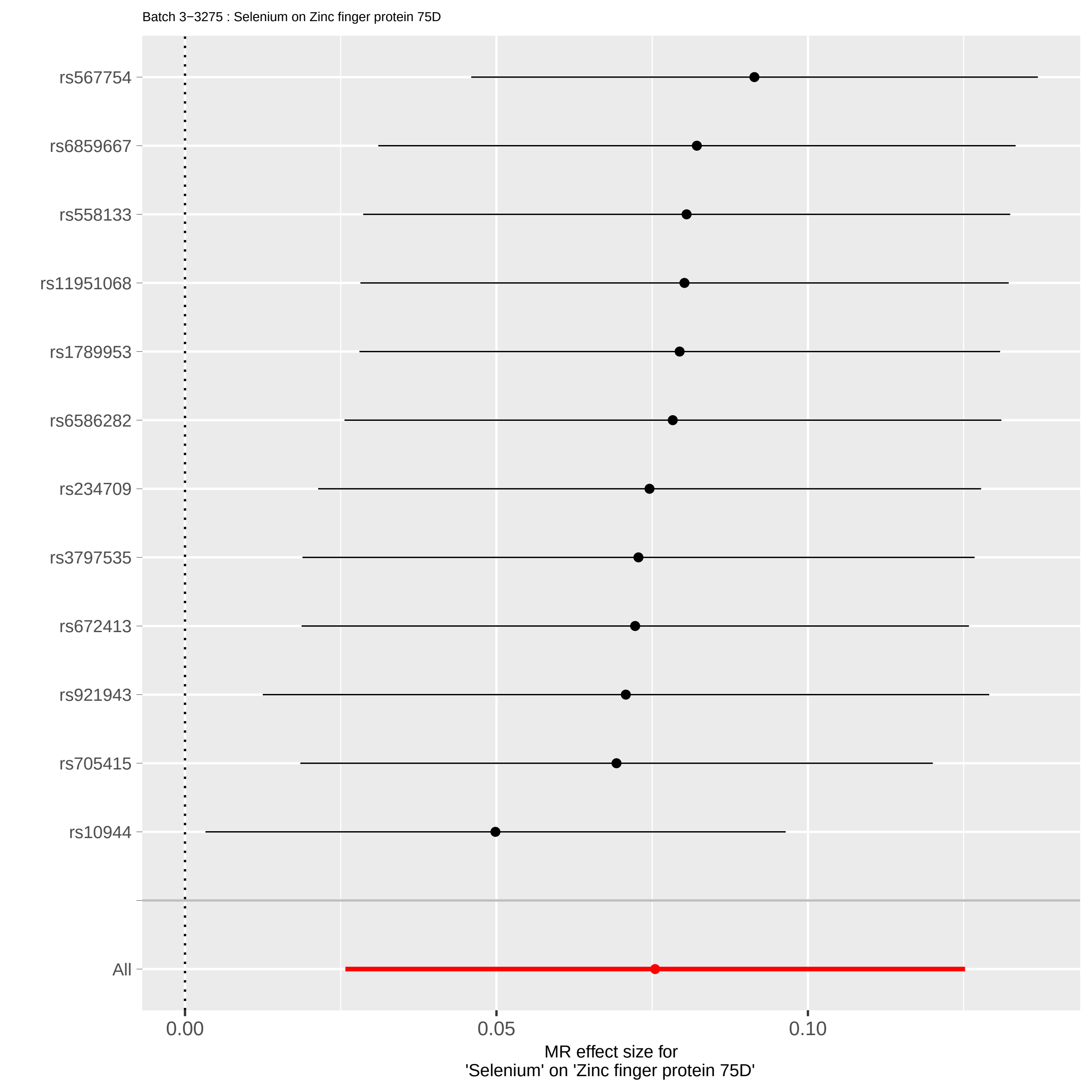
Batch 3−3275 : Selenium on Zinc finger protein 75D
rs567754
rs6859667
rs558133
rs11951068
rs1789953
rs6586282
rs234709
rs3797535
rs672413
rs921943
rs705415
rs10944
All
0.00
0.05
0.10
MR effect size for
'Selenium' on 'Zinc finger protein 75D'

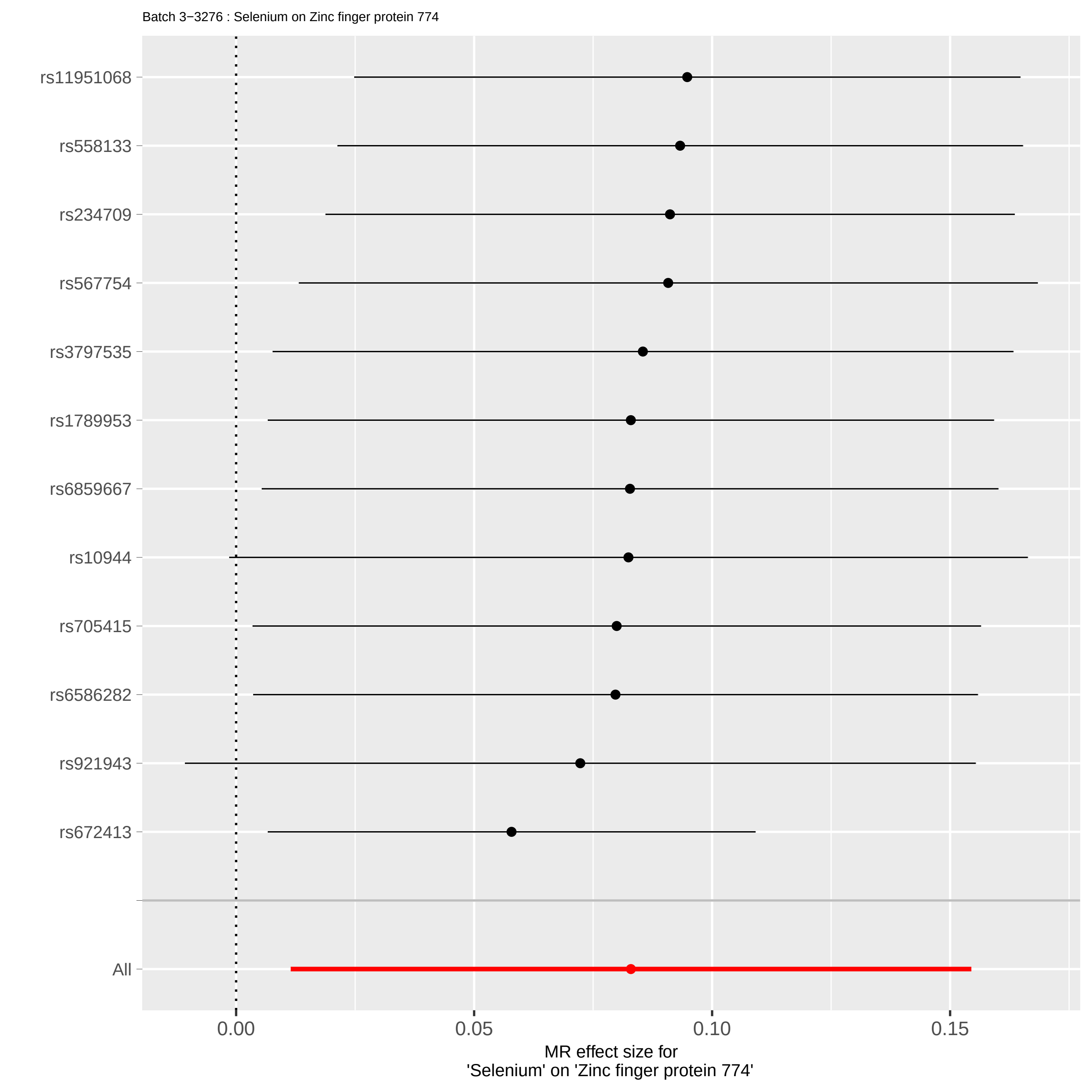

Batch 3−3276 : Selenium on Zinc finger protein 774
rs11951068
rs558133
rs234709
rs567754
rs3797535
rs1789953
rs6859667
rs10944
rs705415
rs6586282
rs921943
rs672413
All
0.00
0.05
0.10
0.15
MR effect size for
'Selenium' on 'Zinc finger protein 774'

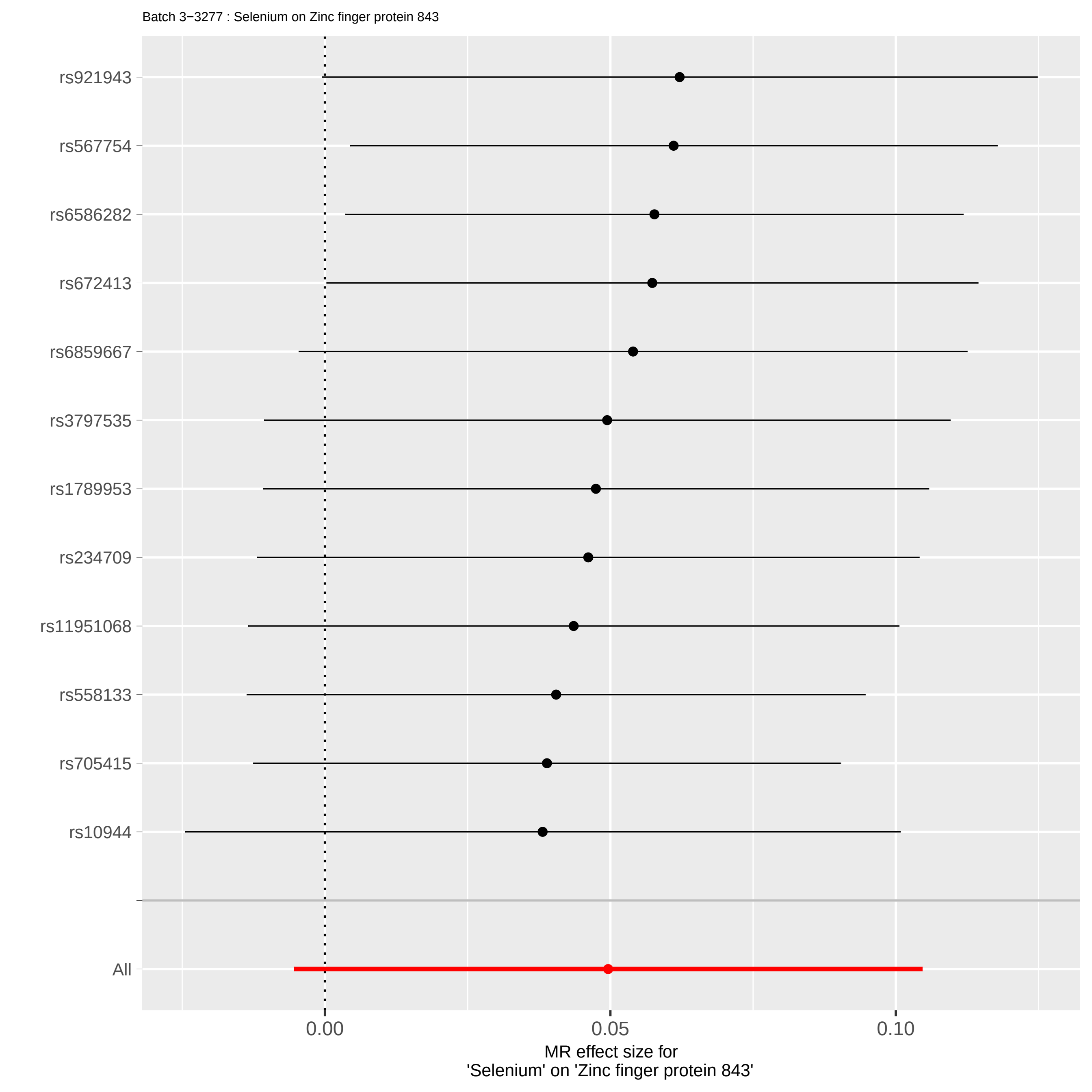

Batch 3−3277 : Selenium on Zinc finger protein 843
rs921943
rs567754
rs6586282
rs672413
rs6859667
rs3797535
rs1789953
rs234709
rs11951068
rs558133
rs705415
rs10944
All
0.00
0.05
0.10
MR effect size for
'Selenium' on 'Zinc finger protein 843'

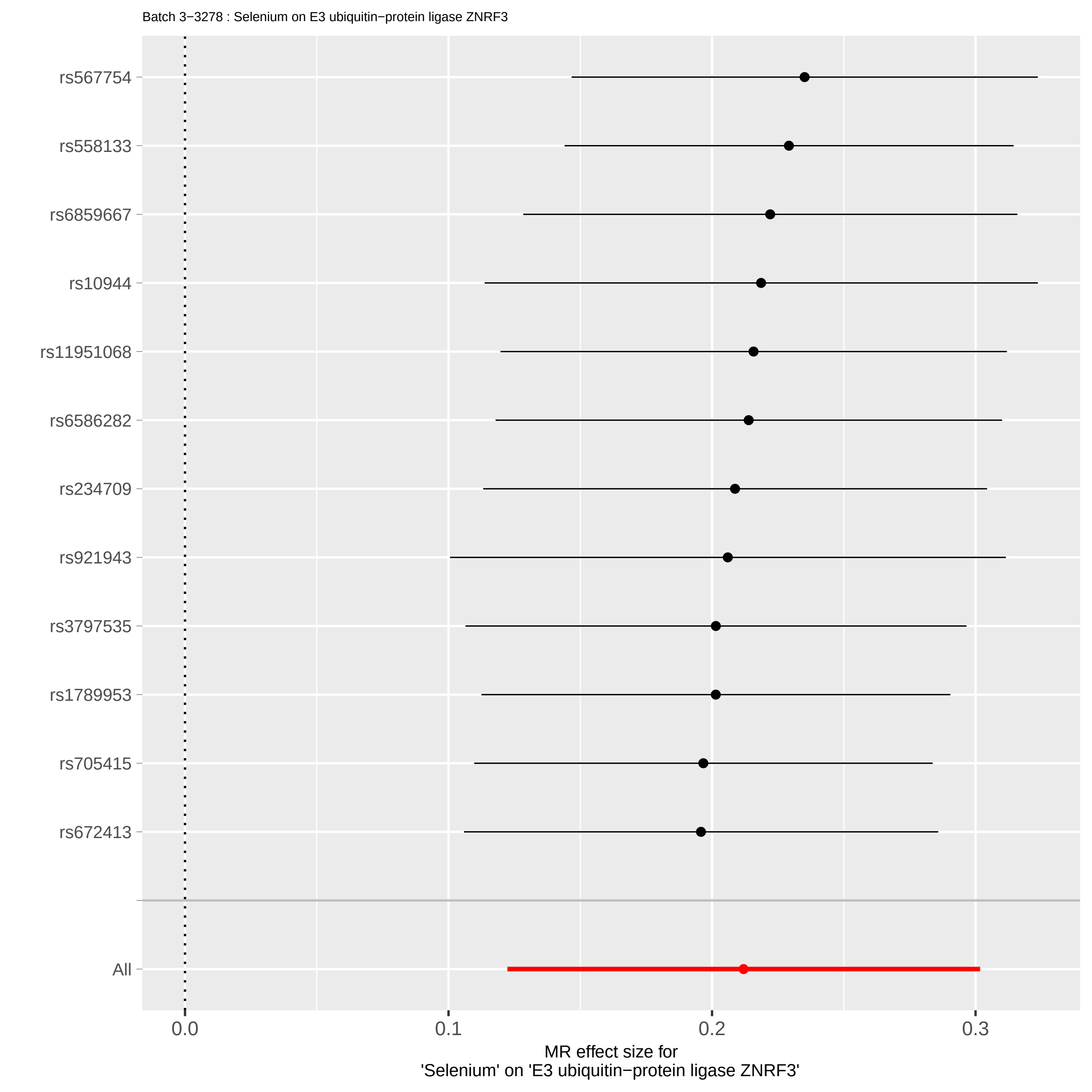
Batch 3−3278 : Selenium on E3 ubiquitin−protein ligase ZNRF3
rs567754
rs558133
rs6859667
rs10944
rs11951068
rs6586282
rs234709
rs921943
rs3797535
rs1789953
rs705415
rs672413
All
0.0
0.1
0.2
0.3
MR effect size for
'Selenium' on 'E3 ubiquitin−protein ligase ZNRF3'

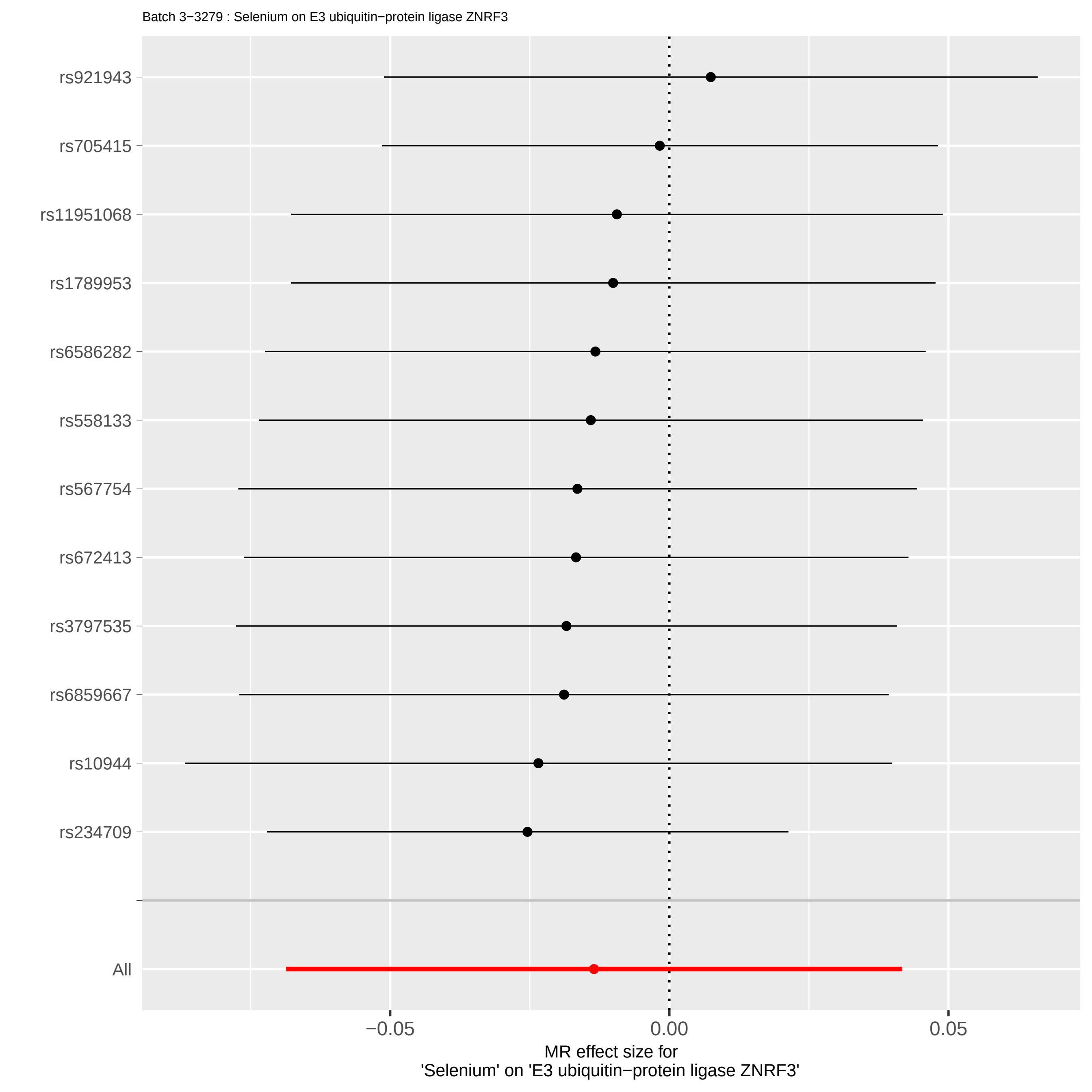

Batch 3−3279 : Selenium on E3 ubiquitin−protein ligase ZNRF3
rs921943
rs705415
rs11951068
rs1789953
rs6586282
rs558133
rs567754
rs672413
rs3797535
rs6859667
rs10944
rs234709
All
−0.05
0.00
0.05
MR effect size for
'Selenium' on 'E3 ubiquitin−protein ligase ZNRF3'

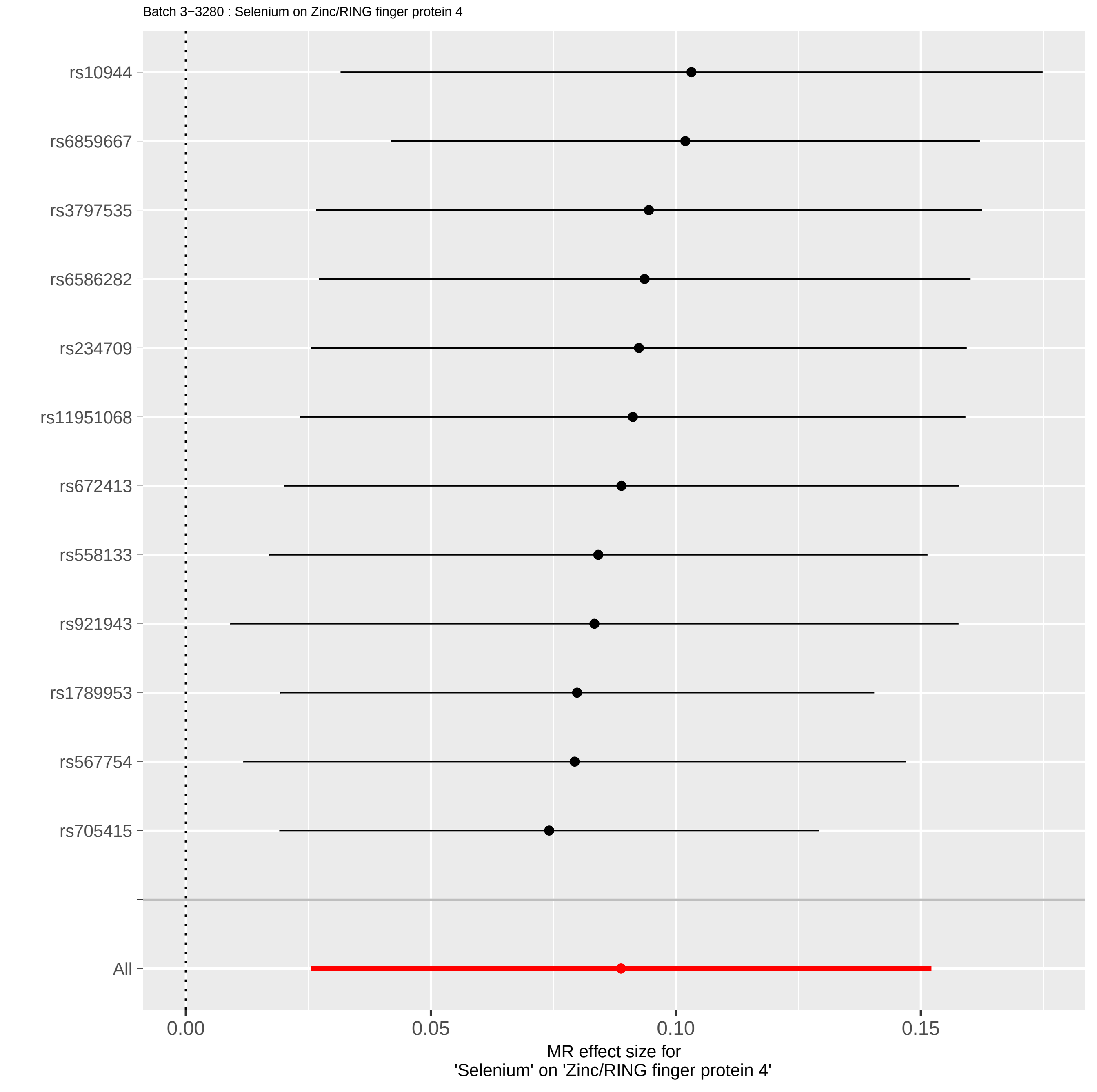
Batch 3−3280 : Selenium on Zinc/RING finger protein 4
rs10944
rs6859667
rs3797535
rs6586282
rs234709
rs11951068
rs672413
rs558133
rs921943
rs1789953
rs567754
rs705415
All
0.00
0.05
0.10
0.15
MR effect size for
'Selenium' on 'Zinc/RING finger protein 4'

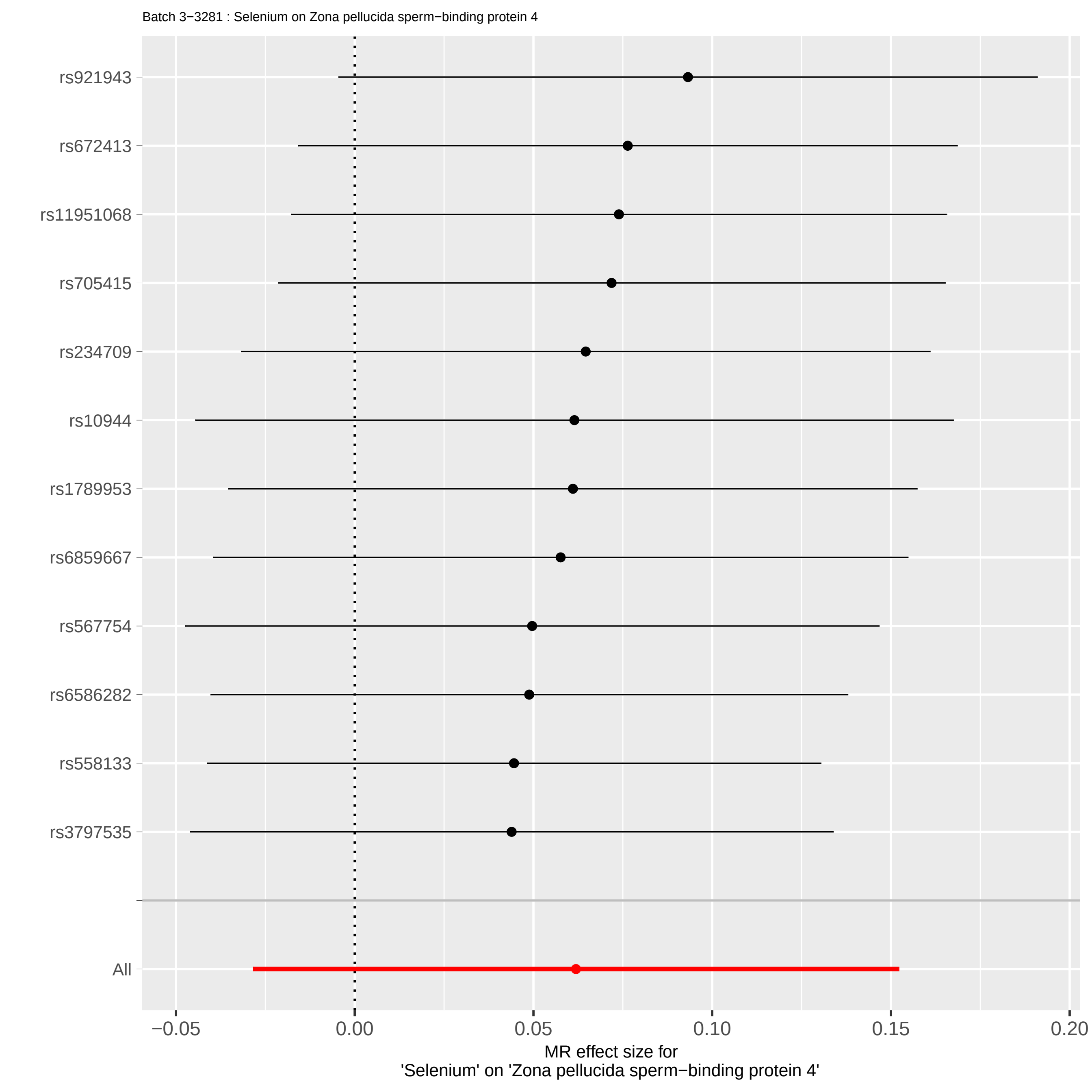

Batch 3−3281 : Selenium on Zona pellucida sperm−binding protein 4
rs921943
rs672413
rs11951068
rs705415
rs234709
rs10944
rs1789953
rs6859667
rs567754
rs6586282
rs558133
rs3797535
All
−0.05
0.00
0.05
0.10
0.15
0.20
MR effect size for
'Selenium' on 'Zona pellucida sperm−binding protein 4'

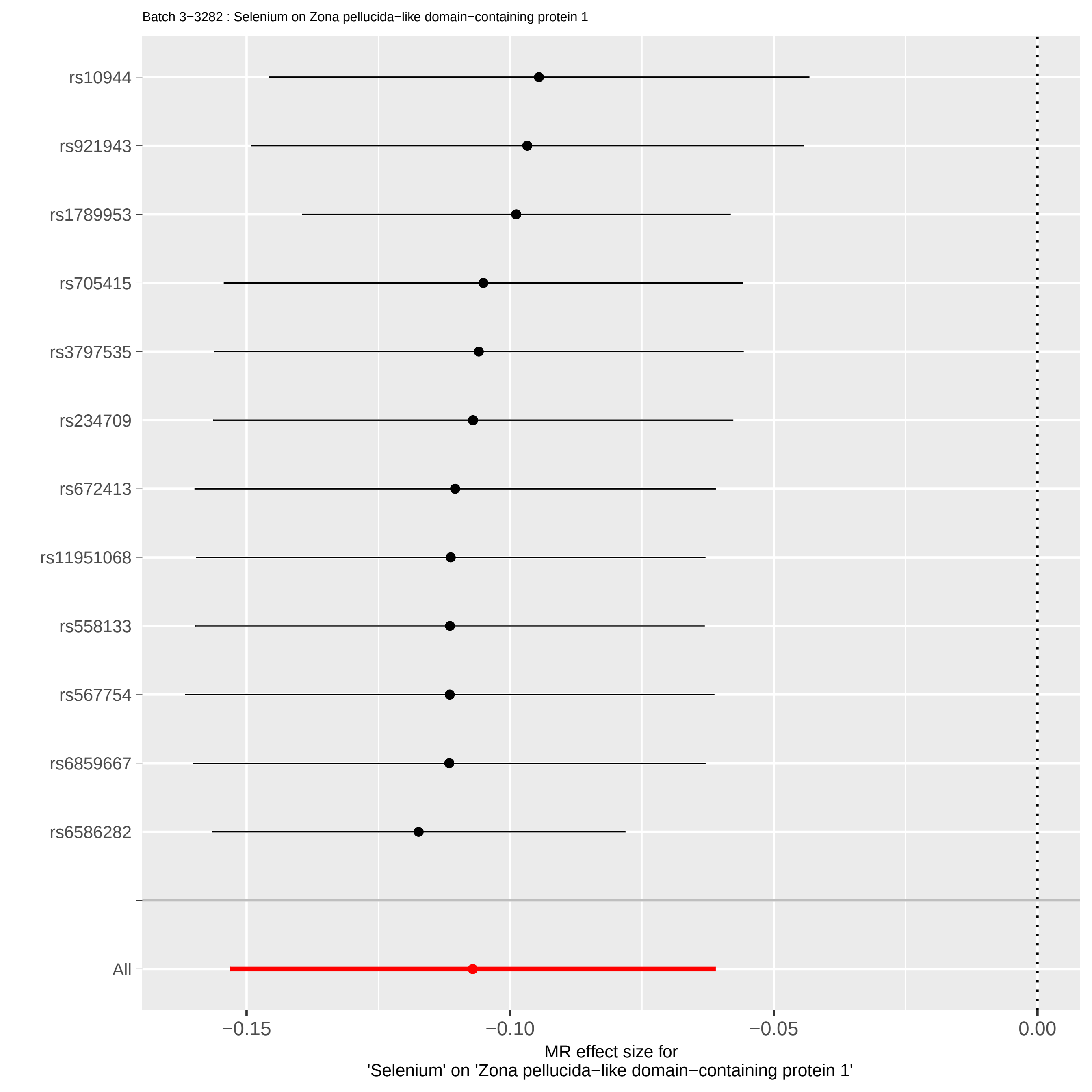

Batch 3−3282 : Selenium on Zona pellucida−like domain−containing protein 1
rs10944
rs921943
rs1789953
rs705415
rs3797535
rs234709
rs672413
rs11951068
rs558133
rs567754
rs6859667
rs6586282
All
−0.15
−0.10
−0.05
0.00
MR effect size for
'Selenium' on 'Zona pellucida−like domain−containing protein 1'

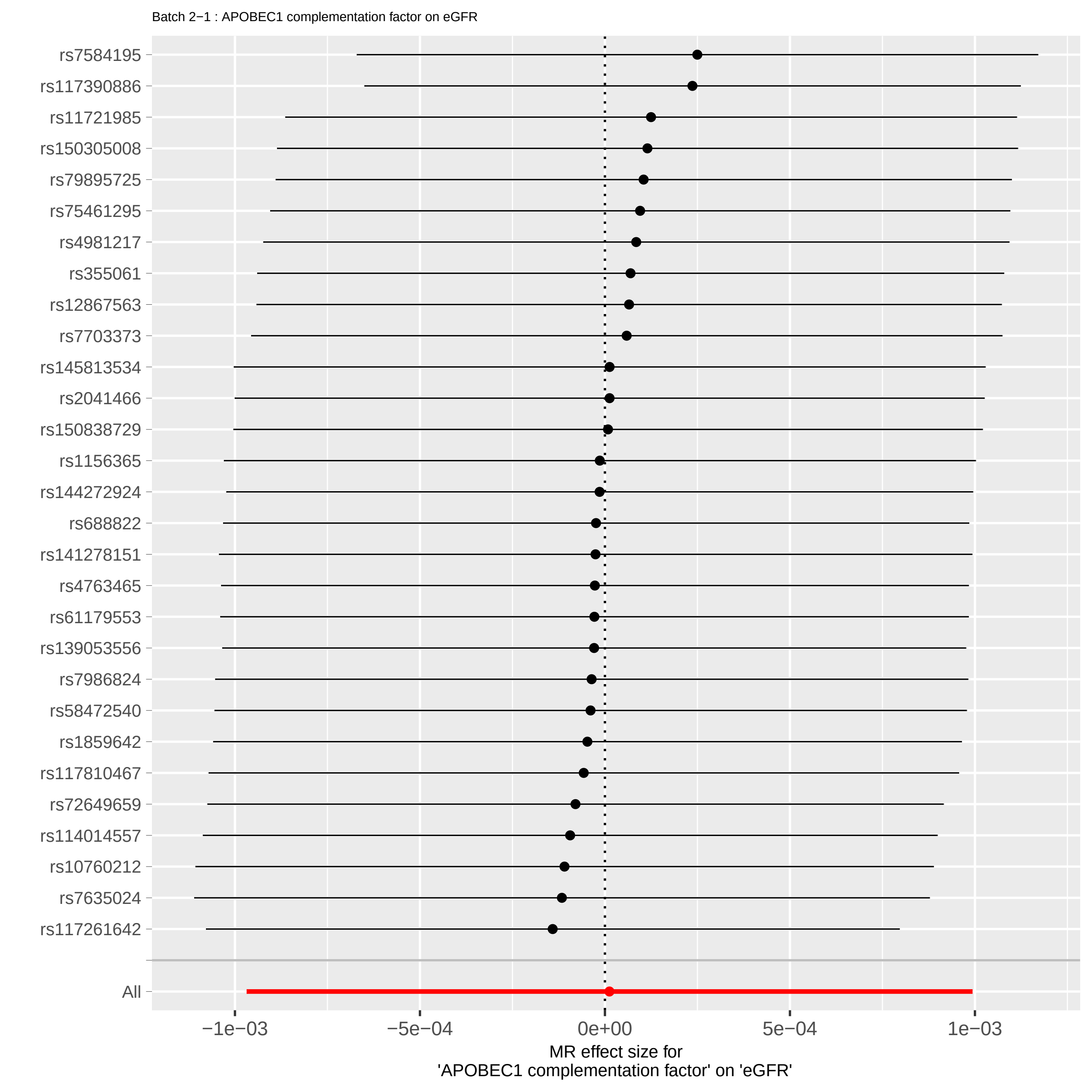

Batch 2−1 : APOBEC1 complementation factor on eGFR
rs7584195
rs117390886
rs11721985
rs150305008
rs79895725
rs75461295
rs4981217
rs355061
rs12867563
rs7703373
rs145813534
rs2041466
rs150838729
rs1156365
rs144272924
rs688822
rs141278151
rs4763465
rs61179553
rs139053556
rs7986824
rs58472540
rs1859642
rs117810467
rs72649659
rs114014557
rs10760212
rs7635024
rs117261642
All
−1e−03
−5e−04
0e+00
5e−04
1e−03
MR effect size for
'APOBEC1 complementation factor' on 'eGFR'

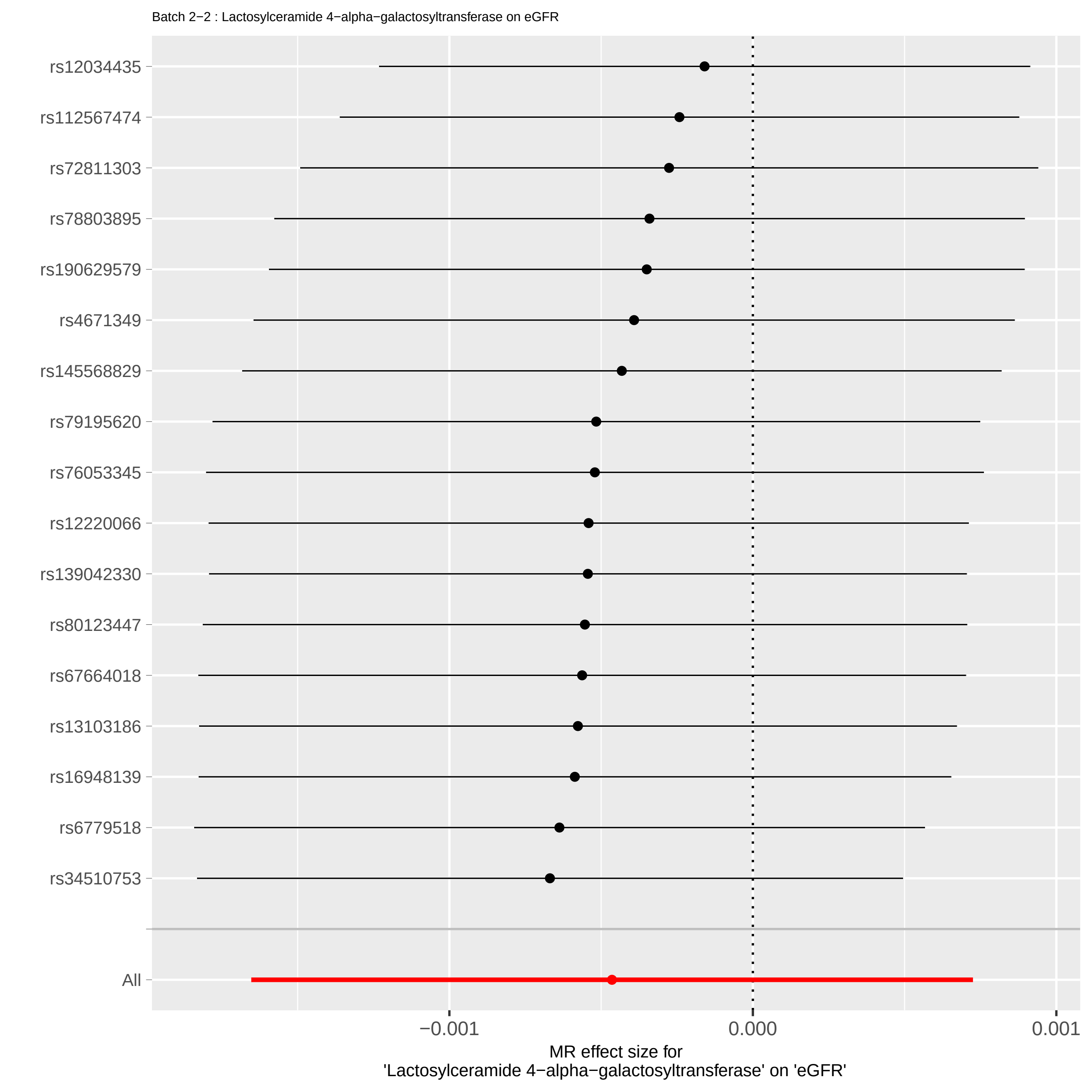

Batch 2−2 : Lactosylceramide 4−alpha−galactosyltransferase on eGFR
rs12034435
rs112567474
rs72811303
rs78803895
rs190629579
rs4671349
rs145568829
rs79195620
rs76053345
rs12220066
rs139042330
rs80123447
rs67664018
rs13103186
rs16948139
rs6779518
rs34510753
All
−0.001
0.000
0.001
MR effect size for
'Lactosylceramide 4−alpha−galactosyltransferase' on 'eGFR'

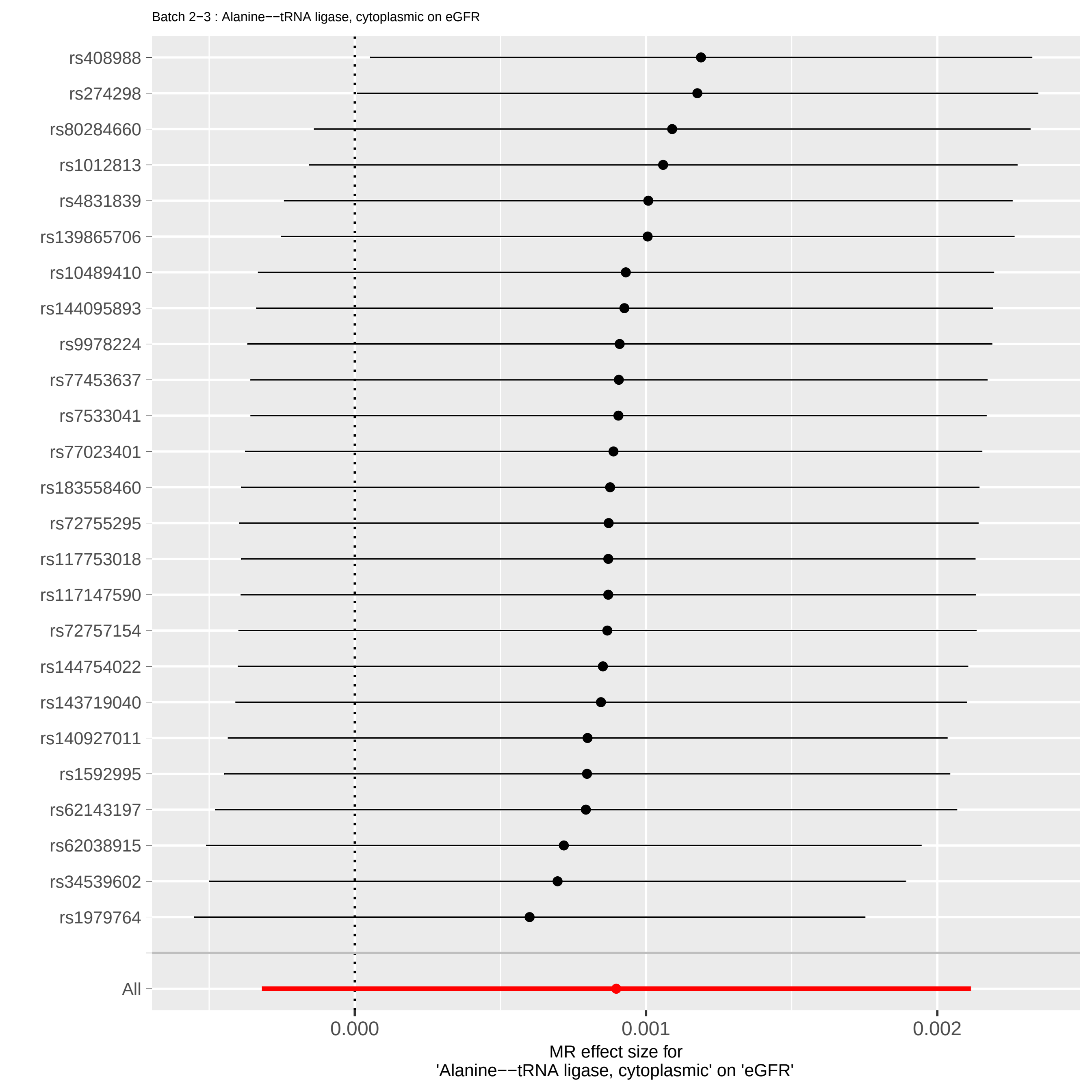

Batch 2−3 : Alanine−−tRNA ligase, cytoplasmic on eGFR
rs408988
rs274298
rs80284660
rs1012813
rs4831839
rs139865706
rs10489410
rs144095893
rs9978224
rs77453637
rs7533041
rs77023401
rs183558460
rs72755295
rs117753018
rs117147590
rs72757154
rs144754022
rs143719040
rs140927011
rs1592995
rs62143197
rs62038915
rs34539602
rs1979764
All
0.000
0.001
0.002
MR effect size for
'Alanine−−tRNA ligase, cytoplasmic' on 'eGFR'

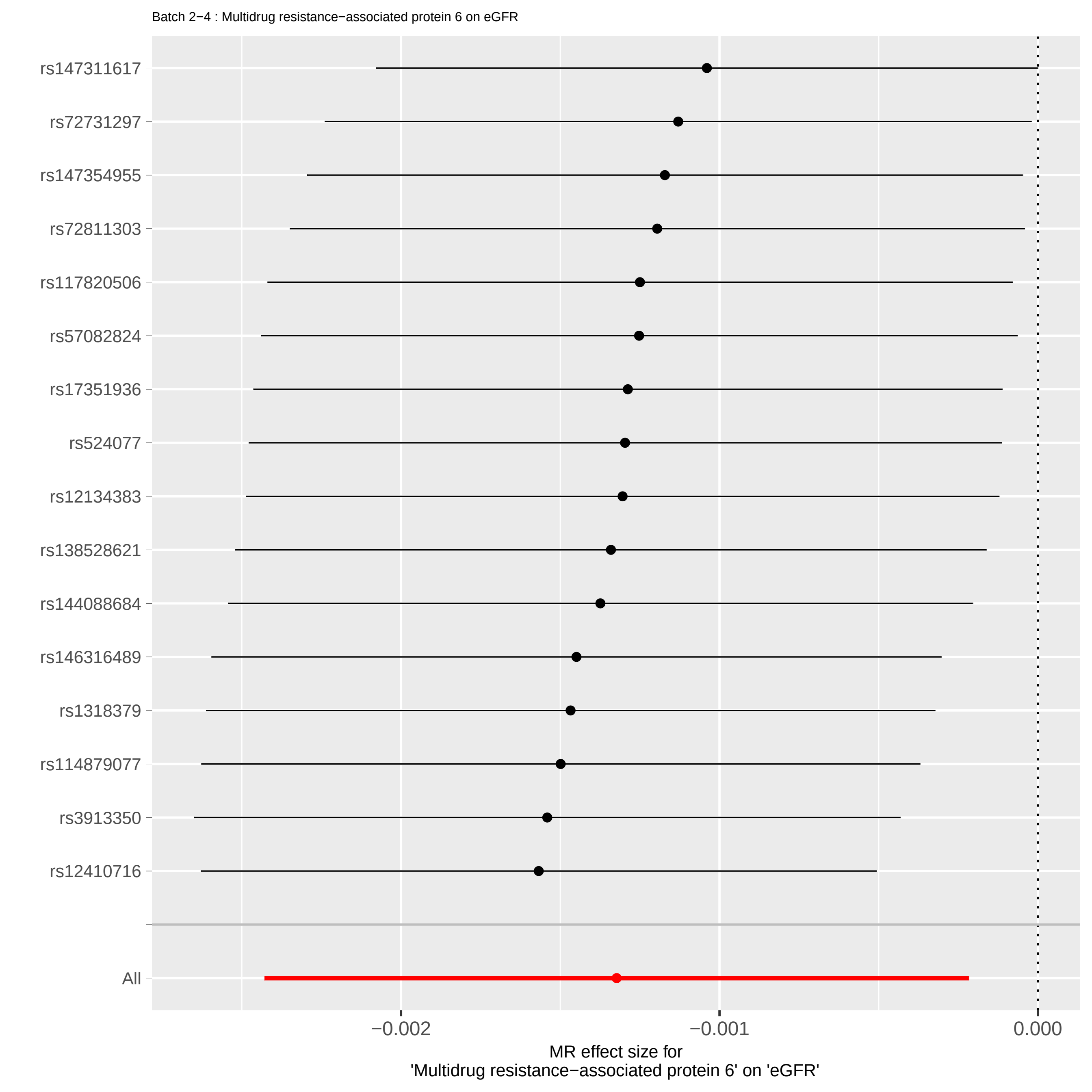

Batch 2−4 : Multidrug resistance−associated protein 6 on eGFR
rs147311617
rs72731297
rs147354955
rs72811303
rs117820506
rs57082824
rs17351936
rs524077
rs12134383
rs138528621
rs144088684
rs146316489
rs1318379
rs114879077
rs3913350
rs12410716
All
−0.002
−0.001
0.000
MR effect size for
'Multidrug resistance−associated protein 6' on 'eGFR'

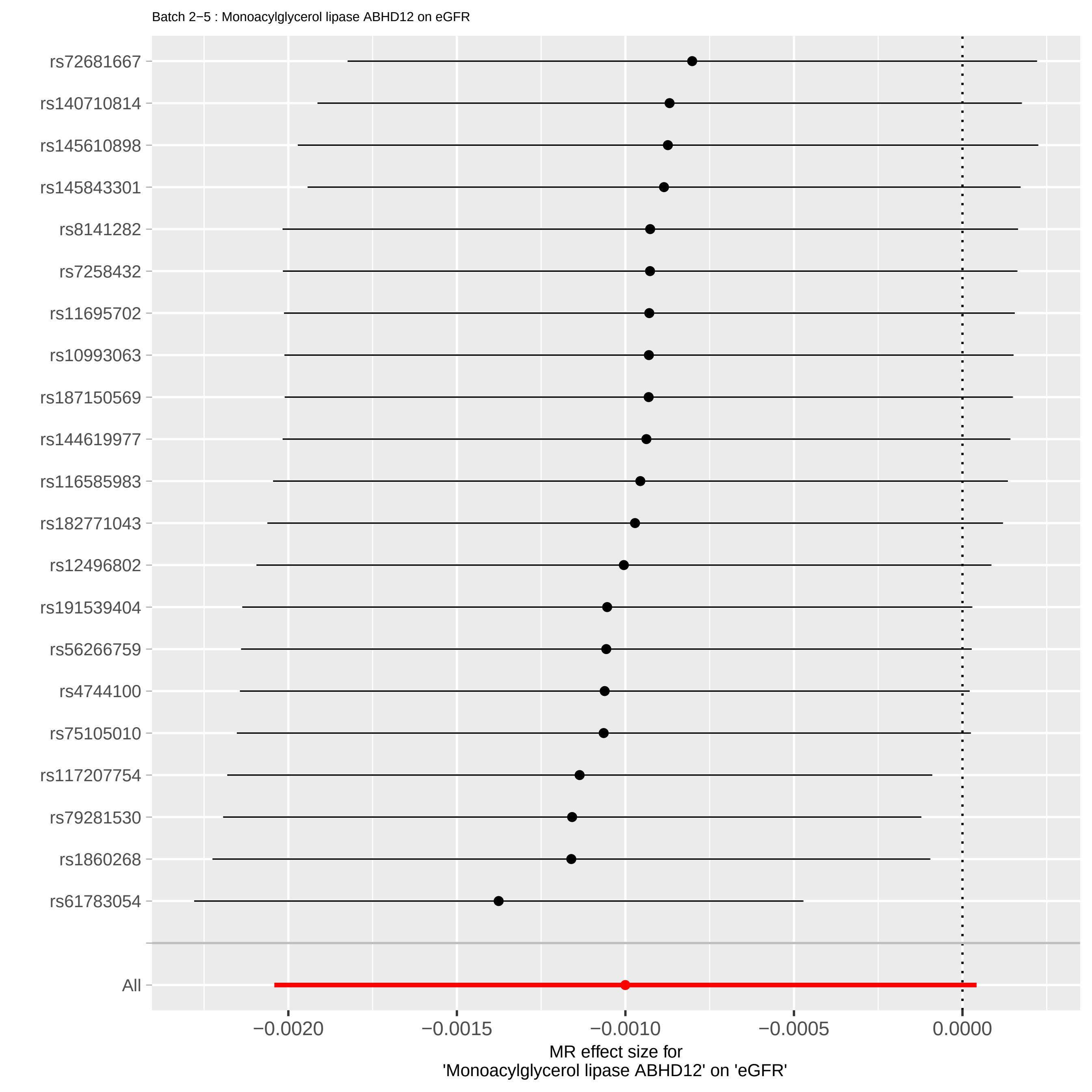

Batch 2−5 : Monoacylglycerol lipase ABHD12 on eGFR
rs72681667
rs140710814
rs145610898
rs145843301
rs8141282
rs7258432
rs11695702
rs10993063
rs187150569
rs144619977
rs116585983
rs182771043
rs12496802
rs191539404
rs56266759
rs4744100
rs75105010
rs117207754
rs79281530
rs1860268
rs61783054
All
−0.0020
−0.0015
−0.0010
−0.0005
0.0000
MR effect size for
'Monoacylglycerol lipase ABHD12' on 'eGFR'

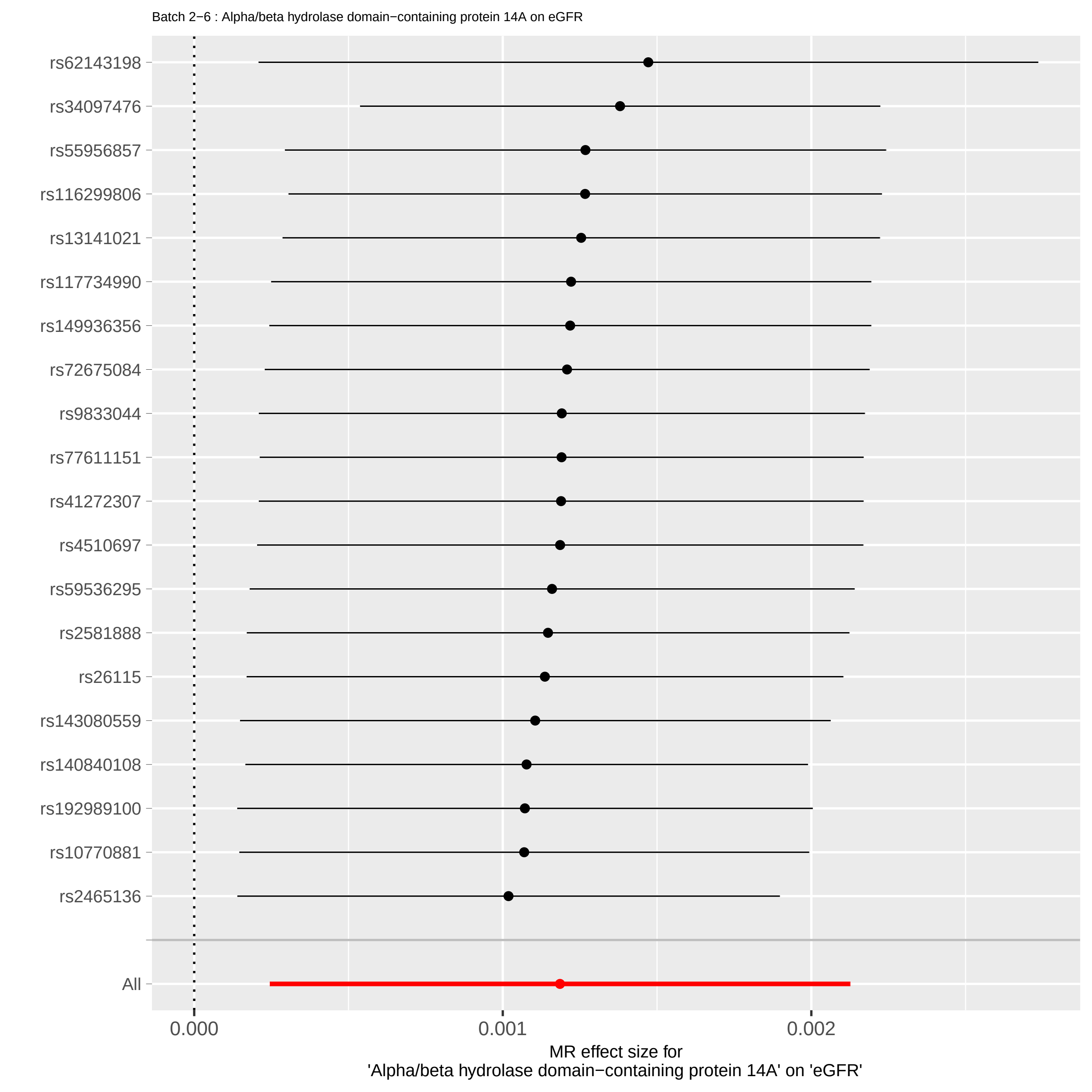

Batch 2−6 : Alpha/beta hydrolase domain−containing protein 14A on eGFR
rs62143198
rs34097476
rs55956857
rs116299806
rs13141021
rs117734990
rs149936356
rs72675084
rs9833044
rs77611151
rs41272307
rs4510697
rs59536295
rs2581888
rs26115
rs143080559
rs140840108
rs192989100
rs10770881
rs2465136
All
0.000
0.001
0.002
MR effect size for
'Alpha/beta hydrolase domain−containing protein 14A' on 'eGFR'

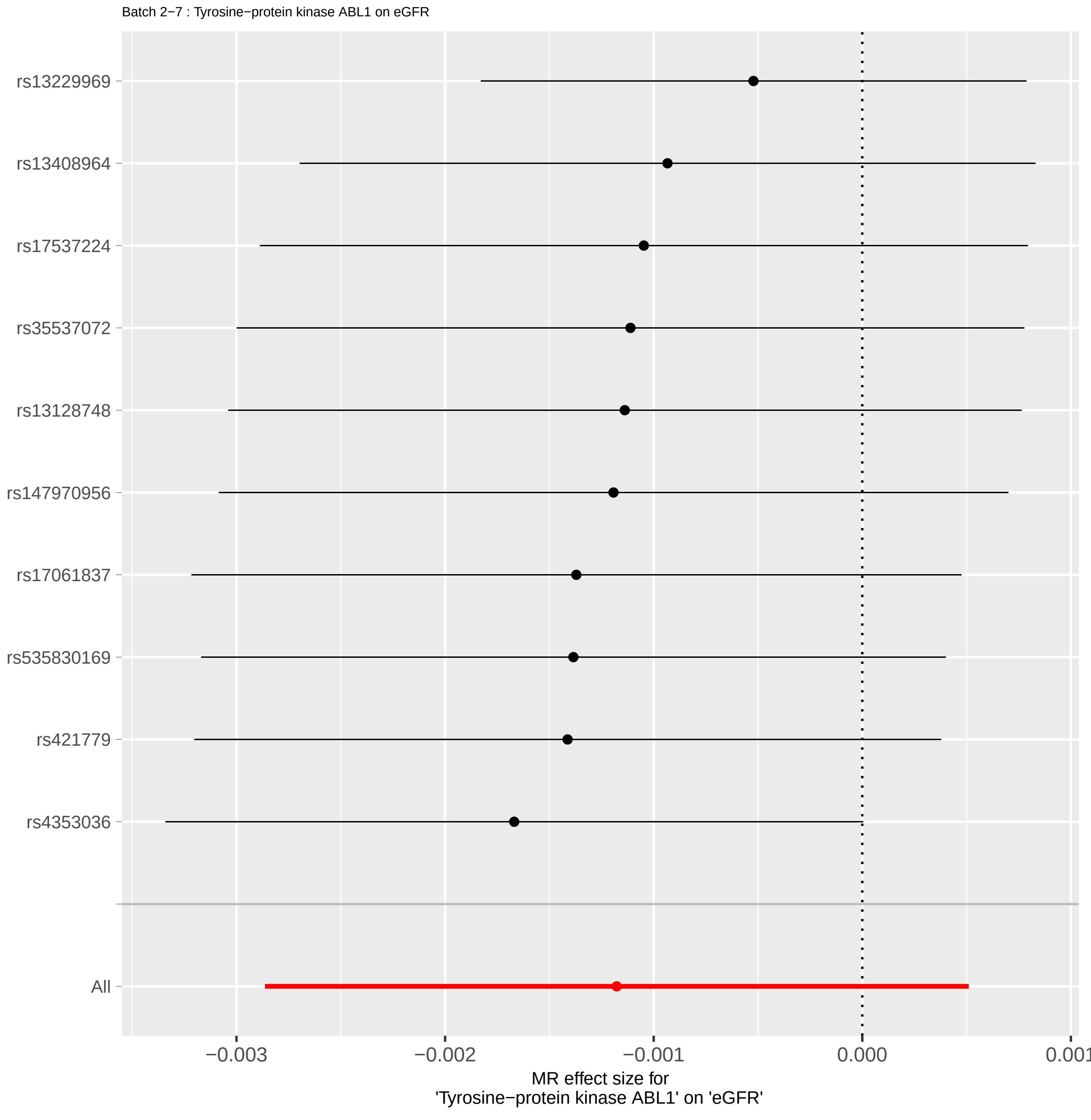

Batch 2−7 : Tyrosine−protein kinase ABL1 on eGFR
rs13229969
rs13408964
rs17537224
rs35537072
rs13128748
rs147970956
rs17061837
rs535830169
rs421779
rs4353036
All
−0.003
−0.002
−0.001
0.000
0.001
MR effect size for
'Tyrosine−protein kinase ABL1' on 'eGFR'

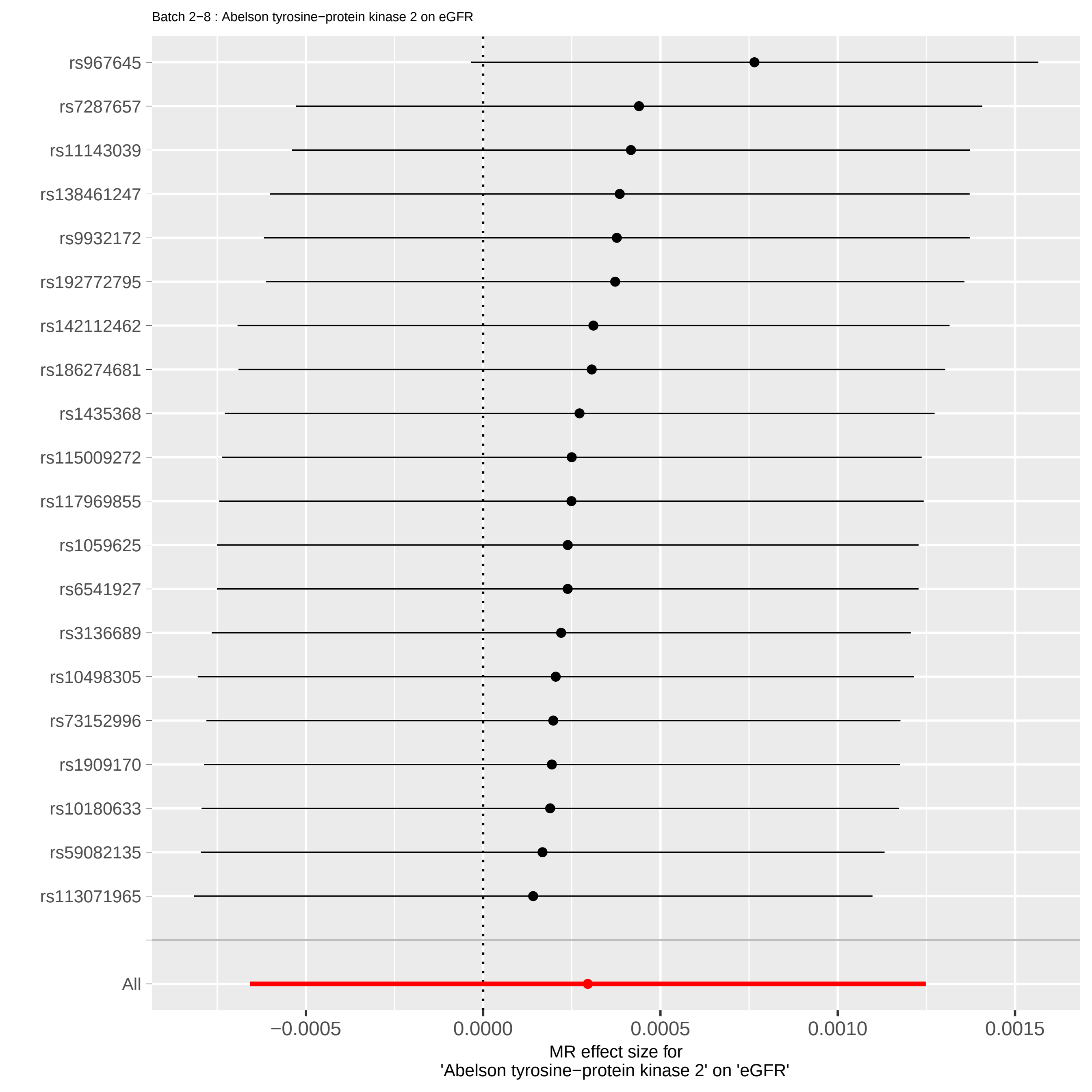

Batch 2−8 : Abelson tyrosine−protein kinase 2 on eGFR
rs967645
rs7287657
rs11143039
rs138461247
rs9932172
rs192772795
rs142112462
rs186274681
rs1435368
rs115009272
rs117969855
rs1059625
rs6541927
rs3136689
rs10498305
rs73152996
rs1909170
rs10180633
rs59082135
rs113071965
All
−0.0005
0.0000
0.0005
0.0010
0.0015
MR effect size for
'Abelson tyrosine−protein kinase 2' on 'eGFR'

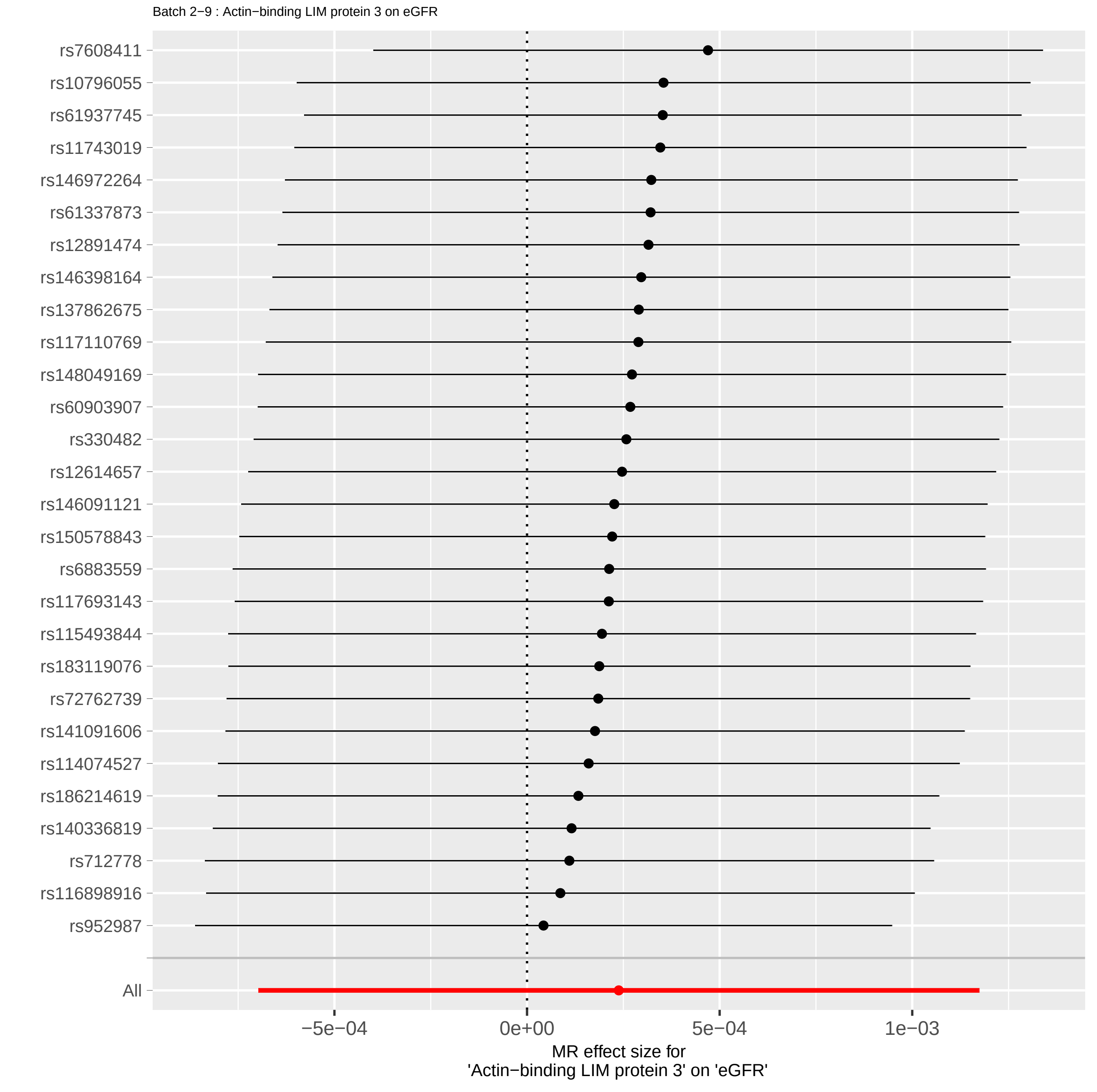
Batch 2−9 : Actin−binding LIM protein 3 on eGFR
rs7608411
rs10796055
rs61937745
rs11743019
rs146972264
rs61337873
rs12891474
rs146398164
rs137862675
rs117110769
rs148049169
rs60903907
rs330482
rs12614657
rs146091121
rs150578843
rs6883559
rs117693143
rs115493844
rs183119076
rs72762739
rs141091606
rs114074527
rs186214619
rs140336819
rs712778
rs116898916
rs952987
All
−5e−04
0e+00
5e−04
1e−03
MR effect size for
'Actin−binding LIM protein 3' on 'eGFR'

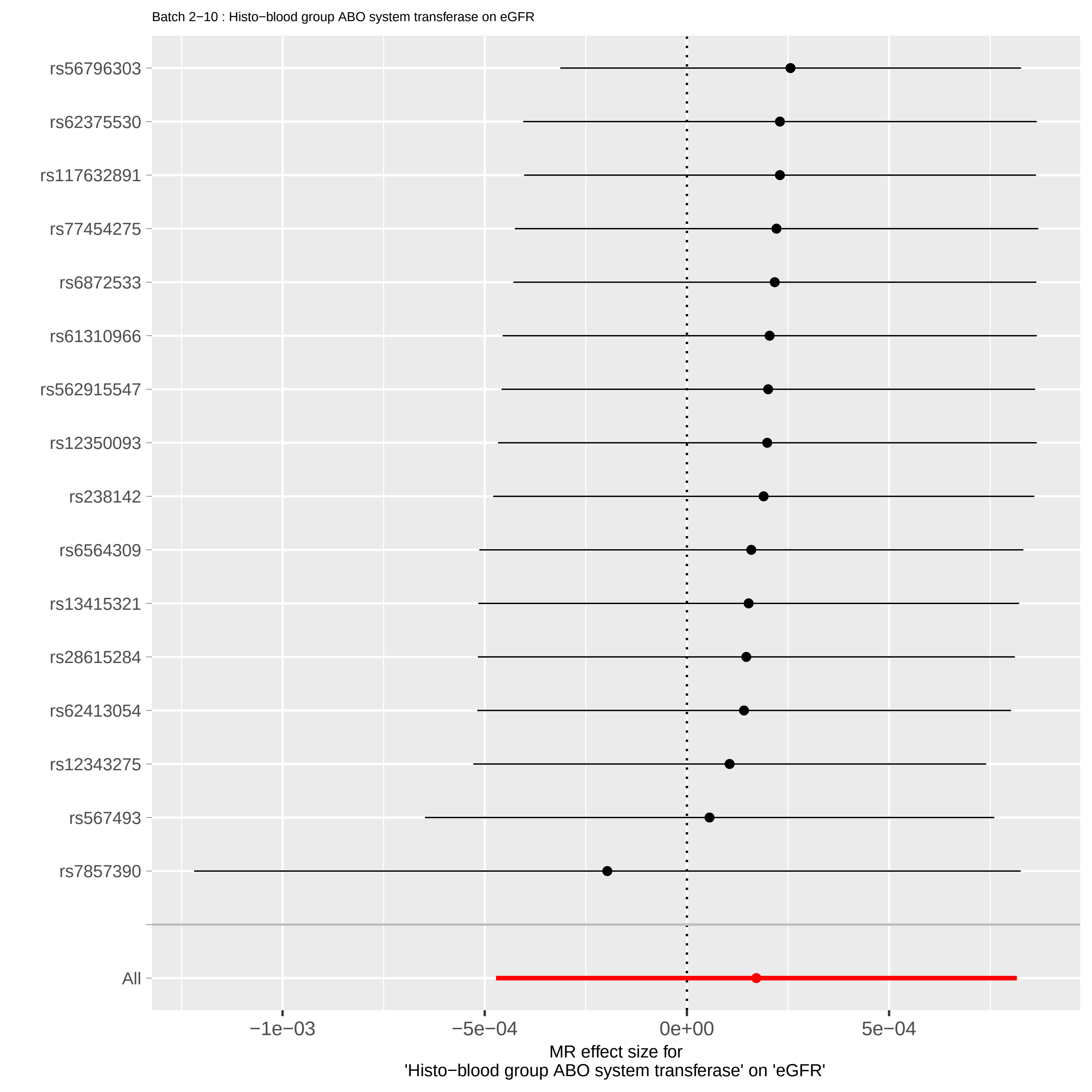

Batch 2−10 : Histo−blood group ABO system transferase on eGFR
rs56796303
rs62375530
rs117632891
rs77454275
rs6872533
rs61310966
rs562915547
rs12350093
rs238142
rs6564309
rs13415321
rs28615284
rs62413054
rs12343275
rs567493
rs7857390
All
−1e−03
−5e−04
0e+00
5e−04
MR effect size for
'Histo−blood group ABO system transferase' on 'eGFR'

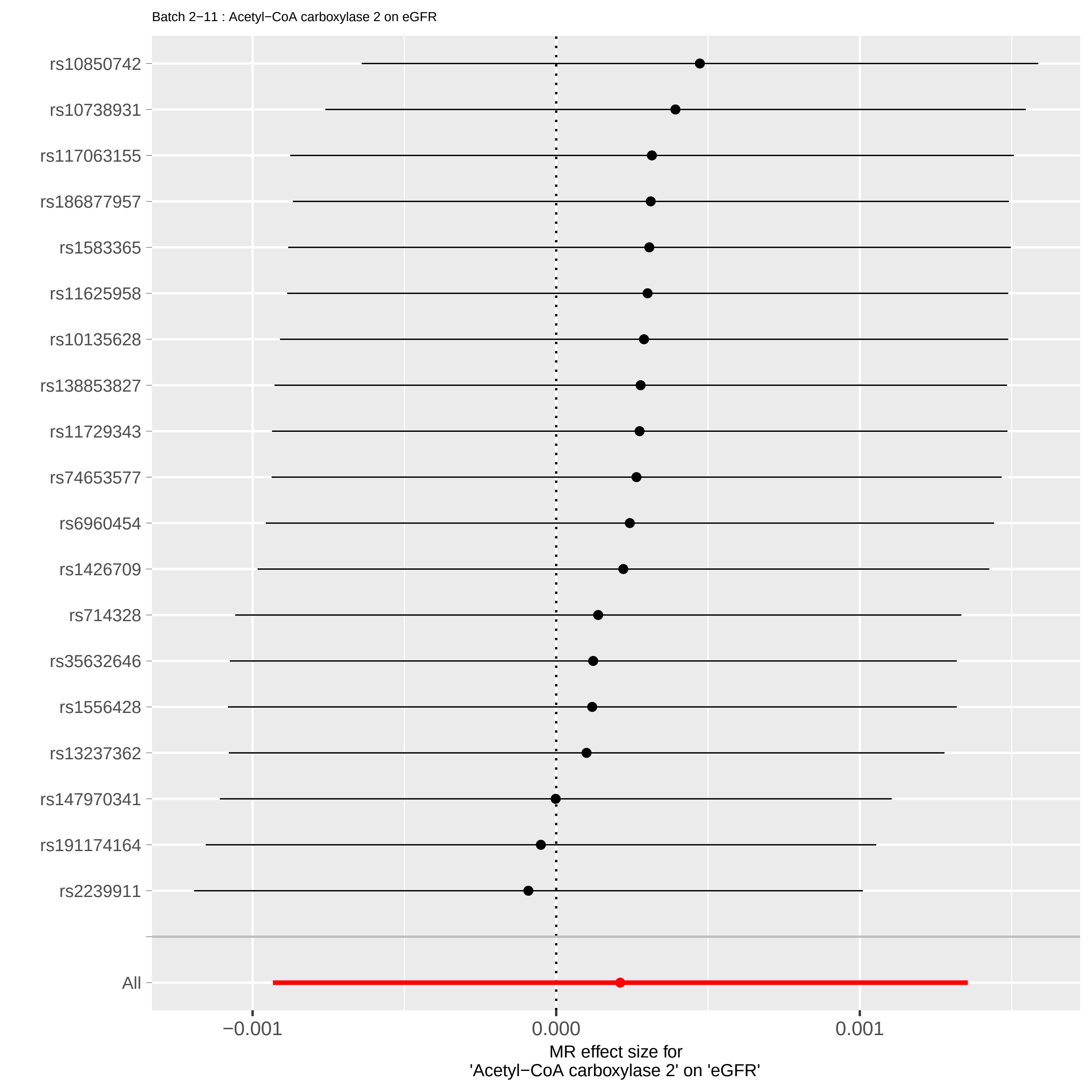

Batch 2−11 : Acetyl−CoA carboxylase 2 on eGFR
rs10850742
rs10738931
rs117063155
rs186877957
rs1583365
rs11625958
rs10135628
rs138853827
rs11729343
rs74653577
rs6960454
rs1426709
rs714328
rs35632646
rs1556428
rs13237362
rs147970341
rs191174164
rs2239911
All
−0.001
0.000
0.001
MR effect size for
'Acetyl−CoA carboxylase 2' on 'eGFR'

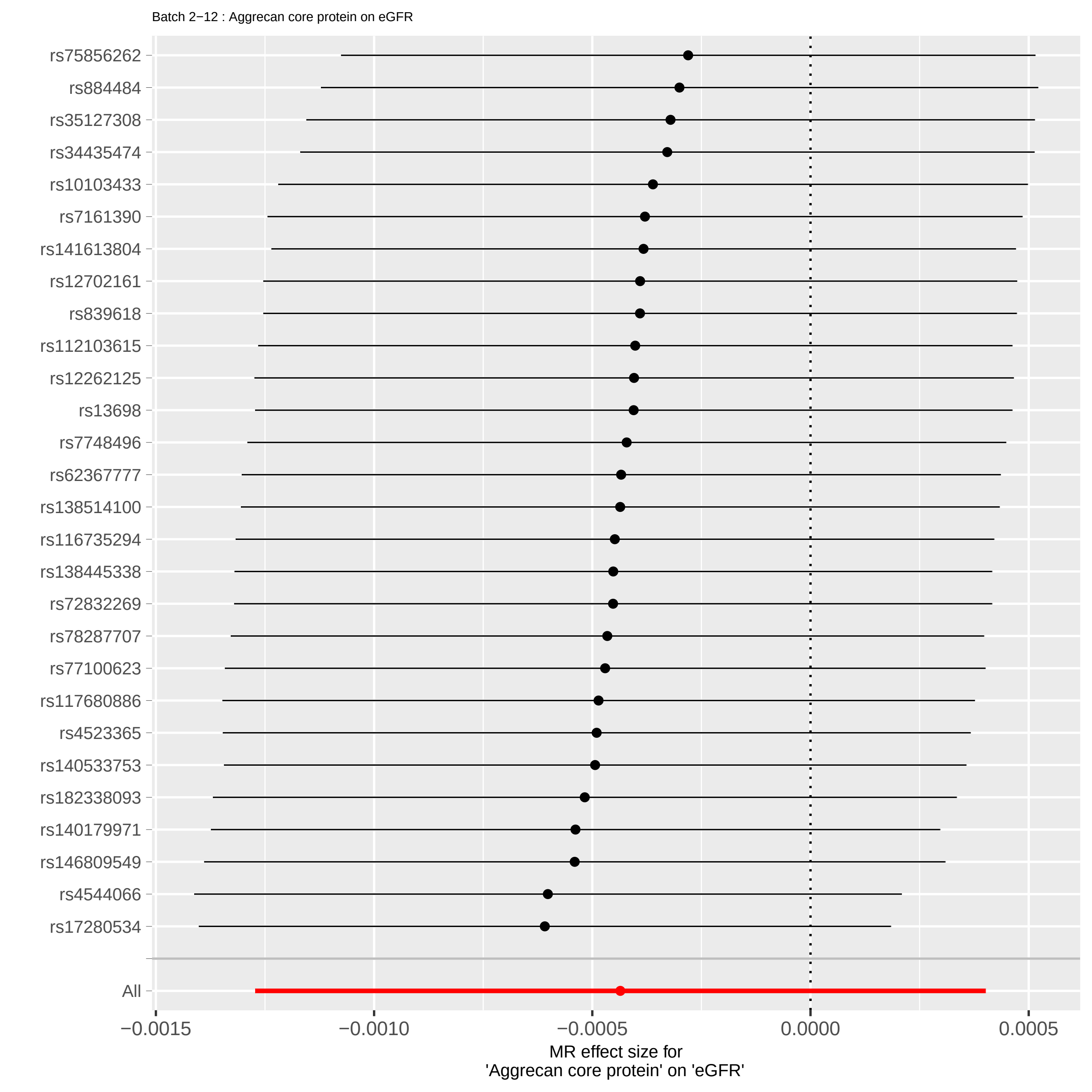

Batch 2−12 : Aggrecan core protein on eGFR
rs75856262
rs884484
rs35127308
rs34435474
rs10103433
rs7161390
rs141613804
rs12702161
rs839618
rs112103615
rs12262125
rs13698
rs7748496
rs62367777
rs138514100
rs116735294
rs138445338
rs72832269
rs78287707
rs77100623
rs117680886
rs4523365
rs140533753
rs182338093
rs140179971
rs146809549
rs4544066
rs17280534
All
−0.0015
−0.0010
−0.0005
0.0000
0.0005
MR effect size for
'Aggrecan core protein' on 'eGFR'

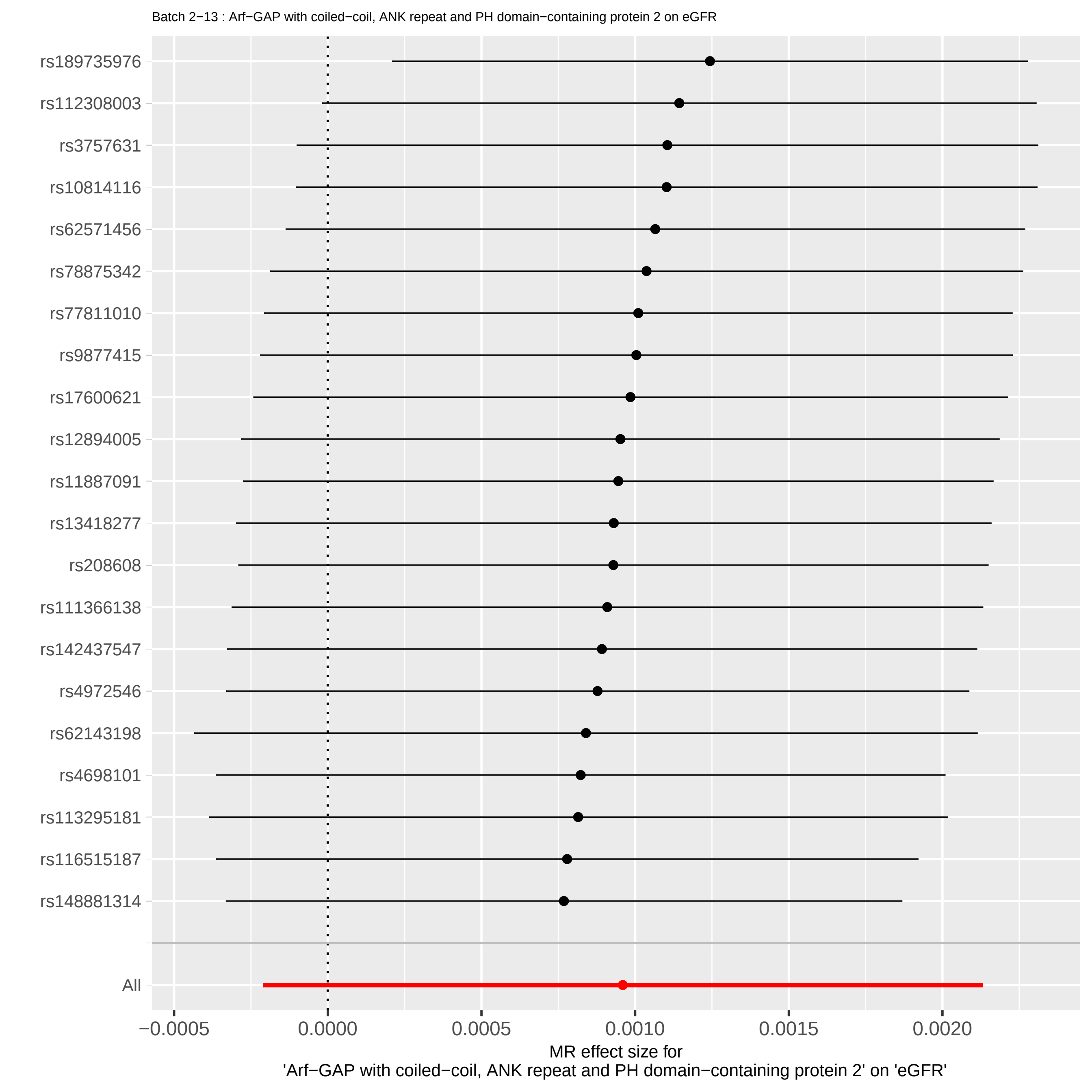

Batch 2−13 : Arf−GAP with coiled−coil, ANK repeat and PH domain−containing protein 2 on eGFR
rs189735976
rs112308003
rs3757631
rs10814116
rs62571456
rs78875342
rs77811010
rs9877415
rs17600621
rs12894005
rs11887091
rs13418277
rs208608
rs111366138
rs142437547
rs4972546
rs62143198
rs4698101
rs113295181
rs116515187
rs148881314
All
−0.0005
0.0000
0.0005
0.0010
0.0015
0.0020
MR effect size for
'Arf−GAP with coiled−coil, ANK repeat and PH domain−containing protein 2' on 'eGFR'

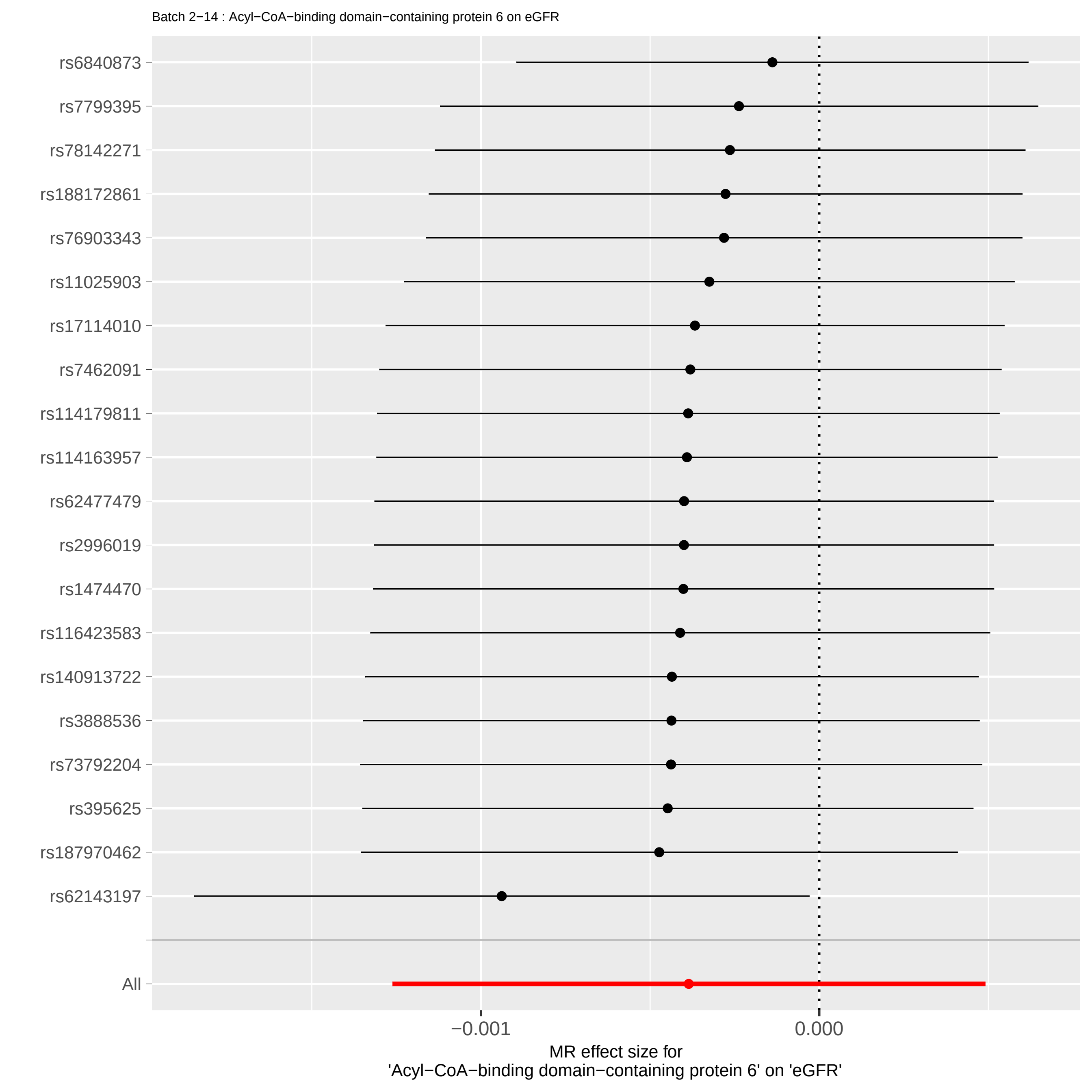

Batch 2−14 : Acyl−CoA−binding domain−containing protein 6 on eGFR
rs6840873
rs7799395
rs78142271
rs188172861
rs76903343
rs11025903
rs17114010
rs7462091
rs114179811
rs114163957
rs62477479
rs2996019
rs1474470
rs116423583
rs140913722
rs3888536
rs73792204
rs395625
rs187970462
rs62143197
All
−0.001
0.000
MR effect size for
'Acyl−CoA−binding domain−containing protein 6' on 'eGFR'

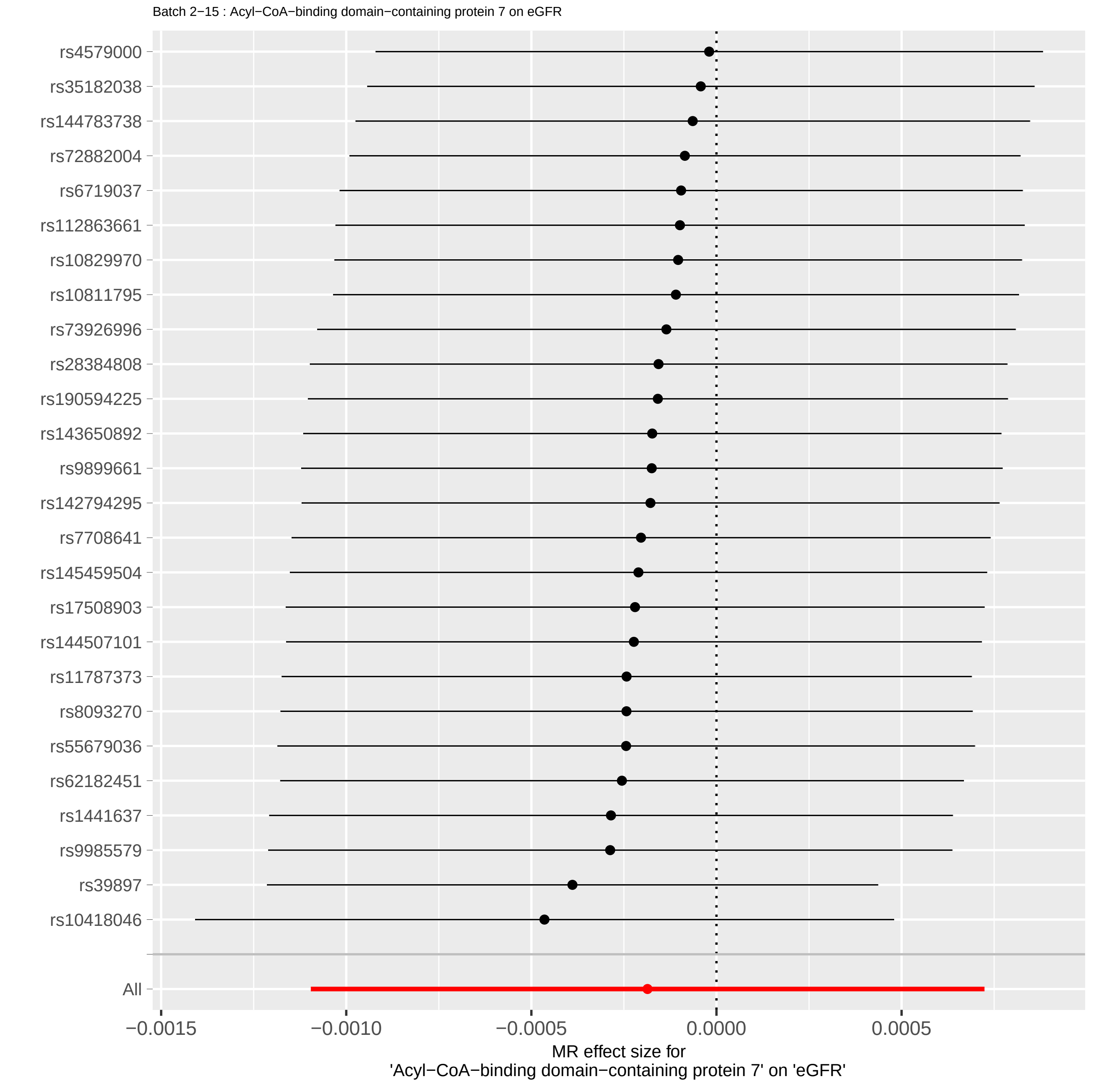

Batch 2−15 : Acyl−CoA−binding domain−containing protein 7 on eGFR
rs4579000
rs35182038
rs144783738
rs72882004
rs6719037
rs112863661
rs10829970
rs10811795
rs73926996
rs28384808
rs190594225
rs143650892
rs9899661
rs142794295
rs7708641
rs145459504
rs17508903
rs144507101
rs11787373
rs8093270
rs55679036
rs62182451
rs1441637
rs9985579
rs39897
rs10418046
All
−0.0015
−0.0010
−0.0005
0.0000
0.0005
MR effect size for
'Acyl−CoA−binding domain−containing protein 7' on 'eGFR'

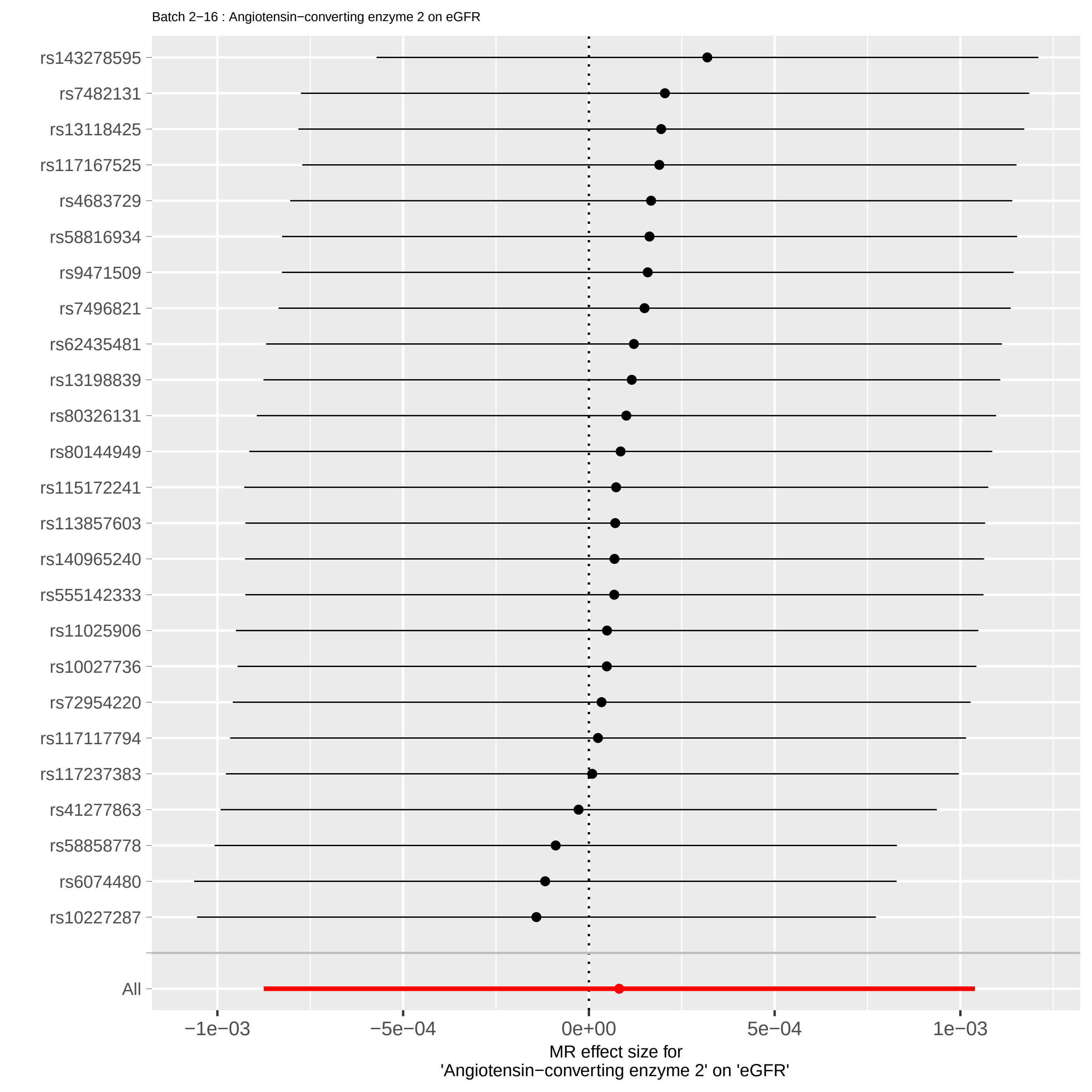

Batch 2−16 : Angiotensin−converting enzyme 2 on eGFR
rs143278595
rs7482131
rs13118425
rs117167525
rs4683729
rs58816934
rs9471509
rs7496821
rs62435481
rs13198839
rs80326131
rs80144949
rs115172241
rs113857603
rs140965240
rs555142333
rs11025906
rs10027736
rs72954220
rs117117794
rs117237383
rs41277863
rs58858778
rs6074480
rs10227287
All
−1e−03
−5e−04
0e+00
5e−04
1e−03
MR effect size for
'Angiotensin−converting enzyme 2' on 'eGFR'

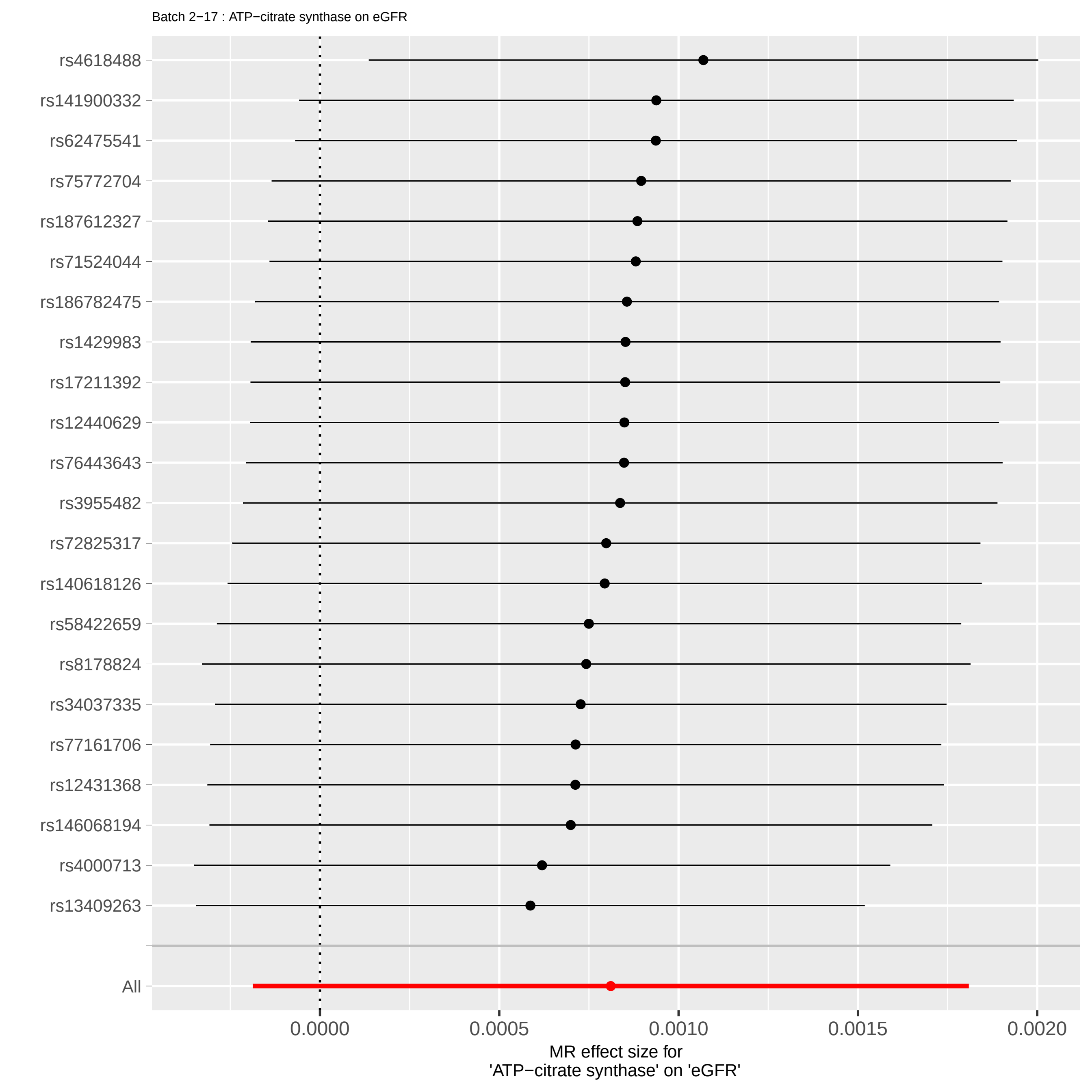

Batch 2−17 : ATP−citrate synthase on eGFR
rs4618488
rs141900332
rs62475541
rs75772704
rs187612327
rs71524044
rs186782475
rs1429983
rs17211392
rs12440629
rs76443643
rs3955482
rs72825317
rs140618126
rs58422659
rs8178824
rs34037335
rs77161706
rs12431368
rs146068194
rs4000713
rs13409263
All
0.0000
0.0005
0.0010
0.0015
0.0020
MR effect size for
'ATP−citrate synthase' on 'eGFR'

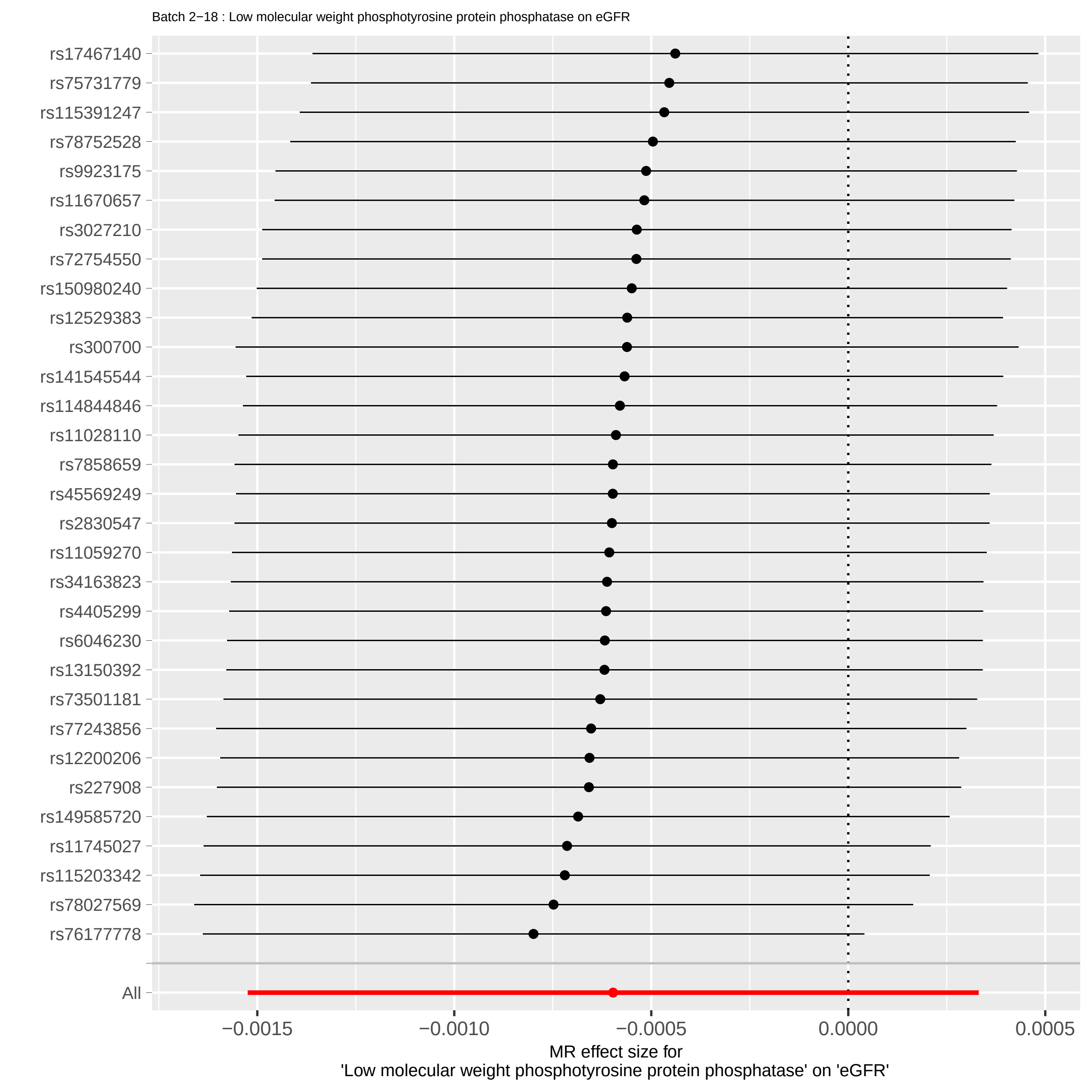

Batch 2−18 : Low molecular weight phosphotyrosine protein phosphatase on eGFR
rs17467140
rs75731779
rs115391247
rs78752528
rs9923175
rs11670657
rs3027210
rs72754550
rs150980240
rs12529383
rs300700
rs141545544
rs114844846
rs11028110
rs7858659
rs45569249
rs2830547
rs11059270
rs34163823
rs4405299
rs6046230
rs13150392
rs73501181
rs77243856
rs12200206
rs227908
rs149585720
rs11745027
rs115203342
rs78027569
rs76177778
All
−0.0015
−0.0010
−0.0005
0.0000
0.0005
MR effect size for
'Low molecular weight phosphotyrosine protein phosphatase' on 'eGFR'

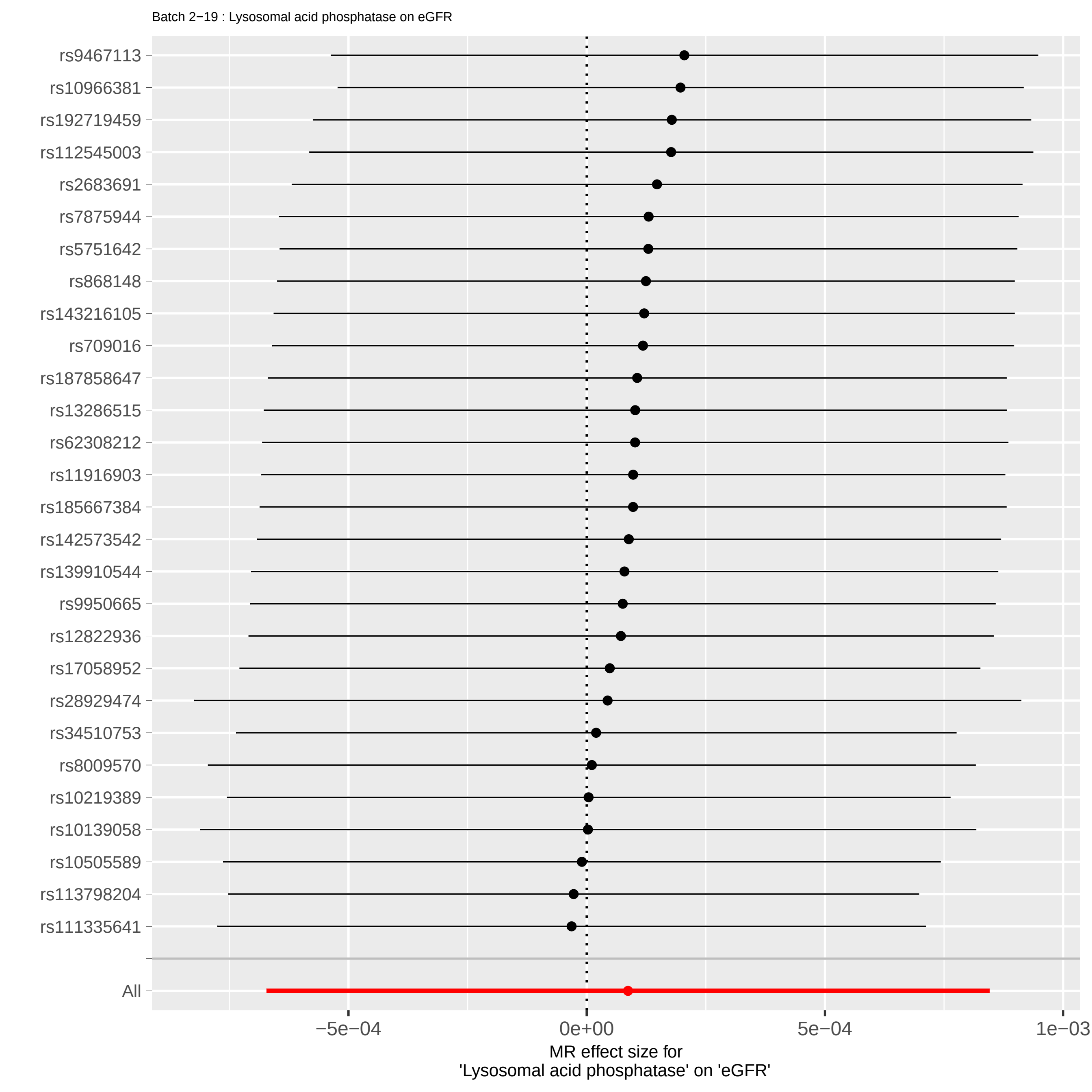

Batch 2−19 : Lysosomal acid phosphatase on eGFR
rs9467113
rs10966381
rs192719459
rs112545003
rs2683691
rs7875944
rs5751642
rs868148
rs143216105
rs709016
rs187858647
rs13286515
rs62308212
rs11916903
rs185667384
rs142573542
rs139910544
rs9950665
rs12822936
rs17058952
rs28929474
rs34510753
rs8009570
rs10219389
rs10139058
rs10505589
rs113798204
rs111335641
All
−5e−04
0e+00
5e−04
1e−03
MR effect size for
'Lysosomal acid phosphatase' on 'eGFR'

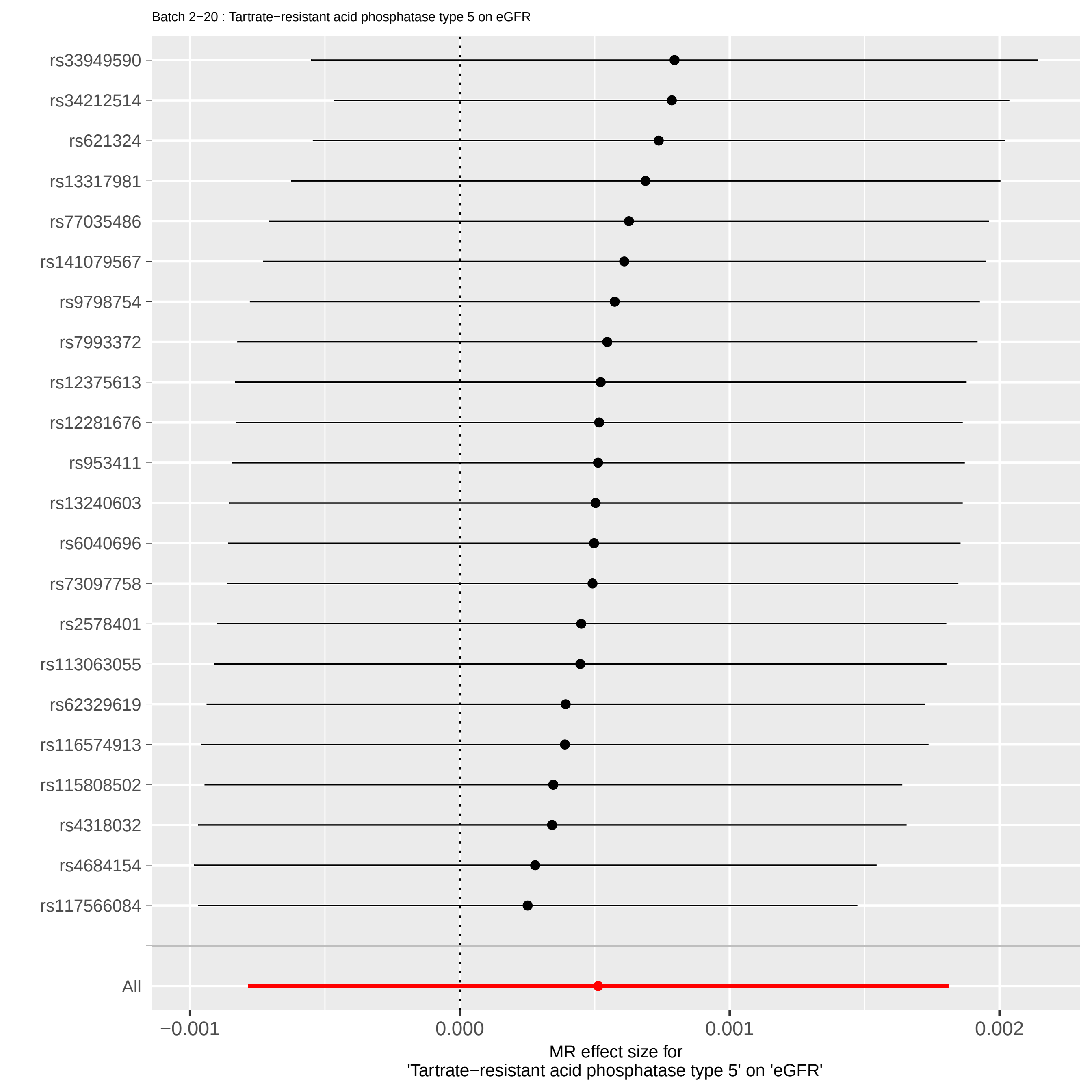

Batch 2−20 : Tartrate−resistant acid phosphatase type 5 on eGFR
rs33949590
rs34212514
rs621324
rs13317981
rs77035486
rs141079567
rs9798754
rs7993372
rs12375613
rs12281676
rs953411
rs13240603
rs6040696
rs73097758
rs2578401
rs113063055
rs62329619
rs116574913
rs115808502
rs4318032
rs4684154
rs117566084
All
−0.001
0.000
0.001
0.002
MR effect size for
'Tartrate−resistant acid phosphatase type 5' on 'eGFR'

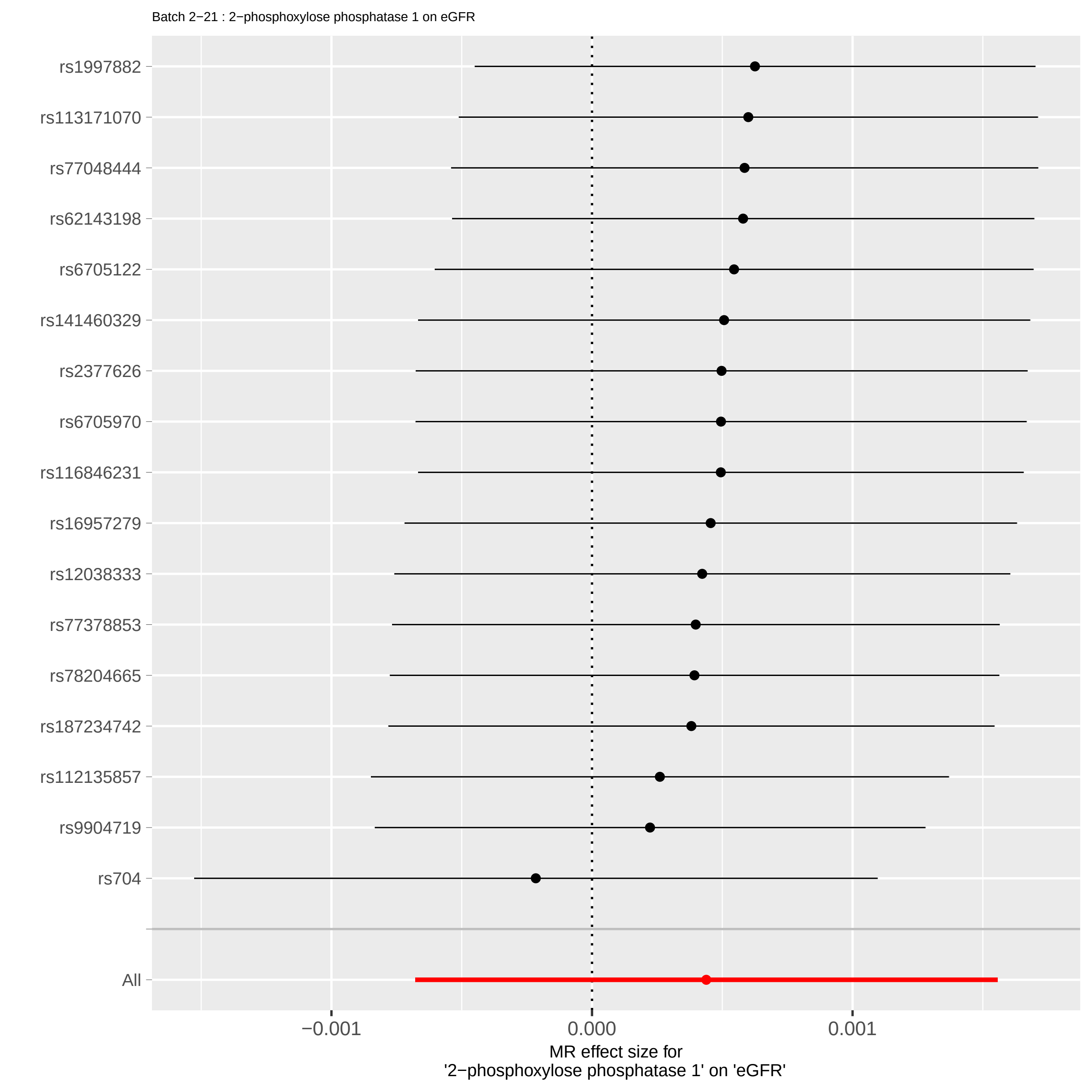
Batch 2−21 : 2−phosphoxylose phosphatase 1 on eGFR
rs1997882
rs113171070
rs77048444
rs62143198
rs6705122
rs141460329
rs2377626
rs6705970
rs116846231
rs16957279
rs12038333
rs77378853
rs78204665
rs187234742
rs112135857
rs9904719
rs704
All
−0.001
0.000
0.001
MR effect size for
'2−phosphoxylose phosphatase 1' on 'eGFR'

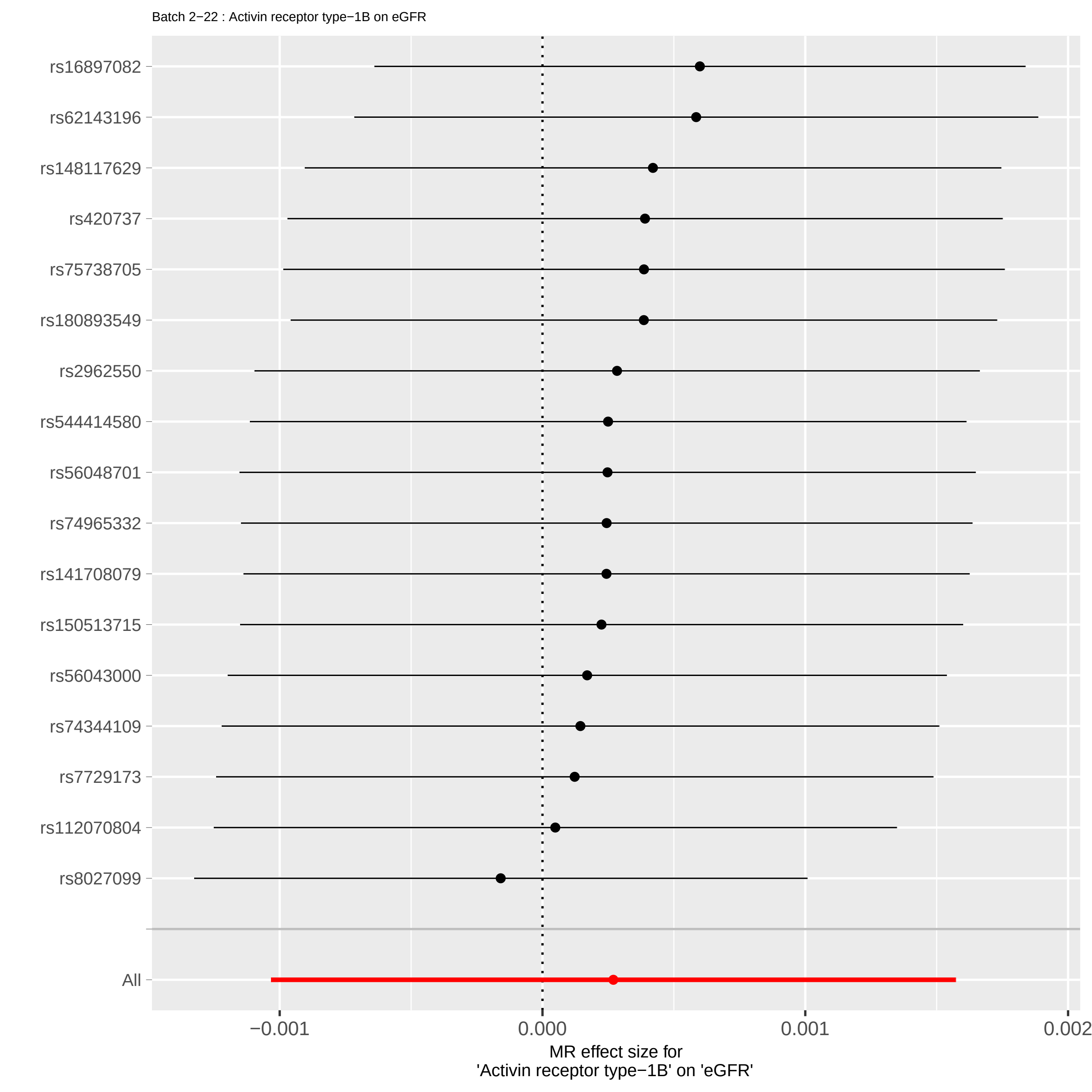

Batch 2−22 : Activin receptor type−1B on eGFR
rs16897082
rs62143196
rs148117629
rs420737
rs75738705
rs180893549
rs2962550
rs544414580
rs56048701
rs74965332
rs141708079
rs150513715
rs56043000
rs74344109
rs7729173
rs112070804
rs8027099
All
−0.001
0.000
0.001
0.002
MR effect size for
'Activin receptor type−1B' on 'eGFR'

Batch 2−23 : Serine/threonine−protein kinase receptor R3 on eGFR

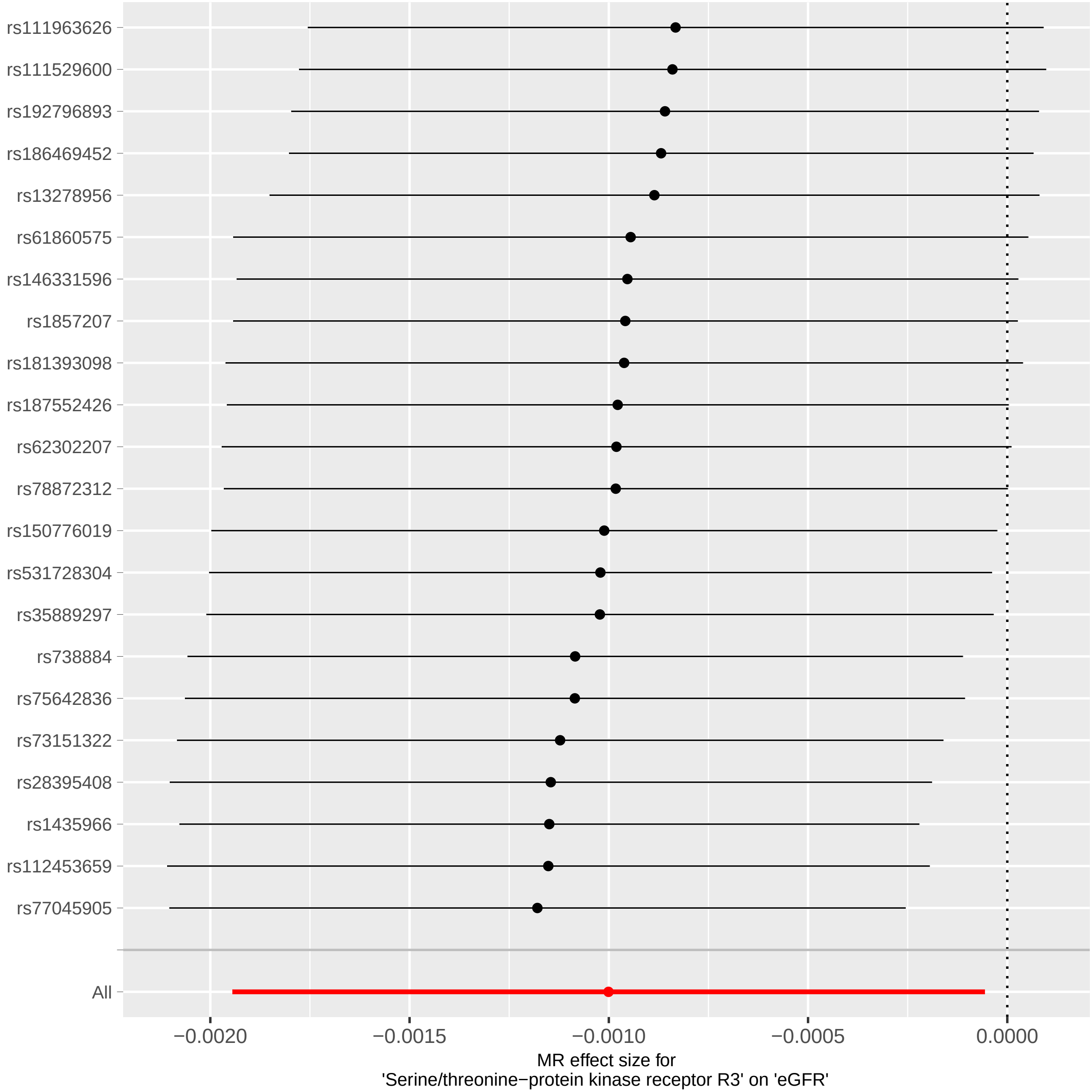

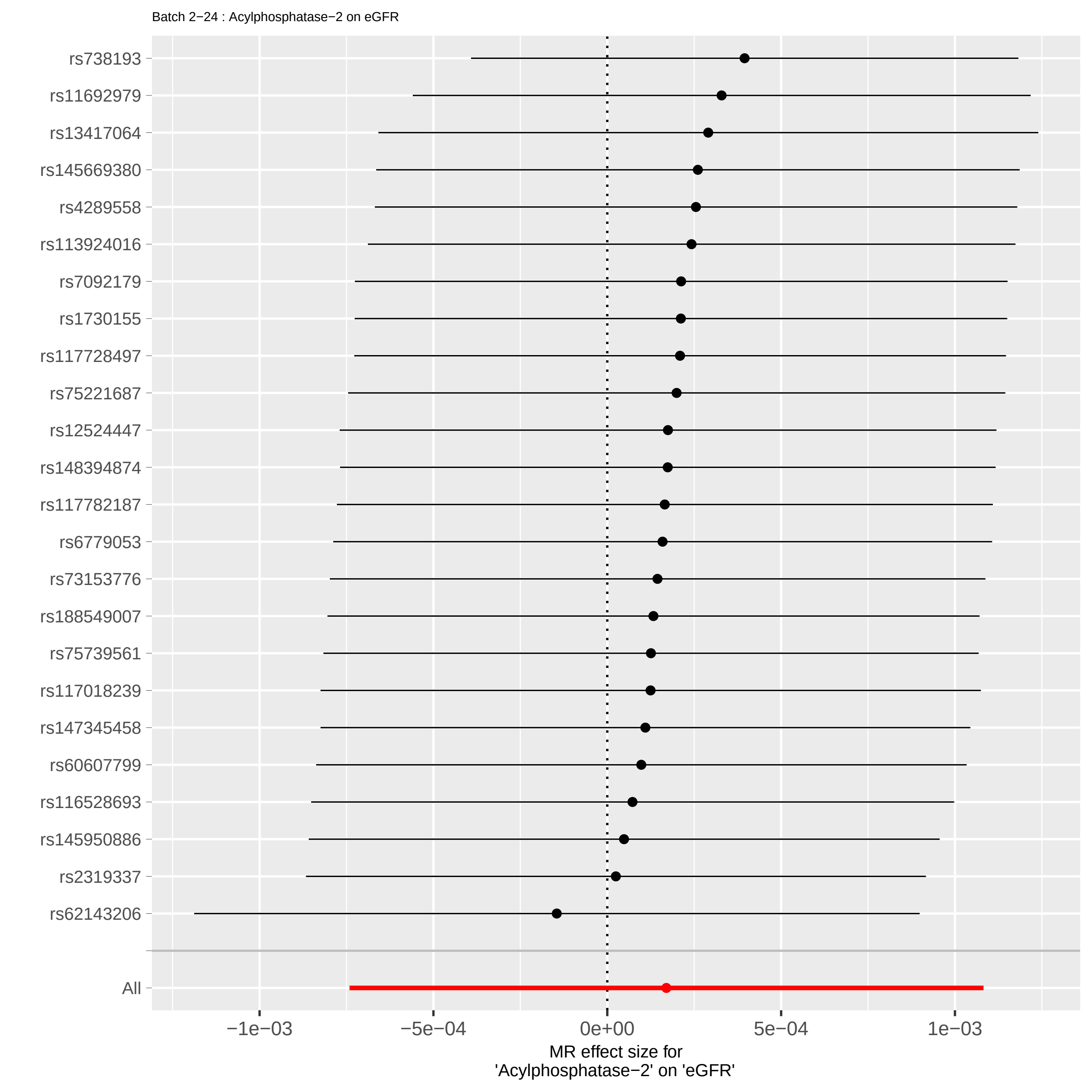

Batch 2−24 : Acylphosphatase−2 on eGFR
rs738193
rs11692979
rs13417064
rs145669380
rs4289558
rs113924016
rs7092179
rs1730155
rs117728497
rs75221687
rs12524447
rs148394874
rs117782187
rs6779053
rs73153776
rs188549007
rs75739561
rs117018239
rs147345458
rs60607799
rs116528693
rs145950886
rs2319337
rs62143206
All
−1e−03
−5e−04
0e+00
5e−04
1e−03
MR effect size for
'Acylphosphatase−2' on 'eGFR'

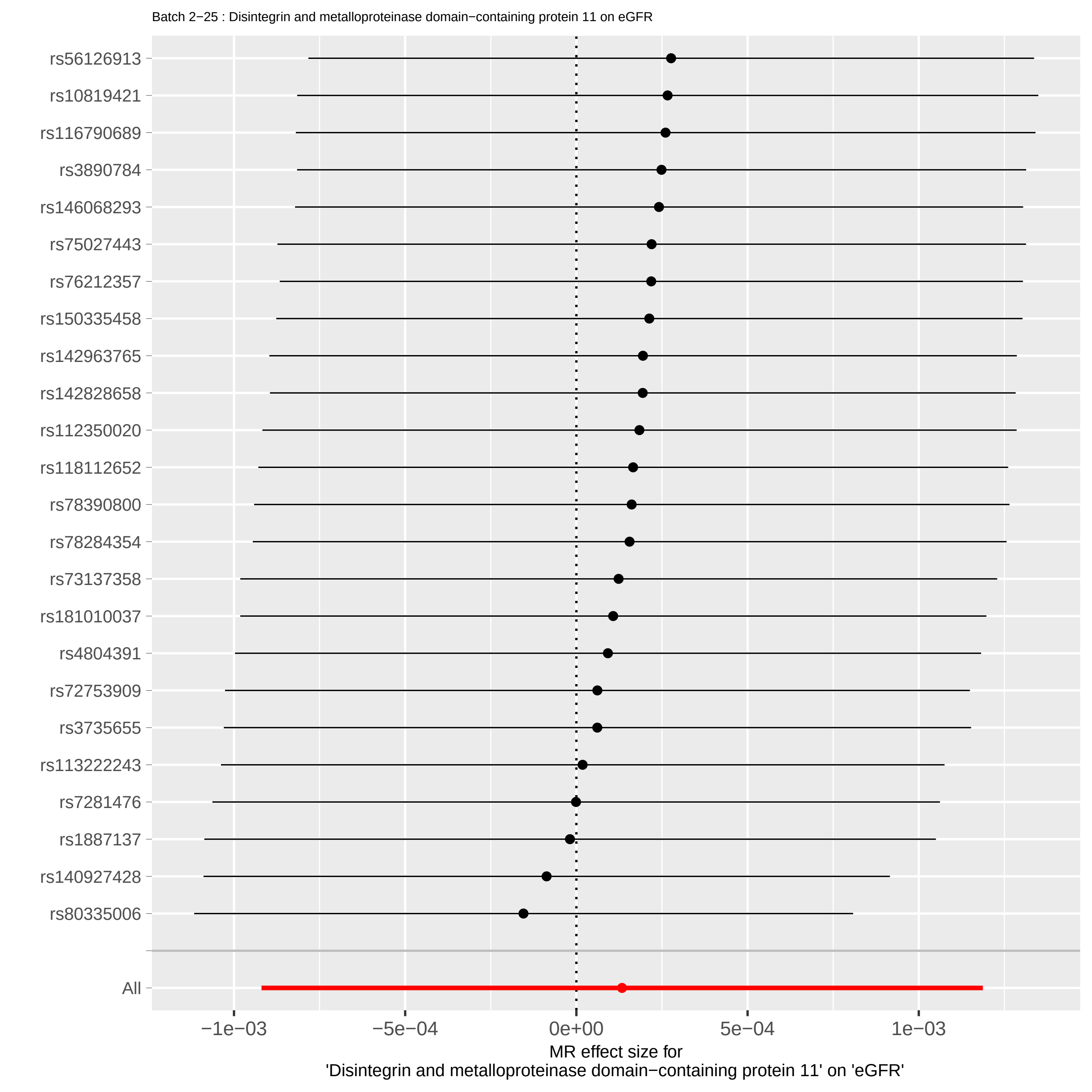

Batch 2−25 : Disintegrin and metalloproteinase domain−containing protein 11 on eGFR
rs56126913
rs10819421
rs116790689
rs3890784
rs146068293
rs75027443
rs76212357
rs150335458
rs142963765
rs142828658
rs112350020
rs118112652
rs78390800
rs78284354
rs73137358
rs181010037
rs4804391
rs72753909
rs3735655
rs113222243
rs7281476
rs1887137
rs140927428
rs80335006
All
−1e−03
−5e−04
0e+00
5e−04
1e−03
MR effect size for
'Disintegrin and metalloproteinase domain−containing protein 11' on 'eGFR'

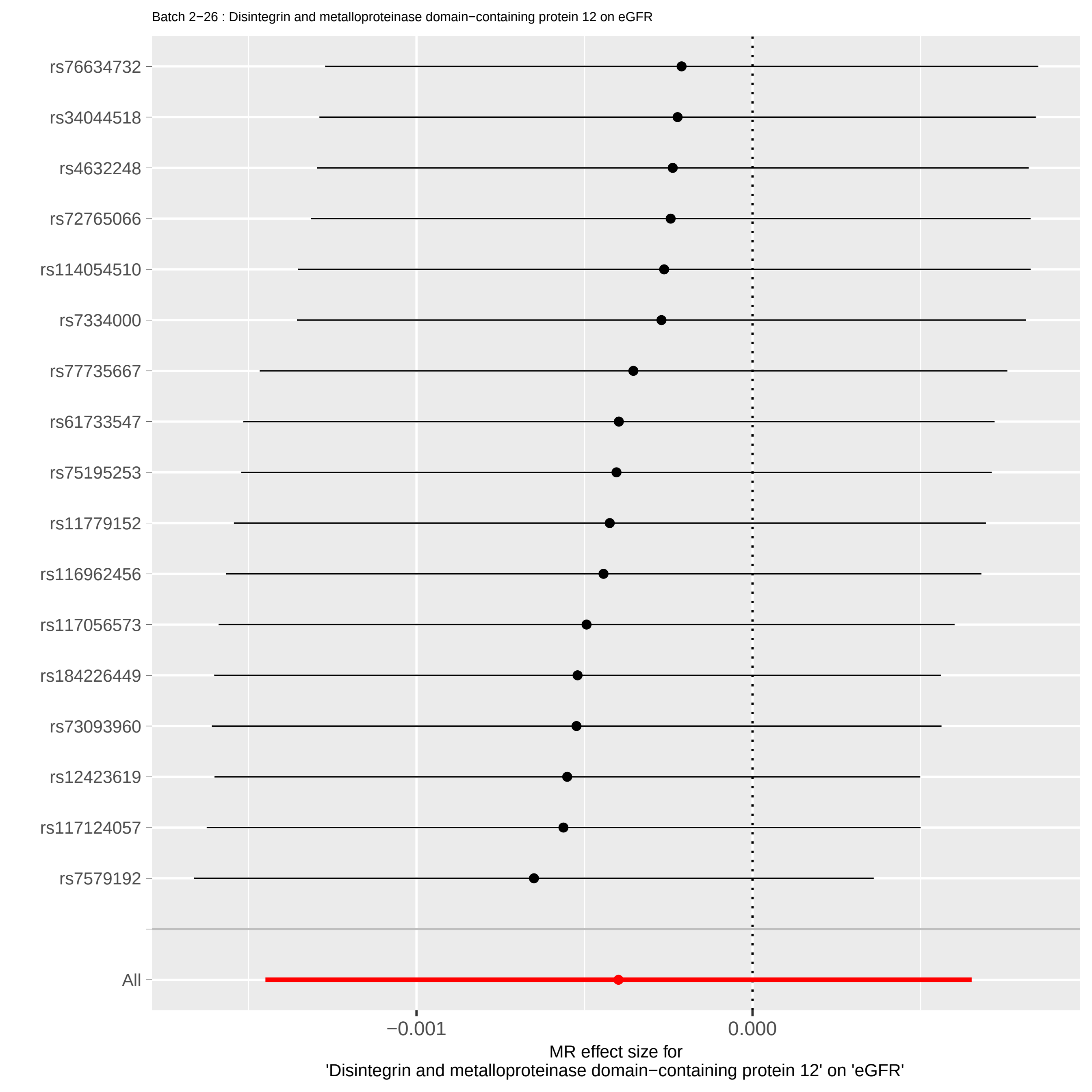
Batch 2−26 : Disintegrin and metalloproteinase domain−containing protein 12 on eGFR
rs76634732
rs34044518
rs4632248
rs72765066
rs114054510
rs7334000
rs77735667
rs61733547
rs75195253
rs11779152
rs116962456
rs117056573
rs184226449
rs73093960
rs12423619
rs117124057
rs7579192
All
−0.001
0.000
MR effect size for
'Disintegrin and metalloproteinase domain−containing protein 12' on 'eGFR'

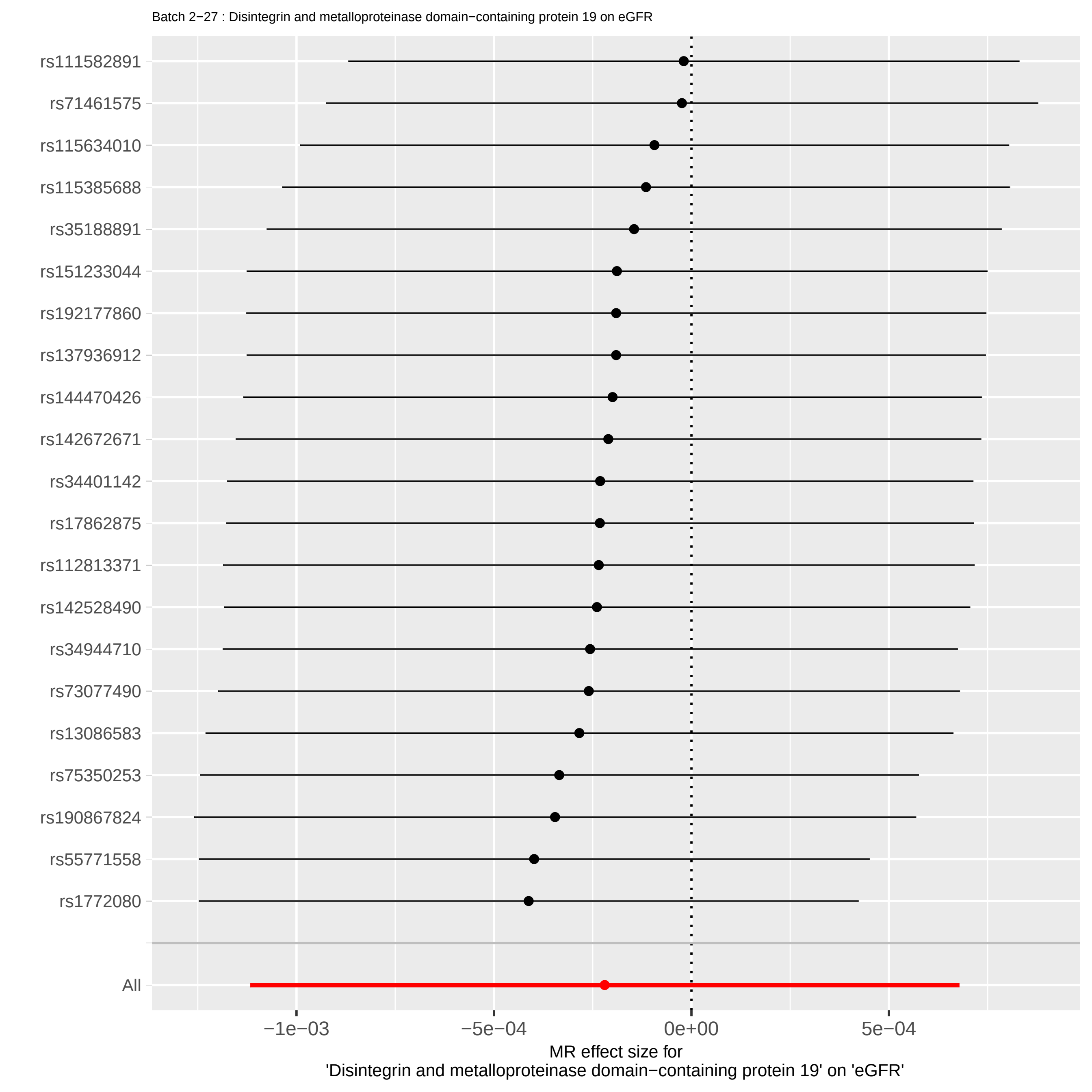
Batch 2−27 : Disintegrin and metalloproteinase domain−containing protein 19 on eGFR
rs111582891
rs71461575
rs115634010
rs115385688
rs35188891
rs151233044
rs192177860
rs137936912
rs144470426
rs142672671
rs34401142
rs17862875
rs112813371
rs142528490
rs34944710
rs73077490
rs13086583
rs75350253
rs190867824
rs55771558
rs1772080
All
−1e−03
−5e−04
0e+00
5e−04
MR effect size for
'Disintegrin and metalloproteinase domain−containing protein 19' on 'eGFR'

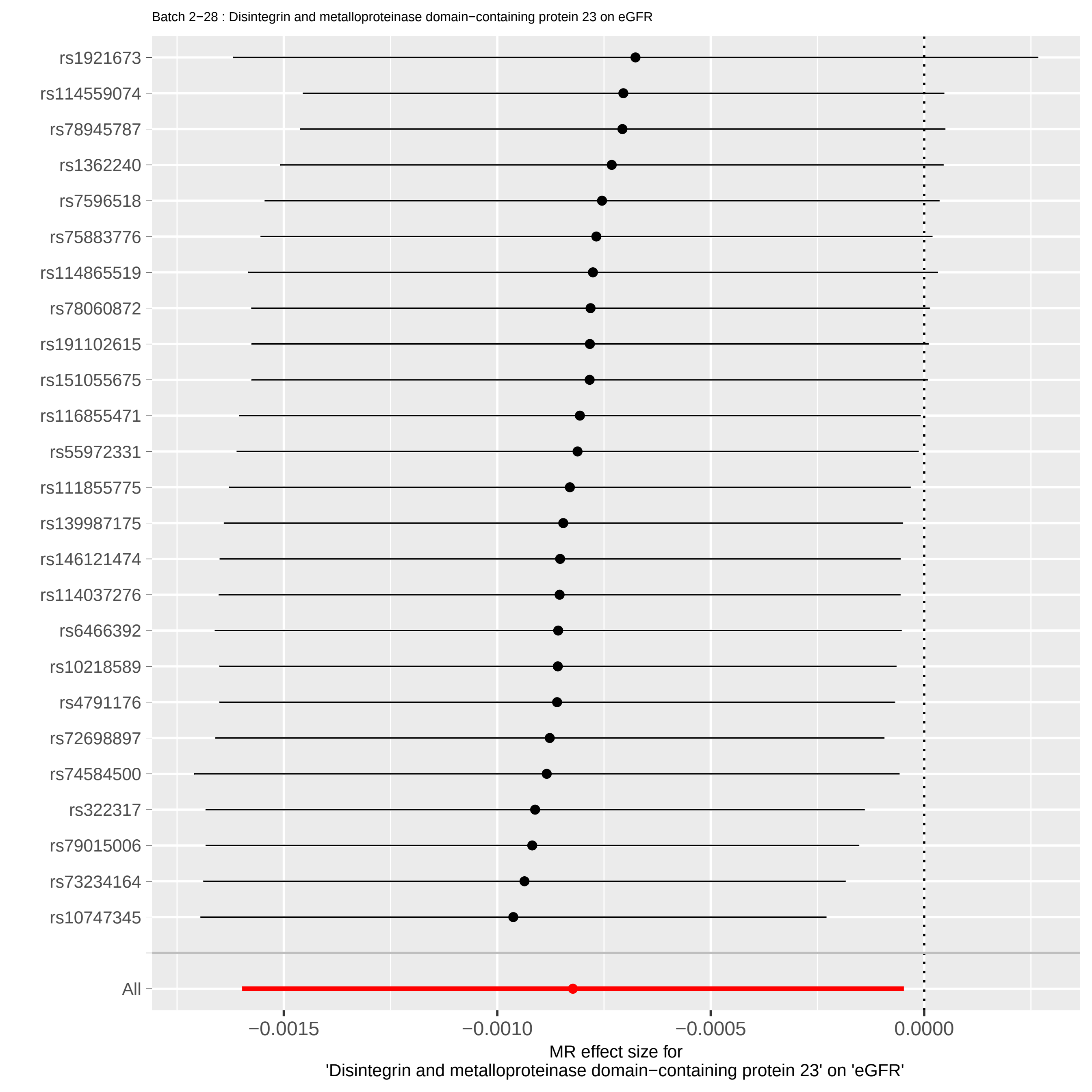
Batch 2−28 : Disintegrin and metalloproteinase domain−containing protein 23 on eGFR
rs1921673
rs114559074
rs78945787
rs1362240
rs7596518
rs75883776
rs114865519
rs78060872
rs191102615
rs151055675
rs116855471
rs55972331
rs111855775
rs139987175
rs146121474
rs114037276
rs6466392
rs10218589
rs4791176
rs72698897
rs74584500
rs322317
rs79015006
rs73234164
rs10747345
All
−0.0015
−0.0010
−0.0005
0.0000
MR effect size for
'Disintegrin and metalloproteinase domain−containing protein 23' on 'eGFR'

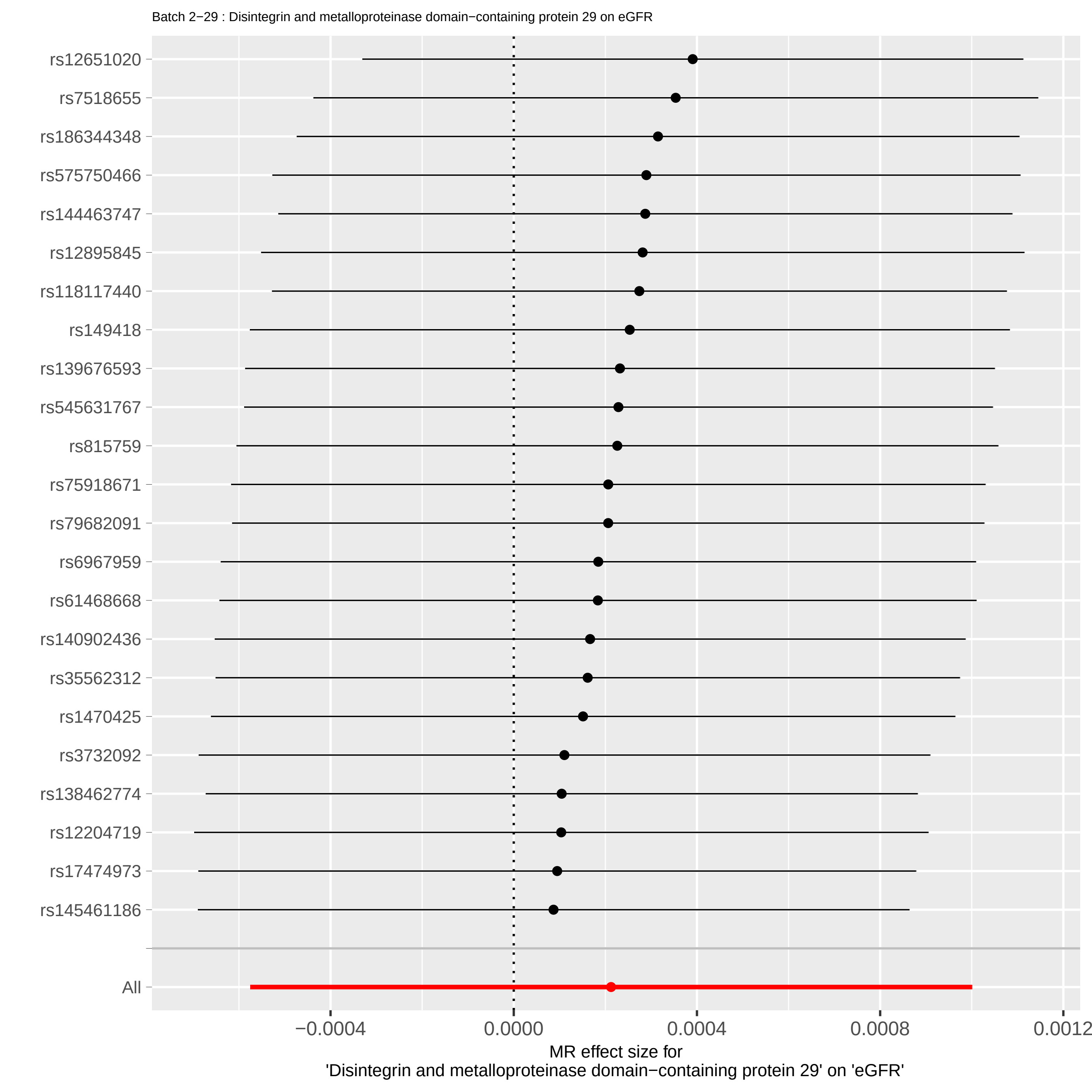

Batch 2−29 : Disintegrin and metalloproteinase domain−containing protein 29 on eGFR
rs12651020
rs7518655
rs186344348
rs575750466
rs144463747
rs12895845
rs118117440
rs149418
rs139676593
rs545631767
rs815759
rs75918671
rs79682091
rs6967959
rs61468668
rs140902436
rs35562312
rs1470425
rs3732092
rs138462774
rs12204719
rs17474973
rs145461186
All
−0.0004
0.0000
0.0004
0.0008
0.0012
MR effect size for
'Disintegrin and metalloproteinase domain−containing protein 29' on 'eGFR'

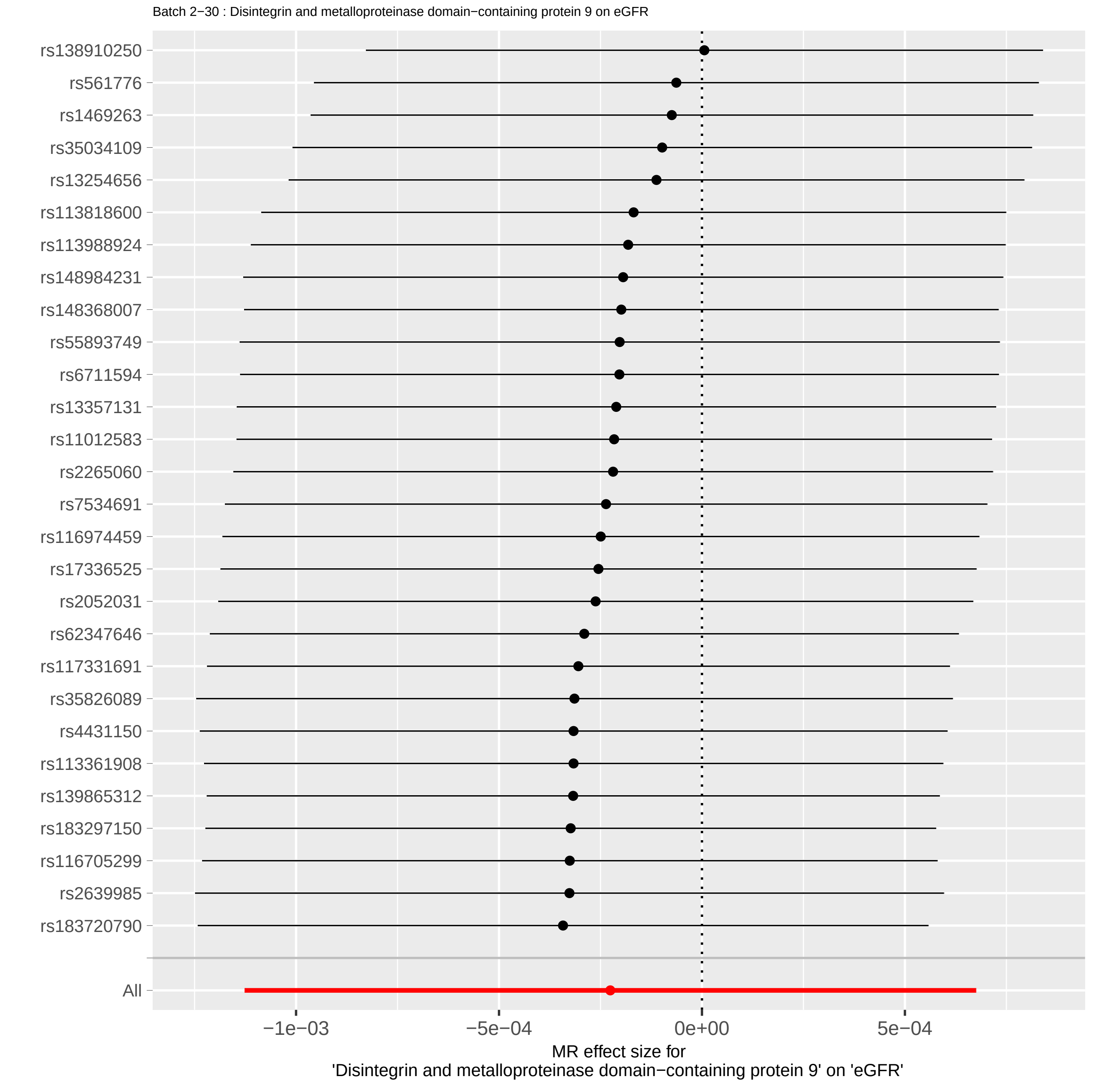

Batch 2−30 : Disintegrin and metalloproteinase domain−containing protein 9 on eGFR
rs138910250
rs561776
rs1469263
rs35034109
rs13254656
rs113818600
rs113988924
rs148984231
rs148368007
rs55893749
rs6711594
rs13357131
rs11012583
rs2265060
rs7534691
rs116974459
rs17336525
rs2052031
rs62347646
rs117331691
rs35826089
rs4431150
rs113361908
rs139865312
rs183297150
rs116705299
rs2639985
rs183720790
All
−1e−03
−5e−04
0e+00
5e−04
MR effect size for
'Disintegrin and metalloproteinase domain−containing protein 9' on 'eGFR'

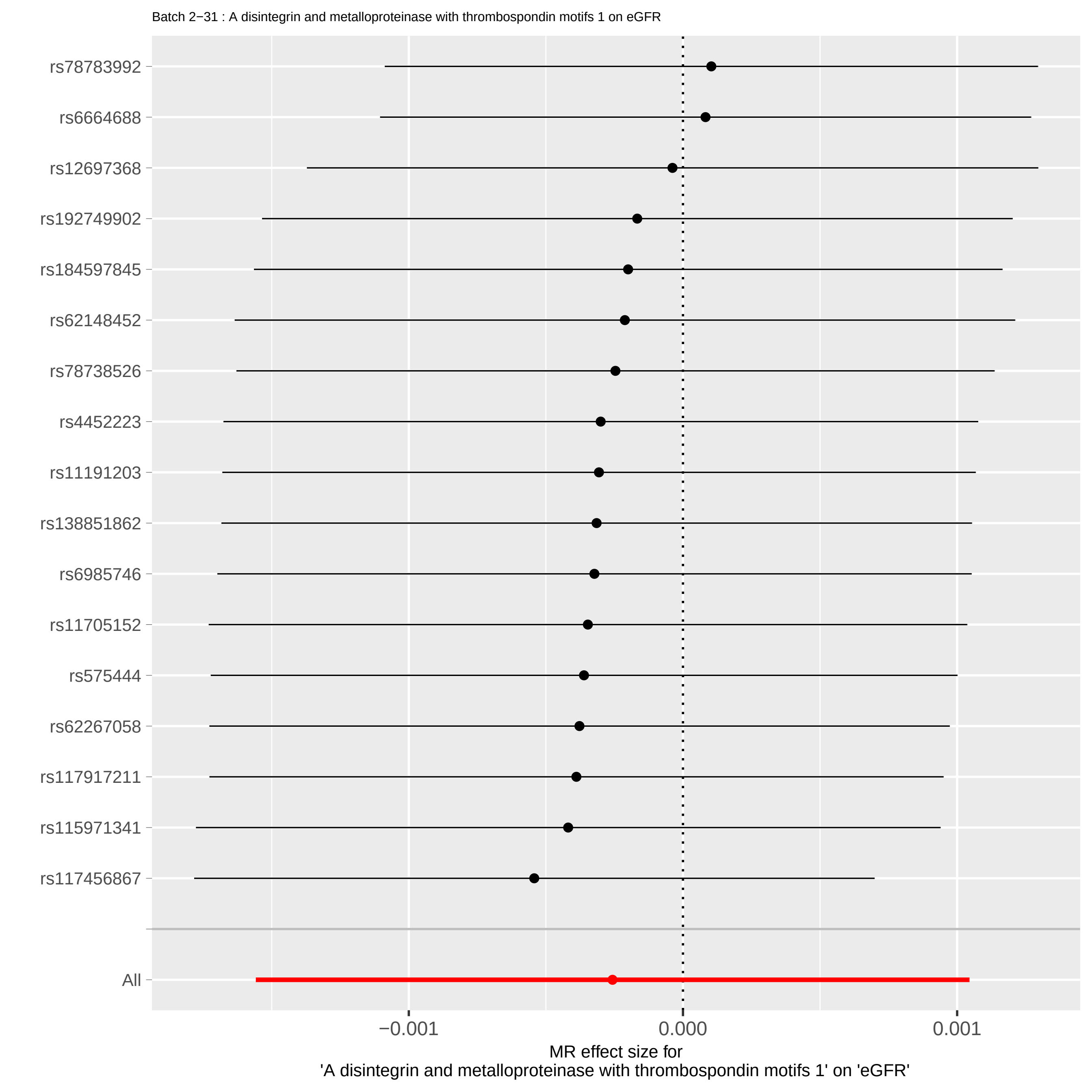

Batch 2−31 : A disintegrin and metalloproteinase with thrombospondin motifs 1 on eGFR
rs78783992
rs6664688
rs12697368
rs192749902
rs184597845
rs62148452
rs78738526
rs4452223
rs11191203
rs138851862
rs6985746
rs11705152
rs575444
rs62267058
rs117917211
rs115971341
rs117456867
All
−0.001
0.000
0.001
MR effect size for
'A disintegrin and metalloproteinase with thrombospondin motifs 1' on 'eGFR'

Batch 2−32 : A disintegrin and metalloproteinase with thrombospondin motifs 13 on eGFR

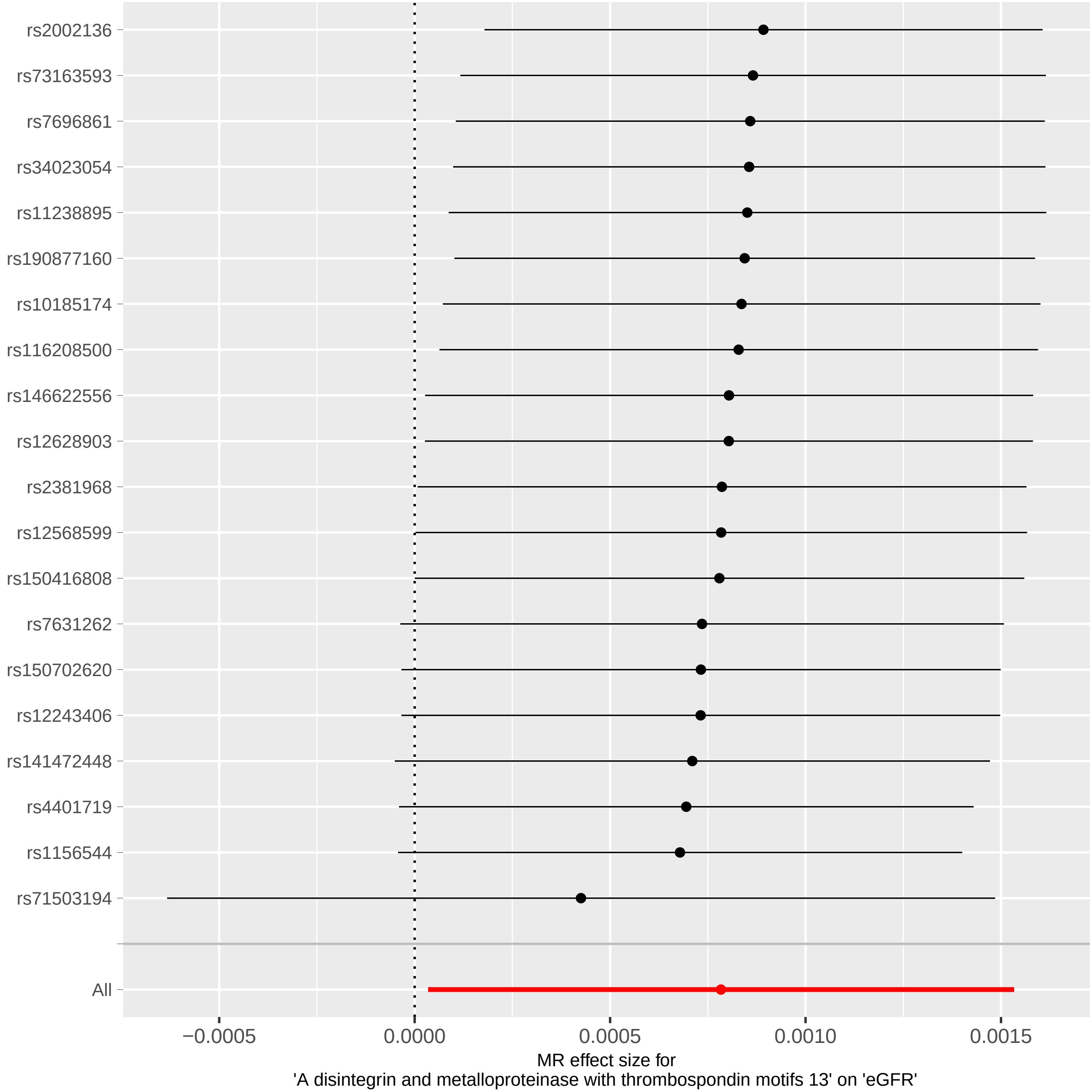

Batch 2−33 : A disintegrin and metalloproteinase with thrombospondin motifs 15 on eGFR

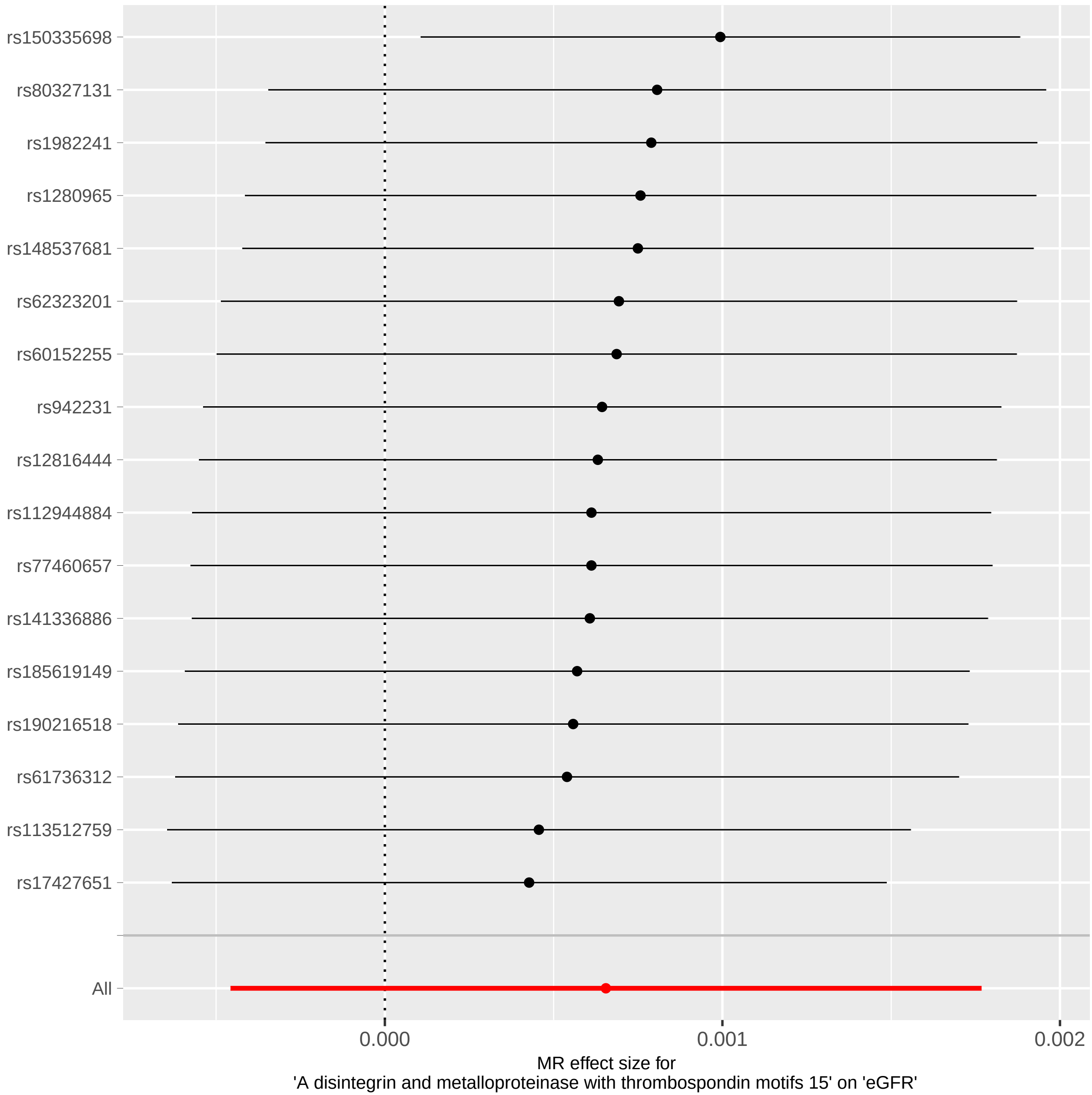

Batch 2−34 : A disintegrin and metalloproteinase with thrombospondin motifs 3 on eGFR

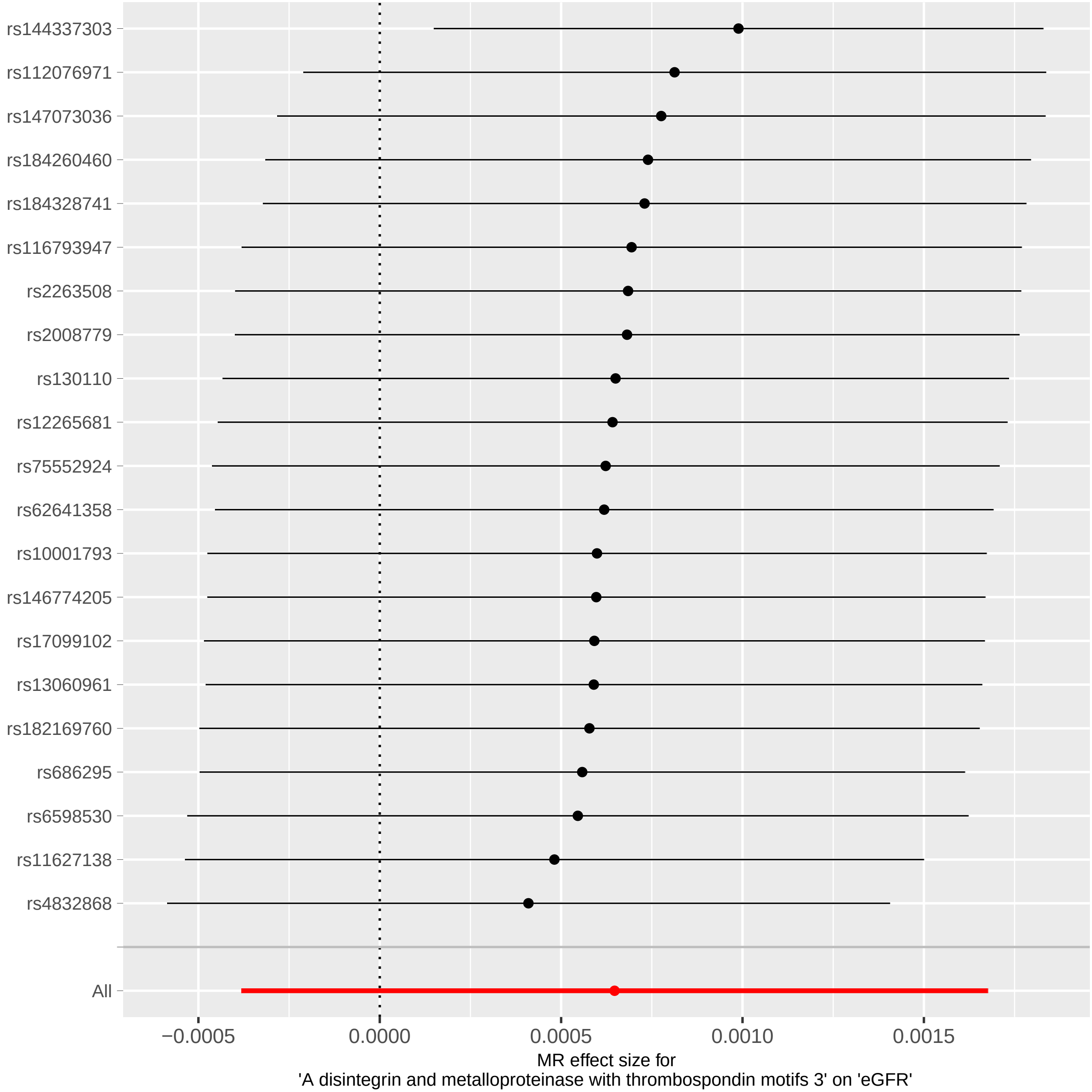

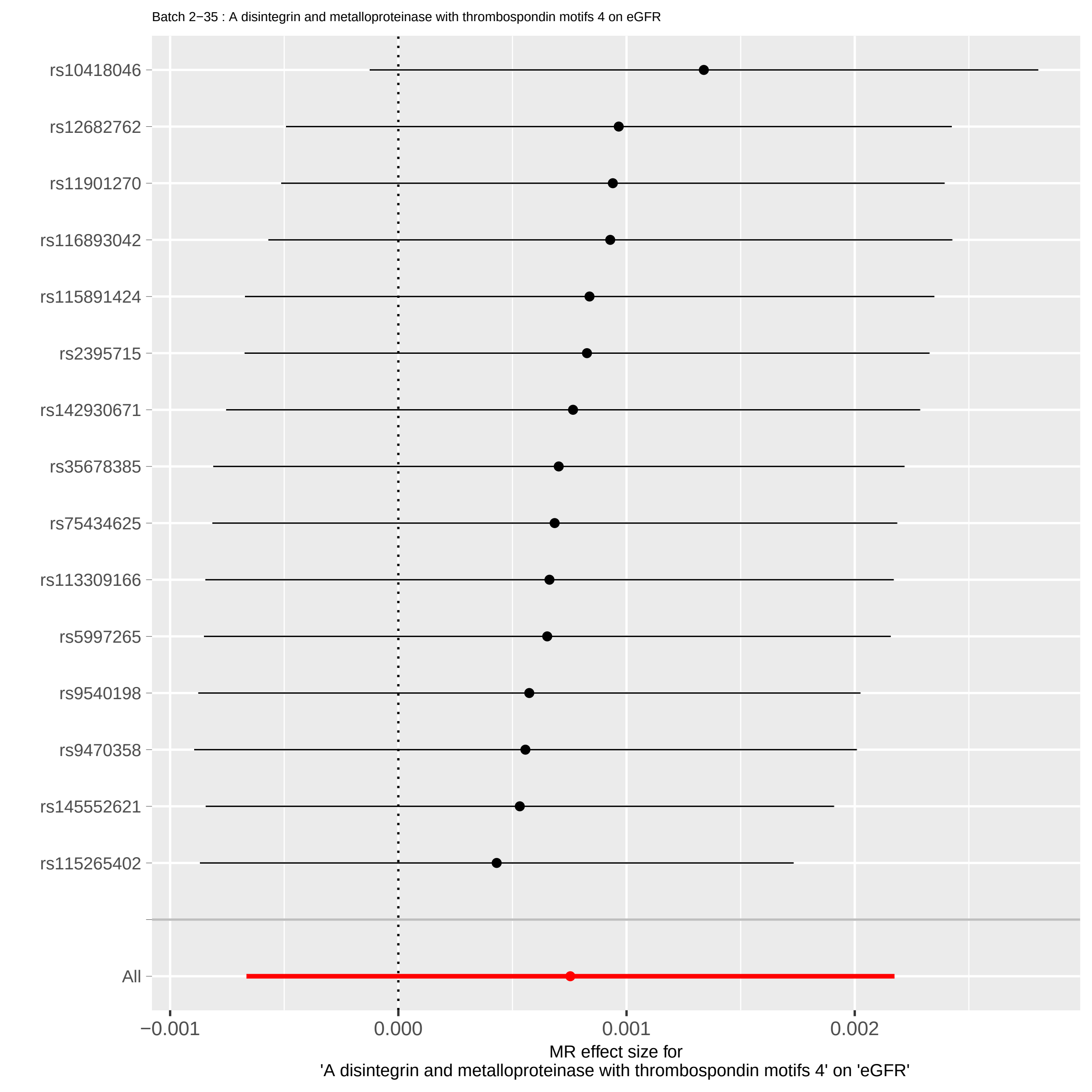

Batch 2−35 : A disintegrin and metalloproteinase with thrombospondin motifs 4 on eGFR
rs10418046
rs12682762
rs11901270
rs116893042
rs115891424
rs2395715
rs142930671
rs35678385
rs75434625
rs113309166
rs5997265
rs9540198
rs9470358
rs145552621
rs115265402
All
−0.001
0.000
0.001
0.002
MR effect size for
'A disintegrin and metalloproteinase with thrombospondin motifs 4' on 'eGFR'

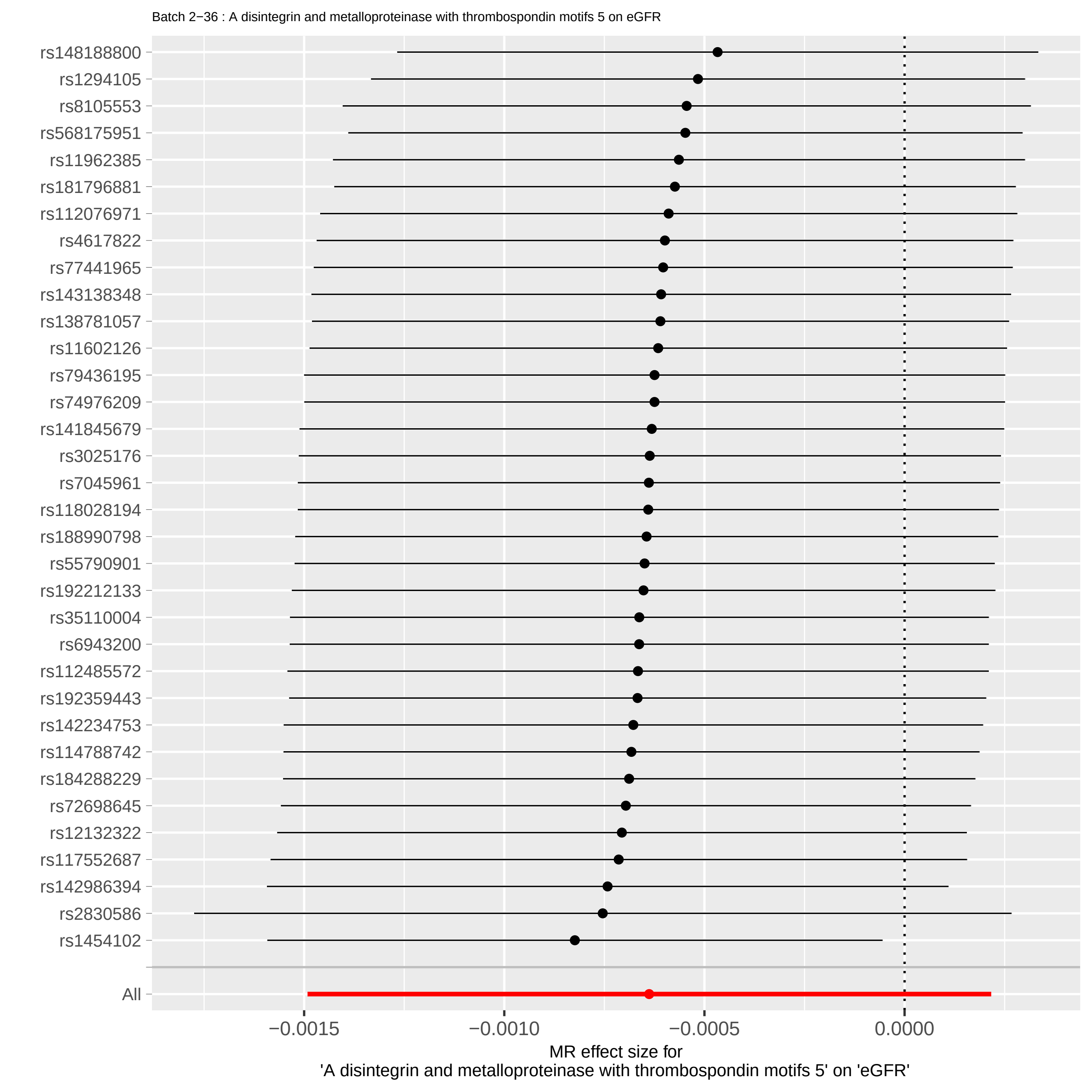

Batch 2−36 : A disintegrin and metalloproteinase with thrombospondin motifs 5 on eGFR
rs148188800
rs1294105
rs8105553
rs568175951
rs11962385
rs181796881
rs112076971
rs4617822
rs77441965
rs143138348
rs138781057
rs11602126
rs79436195
rs74976209
rs141845679
rs3025176
rs7045961
rs118028194
rs188990798
rs55790901
rs192212133
rs35110004
rs6943200
rs112485572
rs192359443
rs142234753
rs114788742
rs184288229
rs72698645
rs12132322
rs117552687
rs142986394
rs2830586
rs1454102
All
−0.0015
−0.0010
−0.0005
0.0000
MR effect size for
'A disintegrin and metalloproteinase with thrombospondin motifs 5' on 'eGFR'

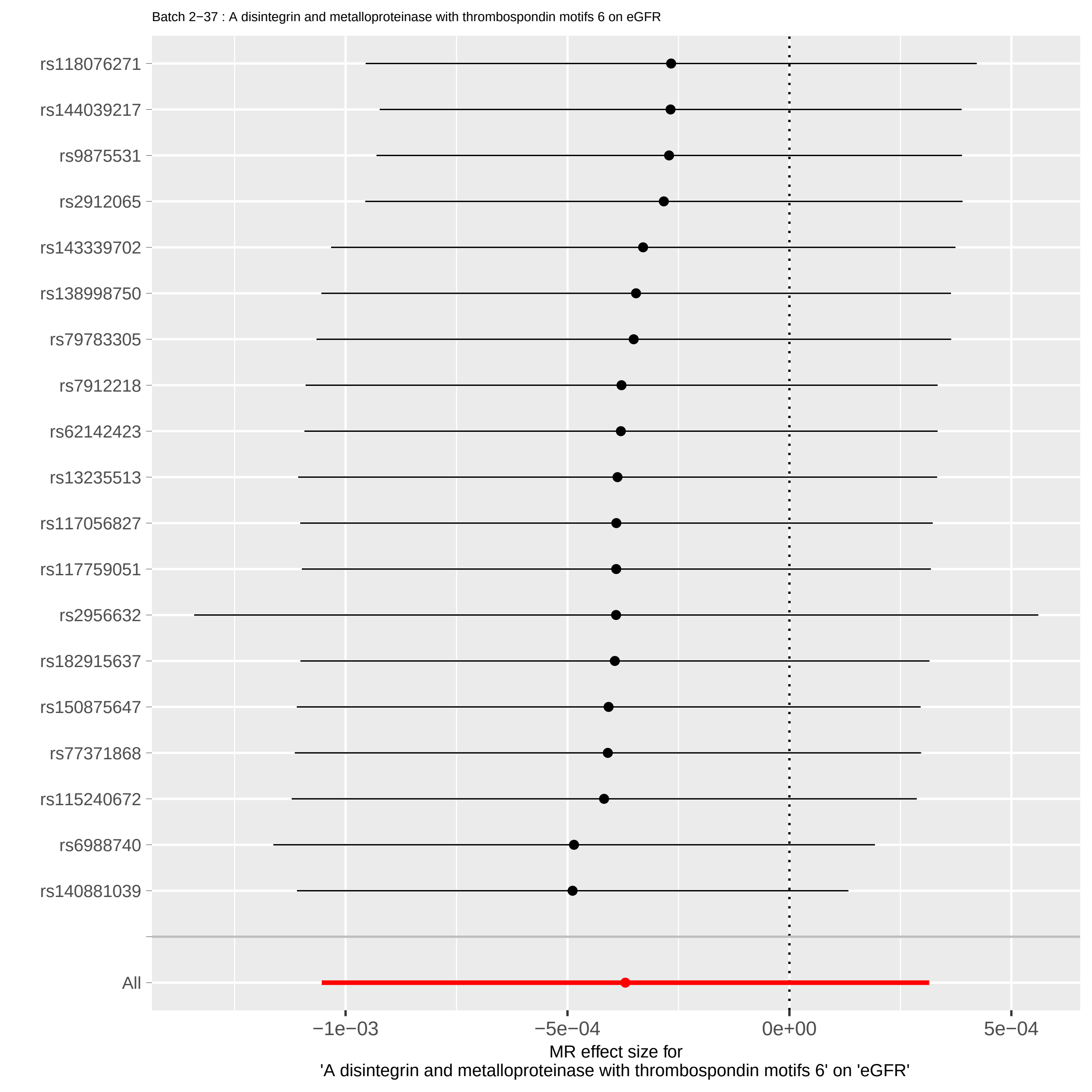

Batch 2−37 : A disintegrin and metalloproteinase with thrombospondin motifs 6 on eGFR
rs118076271
rs144039217
rs9875531
rs2912065
rs143339702
rs138998750
rs79783305
rs7912218
rs62142423
rs13235513
rs117056827
rs117759051
rs2956632
rs182915637
rs150875647
rs77371868
rs115240672
rs6988740
rs140881039
All
−1e−03
−5e−04
0e+00
5e−04
MR effect size for
'A disintegrin and metalloproteinase with thrombospondin motifs 6' on 'eGFR'

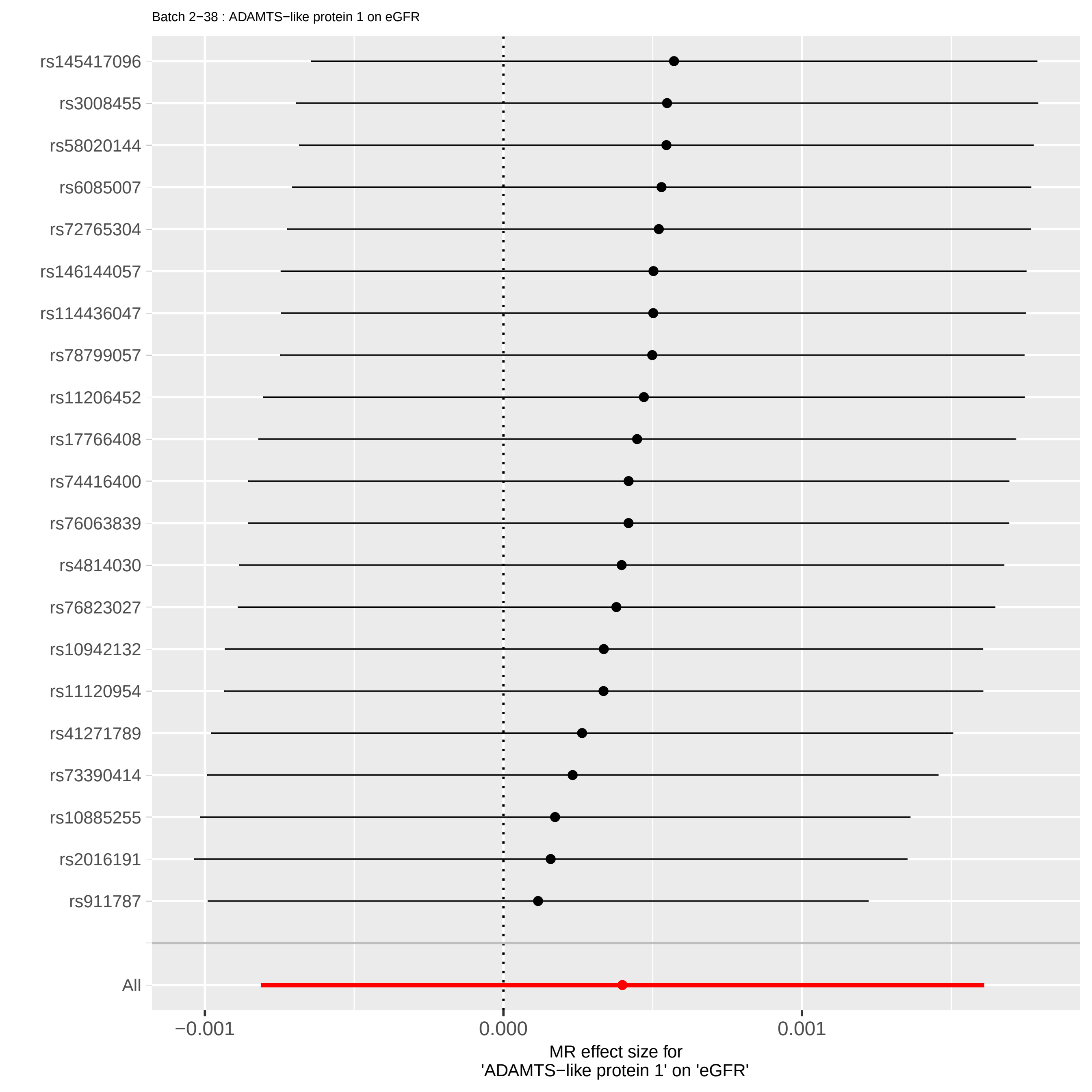

Batch 2−38 : ADAMTS−like protein 1 on eGFR
rs145417096
rs3008455
rs58020144
rs6085007
rs72765304
rs146144057
rs114436047
rs78799057
rs11206452
rs17766408
rs74416400
rs76063839
rs4814030
rs76823027
rs10942132
rs11120954
rs41271789
rs73390414
rs10885255
rs2016191
rs911787
All
−0.001
0.000
0.001
MR effect size for
'ADAMTS−like protein 1' on 'eGFR'

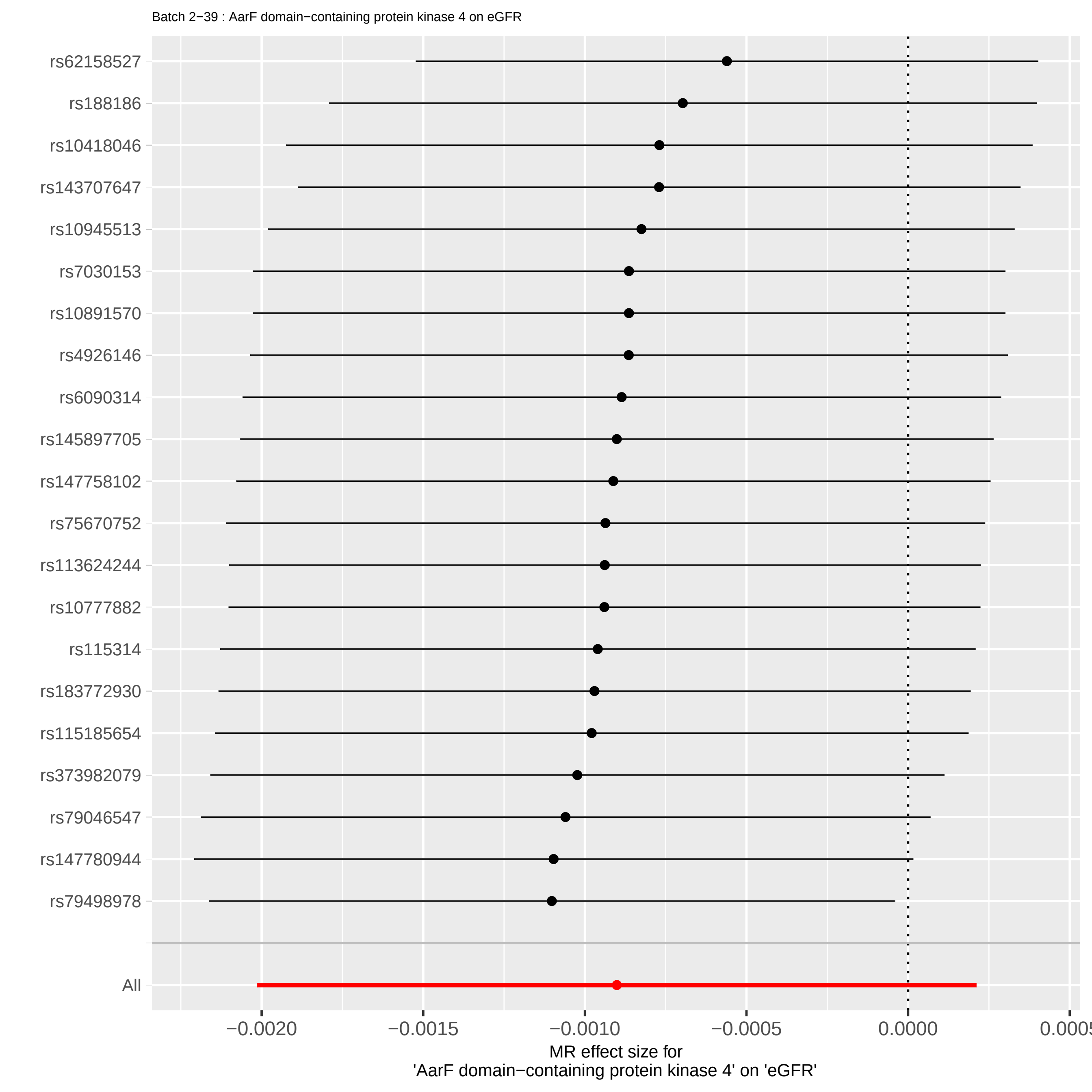

Batch 2−39 : AarF domain−containing protein kinase 4 on eGFR
rs62158527
rs188186
rs10418046
rs143707647
rs10945513
rs7030153
rs10891570
rs4926146
rs6090314
rs145897705
rs147758102
rs75670752
rs113624244
rs10777882
rs115314
rs183772930
rs115185654
rs373982079
rs79046547
rs147780944
rs79498978
All
−0.0020
−0.0015
−0.0010
−0.0005
0.0000
MR effect size for
'AarF domain−containing protein kinase 4' on 'eGFR'

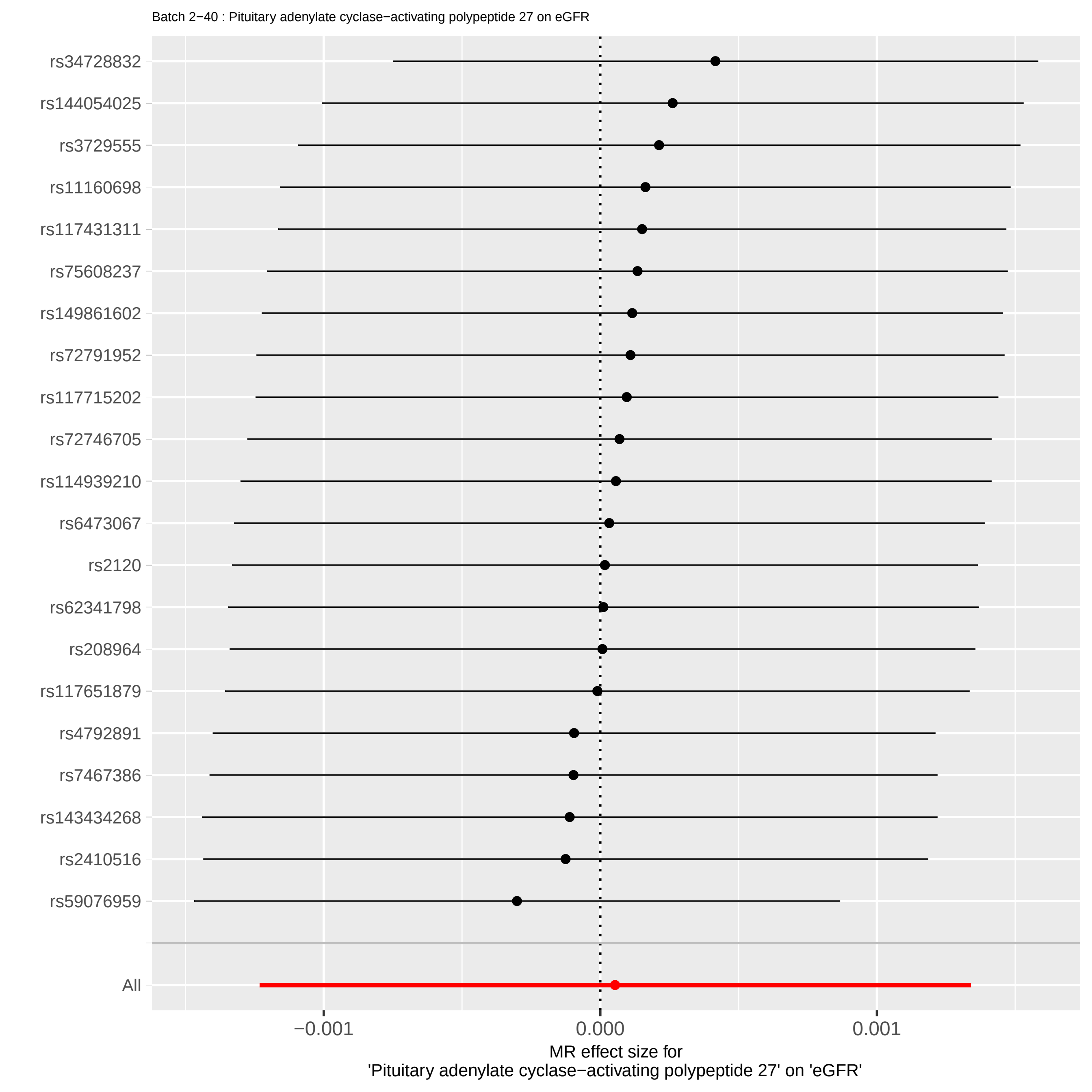
Batch 2−40 : Pituitary adenylate cyclase−activating polypeptide 27 on eGFR
rs34728832
rs144054025
rs3729555
rs11160698
rs117431311
rs75608237
rs149861602
rs72791952
rs117715202
rs72746705
rs114939210
rs6473067
rs2120
rs62341798
rs208964
rs117651879
rs4792891
rs7467386
rs143434268
rs2410516
rs59076959
All
−0.001
0.000
0.001
MR effect size for
'Pituitary adenylate cyclase−activating polypeptide 27' on 'eGFR'

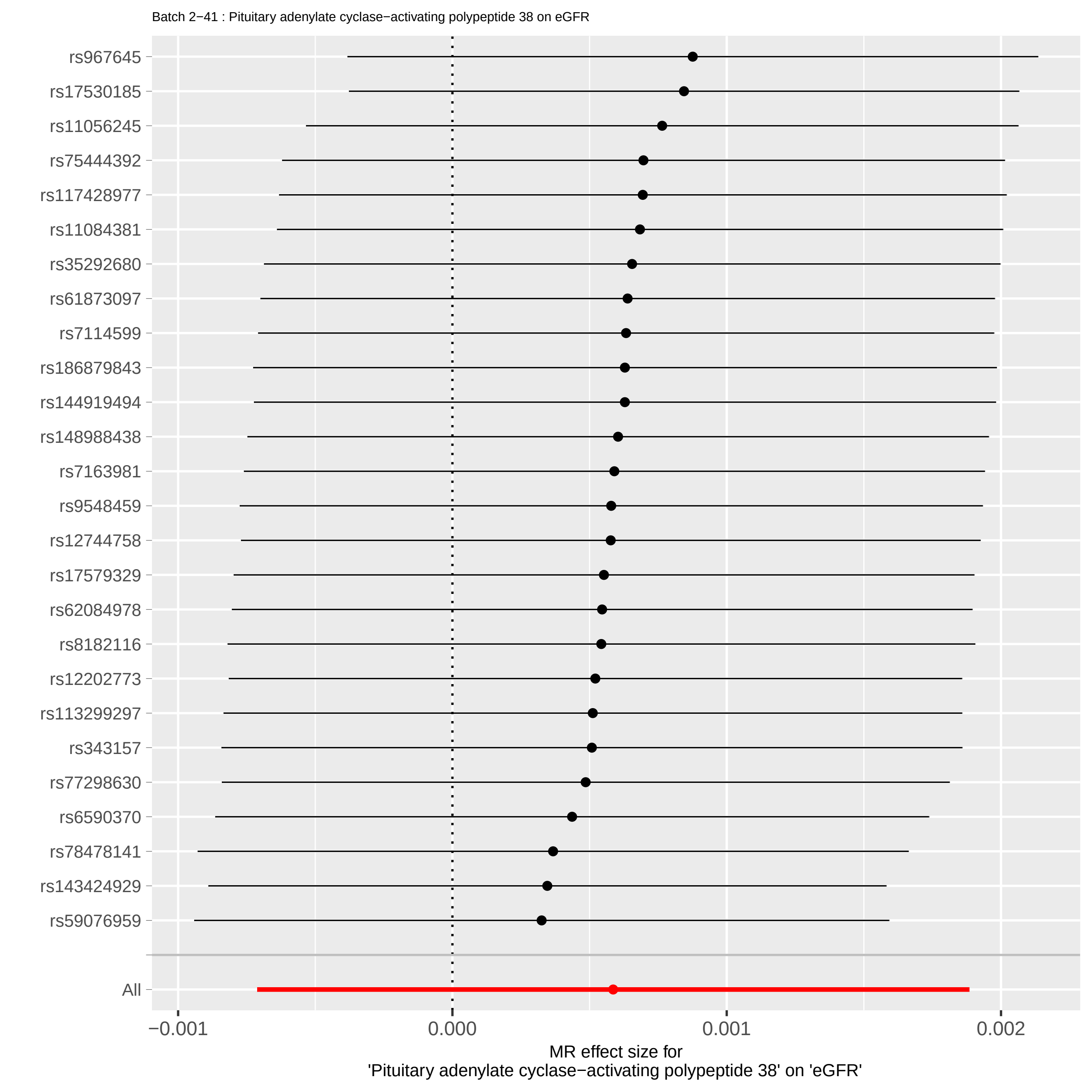
Batch 2−41 : Pituitary adenylate cyclase−activating polypeptide 38 on eGFR
rs967645
rs17530185
rs11056245
rs75444392
rs117428977
rs11084381
rs35292680
rs61873097
rs7114599
rs186879843
rs144919494
rs148988438
rs7163981
rs9548459
rs12744758
rs17579329
rs62084978
rs8182116
rs12202773
rs113299297
rs343157
rs77298630
rs6590370
rs78478141
rs143424929
rs59076959
All
−0.001
0.000
0.001
0.002
MR effect size for
'Pituitary adenylate cyclase−activating polypeptide 38' on 'eGFR'

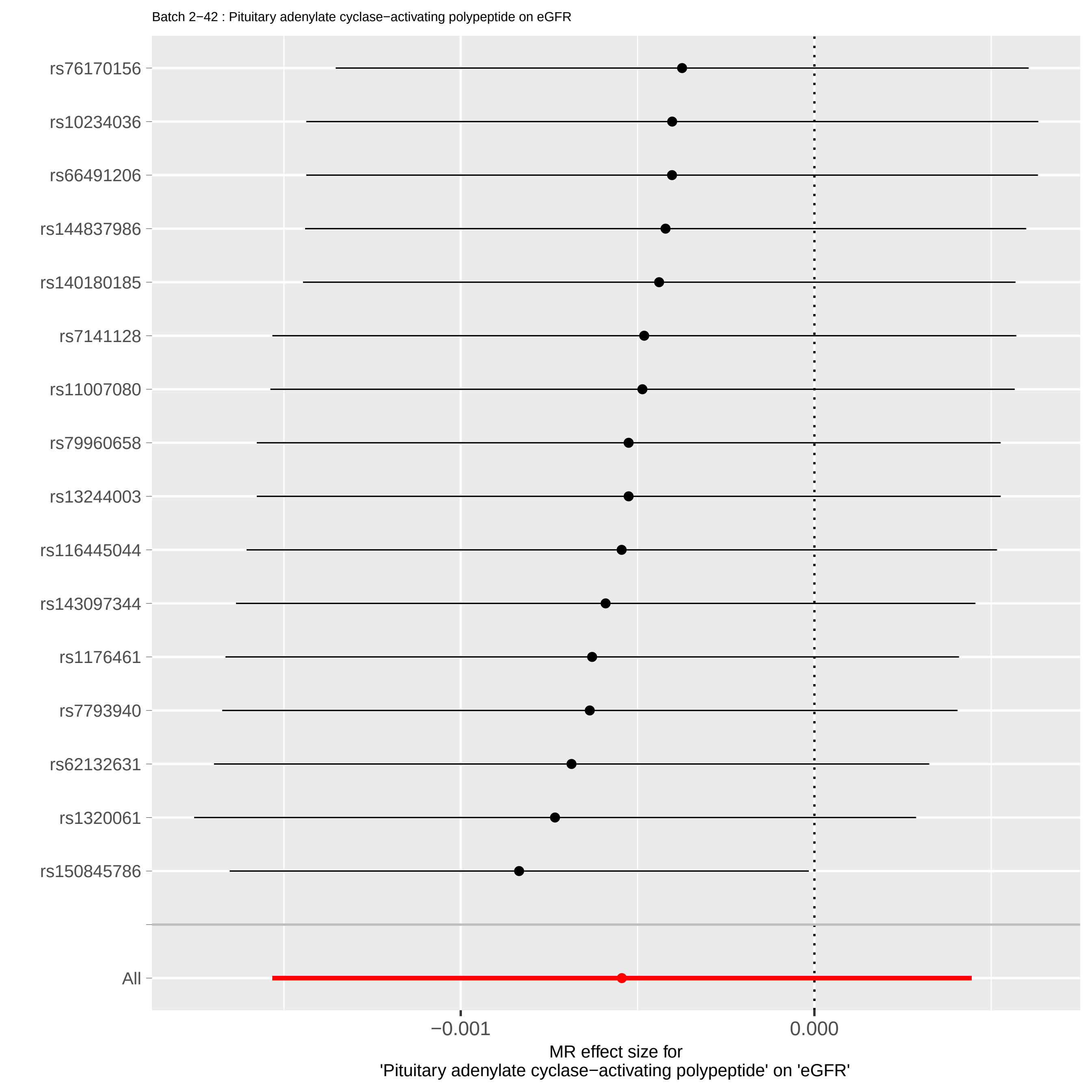

Batch 2−42 : Pituitary adenylate cyclase−activating polypeptide on eGFR
rs76170156
rs10234036
rs66491206
rs144837986
rs140180185
rs7141128
rs11007080
rs79960658
rs13244003
rs116445044
rs143097344
rs1176461
rs7793940
rs62132631
rs1320061
rs150845786
All
−0.001
0.000
MR effect size for
'Pituitary adenylate cyclase−activating polypeptide' on 'eGFR'

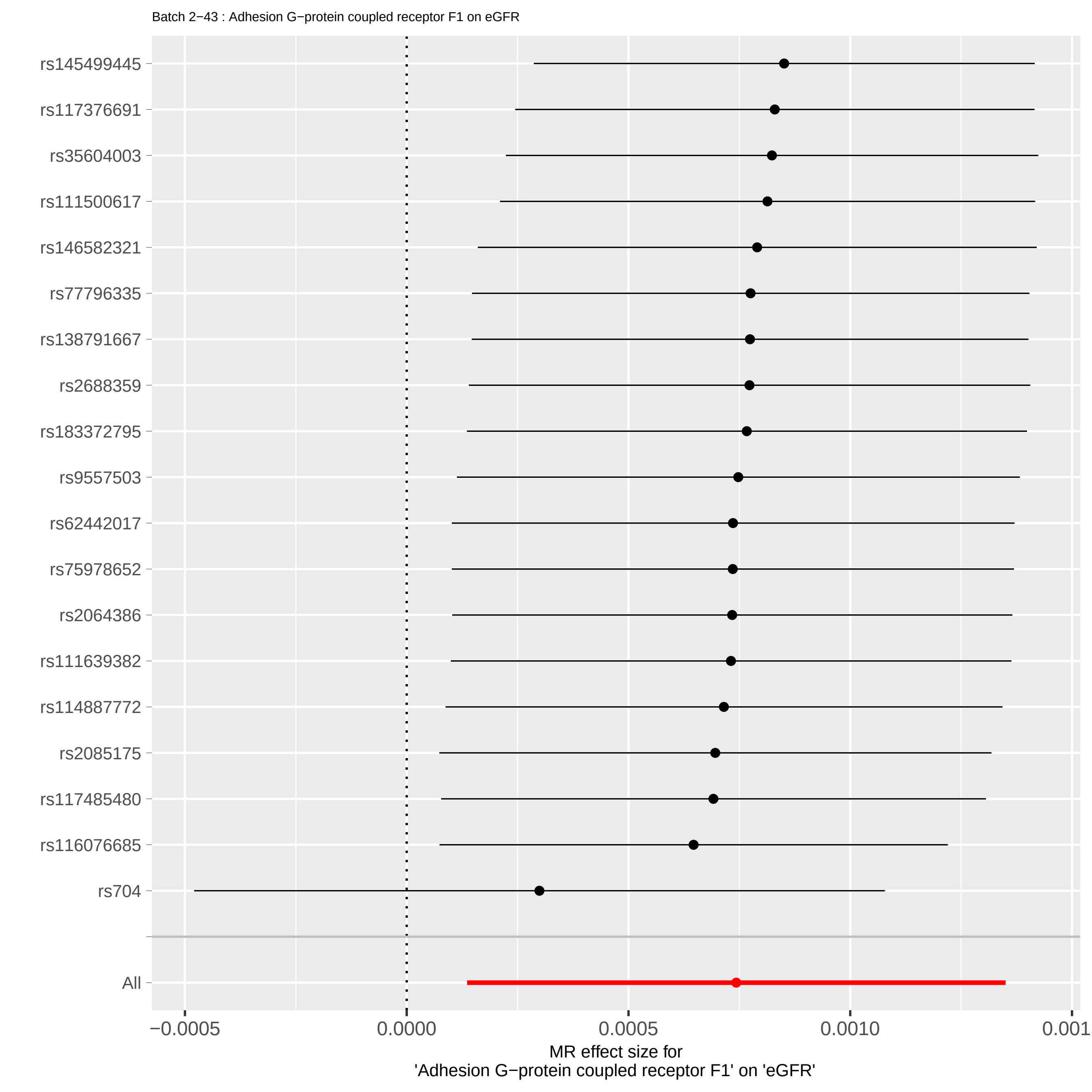

Batch 2−43 : Adhesion G−protein coupled receptor F1 on eGFR
rs145499445
rs117376691
rs35604003
rs111500617
rs146582321
rs77796335
rs138791667
rs2688359
rs183372795
rs9557503
rs62442017
rs75978652
rs2064386
rs111639382
rs114887772
rs2085175
rs117485480
rs116076685
rs704
All
−0.0005
0.0000
0.0005
0.0010
0.001
MR effect size for
'Adhesion G−protein coupled receptor F1' on 'eGFR'

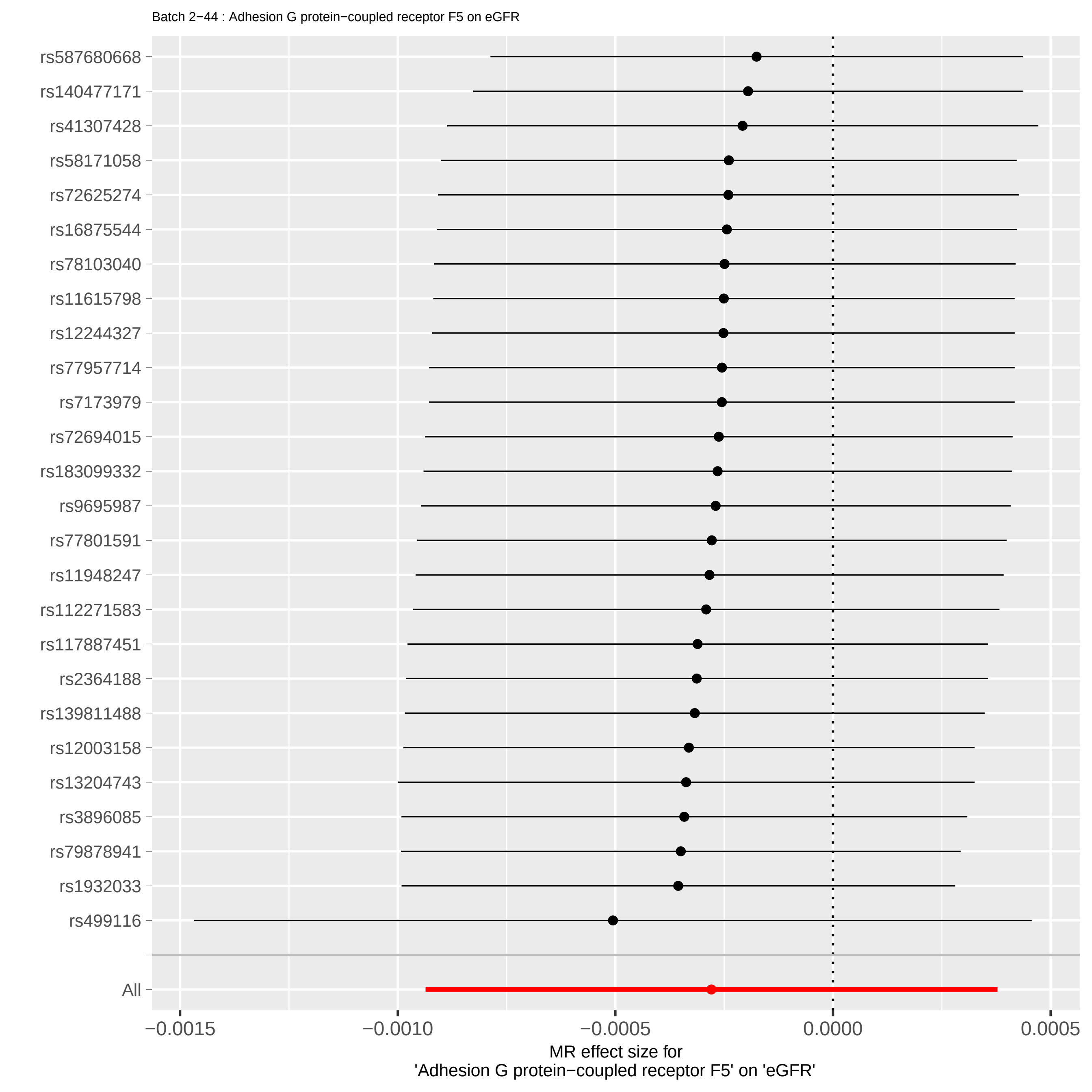

Batch 2−44 : Adhesion G protein−coupled receptor F5 on eGFR
rs587680668
rs140477171
rs41307428
rs58171058
rs72625274
rs16875544
rs78103040
rs11615798
rs12244327
rs77957714
rs7173979
rs72694015
rs183099332
rs9695987
rs77801591
rs11948247
rs112271583
rs117887451
rs2364188
rs139811488
rs12003158
rs13204743
rs3896085
rs79878941
rs1932033
rs499116
All
−0.0015
−0.0010
−0.0005
0.0000
0.0005
MR effect size for
'Adhesion G protein−coupled receptor F5' on 'eGFR'

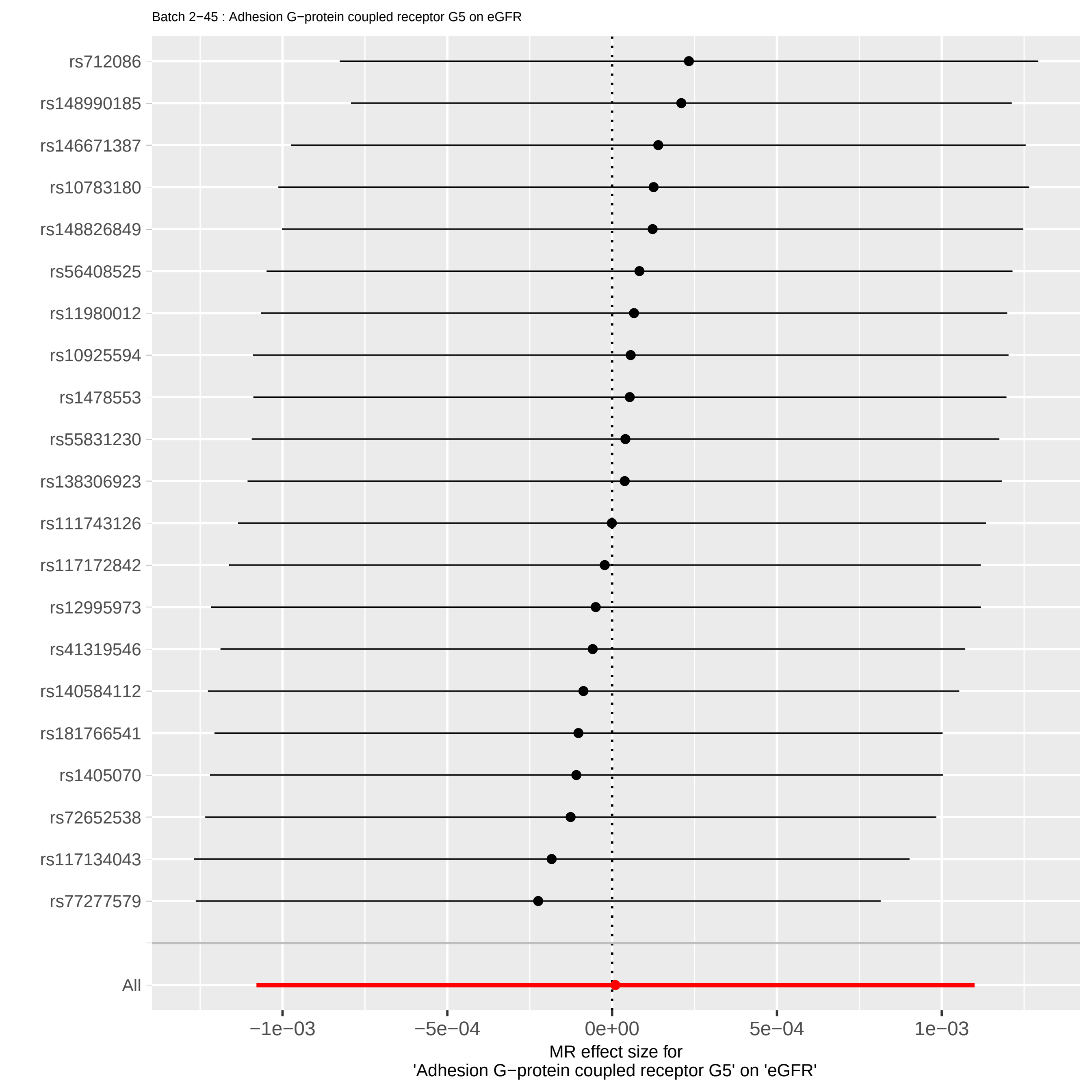

Batch 2−45 : Adhesion G−protein coupled receptor G5 on eGFR
rs712086
rs148990185
rs146671387
rs10783180
rs148826849
rs56408525
rs11980012
rs10925594
rs1478553
rs55831230
rs138306923
rs111743126
rs117172842
rs12995973
rs41319546
rs140584112
rs181766541
rs1405070
rs72652538
rs117134043
rs77277579
All
−1e−03
−5e−04
0e+00
5e−04
1e−03
MR effect size for
'Adhesion G−protein coupled receptor G5' on 'eGFR'

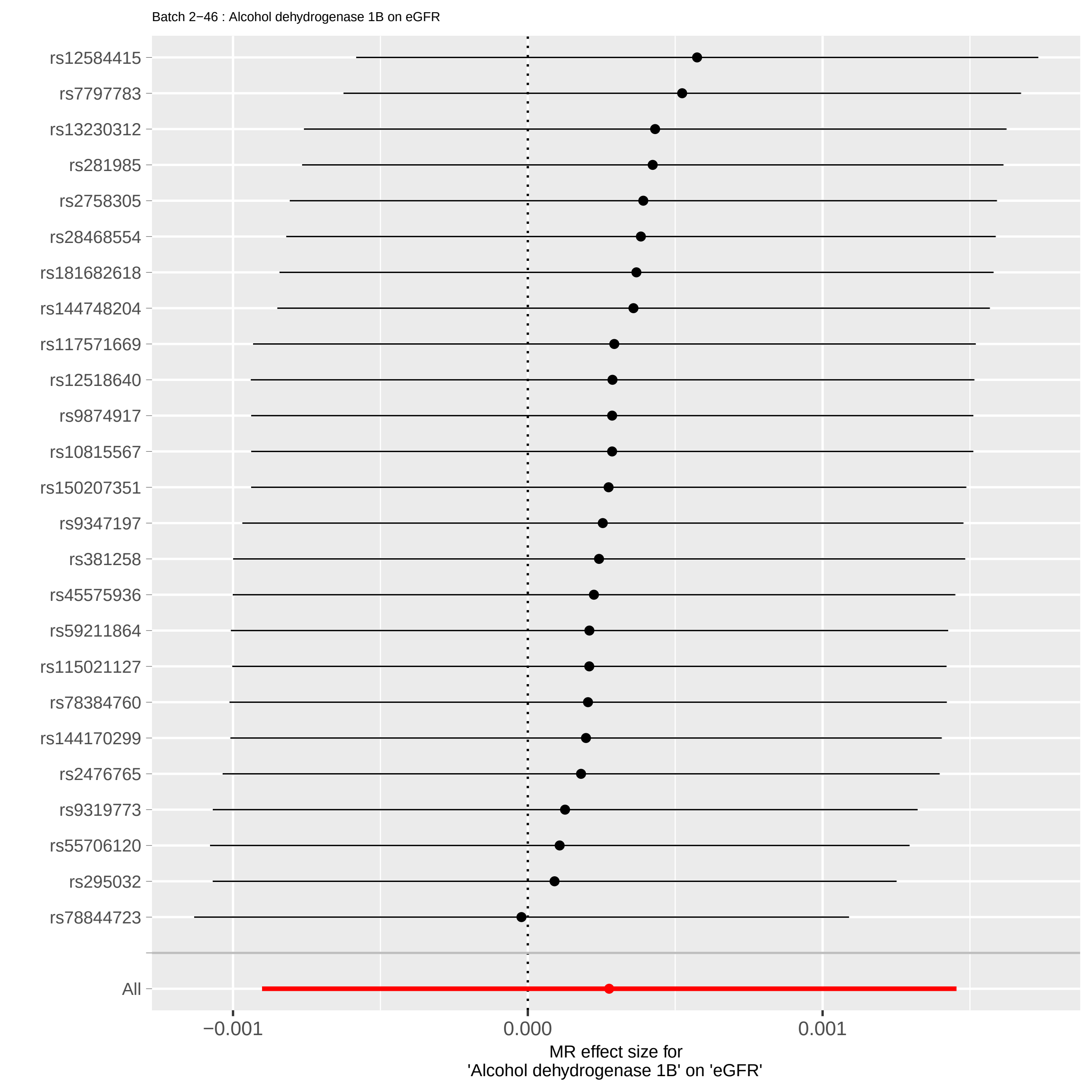
Batch 2−46 : Alcohol dehydrogenase 1B on eGFR
rs12584415
rs7797783
rs13230312
rs281985
rs2758305
rs28468554
rs181682618
rs144748204
rs117571669
rs12518640
rs9874917
rs10815567
rs150207351
rs9347197
rs381258
rs45575936
rs59211864
rs115021127
rs78384760
rs144170299
rs2476765
rs9319773
rs55706120
rs295032
rs78844723
All
−0.001
0.000
0.001
MR effect size for
'Alcohol dehydrogenase 1B' on 'eGFR'

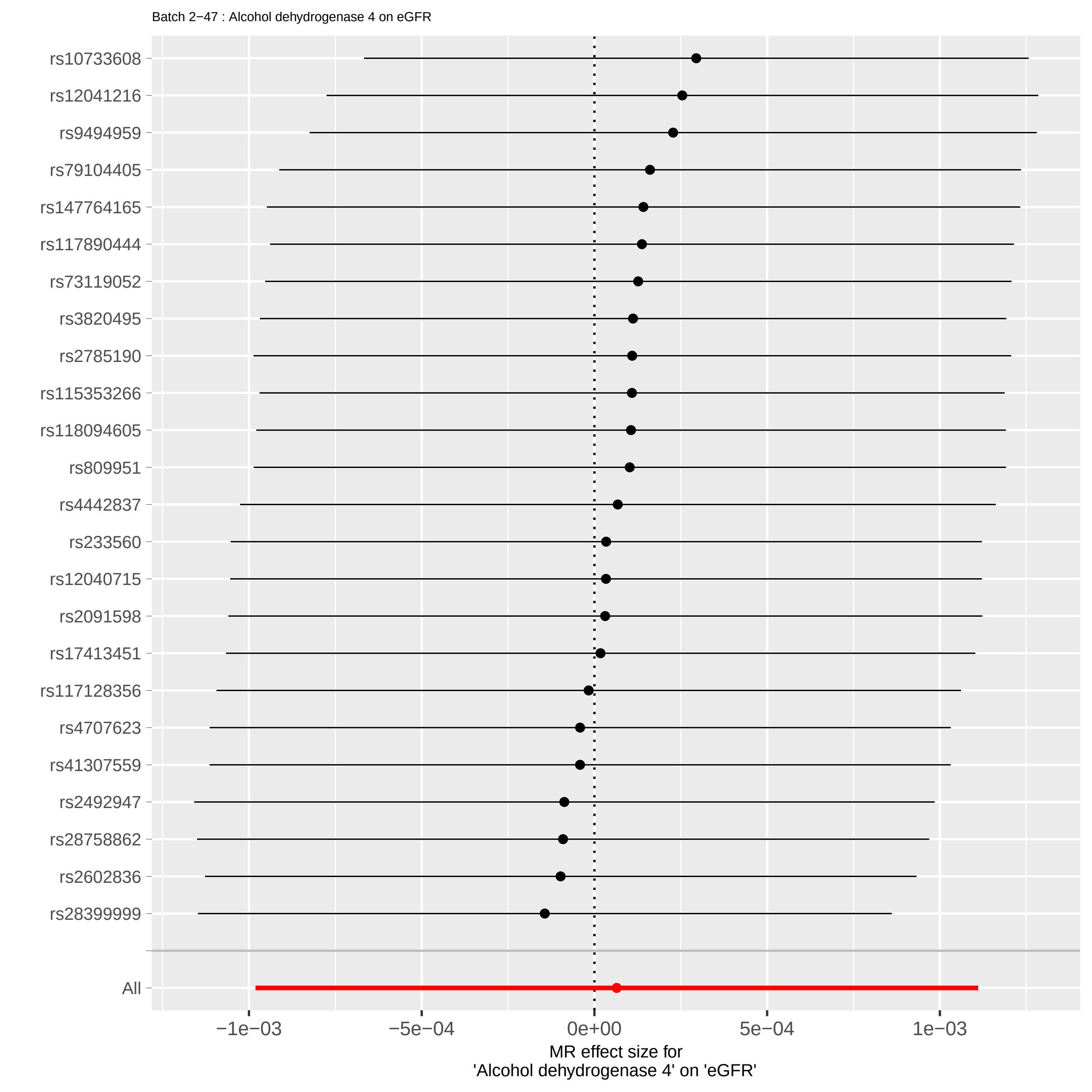

Batch 2−47 : Alcohol dehydrogenase 4 on eGFR
rs10733608
rs12041216
rs9494959
rs79104405
rs147764165
rs117890444
rs73119052
rs3820495
rs2785190
rs115353266
rs118094605
rs809951
rs4442837
rs233560
rs12040715
rs2091598
rs17413451
rs117128356
rs4707623
rs41307559
rs2492947
rs28758862
rs2602836
rs28399999
All
−1e−03
−5e−04
0e+00
5e−04
1e−03
MR effect size for
'Alcohol dehydrogenase 4' on 'eGFR'

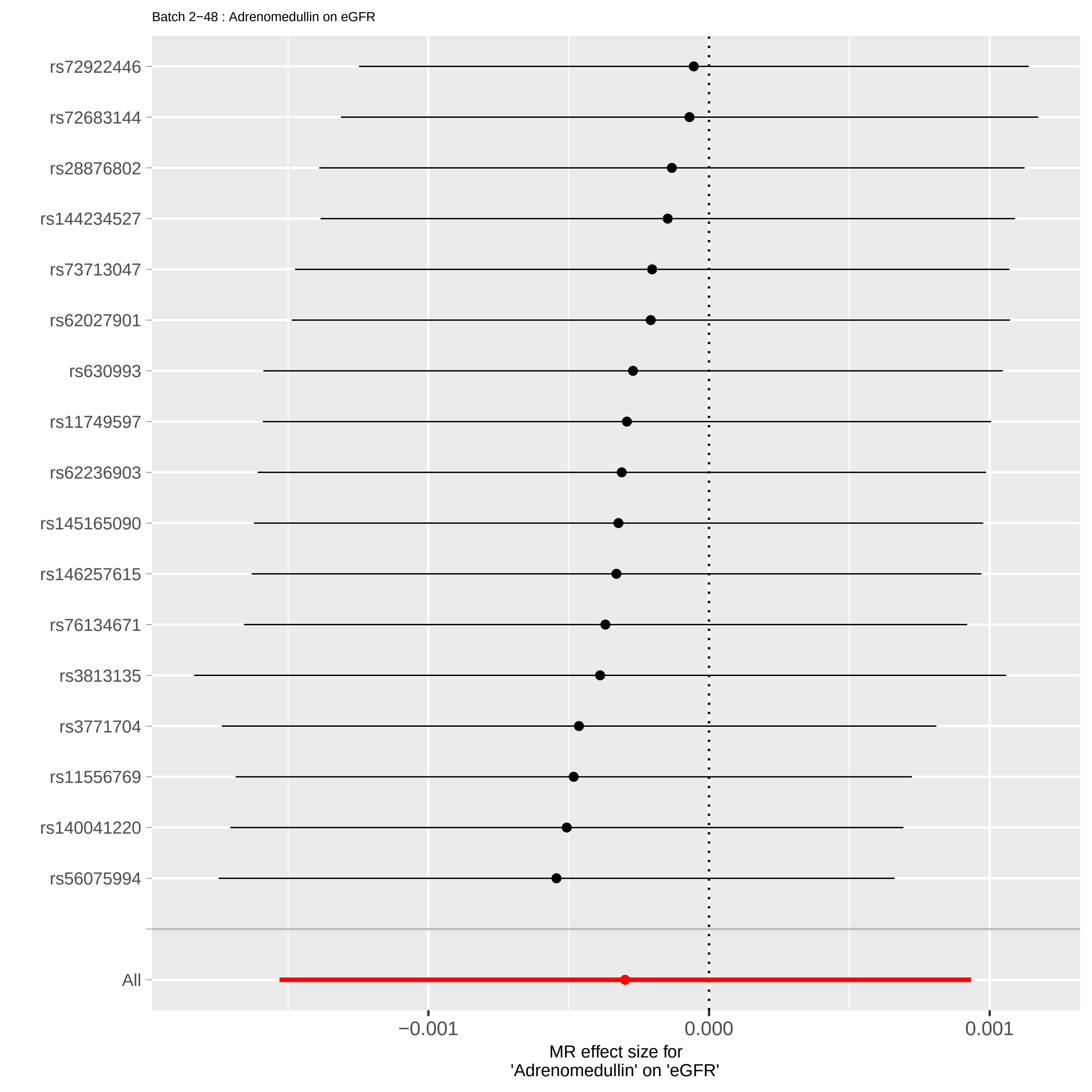

Batch 2−48 : Adrenomedullin on eGFR
rs72922446
rs72683144
rs28876802
rs144234527
rs73713047
rs62027901
rs630993
rs11749597
rs62236903
rs145165090
rs146257615
rs76134671
rs3813135
rs3771704
rs11556769
rs140041220
rs56075994
All
−0.001
0.000
0.001
MR effect size for
'Adrenomedullin' on 'eGFR'

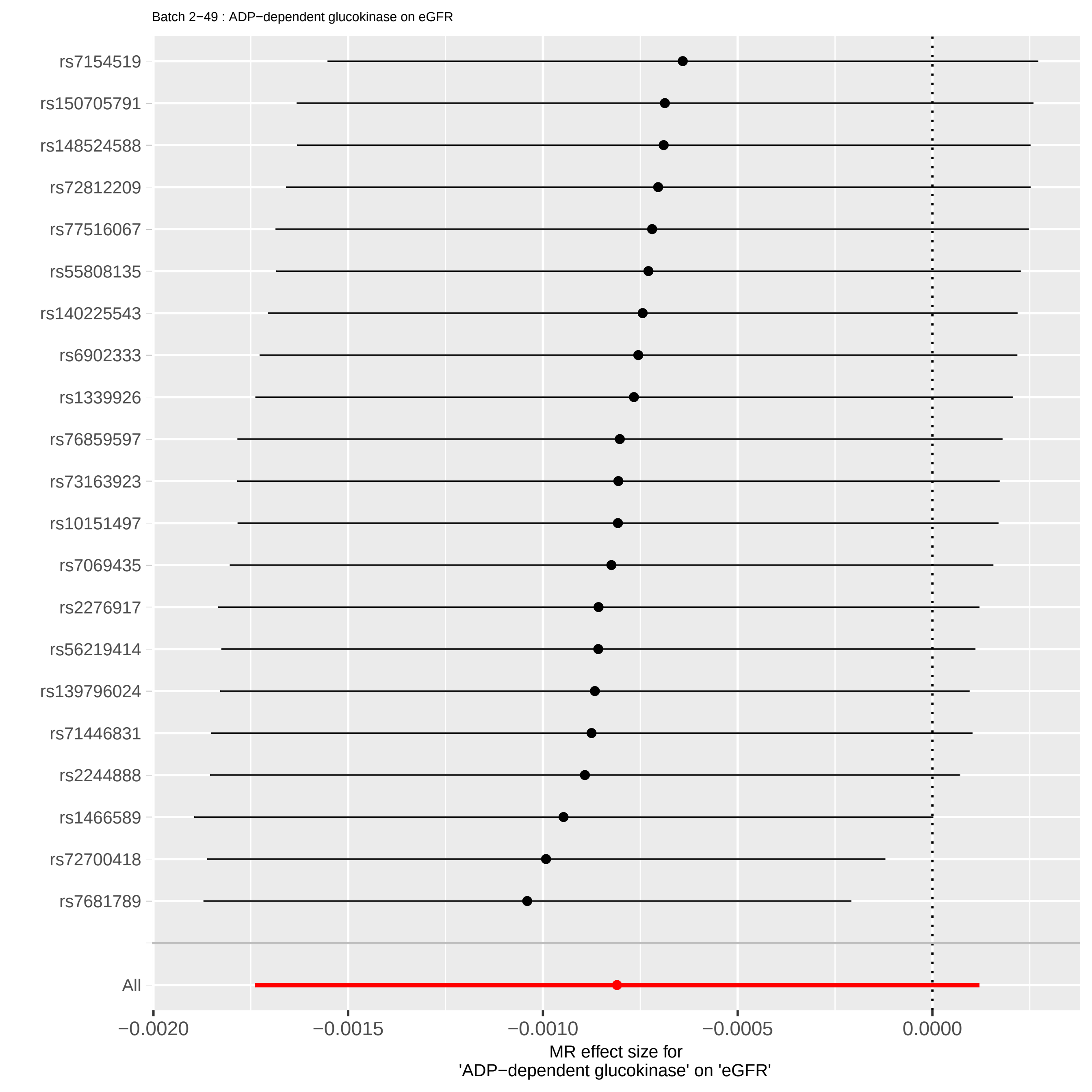

Batch 2−49 : ADP−dependent glucokinase on eGFR
rs7154519
rs150705791
rs148524588
rs72812209
rs77516067
rs55808135
rs140225543
rs6902333
rs1339926
rs76859597
rs73163923
rs10151497
rs7069435
rs2276917
rs56219414
rs139796024
rs71446831
rs2244888
rs1466589
rs72700418
rs7681789
All
−0.0020
−0.0015
−0.0010
−0.0005
0.0000
MR effect size for
'ADP−dependent glucokinase' on 'eGFR'

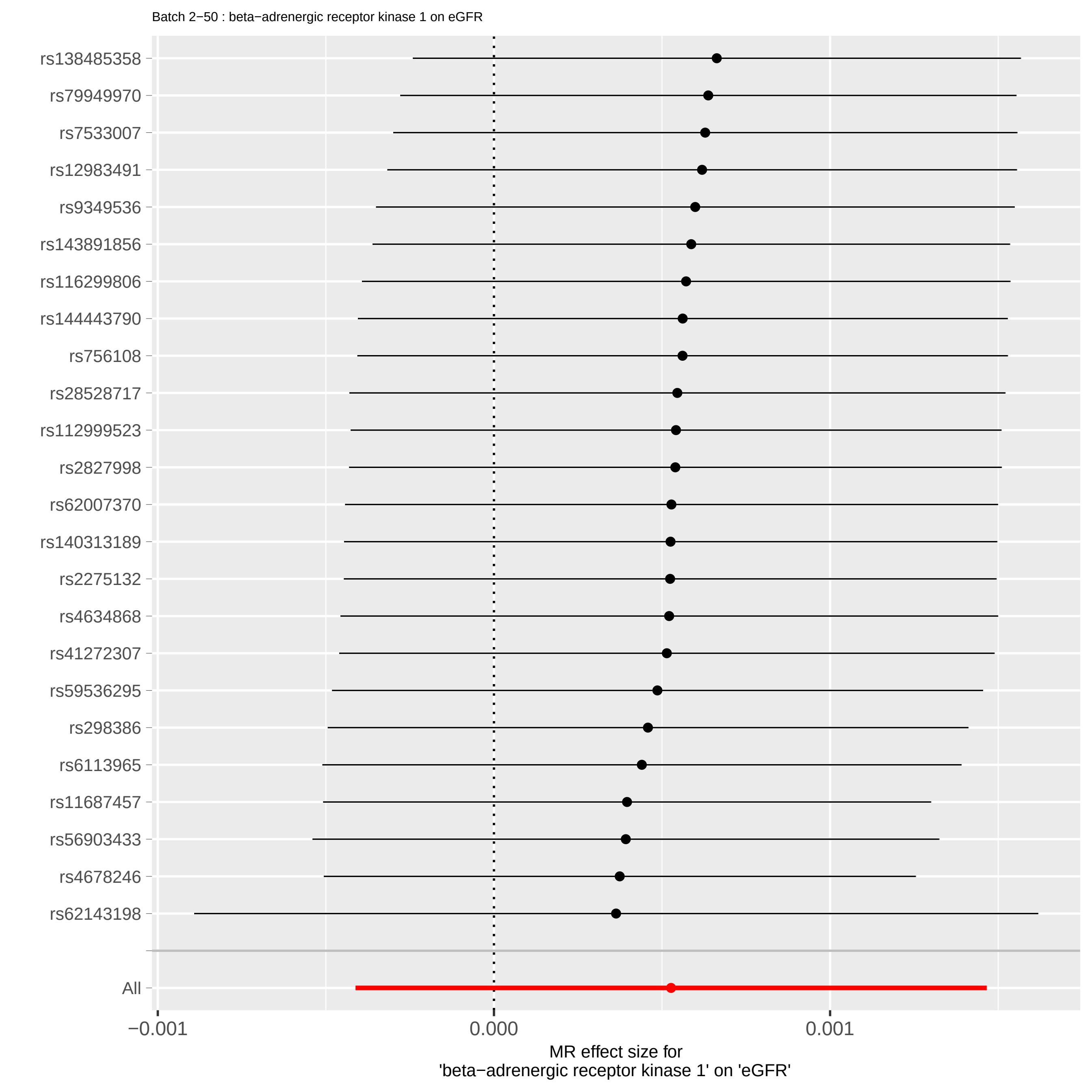

Batch 2−50 : beta−adrenergic receptor kinase 1 on eGFR
rs138485358
rs79949970
rs7533007
rs12983491
rs9349536
rs143891856
rs116299806
rs144443790
rs756108
rs28528717
rs112999523
rs2827998
rs62007370
rs140313189
rs2275132
rs4634868
rs41272307
rs59536295
rs298386
rs6113965
rs11687457
rs56903433
rs4678246
rs62143198
All
−0.001
0.000
0.001
MR effect size for
'beta−adrenergic receptor kinase 1' on 'eGFR'

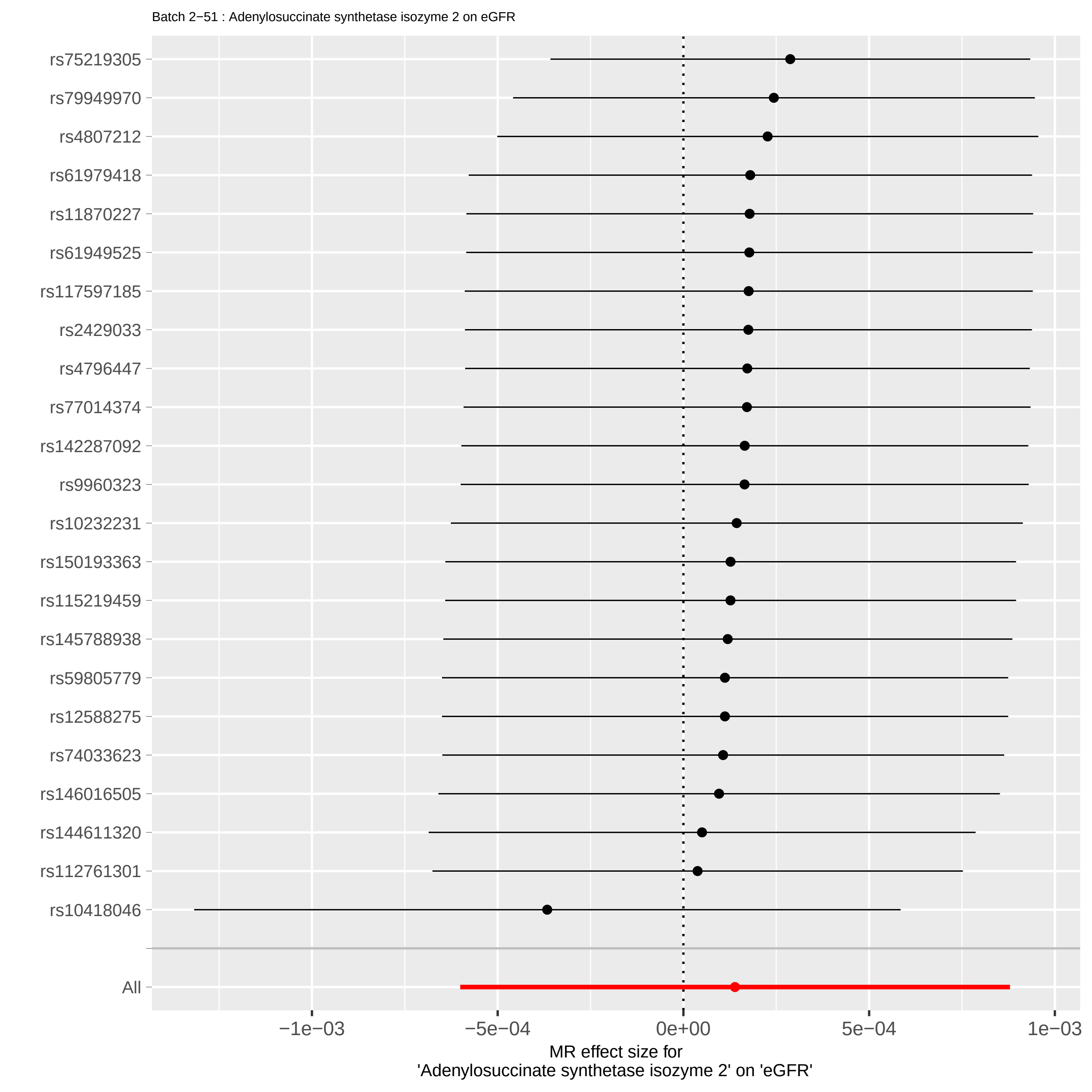
Batch 2−51 : Adenylosuccinate synthetase isozyme 2 on eGFR
rs75219305
rs79949970
rs4807212
rs61979418
rs11870227
rs61949525
rs117597185
rs2429033
rs4796447
rs77014374
rs142287092
rs9960323
rs10232231
rs150193363
rs115219459
rs145788938
rs59805779
rs12588275
rs74033623
rs146016505
rs144611320
rs112761301
rs10418046
All
−1e−03
−5e−04
0e+00
5e−04
1e−03
MR effect size for
'Adenylosuccinate synthetase isozyme 2' on 'eGFR'

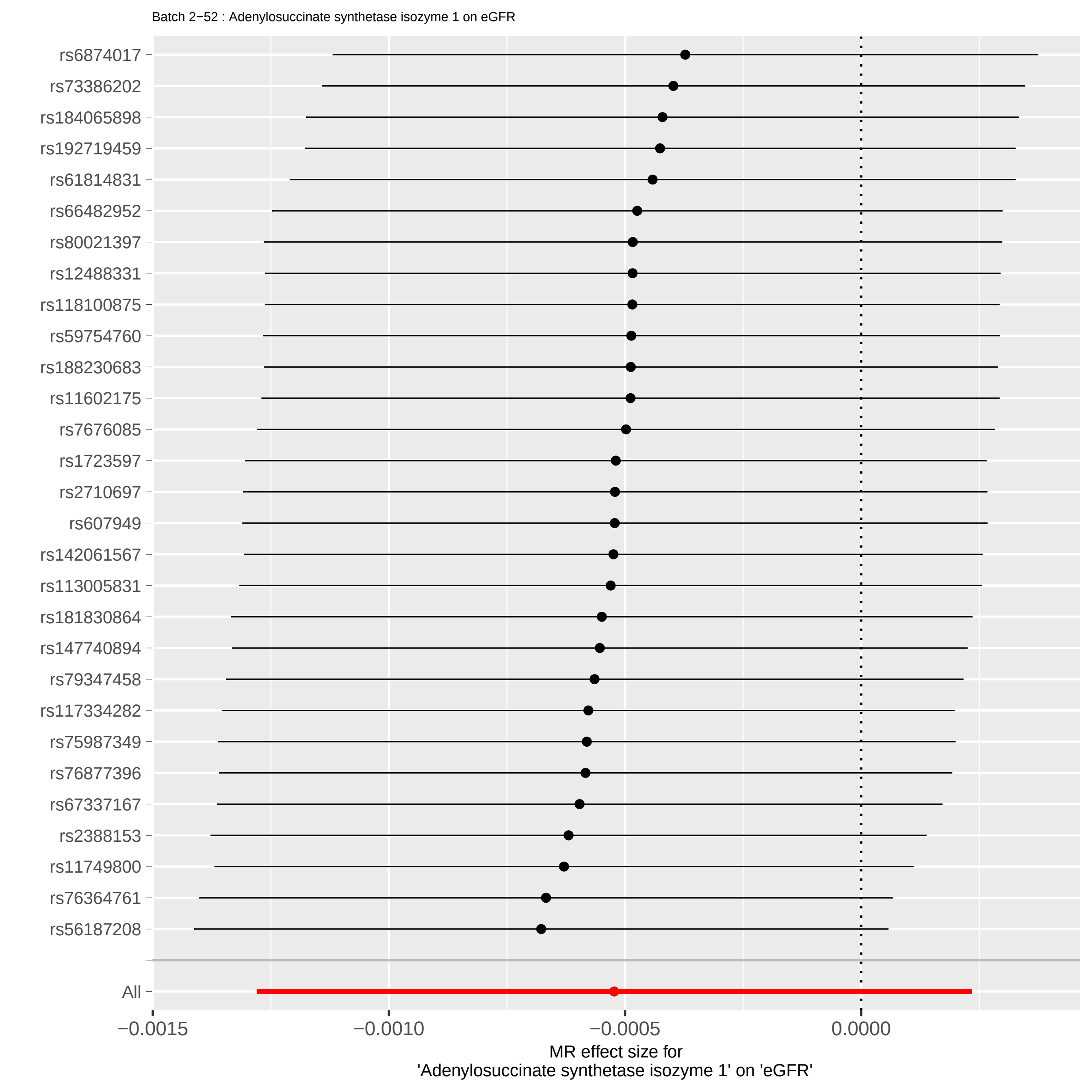

Batch 2−52 : Adenylosuccinate synthetase isozyme 1 on eGFR
rs6874017
rs73386202
rs184065898
rs192719459
rs61814831
rs66482952
rs80021397
rs12488331
rs118100875
rs59754760
rs188230683
rs11602175
rs7676085
rs1723597
rs2710697
rs607949
rs142061567
rs113005831
rs181830864
rs147740894
rs79347458
rs117334282
rs75987349
rs76877396
rs67337167
rs2388153
rs11749800
rs76364761
rs56187208
All
−0.0015
−0.0010
−0.0005
0.0000
MR effect size for
'Adenylosuccinate synthetase isozyme 1' on 'eGFR'

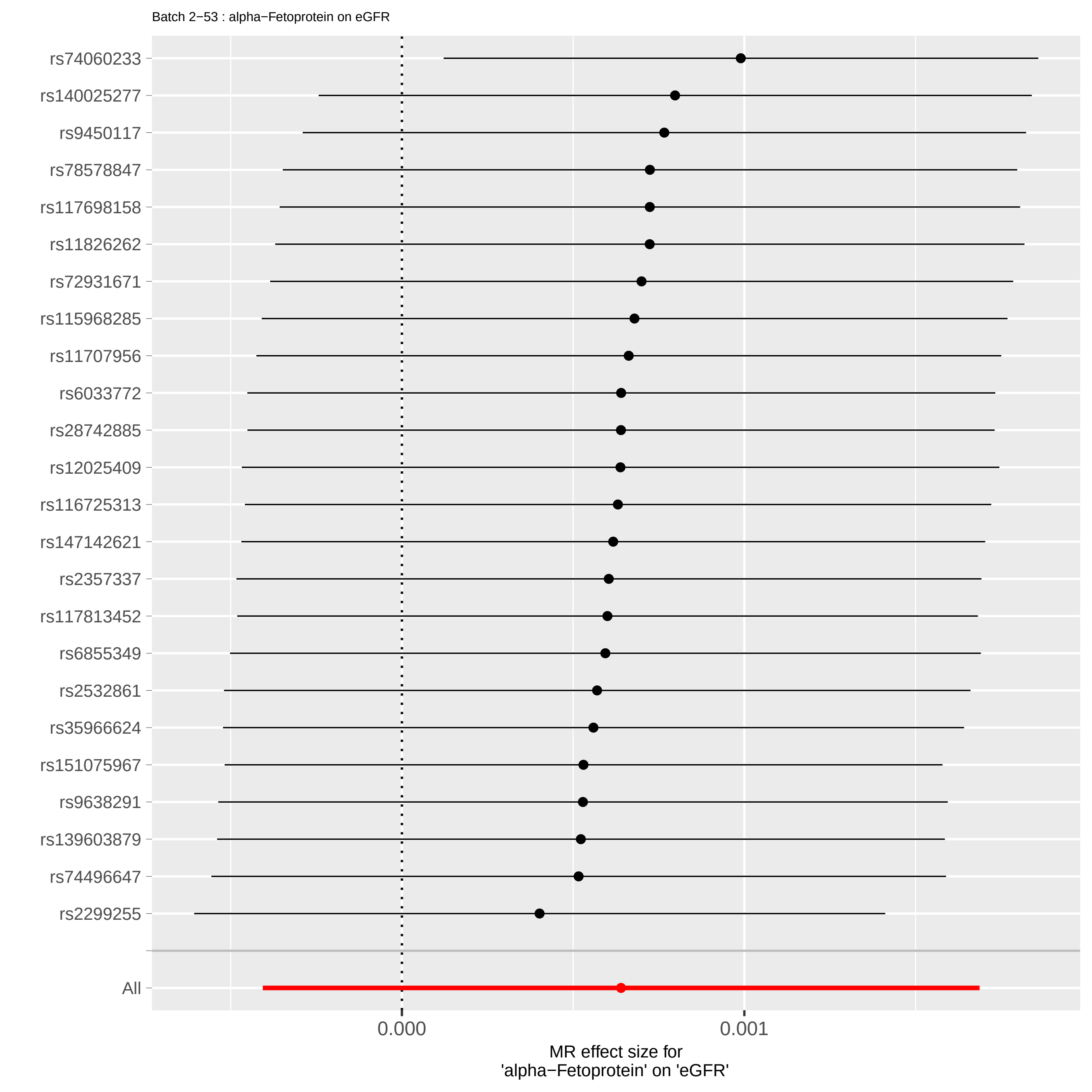

Batch 2−53 : alpha−Fetoprotein on eGFR
rs74060233
rs140025277
rs9450117
rs78578847
rs117698158
rs11826262
rs72931671
rs115968285
rs11707956
rs6033772
rs28742885
rs12025409
rs116725313
rs147142621
rs2357337
rs117813452
rs6855349
rs2532861
rs35966624
rs151075967
rs9638291
rs139603879
rs74496647
rs2299255
All
0.000
0.001
MR effect size for
'alpha−Fetoprotein' on 'eGFR'

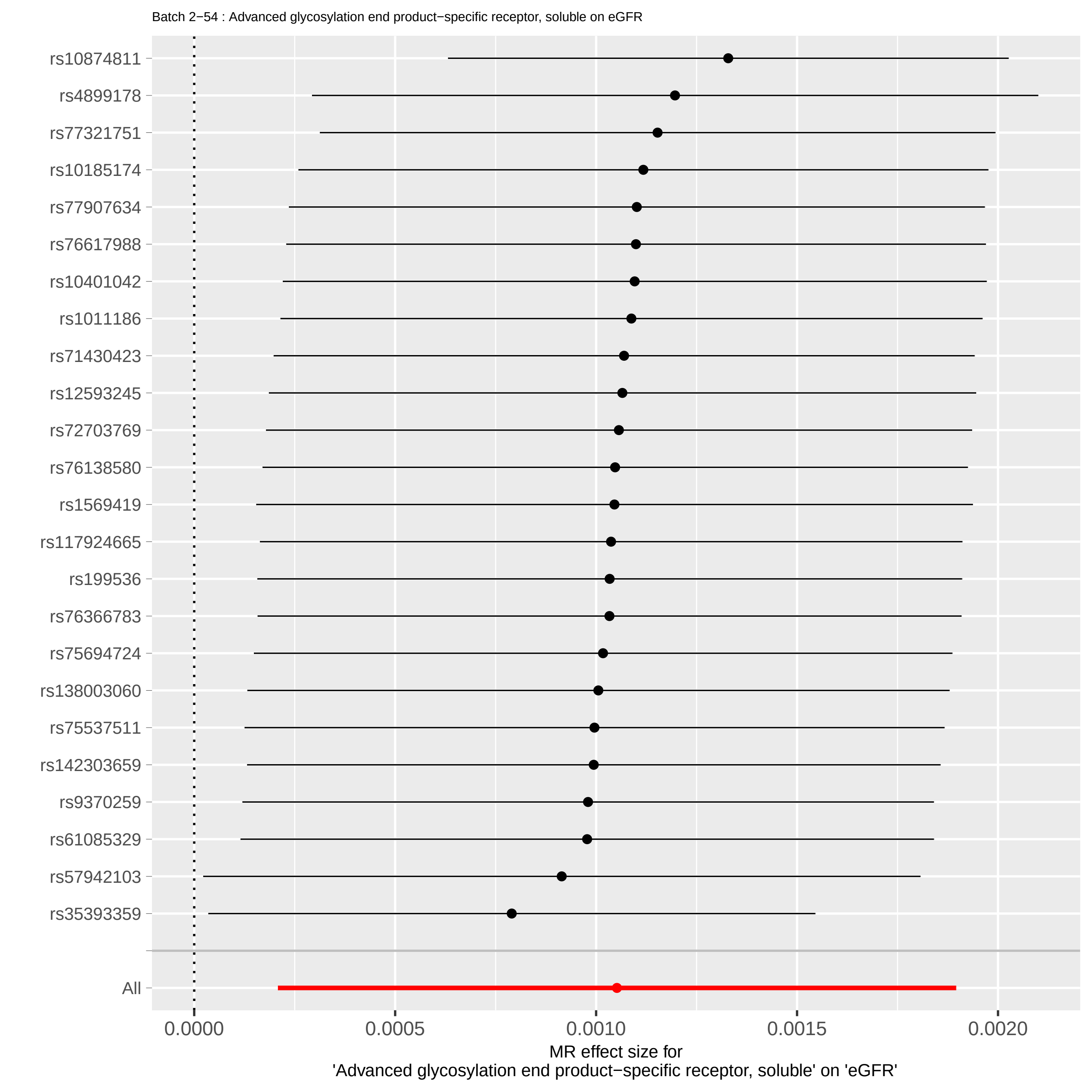

Batch 2−54 : Advanced glycosylation end product−specific receptor, soluble on eGFR
rs10874811
rs4899178
rs77321751
rs10185174
rs77907634
rs76617988
rs10401042
rs1011186
rs71430423
rs12593245
rs72703769
rs76138580
rs1569419
rs117924665
rs199536
rs76366783
rs75694724
rs138003060
rs75537511
rs142303659
rs9370259
rs61085329
rs57942103
rs35393359
All
0.0000
0.0005
0.0010
0.0015
0.0020
MR effect size for
'Advanced glycosylation end product−specific receptor, soluble' on 'eGFR'

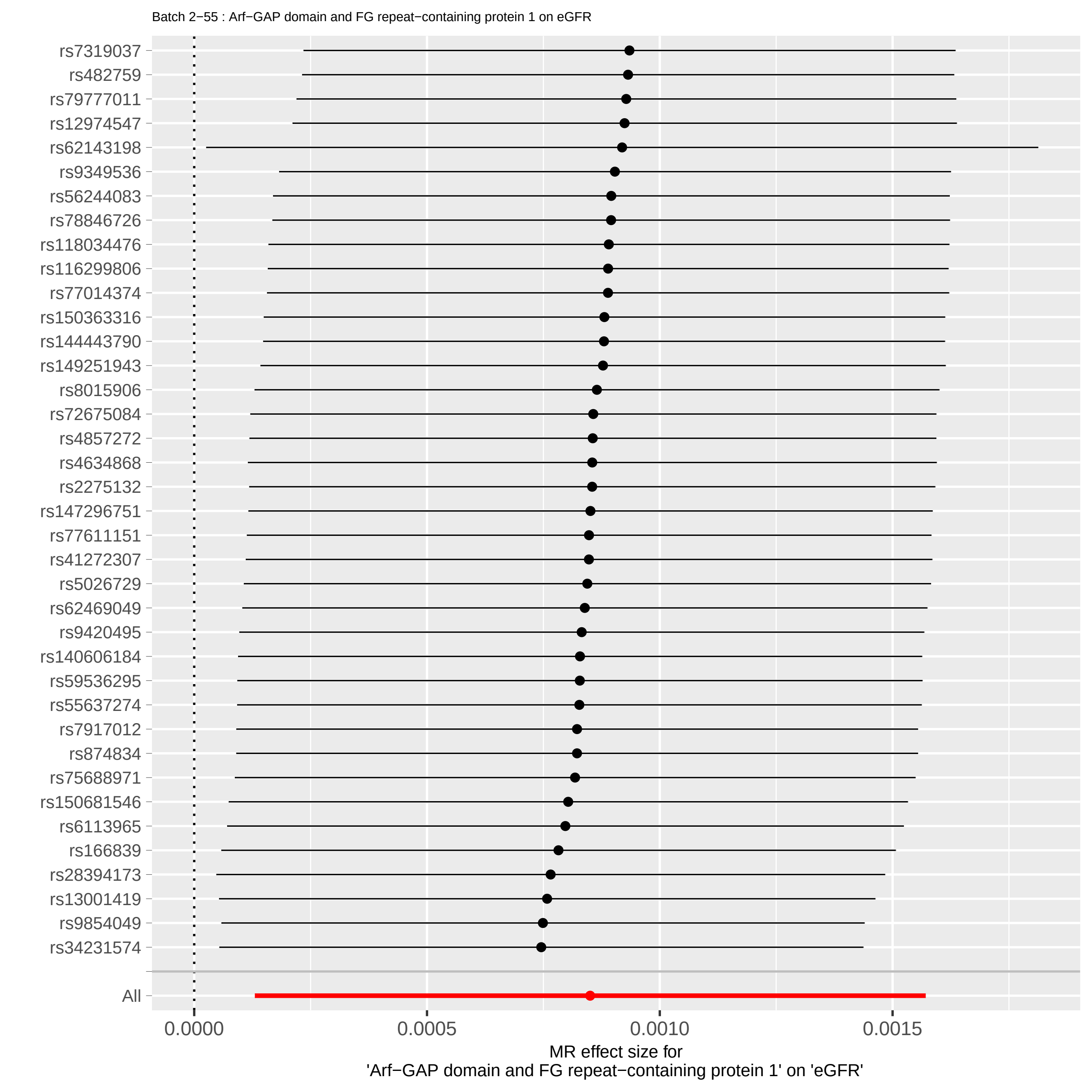

Batch 2−55 : Arf−GAP domain and FG repeat−containing protein 1 on eGFR
rs7319037
rs482759
rs79777011
rs12974547
rs62143198
rs9349536
rs56244083
rs78846726
rs118034476
rs116299806
rs77014374
rs150363316
rs144443790
rs149251943
rs8015906
rs72675084
rs4857272
rs4634868
rs2275132
rs147296751
rs77611151
rs41272307
rs5026729
rs62469049
rs9420495
rs140606184
rs59536295
rs55637274
rs7917012
rs874834
rs75688971
rs150681546
rs6113965
rs166839
rs28394173
rs13001419
rs9854049
rs34231574
All
0.0000
0.0005
0.0010
0.0015
MR effect size for
'Arf−GAP domain and FG repeat−containing protein 1' on 'eGFR'

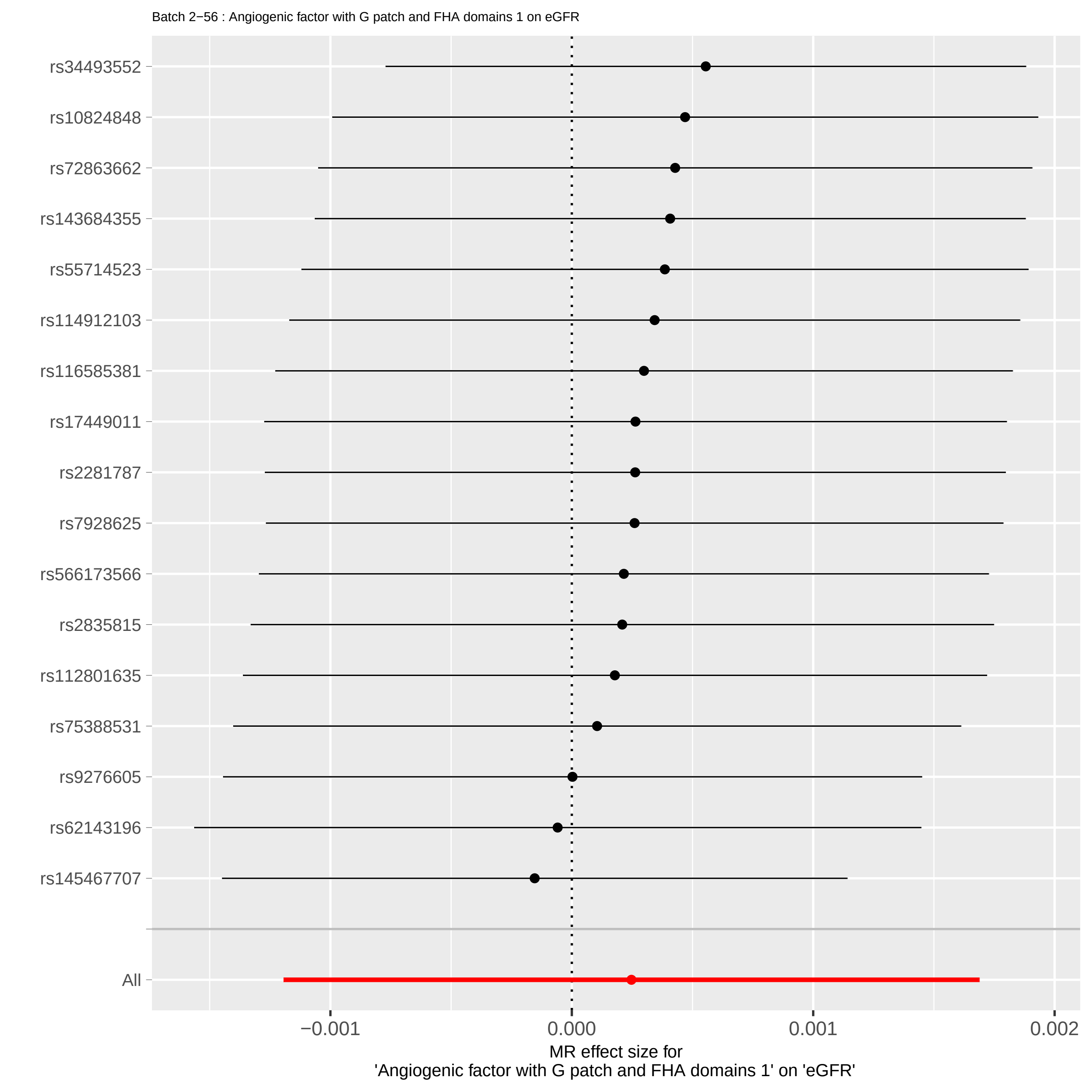
Batch 2−56 : Angiogenic factor with G patch and FHA domains 1 on eGFR
rs34493552
rs10824848
rs72863662
rs143684355
rs55714523
rs114912103
rs116585381
rs17449011
rs2281787
rs7928625
rs566173566
rs2835815
rs112801635
rs75388531
rs9276605
rs62143196
rs145467707
All
−0.001
0.000
0.001
0.002
MR effect size for
'Angiogenic factor with G patch and FHA domains 1' on 'eGFR'

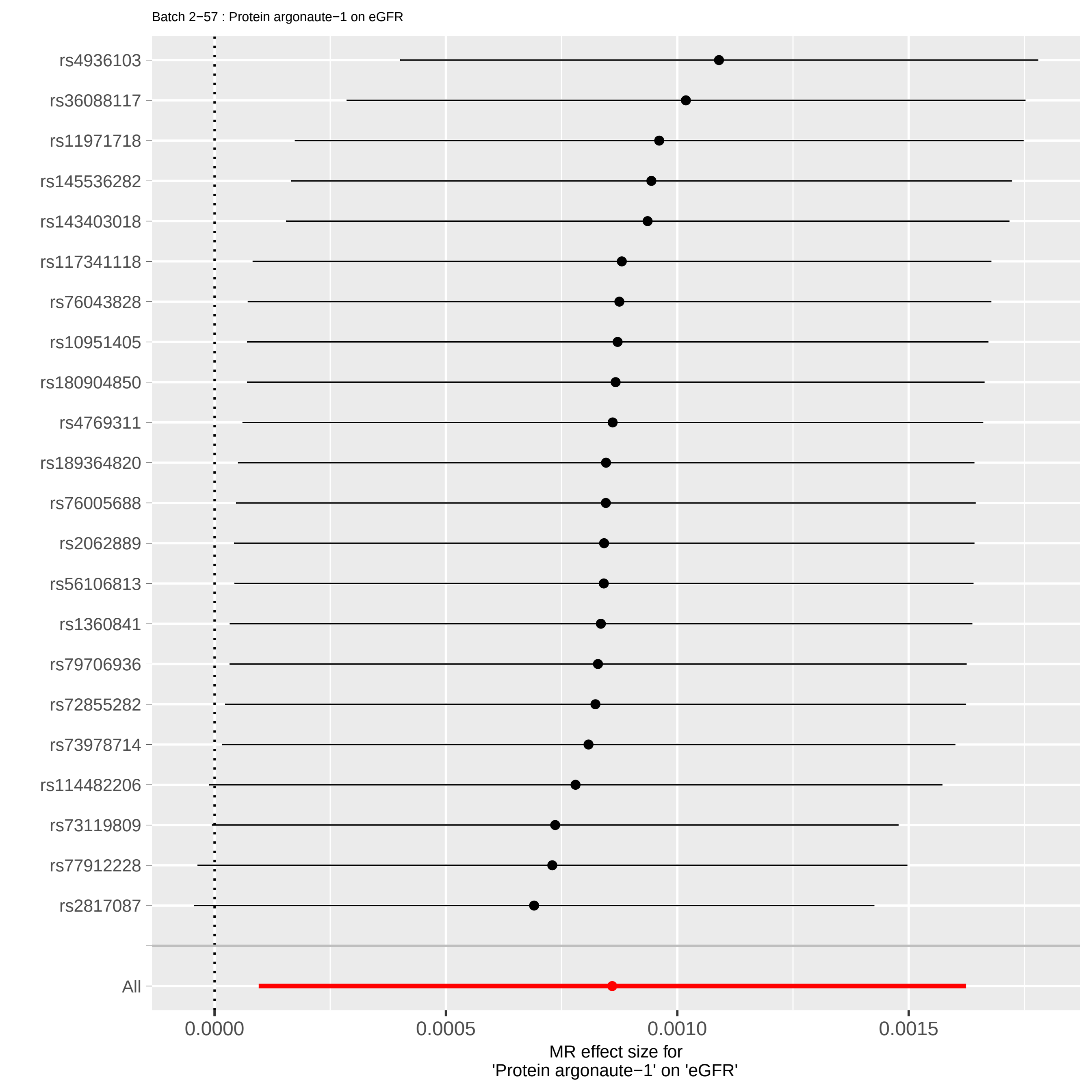
Batch 2−57 : Protein argonaute−1 on eGFR
rs4936103
rs36088117
rs11971718
rs145536282
rs143403018
rs117341118
rs76043828
rs10951405
rs180904850
rs4769311
rs189364820
rs76005688
rs2062889
rs56106813
rs1360841
rs79706936
rs72855282
rs73978714
rs114482206
rs73119809
rs77912228
rs2817087
All
0.0000
0.0005
0.0010
0.0015
MR effect size for
'Protein argonaute−1' on 'eGFR'

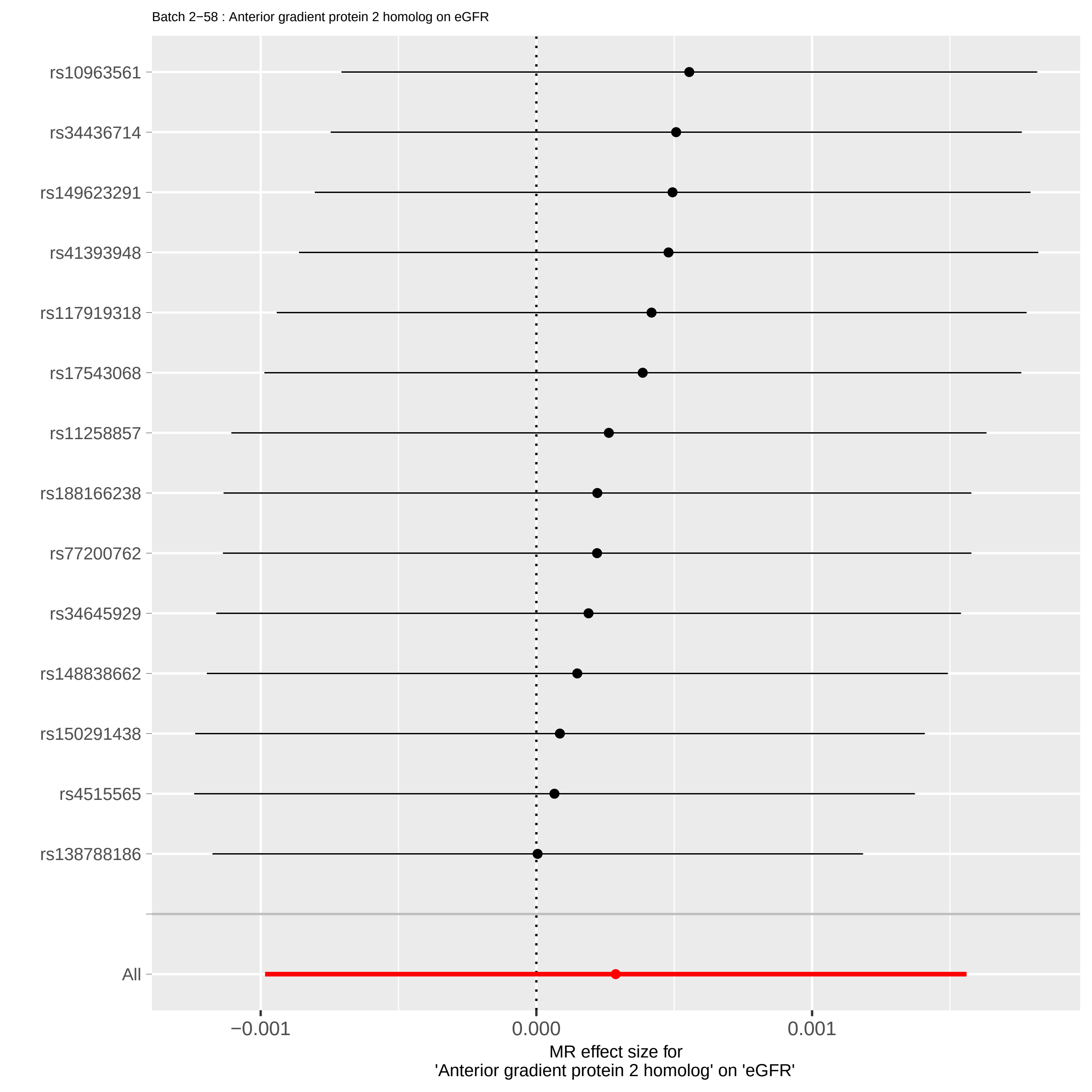

Batch 2−58 : Anterior gradient protein 2 homolog on eGFR
rs10963561
rs34436714
rs149623291
rs41393948
rs117919318
rs17543068
rs11258857
rs188166238
rs77200762
rs34645929
rs148838662
rs150291438
rs4515565
rs138788186
All
−0.001
0.000
0.001
MR effect size for
'Anterior gradient protein 2 homolog' on 'eGFR'

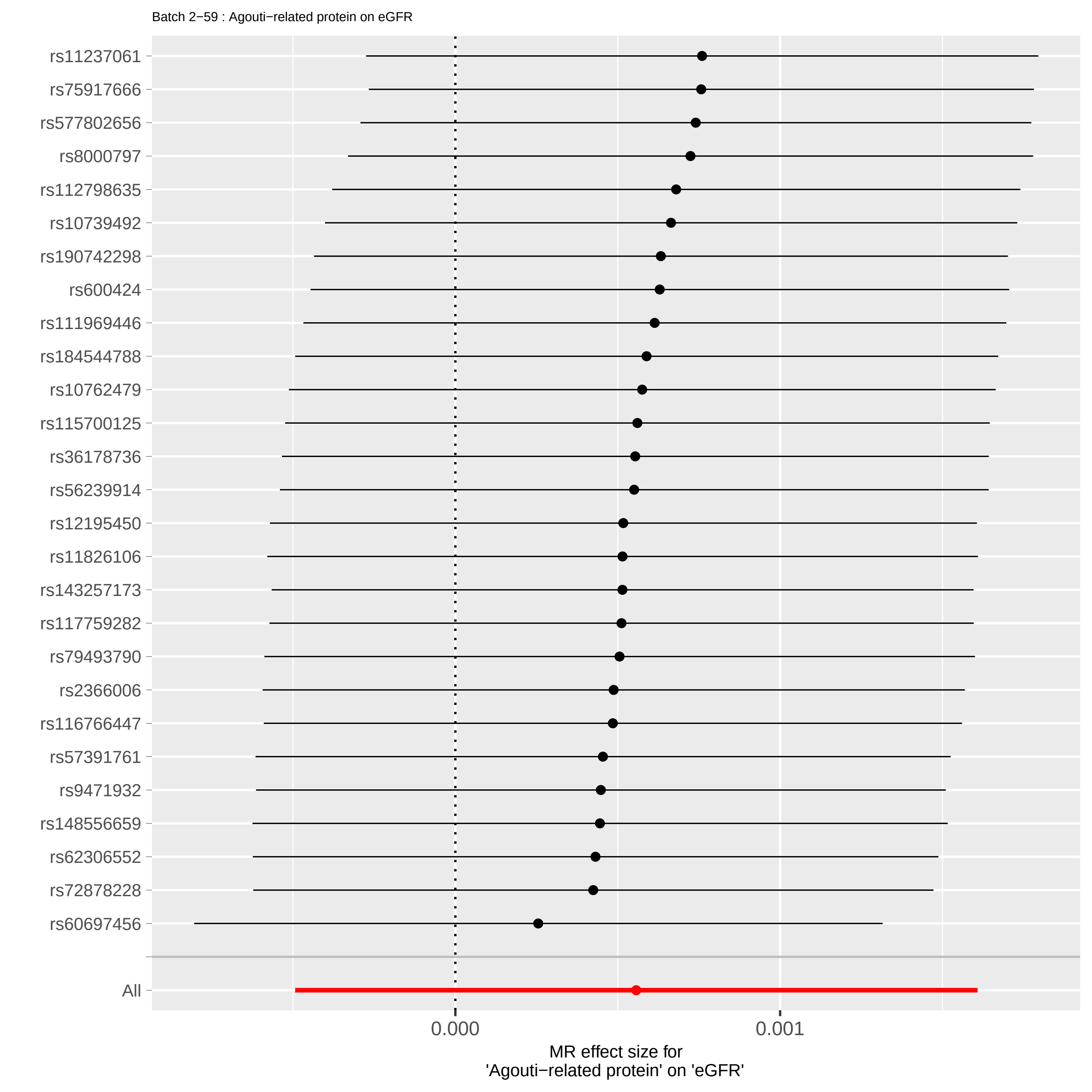

Batch 2−59 : Agouti−related protein on eGFR
rs11237061
rs75917666
rs577802656
rs8000797
rs112798635
rs10739492
rs190742298
rs600424
rs111969446
rs184544788
rs10762479
rs115700125
rs36178736
rs56239914
rs12195450
rs11826106
rs143257173
rs117759282
rs79493790
rs2366006
rs116766447
rs57391761
rs9471932
rs148556659
rs62306552
rs72878228
rs60697456
All
0.000
0.001
MR effect size for
'Agouti−related protein' on 'eGFR'

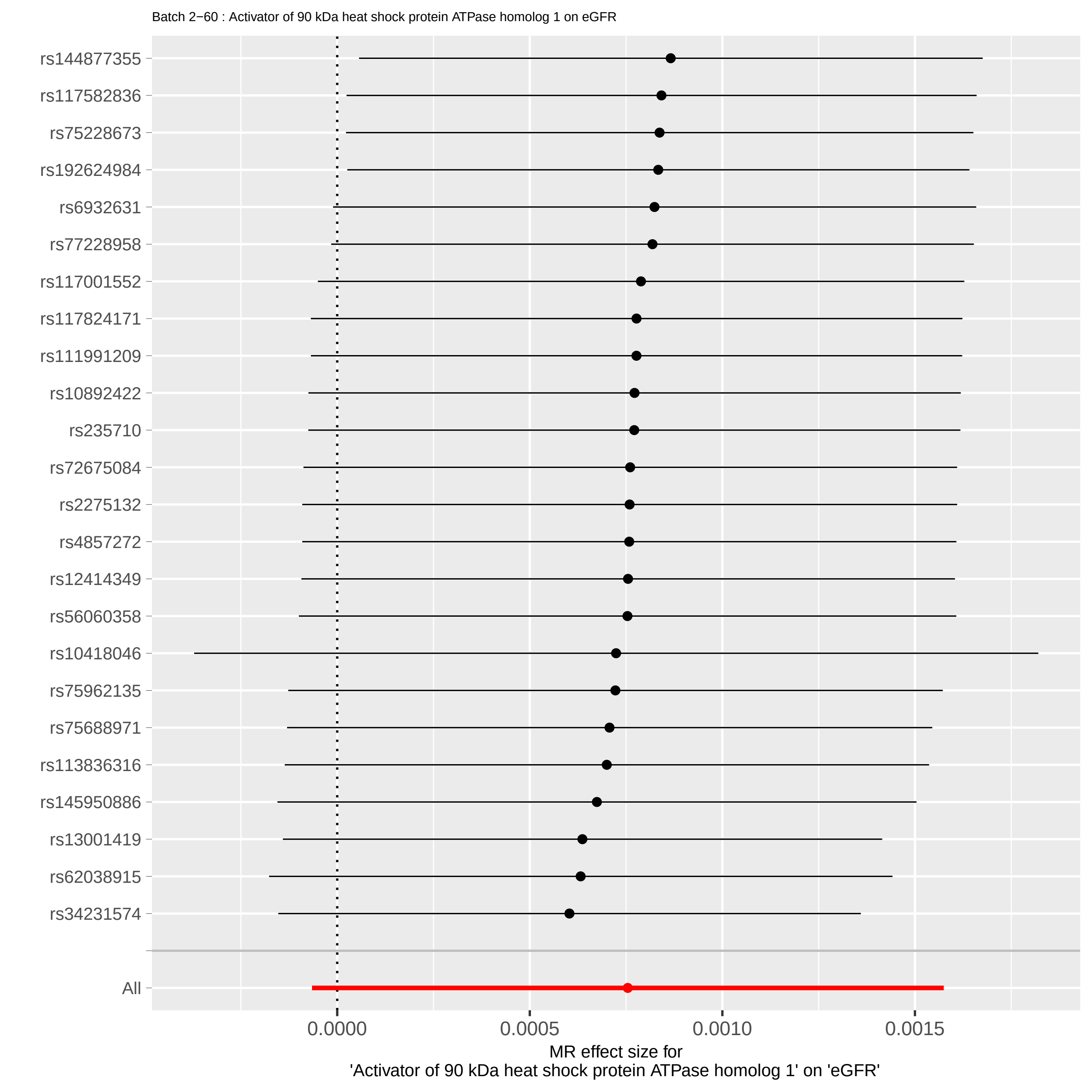
Batch 2−60 : Activator of 90 kDa heat shock protein ATPase homolog 1 on eGFR
rs144877355
rs117582836
rs75228673
rs192624984
rs6932631
rs77228958
rs117001552
rs117824171
rs111991209
rs10892422
rs235710
rs72675084
rs2275132
rs4857272
rs12414349
rs56060358
rs10418046
rs75962135
rs75688971
rs113836316
rs145950886
rs13001419
rs62038915
rs34231574
All
0.0000
0.0005
0.0010
0.0015
MR effect size for
'Activator of 90 kDa heat shock protein ATPase homolog 1' on 'eGFR'

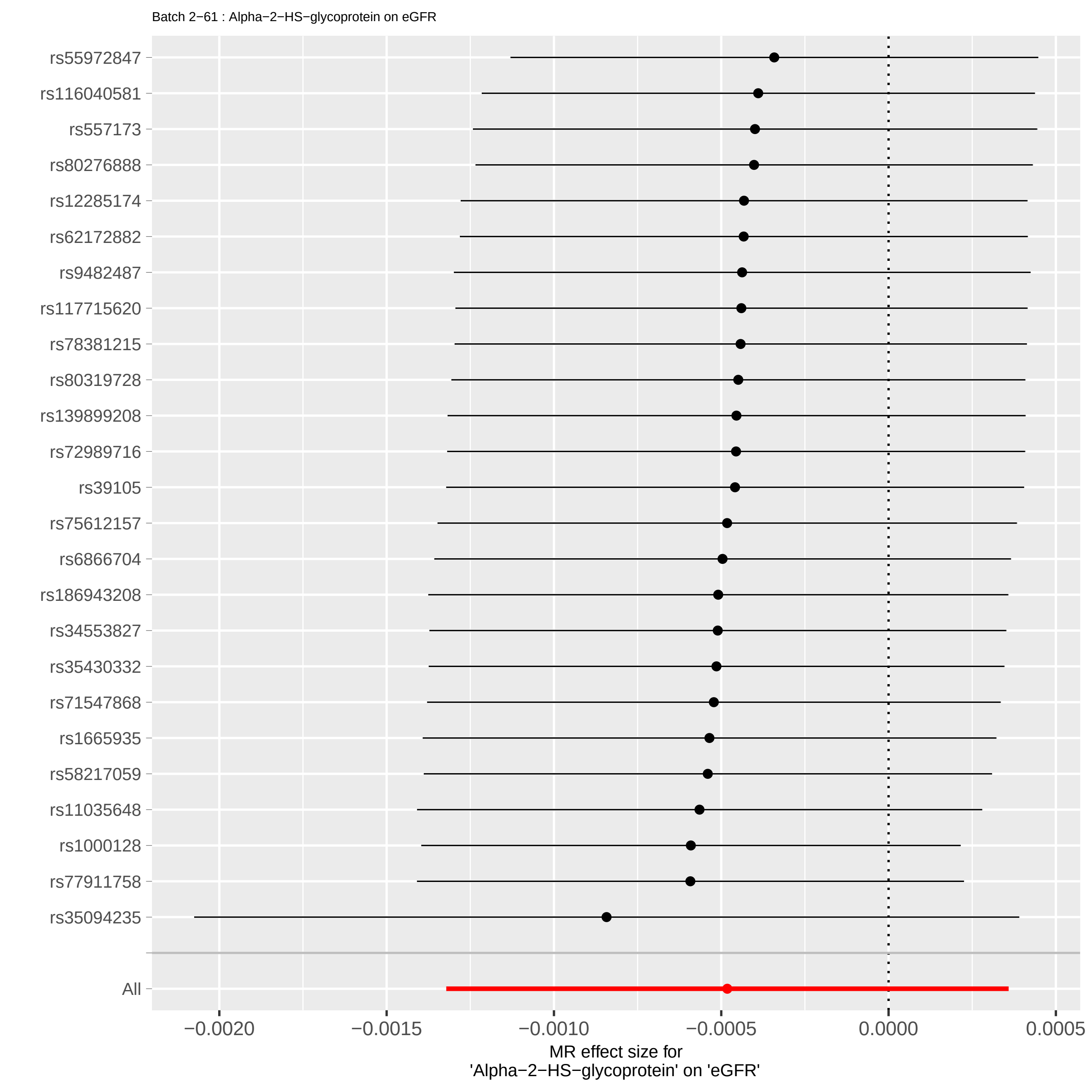
Batch 2−61 : Alpha−2−HS−glycoprotein on eGFR
rs55972847
rs116040581
rs557173
rs80276888
rs12285174
rs62172882
rs9482487
rs117715620
rs78381215
rs80319728
rs139899208
rs72989716
rs39105
rs75612157
rs6866704
rs186943208
rs34553827
rs35430332
rs71547868
rs1665935
rs58217059
rs11035648
rs1000128
rs77911758
rs35094235
All
−0.0020
−0.0015
−0.0010
−0.0005
0.0000
0.0005
MR effect size for
'Alpha−2−HS−glycoprotein' on 'eGFR'

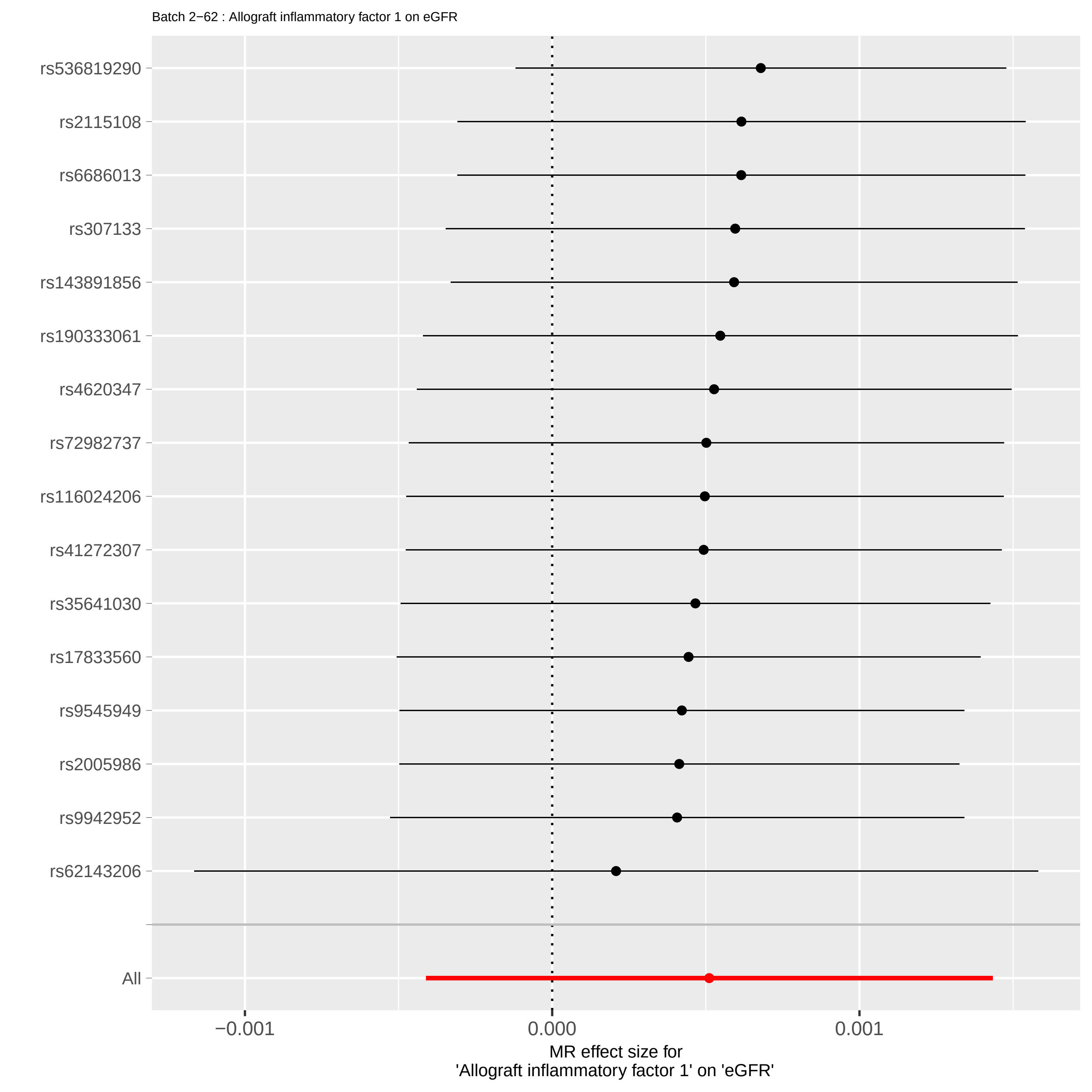

Batch 2−62 : Allograft inflammatory factor 1 on eGFR
rs536819290
rs2115108
rs6686013
rs307133
rs143891856
rs190333061
rs4620347
rs72982737
rs116024206
rs41272307
rs35641030
rs17833560
rs9545949
rs2005986
rs9942952
rs62143206
All
−0.001
0.000
0.001
MR effect size for
'Allograft inflammatory factor 1' on 'eGFR'

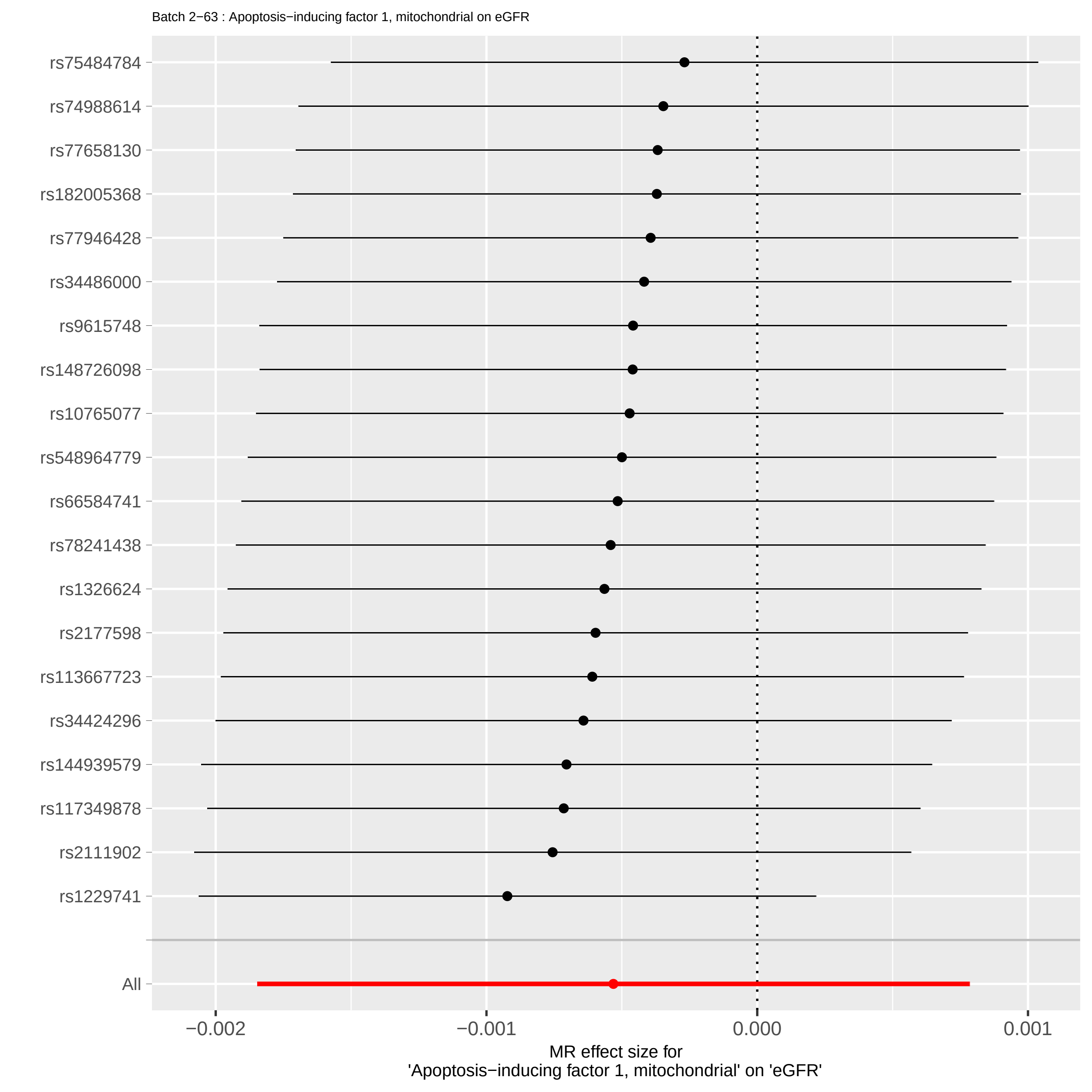

Batch 2−63 : Apoptosis−inducing factor 1, mitochondrial on eGFR
rs75484784
rs74988614
rs77658130
rs182005368
rs77946428
rs34486000
rs9615748
rs148726098
rs10765077
rs548964779
rs66584741
rs78241438
rs1326624
rs2177598
rs113667723
rs34424296
rs144939579
rs117349878
rs2111902
rs1229741
All
−0.002
−0.001
0.000
0.001
MR effect size for
'Apoptosis−inducing factor 1, mitochondrial' on 'eGFR'

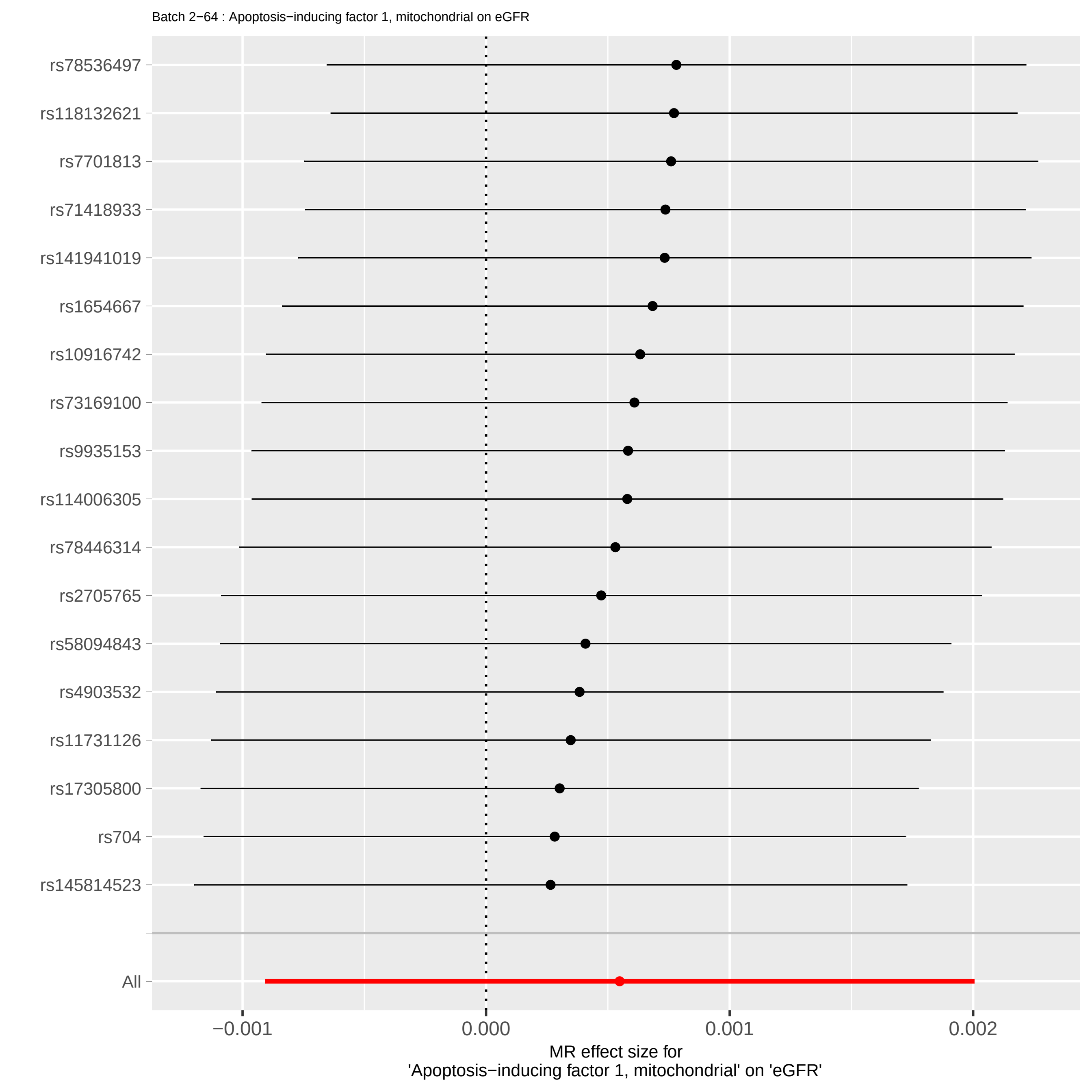

Batch 2−64 : Apoptosis−inducing factor 1, mitochondrial on eGFR
rs78536497
rs118132621
rs7701813
rs71418933
rs141941019
rs1654667
rs10916742
rs73169100
rs9935153
rs114006305
rs78446314
rs2705765
rs58094843
rs4903532
rs11731126
rs17305800
rs704
rs145814523
All
−0.001
0.000
0.001
0.002
MR effect size for
'Apoptosis−inducing factor 1, mitochondrial' on 'eGFR'

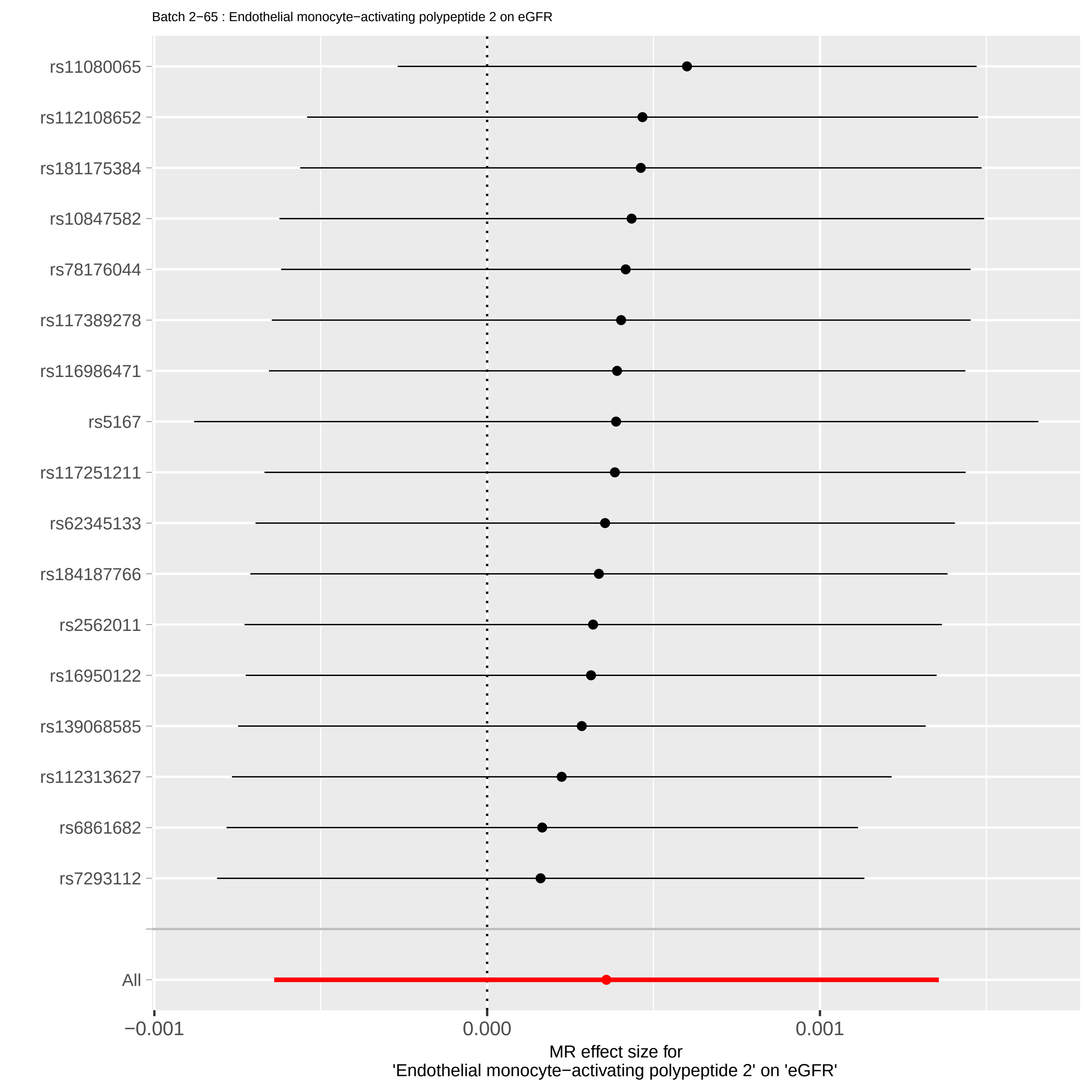

Batch 2−65 : Endothelial monocyte−activating polypeptide 2 on eGFR
rs11080065
rs112108652
rs181175384
rs10847582
rs78176044
rs117389278
rs116986471
rs5167
rs117251211
rs62345133
rs184187766
rs2562011
rs16950122
rs139068585
rs112313627
rs6861682
rs7293112
All
−0.001
0.000
0.001
MR effect size for
'Endothelial monocyte−activating polypeptide 2' on 'eGFR'

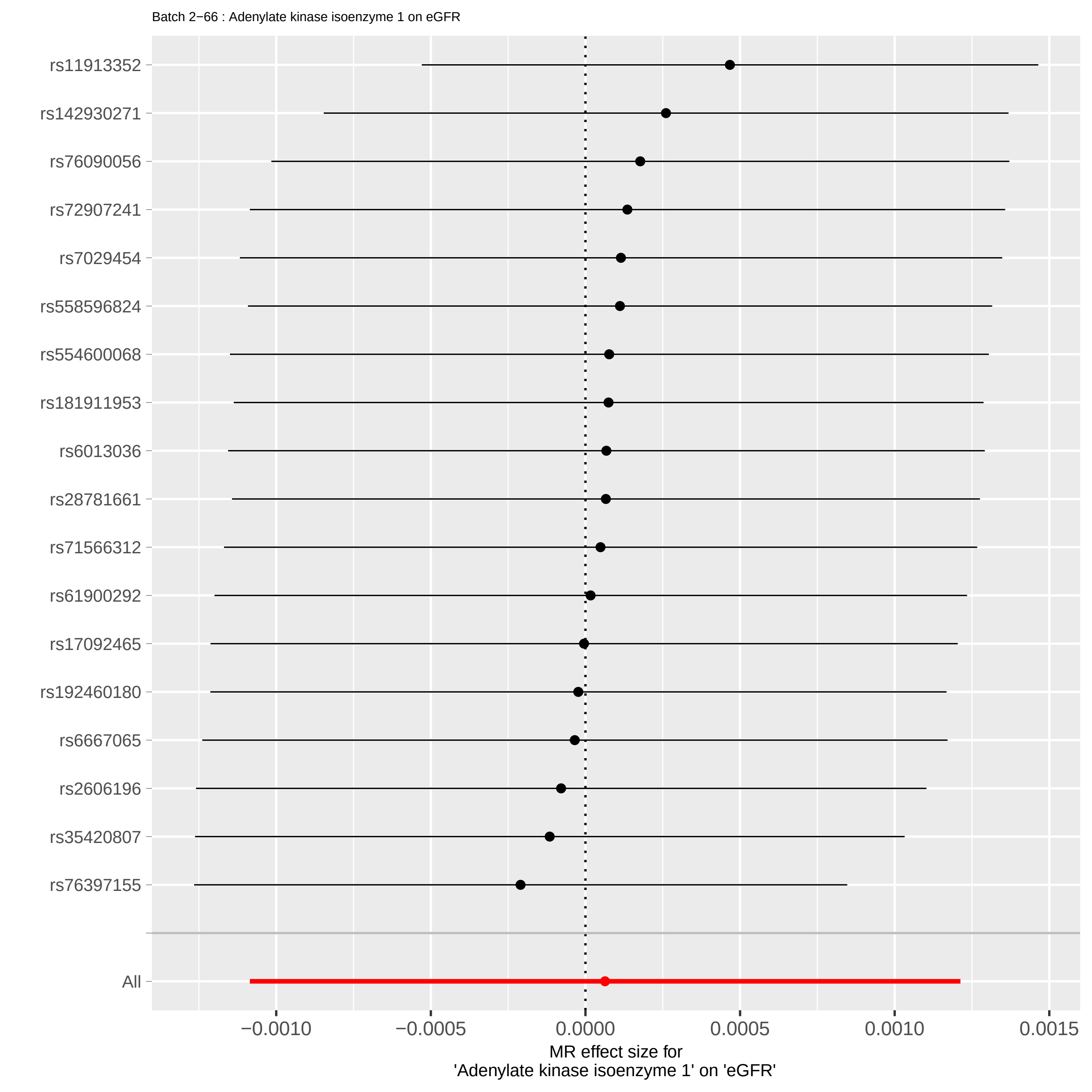

Batch 2−66 : Adenylate kinase isoenzyme 1 on eGFR
rs11913352
rs142930271
rs76090056
rs72907241
rs7029454
rs558596824
rs554600068
rs181911953
rs6013036
rs28781661
rs71566312
rs61900292
rs17092465
rs192460180
rs6667065
rs2606196
rs35420807
rs76397155
All
−0.0010
−0.0005
0.0000
0.0005
0.0010
0.0015
MR effect size for
'Adenylate kinase isoenzyme 1' on 'eGFR'

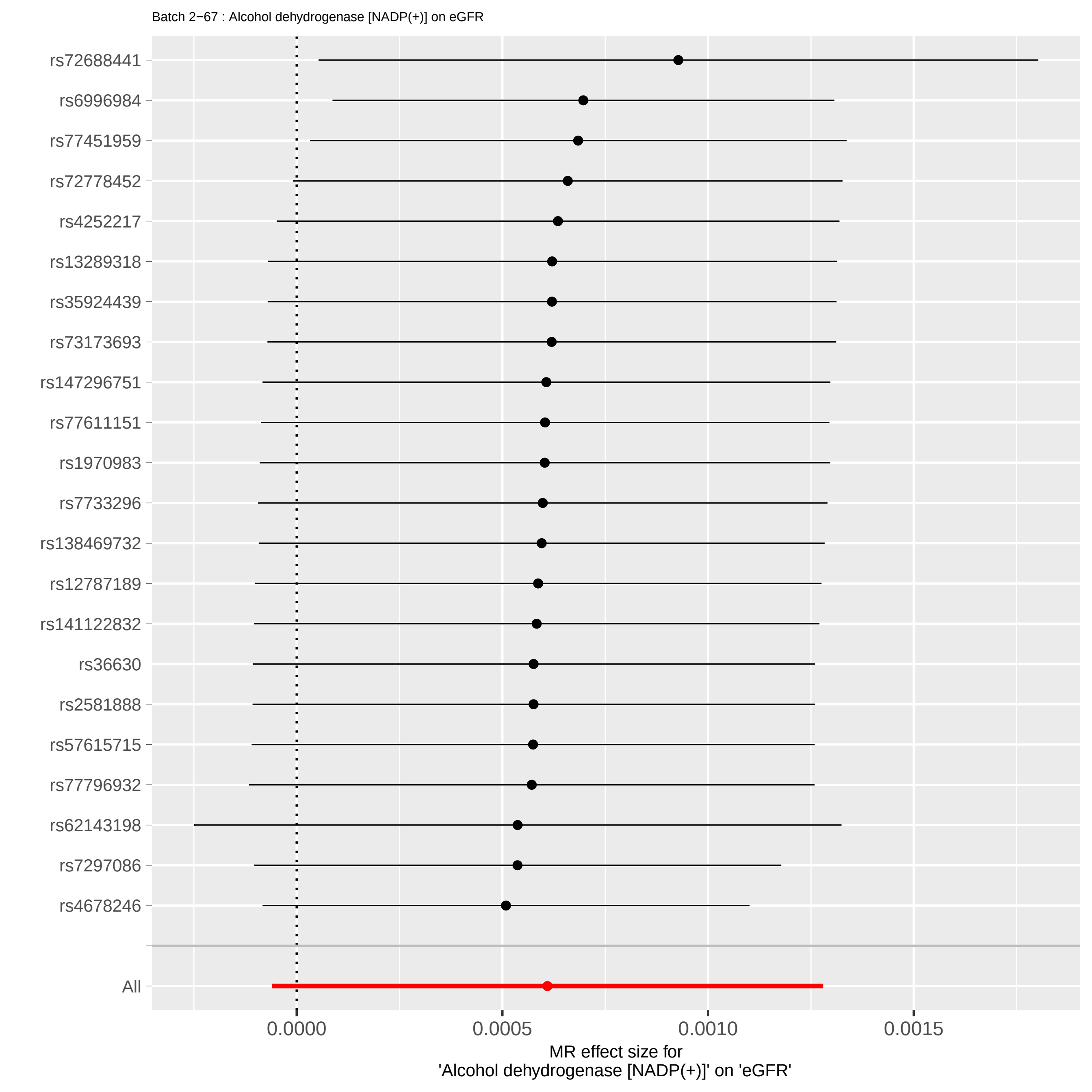

Batch 2−67 : Alcohol dehydrogenase [NADP(+)] on eGFR
rs72688441
rs6996984
rs77451959
rs72778452
rs4252217
rs13289318
rs35924439
rs73173693
rs147296751
rs77611151
rs1970983
rs7733296
rs138469732
rs12787189
rs141122832
rs36630
rs2581888
rs57615715
rs77796932
rs62143198
rs7297086
rs4678246
All
0.0000
0.0005
0.0010
0.0015
MR effect size for
'Alcohol dehydrogenase [NADP(+)]' on 'eGFR'

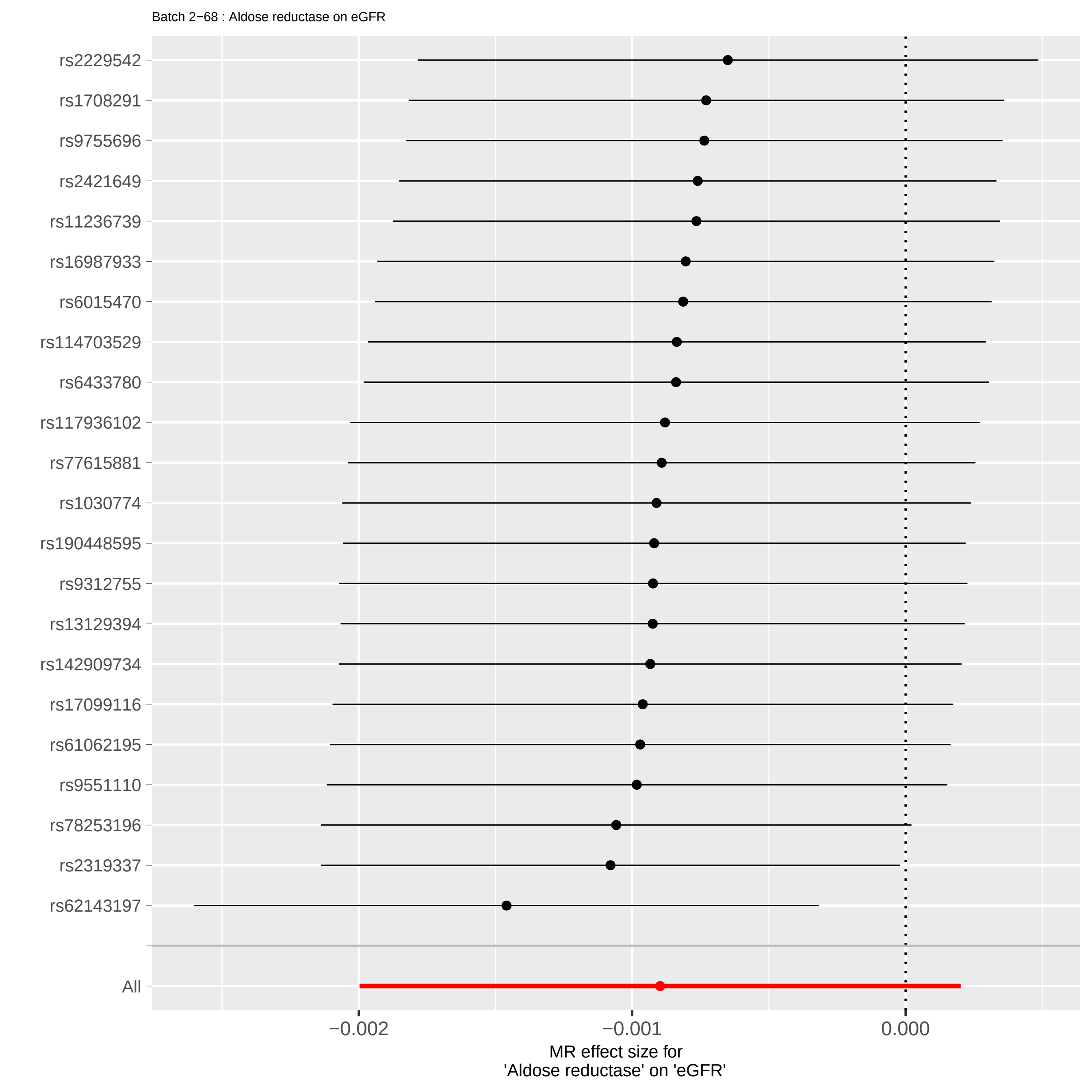

Batch 2−68 : Aldose reductase on eGFR
rs2229542
rs1708291
rs9755696
rs2421649
rs11236739
rs16987933
rs6015470
rs114703529
rs6433780
rs117936102
rs77615881
rs1030774
rs190448595
rs9312755
rs13129394
rs142909734
rs17099116
rs61062195
rs9551110
rs78253196
rs2319337
rs62143197
All
−0.002
−0.001
0.000
MR effect size for
'Aldose reductase' on 'eGFR'

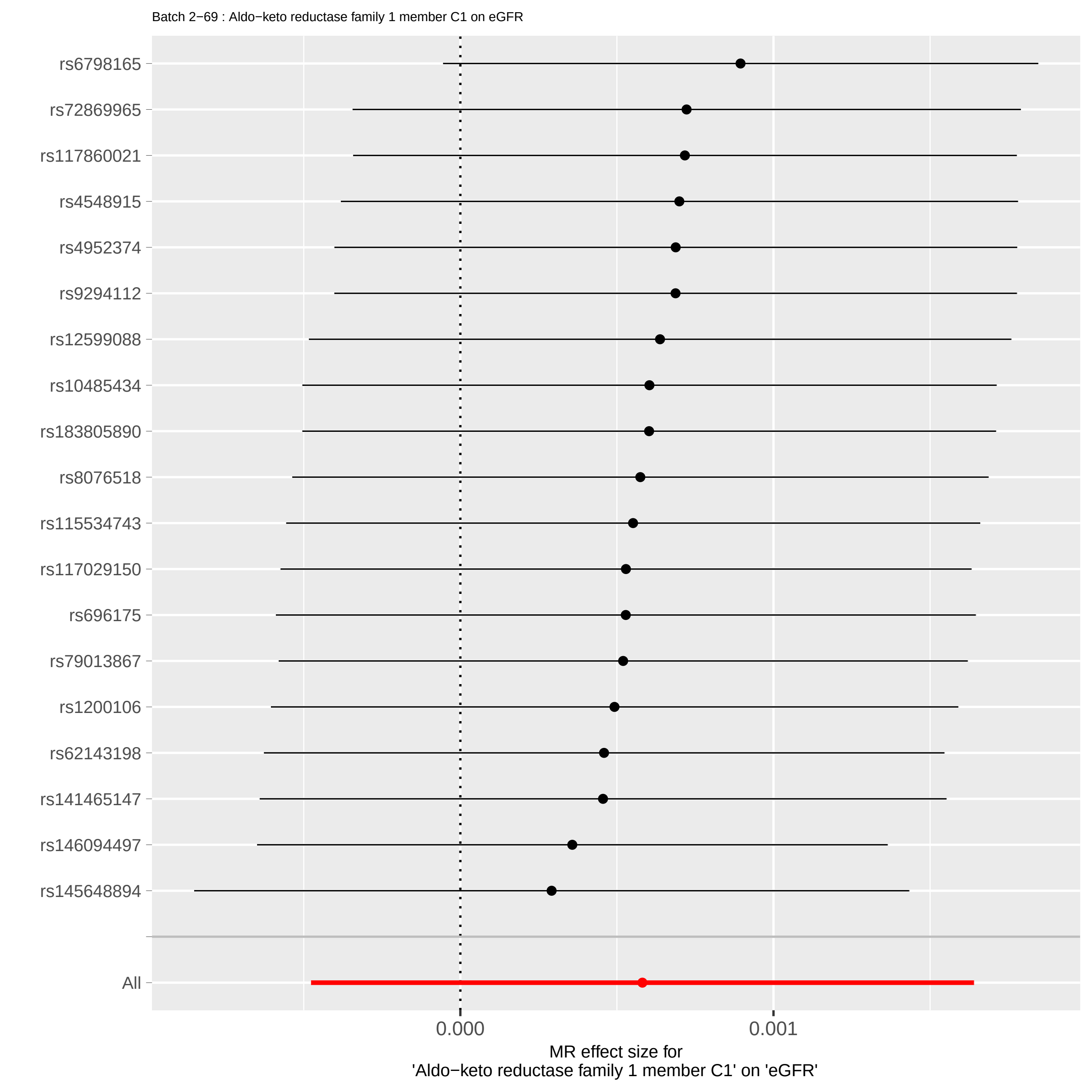

Batch 2−69 : Aldo−keto reductase family 1 member C1 on eGFR
rs6798165
rs72869965
rs117860021
rs4548915
rs4952374
rs9294112
rs12599088
rs10485434
rs183805890
rs8076518
rs115534743
rs117029150
rs696175
rs79013867
rs1200106
rs62143198
rs141465147
rs146094497
rs145648894
All
0.000
0.001
MR effect size for
'Aldo−keto reductase family 1 member C1' on 'eGFR'

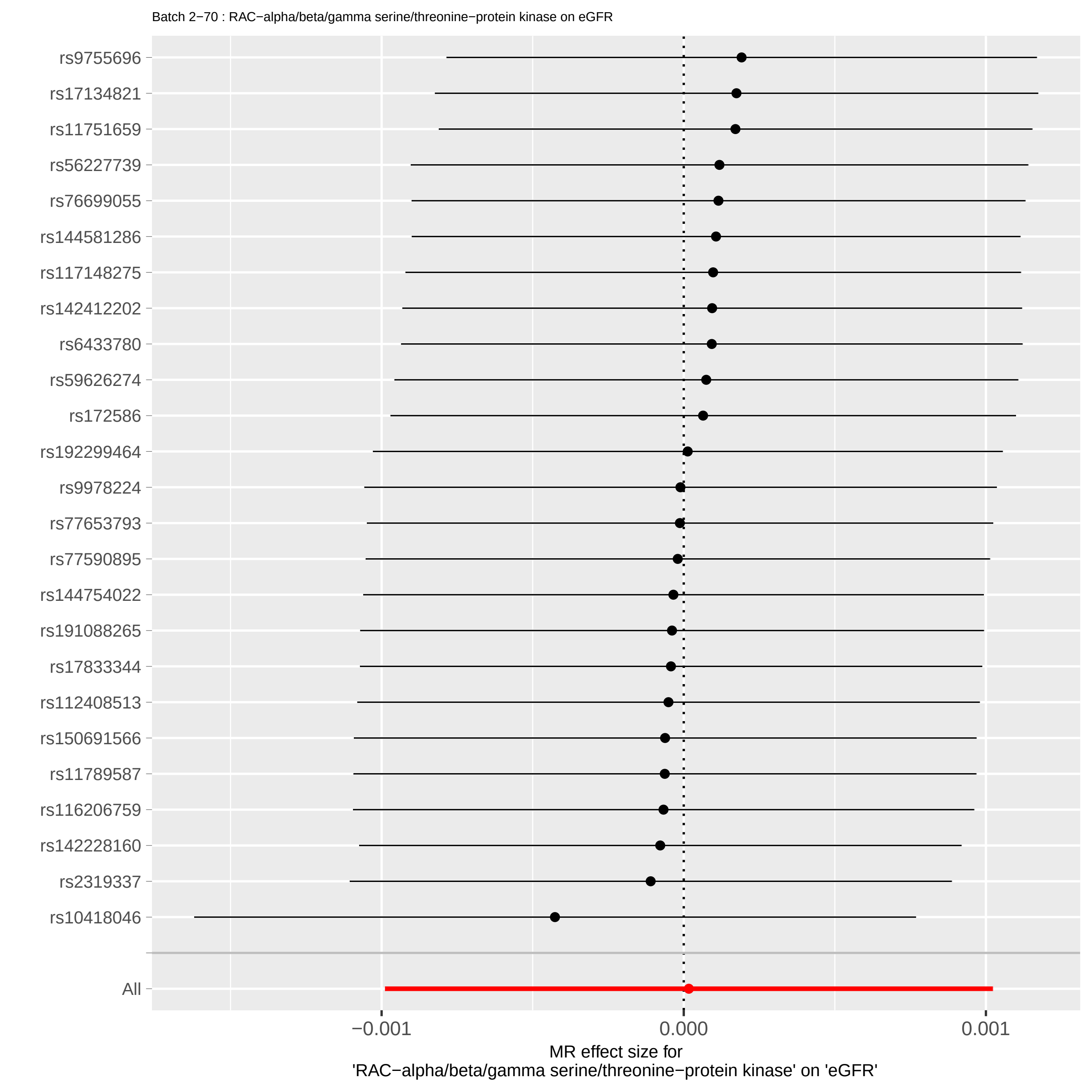

Batch 2−70 : RAC−alpha/beta/gamma serine/threonine−protein kinase on eGFR
rs9755696
rs17134821
rs11751659
rs56227739
rs76699055
rs144581286
rs117148275
rs142412202
rs6433780
rs59626274
rs172586
rs192299464
rs9978224
rs77653793
rs77590895
rs144754022
rs191088265
rs17833344
rs112408513
rs150691566
rs11789587
rs116206759
rs142228160
rs2319337
rs10418046
All
−0.001
0.000
0.001
MR effect size for
'RAC−alpha/beta/gamma serine/threonine−protein kinase' on 'eGFR'

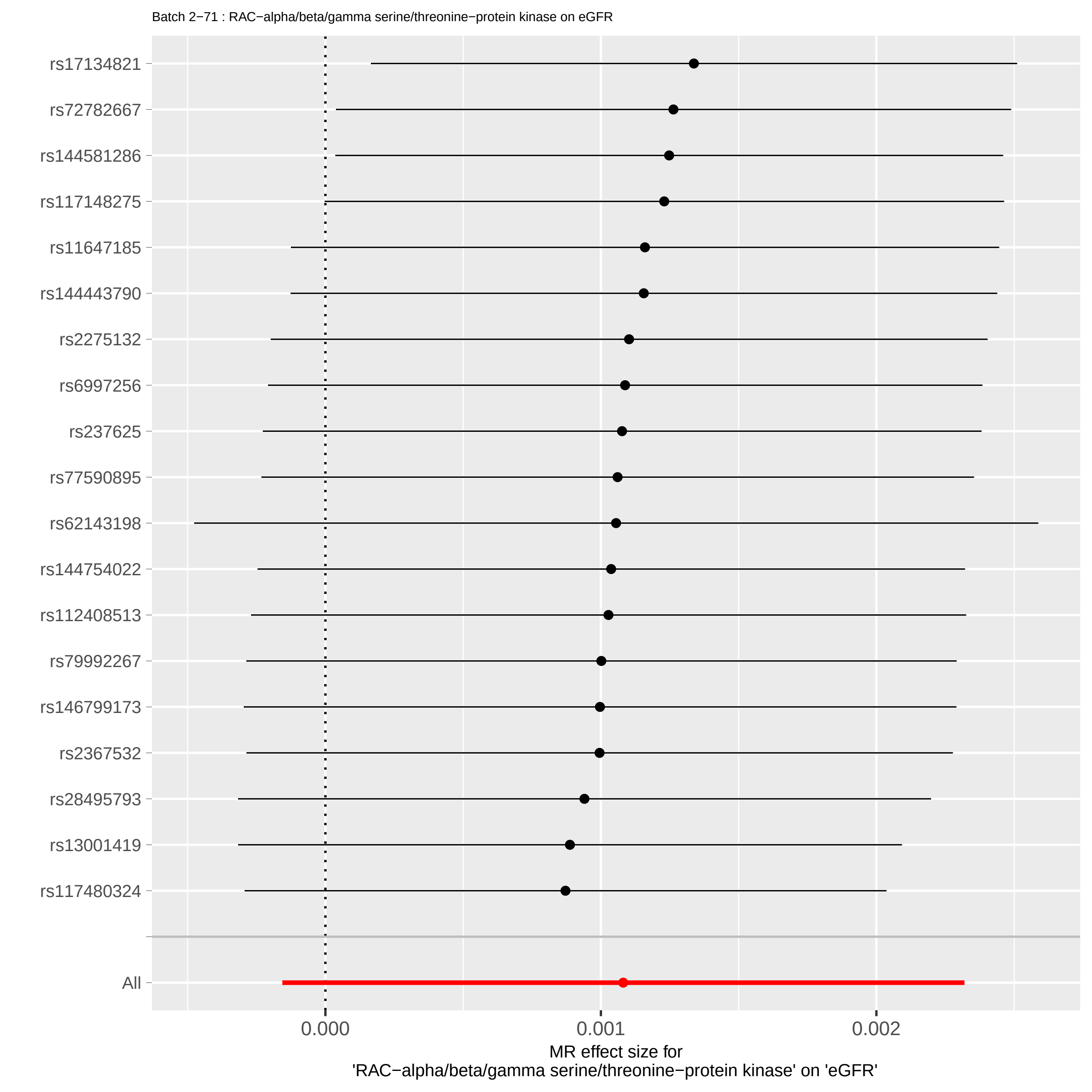

Batch 2−71 : RAC−alpha/beta/gamma serine/threonine−protein kinase on eGFR
rs17134821
rs72782667
rs144581286
rs117148275
rs11647185
rs144443790
rs2275132
rs6997256
rs237625
rs77590895
rs62143198
rs144754022
rs112408513
rs79992267
rs146799173
rs2367532
rs28495793
rs13001419
rs117480324
All
0.000
0.001
0.002
MR effect size for
'RAC−alpha/beta/gamma serine/threonine−protein kinase' on 'eGFR'

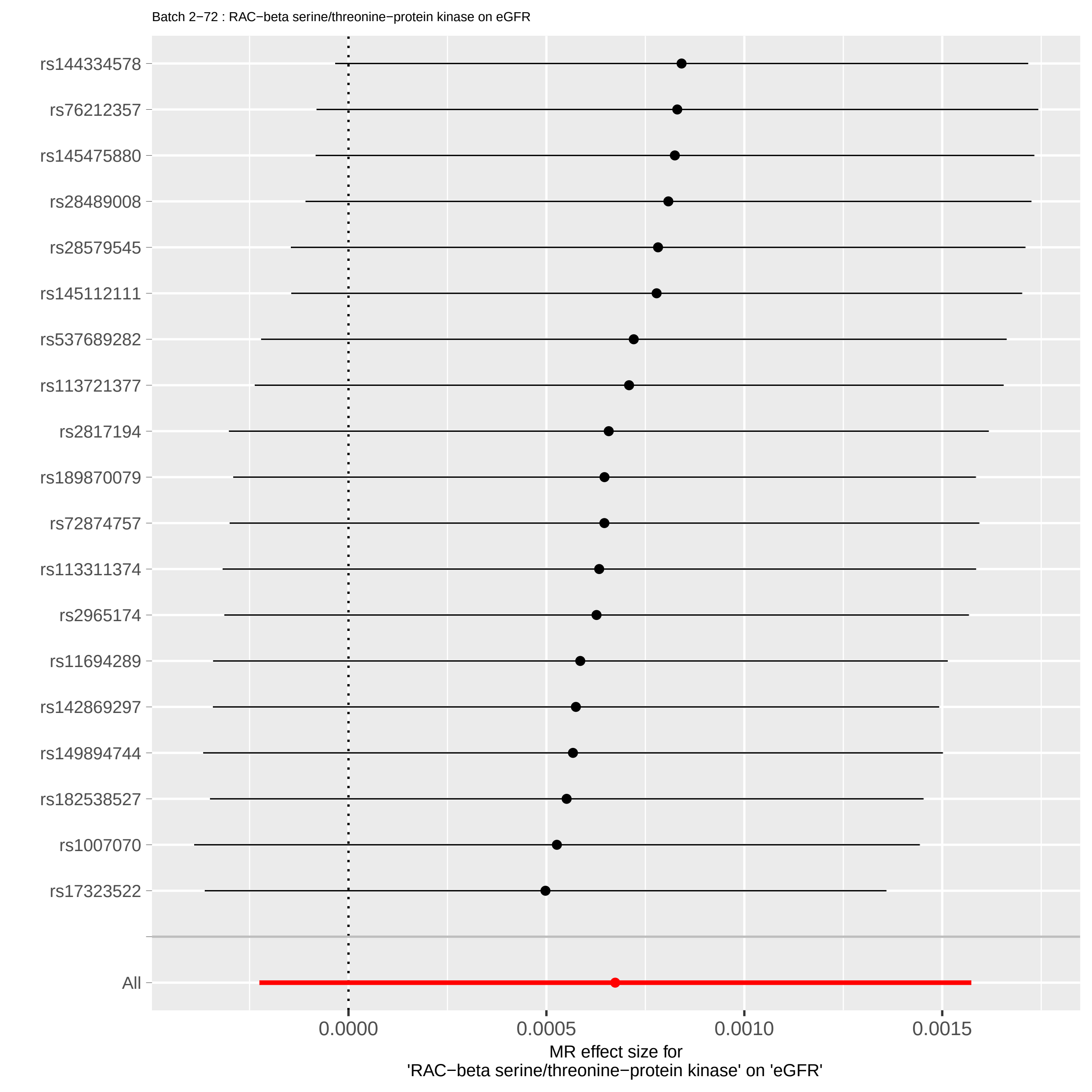
Batch 2−72 : RAC−beta serine/threonine−protein kinase on eGFR
rs144334578
rs76212357
rs145475880
rs28489008
rs28579545
rs145112111
rs537689282
rs113721377
rs2817194
rs189870079
rs72874757
rs113311374
rs2965174
rs11694289
rs142869297
rs149894744
rs182538527
rs1007070
rs17323522
All
0.0000
0.0005
0.0010
0.0015
MR effect size for
'RAC−beta serine/threonine−protein kinase' on 'eGFR'

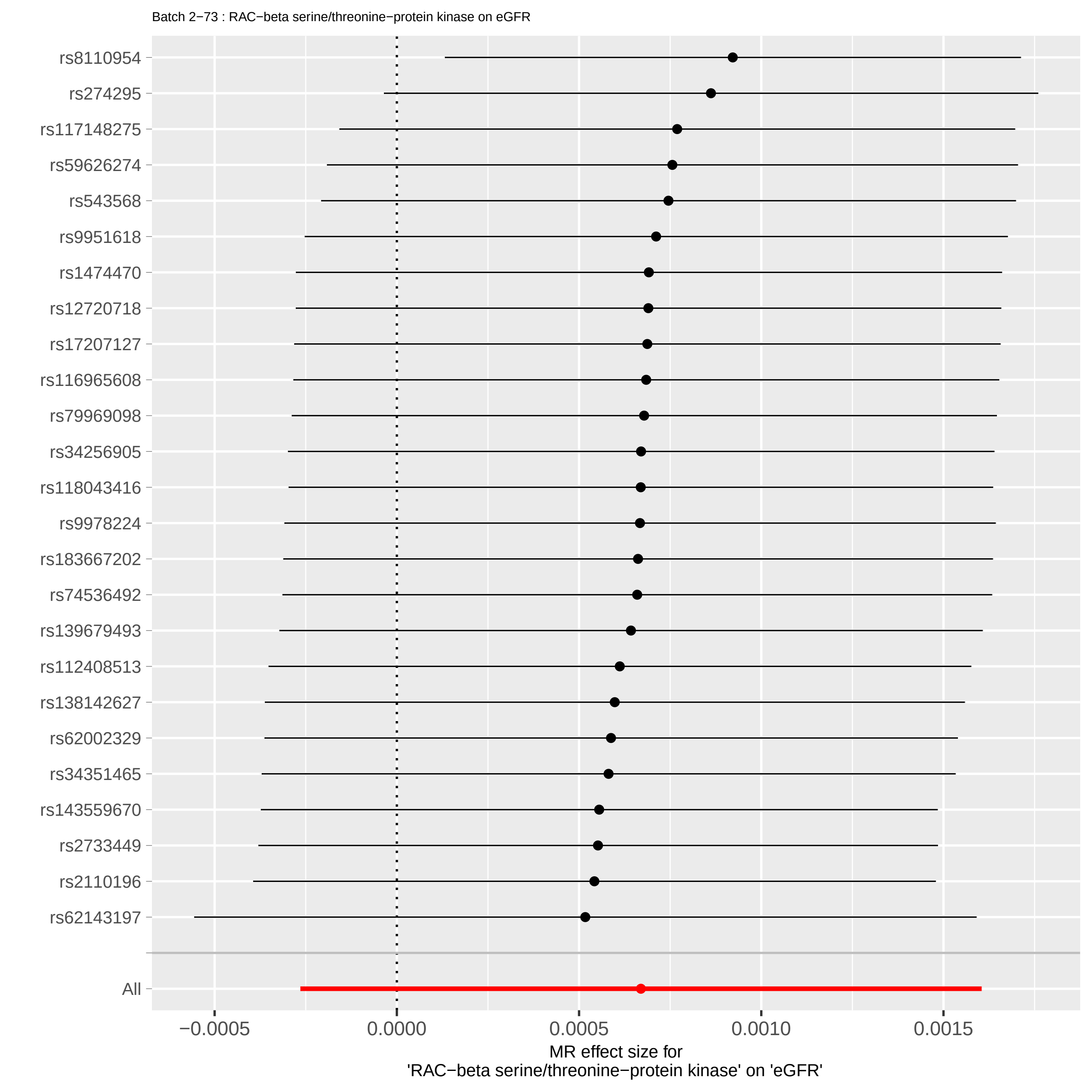

Batch 2−73 : RAC−beta serine/threonine−protein kinase on eGFR
rs8110954
rs274295
rs117148275
rs59626274
rs543568
rs9951618
rs1474470
rs12720718
rs17207127
rs116965608
rs79969098
rs34256905
rs118043416
rs9978224
rs183667202
rs74536492
rs139679493
rs112408513
rs138142627
rs62002329
rs34351465
rs143559670
rs2733449
rs2110196
rs62143197
All
−0.0005
0.0000
0.0005
0.0010
0.0015
MR effect size for
'RAC−beta serine/threonine−protein kinase' on 'eGFR'

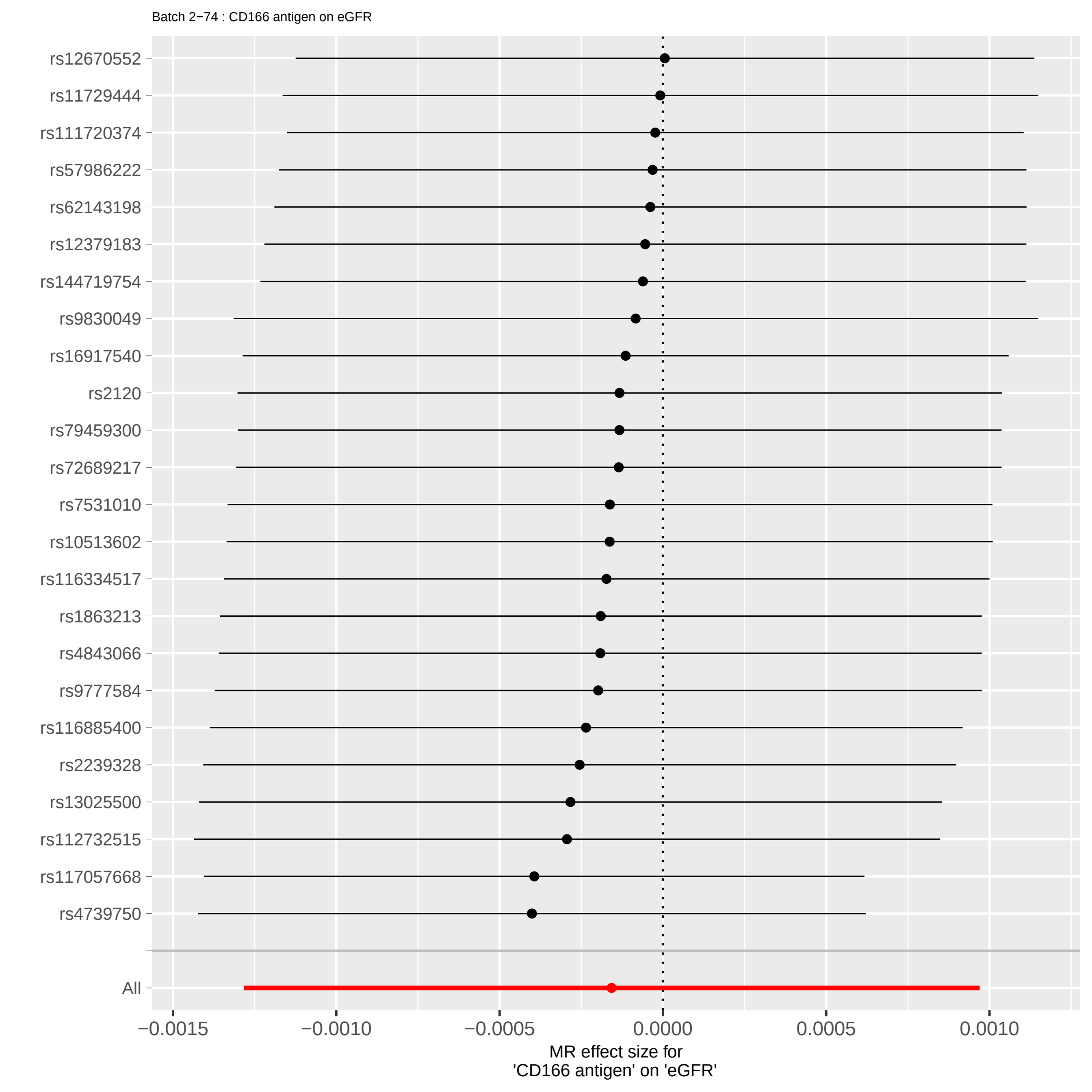

Batch 2−74 : CD166 antigen on eGFR
rs12670552
rs11729444
rs111720374
rs57986222
rs62143198
rs12379183
rs144719754
rs9830049
rs16917540
rs2120
rs79459300
rs72689217
rs7531010
rs10513602
rs116334517
rs1863213
rs4843066
rs9777584
rs116885400
rs2239328
rs13025500
rs112732515
rs117057668
rs4739750
All
−0.0015
−0.0010
−0.0005
0.0000
0.0005
0.0010
MR effect size for
'CD166 antigen' on 'eGFR'

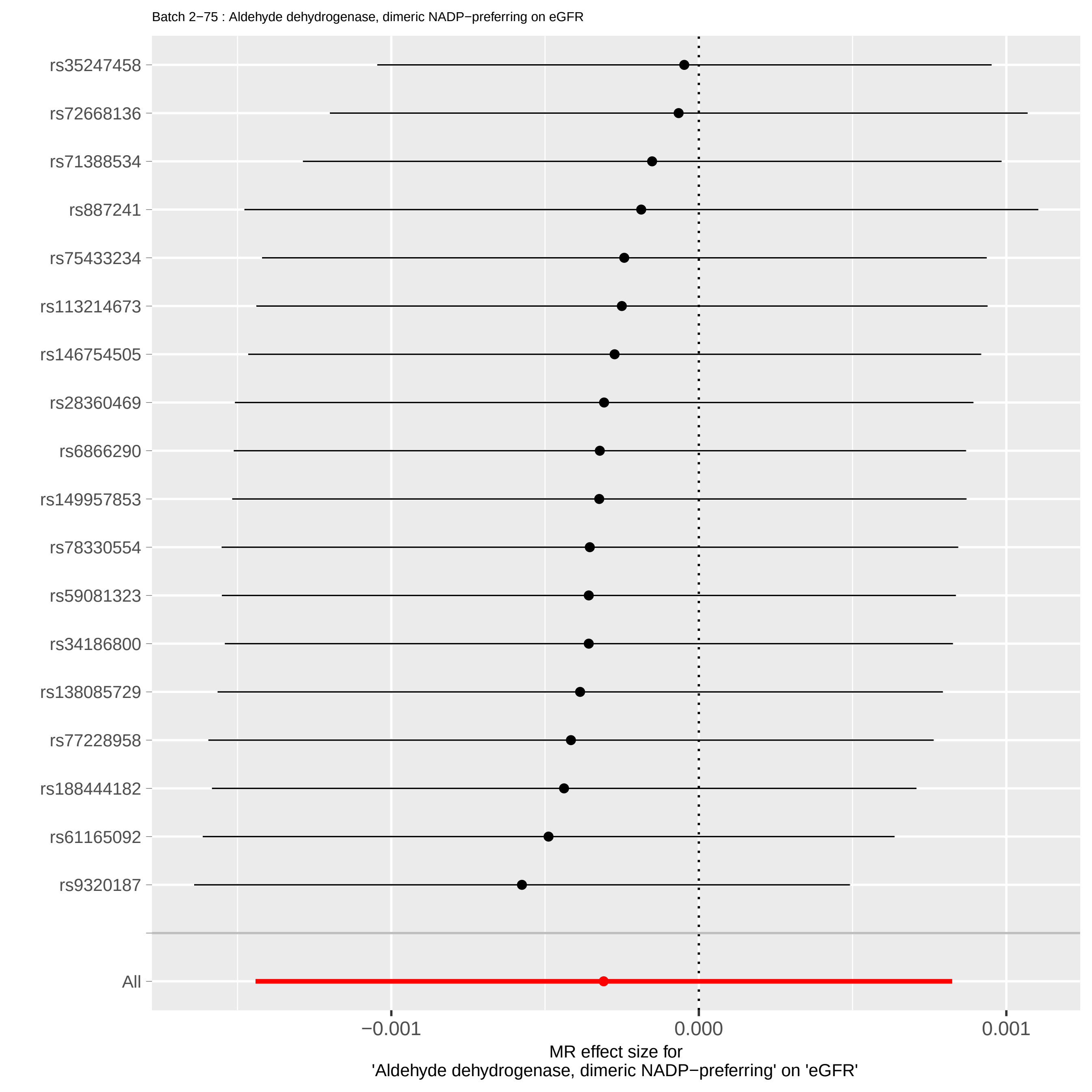
Batch 2−75 : Aldehyde dehydrogenase, dimeric NADP−preferring on eGFR
rs35247458
rs72668136
rs71388534
rs887241
rs75433234
rs113214673
rs146754505
rs28360469
rs6866290
rs149957853
rs78330554
rs59081323
rs34186800
rs138085729
rs77228958
rs188444182
rs61165092
rs9320187
All
−0.001
0.000
0.001
MR effect size for
'Aldehyde dehydrogenase, dimeric NADP−preferring' on 'eGFR'

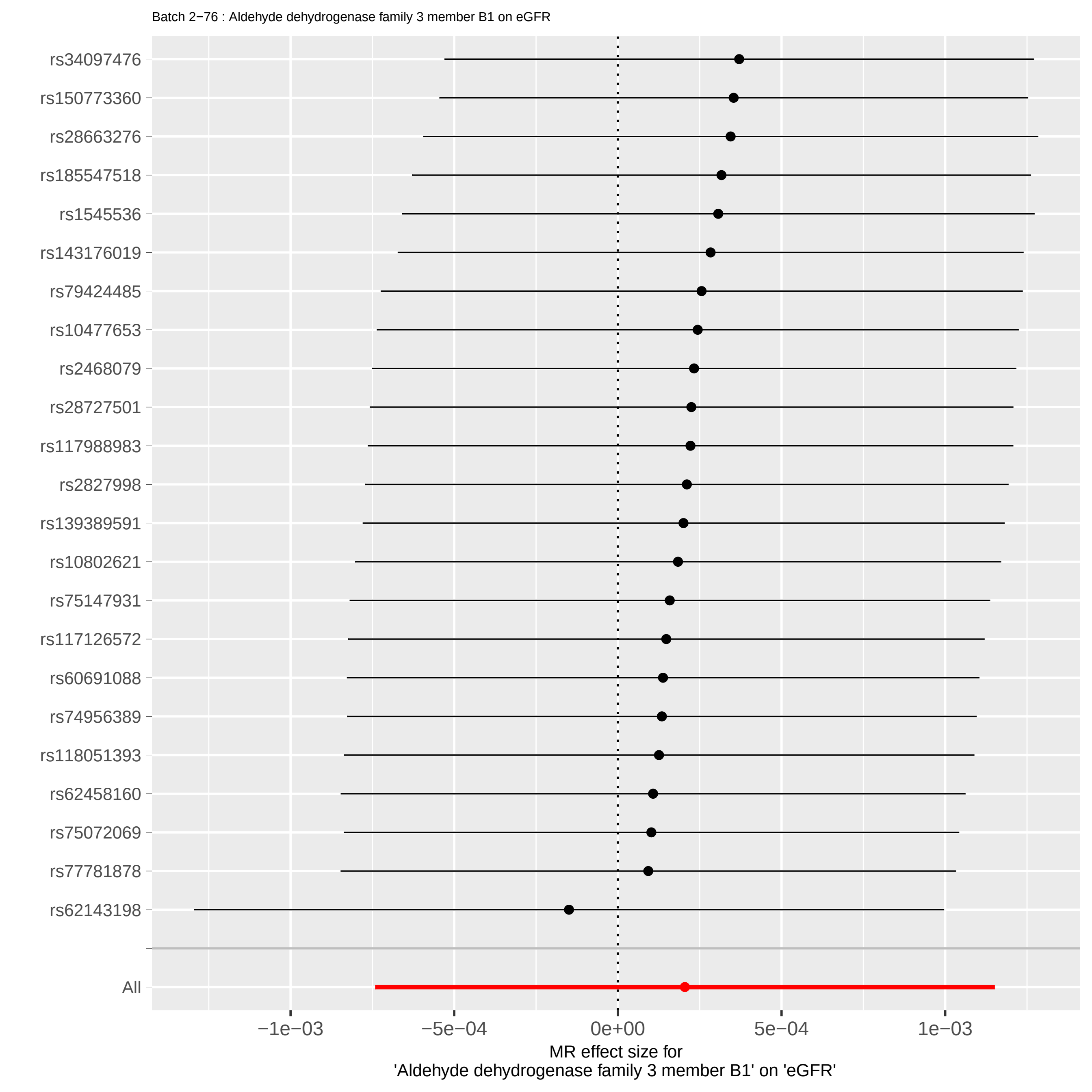
Batch 2−76 : Aldehyde dehydrogenase family 3 member B1 on eGFR
rs34097476
rs150773360
rs28663276
rs185547518
rs1545536
rs143176019
rs79424485
rs10477653
rs2468079
rs28727501
rs117988983
rs2827998
rs139389591
rs10802621
rs75147931
rs117126572
rs60691088
rs74956389
rs118051393
rs62458160
rs75072069
rs77781878
rs62143198
All
−1e−03
−5e−04
0e+00
5e−04
1e−03
MR effect size for
'Aldehyde dehydrogenase family 3 member B1' on 'eGFR'

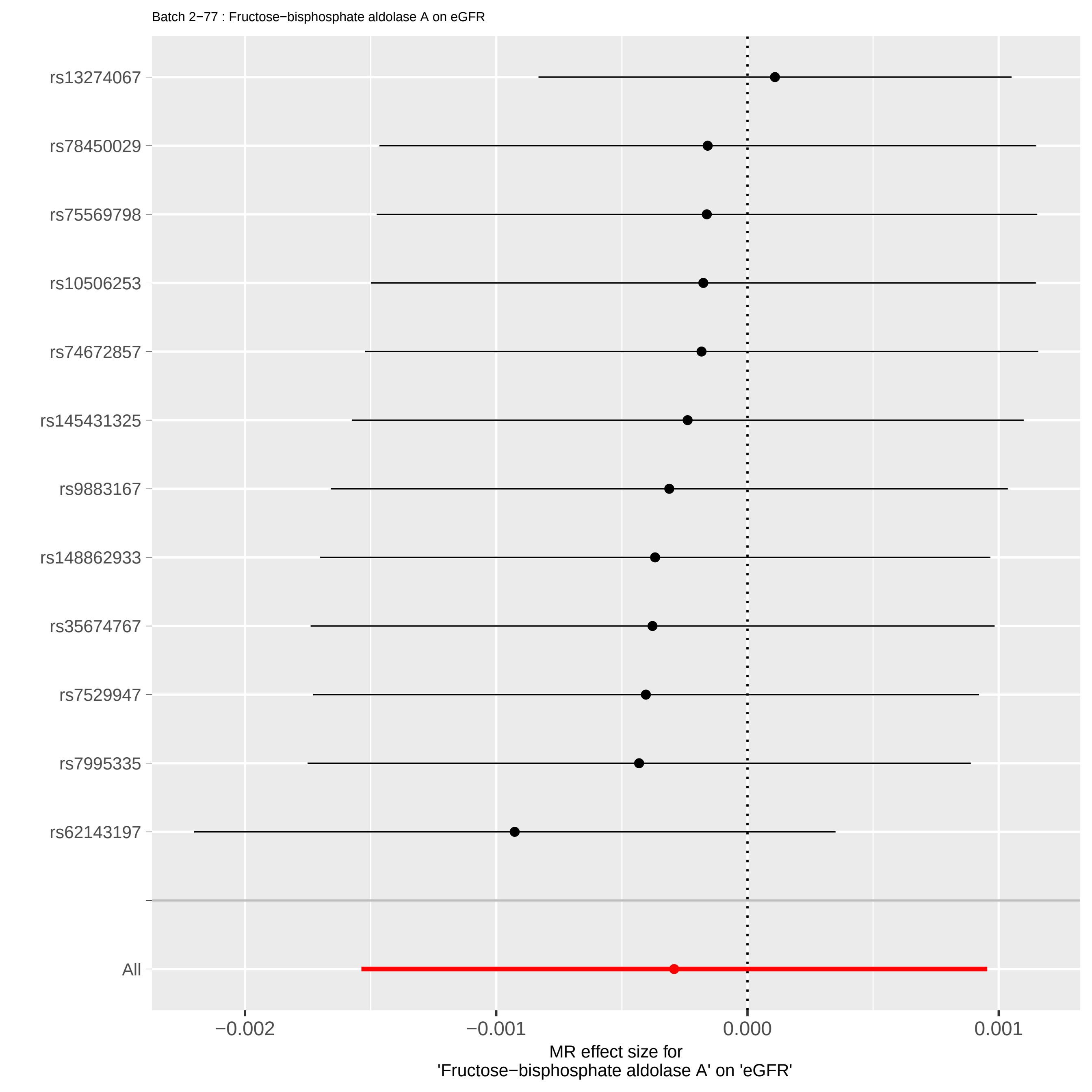
Batch 2−77 : Fructose−bisphosphate aldolase A on eGFR
rs13274067
rs78450029
rs75569798
rs10506253
rs74672857
rs145431325
rs9883167
rs148862933
rs35674767
rs7529947
rs7995335
rs62143197
All
−0.002
−0.001
0.000
0.001
MR effect size for
'Fructose−bisphosphate aldolase A' on 'eGFR'

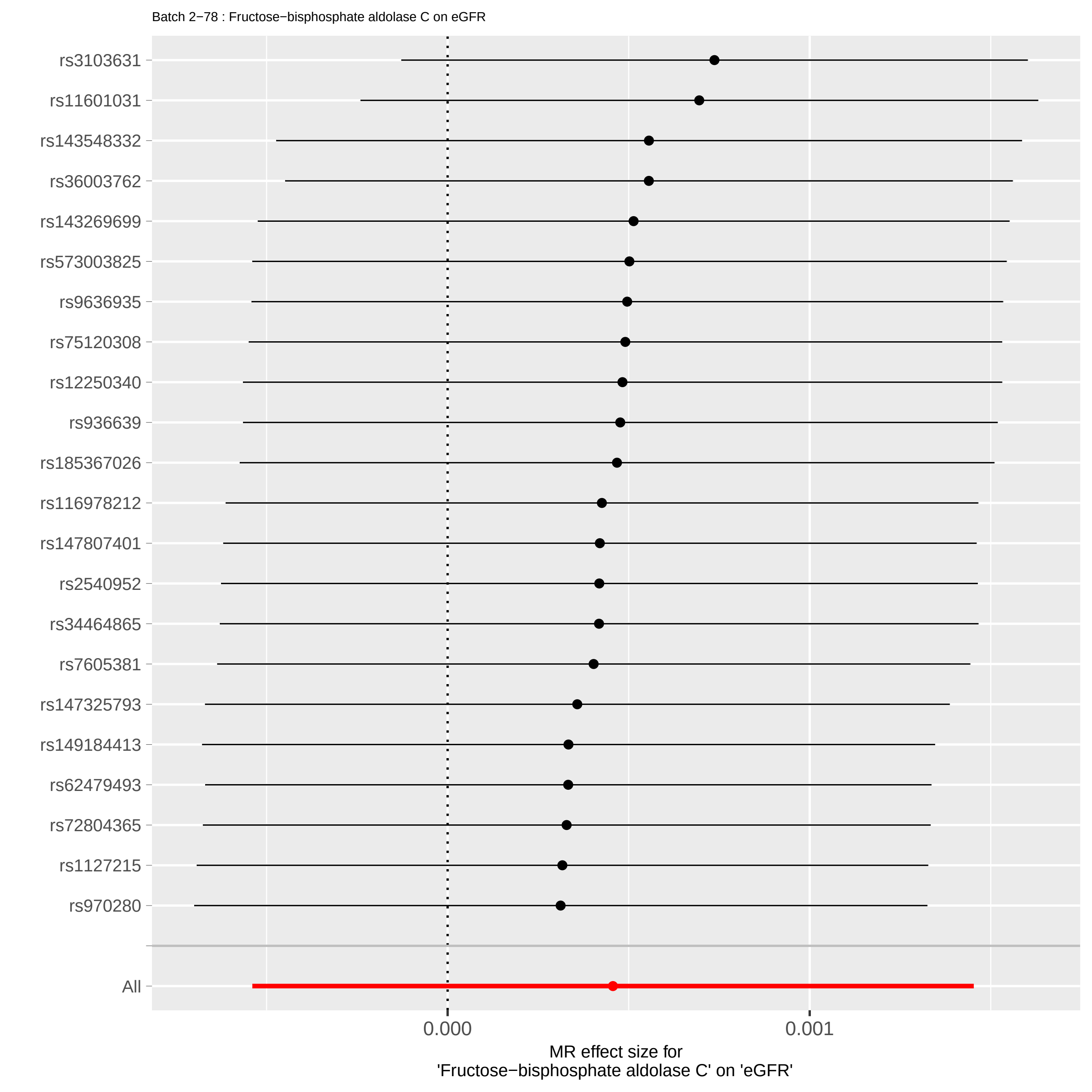

Batch 2−78 : Fructose−bisphosphate aldolase C on eGFR
rs3103631
rs11601031
rs143548332
rs36003762
rs143269699
rs573003825
rs9636935
rs75120308
rs12250340
rs936639
rs185367026
rs116978212
rs147807401
rs2540952
rs34464865
rs7605381
rs147325793
rs149184413
rs62479493
rs72804365
rs1127215
rs970280
All
0.000
0.001
MR effect size for
'Fructose−bisphosphate aldolase C' on 'eGFR'

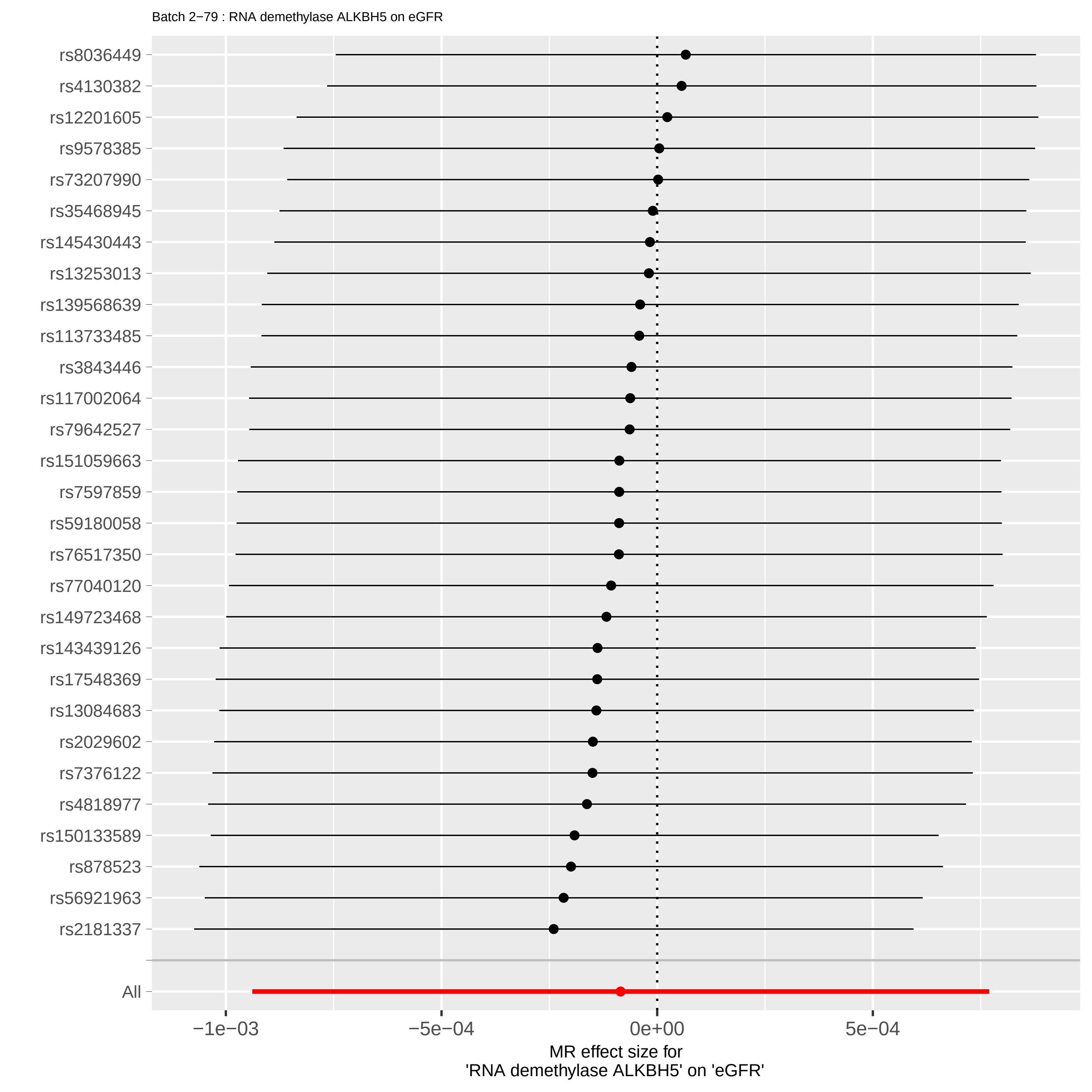

Batch 2−79 : RNA demethylase ALKBH5 on eGFR
rs8036449
rs4130382
rs12201605
rs9578385
rs73207990
rs35468945
rs145430443
rs13253013
rs139568639
rs113733485
rs3843446
rs117002064
rs79642527
rs151059663
rs7597859
rs59180058
rs76517350
rs77040120
rs149723468
rs143439126
rs17548369
rs13084683
rs2029602
rs7376122
rs4818977
rs150133589
rs878523
rs56921963
rs2181337
All
−1e−03
−5e−04
0e+00
5e−04
MR effect size for
'RNA demethylase ALKBH5' on 'eGFR'

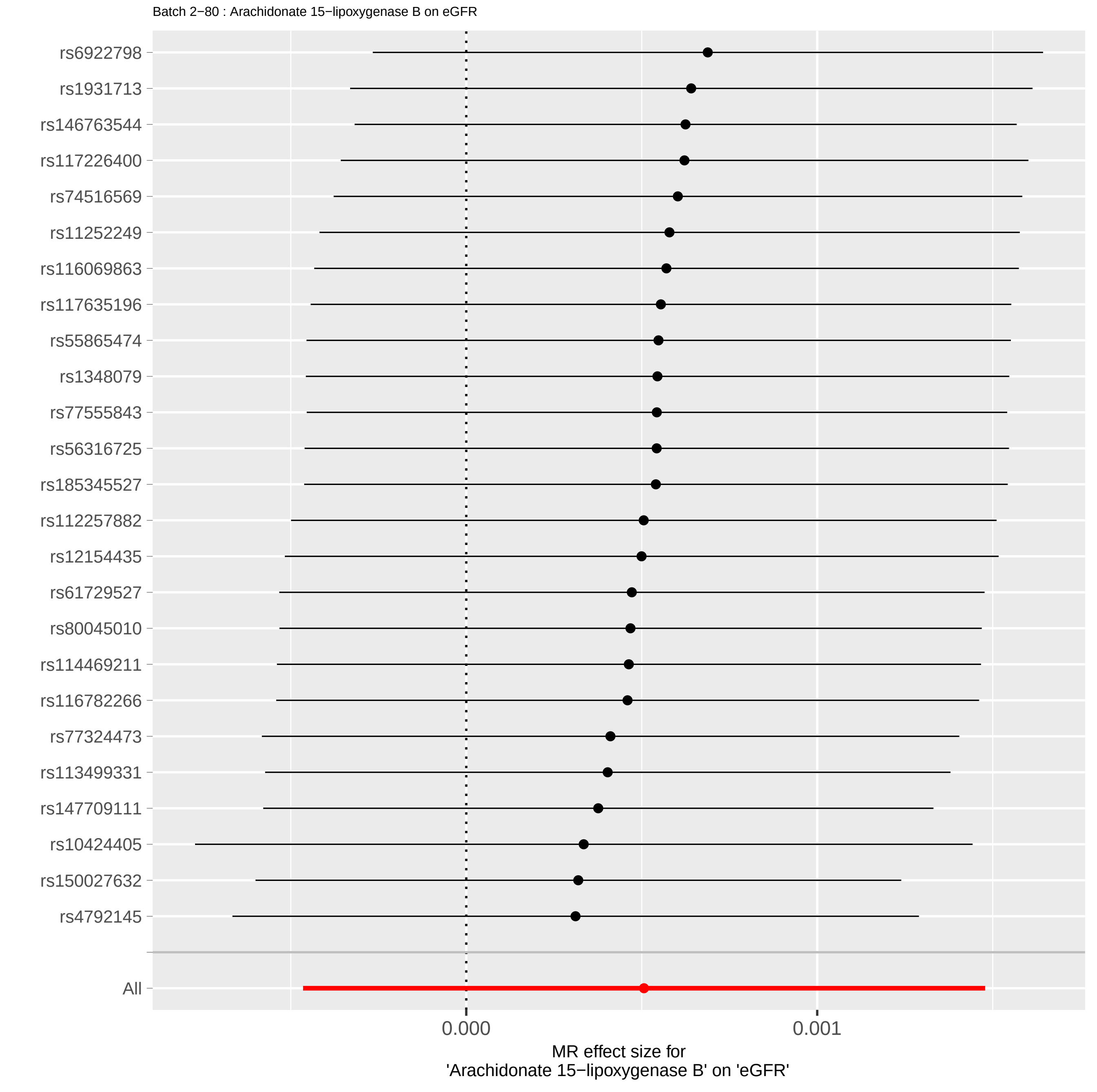

Batch 2−80 : Arachidonate 15−lipoxygenase B on eGFR
rs6922798
rs1931713
rs146763544
rs117226400
rs74516569
rs11252249
rs116069863
rs117635196
rs55865474
rs1348079
rs77555843
rs56316725
rs185345527
rs112257882
rs12154435
rs61729527
rs80045010
rs114469211
rs116782266
rs77324473
rs113499331
rs147709111
rs10424405
rs150027632
rs4792145
All
0.000
0.001
MR effect size for
'Arachidonate 15−lipoxygenase B' on 'eGFR'

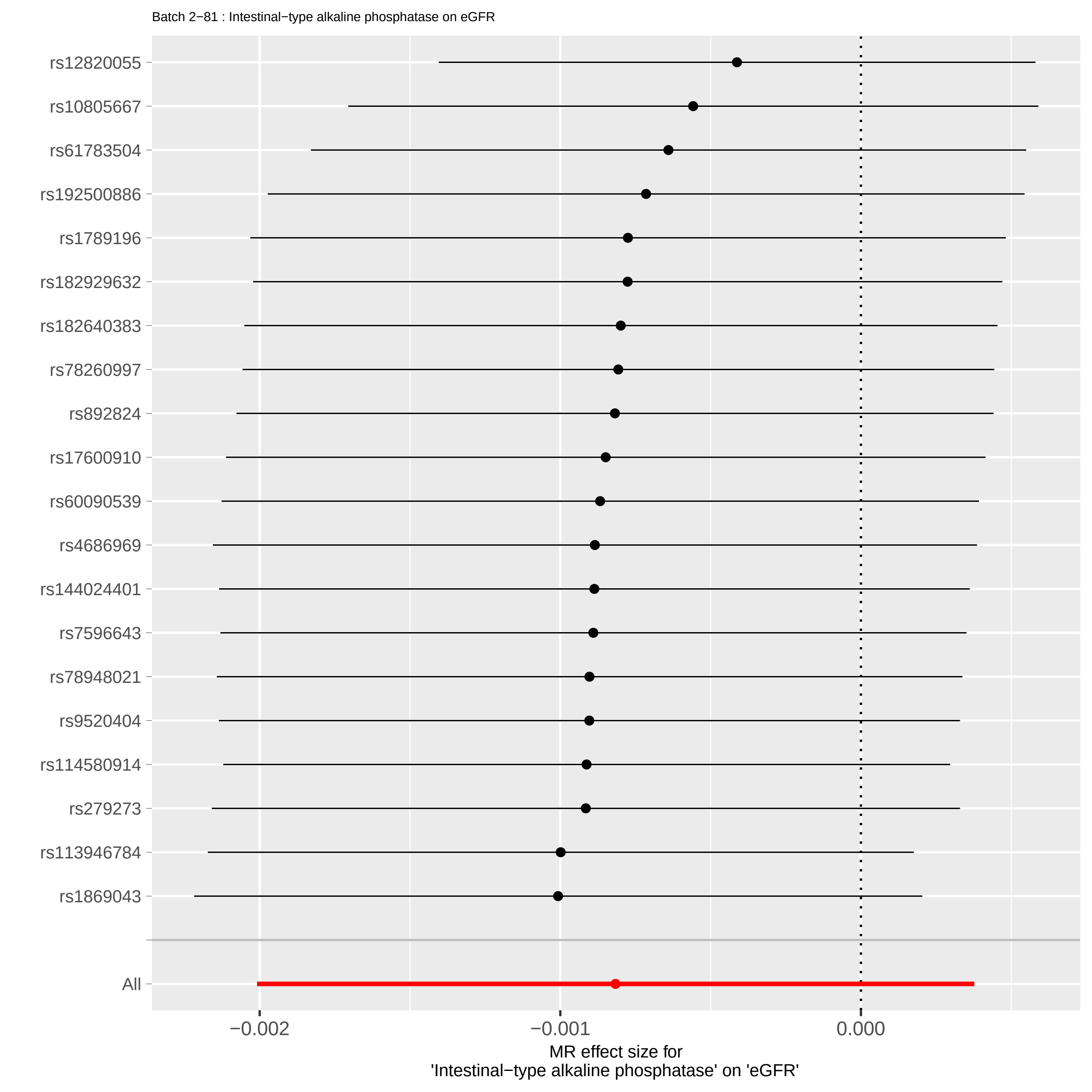
Batch 2−81 : Intestinal−type alkaline phosphatase on eGFR
rs12820055
rs10805667
rs61783504
rs192500886
rs1789196
rs182929632
rs182640383
rs78260997
rs892824
rs17600910
rs60090539
rs4686969
rs144024401
rs7596643
rs78948021
rs9520404
rs114580914
rs279273
rs113946784
rs1869043
All
−0.002
−0.001
0.000
MR effect size for
'Intestinal−type alkaline phosphatase' on 'eGFR'

Batch 2−82 : Ameloblastin on eGFR

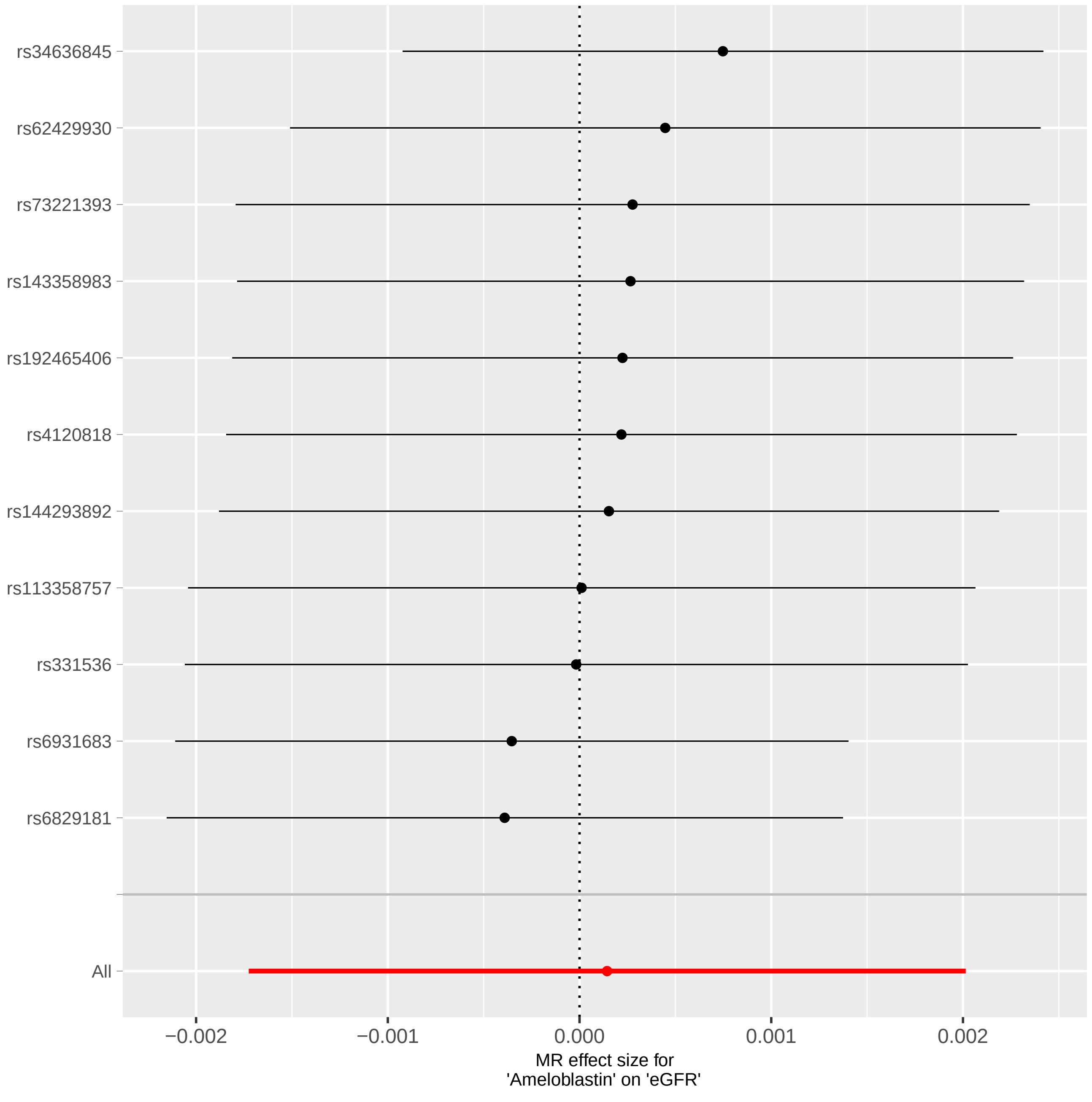

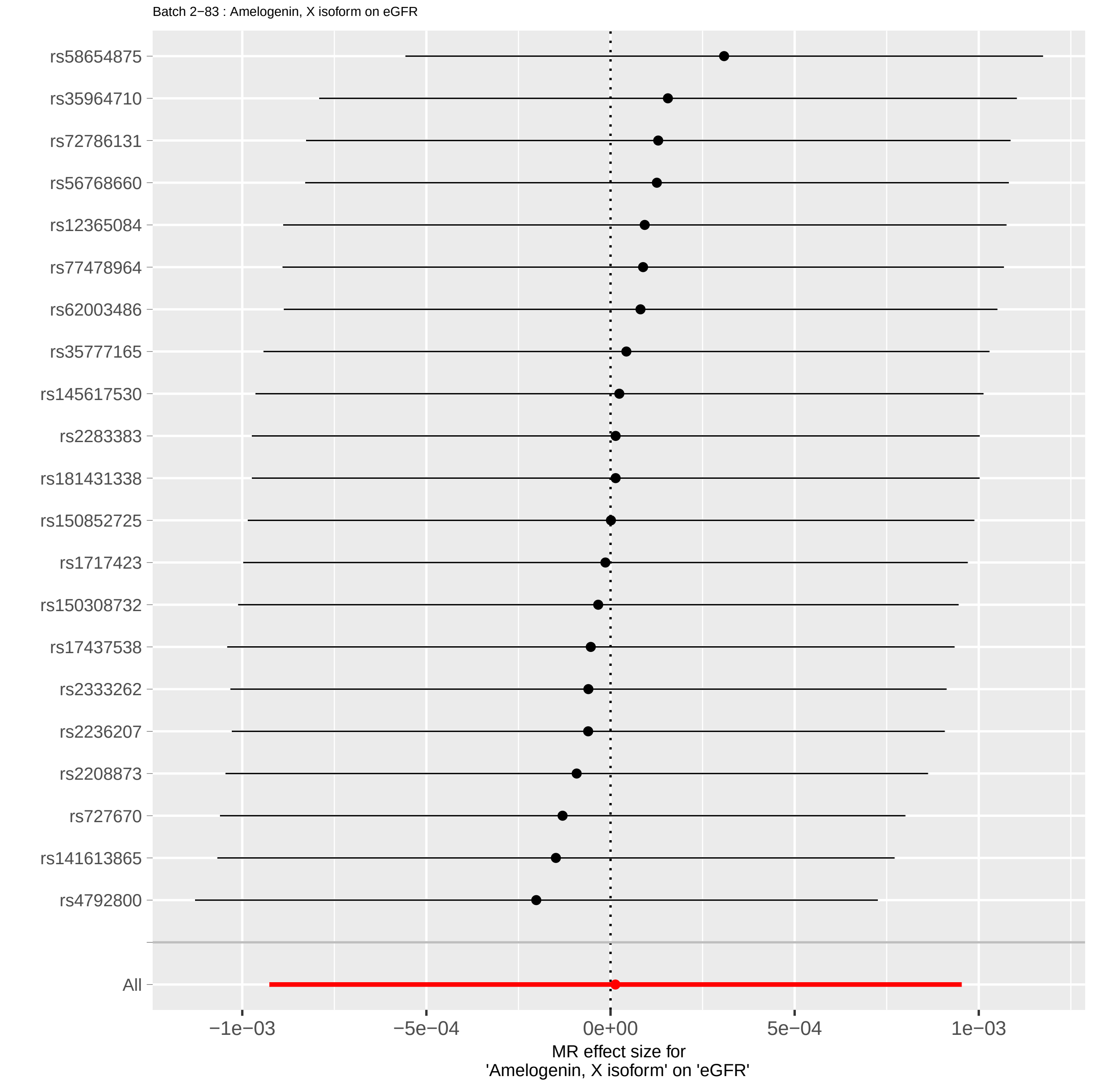

Batch 2−83 : Amelogenin, X isoform on eGFR
rs58654875
rs35964710
rs72786131
rs56768660
rs12365084
rs77478964
rs62003486
rs35777165
rs145617530
rs2283383
rs181431338
rs150852725
rs1717423
rs150308732
rs17437538
rs2333262
rs2236207
rs2208873
rs727670
rs141613865
rs4792800
All
−1e−03
−5e−04
0e+00
5e−04
1e−03
MR effect size for
'Amelogenin, X isoform' on 'eGFR'

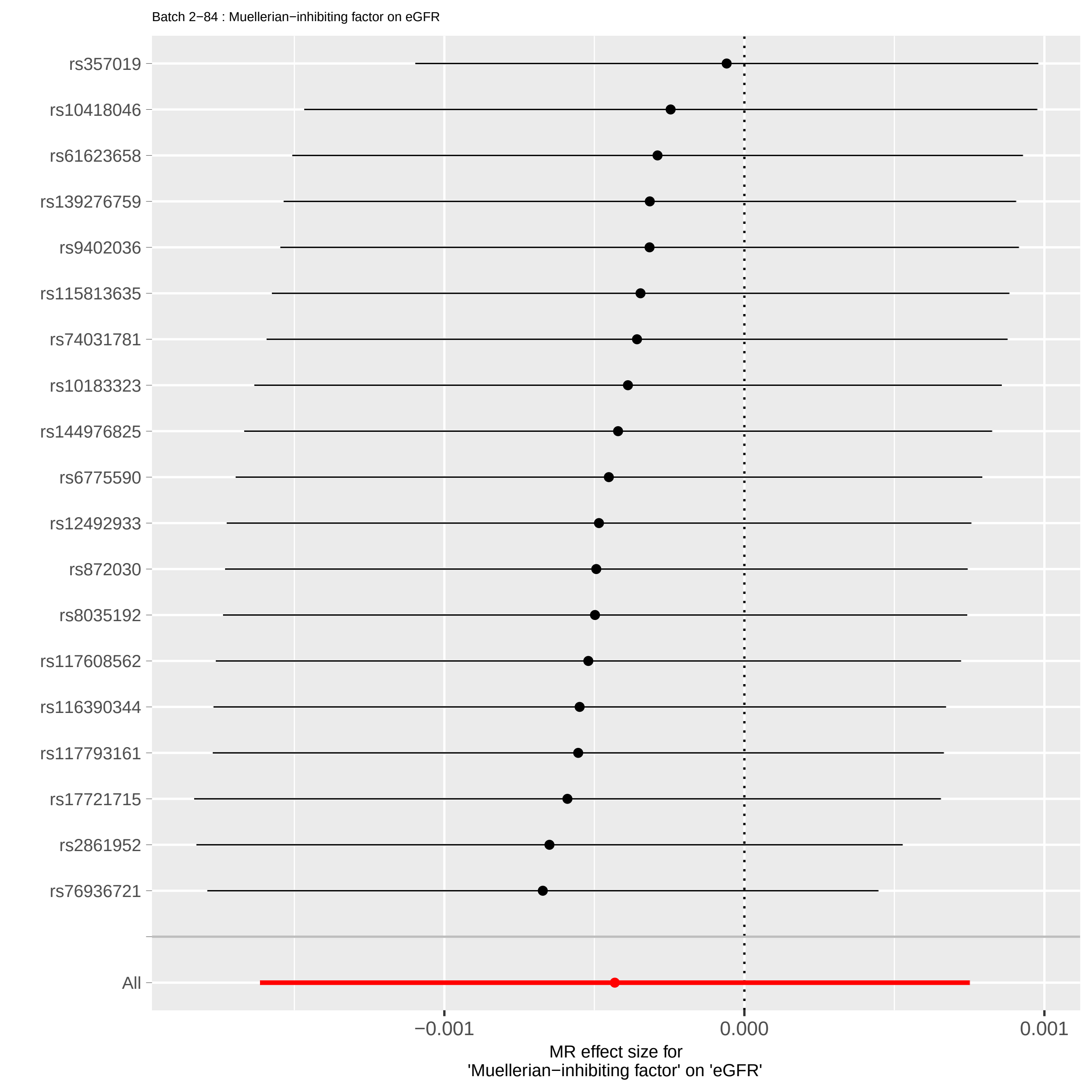
Batch 2−84 : Muellerian−inhibiting factor on eGFR
rs357019
rs10418046
rs61623658
rs139276759
rs9402036
rs115813635
rs74031781
rs10183323
rs144976825
rs6775590
rs12492933
rs872030
rs8035192
rs117608562
rs116390344
rs117793161
rs17721715
rs2861952
rs76936721
All
−0.001
0.000
0.001
MR effect size for
'Muellerian−inhibiting factor' on 'eGFR'

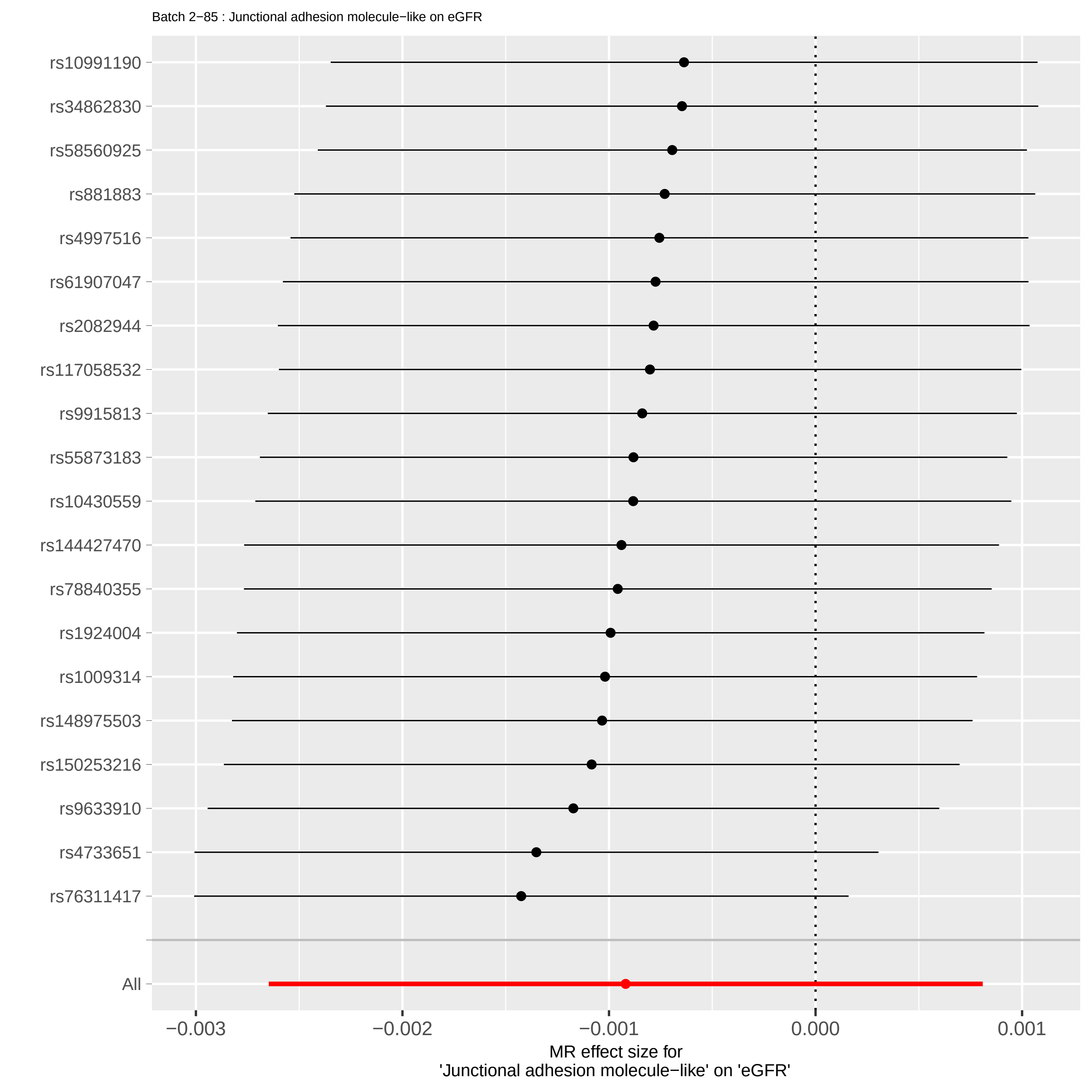

Batch 2−85 : Junctional adhesion molecule−like on eGFR
rs10991190
rs34862830
rs58560925
rs881883
rs4997516
rs61907047
rs2082944
rs117058532
rs9915813
rs55873183
rs10430559
rs144427470
rs78840355
rs1924004
rs1009314
rs148975503
rs150253216
rs9633910
rs4733651
rs76311417
All
−0.003
−0.002
−0.001
0.000
0.001
MR effect size for
'Junctional adhesion molecule−like' on 'eGFR'

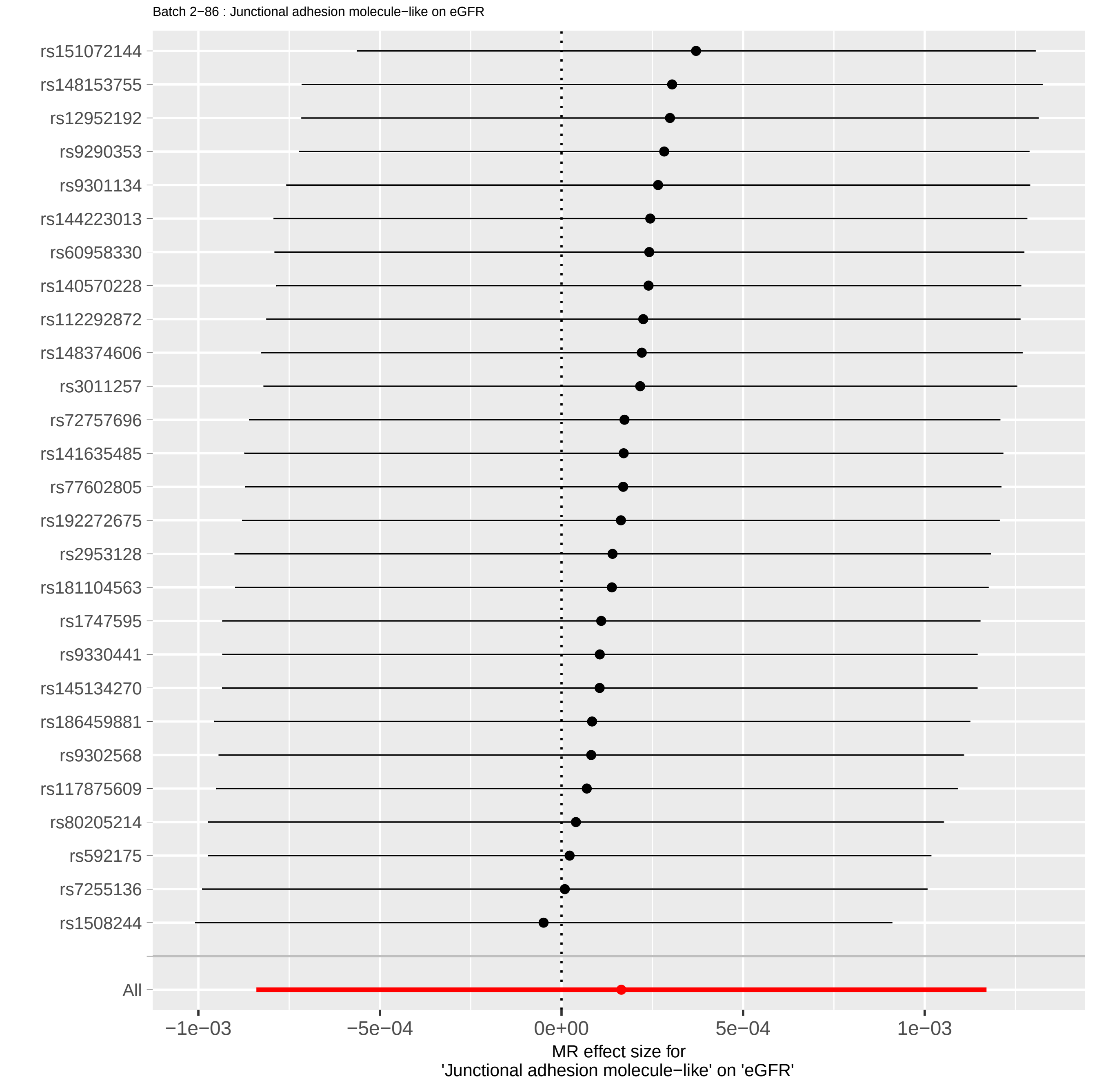
Batch 2−86 : Junctional adhesion molecule−like on eGFR
rs151072144
rs148153755
rs12952192
rs9290353
rs9301134
rs144223013
rs60958330
rs140570228
rs112292872
rs148374606
rs3011257
rs72757696
rs141635485
rs77602805
rs192272675
rs2953128
rs181104563
rs1747595
rs9330441
rs145134270
rs186459881
rs9302568
rs117875609
rs80205214
rs592175
rs7255136
rs1508244
All
−1e−03
−5e−04
0e+00
5e−04
1e−03
MR effect size for
'Junctional adhesion molecule−like' on 'eGFR'

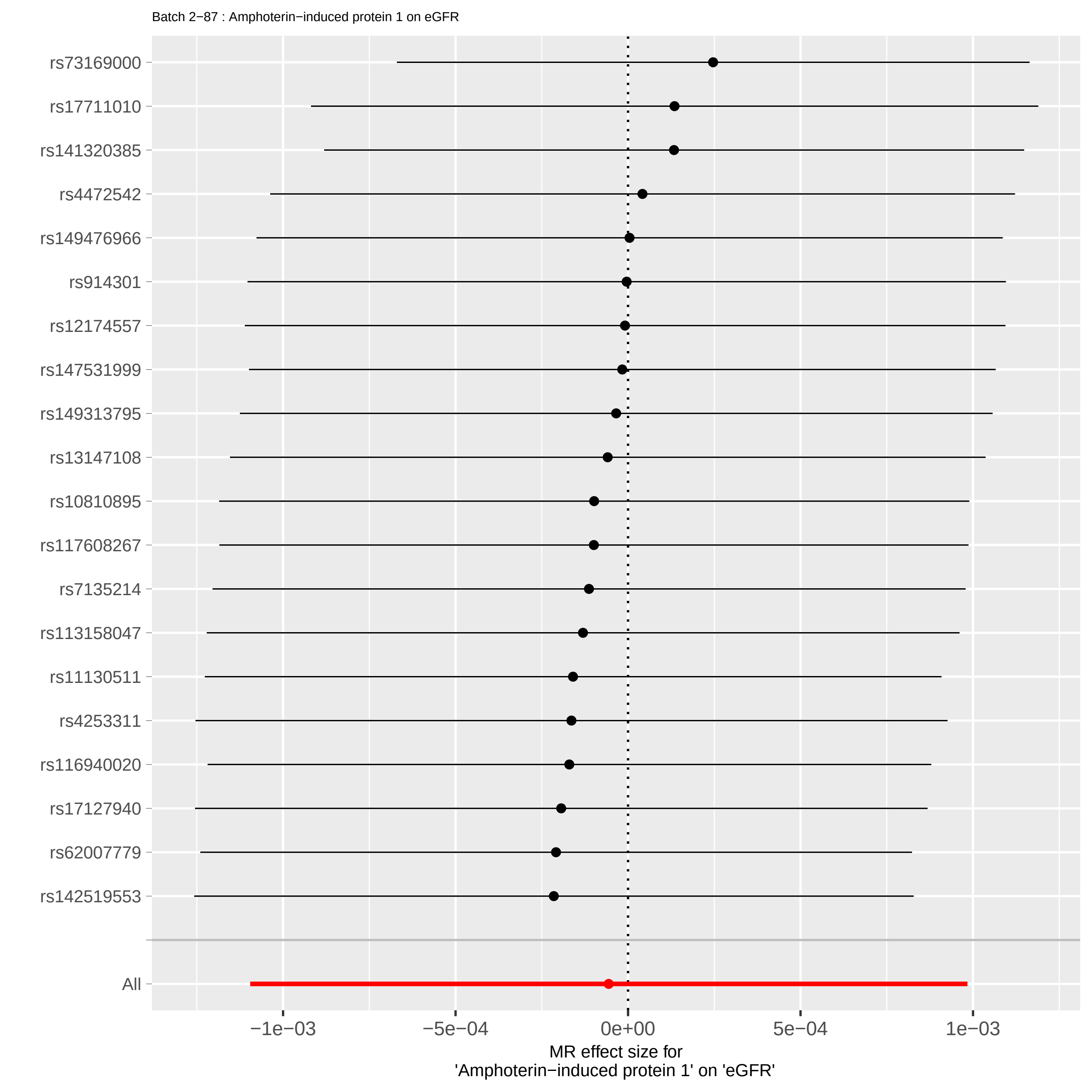

Batch 2−87 : Amphoterin−induced protein 1 on eGFR
rs73169000
rs17711010
rs141320385
rs4472542
rs149476966
rs914301
rs12174557
rs147531999
rs149313795
rs13147108
rs10810895
rs117608267
rs7135214
rs113158047
rs11130511
rs4253311
rs116940020
rs17127940
rs62007779
rs142519553
All
−1e−03
−5e−04
0e+00
5e−04
1e−03
MR effect size for
'Amphoterin−induced protein 1' on 'eGFR'

Batch 2−88 : Protein amnionless on eGFR

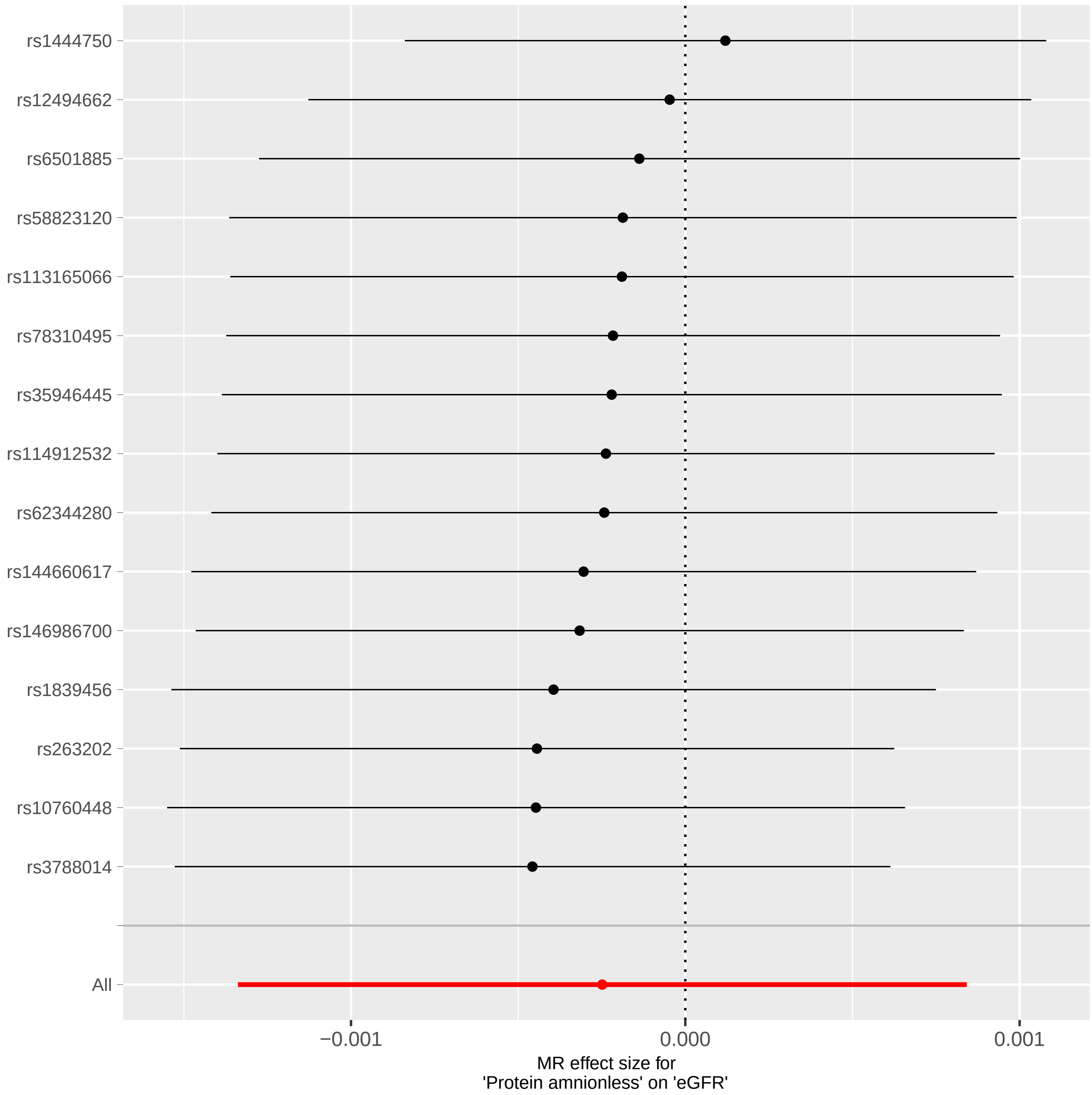

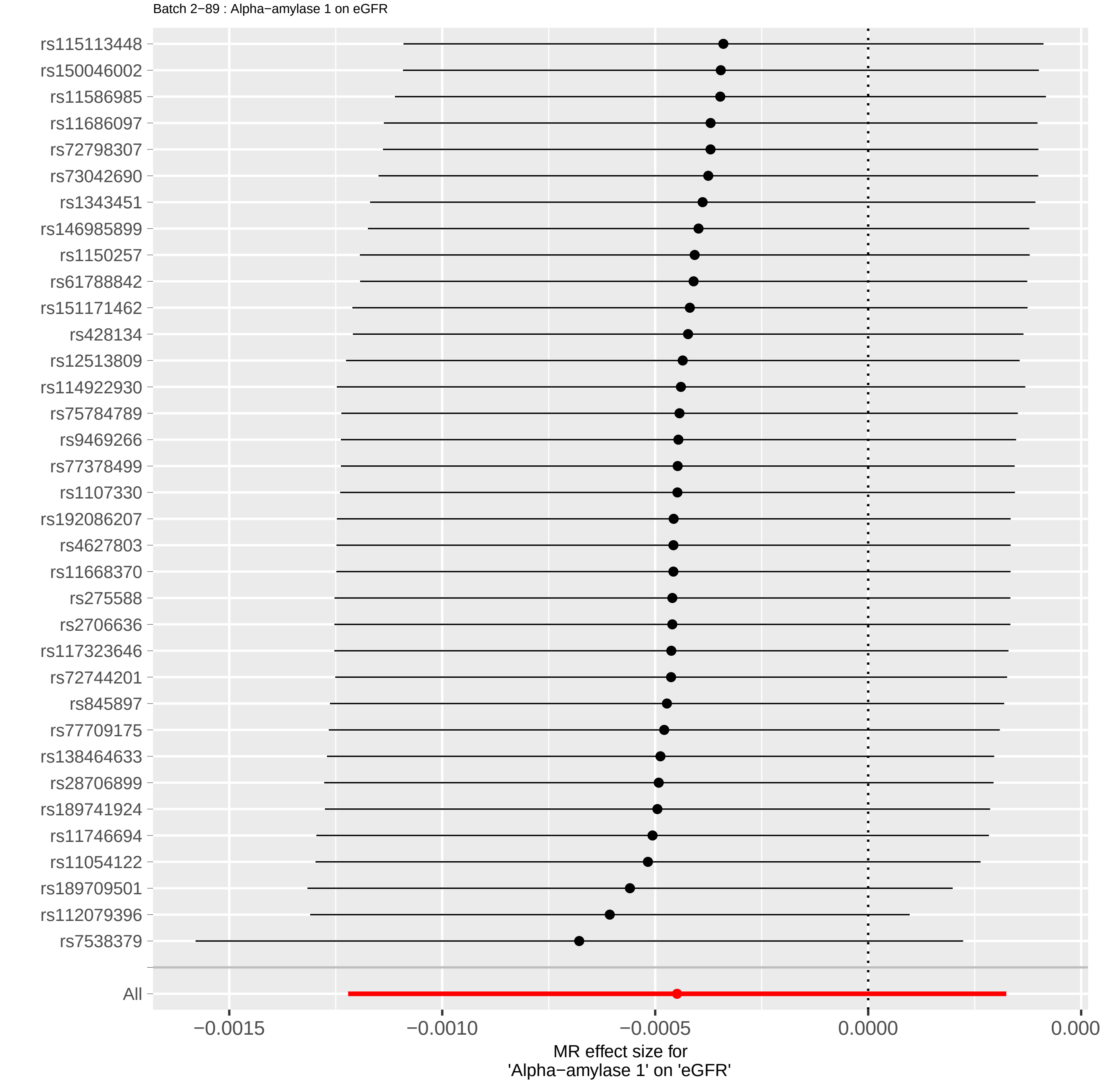

Batch 2−89 : Alpha−amylase 1 on eGFR
rs115113448
rs150046002
rs11586985
rs11686097
rs72798307
rs73042690
rs1343451
rs146985899
rs1150257
rs61788842
rs151171462
rs428134
rs12513809
rs114922930
rs75784789
rs9469266
rs77378499
rs1107330
rs192086207
rs4627803
rs11668370
rs275588
rs2706636
rs117323646
rs72744201
rs845897
rs77709175
rs138464633
rs28706899
rs189741924
rs11746694
rs11054122
rs189709501
rs112079396
rs7538379
All
−0.0015
−0.0010
−0.0005
0.0000
0.000
MR effect size for
'Alpha−amylase 1' on 'eGFR'

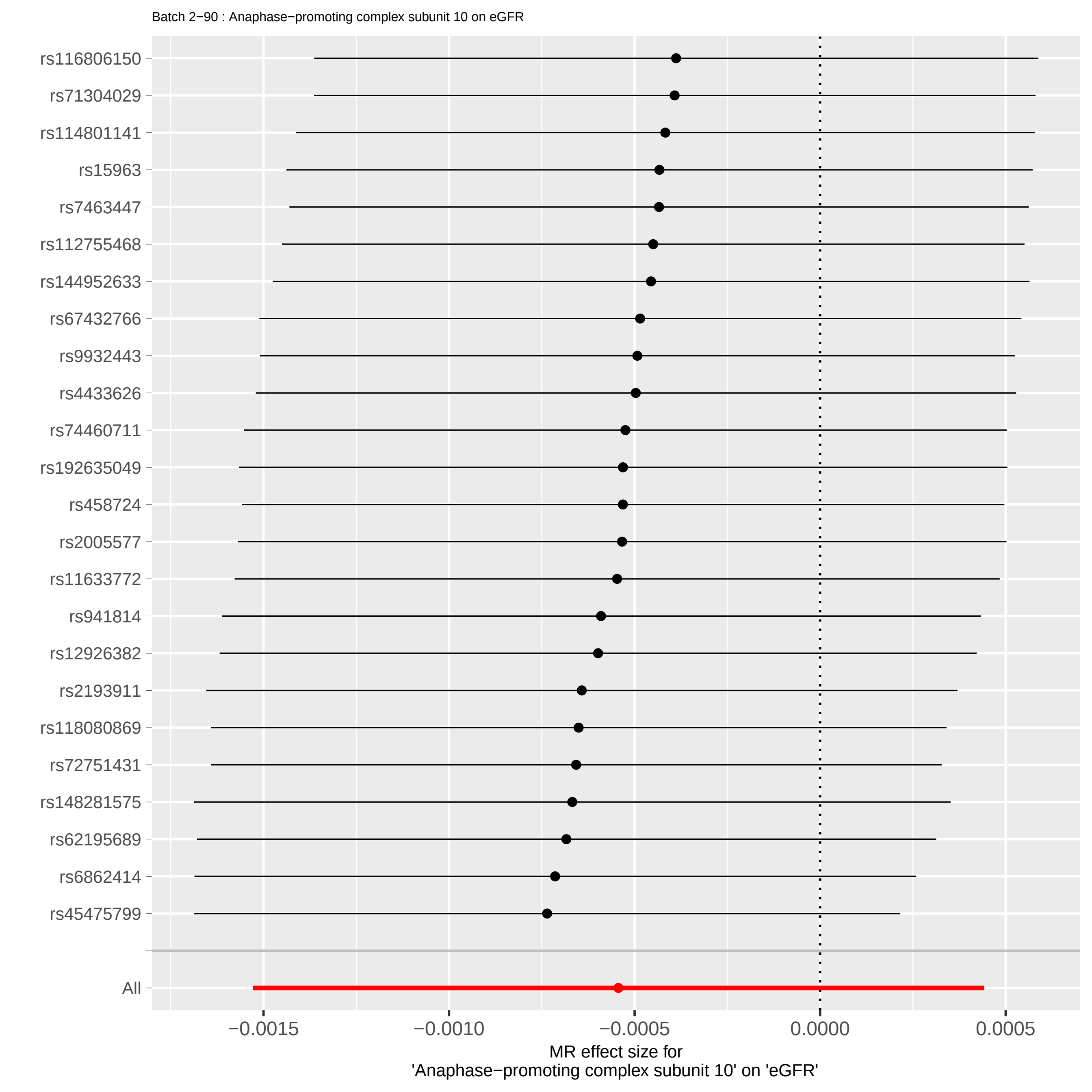

Batch 2−90 : Anaphase−promoting complex subunit 10 on eGFR
rs116806150
rs71304029
rs114801141
rs15963
rs7463447
rs112755468
rs144952633
rs67432766
rs9932443
rs4433626
rs74460711
rs192635049
rs458724
rs2005577
rs11633772
rs941814
rs12926382
rs2193911
rs118080869
rs72751431
rs148281575
rs62195689
rs6862414
rs45475799
All
−0.0015
−0.0010
−0.0005
0.0000
0.0005
MR effect size for
'Anaphase−promoting complex subunit 10' on 'eGFR'

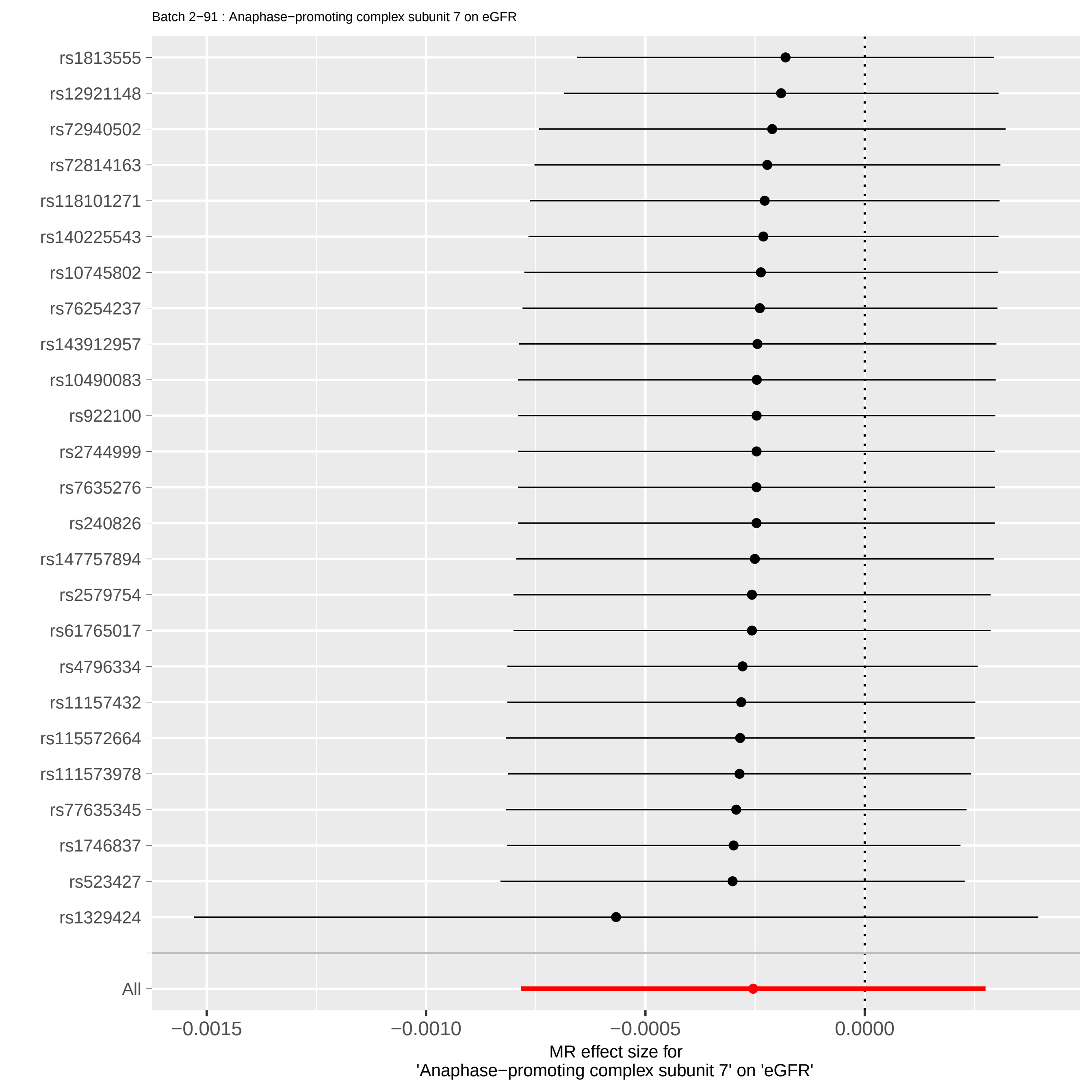

Batch 2−91 : Anaphase−promoting complex subunit 7 on eGFR
rs1813555
rs12921148
rs72940502
rs72814163
rs118101271
rs140225543
rs10745802
rs76254237
rs143912957
rs10490083
rs922100
rs2744999
rs7635276
rs240826
rs147757894
rs2579754
rs61765017
rs4796334
rs11157432
rs115572664
rs111573978
rs77635345
rs1746837
rs523427
rs1329424
All
−0.0015
−0.0010
−0.0005
0.0000
MR effect size for
'Anaphase−promoting complex subunit 7' on 'eGFR'

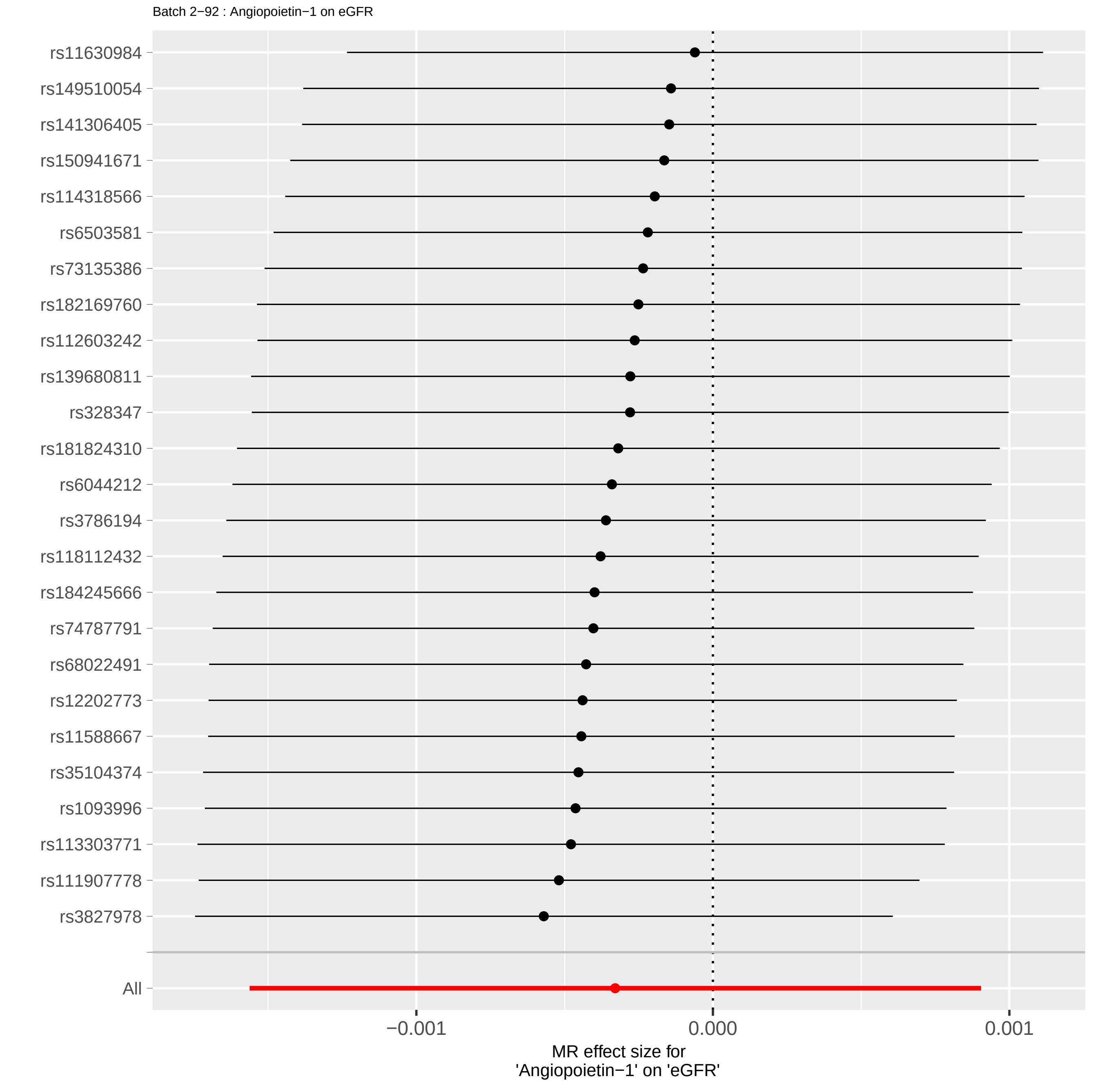

Batch 2−92 : Angiopoietin−1 on eGFR
rs11630984
rs149510054
rs141306405
rs150941671
rs114318566
rs6503581
rs73135386
rs182169760
rs112603242
rs139680811
rs328347
rs181824310
rs6044212
rs3786194
rs118112432
rs184245666
rs74787791
rs68022491
rs12202773
rs11588667
rs35104374
rs1093996
rs113303771
rs111907778
rs3827978
All
−0.001
0.000
0.001
MR effect size for
'Angiopoietin−1' on 'eGFR'

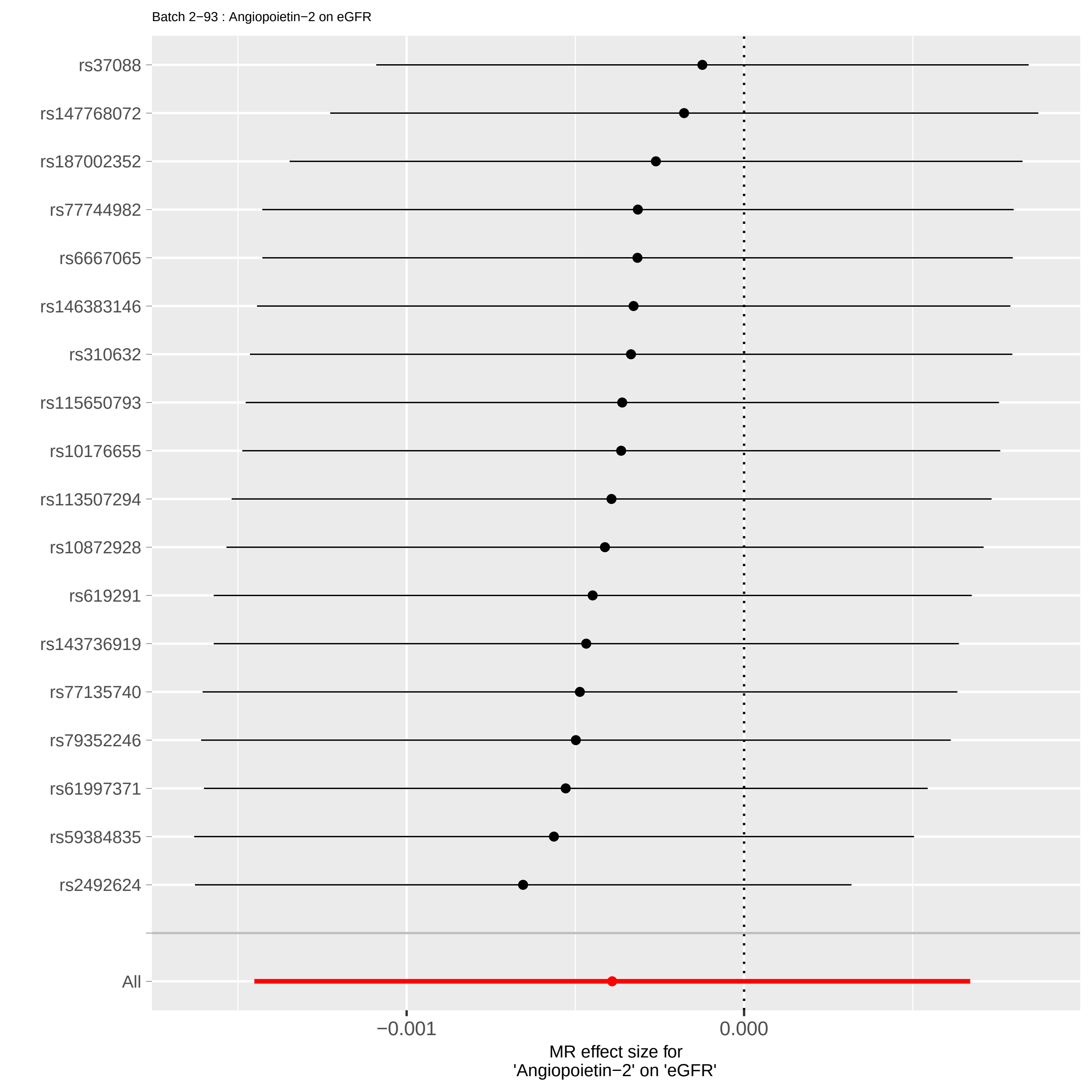
Batch 2−93 : Angiopoietin−2 on eGFR
rs37088
rs147768072
rs187002352
rs77744982
rs6667065
rs146383146
rs310632
rs115650793
rs10176655
rs113507294
rs10872928
rs619291
rs143736919
rs77135740
rs79352246
rs61997371
rs59384835
rs2492624
All
−0.001
0.000
MR effect size for
'Angiopoietin−2' on 'eGFR'

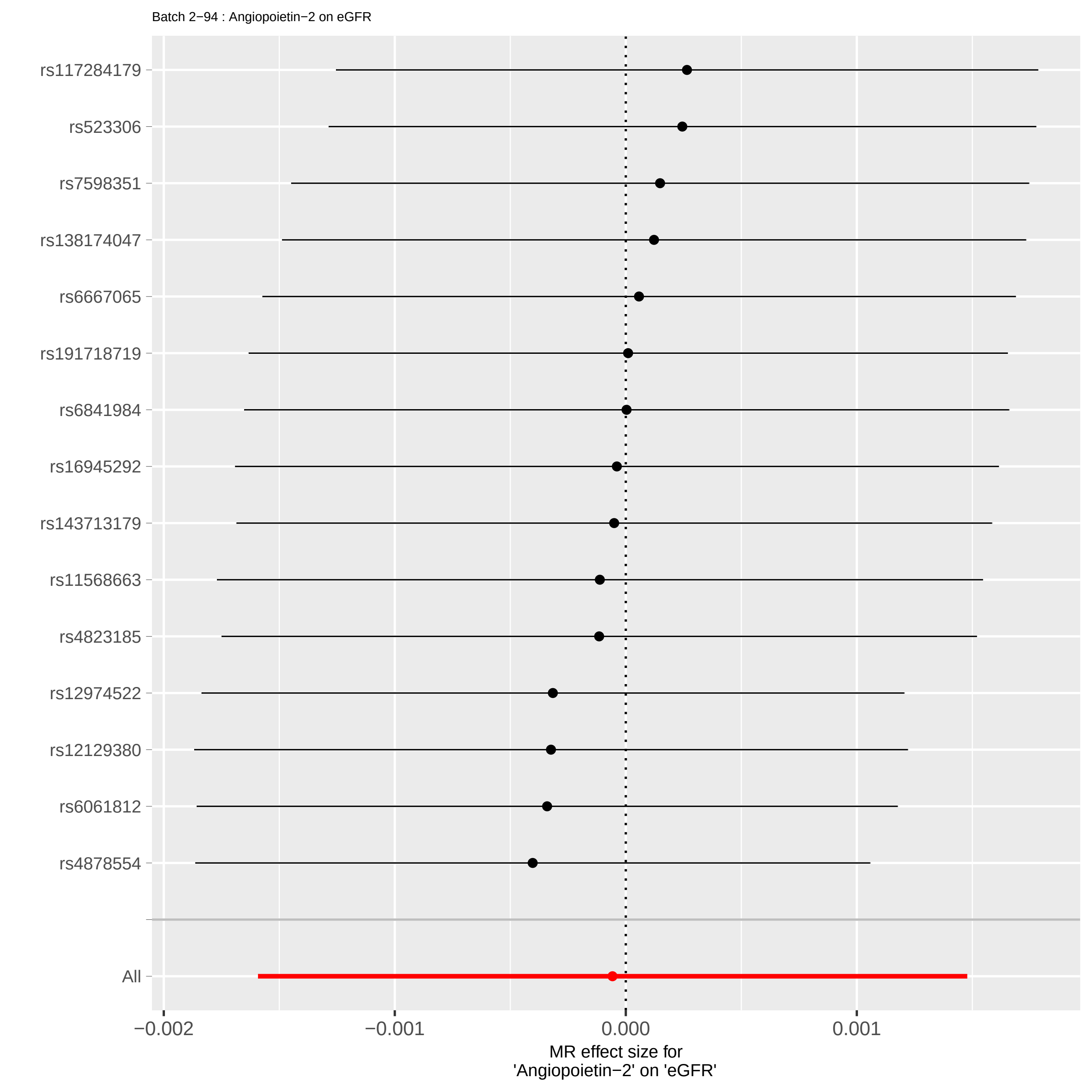

Batch 2−94 : Angiopoietin−2 on eGFR
rs117284179
rs523306
rs7598351
rs138174047
rs6667065
rs191718719
rs6841984
rs16945292
rs143713179
rs11568663
rs4823185
rs12974522
rs12129380
rs6061812
rs4878554
All
−0.002
−0.001
0.000
0.001
MR effect size for
'Angiopoietin−2' on 'eGFR'

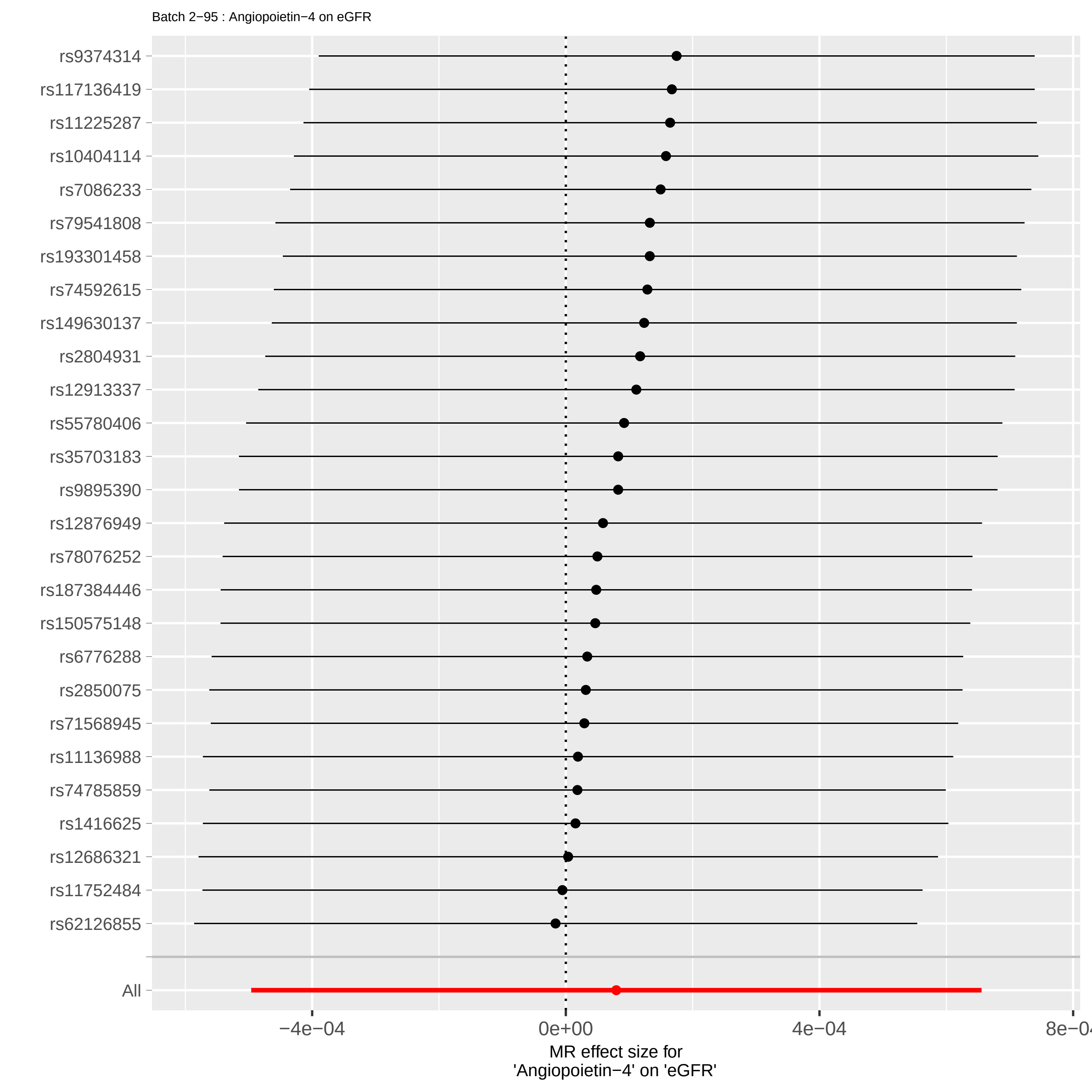

Batch 2−95 : Angiopoietin−4 on eGFR
rs9374314
rs117136419
rs11225287
rs10404114
rs7086233
rs79541808
rs193301458
rs74592615
rs149630137
rs2804931
rs12913337
rs55780406
rs35703183
rs9895390
rs12876949
rs78076252
rs187384446
rs150575148
rs6776288
rs2850075
rs71568945
rs11136988
rs74785859
rs1416625
rs12686321
rs11752484
rs62126855
All
−4e−04
0e+00
4e−04
MR effect size for
'Angiopoietin−4' on 'eGFR'

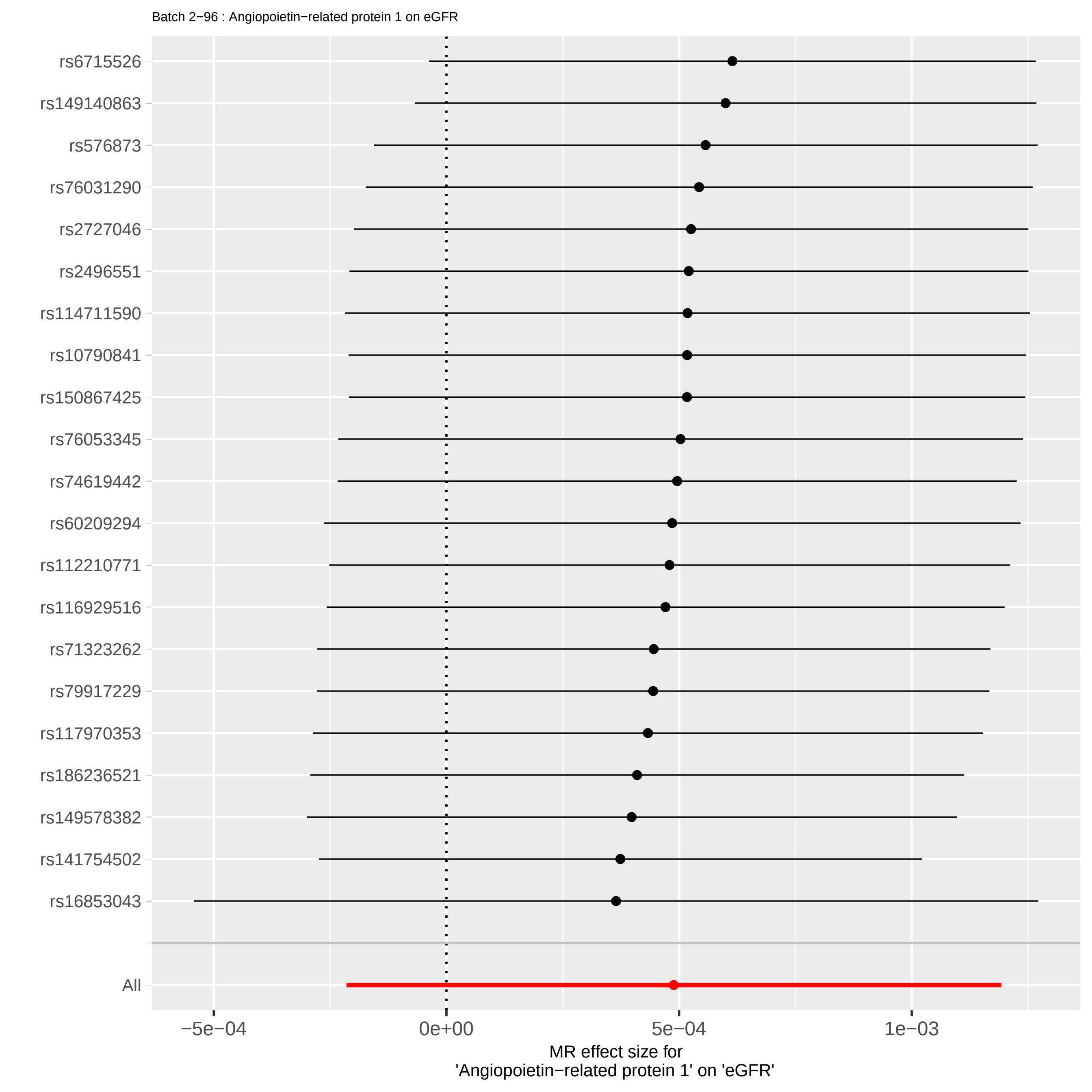

Batch 2−96 : Angiopoietin−related protein 1 on eGFR
rs6715526
rs149140863
rs576873
rs76031290
rs2727046
rs2496551
rs114711590
rs10790841
rs150867425
rs76053345
rs74619442
rs60209294
rs112210771
rs116929516
rs71323262
rs79917229
rs117970353
rs186236521
rs149578382
rs141754502
rs16853043
All
−5e−04
0e+00
5e−04
1e−03
MR effect size for
'Angiopoietin−related protein 1' on 'eGFR'

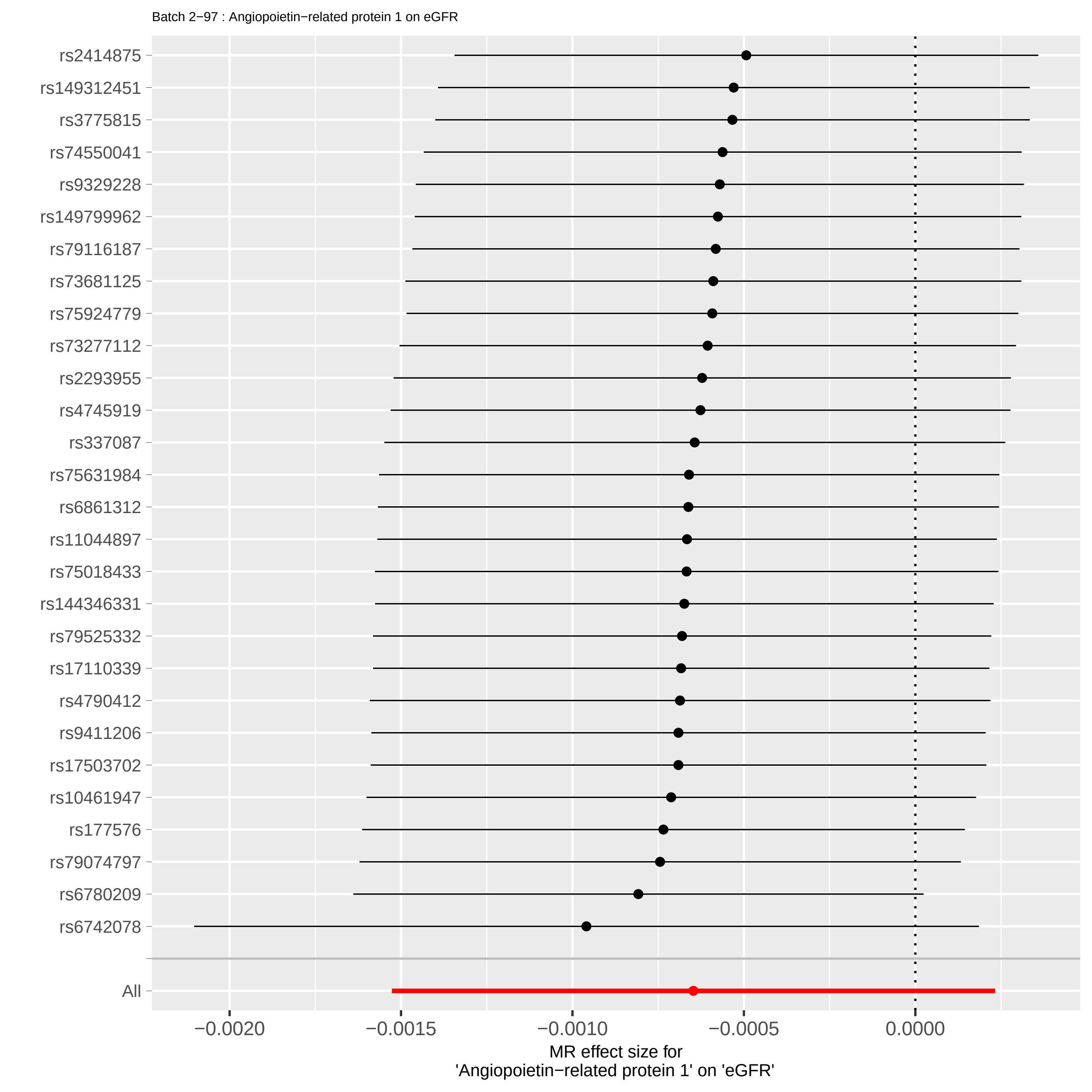

Batch 2−97 : Angiopoietin−related protein 1 on eGFR
rs2414875
rs149312451
rs3775815
rs74550041
rs9329228
rs149799962
rs79116187
rs73681125
rs75924779
rs73277112
rs2293955
rs4745919
rs337087
rs75631984
rs6861312
rs11044897
rs75018433
rs144346331
rs79525332
rs17110339
rs4790412
rs9411206
rs17503702
rs10461947
rs177576
rs79074797
rs6780209
rs6742078
All
−0.0020
−0.0015
−0.0010
−0.0005
0.0000
MR effect size for
'Angiopoietin−related protein 1' on 'eGFR'

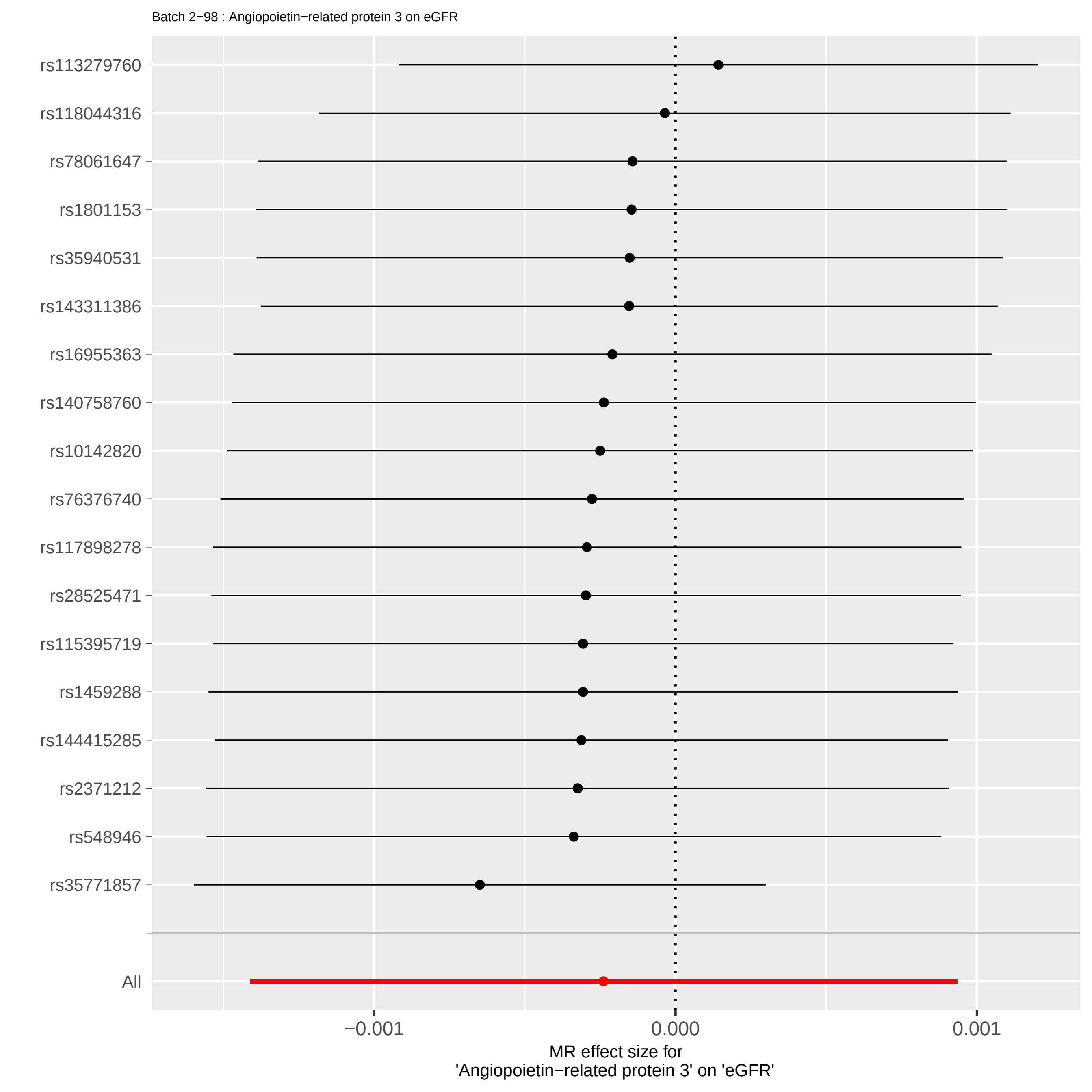
Batch 2−98 : Angiopoietin−related protein 3 on eGFR
rs113279760
rs118044316
rs78061647
rs1801153
rs35940531
rs143311386
rs16955363
rs140758760
rs10142820
rs76376740
rs117898278
rs28525471
rs115395719
rs1459288
rs144415285
rs2371212
rs548946
rs35771857
All
−0.001
0.000
0.001
MR effect size for
'Angiopoietin−related protein 3' on 'eGFR'

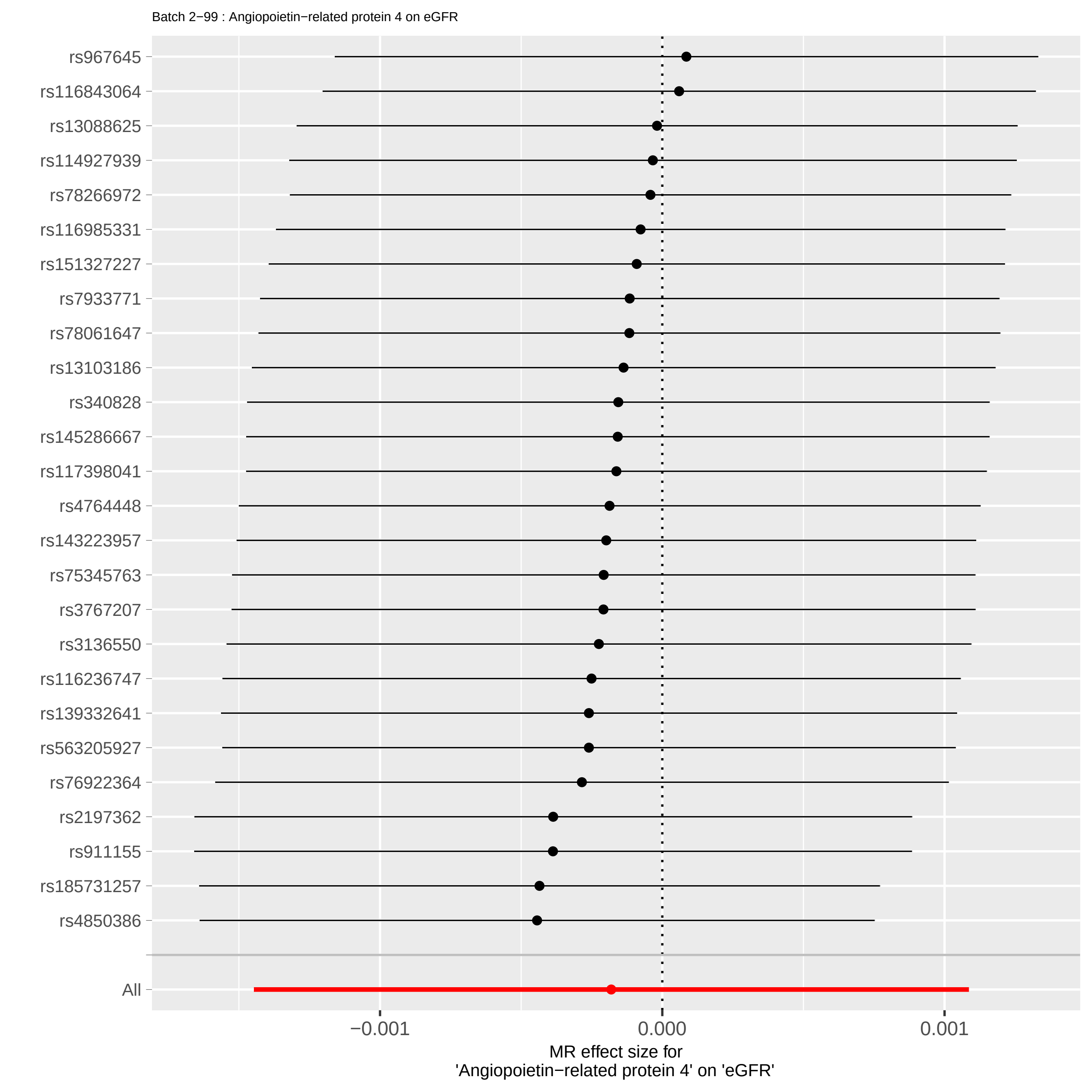
Batch 2−99 : Angiopoietin−related protein 4 on eGFR
rs967645
rs116843064
rs13088625
rs114927939
rs78266972
rs116985331
rs151327227
rs7933771
rs78061647
rs13103186
rs340828
rs145286667
rs117398041
rs4764448
rs143223957
rs75345763
rs3767207
rs3136550
rs116236747
rs139332641
rs563205927
rs76922364
rs2197362
rs911155
rs185731257
rs4850386
All
−0.001
0.000
0.001
MR effect size for
'Angiopoietin−related protein 4' on 'eGFR'

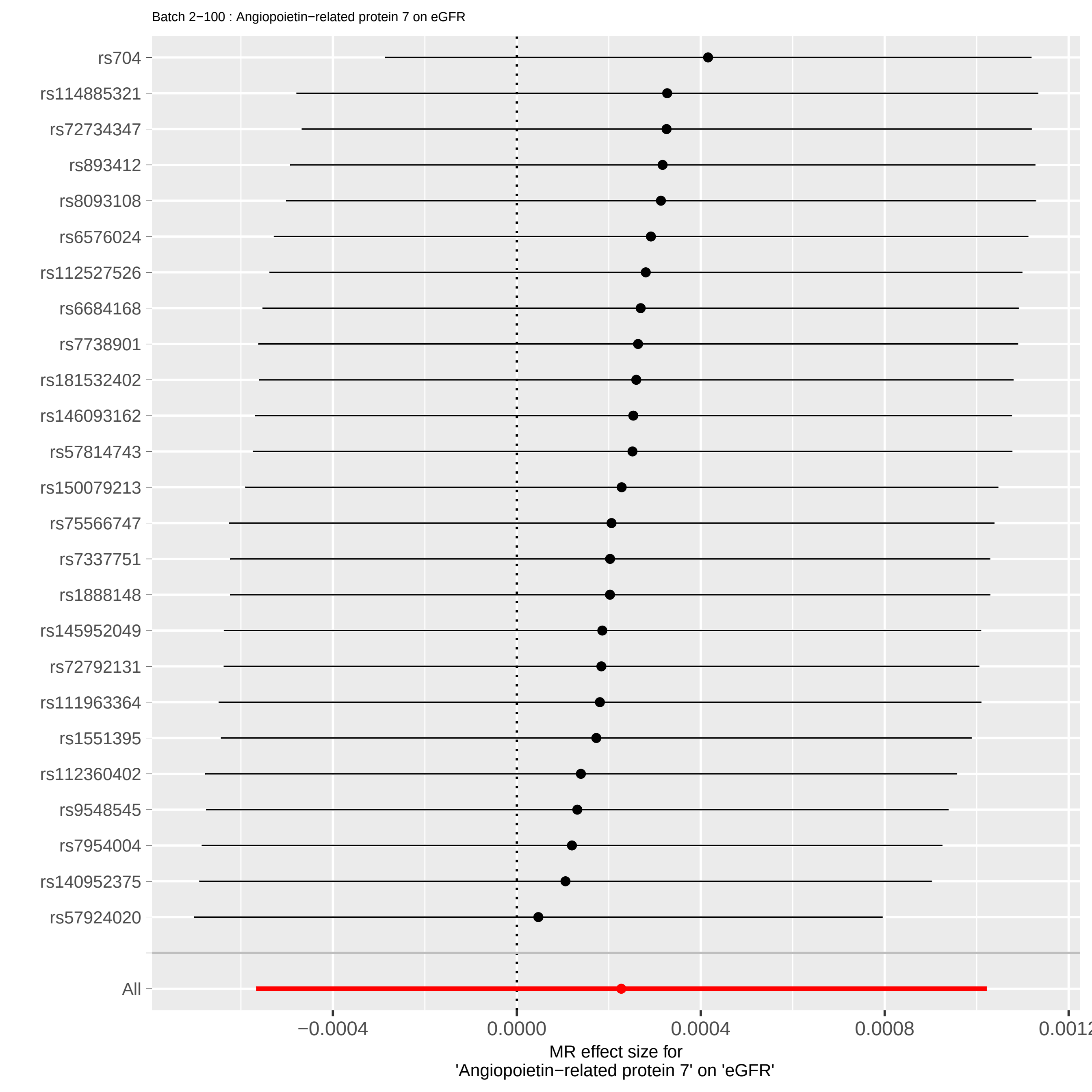

Batch 2−100 : Angiopoietin−related protein 7 on eGFR
rs704
rs114885321
rs72734347
rs893412
rs8093108
rs6576024
rs112527526
rs6684168
rs7738901
rs181532402
rs146093162
rs57814743
rs150079213
rs75566747
rs7337751
rs1888148
rs145952049
rs72792131
rs111963364
rs1551395
rs112360402
rs9548545
rs7954004
rs140952375
rs57924020
All
−0.0004
0.0000
0.0004
0.0008
MR effect size for
'Angiopoietin−related protein 7' on 'eGFR'

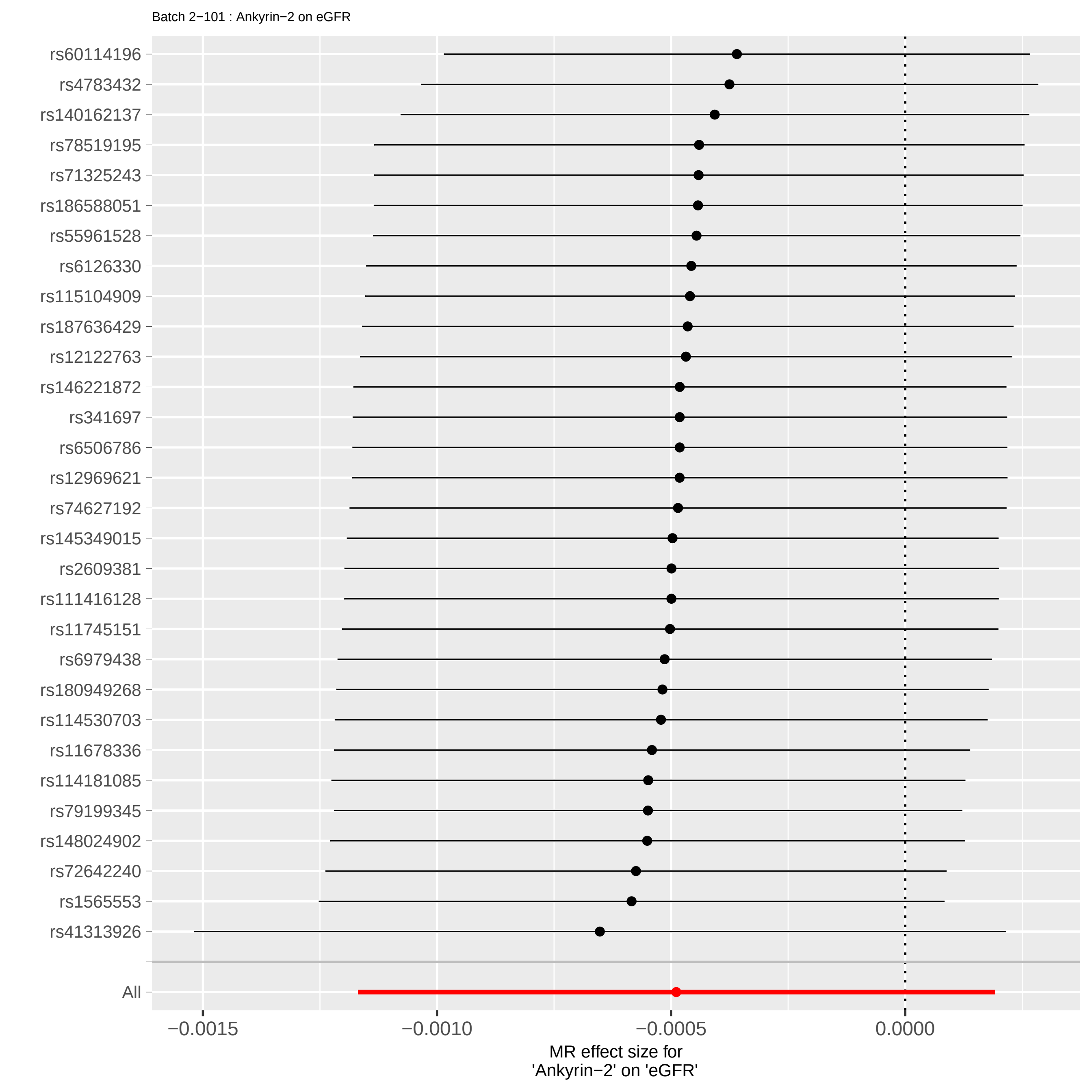

Batch 2−101 : Ankyrin−2 on eGFR
rs60114196
rs4783432
rs140162137
rs78519195
rs71325243
rs186588051
rs55961528
rs6126330
rs115104909
rs187636429
rs12122763
rs146221872
rs341697
rs6506786
rs12969621
rs74627192
rs145349015
rs2609381
rs111416128
rs11745151
rs6979438
rs180949268
rs114530703
rs11678336
rs114181085
rs79199345
rs148024902
rs72642240
rs1565553
rs41313926
All
−0.0015
−0.0010
−0.0005
0.0000
MR effect size for
'Ankyrin−2' on 'eGFR'

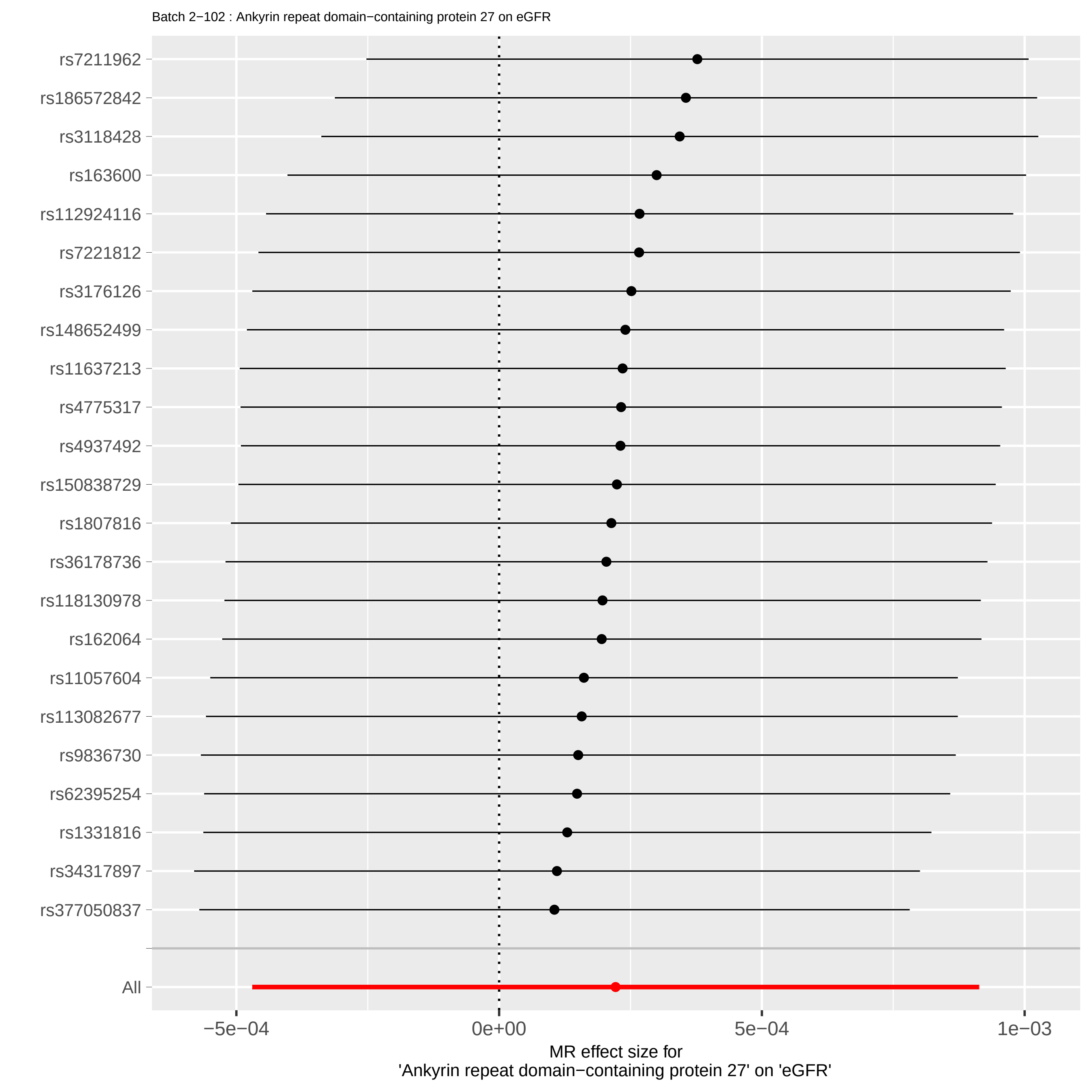

Batch 2−102 : Ankyrin repeat domain−containing protein 27 on eGFR
rs7211962
rs186572842
rs3118428
rs163600
rs112924116
rs7221812
rs3176126
rs148652499
rs11637213
rs4775317
rs4937492
rs150838729
rs1807816
rs36178736
rs118130978
rs162064
rs11057604
rs113082677
rs9836730
rs62395254
rs1331816
rs34317897
rs377050837
All
−5e−04
0e+00
5e−04
1e−03
MR effect size for
'Ankyrin repeat domain−containing protein 27' on 'eGFR'

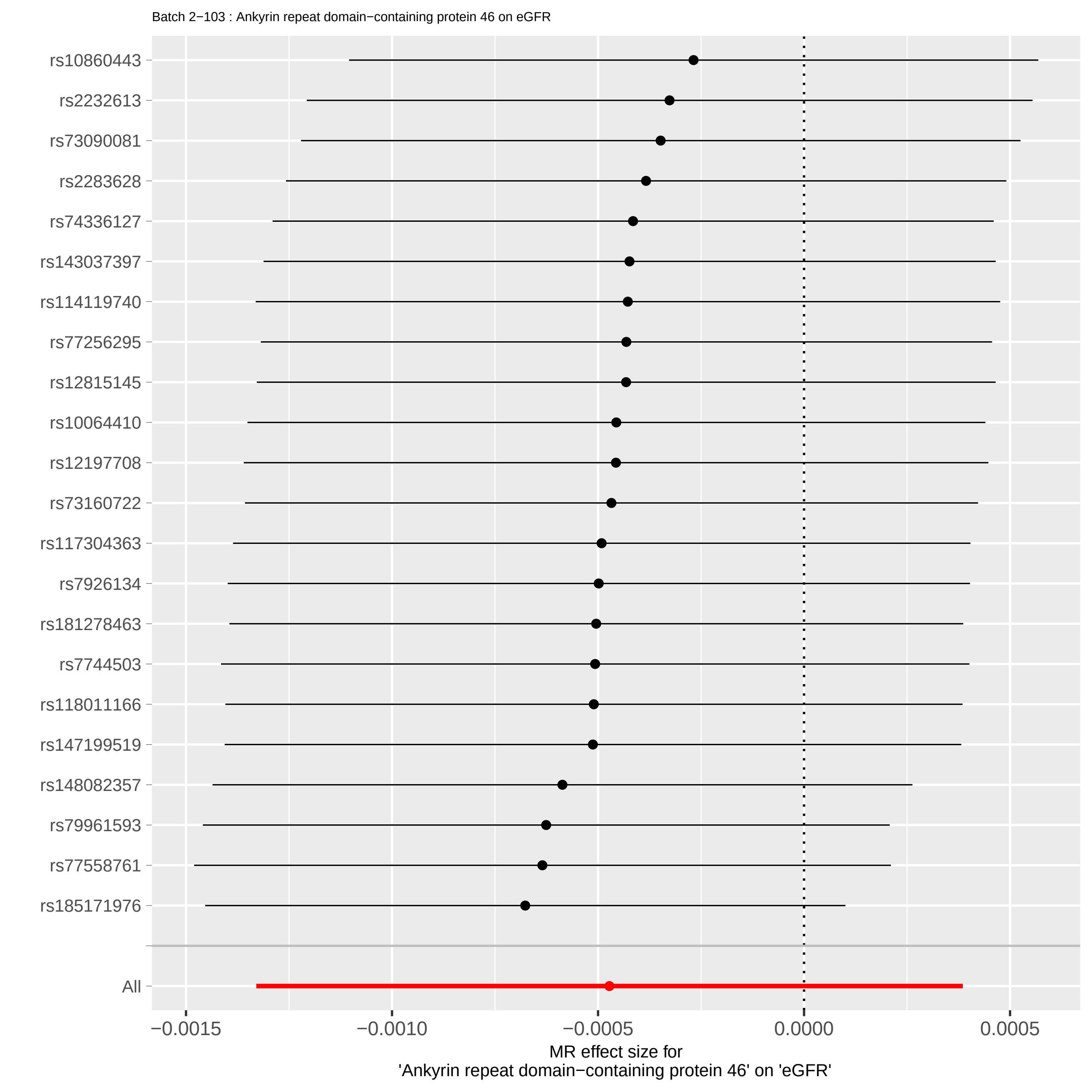

Batch 2−103 : Ankyrin repeat domain−containing protein 46 on eGFR
rs10860443
rs2232613
rs73090081
rs2283628
rs74336127
rs143037397
rs114119740
rs77256295
rs12815145
rs10064410
rs12197708
rs73160722
rs117304363
rs7926134
rs181278463
rs7744503
rs118011166
rs147199519
rs148082357
rs79961593
rs77558761
rs185171976
All
−0.0015
−0.0010
−0.0005
0.0000
0.0005
MR effect size for
'Ankyrin repeat domain−containing protein 46' on 'eGFR'

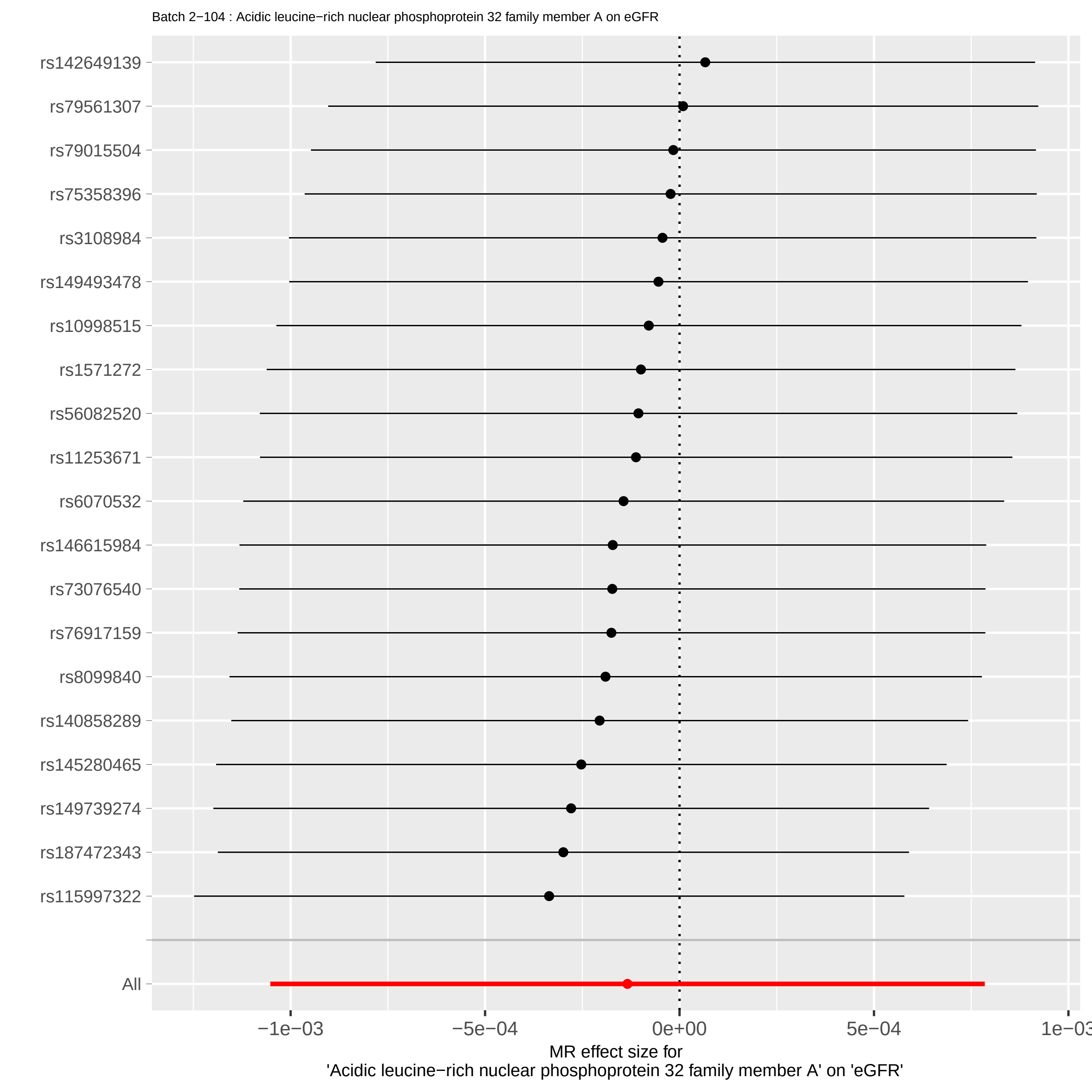

Batch 2−104 : Acidic leucine−rich nuclear phosphoprotein 32 family member A on eGFR
rs142649139
rs79561307
rs79015504
rs75358396
rs3108984
rs149493478
rs10998515
rs1571272
rs56082520
rs11253671
rs6070532
rs146615984
rs73076540
rs76917159
rs8099840
rs140858289
rs145280465
rs149739274
rs187472343
rs115997322
All
−1e−03
−5e−04
0e+00
5e−04
1e−03
MR effect size for
'Acidic leucine−rich nuclear phosphoprotein 32 family member A' on 'eGFR'

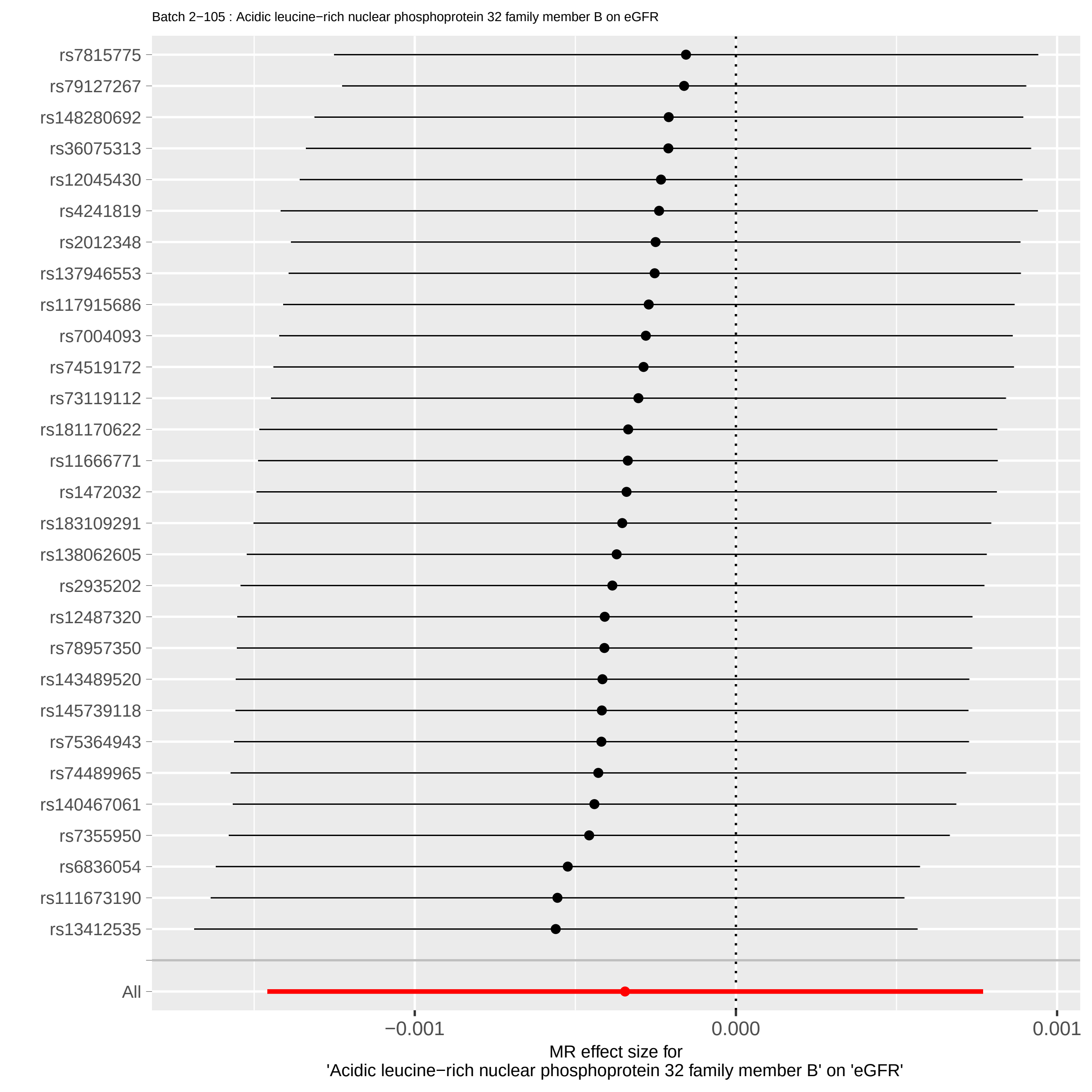
Batch 2−105 : Acidic leucine−rich nuclear phosphoprotein 32 family member B on eGFR
rs7815775
rs79127267
rs148280692
rs36075313
rs12045430
rs4241819
rs2012348
rs137946553
rs117915686
rs7004093
rs74519172
rs73119112
rs181170622
rs11666771
rs1472032
rs183109291
rs138062605
rs2935202
rs12487320
rs78957350
rs143489520
rs145739118
rs75364943
rs74489965
rs140467061
rs7355950
rs6836054
rs111673190
rs13412535
All
−0.001
0.000
0.001
MR effect size for
'Acidic leucine−rich nuclear phosphoprotein 32 family member B' on 'eGFR'

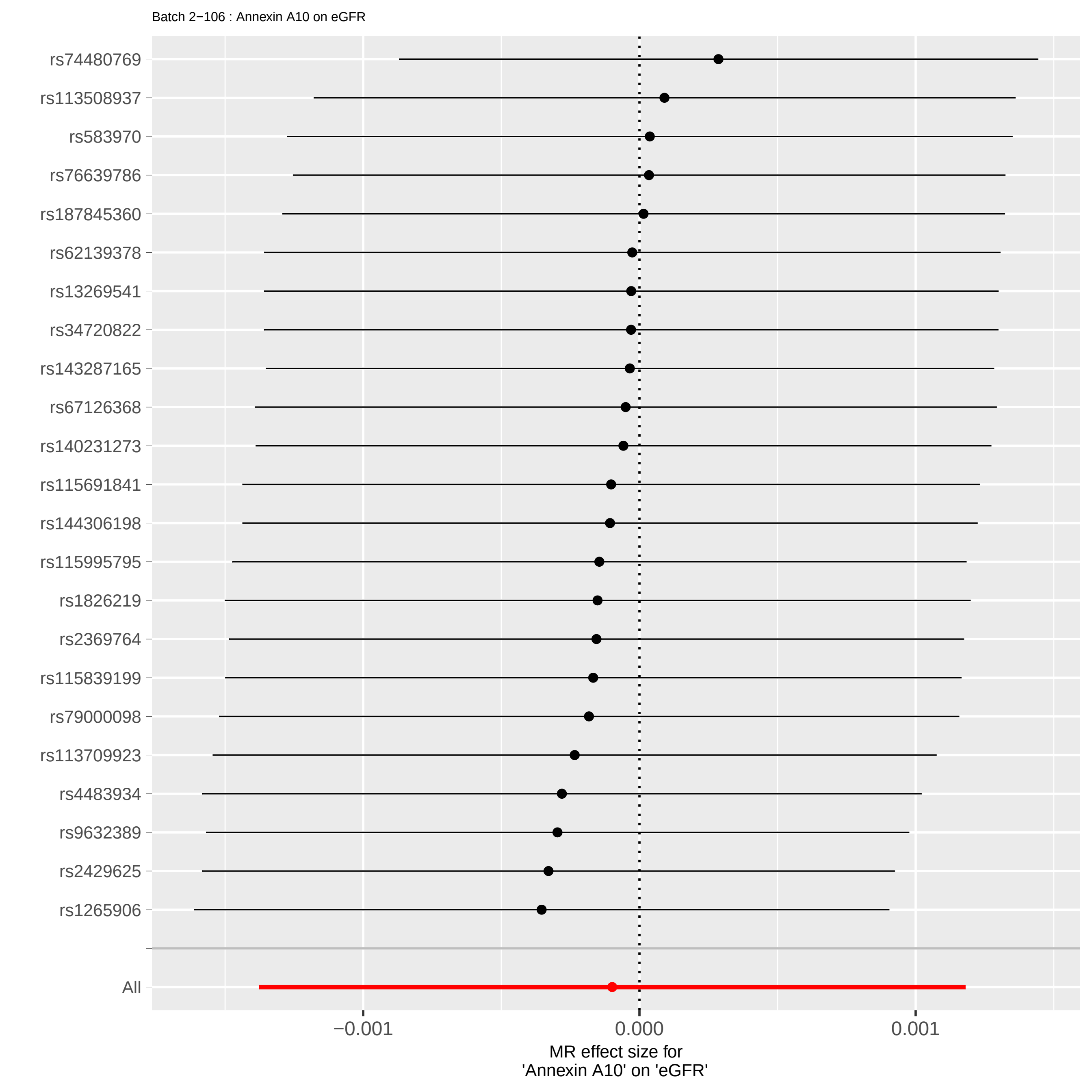

Batch 2−106 : Annexin A10 on eGFR
rs74480769
rs113508937
rs583970
rs76639786
rs187845360
rs62139378
rs13269541
rs34720822
rs143287165
rs67126368
rs140231273
rs115691841
rs144306198
rs115995795
rs1826219
rs2369764
rs115839199
rs79000098
rs113709923
rs4483934
rs9632389
rs2429625
rs1265906
All
−0.001
0.000
0.001
MR effect size for
'Annexin A10' on 'eGFR'

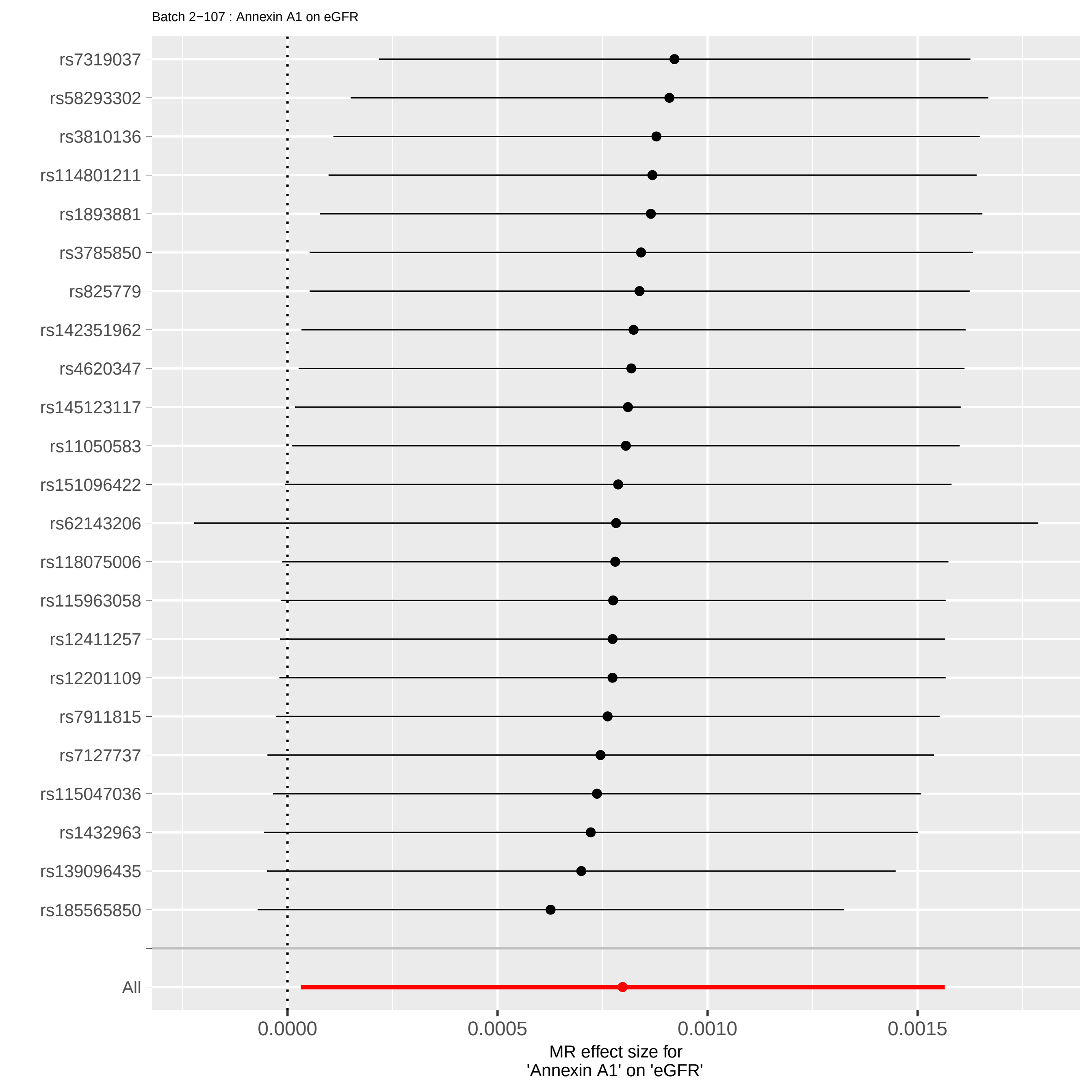

Batch 2−107 : Annexin A1 on eGFR
rs7319037
rs58293302
rs3810136
rs114801211
rs1893881
rs3785850
rs825779
rs142351962
rs4620347
rs145123117
rs11050583
rs151096422
rs62143206
rs118075006
rs115963058
rs12411257
rs12201109
rs7911815
rs7127737
rs115047036
rs1432963
rs139096435
rs185565850
All
0.0000
0.0005
0.0010
0.0015
MR effect size for
'Annexin A1' on 'eGFR'

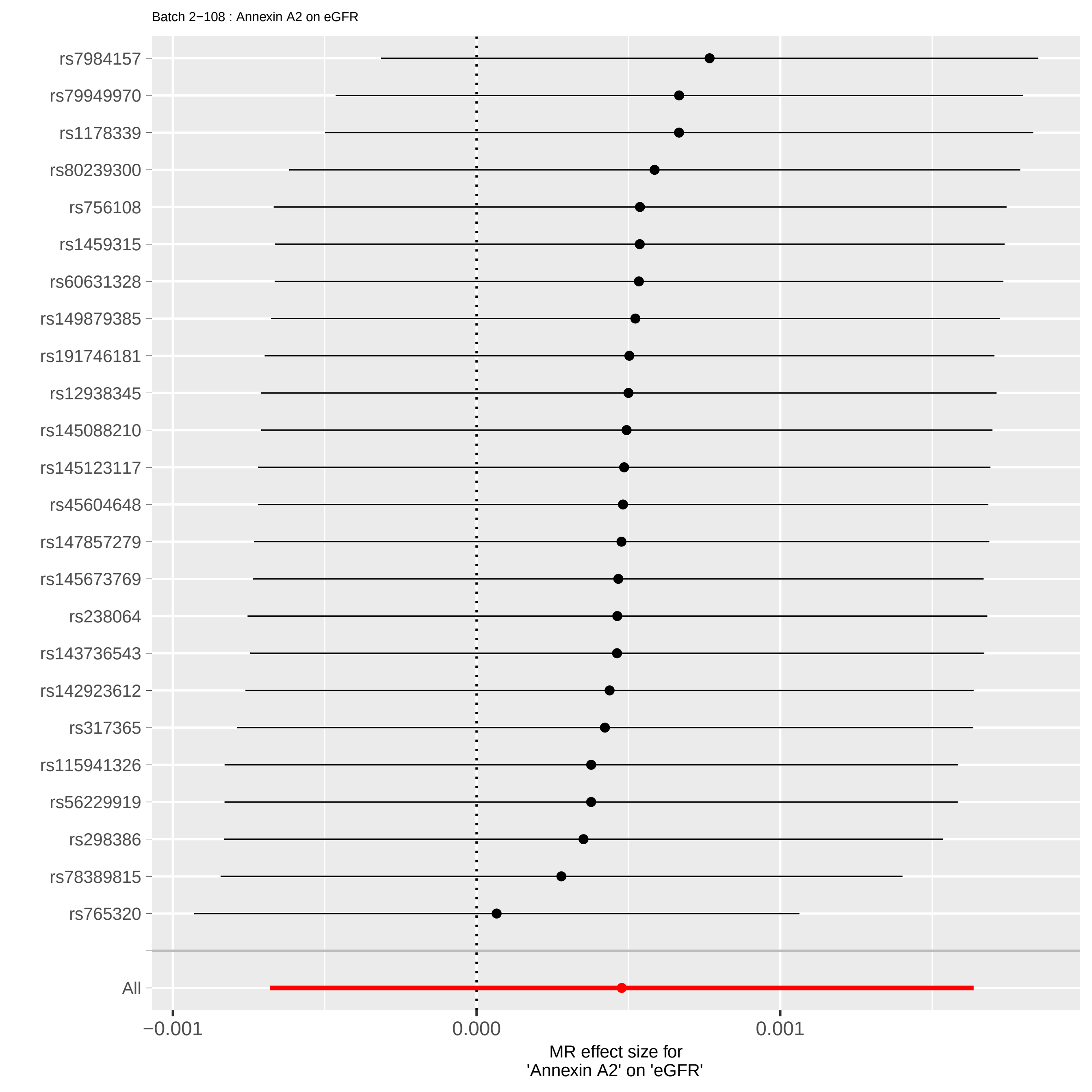

Batch 2−108 : Annexin A2 on eGFR
rs7984157
rs79949970
rs1178339
rs80239300
rs756108
rs1459315
rs60631328
rs149879385
rs191746181
rs12938345
rs145088210
rs145123117
rs45604648
rs147857279
rs145673769
rs238064
rs143736543
rs142923612
rs317365
rs115941326
rs56229919
rs298386
rs78389815
rs765320
All
−0.001
0.000
0.001
MR effect size for
'Annexin A2' on 'eGFR'

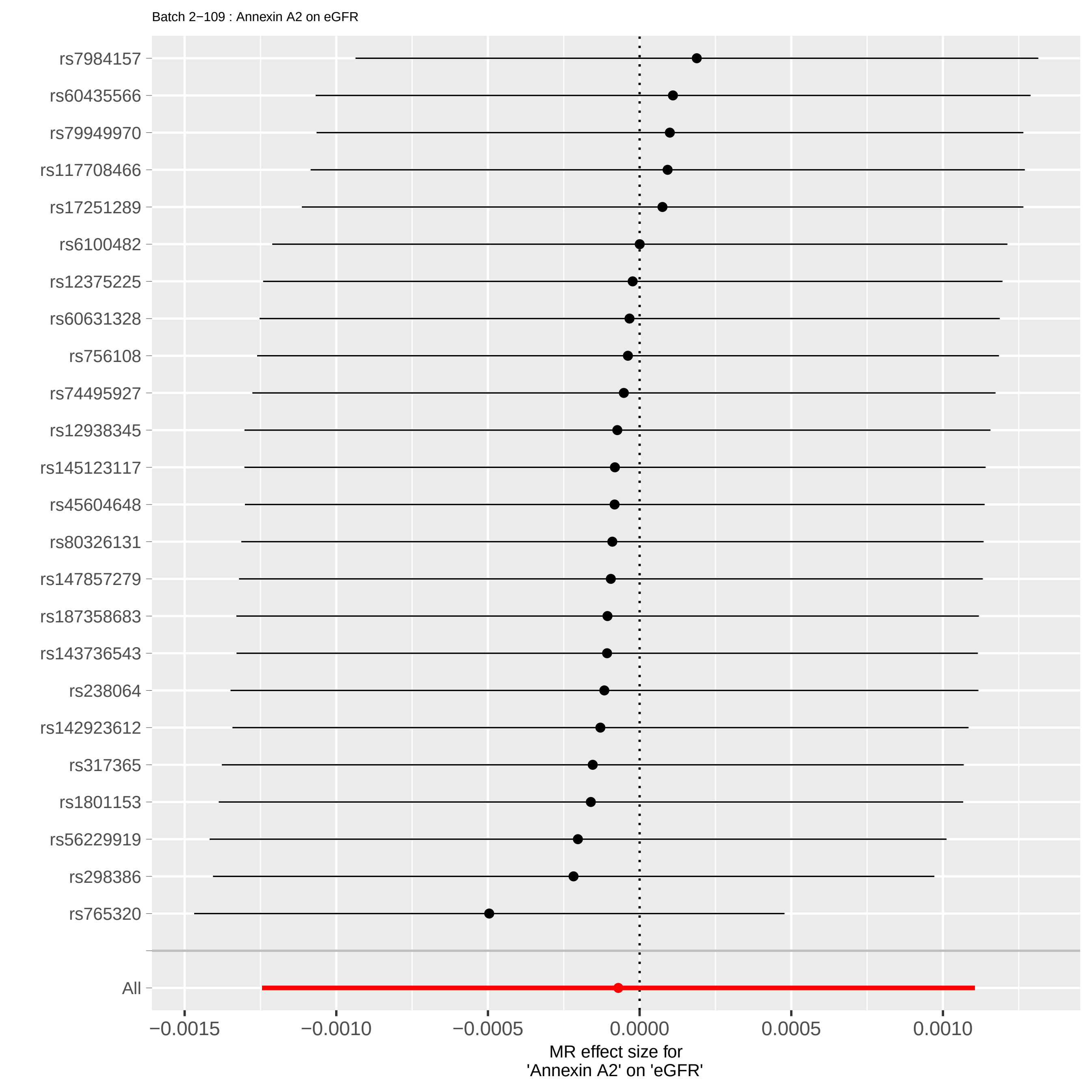

Batch 2−109 : Annexin A2 on eGFR
rs7984157
rs60435566
rs79949970
rs117708466
rs17251289
rs6100482
rs12375225
rs60631328
rs756108
rs74495927
rs12938345
rs145123117
rs45604648
rs80326131
rs147857279
rs187358683
rs143736543
rs238064
rs142923612
rs317365
rs1801153
rs56229919
rs298386
rs765320
All
−0.0015
−0.0010
−0.0005
0.0000
0.0005
0.0010
MR effect size for
'Annexin A2' on 'eGFR'

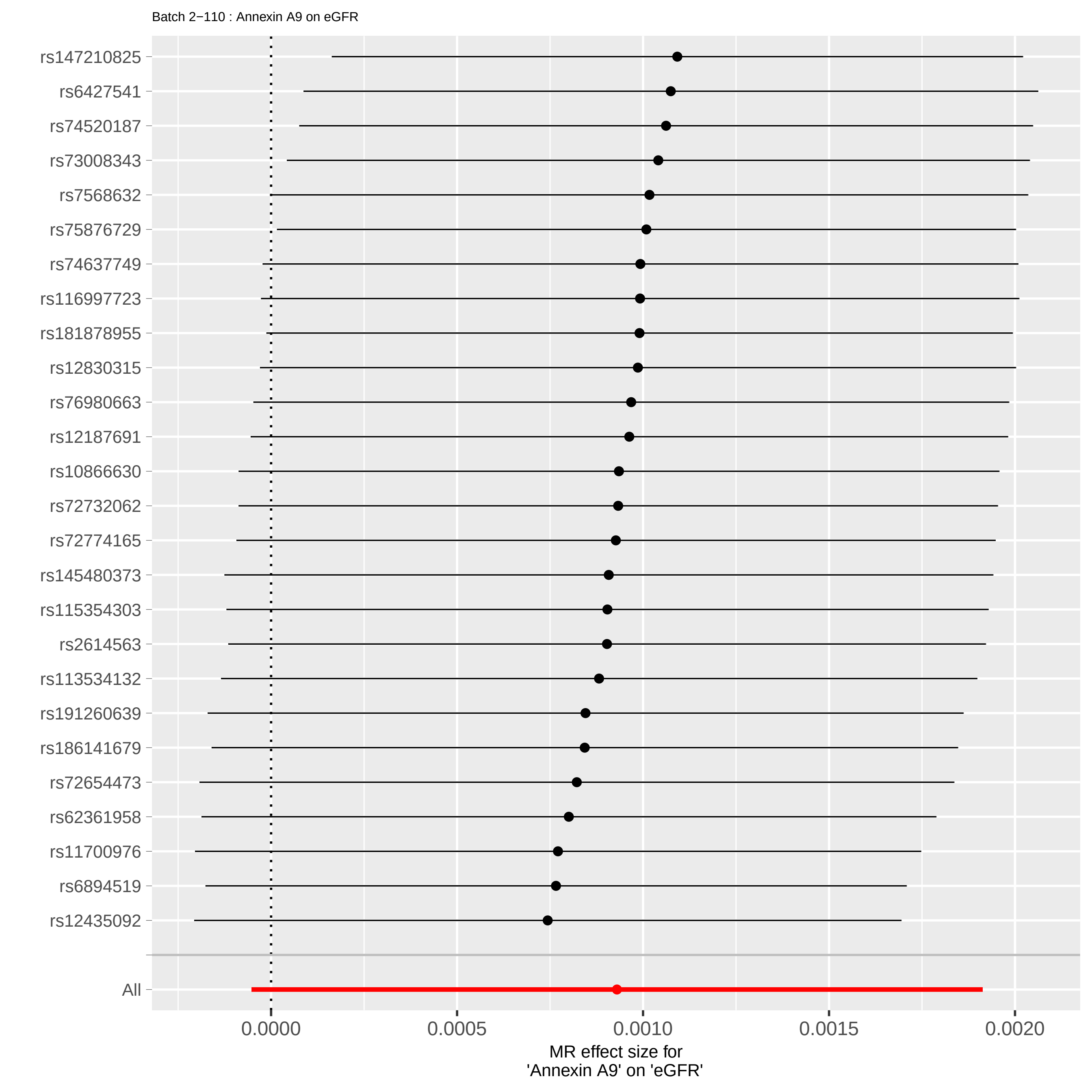

Batch 2−110 : Annexin A9 on eGFR
rs147210825
rs6427541
rs74520187
rs73008343
rs7568632
rs75876729
rs74637749
rs116997723
rs181878955
rs12830315
rs76980663
rs12187691
rs10866630
rs72732062
rs72774165
rs145480373
rs115354303
rs2614563
rs113534132
rs191260639
rs186141679
rs72654473
rs62361958
rs11700976
rs6894519
rs12435092
All
0.0000
0.0005
0.0010
0.0015
0.0020
MR effect size for
'Annexin A9' on 'eGFR'

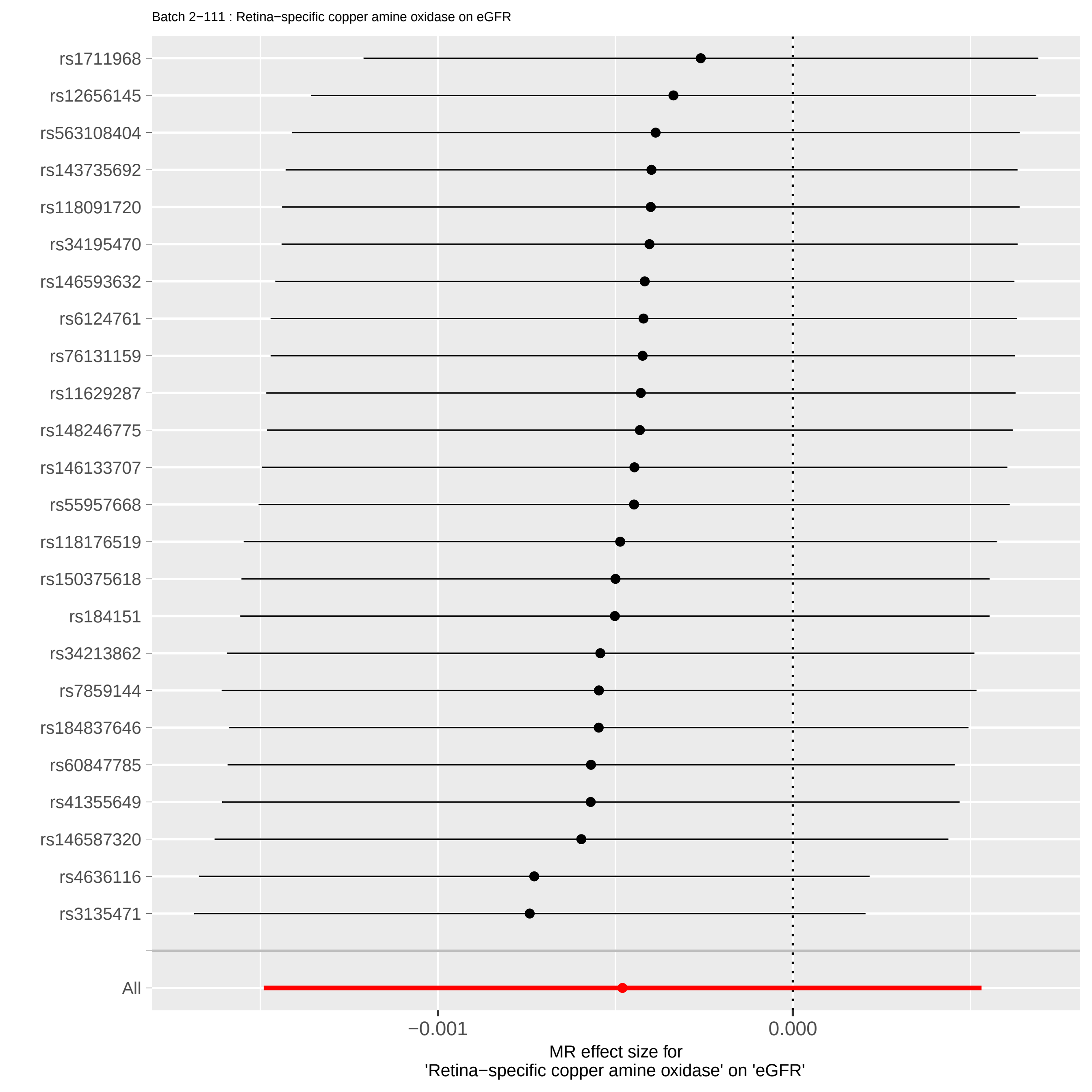

Batch 2−111 : Retina−specific copper amine oxidase on eGFR
rs1711968
rs12656145
rs563108404
rs143735692
rs118091720
rs34195470
rs146593632
rs6124761
rs76131159
rs11629287
rs148246775
rs146133707
rs55957668
rs118176519
rs150375618
rs184151
rs34213862
rs7859144
rs184837646
rs60847785
rs41355649
rs146587320
rs4636116
rs3135471
All
−0.001
0.000
MR effect size for
'Retina−specific copper amine oxidase' on 'eGFR'

Batch 2−112 : AP−4 complex subunit mu−1 on eGFR

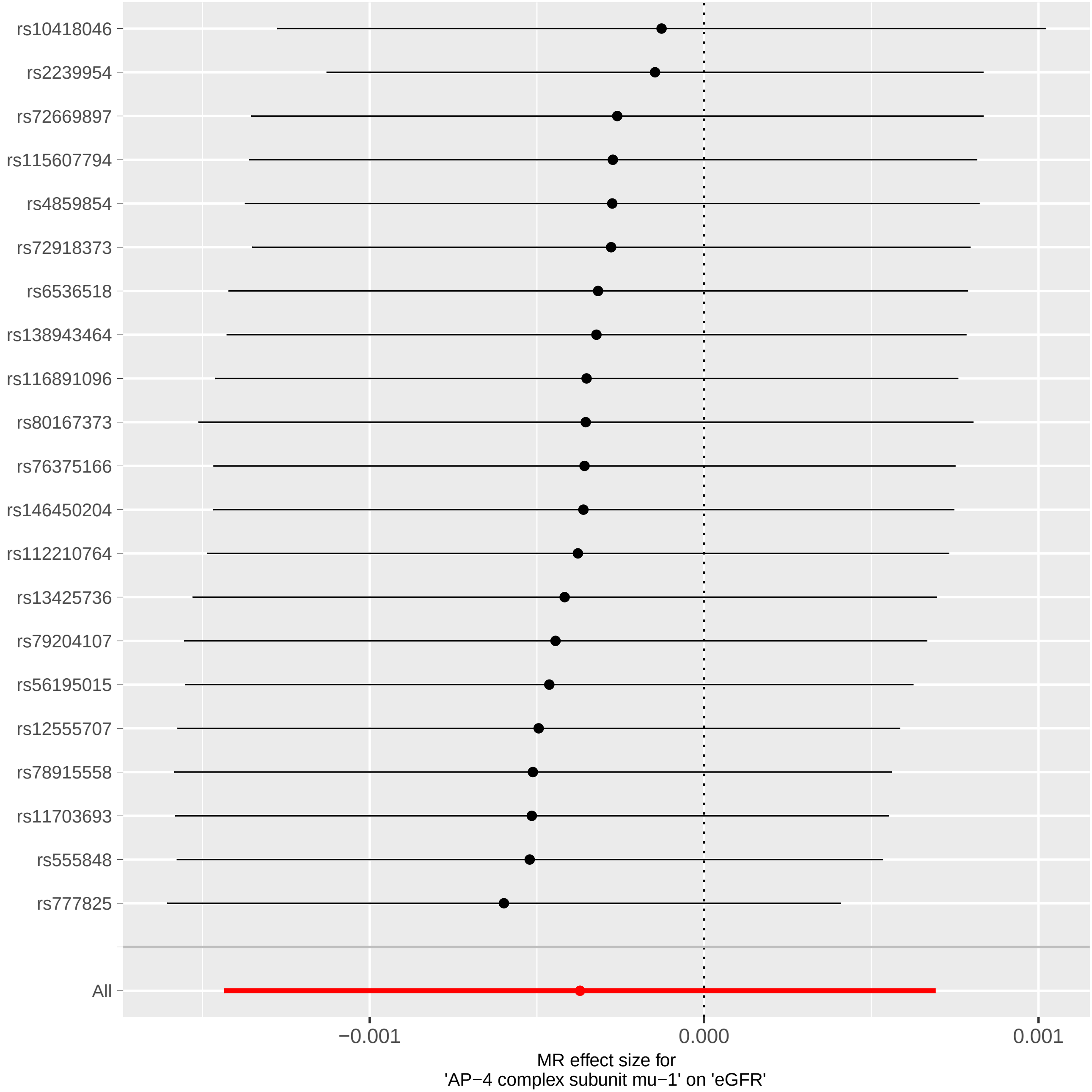

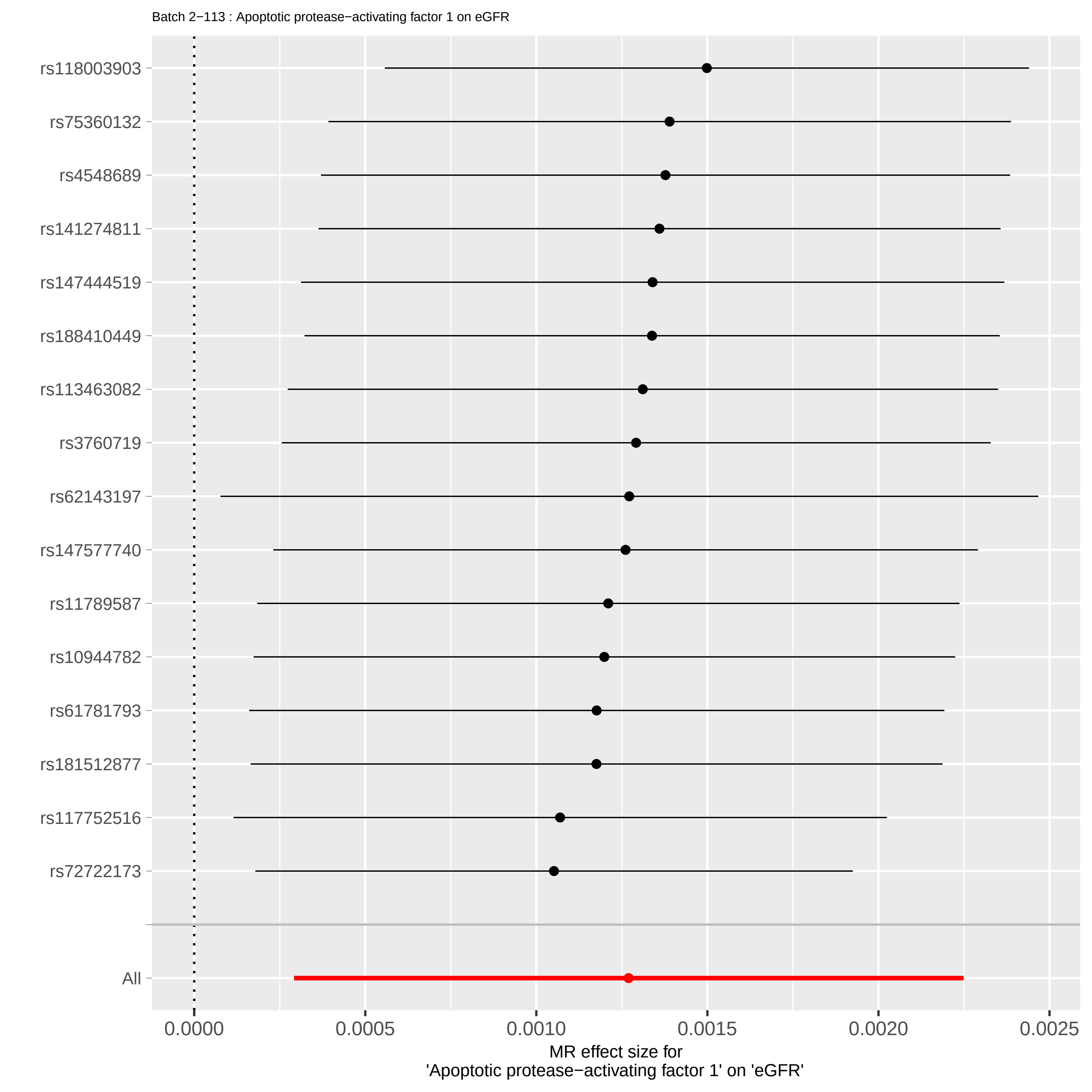
Batch 2−113 : Apoptotic protease−activating factor 1 on eGFR
rs118003903
rs75360132
rs4548689
rs141274811
rs147444519
rs188410449
rs113463082
rs3760719
rs62143197
rs147577740
rs11789587
rs10944782
rs61781793
rs181512877
rs117752516
rs72722173
All
0.0000
0.0005
0.0010
0.0015
0.0020
0.0025
MR effect size for
'Apoptotic protease−activating factor 1' on 'eGFR'

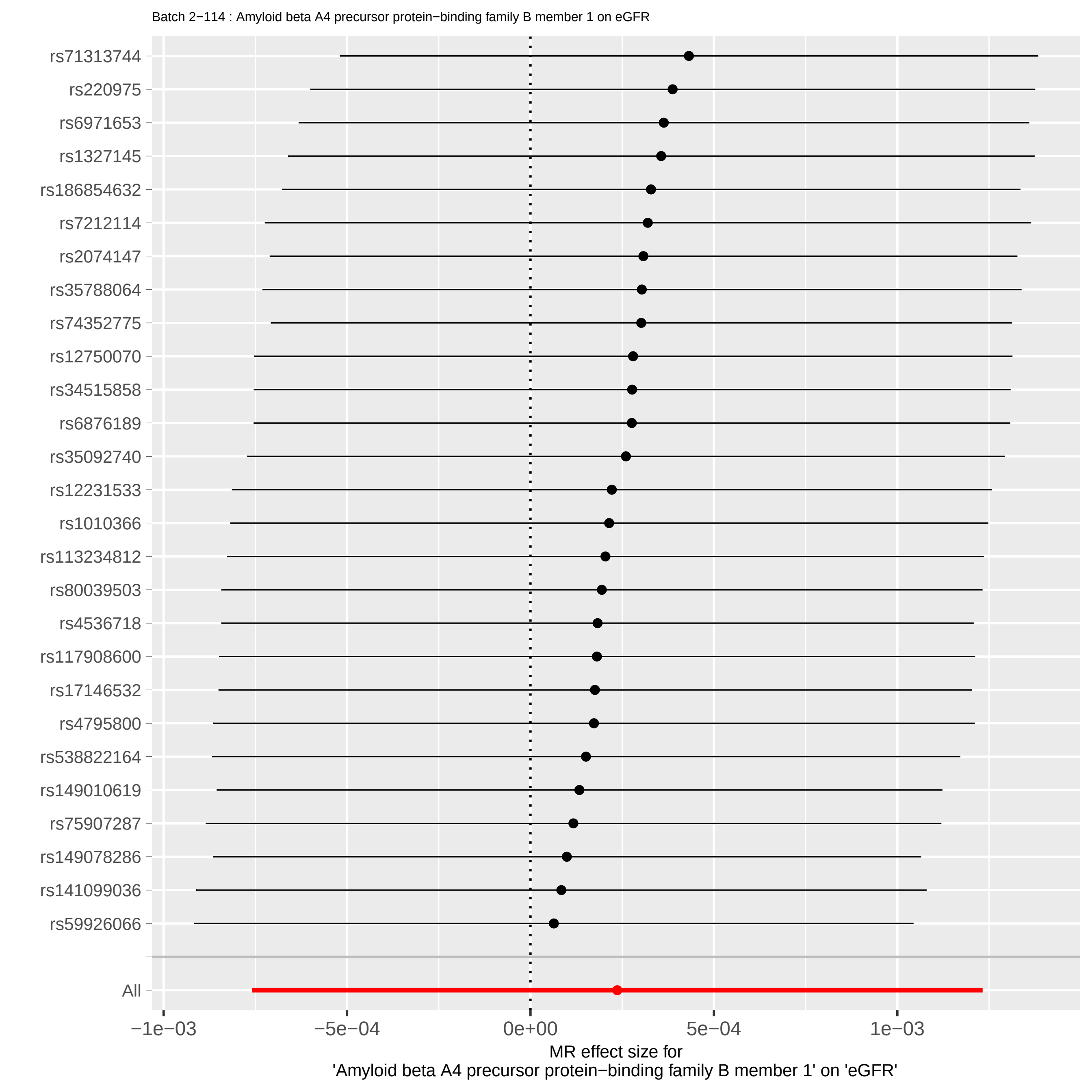

Batch 2−114 : Amyloid beta A4 precursor protein−binding family B member 1 on eGFR
rs71313744
rs220975
rs6971653
rs1327145
rs186854632
rs7212114
rs2074147
rs35788064
rs74352775
rs12750070
rs34515858
rs6876189
rs35092740
rs12231533
rs1010366
rs113234812
rs80039503
rs4536718
rs117908600
rs17146532
rs4795800
rs538822164
rs149010619
rs75907287
rs149078286
rs141099036
rs59926066
All
−1e−03
−5e−04
0e+00
5e−04
1e−03
MR effect size for
'Amyloid beta A4 precursor protein−binding family B member 1' on 'eGFR'

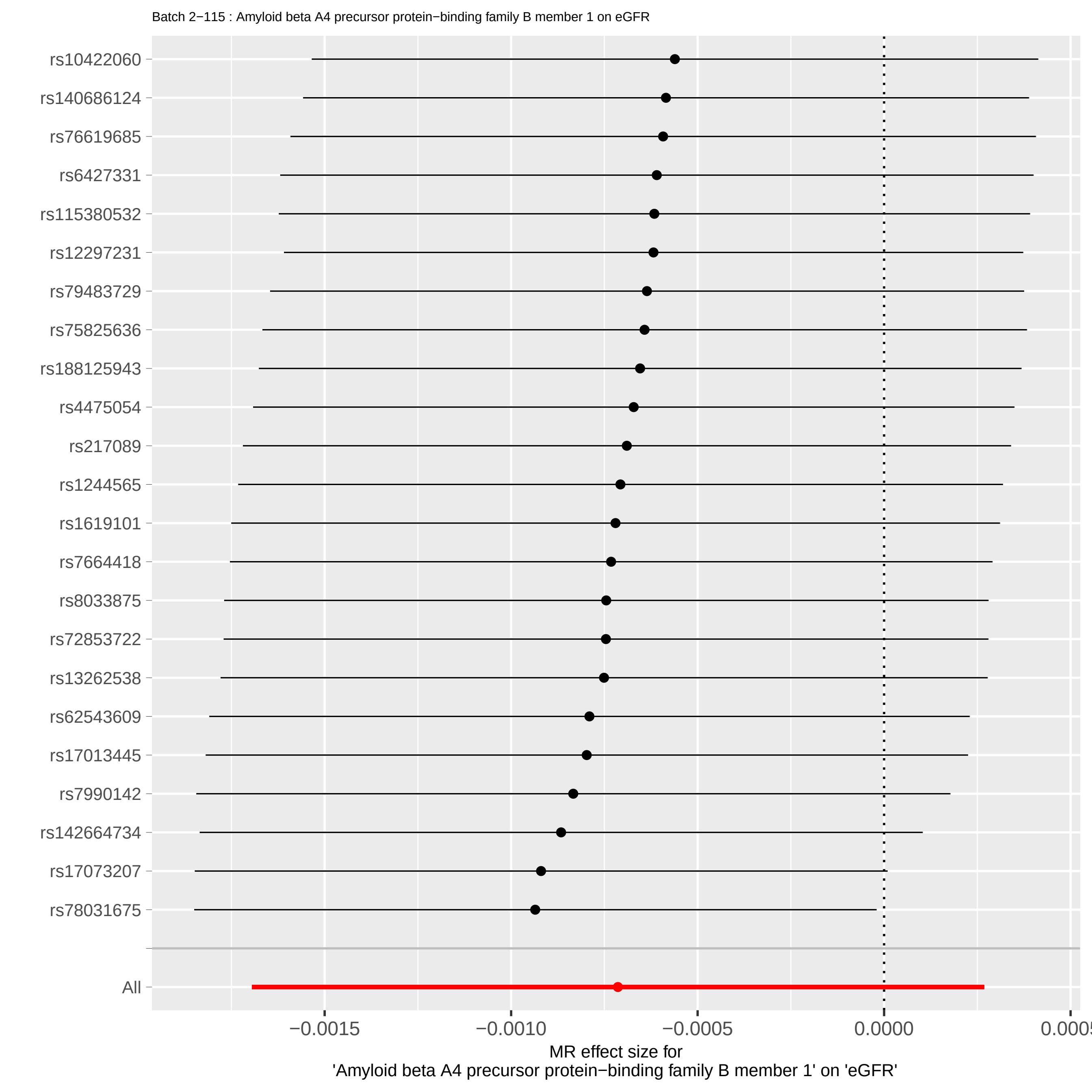

Batch 2−115 : Amyloid beta A4 precursor protein−binding family B member 1 on eGFR
rs10422060
rs140686124
rs76619685
rs6427331
rs115380532
rs12297231
rs79483729
rs75825636
rs188125943
rs4475054
rs217089
rs1244565
rs1619101
rs7664418
rs8033875
rs72853722
rs13262538
rs62543609
rs17013445
rs7990142
rs142664734
rs17073207
rs78031675
All
−0.0015
−0.0010
−0.0005
0.0000
MR effect size for
'Amyloid beta A4 precursor protein−binding family B member 1' on 'eGFR'

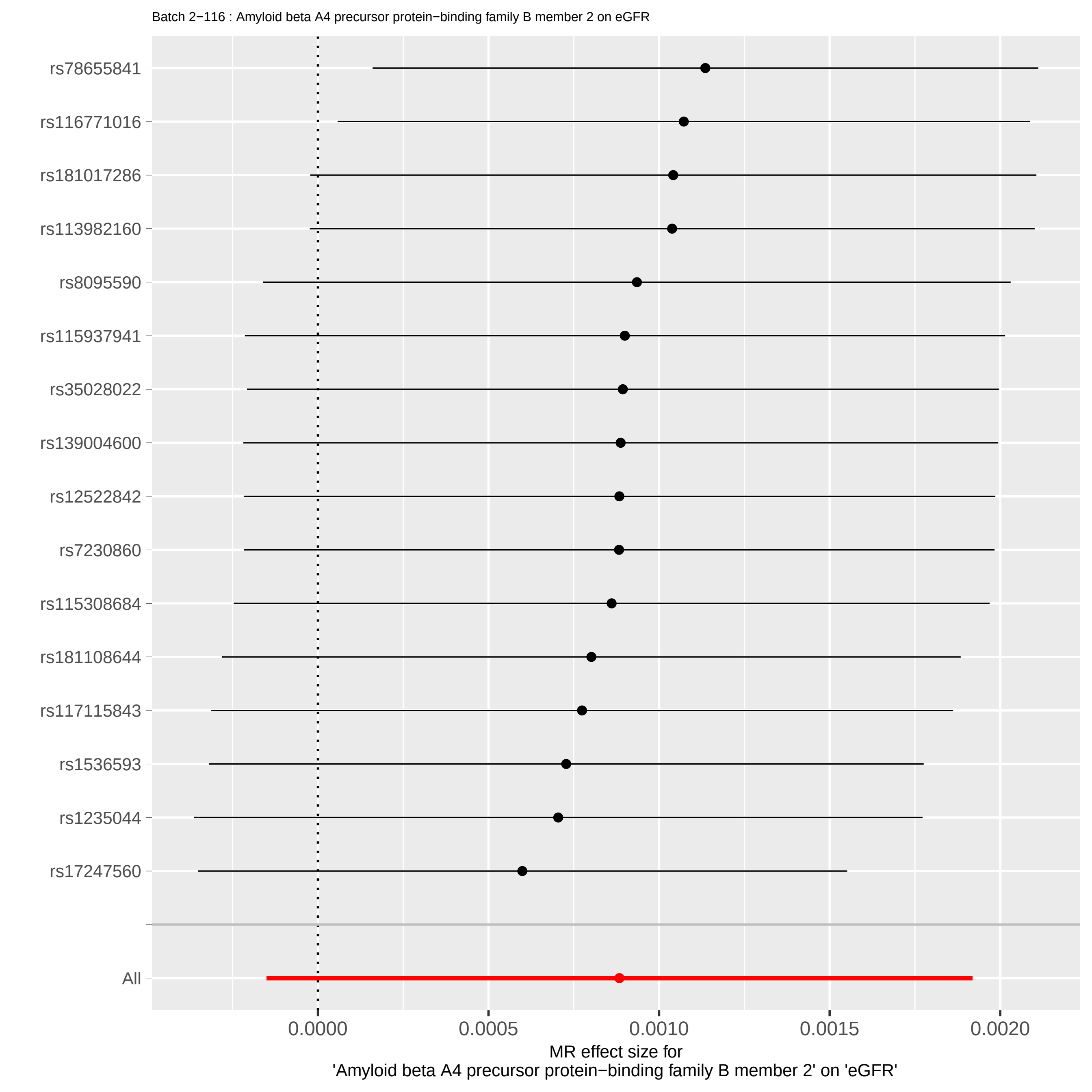

Batch 2−116 : Amyloid beta A4 precursor protein−binding family B member 2 on eGFR
rs78655841
rs116771016
rs181017286
rs113982160
rs8095590
rs115937941
rs35028022
rs139004600
rs12522842
rs7230860
rs115308684
rs181108644
rs117115843
rs1536593
rs1235044
rs17247560
All
0.0000
0.0005
0.0010
0.0015
0.0020
MR effect size for
'Amyloid beta A4 precursor protein−binding family B member 2' on 'eGFR'

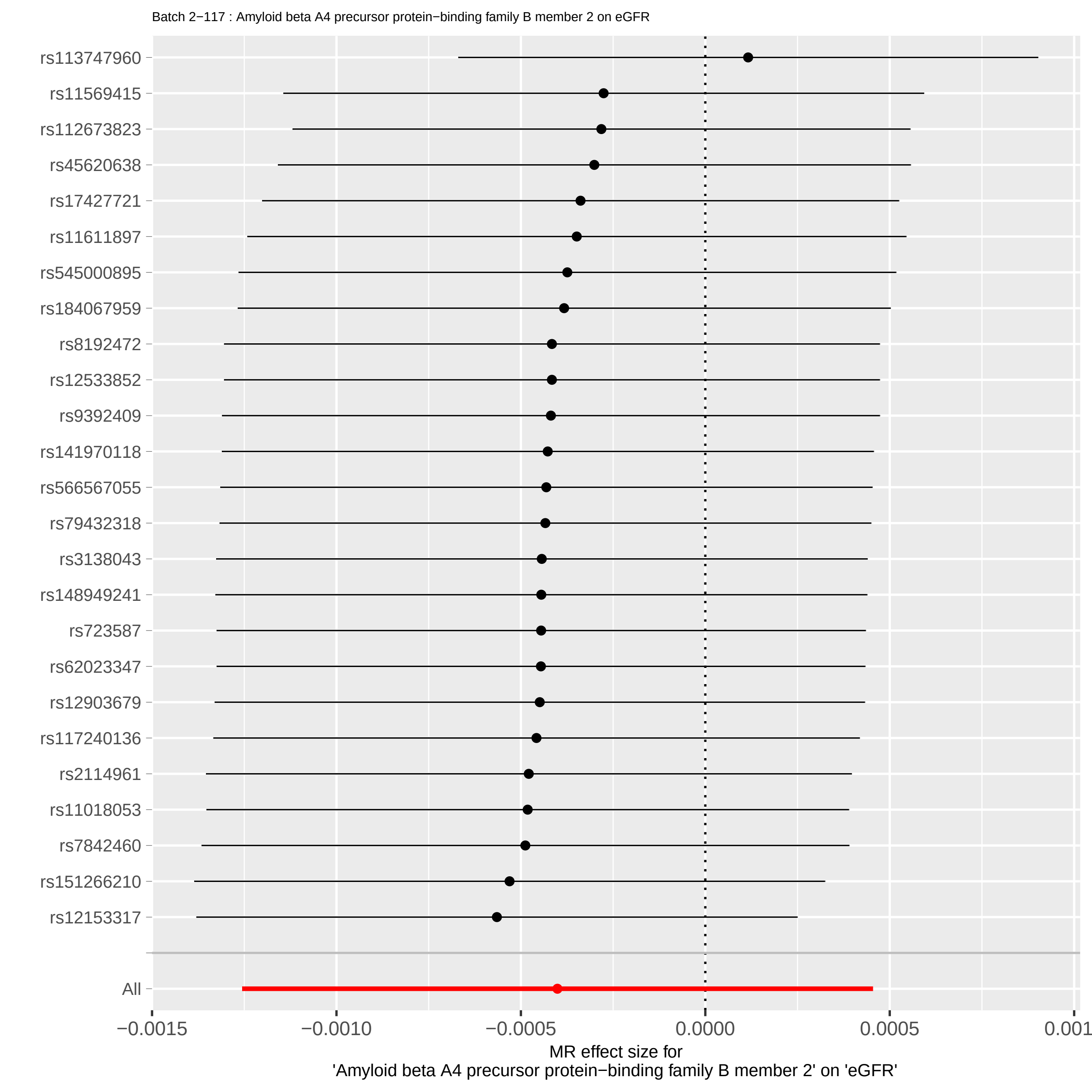
Batch 2−117 : Amyloid beta A4 precursor protein−binding family B member 2 on eGFR
rs113747960
rs11569415
rs112673823
rs45620638
rs17427721
rs11611897
rs545000895
rs184067959
rs8192472
rs12533852
rs9392409
rs141970118
rs566567055
rs79432318
rs3138043
rs148949241
rs723587
rs62023347
rs12903679
rs117240136
rs2114961
rs11018053
rs7842460
rs151266210
rs12153317
All
−0.0015
−0.0010
−0.0005
0.0000
0.0005
0.001
MR effect size for
'Amyloid beta A4 precursor protein−binding family B member 2' on 'eGFR'

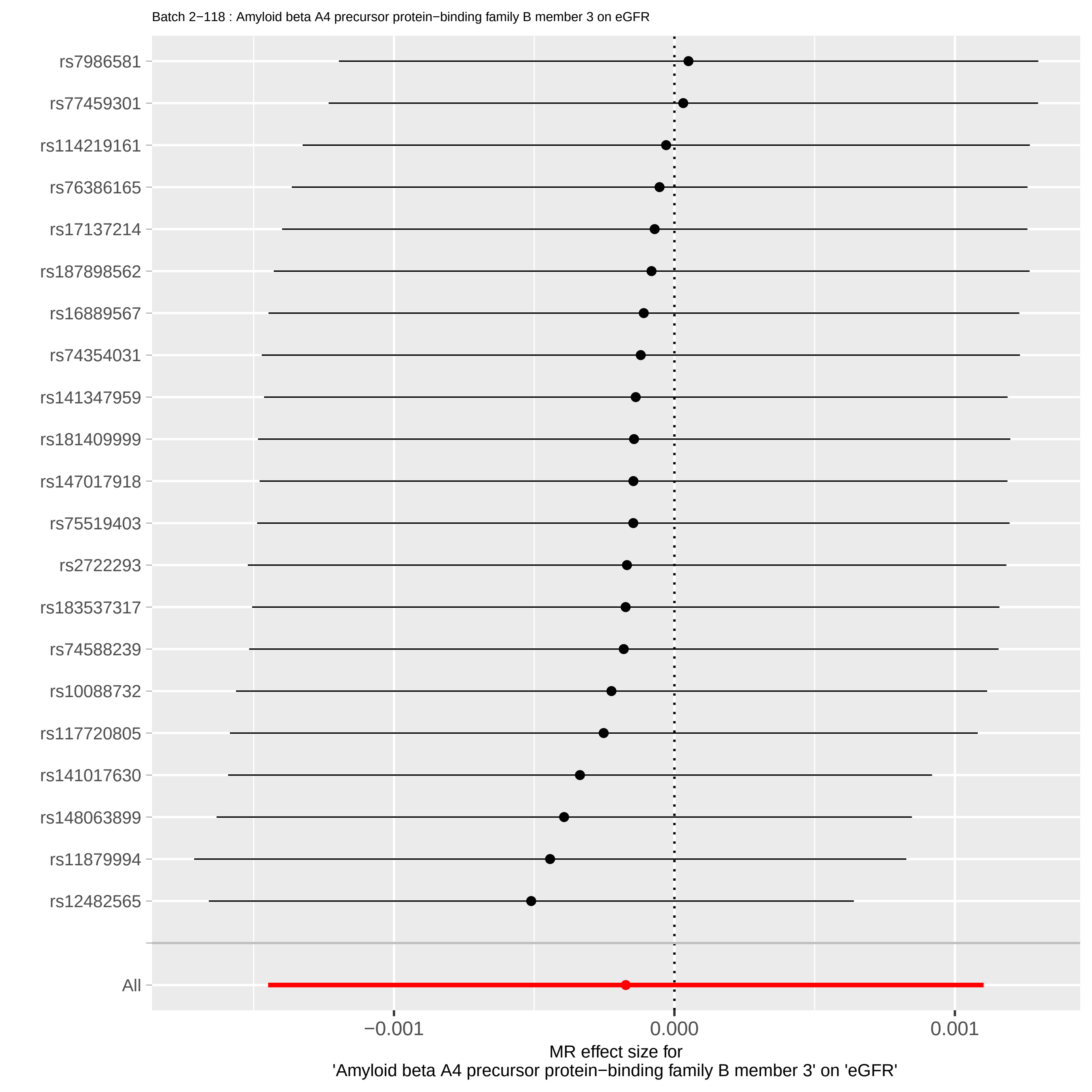

Batch 2−118 : Amyloid beta A4 precursor protein−binding family B member 3 on eGFR
rs7986581
rs77459301
rs114219161
rs76386165
rs17137214
rs187898562
rs16889567
rs74354031
rs141347959
rs181409999
rs147017918
rs75519403
rs2722293
rs183537317
rs74588239
rs10088732
rs117720805
rs141017630
rs148063899
rs11879994
rs12482565
All
−0.001
0.000
0.001
MR effect size for
'Amyloid beta A4 precursor protein−binding family B member 3' on 'eGFR'

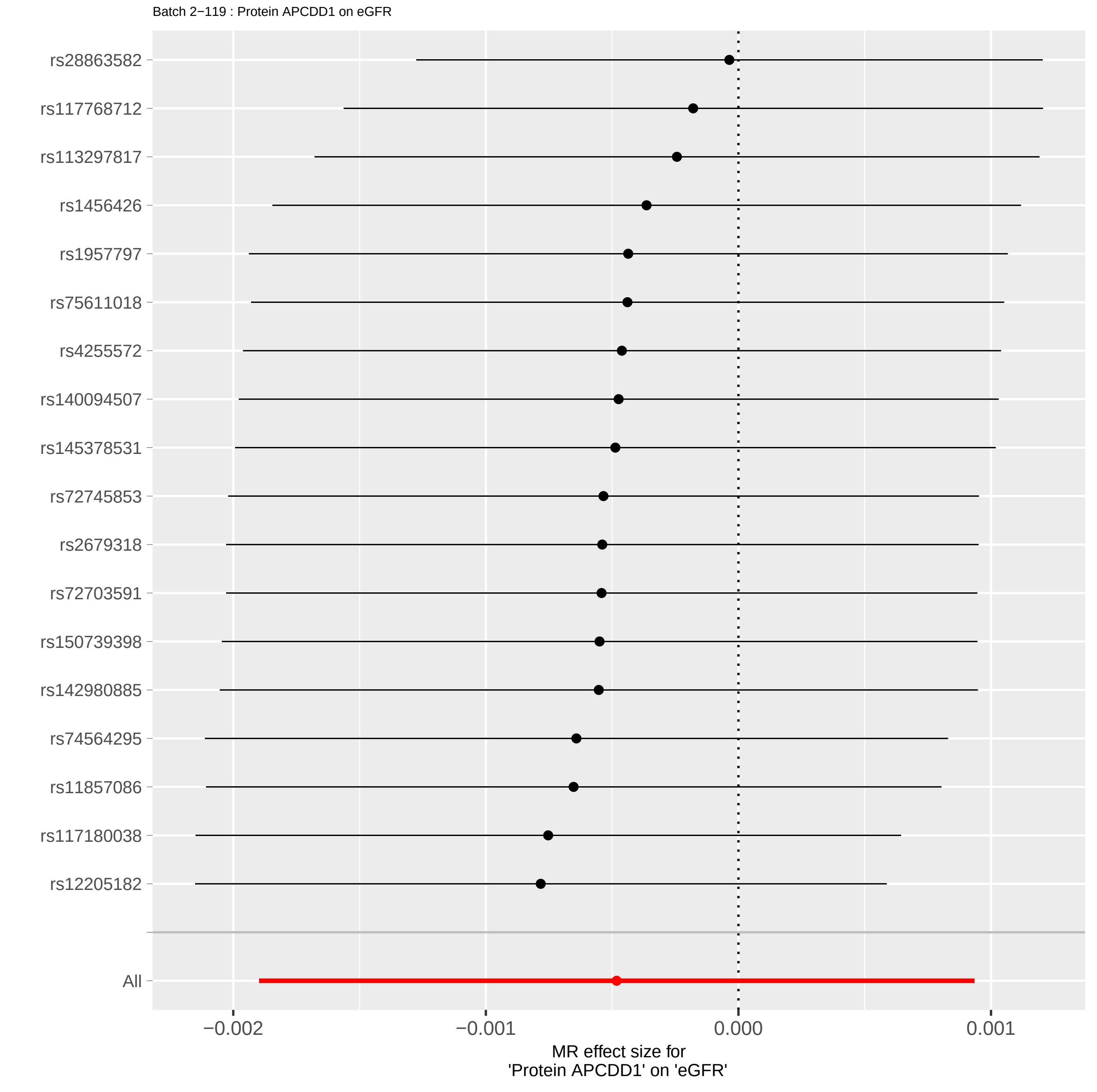

Batch 2−119 : Protein APCDD1 on eGFR
rs28863582
rs117768712
rs113297817
rs1456426
rs1957797
rs75611018
rs4255572
rs140094507
rs145378531
rs72745853
rs2679318
rs72703591
rs150739398
rs142980885
rs74564295
rs11857086
rs117180038
rs12205182
All
−0.002
−0.001
0.000
0.001
MR effect size for
'Protein APCDD1' on 'eGFR'

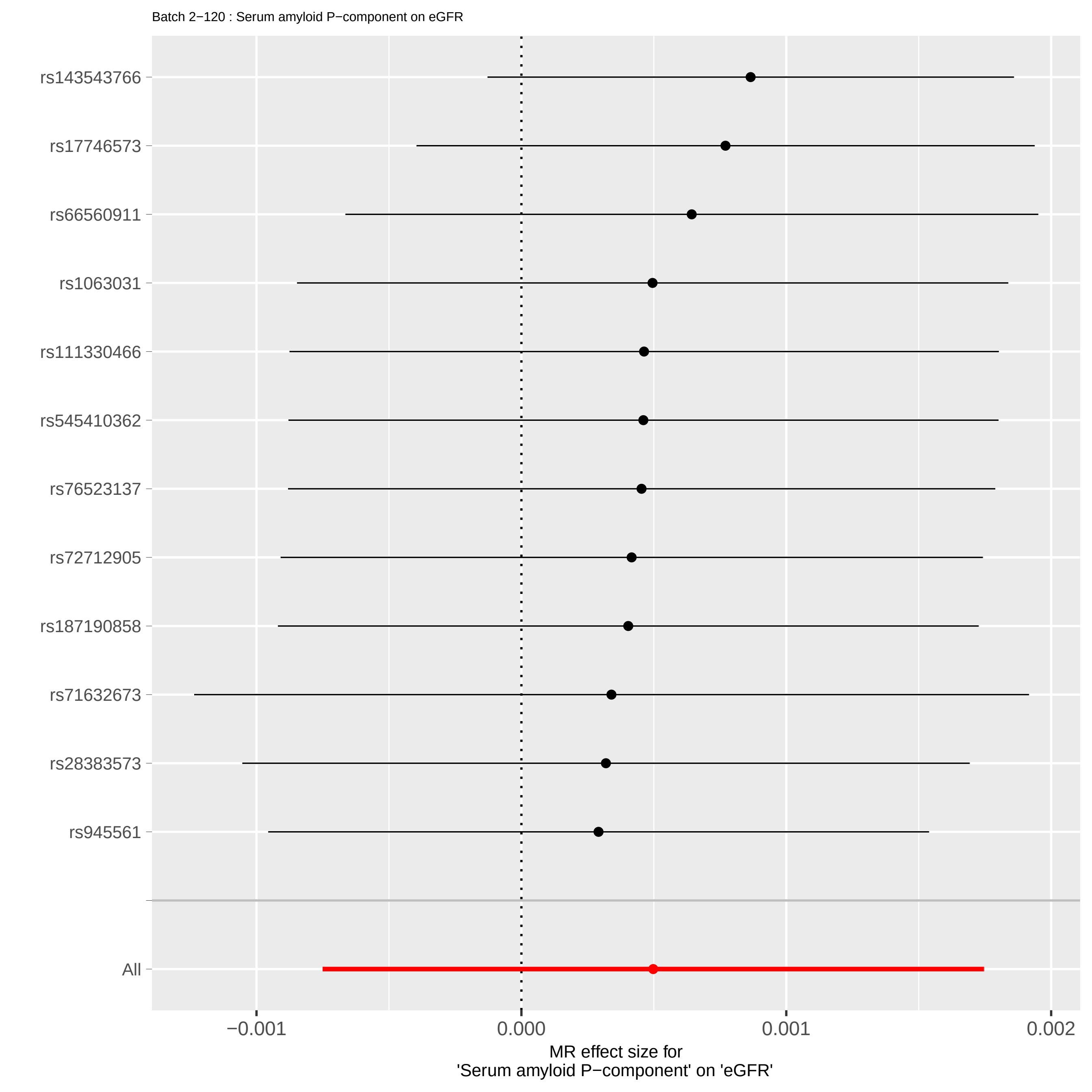
Batch 2−120 : Serum amyloid P−component on eGFR
rs143543766
rs17746573
rs66560911
rs1063031
rs111330466
rs545410362
rs76523137
rs72712905
rs187190858
rs71632673
rs28383573
rs945561
All
−0.001
0.000
0.001
0.002
MR effect size for
'Serum amyloid P−component' on 'eGFR'

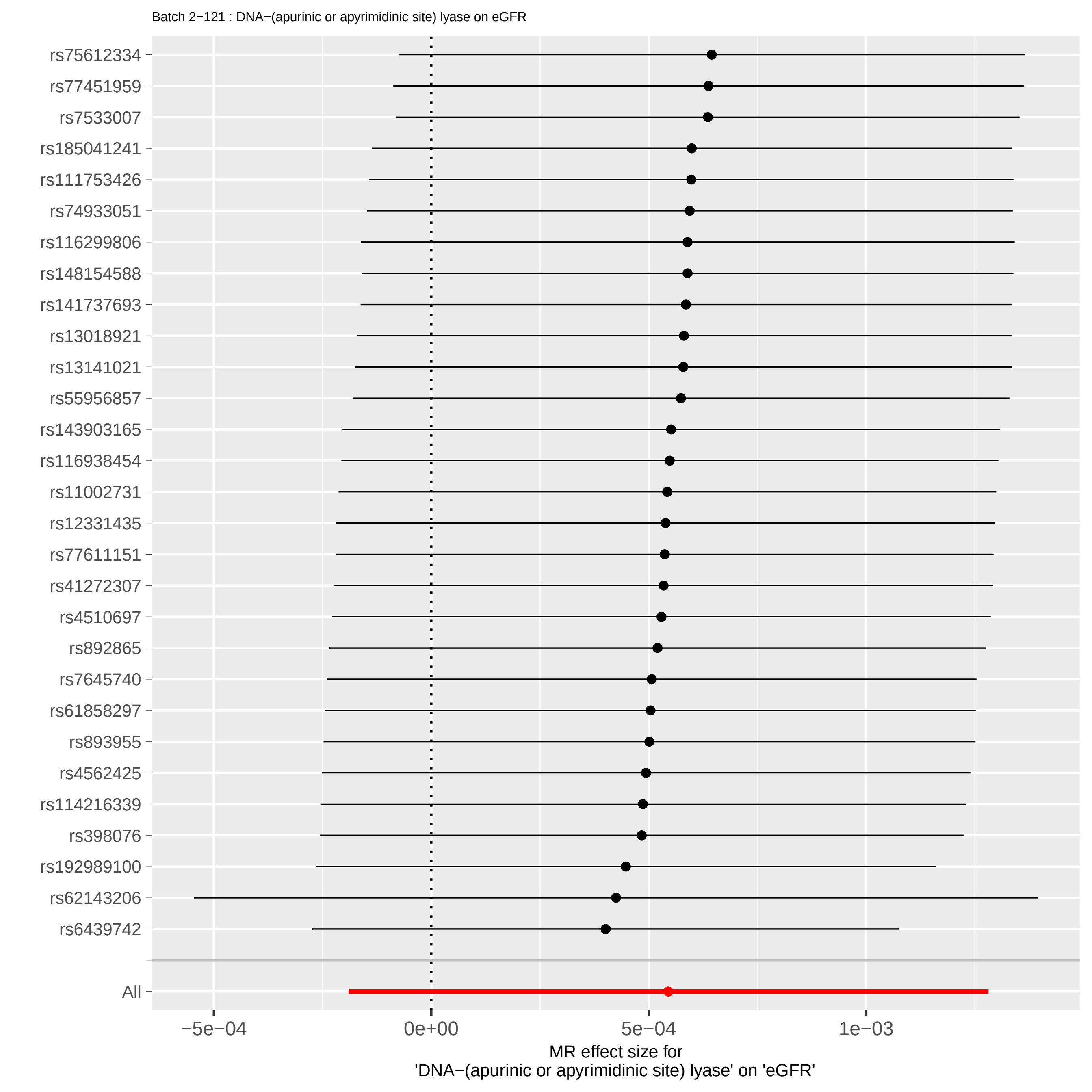
Batch 2−121 : DNA−(apurinic or apyrimidinic site) lyase on eGFR
rs75612334
rs77451959
rs7533007
rs185041241
rs111753426
rs74933051
rs116299806
rs148154588
rs141737693
rs13018921
rs13141021
rs55956857
rs143903165
rs116938454
rs11002731
rs12331435
rs77611151
rs41272307
rs4510697
rs892865
rs7645740
rs61858297
rs893955
rs4562425
rs114216339
rs398076
rs192989100
rs62143206
rs6439742
All
−5e−04
0e+00
5e−04
1e−03
MR effect size for
'DNA−(apurinic or apyrimidinic site) lyase' on 'eGFR'

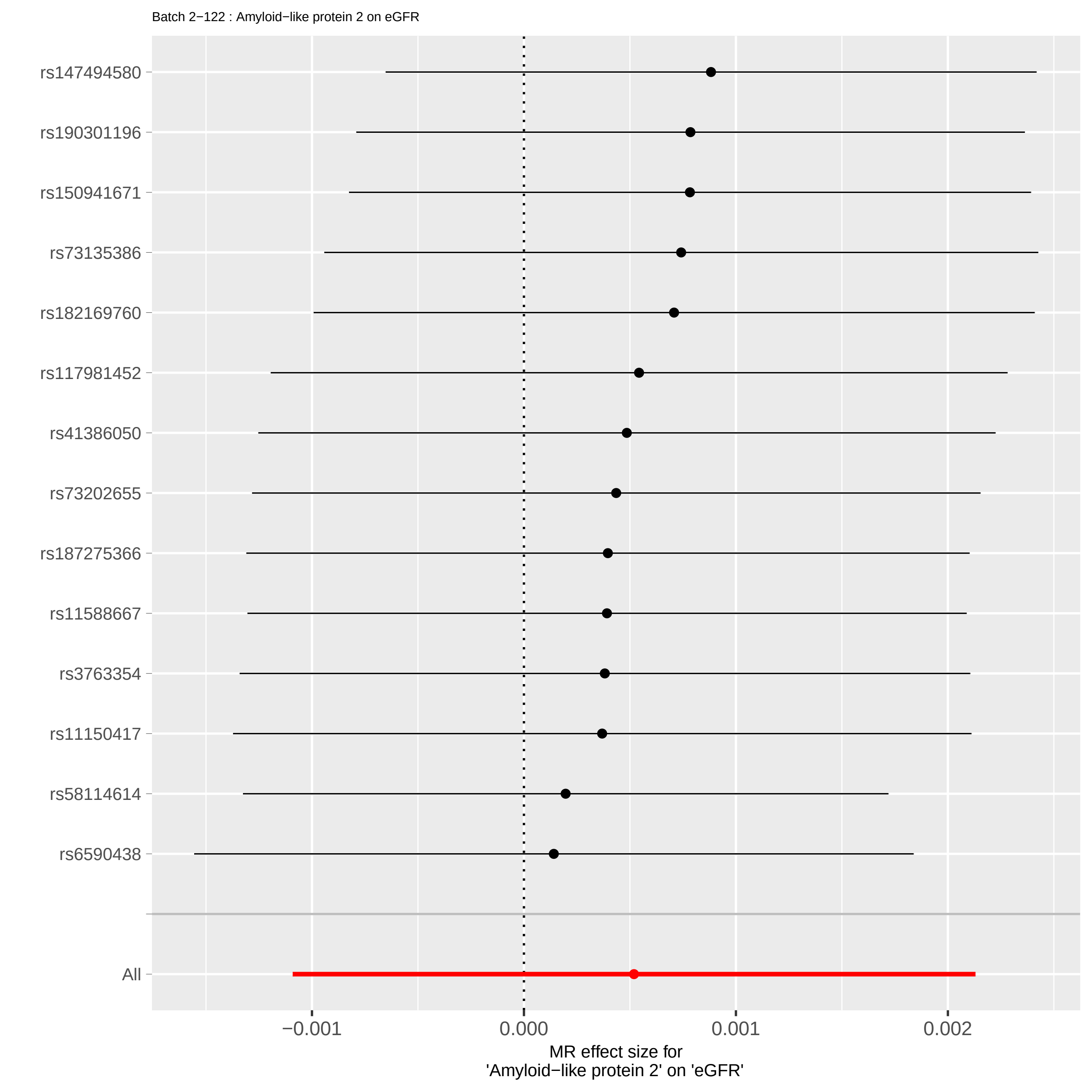
Batch 2−122 : Amyloid−like protein 2 on eGFR
rs147494580
rs190301196
rs150941671
rs73135386
rs182169760
rs117981452
rs41386050
rs73202655
rs187275366
rs11588667
rs3763354
rs11150417
rs58114614
rs6590438
All
−0.001
0.000
0.001
0.002
MR effect size for
'Amyloid−like protein 2' on 'eGFR'

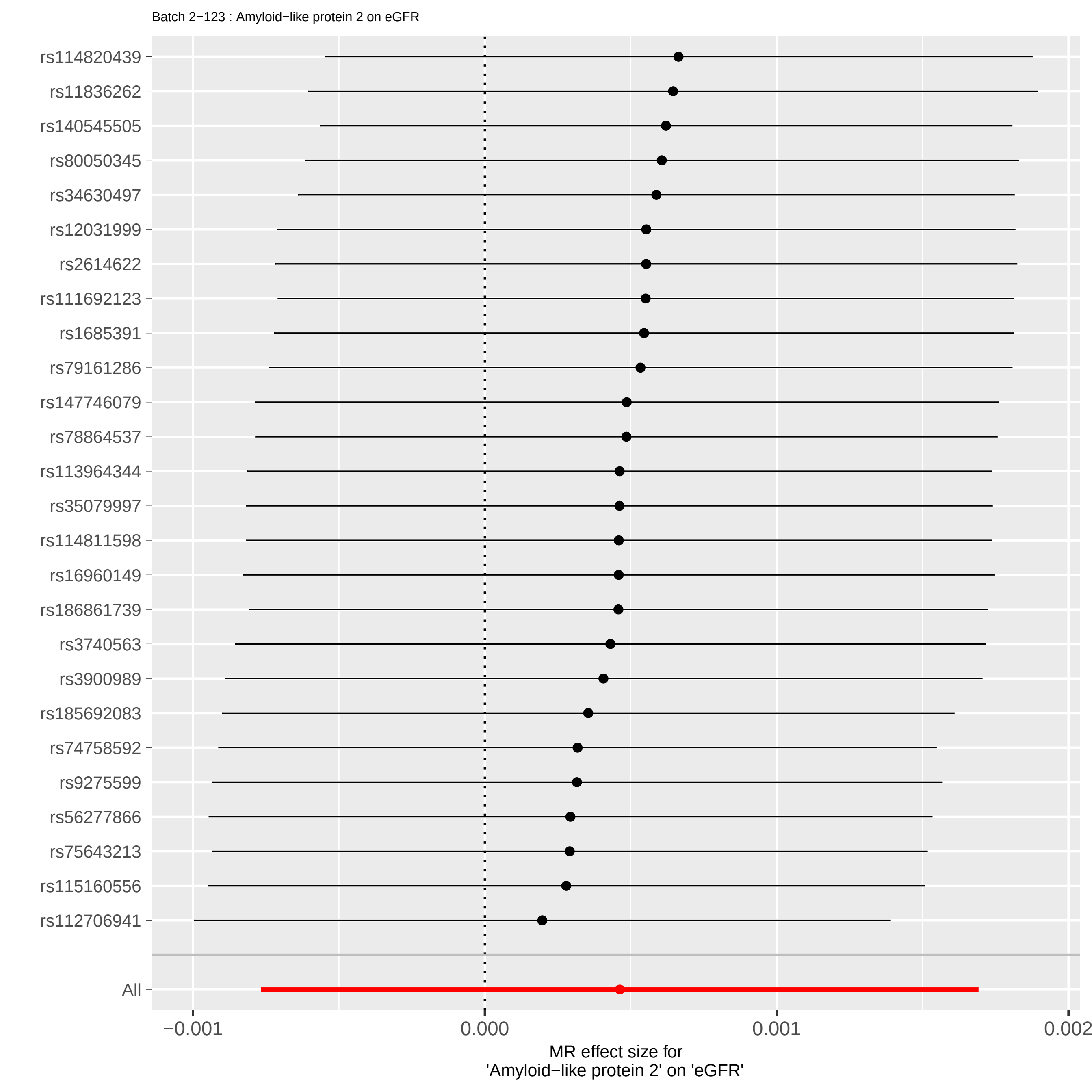

Batch 2−123 : Amyloid−like protein 2 on eGFR
rs114820439
rs11836262
rs140545505
rs80050345
rs34630497
rs12031999
rs2614622
rs111692123
rs1685391
rs79161286
rs147746079
rs78864537
rs113964344
rs35079997
rs114811598
rs16960149
rs186861739
rs3740563
rs3900989
rs185692083
rs74758592
rs9275599
rs56277866
rs75643213
rs115160556
rs112706941
All
−0.001
0.000
0.001
0.002
MR effect size for
'Amyloid−like protein 2' on 'eGFR'

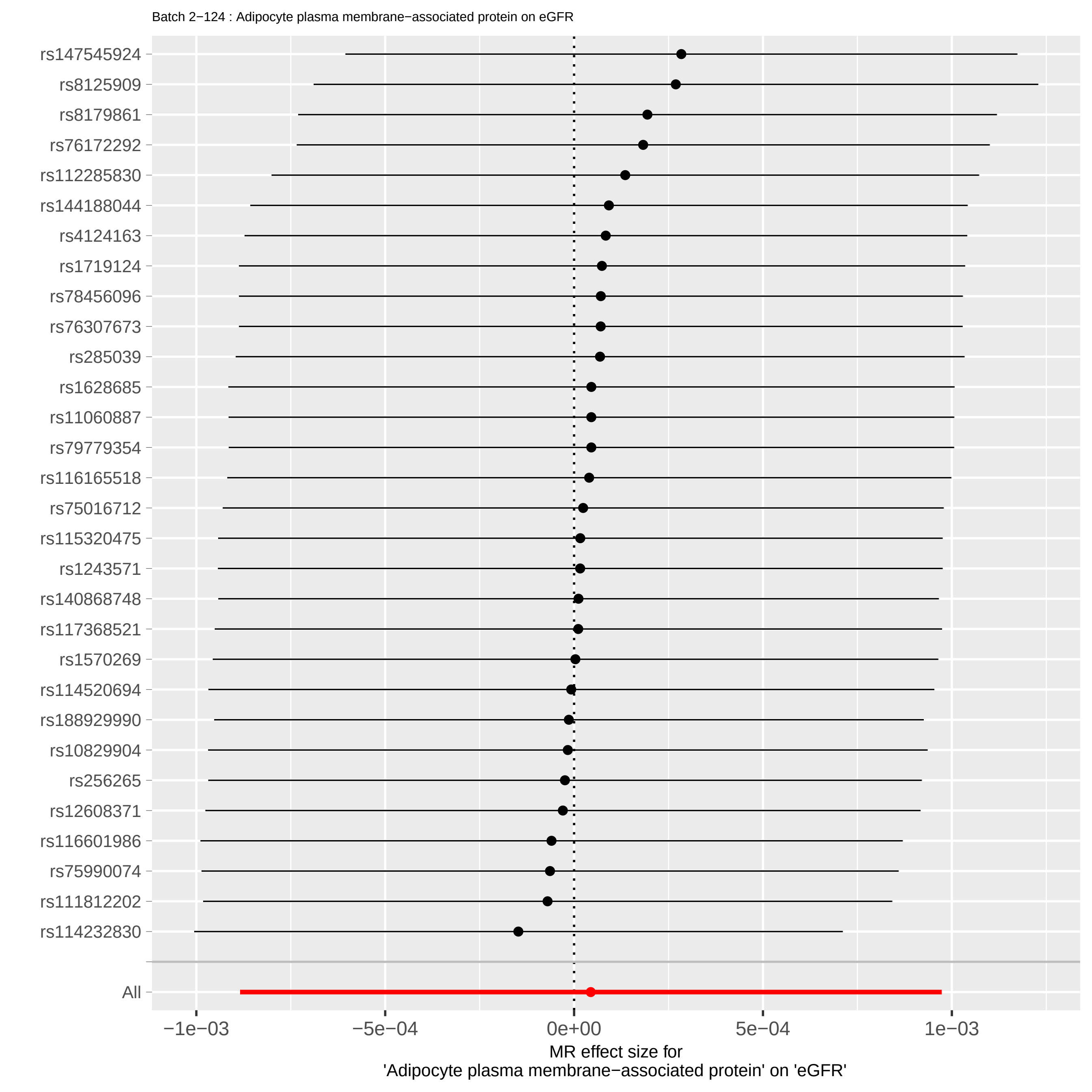
Batch 2−124 : Adipocyte plasma membrane−associated protein on eGFR
rs147545924
rs8125909
rs8179861
rs76172292
rs112285830
rs144188044
rs4124163
rs1719124
rs78456096
rs76307673
rs285039
rs1628685
rs11060887
rs79779354
rs116165518
rs75016712
rs115320475
rs1243571
rs140868748
rs117368521
rs1570269
rs114520694
rs188929990
rs10829904
rs256265
rs12608371
rs116601986
rs75990074
rs111812202
rs114232830
All
−1e−03
−5e−04
0e+00
5e−04
1e−03
MR effect size for
'Adipocyte plasma membrane−associated protein' on 'eGFR'

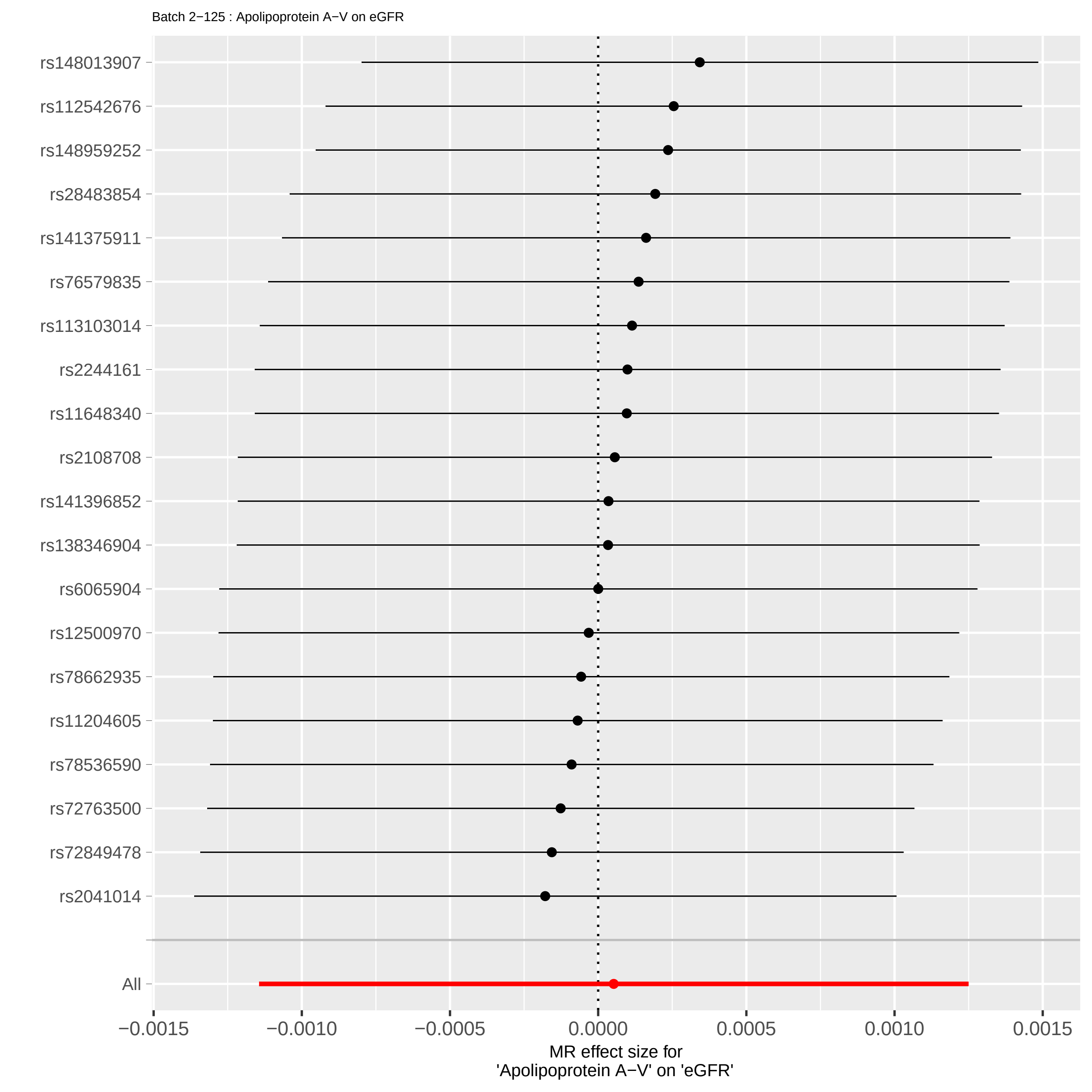

Batch 2−125 : Apolipoprotein A−V on eGFR
rs148013907
rs112542676
rs148959252
rs28483854
rs141375911
rs76579835
rs113103014
rs2244161
rs11648340
rs2108708
rs141396852
rs138346904
rs6065904
rs12500970
rs78662935
rs11204605
rs78536590
rs72763500
rs72849478
rs2041014
All
−0.0015
−0.0010
−0.0005
0.0000
0.0005
0.0010
0.0015
MR effect size for
'Apolipoprotein A−V' on 'eGFR'

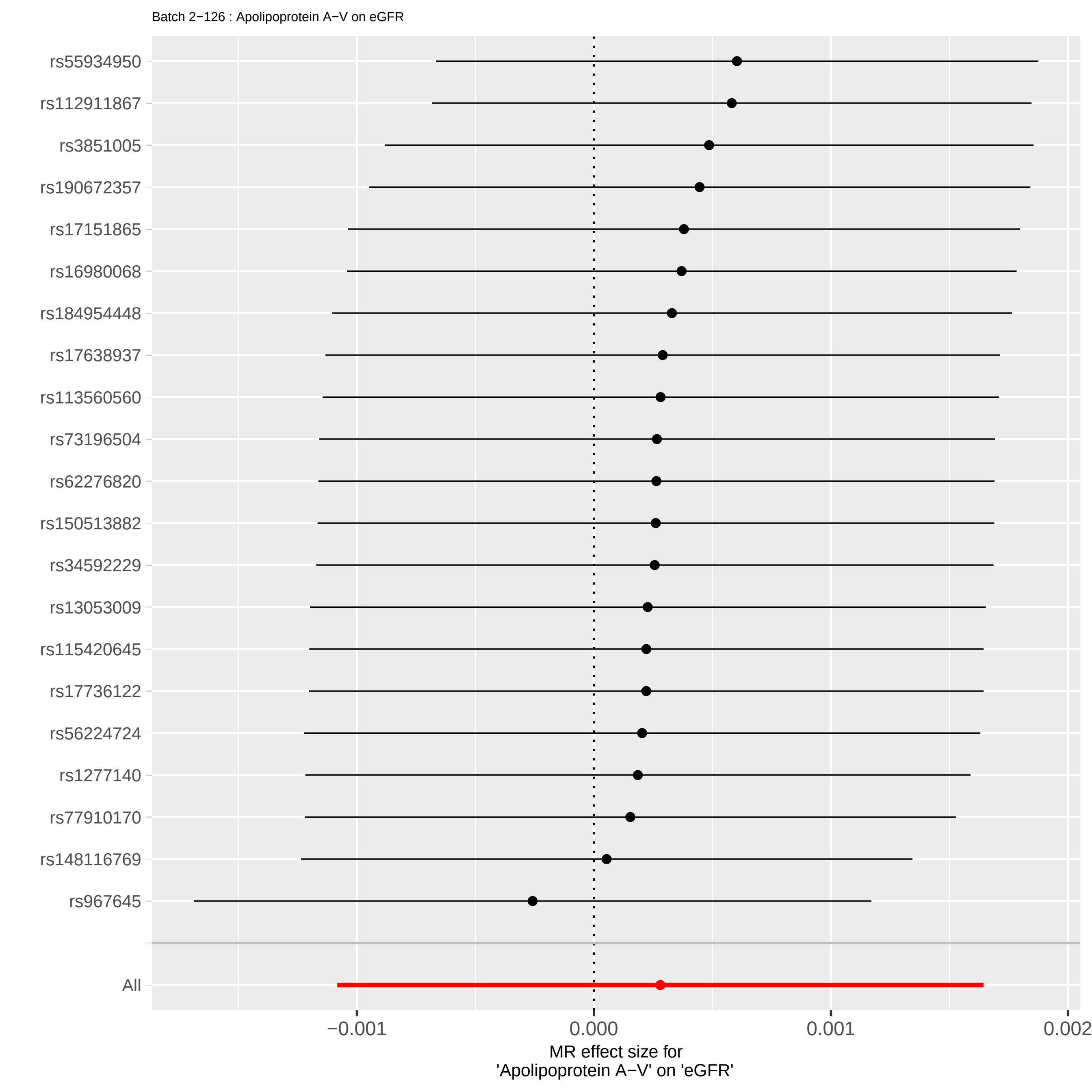

Batch 2−126 : Apolipoprotein A−V on eGFR
rs55934950
rs112911867
rs3851005
rs190672357
rs17151865
rs16980068
rs184954448
rs17638937
rs113560560
rs73196504
rs62276820
rs150513882
rs34592229
rs13053009
rs115420645
rs17736122
rs56224724
rs1277140
rs77910170
rs148116769
rs967645
All
−0.001
0.000
0.001
0.002
MR effect size for
'Apolipoprotein A−V' on 'eGFR'

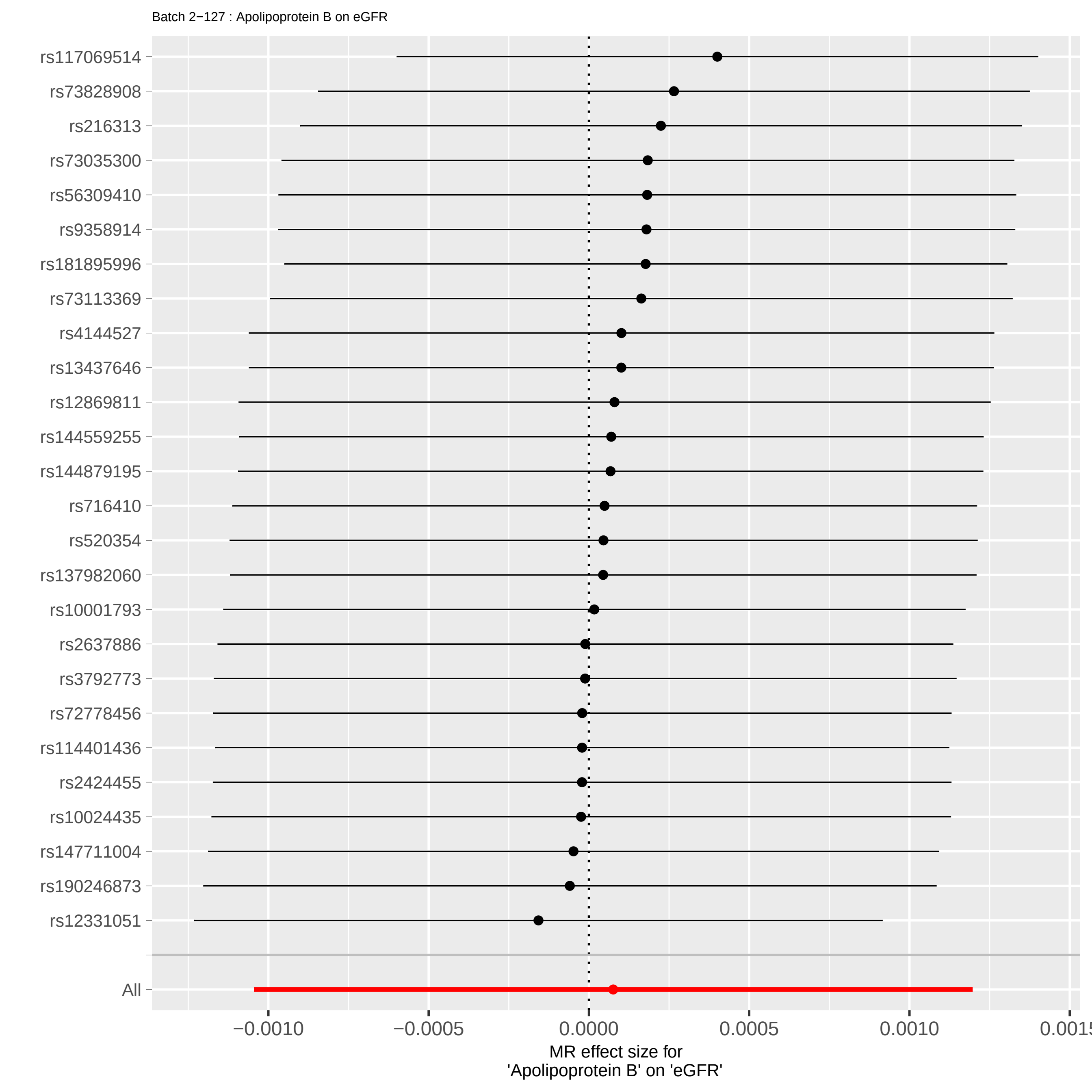

Batch 2−127 : Apolipoprotein B on eGFR
rs117069514
rs73828908
rs216313
rs73035300
rs56309410
rs9358914
rs181895996
rs73113369
rs4144527
rs13437646
rs12869811
rs144559255
rs144879195
rs716410
rs520354
rs137982060
rs10001793
rs2637886
rs3792773
rs72778456
rs114401436
rs2424455
rs10024435
rs147711004
rs190246873
rs12331051
All
−0.0010
−0.0005
0.0000
0.0005
0.0010
MR effect size for
'Apolipoprotein B' on 'eGFR'

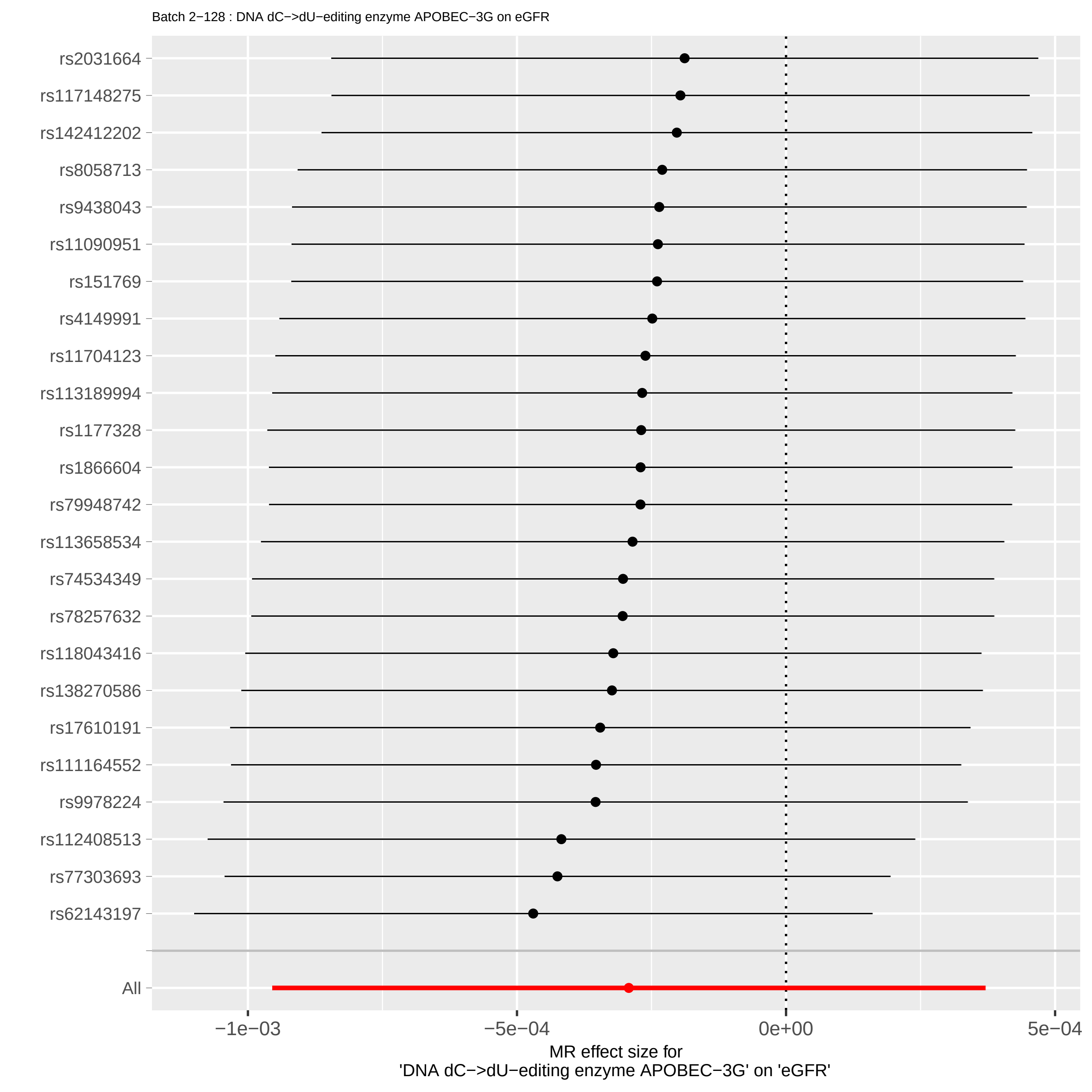

Batch 2−128 : DNA dC−>dU−editing enzyme APOBEC−3G on eGFR
rs2031664
rs117148275
rs142412202
rs8058713
rs9438043
rs11090951
rs151769
rs4149991
rs11704123
rs113189994
rs1177328
rs1866604
rs79948742
rs113658534
rs74534349
rs78257632
rs118043416
rs138270586
rs17610191
rs111164552
rs9978224
rs112408513
rs77303693
rs62143197
All
−1e−03
−5e−04
0e+00
5e−04
MR effect size for
'DNA dC−>dU−editing enzyme APOBEC−3G' on 'eGFR'

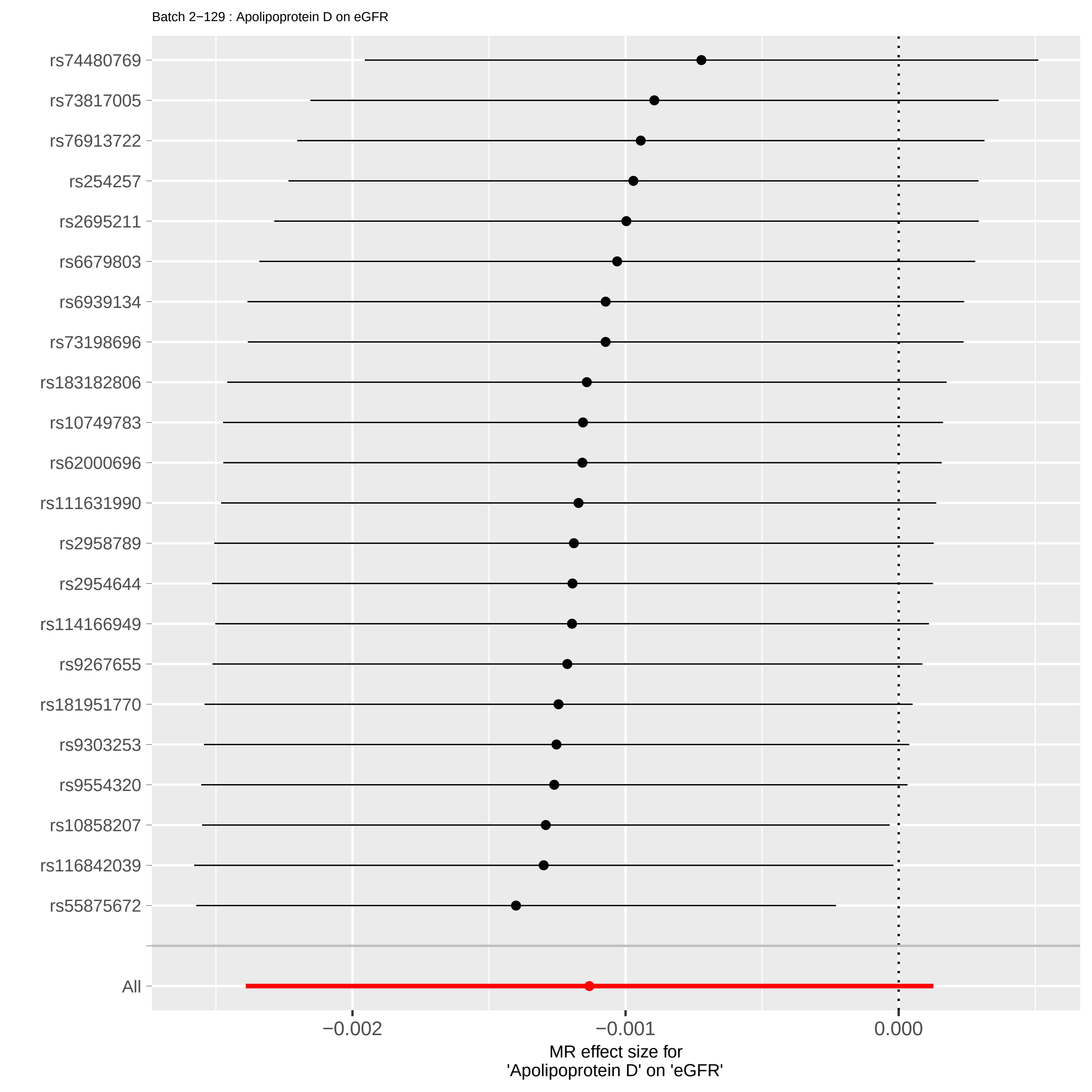
Batch 2−129 : Apolipoprotein D on eGFR
rs74480769
rs73817005
rs76913722
rs254257
rs2695211
rs6679803
rs6939134
rs73198696
rs183182806
rs10749783
rs62000696
rs111631990
rs2958789
rs2954644
rs114166949
rs9267655
rs181951770
rs9303253
rs9554320
rs10858207
rs116842039
rs55875672
All
−0.002
−0.001
0.000
MR effect size for
'Apolipoprotein D' on 'eGFR'

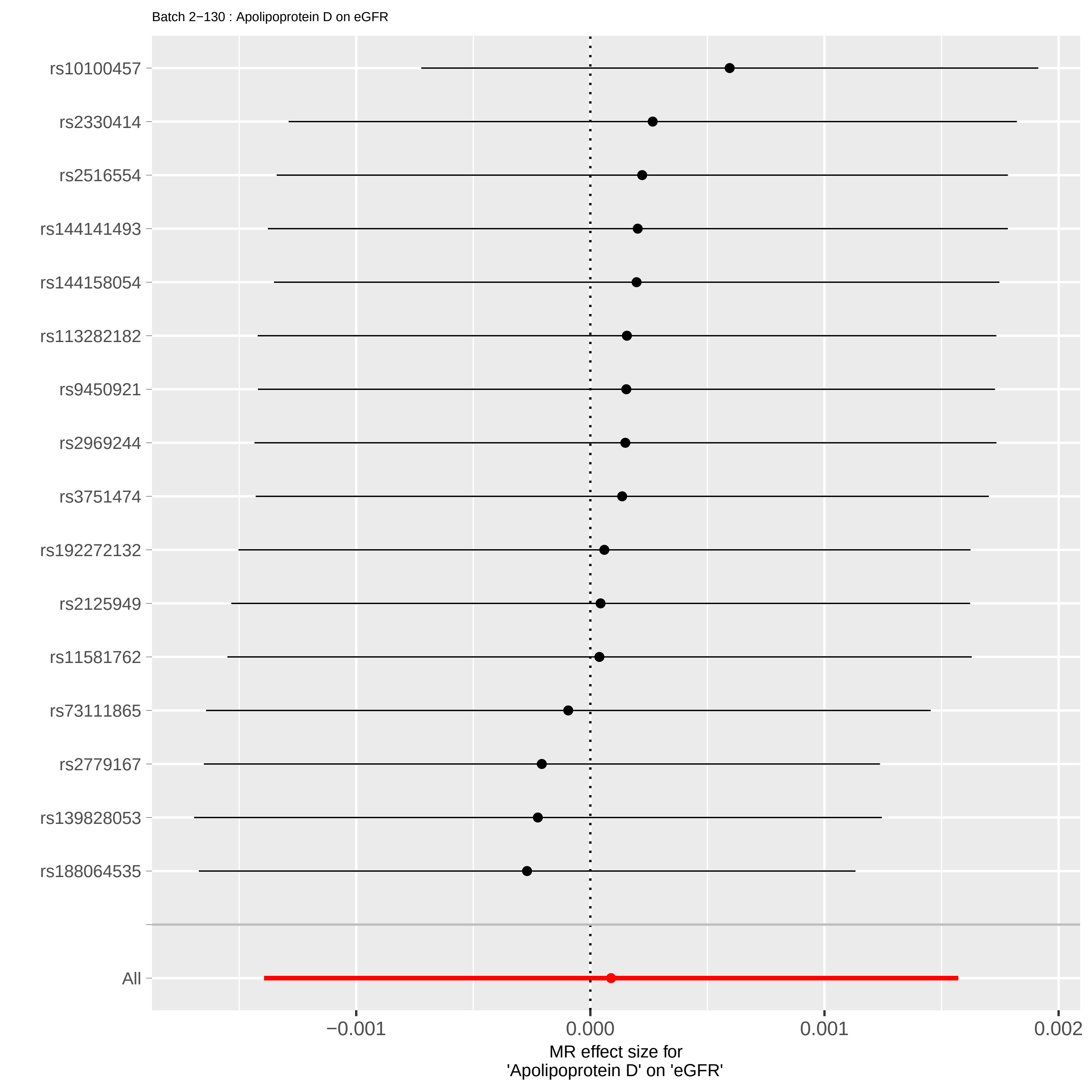

Batch 2−130 : Apolipoprotein D on eGFR
rs10100457
rs2330414
rs2516554
rs144141493
rs144158054
rs113282182
rs9450921
rs2969244
rs3751474
rs192272132
rs2125949
rs11581762
rs73111865
rs2779167
rs139828053
rs188064535
All
−0.001
0.000
0.001
0.002
MR effect size for
'Apolipoprotein D' on 'eGFR'

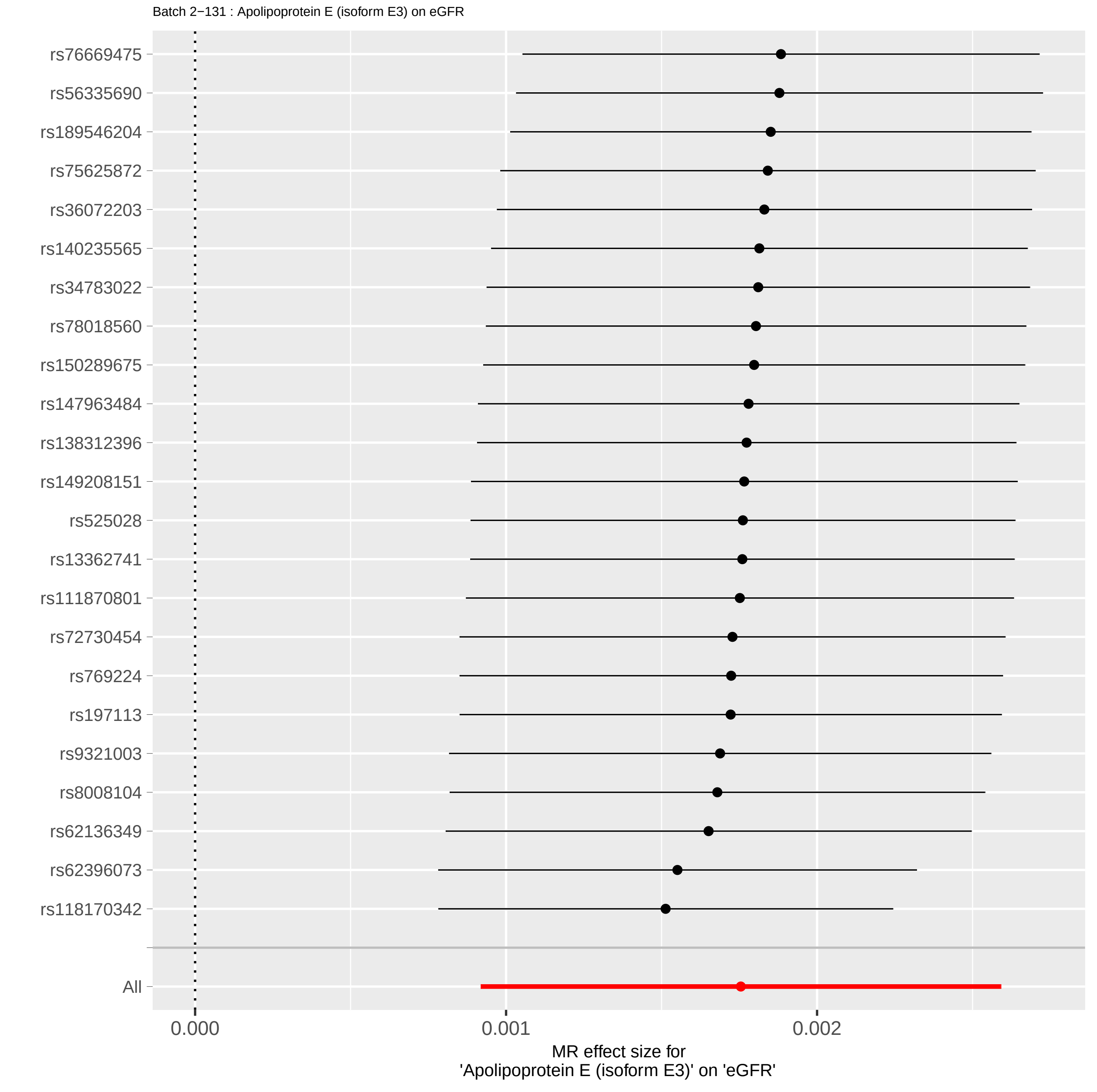
Batch 2−131 : Apolipoprotein E (isoform E3) on eGFR
rs76669475
rs56335690
rs189546204
rs75625872
rs36072203
rs140235565
rs34783022
rs78018560
rs150289675
rs147963484
rs138312396
rs149208151
rs525028
rs13362741
rs111870801
rs72730454
rs769224
rs197113
rs9321003
rs8008104
rs62136349
rs62396073
rs118170342
All
0.000
0.001
0.002
MR effect size for
'Apolipoprotein E (isoform E3)' on 'eGFR'

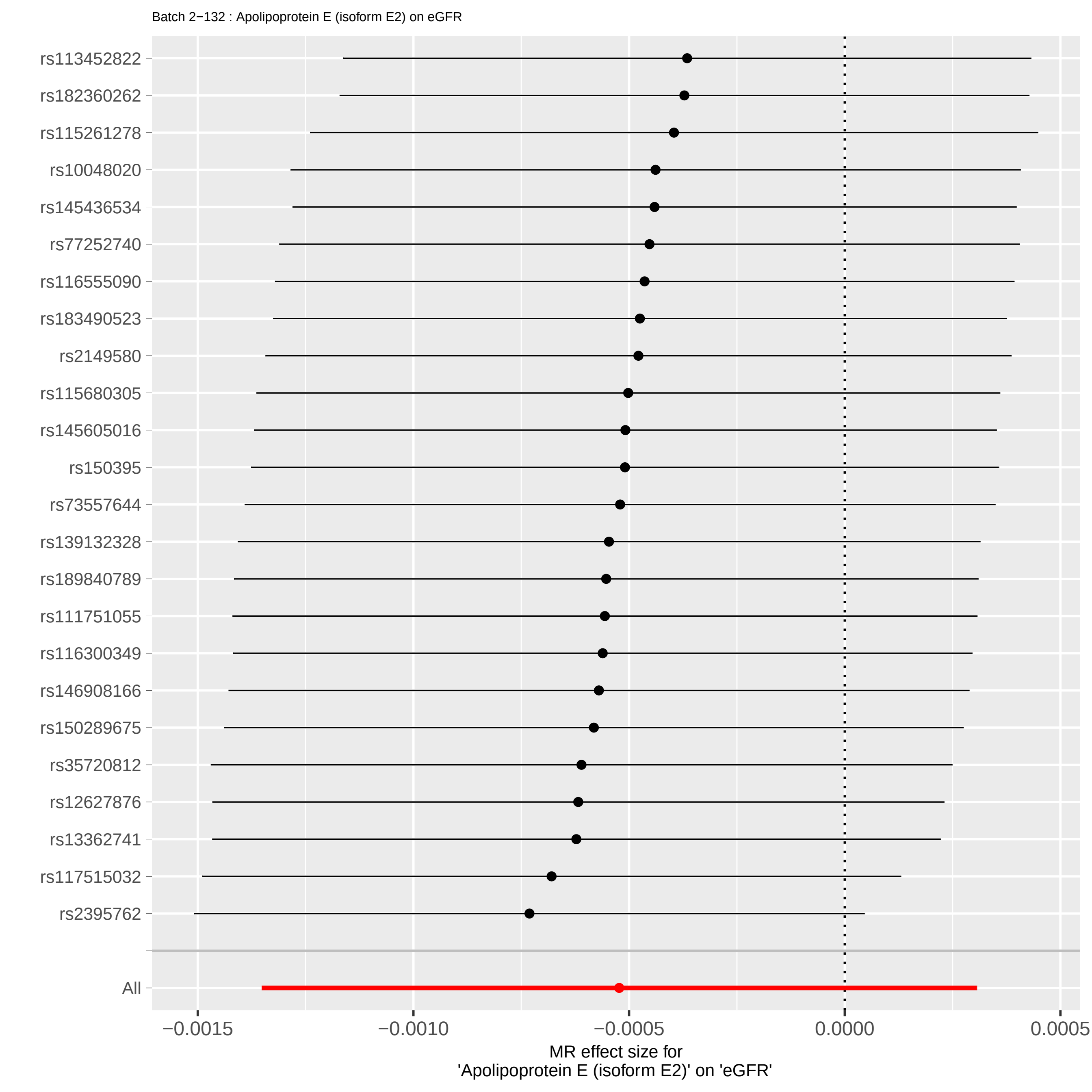

Batch 2−132 : Apolipoprotein E (isoform E2) on eGFR
rs113452822
rs182360262
rs115261278
rs10048020
rs145436534
rs77252740
rs116555090
rs183490523
rs2149580
rs115680305
rs145605016
rs150395
rs73557644
rs139132328
rs189840789
rs111751055
rs116300349
rs146908166
rs150289675
rs35720812
rs12627876
rs13362741
rs117515032
rs2395762
All
−0.0015
−0.0010
−0.0005
0.0000
0.0005
MR effect size for
'Apolipoprotein E (isoform E2)' on 'eGFR'

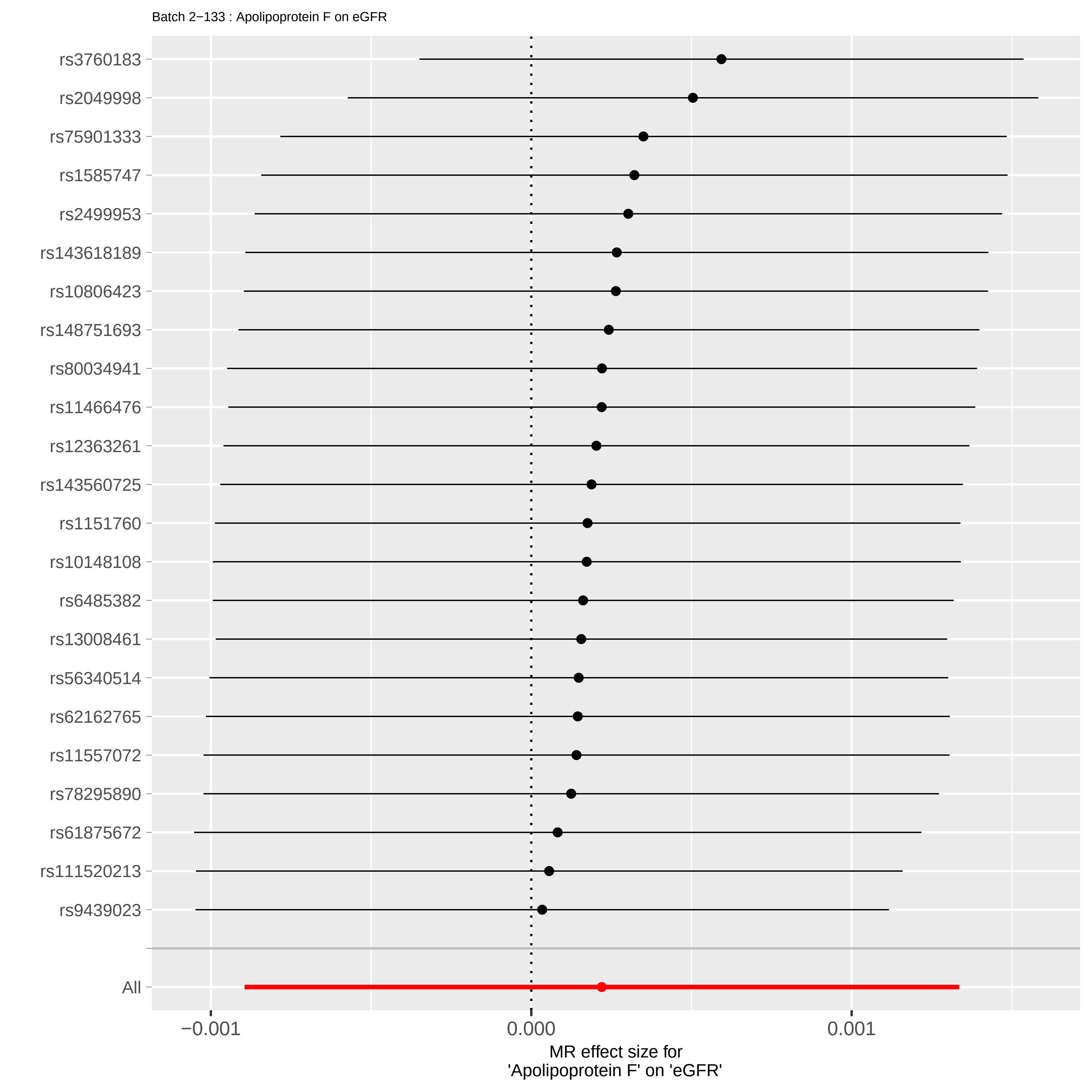

Batch 2−133 : Apolipoprotein F on eGFR
rs3760183
rs2049998
rs75901333
rs1585747
rs2499953
rs143618189
rs10806423
rs148751693
rs80034941
rs11466476
rs12363261
rs143560725
rs1151760
rs10148108
rs6485382
rs13008461
rs56340514
rs62162765
rs11557072
rs78295890
rs61875672
rs111520213
rs9439023
All
−0.001
0.000
0.001
MR effect size for
'Apolipoprotein F' on 'eGFR'

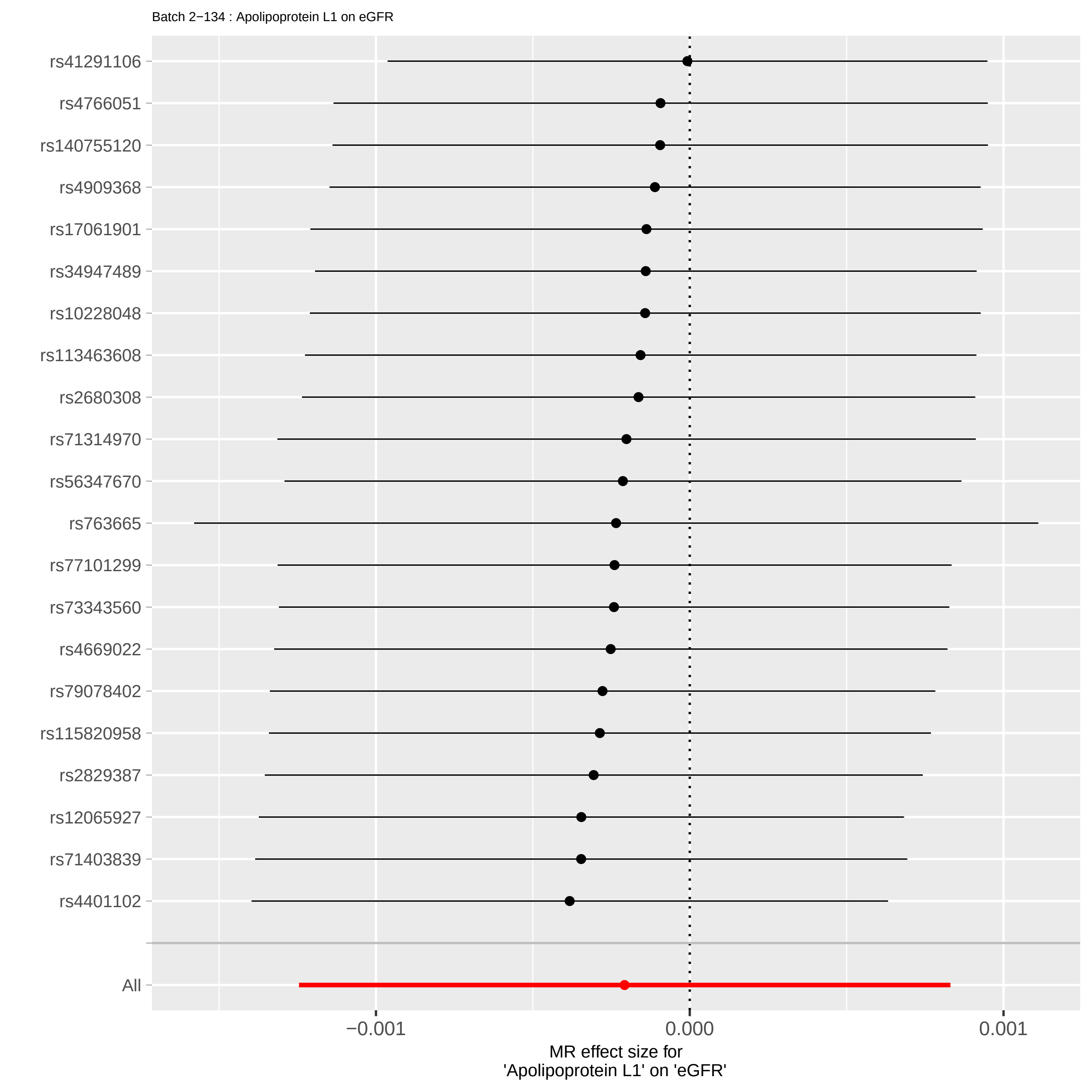

Batch 2−134 : Apolipoprotein L1 on eGFR
rs41291106
rs4766051
rs140755120
rs4909368
rs17061901
rs34947489
rs10228048
rs113463608
rs2680308
rs71314970
rs56347670
rs763665
rs77101299
rs73343560
rs4669022
rs79078402
rs115820958
rs2829387
rs12065927
rs71403839
rs4401102
All
−0.001
0.000
0.001
MR effect size for
'Apolipoprotein L1' on 'eGFR'

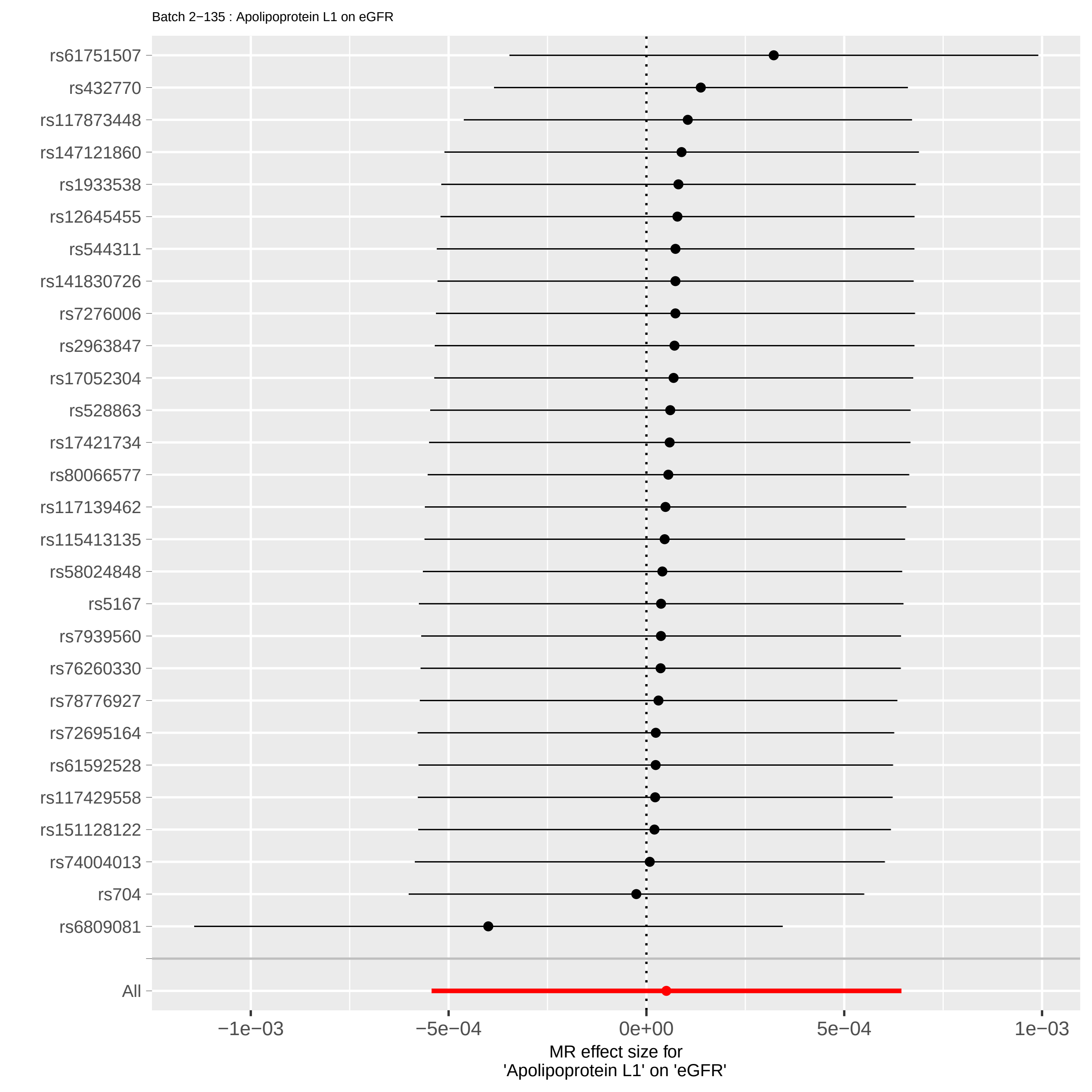

Batch 2−135 : Apolipoprotein L1 on eGFR
rs61751507
rs432770
rs117873448
rs147121860
rs1933538
rs12645455
rs544311
rs141830726
rs7276006
rs2963847
rs17052304
rs528863
rs17421734
rs80066577
rs117139462
rs115413135
rs58024848
rs5167
rs7939560
rs76260330
rs78776927
rs72695164
rs61592528
rs117429558
rs151128122
rs74004013
rs704
rs6809081
All
−1e−03
−5e−04
0e+00
5e−04
1e−03
MR effect size for
'Apolipoprotein L1' on 'eGFR'

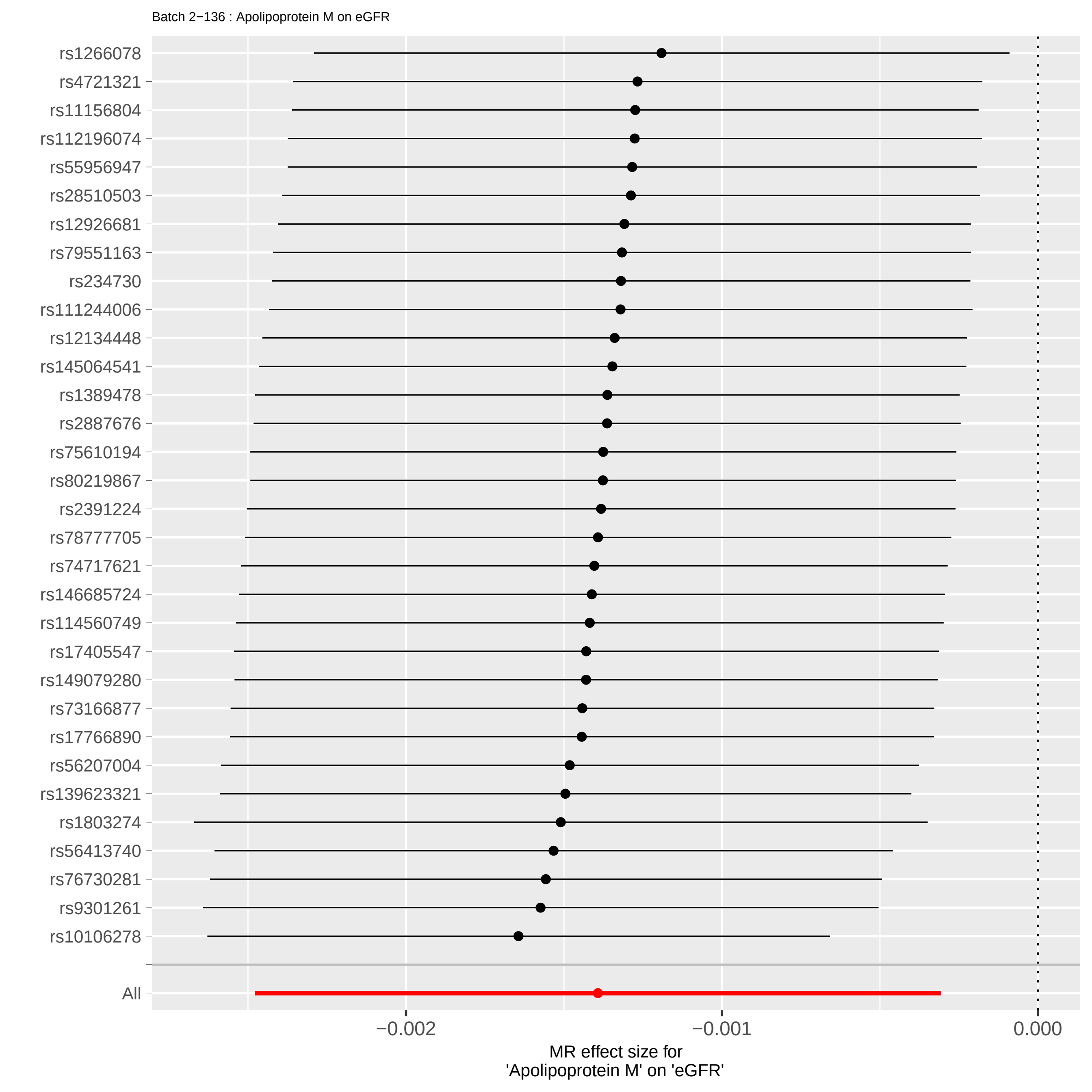

Batch 2−136 : Apolipoprotein M on eGFR
rs1266078
rs4721321
rs11156804
rs112196074
rs55956947
rs28510503
rs12926681
rs79551163
rs234730
rs111244006
rs12134448
rs145064541
rs1389478
rs2887676
rs75610194
rs80219867
rs2391224
rs78777705
rs74717621
rs146685724
rs114560749
rs17405547
rs149079280
rs73166877
rs17766890
rs56207004
rs139623321
rs1803274
rs56413740
rs76730281
rs9301261
rs10106278
All
−0.002
−0.001
0.000
MR effect size for
'Apolipoprotein M' on 'eGFR'

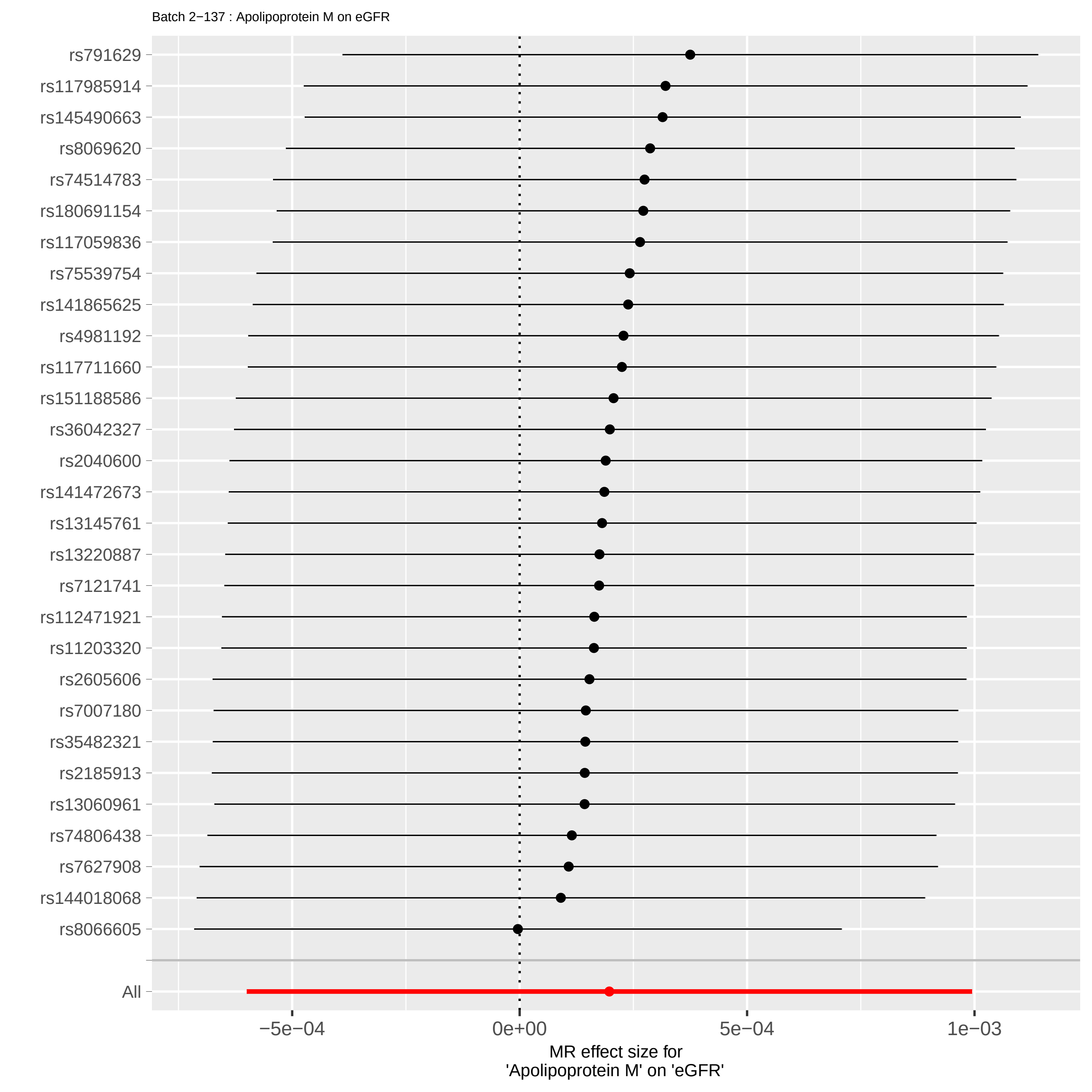
Batch 2−137 : Apolipoprotein M on eGFR
rs791629
rs117985914
rs145490663
rs8069620
rs74514783
rs180691154
rs117059836
rs75539754
rs141865625
rs4981192
rs117711660
rs151188586
rs36042327
rs2040600
rs141472673
rs13145761
rs13220887
rs7121741
rs112471921
rs11203320
rs2605606
rs7007180
rs35482321
rs2185913
rs13060961
rs74806438
rs7627908
rs144018068
rs8066605
All
−5e−04
0e+00
5e−04
1e−03
MR effect size for
'Apolipoprotein M' on 'eGFR'

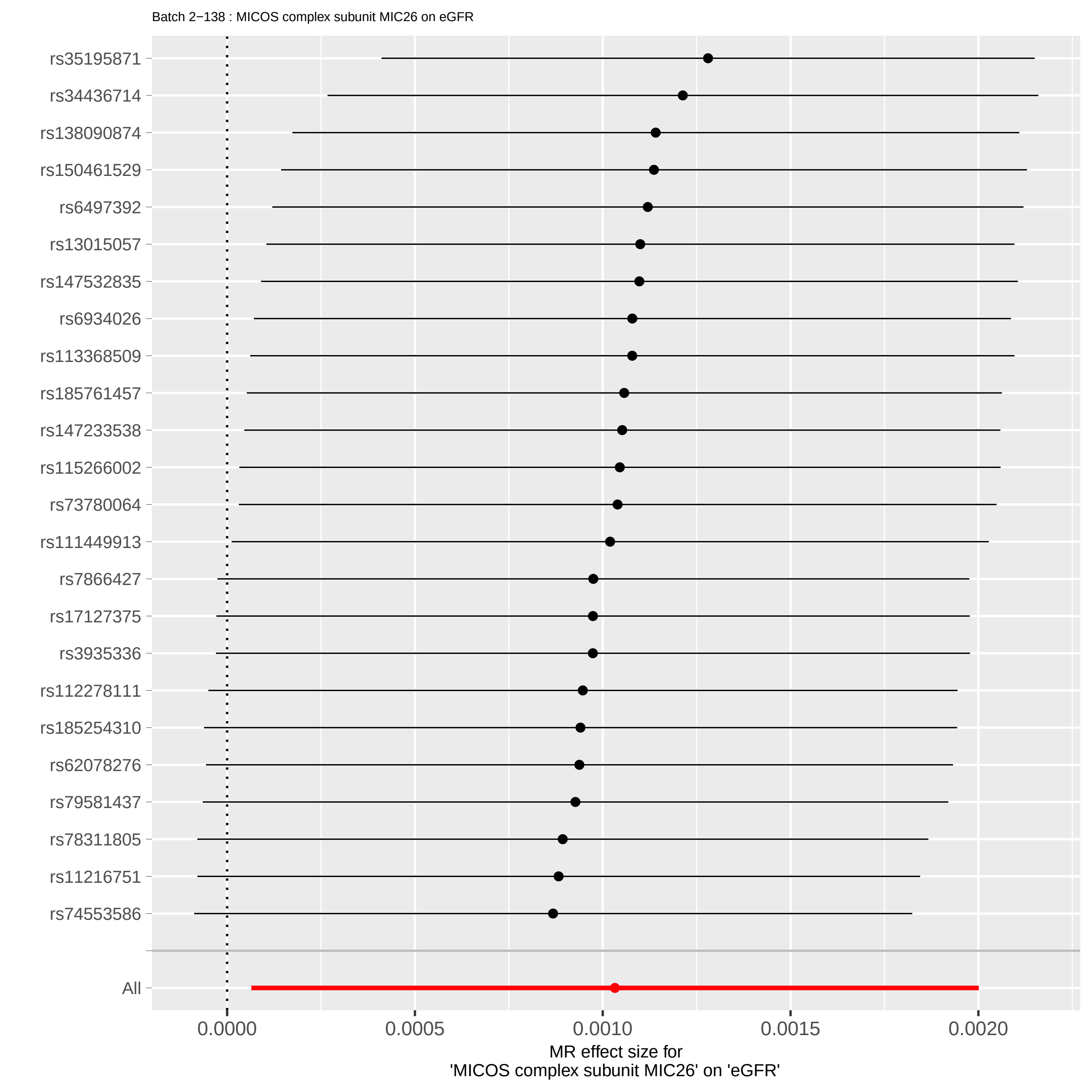

Batch 2−138 : MICOS complex subunit MIC26 on eGFR
rs35195871
rs34436714
rs138090874
rs150461529
rs6497392
rs13015057
rs147532835
rs6934026
rs113368509
rs185761457
rs147233538
rs115266002
rs73780064
rs111449913
rs7866427
rs17127375
rs3935336
rs112278111
rs185254310
rs62078276
rs79581437
rs78311805
rs11216751
rs74553586
All
0.0000
0.0005
0.0010
0.0015
0.0020
MR effect size for
'MICOS complex subunit MIC26' on 'eGFR'

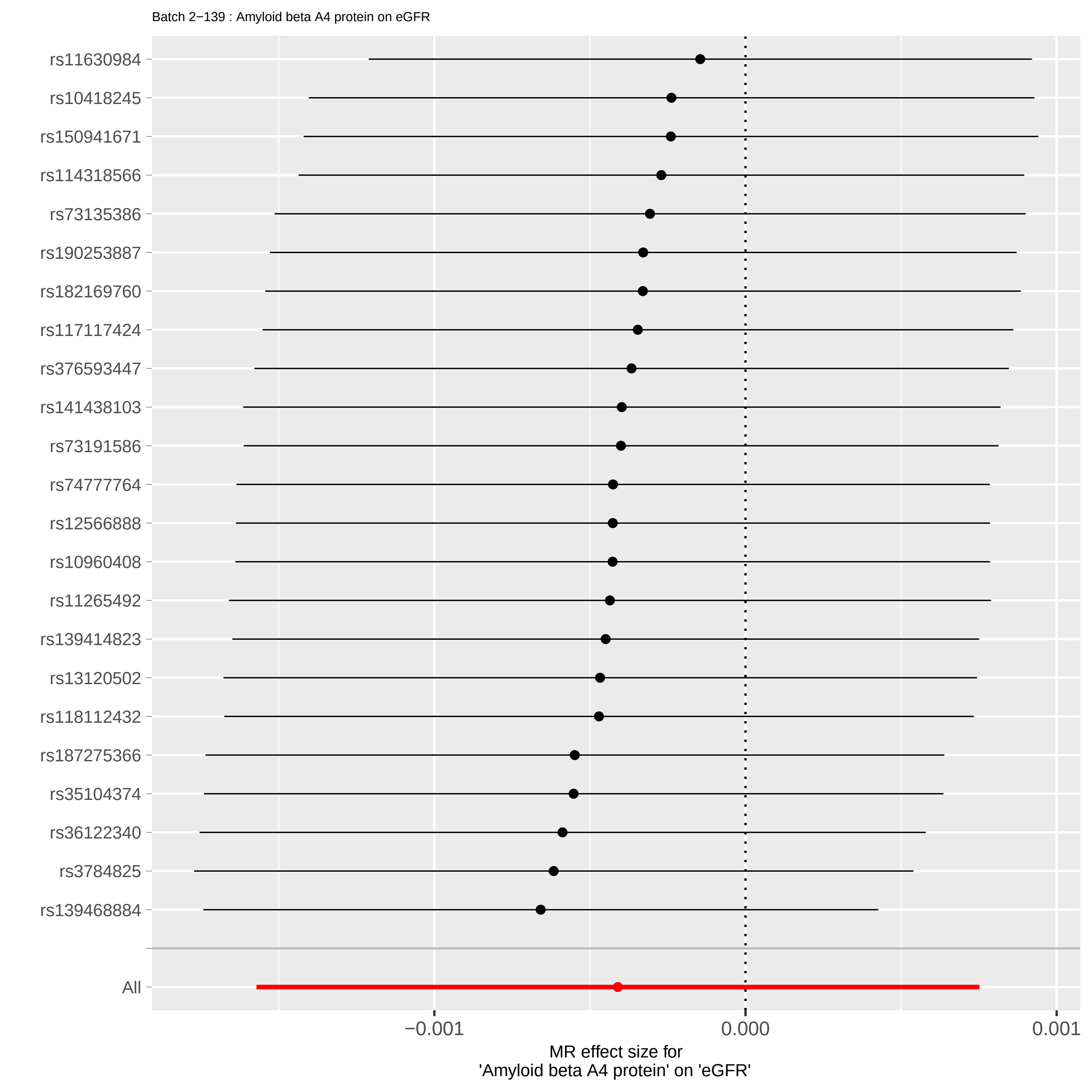

Batch 2−139 : Amyloid beta A4 protein on eGFR
rs11630984
rs10418245
rs150941671
rs114318566
rs73135386
rs190253887
rs182169760
rs117117424
rs376593447
rs141438103
rs73191586
rs74777764
rs12566888
rs10960408
rs11265492
rs139414823
rs13120502
rs118112432
rs187275366
rs35104374
rs36122340
rs3784825
rs139468884
All
−0.001
0.000
0.001
MR effect size for
'Amyloid beta A4 protein' on 'eGFR'

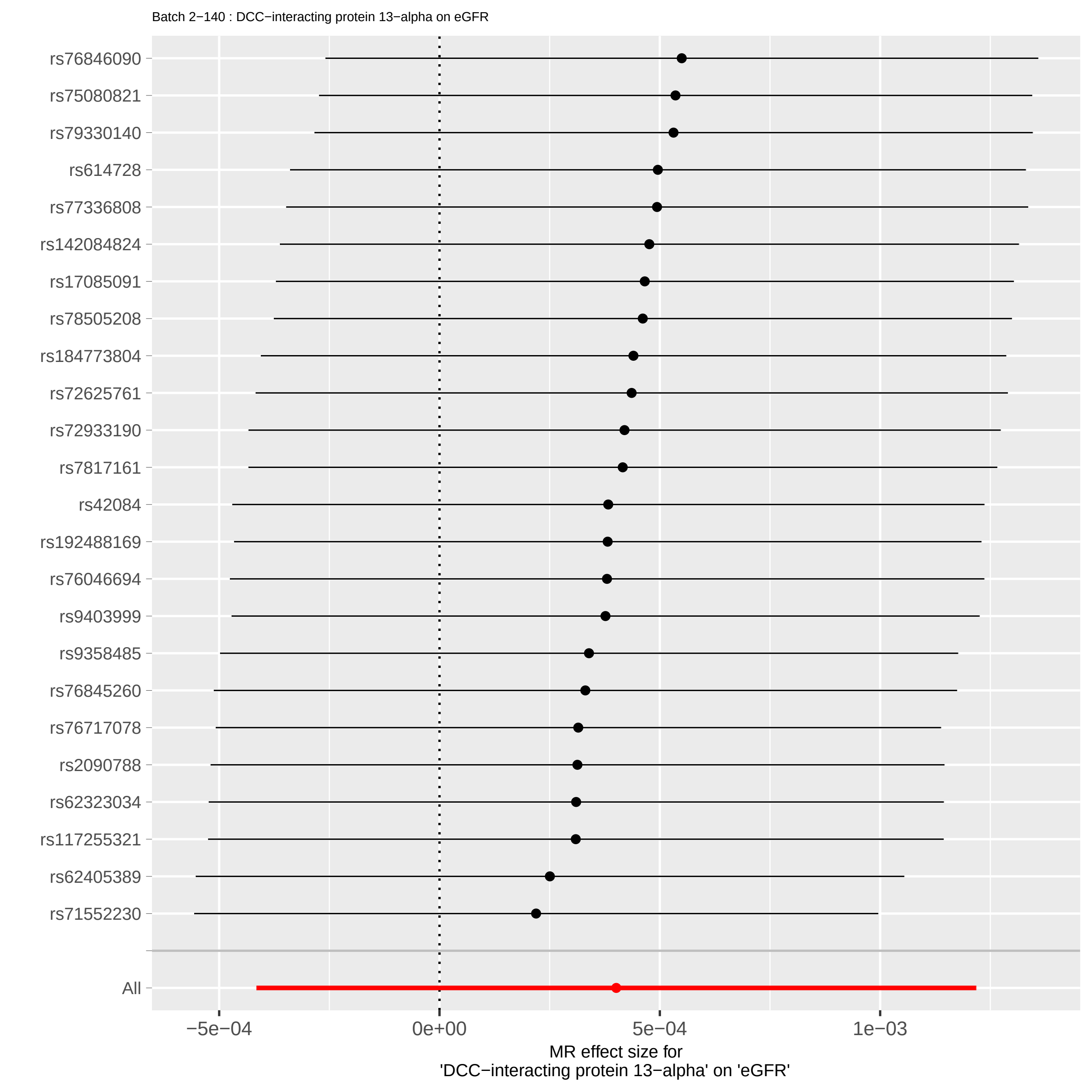

Batch 2−140 : DCC−interacting protein 13−alpha on eGFR
rs76846090
rs75080821
rs79330140
rs614728
rs77336808
rs142084824
rs17085091
rs78505208
rs184773804
rs72625761
rs72933190
rs7817161
rs42084
rs192488169
rs76046694
rs9403999
rs9358485
rs76845260
rs76717078
rs2090788
rs62323034
rs117255321
rs62405389
rs71552230
All
−5e−04
0e+00
5e−04
1e−03
MR effect size for
'DCC−interacting protein 13−alpha' on 'eGFR'

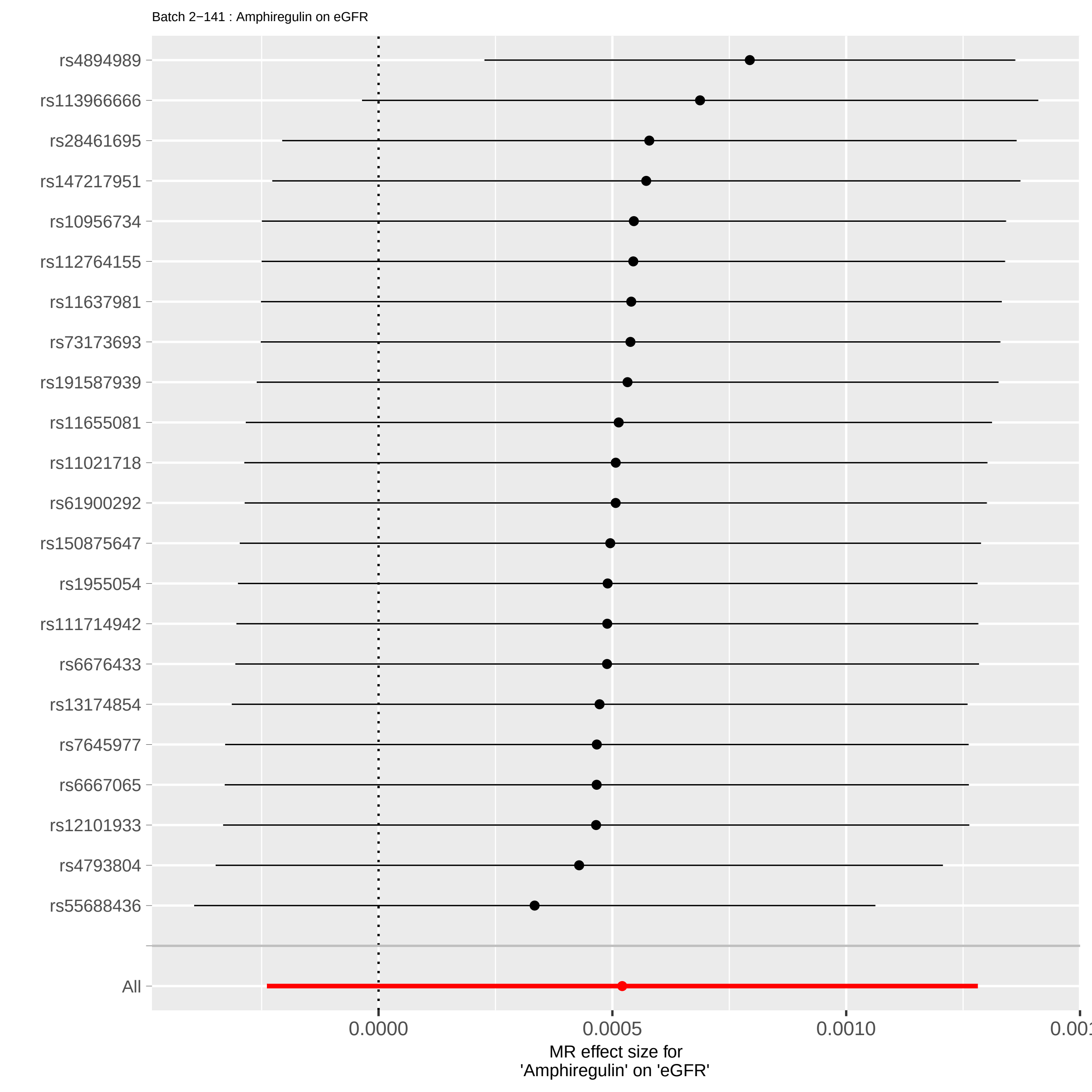
Batch 2−141 : Amphiregulin on eGFR
rs4894989
rs113966666
rs28461695
rs147217951
rs10956734
rs112764155
rs11637981
rs73173693
rs191587939
rs11655081
rs11021718
rs61900292
rs150875647
rs1955054
rs111714942
rs6676433
rs13174854
rs7645977
rs6667065
rs12101933
rs4793804
rs55688436
All
0.0000
0.0005
0.0010
MR effect size for
'Amphiregulin' on 'eGFR'

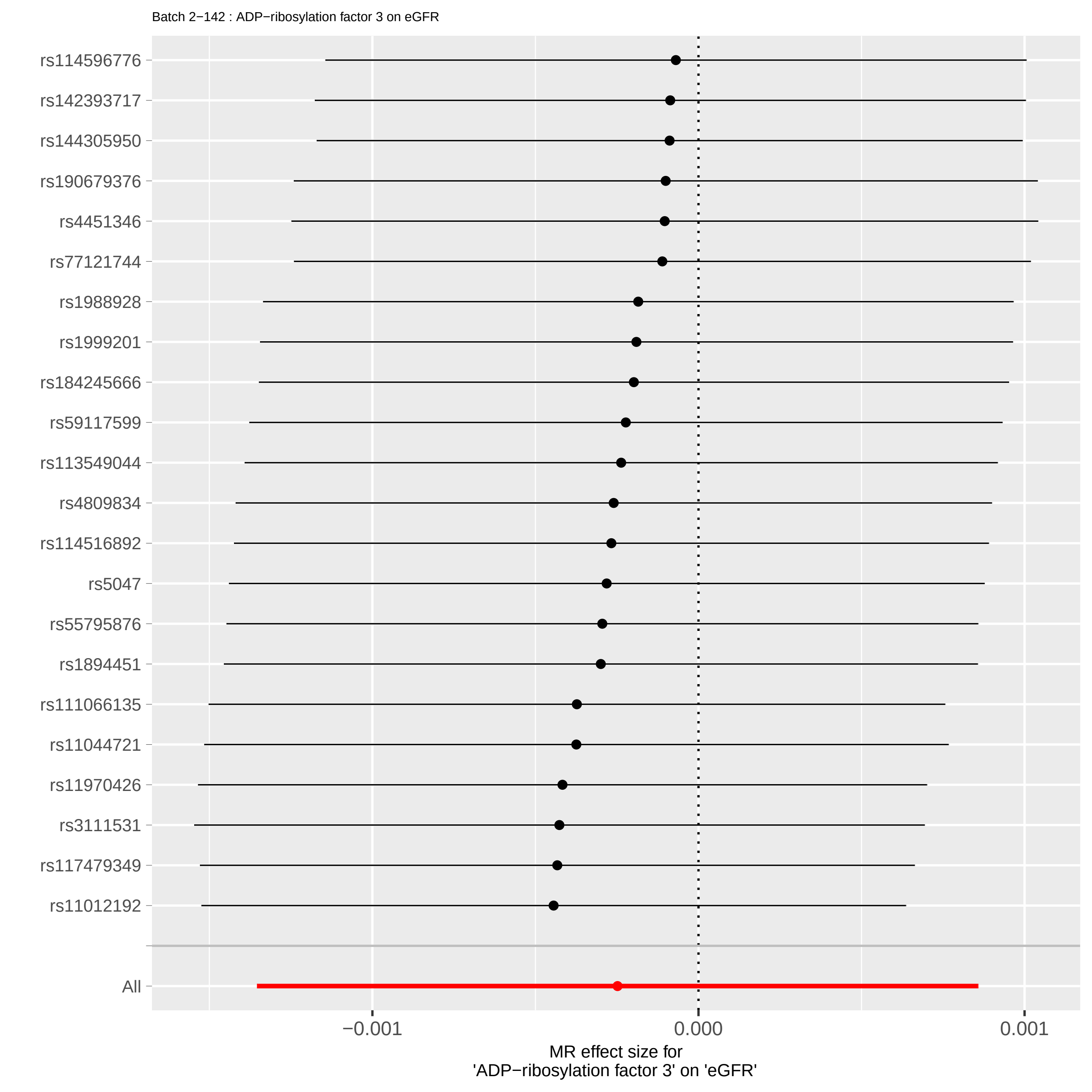

Batch 2−142 : ADP−ribosylation factor 3 on eGFR
rs114596776
rs142393717
rs144305950
rs190679376
rs4451346
rs77121744
rs1988928
rs1999201
rs184245666
rs59117599
rs113549044
rs4809834
rs114516892
rs5047
rs55795876
rs1894451
rs111066135
rs11044721
rs11970426
rs3111531
rs117479349
rs11012192
All
−0.001
0.000
0.001
MR effect size for
'ADP−ribosylation factor 3' on 'eGFR'

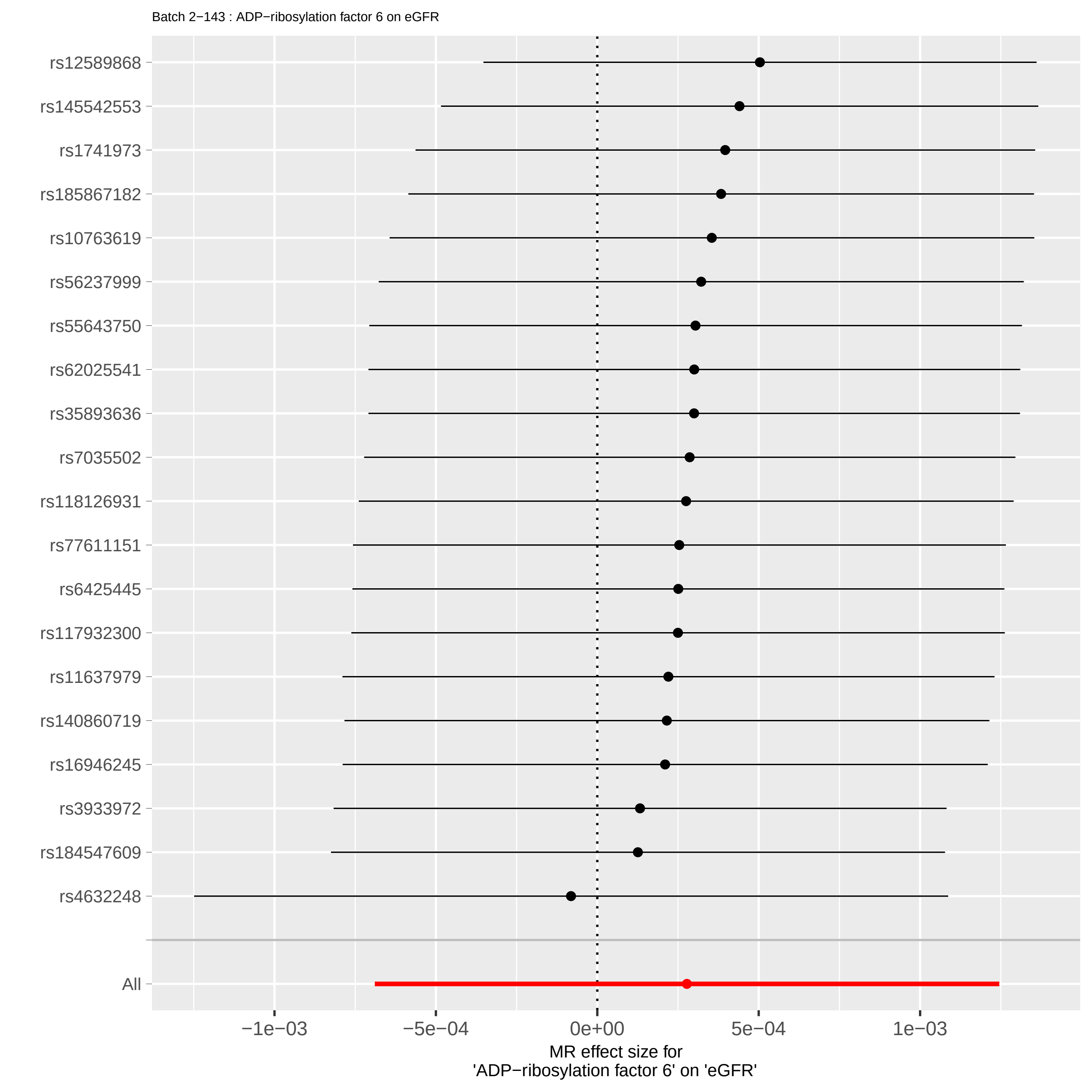

Batch 2−143 : ADP−ribosylation factor 6 on eGFR
rs12589868
rs145542553
rs1741973
rs185867182
rs10763619
rs56237999
rs55643750
rs62025541
rs35893636
rs7035502
rs118126931
rs77611151
rs6425445
rs117932300
rs11637979
rs140860719
rs16946245
rs3933972
rs184547609
rs4632248
All
−1e−03
−5e−04
0e+00
5e−04
1e−03
MR effect size for
'ADP−ribosylation factor 6' on 'eGFR'

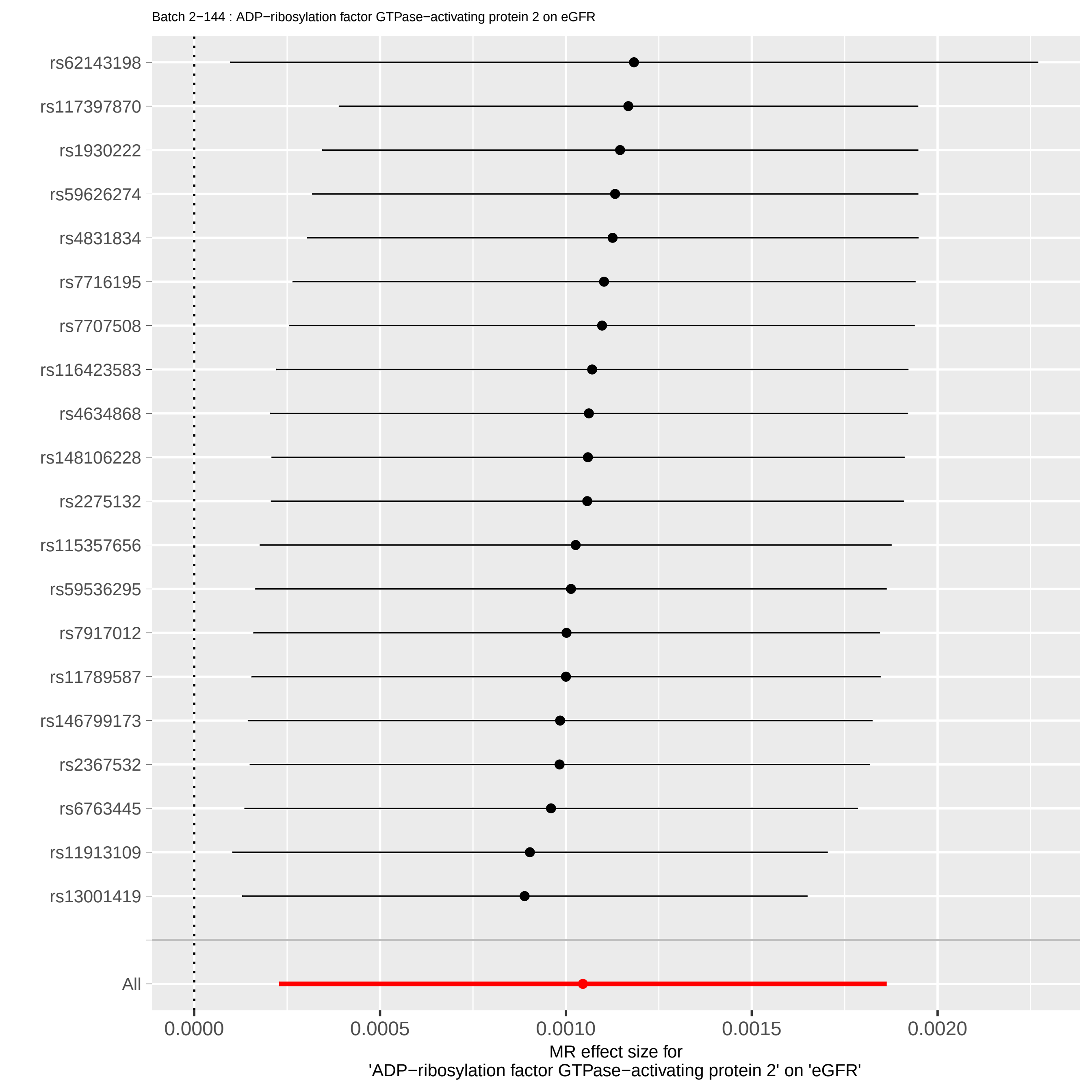

Batch 2−144 : ADP−ribosylation factor GTPase−activating protein 2 on eGFR
rs62143198
rs117397870
rs1930222
rs59626274
rs4831834
rs7716195
rs7707508
rs116423583
rs4634868
rs148106228
rs2275132
rs115357656
rs59536295
rs7917012
rs11789587
rs146799173
rs2367532
rs6763445
rs11913109
rs13001419
All
0.0000
0.0005
0.0010
0.0015
0.0020
MR effect size for
'ADP−ribosylation factor GTPase−activating protein 2' on 'eGFR'

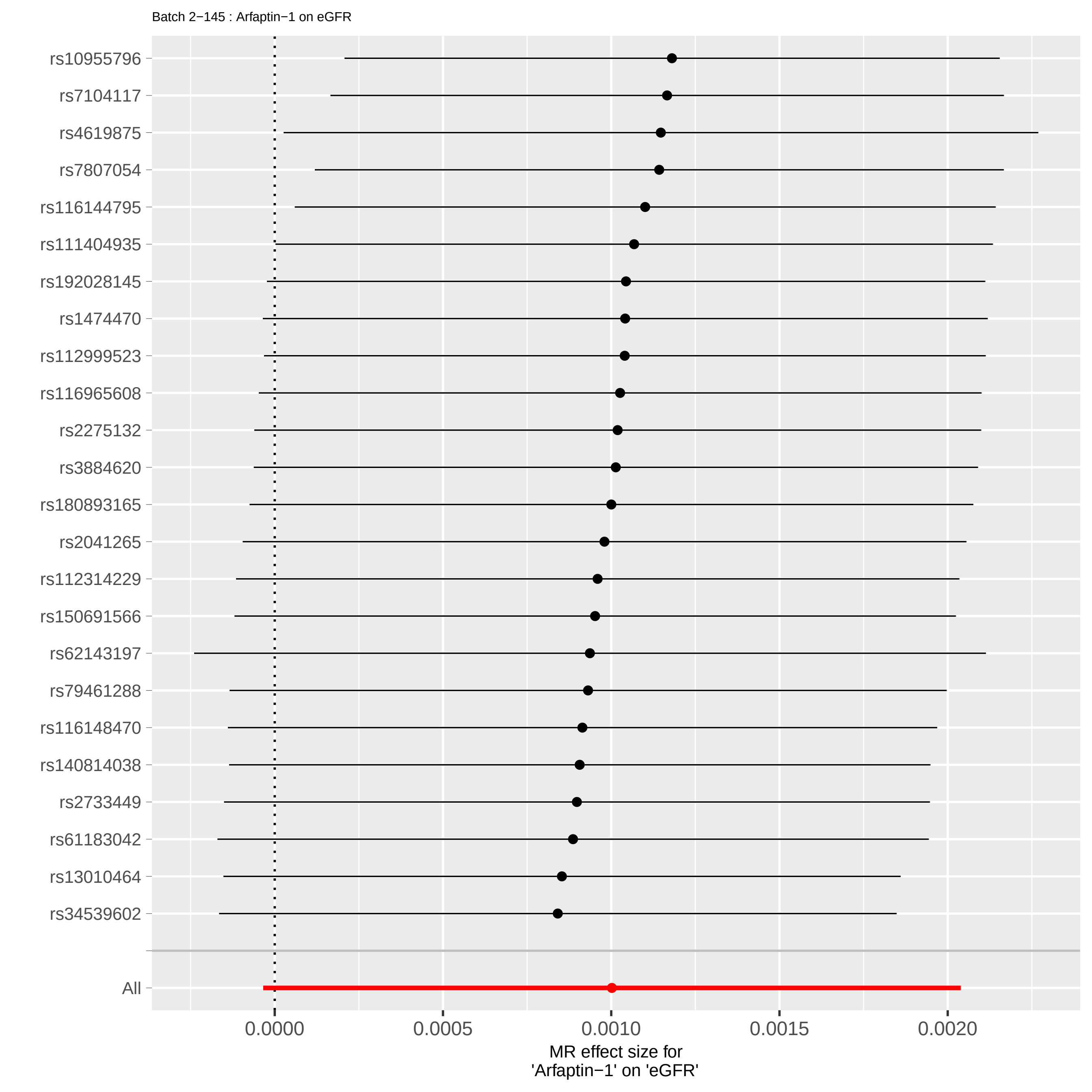

Batch 2−145 : Arfaptin−1 on eGFR
rs10955796
rs7104117
rs4619875
rs7807054
rs116144795
rs111404935
rs192028145
rs1474470
rs112999523
rs116965608
rs2275132
rs3884620
rs180893165
rs2041265
rs112314229
rs150691566
rs62143197
rs79461288
rs116148470
rs140814038
rs2733449
rs61183042
rs13010464
rs34539602
All
0.0000
0.0005
0.0010
0.0015
0.0020
MR effect size for
'Arfaptin−1' on 'eGFR'

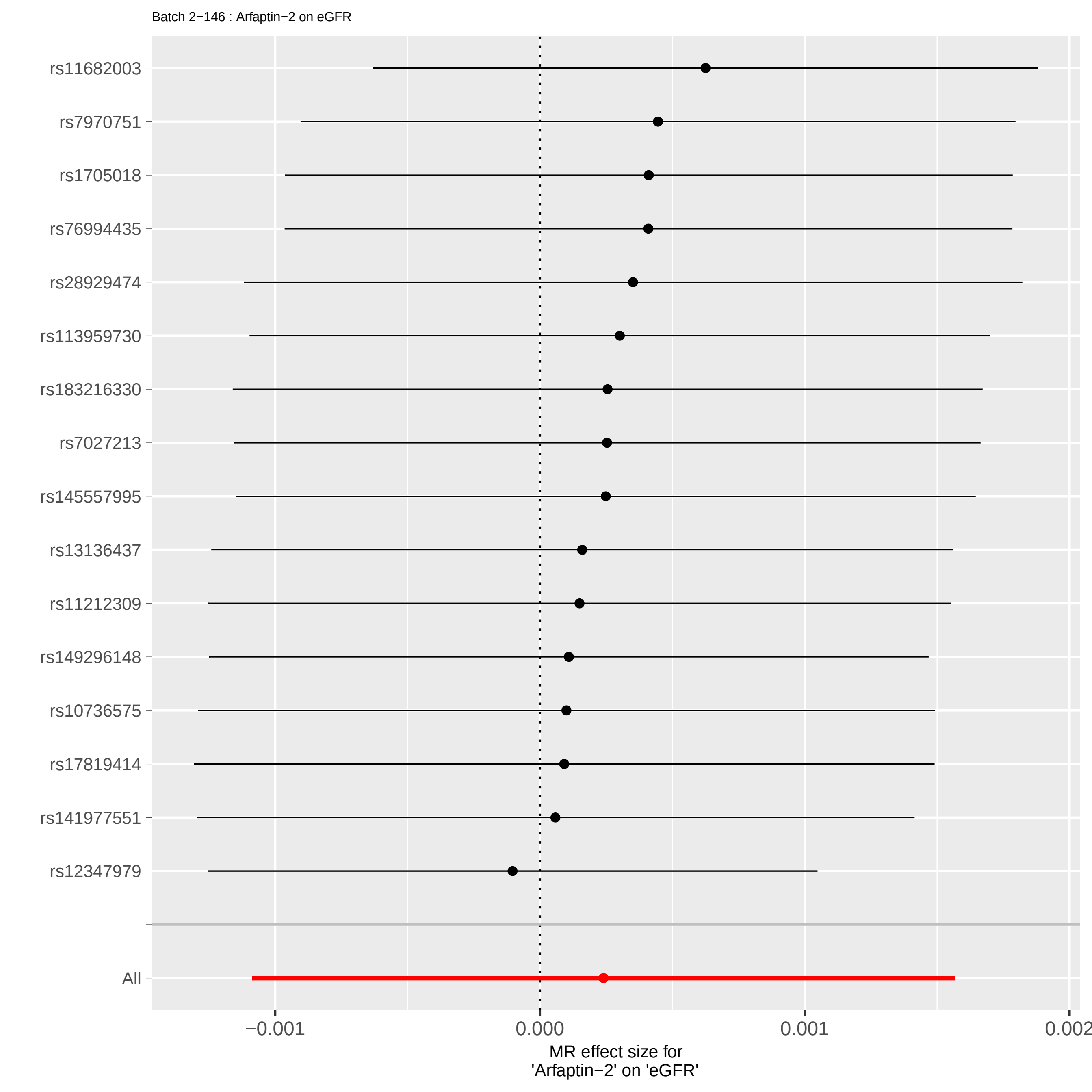

Batch 2−146 : Arfaptin−2 on eGFR
rs11682003
rs7970751
rs1705018
rs76994435
rs28929474
rs113959730
rs183216330
rs7027213
rs145557995
rs13136437
rs11212309
rs149296148
rs10736575
rs17819414
rs141977551
rs12347979
All
−0.001
0.000
0.001
0.002
MR effect size for
'Arfaptin−2' on 'eGFR'

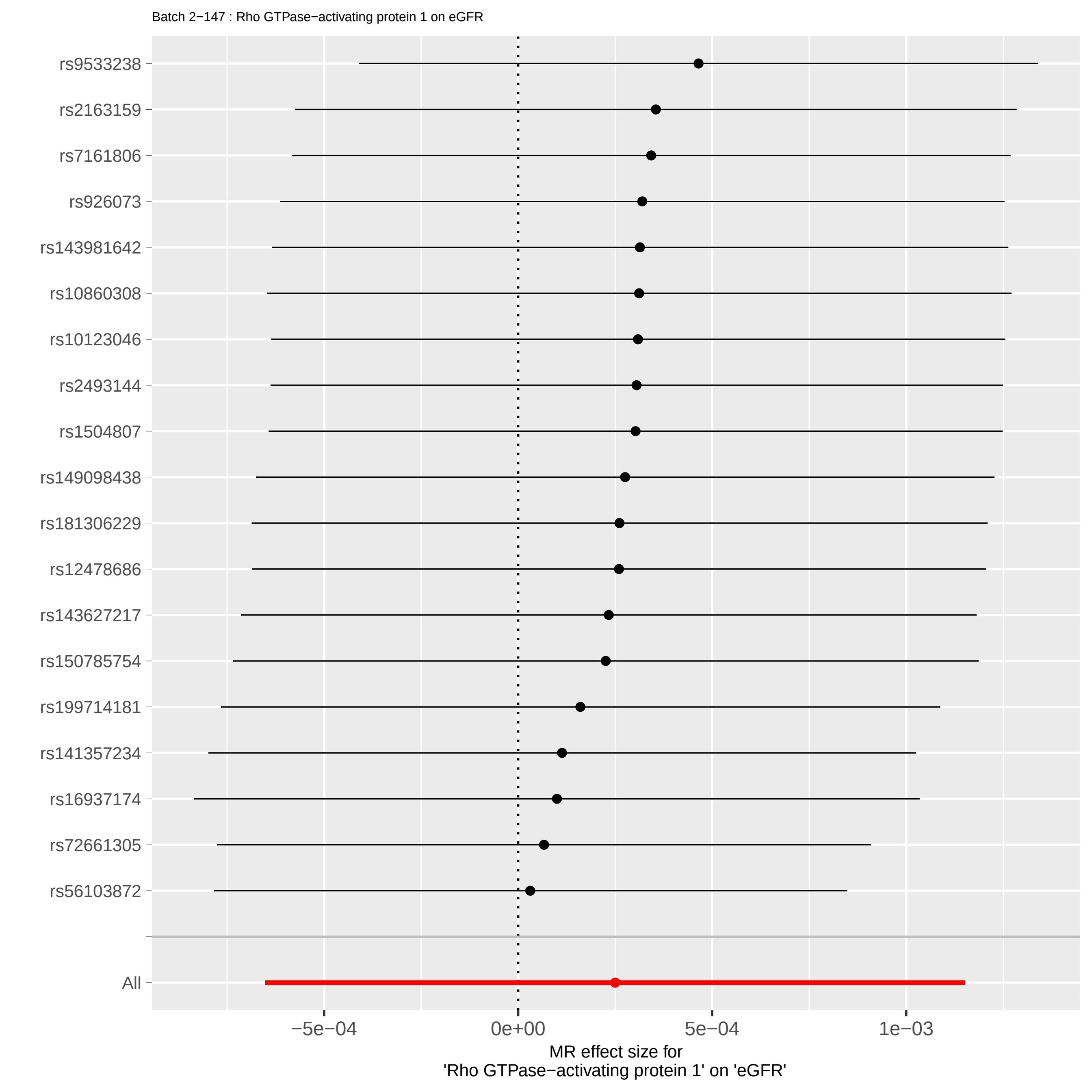

Batch 2−147 : Rho GTPase−activating protein 1 on eGFR
rs9533238
rs2163159
rs7161806
rs926073
rs143981642
rs10860308
rs10123046
rs2493144
rs1504807
rs149098438
rs181306229
rs12478686
rs143627217
rs150785754
rs199714181
rs141357234
rs16937174
rs72661305
rs56103872
All
−5e−04
0e+00
5e−04
1e−03
MR effect size for
'Rho GTPase−activating protein 1' on 'eGFR'

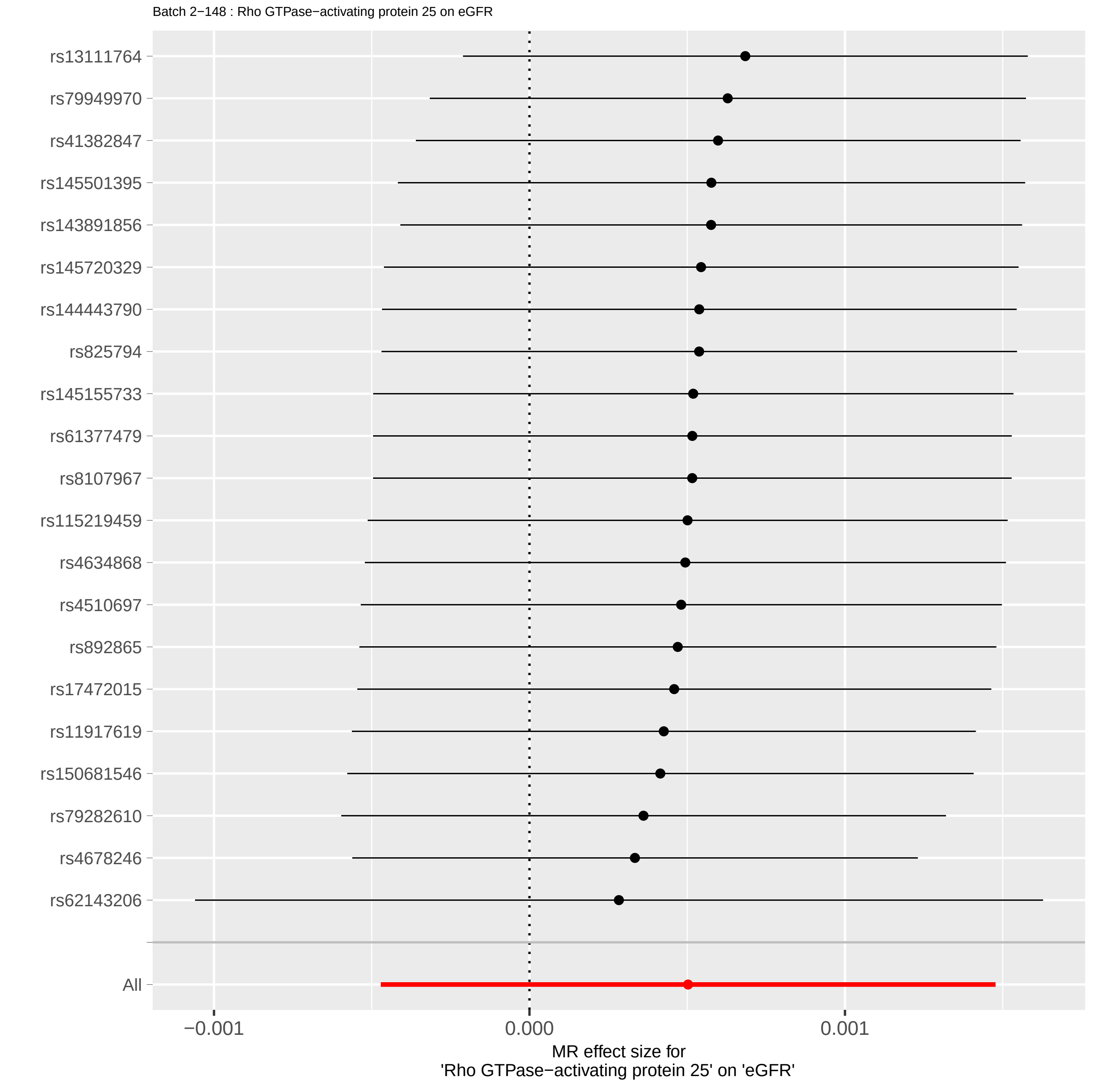

Batch 2−148 : Rho GTPase−activating protein 25 on eGFR
rs13111764
rs79949970
rs41382847
rs145501395
rs143891856
rs145720329
rs144443790
rs825794
rs145155733
rs61377479
rs8107967
rs115219459
rs4634868
rs4510697
rs892865
rs17472015
rs11917619
rs150681546
rs79282610
rs4678246
rs62143206
All
−0.001
0.000
0.001
MR effect size for
'Rho GTPase−activating protein 25' on 'eGFR'

Batch 2−149 : Rho GTPase−activating protein 30 on eGFR

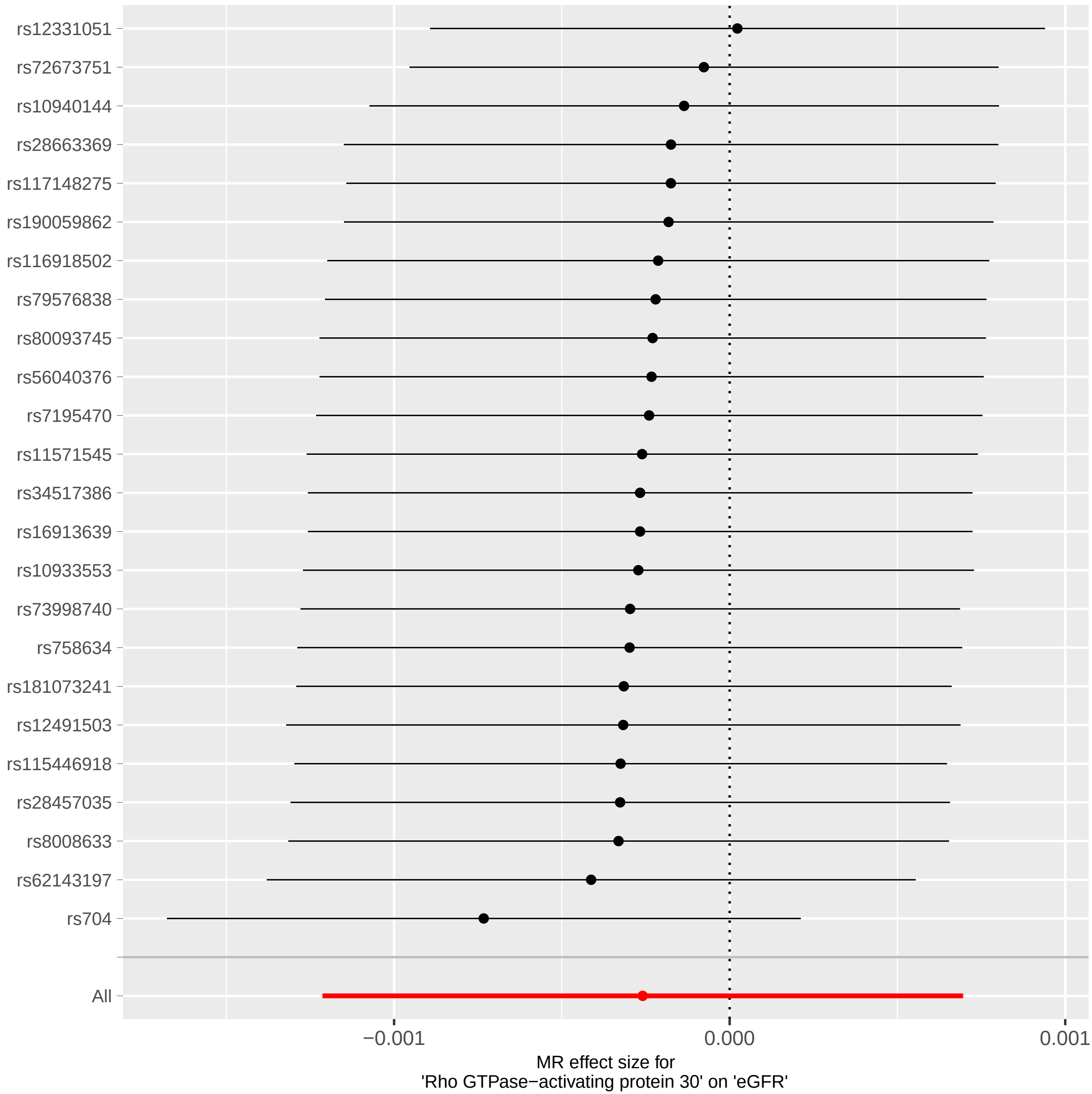

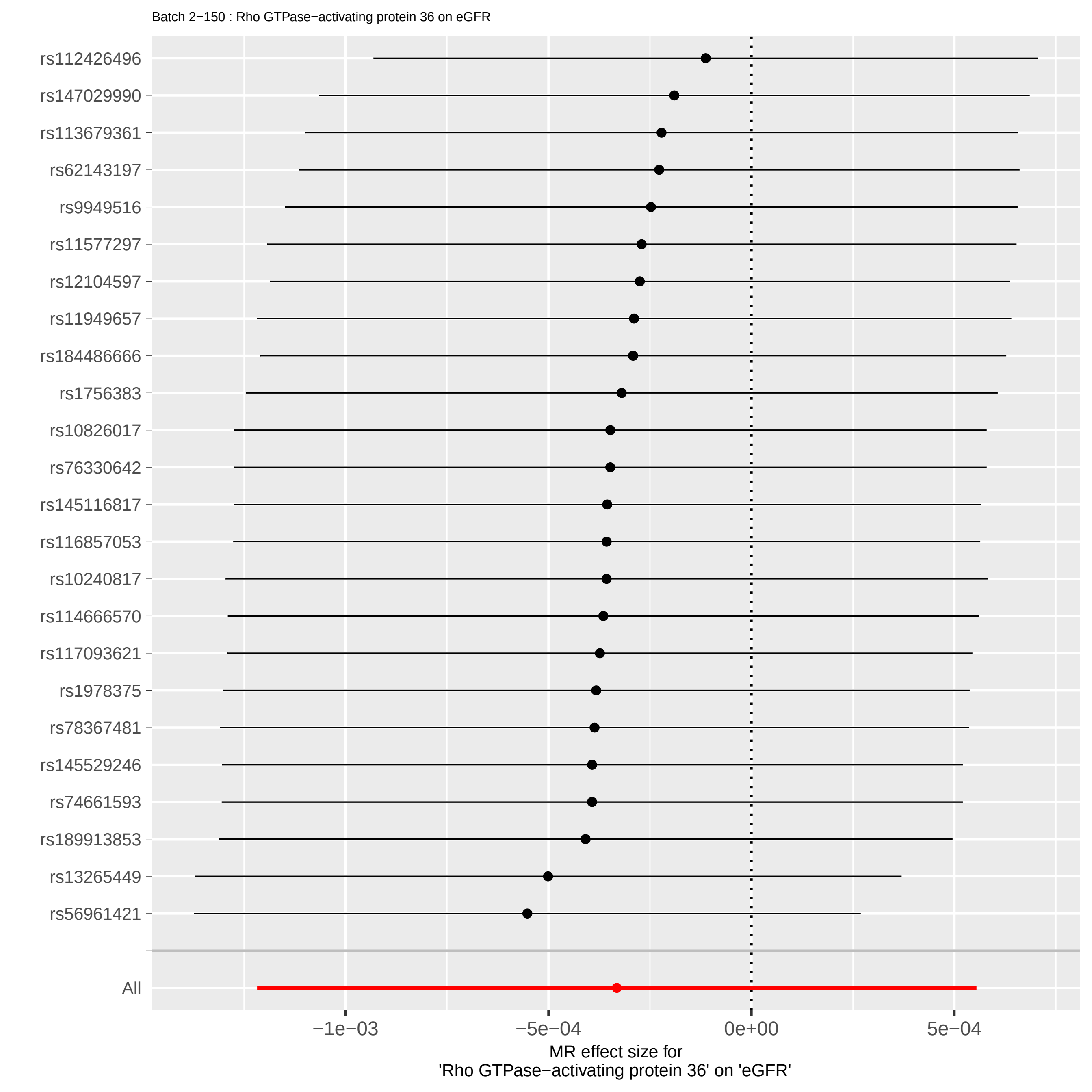

Batch 2−150 : Rho GTPase−activating protein 36 on eGFR
rs112426496
rs147029990
rs113679361
rs62143197
rs9949516
rs11577297
rs12104597
rs11949657
rs184486666
rs1756383
rs10826017
rs76330642
rs145116817
rs116857053
rs10240817
rs114666570
rs117093621
rs1978375
rs78367481
rs145529246
rs74661593
rs189913853
rs13265449
rs56961421
All
−1e−03
−5e−04
0e+00
5e−04
MR effect size for
'Rho GTPase−activating protein 36' on 'eGFR'

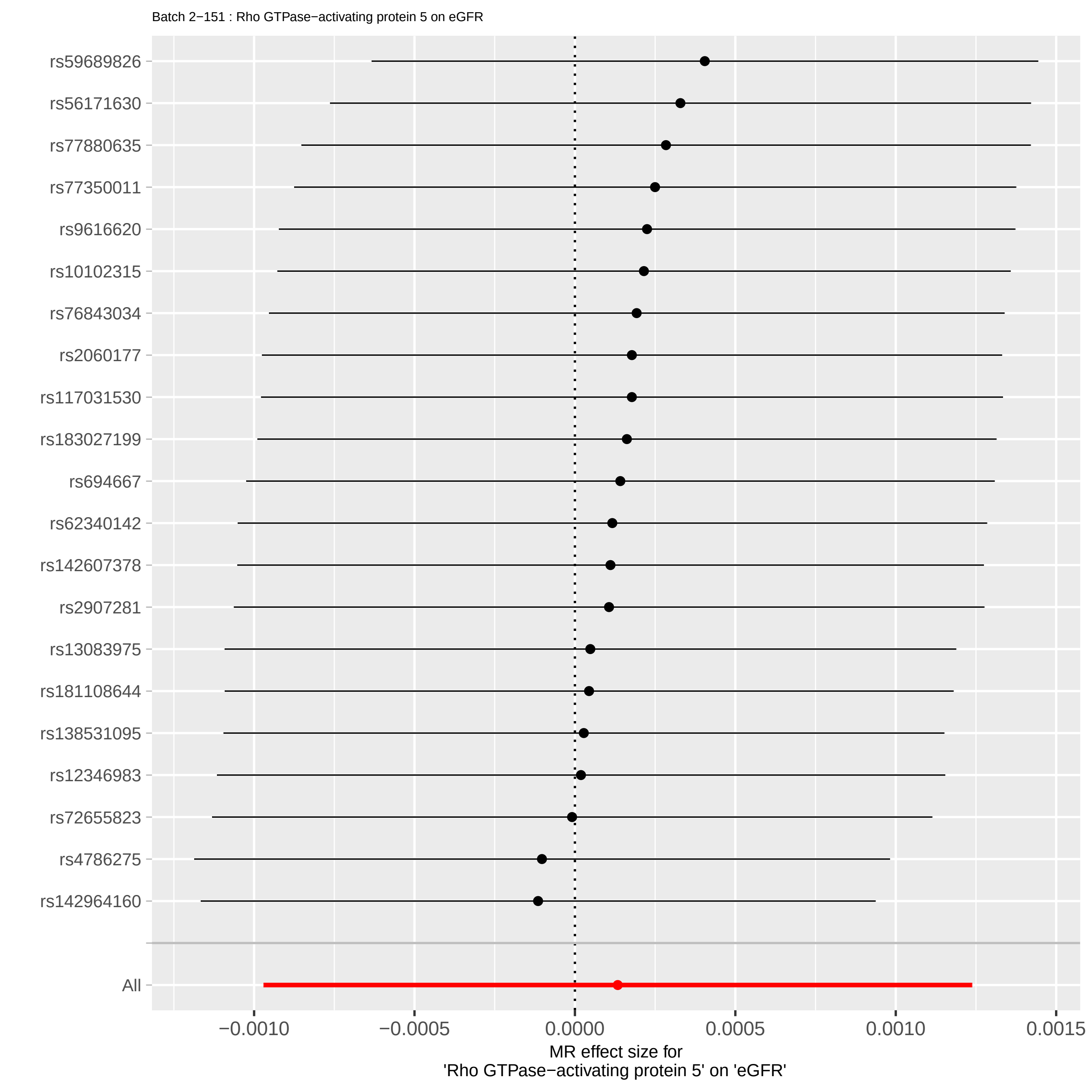

Batch 2−151 : Rho GTPase−activating protein 5 on eGFR
rs59689826
rs56171630
rs77880635
rs77350011
rs9616620
rs10102315
rs76843034
rs2060177
rs117031530
rs183027199
rs694667
rs62340142
rs142607378
rs2907281
rs13083975
rs181108644
rs138531095
rs12346983
rs72655823
rs4786275
rs142964160
All
−0.0010
−0.0005
0.0000
0.0005
0.0010
0.0015
MR effect size for
'Rho GTPase−activating protein 5' on 'eGFR'

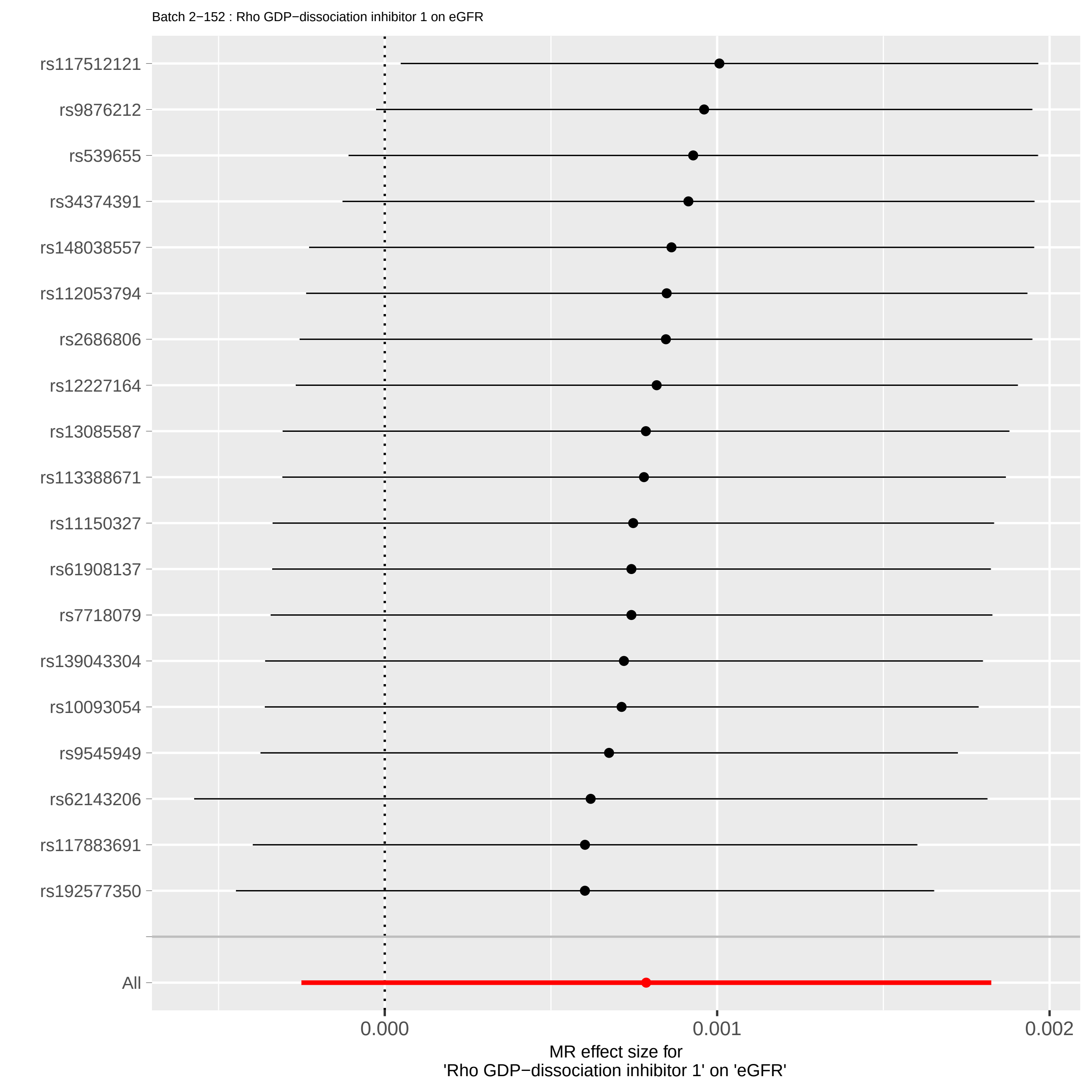

Batch 2−152 : Rho GDP−dissociation inhibitor 1 on eGFR
rs117512121
rs9876212
rs539655
rs34374391
rs148038557
rs112053794
rs2686806
rs12227164
rs13085587
rs113388671
rs11150327
rs61908137
rs7718079
rs139043304
rs10093054
rs9545949
rs62143206
rs117883691
rs192577350
All
0.000
0.001
0.002
MR effect size for
'Rho GDP−dissociation inhibitor 1' on 'eGFR'

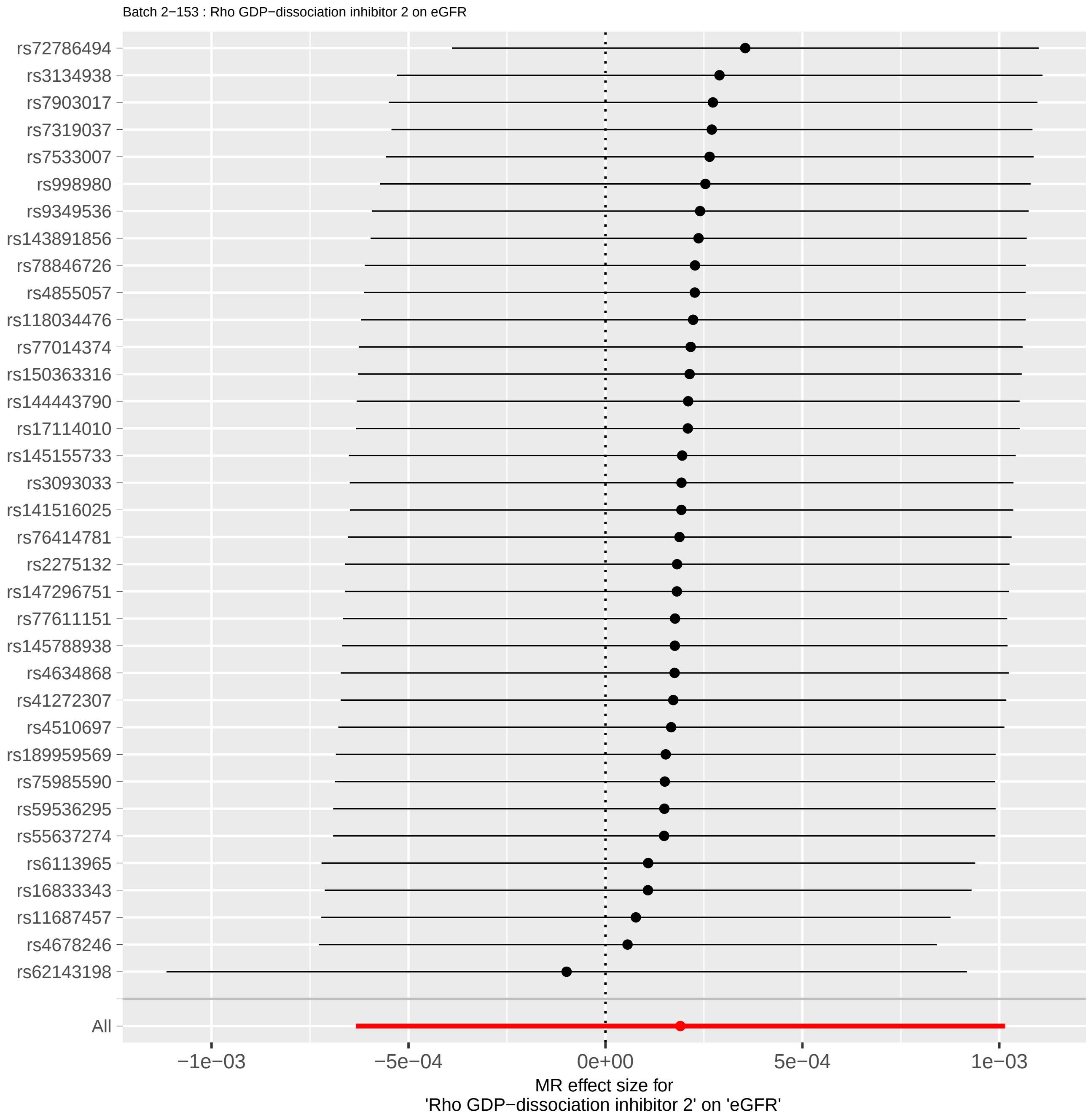

Batch 2−153 : Rho GDP−dissociation inhibitor 2 on eGFR
rs72786494
rs3134938
rs7903017
rs7319037
rs7533007
rs998980
rs9349536
rs143891856
rs78846726
rs4855057
rs118034476
rs77014374
rs150363316
rs144443790
rs17114010
rs145155733
rs3093033
rs141516025
rs76414781
rs2275132
rs147296751
rs77611151
rs145788938
rs4634868
rs41272307
rs4510697
rs189959569
rs75985590
rs59536295
rs55637274
rs6113965
rs16833343
rs11687457
rs4678246
rs62143198
All
−1e−03
−5e−04
0e+00
5e−04
1e−03
MR effect size for
'Rho GDP−dissociation inhibitor 2' on 'eGFR'

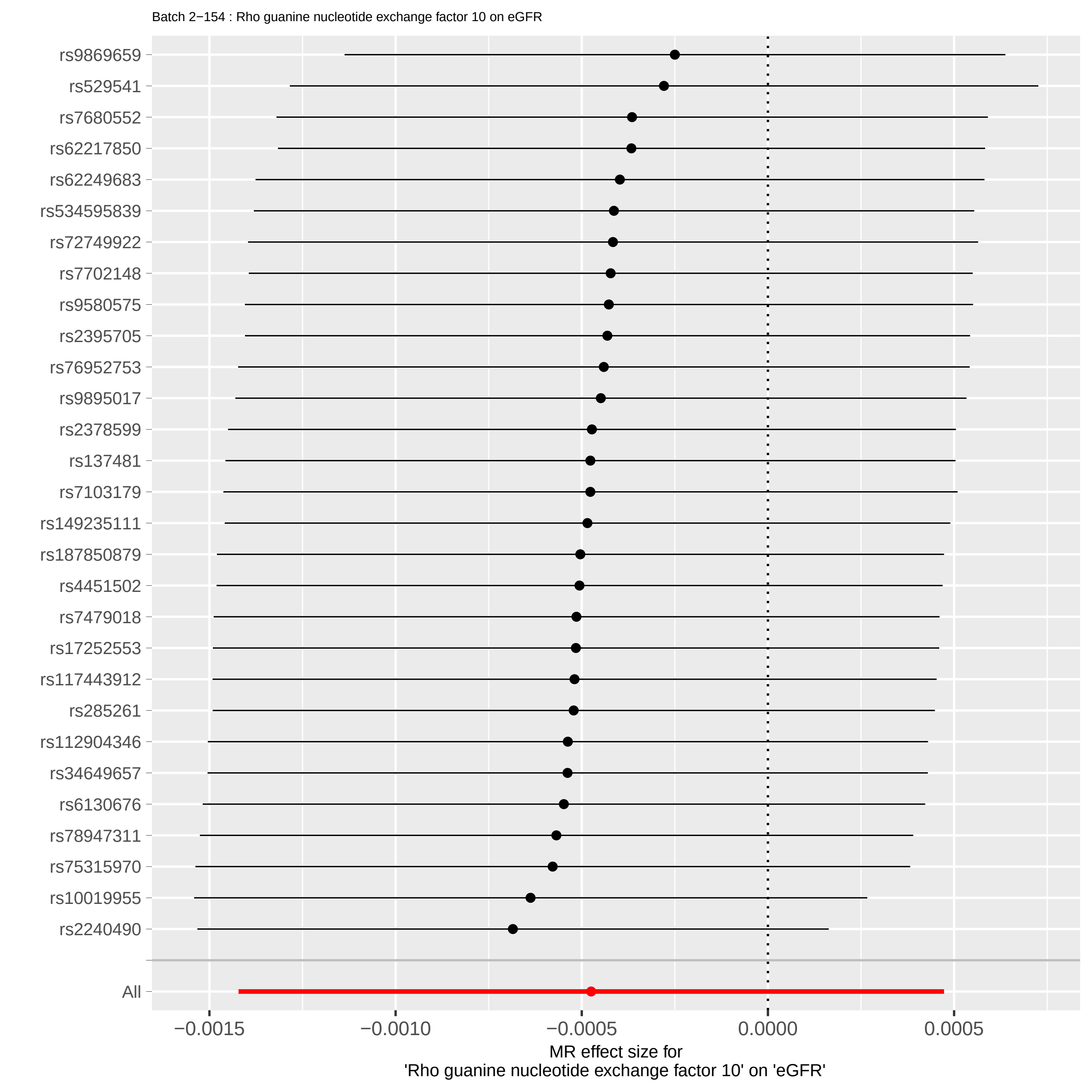

Batch 2−154 : Rho guanine nucleotide exchange factor 10 on eGFR
rs9869659
rs529541
rs7680552
rs62217850
rs62249683
rs534595839
rs72749922
rs7702148
rs9580575
rs2395705
rs76952753
rs9895017
rs2378599
rs137481
rs7103179
rs149235111
rs187850879
rs4451502
rs7479018
rs17252553
rs117443912
rs285261
rs112904346
rs34649657
rs6130676
rs78947311
rs75315970
rs10019955
rs2240490
All
−0.0015
−0.0010
−0.0005
0.0000
0.0005
MR effect size for
'Rho guanine nucleotide exchange factor 10' on 'eGFR'

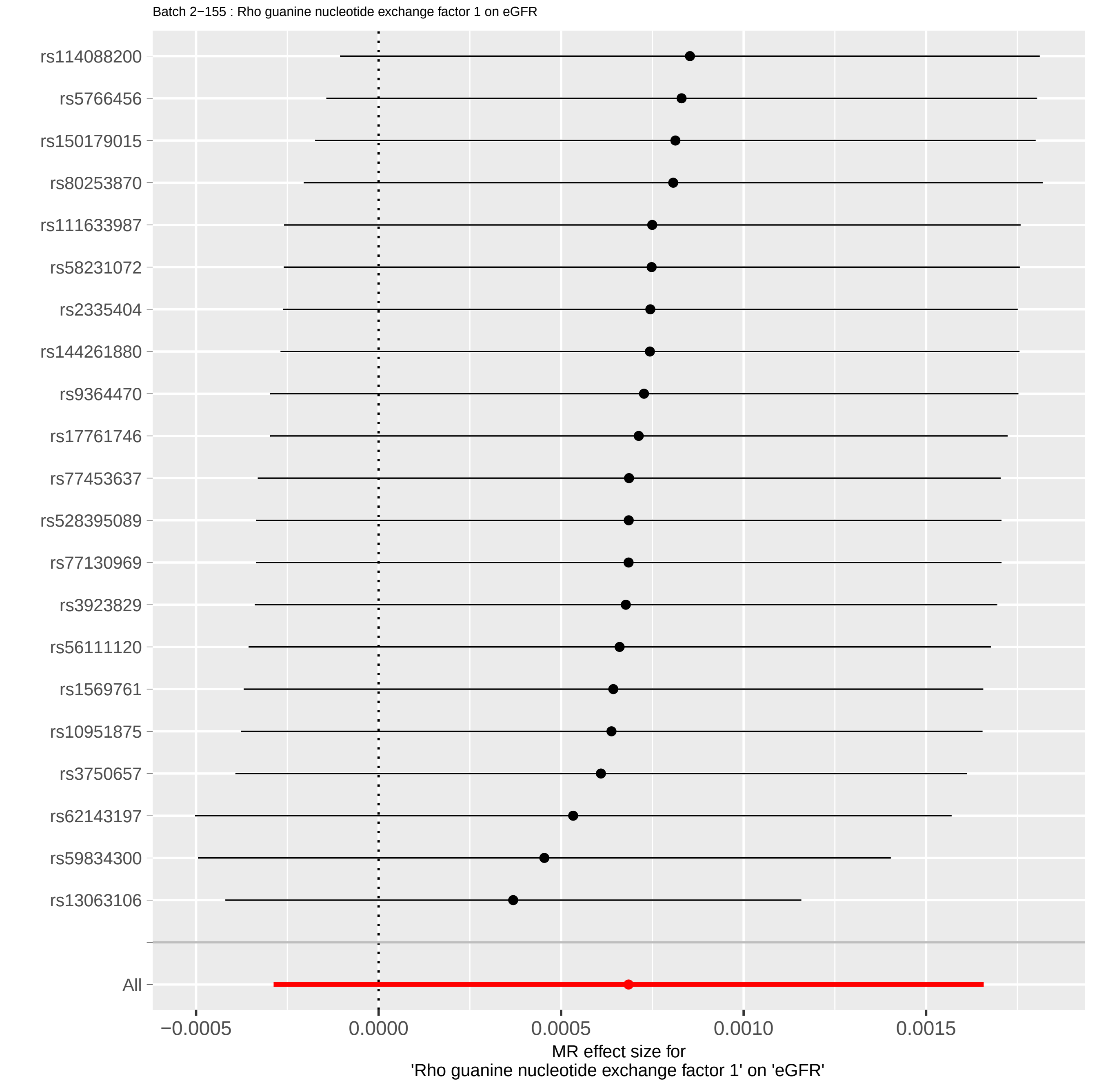
Batch 2−155 : Rho guanine nucleotide exchange factor 1 on eGFR
rs114088200
rs5766456
rs150179015
rs80253870
rs111633987
rs58231072
rs2335404
rs144261880
rs9364470
rs17761746
rs77453637
rs528395089
rs77130969
rs3923829
rs56111120
rs1569761
rs10951875
rs3750657
rs62143197
rs59834300
rs13063106
All
−0.0005
0.0000
0.0005
0.0010
0.0015
MR effect size for
'Rho guanine nucleotide exchange factor 1' on 'eGFR'

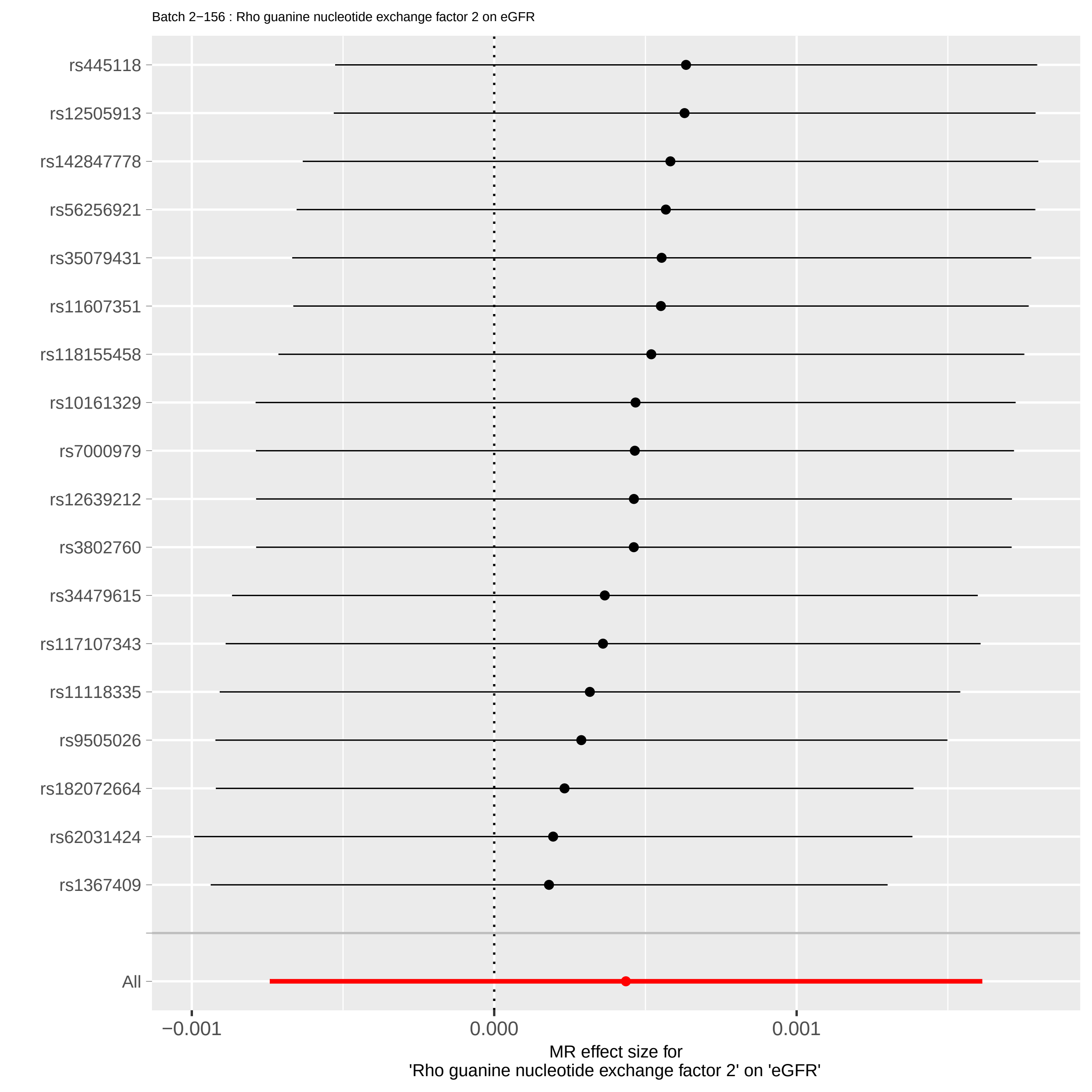
Batch 2−156 : Rho guanine nucleotide exchange factor 2 on eGFR
rs445118
rs12505913
rs142847778
rs56256921
rs35079431
rs11607351
rs118155458
rs10161329
rs7000979
rs12639212
rs3802760
rs34479615
rs117107343
rs11118335
rs9505026
rs182072664
rs62031424
rs1367409
All
−0.001
0.000
0.001
MR effect size for
'Rho guanine nucleotide exchange factor 2' on 'eGFR'

Batch 2−157 : Rho guanine nucleotide exchange factor 25 on eGFR

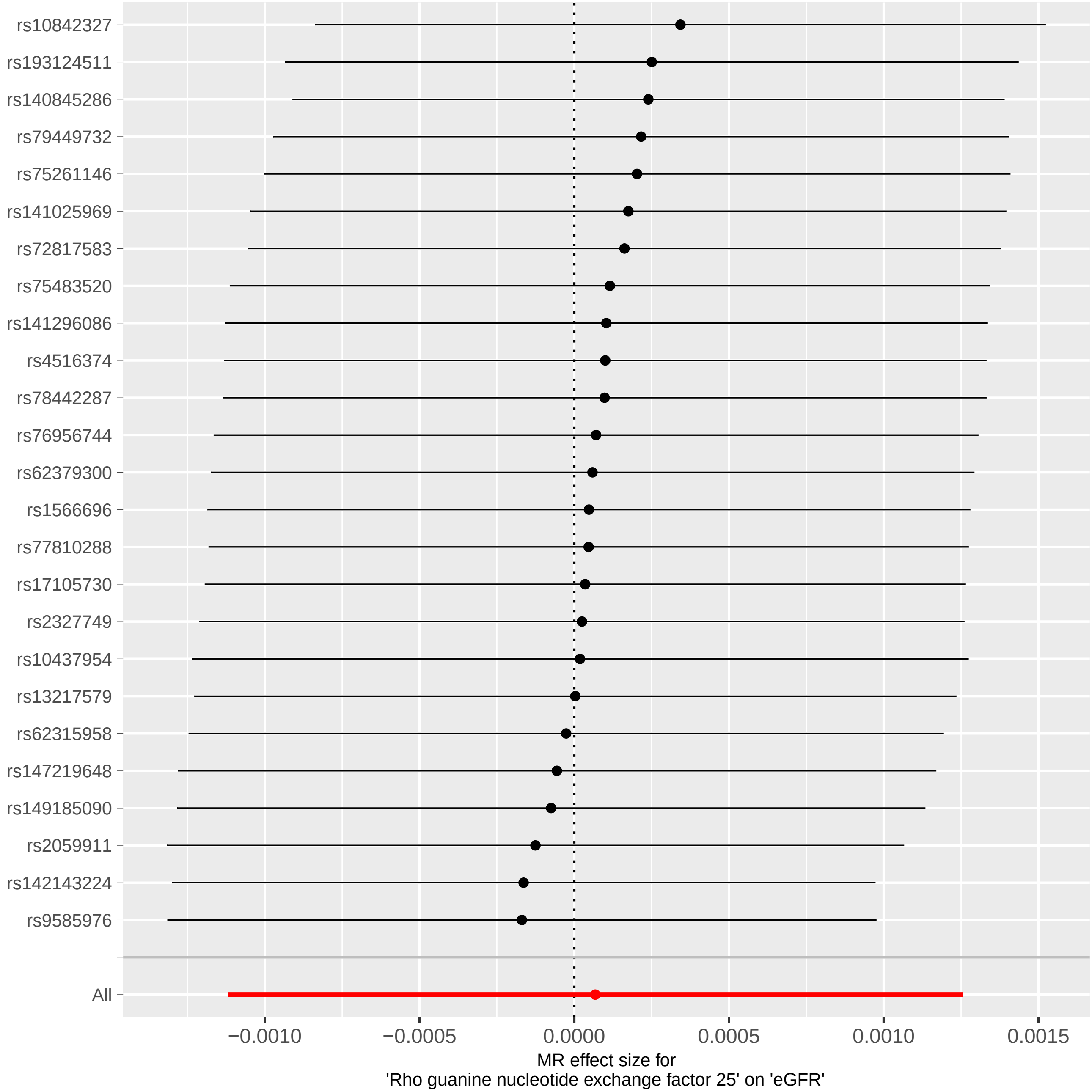

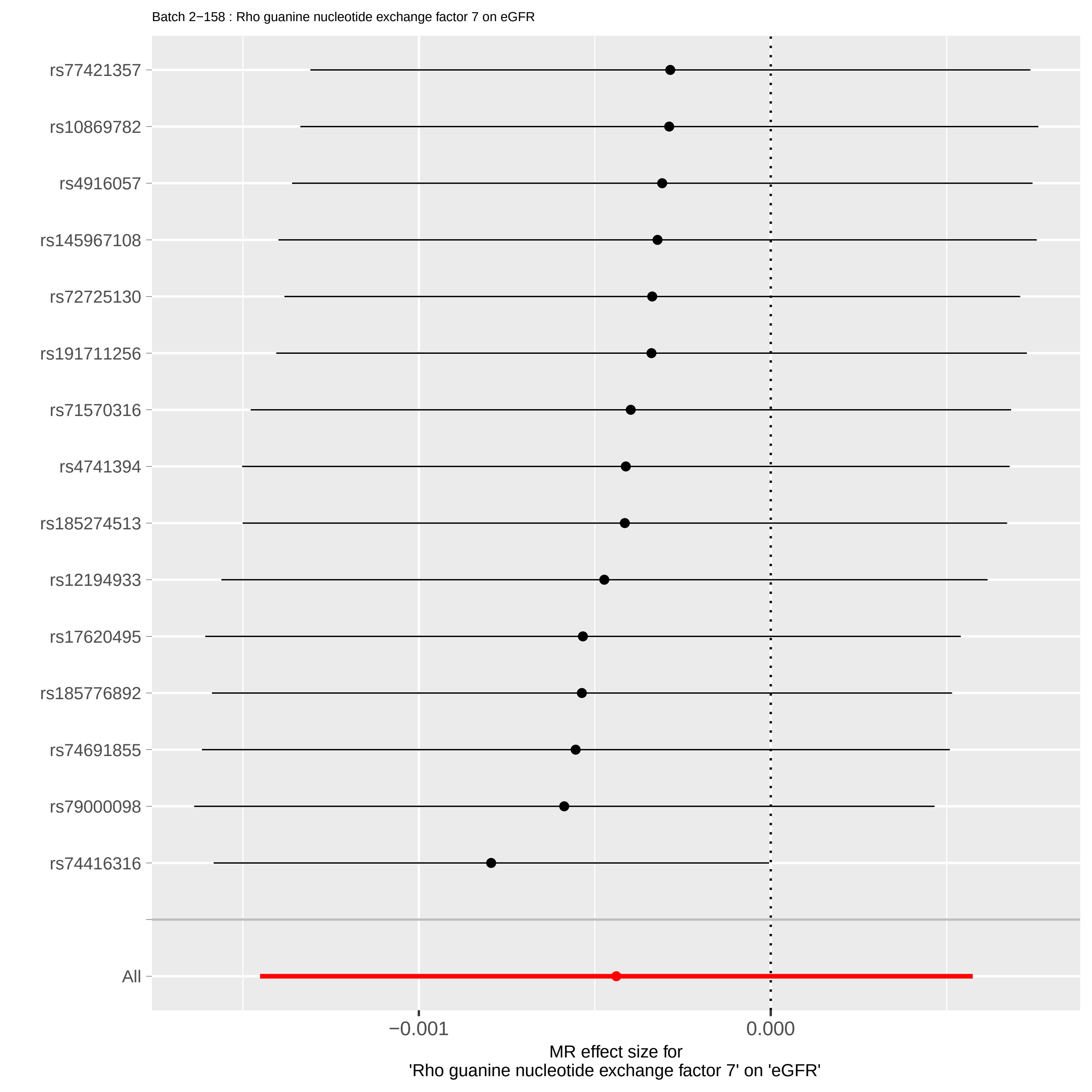
Batch 2−158 : Rho guanine nucleotide exchange factor 7 on eGFR
rs77421357
rs10869782
rs4916057
rs145967108
rs72725130
rs191711256
rs71570316
rs4741394
rs185274513
rs12194933
rs17620495
rs185776892
rs74691855
rs79000098
rs74416316
All
−0.001
0.000
MR effect size for
'Rho guanine nucleotide exchange factor 7' on 'eGFR'

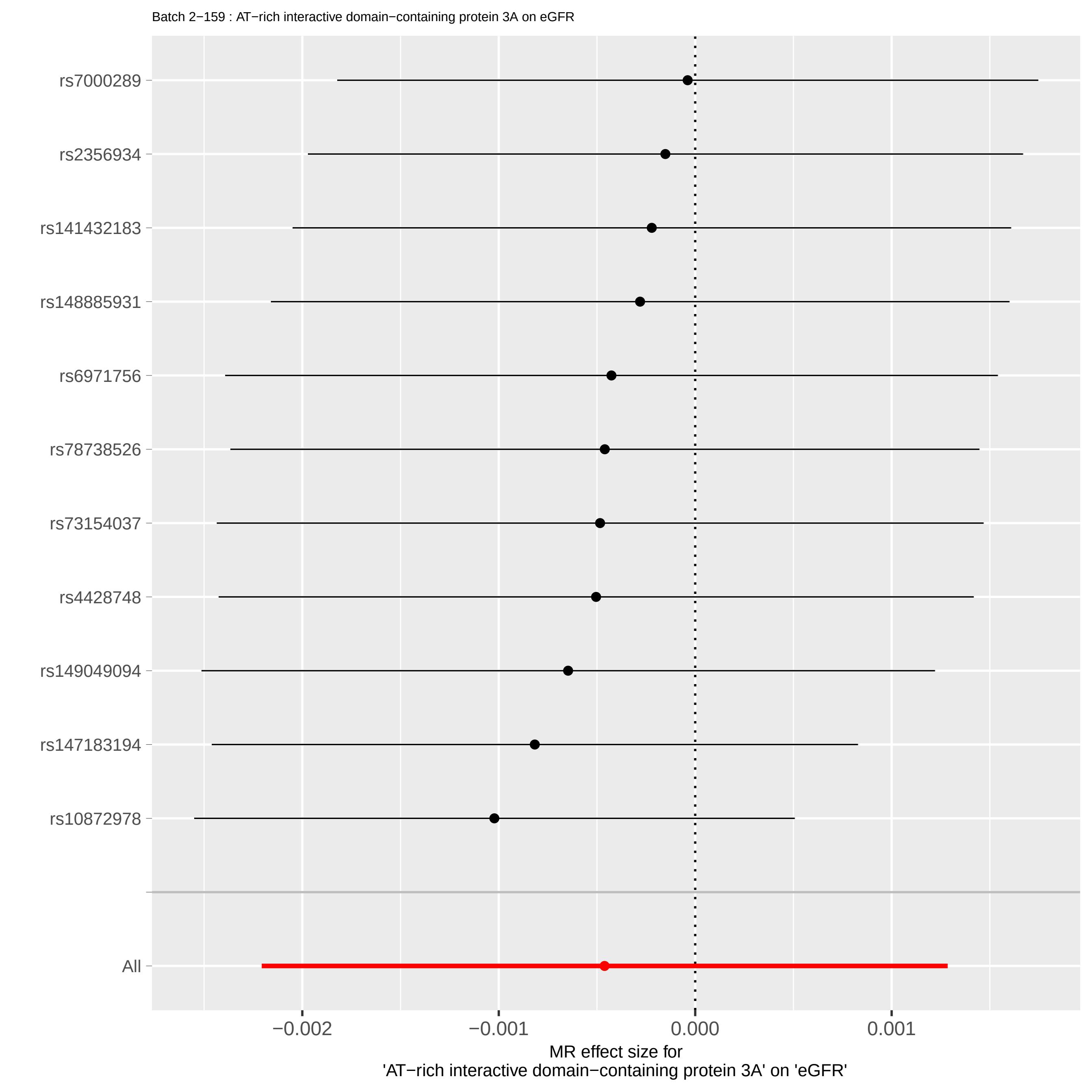

Batch 2−159 : AT−rich interactive domain−containing protein 3A on eGFR
rs7000289
rs2356934
rs141432183
rs148885931
rs6971756
rs78738526
rs73154037
rs4428748
rs149049094
rs147183194
rs10872978
All
−0.002
−0.001
0.000
0.001
MR effect size for
'AT−rich interactive domain−containing protein 3A' on 'eGFR'

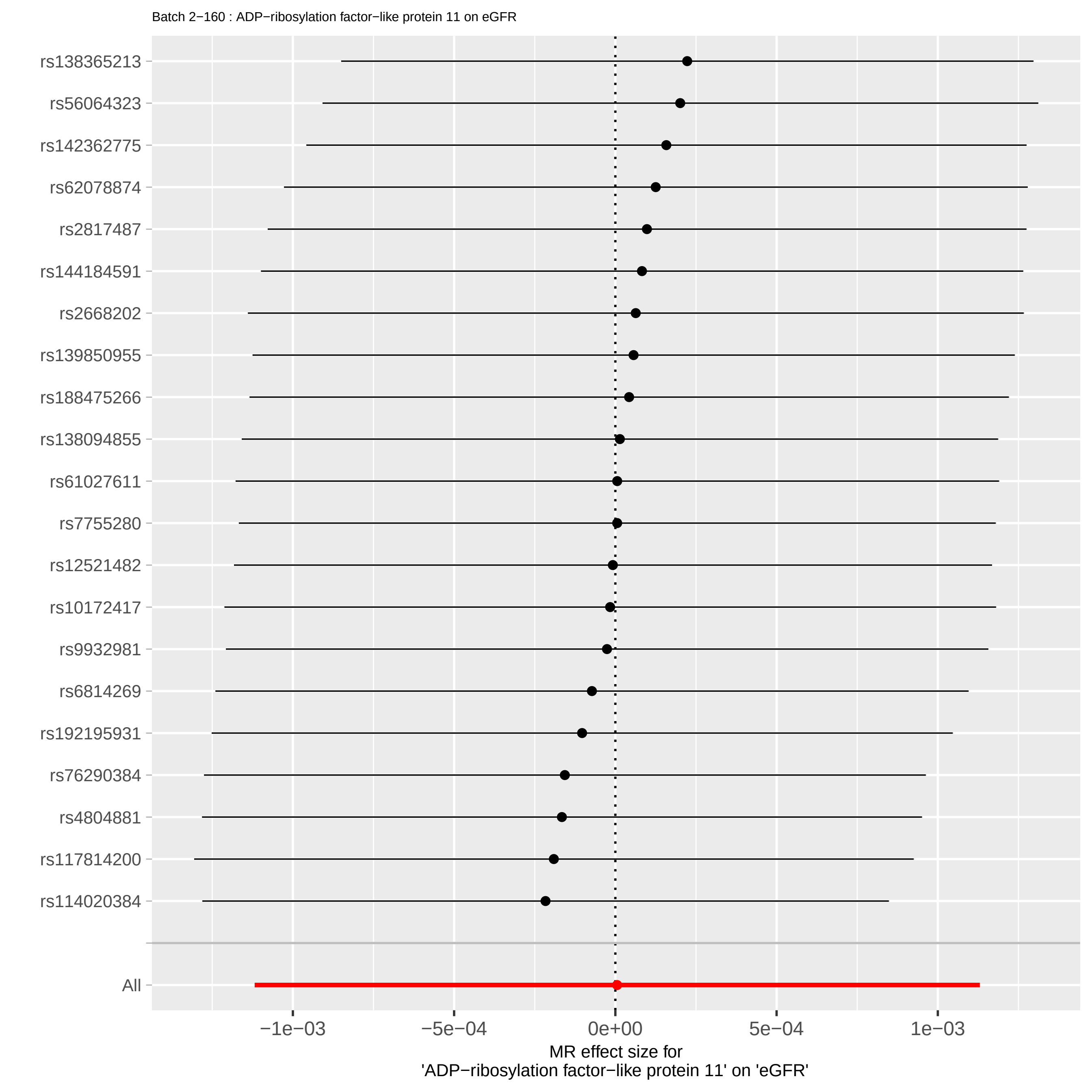

Batch 2−160 : ADP−ribosylation factor−like protein 11 on eGFR
rs138365213
rs56064323
rs142362775
rs62078874
rs2817487
rs144184591
rs2668202
rs139850955
rs188475266
rs138094855
rs61027611
rs7755280
rs12521482
rs10172417
rs9932981
rs6814269
rs192195931
rs76290384
rs4804881
rs117814200
rs114020384
All
−1e−03
−5e−04
0e+00
5e−04
1e−03
MR effect size for
'ADP−ribosylation factor−like protein 11' on 'eGFR'

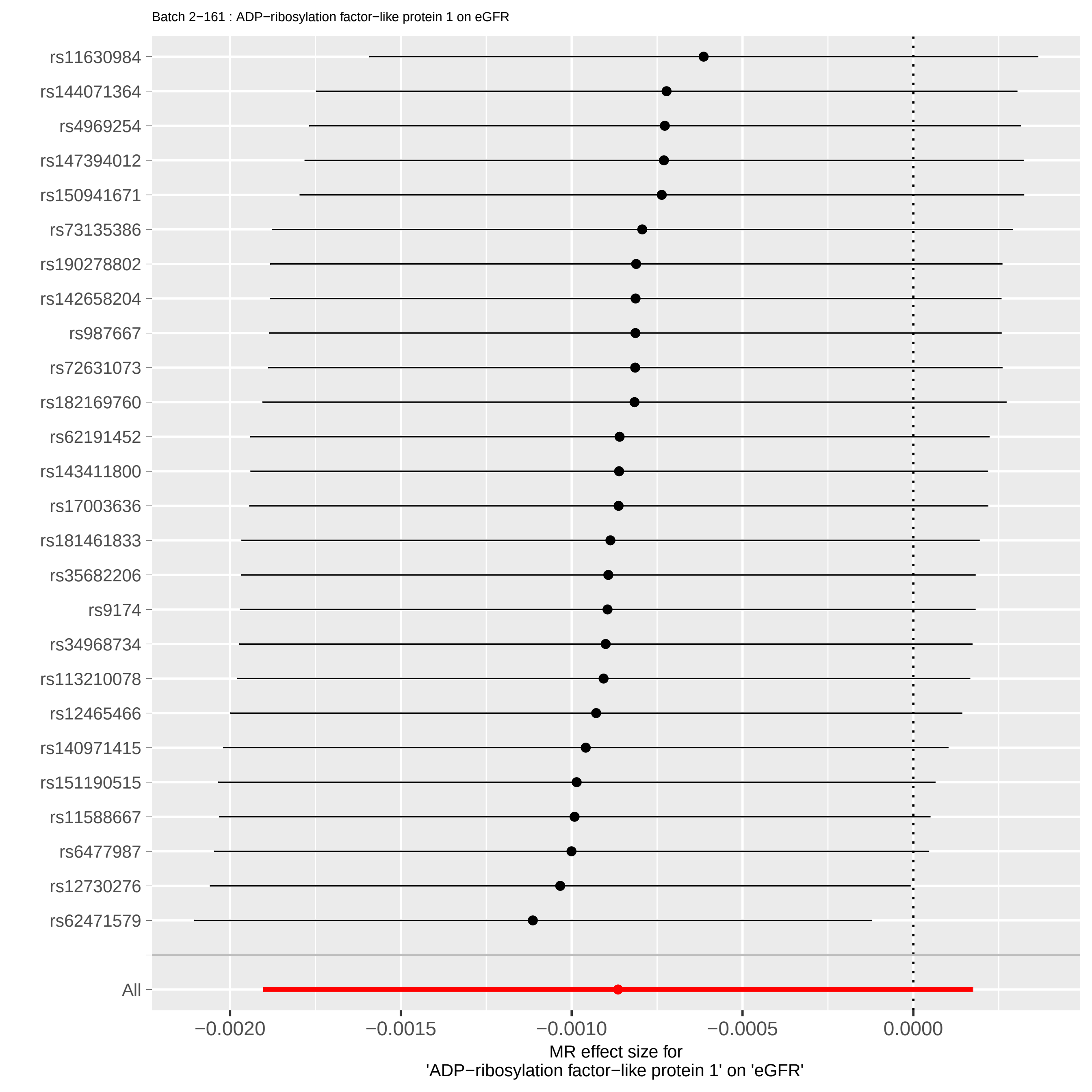
Batch 2−161 : ADP−ribosylation factor−like protein 1 on eGFR
rs11630984
rs144071364
rs4969254
rs147394012
rs150941671
rs73135386
rs190278802
rs142658204
rs987667
rs72631073
rs182169760
rs62191452
rs143411800
rs17003636
rs181461833
rs35682206
rs9174
rs34968734
rs113210078
rs12465466
rs140971415
rs151190515
rs11588667
rs6477987
rs12730276
rs62471579
All
−0.0020
−0.0015
−0.0010
−0.0005
0.0000
MR effect size for
'ADP−ribosylation factor−like protein 1' on 'eGFR'

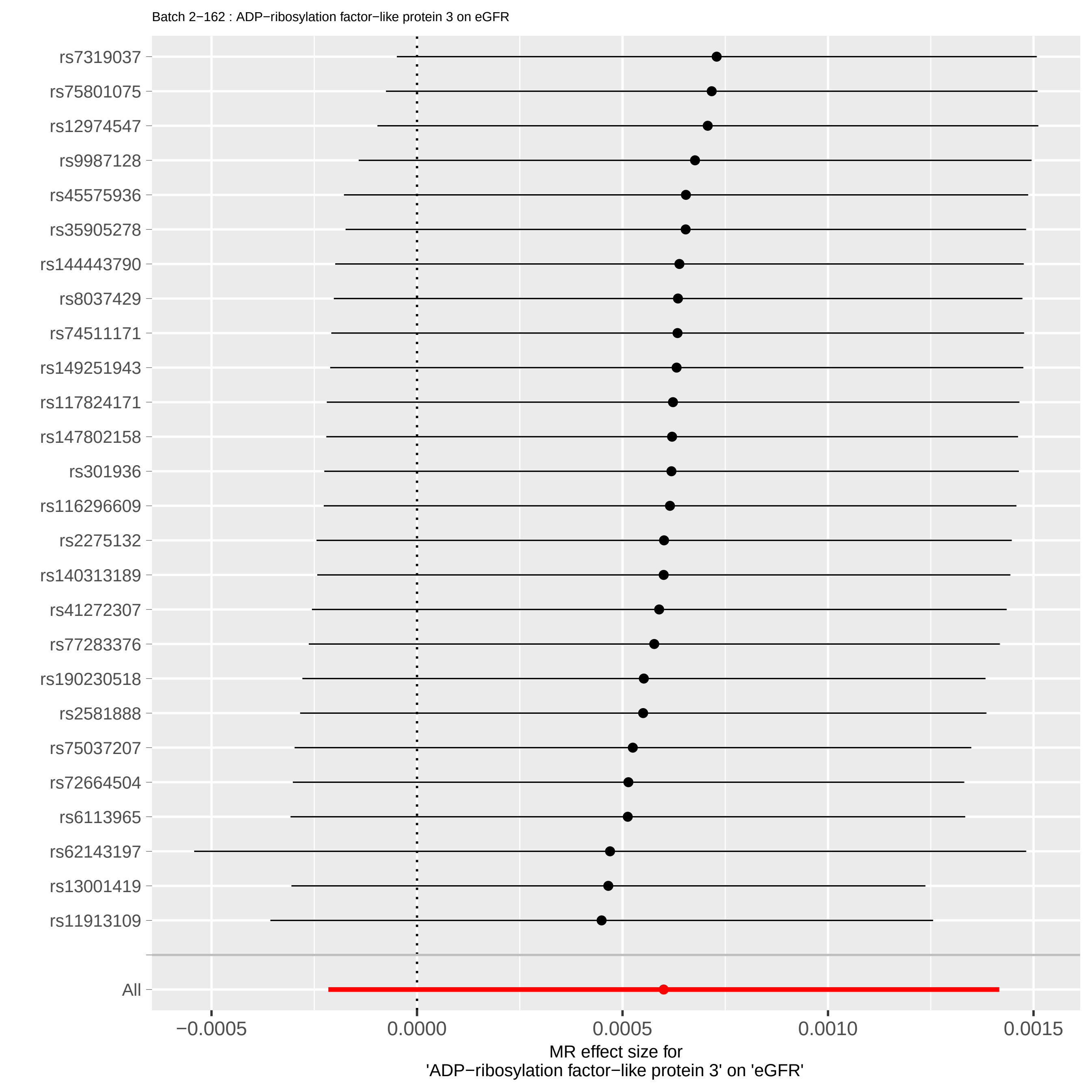
Batch 2−162 : ADP−ribosylation factor−like protein 3 on eGFR
rs7319037
rs75801075
rs12974547
rs9987128
rs45575936
rs35905278
rs144443790
rs8037429
rs74511171
rs149251943
rs117824171
rs147802158
rs301936
rs116296609
rs2275132
rs140313189
rs41272307
rs77283376
rs190230518
rs2581888
rs75037207
rs72664504
rs6113965
rs62143197
rs13001419
rs11913109
All
−0.0005
0.0000
0.0005
0.0010
0.0015
MR effect size for
'ADP−ribosylation factor−like protein 3' on 'eGFR'

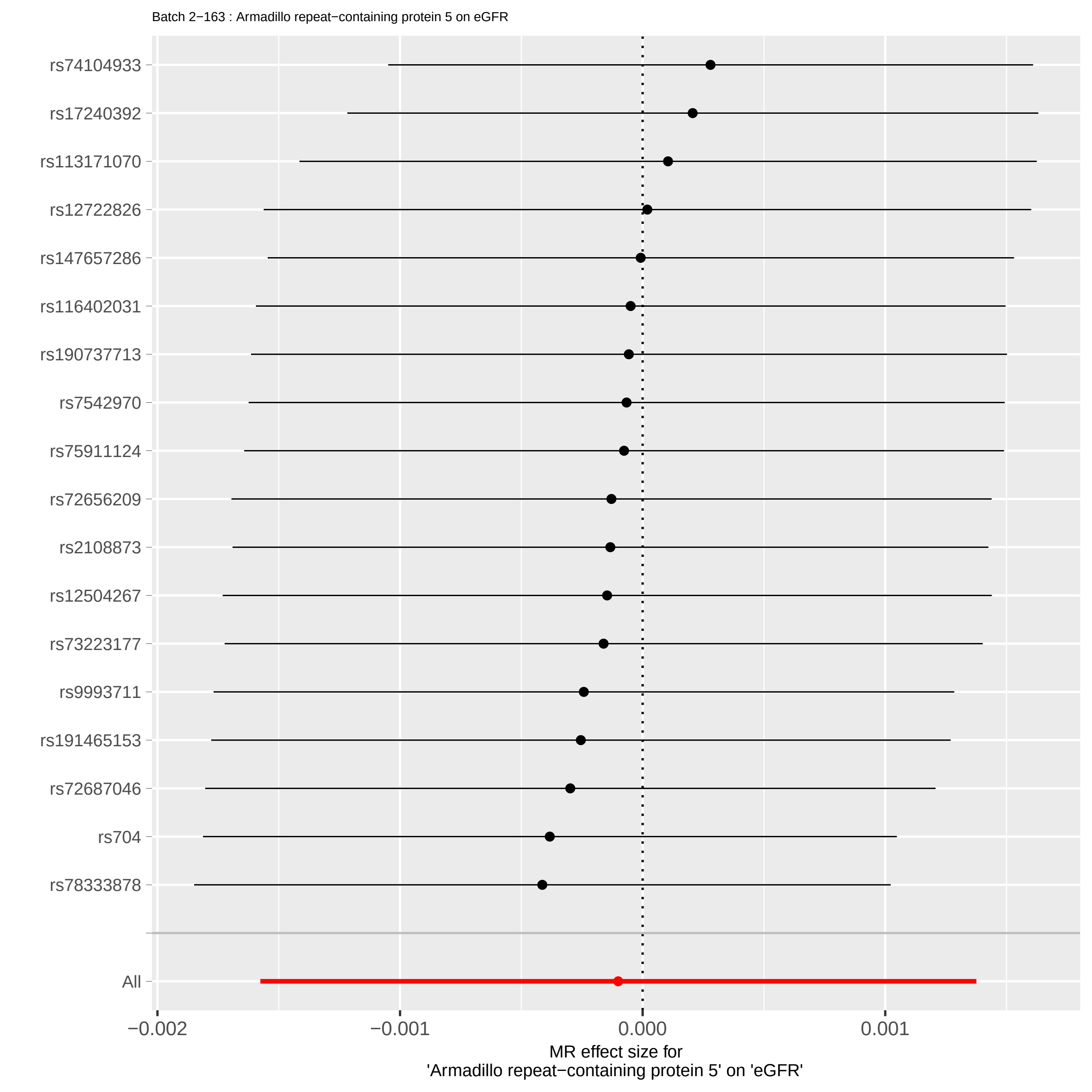

Batch 2−163 : Armadillo repeat−containing protein 5 on eGFR
rs74104933
rs17240392
rs113171070
rs12722826
rs147657286
rs116402031
rs190737713
rs7542970
rs75911124
rs72656209
rs2108873
rs12504267
rs73223177
rs9993711
rs191465153
rs72687046
rs704
rs78333878
All
−0.002
−0.001
0.000
0.001
MR effect size for
'Armadillo repeat−containing protein 5' on 'eGFR'

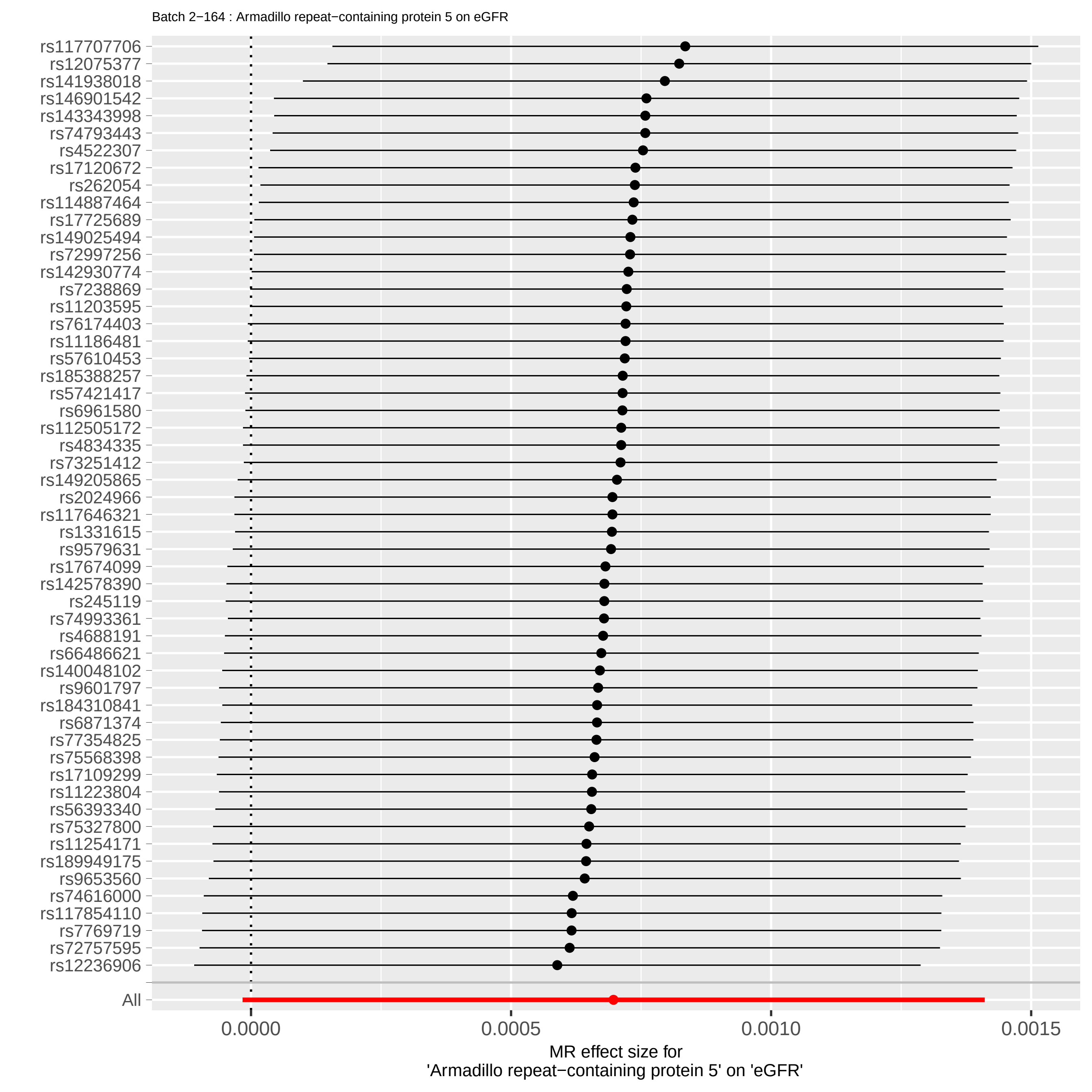

Batch 2−164 : Armadillo repeat−containing protein 5 on eGFR
rs117707706
rs12075377
rs141938018
rs146901542
rs143343998
rs74793443
rs4522307
rs17120672
rs262054
rs114887464
rs17725689
rs149025494
rs72997256
rs142930774
rs7238869
rs11203595
rs76174403
rs11186481
rs57610453
rs185388257
rs57421417
rs6961580
rs112505172
rs4834335
rs73251412
rs149205865
rs2024966
rs117646321
rs1331615
rs9579631
rs17674099
rs142578390
rs245119
rs74993361
rs4688191
rs66486621
rs140048102
rs9601797
rs184310841
rs6871374
rs77354825
rs75568398
rs17109299
rs11223804
rs56393340
rs75327800
rs11254171
rs189949175
rs9653560
rs74616000
rs117854110
rs7769719
rs72757595
rs12236906
All
0.0000
0.0005
0.0010
0.0015
MR effect size for
'Armadillo repeat−containing protein 5' on 'eGFR'

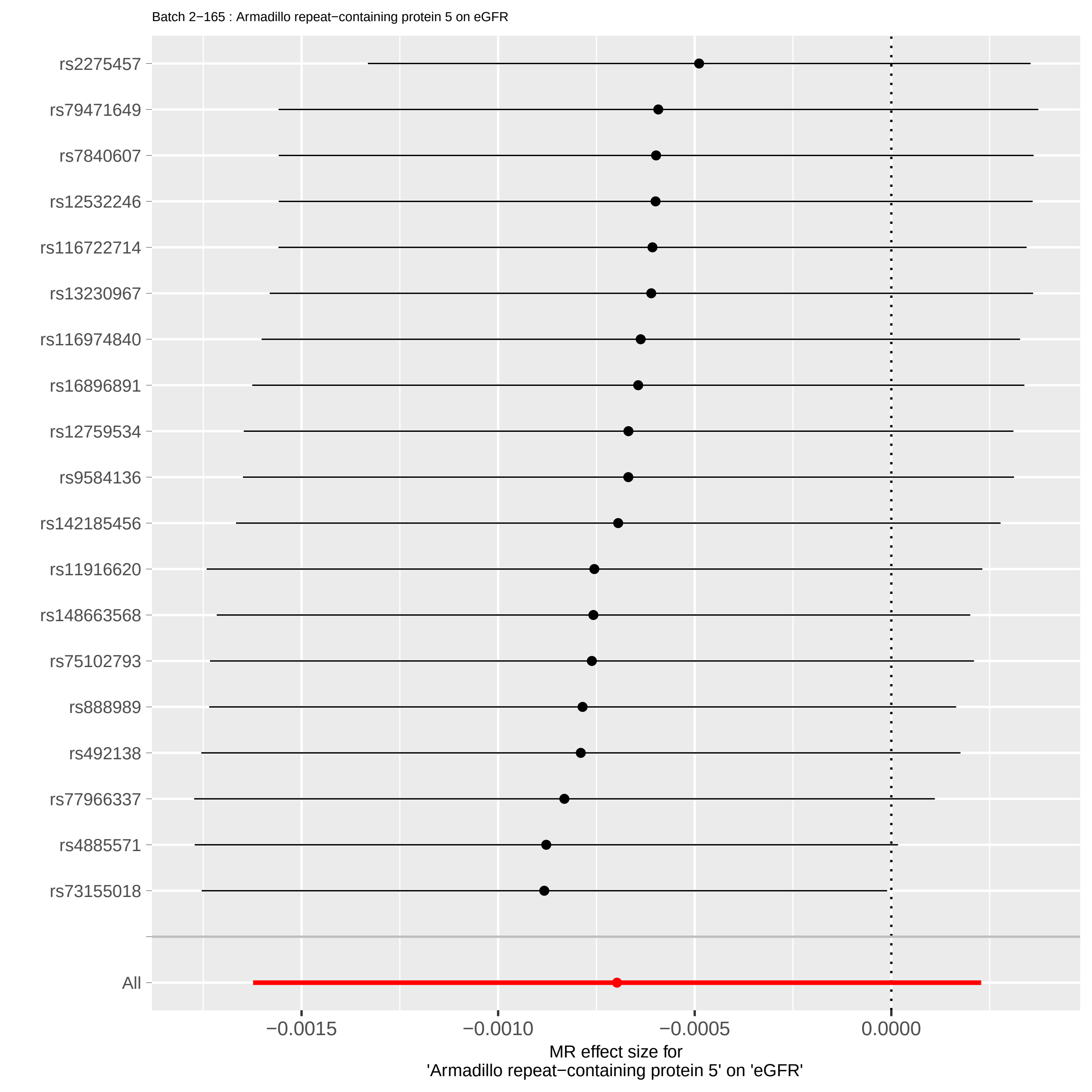
Batch 2−165 : Armadillo repeat−containing protein 5 on eGFR
rs2275457
rs79471649
rs7840607
rs12532246
rs116722714
rs13230967
rs116974840
rs16896891
rs12759534
rs9584136
rs142185456
rs11916620
rs148663568
rs75102793
rs888989
rs492138
rs77966337
rs4885571
rs73155018
All
−0.0015
−0.0010
−0.0005
0.0000
MR effect size for
'Armadillo repeat−containing protein 5' on 'eGFR'

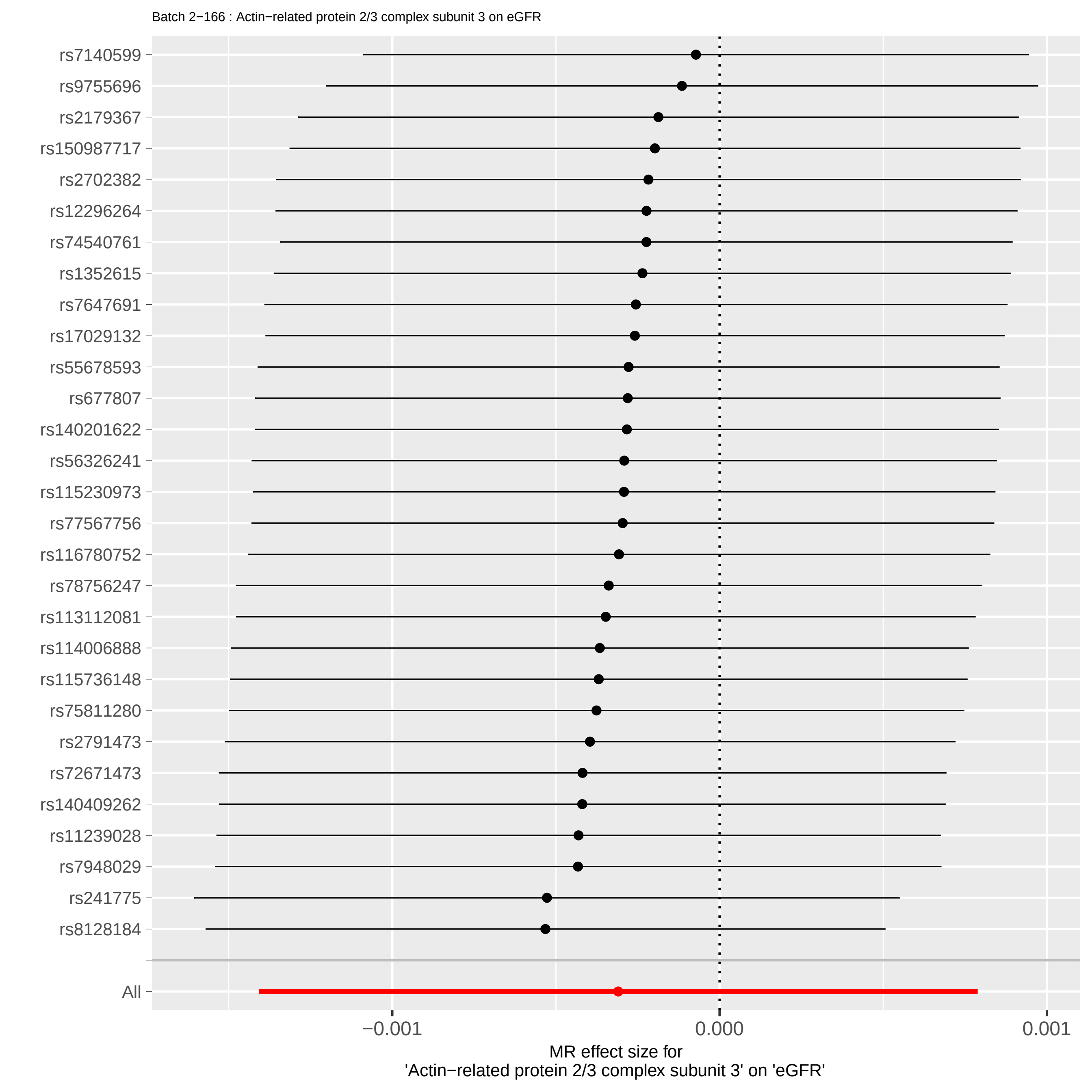

Batch 2−166 : Actin−related protein 2/3 complex subunit 3 on eGFR
rs7140599
rs9755696
rs2179367
rs150987717
rs2702382
rs12296264
rs74540761
rs1352615
rs7647691
rs17029132
rs55678593
rs677807
rs140201622
rs56326241
rs115230973
rs77567756
rs116780752
rs78756247
rs113112081
rs114006888
rs115736148
rs75811280
rs2791473
rs72671473
rs140409262
rs11239028
rs7948029
rs241775
rs8128184
All
−0.001
0.000
0.001
MR effect size for
'Actin−related protein 2/3 complex subunit 3' on 'eGFR'

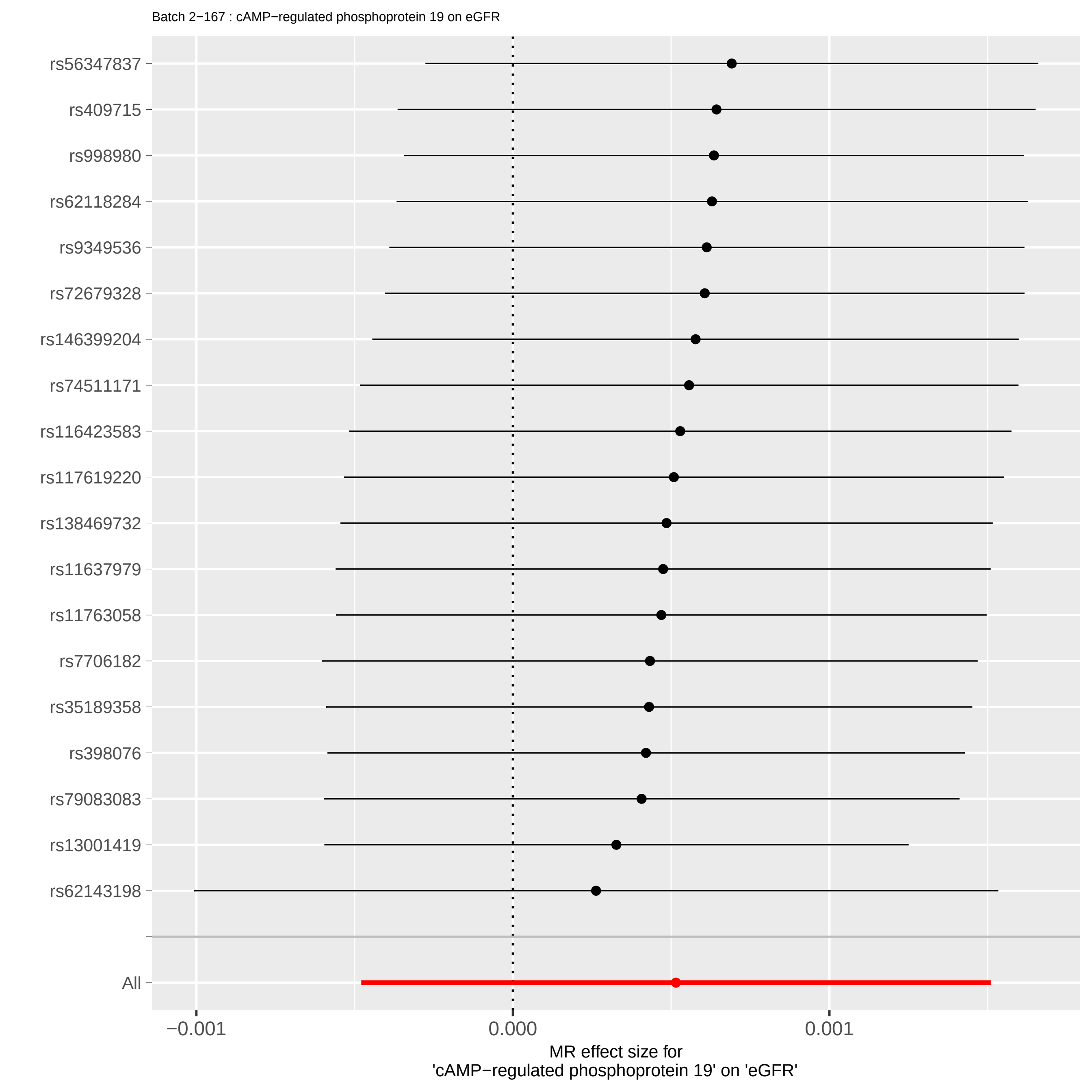

Batch 2−167 : cAMP−regulated phosphoprotein 19 on eGFR
rs56347837
rs409715
rs998980
rs62118284
rs9349536
rs72679328
rs146399204
rs74511171
rs116423583
rs117619220
rs138469732
rs11637979
rs11763058
rs7706182
rs35189358
rs398076
rs79083083
rs13001419
rs62143198
All
−0.001
0.000
0.001
MR effect size for
'cAMP−regulated phosphoprotein 19' on 'eGFR'

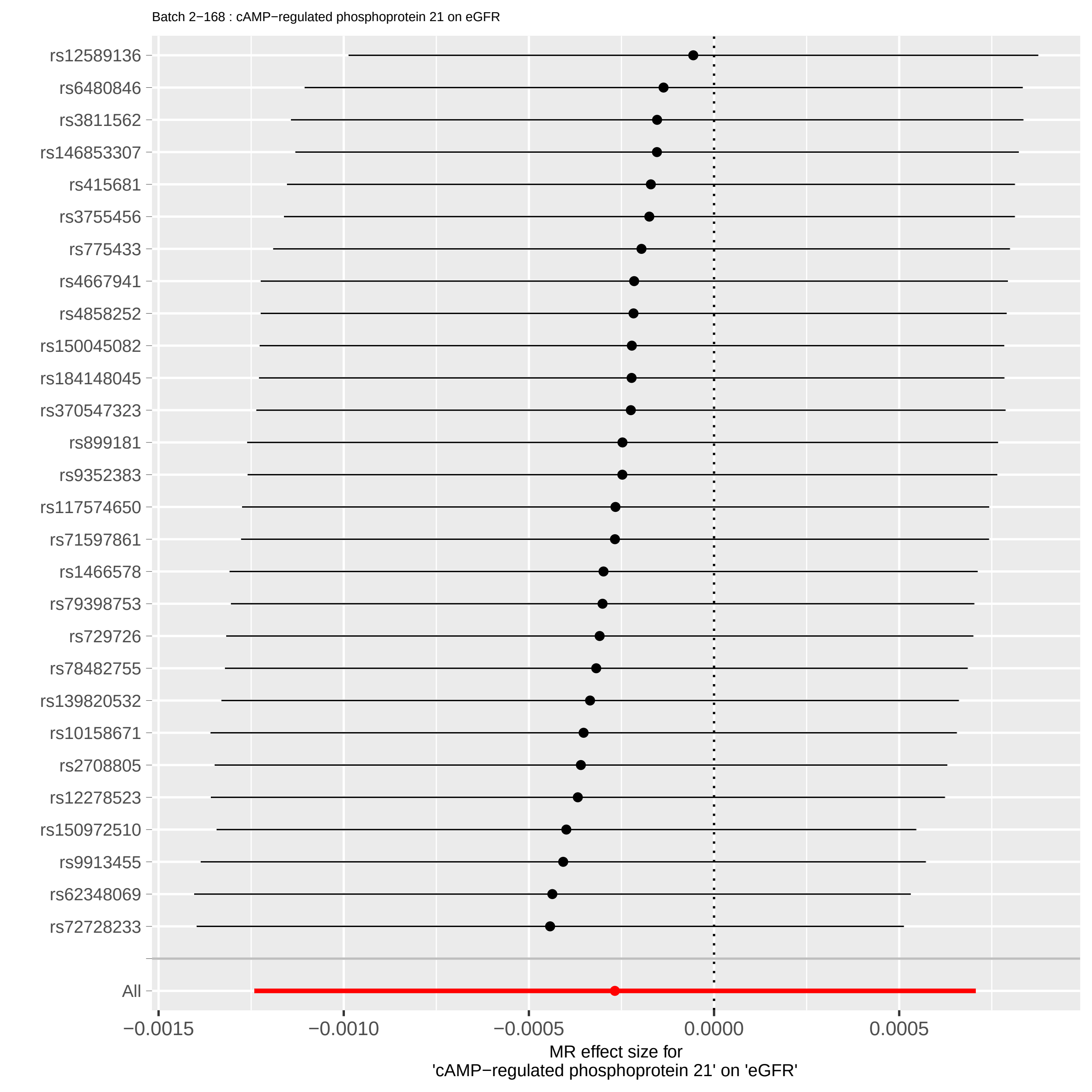
Batch 2−168 : cAMP−regulated phosphoprotein 21 on eGFR
rs12589136
rs6480846
rs3811562
rs146853307
rs415681
rs3755456
rs775433
rs4667941
rs4858252
rs150045082
rs184148045
rs370547323
rs899181
rs9352383
rs117574650
rs71597861
rs1466578
rs79398753
rs729726
rs78482755
rs139820532
rs10158671
rs2708805
rs12278523
rs150972510
rs9913455
rs62348069
rs72728233
All
−0.0015
−0.0010
−0.0005
0.0000
0.0005
MR effect size for
'cAMP−regulated phosphoprotein 21' on 'eGFR'

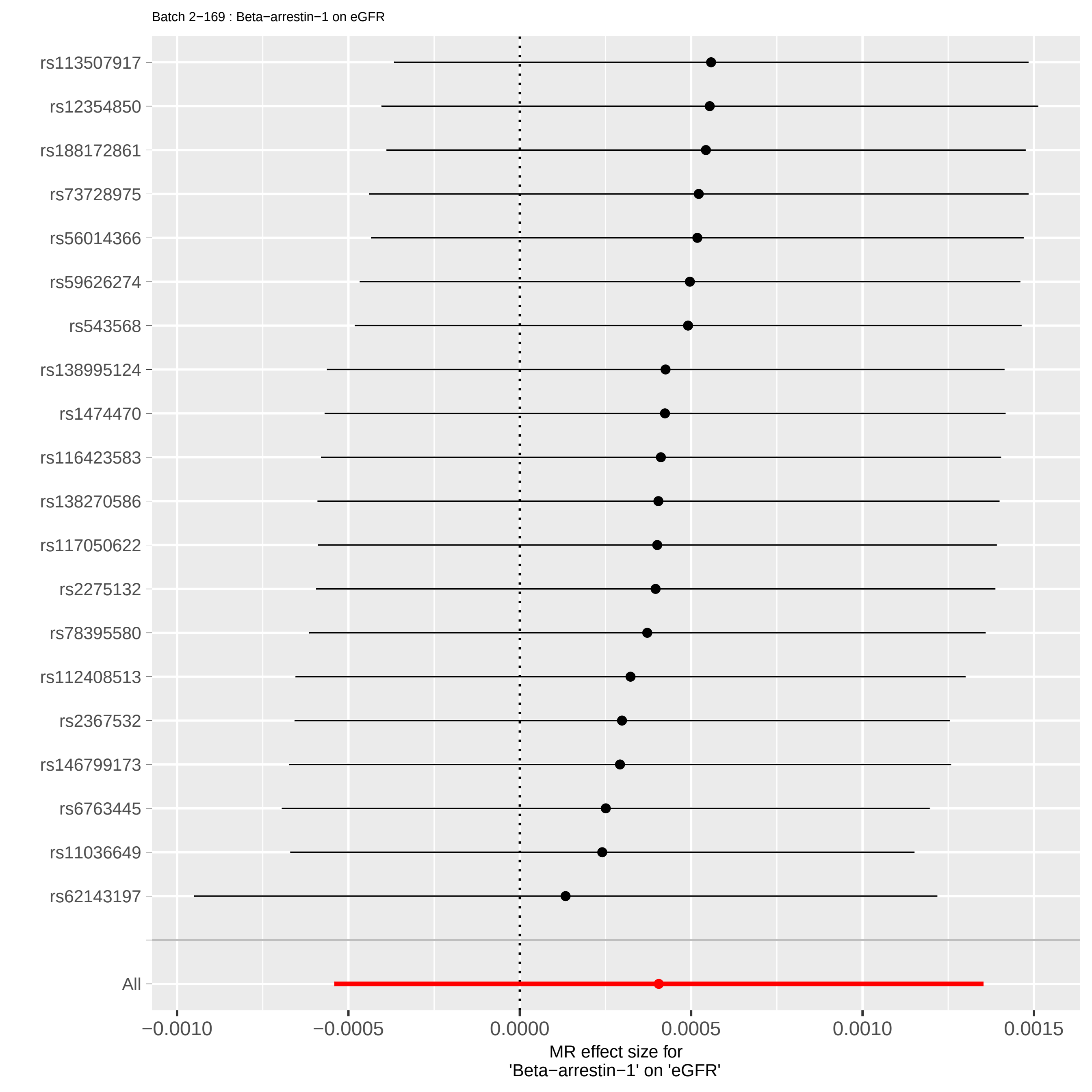

Batch 2−169 : Beta−arrestin−1 on eGFR
rs113507917
rs12354850
rs188172861
rs73728975
rs56014366
rs59626274
rs543568
rs138995124
rs1474470
rs116423583
rs138270586
rs117050622
rs2275132
rs78395580
rs112408513
rs2367532
rs146799173
rs6763445
rs11036649
rs62143197
All
−0.0010
−0.0005
0.0000
0.0005
0.0010
0.0015
MR effect size for
'Beta−arrestin−1' on 'eGFR'

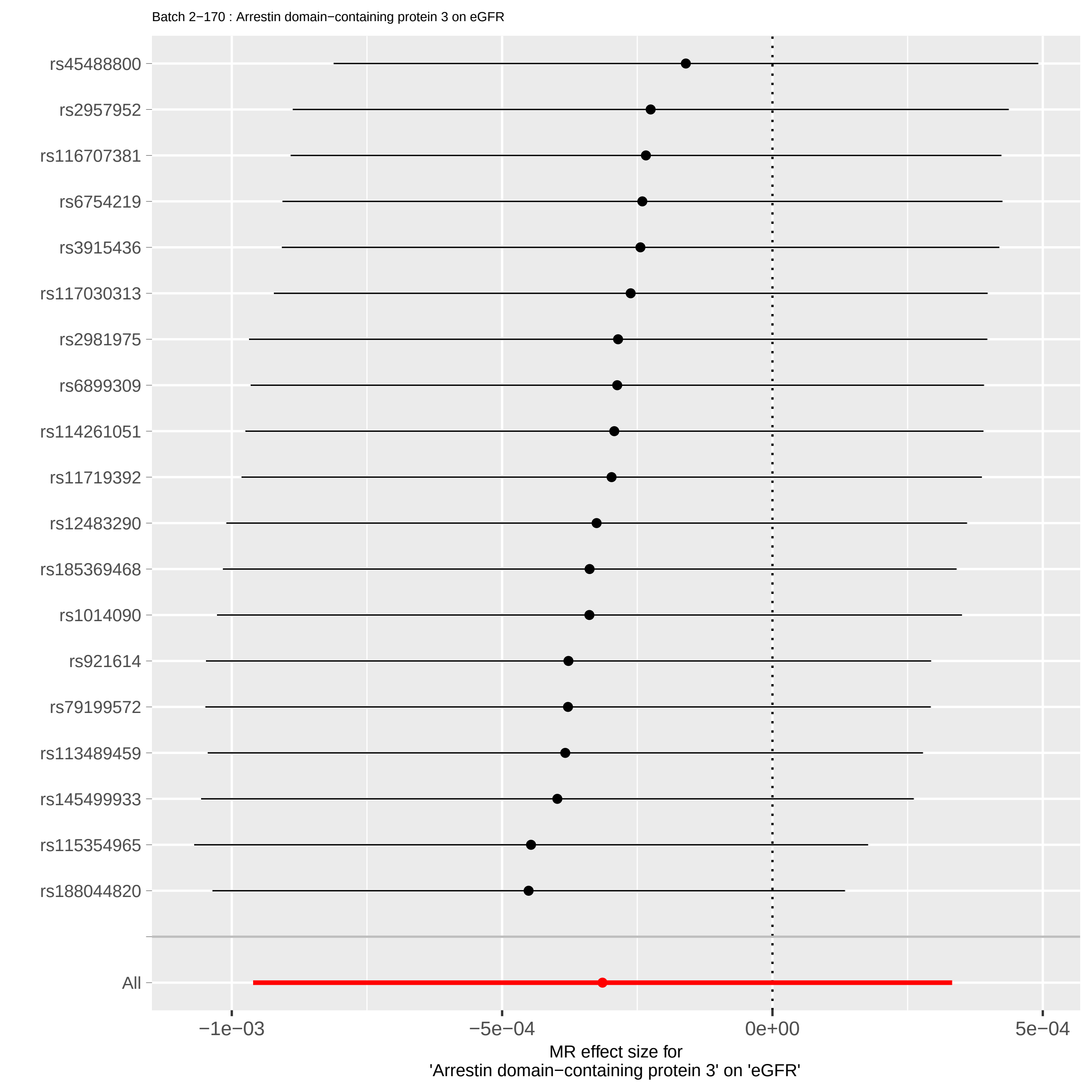

Batch 2−170 : Arrestin domain−containing protein 3 on eGFR
rs45488800
rs2957952
rs116707381
rs6754219
rs3915436
rs117030313
rs2981975
rs6899309
rs114261051
rs11719392
rs12483290
rs185369468
rs1014090
rs921614
rs79199572
rs113489459
rs145499933
rs115354965
rs188044820
All
−1e−03
−5e−04
0e+00
5e−04
MR effect size for
'Arrestin domain−containing protein 3' on 'eGFR'

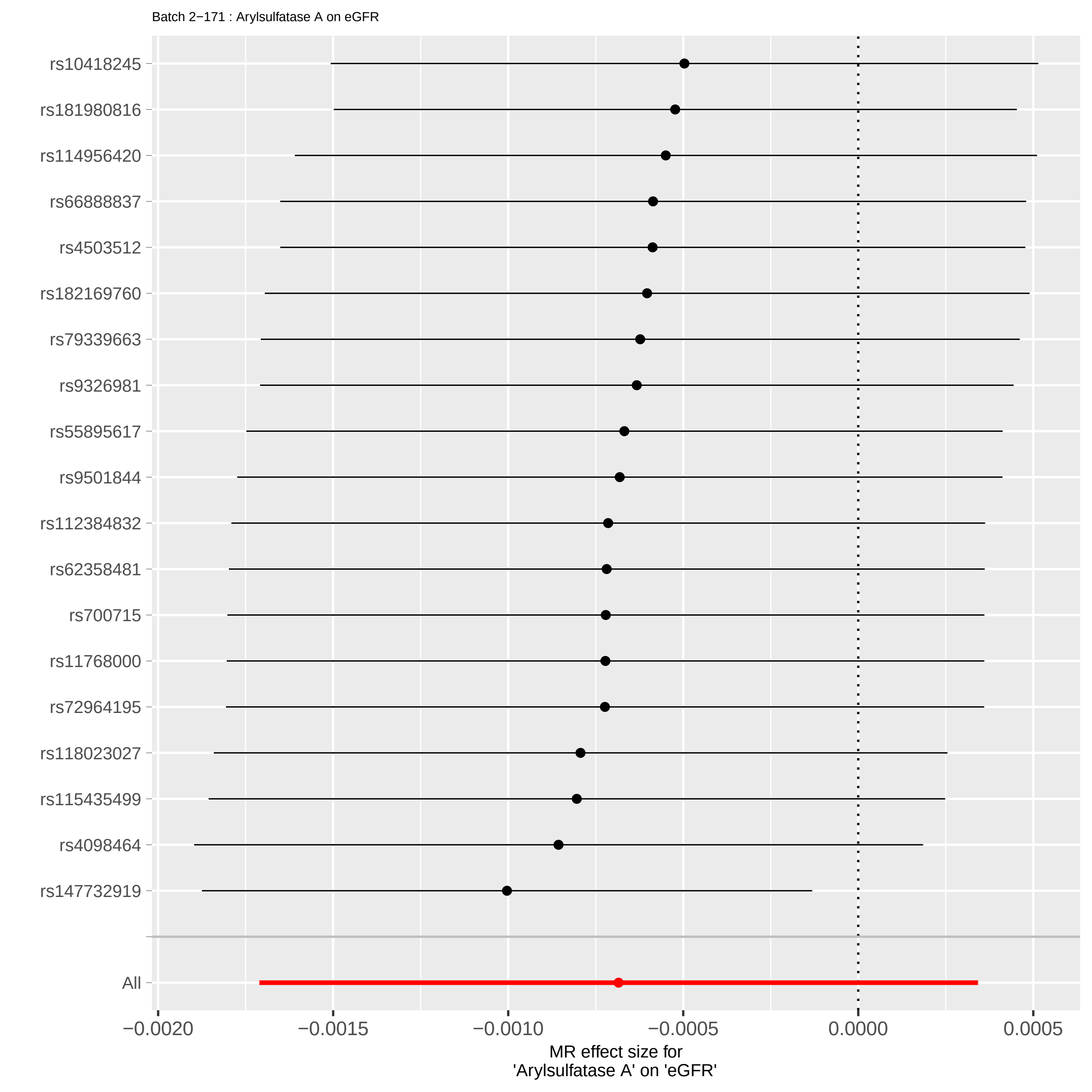

Batch 2−171 : Arylsulfatase A on eGFR
rs10418245
rs181980816
rs114956420
rs66888837
rs4503512
rs182169760
rs79339663
rs9326981
rs55895617
rs9501844
rs112384832
rs62358481
rs700715
rs11768000
rs72964195
rs118023027
rs115435499
rs4098464
rs147732919
All
−0.0020
−0.0015
−0.0010
−0.0005
0.0000
0.0005
MR effect size for
'Arylsulfatase A' on 'eGFR'

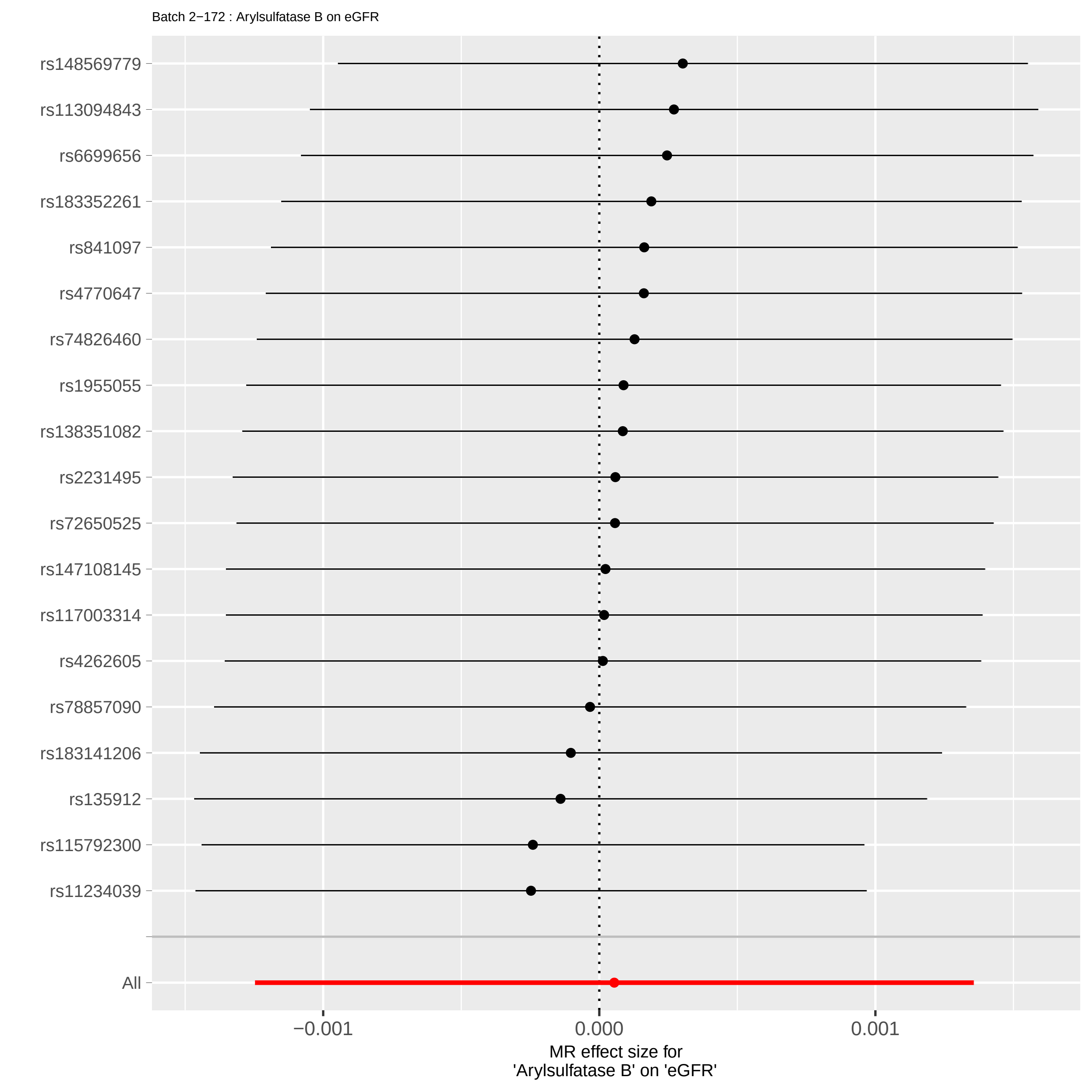

Batch 2−172 : Arylsulfatase B on eGFR
rs148569779
rs113094843
rs6699656
rs183352261
rs841097
rs4770647
rs74826460
rs1955055
rs138351082
rs2231495
rs72650525
rs147108145
rs117003314
rs4262605
rs78857090
rs183141206
rs135912
rs115792300
rs11234039
All
−0.001
0.000
0.001
MR effect size for
'Arylsulfatase B' on 'eGFR'

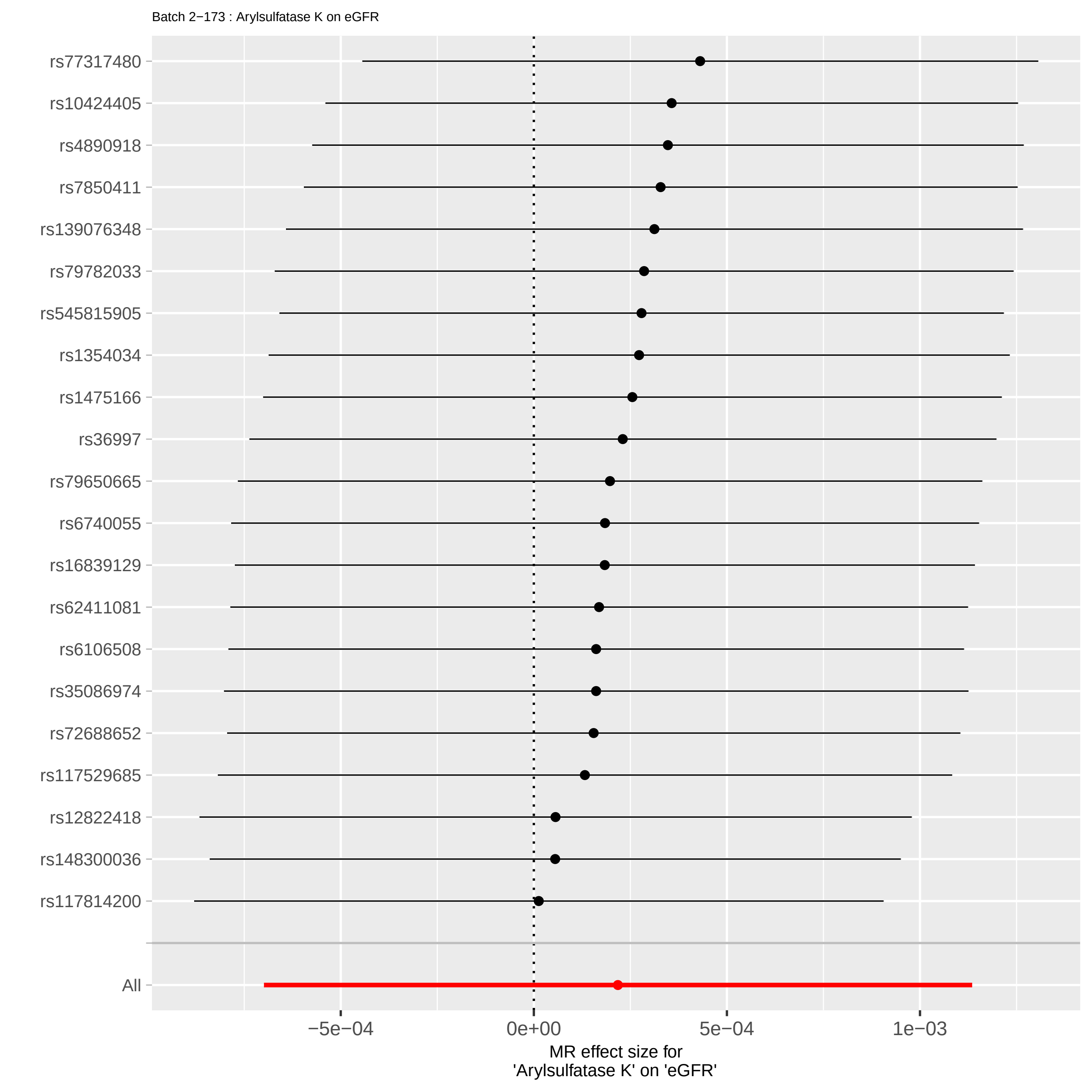

Batch 2−173 : Arylsulfatase K on eGFR
rs77317480
rs10424405
rs4890918
rs7850411
rs139076348
rs79782033
rs545815905
rs1354034
rs1475166
rs36997
rs79650665
rs6740055
rs16839129
rs62411081
rs6106508
rs35086974
rs72688652
rs117529685
rs12822418
rs148300036
rs117814200
All
−5e−04
0e+00
5e−04
1e−03
MR effect size for
'Arylsulfatase K' on 'eGFR'

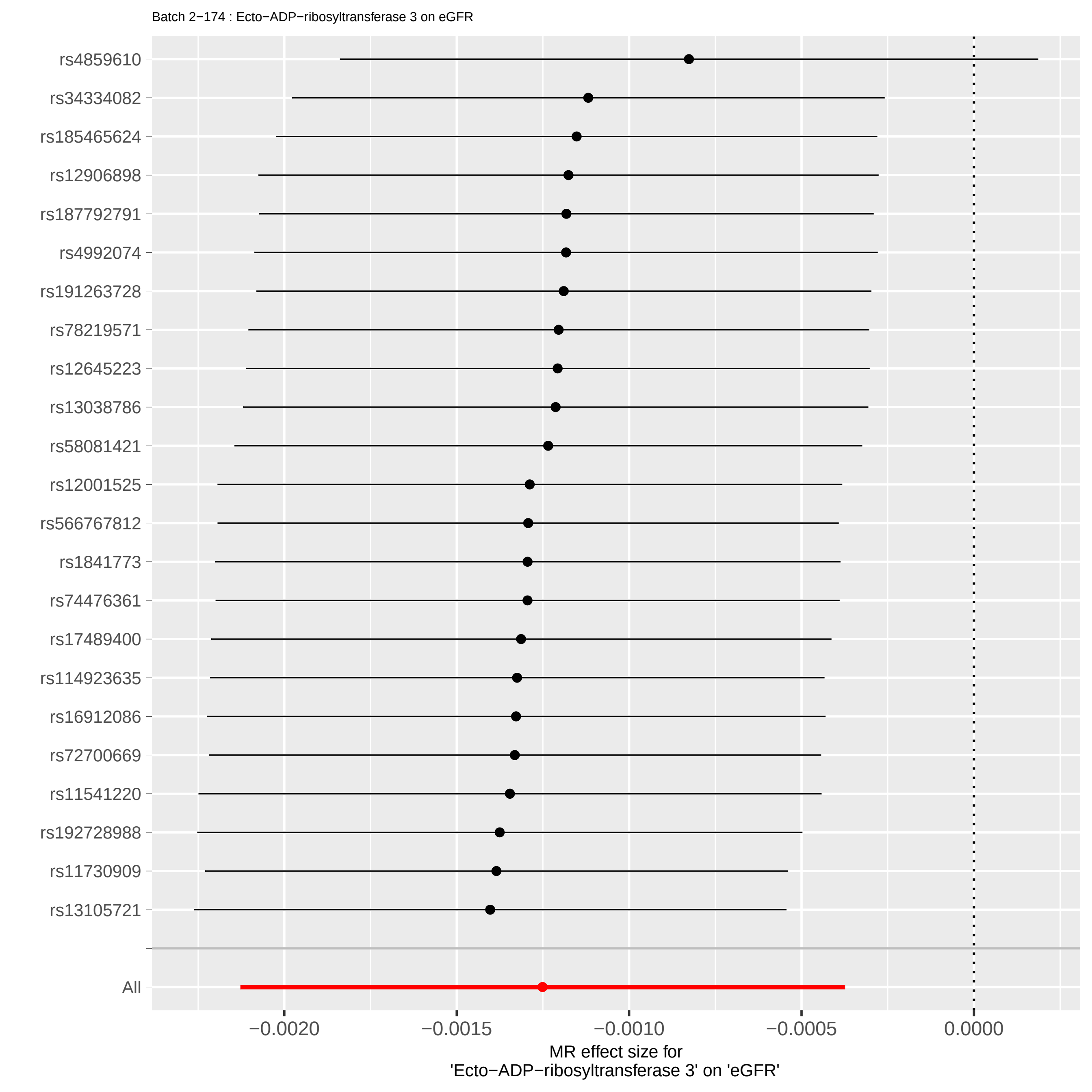

Batch 2−174 : Ecto−ADP−ribosyltransferase 3 on eGFR
rs4859610
rs34334082
rs185465624
rs12906898
rs187792791
rs4992074
rs191263728
rs78219571
rs12645223
rs13038786
rs58081421
rs12001525
rs566767812
rs1841773
rs74476361
rs17489400
rs114923635
rs16912086
rs72700669
rs11541220
rs192728988
rs11730909
rs13105721
All
−0.0020
−0.0015
−0.0010
−0.0005
0.0000
MR effect size for
'Ecto−ADP−ribosyltransferase 3' on 'eGFR'

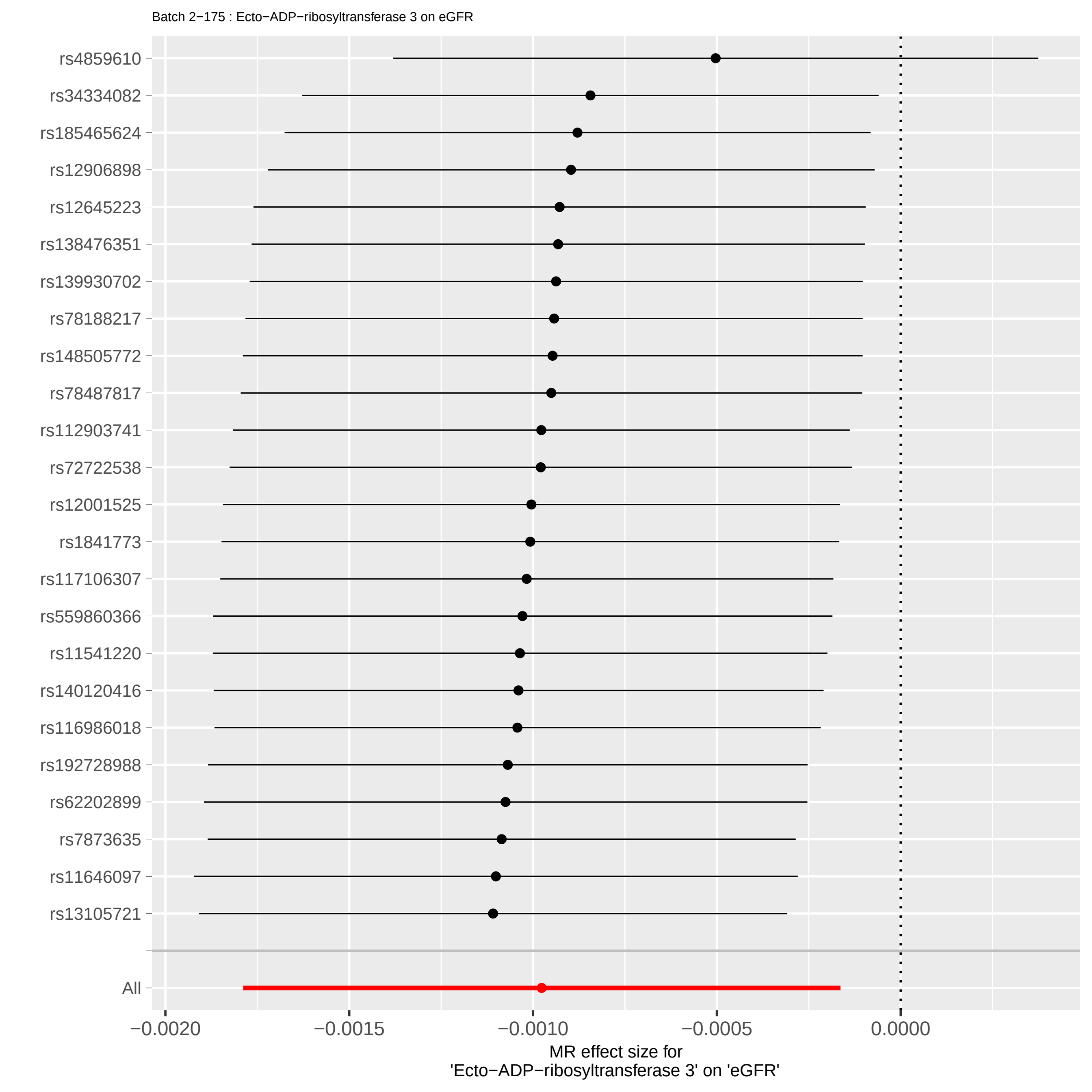

Batch 2−175 : Ecto−ADP−ribosyltransferase 3 on eGFR
rs4859610
rs34334082
rs185465624
rs12906898
rs12645223
rs138476351
rs139930702
rs78188217
rs148505772
rs78487817
rs112903741
rs72722538
rs12001525
rs1841773
rs117106307
rs559860366
rs11541220
rs140120416
rs116986018
rs192728988
rs62202899
rs7873635
rs11646097
rs13105721
All
−0.0020
−0.0015
−0.0010
−0.0005
0.0000
MR effect size for
'Ecto−ADP−ribosyltransferase 3' on 'eGFR'

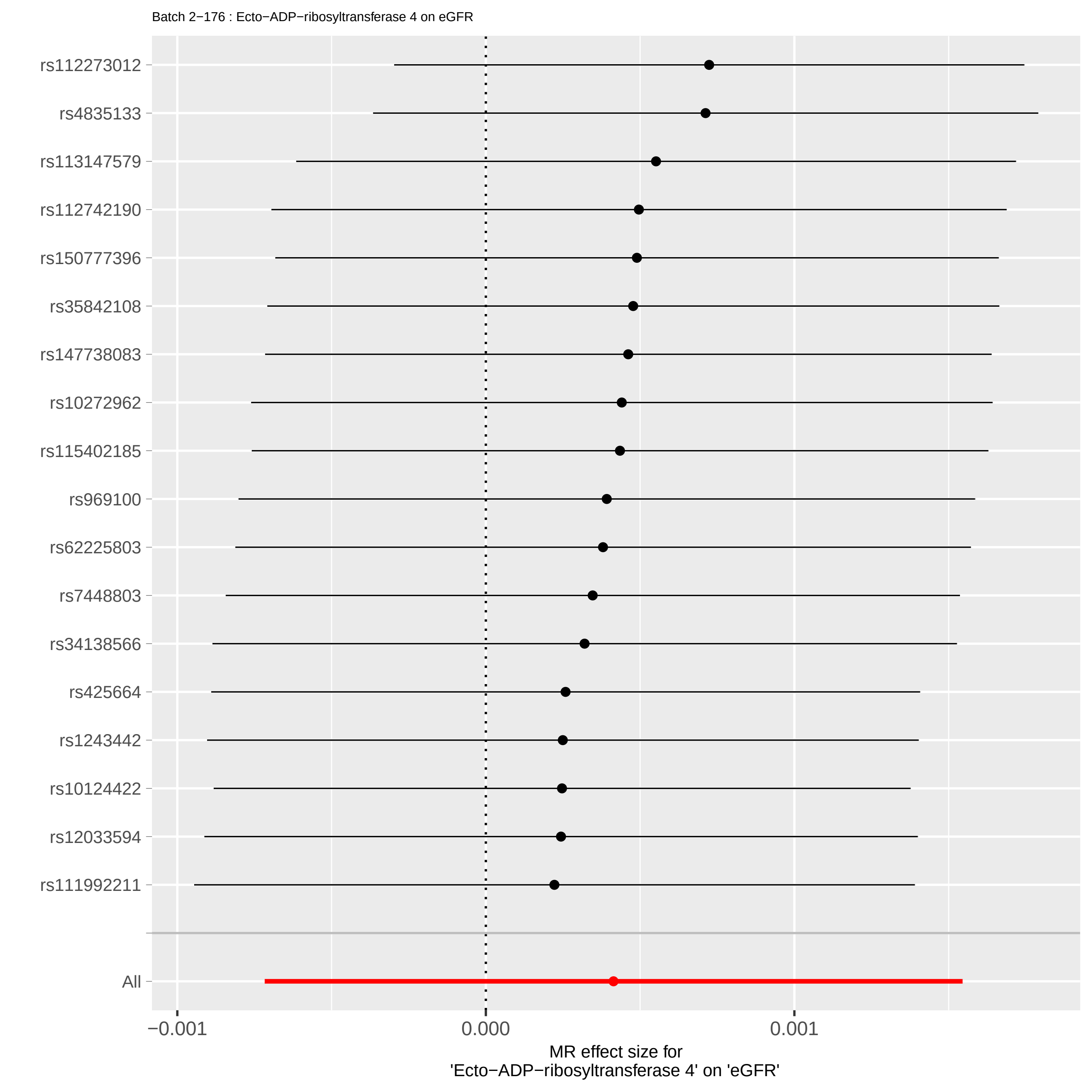
Batch 2−176 : Ecto−ADP−ribosyltransferase 4 on eGFR
rs112273012
rs4835133
rs113147579
rs112742190
rs150777396
rs35842108
rs147738083
rs10272962
rs115402185
rs969100
rs62225803
rs7448803
rs34138566
rs425664
rs1243442
rs10124422
rs12033594
rs111992211
All
−0.001
0.000
0.001
MR effect size for
'Ecto−ADP−ribosyltransferase 4' on 'eGFR'

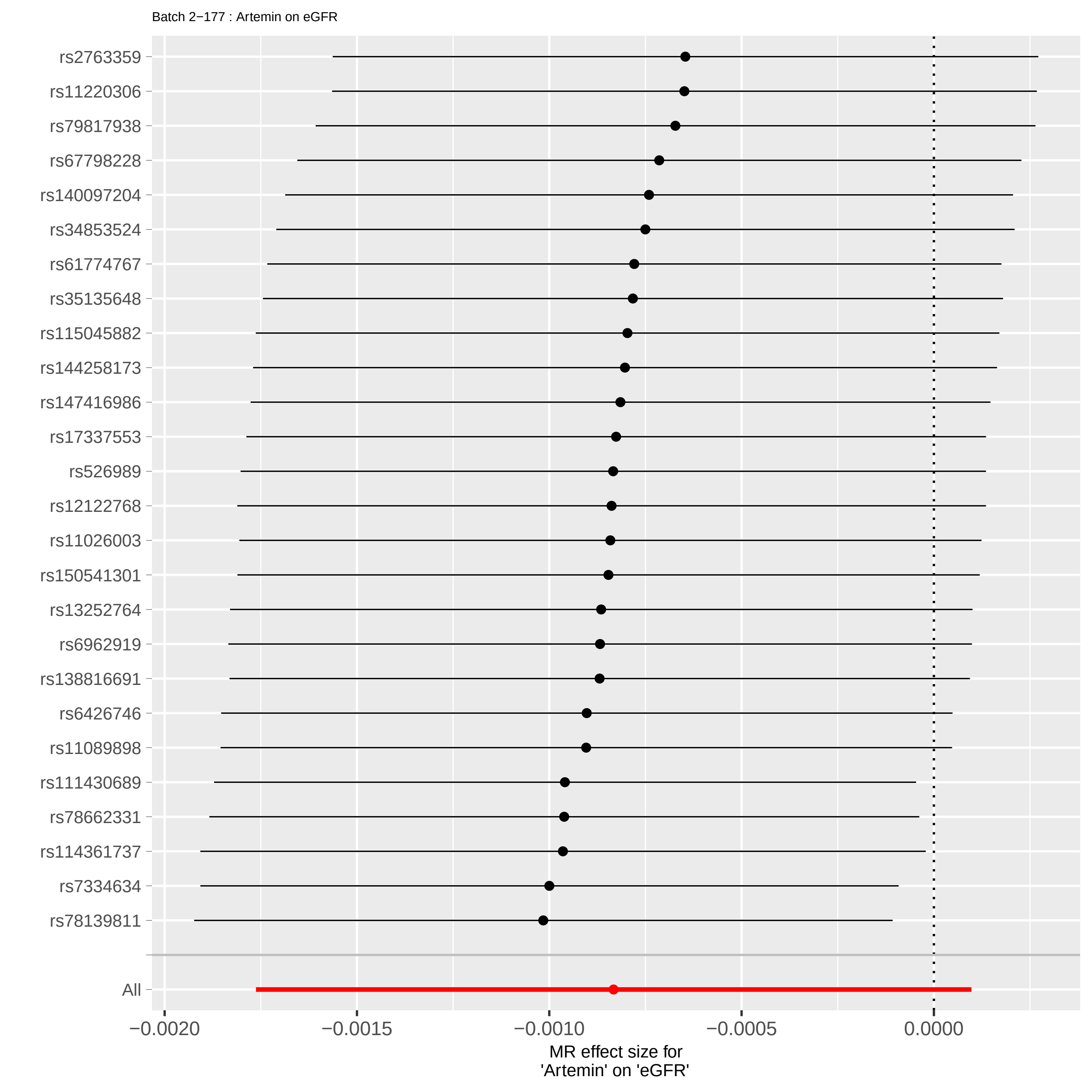
Batch 2−177 : Artemin on eGFR
rs2763359
rs11220306
rs79817938
rs67798228
rs140097204
rs34853524
rs61774767
rs35135648
rs115045882
rs144258173
rs147416986
rs17337553
rs526989
rs12122768
rs11026003
rs150541301
rs13252764
rs6962919
rs138816691
rs6426746
rs11089898
rs111430689
rs78662331
rs114361737
rs7334634
rs78139811
All
−0.0020
−0.0015
−0.0010
−0.0005
0.0000
MR effect size for
'Artemin' on 'eGFR'

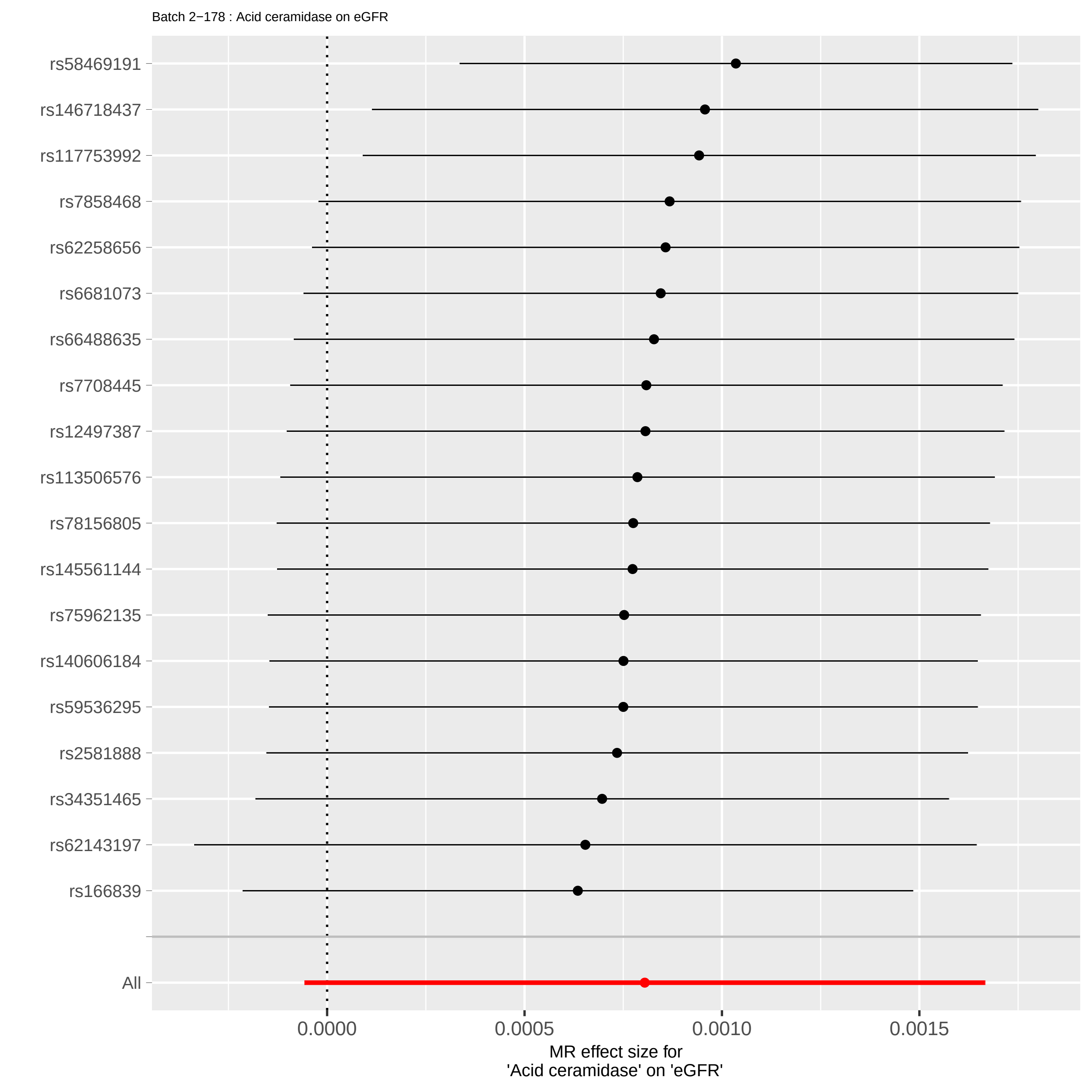

Batch 2−178 : Acid ceramidase on eGFR
rs58469191
rs146718437
rs117753992
rs7858468
rs62258656
rs6681073
rs66488635
rs7708445
rs12497387
rs113506576
rs78156805
rs145561144
rs75962135
rs140606184
rs59536295
rs2581888
rs34351465
rs62143197
rs166839
All
0.0000
0.0005
0.0010
0.0015
MR effect size for
'Acid ceramidase' on 'eGFR'

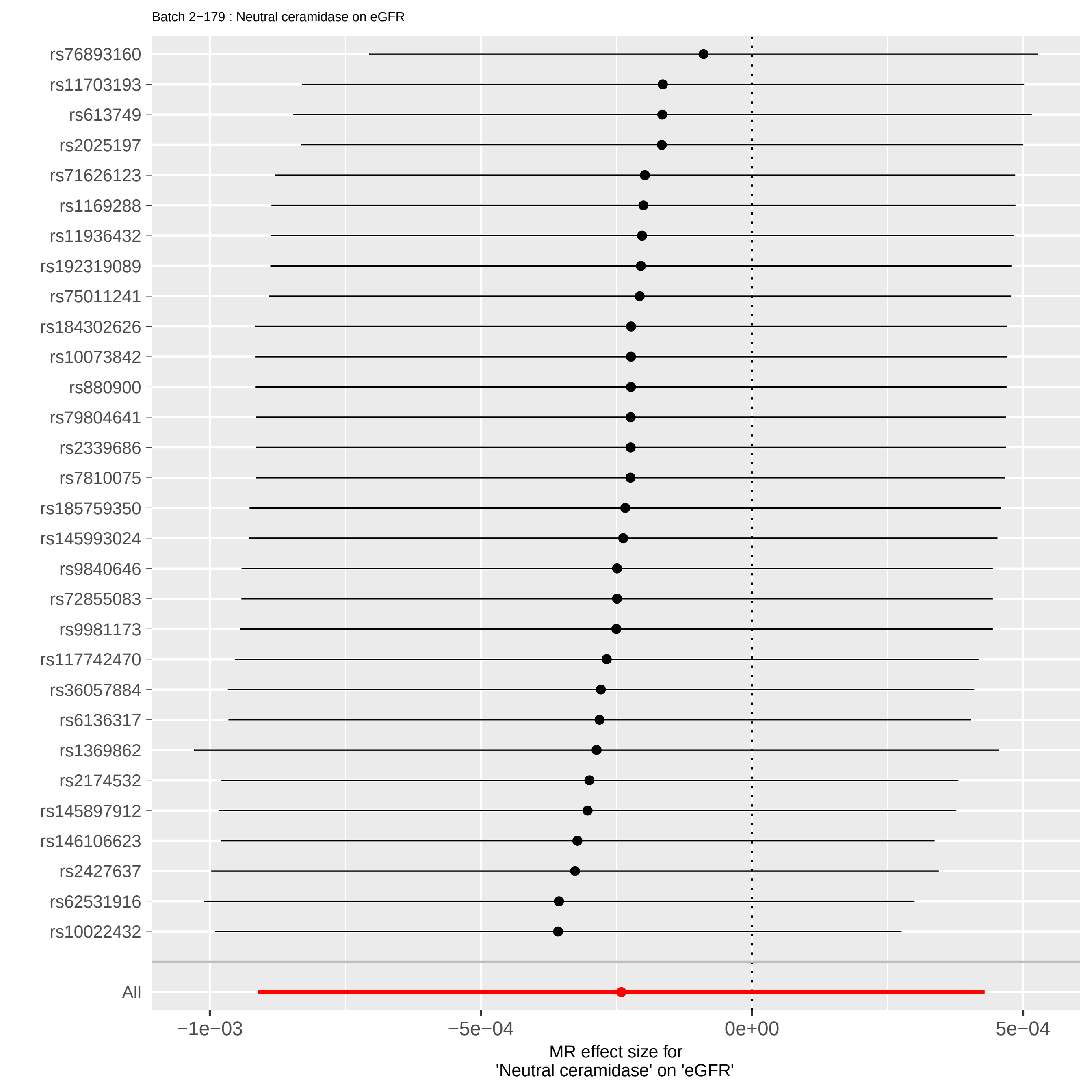
Batch 2−179 : Neutral ceramidase on eGFR
rs76893160
rs11703193
rs613749
rs2025197
rs71626123
rs1169288
rs11936432
rs192319089
rs75011241
rs184302626
rs10073842
rs880900
rs79804641
rs2339686
rs7810075
rs185759350
rs145993024
rs9840646
rs72855083
rs9981173
rs117742470
rs36057884
rs6136317
rs1369862
rs2174532
rs145897912
rs146106623
rs2427637
rs62531916
rs10022432
All
−1e−03
−5e−04
0e+00
5e−04
MR effect size for
'Neutral ceramidase' on 'eGFR'

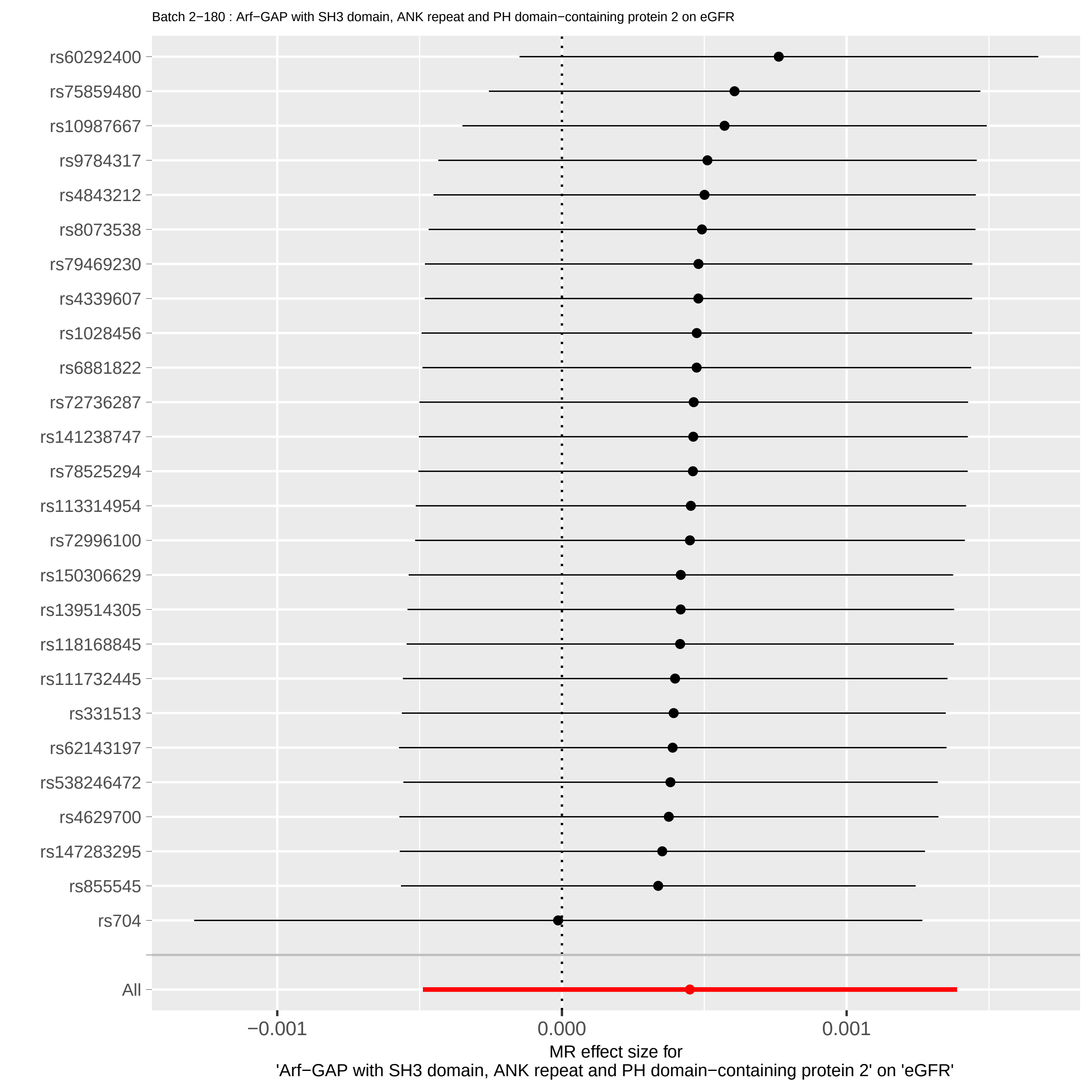
Batch 2−180 : Arf−GAP with SH3 domain, ANK repeat and PH domain−containing protein 2 on eGFR
rs60292400
rs75859480
rs10987667
rs9784317
rs4843212
rs8073538
rs79469230
rs4339607
rs1028456
rs6881822
rs72736287
rs141238747
rs78525294
rs113314954
rs72996100
rs150306629
rs139514305
rs118168845
rs111732445
rs331513
rs62143197
rs538246472
rs4629700
rs147283295
rs855545
rs704
All
−0.001
0.000
0.001
MR effect size for
'Arf−GAP with SH3 domain, ANK repeat and PH domain−containing protein 2' on 'eGFR'

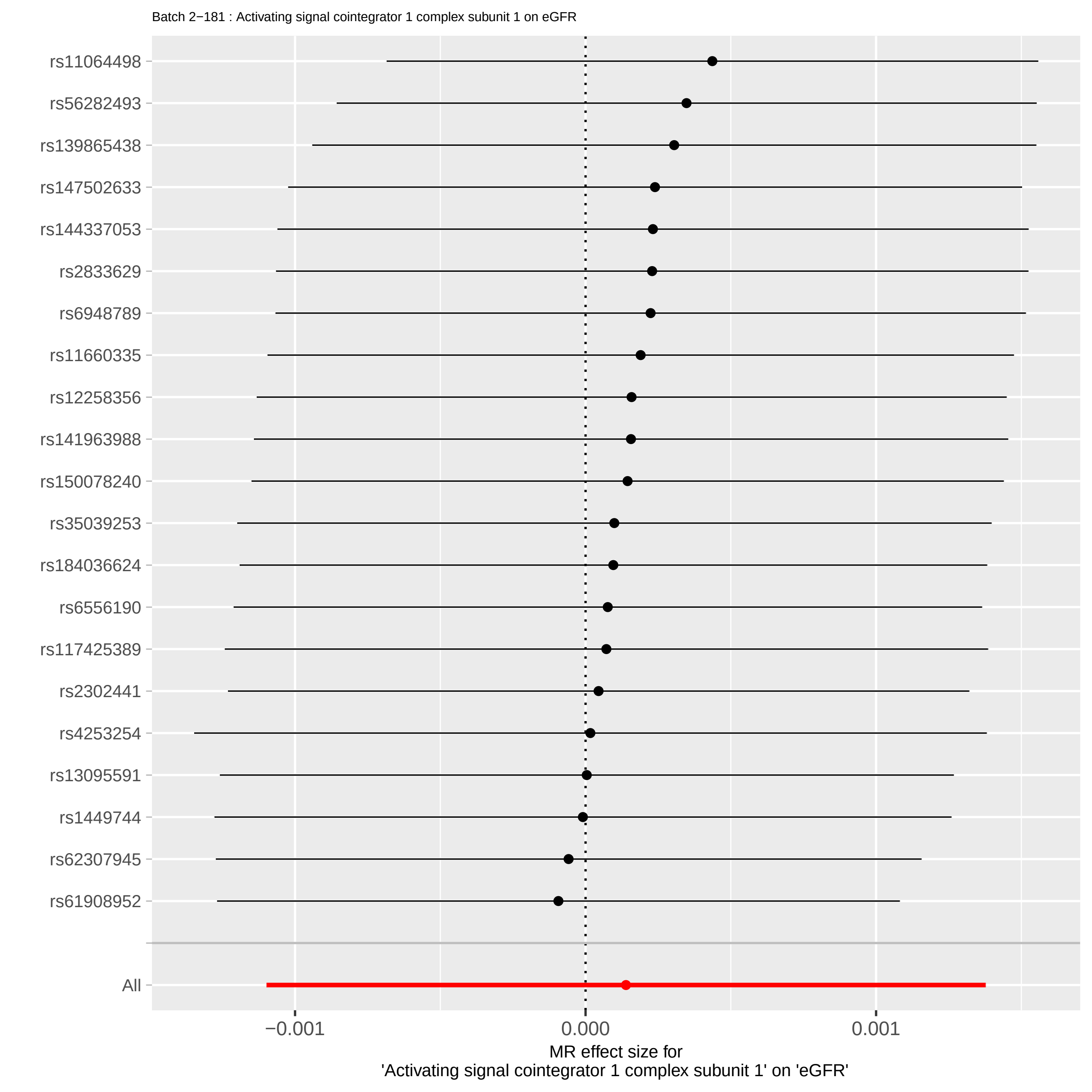

Batch 2−181 : Activating signal cointegrator 1 complex subunit 1 on eGFR
rs11064498
rs56282493
rs139865438
rs147502633
rs144337053
rs2833629
rs6948789
rs11660335
rs12258356
rs141963988
rs150078240
rs35039253
rs184036624
rs6556190
rs117425389
rs2302441
rs4253254
rs13095591
rs1449744
rs62307945
rs61908952
All
−0.001
0.000
0.001
MR effect size for
'Activating signal cointegrator 1 complex subunit 1' on 'eGFR'

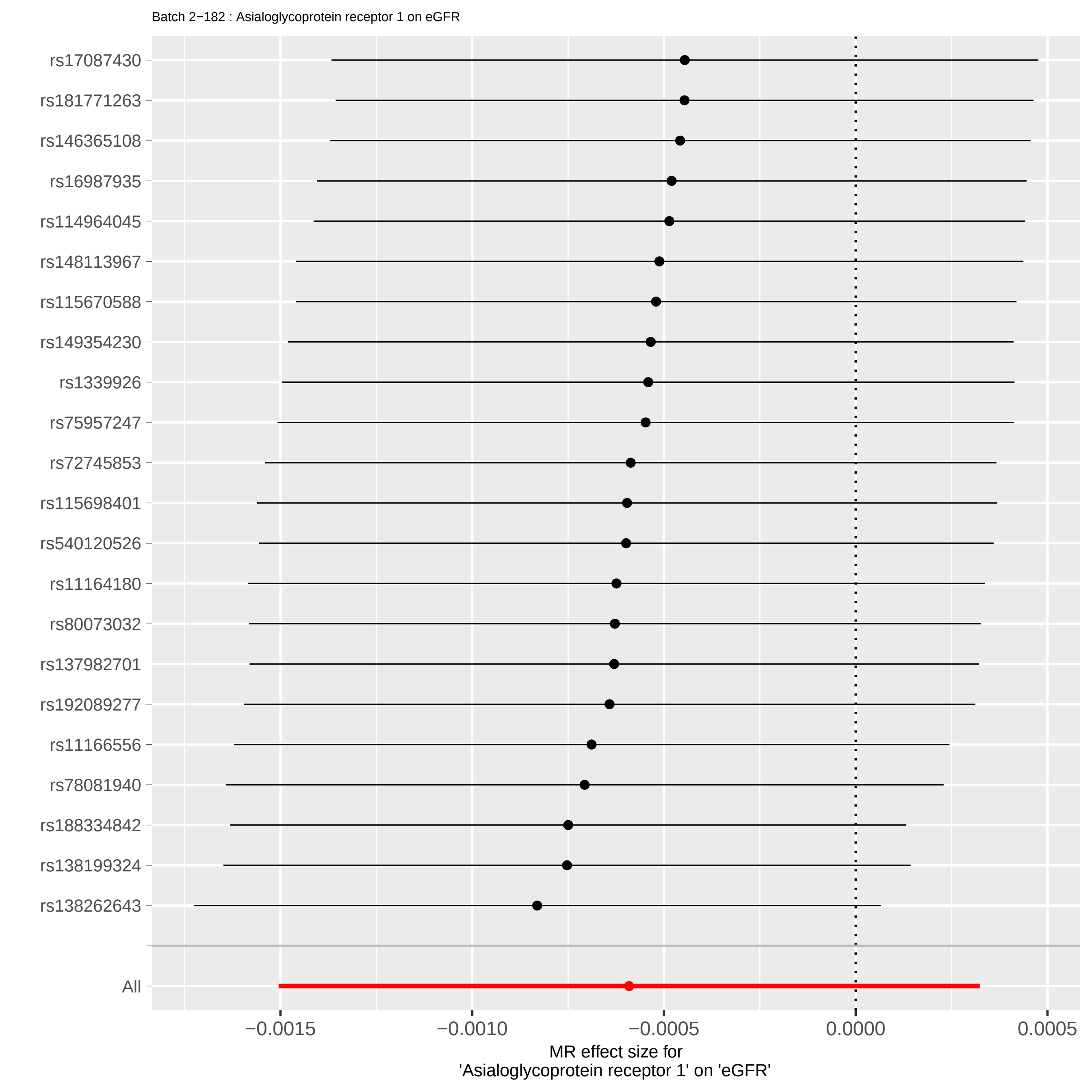

Batch 2−182 : Asialoglycoprotein receptor 1 on eGFR
rs17087430
rs181771263
rs146365108
rs16987935
rs114964045
rs148113967
rs115670588
rs149354230
rs1339926
rs75957247
rs72745853
rs115698401
rs540120526
rs11164180
rs80073032
rs137982701
rs192089277
rs11166556
rs78081940
rs188334842
rs138199324
rs138262643
All
−0.0015
−0.0010
−0.0005
0.0000
0.0005
MR effect size for
'Asialoglycoprotein receptor 1' on 'eGFR'

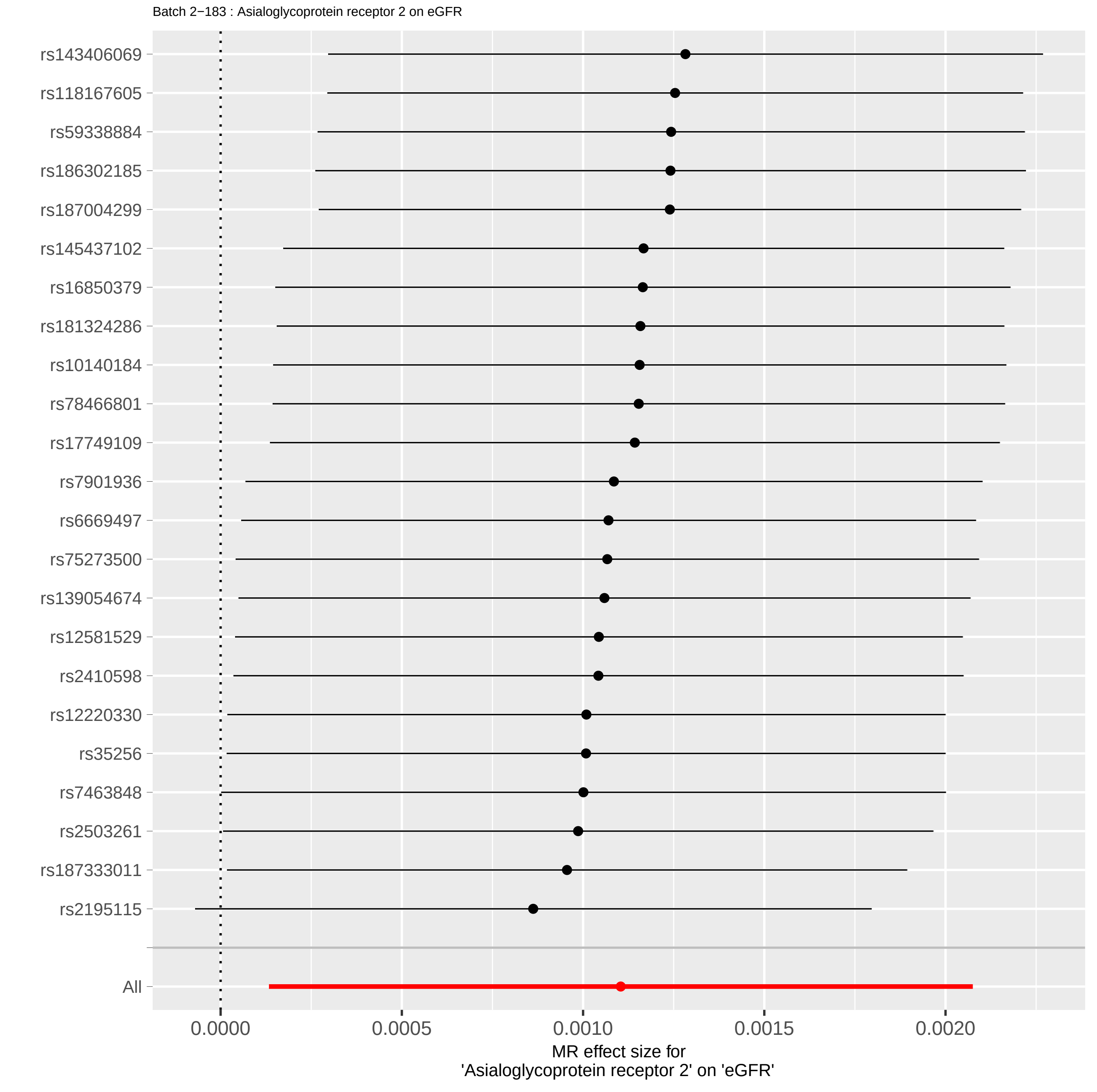

Batch 2−183 : Asialoglycoprotein receptor 2 on eGFR
rs143406069
rs118167605
rs59338884
rs186302185
rs187004299
rs145437102
rs16850379
rs181324286
rs10140184
rs78466801
rs17749109
rs7901936
rs6669497
rs75273500
rs139054674
rs12581529
rs2410598
rs12220330
rs35256
rs7463848
rs2503261
rs187333011
rs2195115
All
0.0000
0.0005
0.0010
0.0015
0.0020
MR effect size for
'Asialoglycoprotein receptor 2' on 'eGFR'

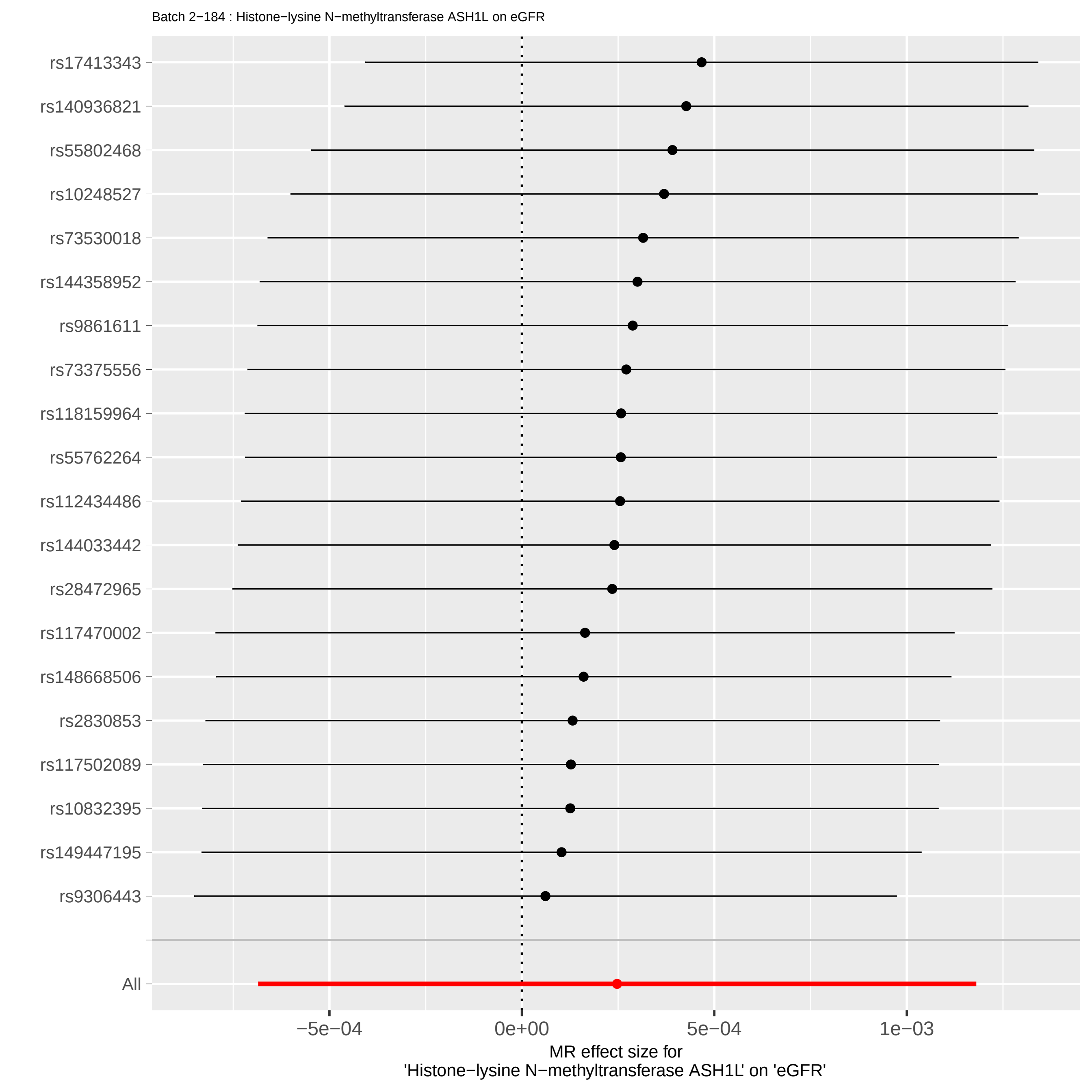

Batch 2−184 : Histone−lysine N−methyltransferase ASH1L on eGFR
rs17413343
rs140936821
rs55802468
rs10248527
rs73530018
rs144358952
rs9861611
rs73375556
rs118159964
rs55762264
rs112434486
rs144033442
rs28472965
rs117470002
rs148668506
rs2830853
rs117502089
rs10832395
rs149447195
rs9306443
All
−5e−04
0e+00
5e−04
1e−03
MR effect size for
'Histone−lysine N−methyltransferase ASH1L' on 'eGFR'

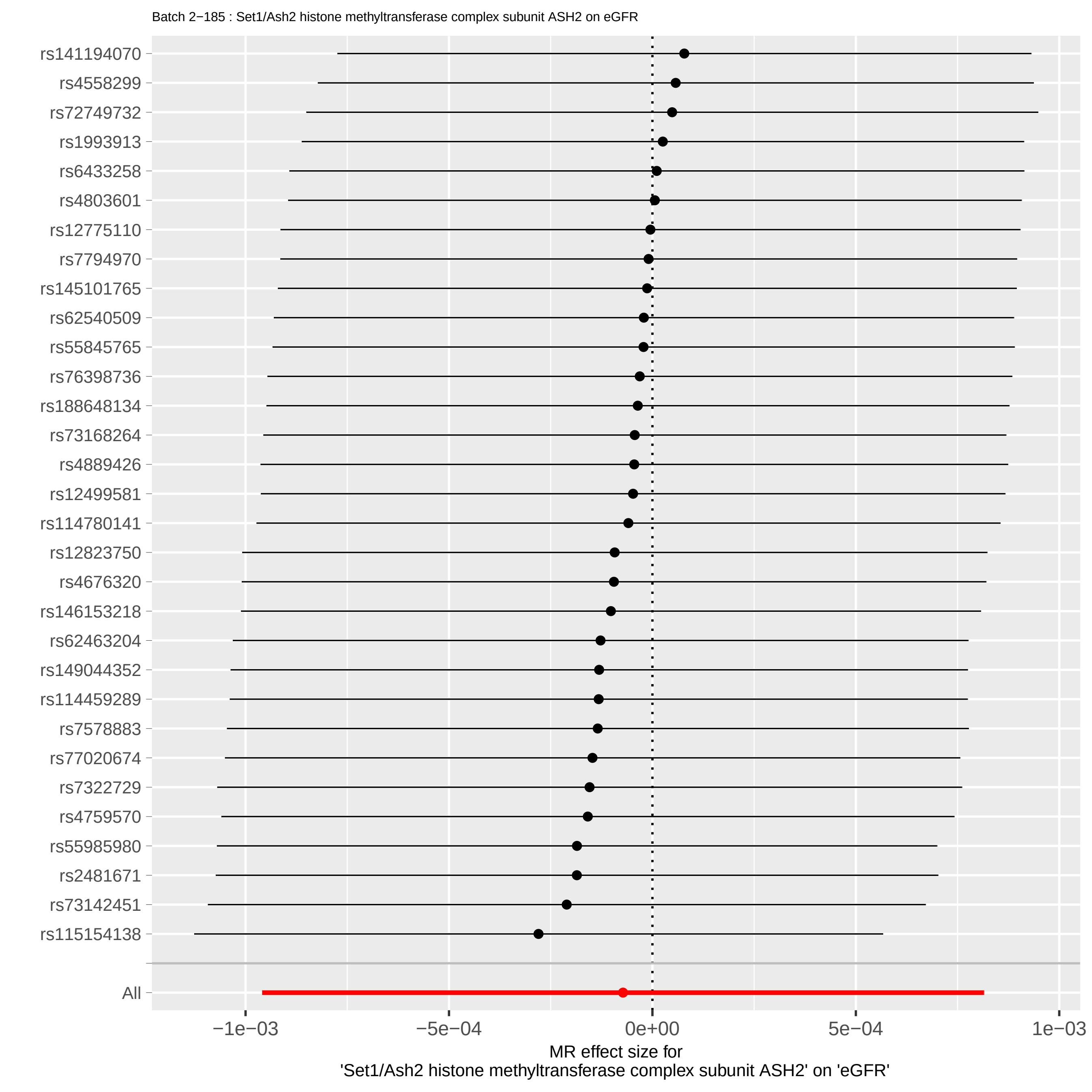

Batch 2−185 : Set1/Ash2 histone methyltransferase complex subunit ASH2 on eGFR
rs141194070
rs4558299
rs72749732
rs1993913
rs6433258
rs4803601
rs12775110
rs7794970
rs145101765
rs62540509
rs55845765
rs76398736
rs188648134
rs73168264
rs4889426
rs12499581
rs114780141
rs12823750
rs4676320
rs146153218
rs62463204
rs149044352
rs114459289
rs7578883
rs77020674
rs7322729
rs4759570
rs55985980
rs2481671
rs73142451
rs115154138
All
−1e−03
−5e−04
0e+00
5e−04
1e−03
MR effect size for
'Set1/Ash2 histone methyltransferase complex subunit ASH2' on 'eGFR'

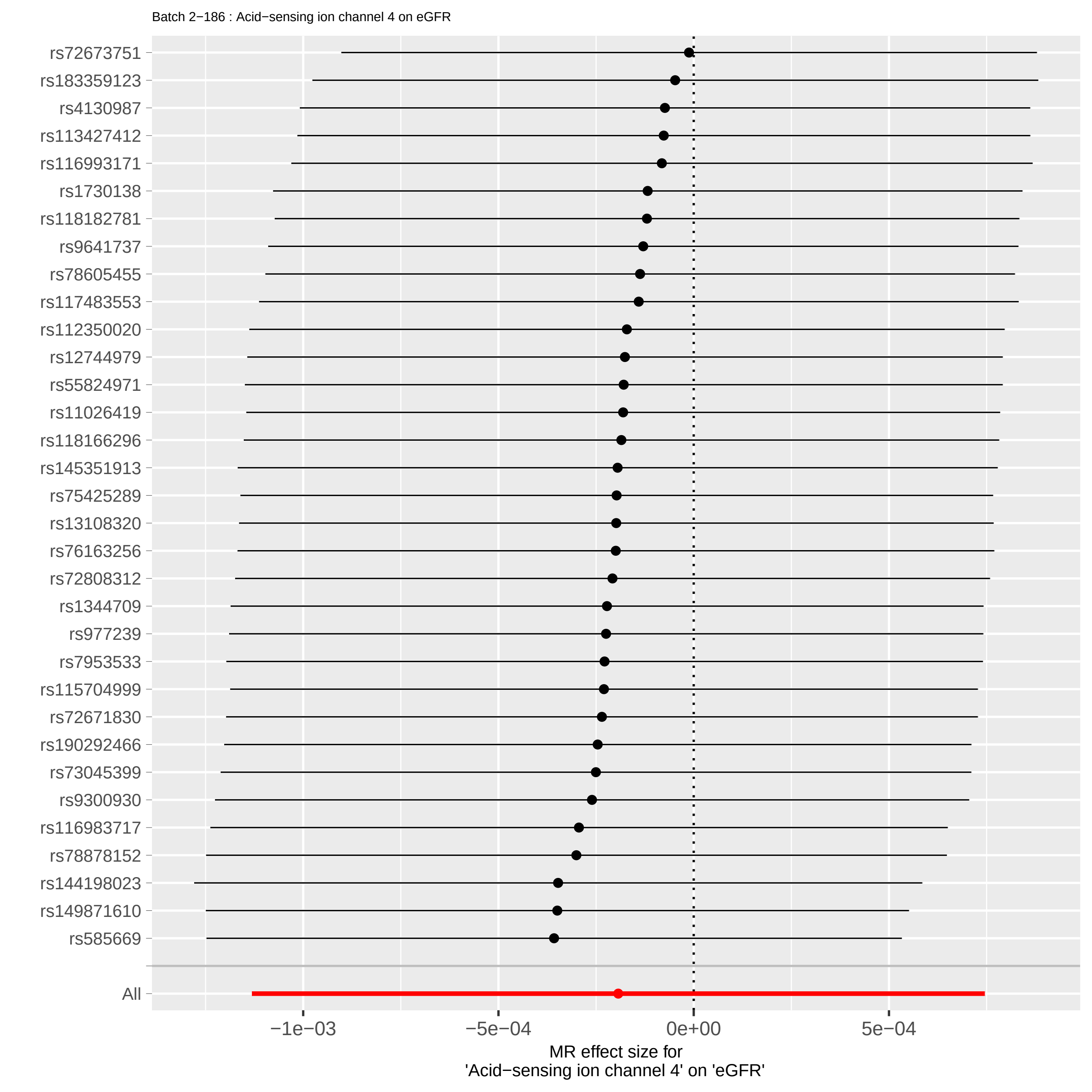

Batch 2−186 : Acid−sensing ion channel 4 on eGFR
rs72673751
rs183359123
rs4130987
rs113427412
rs116993171
rs1730138
rs118182781
rs9641737
rs78605455
rs117483553
rs112350020
rs12744979
rs55824971
rs11026419
rs118166296
rs145351913
rs75425289
rs13108320
rs76163256
rs72808312
rs1344709
rs977239
rs7953533
rs115704999
rs72671830
rs190292466
rs73045399
rs9300930
rs116983717
rs78878152
rs144198023
rs149871610
rs585669
All
−1e−03
−5e−04
0e+00
5e−04
MR effect size for
'Acid−sensing ion channel 4' on 'eGFR'

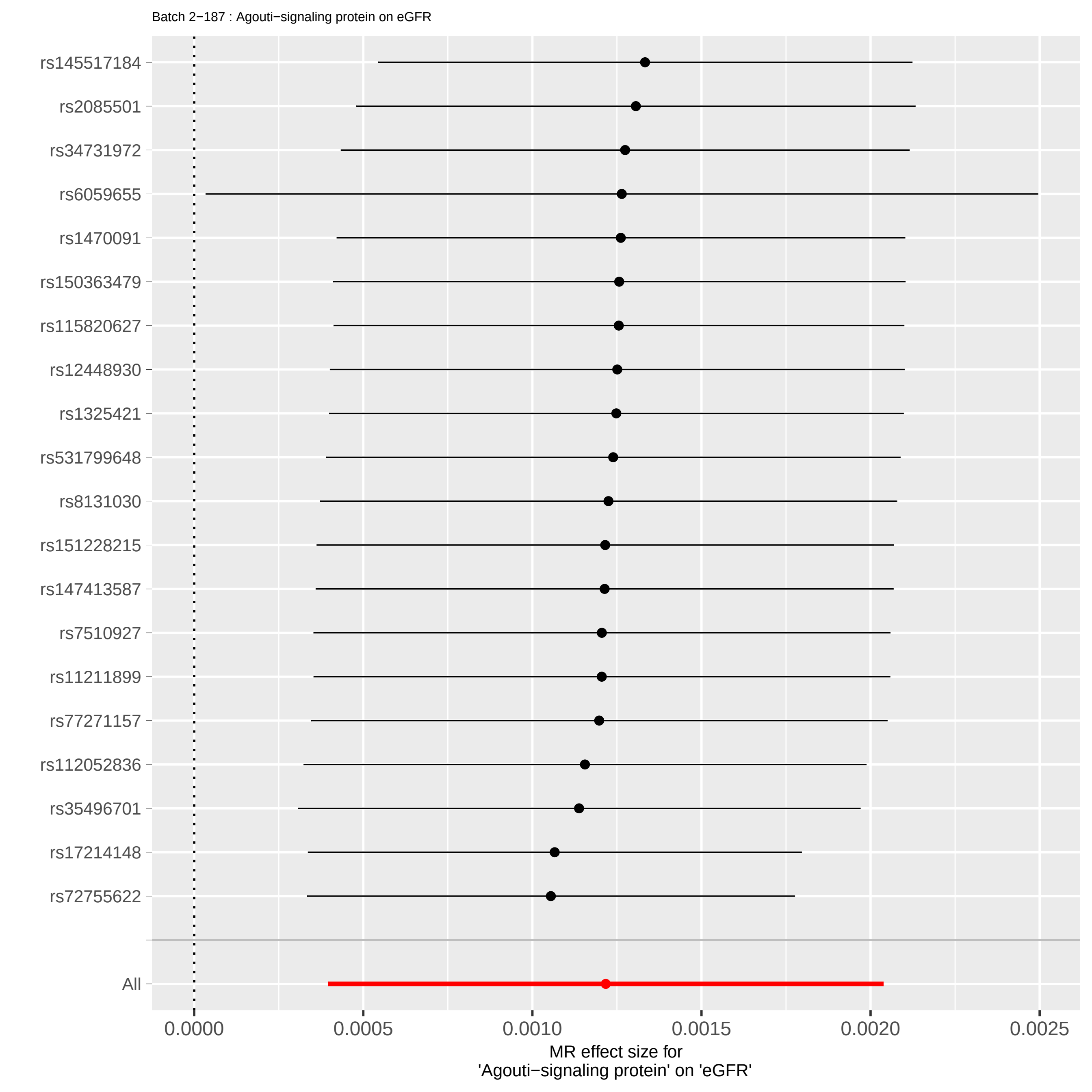

Batch 2−187 : Agouti−signaling protein on eGFR
rs145517184
rs2085501
rs34731972
rs6059655
rs1470091
rs150363479
rs115820627
rs12448930
rs1325421
rs531799648
rs8131030
rs151228215
rs147413587
rs7510927
rs11211899
rs77271157
rs112052836
rs35496701
rs17214148
rs72755622
All
0.0000
0.0005
0.0010
0.0015
0.0020
0.0025
MR effect size for
'Agouti−signaling protein' on 'eGFR'

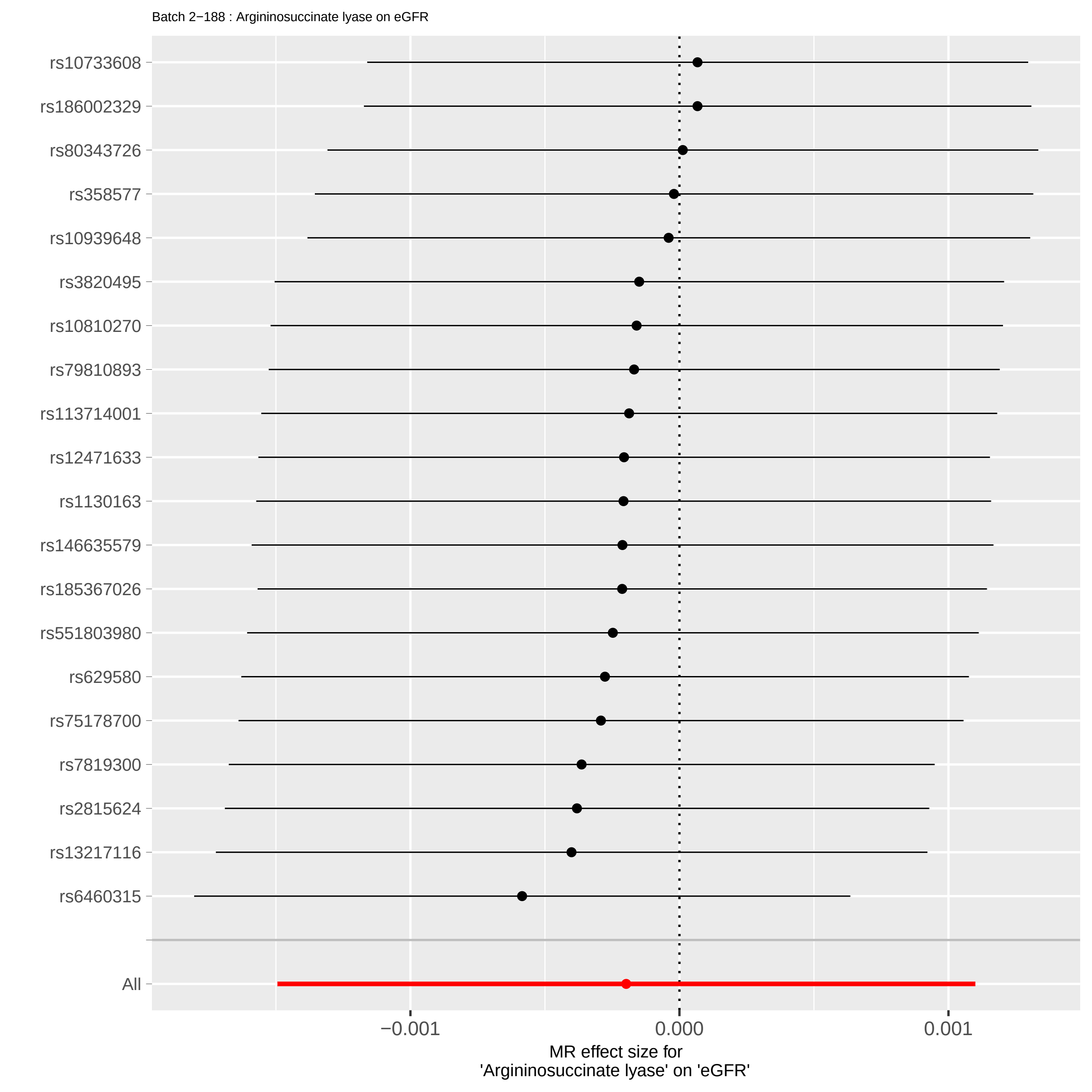

Batch 2−188 : Argininosuccinate lyase on eGFR
rs10733608
rs186002329
rs80343726
rs358577
rs10939648
rs3820495
rs10810270
rs79810893
rs113714001
rs12471633
rs1130163
rs146635579
rs185367026
rs551803980
rs629580
rs75178700
rs7819300
rs2815624
rs13217116
rs6460315
All
−0.001
0.000
0.001
MR effect size for
'Argininosuccinate lyase' on 'eGFR'

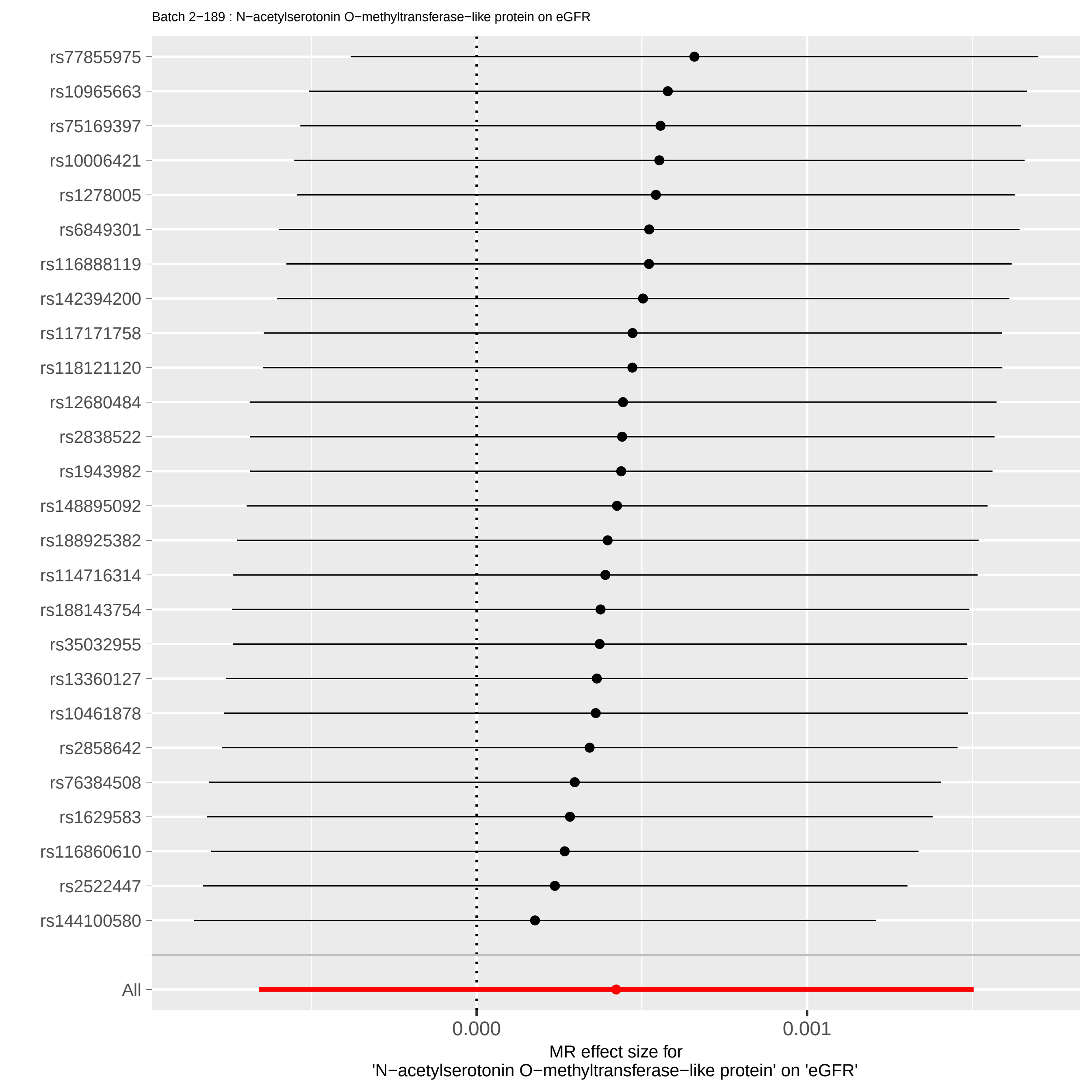

Batch 2−189 : N−acetylserotonin O−methyltransferase−like protein on eGFR
rs77855975
rs10965663
rs75169397
rs10006421
rs1278005
rs6849301
rs116888119
rs142394200
rs117171758
rs118121120
rs12680484
rs2838522
rs1943982
rs148895092
rs188925382
rs114716314
rs188143754
rs35032955
rs13360127
rs10461878
rs2858642
rs76384508
rs1629583
rs116860610
rs2522447
rs144100580
All
0.000
0.001
MR effect size for
'N−acetylserotonin O−methyltransferase−like protein' on 'eGFR'

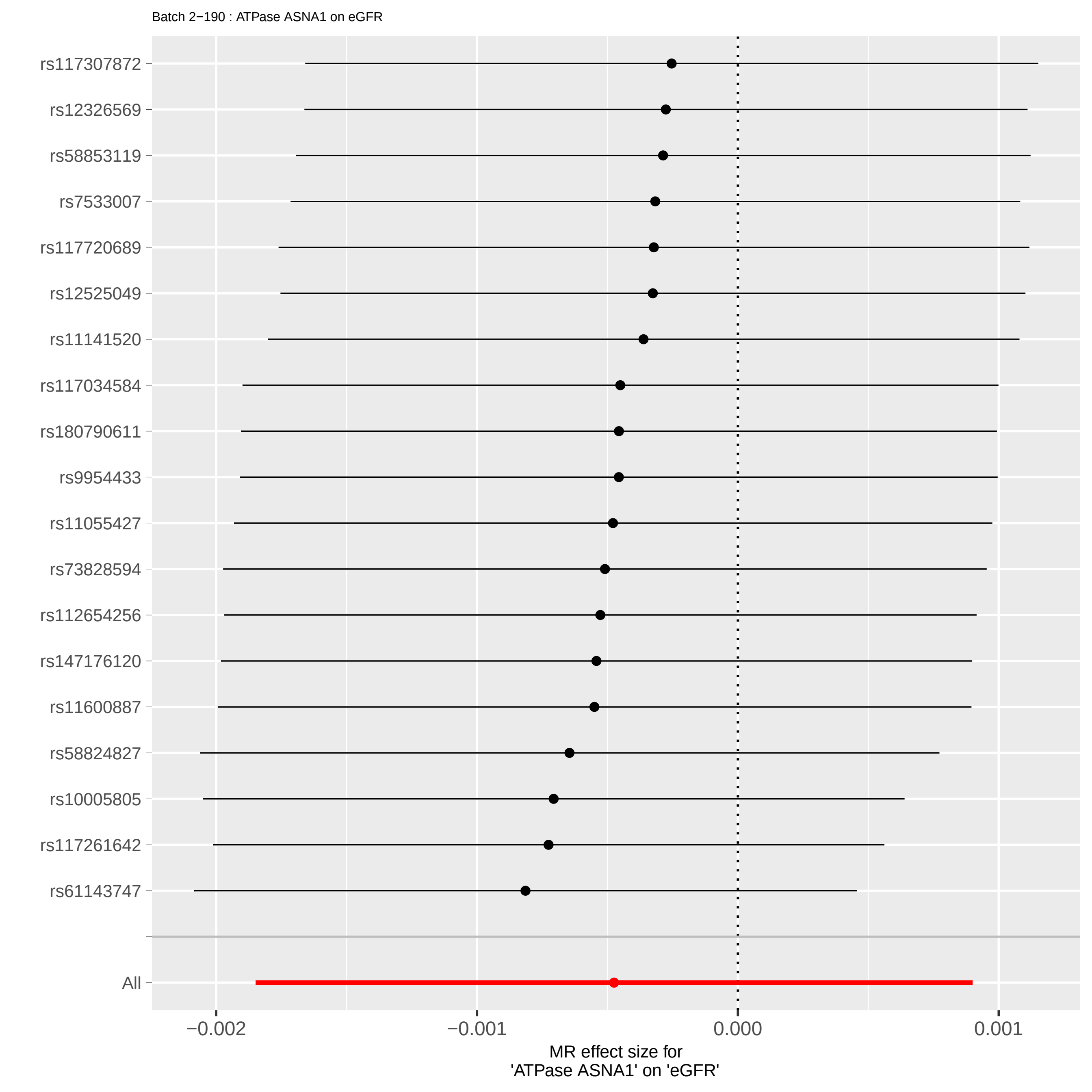

Batch 2−190 : ATPase ASNA1 on eGFR
rs117307872
rs12326569
rs58853119
rs7533007
rs117720689
rs12525049
rs11141520
rs117034584
rs180790611
rs9954433
rs11055427
rs73828594
rs112654256
rs147176120
rs11600887
rs58824827
rs10005805
rs117261642
rs61143747
All
−0.002
−0.001
0.000
0.001
MR effect size for
'ATPase ASNA1' on 'eGFR'

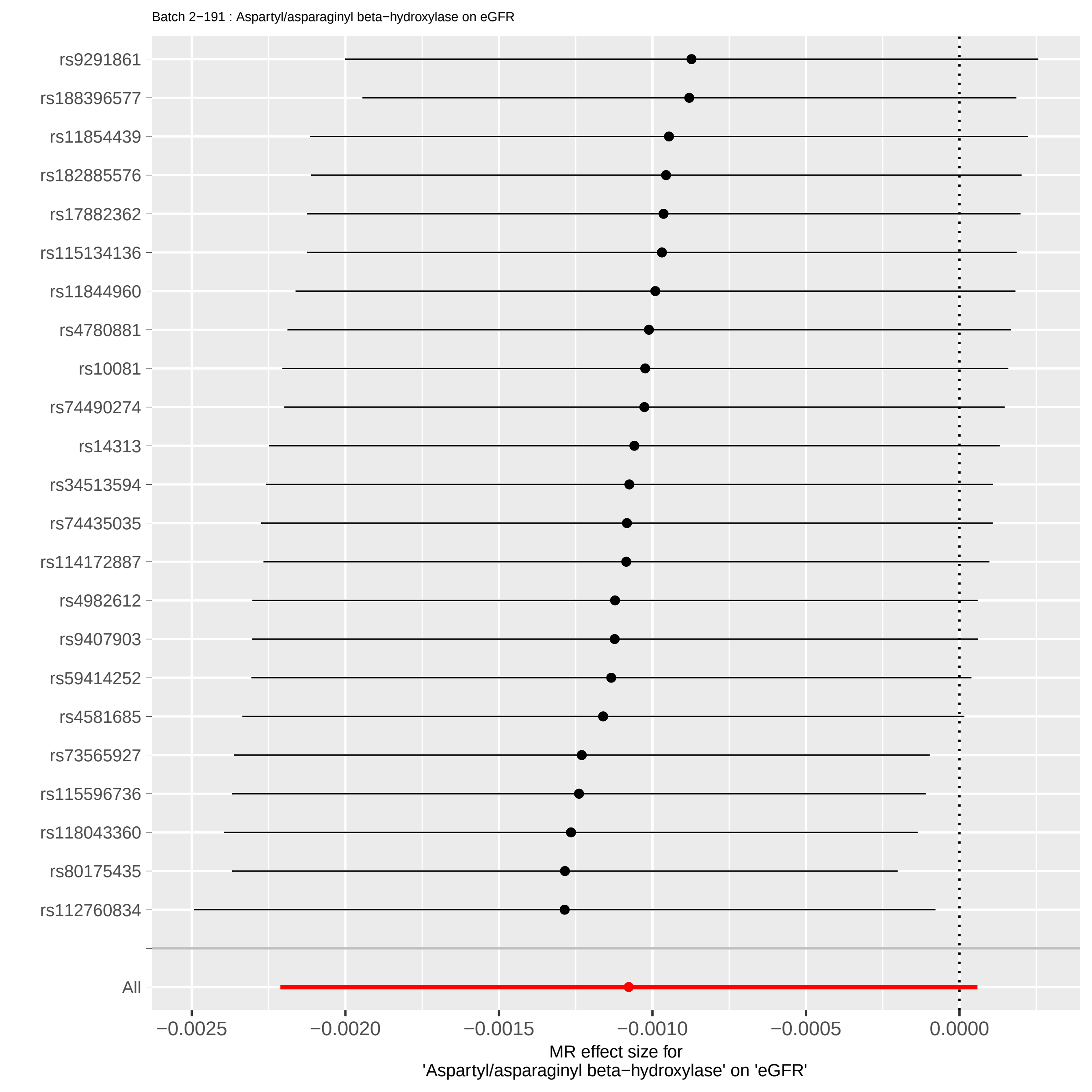

Batch 2−191 : Aspartyl/asparaginyl beta−hydroxylase on eGFR
rs9291861
rs188396577
rs11854439
rs182885576
rs17882362
rs115134136
rs11844960
rs4780881
rs10081
rs74490274
rs14313
rs34513594
rs74435035
rs114172887
rs4982612
rs9407903
rs59414252
rs4581685
rs73565927
rs115596736
rs118043360
rs80175435
rs112760834
All
−0.0025
−0.0020
−0.0015
−0.0010
−0.0005
0.0000
MR effect size for
'Aspartyl/asparaginyl beta−hydroxylase' on 'eGFR'

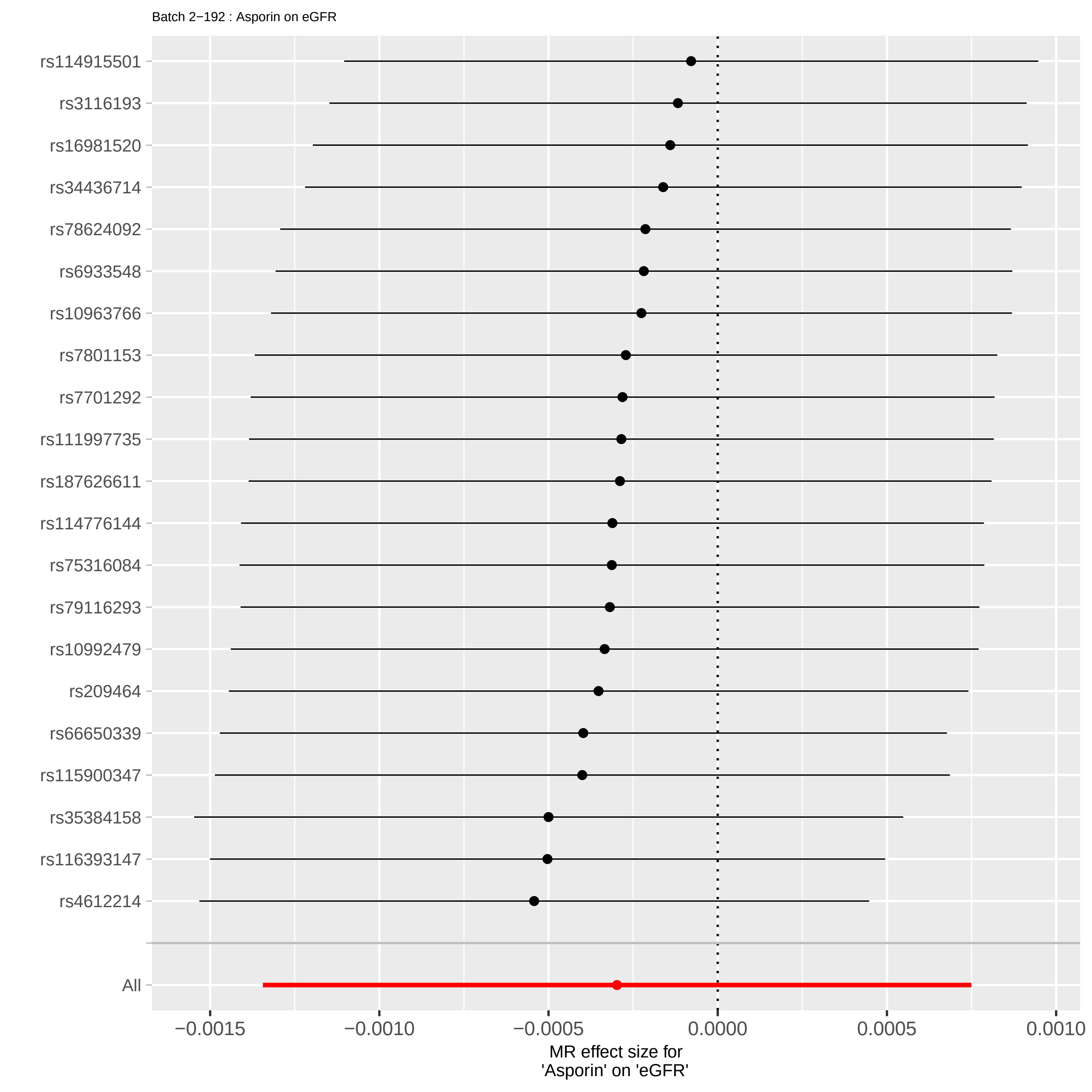
Batch 2−192 : Asporin on eGFR
rs114915501
rs3116193
rs16981520
rs34436714
rs78624092
rs6933548
rs10963766
rs7801153
rs7701292
rs111997735
rs187626611
rs114776144
rs75316084
rs79116293
rs10992479
rs209464
rs66650339
rs115900347
rs35384158
rs116393147
rs4612214
All
−0.0015
−0.0010
−0.0005
0.0000
0.0005
0.0010
MR effect size for
'Asporin' on 'eGFR'

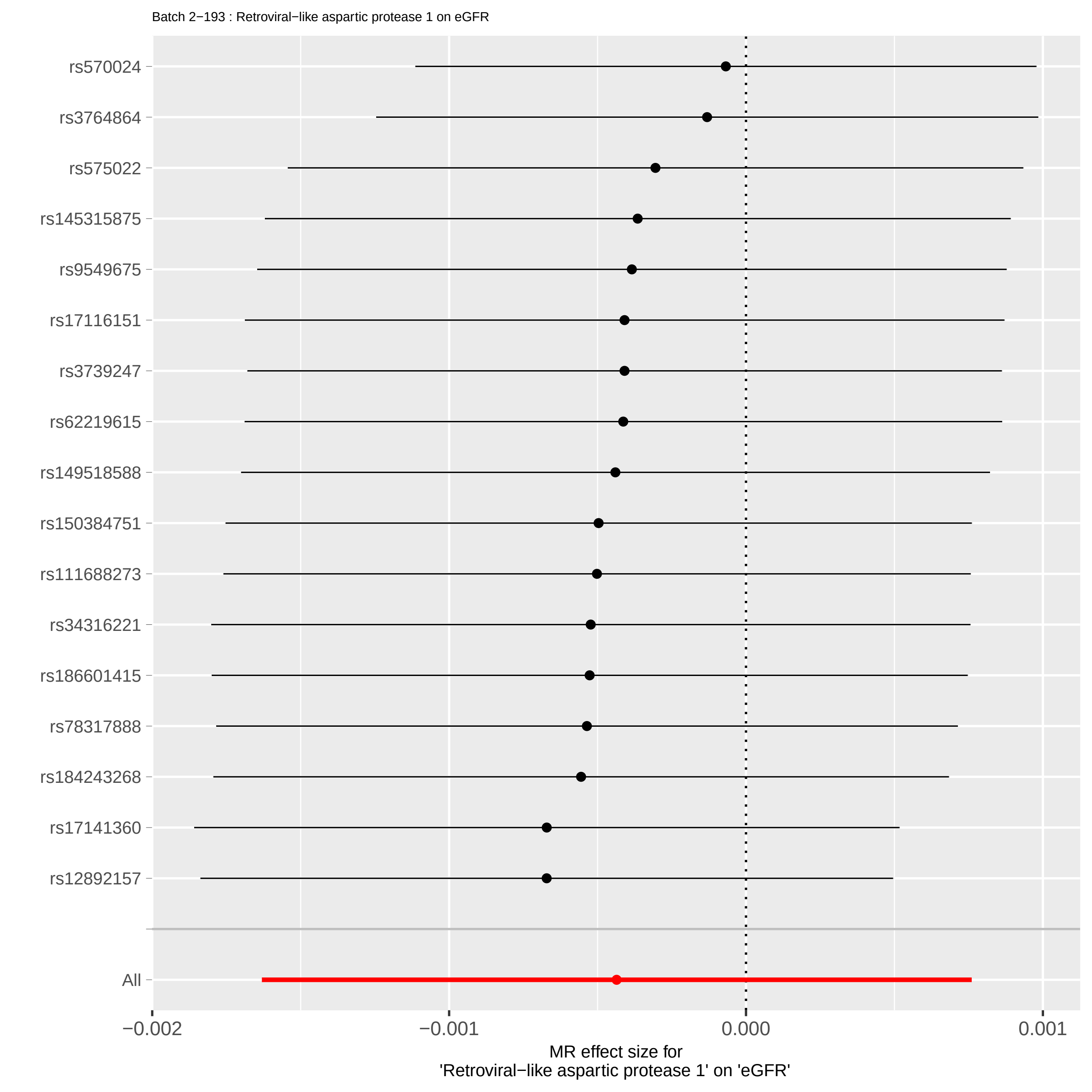

Batch 2−193 : Retroviral−like aspartic protease 1 on eGFR
rs570024
rs3764864
rs575022
rs145315875
rs9549675
rs17116151
rs3739247
rs62219615
rs149518588
rs150384751
rs111688273
rs34316221
rs186601415
rs78317888
rs184243268
rs17141360
rs12892157
All
−0.002
−0.001
0.000
0.001
MR effect size for
'Retroviral−like aspartic protease 1' on 'eGFR'

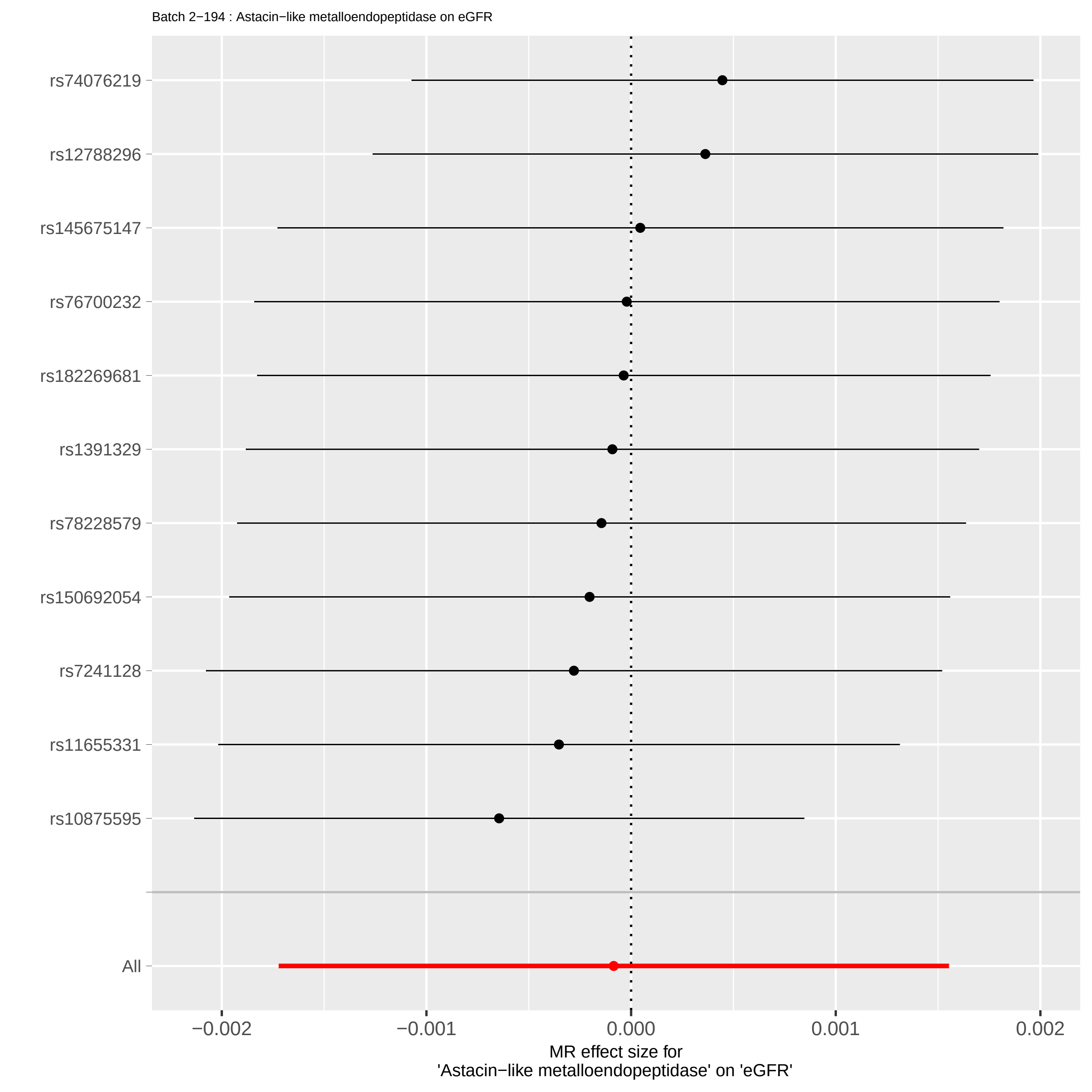

Batch 2−194 : Astacin−like metalloendopeptidase on eGFR
rs74076219
rs12788296
rs145675147
rs76700232
rs182269681
rs1391329
rs78228579
rs150692054
rs7241128
rs11655331
rs10875595
All
−0.002
−0.001
0.000
0.001
0.002
MR effect size for
'Astacin−like metalloendopeptidase' on 'eGFR'

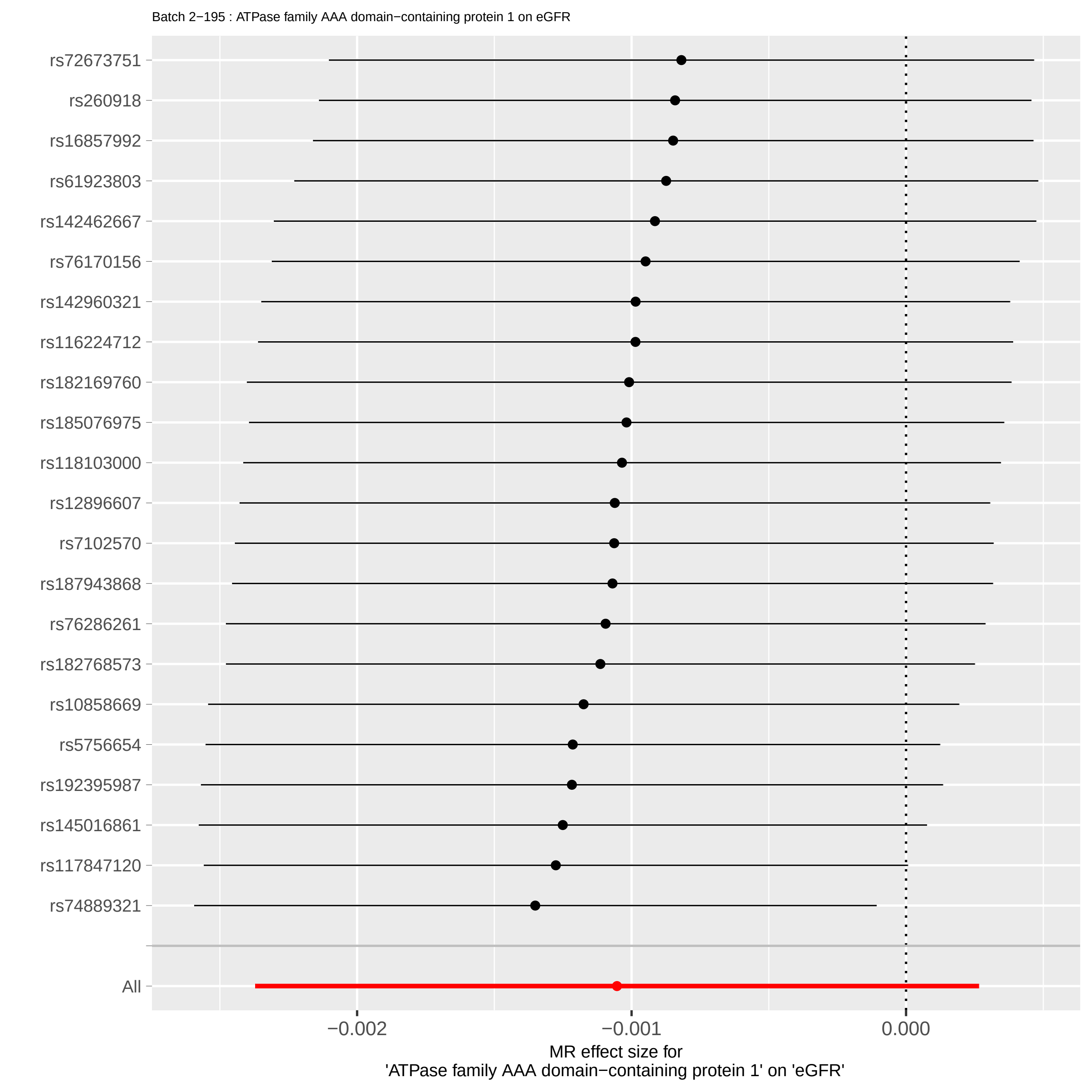

Batch 2−195 : ATPase family AAA domain−containing protein 1 on eGFR
rs72673751
rs260918
rs16857992
rs61923803
rs142462667
rs76170156
rs142960321
rs116224712
rs182169760
rs185076975
rs118103000
rs12896607
rs7102570
rs187943868
rs76286261
rs182768573
rs10858669
rs5756654
rs192395987
rs145016861
rs117847120
rs74889321
All
−0.002
−0.001
0.000
MR effect size for
'ATPase family AAA domain−containing protein 1' on 'eGFR'

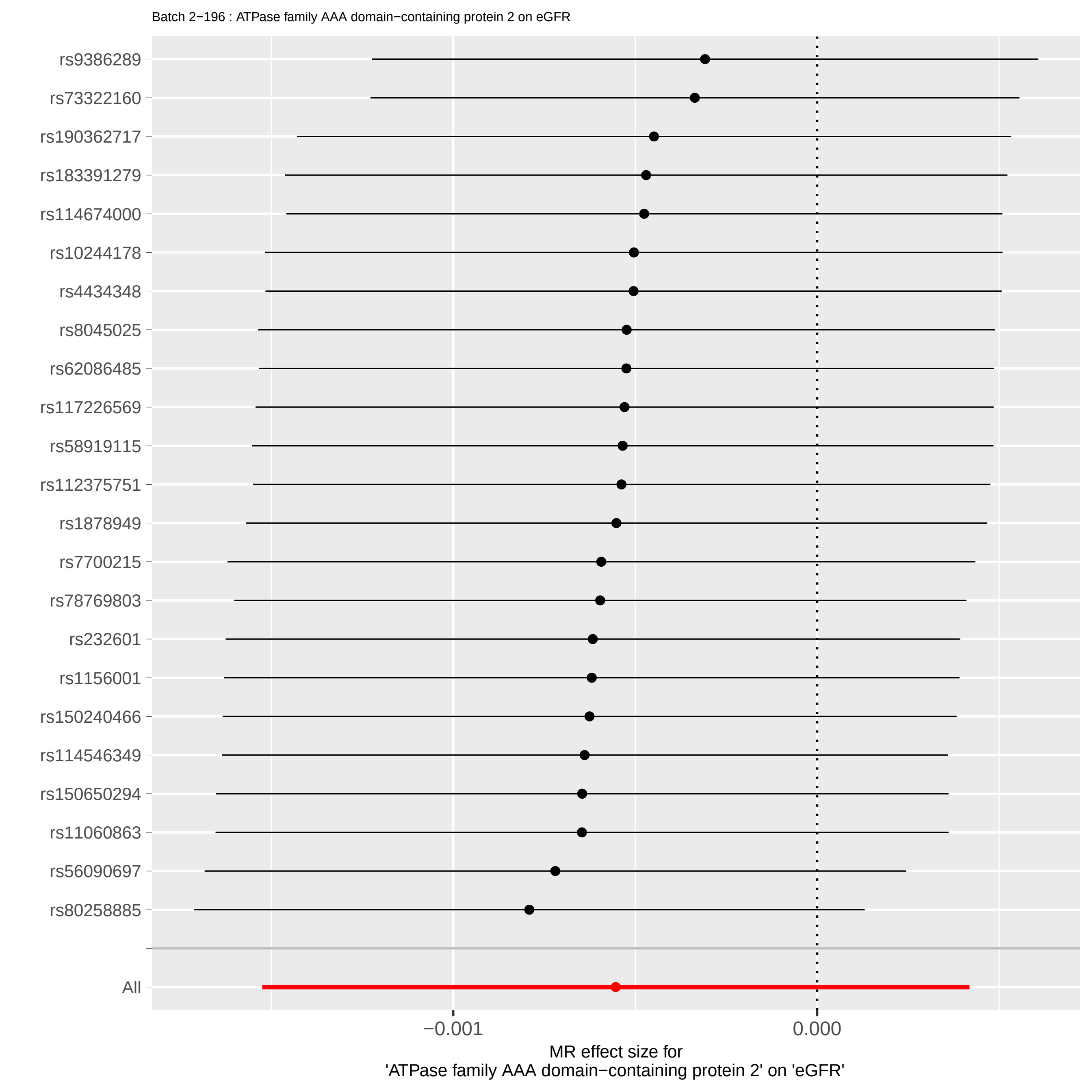
Batch 2−196 : ATPase family AAA domain−containing protein 2 on eGFR
rs9386289
rs73322160
rs190362717
rs183391279
rs114674000
rs10244178
rs4434348
rs8045025
rs62086485
rs117226569
rs58919115
rs112375751
rs1878949
rs7700215
rs78769803
rs232601
rs1156001
rs150240466
rs114546349
rs150650294
rs11060863
rs56090697
rs80258885
All
−0.001
0.000
MR effect size for
'ATPase family AAA domain−containing protein 2' on 'eGFR'

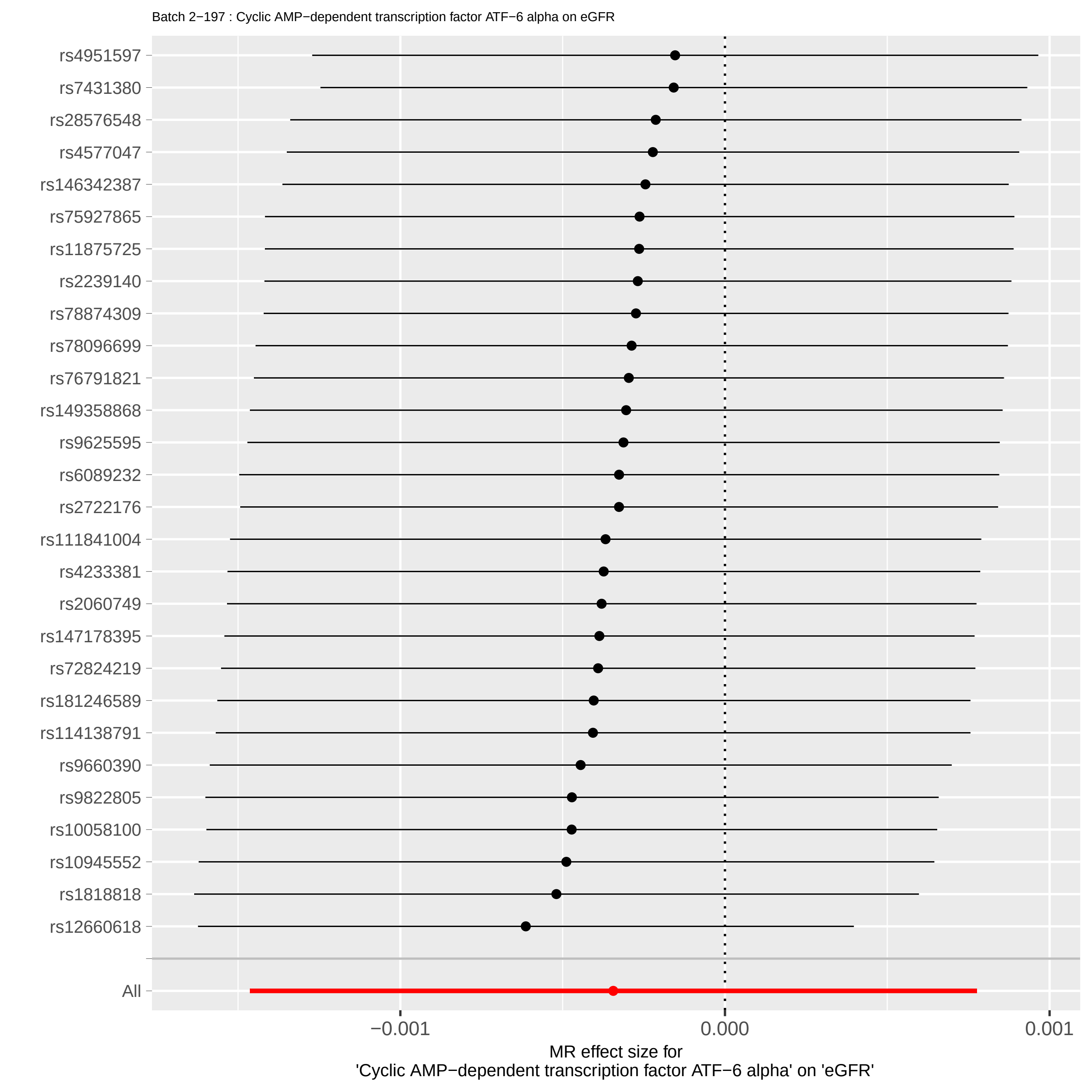

Batch 2−197 : Cyclic AMP−dependent transcription factor ATF−6 alpha on eGFR
rs4951597
rs7431380
rs28576548
rs4577047
rs146342387
rs75927865
rs11875725
rs2239140
rs78874309
rs78096699
rs76791821
rs149358868
rs9625595
rs6089232
rs2722176
rs111841004
rs4233381
rs2060749
rs147178395
rs72824219
rs181246589
rs114138791
rs9660390
rs9822805
rs10058100
rs10945552
rs1818818
rs12660618
All
−0.001
0.000
0.001
MR effect size for
'Cyclic AMP−dependent transcription factor ATF−6 alpha' on 'eGFR'

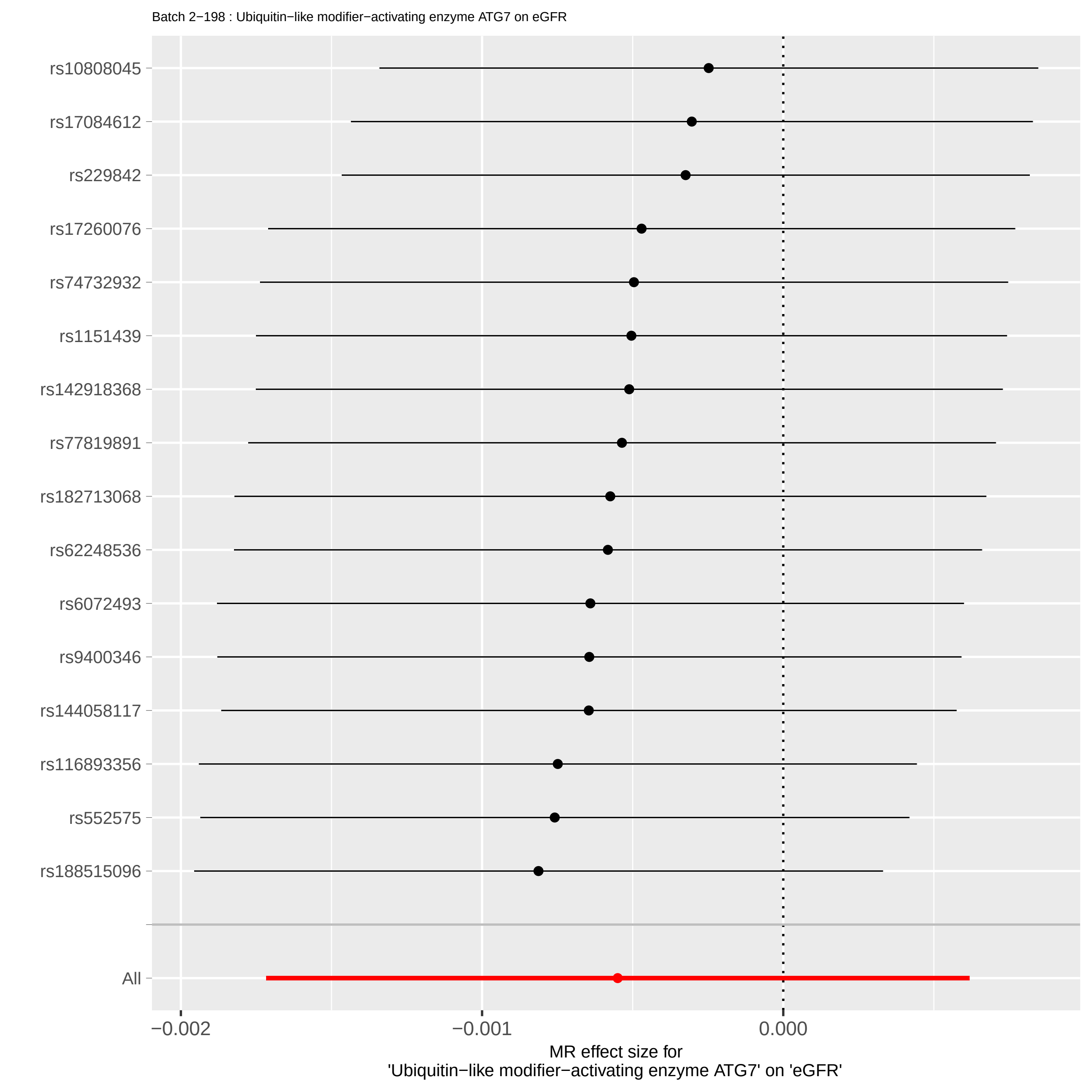
Batch 2−198 : Ubiquitin−like modifier−activating enzyme ATG7 on eGFR
rs10808045
rs17084612
rs229842
rs17260076
rs74732932
rs1151439
rs142918368
rs77819891
rs182713068
rs62248536
rs6072493
rs9400346
rs144058117
rs116893356
rs552575
rs188515096
All
−0.002
−0.001
0.000
MR effect size for
'Ubiquitin−like modifier−activating enzyme ATG7' on 'eGFR'

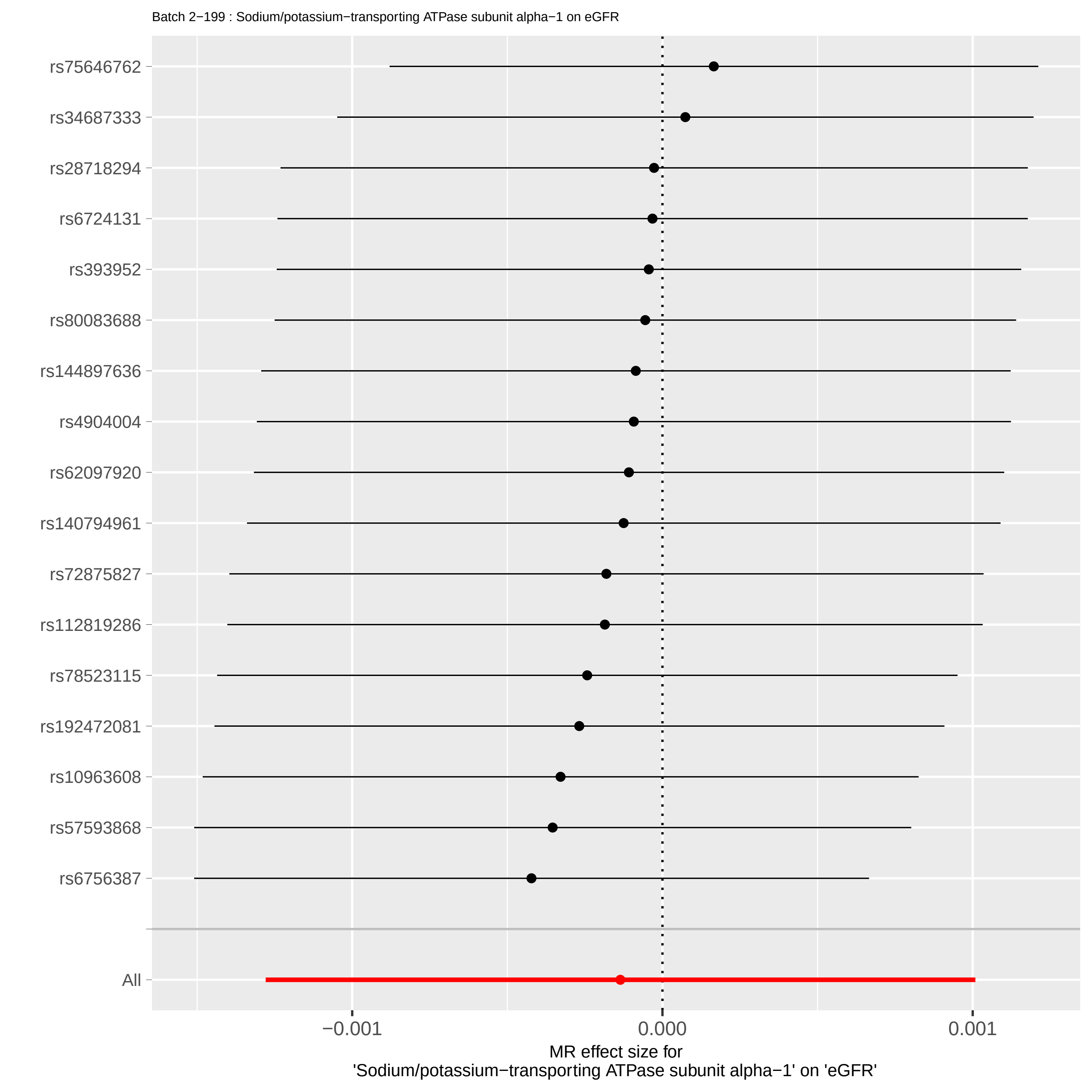

Batch 2−199 : Sodium/potassium−transporting ATPase subunit alpha−1 on eGFR
rs75646762
rs34687333
rs28718294
rs6724131
rs393952
rs80083688
rs144897636
rs4904004
rs62097920
rs140794961
rs72875827
rs112819286
rs78523115
rs192472081
rs10963608
rs57593868
rs6756387
All
−0.001
0.000
0.001
MR effect size for
'Sodium/potassium−transporting ATPase subunit alpha−1' on 'eGFR'

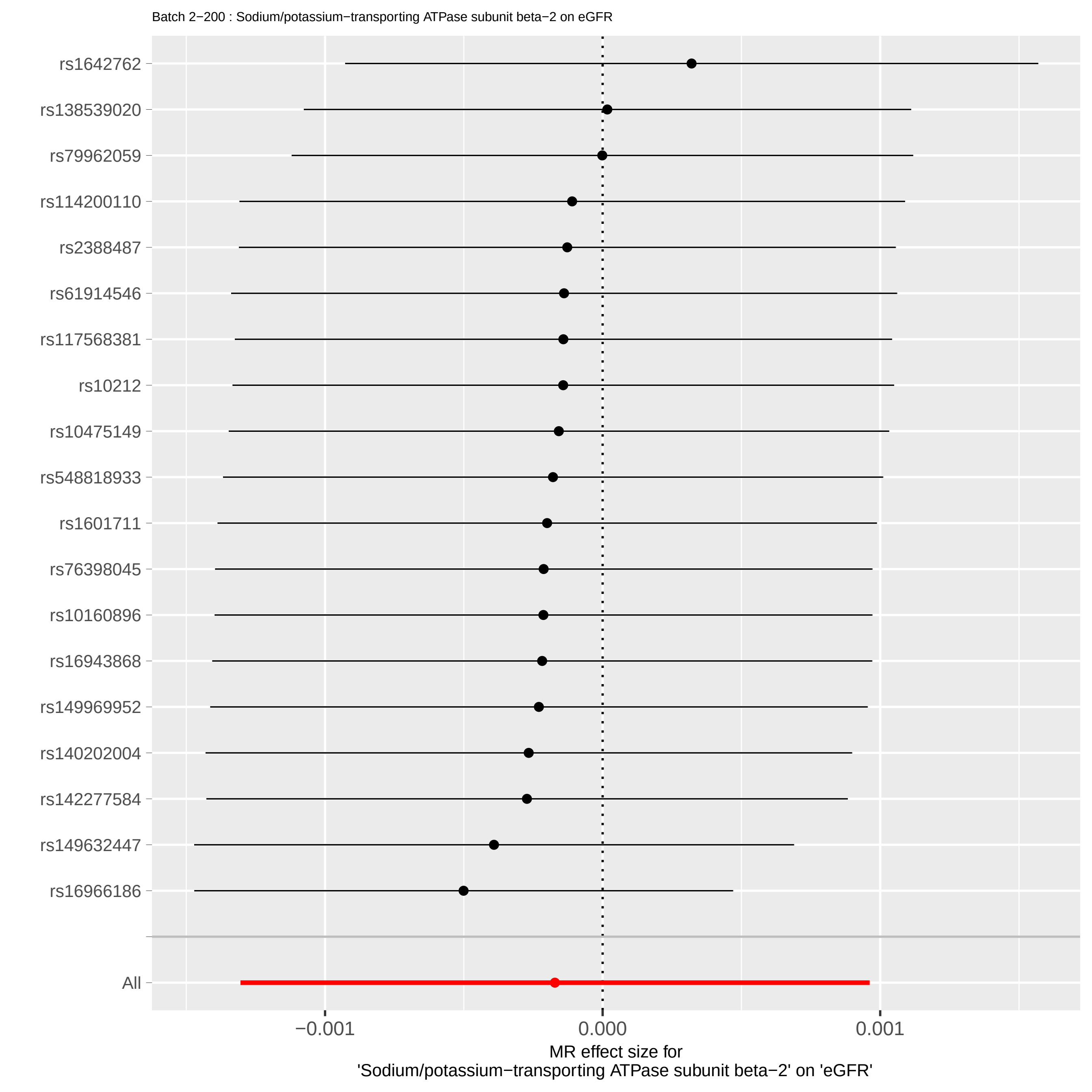
Batch 2−200 : Sodium/potassium−transporting ATPase subunit beta−2 on eGFR
rs1642762
rs138539020
rs79962059
rs114200110
rs2388487
rs61914546
rs117568381
rs10212
rs10475149
rs548818933
rs1601711
rs76398045
rs10160896
rs16943868
rs149969952
rs140202004
rs142277584
rs149632447
rs16966186
All
−0.001
0.000
0.001
MR effect size for
'Sodium/potassium−transporting ATPase subunit beta−2' on 'eGFR'

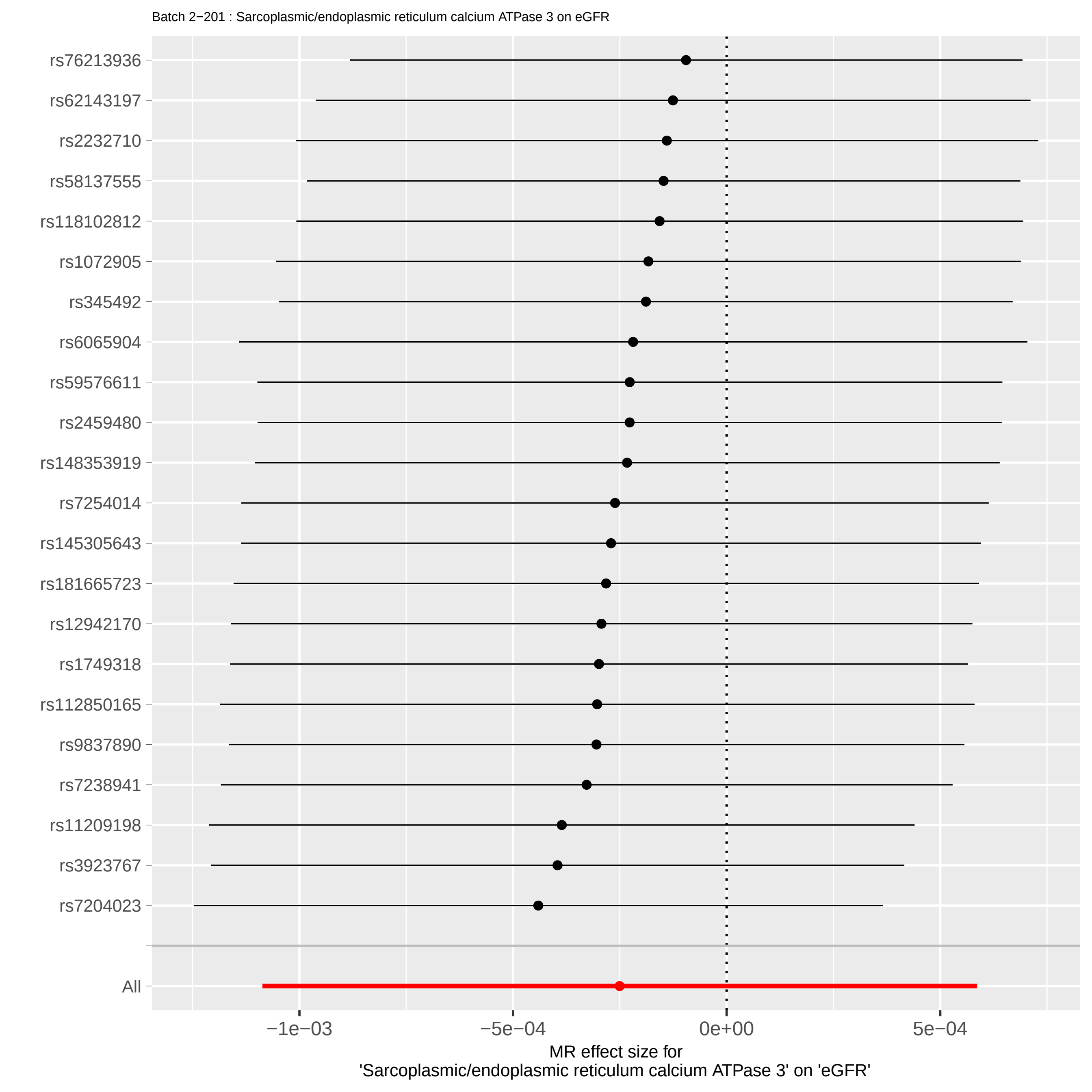

Batch 2−201 : Sarcoplasmic/endoplasmic reticulum calcium ATPase 3 on eGFR
rs76213936
rs62143197
rs2232710
rs58137555
rs118102812
rs1072905
rs345492
rs6065904
rs59576611
rs2459480
rs148353919
rs7254014
rs145305643
rs181665723
rs12942170
rs1749318
rs112850165
rs9837890
rs7238941
rs11209198
rs3923767
rs7204023
All
−1e−03
−5e−04
0e+00
5e−04
MR effect size for
'Sarcoplasmic/endoplasmic reticulum calcium ATPase 3' on 'eGFR'

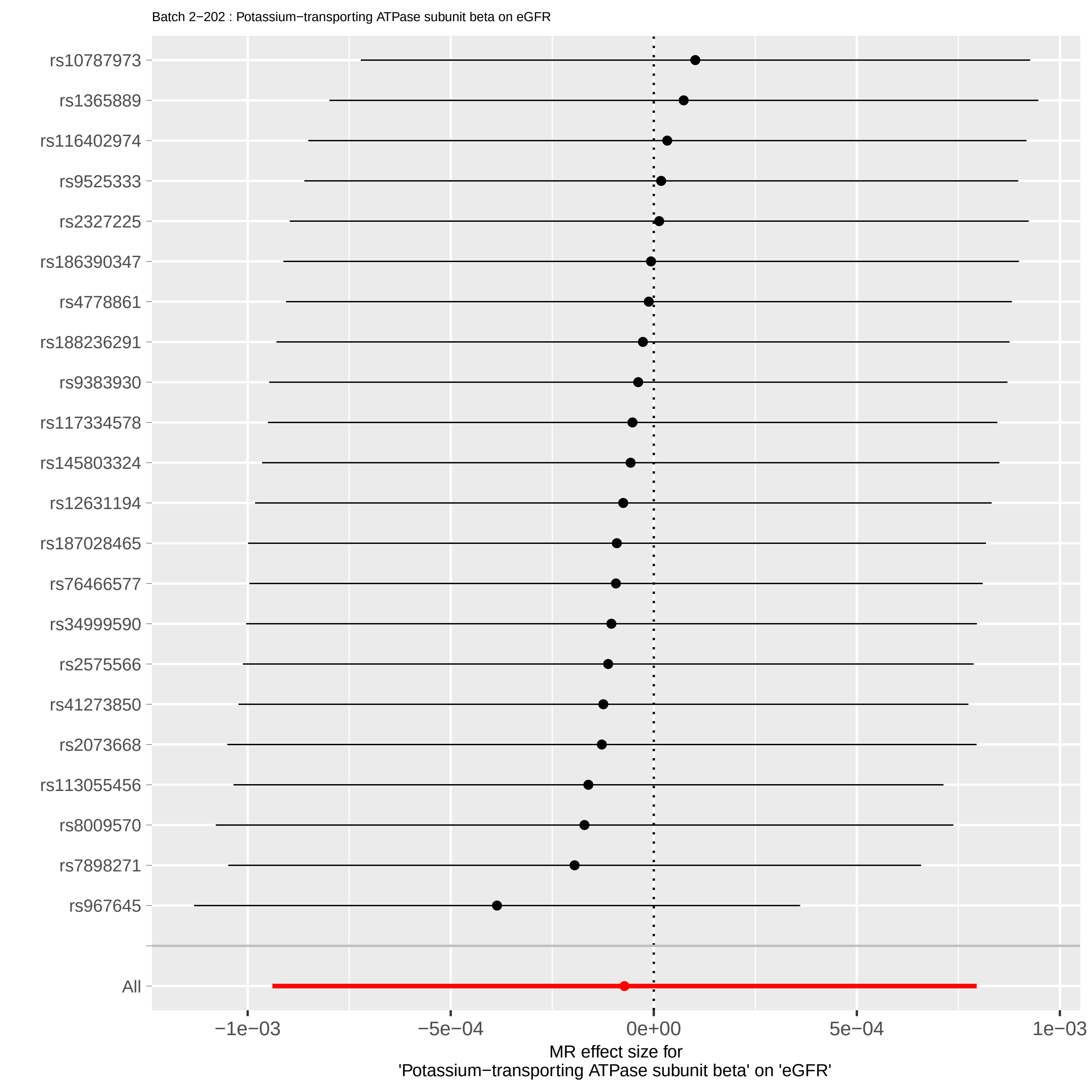
Batch 2−202 : Potassium−transporting ATPase subunit beta on eGFR
rs10787973
rs1365889
rs116402974
rs9525333
rs2327225
rs186390347
rs4778861
rs188236291
rs9383930
rs117334578
rs145803324
rs12631194
rs187028465
rs76466577
rs34999590
rs2575566
rs41273850
rs2073668
rs113055456
rs8009570
rs7898271
rs967645
All
−1e−03
−5e−04
0e+00
5e−04
1e−03
MR effect size for
'Potassium−transporting ATPase subunit beta' on 'eGFR'

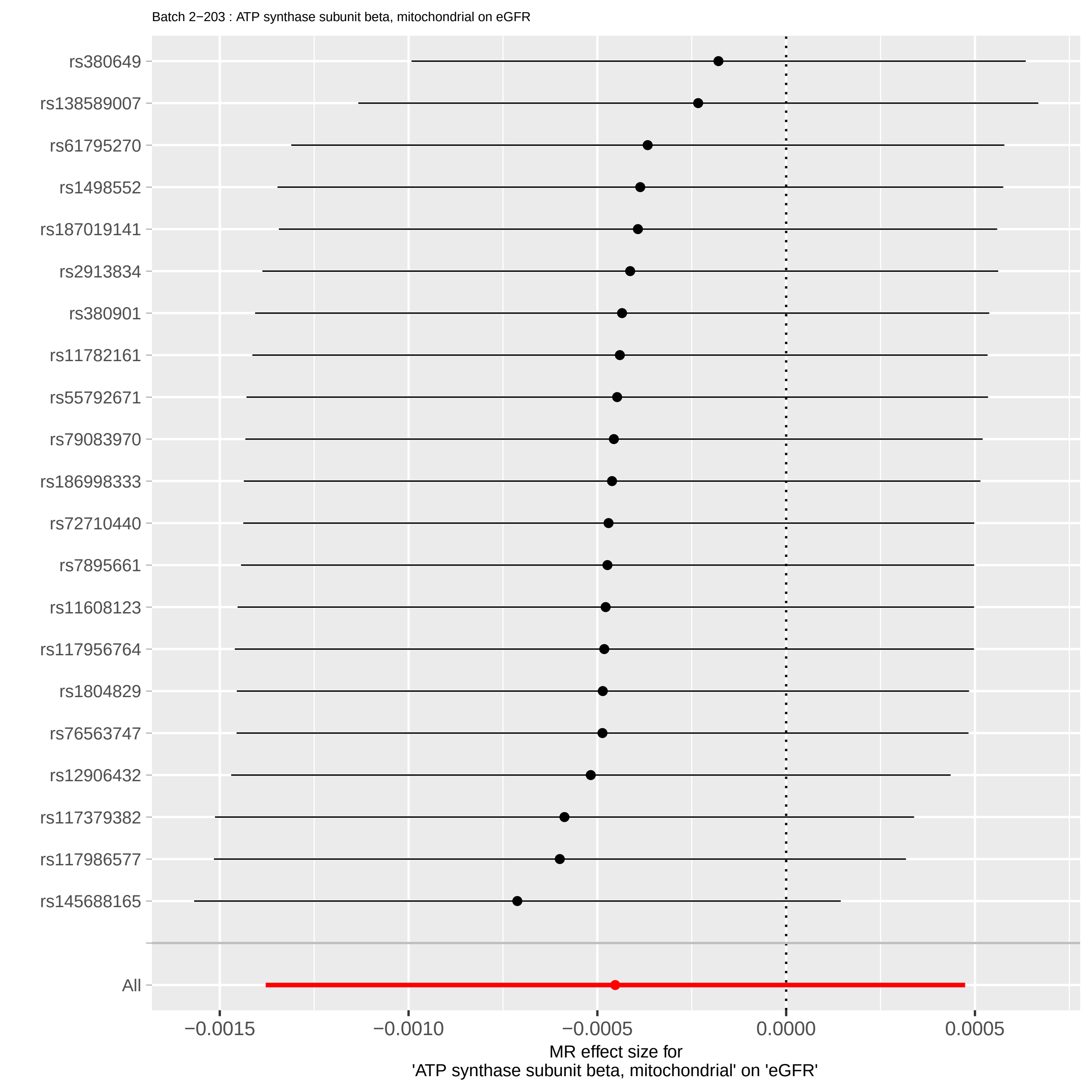

Batch 2−203 : ATP synthase subunit beta, mitochondrial on eGFR
rs380649
rs138589007
rs61795270
rs1498552
rs187019141
rs2913834
rs380901
rs11782161
rs55792671
rs79083970
rs186998333
rs72710440
rs7895661
rs11608123
rs117956764
rs1804829
rs76563747
rs12906432
rs117379382
rs117986577
rs145688165
All
−0.0015
−0.0010
−0.0005
0.0000
0.0005
MR effect size for
'ATP synthase subunit beta, mitochondrial' on 'eGFR'

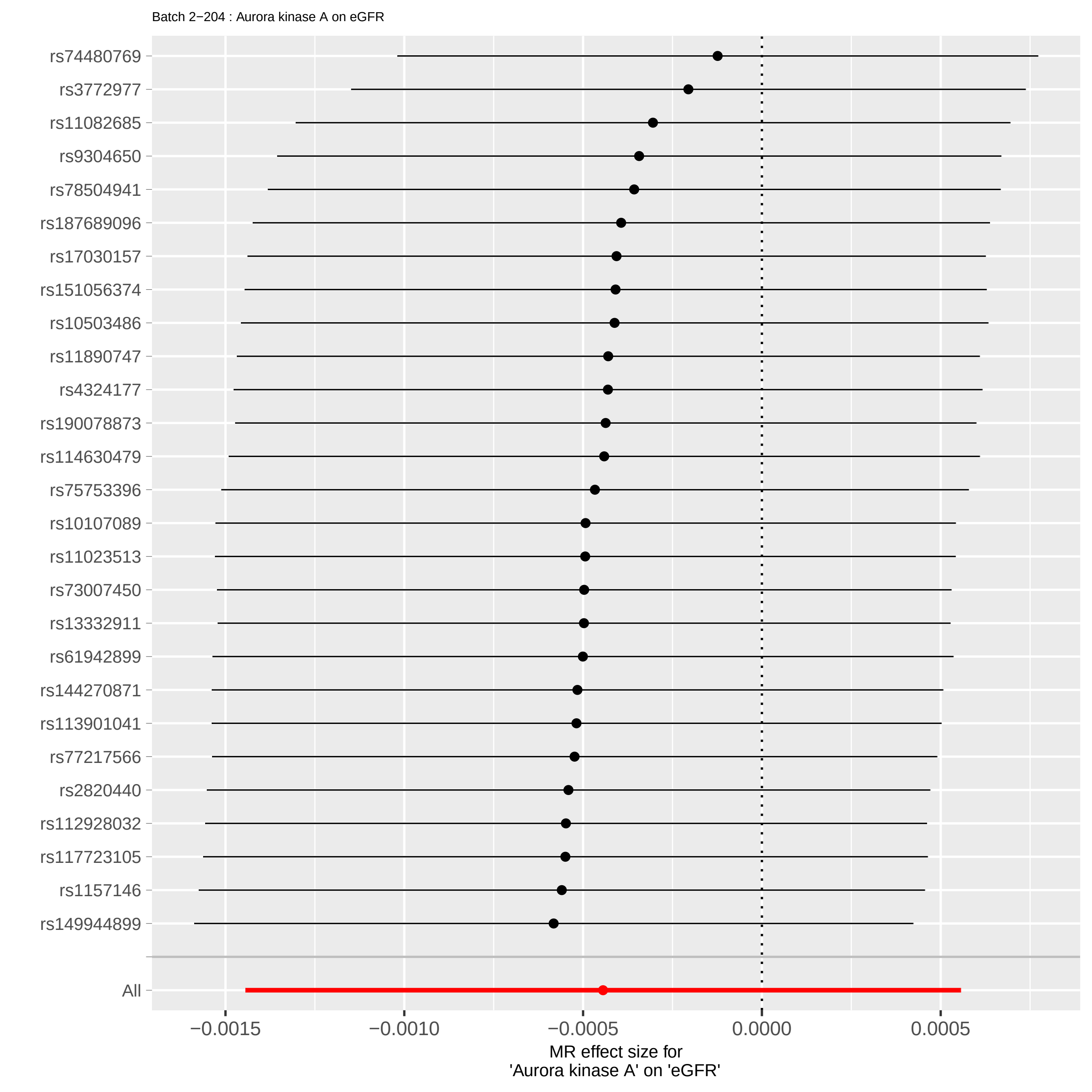

Batch 2−204 : Aurora kinase A on eGFR
rs74480769
rs3772977
rs11082685
rs9304650
rs78504941
rs187689096
rs17030157
rs151056374
rs10503486
rs11890747
rs4324177
rs190078873
rs114630479
rs75753396
rs10107089
rs11023513
rs73007450
rs13332911
rs61942899
rs144270871
rs113901041
rs77217566
rs2820440
rs112928032
rs117723105
rs1157146
rs149944899
All
−0.0015
−0.0010
−0.0005
0.0000
0.0005
MR effect size for
'Aurora kinase A' on 'eGFR'

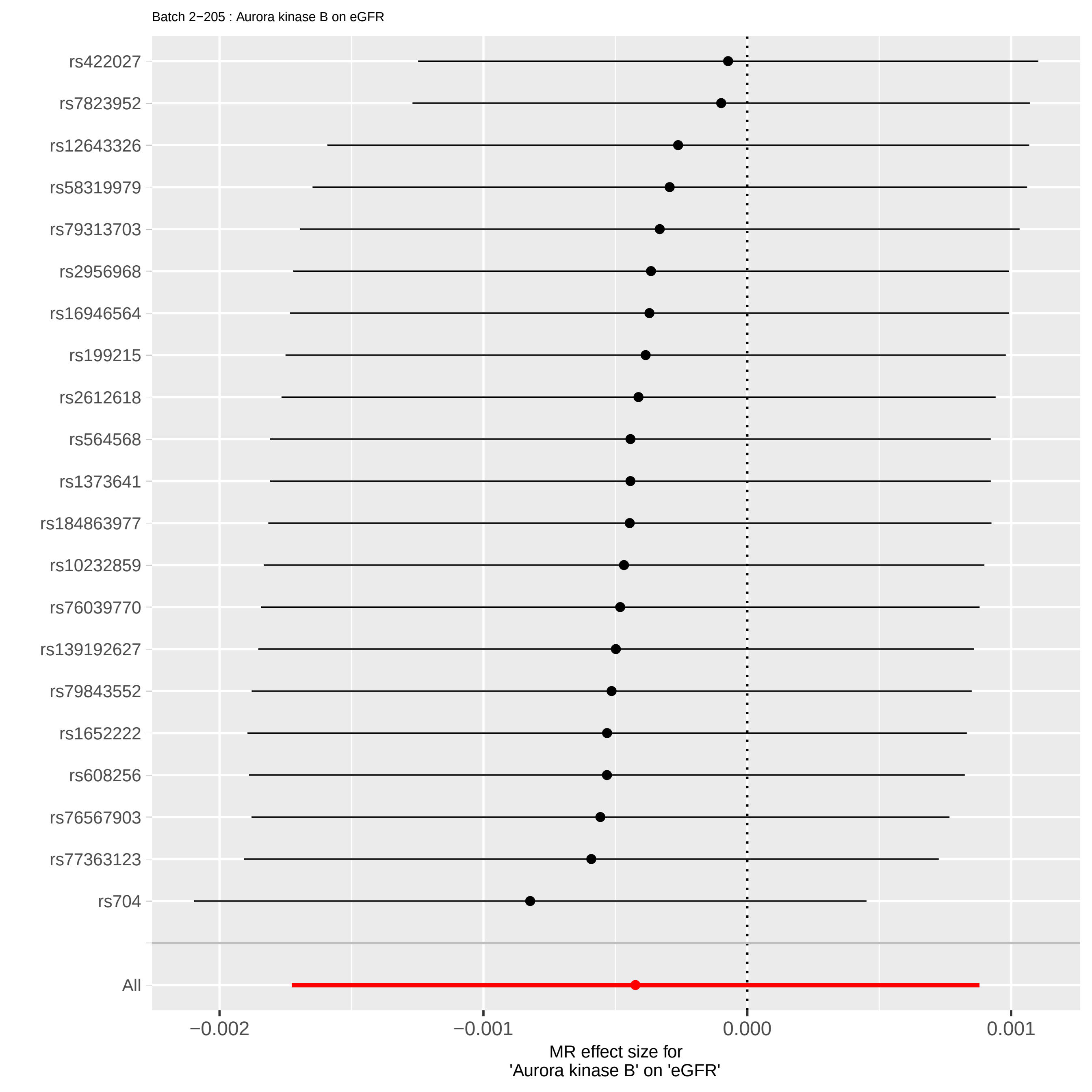

Batch 2−205 : Aurora kinase B on eGFR
rs422027
rs7823952
rs12643326
rs58319979
rs79313703
rs2956968
rs16946564
rs199215
rs2612618
rs564568
rs1373641
rs184863977
rs10232859
rs76039770
rs139192627
rs79843552
rs1652222
rs608256
rs76567903
rs77363123
rs704
All
−0.002
−0.001
0.000
0.001
MR effect size for
'Aurora kinase B' on 'eGFR'

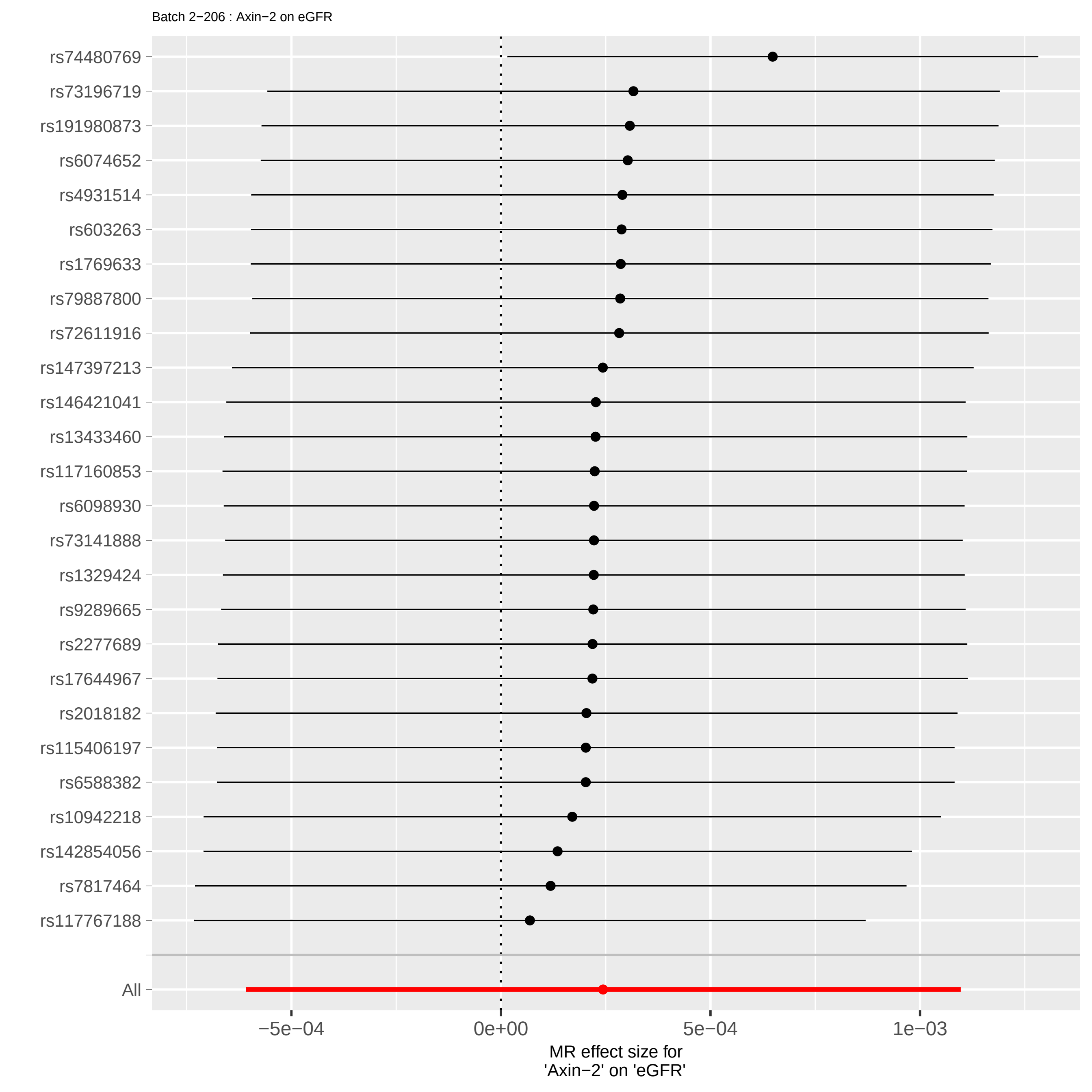

Batch 2−206 : Axin−2 on eGFR
rs74480769
rs73196719
rs191980873
rs6074652
rs4931514
rs603263
rs1769633
rs79887800
rs72611916
rs147397213
rs146421041
rs13433460
rs117160853
rs6098930
rs73141888
rs1329424
rs9289665
rs2277689
rs17644967
rs2018182
rs115406197
rs6588382
rs10942218
rs142854056
rs7817464
rs117767188
All
−5e−04
0e+00
5e−04
1e−03
MR effect size for
'Axin−2' on 'eGFR'

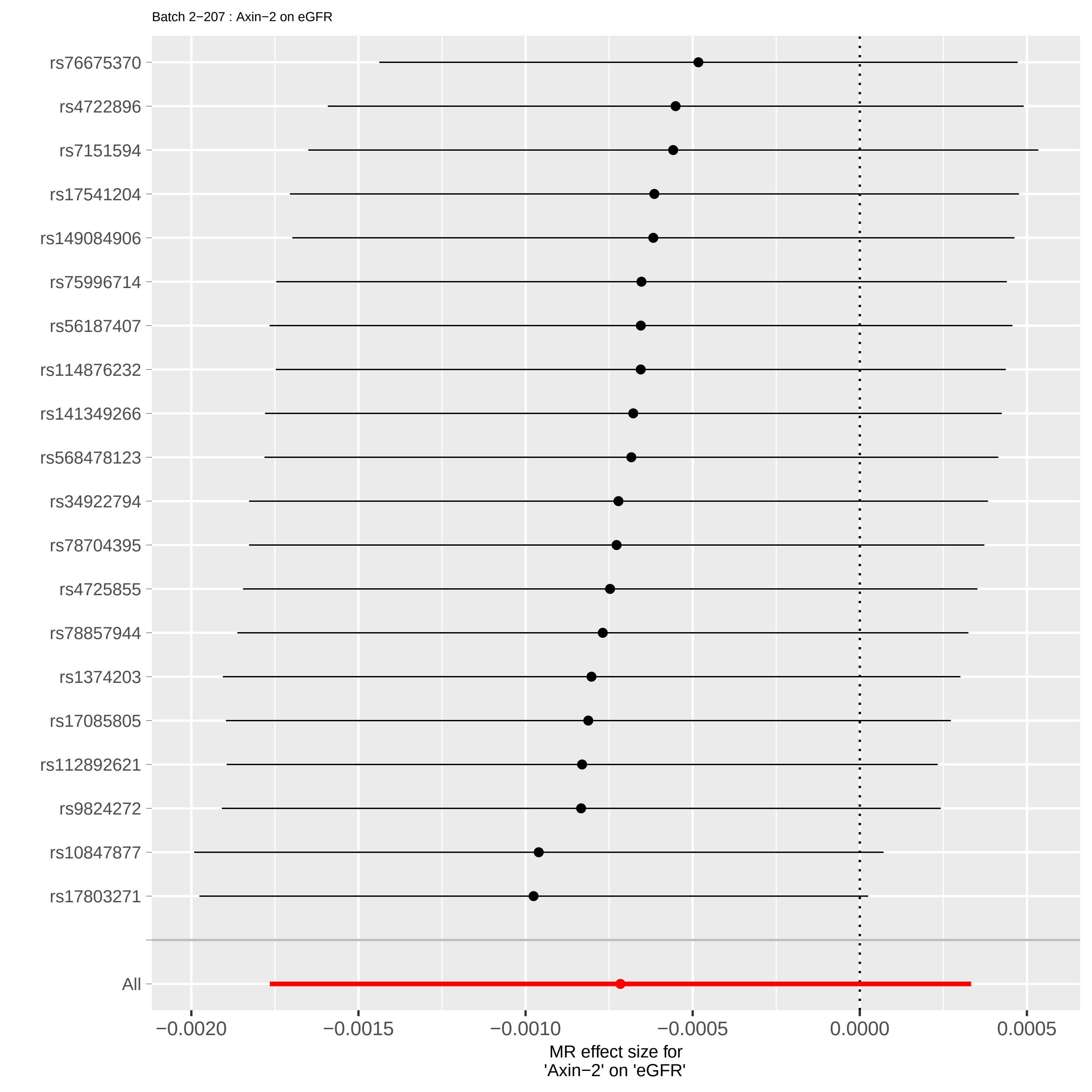

Batch 2−207 : Axin−2 on eGFR
rs76675370
rs4722896
rs7151594
rs17541204
rs149084906
rs75996714
rs56187407
rs114876232
rs141349266
rs568478123
rs34922794
rs78704395
rs4725855
rs78857944
rs1374203
rs17085805
rs112892621
rs9824272
rs10847877
rs17803271
All
−0.0020
−0.0015
−0.0010
−0.0005
0.0000
0.0005
MR effect size for
'Axin−2' on 'eGFR'

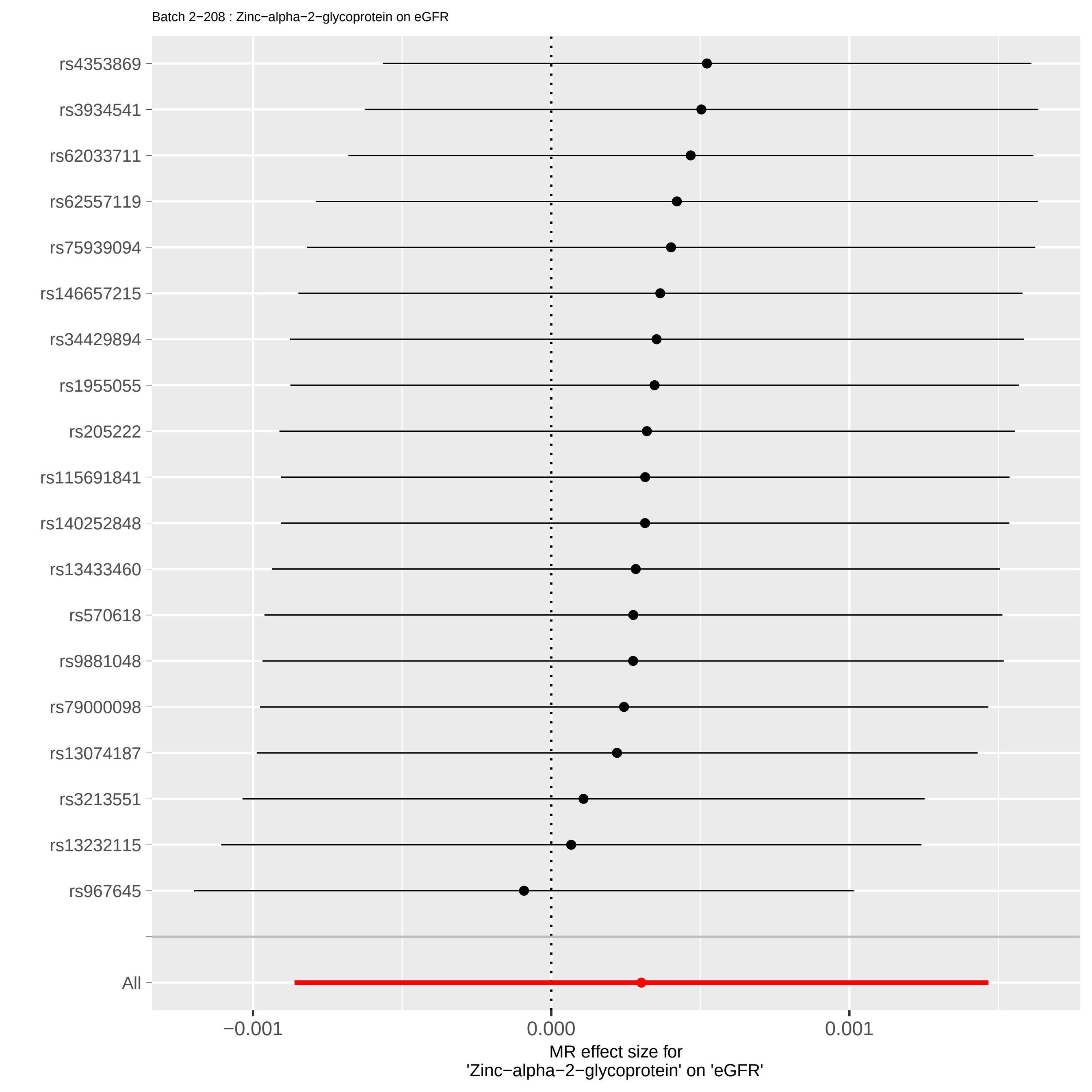

Batch 2−208 : Zinc−alpha−2−glycoprotein on eGFR
rs4353869
rs3934541
rs62033711
rs62557119
rs75939094
rs146657215
rs34429894
rs1955055
rs205222
rs115691841
rs140252848
rs13433460
rs570618
rs9881048
rs79000098
rs13074187
rs3213551
rs13232115
rs967645
All
−0.001
0.000
0.001
MR effect size for
'Zinc−alpha−2−glycoprotein' on 'eGFR'

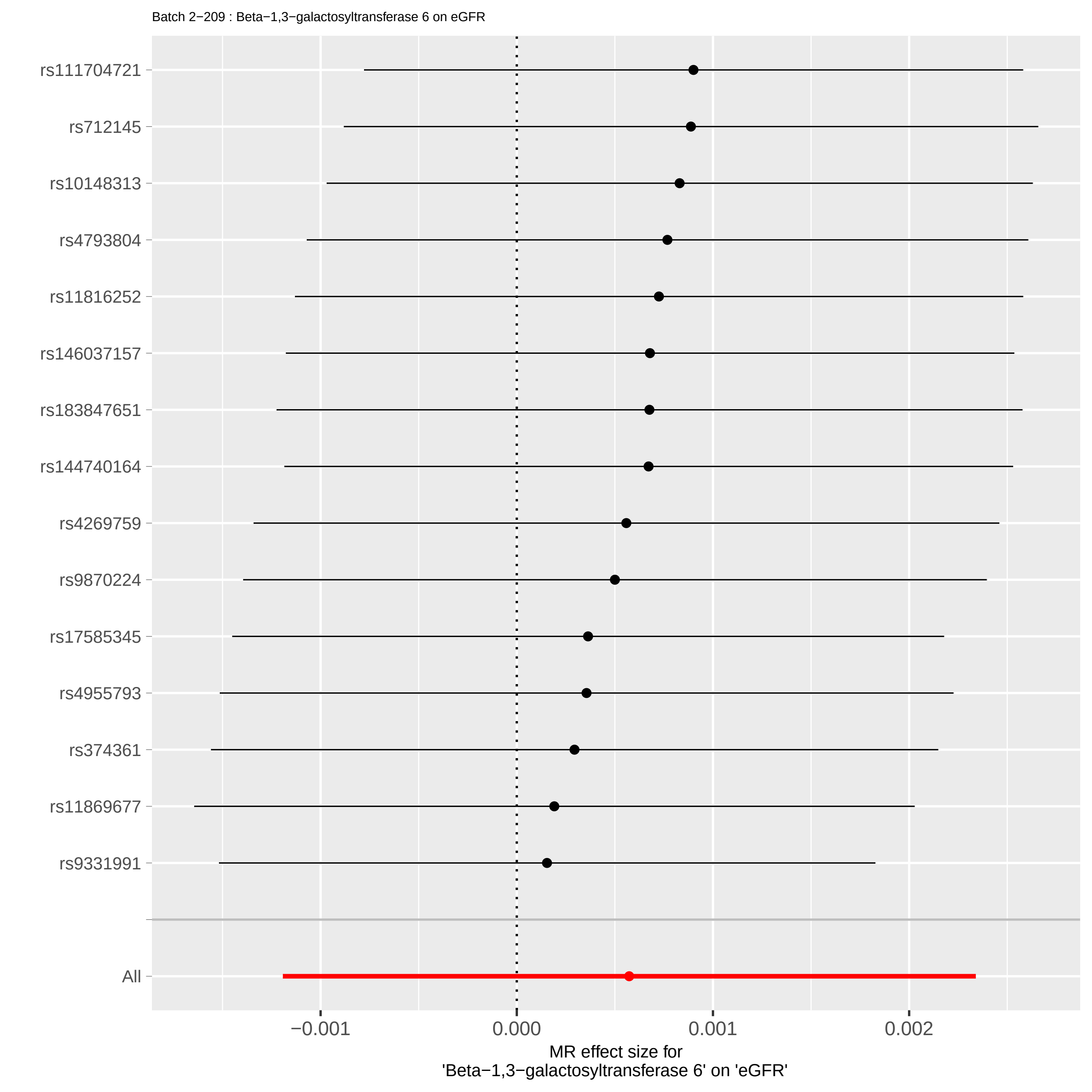

Batch 2−209 : Beta−1,3−galactosyltransferase 6 on eGFR
rs111704721
rs712145
rs10148313
rs4793804
rs11816252
rs146037157
rs183847651
rs144740164
rs4269759
rs9870224
rs17585345
rs4955793
rs374361
rs11869677
rs9331991
All
−0.001
0.000
0.001
0.002
MR effect size for
'Beta−1,3−galactosyltransferase 6' on 'eGFR'

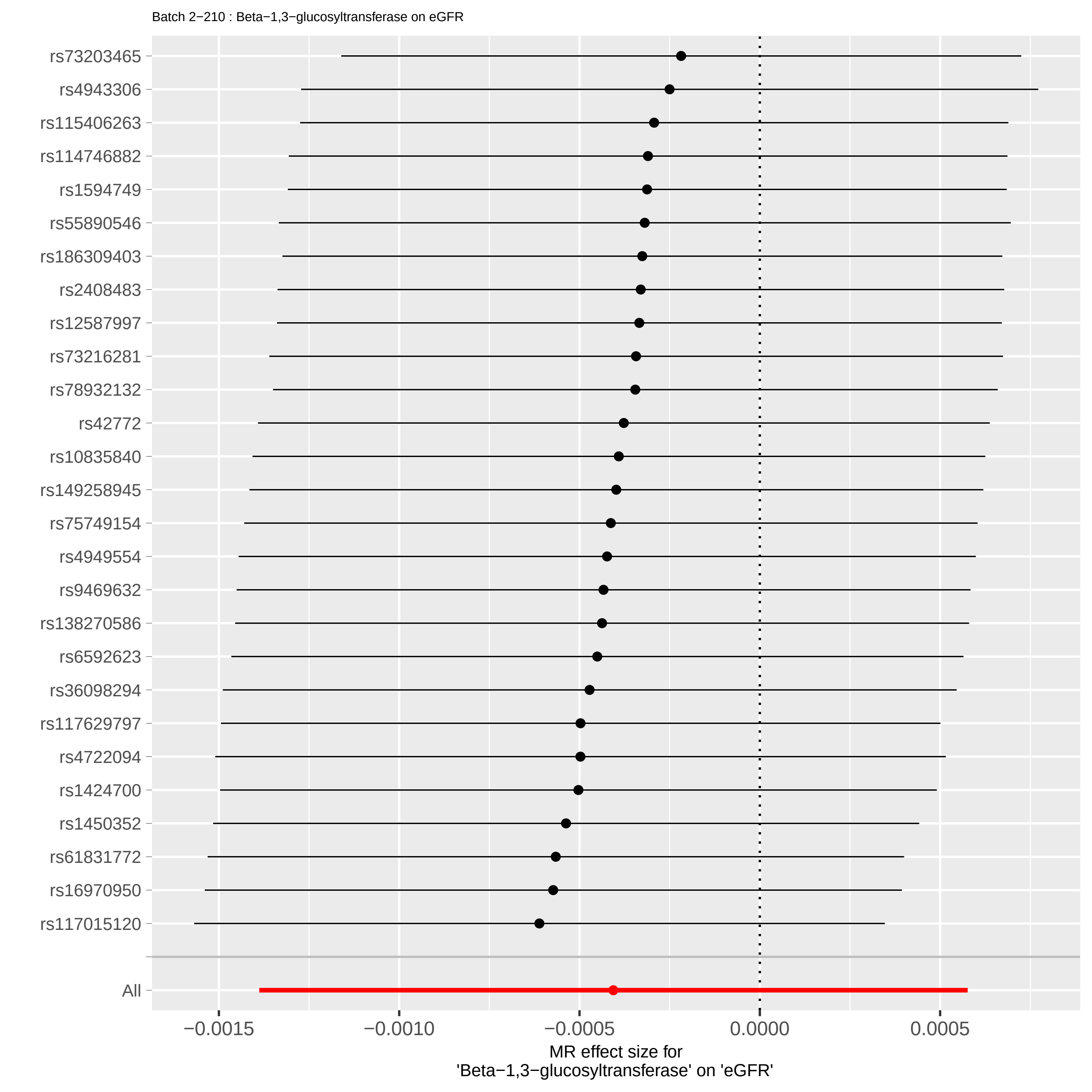
Batch 2−210 : Beta−1,3−glucosyltransferase on eGFR
rs73203465
rs4943306
rs115406263
rs114746882
rs1594749
rs55890546
rs186309403
rs2408483
rs12587997
rs73216281
rs78932132
rs42772
rs10835840
rs149258945
rs75749154
rs4949554
rs9469632
rs138270586
rs6592623
rs36098294
rs117629797
rs4722094
rs1424700
rs1450352
rs61831772
rs16970950
rs117015120
All
−0.0015
−0.0010
−0.0005
0.0000
0.0005
MR effect size for
'Beta−1,3−glucosyltransferase' on 'eGFR'

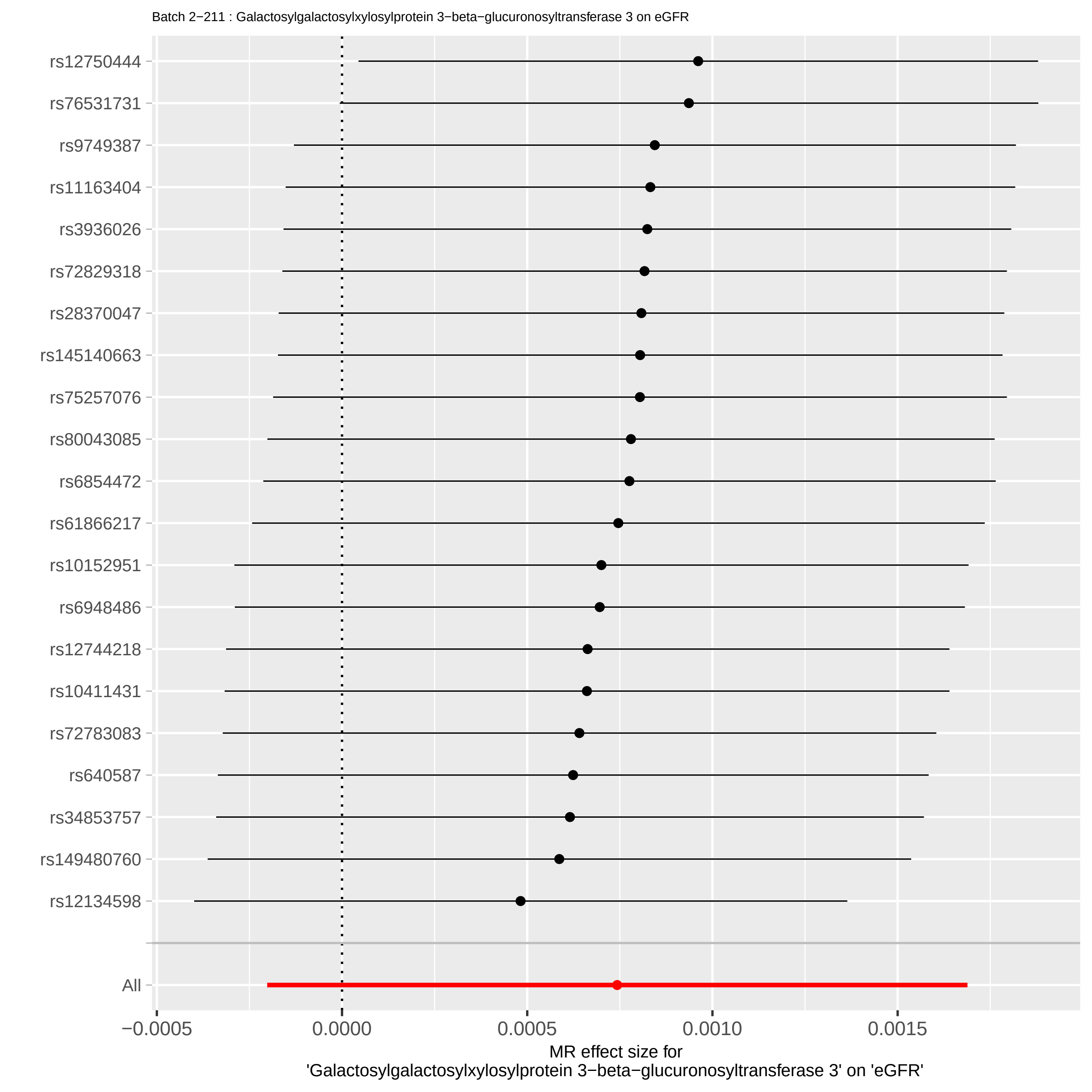

Batch 2−211 : Galactosylgalactosylxylosylprotein 3−beta−glucuronosyltransferase 3 on eGFR
rs12750444
rs76531731
rs9749387
rs11163404
rs3936026
rs72829318
rs28370047
rs145140663
rs75257076
rs80043085
rs6854472
rs61866217
rs10152951
rs6948486
rs12744218
rs10411431
rs72783083
rs640587
rs34853757
rs149480760
rs12134598
All
−0.0005
0.0000
0.0005
0.0010
0.0015
MR effect size for
'Galactosylgalactosylxylosylprotein 3−beta−glucuronosyltransferase 3' on 'eGFR'

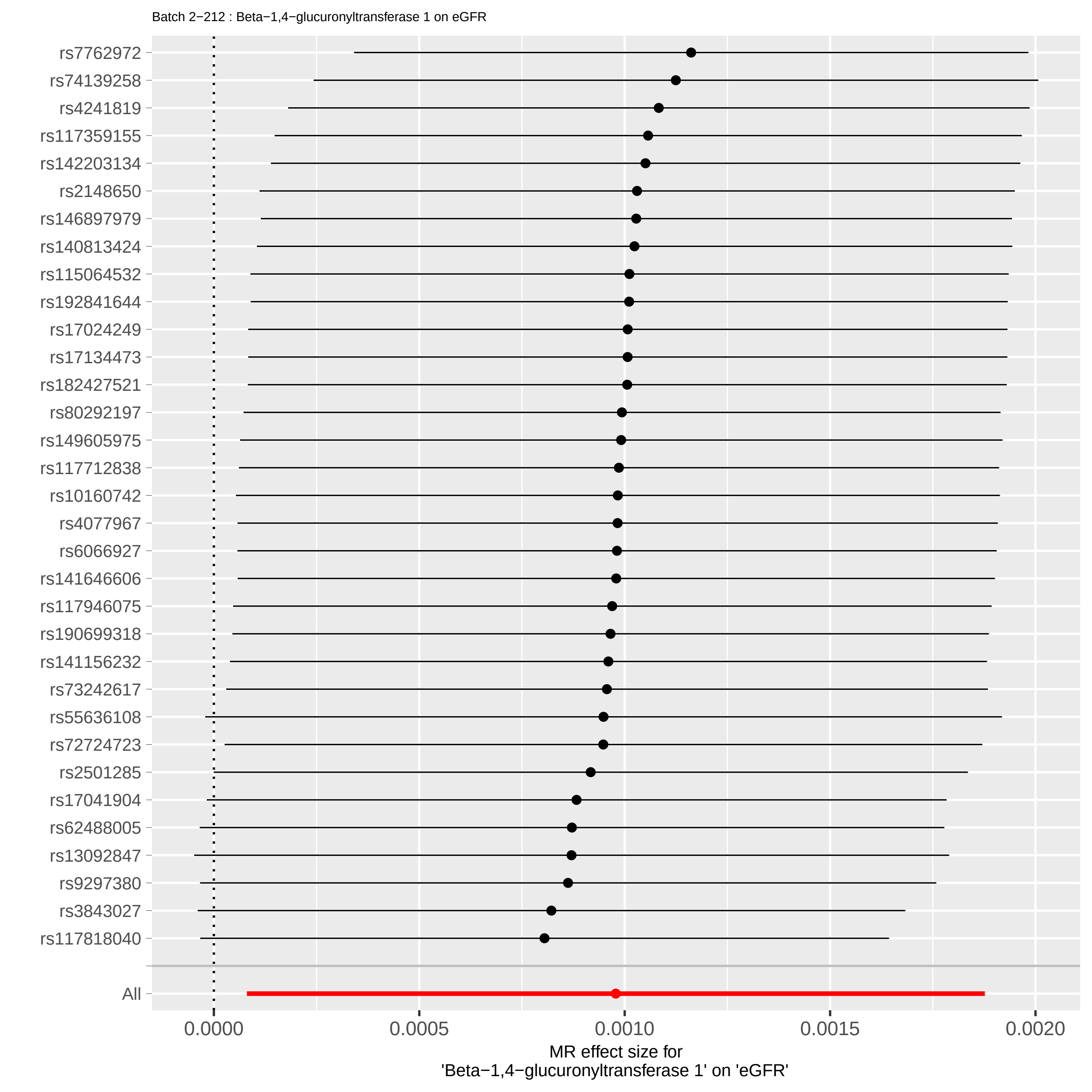

Batch 2−212 : Beta−1,4−glucuronyltransferase 1 on eGFR
rs7762972
rs74139258
rs4241819
rs117359155
rs142203134
rs2148650
rs146897979
rs140813424
rs115064532
rs192841644
rs17024249
rs17134473
rs182427521
rs80292197
rs149605975
rs117712838
rs10160742
rs4077967
rs6066927
rs141646606
rs117946075
rs190699318
rs141156232
rs73242617
rs55636108
rs72724723
rs2501285
rs17041904
rs62488005
rs13092847
rs9297380
rs3843027
rs117818040
All
0.0000
0.0005
0.0010
0.0015
0.0020
MR effect size for
'Beta−1,4−glucuronyltransferase 1' on 'eGFR'

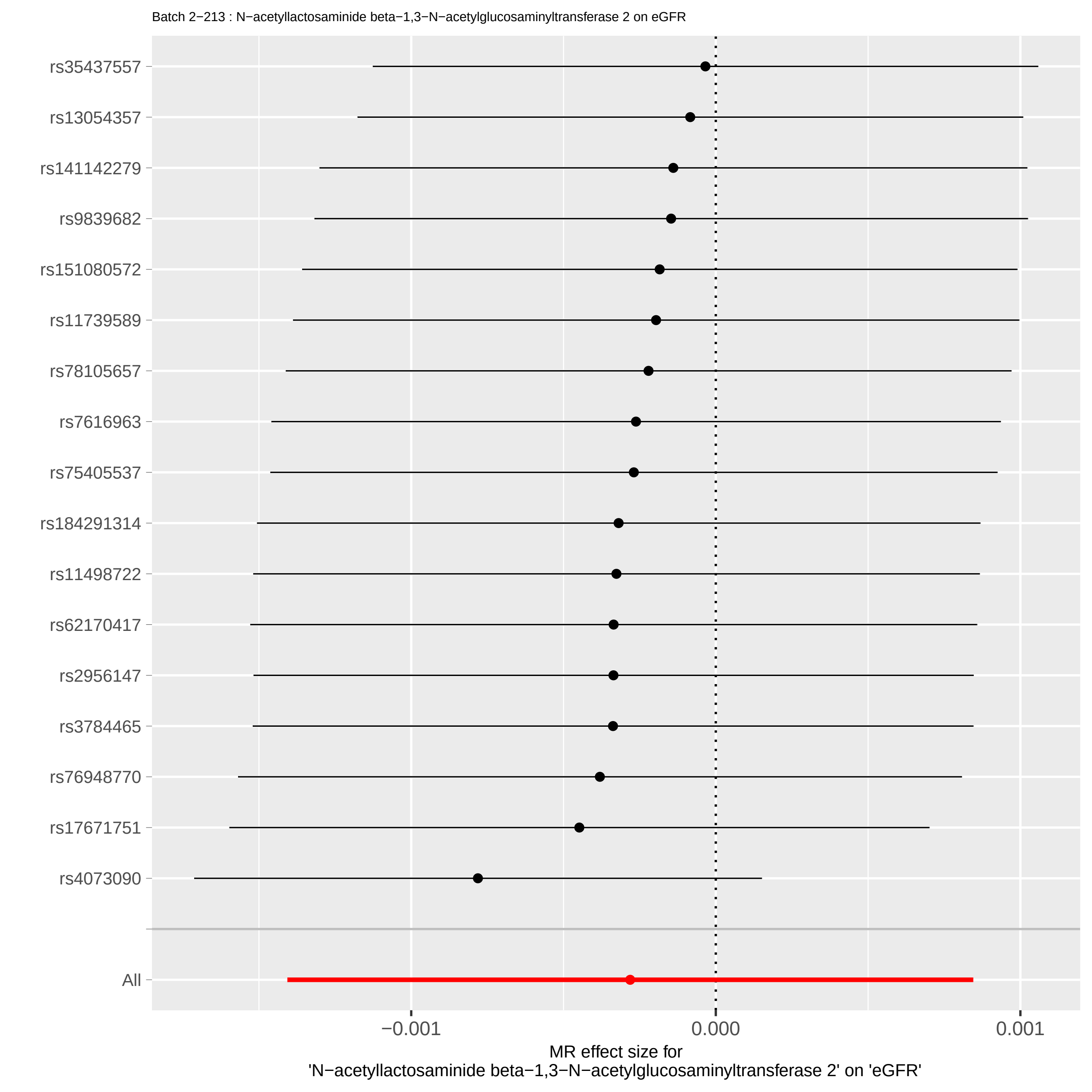
Batch 2−213 : N−acetyllactosaminide beta−1,3−N−acetylglucosaminyltransferase 2 on eGFR
rs35437557
rs13054357
rs141142279
rs9839682
rs151080572
rs11739589
rs78105657
rs7616963
rs75405537
rs184291314
rs11498722
rs62170417
rs2956147
rs3784465
rs76948770
rs17671751
rs4073090
All
−0.001
0.000
0.001
MR effect size for
'N−acetyllactosaminide beta−1,3−N−acetylglucosaminyltransferase 2' on 'eGFR'

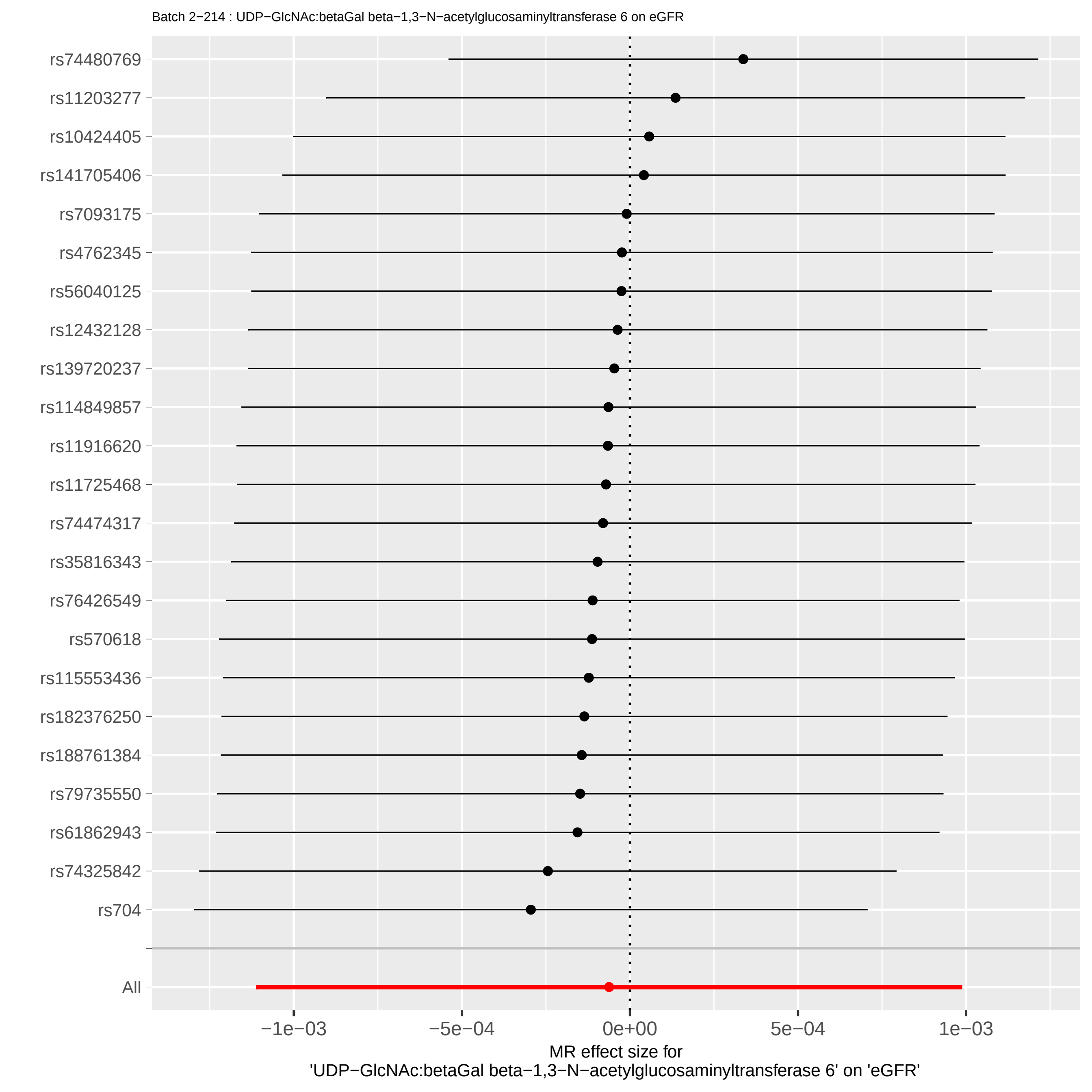

Batch 2−214 : UDP−GlcNAc:betaGal beta−1,3−N−acetylglucosaminyltransferase 6 on eGFR
rs74480769
rs11203277
rs10424405
rs141705406
rs7093175
rs4762345
rs56040125
rs12432128
rs139720237
rs114849857
rs11916620
rs11725468
rs74474317
rs35816343
rs76426549
rs570618
rs115553436
rs182376250
rs188761384
rs79735550
rs61862943
rs74325842
rs704
All
−1e−03
−5e−04
0e+00
5e−04
1e−03
MR effect size for
'UDP−GlcNAc:betaGal beta−1,3−N−acetylglucosaminyltransferase 6' on 'eGFR'

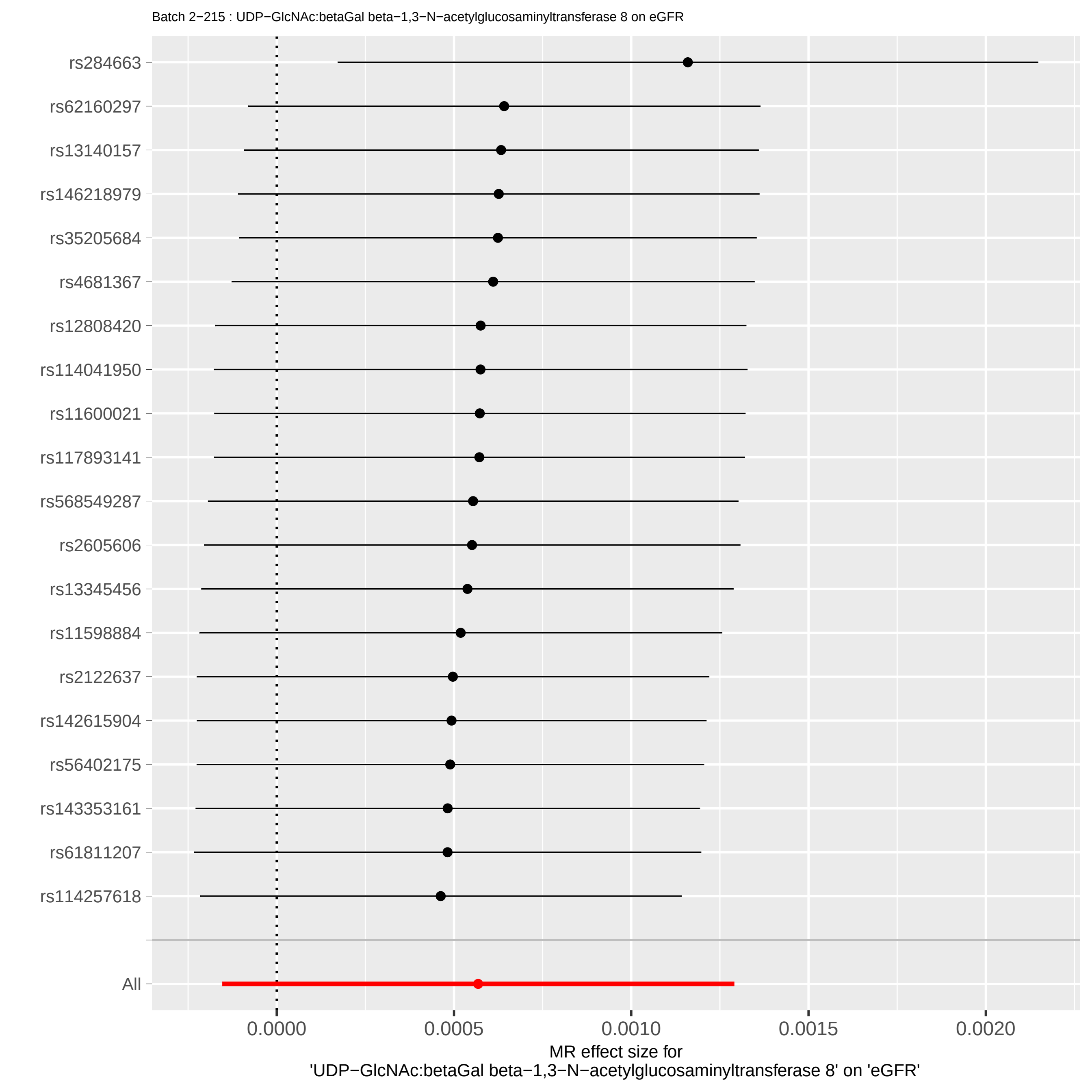

Batch 2−215 : UDP−GlcNAc:betaGal beta−1,3−N−acetylglucosaminyltransferase 8 on eGFR
rs284663
rs62160297
rs13140157
rs146218979
rs35205684
rs4681367
rs12808420
rs114041950
rs11600021
rs117893141
rs568549287
rs2605606
rs13345456
rs11598884
rs2122637
rs142615904
rs56402175
rs143353161
rs61811207
rs114257618
All
0.0000
0.0005
0.0010
0.0015
0.0020
MR effect size for
'UDP−GlcNAc:betaGal beta−1,3−N−acetylglucosaminyltransferase 8' on 'eGFR'

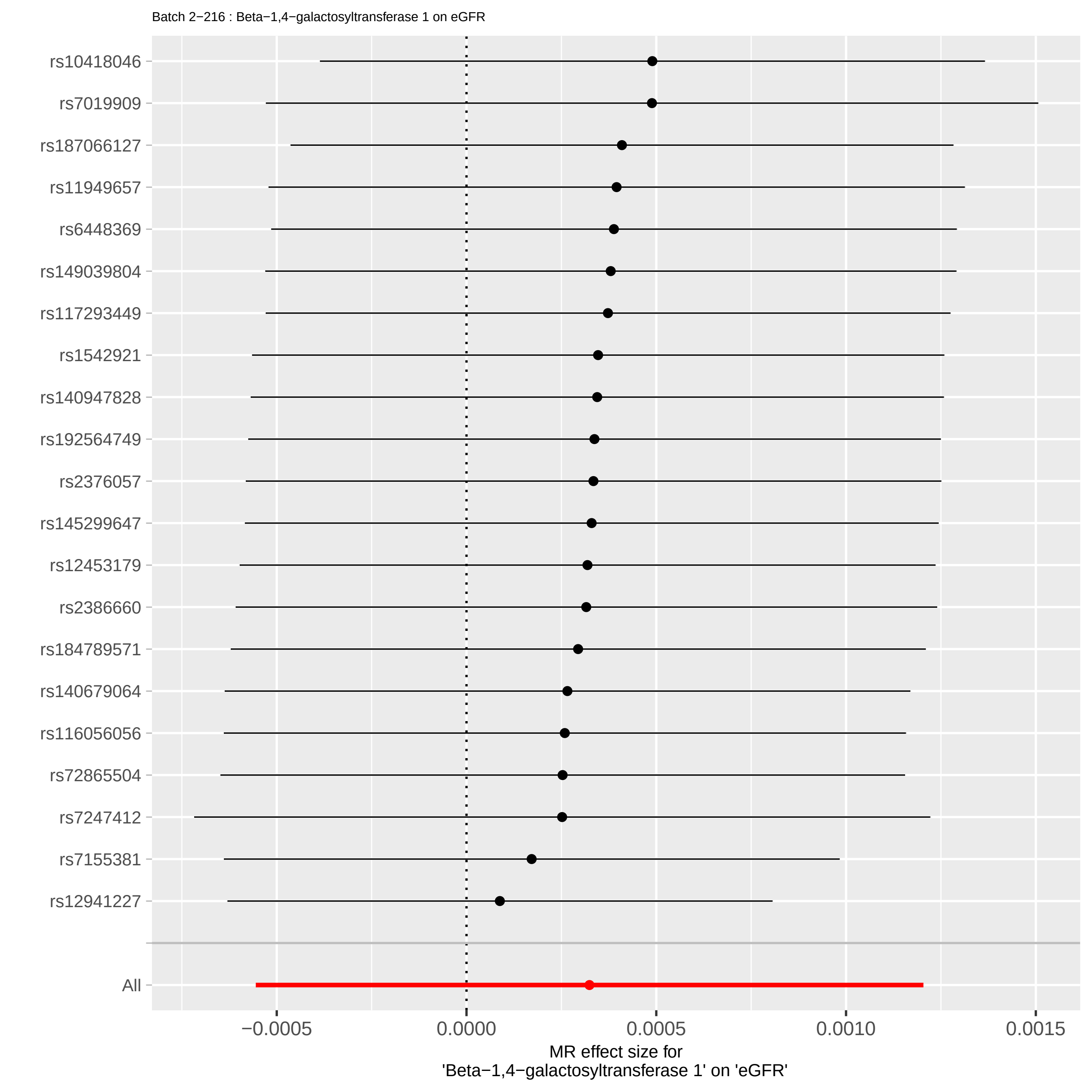
Batch 2−216 : Beta−1,4−galactosyltransferase 1 on eGFR
rs10418046
rs7019909
rs187066127
rs11949657
rs6448369
rs149039804
rs117293449
rs1542921
rs140947828
rs192564749
rs2376057
rs145299647
rs12453179
rs2386660
rs184789571
rs140679064
rs116056056
rs72865504
rs7247412
rs7155381
rs12941227
All
−0.0005
0.0000
0.0005
0.0010
0.0015
MR effect size for
'Beta−1,4−galactosyltransferase 1' on 'eGFR'

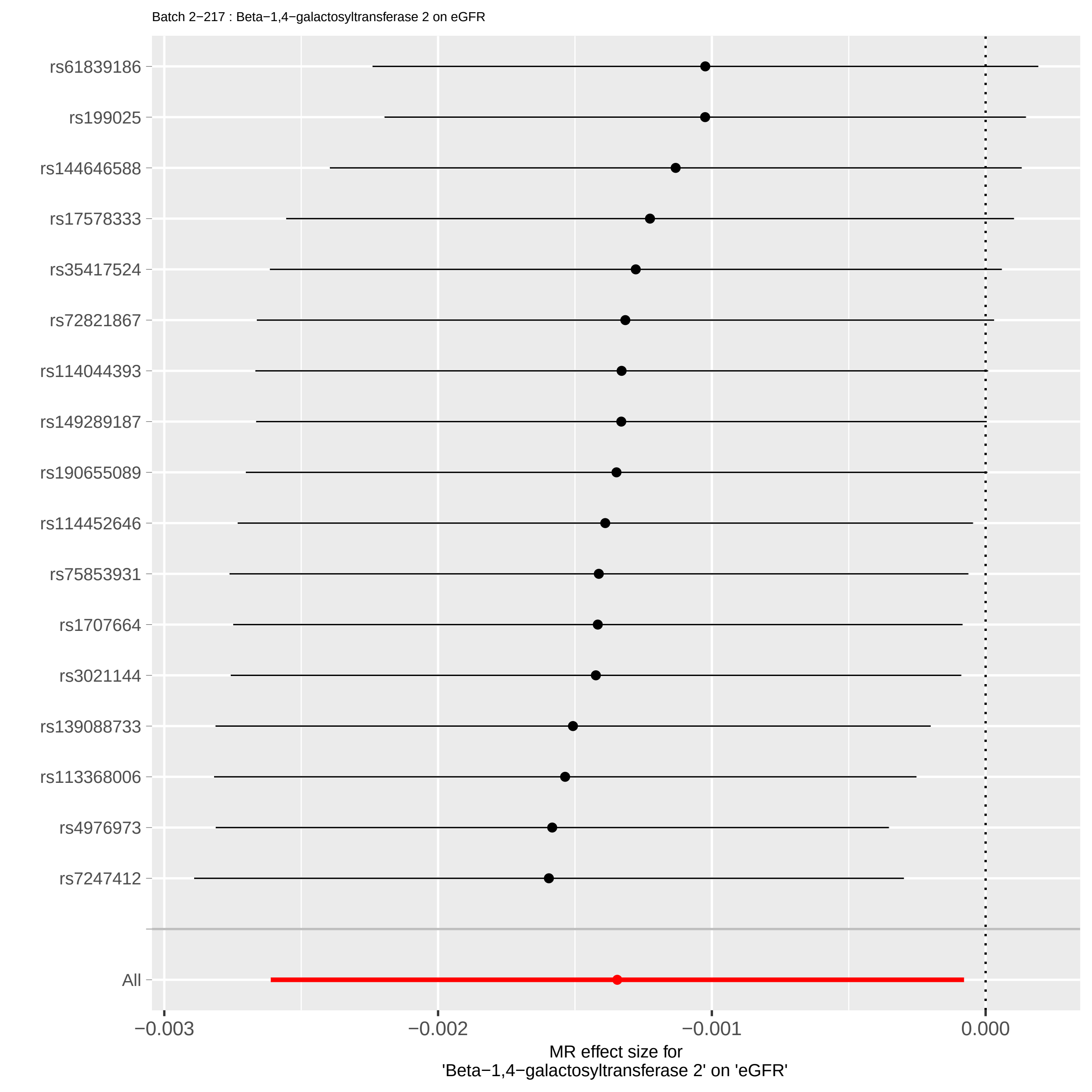
Batch 2−217 : Beta−1,4−galactosyltransferase 2 on eGFR
rs61839186
rs199025
rs144646588
rs17578333
rs35417524
rs72821867
rs114044393
rs149289187
rs190655089
rs114452646
rs75853931
rs1707664
rs3021144
rs139088733
rs113368006
rs4976973
rs7247412
All
−0.003
−0.002
−0.001
0.000
MR effect size for
'Beta−1,4−galactosyltransferase 2' on 'eGFR'

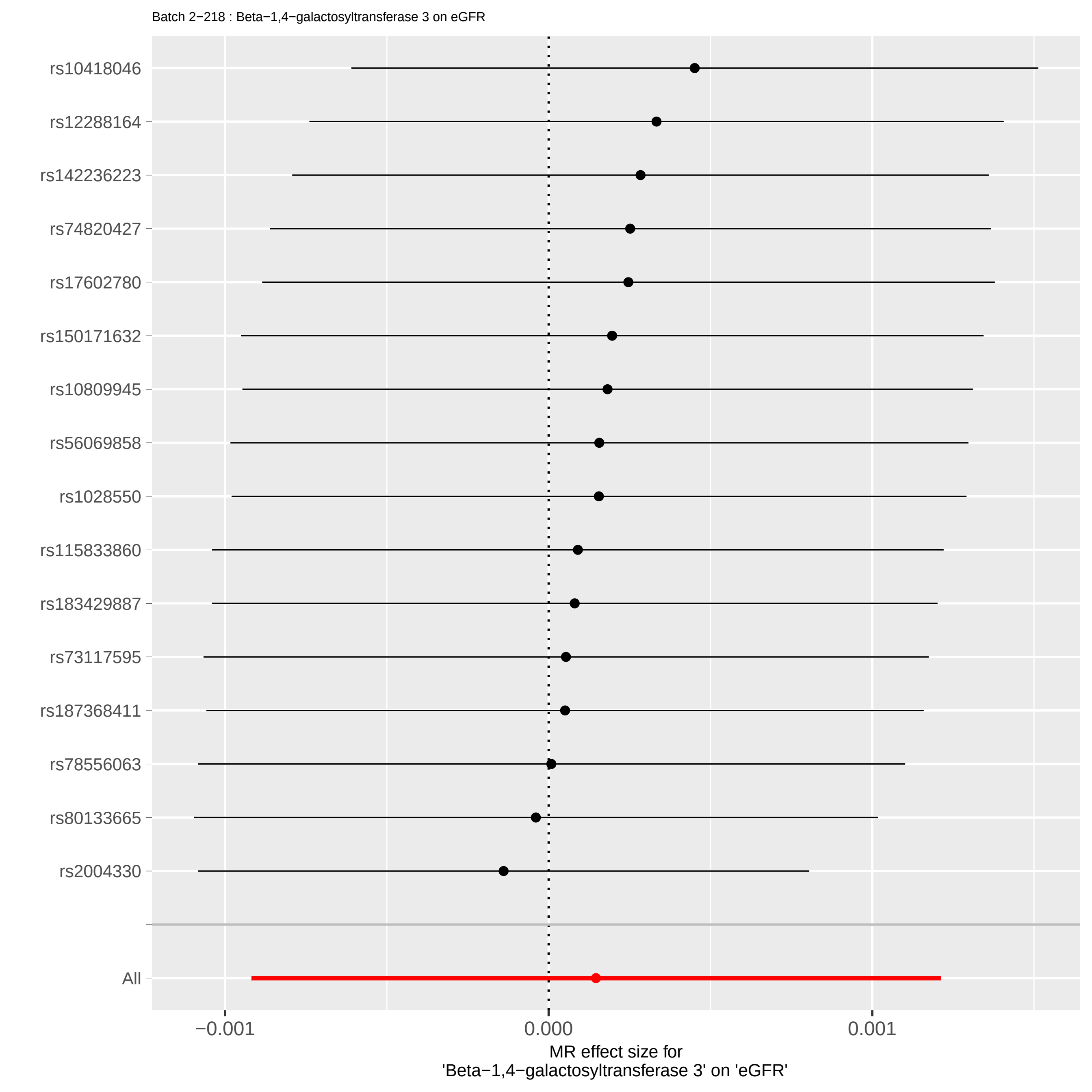

Batch 2−218 : Beta−1,4−galactosyltransferase 3 on eGFR
rs10418046
rs12288164
rs142236223
rs74820427
rs17602780
rs150171632
rs10809945
rs56069858
rs1028550
rs115833860
rs183429887
rs73117595
rs187368411
rs78556063
rs80133665
rs2004330
All
−0.001
0.000
0.001
MR effect size for
'Beta−1,4−galactosyltransferase 3' on 'eGFR'

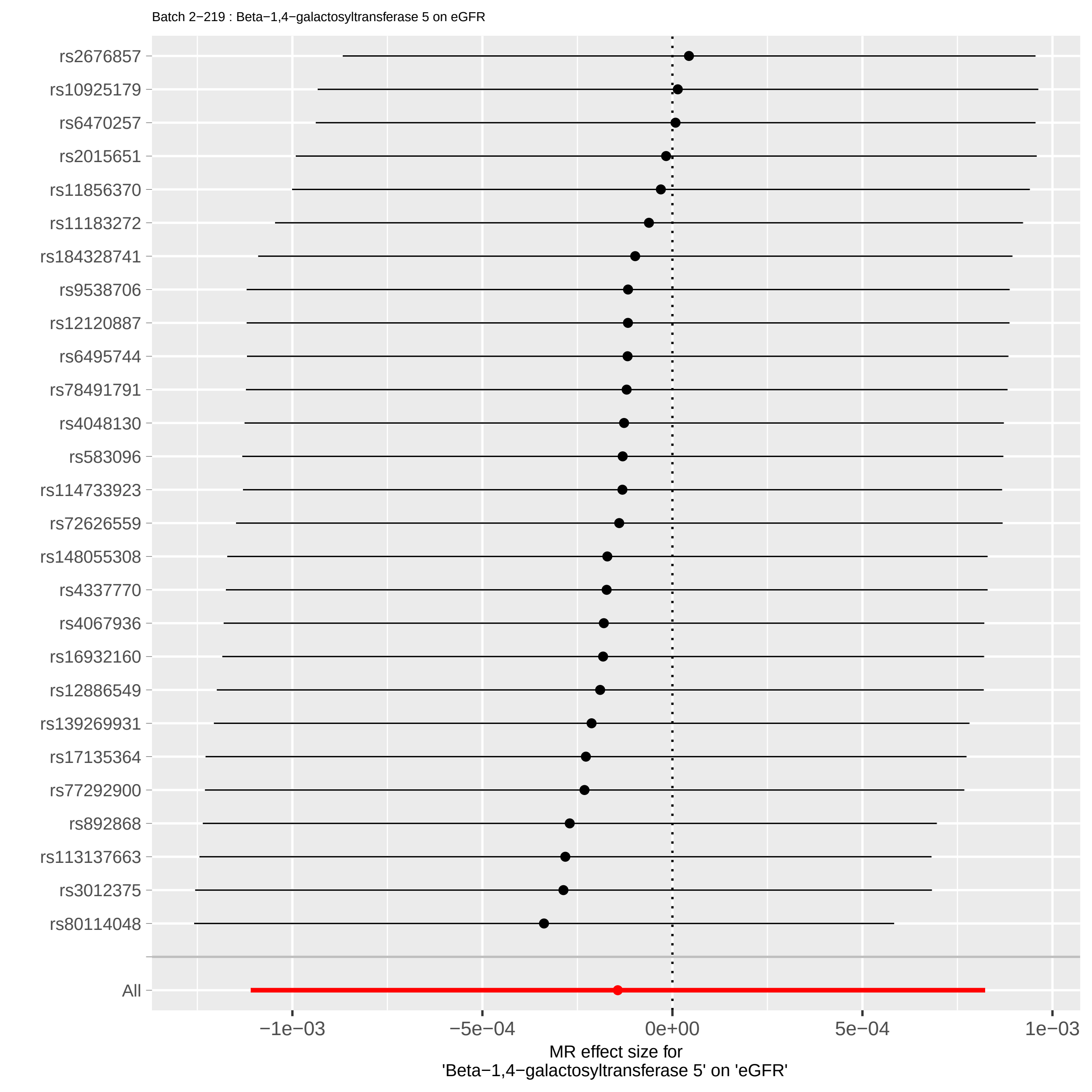

Batch 2−219 : Beta−1,4−galactosyltransferase 5 on eGFR
rs2676857
rs10925179
rs6470257
rs2015651
rs11856370
rs11183272
rs184328741
rs9538706
rs12120887
rs6495744
rs78491791
rs4048130
rs583096
rs114733923
rs72626559
rs148055308
rs4337770
rs4067936
rs16932160
rs12886549
rs139269931
rs17135364
rs77292900
rs892868
rs113137663
rs3012375
rs80114048
All
−1e−03
−5e−04
0e+00
5e−04
1e−03
MR effect size for
'Beta−1,4−galactosyltransferase 5' on 'eGFR'

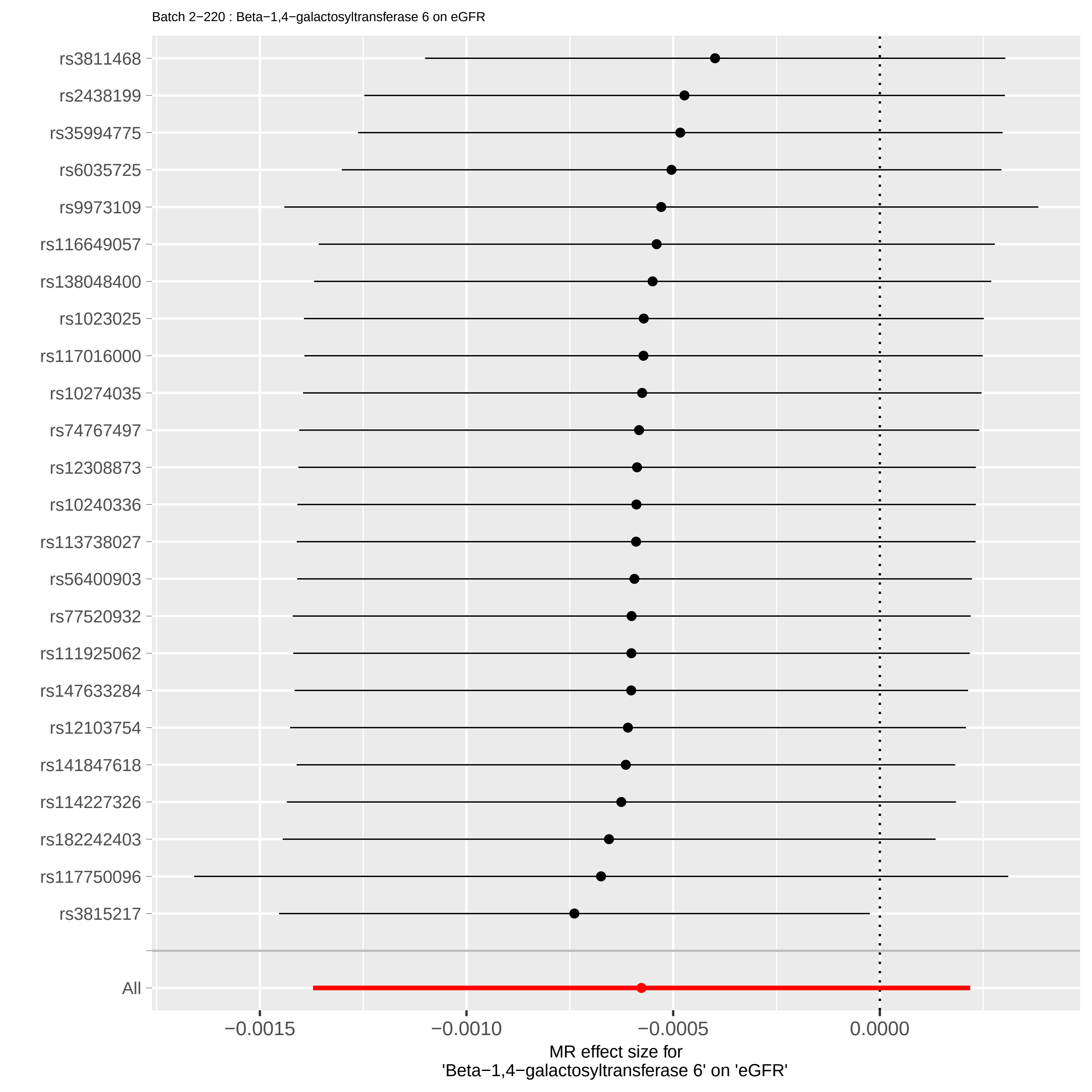

Batch 2−220 : Beta−1,4−galactosyltransferase 6 on eGFR
rs3811468
rs2438199
rs35994775
rs6035725
rs9973109
rs116649057
rs138048400
rs1023025
rs117016000
rs10274035
rs74767497
rs12308873
rs10240336
rs113738027
rs56400903
rs77520932
rs111925062
rs147633284
rs12103754
rs141847618
rs114227326
rs182242403
rs117750096
rs3815217
All
−0.0015
−0.0010
−0.0005
0.0000
MR effect size for
'Beta−1,4−galactosyltransferase 6' on 'eGFR'

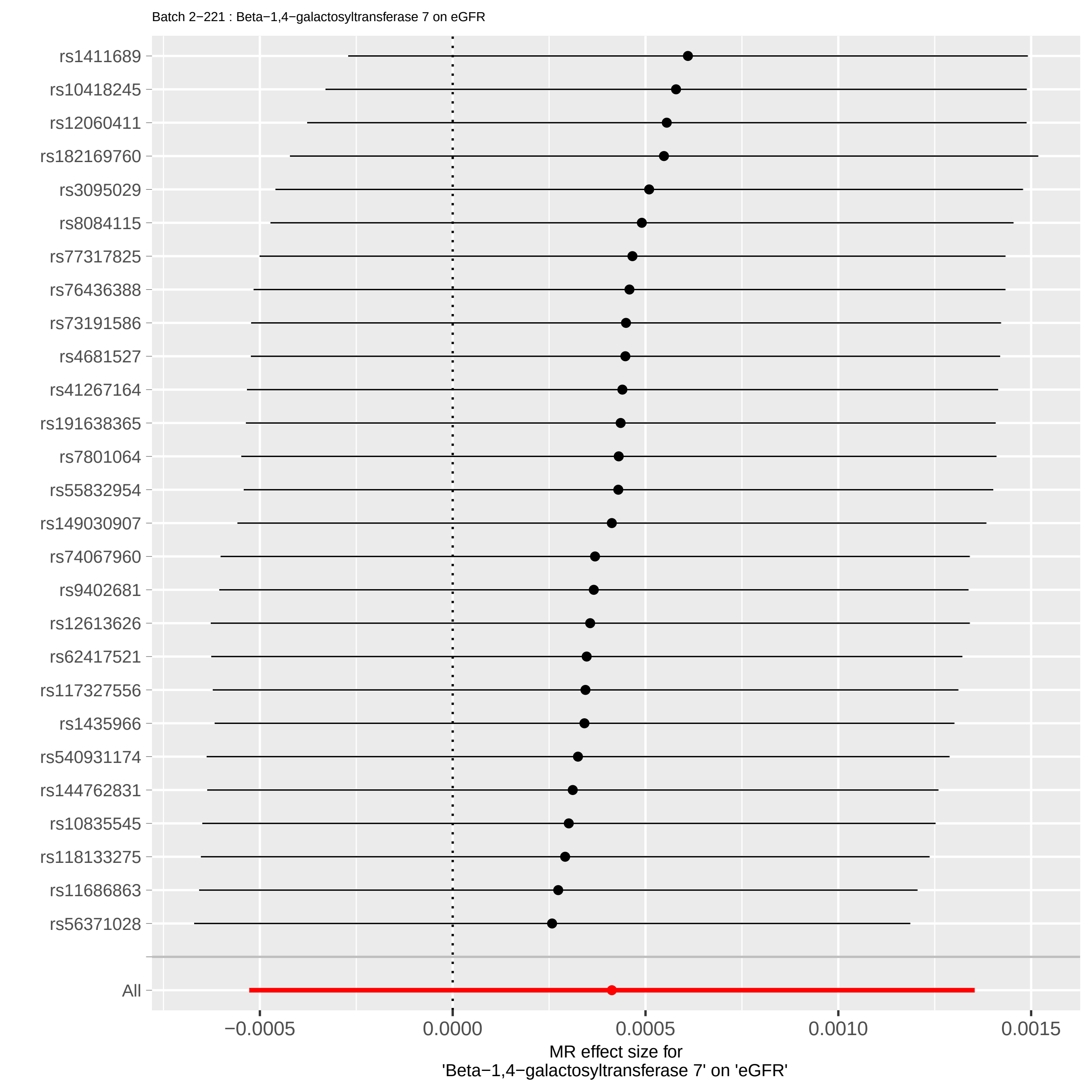

Batch 2−221 : Beta−1,4−galactosyltransferase 7 on eGFR
rs1411689
rs10418245
rs12060411
rs182169760
rs3095029
rs8084115
rs77317825
rs76436388
rs73191586
rs4681527
rs41267164
rs191638365
rs7801064
rs55832954
rs149030907
rs74067960
rs9402681
rs12613626
rs62417521
rs117327556
rs1435966
rs540931174
rs144762831
rs10835545
rs118133275
rs11686863
rs56371028
All
−0.0005
0.0000
0.0005
0.0010
0.0015
MR effect size for
'Beta−1,4−galactosyltransferase 7' on 'eGFR'

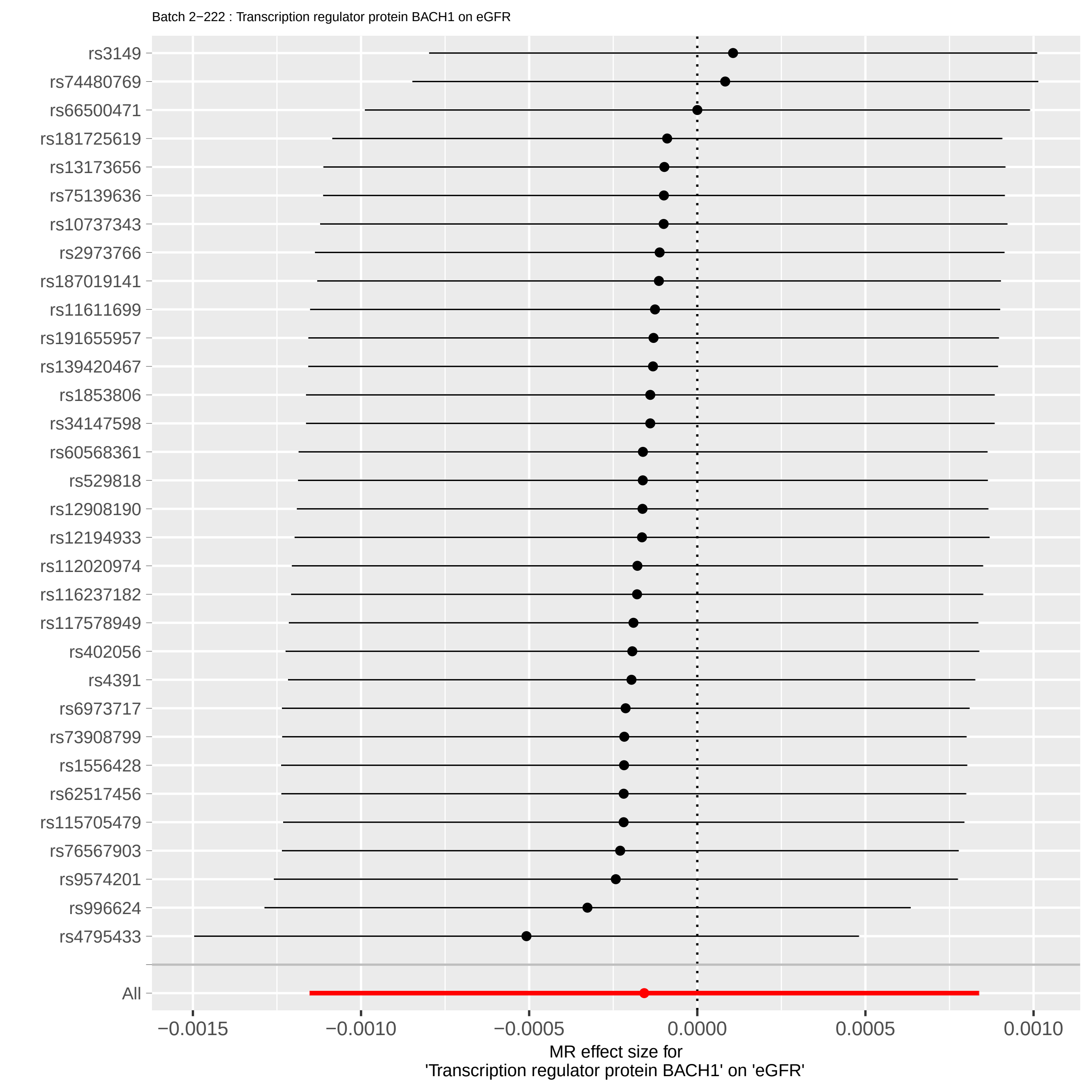

Batch 2−222 : Transcription regulator protein BACH1 on eGFR
rs3149
rs74480769
rs66500471
rs181725619
rs13173656
rs75139636
rs10737343
rs2973766
rs187019141
rs11611699
rs191655957
rs139420467
rs1853806
rs34147598
rs60568361
rs529818
rs12908190
rs12194933
rs112020974
rs116237182
rs117578949
rs402056
rs4391
rs6973717
rs73908799
rs1556428
rs62517456
rs115705479
rs76567903
rs9574201
rs996624
rs4795433
All
−0.0015
−0.0010
−0.0005
0.0000
0.0005
0.0010
MR effect size for
'Transcription regulator protein BACH1' on 'eGFR'

Batch 2−223 : Transcription regulator protein BACH2 on eGFR

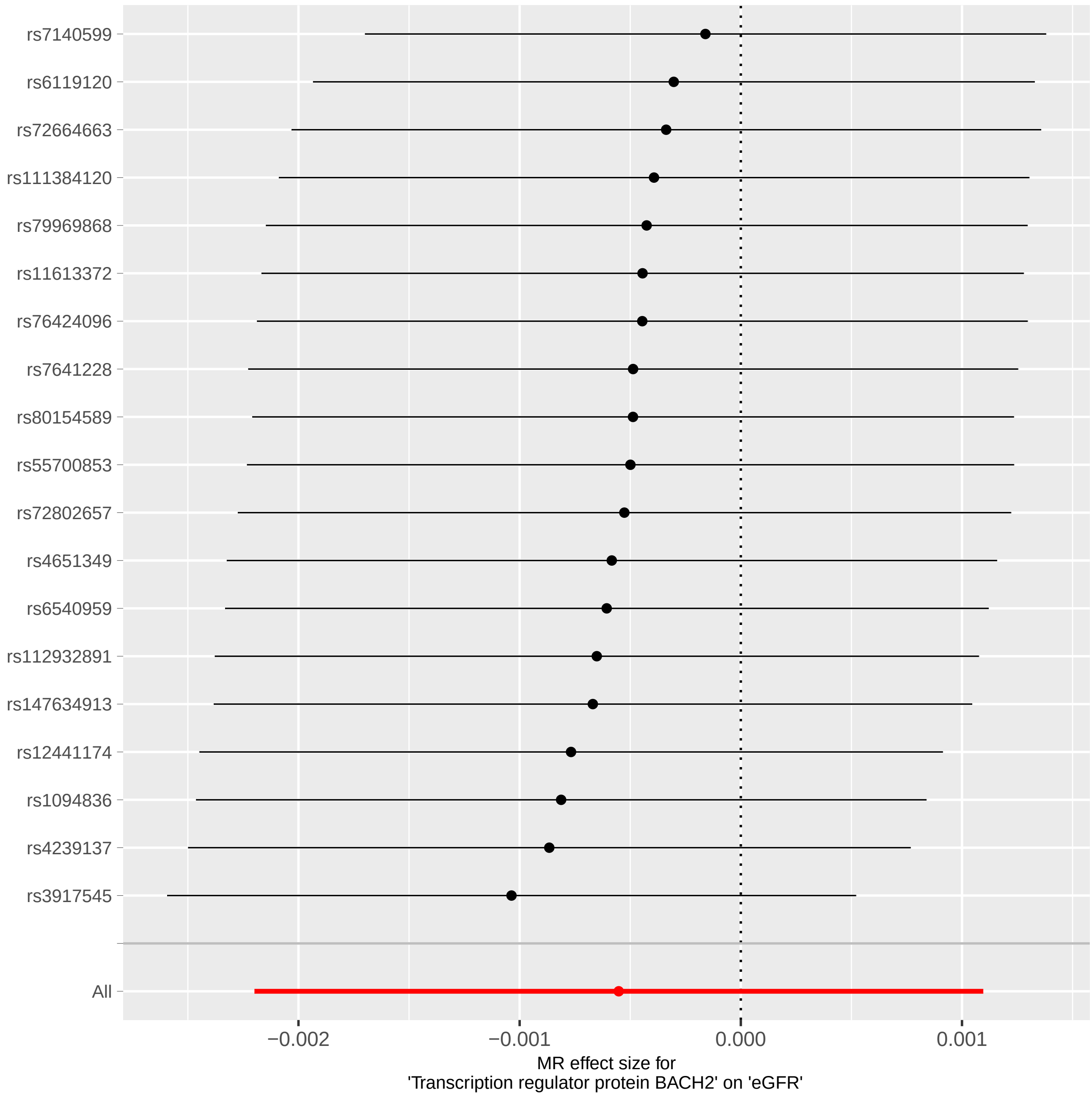

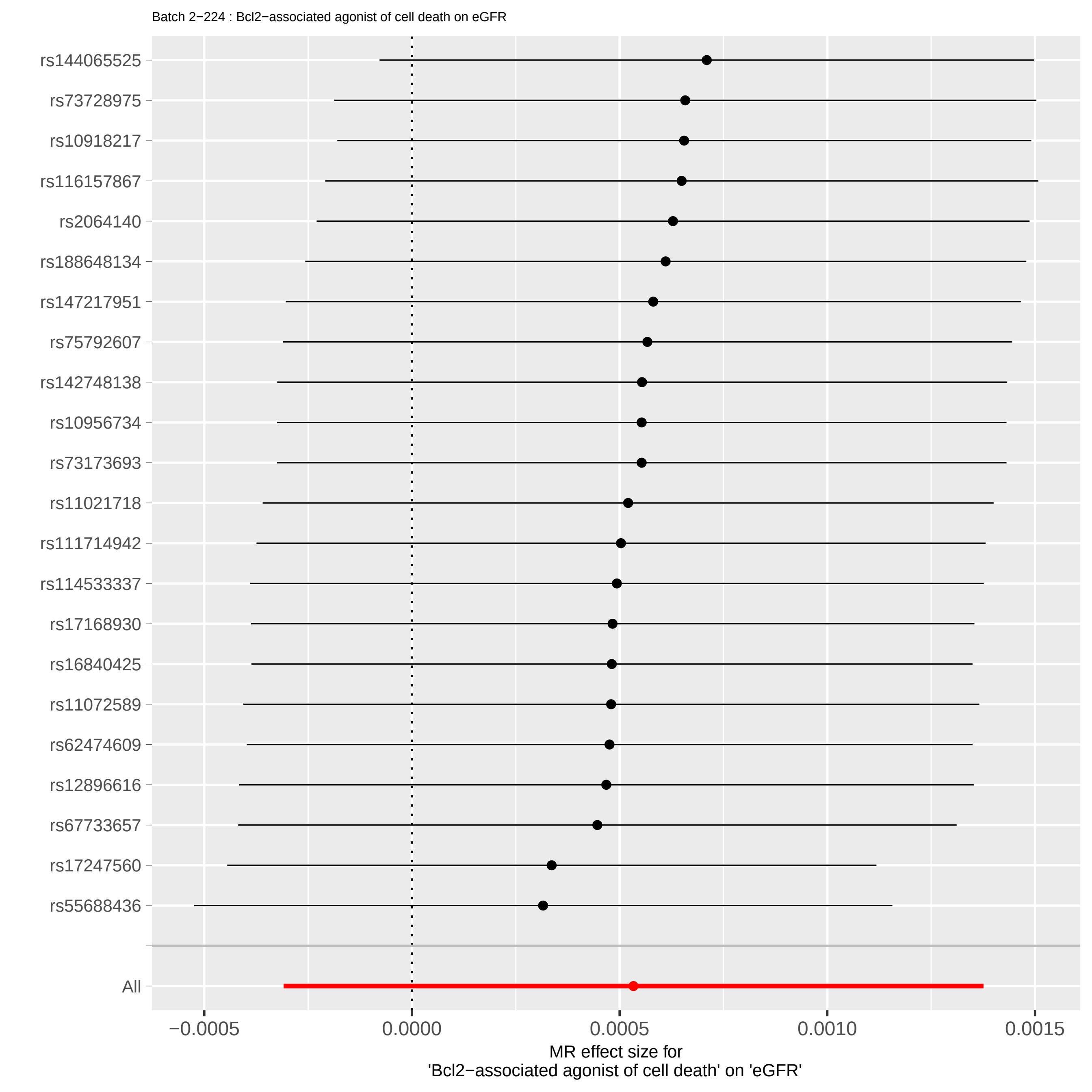
Batch 2−224 : Bcl2−associated agonist of cell death on eGFR
rs144065525
rs73728975
rs10918217
rs116157867
rs2064140
rs188648134
rs147217951
rs75792607
rs142748138
rs10956734
rs73173693
rs11021718
rs111714942
rs114533337
rs17168930
rs16840425
rs11072589
rs62474609
rs12896616
rs67733657
rs17247560
rs55688436
All
−0.0005
0.0000
0.0005
0.0010
0.0015
MR effect size for
'Bcl2−associated agonist of cell death' on 'eGFR'

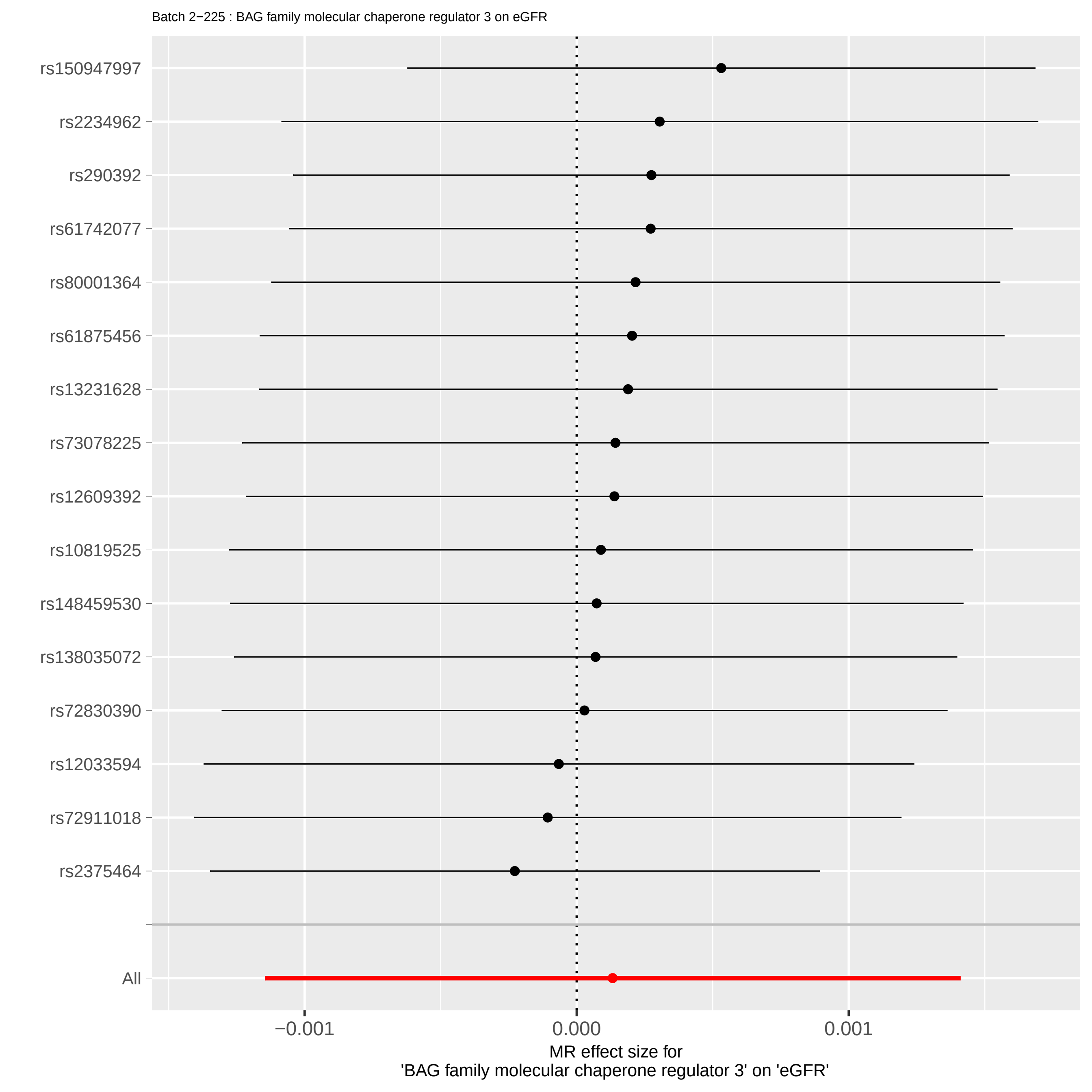

Batch 2−225 : BAG family molecular chaperone regulator 3 on eGFR
rs150947997
rs2234962
rs290392
rs61742077
rs80001364
rs61875456
rs13231628
rs73078225
rs12609392
rs10819525
rs148459530
rs138035072
rs72830390
rs12033594
rs72911018
rs2375464
All
−0.001
0.000
0.001
MR effect size for
'BAG family molecular chaperone regulator 3' on 'eGFR'

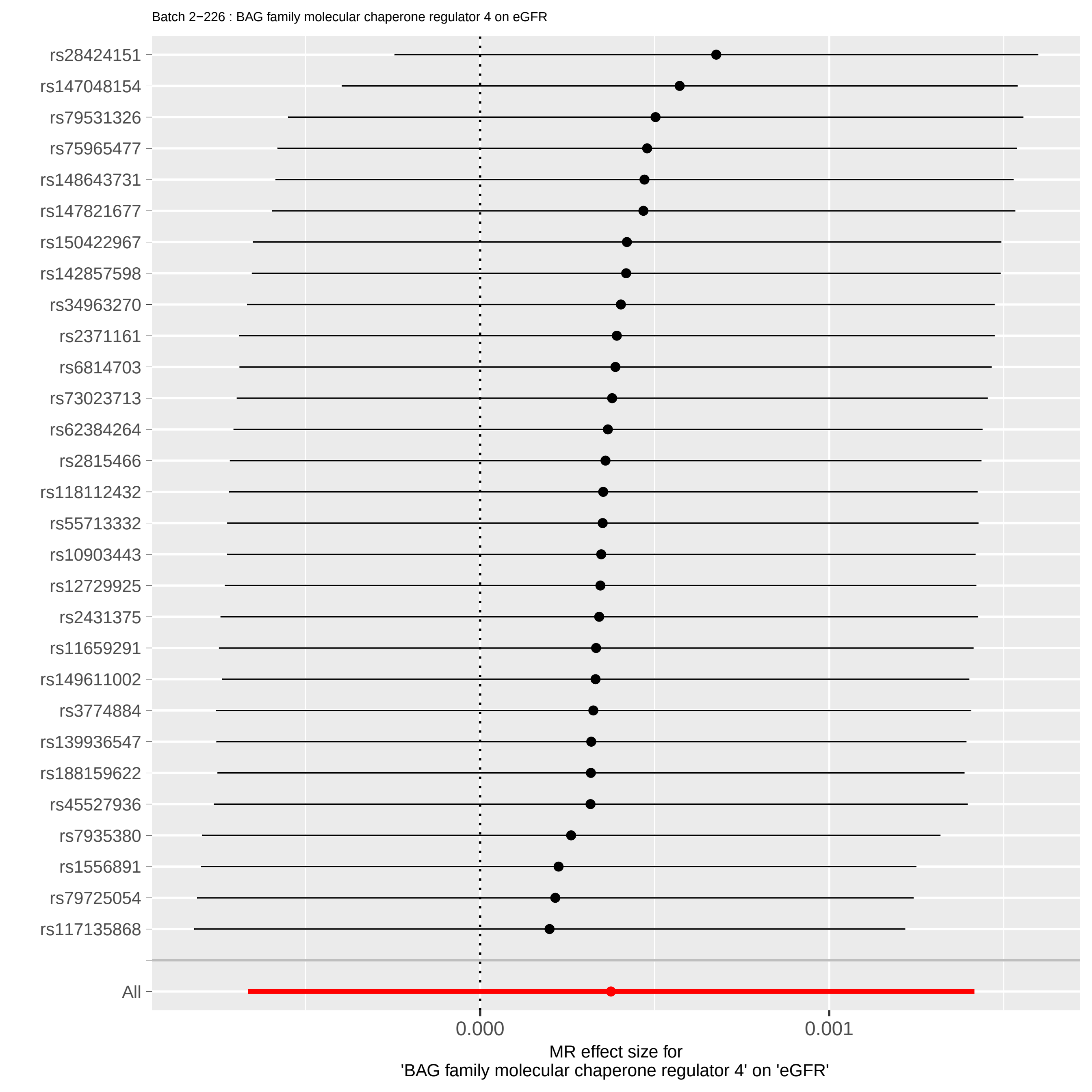
Batch 2−226 : BAG family molecular chaperone regulator 4 on eGFR
rs28424151
rs147048154
rs79531326
rs75965477
rs148643731
rs147821677
rs150422967
rs142857598
rs34963270
rs2371161
rs6814703
rs73023713
rs62384264
rs2815466
rs118112432
rs55713332
rs10903443
rs12729925
rs2431375
rs11659291
rs149611002
rs3774884
rs139936547
rs188159622
rs45527936
rs7935380
rs1556891
rs79725054
rs117135868
All
0.000
0.001
MR effect size for
'BAG family molecular chaperone regulator 4' on 'eGFR'

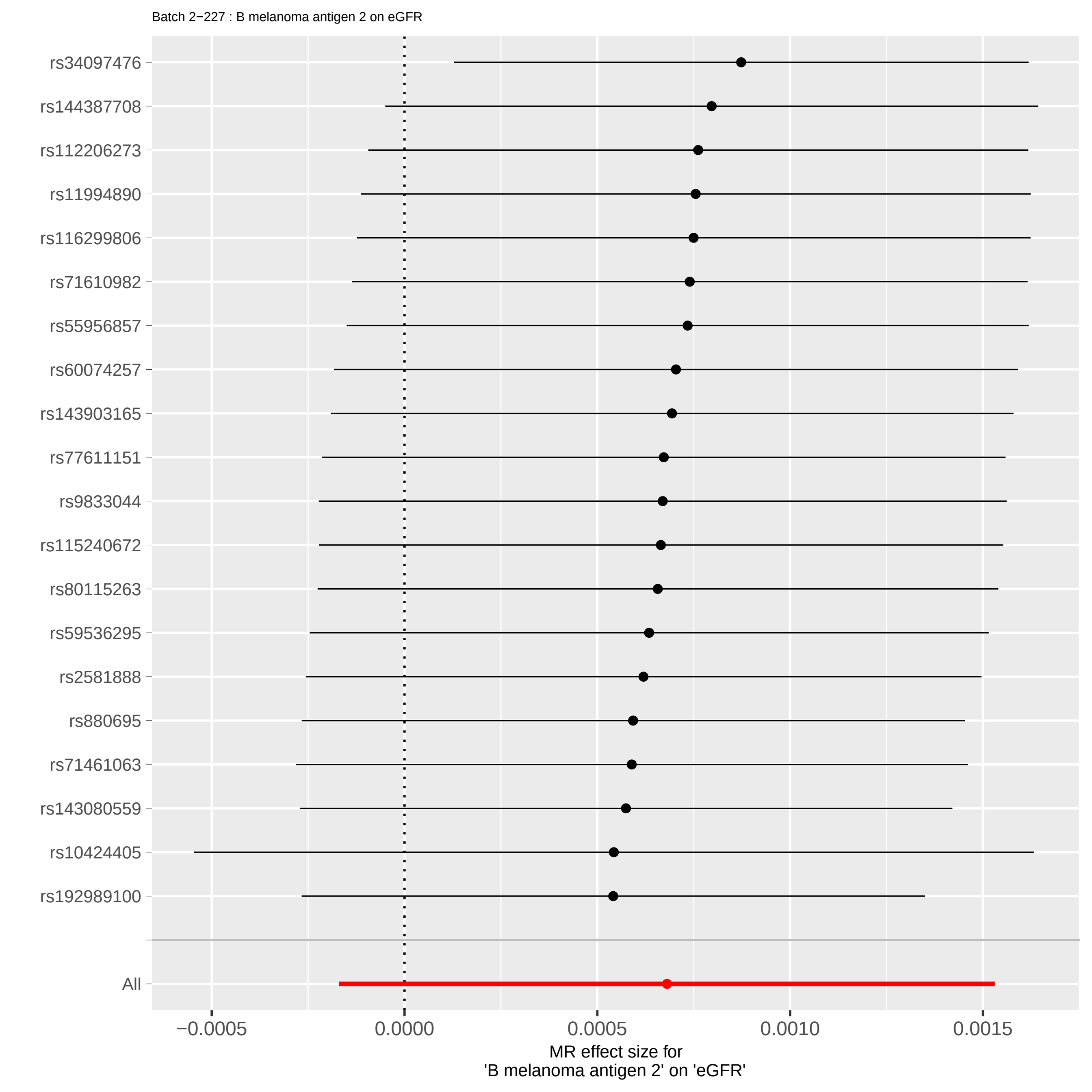

Batch 2−227 : B melanoma antigen 2 on eGFR
rs34097476
rs144387708
rs112206273
rs11994890
rs116299806
rs71610982
rs55956857
rs60074257
rs143903165
rs77611151
rs9833044
rs115240672
rs80115263
rs59536295
rs2581888
rs880695
rs71461063
rs143080559
rs10424405
rs192989100
All
−0.0005
0.0000
0.0005
0.0010
0.0015
MR effect size for
'B melanoma antigen 2' on 'eGFR'

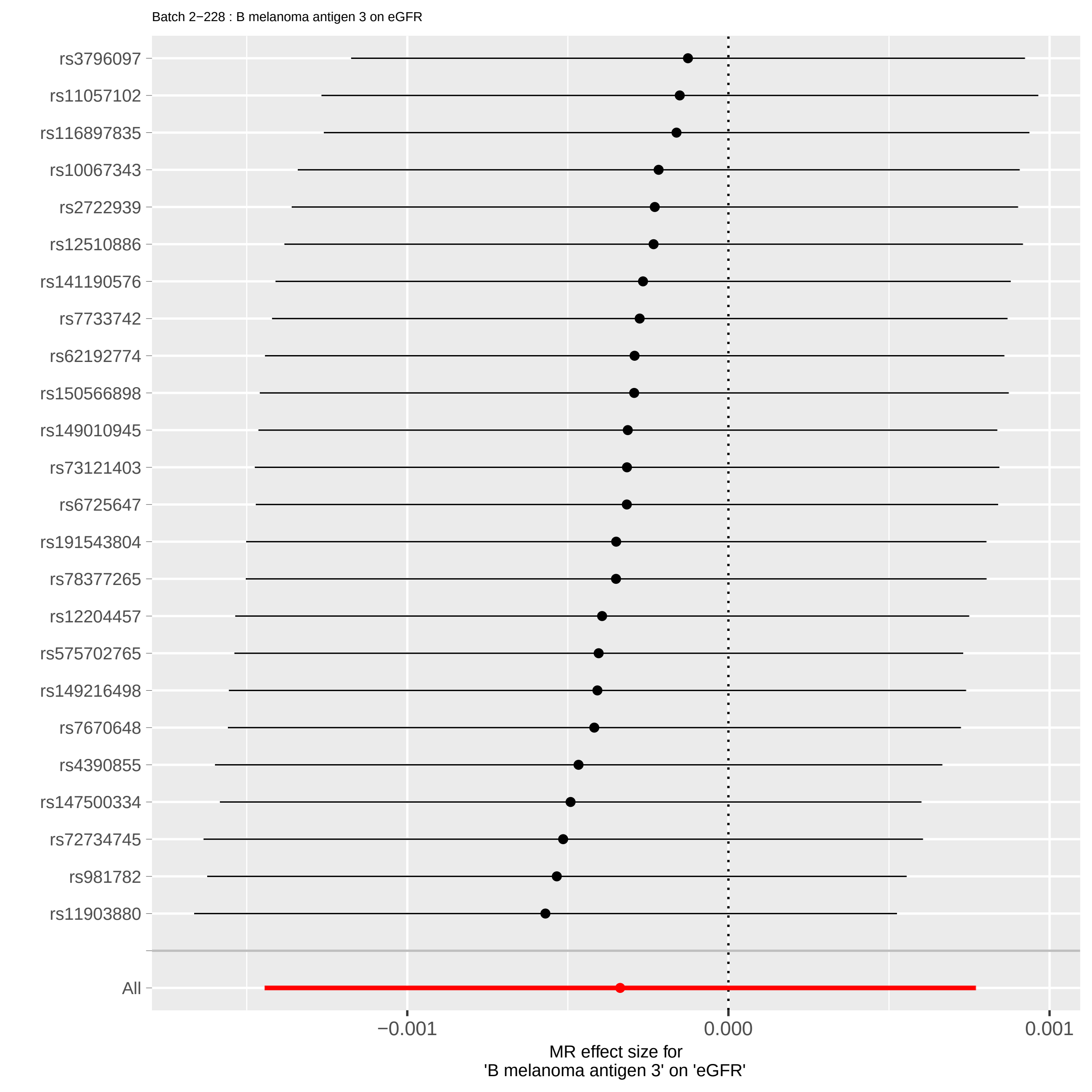
Batch 2−228 : B melanoma antigen 3 on eGFR
rs3796097
rs11057102
rs116897835
rs10067343
rs2722939
rs12510886
rs141190576
rs7733742
rs62192774
rs150566898
rs149010945
rs73121403
rs6725647
rs191543804
rs78377265
rs12204457
rs575702765
rs149216498
rs7670648
rs4390855
rs147500334
rs72734745
rs981782
rs11903880
All
−0.001
0.000
0.001
MR effect size for
'B melanoma antigen 3' on 'eGFR'

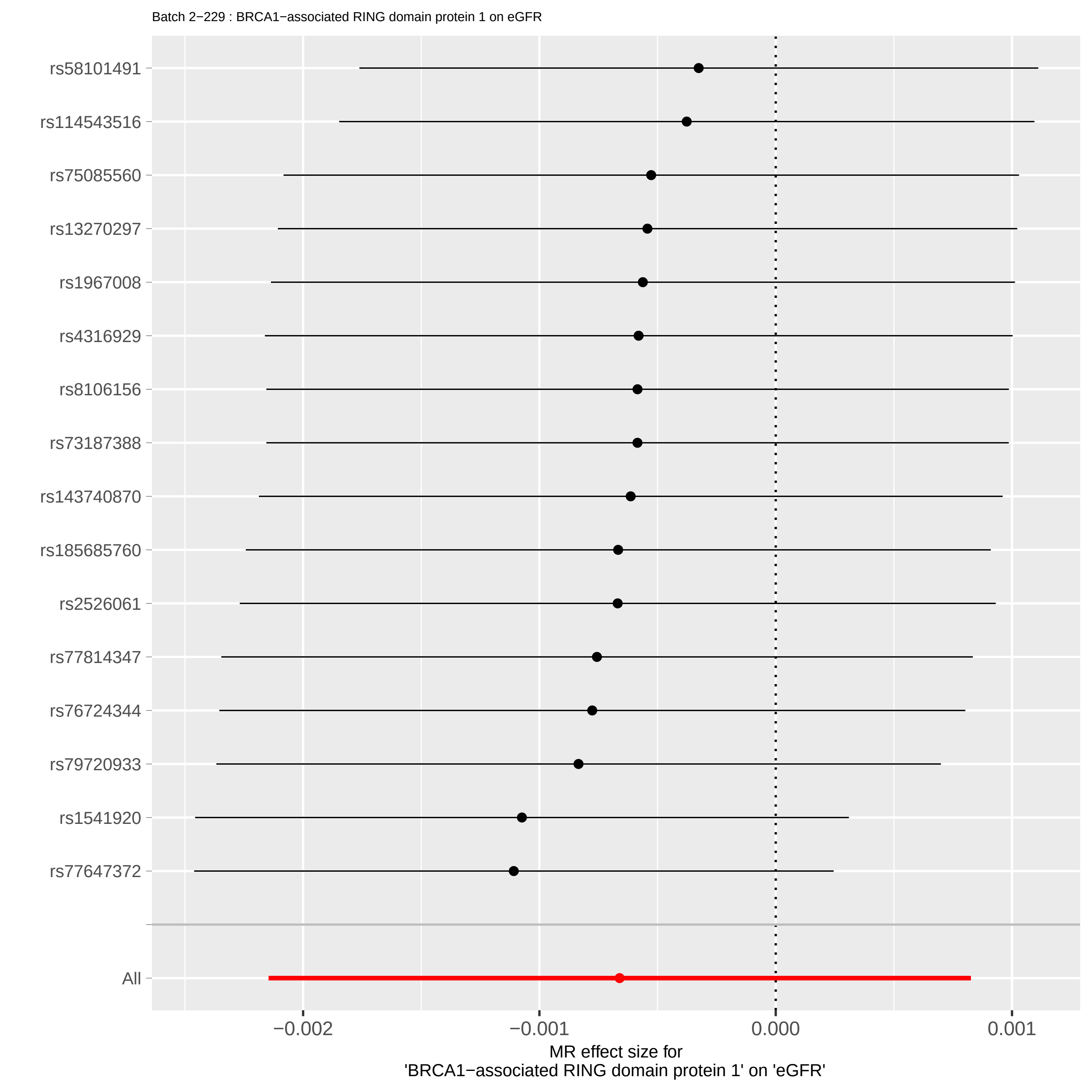

Batch 2−229 : BRCA1−associated RING domain protein 1 on eGFR
rs58101491
rs114543516
rs75085560
rs13270297
rs1967008
rs4316929
rs8106156
rs73187388
rs143740870
rs185685760
rs2526061
rs77814347
rs76724344
rs79720933
rs1541920
rs77647372
All
−0.002
−0.001
0.000
0.001
MR effect size for
'BRCA1−associated RING domain protein 1' on 'eGFR'

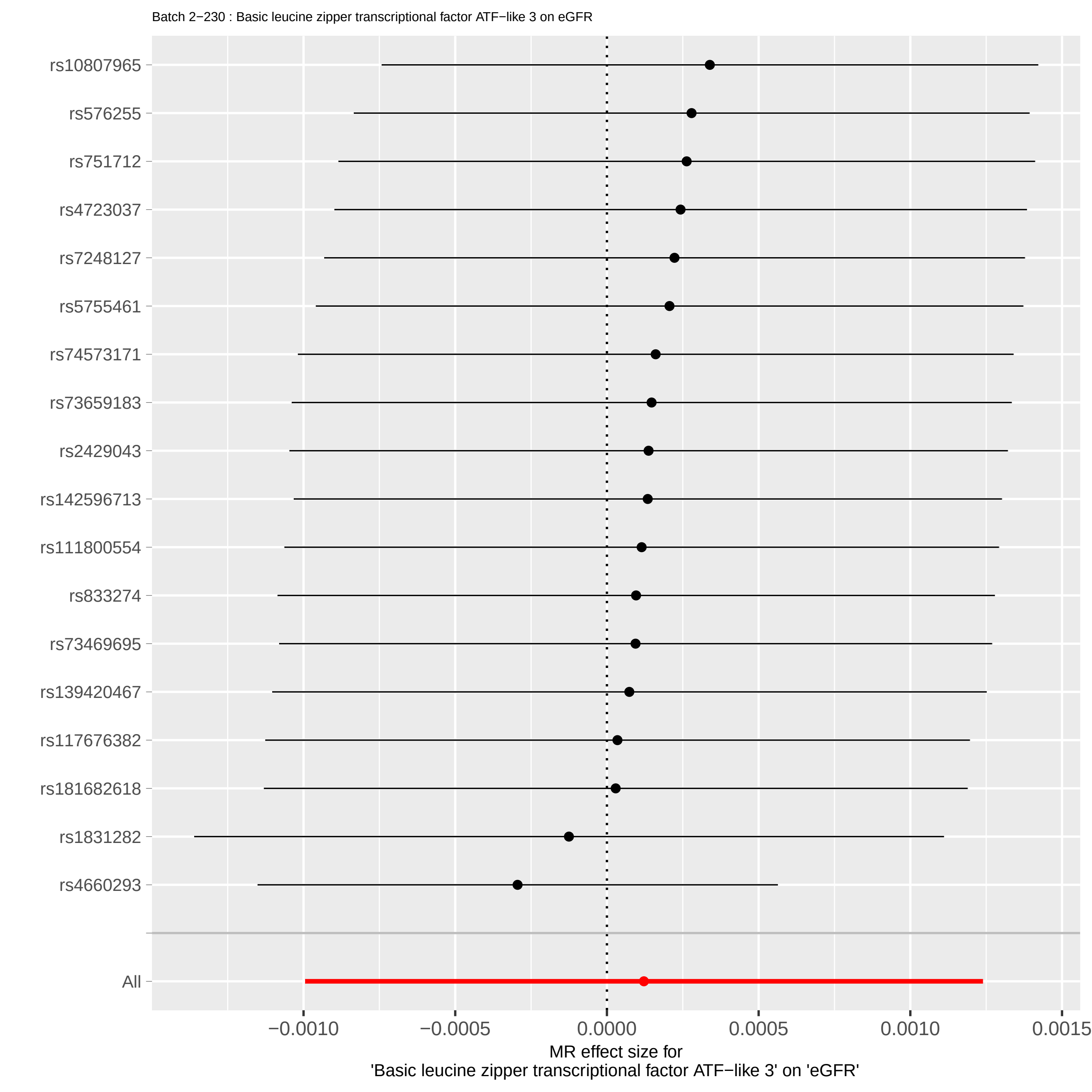

Batch 2−230 : Basic leucine zipper transcriptional factor ATF−like 3 on eGFR
rs10807965
rs576255
rs751712
rs4723037
rs7248127
rs5755461
rs74573171
rs73659183
rs2429043
rs142596713
rs111800554
rs833274
rs73469695
rs139420467
rs117676382
rs181682618
rs1831282
rs4660293
All
−0.0010
−0.0005
0.0000
0.0005
0.0010
0.0015
MR effect size for
'Basic leucine zipper transcriptional factor ATF−like 3' on 'eGFR'

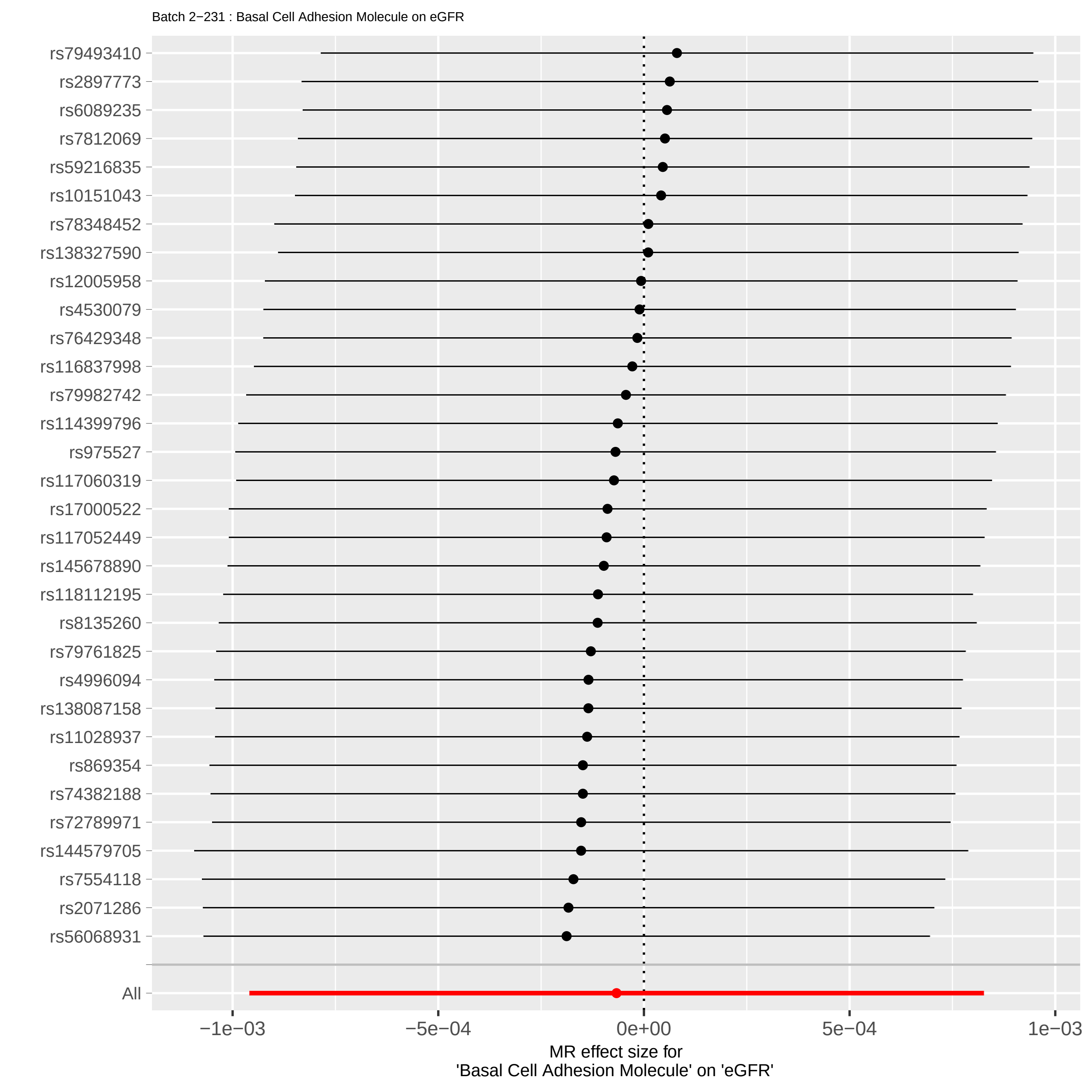

Batch 2−231 : Basal Cell Adhesion Molecule on eGFR
rs79493410
rs2897773
rs6089235
rs7812069
rs59216835
rs10151043
rs78348452
rs138327590
rs12005958
rs4530079
rs76429348
rs116837998
rs79982742
rs114399796
rs975527
rs117060319
rs17000522
rs117052449
rs145678890
rs118112195
rs8135260
rs79761825
rs4996094
rs138087158
rs11028937
rs869354
rs74382188
rs72789971
rs144579705
rs7554118
rs2071286
rs56068931
All
−1e−03
−5e−04
0e+00
5e−04
1e−03
MR effect size for
'Basal Cell Adhesion Molecule' on 'eGFR'

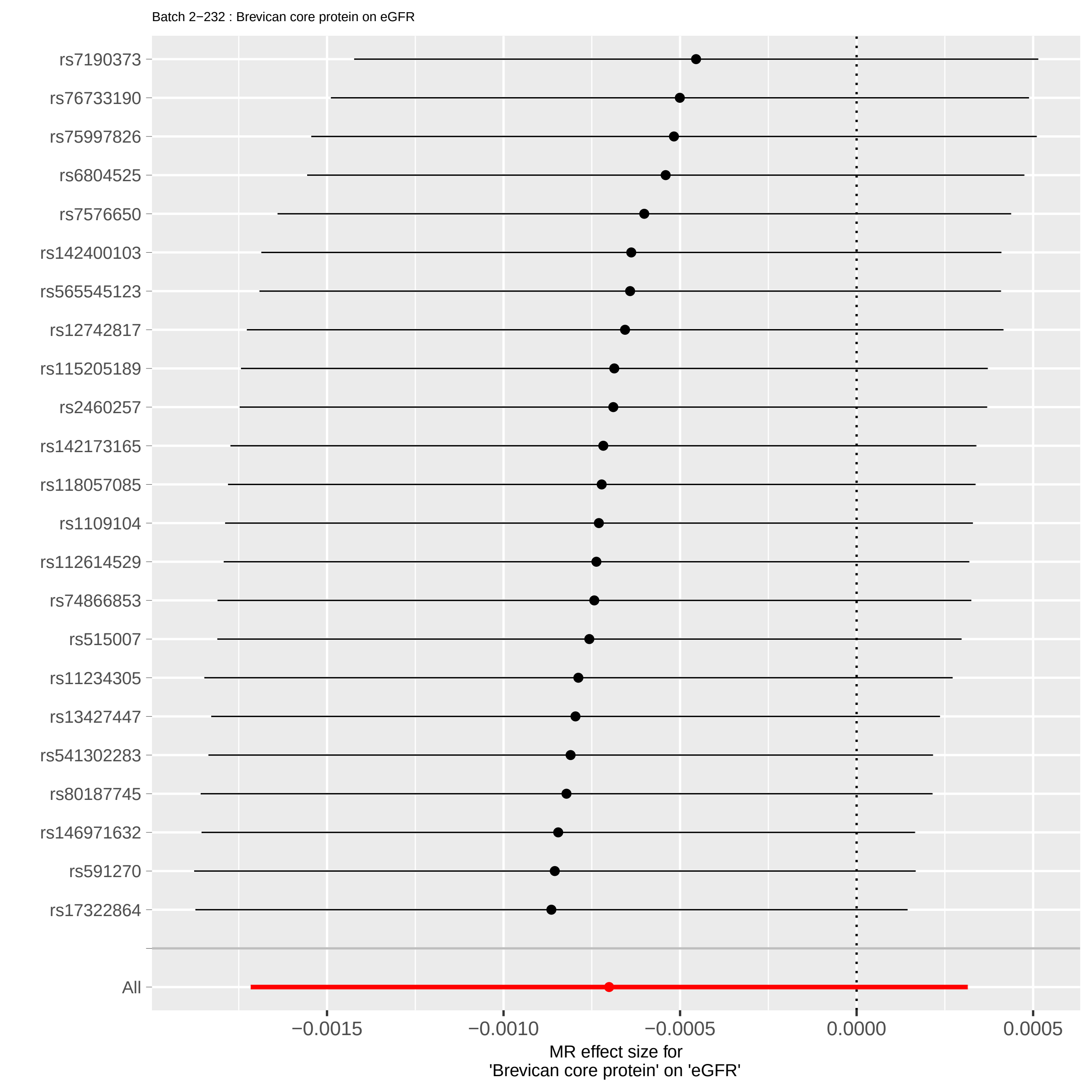

Batch 2−232 : Brevican core protein on eGFR
rs7190373
rs76733190
rs75997826
rs6804525
rs7576650
rs142400103
rs565545123
rs12742817
rs115205189
rs2460257
rs142173165
rs118057085
rs1109104
rs112614529
rs74866853
rs515007
rs11234305
rs13427447
rs541302283
rs80187745
rs146971632
rs591270
rs17322864
All
−0.0015
−0.0010
−0.0005
0.0000
0.0005
MR effect size for
'Brevican core protein' on 'eGFR'

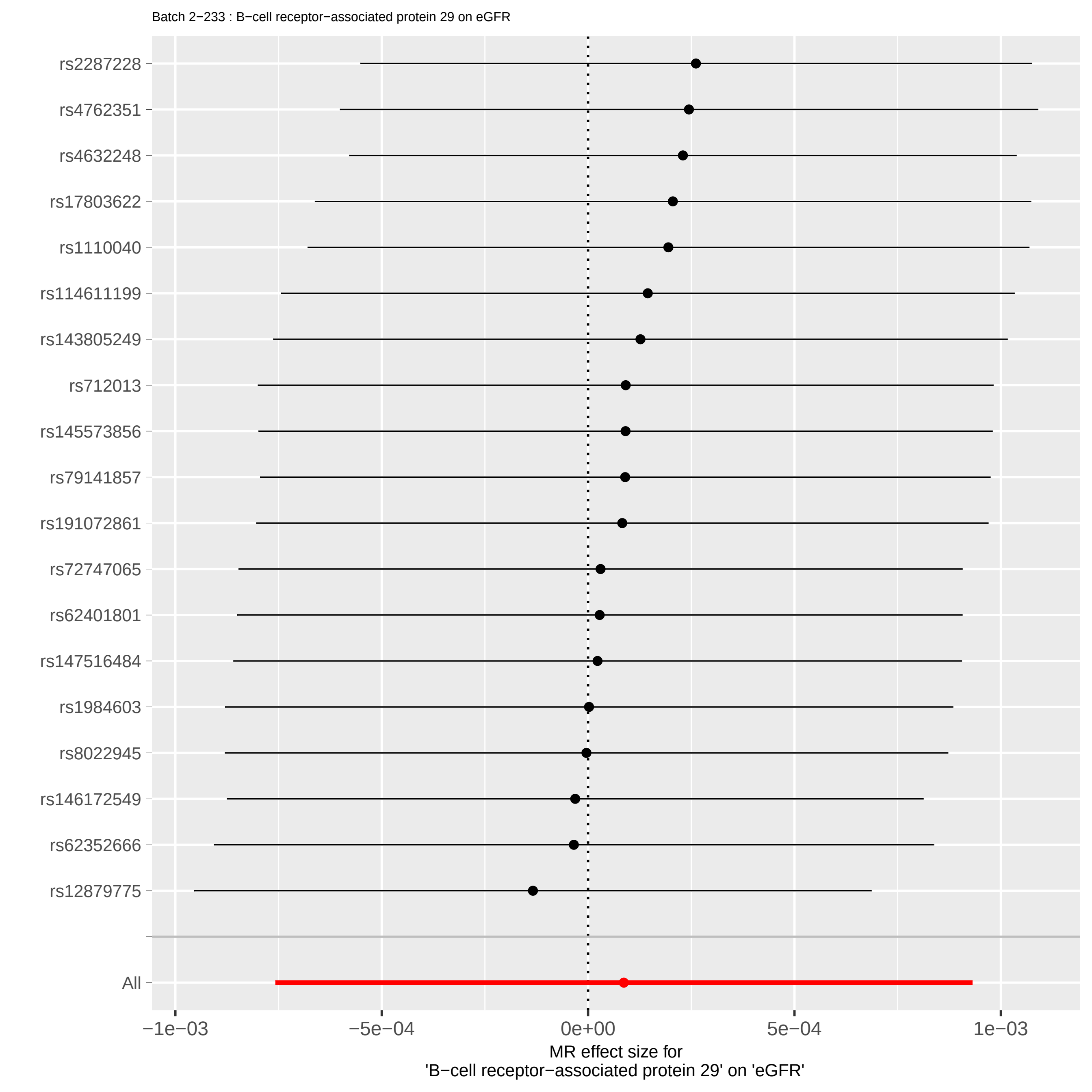

Batch 2−233 : B−cell receptor−associated protein 29 on eGFR
rs2287228
rs4762351
rs4632248
rs17803622
rs1110040
rs114611199
rs143805249
rs712013
rs145573856
rs79141857
rs191072861
rs72747065
rs62401801
rs147516484
rs1984603
rs8022945
rs146172549
rs62352666
rs12879775
All
−1e−03
−5e−04
0e+00
5e−04
1e−03
MR effect size for
'B−cell receptor−associated protein 29' on 'eGFR'

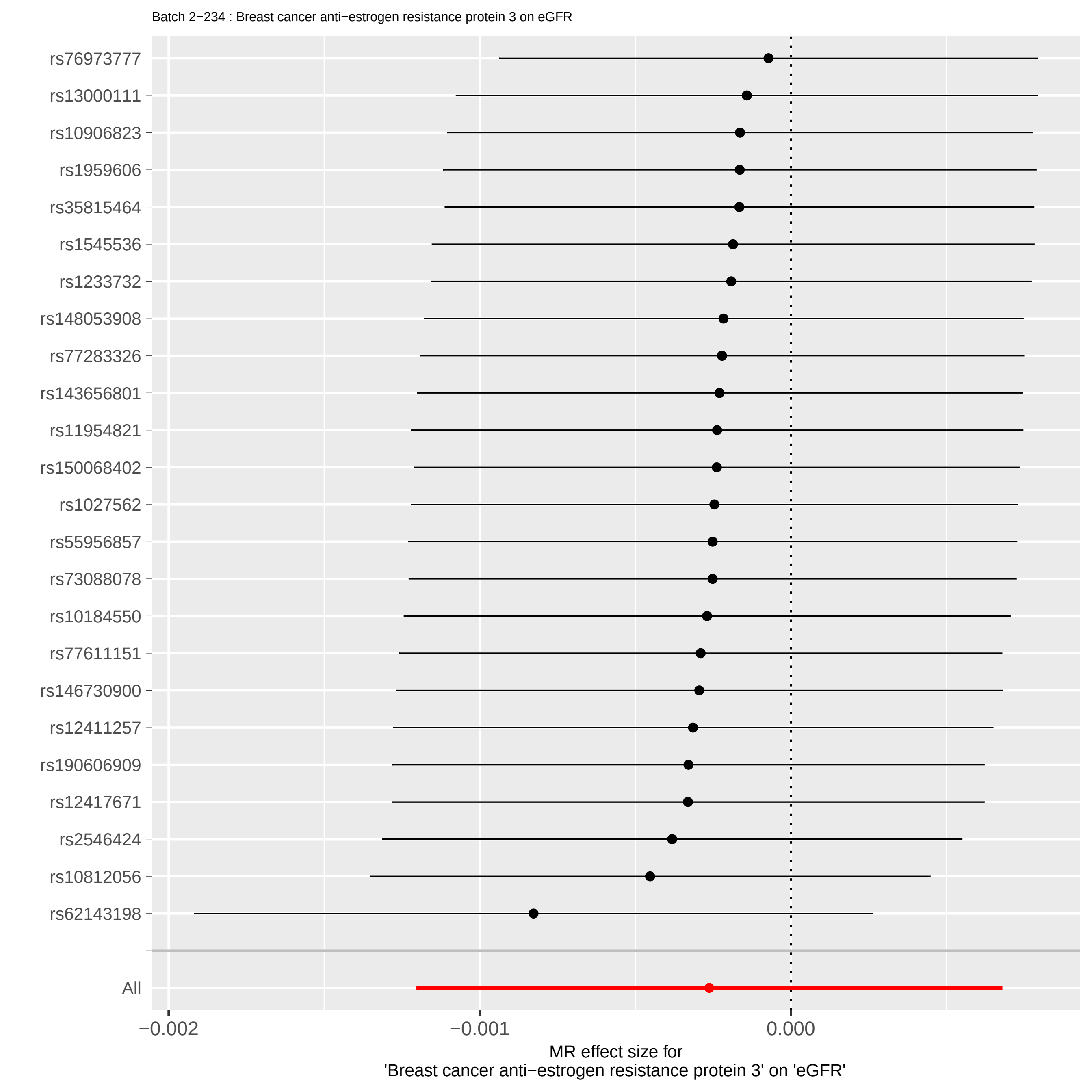

Batch 2−234 : Breast cancer anti−estrogen resistance protein 3 on eGFR
rs76973777
rs13000111
rs10906823
rs1959606
rs35815464
rs1545536
rs1233732
rs148053908
rs77283326
rs143656801
rs11954821
rs150068402
rs1027562
rs55956857
rs73088078
rs10184550
rs77611151
rs146730900
rs12411257
rs190606909
rs12417671
rs2546424
rs10812056
rs62143198
All
−0.002
−0.001
0.000
MR effect size for
'Breast cancer anti−estrogen resistance protein 3' on 'eGFR'

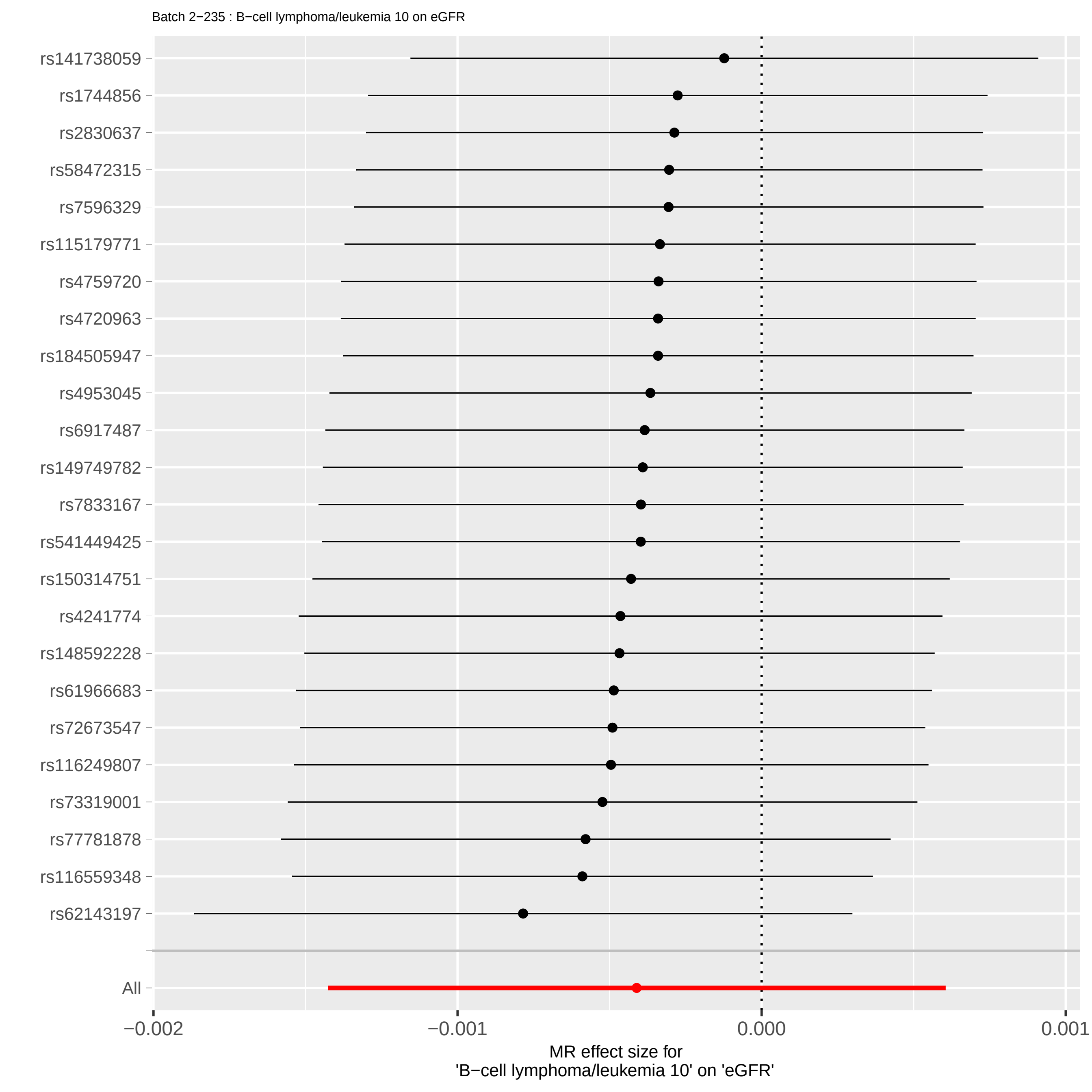

Batch 2−235 : B−cell lymphoma/leukemia 10 on eGFR
rs141738059
rs1744856
rs2830637
rs58472315
rs7596329
rs115179771
rs4759720
rs4720963
rs184505947
rs4953045
rs6917487
rs149749782
rs7833167
rs541449425
rs150314751
rs4241774
rs148592228
rs61966683
rs72673547
rs116249807
rs73319001
rs77781878
rs116559348
rs62143197
All
−0.002
−0.001
0.000
0.001
MR effect size for
'B−cell lymphoma/leukemia 10' on 'eGFR'

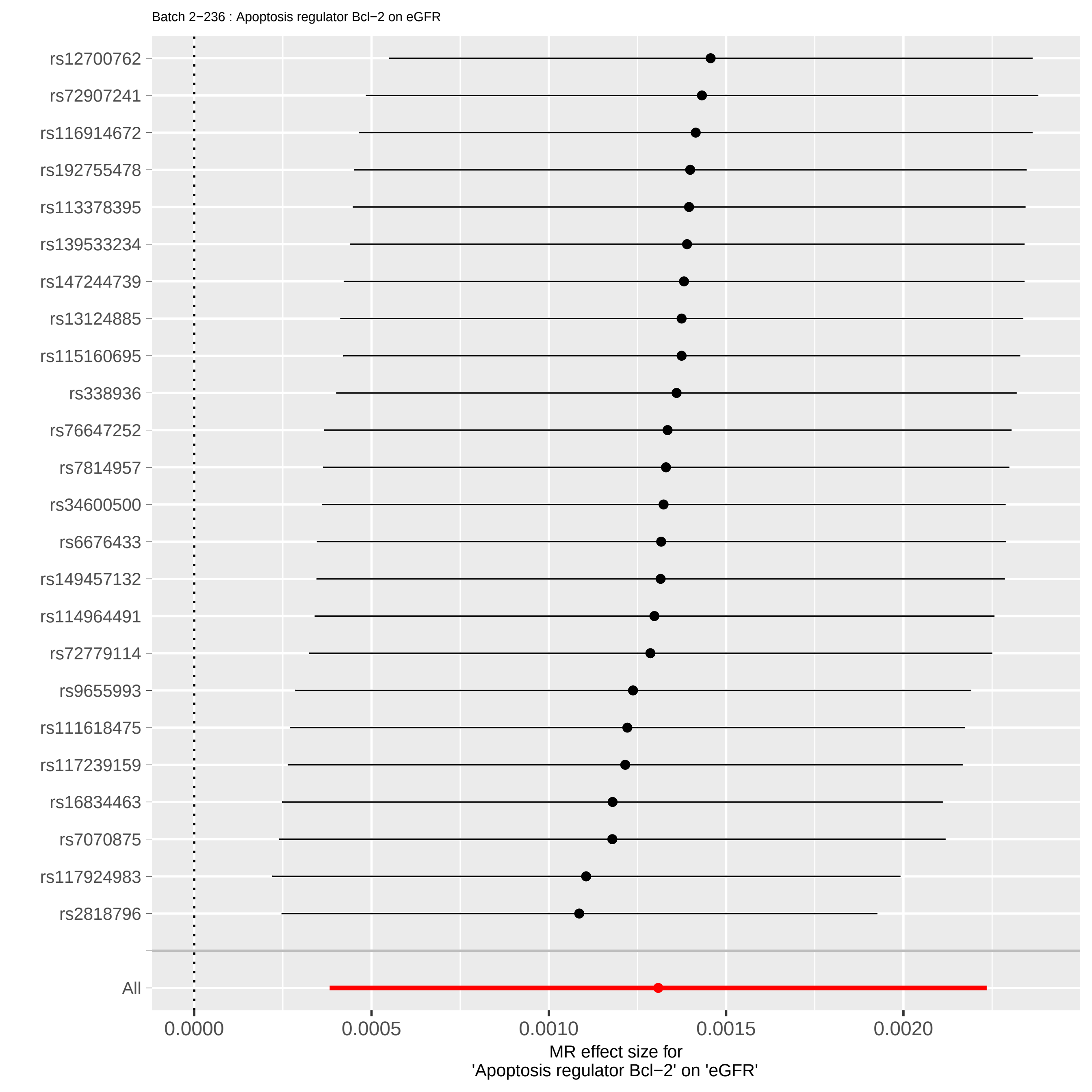
Batch 2−236 : Apoptosis regulator Bcl−2 on eGFR
rs12700762
rs72907241
rs116914672
rs192755478
rs113378395
rs139533234
rs147244739
rs13124885
rs115160695
rs338936
rs76647252
rs7814957
rs34600500
rs6676433
rs149457132
rs114964491
rs72779114
rs9655993
rs111618475
rs117239159
rs16834463
rs7070875
rs117924983
rs2818796
All
0.0000
0.0005
0.0010
0.0015
0.0020
MR effect size for
'Apoptosis regulator Bcl−2' on 'eGFR'

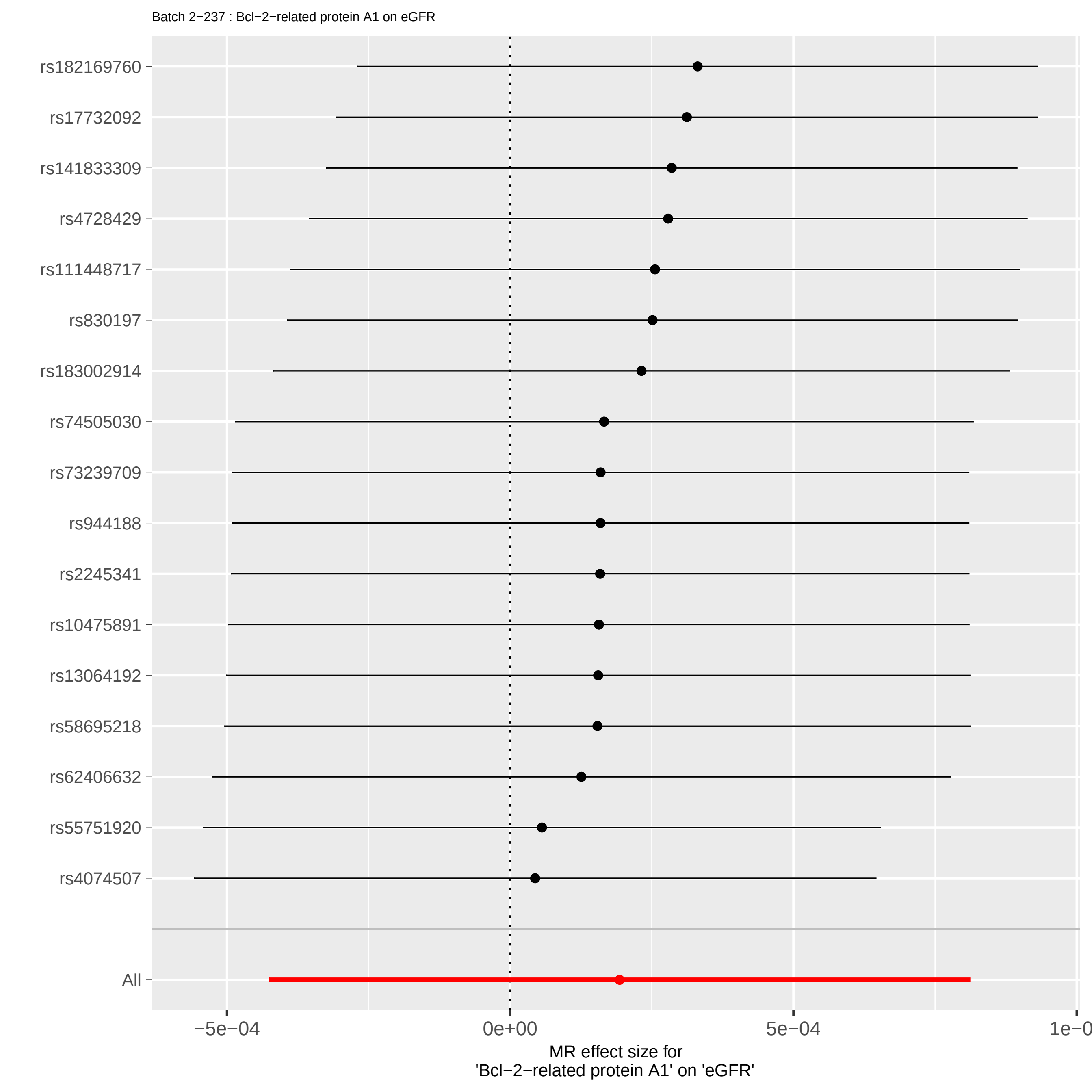

Batch 2−237 : Bcl−2−related protein A1 on eGFR
rs182169760
rs17732092
rs141833309
rs4728429
rs111448717
rs830197
rs183002914
rs74505030
rs73239709
rs944188
rs2245341
rs10475891
rs13064192
rs58695218
rs62406632
rs55751920
rs4074507
All
−5e−04
0e+00
5e−04
1e−0
MR effect size for
'Bcl−2−related protein A1' on 'eGFR'

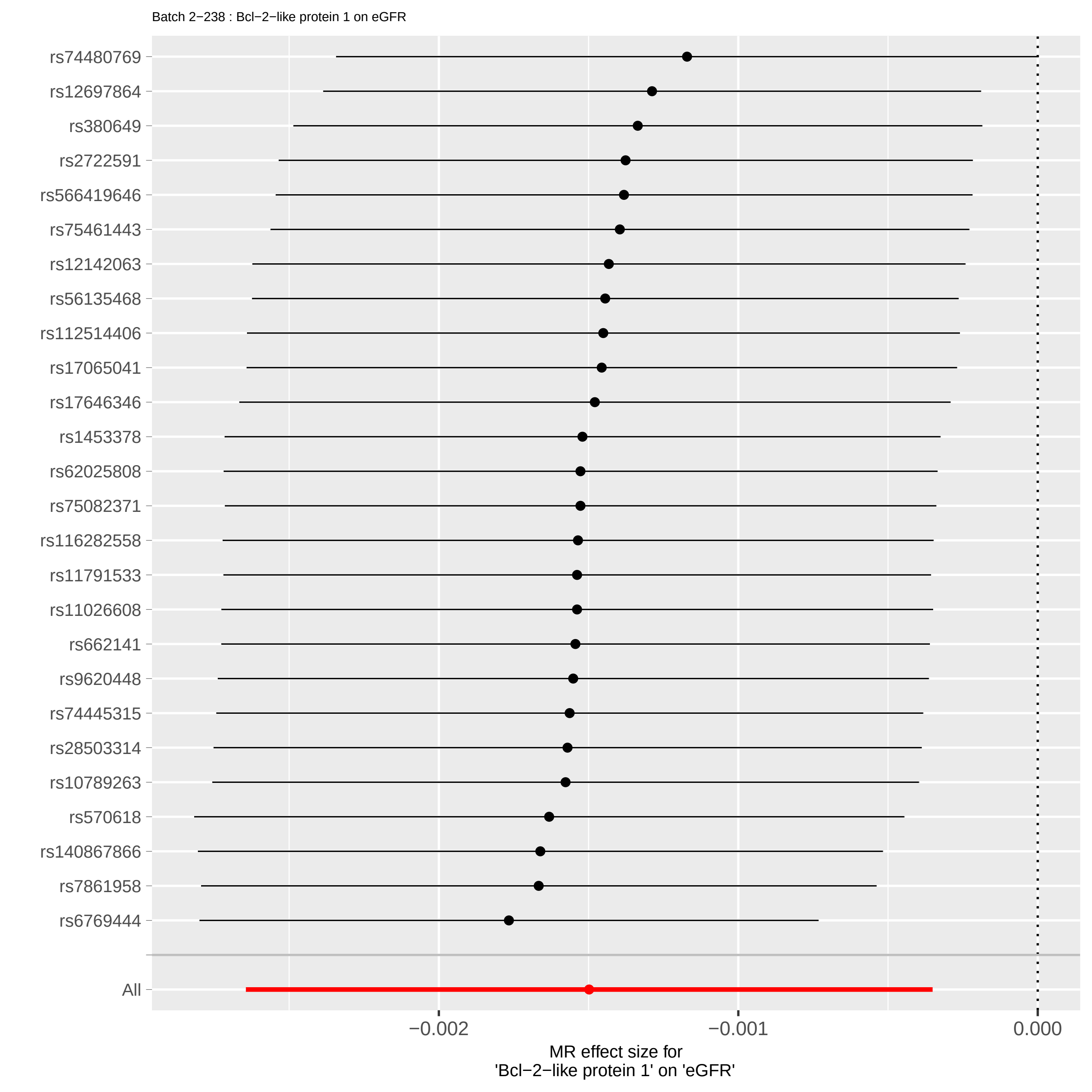

Batch 2−238 : Bcl−2−like protein 1 on eGFR
rs74480769
rs12697864
rs380649
rs2722591
rs566419646
rs75461443
rs12142063
rs56135468
rs112514406
rs17065041
rs17646346
rs1453378
rs62025808
rs75082371
rs116282558
rs11791533
rs11026608
rs662141
rs9620448
rs74445315
rs28503314
rs10789263
rs570618
rs140867866
rs7861958
rs6769444
All
−0.002
−0.001
0.000
MR effect size for
'Bcl−2−like protein 1' on 'eGFR'

Batch 2−239 : Bcl−2−like protein 2 on eGFR

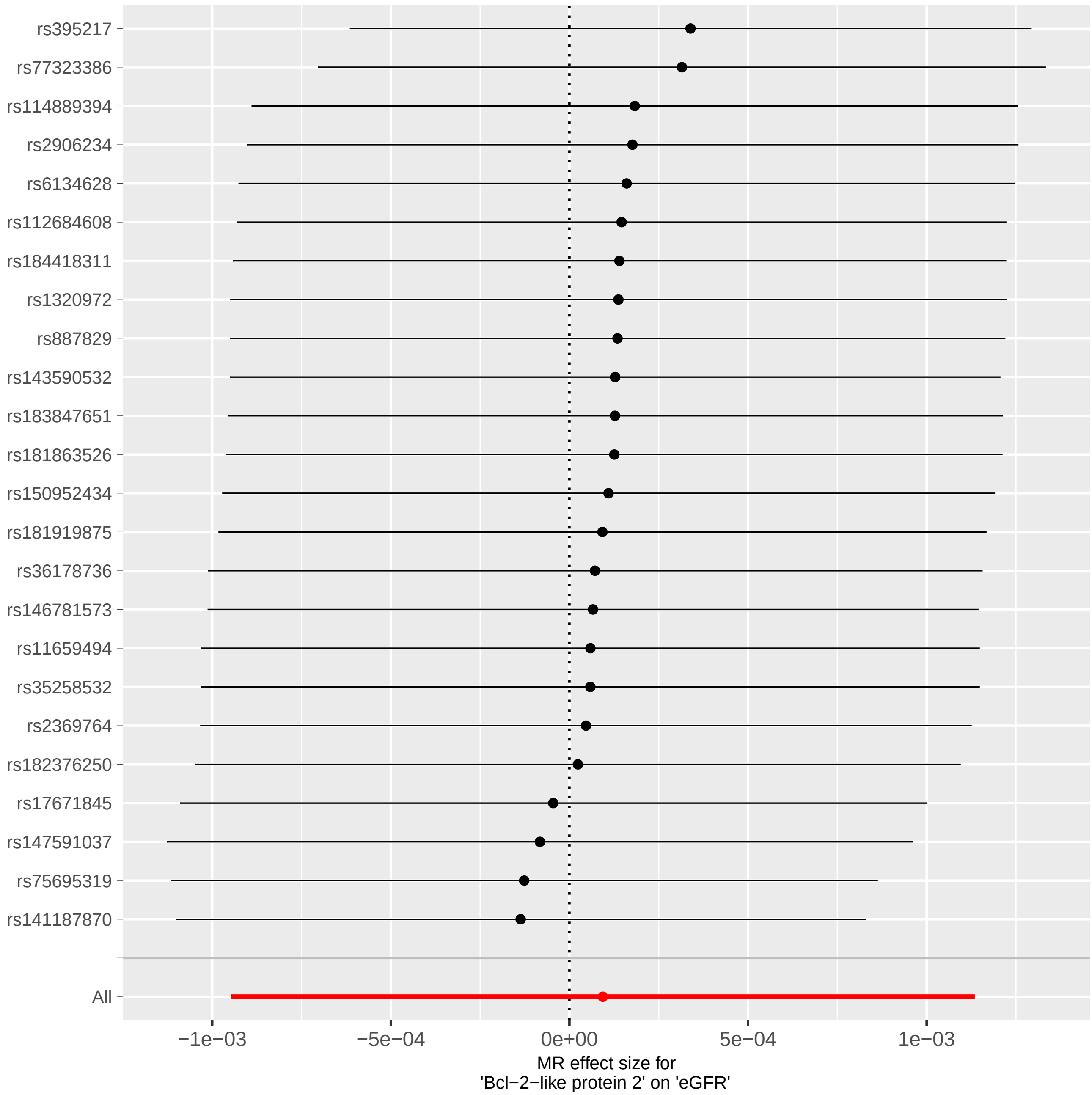

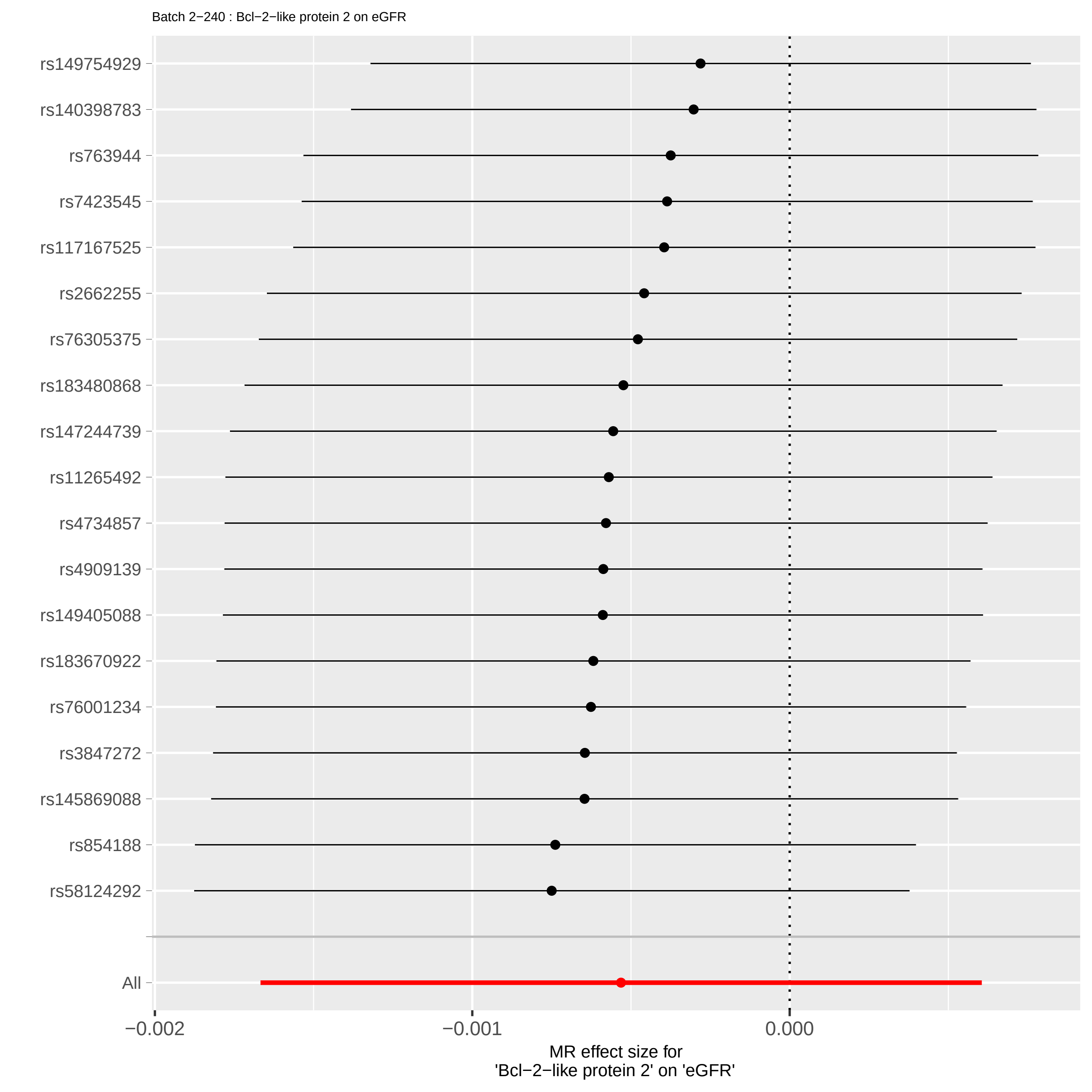

Batch 2−240 : Bcl−2−like protein 2 on eGFR
rs149754929
rs140398783
rs763944
rs7423545
rs117167525
rs2662255
rs76305375
rs183480868
rs147244739
rs11265492
rs4734857
rs4909139
rs149405088
rs183670922
rs76001234
rs3847272
rs145869088
rs854188
rs58124292
All
−0.002
−0.001
0.000
MR effect size for
'Bcl−2−like protein 2' on 'eGFR'

Batch 2−241 : B−cell lymphoma 6 protein on eGFR

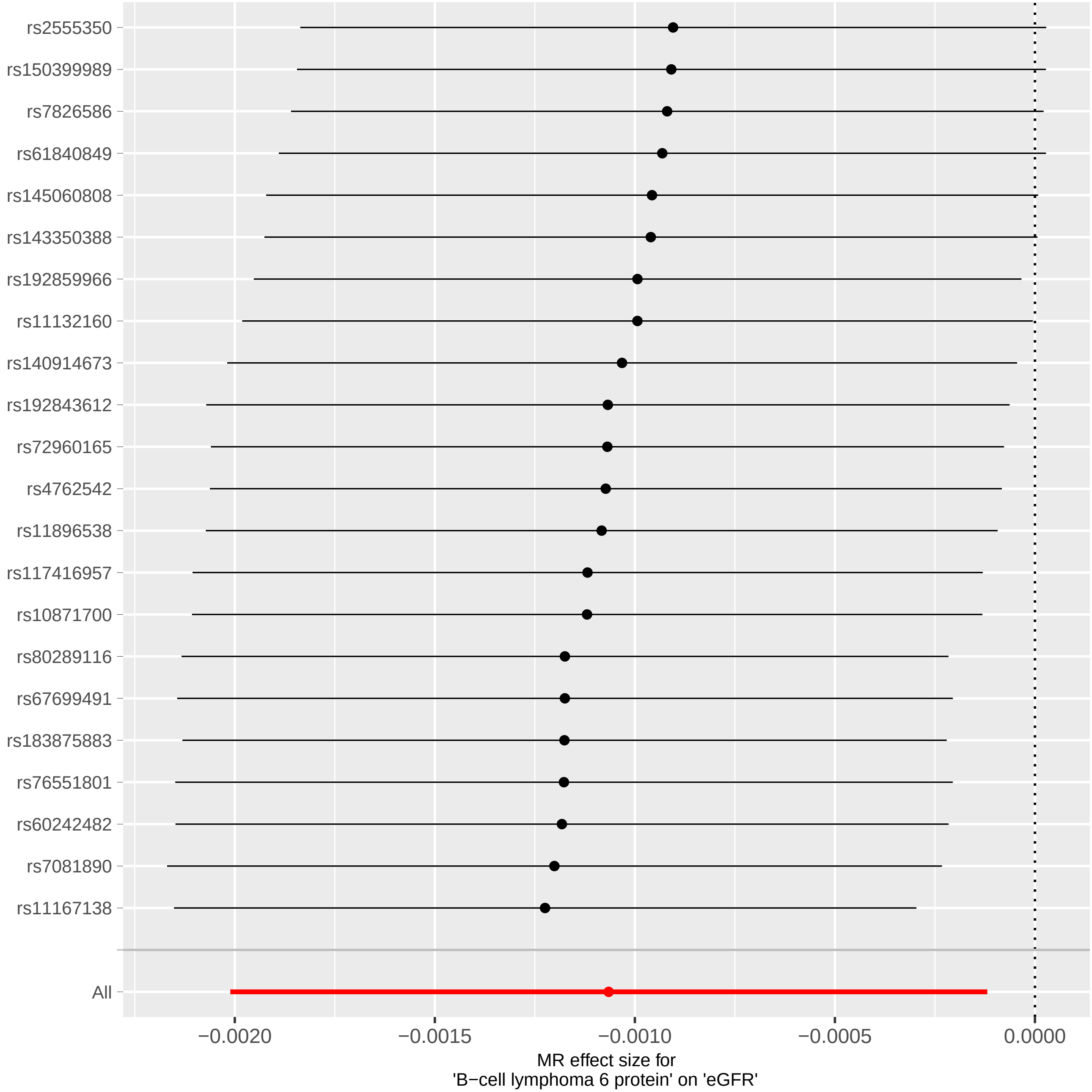

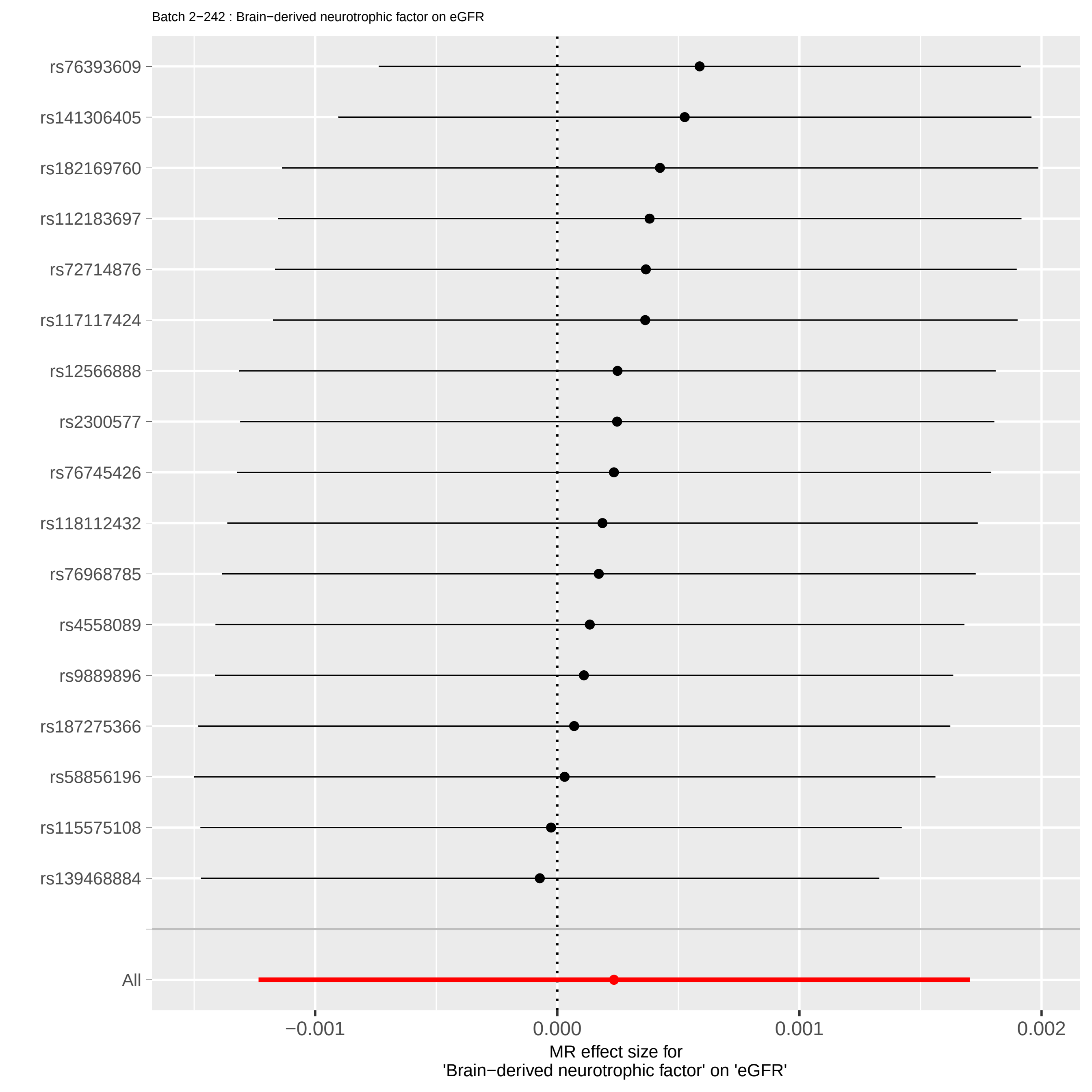

Batch 2−242 : Brain−derived neurotrophic factor on eGFR
rs76393609
rs141306405
rs182169760
rs112183697
rs72714876
rs117117424
rs12566888
rs2300577
rs76745426
rs118112432
rs76968785
rs4558089
rs9889896
rs187275366
rs58856196
rs115575108
rs139468884
All
−0.001
0.000
0.001
0.002
MR effect size for
'Brain−derived neurotrophic factor' on 'eGFR'

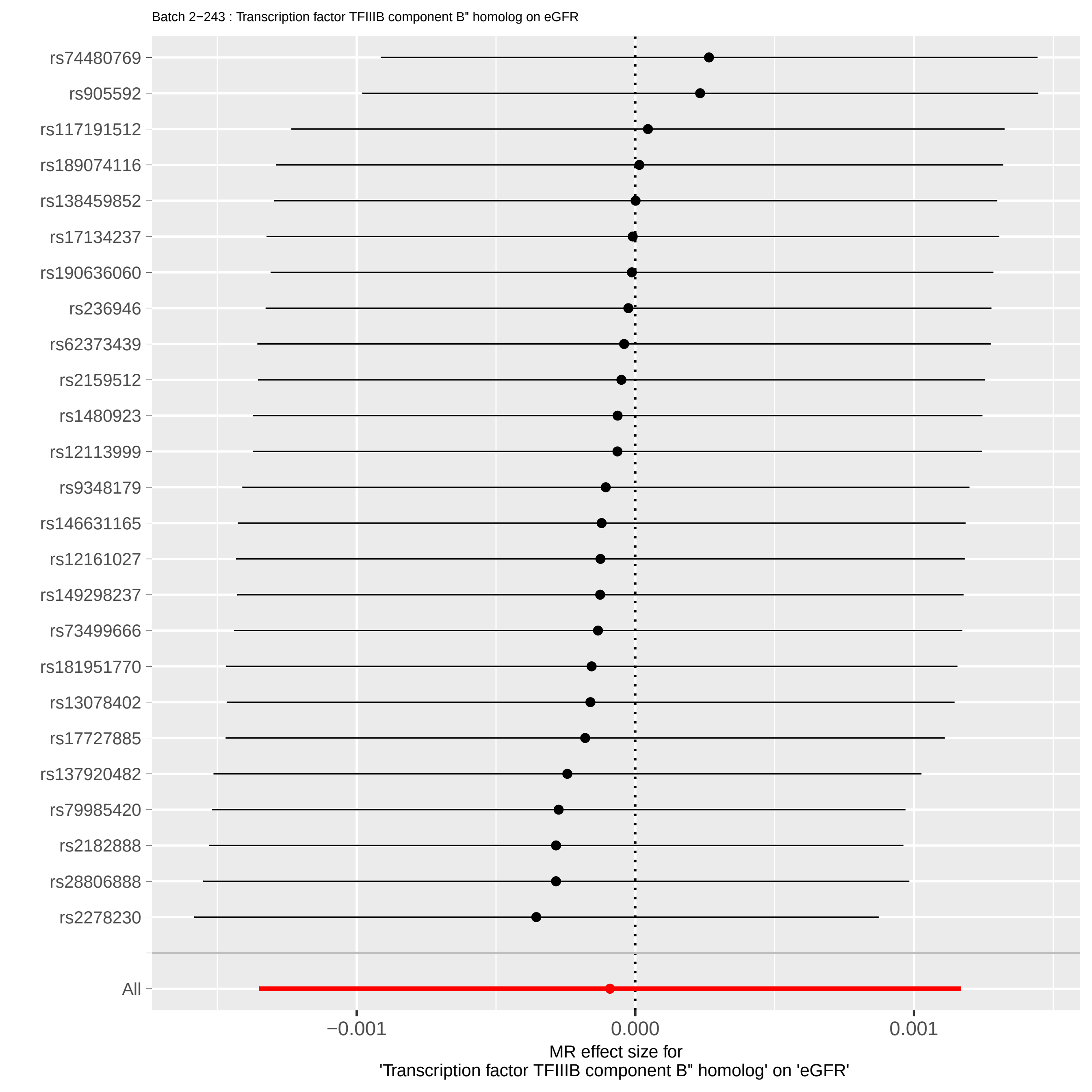

Batch 2−243 : Transcription factor TFIIIB component B" homolog on eGFR
rs74480769
rs905592
rs117191512
rs189074116
rs138459852
rs17134237
rs190636060
rs236946
rs62373439
rs2159512
rs1480923
rs12113999
rs9348179
rs146631165
rs12161027
rs149298237
rs73499666
rs181951770
rs13078402
rs17727885
rs137920482
rs79985420
rs2182888
rs28806888
rs2278230
All
−0.001
0.000
0.001
MR effect size for
'Transcription factor TFIIIB component B" homolog' on 'eGFR'

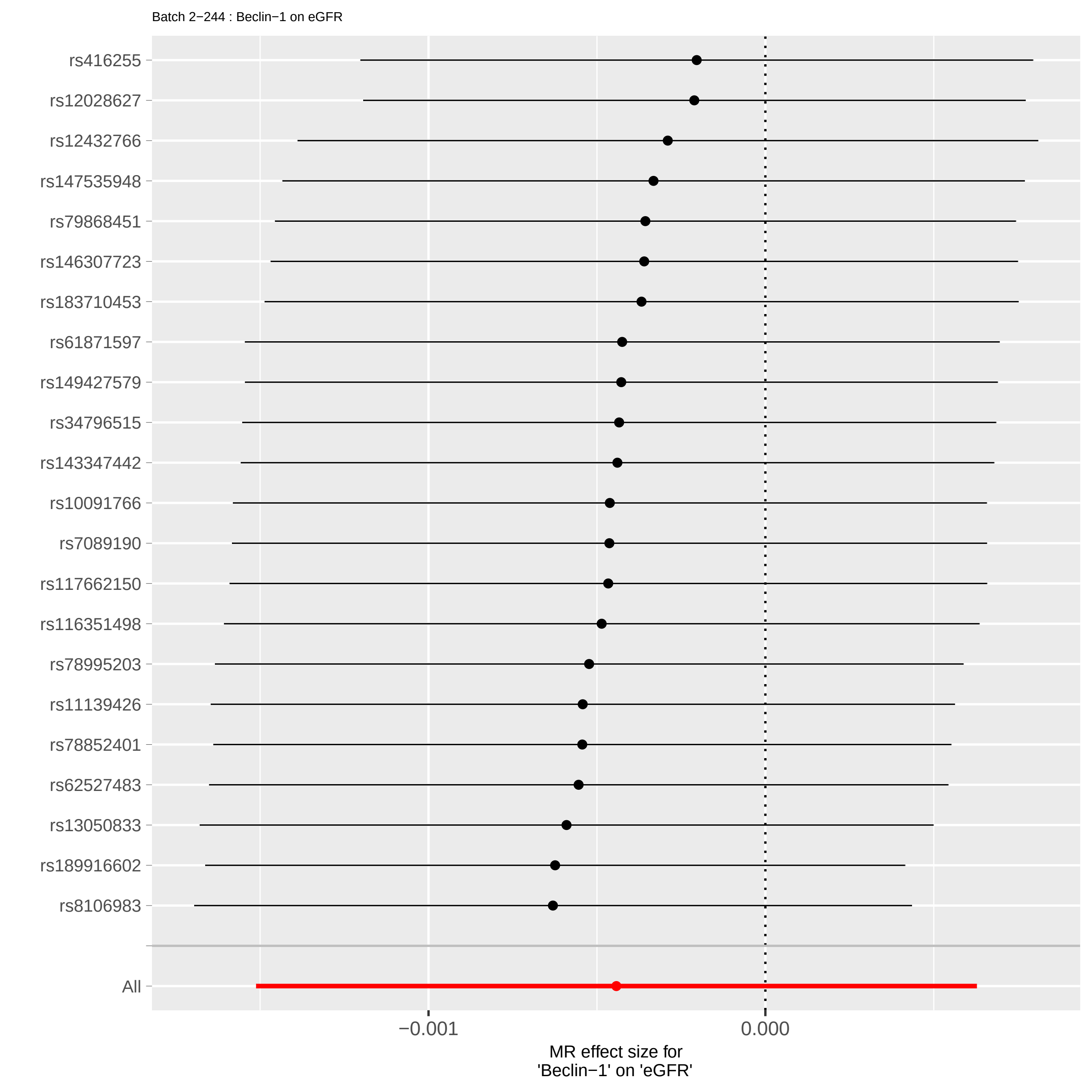

Batch 2−244 : Beclin−1 on eGFR
rs416255
rs12028627
rs12432766
rs147535948
rs79868451
rs146307723
rs183710453
rs61871597
rs149427579
rs34796515
rs143347442
rs10091766
rs7089190
rs117662150
rs116351498
rs78995203
rs11139426
rs78852401
rs62527483
rs13050833
rs189916602
rs8106983
All
−0.001
0.000
MR effect size for
'Beclin−1' on 'eGFR'

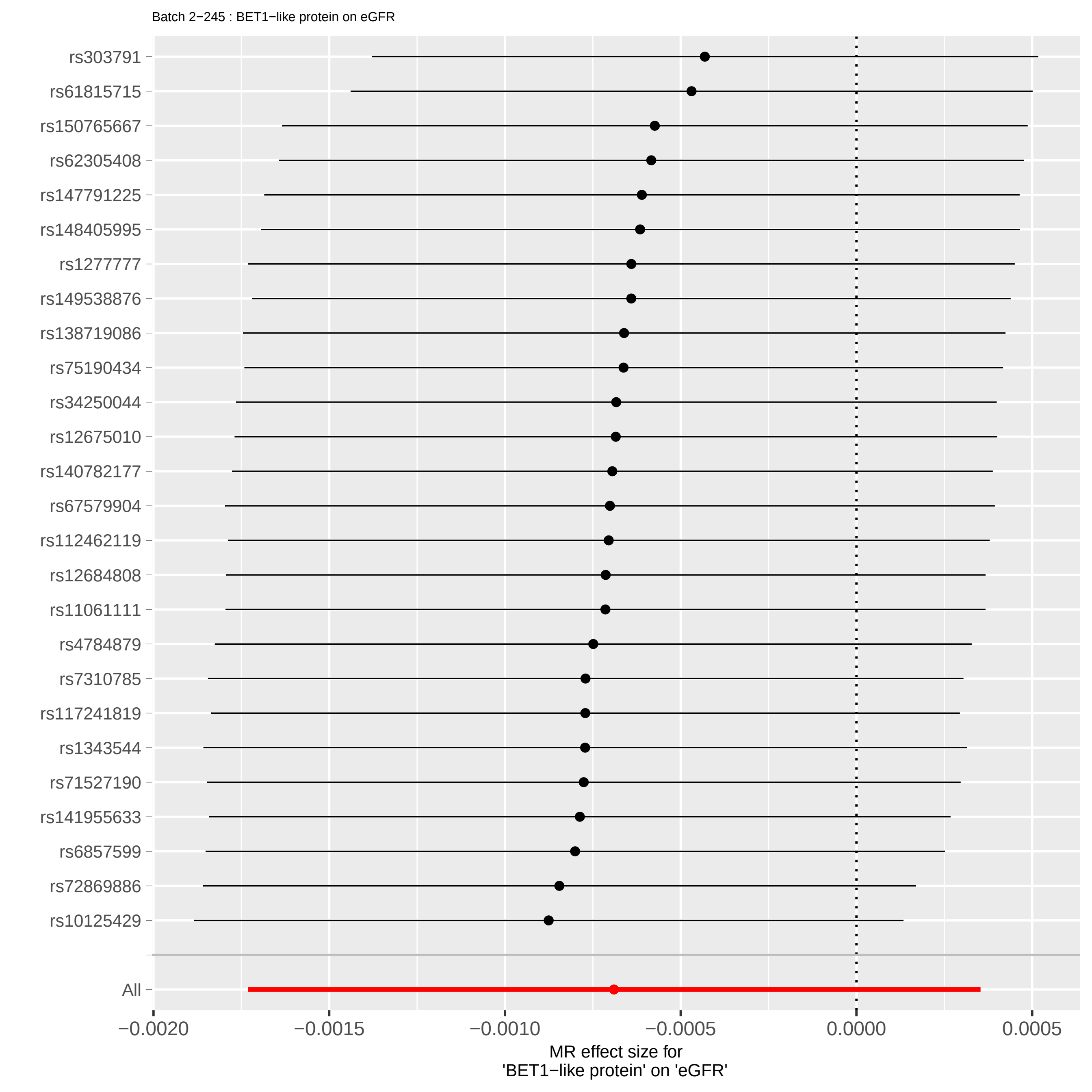

Batch 2−245 : BET1−like protein on eGFR
rs303791
rs61815715
rs150765667
rs62305408
rs147791225
rs148405995
rs1277777
rs149538876
rs138719086
rs75190434
rs34250044
rs12675010
rs140782177
rs67579904
rs112462119
rs12684808
rs11061111
rs4784879
rs7310785
rs117241819
rs1343544
rs71527190
rs141955633
rs6857599
rs72869886
rs10125429
All
−0.0020
−0.0015
−0.0010
−0.0005
0.0000
0.0005
MR effect size for
'BET1−like protein' on 'eGFR'

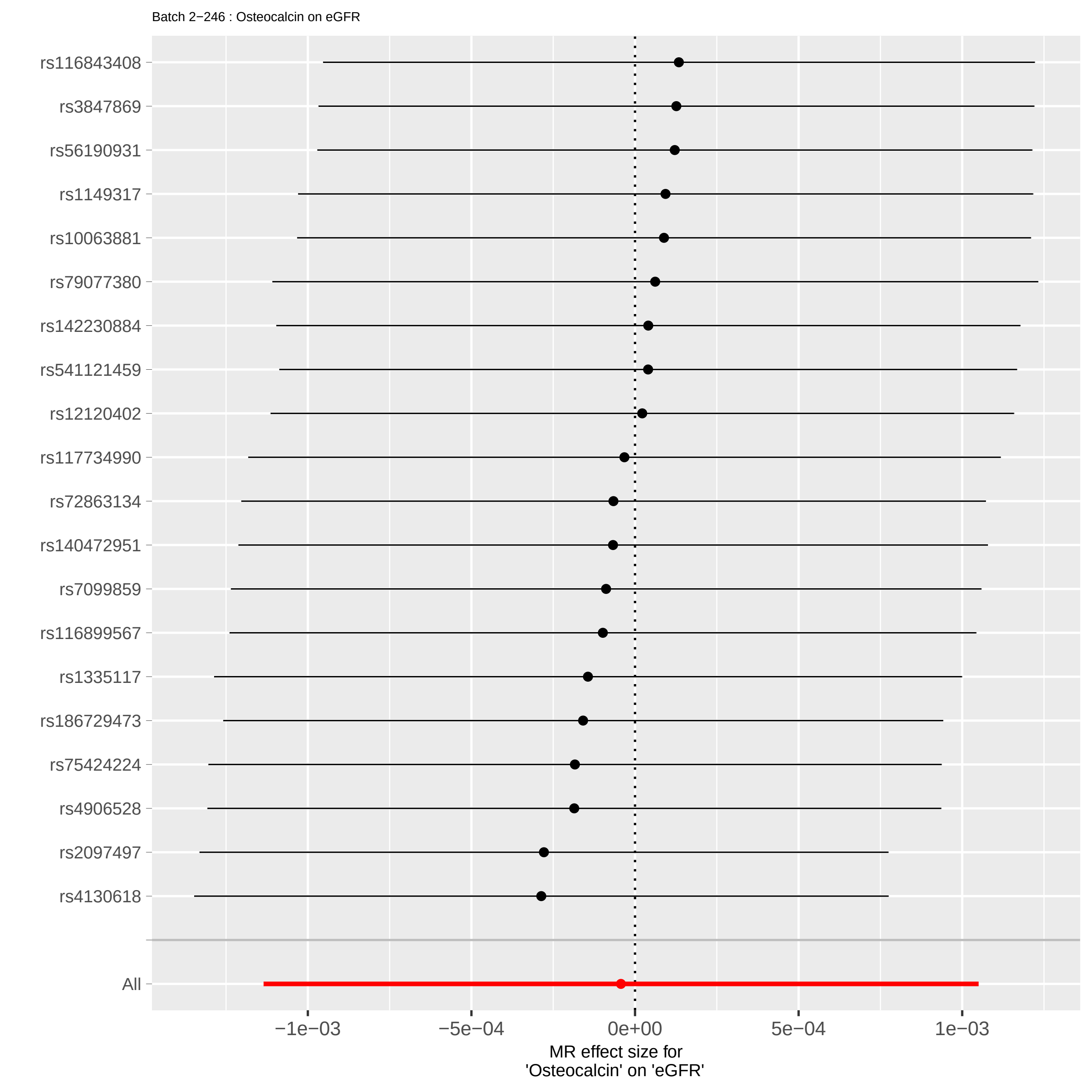

Batch 2−246 : Osteocalcin on eGFR
rs116843408
rs3847869
rs56190931
rs1149317
rs10063881
rs79077380
rs142230884
rs541121459
rs12120402
rs117734990
rs72863134
rs140472951
rs7099859
rs116899567
rs1335117
rs186729473
rs75424224
rs4906528
rs2097497
rs4130618
All
−1e−03
−5e−04
0e+00
5e−04
1e−03
MR effect size for
'Osteocalcin' on 'eGFR'

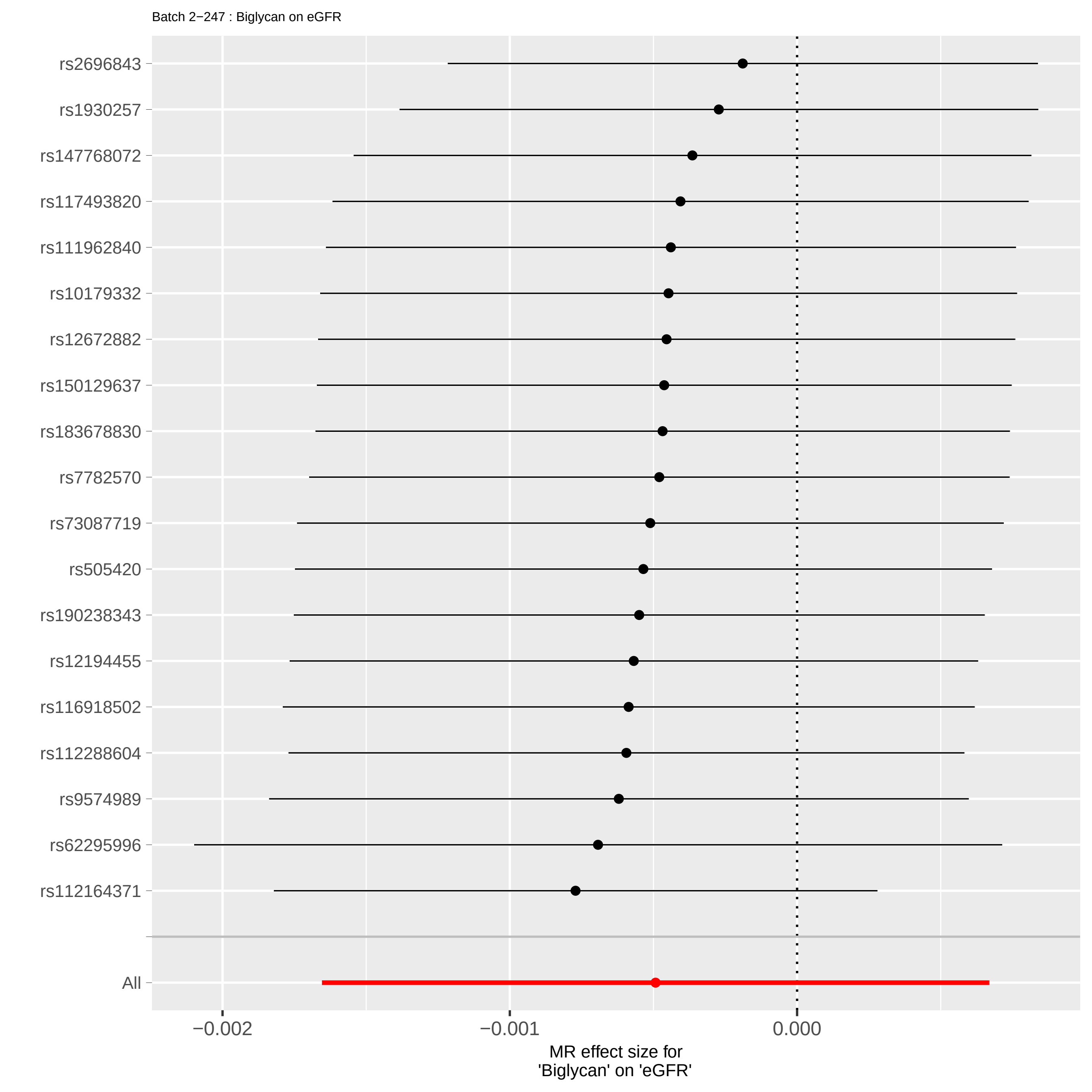

Batch 2−247 : Biglycan on eGFR
rs2696843
rs1930257
rs147768072
rs117493820
rs111962840
rs10179332
rs12672882
rs150129637
rs183678830
rs7782570
rs73087719
rs505420
rs190238343
rs12194455
rs116918502
rs112288604
rs9574989
rs62295996
rs112164371
All
−0.002
−0.001
0.000
MR effect size for
'Biglycan' on 'eGFR'

Batch 2−248 : Biglycan on eGFR

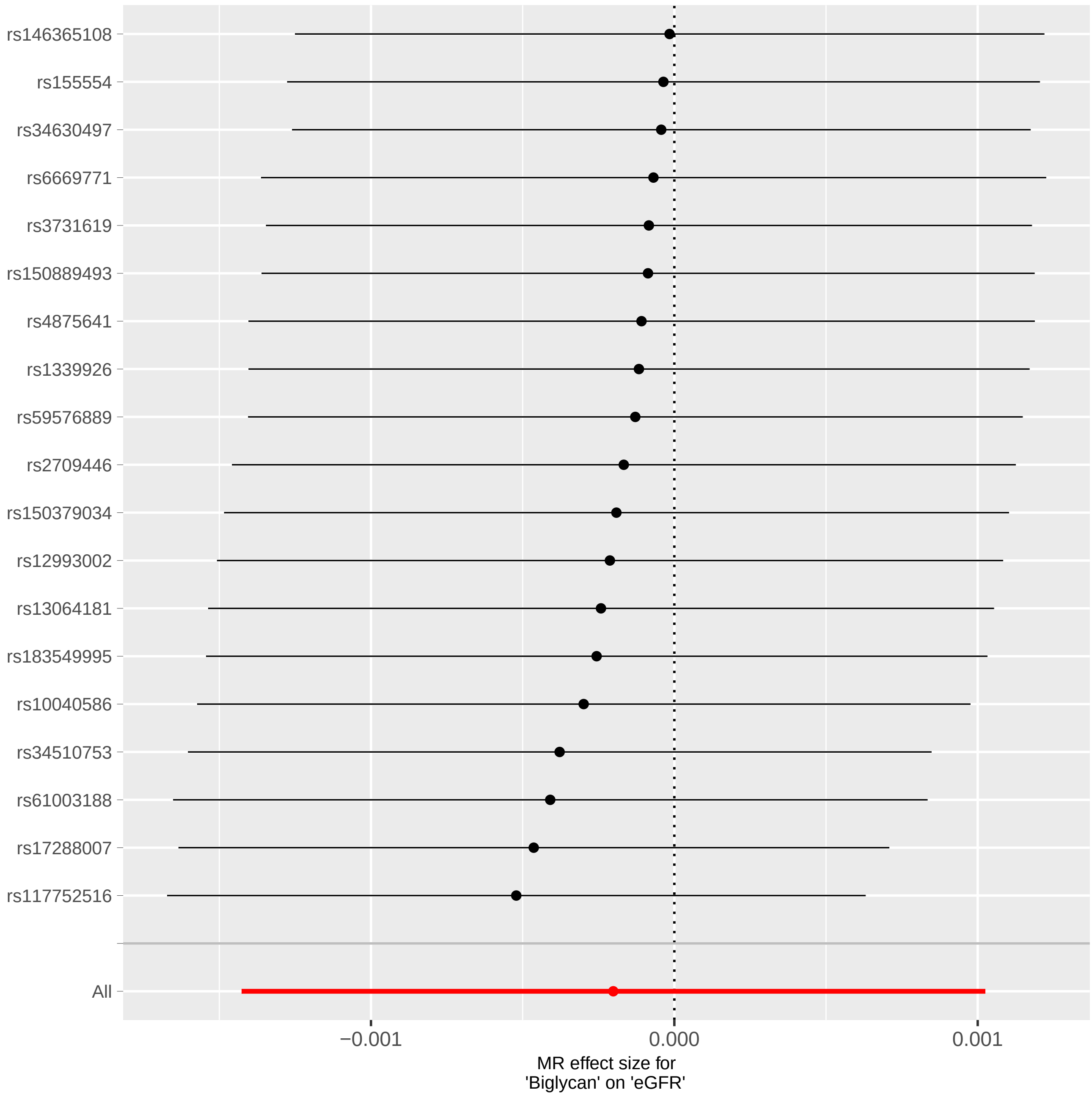

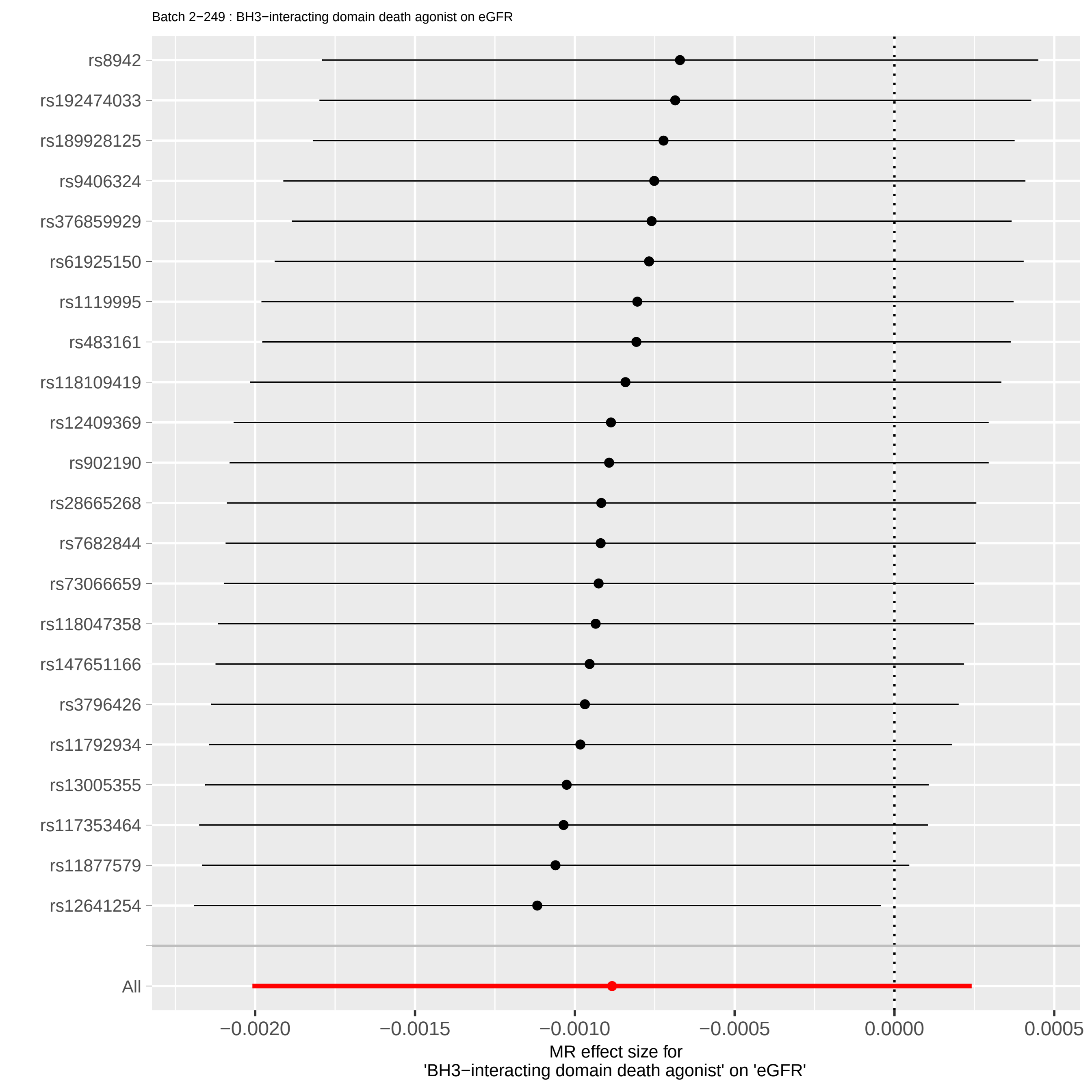

Batch 2−249 : BH3−interacting domain death agonist on eGFR
rs8942
rs192474033
rs189928125
rs9406324
rs376859929
rs61925150
rs1119995
rs483161
rs118109419
rs12409369
rs902190
rs28665268
rs7682844
rs73066659
rs118047358
rs147651166
rs3796426
rs11792934
rs13005355
rs117353464
rs11877579
rs12641254
All
−0.0020
−0.0015
−0.0010
−0.0005
0.0000
0.0005
MR effect size for
'BH3−interacting domain death agonist' on 'eGFR'

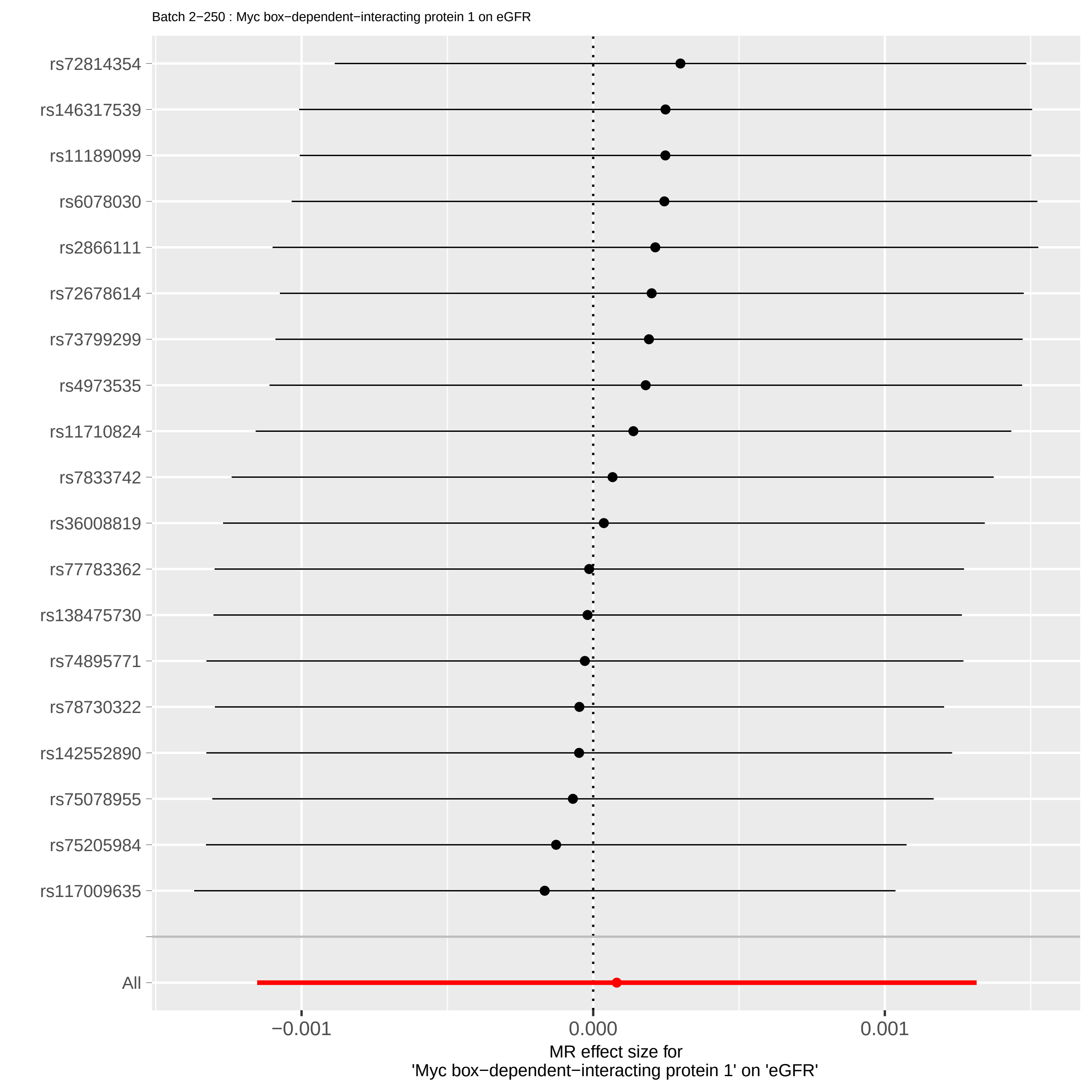
Batch 2−250 : Myc box−dependent−interacting protein 1 on eGFR
rs72814354
rs146317539
rs11189099
rs6078030
rs2866111
rs72678614
rs73799299
rs4973535
rs11710824
rs7833742
rs36008819
rs77783362
rs138475730
rs74895771
rs78730322
rs142552890
rs75078955
rs75205984
rs117009635
All
−0.001
0.000
0.001
MR effect size for
'Myc box−dependent−interacting protein 1' on 'eGFR'

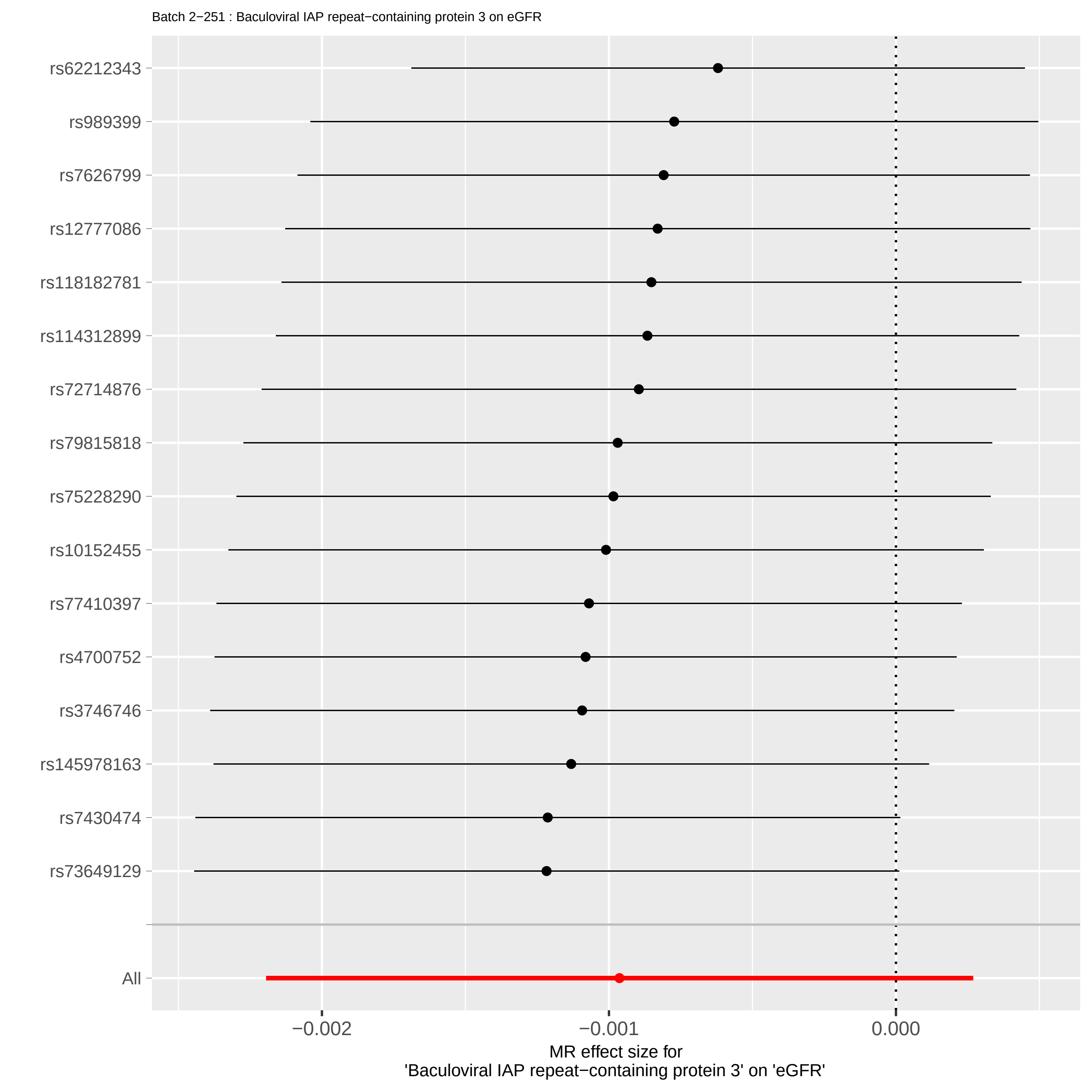
Batch 2−251 : Baculoviral IAP repeat−containing protein 3 on eGFR
rs62212343
rs989399
rs7626799
rs12777086
rs118182781
rs114312899
rs72714876
rs79815818
rs75228290
rs10152455
rs77410397
rs4700752
rs3746746
rs145978163
rs7430474
rs73649129
All
−0.002
−0.001
0.000
MR effect size for
'Baculoviral IAP repeat−containing protein 3' on 'eGFR'

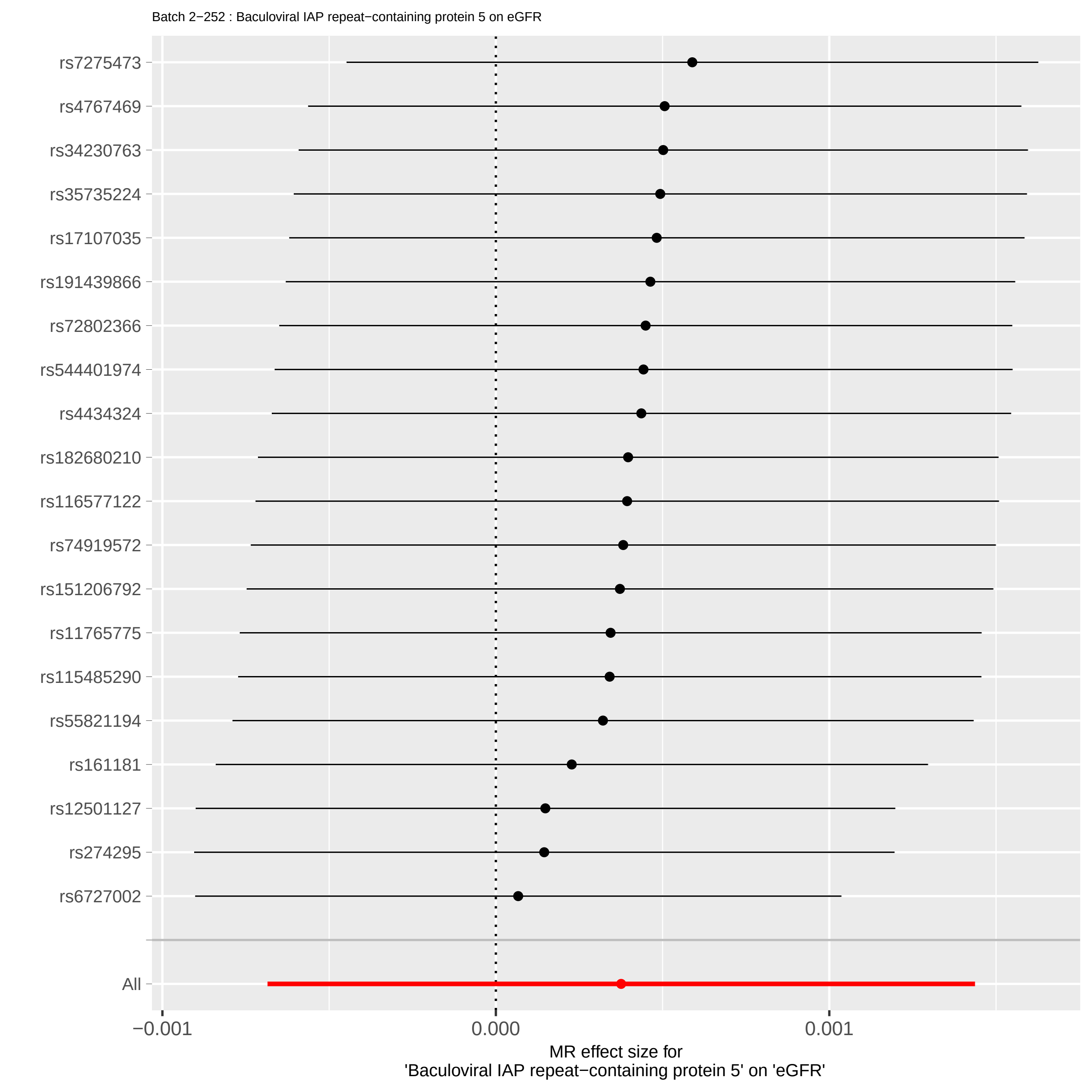

Batch 2−252 : Baculoviral IAP repeat−containing protein 5 on eGFR
rs7275473
rs4767469
rs34230763
rs35735224
rs17107035
rs191439866
rs72802366
rs544401974
rs4434324
rs182680210
rs116577122
rs74919572
rs151206792
rs11765775
rs115485290
rs55821194
rs161181
rs12501127
rs274295
rs6727002
All
−0.001
0.000
0.001
MR effect size for
'Baculoviral IAP repeat−containing protein 5' on 'eGFR'

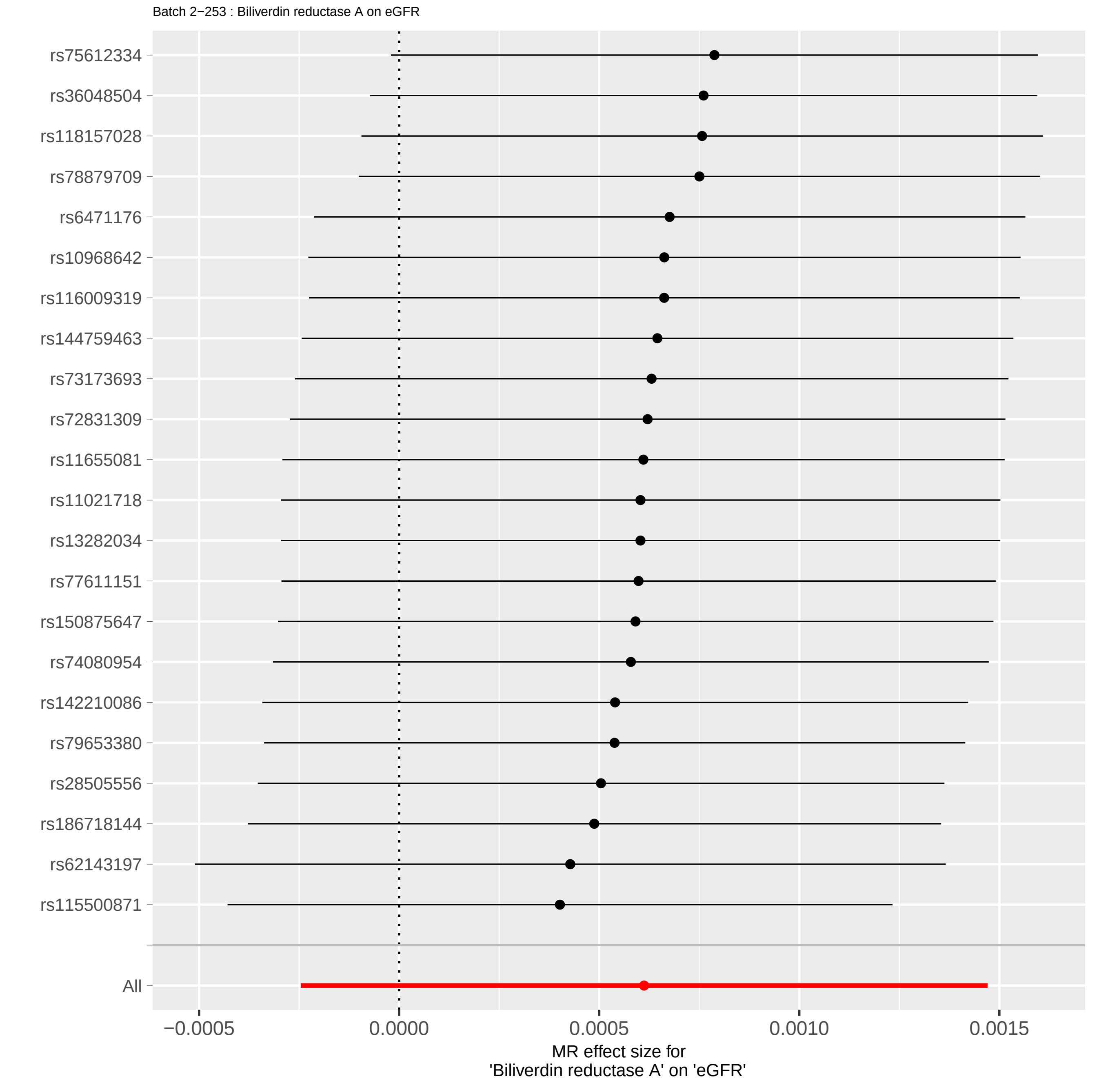
Batch 2−253 : Biliverdin reductase A on eGFR
rs75612334
rs36048504
rs118157028
rs78879709
rs6471176
rs10968642
rs116009319
rs144759463
rs73173693
rs72831309
rs11655081
rs11021718
rs13282034
rs77611151
rs150875647
rs74080954
rs142210086
rs79653380
rs28505556
rs186718144
rs62143197
rs115500871
All
−0.0005
0.0000
0.0005
0.0010
0.0015
MR effect size for
'Biliverdin reductase A' on 'eGFR'

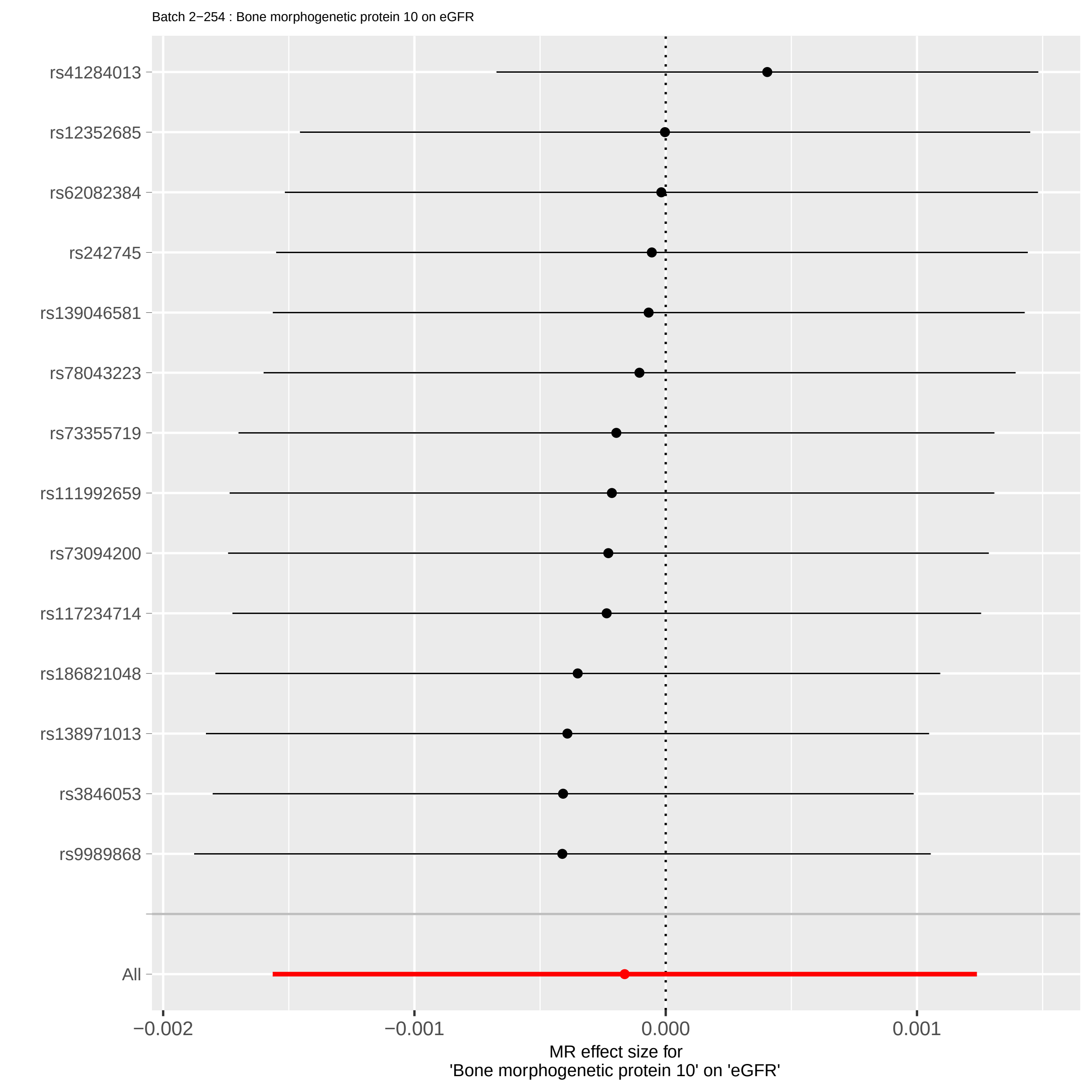

Batch 2−254 : Bone morphogenetic protein 10 on eGFR
rs41284013
rs12352685
rs62082384
rs242745
rs139046581
rs78043223
rs73355719
rs111992659
rs73094200
rs117234714
rs186821048
rs138971013
rs3846053
rs9989868
All
−0.002
−0.001
0.000
0.001
MR effect size for
'Bone morphogenetic protein 10' on 'eGFR'

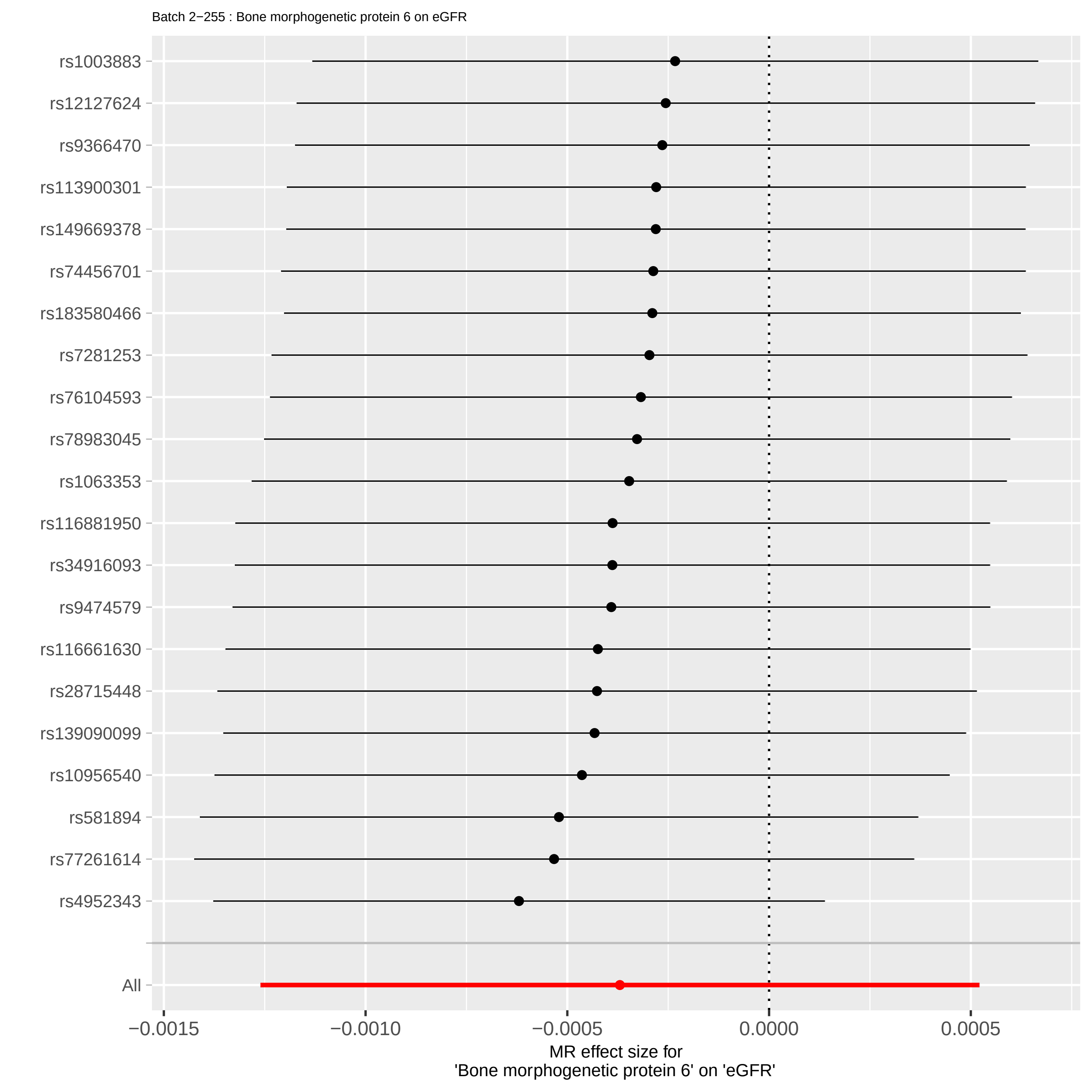

Batch 2−255 : Bone morphogenetic protein 6 on eGFR
rs1003883
rs12127624
rs9366470
rs113900301
rs149669378
rs74456701
rs183580466
rs7281253
rs76104593
rs78983045
rs1063353
rs116881950
rs34916093
rs9474579
rs116661630
rs28715448
rs139090099
rs10956540
rs581894
rs77261614
rs4952343
All
−0.0015
−0.0010
−0.0005
0.0000
0.0005
MR effect size for
'Bone morphogenetic protein 6' on 'eGFR'

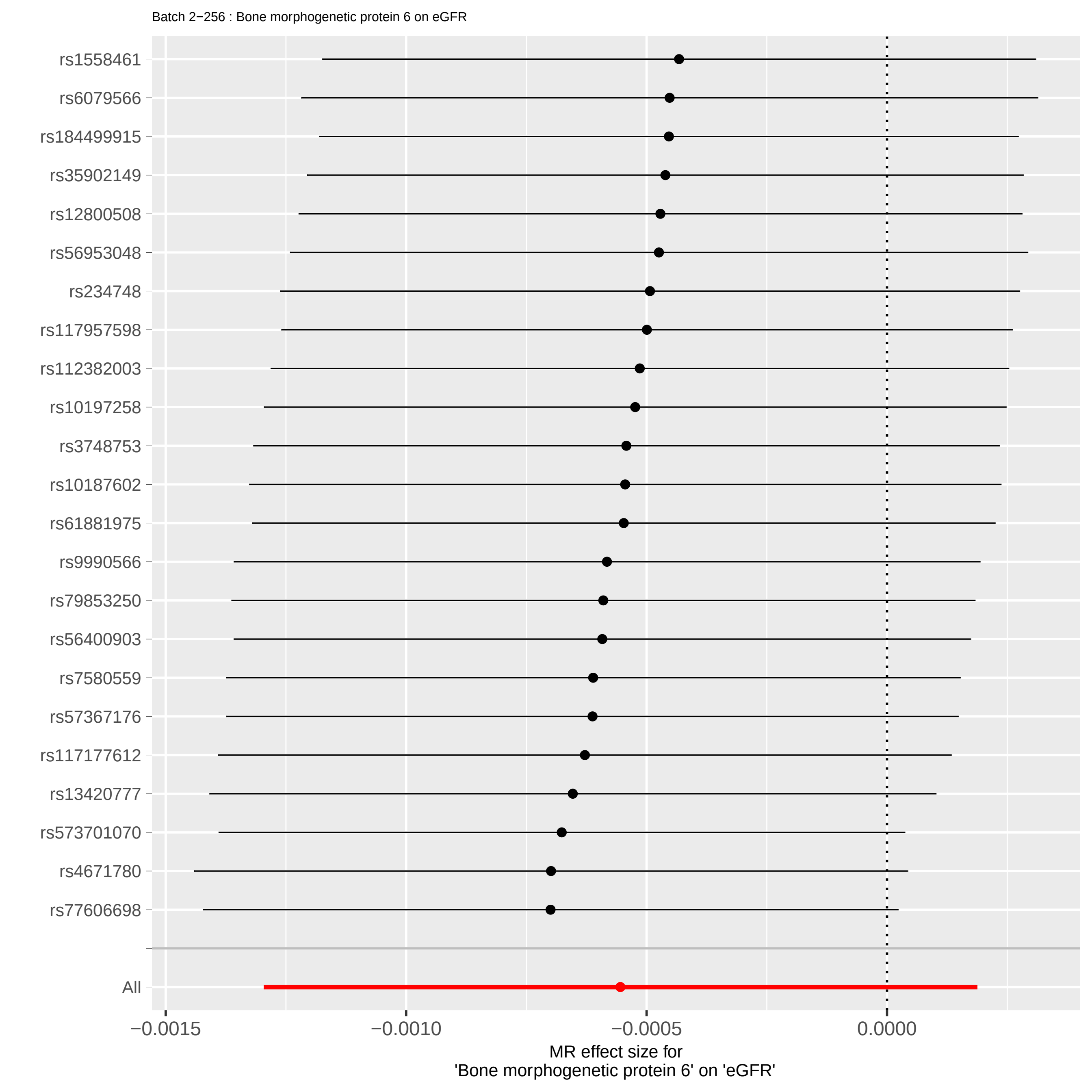

Batch 2−256 : Bone morphogenetic protein 6 on eGFR
rs1558461
rs6079566
rs184499915
rs35902149
rs12800508
rs56953048
rs234748
rs117957598
rs112382003
rs10197258
rs3748753
rs10187602
rs61881975
rs9990566
rs79853250
rs56400903
rs7580559
rs57367176
rs117177612
rs13420777
rs573701070
rs4671780
rs77606698
All
−0.0015
−0.0010
−0.0005
0.0000
MR effect size for
'Bone morphogenetic protein 6' on 'eGFR'

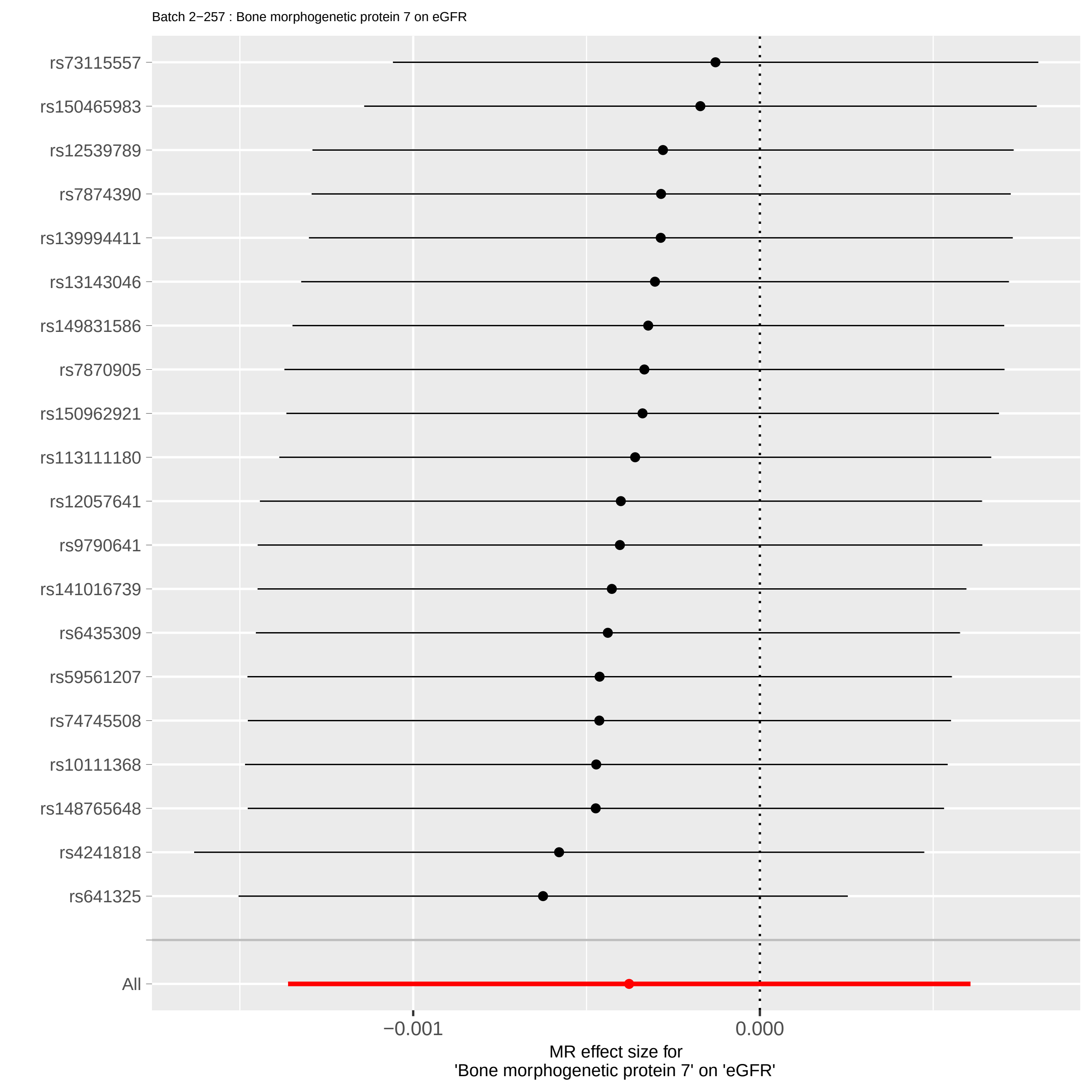

Batch 2−257 : Bone morphogenetic protein 7 on eGFR
rs73115557
rs150465983
rs12539789
rs7874390
rs139994411
rs13143046
rs149831586
rs7870905
rs150962921
rs113111180
rs12057641
rs9790641
rs141016739
rs6435309
rs59561207
rs74745508
rs10111368
rs148765648
rs4241818
rs641325
All
−0.001
0.000
MR effect size for
'Bone morphogenetic protein 7' on 'eGFR'

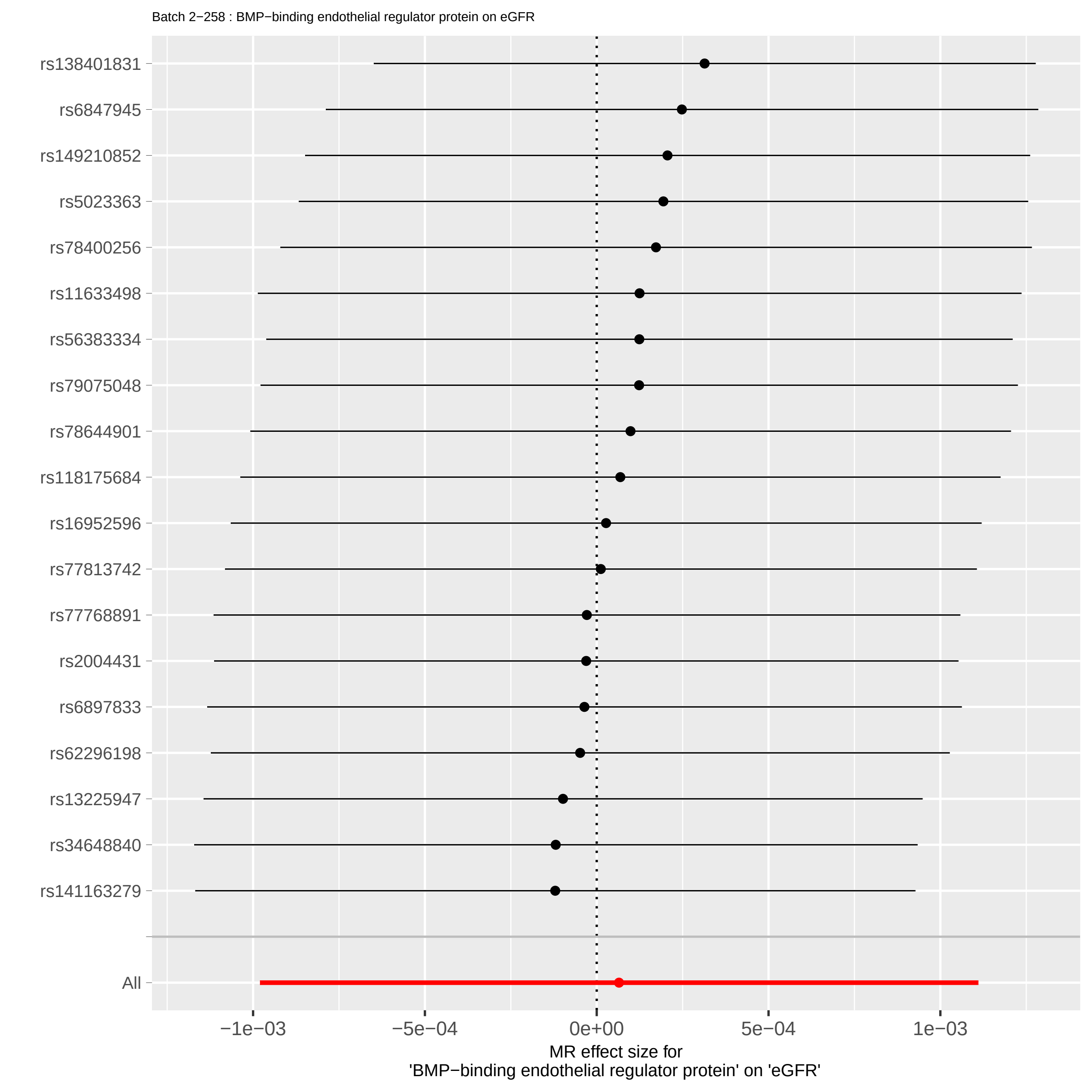

Batch 2−258 : BMP−binding endothelial regulator protein on eGFR
rs138401831
rs6847945
rs149210852
rs5023363
rs78400256
rs11633498
rs56383334
rs79075048
rs78644901
rs118175684
rs16952596
rs77813742
rs77768891
rs2004431
rs6897833
rs62296198
rs13225947
rs34648840
rs141163279
All
−1e−03
−5e−04
0e+00
5e−04
1e−03
MR effect size for
'BMP−binding endothelial regulator protein' on 'eGFR'

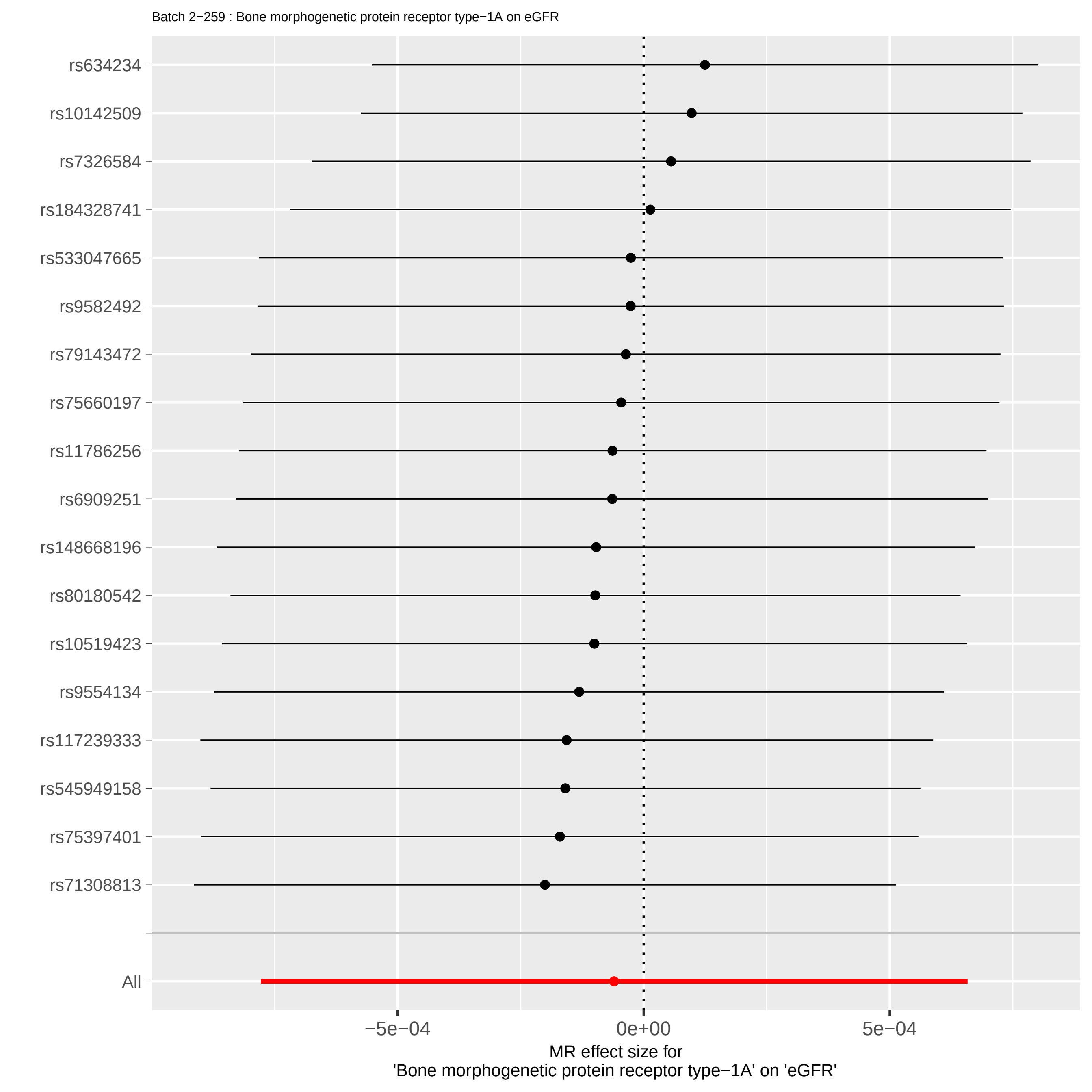

Batch 2−259 : Bone morphogenetic protein receptor type−1A on eGFR
rs634234
rs10142509
rs7326584
rs184328741
rs533047665
rs9582492
rs79143472
rs75660197
rs11786256
rs6909251
rs148668196
rs80180542
rs10519423
rs9554134
rs117239333
rs545949158
rs75397401
rs71308813
All
−5e−04
0e+00
5e−04
MR effect size for
'Bone morphogenetic protein receptor type−1A' on 'eGFR'

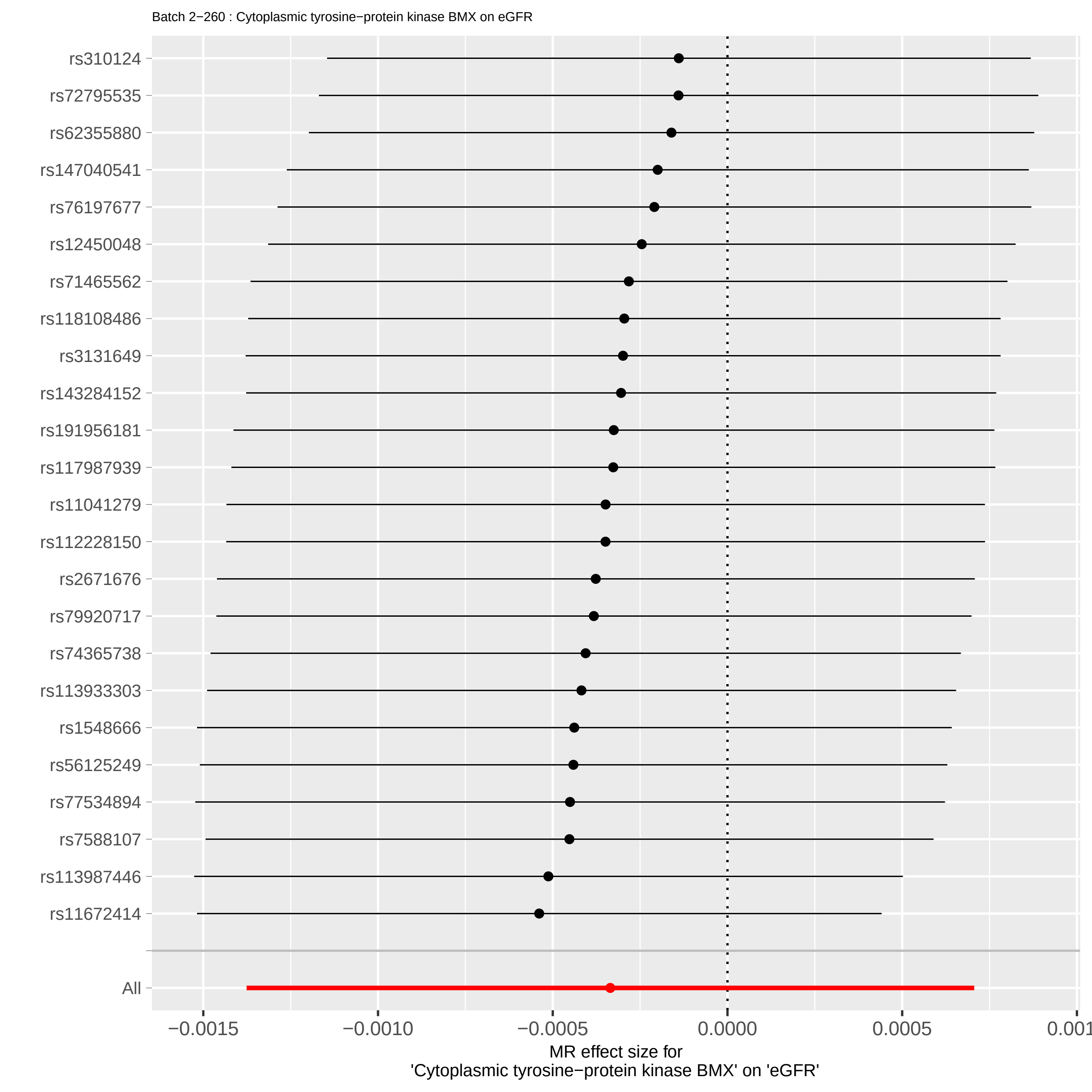

Batch 2−260 : Cytoplasmic tyrosine−protein kinase BMX on eGFR
rs310124
rs72795535
rs62355880
rs147040541
rs76197677
rs12450048
rs71465562
rs118108486
rs3131649
rs143284152
rs191956181
rs117987939
rs11041279
rs112228150
rs2671676
rs79920717
rs74365738
rs113933303
rs1548666
rs56125249
rs77534894
rs7588107
rs113987446
rs11672414
All
−0.0015
−0.0010
−0.0005
0.0000
0.0005
0.001
MR effect size for
'Cytoplasmic tyrosine−protein kinase BMX' on 'eGFR'

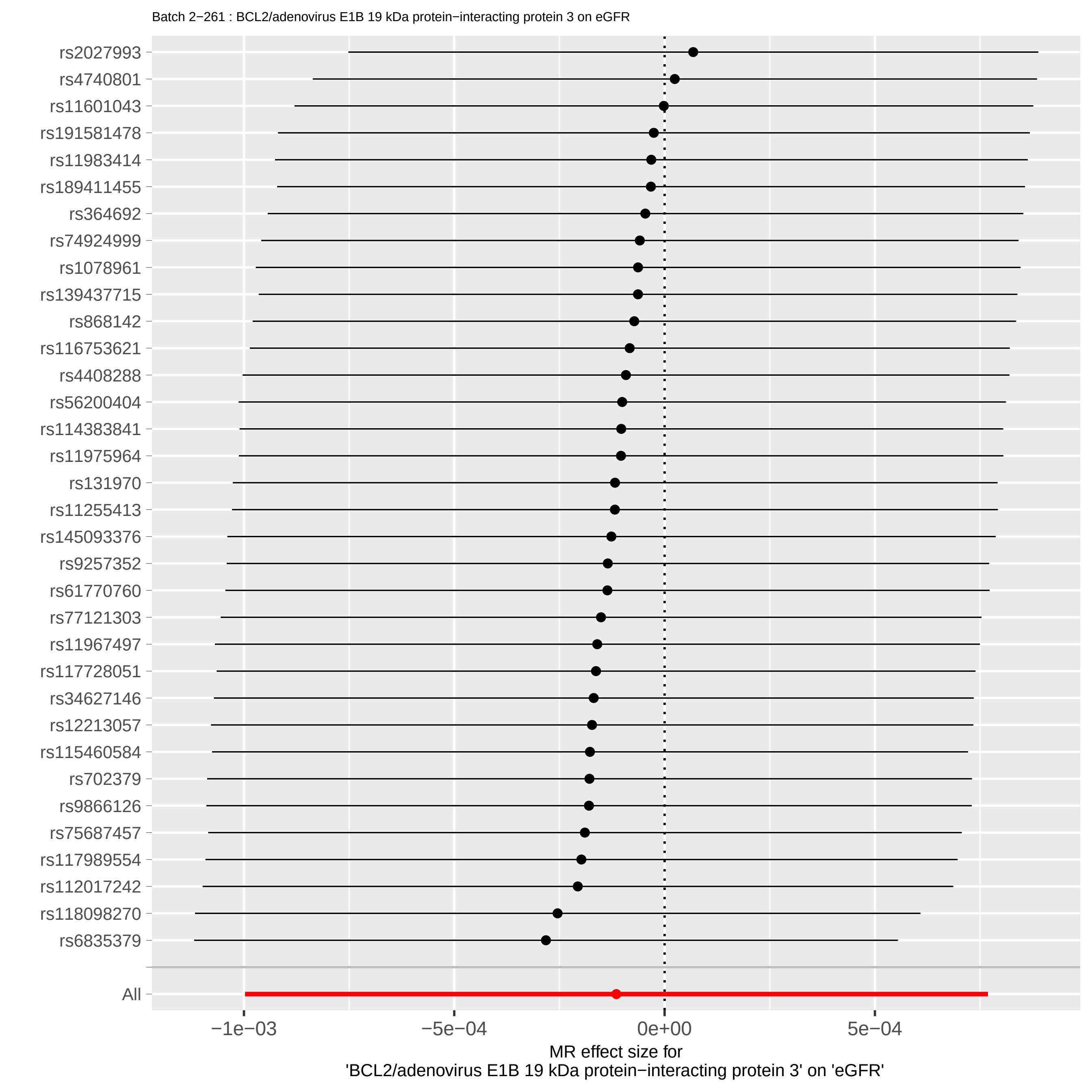

Batch 2−261 : BCL2/adenovirus E1B 19 kDa protein−interacting protein 3 on eGFR
rs2027993
rs4740801
rs11601043
rs191581478
rs11983414
rs189411455
rs364692
rs74924999
rs1078961
rs139437715
rs868142
rs116753621
rs4408288
rs56200404
rs114383841
rs11975964
rs131970
rs11255413
rs145093376
rs9257352
rs61770760
rs77121303
rs11967497
rs117728051
rs34627146
rs12213057
rs115460584
rs702379
rs9866126
rs75687457
rs117989554
rs112017242
rs118098270
rs6835379
All
−1e−03
−5e−04
0e+00
5e−04
MR effect size for
'BCL2/adenovirus E1B 19 kDa protein−interacting protein 3' on 'eGFR'

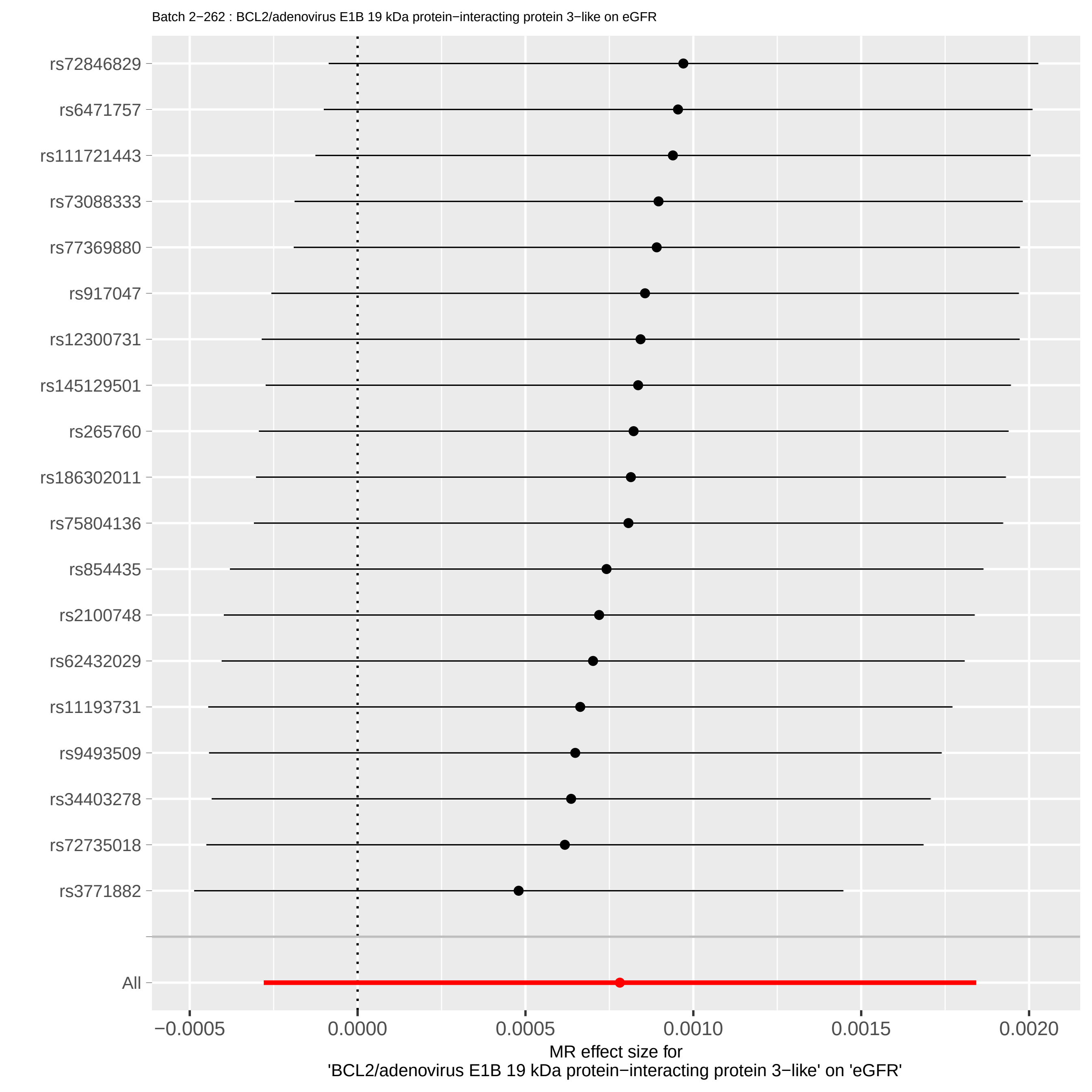

Batch 2−262 : BCL2/adenovirus E1B 19 kDa protein−interacting protein 3−like on eGFR
rs72846829
rs6471757
rs111721443
rs73088333
rs77369880
rs917047
rs12300731
rs145129501
rs265760
rs186302011
rs75804136
rs854435
rs2100748
rs62432029
rs11193731
rs9493509
rs34403278
rs72735018
rs3771882
All
−0.0005
0.0000
0.0005
0.0010
0.0015
0.0020
MR effect size for
'BCL2/adenovirus E1B 19 kDa protein−interacting protein 3−like' on 'eGFR'

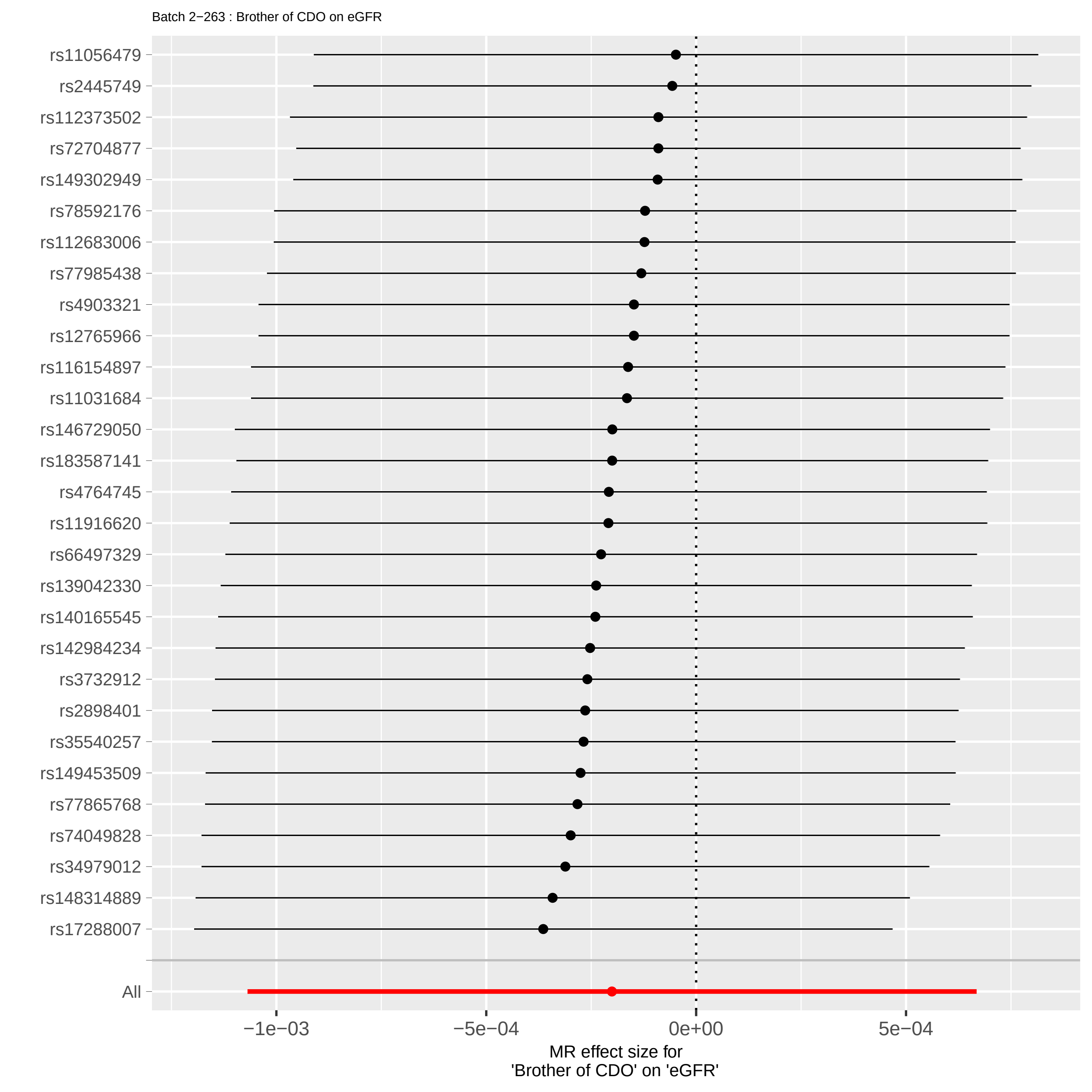

Batch 2−263 : Brother of CDO on eGFR
rs11056479
rs2445749
rs112373502
rs72704877
rs149302949
rs78592176
rs112683006
rs77985438
rs4903321
rs12765966
rs116154897
rs11031684
rs146729050
rs183587141
rs4764745
rs11916620
rs66497329
rs139042330
rs140165545
rs142984234
rs3732912
rs2898401
rs35540257
rs149453509
rs77865768
rs74049828
rs34979012
rs148314889
rs17288007
All
−1e−03
−5e−04
0e+00
5e−04
MR effect size for
'Brother of CDO' on 'eGFR'

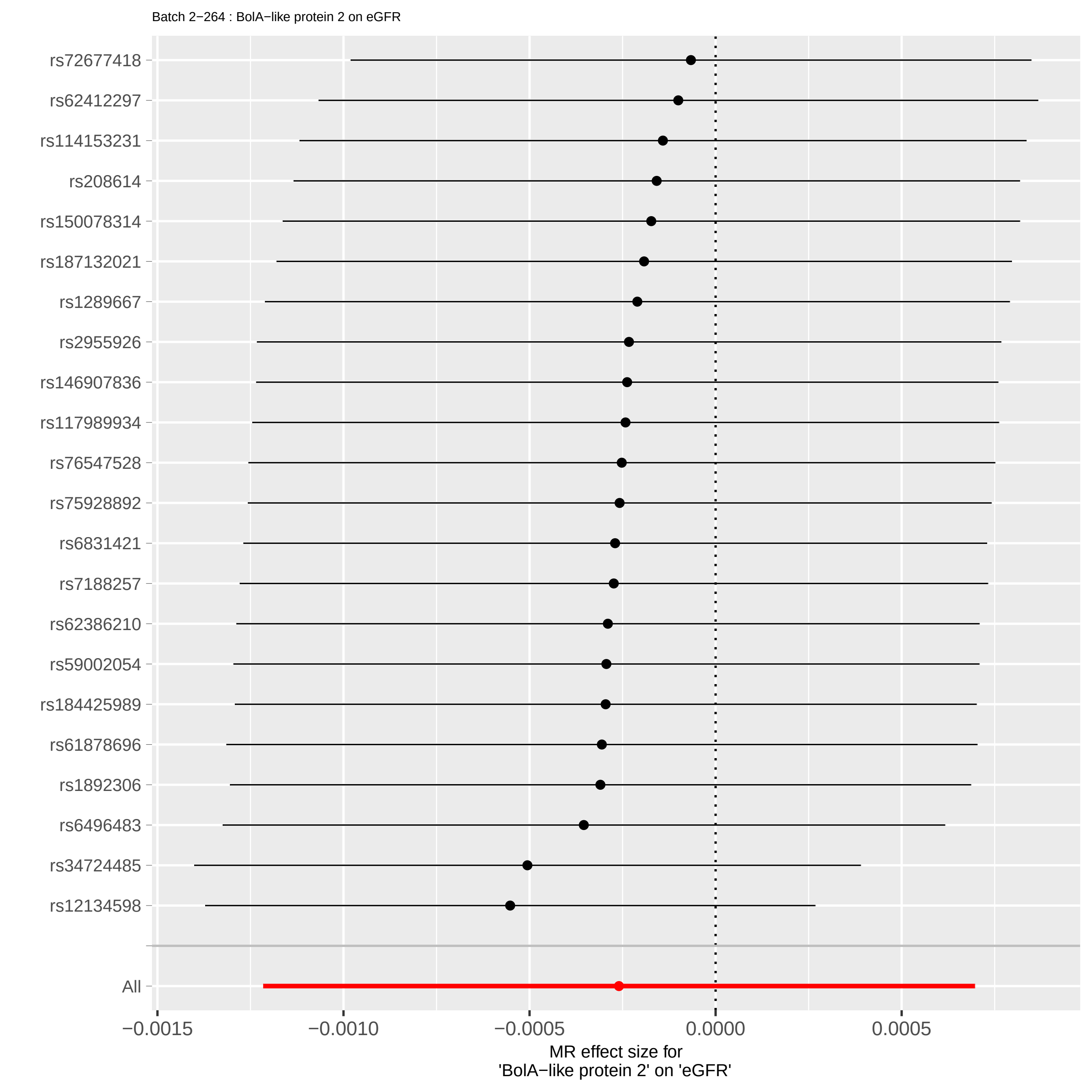

Batch 2−264 : BolA−like protein 2 on eGFR
rs72677418
rs62412297
rs114153231
rs208614
rs150078314
rs187132021
rs1289667
rs2955926
rs146907836
rs117989934
rs76547528
rs75928892
rs6831421
rs7188257
rs62386210
rs59002054
rs184425989
rs61878696
rs1892306
rs6496483
rs34724485
rs12134598
All
−0.0015
−0.0010
−0.0005
0.0000
0.0005
MR effect size for
'BolA−like protein 2' on 'eGFR'

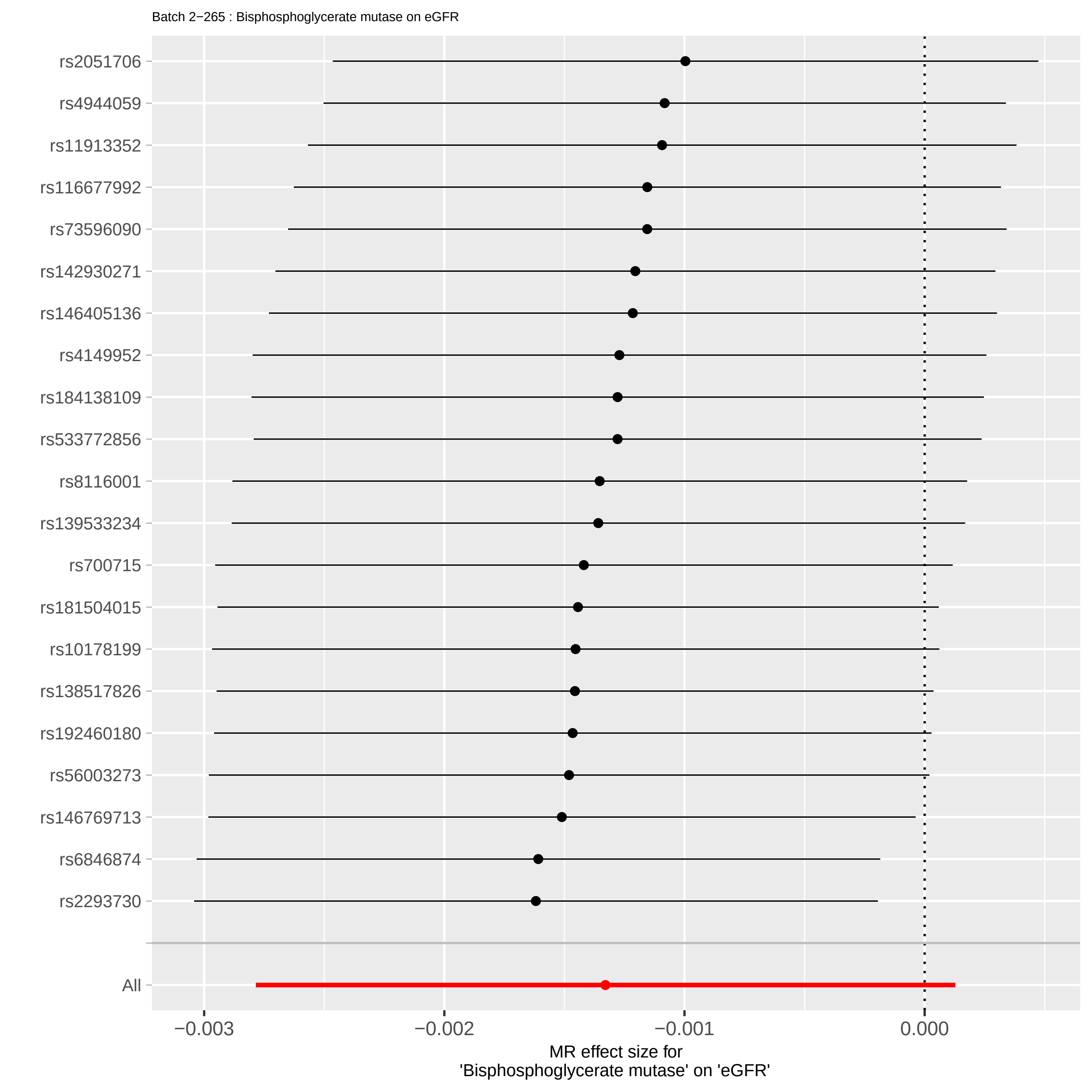

Batch 2−265 : Bisphosphoglycerate mutase on eGFR
rs2051706
rs4944059
rs11913352
rs116677992
rs73596090
rs142930271
rs146405136
rs4149952
rs184138109
rs533772856
rs8116001
rs139533234
rs700715
rs181504015
rs10178199
rs138517826
rs192460180
rs56003273
rs146769713
rs6846874
rs2293730
All
−0.003
−0.002
−0.001
0.000
MR effect size for
'Bisphosphoglycerate mutase' on 'eGFR'

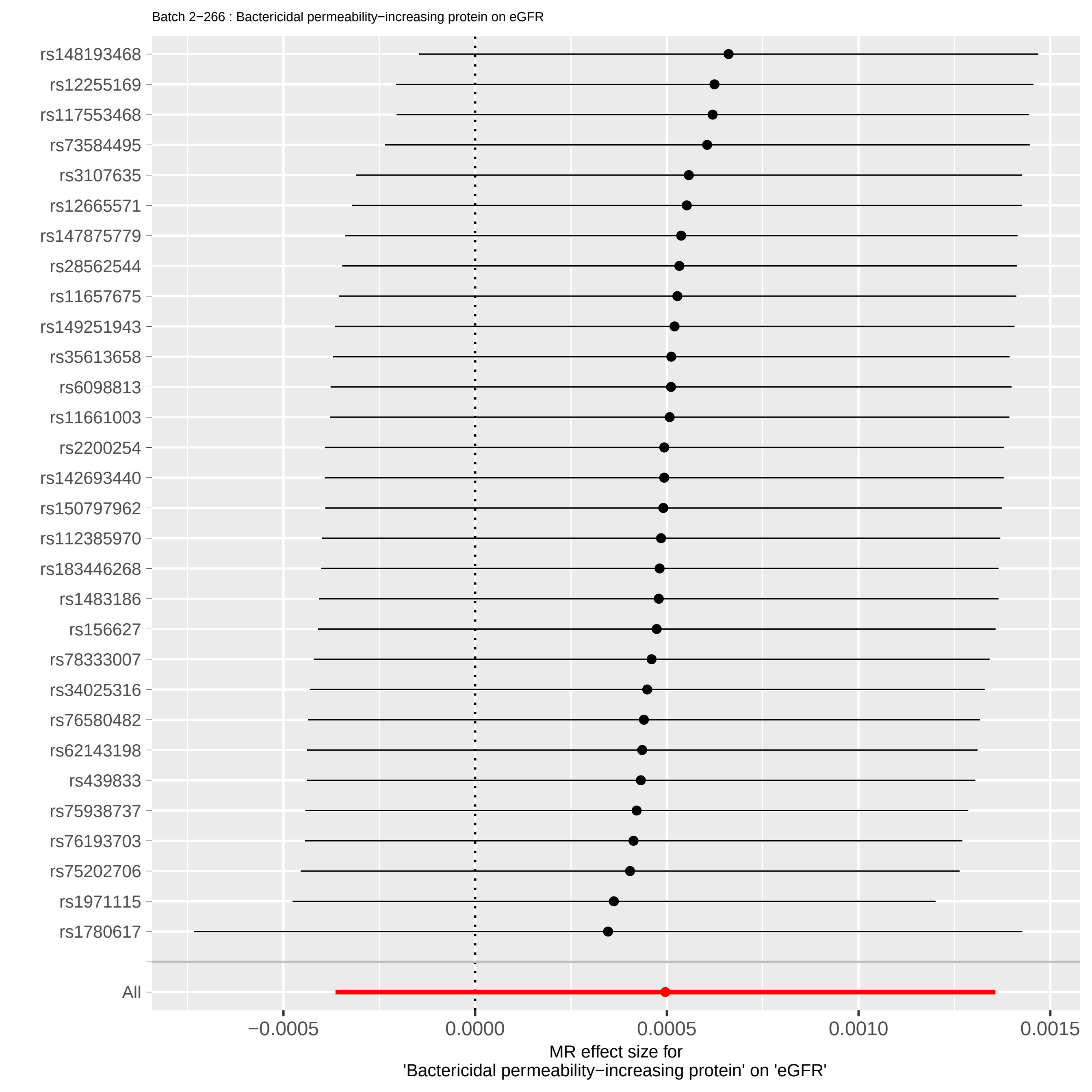

Batch 2−266 : Bactericidal permeability−increasing protein on eGFR
rs148193468
rs12255169
rs117553468
rs73584495
rs3107635
rs12665571
rs147875779
rs28562544
rs11657675
rs149251943
rs35613658
rs6098813
rs11661003
rs2200254
rs142693440
rs150797962
rs112385970
rs183446268
rs1483186
rs156627
rs78333007
rs34025316
rs76580482
rs62143198
rs439833
rs75938737
rs76193703
rs75202706
rs1971115
rs1780617
All
−0.0005
0.0000
0.0005
0.0010
0.0015
MR effect size for
'Bactericidal permeability−increasing protein' on 'eGFR'

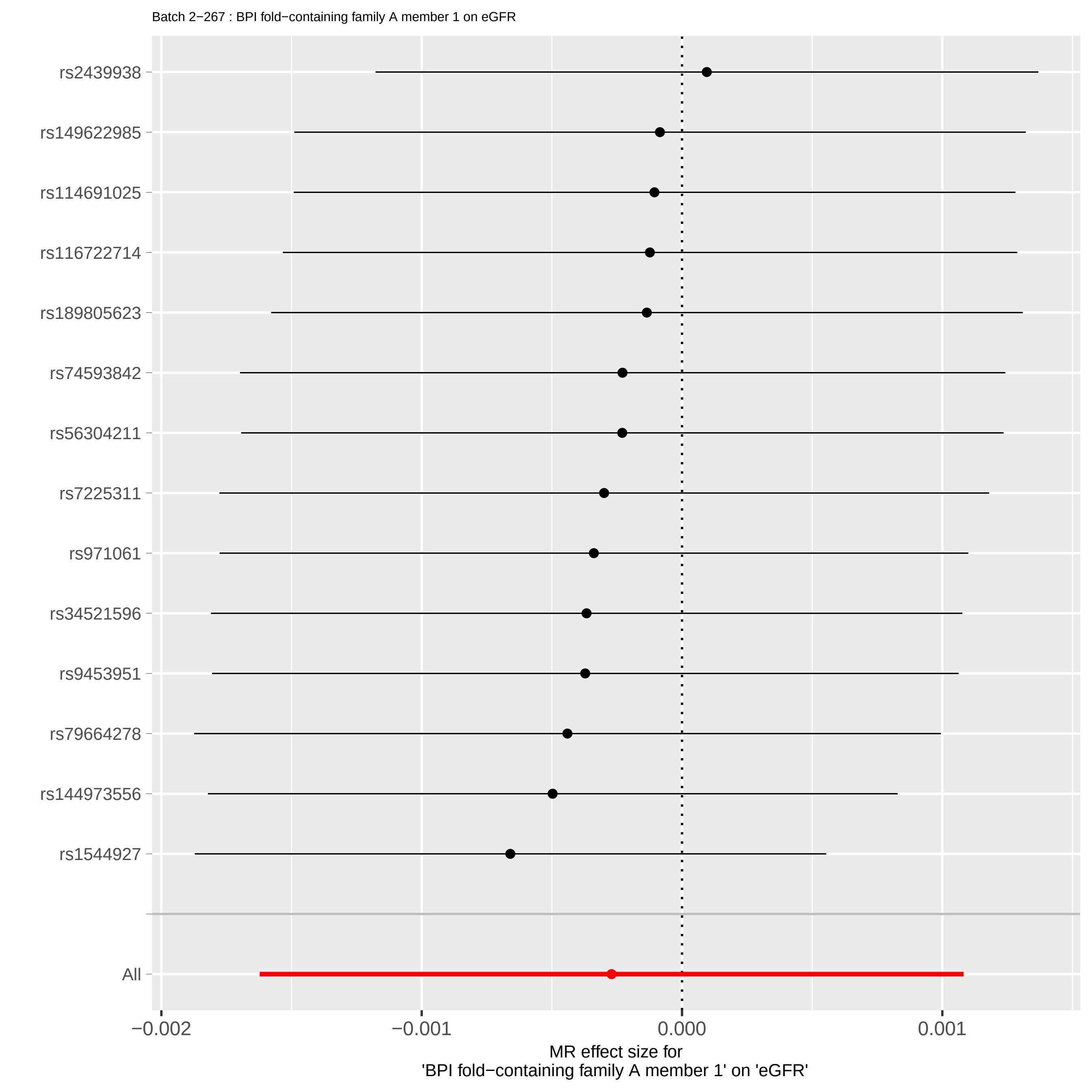

Batch 2−267 : BPI fold−containing family A member 1 on eGFR
rs2439938
rs149622985
rs114691025
rs116722714
rs189805623
rs74593842
rs56304211
rs7225311
rs971061
rs34521596
rs9453951
rs79664278
rs144973556
rs1544927
All
−0.002
−0.001
0.000
0.001
MR effect size for
'BPI fold−containing family A member 1' on 'eGFR'

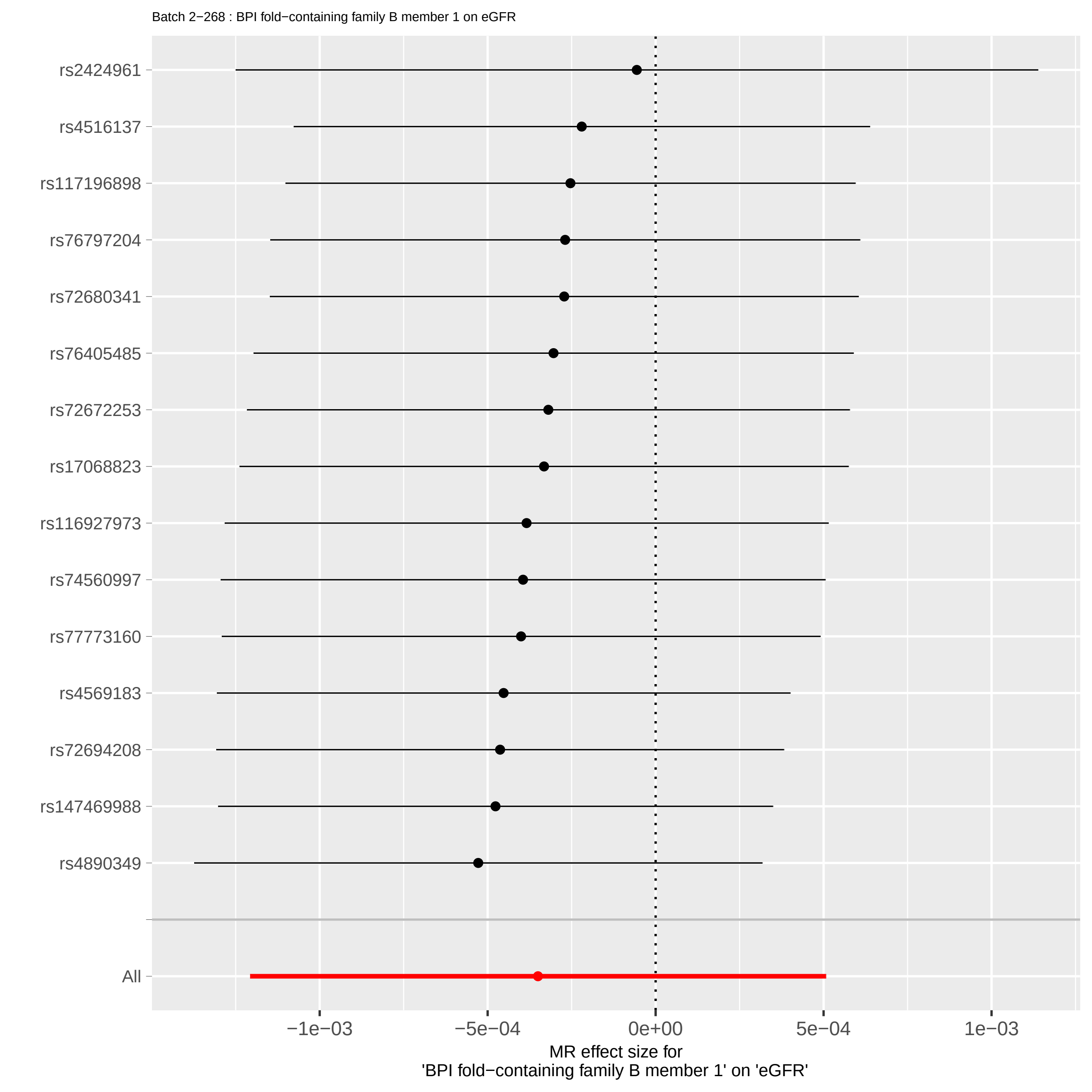
Batch 2−268 : BPI fold−containing family B member 1 on eGFR
rs2424961
rs4516137
rs117196898
rs76797204
rs72680341
rs76405485
rs72672253
rs17068823
rs116927973
rs74560997
rs77773160
rs4569183
rs72694208
rs147469988
rs4890349
All
−1e−03
−5e−04
0e+00
5e−04
1e−03
MR effect size for
'BPI fold−containing family B member 1' on 'eGFR'

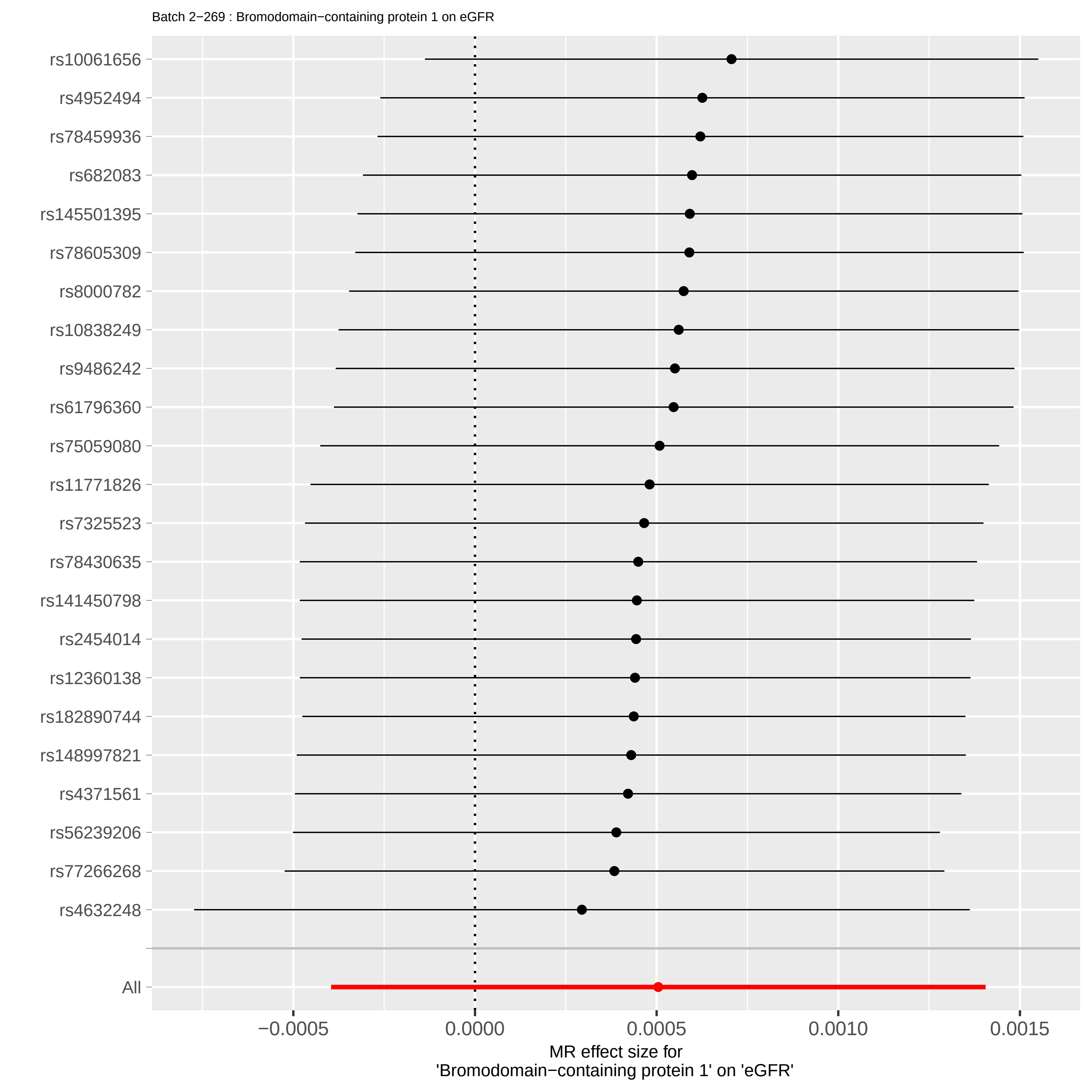

Batch 2−269 : Bromodomain−containing protein 1 on eGFR
rs10061656
rs4952494
rs78459936
rs682083
rs145501395
rs78605309
rs8000782
rs10838249
rs9486242
rs61796360
rs75059080
rs11771826
rs7325523
rs78430635
rs141450798
rs2454014
rs12360138
rs182890744
rs148997821
rs4371561
rs56239206
rs77266268
rs4632248
All
−0.0005
0.0000
0.0005
0.0010
0.0015
MR effect size for
'Bromodomain−containing protein 1' on 'eGFR'

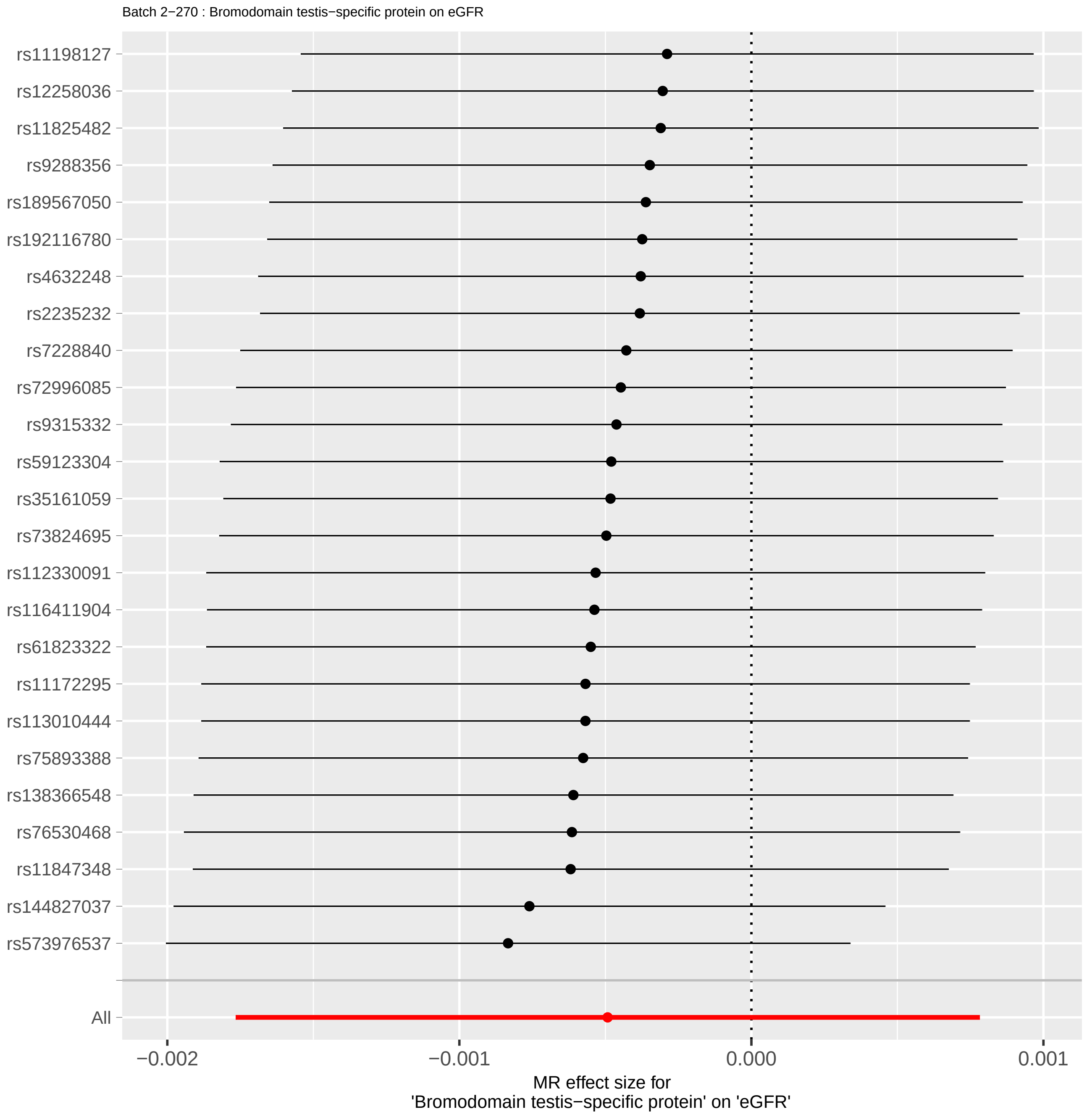

Batch 2−270 : Bromodomain testis−specific protein on eGFR
rs11198127
rs12258036
rs11825482
rs9288356
rs189567050
rs192116780
rs4632248
rs2235232
rs7228840
rs72996085
rs9315332
rs59123304
rs35161059
rs73824695
rs112330091
rs116411904
rs61823322
rs11172295
rs113010444
rs75893388
rs138366548
rs76530468
rs11847348
rs144827037
rs573976537
All
−0.002
−0.001
0.000
0.001
MR effect size for
'Bromodomain testis−specific protein' on 'eGFR'

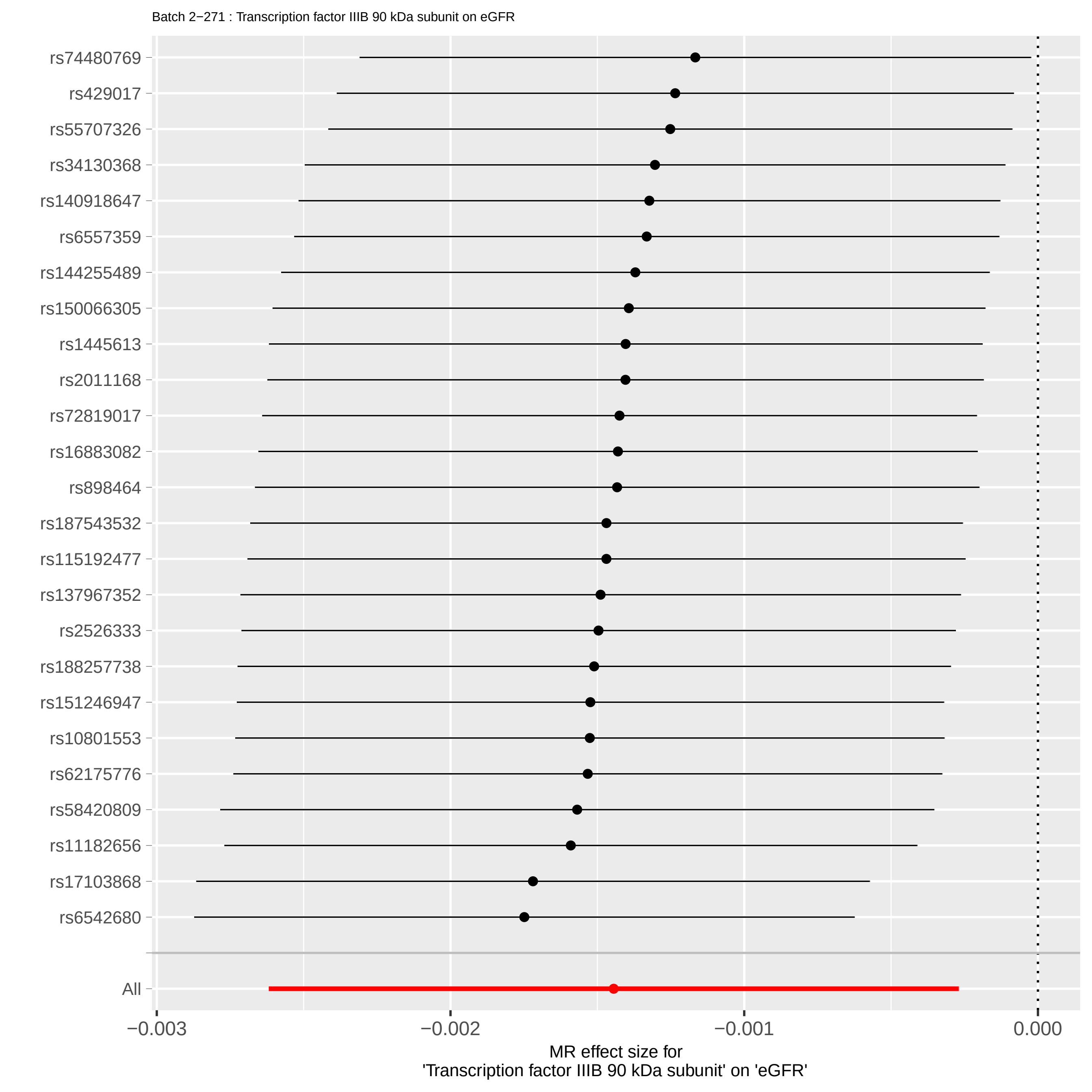

Batch 2−271 : Transcription factor IIIB 90 kDa subunit on eGFR
rs74480769
rs429017
rs55707326
rs34130368
rs140918647
rs6557359
rs144255489
rs150066305
rs1445613
rs2011168
rs72819017
rs16883082
rs898464
rs187543532
rs115192477
rs137967352
rs2526333
rs188257738
rs151246947
rs10801553
rs62175776
rs58420809
rs11182656
rs17103868
rs6542680
All
−0.003
−0.002
−0.001
0.000
MR effect size for
'Transcription factor IIIB 90 kDa subunit' on 'eGFR'

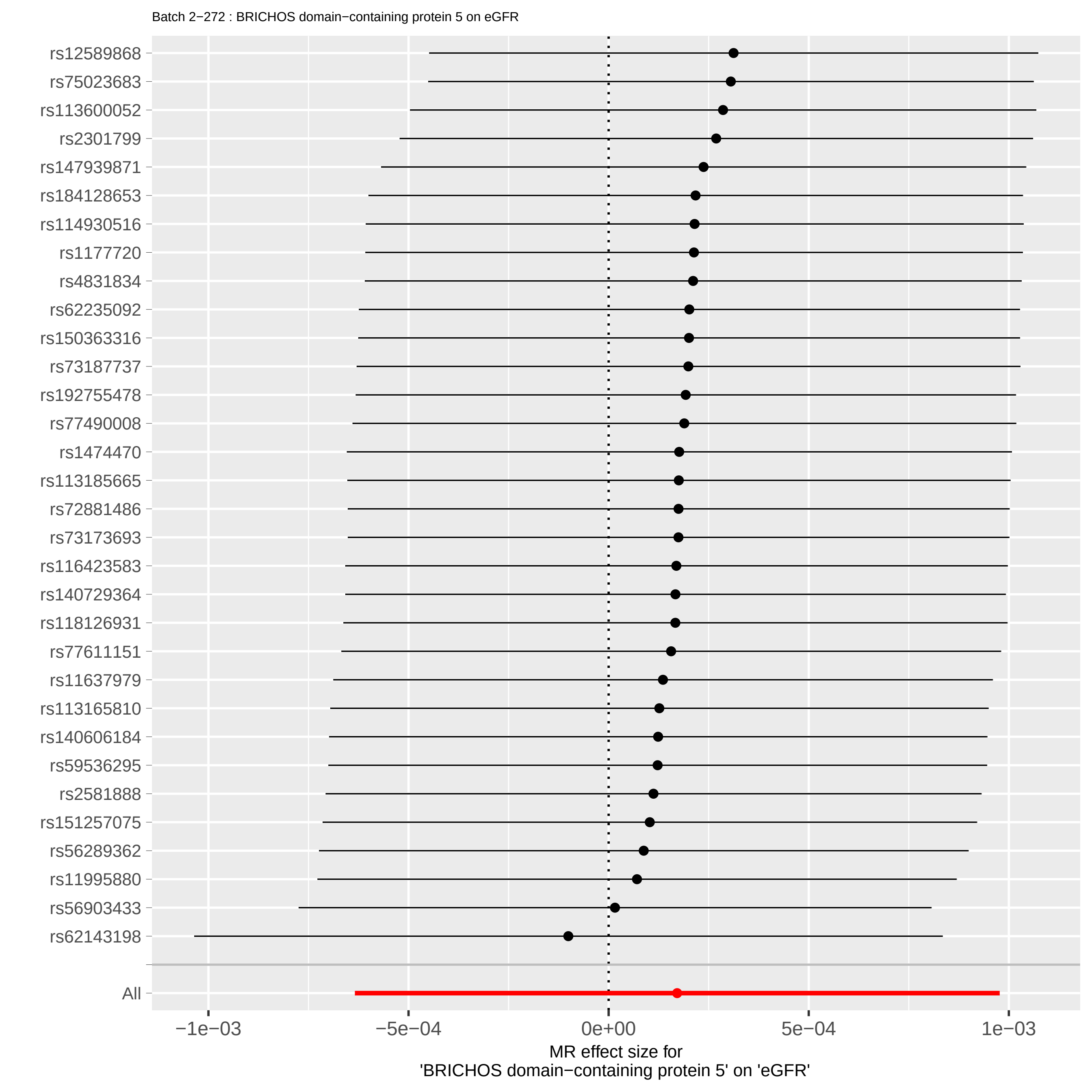

Batch 2−272 : BRICHOS domain−containing protein 5 on eGFR
rs12589868
rs75023683
rs113600052
rs2301799
rs147939871
rs184128653
rs114930516
rs1177720
rs4831834
rs62235092
rs150363316
rs73187737
rs192755478
rs77490008
rs1474470
rs113185665
rs72881486
rs73173693
rs116423583
rs140729364
rs118126931
rs77611151
rs11637979
rs113165810
rs140606184
rs59536295
rs2581888
rs151257075
rs56289362
rs11995880
rs56903433
rs62143198
All
−1e−03
−5e−04
0e+00
5e−04
1e−03
MR effect size for
'BRICHOS domain−containing protein 5' on 'eGFR'

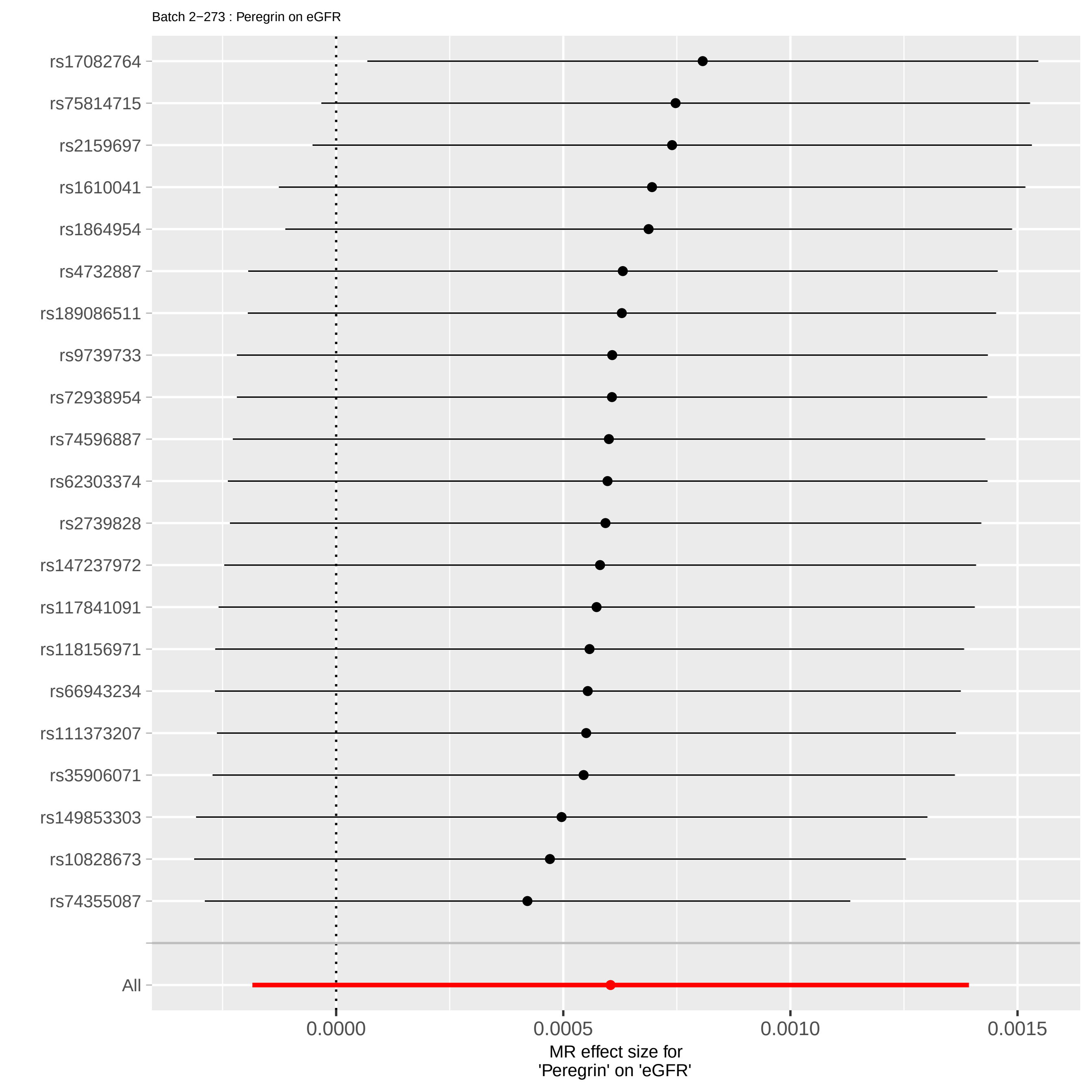

Batch 2−273 : Peregrin on eGFR
rs17082764
rs75814715
rs2159697
rs1610041
rs1864954
rs4732887
rs189086511
rs9739733
rs72938954
rs74596887
rs62303374
rs2739828
rs147237972
rs117841091
rs118156971
rs66943234
rs111373207
rs35906071
rs149853303
rs10828673
rs74355087
All
0.0000
0.0005
0.0010
0.0015
MR effect size for
'Peregrin' on 'eGFR'

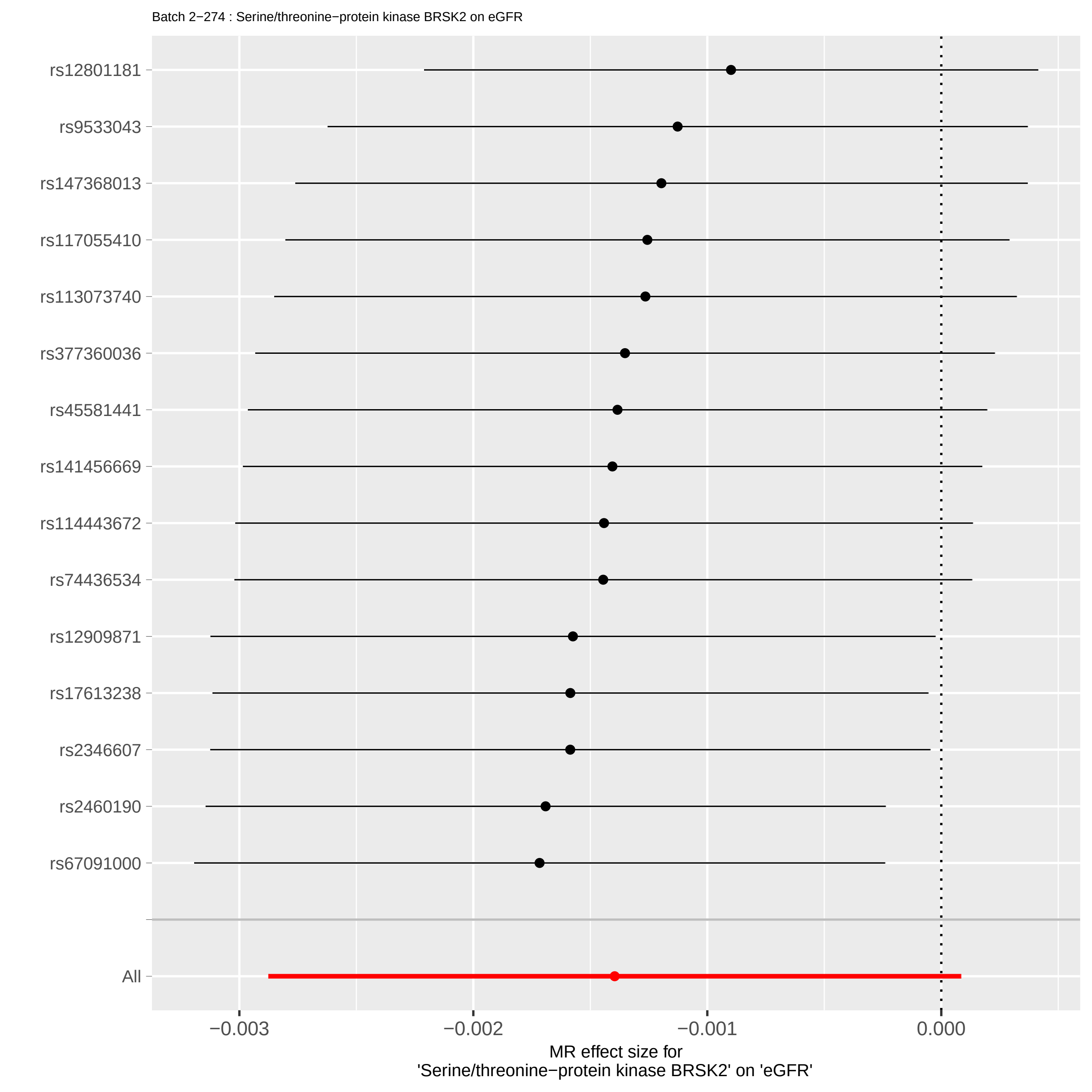
Batch 2−274 : Serine/threonine−protein kinase BRSK2 on eGFR
rs12801181
rs9533043
rs147368013
rs117055410
rs113073740
rs377360036
rs45581441
rs141456669
rs114443672
rs74436534
rs12909871
rs17613238
rs2346607
rs2460190
rs67091000
All
−0.003
−0.002
−0.001
0.000
MR effect size for
'Serine/threonine−protein kinase BRSK2' on 'eGFR'

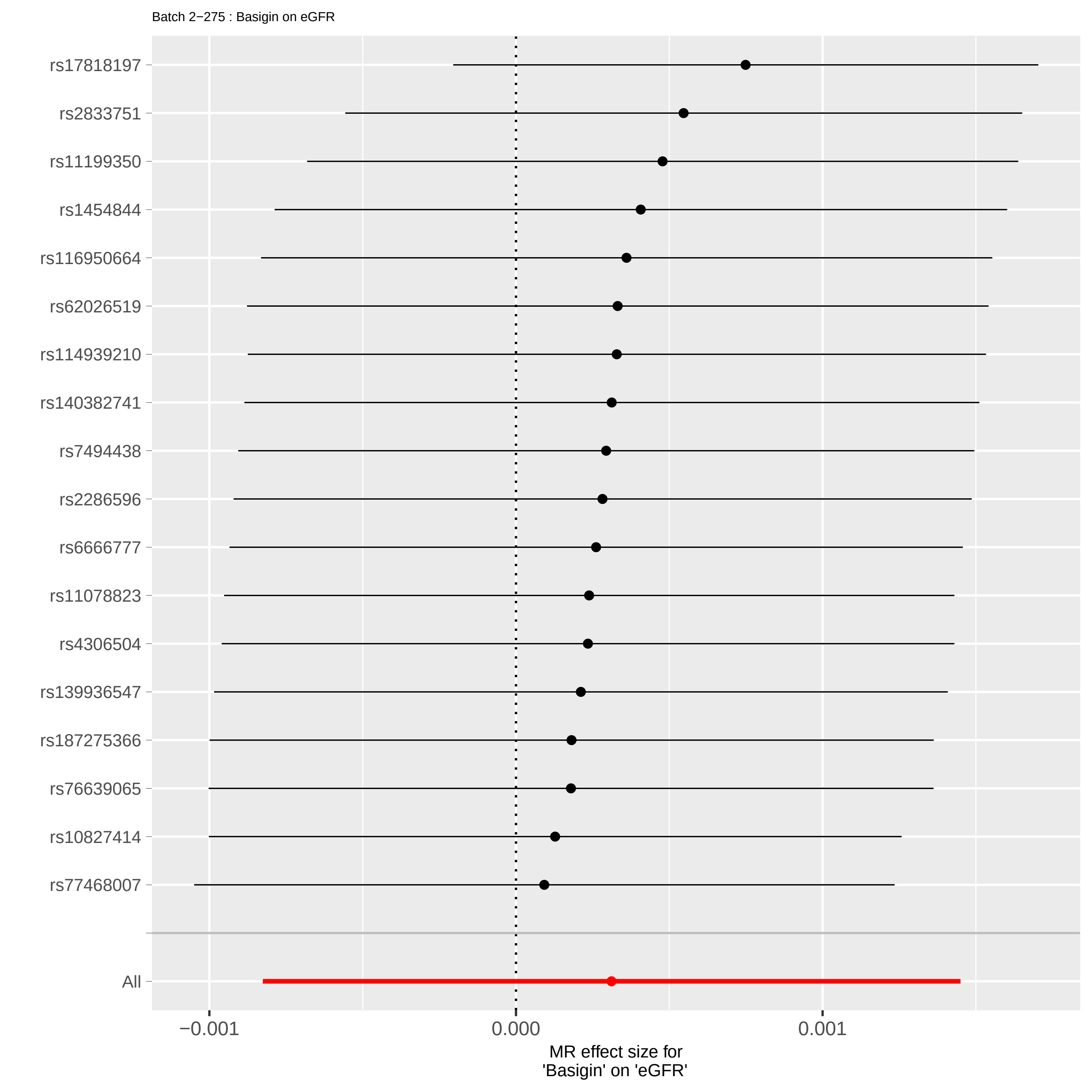
Batch 2−275 : Basigin on eGFR
rs17818197
rs2833751
rs11199350
rs1454844
rs116950664
rs62026519
rs114939210
rs140382741
rs7494438
rs2286596
rs6666777
rs11078823
rs4306504
rs139936547
rs187275366
rs76639065
rs10827414
rs77468007
All
−0.001
0.000
0.001
MR effect size for
'Basigin' on 'eGFR'

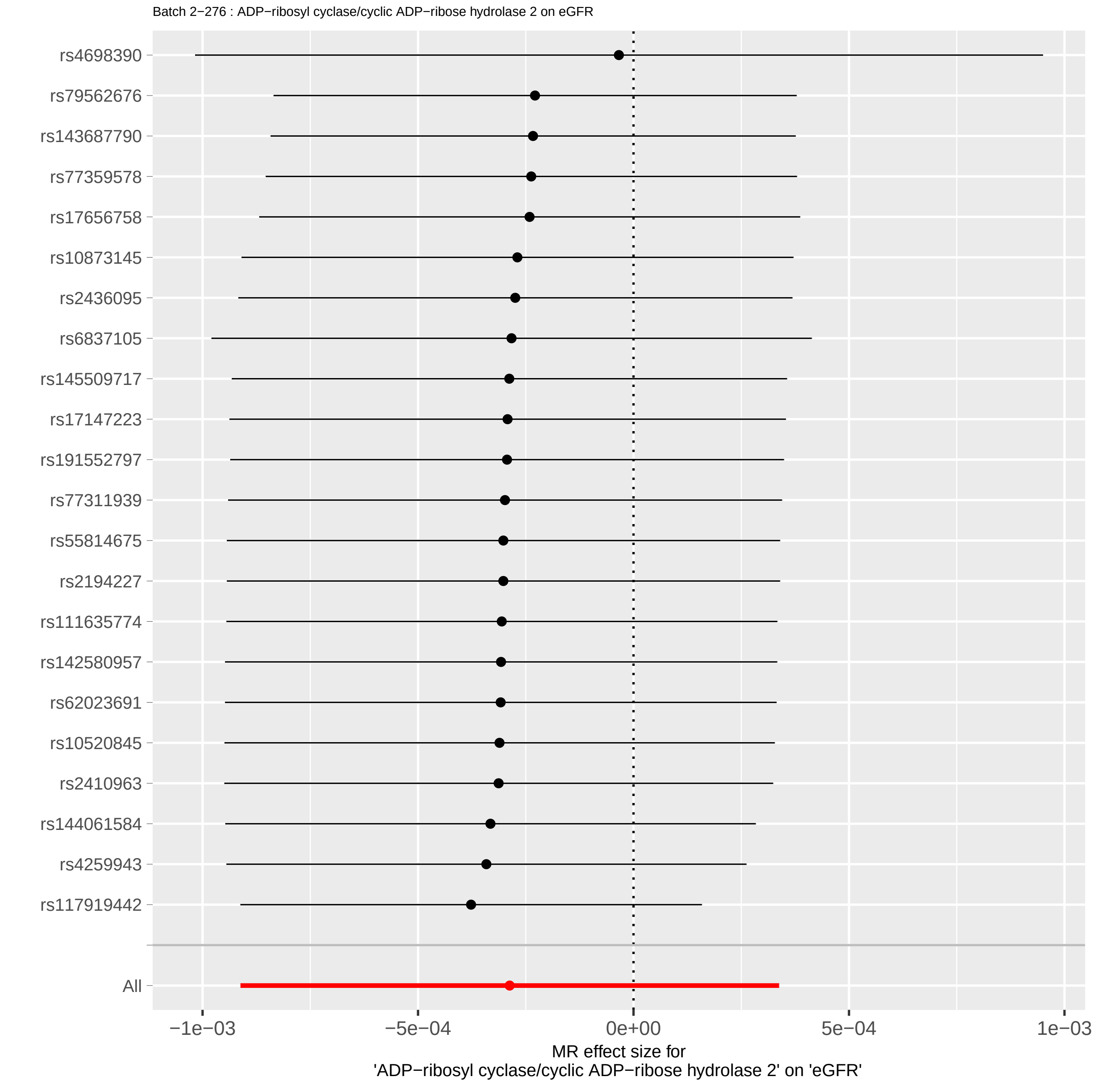
Batch 2−276 : ADP−ribosyl cyclase/cyclic ADP−ribose hydrolase 2 on eGFR
rs4698390
rs79562676
rs143687790
rs77359578
rs17656758
rs10873145
rs2436095
rs6837105
rs145509717
rs17147223
rs191552797
rs77311939
rs55814675
rs2194227
rs111635774
rs142580957
rs62023691
rs10520845
rs2410963
rs144061584
rs4259943
rs117919442
All
−1e−03
−5e−04
0e+00
5e−04
1e−03
MR effect size for
'ADP−ribosyl cyclase/cyclic ADP−ribose hydrolase 2' on 'eGFR'

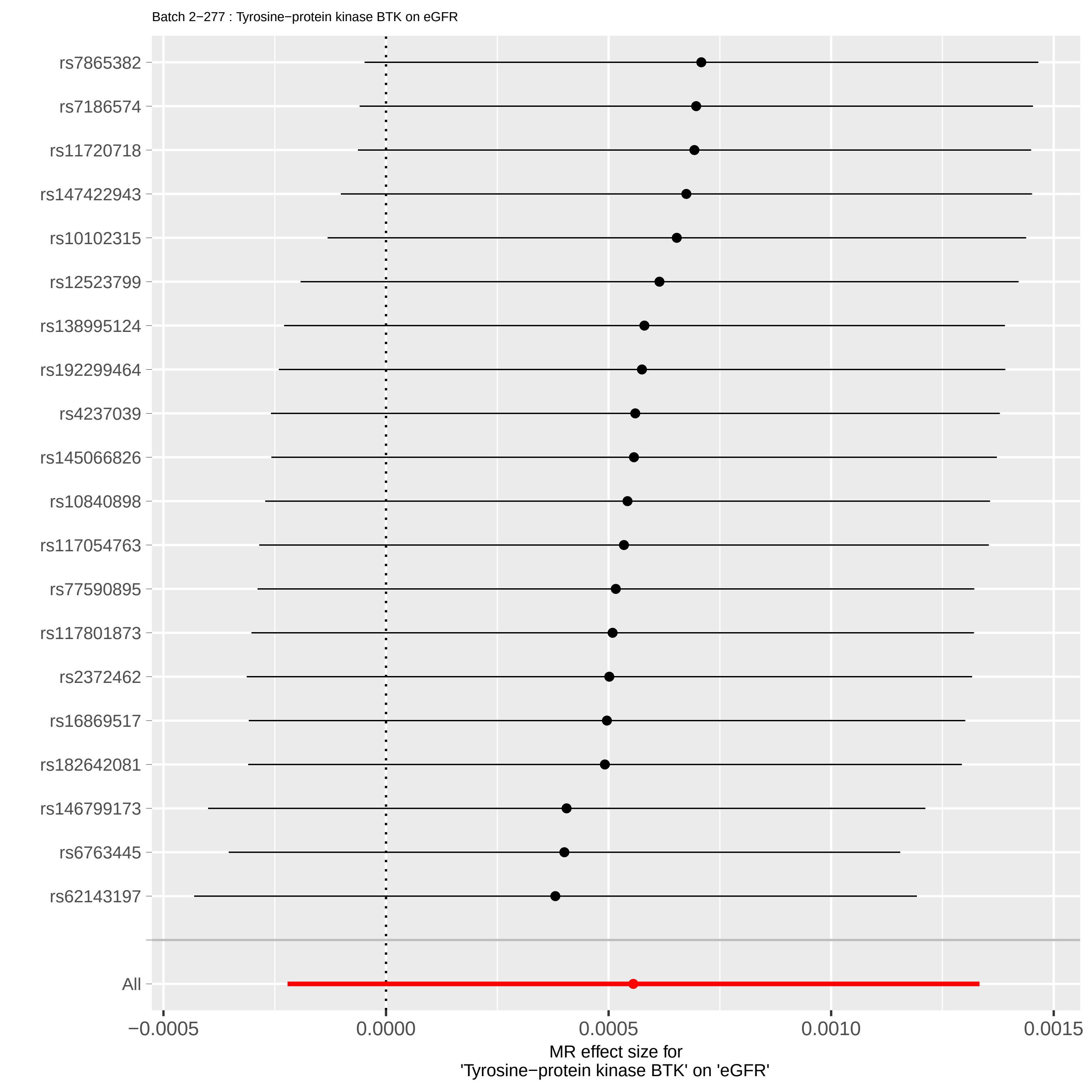

Batch 2−277 : Tyrosine−protein kinase BTK on eGFR
rs7865382
rs7186574
rs11720718
rs147422943
rs10102315
rs12523799
rs138995124
rs192299464
rs4237039
rs145066826
rs10840898
rs117054763
rs77590895
rs117801873
rs2372462
rs16869517
rs182642081
rs146799173
rs6763445
rs62143197
All
−0.0005
0.0000
0.0005
0.0010
0.0015
MR effect size for
'Tyrosine−protein kinase BTK' on 'eGFR'

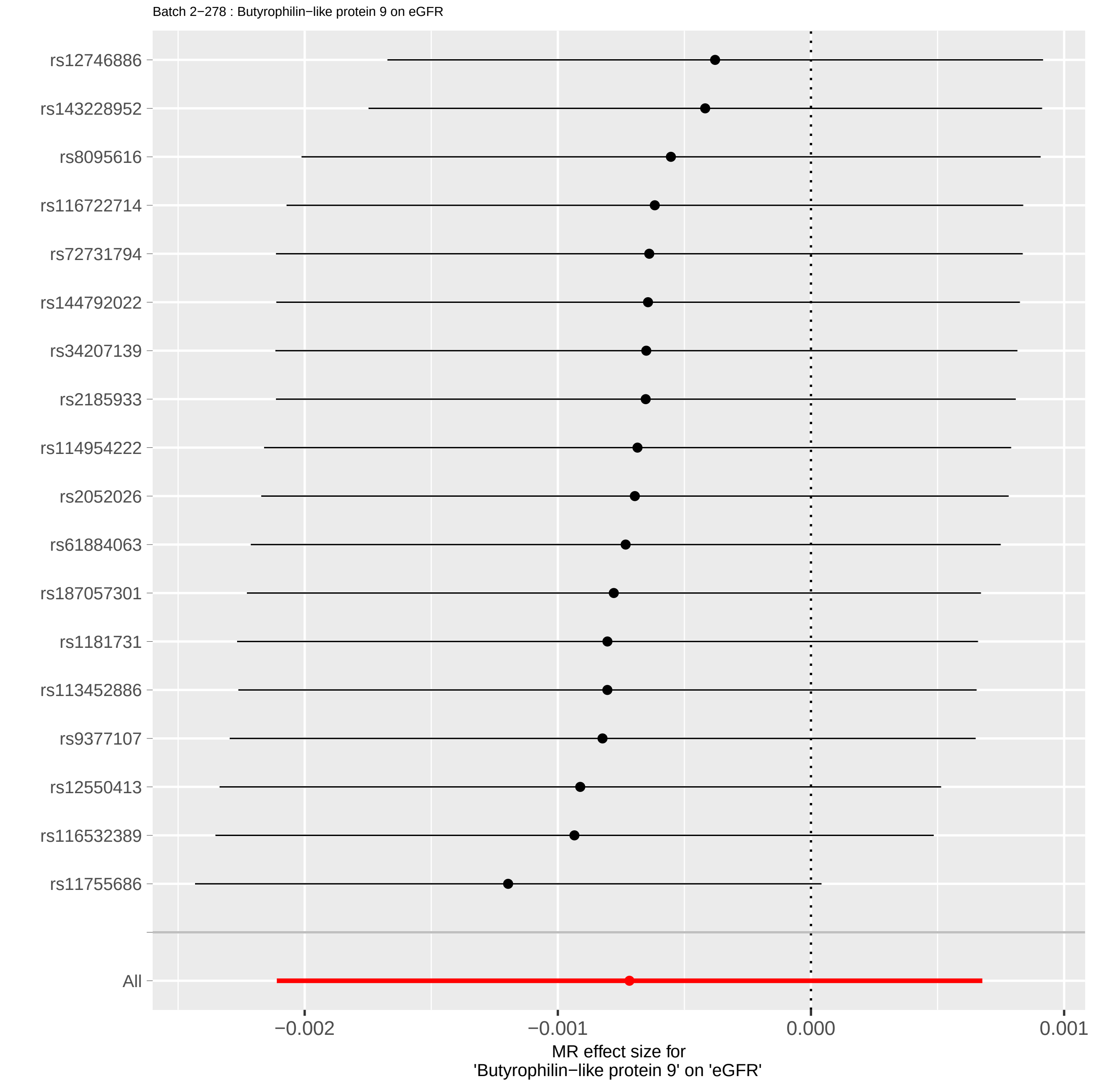

Batch 2−278 : Butyrophilin−like protein 9 on eGFR
rs12746886
rs143228952
rs8095616
rs116722714
rs72731794
rs144792022
rs34207139
rs2185933
rs114954222
rs2052026
rs61884063
rs187057301
rs1181731
rs113452886
rs9377107
rs12550413
rs116532389
rs11755686
All
−0.002
−0.001
0.000
0.001
MR effect size for
'Butyrophilin−like protein 9' on 'eGFR'

Batch 2−279 : Mitotic checkpoint serine/threonine−protein kinase BUB1 on eGFR

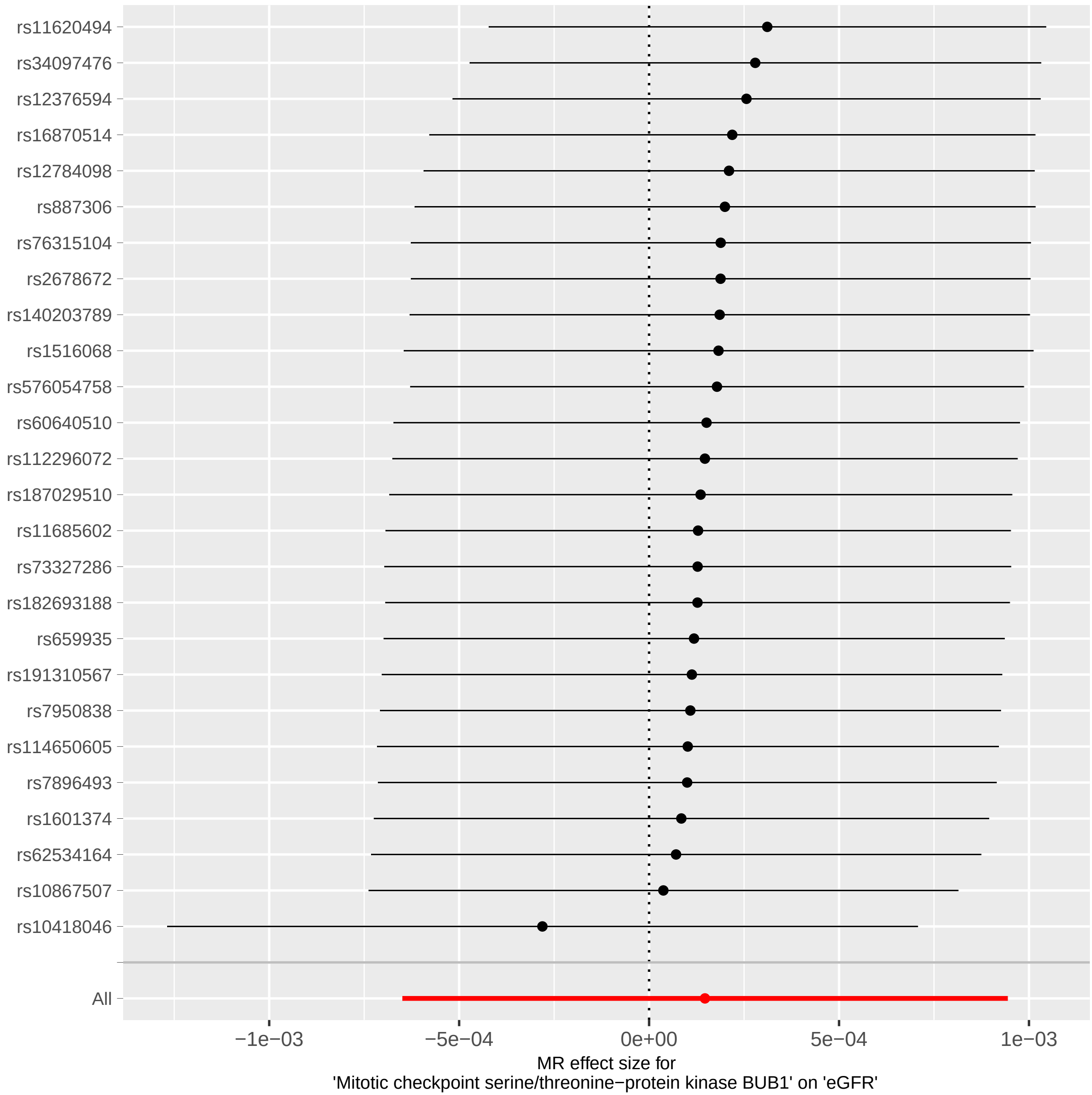

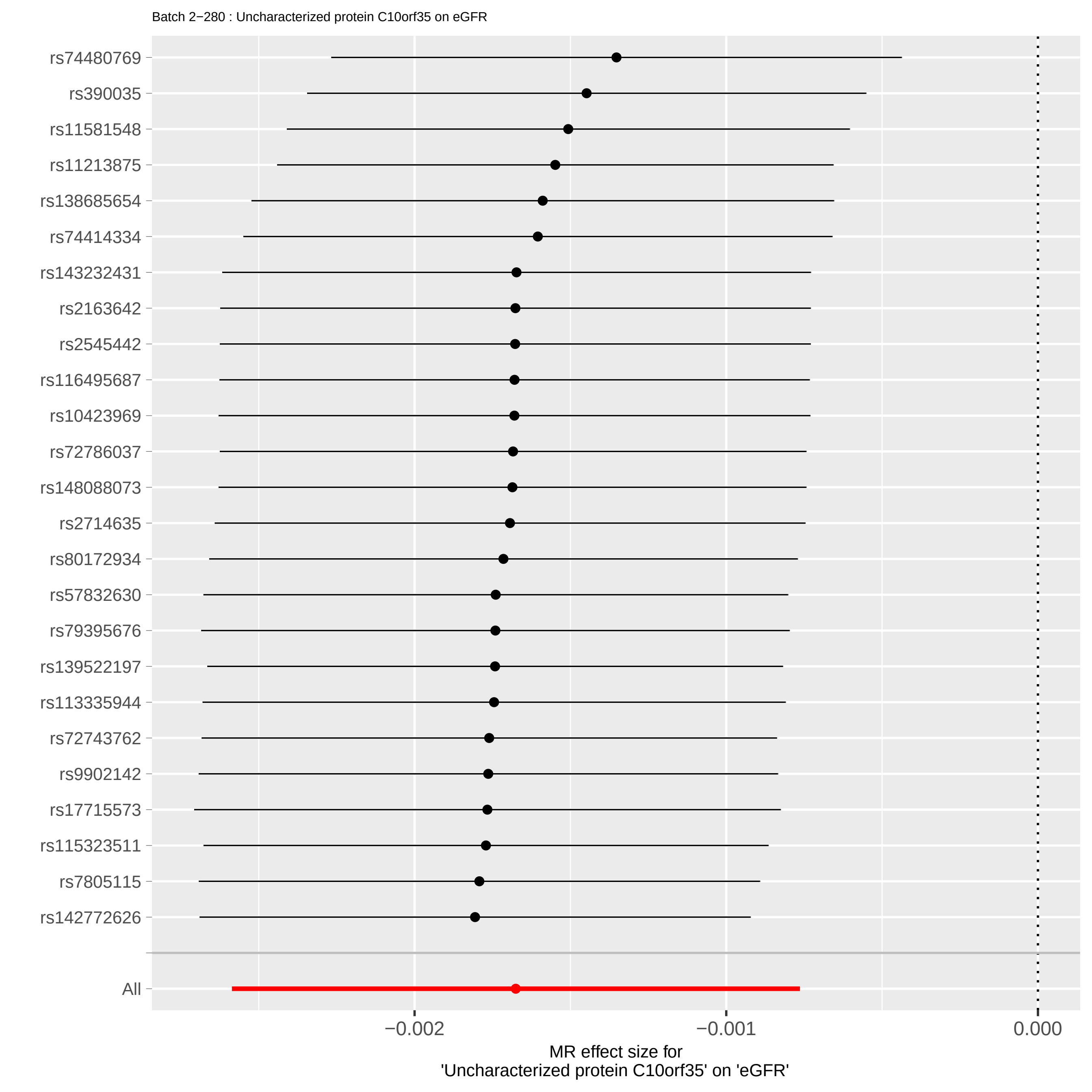

Batch 2−280 : Uncharacterized protein C10orf35 on eGFR
rs74480769
rs390035
rs11581548
rs11213875
rs138685654
rs74414334
rs143232431
rs2163642
rs2545442
rs116495687
rs10423969
rs72786037
rs148088073
rs2714635
rs80172934
rs57832630
rs79395676
rs139522197
rs113335944
rs72743762
rs9902142
rs17715573
rs115323511
rs7805115
rs142772626
All
−0.002
−0.001
0.000
MR effect size for
'Uncharacterized protein C10orf35' on 'eGFR'

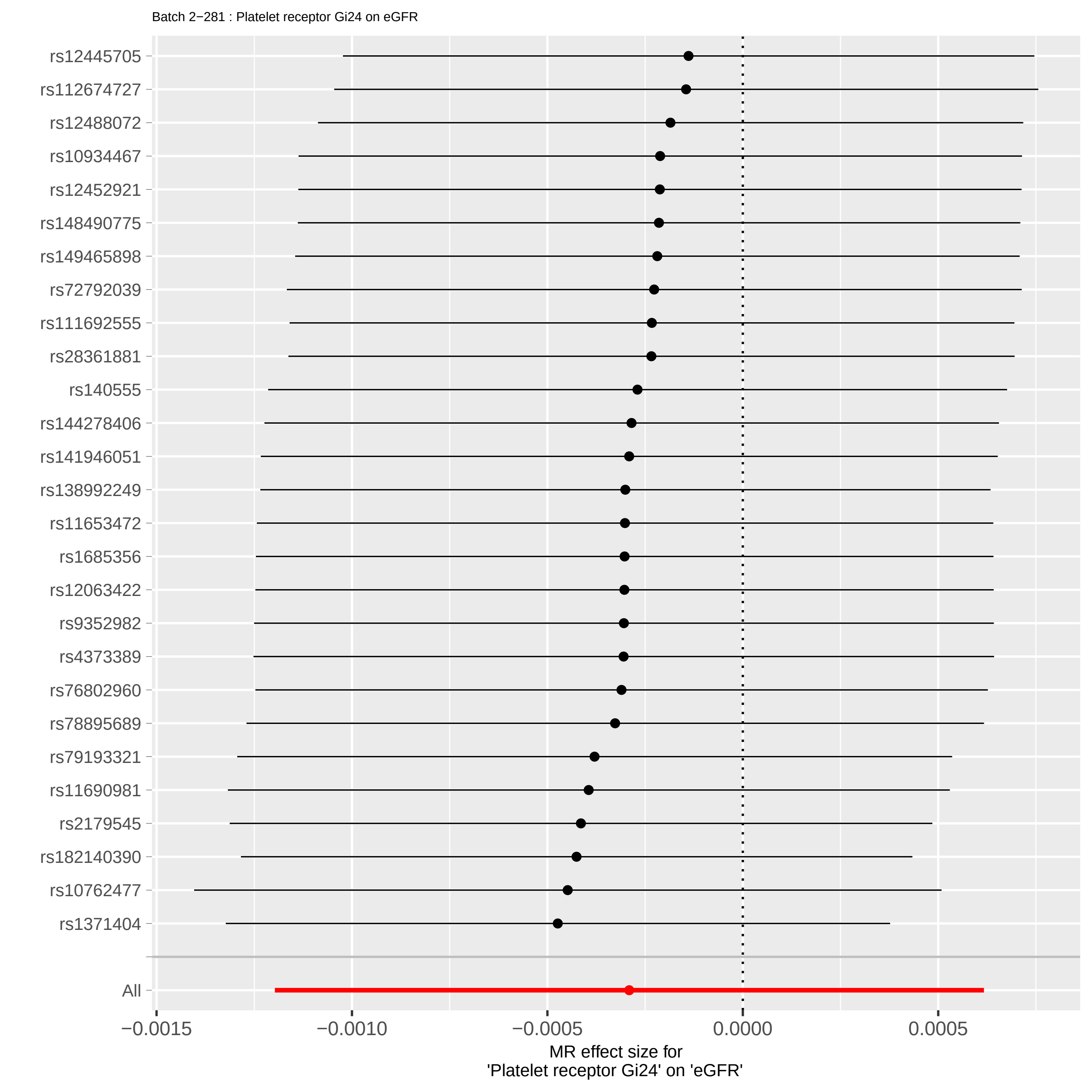
Batch 2−281 : Platelet receptor Gi24 on eGFR
rs12445705
rs112674727
rs12488072
rs10934467
rs12452921
rs148490775
rs149465898
rs72792039
rs111692555
rs28361881
rs140555
rs144278406
rs141946051
rs138992249
rs11653472
rs1685356
rs12063422
rs9352982
rs4373389
rs76802960
rs78895689
rs79193321
rs11690981
rs2179545
rs182140390
rs10762477
rs1371404
All
−0.0015
−0.0010
−0.0005
0.0000
0.0005
MR effect size for
'Platelet receptor Gi24' on 'eGFR'

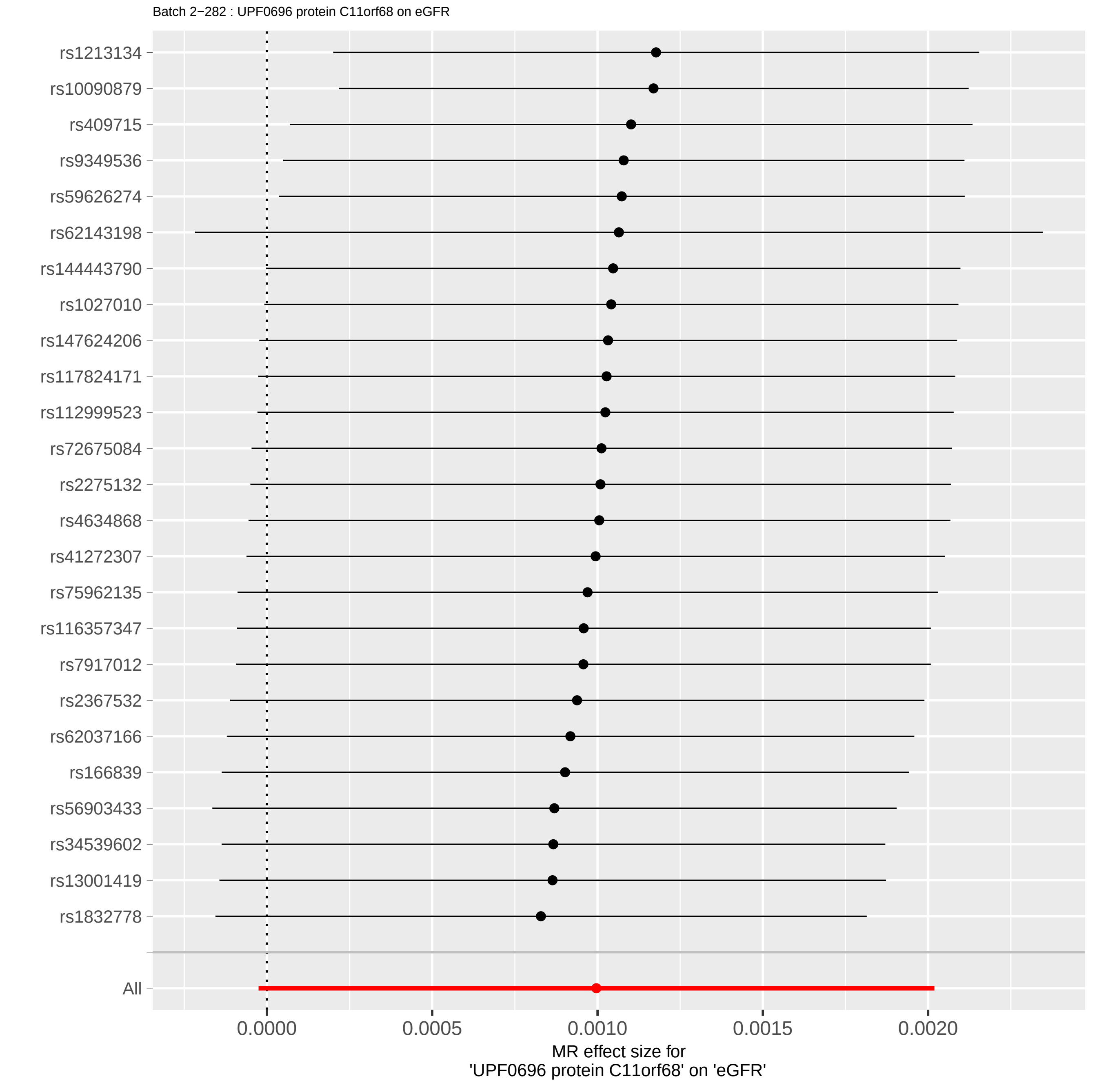

Batch 2−282 : UPF0696 protein C11orf68 on eGFR
rs1213134
rs10090879
rs409715
rs9349536
rs59626274
rs62143198
rs144443790
rs1027010
rs147624206
rs117824171
rs112999523
rs72675084
rs2275132
rs4634868
rs41272307
rs75962135
rs116357347
rs7917012
rs2367532
rs62037166
rs166839
rs56903433
rs34539602
rs13001419
rs1832778
All
0.0000
0.0005
0.0010
0.0015
0.0020
MR effect size for
'UPF0696 protein C11orf68' on 'eGFR'

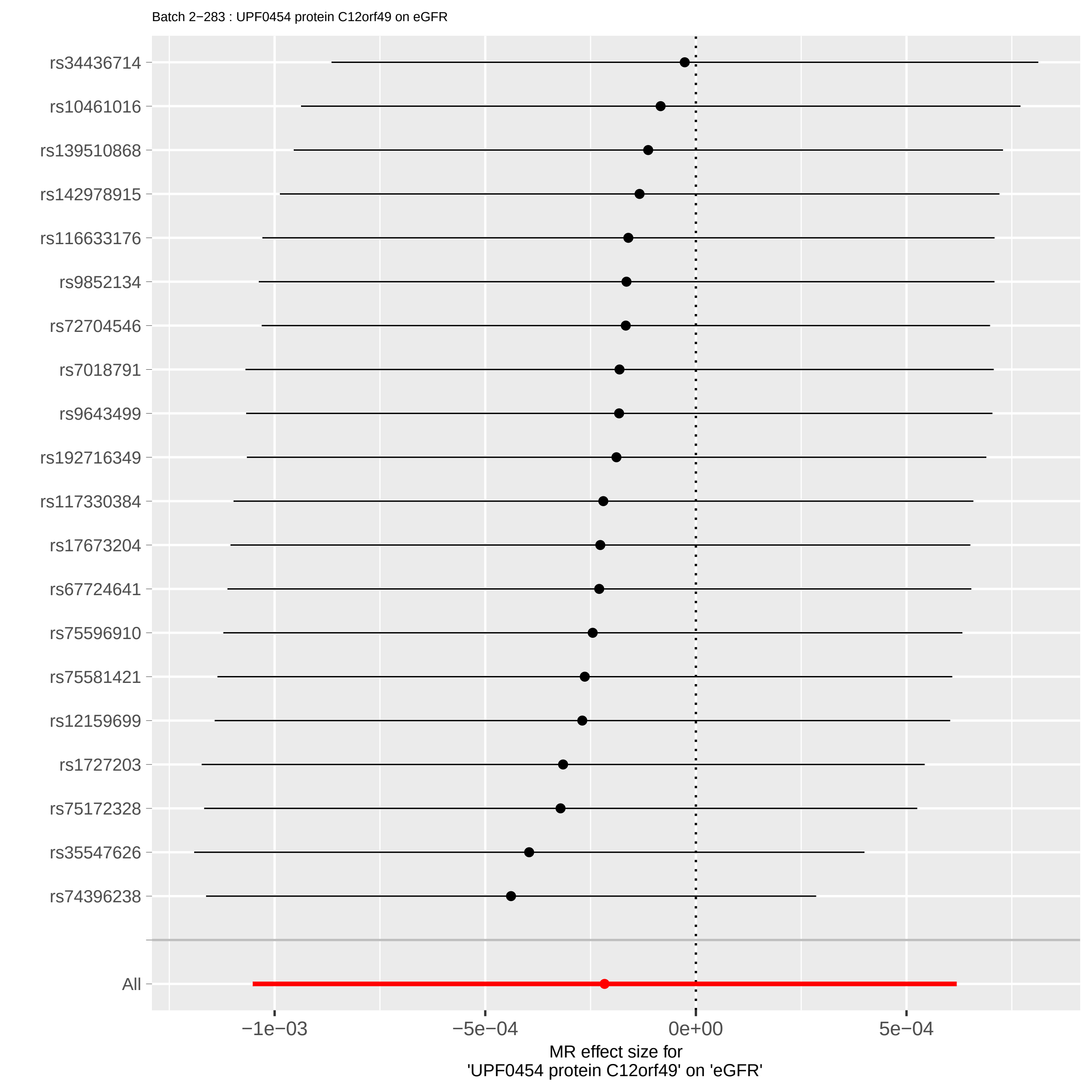

Batch 2−283 : UPF0454 protein C12orf49 on eGFR
rs34436714
rs10461016
rs139510868
rs142978915
rs116633176
rs9852134
rs72704546
rs7018791
rs9643499
rs192716349
rs117330384
rs17673204
rs67724641
rs75596910
rs75581421
rs12159699
rs1727203
rs75172328
rs35547626
rs74396238
All
−1e−03
−5e−04
0e+00
5e−04
MR effect size for
'UPF0454 protein C12orf49' on 'eGFR'

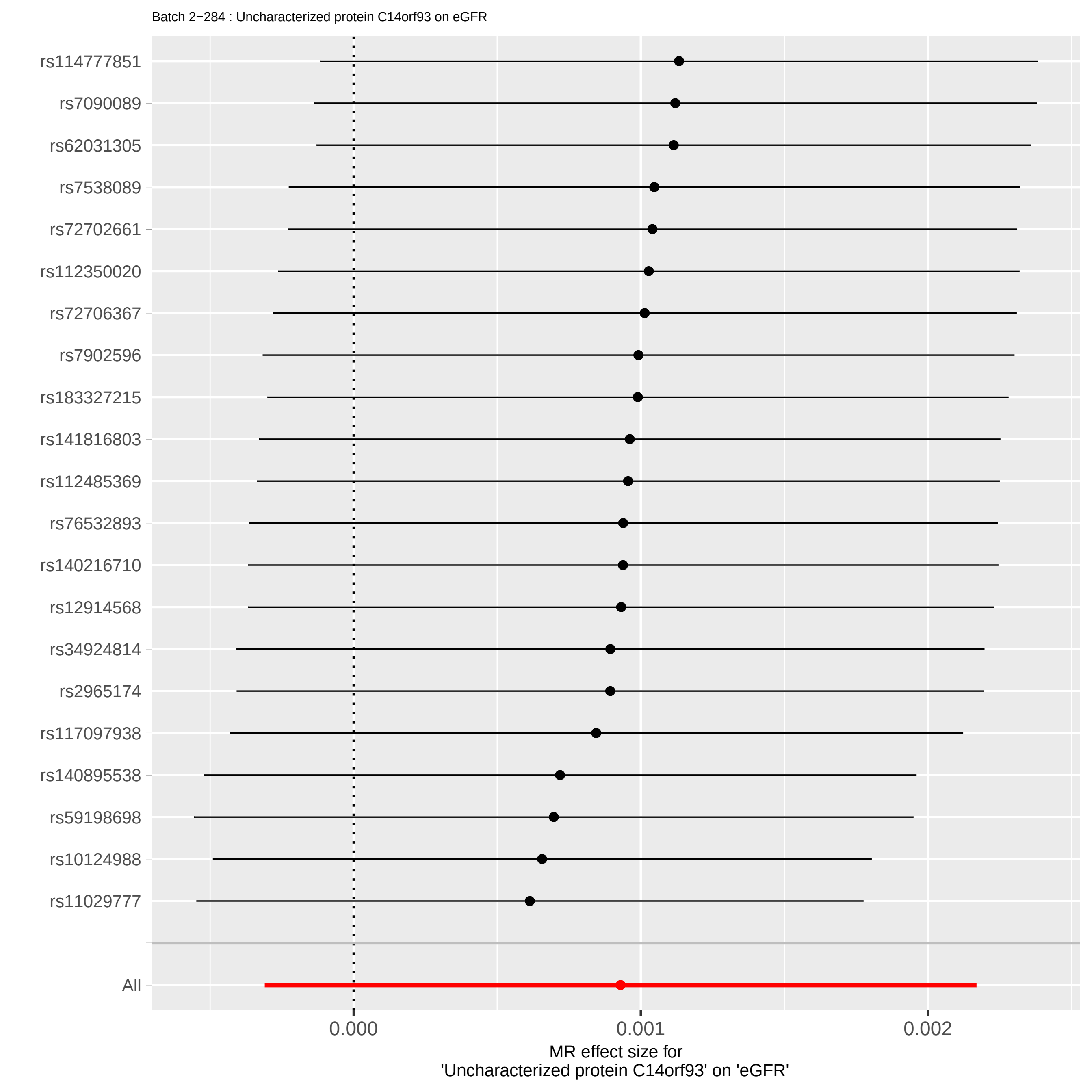
Batch 2−284 : Uncharacterized protein C14orf93 on eGFR
rs114777851
rs7090089
rs62031305
rs7538089
rs72702661
rs112350020
rs72706367
rs7902596
rs183327215
rs141816803
rs112485369
rs76532893
rs140216710
rs12914568
rs34924814
rs2965174
rs117097938
rs140895538
rs59198698
rs10124988
rs11029777
All
0.000
0.001
0.002
MR effect size for
'Uncharacterized protein C14orf93' on 'eGFR'

Batch 2−285 : Transmembrane protein C16orf54 on eGFR

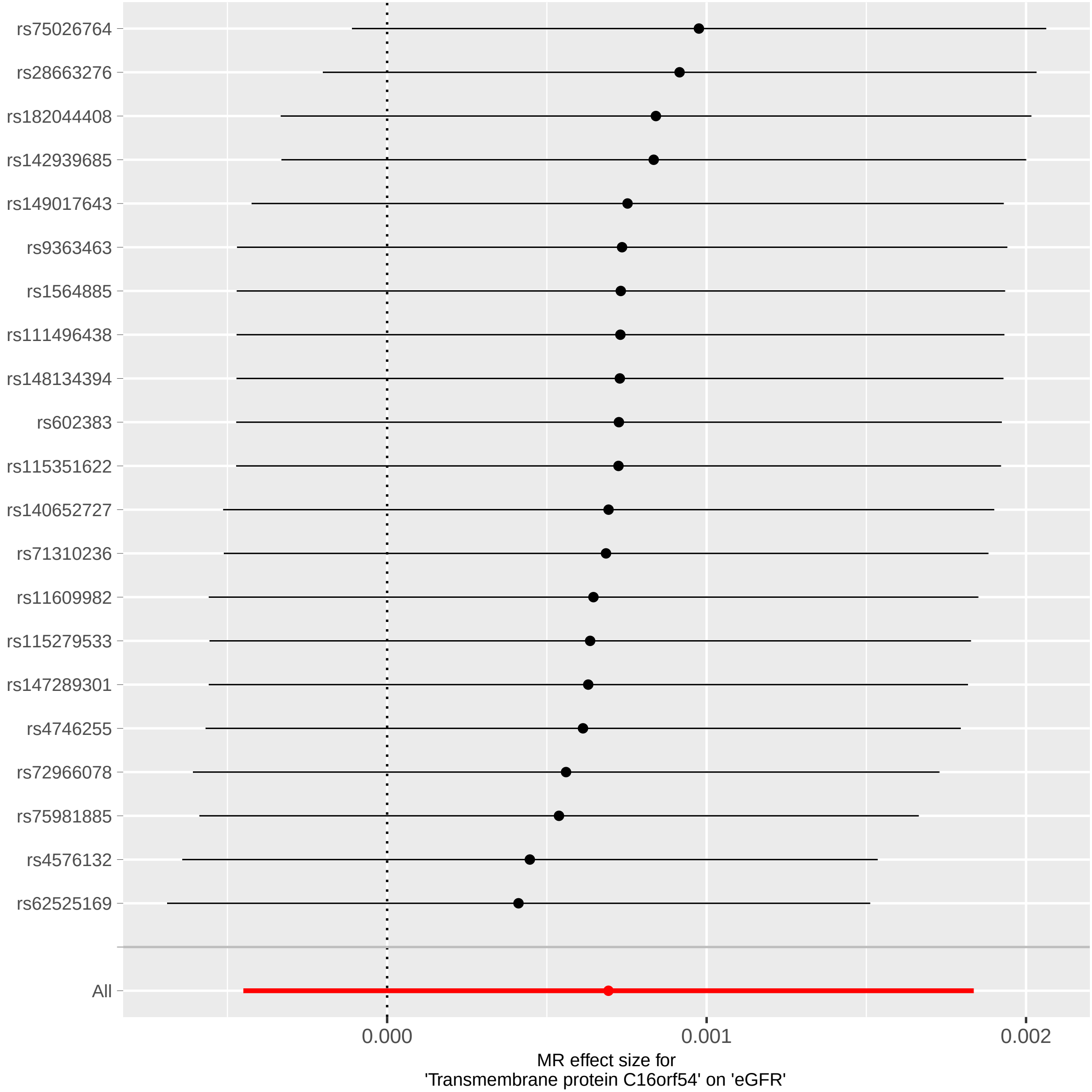

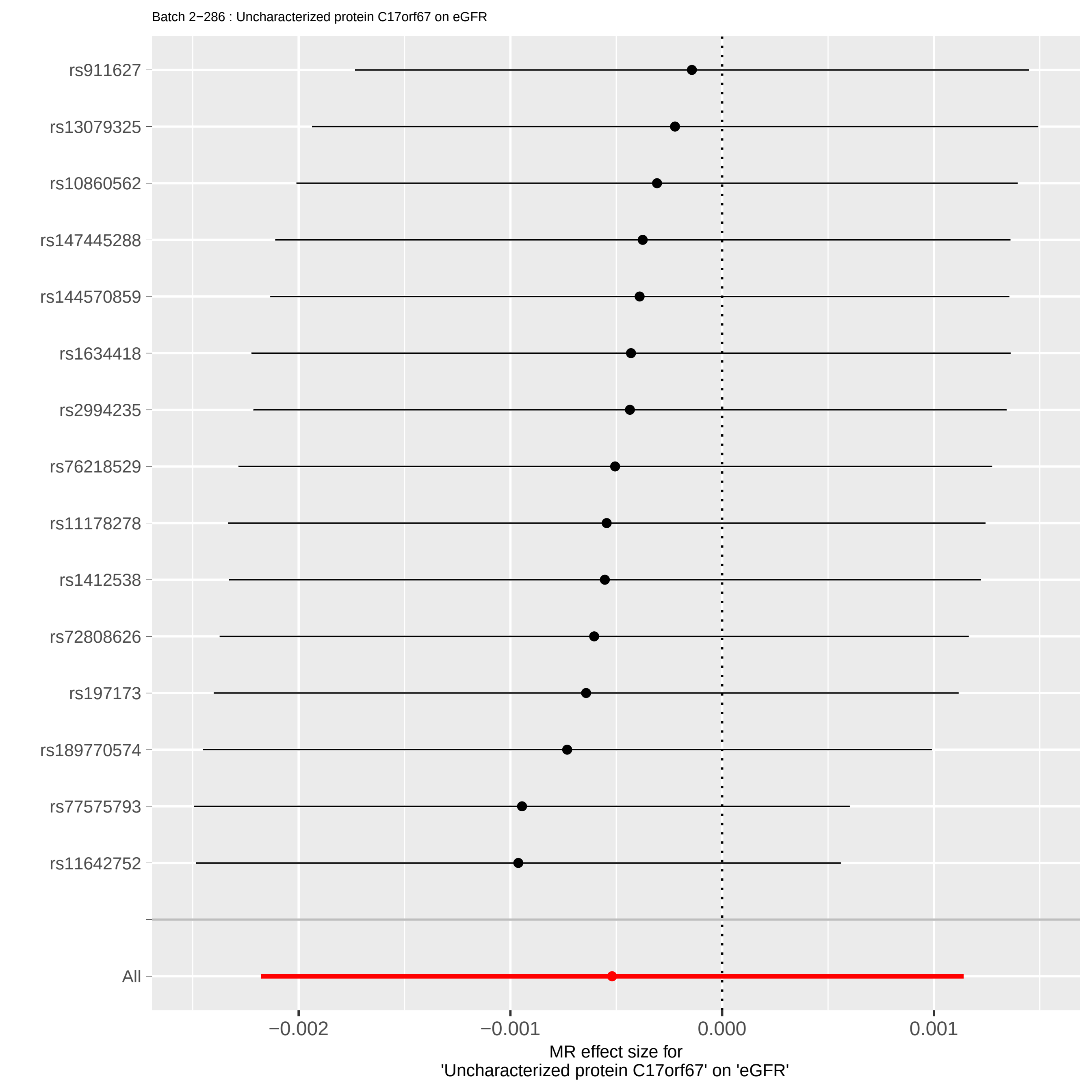

Batch 2−286 : Uncharacterized protein C17orf67 on eGFR
rs911627
rs13079325
rs10860562
rs147445288
rs144570859
rs1634418
rs2994235
rs76218529
rs11178278
rs1412538
rs72808626
rs197173
rs189770574
rs77575793
rs11642752
All
−0.002
−0.001
0.000
0.001
MR effect size for
'Uncharacterized protein C17orf67' on 'eGFR'

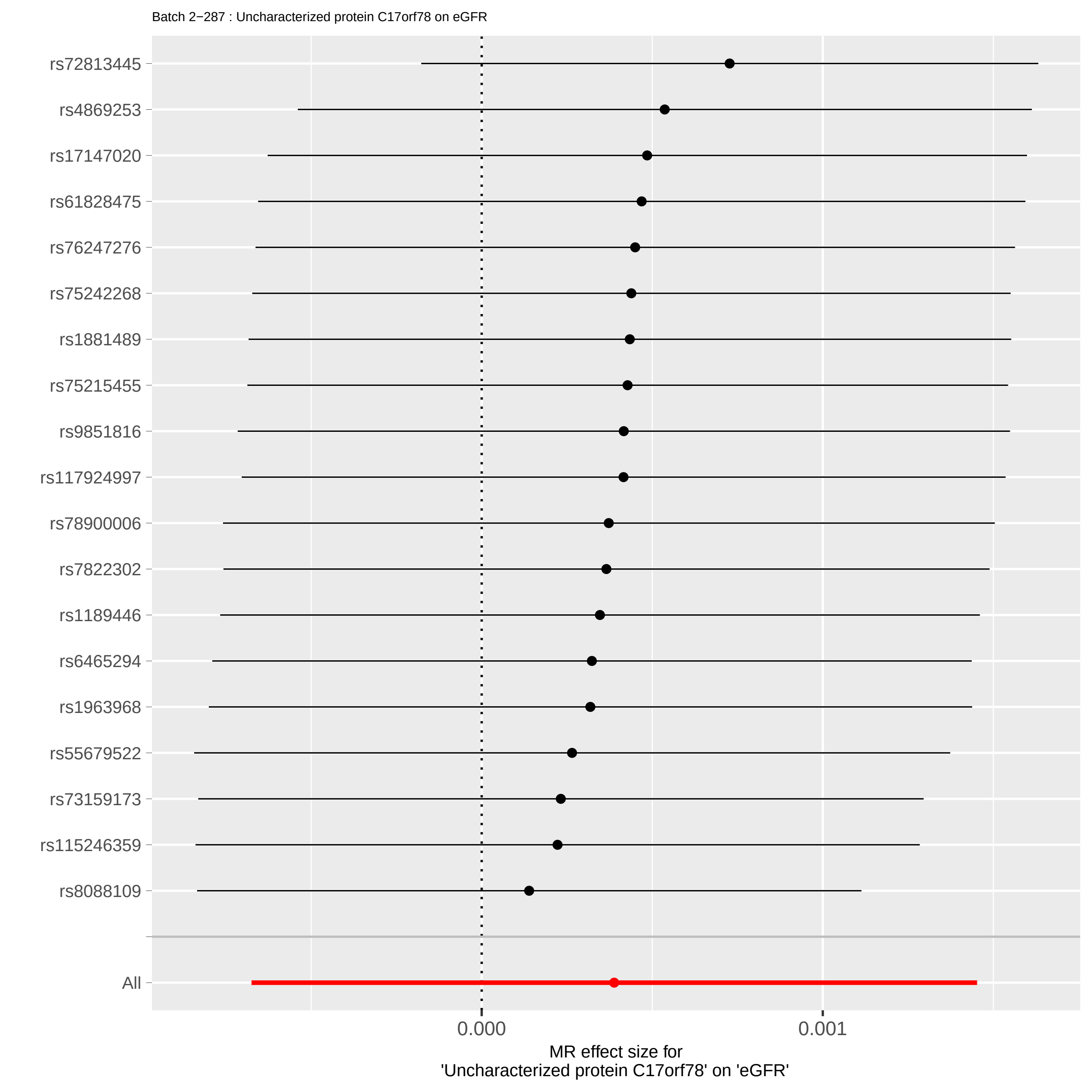
Batch 2−287 : Uncharacterized protein C17orf78 on eGFR
rs72813445
rs4869253
rs17147020
rs61828475
rs76247276
rs75242268
rs1881489
rs75215455
rs9851816
rs117924997
rs78900006
rs7822302
rs1189446
rs6465294
rs1963968
rs55679522
rs73159173
rs115246359
rs8088109
All
0.000
0.001
MR effect size for
'Uncharacterized protein C17orf78' on 'eGFR'

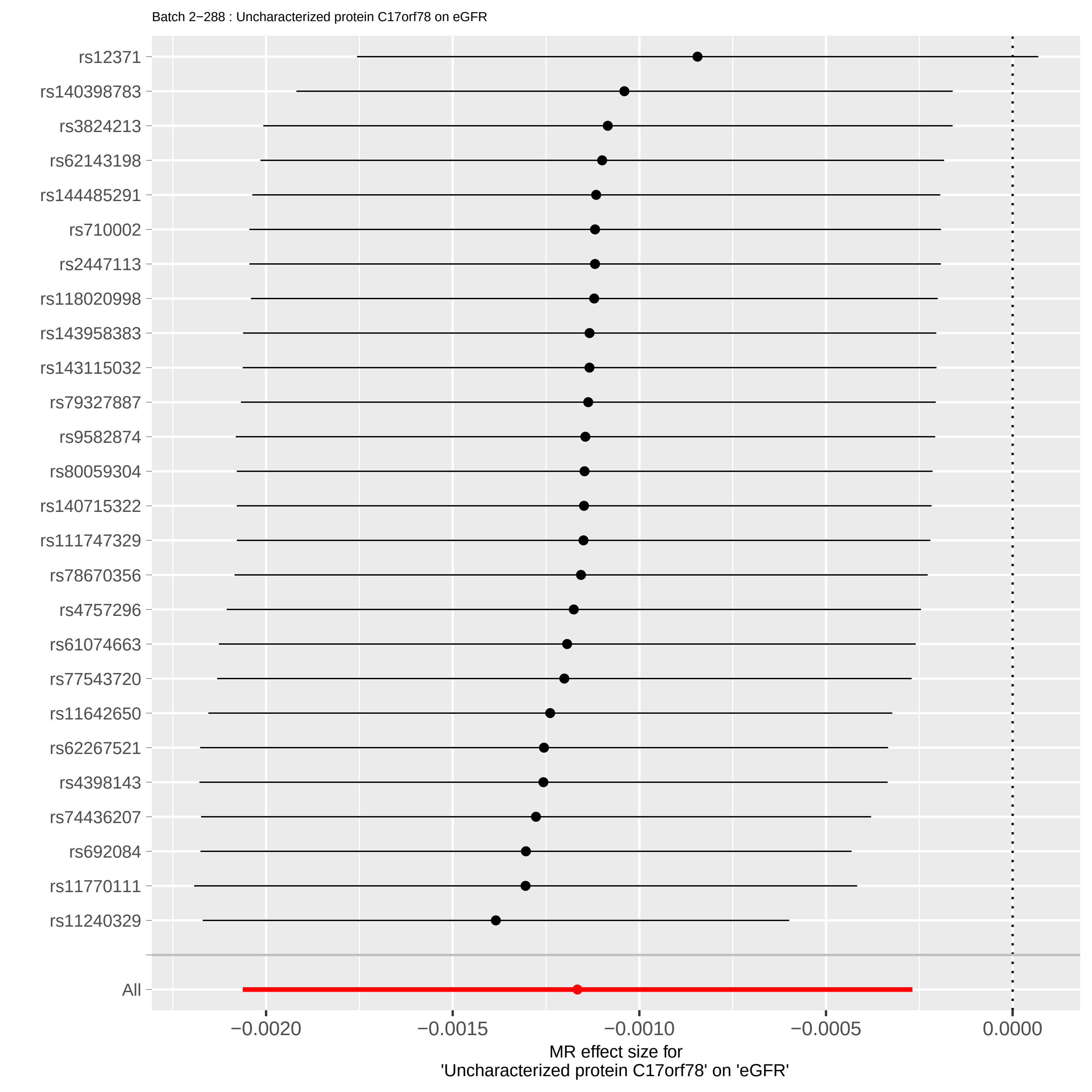

Batch 2−288 : Uncharacterized protein C17orf78 on eGFR
rs12371
rs140398783
rs3824213
rs62143198
rs144485291
rs710002
rs2447113
rs118020998
rs143958383
rs143115032
rs79327887
rs9582874
rs80059304
rs140715322
rs111747329
rs78670356
rs4757296
rs61074663
rs77543720
rs11642650
rs62267521
rs4398143
rs74436207
rs692084
rs11770111
rs11240329
All
−0.0020
−0.0015
−0.0010
−0.0005
0.0000
MR effect size for
'Uncharacterized protein C17orf78' on 'eGFR'

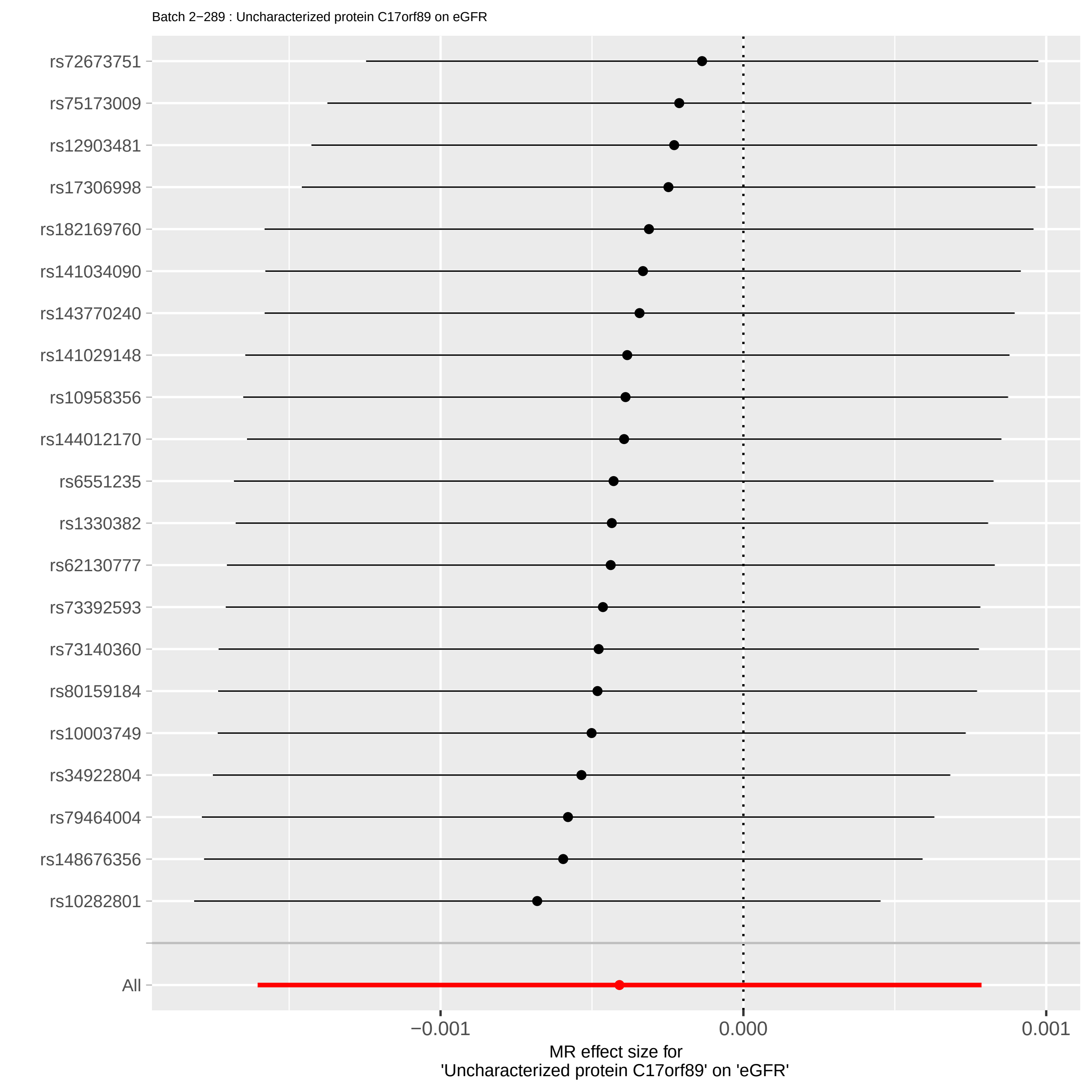

Batch 2−289 : Uncharacterized protein C17orf89 on eGFR
rs72673751
rs75173009
rs12903481
rs17306998
rs182169760
rs141034090
rs143770240
rs141029148
rs10958356
rs144012170
rs6551235
rs1330382
rs62130777
rs73392593
rs73140360
rs80159184
rs10003749
rs34922804
rs79464004
rs148676356
rs10282801
All
−0.001
0.000
0.001
MR effect size for
'Uncharacterized protein C17orf89' on 'eGFR'

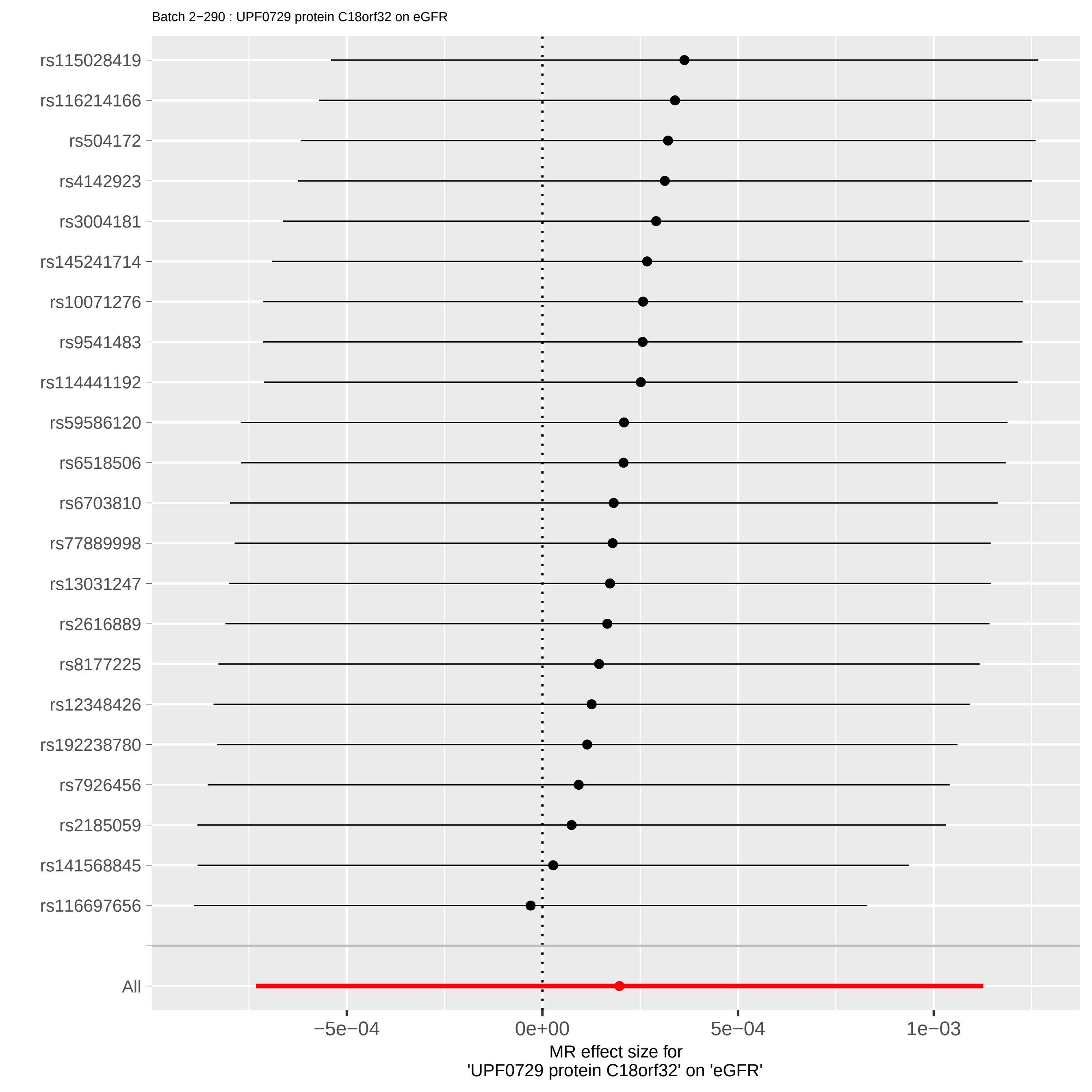

Batch 2−290 : UPF0729 protein C18orf32 on eGFR
rs115028419
rs116214166
rs504172
rs4142923
rs3004181
rs145241714
rs10071276
rs9541483
rs114441192
rs59586120
rs6518506
rs6703810
rs77889998
rs13031247
rs2616889
rs8177225
rs12348426
rs192238780
rs7926456
rs2185059
rs141568845
rs116697656
All
−5e−04
0e+00
5e−04
1e−03
MR effect size for
'UPF0729 protein C18orf32' on 'eGFR'

Batch 2−291 : Uncharacterized protein C19orf18 on eGFR

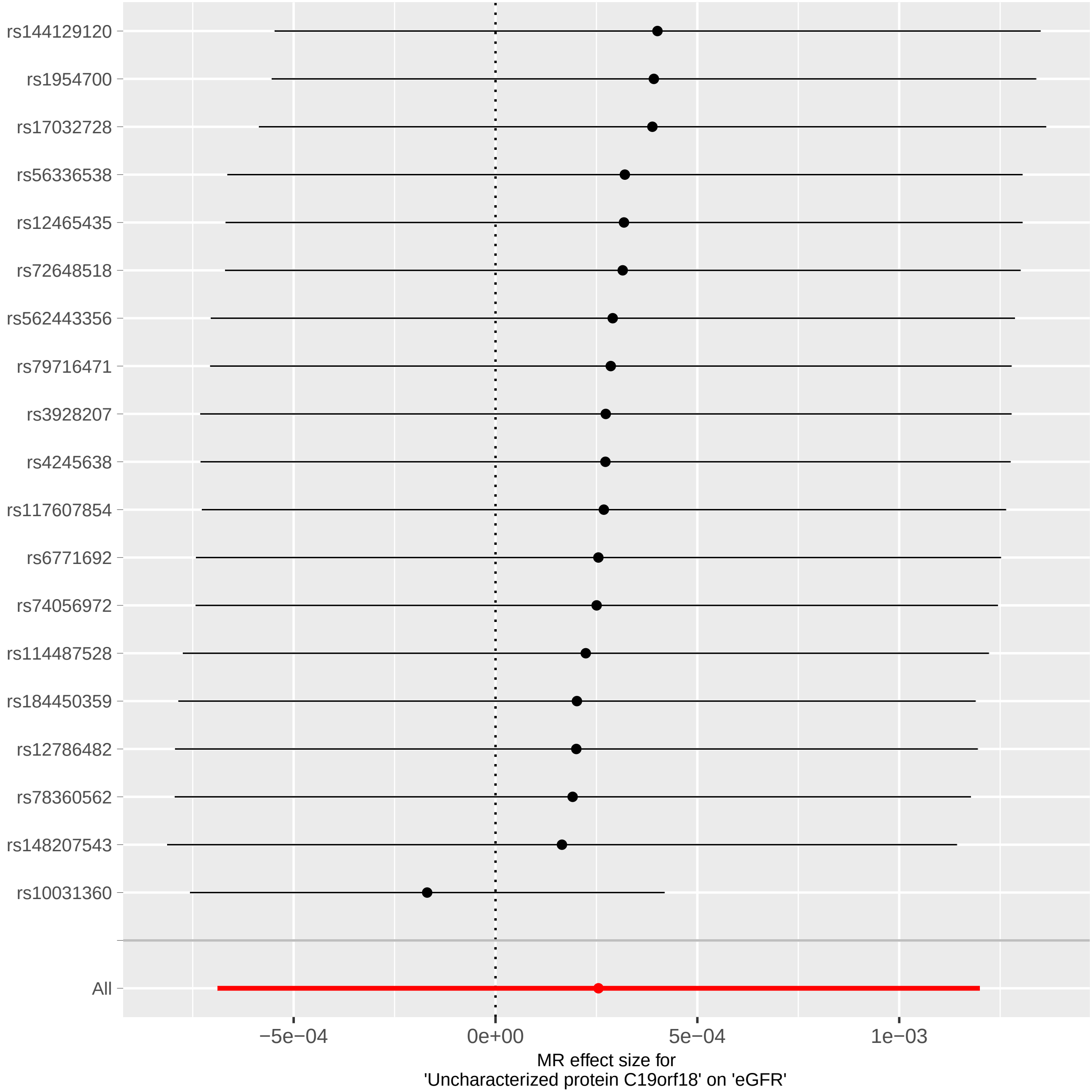

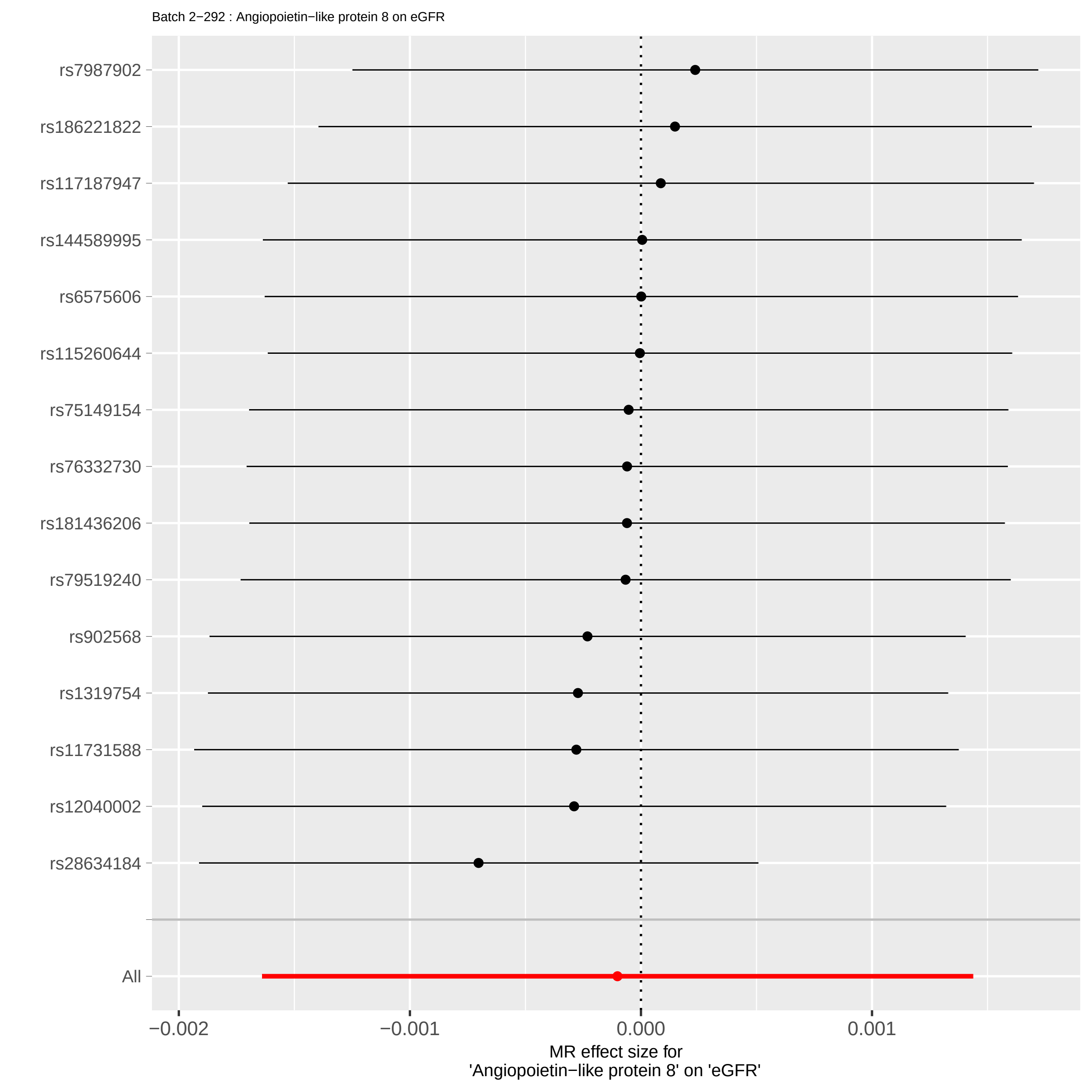

Batch 2−292 : Angiopoietin−like protein 8 on eGFR
rs7987902
rs186221822
rs117187947
rs144589995
rs6575606
rs115260644
rs75149154
rs76332730
rs181436206
rs79519240
rs902568
rs1319754
rs11731588
rs12040002
rs28634184
All
−0.002
−0.001
0.000
0.001
MR effect size for
'Angiopoietin−like protein 8' on 'eGFR'

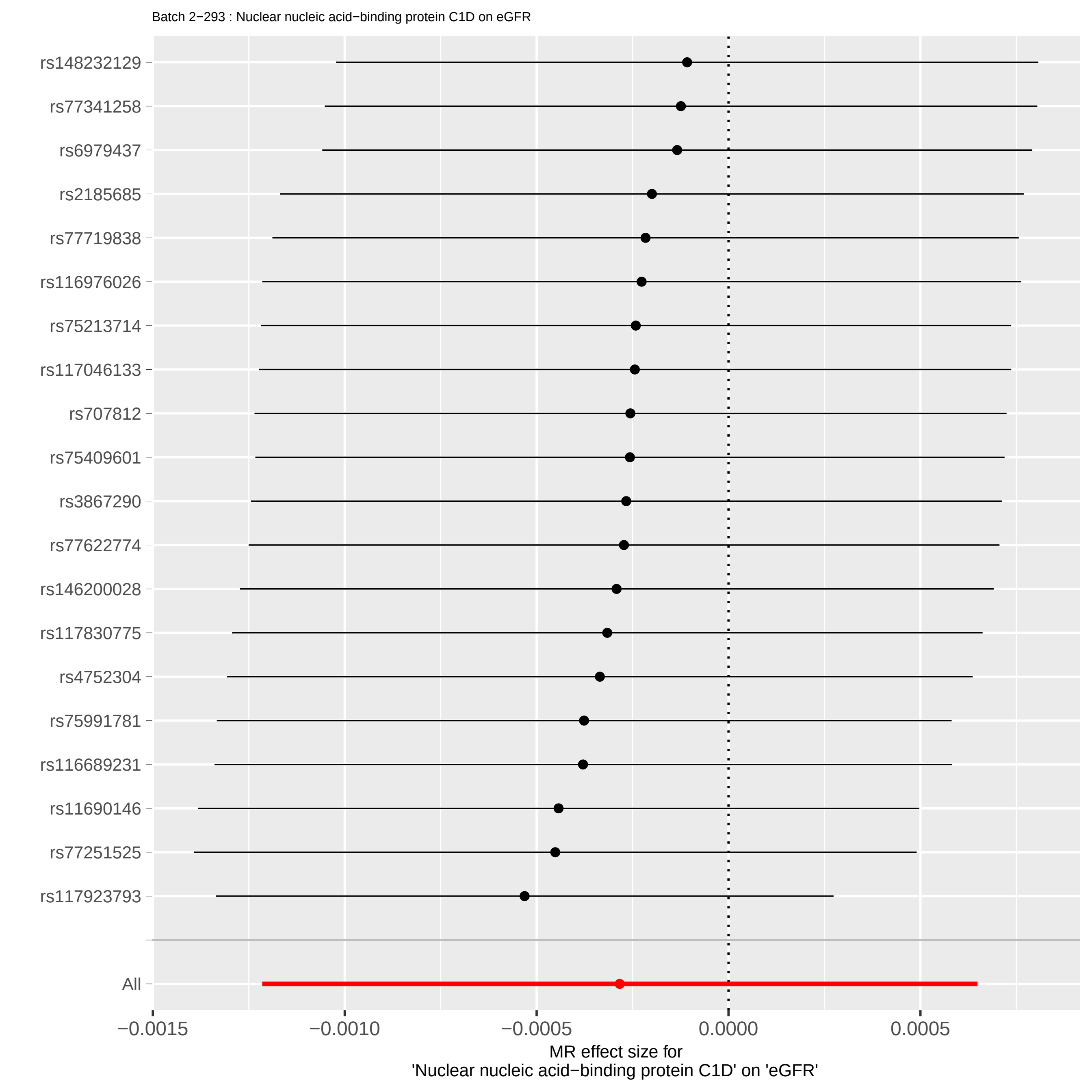

Batch 2−293 : Nuclear nucleic acid−binding protein C1D on eGFR
rs148232129
rs77341258
rs6979437
rs2185685
rs77719838
rs116976026
rs75213714
rs117046133
rs707812
rs75409601
rs3867290
rs77622774
rs146200028
rs117830775
rs4752304
rs75991781
rs116689231
rs11690146
rs77251525
rs117923793
All
−0.0015
−0.0010
−0.0005
0.0000
0.0005
MR effect size for
'Nuclear nucleic acid−binding protein C1D' on 'eGFR'

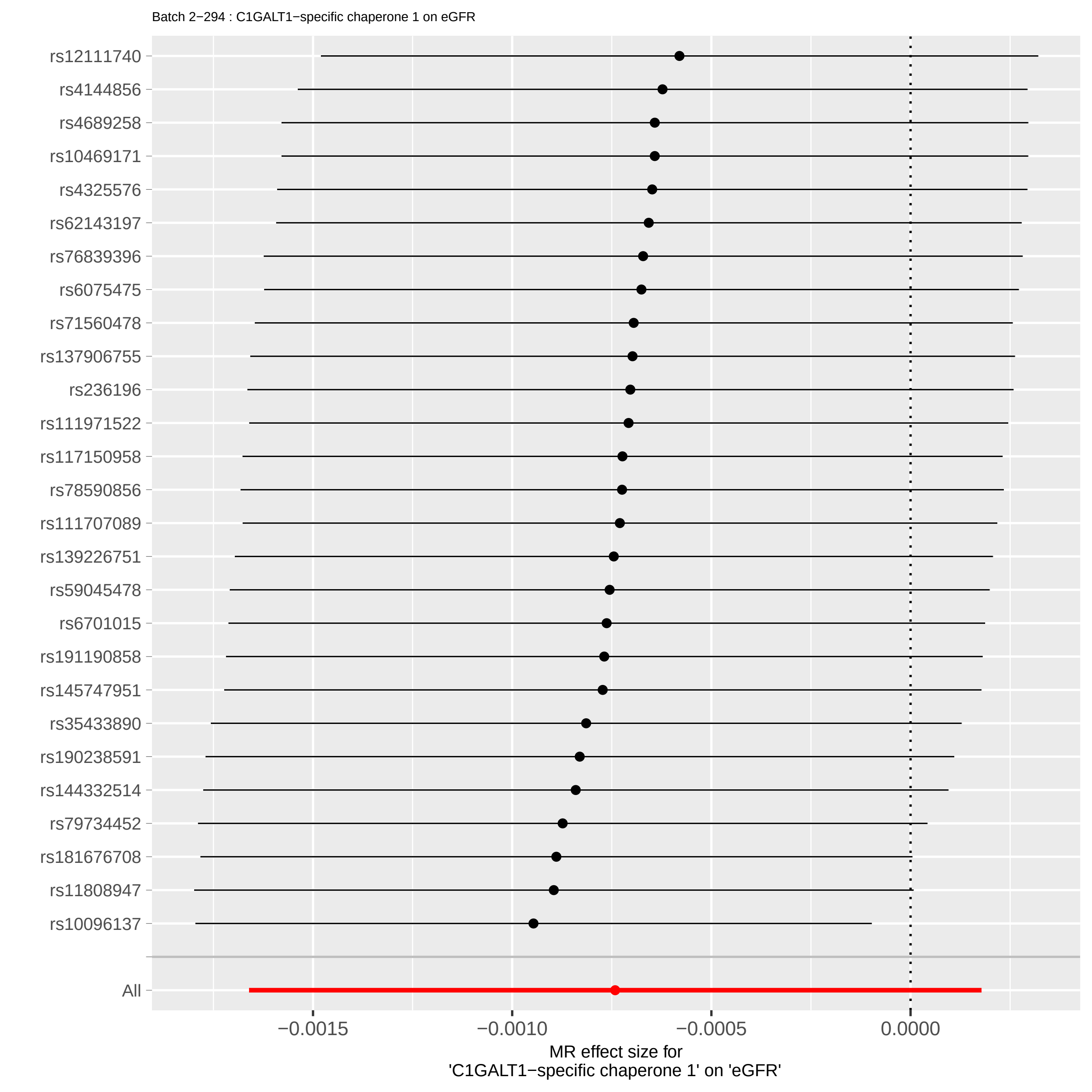

Batch 2−294 : C1GALT1−specific chaperone 1 on eGFR
rs12111740
rs4144856
rs4689258
rs10469171
rs4325576
rs62143197
rs76839396
rs6075475
rs71560478
rs137906755
rs236196
rs111971522
rs117150958
rs78590856
rs111707089
rs139226751
rs59045478
rs6701015
rs191190858
rs145747951
rs35433890
rs190238591
rs144332514
rs79734452
rs181676708
rs11808947
rs10096137
All
−0.0015
−0.0010
−0.0005
0.0000
MR effect size for
'C1GALT1−specific chaperone 1' on 'eGFR'

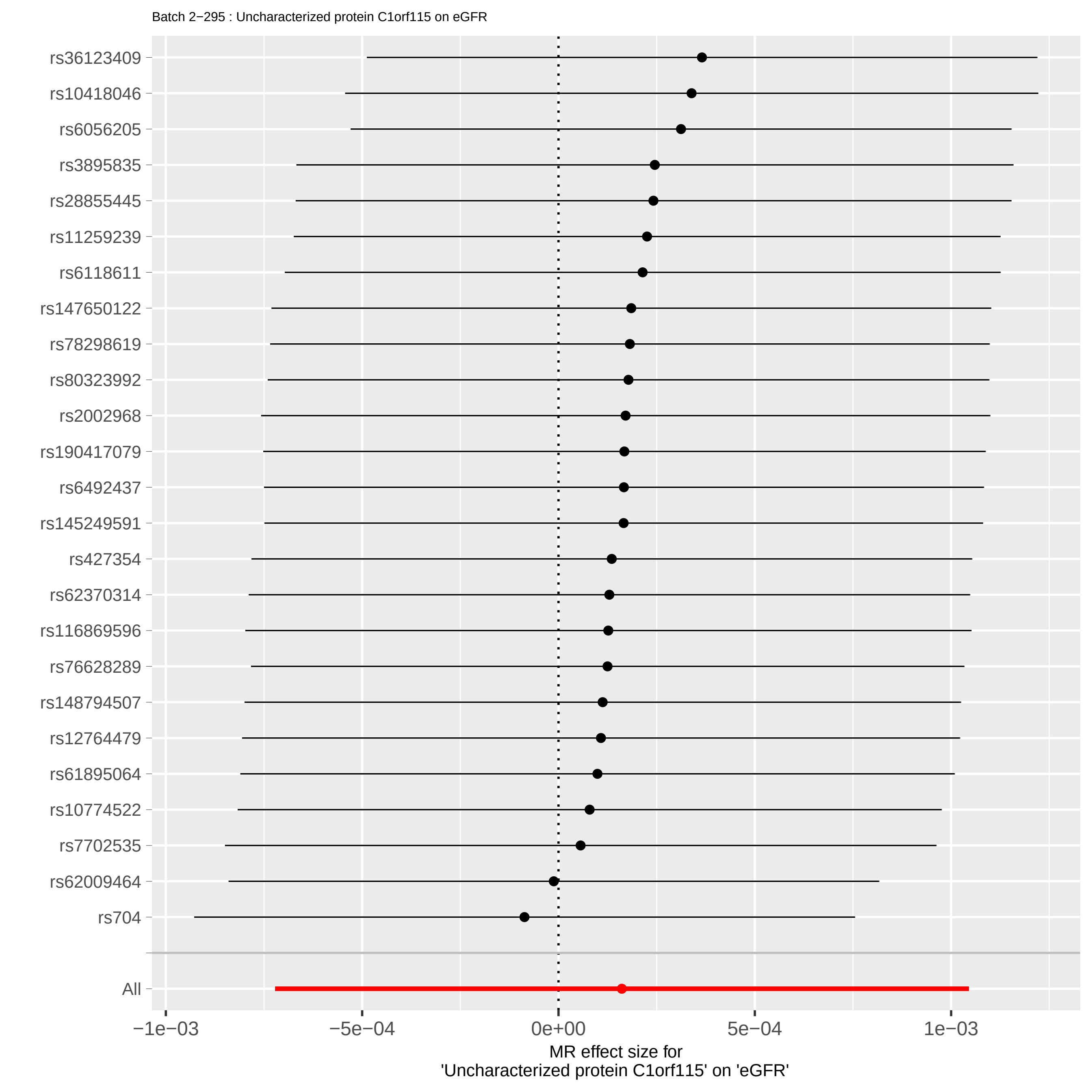
Batch 2−295 : Uncharacterized protein C1orf115 on eGFR
rs36123409
rs10418046
rs6056205
rs3895835
rs28855445
rs11259239
rs6118611
rs147650122
rs78298619
rs80323992
rs2002968
rs190417079
rs6492437
rs145249591
rs427354
rs62370314
rs116869596
rs76628289
rs148794507
rs12764479
rs61895064
rs10774522
rs7702535
rs62009464
rs704
All
−1e−03
−5e−04
0e+00
5e−04
1e−03
MR effect size for
'Uncharacterized protein C1orf115' on 'eGFR'

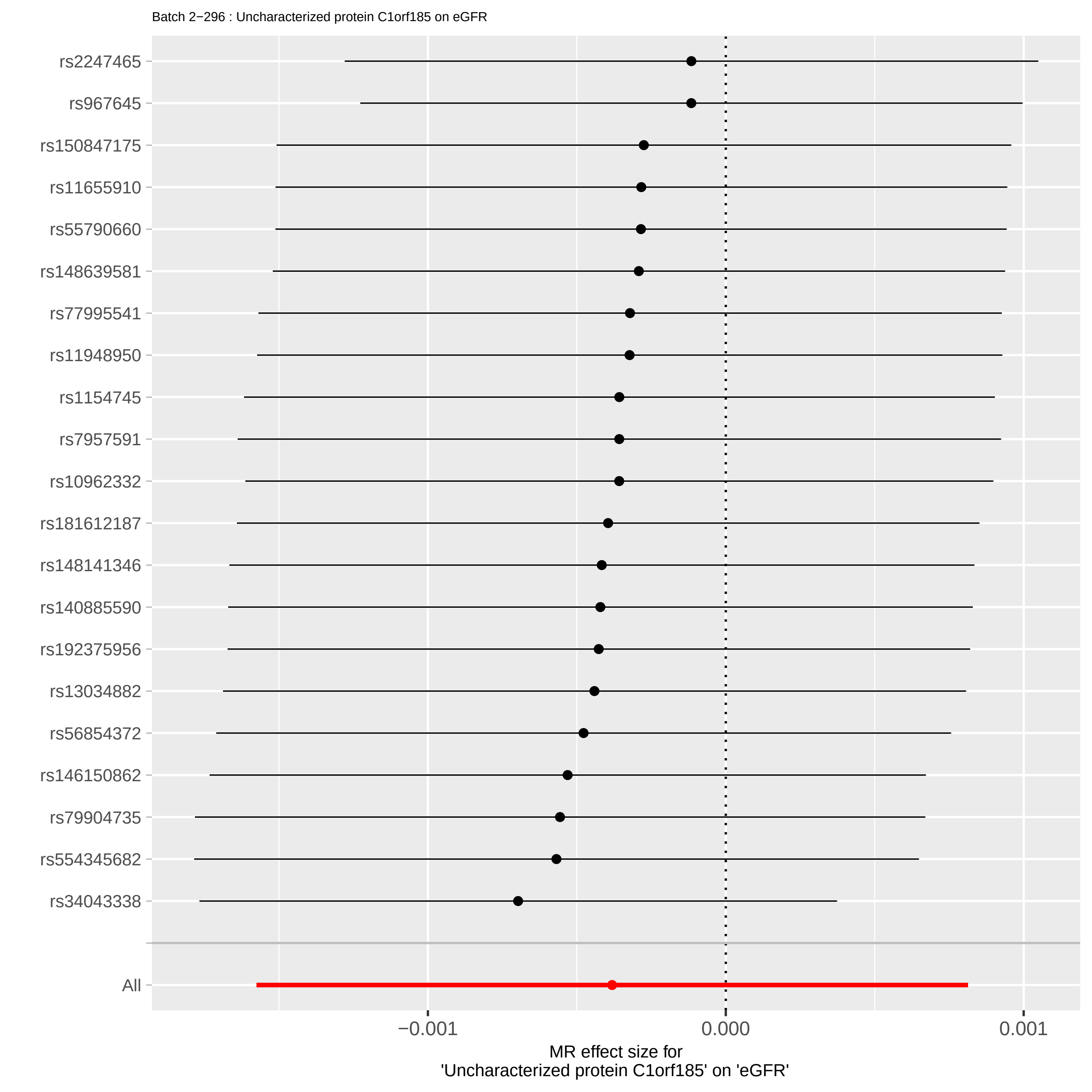

Batch 2−296 : Uncharacterized protein C1orf185 on eGFR
rs2247465
rs967645
rs150847175
rs11655910
rs55790660
rs148639581
rs77995541
rs11948950
rs1154745
rs7957591
rs10962332
rs181612187
rs148141346
rs140885590
rs192375956
rs13034882
rs56854372
rs146150862
rs79904735
rs554345682
rs34043338
All
−0.001
0.000
0.001
MR effect size for
'Uncharacterized protein C1orf185' on 'eGFR'

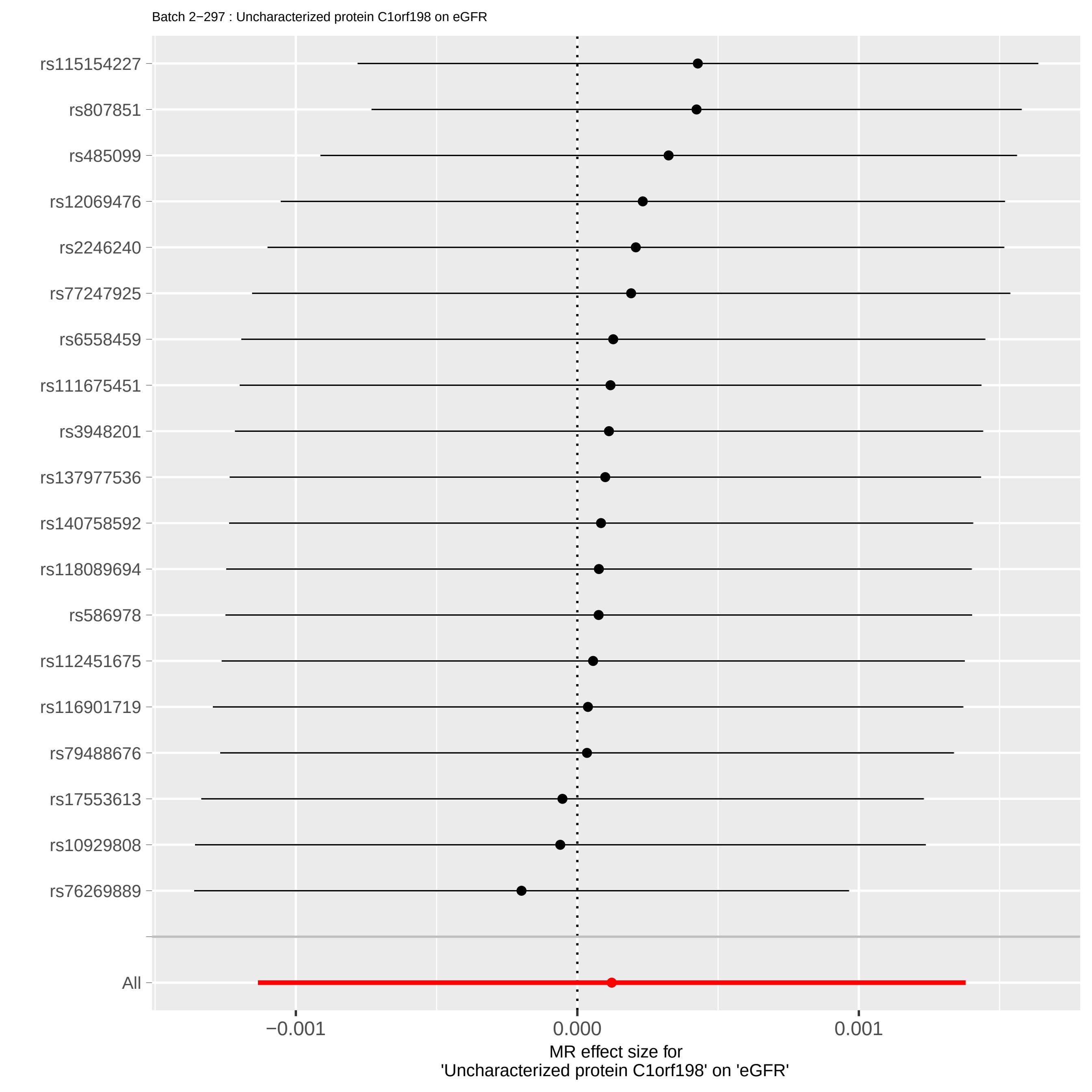
Batch 2−297 : Uncharacterized protein C1orf198 on eGFR
rs115154227
rs807851
rs485099
rs12069476
rs2246240
rs77247925
rs6558459
rs111675451
rs3948201
rs137977536
rs140758592
rs118089694
rs586978
rs112451675
rs116901719
rs79488676
rs17553613
rs10929808
rs76269889
All
−0.001
0.000
0.001
MR effect size for
'Uncharacterized protein C1orf198' on 'eGFR'

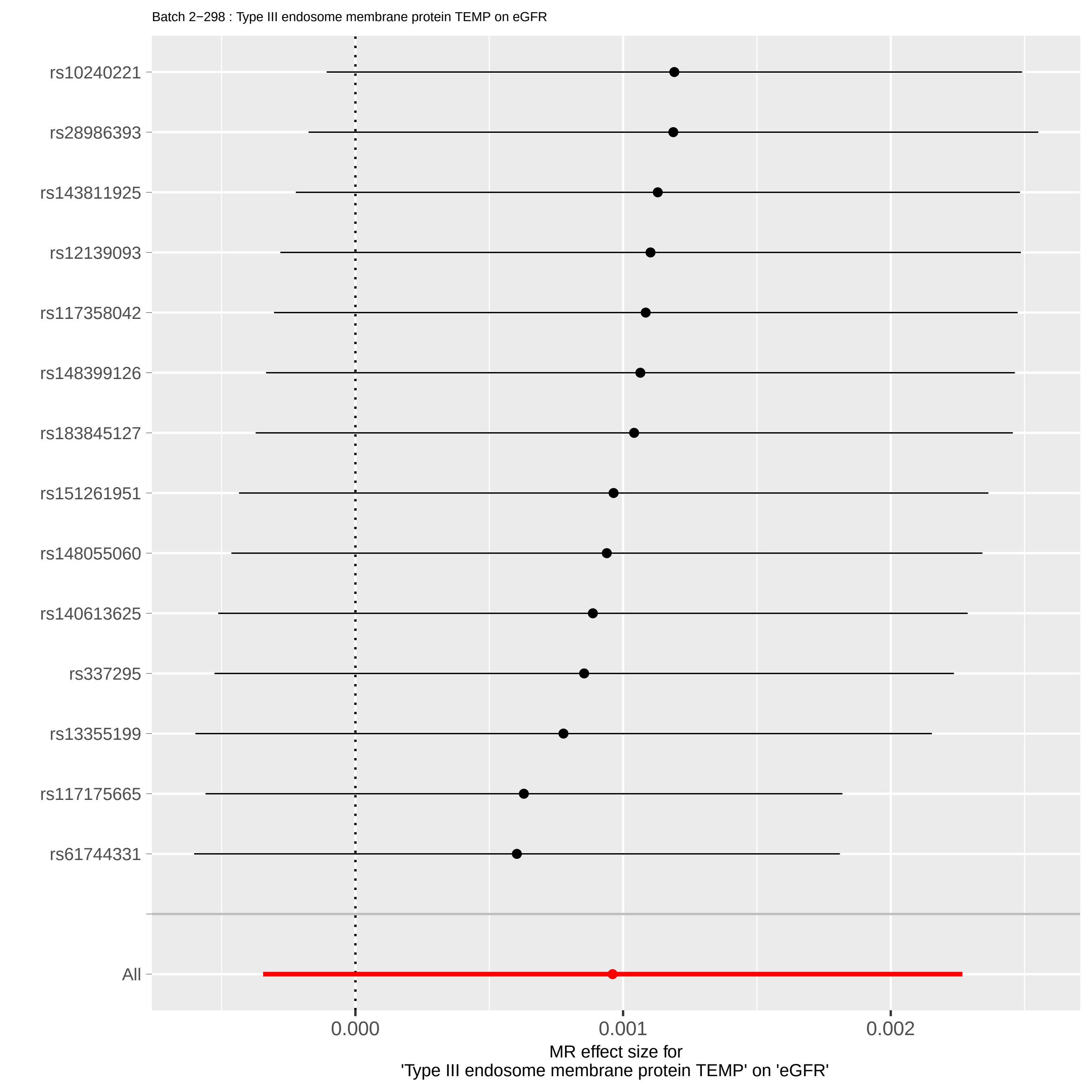
Batch 2−298 : Type III endosome membrane protein TEMP on eGFR
rs10240221
rs28986393
rs143811925
rs12139093
rs117358042
rs148399126
rs183845127
rs151261951
rs148055060
rs140613625
rs337295
rs13355199
rs117175665
rs61744331
All
0.000
0.001
0.002
MR effect size for
'Type III endosome membrane protein TEMP' on 'eGFR'

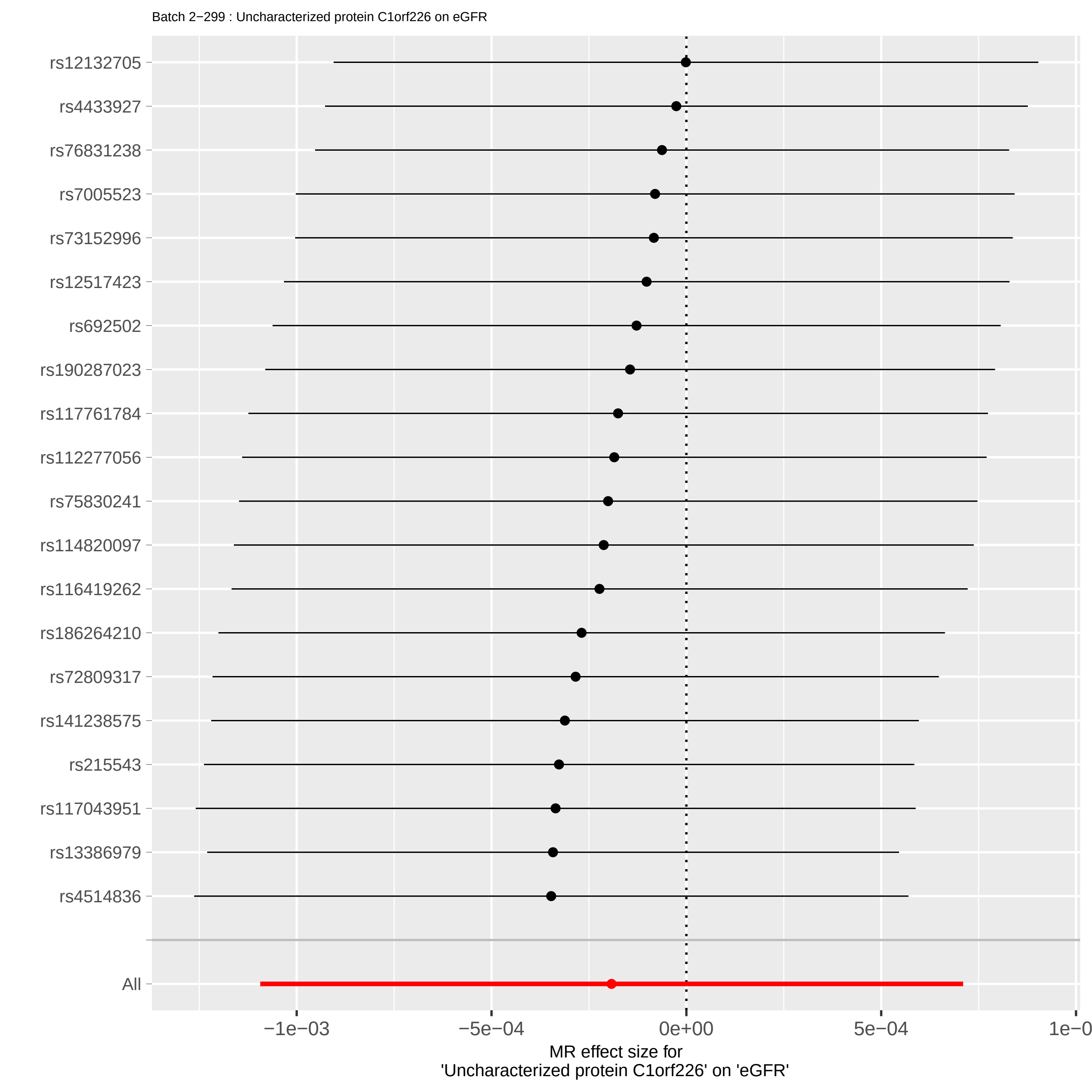

Batch 2−299 : Uncharacterized protein C1orf226 on eGFR
rs12132705
rs4433927
rs76831238
rs7005523
rs73152996
rs12517423
rs692502
rs190287023
rs117761784
rs112277056
rs75830241
rs114820097
rs116419262
rs186264210
rs72809317
rs141238575
rs215543
rs117043951
rs13386979
rs4514836
All
−1e−03
−5e−04
0e+00
5e−04
1e−0
MR effect size for
'Uncharacterized protein C1orf226' on 'eGFR'

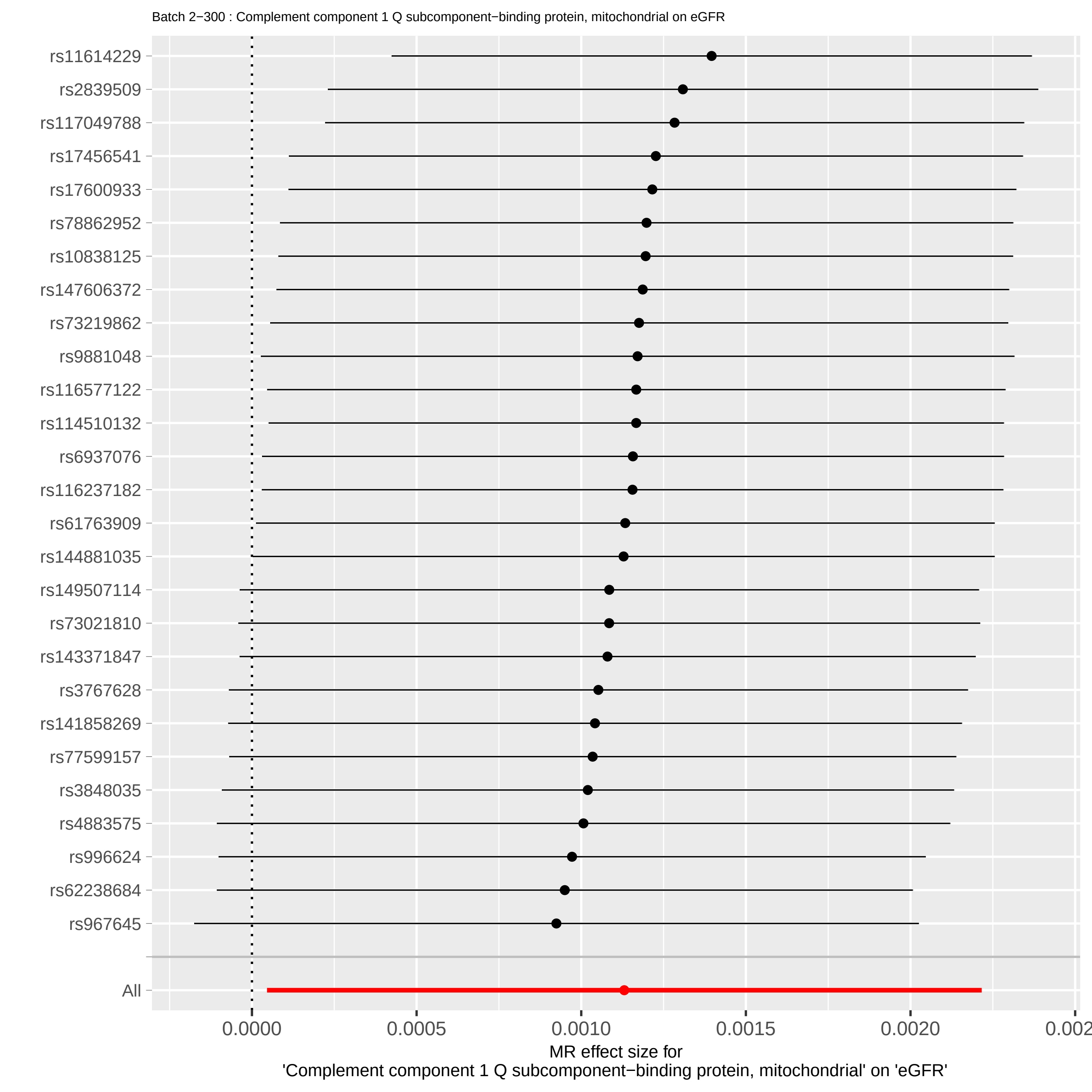

Batch 2−300 : Complement component 1 Q subcomponent−binding protein, mitochondrial on eGFR
rs11614229
rs2839509
rs117049788
rs17456541
rs17600933
rs78862952
rs10838125
rs147606372
rs73219862
rs9881048
rs116577122
rs114510132
rs6937076
rs116237182
rs61763909
rs144881035
rs149507114
rs73021810
rs143371847
rs3767628
rs141858269
rs77599157
rs3848035
rs4883575
rs996624
rs62238684
rs967645
All
0.0000
0.0005
0.0010
0.0015
0.0020
MR effect size for
'Complement component 1 Q subcomponent−binding protein, mitochondrial' on 'eGFR'

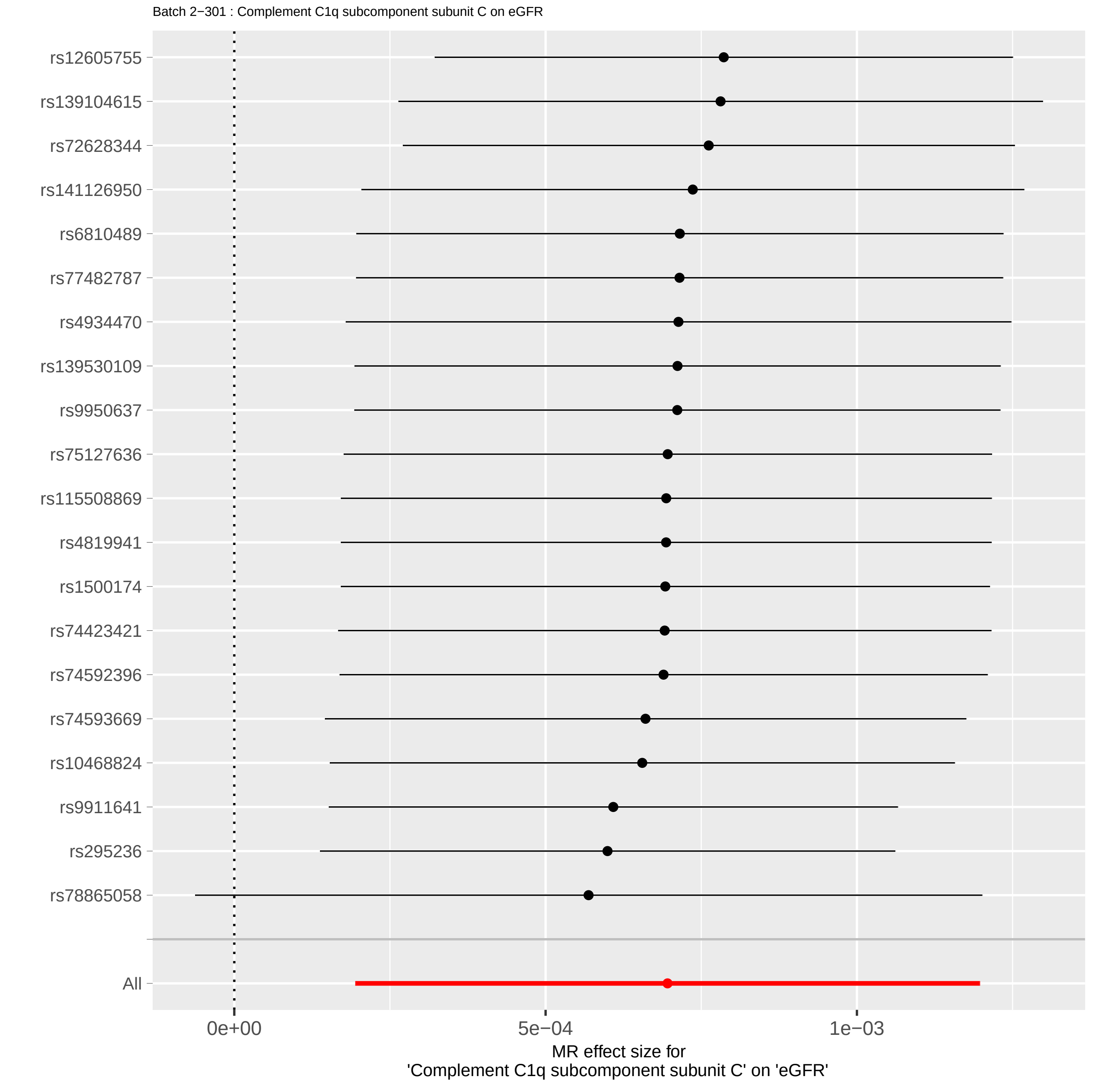

Batch 2−301 : Complement C1q subcomponent subunit C on eGFR
rs12605755
rs139104615
rs72628344
rs141126950
rs6810489
rs77482787
rs4934470
rs139530109
rs9950637
rs75127636
rs115508869
rs4819941
rs1500174
rs74423421
rs74592396
rs74593669
rs10468824
rs9911641
rs295236
rs78865058
All
0e+00
5e−04
1e−03
MR effect size for
'Complement C1q subcomponent subunit C' on 'eGFR'

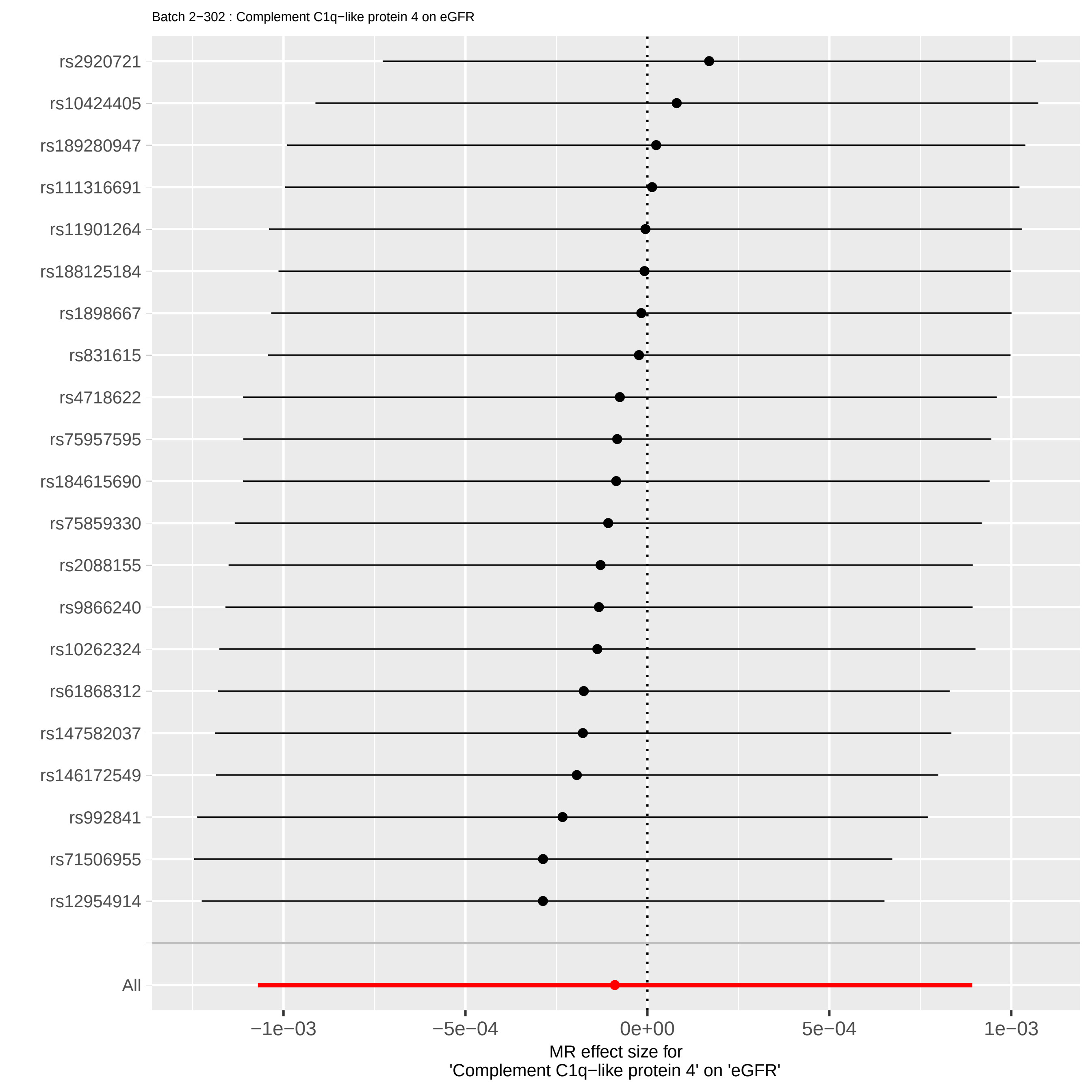
Batch 2−302 : Complement C1q−like protein 4 on eGFR
rs2920721
rs10424405
rs189280947
rs111316691
rs11901264
rs188125184
rs1898667
rs831615
rs4718622
rs75957595
rs184615690
rs75859330
rs2088155
rs9866240
rs10262324
rs61868312
rs147582037
rs146172549
rs992841
rs71506955
rs12954914
All
−1e−03
−5e−04
0e+00
5e−04
1e−03
MR effect size for
'Complement C1q−like protein 4' on 'eGFR'

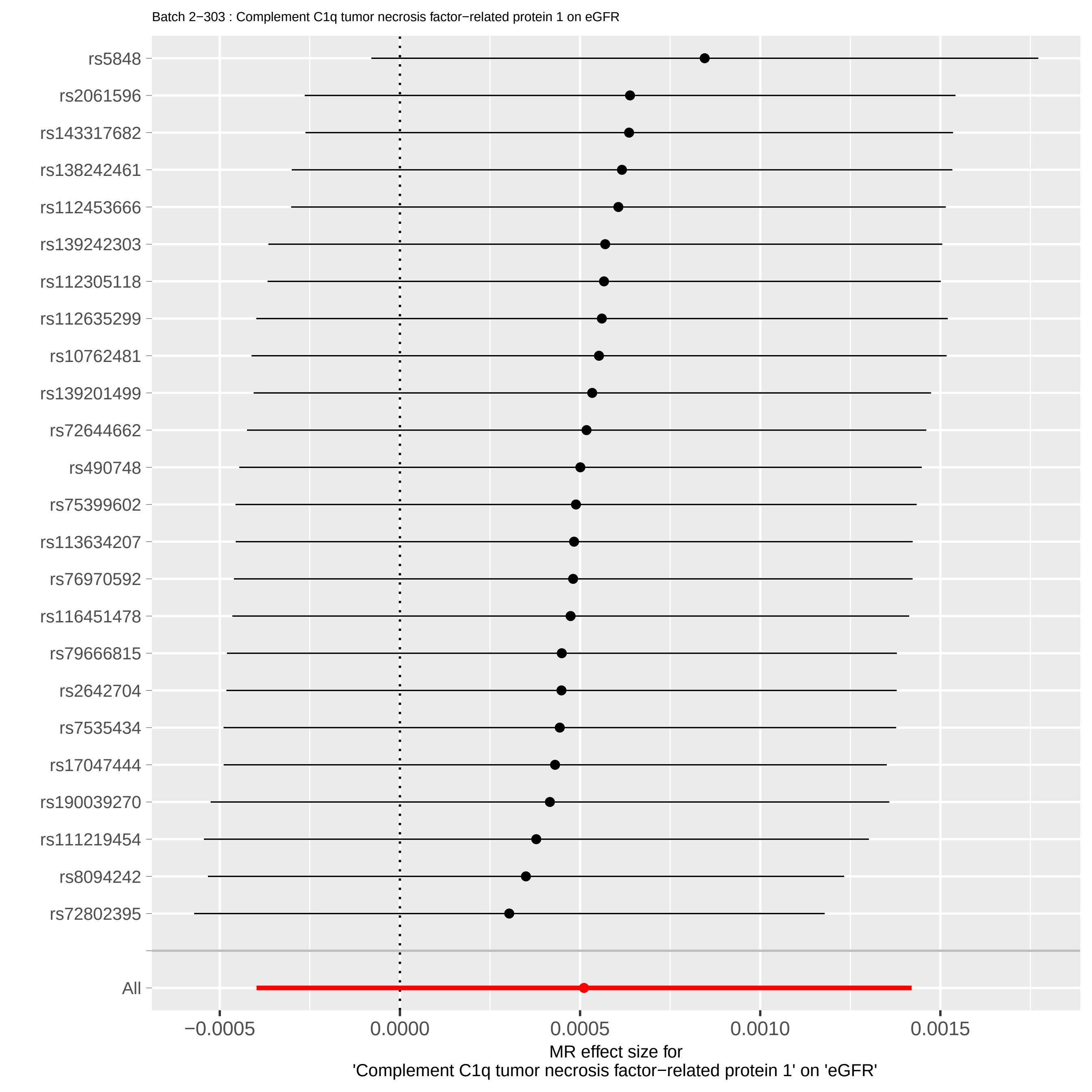
Batch 2−303 : Complement C1q tumor necrosis factor−related protein 1 on eGFR
rs5848
rs2061596
rs143317682
rs138242461
rs112453666
rs139242303
rs112305118
rs112635299
rs10762481
rs139201499
rs72644662
rs490748
rs75399602
rs113634207
rs76970592
rs116451478
rs79666815
rs2642704
rs7535434
rs17047444
rs190039270
rs111219454
rs8094242
rs72802395
All
−0.0005
0.0000
0.0005
0.0010
0.0015
MR effect size for
'Complement C1q tumor necrosis factor−related protein 1' on 'eGFR'

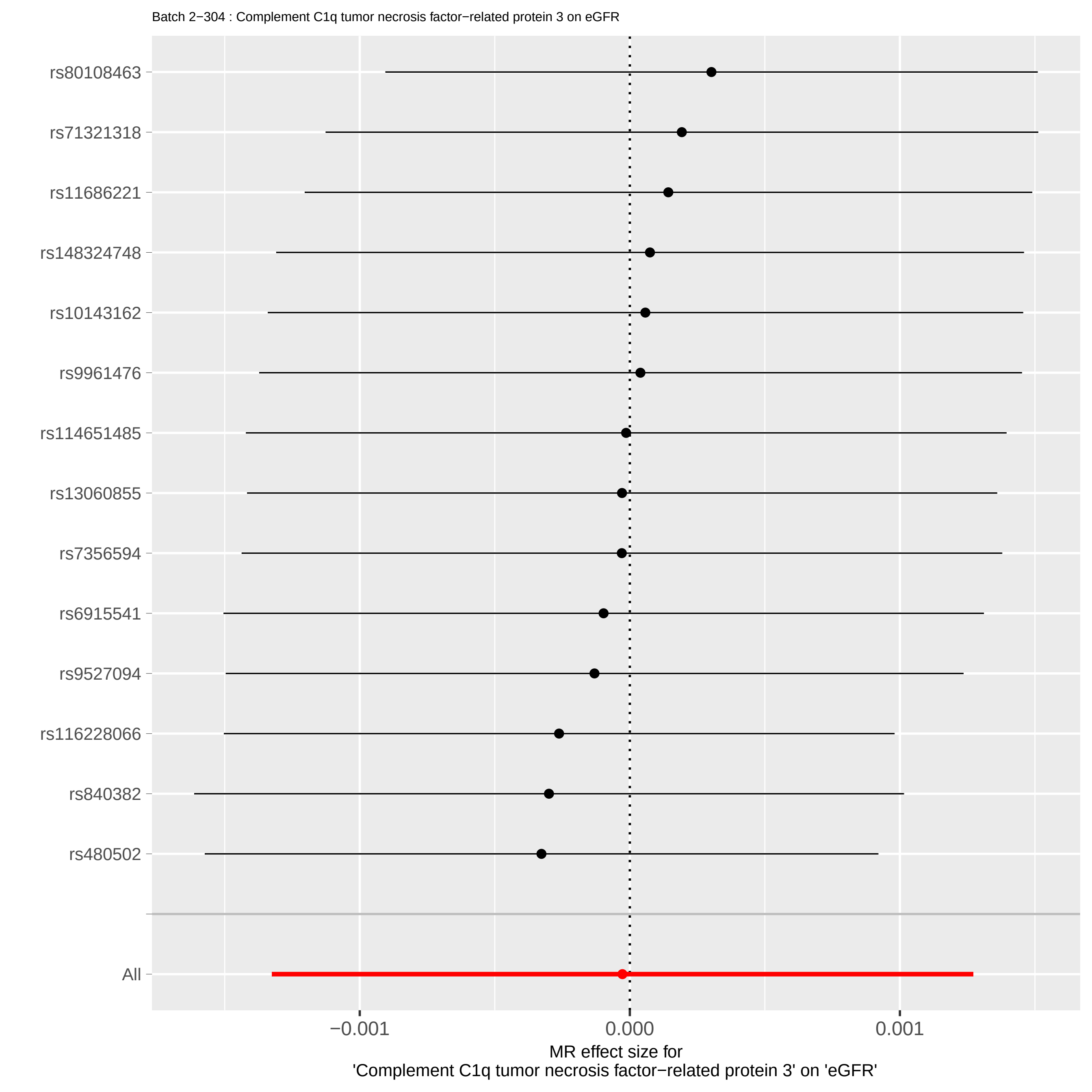
Batch 2−304 : Complement C1q tumor necrosis factor−related protein 3 on eGFR
rs80108463
rs71321318
rs11686221
rs148324748
rs10143162
rs9961476
rs114651485
rs13060855
rs7356594
rs6915541
rs9527094
rs116228066
rs840382
rs480502
All
−0.001
0.000
0.001
MR effect size for
'Complement C1q tumor necrosis factor−related protein 3' on 'eGFR'

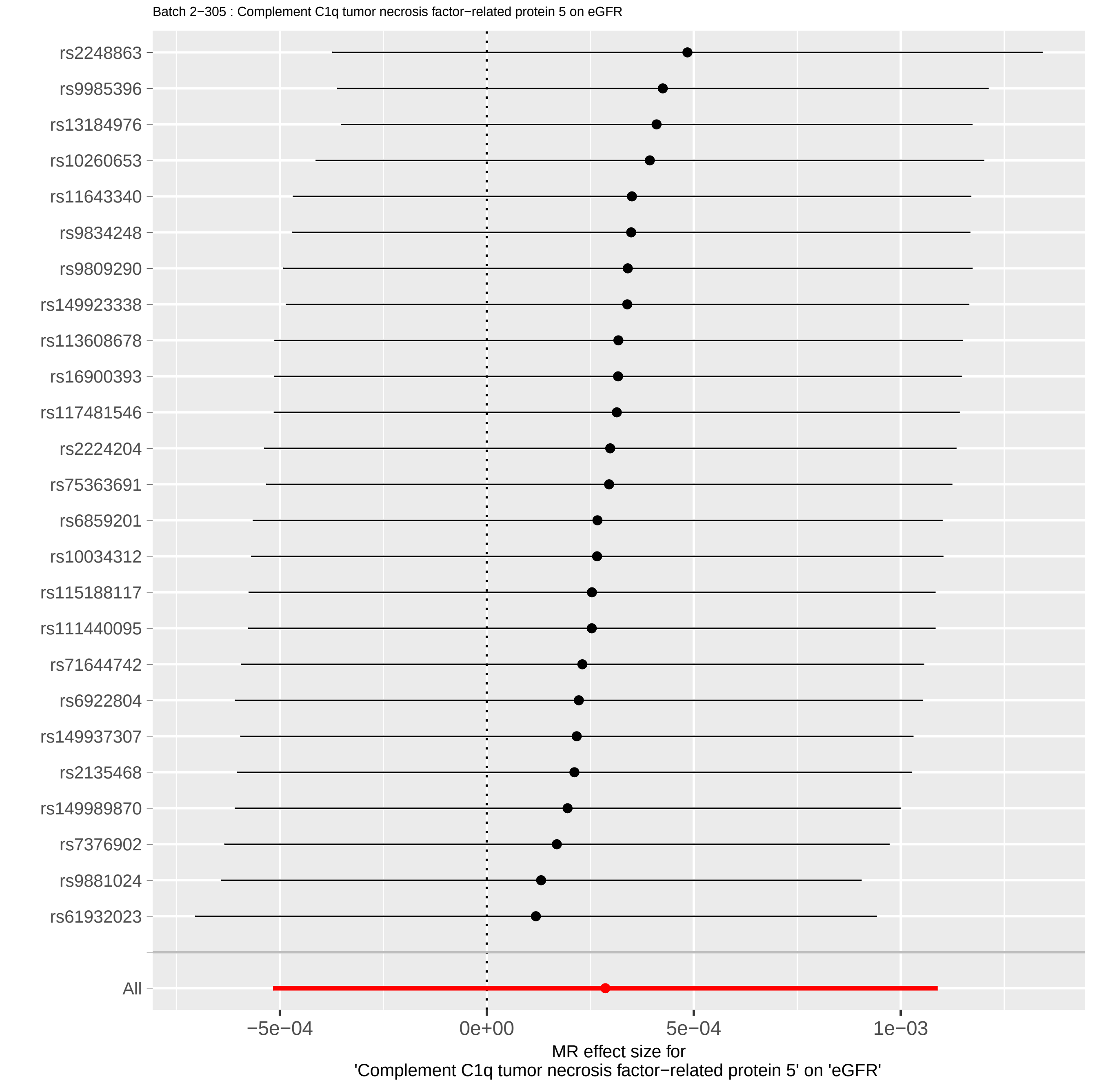

Batch 2−305 : Complement C1q tumor necrosis factor−related protein 5 on eGFR
rs2248863
rs9985396
rs13184976
rs10260653
rs11643340
rs9834248
rs9809290
rs149923338
rs113608678
rs16900393
rs117481546
rs2224204
rs75363691
rs6859201
rs10034312
rs115188117
rs111440095
rs71644742
rs6922804
rs149937307
rs2135468
rs149989870
rs7376902
rs9881024
rs61932023
All
−5e−04
0e+00
5e−04
1e−03
MR effect size for
'Complement C1q tumor necrosis factor−related protein 5' on 'eGFR'

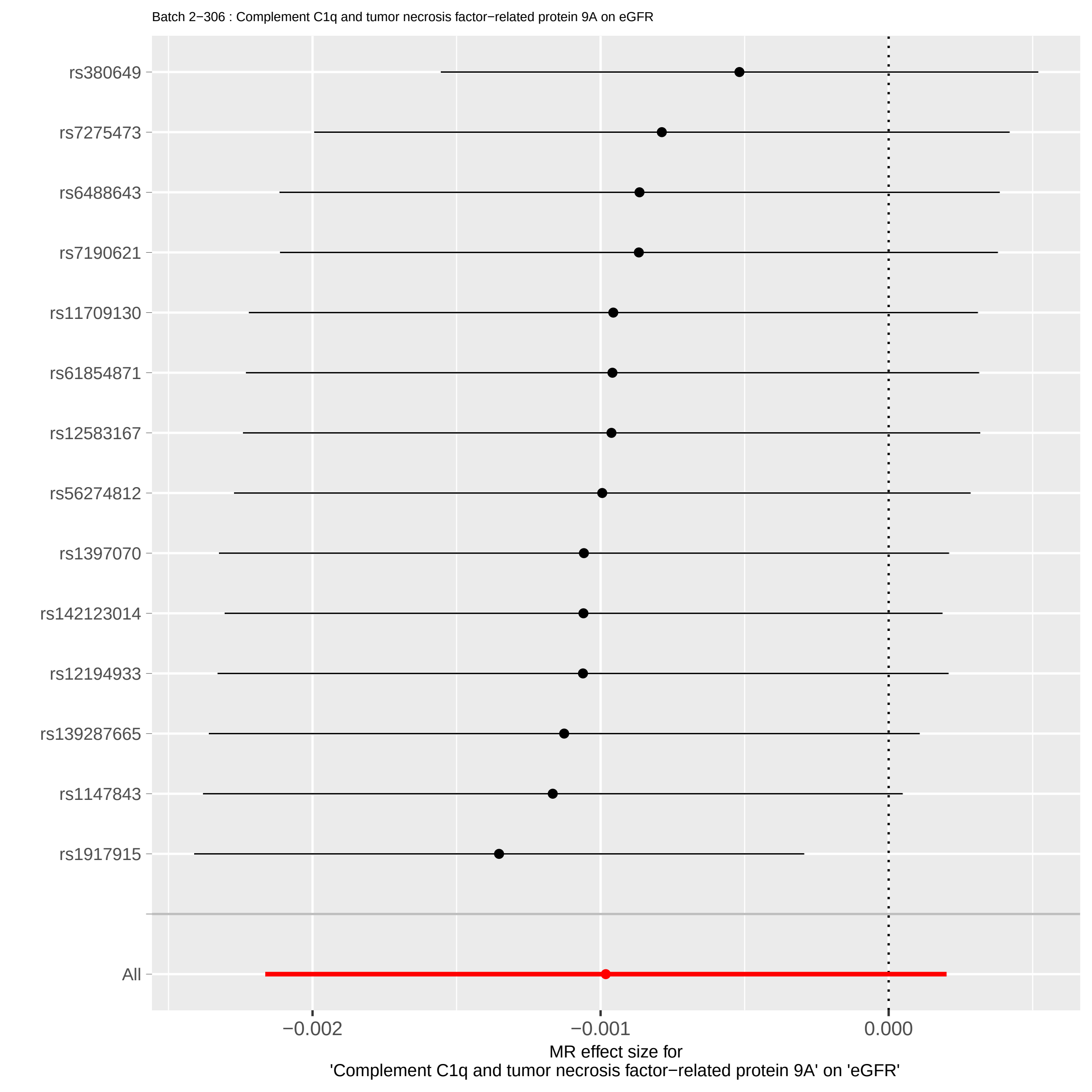

Batch 2−306 : Complement C1q and tumor necrosis factor−related protein 9A on eGFR
rs380649
rs7275473
rs6488643
rs7190621
rs11709130
rs61854871
rs12583167
rs56274812
rs1397070
rs142123014
rs12194933
rs139287665
rs1147843
rs1917915
All
−0.002
−0.001
0.000
MR effect size for
'Complement C1q and tumor necrosis factor−related protein 9A' on 'eGFR'

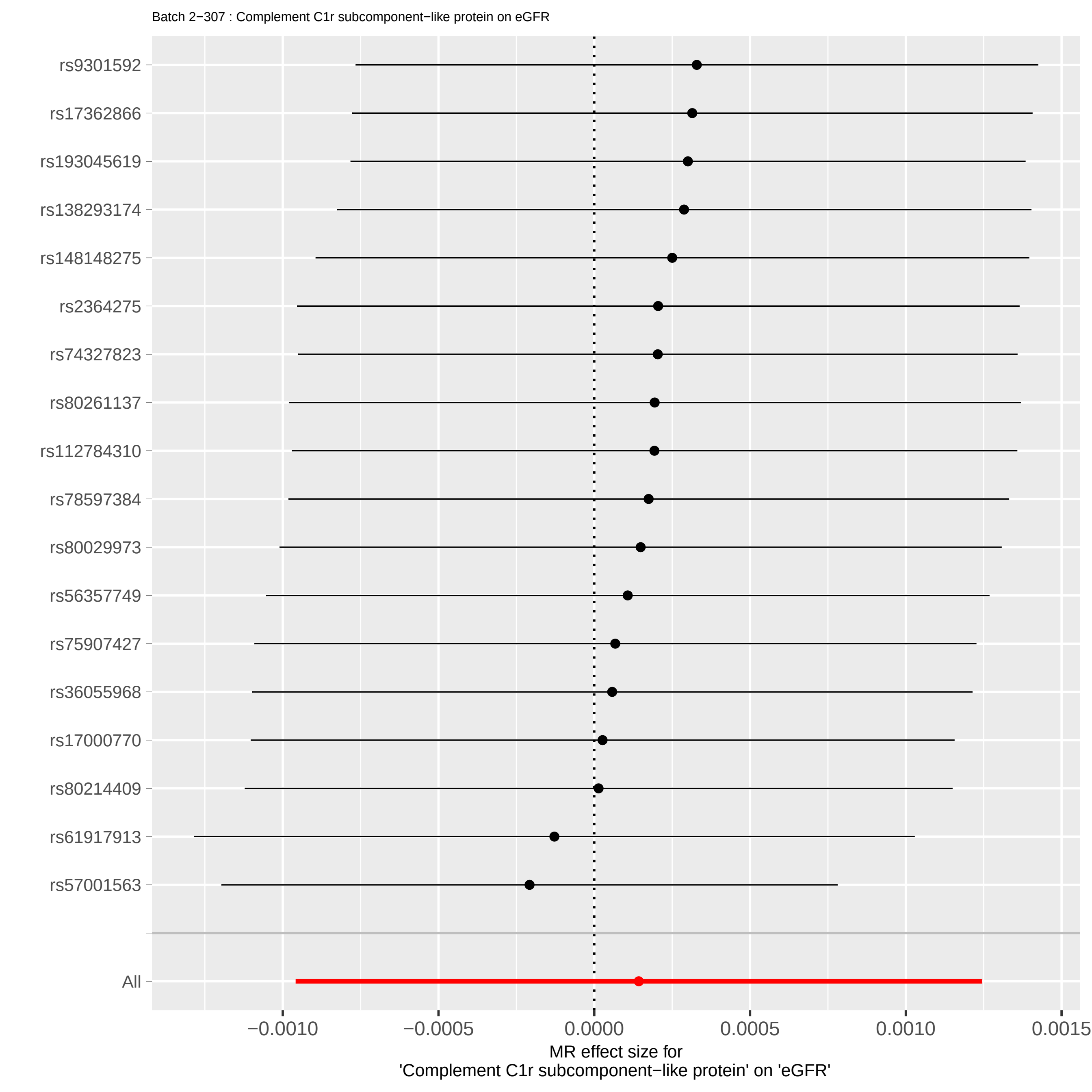

Batch 2−307 : Complement C1r subcomponent−like protein on eGFR
rs9301592
rs17362866
rs193045619
rs138293174
rs148148275
rs2364275
rs74327823
rs80261137
rs112784310
rs78597384
rs80029973
rs56357749
rs75907427
rs36055968
rs17000770
rs80214409
rs61917913
rs57001563
All
−0.0010
−0.0005
0.0000
0.0005
0.0010
0.0015
MR effect size for
'Complement C1r subcomponent−like protein' on 'eGFR'

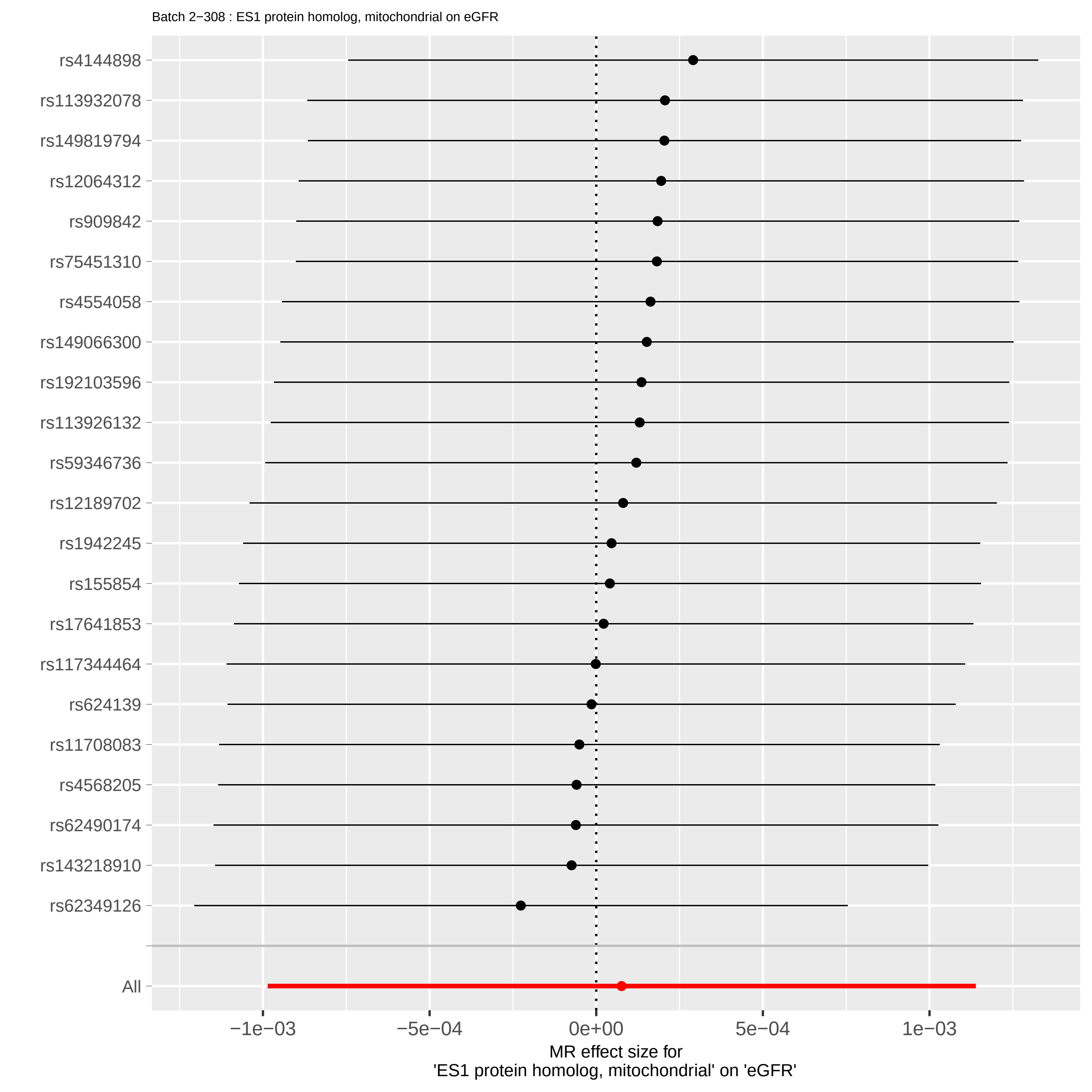

Batch 2−308 : ES1 protein homolog, mitochondrial on eGFR
rs4144898
rs113932078
rs149819794
rs12064312
rs909842
rs75451310
rs4554058
rs149066300
rs192103596
rs113926132
rs59346736
rs12189702
rs1942245
rs155854
rs17641853
rs117344464
rs624139
rs11708083
rs4568205
rs62490174
rs143218910
rs62349126
All
−1e−03
−5e−04
0e+00
5e−04
1e−03
MR effect size for
'ES1 protein homolog, mitochondrial' on 'eGFR'

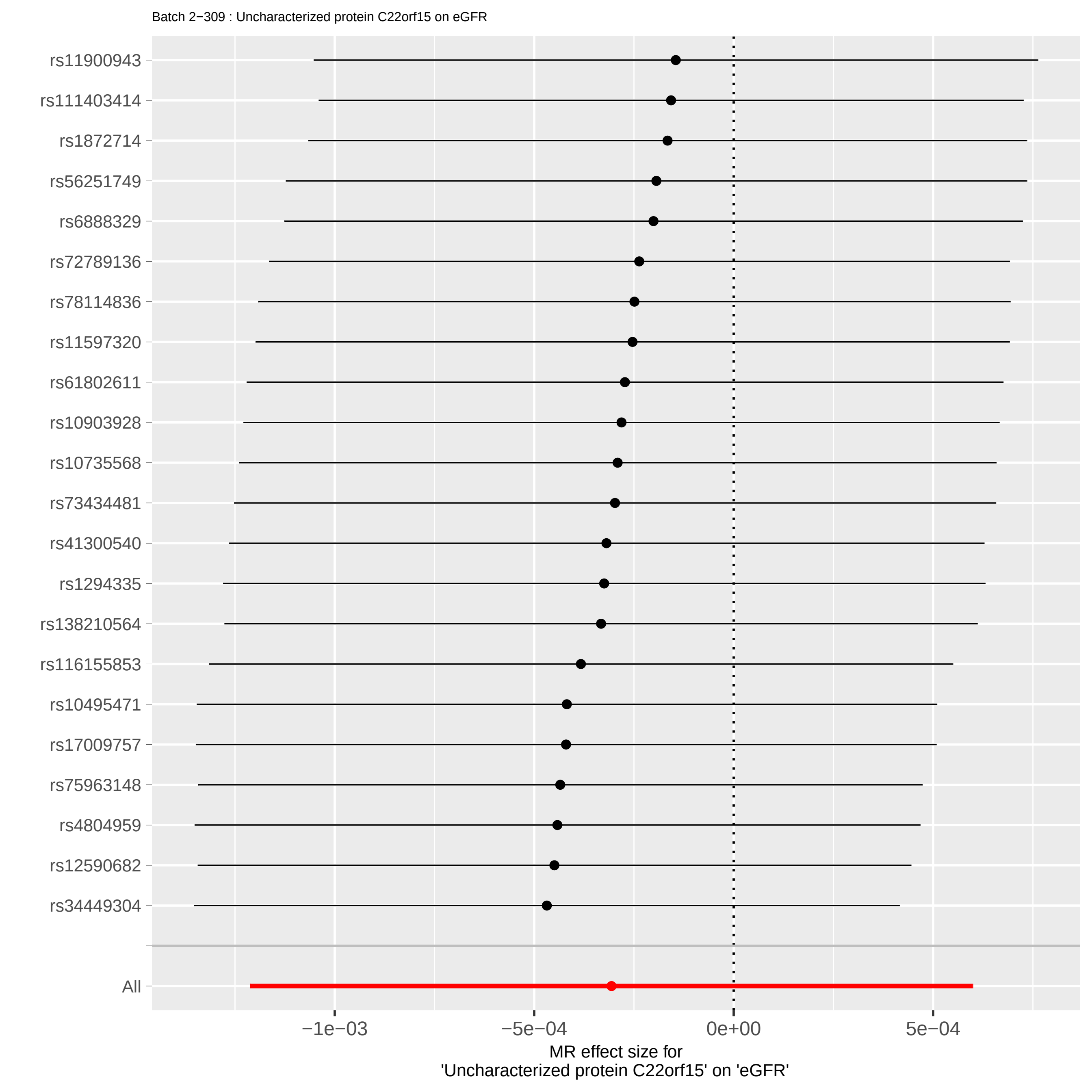

Batch 2−309 : Uncharacterized protein C22orf15 on eGFR
rs11900943
rs111403414
rs1872714
rs56251749
rs6888329
rs72789136
rs78114836
rs11597320
rs61802611
rs10903928
rs10735568
rs73434481
rs41300540
rs1294335
rs138210564
rs116155853
rs10495471
rs17009757
rs75963148
rs4804959
rs12590682
rs34449304
All
−1e−03
−5e−04
0e+00
5e−04
MR effect size for
'Uncharacterized protein C22orf15' on 'eGFR'

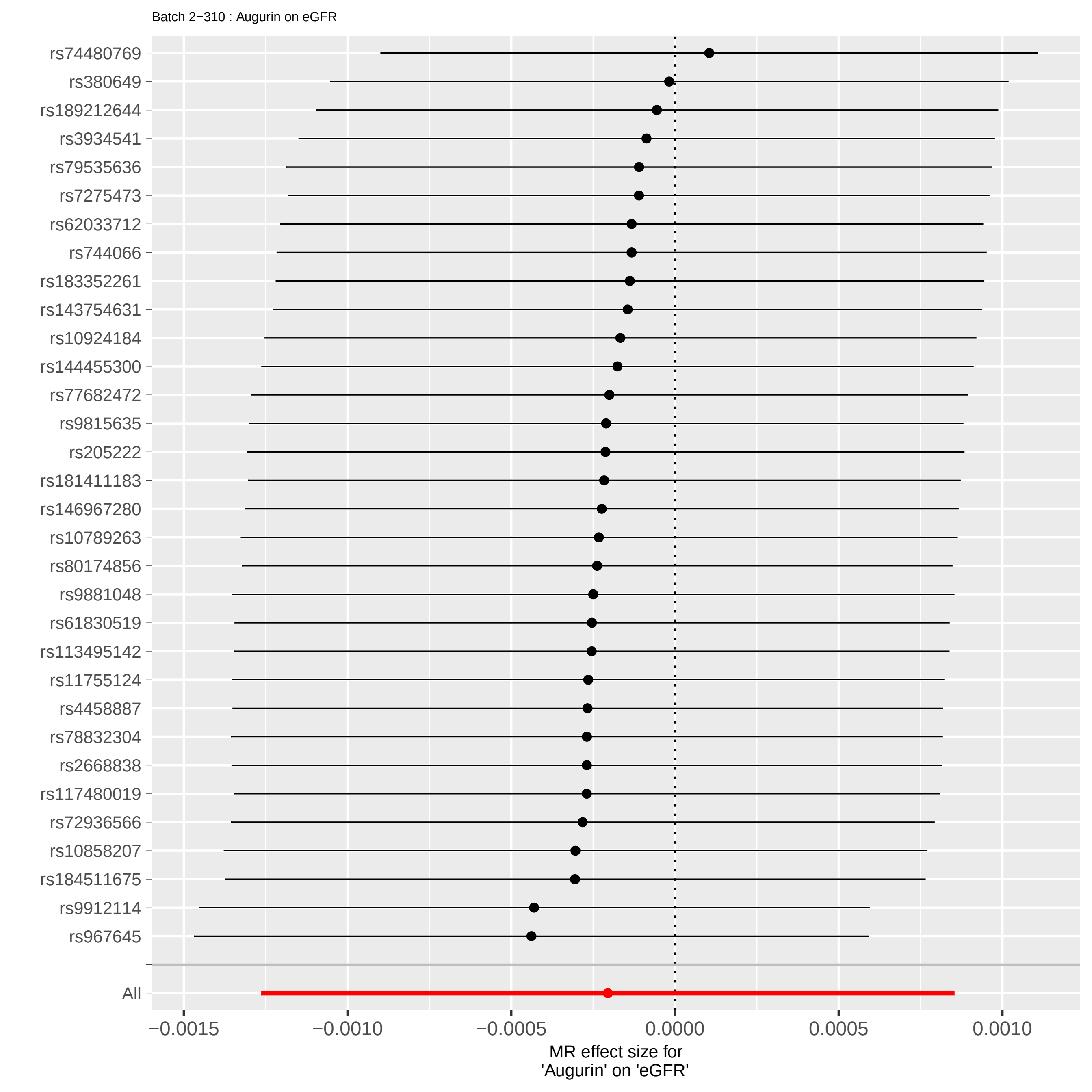
Batch 2−310 : Augurin on eGFR
rs74480769
rs380649
rs189212644
rs3934541
rs79535636
rs7275473
rs62033712
rs744066
rs183352261
rs143754631
rs10924184
rs144455300
rs77682472
rs9815635
rs205222
rs181411183
rs146967280
rs10789263
rs80174856
rs9881048
rs61830519
rs113495142
rs11755124
rs4458887
rs78832304
rs2668838
rs117480019
rs72936566
rs10858207
rs184511675
rs9912114
rs967645
All
−0.0015
−0.0010
−0.0005
0.0000
0.0005
0.0010
MR effect size for
'Augurin' on 'eGFR'

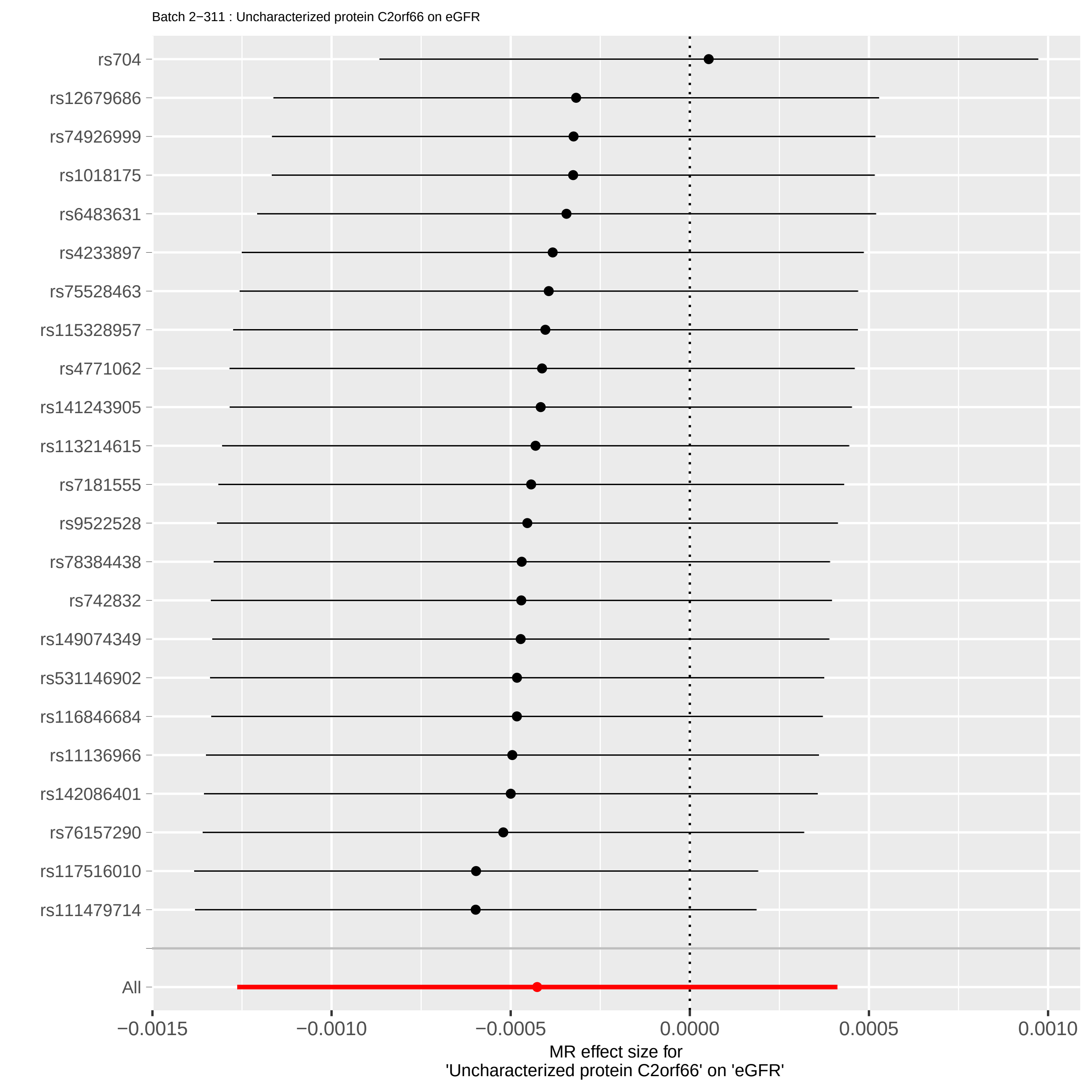
Batch 2−311 : Uncharacterized protein C2orf66 on eGFR
rs704
rs12679686
rs74926999
rs1018175
rs6483631
rs4233897
rs75528463
rs115328957
rs4771062
rs141243905
rs113214615
rs7181555
rs9522528
rs78384438
rs742832
rs149074349
rs531146902
rs116846684
rs11136966
rs142086401
rs76157290
rs117516010
rs111479714
All
−0.0015
−0.0010
−0.0005
0.0000
0.0005
0.0010
MR effect size for
'Uncharacterized protein C2orf66' on 'eGFR'

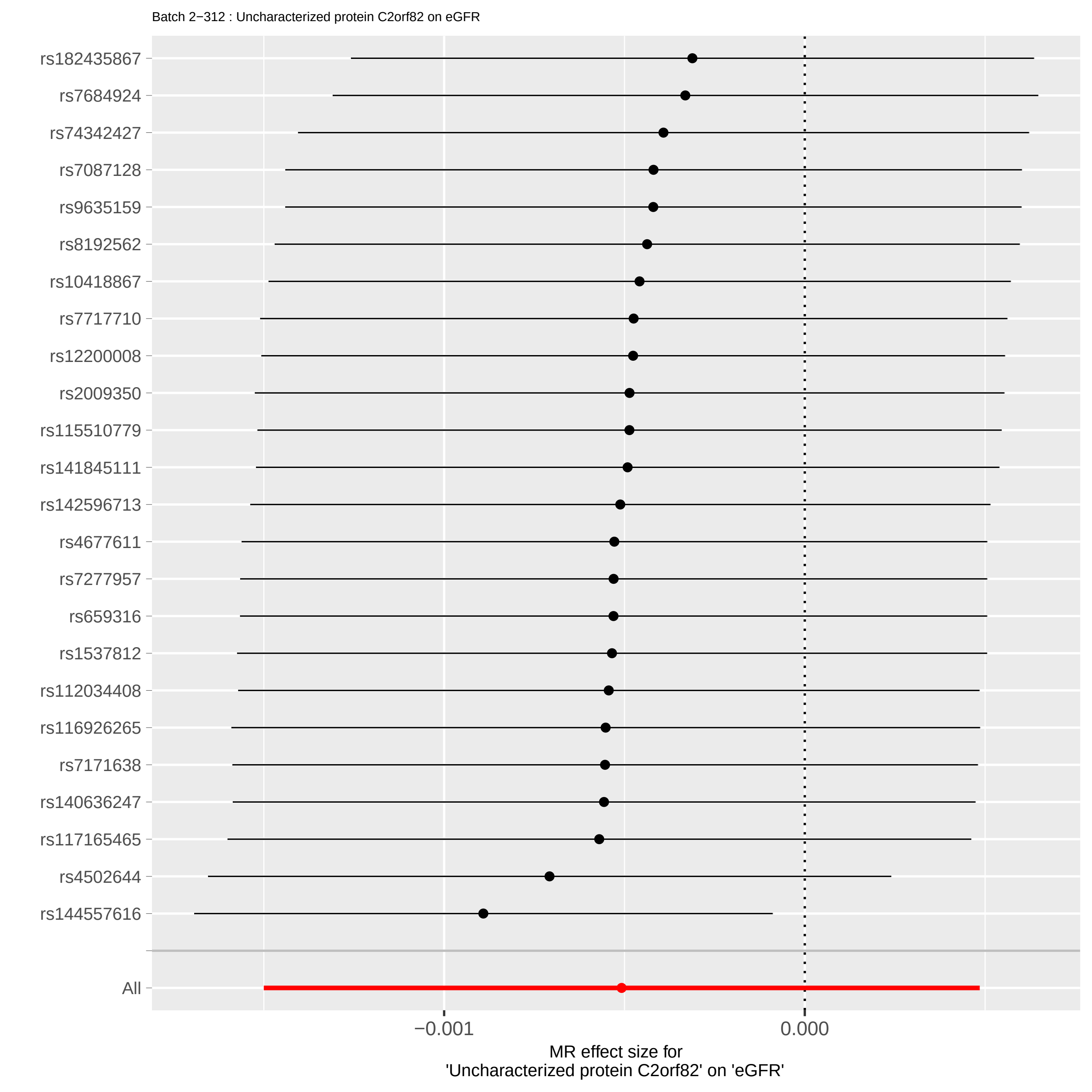

Batch 2−312 : Uncharacterized protein C2orf82 on eGFR
rs182435867
rs7684924
rs74342427
rs7087128
rs9635159
rs8192562
rs10418867
rs7717710
rs12200008
rs2009350
rs115510779
rs141845111
rs142596713
rs4677611
rs7277957
rs659316
rs1537812
rs112034408
rs116926265
rs7171638
rs140636247
rs117165465
rs4502644
rs144557616
All
−0.001
0.000
MR effect size for
'Uncharacterized protein C2orf82' on 'eGFR'

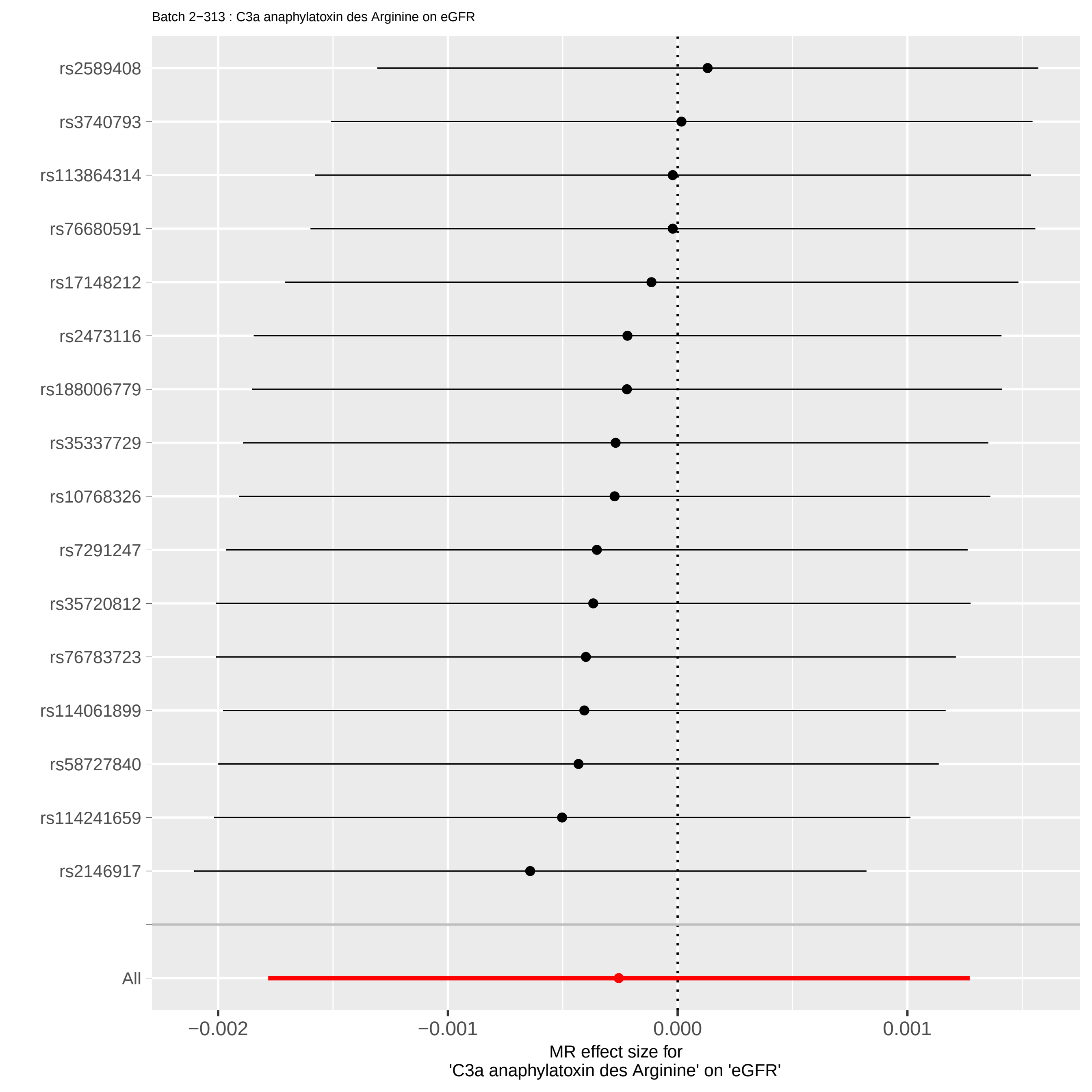
Batch 2−313 : C3a anaphylatoxin des Arginine on eGFR
rs2589408
rs3740793
rs113864314
rs76680591
rs17148212
rs2473116
rs188006779
rs35337729
rs10768326
rs7291247
rs35720812
rs76783723
rs114061899
rs58727840
rs114241659
rs2146917
All
−0.002
−0.001
0.000
0.001
MR effect size for
'C3a anaphylatoxin des Arginine' on 'eGFR'

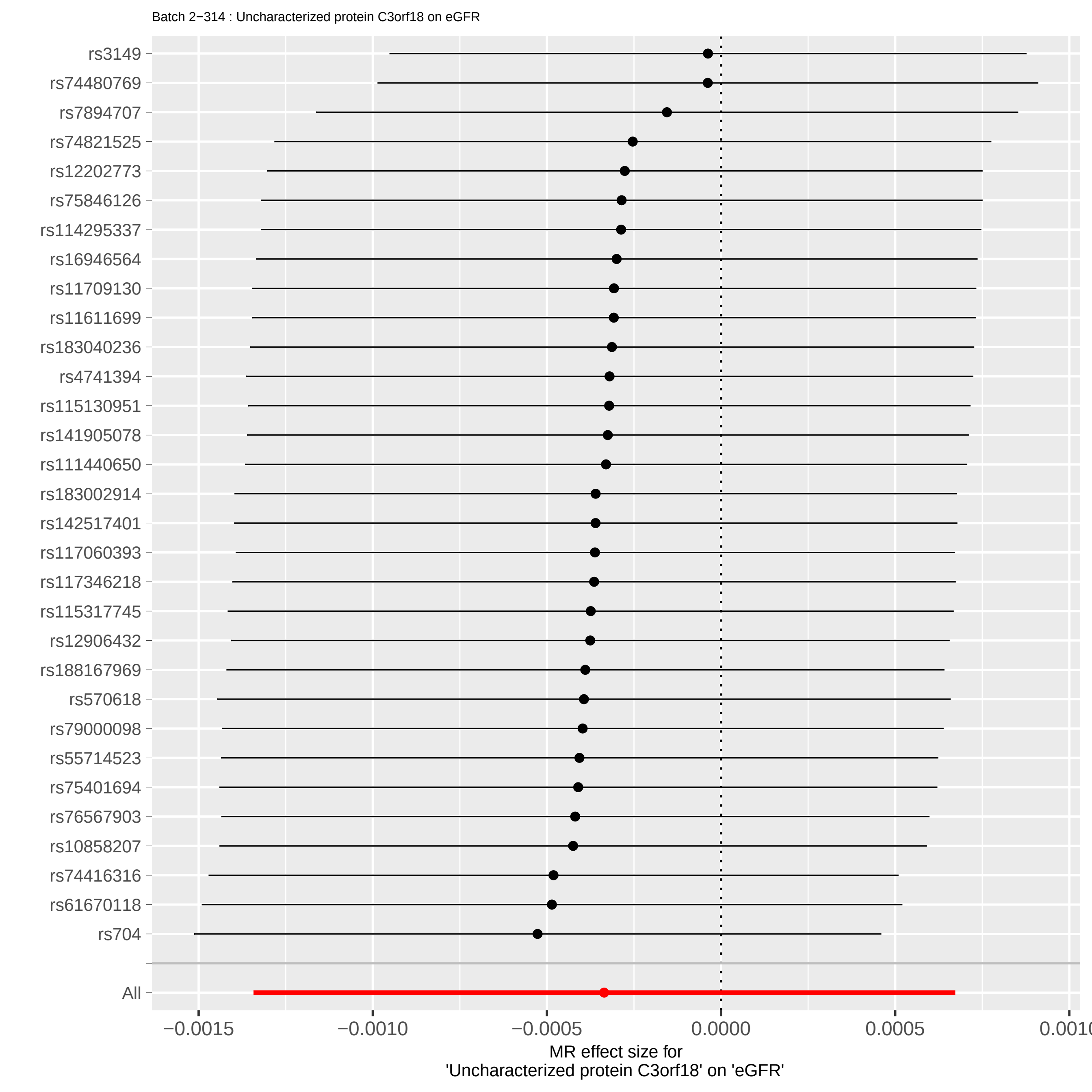

Batch 2−314 : Uncharacterized protein C3orf18 on eGFR
rs3149
rs74480769
rs7894707
rs74821525
rs12202773
rs75846126
rs114295337
rs16946564
rs11709130
rs11611699
rs183040236
rs4741394
rs115130951
rs141905078
rs111440650
rs183002914
rs142517401
rs117060393
rs117346218
rs115317745
rs12906432
rs188167969
rs570618
rs79000098
rs55714523
rs75401694
rs76567903
rs10858207
rs74416316
rs61670118
rs704
All
−0.0015
−0.0010
−0.0005
0.0000
0.0005
MR effect size for
'Uncharacterized protein C3orf18' on 'eGFR'

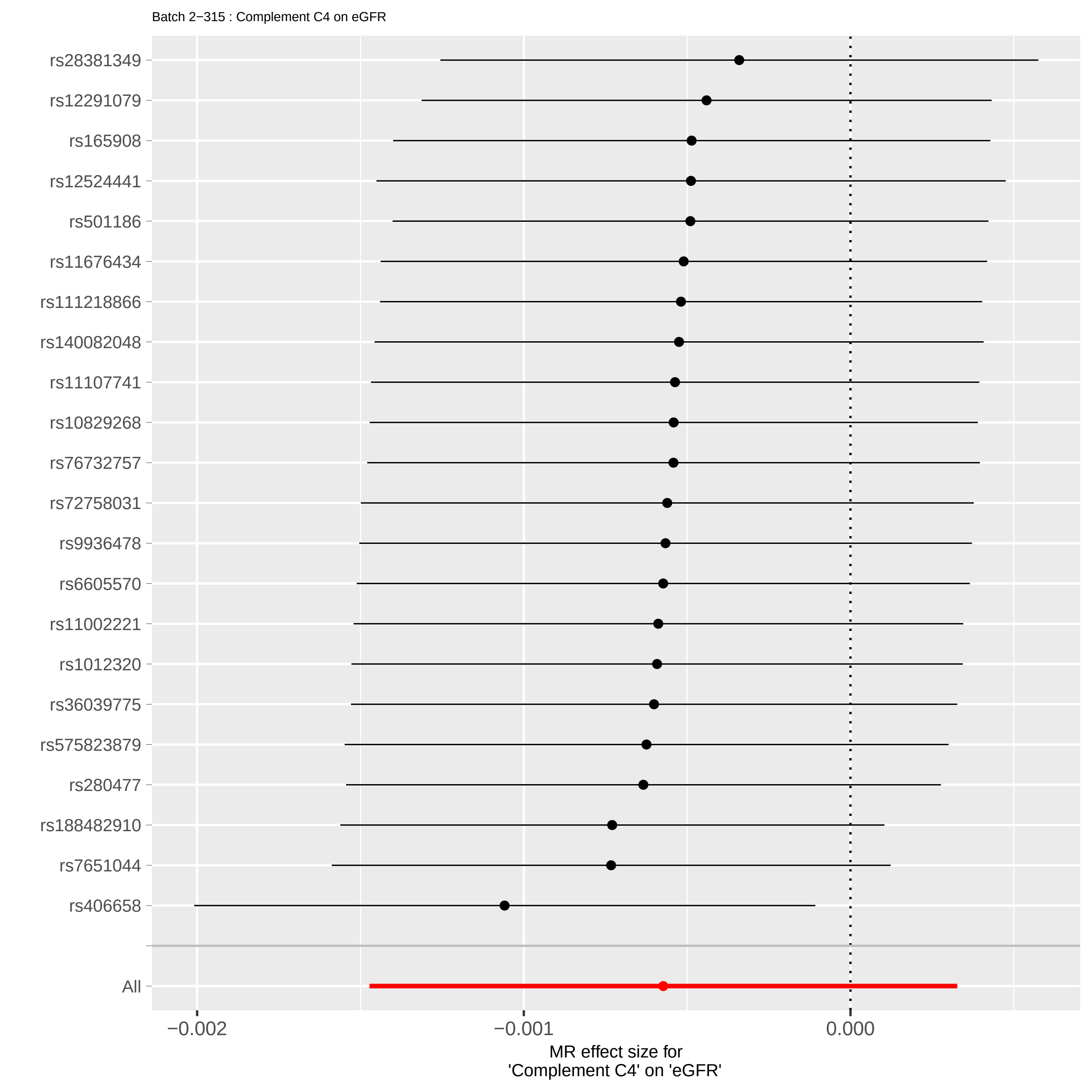

Batch 2−315 : Complement C4 on eGFR
rs28381349
rs12291079
rs165908
rs12524441
rs501186
rs11676434
rs111218866
rs140082048
rs11107741
rs10829268
rs76732757
rs72758031
rs9936478
rs6605570
rs11002221
rs1012320
rs36039775
rs575823879
rs280477
rs188482910
rs7651044
rs406658
All
−0.002
−0.001
0.000
MR effect size for
'Complement C4' on 'eGFR'

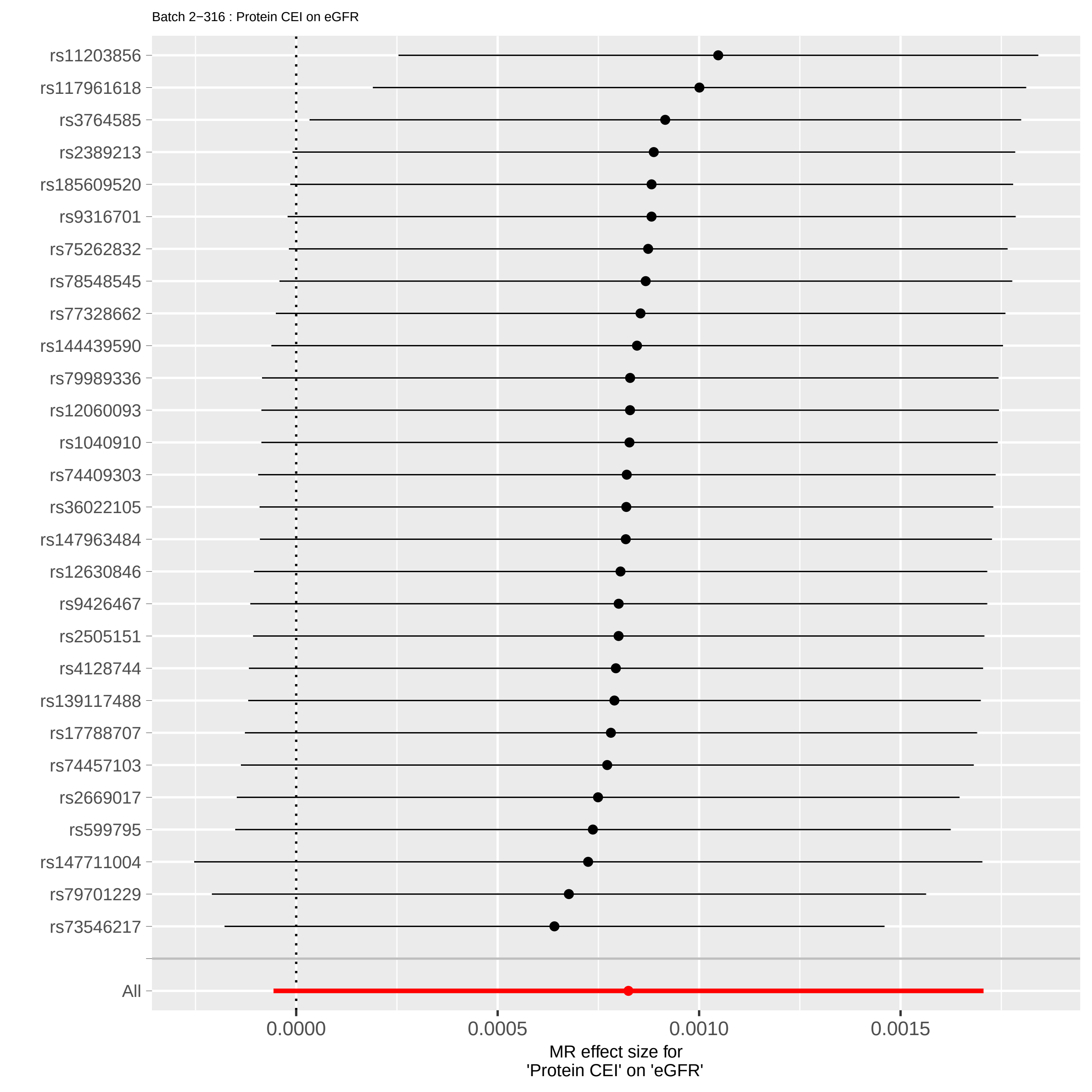
Batch 2−316 : Protein CEI on eGFR
rs11203856
rs117961618
rs3764585
rs2389213
rs185609520
rs9316701
rs75262832
rs78548545
rs77328662
rs144439590
rs79989336
rs12060093
rs1040910
rs74409303
rs36022105
rs147963484
rs12630846
rs9426467
rs2505151
rs4128744
rs139117488
rs17788707
rs74457103
rs2669017
rs599795
rs147711004
rs79701229
rs73546217
All
0.0000
0.0005
0.0010
0.0015
MR effect size for
'Protein CEI' on 'eGFR'

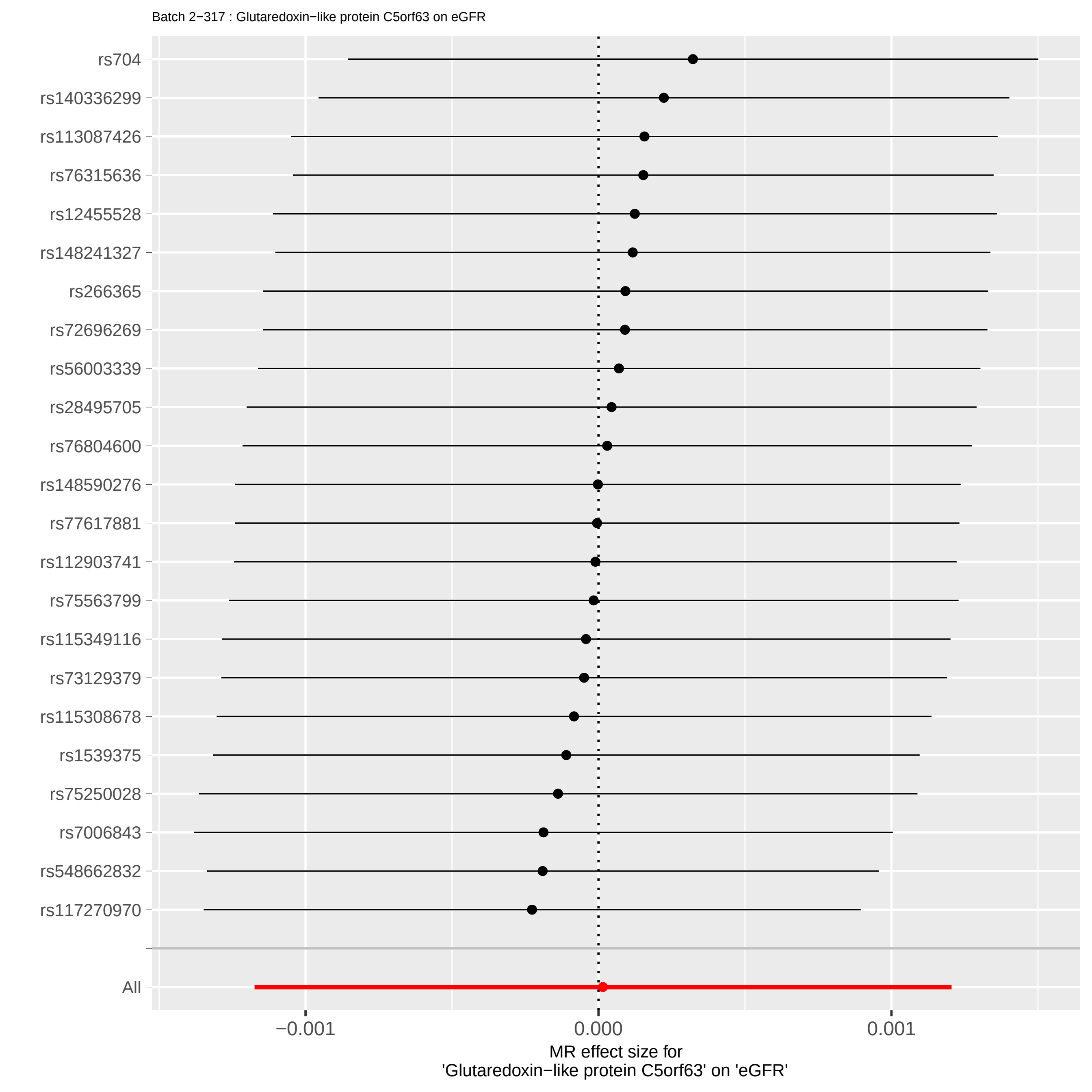
Batch 2−317 : Glutaredoxin−like protein C5orf63 on eGFR
rs704
rs140336299
rs113087426
rs76315636
rs12455528
rs148241327
rs266365
rs72696269
rs56003339
rs28495705
rs76804600
rs148590276
rs77617881
rs112903741
rs75563799
rs115349116
rs73129379
rs115308678
rs1539375
rs75250028
rs7006843
rs548662832
rs117270970
All
−0.001
0.000
0.001
MR effect size for
'Glutaredoxin−like protein C5orf63' on 'eGFR'

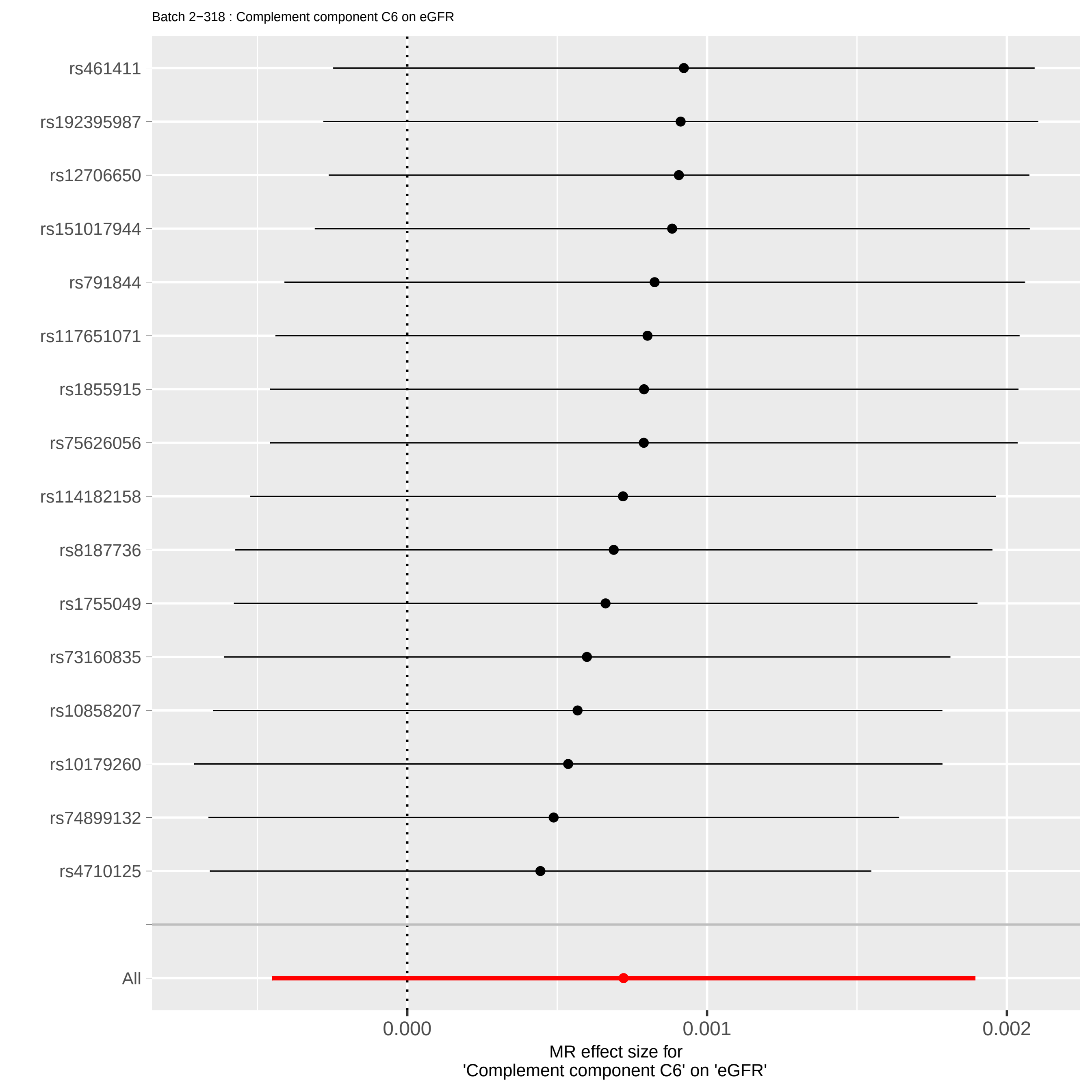
Batch 2−318 : Complement component C6 on eGFR
rs461411
rs192395987
rs12706650
rs151017944
rs791844
rs117651071
rs1855915
rs75626056
rs114182158
rs8187736
rs1755049
rs73160835
rs10858207
rs10179260
rs74899132
rs4710125
All
0.000
0.001
0.002
MR effect size for
'Complement component C6' on 'eGFR'

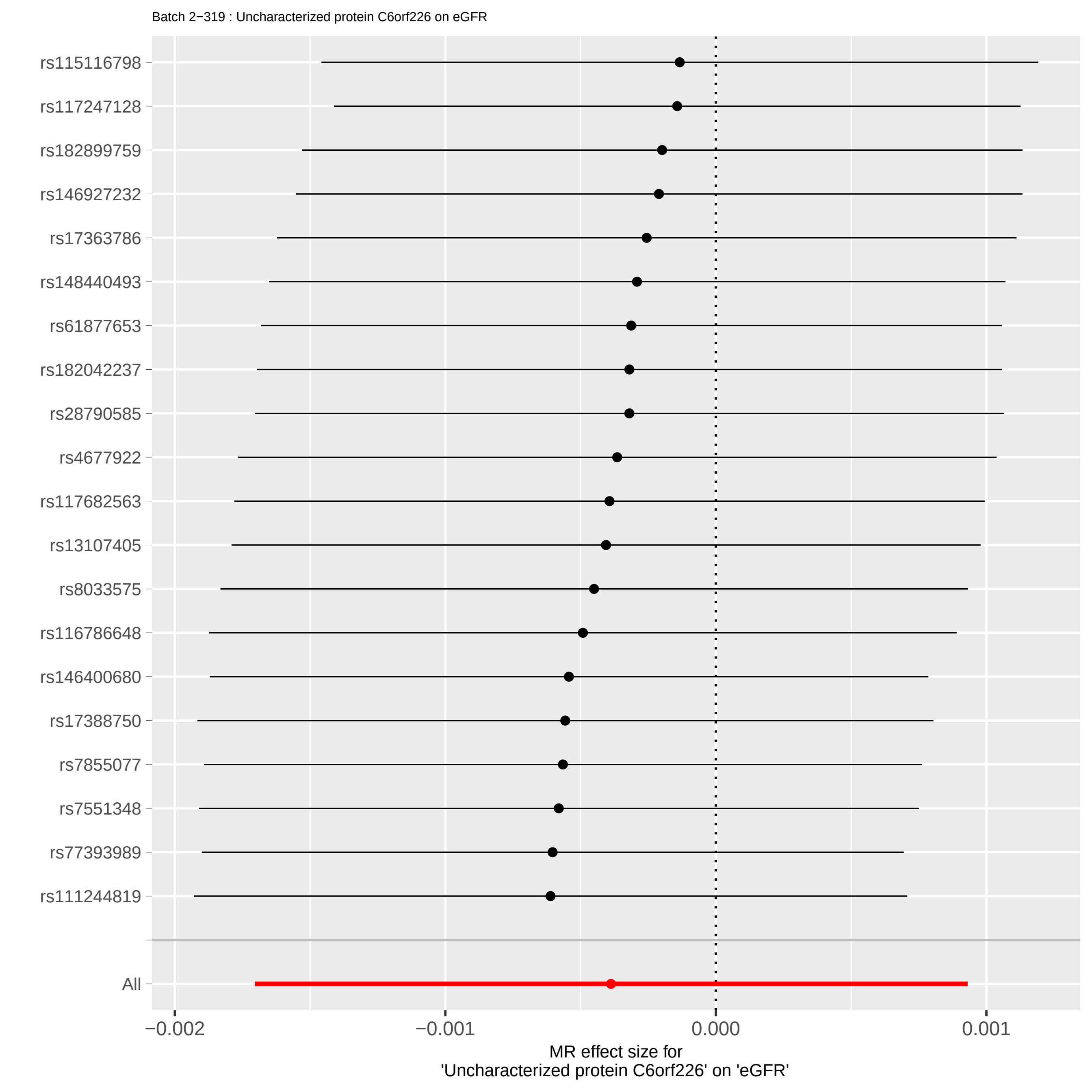

Batch 2−319 : Uncharacterized protein C6orf226 on eGFR
rs115116798
rs117247128
rs182899759
rs146927232
rs17363786
rs148440493
rs61877653
rs182042237
rs28790585
rs4677922
rs117682563
rs13107405
rs8033575
rs116786648
rs146400680
rs17388750
rs7855077
rs7551348
rs77393989
rs111244819
All
−0.002
−0.001
0.000
0.001
MR effect size for
'Uncharacterized protein C6orf226' on 'eGFR'

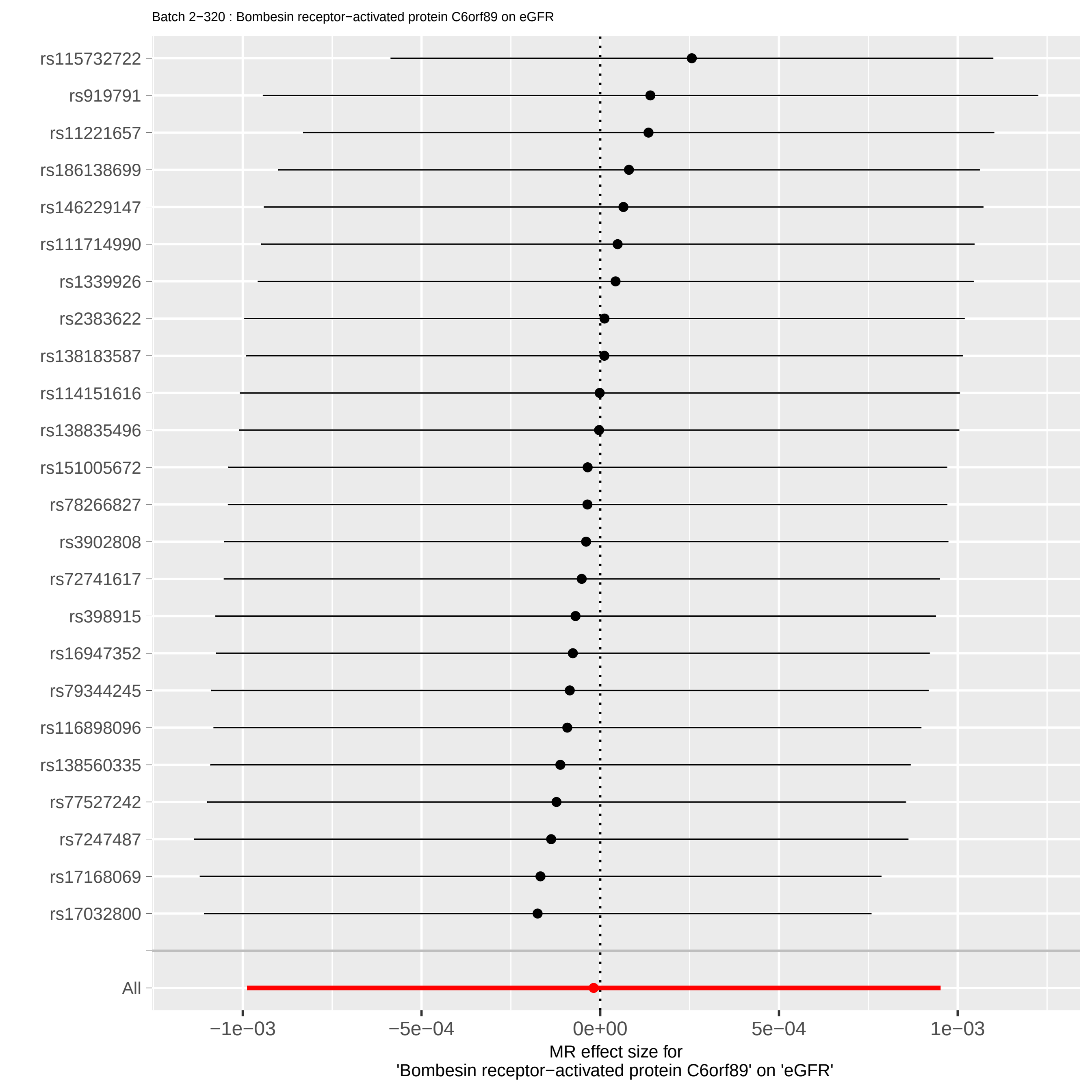

Batch 2−320 : Bombesin receptor−activated protein C6orf89 on eGFR
rs115732722
rs919791
rs11221657
rs186138699
rs146229147
rs111714990
rs1339926
rs2383622
rs138183587
rs114151616
rs138835496
rs151005672
rs78266827
rs3902808
rs72741617
rs398915
rs16947352
rs79344245
rs116898096
rs138560335
rs77527242
rs7247487
rs17168069
rs17032800
All
−1e−03
−5e−04
0e+00
5e−04
1e−03
MR effect size for
'Bombesin receptor−activated protein C6orf89' on 'eGFR'

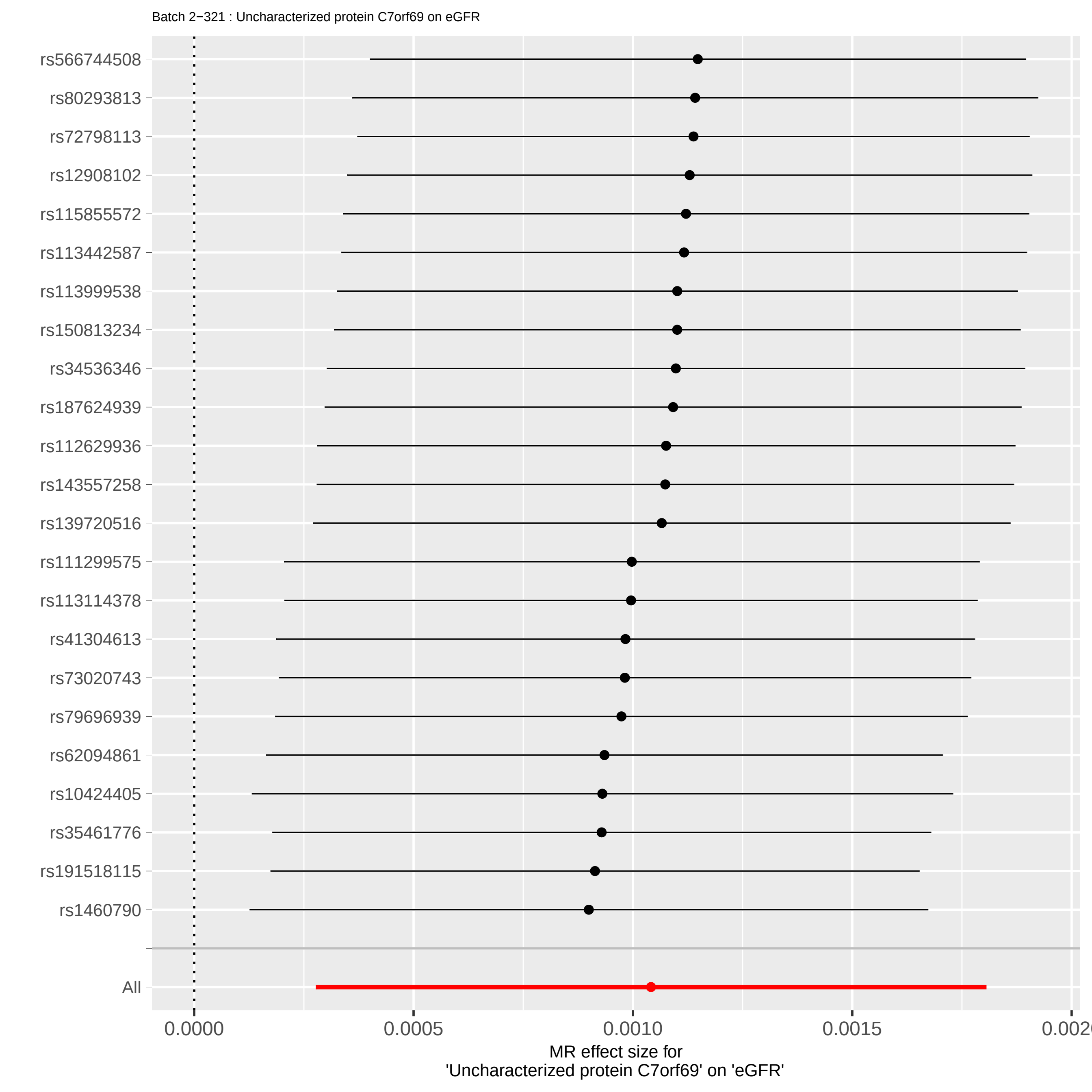

Batch 2−321 : Uncharacterized protein C7orf69 on eGFR
rs566744508
rs80293813
rs72798113
rs12908102
rs115855572
rs113442587
rs113999538
rs150813234
rs34536346
rs187624939
rs112629936
rs143557258
rs139720516
rs111299575
rs113114378
rs41304613
rs73020743
rs79696939
rs62094861
rs10424405
rs35461776
rs191518115
rs1460790
All
0.0000
0.0005
0.0010
0.0015
MR effect size for
'Uncharacterized protein C7orf69' on 'eGFR'

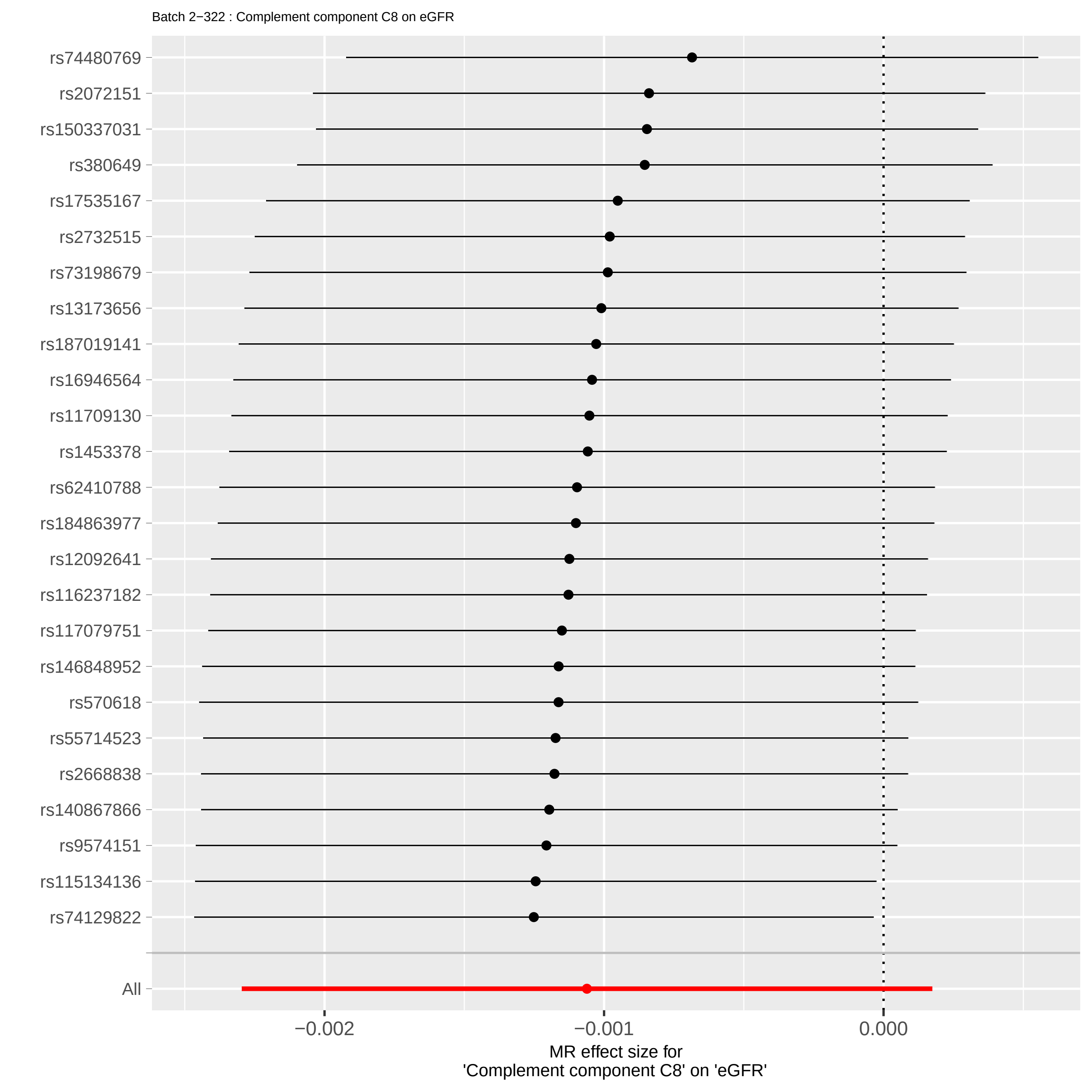

Batch 2−322 : Complement component C8 on eGFR
rs74480769
rs2072151
rs150337031
rs380649
rs17535167
rs2732515
rs73198679
rs13173656
rs187019141
rs16946564
rs11709130
rs1453378
rs62410788
rs184863977
rs12092641
rs116237182
rs117079751
rs146848952
rs570618
rs55714523
rs2668838
rs140867866
rs9574151
rs115134136
rs74129822
All
−0.002
−0.001
0.000
MR effect size for
'Complement component C8' on 'eGFR'

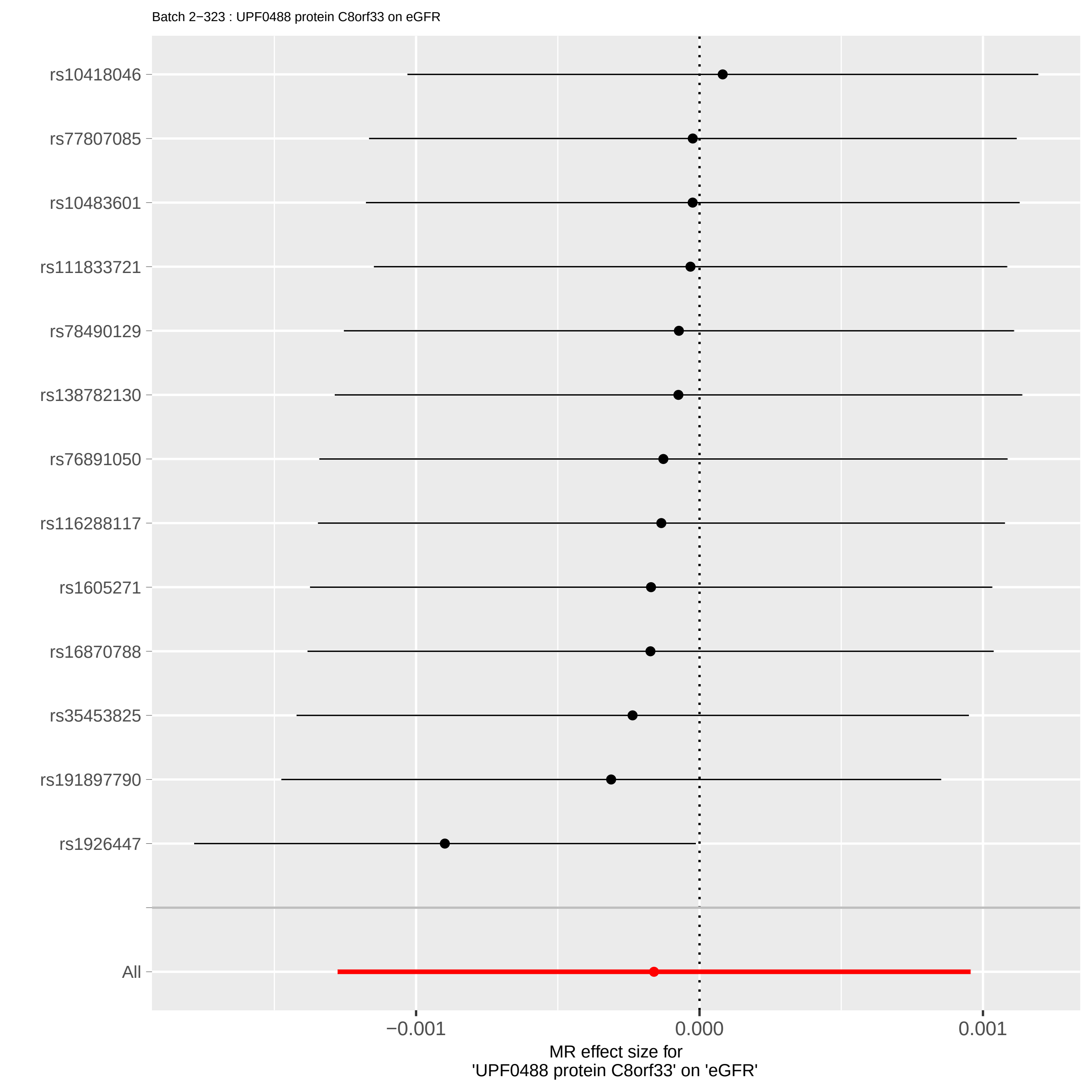

Batch 2−323 : UPF0488 protein C8orf33 on eGFR
rs10418046
rs77807085
rs10483601
rs111833721
rs78490129
rs138782130
rs76891050
rs116288117
rs1605271
rs16870788
rs35453825
rs191897790
rs1926447
All
−0.001
0.000
0.001
MR effect size for
'UPF0488 protein C8orf33' on 'eGFR'

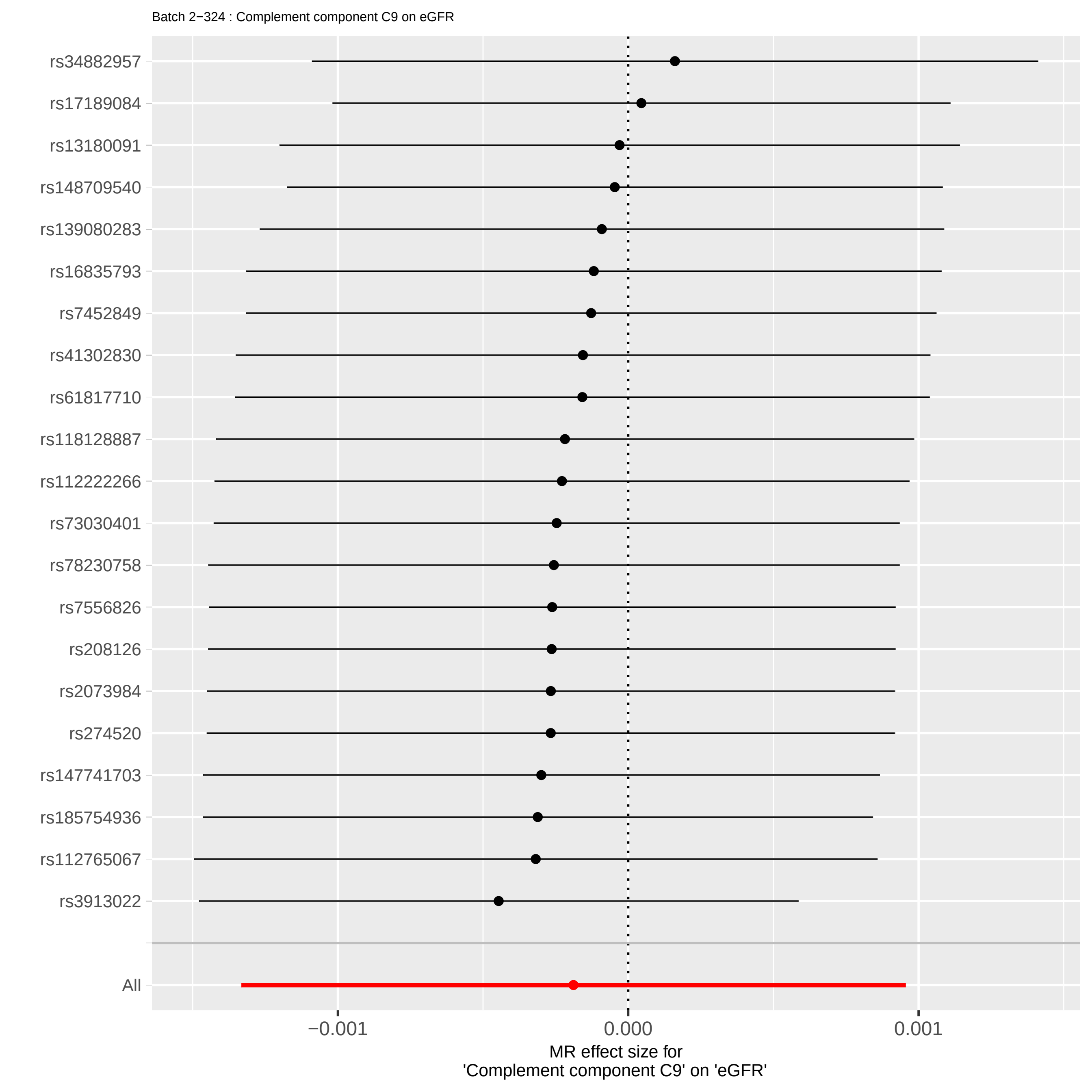

Batch 2−324 : Complement component C9 on eGFR
rs34882957
rs17189084
rs13180091
rs148709540
rs139080283
rs16835793
rs7452849
rs41302830
rs61817710
rs118128887
rs112222266
rs73030401
rs78230758
rs7556826
rs208126
rs2073984
rs274520
rs147741703
rs185754936
rs112765067
rs3913022
All
−0.001
0.000
0.001
MR effect size for
'Complement component C9' on 'eGFR'

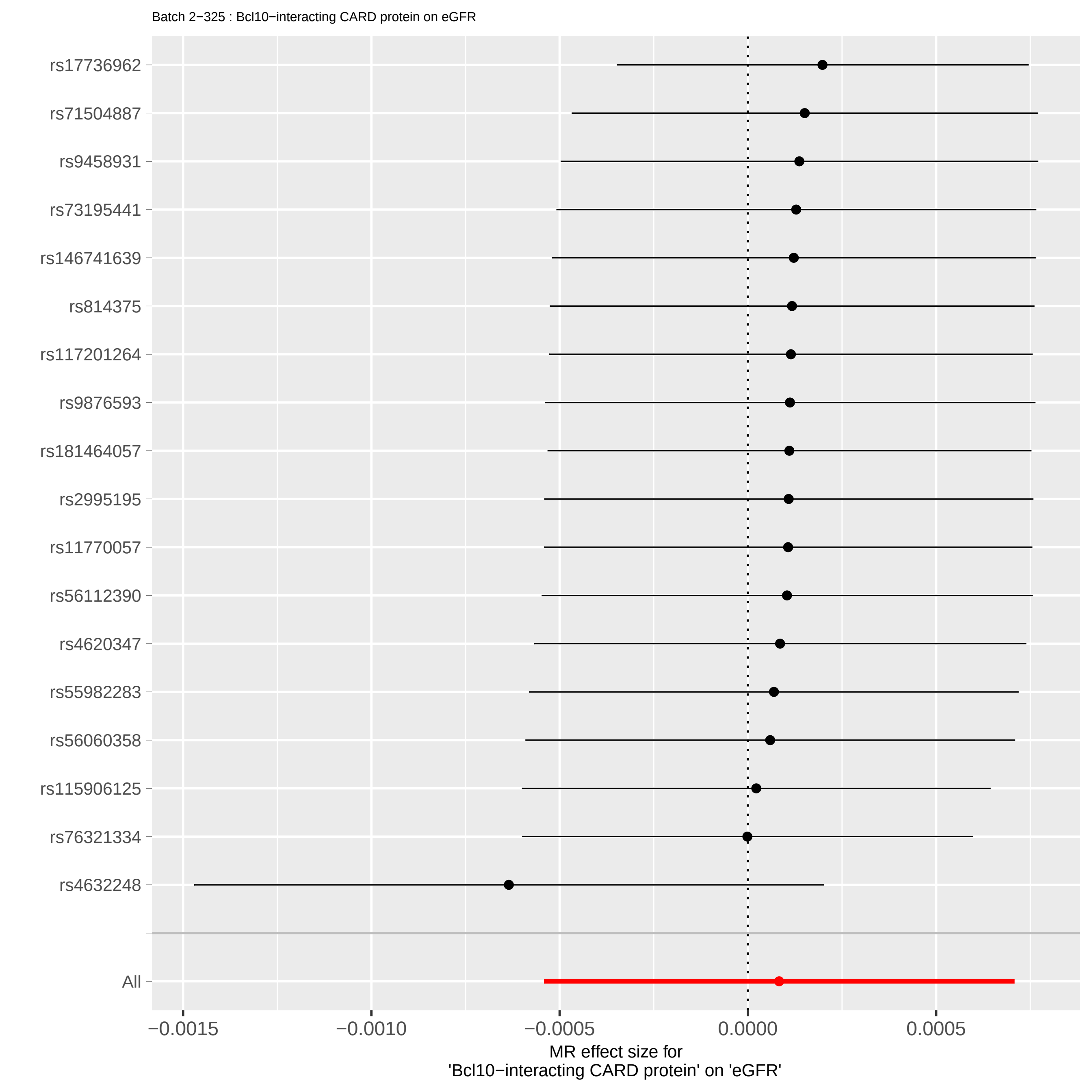
Batch 2−325 : Bcl10−interacting CARD protein on eGFR
rs17736962
rs71504887
rs9458931
rs73195441
rs146741639
rs814375
rs117201264
rs9876593
rs181464057
rs2995195
rs11770057
rs56112390
rs4620347
rs55982283
rs56060358
rs115906125
rs76321334
rs4632248
All
−0.0015
−0.0010
−0.0005
0.0000
0.0005
MR effect size for
'Bcl10−interacting CARD protein' on 'eGFR'

Batch 2−326 : Carbonic anhydrase−related protein 10 on eGFR

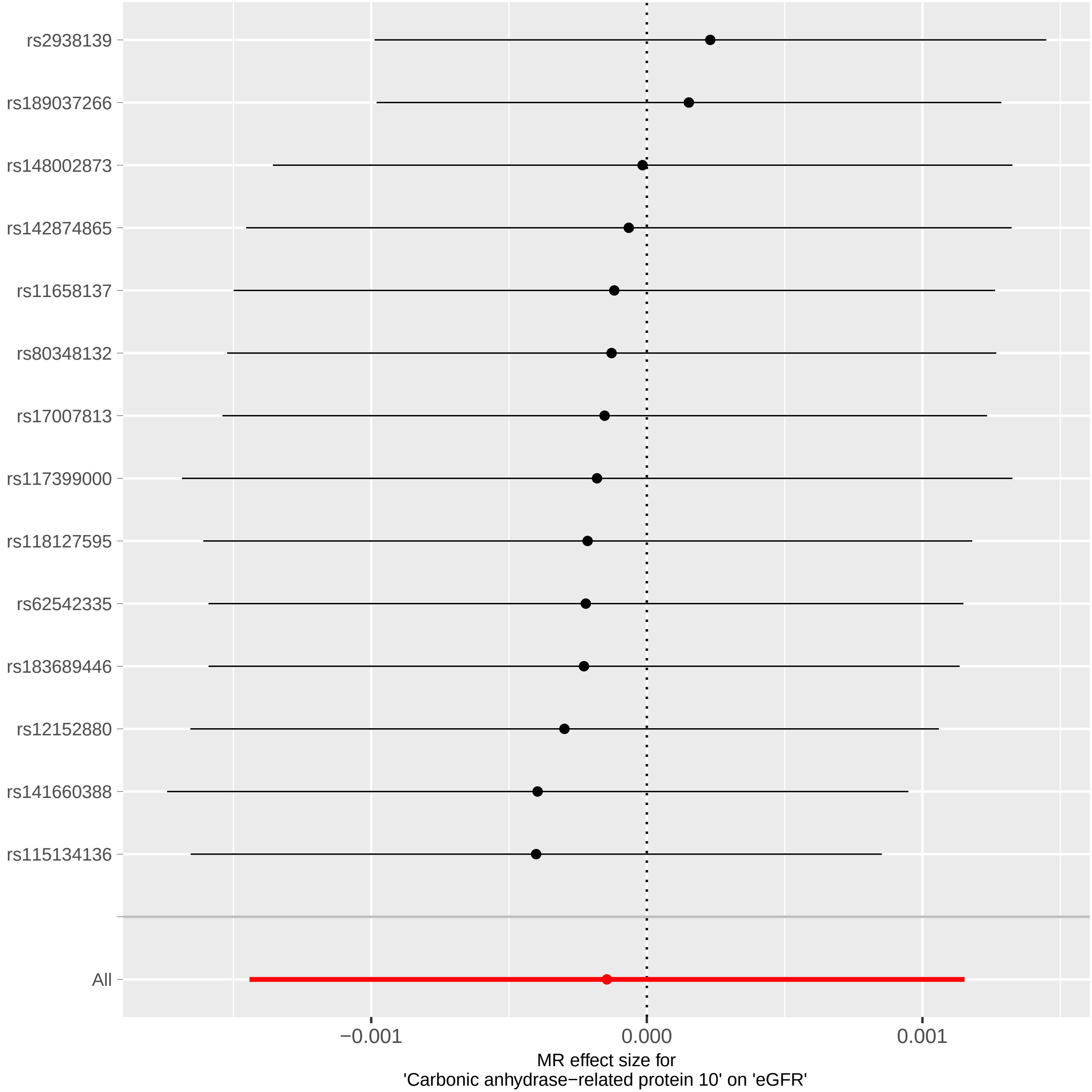

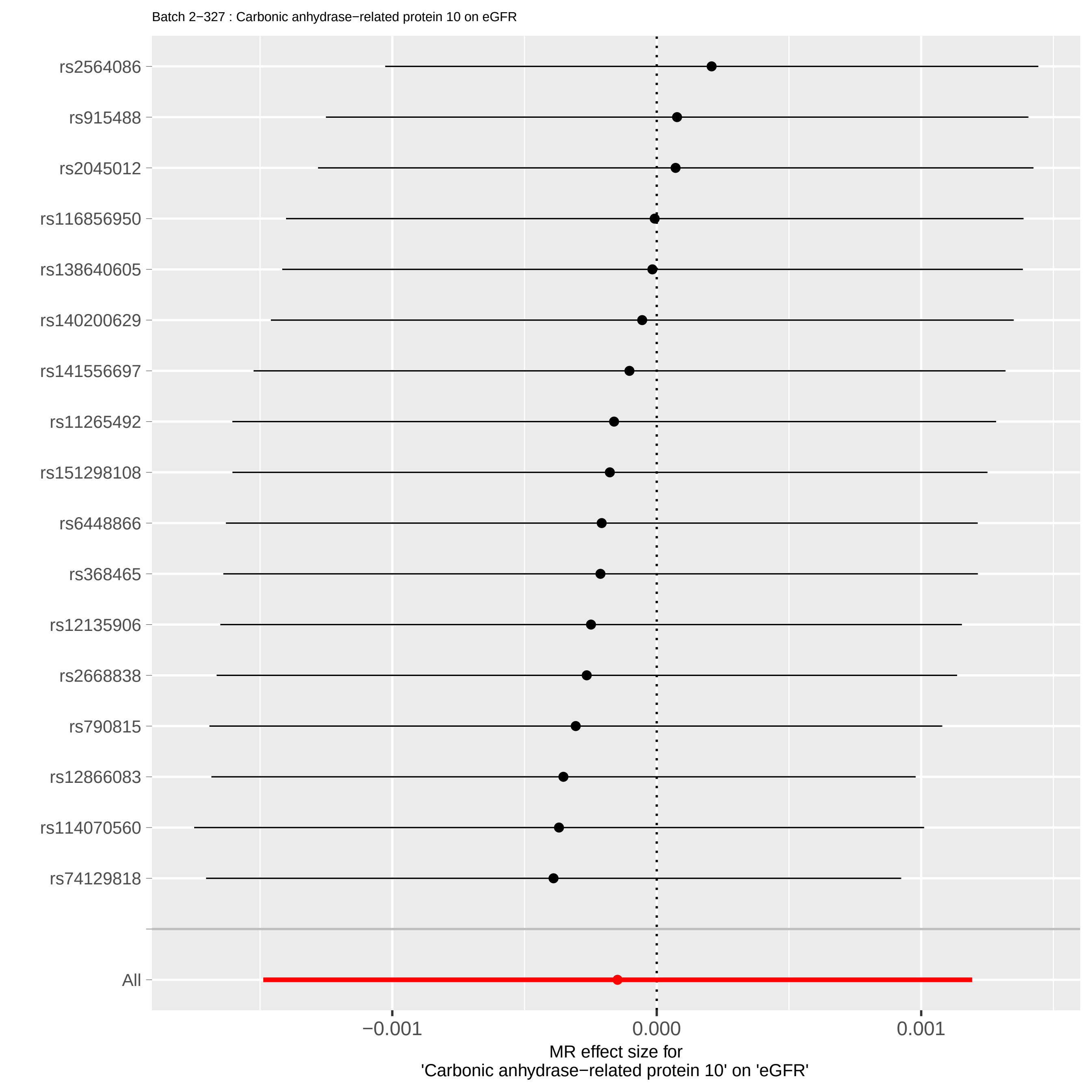

Batch 2−327 : Carbonic anhydrase−related protein 10 on eGFR
rs2564086
rs915488
rs2045012
rs116856950
rs138640605
rs140200629
rs141556697
rs11265492
rs151298108
rs6448866
rs368465
rs12135906
rs2668838
rs790815
rs12866083
rs114070560
rs74129818
All
−0.001
0.000
0.001
MR effect size for
'Carbonic anhydrase−related protein 10' on 'eGFR'

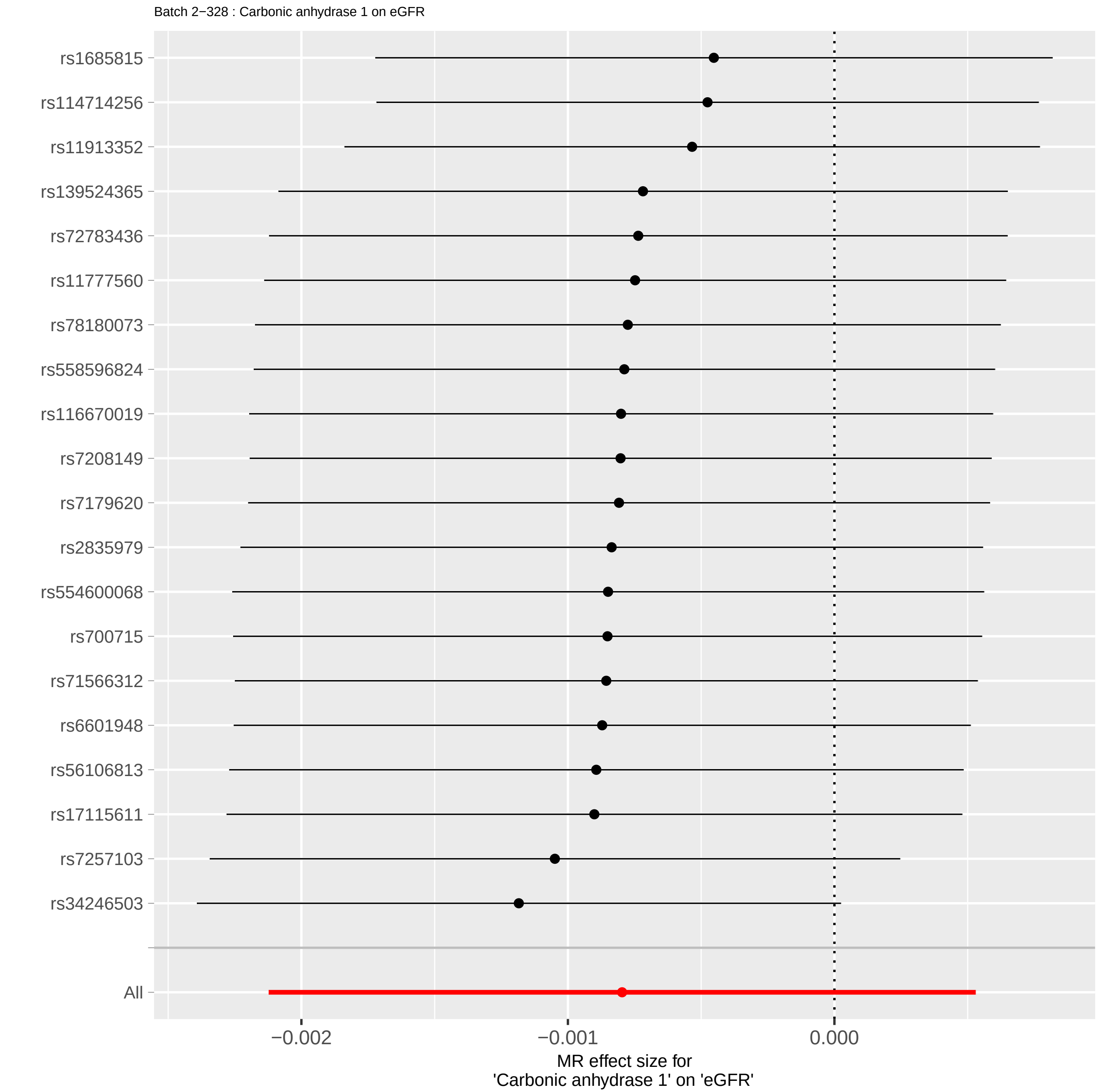
Batch 2−328 : Carbonic anhydrase 1 on eGFR
rs1685815
rs114714256
rs11913352
rs139524365
rs72783436
rs11777560
rs78180073
rs558596824
rs116670019
rs7208149
rs7179620
rs2835979
rs554600068
rs700715
rs71566312
rs6601948
rs56106813
rs17115611
rs7257103
rs34246503
All
−0.002
−0.001
0.000
MR effect size for
'Carbonic anhydrase 1' on 'eGFR'

Batch 2−329 : Carbonic anhydrase 2 on eGFR

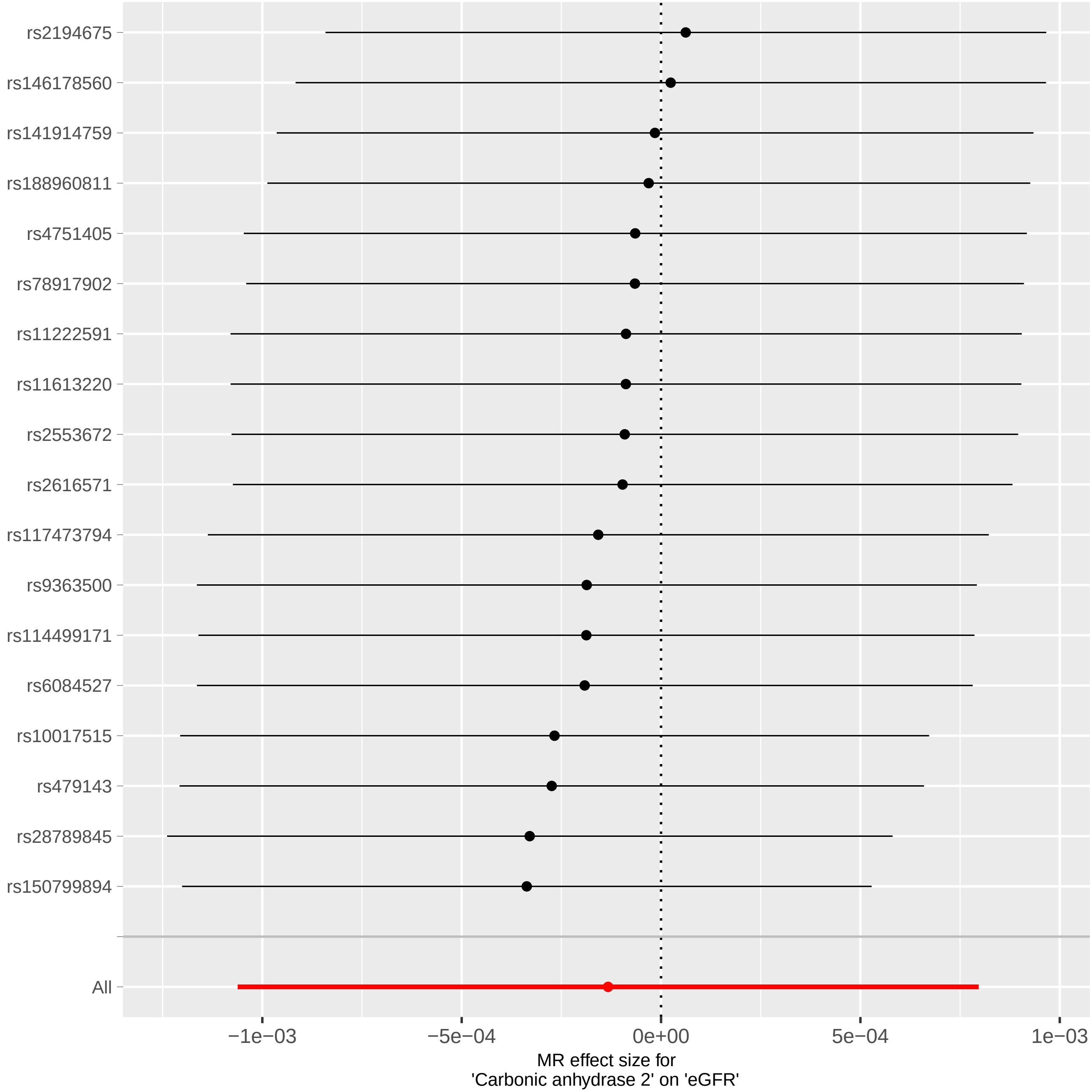

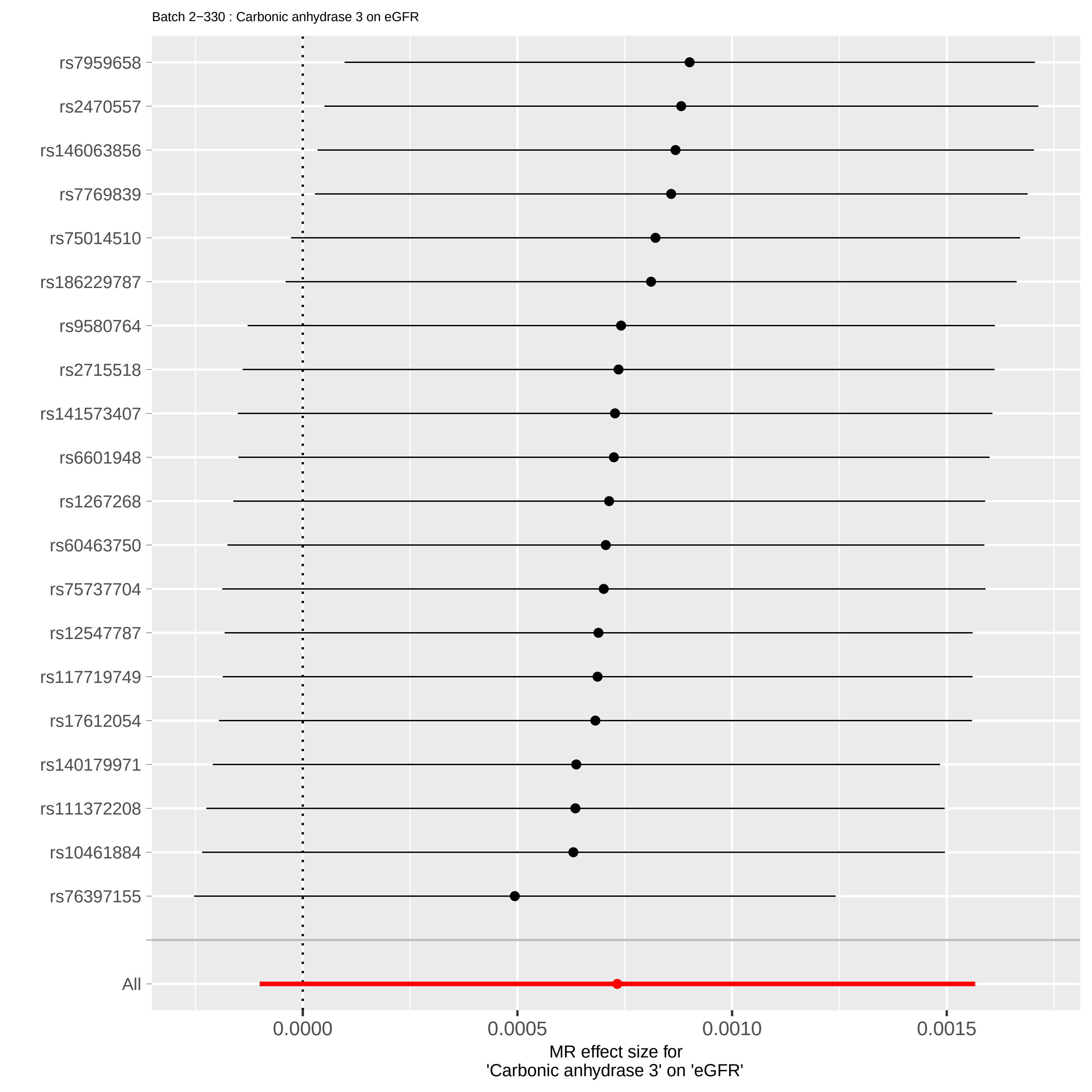

Batch 2−330 : Carbonic anhydrase 3 on eGFR
rs7959658
rs2470557
rs146063856
rs7769839
rs75014510
rs186229787
rs9580764
rs2715518
rs141573407
rs6601948
rs1267268
rs60463750
rs75737704
rs12547787
rs117719749
rs17612054
rs140179971
rs111372208
rs10461884
rs76397155
All
0.0000
0.0005
0.0010
0.0015
MR effect size for
'Carbonic anhydrase 3' on 'eGFR'

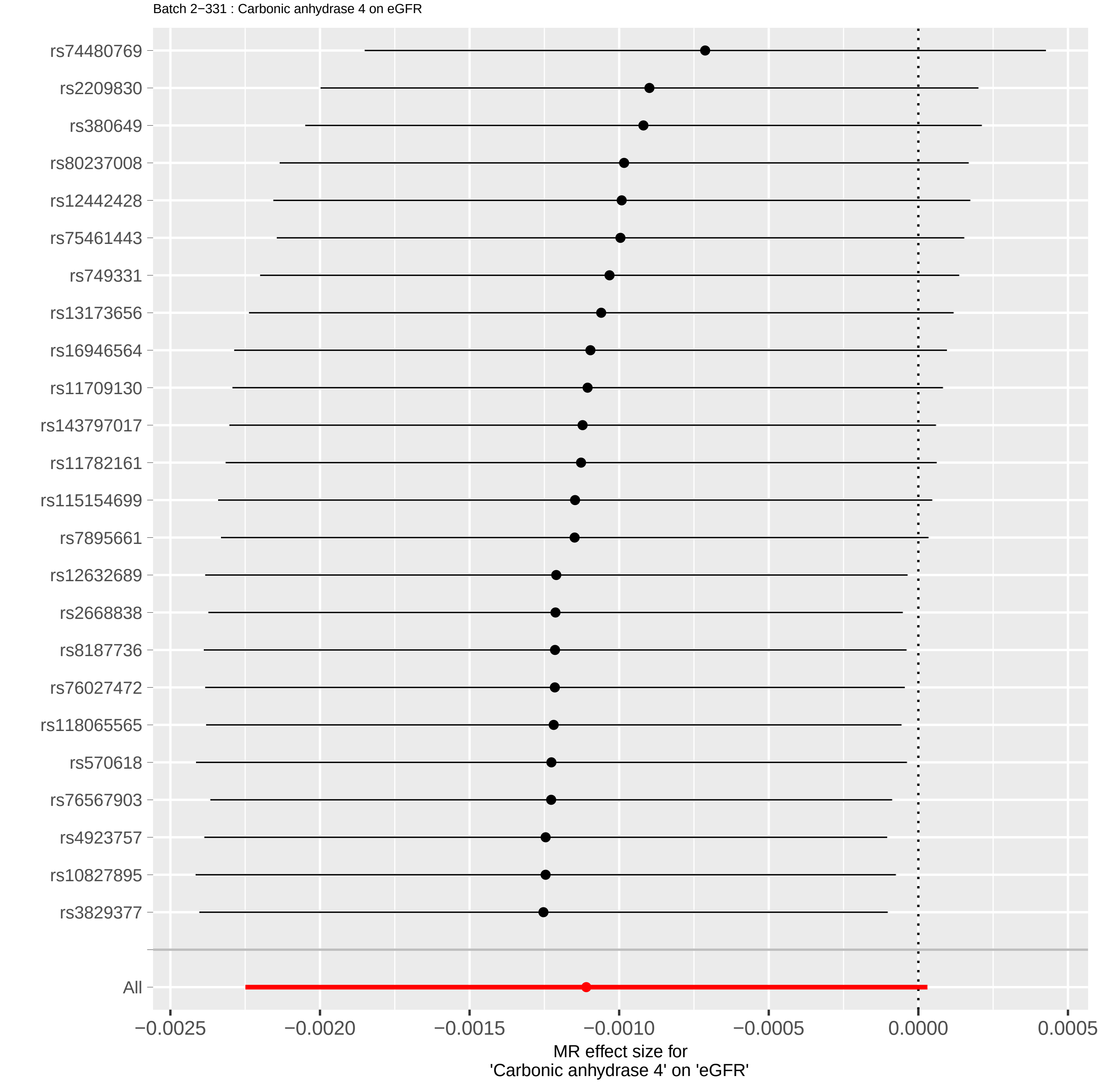

Batch 2−331 : Carbonic anhydrase 4 on eGFR
rs74480769
rs2209830
rs380649
rs80237008
rs12442428
rs75461443
rs749331
rs13173656
rs16946564
rs11709130
rs143797017
rs11782161
rs115154699
rs7895661
rs12632689
rs2668838
rs8187736
rs76027472
rs118065565
rs570618
rs76567903
rs4923757
rs10827895
rs3829377
All
−0.0025
−0.0020
−0.0015
−0.0010
−0.0005
0.0000
0.0005
MR effect size for
'Carbonic anhydrase 4' on 'eGFR'

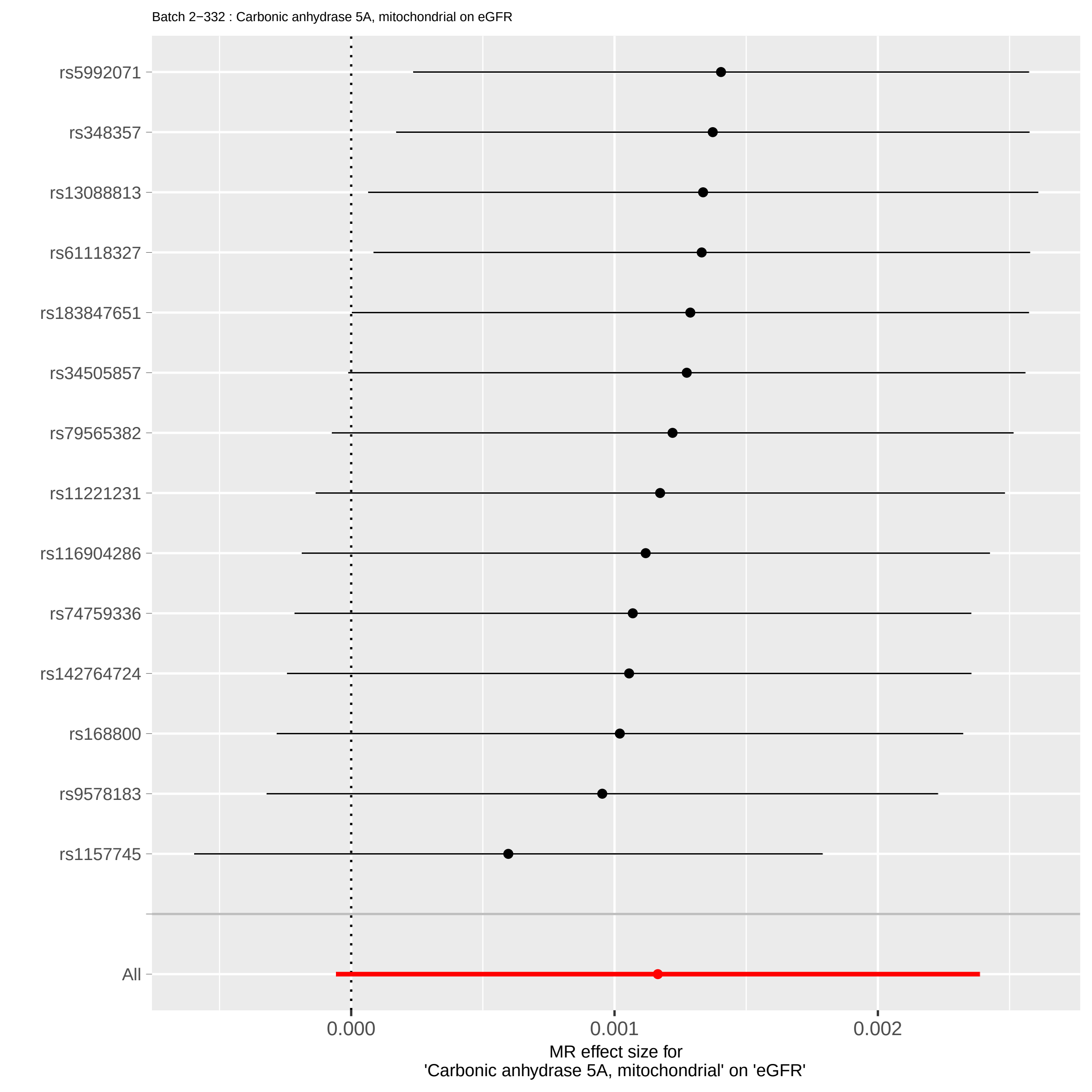

Batch 2−332 : Carbonic anhydrase 5A, mitochondrial on eGFR
rs5992071
rs348357
rs13088813
rs61118327
rs183847651
rs34505857
rs79565382
rs11221231
rs116904286
rs74759336
rs142764724
rs168800
rs9578183
rs1157745
All
0.000
0.001
0.002
MR effect size for
'Carbonic anhydrase 5A, mitochondrial' on 'eGFR'

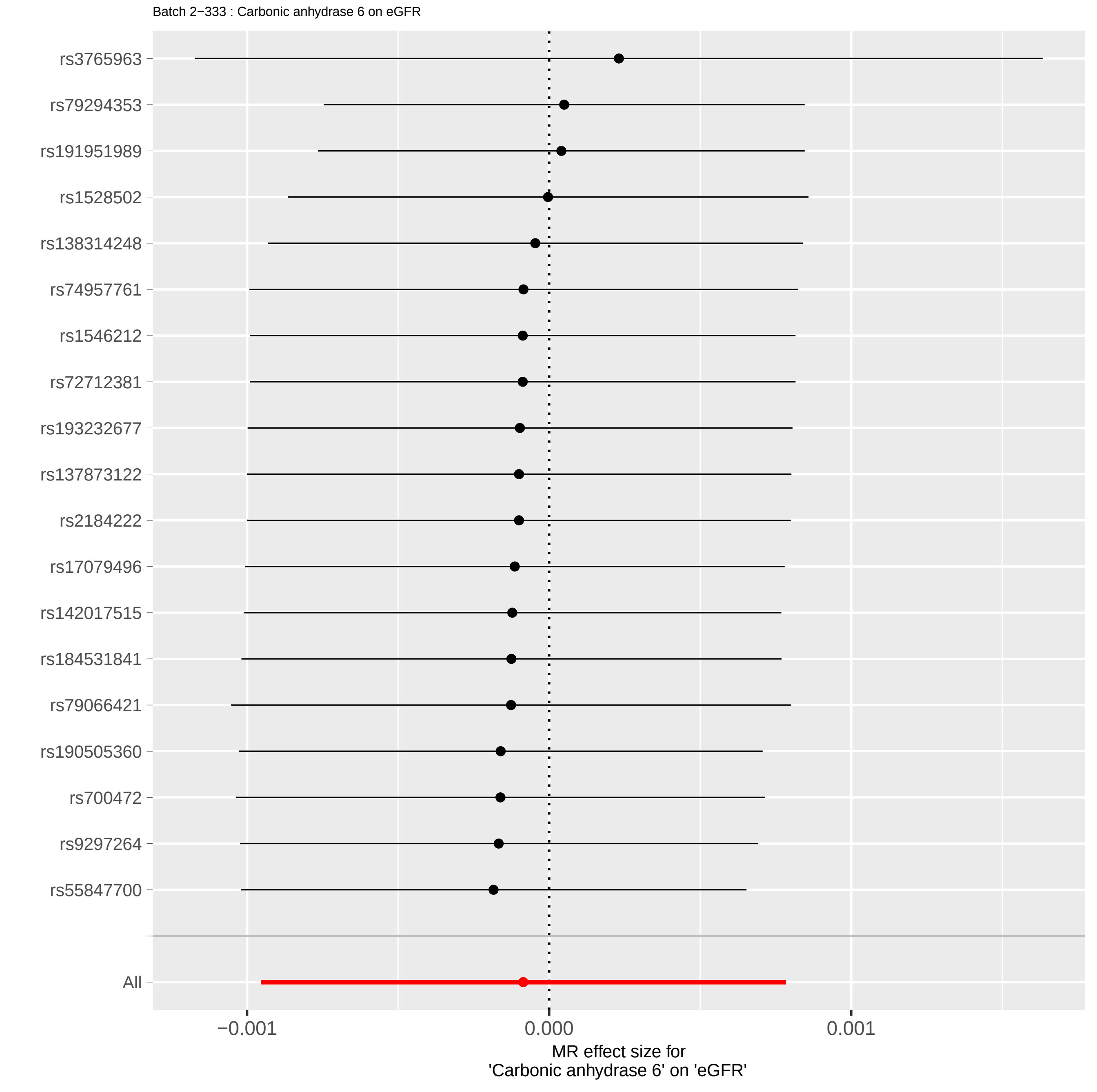

Batch 2−333 : Carbonic anhydrase 6 on eGFR
rs3765963
rs79294353
rs191951989
rs1528502
rs138314248
rs74957761
rs1546212
rs72712381
rs193232677
rs137873122
rs2184222
rs17079496
rs142017515
rs184531841
rs79066421
rs190505360
rs700472
rs9297264
rs55847700
All
−0.001
0.000
0.001
MR effect size for
'Carbonic anhydrase 6' on 'eGFR'

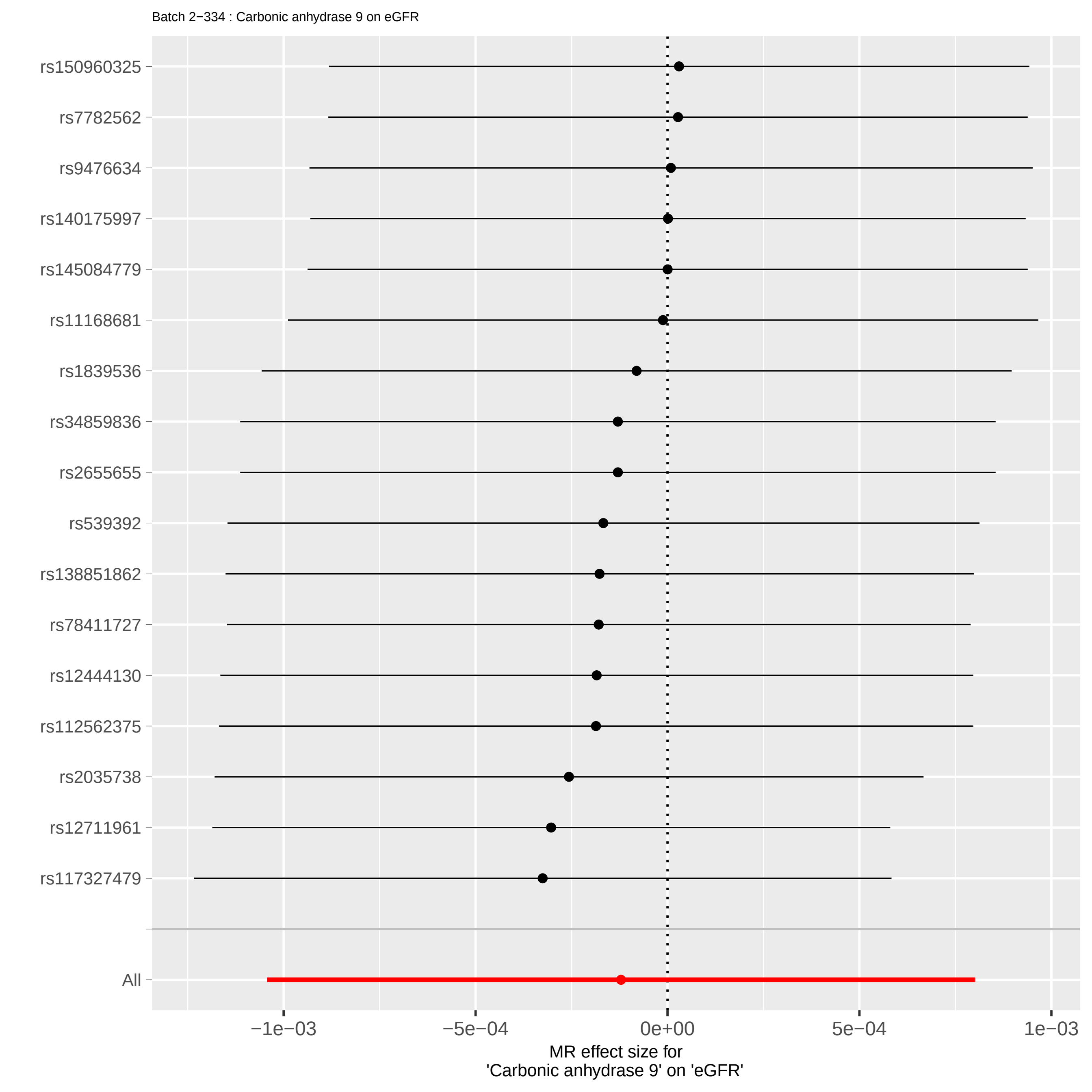

Batch 2−334 : Carbonic anhydrase 9 on eGFR
rs150960325
rs7782562
rs9476634
rs140175997
rs145084779
rs11168681
rs1839536
rs34859836
rs2655655
rs539392
rs138851862
rs78411727
rs12444130
rs112562375
rs2035738
rs12711961
rs117327479
All
−1e−03
−5e−04
0e+00
5e−04
1e−03
MR effect size for
'Carbonic anhydrase 9' on 'eGFR'

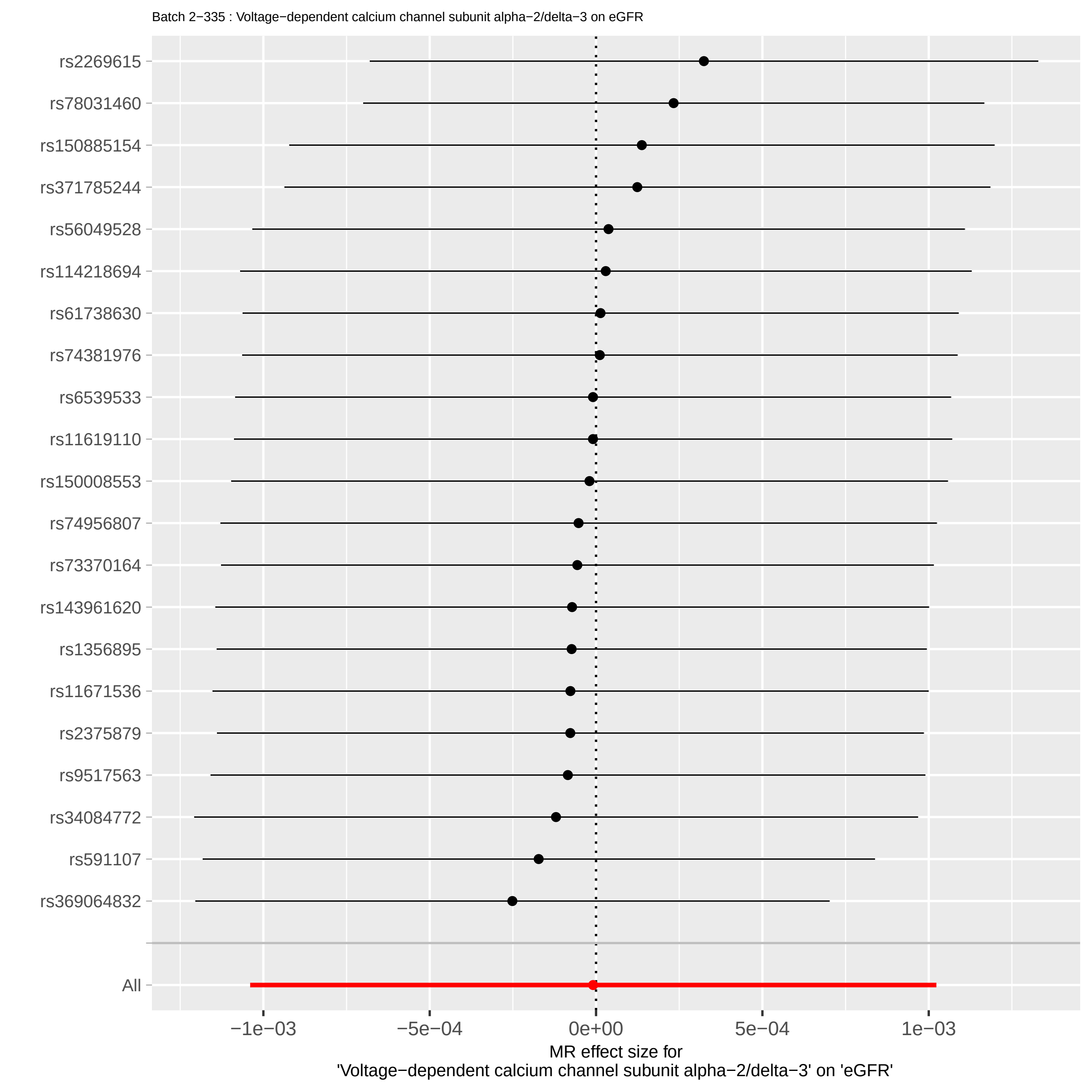

Batch 2−335 : Voltage−dependent calcium channel subunit alpha−2/delta−3 on eGFR
rs2269615
rs78031460
rs150885154
rs371785244
rs56049528
rs114218694
rs61738630
rs74381976
rs6539533
rs11619110
rs150008553
rs74956807
rs73370164
rs143961620
rs1356895
rs11671536
rs2375879
rs9517563
rs34084772
rs591107
rs369064832
All
−1e−03
−5e−04
0e+00
5e−04
1e−03
MR effect size for
'Voltage−dependent calcium channel subunit alpha−2/delta−3' on 'eGFR'

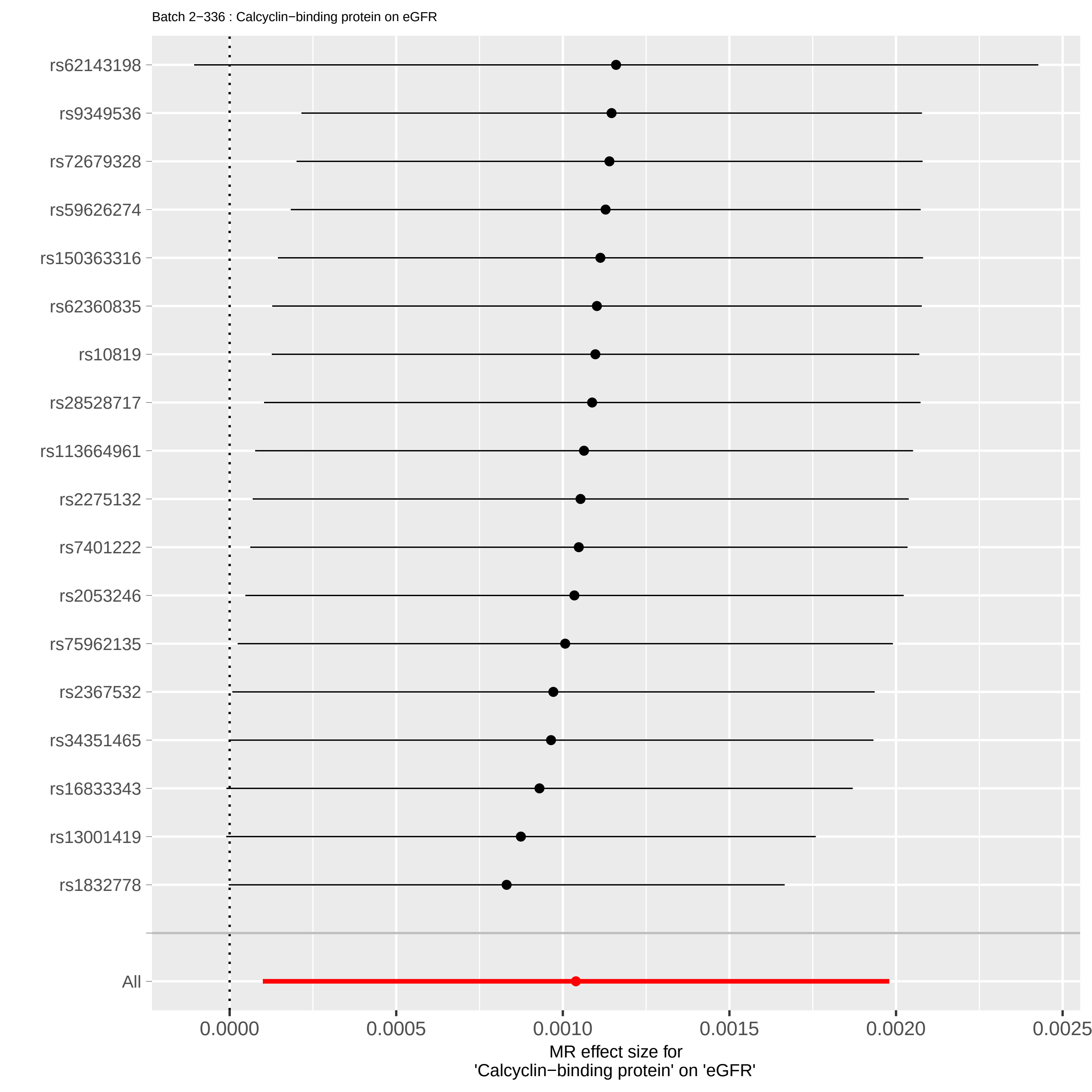

Batch 2−336 : Calcyclin−binding protein on eGFR
rs62143198
rs9349536
rs72679328
rs59626274
rs150363316
rs62360835
rs10819
rs28528717
rs113664961
rs2275132
rs7401222
rs2053246
rs75962135
rs2367532
rs34351465
rs16833343
rs13001419
rs1832778
All
0.0000
0.0005
0.0010
0.0015
0.0020
0.0025
MR effect size for
'Calcyclin−binding protein' on 'eGFR'

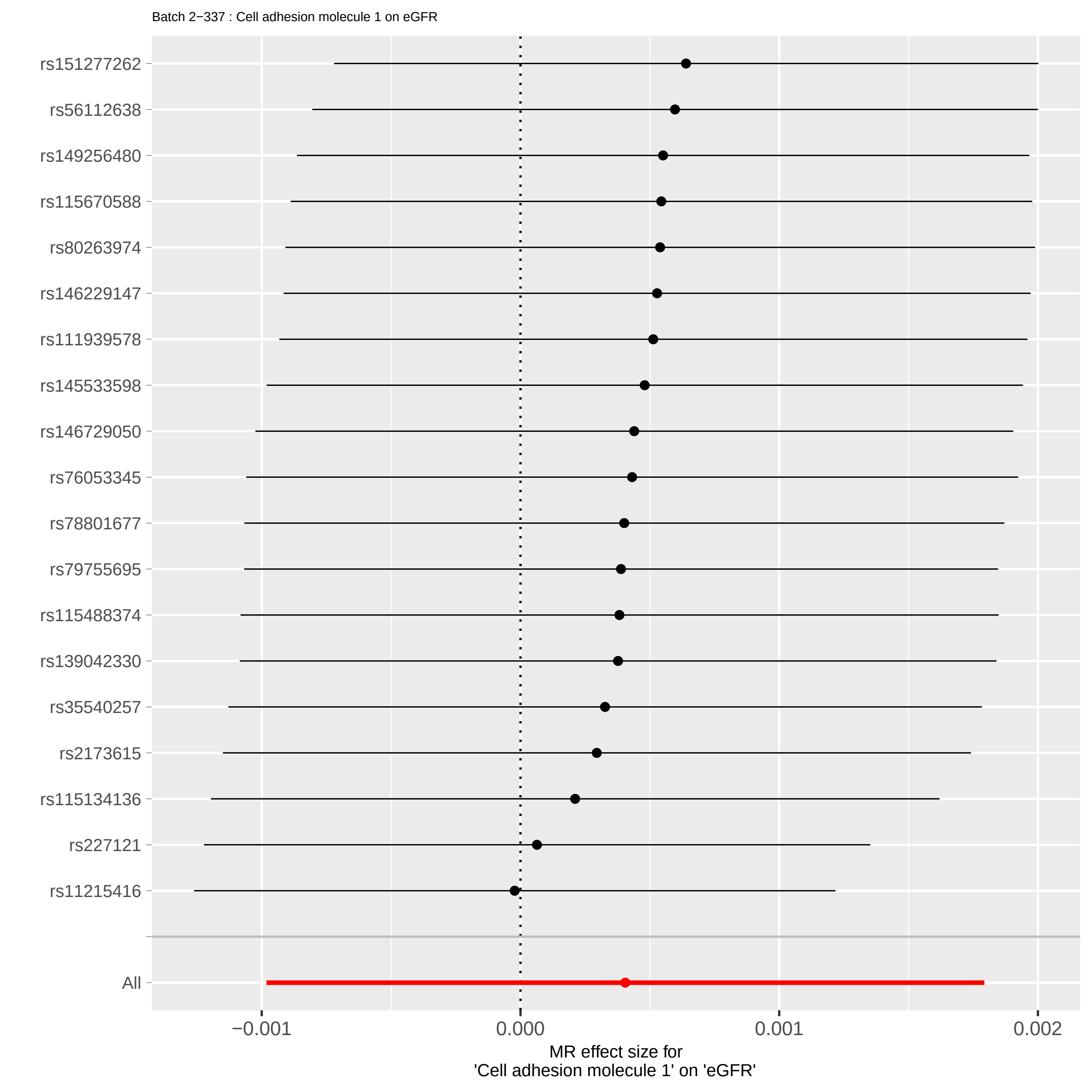

Batch 2−337 : Cell adhesion molecule 1 on eGFR
rs151277262
rs56112638
rs149256480
rs115670588
rs80263974
rs146229147
rs111939578
rs145533598
rs146729050
rs76053345
rs78801677
rs79755695
rs115488374
rs139042330
rs35540257
rs2173615
rs115134136
rs227121
rs11215416
All
−0.001
0.000
0.001
0.002
MR effect size for
'Cell adhesion molecule 1' on 'eGFR'

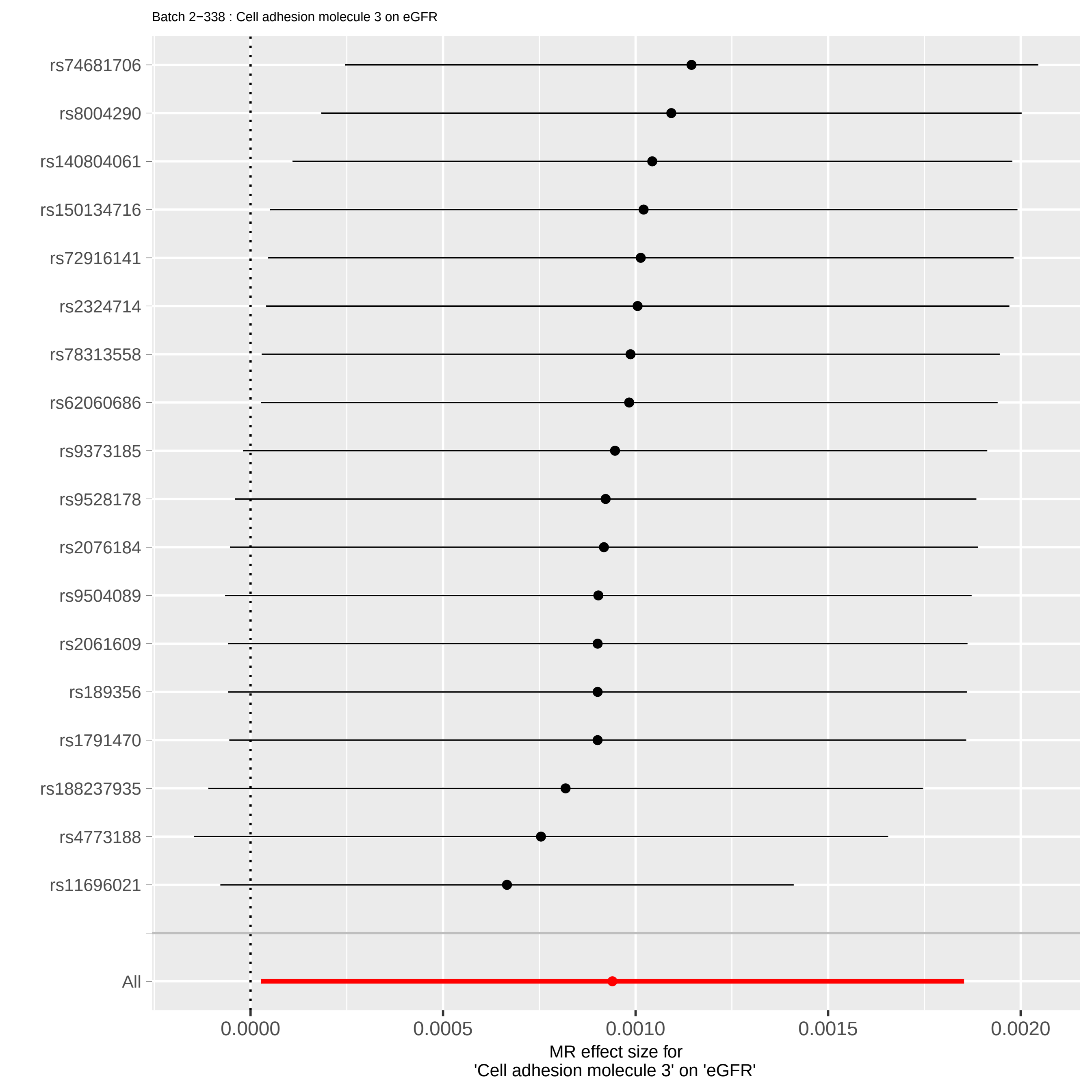
Batch 2−338 : Cell adhesion molecule 3 on eGFR
rs74681706
rs8004290
rs140804061
rs150134716
rs72916141
rs2324714
rs78313558
rs62060686
rs9373185
rs9528178
rs2076184
rs9504089
rs2061609
rs189356
rs1791470
rs188237935
rs4773188
rs11696021
All
0.0000
0.0005
0.0010
0.0015
0.0020
MR effect size for
'Cell adhesion molecule 3' on 'eGFR'

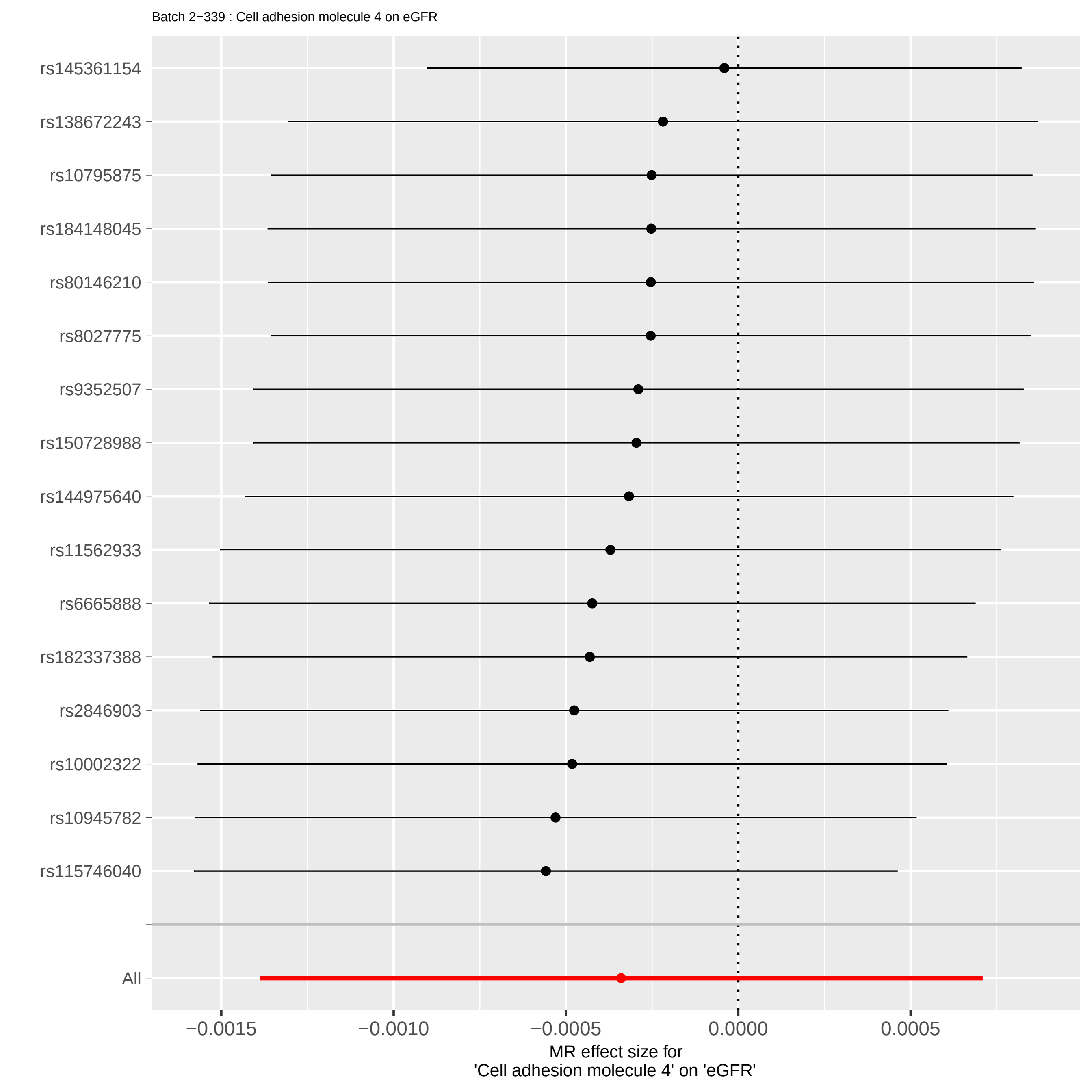

Batch 2−339 : Cell adhesion molecule 4 on eGFR
rs145361154
rs138672243
rs10795875
rs184148045
rs80146210
rs8027775
rs9352507
rs150728988
rs144975640
rs11562933
rs6665888
rs182337388
rs2846903
rs10002322
rs10945782
rs115746040
All
−0.0015
−0.0010
−0.0005
0.0000
0.0005
MR effect size for
'Cell adhesion molecule 4' on 'eGFR'

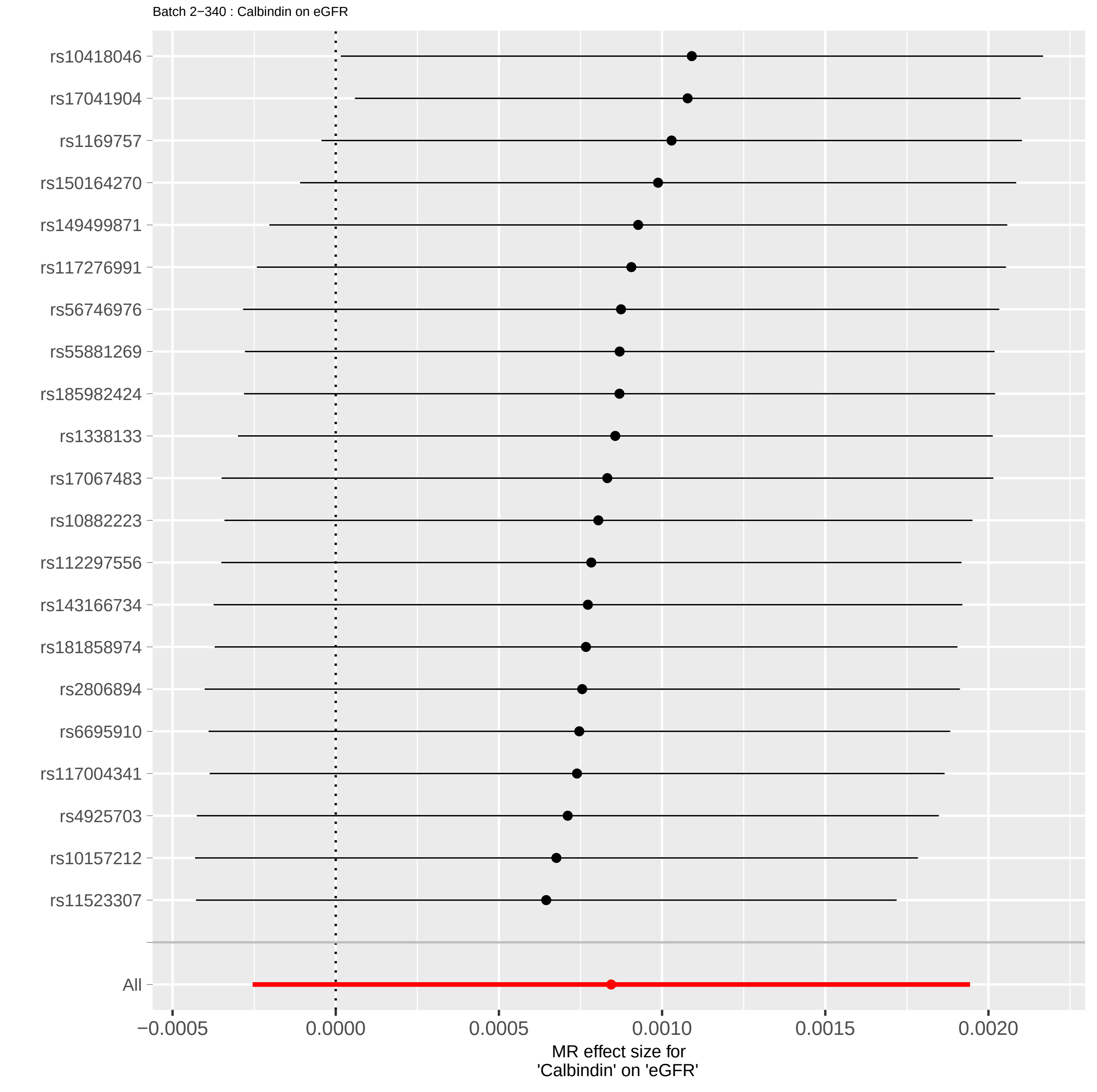

Batch 2−340 : Calbindin on eGFR
rs10418046
rs17041904
rs1169757
rs150164270
rs149499871
rs117276991
rs56746976
rs55881269
rs185982424
rs1338133
rs17067483
rs10882223
rs112297556
rs143166734
rs181858974
rs2806894
rs6695910
rs117004341
rs4925703
rs10157212
rs11523307
All
−0.0005
0.0000
0.0005
0.0010
0.0015
0.0020
MR effect size for
'Calbindin' on 'eGFR'

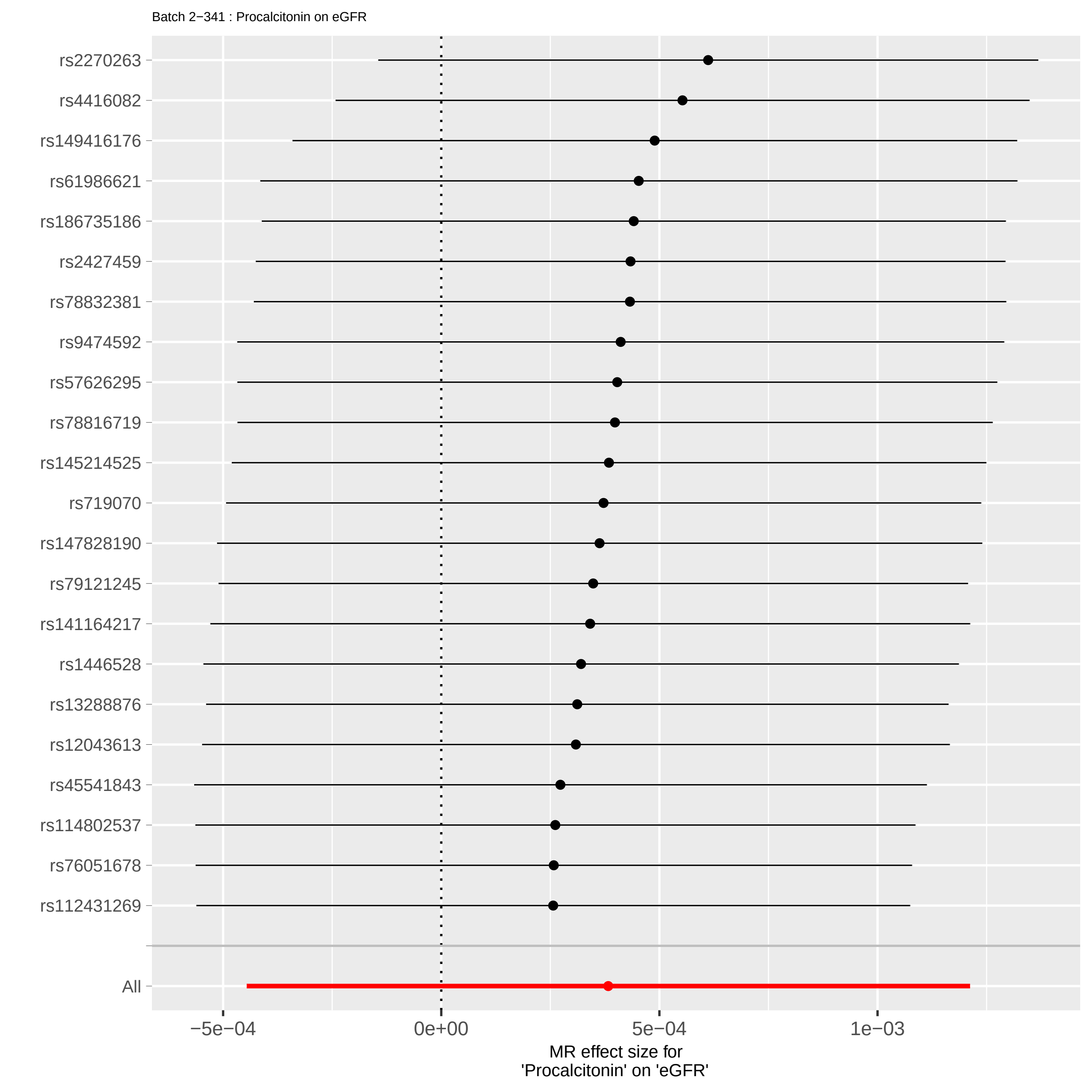
Batch 2−341 : Procalcitonin on eGFR
rs2270263
rs4416082
rs149416176
rs61986621
rs186735186
rs2427459
rs78832381
rs9474592
rs57626295
rs78816719
rs145214525
rs719070
rs147828190
rs79121245
rs141164217
rs1446528
rs13288876
rs12043613
rs45541843
rs114802537
rs76051678
rs112431269
All
−5e−04
0e+00
5e−04
1e−03
MR effect size for
'Procalcitonin' on 'eGFR'

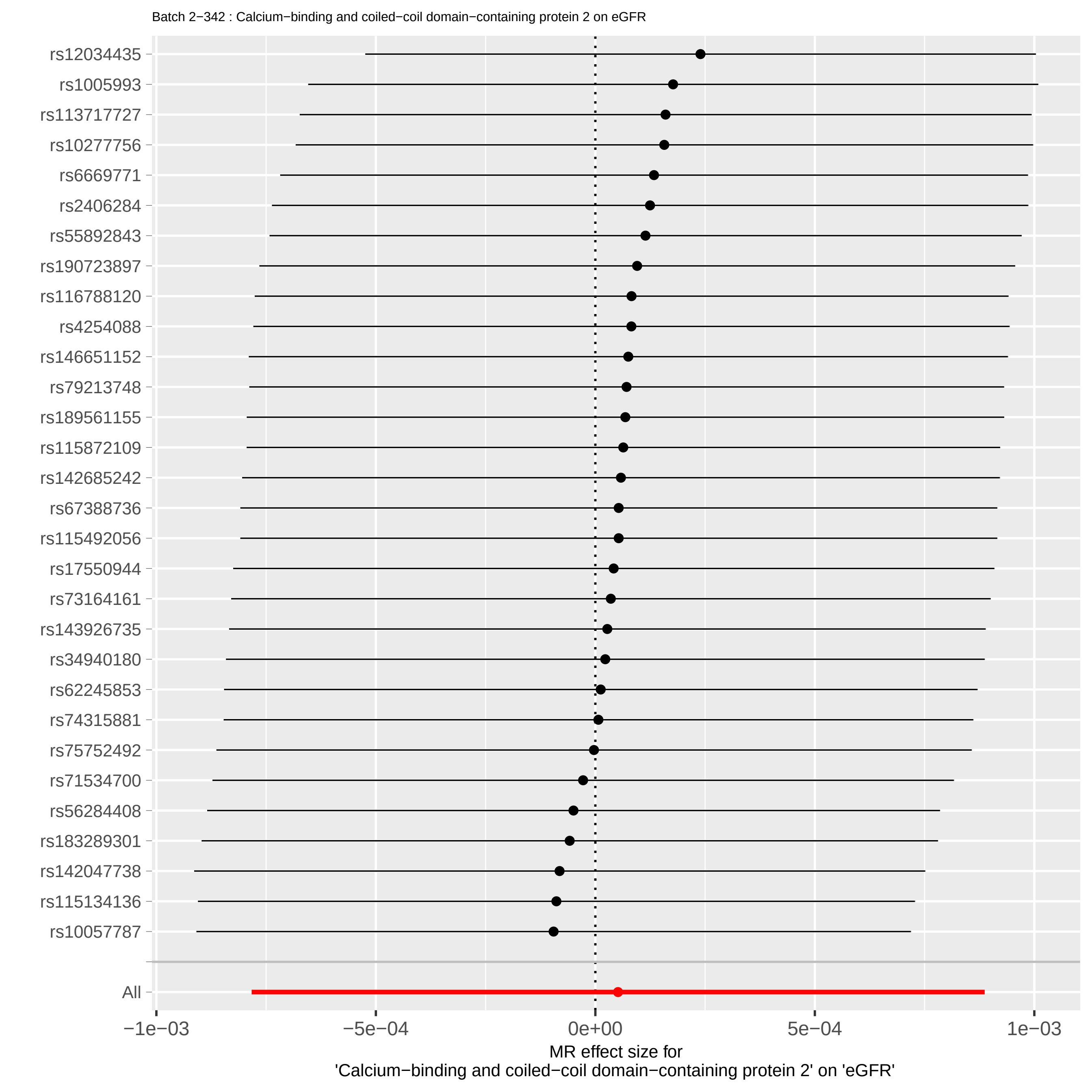

Batch 2−342 : Calcium−binding and coiled−coil domain−containing protein 2 on eGFR
rs12034435
rs1005993
rs113717727
rs10277756
rs6669771
rs2406284
rs55892843
rs190723897
rs116788120
rs4254088
rs146651152
rs79213748
rs189561155
rs115872109
rs142685242
rs67388736
rs115492056
rs17550944
rs73164161
rs143926735
rs34940180
rs62245853
rs74315881
rs75752492
rs71534700
rs56284408
rs183289301
rs142047738
rs115134136
rs10057787
All
−1e−03
−5e−04
0e+00
5e−04
1e−03
MR effect size for
'Calcium−binding and coiled−coil domain−containing protein 2' on 'eGFR'

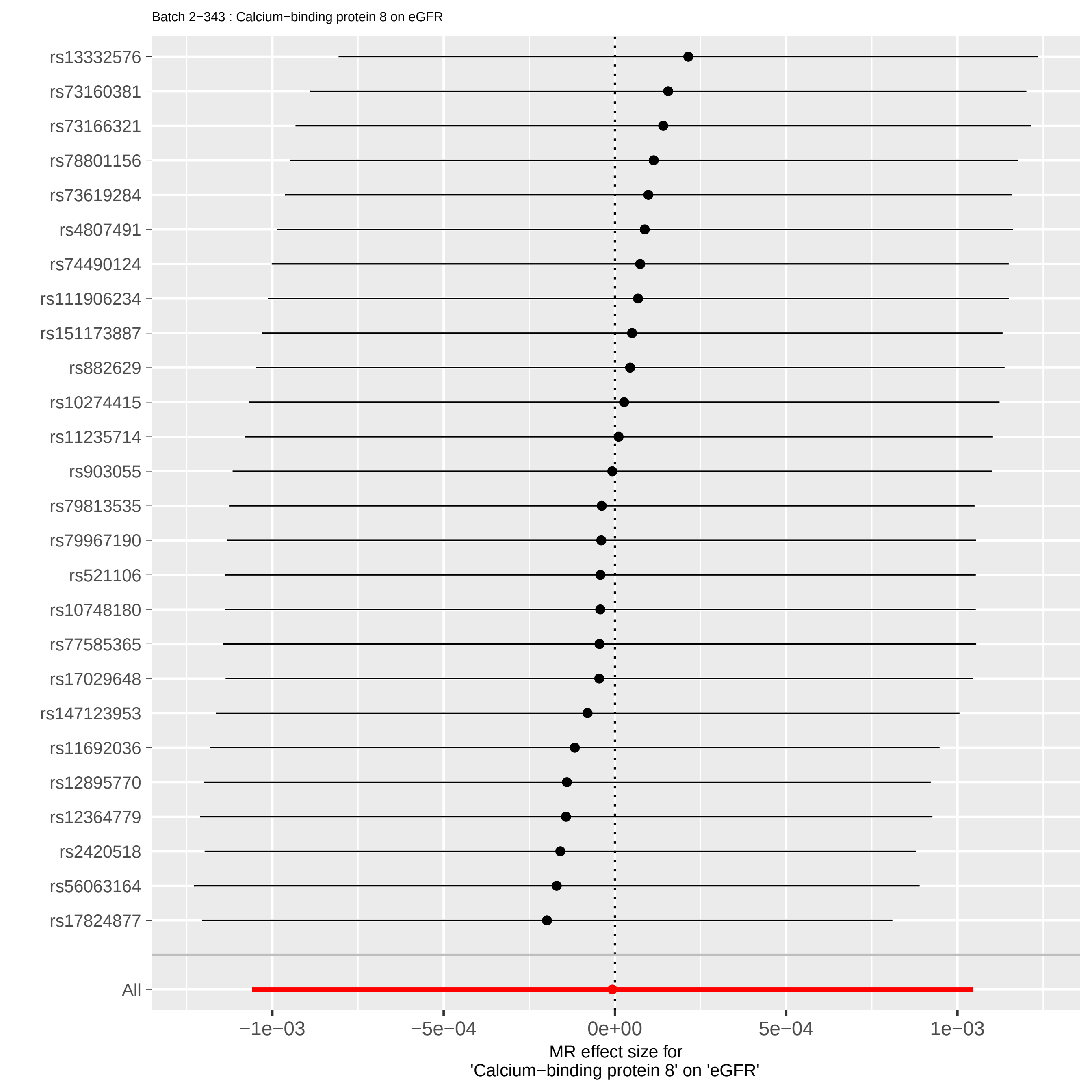

Batch 2−343 : Calcium−binding protein 8 on eGFR
rs13332576
rs73160381
rs73166321
rs78801156
rs73619284
rs4807491
rs74490124
rs111906234
rs151173887
rs882629
rs10274415
rs11235714
rs903055
rs79813535
rs79967190
rs521106
rs10748180
rs77585365
rs17029648
rs147123953
rs11692036
rs12895770
rs12364779
rs2420518
rs56063164
rs17824877
All
−1e−03
−5e−04
0e+00
5e−04
1e−03
MR effect size for
'Calcium−binding protein 8' on 'eGFR'

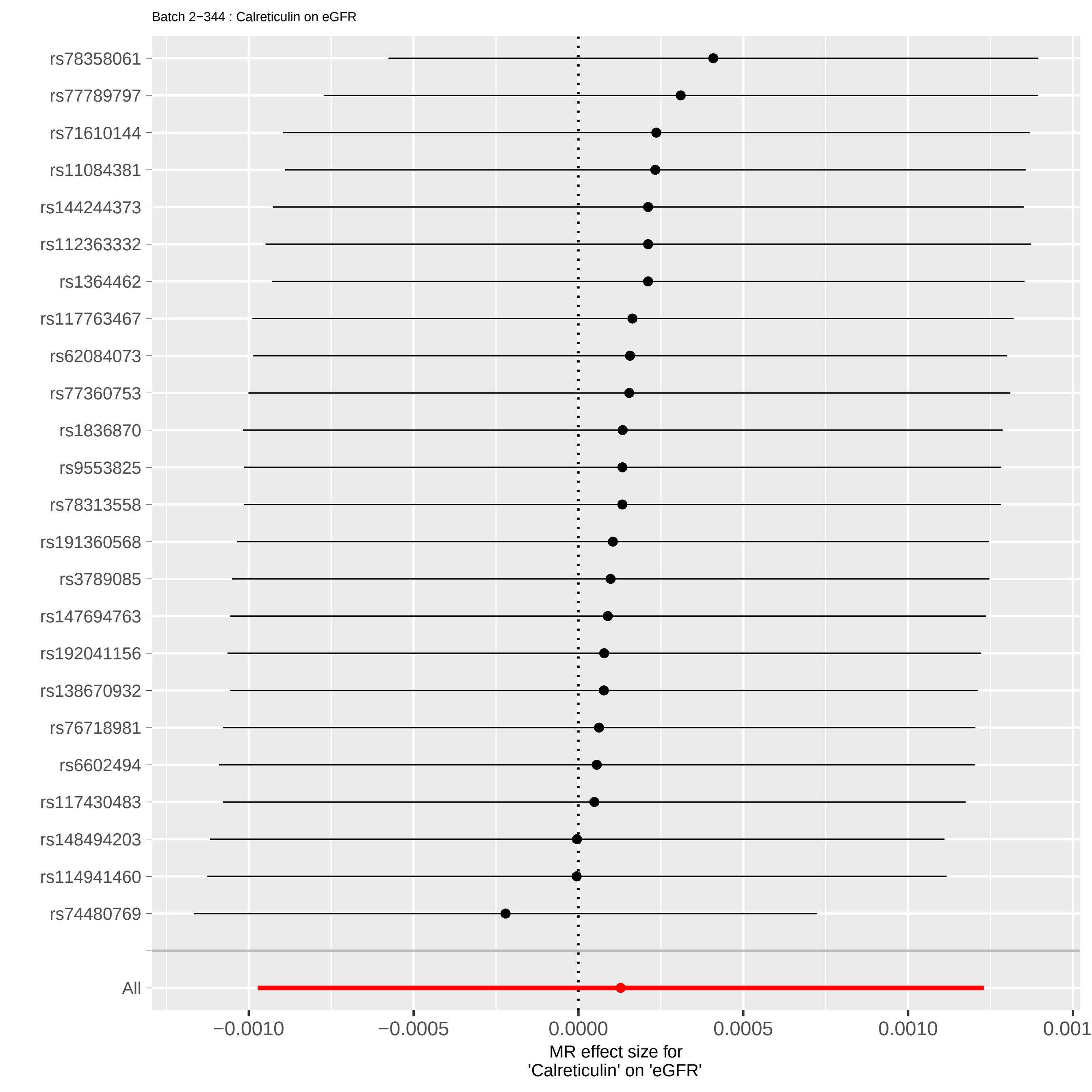

Batch 2−344 : Calreticulin on eGFR
rs78358061
rs77789797
rs71610144
rs11084381
rs144244373
rs112363332
rs1364462
rs117763467
rs62084073
rs77360753
rs1836870
rs9553825
rs78313558
rs191360568
rs3789085
rs147694763
rs192041156
rs138670932
rs76718981
rs6602494
rs117430483
rs148494203
rs114941460
rs74480769
All
−0.0010
−0.0005
0.0000
0.0005
0.0010
0.001
MR effect size for
'Calreticulin' on 'eGFR'

Batch 2−345 : Neuron−specific vesicular protein calcyon on eGFR

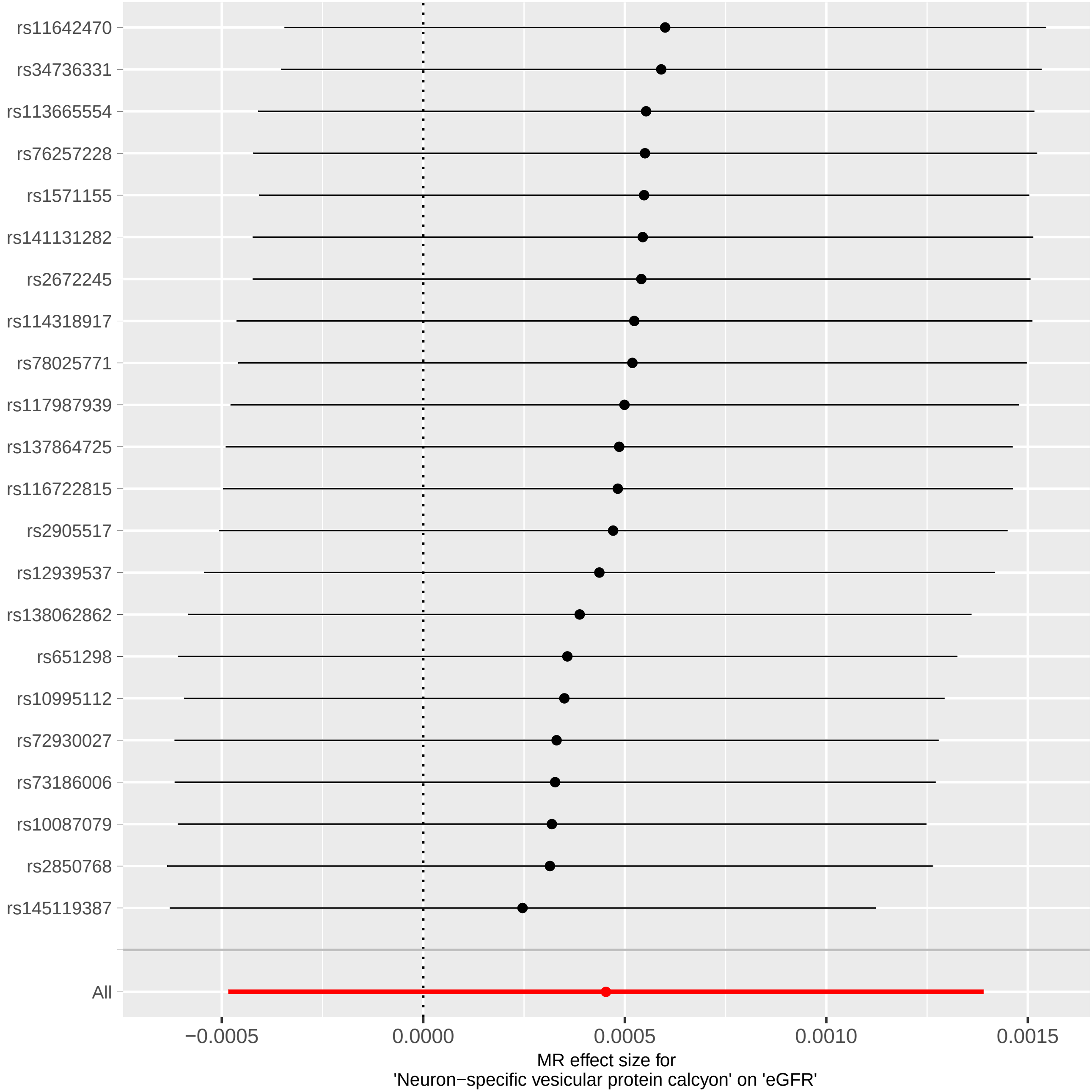

Batch 2−346 : Calcium/calmodulin−dependent protein kinase type 1 on eGFR

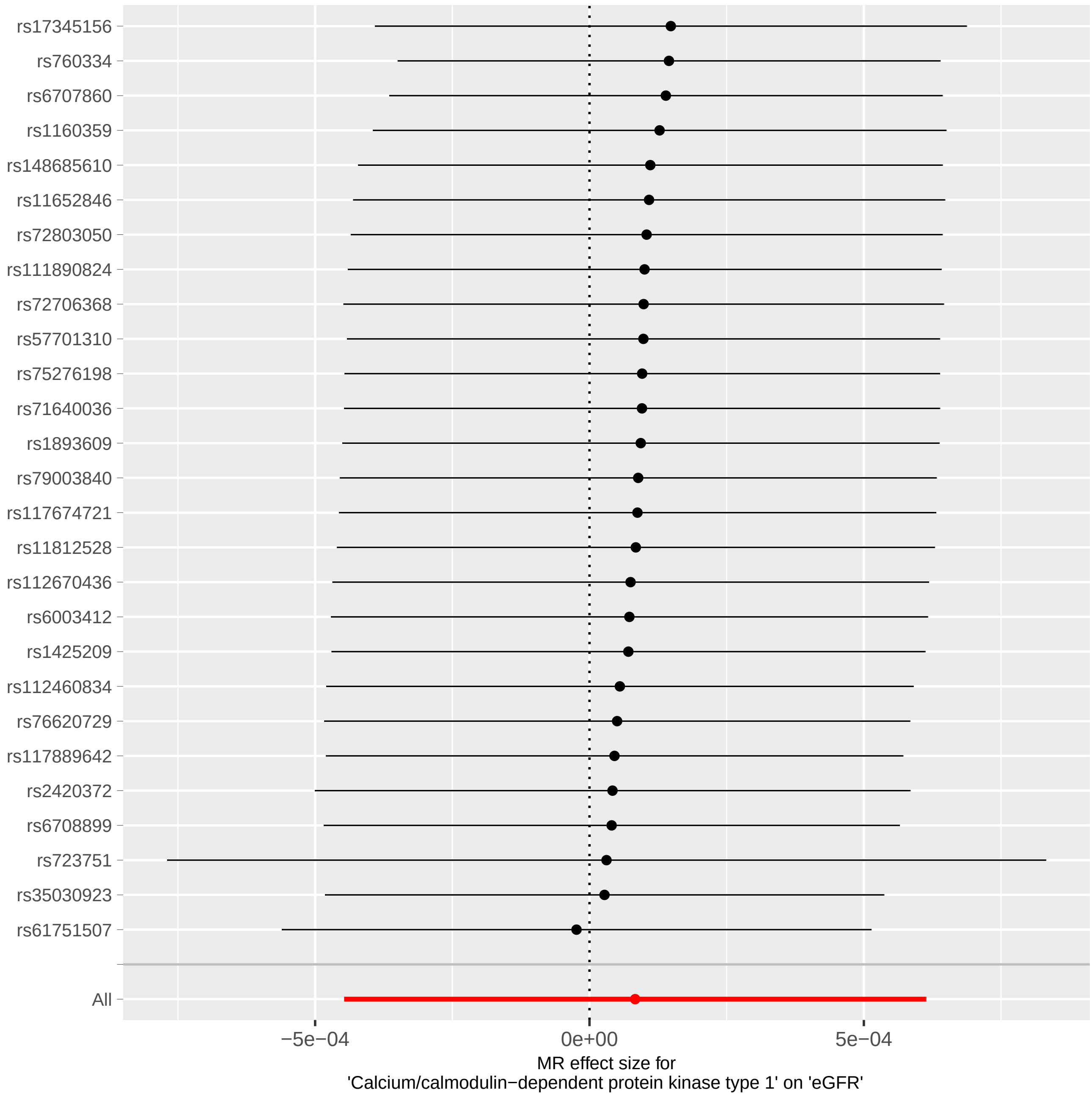

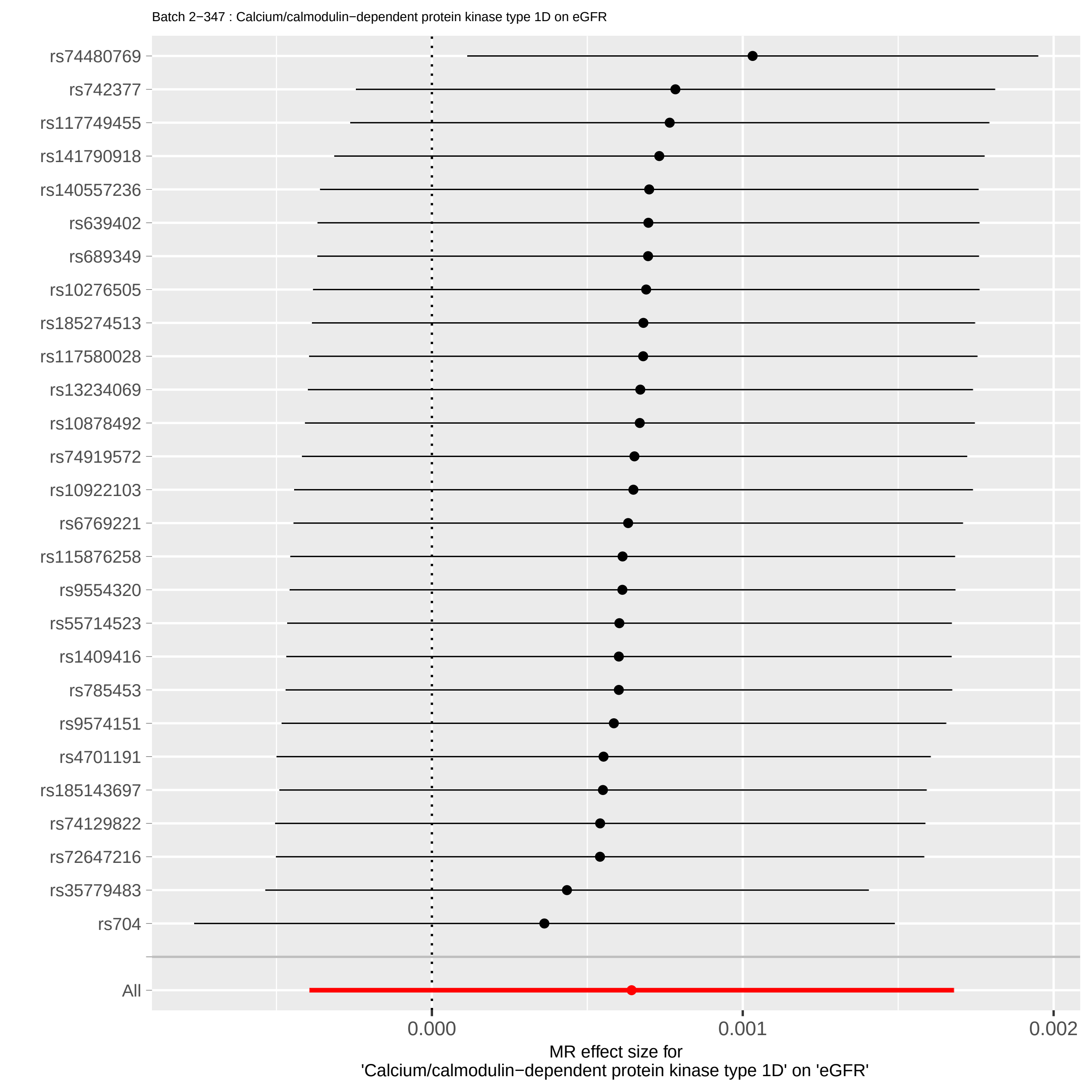
Batch 2−347 : Calcium/calmodulin−dependent protein kinase type 1D on eGFR
rs74480769
rs742377
rs117749455
rs141790918
rs140557236
rs639402
rs689349
rs10276505
rs185274513
rs117580028
rs13234069
rs10878492
rs74919572
rs10922103
rs6769221
rs115876258
rs9554320
rs55714523
rs1409416
rs785453
rs9574151
rs4701191
rs185143697
rs74129822
rs72647216
rs35779483
rs704
All
0.000
0.001
0.002
MR effect size for
'Calcium/calmodulin−dependent protein kinase type 1D' on 'eGFR'

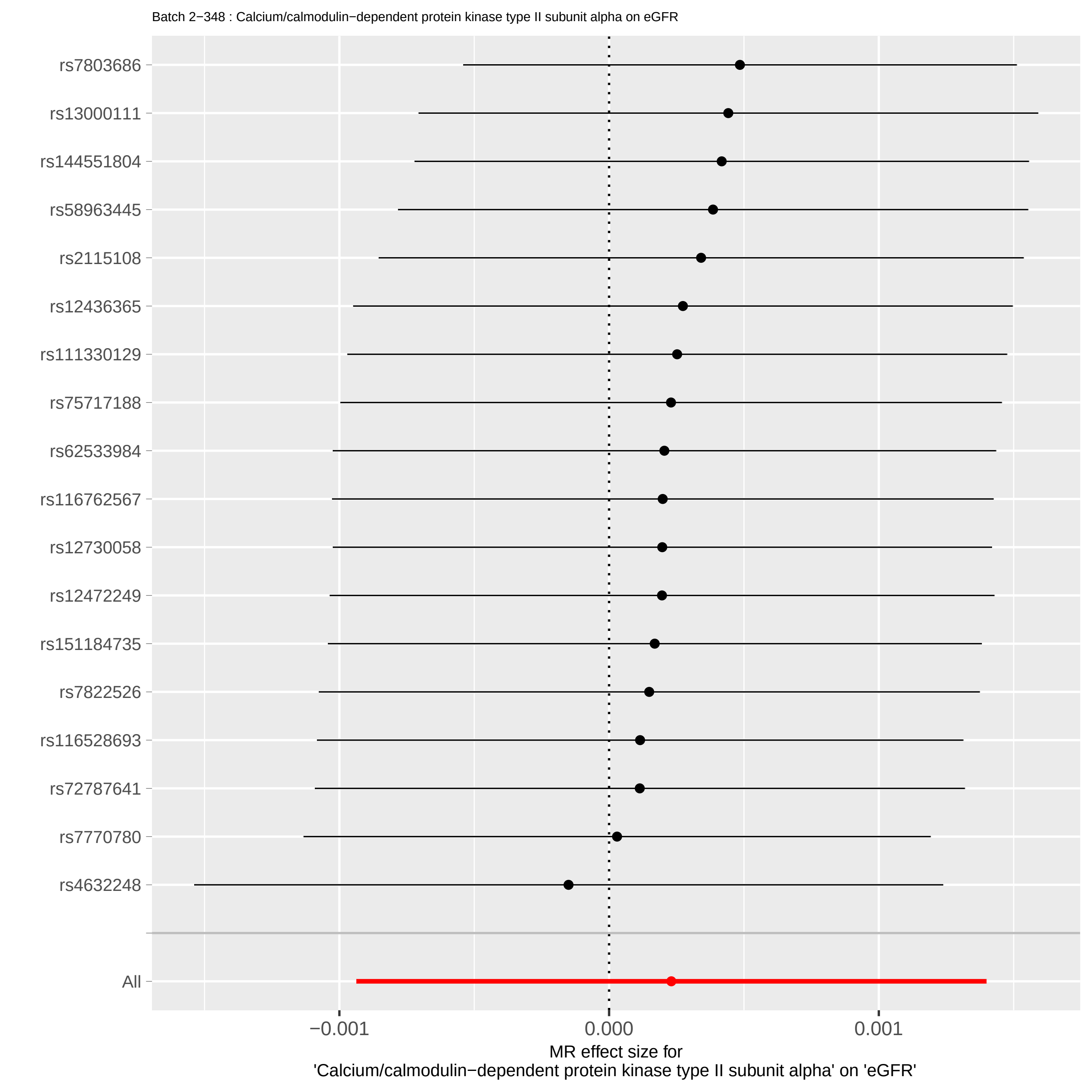

Batch 2−348 : Calcium/calmodulin−dependent protein kinase type II subunit alpha on eGFR
rs7803686
rs13000111
rs144551804
rs58963445
rs2115108
rs12436365
rs111330129
rs75717188
rs62533984
rs116762567
rs12730058
rs12472249
rs151184735
rs7822526
rs116528693
rs72787641
rs7770780
rs4632248
All
−0.001
0.000
0.001
MR effect size for
'Calcium/calmodulin−dependent protein kinase type II subunit alpha' on 'eGFR'

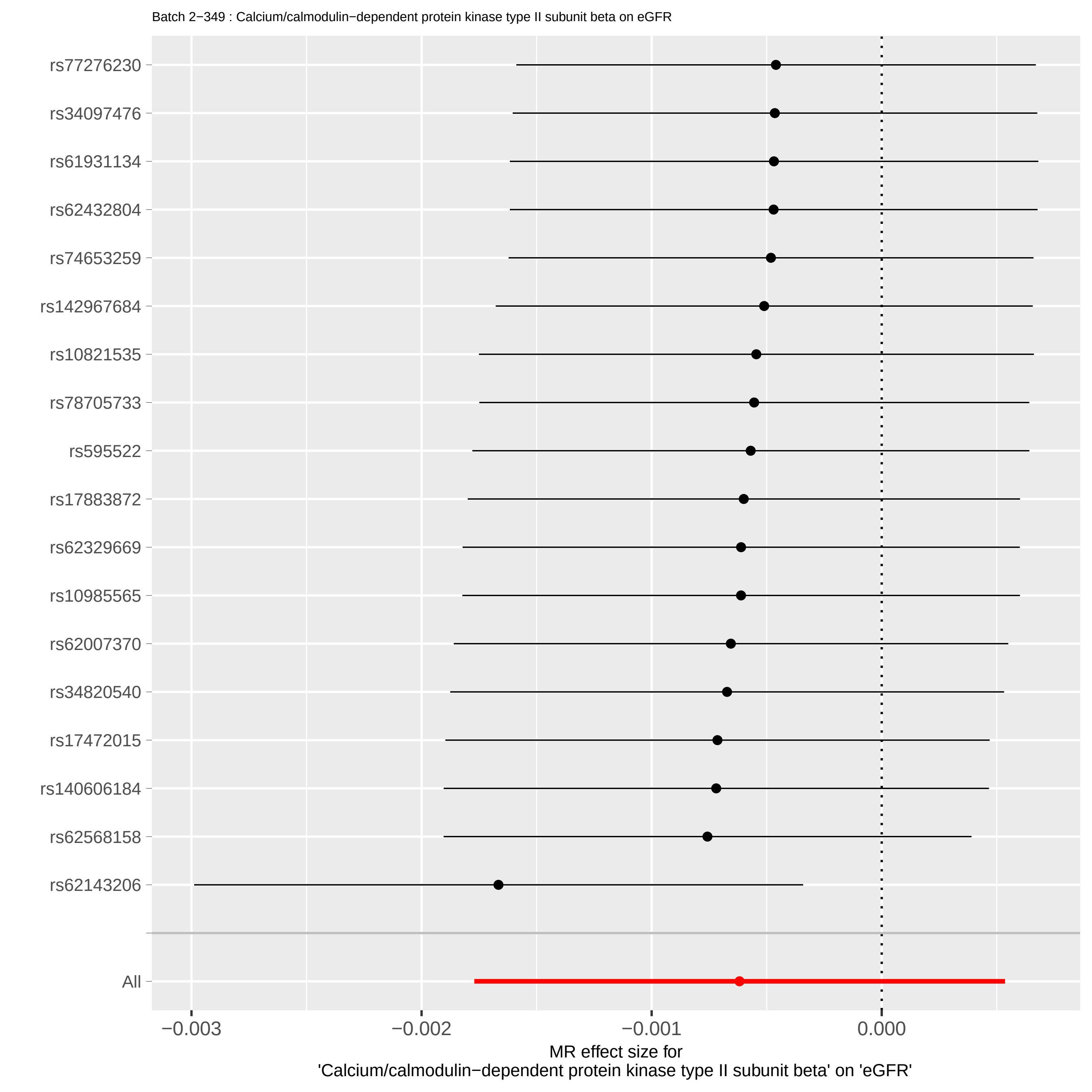

Batch 2−349 : Calcium/calmodulin−dependent protein kinase type II subunit beta on eGFR
rs77276230
rs34097476
rs61931134
rs62432804
rs74653259
rs142967684
rs10821535
rs78705733
rs595522
rs17883872
rs62329669
rs10985565
rs62007370
rs34820540
rs17472015
rs140606184
rs62568158
rs62143206
All
−0.003
−0.002
−0.001
0.000
MR effect size for
'Calcium/calmodulin−dependent protein kinase type II subunit beta' on 'eGFR'

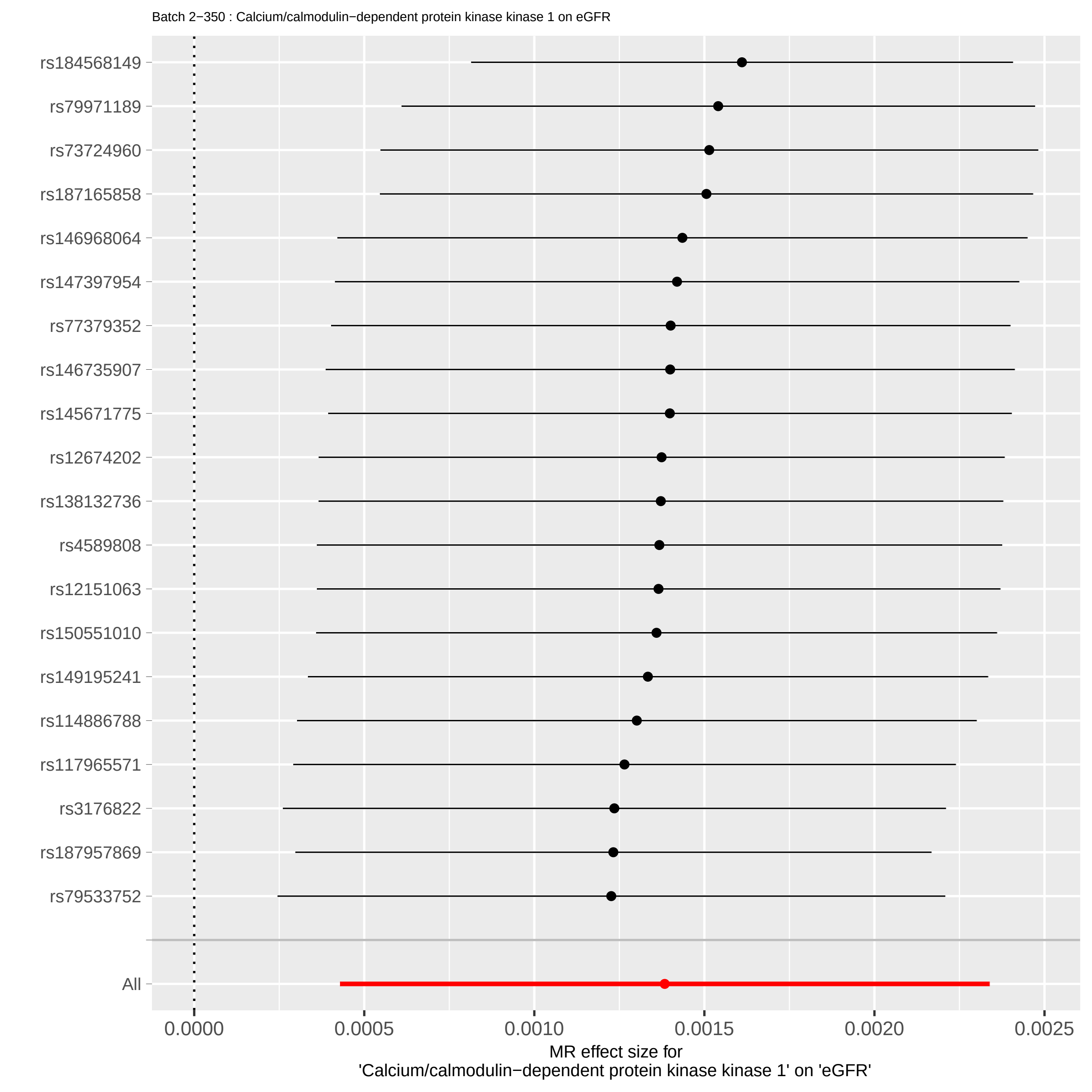

Batch 2−350 : Calcium/calmodulin−dependent protein kinase kinase 1 on eGFR
rs184568149
rs79971189
rs73724960
rs187165858
rs146968064
rs147397954
rs77379352
rs146735907
rs145671775
rs12674202
rs138132736
rs4589808
rs12151063
rs150551010
rs149195241
rs114886788
rs117965571
rs3176822
rs187957869
rs79533752
All
0.0000
0.0005
0.0010
0.0015
0.0020
0.0025
MR effect size for
'Calcium/calmodulin−dependent protein kinase kinase 1' on 'eGFR'

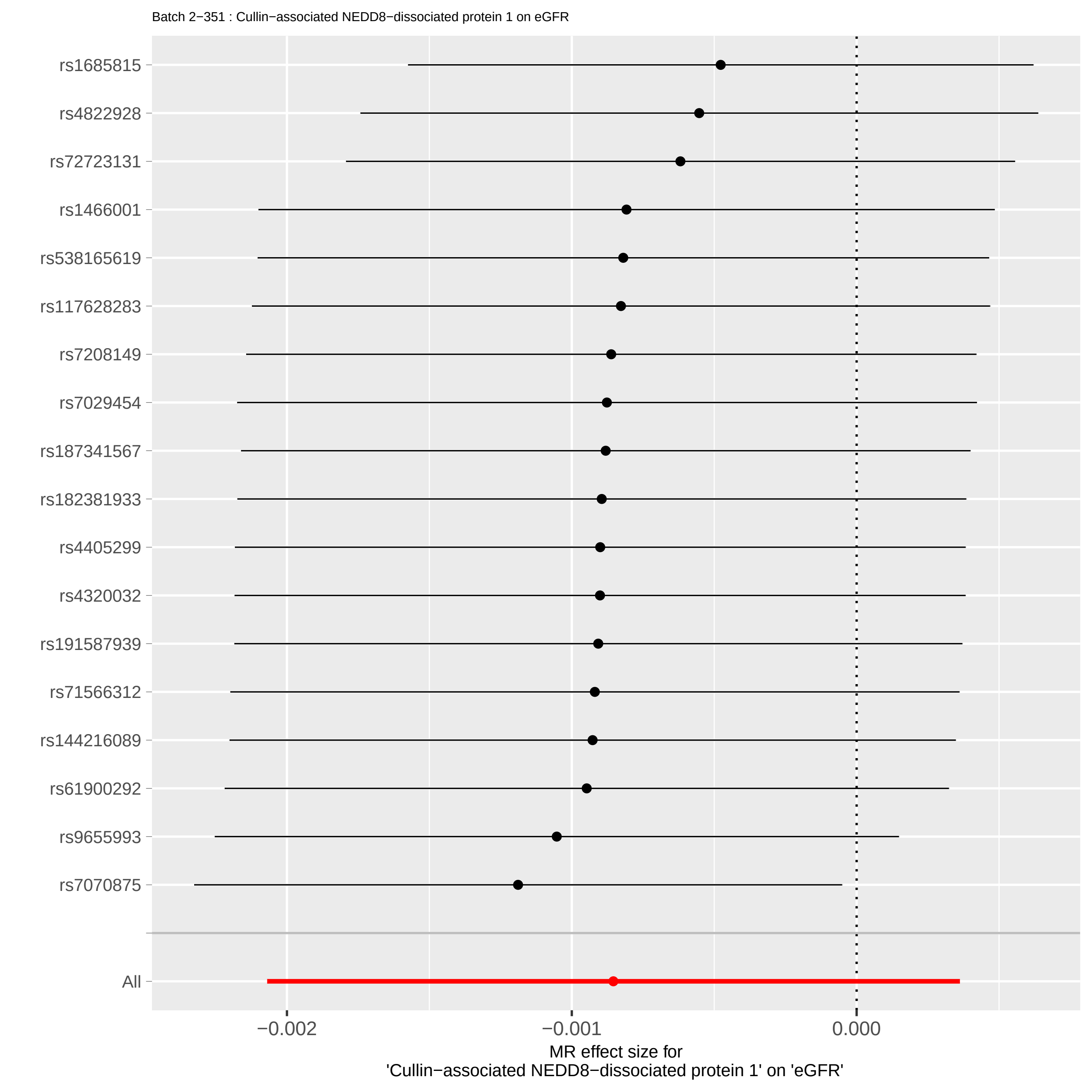

Batch 2−351 : Cullin−associated NEDD8−dissociated protein 1 on eGFR
rs1685815
rs4822928
rs72723131
rs1466001
rs538165619
rs117628283
rs7208149
rs7029454
rs187341567
rs182381933
rs4405299
rs4320032
rs191587939
rs71566312
rs144216089
rs61900292
rs9655993
rs7070875
All
−0.002
−0.001
0.000
MR effect size for
'Cullin−associated NEDD8−dissociated protein 1' on 'eGFR'

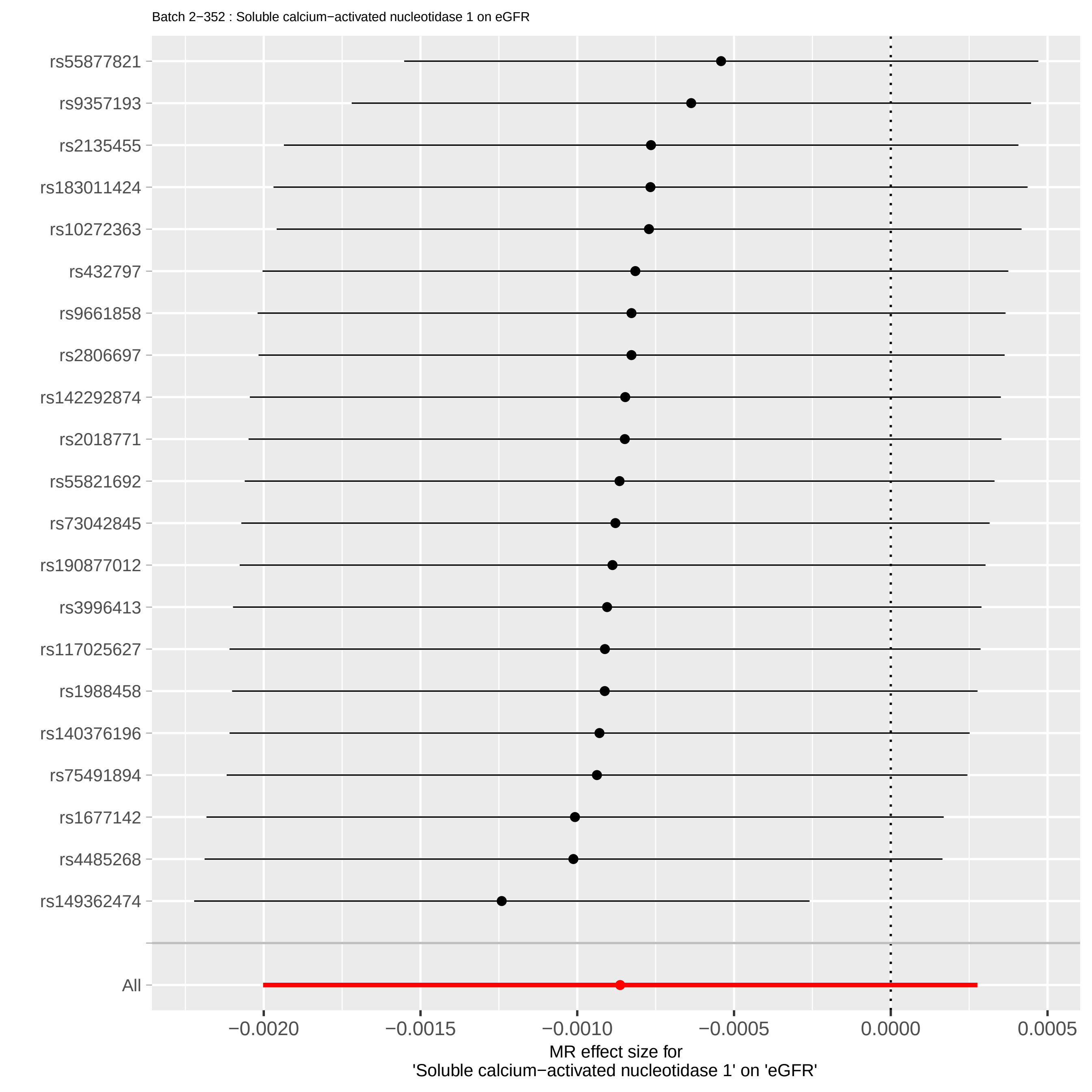
Batch 2−352 : Soluble calcium−activated nucleotidase 1 on eGFR
rs55877821
rs9357193
rs2135455
rs183011424
rs10272363
rs432797
rs9661858
rs2806697
rs142292874
rs2018771
rs55821692
rs73042845
rs190877012
rs3996413
rs117025627
rs1988458
rs140376196
rs75491894
rs1677142
rs4485268
rs149362474
All
−0.0020
−0.0015
−0.0010
−0.0005
0.0000
0.0005
MR effect size for
'Soluble calcium−activated nucleotidase 1' on 'eGFR'

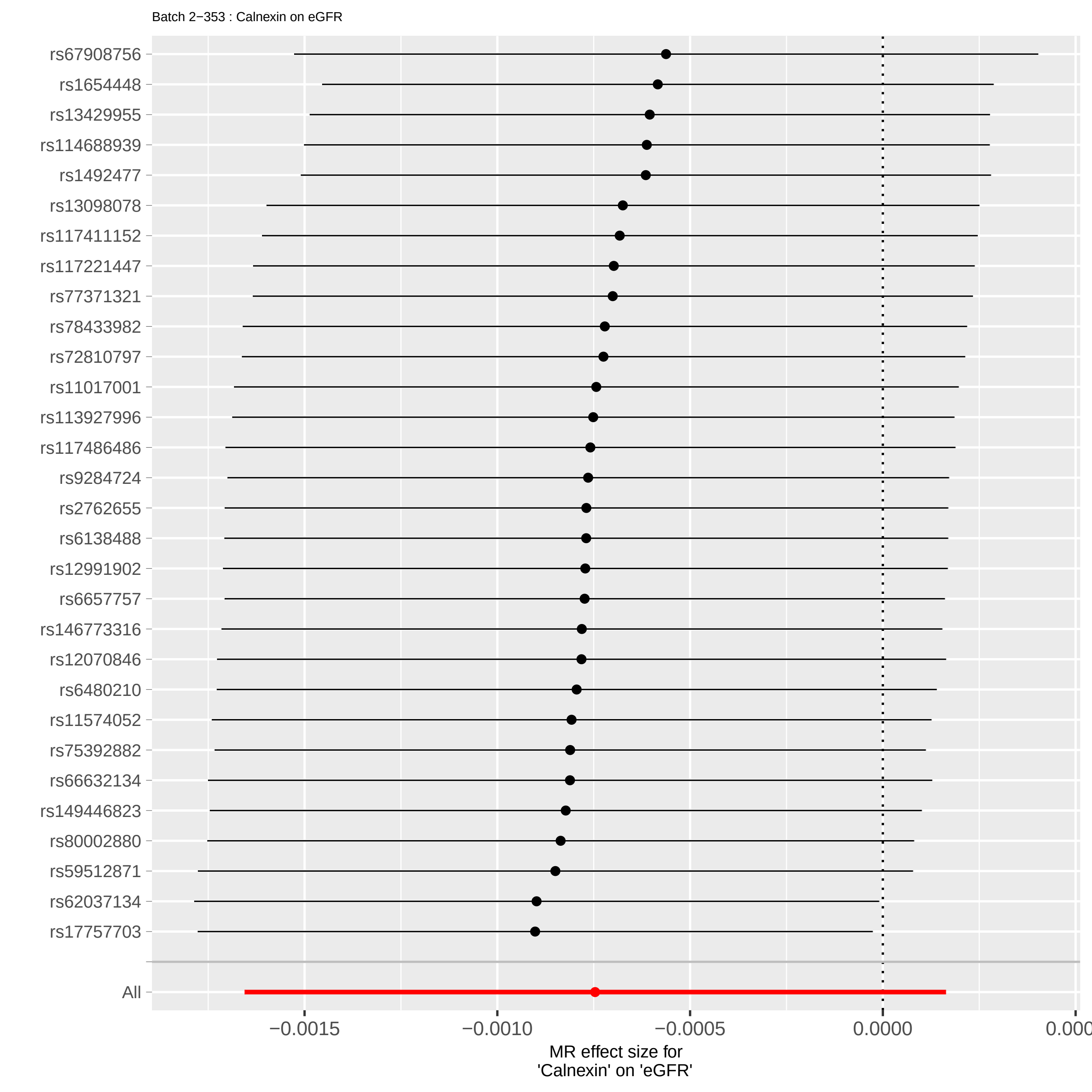

Batch 2−353 : Calnexin on eGFR
rs67908756
rs1654448
rs13429955
rs114688939
rs1492477
rs13098078
rs117411152
rs117221447
rs77371321
rs78433982
rs72810797
rs11017001
rs113927996
rs117486486
rs9284724
rs2762655
rs6138488
rs12991902
rs6657757
rs146773316
rs12070846
rs6480210
rs11574052
rs75392882
rs66632134
rs149446823
rs80002880
rs59512871
rs62037134
rs17757703
All
−0.0015
−0.0010
−0.0005
0.0000
MR effect size for
'Calnexin' on 'eGFR'

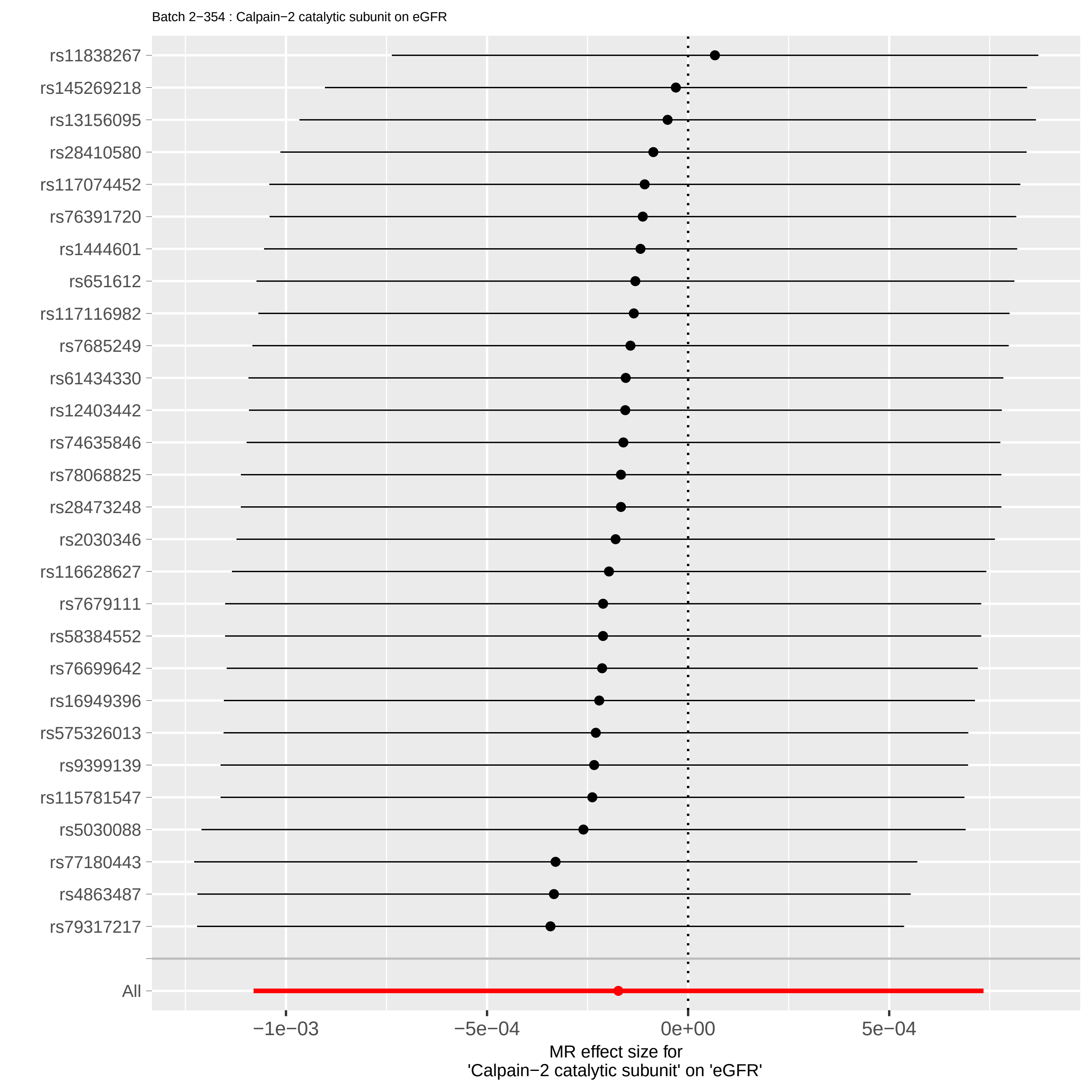
Batch 2−354 : Calpain−2 catalytic subunit on eGFR
rs11838267
rs145269218
rs13156095
rs28410580
rs117074452
rs76391720
rs1444601
rs651612
rs117116982
rs7685249
rs61434330
rs12403442
rs74635846
rs78068825
rs28473248
rs2030346
rs116628627
rs7679111
rs58384552
rs76699642
rs16949396
rs575326013
rs9399139
rs115781547
rs5030088
rs77180443
rs4863487
rs79317217
All
−1e−03
−5e−04
0e+00
5e−04
MR effect size for
'Calpain−2 catalytic subunit' on 'eGFR'

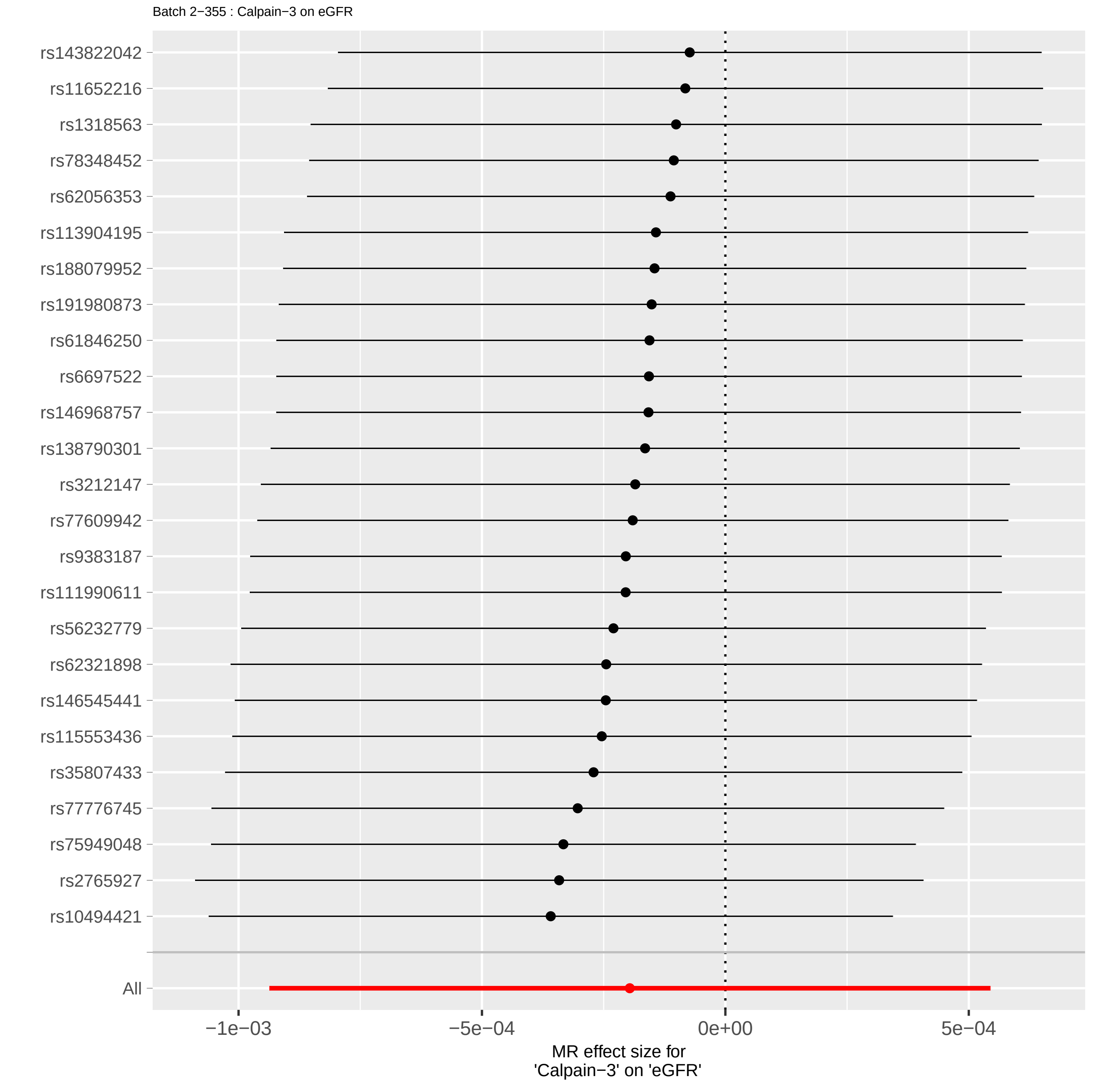

Batch 2−355 : Calpain−3 on eGFR
rs143822042
rs11652216
rs1318563
rs78348452
rs62056353
rs113904195
rs188079952
rs191980873
rs61846250
rs6697522
rs146968757
rs138790301
rs3212147
rs77609942
rs9383187
rs111990611
rs56232779
rs62321898
rs146545441
rs115553436
rs35807433
rs77776745
rs75949048
rs2765927
rs10494421
All
−1e−03
−5e−04
0e+00
5e−04
MR effect size for
'Calpain−3' on 'eGFR'

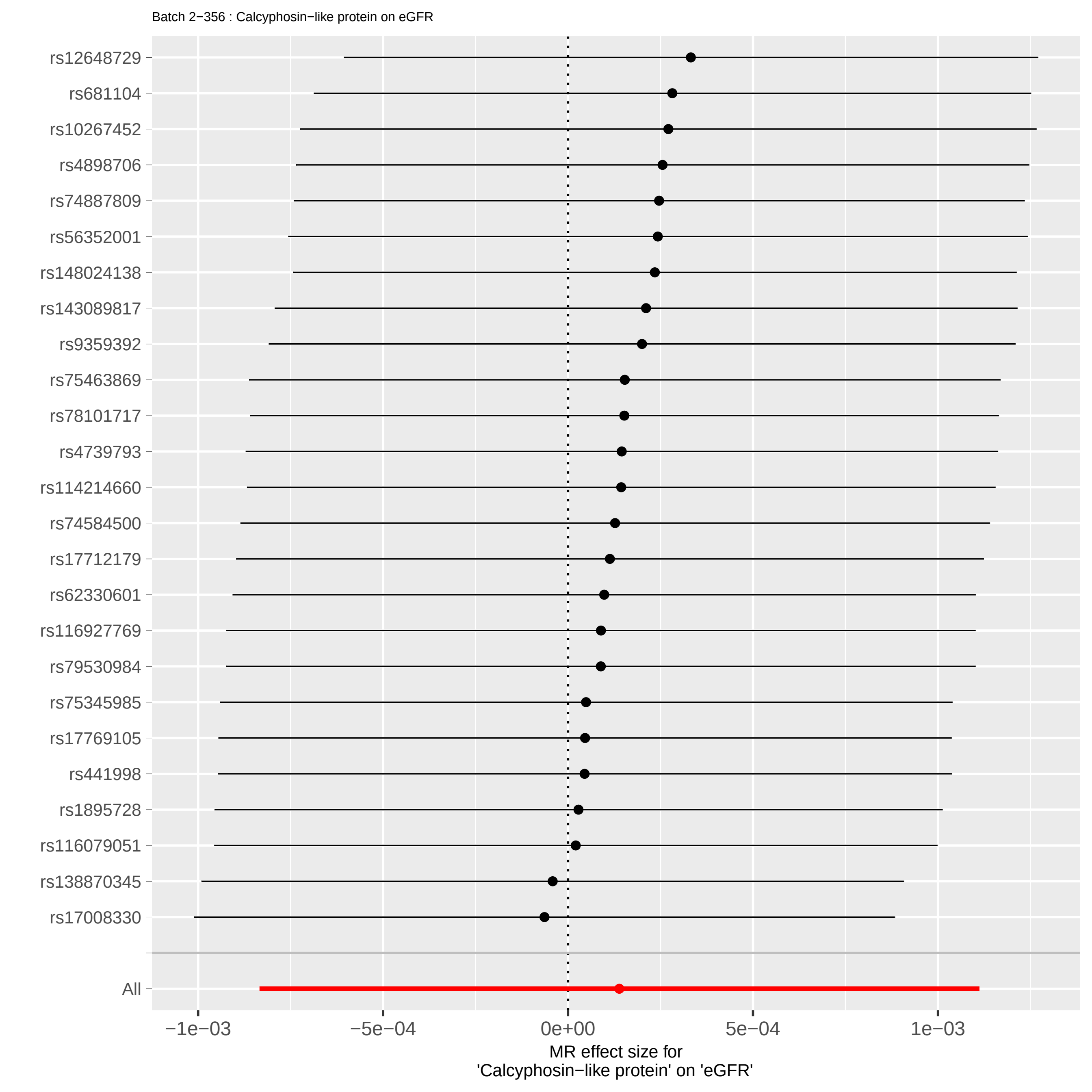

Batch 2−356 : Calcyphosin−like protein on eGFR
rs12648729
rs681104
rs10267452
rs4898706
rs74887809
rs56352001
rs148024138
rs143089817
rs9359392
rs75463869
rs78101717
rs4739793
rs114214660
rs74584500
rs17712179
rs62330601
rs116927769
rs79530984
rs75345985
rs17769105
rs441998
rs1895728
rs116079051
rs138870345
rs17008330
All
−1e−03
−5e−04
0e+00
5e−04
1e−03
MR effect size for
'Calcyphosin−like protein' on 'eGFR'

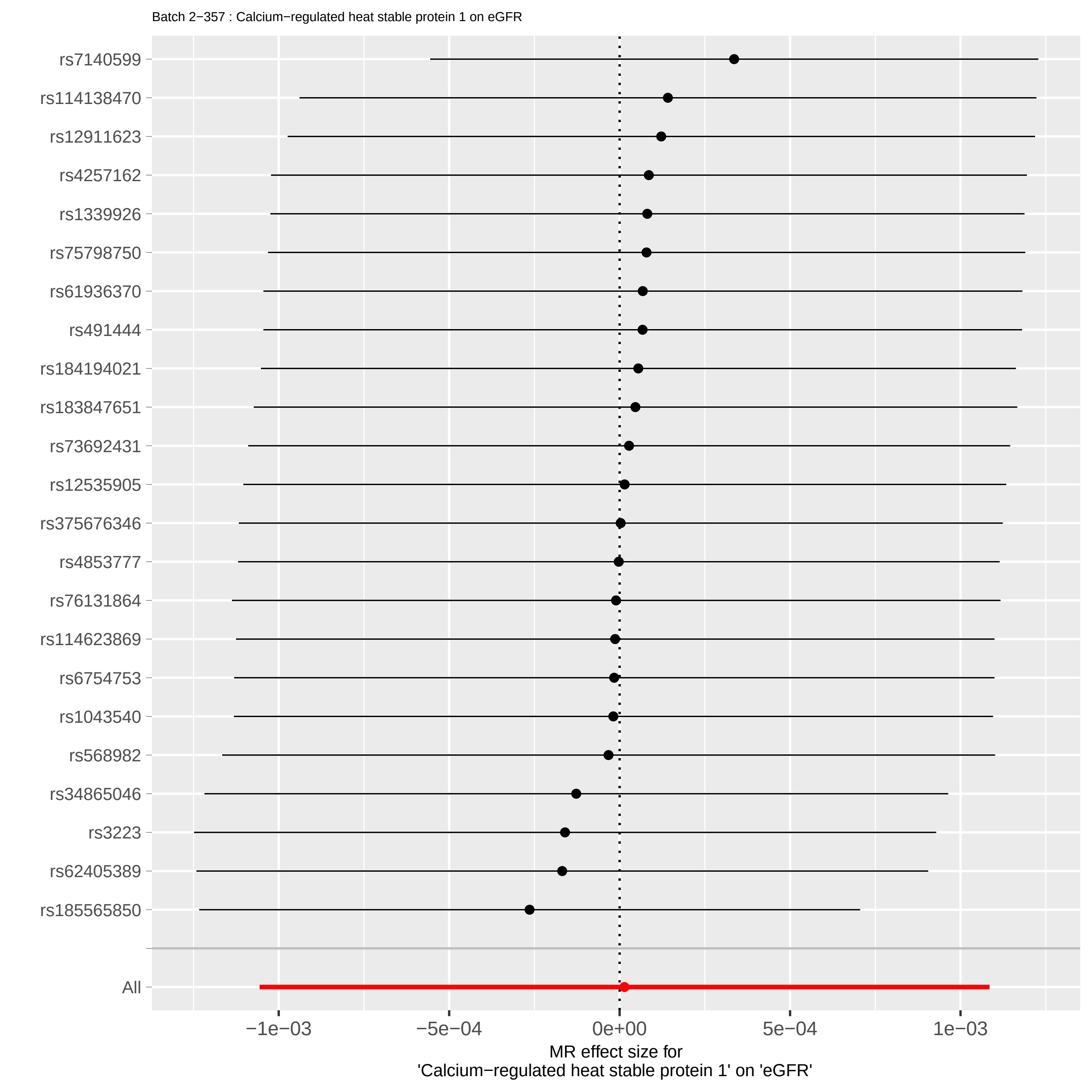

Batch 2−357 : Calcium−regulated heat stable protein 1 on eGFR
rs7140599
rs114138470
rs12911623
rs4257162
rs1339926
rs75798750
rs61936370
rs491444
rs184194021
rs183847651
rs73692431
rs12535905
rs375676346
rs4853777
rs76131864
rs114623869
rs6754753
rs1043540
rs568982
rs34865046
rs3223
rs62405389
rs185565850
All
−1e−03
−5e−04
0e+00
5e−04
1e−03
MR effect size for
'Calcium−regulated heat stable protein 1' on 'eGFR'

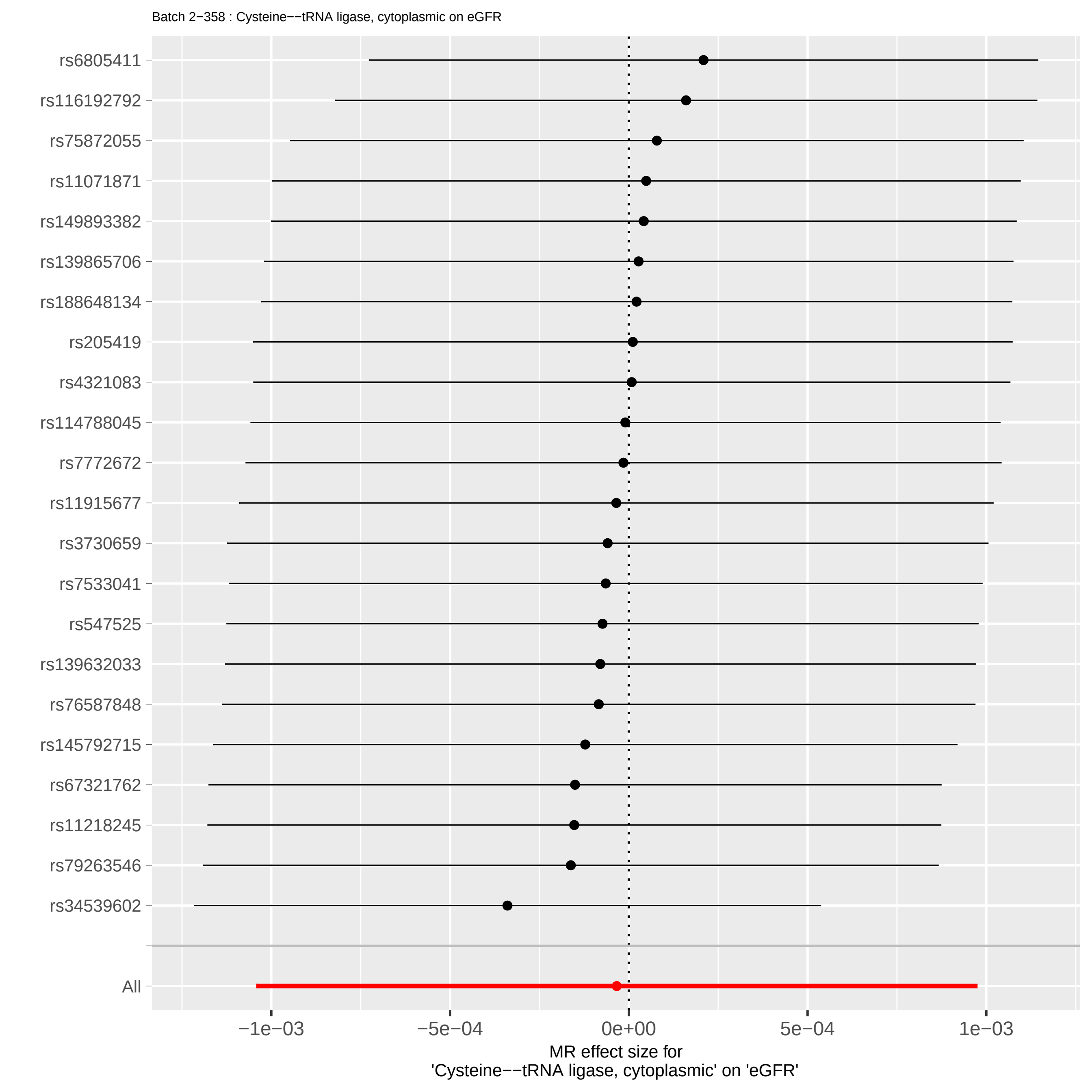

Batch 2−358 : Cysteine−−tRNA ligase, cytoplasmic on eGFR
rs6805411
rs116192792
rs75872055
rs11071871
rs149893382
rs139865706
rs188648134
rs205419
rs4321083
rs114788045
rs7772672
rs11915677
rs3730659
rs7533041
rs547525
rs139632033
rs76587848
rs145792715
rs67321762
rs11218245
rs79263546
rs34539602
All
−1e−03
−5e−04
0e+00
5e−04
1e−03
MR effect size for
'Cysteine−−tRNA ligase, cytoplasmic' on 'eGFR'

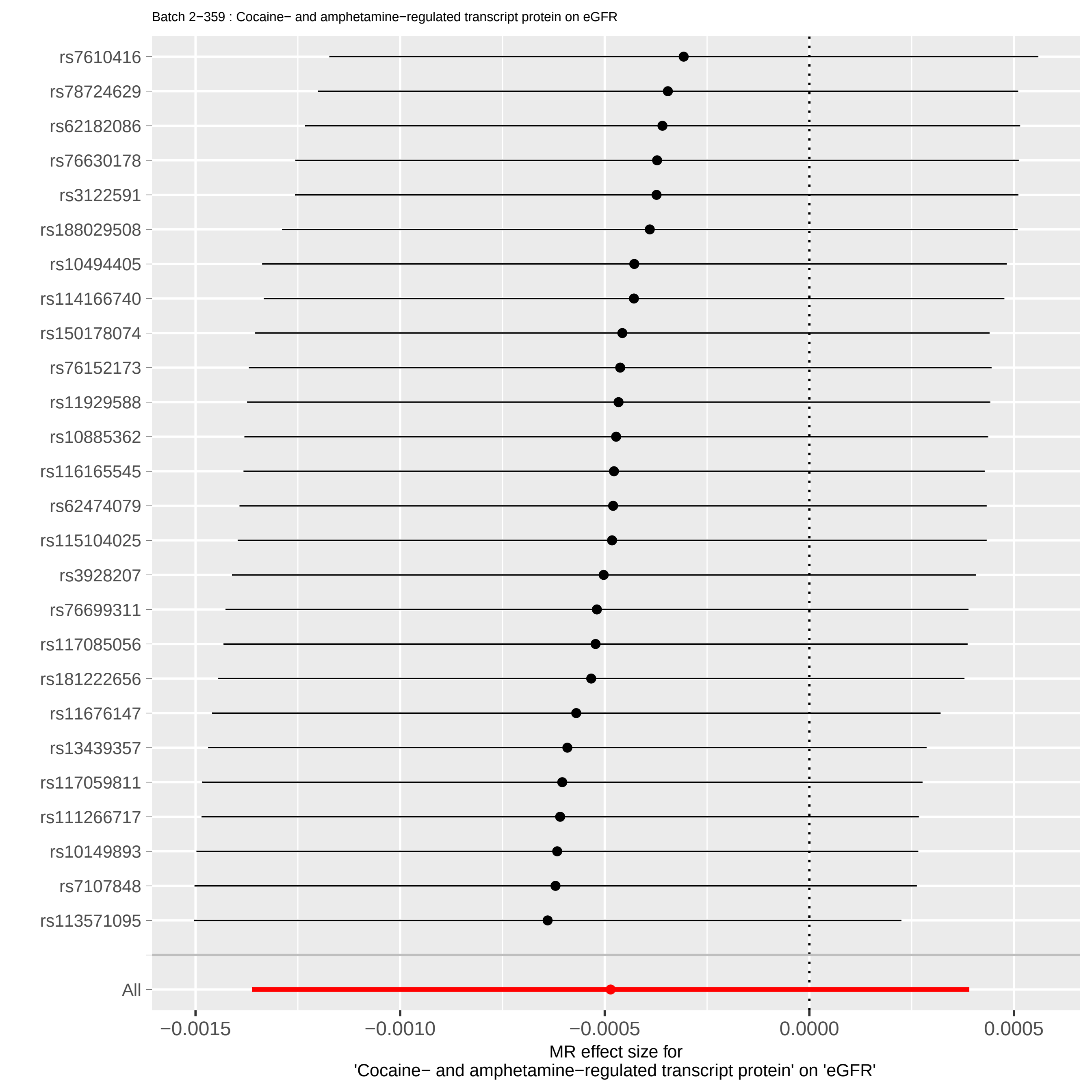

Batch 2−359 : Cocaine− and amphetamine−regulated transcript protein on eGFR
rs7610416
rs78724629
rs62182086
rs76630178
rs3122591
rs188029508
rs10494405
rs114166740
rs150178074
rs76152173
rs11929588
rs10885362
rs116165545
rs62474079
rs115104025
rs3928207
rs76699311
rs117085056
rs181222656
rs11676147
rs13439357
rs117059811
rs111266717
rs10149893
rs7107848
rs113571095
All
−0.0015
−0.0010
−0.0005
0.0000
0.0005
MR effect size for
'Cocaine− and amphetamine−regulated transcript protein' on 'eGFR'

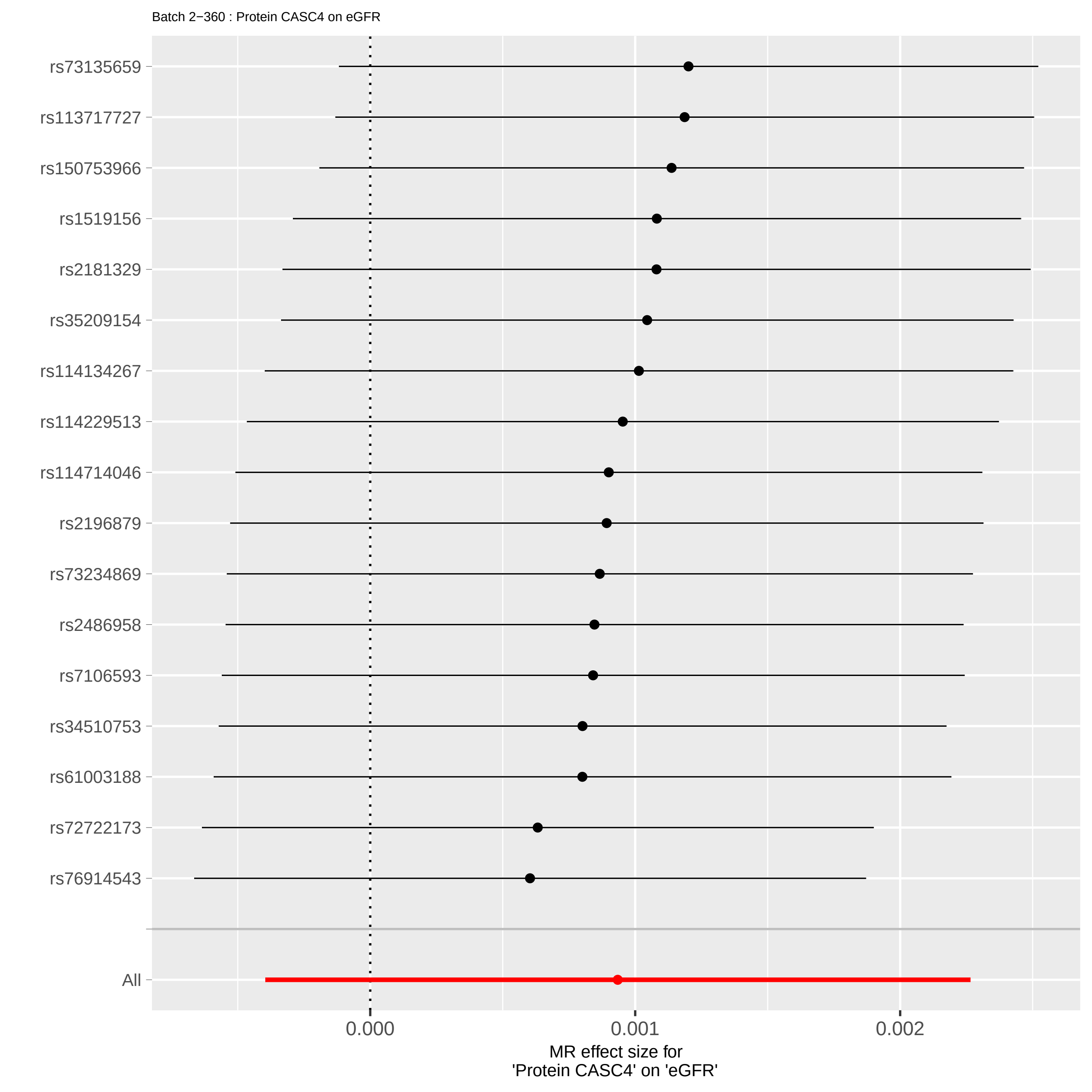

Batch 2−360 : Protein CASC4 on eGFR
rs73135659
rs113717727
rs150753966
rs1519156
rs2181329
rs35209154
rs114134267
rs114229513
rs114714046
rs2196879
rs73234869
rs2486958
rs7106593
rs34510753
rs61003188
rs72722173
rs76914543
All
0.000
0.001
0.002
MR effect size for
'Protein CASC4' on 'eGFR'

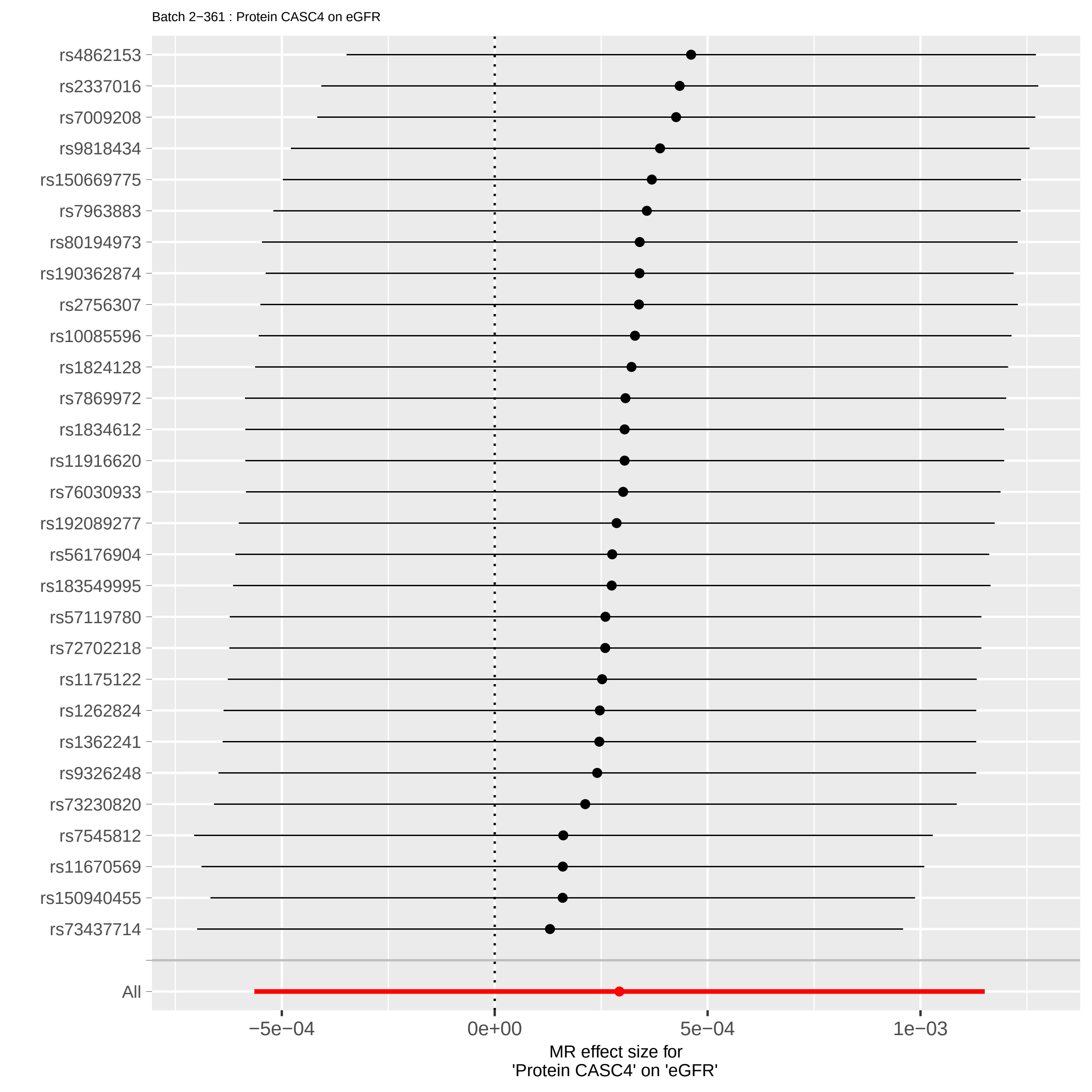

Batch 2−361 : Protein CASC4 on eGFR
rs4862153
rs2337016
rs7009208
rs9818434
rs150669775
rs7963883
rs80194973
rs190362874
rs2756307
rs10085596
rs1824128
rs7869972
rs1834612
rs11916620
rs76030933
rs192089277
rs56176904
rs183549995
rs57119780
rs72702218
rs1175122
rs1262824
rs1362241
rs9326248
rs73230820
rs7545812
rs11670569
rs150940455
rs73437714
All
−5e−04
0e+00
5e−04
1e−03
MR effect size for
'Protein CASC4' on 'eGFR'

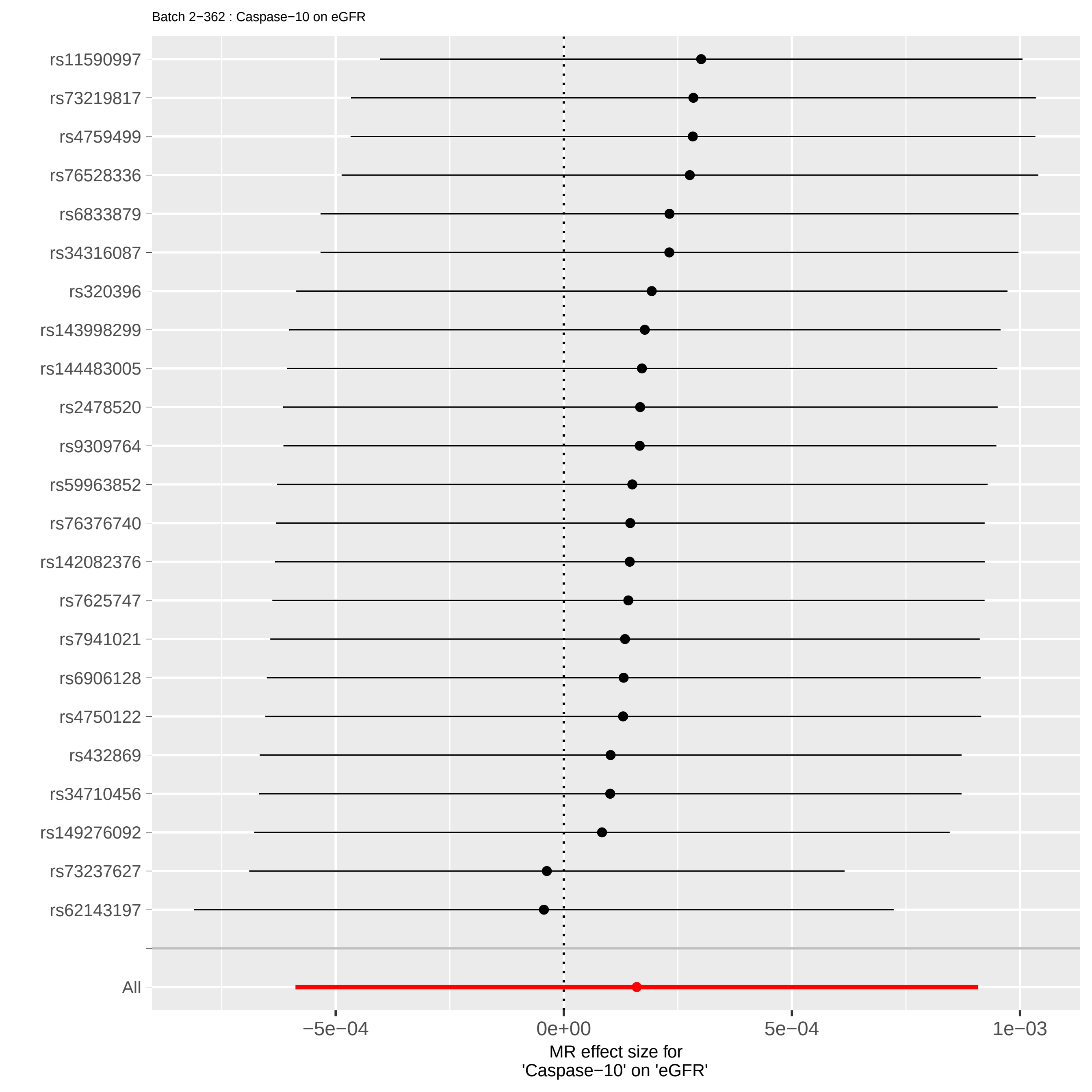
Batch 2−362 : Caspase−10 on eGFR
rs11590997
rs73219817
rs4759499
rs76528336
rs6833879
rs34316087
rs320396
rs143998299
rs144483005
rs2478520
rs9309764
rs59963852
rs76376740
rs142082376
rs7625747
rs7941021
rs6906128
rs4750122
rs432869
rs34710456
rs149276092
rs73237627
rs62143197
All
−5e−04
0e+00
5e−04
1e−03
MR effect size for
'Caspase−10' on 'eGFR'

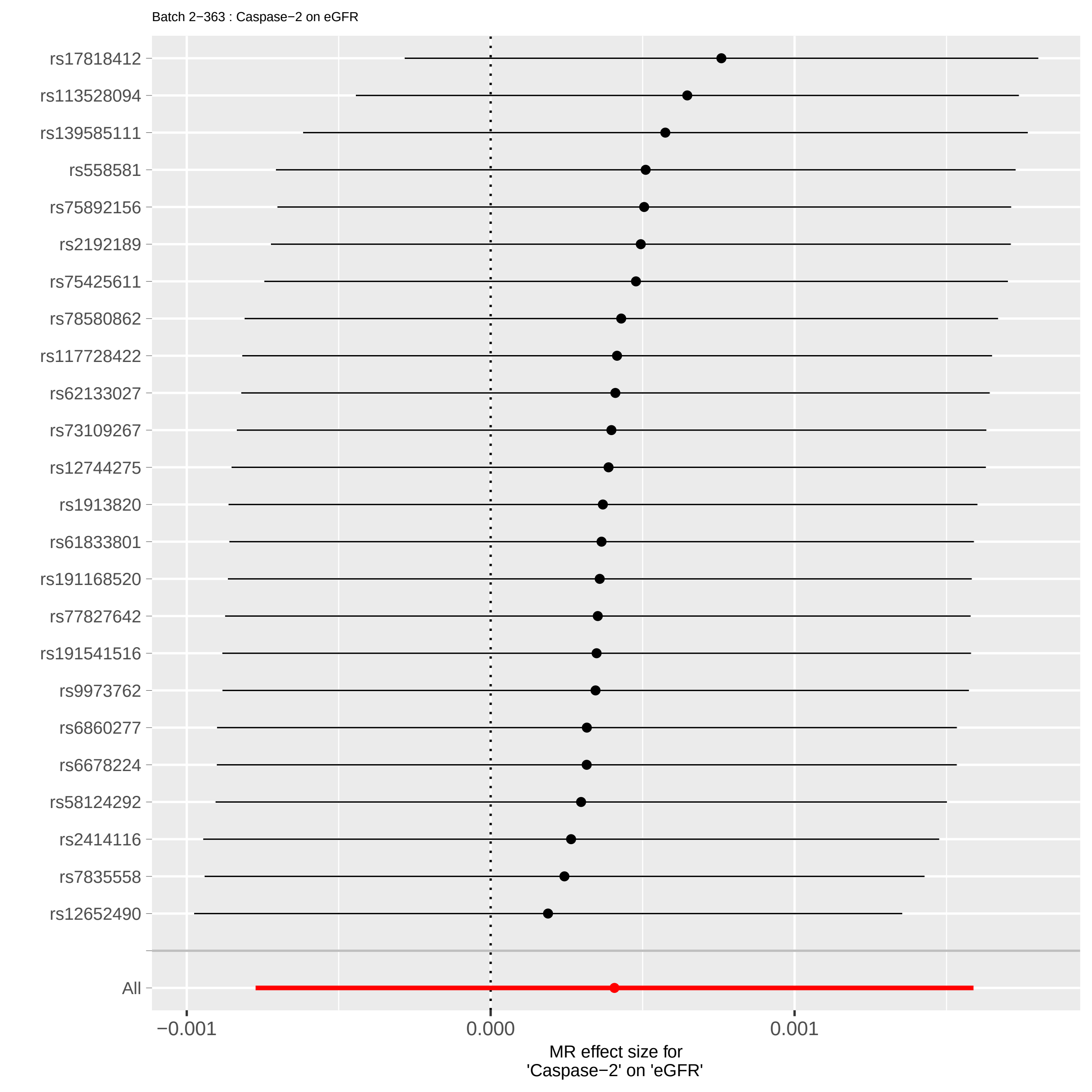

Batch 2−363 : Caspase−2 on eGFR
rs17818412
rs113528094
rs139585111
rs558581
rs75892156
rs2192189
rs75425611
rs78580862
rs117728422
rs62133027
rs73109267
rs12744275
rs1913820
rs61833801
rs191168520
rs77827642
rs191541516
rs9973762
rs6860277
rs6678224
rs58124292
rs2414116
rs7835558
rs12652490
All
−0.001
0.000
0.001
MR effect size for
'Caspase−2' on 'eGFR'

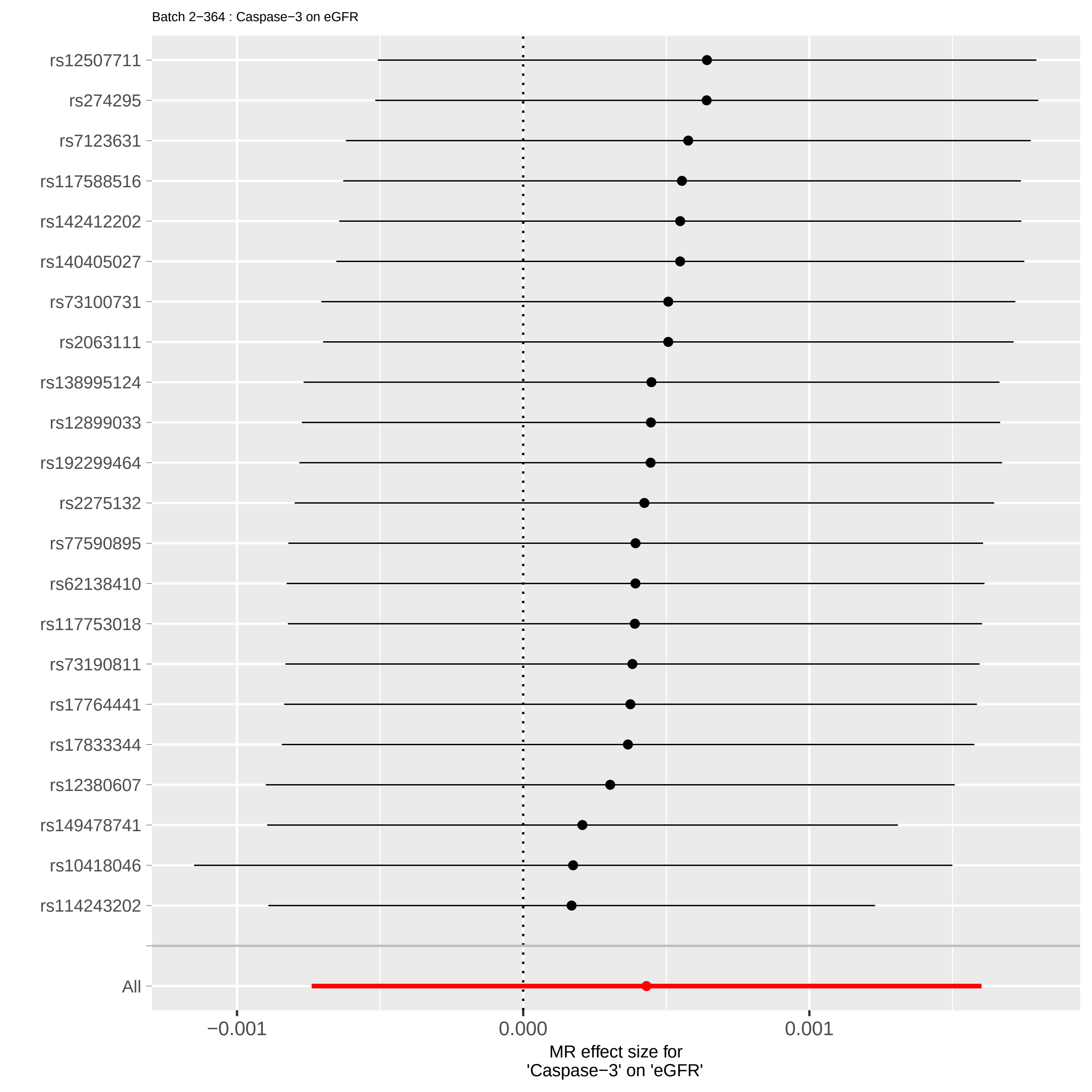
Batch 2−364 : Caspase−3 on eGFR
rs12507711
rs274295
rs7123631
rs117588516
rs142412202
rs140405027
rs73100731
rs2063111
rs138995124
rs12899033
rs192299464
rs2275132
rs77590895
rs62138410
rs117753018
rs73190811
rs17764441
rs17833344
rs12380607
rs149478741
rs10418046
rs114243202
All
−0.001
0.000
0.001
MR effect size for
'Caspase−3' on 'eGFR'

Batch 2−365 : Cas scaffolding protein family member 4 on eGFR

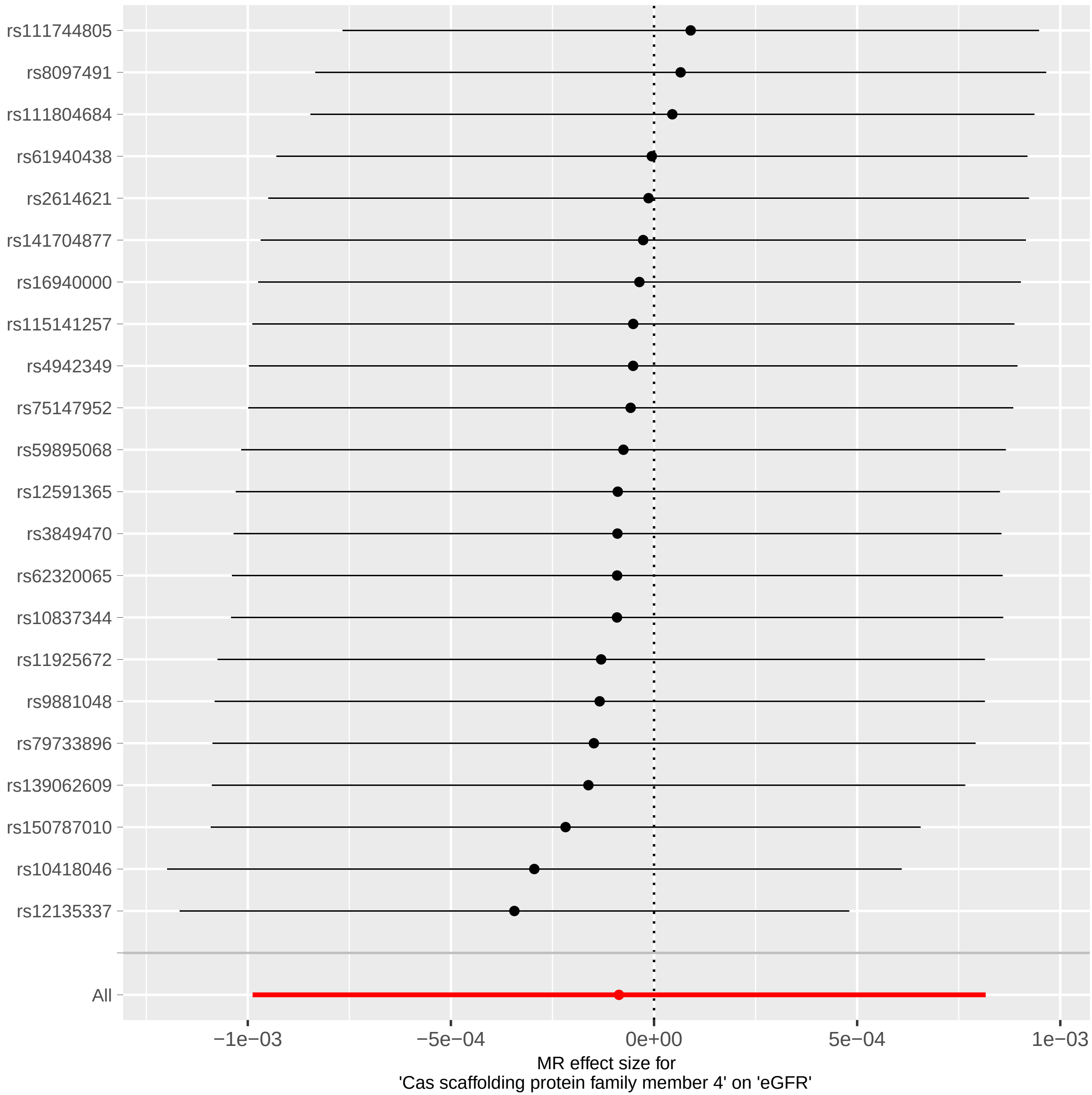

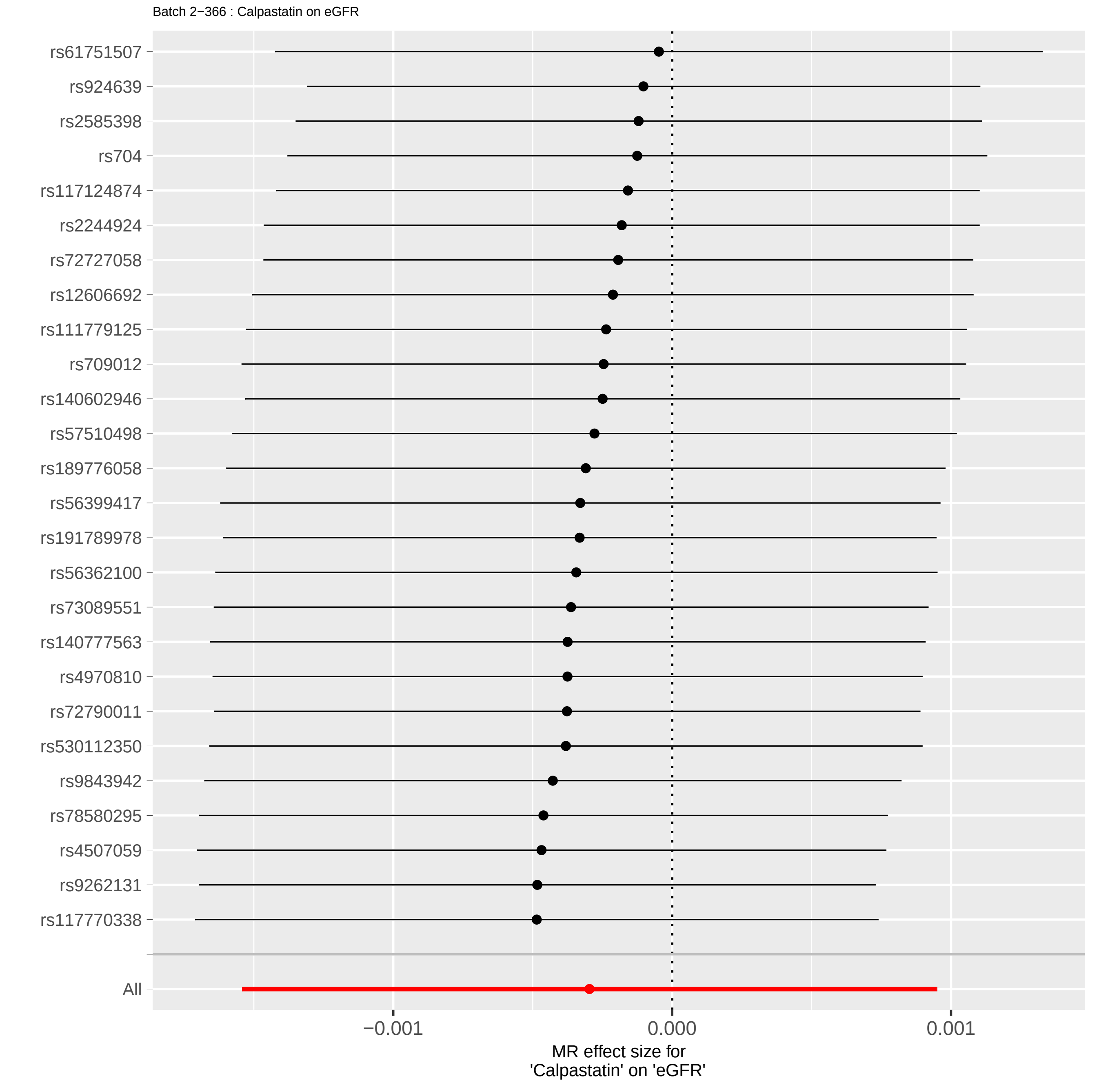

Batch 2−366 : Calpastatin on eGFR
rs61751507
rs924639
rs2585398
rs704
rs117124874
rs2244924
rs72727058
rs12606692
rs111779125
rs709012
rs140602946
rs57510498
rs189776058
rs56399417
rs191789978
rs56362100
rs73089551
rs140777563
rs4970810
rs72790011
rs530112350
rs9843942
rs78580295
rs4507059
rs9262131
rs117770338
All
−0.001
0.000
0.001
MR effect size for
'Calpastatin' on 'eGFR'

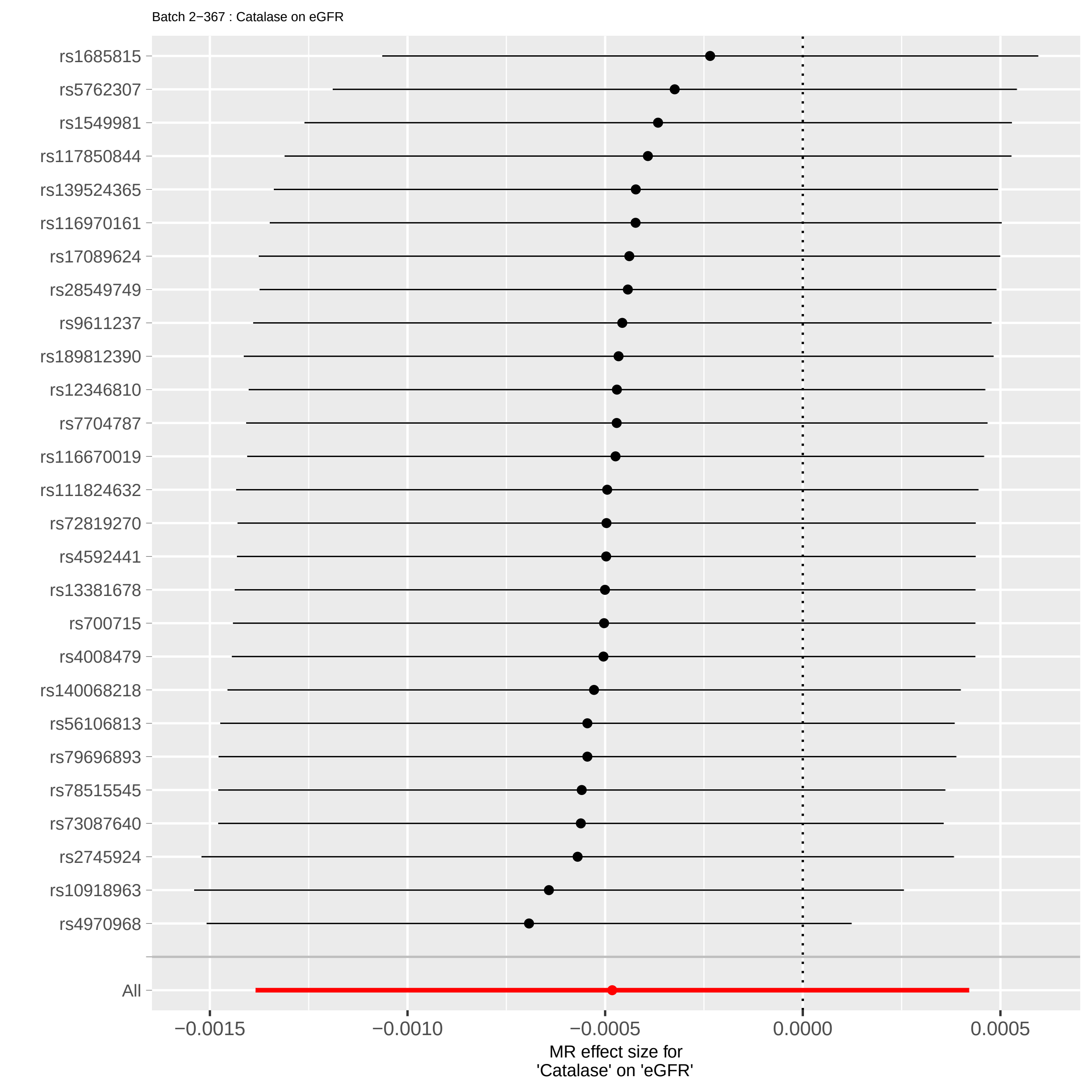

Batch 2−367 : Catalase on eGFR
rs1685815
rs5762307
rs1549981
rs117850844
rs139524365
rs116970161
rs17089624
rs28549749
rs9611237
rs189812390
rs12346810
rs7704787
rs116670019
rs111824632
rs72819270
rs4592441
rs13381678
rs700715
rs4008479
rs140068218
rs56106813
rs79696893
rs78515545
rs73087640
rs2745924
rs10918963
rs4970968
All
−0.0015
−0.0010
−0.0005
0.0000
0.0005
MR effect size for
'Catalase' on 'eGFR'

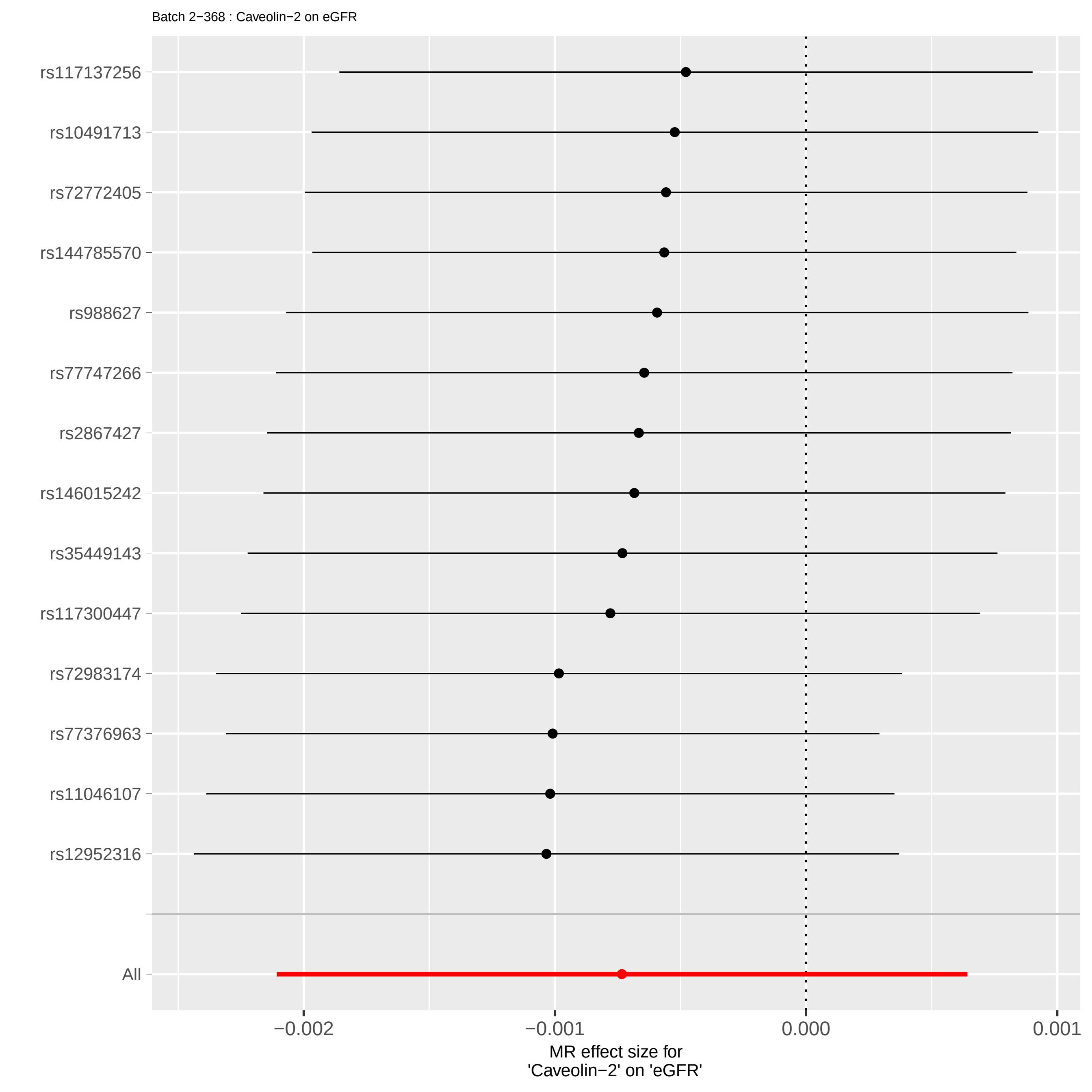
Batch 2−368 : Caveolin−2 on eGFR
rs117137256
rs10491713
rs72772405
rs144785570
rs988627
rs77747266
rs2867427
rs146015242
rs35449143
rs117300447
rs72983174
rs77376963
rs11046107
rs12952316
All
−0.002
−0.001
0.000
0.001
MR effect size for
'Caveolin−2' on 'eGFR'

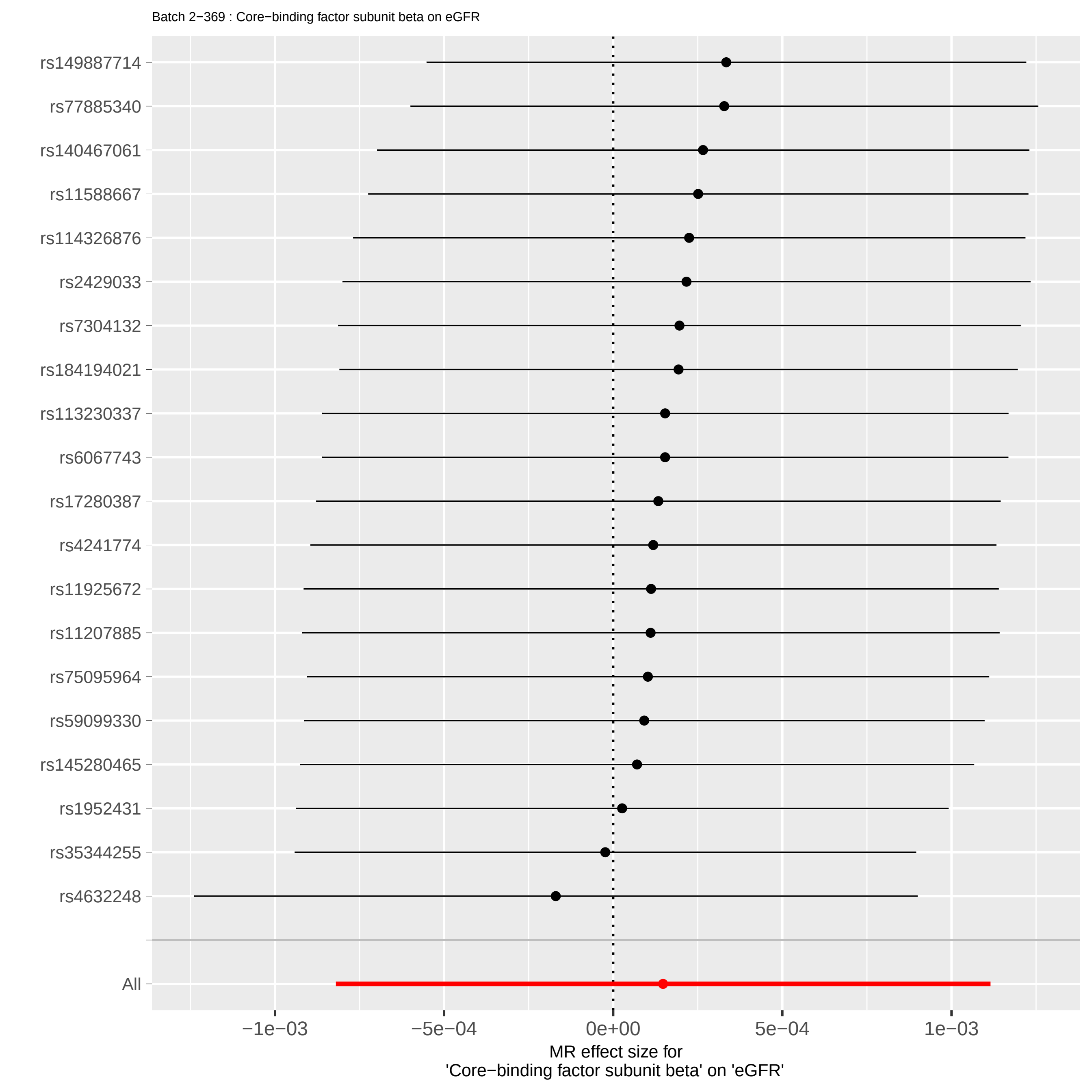

Batch 2−369 : Core−binding factor subunit beta on eGFR
rs149887714
rs77885340
rs140467061
rs11588667
rs114326876
rs2429033
rs7304132
rs184194021
rs113230337
rs6067743
rs17280387
rs4241774
rs11925672
rs11207885
rs75095964
rs59099330
rs145280465
rs1952431
rs35344255
rs4632248
All
−1e−03
−5e−04
0e+00
5e−04
1e−03
MR effect size for
'Core−binding factor subunit beta' on 'eGFR'

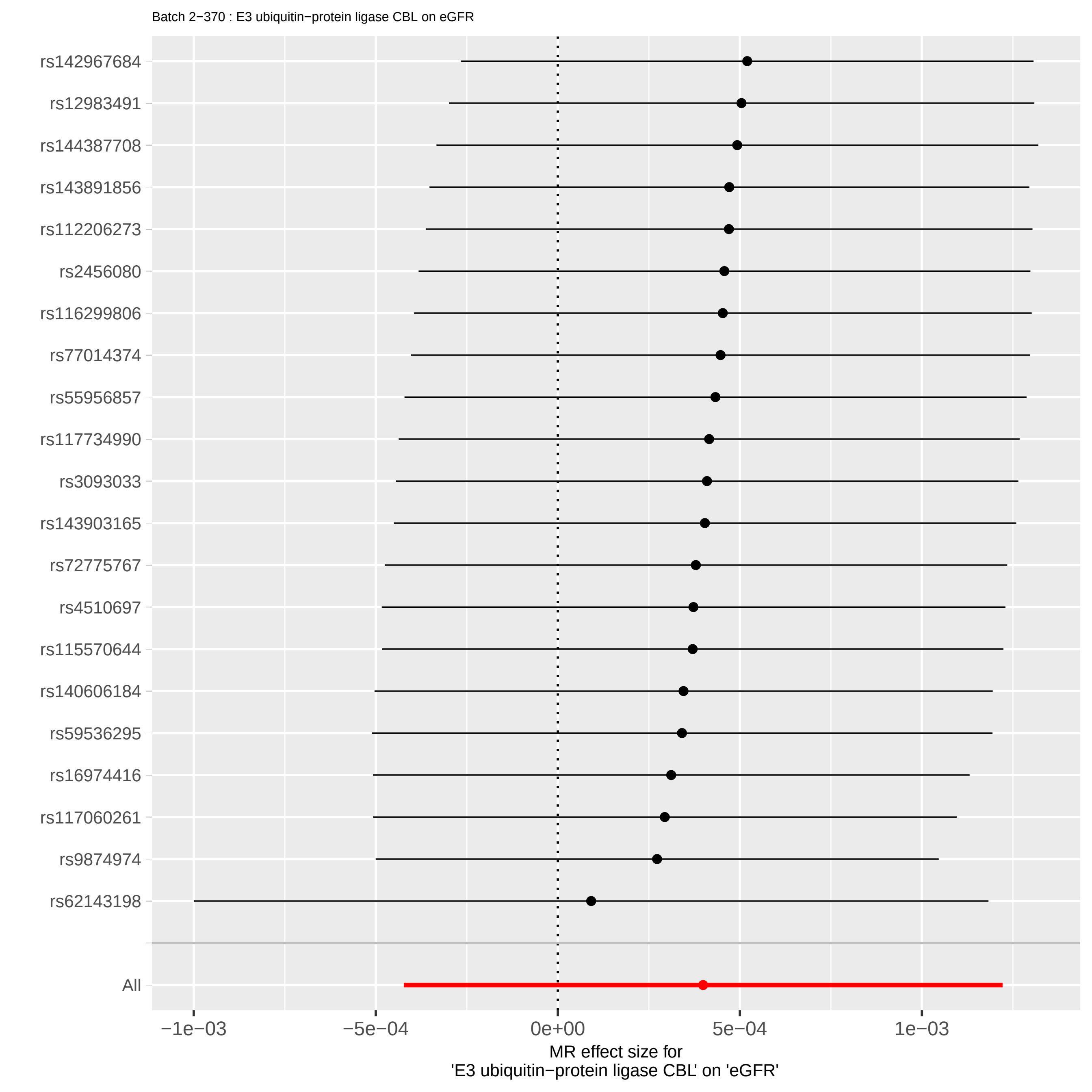

Batch 2−370 : E3 ubiquitin−protein ligase CBL on eGFR
rs142967684
rs12983491
rs144387708
rs143891856
rs112206273
rs2456080
rs116299806
rs77014374
rs55956857
rs117734990
rs3093033
rs143903165
rs72775767
rs4510697
rs115570644
rs140606184
rs59536295
rs16974416
rs117060261
rs9874974
rs62143198
All
−1e−03
−5e−04
0e+00
5e−04
1e−03
MR effect size for
'E3 ubiquitin−protein ligase CBL' on 'eGFR'

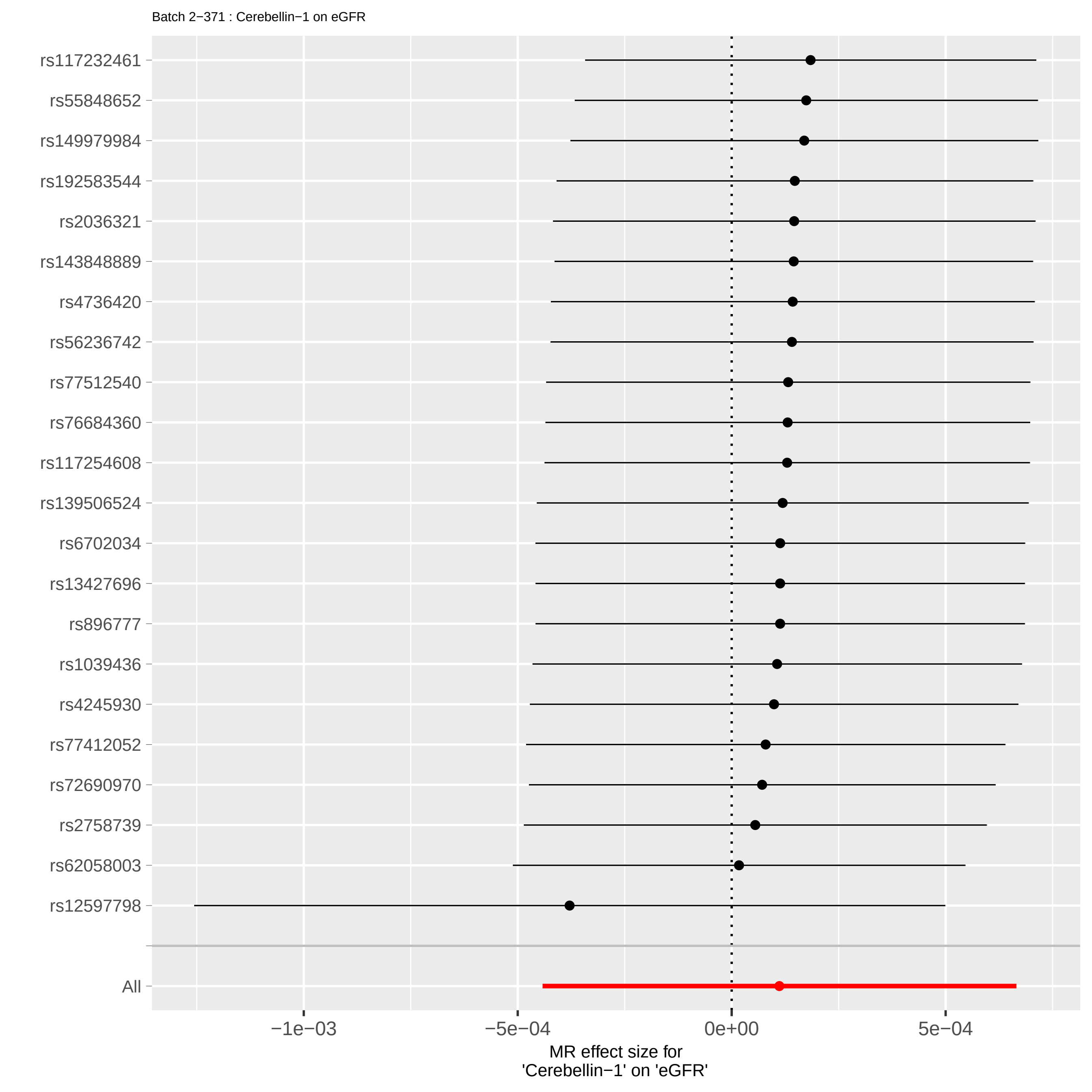

Batch 2−371 : Cerebellin−1 on eGFR
rs117232461
rs55848652
rs149979984
rs192583544
rs2036321
rs143848889
rs4736420
rs56236742
rs77512540
rs76684360
rs117254608
rs139506524
rs6702034
rs13427696
rs896777
rs1039436
rs4245930
rs77412052
rs72690970
rs2758739
rs62058003
rs12597798
All
−1e−03
−5e−04
0e+00
5e−04
MR effect size for
'Cerebellin−1' on 'eGFR'

Batch 2−372 : Cerebellin−4 on eGFR

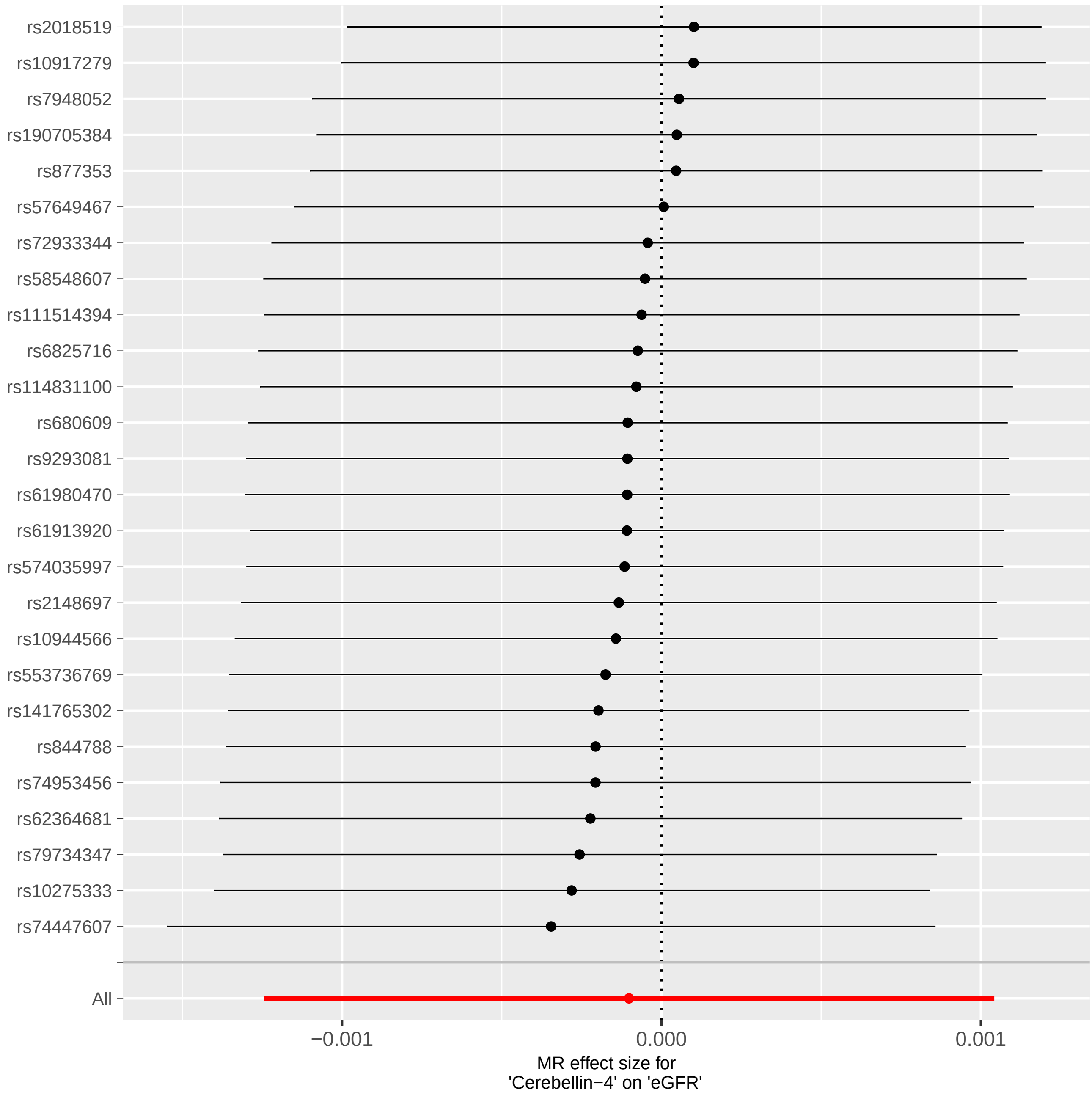

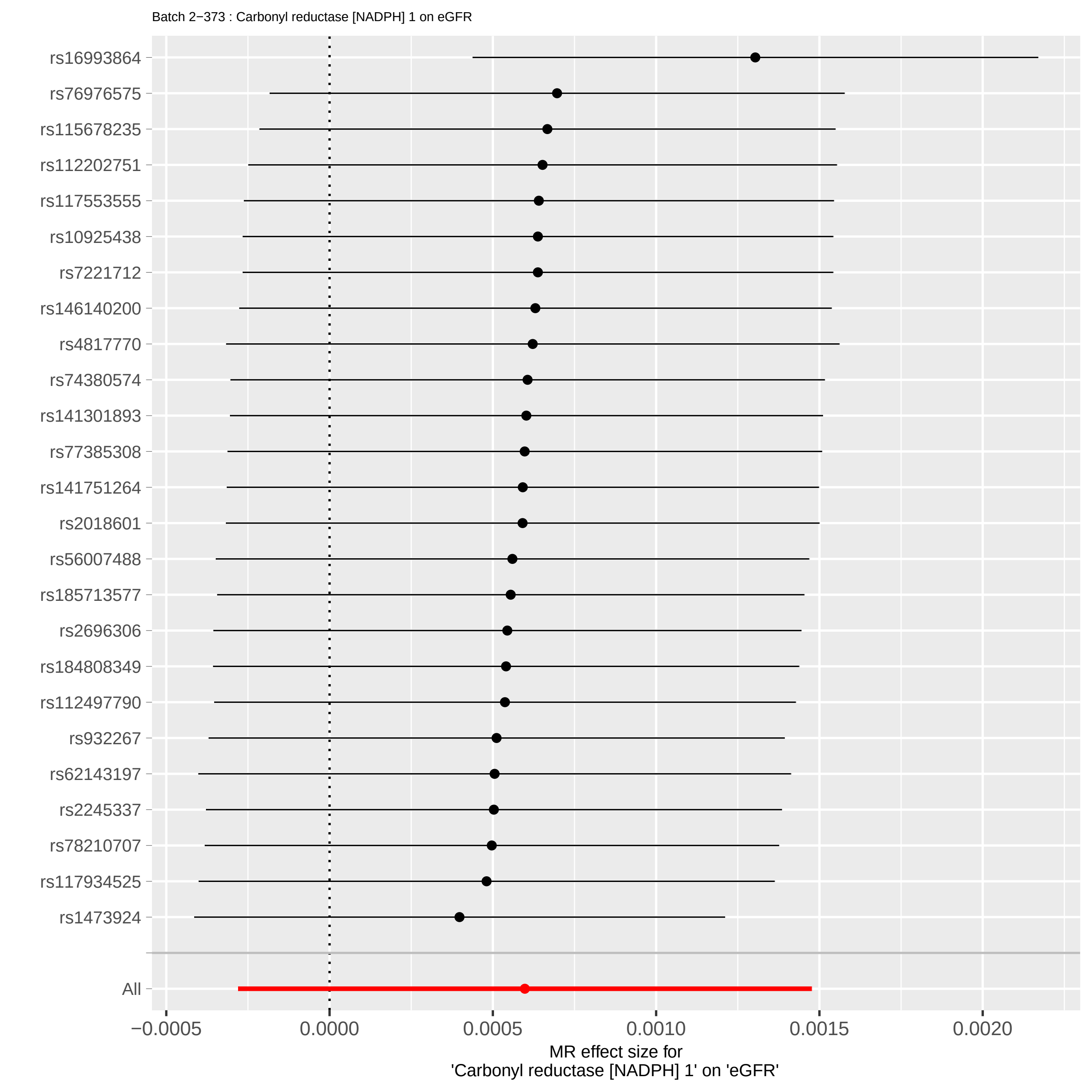

Batch 2−373 : Carbonyl reductase [NADPH] 1 on eGFR
rs16993864
rs76976575
rs115678235
rs112202751
rs117553555
rs10925438
rs7221712
rs146140200
rs4817770
rs74380574
rs141301893
rs77385308
rs141751264
rs2018601
rs56007488
rs185713577
rs2696306
rs184808349
rs112497790
rs932267
rs62143197
rs2245337
rs78210707
rs117934525
rs1473924
All
−0.0005
0.0000
0.0005
0.0010
0.0015
0.0020
MR effect size for
'Carbonyl reductase [NADPH] 1' on 'eGFR'

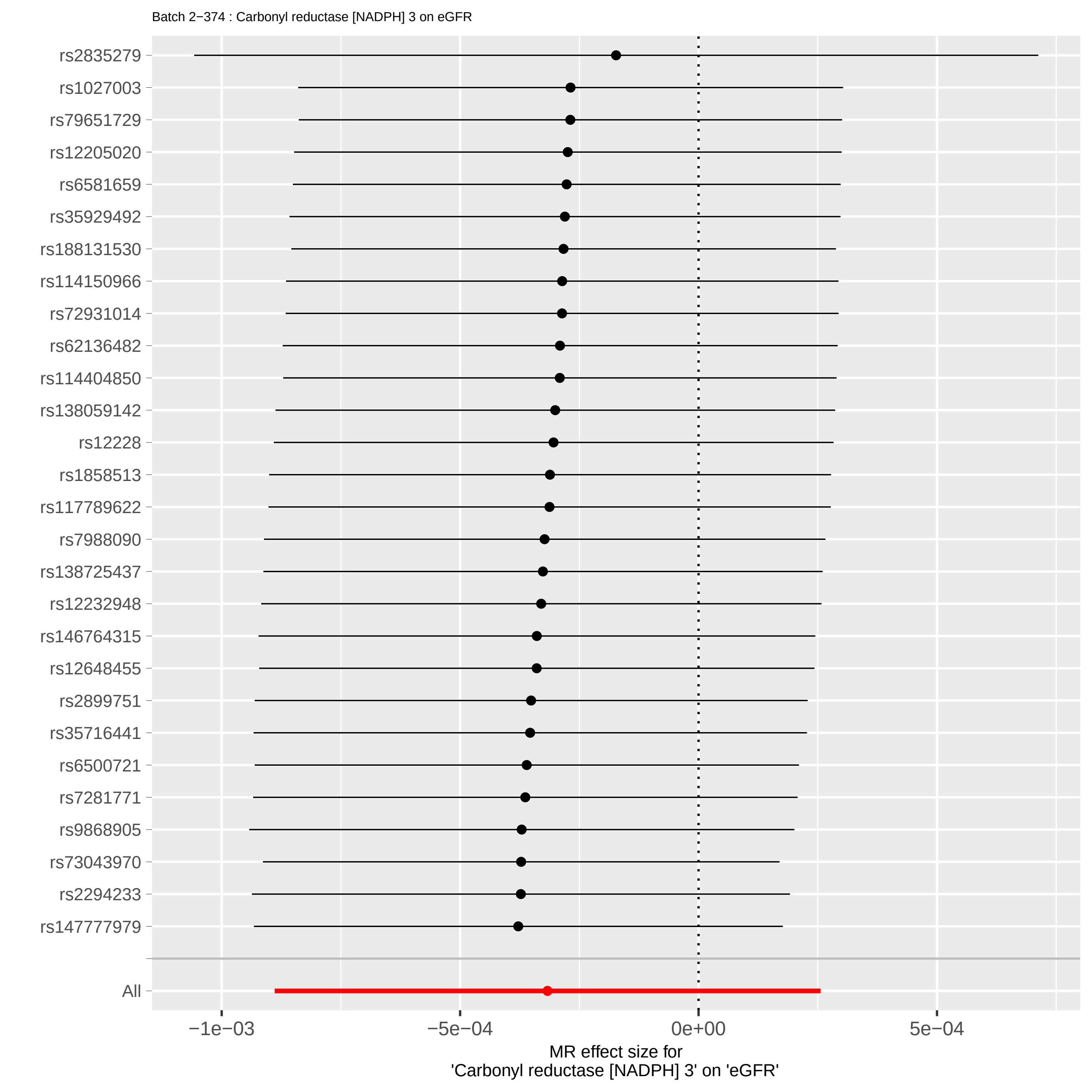

Batch 2−374 : Carbonyl reductase [NADPH] 3 on eGFR
rs2835279
rs1027003
rs79651729
rs12205020
rs6581659
rs35929492
rs188131530
rs114150966
rs72931014
rs62136482
rs114404850
rs138059142
rs12228
rs1858513
rs117789622
rs7988090
rs138725437
rs12232948
rs146764315
rs12648455
rs2899751
rs35716441
rs6500721
rs7281771
rs9868905
rs73043970
rs2294233
rs147777979
All
−1e−03
−5e−04
0e+00
5e−04
MR effect size for
'Carbonyl reductase [NADPH] 3' on 'eGFR'

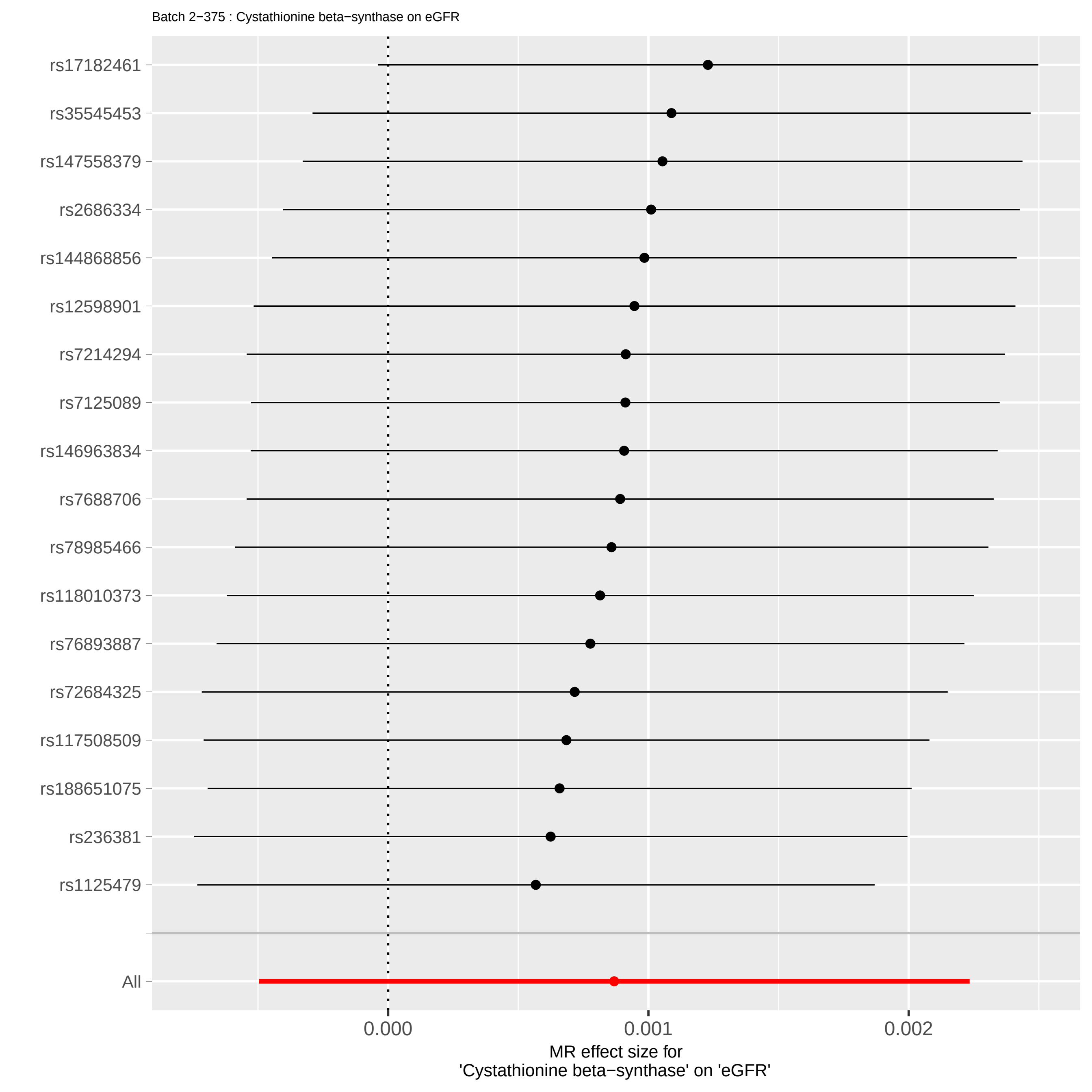

Batch 2−375 : Cystathionine beta−synthase on eGFR
rs17182461
rs35545453
rs147558379
rs2686334
rs144868856
rs12598901
rs7214294
rs7125089
rs146963834
rs7688706
rs78985466
rs118010373
rs76893887
rs72684325
rs117508509
rs188651075
rs236381
rs1125479
All
0.000
0.001
0.002
MR effect size for
'Cystathionine beta−synthase' on 'eGFR'

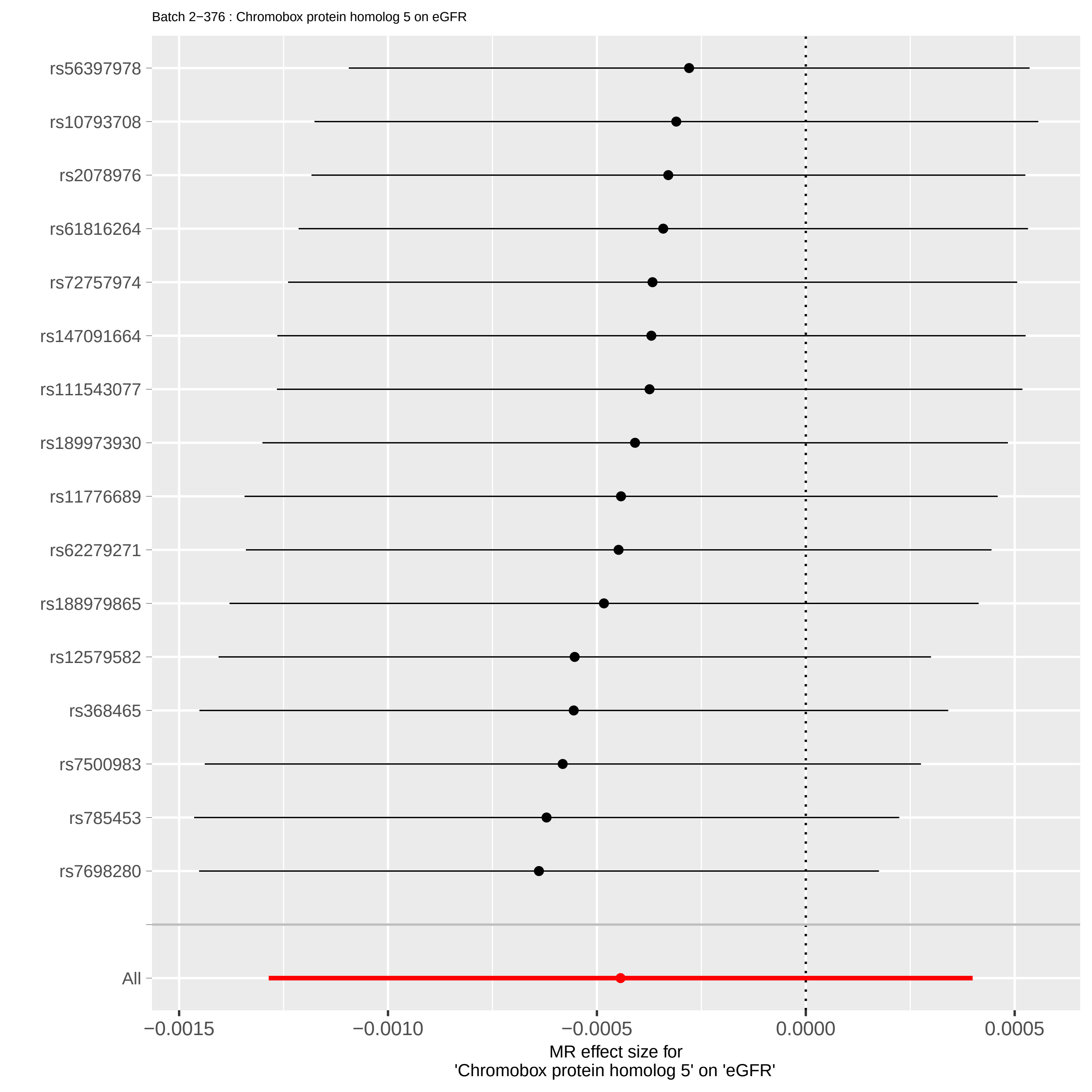

Batch 2−376 : Chromobox protein homolog 5 on eGFR
rs56397978
rs10793708
rs2078976
rs61816264
rs72757974
rs147091664
rs111543077
rs189973930
rs11776689
rs62279271
rs188979865
rs12579582
rs368465
rs7500983
rs785453
rs7698280
All
−0.0015
−0.0010
−0.0005
0.0000
0.0005
MR effect size for
'Chromobox protein homolog 5' on 'eGFR'

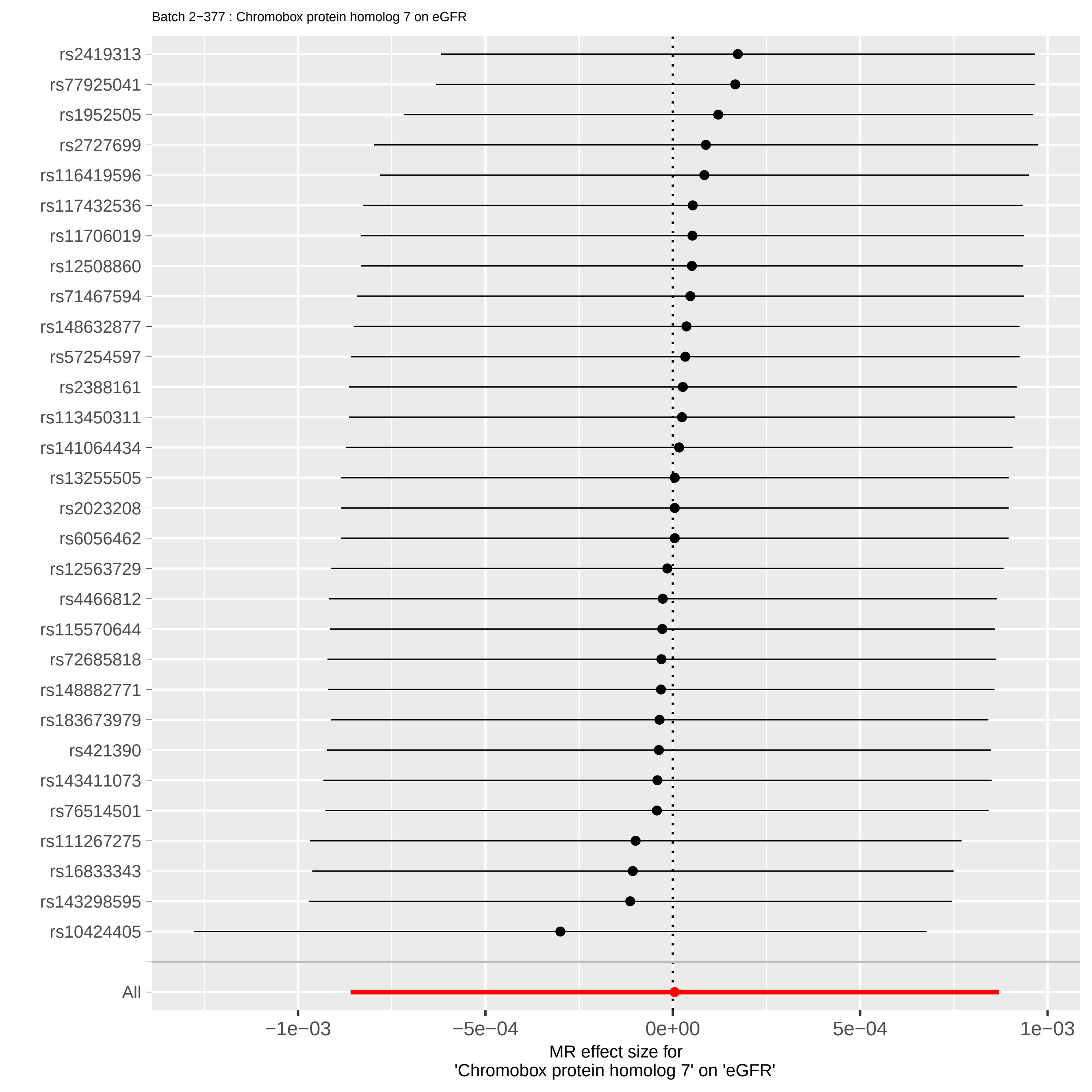
Batch 2−377 : Chromobox protein homolog 7 on eGFR
rs2419313
rs77925041
rs1952505
rs2727699
rs116419596
rs117432536
rs11706019
rs12508860
rs71467594
rs148632877
rs57254597
rs2388161
rs113450311
rs141064434
rs13255505
rs2023208
rs6056462
rs12563729
rs4466812
rs115570644
rs72685818
rs148882771
rs183673979
rs421390
rs143411073
rs76514501
rs111267275
rs16833343
rs143298595
rs10424405
All
−1e−03
−5e−04
0e+00
5e−04
1e−03
MR effect size for
'Chromobox protein homolog 7' on 'eGFR'

Batch 2−378 : Kynurenine−−oxoglutarate transaminase 3 on eGFR

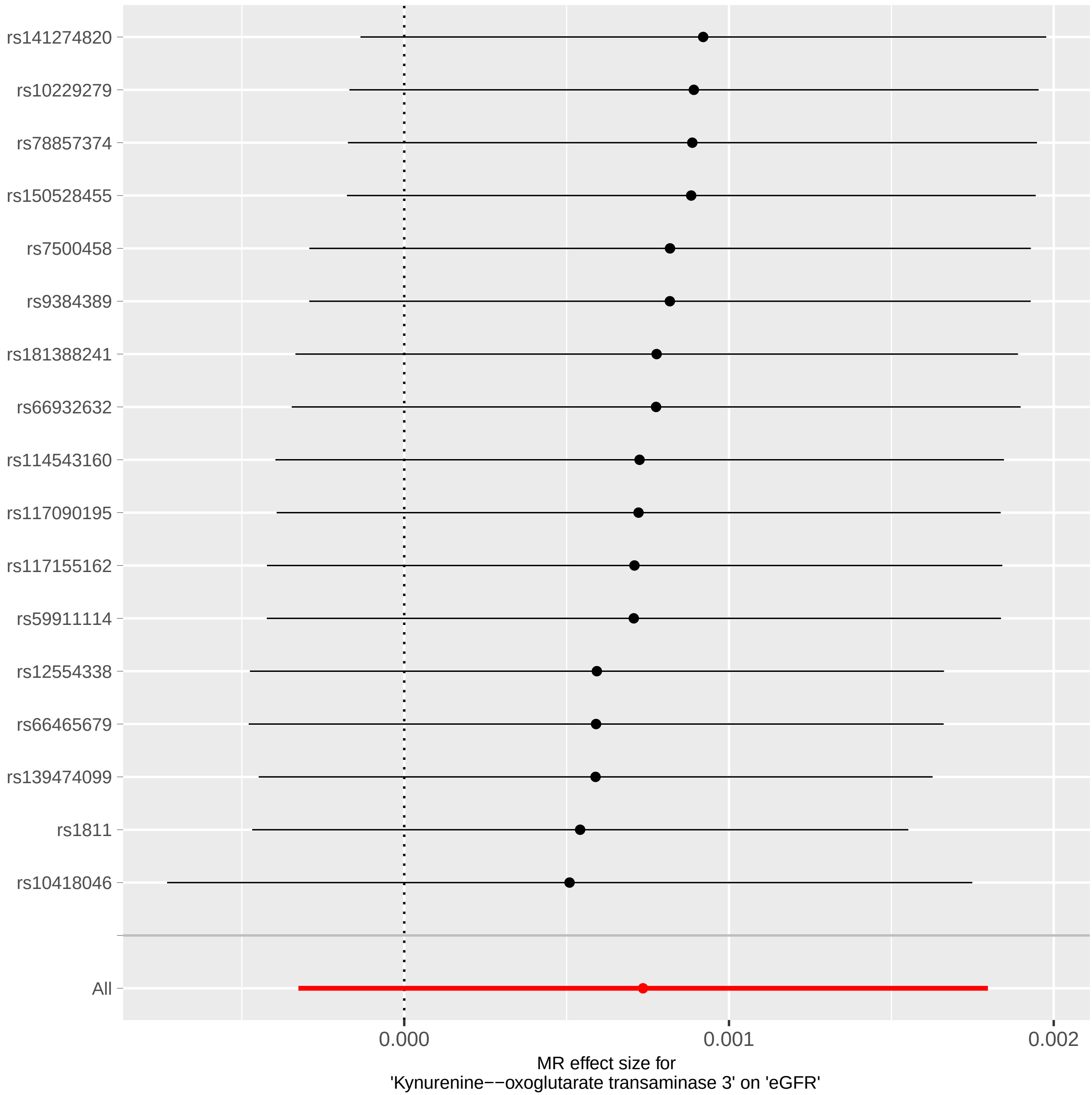

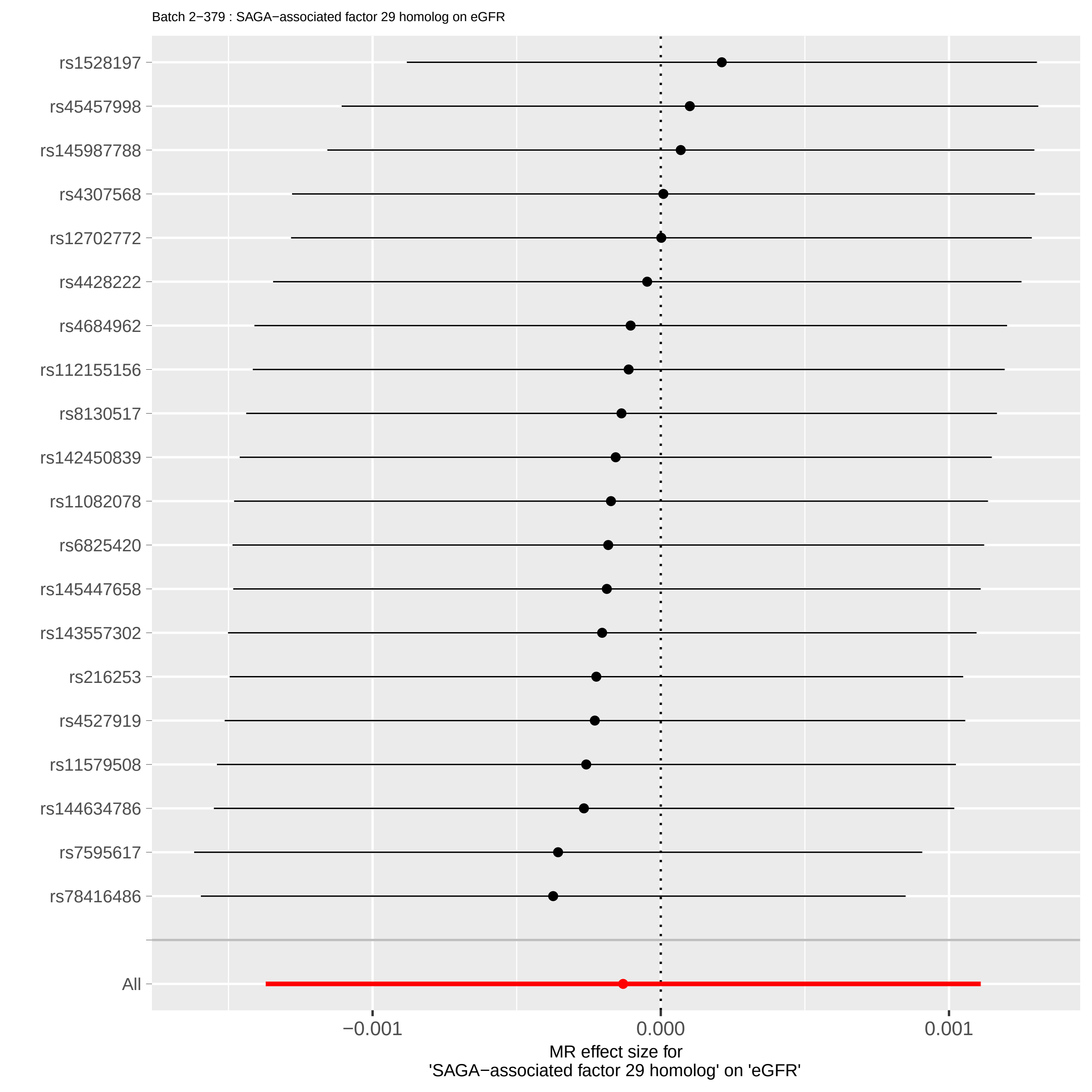

Batch 2−379 : SAGA−associated factor 29 homolog on eGFR
rs1528197
rs45457998
rs145987788
rs4307568
rs12702772
rs4428222
rs4684962
rs112155156
rs8130517
rs142450839
rs11082078
rs6825420
rs145447658
rs143557302
rs216253
rs4527919
rs11579508
rs144634786
rs7595617
rs78416486
All
−0.001
0.000
0.001
MR effect size for
'SAGA−associated factor 29 homolog' on 'eGFR'

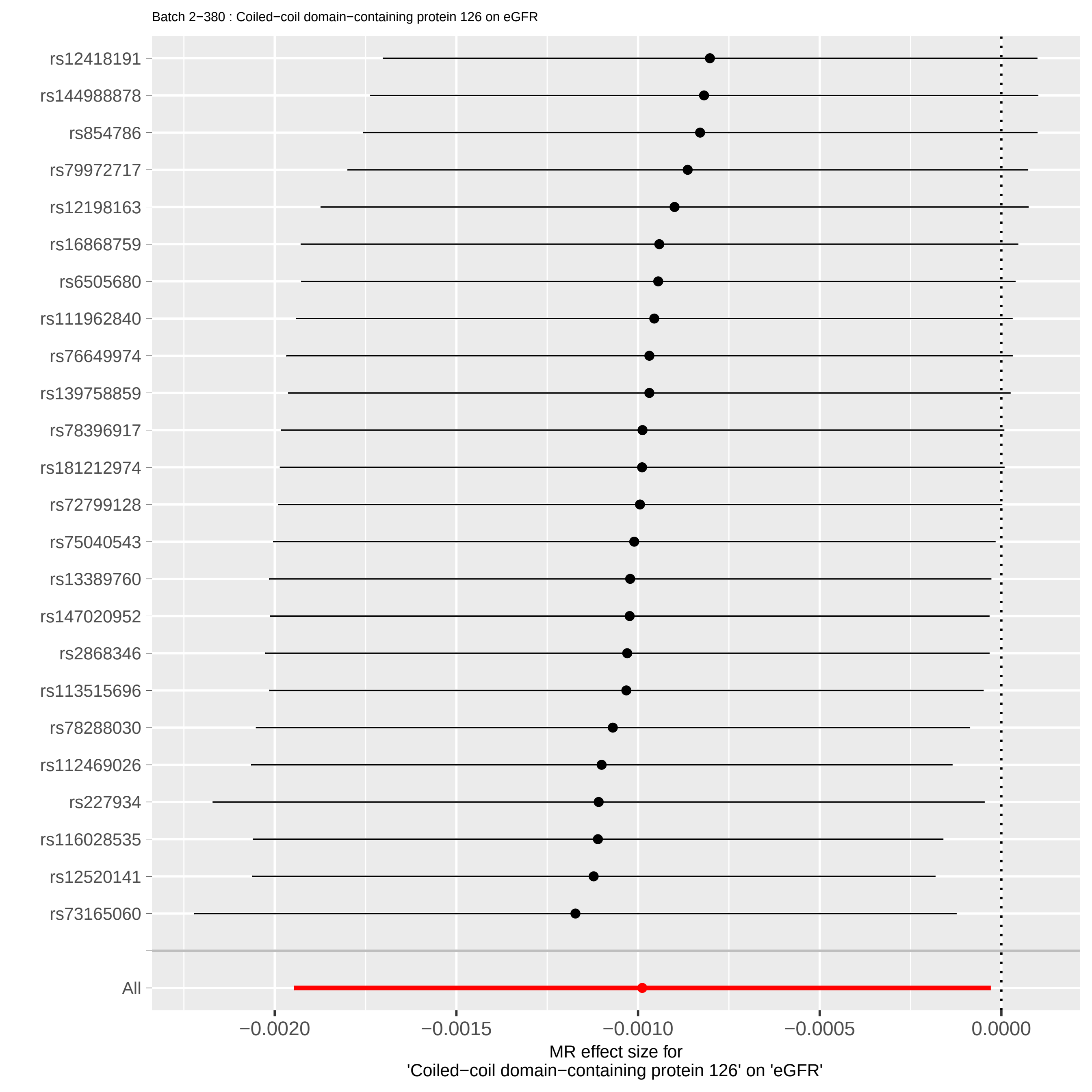
Batch 2−380 : Coiled−coil domain−containing protein 126 on eGFR
rs12418191
rs144988878
rs854786
rs79972717
rs12198163
rs16868759
rs6505680
rs111962840
rs76649974
rs139758859
rs78396917
rs181212974
rs72799128
rs75040543
rs13389760
rs147020952
rs2868346
rs113515696
rs78288030
rs112469026
rs227934
rs116028535
rs12520141
rs73165060
All
−0.0020
−0.0015
−0.0010
−0.0005
0.0000
MR effect size for
'Coiled−coil domain−containing protein 126' on 'eGFR'

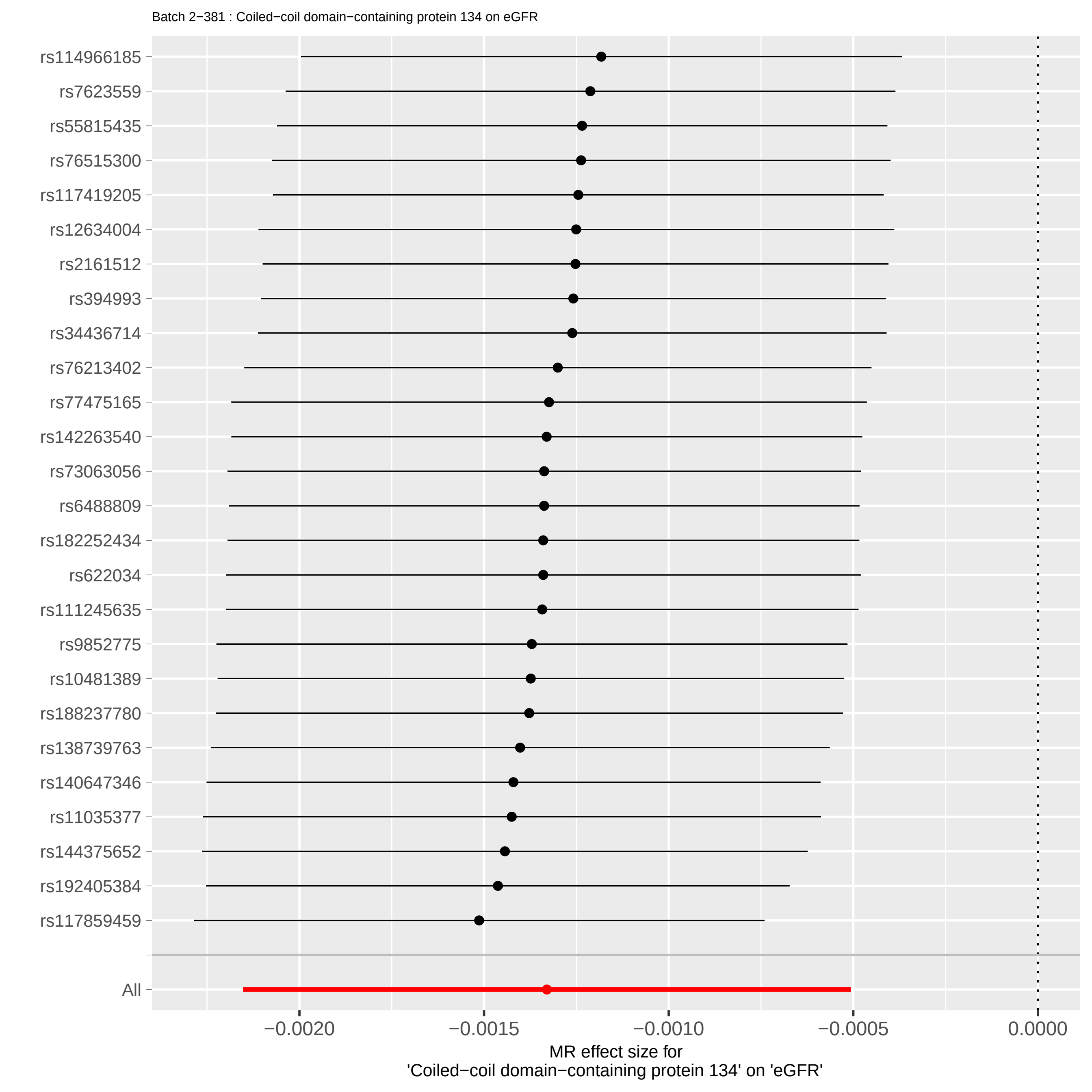
Batch 2−381 : Coiled−coil domain−containing protein 134 on eGFR
rs114966185
rs7623559
rs55815435
rs76515300
rs117419205
rs12634004
rs2161512
rs394993
rs34436714
rs76213402
rs77475165
rs142263540
rs73063056
rs6488809
rs182252434
rs622034
rs111245635
rs9852775
rs10481389
rs188237780
rs138739763
rs140647346
rs11035377
rs144375652
rs192405384
rs117859459
All
−0.0020
−0.0015
−0.0010
−0.0005
0.0000
MR effect size for
'Coiled−coil domain−containing protein 134' on 'eGFR'

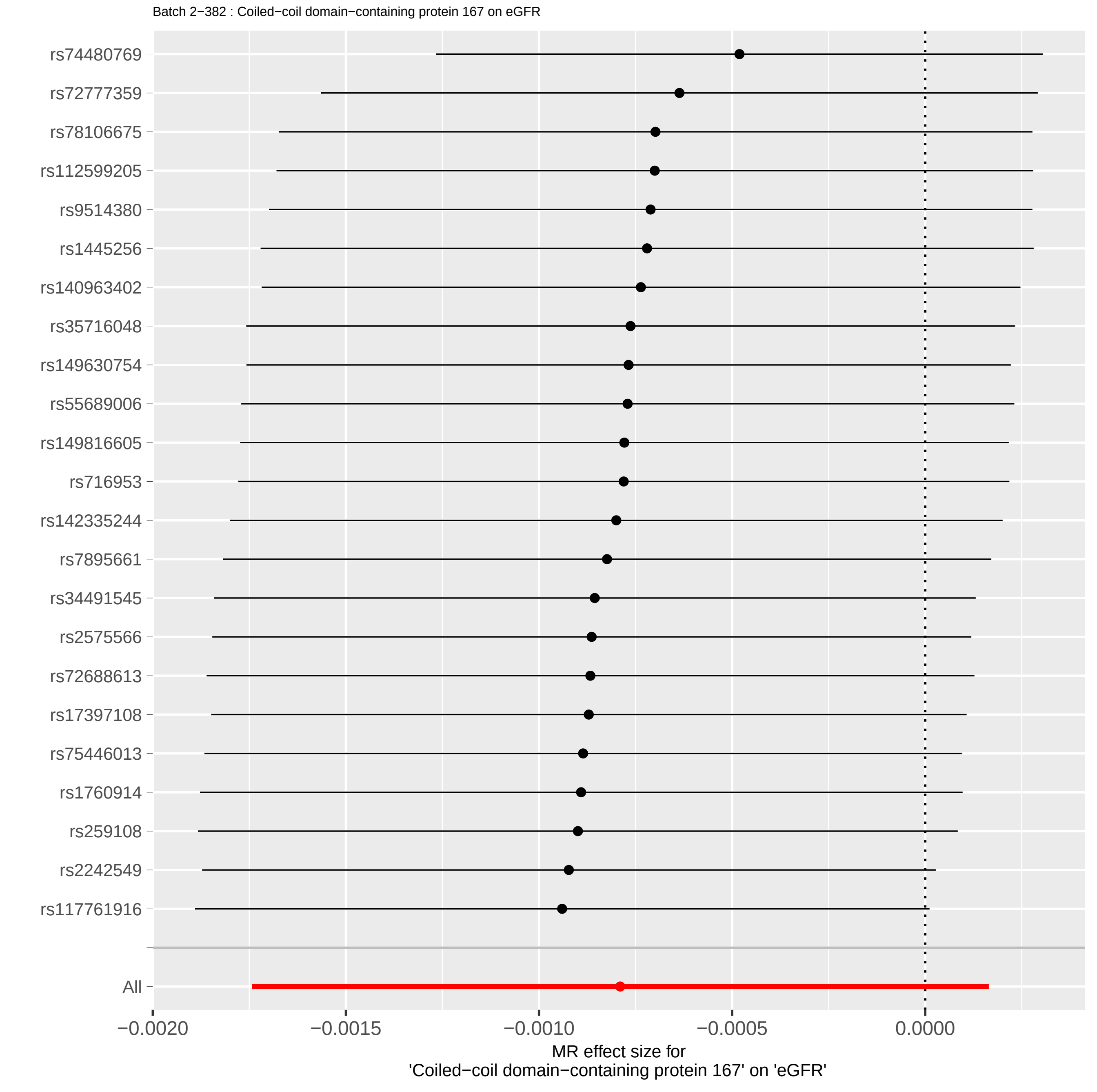

Batch 2−382 : Coiled−coil domain−containing protein 167 on eGFR
rs74480769
rs72777359
rs78106675
rs112599205
rs9514380
rs1445256
rs140963402
rs35716048
rs149630754
rs55689006
rs149816605
rs716953
rs142335244
rs7895661
rs34491545
rs2575566
rs72688613
rs17397108
rs75446013
rs1760914
rs259108
rs2242549
rs117761916
All
−0.0020
−0.0015
−0.0010
−0.0005
0.0000
MR effect size for
'Coiled−coil domain−containing protein 167' on 'eGFR'

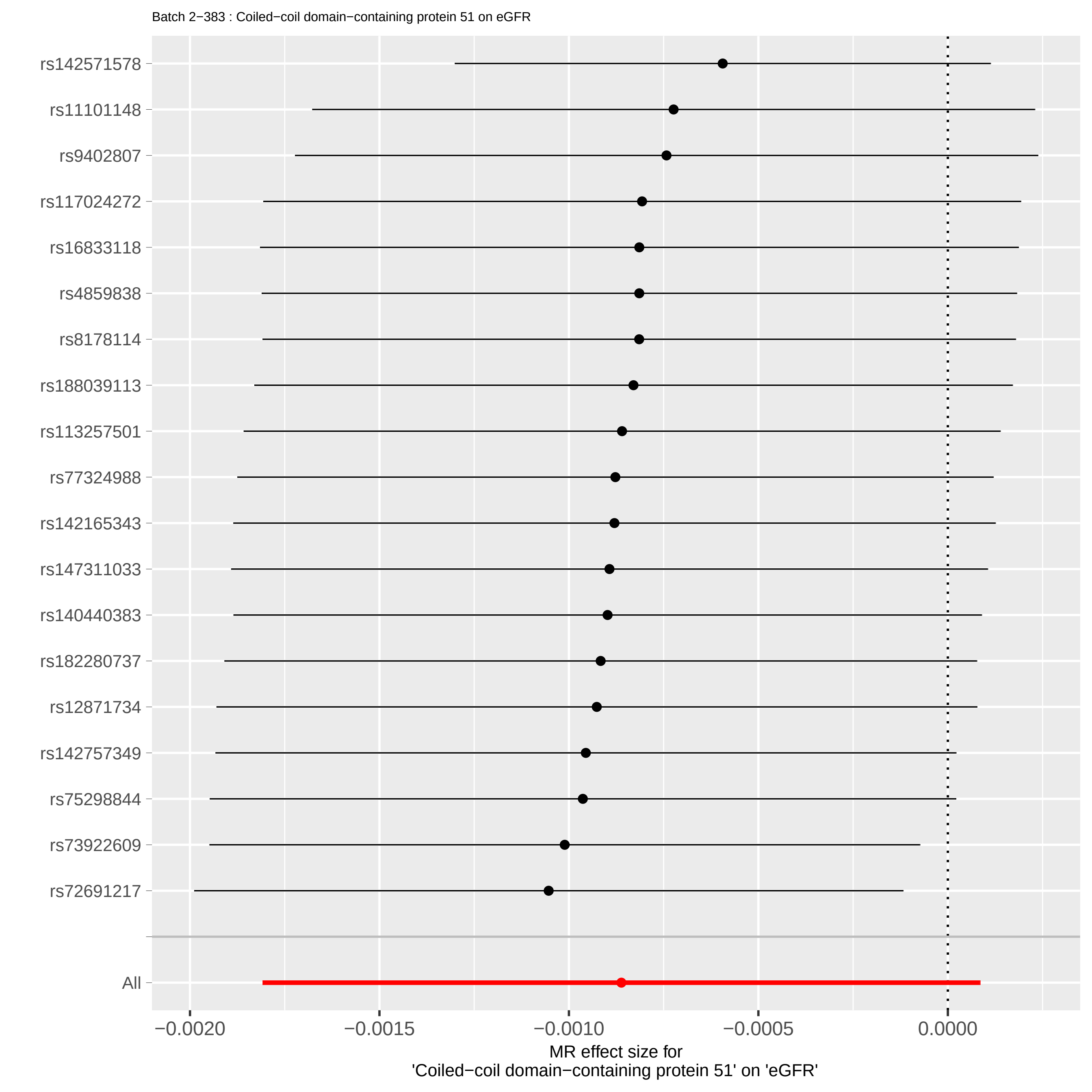

Batch 2−383 : Coiled−coil domain−containing protein 51 on eGFR
rs142571578
rs11101148
rs9402807
rs117024272
rs16833118
rs4859838
rs8178114
rs188039113
rs113257501
rs77324988
rs142165343
rs147311033
rs140440383
rs182280737
rs12871734
rs142757349
rs75298844
rs73922609
rs72691217
All
−0.0020
−0.0015
−0.0010
−0.0005
0.0000
MR effect size for
'Coiled−coil domain−containing protein 51' on 'eGFR'

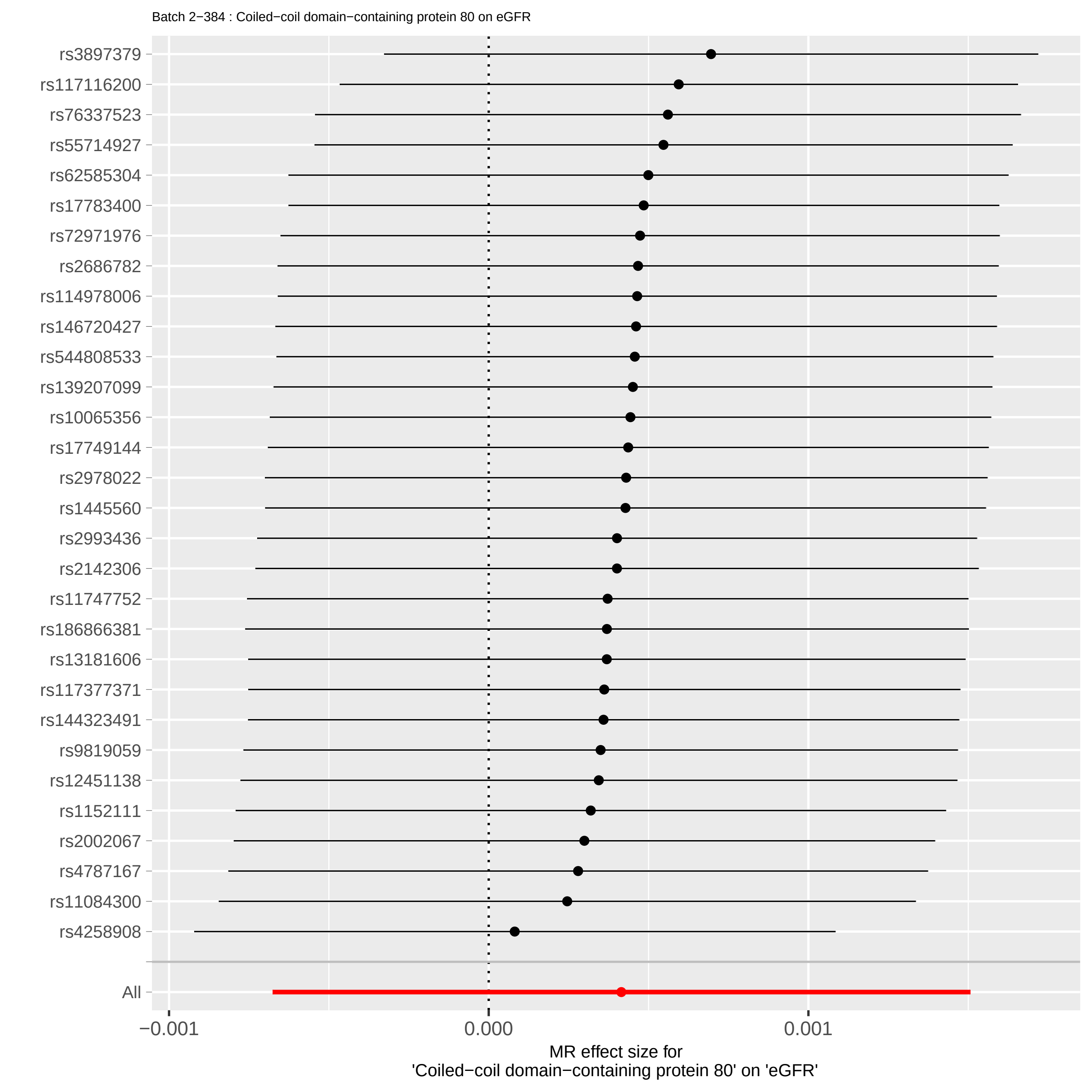

Batch 2−384 : Coiled−coil domain−containing protein 80 on eGFR
rs3897379
rs117116200
rs76337523
rs55714927
rs62585304
rs17783400
rs72971976
rs2686782
rs114978006
rs146720427
rs544808533
rs139207099
rs10065356
rs17749144
rs2978022
rs1445560
rs2993436
rs2142306
rs11747752
rs186866381
rs13181606
rs117377371
rs144323491
rs9819059
rs12451138
rs1152111
rs2002067
rs4787167
rs11084300
rs4258908
All
−0.001
0.000
0.001
MR effect size for
'Coiled−coil domain−containing protein 80' on 'eGFR'

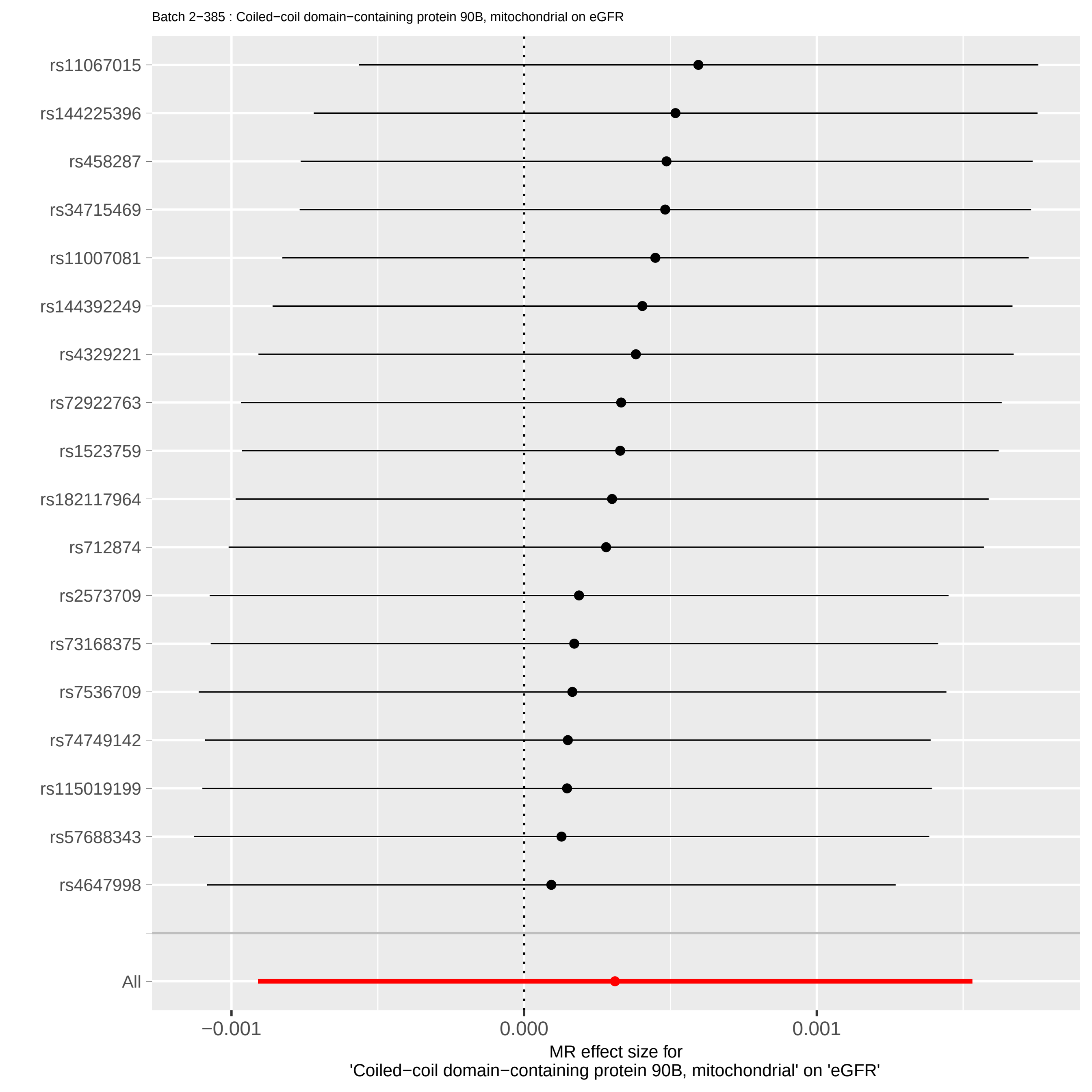
Batch 2−385 : Coiled−coil domain−containing protein 90B, mitochondrial on eGFR
rs11067015
rs144225396
rs458287
rs34715469
rs11007081
rs144392249
rs4329221
rs72922763
rs1523759
rs182117964
rs712874
rs2573709
rs73168375
rs7536709
rs74749142
rs115019199
rs57688343
rs4647998
All
−0.001
0.000
0.001
MR effect size for
'Coiled−coil domain−containing protein 90B, mitochondrial' on 'eGFR'

Batch 2−386 : C−C motif chemokine 1 on eGFR

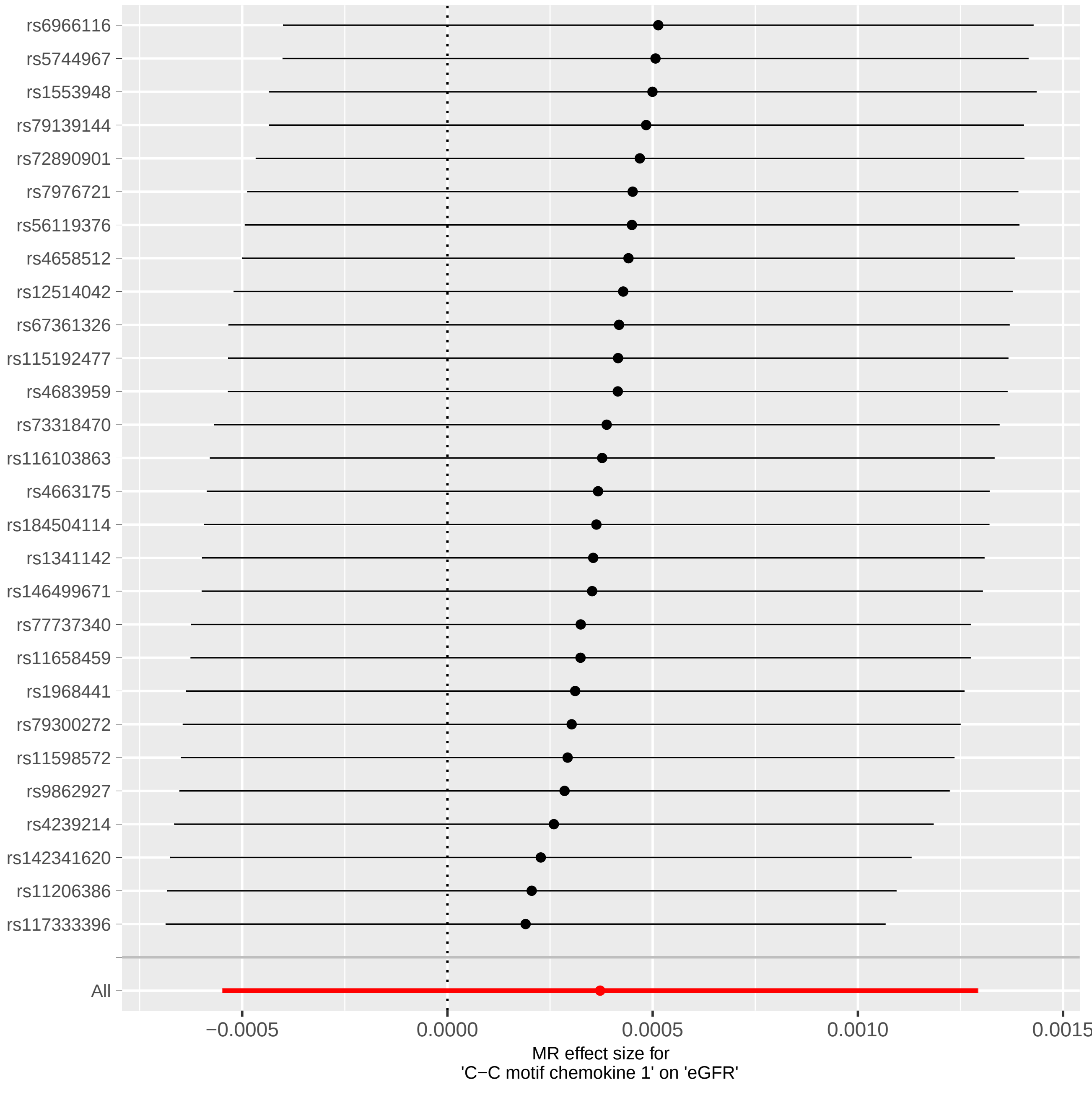

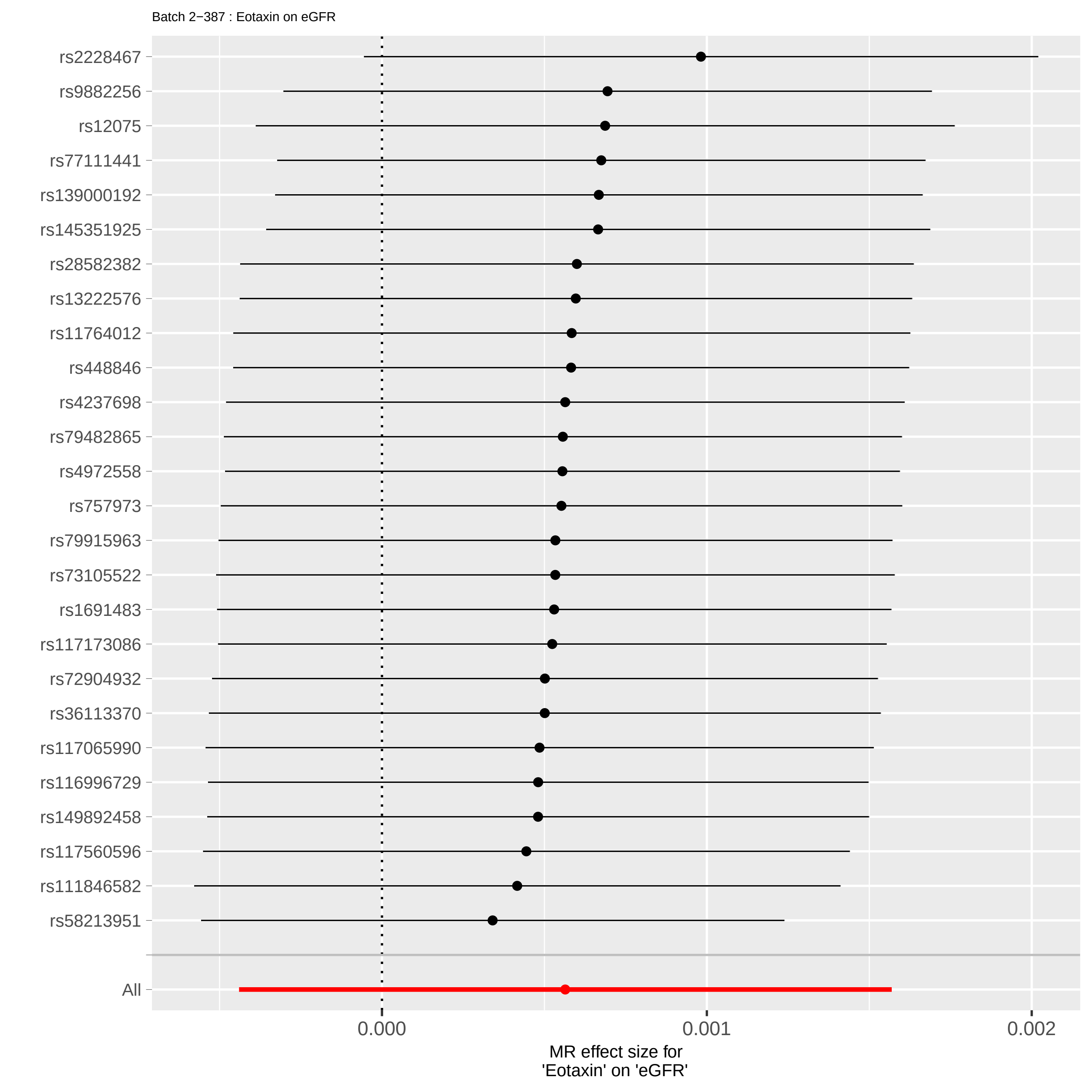

Batch 2−387 : Eotaxin on eGFR
rs2228467
rs9882256
rs12075
rs77111441
rs139000192
rs145351925
rs28582382
rs13222576
rs11764012
rs448846
rs4237698
rs79482865
rs4972558
rs757973
rs79915963
rs73105522
rs1691483
rs117173086
rs72904932
rs36113370
rs117065990
rs116996729
rs149892458
rs117560596
rs111846582
rs58213951
All
0.000
0.001
0.002
MR effect size for
'Eotaxin' on 'eGFR'

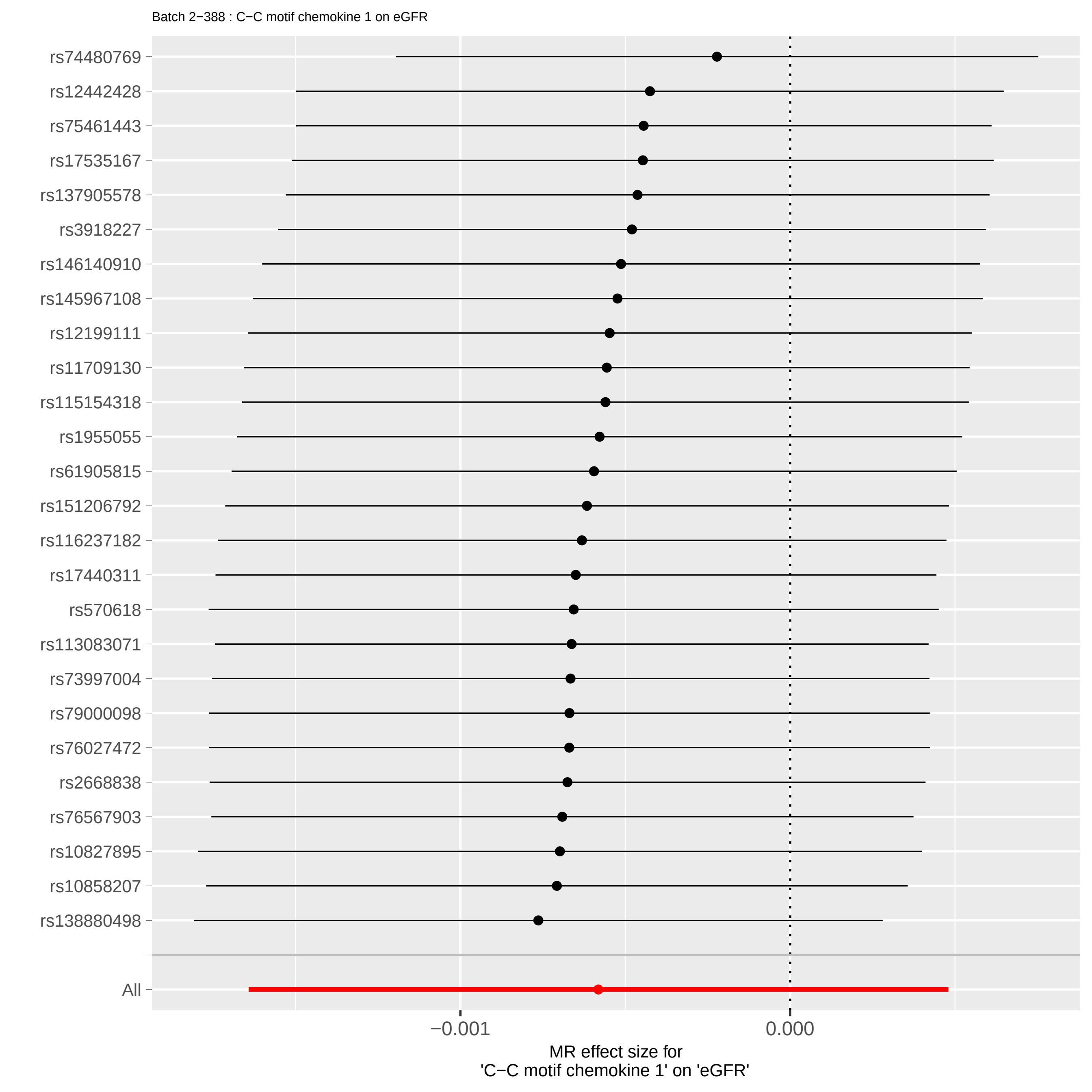

Batch 2−388 : C−C motif chemokine 1 on eGFR
rs74480769
rs12442428
rs75461443
rs17535167
rs137905578
rs3918227
rs146140910
rs145967108
rs12199111
rs11709130
rs115154318
rs1955055
rs61905815
rs151206792
rs116237182
rs17440311
rs570618
rs113083071
rs73997004
rs79000098
rs76027472
rs2668838
rs76567903
rs10827895
rs10858207
rs138880498
All
−0.001
0.000
MR effect size for
'C−C motif chemokine 1' on 'eGFR'

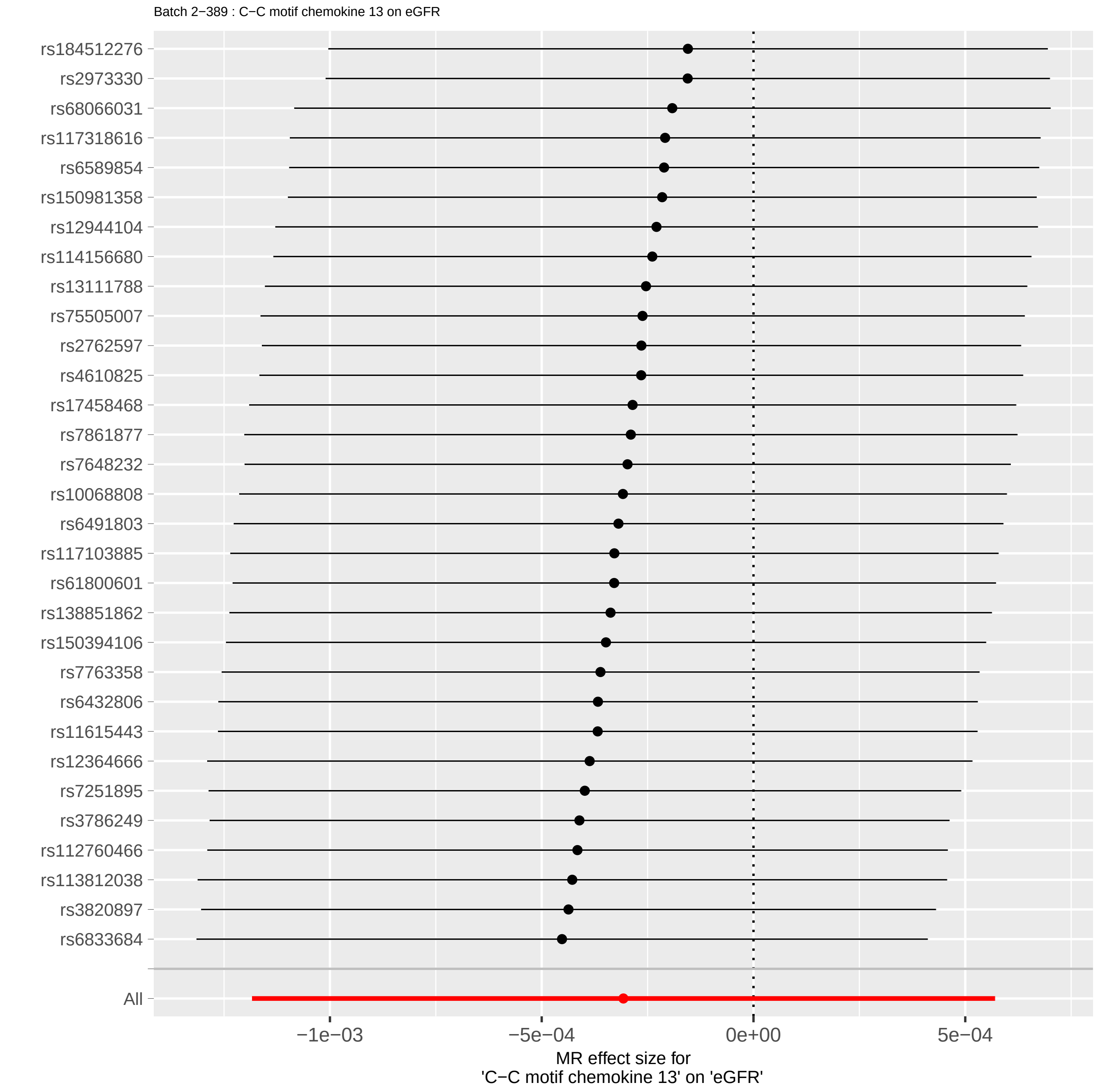

Batch 2−389 : C−C motif chemokine 13 on eGFR
rs184512276
rs2973330
rs68066031
rs117318616
rs6589854
rs150981358
rs12944104
rs114156680
rs13111788
rs75505007
rs2762597
rs4610825
rs17458468
rs7861877
rs7648232
rs10068808
rs6491803
rs117103885
rs61800601
rs138851862
rs150394106
rs7763358
rs6432806
rs11615443
rs12364666
rs7251895
rs3786249
rs112760466
rs113812038
rs3820897
rs6833684
All
−1e−03
−5e−04
0e+00
5e−04
MR effect size for
'C−C motif chemokine 13' on 'eGFR'

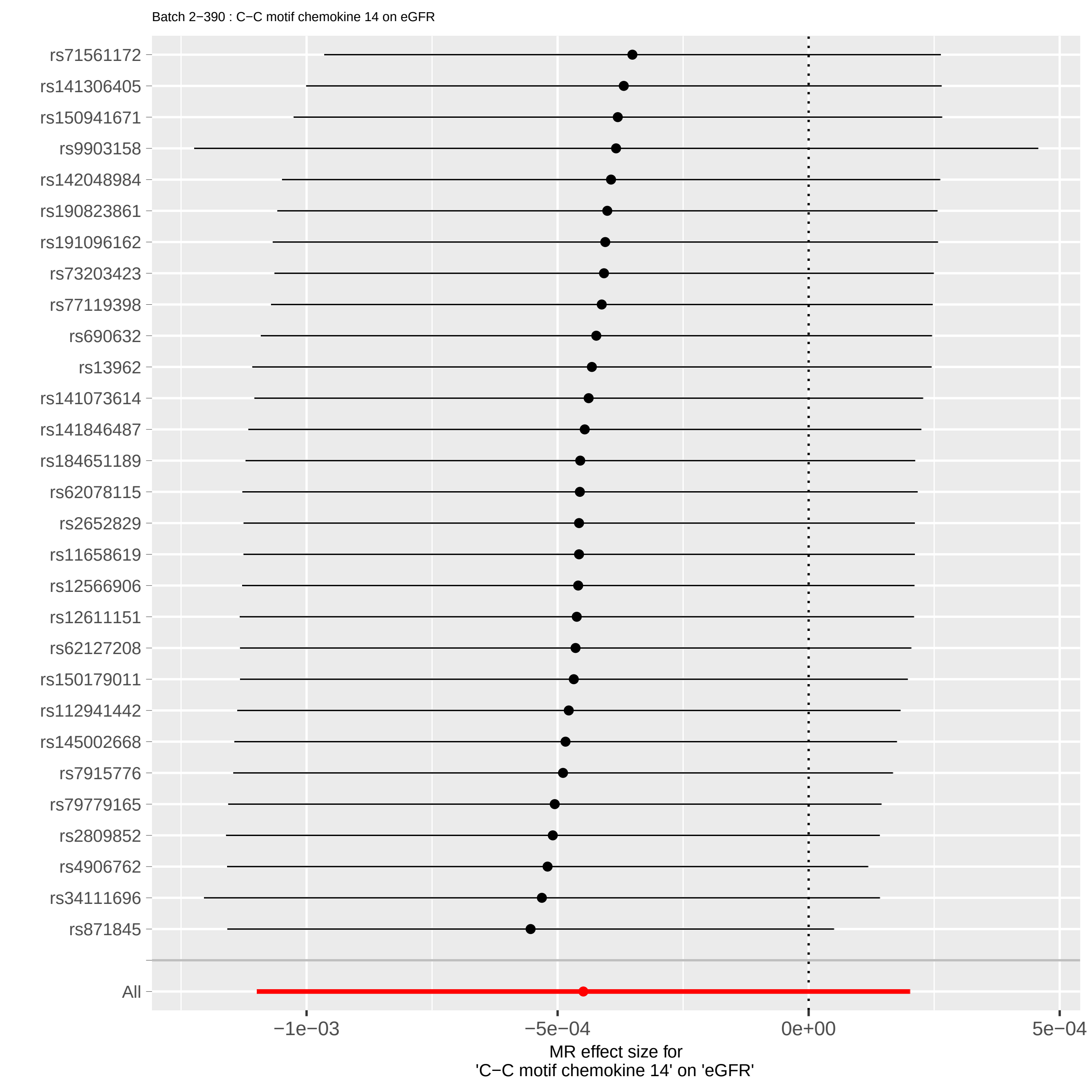

Batch 2−390 : C−C motif chemokine 14 on eGFR
rs71561172
rs141306405
rs150941671
rs9903158
rs142048984
rs190823861
rs191096162
rs73203423
rs77119398
rs690632
rs13962
rs141073614
rs141846487
rs184651189
rs62078115
rs2652829
rs11658619
rs12566906
rs12611151
rs62127208
rs150179011
rs112941442
rs145002668
rs7915776
rs79779165
rs2809852
rs4906762
rs34111696
rs871845
All
−1e−03
−5e−04
0e+00
5e−04
MR effect size for
'C−C motif chemokine 14' on 'eGFR'

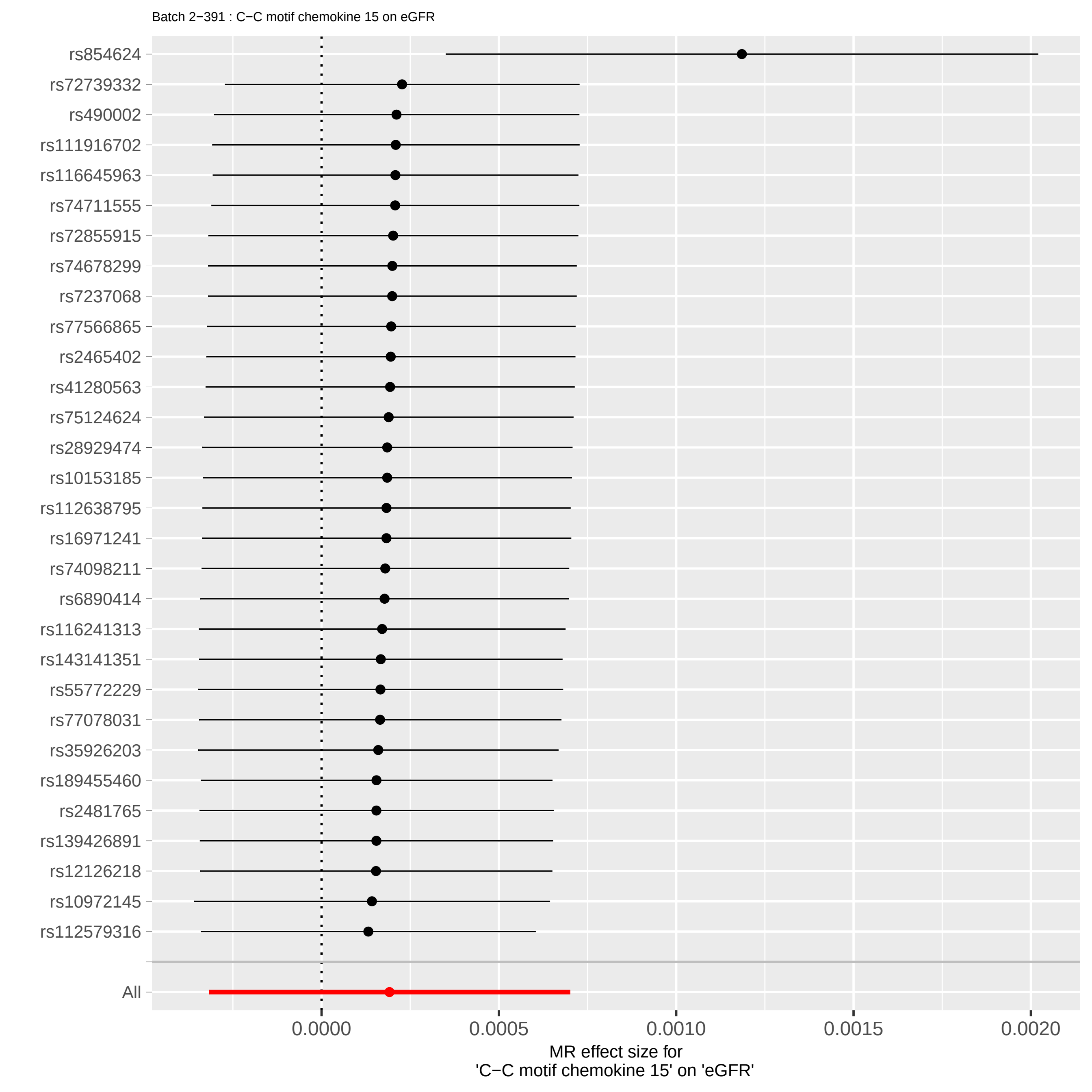

Batch 2−391 : C−C motif chemokine 15 on eGFR
rs854624
rs72739332
rs490002
rs111916702
rs116645963
rs74711555
rs72855915
rs74678299
rs7237068
rs77566865
rs2465402
rs41280563
rs75124624
rs28929474
rs10153185
rs112638795
rs16971241
rs74098211
rs6890414
rs116241313
rs143141351
rs55772229
rs77078031
rs35926203
rs189455460
rs2481765
rs139426891
rs12126218
rs10972145
rs112579316
All
0.0000
0.0005
0.0010
0.0015
0.0020
MR effect size for
'C−C motif chemokine 15' on 'eGFR'

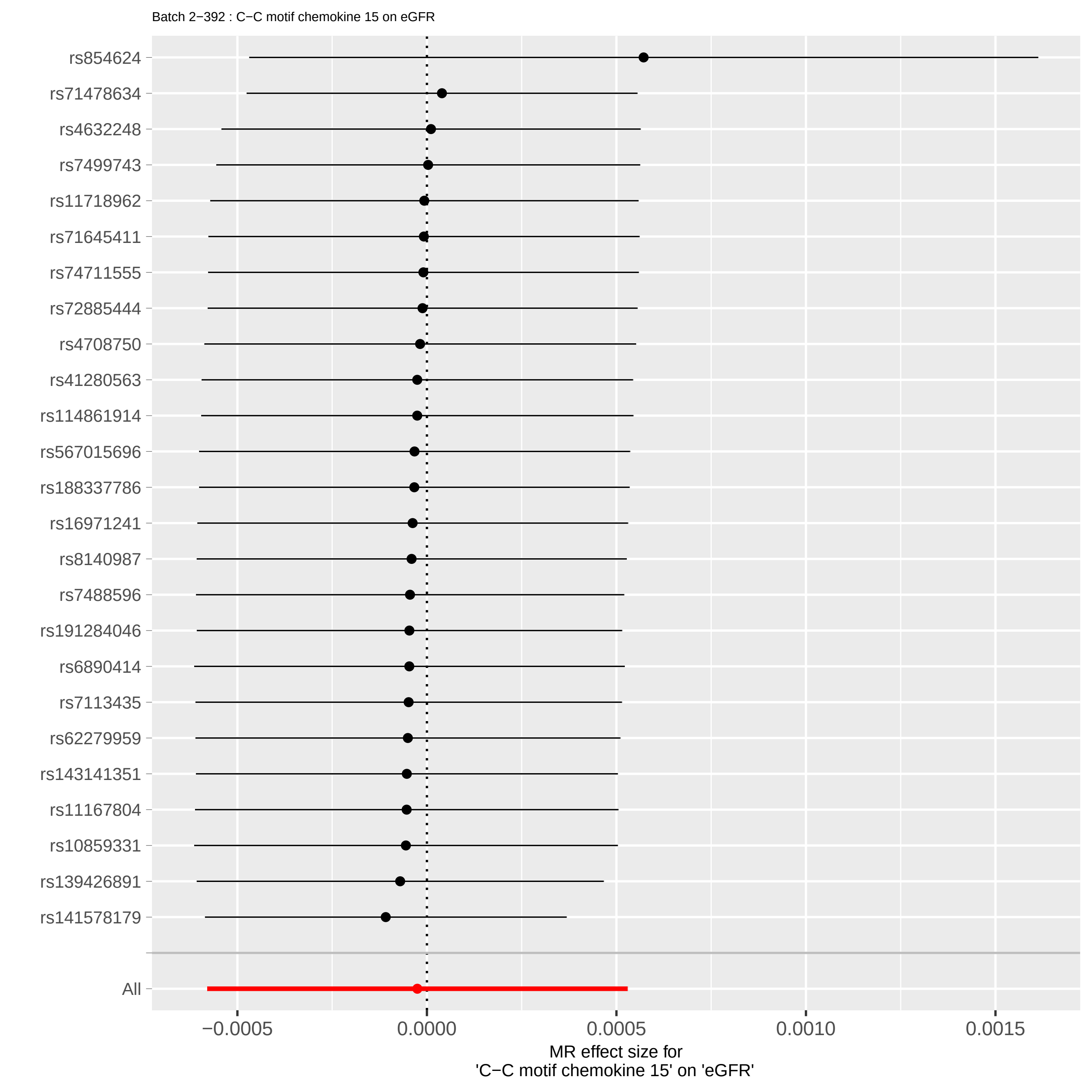

Batch 2−392 : C−C motif chemokine 15 on eGFR
rs854624
rs71478634
rs4632248
rs7499743
rs11718962
rs71645411
rs74711555
rs72885444
rs4708750
rs41280563
rs114861914
rs567015696
rs188337786
rs16971241
rs8140987
rs7488596
rs191284046
rs6890414
rs7113435
rs62279959
rs143141351
rs11167804
rs10859331
rs139426891
rs141578179
All
−0.0005
0.0000
0.0005
0.0010
0.0015
MR effect size for
'C−C motif chemokine 15' on 'eGFR'

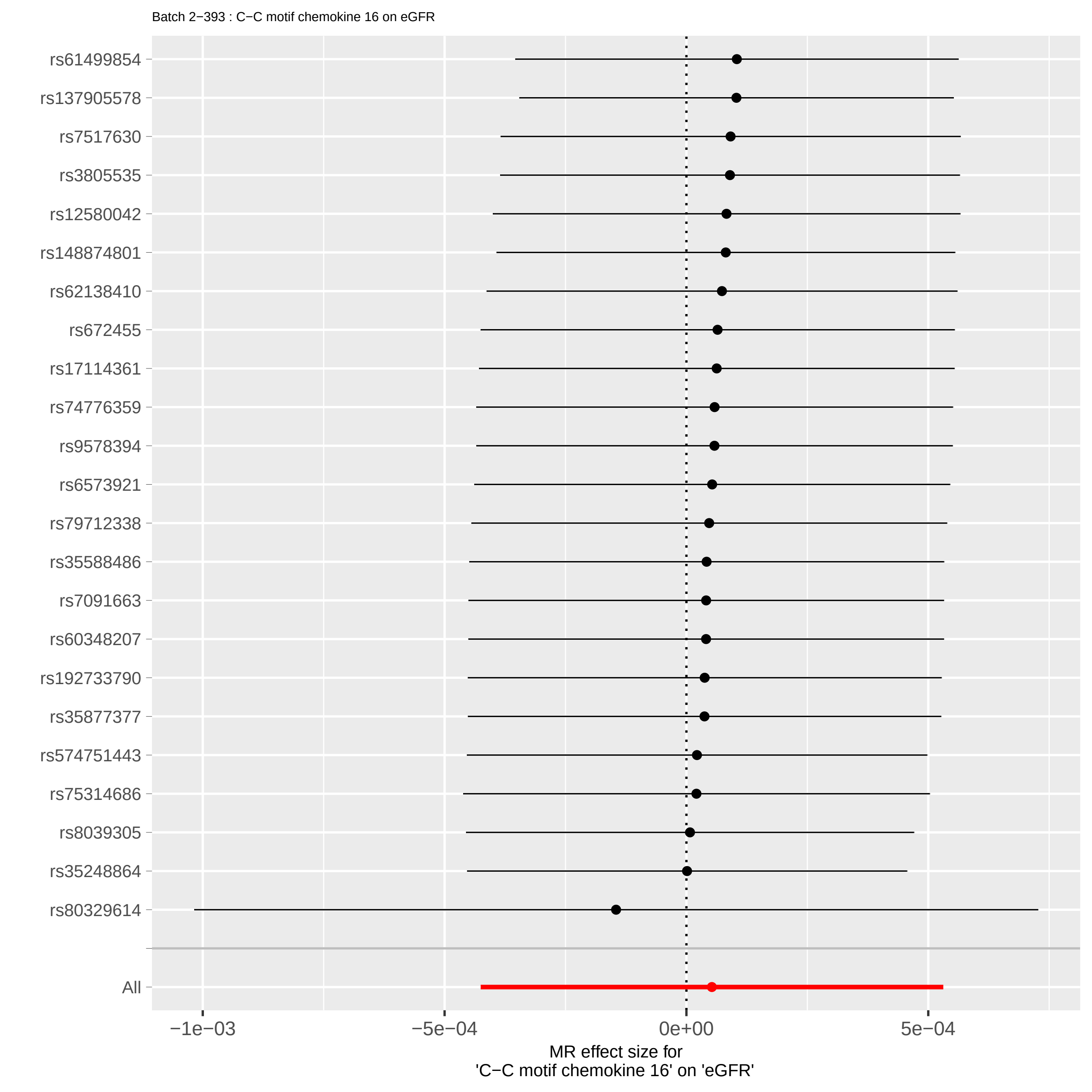

Batch 2−393 : C−C motif chemokine 16 on eGFR
rs61499854
rs137905578
rs7517630
rs3805535
rs12580042
rs148874801
rs62138410
rs672455
rs17114361
rs74776359
rs9578394
rs6573921
rs79712338
rs35588486
rs7091663
rs60348207
rs192733790
rs35877377
rs574751443
rs75314686
rs8039305
rs35248864
rs80329614
All
−1e−03
−5e−04
0e+00
5e−04
MR effect size for
'C−C motif chemokine 16' on 'eGFR'

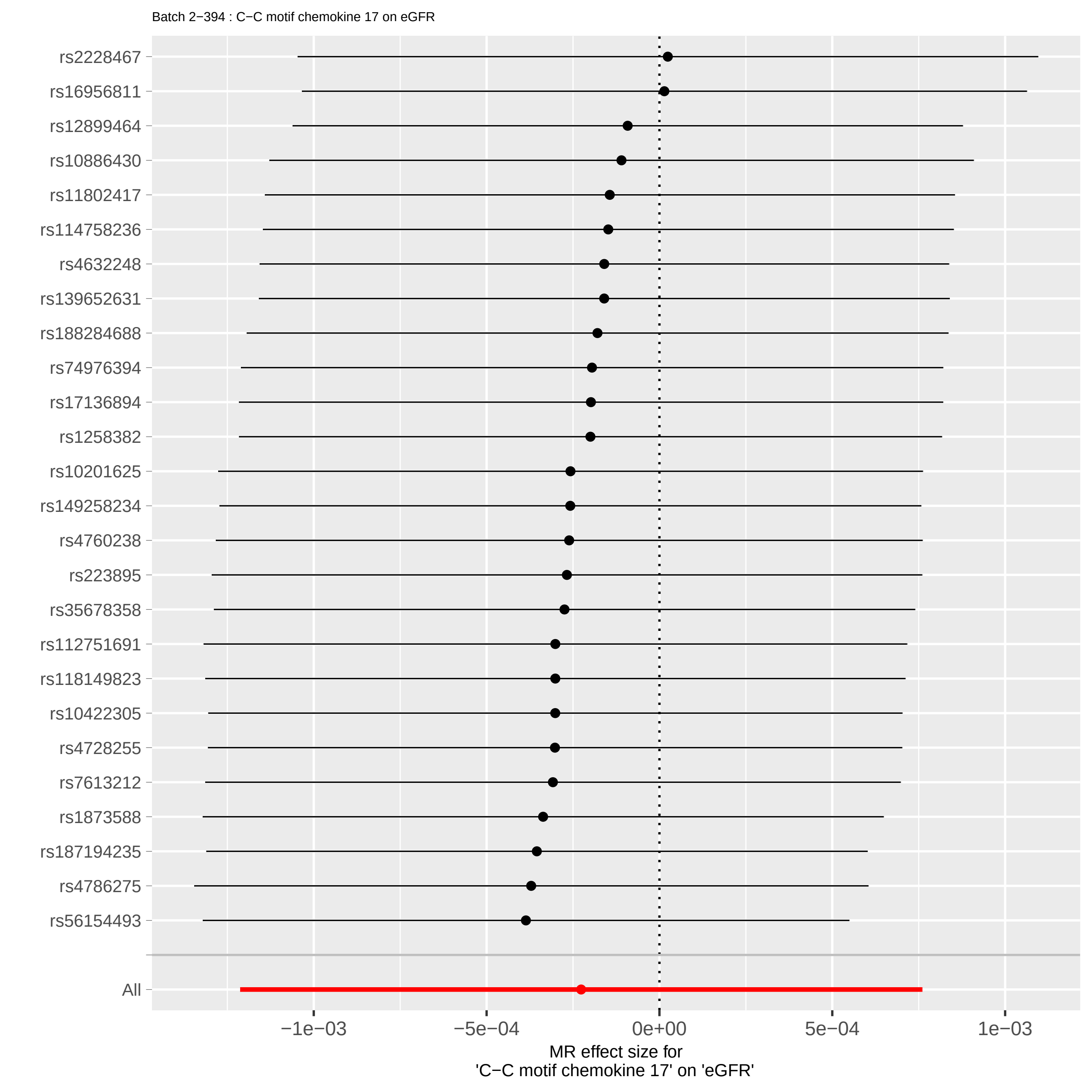

Batch 2−394 : C−C motif chemokine 17 on eGFR
rs2228467
rs16956811
rs12899464
rs10886430
rs11802417
rs114758236
rs4632248
rs139652631
rs188284688
rs74976394
rs17136894
rs1258382
rs10201625
rs149258234
rs4760238
rs223895
rs35678358
rs112751691
rs118149823
rs10422305
rs4728255
rs7613212
rs1873588
rs187194235
rs4786275
rs56154493
All
−1e−03
−5e−04
0e+00
5e−04
1e−03
MR effect size for
'C−C motif chemokine 17' on 'eGFR'

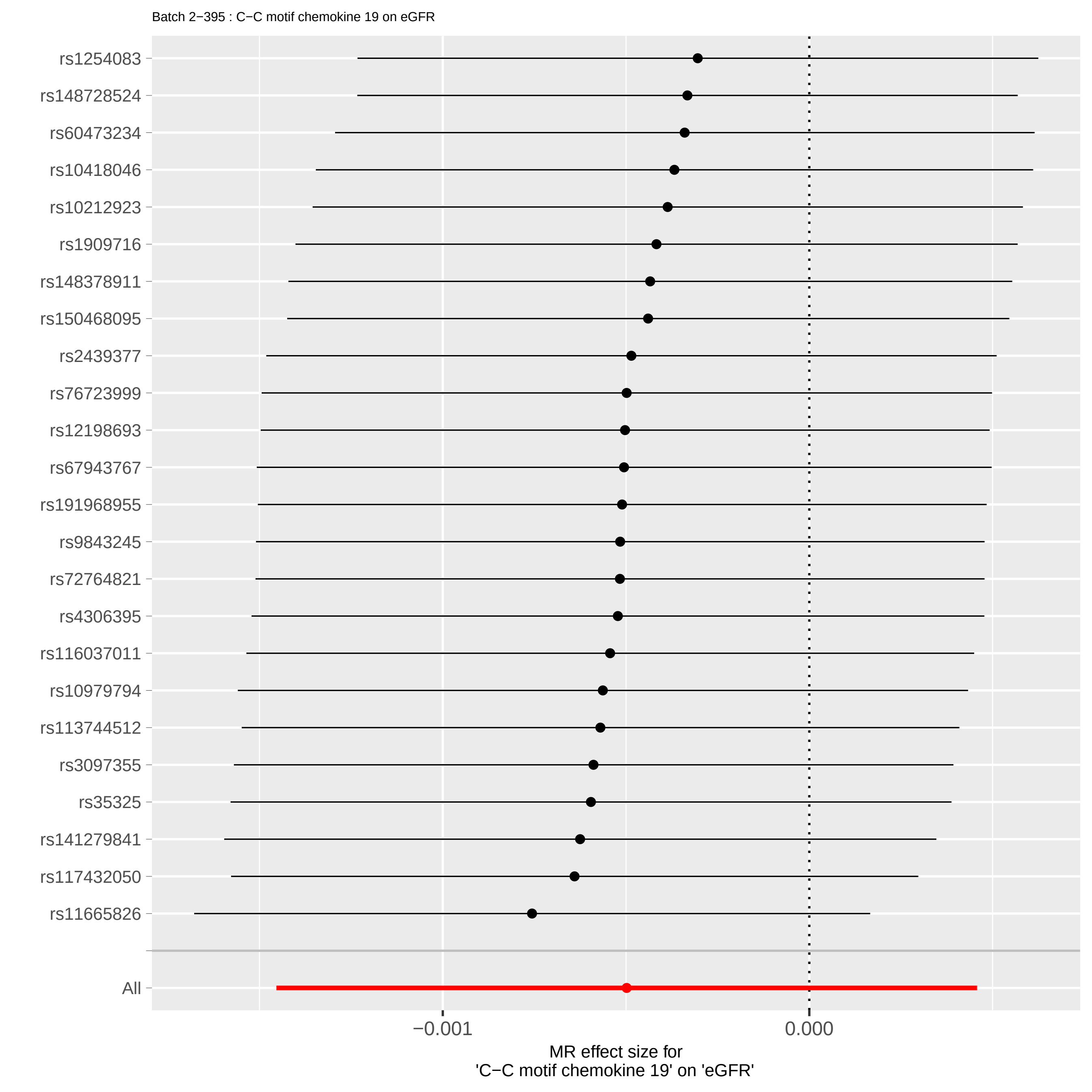
Batch 2−395 : C−C motif chemokine 19 on eGFR
rs1254083
rs148728524
rs60473234
rs10418046
rs10212923
rs1909716
rs148378911
rs150468095
rs2439377
rs76723999
rs12198693
rs67943767
rs191968955
rs9843245
rs72764821
rs4306395
rs116037011
rs10979794
rs113744512
rs3097355
rs35325
rs141279841
rs117432050
rs11665826
All
−0.001
0.000
MR effect size for
'C−C motif chemokine 19' on 'eGFR'

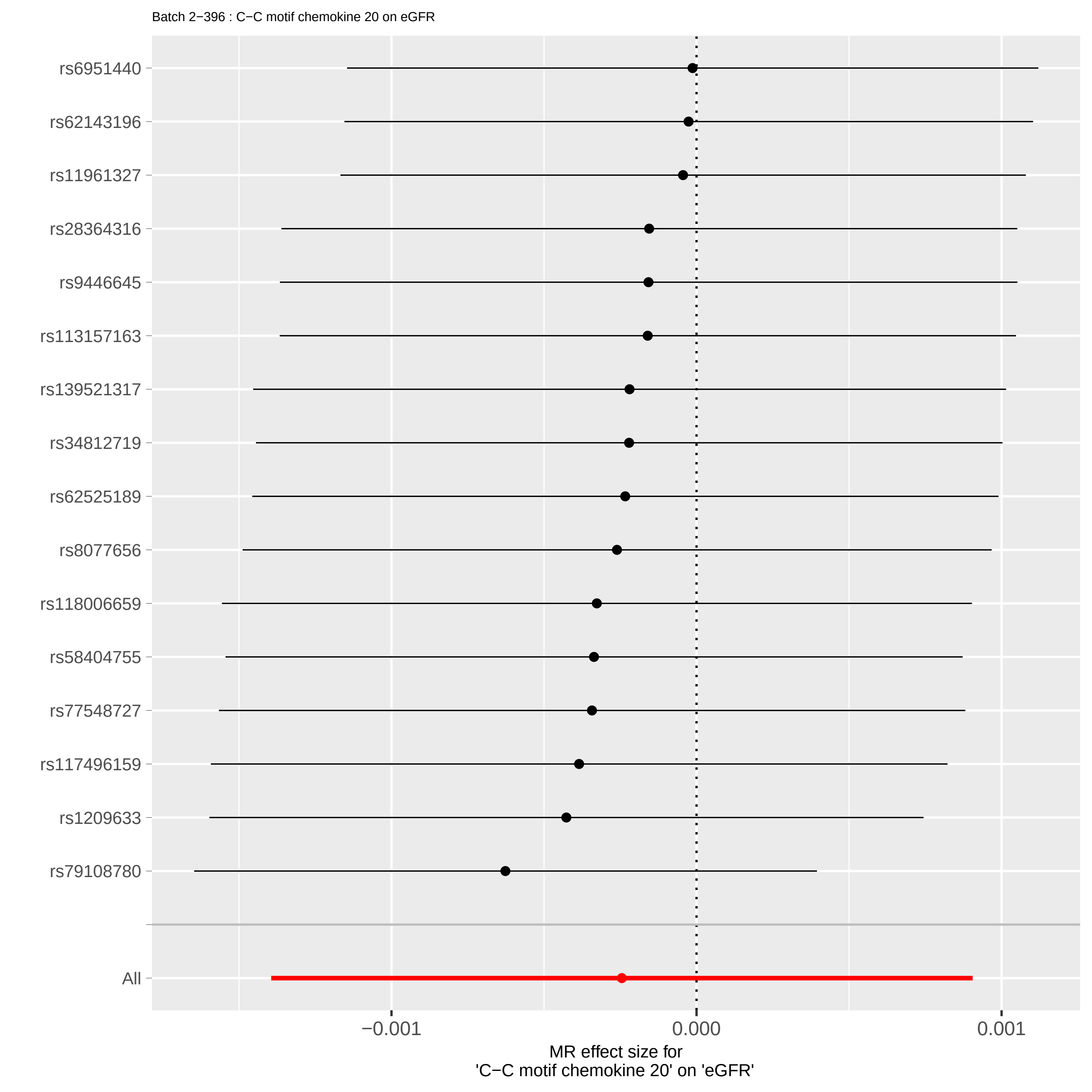

Batch 2−396 : C−C motif chemokine 20 on eGFR
rs6951440
rs62143196
rs11961327
rs28364316
rs9446645
rs113157163
rs139521317
rs34812719
rs62525189
rs8077656
rs118006659
rs58404755
rs77548727
rs117496159
rs1209633
rs79108780
All
−0.001
0.000
0.001
MR effect size for
'C−C motif chemokine 20' on 'eGFR'

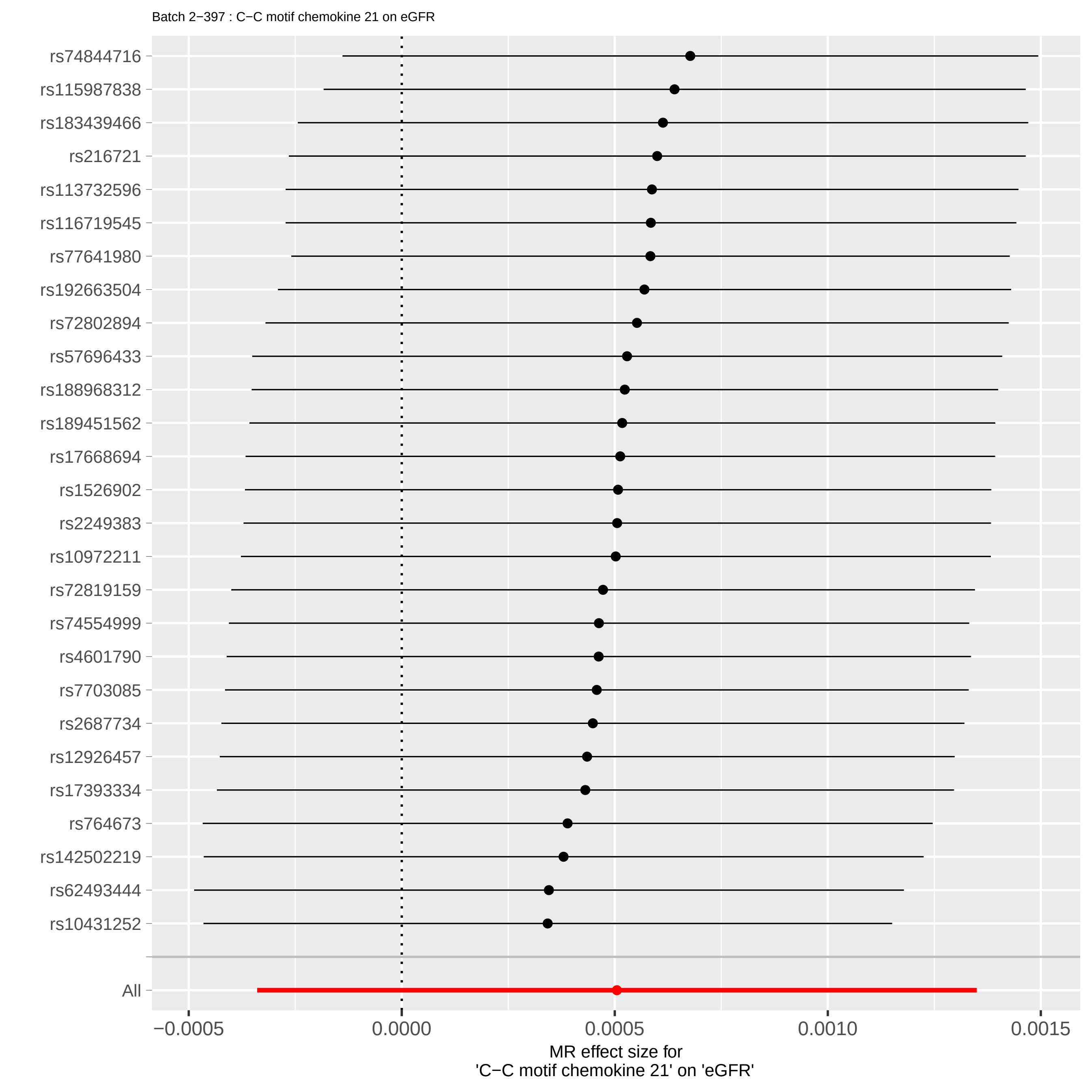

Batch 2−397 : C−C motif chemokine 21 on eGFR
rs74844716
rs115987838
rs183439466
rs216721
rs113732596
rs116719545
rs77641980
rs192663504
rs72802894
rs57696433
rs188968312
rs189451562
rs17668694
rs1526902
rs2249383
rs10972211
rs72819159
rs74554999
rs4601790
rs7703085
rs2687734
rs12926457
rs17393334
rs764673
rs142502219
rs62493444
rs10431252
All
−0.0005
0.0000
0.0005
0.0010
0.0015
MR effect size for
'C−C motif chemokine 21' on 'eGFR'

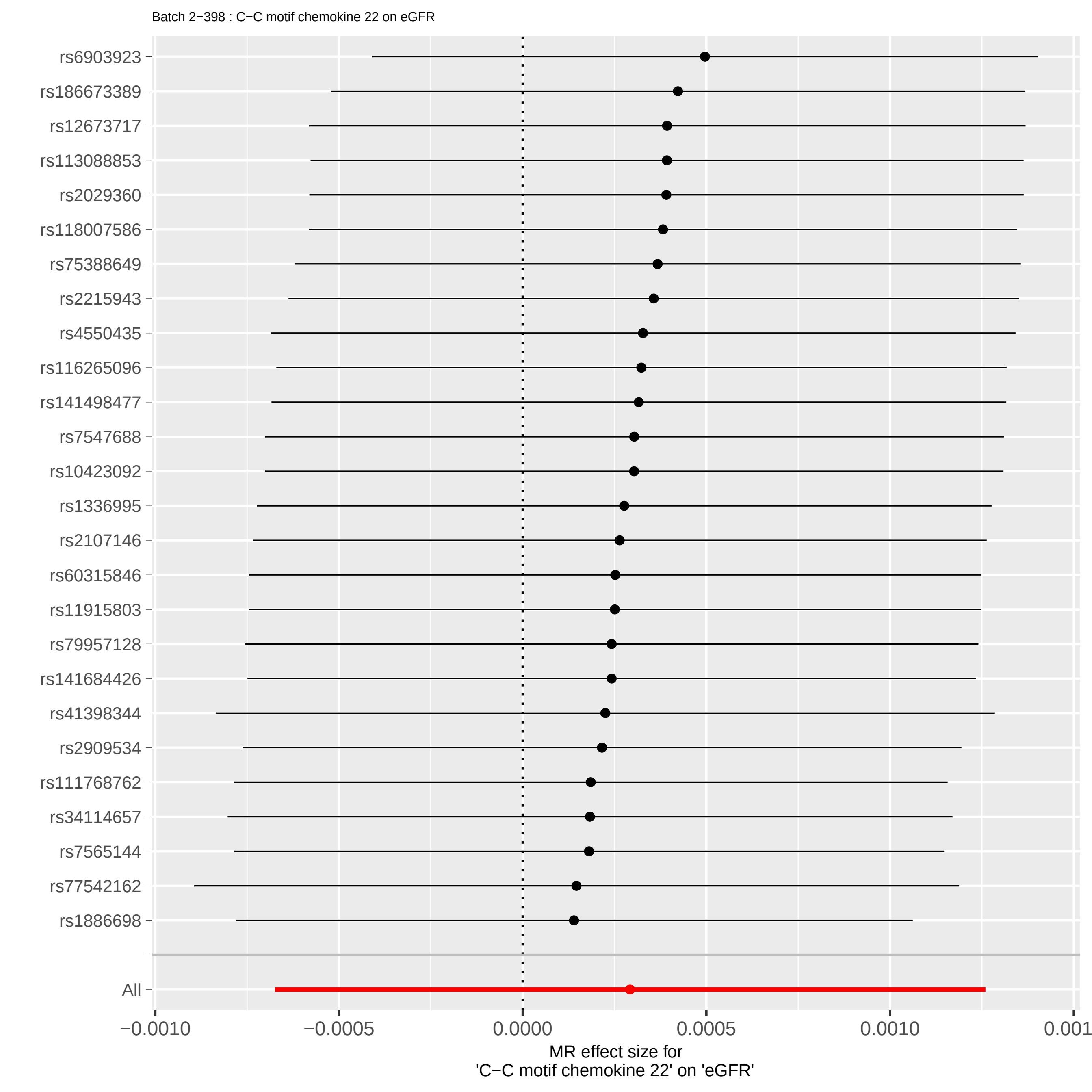

Batch 2−398 : C−C motif chemokine 22 on eGFR
rs6903923
rs186673389
rs12673717
rs113088853
rs2029360
rs118007586
rs75388649
rs2215943
rs4550435
rs116265096
rs141498477
rs7547688
rs10423092
rs1336995
rs2107146
rs60315846
rs11915803
rs79957128
rs141684426
rs41398344
rs2909534
rs111768762
rs34114657
rs7565144
rs77542162
rs1886698
All
−0.0010
−0.0005
0.0000
0.0005
0.0010
0.001
MR effect size for
'C−C motif chemokine 22' on 'eGFR'

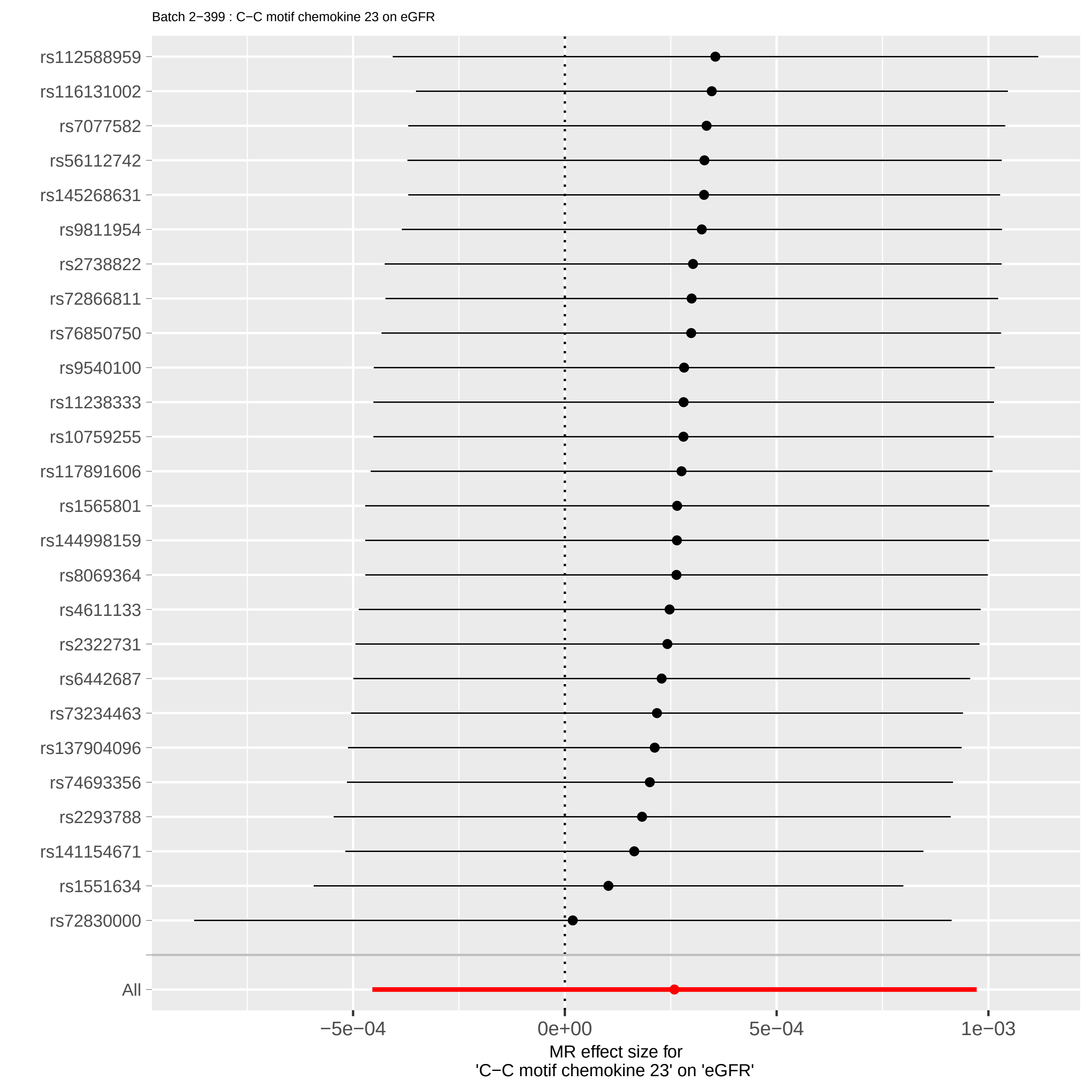
Batch 2−399 : C−C motif chemokine 23 on eGFR
rs112588959
rs116131002
rs7077582
rs56112742
rs145268631
rs9811954
rs2738822
rs72866811
rs76850750
rs9540100
rs11238333
rs10759255
rs117891606
rs1565801
rs144998159
rs8069364
rs4611133
rs2322731
rs6442687
rs73234463
rs137904096
rs74693356
rs2293788
rs141154671
rs1551634
rs72830000
All
−5e−04
0e+00
5e−04
1e−03
MR effect size for
'C−C motif chemokine 23' on 'eGFR'

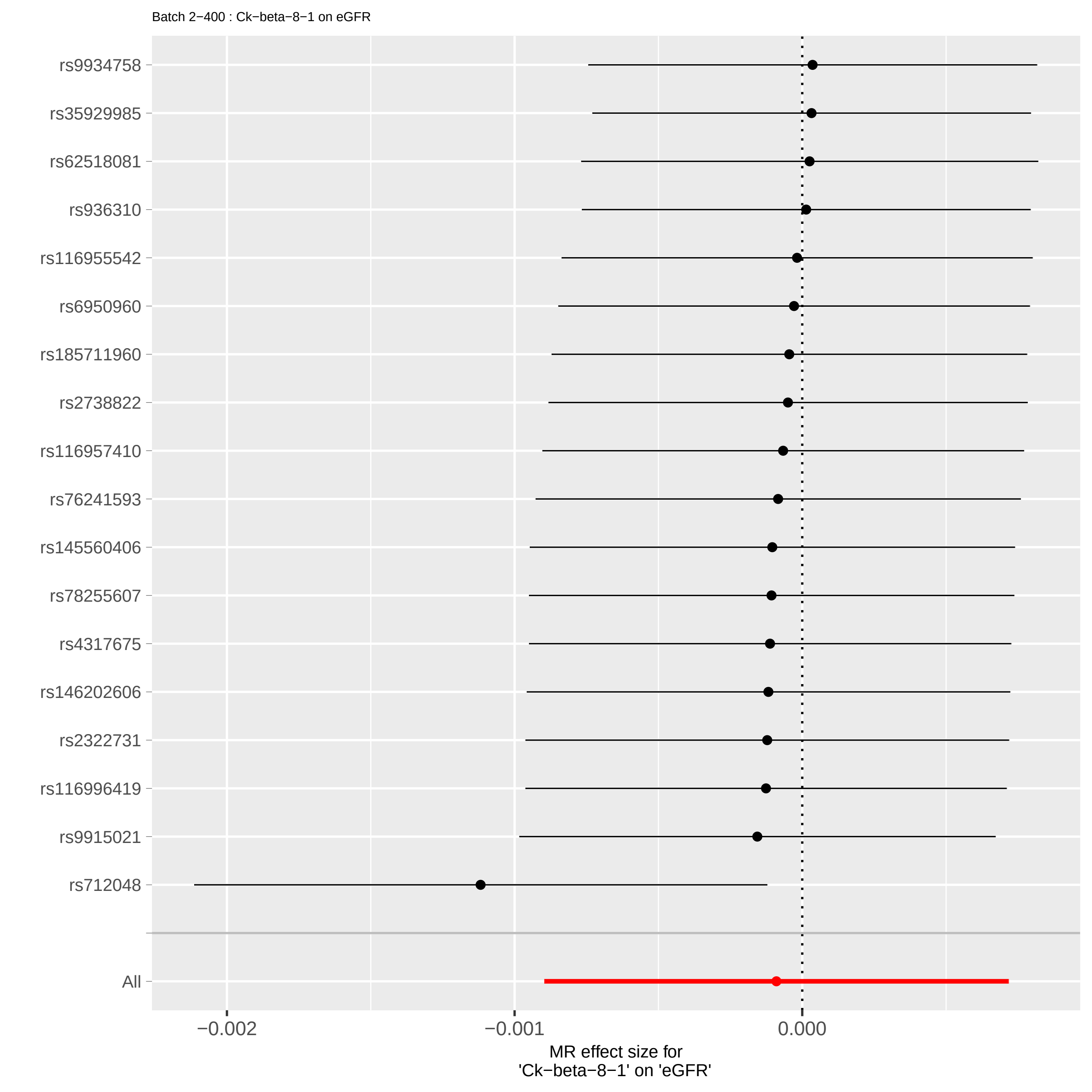

Batch 2−400 : Ck−beta−8−1 on eGFR
rs9934758
rs35929985
rs62518081
rs936310
rs116955542
rs6950960
rs185711960
rs2738822
rs116957410
rs76241593
rs145560406
rs78255607
rs4317675
rs146202606
rs2322731
rs116996419
rs9915021
rs712048
All
−0.002
−0.001
0.000
MR effect size for
'Ck−beta−8−1' on 'eGFR'

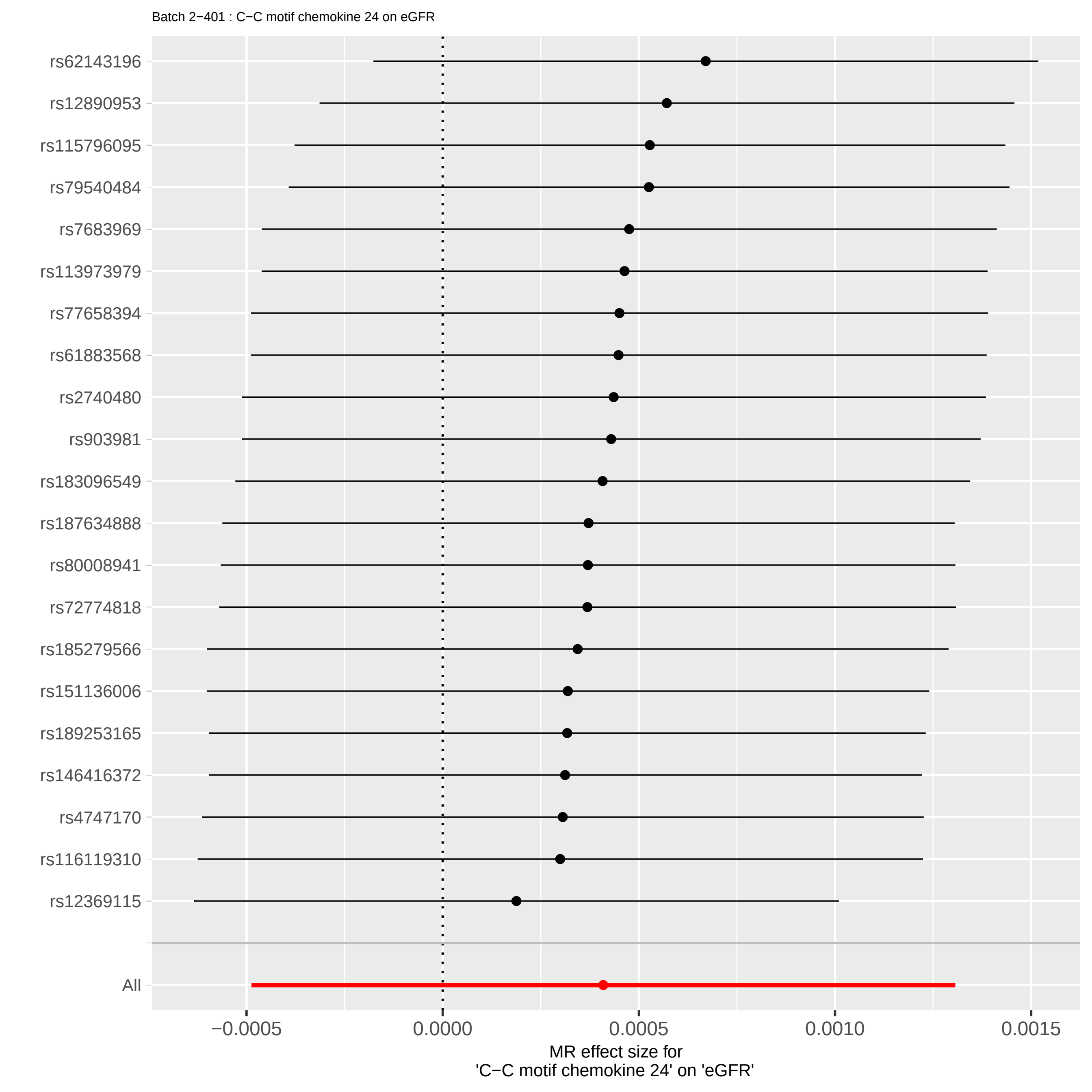
Batch 2−401 : C−C motif chemokine 24 on eGFR
rs62143196
rs12890953
rs115796095
rs79540484
rs7683969
rs113973979
rs77658394
rs61883568
rs2740480
rs903981
rs183096549
rs187634888
rs80008941
rs72774818
rs185279566
rs151136006
rs189253165
rs146416372
rs4747170
rs116119310
rs12369115
All
−0.0005
0.0000
0.0005
0.0010
0.0015
MR effect size for
'C−C motif chemokine 24' on 'eGFR'

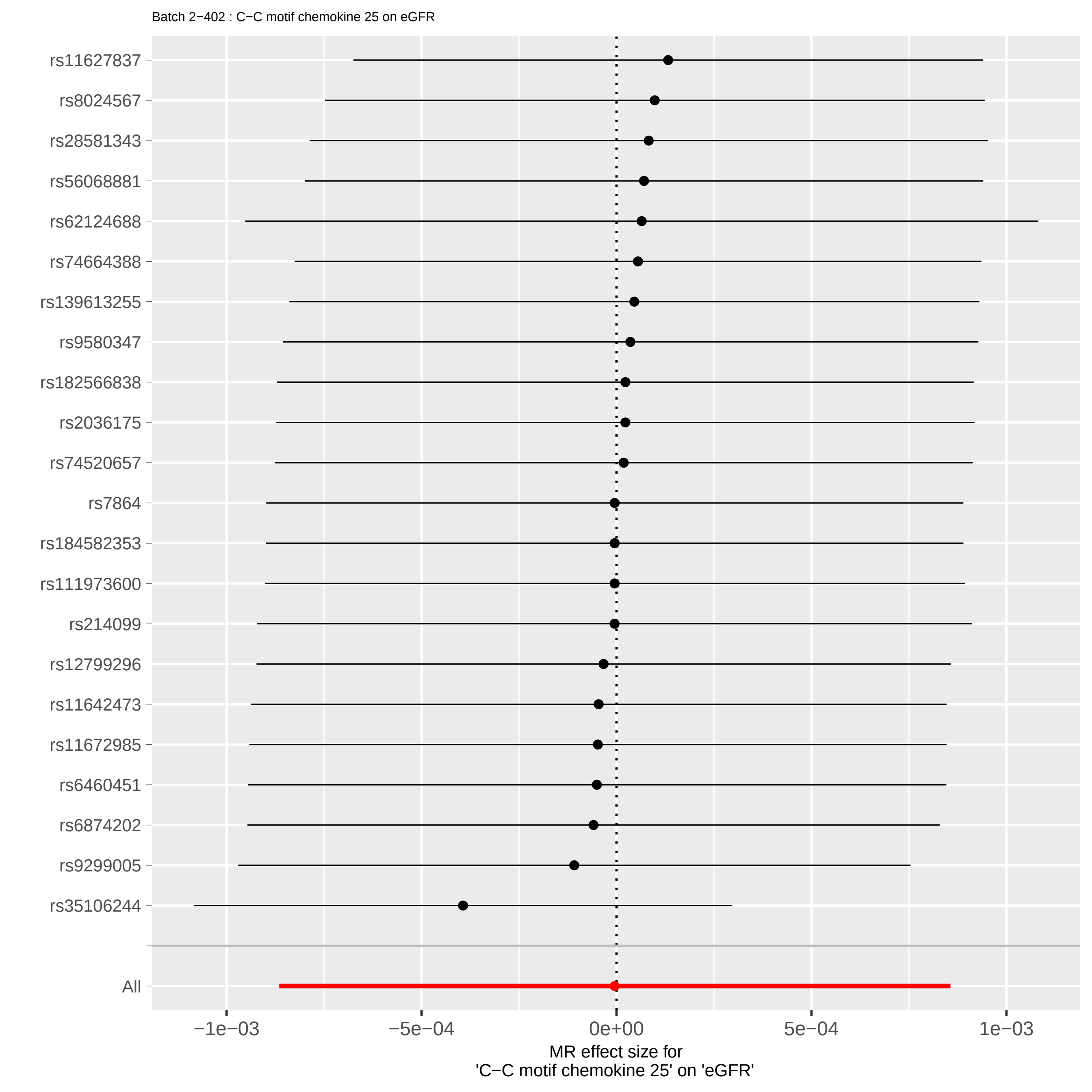
Batch 2−402 : C−C motif chemokine 25 on eGFR
rs11627837
rs8024567
rs28581343
rs56068881
rs62124688
rs74664388
rs139613255
rs9580347
rs182566838
rs2036175
rs74520657
rs7864
rs184582353
rs111973600
rs214099
rs12799296
rs11642473
rs11672985
rs6460451
rs6874202
rs9299005
rs35106244
All
−1e−03
−5e−04
0e+00
5e−04
1e−03
MR effect size for
'C−C motif chemokine 25' on 'eGFR'

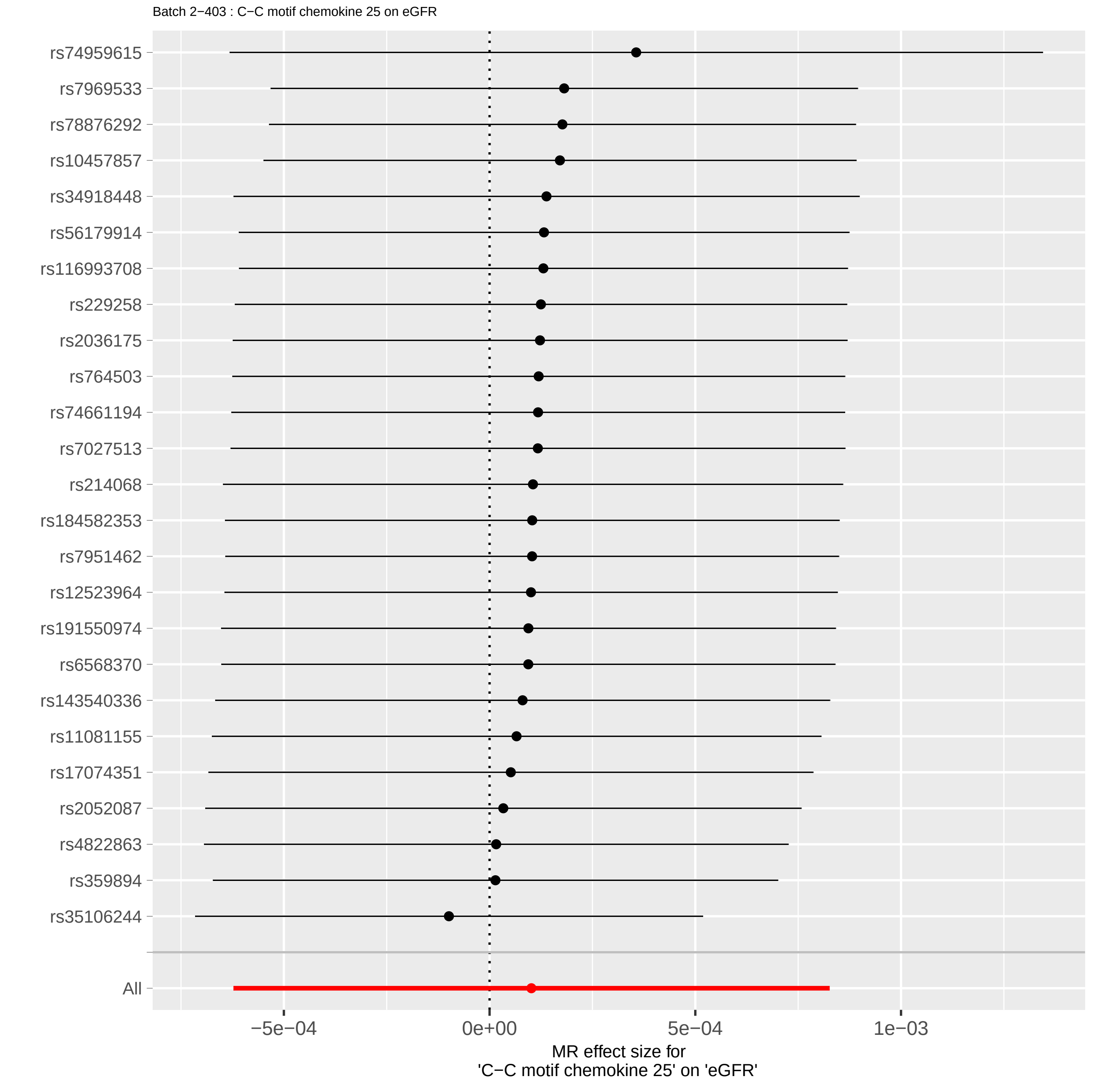
Batch 2−403 : C−C motif chemokine 25 on eGFR
rs74959615
rs7969533
rs78876292
rs10457857
rs34918448
rs56179914
rs116993708
rs229258
rs2036175
rs764503
rs74661194
rs7027513
rs214068
rs184582353
rs7951462
rs12523964
rs191550974
rs6568370
rs143540336
rs11081155
rs17074351
rs2052087
rs4822863
rs359894
rs35106244
All
−5e−04
0e+00
5e−04
1e−03
MR effect size for
'C−C motif chemokine 25' on 'eGFR'

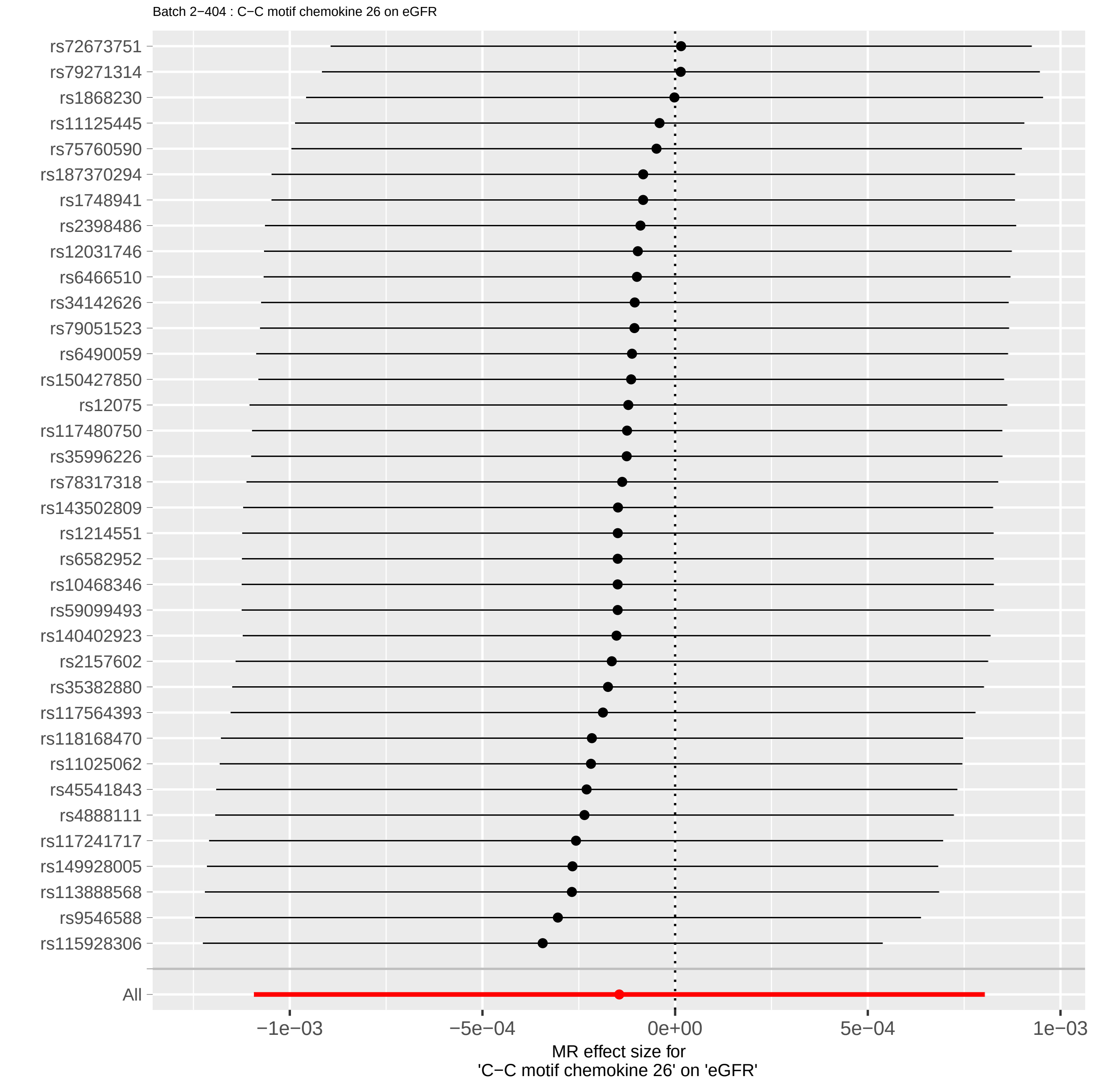
Batch 2−404 : C−C motif chemokine 26 on eGFR
rs72673751
rs79271314
rs1868230
rs11125445
rs75760590
rs187370294
rs1748941
rs2398486
rs12031746
rs6466510
rs34142626
rs79051523
rs6490059
rs150427850
rs12075
rs117480750
rs35996226
rs78317318
rs143502809
rs1214551
rs6582952
rs10468346
rs59099493
rs140402923
rs2157602
rs35382880
rs117564393
rs118168470
rs11025062
rs45541843
rs4888111
rs117241717
rs149928005
rs113888568
rs9546588
rs115928306
All
−1e−03
−5e−04
0e+00
5e−04
1e−03
MR effect size for
'C−C motif chemokine 26' on 'eGFR'

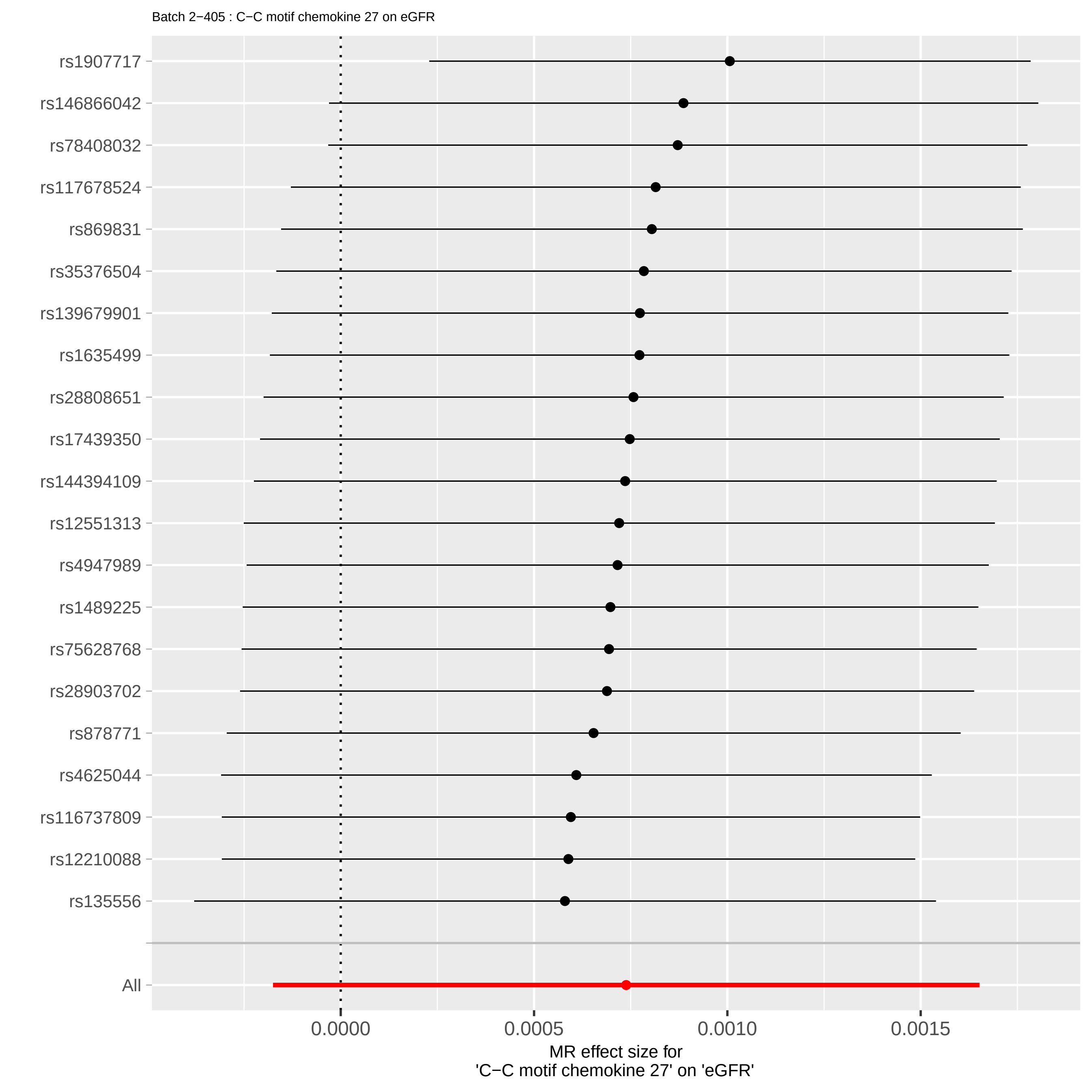
Batch 2−405 : C−C motif chemokine 27 on eGFR
rs1907717
rs146866042
rs78408032
rs117678524
rs869831
rs35376504
rs139679901
rs1635499
rs28808651
rs17439350
rs144394109
rs12551313
rs4947989
rs1489225
rs75628768
rs28903702
rs878771
rs4625044
rs116737809
rs12210088
rs135556
All
0.0000
0.0005
0.0010
0.0015
MR effect size for
'C−C motif chemokine 27' on 'eGFR'

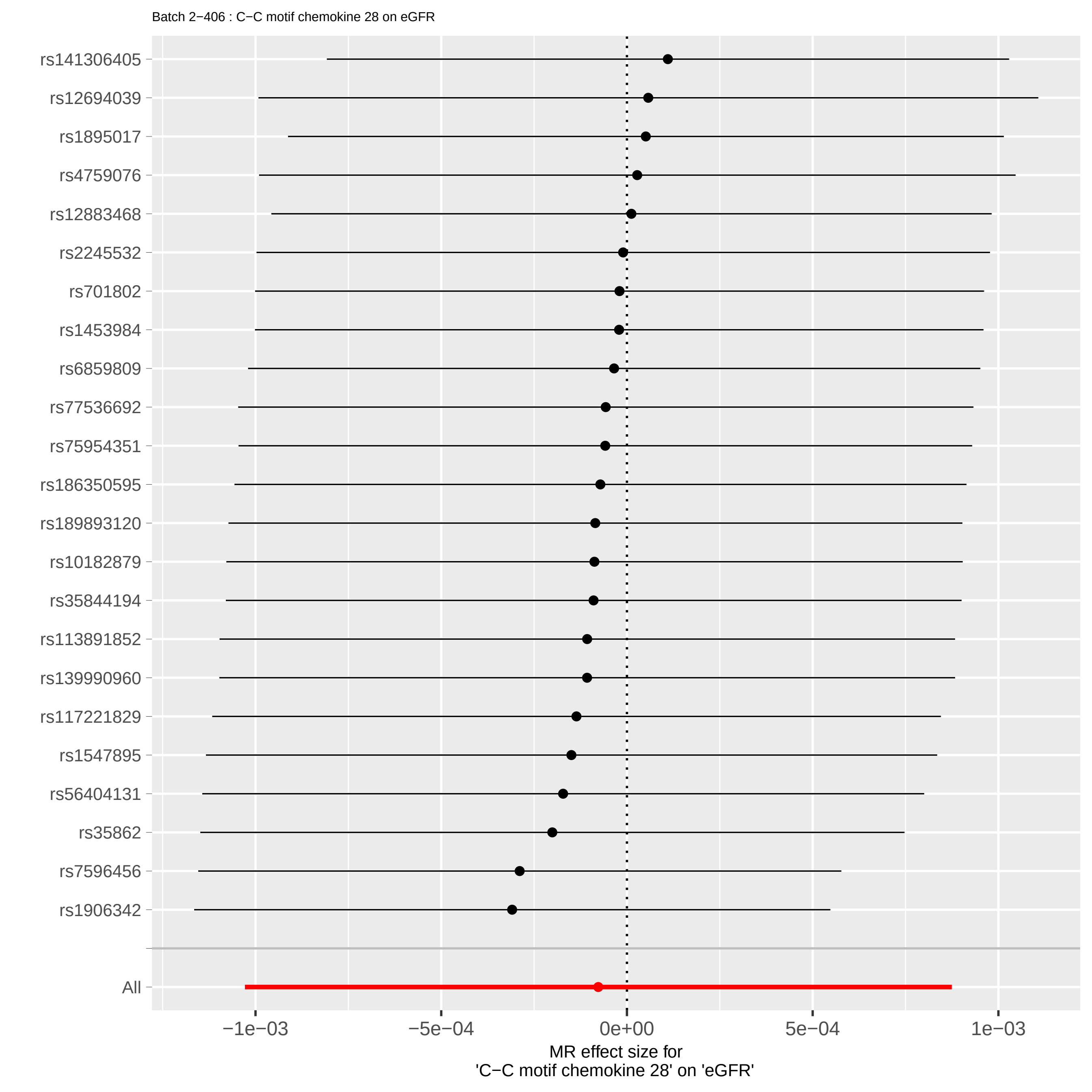

Batch 2−406 : C−C motif chemokine 28 on eGFR
rs141306405
rs12694039
rs1895017
rs4759076
rs12883468
rs2245532
rs701802
rs1453984
rs6859809
rs77536692
rs75954351
rs186350595
rs189893120
rs10182879
rs35844194
rs113891852
rs139990960
rs117221829
rs1547895
rs56404131
rs35862
rs7596456
rs1906342
All
−1e−03
−5e−04
0e+00
5e−04
1e−03
MR effect size for
'C−C motif chemokine 28' on 'eGFR'

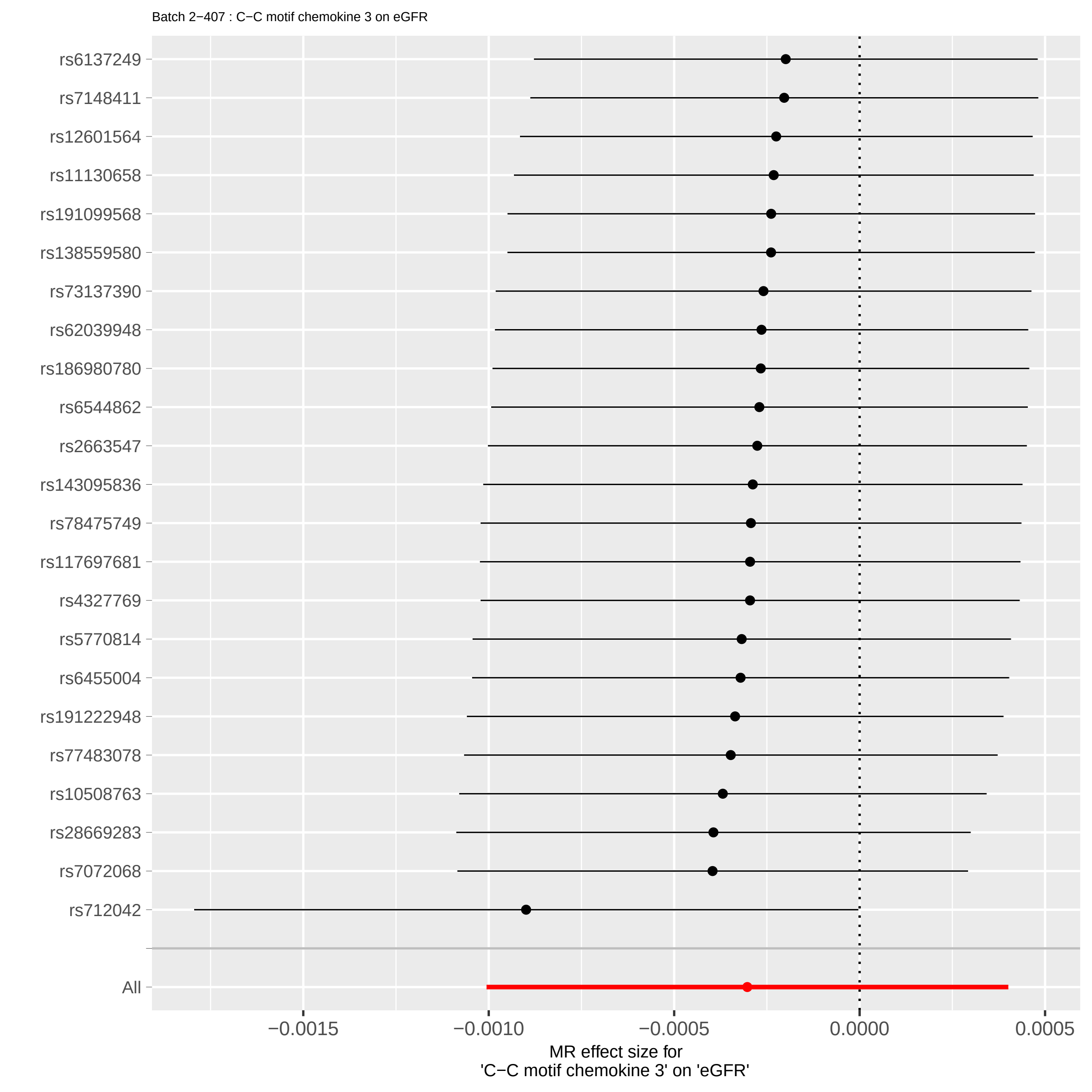

Batch 2−407 : C−C motif chemokine 3 on eGFR
rs6137249
rs7148411
rs12601564
rs11130658
rs191099568
rs138559580
rs73137390
rs62039948
rs186980780
rs6544862
rs2663547
rs143095836
rs78475749
rs117697681
rs4327769
rs5770814
rs6455004
rs191222948
rs77483078
rs10508763
rs28669283
rs7072068
rs712042
All
−0.0015
−0.0010
−0.0005
0.0000
0.0005
MR effect size for
'C−C motif chemokine 3' on 'eGFR'

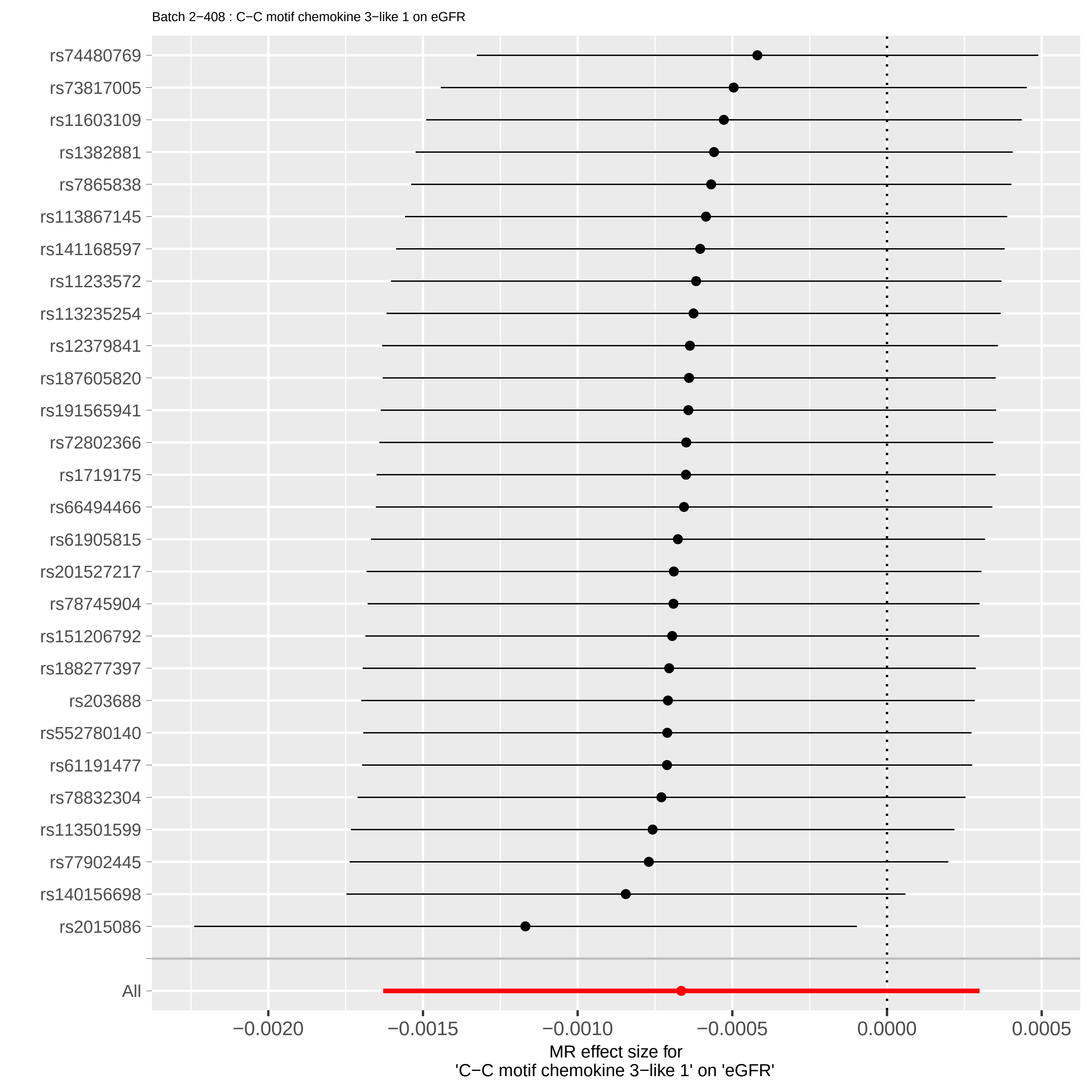

Batch 2−408 : C−C motif chemokine 3−like 1 on eGFR
rs74480769
rs73817005
rs11603109
rs1382881
rs7865838
rs113867145
rs141168597
rs11233572
rs113235254
rs12379841
rs187605820
rs191565941
rs72802366
rs1719175
rs66494466
rs61905815
rs201527217
rs78745904
rs151206792
rs188277397
rs203688
rs552780140
rs61191477
rs78832304
rs113501599
rs77902445
rs140156698
rs2015086
All
−0.0020
−0.0015
−0.0010
−0.0005
0.0000
0.0005
MR effect size for
'C−C motif chemokine 3−like 1' on 'eGFR'

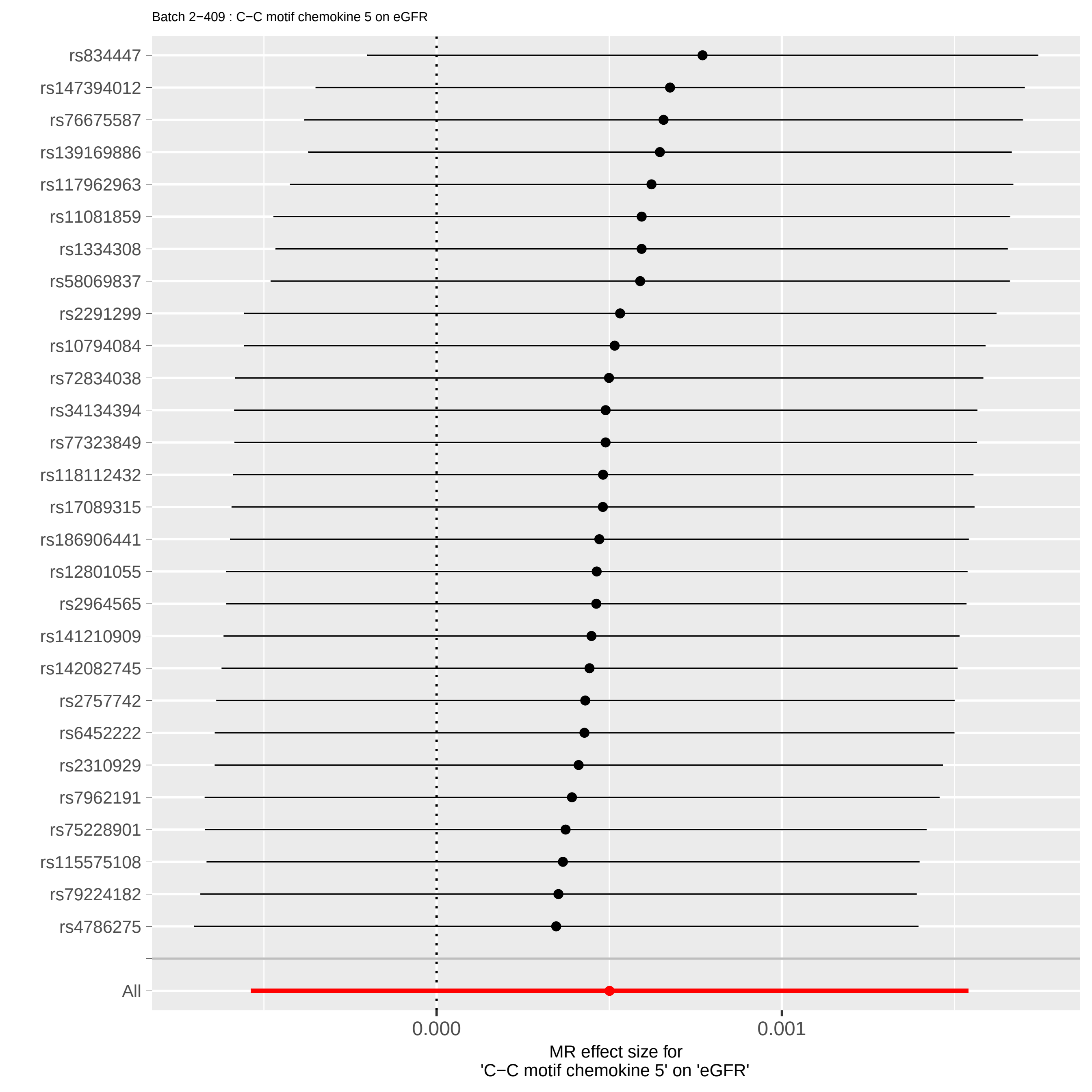

Batch 2−409 : C−C motif chemokine 5 on eGFR
rs834447
rs147394012
rs76675587
rs139169886
rs117962963
rs11081859
rs1334308
rs58069837
rs2291299
rs10794084
rs72834038
rs34134394
rs77323849
rs118112432
rs17089315
rs186906441
rs12801055
rs2964565
rs141210909
rs142082745
rs2757742
rs6452222
rs2310929
rs7962191
rs75228901
rs115575108
rs79224182
rs4786275
All
0.000
0.001
MR effect size for
'C−C motif chemokine 5' on 'eGFR'

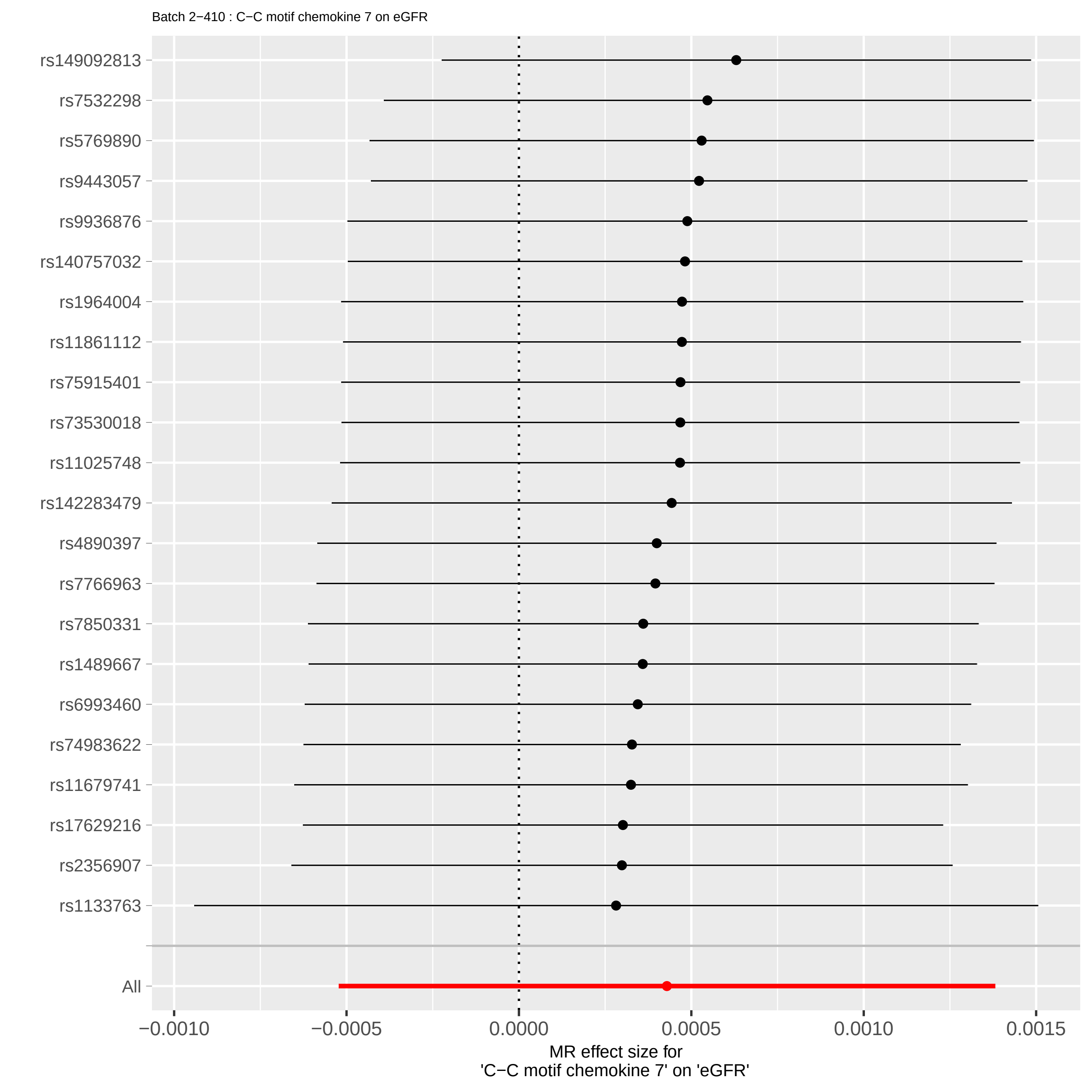

Batch 2−410 : C−C motif chemokine 7 on eGFR
rs149092813
rs7532298
rs5769890
rs9443057
rs9936876
rs140757032
rs1964004
rs11861112
rs75915401
rs73530018
rs11025748
rs142283479
rs4890397
rs7766963
rs7850331
rs1489667
rs6993460
rs74983622
rs11679741
rs17629216
rs2356907
rs1133763
All
−0.0010
−0.0005
0.0000
0.0005
0.0010
0.0015
MR effect size for
'C−C motif chemokine 7' on 'eGFR'

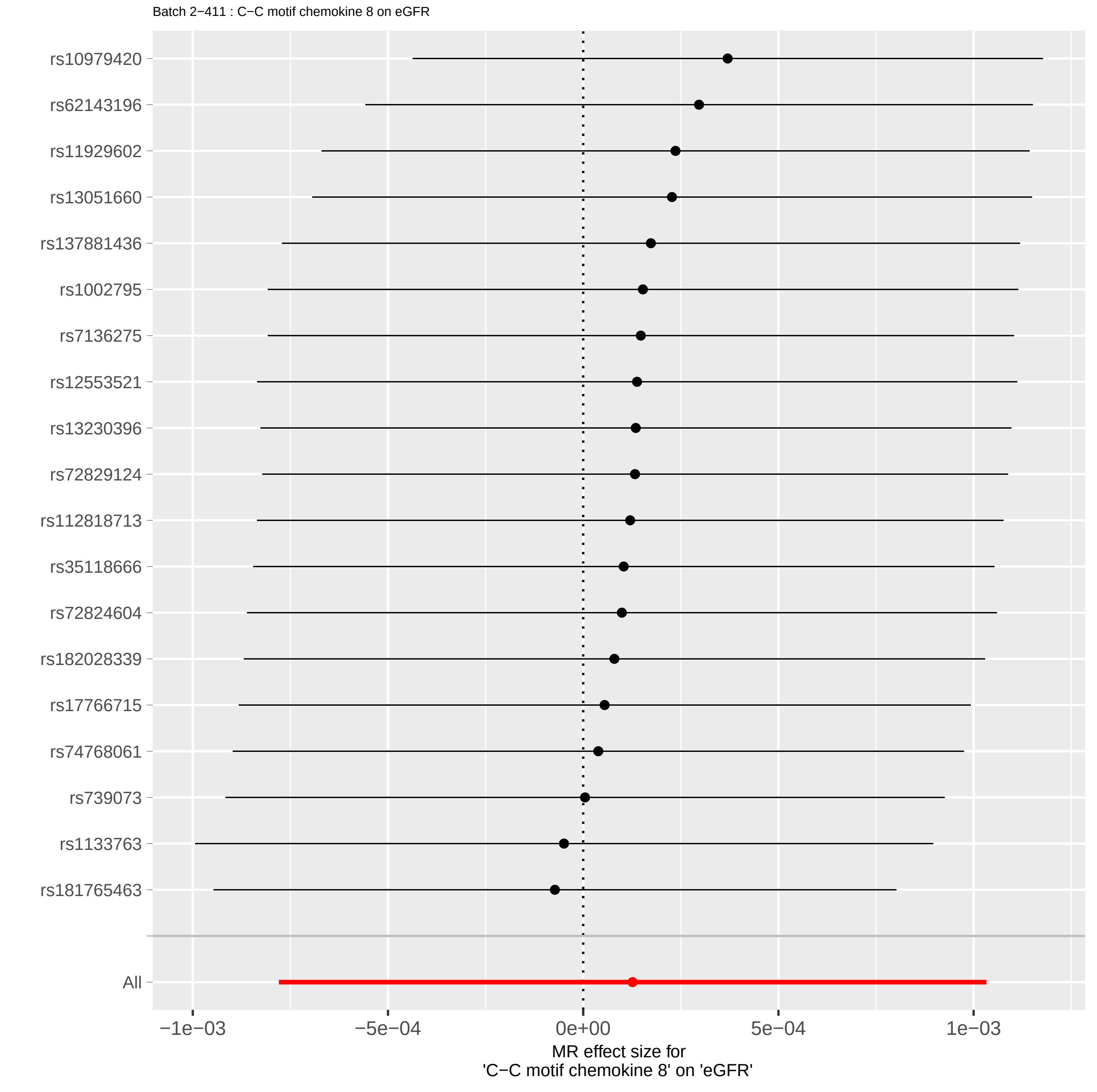

Batch 2−411 : C−C motif chemokine 8 on eGFR
rs10979420
rs62143196
rs11929602
rs13051660
rs137881436
rs1002795
rs7136275
rs12553521
rs13230396
rs72829124
rs112818713
rs35118666
rs72824604
rs182028339
rs17766715
rs74768061
rs739073
rs1133763
rs181765463
All
−1e−03
−5e−04
0e+00
5e−04
1e−03
MR effect size for
'C−C motif chemokine 8' on 'eGFR'

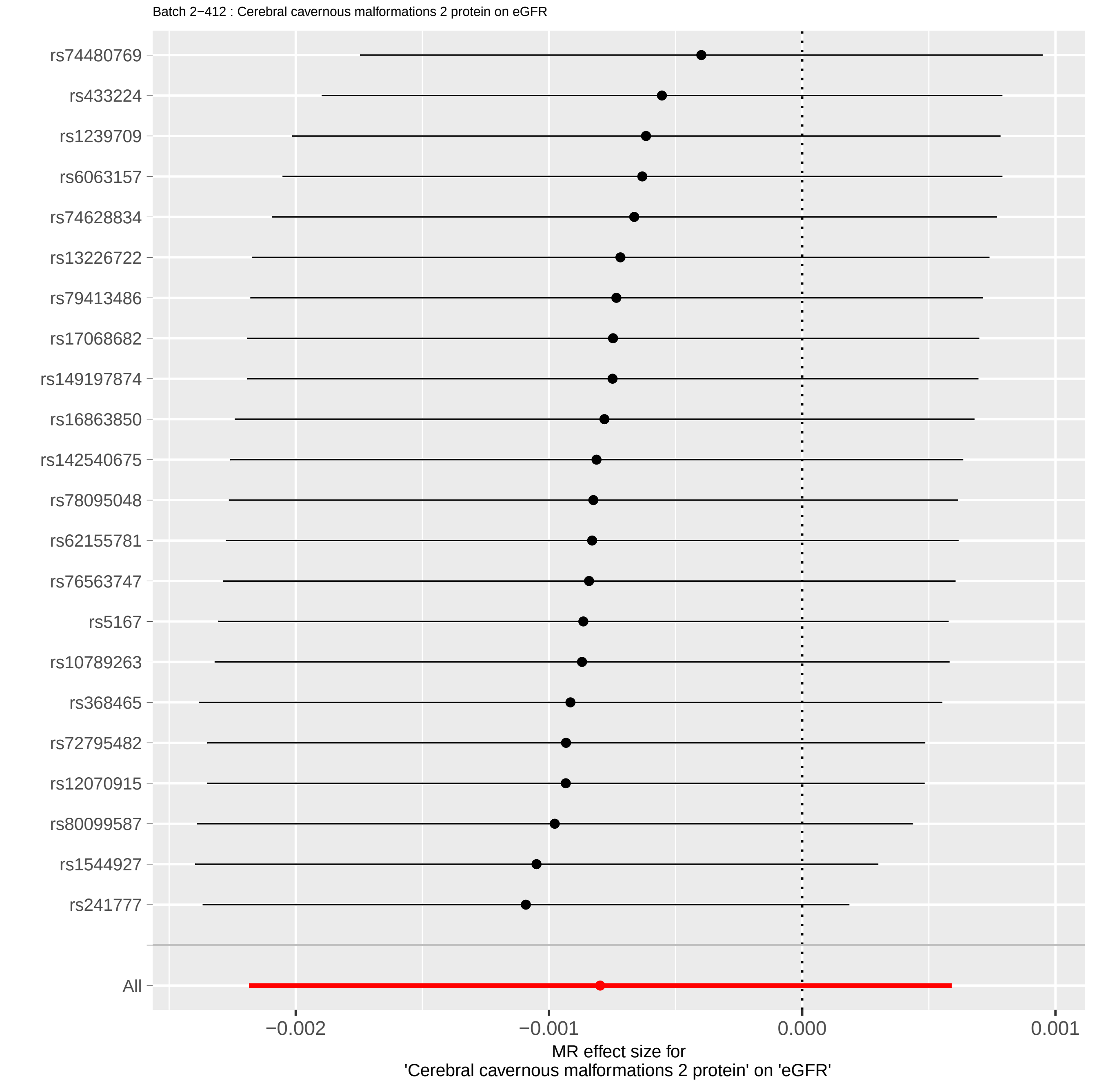

Batch 2−412 : Cerebral cavernous malformations 2 protein on eGFR
rs74480769
rs433224
rs1239709
rs6063157
rs74628834
rs13226722
rs79413486
rs17068682
rs149197874
rs16863850
rs142540675
rs78095048
rs62155781
rs76563747
rs5167
rs10789263
rs368465
rs72795482
rs12070915
rs80099587
rs1544927
rs241777
All
−0.002
−0.001
0.000
0.001
MR effect size for
'Cerebral cavernous malformations 2 protein' on 'eGFR'

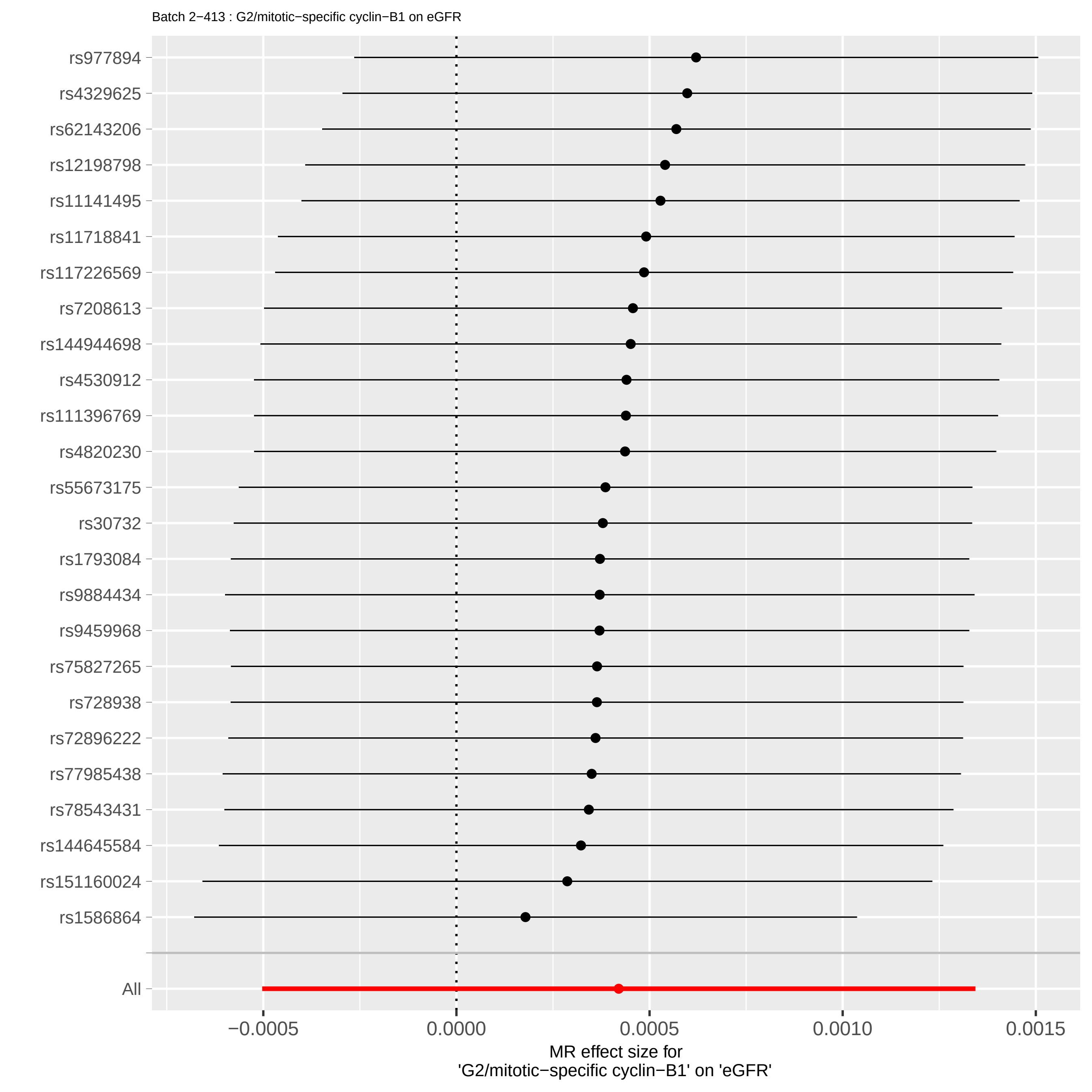

Batch 2−413 : G2/mitotic−specific cyclin−B1 on eGFR
rs977894
rs4329625
rs62143206
rs12198798
rs11141495
rs11718841
rs117226569
rs7208613
rs144944698
rs4530912
rs111396769
rs4820230
rs55673175
rs30732
rs1793084
rs9884434
rs9459968
rs75827265
rs728938
rs72896222
rs77985438
rs78543431
rs144645584
rs151160024
rs1586864
All
−0.0005
0.0000
0.0005
0.0010
0.0015
MR effect size for
'G2/mitotic−specific cyclin−B1' on 'eGFR'

Batch 2−414 : E3 ubiquitin−protein ligase CCNB1IP1 on eGFR

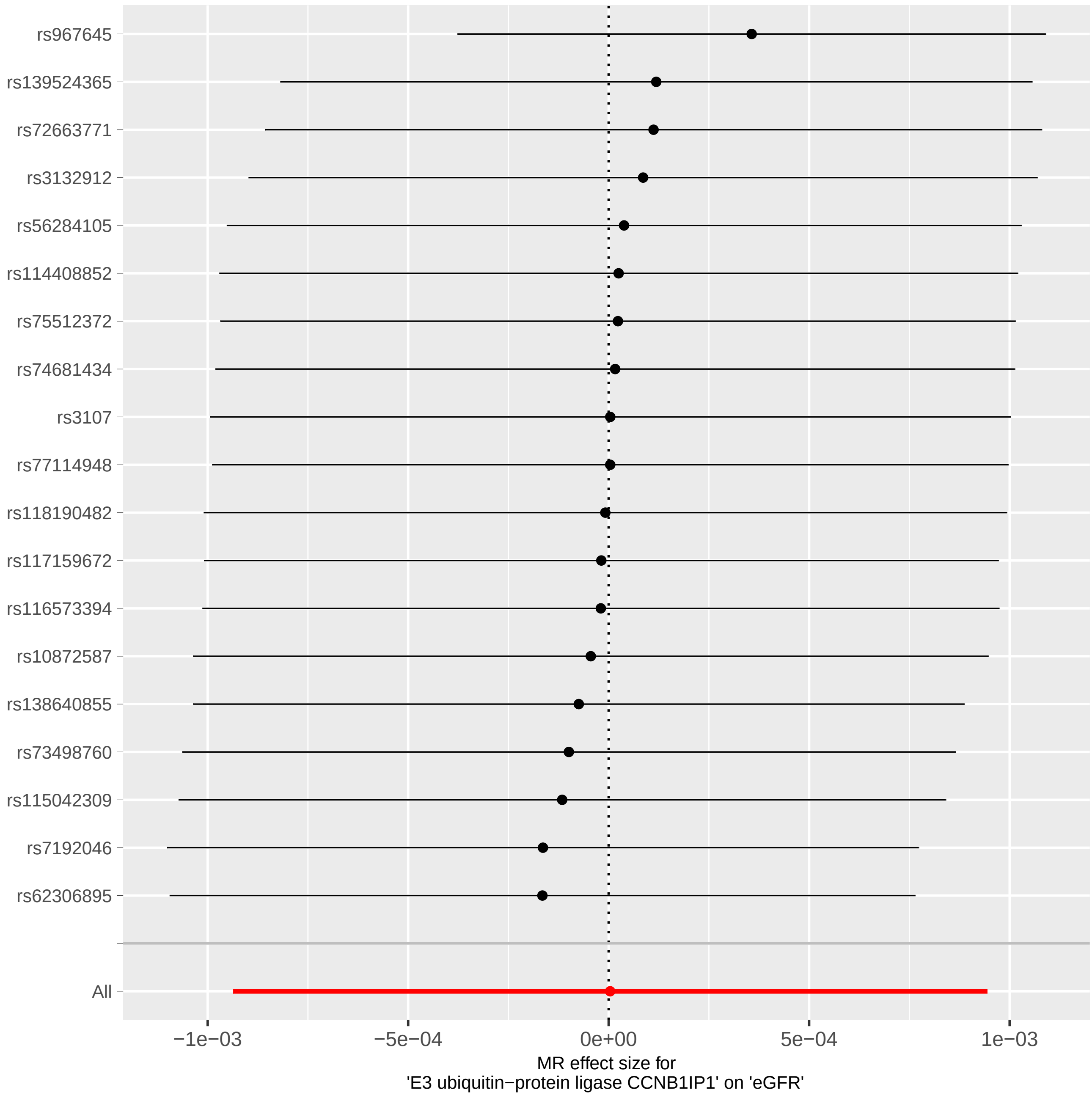

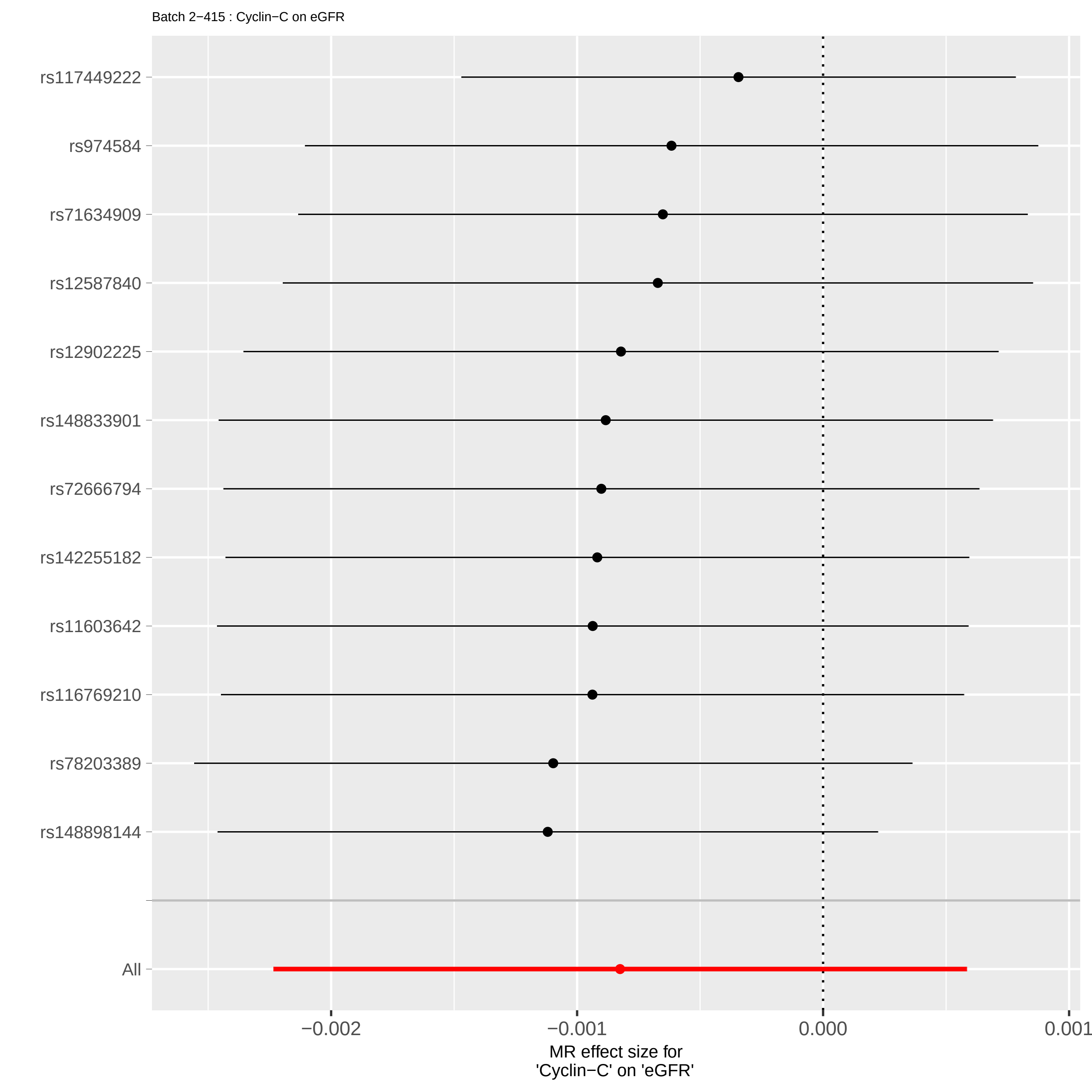

Batch 2−415 : Cyclin−C on eGFR
rs117449222
rs974584
rs71634909
rs12587840
rs12902225
rs148833901
rs72666794
rs142255182
rs11603642
rs116769210
rs78203389
rs148898144
All
−0.002
−0.001
0.000
0.001
MR effect size for
'Cyclin−C' on 'eGFR'

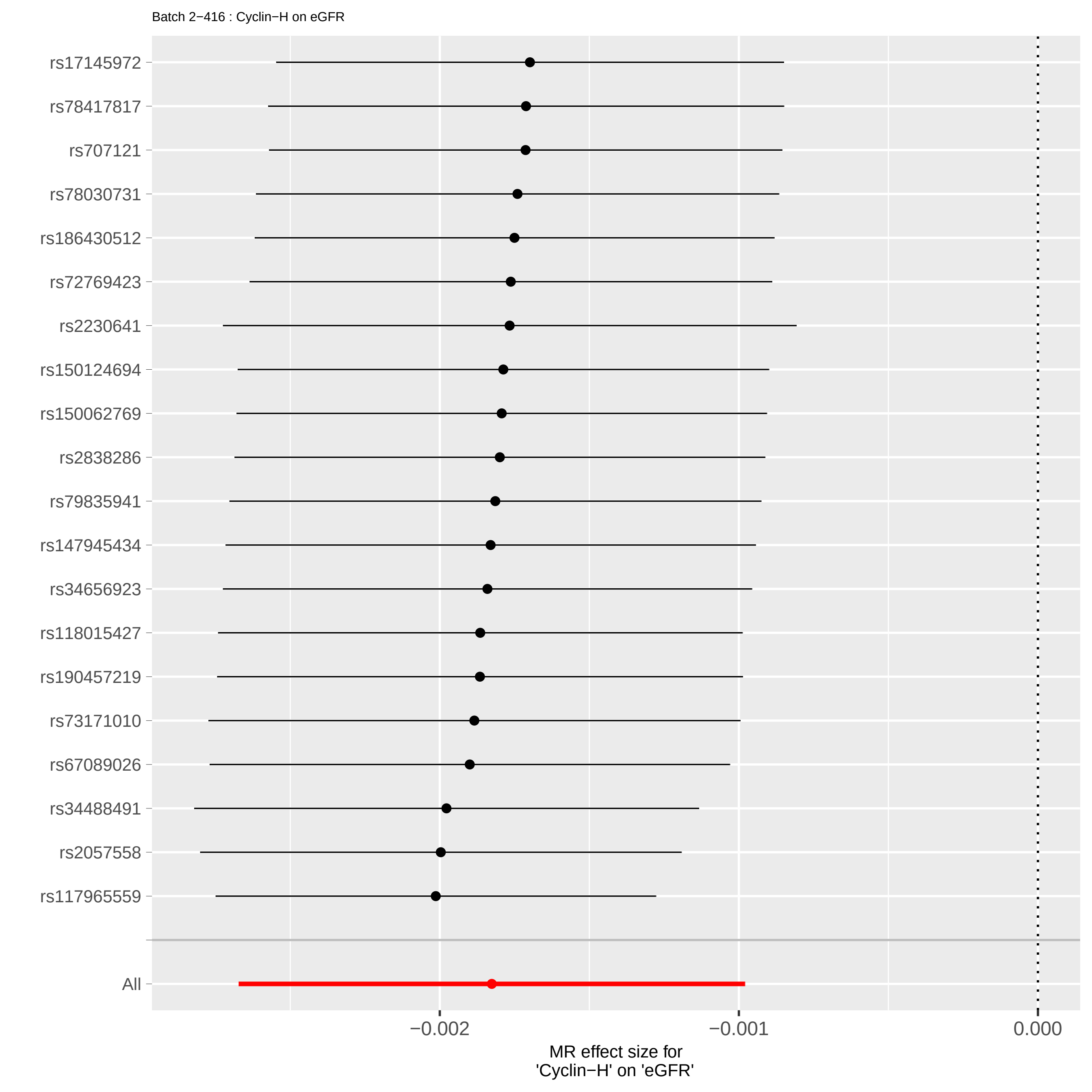

Batch 2−416 : Cyclin−H on eGFR
rs17145972
rs78417817
rs707121
rs78030731
rs186430512
rs72769423
rs2230641
rs150124694
rs150062769
rs2838286
rs79835941
rs147945434
rs34656923
rs118015427
rs190457219
rs73171010
rs67089026
rs34488491
rs2057558
rs117965559
All
−0.002
−0.001
0.000
MR effect size for
'Cyclin−H' on 'eGFR'

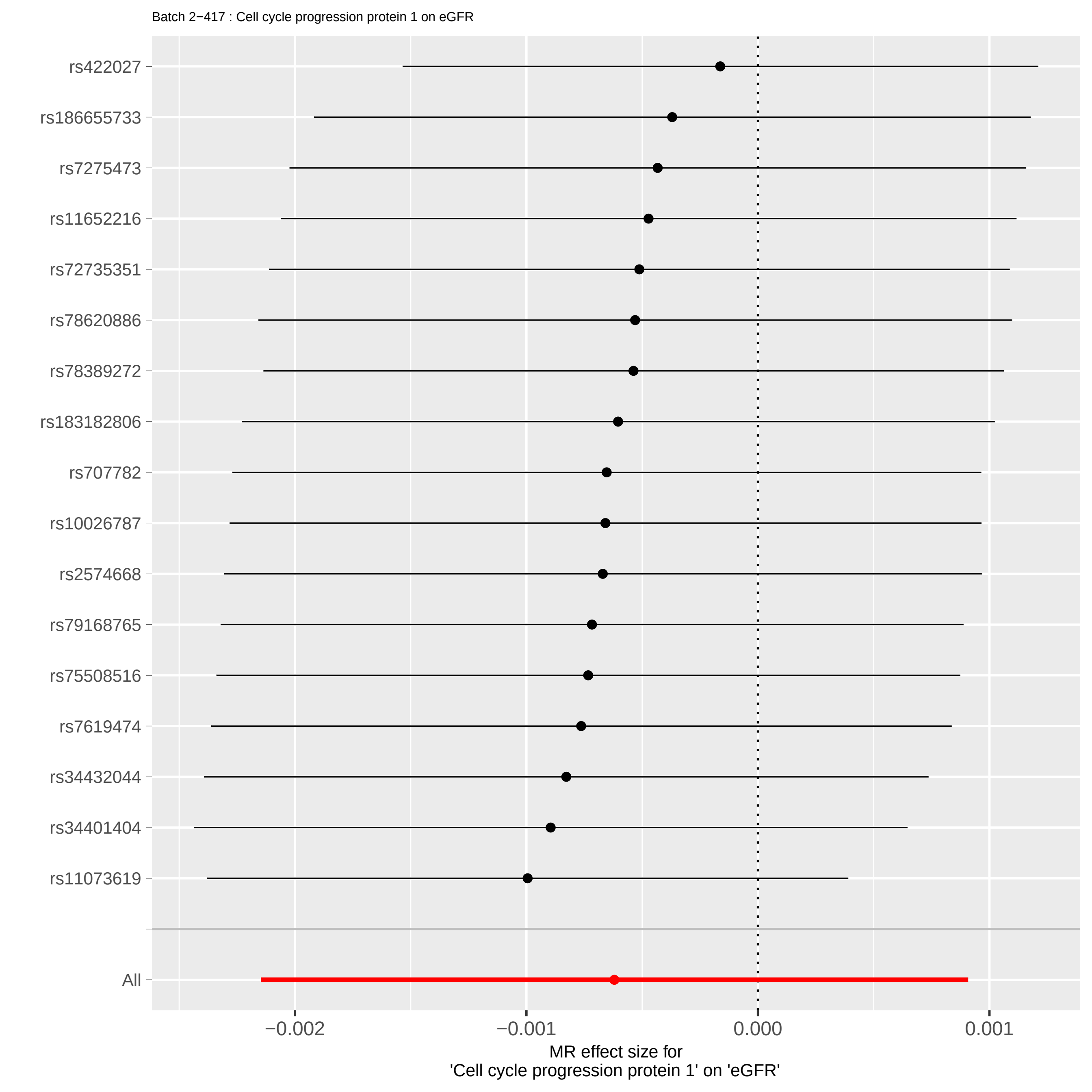
Batch 2−417 : Cell cycle progression protein 1 on eGFR
rs422027
rs186655733
rs7275473
rs11652216
rs72735351
rs78620886
rs78389272
rs183182806
rs707782
rs10026787
rs2574668
rs79168765
rs75508516
rs7619474
rs34432044
rs34401404
rs11073619
All
−0.002
−0.001
0.000
0.001
MR effect size for
'Cell cycle progression protein 1' on 'eGFR'

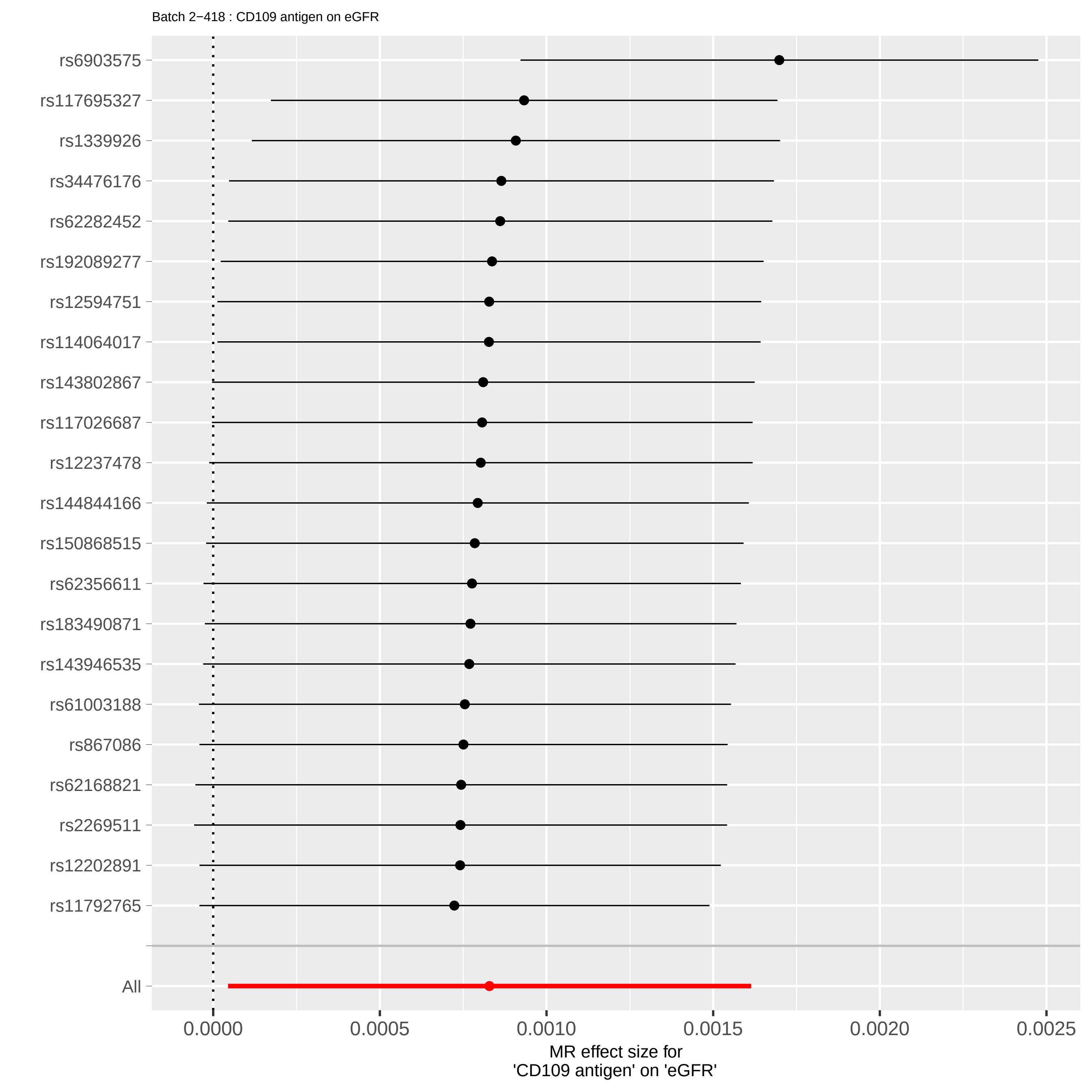

Batch 2−418 : CD109 antigen on eGFR
rs6903575
rs117695327
rs1339926
rs34476176
rs62282452
rs192089277
rs12594751
rs114064017
rs143802867
rs117026687
rs12237478
rs144844166
rs150868515
rs62356611
rs183490871
rs143946535
rs61003188
rs867086
rs62168821
rs2269511
rs12202891
rs11792765
All
0.0000
0.0005
0.0010
0.0015
0.0020
0.0025
MR effect size for
'CD109 antigen' on 'eGFR'

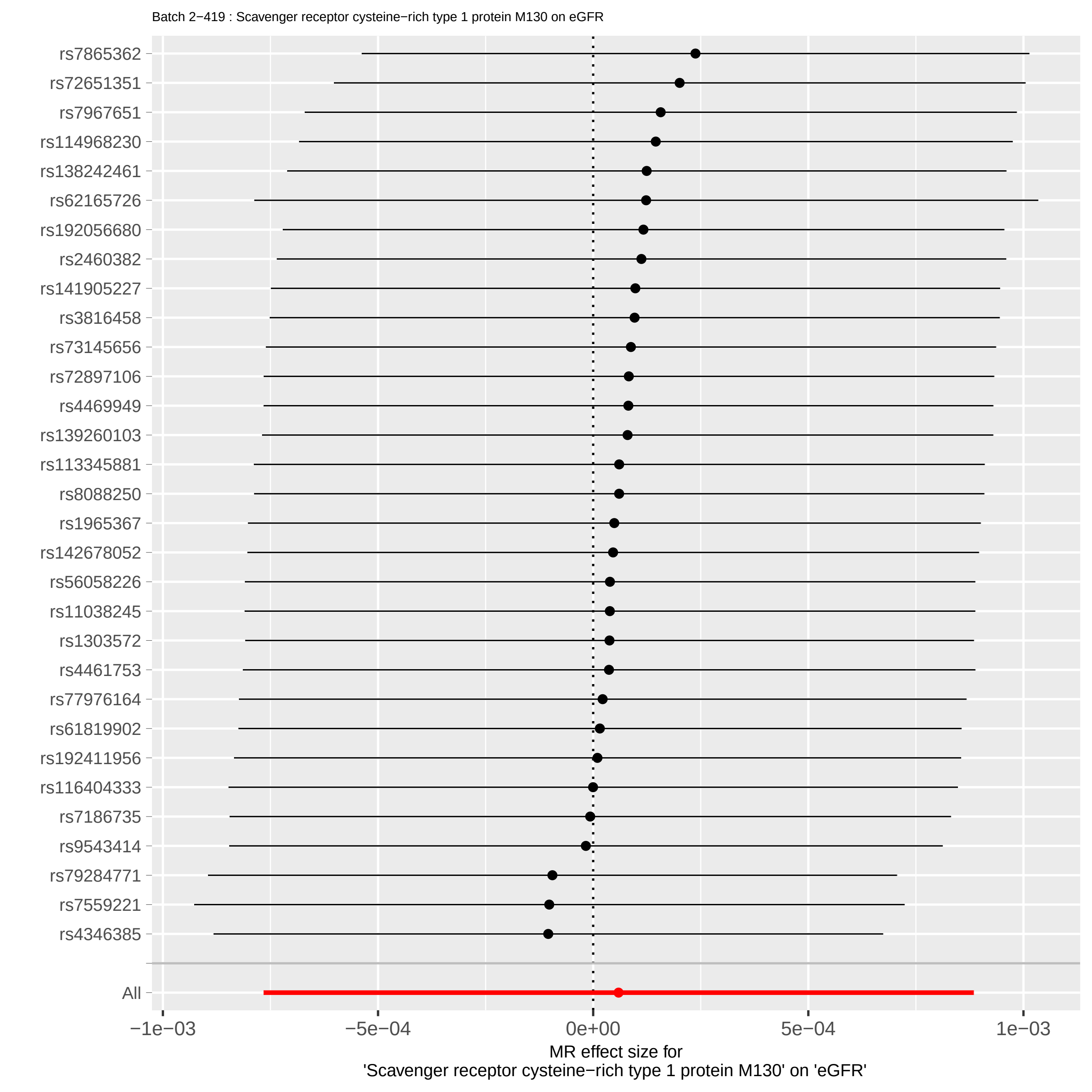

Batch 2−419 : Scavenger receptor cysteine−rich type 1 protein M130 on eGFR
rs7865362
rs72651351
rs7967651
rs114968230
rs138242461
rs62165726
rs192056680
rs2460382
rs141905227
rs3816458
rs73145656
rs72897106
rs4469949
rs139260103
rs113345881
rs8088250
rs1965367
rs142678052
rs56058226
rs11038245
rs1303572
rs4461753
rs77976164
rs61819902
rs192411956
rs116404333
rs7186735
rs9543414
rs79284771
rs7559221
rs4346385
All
−1e−03
−5e−04
0e+00
5e−04
1e−03
MR effect size for
'Scavenger receptor cysteine−rich type 1 protein M130' on 'eGFR'

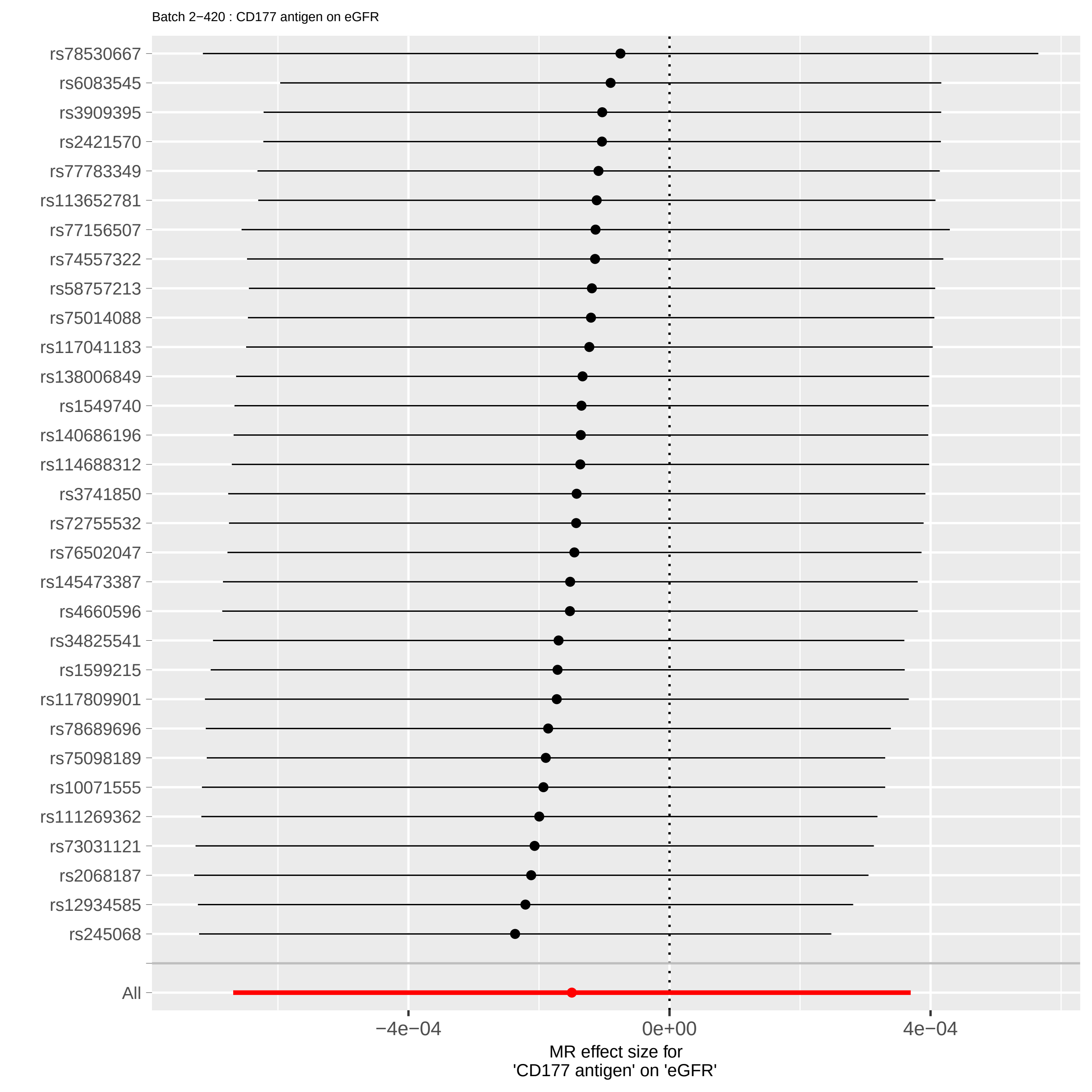

Batch 2−420 : CD177 antigen on eGFR
rs78530667
rs6083545
rs3909395
rs2421570
rs77783349
rs113652781
rs77156507
rs74557322
rs58757213
rs75014088
rs117041183
rs138006849
rs1549740
rs140686196
rs114688312
rs3741850
rs72755532
rs76502047
rs145473387
rs4660596
rs34825541
rs1599215
rs117809901
rs78689696
rs75098189
rs10071555
rs111269362
rs73031121
rs2068187
rs12934585
rs245068
All
−4e−04
0e+00
4e−04
MR effect size for
'CD177 antigen' on 'eGFR'

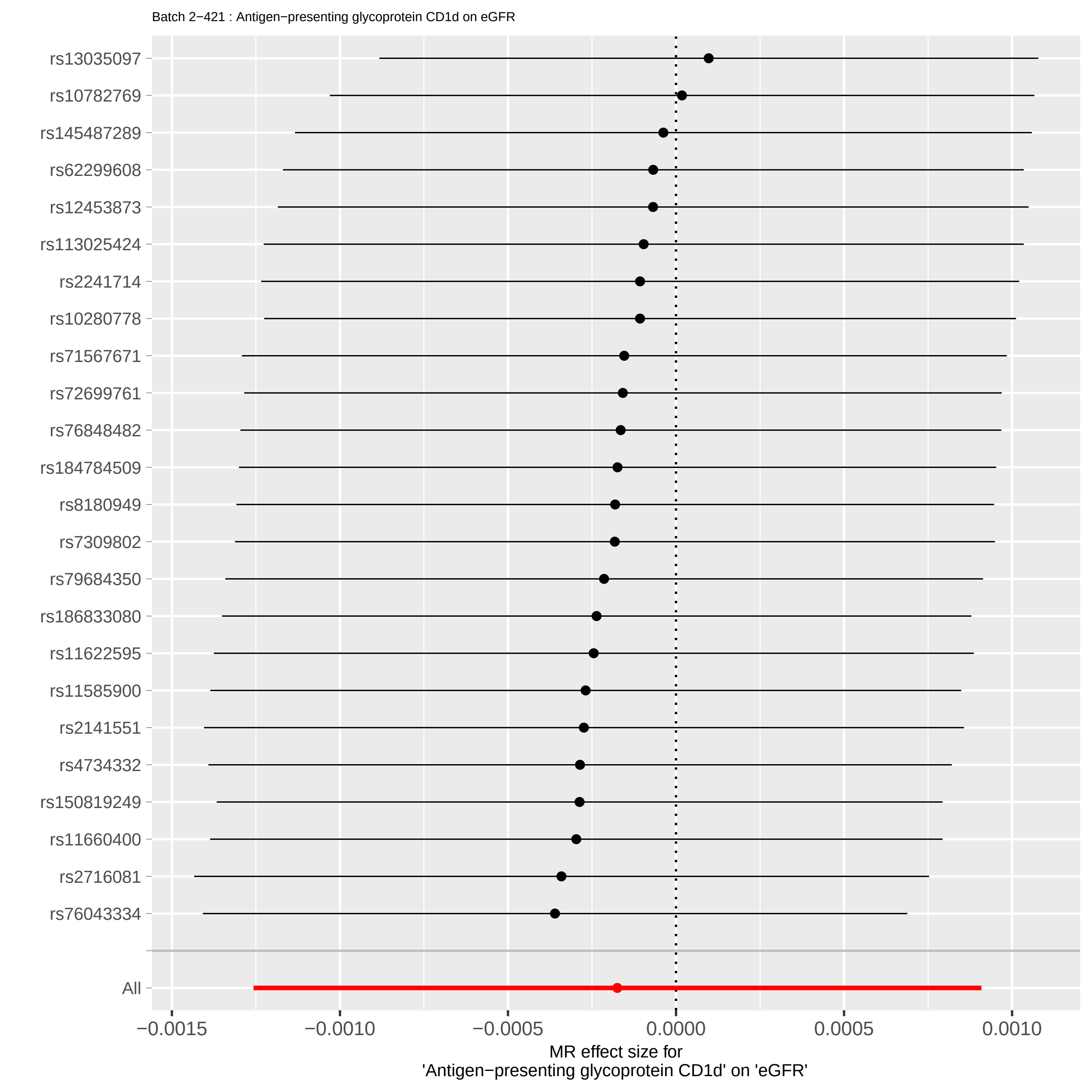

Batch 2−421 : Antigen−presenting glycoprotein CD1d on eGFR
rs13035097
rs10782769
rs145487289
rs62299608
rs12453873
rs113025424
rs2241714
rs10280778
rs71567671
rs72699761
rs76848482
rs184784509
rs8180949
rs7309802
rs79684350
rs186833080
rs11622595
rs11585900
rs2141551
rs4734332
rs150819249
rs11660400
rs2716081
rs76043334
All
−0.0015
−0.0010
−0.0005
0.0000
0.0005
0.0010
MR effect size for
'Antigen−presenting glycoprotein CD1d' on 'eGFR'

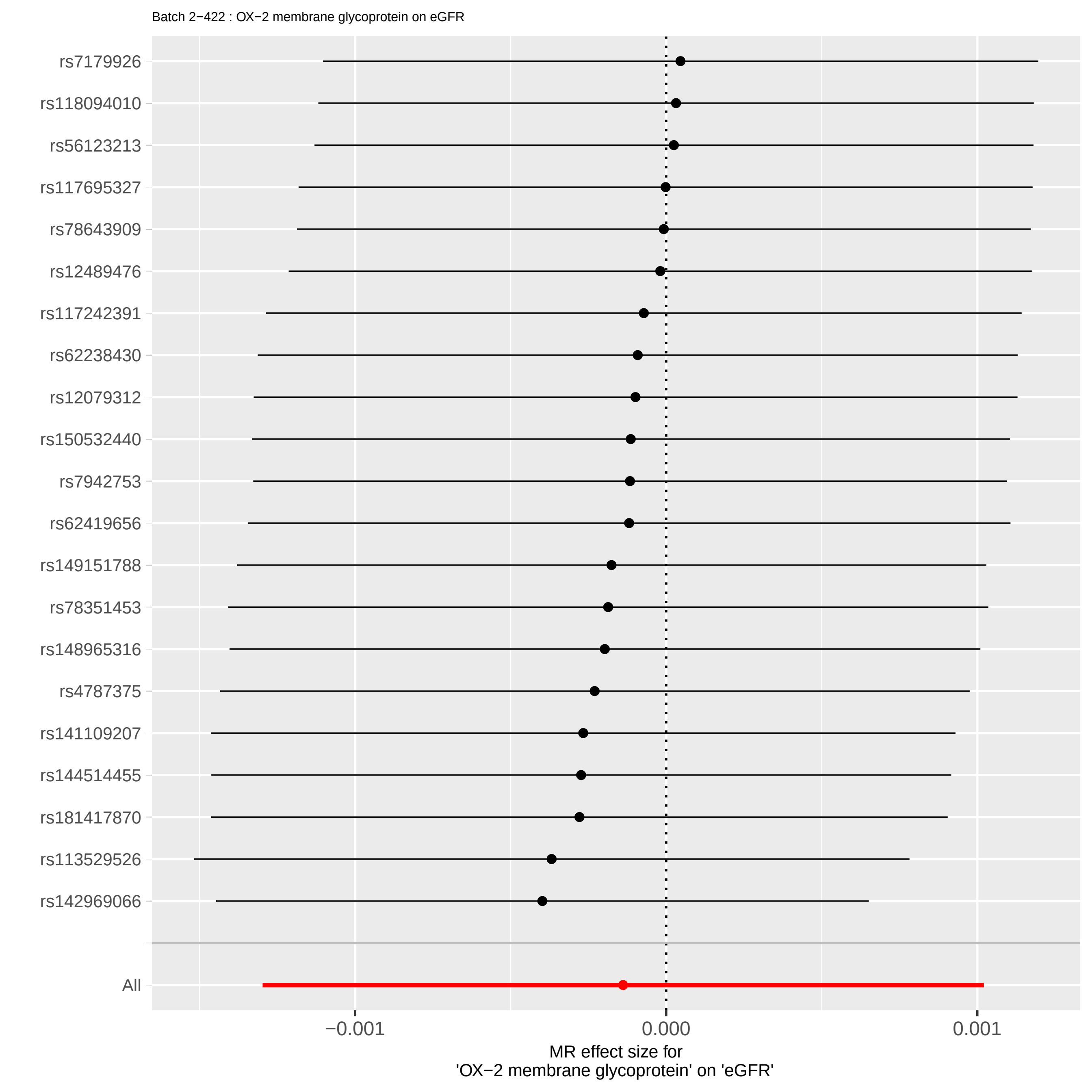

Batch 2−422 : OX−2 membrane glycoprotein on eGFR
rs7179926
rs118094010
rs56123213
rs117695327
rs78643909
rs12489476
rs117242391
rs62238430
rs12079312
rs150532440
rs7942753
rs62419656
rs149151788
rs78351453
rs148965316
rs4787375
rs141109207
rs144514455
rs181417870
rs113529526
rs142969066
All
−0.001
0.000
0.001
MR effect size for
'OX−2 membrane glycoprotein' on 'eGFR'

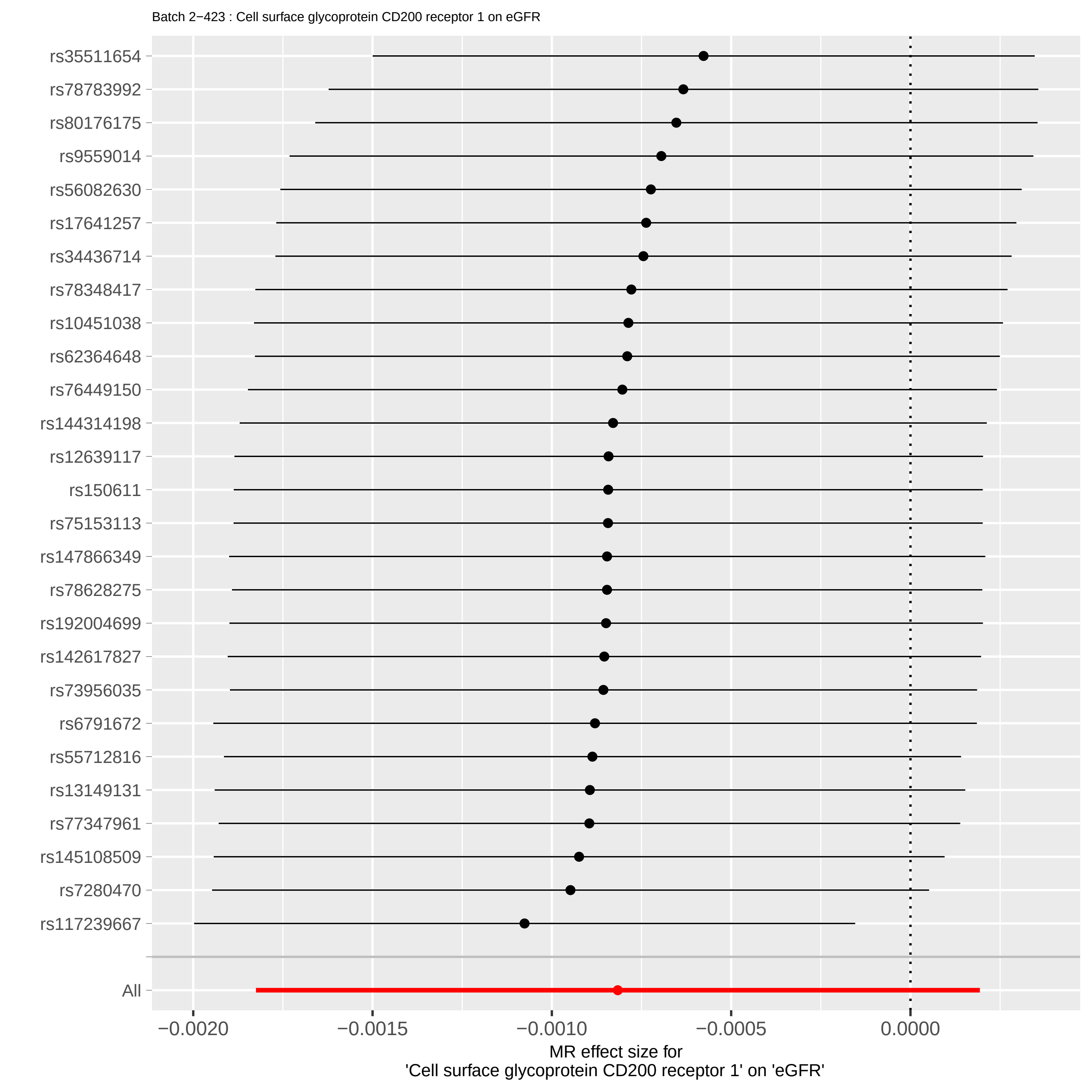
Batch 2−423 : Cell surface glycoprotein CD200 receptor 1 on eGFR
rs35511654
rs78783992
rs80176175
rs9559014
rs56082630
rs17641257
rs34436714
rs78348417
rs10451038
rs62364648
rs76449150
rs144314198
rs12639117
rs150611
rs75153113
rs147866349
rs78628275
rs192004699
rs142617827
rs73956035
rs6791672
rs55712816
rs13149131
rs77347961
rs145108509
rs7280470
rs117239667
All
−0.0020
−0.0015
−0.0010
−0.0005
0.0000
MR effect size for
'Cell surface glycoprotein CD200 receptor 1' on 'eGFR'

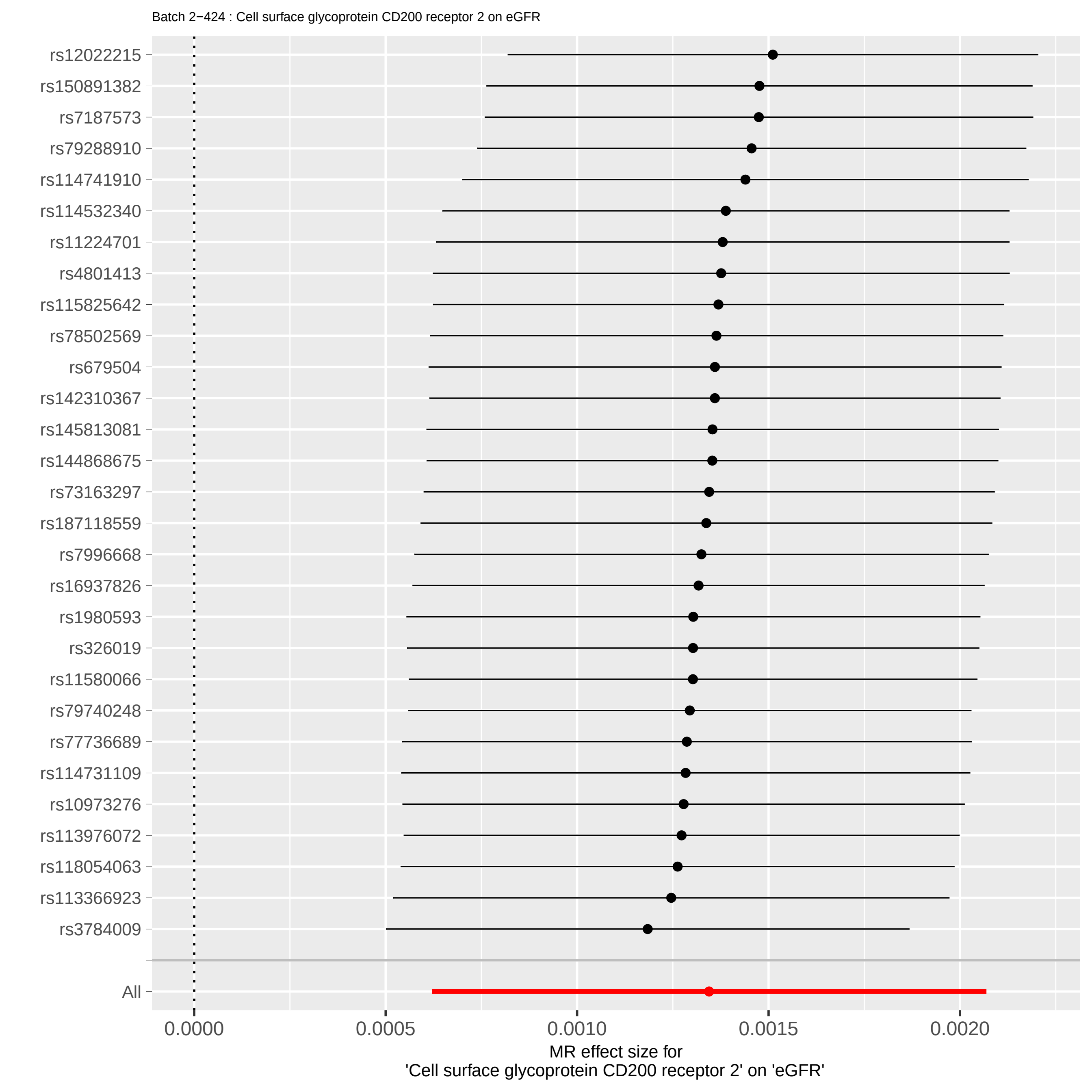

Batch 2−424 : Cell surface glycoprotein CD200 receptor 2 on eGFR
rs12022215
rs150891382
rs7187573
rs79288910
rs114741910
rs114532340
rs11224701
rs4801413
rs115825642
rs78502569
rs679504
rs142310367
rs145813081
rs144868675
rs73163297
rs187118559
rs7996668
rs16937826
rs1980593
rs326019
rs11580066
rs79740248
rs77736689
rs114731109
rs10973276
rs113976072
rs118054063
rs113366923
rs3784009
All
0.0000
0.0005
0.0010
0.0015
0.0020
MR effect size for
'Cell surface glycoprotein CD200 receptor 2' on 'eGFR'

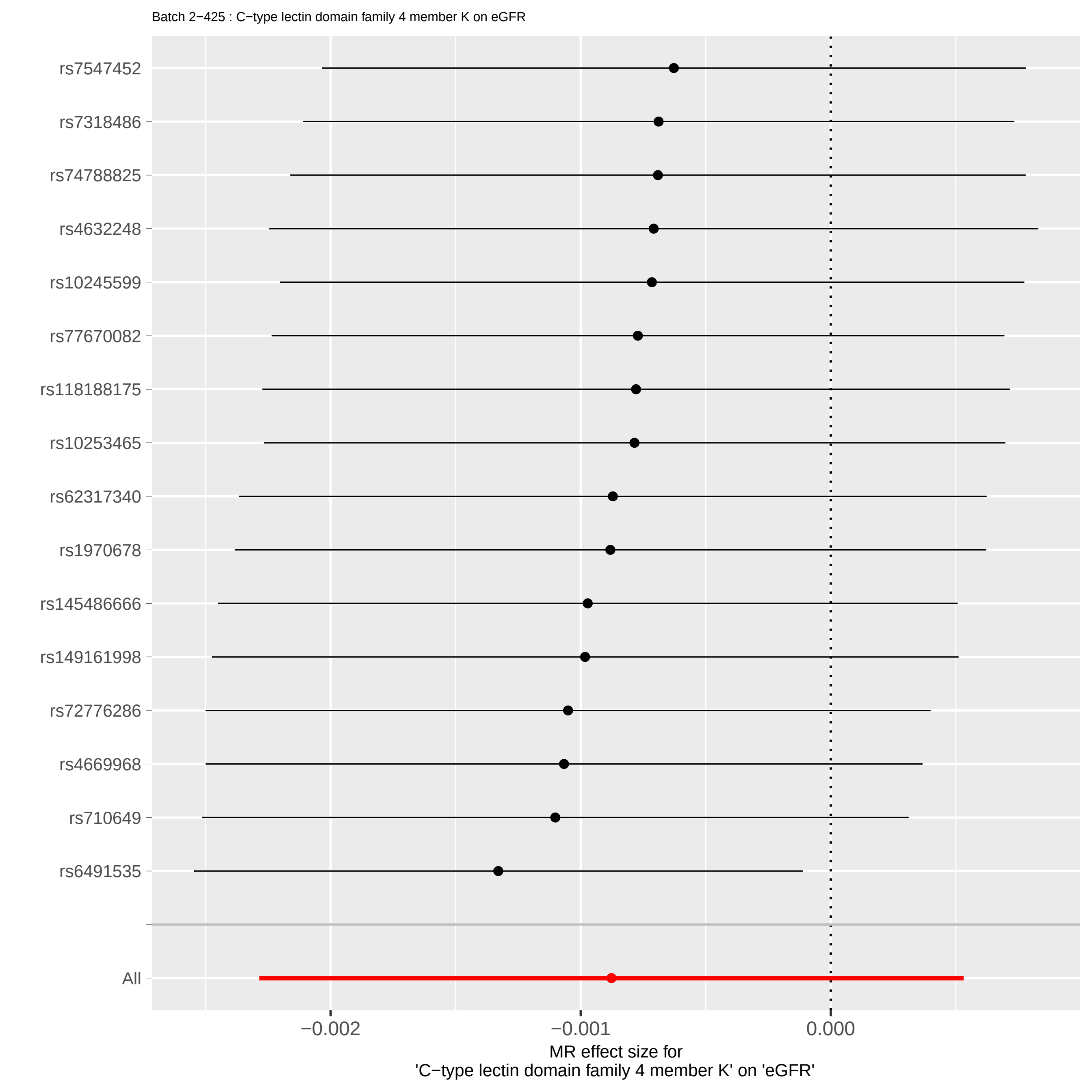

Batch 2−425 : C−type lectin domain family 4 member K on eGFR
rs7547452
rs7318486
rs74788825
rs4632248
rs10245599
rs77670082
rs118188175
rs10253465
rs62317340
rs1970678
rs145486666
rs149161998
rs72776286
rs4669968
rs710649
rs6491535
All
−0.002
−0.001
0.000
MR effect size for
'C−type lectin domain family 4 member K' on 'eGFR'

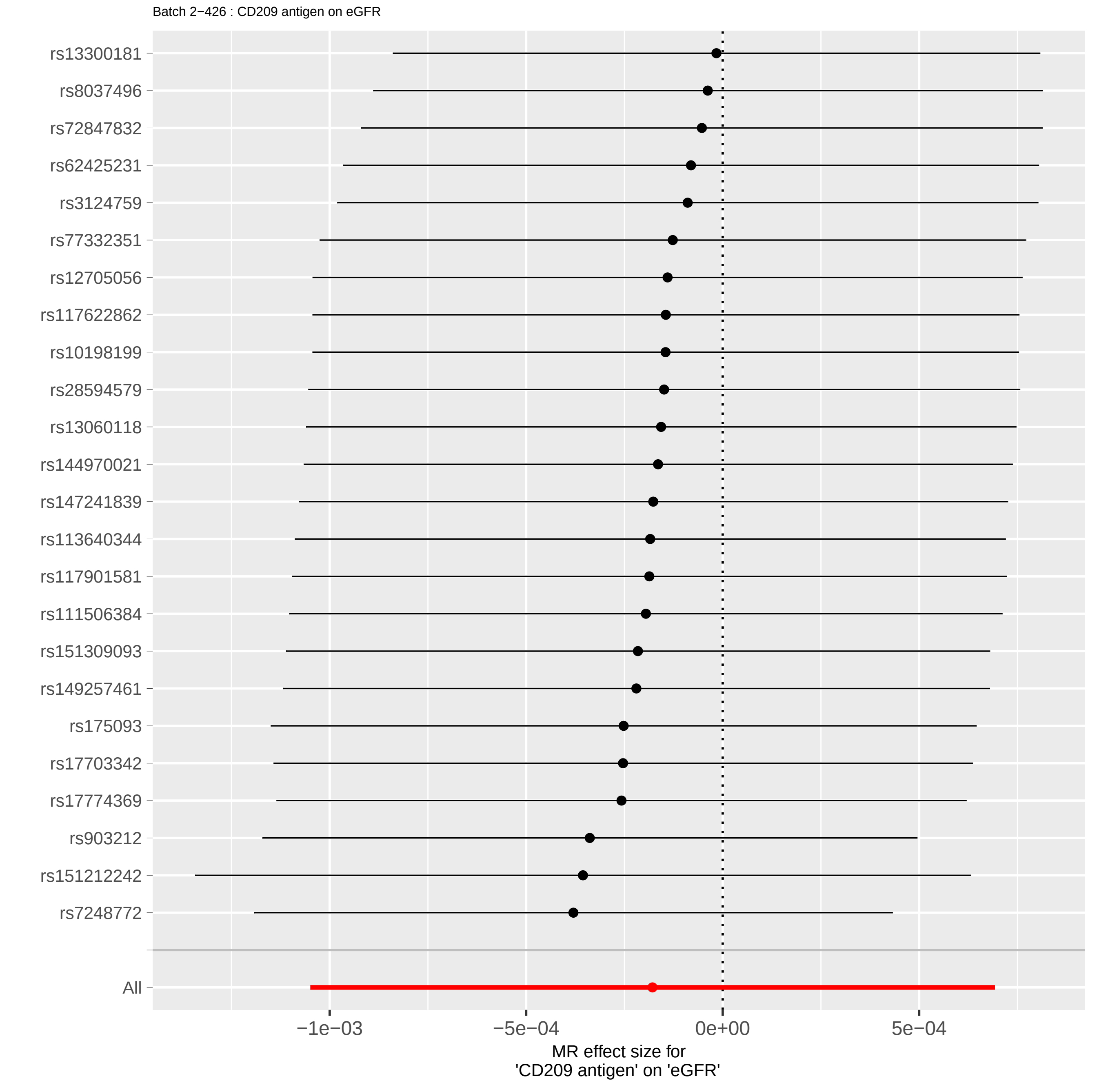

Batch 2−426 : CD209 antigen on eGFR
rs13300181
rs8037496
rs72847832
rs62425231
rs3124759
rs77332351
rs12705056
rs117622862
rs10198199
rs28594579
rs13060118
rs144970021
rs147241839
rs113640344
rs117901581
rs111506384
rs151309093
rs149257461
rs175093
rs17703342
rs17774369
rs903212
rs151212242
rs7248772
All
−1e−03
−5e−04
0e+00
5e−04
MR effect size for
'CD209 antigen' on 'eGFR'

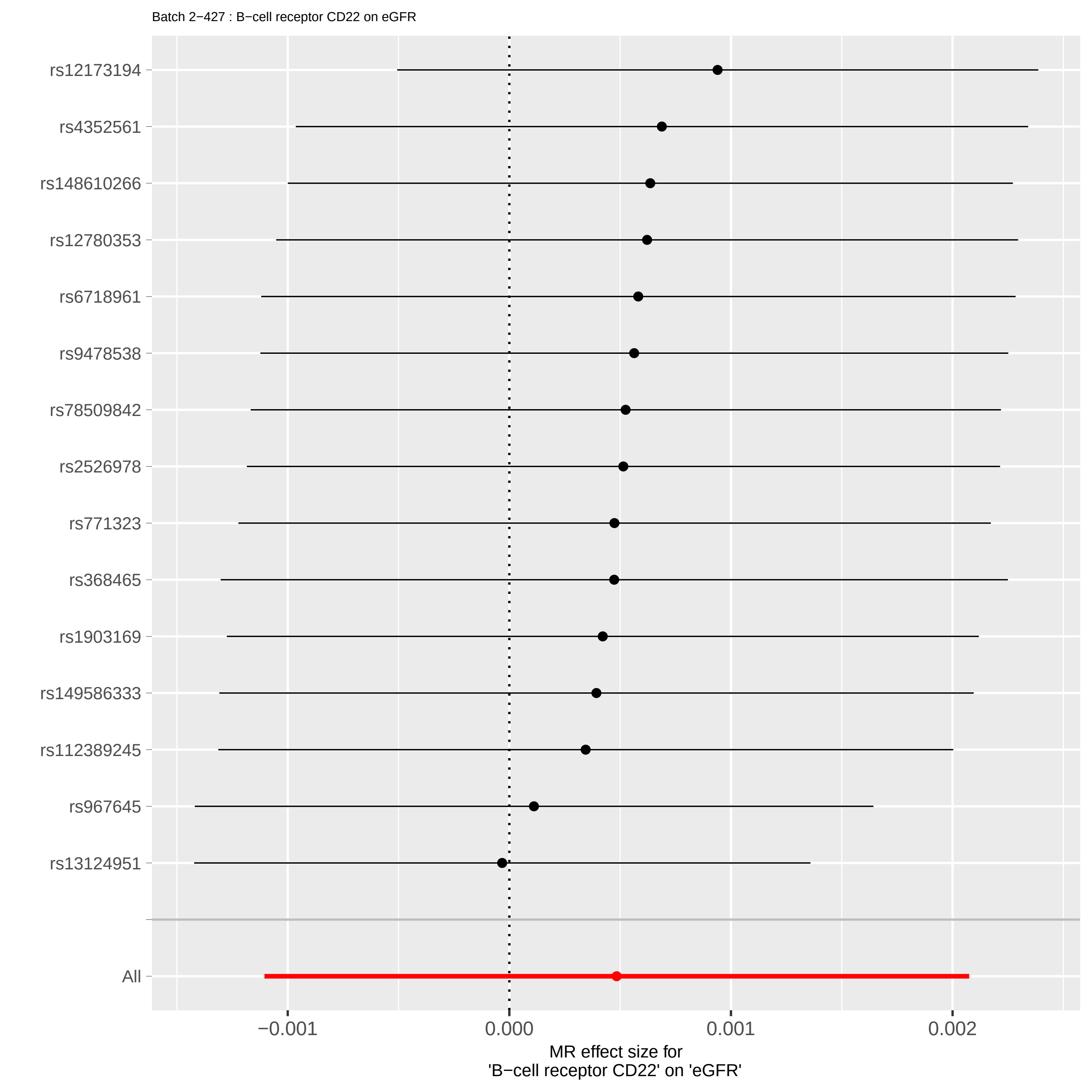

Batch 2−427 : B−cell receptor CD22 on eGFR
rs12173194
rs4352561
rs148610266
rs12780353
rs6718961
rs9478538
rs78509842
rs2526978
rs771323
rs368465
rs1903169
rs149586333
rs112389245
rs967645
rs13124951
All
−0.001
0.000
0.001
0.002
MR effect size for
'B−cell receptor CD22' on 'eGFR'

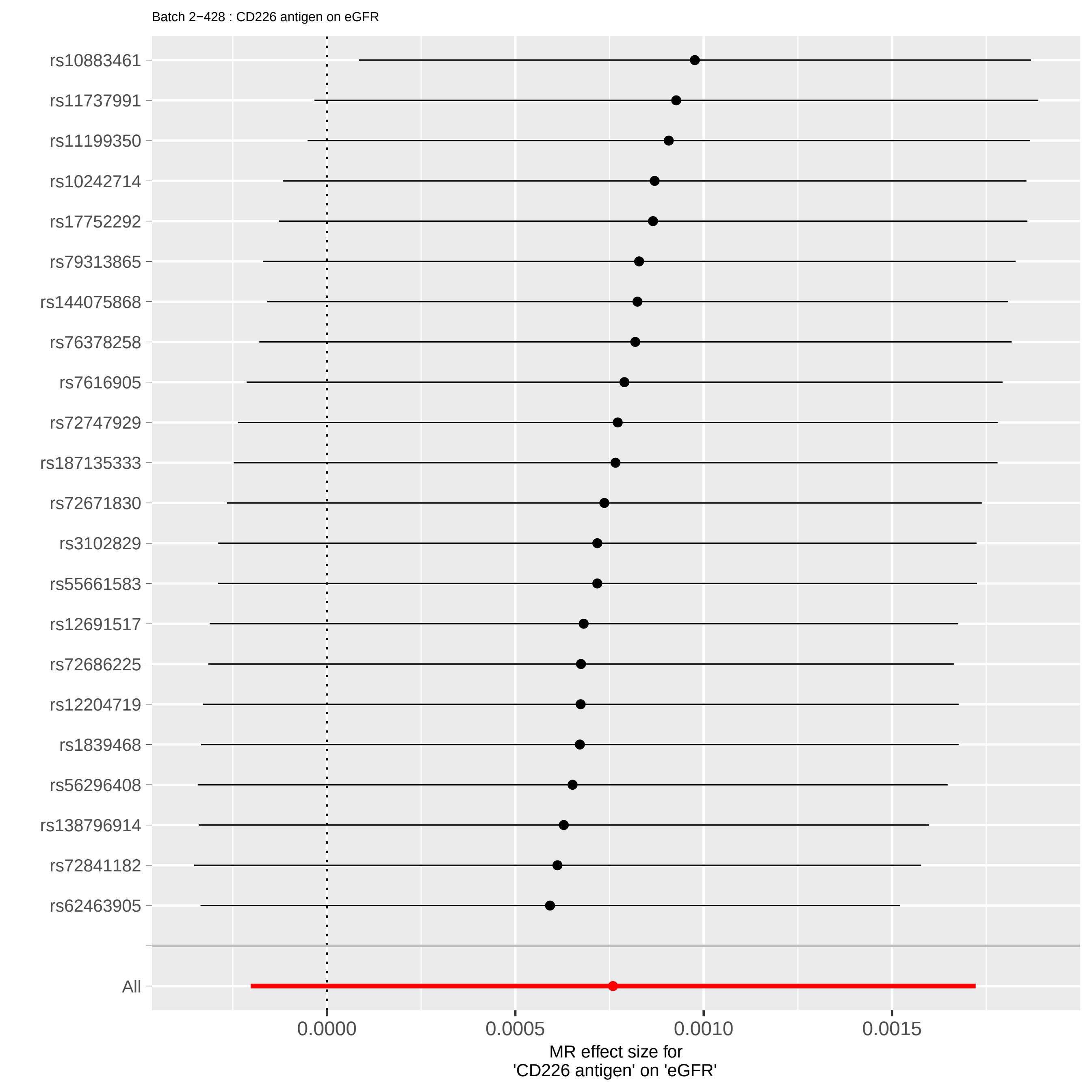

Batch 2−428 : CD226 antigen on eGFR
rs10883461
rs11737991
rs11199350
rs10242714
rs17752292
rs79313865
rs144075868
rs76378258
rs7616905
rs72747929
rs187135333
rs72671830
rs3102829
rs55661583
rs12691517
rs72686225
rs12204719
rs1839468
rs56296408
rs138796914
rs72841182
rs62463905
All
0.0000
0.0005
0.0010
0.0015
MR effect size for
'CD226 antigen' on 'eGFR'

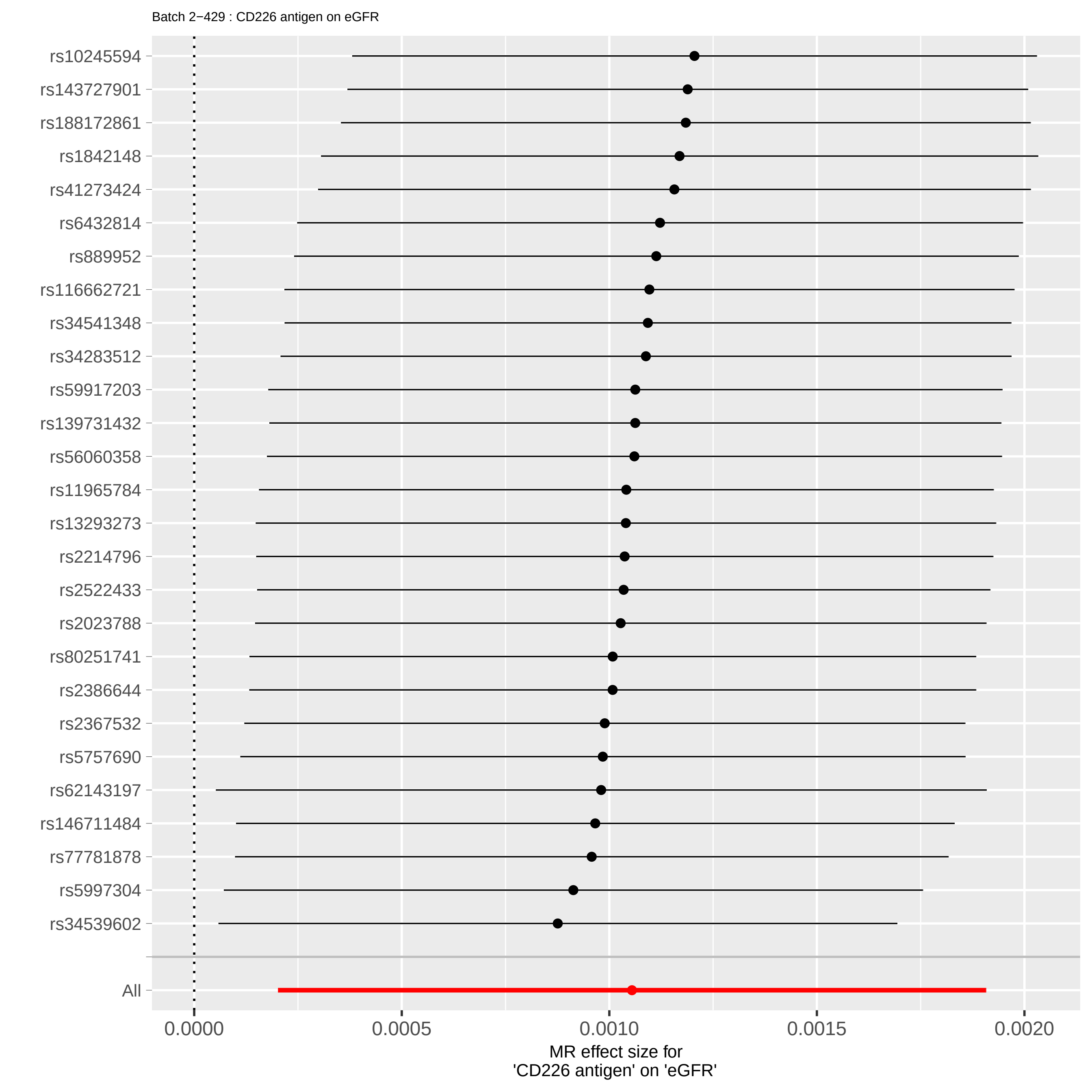
Batch 2−429 : CD226 antigen on eGFR
rs10245594
rs143727901
rs188172861
rs1842148
rs41273424
rs6432814
rs889952
rs116662721
rs34541348
rs34283512
rs59917203
rs139731432
rs56060358
rs11965784
rs13293273
rs2214796
rs2522433
rs2023788
rs80251741
rs2386644
rs2367532
rs5757690
rs62143197
rs146711484
rs77781878
rs5997304
rs34539602
All
0.0000
0.0005
0.0010
0.0015
0.0020
MR effect size for
'CD226 antigen' on 'eGFR'

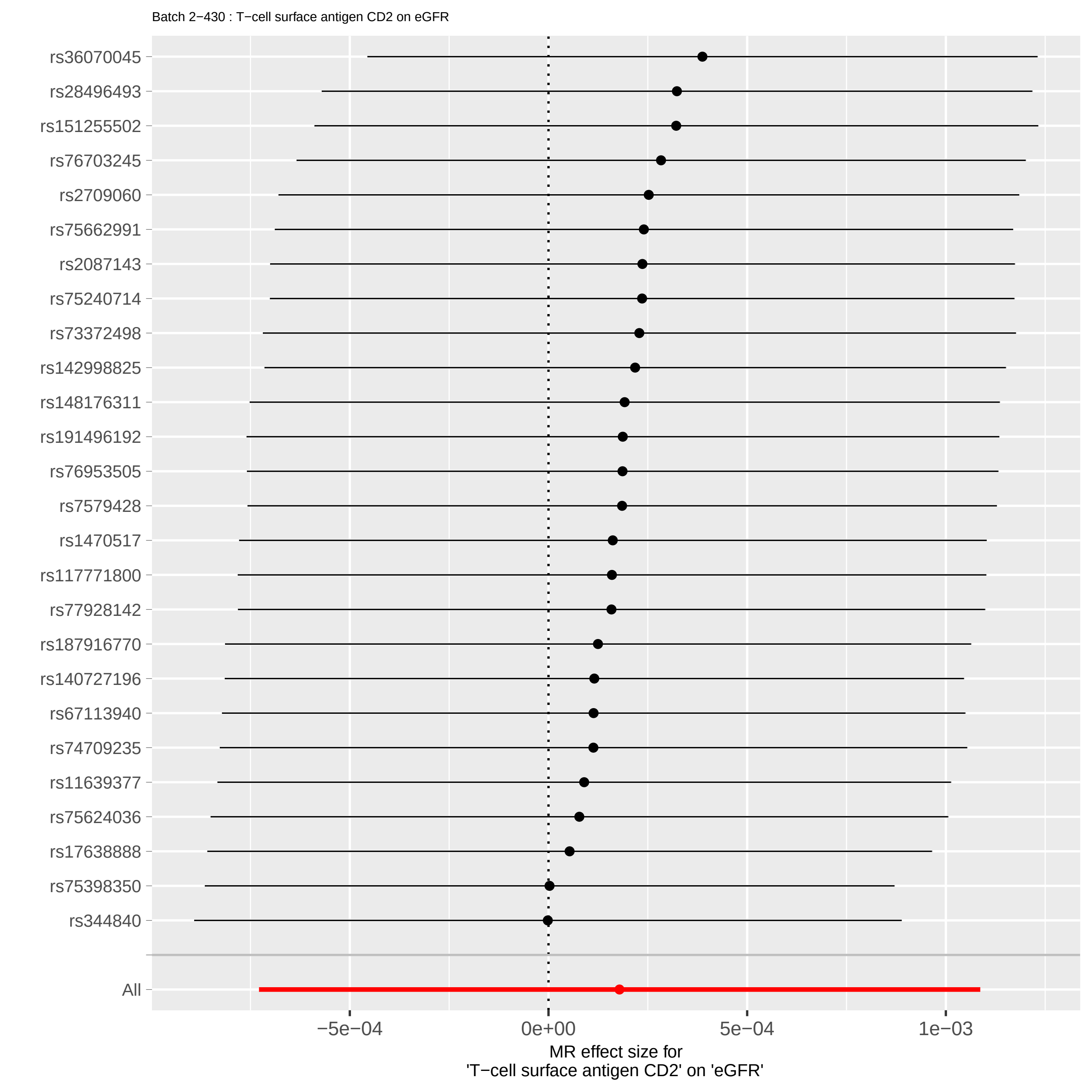

Batch 2−430 : T−cell surface antigen CD2 on eGFR
rs36070045
rs28496493
rs151255502
rs76703245
rs2709060
rs75662991
rs2087143
rs75240714
rs73372498
rs142998825
rs148176311
rs191496192
rs76953505
rs7579428
rs1470517
rs117771800
rs77928142
rs187916770
rs140727196
rs67113940
rs74709235
rs11639377
rs75624036
rs17638888
rs75398350
rs344840
All
−5e−04
0e+00
5e−04
1e−03
MR effect size for
'T−cell surface antigen CD2' on 'eGFR'

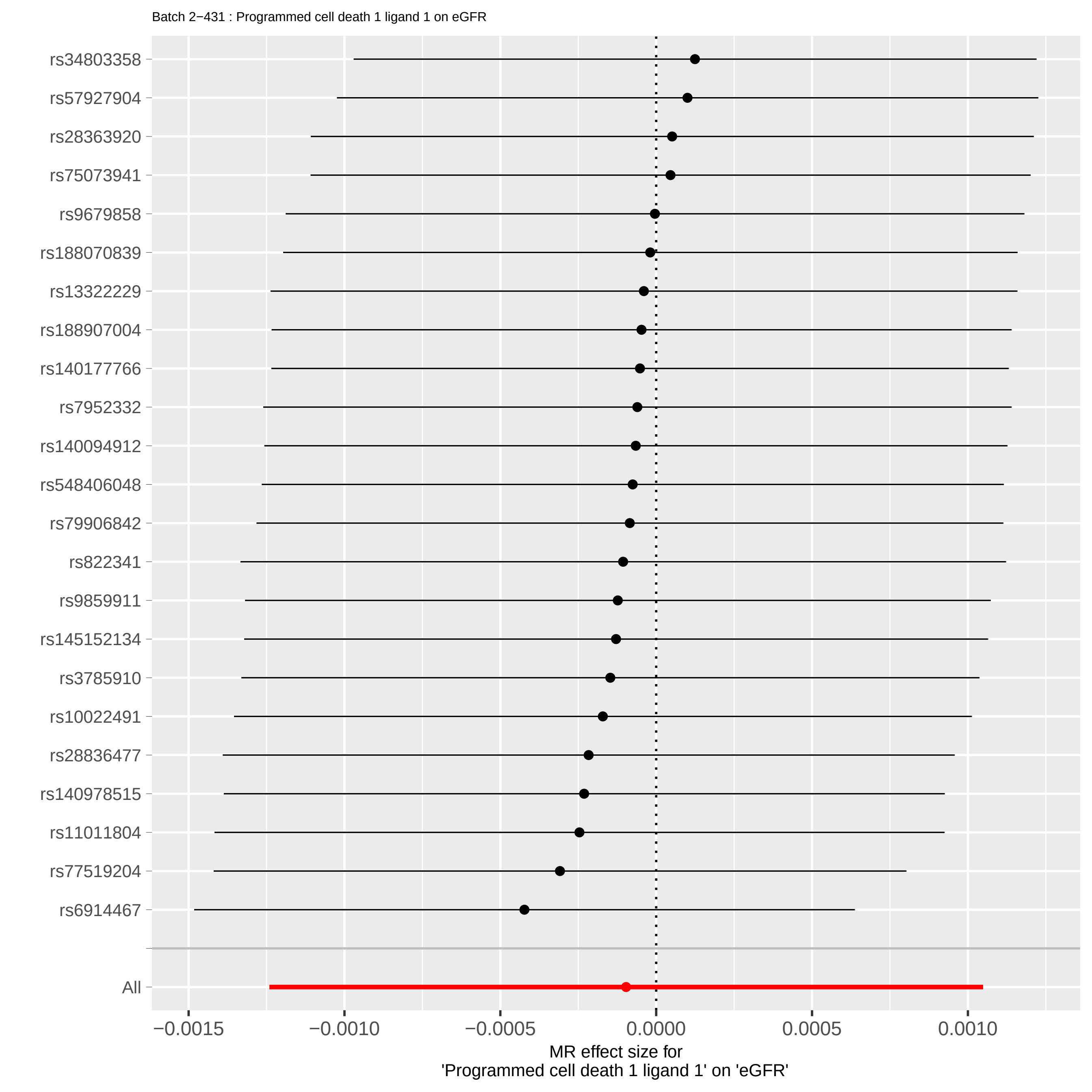
Batch 2−431 : Programmed cell death 1 ligand 1 on eGFR
rs34803358
rs57927904
rs28363920
rs75073941
rs9679858
rs188070839
rs13322229
rs188907004
rs140177766
rs7952332
rs140094912
rs548406048
rs79906842
rs822341
rs9859911
rs145152134
rs3785910
rs10022491
rs28836477
rs140978515
rs11011804
rs77519204
rs6914467
All
−0.0015
−0.0010
−0.0005
0.0000
0.0005
0.0010
MR effect size for
'Programmed cell death 1 ligand 1' on 'eGFR'

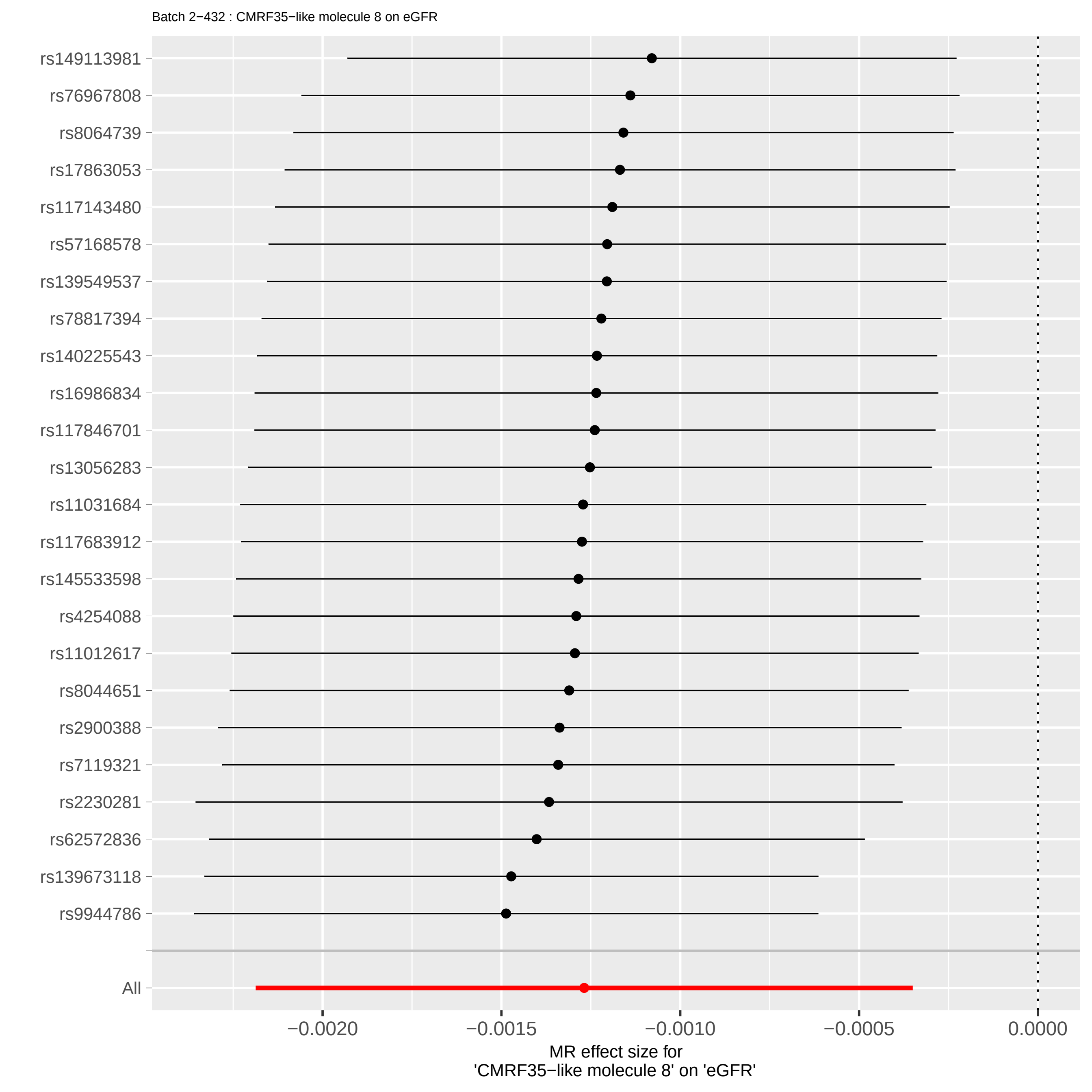

Batch 2−432 : CMRF35−like molecule 8 on eGFR
rs149113981
rs76967808
rs8064739
rs17863053
rs117143480
rs57168578
rs139549537
rs78817394
rs140225543
rs16986834
rs117846701
rs13056283
rs11031684
rs117683912
rs145533598
rs4254088
rs11012617
rs8044651
rs2900388
rs7119321
rs2230281
rs62572836
rs139673118
rs9944786
All
−0.0020
−0.0015
−0.0010
−0.0005
0.0000
MR effect size for
'CMRF35−like molecule 8' on 'eGFR'

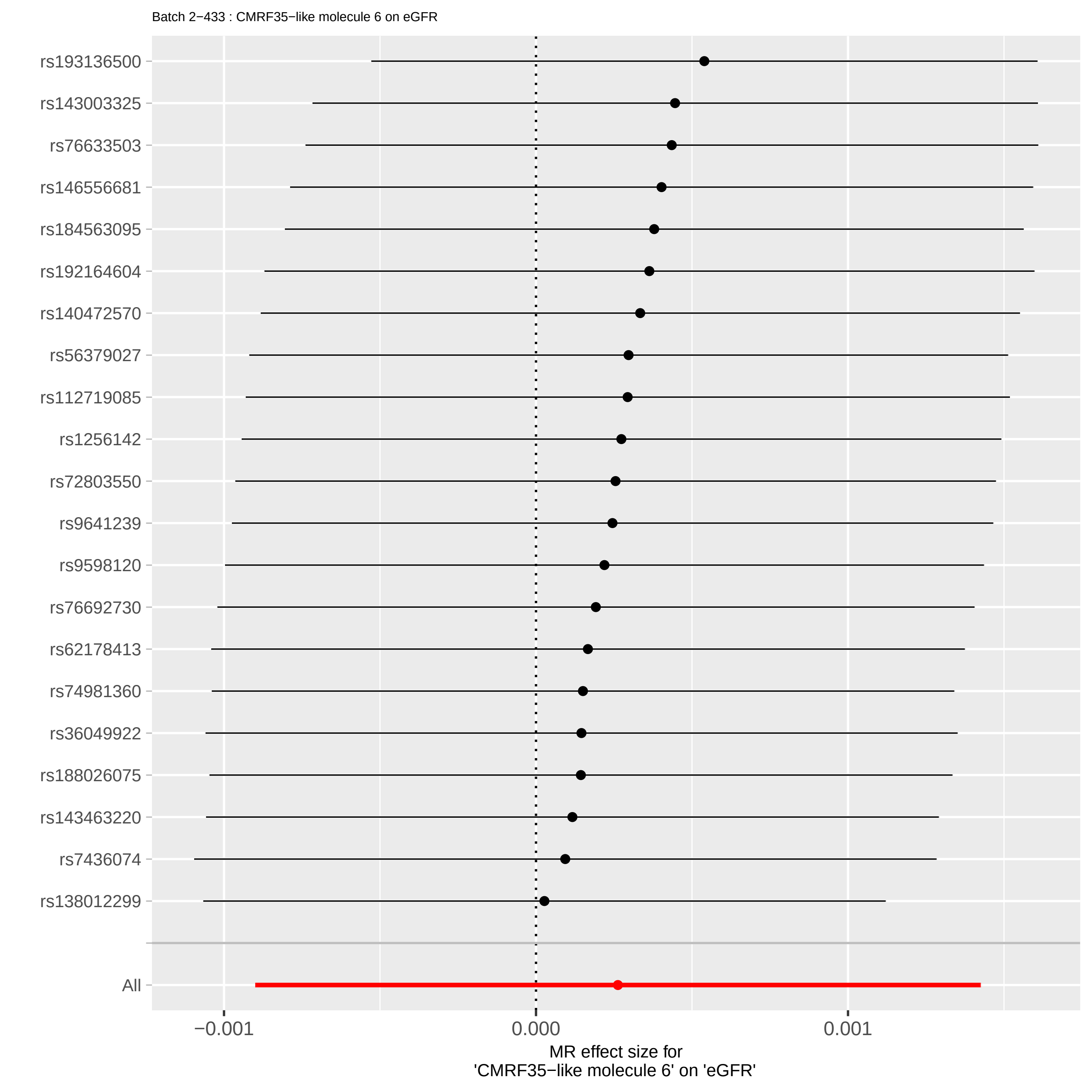

Batch 2−433 : CMRF35−like molecule 6 on eGFR
rs193136500
rs143003325
rs76633503
rs146556681
rs184563095
rs192164604
rs140472570
rs56379027
rs112719085
rs1256142
rs72803550
rs9641239
rs9598120
rs76692730
rs62178413
rs74981360
rs36049922
rs188026075
rs143463220
rs7436074
rs138012299
All
−0.001
0.000
0.001
MR effect size for
'CMRF35−like molecule 6' on 'eGFR'

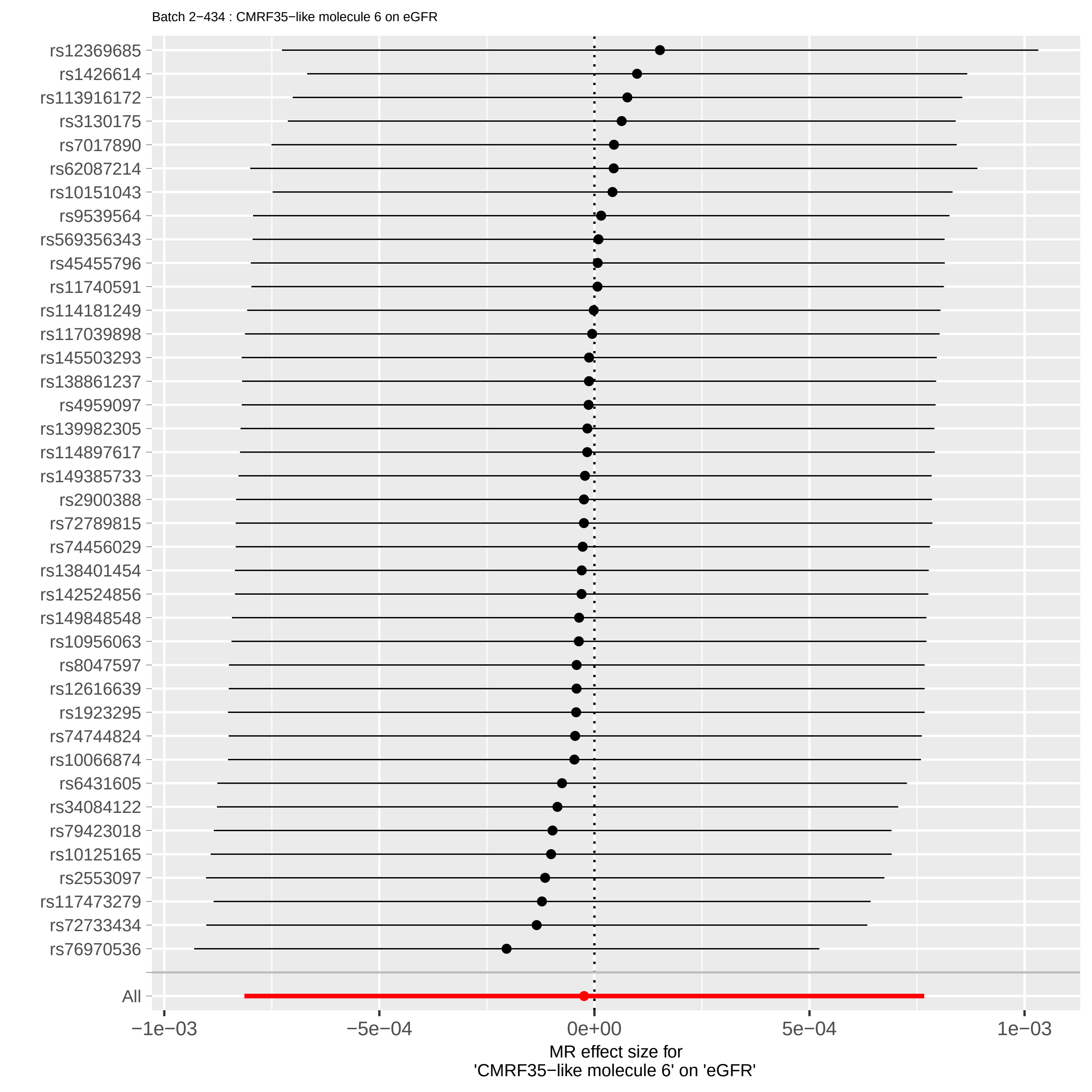
Batch 2−434 : CMRF35−like molecule 6 on eGFR
rs12369685
rs1426614
rs113916172
rs3130175
rs7017890
rs62087214
rs10151043
rs9539564
rs569356343
rs45455796
rs11740591
rs114181249
rs117039898
rs145503293
rs138861237
rs4959097
rs139982305
rs114897617
rs149385733
rs2900388
rs72789815
rs74456029
rs138401454
rs142524856
rs149848548
rs10956063
rs8047597
rs12616639
rs1923295
rs74744824
rs10066874
rs6431605
rs34084122
rs79423018
rs10125165
rs2553097
rs117473279
rs72733434
rs76970536
All
−1e−03
−5e−04
0e+00
5e−04
1e−03
MR effect size for
'CMRF35−like molecule 6' on 'eGFR'

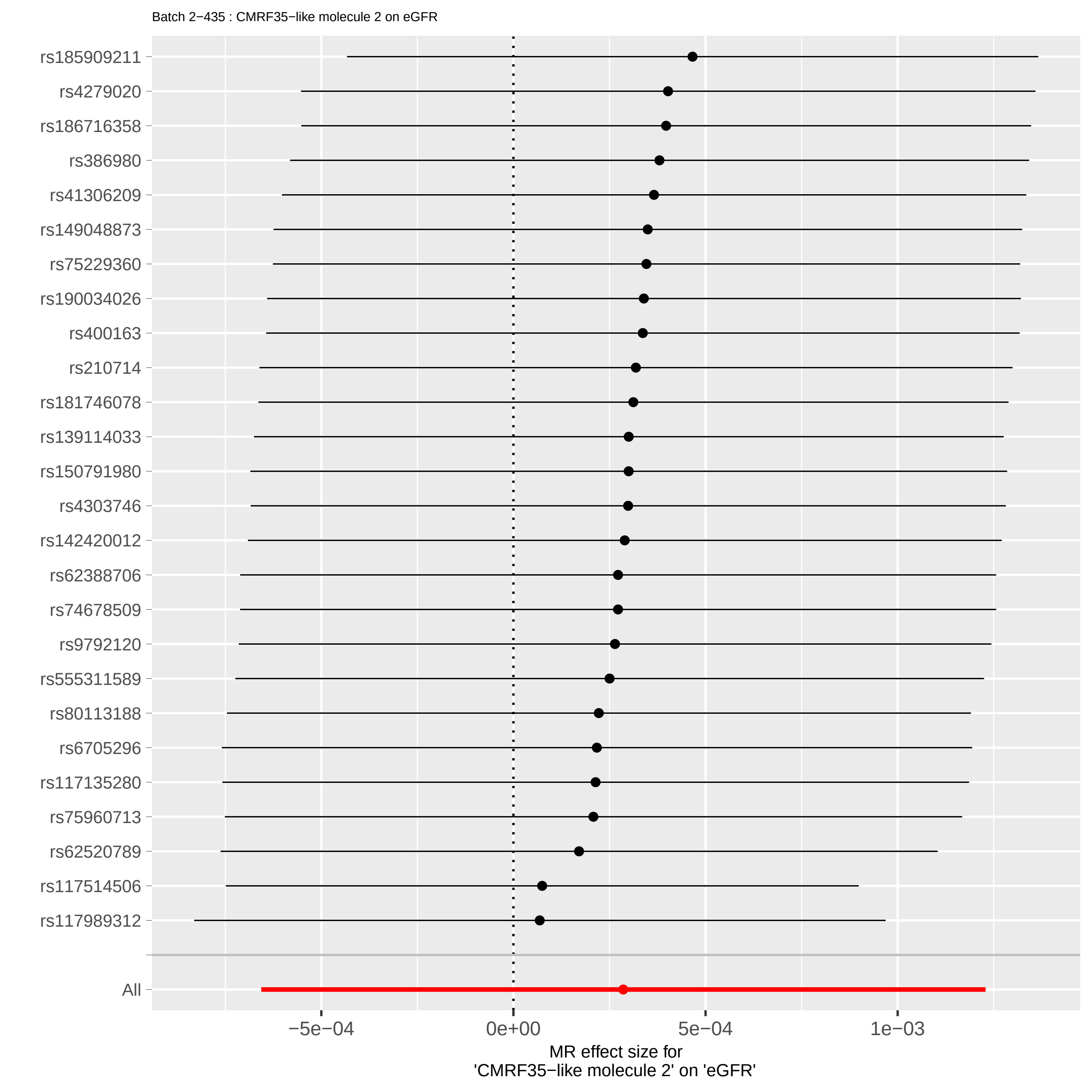

Batch 2−435 : CMRF35−like molecule 2 on eGFR
rs185909211
rs4279020
rs186716358
rs386980
rs41306209
rs149048873
rs75229360
rs190034026
rs400163
rs210714
rs181746078
rs139114033
rs150791980
rs4303746
rs142420012
rs62388706
rs74678509
rs9792120
rs555311589
rs80113188
rs6705296
rs117135280
rs75960713
rs62520789
rs117514506
rs117989312
All
−5e−04
0e+00
5e−04
1e−03
MR effect size for
'CMRF35−like molecule 2' on 'eGFR'

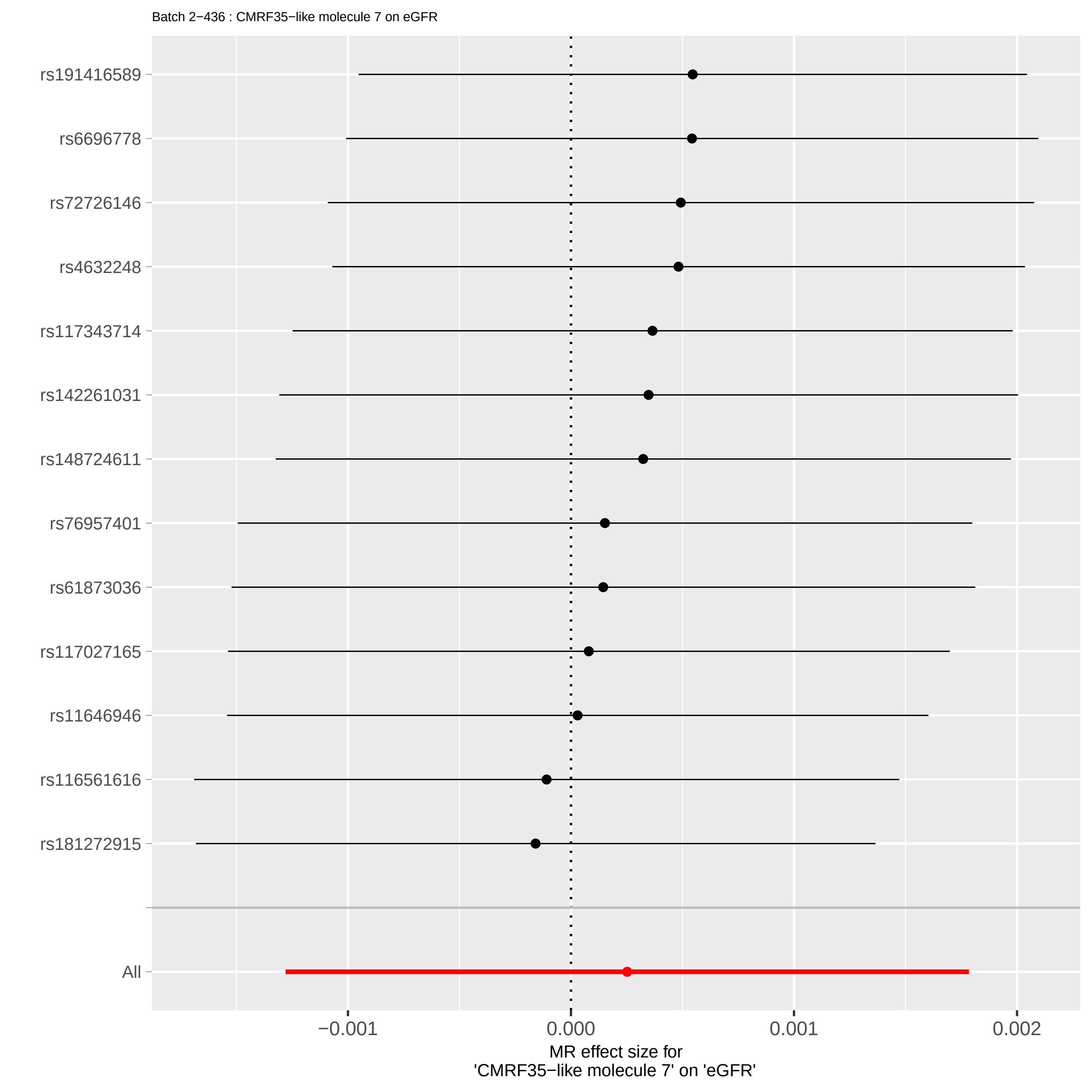
Batch 2−436 : CMRF35−like molecule 7 on eGFR
rs191416589
rs6696778
rs72726146
rs4632248
rs117343714
rs142261031
rs148724611
rs76957401
rs61873036
rs117027165
rs11646946
rs116561616
rs181272915
All
−0.001
0.000
0.001
0.002
MR effect size for
'CMRF35−like molecule 7' on 'eGFR'

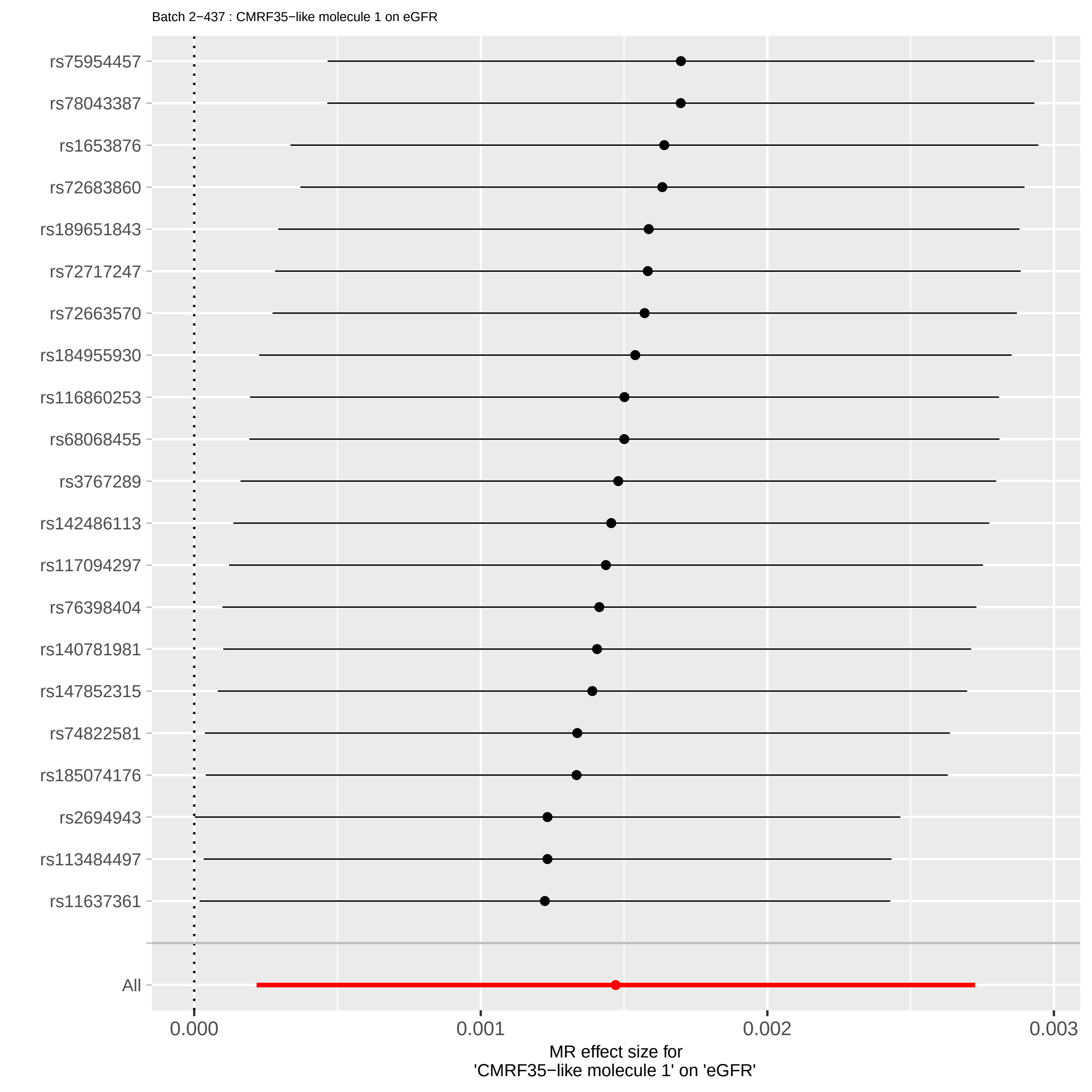

Batch 2−437 : CMRF35−like molecule 1 on eGFR
rs75954457
rs78043387
rs1653876
rs72683860
rs189651843
rs72717247
rs72663570
rs184955930
rs116860253
rs68068455
rs3767289
rs142486113
rs117094297
rs76398404
rs140781981
rs147852315
rs74822581
rs185074176
rs2694943
rs113484497
rs11637361
All
0.000
0.001
0.002
0.003
MR effect size for
'CMRF35−like molecule 1' on 'eGFR'

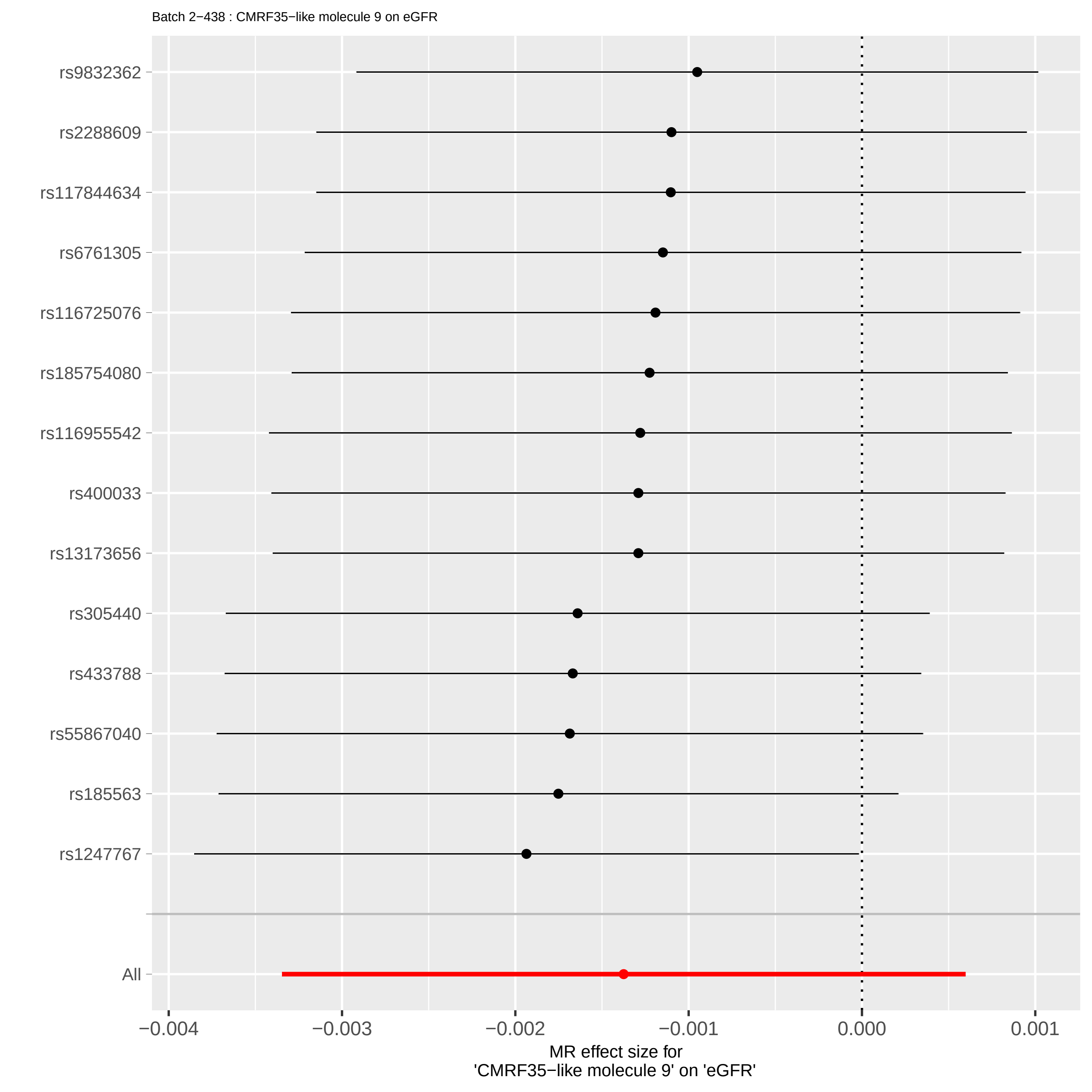

Batch 2−438 : CMRF35−like molecule 9 on eGFR
rs9832362
rs2288609
rs117844634
rs6761305
rs116725076
rs185754080
rs116955542
rs400033
rs13173656
rs305440
rs433788
rs55867040
rs185563
rs1247767
All
−0.004
−0.003
−0.002
−0.001
0.000
0.001
MR effect size for
'CMRF35−like molecule 9' on 'eGFR'

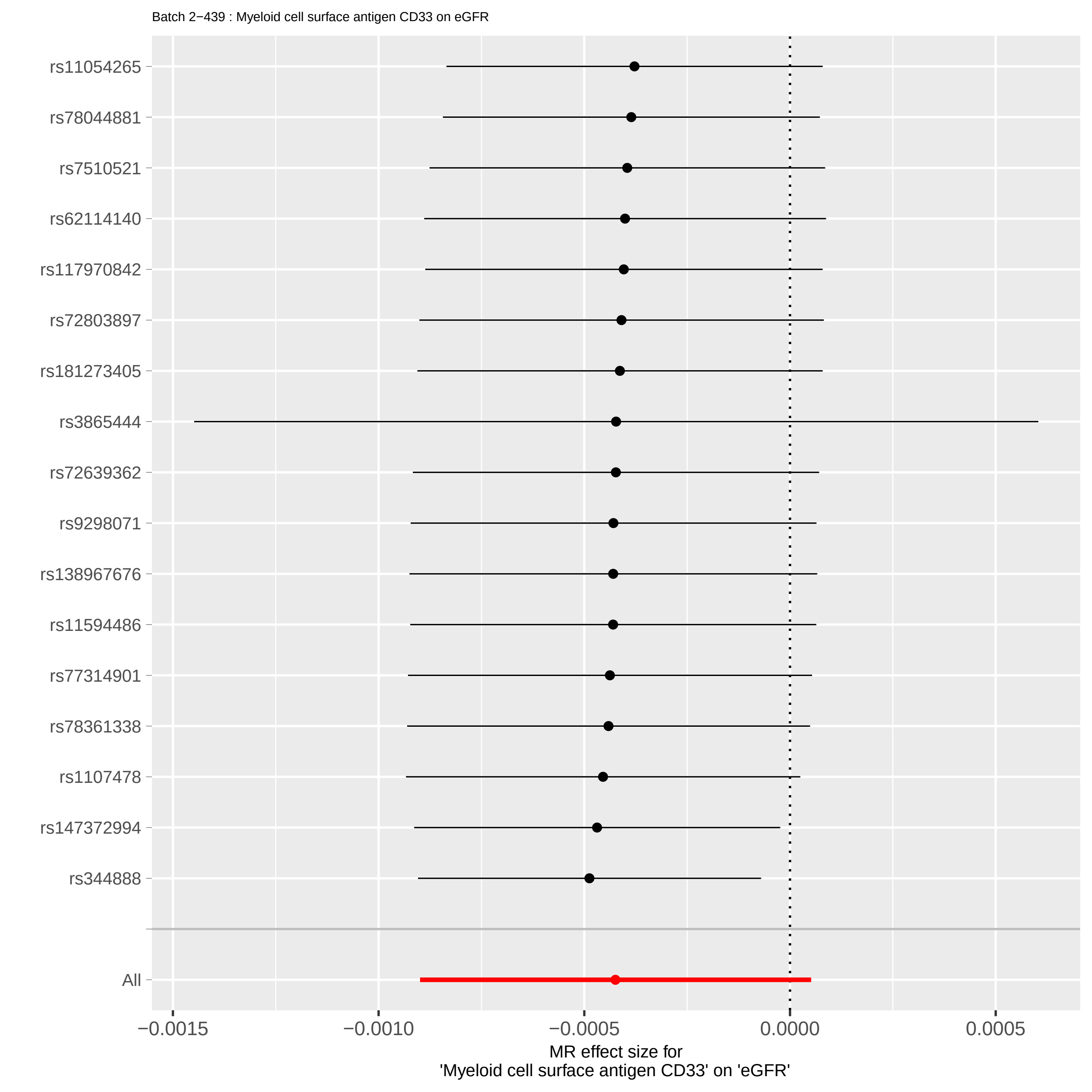

Batch 2−439 : Myeloid cell surface antigen CD33 on eGFR
rs11054265
rs78044881
rs7510521
rs62114140
rs117970842
rs72803897
rs181273405
rs3865444
rs72639362
rs9298071
rs138967676
rs11594486
rs77314901
rs78361338
rs1107478
rs147372994
rs344888
All
−0.0015
−0.0010
−0.0005
0.0000
0.0005
MR effect size for
'Myeloid cell surface antigen CD33' on 'eGFR'

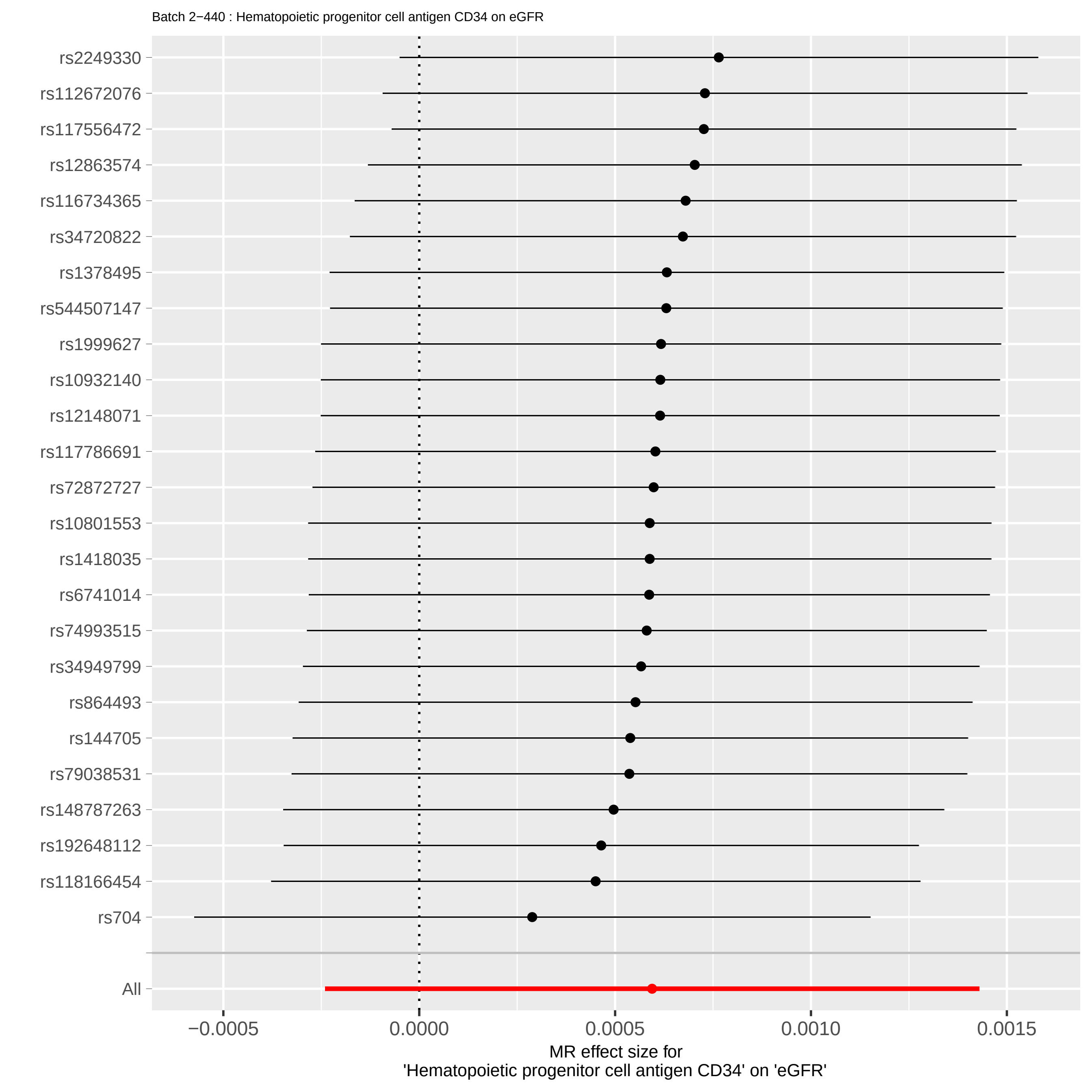

Batch 2−440 : Hematopoietic progenitor cell antigen CD34 on eGFR
rs2249330
rs112672076
rs117556472
rs12863574
rs116734365
rs34720822
rs1378495
rs544507147
rs1999627
rs10932140
rs12148071
rs117786691
rs72872727
rs10801553
rs1418035
rs6741014
rs74993515
rs34949799
rs864493
rs144705
rs79038531
rs148787263
rs192648112
rs118166454
rs704
All
−0.0005
0.0000
0.0005
0.0010
0.0015
MR effect size for
'Hematopoietic progenitor cell antigen CD34' on 'eGFR'

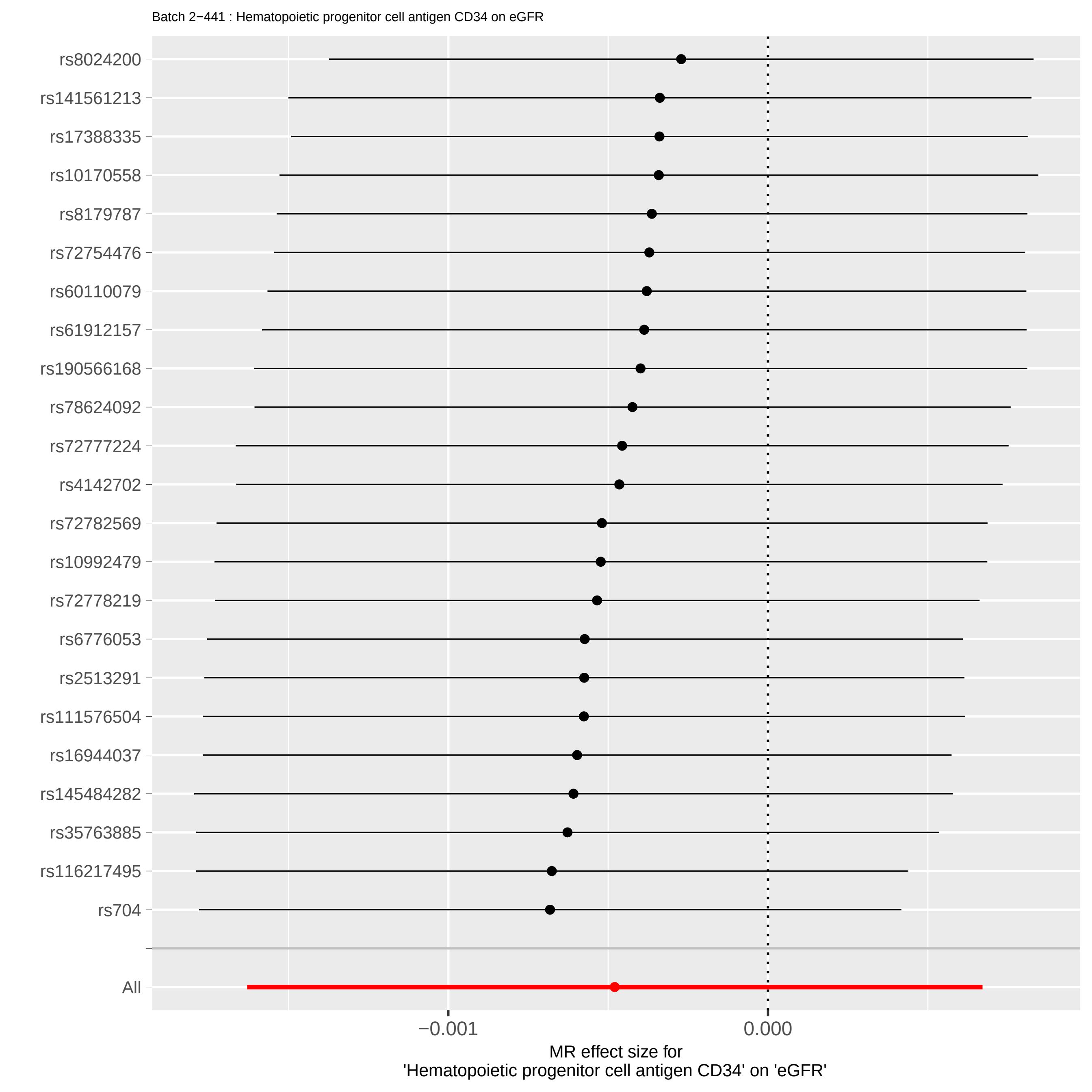
Batch 2−441 : Hematopoietic progenitor cell antigen CD34 on eGFR
rs8024200
rs141561213
rs17388335
rs10170558
rs8179787
rs72754476
rs60110079
rs61912157
rs190566168
rs78624092
rs72777224
rs4142702
rs72782569
rs10992479
rs72778219
rs6776053
rs2513291
rs111576504
rs16944037
rs145484282
rs35763885
rs116217495
rs704
All
−0.001
0.000
MR effect size for
'Hematopoietic progenitor cell antigen CD34' on 'eGFR'

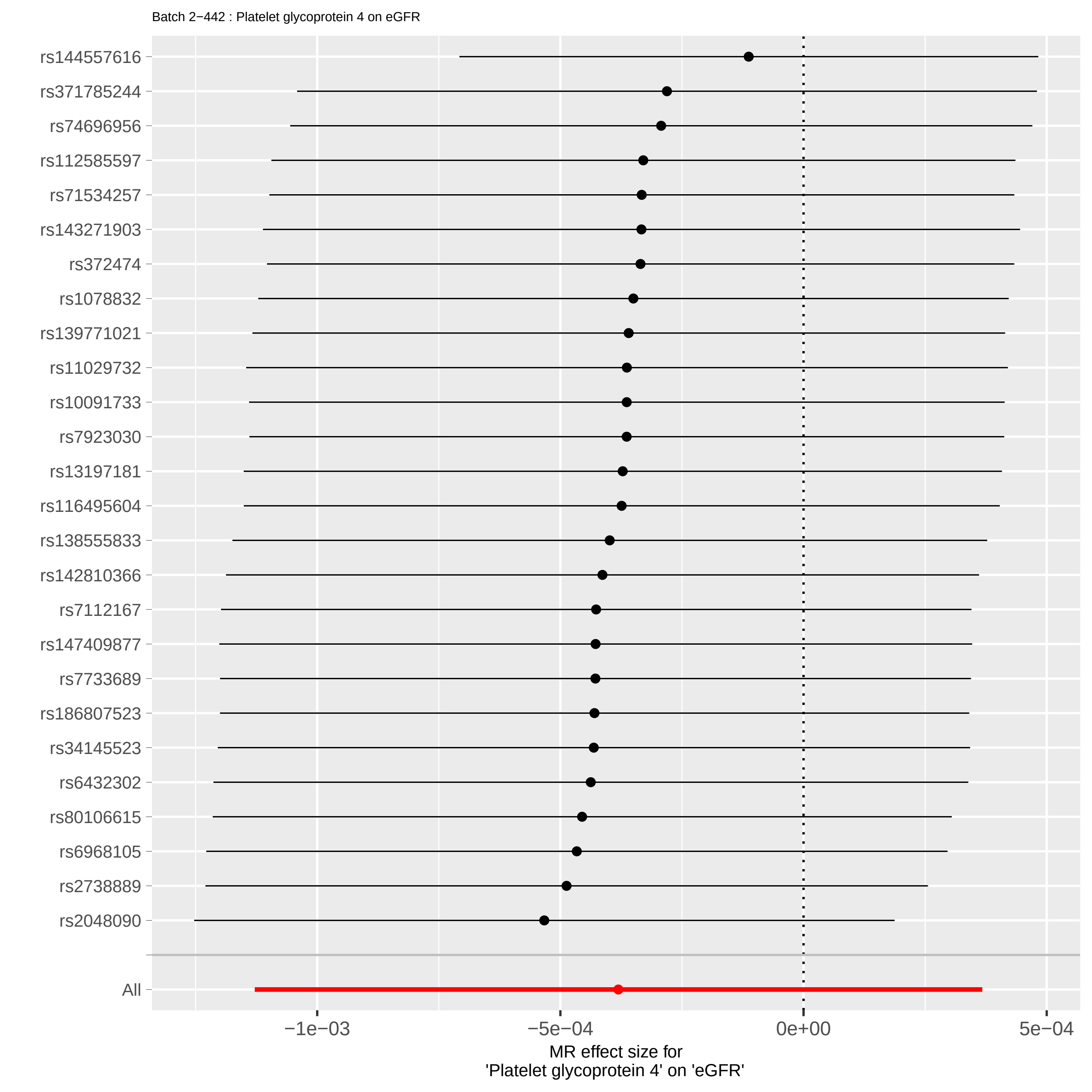

Batch 2−442 : Platelet glycoprotein 4 on eGFR
rs144557616
rs371785244
rs74696956
rs112585597
rs71534257
rs143271903
rs372474
rs1078832
rs139771021
rs11029732
rs10091733
rs7923030
rs13197181
rs116495604
rs138555833
rs142810366
rs7112167
rs147409877
rs7733689
rs186807523
rs34145523
rs6432302
rs80106615
rs6968105
rs2738889
rs2048090
All
−1e−03
−5e−04
0e+00
5e−04
MR effect size for
'Platelet glycoprotein 4' on 'eGFR'

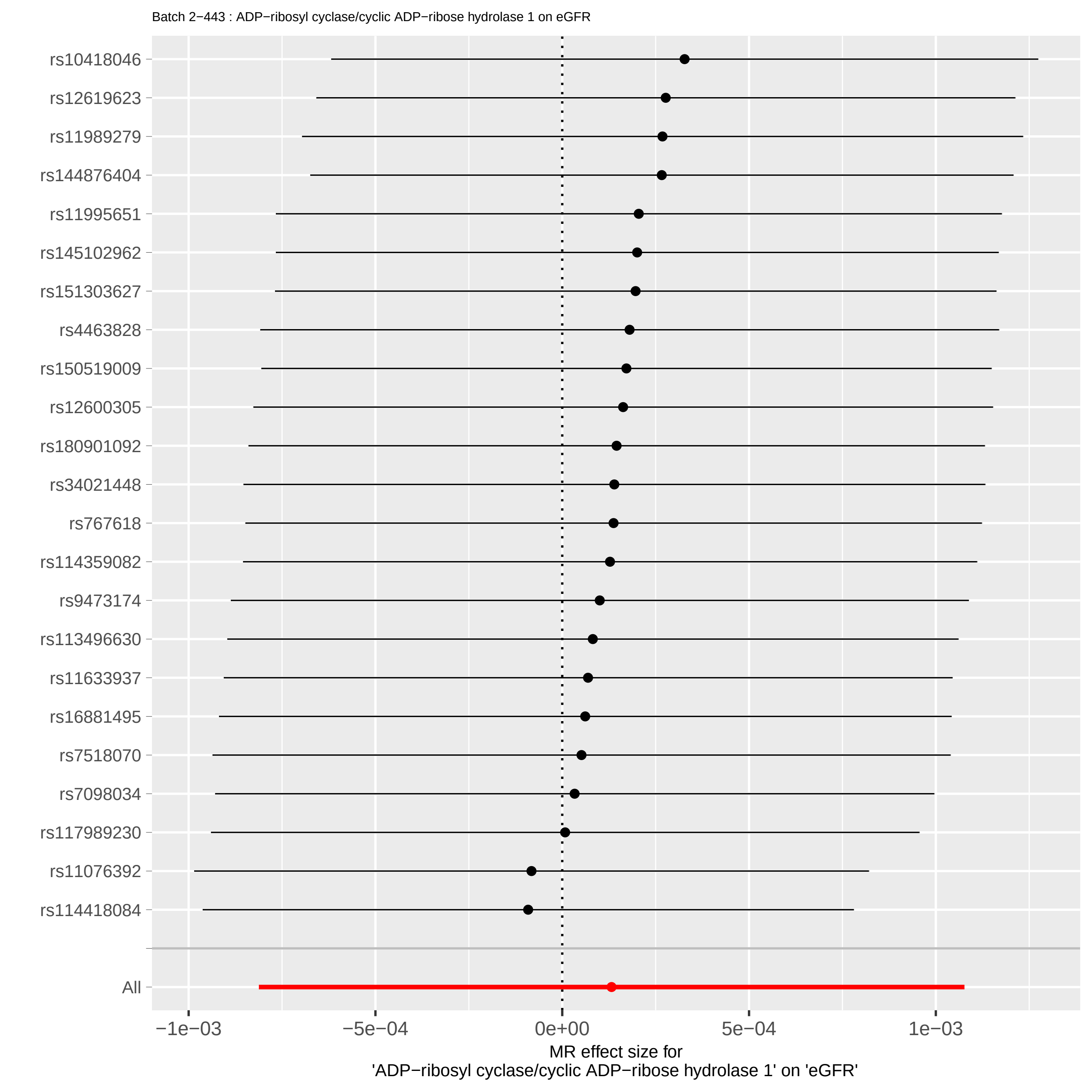

Batch 2−443 : ADP−ribosyl cyclase/cyclic ADP−ribose hydrolase 1 on eGFR
rs10418046
rs12619623
rs11989279
rs144876404
rs11995651
rs145102962
rs151303627
rs4463828
rs150519009
rs12600305
rs180901092
rs34021448
rs767618
rs114359082
rs9473174
rs113496630
rs11633937
rs16881495
rs7518070
rs7098034
rs117989230
rs11076392
rs114418084
All
−1e−03
−5e−04
0e+00
5e−04
1e−03
MR effect size for
'ADP−ribosyl cyclase/cyclic ADP−ribose hydrolase 1' on 'eGFR'

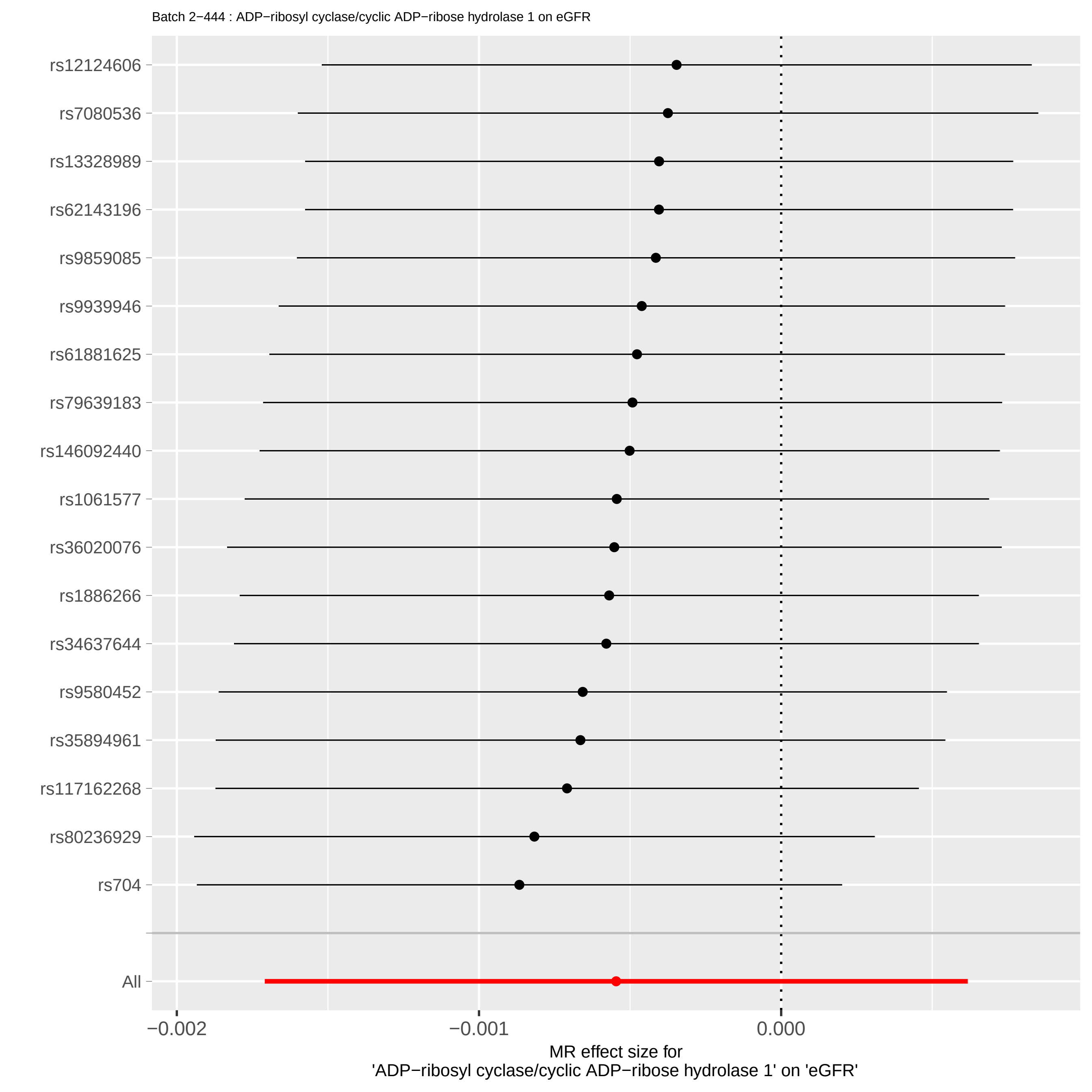

Batch 2−444 : ADP−ribosyl cyclase/cyclic ADP−ribose hydrolase 1 on eGFR
rs12124606
rs7080536
rs13328989
rs62143196
rs9859085
rs9939946
rs61881625
rs79639183
rs146092440
rs1061577
rs36020076
rs1886266
rs34637644
rs9580452
rs35894961
rs117162268
rs80236929
rs704
All
−0.002
−0.001
0.000
MR effect size for
'ADP−ribosyl cyclase/cyclic ADP−ribose hydrolase 1' on 'eGFR'

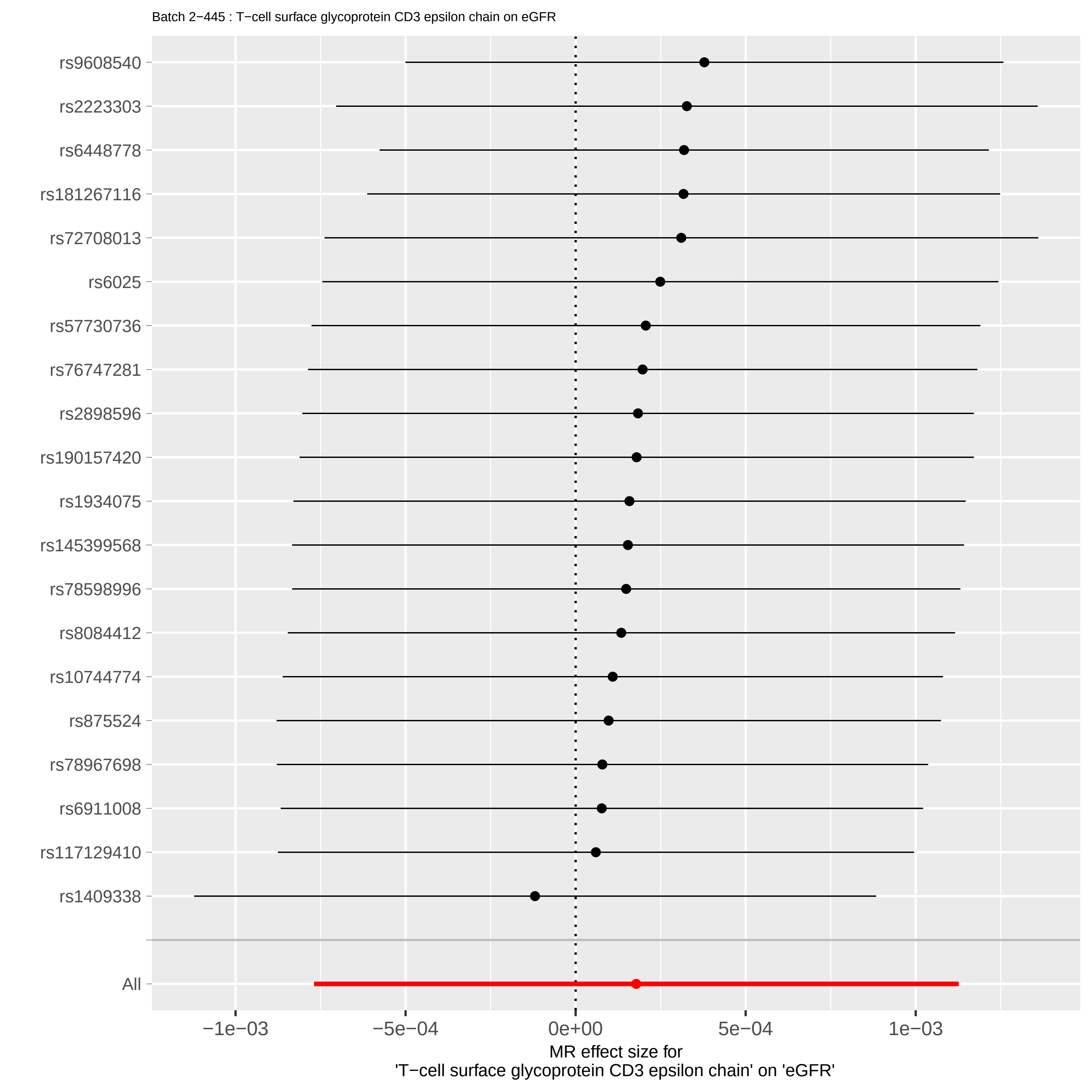

Batch 2−445 : T−cell surface glycoprotein CD3 epsilon chain on eGFR
rs9608540
rs2223303
rs6448778
rs181267116
rs72708013
rs6025
rs57730736
rs76747281
rs2898596
rs190157420
rs1934075
rs145399568
rs78598996
rs8084412
rs10744774
rs875524
rs78967698
rs6911008
rs117129410
rs1409338
All
−1e−03
−5e−04
0e+00
5e−04
1e−03
MR effect size for
'T−cell surface glycoprotein CD3 epsilon chain' on 'eGFR'

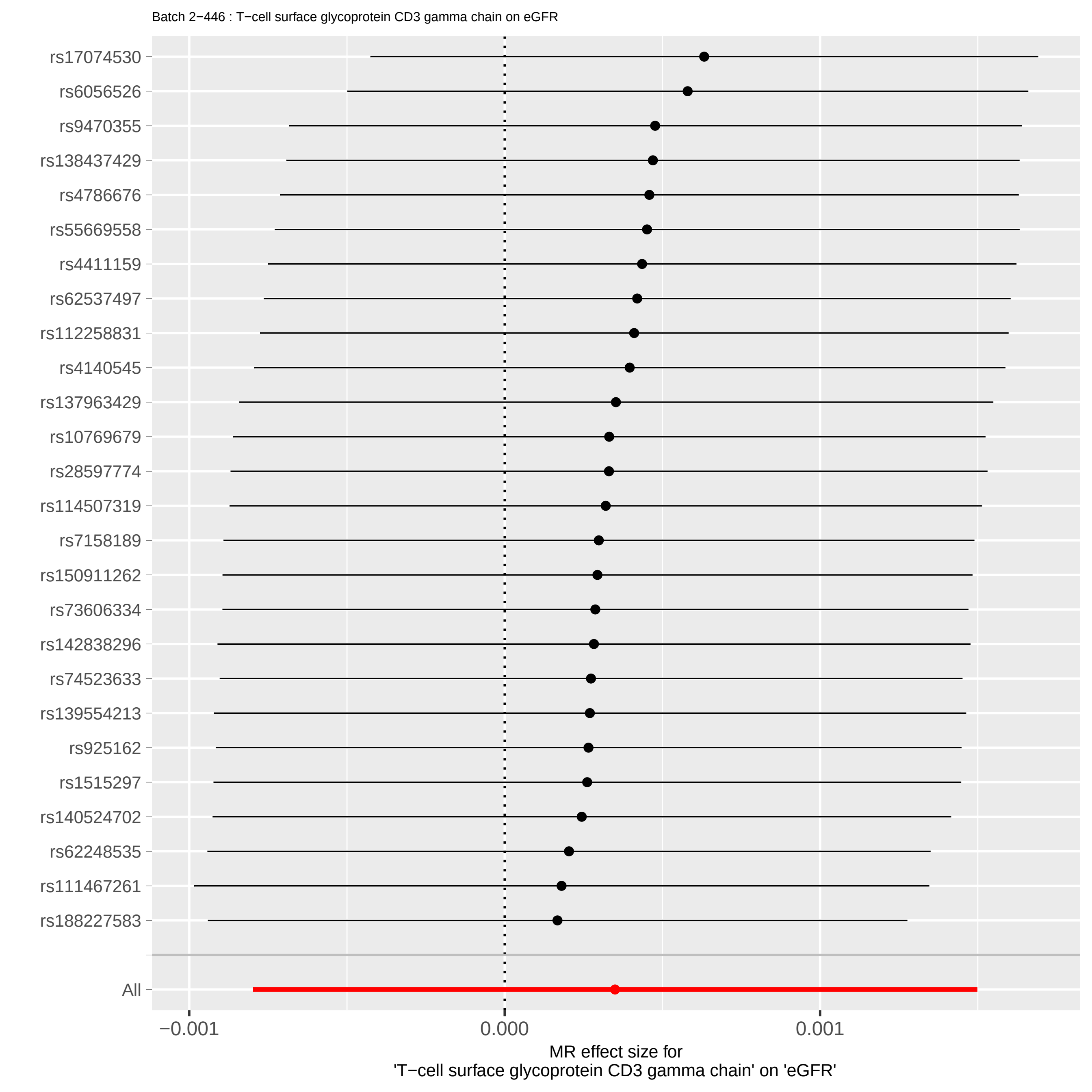

Batch 2−446 : T−cell surface glycoprotein CD3 gamma chain on eGFR
rs17074530
rs6056526
rs9470355
rs138437429
rs4786676
rs55669558
rs4411159
rs62537497
rs112258831
rs4140545
rs137963429
rs10769679
rs28597774
rs114507319
rs7158189
rs150911262
rs73606334
rs142838296
rs74523633
rs139554213
rs925162
rs1515297
rs140524702
rs62248535
rs111467261
rs188227583
All
−0.001
0.000
0.001
MR effect size for
'T−cell surface glycoprotein CD3 gamma chain' on 'eGFR'

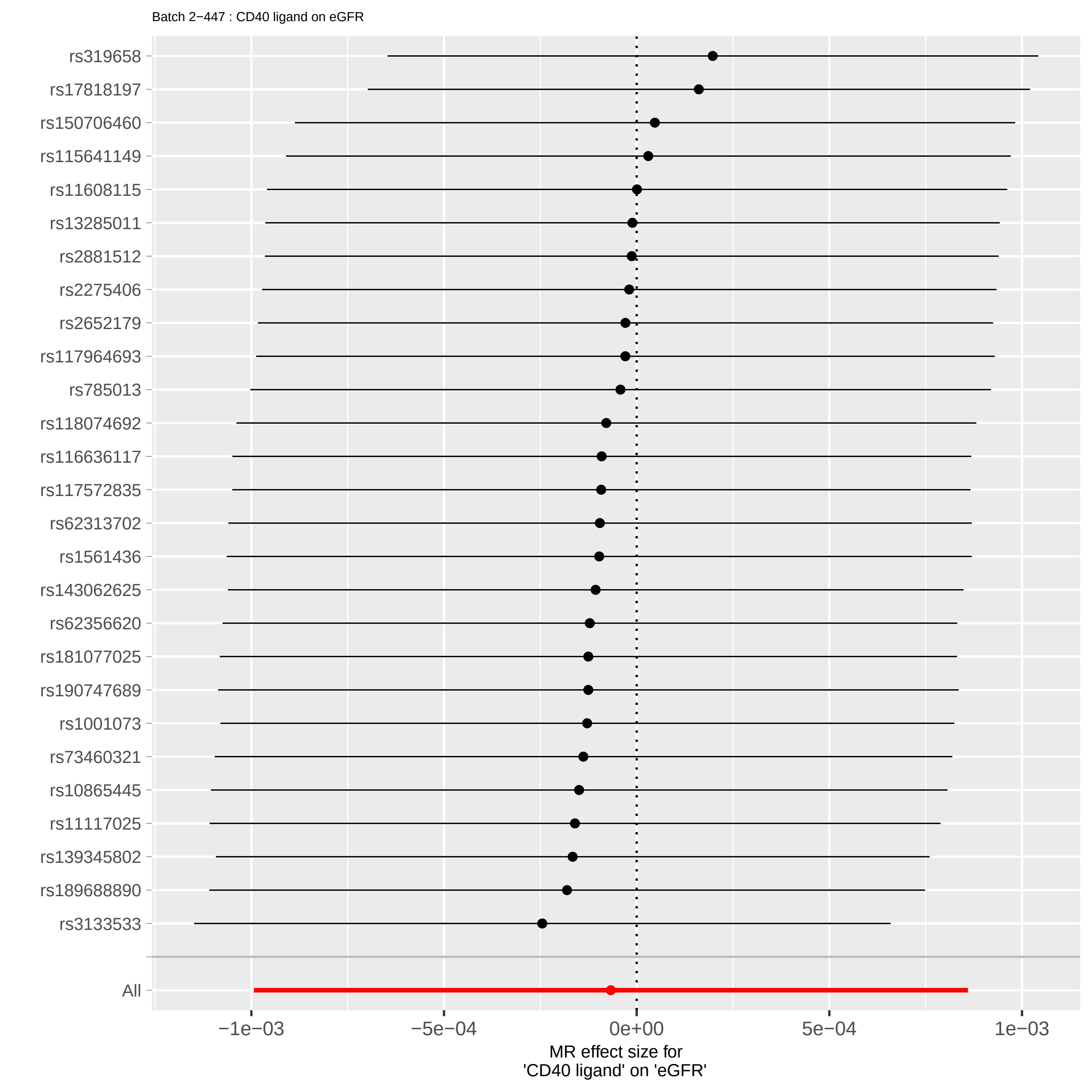

Batch 2−447 : CD40 ligand on eGFR
rs319658
rs17818197
rs150706460
rs115641149
rs11608115
rs13285011
rs2881512
rs2275406
rs2652179
rs117964693
rs785013
rs118074692
rs116636117
rs117572835
rs62313702
rs1561436
rs143062625
rs62356620
rs181077025
rs190747689
rs1001073
rs73460321
rs10865445
rs11117025
rs139345802
rs189688890
rs3133533
All
−1e−03
−5e−04
0e+00
5e−04
1e−03
MR effect size for
'CD40 ligand' on 'eGFR'

Batch 2−448 : T−cell surface glycoprotein CD4 on eGFR

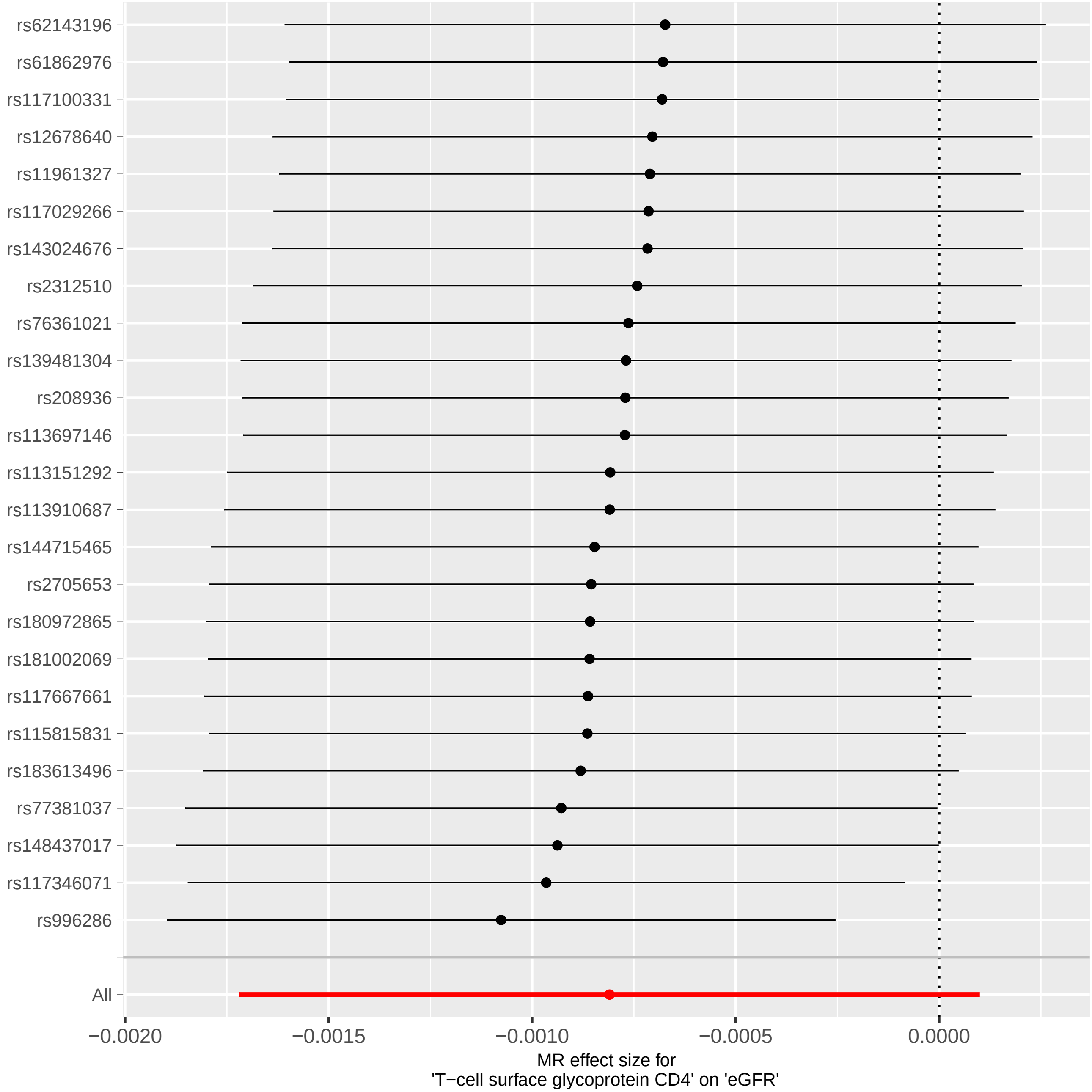

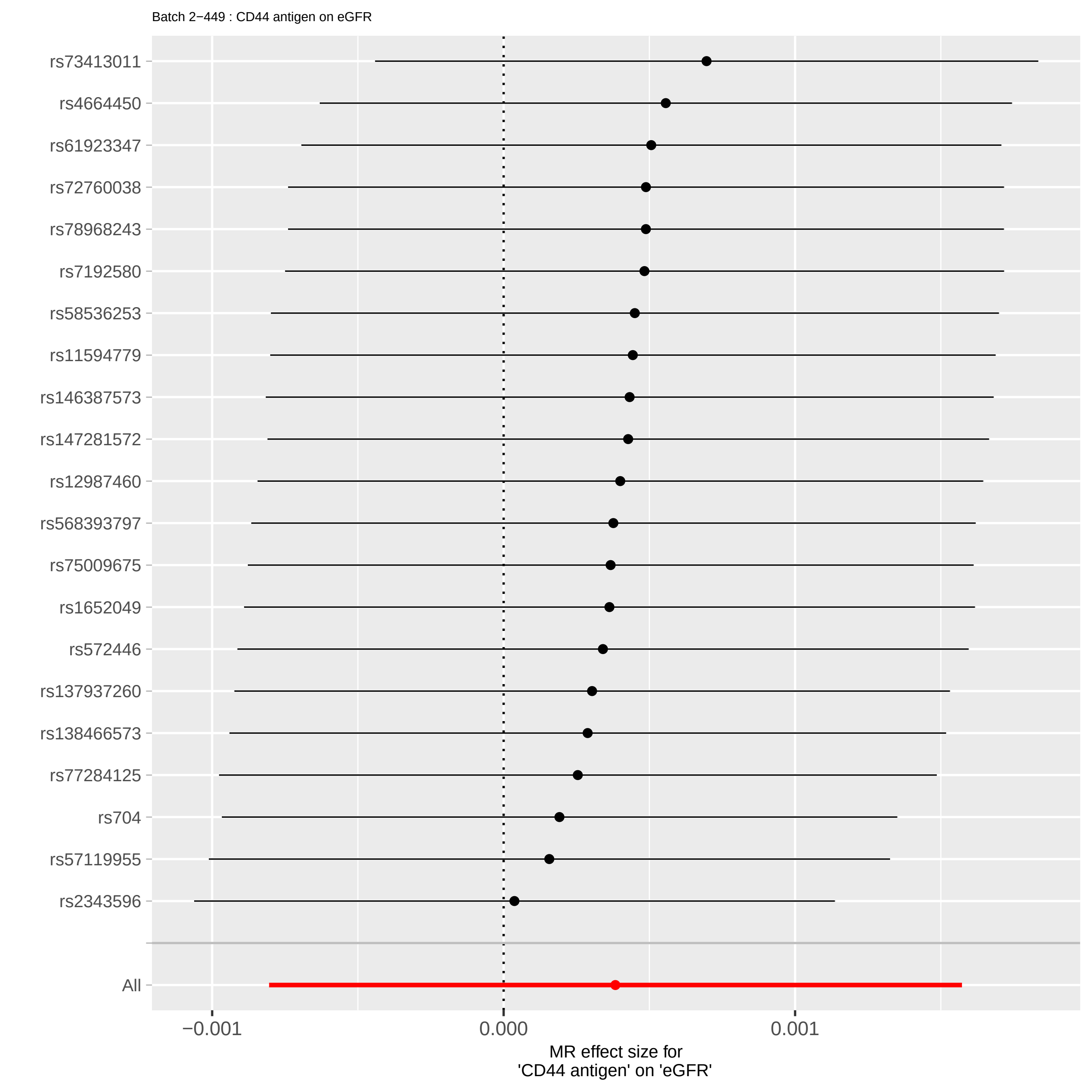
Batch 2−449 : CD44 antigen on eGFR
rs73413011
rs4664450
rs61923347
rs72760038
rs78968243
rs7192580
rs58536253
rs11594779
rs146387573
rs147281572
rs12987460
rs568393797
rs75009675
rs1652049
rs572446
rs137937260
rs138466573
rs77284125
rs704
rs57119955
rs2343596
All
−0.001
0.000
0.001
MR effect size for
'CD44 antigen' on 'eGFR'

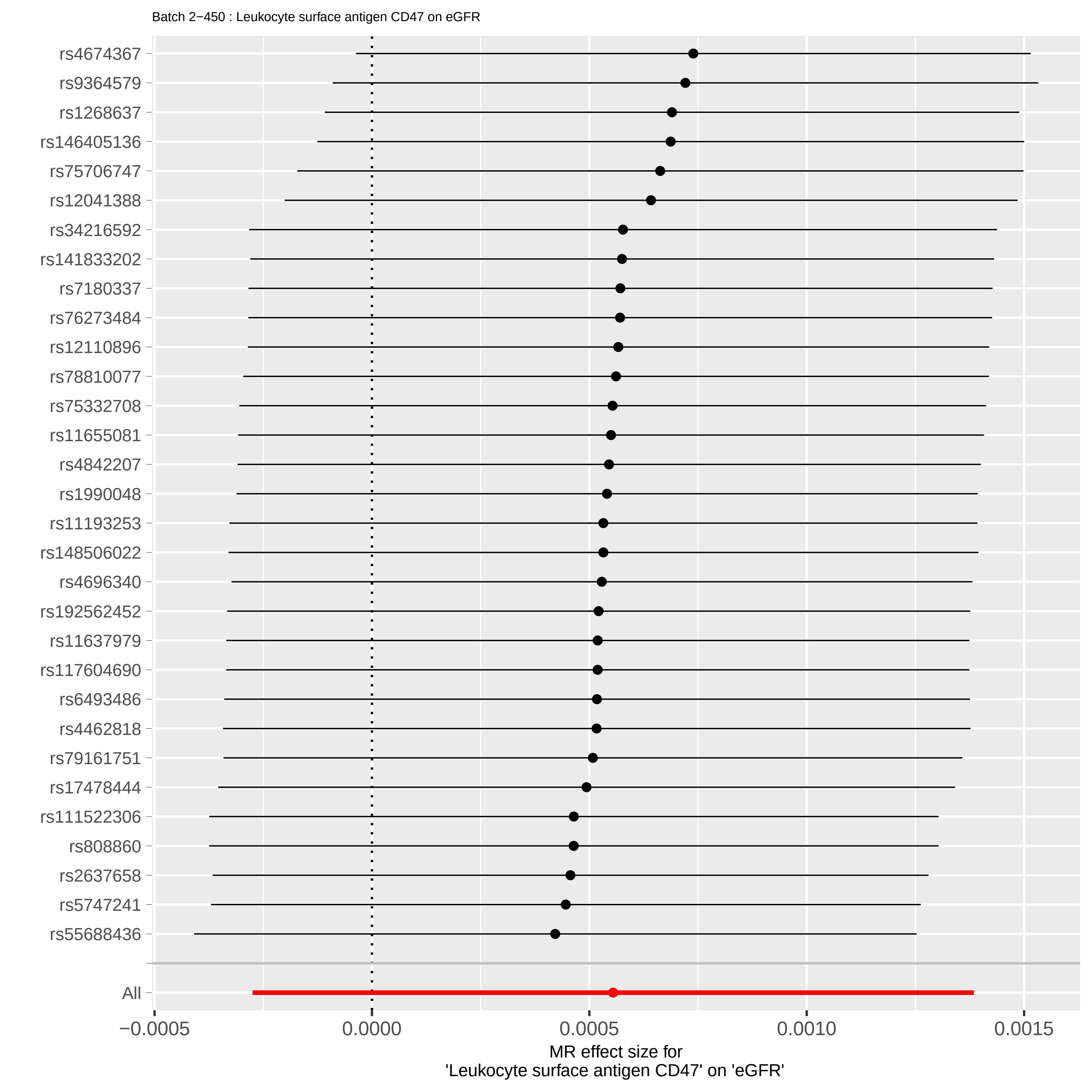

Batch 2−450 : Leukocyte surface antigen CD47 on eGFR
rs4674367
rs9364579
rs1268637
rs146405136
rs75706747
rs12041388
rs34216592
rs141833202
rs7180337
rs76273484
rs12110896
rs78810077
rs75332708
rs11655081
rs4842207
rs1990048
rs11193253
rs148506022
rs4696340
rs192562452
rs11637979
rs117604690
rs6493486
rs4462818
rs79161751
rs17478444
rs111522306
rs808860
rs2637658
rs5747241
rs55688436
All
−0.0005
0.0000
0.0005
0.0010
0.0015
MR effect size for
'Leukocyte surface antigen CD47' on 'eGFR'

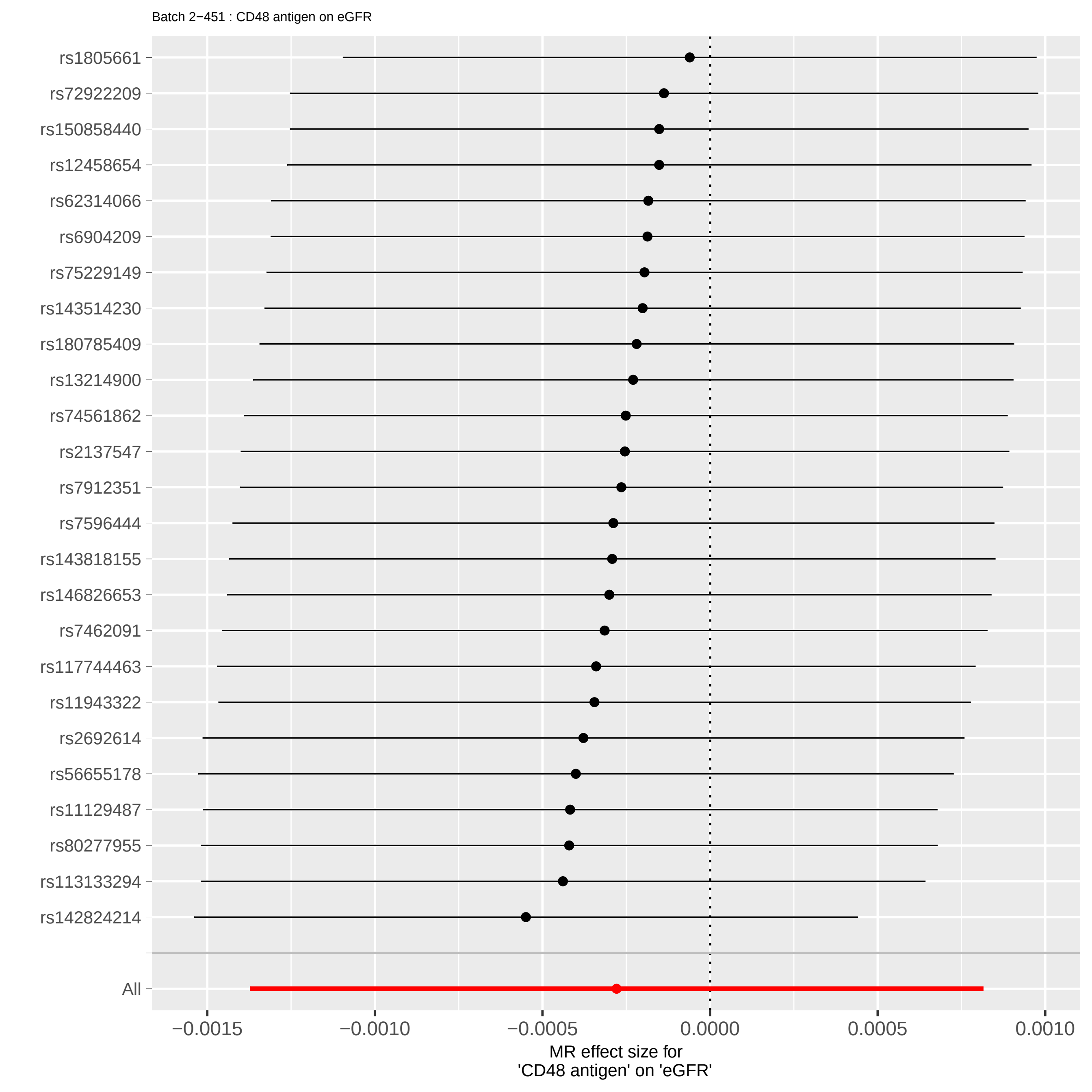
Batch 2−451 : CD48 antigen on eGFR
rs1805661
rs72922209
rs150858440
rs12458654
rs62314066
rs6904209
rs75229149
rs143514230
rs180785409
rs13214900
rs74561862
rs2137547
rs7912351
rs7596444
rs143818155
rs146826653
rs7462091
rs117744463
rs11943322
rs2692614
rs56655178
rs11129487
rs80277955
rs113133294
rs142824214
All
−0.0015
−0.0010
−0.0005
0.0000
0.0005
0.0010
MR effect size for
'CD48 antigen' on 'eGFR'

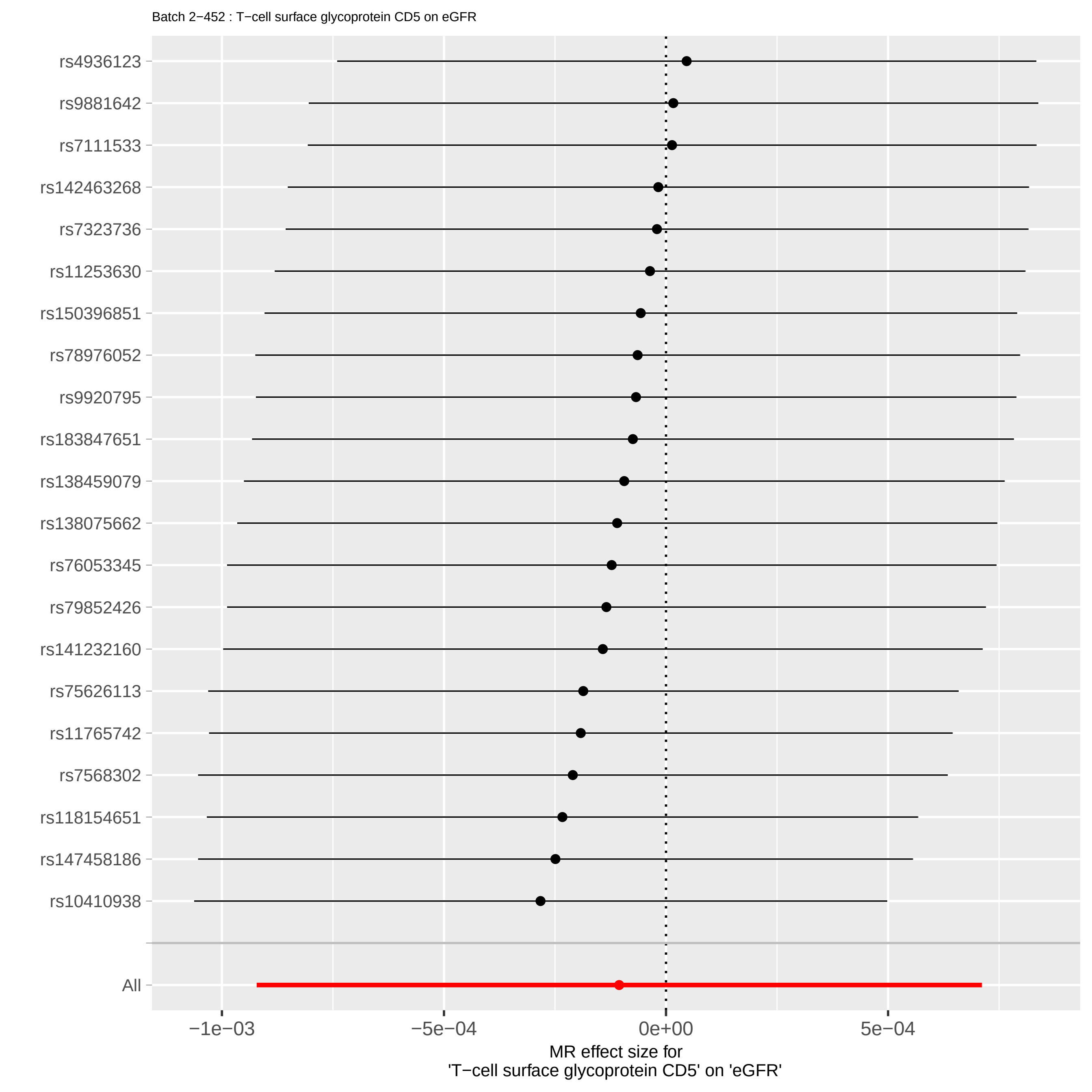

Batch 2−452 : T−cell surface glycoprotein CD5 on eGFR
rs4936123
rs9881642
rs7111533
rs142463268
rs7323736
rs11253630
rs150396851
rs78976052
rs9920795
rs183847651
rs138459079
rs138075662
rs76053345
rs79852426
rs141232160
rs75626113
rs11765742
rs7568302
rs118154651
rs147458186
rs10410938
All
−1e−03
−5e−04
0e+00
5e−04
MR effect size for
'T−cell surface glycoprotein CD5' on 'eGFR'

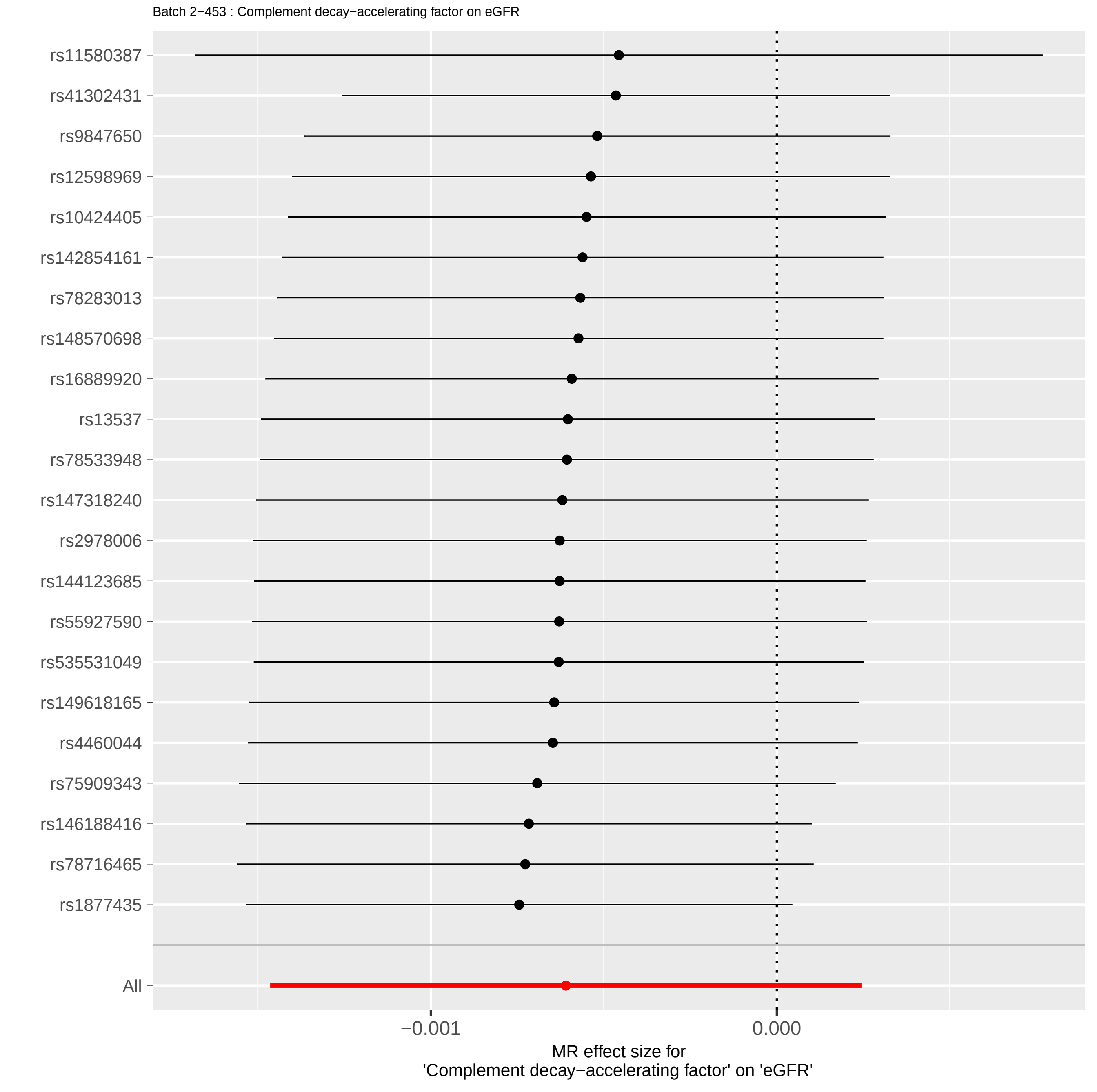

Batch 2−453 : Complement decay−accelerating factor on eGFR
rs11580387
rs41302431
rs9847650
rs12598969
rs10424405
rs142854161
rs78283013
rs148570698
rs16889920
rs13537
rs78533948
rs147318240
rs2978006
rs144123685
rs55927590
rs535531049
rs149618165
rs4460044
rs75909343
rs146188416
rs78716465
rs1877435
All
−0.001
0.000
MR effect size for
'Complement decay−accelerating factor' on 'eGFR'

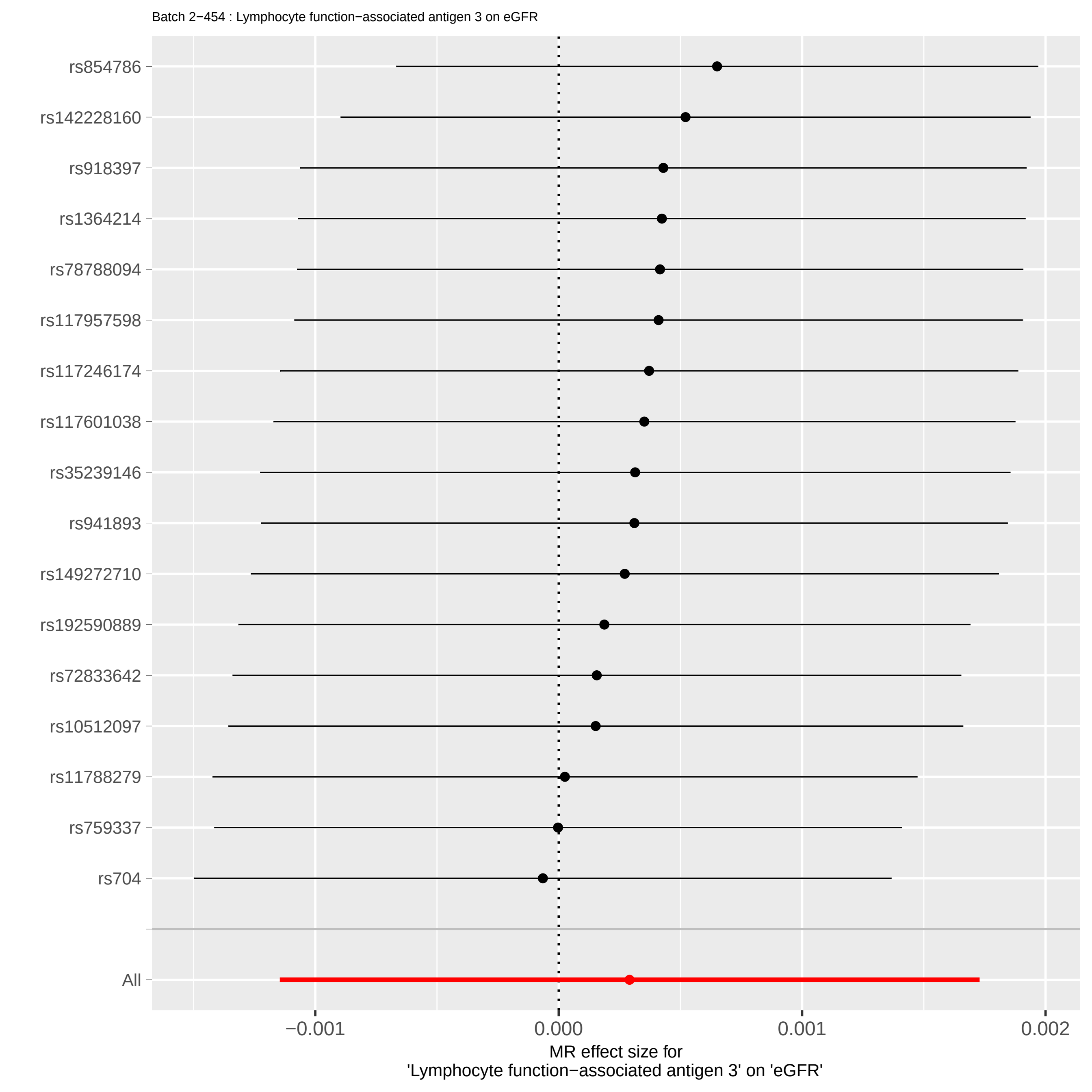

Batch 2−454 : Lymphocyte function−associated antigen 3 on eGFR
rs854786
rs142228160
rs918397
rs1364214
rs78788094
rs117957598
rs117246174
rs117601038
rs35239146
rs941893
rs149272710
rs192590889
rs72833642
rs10512097
rs11788279
rs759337
rs704
All
−0.001
0.000
0.001
0.002
MR effect size for
'Lymphocyte function−associated antigen 3' on 'eGFR'

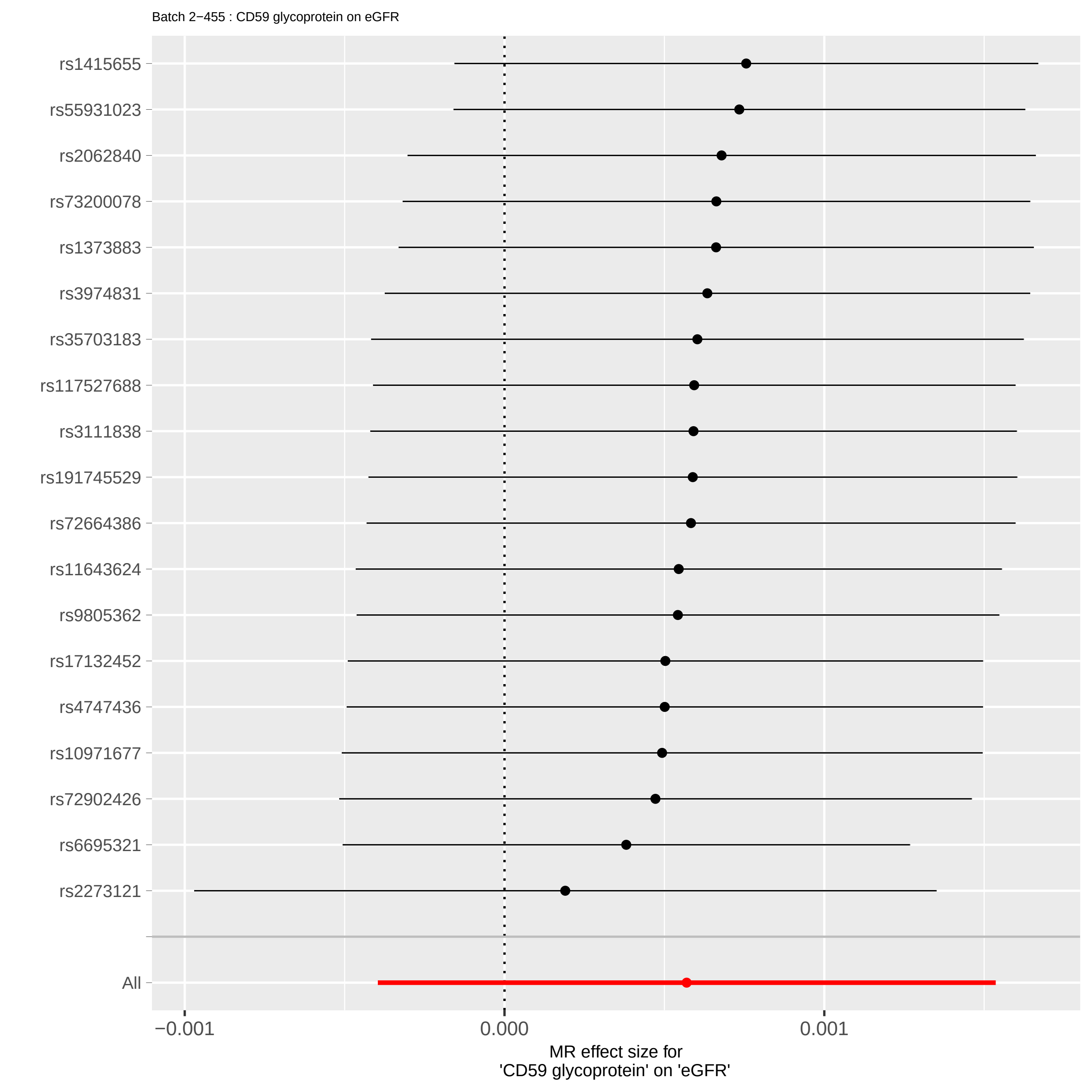
Batch 2−455 : CD59 glycoprotein on eGFR
rs1415655
rs55931023
rs2062840
rs73200078
rs1373883
rs3974831
rs35703183
rs117527688
rs3111838
rs191745529
rs72664386
rs11643624
rs9805362
rs17132452
rs4747436
rs10971677
rs72902426
rs6695321
rs2273121
All
−0.001
0.000
0.001
MR effect size for
'CD59 glycoprotein' on 'eGFR'

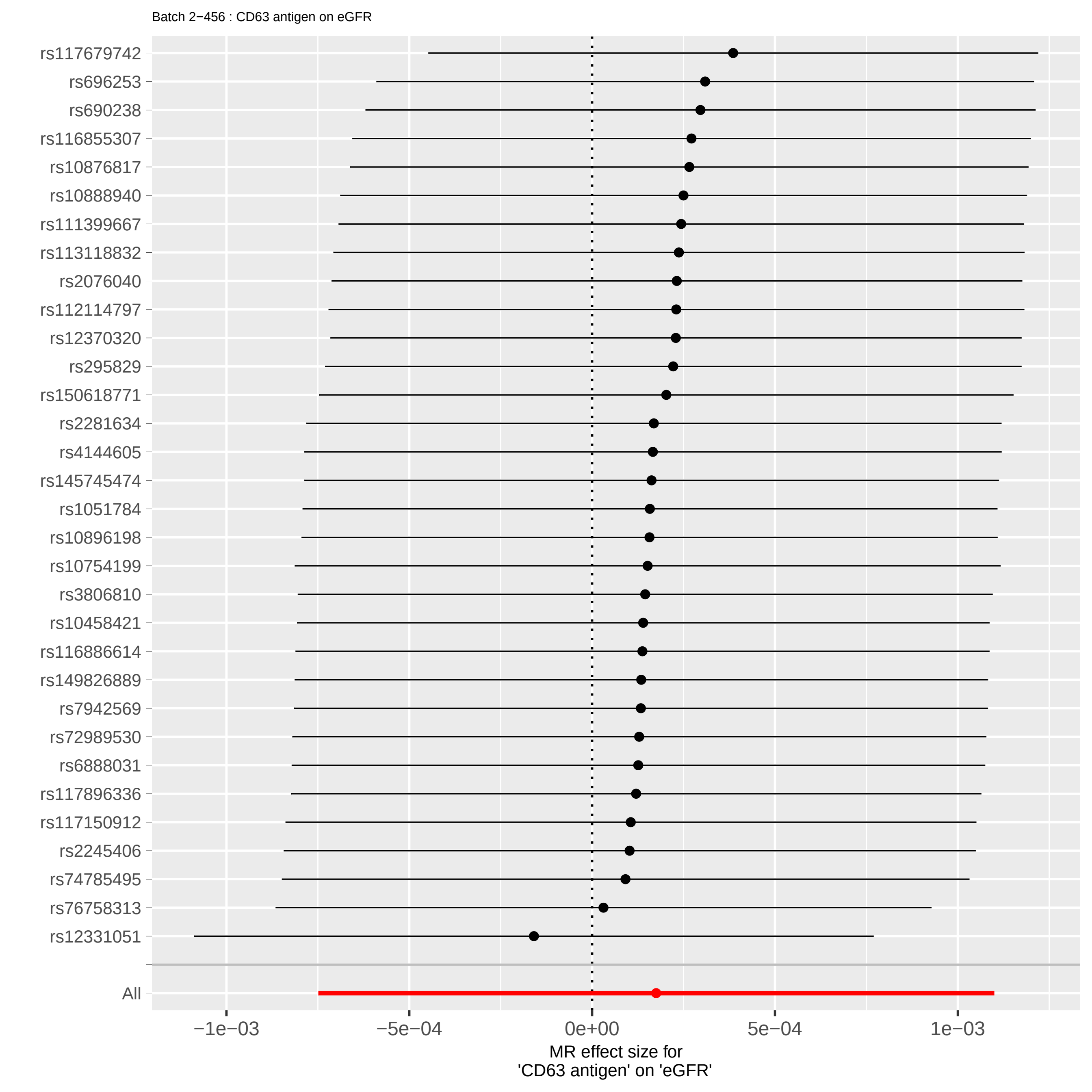

Batch 2−456 : CD63 antigen on eGFR
rs117679742
rs696253
rs690238
rs116855307
rs10876817
rs10888940
rs111399667
rs113118832
rs2076040
rs112114797
rs12370320
rs295829
rs150618771
rs2281634
rs4144605
rs145745474
rs1051784
rs10896198
rs10754199
rs3806810
rs10458421
rs116886614
rs149826889
rs7942569
rs72989530
rs6888031
rs117896336
rs117150912
rs2245406
rs74785495
rs76758313
rs12331051
All
−1e−03
−5e−04
0e+00
5e−04
1e−03
MR effect size for
'CD63 antigen' on 'eGFR'

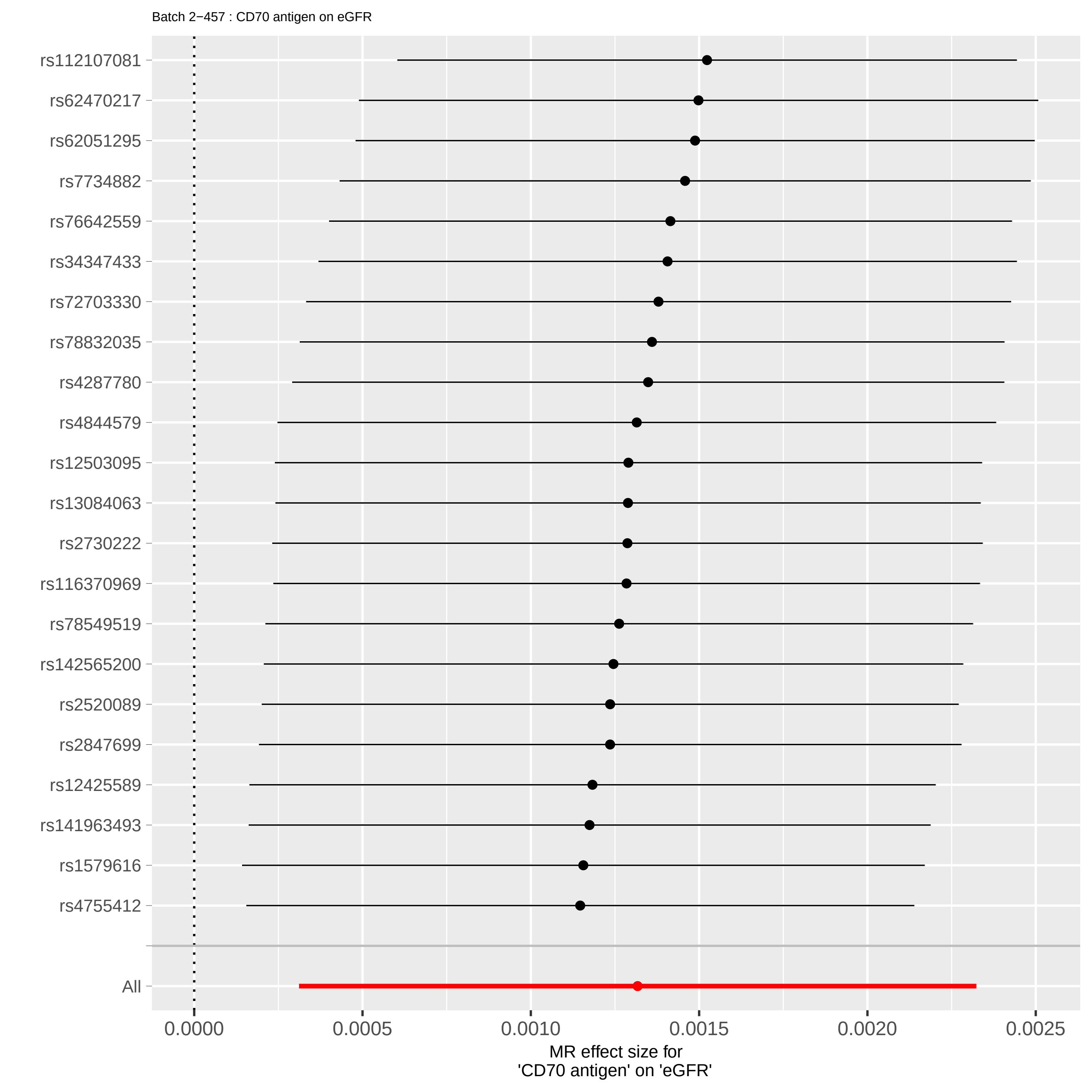

Batch 2−457 : CD70 antigen on eGFR
rs112107081
rs62470217
rs62051295
rs7734882
rs76642559
rs34347433
rs72703330
rs78832035
rs4287780
rs4844579
rs12503095
rs13084063
rs2730222
rs116370969
rs78549519
rs142565200
rs2520089
rs2847699
rs12425589
rs141963493
rs1579616
rs4755412
All
0.0000
0.0005
0.0010
0.0015
0.0020
0.0025
MR effect size for
'CD70 antigen' on 'eGFR'

Batch 2−458 : CD70 antigen on eGFR

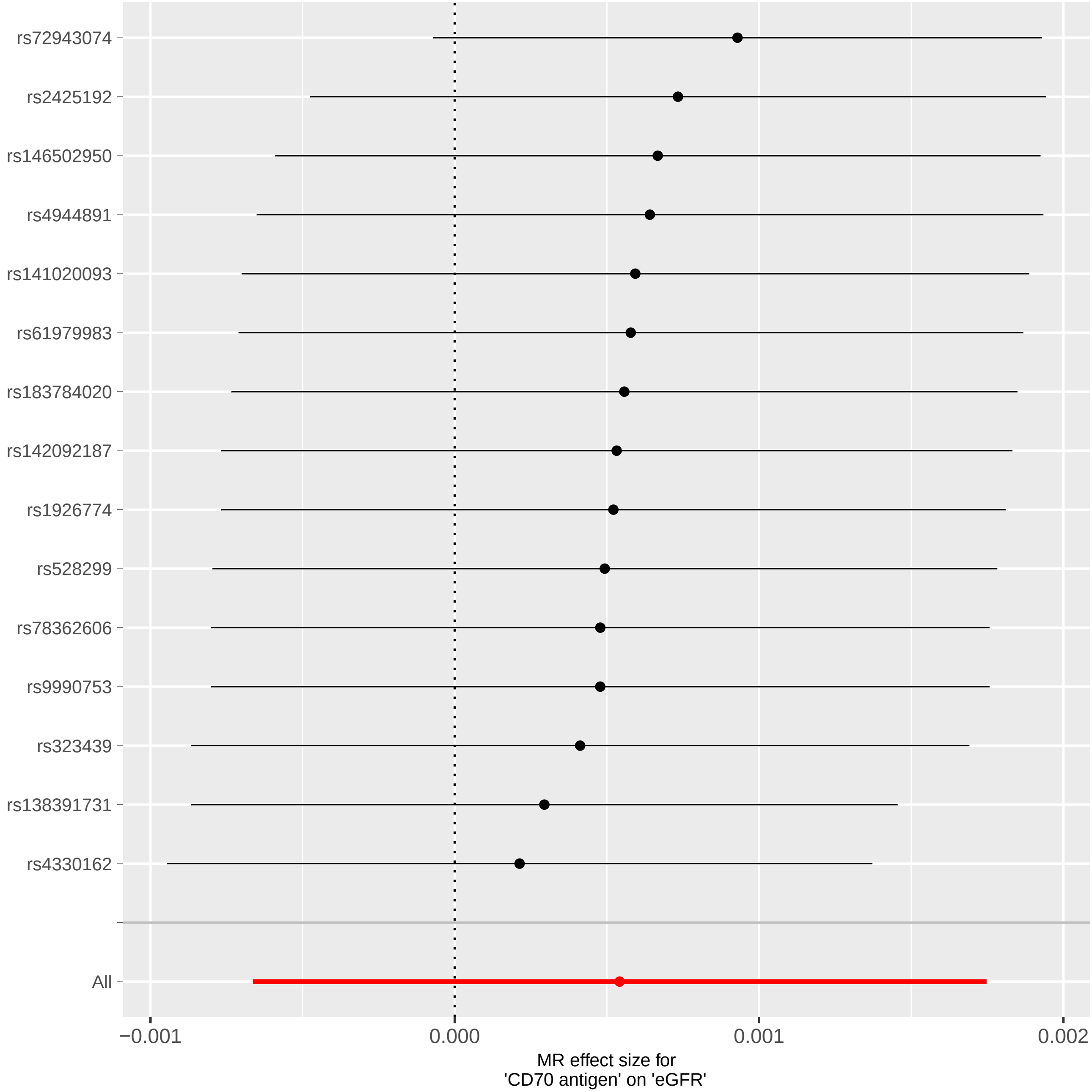

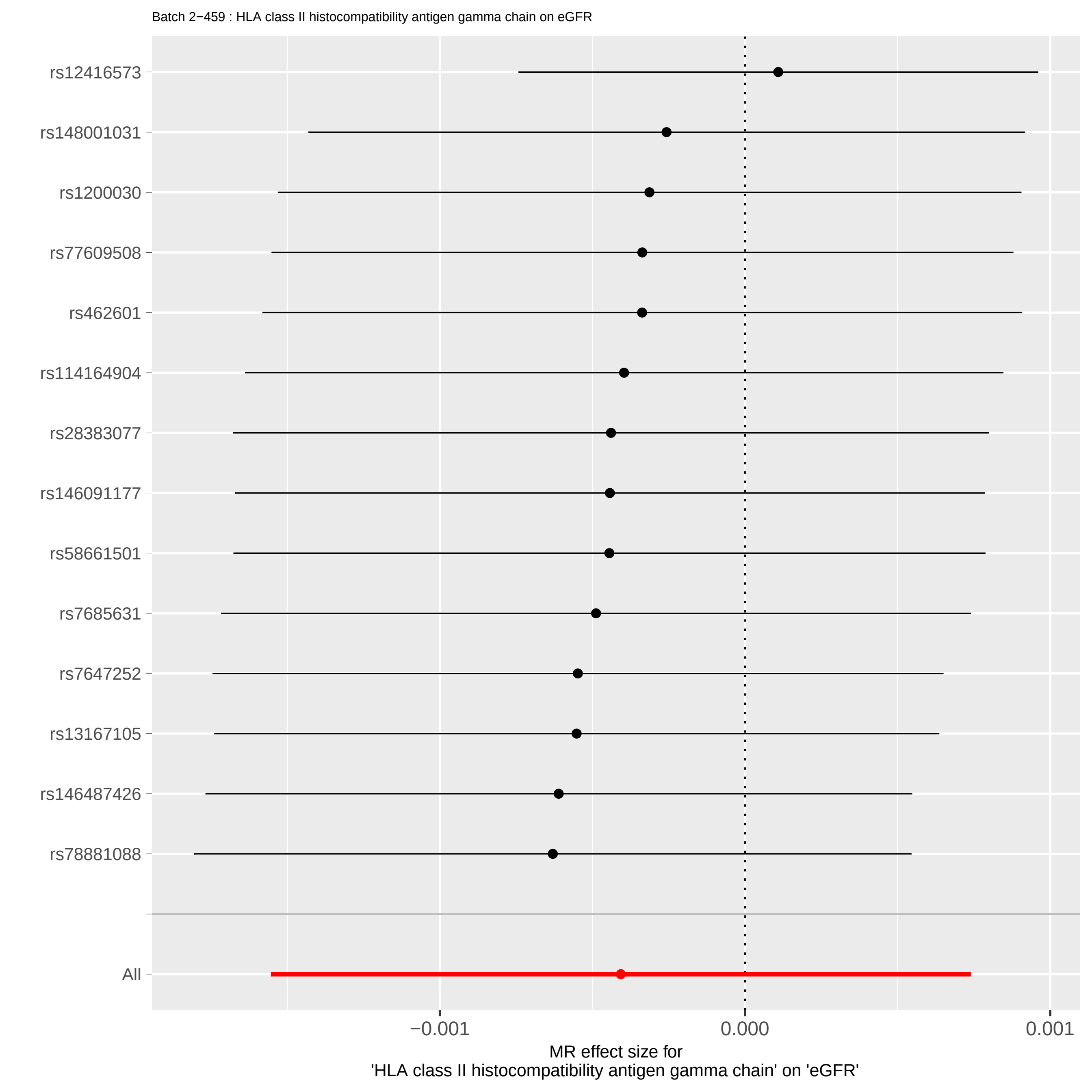

Batch 2−459 : HLA class II histocompatibility antigen gamma chain on eGFR
rs12416573
rs148001031
rs1200030
rs77609508
rs462601
rs114164904
rs28383077
rs146091177
rs58661501
rs7685631
rs7647252
rs13167105
rs146487426
rs78881088
All
−0.001
0.000
0.001
MR effect size for
'HLA class II histocompatibility antigen gamma chain' on 'eGFR'

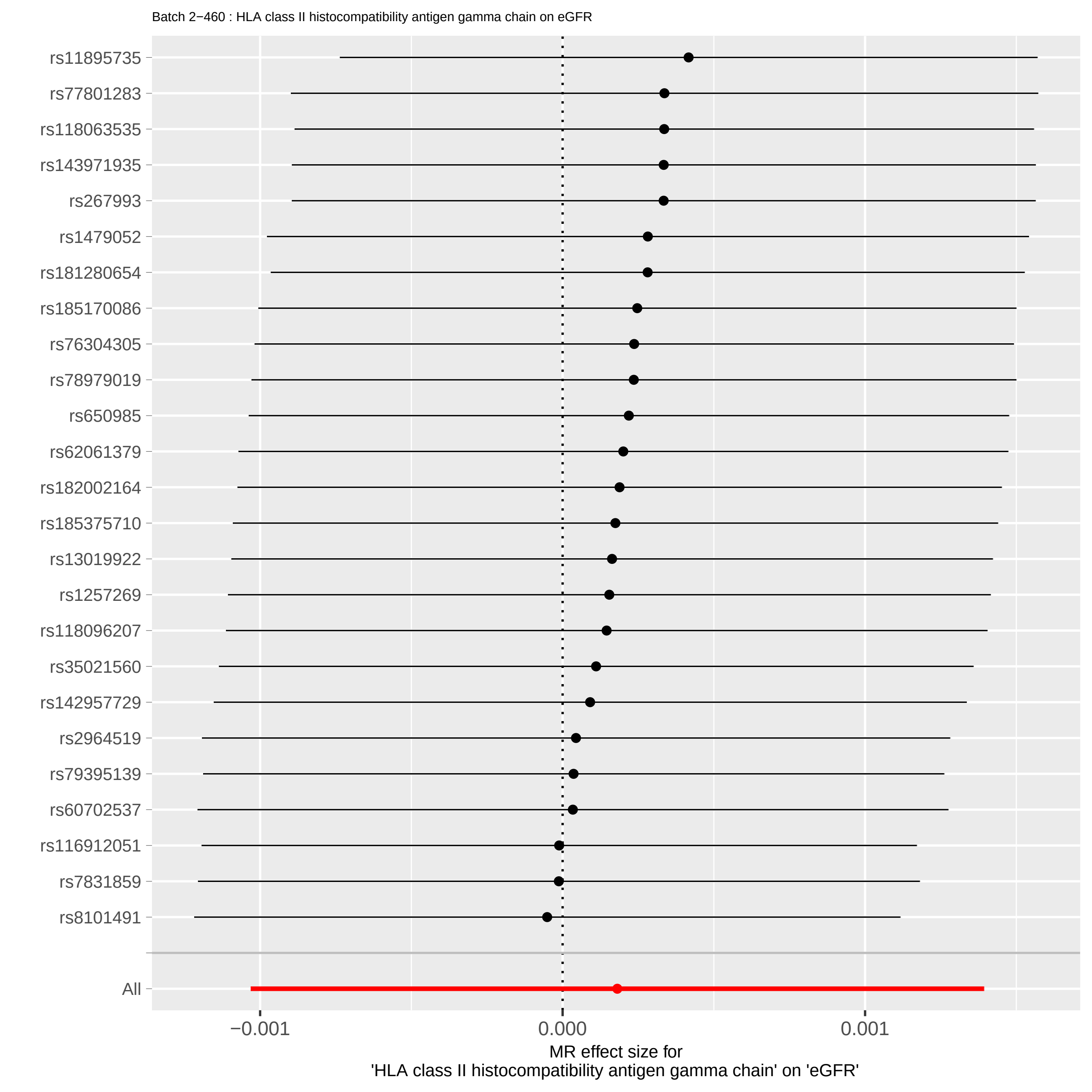
Batch 2−460 : HLA class II histocompatibility antigen gamma chain on eGFR
rs11895735
rs77801283
rs118063535
rs143971935
rs267993
rs1479052
rs181280654
rs185170086
rs76304305
rs78979019
rs650985
rs62061379
rs182002164
rs185375710
rs13019922
rs1257269
rs118096207
rs35021560
rs142957729
rs2964519
rs79395139
rs60702537
rs116912051
rs7831859
rs8101491
All
−0.001
0.000
0.001
MR effect size for
'HLA class II histocompatibility antigen gamma chain' on 'eGFR'

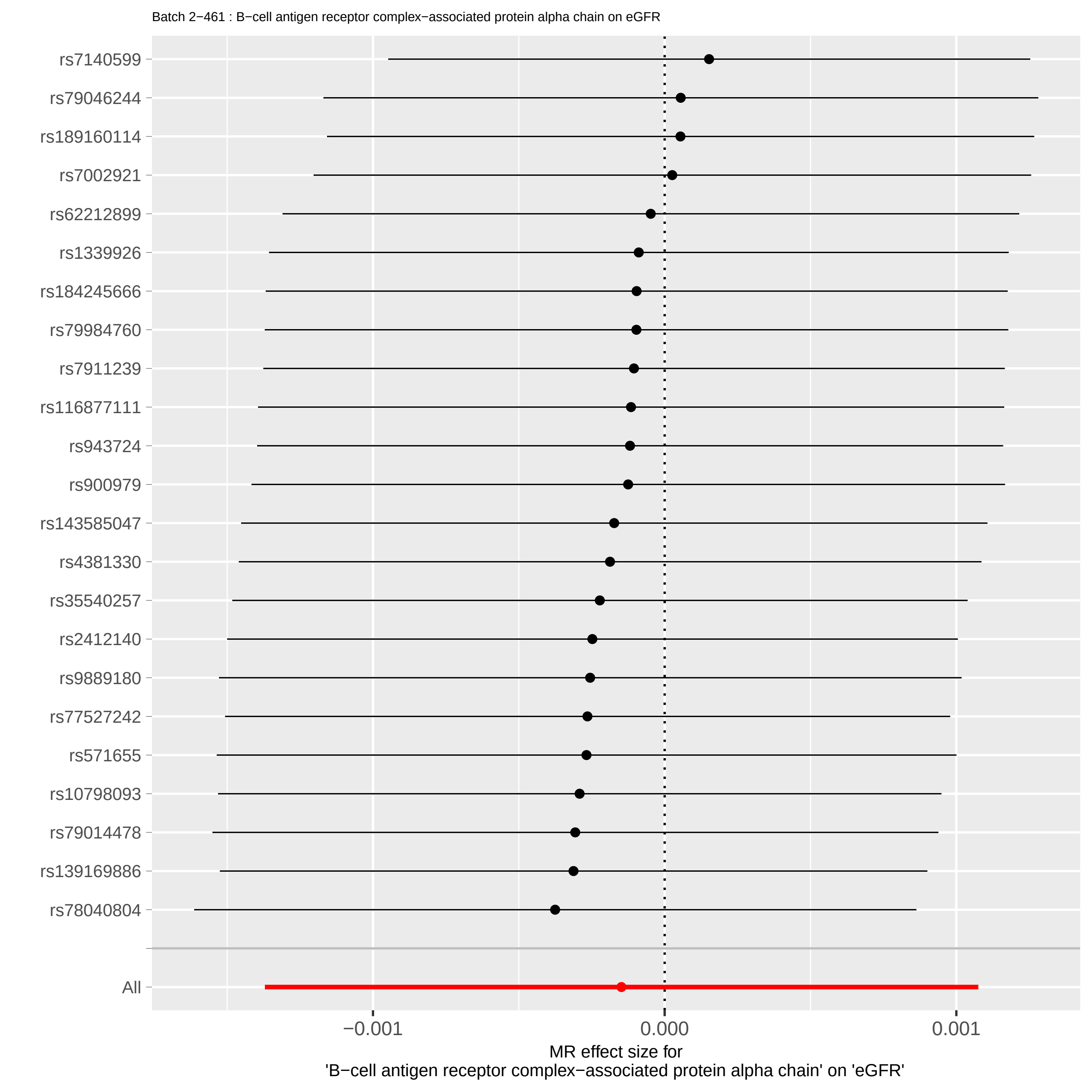
Batch 2−461 : B−cell antigen receptor complex−associated protein alpha chain on eGFR
rs7140599
rs79046244
rs189160114
rs7002921
rs62212899
rs1339926
rs184245666
rs79984760
rs7911239
rs116877111
rs943724
rs900979
rs143585047
rs4381330
rs35540257
rs2412140
rs9889180
rs77527242
rs571655
rs10798093
rs79014478
rs139169886
rs78040804
All
−0.001
0.000
0.001
MR effect size for
'B−cell antigen receptor complex−associated protein alpha chain' on 'eGFR'

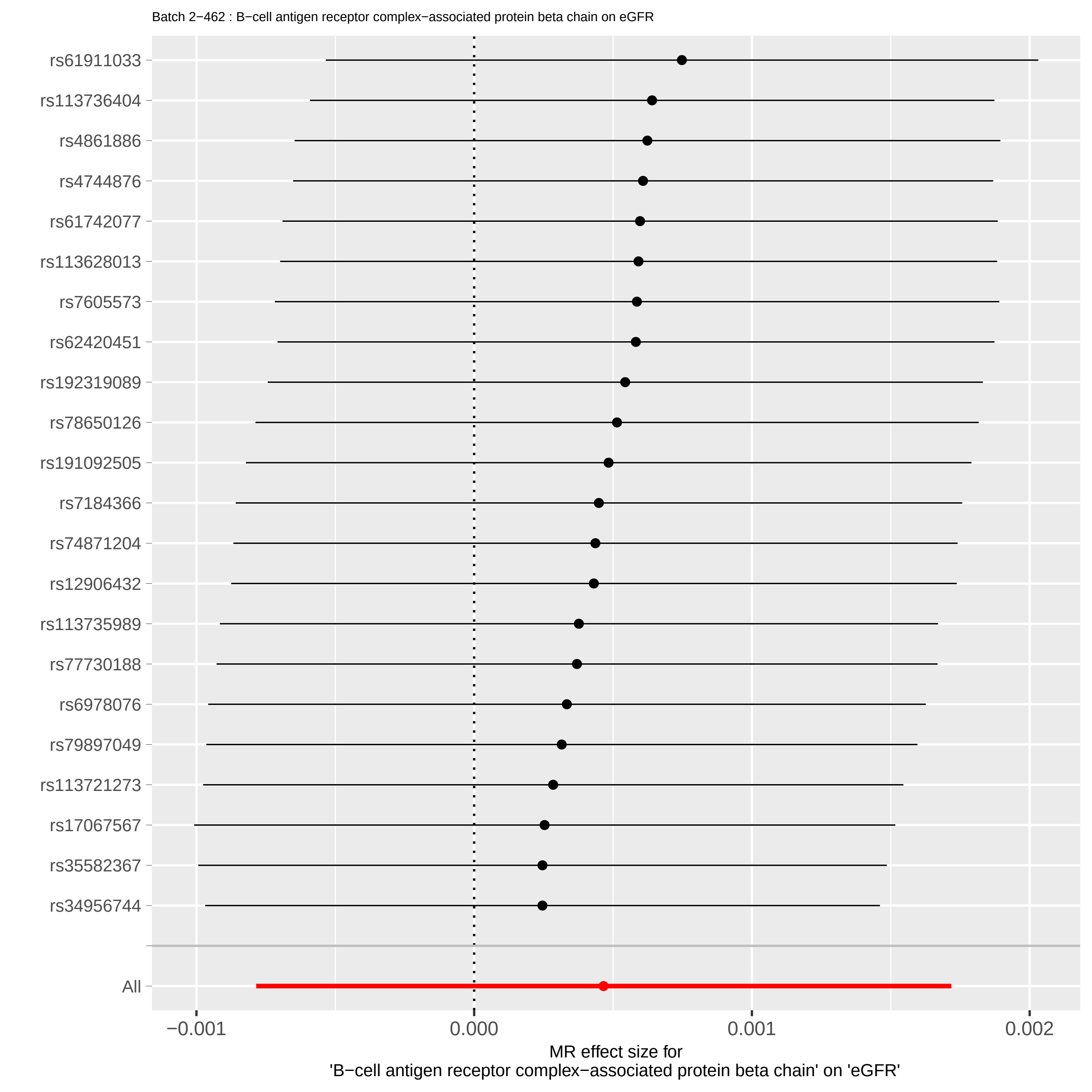

Batch 2−462 : B−cell antigen receptor complex−associated protein beta chain on eGFR
rs61911033
rs113736404
rs4861886
rs4744876
rs61742077
rs113628013
rs7605573
rs62420451
rs192319089
rs78650126
rs191092505
rs7184366
rs74871204
rs12906432
rs113735989
rs77730188
rs6978076
rs79897049
rs113721273
rs17067567
rs35582367
rs34956744
All
−0.001
0.000
0.001
0.002
MR effect size for
'B−cell antigen receptor complex−associated protein beta chain' on 'eGFR'

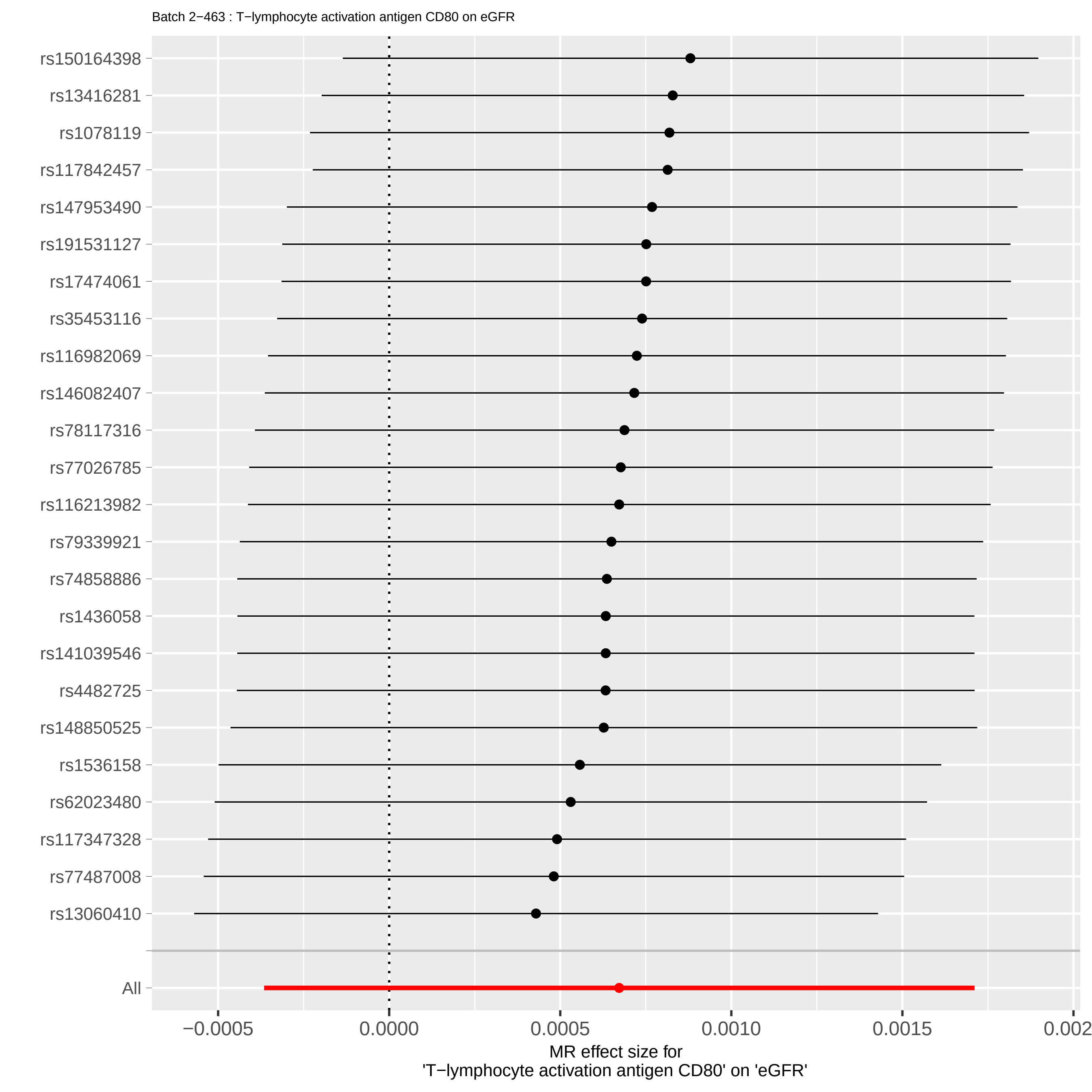
Batch 2−463 : T−lymphocyte activation antigen CD80 on eGFR
rs150164398
rs13416281
rs1078119
rs117842457
rs147953490
rs191531127
rs17474061
rs35453116
rs116982069
rs146082407
rs78117316
rs77026785
rs116213982
rs79339921
rs74858886
rs1436058
rs141039546
rs4482725
rs148850525
rs1536158
rs62023480
rs117347328
rs77487008
rs13060410
All
−0.0005
0.0000
0.0005
0.0010
0.0015
0.002
MR effect size for
'T−lymphocyte activation antigen CD80' on 'eGFR'

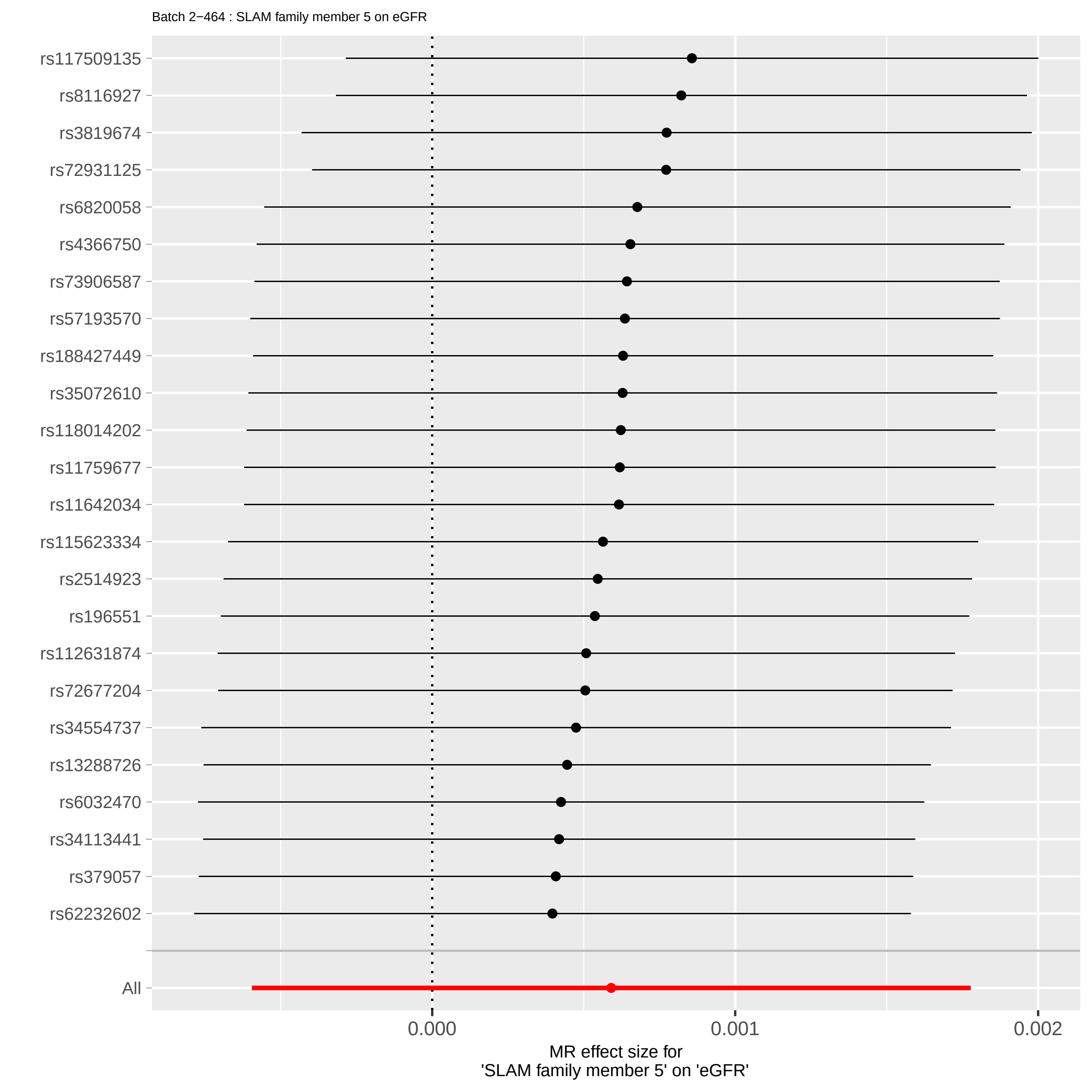

Batch 2−464 : SLAM family member 5 on eGFR
rs117509135
rs8116927
rs3819674
rs72931125
rs6820058
rs4366750
rs73906587
rs57193570
rs188427449
rs35072610
rs118014202
rs11759677
rs11642034
rs115623334
rs2514923
rs196551
rs112631874
rs72677204
rs34554737
rs13288726
rs6032470
rs34113441
rs379057
rs62232602
All
0.000
0.001
0.002
MR effect size for
'SLAM family member 5' on 'eGFR'

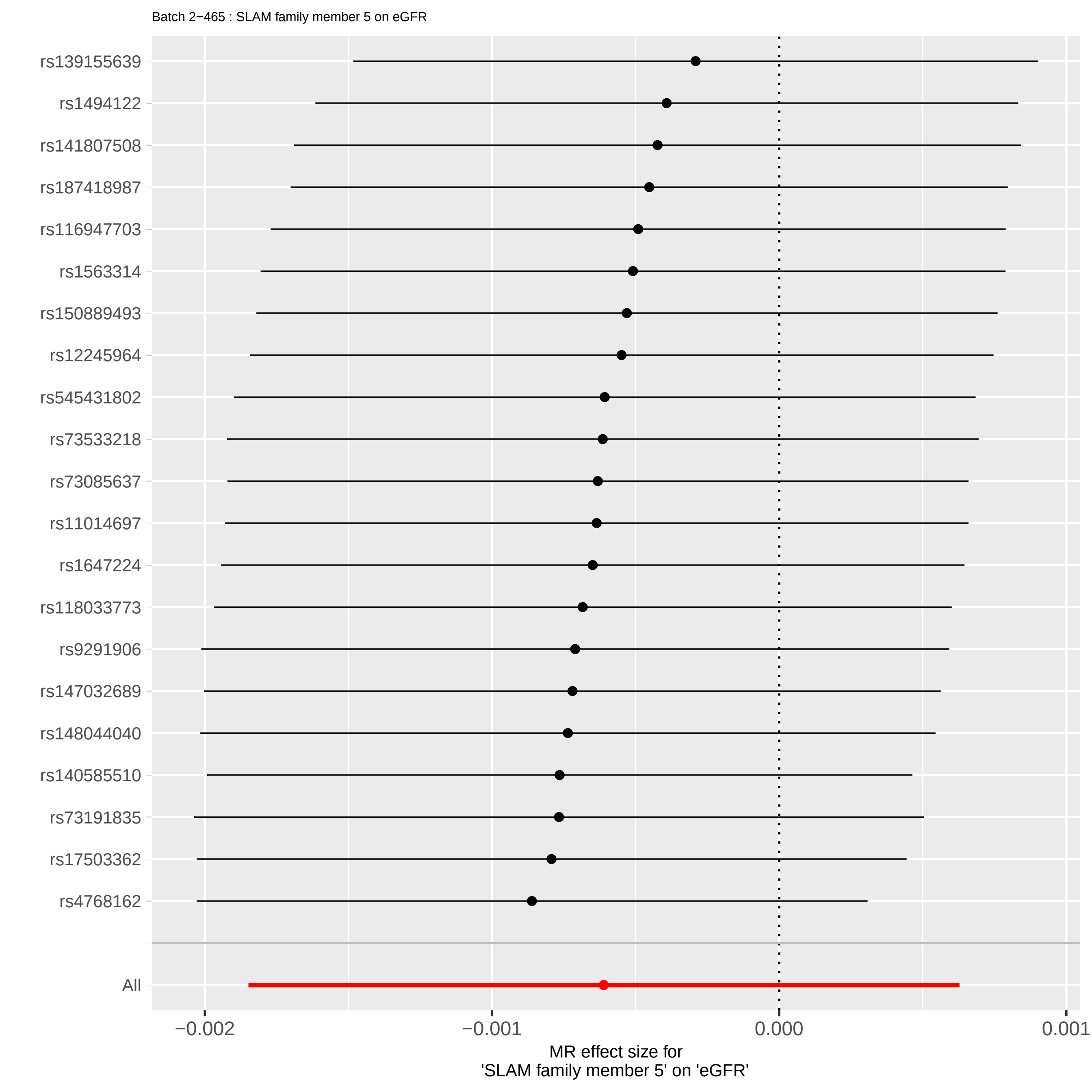

Batch 2−465 : SLAM family member 5 on eGFR
rs139155639
rs1494122
rs141807508
rs187418987
rs116947703
rs1563314
rs150889493
rs12245964
rs545431802
rs73533218
rs73085637
rs11014697
rs1647224
rs118033773
rs9291906
rs147032689
rs148044040
rs140585510
rs73191835
rs17503362
rs4768162
All
−0.002
−0.001
0.000
0.001
MR effect size for
'SLAM family member 5' on 'eGFR'

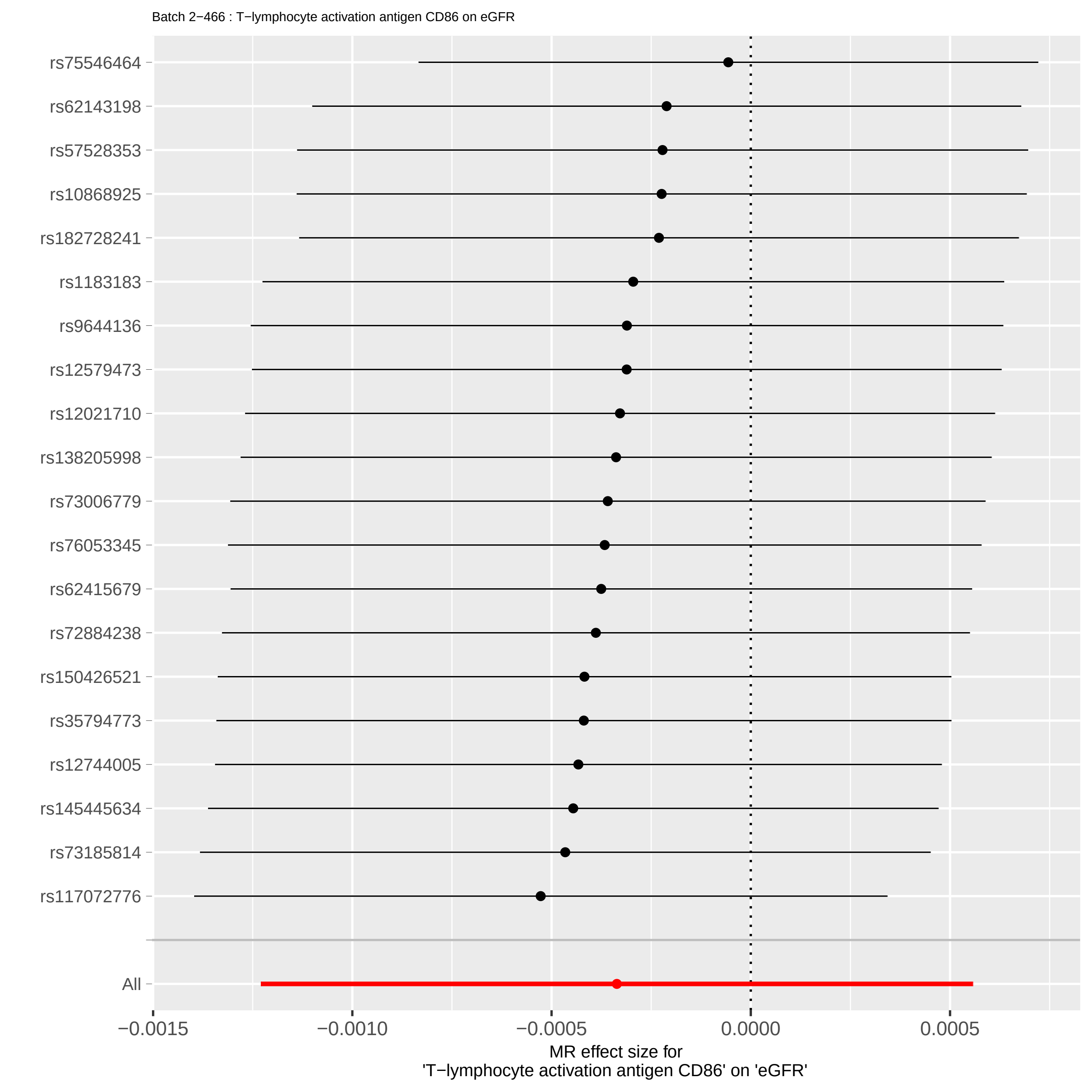

Batch 2−466 : T−lymphocyte activation antigen CD86 on eGFR
rs75546464
rs62143198
rs57528353
rs10868925
rs182728241
rs1183183
rs9644136
rs12579473
rs12021710
rs138205998
rs73006779
rs76053345
rs62415679
rs72884238
rs150426521
rs35794773
rs12744005
rs145445634
rs73185814
rs117072776
All
−0.0015
−0.0010
−0.0005
0.0000
0.0005
MR effect size for
'T−lymphocyte activation antigen CD86' on 'eGFR'

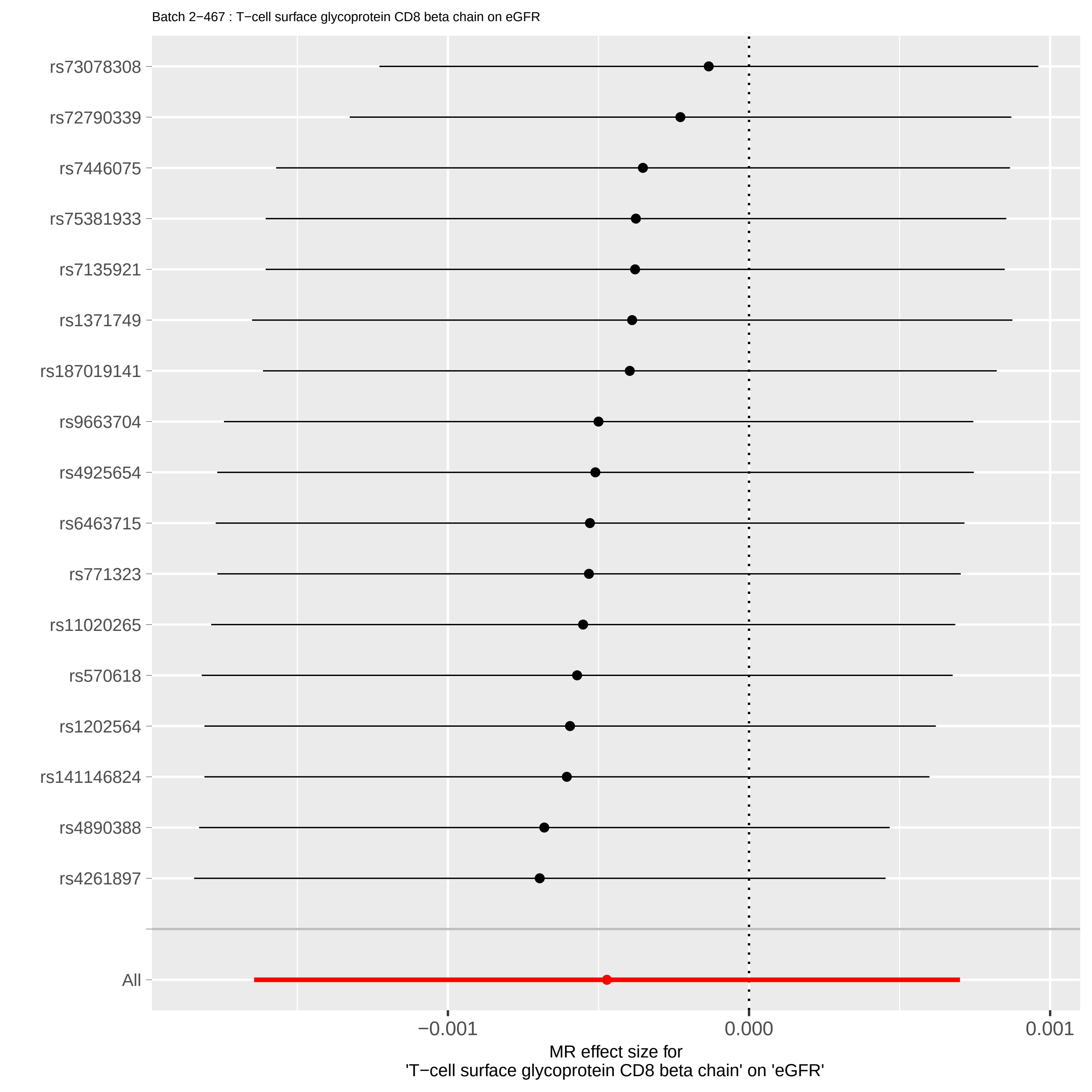
Batch 2−467 : T−cell surface glycoprotein CD8 beta chain on eGFR
rs73078308
rs72790339
rs7446075
rs75381933
rs7135921
rs1371749
rs187019141
rs9663704
rs4925654
rs6463715
rs771323
rs11020265
rs570618
rs1202564
rs141146824
rs4890388
rs4261897
All
−0.001
0.000
0.001
MR effect size for
'T−cell surface glycoprotein CD8 beta chain' on 'eGFR'

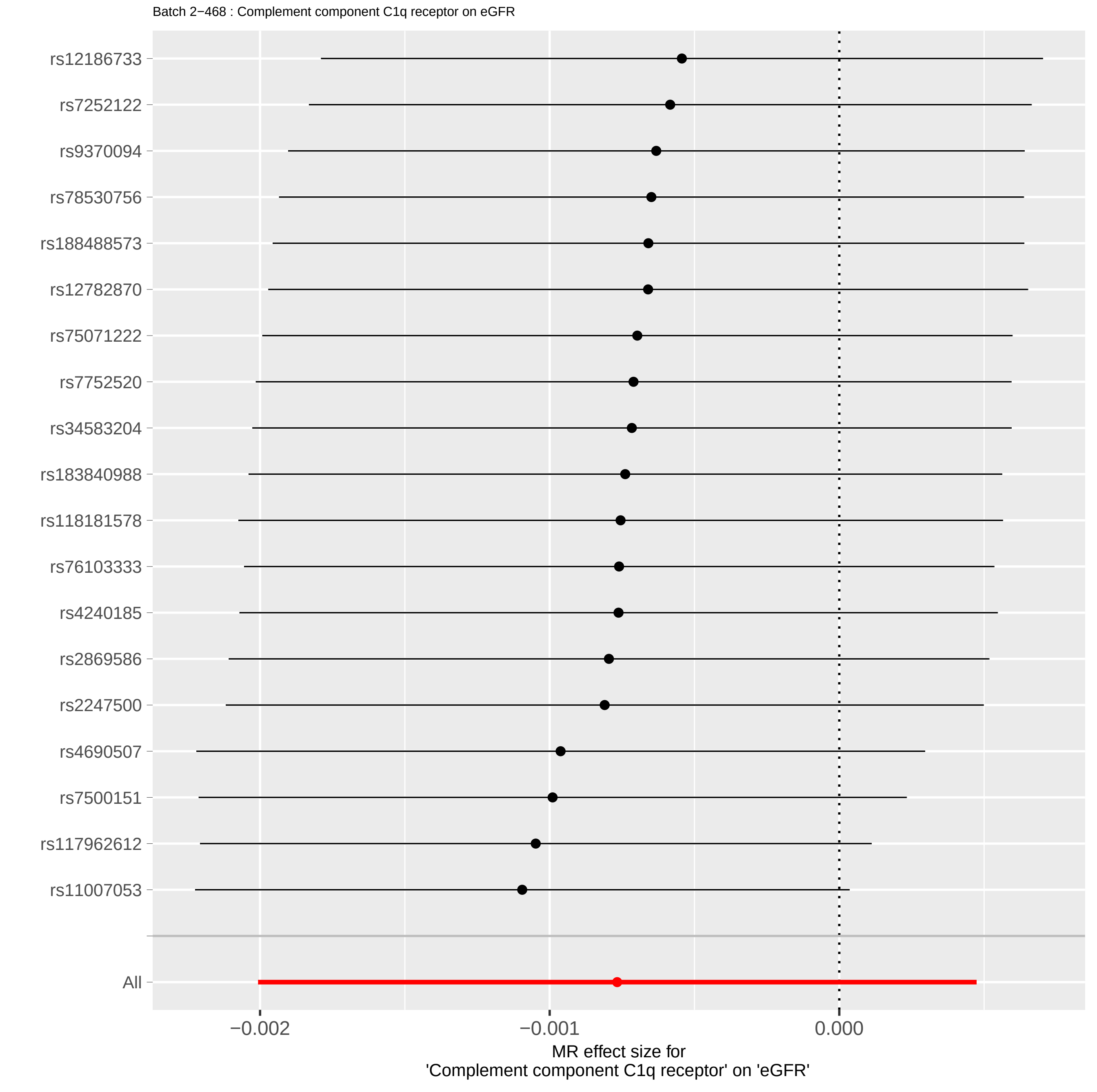

Batch 2−468 : Complement component C1q receptor on eGFR
rs12186733
rs7252122
rs9370094
rs78530756
rs188488573
rs12782870
rs75071222
rs7752520
rs34583204
rs183840988
rs118181578
rs76103333
rs4240185
rs2869586
rs2247500
rs4690507
rs7500151
rs117962612
rs11007053
All
−0.002
−0.001
0.000
MR effect size for
'Complement component C1q receptor' on 'eGFR'

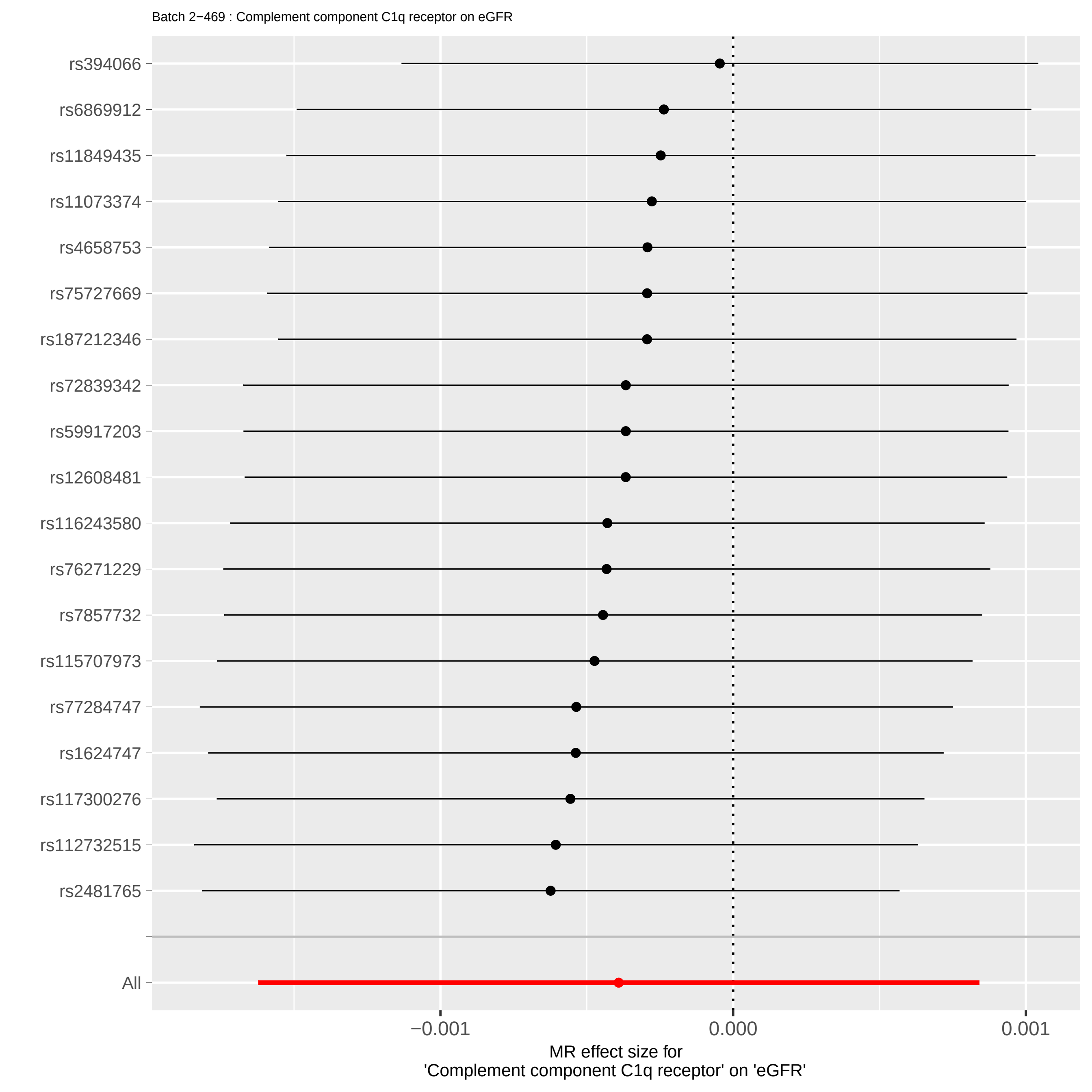

Batch 2−469 : Complement component C1q receptor on eGFR
rs394066
rs6869912
rs11849435
rs11073374
rs4658753
rs75727669
rs187212346
rs72839342
rs59917203
rs12608481
rs116243580
rs76271229
rs7857732
rs115707973
rs77284747
rs1624747
rs117300276
rs112732515
rs2481765
All
−0.001
0.000
0.001
MR effect size for
'Complement component C1q receptor' on 'eGFR'

Batch 2−470 : T−cell surface protein tactile on eGFR

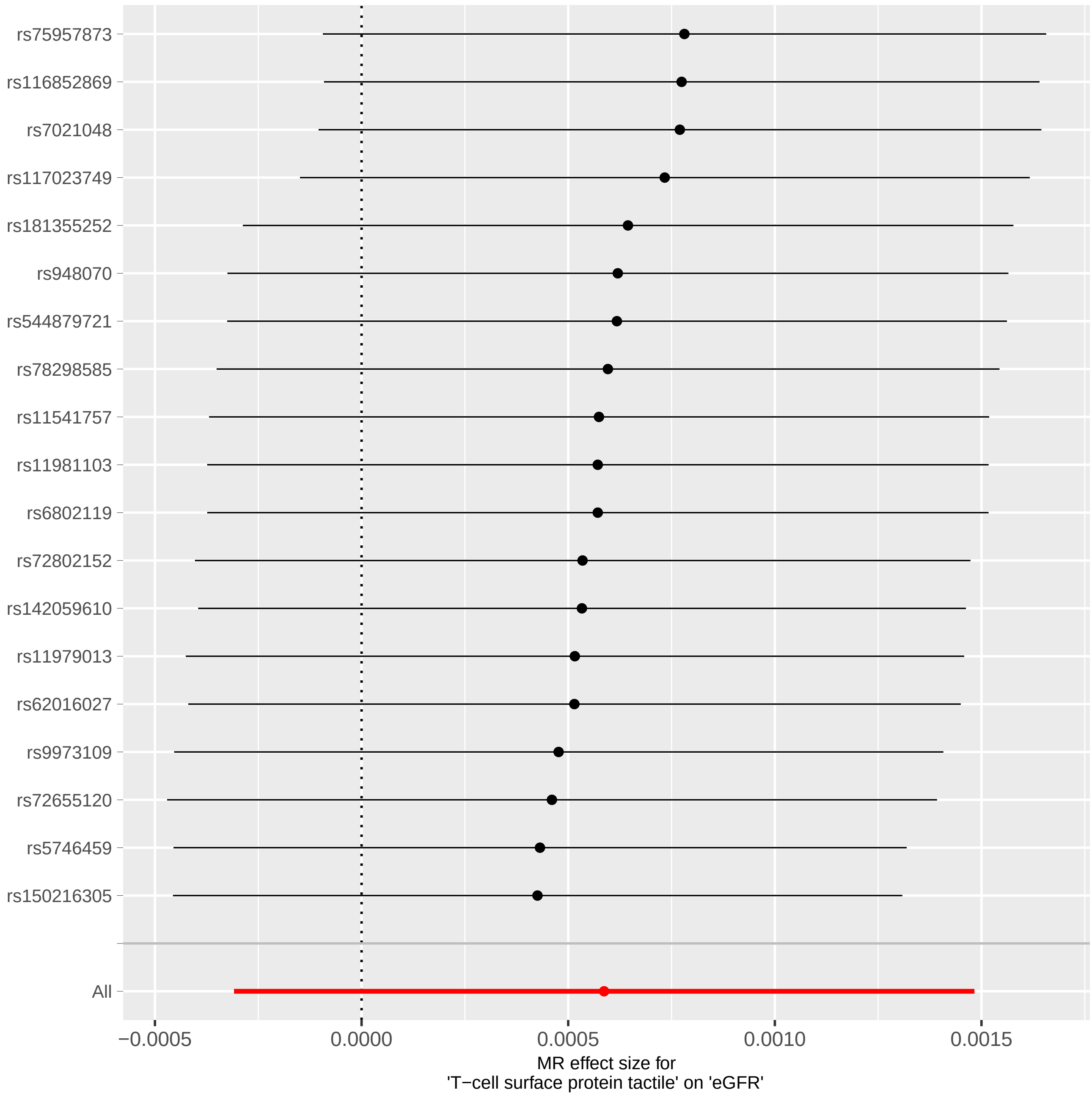

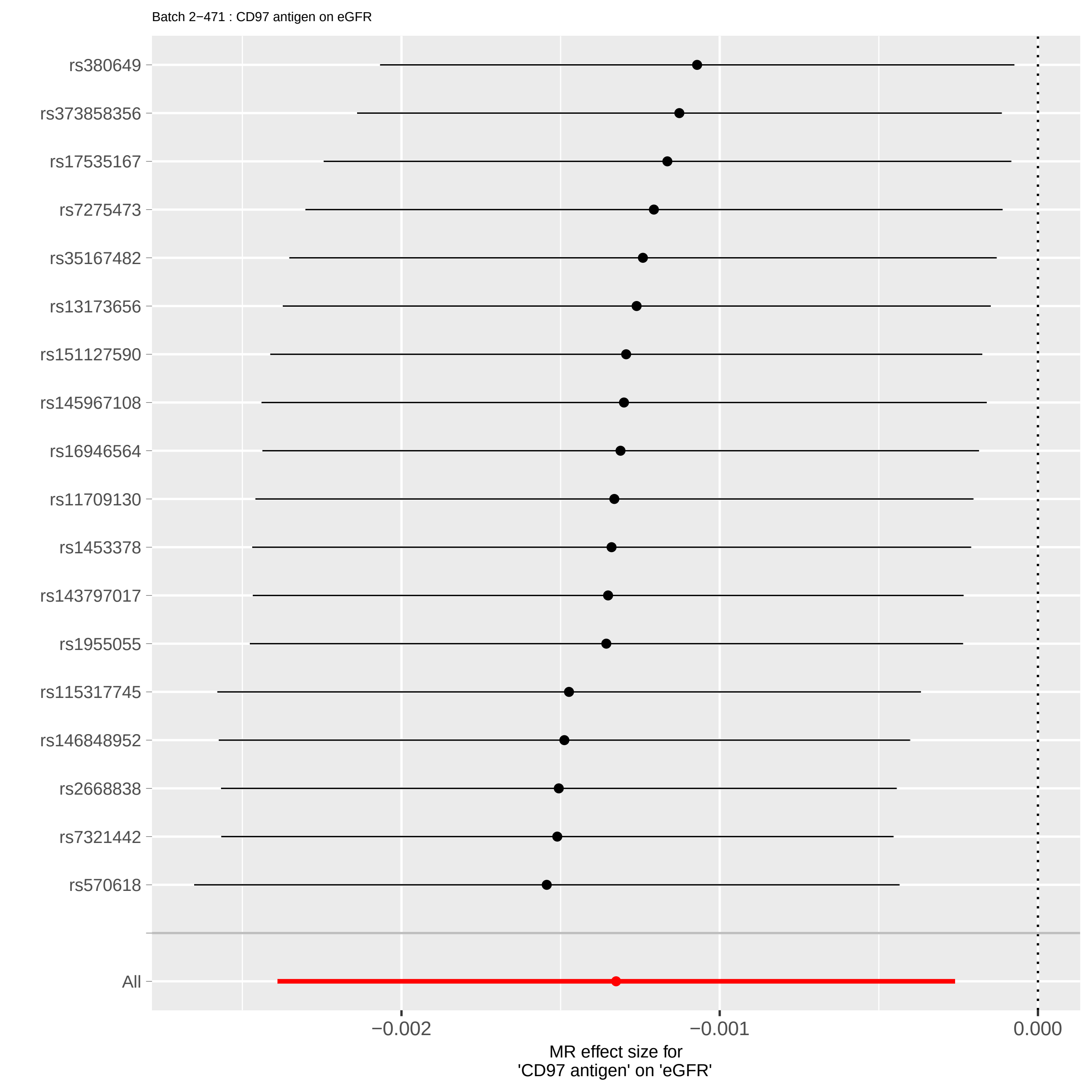

Batch 2−471 : CD97 antigen on eGFR
rs380649
rs373858356
rs17535167
rs7275473
rs35167482
rs13173656
rs151127590
rs145967108
rs16946564
rs11709130
rs1453378
rs143797017
rs1955055
rs115317745
rs146848952
rs2668838
rs7321442
rs570618
All
−0.002
−0.001
0.000
MR effect size for
'CD97 antigen' on 'eGFR'

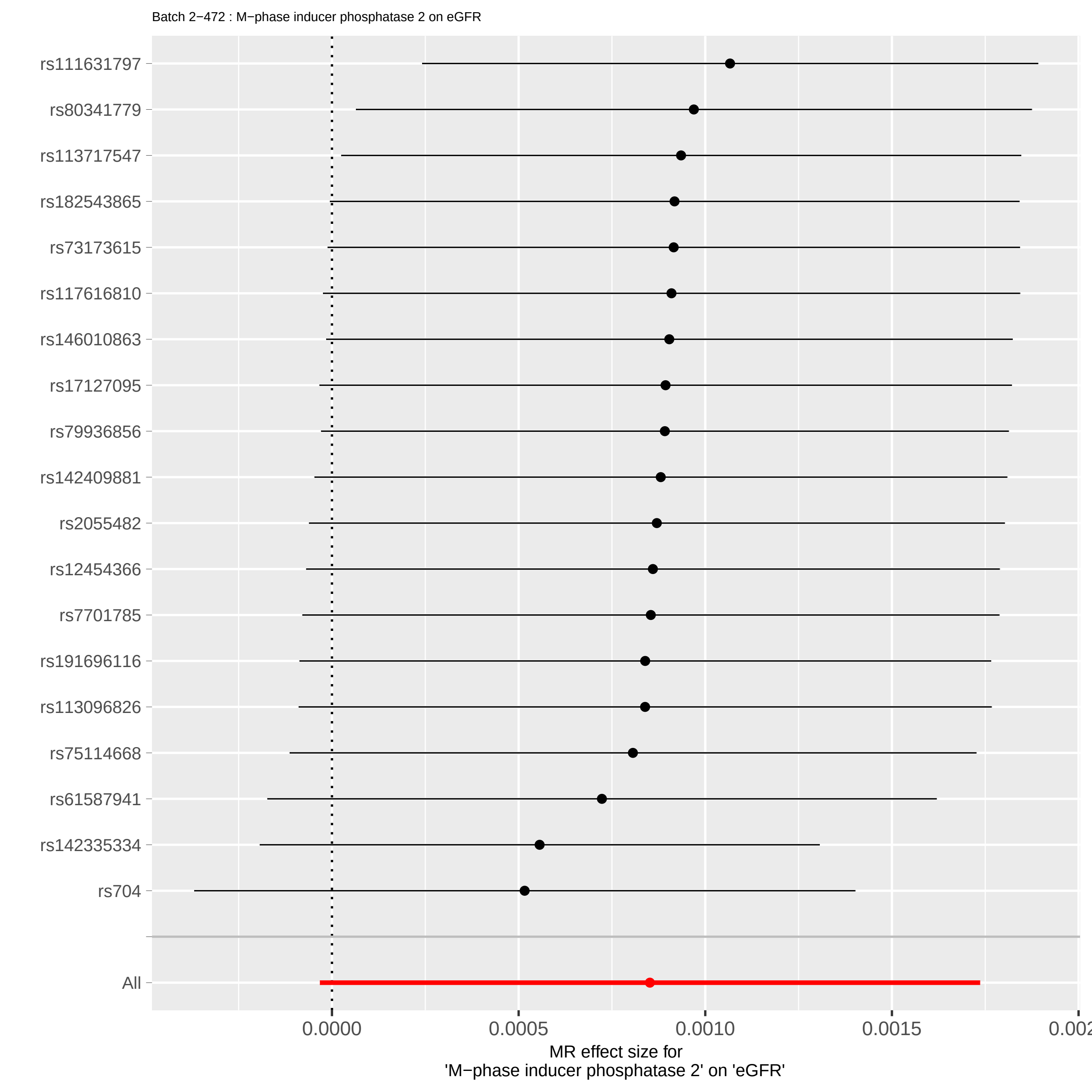

Batch 2−472 : M−phase inducer phosphatase 2 on eGFR
rs111631797
rs80341779
rs113717547
rs182543865
rs73173615
rs117616810
rs146010863
rs17127095
rs79936856
rs142409881
rs2055482
rs12454366
rs7701785
rs191696116
rs113096826
rs75114668
rs61587941
rs142335334
rs704
All
0.0000
0.0005
0.0010
0.0015
MR effect size for
'M−phase inducer phosphatase 2' on 'eGFR'

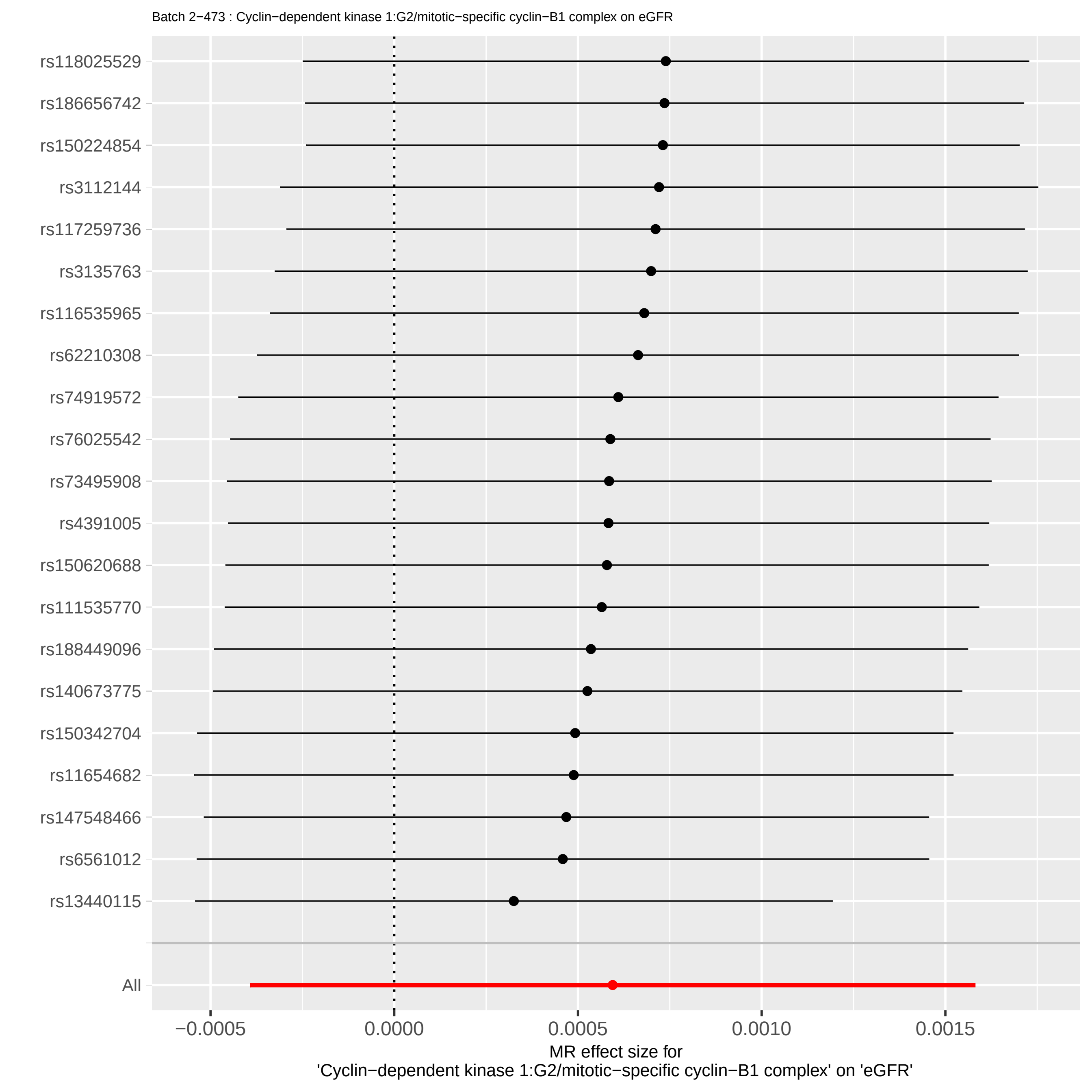

Batch 2−473 : Cyclin−dependent kinase 1:G2/mitotic−specific cyclin−B1 complex on eGFR
rs118025529
rs186656742
rs150224854
rs3112144
rs117259736
rs3135763
rs116535965
rs62210308
rs74919572
rs76025542
rs73495908
rs4391005
rs150620688
rs111535770
rs188449096
rs140673775
rs150342704
rs11654682
rs147548466
rs6561012
rs13440115
All
−0.0005
0.0000
0.0005
0.0010
0.0015
MR effect size for
'Cyclin−dependent kinase 1:G2/mitotic−specific cyclin−B1 complex' on 'eGFR'

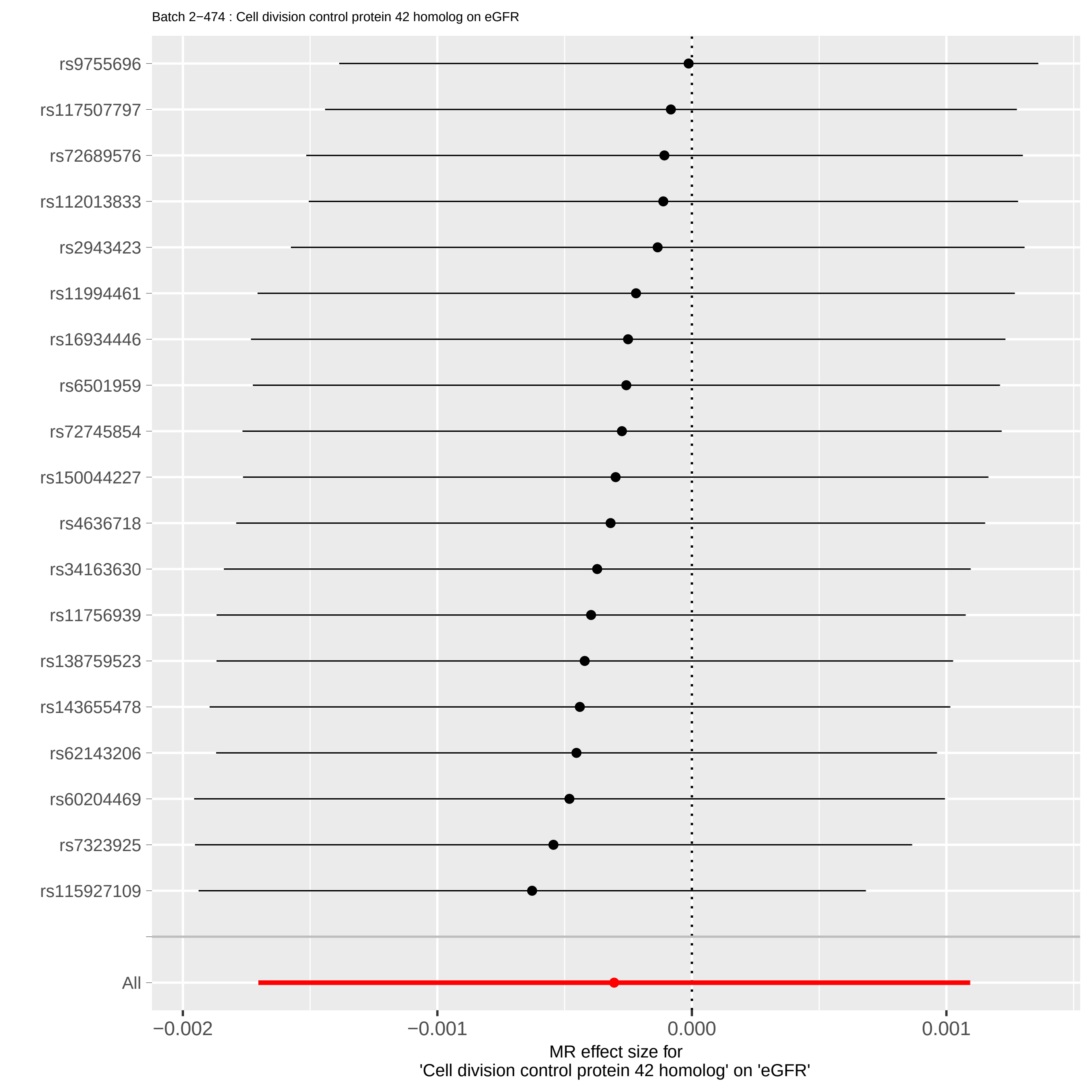

Batch 2−474 : Cell division control protein 42 homolog on eGFR
rs9755696
rs117507797
rs72689576
rs112013833
rs2943423
rs11994461
rs16934446
rs6501959
rs72745854
rs150044227
rs4636718
rs34163630
rs11756939
rs138759523
rs143655478
rs62143206
rs60204469
rs7323925
rs115927109
All
−0.002
−0.001
0.000
0.001
MR effect size for
'Cell division control protein 42 homolog' on 'eGFR'

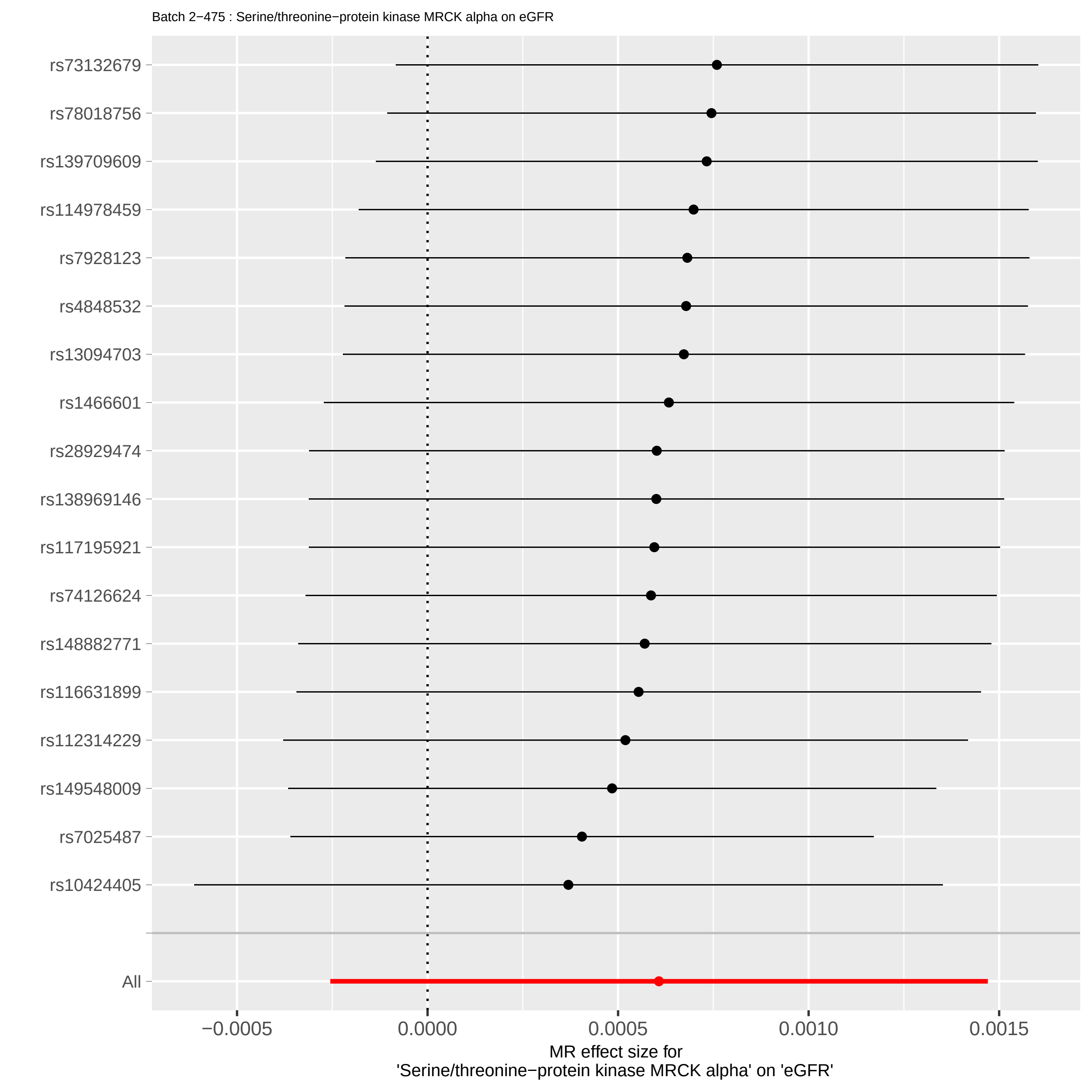
Batch 2−475 : Serine/threonine−protein kinase MRCK alpha on eGFR
rs73132679
rs78018756
rs139709609
rs114978459
rs7928123
rs4848532
rs13094703
rs1466601
rs28929474
rs138969146
rs117195921
rs74126624
rs148882771
rs116631899
rs112314229
rs149548009
rs7025487
rs10424405
All
−0.0005
0.0000
0.0005
0.0010
0.0015
MR effect size for
'Serine/threonine−protein kinase MRCK alpha' on 'eGFR'

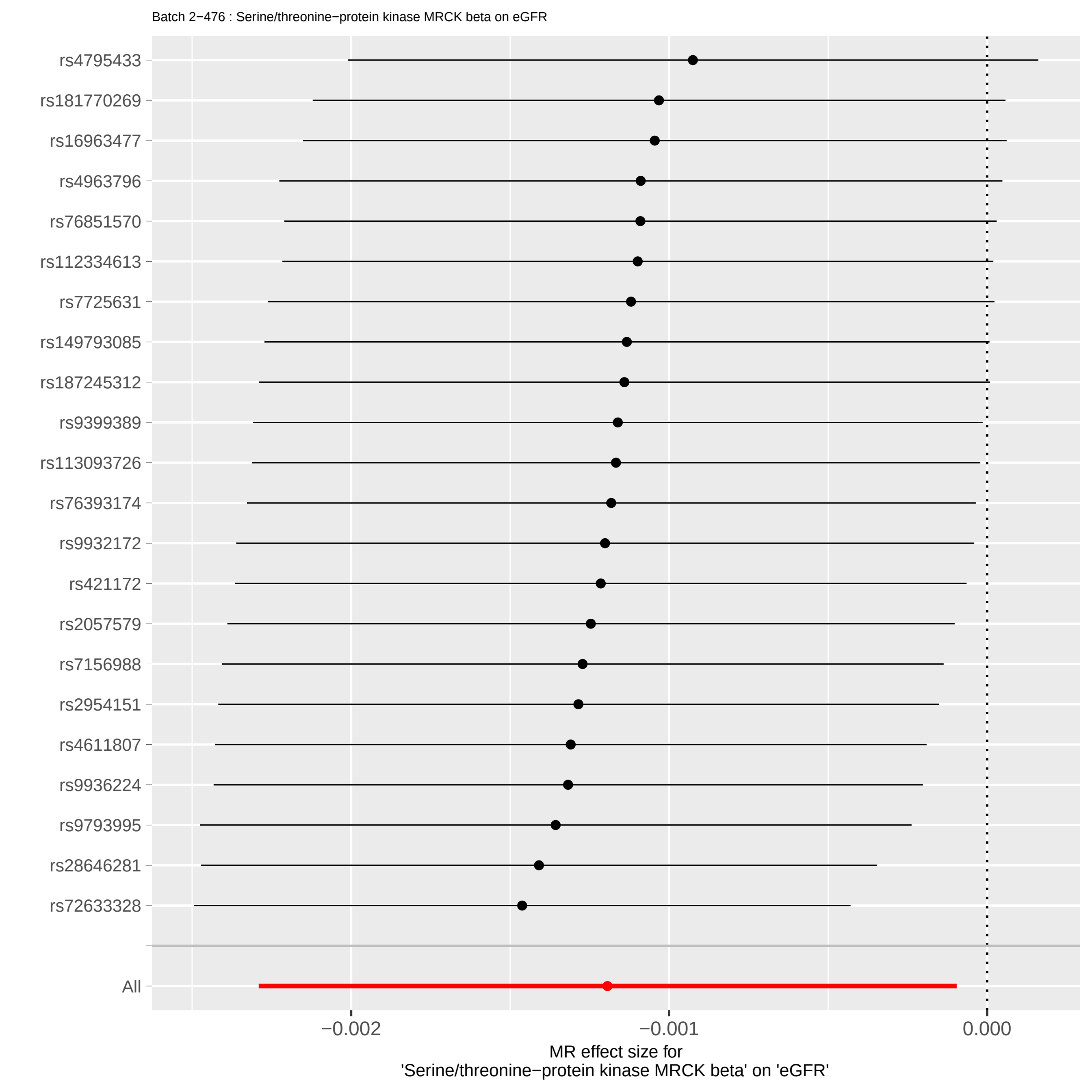

Batch 2−476 : Serine/threonine−protein kinase MRCK beta on eGFR
rs4795433
rs181770269
rs16963477
rs4963796
rs76851570
rs112334613
rs7725631
rs149793085
rs187245312
rs9399389
rs113093726
rs76393174
rs9932172
rs421172
rs2057579
rs7156988
rs2954151
rs4611807
rs9936224
rs9793995
rs28646281
rs72633328
All
−0.002
−0.001
0.000
MR effect size for
'Serine/threonine−protein kinase MRCK beta' on 'eGFR'

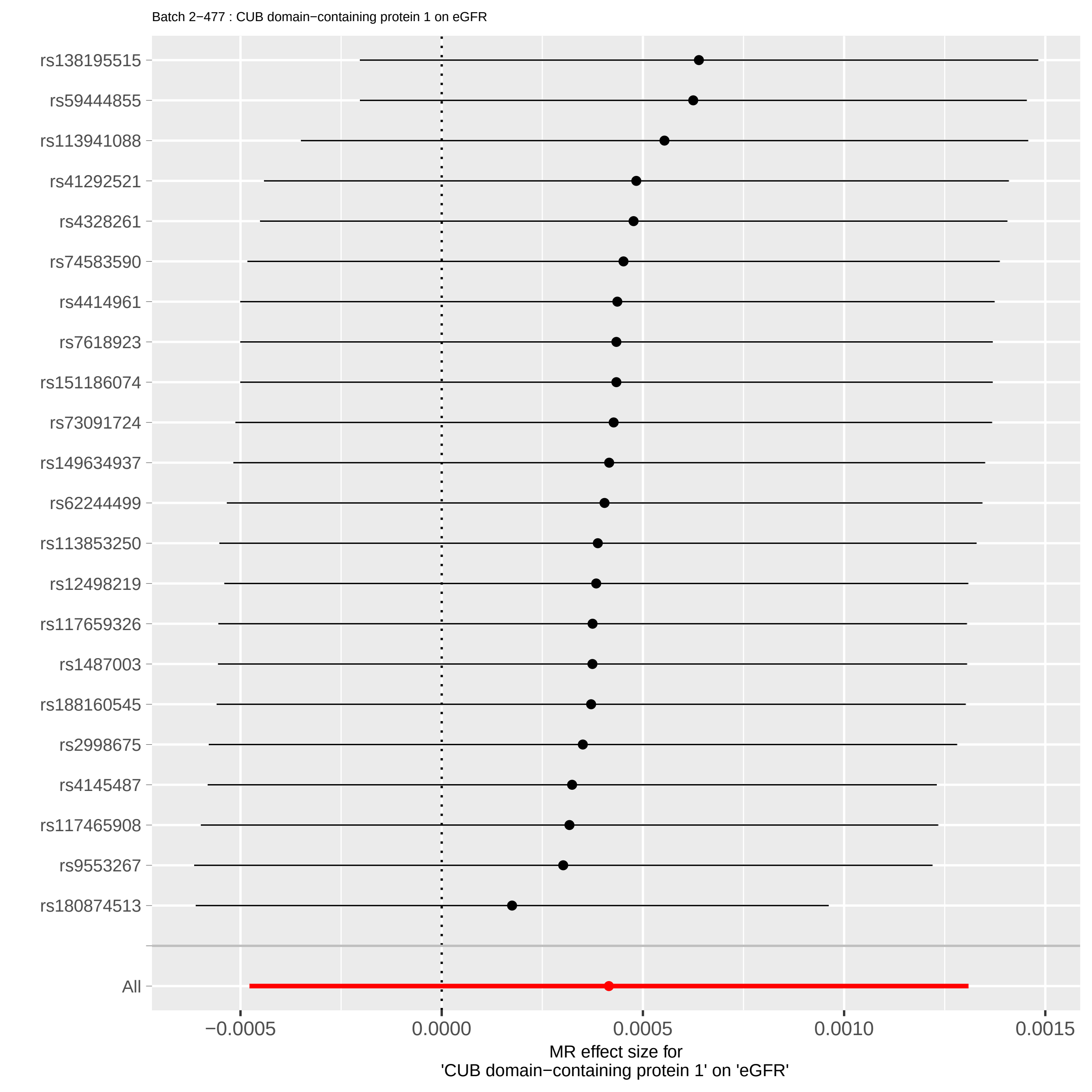

Batch 2−477 : CUB domain−containing protein 1 on eGFR
rs138195515
rs59444855
rs113941088
rs41292521
rs4328261
rs74583590
rs4414961
rs7618923
rs151186074
rs73091724
rs149634937
rs62244499
rs113853250
rs12498219
rs117659326
rs1487003
rs188160545
rs2998675
rs4145487
rs117465908
rs9553267
rs180874513
All
−0.0005
0.0000
0.0005
0.0010
0.0015
MR effect size for
'CUB domain−containing protein 1' on 'eGFR'

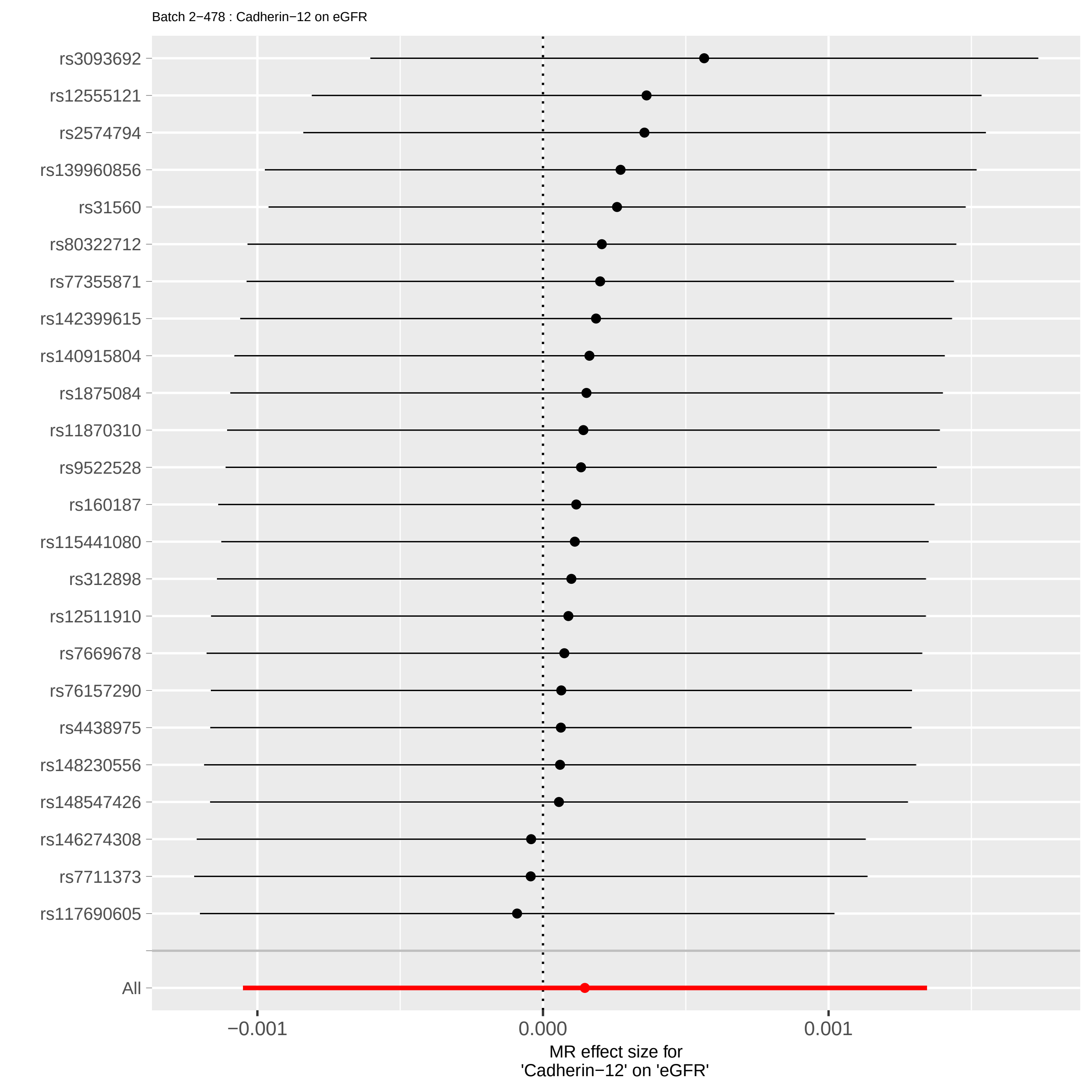

Batch 2−478 : Cadherin−12 on eGFR
rs3093692
rs12555121
rs2574794
rs139960856
rs31560
rs80322712
rs77355871
rs142399615
rs140915804
rs1875084
rs11870310
rs9522528
rs160187
rs115441080
rs312898
rs12511910
rs7669678
rs76157290
rs4438975
rs148230556
rs148547426
rs146274308
rs7711373
rs117690605
All
−0.001
0.000
0.001
MR effect size for
'Cadherin−12' on 'eGFR'

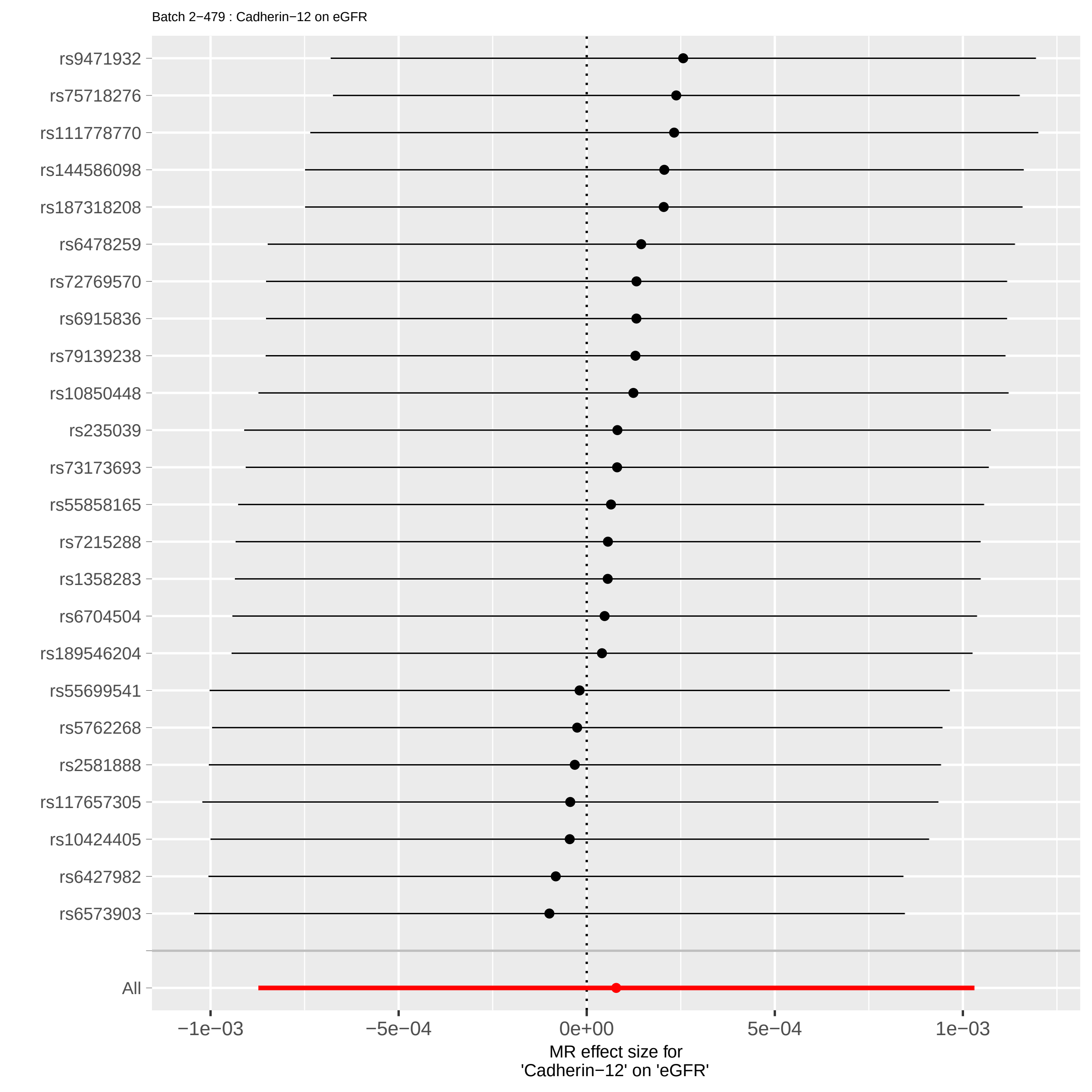

Batch 2−479 : Cadherin−12 on eGFR
rs9471932
rs75718276
rs111778770
rs144586098
rs187318208
rs6478259
rs72769570
rs6915836
rs79139238
rs10850448
rs235039
rs73173693
rs55858165
rs7215288
rs1358283
rs6704504
rs189546204
rs55699541
rs5762268
rs2581888
rs117657305
rs10424405
rs6427982
rs6573903
All
−1e−03
−5e−04
0e+00
5e−04
1e−03
MR effect size for
'Cadherin−12' on 'eGFR'

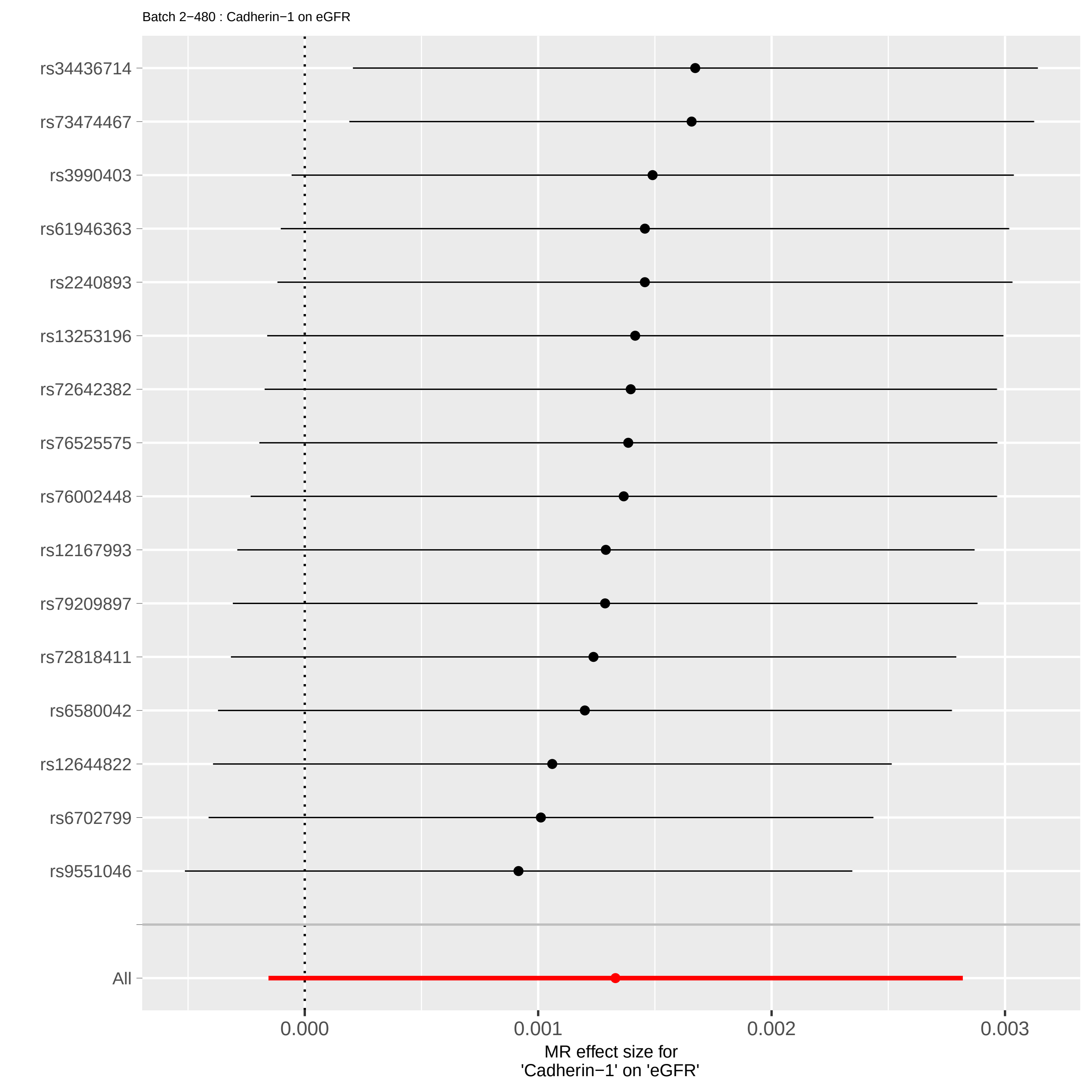

Batch 2−480 : Cadherin−1 on eGFR
rs34436714
rs73474467
rs3990403
rs61946363
rs2240893
rs13253196
rs72642382
rs76525575
rs76002448
rs12167993
rs79209897
rs72818411
rs6580042
rs12644822
rs6702799
rs9551046
All
0.000
0.001
0.002
0.003
MR effect size for
'Cadherin−1' on 'eGFR'

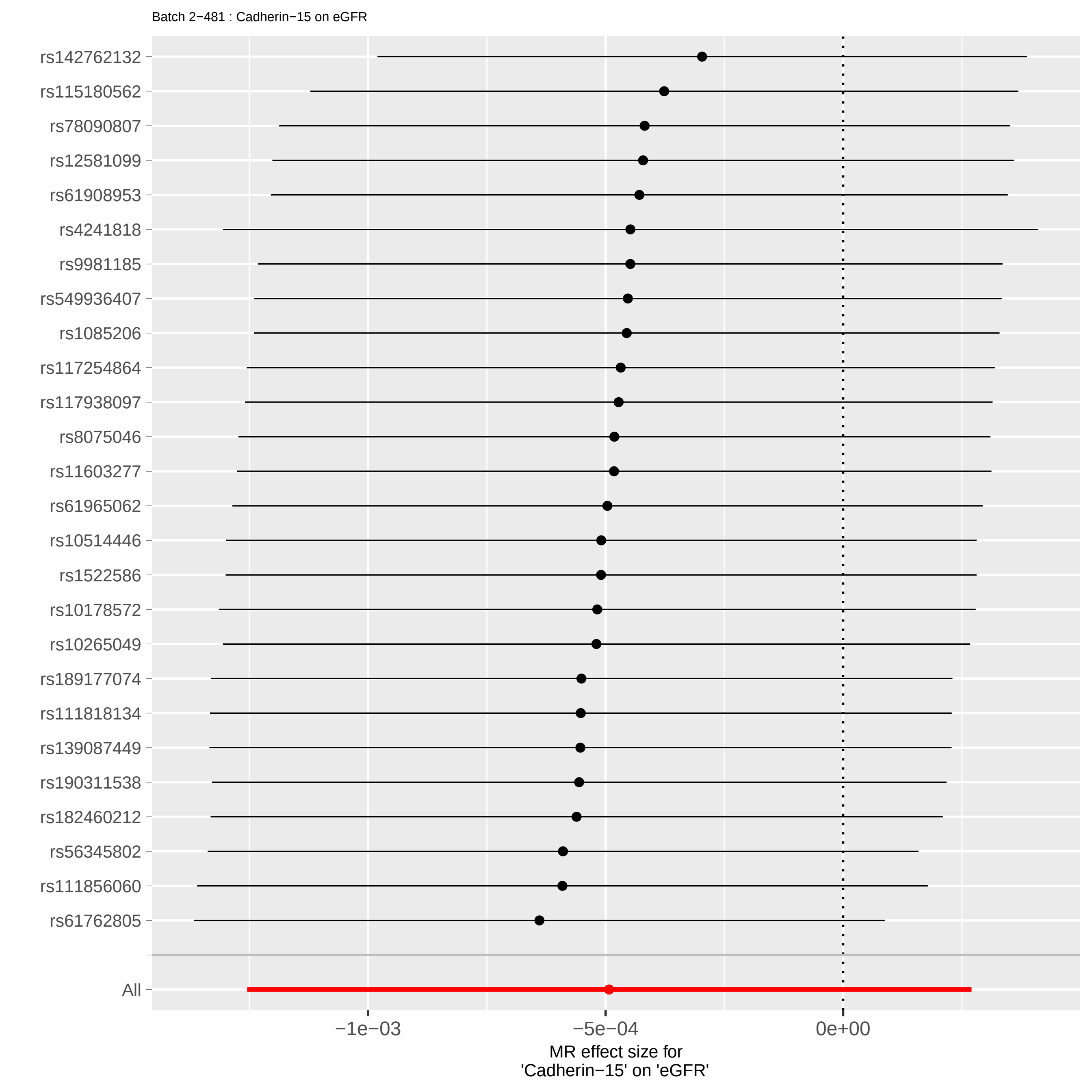

Batch 2−481 : Cadherin−15 on eGFR
rs142762132
rs115180562
rs78090807
rs12581099
rs61908953
rs4241818
rs9981185
rs549936407
rs1085206
rs117254864
rs117938097
rs8075046
rs11603277
rs61965062
rs10514446
rs1522586
rs10178572
rs10265049
rs189177074
rs111818134
rs139087449
rs190311538
rs182460212
rs56345802
rs111856060
rs61762805
All
−1e−03
−5e−04
0e+00
MR effect size for
'Cadherin−15' on 'eGFR'

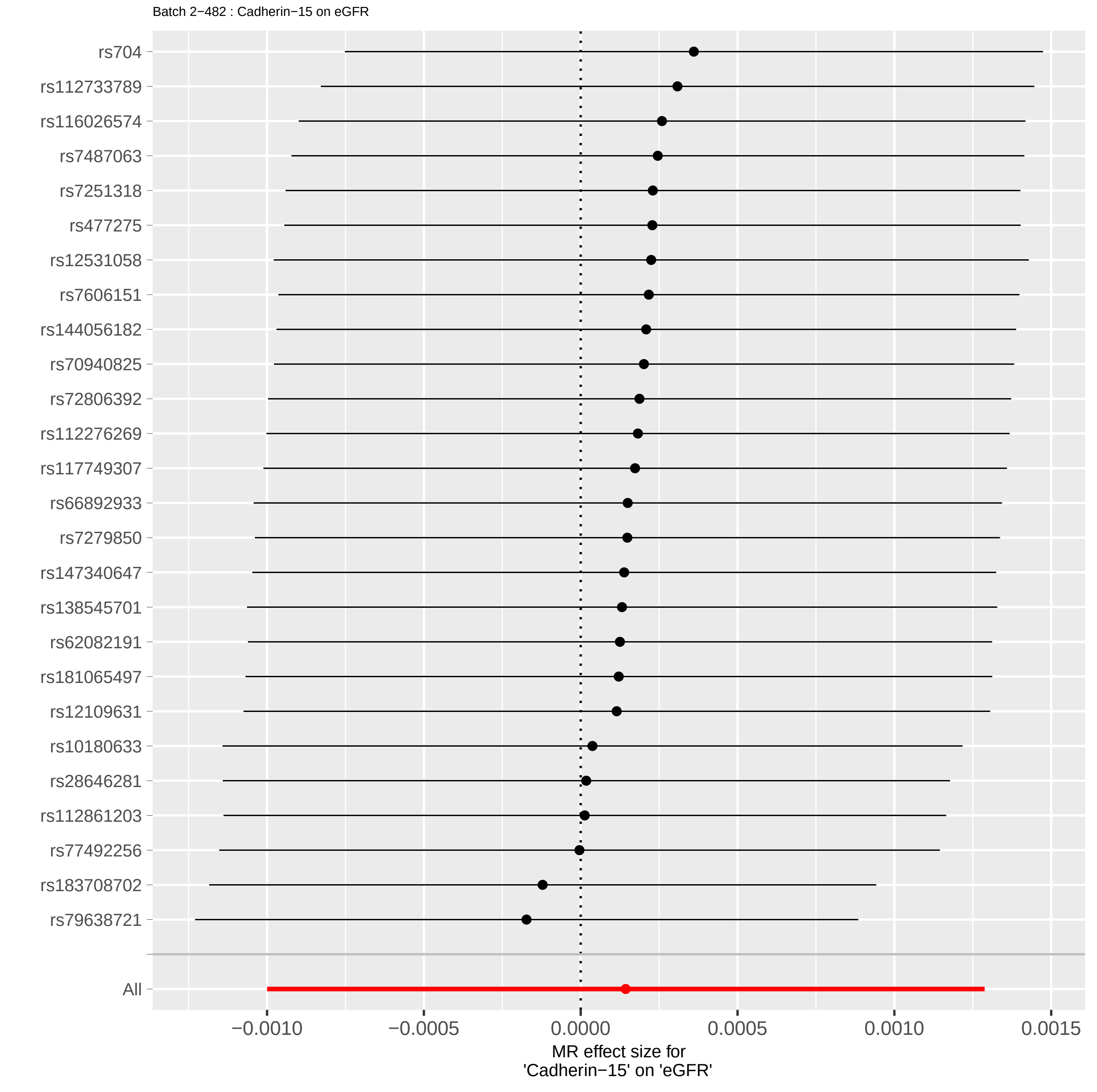

Batch 2−482 : Cadherin−15 on eGFR
rs704
rs112733789
rs116026574
rs7487063
rs7251318
rs477275
rs12531058
rs7606151
rs144056182
rs70940825
rs72806392
rs112276269
rs117749307
rs66892933
rs7279850
rs147340647
rs138545701
rs62082191
rs181065497
rs12109631
rs10180633
rs28646281
rs112861203
rs77492256
rs183708702
rs79638721
All
−0.0010
−0.0005
0.0000
0.0005
0.0010
0.0015
MR effect size for
'Cadherin−15' on 'eGFR'

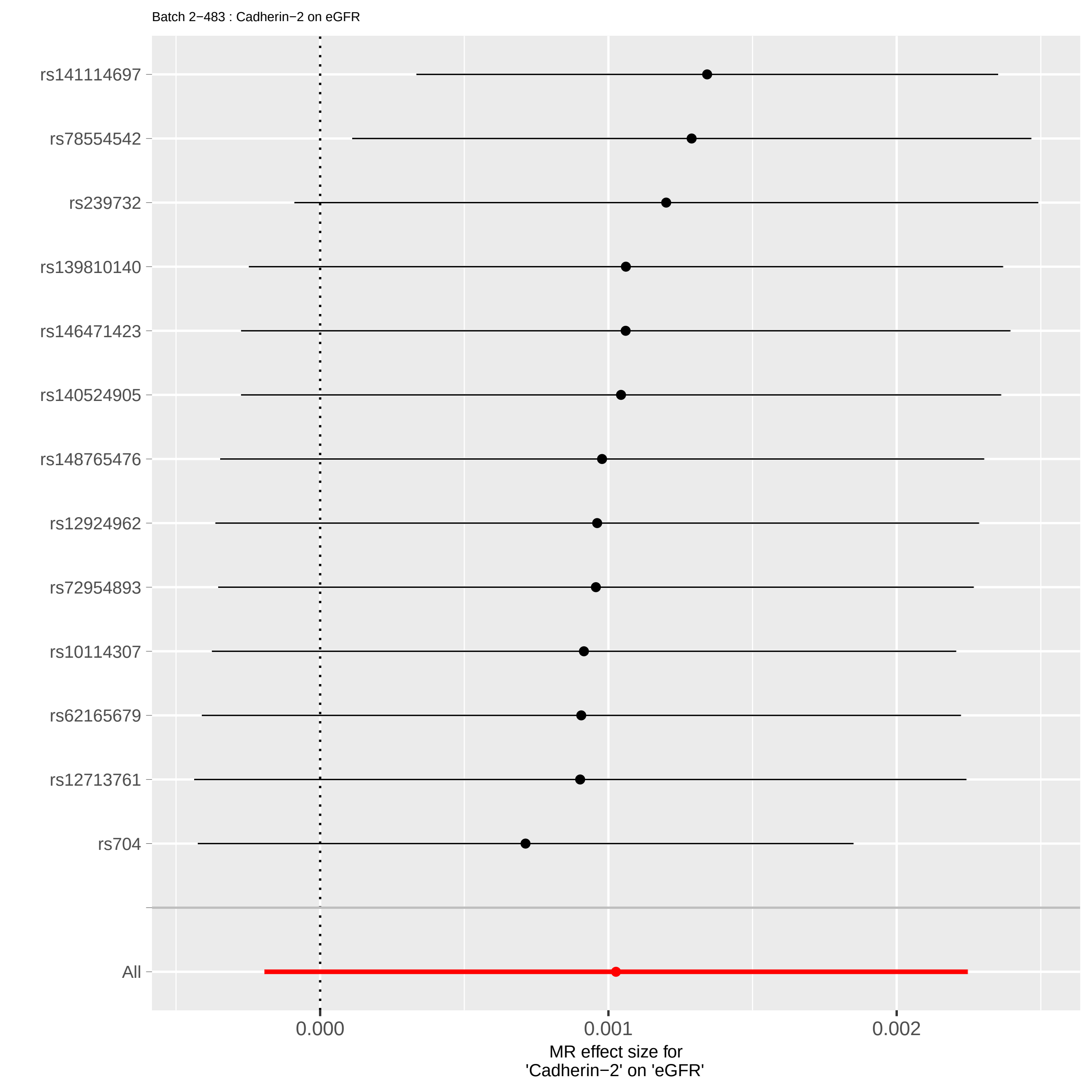

Batch 2−483 : Cadherin−2 on eGFR
rs141114697
rs78554542
rs239732
rs139810140
rs146471423
rs140524905
rs148765476
rs12924962
rs72954893
rs10114307
rs62165679
rs12713761
rs704
All
0.000
0.001
0.002
MR effect size for
'Cadherin−2' on 'eGFR'

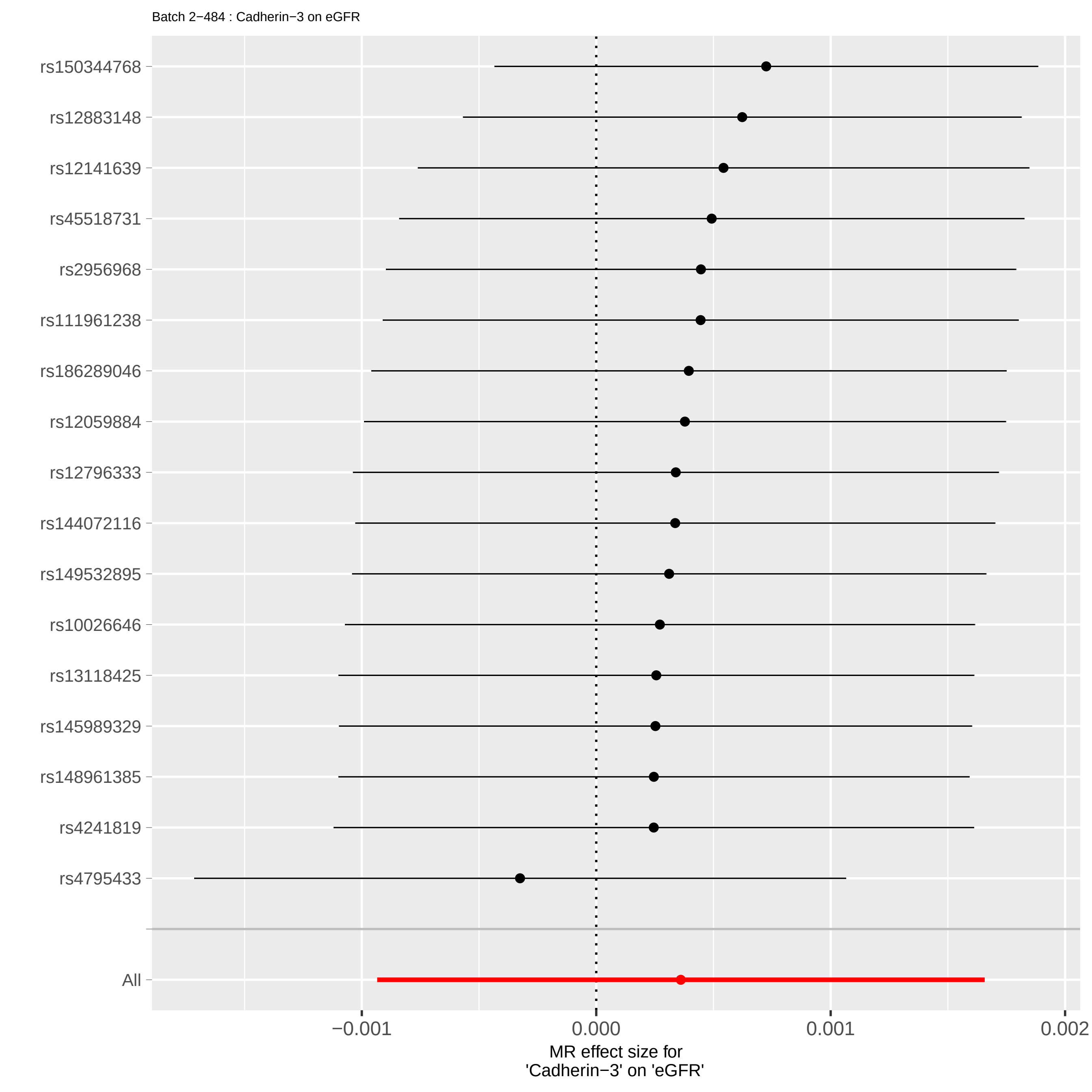

Batch 2−484 : Cadherin−3 on eGFR
rs150344768
rs12883148
rs12141639
rs45518731
rs2956968
rs111961238
rs186289046
rs12059884
rs12796333
rs144072116
rs149532895
rs10026646
rs13118425
rs145989329
rs148961385
rs4241819
rs4795433
All
−0.001
0.000
0.001
0.002
MR effect size for
'Cadherin−3' on 'eGFR'

Batch 2−485 : Cadherin−5 on eGFR

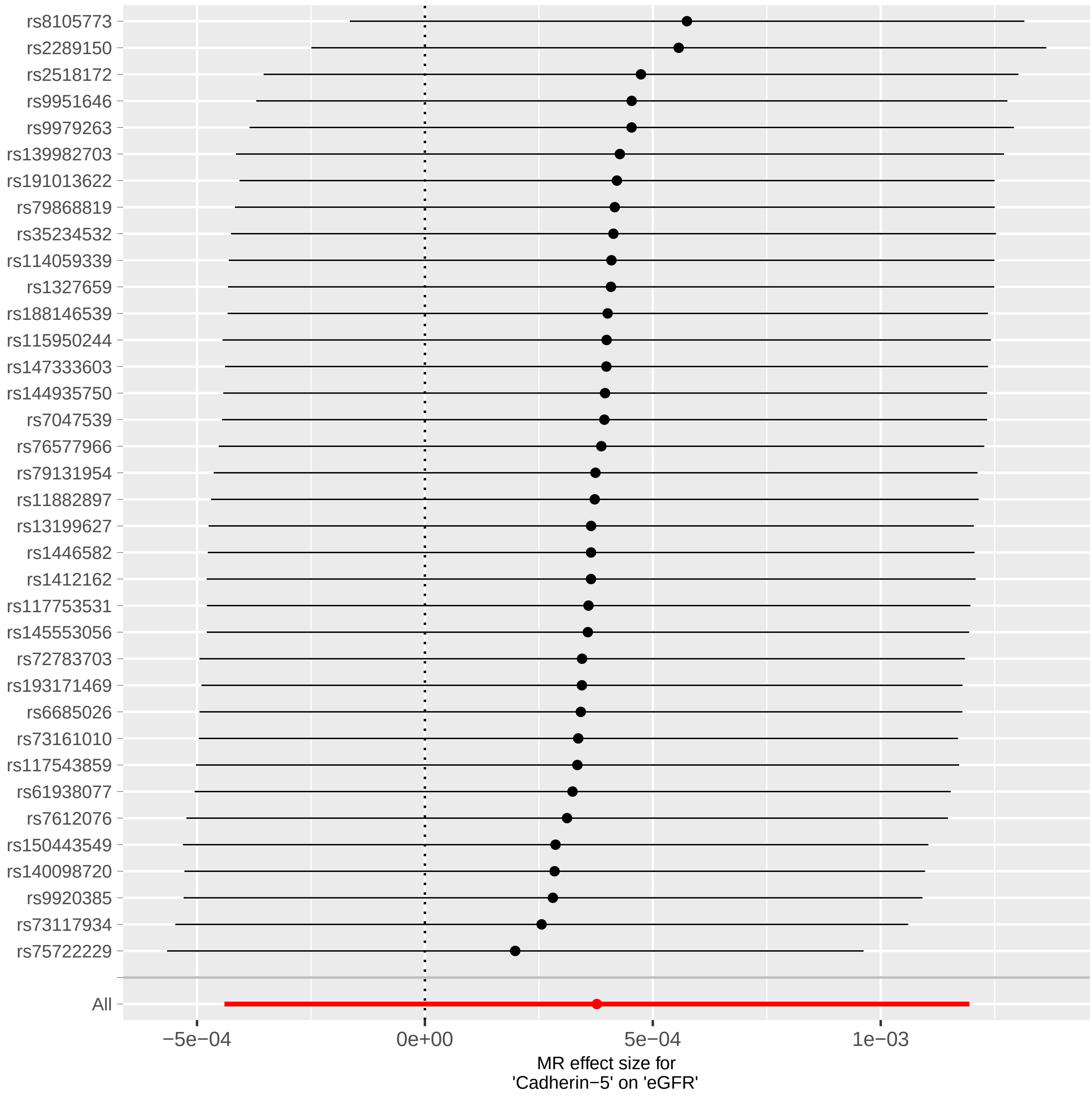

Batch 2−486 : Cadherin−7 on eGFR

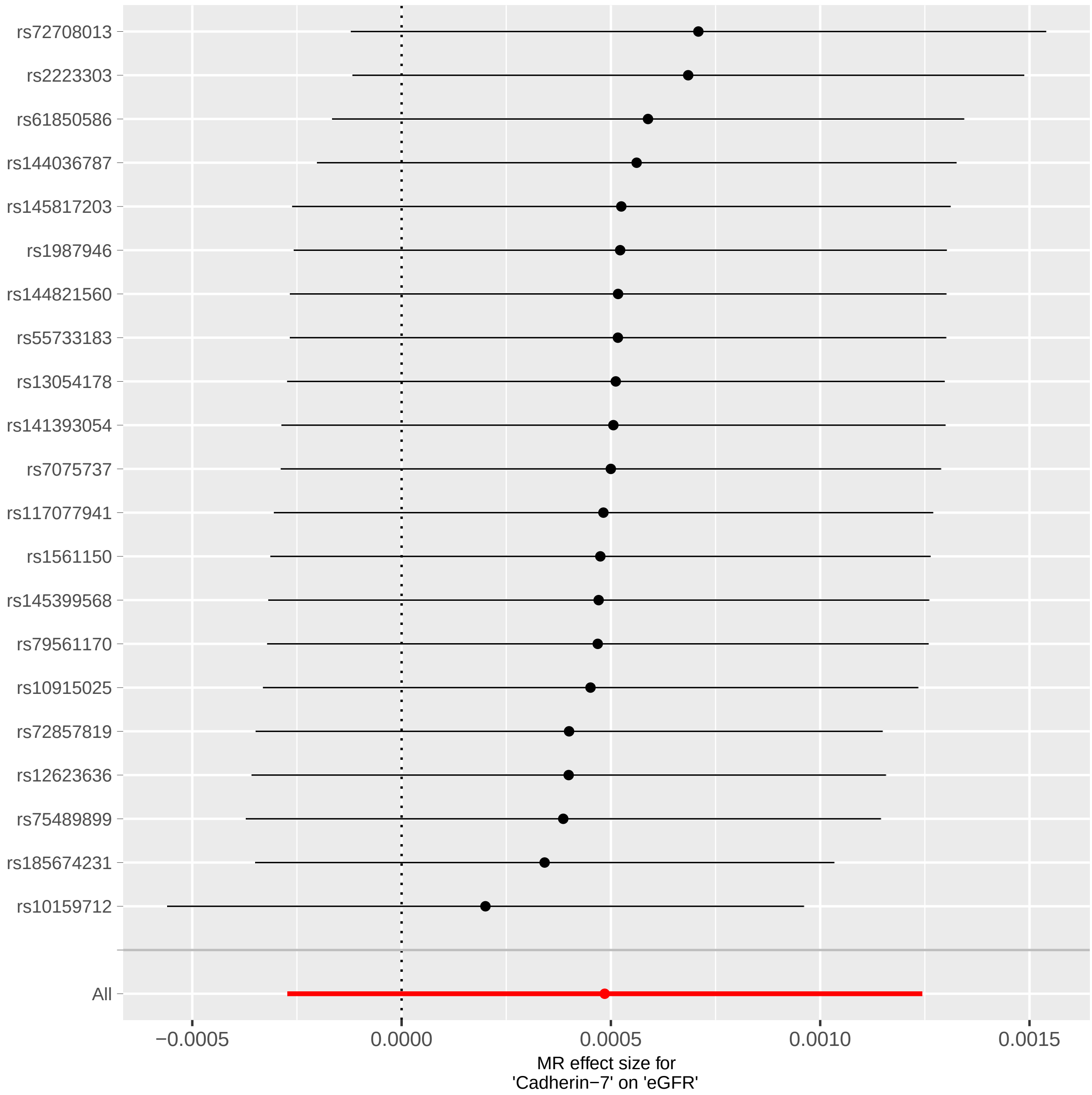

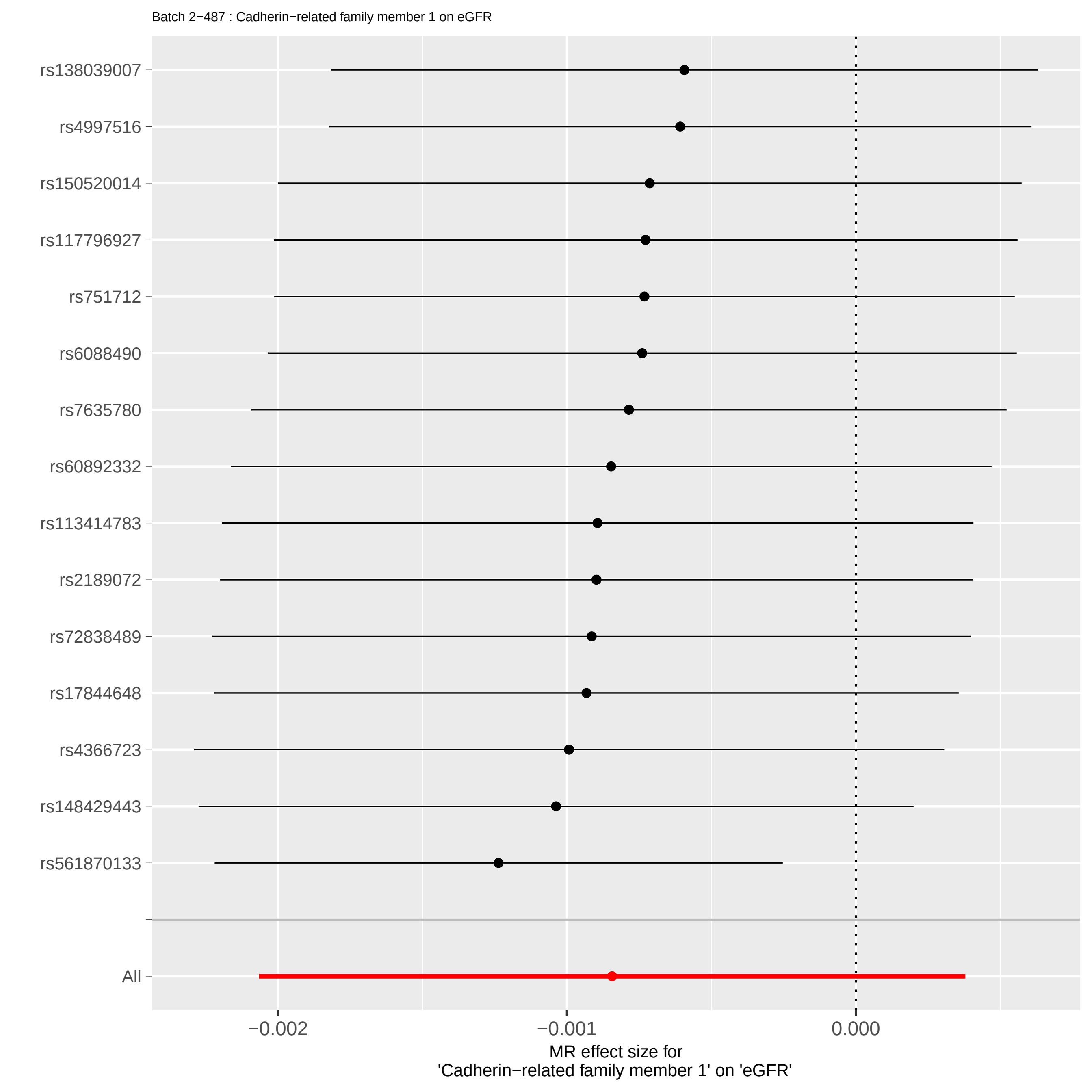
Batch 2−487 : Cadherin−related family member 1 on eGFR
rs138039007
rs4997516
rs150520014
rs117796927
rs751712
rs6088490
rs7635780
rs60892332
rs113414783
rs2189072
rs72838489
rs17844648
rs4366723
rs148429443
rs561870133
All
−0.002
−0.001
0.000
MR effect size for
'Cadherin−related family member 1' on 'eGFR'

Batch 2−488 : Cadherin−related family member 3 on eGFR

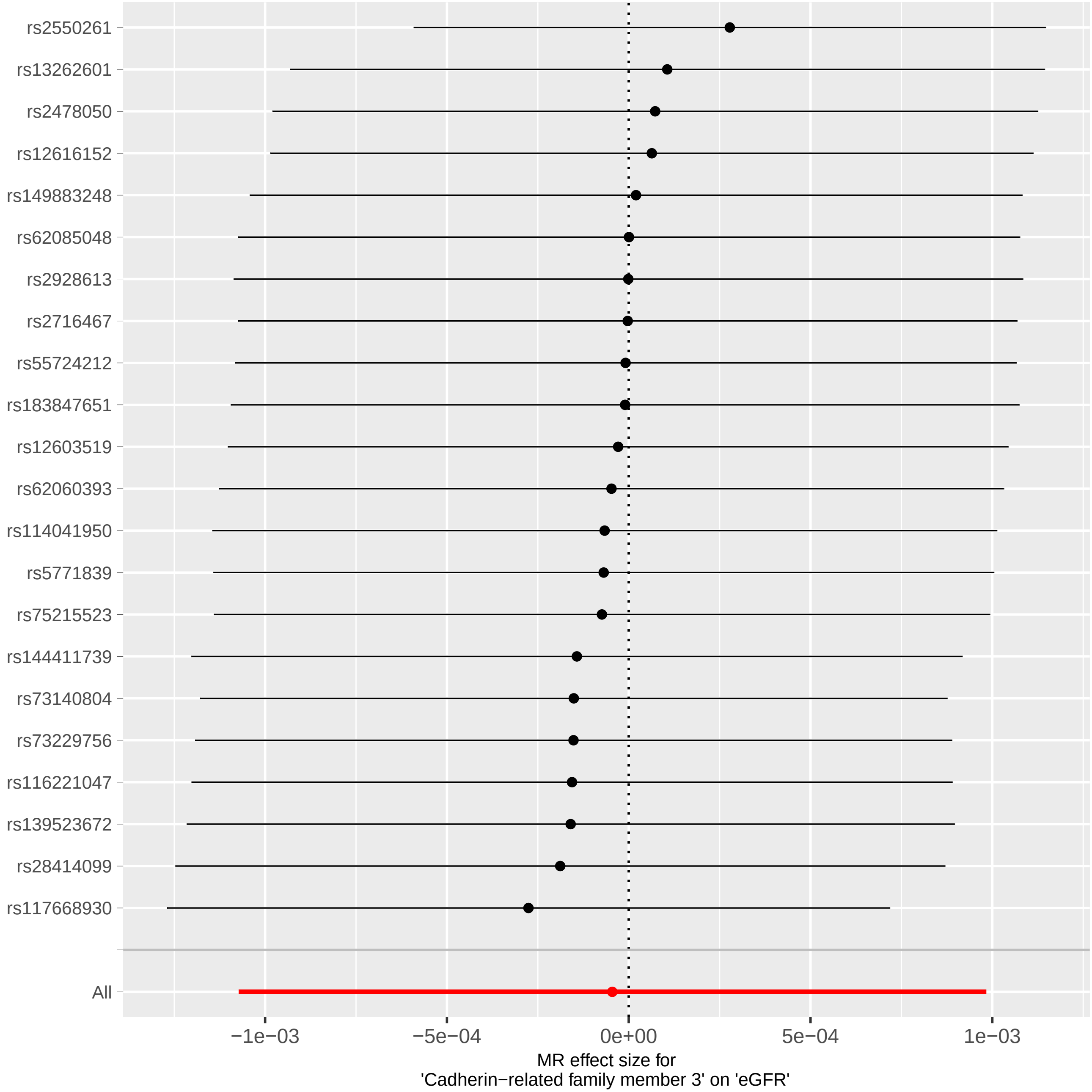

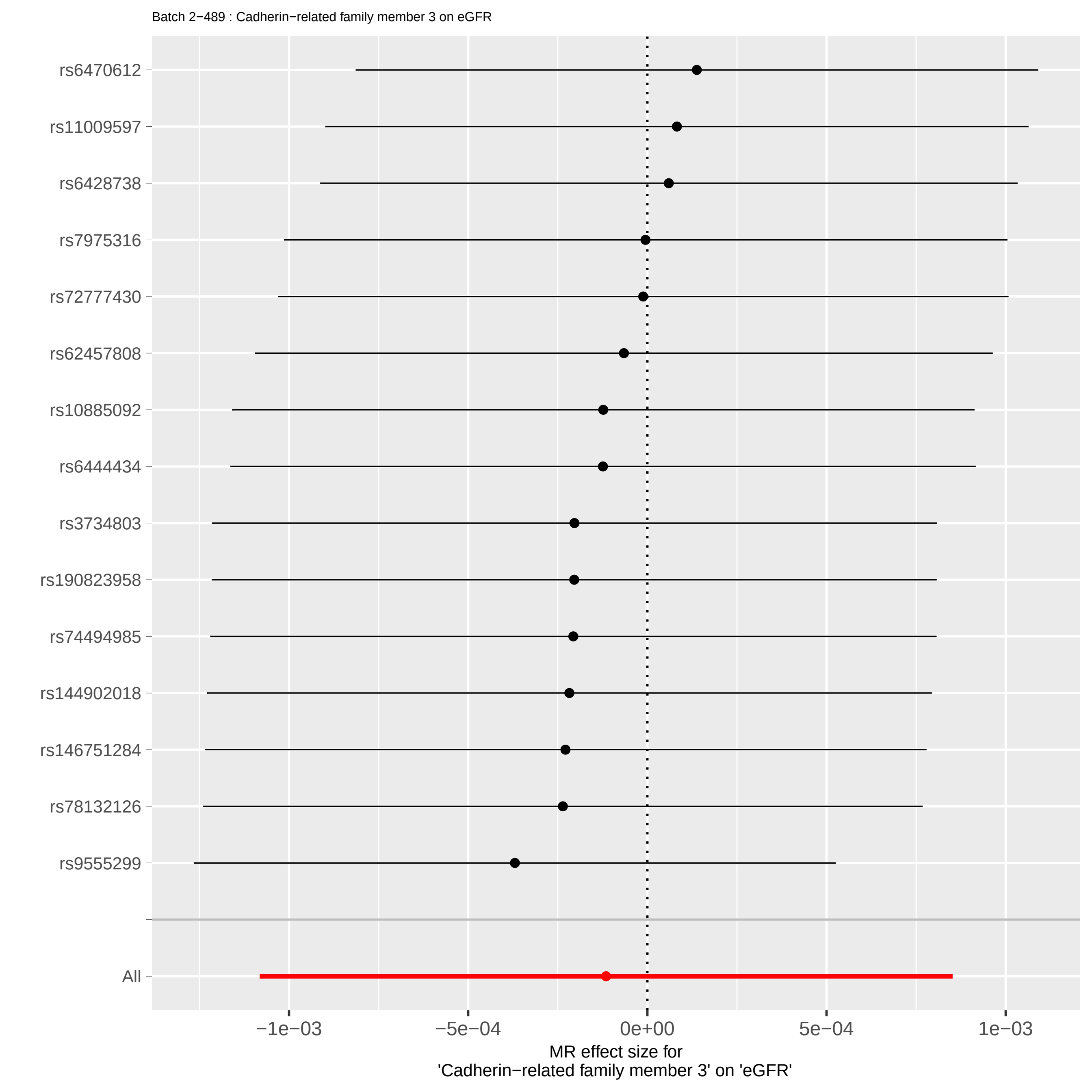

Batch 2−489 : Cadherin−related family member 3 on eGFR
rs6470612
rs11009597
rs6428738
rs7975316
rs72777430
rs62457808
rs10885092
rs6444434
rs3734803
rs190823958
rs74494985
rs144902018
rs146751284
rs78132126
rs9555299
All
−1e−03
−5e−04
0e+00
5e−04
1e−03
MR effect size for
'Cadherin−related family member 3' on 'eGFR'

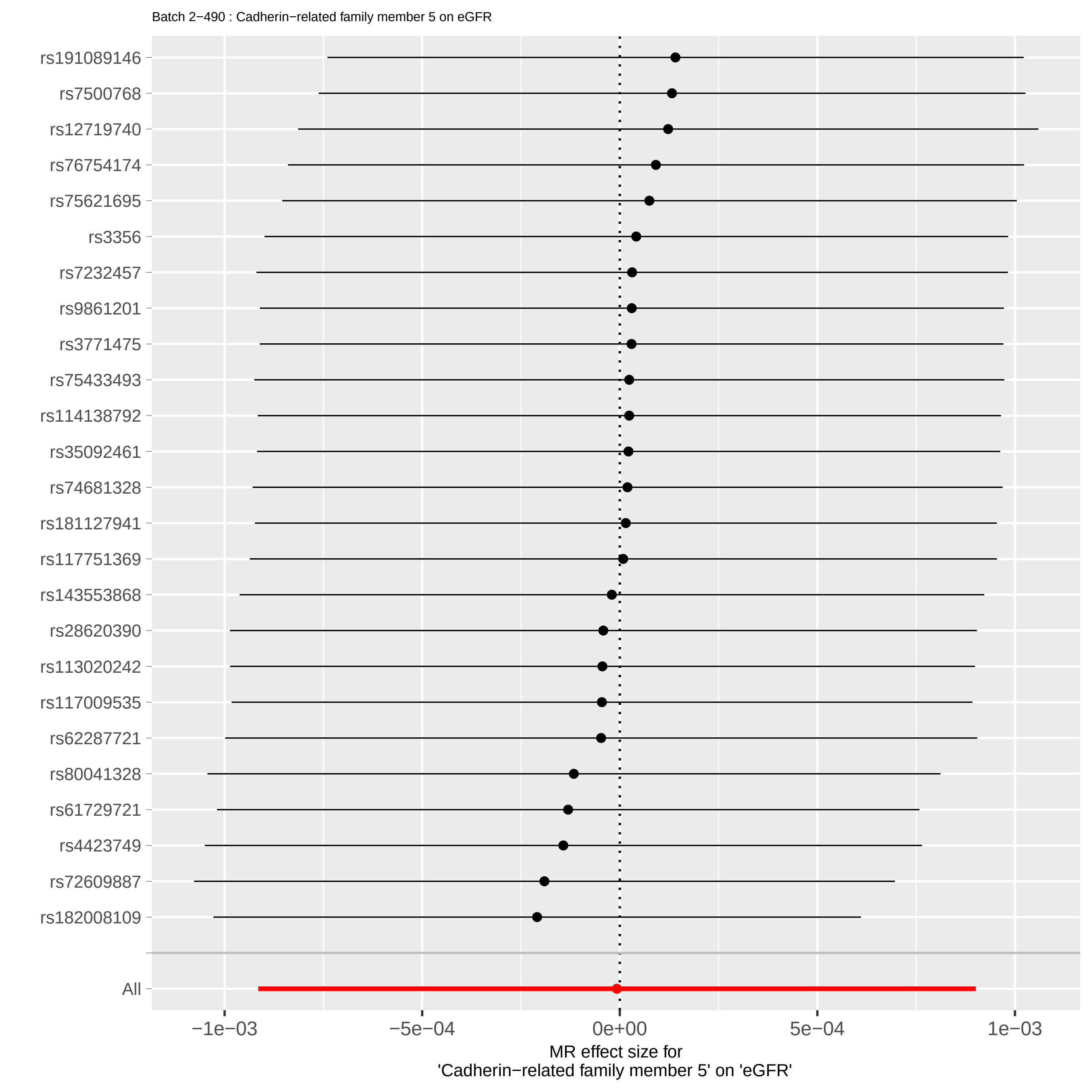

Batch 2−490 : Cadherin−related family member 5 on eGFR
rs191089146
rs7500768
rs12719740
rs76754174
rs75621695
rs3356
rs7232457
rs9861201
rs3771475
rs75433493
rs114138792
rs35092461
rs74681328
rs181127941
rs117751369
rs143553868
rs28620390
rs113020242
rs117009535
rs62287721
rs80041328
rs61729721
rs4423749
rs72609887
rs182008109
All
−1e−03
−5e−04
0e+00
5e−04
1e−03
MR effect size for
'Cadherin−related family member 5' on 'eGFR'

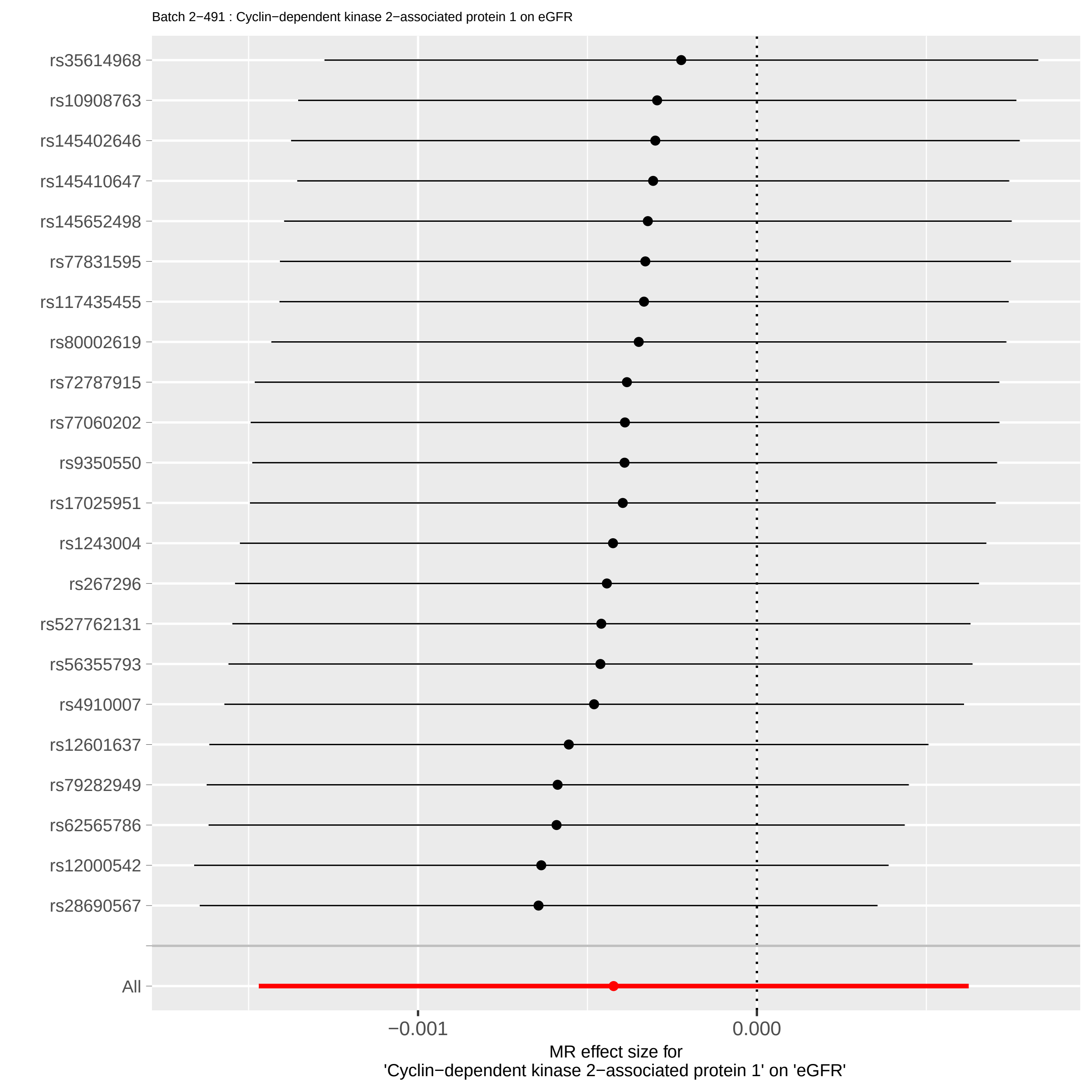

Batch 2−491 : Cyclin−dependent kinase 2−associated protein 1 on eGFR
rs35614968
rs10908763
rs145402646
rs145410647
rs145652498
rs77831595
rs117435455
rs80002619
rs72787915
rs77060202
rs9350550
rs17025951
rs1243004
rs267296
rs527762131
rs56355793
rs4910007
rs12601637
rs79282949
rs62565786
rs12000542
rs28690567
All
−0.001
0.000
MR effect size for
'Cyclin−dependent kinase 2−associated protein 1' on 'eGFR'

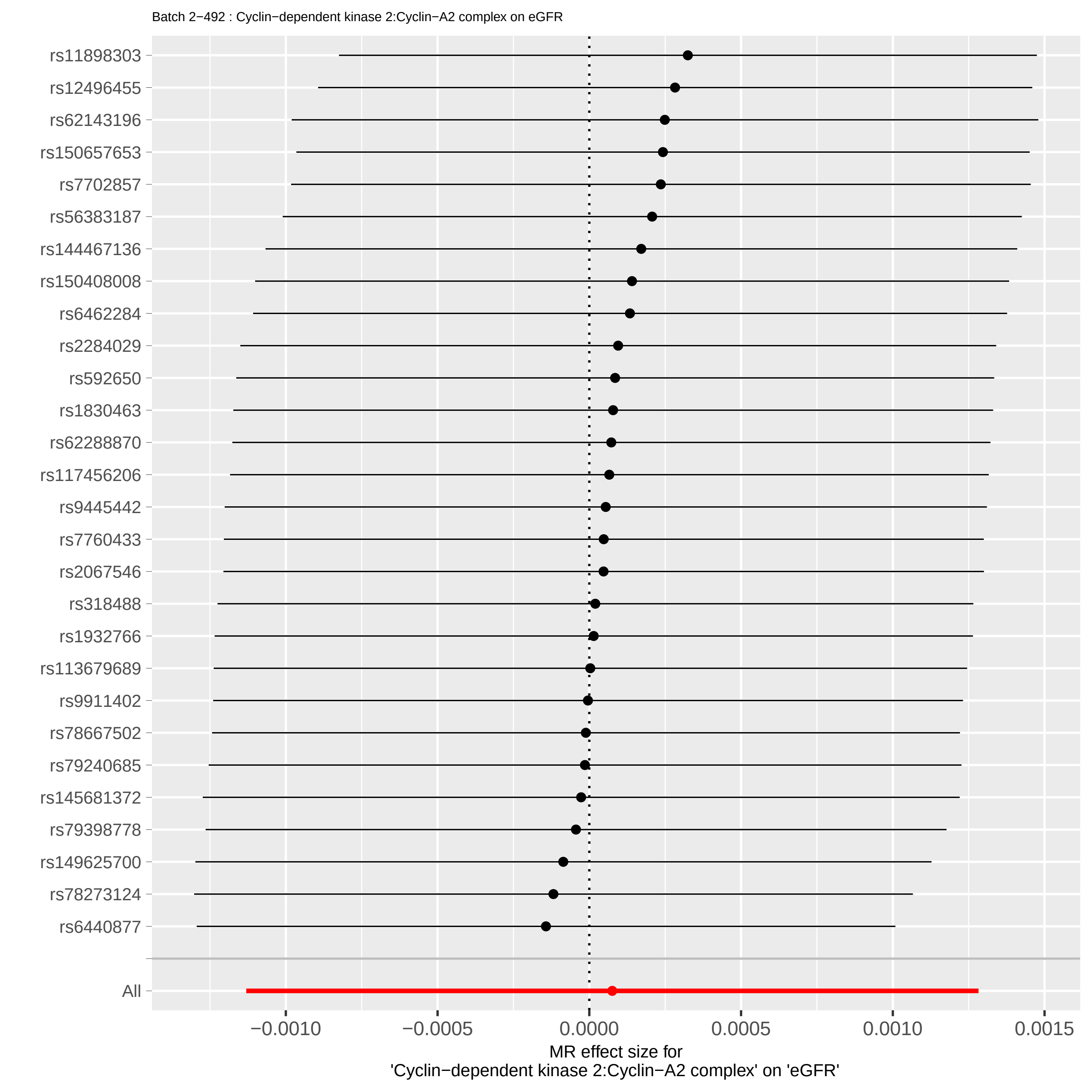

Batch 2−492 : Cyclin−dependent kinase 2:Cyclin−A2 complex on eGFR
rs11898303
rs12496455
rs62143196
rs150657653
rs7702857
rs56383187
rs144467136
rs150408008
rs6462284
rs2284029
rs592650
rs1830463
rs62288870
rs117456206
rs9445442
rs7760433
rs2067546
rs318488
rs1932766
rs113679689
rs9911402
rs78667502
rs79240685
rs145681372
rs79398778
rs149625700
rs78273124
rs6440877
All
−0.0010
−0.0005
0.0000
0.0005
0.0010
0.0015
MR effect size for
'Cyclin−dependent kinase 2:Cyclin−A2 complex' on 'eGFR'

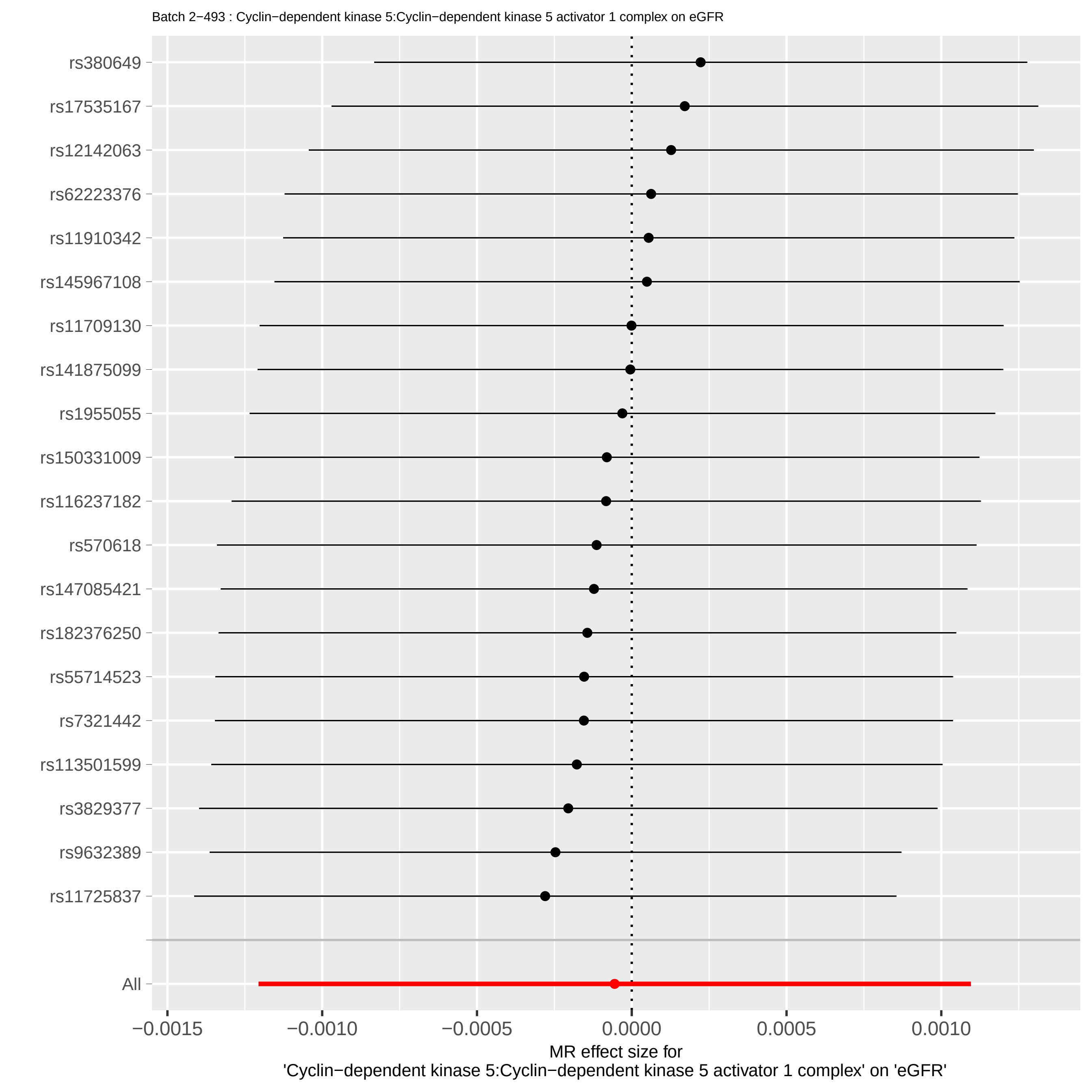

Batch 2−493 : Cyclin−dependent kinase 5:Cyclin−dependent kinase 5 activator 1 complex on eGFR
rs380649
rs17535167
rs12142063
rs62223376
rs11910342
rs145967108
rs11709130
rs141875099
rs1955055
rs150331009
rs116237182
rs570618
rs147085421
rs182376250
rs55714523
rs7321442
rs113501599
rs3829377
rs9632389
rs11725837
All
−0.0015
−0.0010
−0.0005
0.0000
0.0005
0.0010
MR effect size for
'Cyclin−dependent kinase 5:Cyclin−dependent kinase 5 activator 1 complex' on 'eGFR'

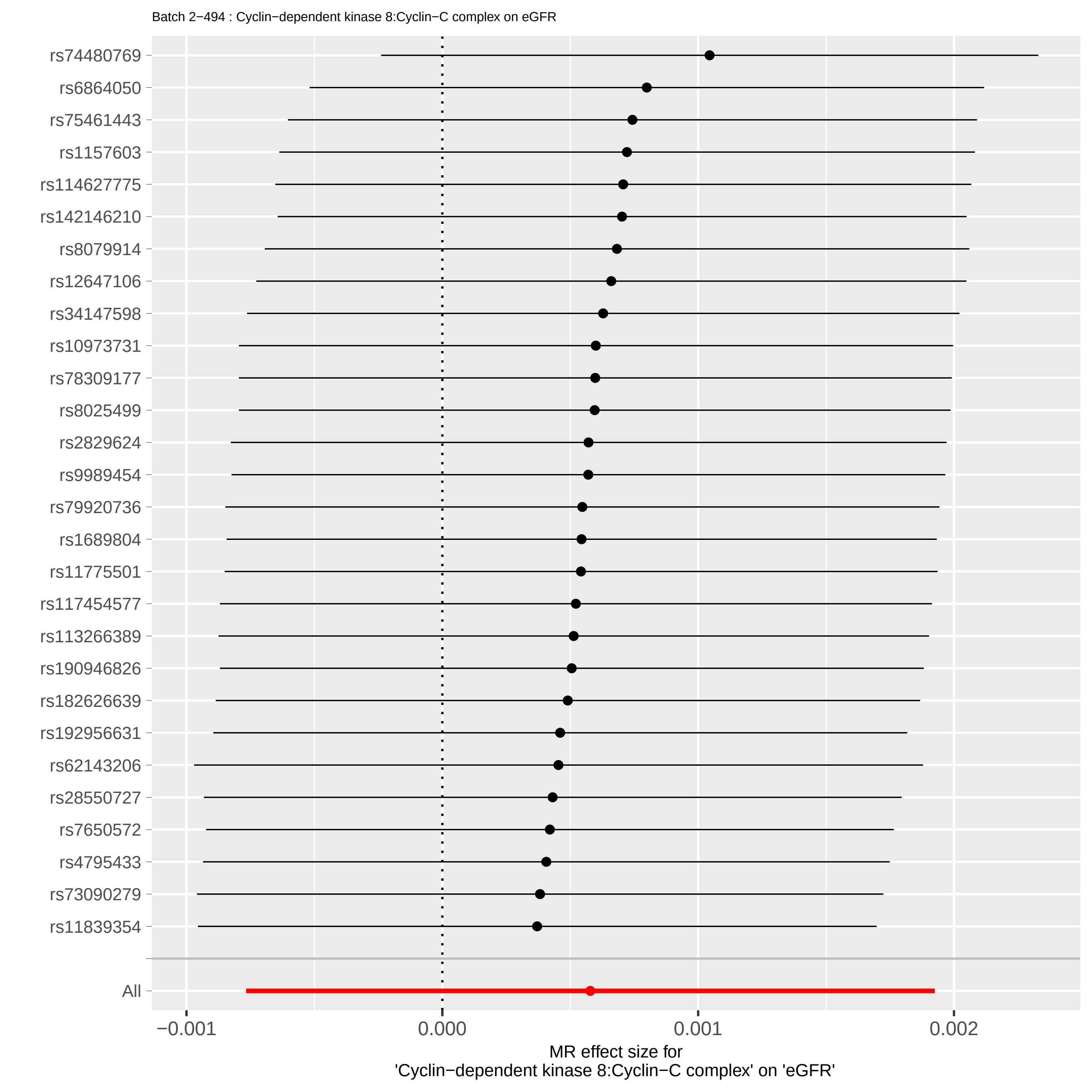

Batch 2−494 : Cyclin−dependent kinase 8:Cyclin−C complex on eGFR
rs74480769
rs6864050
rs75461443
rs1157603
rs114627775
rs142146210
rs8079914
rs12647106
rs34147598
rs10973731
rs78309177
rs8025499
rs2829624
rs9989454
rs79920736
rs1689804
rs11775501
rs117454577
rs113266389
rs190946826
rs182626639
rs192956631
rs62143206
rs28550727
rs7650572
rs4795433
rs73090279
rs11839354
All
−0.001
0.000
0.001
0.002
MR effect size for
'Cyclin−dependent kinase 8:Cyclin−C complex' on 'eGFR'

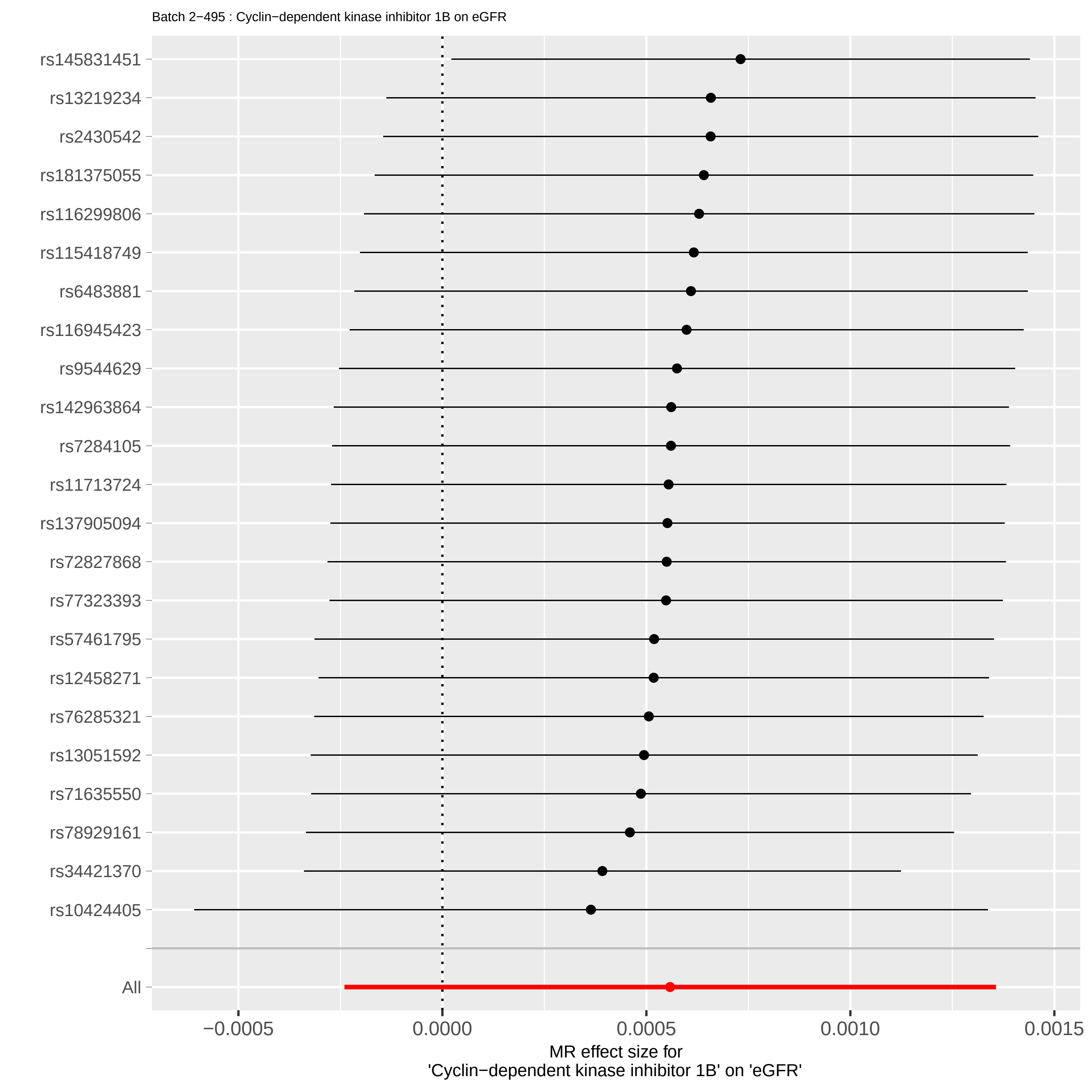
Batch 2−495 : Cyclin−dependent kinase inhibitor 1B on eGFR
rs145831451
rs13219234
rs2430542
rs181375055
rs116299806
rs115418749
rs6483881
rs116945423
rs9544629
rs142963864
rs7284105
rs11713724
rs137905094
rs72827868
rs77323393
rs57461795
rs12458271
rs76285321
rs13051592
rs71635550
rs78929161
rs34421370
rs10424405
All
−0.0005
0.0000
0.0005
0.0010
0.0015
MR effect size for
'Cyclin−dependent kinase inhibitor 1B' on 'eGFR'

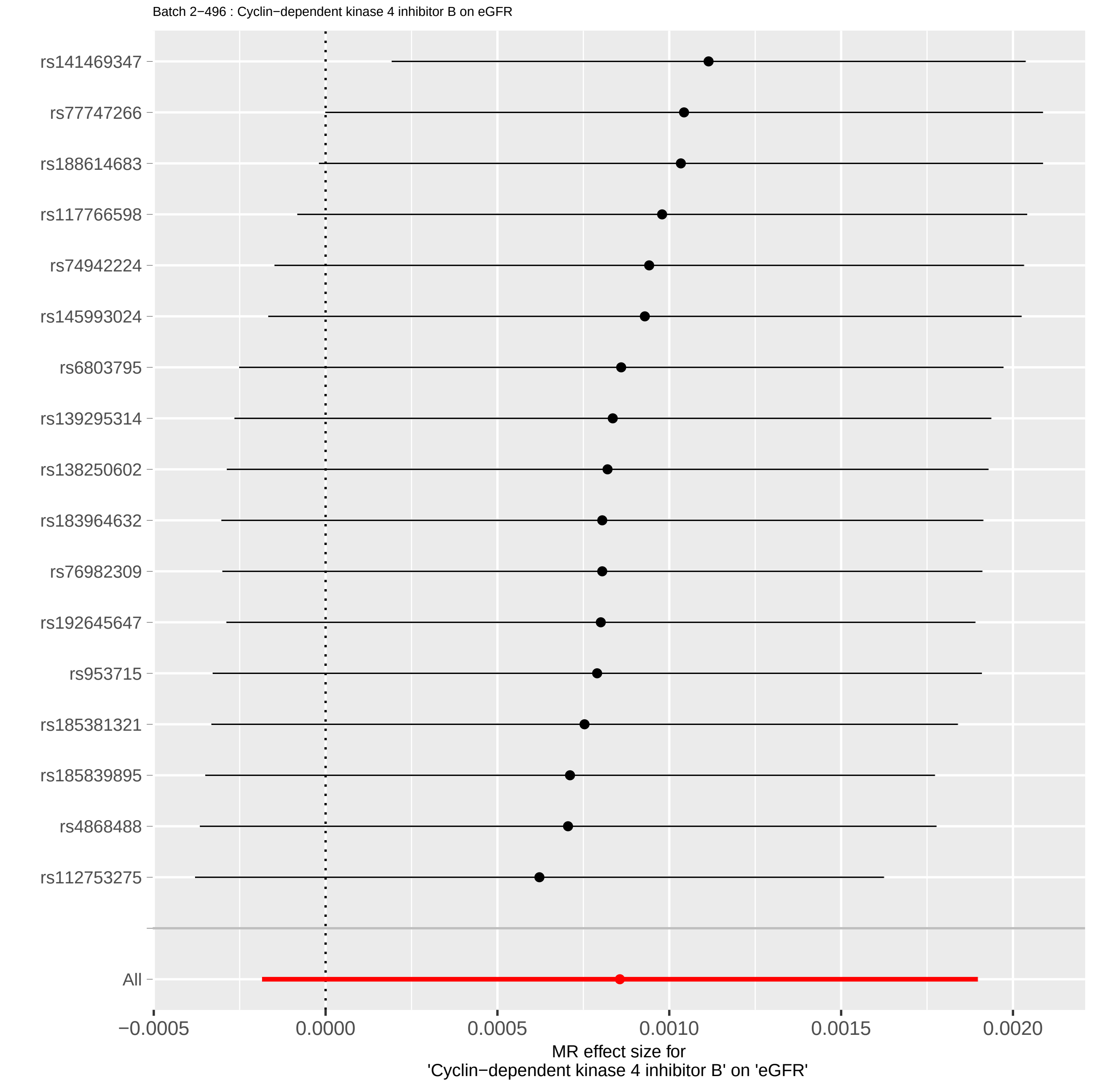

Batch 2−496 : Cyclin−dependent kinase 4 inhibitor B on eGFR
rs141469347
rs77747266
rs188614683
rs117766598
rs74942224
rs145993024
rs6803795
rs139295314
rs138250602
rs183964632
rs76982309
rs192645647
rs953715
rs185381321
rs185839895
rs4868488
rs112753275
All
−0.0005
0.0000
0.0005
0.0010
0.0015
0.0020
MR effect size for
'Cyclin−dependent kinase 4 inhibitor B' on 'eGFR'

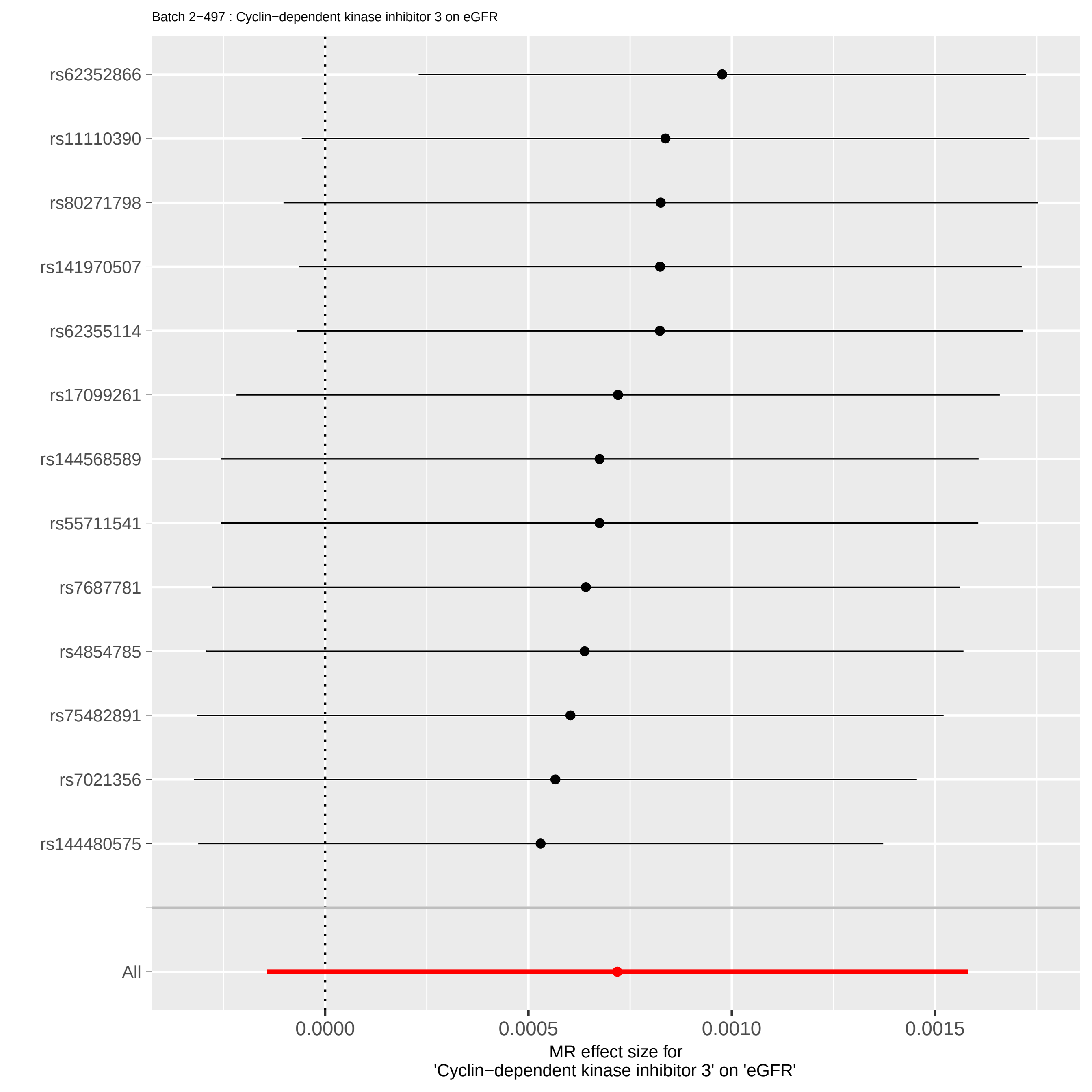

Batch 2−497 : Cyclin−dependent kinase inhibitor 3 on eGFR
rs62352866
rs11110390
rs80271798
rs141970507
rs62355114
rs17099261
rs144568589
rs55711541
rs7687781
rs4854785
rs75482891
rs7021356
rs144480575
All
0.0000
0.0005
0.0010
0.0015
MR effect size for
'Cyclin−dependent kinase inhibitor 3' on 'eGFR'

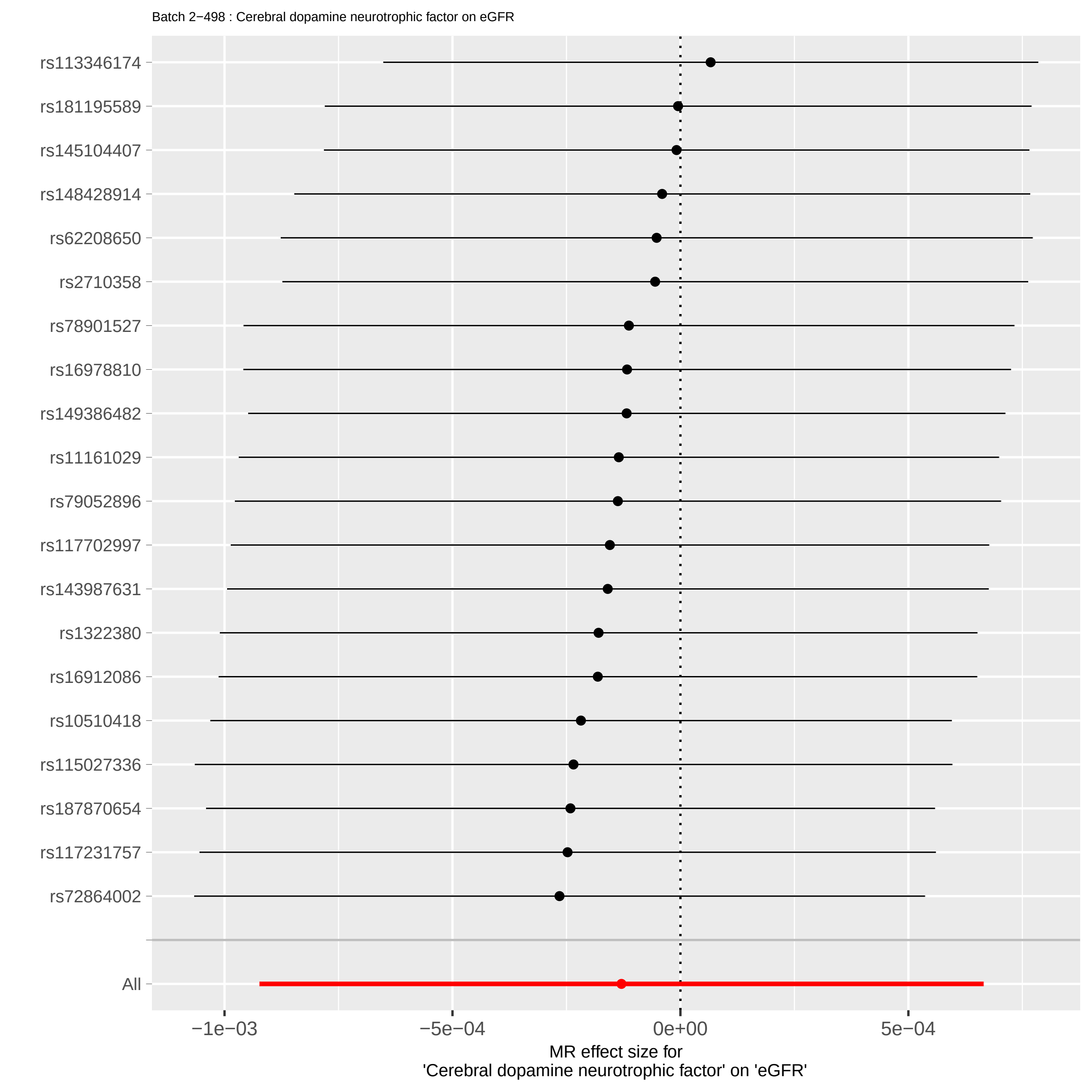

Batch 2−498 : Cerebral dopamine neurotrophic factor on eGFR
rs113346174
rs181195589
rs145104407
rs148428914
rs62208650
rs2710358
rs78901527
rs16978810
rs149386482
rs11161029
rs79052896
rs117702997
rs143987631
rs1322380
rs16912086
rs10510418
rs115027336
rs187870654
rs117231757
rs72864002
All
−1e−03
−5e−04
0e+00
5e−04
MR effect size for
'Cerebral dopamine neurotrophic factor' on 'eGFR'

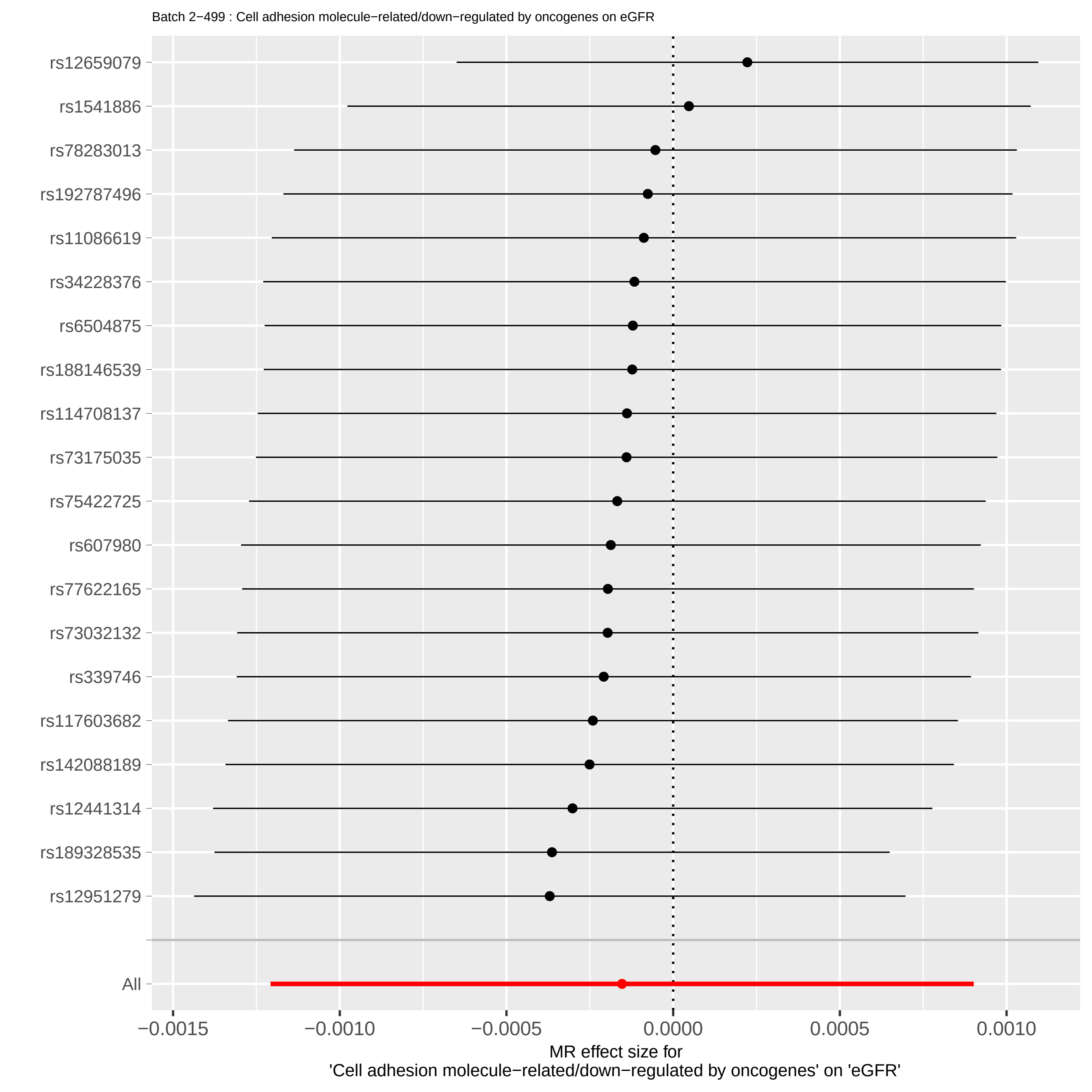
Batch 2−499 : Cell adhesion molecule−related/down−regulated by oncogenes on eGFR
rs12659079
rs1541886
rs78283013
rs192787496
rs11086619
rs34228376
rs6504875
rs188146539
rs114708137
rs73175035
rs75422725
rs607980
rs77622165
rs73032132
rs339746
rs117603682
rs142088189
rs12441314
rs189328535
rs12951279
All
−0.0015
−0.0010
−0.0005
0.0000
0.0005
0.0010
MR effect size for
'Cell adhesion molecule−related/down−regulated by oncogenes' on 'eGFR'

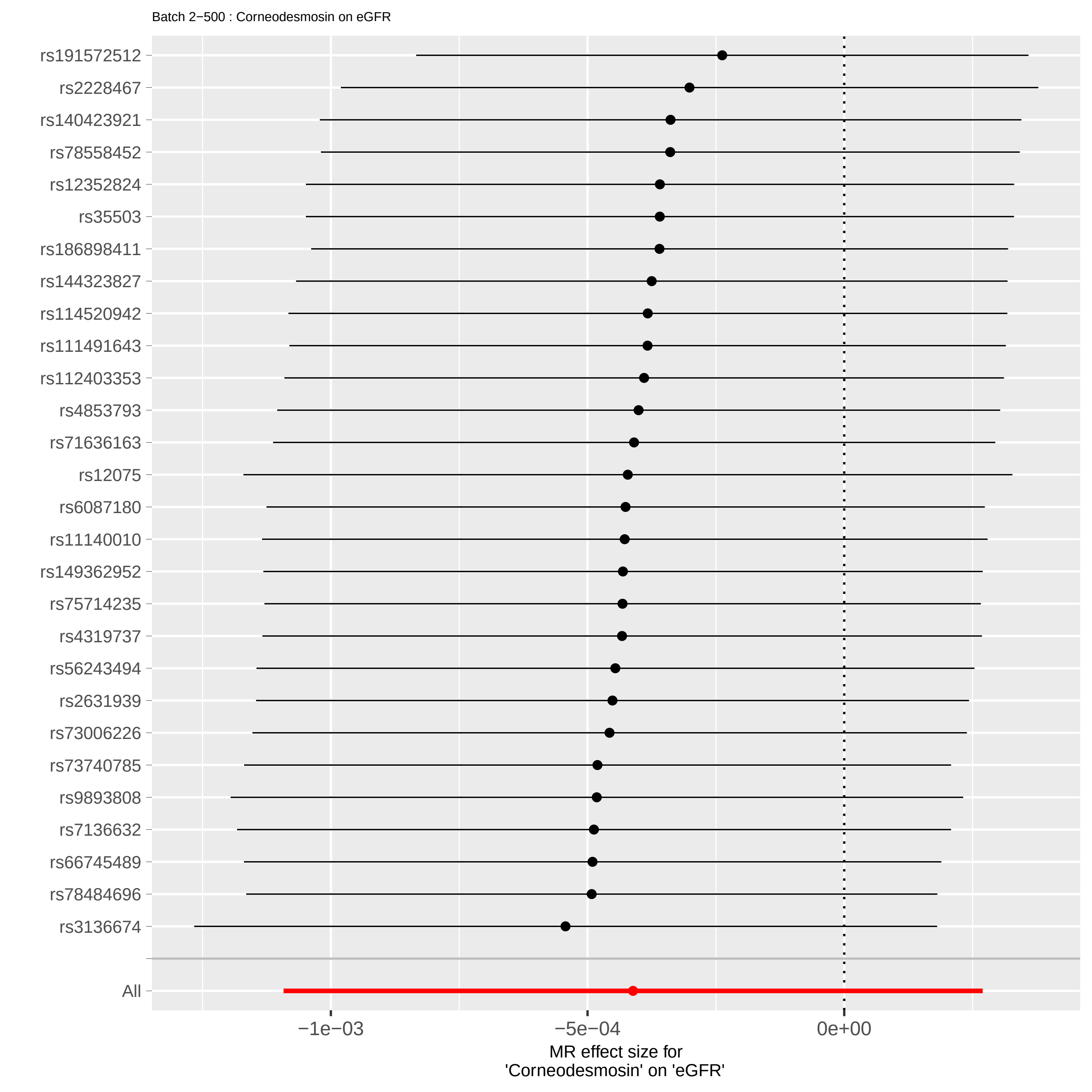

Batch 2−500 : Corneodesmosin on eGFR
rs191572512
rs2228467
rs140423921
rs78558452
rs12352824
rs35503
rs186898411
rs144323827
rs114520942
rs111491643
rs112403353
rs4853793
rs71636163
rs12075
rs6087180
rs11140010
rs149362952
rs75714235
rs4319737
rs56243494
rs2631939
rs73006226
rs73740785
rs9893808
rs7136632
rs66745489
rs78484696
rs3136674
All
−1e−03
−5e−04
0e+00
MR effect size for
'Corneodesmosin' on 'eGFR'

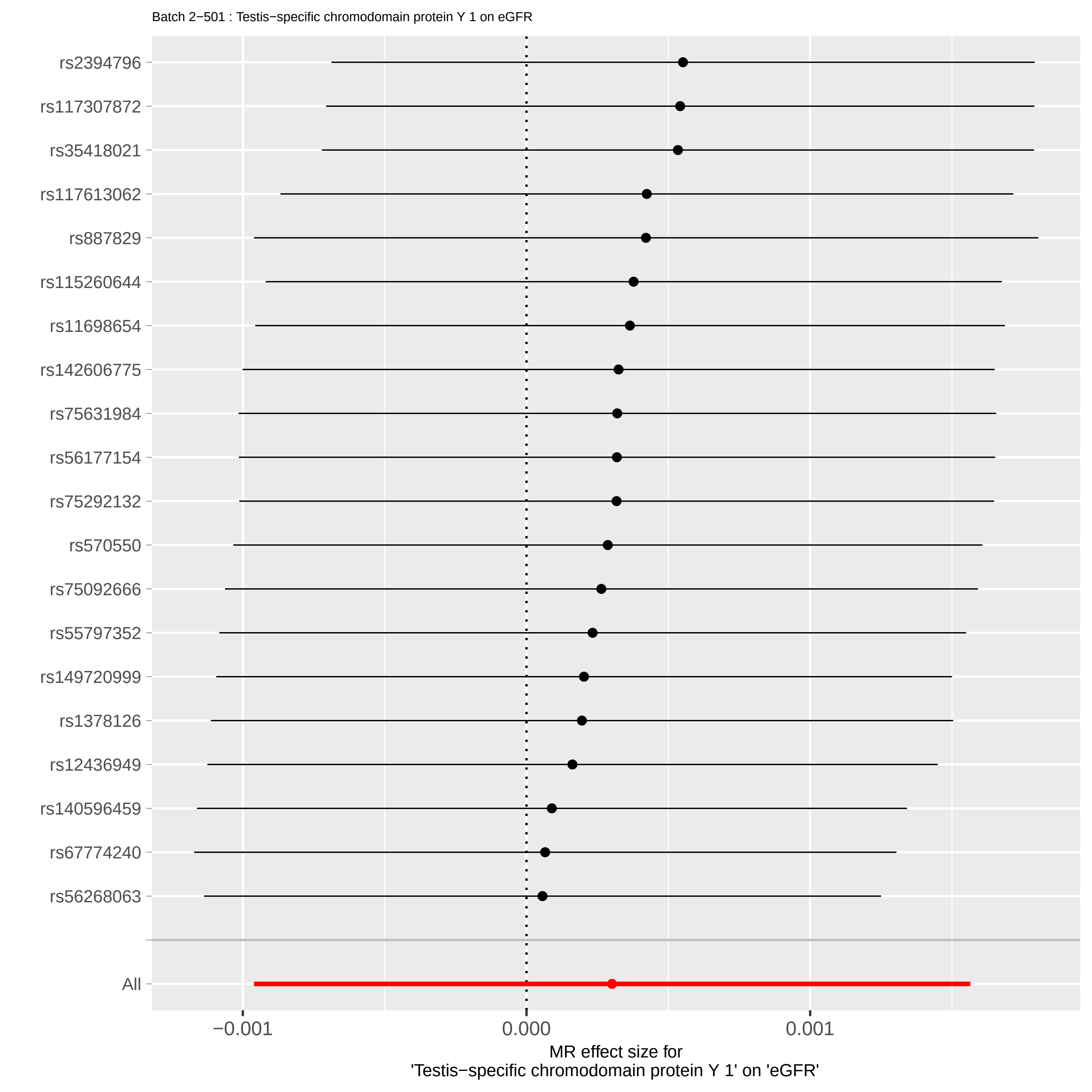

Batch 2−501 : Testis−specific chromodomain protein Y 1 on eGFR
rs2394796
rs117307872
rs35418021
rs117613062
rs887829
rs115260644
rs11698654
rs142606775
rs75631984
rs56177154
rs75292132
rs570550
rs75092666
rs55797352
rs149720999
rs1378126
rs12436949
rs140596459
rs67774240
rs56268063
All
−0.001
0.000
0.001
MR effect size for
'Testis−specific chromodomain protein Y 1' on 'eGFR'

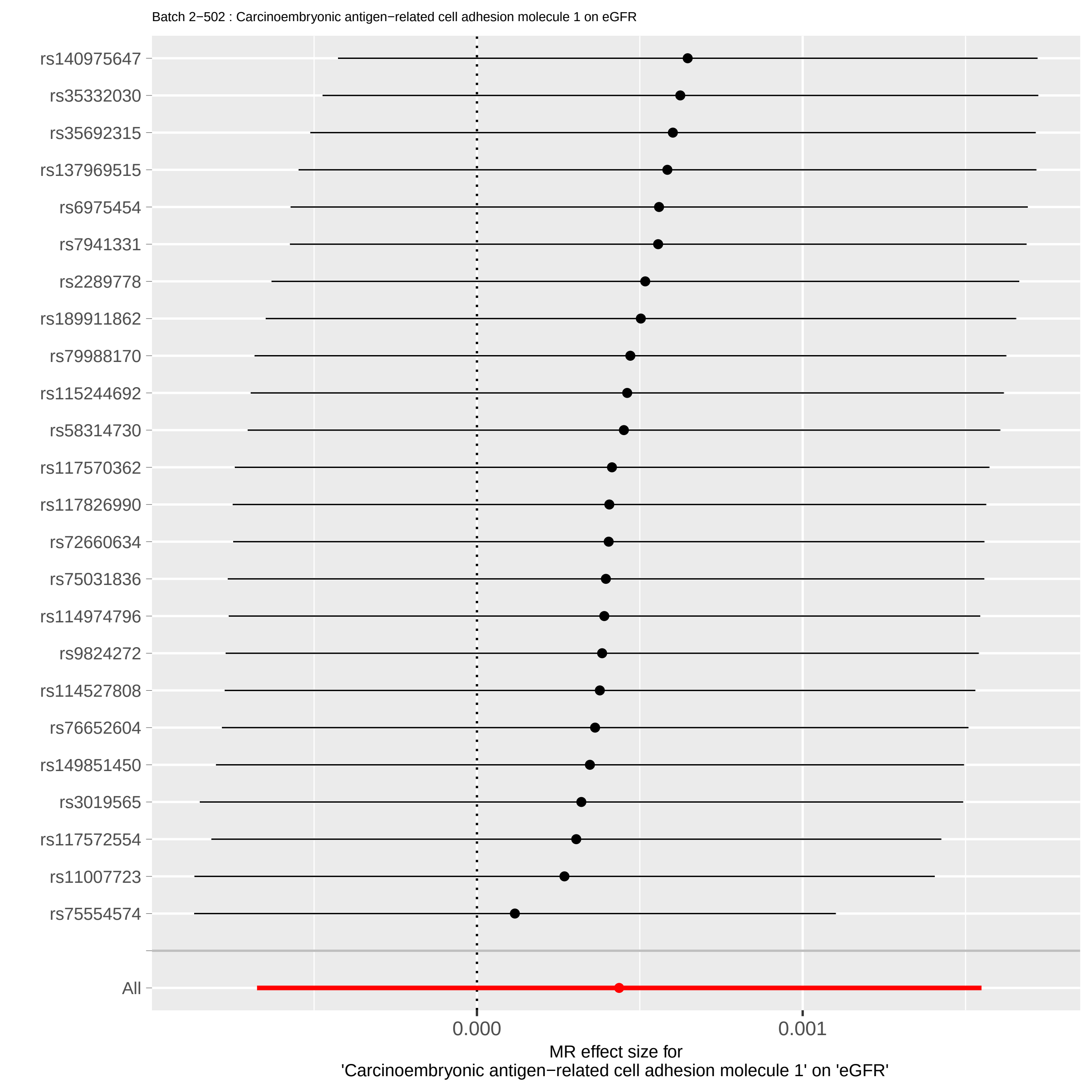
Batch 2−502 : Carcinoembryonic antigen−related cell adhesion molecule 1 on eGFR
rs140975647
rs35332030
rs35692315
rs137969515
rs6975454
rs7941331
rs2289778
rs189911862
rs79988170
rs115244692
rs58314730
rs117570362
rs117826990
rs72660634
rs75031836
rs114974796
rs9824272
rs114527808
rs76652604
rs149851450
rs3019565
rs117572554
rs11007723
rs75554574
All
0.000
0.001
MR effect size for
'Carcinoembryonic antigen−related cell adhesion molecule 1' on 'eGFR'

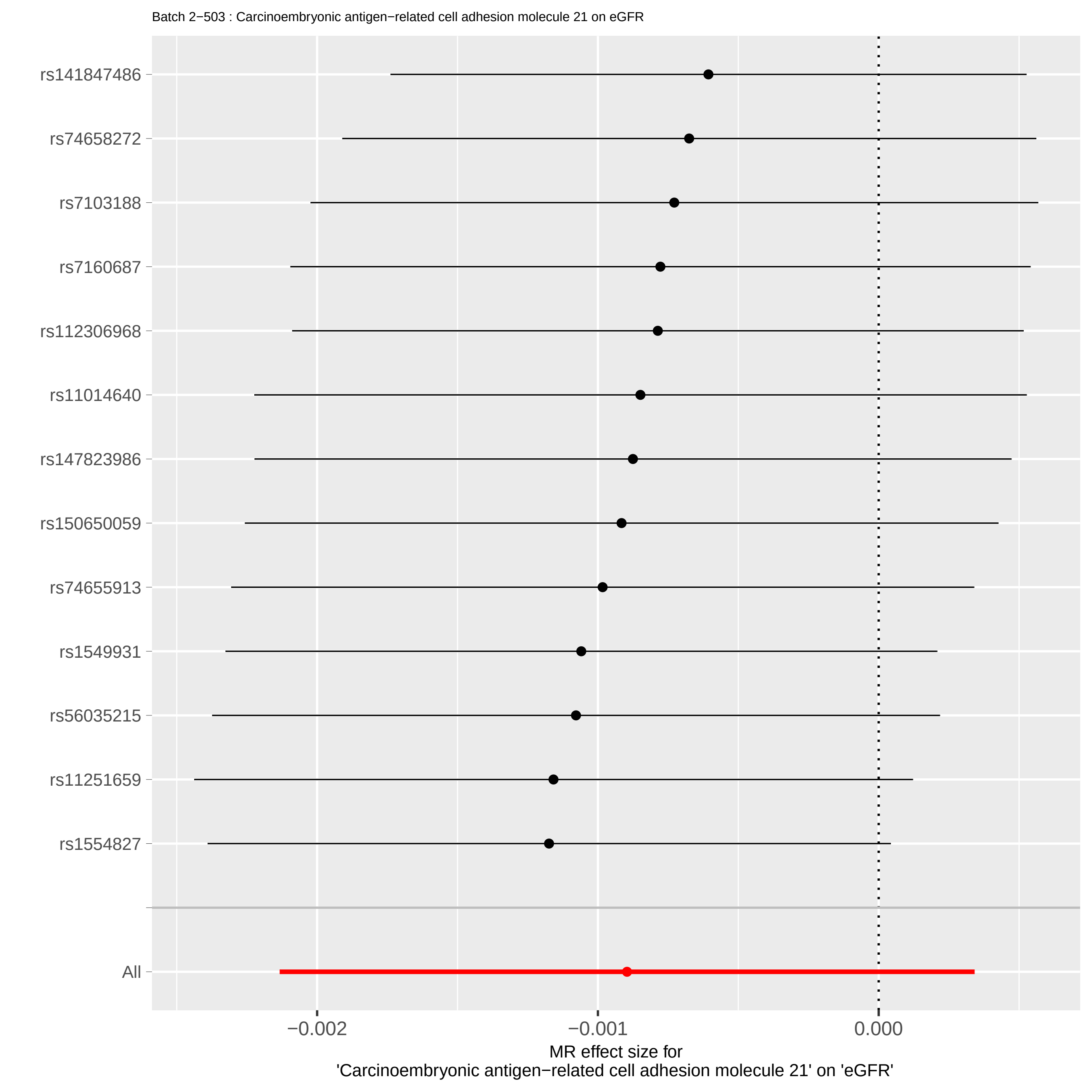

Batch 2−503 : Carcinoembryonic antigen−related cell adhesion molecule 21 on eGFR
rs141847486
rs74658272
rs7103188
rs7160687
rs112306968
rs11014640
rs147823986
rs150650059
rs74655913
rs1549931
rs56035215
rs11251659
rs1554827
All
−0.002
−0.001
0.000
MR effect size for
'Carcinoembryonic antigen−related cell adhesion molecule 21' on 'eGFR'

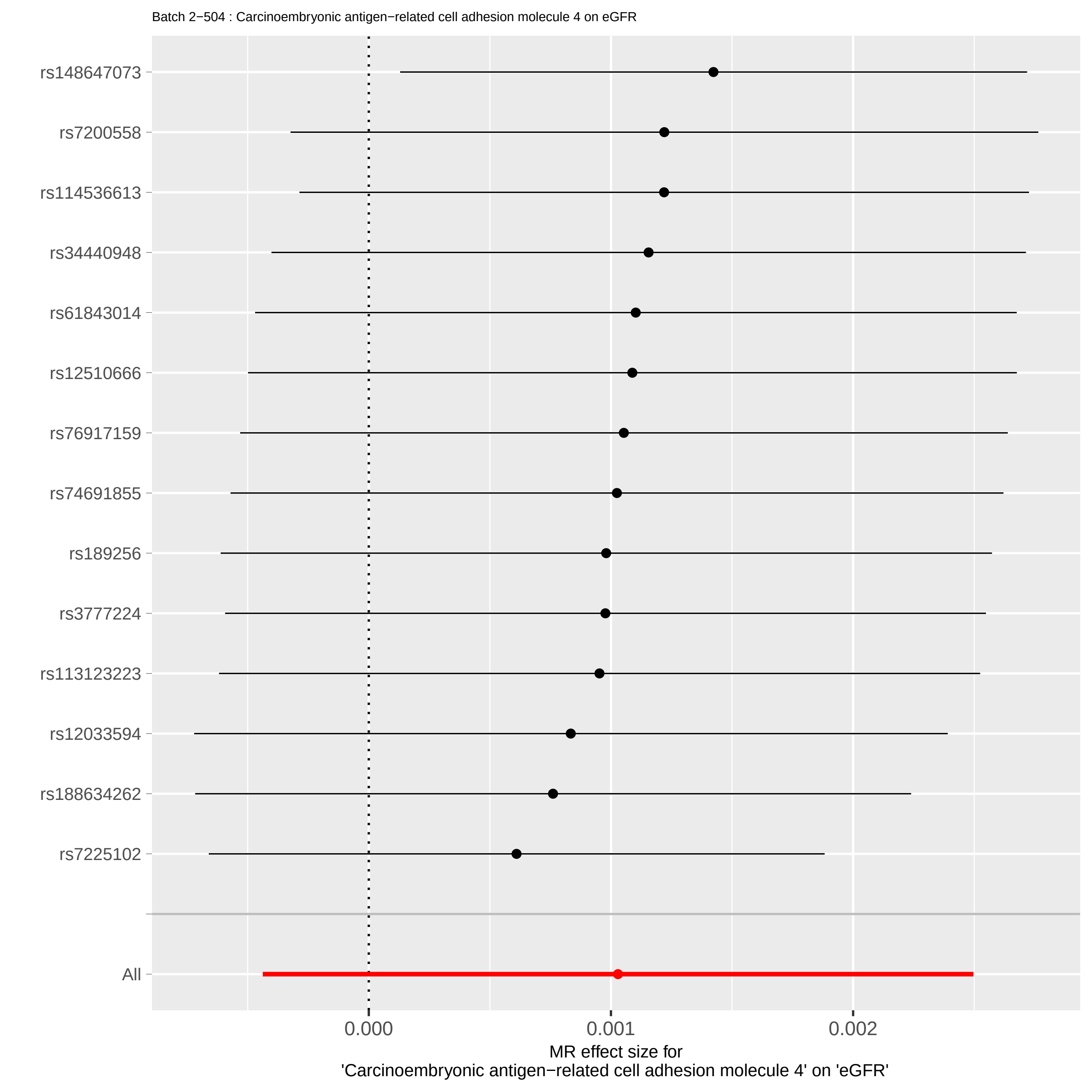

Batch 2−504 : Carcinoembryonic antigen−related cell adhesion molecule 4 on eGFR
rs148647073
rs7200558
rs114536613
rs34440948
rs61843014
rs12510666
rs76917159
rs74691855
rs189256
rs3777224
rs113123223
rs12033594
rs188634262
rs7225102
All
0.000
0.001
0.002
MR effect size for
'Carcinoembryonic antigen−related cell adhesion molecule 4' on 'eGFR'

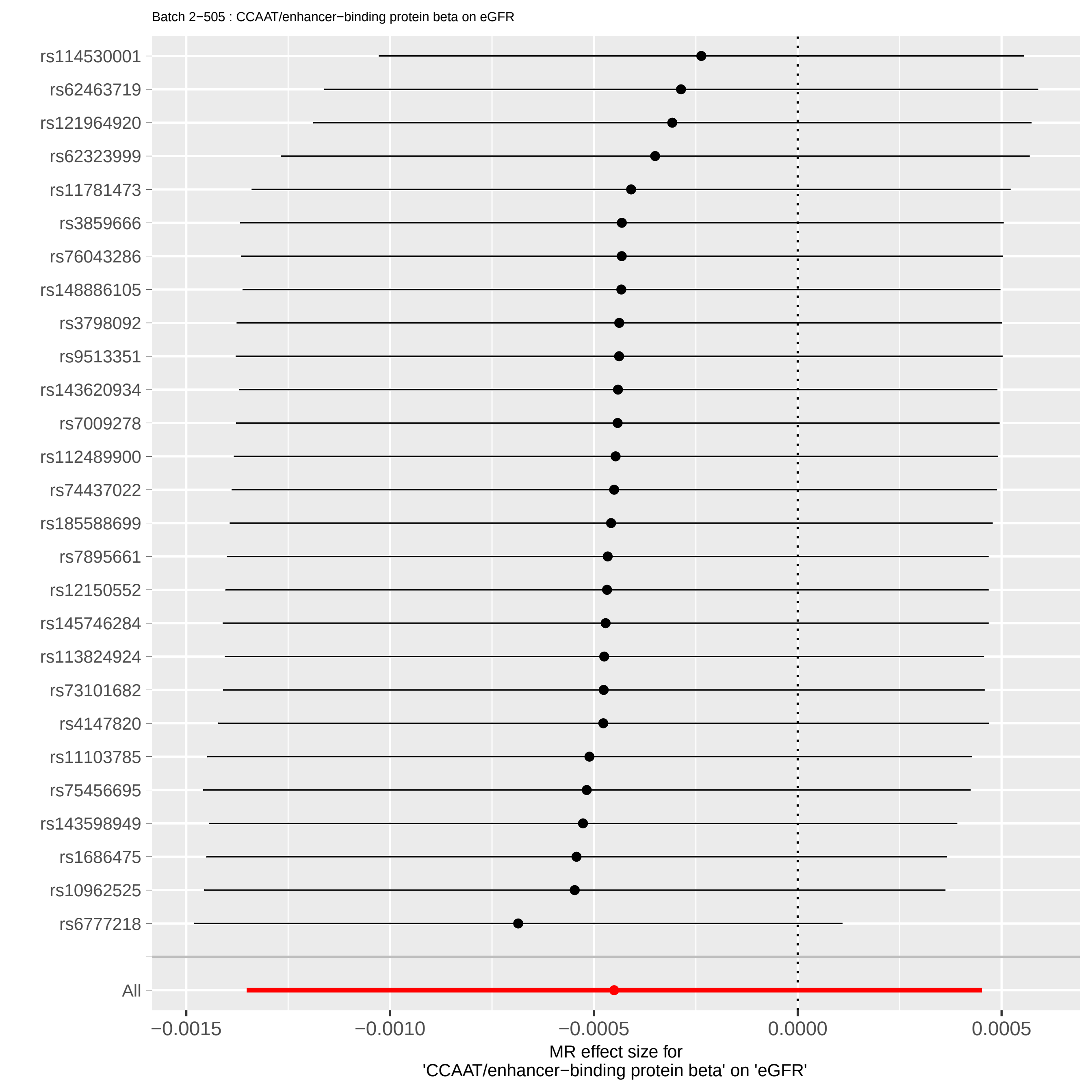

Batch 2−505 : CCAAT/enhancer−binding protein beta on eGFR
rs114530001
rs62463719
rs121964920
rs62323999
rs11781473
rs3859666
rs76043286
rs148886105
rs3798092
rs9513351
rs143620934
rs7009278
rs112489900
rs74437022
rs185588699
rs7895661
rs12150552
rs145746284
rs113824924
rs73101682
rs4147820
rs11103785
rs75456695
rs143598949
rs1686475
rs10962525
rs6777218
All
−0.0015
−0.0010
−0.0005
0.0000
0.0005
MR effect size for
'CCAAT/enhancer−binding protein beta' on 'eGFR'

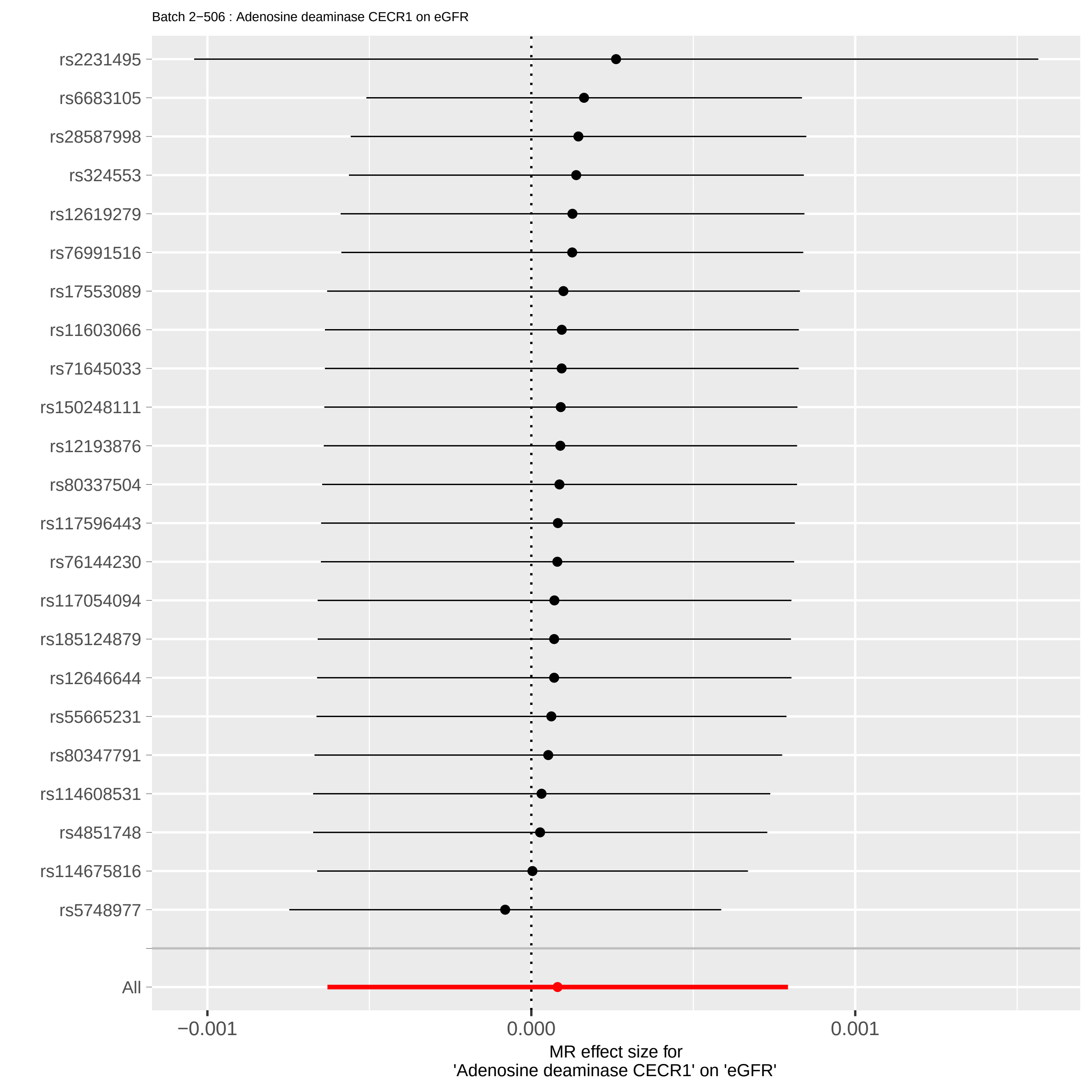

Batch 2−506 : Adenosine deaminase CECR1 on eGFR
rs2231495
rs6683105
rs28587998
rs324553
rs12619279
rs76991516
rs17553089
rs11603066
rs71645033
rs150248111
rs12193876
rs80337504
rs117596443
rs76144230
rs117054094
rs185124879
rs12646644
rs55665231
rs80347791
rs114608531
rs4851748
rs114675816
rs5748977
All
−0.001
0.000
0.001
MR effect size for
'Adenosine deaminase CECR1' on 'eGFR'

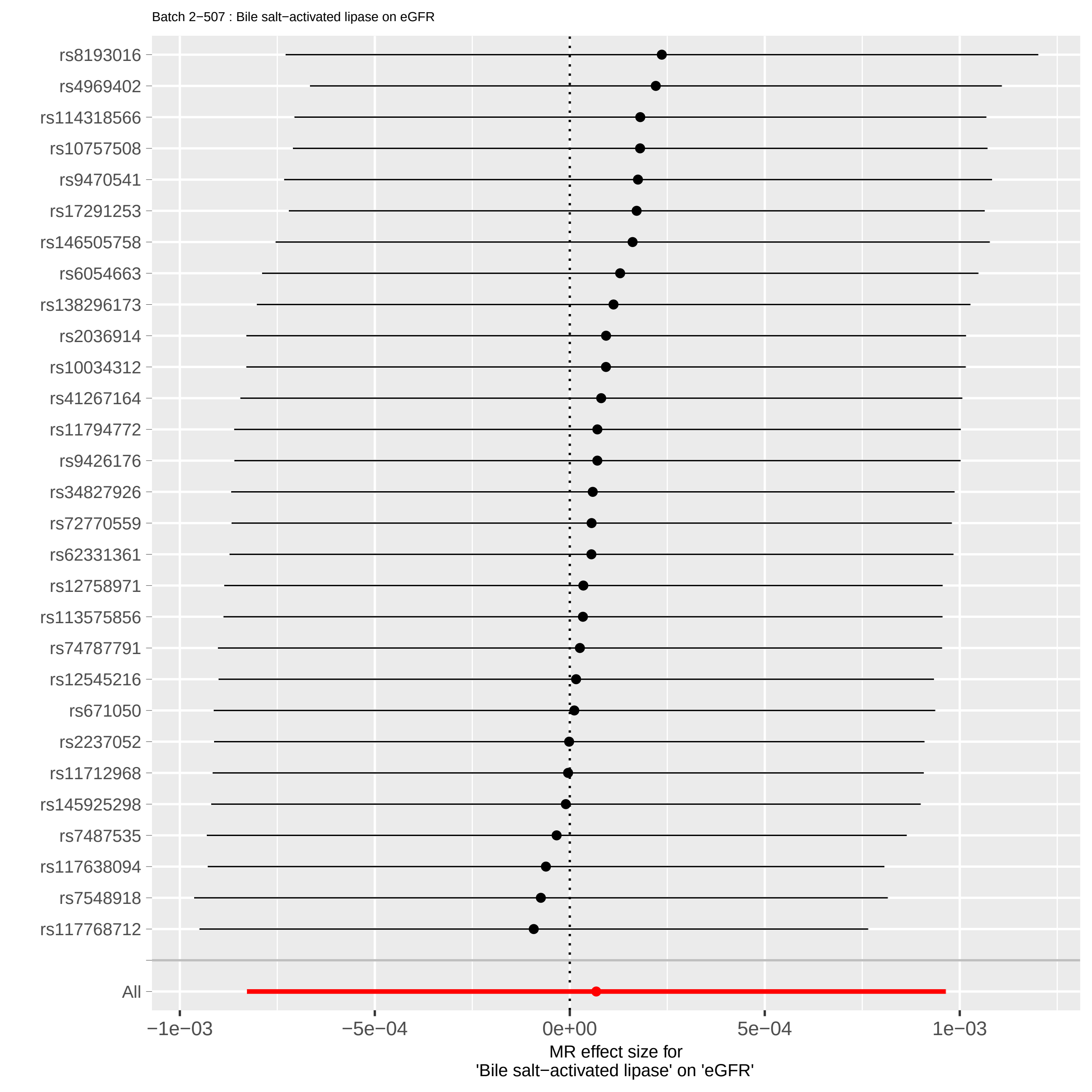

Batch 2−507 : Bile salt−activated lipase on eGFR
rs8193016
rs4969402
rs114318566
rs10757508
rs9470541
rs17291253
rs146505758
rs6054663
rs138296173
rs2036914
rs10034312
rs41267164
rs11794772
rs9426176
rs34827926
rs72770559
rs62331361
rs12758971
rs113575856
rs74787791
rs12545216
rs671050
rs2237052
rs11712968
rs145925298
rs7487535
rs117638094
rs7548918
rs117768712
All
−1e−03
−5e−04
0e+00
5e−04
1e−03
MR effect size for
'Bile salt−activated lipase' on 'eGFR'

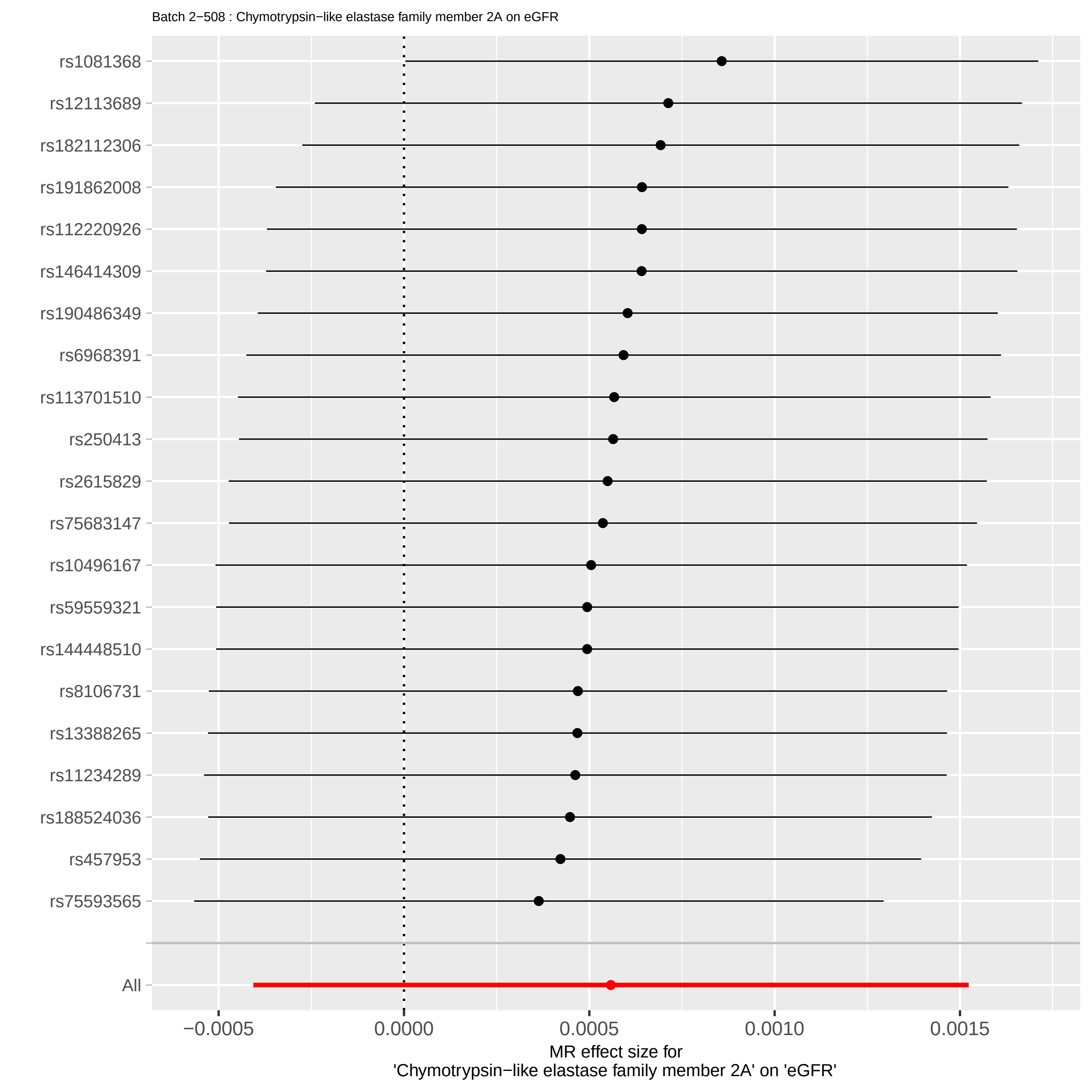
Batch 2−508 : Chymotrypsin−like elastase family member 2A on eGFR
rs1081368
rs12113689
rs182112306
rs191862008
rs112220926
rs146414309
rs190486349
rs6968391
rs113701510
rs250413
rs2615829
rs75683147
rs10496167
rs59559321
rs144448510
rs8106731
rs13388265
rs11234289
rs188524036
rs457953
rs75593565
All
−0.0005
0.0000
0.0005
0.0010
0.0015
MR effect size for
'Chymotrypsin−like elastase family member 2A' on 'eGFR'

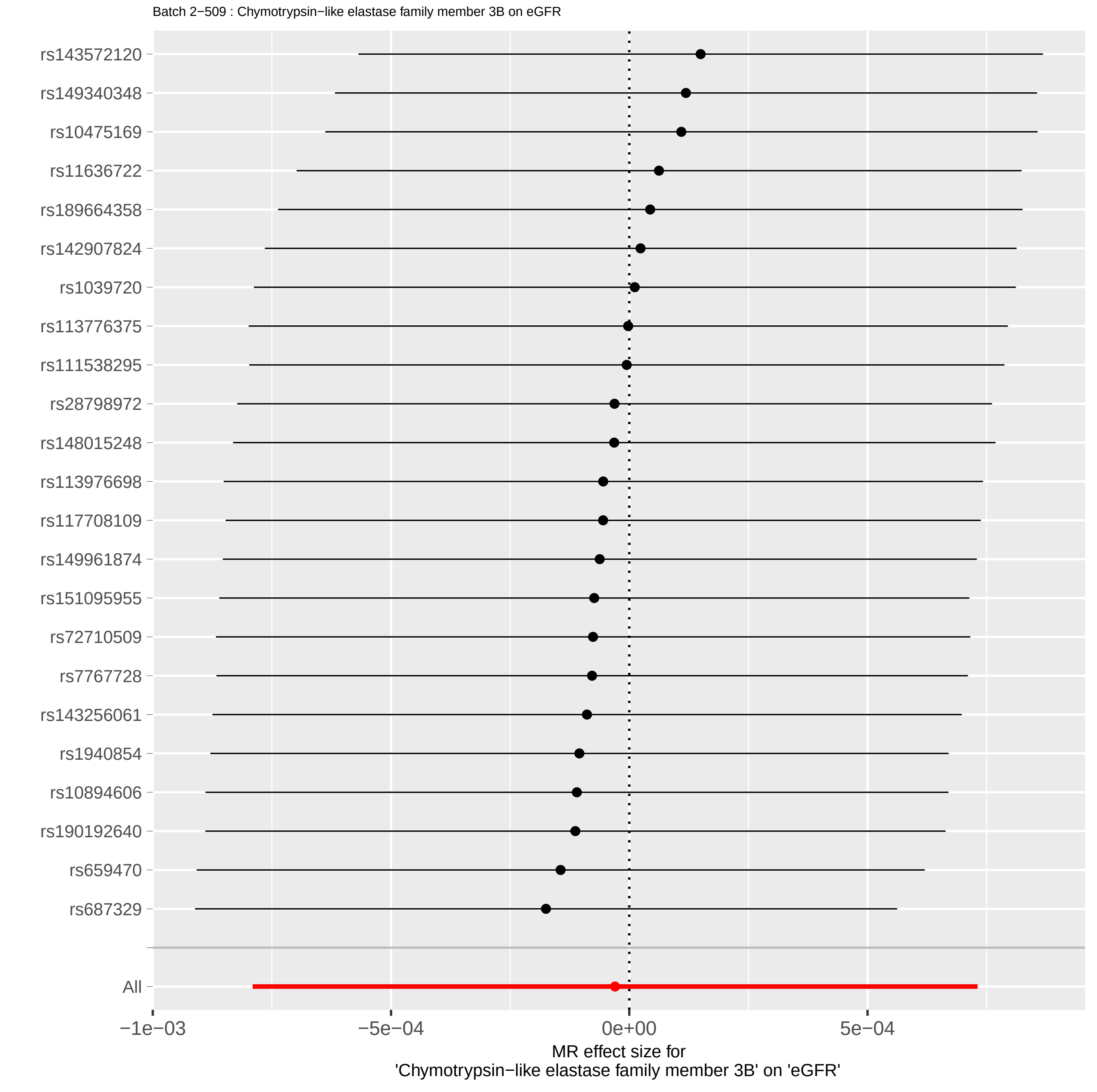

Batch 2−509 : Chymotrypsin−like elastase family member 3B on eGFR
rs143572120
rs149340348
rs10475169
rs11636722
rs189664358
rs142907824
rs1039720
rs113776375
rs111538295
rs28798972
rs148015248
rs113976698
rs117708109
rs149961874
rs151095955
rs72710509
rs7767728
rs143256061
rs1940854
rs10894606
rs190192640
rs659470
rs687329
All
−1e−03
−5e−04
0e+00
5e−04
MR effect size for
'Chymotrypsin−like elastase family member 3B' on 'eGFR'

Batch 2−510 : CUGBP Elav−like family member 2 on eGFR

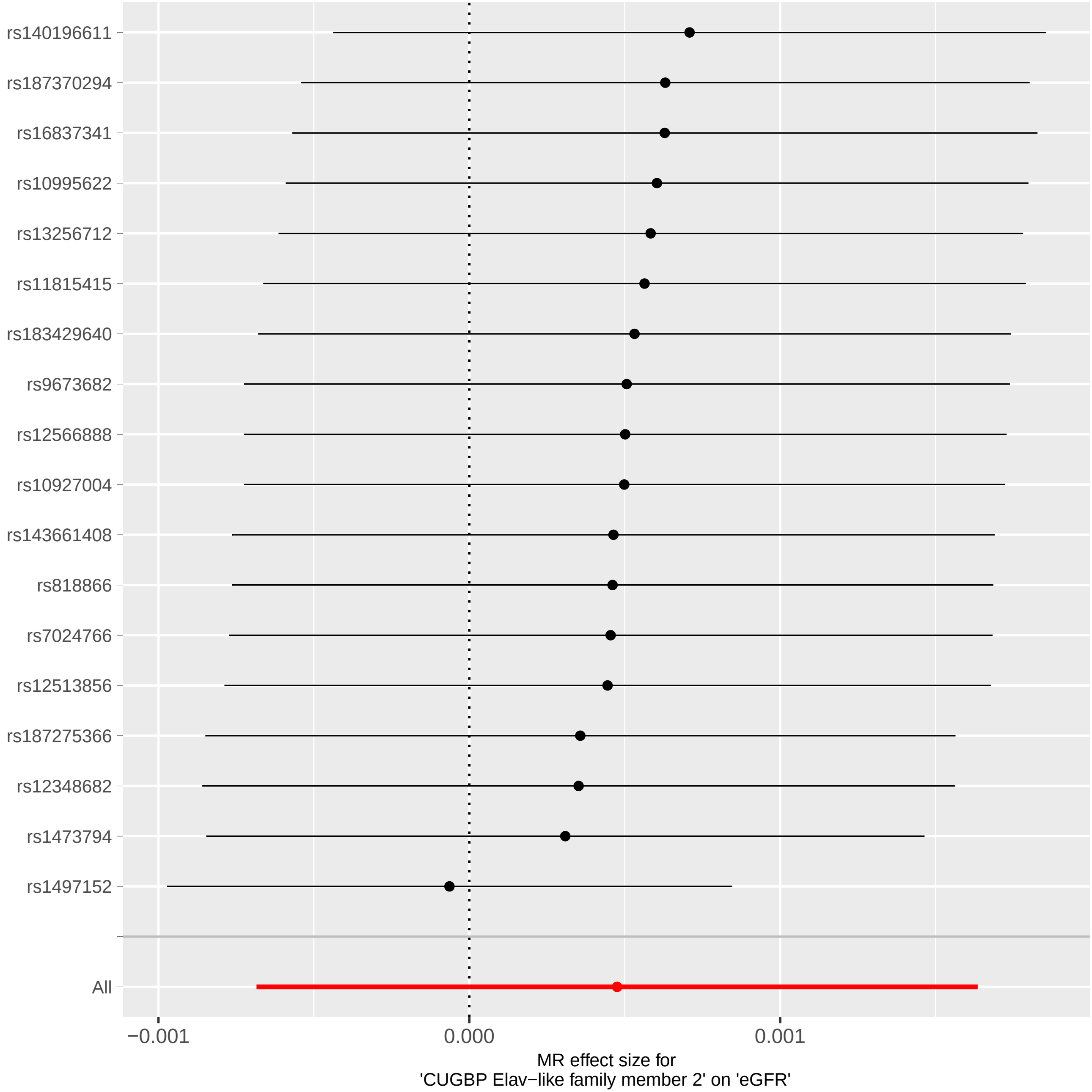

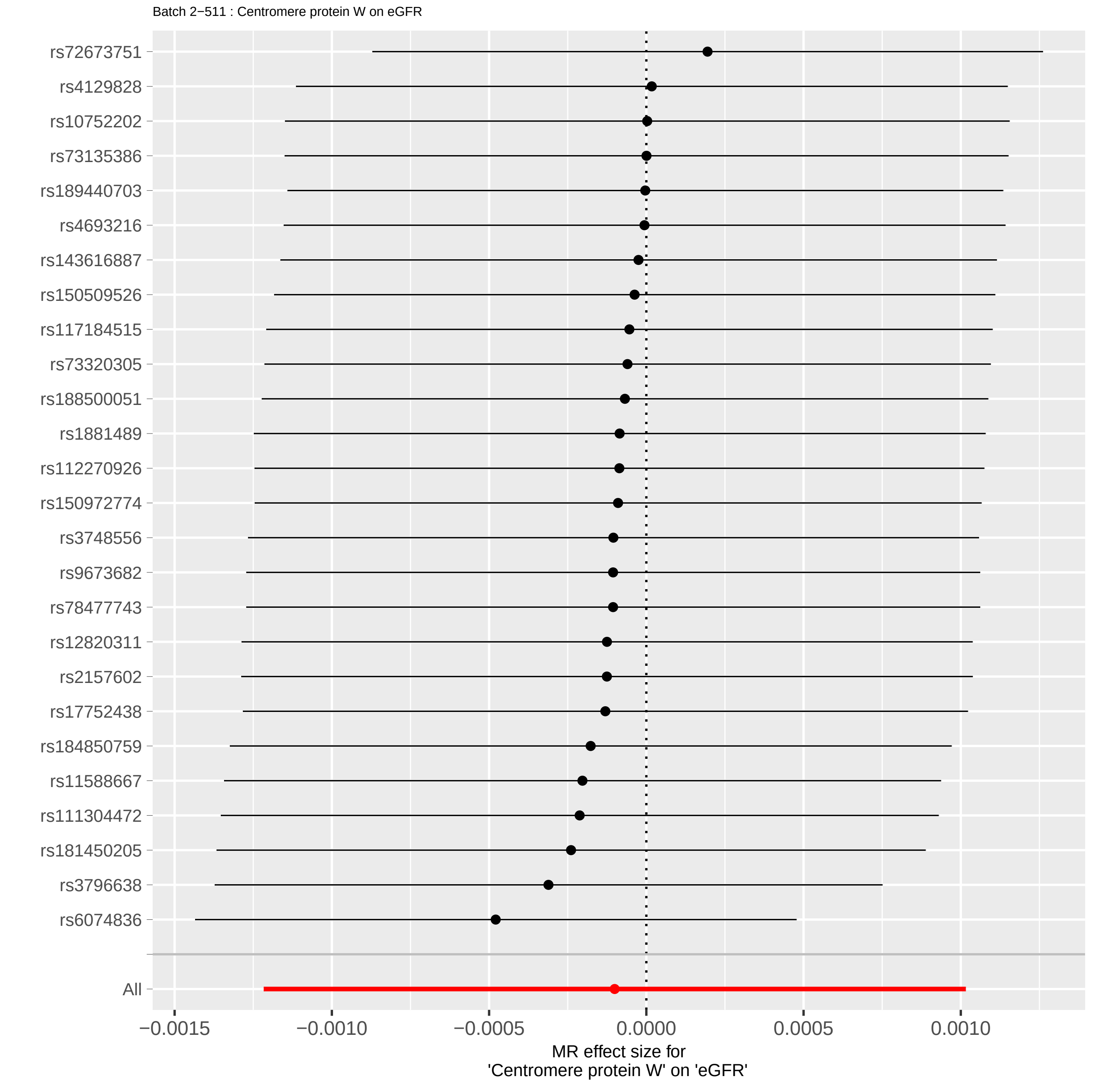

Batch 2−511 : Centromere protein W on eGFR
rs72673751
rs4129828
rs10752202
rs73135386
rs189440703
rs4693216
rs143616887
rs150509526
rs117184515
rs73320305
rs188500051
rs1881489
rs112270926
rs150972774
rs3748556
rs9673682
rs78477743
rs12820311
rs2157602
rs17752438
rs184850759
rs11588667
rs111304472
rs181450205
rs3796638
rs6074836
All
−0.0015
−0.0010
−0.0005
0.0000
0.0005
0.0010
MR effect size for
'Centromere protein W' on 'eGFR'

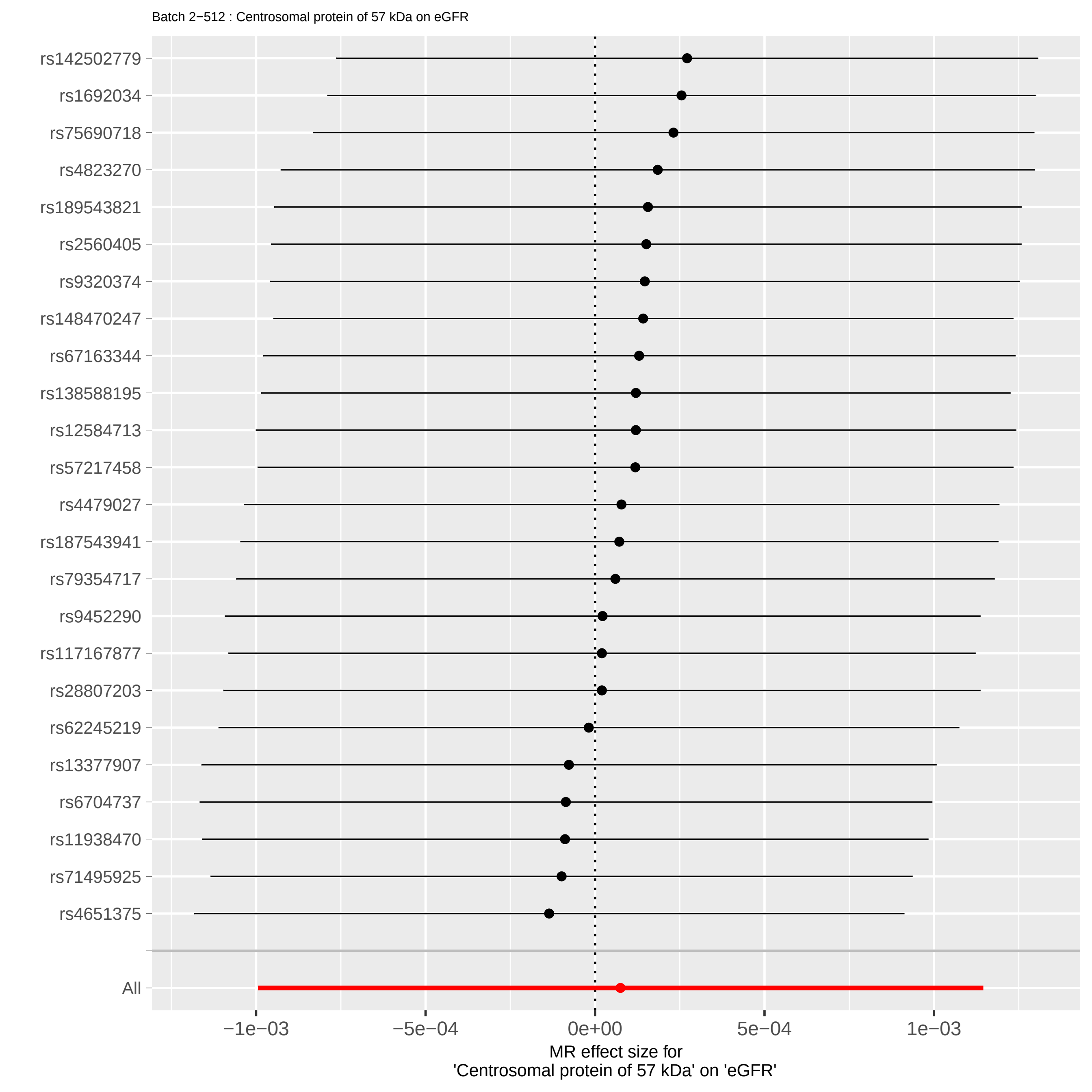

Batch 2−512 : Centrosomal protein of 57 kDa on eGFR
rs142502779
rs1692034
rs75690718
rs4823270
rs189543821
rs2560405
rs9320374
rs148470247
rs67163344
rs138588195
rs12584713
rs57217458
rs4479027
rs187543941
rs79354717
rs9452290
rs117167877
rs28807203
rs62245219
rs13377907
rs6704737
rs11938470
rs71495925
rs4651375
All
−1e−03
−5e−04
0e+00
5e−04
1e−03
MR effect size for
'Centrosomal protein of 57 kDa' on 'eGFR'

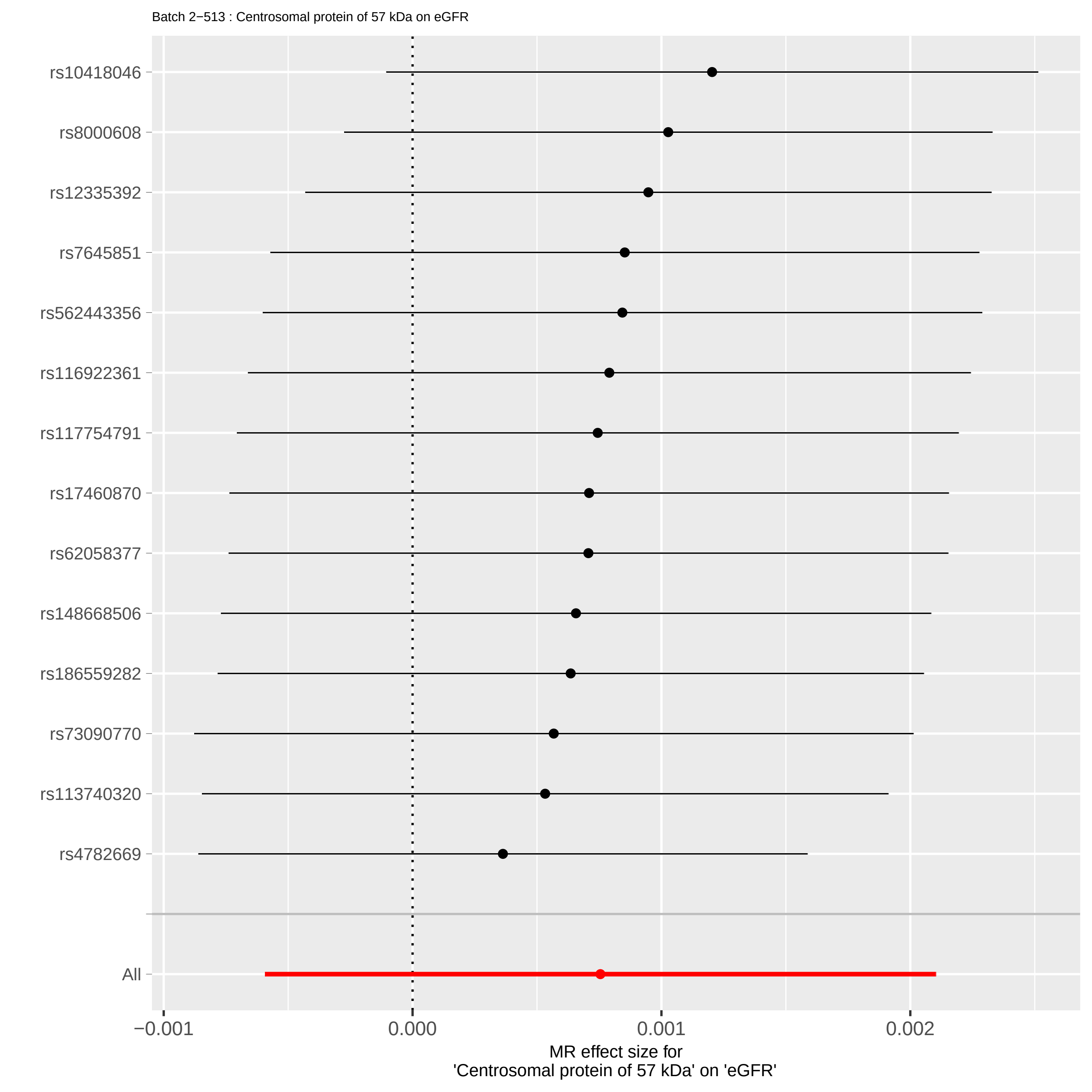

Batch 2−513 : Centrosomal protein of 57 kDa on eGFR
rs10418046
rs8000608
rs12335392
rs7645851
rs562443356
rs116922361
rs117754791
rs17460870
rs62058377
rs148668506
rs186559282
rs73090770
rs113740320
rs4782669
All
−0.001
0.000
0.001
0.002
MR effect size for
'Centrosomal protein of 57 kDa' on 'eGFR'

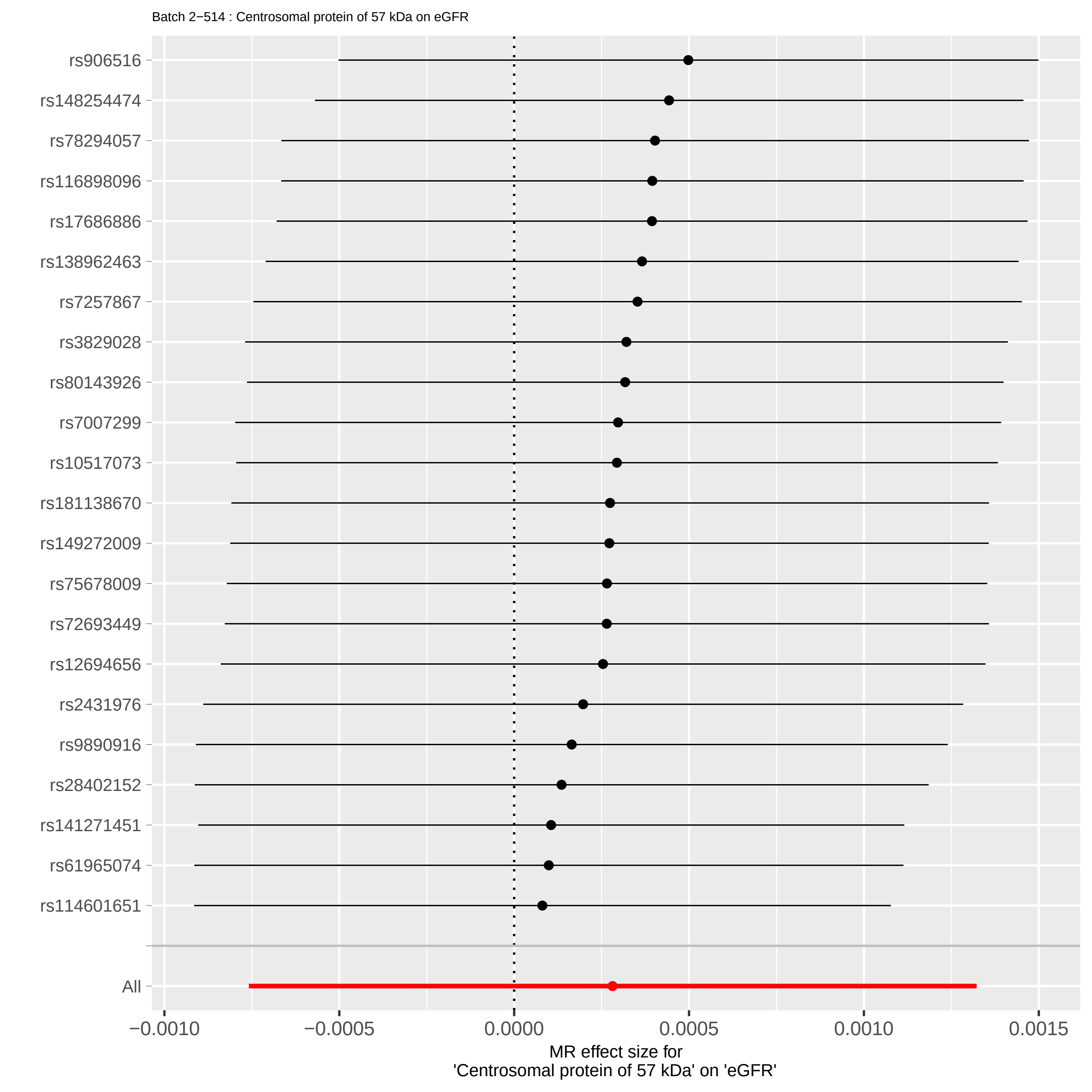

Batch 2−514 : Centrosomal protein of 57 kDa on eGFR
rs906516
rs148254474
rs78294057
rs116898096
rs17686886
rs138962463
rs7257867
rs3829028
rs80143926
rs7007299
rs10517073
rs181138670
rs149272009
rs75678009
rs72693449
rs12694656
rs2431976
rs9890916
rs28402152
rs141271451
rs61965074
rs114601651
All
−0.0010
−0.0005
0.0000
0.0005
0.0010
0.0015
MR effect size for
'Centrosomal protein of 57 kDa' on 'eGFR'

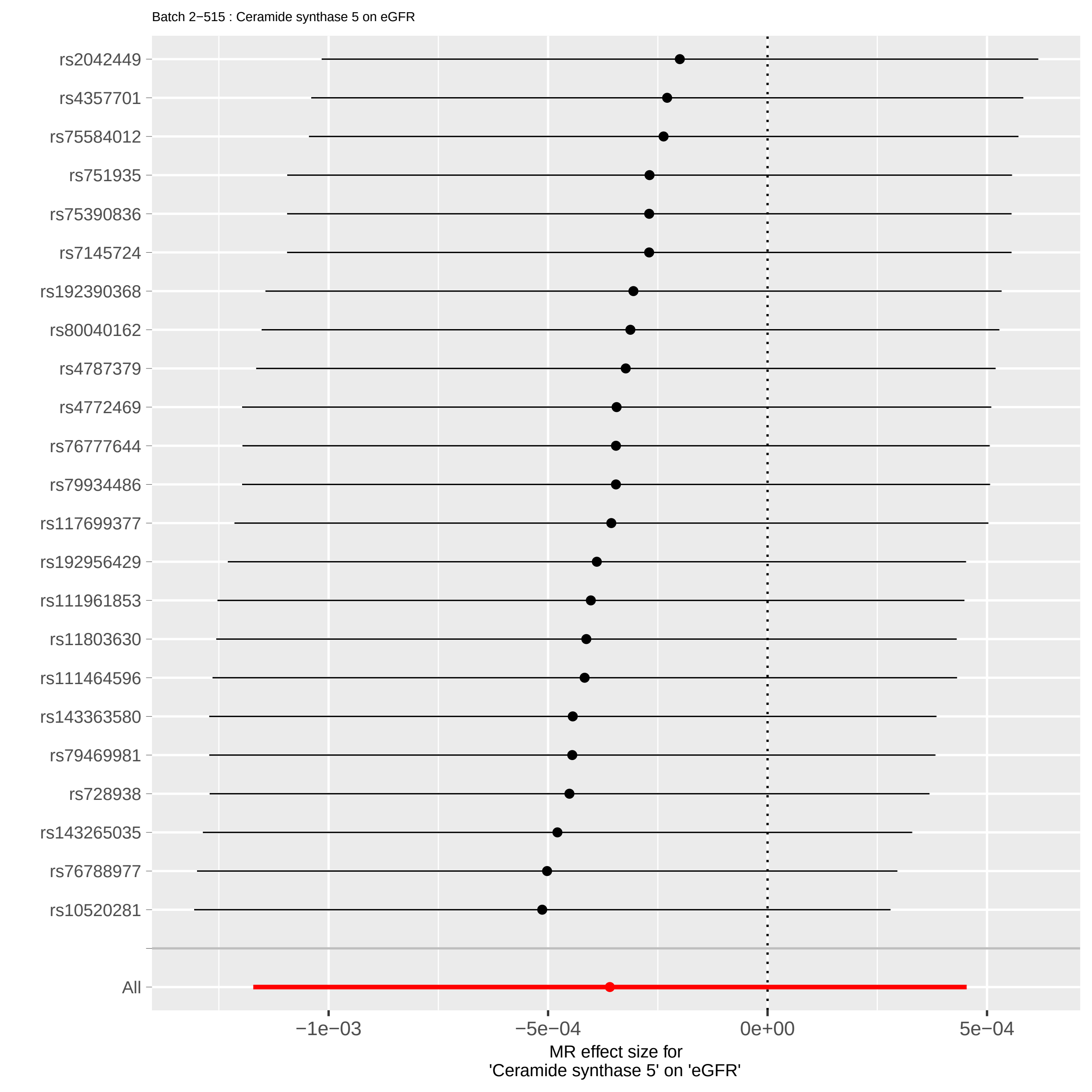

Batch 2−515 : Ceramide synthase 5 on eGFR
rs2042449
rs4357701
rs75584012
rs751935
rs75390836
rs7145724
rs192390368
rs80040162
rs4787379
rs4772469
rs76777644
rs79934486
rs117699377
rs192956429
rs111961853
rs11803630
rs111464596
rs143363580
rs79469981
rs728938
rs143265035
rs76788977
rs10520281
All
−1e−03
−5e−04
0e+00
5e−04
MR effect size for
'Ceramide synthase 5' on 'eGFR'

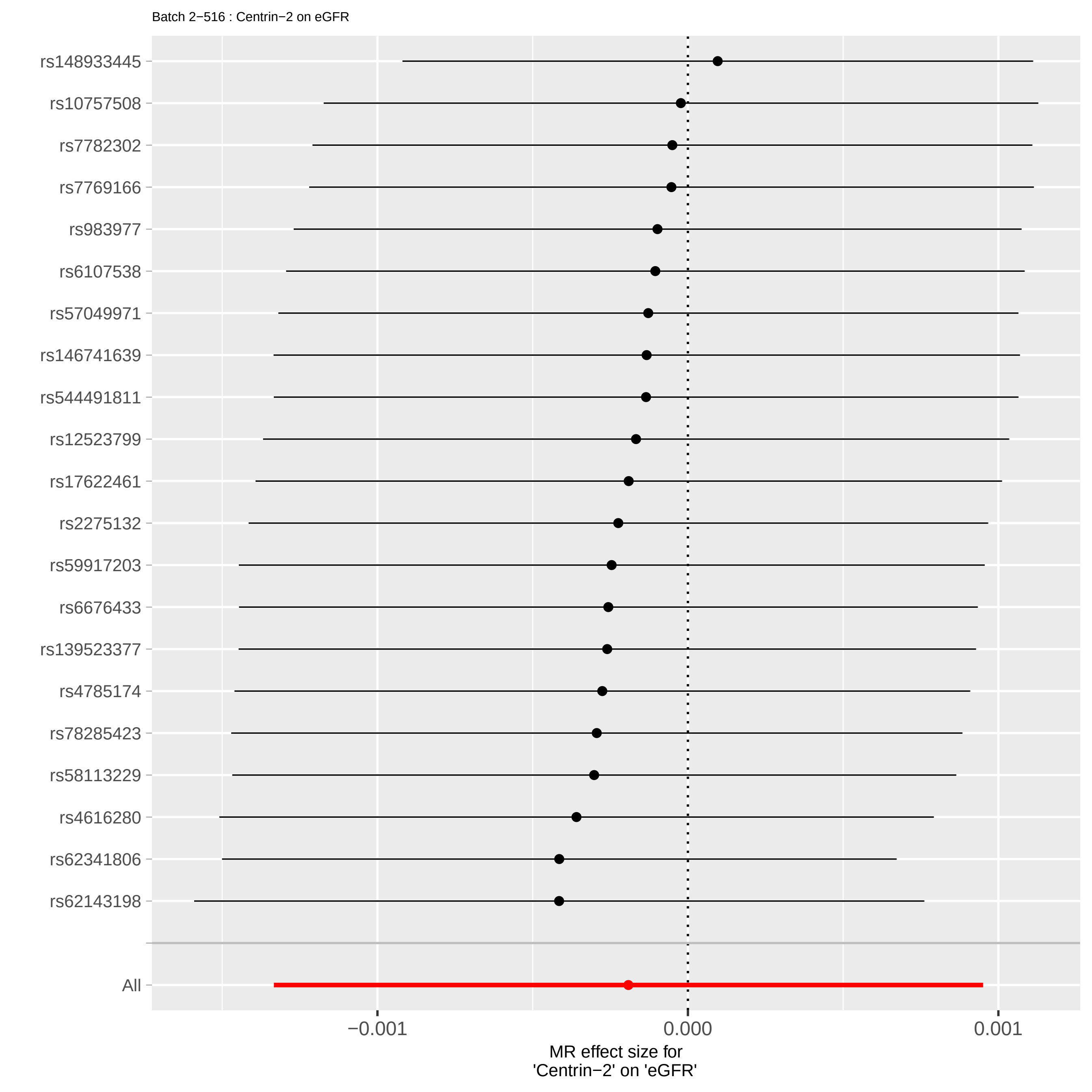

Batch 2−516 : Centrin−2 on eGFR
rs148933445
rs10757508
rs7782302
rs7769166
rs983977
rs6107538
rs57049971
rs146741639
rs544491811
rs12523799
rs17622461
rs2275132
rs59917203
rs6676433
rs139523377
rs4785174
rs78285423
rs58113229
rs4616280
rs62341806
rs62143198
All
−0.001
0.000
0.001
MR effect size for
'Centrin−2' on 'eGFR'

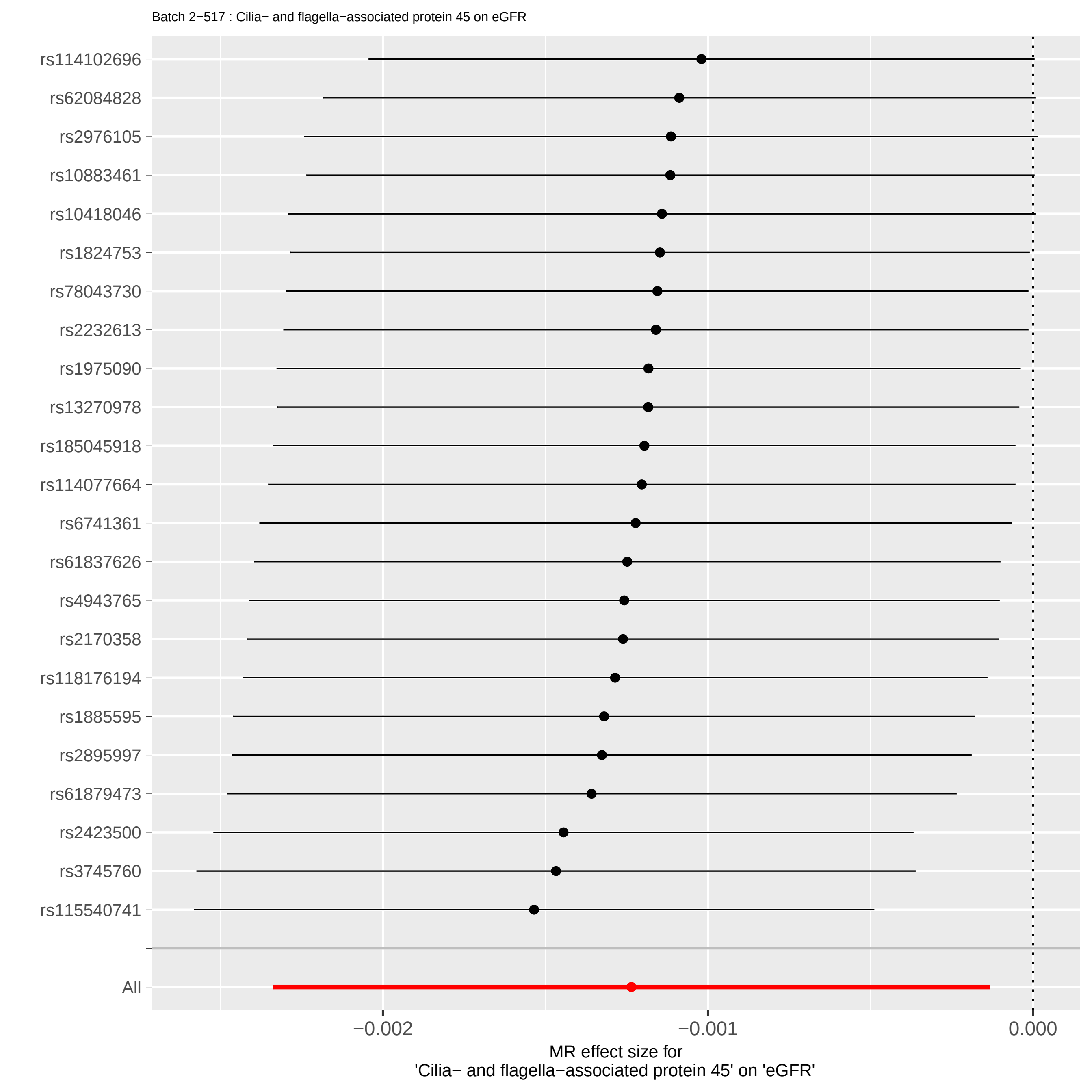

Batch 2−517 : Cilia− and flagella−associated protein 45 on eGFR
rs114102696
rs62084828
rs2976105
rs10883461
rs10418046
rs1824753
rs78043730
rs2232613
rs1975090
rs13270978
rs185045918
rs114077664
rs6741361
rs61837626
rs4943765
rs2170358
rs118176194
rs1885595
rs2895997
rs61879473
rs2423500
rs3745760
rs115540741
All
−0.002
−0.001
0.000
MR effect size for
'Cilia− and flagella−associated protein 45' on 'eGFR'

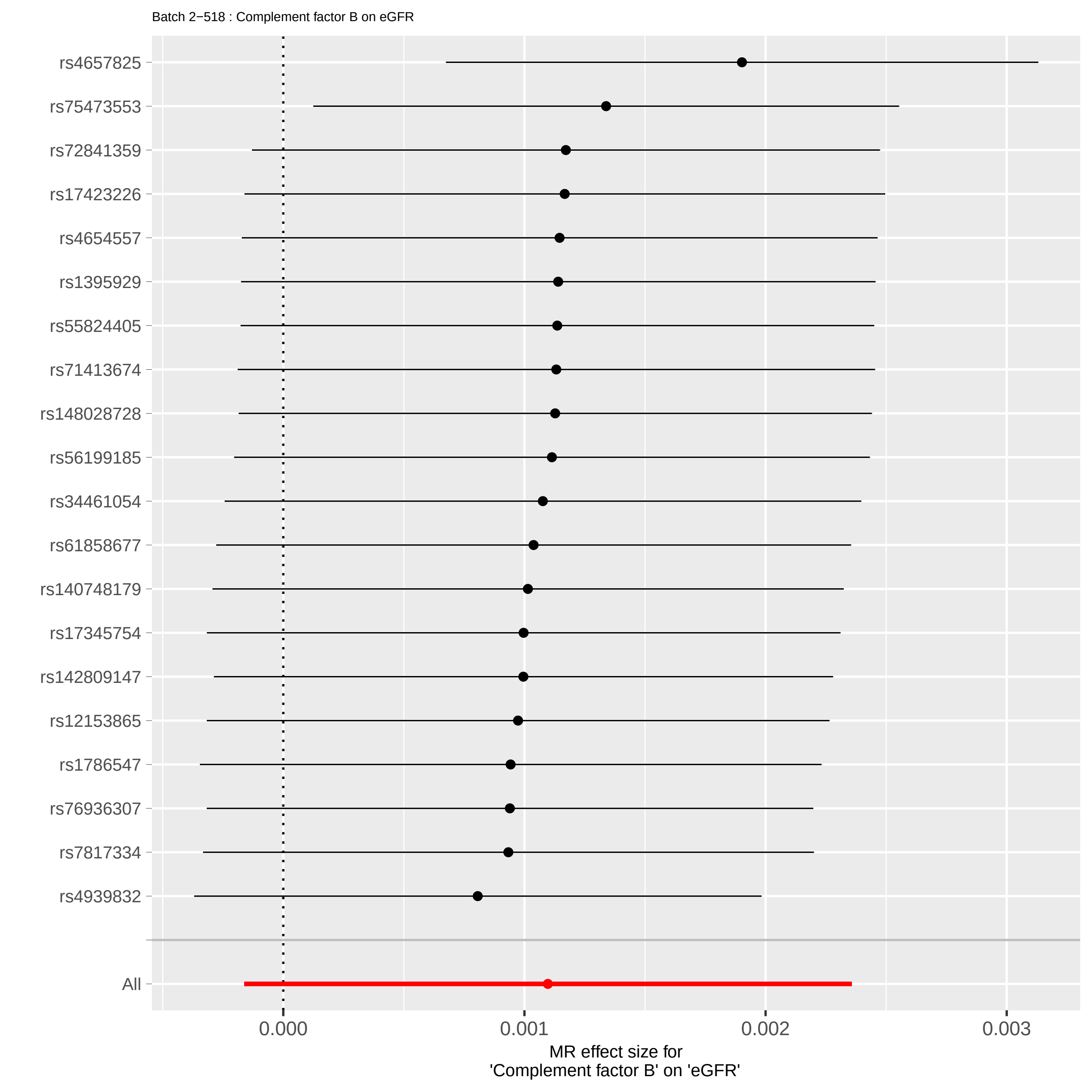

Batch 2−518 : Complement factor B on eGFR
rs4657825
rs75473553
rs72841359
rs17423226
rs4654557
rs1395929
rs55824405
rs71413674
rs148028728
rs56199185
rs34461054
rs61858677
rs140748179
rs17345754
rs142809147
rs12153865
rs1786547
rs76936307
rs7817334
rs4939832
All
0.000
0.001
0.002
0.003
MR effect size for
'Complement factor B' on 'eGFR'

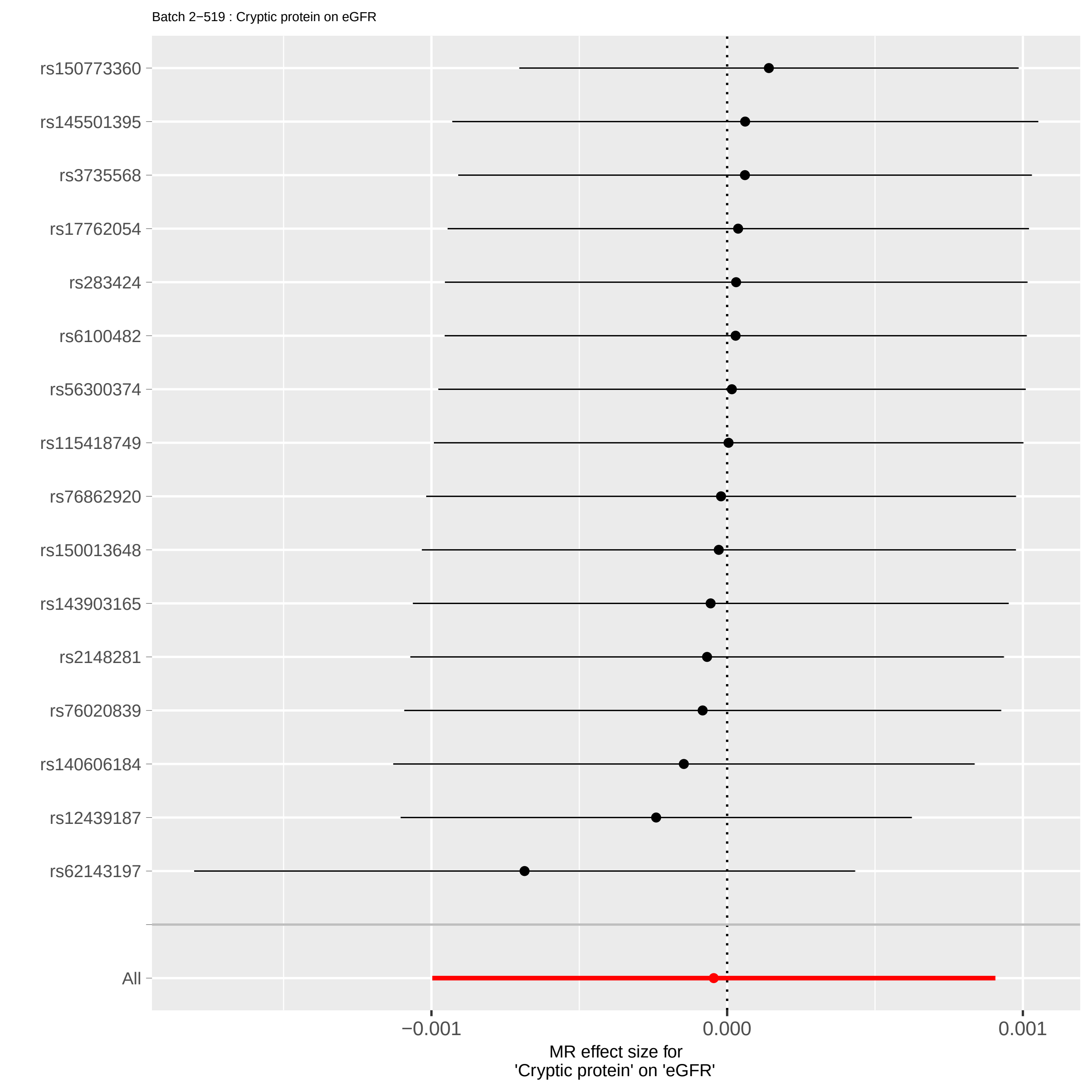

Batch 2−519 : Cryptic protein on eGFR
rs150773360
rs145501395
rs3735568
rs17762054
rs283424
rs6100482
rs56300374
rs115418749
rs76862920
rs150013648
rs143903165
rs2148281
rs76020839
rs140606184
rs12439187
rs62143197
All
−0.001
0.000
0.001
MR effect size for
'Cryptic protein' on 'eGFR'

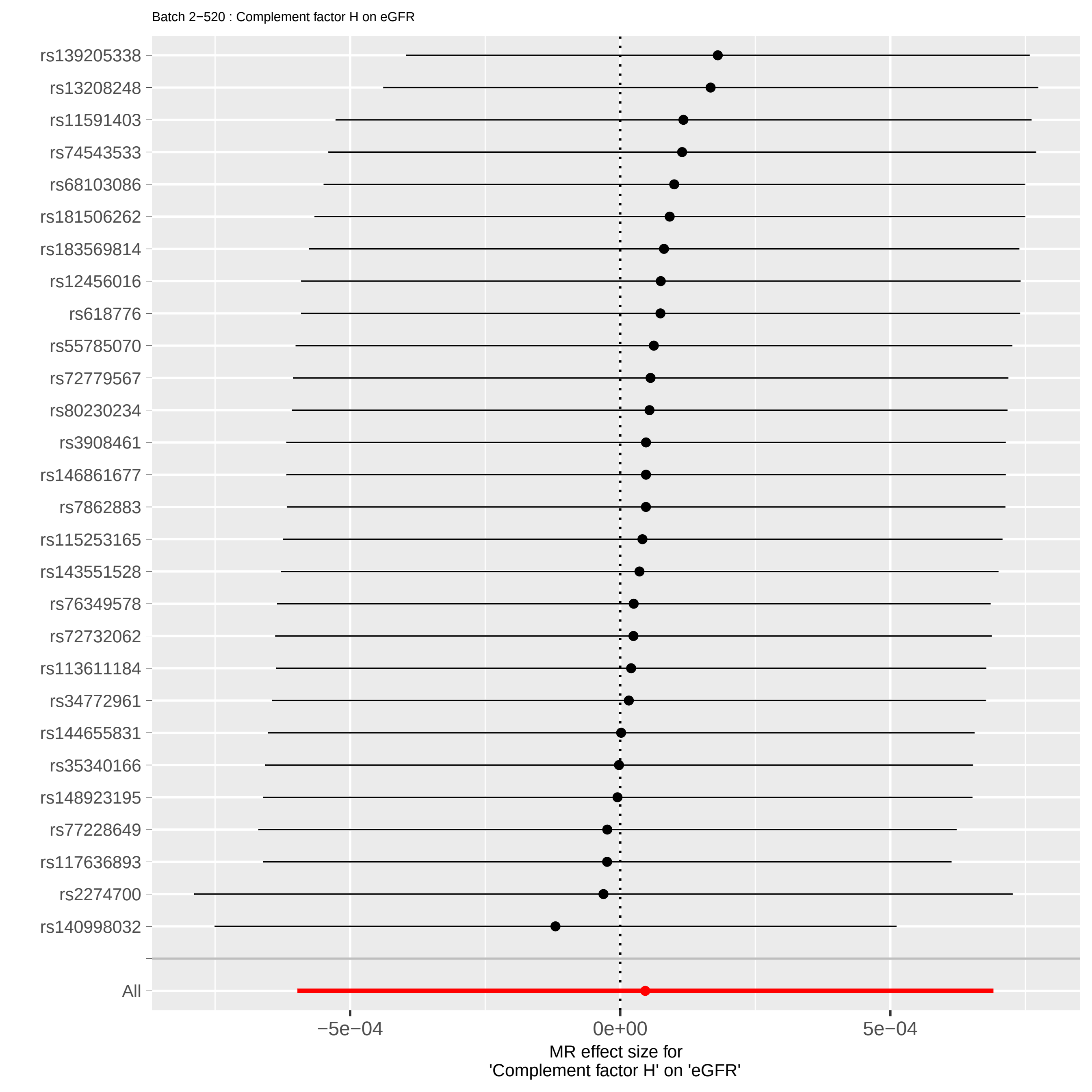

Batch 2−520 : Complement factor H on eGFR
rs139205338
rs13208248
rs11591403
rs74543533
rs68103086
rs181506262
rs183569814
rs12456016
rs618776
rs55785070
rs72779567
rs80230234
rs3908461
rs146861677
rs7862883
rs115253165
rs143551528
rs76349578
rs72732062
rs113611184
rs34772961
rs144655831
rs35340166
rs148923195
rs77228649
rs117636893
rs2274700
rs140998032
All
−5e−04
0e+00
5e−04
MR effect size for
'Complement factor H' on 'eGFR'

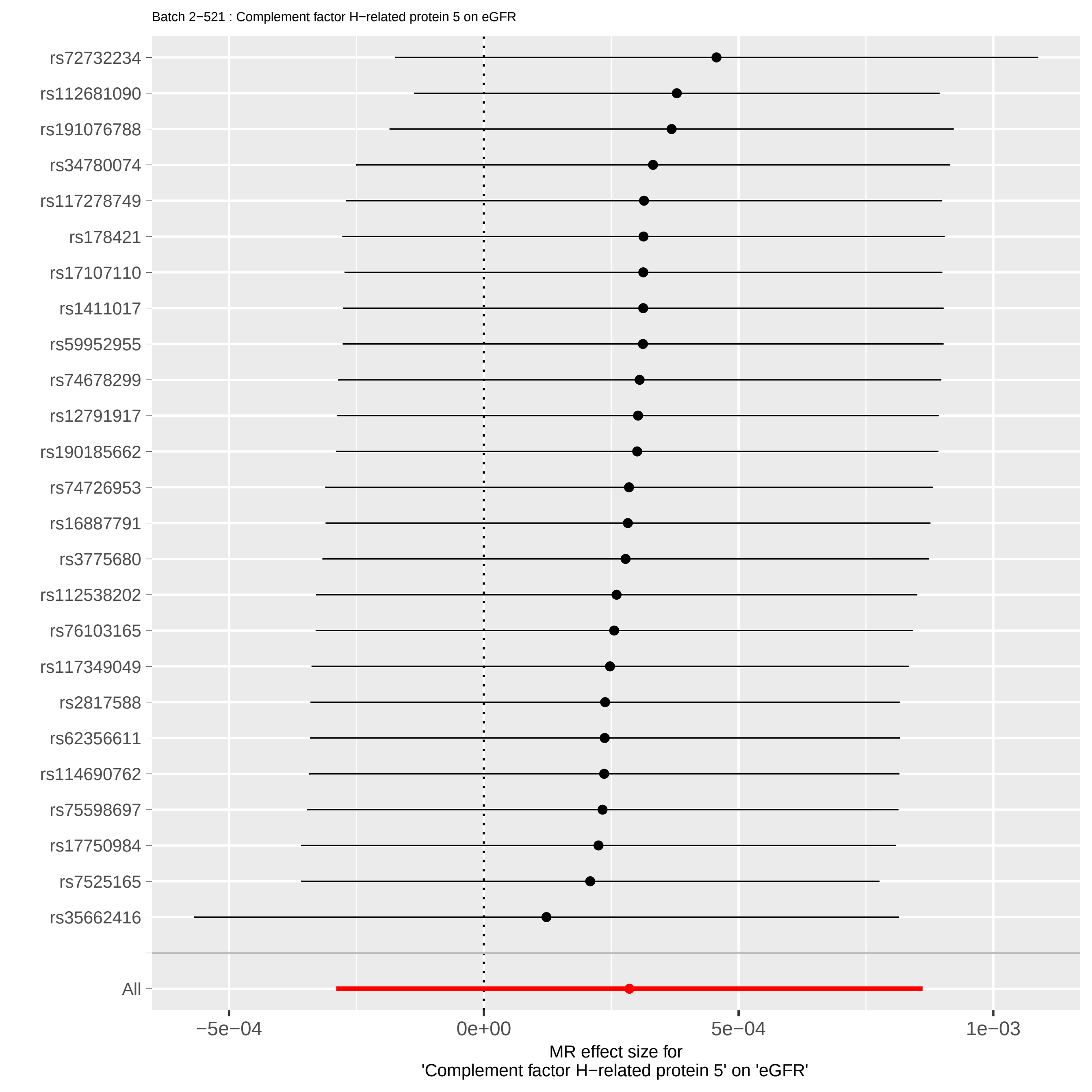
Batch 2−521 : Complement factor H−related protein 5 on eGFR
rs72732234
rs112681090
rs191076788
rs34780074
rs117278749
rs178421
rs17107110
rs1411017
rs59952955
rs74678299
rs12791917
rs190185662
rs74726953
rs16887791
rs3775680
rs112538202
rs76103165
rs117349049
rs2817588
rs62356611
rs114690762
rs75598697
rs17750984
rs7525165
rs35662416
All
−5e−04
0e+00
5e−04
1e−03
MR effect size for
'Complement factor H−related protein 5' on 'eGFR'

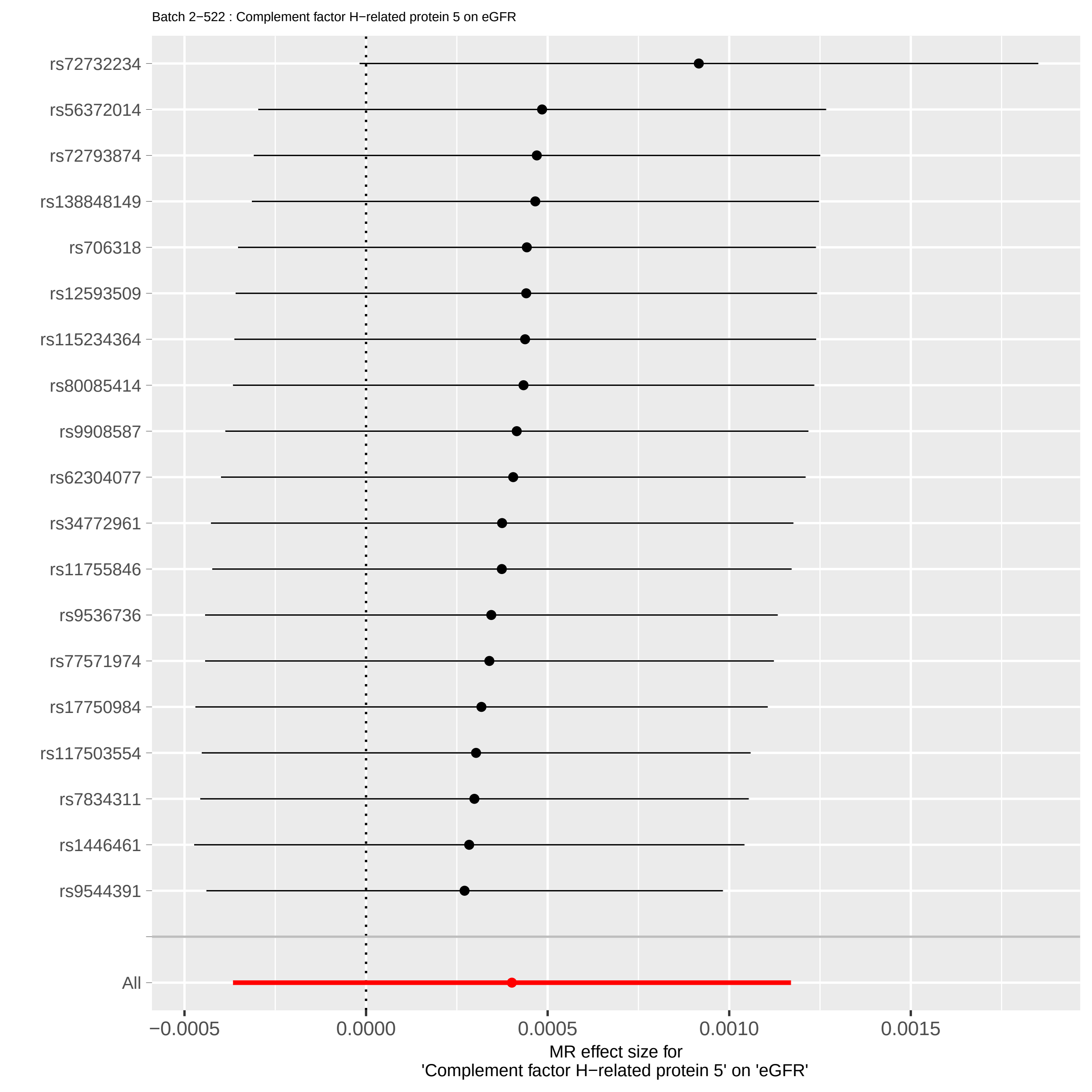
Batch 2−522 : Complement factor H−related protein 5 on eGFR
rs72732234
rs56372014
rs72793874
rs138848149
rs706318
rs12593509
rs115234364
rs80085414
rs9908587
rs62304077
rs34772961
rs11755846
rs9536736
rs77571974
rs17750984
rs117503554
rs7834311
rs1446461
rs9544391
All
−0.0005
0.0000
0.0005
0.0010
0.0015
MR effect size for
'Complement factor H−related protein 5' on 'eGFR'

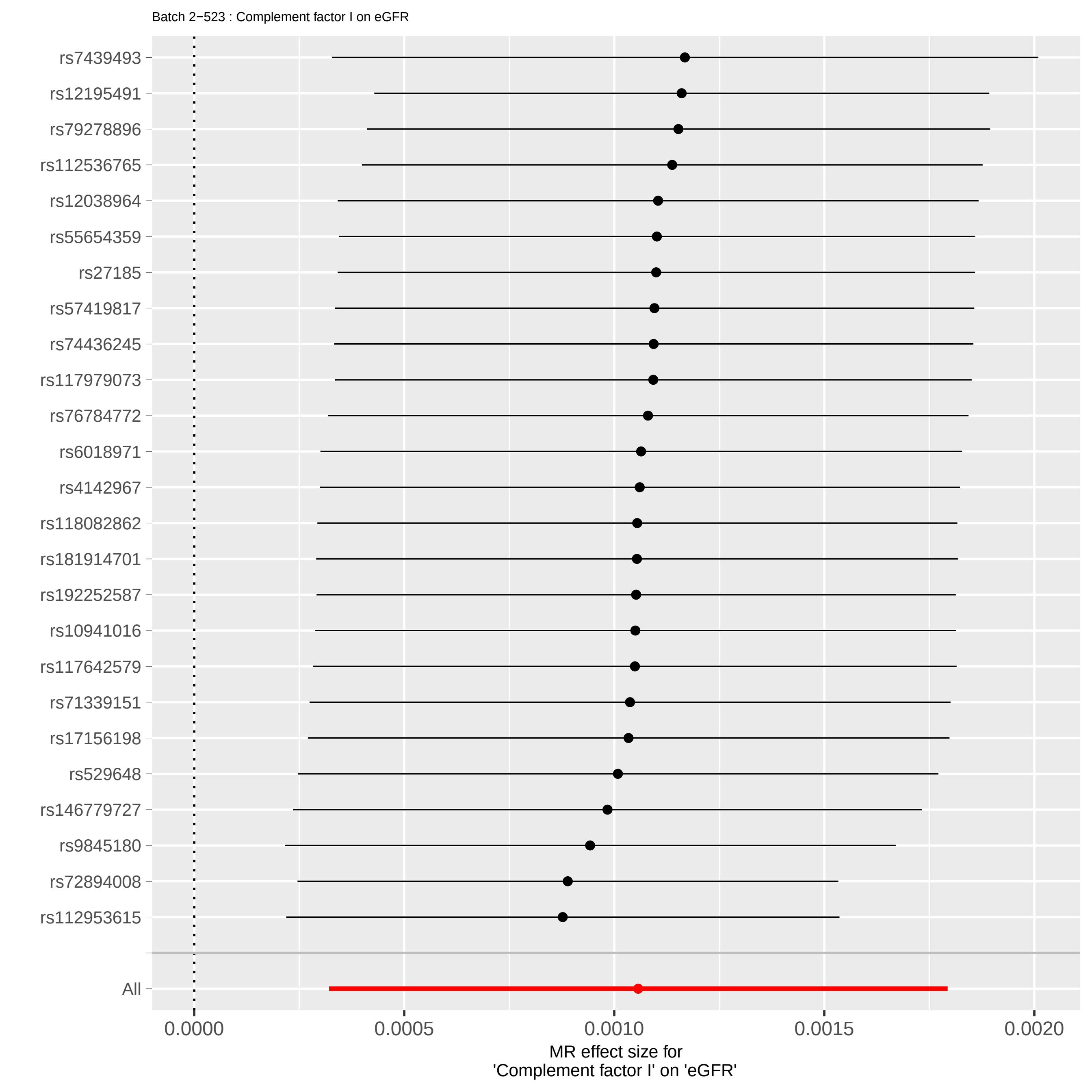
Batch 2−523 : Complement factor I on eGFR
rs7439493
rs12195491
rs79278896
rs112536765
rs12038964
rs55654359
rs27185
rs57419817
rs74436245
rs117979073
rs76784772
rs6018971
rs4142967
rs118082862
rs181914701
rs192252587
rs10941016
rs117642579
rs71339151
rs17156198
rs529648
rs146779727
rs9845180
rs72894008
rs112953615
All
0.0000
0.0005
0.0010
0.0015
0.0020
MR effect size for
'Complement factor I' on 'eGFR'

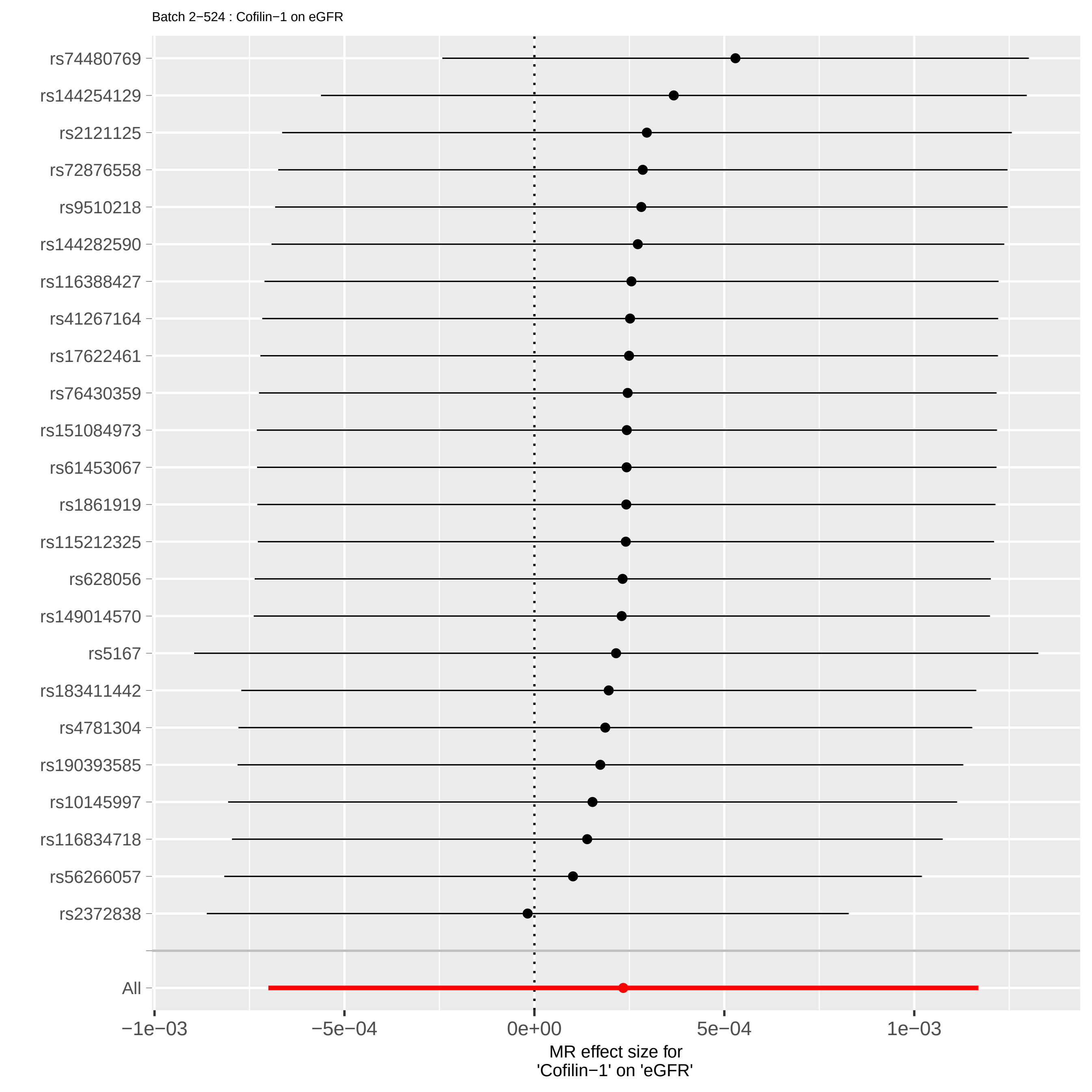

Batch 2−524 : Cofilin−1 on eGFR
rs74480769
rs144254129
rs2121125
rs72876558
rs9510218
rs144282590
rs116388427
rs41267164
rs17622461
rs76430359
rs151084973
rs61453067
rs1861919
rs115212325
rs628056
rs149014570
rs5167
rs183411442
rs4781304
rs190393585
rs10145997
rs116834718
rs56266057
rs2372838
All
−1e−03
−5e−04
0e+00
5e−04
1e−03
MR effect size for
'Cofilin−1' on 'eGFR'

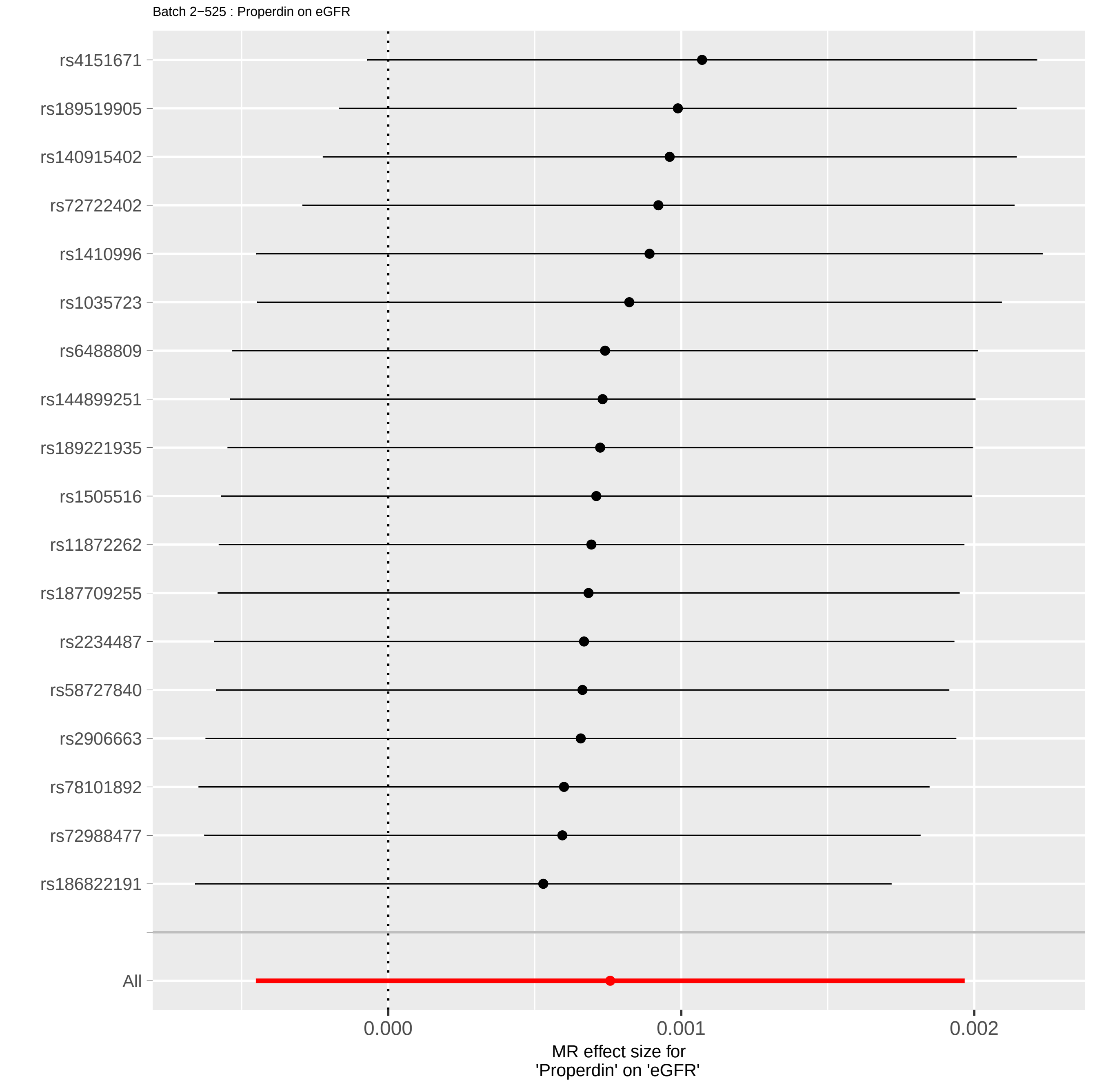

Batch 2−525 : Properdin on eGFR
rs4151671
rs189519905
rs140915402
rs72722402
rs1410996
rs1035723
rs6488809
rs144899251
rs189221935
rs1505516
rs11872262
rs187709255
rs2234487
rs58727840
rs2906663
rs78101892
rs72988477
rs186822191
All
0.000
0.001
0.002
MR effect size for
'Properdin' on 'eGFR'

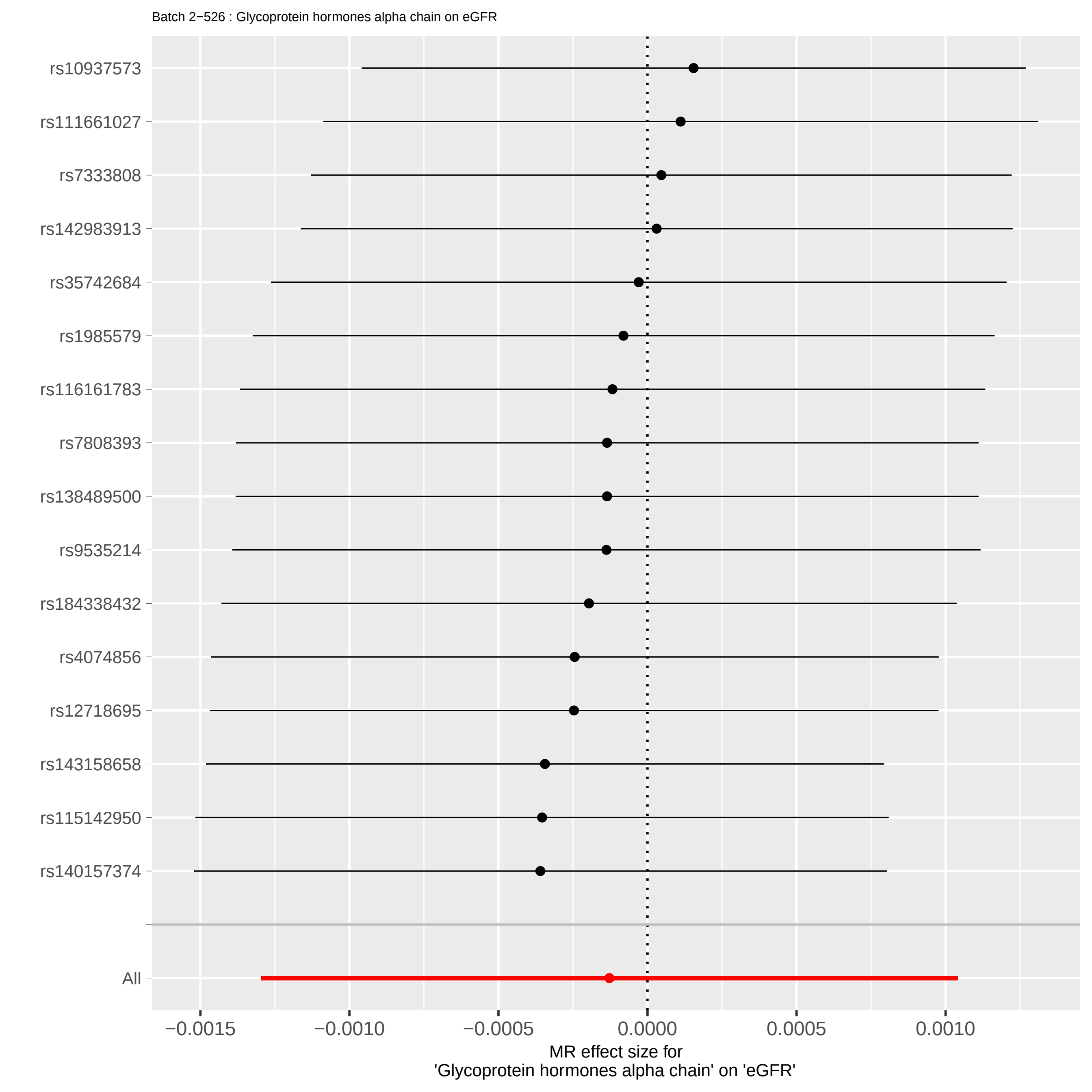

Batch 2−526 : Glycoprotein hormones alpha chain on eGFR
rs10937573
rs111661027
rs7333808
rs142983913
rs35742684
rs1985579
rs116161783
rs7808393
rs138489500
rs9535214
rs184338432
rs4074856
rs12718695
rs143158658
rs115142950
rs140157374
All
−0.0015
−0.0010
−0.0005
0.0000
0.0005
0.0010
MR effect size for
'Glycoprotein hormones alpha chain' on 'eGFR'

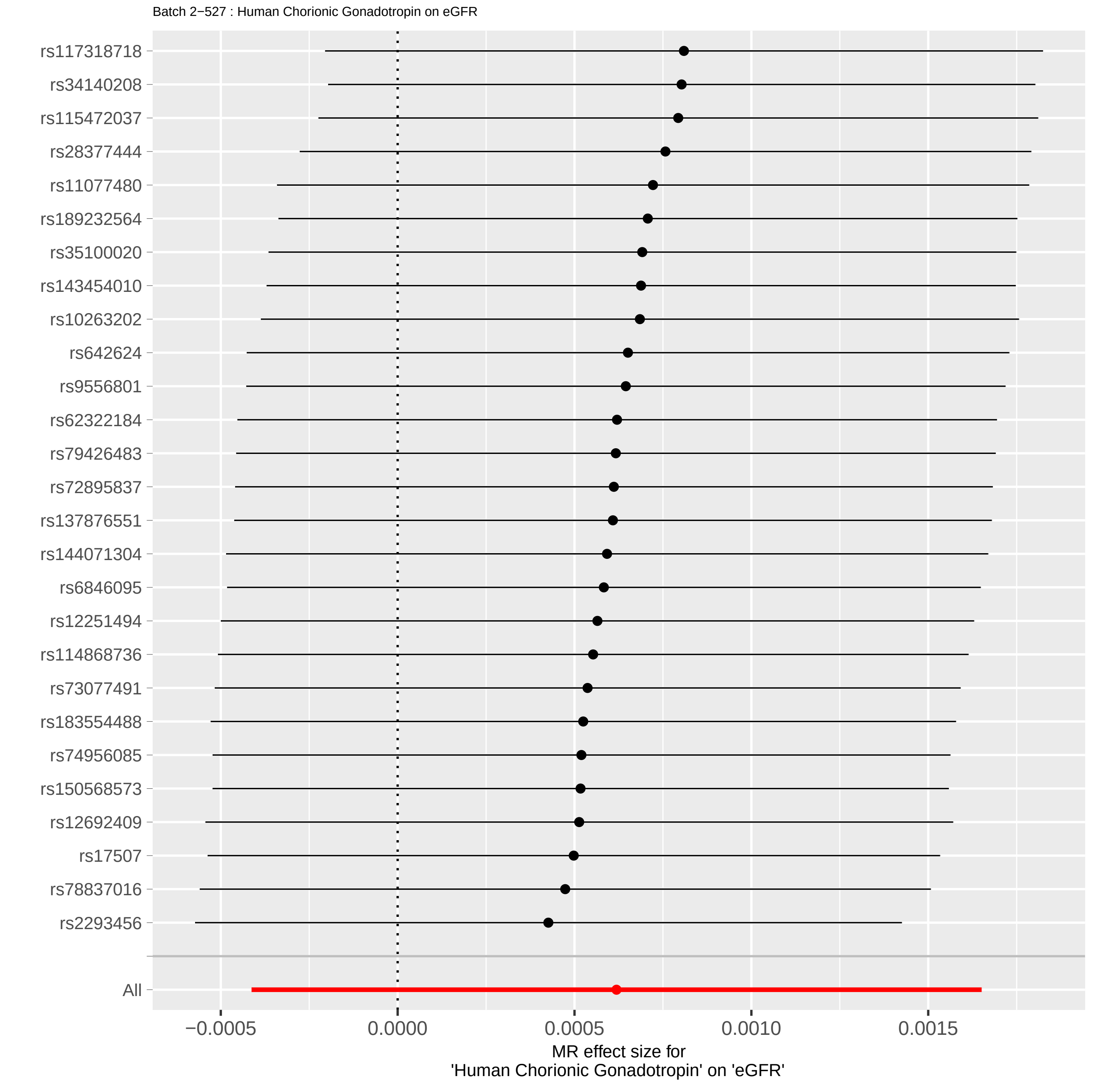
Batch 2−527 : Human Chorionic Gonadotropin on eGFR
rs117318718
rs34140208
rs115472037
rs28377444
rs11077480
rs189232564
rs35100020
rs143454010
rs10263202
rs642624
rs9556801
rs62322184
rs79426483
rs72895837
rs137876551
rs144071304
rs6846095
rs12251494
rs114868736
rs73077491
rs183554488
rs74956085
rs150568573
rs12692409
rs17507
rs78837016
rs2293456
All
−0.0005
0.0000
0.0005
0.0010
0.0015
MR effect size for
'Human Chorionic Gonadotropin' on 'eGFR'

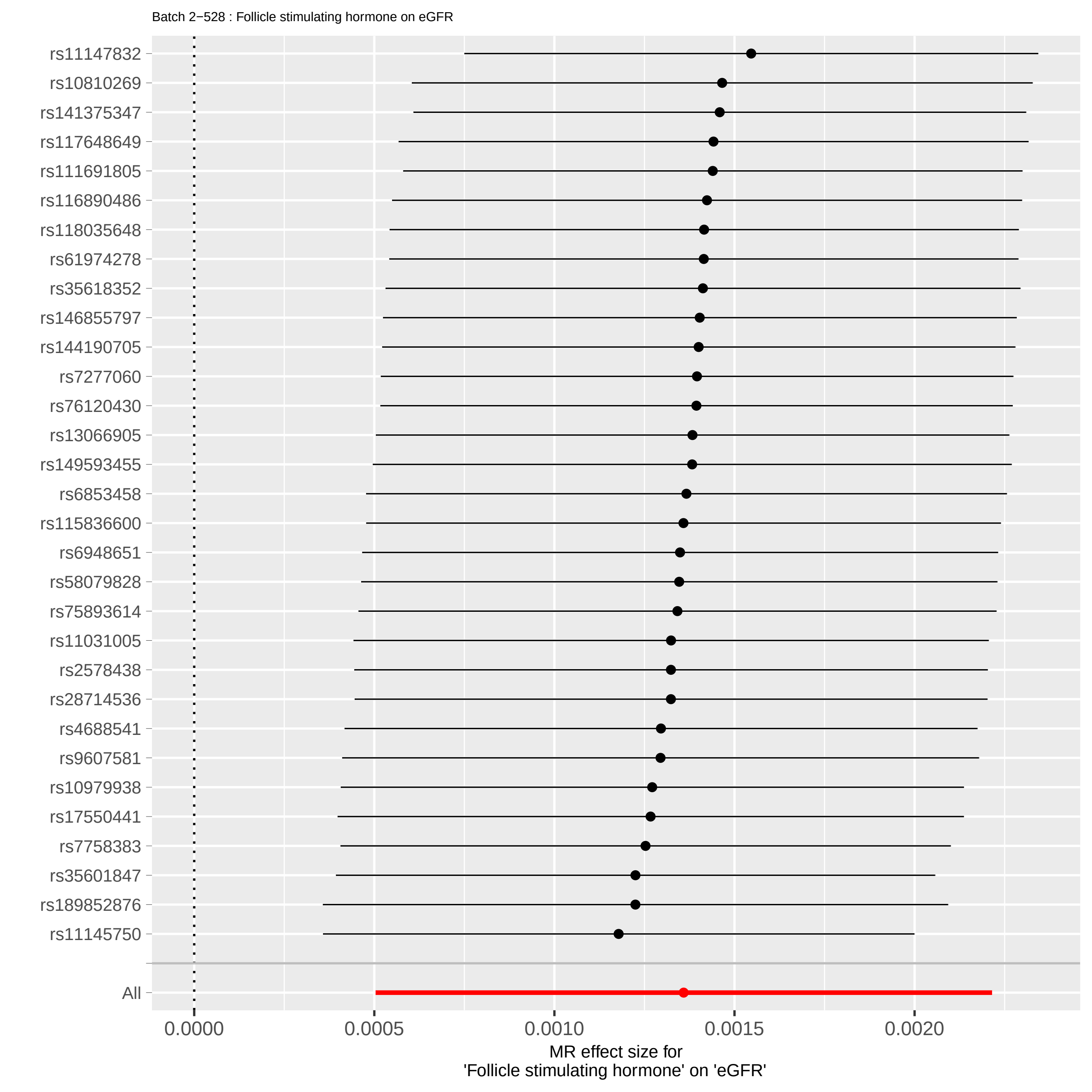

Batch 2−528 : Follicle stimulating hormone on eGFR
rs11147832
rs10810269
rs141375347
rs117648649
rs111691805
rs116890486
rs118035648
rs61974278
rs35618352
rs146855797
rs144190705
rs7277060
rs76120430
rs13066905
rs149593455
rs6853458
rs115836600
rs6948651
rs58079828
rs75893614
rs11031005
rs2578438
rs28714536
rs4688541
rs9607581
rs10979938
rs17550441
rs7758383
rs35601847
rs189852876
rs11145750
All
0.0000
0.0005
0.0010
0.0015
0.0020
MR effect size for
'Follicle stimulating hormone' on 'eGFR'

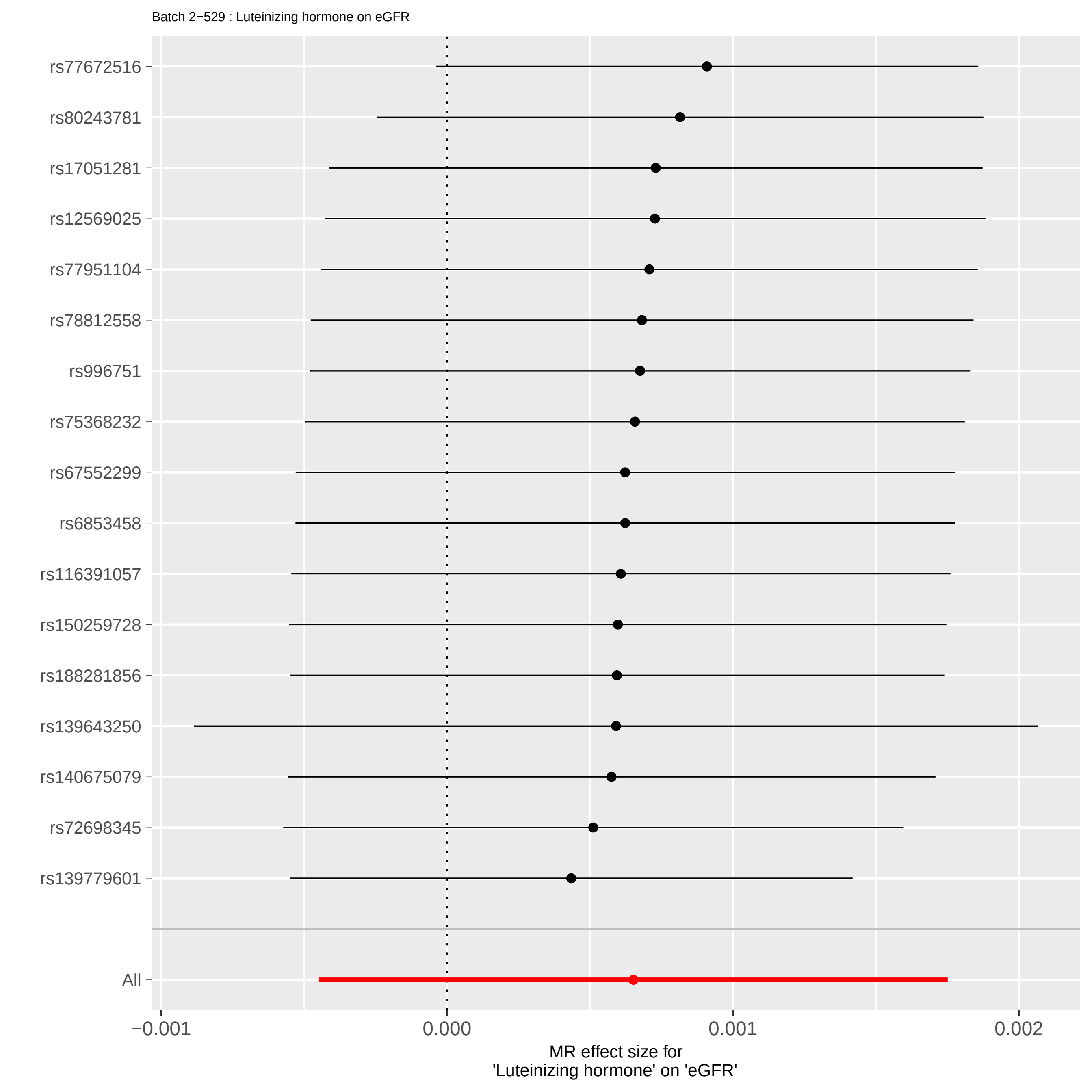

Batch 2−529 : Luteinizing hormone on eGFR
rs77672516
rs80243781
rs17051281
rs12569025
rs77951104
rs78812558
rs996751
rs75368232
rs67552299
rs6853458
rs116391057
rs150259728
rs188281856
rs139643250
rs140675079
rs72698345
rs139779601
All
−0.001
0.000
0.001
0.002
MR effect size for
'Luteinizing hormone' on 'eGFR'

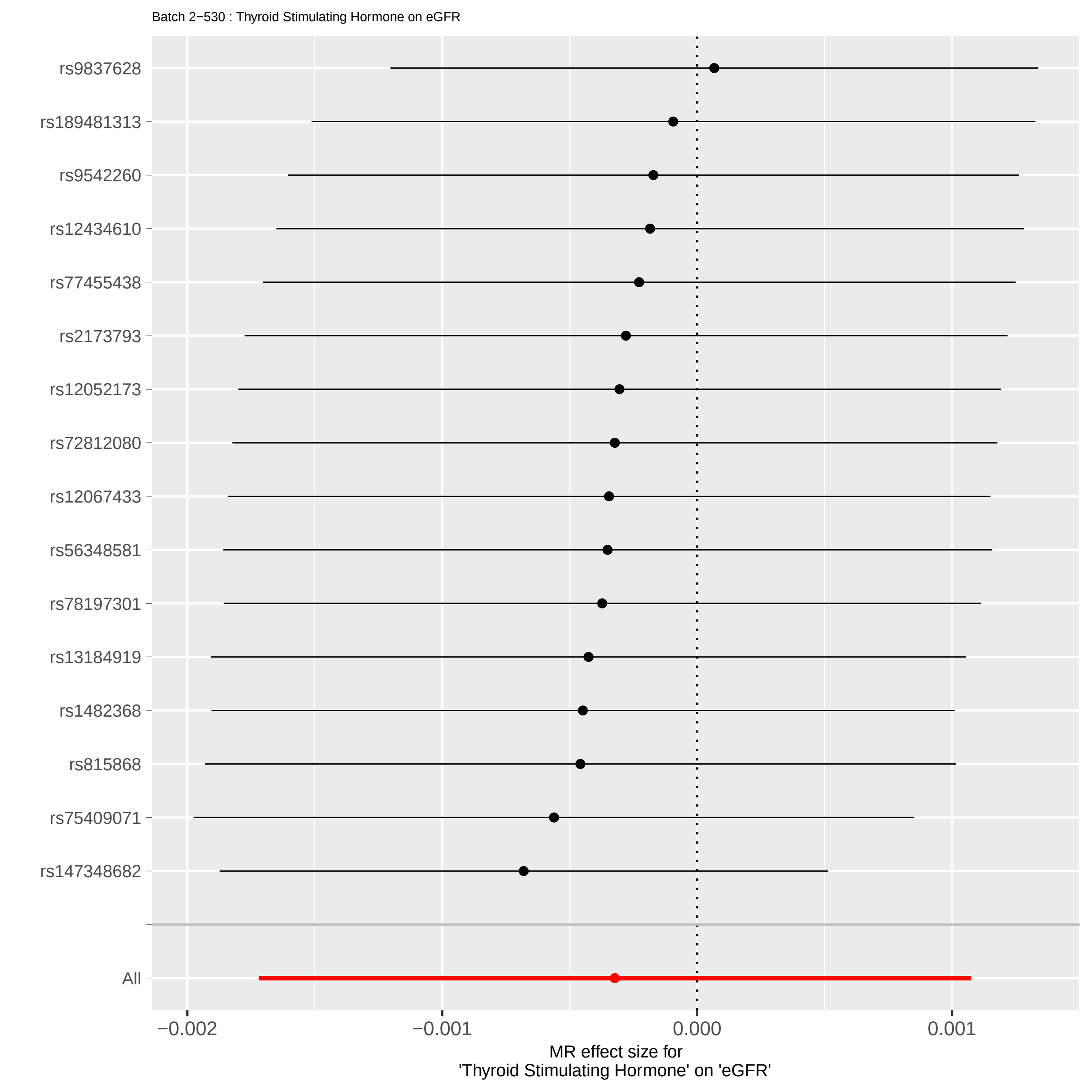

Batch 2−530 : Thyroid Stimulating Hormone on eGFR
rs9837628
rs189481313
rs9542260
rs12434610
rs77455438
rs2173793
rs12052173
rs72812080
rs12067433
rs56348581
rs78197301
rs13184919
rs1482368
rs815868
rs75409071
rs147348682
All
−0.002
−0.001
0.000
0.001
MR effect size for
'Thyroid Stimulating Hormone' on 'eGFR'

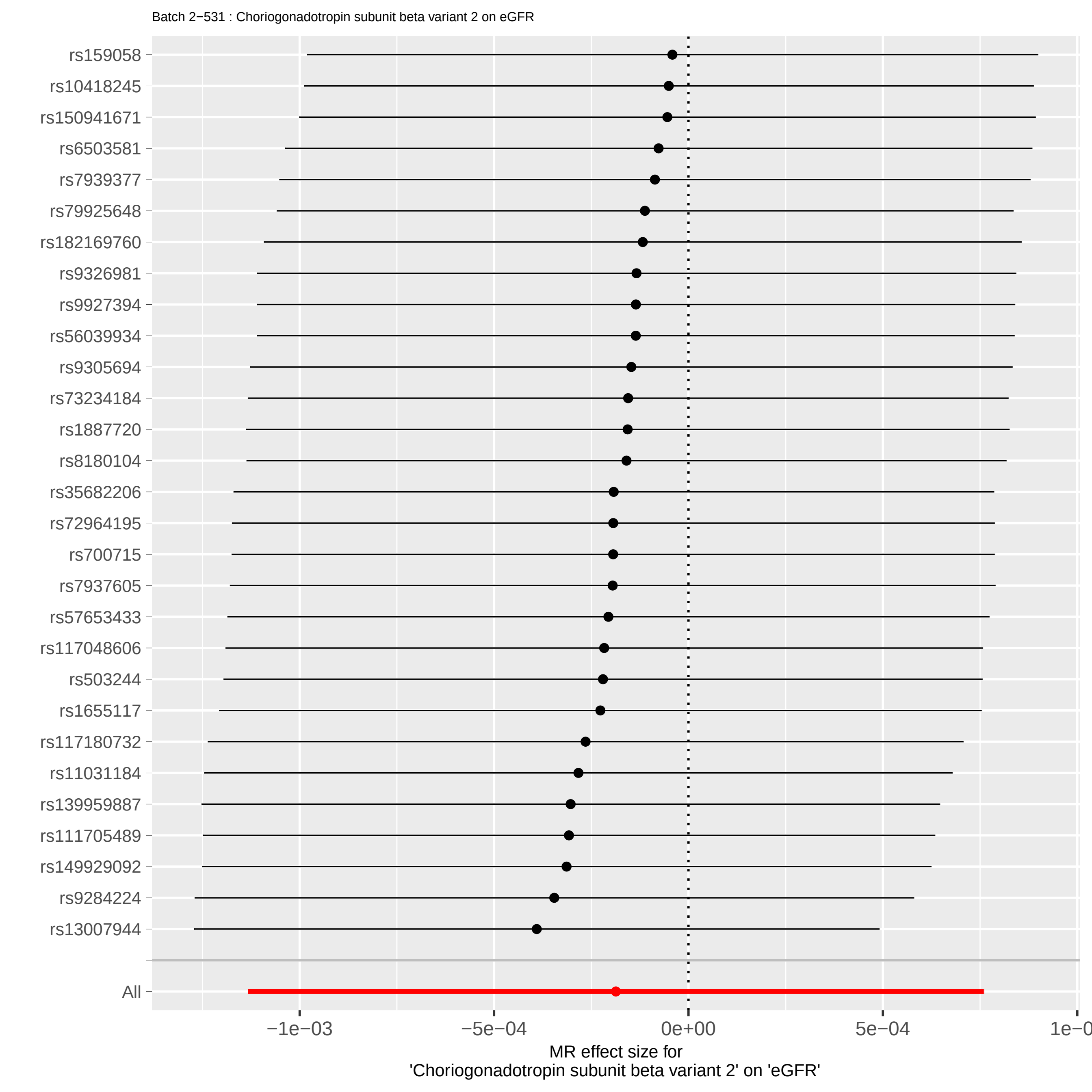

Batch 2−531 : Choriogonadotropin subunit beta variant 2 on eGFR
rs159058
rs10418245
rs150941671
rs6503581
rs7939377
rs79925648
rs182169760
rs9326981
rs9927394
rs56039934
rs9305694
rs73234184
rs1887720
rs8180104
rs35682206
rs72964195
rs700715
rs7937605
rs57653433
rs117048606
rs503244
rs1655117
rs117180732
rs11031184
rs139959887
rs111705489
rs149929092
rs9284224
rs13007944
All
−1e−03
−5e−04
0e+00
5e−04
MR effect size for
'Choriogonadotropin subunit beta variant 2' on 'eGFR'

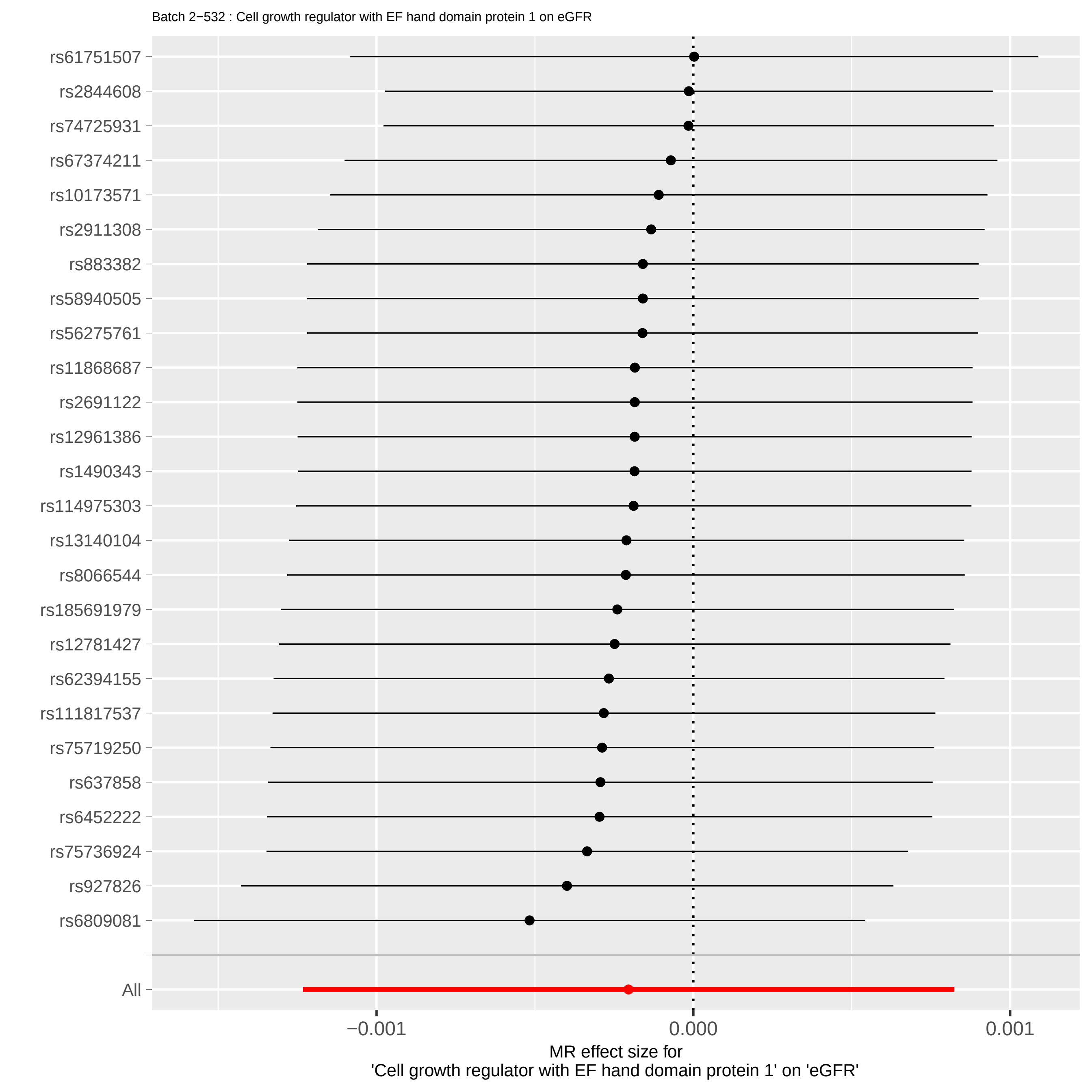

Batch 2−532 : Cell growth regulator with EF hand domain protein 1 on eGFR
rs61751507
rs2844608
rs74725931
rs67374211
rs10173571
rs2911308
rs883382
rs58940505
rs56275761
rs11868687
rs2691122
rs12961386
rs1490343
rs114975303
rs13140104
rs8066544
rs185691979
rs12781427
rs62394155
rs111817537
rs75719250
rs637858
rs6452222
rs75736924
rs927826
rs6809081
All
−0.001
0.000
0.001
MR effect size for
'Cell growth regulator with EF hand domain protein 1' on 'eGFR'

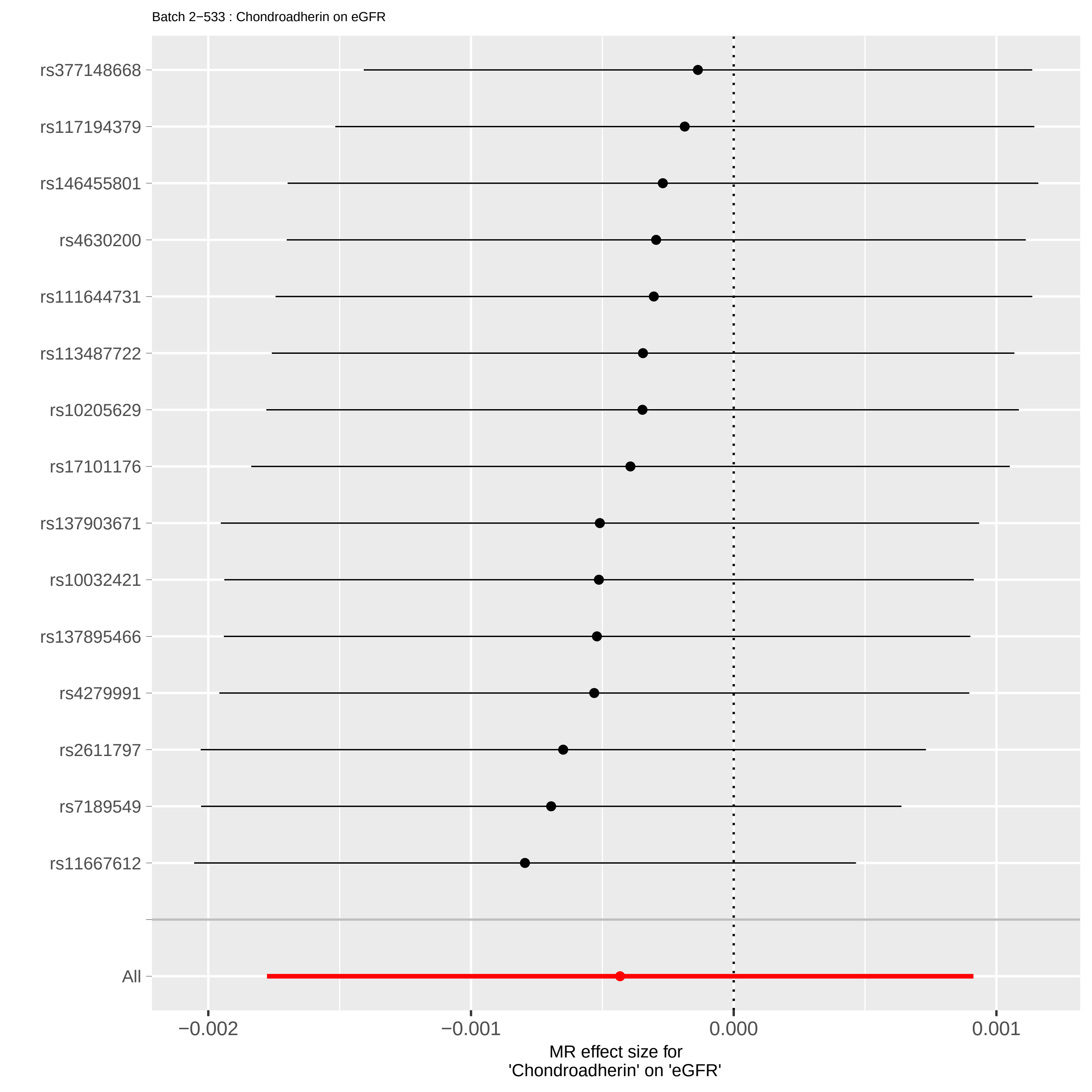

Batch 2−533 : Chondroadherin on eGFR
rs377148668
rs117194379
rs146455801
rs4630200
rs111644731
rs113487722
rs10205629
rs17101176
rs137903671
rs10032421
rs137895466
rs4279991
rs2611797
rs7189549
rs11667612
All
−0.002
−0.001
0.000
0.001
MR effect size for
'Chondroadherin' on 'eGFR'

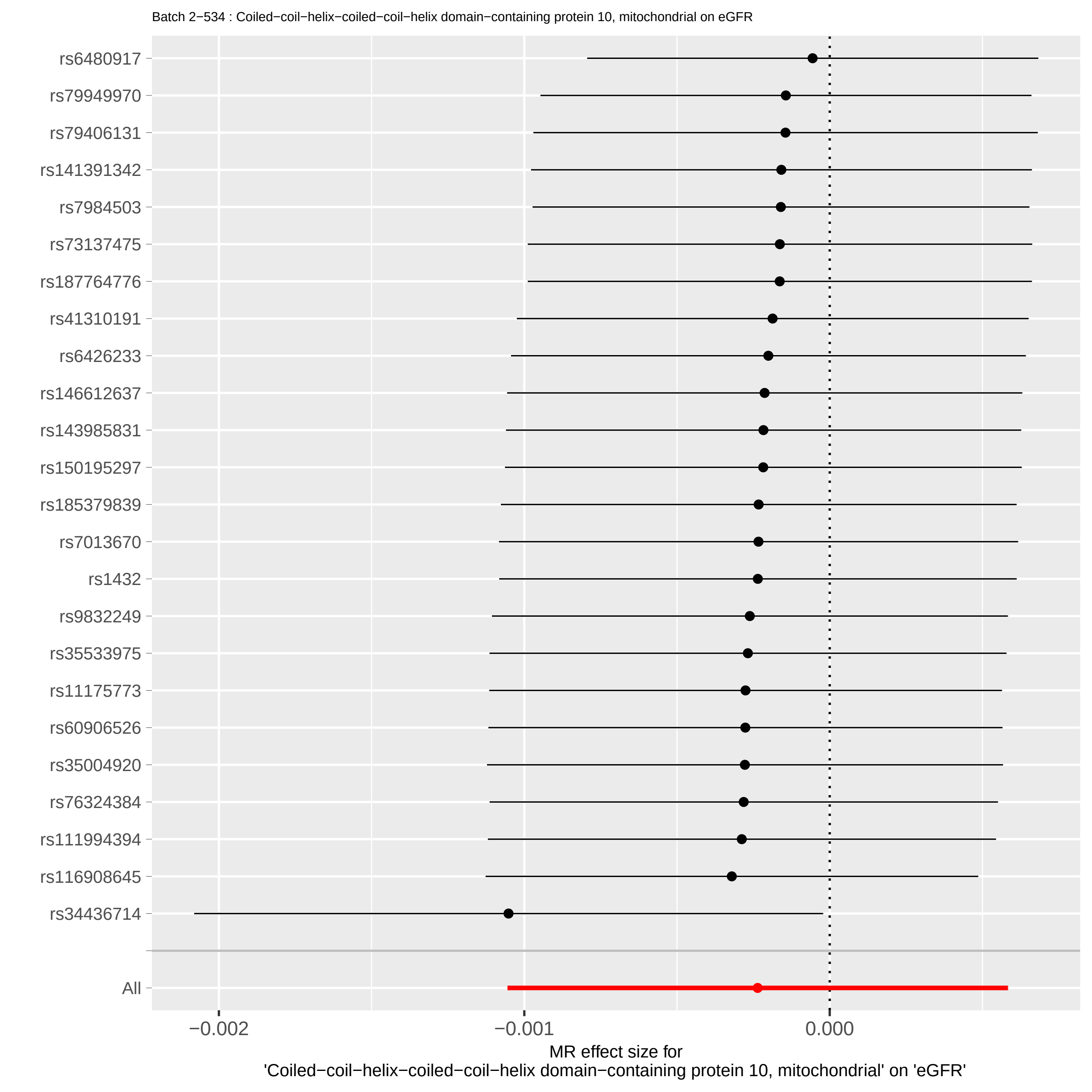

Batch 2−534 : Coiled−coil−helix−coiled−coil−helix domain−containing protein 10, mitochondrial on eGFR
rs6480917
rs79949970
rs79406131
rs141391342
rs7984503
rs73137475
rs187764776
rs41310191
rs6426233
rs146612637
rs143985831
rs150195297
rs185379839
rs7013670
rs1432
rs9832249
rs35533975
rs11175773
rs60906526
rs35004920
rs76324384
rs111994394
rs116908645
rs34436714
All
−0.002
−0.001
0.000
MR effect size for
'Coiled−coil−helix−coiled−coil−helix domain−containing protein 10, mitochondrial' on 'eGFR'

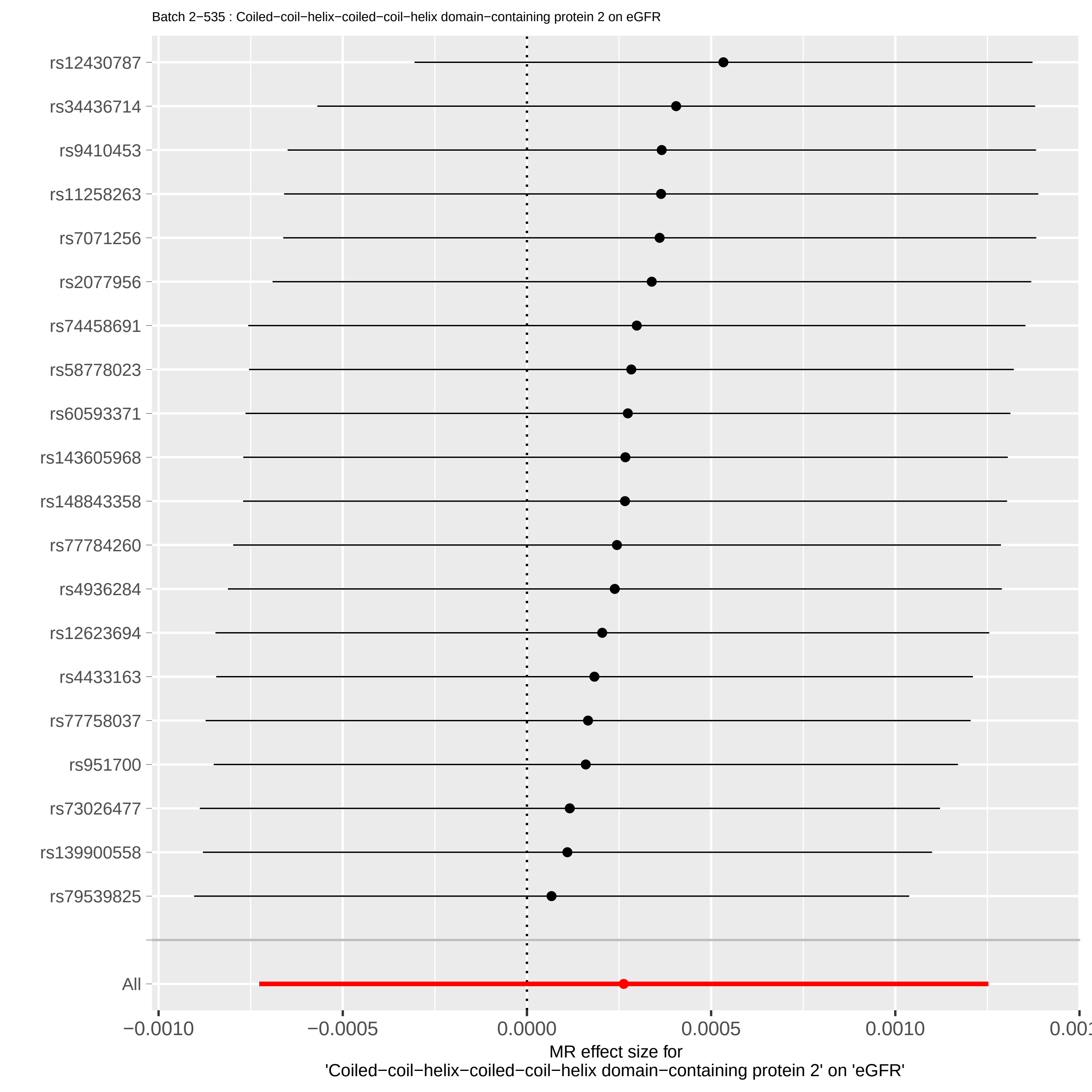
Batch 2−535 : Coiled−coil−helix−coiled−coil−helix domain−containing protein 2 on eGFR
rs12430787
rs34436714
rs9410453
rs11258263
rs7071256
rs2077956
rs74458691
rs58778023
rs60593371
rs143605968
rs148843358
rs77784260
rs4936284
rs12623694
rs4433163
rs77758037
rs951700
rs73026477
rs139900558
rs79539825
All
−0.0010
−0.0005
0.0000
0.0005
0.0010
MR effect size for
'Coiled−coil−helix−coiled−coil−helix domain−containing protein 2' on 'eGFR'

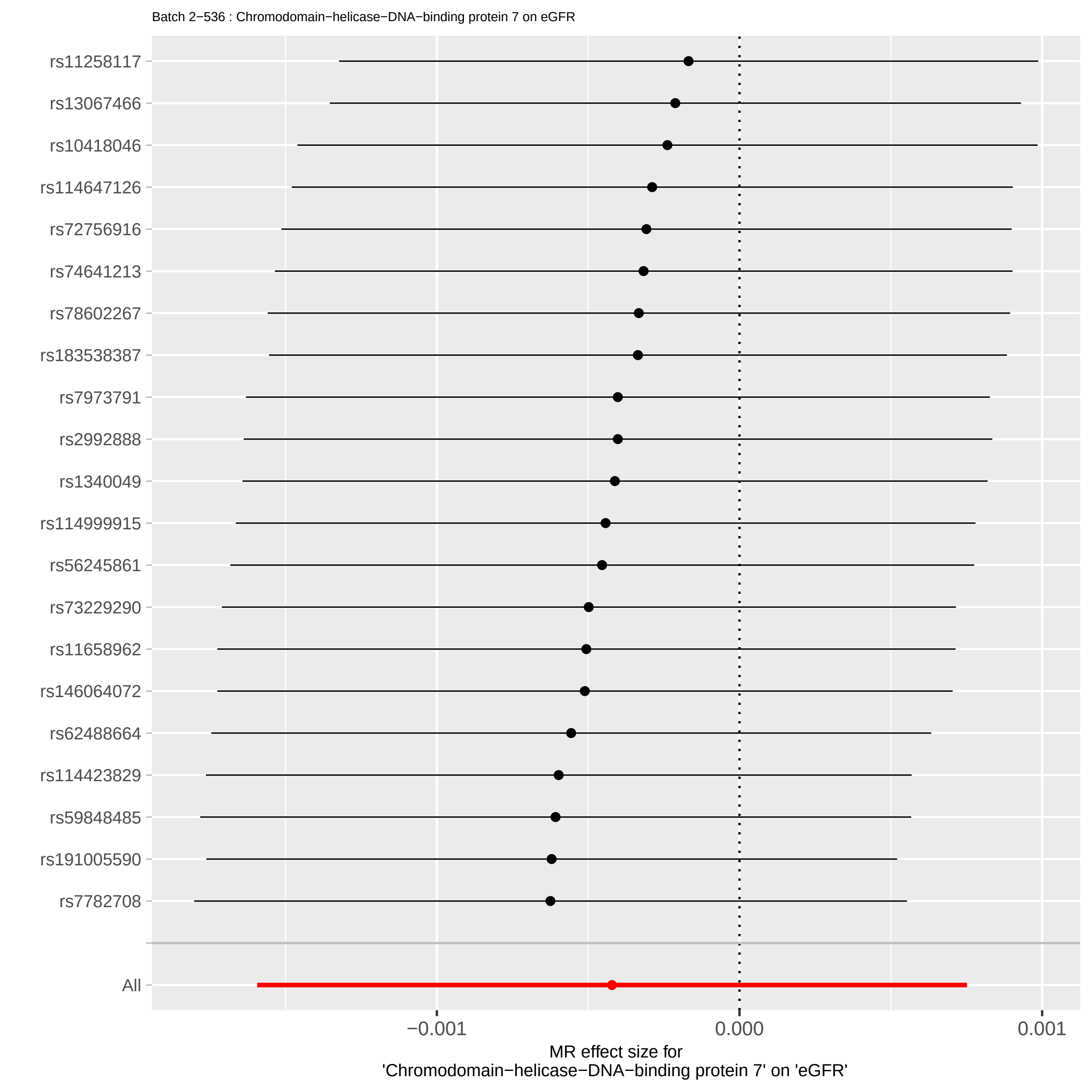

Batch 2−536 : Chromodomain−helicase−DNA−binding protein 7 on eGFR
rs11258117
rs13067466
rs10418046
rs114647126
rs72756916
rs74641213
rs78602267
rs183538387
rs7973791
rs2992888
rs1340049
rs114999915
rs56245861
rs73229290
rs11658962
rs146064072
rs62488664
rs114423829
rs59848485
rs191005590
rs7782708
All
−0.001
0.000
0.001
MR effect size for
'Chromodomain−helicase−DNA−binding protein 7' on 'eGFR'

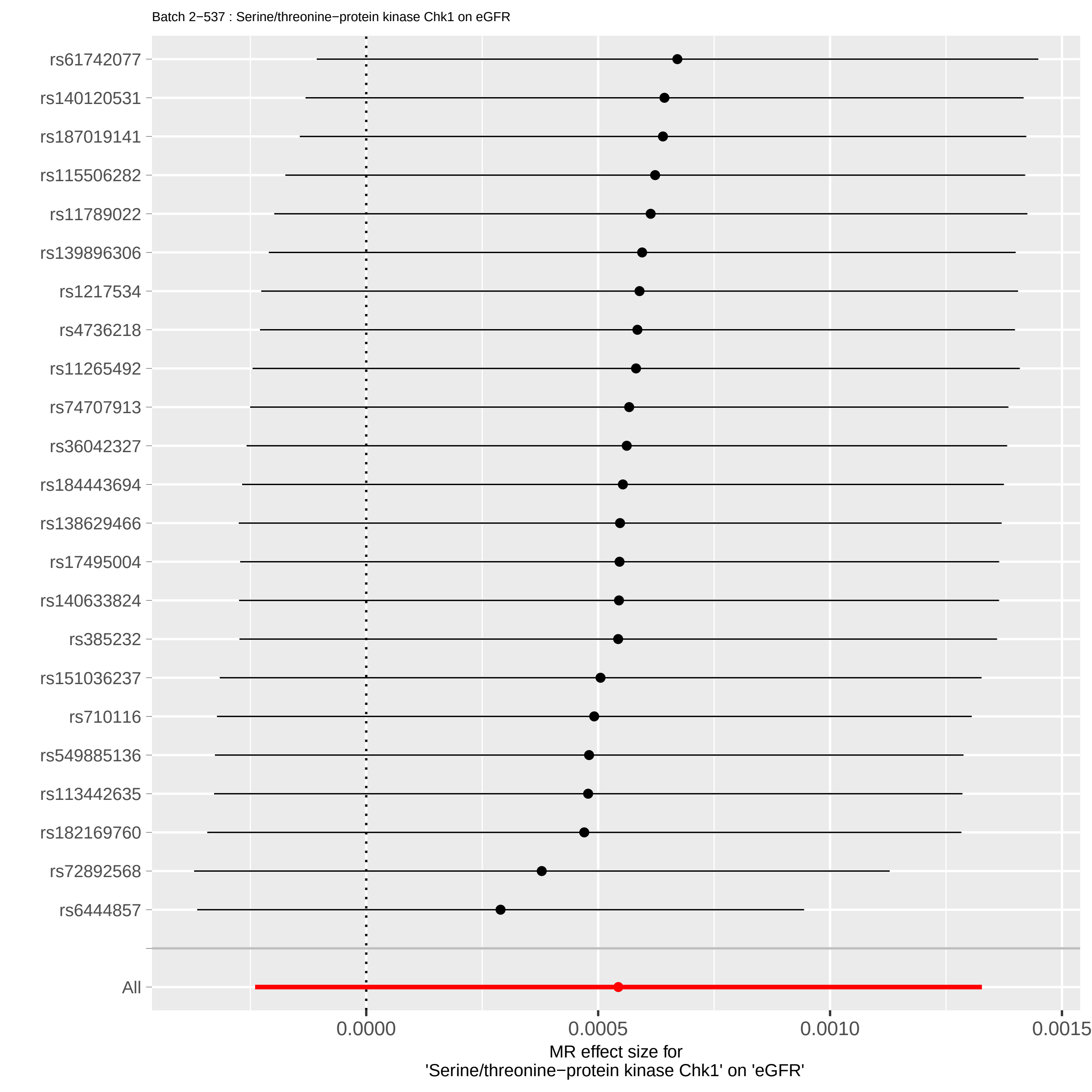
Batch 2−537 : Serine/threonine−protein kinase Chk1 on eGFR
rs61742077
rs140120531
rs187019141
rs115506282
rs11789022
rs139896306
rs1217534
rs4736218
rs11265492
rs74707913
rs36042327
rs184443694
rs138629466
rs17495004
rs140633824
rs385232
rs151036237
rs710116
rs549885136
rs113442635
rs182169760
rs72892568
rs6444857
All
0.0000
0.0005
0.0010
0.0015
MR effect size for
'Serine/threonine−protein kinase Chk1' on 'eGFR'

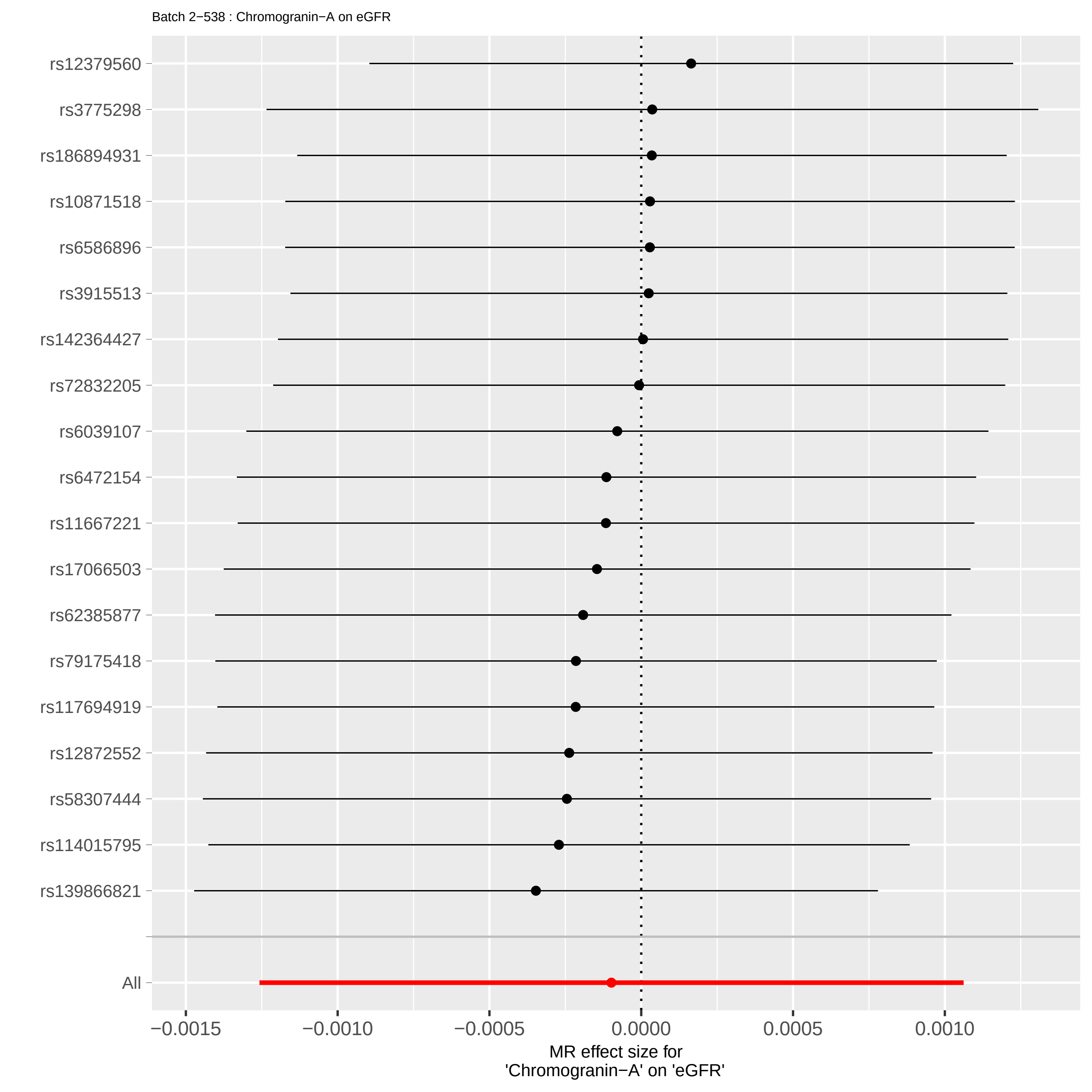

Batch 2−538 : Chromogranin−A on eGFR
rs12379560
rs3775298
rs186894931
rs10871518
rs6586896
rs3915513
rs142364427
rs72832205
rs6039107
rs6472154
rs11667221
rs17066503
rs62385877
rs79175418
rs117694919
rs12872552
rs58307444
rs114015795
rs139866821
All
−0.0015
−0.0010
−0.0005
0.0000
0.0005
0.0010
MR effect size for
'Chromogranin−A' on 'eGFR'

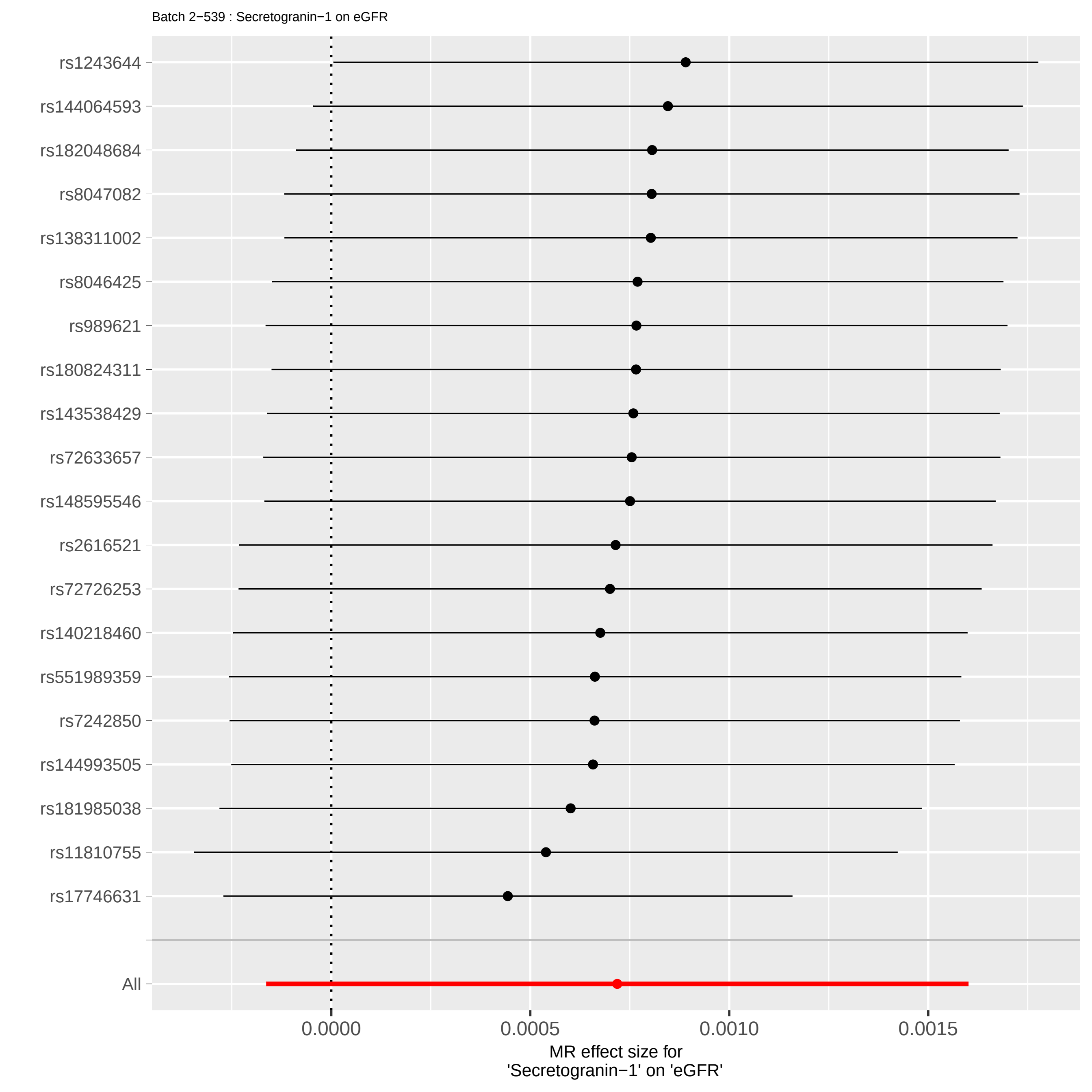

Batch 2−539 : Secretogranin−1 on eGFR
rs1243644
rs144064593
rs182048684
rs8047082
rs138311002
rs8046425
rs989621
rs180824311
rs143538429
rs72633657
rs148595546
rs2616521
rs72726253
rs140218460
rs551989359
rs7242850
rs144993505
rs181985038
rs11810755
rs17746631
All
0.0000
0.0005
0.0010
0.0015
MR effect size for
'Secretogranin−1' on 'eGFR'

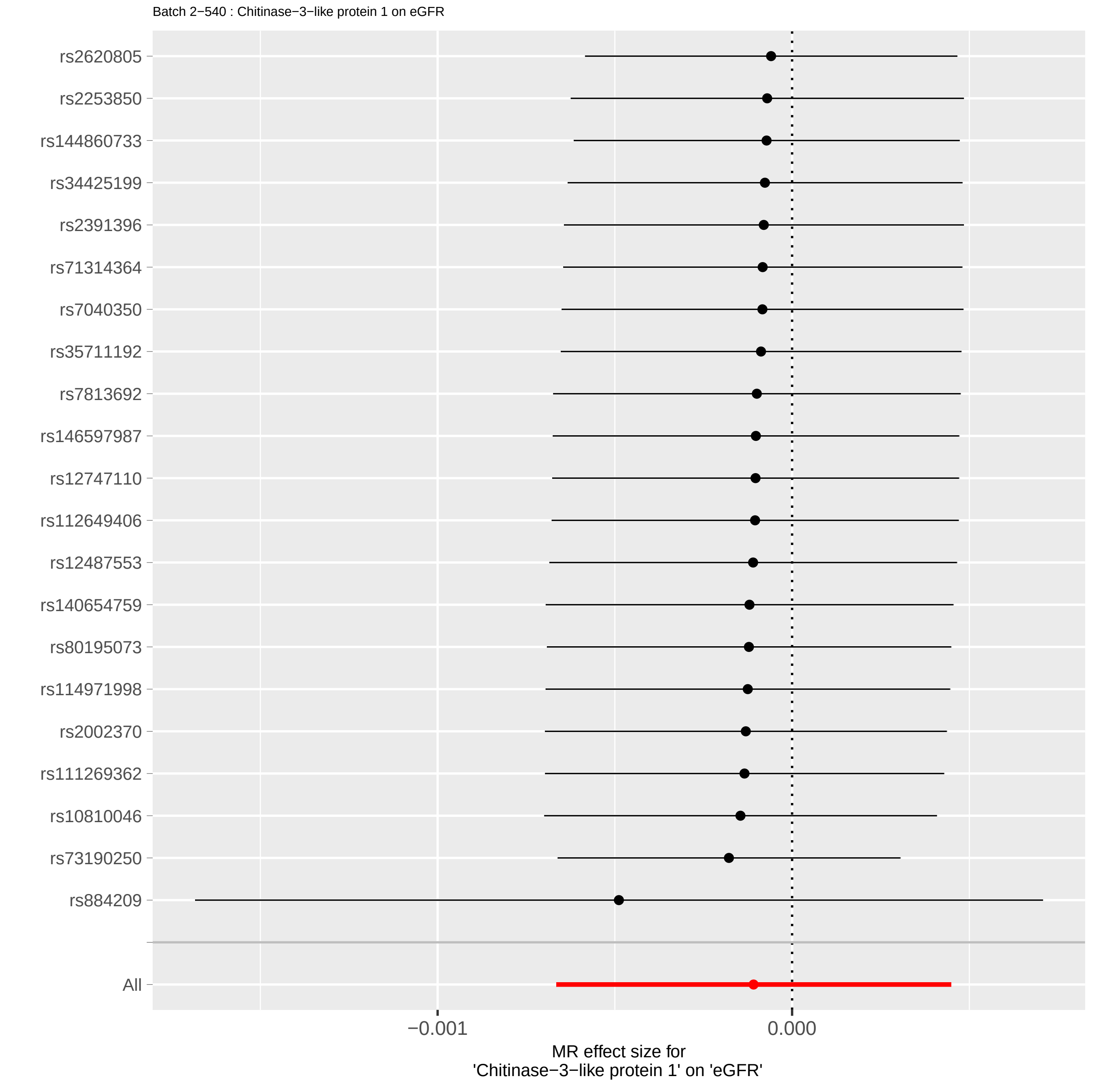

Batch 2−540 : Chitinase−3−like protein 1 on eGFR
rs2620805
rs2253850
rs144860733
rs34425199
rs2391396
rs71314364
rs7040350
rs35711192
rs7813692
rs146597987
rs12747110
rs112649406
rs12487553
rs140654759
rs80195073
rs114971998
rs2002370
rs111269362
rs10810046
rs73190250
rs884209
All
−0.001
0.000
MR effect size for
'Chitinase−3−like protein 1' on 'eGFR'

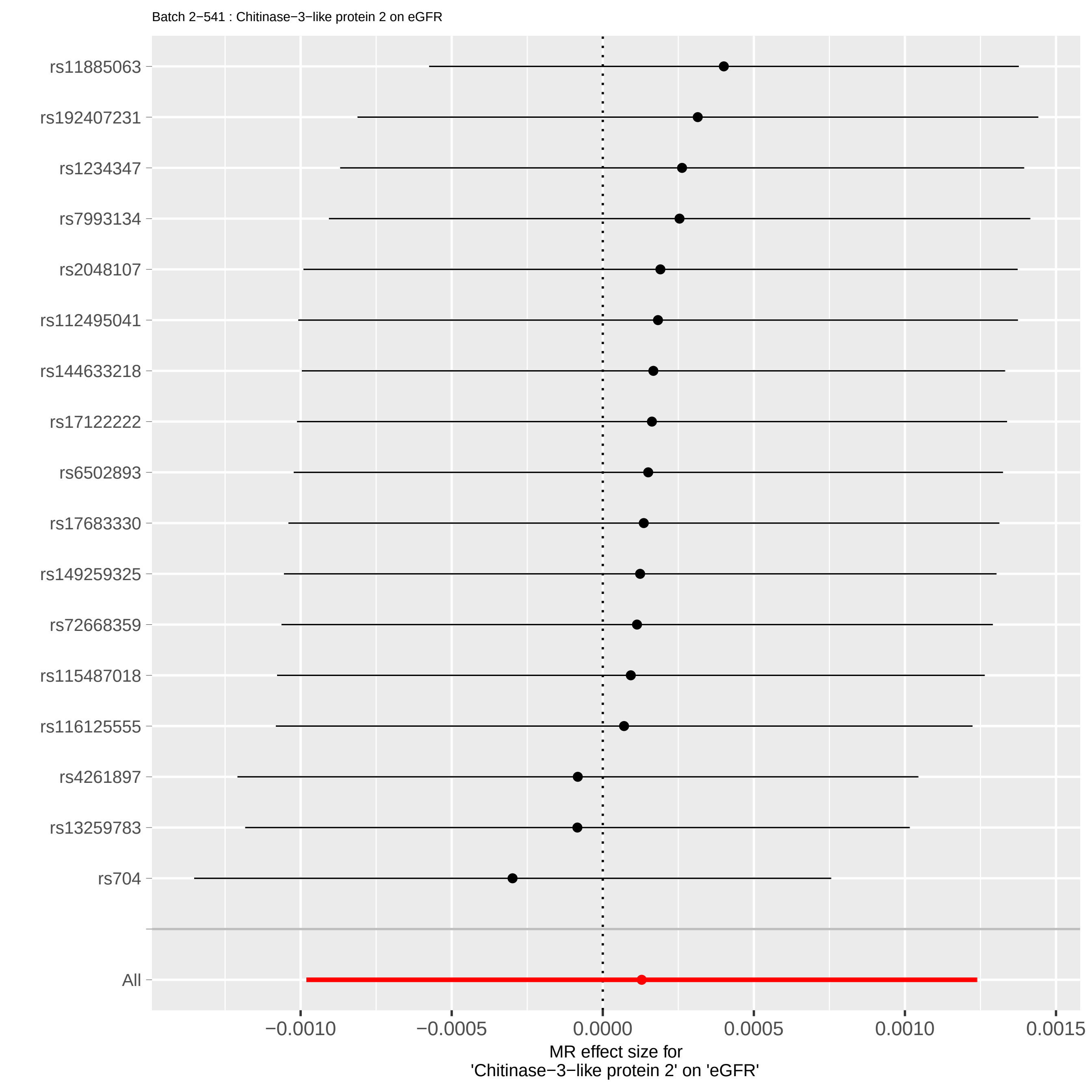

Batch 2−541 : Chitinase−3−like protein 2 on eGFR
rs11885063
rs192407231
rs1234347
rs7993134
rs2048107
rs112495041
rs144633218
rs17122222
rs6502893
rs17683330
rs149259325
rs72668359
rs115487018
rs116125555
rs4261897
rs13259783
rs704
All
−0.0010
−0.0005
0.0000
0.0005
0.0010
0.0015
MR effect size for
'Chitinase−3−like protein 2' on 'eGFR'

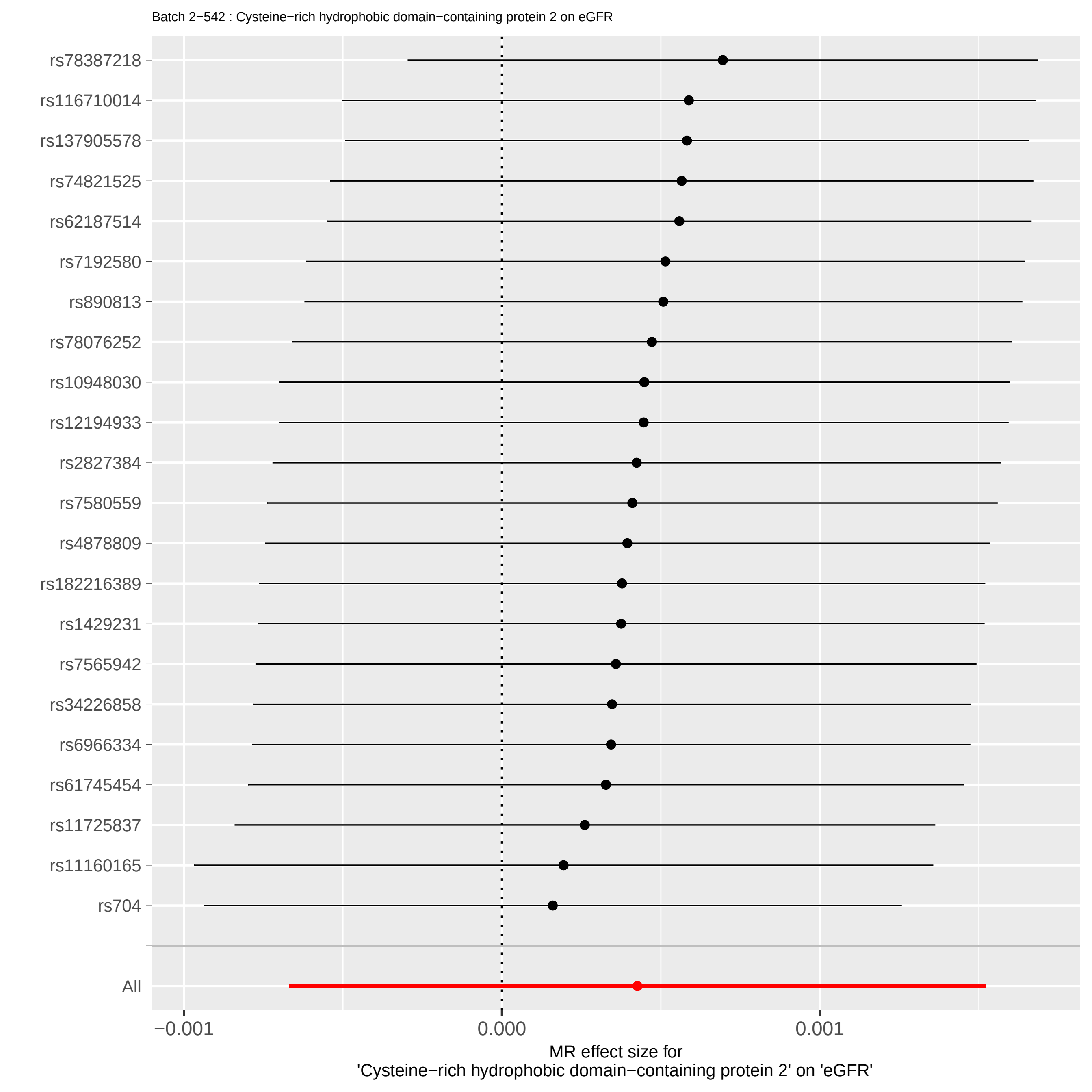

Batch 2−542 : Cysteine−rich hydrophobic domain−containing protein 2 on eGFR
rs78387218
rs116710014
rs137905578
rs74821525
rs62187514
rs7192580
rs890813
rs78076252
rs10948030
rs12194933
rs2827384
rs7580559
rs4878809
rs182216389
rs1429231
rs7565942
rs34226858
rs6966334
rs61745454
rs11725837
rs11160165
rs704
All
−0.001
0.000
0.001
MR effect size for
'Cysteine−rich hydrophobic domain−containing protein 2' on 'eGFR'

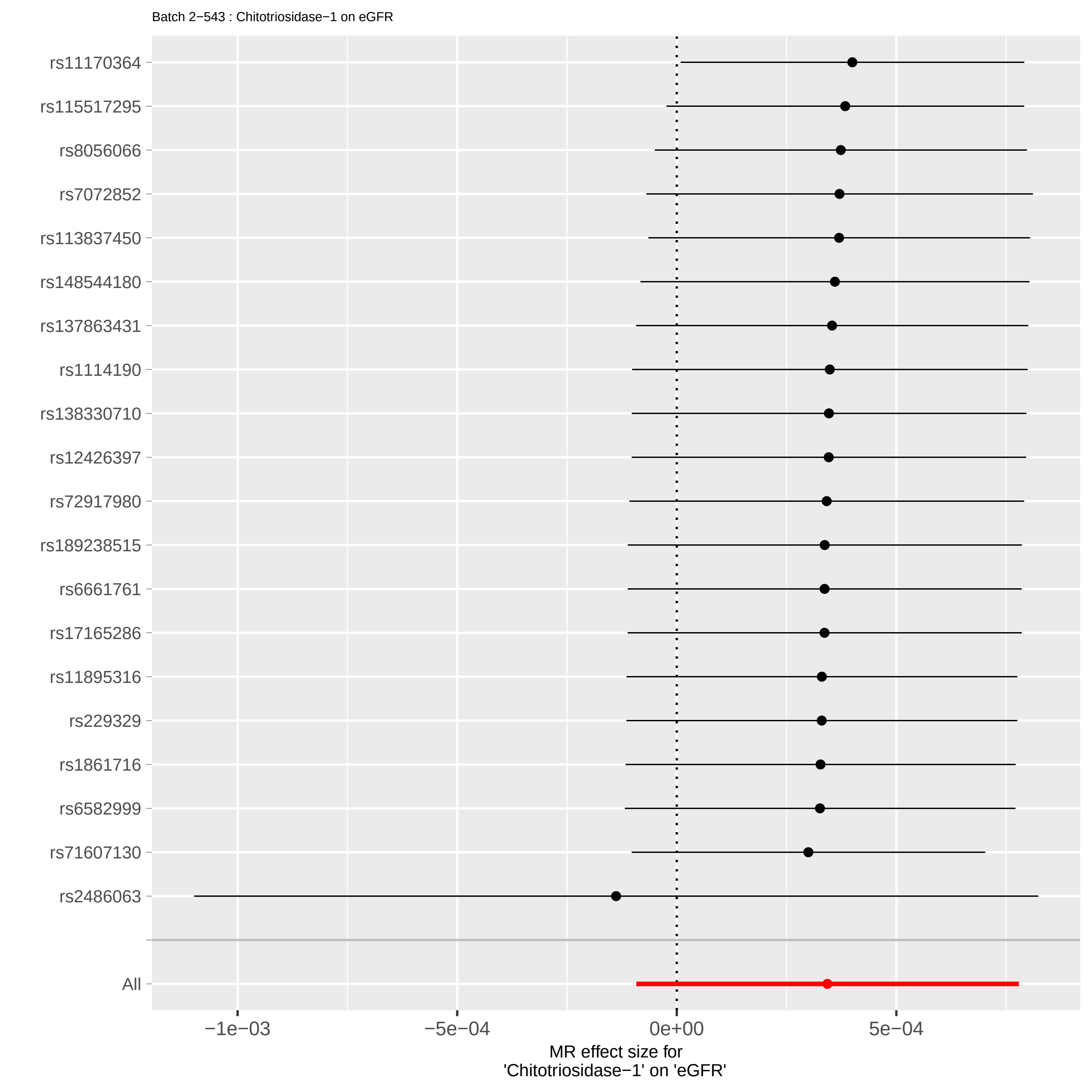
Batch 2−543 : Chitotriosidase−1 on eGFR
rs11170364
rs115517295
rs8056066
rs7072852
rs113837450
rs148544180
rs137863431
rs1114190
rs138330710
rs12426397
rs72917980
rs189238515
rs6661761
rs17165286
rs11895316
rs229329
rs1861716
rs6582999
rs71607130
rs2486063
All
−1e−03
−5e−04
0e+00
5e−04
MR effect size for
'Chitotriosidase−1' on 'eGFR'

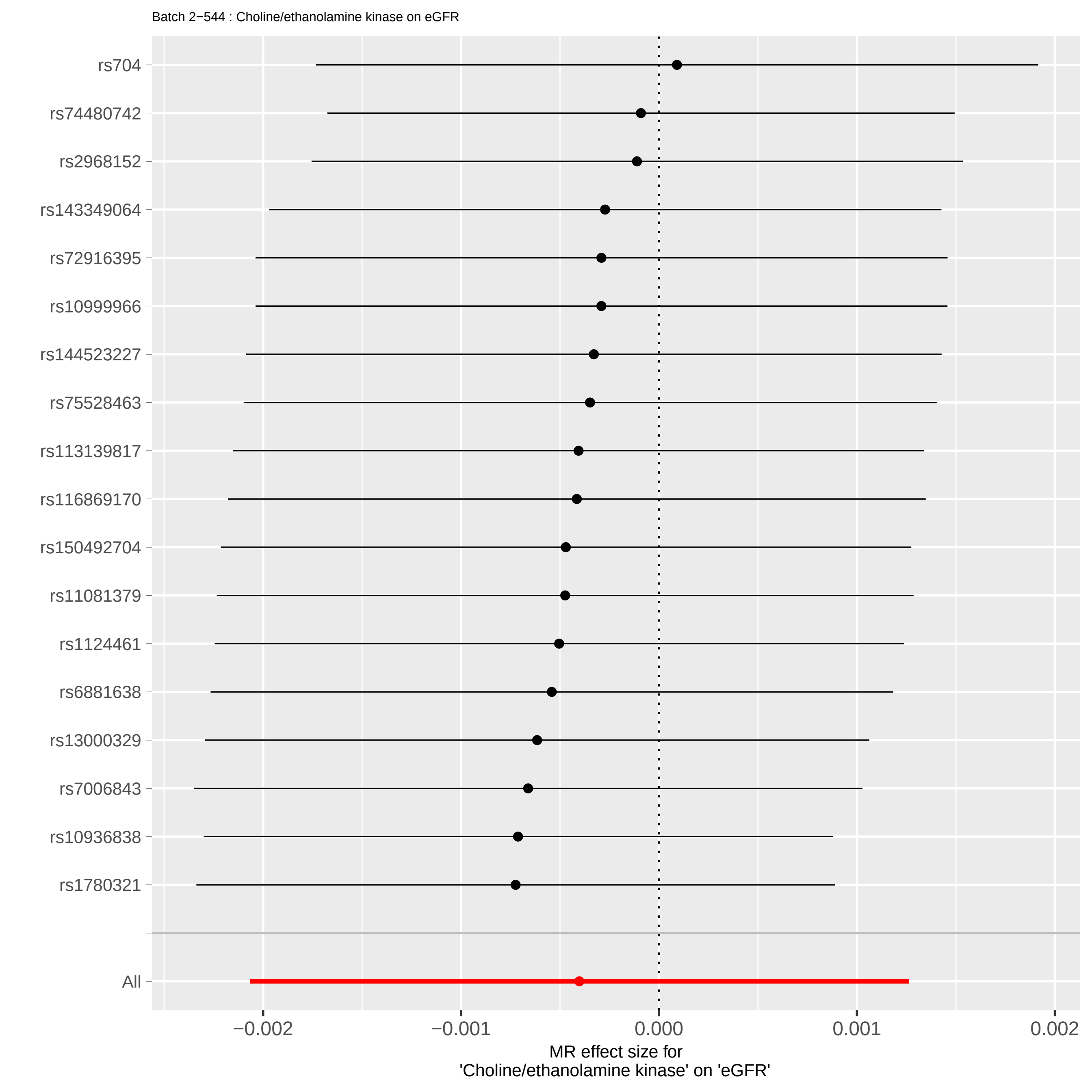
Batch 2−544 : Choline/ethanolamine kinase on eGFR
rs704
rs74480742
rs2968152
rs143349064
rs72916395
rs10999966
rs144523227
rs75528463
rs113139817
rs116869170
rs150492704
rs11081379
rs1124461
rs6881638
rs13000329
rs7006843
rs10936838
rs1780321
All
−0.002
−0.001
0.000
0.001
0.002
MR effect size for
'Choline/ethanolamine kinase' on 'eGFR'

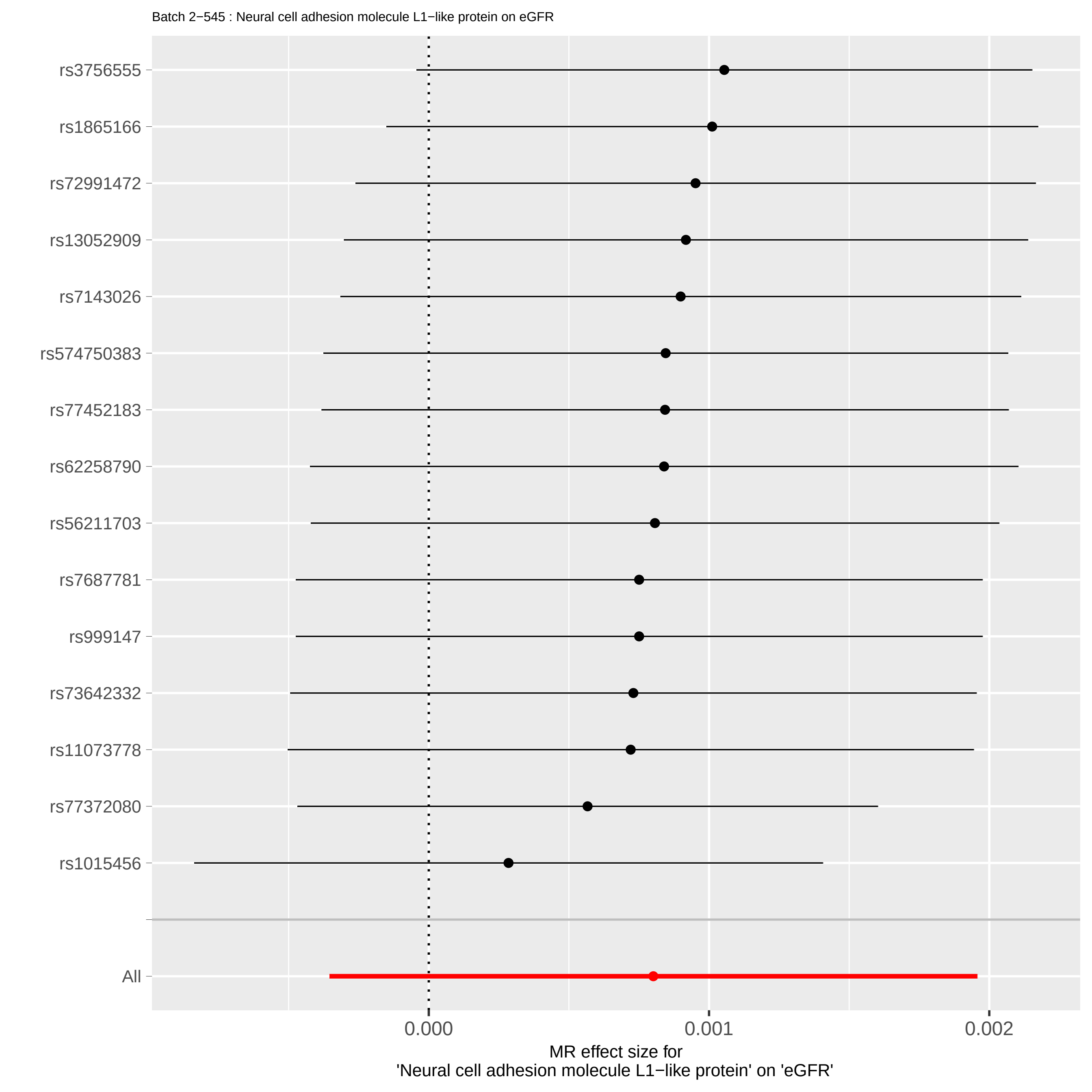

Batch 2−545 : Neural cell adhesion molecule L1−like protein on eGFR
rs3756555
rs1865166
rs72991472
rs13052909
rs7143026
rs574750383
rs77452183
rs62258790
rs56211703
rs7687781
rs999147
rs73642332
rs11073778
rs77372080
rs1015456
All
0.000
0.001
0.002
MR effect size for
'Neural cell adhesion molecule L1−like protein' on 'eGFR'

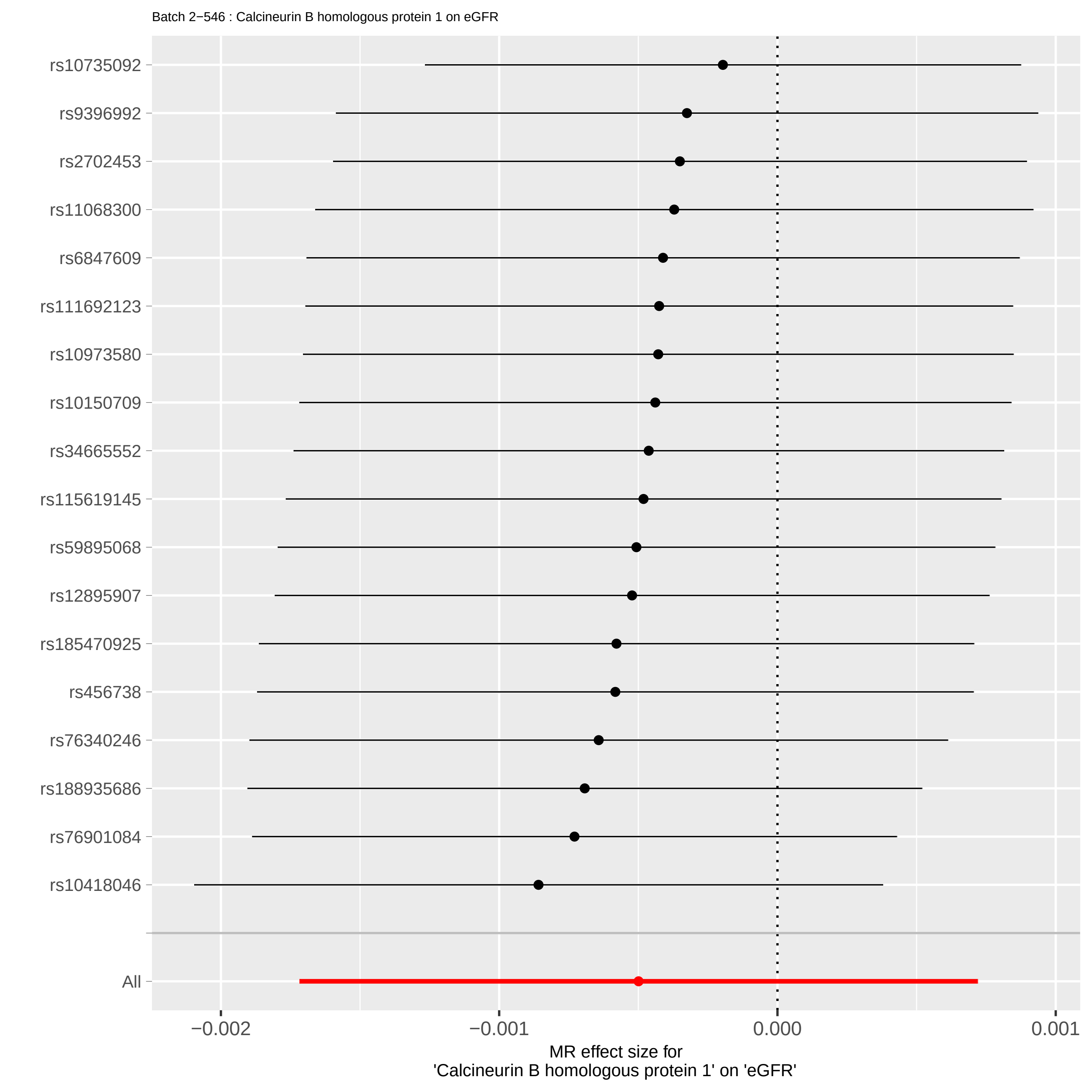

Batch 2−546 : Calcineurin B homologous protein 1 on eGFR
rs10735092
rs9396992
rs2702453
rs11068300
rs6847609
rs111692123
rs10973580
rs10150709
rs34665552
rs115619145
rs59895068
rs12895907
rs185470925
rs456738
rs76340246
rs188935686
rs76901084
rs10418046
All
−0.002
−0.001
0.000
0.001
MR effect size for
'Calcineurin B homologous protein 1' on 'eGFR'

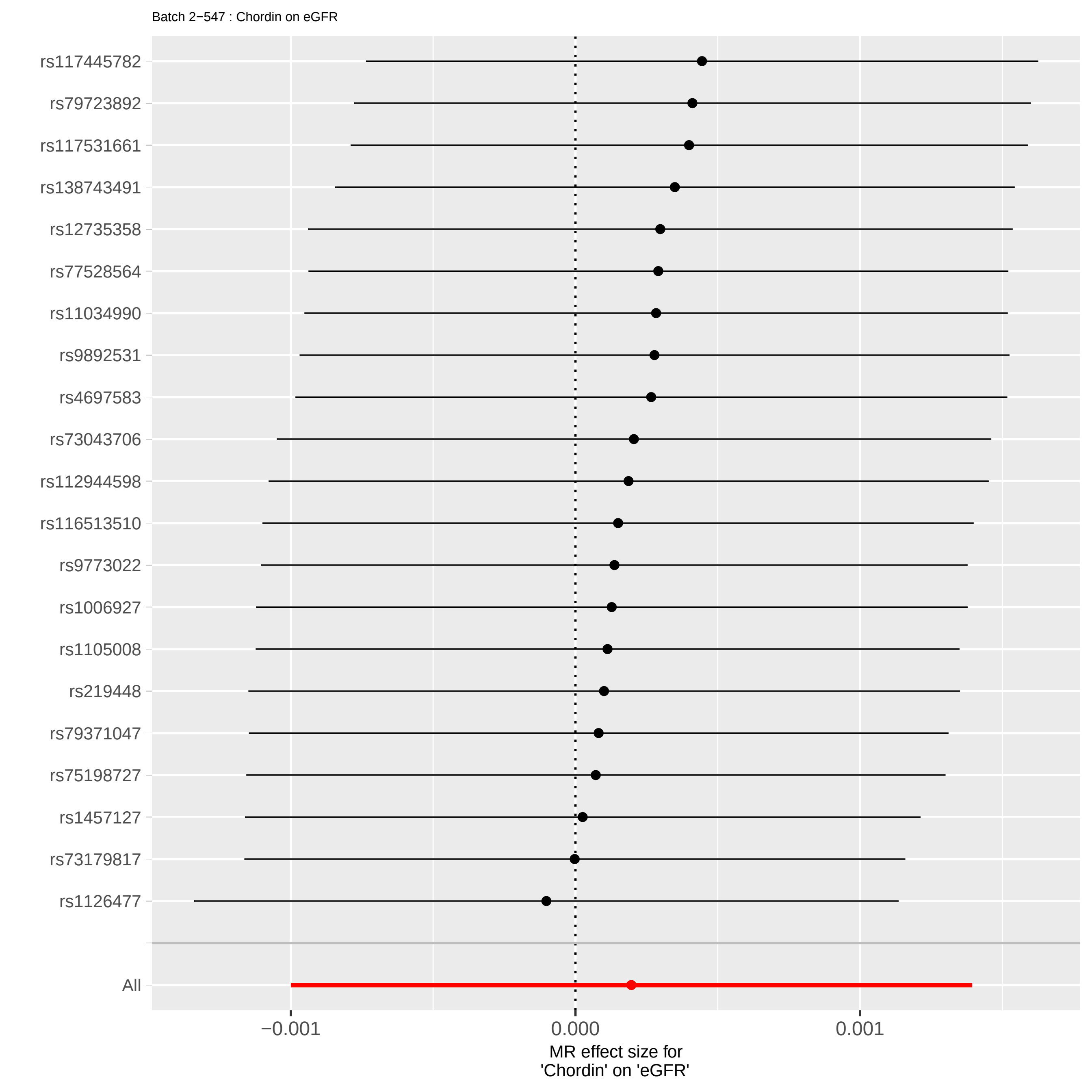

Batch 2−547 : Chordin on eGFR
rs117445782
rs79723892
rs117531661
rs138743491
rs12735358
rs77528564
rs11034990
rs9892531
rs4697583
rs73043706
rs112944598
rs116513510
rs9773022
rs1006927
rs1105008
rs219448
rs79371047
rs75198727
rs1457127
rs73179817
rs1126477
All
−0.001
0.000
0.001
MR effect size for
'Chordin' on 'eGFR'

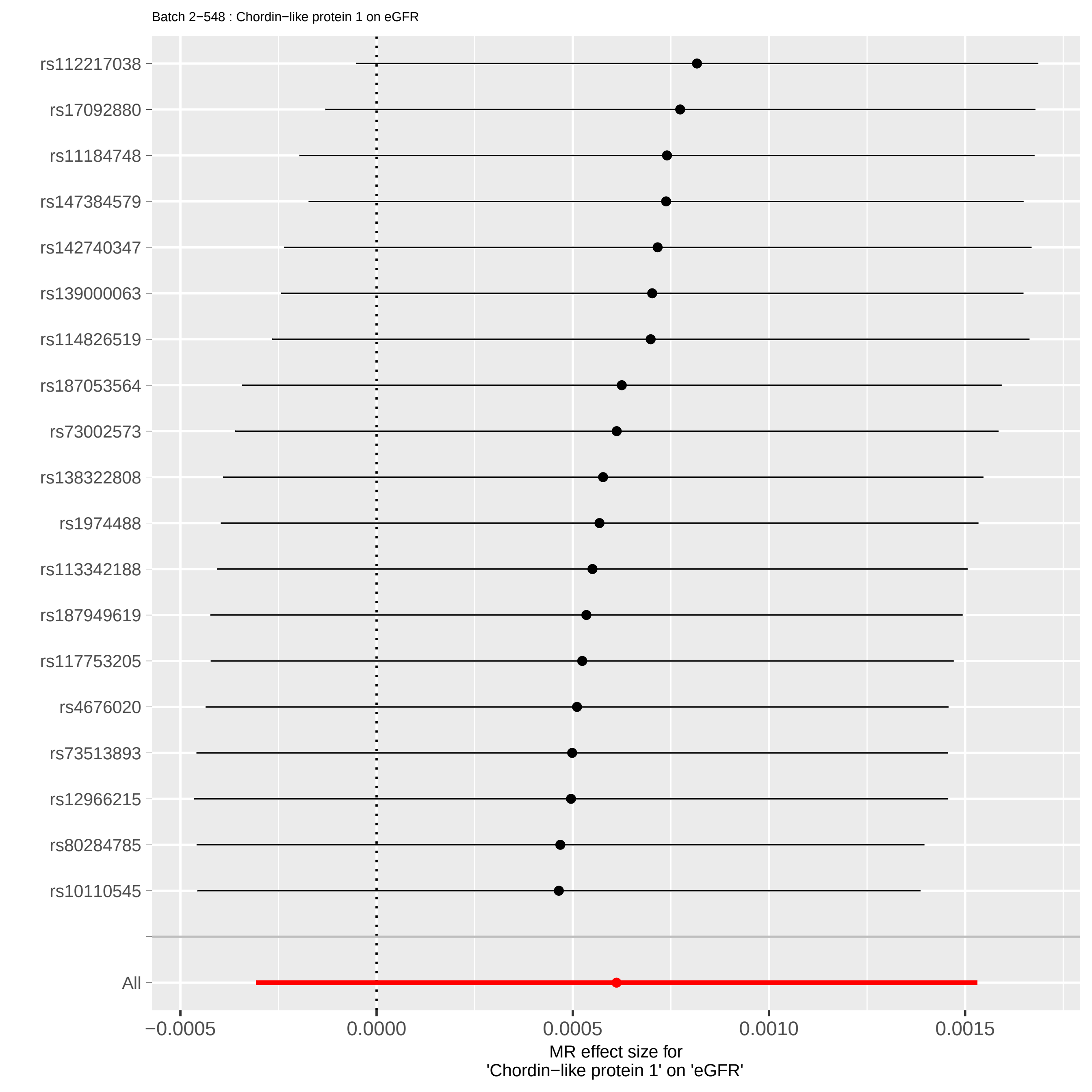

Batch 2−548 : Chordin−like protein 1 on eGFR
rs112217038
rs17092880
rs11184748
rs147384579
rs142740347
rs139000063
rs114826519
rs187053564
rs73002573
rs138322808
rs1974488
rs113342188
rs187949619
rs117753205
rs4676020
rs73513893
rs12966215
rs80284785
rs10110545
All
−0.0005
0.0000
0.0005
0.0010
0.0015
MR effect size for
'Chordin−like protein 1' on 'eGFR'

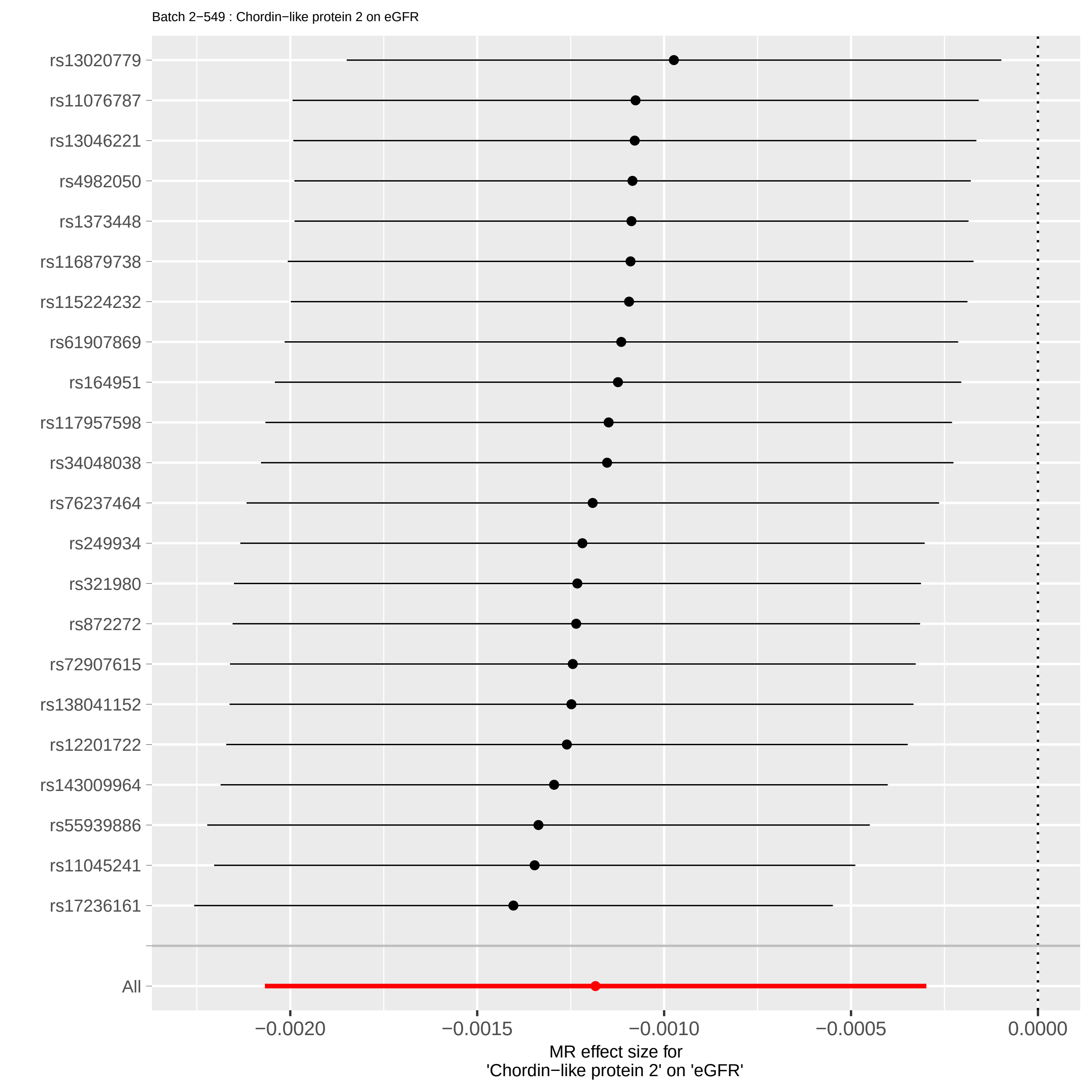
Batch 2−549 : Chordin−like protein 2 on eGFR
rs13020779
rs11076787
rs13046221
rs4982050
rs1373448
rs116879738
rs115224232
rs61907869
rs164951
rs117957598
rs34048038
rs76237464
rs249934
rs321980
rs872272
rs72907615
rs138041152
rs12201722
rs143009964
rs55939886
rs11045241
rs17236161
All
−0.0020
−0.0015
−0.0010
−0.0005
0.0000
MR effect size for
'Chordin−like protein 2' on 'eGFR'

Batch 2−550 : Carbohydrate sulfotransferase 11 on eGFR

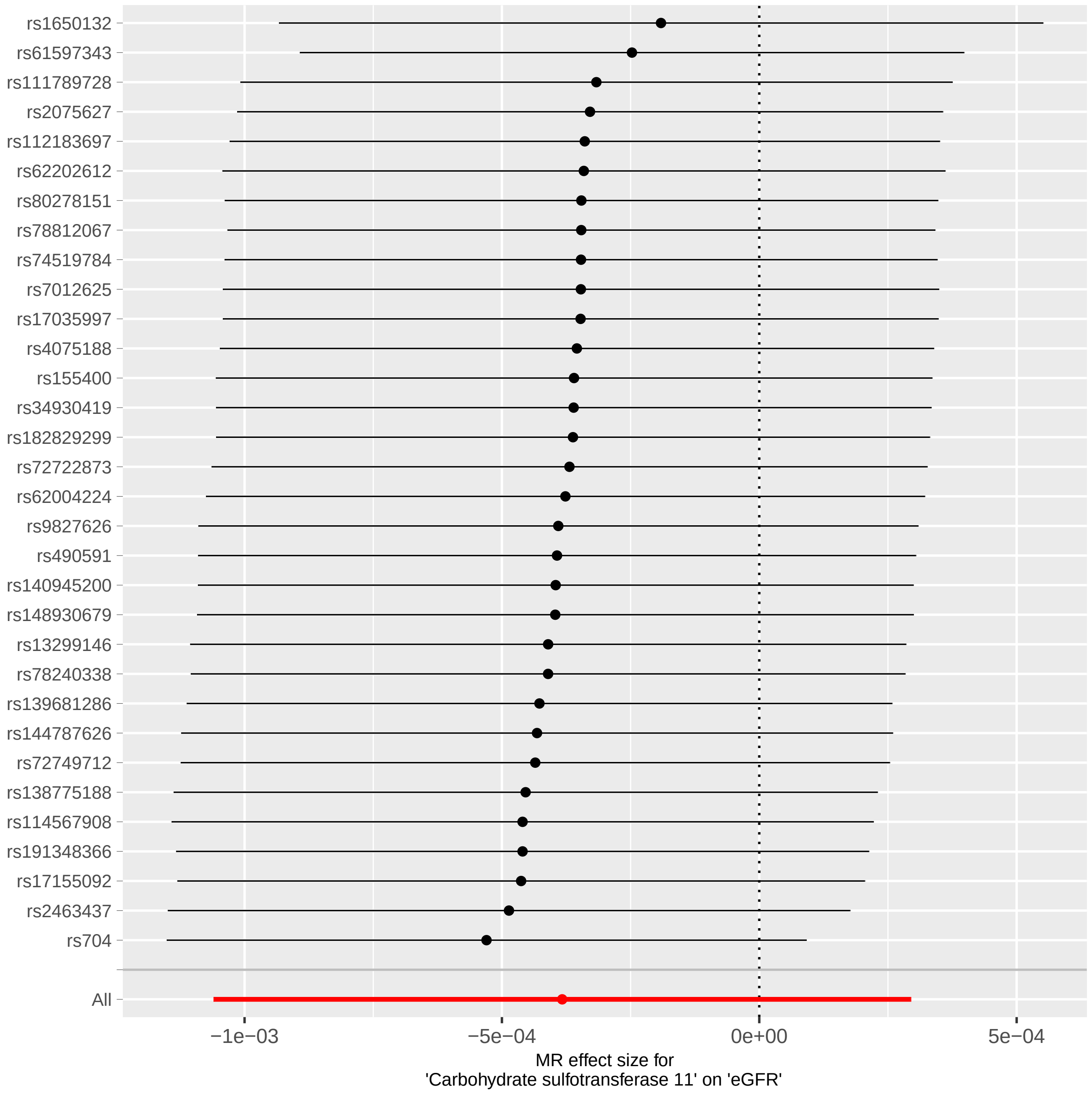

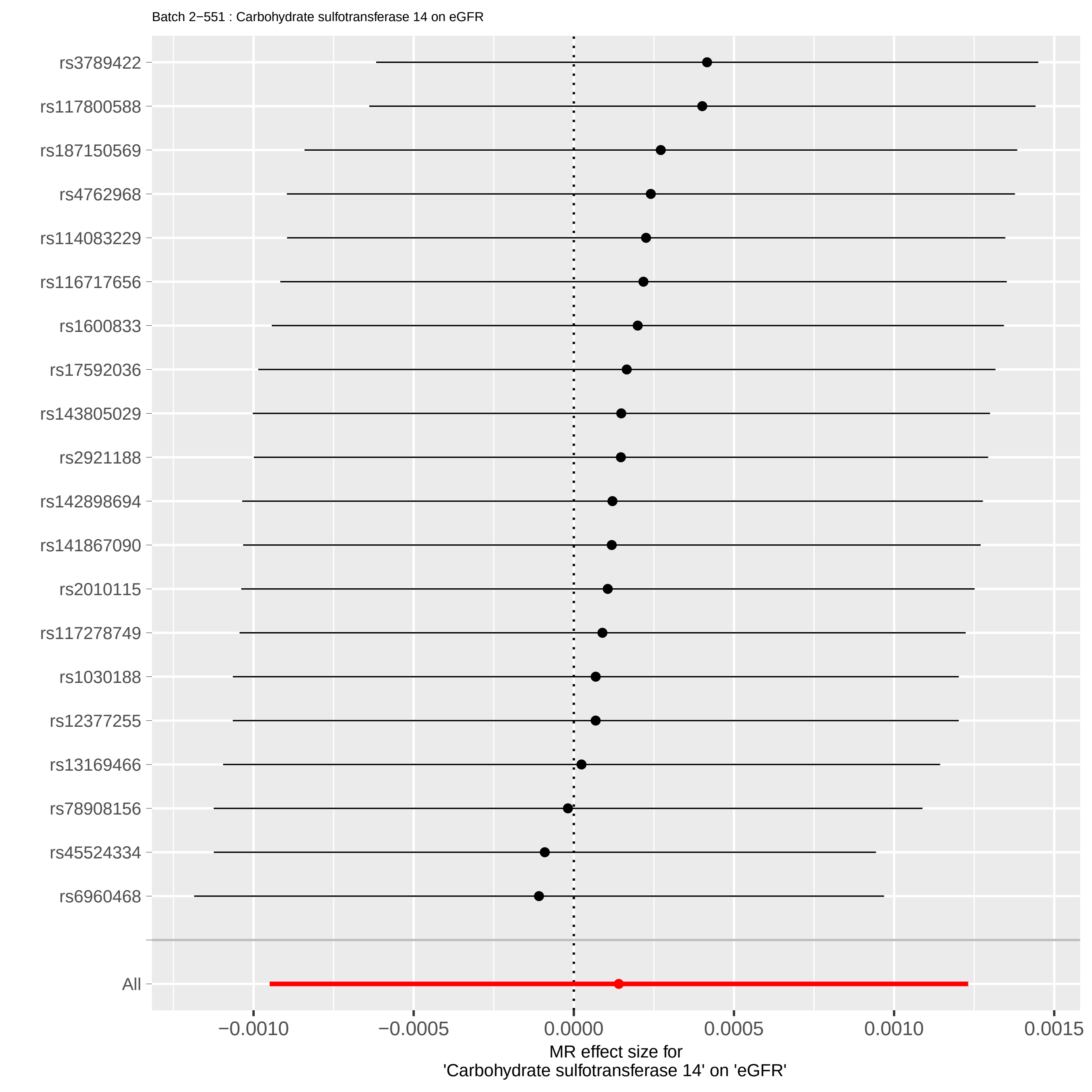

Batch 2−551 : Carbohydrate sulfotransferase 14 on eGFR
rs3789422
rs117800588
rs187150569
rs4762968
rs114083229
rs116717656
rs1600833
rs17592036
rs143805029
rs2921188
rs142898694
rs141867090
rs2010115
rs117278749
rs1030188
rs12377255
rs13169466
rs78908156
rs45524334
rs6960468
All
−0.0010
−0.0005
0.0000
0.0005
0.0010
0.0015
MR effect size for
'Carbohydrate sulfotransferase 14' on 'eGFR'

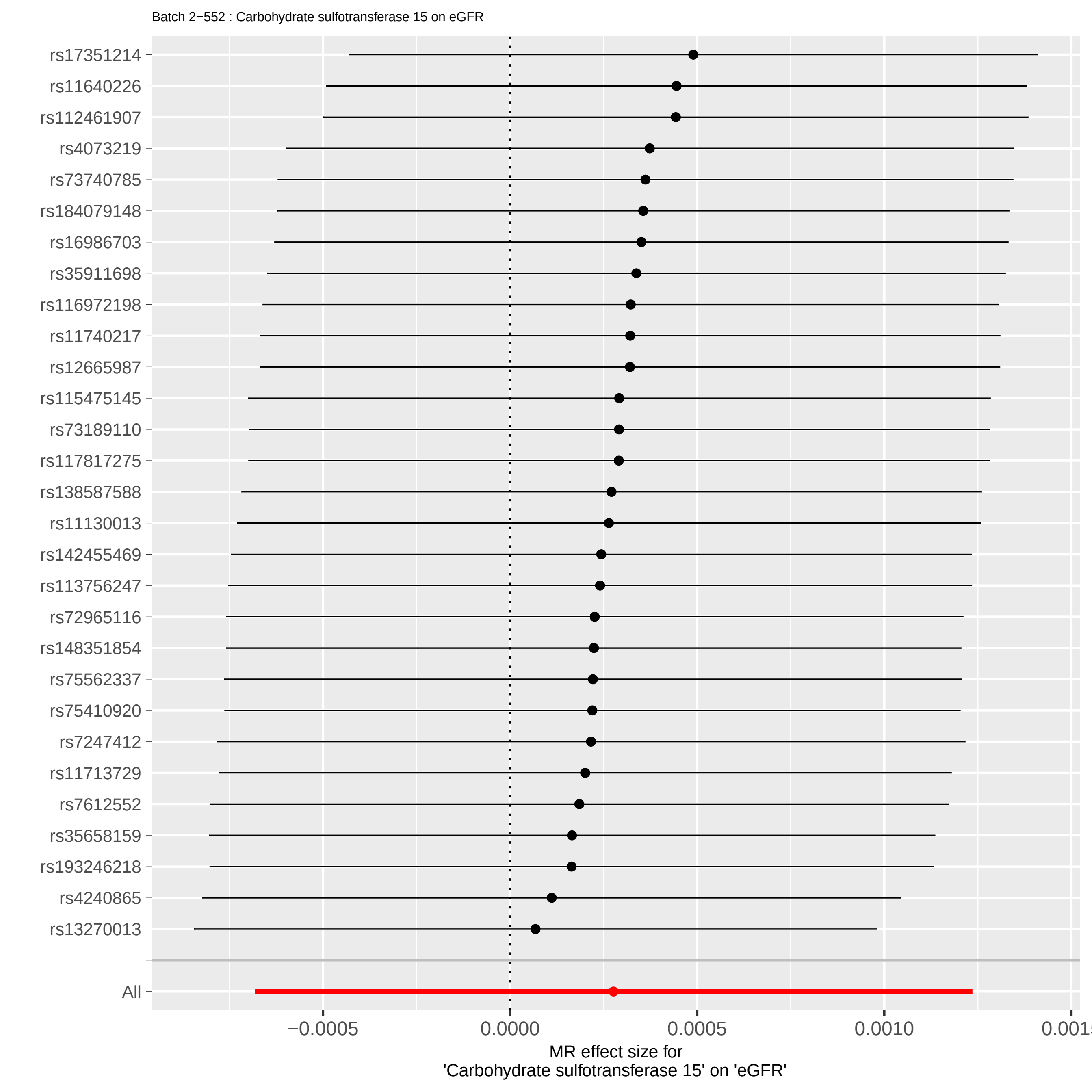

Batch 2−552 : Carbohydrate sulfotransferase 15 on eGFR
rs17351214
rs11640226
rs112461907
rs4073219
rs73740785
rs184079148
rs16986703
rs35911698
rs116972198
rs11740217
rs12665987
rs115475145
rs73189110
rs117817275
rs138587588
rs11130013
rs142455469
rs113756247
rs72965116
rs148351854
rs75562337
rs75410920
rs7247412
rs11713729
rs7612552
rs35658159
rs193246218
rs4240865
rs13270013
All
−0.0005
0.0000
0.0005
0.0010
MR effect size for
'Carbohydrate sulfotransferase 15' on 'eGFR'

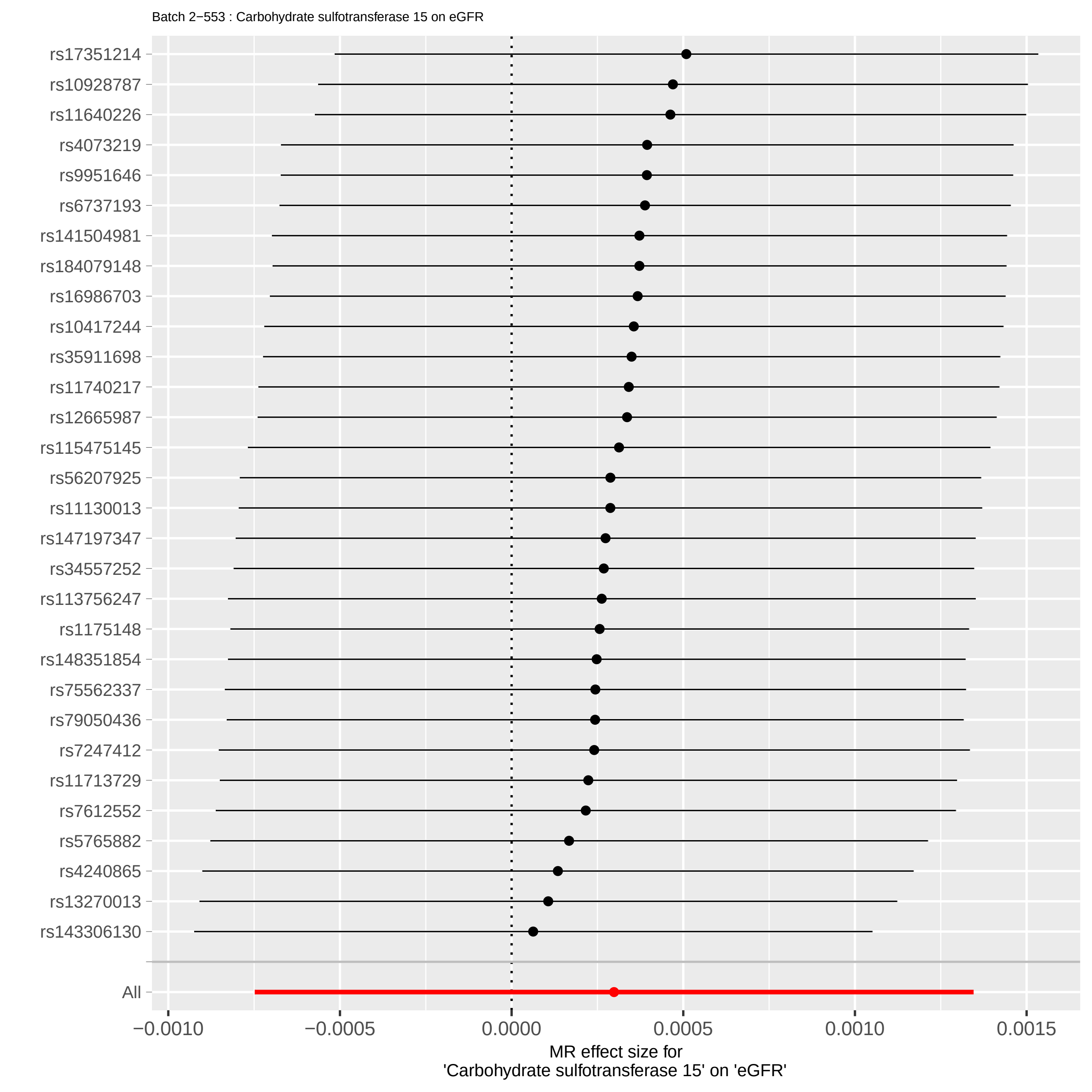

Batch 2−553 : Carbohydrate sulfotransferase 15 on eGFR
rs17351214
rs10928787
rs11640226
rs4073219
rs9951646
rs6737193
rs141504981
rs184079148
rs16986703
rs10417244
rs35911698
rs11740217
rs12665987
rs115475145
rs56207925
rs11130013
rs147197347
rs34557252
rs113756247
rs1175148
rs148351854
rs75562337
rs79050436
rs7247412
rs11713729
rs7612552
rs5765882
rs4240865
rs13270013
rs143306130
All
−0.0010
−0.0005
0.0000
0.0005
0.0010
0.0015
MR effect size for
'Carbohydrate sulfotransferase 15' on 'eGFR'

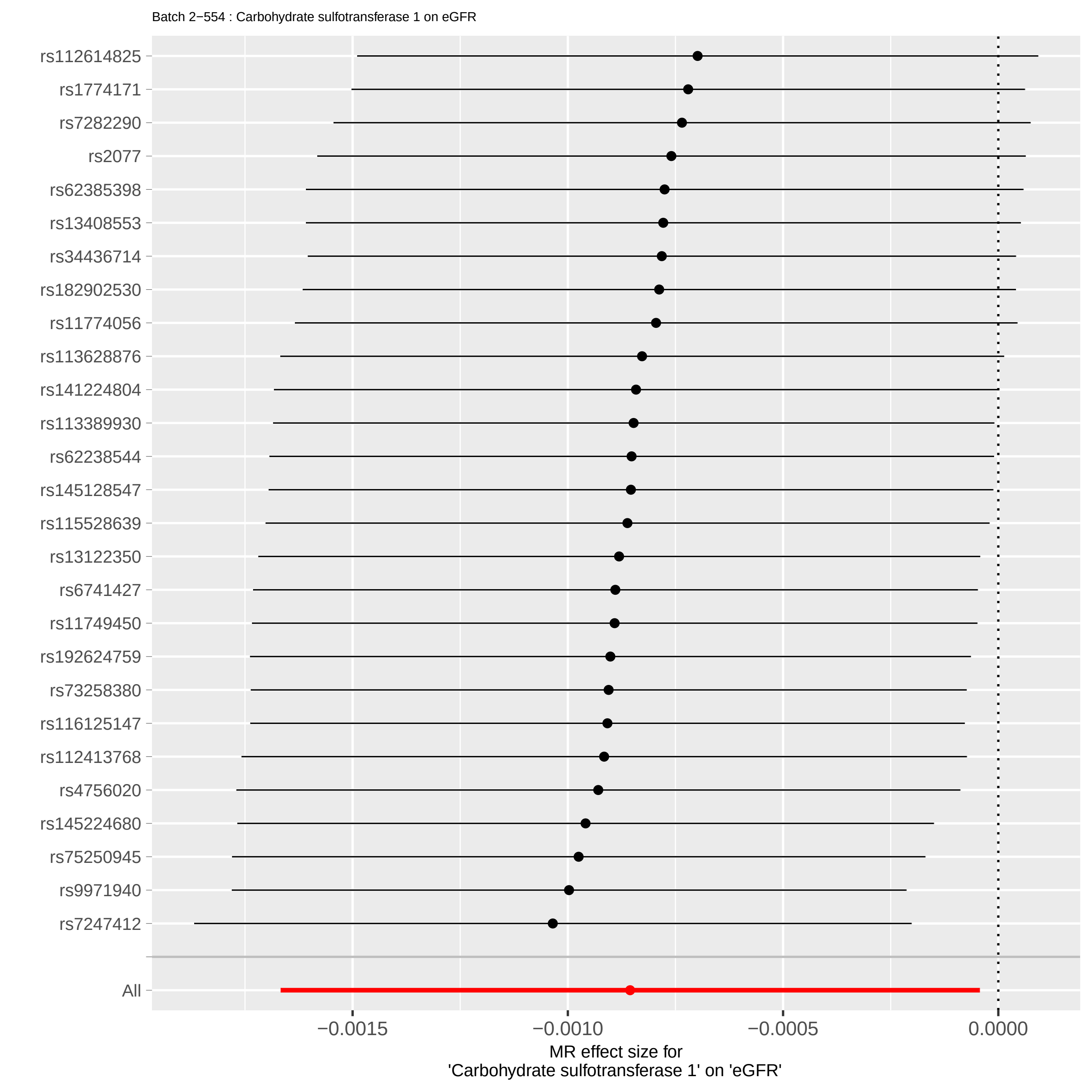

Batch 2−554 : Carbohydrate sulfotransferase 1 on eGFR
rs112614825
rs1774171
rs7282290
rs2077
rs62385398
rs13408553
rs34436714
rs182902530
rs11774056
rs113628876
rs141224804
rs113389930
rs62238544
rs145128547
rs115528639
rs13122350
rs6741427
rs11749450
rs192624759
rs73258380
rs116125147
rs112413768
rs4756020
rs145224680
rs75250945
rs9971940
rs7247412
All
−0.0015
−0.0010
−0.0005
0.0000
MR effect size for
'Carbohydrate sulfotransferase 1' on 'eGFR'

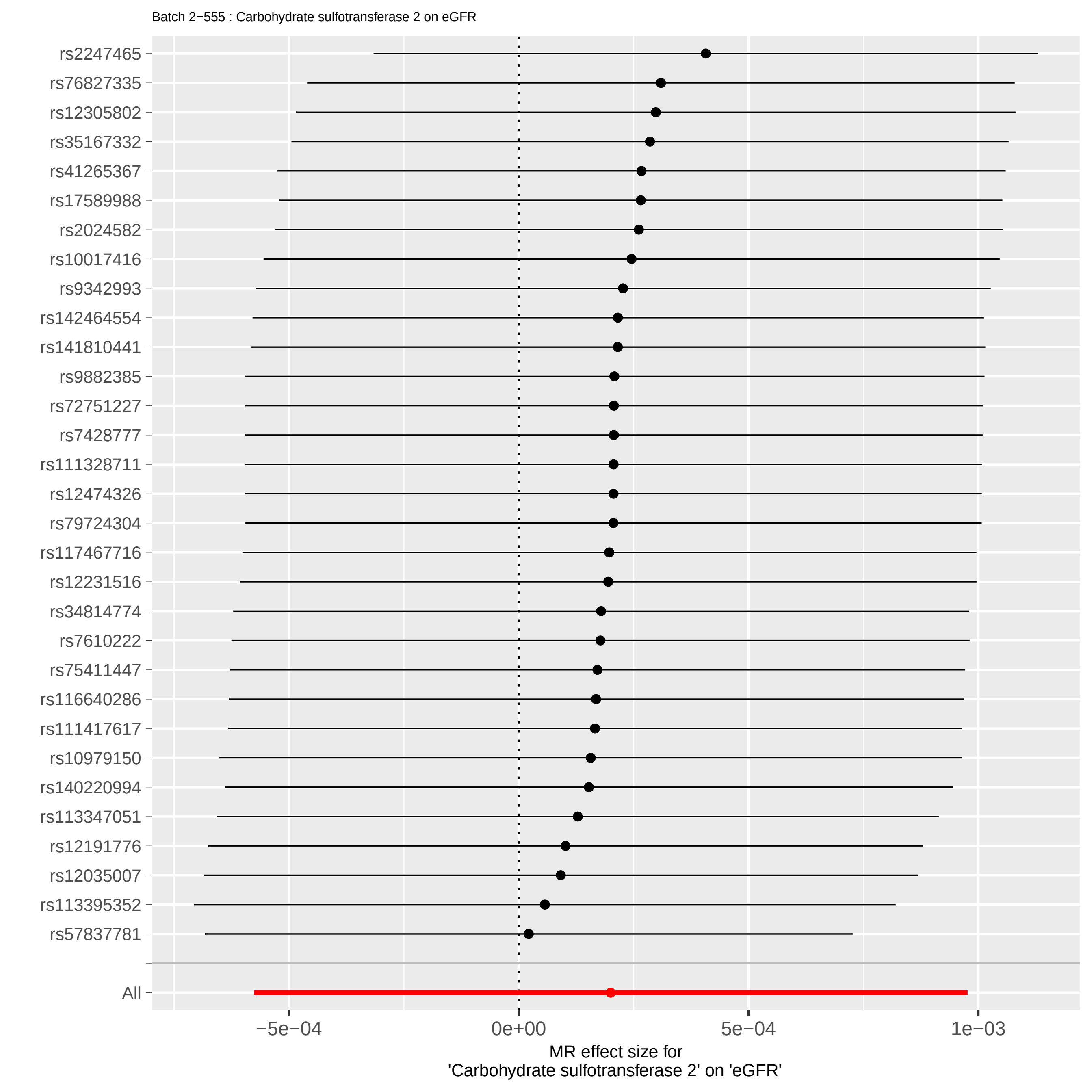

Batch 2−555 : Carbohydrate sulfotransferase 2 on eGFR
rs2247465
rs76827335
rs12305802
rs35167332
rs41265367
rs17589988
rs2024582
rs10017416
rs9342993
rs142464554
rs141810441
rs9882385
rs72751227
rs7428777
rs111328711
rs12474326
rs79724304
rs117467716
rs12231516
rs34814774
rs7610222
rs75411447
rs116640286
rs111417617
rs10979150
rs140220994
rs113347051
rs12191776
rs12035007
rs113395352
rs57837781
All
−5e−04
0e+00
5e−04
1e−03
MR effect size for
'Carbohydrate sulfotransferase 2' on 'eGFR'

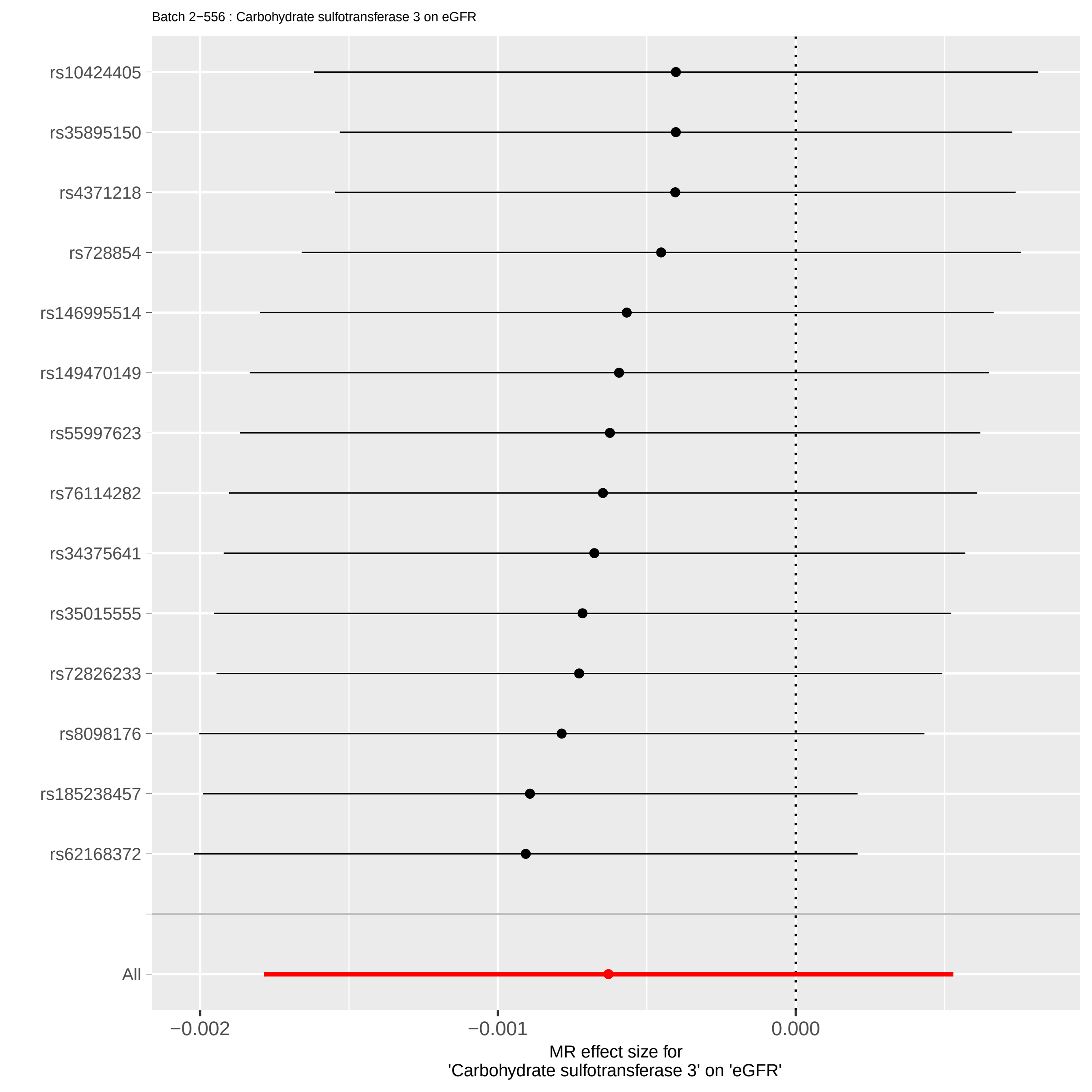

Batch 2−556 : Carbohydrate sulfotransferase 3 on eGFR
rs10424405
rs35895150
rs4371218
rs728854
rs146995514
rs149470149
rs55997623
rs76114282
rs34375641
rs35015555
rs72826233
rs8098176
rs185238457
rs62168372
All
−0.002
−0.001
0.000
MR effect size for
'Carbohydrate sulfotransferase 3' on 'eGFR'

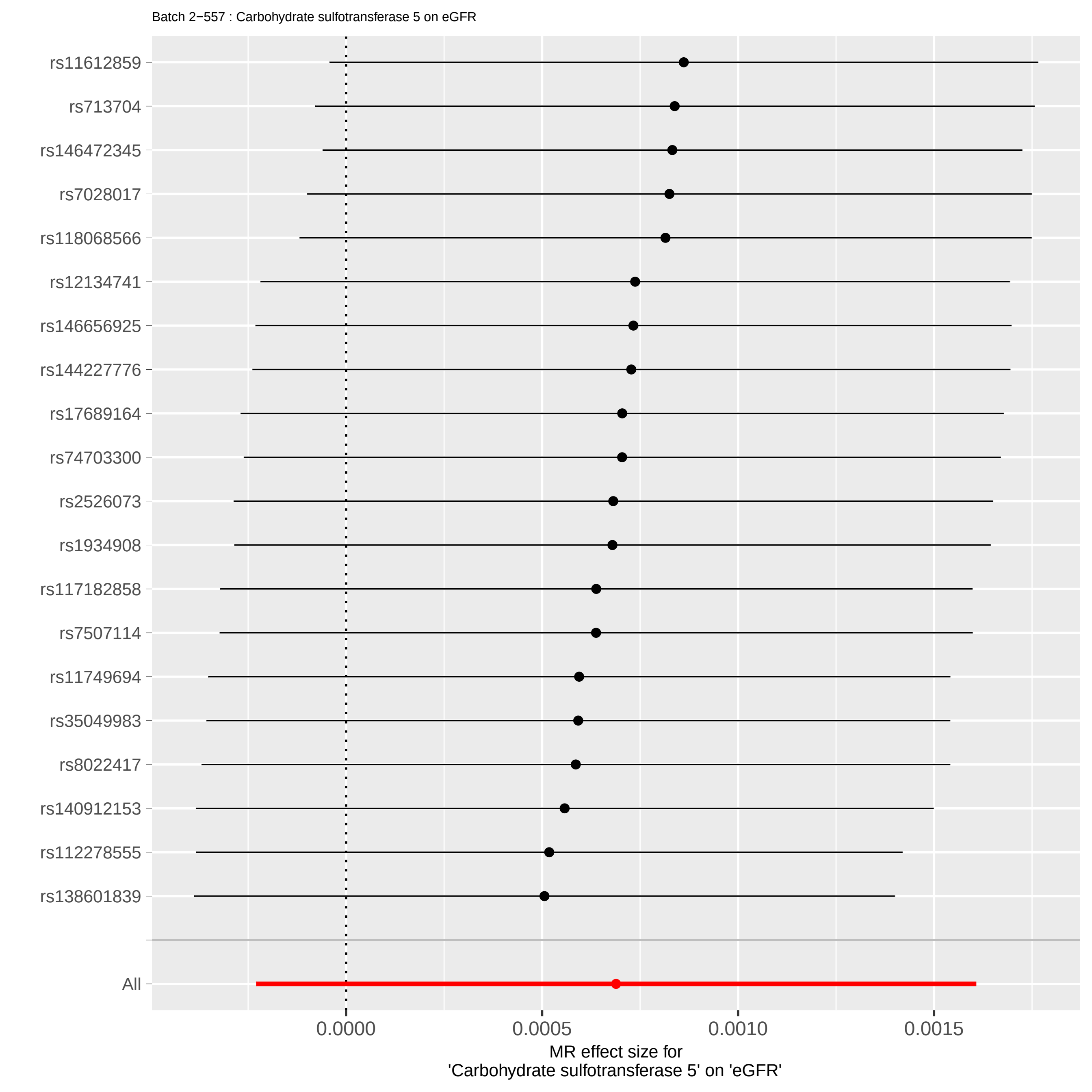
Batch 2−557 : Carbohydrate sulfotransferase 5 on eGFR
rs11612859
rs713704
rs146472345
rs7028017
rs118068566
rs12134741
rs146656925
rs144227776
rs17689164
rs74703300
rs2526073
rs1934908
rs117182858
rs7507114
rs11749694
rs35049983
rs8022417
rs140912153
rs112278555
rs138601839
All
0.0000
0.0005
0.0010
0.0015
MR effect size for
'Carbohydrate sulfotransferase 5' on 'eGFR'

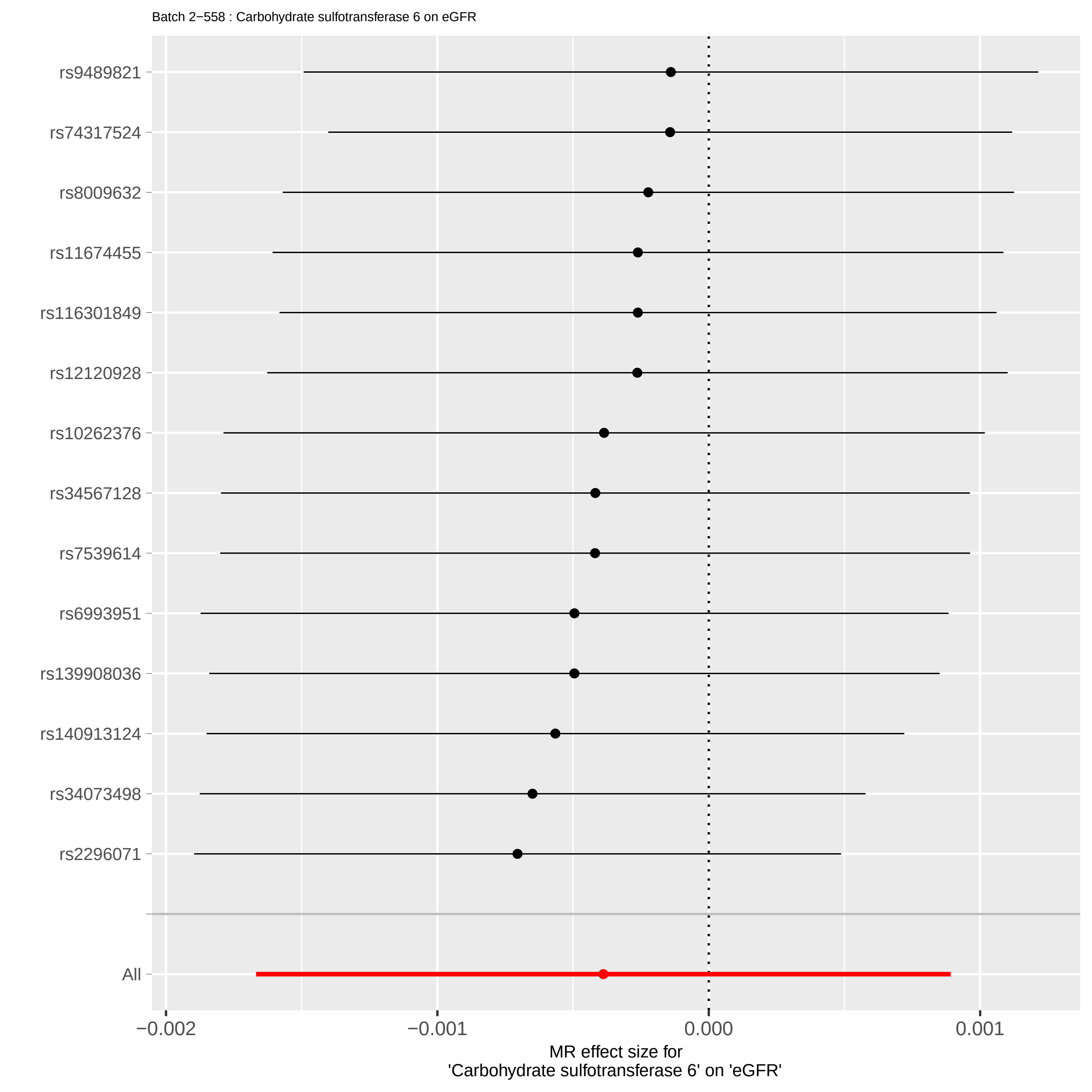

Batch 2−558 : Carbohydrate sulfotransferase 6 on eGFR
rs9489821
rs74317524
rs8009632
rs11674455
rs116301849
rs12120928
rs10262376
rs34567128
rs7539614
rs6993951
rs139908036
rs140913124
rs34073498
rs2296071
All
−0.002
−0.001
0.000
0.001
MR effect size for
'Carbohydrate sulfotransferase 6' on 'eGFR'

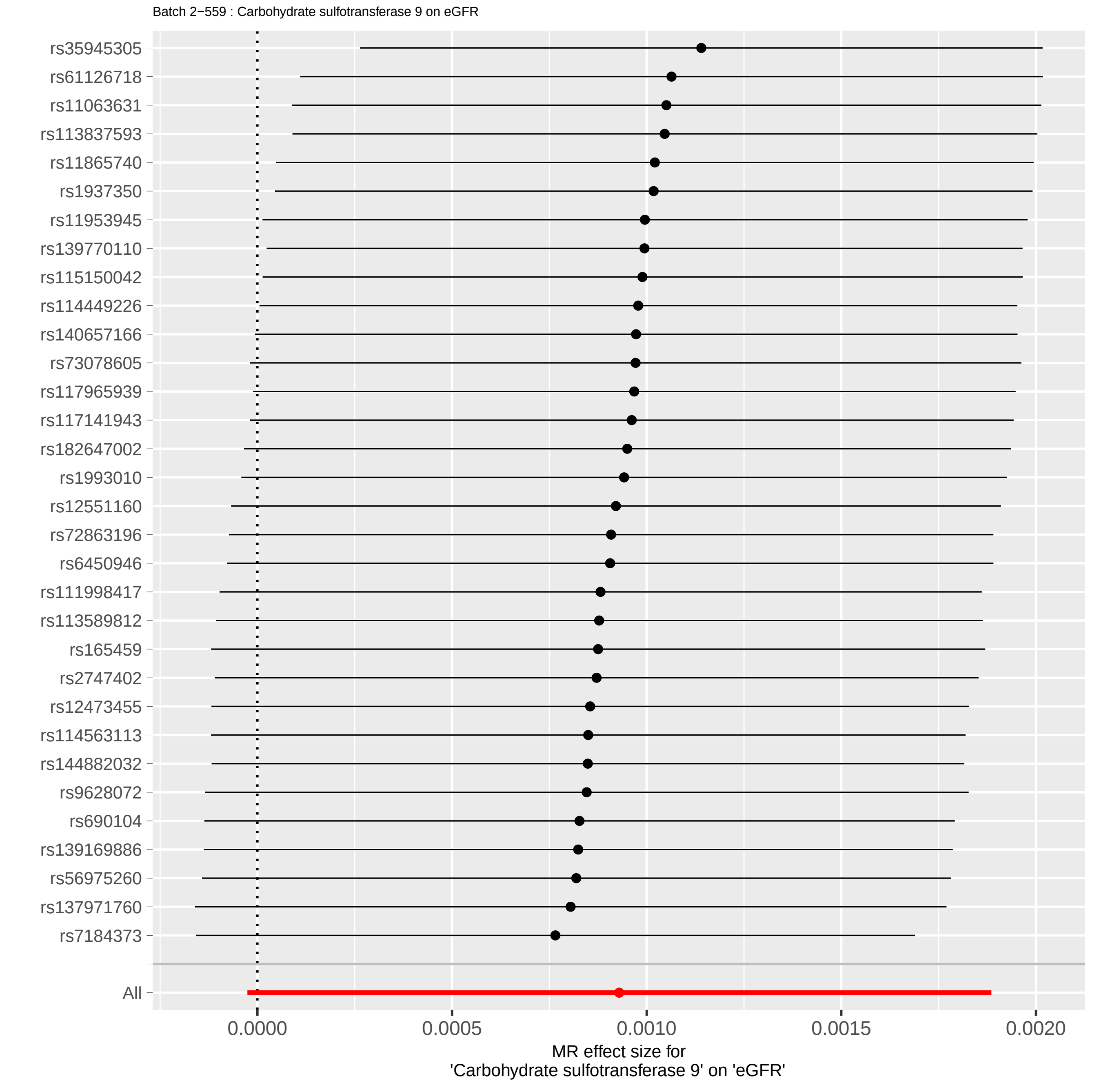

Batch 2−559 : Carbohydrate sulfotransferase 9 on eGFR
rs35945305
rs61126718
rs11063631
rs113837593
rs11865740
rs1937350
rs11953945
rs139770110
rs115150042
rs114449226
rs140657166
rs73078605
rs117965939
rs117141943
rs182647002
rs1993010
rs12551160
rs72863196
rs6450946
rs111998417
rs113589812
rs165459
rs2747402
rs12473455
rs114563113
rs144882032
rs9628072
rs690104
rs139169886
rs56975260
rs137971760
rs7184373
All
0.0000
0.0005
0.0010
0.0015
0.0020
MR effect size for
'Carbohydrate sulfotransferase 9' on 'eGFR'

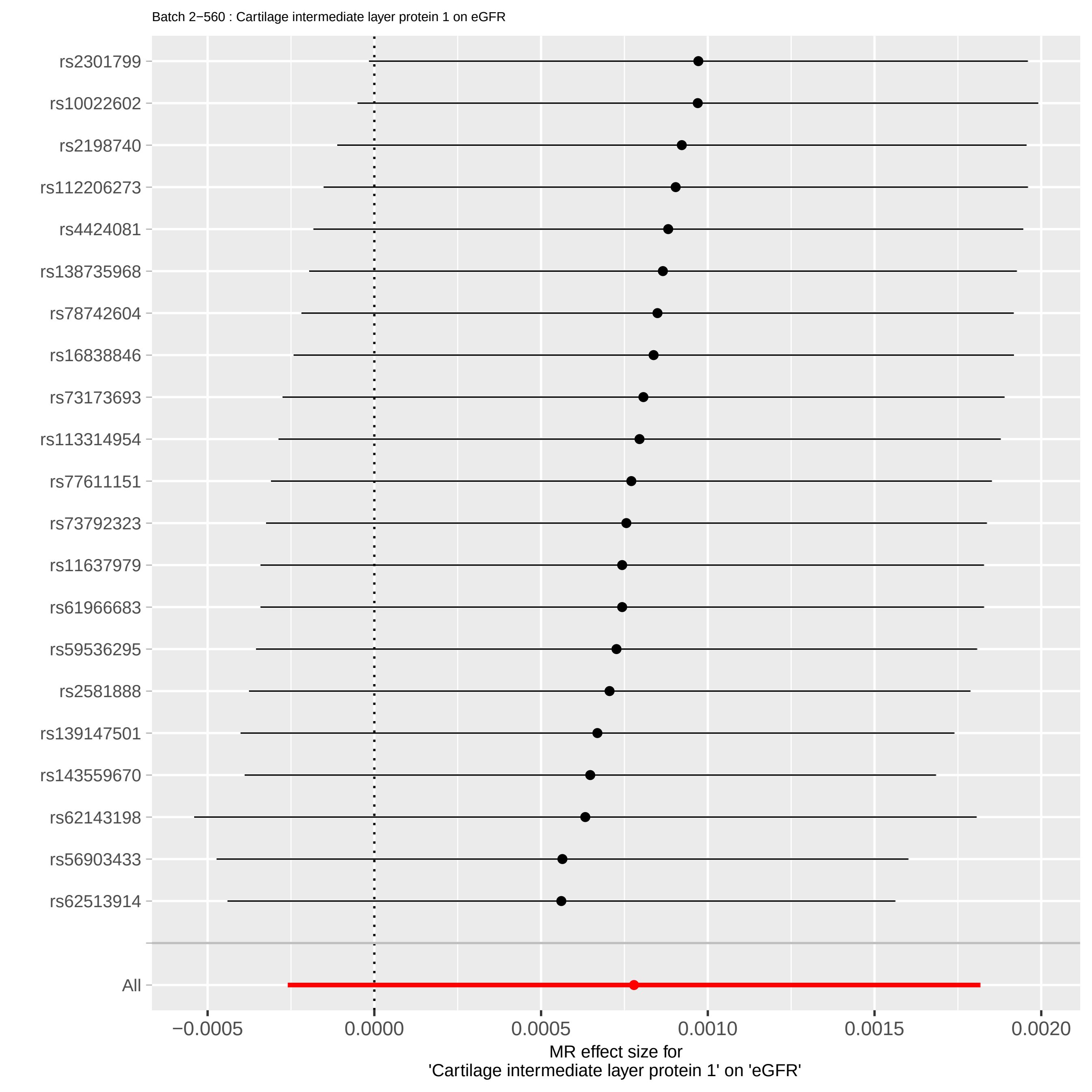

Batch 2−560 : Cartilage intermediate layer protein 1 on eGFR
rs2301799
rs10022602
rs2198740
rs112206273
rs4424081
rs138735968
rs78742604
rs16838846
rs73173693
rs113314954
rs77611151
rs73792323
rs11637979
rs61966683
rs59536295
rs2581888
rs139147501
rs143559670
rs62143198
rs56903433
rs62513914
All
−0.0005
0.0000
0.0005
0.0010
0.0015
0.0020
MR effect size for
'Cartilage intermediate layer protein 1' on 'eGFR'

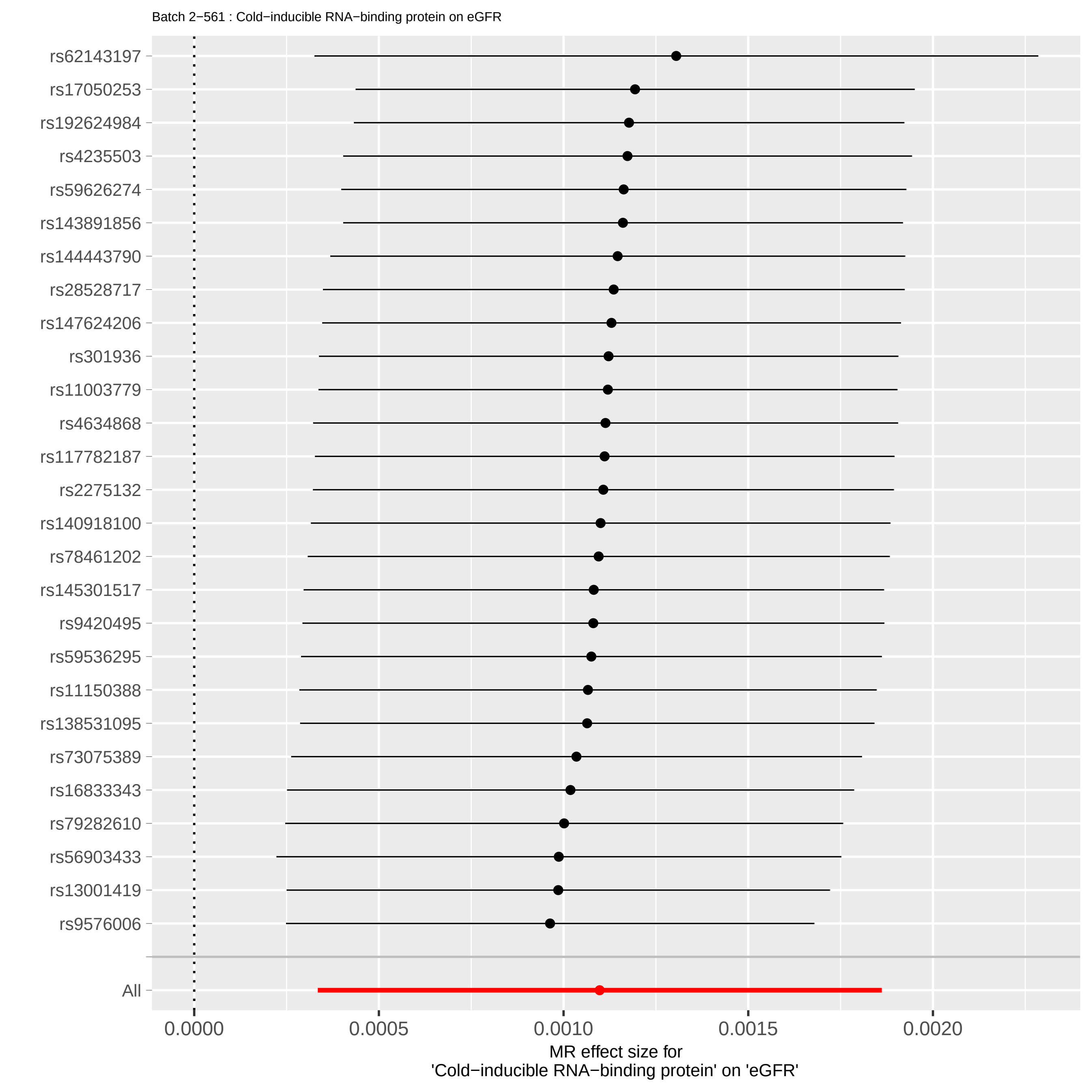

Batch 2−561 : Cold−inducible RNA−binding protein on eGFR
rs62143197
rs17050253
rs192624984
rs4235503
rs59626274
rs143891856
rs144443790
rs28528717
rs147624206
rs301936
rs11003779
rs4634868
rs117782187
rs2275132
rs140918100
rs78461202
rs145301517
rs9420495
rs59536295
rs11150388
rs138531095
rs73075389
rs16833343
rs79282610
rs56903433
rs13001419
rs9576006
All
0.0000
0.0005
0.0010
0.0015
0.0020
MR effect size for
'Cold−inducible RNA−binding protein' on 'eGFR'

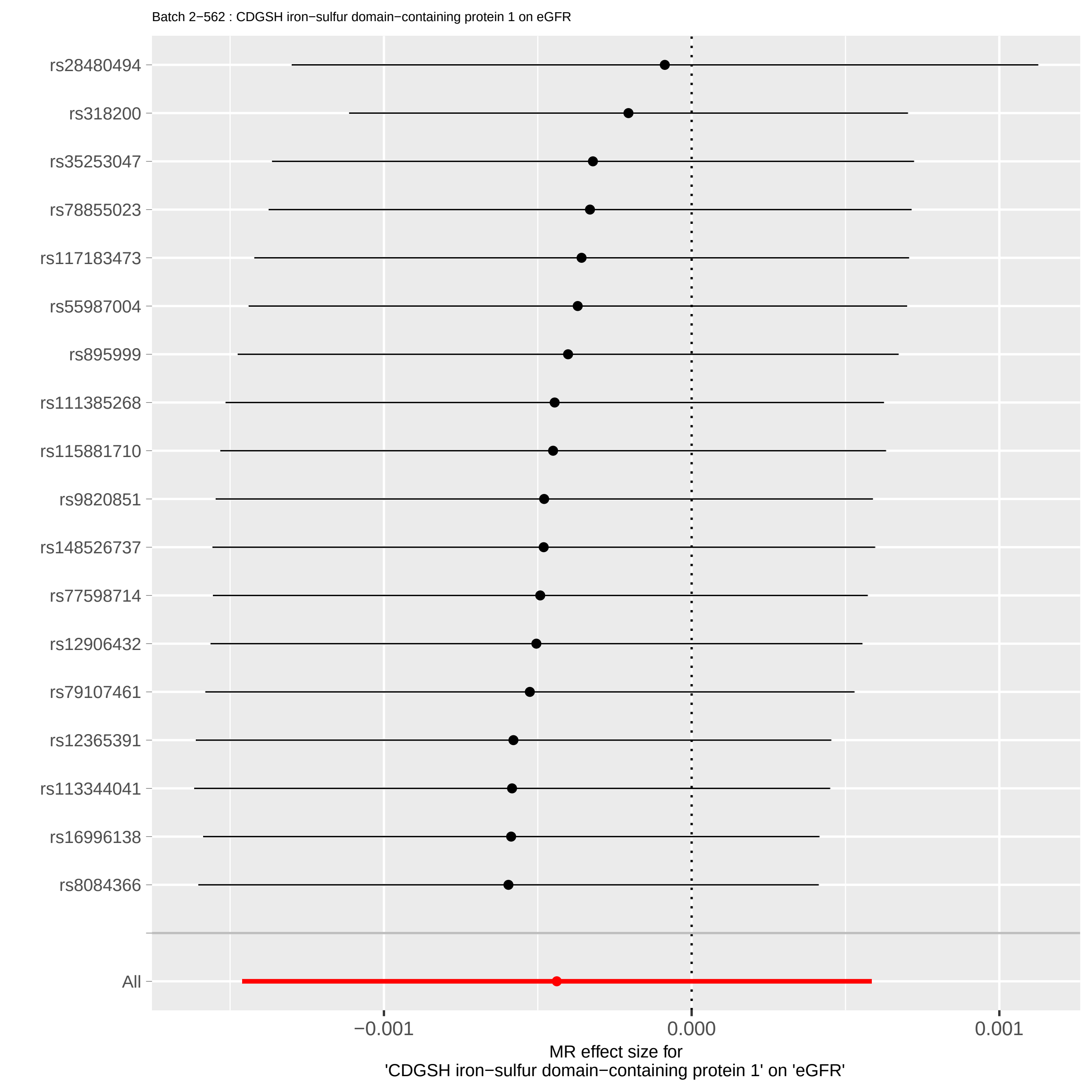

Batch 2−562 : CDGSH iron−sulfur domain−containing protein 1 on eGFR
rs28480494
rs318200
rs35253047
rs78855023
rs117183473
rs55987004
rs895999
rs111385268
rs115881710
rs9820851
rs148526737
rs77598714
rs12906432
rs79107461
rs12365391
rs113344041
rs16996138
rs8084366
All
−0.001
0.000
0.001
MR effect size for
'CDGSH iron−sulfur domain−containing protein 1' on 'eGFR'

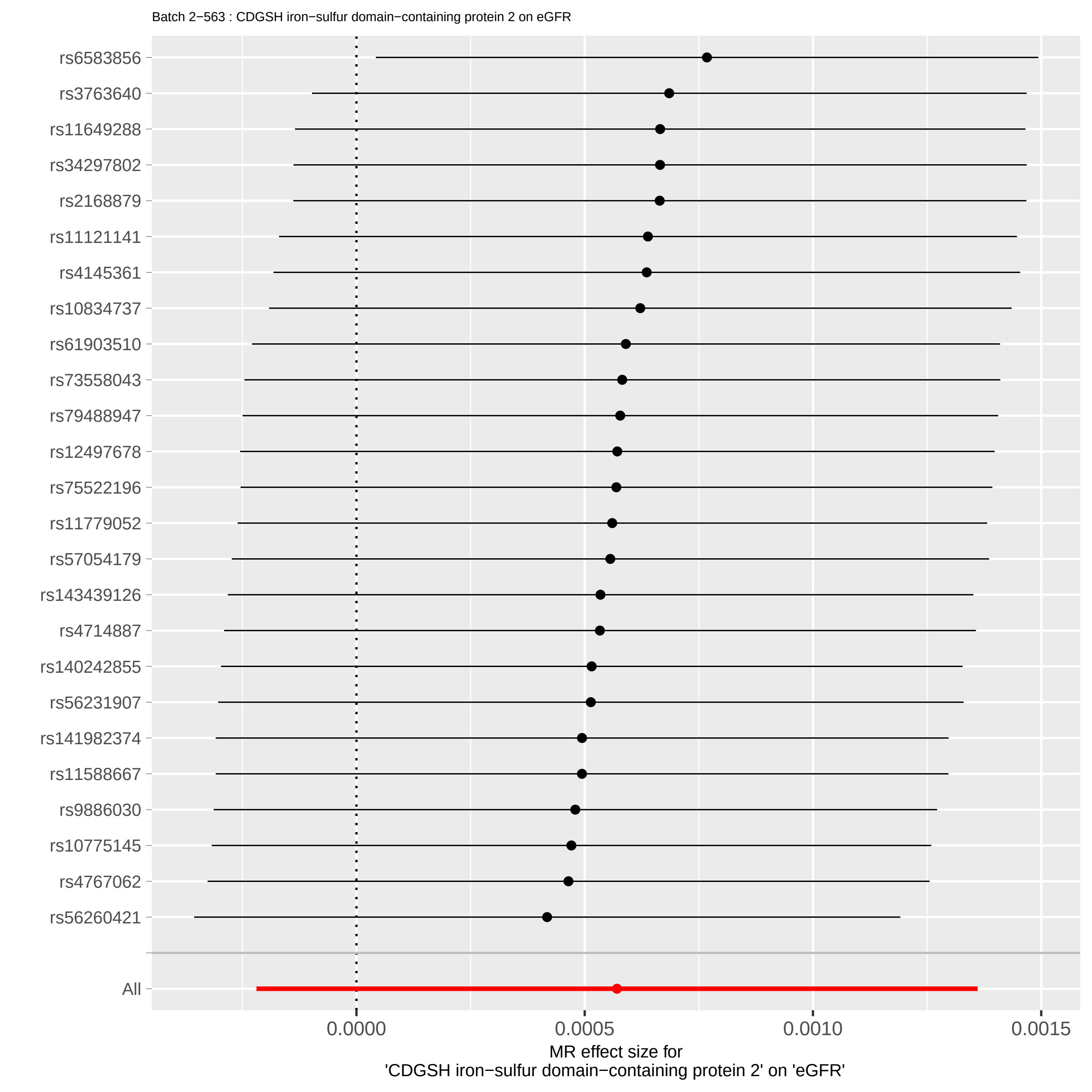
Batch 2−563 : CDGSH iron−sulfur domain−containing protein 2 on eGFR
rs6583856
rs3763640
rs11649288
rs34297802
rs2168879
rs11121141
rs4145361
rs10834737
rs61903510
rs73558043
rs79488947
rs12497678
rs75522196
rs11779052
rs57054179
rs143439126
rs4714887
rs140242855
rs56231907
rs141982374
rs11588667
rs9886030
rs10775145
rs4767062
rs56260421
All
0.0000
0.0005
0.0010
0.0015
MR effect size for
'CDGSH iron−sulfur domain−containing protein 2' on 'eGFR'

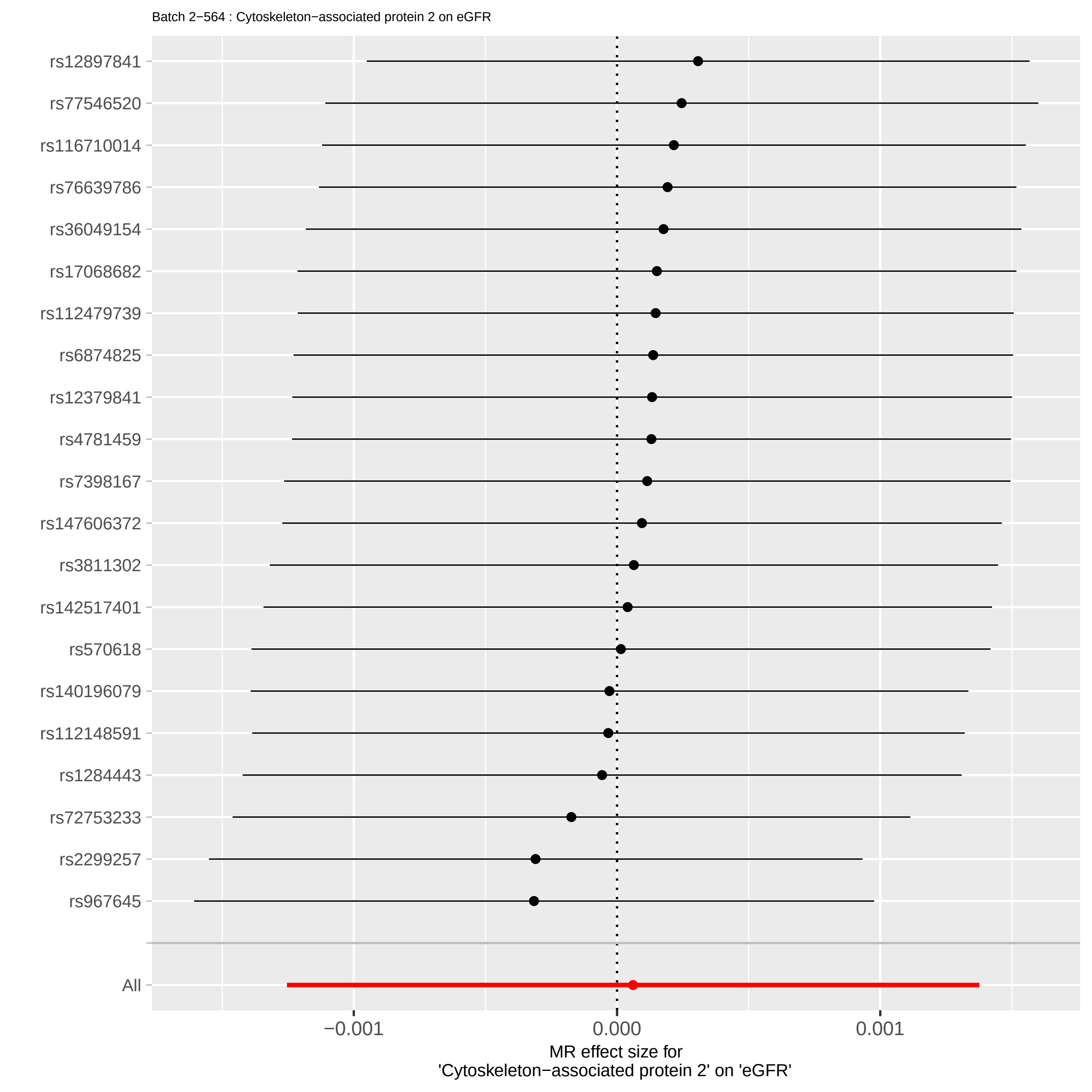
Batch 2−564 : Cytoskeleton−associated protein 2 on eGFR
rs12897841
rs77546520
rs116710014
rs76639786
rs36049154
rs17068682
rs112479739
rs6874825
rs12379841
rs4781459
rs7398167
rs147606372
rs3811302
rs142517401
rs570618
rs140196079
rs112148591
rs1284443
rs72753233
rs2299257
rs967645
All
−0.001
0.000
0.001
MR effect size for
'Cytoskeleton−associated protein 2' on 'eGFR'

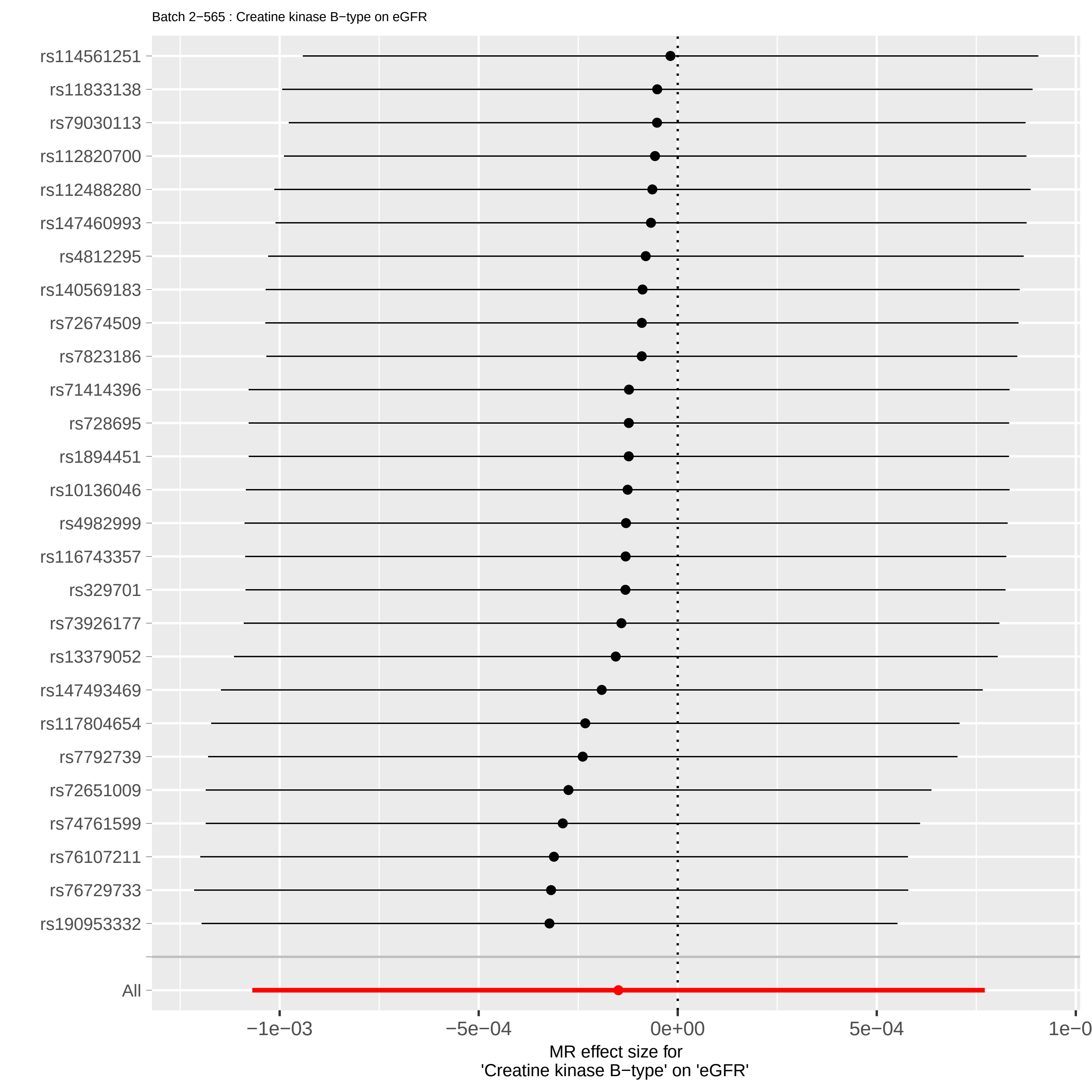

Batch 2−565 : Creatine kinase B−type on eGFR
rs114561251
rs11833138
rs79030113
rs112820700
rs112488280
rs147460993
rs4812295
rs140569183
rs72674509
rs7823186
rs71414396
rs728695
rs1894451
rs10136046
rs4982999
rs116743357
rs329701
rs73926177
rs13379052
rs147493469
rs117804654
rs7792739
rs72651009
rs74761599
rs76107211
rs76729733
rs190953332
All
−1e−03
−5e−04
0e+00
5e−04
1e−0
MR effect size for
'Creatine kinase B−type' on 'eGFR'

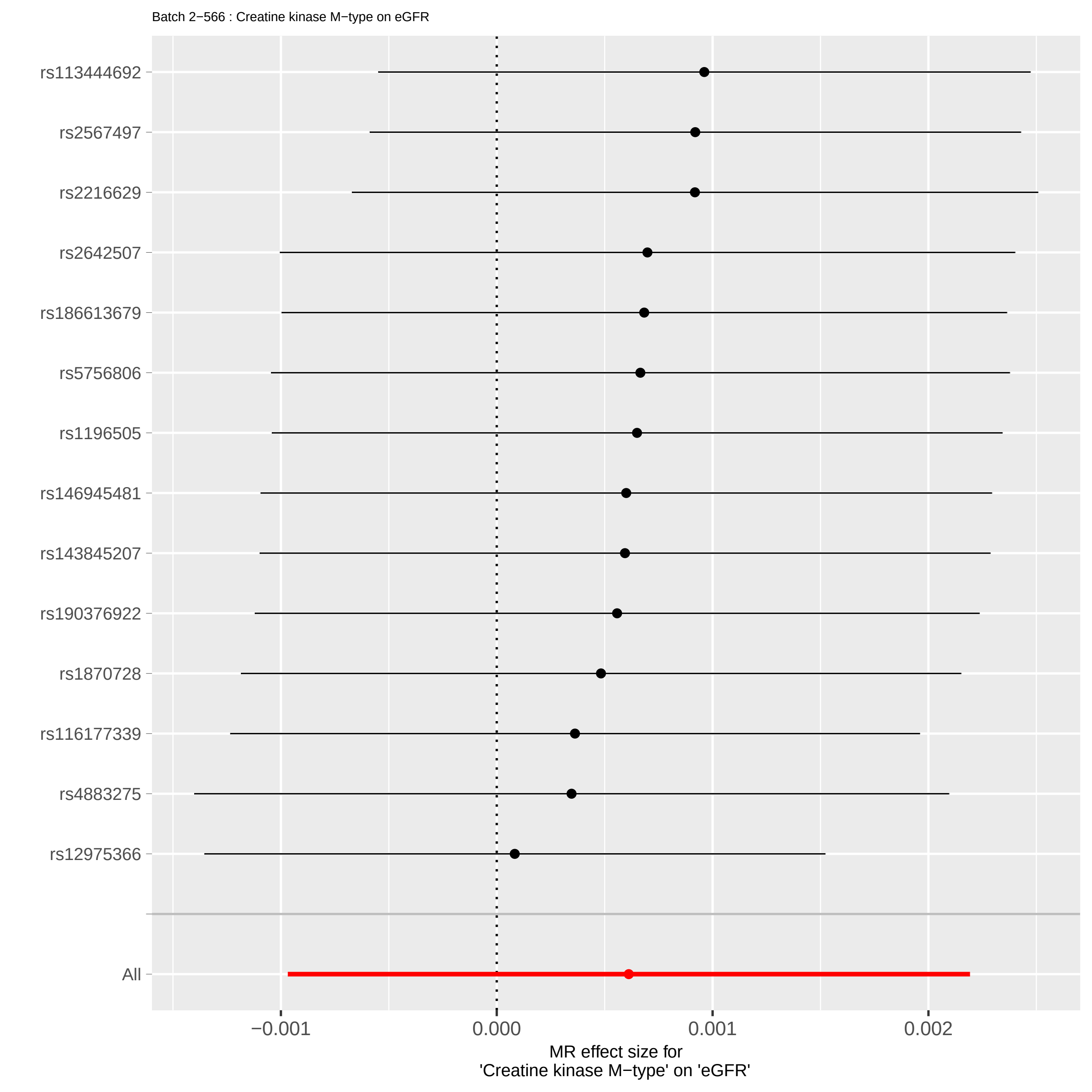

Batch 2−566 : Creatine kinase M−type on eGFR
rs113444692
rs2567497
rs2216629
rs2642507
rs186613679
rs5756806
rs1196505
rs146945481
rs143845207
rs190376922
rs1870728
rs116177339
rs4883275
rs12975366
All
−0.001
0.000
0.001
0.002
MR effect size for
'Creatine kinase M−type' on 'eGFR'

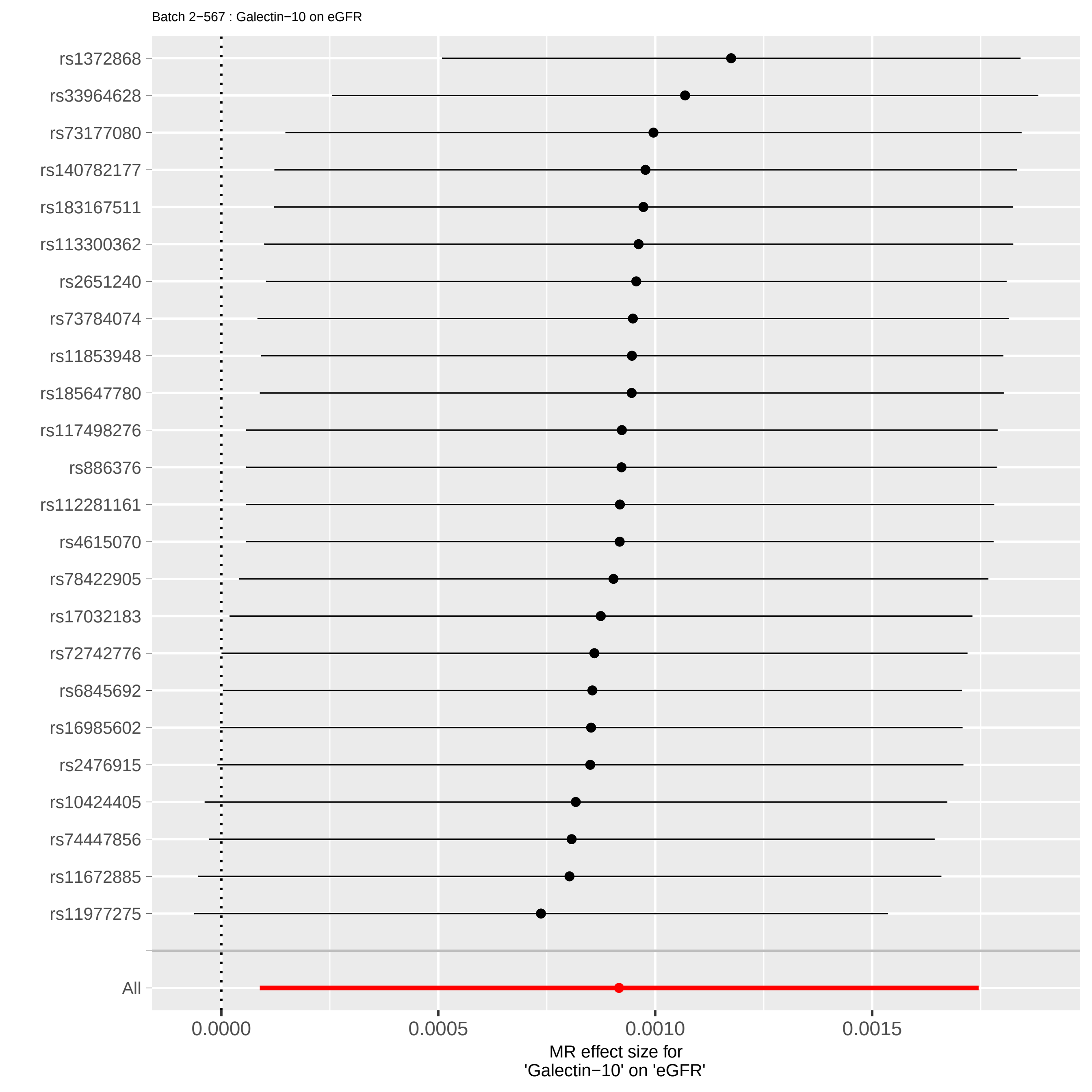

Batch 2−567 : Galectin−10 on eGFR
rs1372868
rs33964628
rs73177080
rs140782177
rs183167511
rs113300362
rs2651240
rs73784074
rs11853948
rs185647780
rs117498276
rs886376
rs112281161
rs4615070
rs78422905
rs17032183
rs72742776
rs6845692
rs16985602
rs2476915
rs10424405
rs74447856
rs11672885
rs11977275
All
0.0000
0.0005
0.0010
0.0015
MR effect size for
'Galectin−10' on 'eGFR'

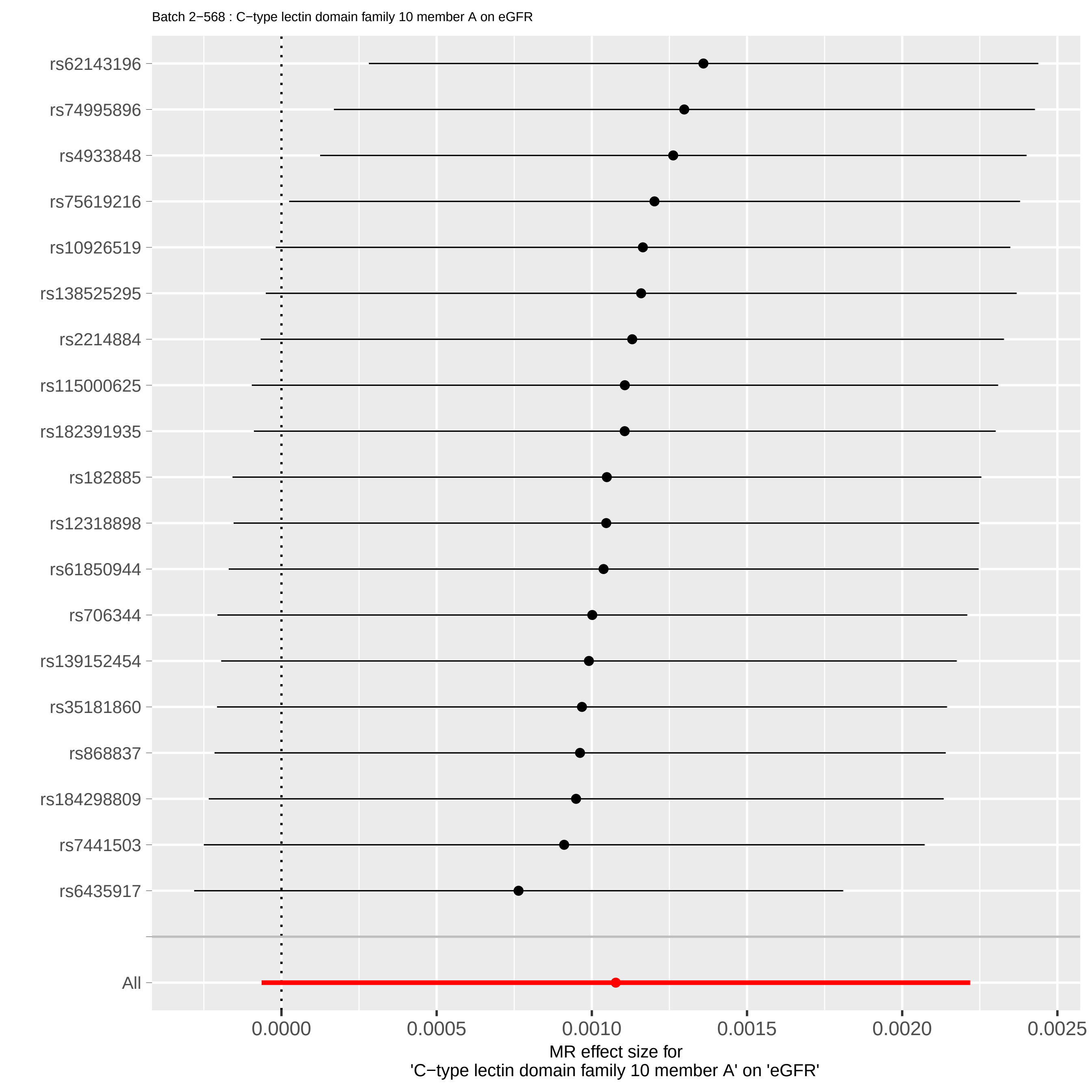

Batch 2−568 : C−type lectin domain family 10 member A on eGFR
rs62143196
rs74995896
rs4933848
rs75619216
rs10926519
rs138525295
rs2214884
rs115000625
rs182391935
rs182885
rs12318898
rs61850944
rs706344
rs139152454
rs35181860
rs868837
rs184298809
rs7441503
rs6435917
All
0.0000
0.0005
0.0010
0.0015
0.0020
0.0025
MR effect size for
'C−type lectin domain family 10 member A' on 'eGFR'

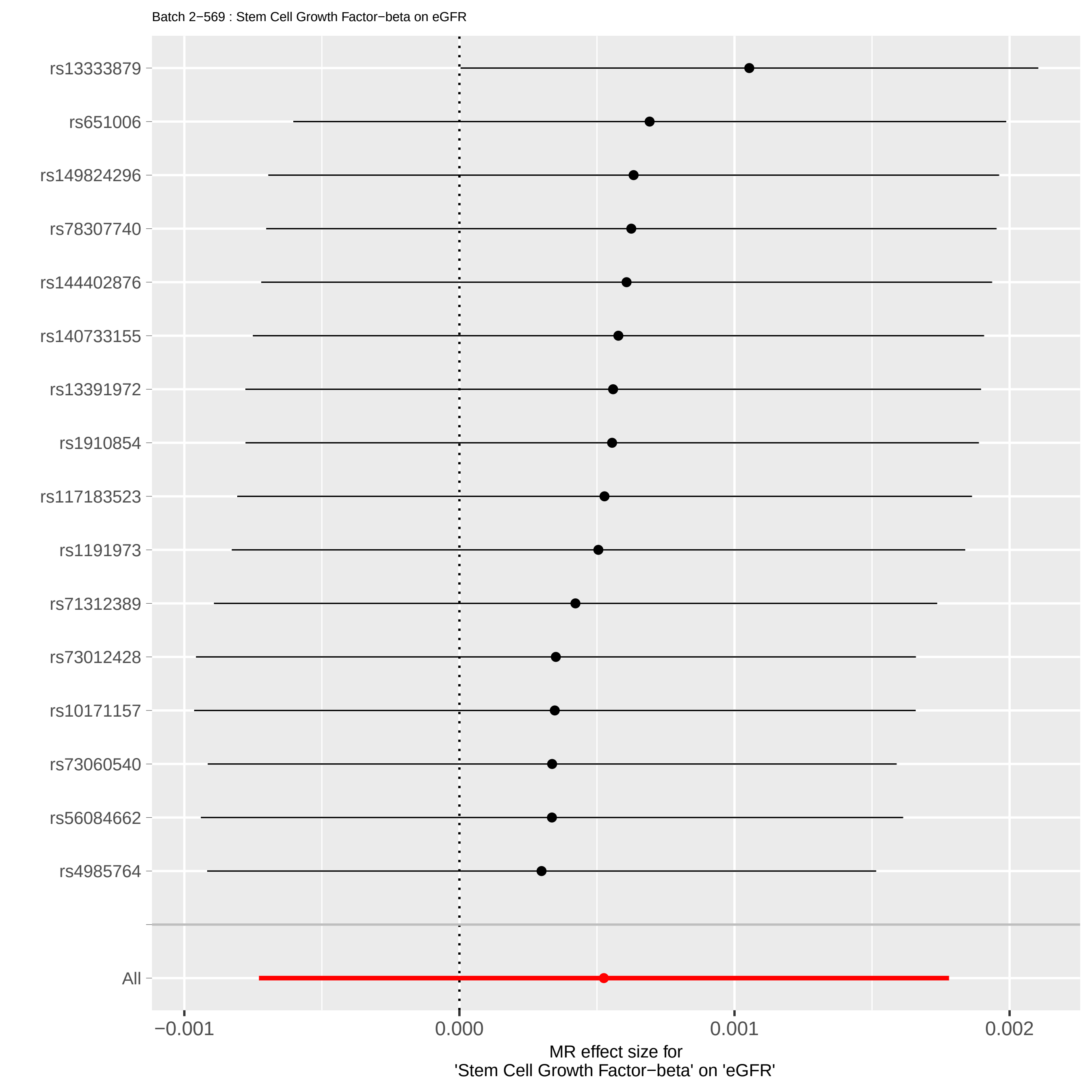

Batch 2−569 : Stem Cell Growth Factor−beta on eGFR
rs13333879
rs651006
rs149824296
rs78307740
rs144402876
rs140733155
rs13391972
rs1910854
rs117183523
rs1191973
rs71312389
rs73012428
rs10171157
rs73060540
rs56084662
rs4985764
All
−0.001
0.000
0.001
0.002
MR effect size for
'Stem Cell Growth Factor−beta' on 'eGFR'

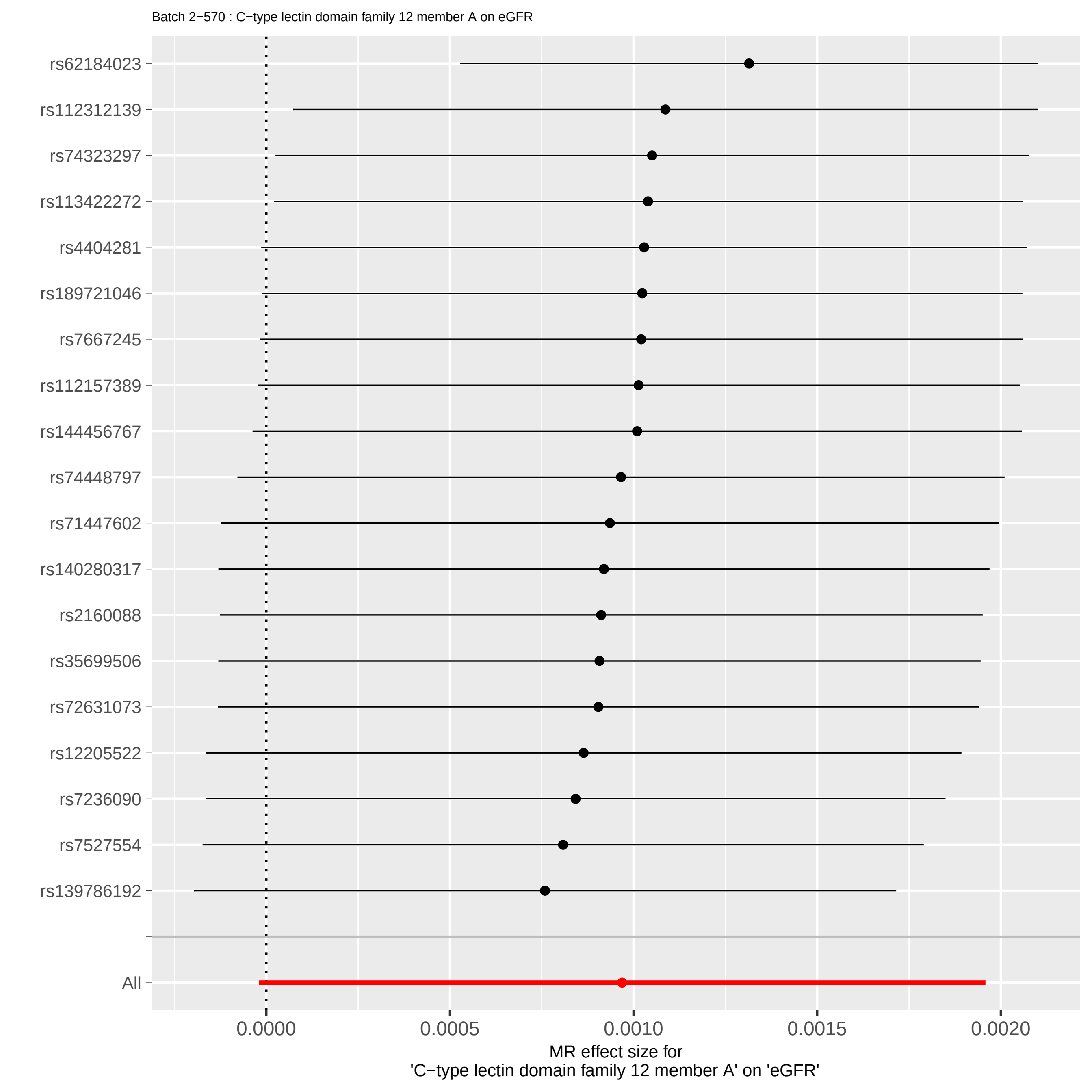

Batch 2−570 : C−type lectin domain family 12 member A on eGFR
rs62184023
rs112312139
rs74323297
rs113422272
rs4404281
rs189721046
rs7667245
rs112157389
rs144456767
rs74448797
rs71447602
rs140280317
rs2160088
rs35699506
rs72631073
rs12205522
rs7236090
rs7527554
rs139786192
All
0.0000
0.0005
0.0010
0.0015
0.0020
MR effect size for
'C−type lectin domain family 12 member A' on 'eGFR'

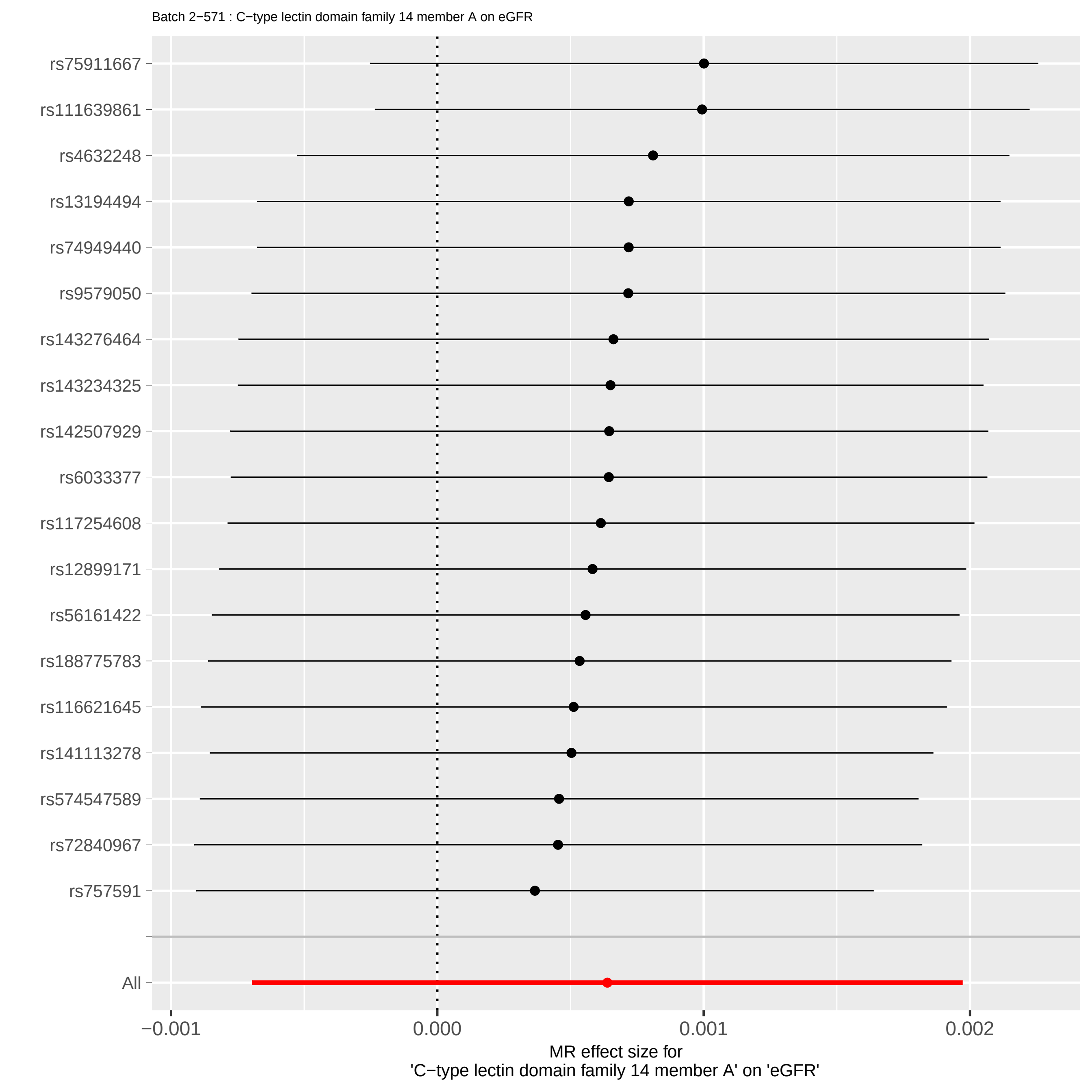
Batch 2−571 : C−type lectin domain family 14 member A on eGFR
rs75911667
rs111639861
rs4632248
rs13194494
rs74949440
rs9579050
rs143276464
rs143234325
rs142507929
rs6033377
rs117254608
rs12899171
rs56161422
rs188775783
rs116621645
rs141113278
rs574547589
rs72840967
rs757591
All
−0.001
0.000
0.001
0.002
MR effect size for
'C−type lectin domain family 14 member A' on 'eGFR'

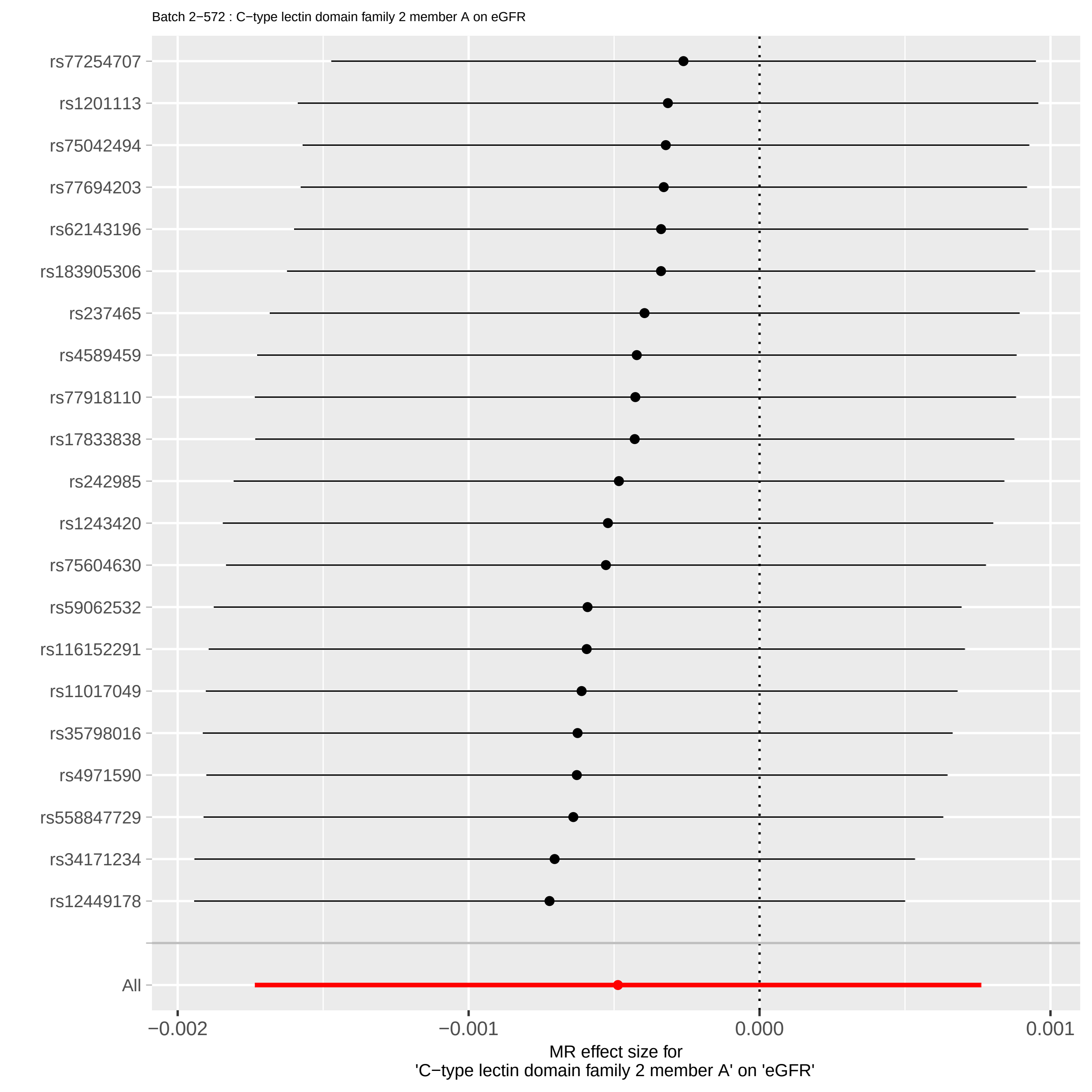

Batch 2−572 : C−type lectin domain family 2 member A on eGFR
rs77254707
rs1201113
rs75042494
rs77694203
rs62143196
rs183905306
rs237465
rs4589459
rs77918110
rs17833838
rs242985
rs1243420
rs75604630
rs59062532
rs116152291
rs11017049
rs35798016
rs4971590
rs558847729
rs34171234
rs12449178
All
−0.002
−0.001
0.000
0.001
MR effect size for
'C−type lectin domain family 2 member A' on 'eGFR'

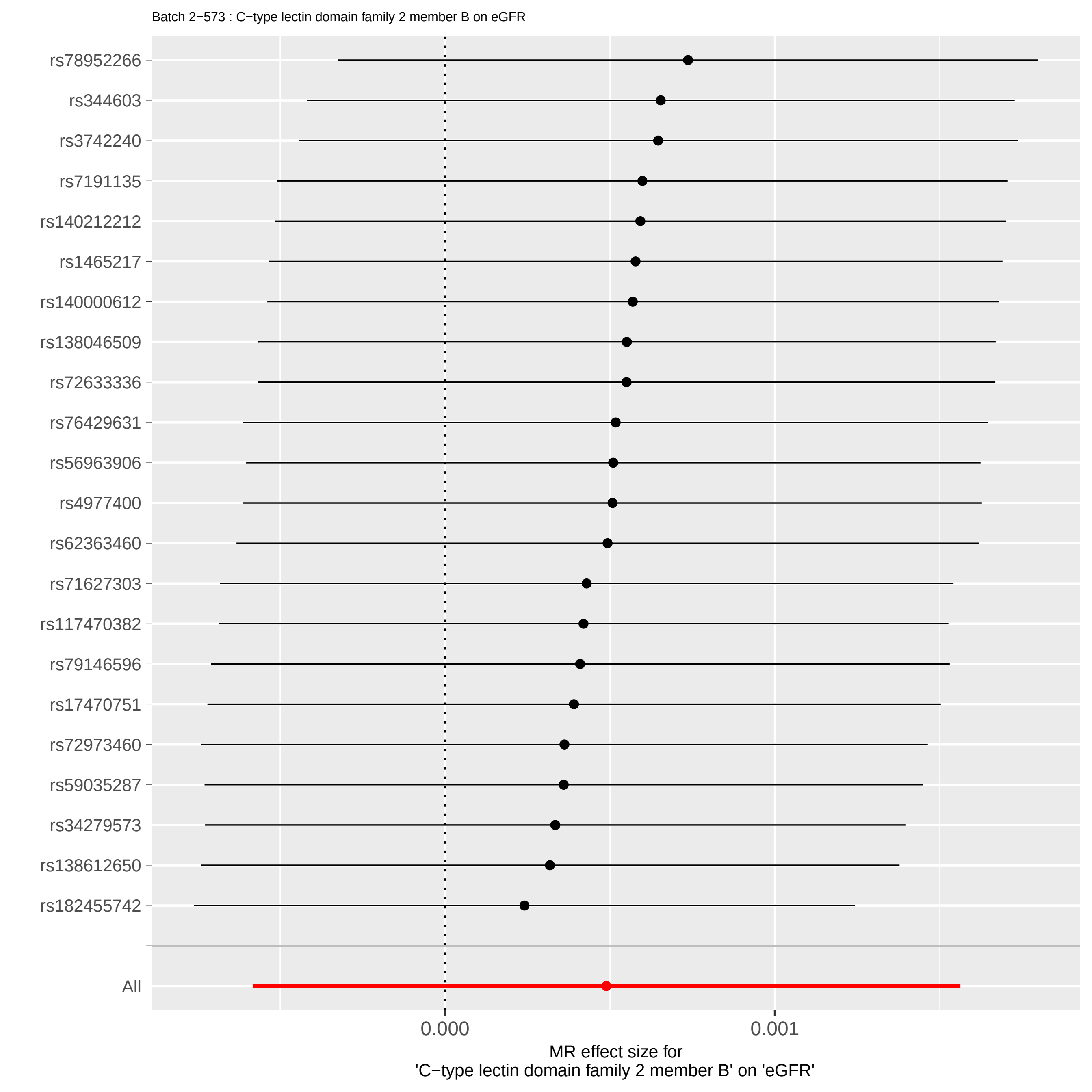

Batch 2−573 : C−type lectin domain family 2 member B on eGFR
rs78952266
rs344603
rs3742240
rs7191135
rs140212212
rs1465217
rs140000612
rs138046509
rs72633336
rs76429631
rs56963906
rs4977400
rs62363460
rs71627303
rs117470382
rs79146596
rs17470751
rs72973460
rs59035287
rs34279573
rs138612650
rs182455742
All
0.000
0.001
MR effect size for
'C−type lectin domain family 2 member B' on 'eGFR'

Batch 2−574 : C−type lectin domain family 2 member D on eGFR

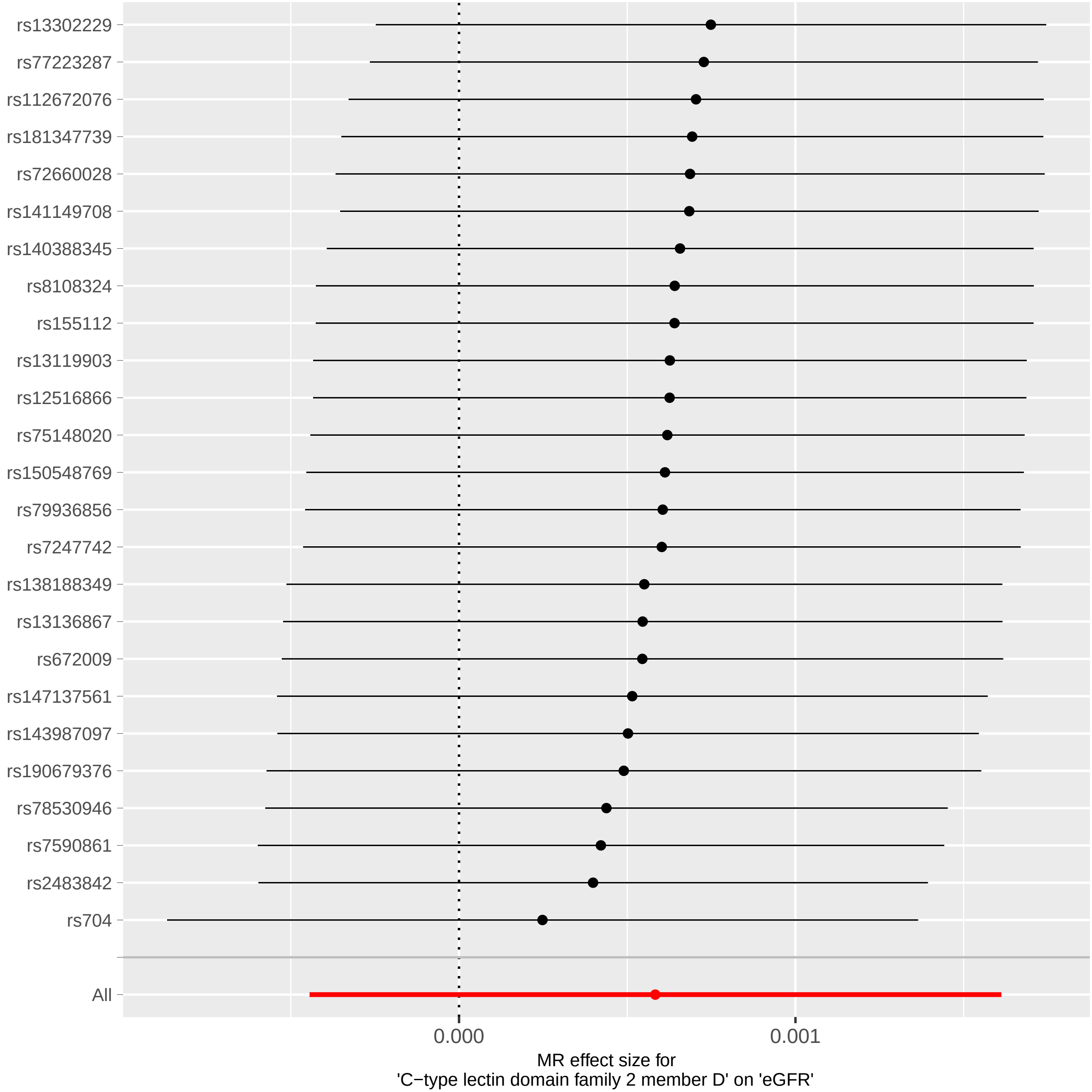

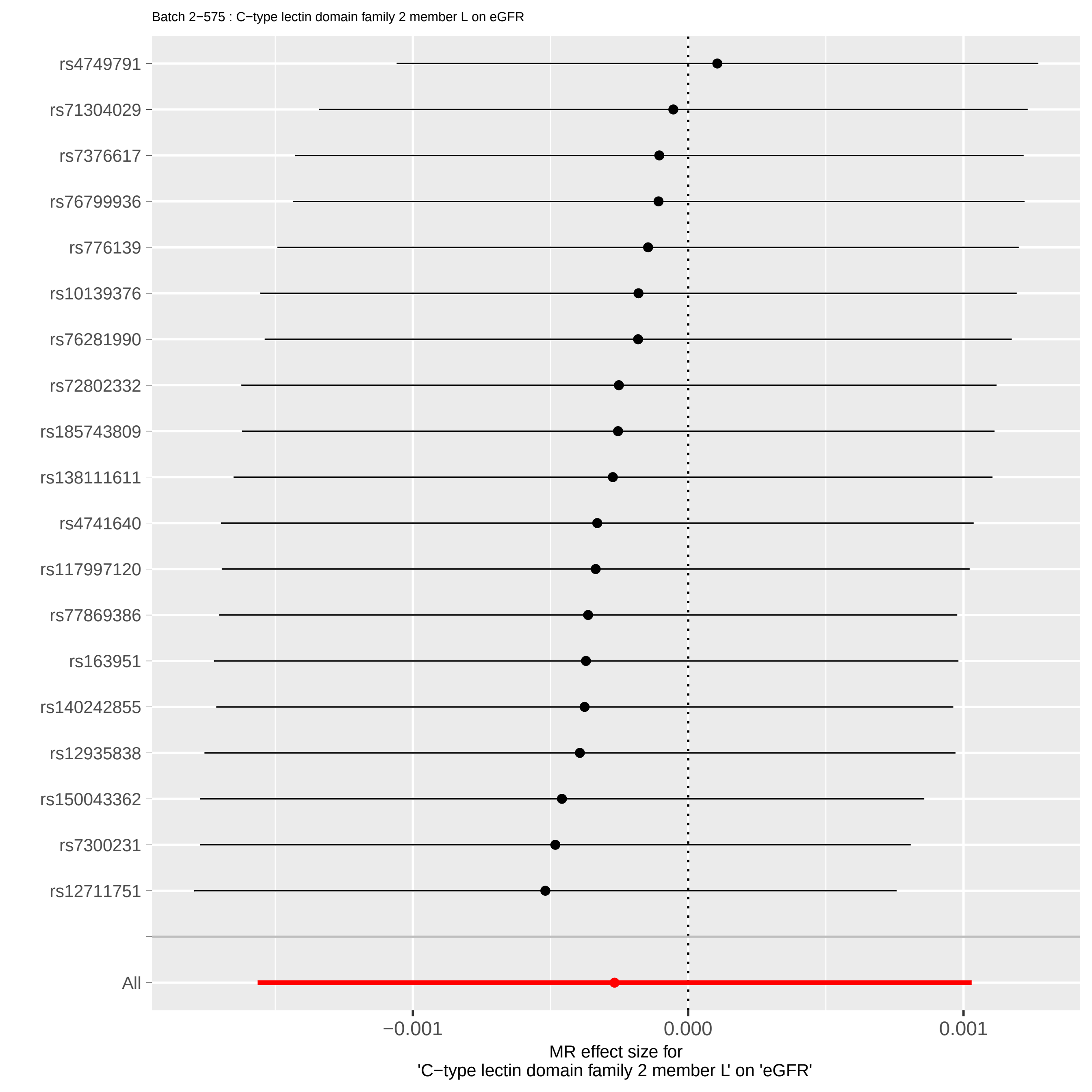

Batch 2−575 : C−type lectin domain family 2 member L on eGFR
rs4749791
rs71304029
rs7376617
rs76799936
rs776139
rs10139376
rs76281990
rs72802332
rs185743809
rs138111611
rs4741640
rs117997120
rs77869386
rs163951
rs140242855
rs12935838
rs150043362
rs7300231
rs12711751
All
−0.001
0.000
0.001
MR effect size for
'C−type lectin domain family 2 member L' on 'eGFR'

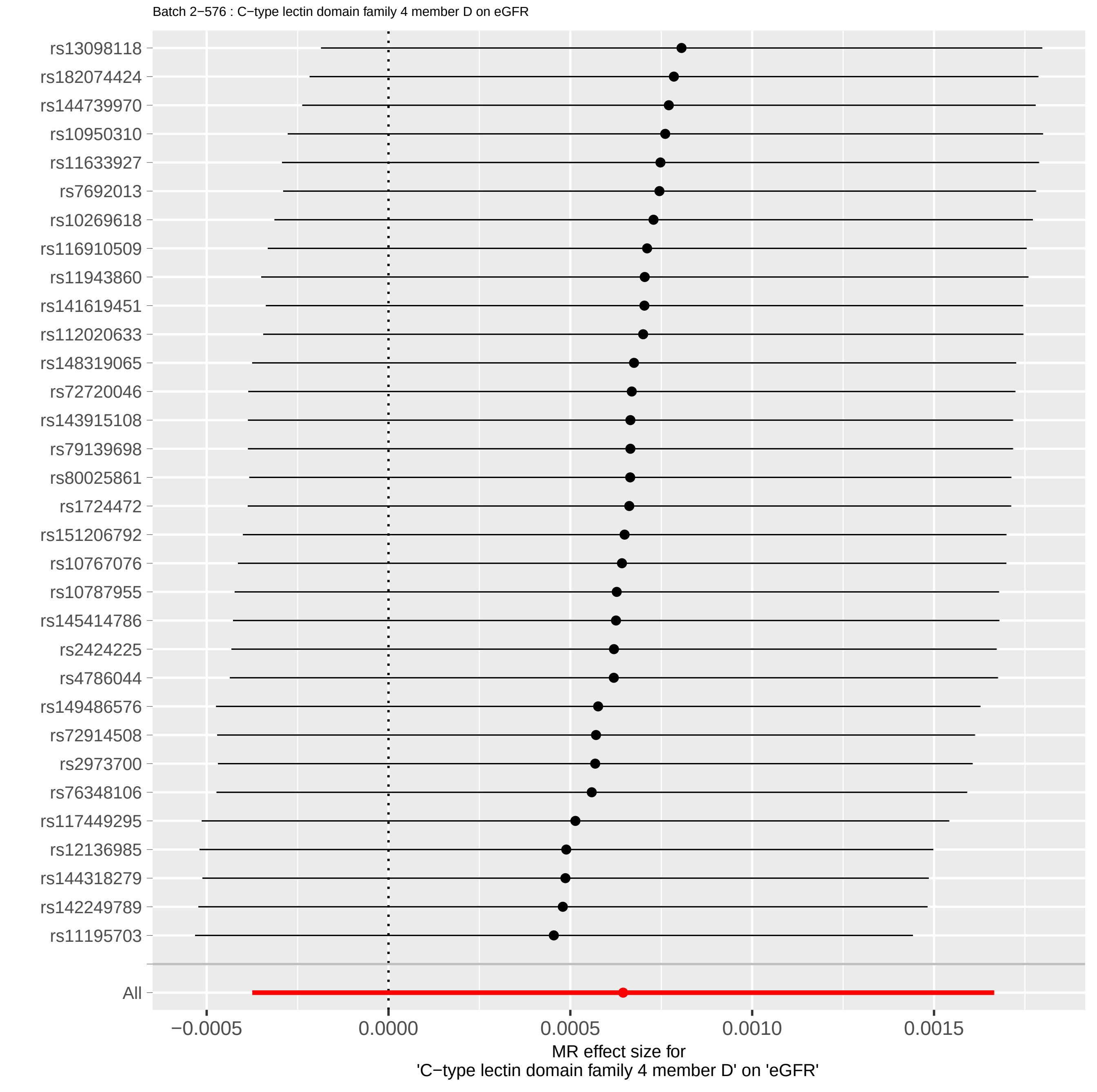

Batch 2−576 : C−type lectin domain family 4 member D on eGFR
rs13098118
rs182074424
rs144739970
rs10950310
rs11633927
rs7692013
rs10269618
rs116910509
rs11943860
rs141619451
rs112020633
rs148319065
rs72720046
rs143915108
rs79139698
rs80025861
rs1724472
rs151206792
rs10767076
rs10787955
rs145414786
rs2424225
rs4786044
rs149486576
rs72914508
rs2973700
rs76348106
rs117449295
rs12136985
rs144318279
rs142249789
rs11195703
All
−0.0005
0.0000
0.0005
0.0010
0.0015
MR effect size for
'C−type lectin domain family 4 member D' on 'eGFR'

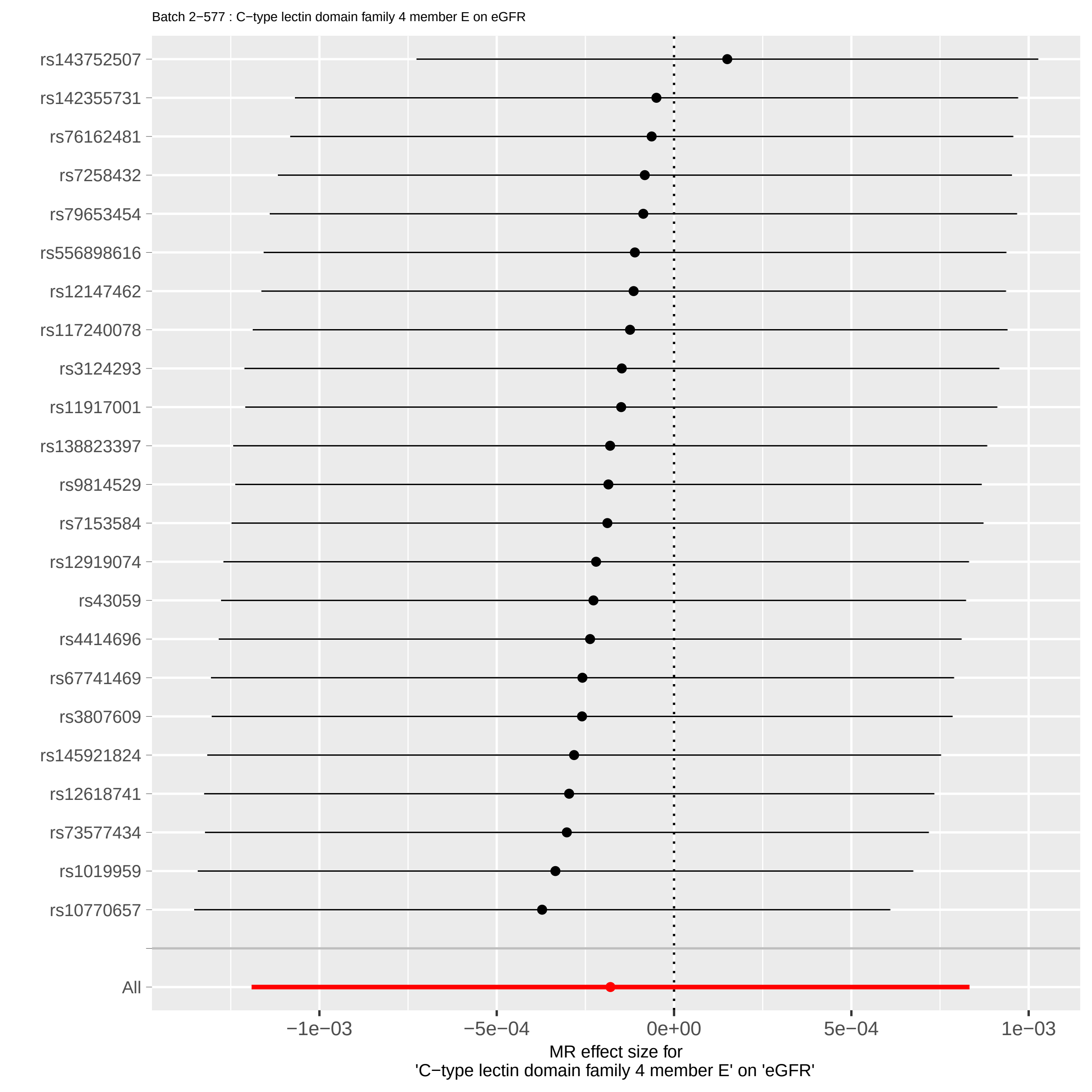

Batch 2−577 : C−type lectin domain family 4 member E on eGFR
rs143752507
rs142355731
rs76162481
rs7258432
rs79653454
rs556898616
rs12147462
rs117240078
rs3124293
rs11917001
rs138823397
rs9814529
rs7153584
rs12919074
rs43059
rs4414696
rs67741469
rs3807609
rs145921824
rs12618741
rs73577434
rs1019959
rs10770657
All
−1e−03
−5e−04
0e+00
5e−04
1e−03
MR effect size for
'C−type lectin domain family 4 member E' on 'eGFR'

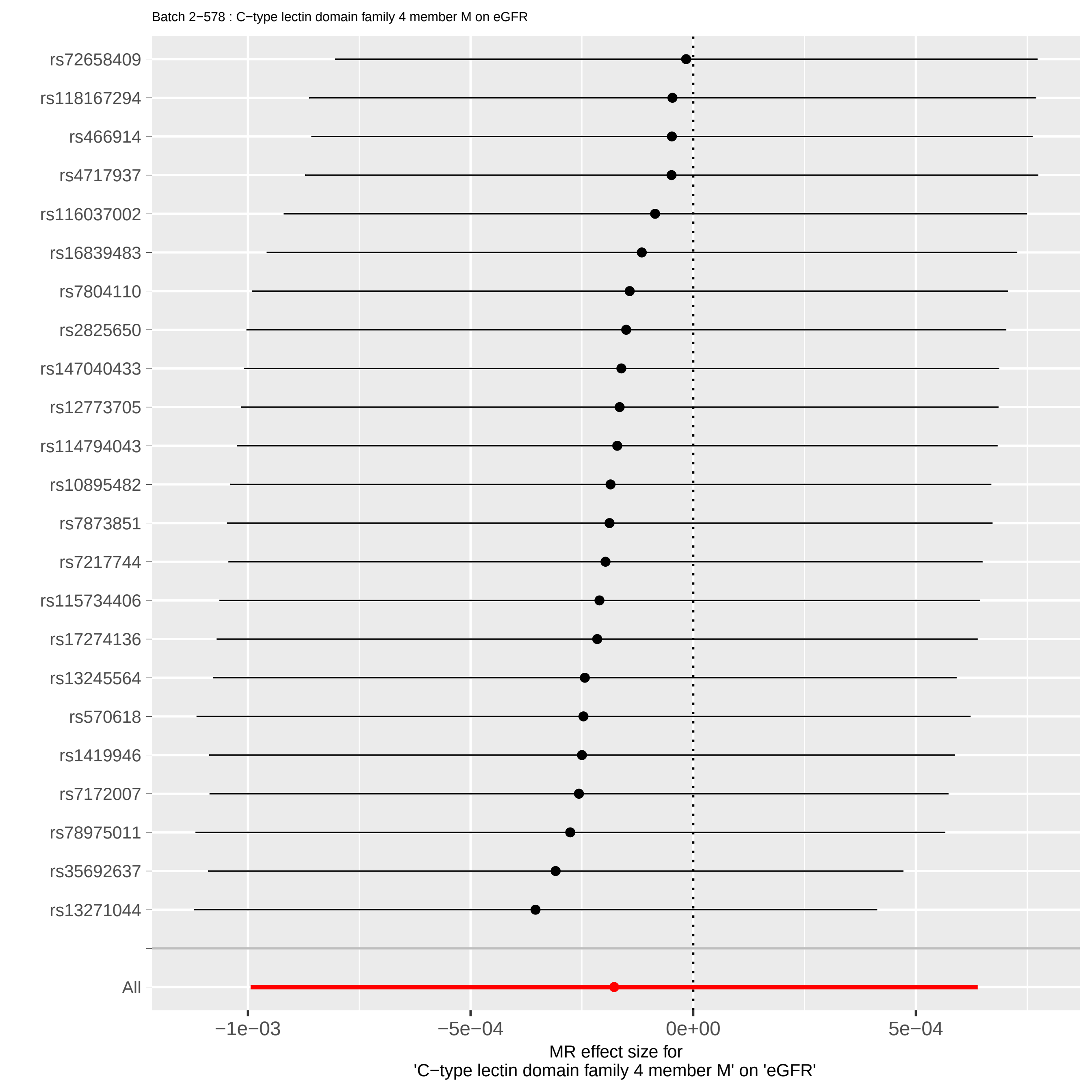

Batch 2−578 : C−type lectin domain family 4 member M on eGFR
rs72658409
rs118167294
rs466914
rs4717937
rs116037002
rs16839483
rs7804110
rs2825650
rs147040433
rs12773705
rs114794043
rs10895482
rs7873851
rs7217744
rs115734406
rs17274136
rs13245564
rs570618
rs1419946
rs7172007
rs78975011
rs35692637
rs13271044
All
−1e−03
−5e−04
0e+00
5e−04
MR effect size for
'C−type lectin domain family 4 member M' on 'eGFR'

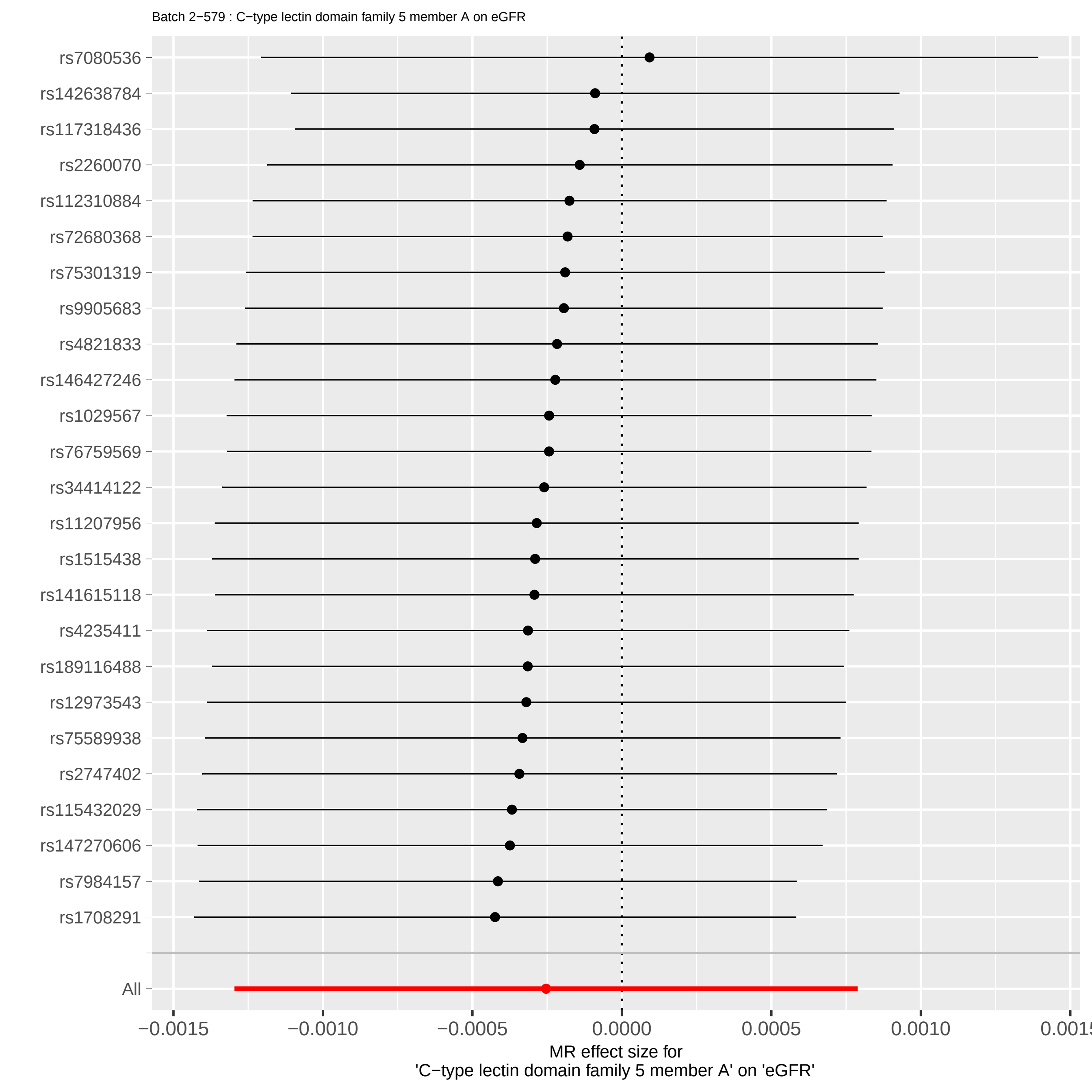

Batch 2−579 : C−type lectin domain family 5 member A on eGFR
rs7080536
rs142638784
rs117318436
rs2260070
rs112310884
rs72680368
rs75301319
rs9905683
rs4821833
rs146427246
rs1029567
rs76759569
rs34414122
rs11207956
rs1515438
rs141615118
rs4235411
rs189116488
rs12973543
rs75589938
rs2747402
rs115432029
rs147270606
rs7984157
rs1708291
All
−0.0015
−0.0010
−0.0005
0.0000
0.0005
0.0010
MR effect size for
'C−type lectin domain family 5 member A' on 'eGFR'

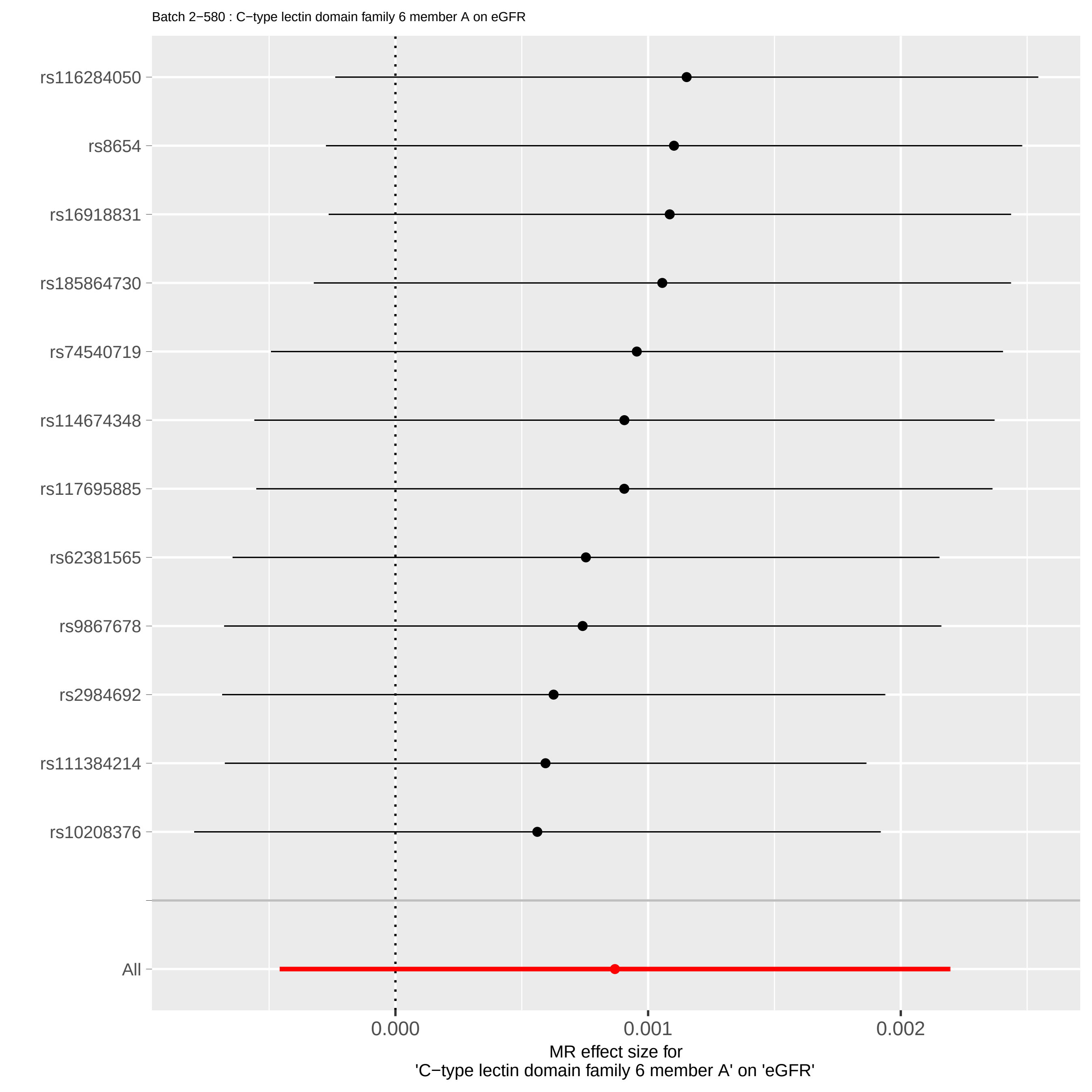

Batch 2−580 : C−type lectin domain family 6 member A on eGFR
rs116284050
rs8654
rs16918831
rs185864730
rs74540719
rs114674348
rs117695885
rs62381565
rs9867678
rs2984692
rs111384214
rs10208376
All
0.000
0.001
0.002
MR effect size for
'C−type lectin domain family 6 member A' on 'eGFR'

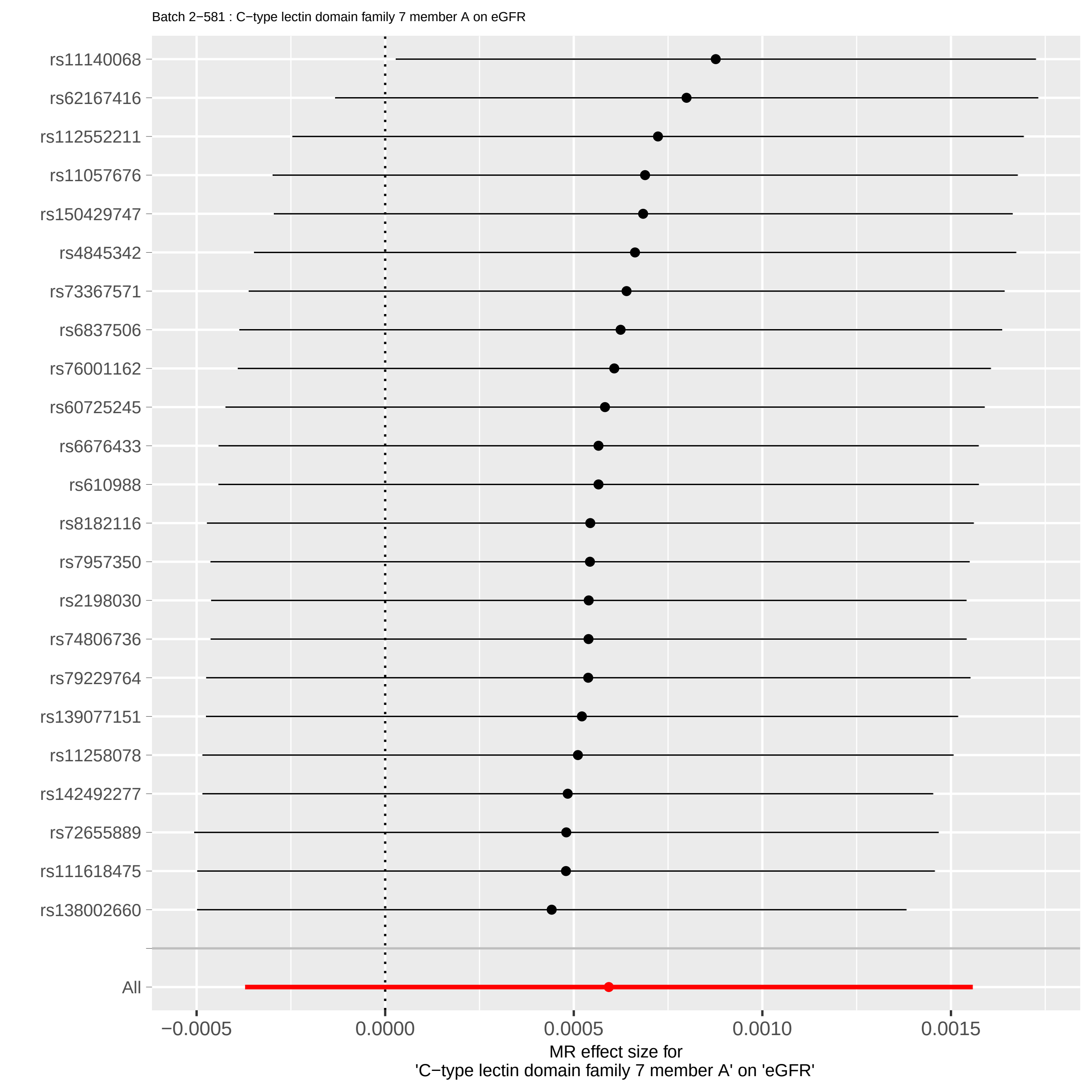
Batch 2−581 : C−type lectin domain family 7 member A on eGFR
rs11140068
rs62167416
rs112552211
rs11057676
rs150429747
rs4845342
rs73367571
rs6837506
rs76001162
rs60725245
rs6676433
rs610988
rs8182116
rs7957350
rs2198030
rs74806736
rs79229764
rs139077151
rs11258078
rs142492277
rs72655889
rs111618475
rs138002660
All
−0.0005
0.0000
0.0005
0.0010
0.0015
MR effect size for
'C−type lectin domain family 7 member A' on 'eGFR'

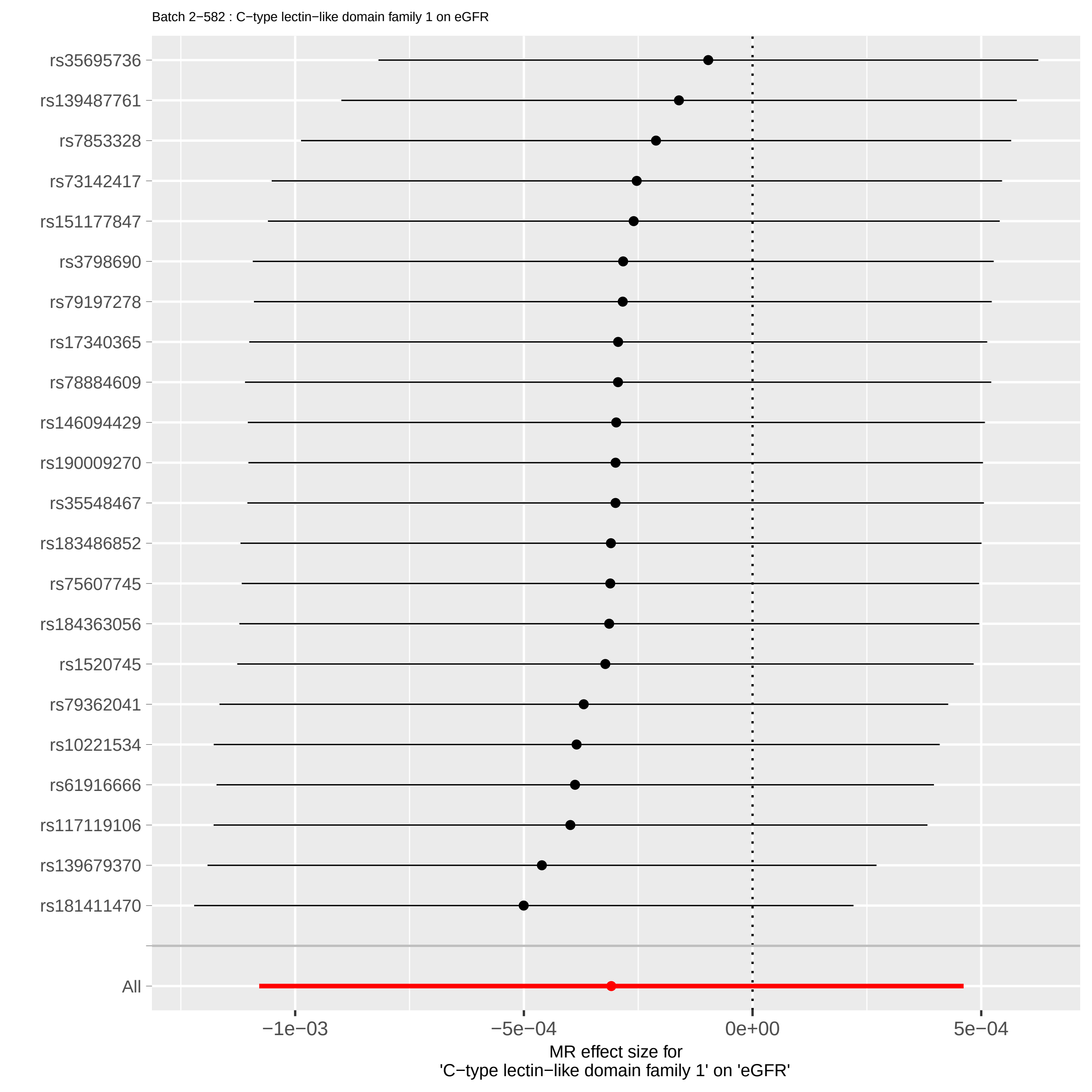

Batch 2−582 : C−type lectin−like domain family 1 on eGFR
rs35695736
rs139487761
rs7853328
rs73142417
rs151177847
rs3798690
rs79197278
rs17340365
rs78884609
rs146094429
rs190009270
rs35548467
rs183486852
rs75607745
rs184363056
rs1520745
rs79362041
rs10221534
rs61916666
rs117119106
rs139679370
rs181411470
All
−1e−03
−5e−04
0e+00
5e−04
MR effect size for
'C−type lectin−like domain family 1' on 'eGFR'

Batch 2−583 : Chloride intracellular channel protein 4 on eGFR

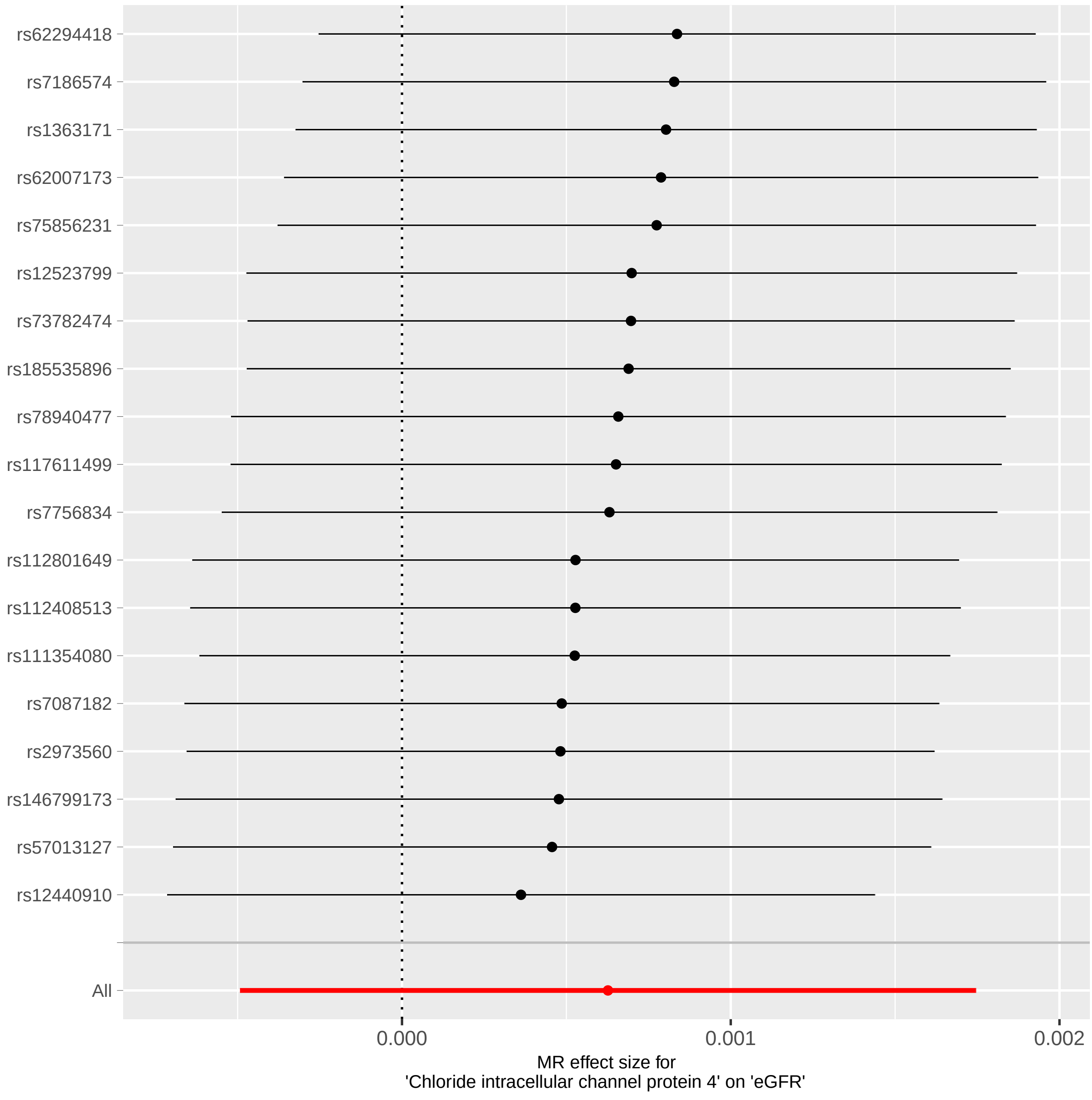

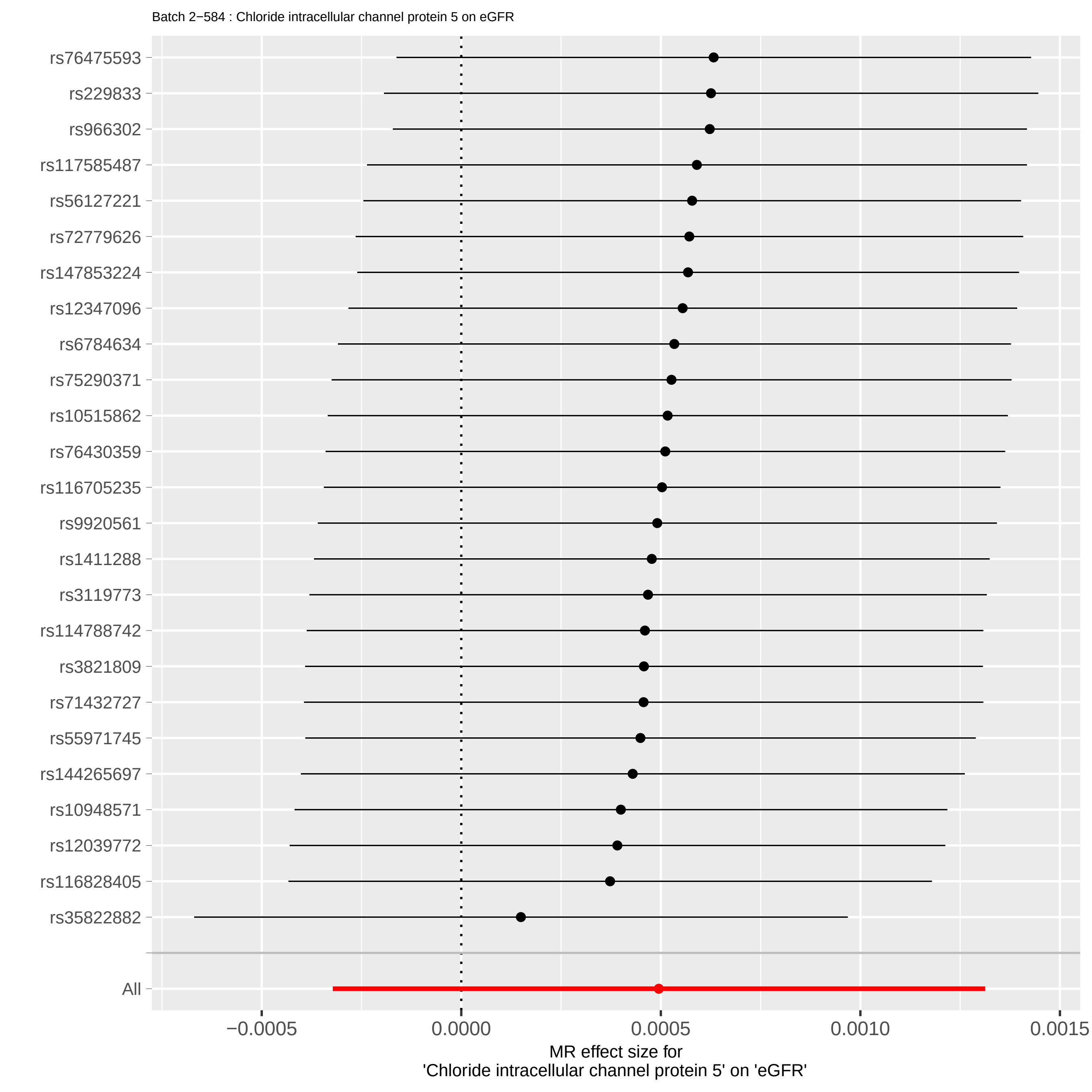

Batch 2−584 : Chloride intracellular channel protein 5 on eGFR
rs76475593
rs229833
rs966302
rs117585487
rs56127221
rs72779626
rs147853224
rs12347096
rs6784634
rs75290371
rs10515862
rs76430359
rs116705235
rs9920561
rs1411288
rs3119773
rs114788742
rs3821809
rs71432727
rs55971745
rs144265697
rs10948571
rs12039772
rs116828405
rs35822882
All
−0.0005
0.0000
0.0005
0.0010
0.0015
MR effect size for
'Chloride intracellular channel protein 5' on 'eGFR'

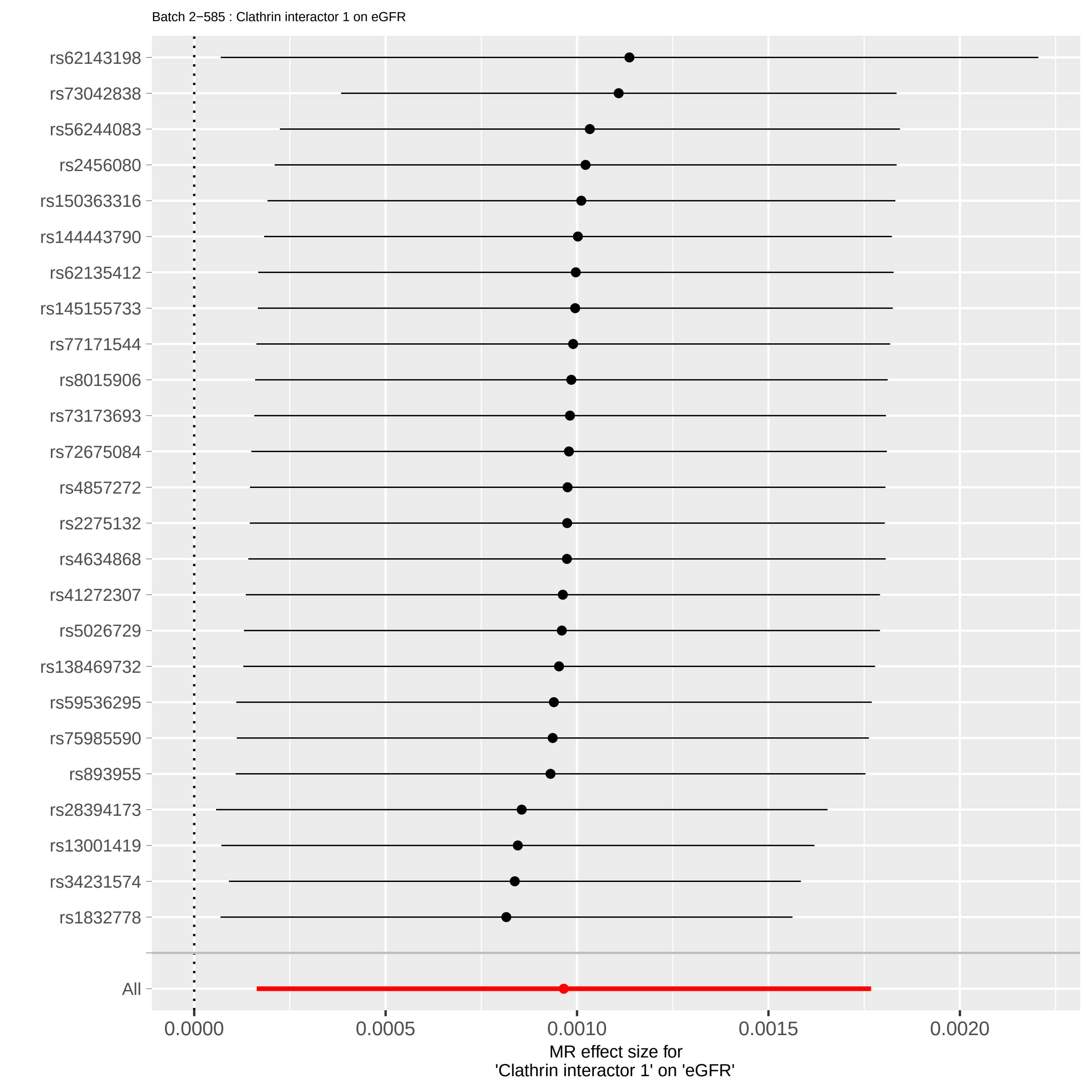
Batch 2−585 : Clathrin interactor 1 on eGFR
rs62143198
rs73042838
rs56244083
rs2456080
rs150363316
rs144443790
rs62135412
rs145155733
rs77171544
rs8015906
rs73173693
rs72675084
rs4857272
rs2275132
rs4634868
rs41272307
rs5026729
rs138469732
rs59536295
rs75985590
rs893955
rs28394173
rs13001419
rs34231574
rs1832778
All
0.0000
0.0005
0.0010
0.0015
0.0020
MR effect size for
'Clathrin interactor 1' on 'eGFR'

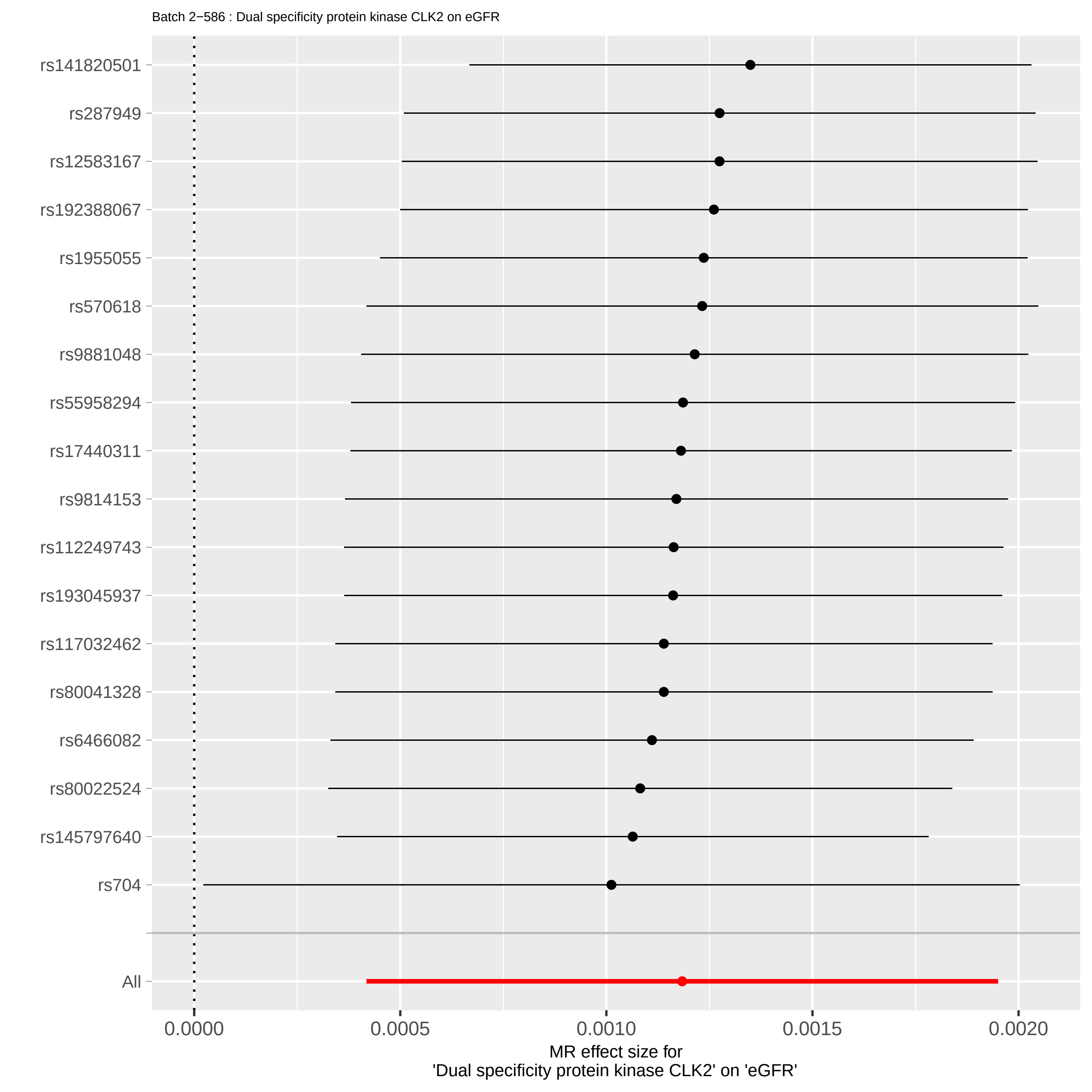
Batch 2−586 : Dual specificity protein kinase CLK2 on eGFR
rs141820501
rs287949
rs12583167
rs192388067
rs1955055
rs570618
rs9881048
rs55958294
rs17440311
rs9814153
rs112249743
rs193045937
rs117032462
rs80041328
rs6466082
rs80022524
rs145797640
rs704
All
0.0000
0.0005
0.0010
0.0015
0.0020
MR effect size for
'Dual specificity protein kinase CLK2' on 'eGFR'

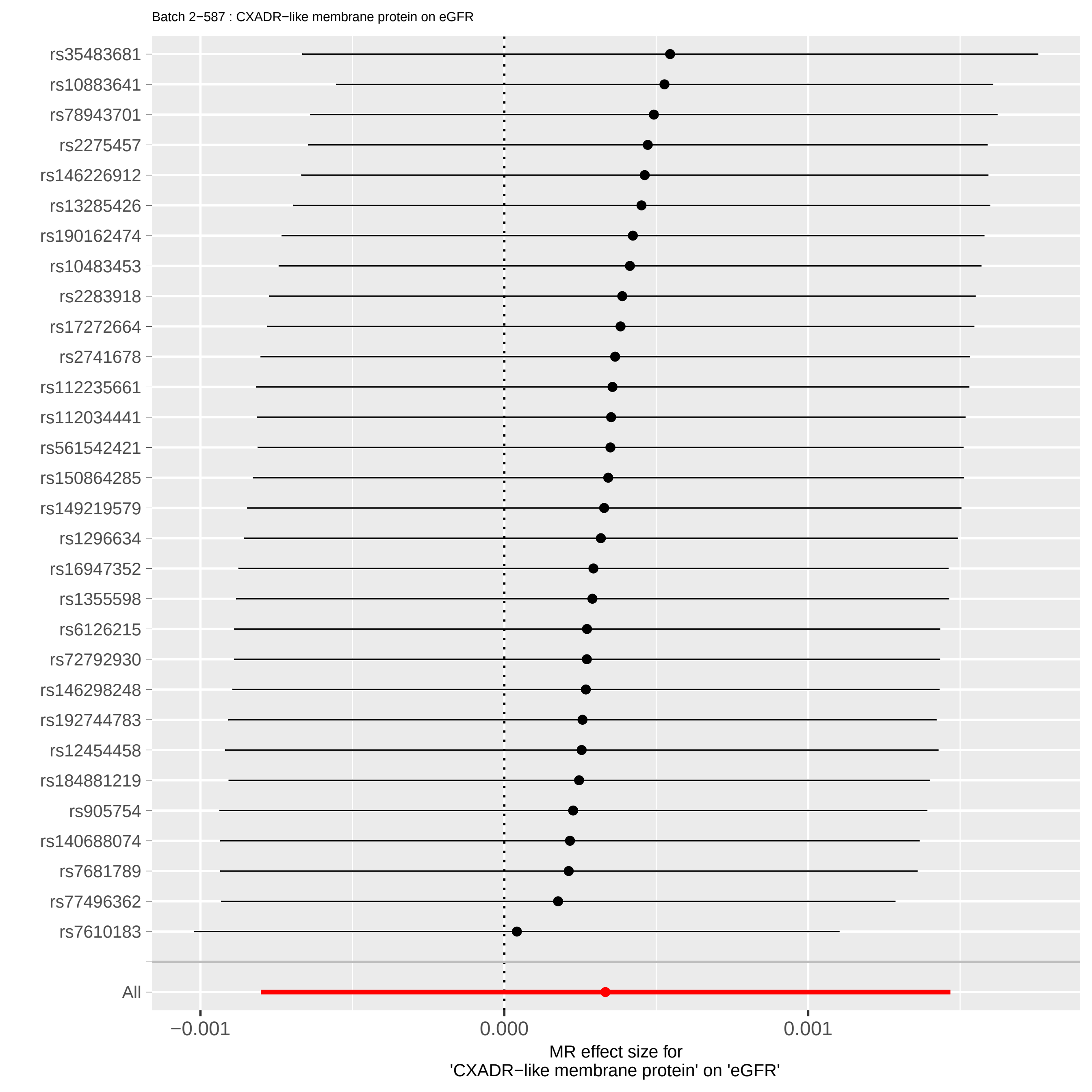
Batch 2−587 : CXADR−like membrane protein on eGFR
rs35483681
rs10883641
rs78943701
rs2275457
rs146226912
rs13285426
rs190162474
rs10483453
rs2283918
rs17272664
rs2741678
rs112235661
rs112034441
rs561542421
rs150864285
rs149219579
rs1296634
rs16947352
rs1355598
rs6126215
rs72792930
rs146298248
rs192744783
rs12454458
rs184881219
rs905754
rs140688074
rs7681789
rs77496362
rs7610183
All
−0.001
0.000
0.001
MR effect size for
'CXADR−like membrane protein' on 'eGFR'

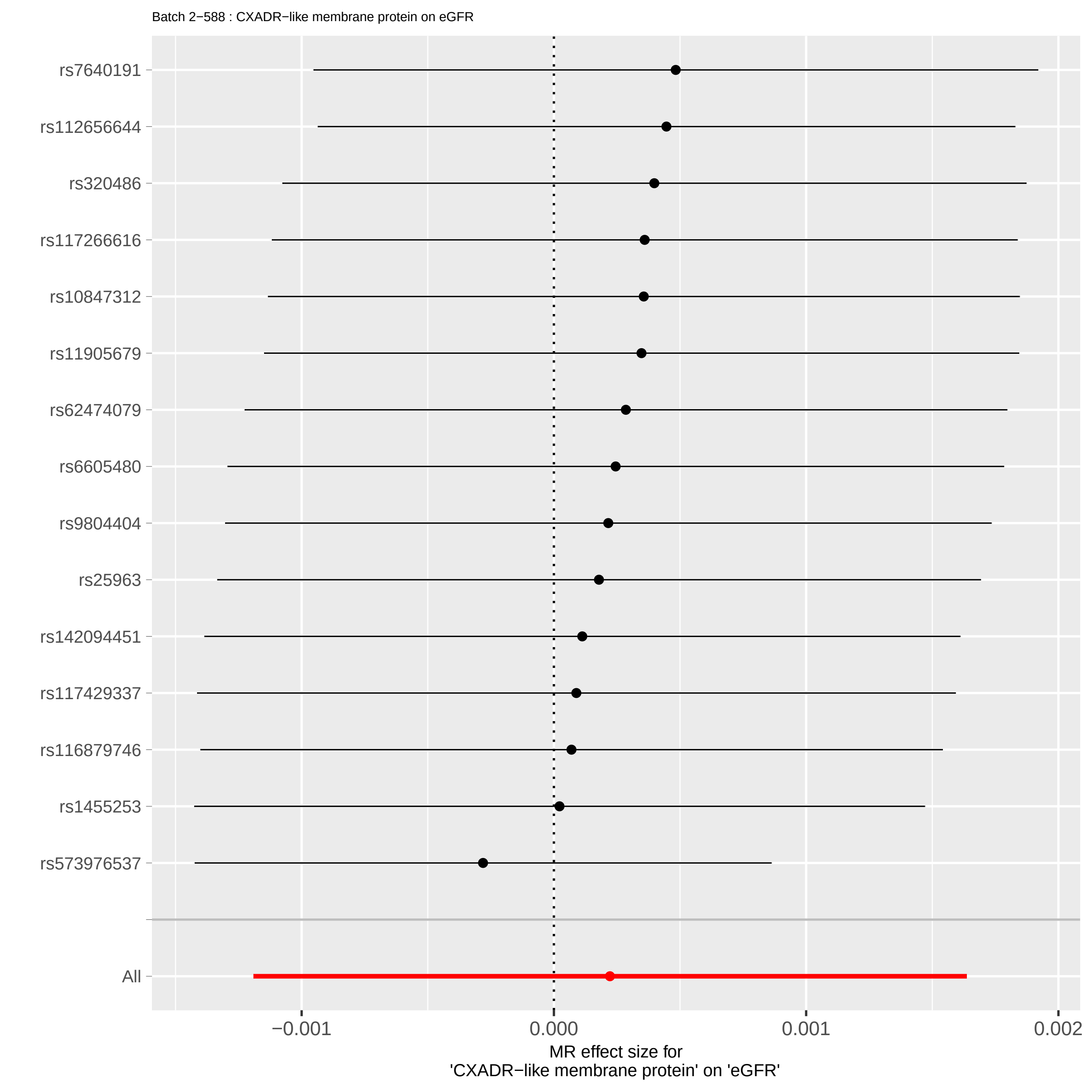

Batch 2−588 : CXADR−like membrane protein on eGFR
rs7640191
rs112656644
rs320486
rs117266616
rs10847312
rs11905679
rs62474079
rs6605480
rs9804404
rs25963
rs142094451
rs117429337
rs116879746
rs1455253
rs573976537
All
−0.001
0.000
0.001
0.002
MR effect size for
'CXADR−like membrane protein' on 'eGFR'

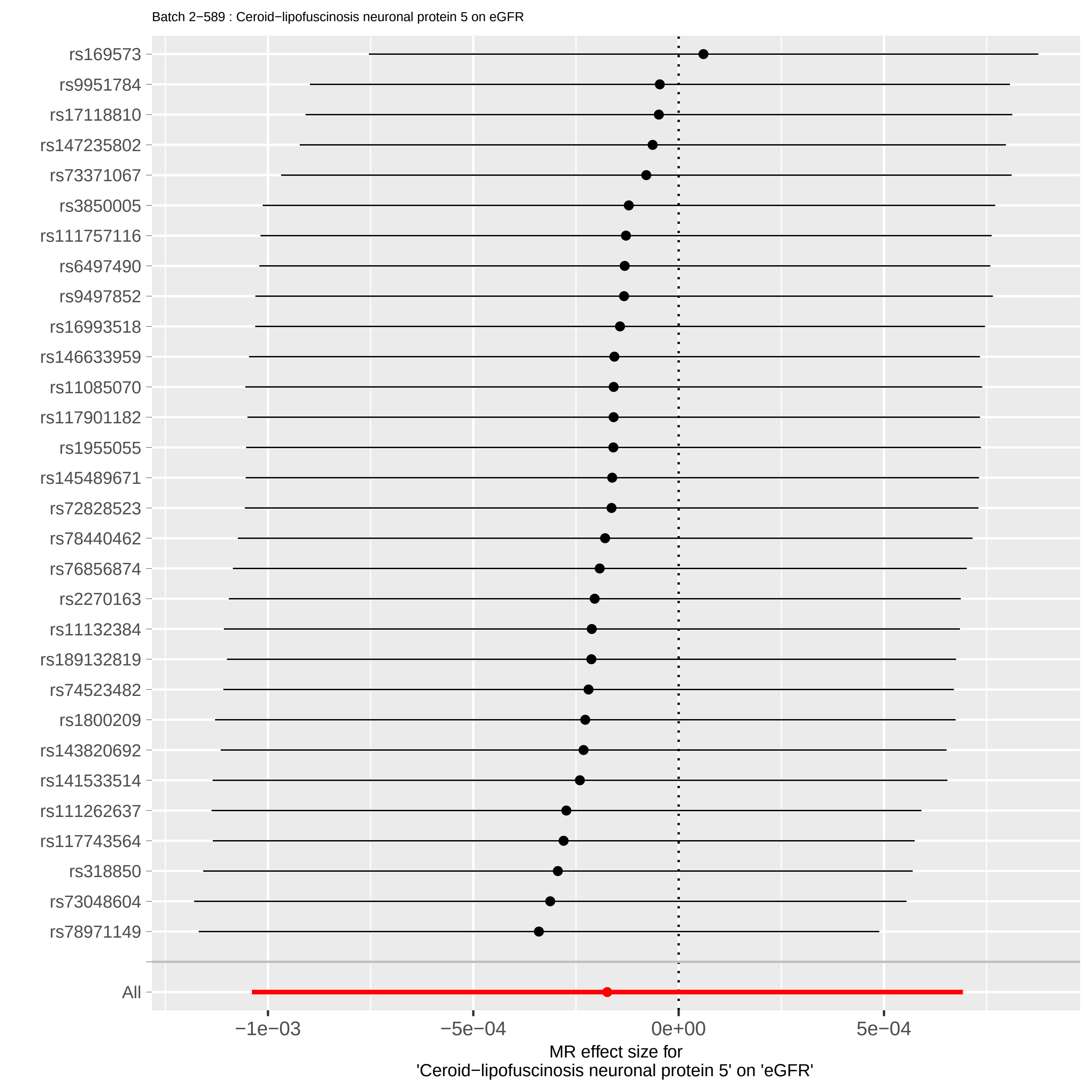

Batch 2−589 : Ceroid−lipofuscinosis neuronal protein 5 on eGFR
rs169573
rs9951784
rs17118810
rs147235802
rs73371067
rs3850005
rs111757116
rs6497490
rs9497852
rs16993518
rs146633959
rs11085070
rs117901182
rs1955055
rs145489671
rs72828523
rs78440462
rs76856874
rs2270163
rs11132384
rs189132819
rs74523482
rs1800209
rs143820692
rs141533514
rs111262637
rs117743564
rs318850
rs73048604
rs78971149
All
−1e−03
−5e−04
0e+00
5e−04
MR effect size for
'Ceroid−lipofuscinosis neuronal protein 5' on 'eGFR'

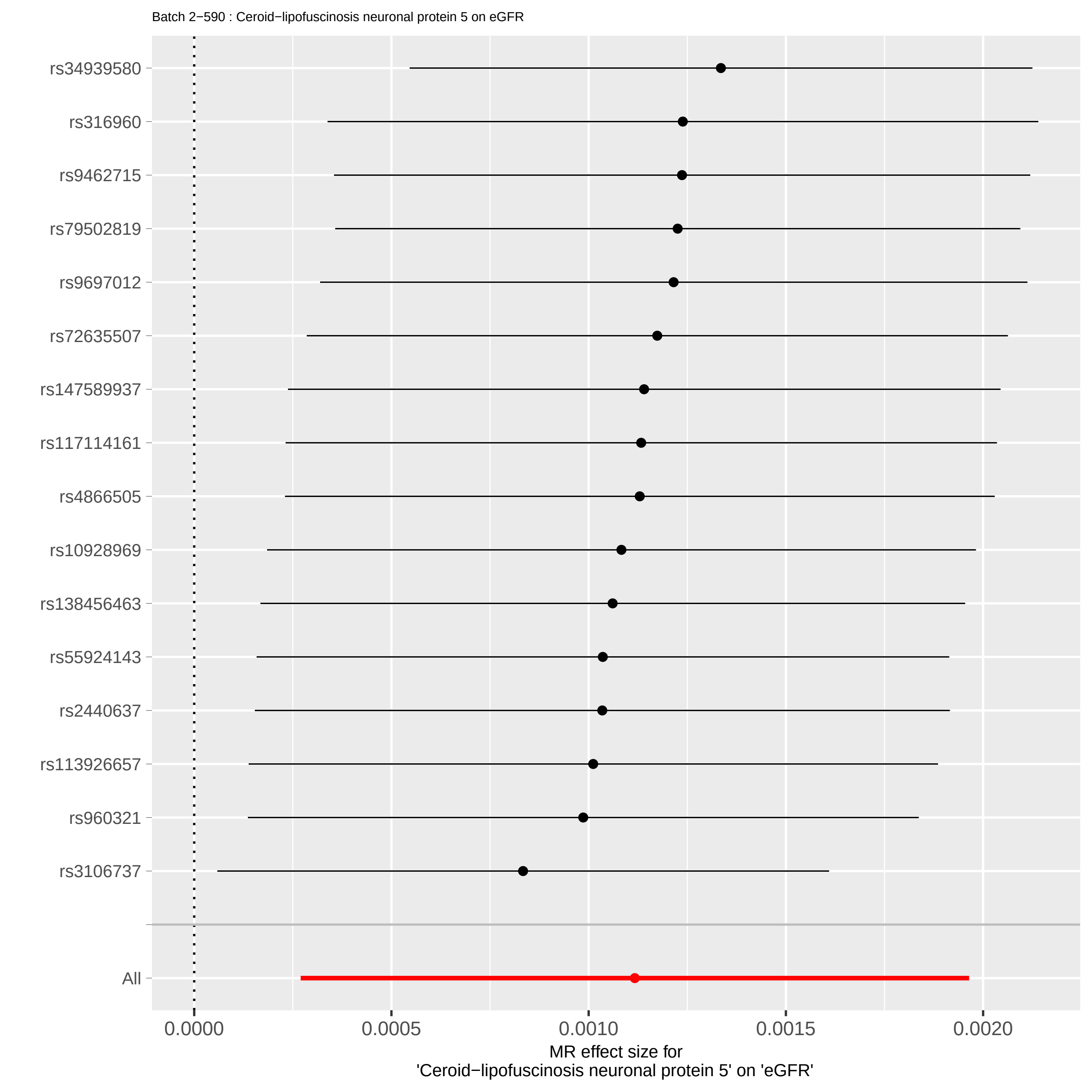

Batch 2−590 : Ceroid−lipofuscinosis neuronal protein 5 on eGFR
rs34939580
rs316960
rs9462715
rs79502819
rs9697012
rs72635507
rs147589937
rs117114161
rs4866505
rs10928969
rs138456463
rs55924143
rs2440637
rs113926657
rs960321
rs3106737
All
0.0000
0.0005
0.0010
0.0015
0.0020
MR effect size for
'Ceroid−lipofuscinosis neuronal protein 5' on 'eGFR'

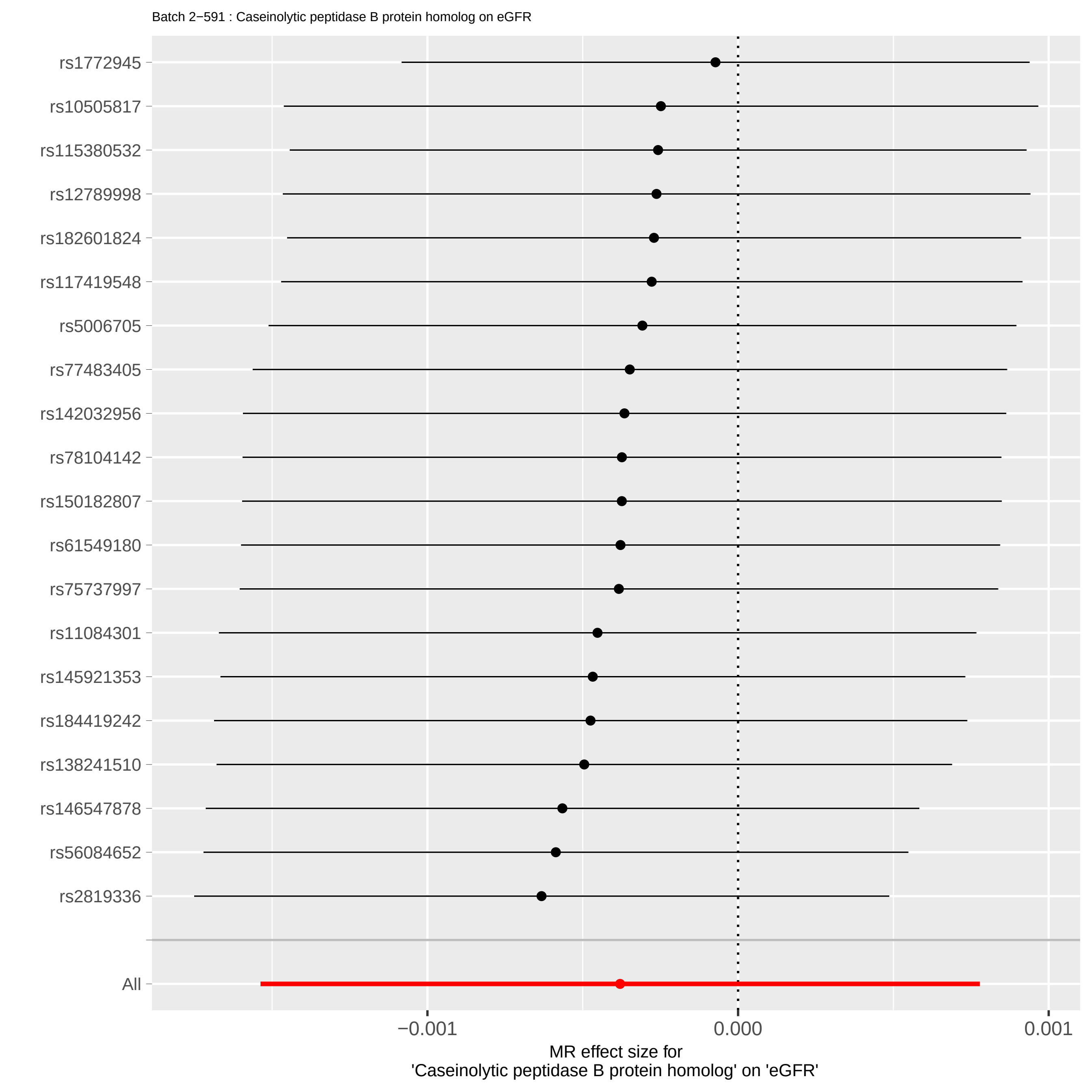

Batch 2−591 : Caseinolytic peptidase B protein homolog on eGFR
rs1772945
rs10505817
rs115380532
rs12789998
rs182601824
rs117419548
rs5006705
rs77483405
rs142032956
rs78104142
rs150182807
rs61549180
rs75737997
rs11084301
rs145921353
rs184419242
rs138241510
rs146547878
rs56084652
rs2819336
All
−0.001
0.000
0.001
MR effect size for
'Caseinolytic peptidase B protein homolog' on 'eGFR'

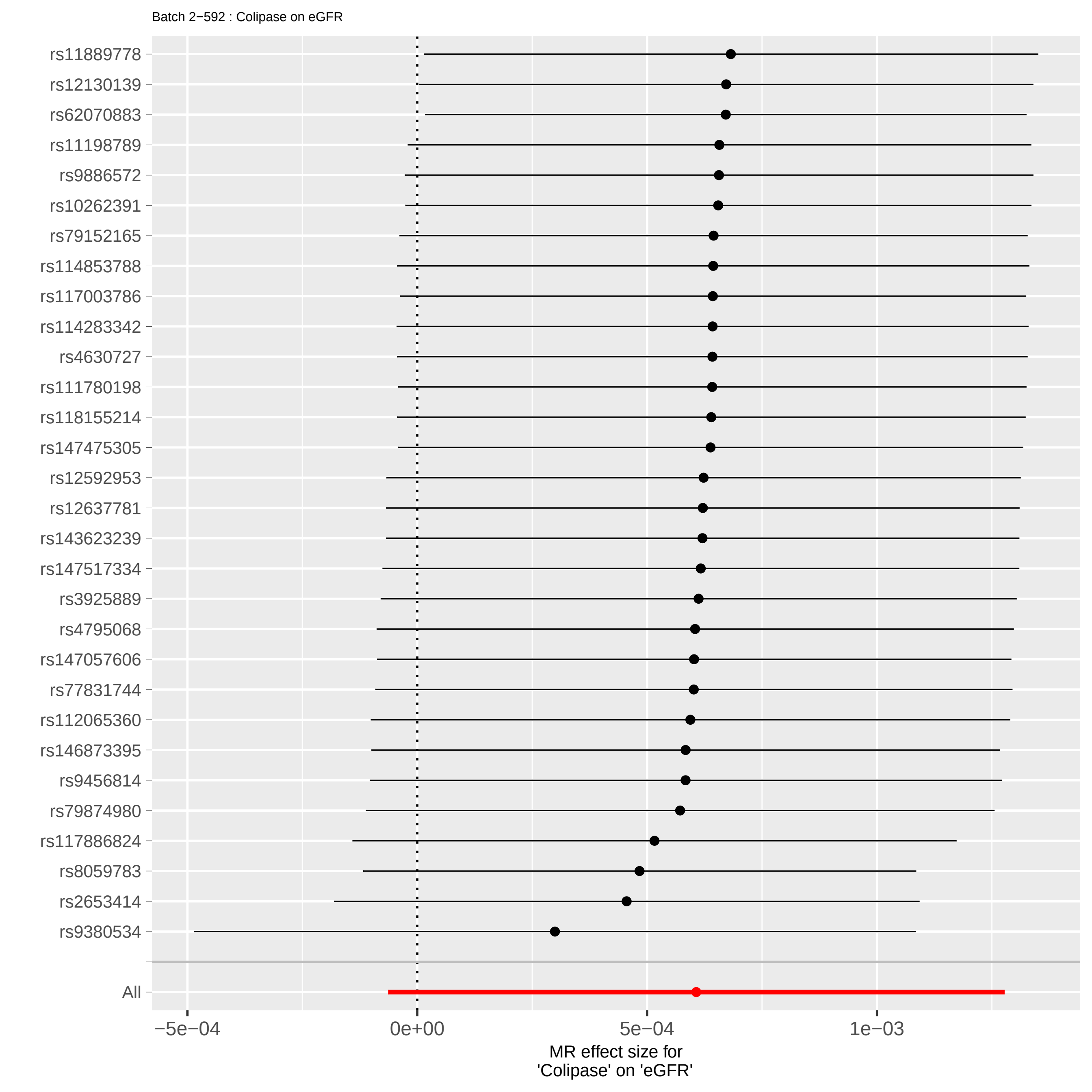

Batch 2−592 : Colipase on eGFR
rs11889778
rs12130139
rs62070883
rs11198789
rs9886572
rs10262391
rs79152165
rs114853788
rs117003786
rs114283342
rs4630727
rs111780198
rs118155214
rs147475305
rs12592953
rs12637781
rs143623239
rs147517334
rs3925889
rs4795068
rs147057606
rs77831744
rs112065360
rs146873395
rs9456814
rs79874980
rs117886824
rs8059783
rs2653414
rs9380534
All
−5e−04
0e+00
5e−04
1e−03
MR effect size for
'Colipase' on 'eGFR'

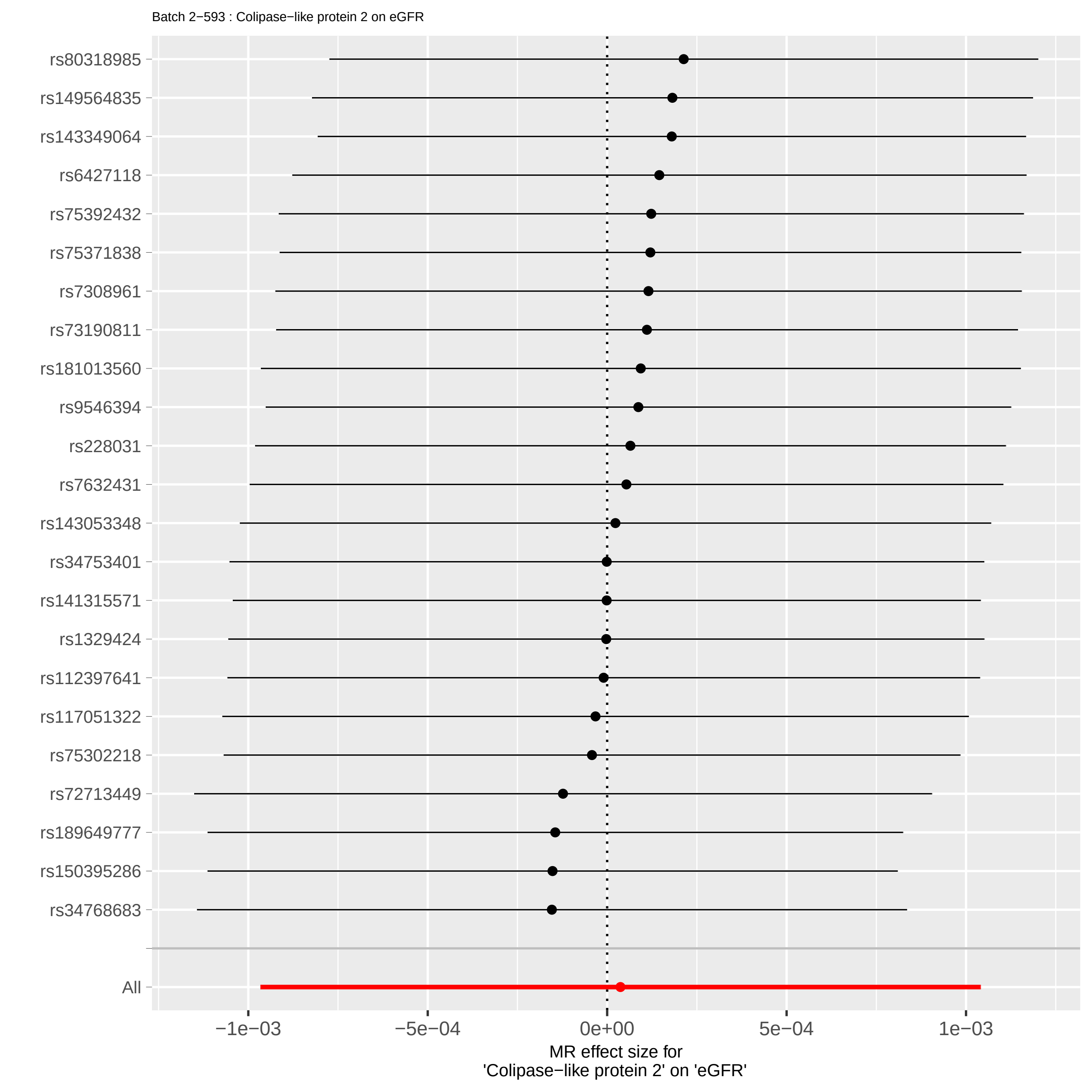

Batch 2−593 : Colipase−like protein 2 on eGFR
rs80318985
rs149564835
rs143349064
rs6427118
rs75392432
rs75371838
rs7308961
rs73190811
rs181013560
rs9546394
rs228031
rs7632431
rs143053348
rs34753401
rs141315571
rs1329424
rs112397641
rs117051322
rs75302218
rs72713449
rs189649777
rs150395286
rs34768683
All
−1e−03
−5e−04
0e+00
5e−04
1e−03
MR effect size for
'Colipase−like protein 2' on 'eGFR'

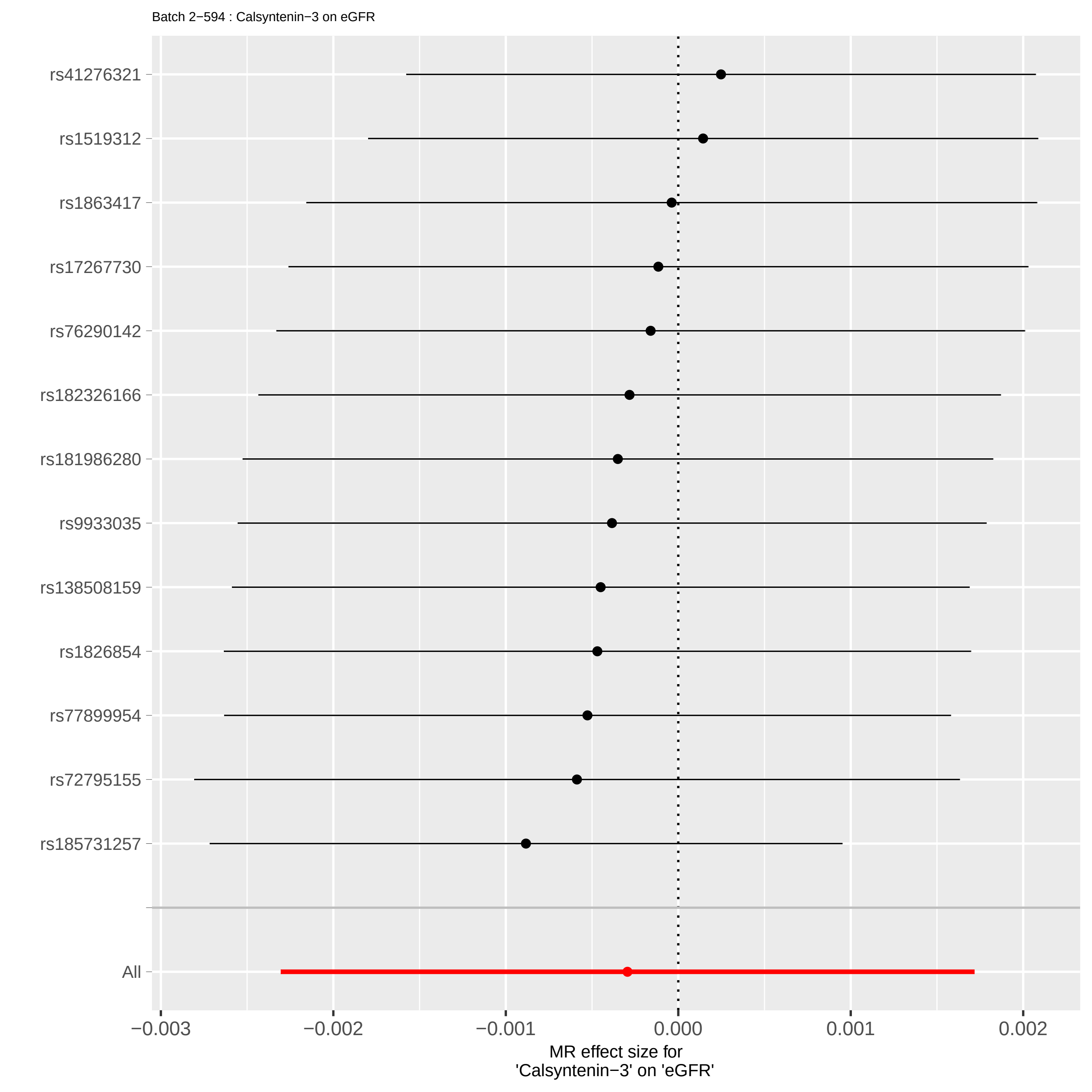
Batch 2−594 : Calsyntenin−3 on eGFR
rs41276321
rs1519312
rs1863417
rs17267730
rs76290142
rs182326166
rs181986280
rs9933035
rs138508159
rs1826854
rs77899954
rs72795155
rs185731257
All
−0.003
−0.002
−0.001
0.000
0.001
0.002
MR effect size for
'Calsyntenin−3' on 'eGFR'

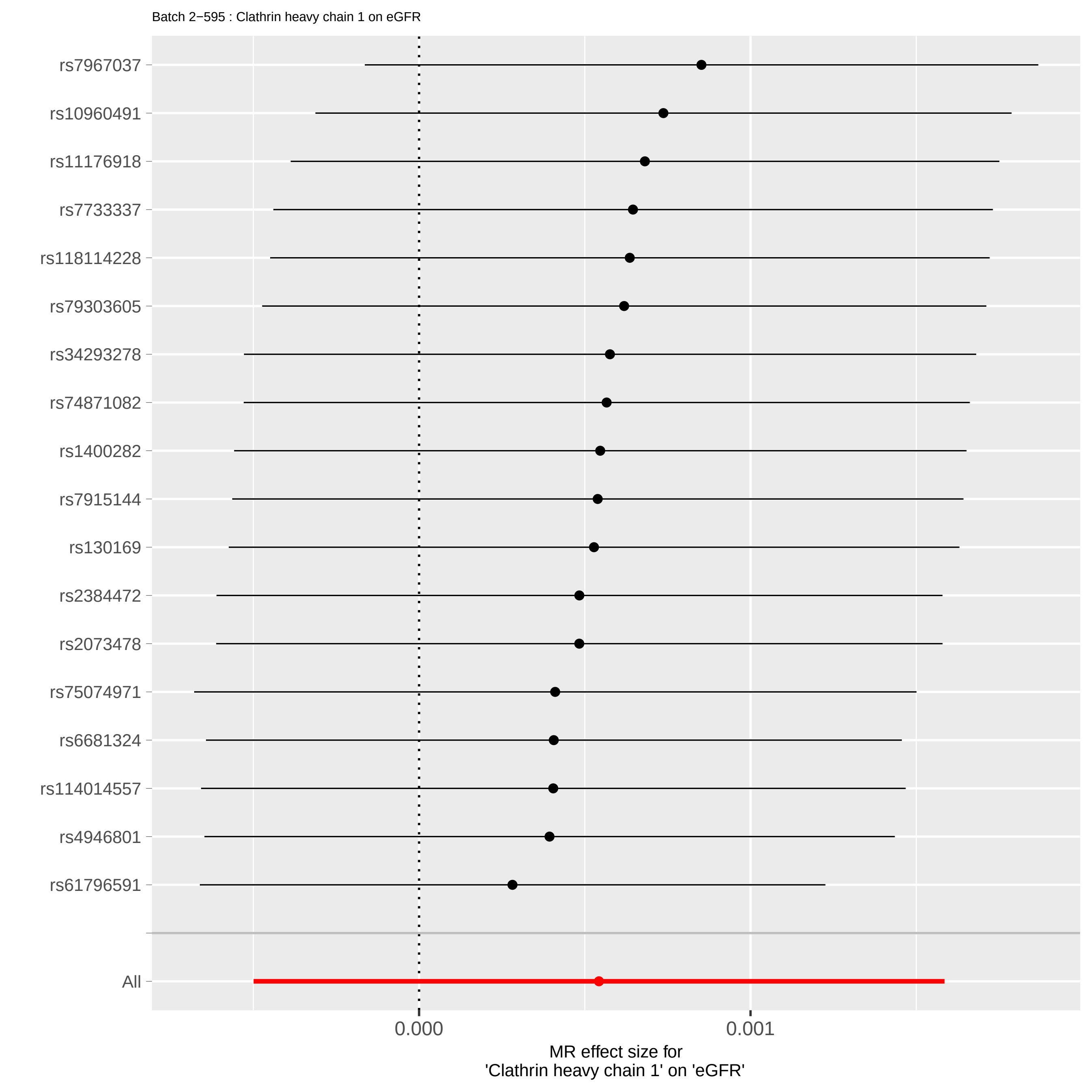

Batch 2−595 : Clathrin heavy chain 1 on eGFR
rs7967037
rs10960491
rs11176918
rs7733337
rs118114228
rs79303605
rs34293278
rs74871082
rs1400282
rs7915144
rs130169
rs2384472
rs2073478
rs75074971
rs6681324
rs114014557
rs4946801
rs61796591
All
0.000
0.001
MR effect size for
'Clathrin heavy chain 1' on 'eGFR'

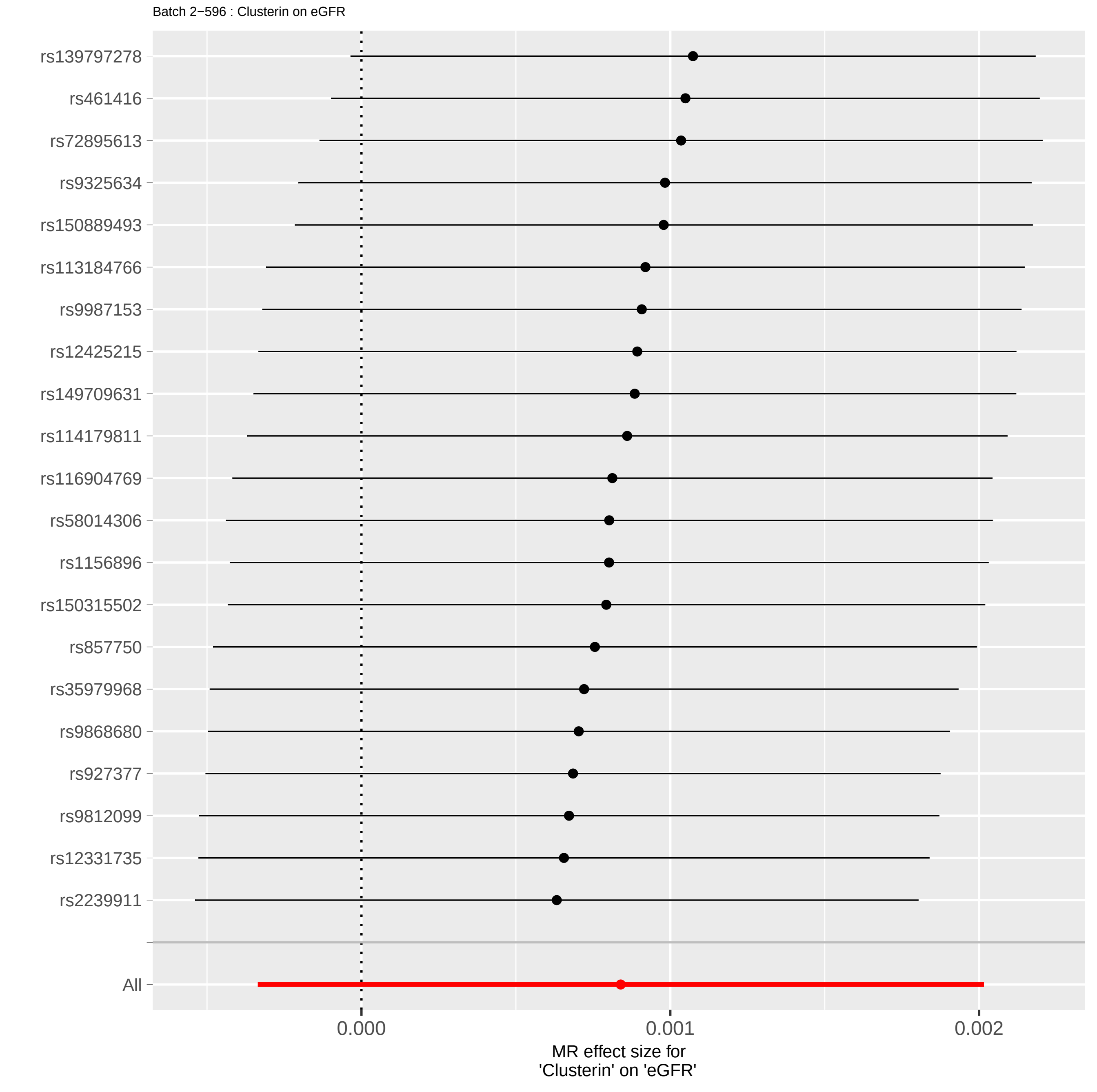
Batch 2−596 : Clusterin on eGFR
rs139797278
rs461416
rs72895613
rs9325634
rs150889493
rs113184766
rs9987153
rs12425215
rs149709631
rs114179811
rs116904769
rs58014306
rs1156896
rs150315502
rs857750
rs35979968
rs9868680
rs927377
rs9812099
rs12331735
rs2239911
All
0.000
0.001
0.002
MR effect size for
'Clusterin' on 'eGFR'

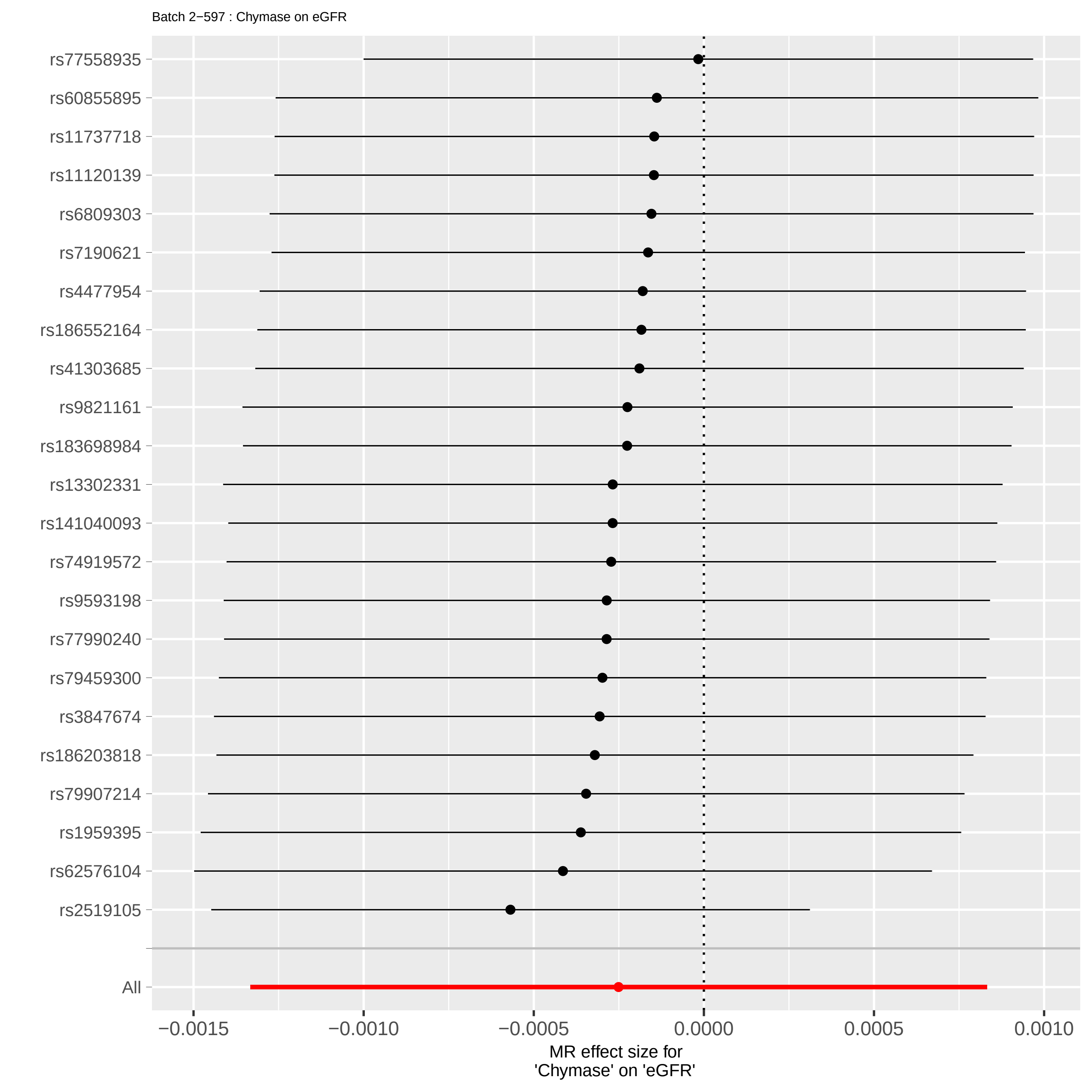

Batch 2−597 : Chymase on eGFR
rs77558935
rs60855895
rs11737718
rs11120139
rs6809303
rs7190621
rs4477954
rs186552164
rs41303685
rs9821161
rs183698984
rs13302331
rs141040093
rs74919572
rs9593198
rs77990240
rs79459300
rs3847674
rs186203818
rs79907214
rs1959395
rs62576104
rs2519105
All
−0.0015
−0.0010
−0.0005
0.0000
0.0005
0.0010
MR effect size for
'Chymase' on 'eGFR'

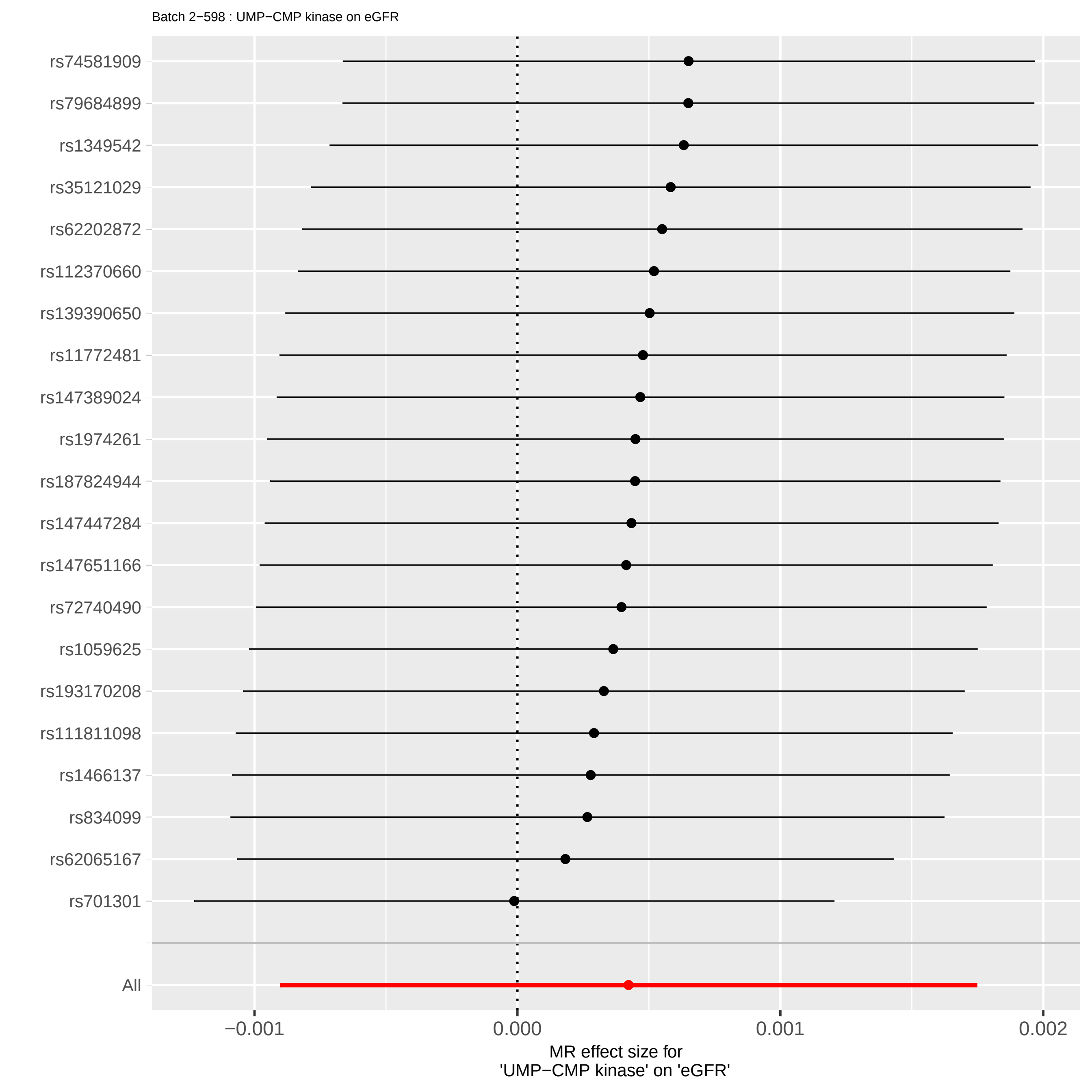
Batch 2−598 : UMP−CMP kinase on eGFR
rs74581909
rs79684899
rs1349542
rs35121029
rs62202872
rs112370660
rs139390650
rs11772481
rs147389024
rs1974261
rs187824944
rs147447284
rs147651166
rs72740490
rs1059625
rs193170208
rs111811098
rs1466137
rs834099
rs62065167
rs701301
All
−0.001
0.000
0.001
0.002
MR effect size for
'UMP−CMP kinase' on 'eGFR'

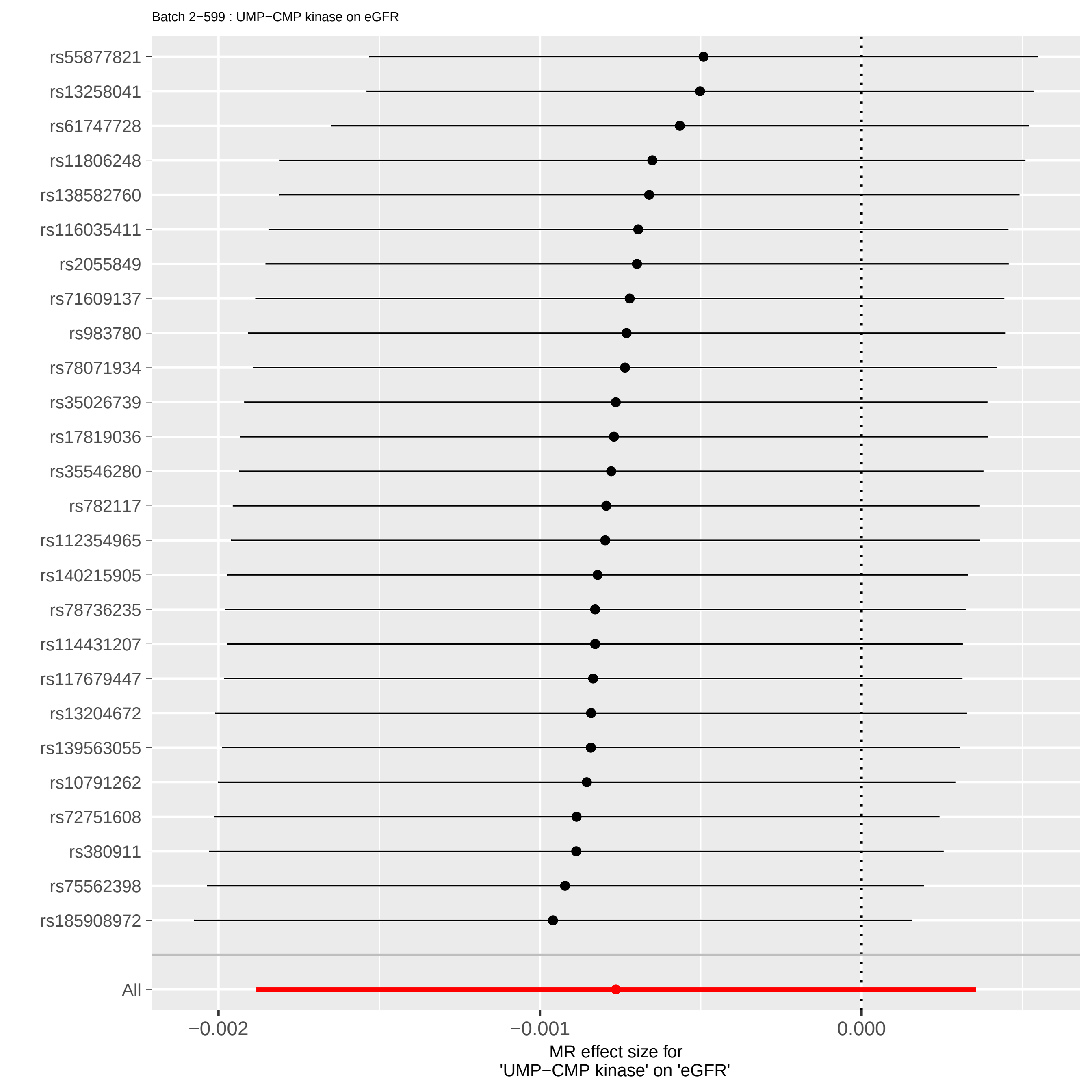
Batch 2−599 : UMP−CMP kinase on eGFR
rs55877821
rs13258041
rs61747728
rs11806248
rs138582760
rs116035411
rs2055849
rs71609137
rs983780
rs78071934
rs35026739
rs17819036
rs35546280
rs782117
rs112354965
rs140215905
rs78736235
rs114431207
rs117679447
rs13204672
rs139563055
rs10791262
rs72751608
rs380911
rs75562398
rs185908972
All
−0.002
−0.001
0.000
MR effect size for
'UMP−CMP kinase' on 'eGFR'

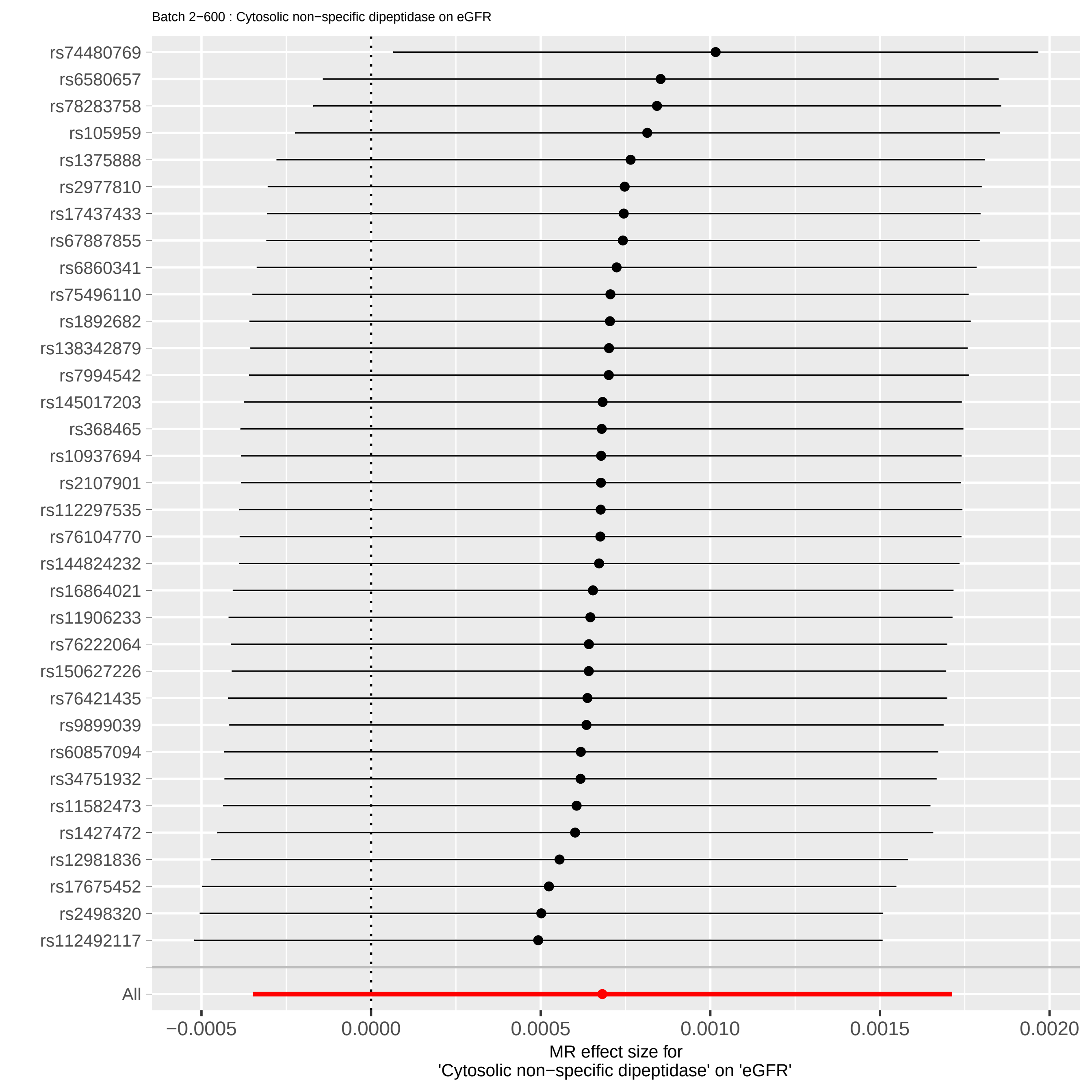
Batch 2−600 : Cytosolic non−specific dipeptidase on eGFR
rs74480769
rs6580657
rs78283758
rs105959
rs1375888
rs2977810
rs17437433
rs67887855
rs6860341
rs75496110
rs1892682
rs138342879
rs7994542
rs145017203
rs368465
rs10937694
rs2107901
rs112297535
rs76104770
rs144824232
rs16864021
rs11906233
rs76222064
rs150627226
rs76421435
rs9899039
rs60857094
rs34751932
rs11582473
rs1427472
rs12981836
rs17675452
rs2498320
rs112492117
All
−0.0005
0.0000
0.0005
0.0010
0.0015
0.0020
MR effect size for
'Cytosolic non−specific dipeptidase' on 'eGFR'

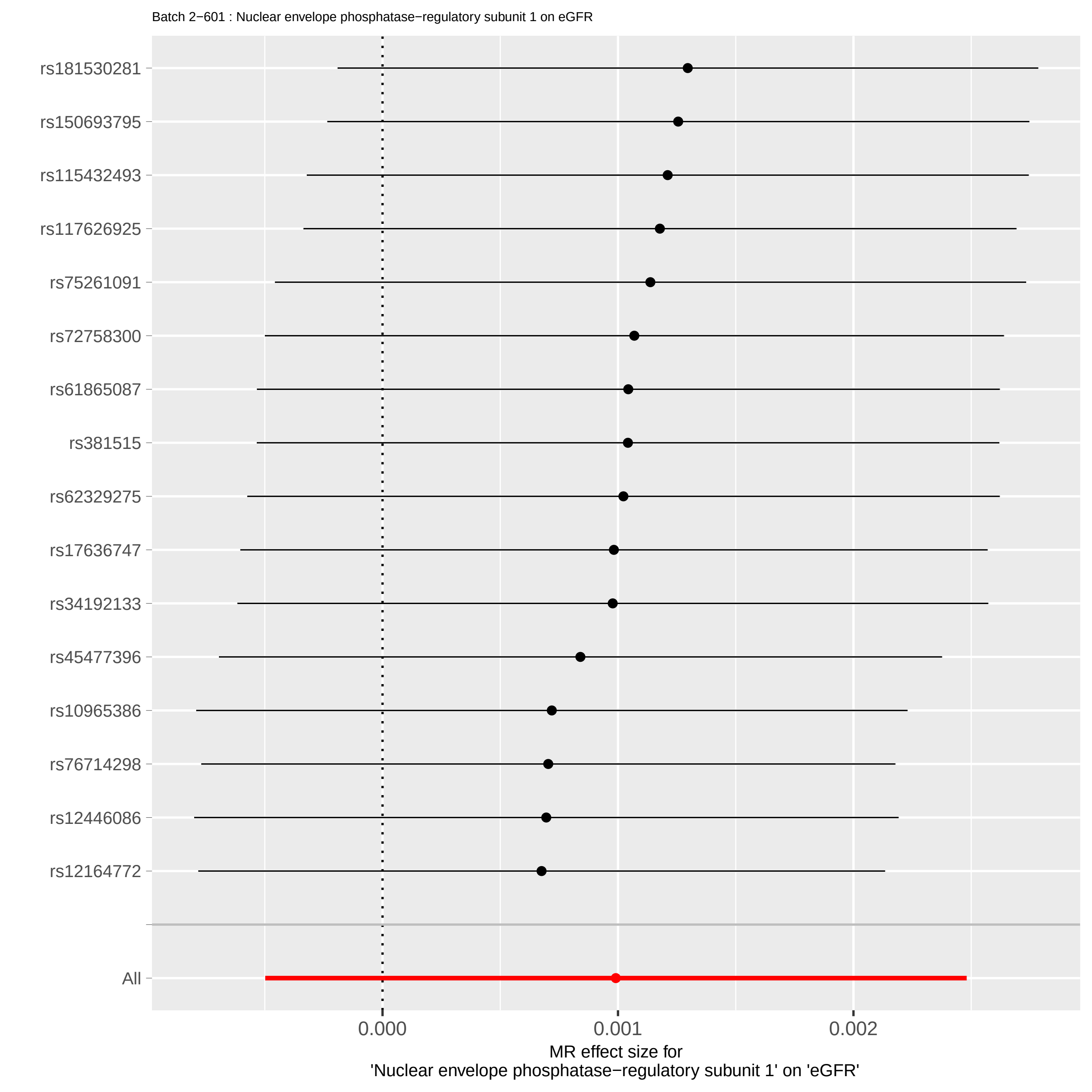

Batch 2−601 : Nuclear envelope phosphatase−regulatory subunit 1 on eGFR
rs181530281
rs150693795
rs115432493
rs117626925
rs75261091
rs72758300
rs61865087
rs381515
rs62329275
rs17636747
rs34192133
rs45477396
rs10965386
rs76714298
rs12446086
rs12164772
All
0.000
0.001
0.002
MR effect size for
'Nuclear envelope phosphatase−regulatory subunit 1' on 'eGFR'

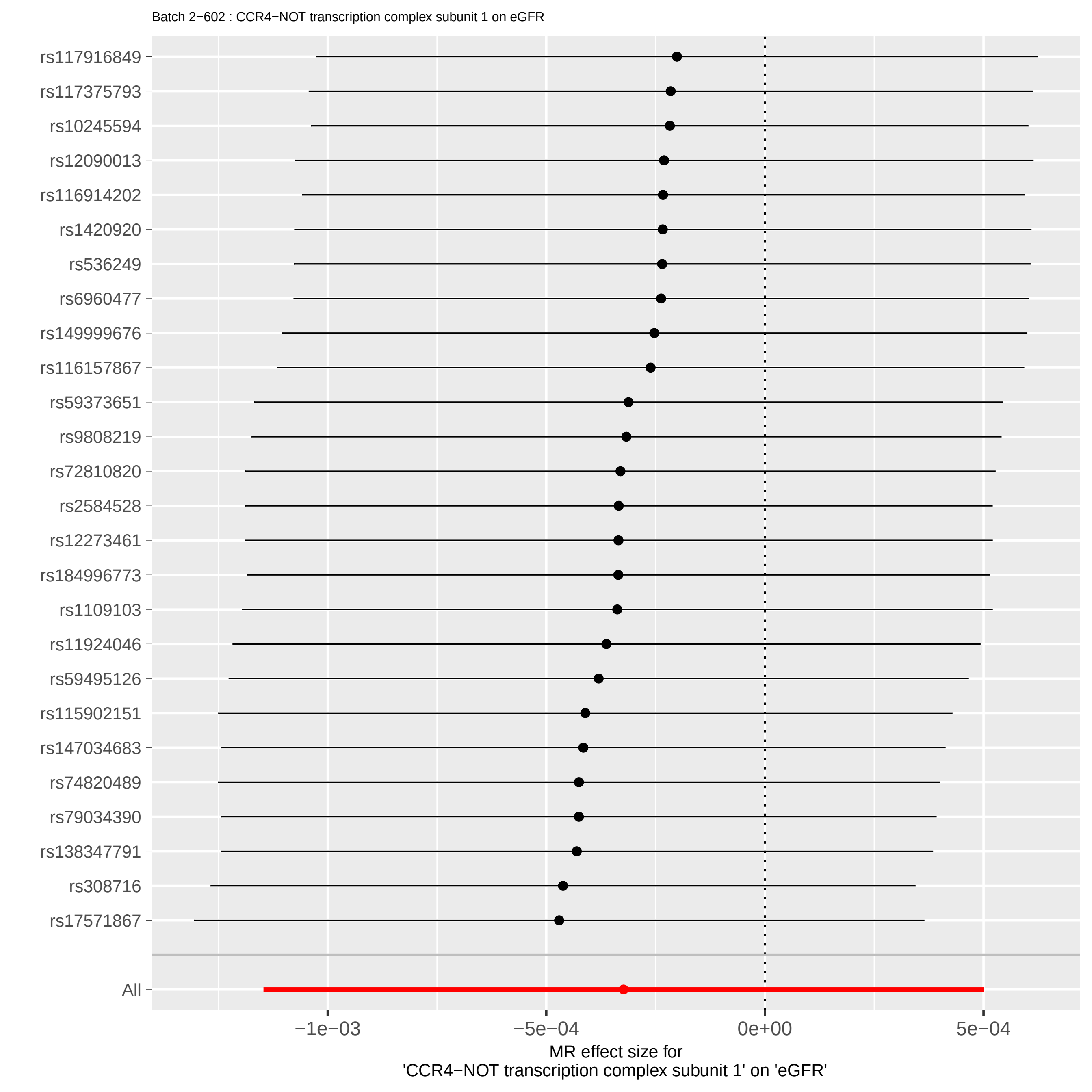

Batch 2−602 : CCR4−NOT transcription complex subunit 1 on eGFR
rs117916849
rs117375793
rs10245594
rs12090013
rs116914202
rs1420920
rs536249
rs6960477
rs149999676
rs116157867
rs59373651
rs9808219
rs72810820
rs2584528
rs12273461
rs184996773
rs1109103
rs11924046
rs59495126
rs115902151
rs147034683
rs74820489
rs79034390
rs138347791
rs308716
rs17571867
All
−1e−03
−5e−04
0e+00
5e−04
MR effect size for
'CCR4−NOT transcription complex subunit 1' on 'eGFR'

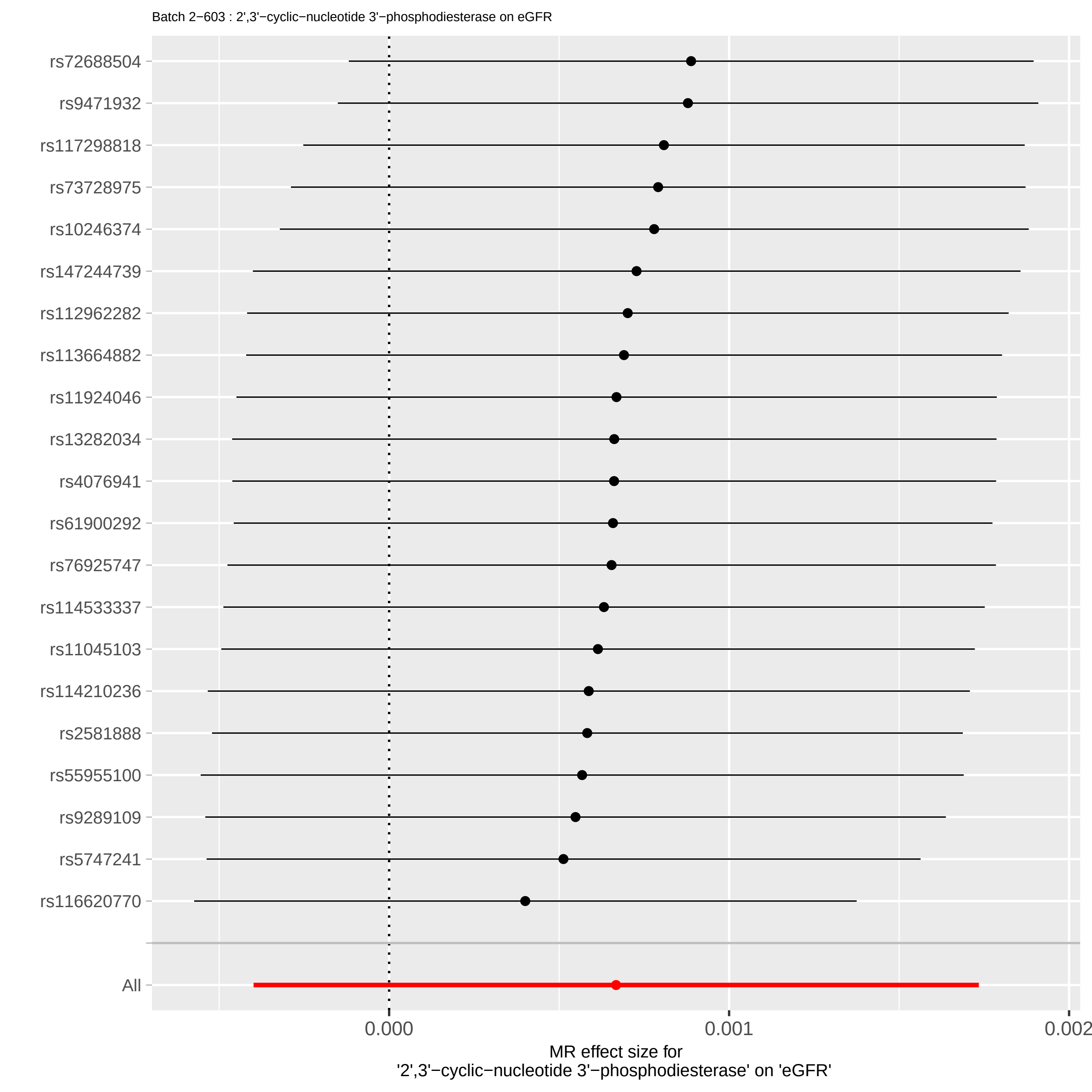

Batch 2−603 : 2',3'−cyclic−nucleotide 3'−phosphodiesterase on eGFR
rs72688504
rs9471932
rs117298818
rs73728975
rs10246374
rs147244739
rs112962282
rs113664882
rs11924046
rs13282034
rs4076941
rs61900292
rs76925747
rs114533337
rs11045103
rs114210236
rs2581888
rs55955100
rs9289109
rs5747241
rs116620770
All
0.000
0.001
0.002
MR effect size for
'2',3'−cyclic−nucleotide 3'−phosphodiesterase' on 'eGFR'

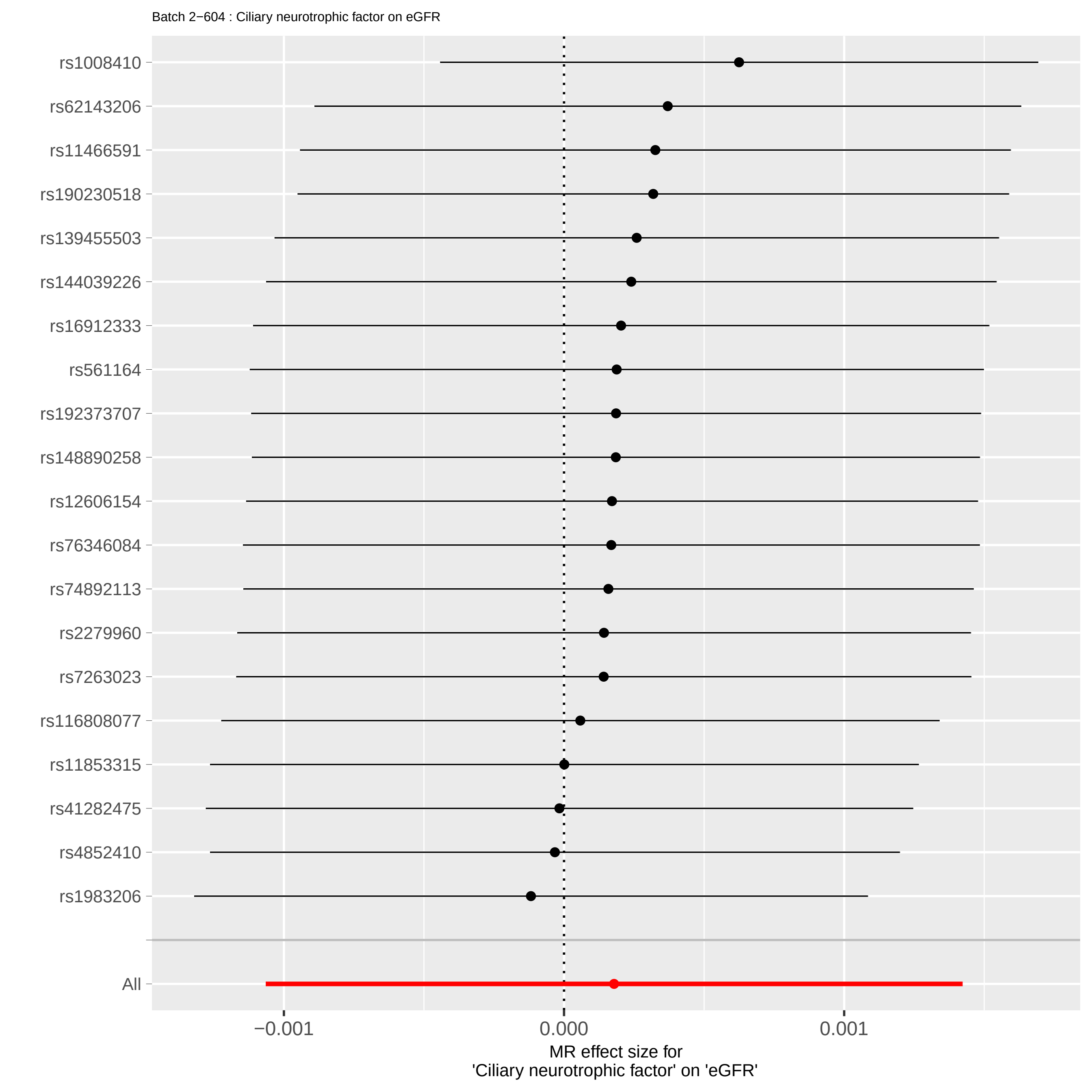
Batch 2−604 : Ciliary neurotrophic factor on eGFR
rs1008410
rs62143206
rs11466591
rs190230518
rs139455503
rs144039226
rs16912333
rs561164
rs192373707
rs148890258
rs12606154
rs76346084
rs74892113
rs2279960
rs7263023
rs116808077
rs11853315
rs41282475
rs4852410
rs1983206
All
−0.001
0.000
0.001
MR effect size for
'Ciliary neurotrophic factor' on 'eGFR'

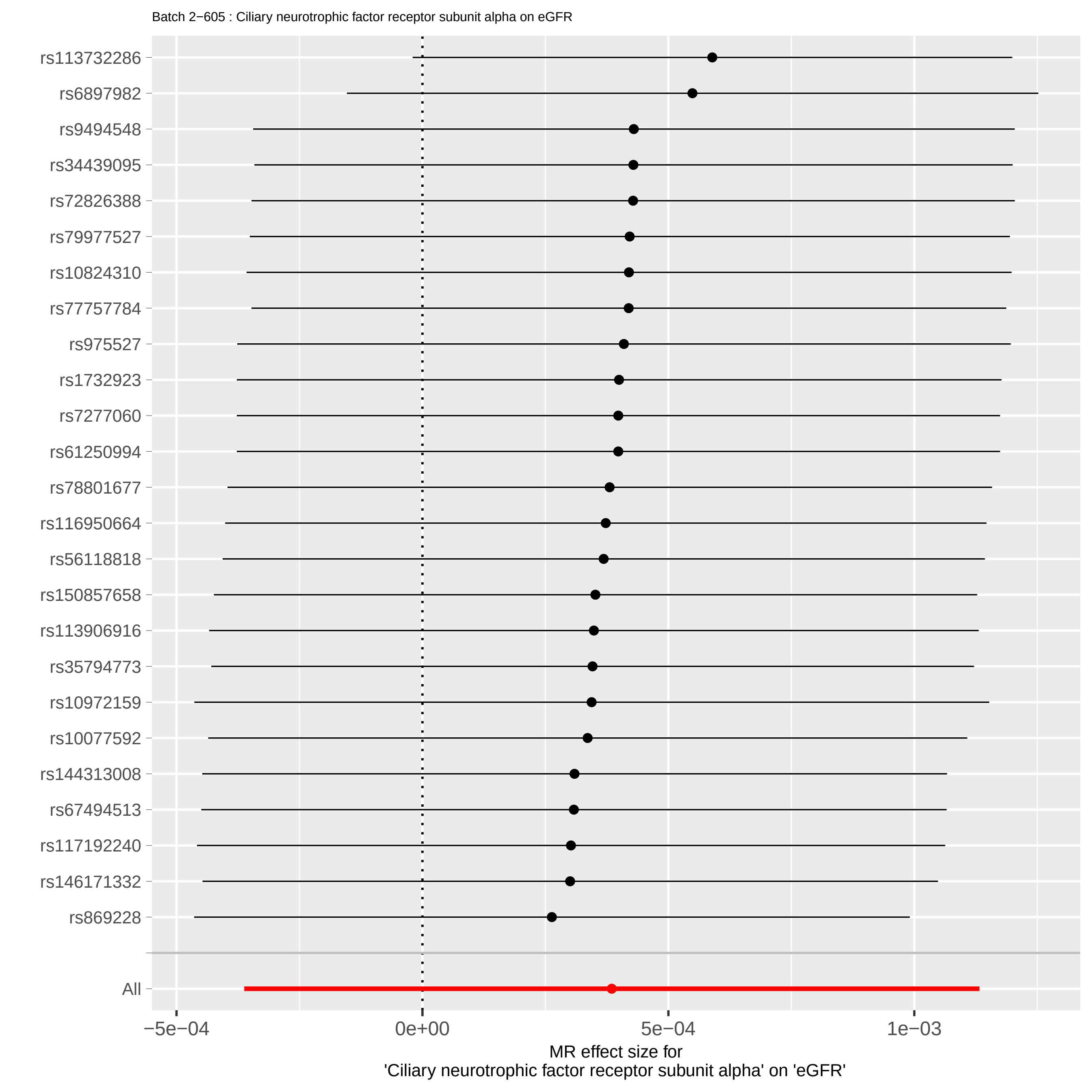

Batch 2−605 : Ciliary neurotrophic factor receptor subunit alpha on eGFR
rs113732286
rs6897982
rs9494548
rs34439095
rs72826388
rs79977527
rs10824310
rs77757784
rs975527
rs1732923
rs7277060
rs61250994
rs78801677
rs116950664
rs56118818
rs150857658
rs113906916
rs35794773
rs10972159
rs10077592
rs144313008
rs67494513
rs117192240
rs146171332
rs869228
All
−5e−04
0e+00
5e−04
1e−03
MR effect size for
'Ciliary neurotrophic factor receptor subunit alpha' on 'eGFR'

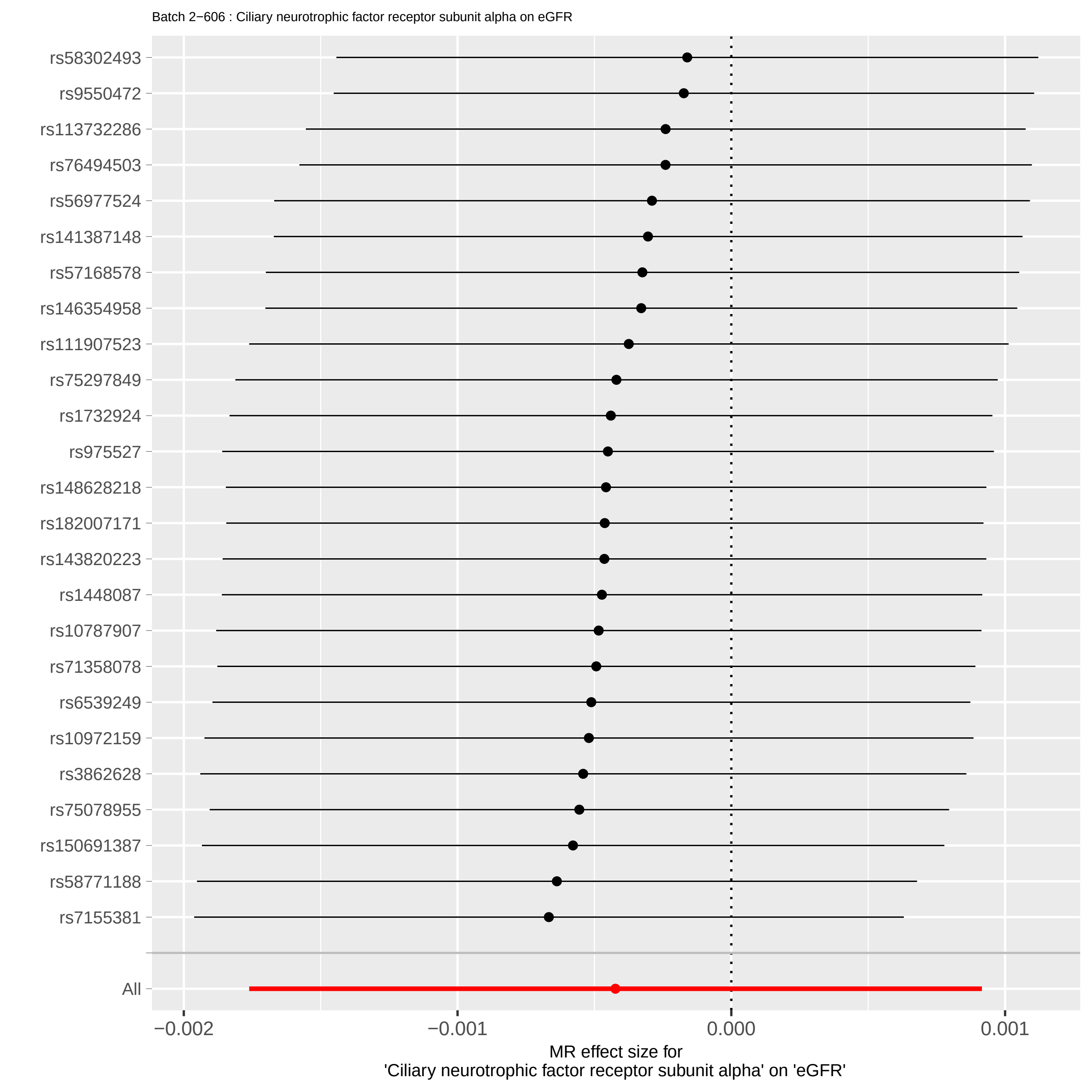

Batch 2−606 : Ciliary neurotrophic factor receptor subunit alpha on eGFR
rs58302493
rs9550472
rs113732286
rs76494503
rs56977524
rs141387148
rs57168578
rs146354958
rs111907523
rs75297849
rs1732924
rs975527
rs148628218
rs182007171
rs143820223
rs1448087
rs10787907
rs71358078
rs6539249
rs10972159
rs3862628
rs75078955
rs150691387
rs58771188
rs7155381
All
−0.002
−0.001
0.000
0.001
MR effect size for
'Ciliary neurotrophic factor receptor subunit alpha' on 'eGFR'

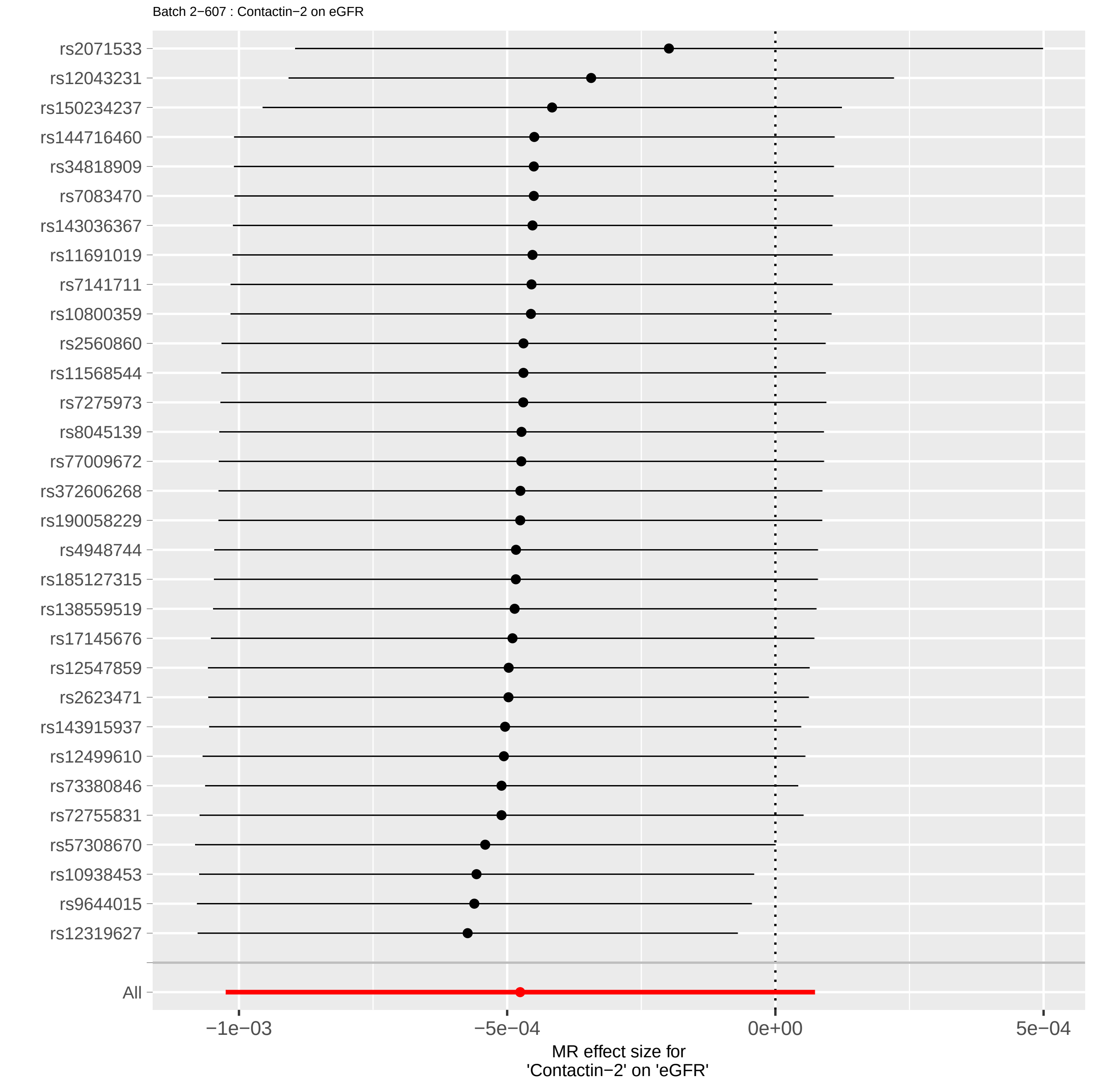
Batch 2−607 : Contactin−2 on eGFR
rs2071533
rs12043231
rs150234237
rs144716460
rs34818909
rs7083470
rs143036367
rs11691019
rs7141711
rs10800359
rs2560860
rs11568544
rs7275973
rs8045139
rs77009672
rs372606268
rs190058229
rs4948744
rs185127315
rs138559519
rs17145676
rs12547859
rs2623471
rs143915937
rs12499610
rs73380846
rs72755831
rs57308670
rs10938453
rs9644015
rs12319627
All
−1e−03
−5e−04
0e+00
5e−04
MR effect size for
'Contactin−2' on 'eGFR'

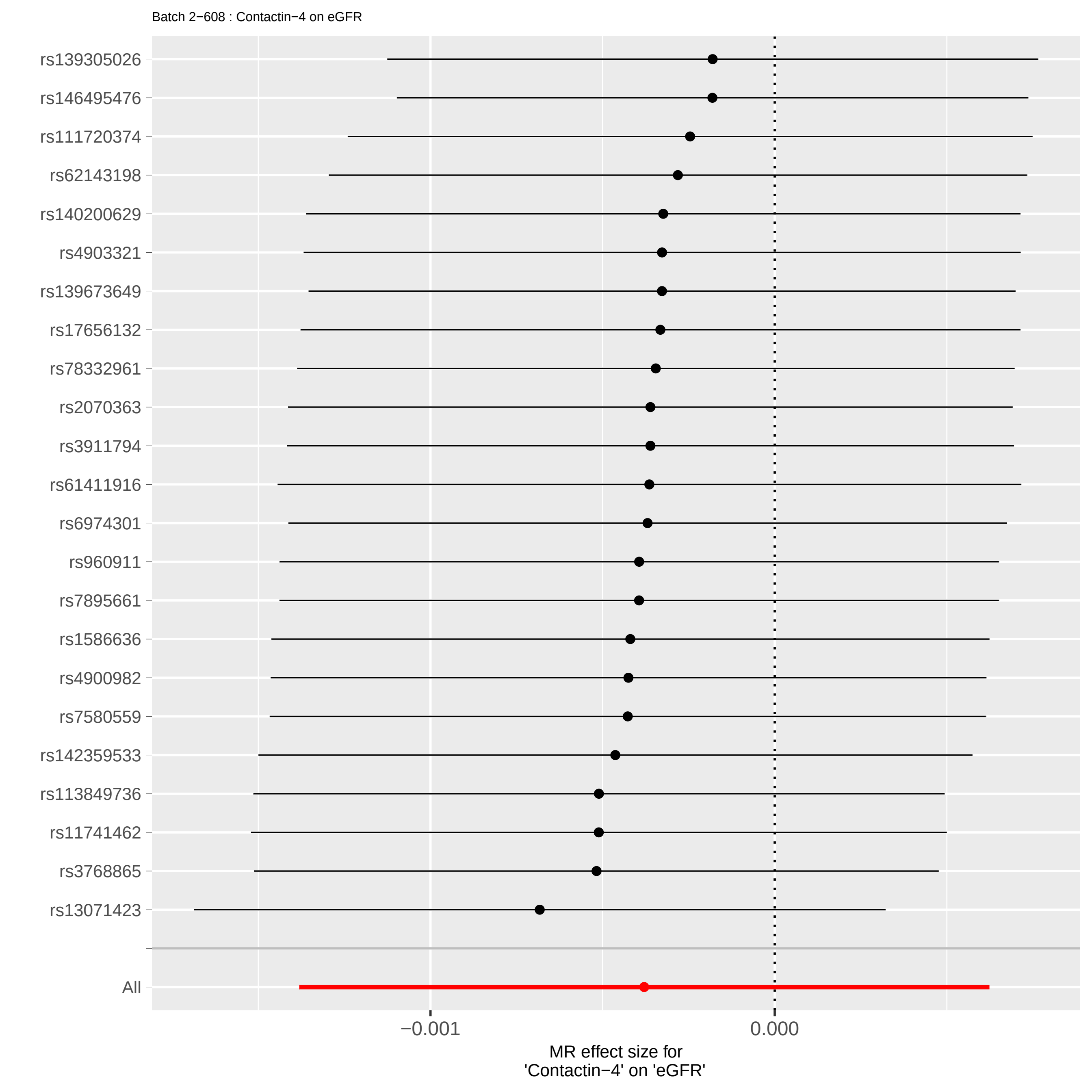
Batch 2−608 : Contactin−4 on eGFR
rs139305026
rs146495476
rs111720374
rs62143198
rs140200629
rs4903321
rs139673649
rs17656132
rs78332961
rs2070363
rs3911794
rs61411916
rs6974301
rs960911
rs7895661
rs1586636
rs4900982
rs7580559
rs142359533
rs113849736
rs11741462
rs3768865
rs13071423
All
−0.001
0.000
MR effect size for
'Contactin−4' on 'eGFR'

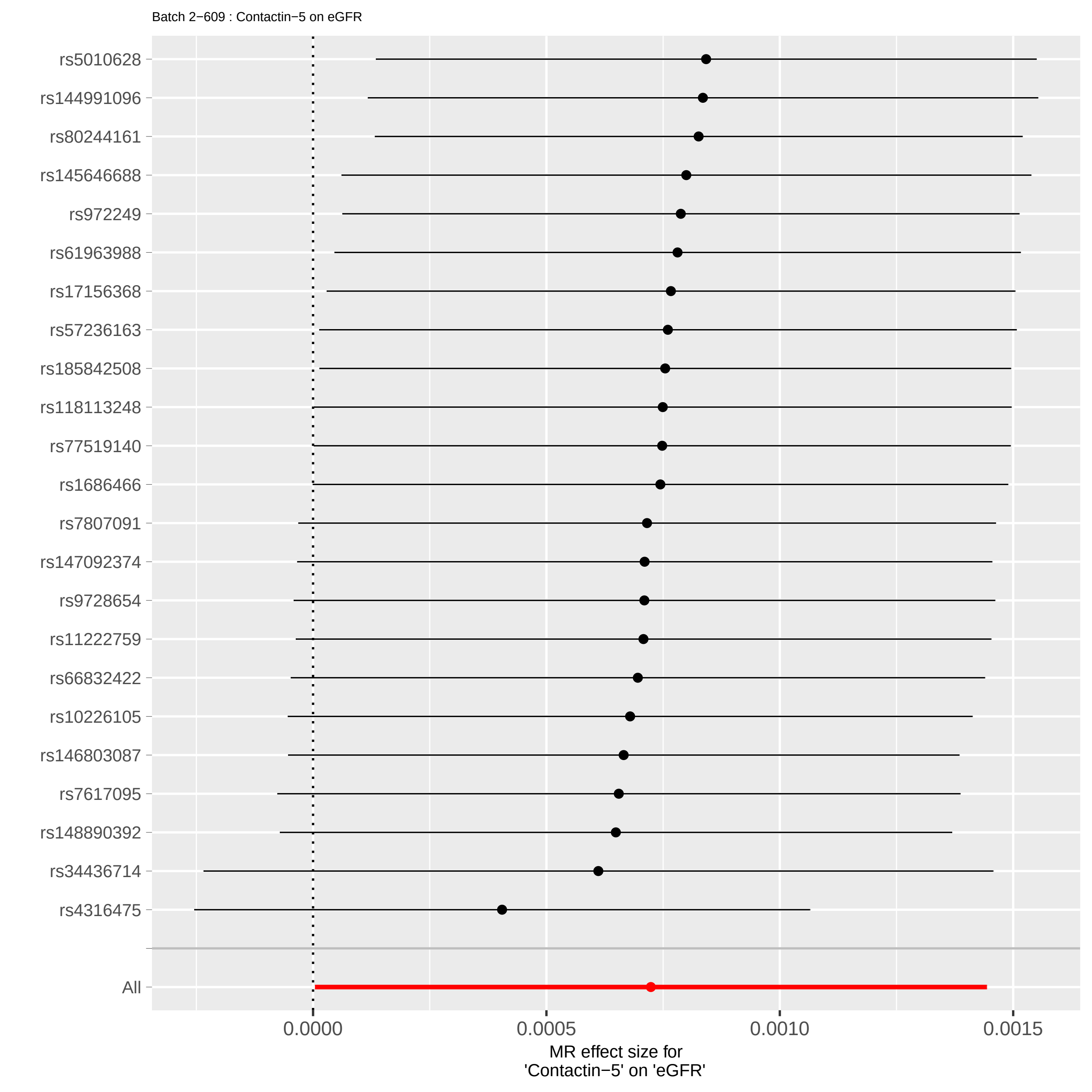

Batch 2−609 : Contactin−5 on eGFR
rs5010628
rs144991096
rs80244161
rs145646688
rs972249
rs61963988
rs17156368
rs57236163
rs185842508
rs118113248
rs77519140
rs1686466
rs7807091
rs147092374
rs9728654
rs11222759
rs66832422
rs10226105
rs146803087
rs7617095
rs148890392
rs34436714
rs4316475
All
0.0000
0.0005
0.0010
0.0015
MR effect size for
'Contactin−5' on 'eGFR'

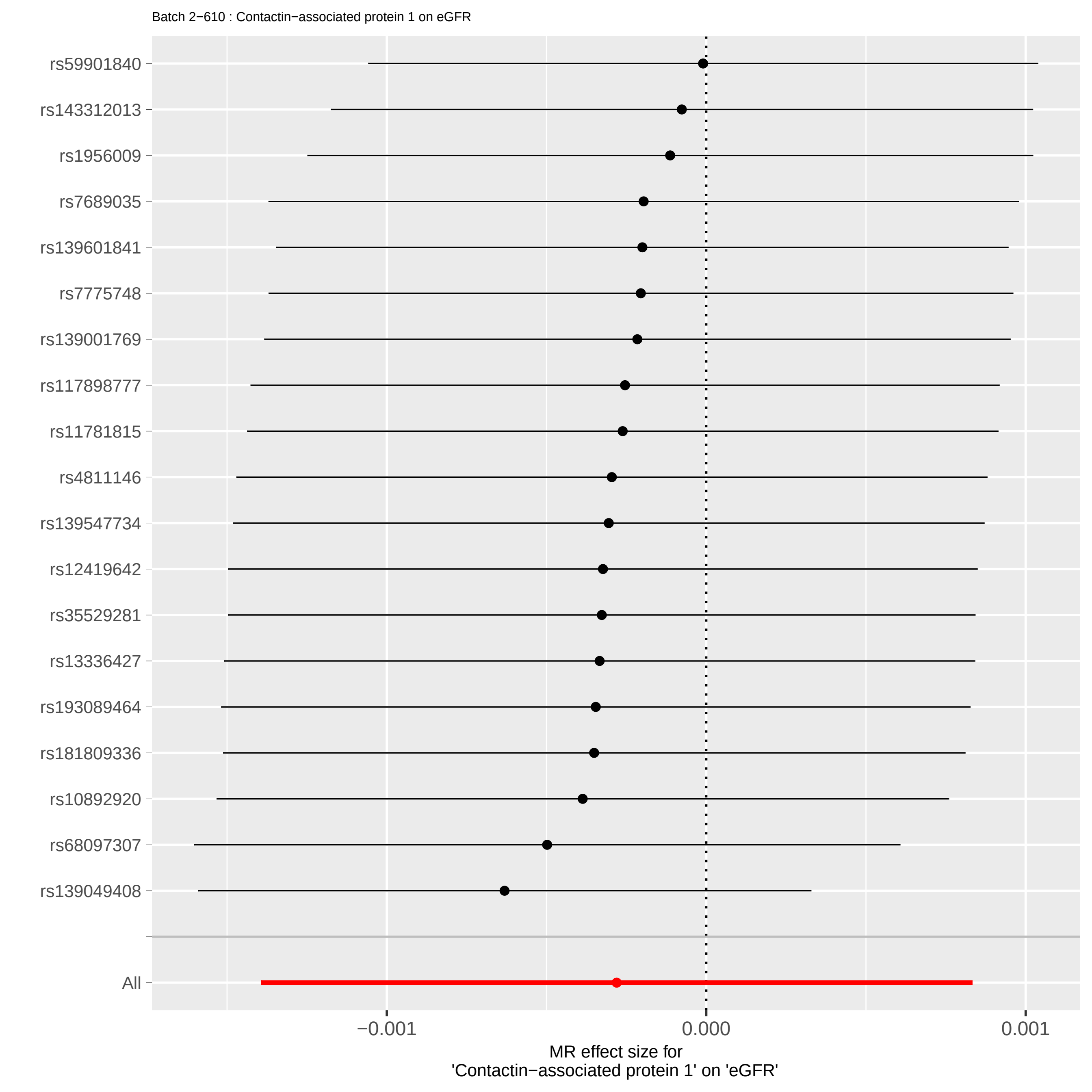

Batch 2−610 : Contactin−associated protein 1 on eGFR
rs59901840
rs143312013
rs1956009
rs7689035
rs139601841
rs7775748
rs139001769
rs117898777
rs11781815
rs4811146
rs139547734
rs12419642
rs35529281
rs13336427
rs193089464
rs181809336
rs10892920
rs68097307
rs139049408
All
−0.001
0.000
0.001
MR effect size for
'Contactin−associated protein 1' on 'eGFR'

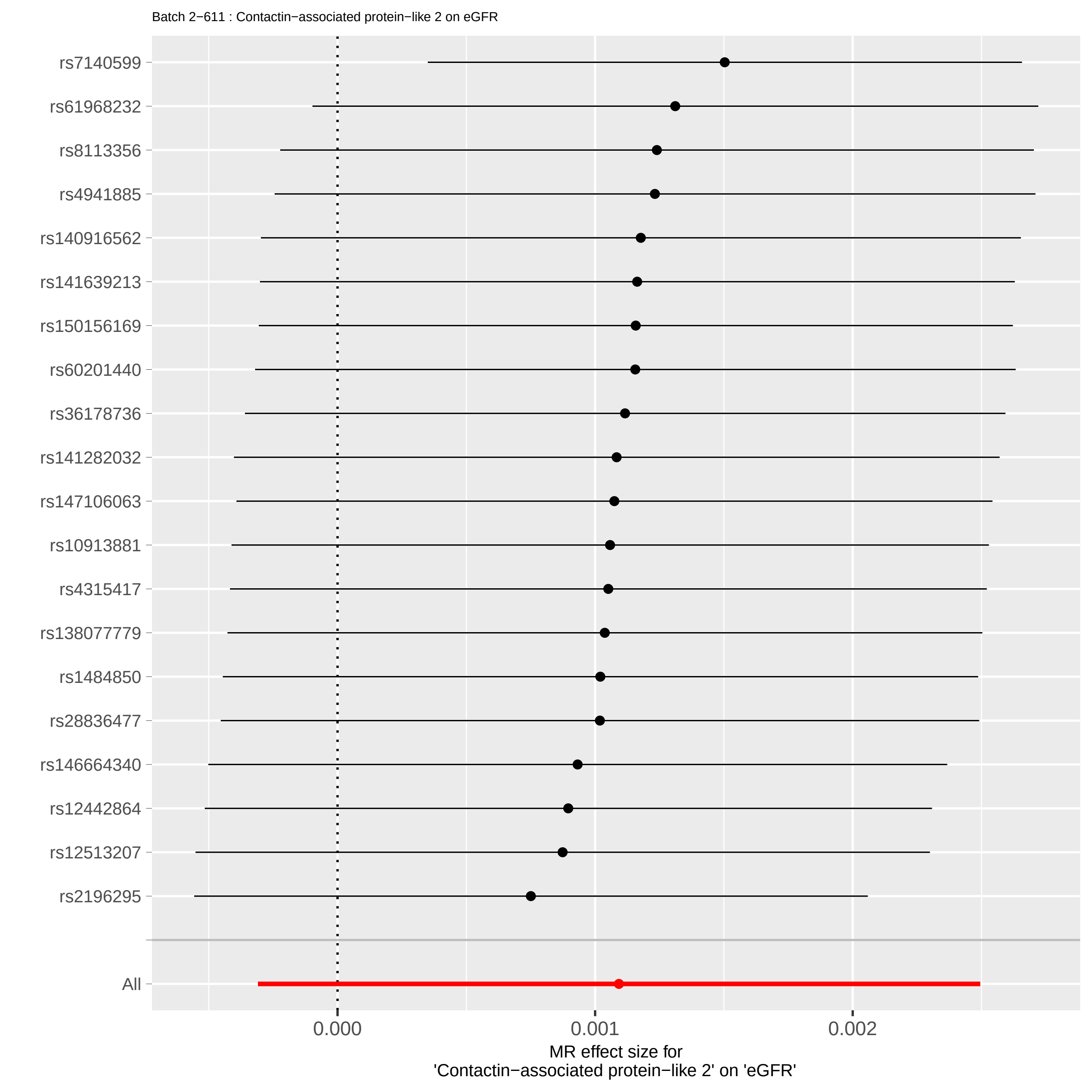
Batch 2−611 : Contactin−associated protein−like 2 on eGFR
rs7140599
rs61968232
rs8113356
rs4941885
rs140916562
rs141639213
rs150156169
rs60201440
rs36178736
rs141282032
rs147106063
rs10913881
rs4315417
rs138077779
rs1484850
rs28836477
rs146664340
rs12442864
rs12513207
rs2196295
All
0.000
0.001
0.002
MR effect size for
'Contactin−associated protein−like 2' on 'eGFR'

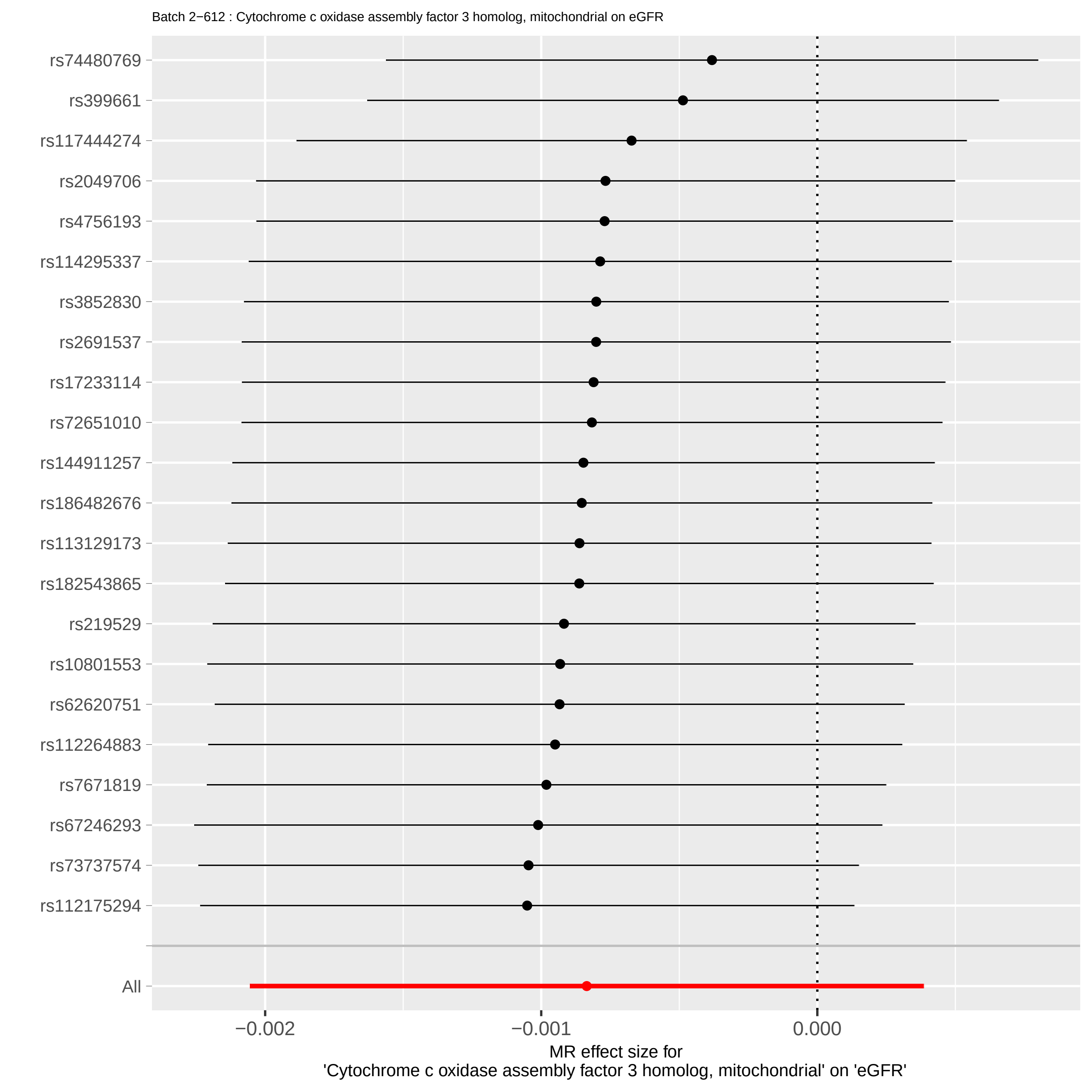

Batch 2−612 : Cytochrome c oxidase assembly factor 3 homolog, mitochondrial on eGFR
rs74480769
rs399661
rs117444274
rs2049706
rs4756193
rs114295337
rs3852830
rs2691537
rs17233114
rs72651010
rs144911257
rs186482676
rs113129173
rs182543865
rs219529
rs10801553
rs62620751
rs112264883
rs7671819
rs67246293
rs73737574
rs112175294
All
−0.002
−0.001
0.000
MR effect size for
'Cytochrome c oxidase assembly factor 3 homolog, mitochondrial' on 'eGFR'

Batch 2−613 : Cochlin on eGFR

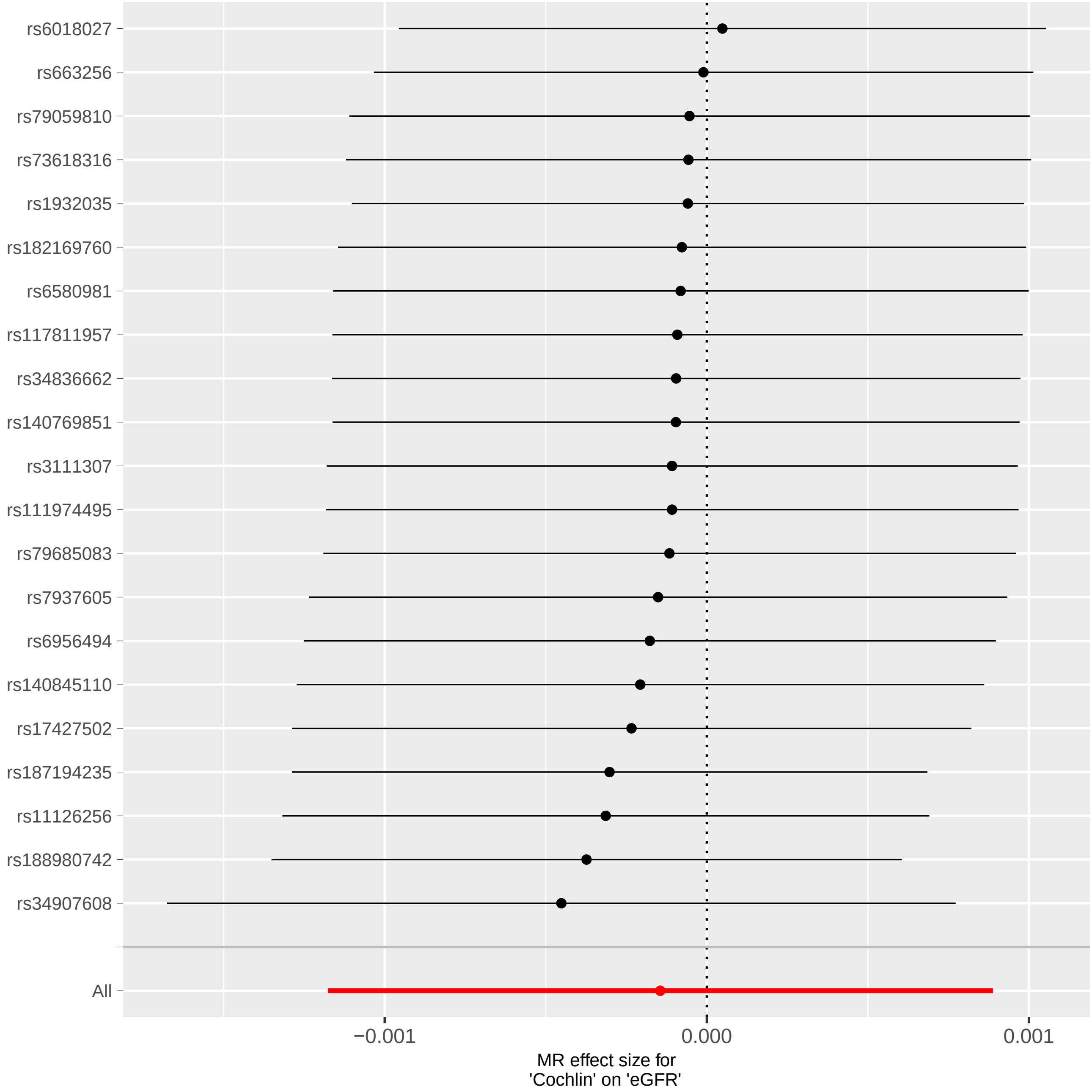

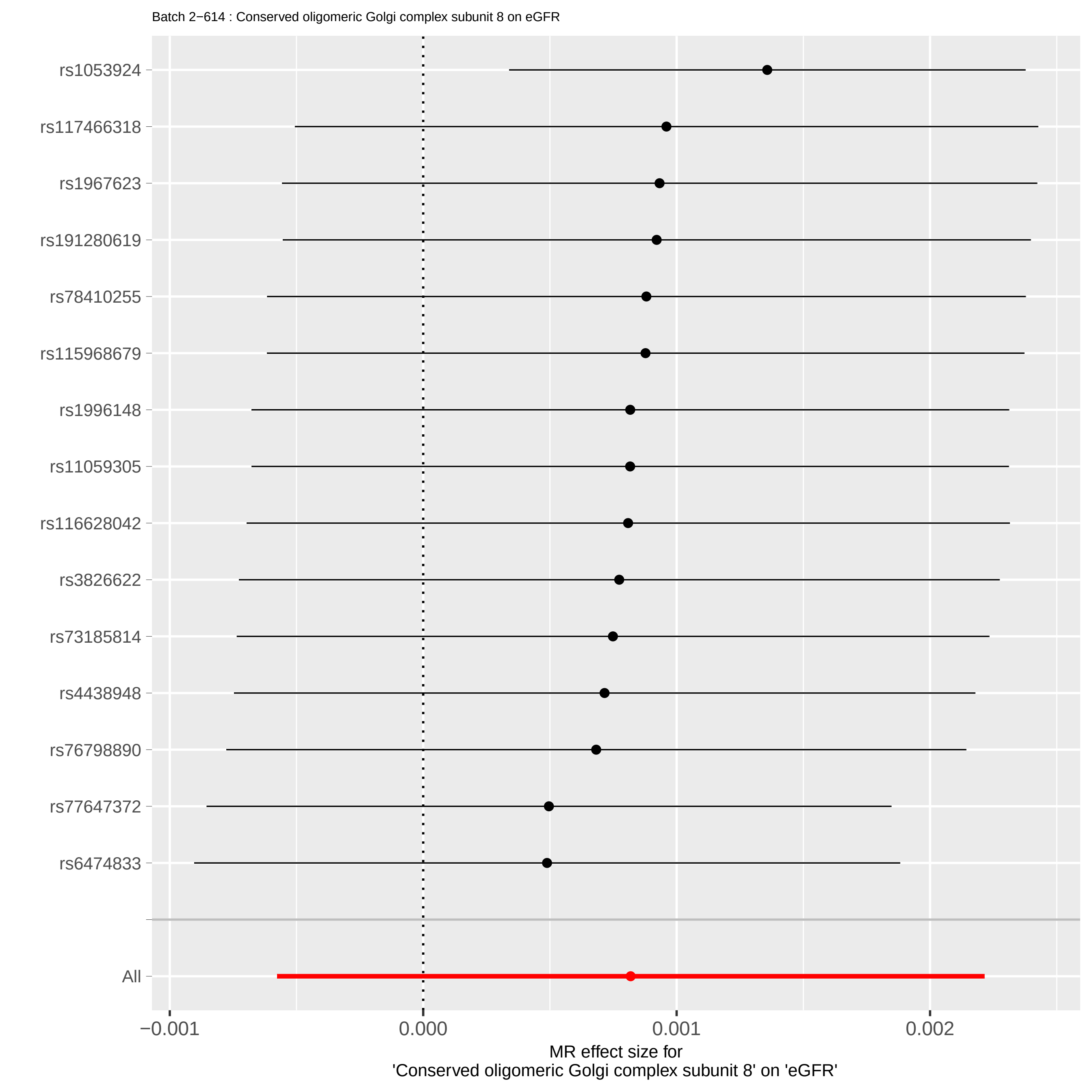
Batch 2−614 : Conserved oligomeric Golgi complex subunit 8 on eGFR
rs1053924
rs117466318
rs1967623
rs191280619
rs78410255
rs115968679
rs1996148
rs11059305
rs116628042
rs3826622
rs73185814
rs4438948
rs76798890
rs77647372
rs6474833
All
−0.001
0.000
0.001
0.002
MR effect size for
'Conserved oligomeric Golgi complex subunit 8' on 'eGFR'

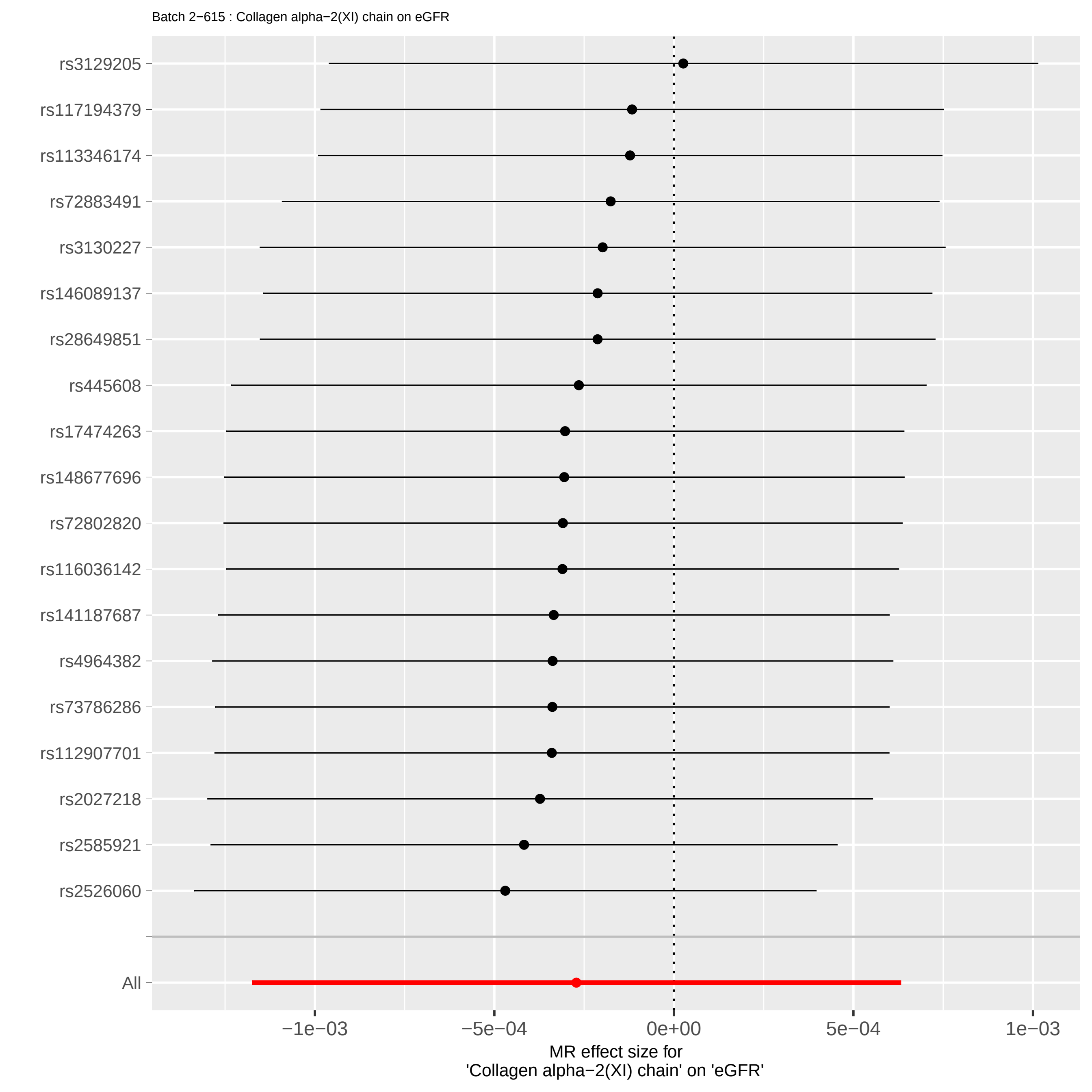

Batch 2−615 : Collagen alpha−2(XI) chain on eGFR
rs3129205
rs117194379
rs113346174
rs72883491
rs3130227
rs146089137
rs28649851
rs445608
rs17474263
rs148677696
rs72802820
rs116036142
rs141187687
rs4964382
rs73786286
rs112907701
rs2027218
rs2585921
rs2526060
All
−1e−03
−5e−04
0e+00
5e−04
1e−03
MR effect size for
'Collagen alpha−2(XI) chain' on 'eGFR'

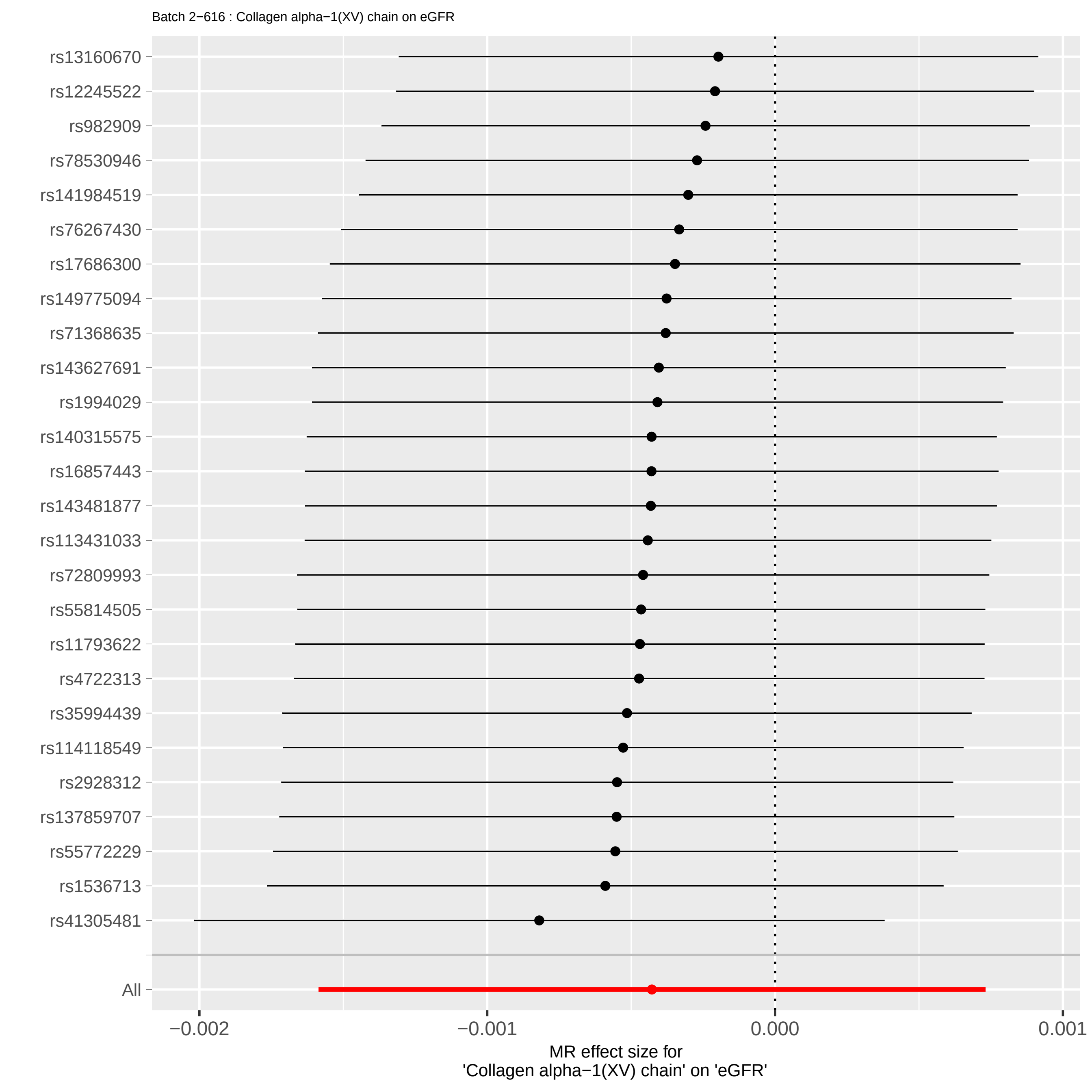

Batch 2−616 : Collagen alpha−1(XV) chain on eGFR
rs13160670
rs12245522
rs982909
rs78530946
rs141984519
rs76267430
rs17686300
rs149775094
rs71368635
rs143627691
rs1994029
rs140315575
rs16857443
rs143481877
rs113431033
rs72809993
rs55814505
rs11793622
rs4722313
rs35994439
rs114118549
rs2928312
rs137859707
rs55772229
rs1536713
rs41305481
All
−0.002
−0.001
0.000
0.001
MR effect size for
'Collagen alpha−1(XV) chain' on 'eGFR'

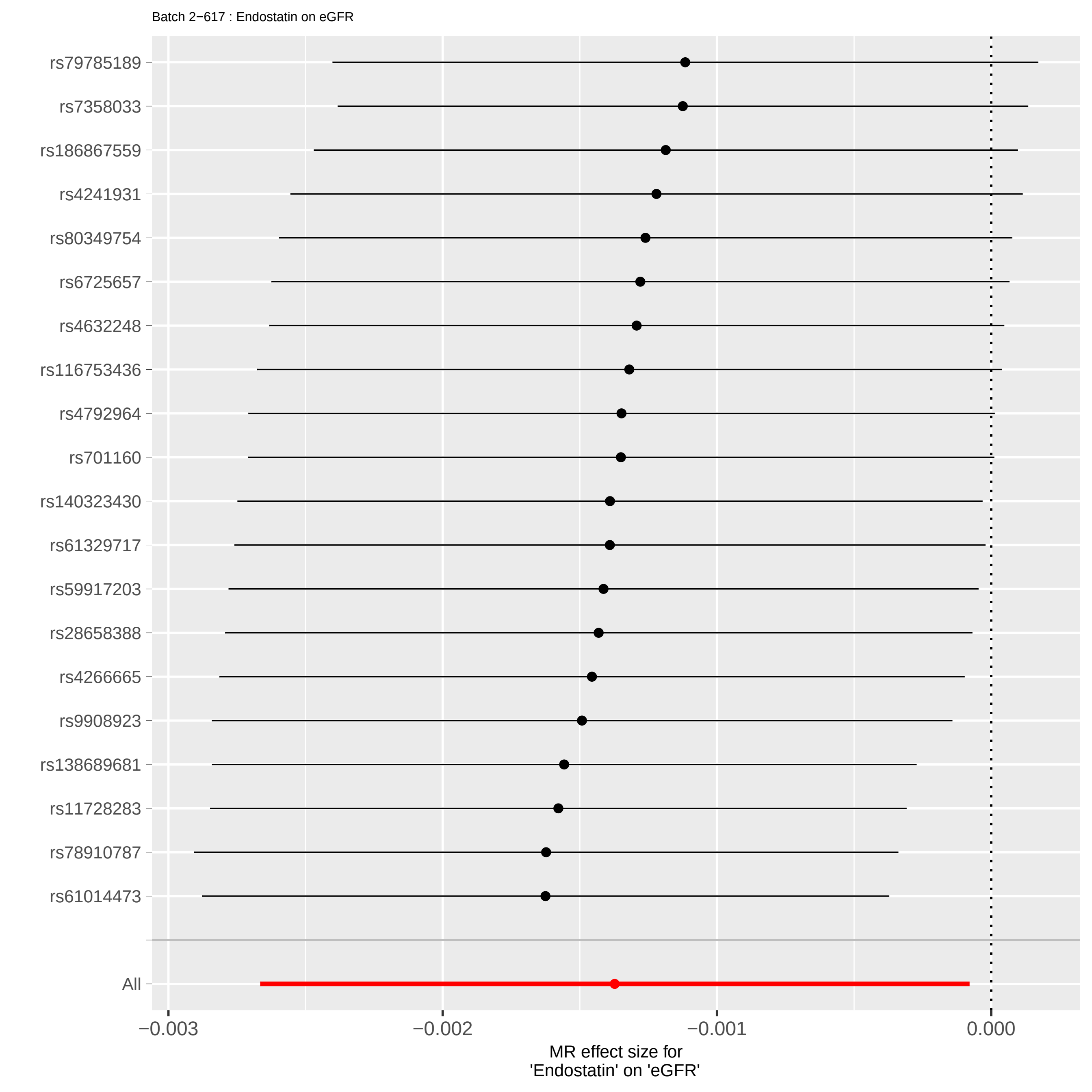

Batch 2−617 : Endostatin on eGFR
rs79785189
rs7358033
rs186867559
rs4241931
rs80349754
rs6725657
rs4632248
rs116753436
rs4792964
rs701160
rs140323430
rs61329717
rs59917203
rs28658388
rs4266665
rs9908923
rs138689681
rs11728283
rs78910787
rs61014473
All
−0.003
−0.002
−0.001
0.000
MR effect size for
'Endostatin' on 'eGFR'

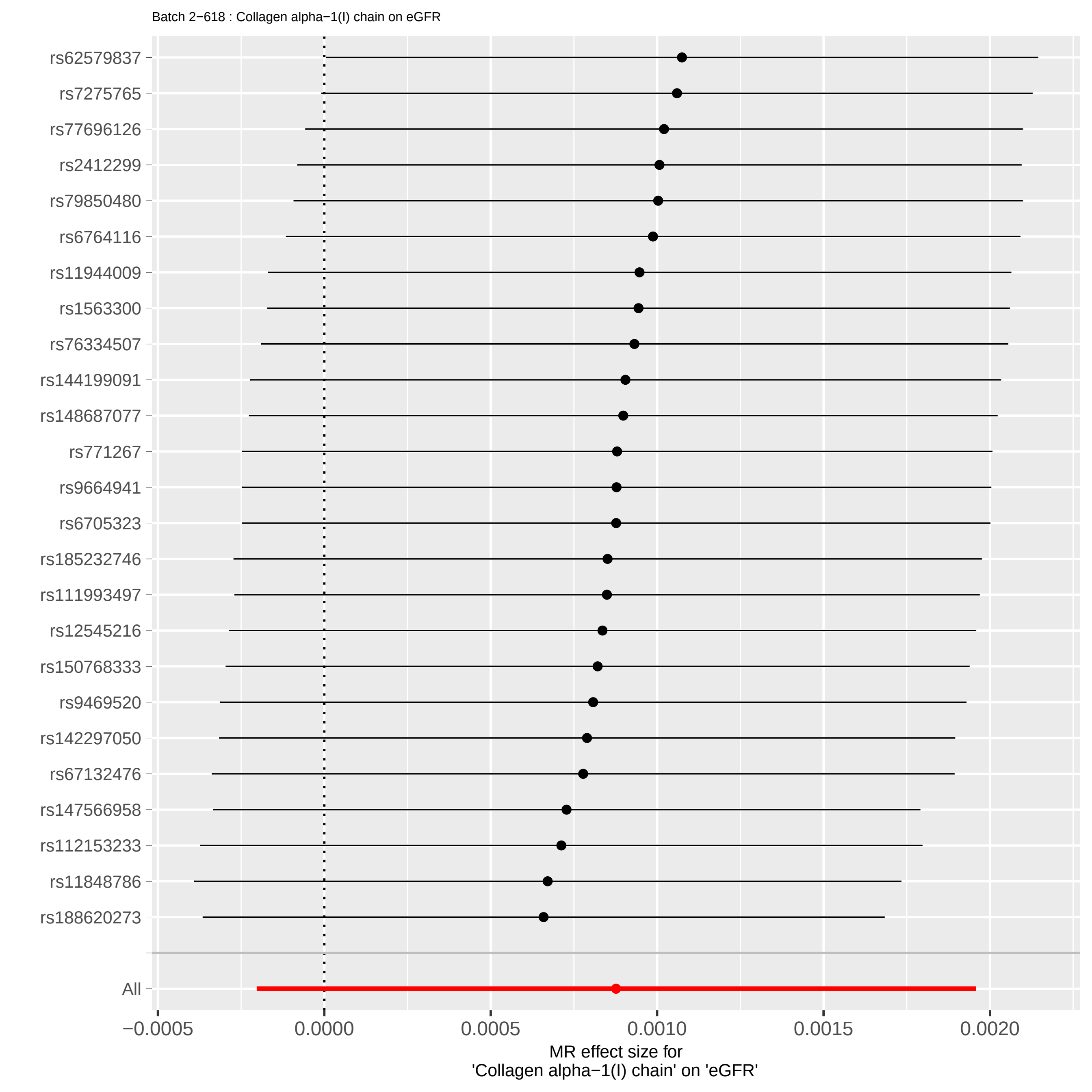

Batch 2−618 : Collagen alpha−1(I) chain on eGFR
rs62579837
rs7275765
rs77696126
rs2412299
rs79850480
rs6764116
rs11944009
rs1563300
rs76334507
rs144199091
rs148687077
rs771267
rs9664941
rs6705323
rs185232746
rs111993497
rs12545216
rs150768333
rs9469520
rs142297050
rs67132476
rs147566958
rs112153233
rs11848786
rs188620273
All
−0.0005
0.0000
0.0005
0.0010
0.0015
0.0020
MR effect size for
'Collagen alpha−1(I) chain' on 'eGFR'

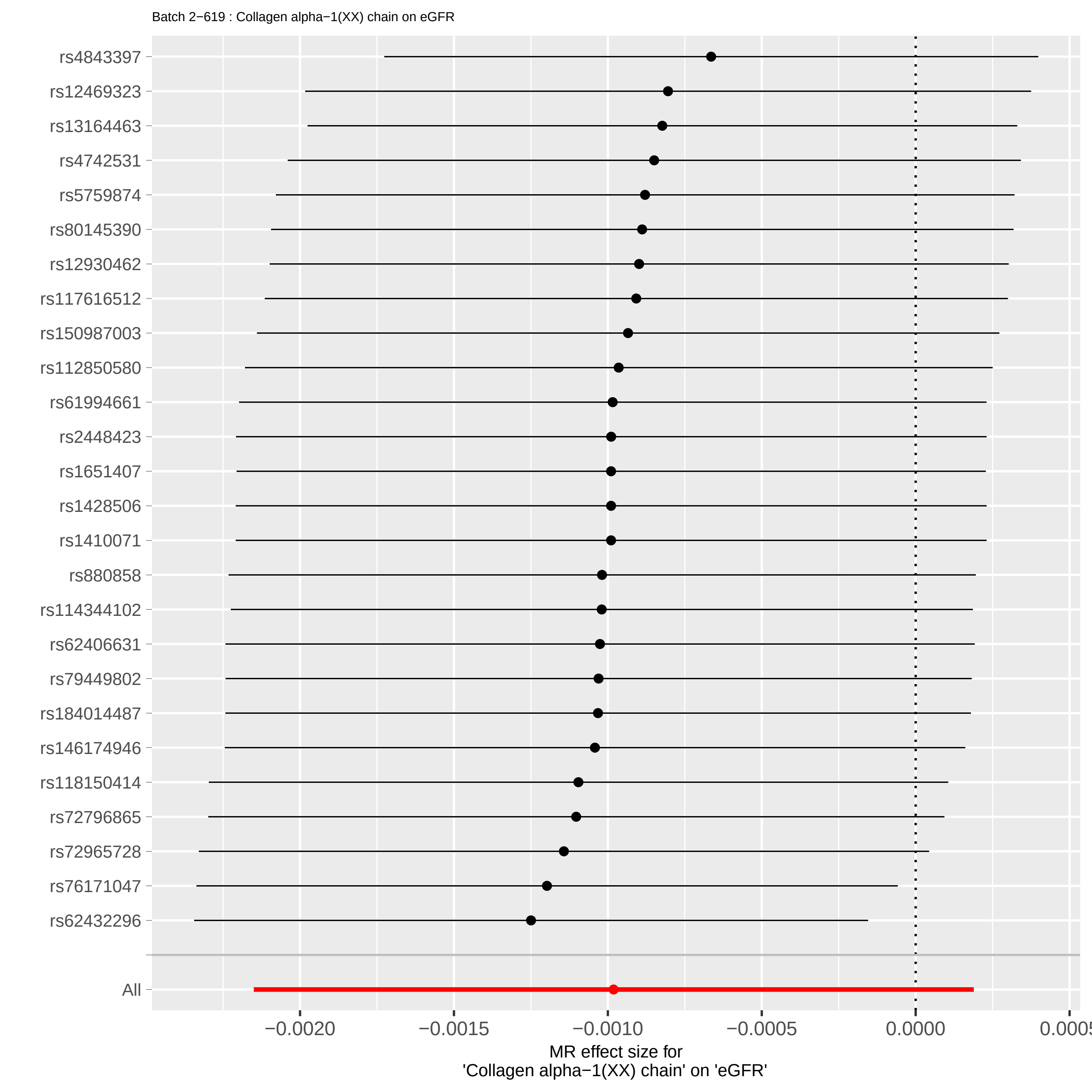

Batch 2−619 : Collagen alpha−1(XX) chain on eGFR
rs4843397
rs12469323
rs13164463
rs4742531
rs5759874
rs80145390
rs12930462
rs117616512
rs150987003
rs112850580
rs61994661
rs2448423
rs1651407
rs1428506
rs1410071
rs880858
rs114344102
rs62406631
rs79449802
rs184014487
rs146174946
rs118150414
rs72796865
rs72965728
rs76171047
rs62432296
All
−0.0020
−0.0015
−0.0010
−0.0005
0.0000
MR effect size for
'Collagen alpha−1(XX) chain' on 'eGFR'

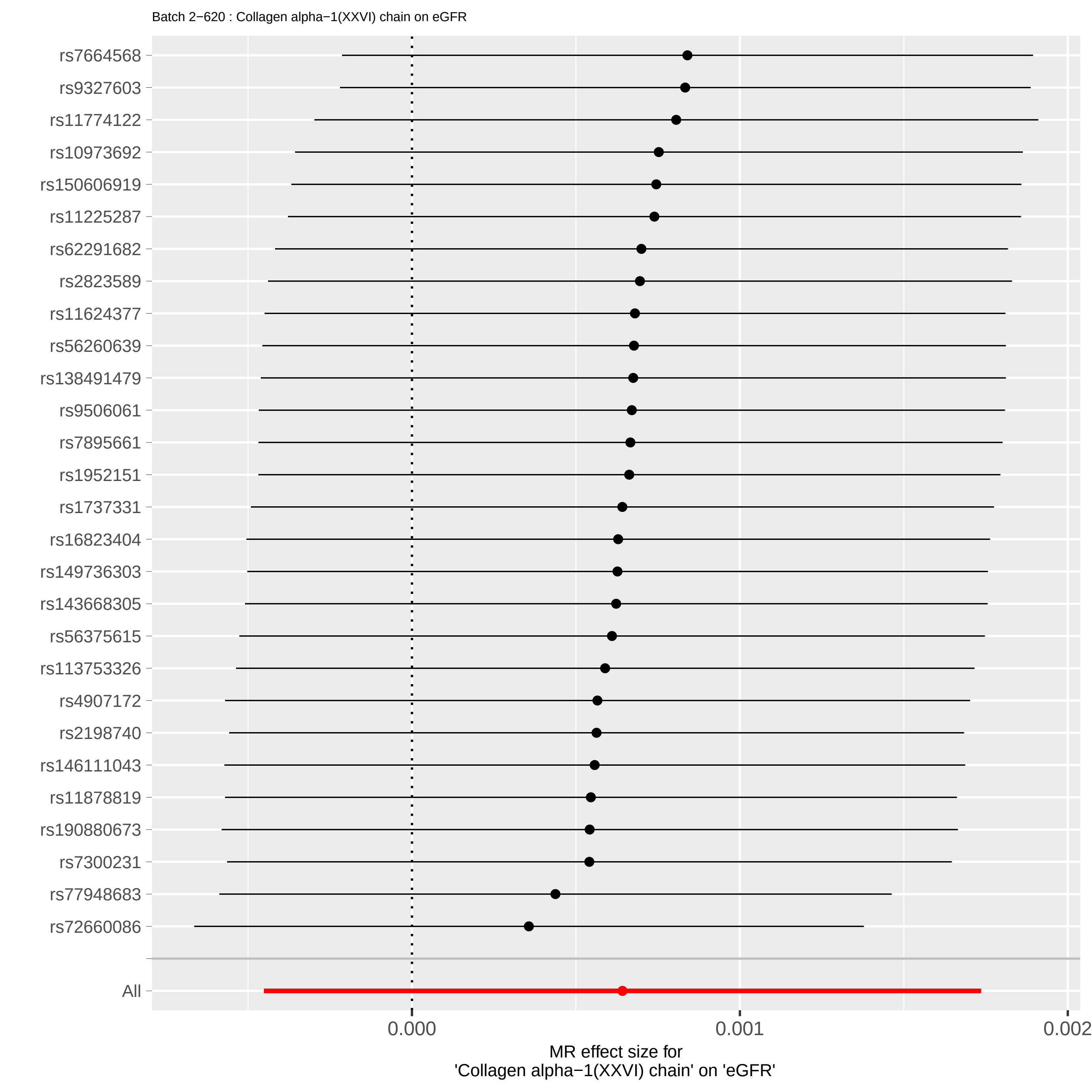

Batch 2−620 : Collagen alpha−1(XXVI) chain on eGFR
rs7664568
rs9327603
rs11774122
rs10973692
rs150606919
rs11225287
rs62291682
rs2823589
rs11624377
rs56260639
rs138491479
rs9506061
rs7895661
rs1952151
rs1737331
rs16823404
rs149736303
rs143668305
rs56375615
rs113753326
rs4907172
rs2198740
rs146111043
rs11878819
rs190880673
rs7300231
rs77948683
rs72660086
All
0.000
0.001
0.002
MR effect size for
'Collagen alpha−1(XXVI) chain' on 'eGFR'

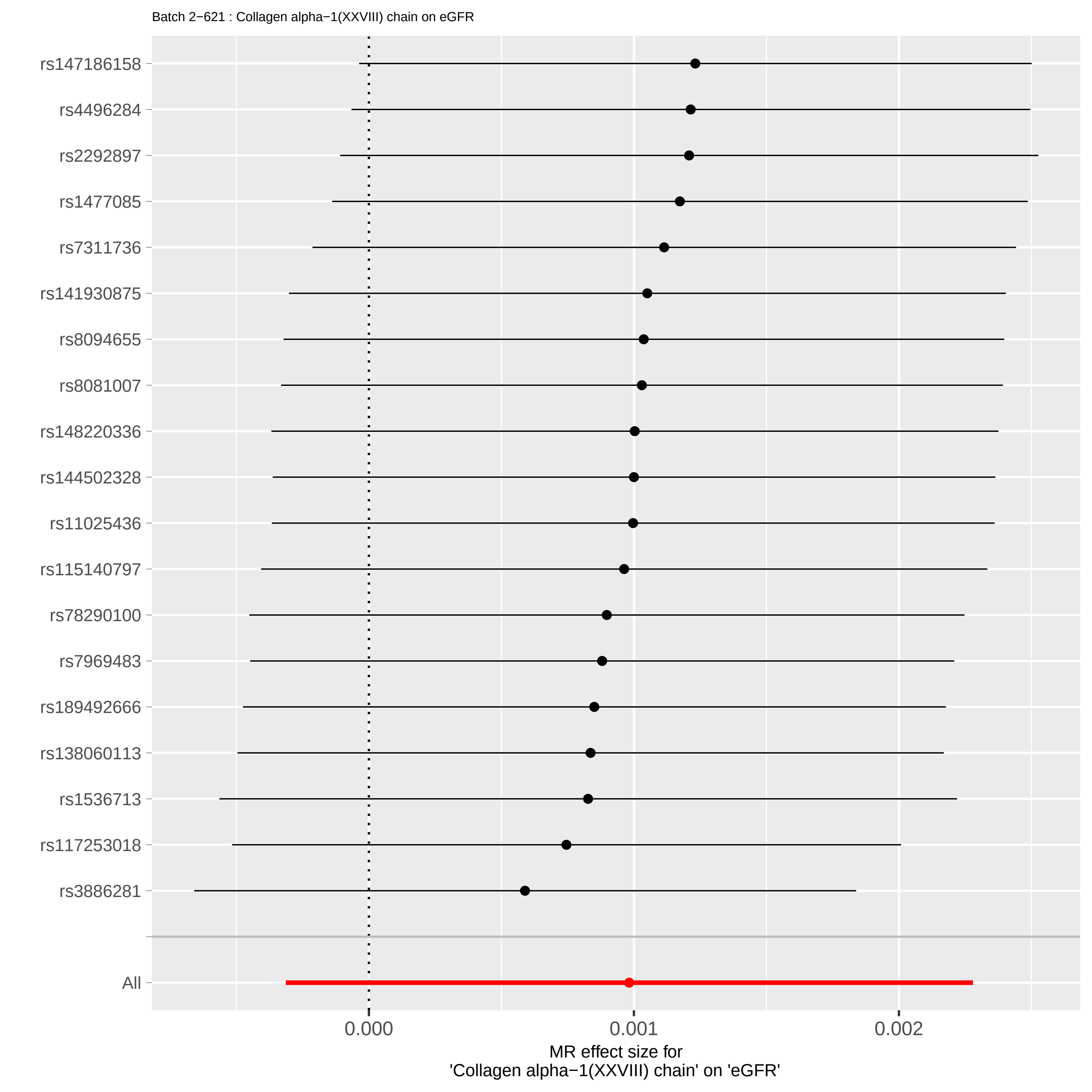

Batch 2−621 : Collagen alpha−1(XXVIII) chain on eGFR
rs147186158
rs4496284
rs2292897
rs1477085
rs7311736
rs141930875
rs8094655
rs8081007
rs148220336
rs144502328
rs11025436
rs115140797
rs78290100
rs7969483
rs189492666
rs138060113
rs1536713
rs117253018
rs3886281
All
0.000
0.001
0.002
MR effect size for
'Collagen alpha−1(XXVIII) chain' on 'eGFR'

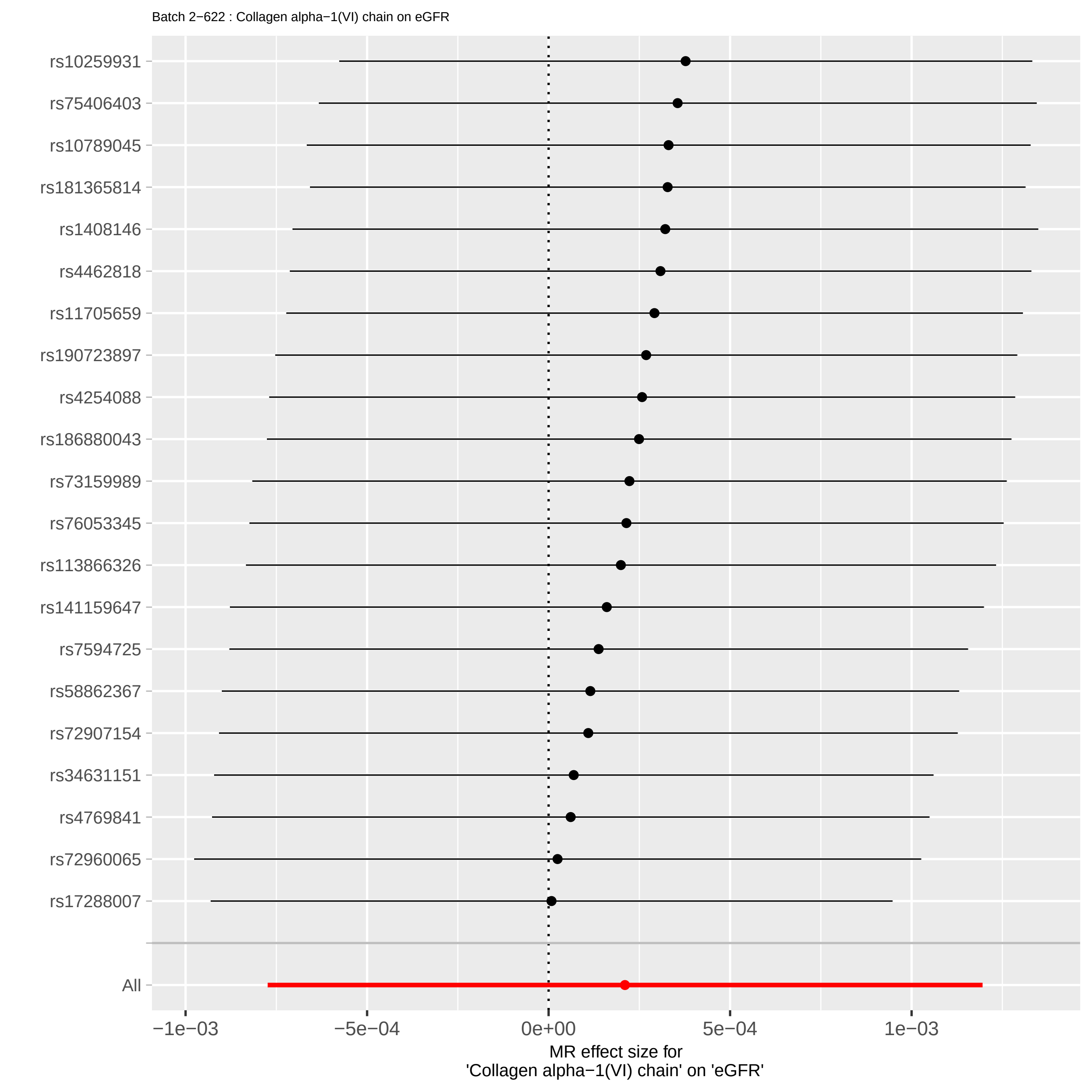

Batch 2−622 : Collagen alpha−1(VI) chain on eGFR
rs10259931
rs75406403
rs10789045
rs181365814
rs1408146
rs4462818
rs11705659
rs190723897
rs4254088
rs186880043
rs73159989
rs76053345
rs113866326
rs141159647
rs7594725
rs58862367
rs72907154
rs34631151
rs4769841
rs72960065
rs17288007
All
−1e−03
−5e−04
0e+00
5e−04
1e−03
MR effect size for
'Collagen alpha−1(VI) chain' on 'eGFR'

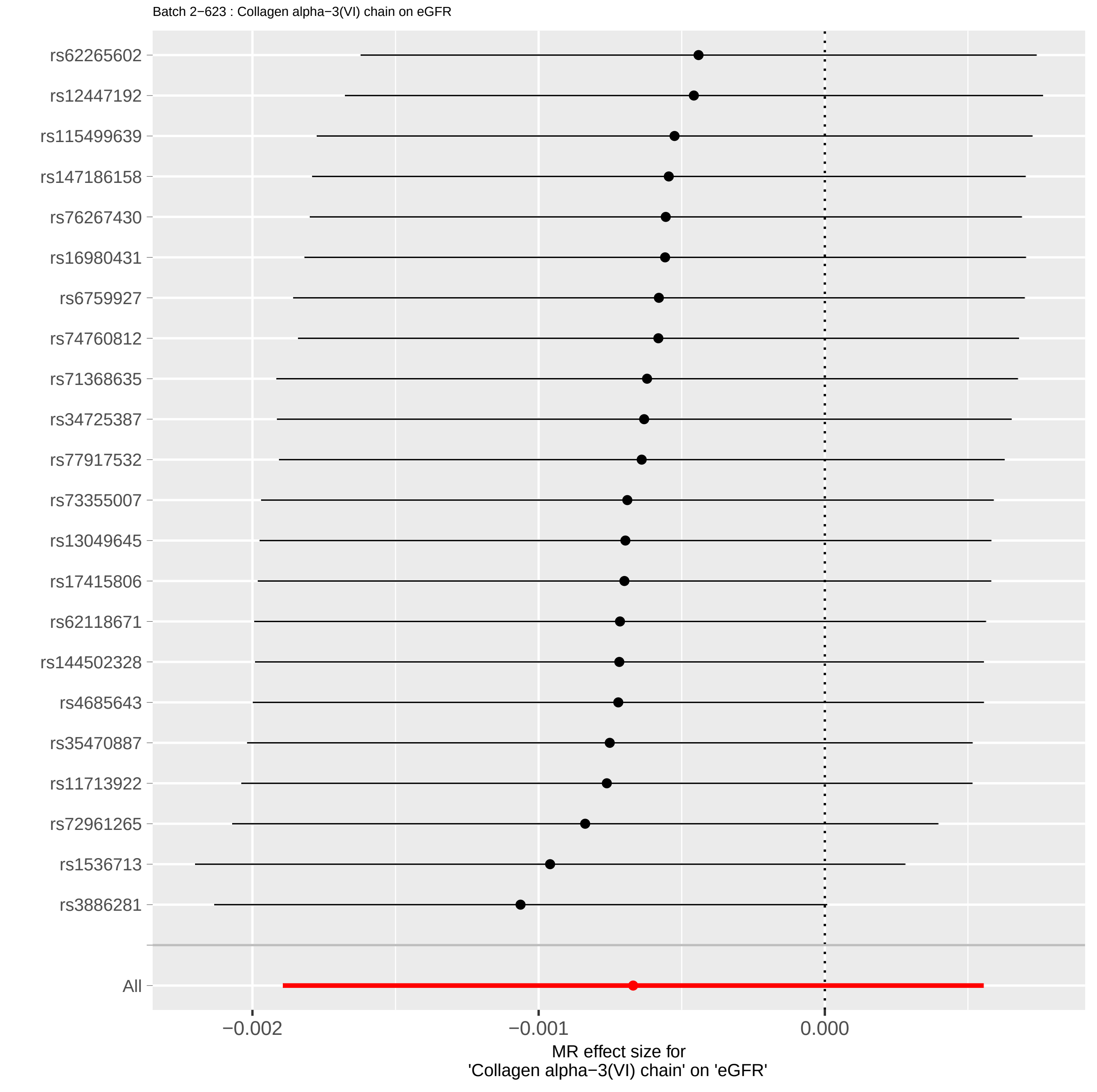
Batch 2−623 : Collagen alpha−3(VI) chain on eGFR
rs62265602
rs12447192
rs115499639
rs147186158
rs76267430
rs16980431
rs6759927
rs74760812
rs71368635
rs34725387
rs77917532
rs73355007
rs13049645
rs17415806
rs62118671
rs144502328
rs4685643
rs35470887
rs11713922
rs72961265
rs1536713
rs3886281
All
−0.002
−0.001
0.000
MR effect size for
'Collagen alpha−3(VI) chain' on 'eGFR'

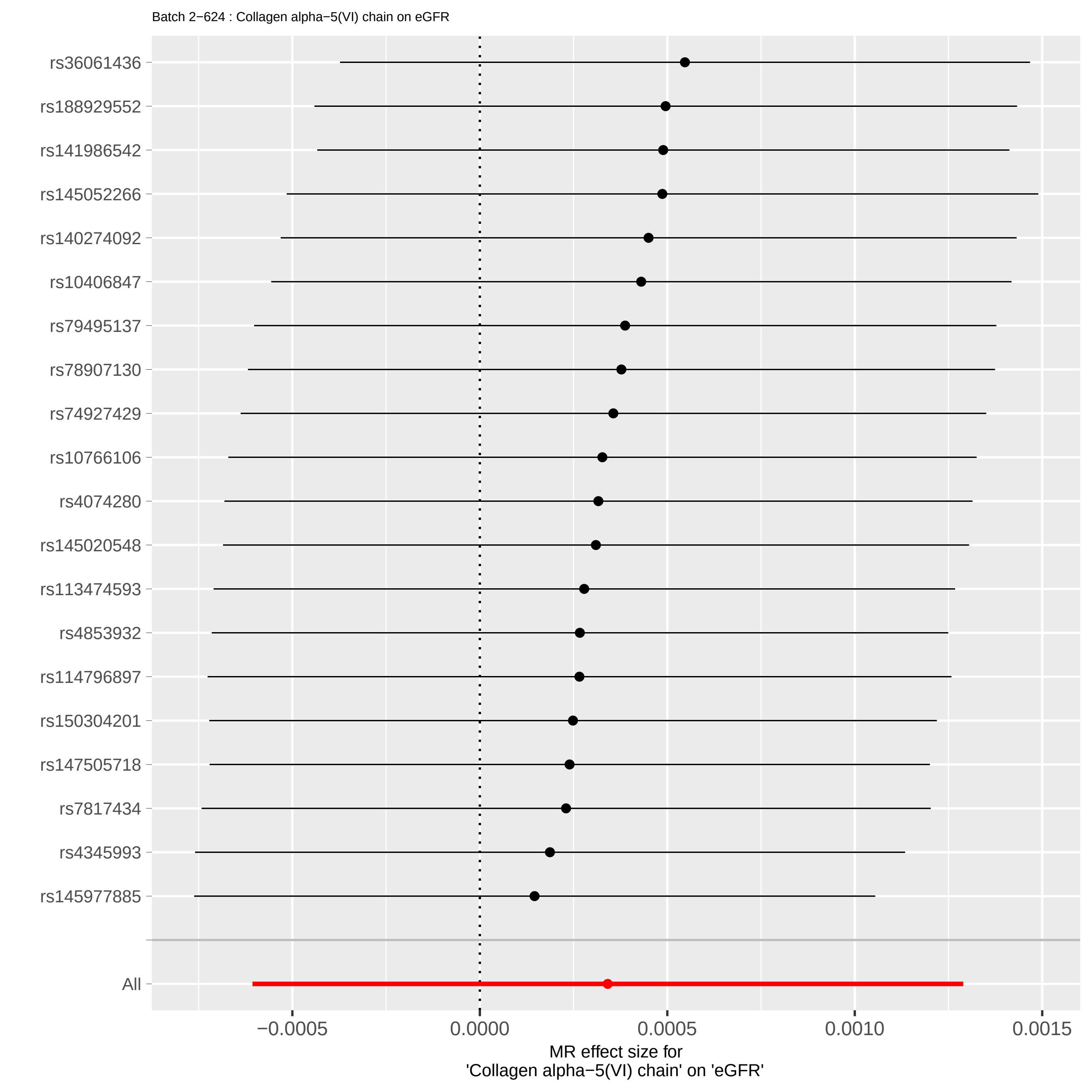

Batch 2−624 : Collagen alpha−5(VI) chain on eGFR
rs36061436
rs188929552
rs141986542
rs145052266
rs140274092
rs10406847
rs79495137
rs78907130
rs74927429
rs10766106
rs4074280
rs145020548
rs113474593
rs4853932
rs114796897
rs150304201
rs147505718
rs7817434
rs4345993
rs145977885
All
−0.0005
0.0000
0.0005
0.0010
0.0015
MR effect size for
'Collagen alpha−5(VI) chain' on 'eGFR'

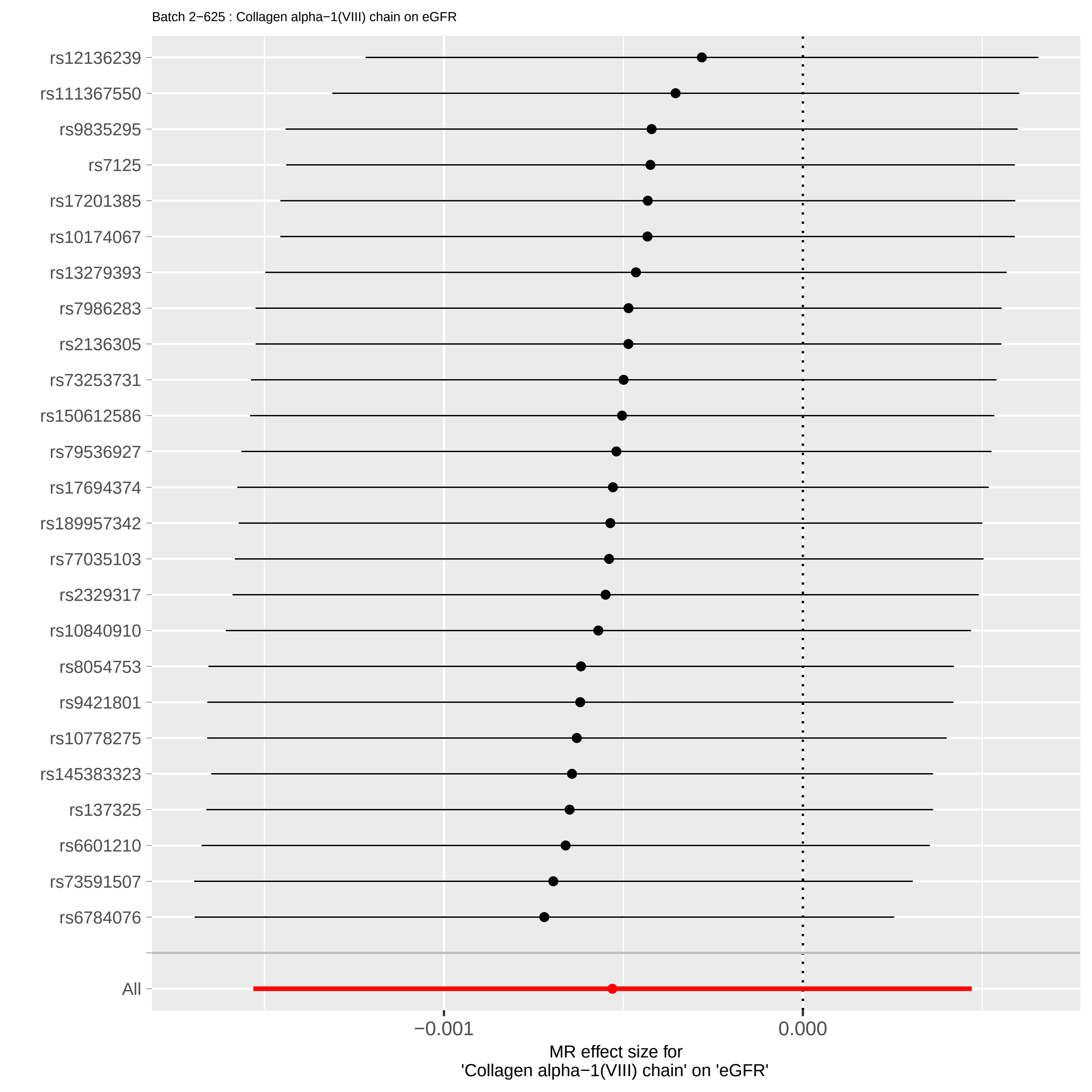

Batch 2−625 : Collagen alpha−1(VIII) chain on eGFR
rs12136239
rs111367550
rs9835295
rs7125
rs17201385
rs10174067
rs13279393
rs7986283
rs2136305
rs73253731
rs150612586
rs79536927
rs17694374
rs189957342
rs77035103
rs2329317
rs10840910
rs8054753
rs9421801
rs10778275
rs145383323
rs137325
rs6601210
rs73591507
rs6784076
All
−0.001
0.000
MR effect size for
'Collagen alpha−1(VIII) chain' on 'eGFR'

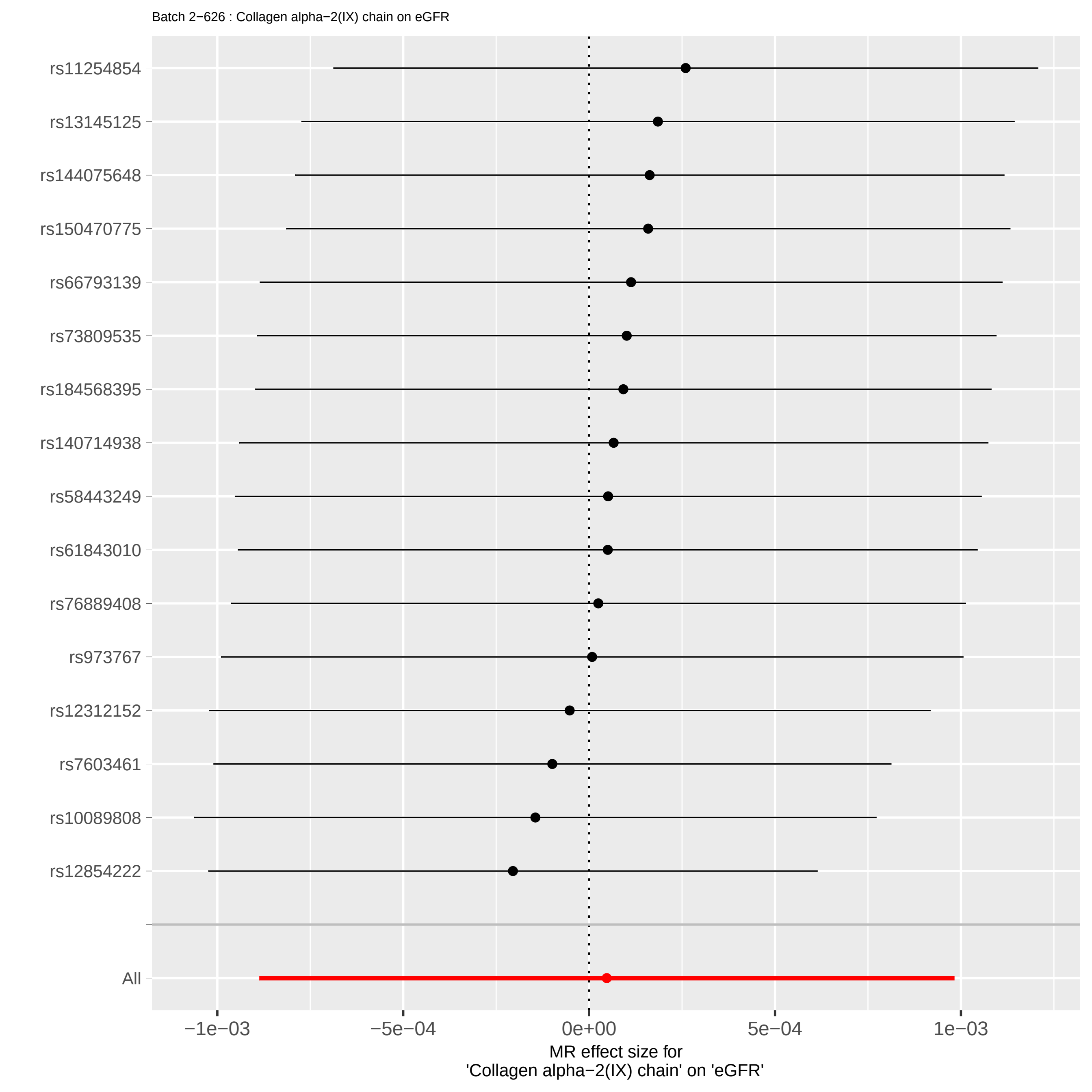

Batch 2−626 : Collagen alpha−2(IX) chain on eGFR
rs11254854
rs13145125
rs144075648
rs150470775
rs66793139
rs73809535
rs184568395
rs140714938
rs58443249
rs61843010
rs76889408
rs973767
rs12312152
rs7603461
rs10089808
rs12854222
All
−1e−03
−5e−04
0e+00
5e−04
1e−03
MR effect size for
'Collagen alpha−2(IX) chain' on 'eGFR'

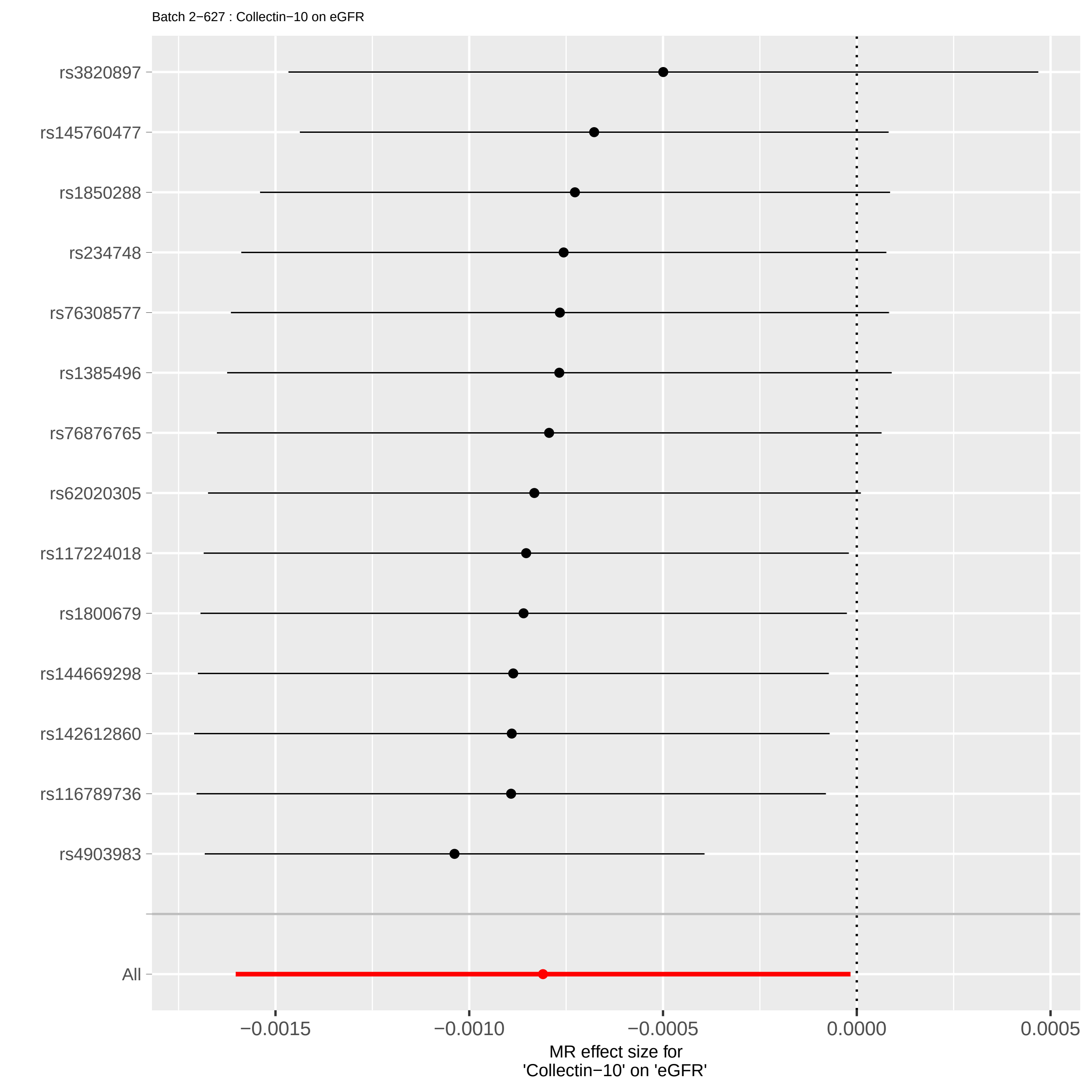

Batch 2−627 : Collectin−10 on eGFR
rs3820897
rs145760477
rs1850288
rs234748
rs76308577
rs1385496
rs76876765
rs62020305
rs117224018
rs1800679
rs144669298
rs142612860
rs116789736
rs4903983
All
−0.0015
−0.0010
−0.0005
0.0000
0.0005
MR effect size for
'Collectin−10' on 'eGFR'

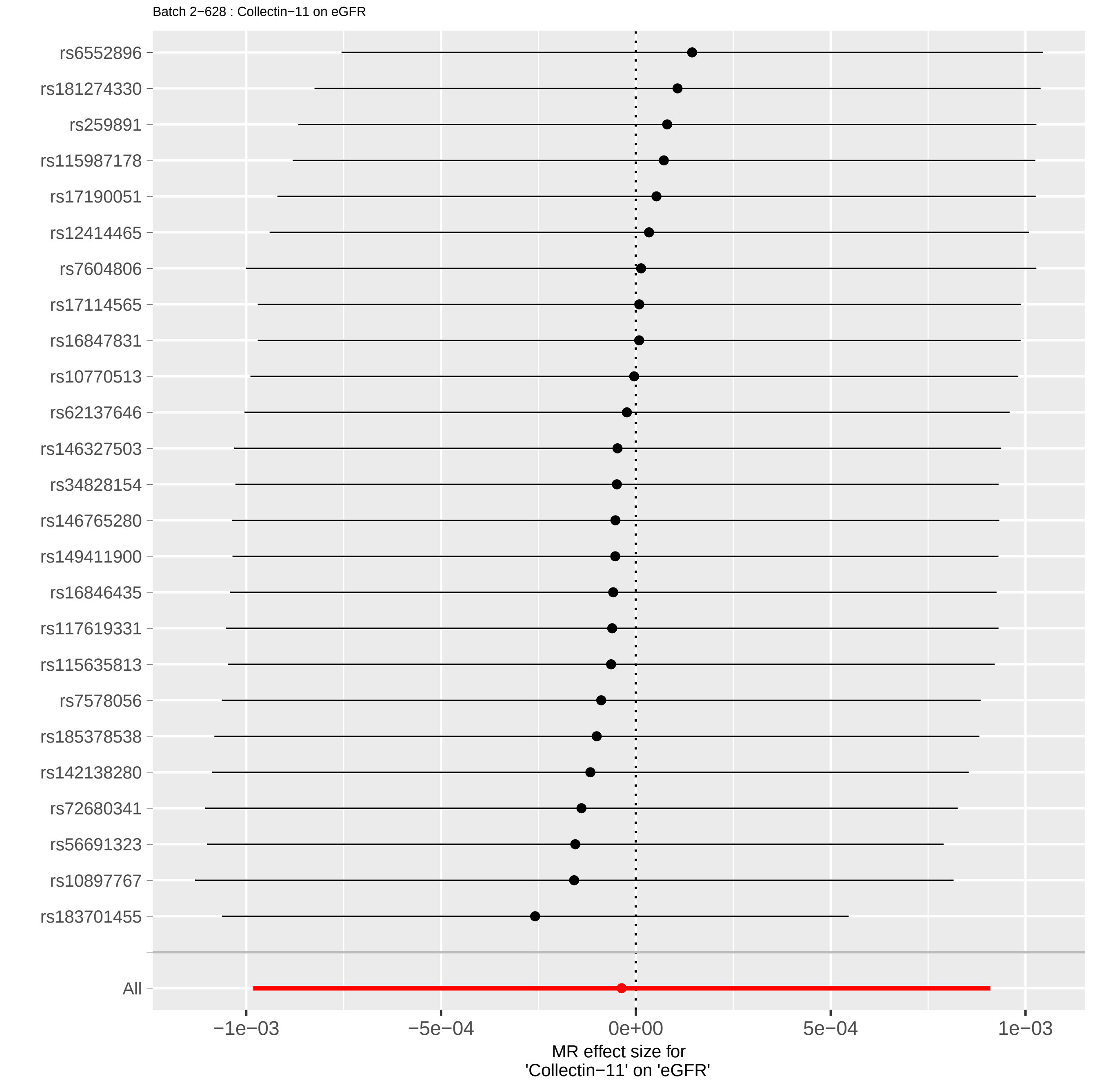
Batch 2−628 : Collectin−11 on eGFR
rs6552896
rs181274330
rs259891
rs115987178
rs17190051
rs12414465
rs7604806
rs17114565
rs16847831
rs10770513
rs62137646
rs146327503
rs34828154
rs146765280
rs149411900
rs16846435
rs117619331
rs115635813
rs7578056
rs185378538
rs142138280
rs72680341
rs56691323
rs10897767
rs183701455
All
−1e−03
−5e−04
0e+00
5e−04
1e−03
MR effect size for
'Collectin−11' on 'eGFR'

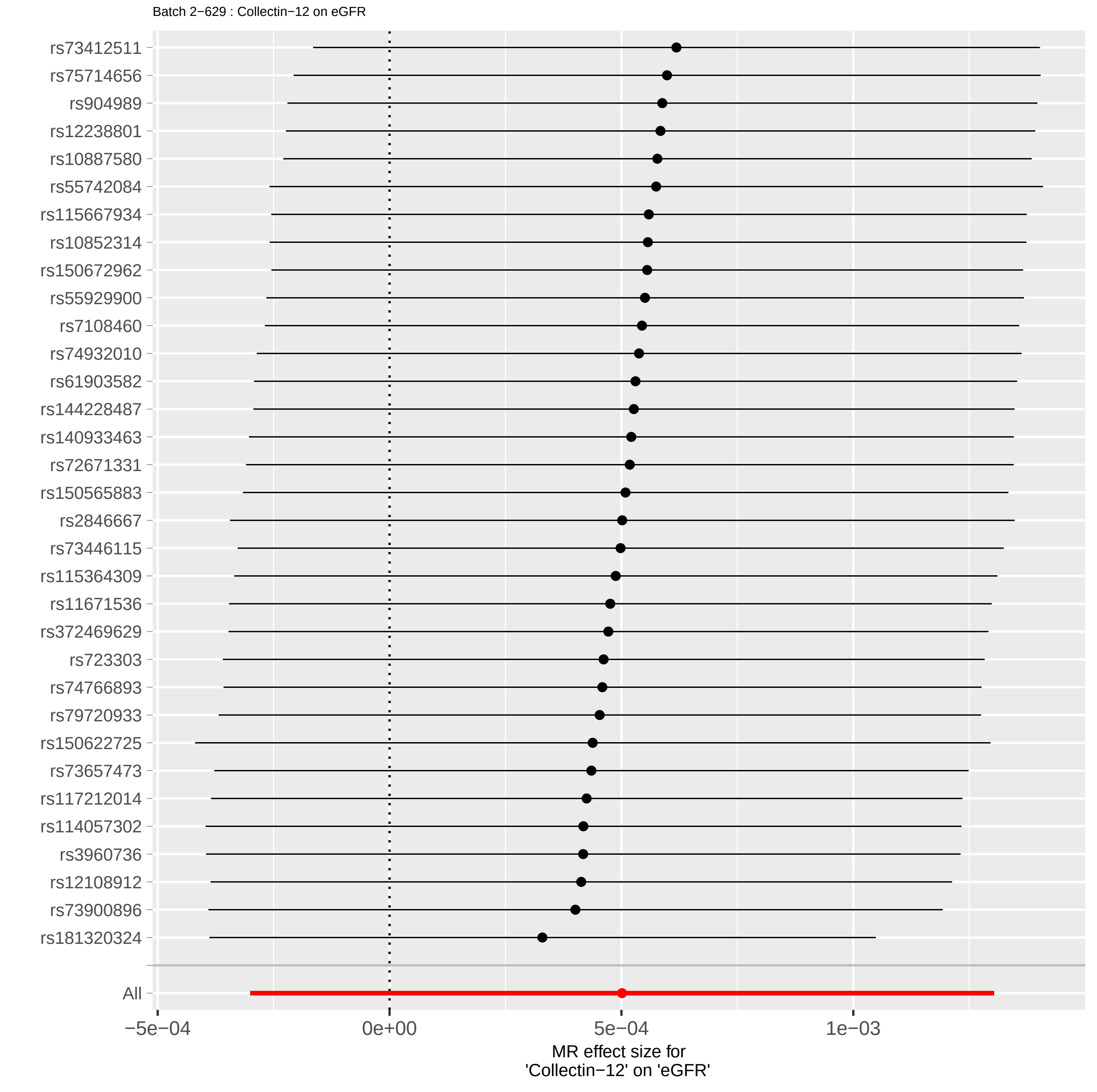

Batch 2−629 : Collectin−12 on eGFR
rs73412511
rs75714656
rs904989
rs12238801
rs10887580
rs55742084
rs115667934
rs10852314
rs150672962
rs55929900
rs7108460
rs74932010
rs61903582
rs144228487
rs140933463
rs72671331
rs150565883
rs2846667
rs73446115
rs115364309
rs11671536
rs372469629
rs723303
rs74766893
rs79720933
rs150622725
rs73657473
rs117212014
rs114057302
rs3960736
rs12108912
rs73900896
rs181320324
All
−5e−04
0e+00
5e−04
1e−03
MR effect size for
'Collectin−12' on 'eGFR'

Batch 2−630 : Collectin−12 on eGFR

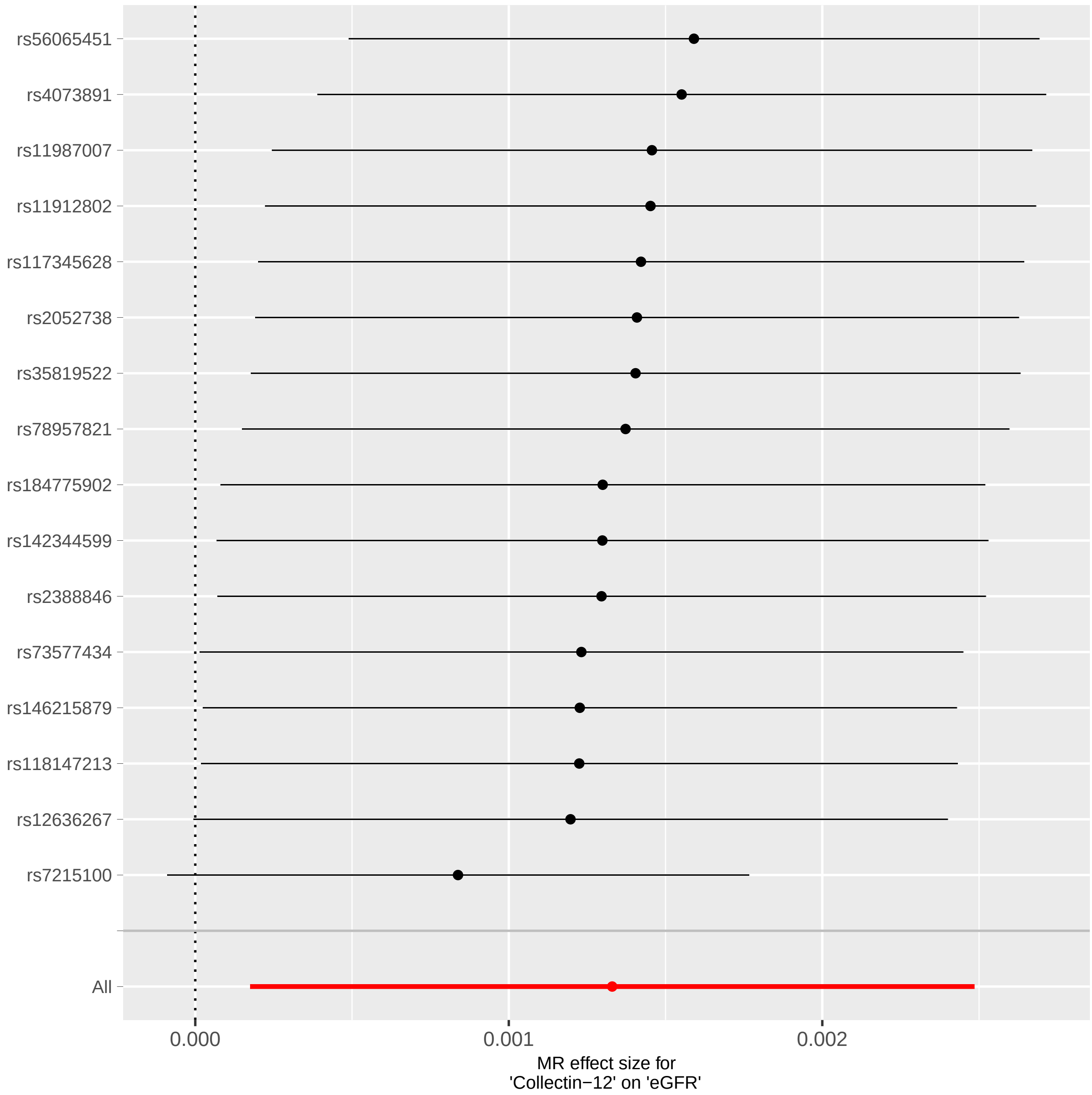

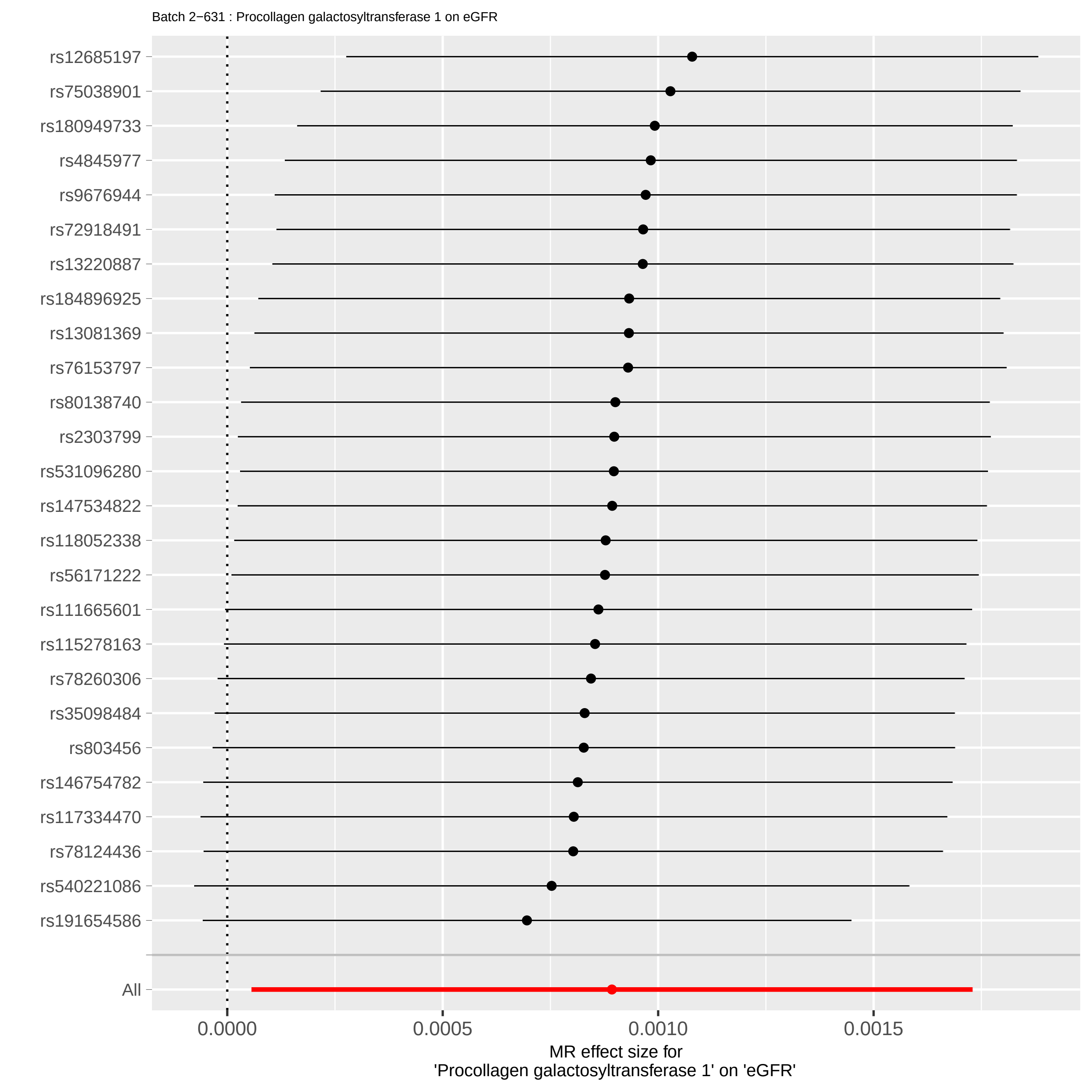

Batch 2−631 : Procollagen galactosyltransferase 1 on eGFR
rs12685197
rs75038901
rs180949733
rs4845977
rs9676944
rs72918491
rs13220887
rs184896925
rs13081369
rs76153797
rs80138740
rs2303799
rs531096280
rs147534822
rs118052338
rs56171222
rs111665601
rs115278163
rs78260306
rs35098484
rs803456
rs146754782
rs117334470
rs78124436
rs540221086
rs191654586
All
0.0000
0.0005
0.0010
0.0015
MR effect size for
'Procollagen galactosyltransferase 1' on 'eGFR'

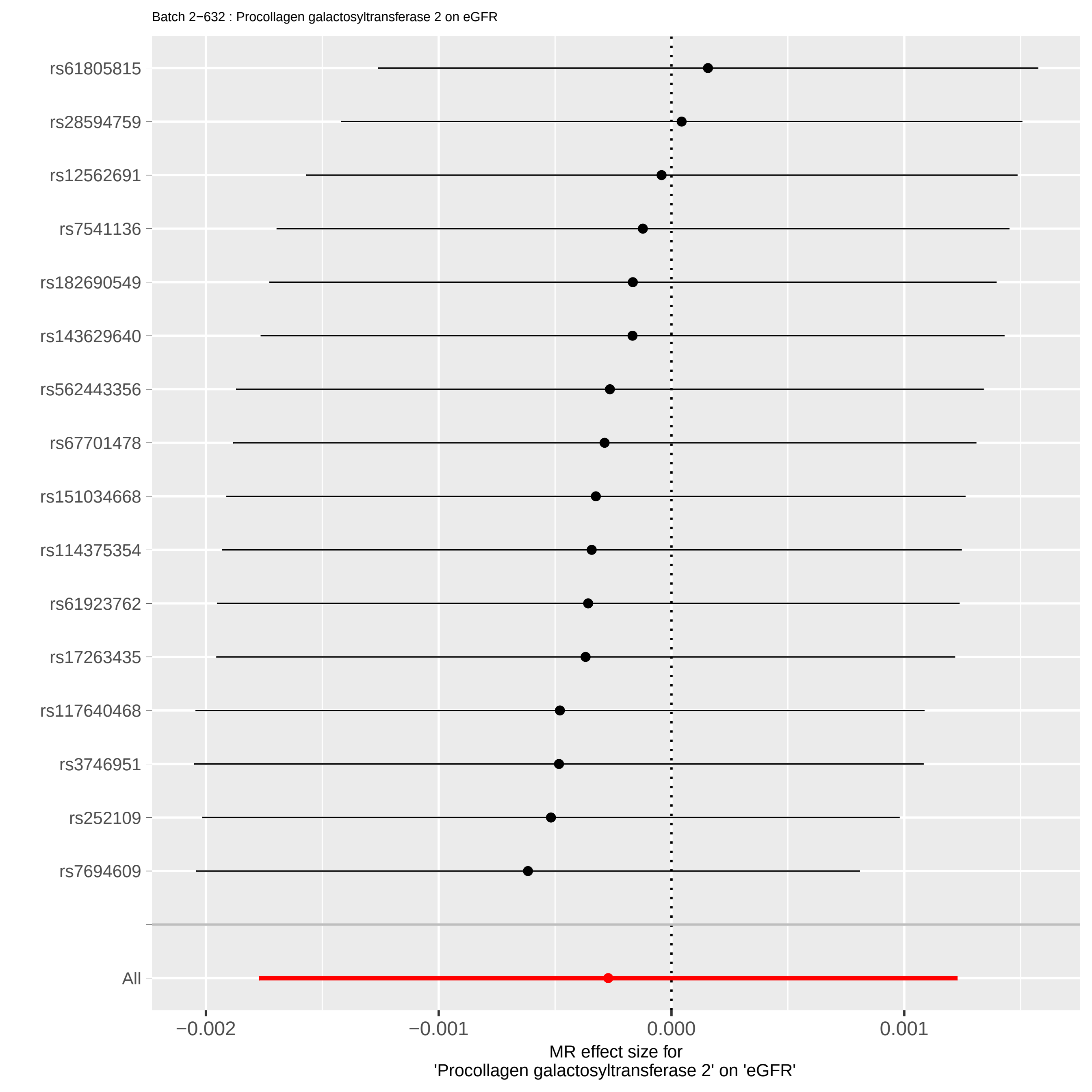
Batch 2−632 : Procollagen galactosyltransferase 2 on eGFR
rs61805815
rs28594759
rs12562691
rs7541136
rs182690549
rs143629640
rs562443356
rs67701478
rs151034668
rs114375354
rs61923762
rs17263435
rs117640468
rs3746951
rs252109
rs7694609
All
−0.002
−0.001
0.000
0.001
MR effect size for
'Procollagen galactosyltransferase 2' on 'eGFR'

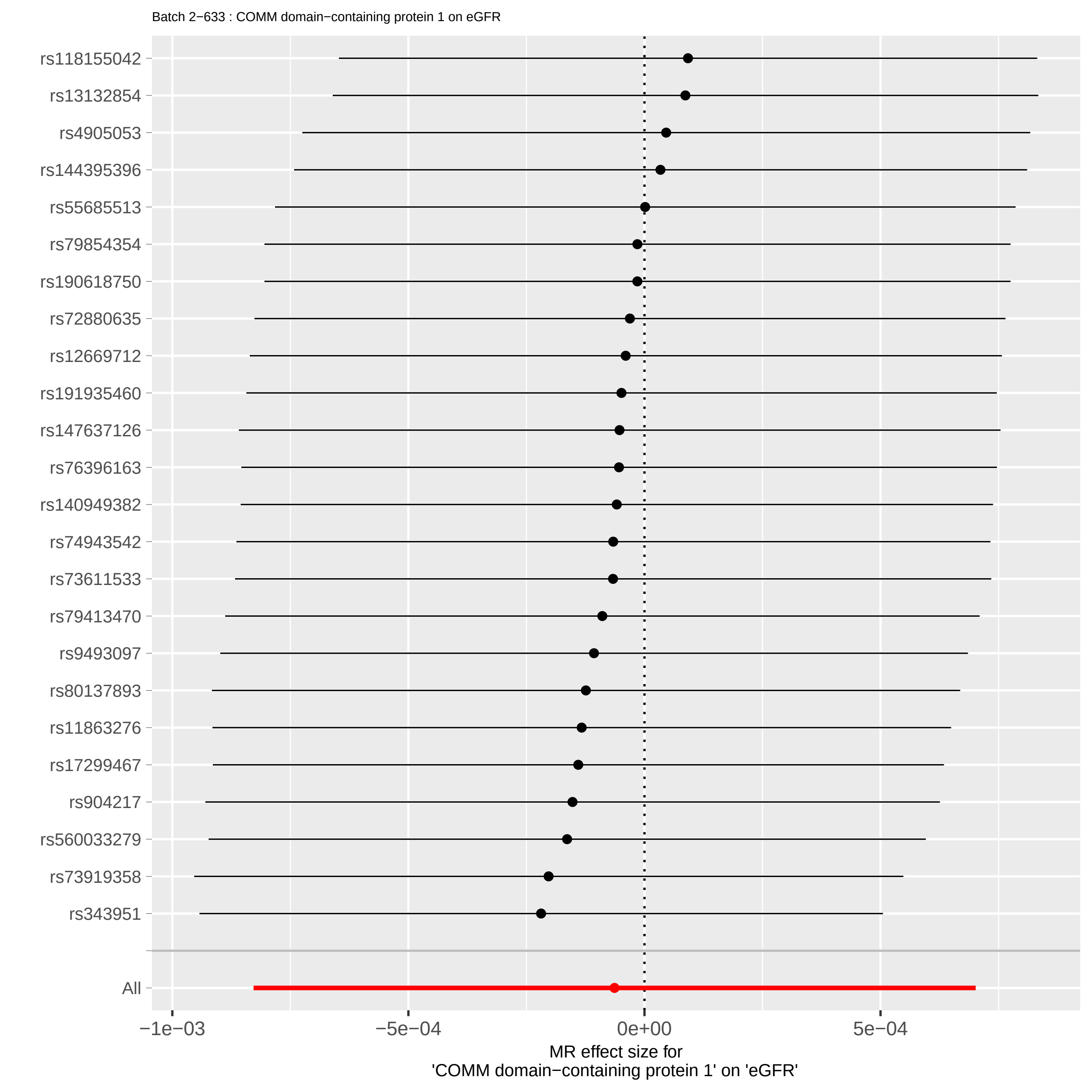

Batch 2−633 : COMM domain−containing protein 1 on eGFR
rs118155042
rs13132854
rs4905053
rs144395396
rs55685513
rs79854354
rs190618750
rs72880635
rs12669712
rs191935460
rs147637126
rs76396163
rs140949382
rs74943542
rs73611533
rs79413470
rs9493097
rs80137893
rs11863276
rs17299467
rs904217
rs560033279
rs73919358
rs343951
All
−1e−03
−5e−04
0e+00
5e−04
MR effect size for
'COMM domain−containing protein 1' on 'eGFR'

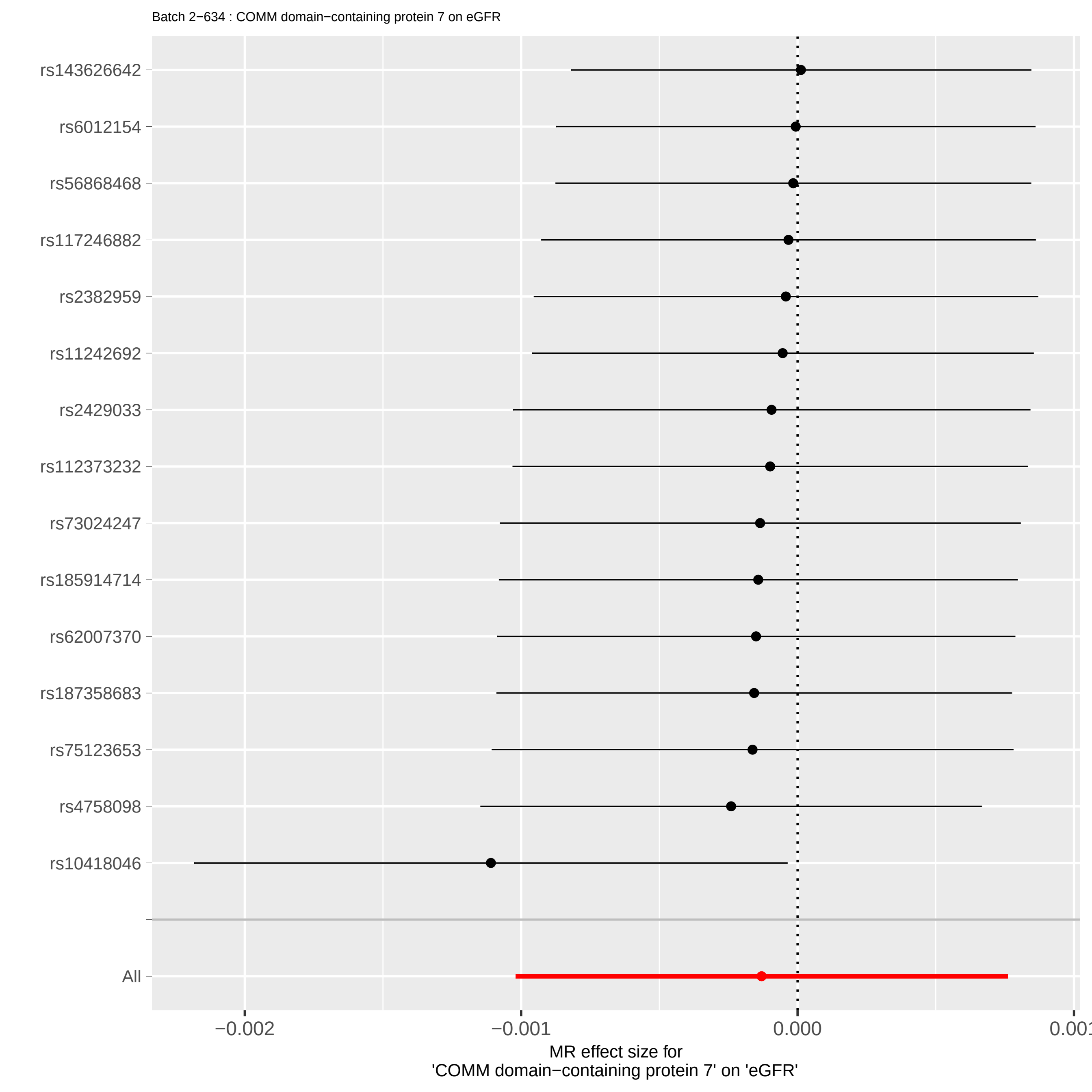

Batch 2−634 : COMM domain−containing protein 7 on eGFR
rs143626642
rs6012154
rs56868468
rs117246882
rs2382959
rs11242692
rs2429033
rs112373232
rs73024247
rs185914714
rs62007370
rs187358683
rs75123653
rs4758098
rs10418046
All
−0.002
−0.001
0.000
MR effect size for
'COMM domain−containing protein 7' on 'eGFR'

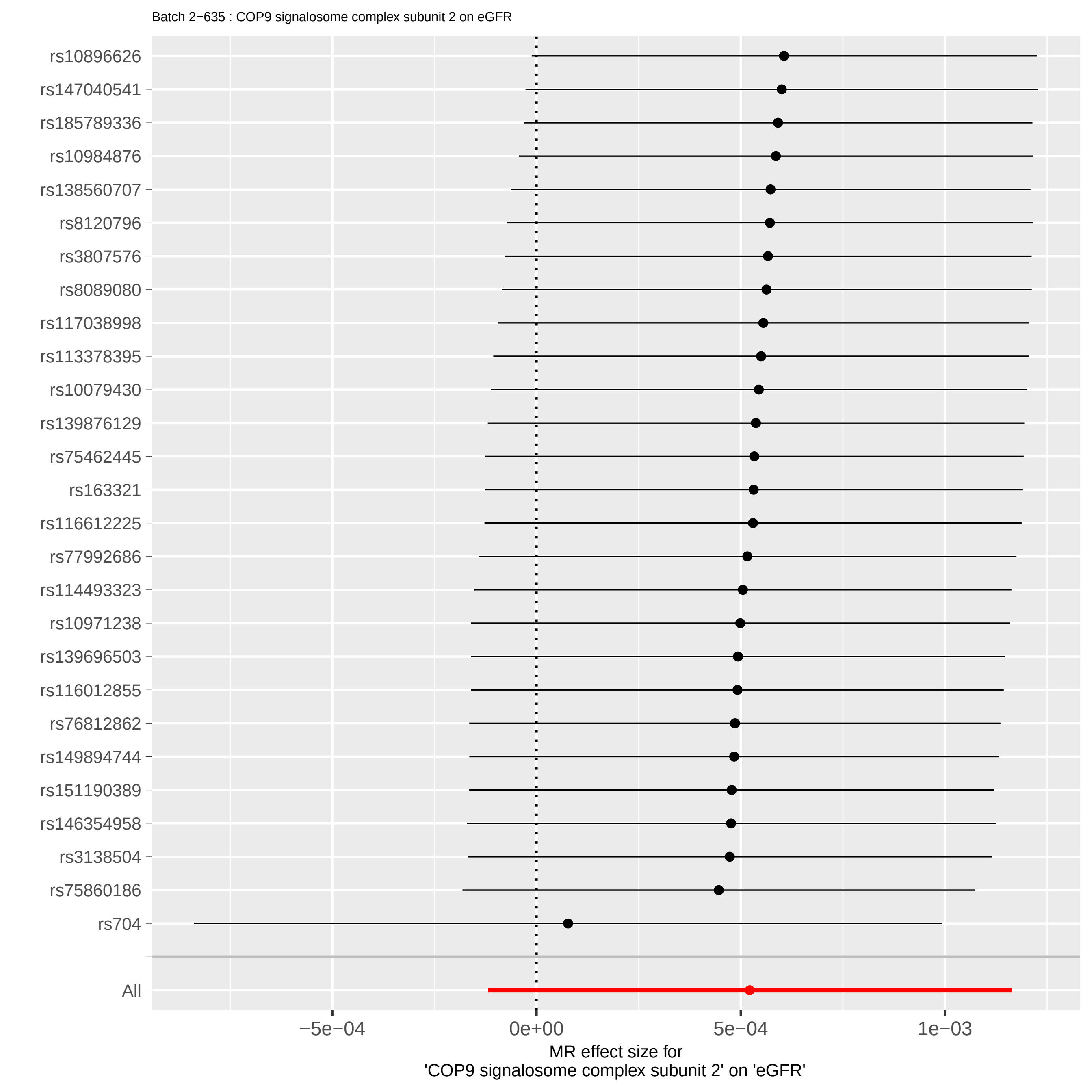

Batch 2−635 : COP9 signalosome complex subunit 2 on eGFR
rs10896626
rs147040541
rs185789336
rs10984876
rs138560707
rs8120796
rs3807576
rs8089080
rs117038998
rs113378395
rs10079430
rs139876129
rs75462445
rs163321
rs116612225
rs77992686
rs114493323
rs10971238
rs139696503
rs116012855
rs76812862
rs149894744
rs151190389
rs146354958
rs3138504
rs75860186
rs704
All
−5e−04
0e+00
5e−04
1e−03
MR effect size for
'COP9 signalosome complex subunit 2' on 'eGFR'

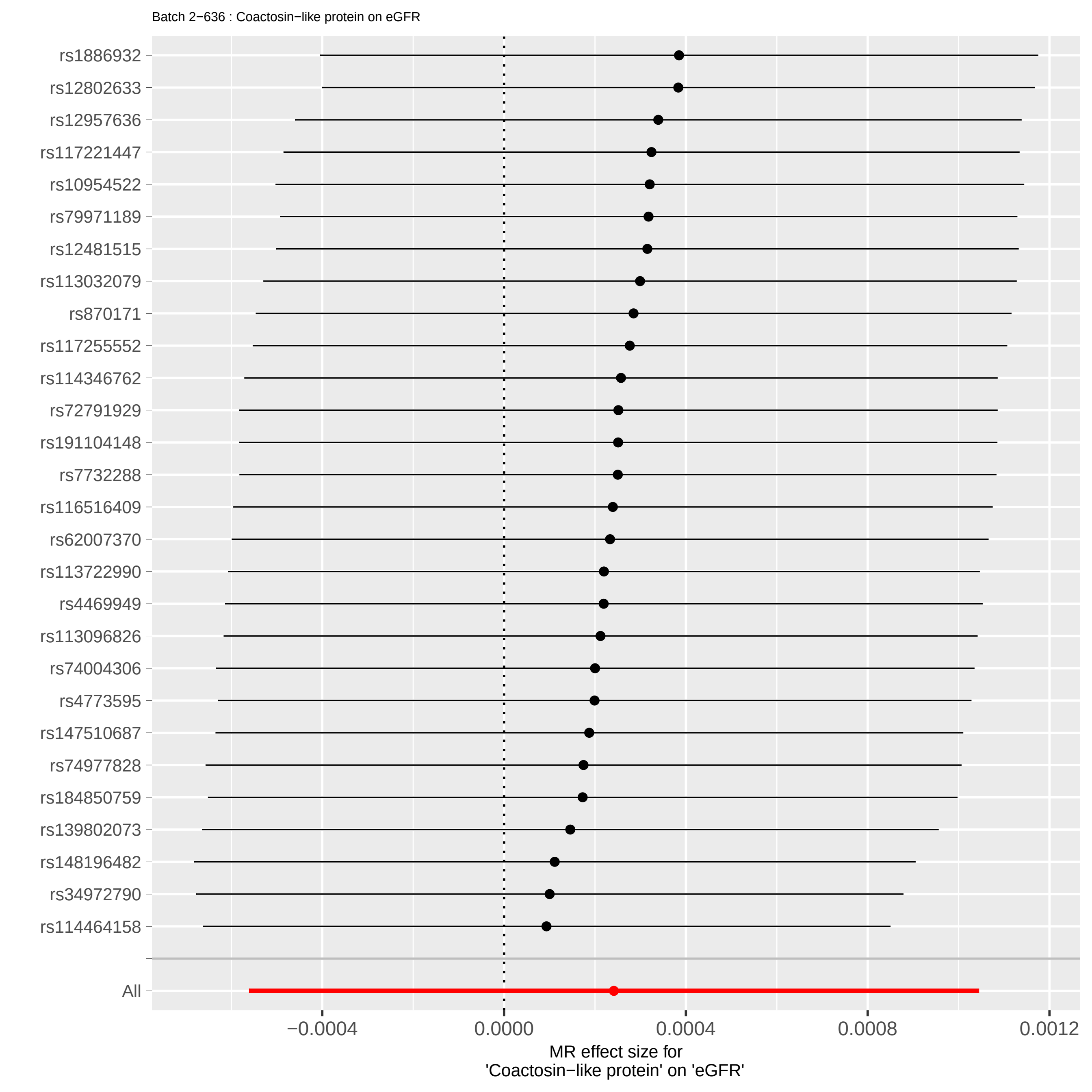

Batch 2−636 : Coactosin−like protein on eGFR
rs1886932
rs12802633
rs12957636
rs117221447
rs10954522
rs79971189
rs12481515
rs113032079
rs870171
rs117255552
rs114346762
rs72791929
rs191104148
rs7732288
rs116516409
rs62007370
rs113722990
rs4469949
rs113096826
rs74004306
rs4773595
rs147510687
rs74977828
rs184850759
rs139802073
rs148196482
rs34972790
rs114464158
All
−0.0004
0.0000
0.0004
0.0008
0.0012
MR effect size for
'Coactosin−like protein' on 'eGFR'

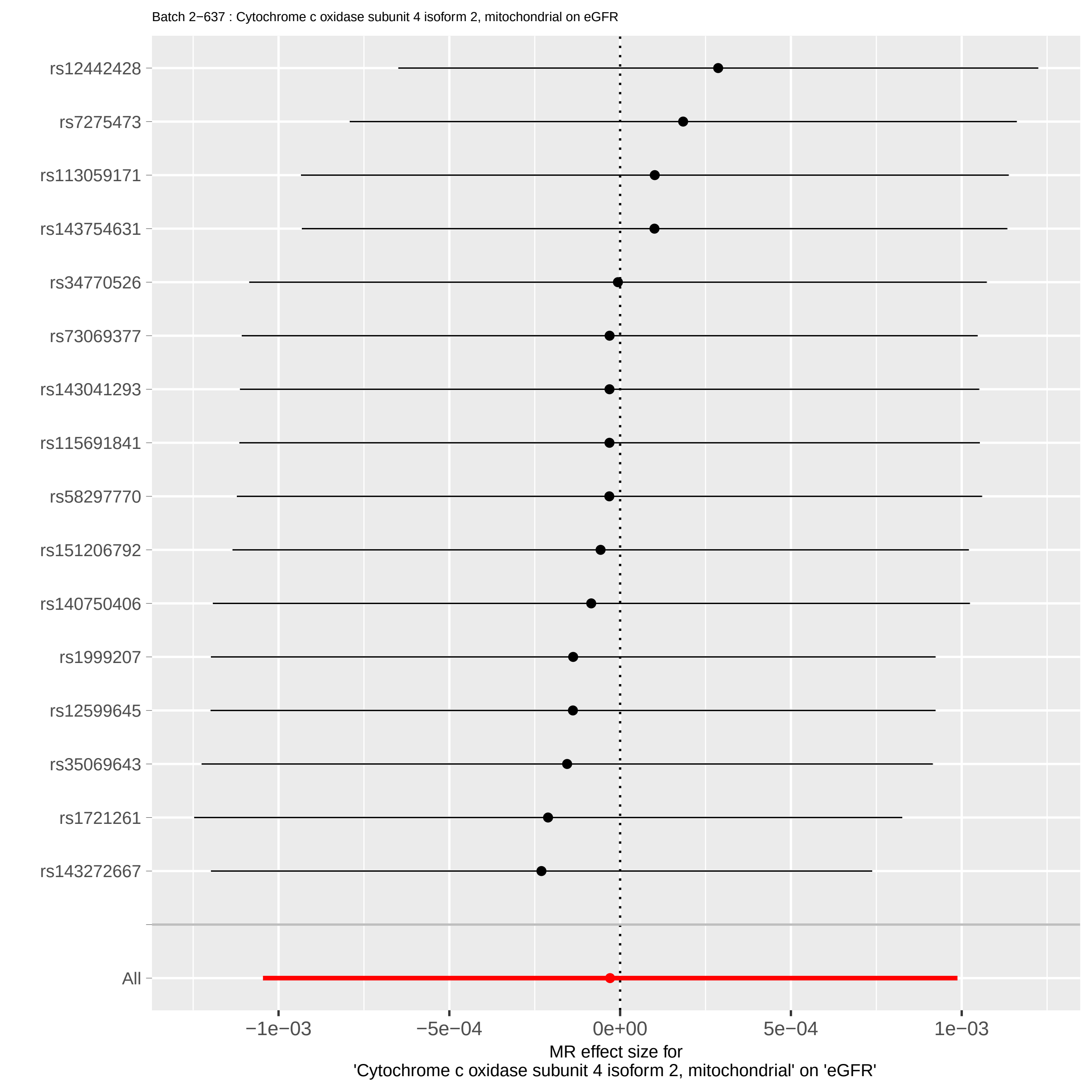

Batch 2−637 : Cytochrome c oxidase subunit 4 isoform 2, mitochondrial on eGFR
rs12442428
rs7275473
rs113059171
rs143754631
rs34770526
rs73069377
rs143041293
rs115691841
rs58297770
rs151206792
rs140750406
rs1999207
rs12599645
rs35069643
rs1721261
rs143272667
All
−1e−03
−5e−04
0e+00
5e−04
1e−03
MR effect size for
'Cytochrome c oxidase subunit 4 isoform 2, mitochondrial' on 'eGFR'

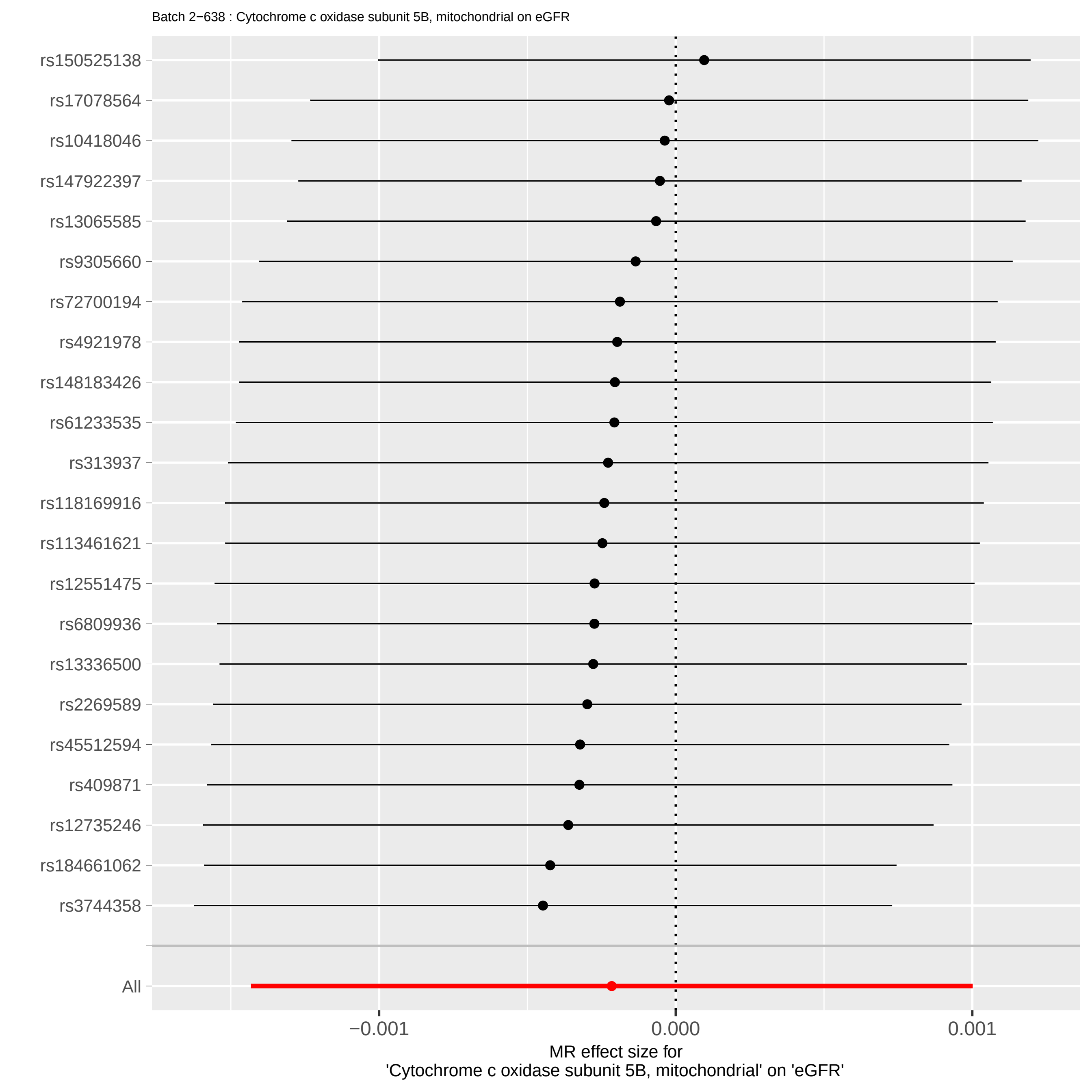

Batch 2−638 : Cytochrome c oxidase subunit 5B, mitochondrial on eGFR
rs150525138
rs17078564
rs10418046
rs147922397
rs13065585
rs9305660
rs72700194
rs4921978
rs148183426
rs61233535
rs313937
rs118169916
rs113461621
rs12551475
rs6809936
rs13336500
rs2269589
rs45512594
rs409871
rs12735246
rs184661062
rs3744358
All
−0.001
0.000
0.001
MR effect size for
'Cytochrome c oxidase subunit 5B, mitochondrial' on 'eGFR'

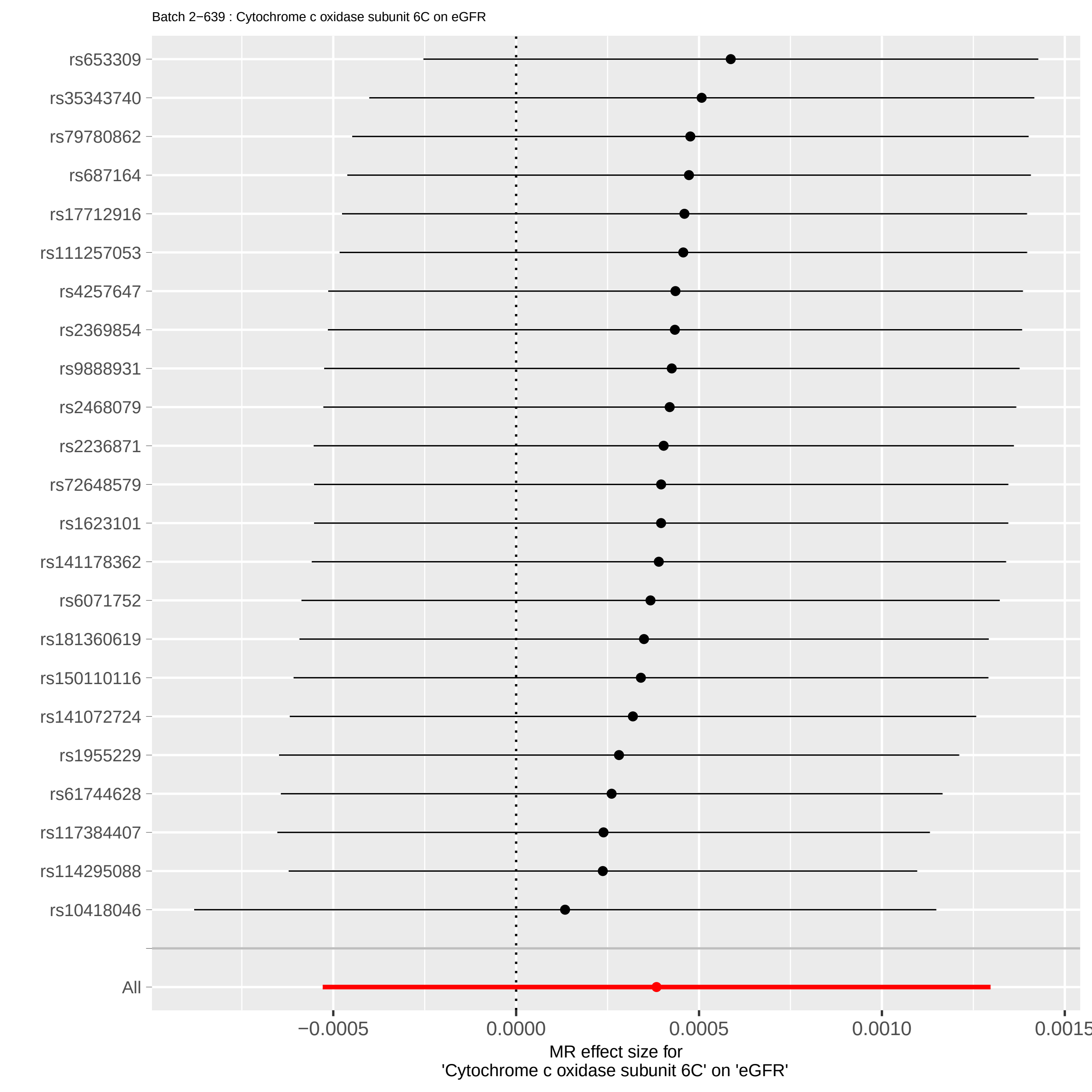

Batch 2−639 : Cytochrome c oxidase subunit 6C on eGFR
rs653309
rs35343740
rs79780862
rs687164
rs17712916
rs111257053
rs4257647
rs2369854
rs9888931
rs2468079
rs2236871
rs72648579
rs1623101
rs141178362
rs6071752
rs181360619
rs150110116
rs141072724
rs1955229
rs61744628
rs117384407
rs114295088
rs10418046
All
−0.0005
0.0000
0.0005
0.0010
0.0015
MR effect size for
'Cytochrome c oxidase subunit 6C' on 'eGFR'

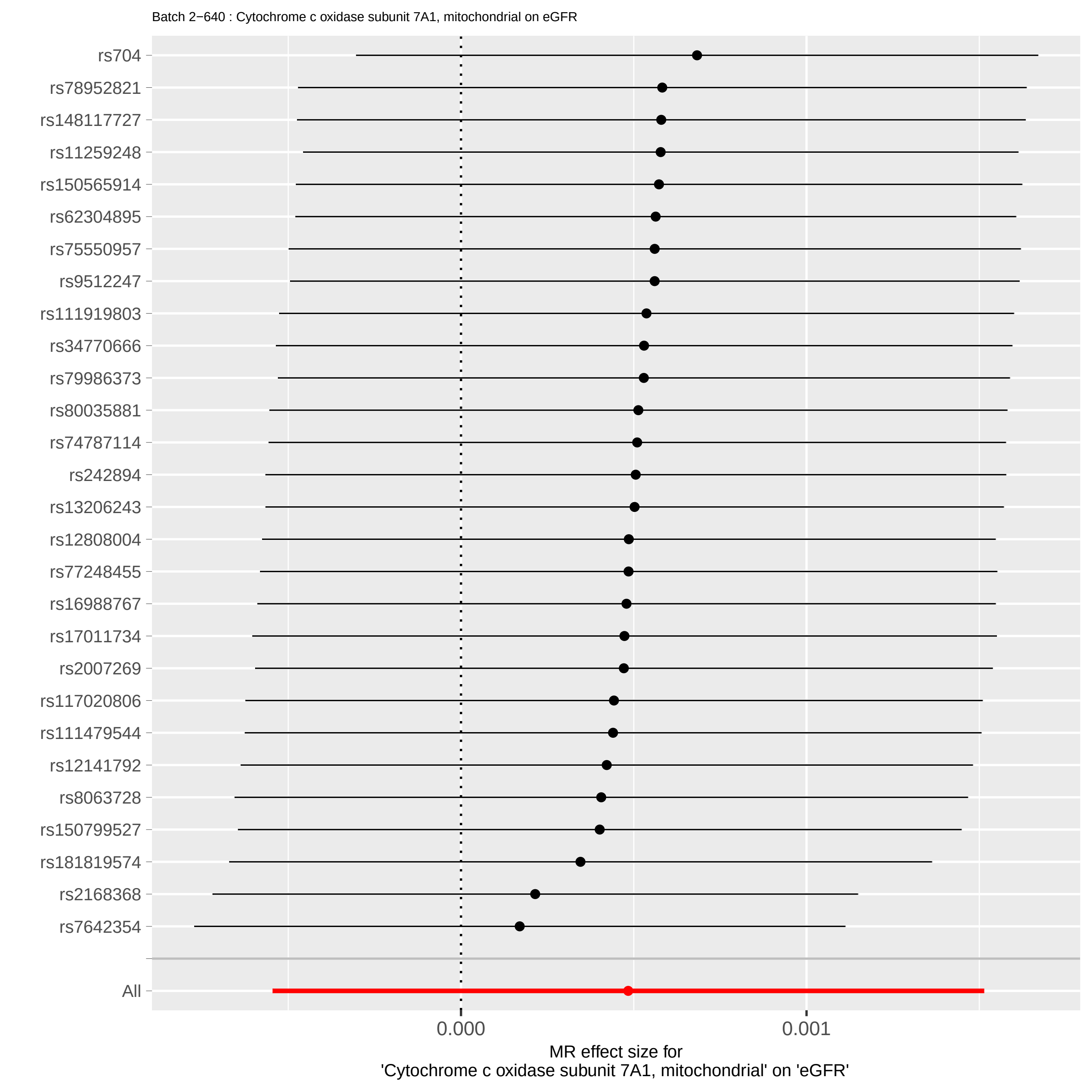

Batch 2−640 : Cytochrome c oxidase subunit 7A1, mitochondrial on eGFR
rs704
rs78952821
rs148117727
rs11259248
rs150565914
rs62304895
rs75550957
rs9512247
rs111919803
rs34770666
rs79986373
rs80035881
rs74787114
rs242894
rs13206243
rs12808004
rs77248455
rs16988767
rs17011734
rs2007269
rs117020806
rs111479544
rs12141792
rs8063728
rs150799527
rs181819574
rs2168368
rs7642354
All
0.000
0.001
MR effect size for
'Cytochrome c oxidase subunit 7A1, mitochondrial' on 'eGFR'

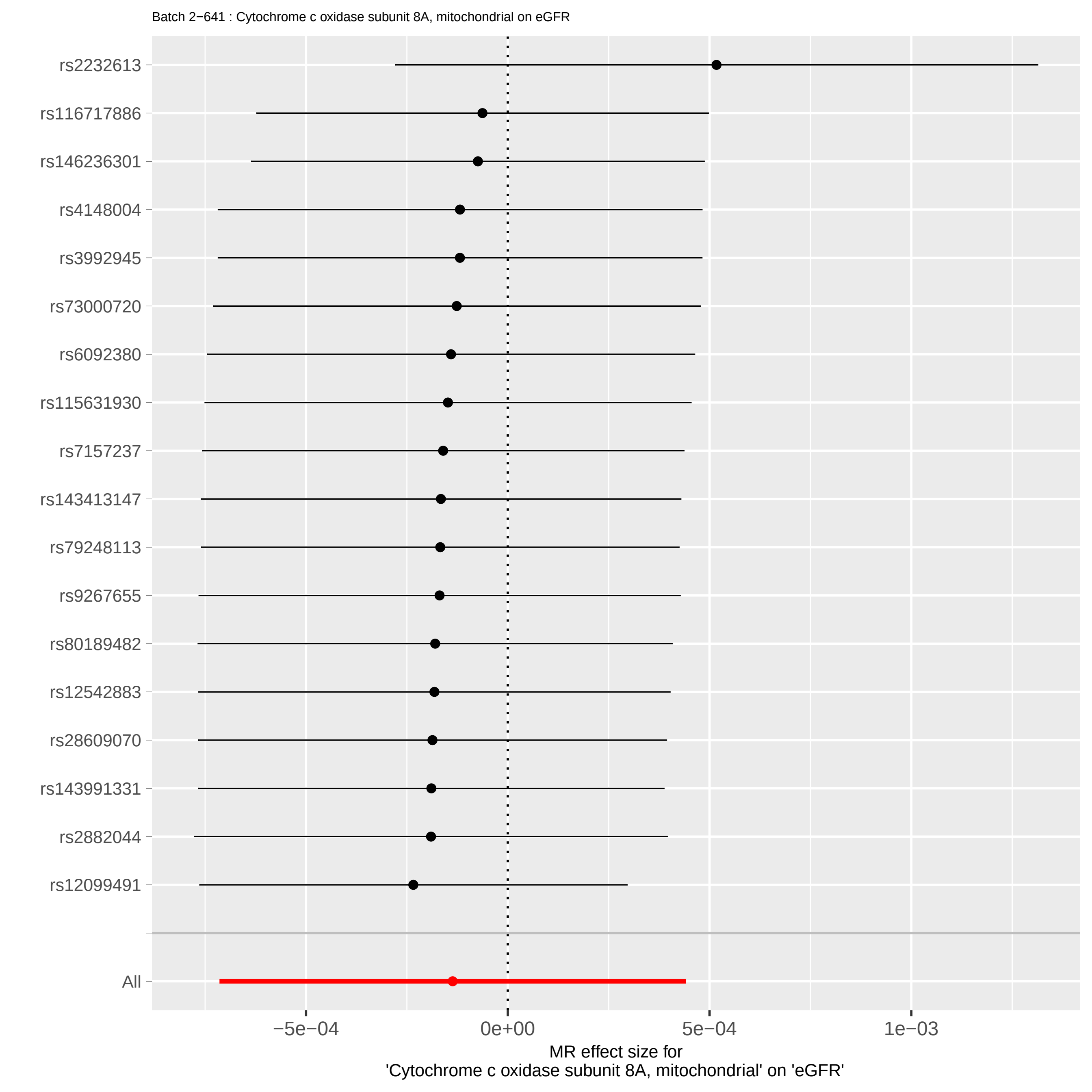

Batch 2−641 : Cytochrome c oxidase subunit 8A, mitochondrial on eGFR
rs2232613
rs116717886
rs146236301
rs4148004
rs3992945
rs73000720
rs6092380
rs115631930
rs7157237
rs143413147
rs79248113
rs9267655
rs80189482
rs12542883
rs28609070
rs143991331
rs2882044
rs12099491
All
−5e−04
0e+00
5e−04
1e−03
MR effect size for
'Cytochrome c oxidase subunit 8A, mitochondrial' on 'eGFR'

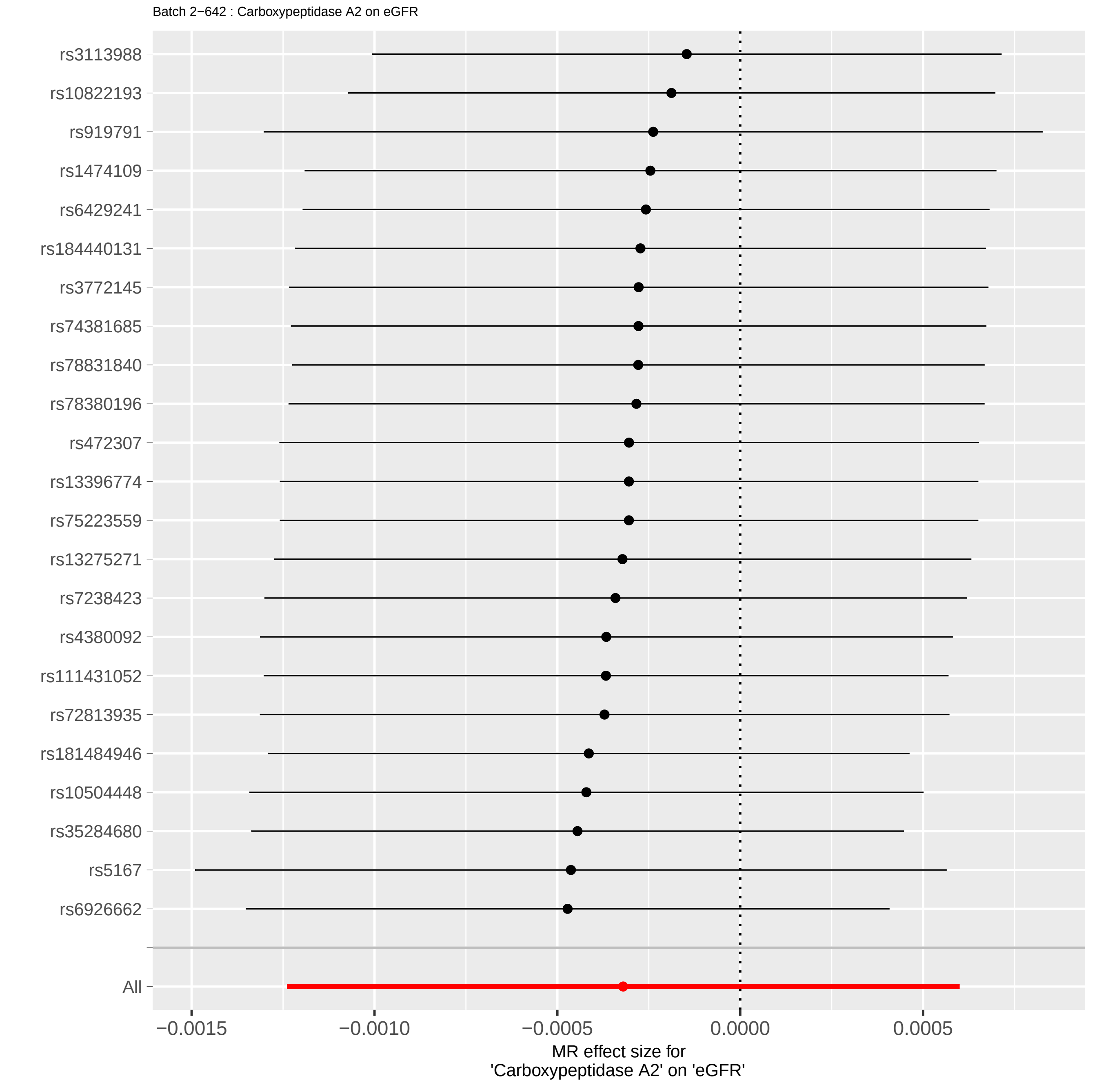
Batch 2−642 : Carboxypeptidase A2 on eGFR
rs3113988
rs10822193
rs919791
rs1474109
rs6429241
rs184440131
rs3772145
rs74381685
rs78831840
rs78380196
rs472307
rs13396774
rs75223559
rs13275271
rs7238423
rs4380092
rs111431052
rs72813935
rs181484946
rs10504448
rs35284680
rs5167
rs6926662
All
−0.0015
−0.0010
−0.0005
0.0000
0.0005
MR effect size for
'Carboxypeptidase A2' on 'eGFR'

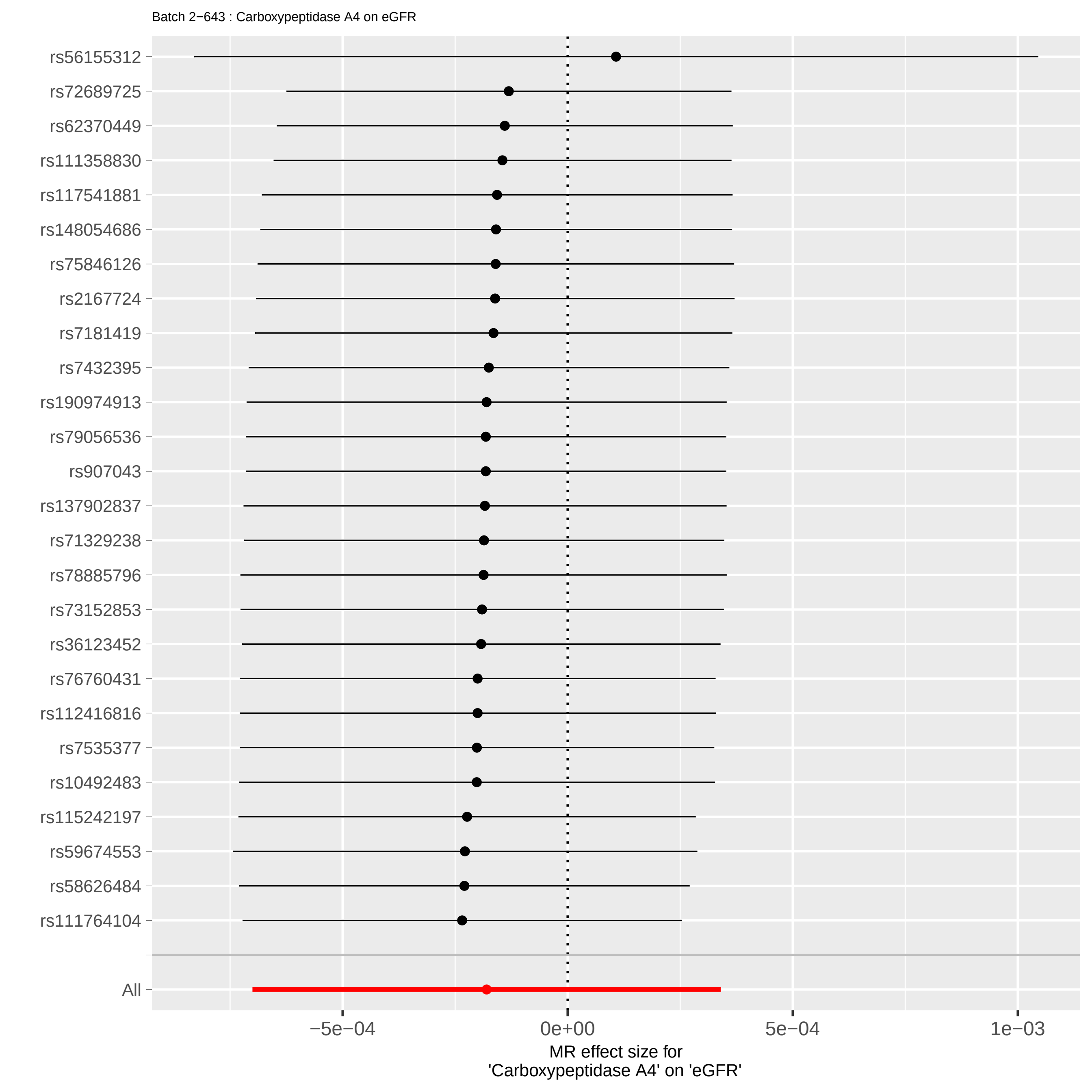

Batch 2−643 : Carboxypeptidase A4 on eGFR
rs56155312
rs72689725
rs62370449
rs111358830
rs117541881
rs148054686
rs75846126
rs2167724
rs7181419
rs7432395
rs190974913
rs79056536
rs907043
rs137902837
rs71329238
rs78885796
rs73152853
rs36123452
rs76760431
rs112416816
rs7535377
rs10492483
rs115242197
rs59674553
rs58626484
rs111764104
All
−5e−04
0e+00
5e−04
1e−03
MR effect size for
'Carboxypeptidase A4' on 'eGFR'

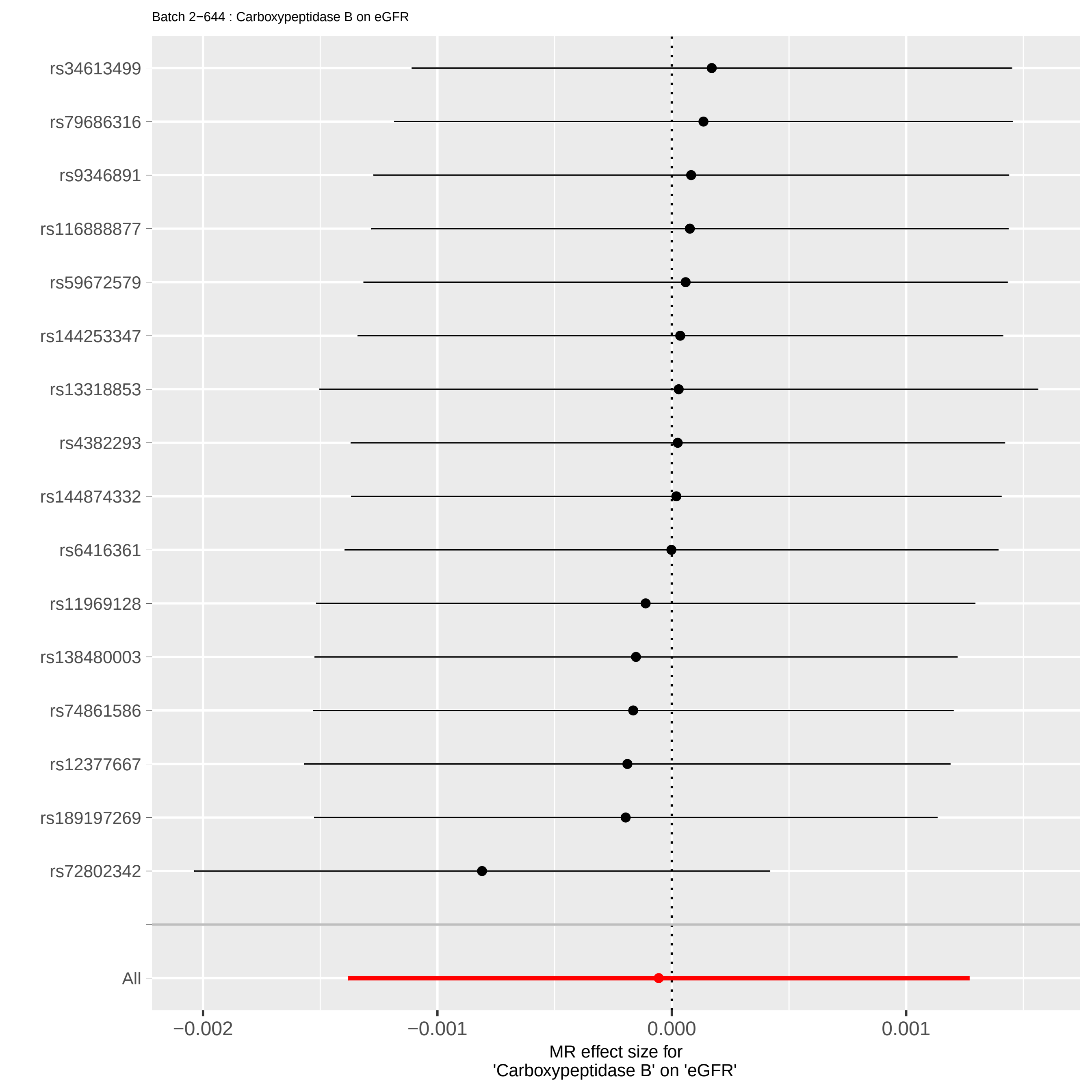
Batch 2−644 : Carboxypeptidase B on eGFR
rs34613499
rs79686316
rs9346891
rs116888877
rs59672579
rs144253347
rs13318853
rs4382293
rs144874332
rs6416361
rs11969128
rs138480003
rs74861586
rs12377667
rs189197269
rs72802342
All
−0.002
−0.001
0.000
0.001
MR effect size for
'Carboxypeptidase B' on 'eGFR'

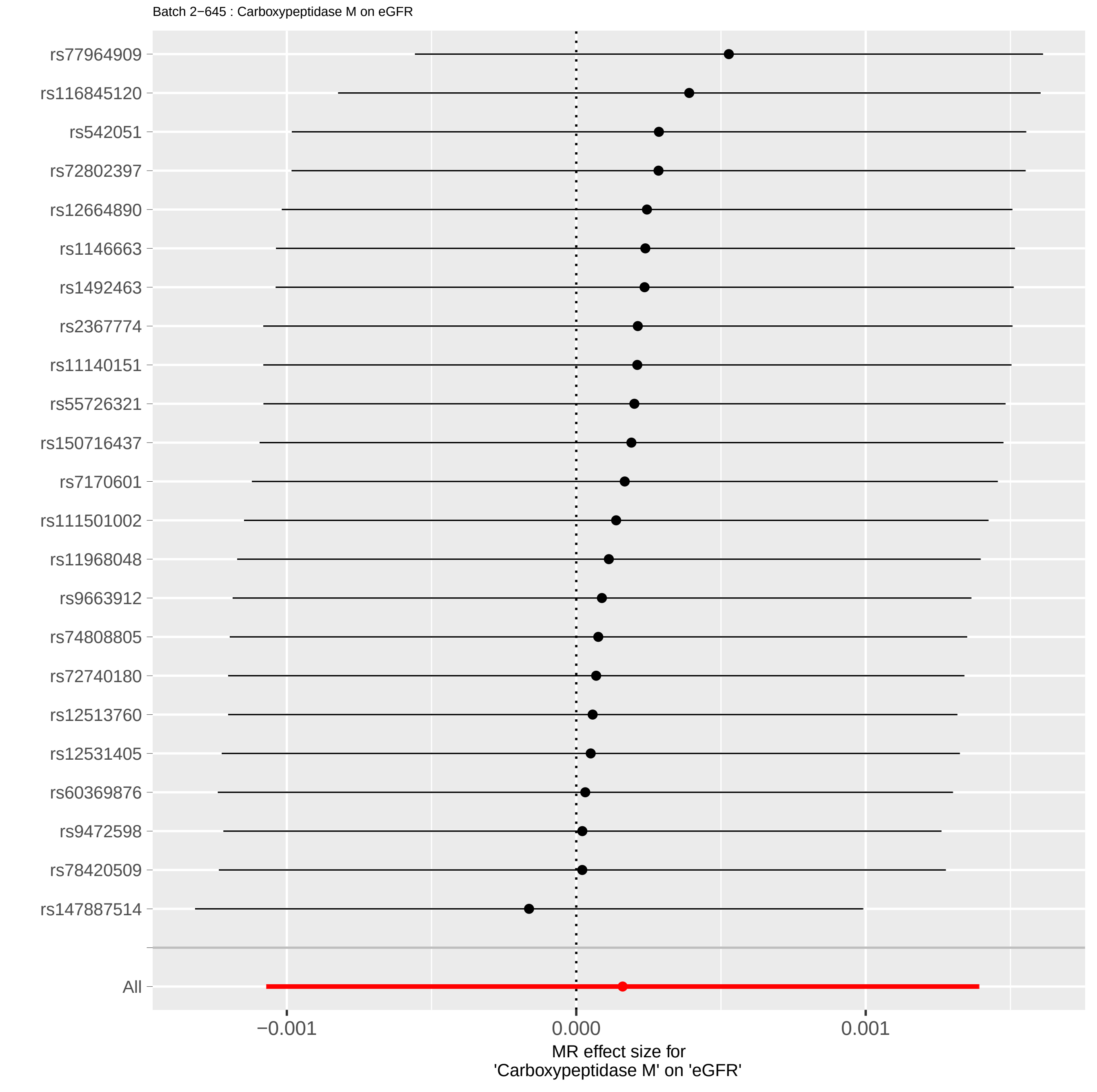

Batch 2−645 : Carboxypeptidase M on eGFR
rs77964909
rs116845120
rs542051
rs72802397
rs12664890
rs1146663
rs1492463
rs2367774
rs11140151
rs55726321
rs150716437
rs7170601
rs111501002
rs11968048
rs9663912
rs74808805
rs72740180
rs12513760
rs12531405
rs60369876
rs9472598
rs78420509
rs147887514
All
−0.001
0.000
0.001
MR effect size for
'Carboxypeptidase M' on 'eGFR'

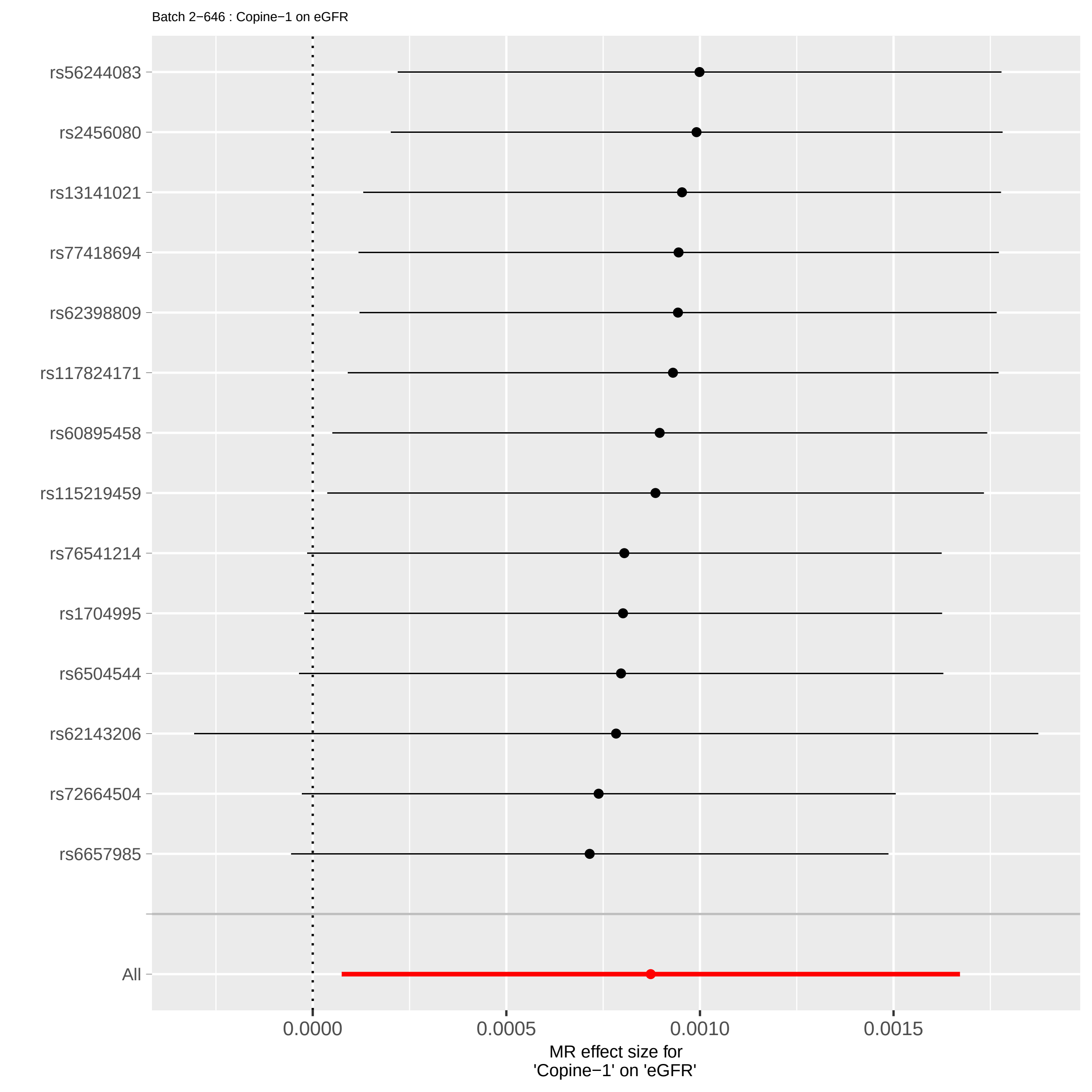

Batch 2−646 : Copine−1 on eGFR
rs56244083
rs2456080
rs13141021
rs77418694
rs62398809
rs117824171
rs60895458
rs115219459
rs76541214
rs1704995
rs6504544
rs62143206
rs72664504
rs6657985
All
0.0000
0.0005
0.0010
0.0015
MR effect size for
'Copine−1' on 'eGFR'

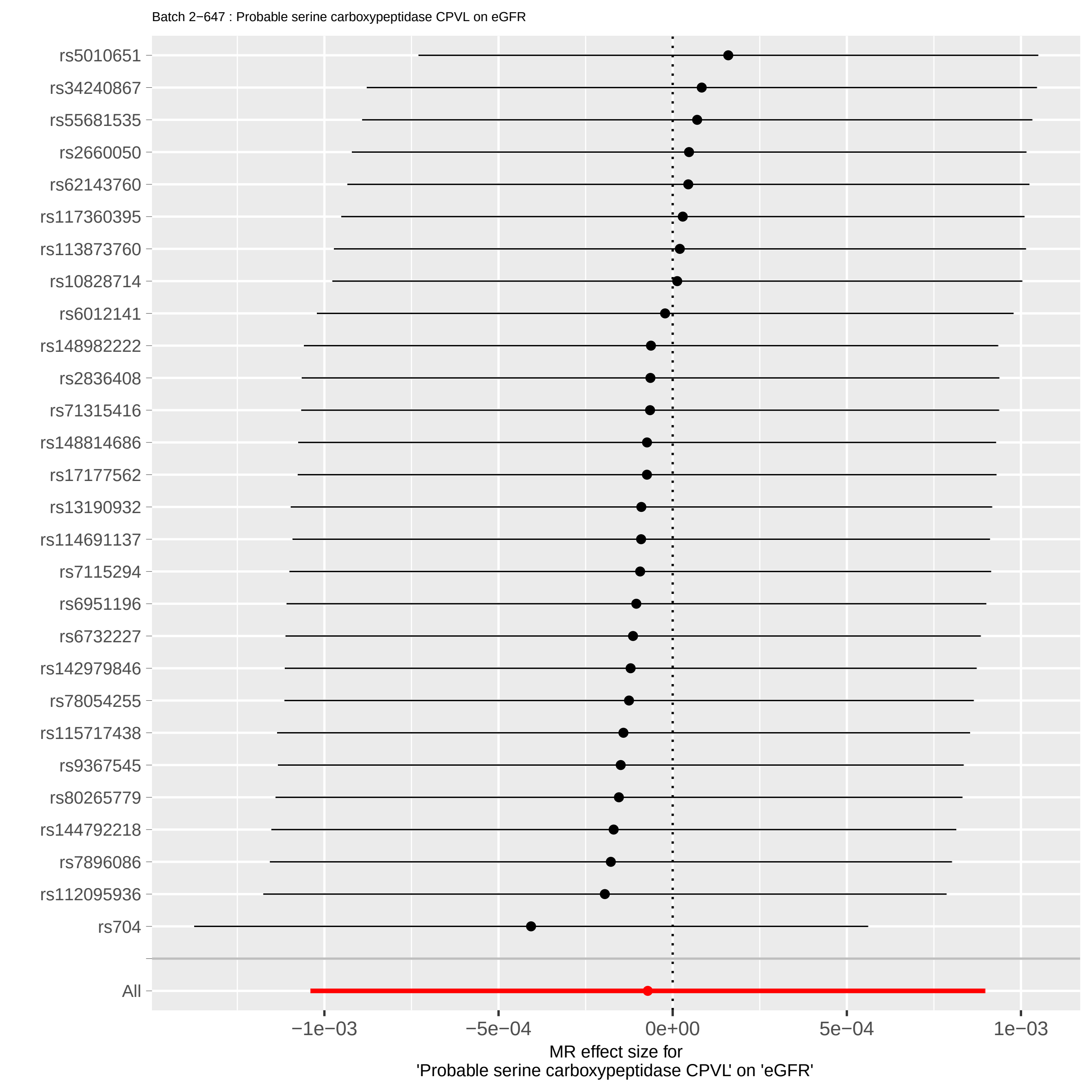

Batch 2−647 : Probable serine carboxypeptidase CPVL on eGFR
rs5010651
rs34240867
rs55681535
rs2660050
rs62143760
rs117360395
rs113873760
rs10828714
rs6012141
rs148982222
rs2836408
rs71315416
rs148814686
rs17177562
rs13190932
rs114691137
rs7115294
rs6951196
rs6732227
rs142979846
rs78054255
rs115717438
rs9367545
rs80265779
rs144792218
rs7896086
rs112095936
rs704
All
−1e−03
−5e−04
0e+00
5e−04
1e−03
MR effect size for
'Probable serine carboxypeptidase CPVL' on 'eGFR'

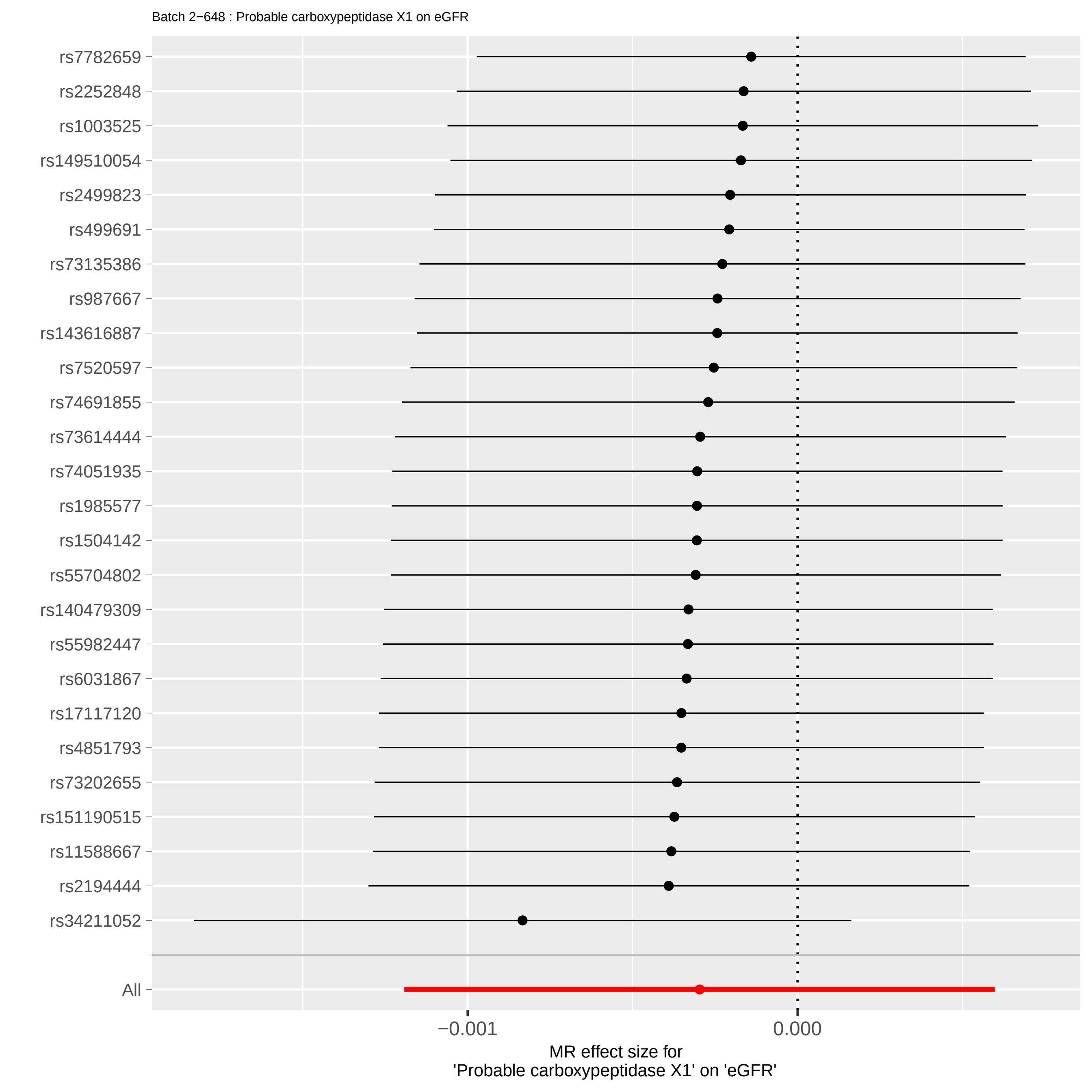
Batch 2−648 : Probable carboxypeptidase X1 on eGFR
rs7782659
rs2252848
rs1003525
rs149510054
rs2499823
rs499691
rs73135386
rs987667
rs143616887
rs7520597
rs74691855
rs73614444
rs74051935
rs1985577
rs1504142
rs55704802
rs140479309
rs55982447
rs6031867
rs17117120
rs4851793
rs73202655
rs151190515
rs11588667
rs2194444
rs34211052
All
−0.001
0.000
MR effect size for
'Probable carboxypeptidase X1' on 'eGFR'

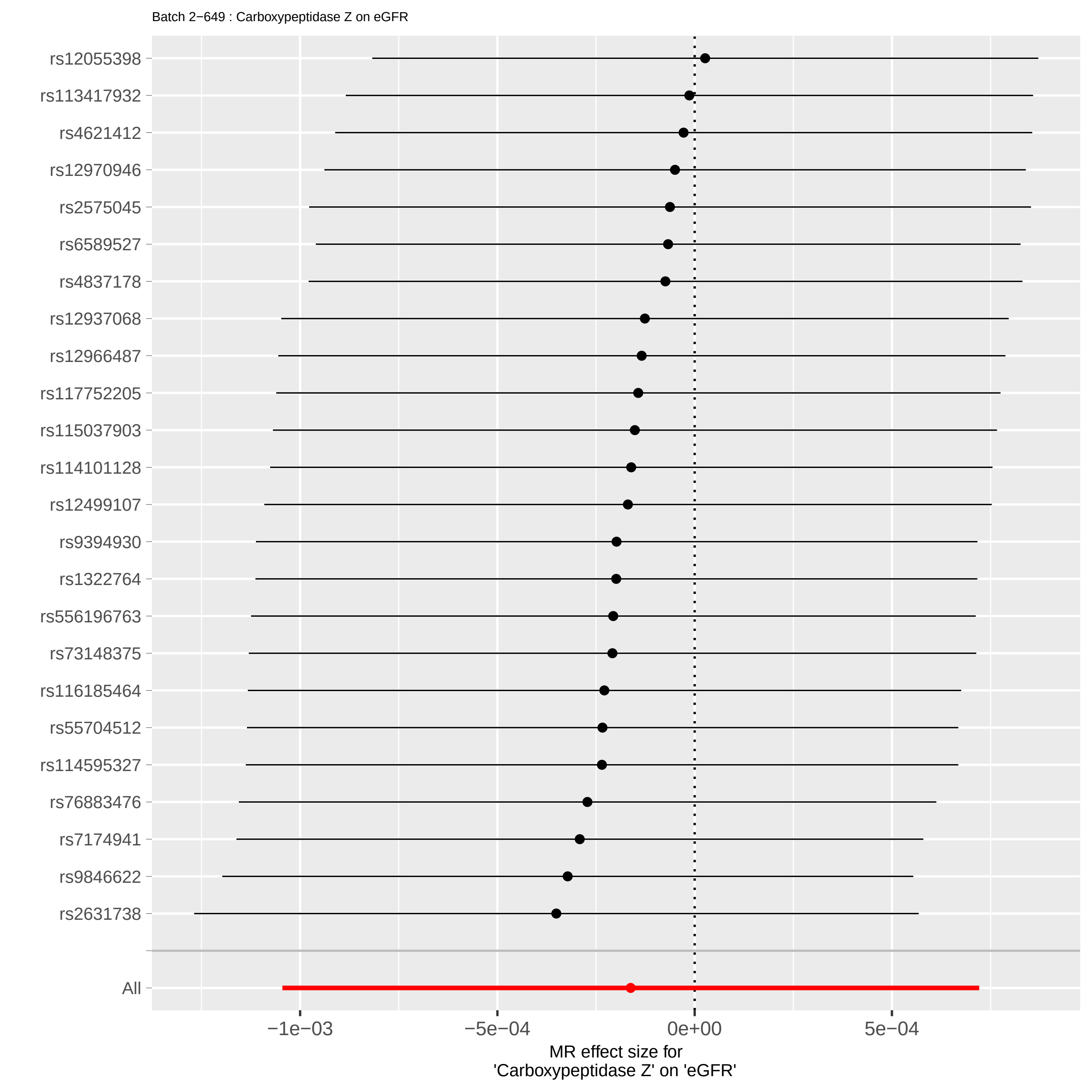

Batch 2−649 : Carboxypeptidase Z on eGFR
rs12055398
rs113417932
rs4621412
rs12970946
rs2575045
rs6589527
rs4837178
rs12937068
rs12966487
rs117752205
rs115037903
rs114101128
rs12499107
rs9394930
rs1322764
rs556196763
rs73148375
rs116185464
rs55704512
rs114595327
rs76883476
rs7174941
rs9846622
rs2631738
All
−1e−03
−5e−04
0e+00
5e−04
MR effect size for
'Carboxypeptidase Z' on 'eGFR'

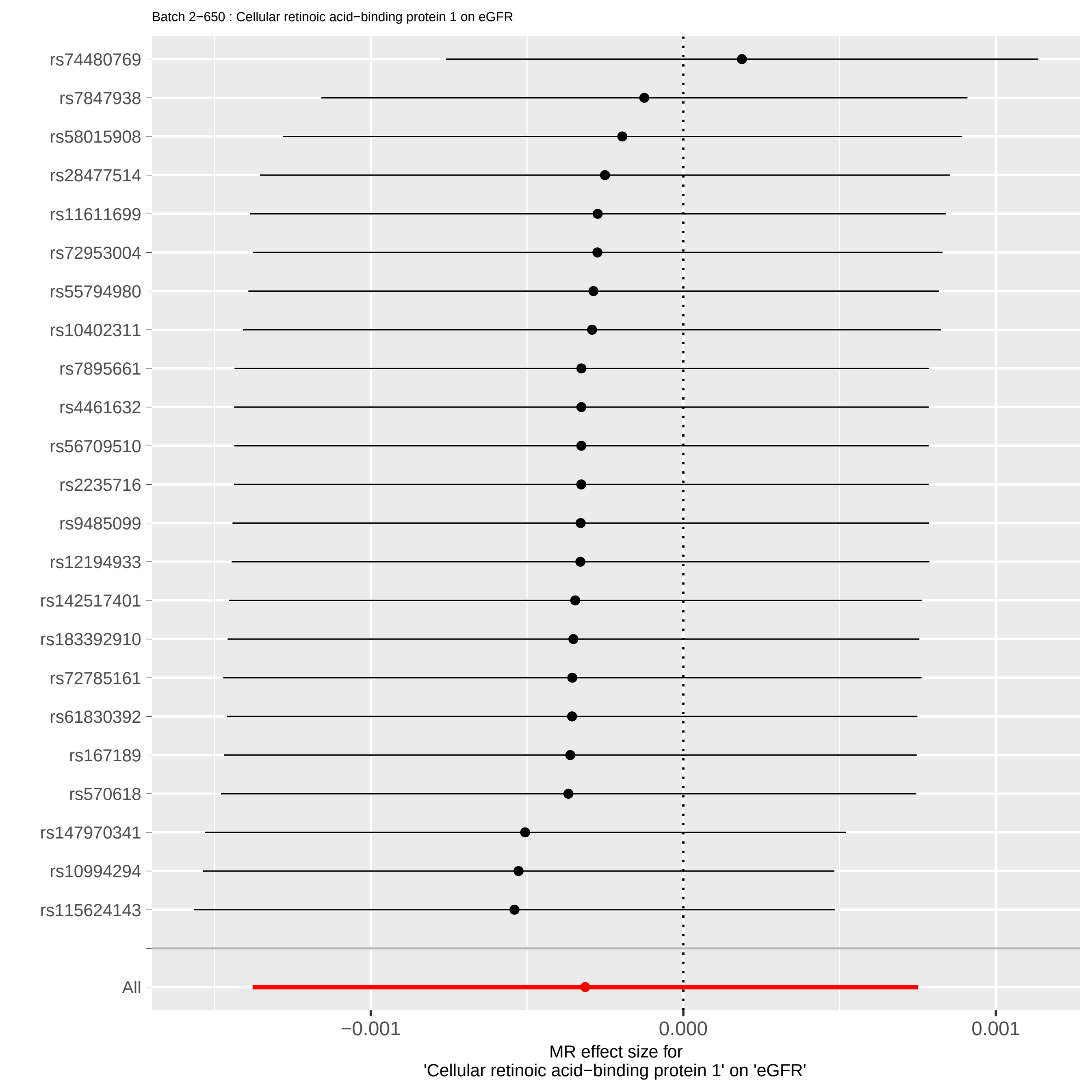

Batch 2−650 : Cellular retinoic acid−binding protein 1 on eGFR
rs74480769
rs7847938
rs58015908
rs28477514
rs11611699
rs72953004
rs55794980
rs10402311
rs7895661
rs4461632
rs56709510
rs2235716
rs9485099
rs12194933
rs142517401
rs183392910
rs72785161
rs61830392
rs167189
rs570618
rs147970341
rs10994294
rs115624143
All
−0.001
0.000
0.001
MR effect size for
'Cellular retinoic acid−binding protein 1' on 'eGFR'

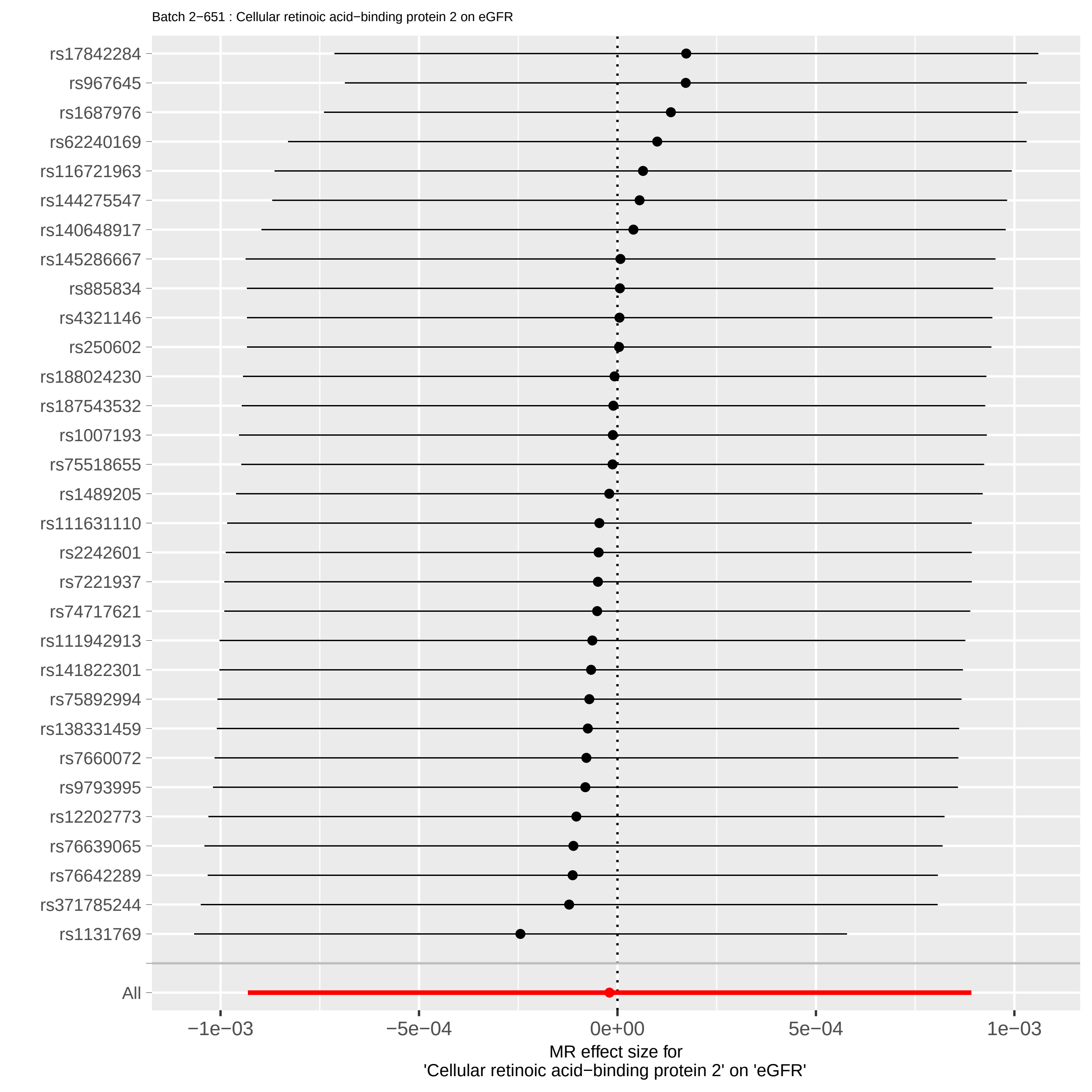
Batch 2−651 : Cellular retinoic acid−binding protein 2 on eGFR
rs17842284
rs967645
rs1687976
rs62240169
rs116721963
rs144275547
rs140648917
rs145286667
rs885834
rs4321146
rs250602
rs188024230
rs187543532
rs1007193
rs75518655
rs1489205
rs111631110
rs2242601
rs7221937
rs74717621
rs111942913
rs141822301
rs75892994
rs138331459
rs7660072
rs9793995
rs12202773
rs76639065
rs76642289
rs371785244
rs1131769
All
−1e−03
−5e−04
0e+00
5e−04
1e−03
MR effect size for
'Cellular retinoic acid−binding protein 2' on 'eGFR'

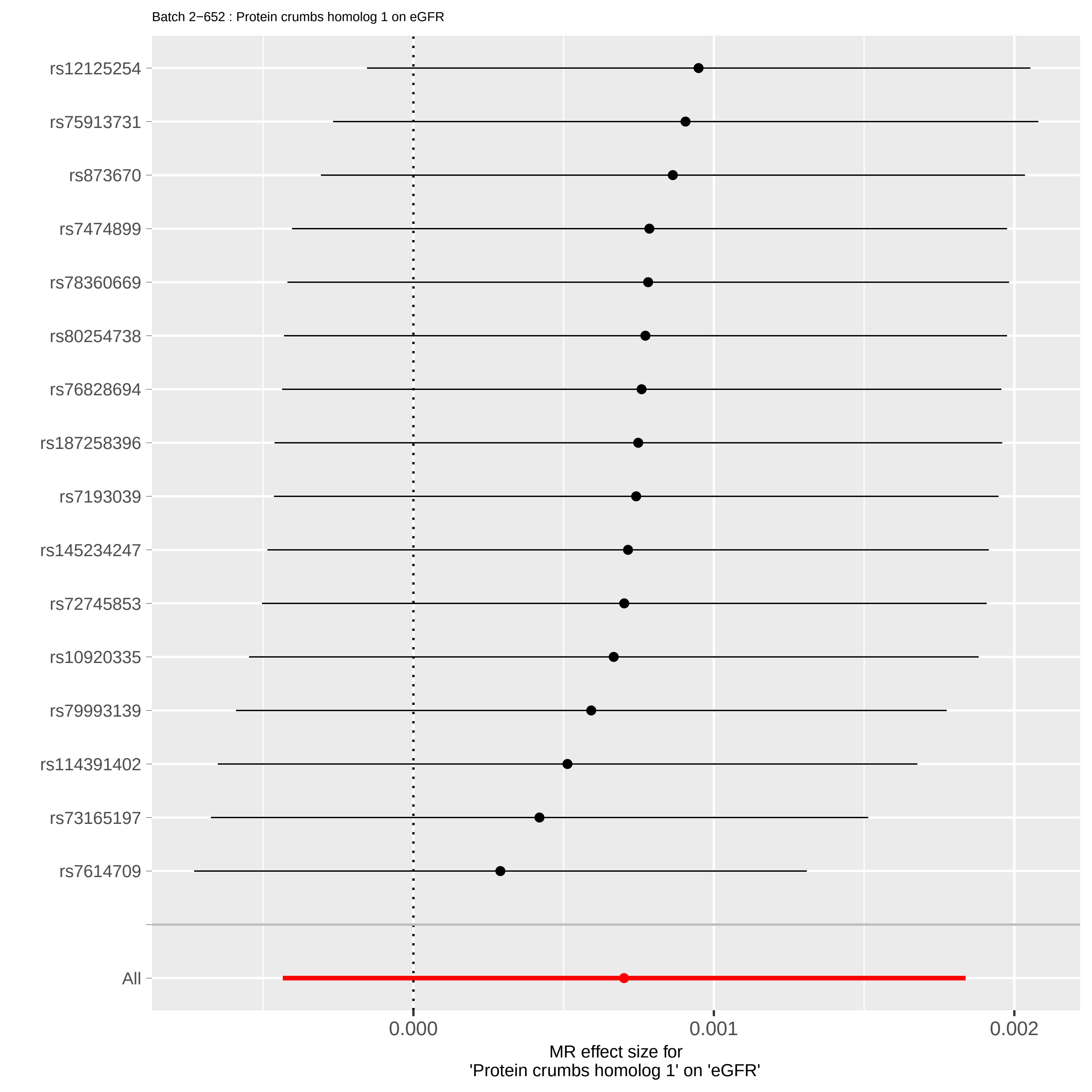

Batch 2−652 : Protein crumbs homolog 1 on eGFR
rs12125254
rs75913731
rs873670
rs7474899
rs78360669
rs80254738
rs76828694
rs187258396
rs7193039
rs145234247
rs72745853
rs10920335
rs79993139
rs114391402
rs73165197
rs7614709
All
0.000
0.001
0.002
MR effect size for
'Protein crumbs homolog 1' on 'eGFR'

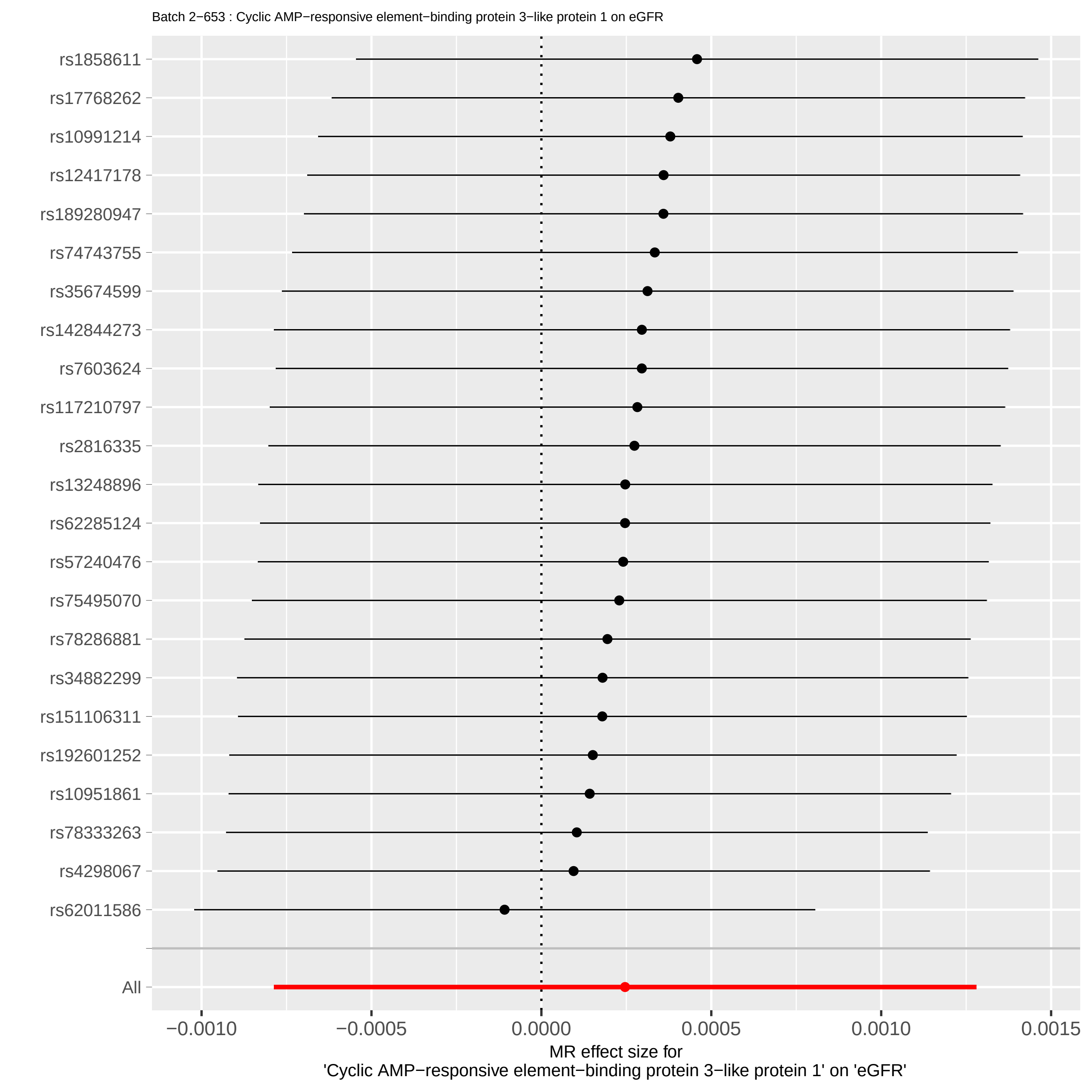

Batch 2−653 : Cyclic AMP−responsive element−binding protein 3−like protein 1 on eGFR
rs1858611
rs17768262
rs10991214
rs12417178
rs189280947
rs74743755
rs35674599
rs142844273
rs7603624
rs117210797
rs2816335
rs13248896
rs62285124
rs57240476
rs75495070
rs78286881
rs34882299
rs151106311
rs192601252
rs10951861
rs78333263
rs4298067
rs62011586
All
−0.0010
−0.0005
0.0000
0.0005
0.0010
0.0015
MR effect size for
'Cyclic AMP−responsive element−binding protein 3−like protein 1' on 'eGFR'

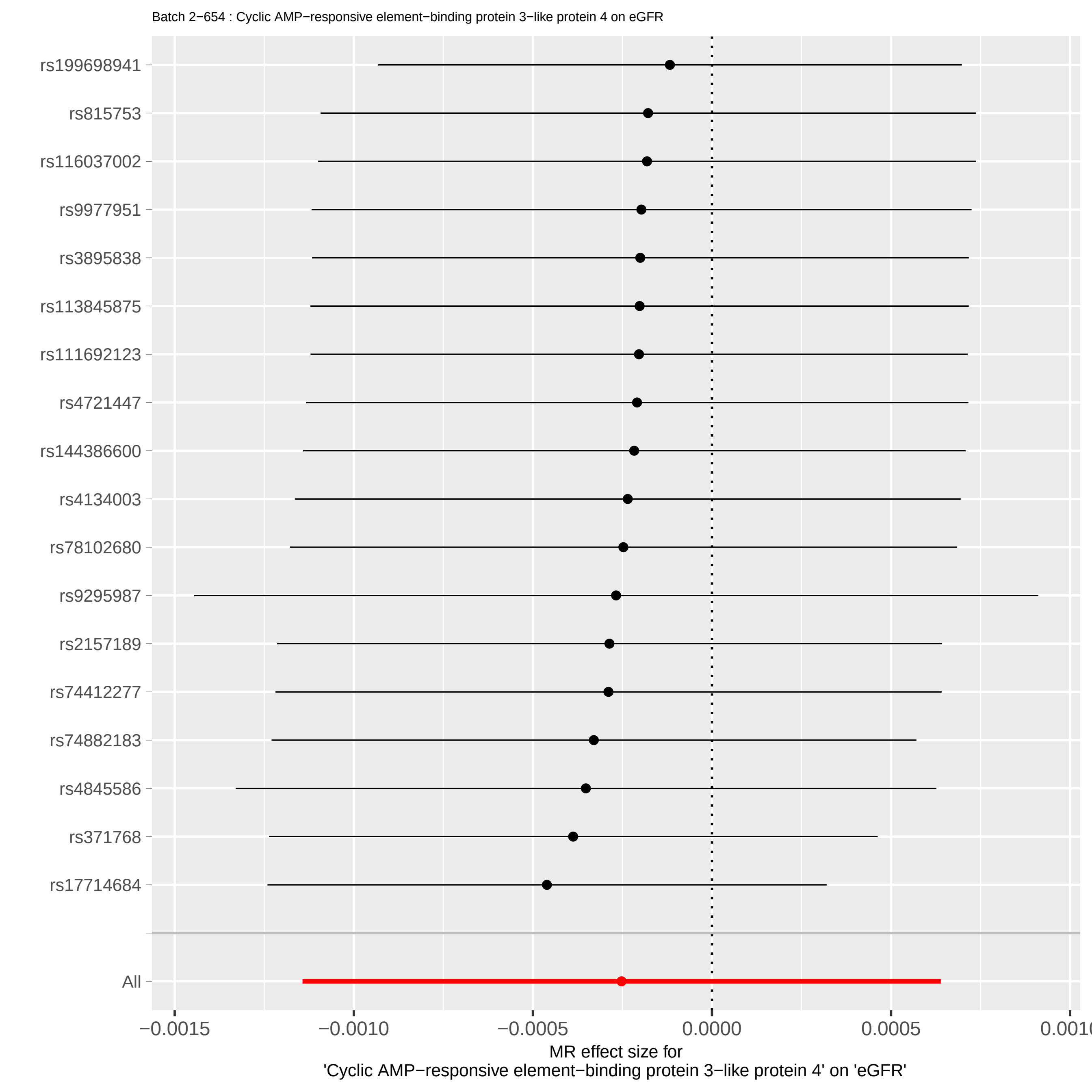

Batch 2−654 : Cyclic AMP−responsive element−binding protein 3−like protein 4 on eGFR
rs199698941
rs815753
rs116037002
rs9977951
rs3895838
rs113845875
rs111692123
rs4721447
rs144386600
rs4134003
rs78102680
rs9295987
rs2157189
rs74412277
rs74882183
rs4845586
rs371768
rs17714684
All
−0.0015
−0.0010
−0.0005
0.0000
0.0005
MR effect size for
'Cyclic AMP−responsive element−binding protein 3−like protein 4' on 'eGFR'

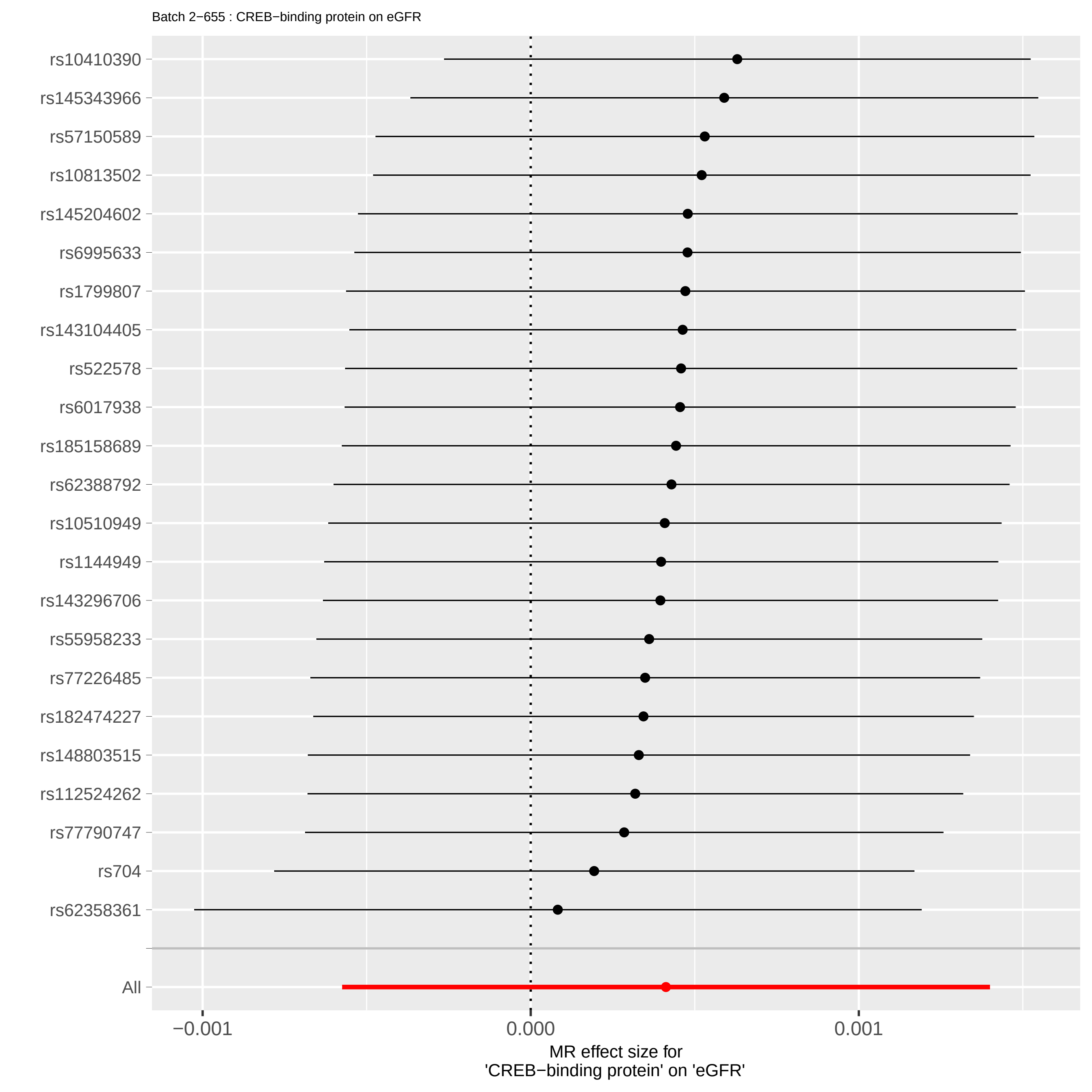

Batch 2−655 : CREB−binding protein on eGFR
rs10410390
rs145343966
rs57150589
rs10813502
rs145204602
rs6995633
rs1799807
rs143104405
rs522578
rs6017938
rs185158689
rs62388792
rs10510949
rs1144949
rs143296706
rs55958233
rs77226485
rs182474227
rs148803515
rs112524262
rs77790747
rs704
rs62358361
All
−0.001
0.000
0.001
MR effect size for
'CREB−binding protein' on 'eGFR'

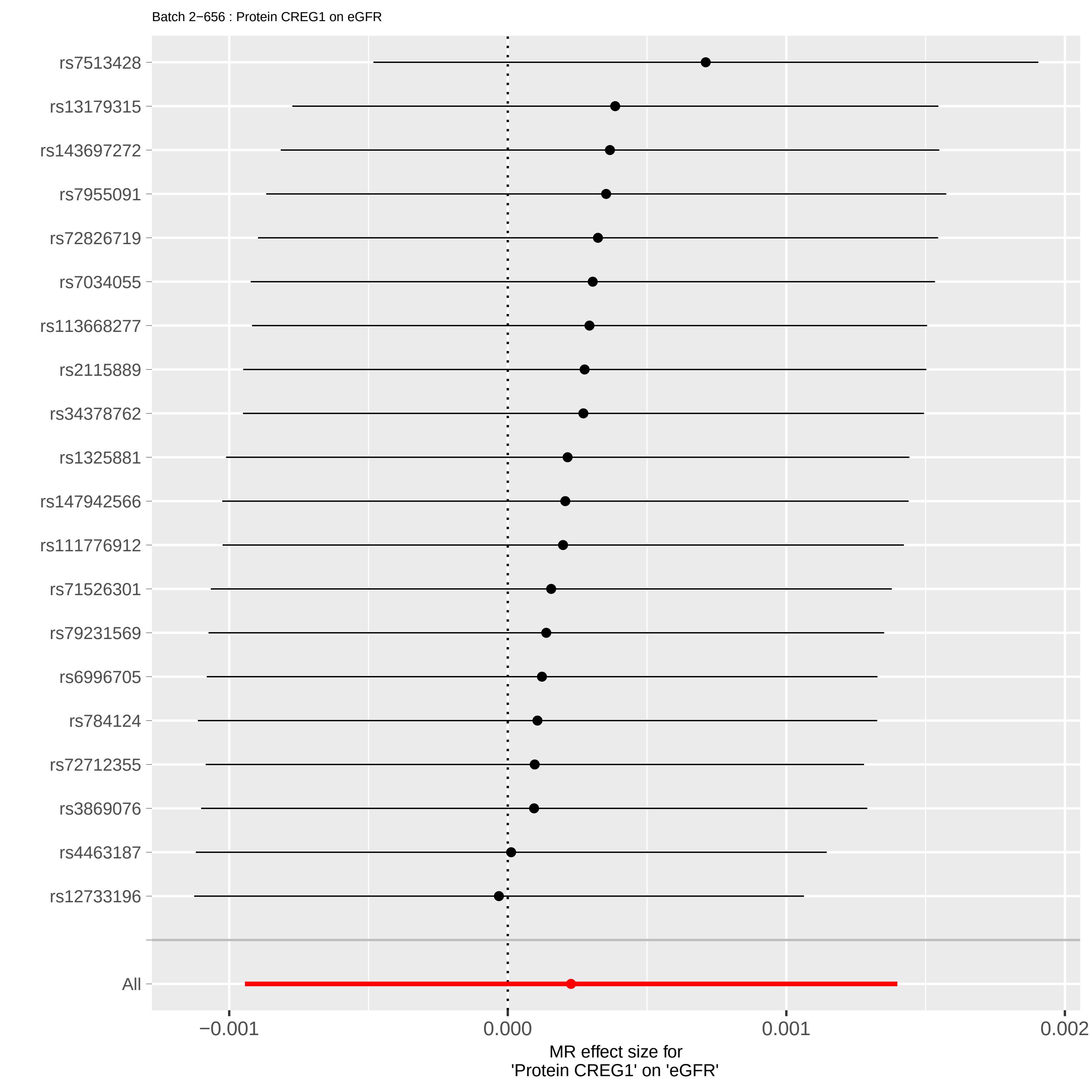
Batch 2−656 : Protein CREG1 on eGFR
rs7513428
rs13179315
rs143697272
rs7955091
rs72826719
rs7034055
rs113668277
rs2115889
rs34378762
rs1325881
rs147942566
rs111776912
rs71526301
rs79231569
rs6996705
rs784124
rs72712355
rs3869076
rs4463187
rs12733196
All
−0.001
0.000
0.001
0.002
MR effect size for
'Protein CREG1' on 'eGFR'

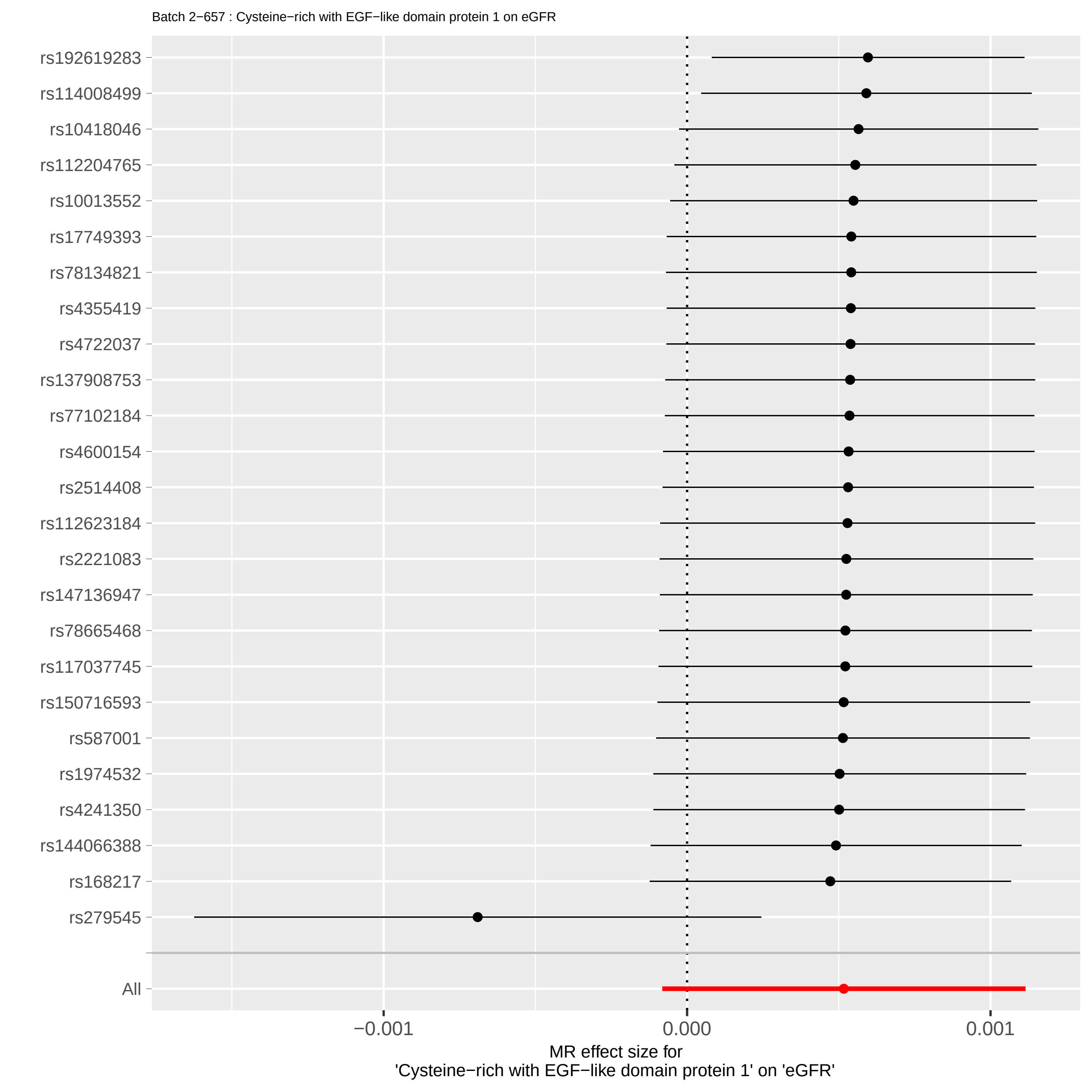
Batch 2−657 : Cysteine−rich with EGF−like domain protein 1 on eGFR
rs192619283
rs114008499
rs10418046
rs112204765
rs10013552
rs17749393
rs78134821
rs4355419
rs4722037
rs137908753
rs77102184
rs4600154
rs2514408
rs112623184
rs2221083
rs147136947
rs78665468
rs117037745
rs150716593
rs587001
rs1974532
rs4241350
rs144066388
rs168217
rs279545
All
−0.001
0.000
0.001
MR effect size for
'Cysteine−rich with EGF−like domain protein 1' on 'eGFR'

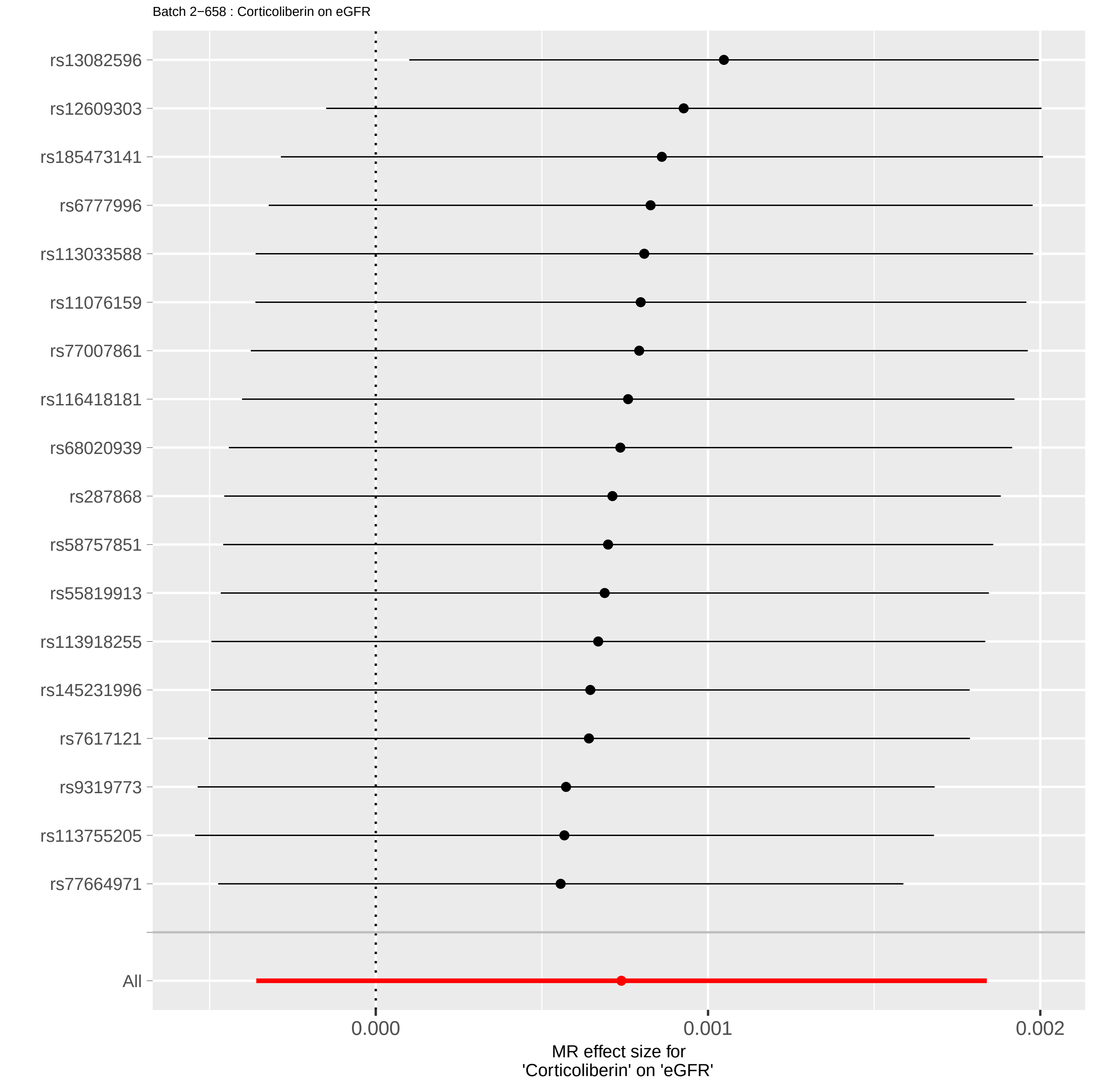
Batch 2−658 : Corticoliberin on eGFR
rs13082596
rs12609303
rs185473141
rs6777996
rs113033588
rs11076159
rs77007861
rs116418181
rs68020939
rs287868
rs58757851
rs55819913
rs113918255
rs145231996
rs7617121
rs9319773
rs113755205
rs77664971
All
0.000
0.001
0.002
MR effect size for
'Corticoliberin' on 'eGFR'

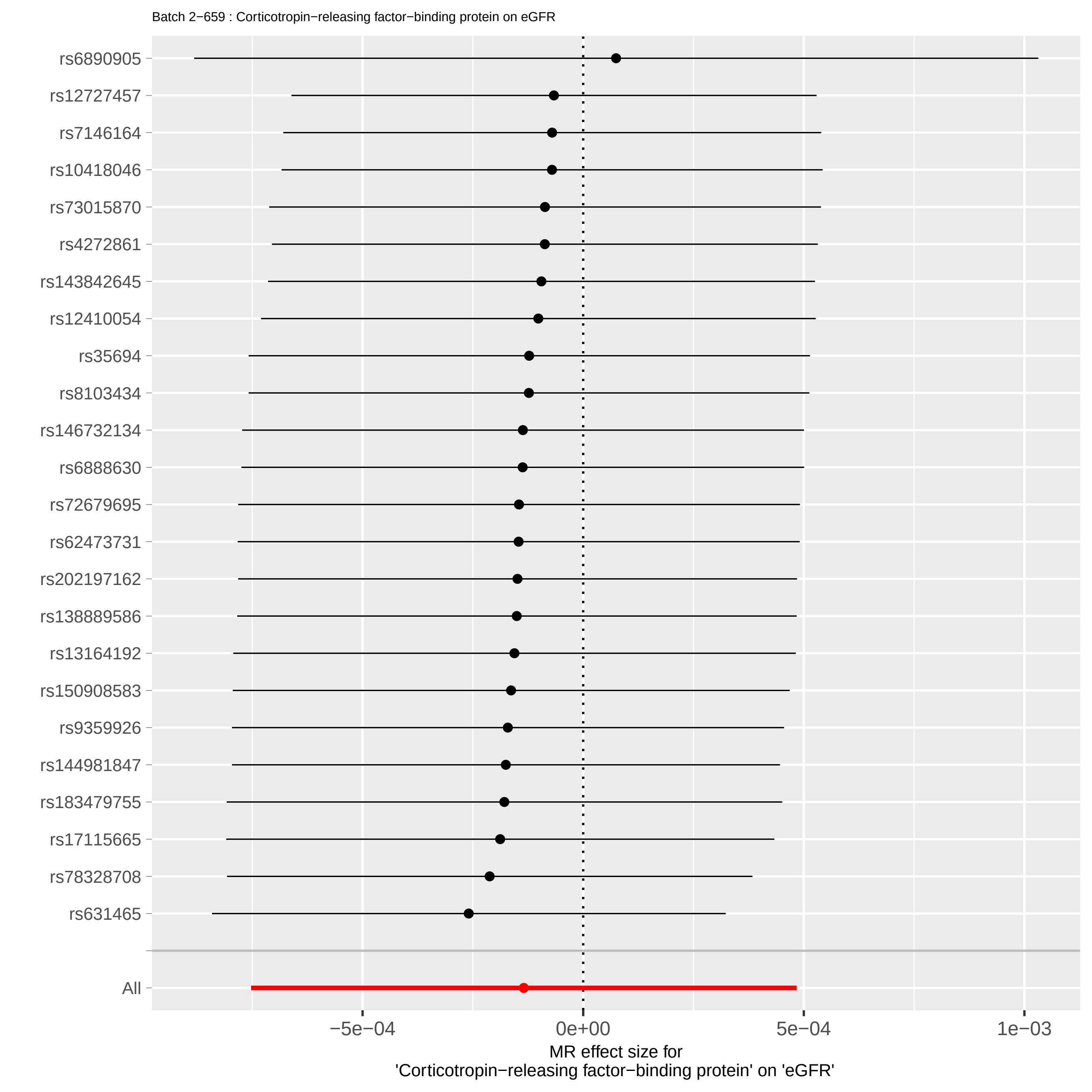

Batch 2−659 : Corticotropin−releasing factor−binding protein on eGFR
rs6890905
rs12727457
rs7146164
rs10418046
rs73015870
rs4272861
rs143842645
rs12410054
rs35694
rs8103434
rs146732134
rs6888630
rs72679695
rs62473731
rs202197162
rs138889586
rs13164192
rs150908583
rs9359926
rs144981847
rs183479755
rs17115665
rs78328708
rs631465
All
−5e−04
0e+00
5e−04
1e−03
MR effect size for
'Corticotropin−releasing factor−binding protein' on 'eGFR'

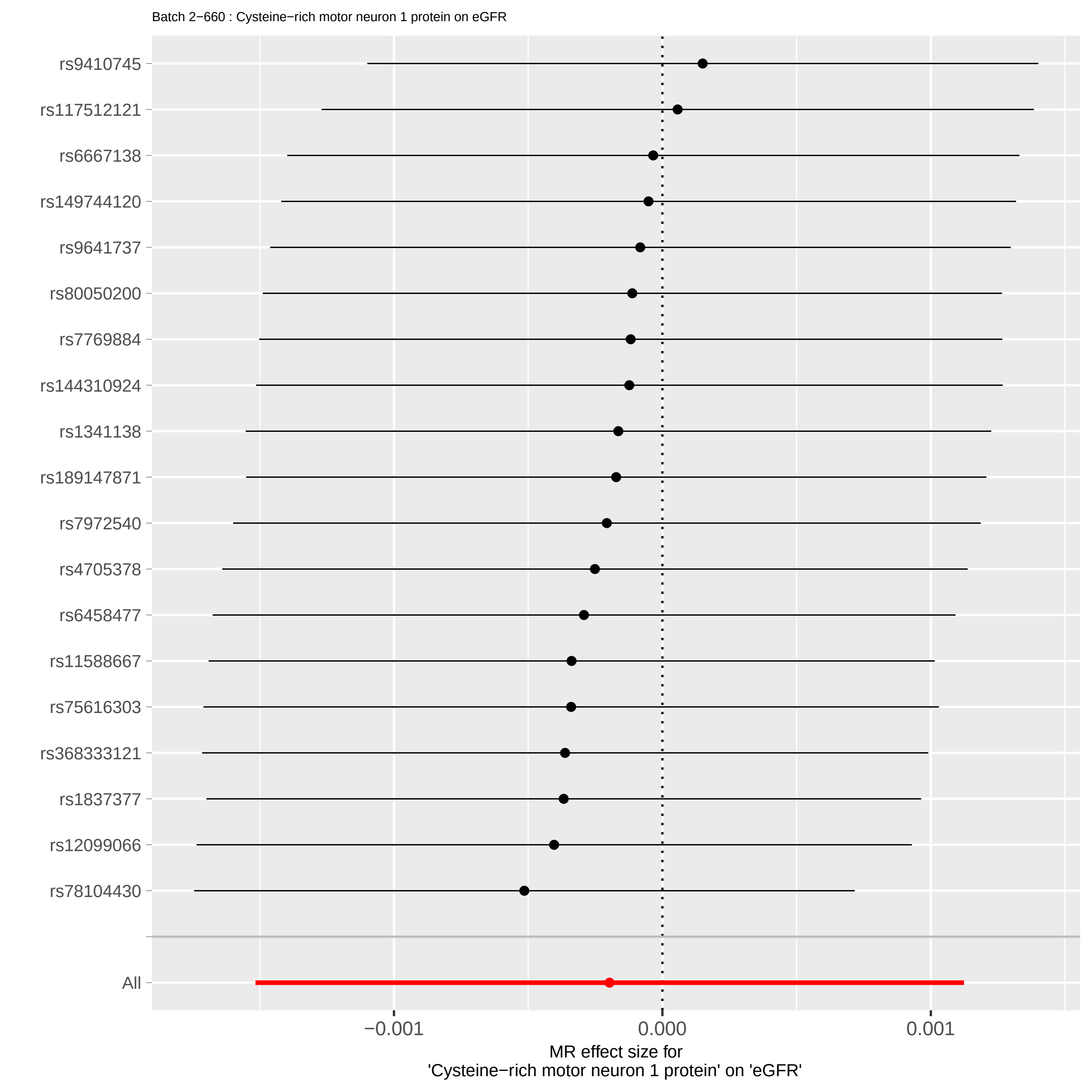

Batch 2−660 : Cysteine−rich motor neuron 1 protein on eGFR
rs9410745
rs117512121
rs6667138
rs149744120
rs9641737
rs80050200
rs7769884
rs144310924
rs1341138
rs189147871
rs7972540
rs4705378
rs6458477
rs11588667
rs75616303
rs368333121
rs1837377
rs12099066
rs78104430
All
−0.001
0.000
0.001
MR effect size for
'Cysteine−rich motor neuron 1 protein' on 'eGFR'

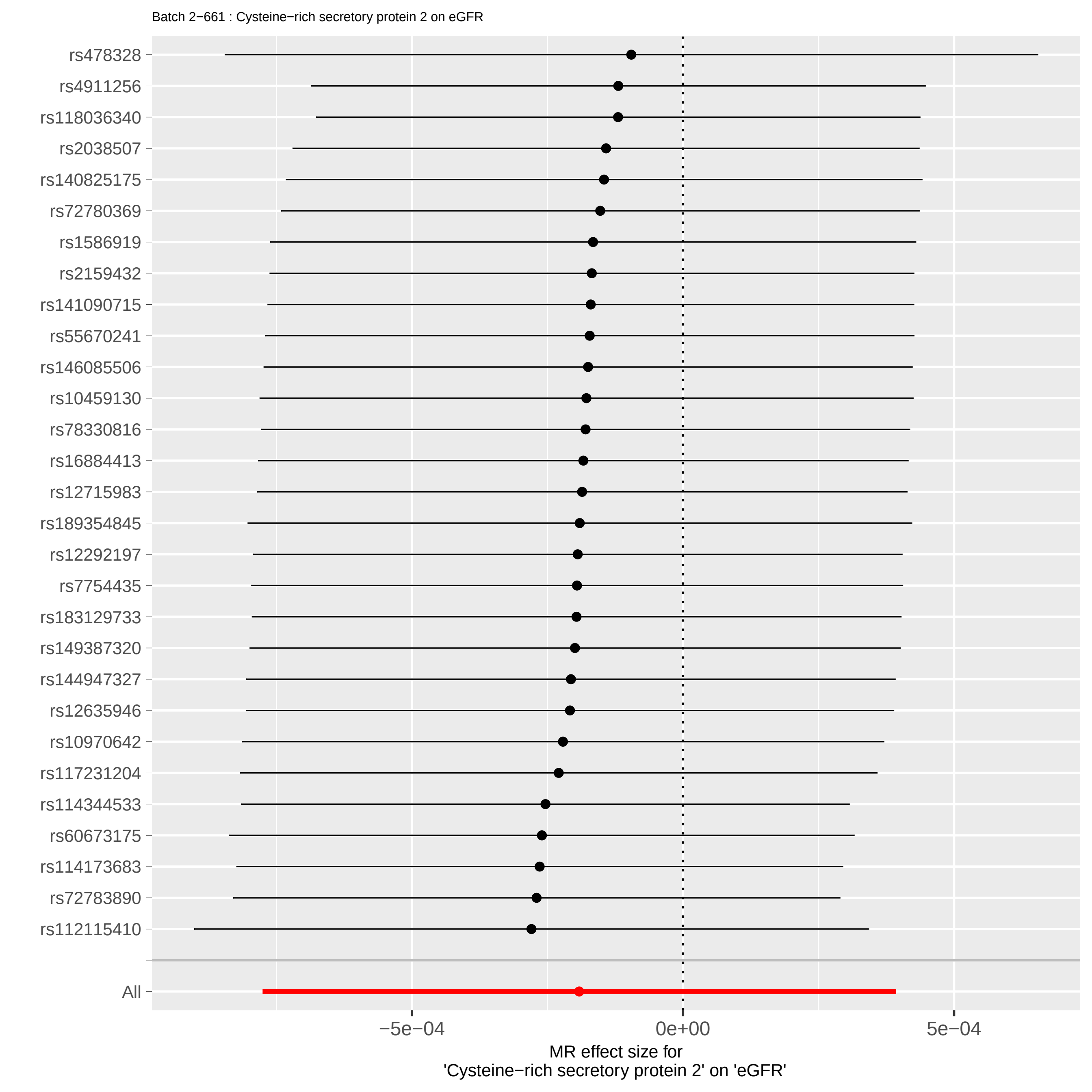
Batch 2−661 : Cysteine−rich secretory protein 2 on eGFR
rs478328
rs4911256
rs118036340
rs2038507
rs140825175
rs72780369
rs1586919
rs2159432
rs141090715
rs55670241
rs146085506
rs10459130
rs78330816
rs16884413
rs12715983
rs189354845
rs12292197
rs7754435
rs183129733
rs149387320
rs144947327
rs12635946
rs10970642
rs117231204
rs114344533
rs60673175
rs114173683
rs72783890
rs112115410
All
−5e−04
0e+00
5e−04
MR effect size for
'Cysteine−rich secretory protein 2' on 'eGFR'

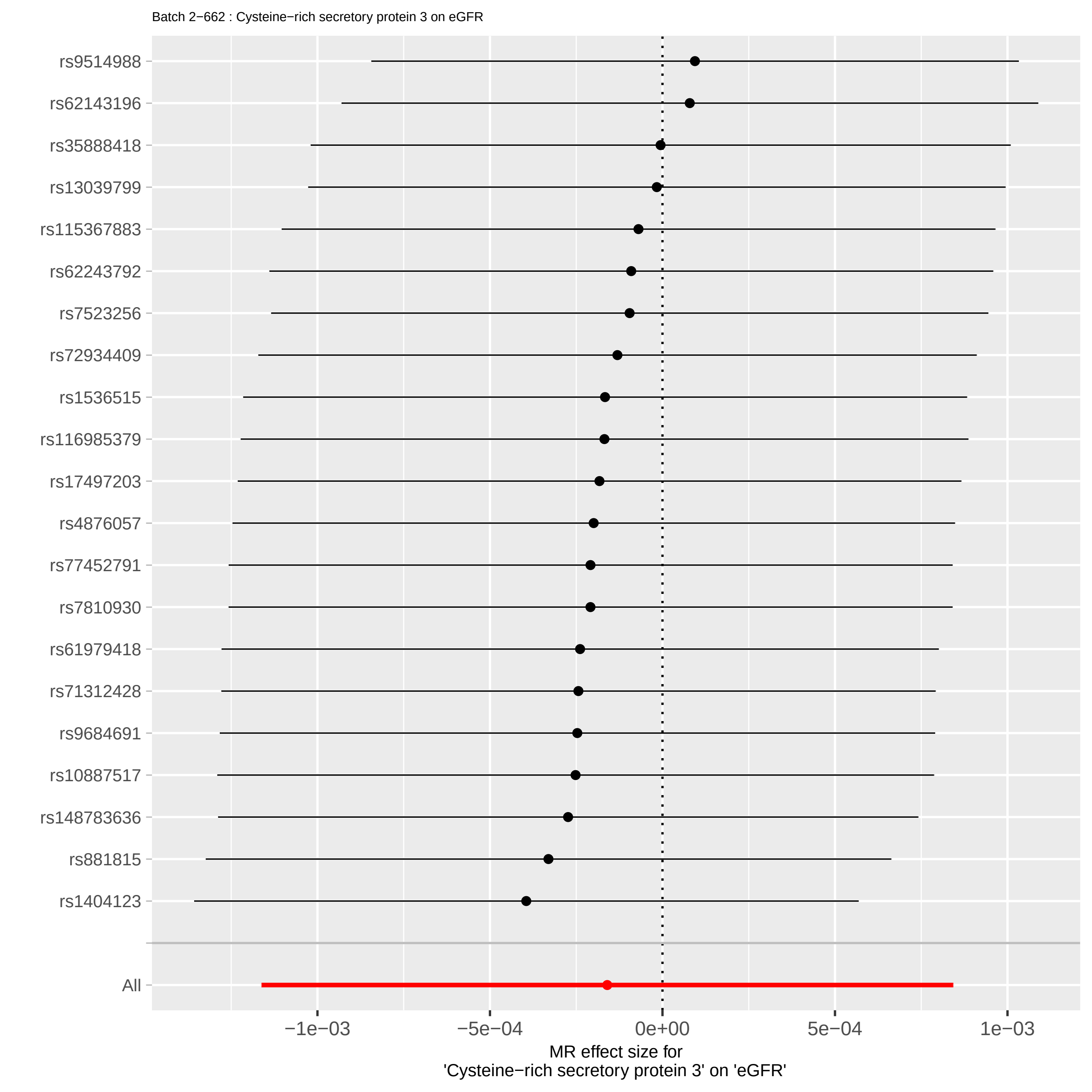
Batch 2−662 : Cysteine−rich secretory protein 3 on eGFR
rs9514988
rs62143196
rs35888418
rs13039799
rs115367883
rs62243792
rs7523256
rs72934409
rs1536515
rs116985379
rs17497203
rs4876057
rs77452791
rs7810930
rs61979418
rs71312428
rs9684691
rs10887517
rs148783636
rs881815
rs1404123
All
−1e−03
−5e−04
0e+00
5e−04
1e−03
MR effect size for
'Cysteine−rich secretory protein 3' on 'eGFR'

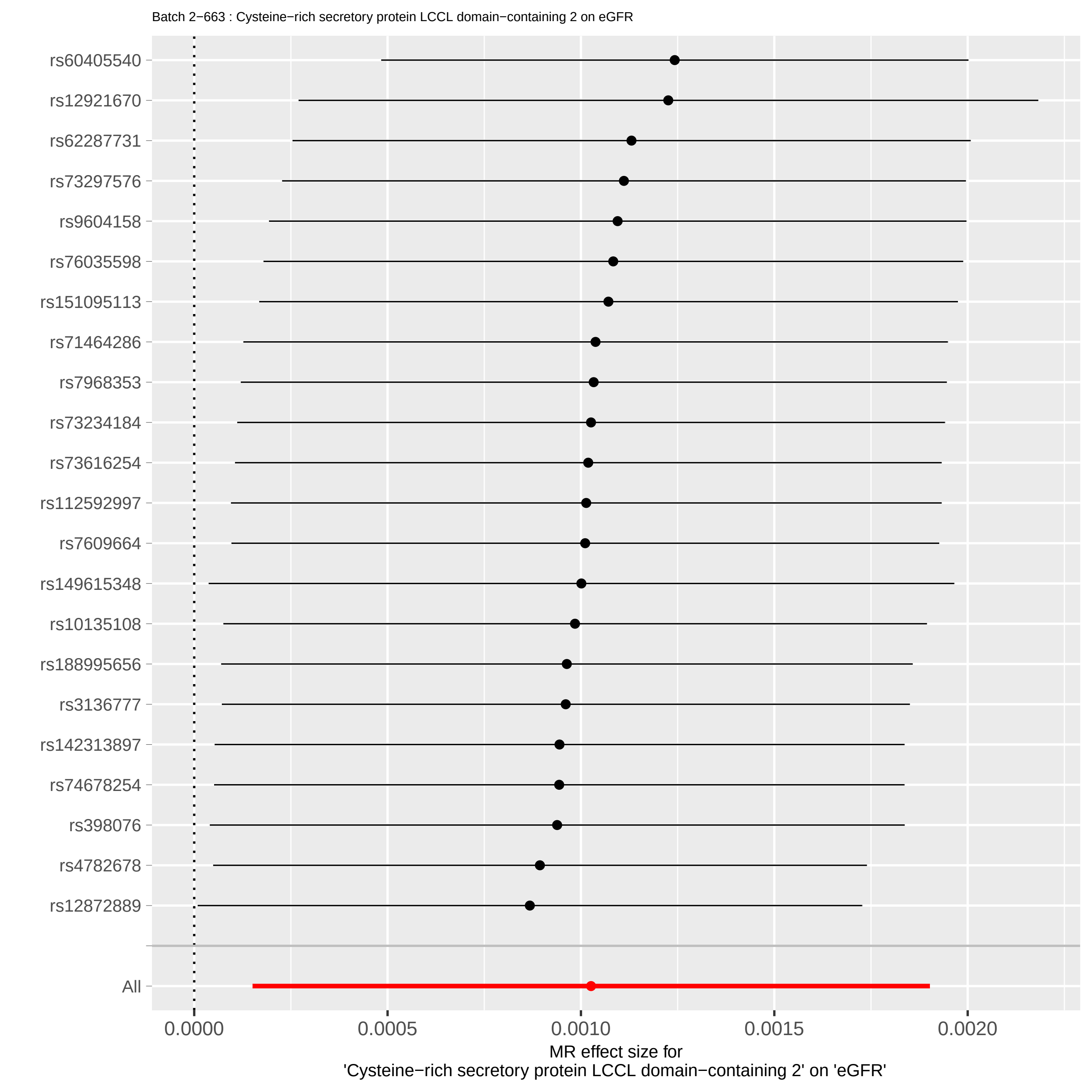

Batch 2−663 : Cysteine−rich secretory protein LCCL domain−containing 2 on eGFR
rs60405540
rs12921670
rs62287731
rs73297576
rs9604158
rs76035598
rs151095113
rs71464286
rs7968353
rs73234184
rs73616254
rs112592997
rs7609664
rs149615348
rs10135108
rs188995656
rs3136777
rs142313897
rs74678254
rs398076
rs4782678
rs12872889
All
0.0000
0.0005
0.0010
0.0015
0.0020
MR effect size for
'Cysteine−rich secretory protein LCCL domain−containing 2' on 'eGFR'

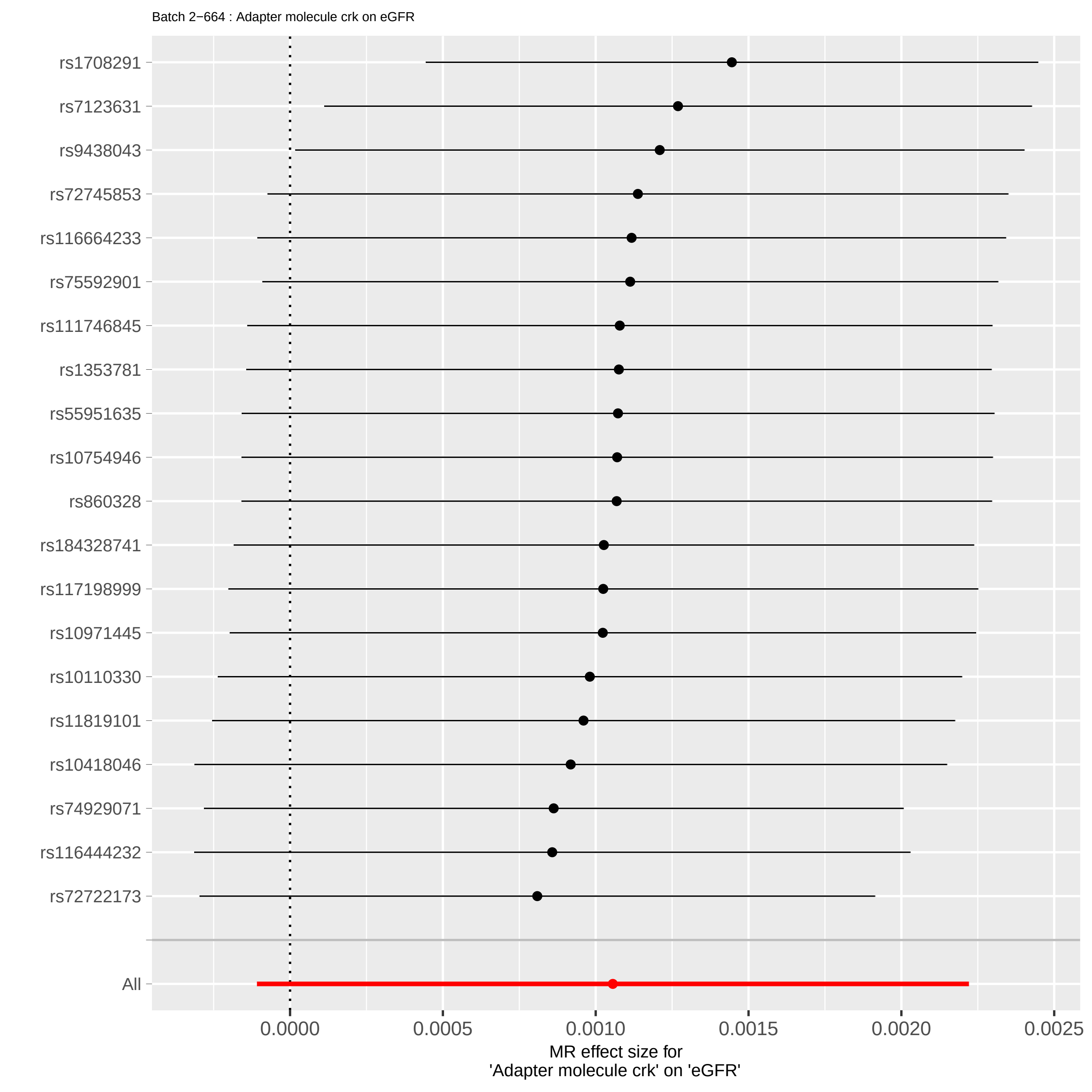

Batch 2−664 : Adapter molecule crk on eGFR
rs1708291
rs7123631
rs9438043
rs72745853
rs116664233
rs75592901
rs111746845
rs1353781
rs55951635
rs10754946
rs860328
rs184328741
rs117198999
rs10971445
rs10110330
rs11819101
rs10418046
rs74929071
rs116444232
rs72722173
All
0.0000
0.0005
0.0010
0.0015
0.0020
0.0025
MR effect size for
'Adapter molecule crk' on 'eGFR'

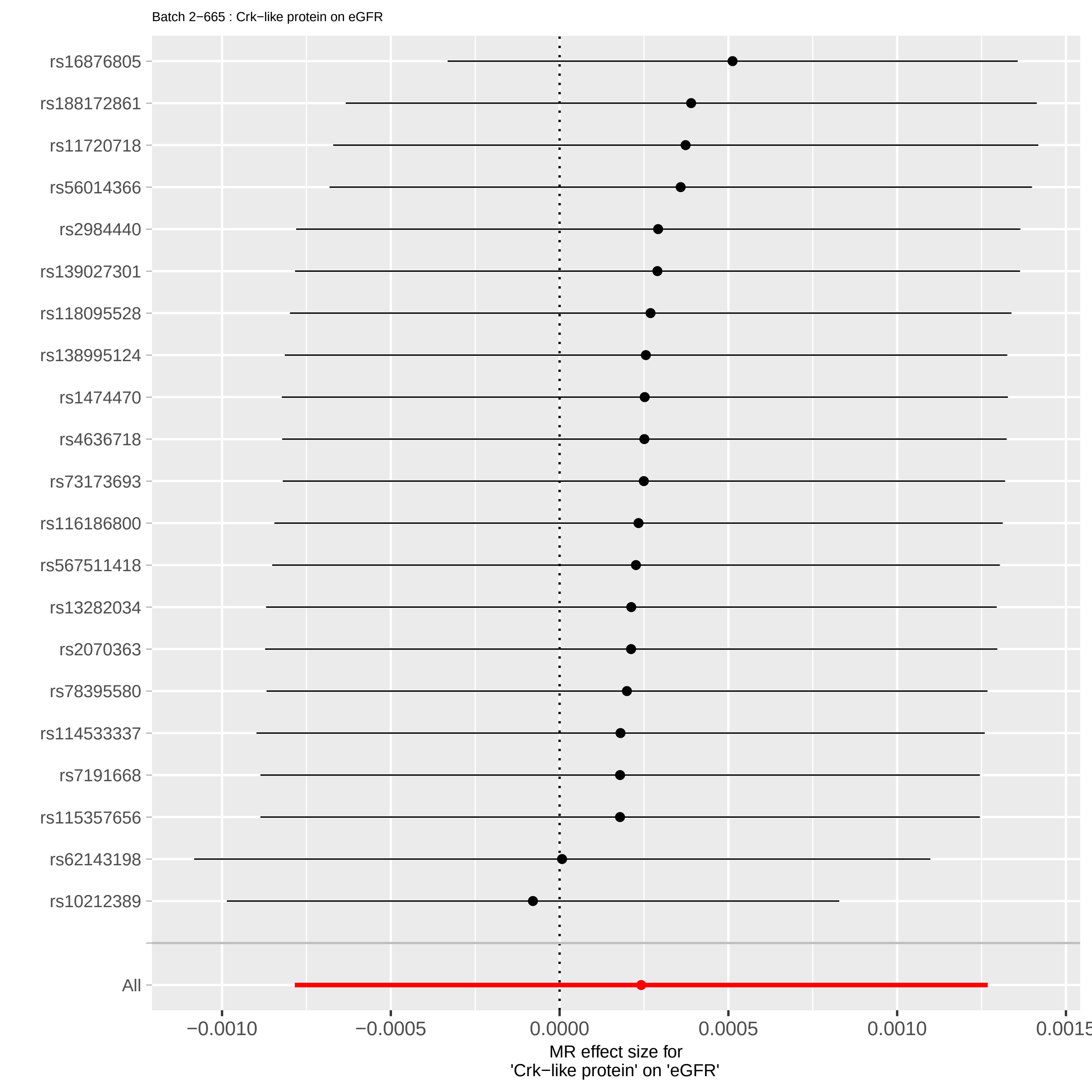

Batch 2−665 : Crk−like protein on eGFR
rs16876805
rs188172861
rs11720718
rs56014366
rs2984440
rs139027301
rs118095528
rs138995124
rs1474470
rs4636718
rs73173693
rs116186800
rs567511418
rs13282034
rs2070363
rs78395580
rs114533337
rs7191668
rs115357656
rs62143198
rs10212389
All
−0.0010
−0.0005
0.0000
0.0005
0.0010
MR effect size for
'Crk−like protein' on 'eGFR'

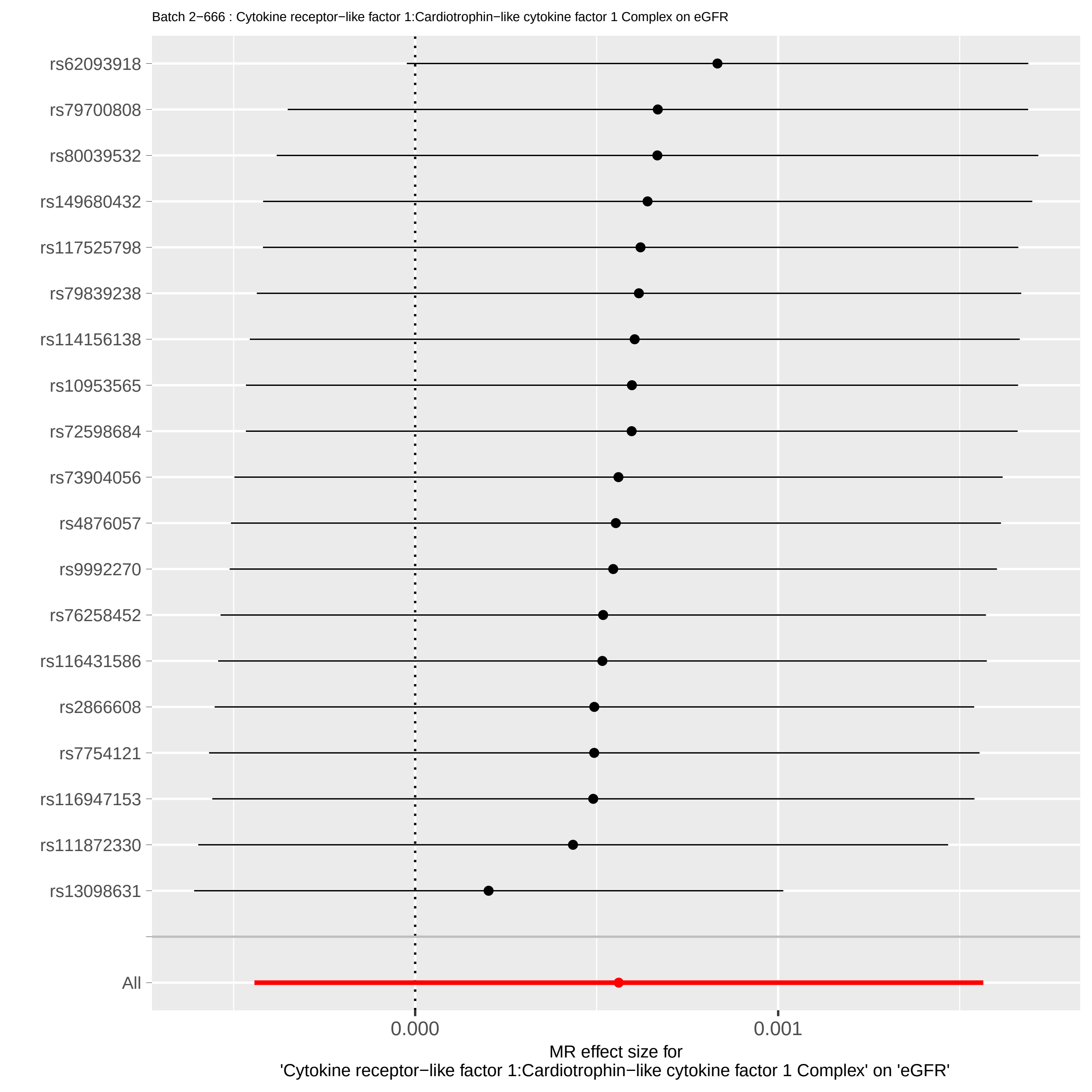

Batch 2−666 : Cytokine receptor−like factor 1:Cardiotrophin−like cytokine factor 1 Complex on eGFR
rs62093918
rs79700808
rs80039532
rs149680432
rs117525798
rs79839238
rs114156138
rs10953565
rs72598684
rs73904056
rs4876057
rs9992270
rs76258452
rs116431586
rs2866608
rs7754121
rs116947153
rs111872330
rs13098631
All
0.000
0.001
MR effect size for
'Cytokine receptor−like factor 1:Cardiotrophin−like cytokine factor 1 Complex' on 'eGFR'

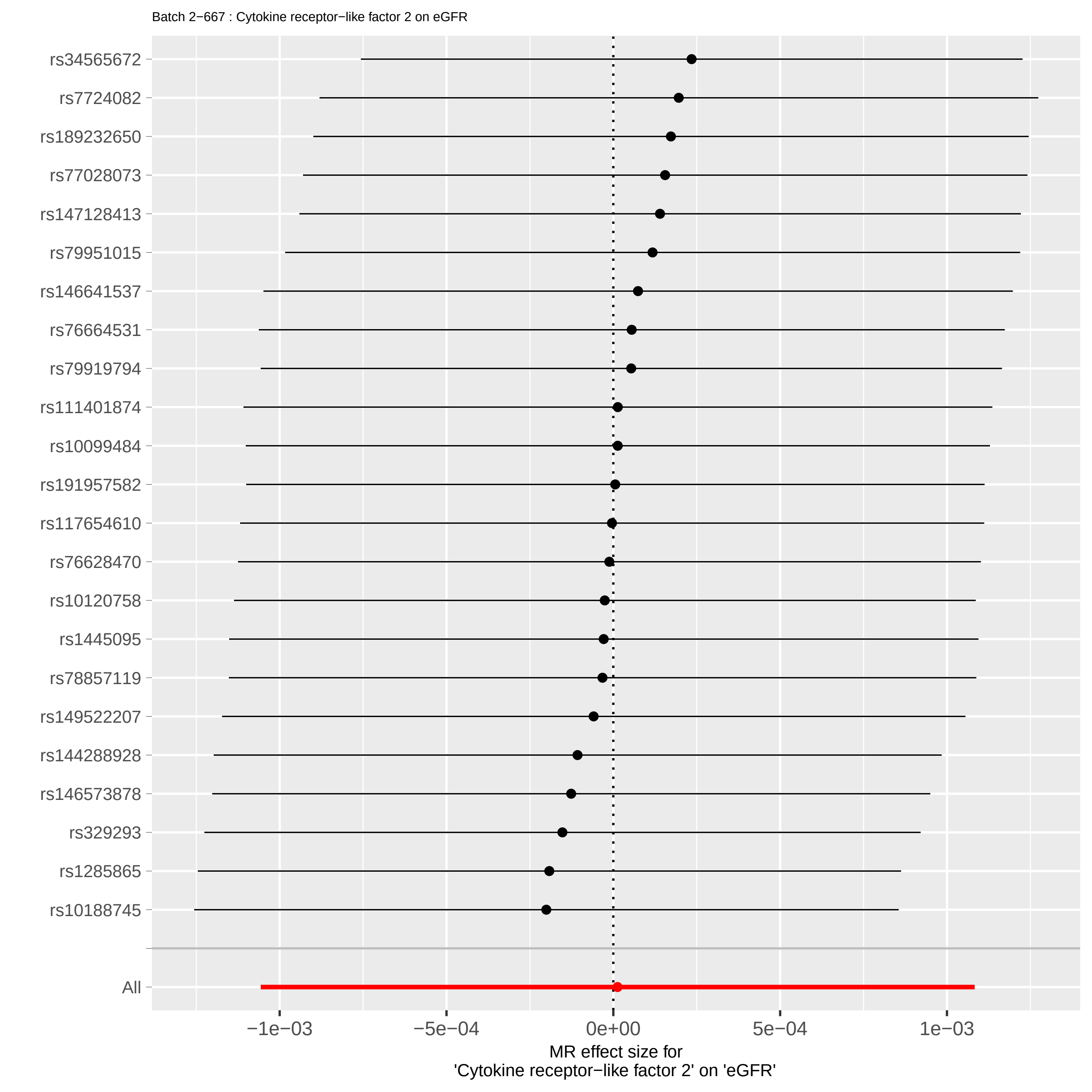

Batch 2−667 : Cytokine receptor−like factor 2 on eGFR
rs34565672
rs7724082
rs189232650
rs77028073
rs147128413
rs79951015
rs146641537
rs76664531
rs79919794
rs111401874
rs10099484
rs191957582
rs117654610
rs76628470
rs10120758
rs1445095
rs78857119
rs149522207
rs144288928
rs146573878
rs329293
rs1285865
rs10188745
All
−1e−03
−5e−04
0e+00
5e−04
1e−03
MR effect size for
'Cytokine receptor−like factor 2' on 'eGFR'

Batch 2−668 : Cytokine receptor−like factor 2 on eGFR

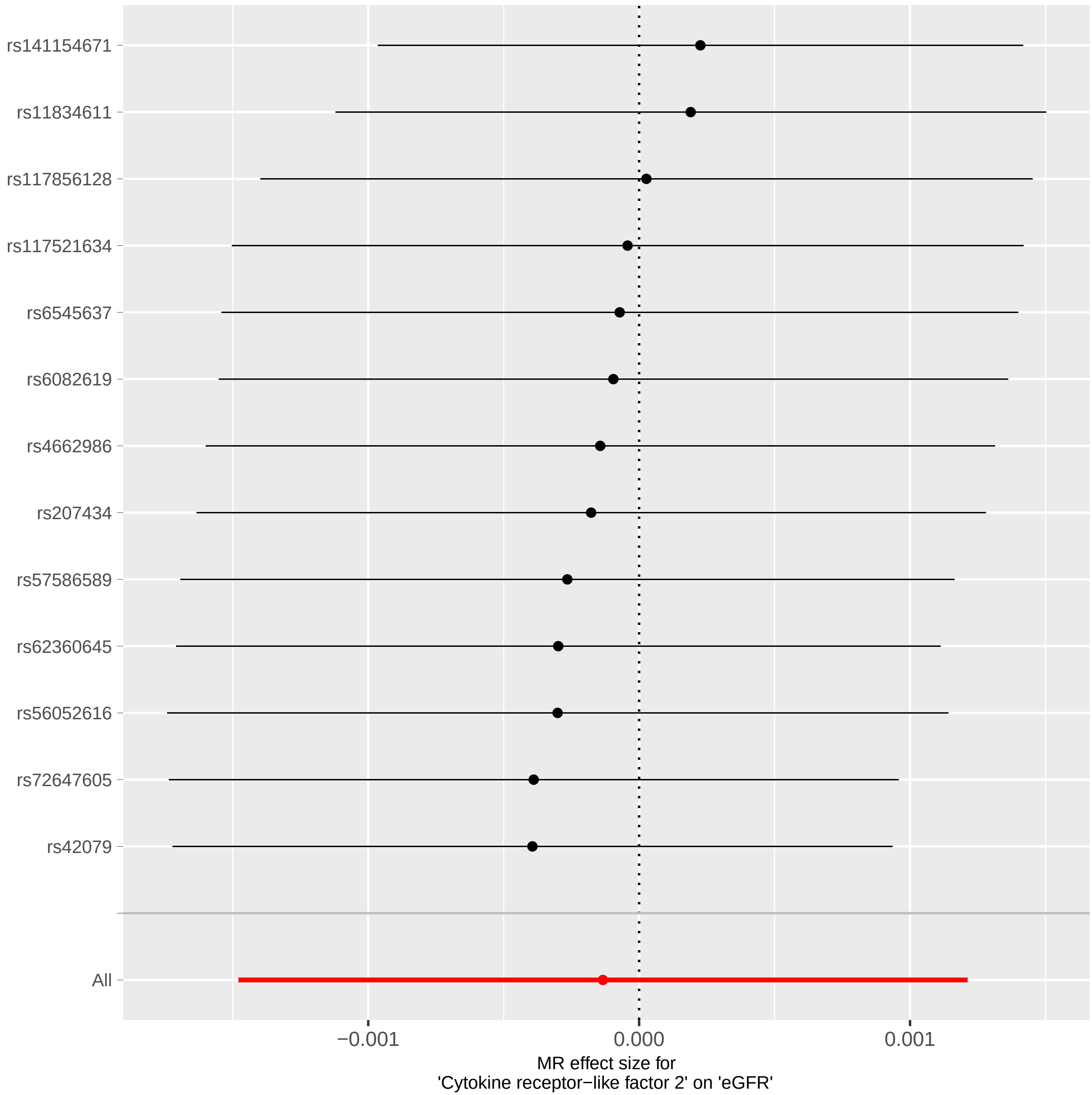

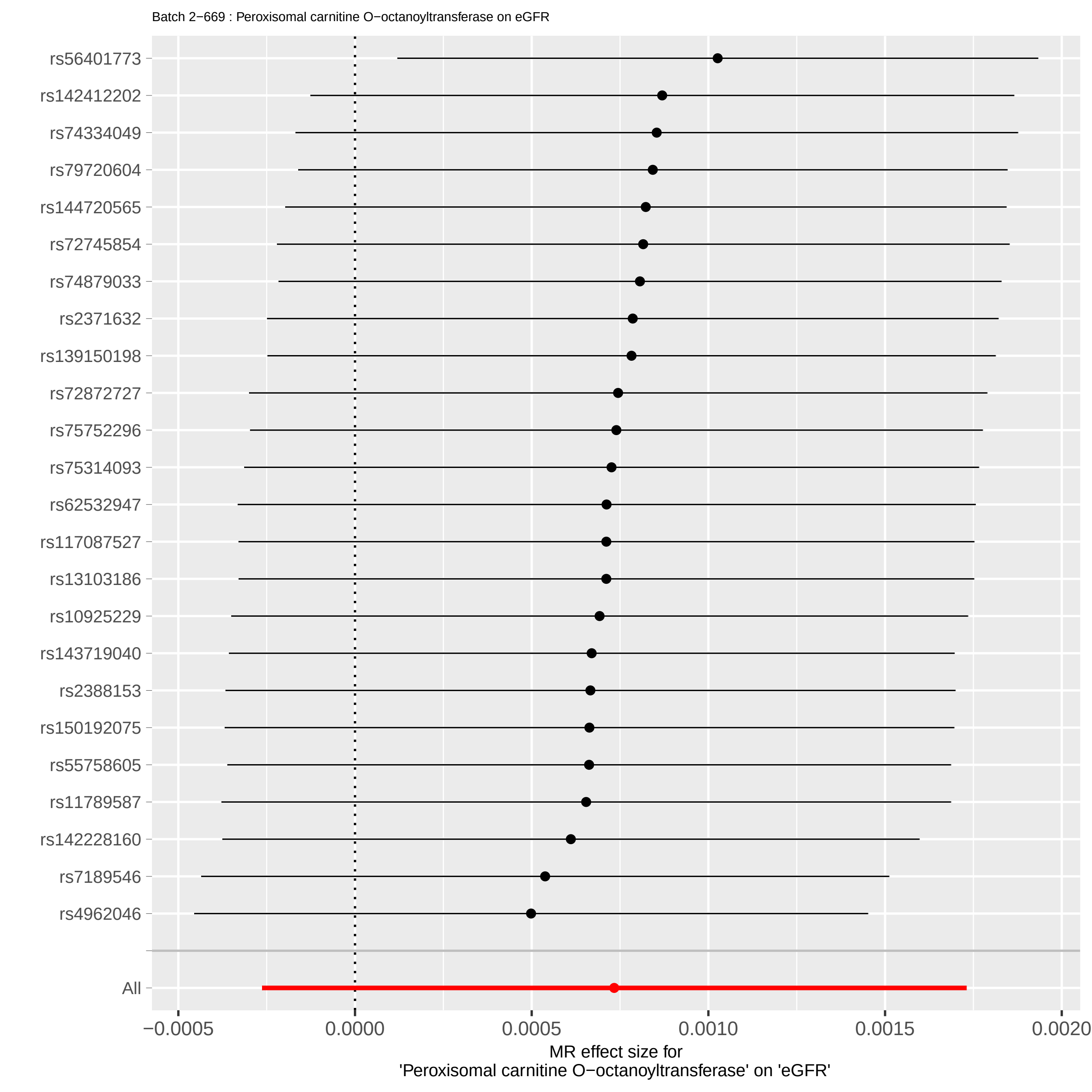

Batch 2−669 : Peroxisomal carnitine O−octanoyltransferase on eGFR
rs56401773
rs142412202
rs74334049
rs79720604
rs144720565
rs72745854
rs74879033
rs2371632
rs139150198
rs72872727
rs75752296
rs75314093
rs62532947
rs117087527
rs13103186
rs10925229
rs143719040
rs2388153
rs150192075
rs55758605
rs11789587
rs142228160
rs7189546
rs4962046
All
−0.0005
0.0000
0.0005
0.0010
0.0015
0.0020
MR effect size for
'Peroxisomal carnitine O−octanoyltransferase' on 'eGFR'

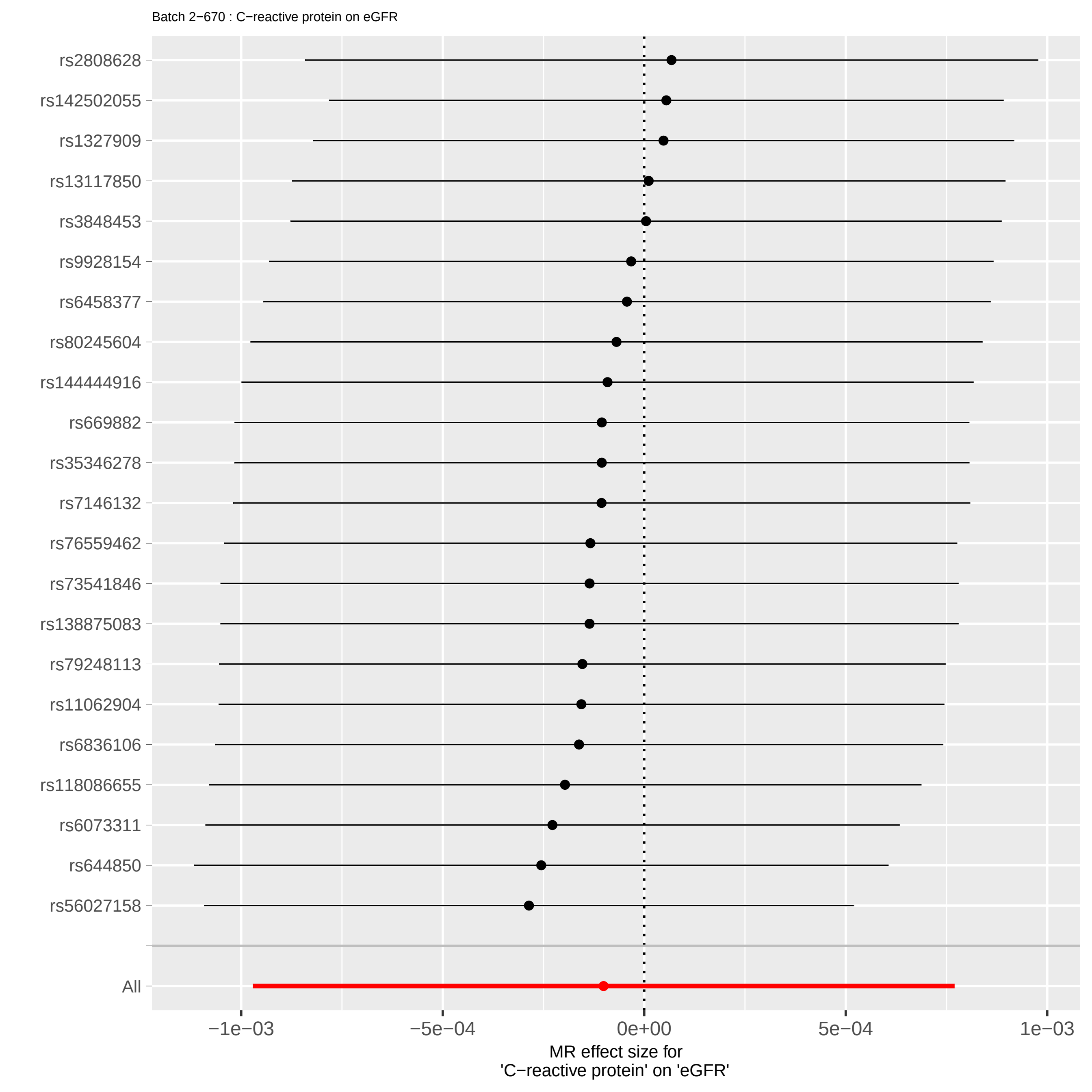
Batch 2−670 : C−reactive protein on eGFR
rs2808628
rs142502055
rs1327909
rs13117850
rs3848453
rs9928154
rs6458377
rs80245604
rs144444916
rs669882
rs35346278
rs7146132
rs76559462
rs73541846
rs138875083
rs79248113
rs11062904
rs6836106
rs118086655
rs6073311
rs644850
rs56027158
All
−1e−03
−5e−04
0e+00
5e−04
1e−03
MR effect size for
'C−reactive protein' on 'eGFR'

Batch 2−671 : Cytotoxic and regulatory T−cell molecule on eGFR

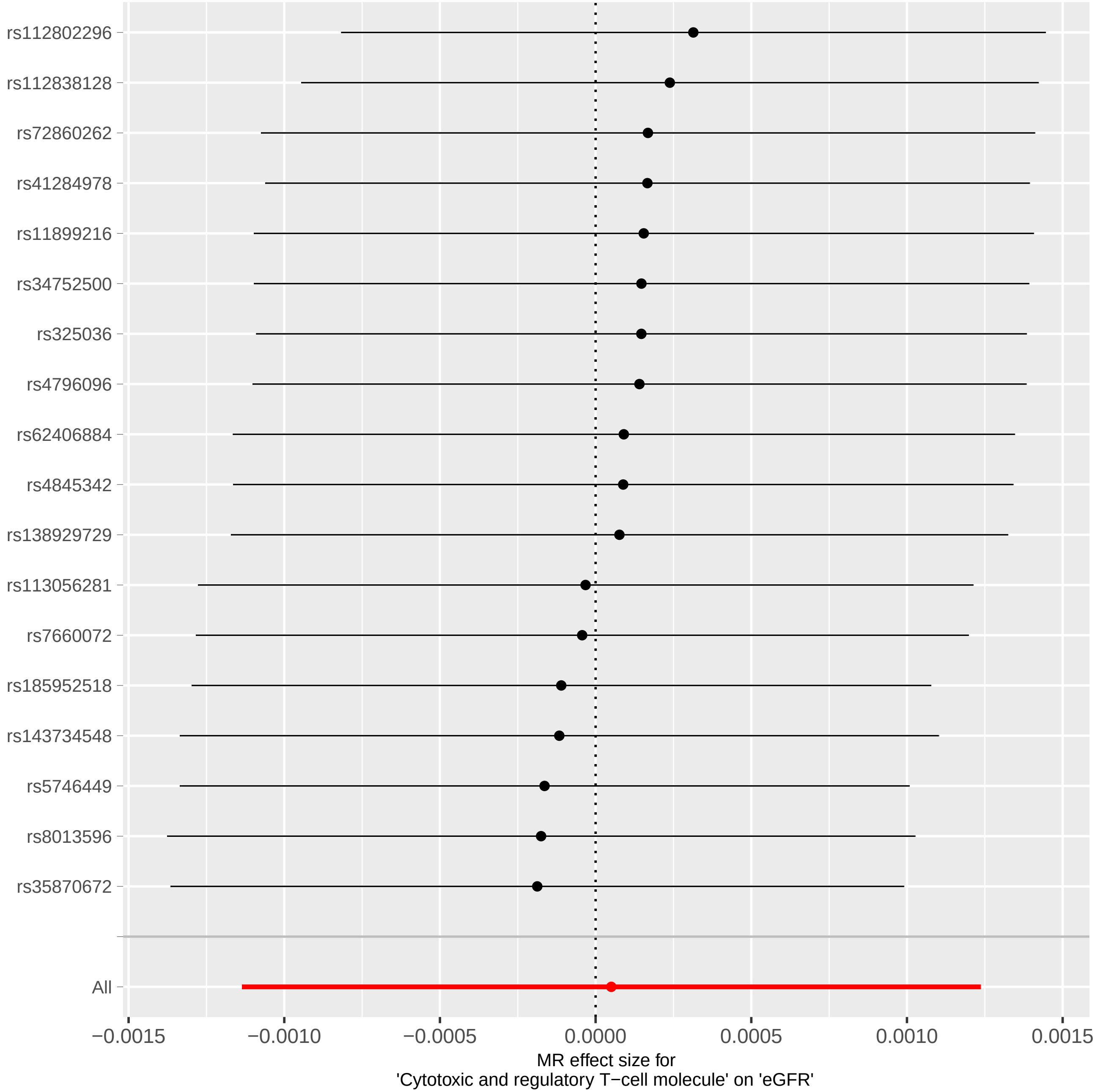

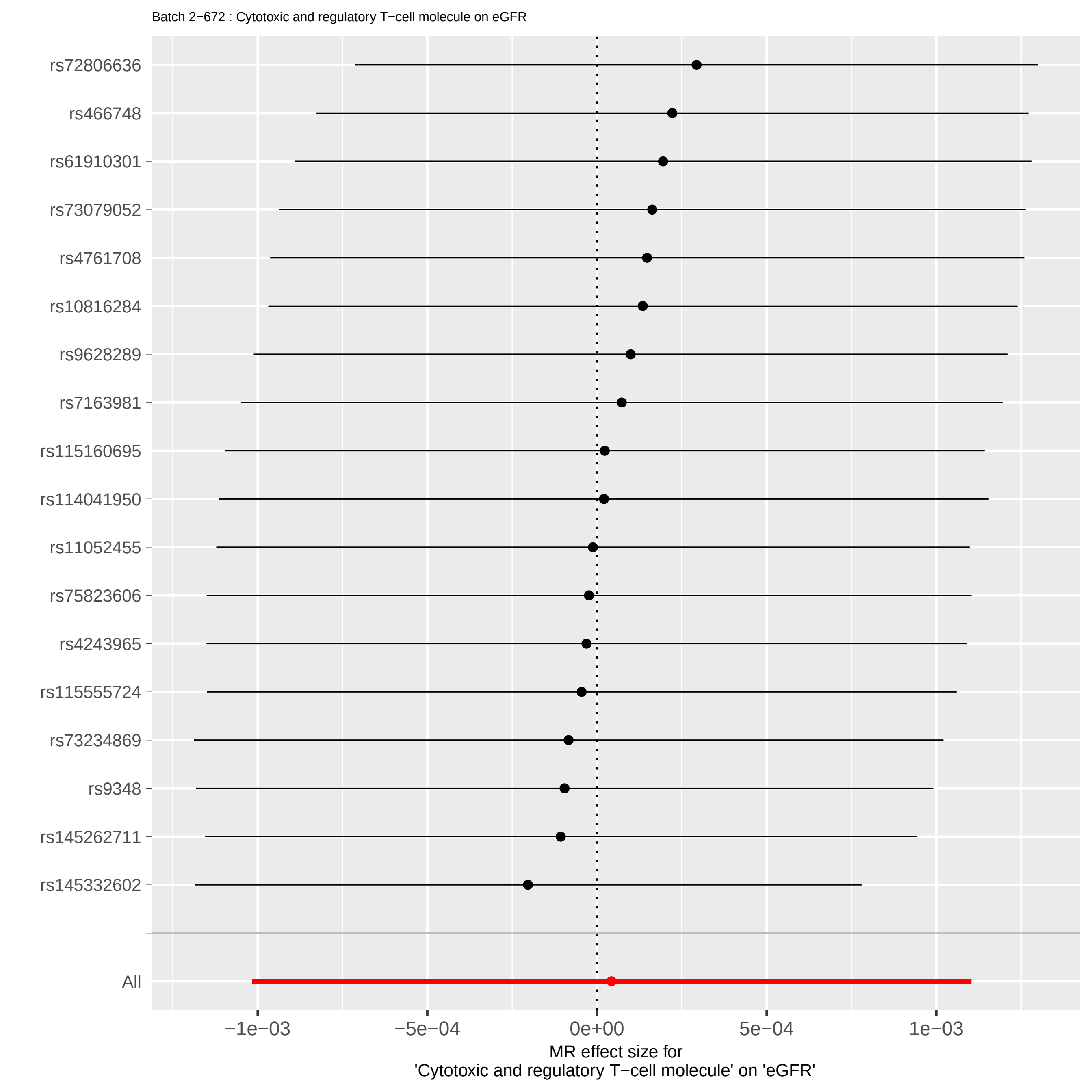

Batch 2−672 : Cytotoxic and regulatory T−cell molecule on eGFR
rs72806636
rs466748
rs61910301
rs73079052
rs4761708
rs10816284
rs9628289
rs7163981
rs115160695
rs114041950
rs11052455
rs75823606
rs4243965
rs115555724
rs73234869
rs9348
rs145262711
rs145332602
All
−1e−03
−5e−04
0e+00
5e−04
1e−03
MR effect size for
'Cytotoxic and regulatory T−cell molecule' on 'eGFR'

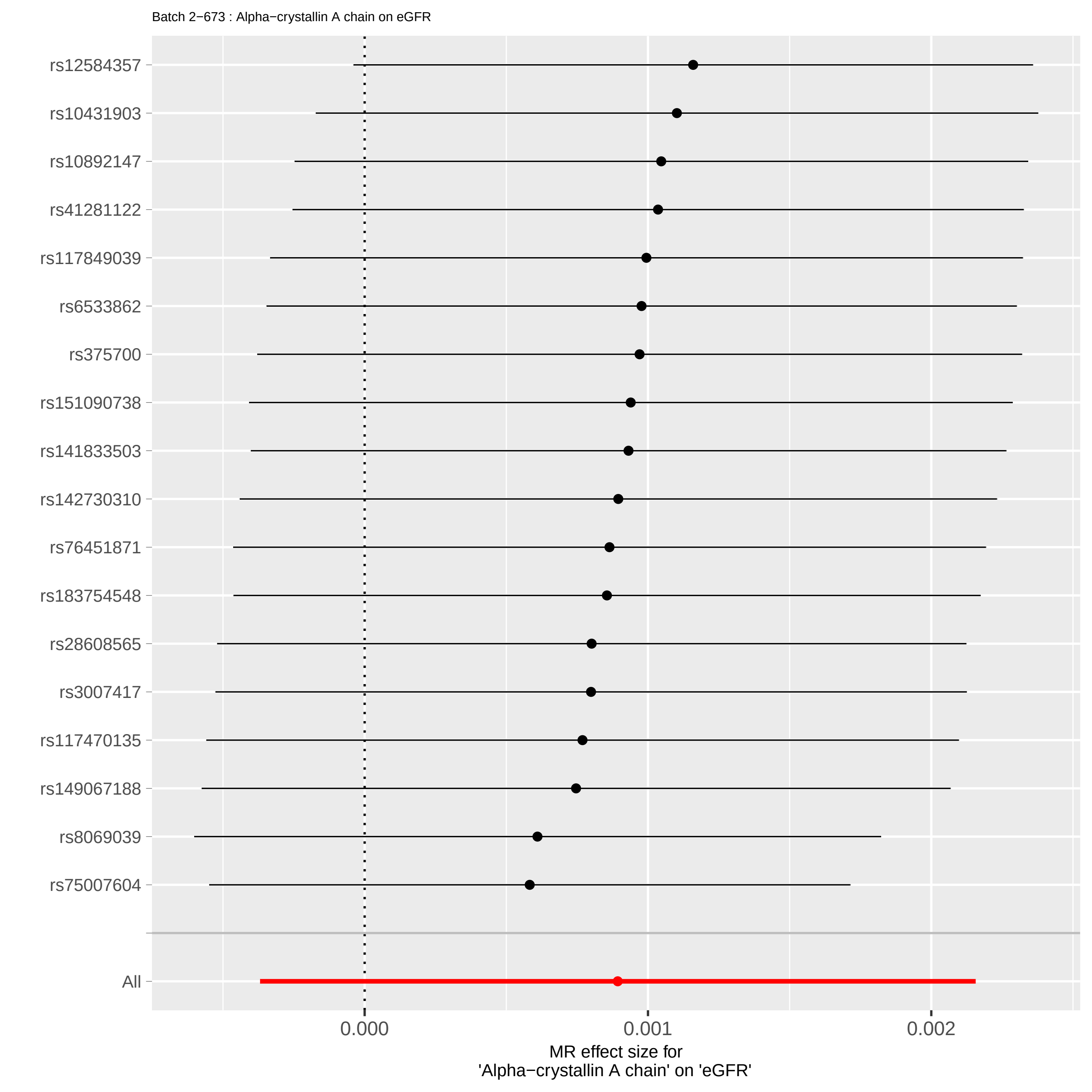
Batch 2−673 : Alpha−crystallin A chain on eGFR
rs12584357
rs10431903
rs10892147
rs41281122
rs117849039
rs6533862
rs375700
rs151090738
rs141833503
rs142730310
rs76451871
rs183754548
rs28608565
rs3007417
rs117470135
rs149067188
rs8069039
rs75007604
All
0.000
0.001
0.002
MR effect size for
'Alpha−crystallin A chain' on 'eGFR'

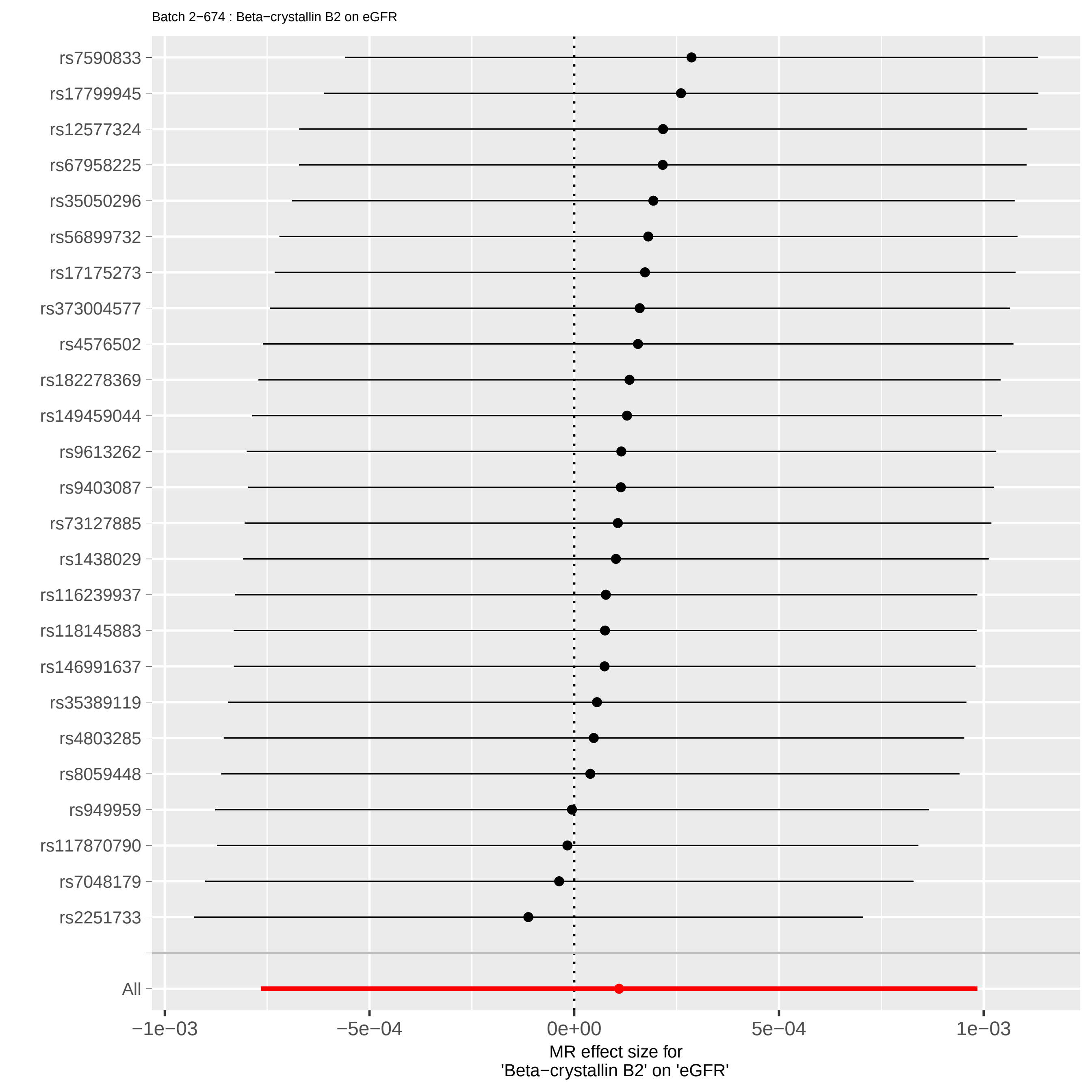

Batch 2−674 : Beta−crystallin B2 on eGFR
rs7590833
rs17799945
rs12577324
rs67958225
rs35050296
rs56899732
rs17175273
rs373004577
rs4576502
rs182278369
rs149459044
rs9613262
rs9403087
rs73127885
rs1438029
rs116239937
rs118145883
rs146991637
rs35389119
rs4803285
rs8059448
rs949959
rs117870790
rs7048179
rs2251733
All
−1e−03
−5e−04
0e+00
5e−04
1e−03
MR effect size for
'Beta−crystallin B2' on 'eGFR'

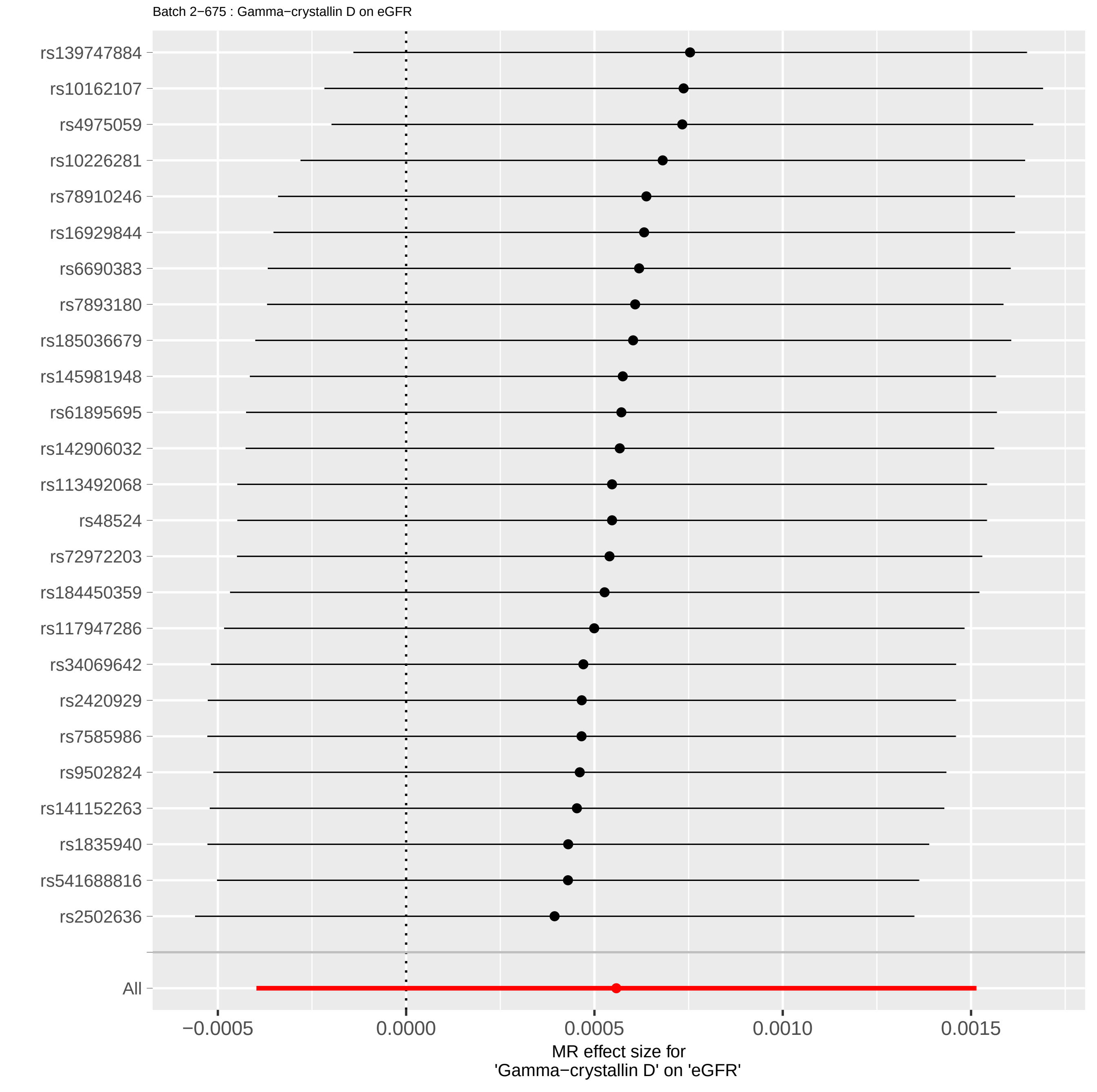
Batch 2−675 : Gamma−crystallin D on eGFR
rs139747884
rs10162107
rs4975059
rs10226281
rs78910246
rs16929844
rs6690383
rs7893180
rs185036679
rs145981948
rs61895695
rs142906032
rs113492068
rs48524
rs72972203
rs184450359
rs117947286
rs34069642
rs2420929
rs7585986
rs9502824
rs141152263
rs1835940
rs541688816
rs2502636
All
−0.0005
0.0000
0.0005
0.0010
0.0015
MR effect size for
'Gamma−crystallin D' on 'eGFR'

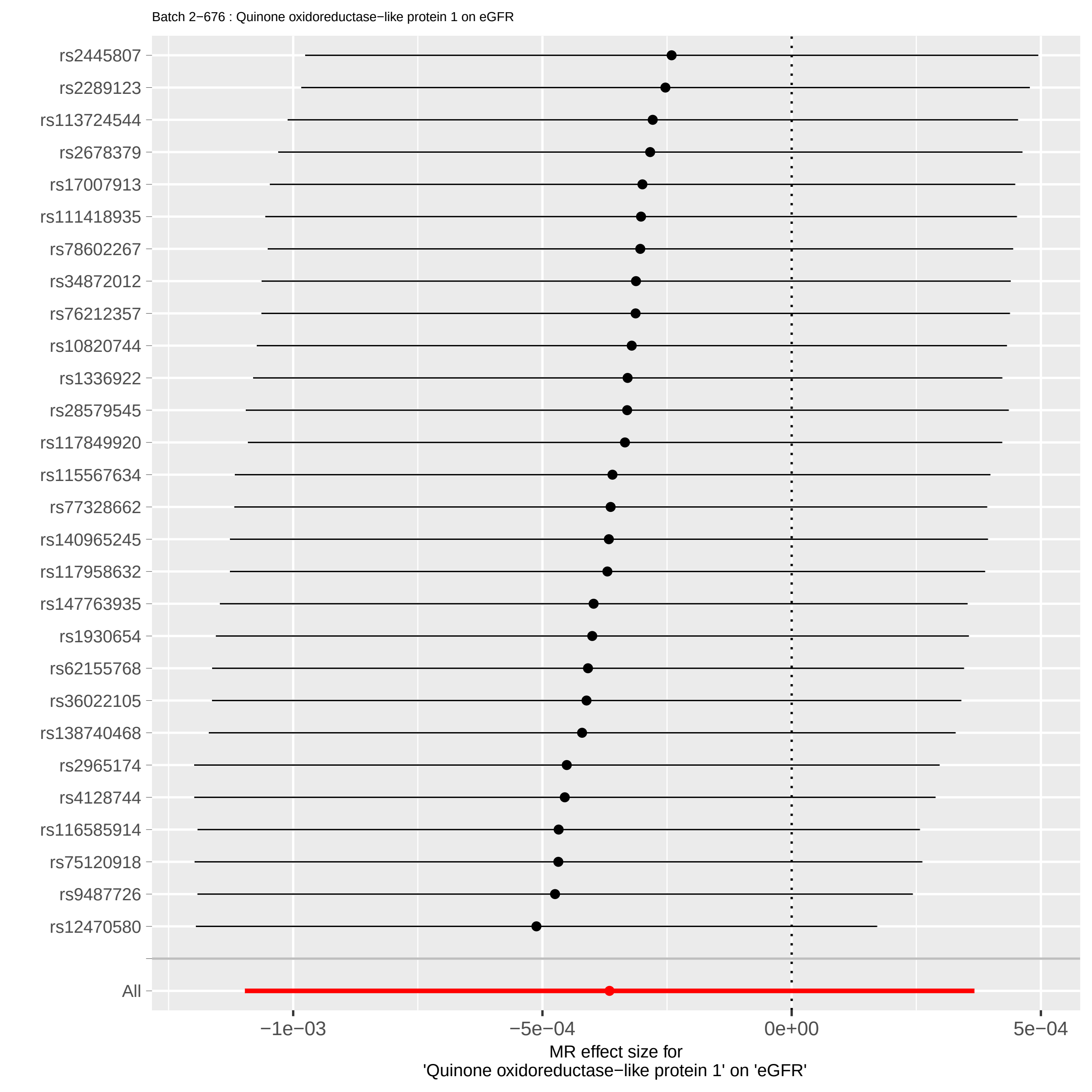

Batch 2−676 : Quinone oxidoreductase−like protein 1 on eGFR
rs2445807
rs2289123
rs113724544
rs2678379
rs17007913
rs111418935
rs78602267
rs34872012
rs76212357
rs10820744
rs1336922
rs28579545
rs117849920
rs115567634
rs77328662
rs140965245
rs117958632
rs147763935
rs1930654
rs62155768
rs36022105
rs138740468
rs2965174
rs4128744
rs116585914
rs75120918
rs9487726
rs12470580
All
−1e−03
−5e−04
0e+00
5e−04
MR effect size for
'Quinone oxidoreductase−like protein 1' on 'eGFR'

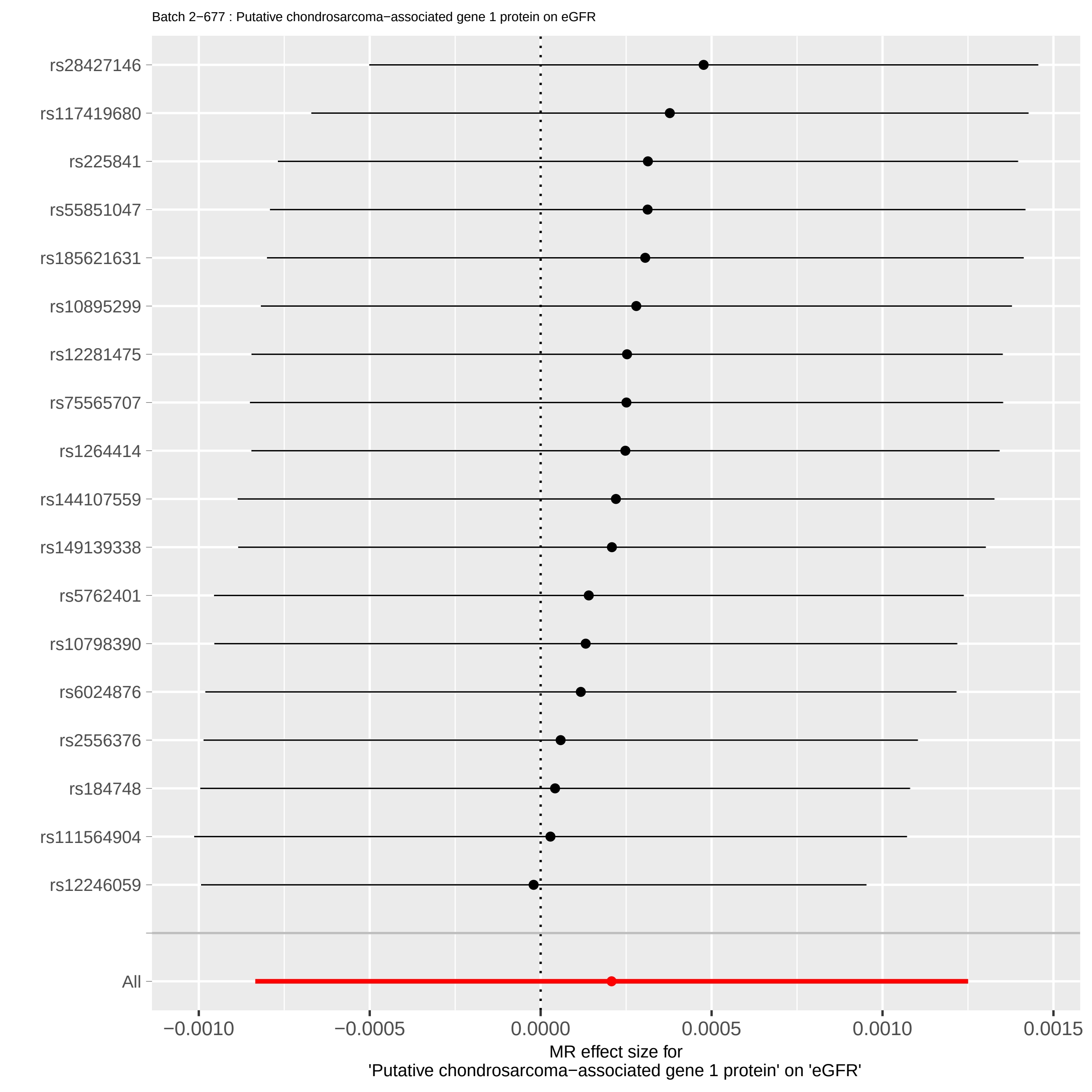

Batch 2−677 : Putative chondrosarcoma−associated gene 1 protein on eGFR
rs28427146
rs117419680
rs225841
rs55851047
rs185621631
rs10895299
rs12281475
rs75565707
rs1264414
rs144107559
rs149139338
rs5762401
rs10798390
rs6024876
rs2556376
rs184748
rs111564904
rs12246059
All
−0.0010
−0.0005
0.0000
0.0005
0.0010
0.0015
MR effect size for
'Putative chondrosarcoma−associated gene 1 protein' on 'eGFR'

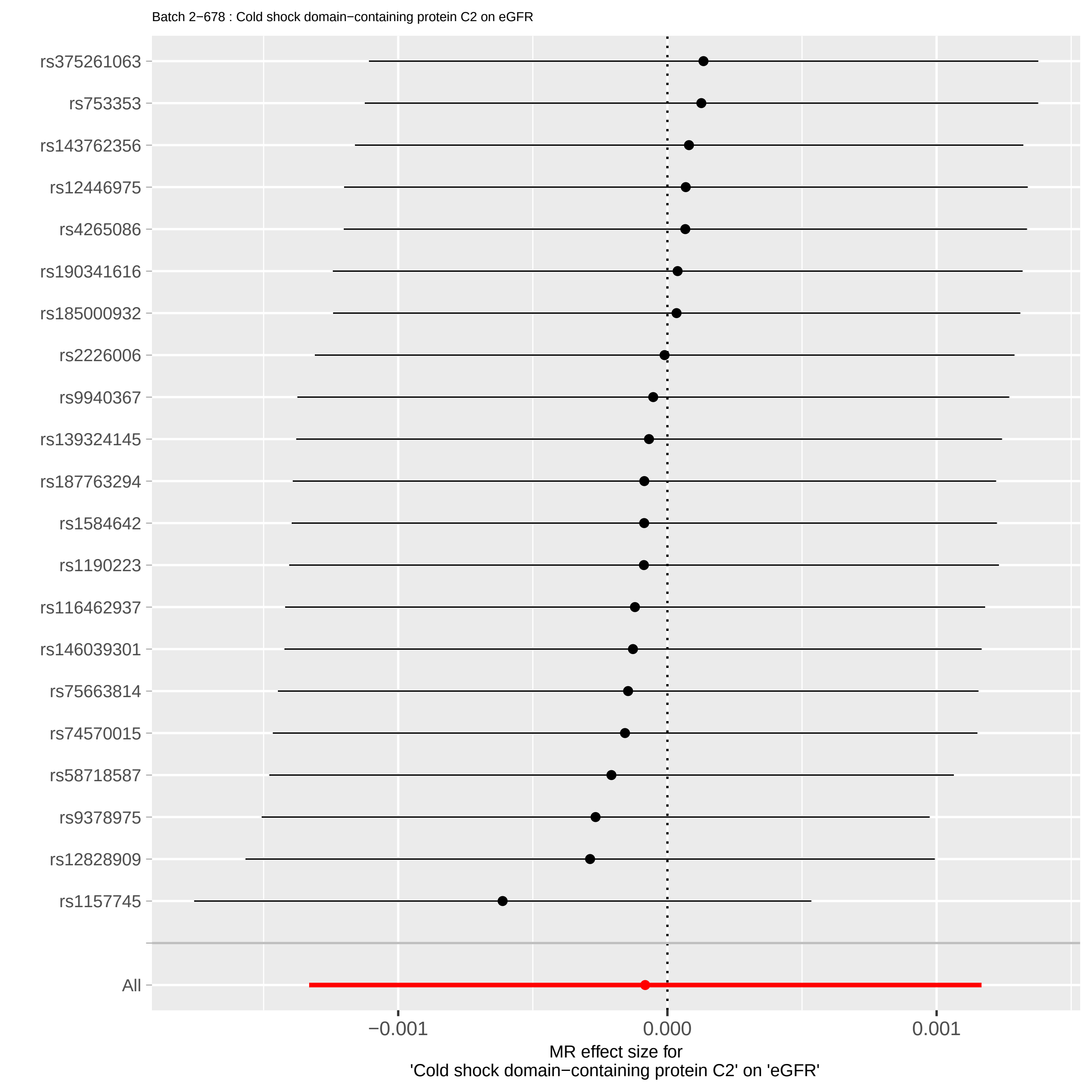

Batch 2−678 : Cold shock domain−containing protein C2 on eGFR
rs375261063
rs753353
rs143762356
rs12446975
rs4265086
rs190341616
rs185000932
rs2226006
rs9940367
rs139324145
rs187763294
rs1584642
rs1190223
rs116462937
rs146039301
rs75663814
rs74570015
rs58718587
rs9378975
rs12828909
rs1157745
All
−0.001
0.000
0.001
MR effect size for
'Cold shock domain−containing protein C2' on 'eGFR'

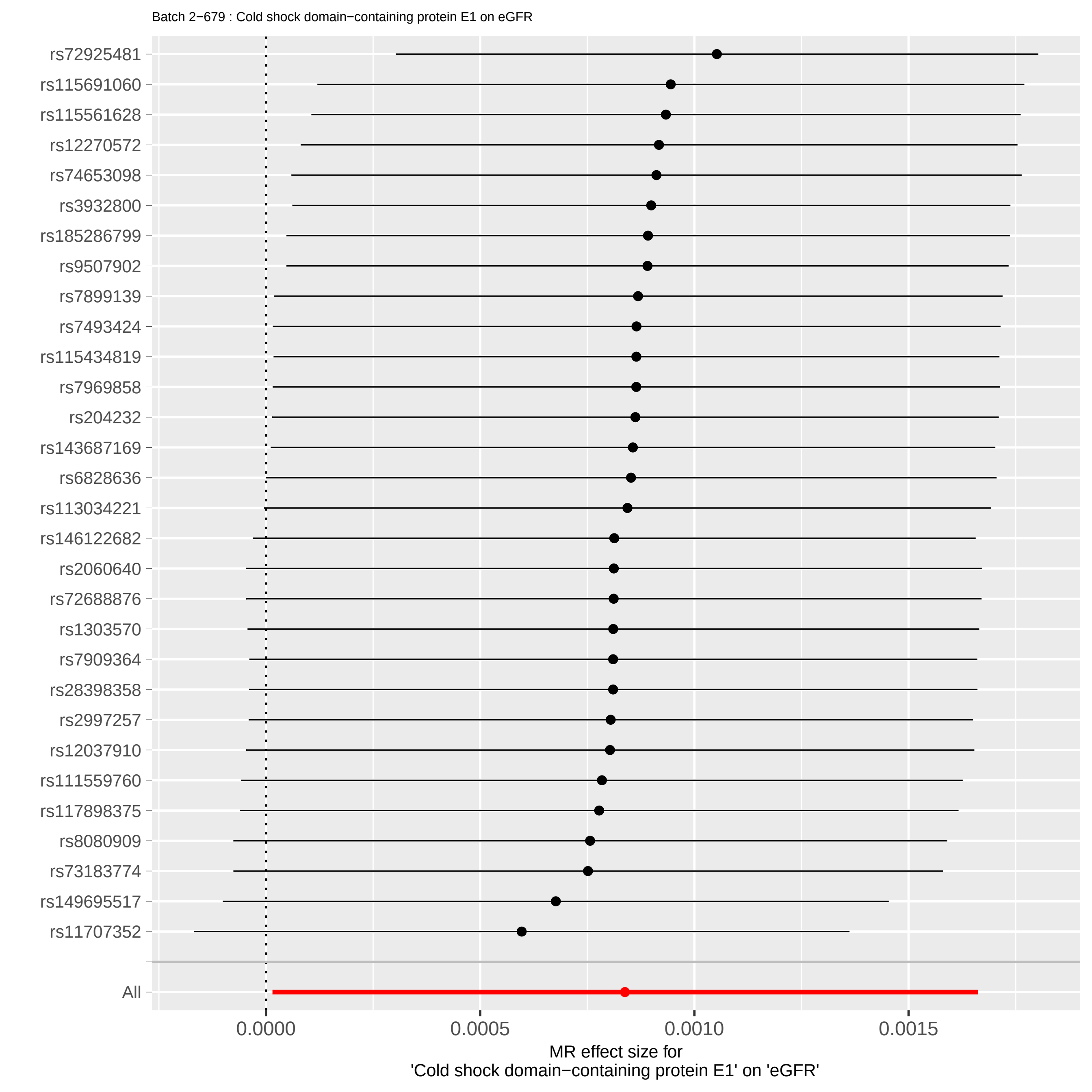

Batch 2−679 : Cold shock domain−containing protein E1 on eGFR
rs72925481
rs115691060
rs115561628
rs12270572
rs74653098
rs3932800
rs185286799
rs9507902
rs7899139
rs7493424
rs115434819
rs7969858
rs204232
rs143687169
rs6828636
rs113034221
rs146122682
rs2060640
rs72688876
rs1303570
rs7909364
rs28398358
rs2997257
rs12037910
rs111559760
rs117898375
rs8080909
rs73183774
rs149695517
rs11707352
All
0.0000
0.0005
0.0010
0.0015
MR effect size for
'Cold shock domain−containing protein E1' on 'eGFR'

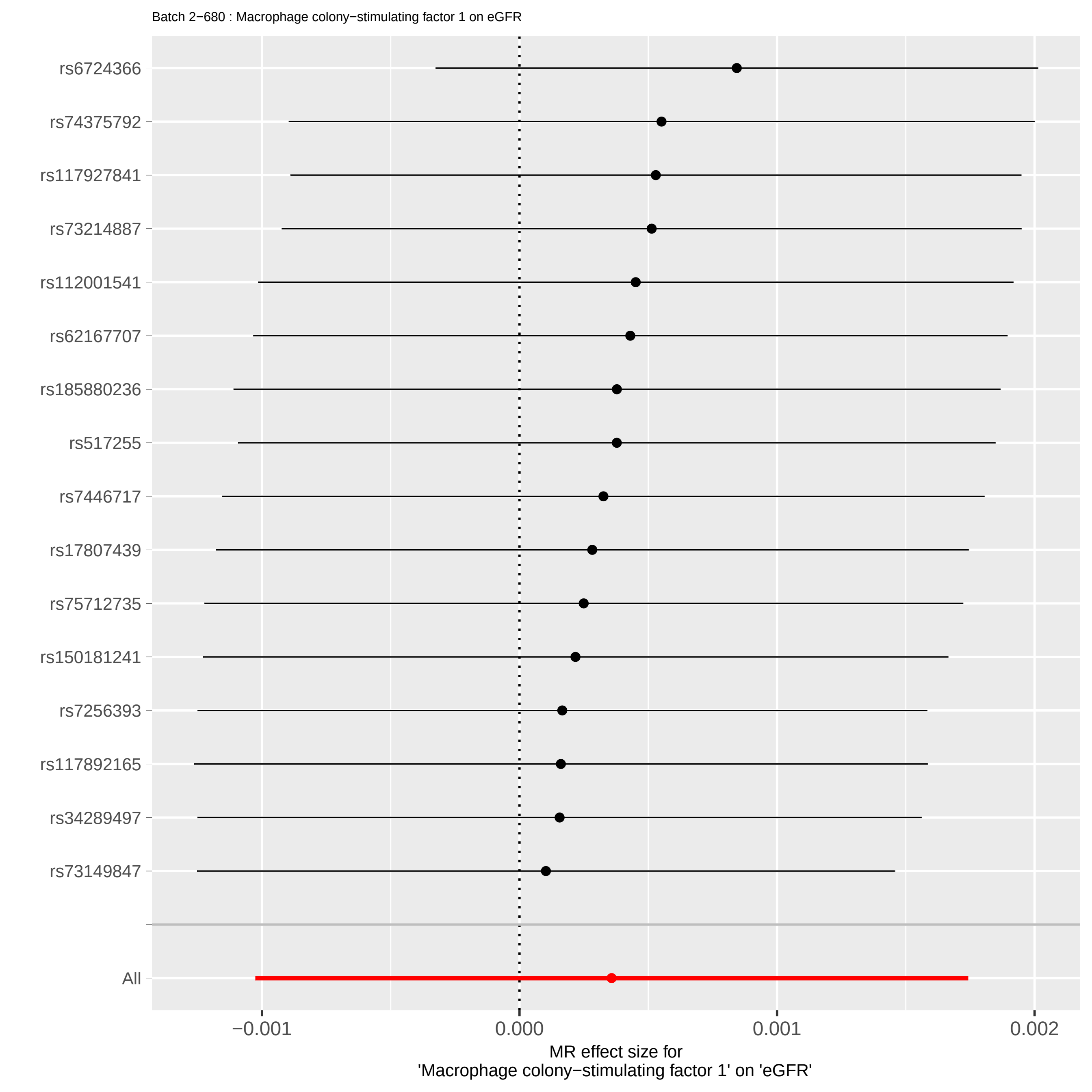

Batch 2−680 : Macrophage colony−stimulating factor 1 on eGFR
rs6724366
rs74375792
rs117927841
rs73214887
rs112001541
rs62167707
rs185880236
rs517255
rs7446717
rs17807439
rs75712735
rs150181241
rs7256393
rs117892165
rs34289497
rs73149847
All
−0.001
0.000
0.001
0.002
MR effect size for
'Macrophage colony−stimulating factor 1' on 'eGFR'

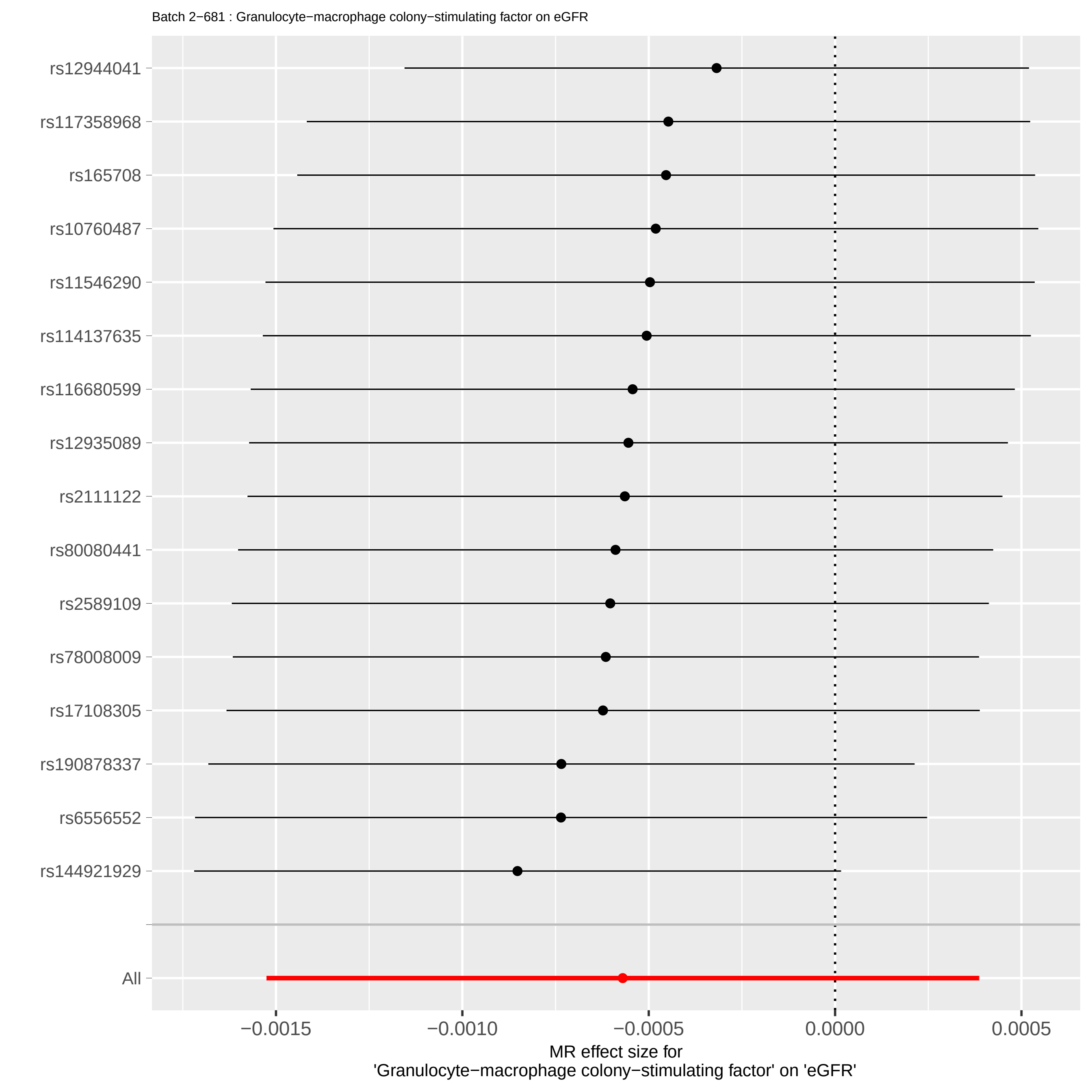
Batch 2−681 : Granulocyte−macrophage colony−stimulating factor on eGFR
rs12944041
rs117358968
rs165708
rs10760487
rs11546290
rs114137635
rs116680599
rs12935089
rs2111122
rs80080441
rs2589109
rs78008009
rs17108305
rs190878337
rs6556552
rs144921929
All
−0.0015
−0.0010
−0.0005
0.0000
0.0005
MR effect size for
'Granulocyte−macrophage colony−stimulating factor' on 'eGFR'

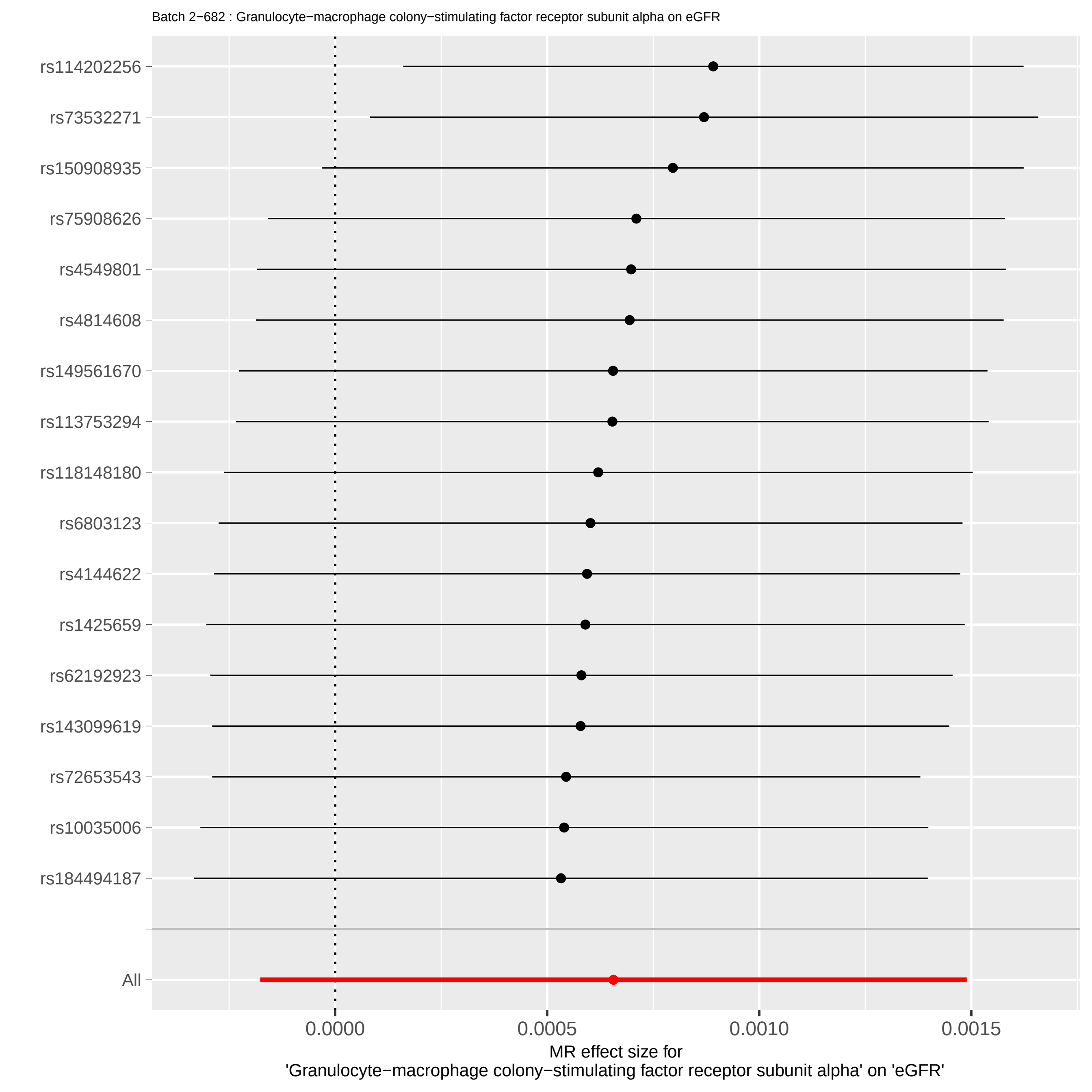
Batch 2−682 : Granulocyte−macrophage colony−stimulating factor receptor subunit alpha on eGFR
rs114202256
rs73532271
rs150908935
rs75908626
rs4549801
rs4814608
rs149561670
rs113753294
rs118148180
rs6803123
rs4144622
rs1425659
rs62192923
rs143099619
rs72653543
rs10035006
rs184494187
All
0.0000
0.0005
0.0010
0.0015
MR effect size for
'Granulocyte−macrophage colony−stimulating factor receptor subunit alpha' on 'eGFR'

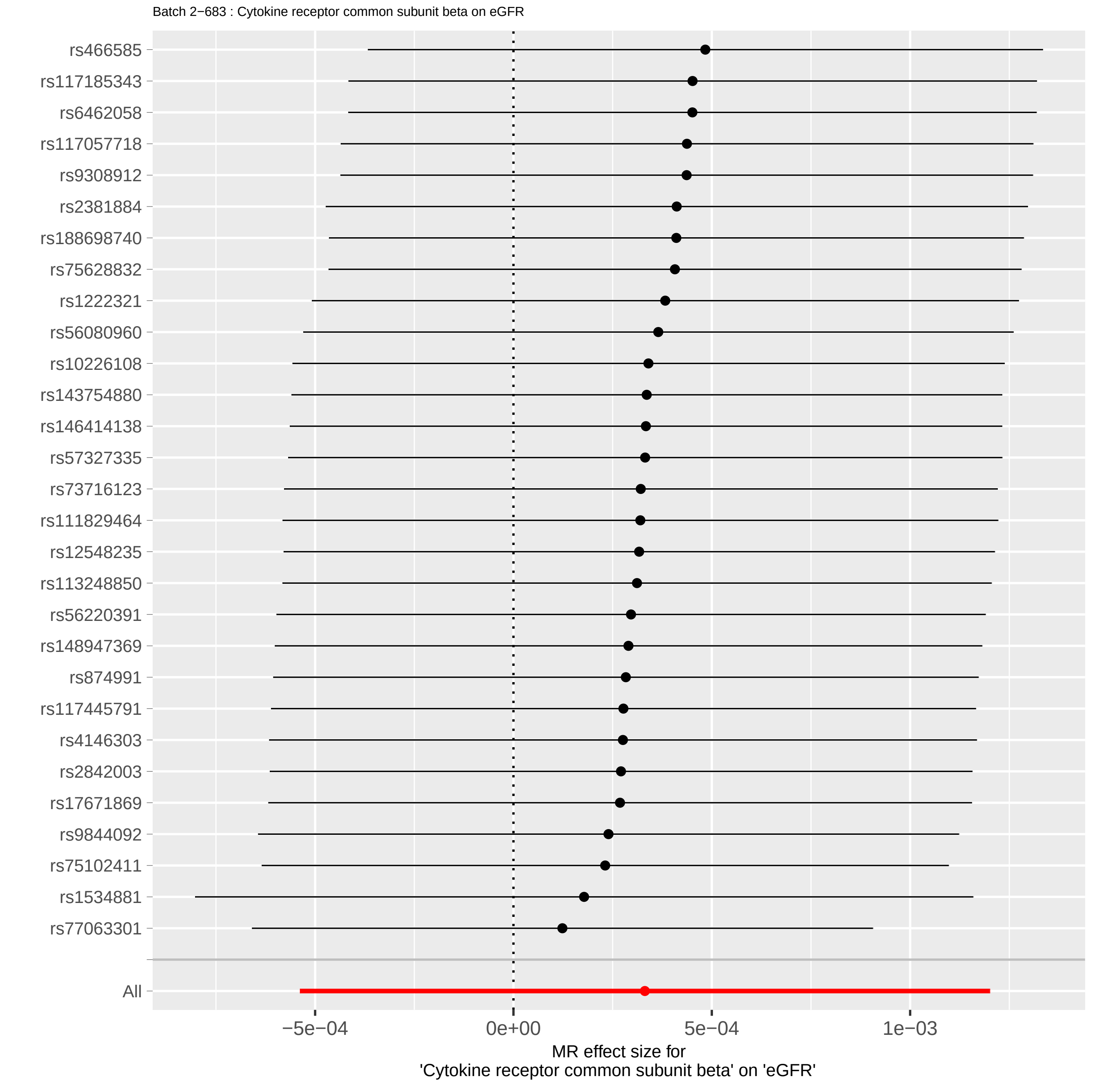

Batch 2−683 : Cytokine receptor common subunit beta on eGFR
rs466585
rs117185343
rs6462058
rs117057718
rs9308912
rs2381884
rs188698740
rs75628832
rs1222321
rs56080960
rs10226108
rs143754880
rs146414138
rs57327335
rs73716123
rs111829464
rs12548235
rs113248850
rs56220391
rs148947369
rs874991
rs117445791
rs4146303
rs2842003
rs17671869
rs9844092
rs75102411
rs1534881
rs77063301
All
−5e−04
0e+00
5e−04
1e−03
MR effect size for
'Cytokine receptor common subunit beta' on 'eGFR'

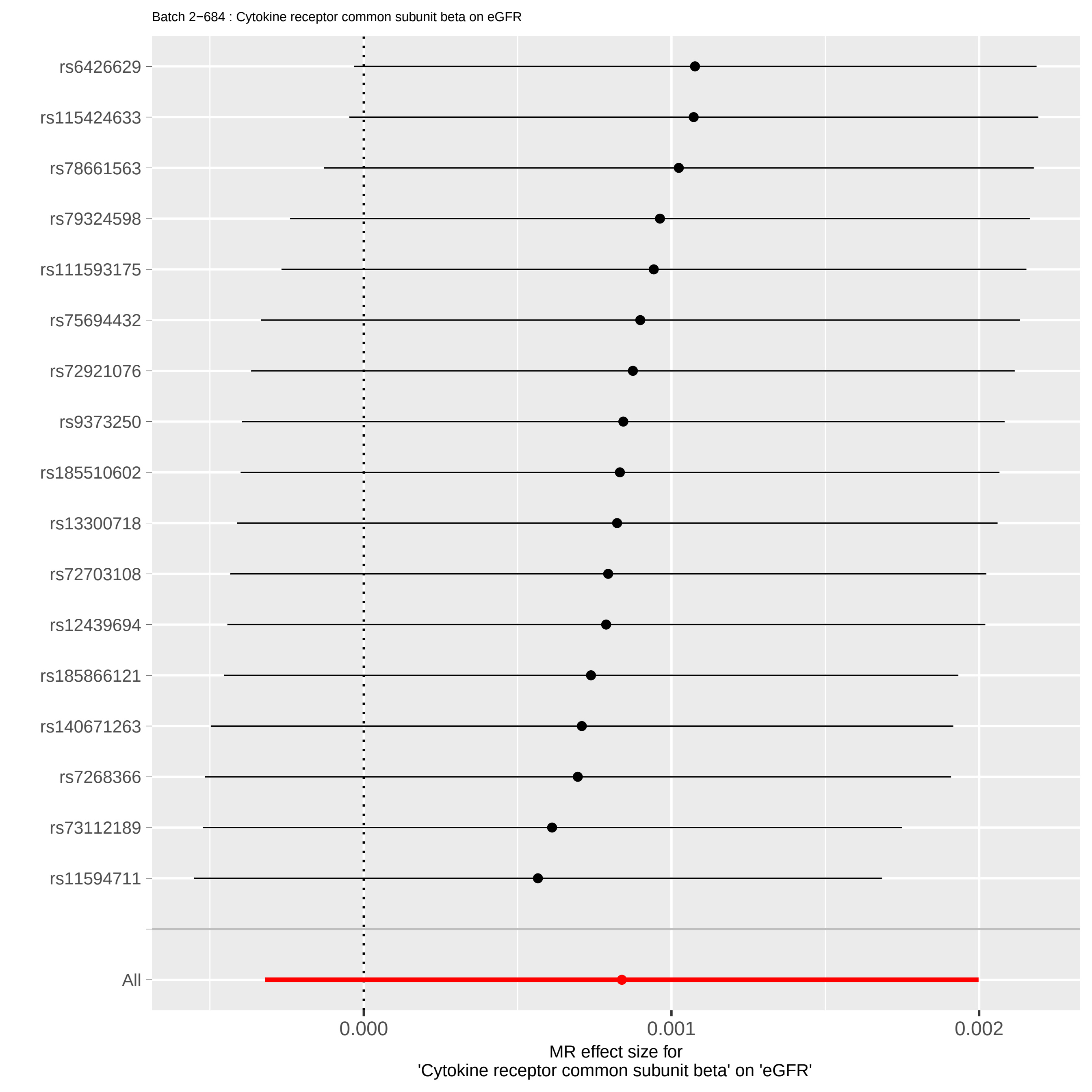
Batch 2−684 : Cytokine receptor common subunit beta on eGFR
rs6426629
rs115424633
rs78661563
rs79324598
rs111593175
rs75694432
rs72921076
rs9373250
rs185510602
rs13300718
rs72703108
rs12439694
rs185866121
rs140671263
rs7268366
rs73112189
rs11594711
All
0.000
0.001
0.002
MR effect size for
'Cytokine receptor common subunit beta' on 'eGFR'

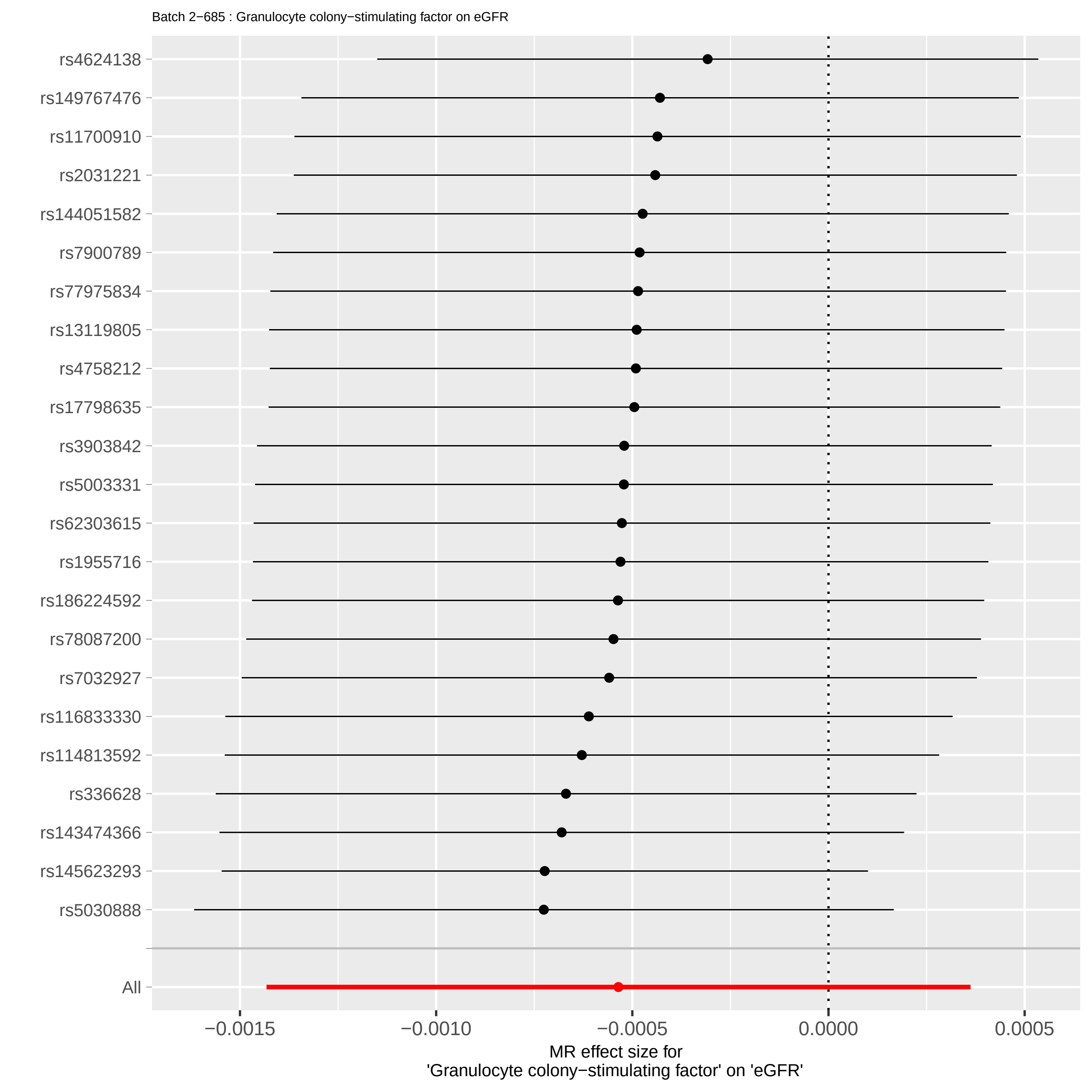

Batch 2−685 : Granulocyte colony−stimulating factor on eGFR
rs4624138
rs149767476
rs11700910
rs2031221
rs144051582
rs7900789
rs77975834
rs13119805
rs4758212
rs17798635
rs3903842
rs5003331
rs62303615
rs1955716
rs186224592
rs78087200
rs7032927
rs116833330
rs114813592
rs336628
rs143474366
rs145623293
rs5030888
All
−0.0015
−0.0010
−0.0005
0.0000
0.0005
MR effect size for
'Granulocyte colony−stimulating factor' on 'eGFR'

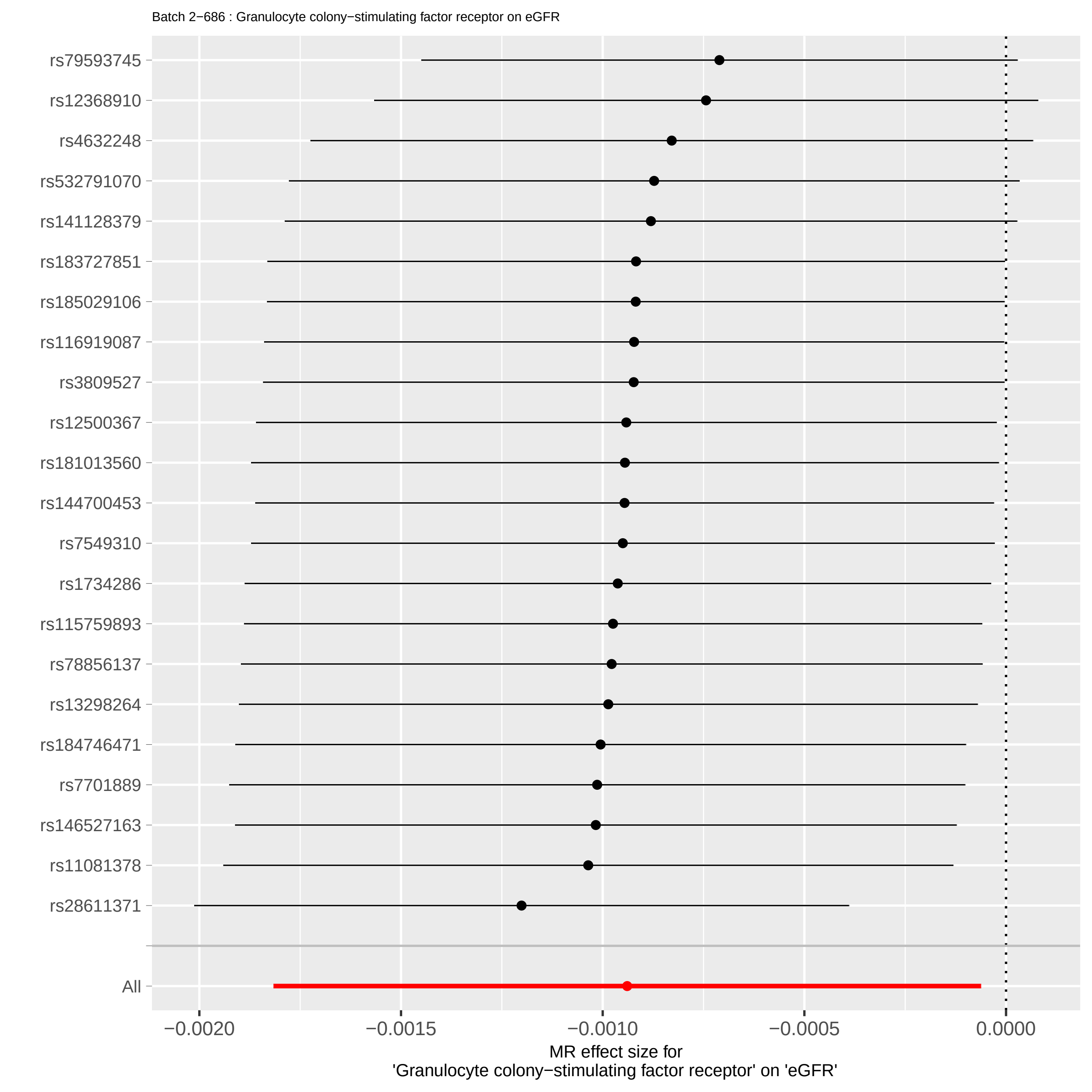
Batch 2−686 : Granulocyte colony−stimulating factor receptor on eGFR
rs79593745
rs12368910
rs4632248
rs532791070
rs141128379
rs183727851
rs185029106
rs116919087
rs3809527
rs12500367
rs181013560
rs144700453
rs7549310
rs1734286
rs115759893
rs78856137
rs13298264
rs184746471
rs7701889
rs146527163
rs11081378
rs28611371
All
−0.0020
−0.0015
−0.0010
−0.0005
0.0000
MR effect size for
'Granulocyte colony−stimulating factor receptor' on 'eGFR'

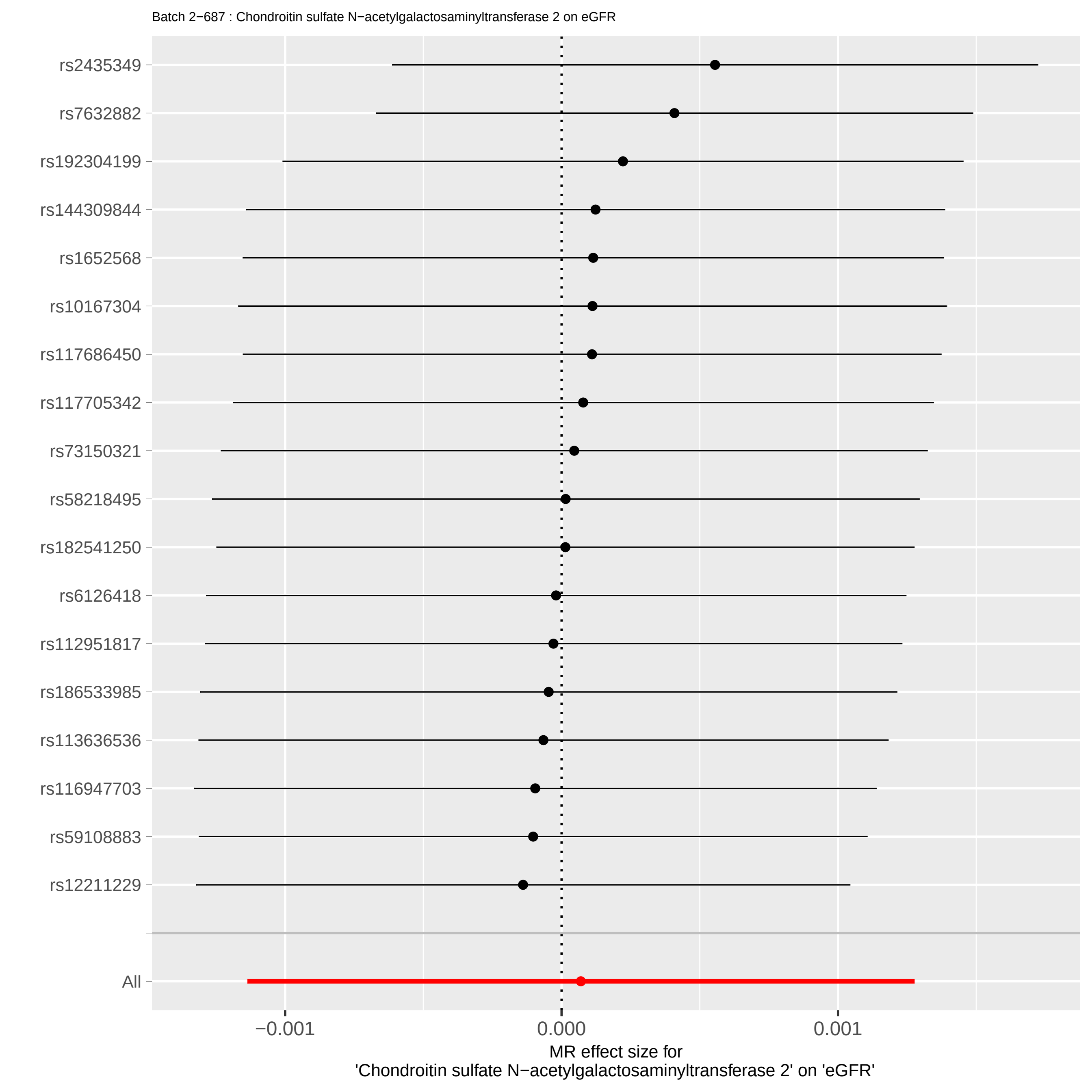

Batch 2−687 : Chondroitin sulfate N−acetylgalactosaminyltransferase 2 on eGFR
rs2435349
rs7632882
rs192304199
rs144309844
rs1652568
rs10167304
rs117686450
rs117705342
rs73150321
rs58218495
rs182541250
rs6126418
rs112951817
rs186533985
rs113636536
rs116947703
rs59108883
rs12211229
All
−0.001
0.000
0.001
MR effect size for
'Chondroitin sulfate N−acetylgalactosaminyltransferase 2' on 'eGFR'

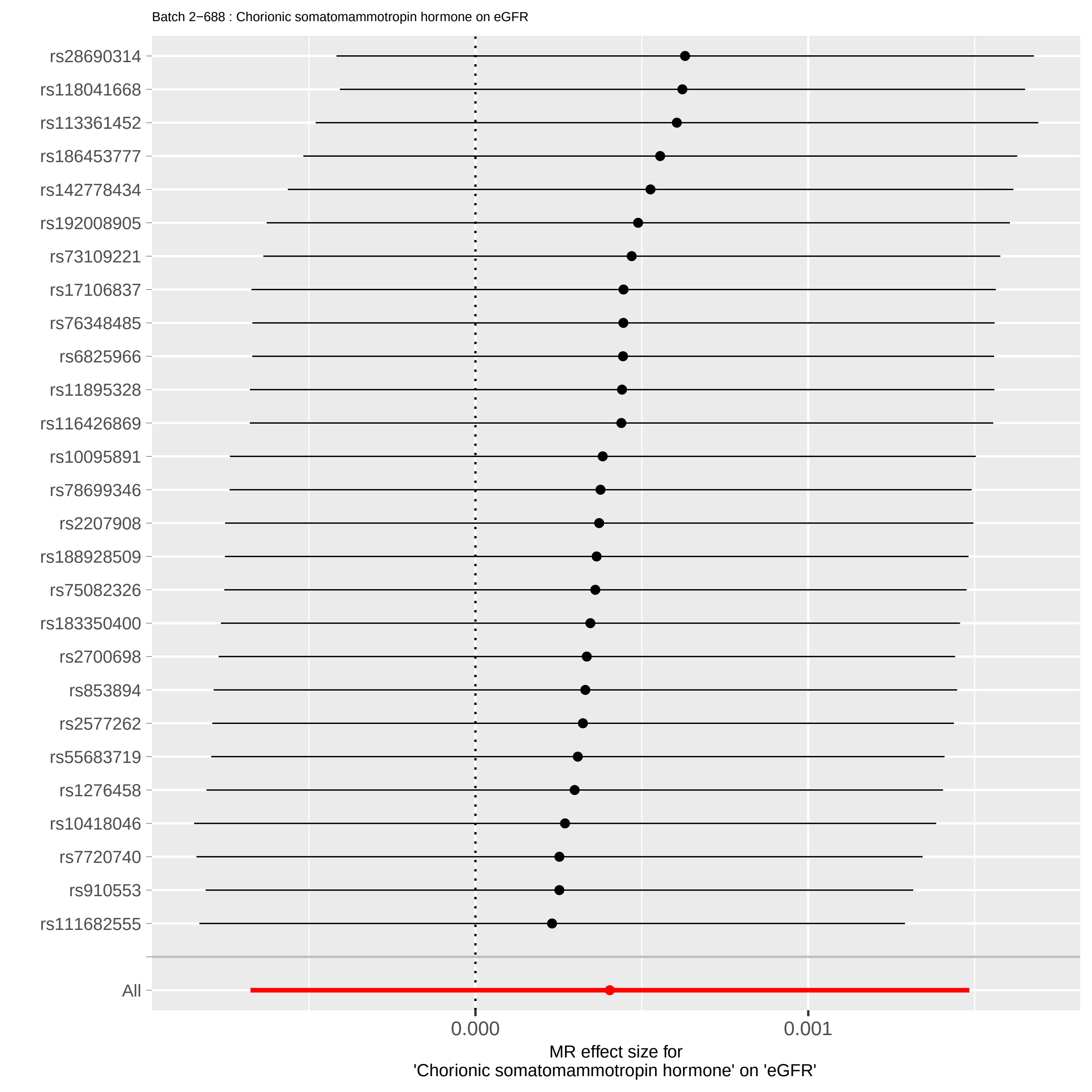

Batch 2−688 : Chorionic somatomammotropin hormone on eGFR
rs28690314
rs118041668
rs113361452
rs186453777
rs142778434
rs192008905
rs73109221
rs17106837
rs76348485
rs6825966
rs11895328
rs116426869
rs10095891
rs78699346
rs2207908
rs188928509
rs75082326
rs183350400
rs2700698
rs853894
rs2577262
rs55683719
rs1276458
rs10418046
rs7720740
rs910553
rs111682555
All
0.000
0.001
MR effect size for
'Chorionic somatomammotropin hormone' on 'eGFR'

Batch 2−689 : Tyrosine−protein kinase CSK on eGFR

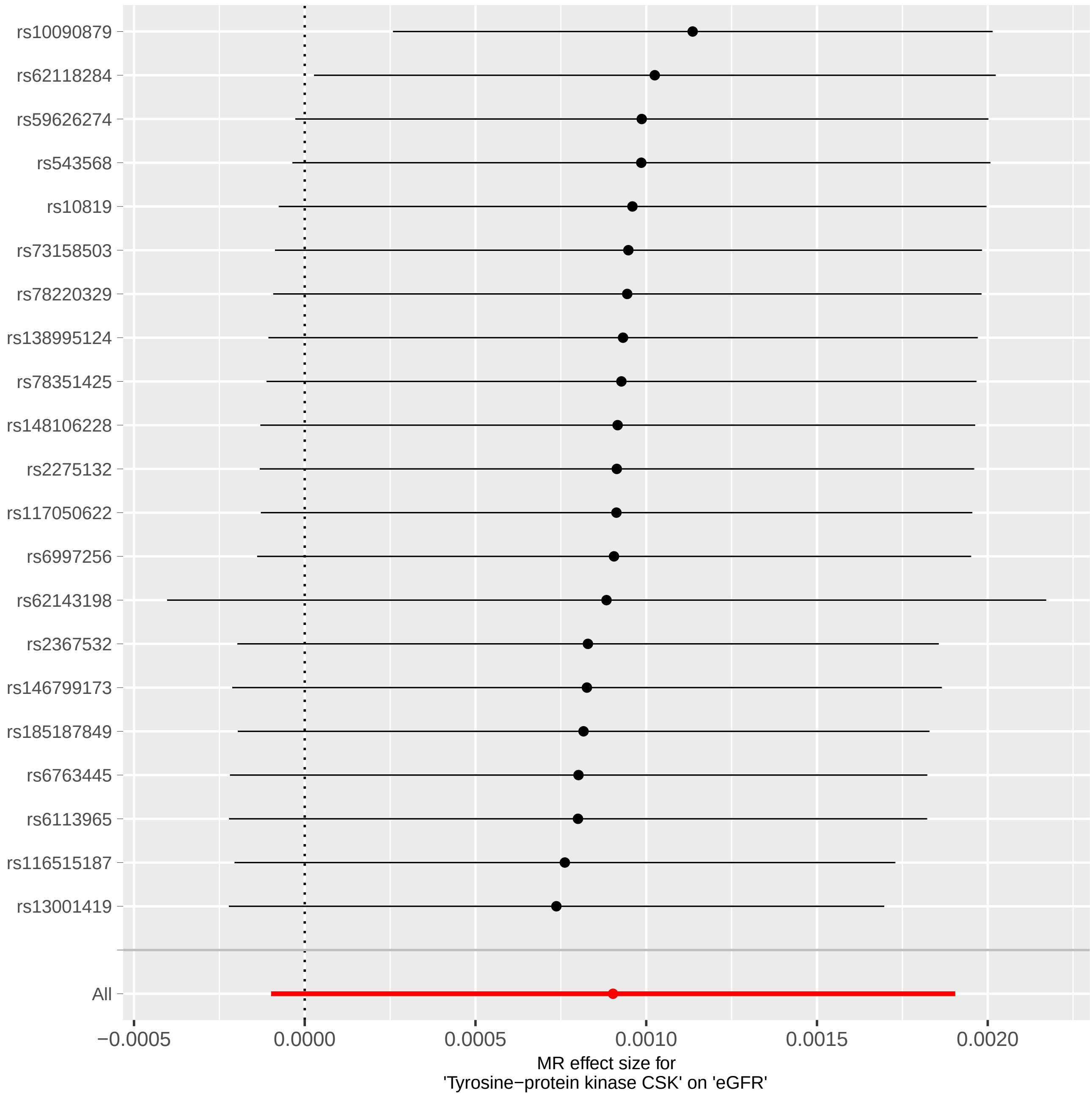

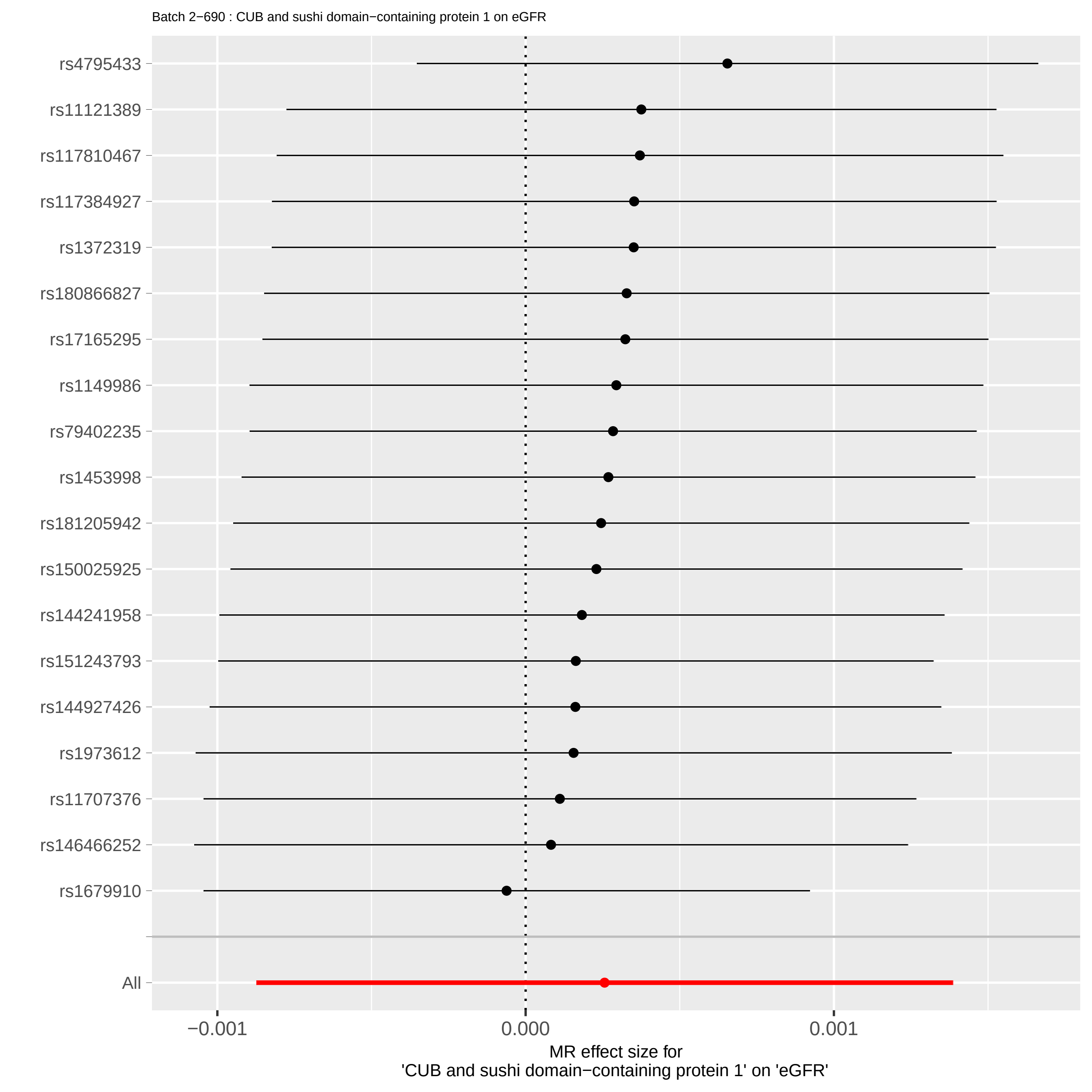

Batch 2−690 : CUB and sushi domain−containing protein 1 on eGFR
rs4795433
rs11121389
rs117810467
rs117384927
rs1372319
rs180866827
rs17165295
rs1149986
rs79402235
rs1453998
rs181205942
rs150025925
rs144241958
rs151243793
rs144927426
rs1973612
rs11707376
rs146466252
rs1679910
All
−0.001
0.000
0.001
MR effect size for
'CUB and sushi domain−containing protein 1' on 'eGFR'

Batch 2−691 : CUB and sushi domain−containing protein 2 on eGFR

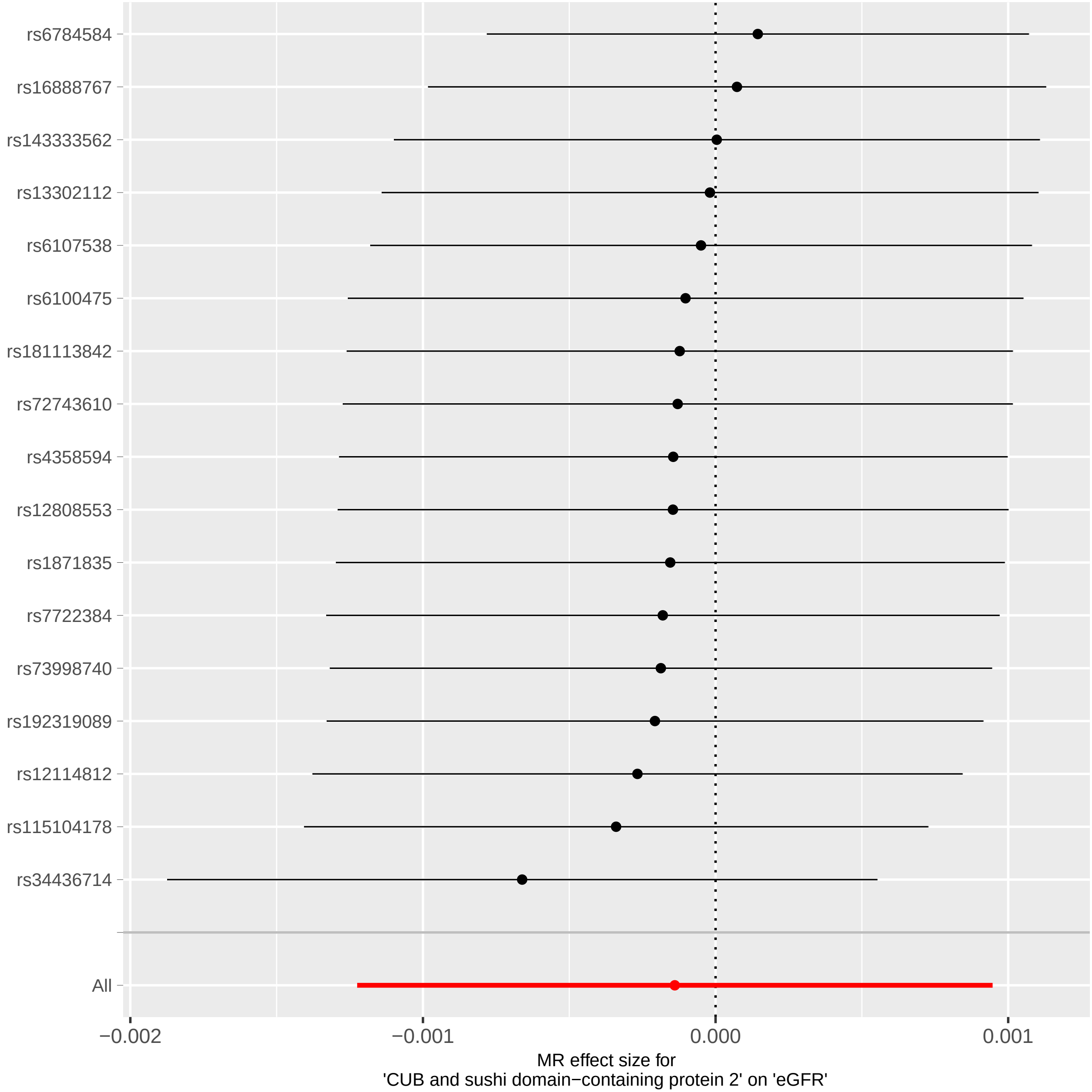

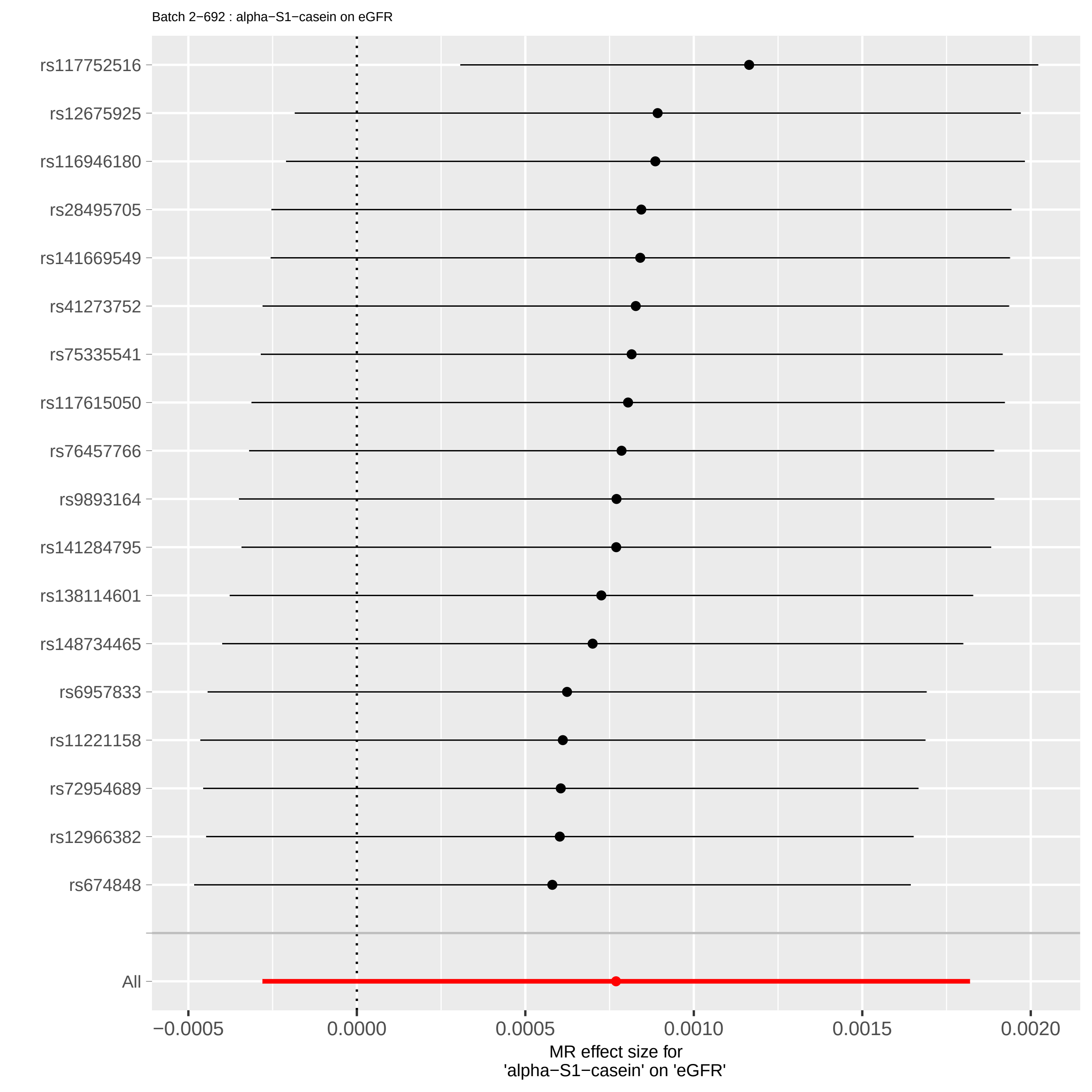

Batch 2−692 : alpha−S1−casein on eGFR
rs117752516
rs12675925
rs116946180
rs28495705
rs141669549
rs41273752
rs75335541
rs117615050
rs76457766
rs9893164
rs141284795
rs138114601
rs148734465
rs6957833
rs11221158
rs72954689
rs12966382
rs674848
All
−0.0005
0.0000
0.0005
0.0010
0.0015
0.0020
MR effect size for
'alpha−S1−casein' on 'eGFR'

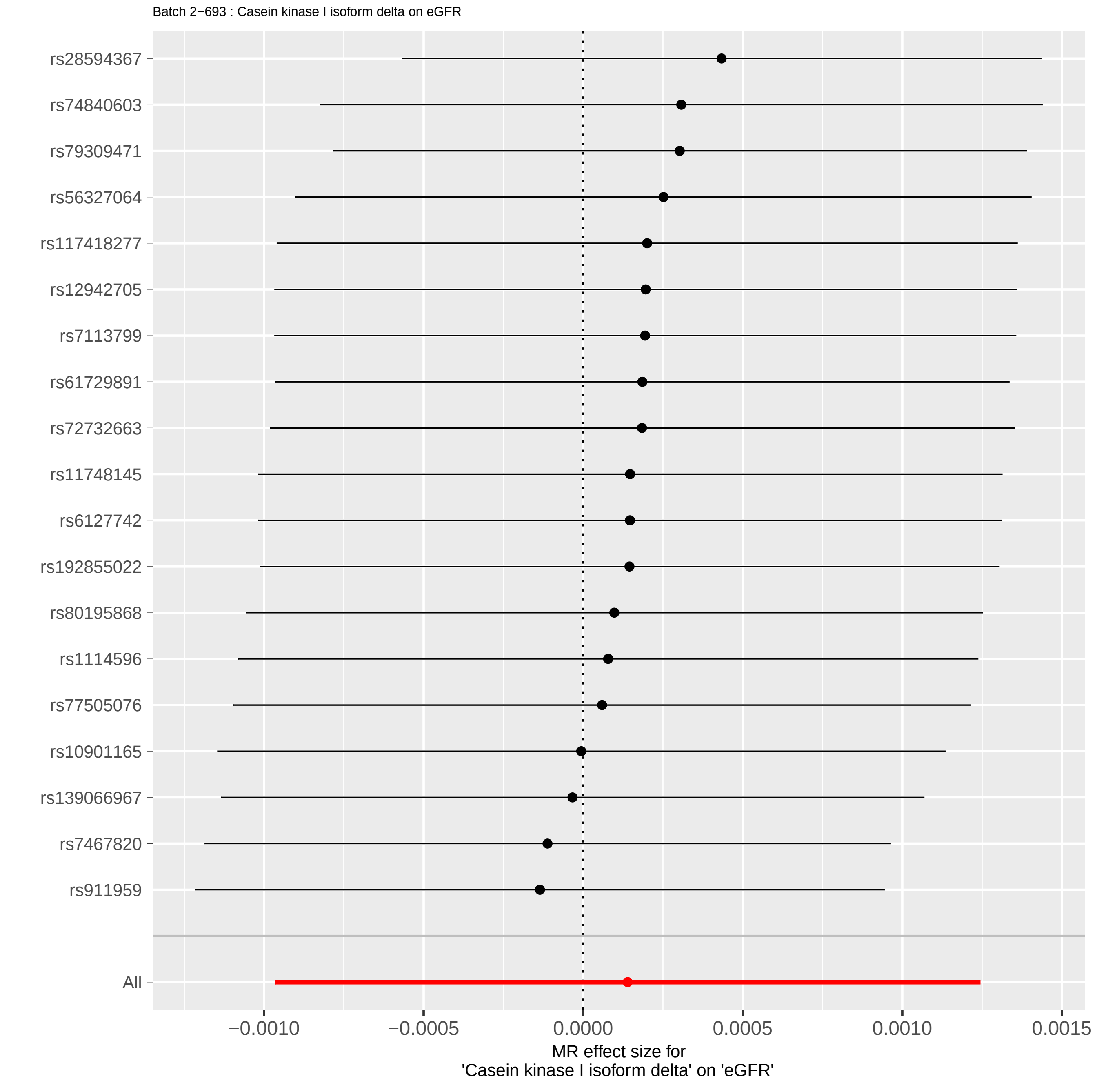

Batch 2−693 : Casein kinase I isoform delta on eGFR
rs28594367
rs74840603
rs79309471
rs56327064
rs117418277
rs12942705
rs7113799
rs61729891
rs72732663
rs11748145
rs6127742
rs192855022
rs80195868
rs1114596
rs77505076
rs10901165
rs139066967
rs7467820
rs911959
All
−0.0010
−0.0005
0.0000
0.0005
0.0010
0.0015
MR effect size for
'Casein kinase I isoform delta' on 'eGFR'

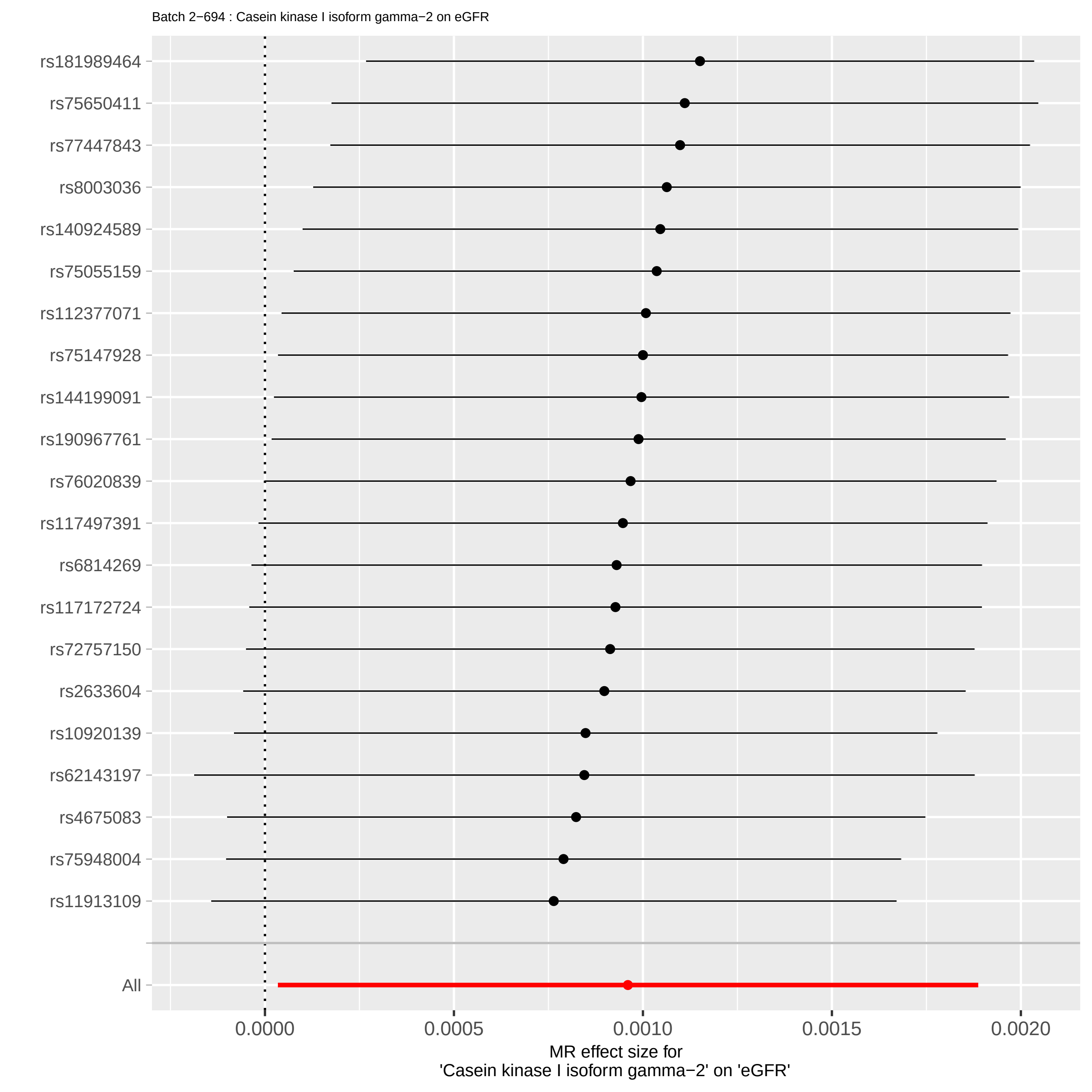

Batch 2−694 : Casein kinase I isoform gamma−2 on eGFR
rs181989464
rs75650411
rs77447843
rs8003036
rs140924589
rs75055159
rs112377071
rs75147928
rs144199091
rs190967761
rs76020839
rs117497391
rs6814269
rs117172724
rs72757150
rs2633604
rs10920139
rs62143197
rs4675083
rs75948004
rs11913109
All
0.0000
0.0005
0.0010
0.0015
0.0020
MR effect size for
'Casein kinase I isoform gamma−2' on 'eGFR'

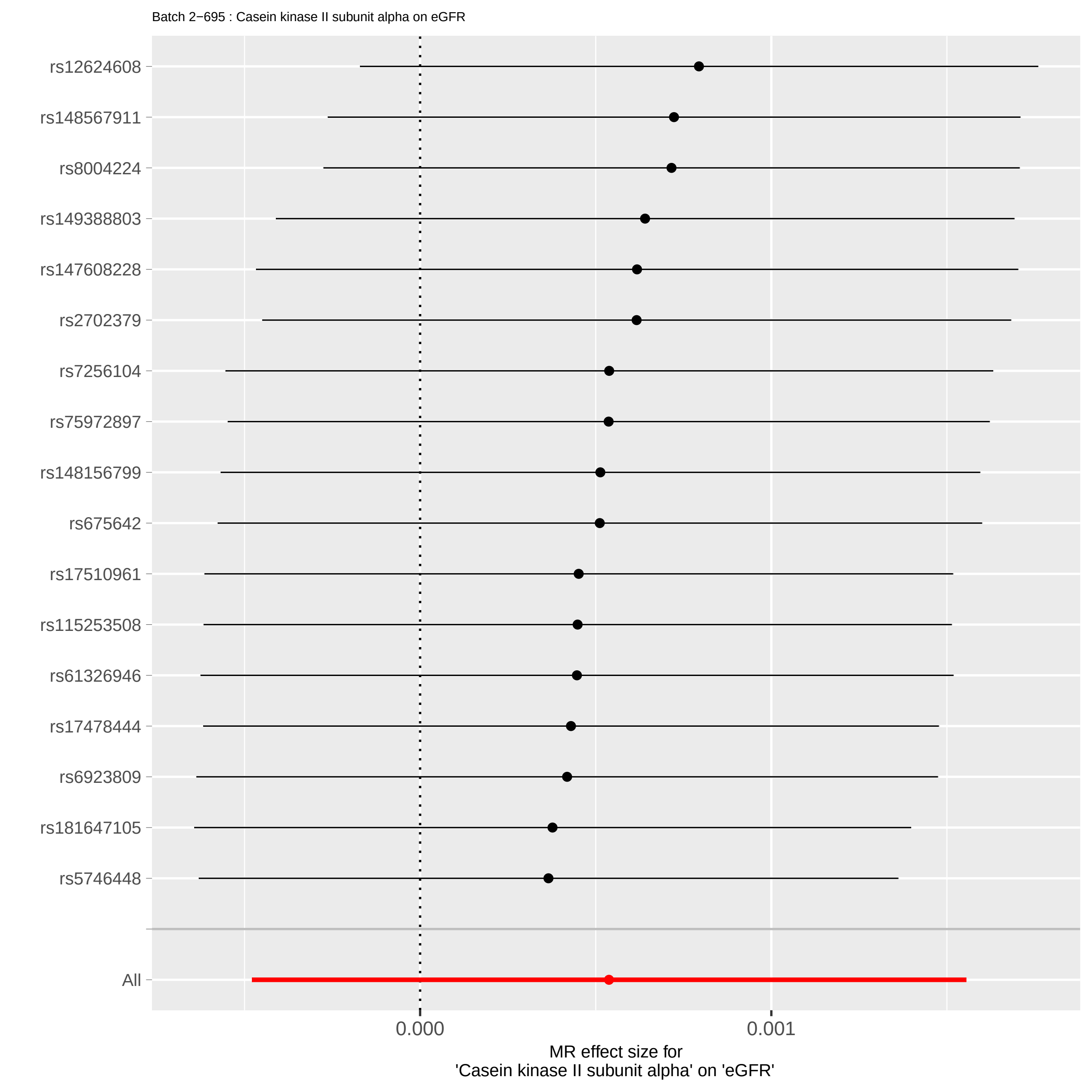

Batch 2−695 : Casein kinase II subunit alpha on eGFR
rs12624608
rs148567911
rs8004224
rs149388803
rs147608228
rs2702379
rs7256104
rs75972897
rs148156799
rs675642
rs17510961
rs115253508
rs61326946
rs17478444
rs6923809
rs181647105
rs5746448
All
0.000
0.001
MR effect size for
'Casein kinase II subunit alpha' on 'eGFR'

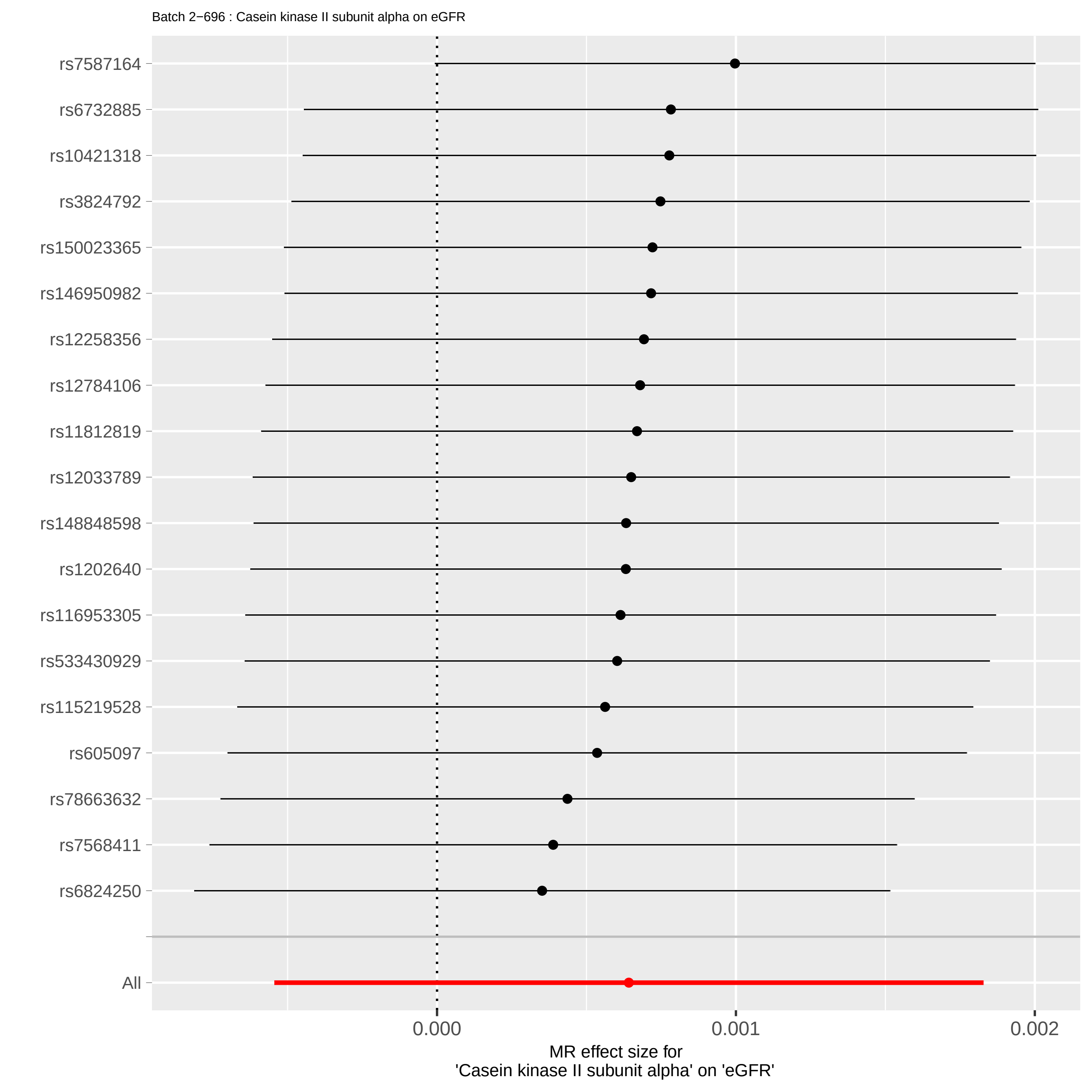

Batch 2−696 : Casein kinase II subunit alpha on eGFR
rs7587164
rs6732885
rs10421318
rs3824792
rs150023365
rs146950982
rs12258356
rs12784106
rs11812819
rs12033789
rs148848598
rs1202640
rs116953305
rs533430929
rs115219528
rs605097
rs78663632
rs7568411
rs6824250
All
0.000
0.001
0.002
MR effect size for
'Casein kinase II subunit alpha' on 'eGFR'

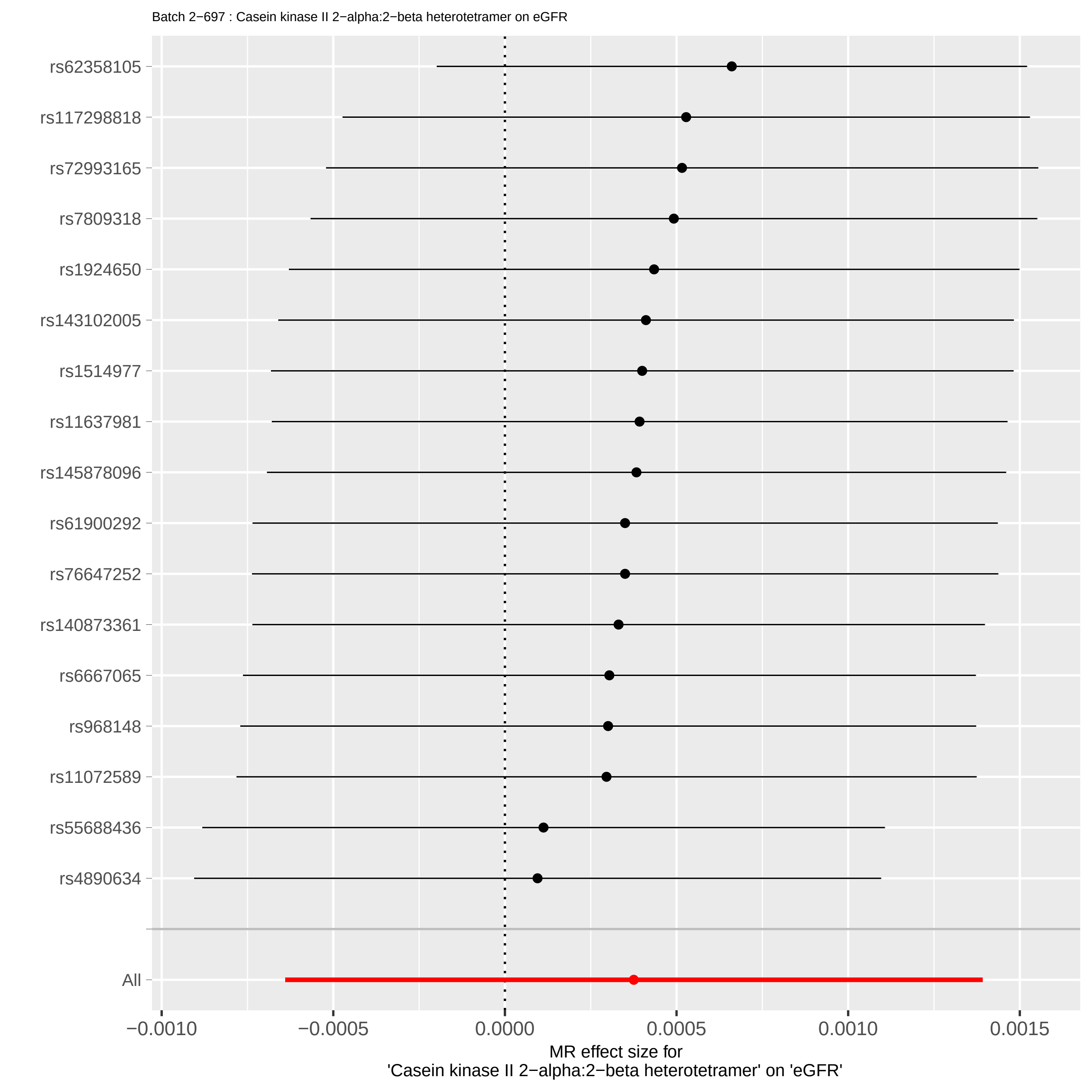

Batch 2−697 : Casein kinase II 2−alpha:2−beta heterotetramer on eGFR
rs62358105
rs117298818
rs72993165
rs7809318
rs1924650
rs143102005
rs1514977
rs11637981
rs145878096
rs61900292
rs76647252
rs140873361
rs6667065
rs968148
rs11072589
rs55688436
rs4890634
All
−0.0010
−0.0005
0.0000
0.0005
0.0010
0.0015
MR effect size for
'Casein kinase II 2−alpha:2−beta heterotetramer' on 'eGFR'

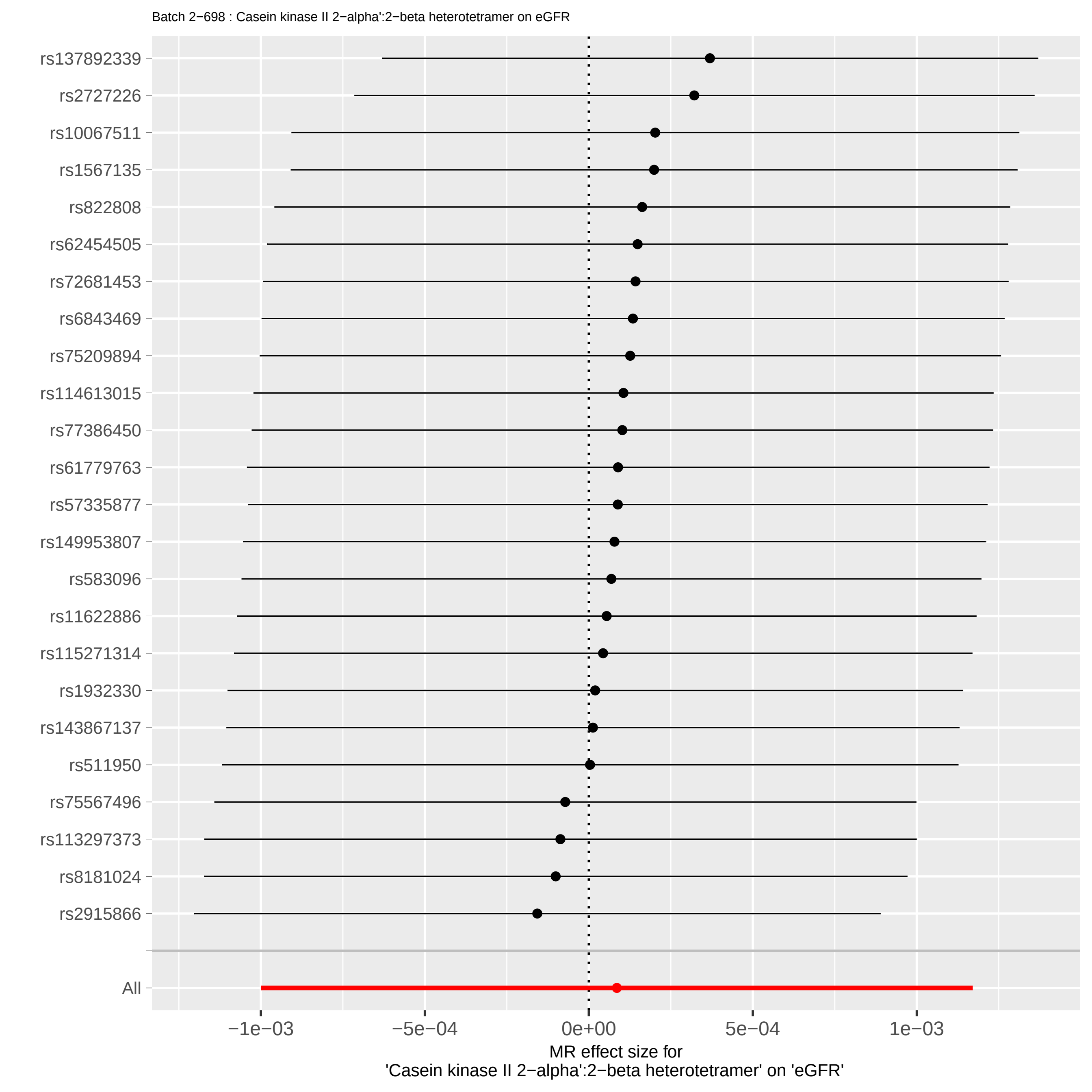
Batch 2−698 : Casein kinase II 2−alpha':2−beta heterotetramer on eGFR
rs137892339
rs2727226
rs10067511
rs1567135
rs822808
rs62454505
rs72681453
rs6843469
rs75209894
rs114613015
rs77386450
rs61779763
rs57335877
rs149953807
rs583096
rs11622886
rs115271314
rs1932330
rs143867137
rs511950
rs75567496
rs113297373
rs8181024
rs2915866
All
−1e−03
−5e−04
0e+00
5e−04
1e−03
MR effect size for
'Casein kinase II 2−alpha':2−beta heterotetramer' on 'eGFR'

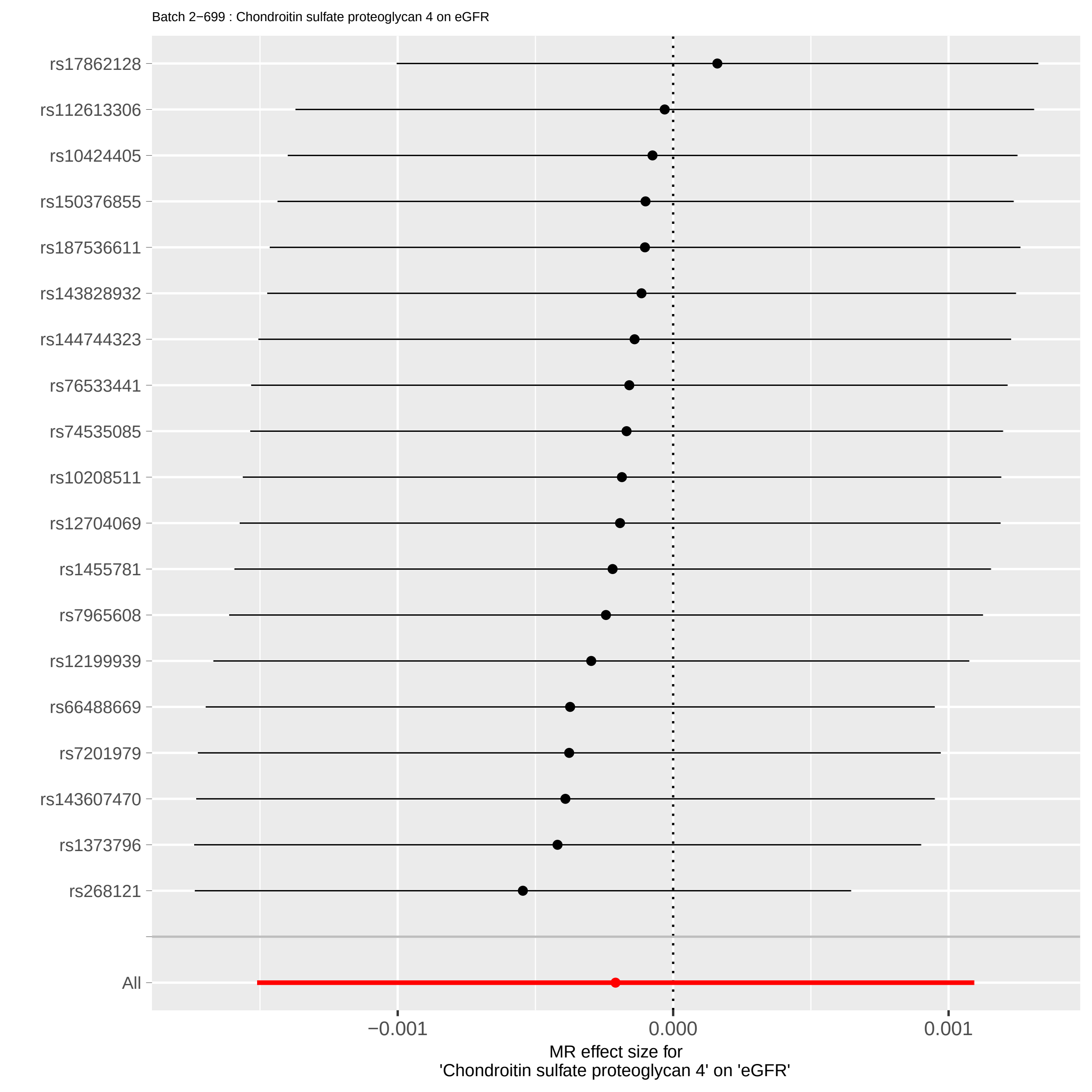

Batch 2−699 : Chondroitin sulfate proteoglycan 4 on eGFR
rs17862128
rs112613306
rs10424405
rs150376855
rs187536611
rs143828932
rs144744323
rs76533441
rs74535085
rs10208511
rs12704069
rs1455781
rs7965608
rs12199939
rs66488669
rs7201979
rs143607470
rs1373796
rs268121
All
−0.001
0.000
0.001
MR effect size for
'Chondroitin sulfate proteoglycan 4' on 'eGFR'

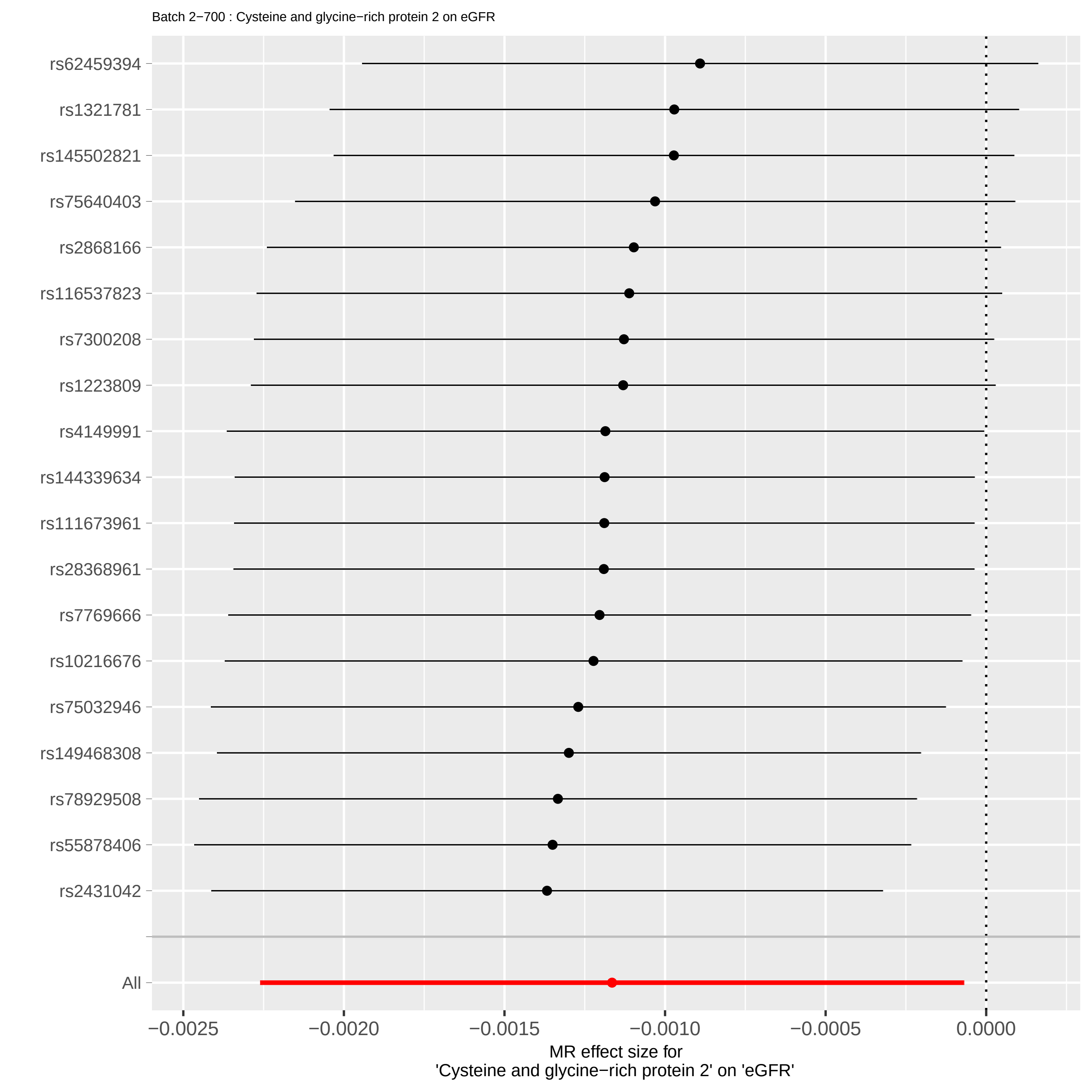

Batch 2−700 : Cysteine and glycine−rich protein 2 on eGFR
rs62459394
rs1321781
rs145502821
rs75640403
rs2868166
rs116537823
rs7300208
rs1223809
rs4149991
rs144339634
rs111673961
rs28368961
rs7769666
rs10216676
rs75032946
rs149468308
rs78929508
rs55878406
rs2431042
All
−0.0025
−0.0020
−0.0015
−0.0010
−0.0005
0.0000
MR effect size for
'Cysteine and glycine−rich protein 2' on 'eGFR'

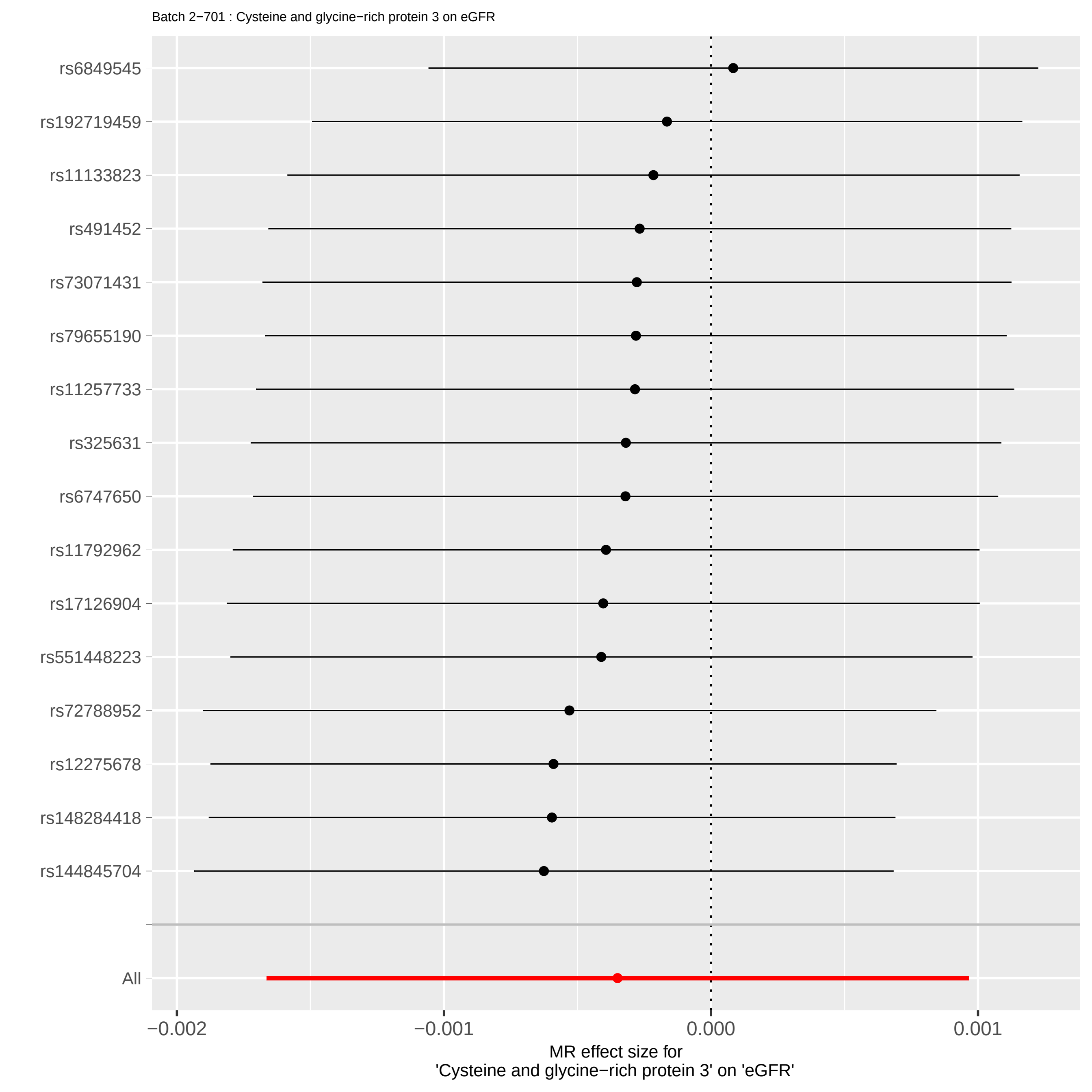

Batch 2−701 : Cysteine and glycine−rich protein 3 on eGFR
rs6849545
rs192719459
rs11133823
rs491452
rs73071431
rs79655190
rs11257733
rs325631
rs6747650
rs11792962
rs17126904
rs551448223
rs72788952
rs12275678
rs148284418
rs144845704
All
−0.002
−0.001
0.000
0.001
MR effect size for
'Cysteine and glycine−rich protein 3' on 'eGFR'

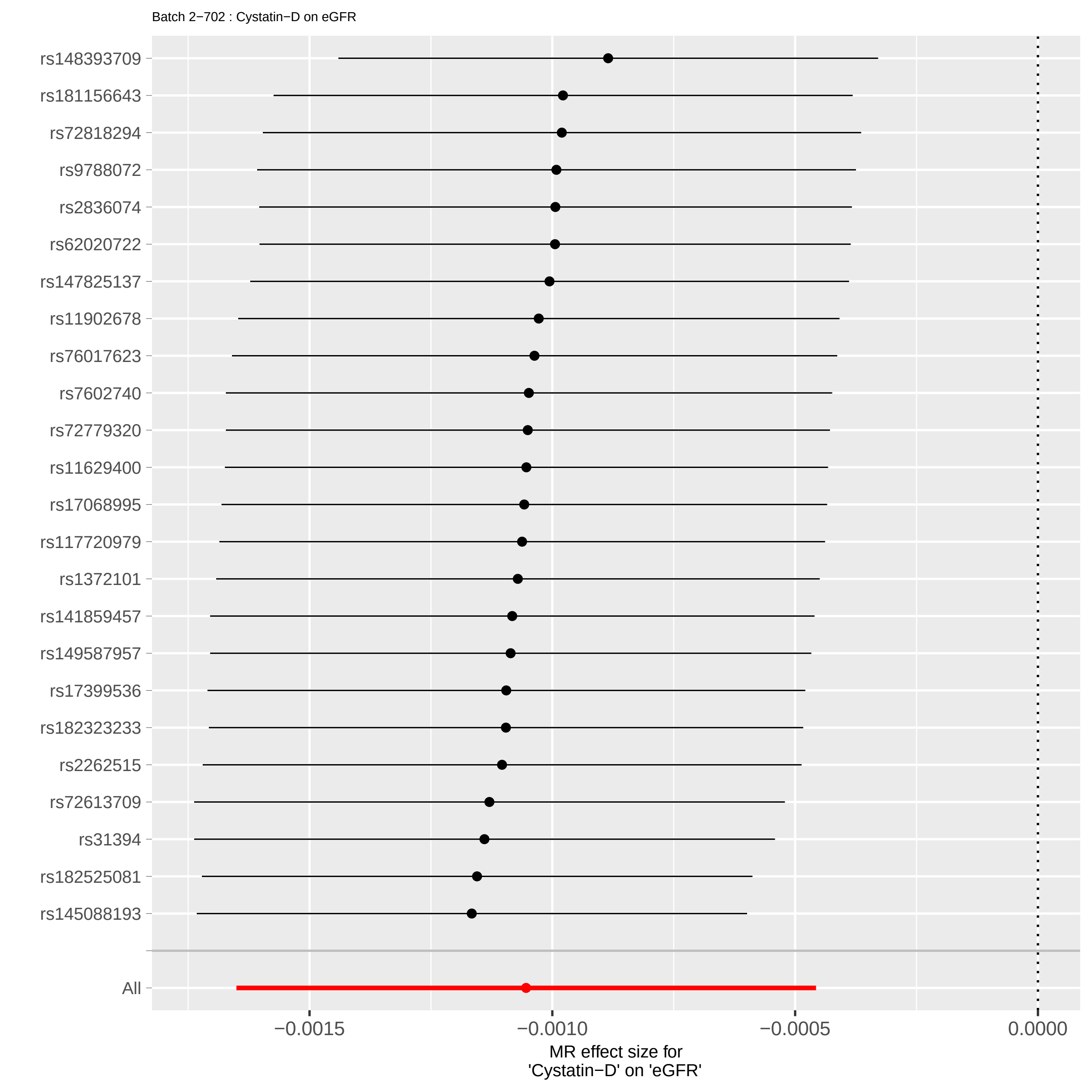
Batch 2−702 : Cystatin−D on eGFR
rs148393709
rs181156643
rs72818294
rs9788072
rs2836074
rs62020722
rs147825137
rs11902678
rs76017623
rs7602740
rs72779320
rs11629400
rs17068995
rs117720979
rs1372101
rs141859457
rs149587957
rs17399536
rs182323233
rs2262515
rs72613709
rs31394
rs182525081
rs145088193
All
−0.0015
−0.0010
−0.0005
0.0000
MR effect size for
'Cystatin−D' on 'eGFR'

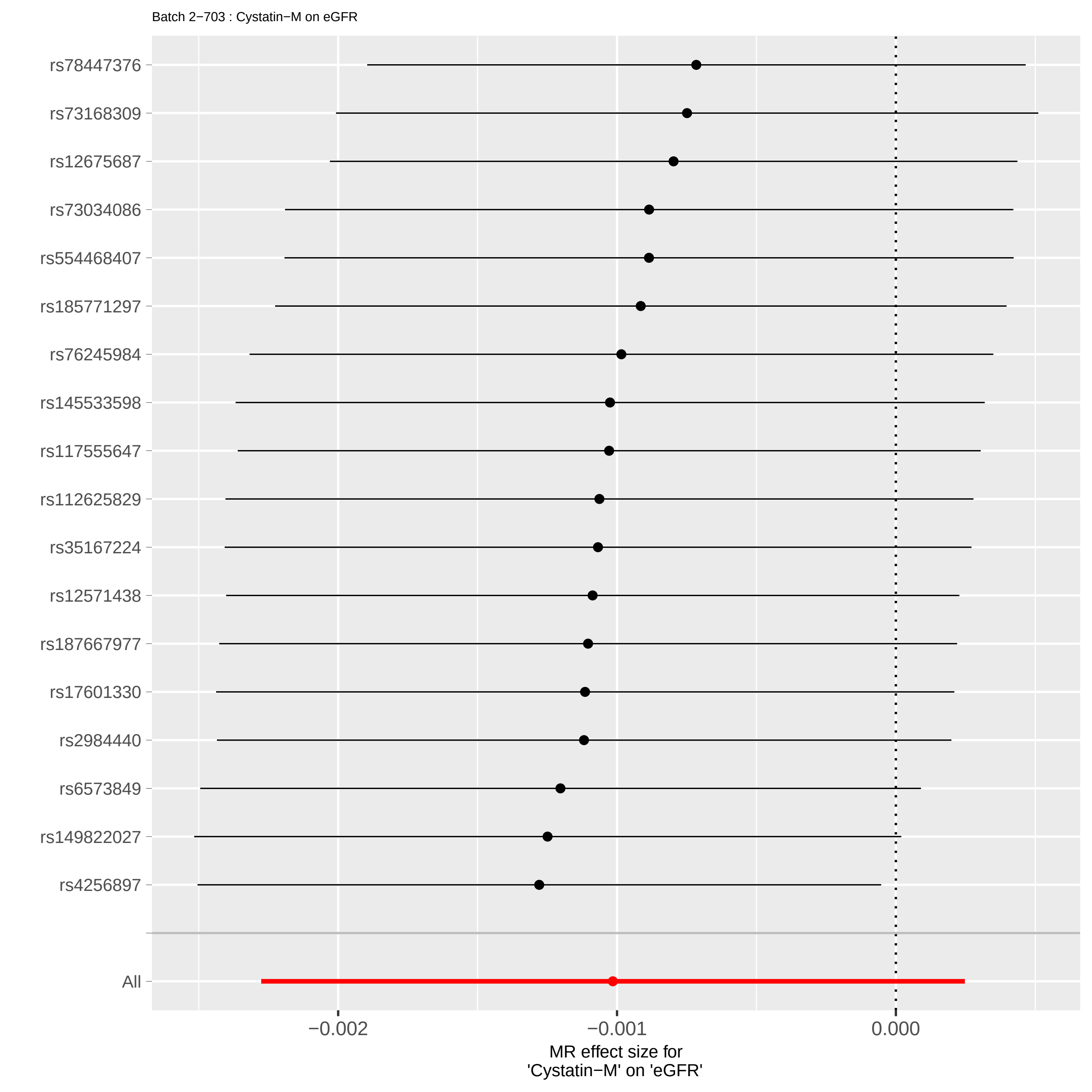

Batch 2−703 : Cystatin−M on eGFR
rs78447376
rs73168309
rs12675687
rs73034086
rs554468407
rs185771297
rs76245984
rs145533598
rs117555647
rs112625829
rs35167224
rs12571438
rs187667977
rs17601330
rs2984440
rs6573849
rs149822027
rs4256897
All
−0.002
−0.001
0.000
MR effect size for
'Cystatin−M' on 'eGFR'

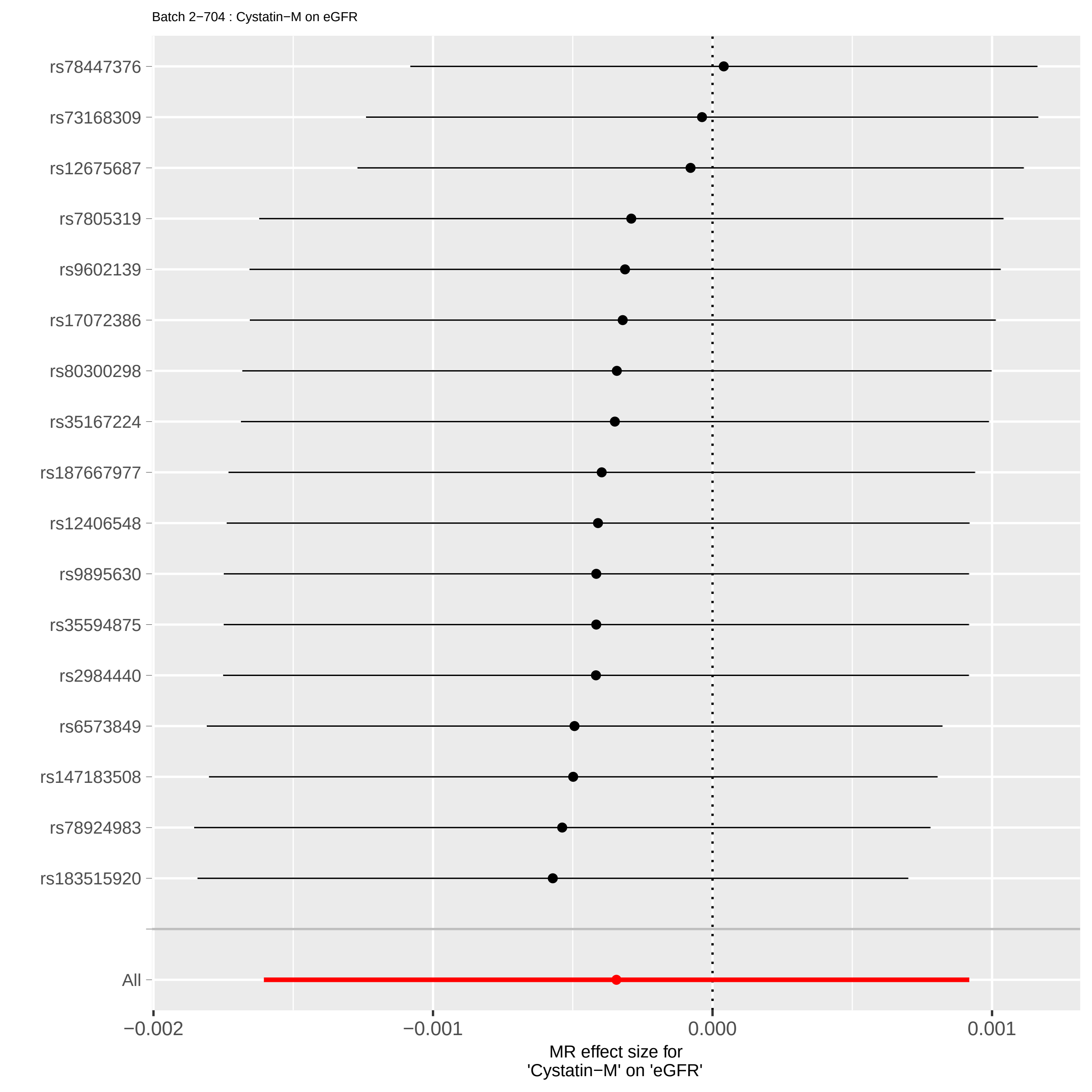

Batch 2−704 : Cystatin−M on eGFR
rs78447376
rs73168309
rs12675687
rs7805319
rs9602139
rs17072386
rs80300298
rs35167224
rs187667977
rs12406548
rs9895630
rs35594875
rs2984440
rs6573849
rs147183508
rs78924983
rs183515920
All
−0.002
−0.001
0.000
0.001
MR effect size for
'Cystatin−M' on 'eGFR'

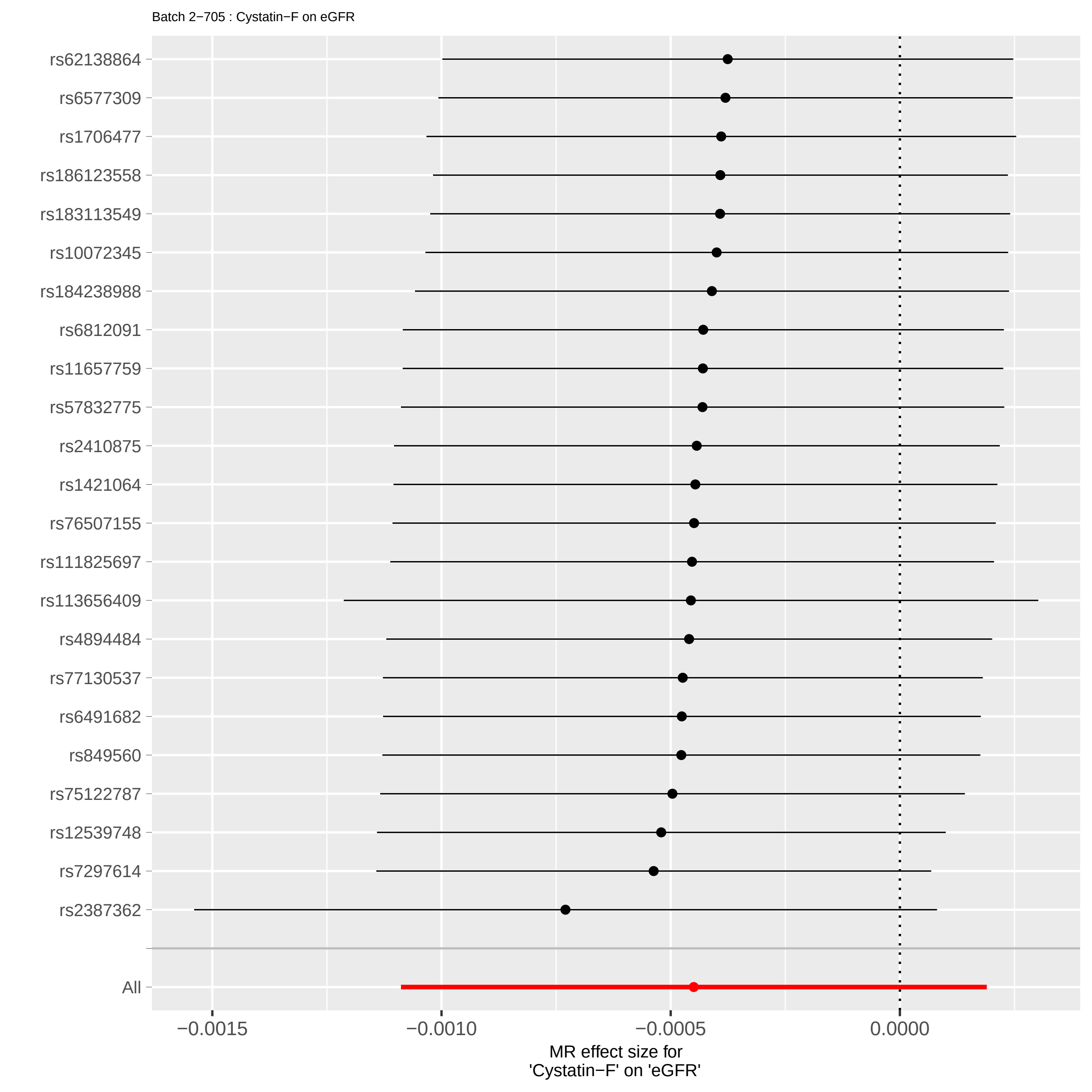
Batch 2−705 : Cystatin−F on eGFR
rs62138864
rs6577309
rs1706477
rs186123558
rs183113549
rs10072345
rs184238988
rs6812091
rs11657759
rs57832775
rs2410875
rs1421064
rs76507155
rs111825697
rs113656409
rs4894484
rs77130537
rs6491682
rs849560
rs75122787
rs12539748
rs7297614
rs2387362
All
−0.0015
−0.0010
−0.0005
0.0000
MR effect size for
'Cystatin−F' on 'eGFR'

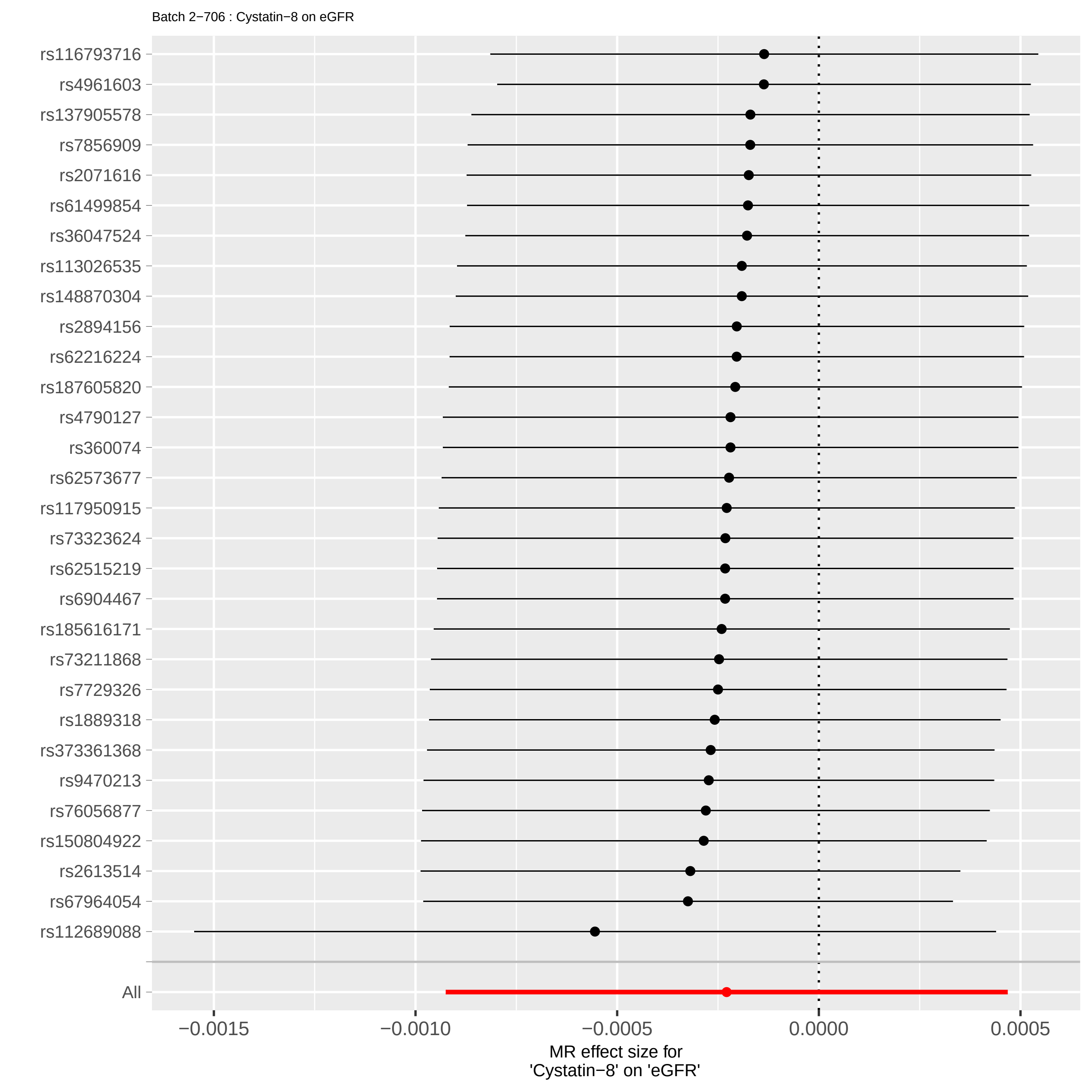

Batch 2−706 : Cystatin−8 on eGFR
rs116793716
rs4961603
rs137905578
rs7856909
rs2071616
rs61499854
rs36047524
rs113026535
rs148870304
rs2894156
rs62216224
rs187605820
rs4790127
rs360074
rs62573677
rs117950915
rs73323624
rs62515219
rs6904467
rs185616171
rs73211868
rs7729326
rs1889318
rs373361368
rs9470213
rs76056877
rs150804922
rs2613514
rs67964054
rs112689088
All
−0.0015
−0.0010
−0.0005
0.0000
0.0005
MR effect size for
'Cystatin−8' on 'eGFR'

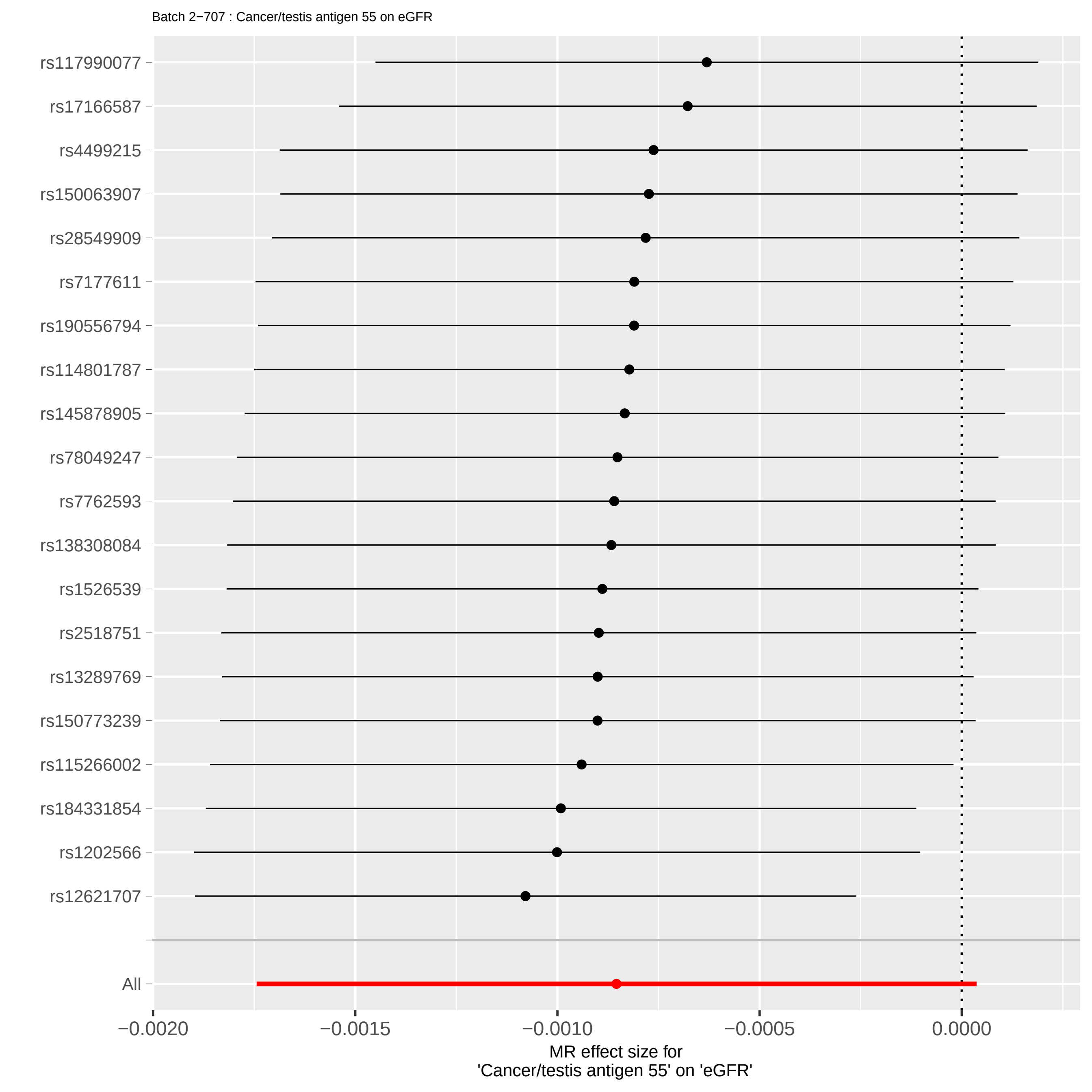

Batch 2−707 : Cancer/testis antigen 55 on eGFR
rs117990077
rs17166587
rs4499215
rs150063907
rs28549909
rs7177611
rs190556794
rs114801787
rs145878905
rs78049247
rs7762593
rs138308084
rs1526539
rs2518751
rs13289769
rs150773239
rs115266002
rs184331854
rs1202566
rs12621707
All
−0.0020
−0.0015
−0.0010
−0.0005
0.0000
MR effect size for
'Cancer/testis antigen 55' on 'eGFR'

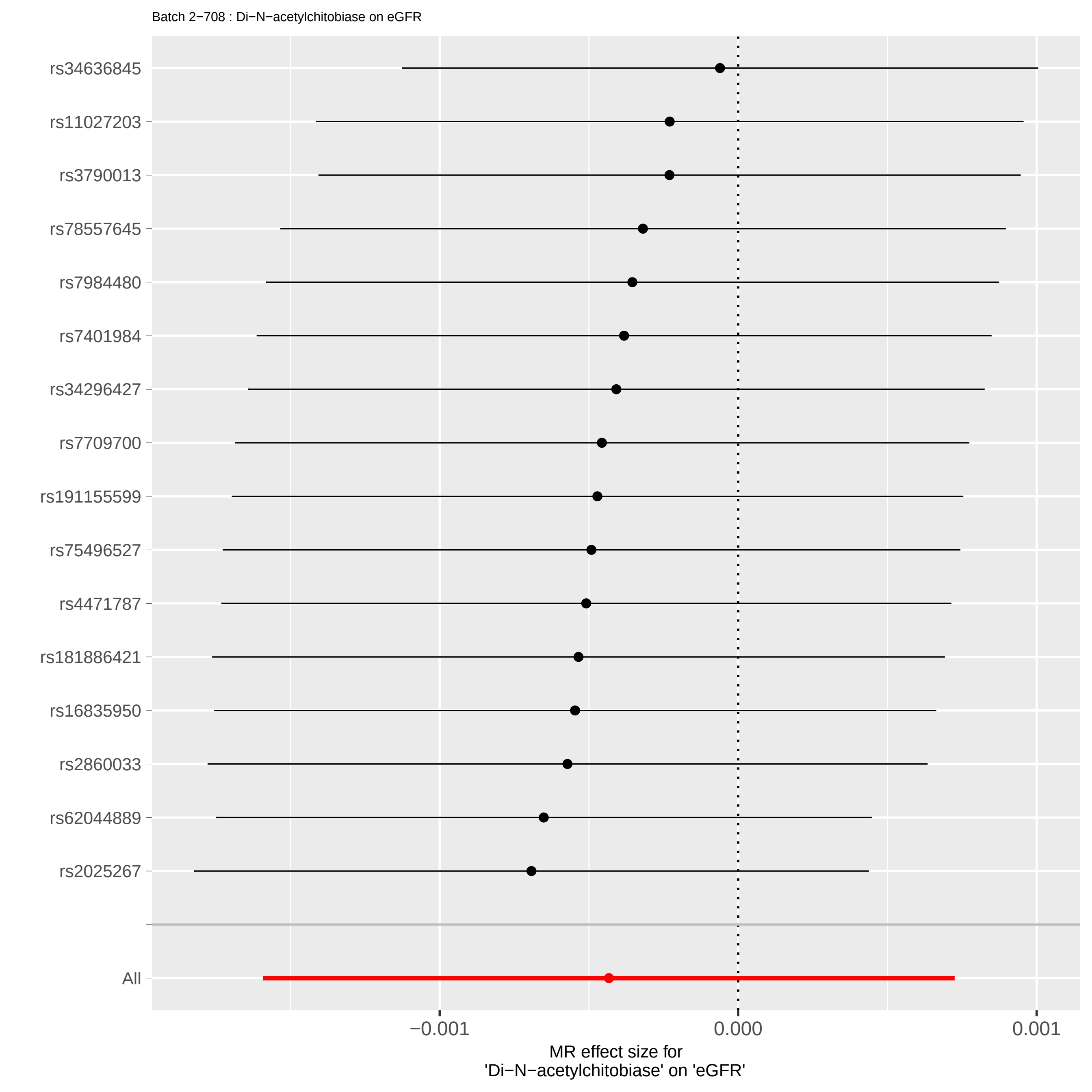
Batch 2−708 : Di−N−acetylchitobiase on eGFR
rs34636845
rs11027203
rs3790013
rs78557645
rs7984480
rs7401984
rs34296427
rs7709700
rs191155599
rs75496527
rs4471787
rs181886421
rs16835950
rs2860033
rs62044889
rs2025267
All
−0.001
0.000
0.001
MR effect size for
'Di−N−acetylchitobiase' on 'eGFR'

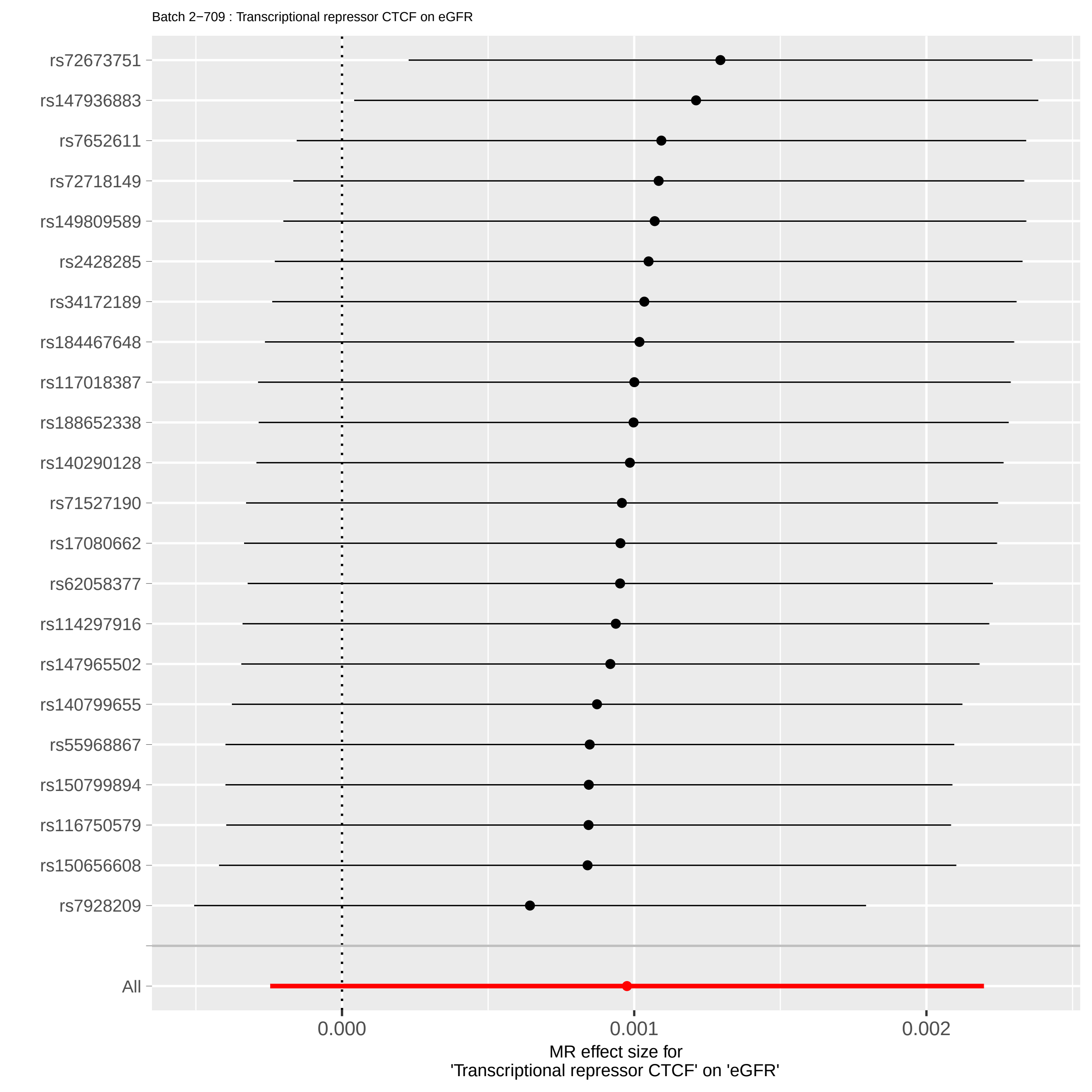

Batch 2−709 : Transcriptional repressor CTCF on eGFR
rs72673751
rs147936883
rs7652611
rs72718149
rs149809589
rs2428285
rs34172189
rs184467648
rs117018387
rs188652338
rs140290128
rs71527190
rs17080662
rs62058377
rs114297916
rs147965502
rs140799655
rs55968867
rs150799894
rs116750579
rs150656608
rs7928209
All
0.000
0.001
0.002
MR effect size for
'Transcriptional repressor CTCF' on 'eGFR'

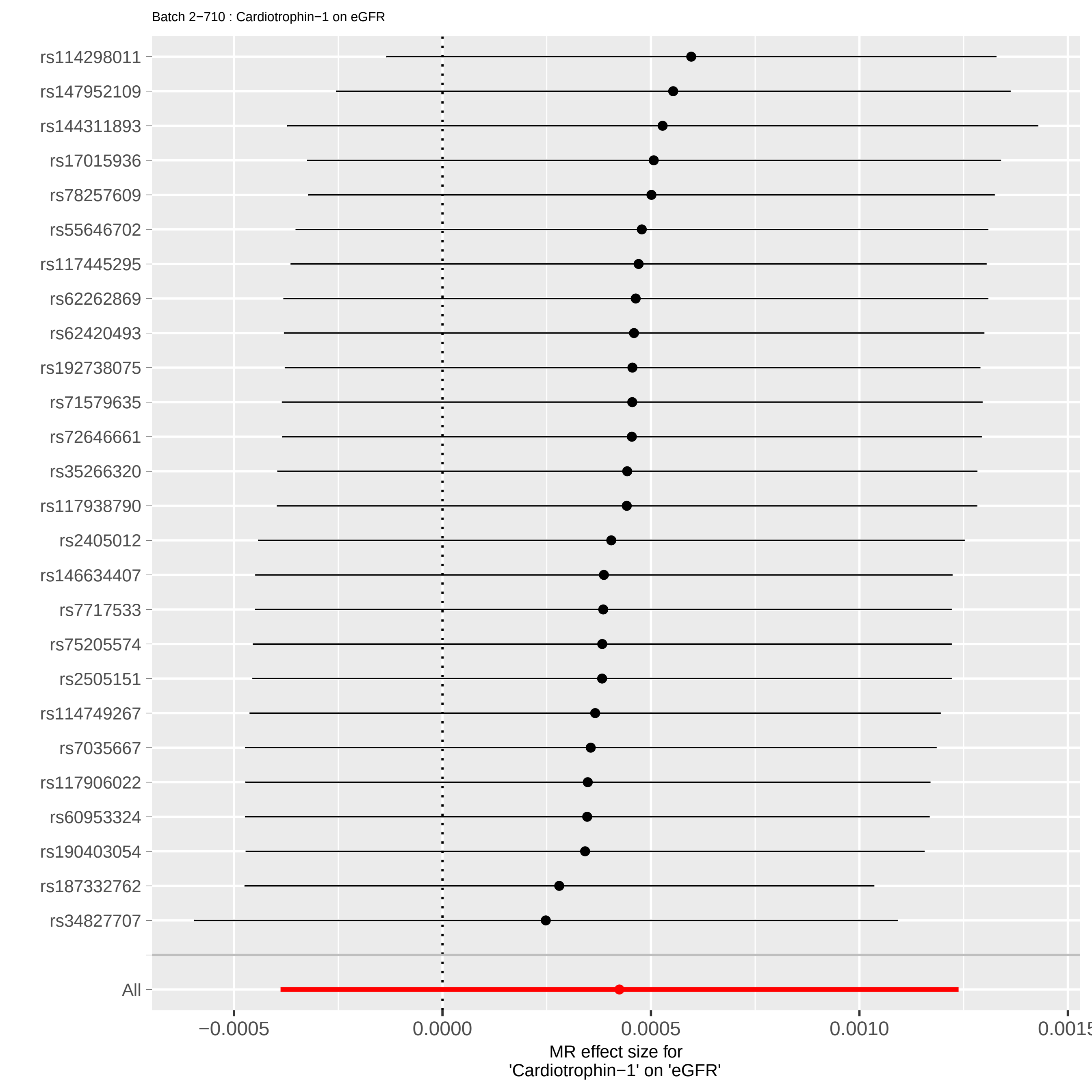

Batch 2−710 : Cardiotrophin−1 on eGFR
rs114298011
rs147952109
rs144311893
rs17015936
rs78257609
rs55646702
rs117445295
rs62262869
rs62420493
rs192738075
rs71579635
rs72646661
rs35266320
rs117938790
rs2405012
rs146634407
rs7717533
rs75205574
rs2505151
rs114749267
rs7035667
rs117906022
rs60953324
rs190403054
rs187332762
rs34827707
All
−0.0005
0.0000
0.0005
0.0010
MR effect size for
'Cardiotrophin−1' on 'eGFR'

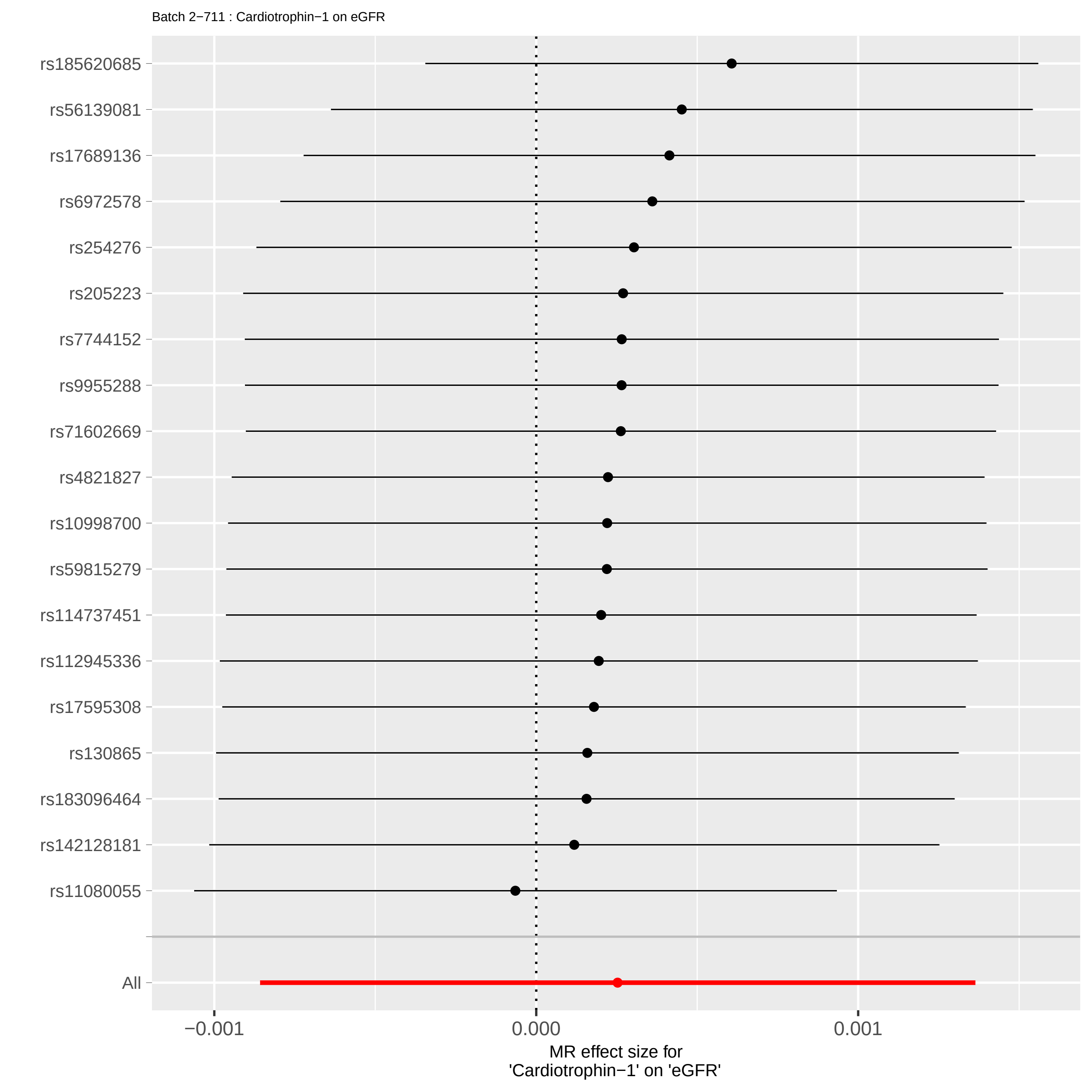
Batch 2−711 : Cardiotrophin−1 on eGFR
rs185620685
rs56139081
rs17689136
rs6972578
rs254276
rs205223
rs7744152
rs9955288
rs71602669
rs4821827
rs10998700
rs59815279
rs114737451
rs112945336
rs17595308
rs130865
rs183096464
rs142128181
rs11080055
All
−0.001
0.000
0.001
MR effect size for
'Cardiotrophin−1' on 'eGFR'

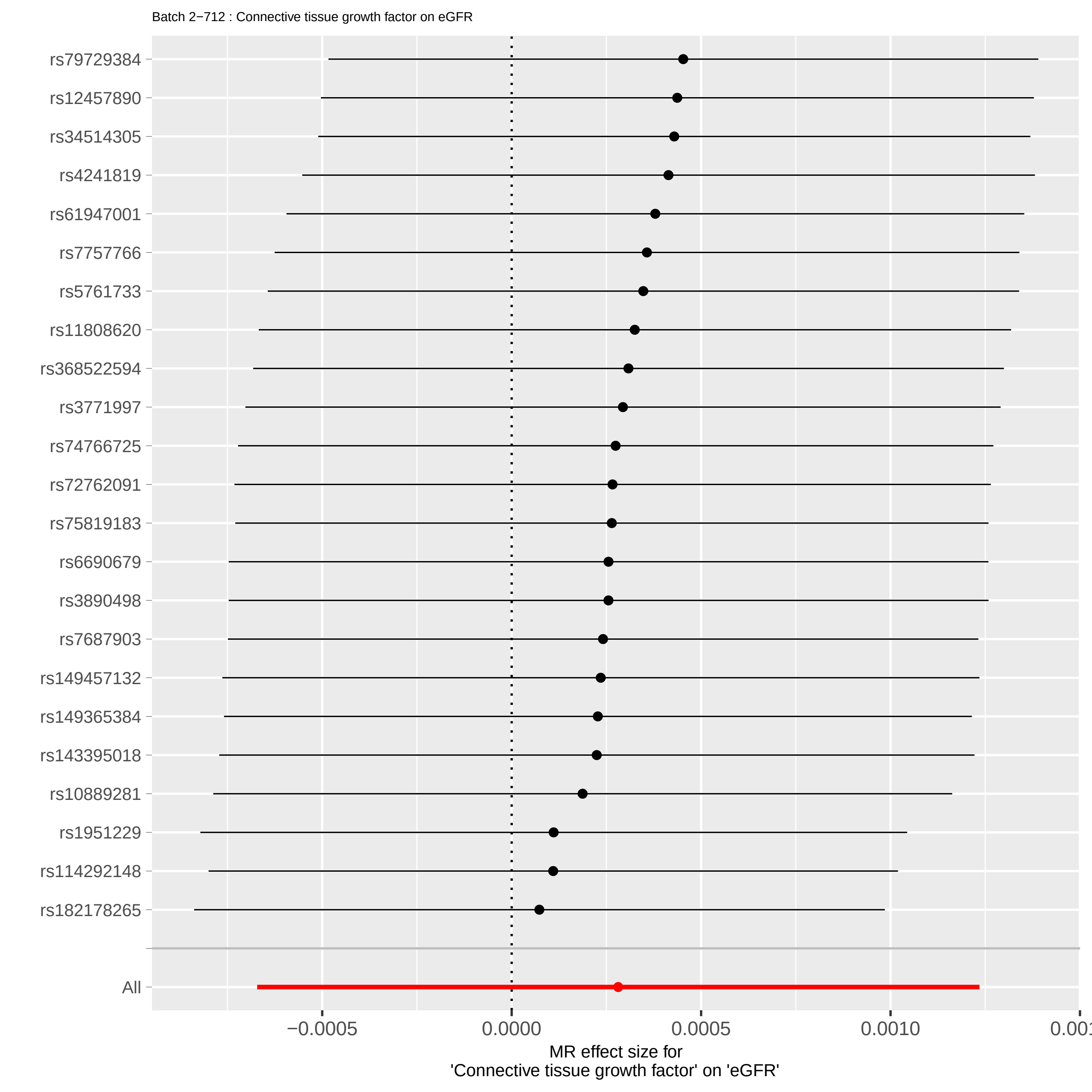

Batch 2−712 : Connective tissue growth factor on eGFR
rs79729384
rs12457890
rs34514305
rs4241819
rs61947001
rs7757766
rs5761733
rs11808620
rs368522594
rs3771997
rs74766725
rs72762091
rs75819183
rs6690679
rs3890498
rs7687903
rs149457132
rs149365384
rs143395018
rs10889281
rs1951229
rs114292148
rs182178265
All
−0.0005
0.0000
0.0005
0.0010
MR effect size for
'Connective tissue growth factor' on 'eGFR'

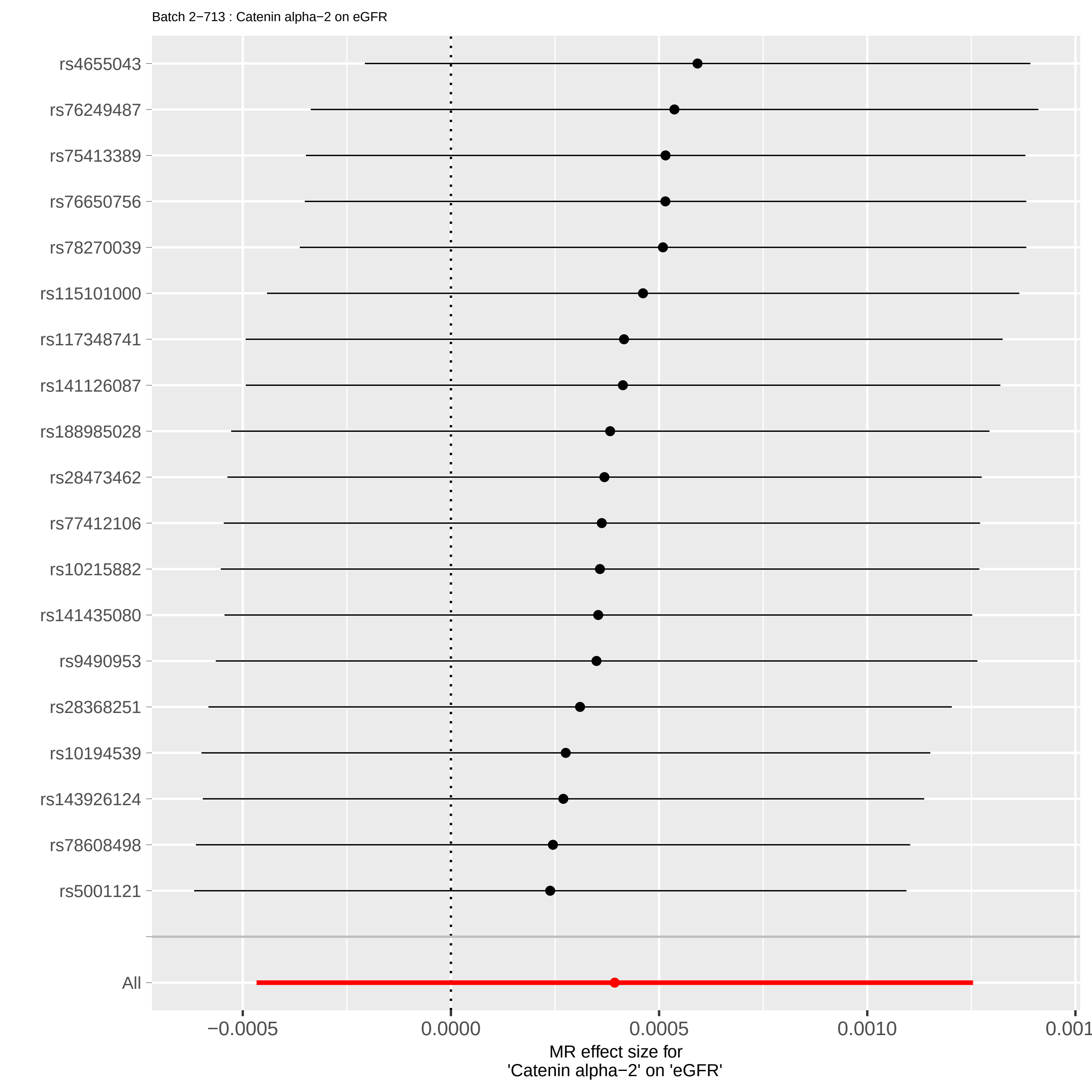
Batch 2−713 : Catenin alpha−2 on eGFR
rs4655043
rs76249487
rs75413389
rs76650756
rs78270039
rs115101000
rs117348741
rs141126087
rs188985028
rs28473462
rs77412106
rs10215882
rs141435080
rs9490953
rs28368251
rs10194539
rs143926124
rs78608498
rs5001121
All
−0.0005
0.0000
0.0005
0.0010
0.001
MR effect size for
'Catenin alpha−2' on 'eGFR'

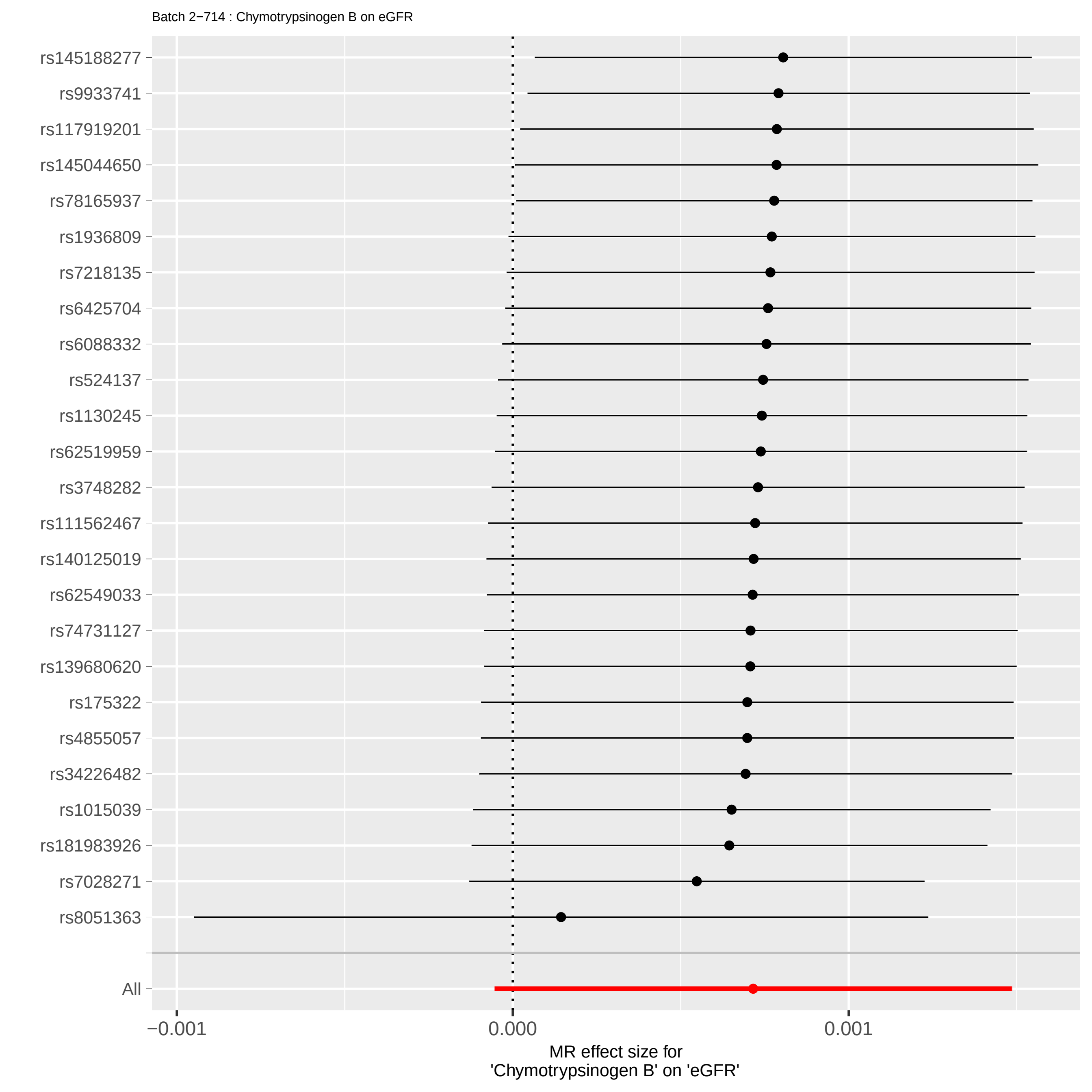
Batch 2−714 : Chymotrypsinogen B on eGFR
rs145188277
rs9933741
rs117919201
rs145044650
rs78165937
rs1936809
rs7218135
rs6425704
rs6088332
rs524137
rs1130245
rs62519959
rs3748282
rs111562467
rs140125019
rs62549033
rs74731127
rs139680620
rs175322
rs4855057
rs34226482
rs1015039
rs181983926
rs7028271
rs8051363
All
−0.001
0.000
0.001
MR effect size for
'Chymotrypsinogen B' on 'eGFR'

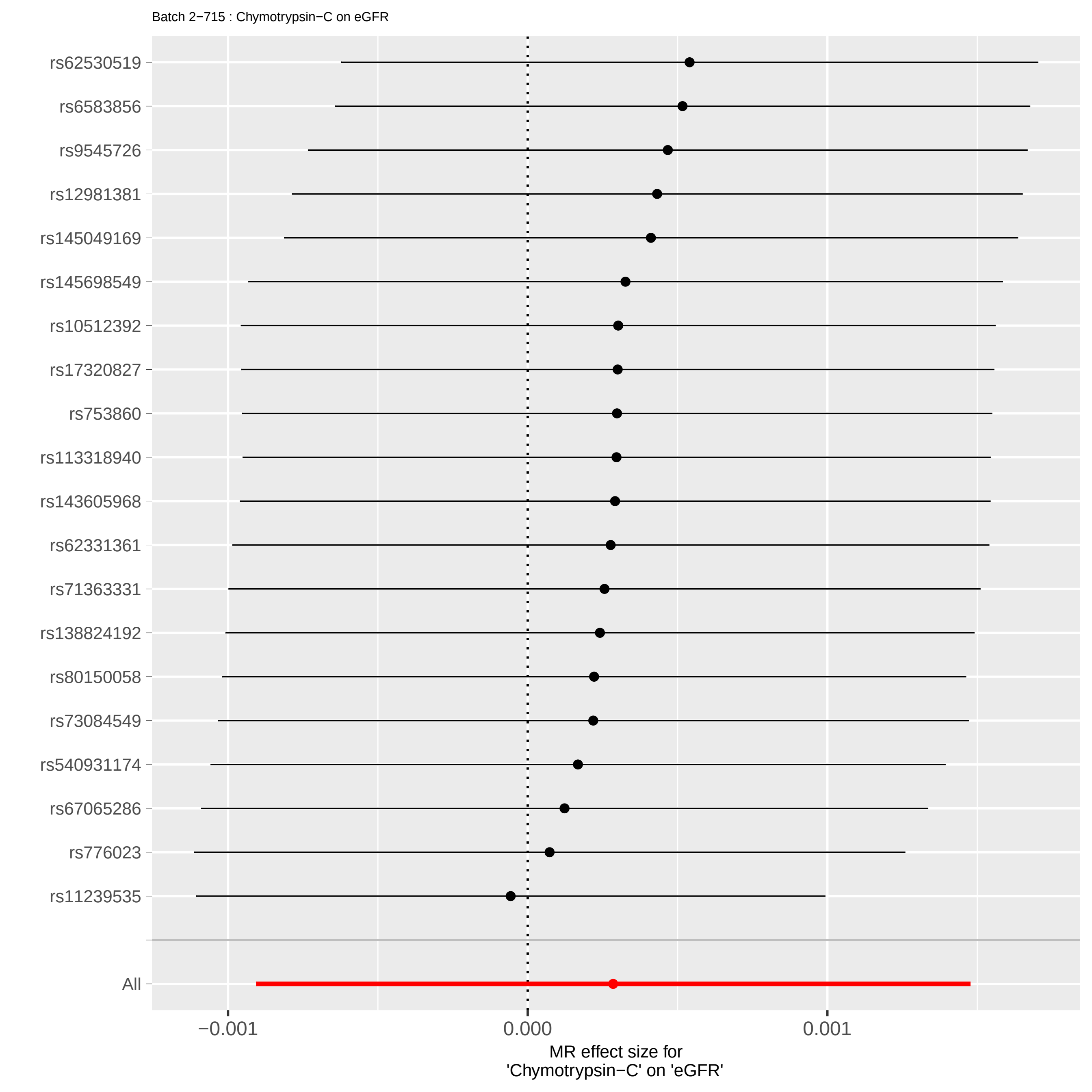
Batch 2−715 : Chymotrypsin−C on eGFR
rs62530519
rs6583856
rs9545726
rs12981381
rs145049169
rs145698549
rs10512392
rs17320827
rs753860
rs113318940
rs143605968
rs62331361
rs71363331
rs138824192
rs80150058
rs73084549
rs540931174
rs67065286
rs776023
rs11239535
All
−0.001
0.000
0.001
MR effect size for
'Chymotrypsin−C' on 'eGFR'

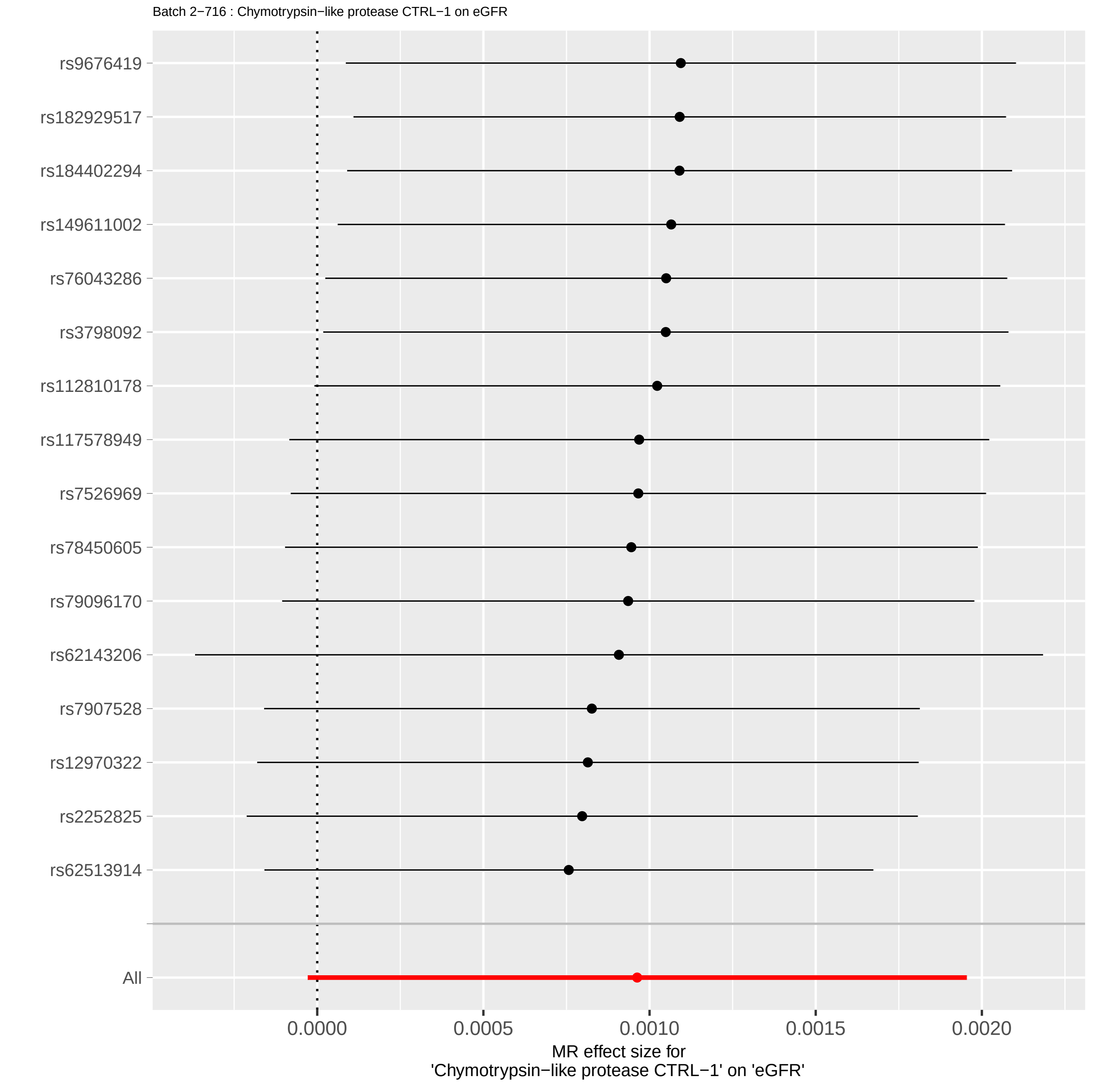

Batch 2−716 : Chymotrypsin−like protease CTRL−1 on eGFR
rs9676419
rs182929517
rs184402294
rs149611002
rs76043286
rs3798092
rs112810178
rs117578949
rs7526969
rs78450605
rs79096170
rs62143206
rs7907528
rs12970322
rs2252825
rs62513914
All
0.0000
0.0005
0.0010
0.0015
0.0020
MR effect size for
'Chymotrypsin−like protease CTRL−1' on 'eGFR'

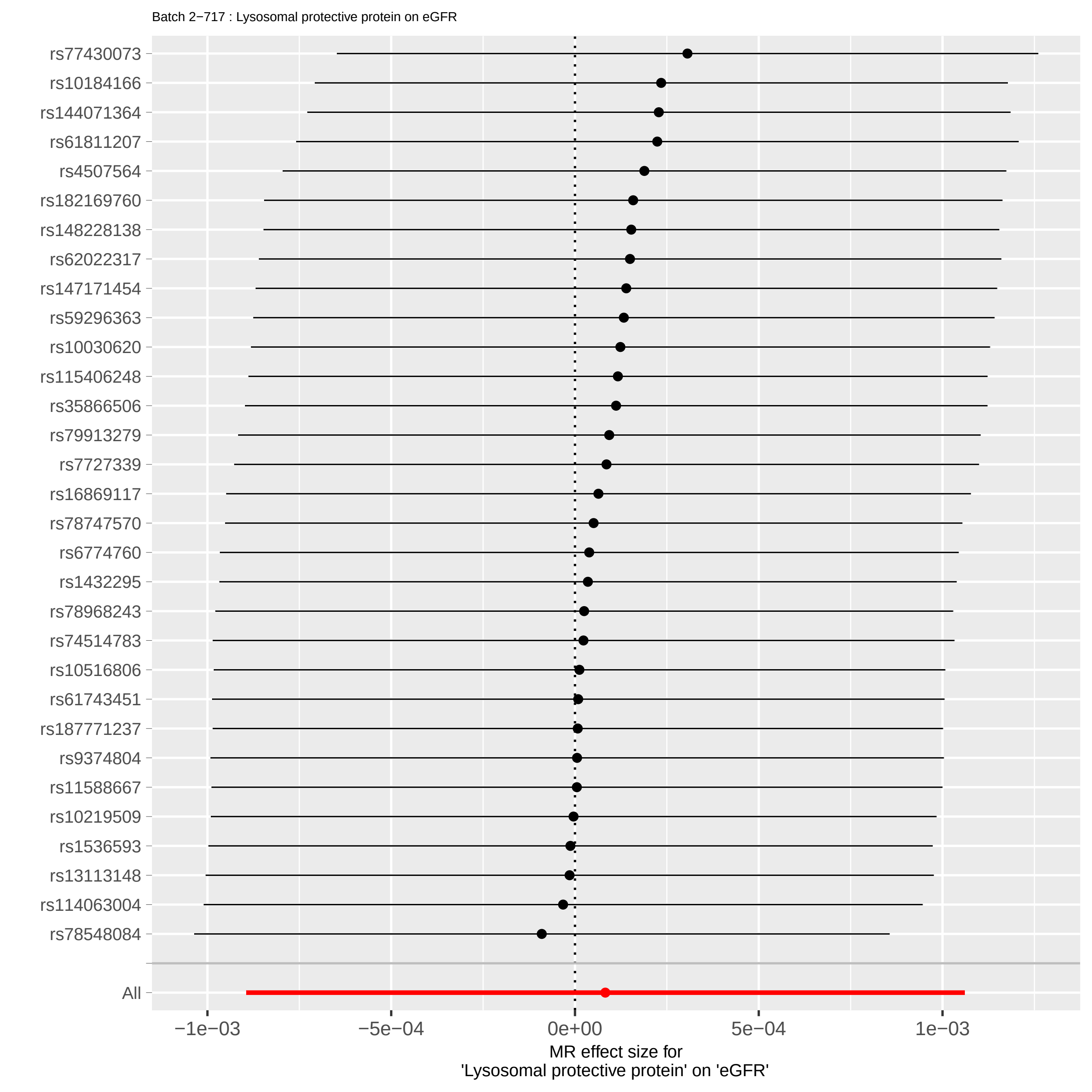
Batch 2−717 : Lysosomal protective protein on eGFR
rs77430073
rs10184166
rs144071364
rs61811207
rs4507564
rs182169760
rs148228138
rs62022317
rs147171454
rs59296363
rs10030620
rs115406248
rs35866506
rs79913279
rs7727339
rs16869117
rs78747570
rs6774760
rs1432295
rs78968243
rs74514783
rs10516806
rs61743451
rs187771237
rs9374804
rs11588667
rs10219509
rs1536593
rs13113148
rs114063004
rs78548084
All
−1e−03
−5e−04
0e+00
5e−04
1e−03
MR effect size for
'Lysosomal protective protein' on 'eGFR'

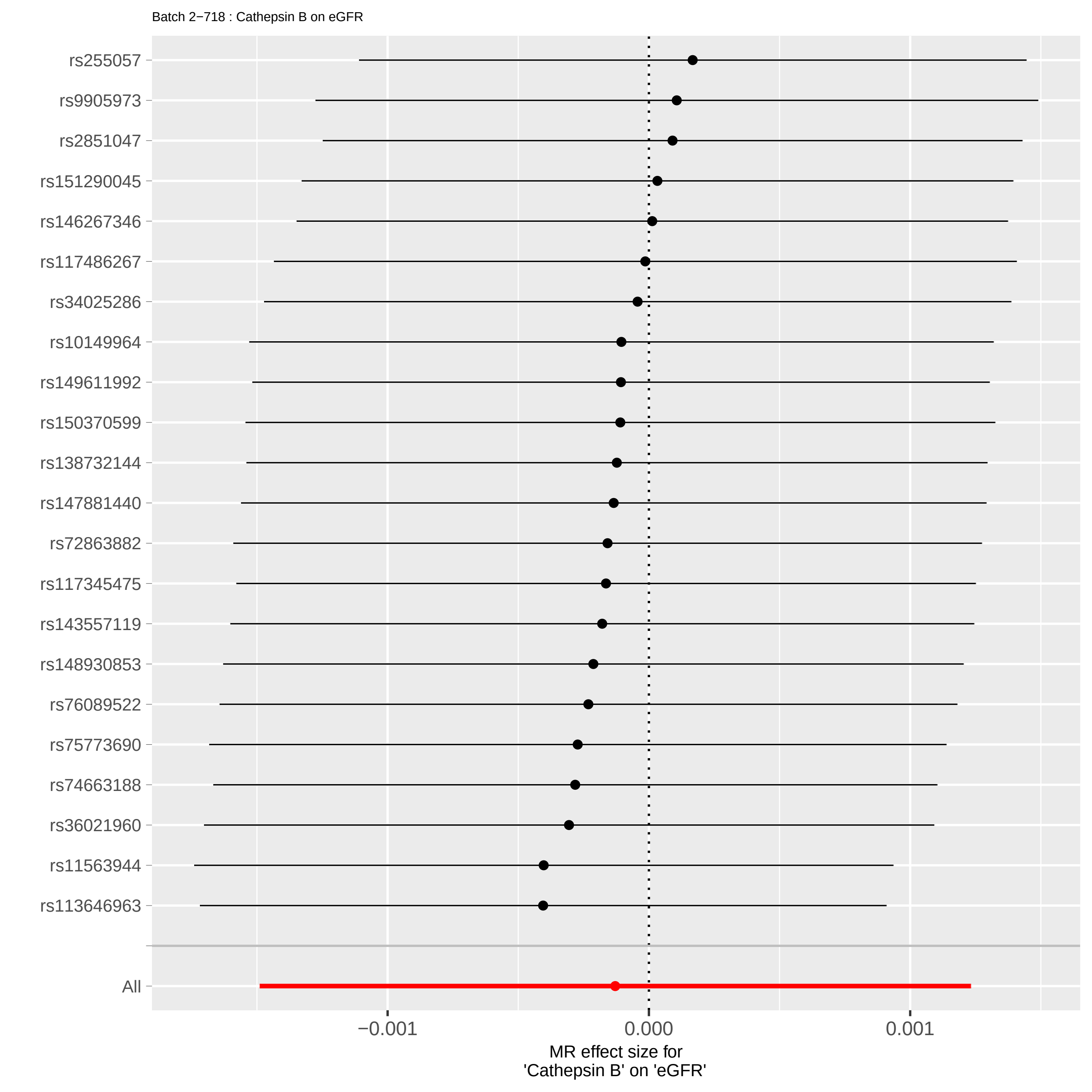

Batch 2−718 : Cathepsin B on eGFR
rs255057
rs9905973
rs2851047
rs151290045
rs146267346
rs117486267
rs34025286
rs10149964
rs149611992
rs150370599
rs138732144
rs147881440
rs72863882
rs117345475
rs143557119
rs148930853
rs76089522
rs75773690
rs74663188
rs36021960
rs11563944
rs113646963
All
−0.001
0.000
0.001
MR effect size for
'Cathepsin B' on 'eGFR'

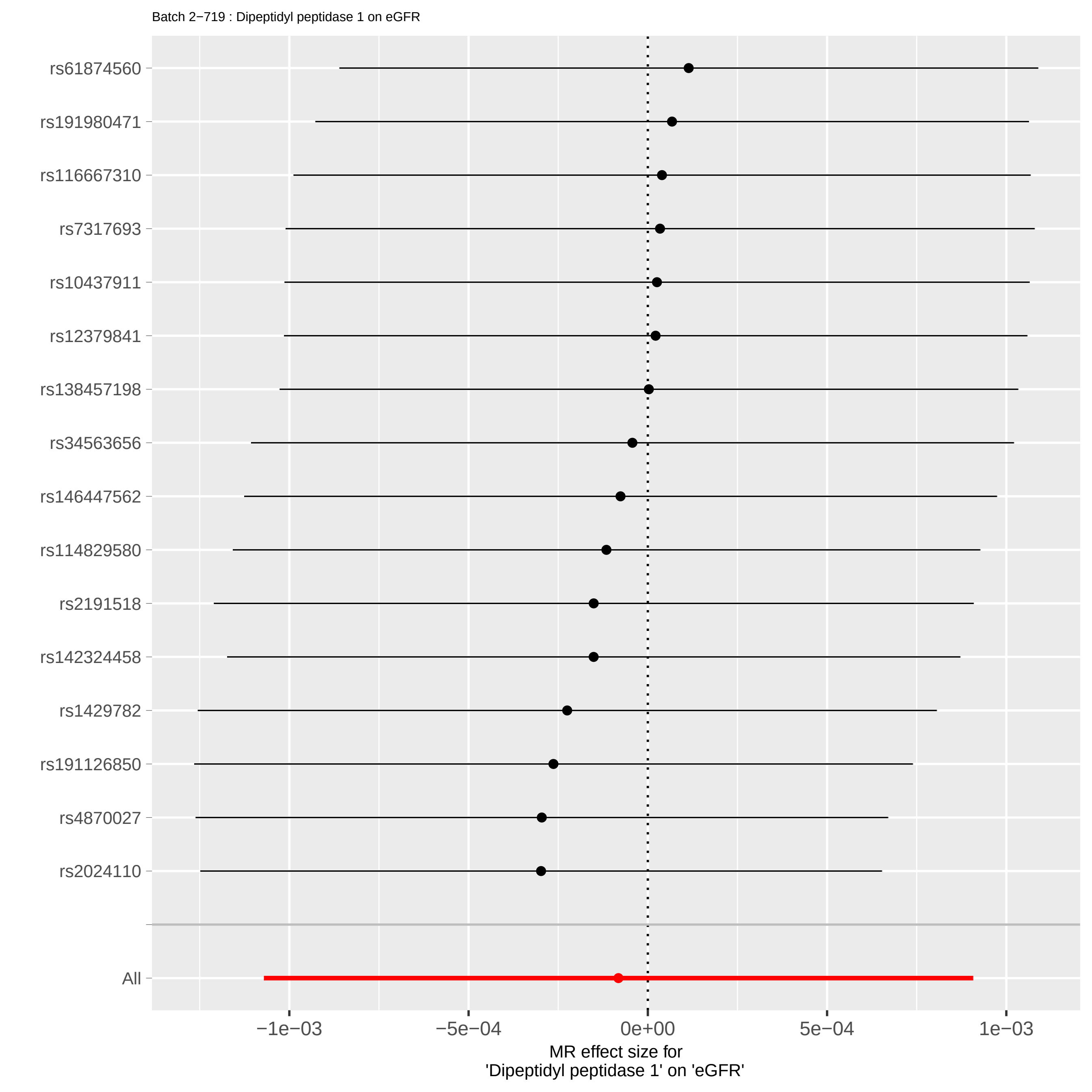

Batch 2−719 : Dipeptidyl peptidase 1 on eGFR
rs61874560
rs191980471
rs116667310
rs7317693
rs10437911
rs12379841
rs138457198
rs34563656
rs146447562
rs114829580
rs2191518
rs142324458
rs1429782
rs191126850
rs4870027
rs2024110
All
−1e−03
−5e−04
0e+00
5e−04
1e−03
MR effect size for
'Dipeptidyl peptidase 1' on 'eGFR'

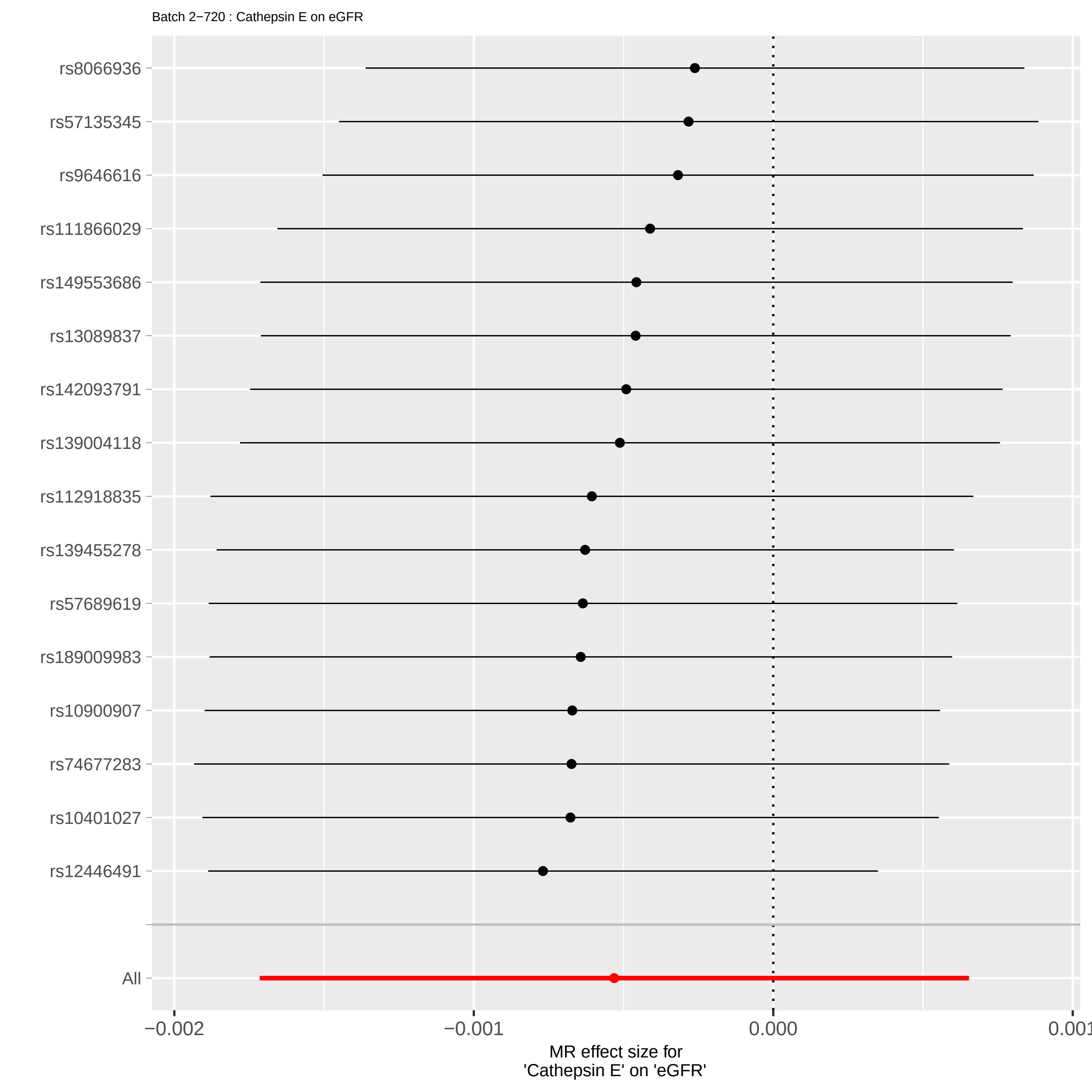
Batch 2−720 : Cathepsin E on eGFR
rs8066936
rs57135345
rs9646616
rs111866029
rs149553686
rs13089837
rs142093791
rs139004118
rs112918835
rs139455278
rs57689619
rs189009983
rs10900907
rs74677283
rs10401027
rs12446491
All
−0.002
−0.001
0.000
0.001
MR effect size for
'Cathepsin E' on 'eGFR'

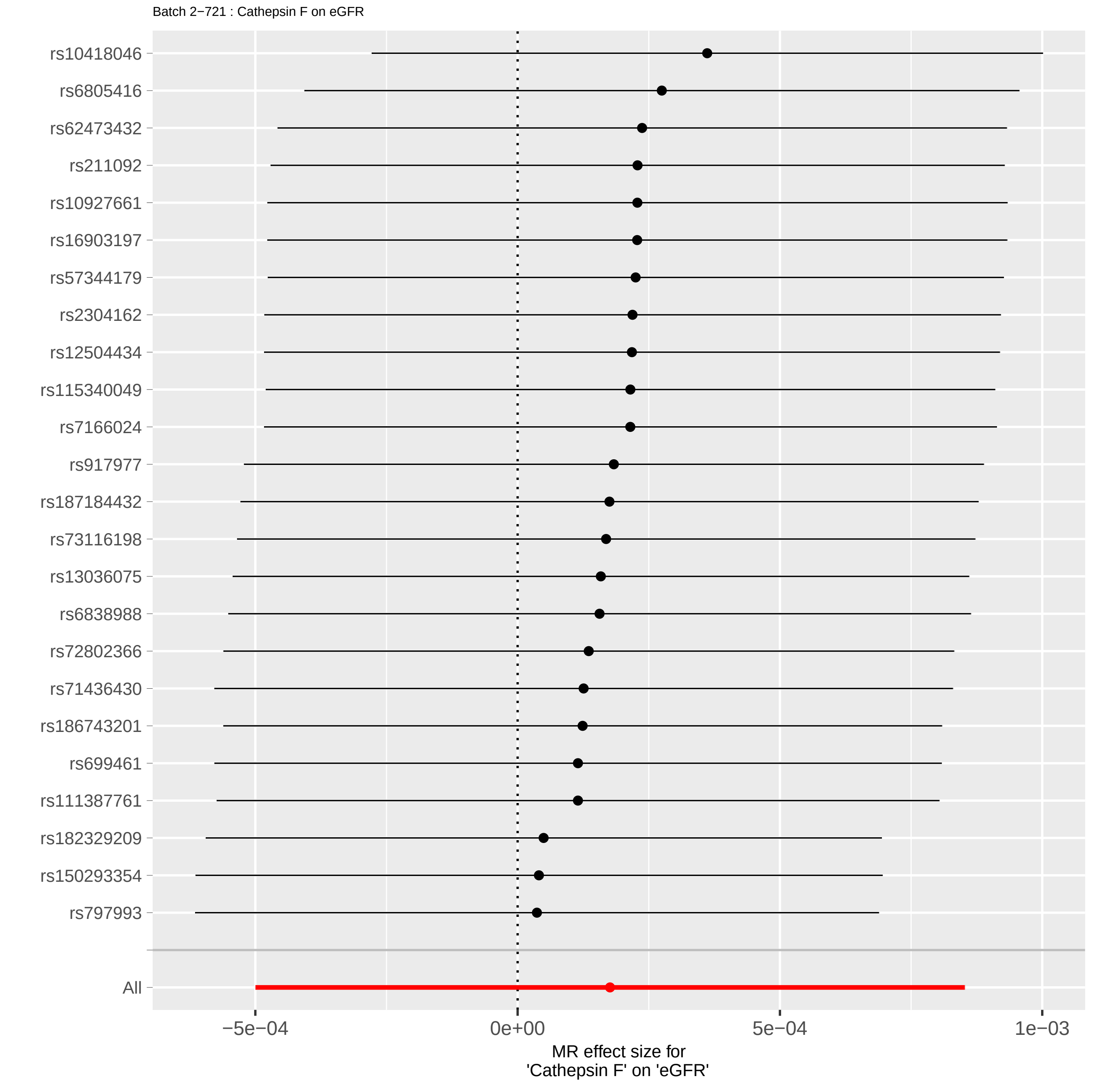

Batch 2−721 : Cathepsin F on eGFR
rs10418046
rs6805416
rs62473432
rs211092
rs10927661
rs16903197
rs57344179
rs2304162
rs12504434
rs115340049
rs7166024
rs917977
rs187184432
rs73116198
rs13036075
rs6838988
rs72802366
rs71436430
rs186743201
rs699461
rs111387761
rs182329209
rs150293354
rs797993
All
−5e−04
0e+00
5e−04
1e−03
MR effect size for
'Cathepsin F' on 'eGFR'

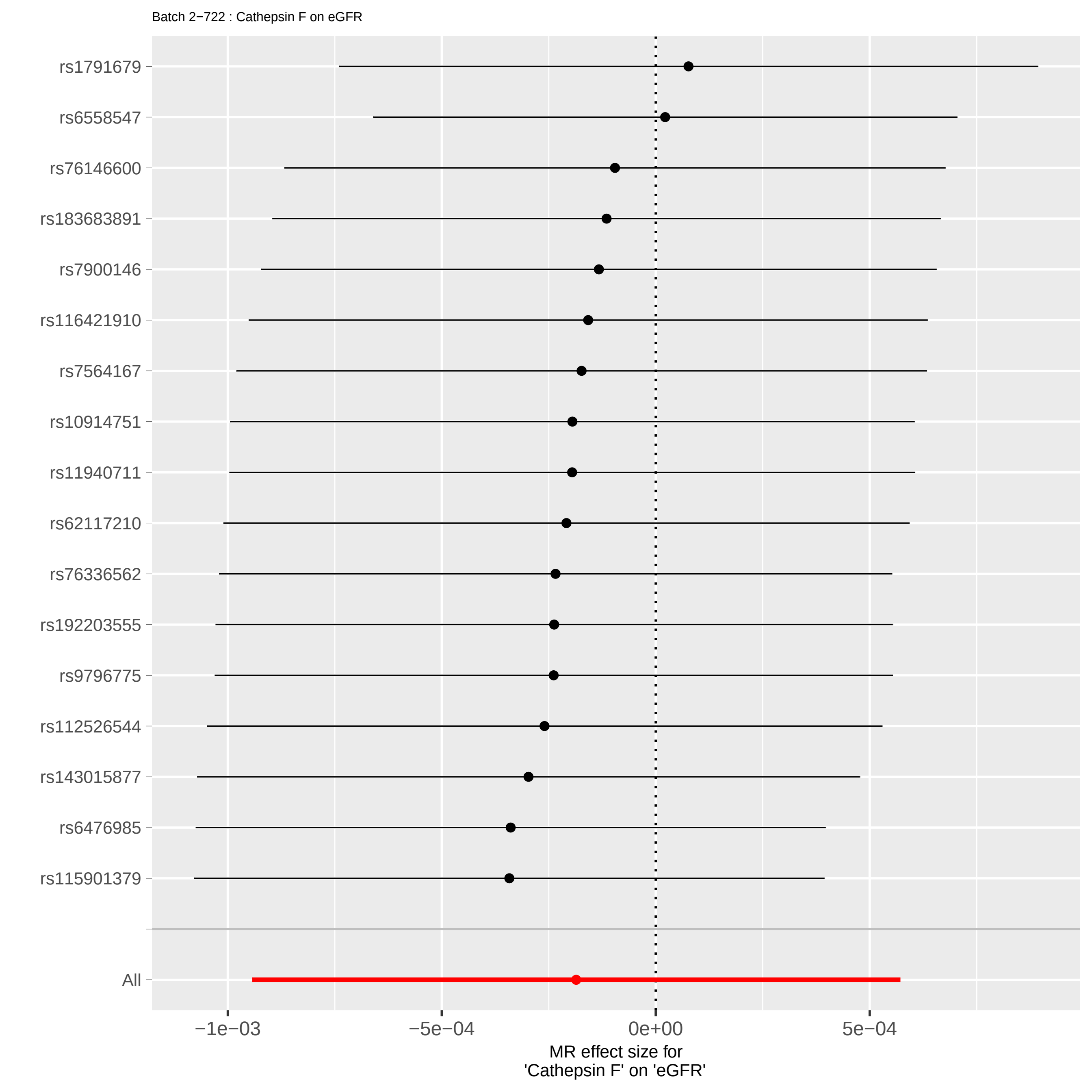

Batch 2−722 : Cathepsin F on eGFR
rs1791679
rs6558547
rs76146600
rs183683891
rs7900146
rs116421910
rs7564167
rs10914751
rs11940711
rs62117210
rs76336562
rs192203555
rs9796775
rs112526544
rs143015877
rs6476985
rs115901379
All
−1e−03
−5e−04
0e+00
5e−04
MR effect size for
'Cathepsin F' on 'eGFR'

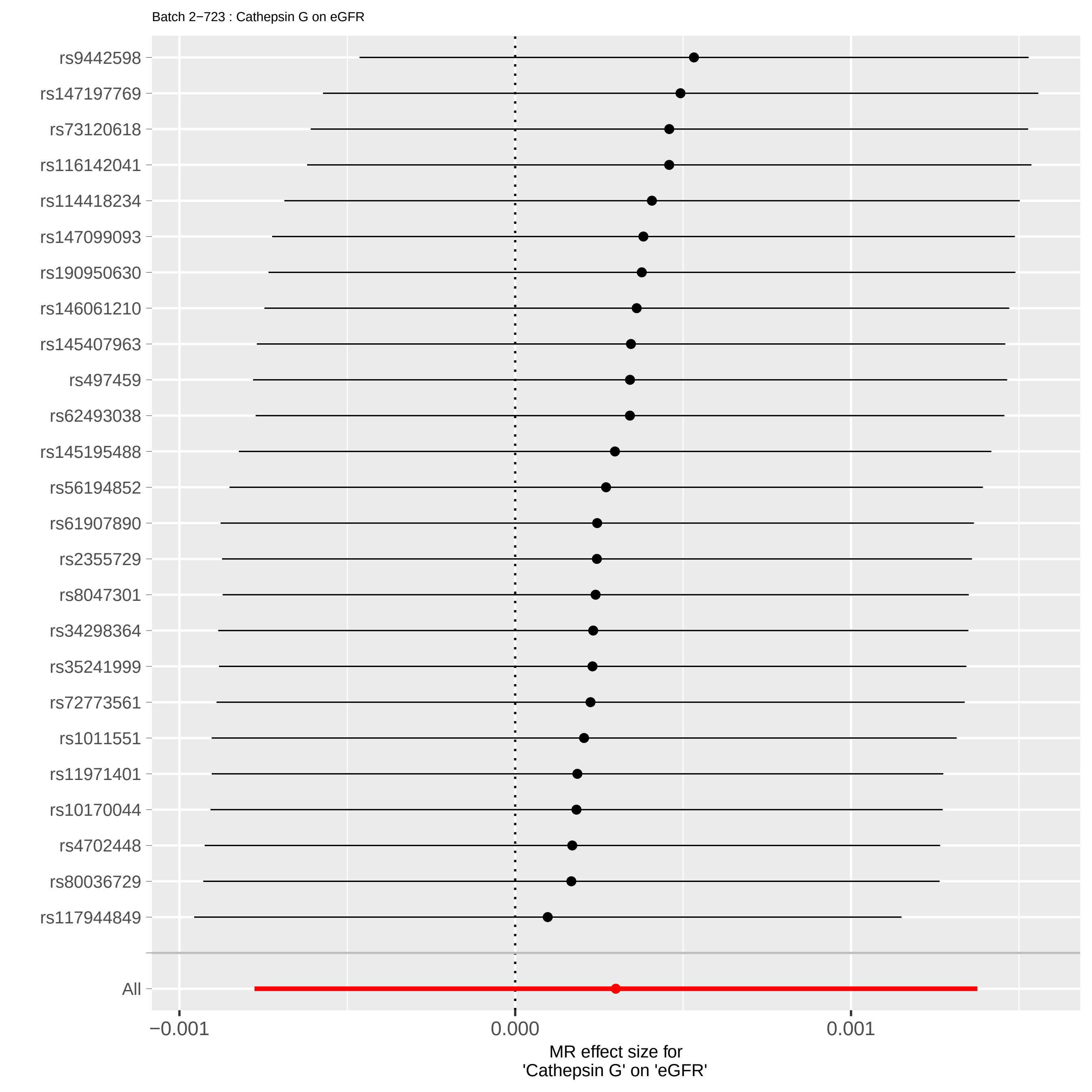

Batch 2−723 : Cathepsin G on eGFR
rs9442598
rs147197769
rs73120618
rs116142041
rs114418234
rs147099093
rs190950630
rs146061210
rs145407963
rs497459
rs62493038
rs145195488
rs56194852
rs61907890
rs2355729
rs8047301
rs34298364
rs35241999
rs72773561
rs1011551
rs11971401
rs10170044
rs4702448
rs80036729
rs117944849
All
−0.001
0.000
0.001
MR effect size for
'Cathepsin G' on 'eGFR'

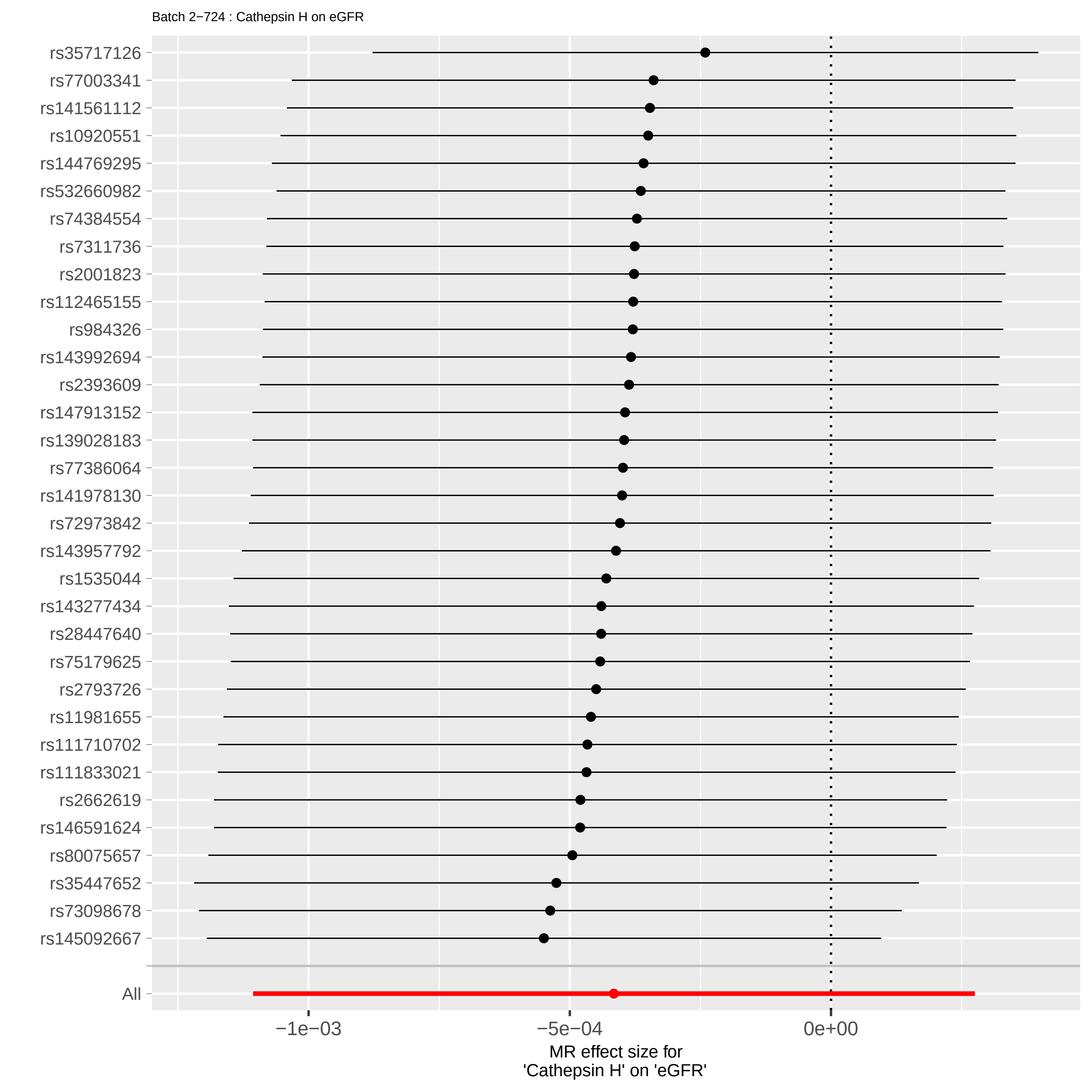

Batch 2−724 : Cathepsin H on eGFR
rs35717126
rs77003341
rs141561112
rs10920551
rs144769295
rs532660982
rs74384554
rs7311736
rs2001823
rs112465155
rs984326
rs143992694
rs2393609
rs147913152
rs139028183
rs77386064
rs141978130
rs72973842
rs143957792
rs1535044
rs143277434
rs28447640
rs75179625
rs2793726
rs11981655
rs111710702
rs111833021
rs2662619
rs146591624
rs80075657
rs35447652
rs73098678
rs145092667
All
−1e−03
−5e−04
0e+00
MR effect size for
'Cathepsin H' on 'eGFR'

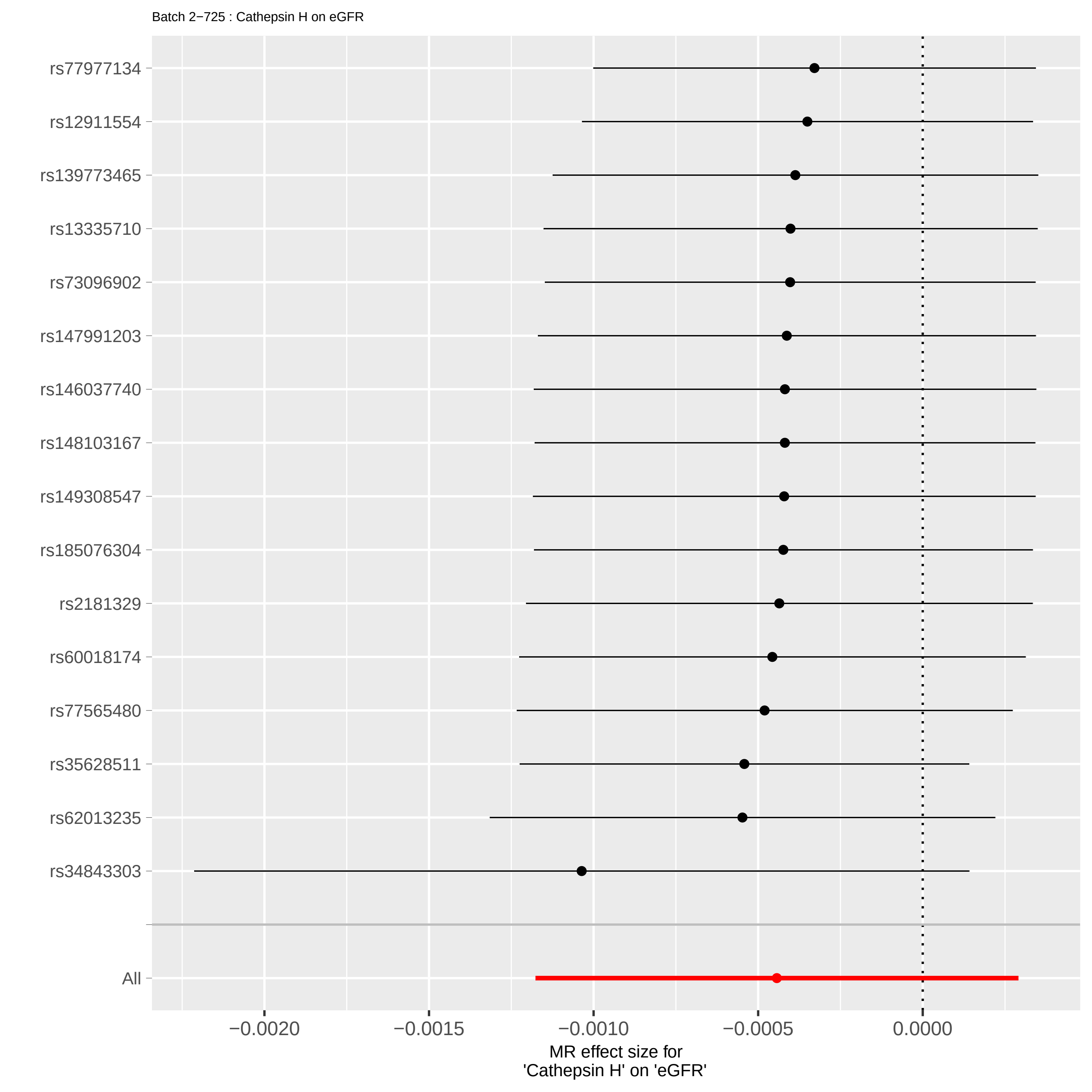

Batch 2−725 : Cathepsin H on eGFR
rs77977134
rs12911554
rs139773465
rs13335710
rs73096902
rs147991203
rs146037740
rs148103167
rs149308547
rs185076304
rs2181329
rs60018174
rs77565480
rs35628511
rs62013235
rs34843303
All
−0.0020
−0.0015
−0.0010
−0.0005
0.0000
MR effect size for
'Cathepsin H' on 'eGFR'

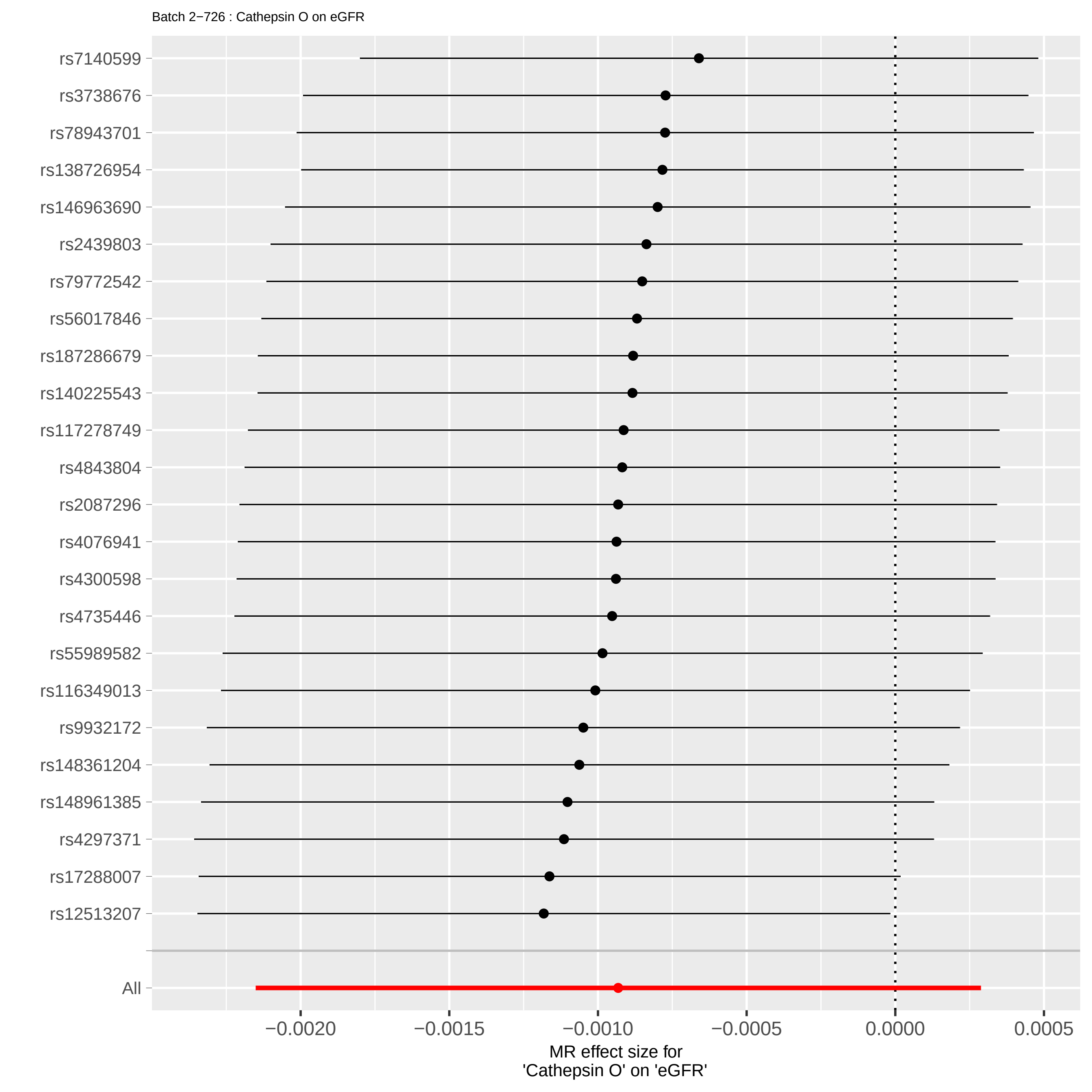
Batch 2−726 : Cathepsin O on eGFR
rs7140599
rs3738676
rs78943701
rs138726954
rs146963690
rs2439803
rs79772542
rs56017846
rs187286679
rs140225543
rs117278749
rs4843804
rs2087296
rs4076941
rs4300598
rs4735446
rs55989582
rs116349013
rs9932172
rs148361204
rs148961385
rs4297371
rs17288007
rs12513207
All
−0.0020
−0.0015
−0.0010
−0.0005
0.0000
0.0005
MR effect size for
'Cathepsin O' on 'eGFR'

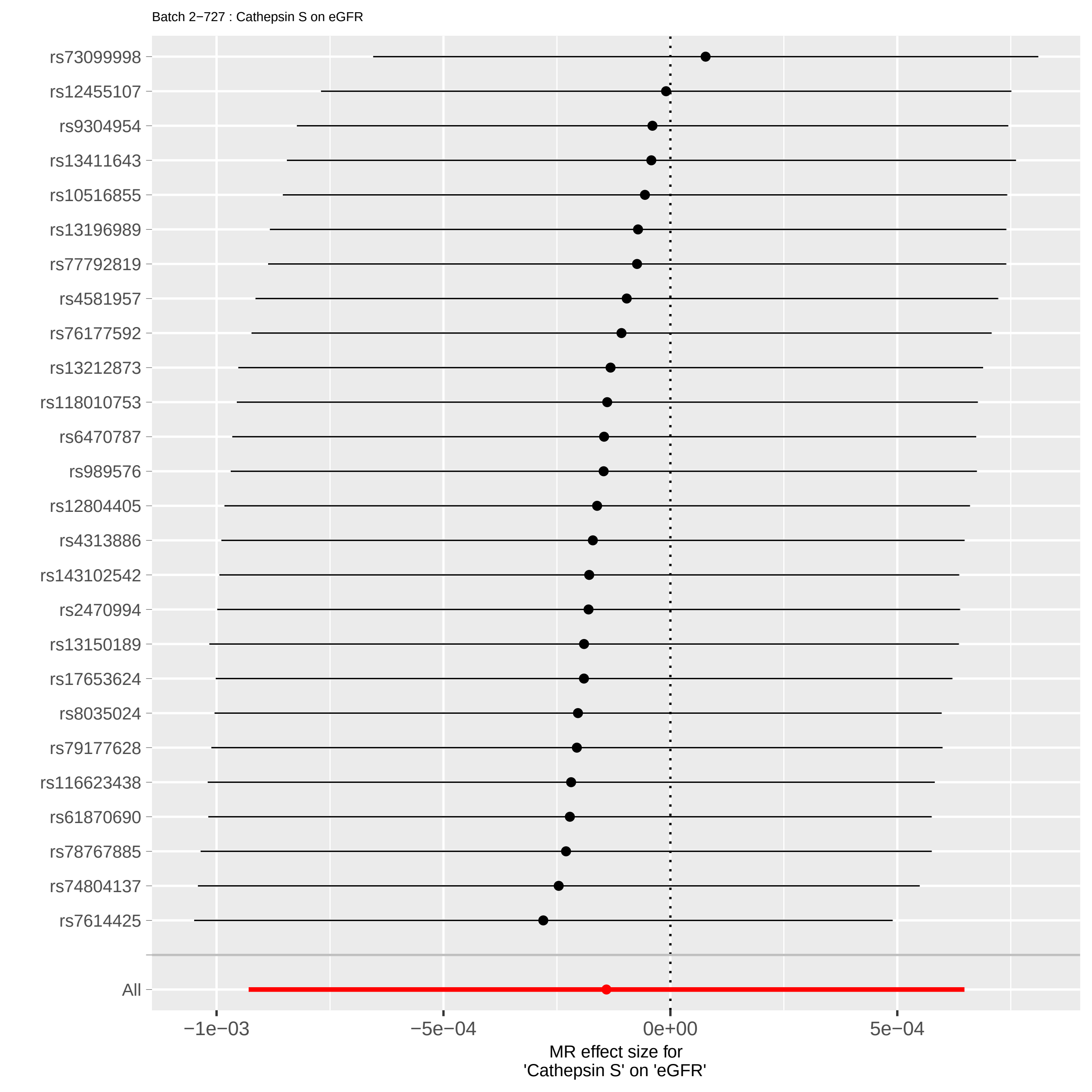

Batch 2−727 : Cathepsin S on eGFR
rs73099998
rs12455107
rs9304954
rs13411643
rs10516855
rs13196989
rs77792819
rs4581957
rs76177592
rs13212873
rs118010753
rs6470787
rs989576
rs12804405
rs4313886
rs143102542
rs2470994
rs13150189
rs17653624
rs8035024
rs79177628
rs116623438
rs61870690
rs78767885
rs74804137
rs7614425
All
−1e−03
−5e−04
0e+00
5e−04
MR effect size for
'Cathepsin S' on 'eGFR'

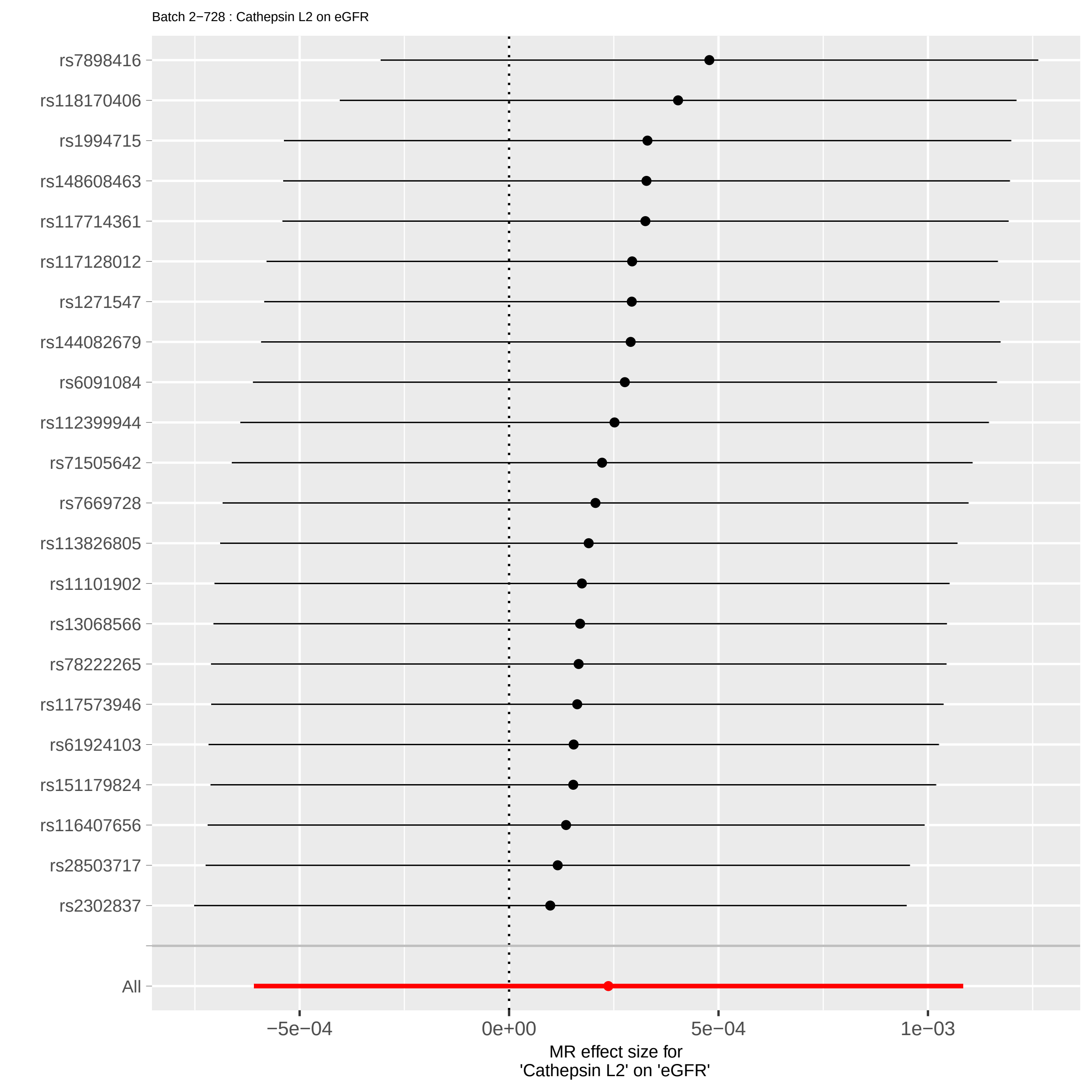
Batch 2−728 : Cathepsin L2 on eGFR
rs7898416
rs118170406
rs1994715
rs148608463
rs117714361
rs117128012
rs1271547
rs144082679
rs6091084
rs112399944
rs71505642
rs7669728
rs113826805
rs11101902
rs13068566
rs78222265
rs117573946
rs61924103
rs151179824
rs116407656
rs28503717
rs2302837
All
−5e−04
0e+00
5e−04
1e−03
MR effect size for
'Cathepsin L2' on 'eGFR'

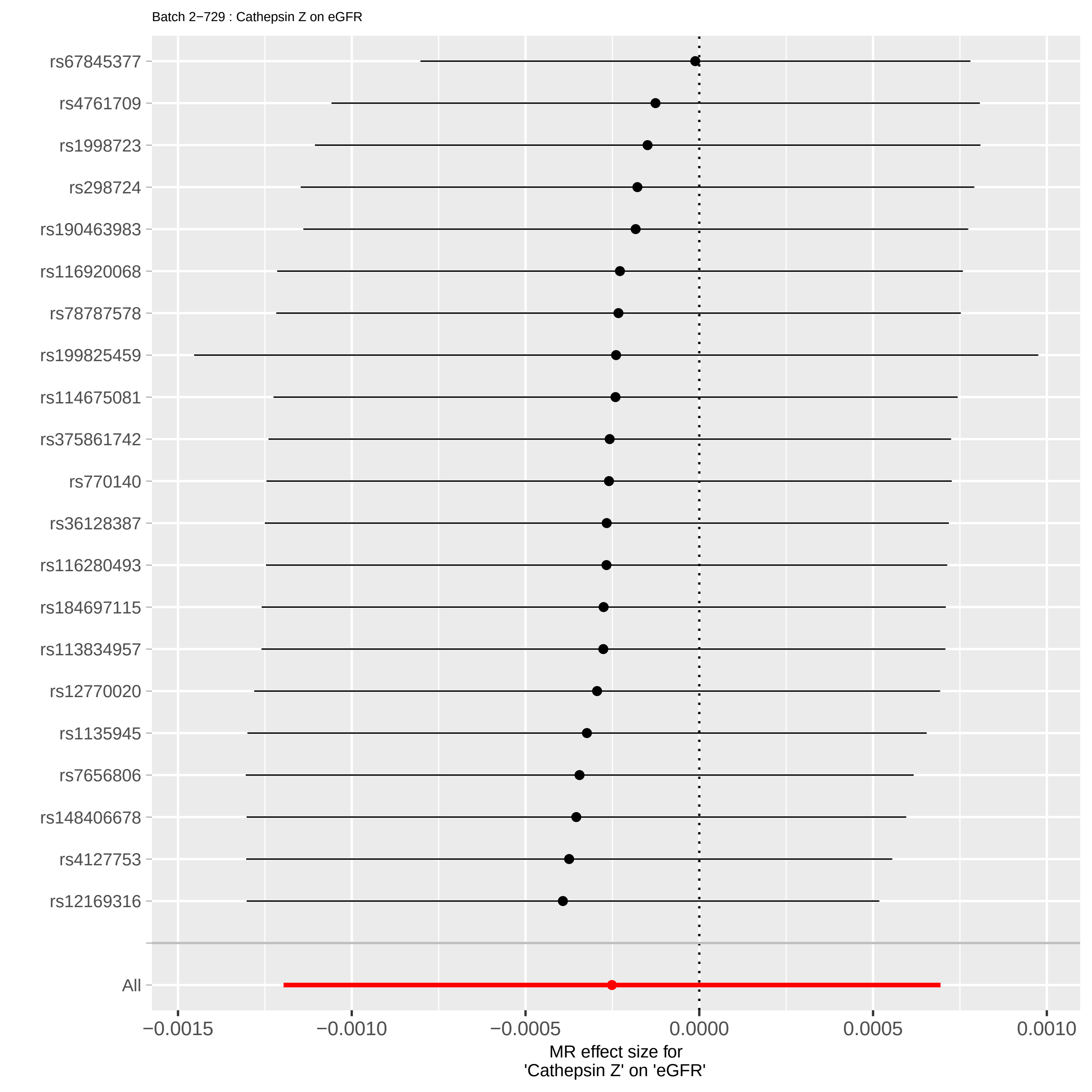

Batch 2−729 : Cathepsin Z on eGFR
rs67845377
rs4761709
rs1998723
rs298724
rs190463983
rs116920068
rs78787578
rs199825459
rs114675081
rs375861742
rs770140
rs36128387
rs116280493
rs184697115
rs113834957
rs12770020
rs1135945
rs7656806
rs148406678
rs4127753
rs12169316
All
−0.0015
−0.0010
−0.0005
0.0000
0.0005
0.0010
MR effect size for
'Cathepsin Z' on 'eGFR'

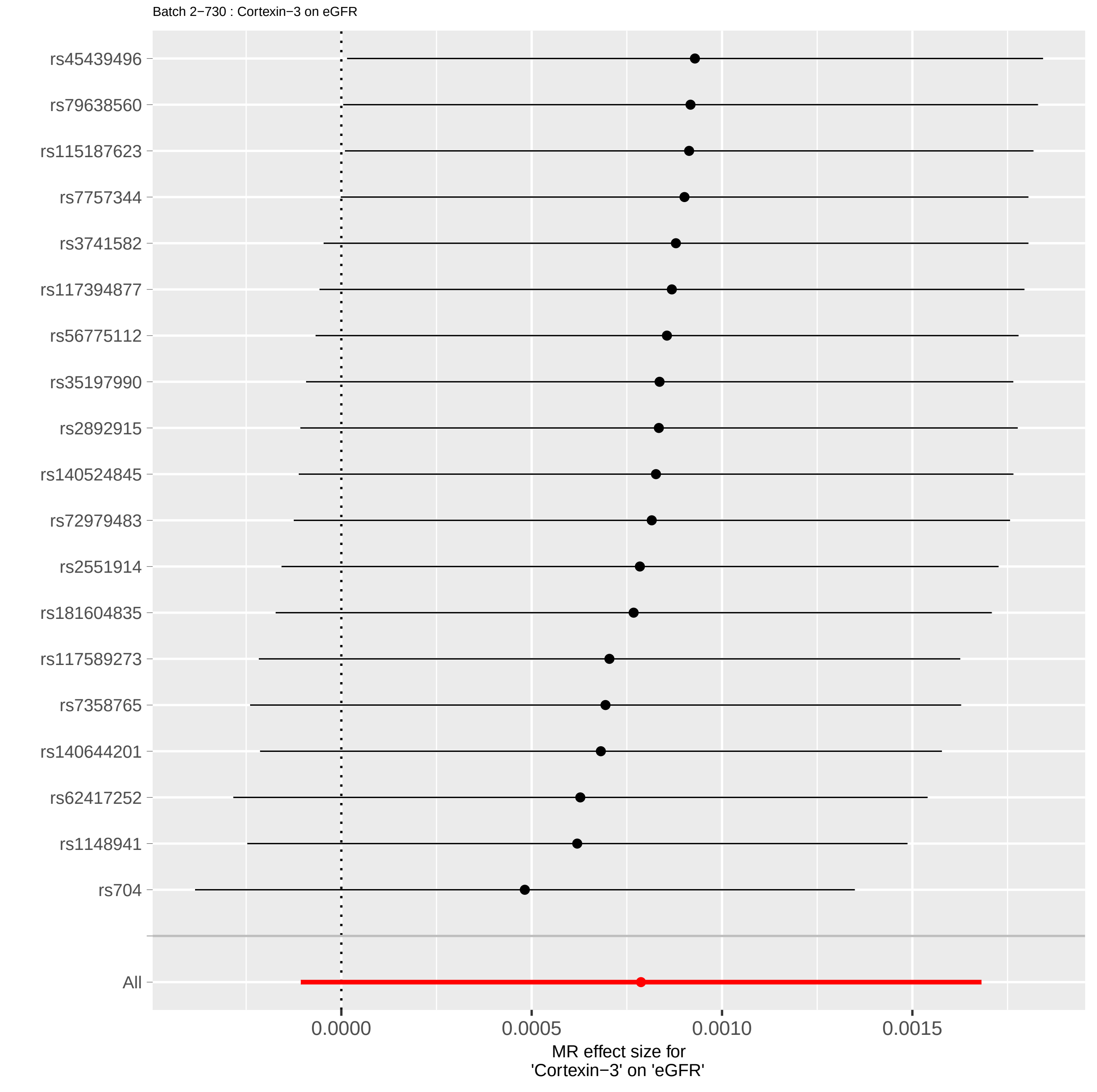

Batch 2−730 : Cortexin−3 on eGFR
rs45439496
rs79638560
rs115187623
rs7757344
rs3741582
rs117394877
rs56775112
rs35197990
rs2892915
rs140524845
rs72979483
rs2551914
rs181604835
rs117589273
rs7358765
rs140644201
rs62417252
rs1148941
rs704
All
0.0000
0.0005
0.0010
0.0015
MR effect size for
'Cortexin−3' on 'eGFR'

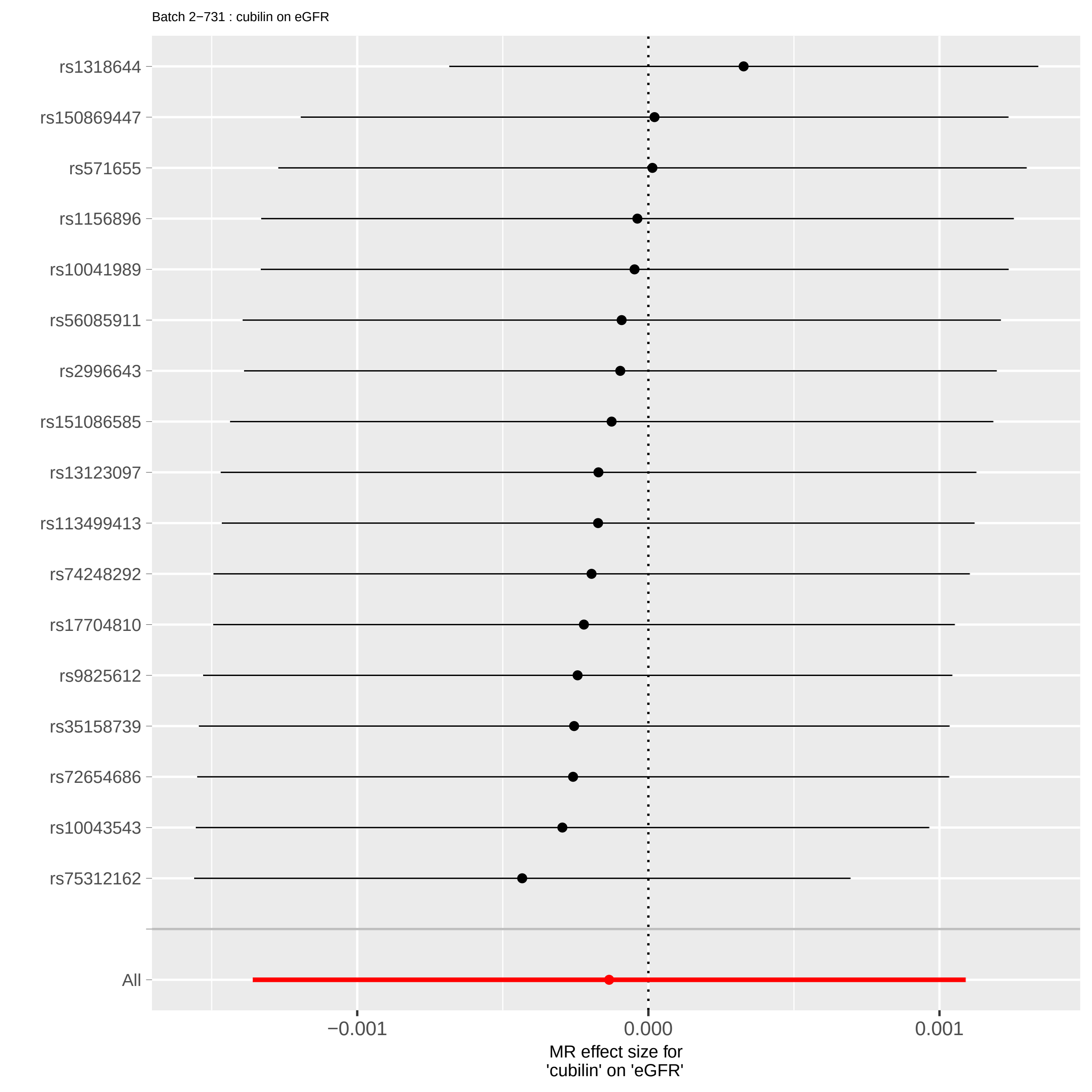

Batch 2−731 : cubilin on eGFR
rs1318644
rs150869447
rs571655
rs1156896
rs10041989
rs56085911
rs2996643
rs151086585
rs13123097
rs113499413
rs74248292
rs17704810
rs9825612
rs35158739
rs72654686
rs10043543
rs75312162
All
−0.001
0.000
0.001
MR effect size for
'cubilin' on 'eGFR'

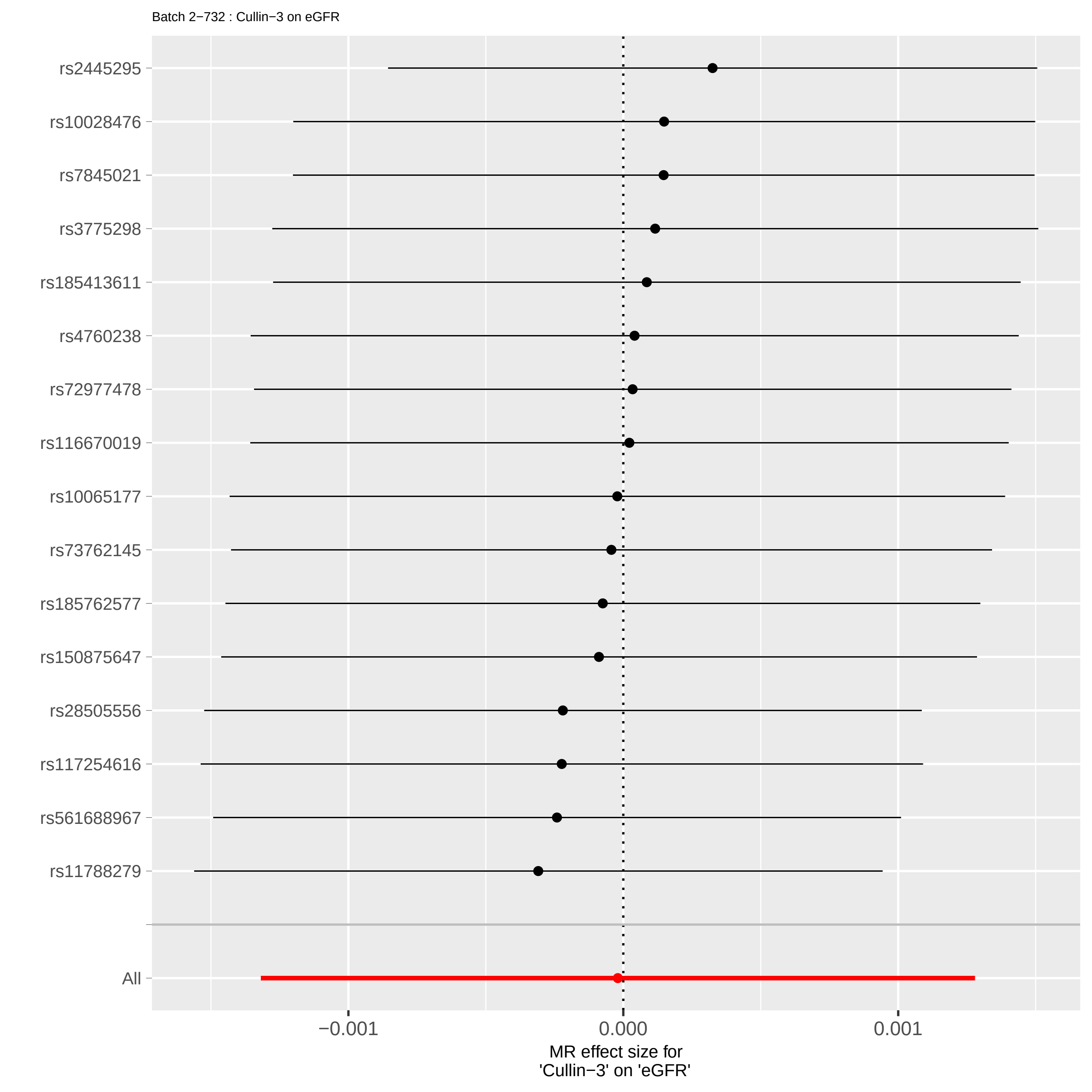
Batch 2−732 : Cullin−3 on eGFR
rs2445295
rs10028476
rs7845021
rs3775298
rs185413611
rs4760238
rs72977478
rs116670019
rs10065177
rs73762145
rs185762577
rs150875647
rs28505556
rs117254616
rs561688967
rs11788279
All
−0.001
0.000
0.001
MR effect size for
'Cullin−3' on 'eGFR'

Batch 2−733 : Cullin−4B on eGFR

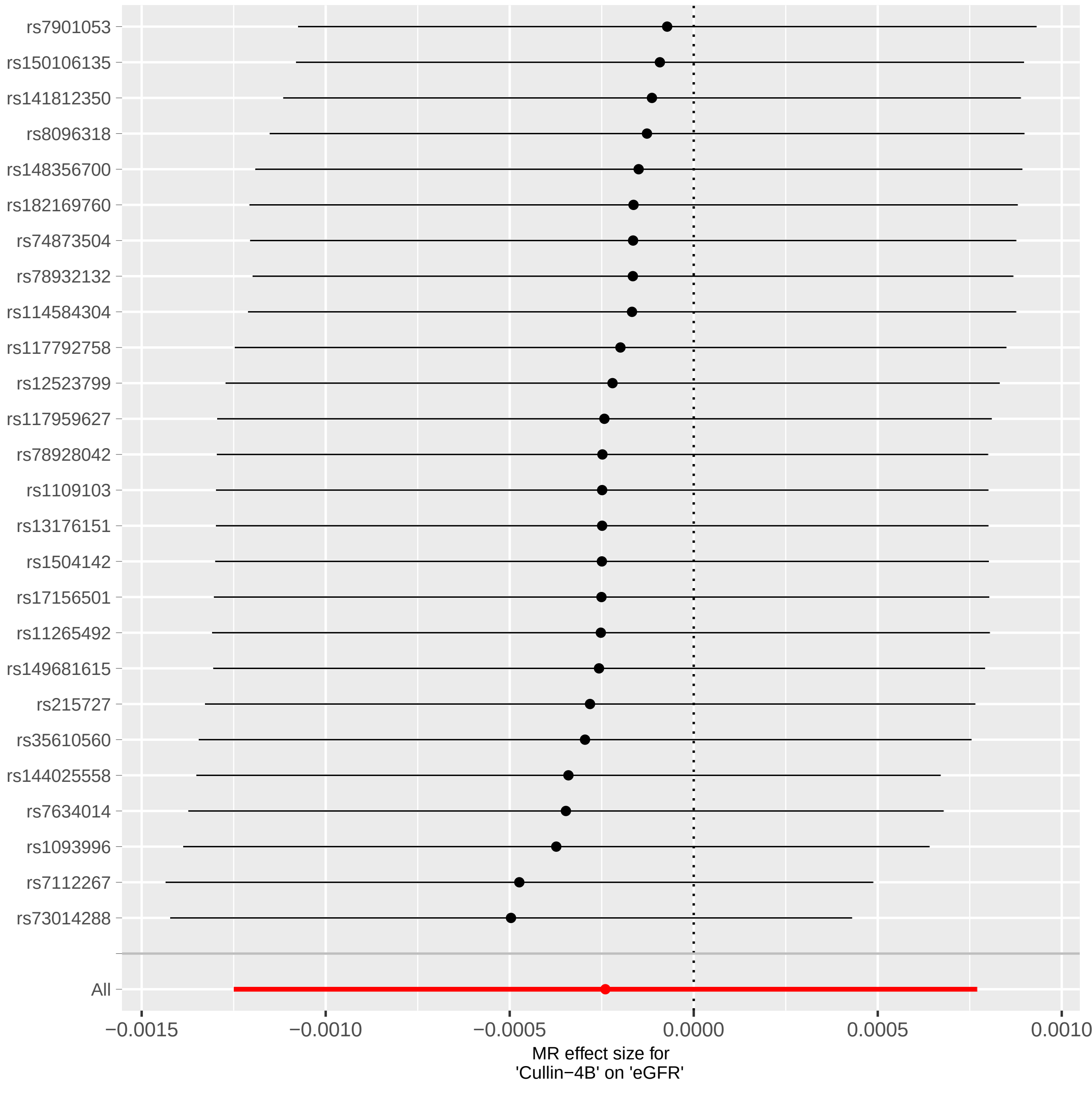

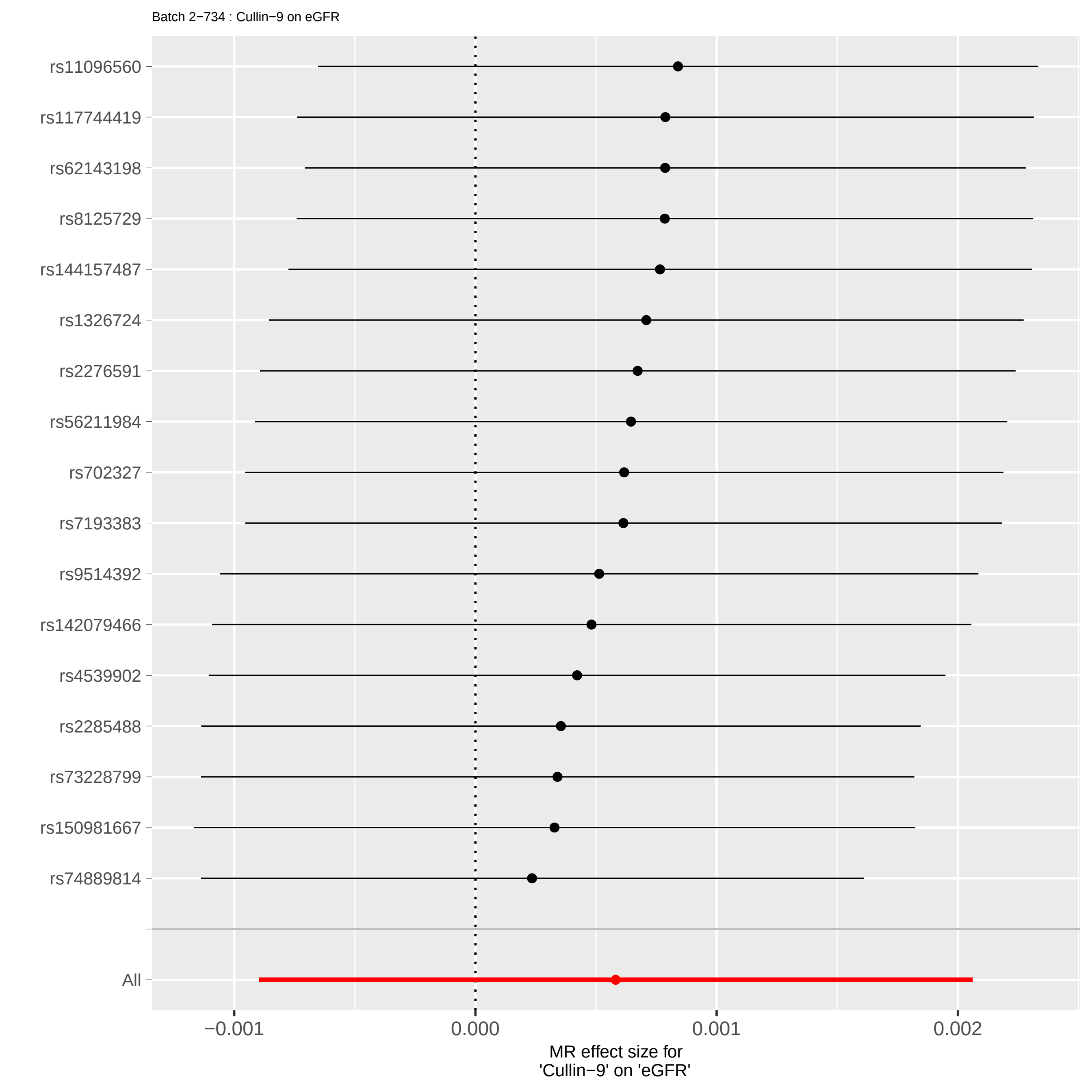

Batch 2−734 : Cullin−9 on eGFR
rs11096560
rs117744419
rs62143198
rs8125729
rs144157487
rs1326724
rs2276591
rs56211984
rs702327
rs7193383
rs9514392
rs142079466
rs4539902
rs2285488
rs73228799
rs150981667
rs74889814
All
−0.001
0.000
0.001
0.002
MR effect size for
'Cullin−9' on 'eGFR'

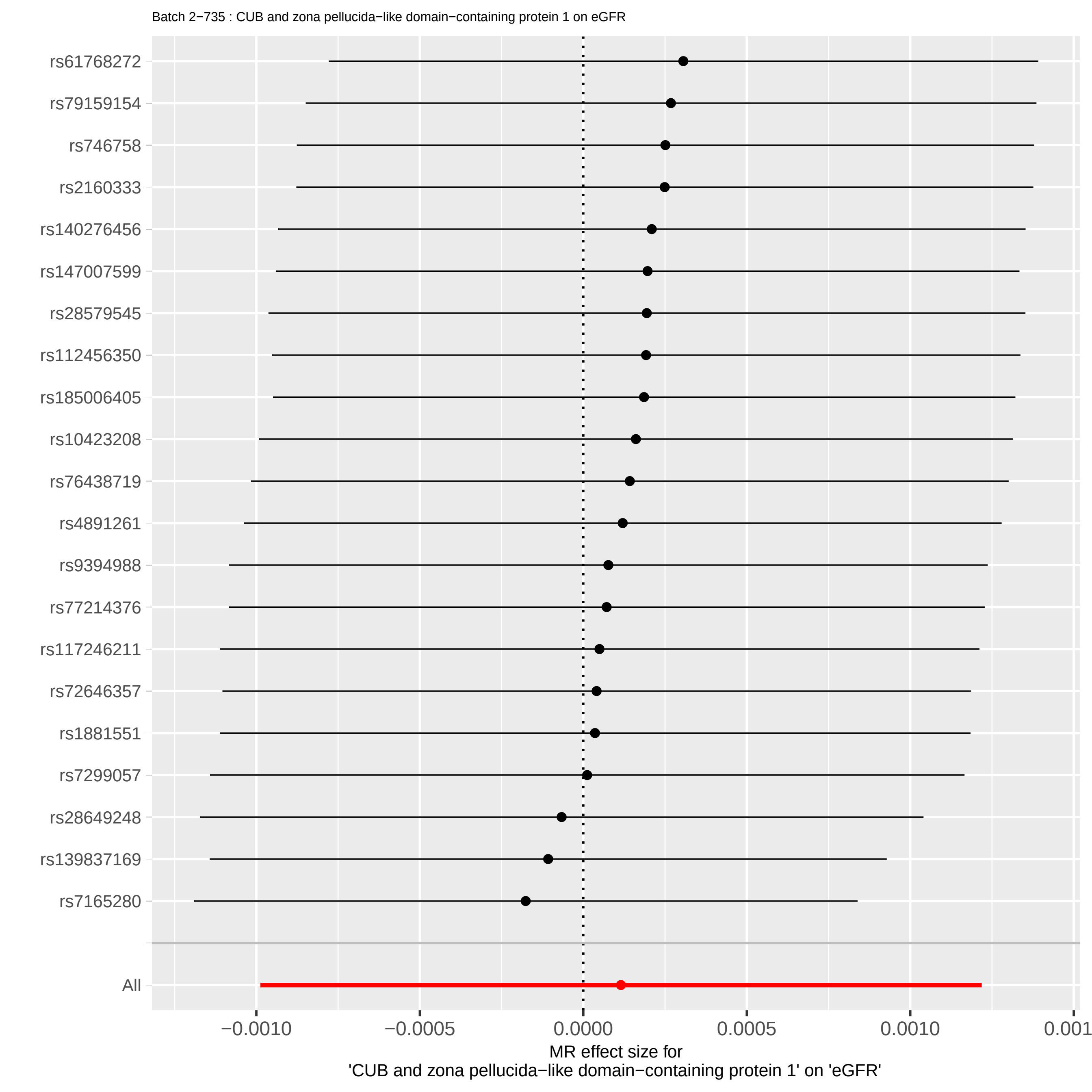

Batch 2−735 : CUB and zona pellucida−like domain−containing protein 1 on eGFR
rs61768272
rs79159154
rs746758
rs2160333
rs140276456
rs147007599
rs28579545
rs112456350
rs185006405
rs10423208
rs76438719
rs4891261
rs9394988
rs77214376
rs117246211
rs72646357
rs1881551
rs7299057
rs28649248
rs139837169
rs7165280
All
−0.0010
−0.0005
0.0000
0.0005
0.0010
0.001
MR effect size for
'CUB and zona pellucida−like domain−containing protein 1' on 'eGFR'

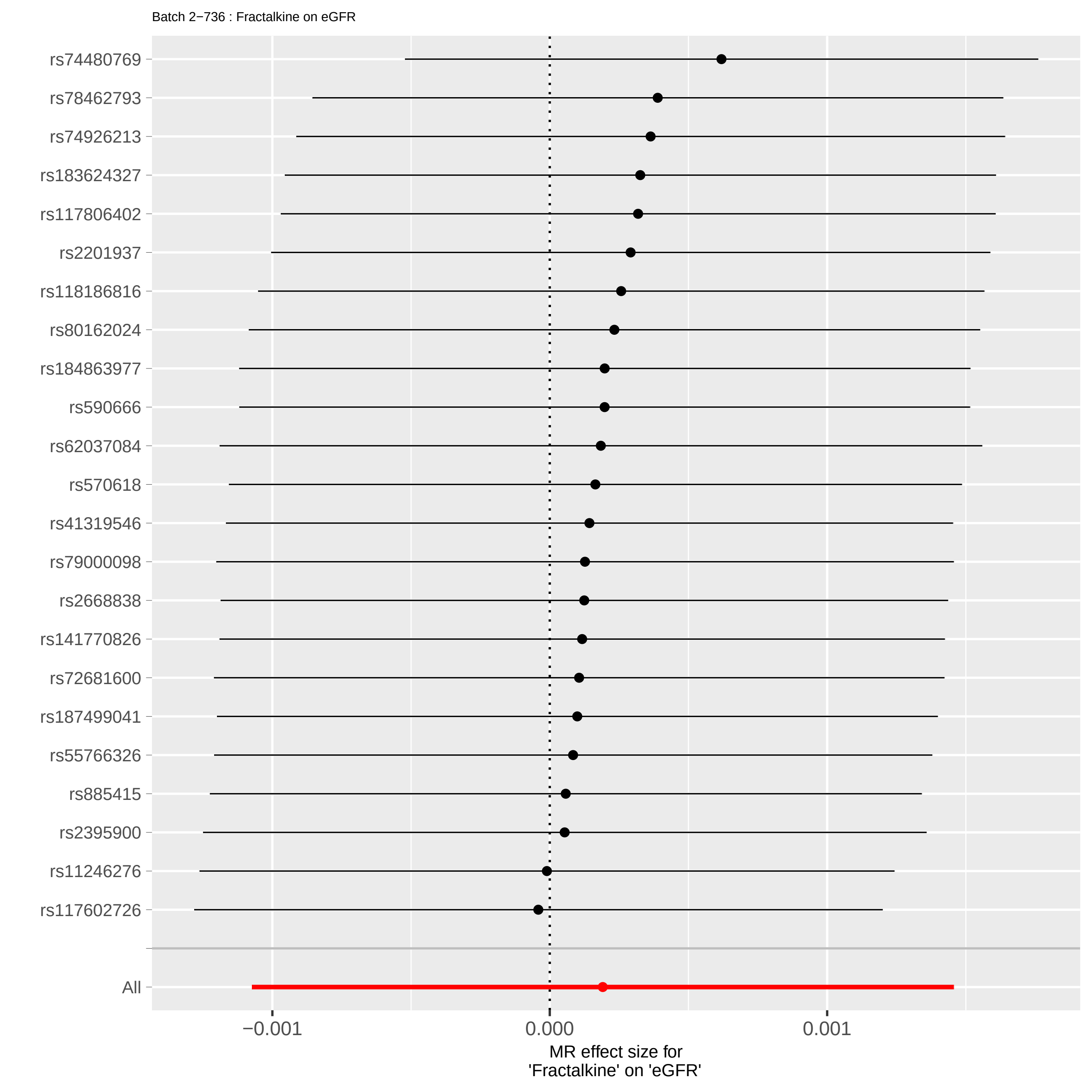

Batch 2−736 : Fractalkine on eGFR
rs74480769
rs78462793
rs74926213
rs183624327
rs117806402
rs2201937
rs118186816
rs80162024
rs184863977
rs590666
rs62037084
rs570618
rs41319546
rs79000098
rs2668838
rs141770826
rs72681600
rs187499041
rs55766326
rs885415
rs2395900
rs11246276
rs117602726
All
−0.001
0.000
0.001
MR effect size for
'Fractalkine' on 'eGFR'

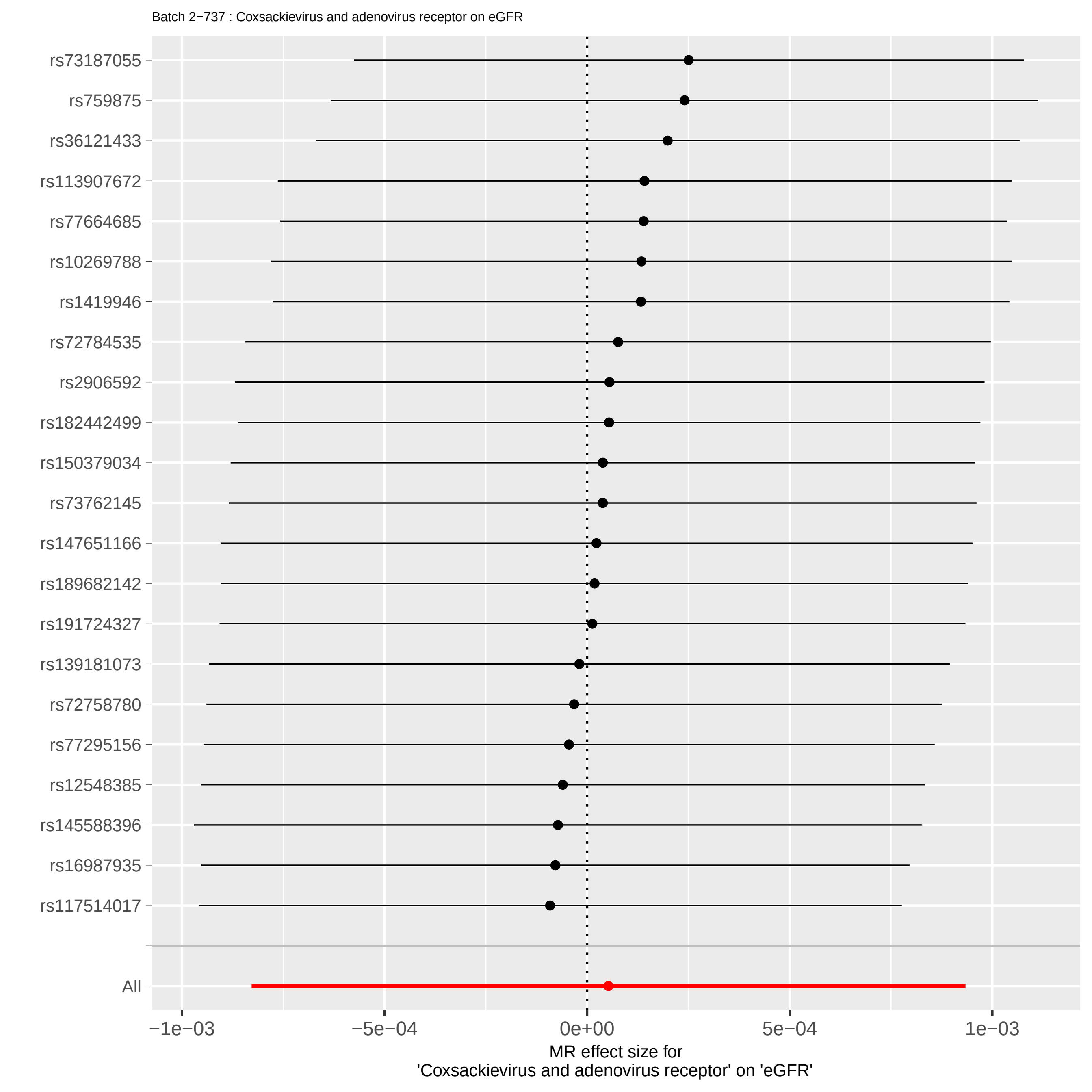
Batch 2−737 : Coxsackievirus and adenovirus receptor on eGFR
rs73187055
rs759875
rs36121433
rs113907672
rs77664685
rs10269788
rs1419946
rs72784535
rs2906592
rs182442499
rs150379034
rs73762145
rs147651166
rs189682142
rs191724327
rs139181073
rs72758780
rs77295156
rs12548385
rs145588396
rs16987935
rs117514017
All
−1e−03
−5e−04
0e+00
5e−04
1e−03
MR effect size for
'Coxsackievirus and adenovirus receptor' on 'eGFR'

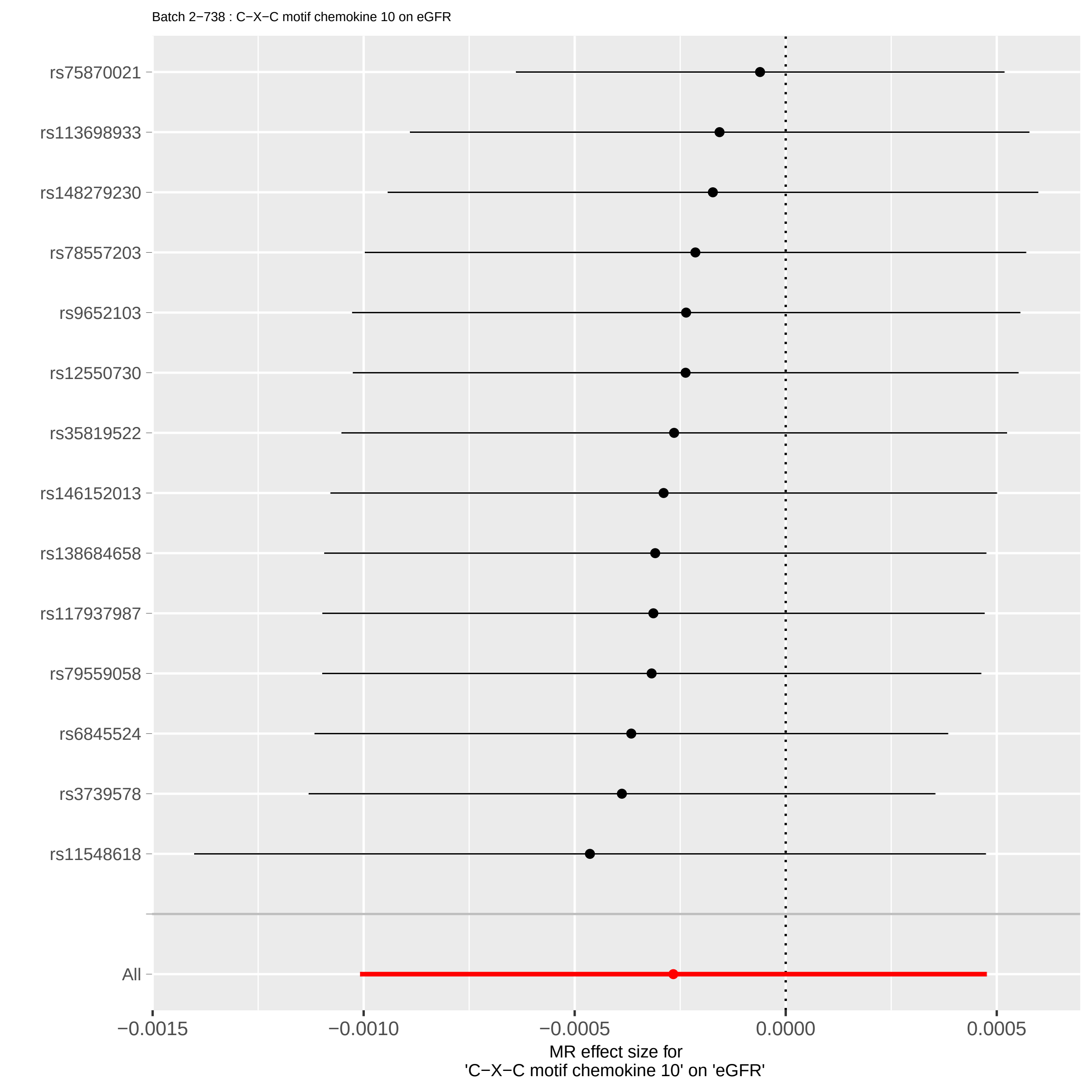

Batch 2−738 : C−X−C motif chemokine 10 on eGFR
rs75870021
rs113698933
rs148279230
rs78557203
rs9652103
rs12550730
rs35819522
rs146152013
rs138684658
rs117937987
rs79559058
rs6845524
rs3739578
rs11548618
All
−0.0015
−0.0010
−0.0005
0.0000
0.0005
MR effect size for
'C−X−C motif chemokine 10' on 'eGFR'

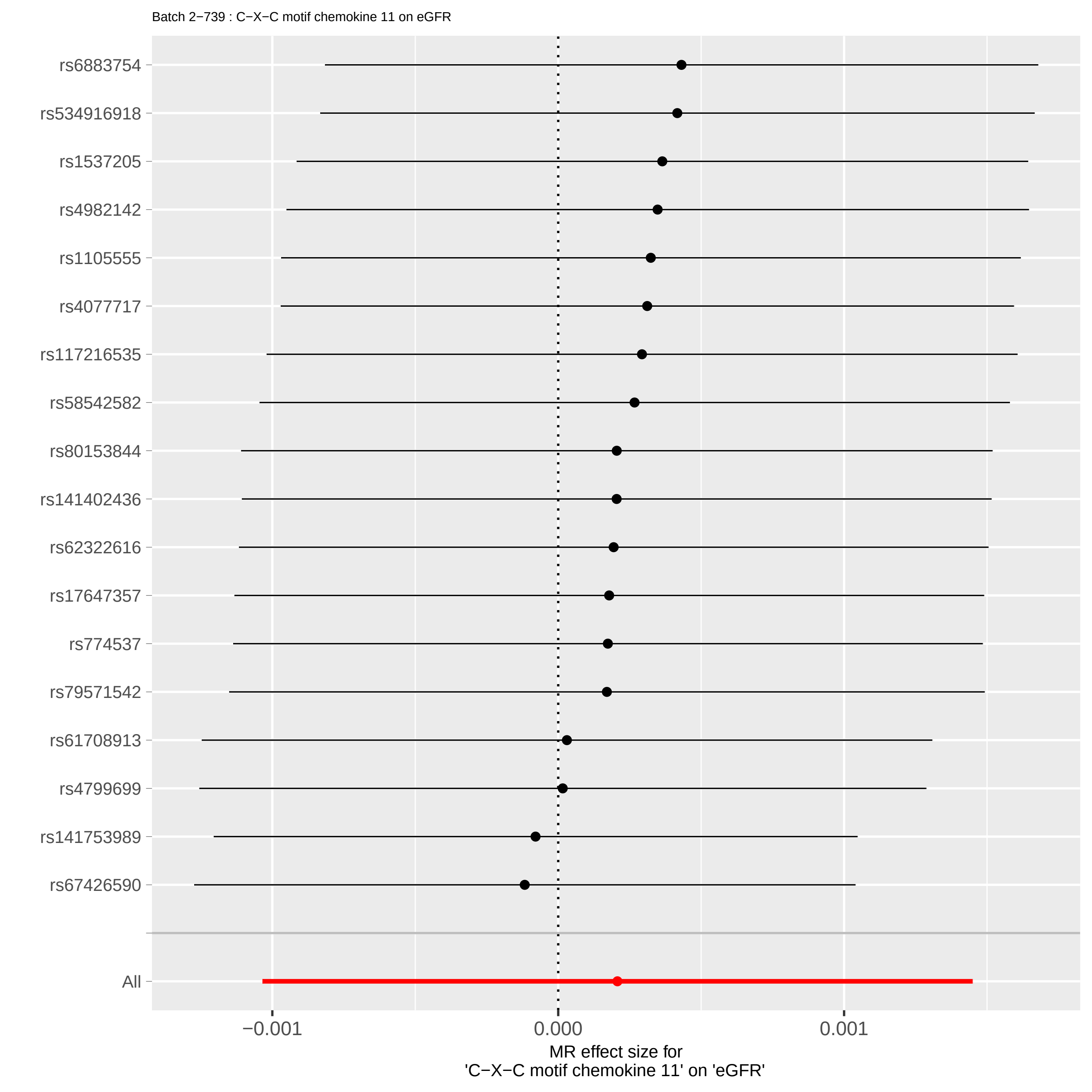

Batch 2−739 : C−X−C motif chemokine 11 on eGFR
rs6883754
rs534916918
rs1537205
rs4982142
rs1105555
rs4077717
rs117216535
rs58542582
rs80153844
rs141402436
rs62322616
rs17647357
rs774537
rs79571542
rs61708913
rs4799699
rs141753989
rs67426590
All
−0.001
0.000
0.001
MR effect size for
'C−X−C motif chemokine 11' on 'eGFR'

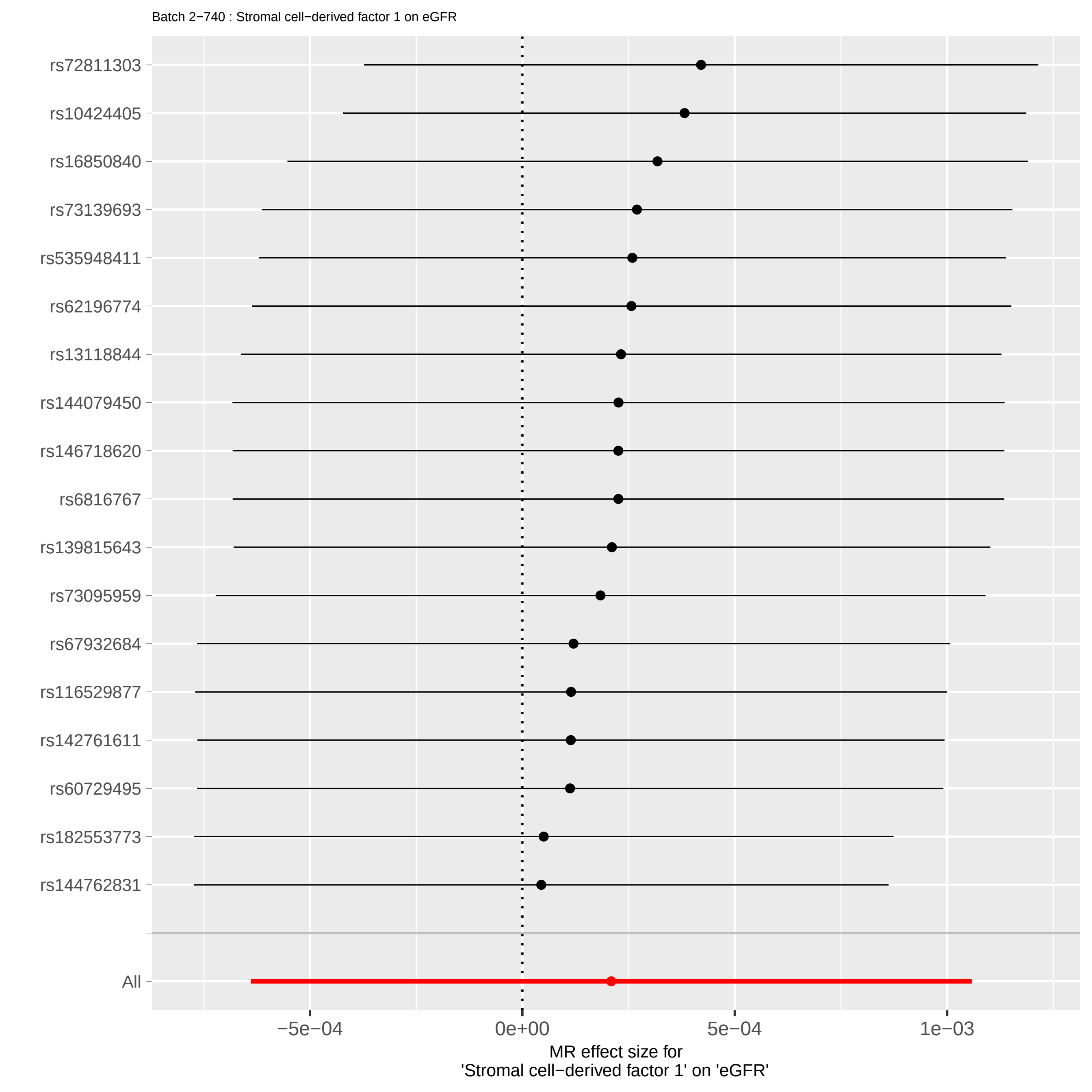
Batch 2−740 : Stromal cell−derived factor 1 on eGFR
rs72811303
rs10424405
rs16850840
rs73139693
rs535948411
rs62196774
rs13118844
rs144079450
rs146718620
rs6816767
rs139815643
rs73095959
rs67932684
rs116529877
rs142761611
rs60729495
rs182553773
rs144762831
All
−5e−04
0e+00
5e−04
1e−03
MR effect size for
'Stromal cell−derived factor 1' on 'eGFR'

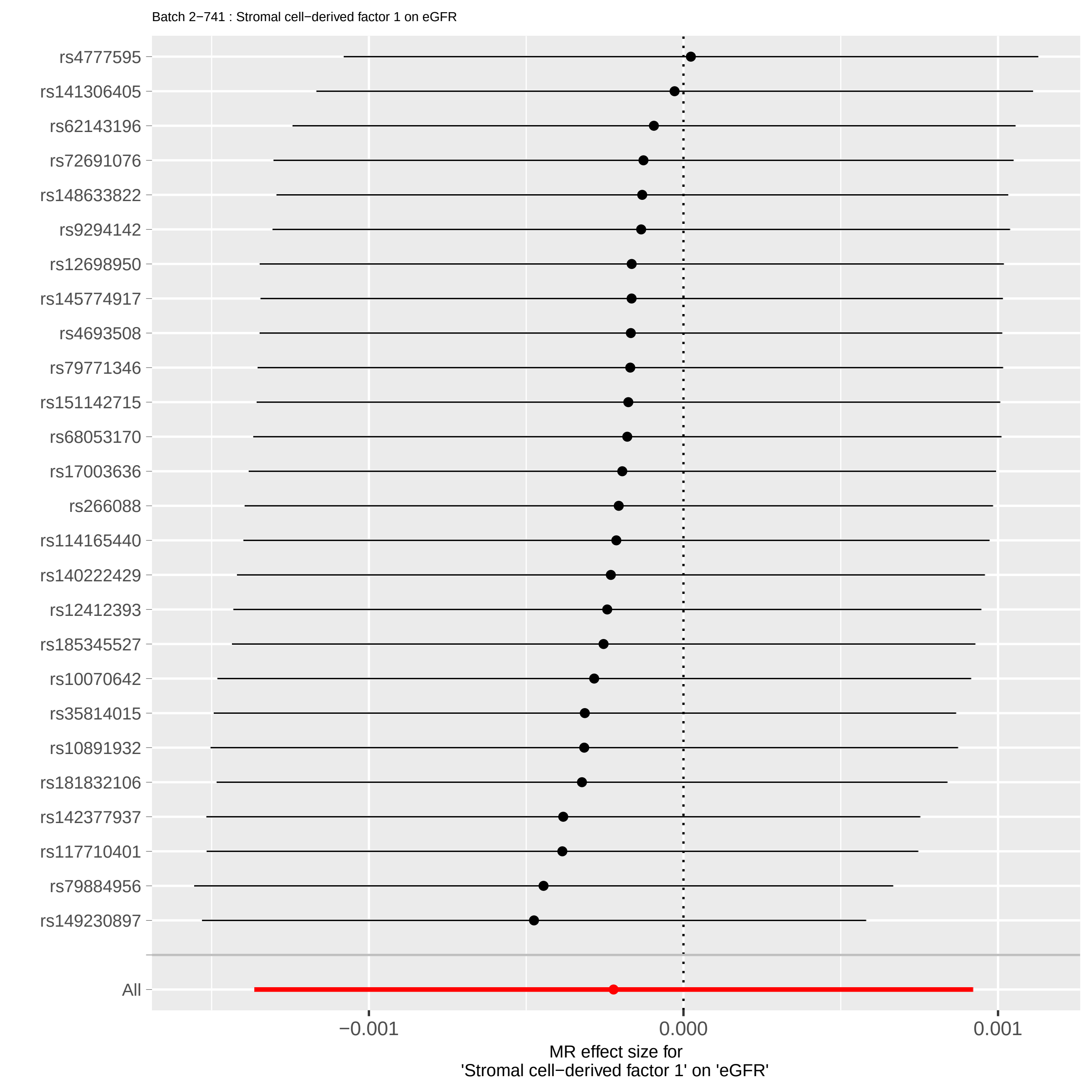
Batch 2−741 : Stromal cell−derived factor 1 on eGFR
rs4777595
rs141306405
rs62143196
rs72691076
rs148633822
rs9294142
rs12698950
rs145774917
rs4693508
rs79771346
rs151142715
rs68053170
rs17003636
rs266088
rs114165440
rs140222429
rs12412393
rs185345527
rs10070642
rs35814015
rs10891932
rs181832106
rs142377937
rs117710401
rs79884956
rs149230897
All
−0.001
0.000
0.001
MR effect size for
'Stromal cell−derived factor 1' on 'eGFR'

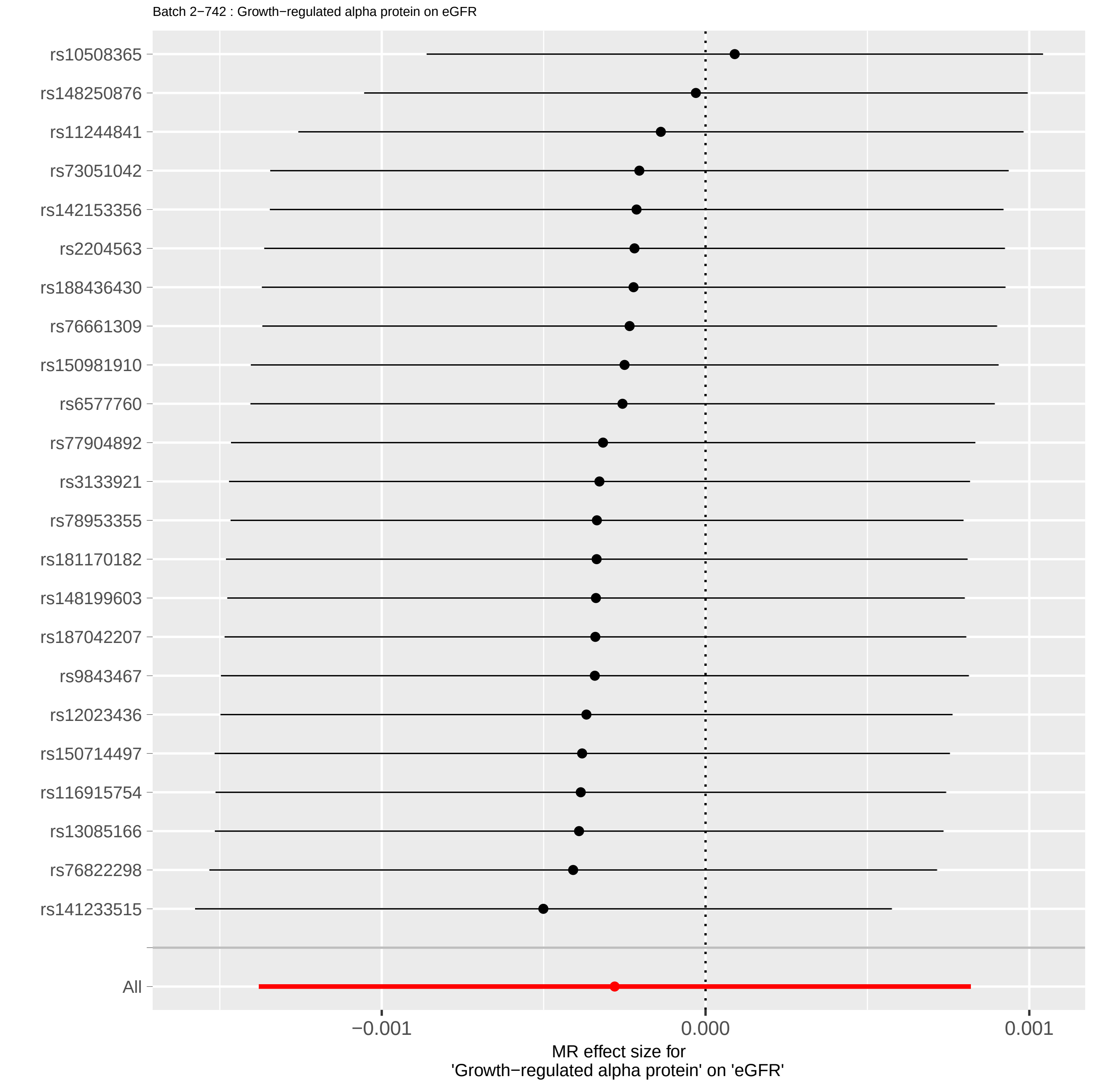

Batch 2−742 : Growth−regulated alpha protein on eGFR
rs10508365
rs148250876
rs11244841
rs73051042
rs142153356
rs2204563
rs188436430
rs76661309
rs150981910
rs6577760
rs77904892
rs3133921
rs78953355
rs181170182
rs148199603
rs187042207
rs9843467
rs12023436
rs150714497
rs116915754
rs13085166
rs76822298
rs141233515
All
−0.001
0.000
0.001
MR effect size for
'Growth−regulated alpha protein' on 'eGFR'

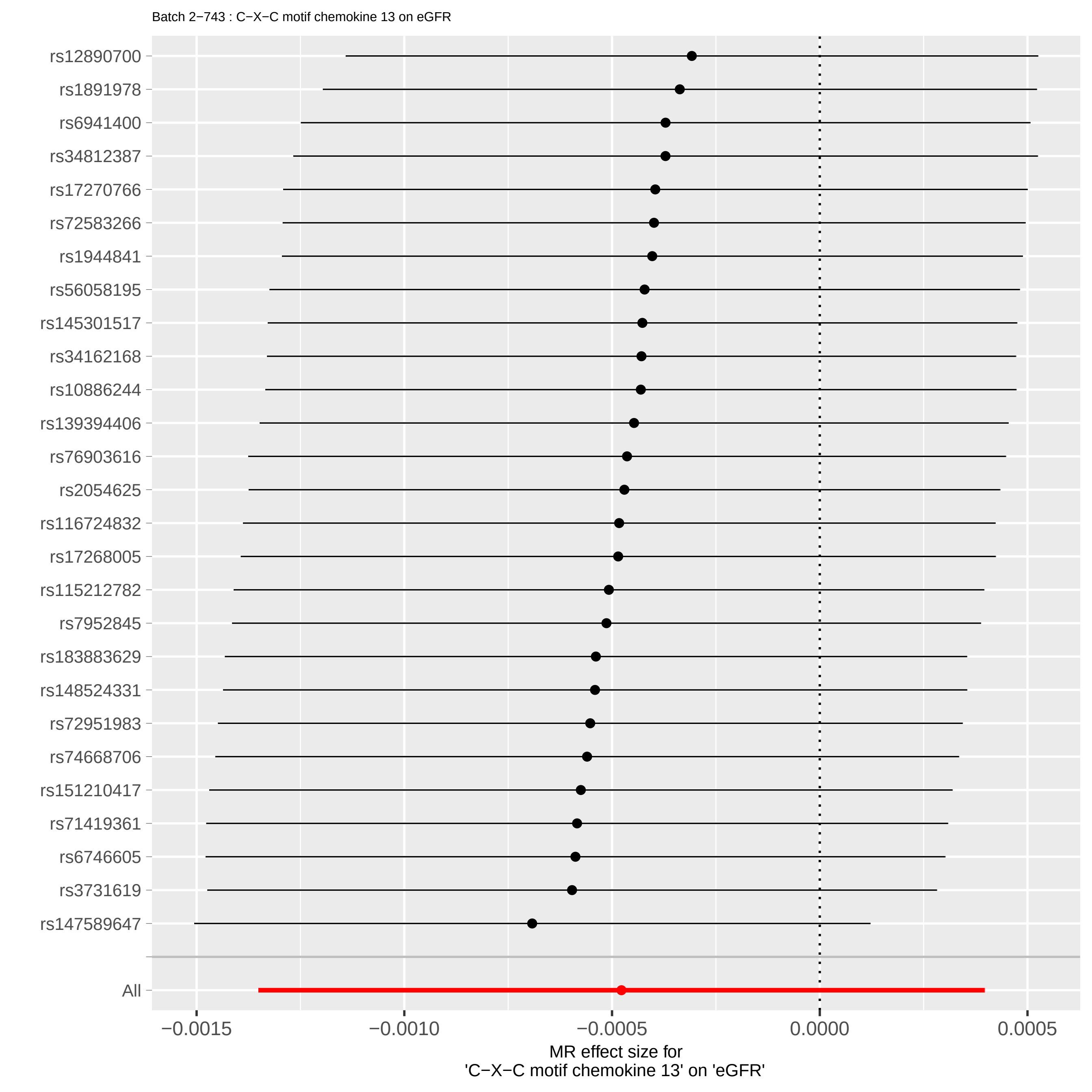

Batch 2−743 : C−X−C motif chemokine 13 on eGFR
rs12890700
rs1891978
rs6941400
rs34812387
rs17270766
rs72583266
rs1944841
rs56058195
rs145301517
rs34162168
rs10886244
rs139394406
rs76903616
rs2054625
rs116724832
rs17268005
rs115212782
rs7952845
rs183883629
rs148524331
rs72951983
rs74668706
rs151210417
rs71419361
rs6746605
rs3731619
rs147589647
All
−0.0015
−0.0010
−0.0005
0.0000
0.0005
MR effect size for
'C−X−C motif chemokine 13' on 'eGFR'

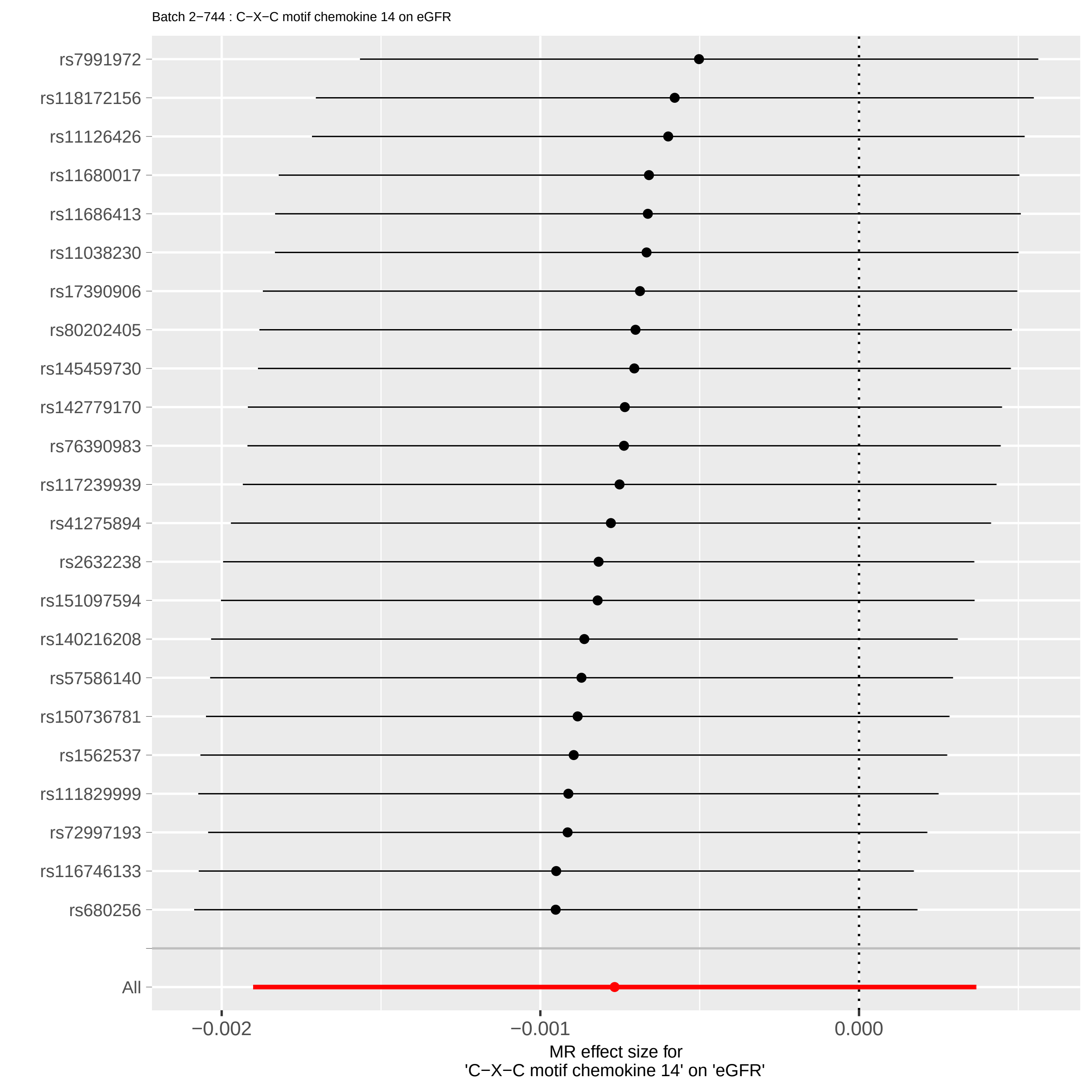
Batch 2−744 : C−X−C motif chemokine 14 on eGFR
rs7991972
rs118172156
rs11126426
rs11680017
rs11686413
rs11038230
rs17390906
rs80202405
rs145459730
rs142779170
rs76390983
rs117239939
rs41275894
rs2632238
rs151097594
rs140216208
rs57586140
rs150736781
rs1562537
rs111829999
rs72997193
rs116746133
rs680256
All
−0.002
−0.001
0.000
MR effect size for
'C−X−C motif chemokine 14' on 'eGFR'

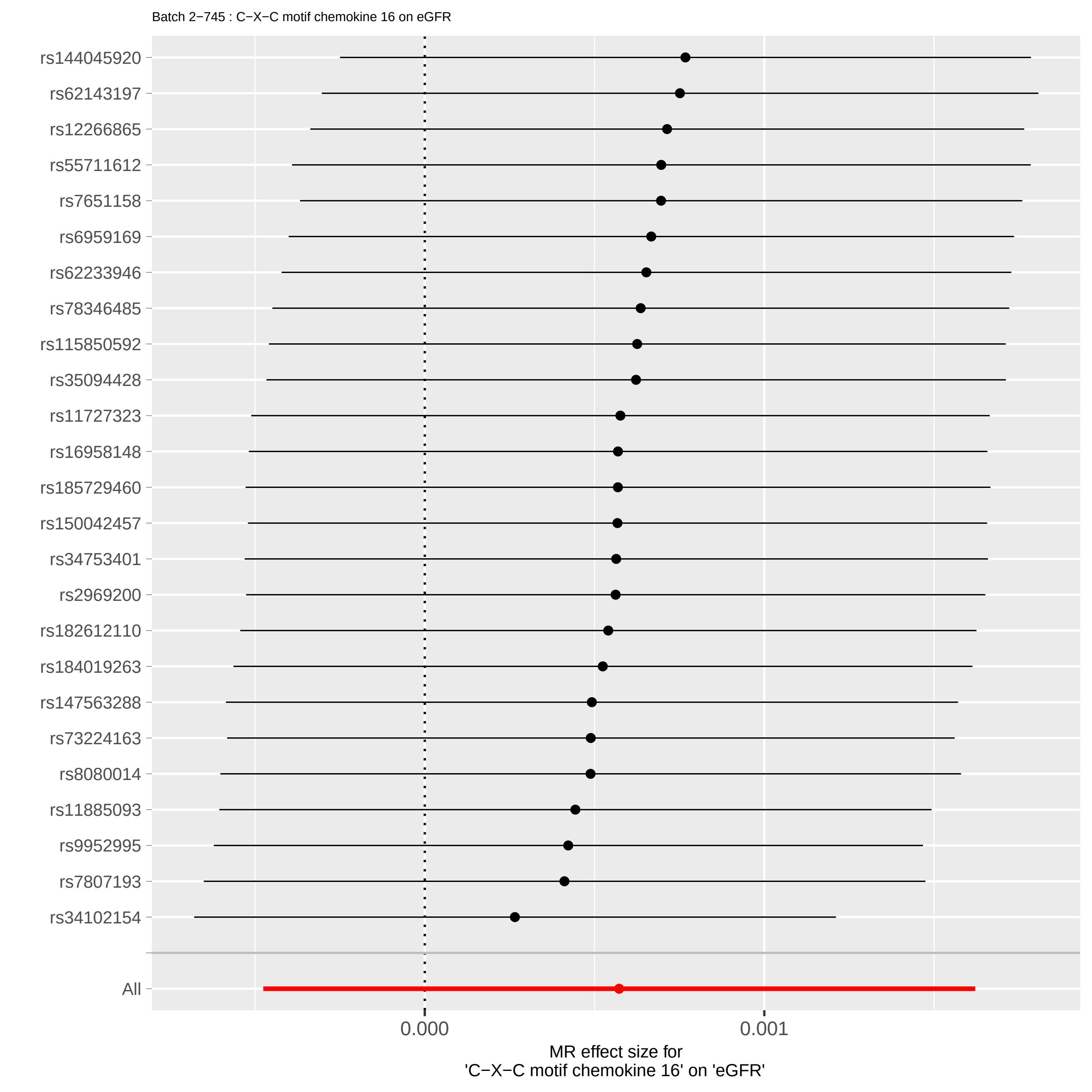

Batch 2−745 : C−X−C motif chemokine 16 on eGFR
rs144045920
rs62143197
rs12266865
rs55711612
rs7651158
rs6959169
rs62233946
rs78346485
rs115850592
rs35094428
rs11727323
rs16958148
rs185729460
rs150042457
rs34753401
rs2969200
rs182612110
rs184019263
rs147563288
rs73224163
rs8080014
rs11885093
rs9952995
rs7807193
rs34102154
All
0.000
0.001
MR effect size for
'C−X−C motif chemokine 16' on 'eGFR'

Batch 2−746 : Gro−beta/gamma on eGFR

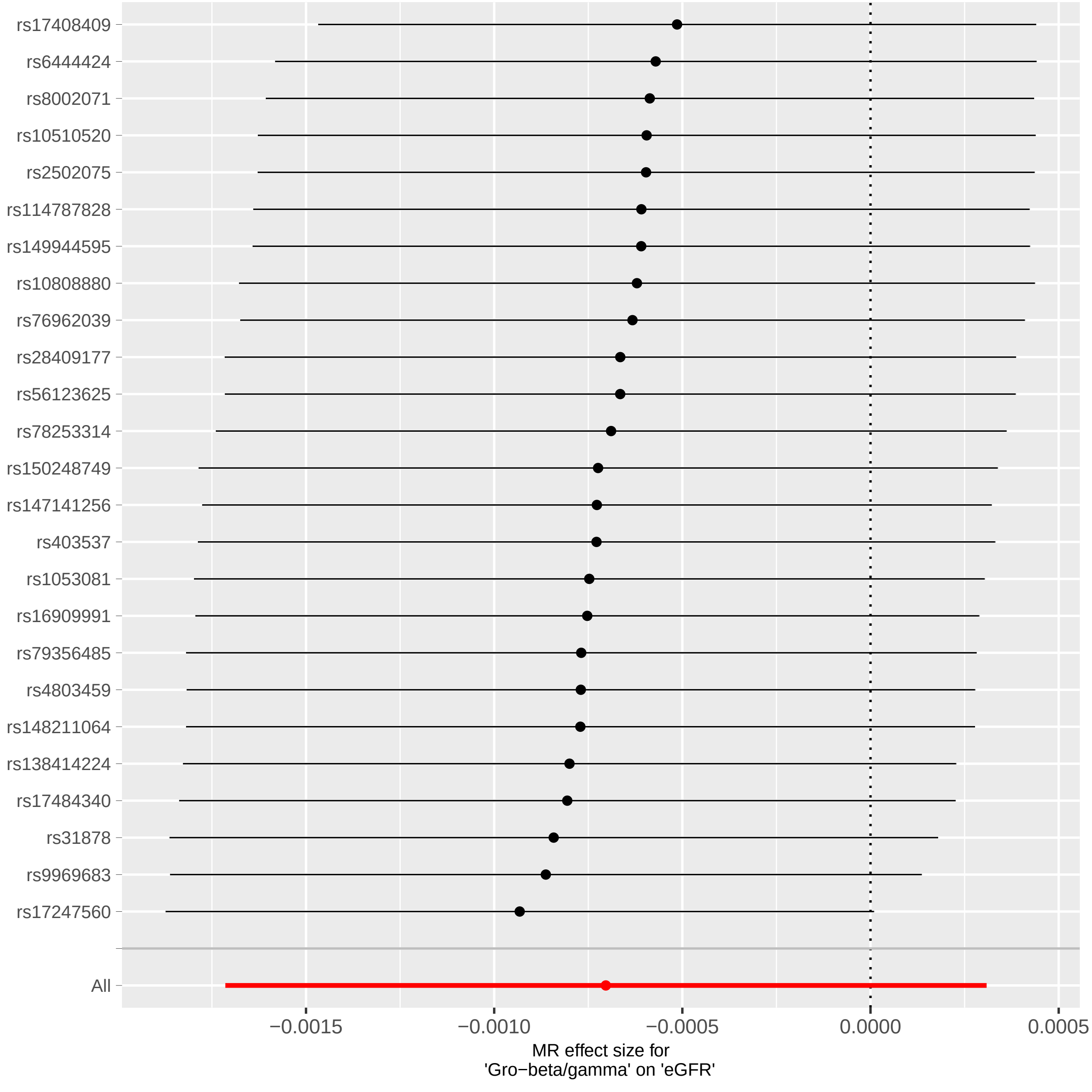

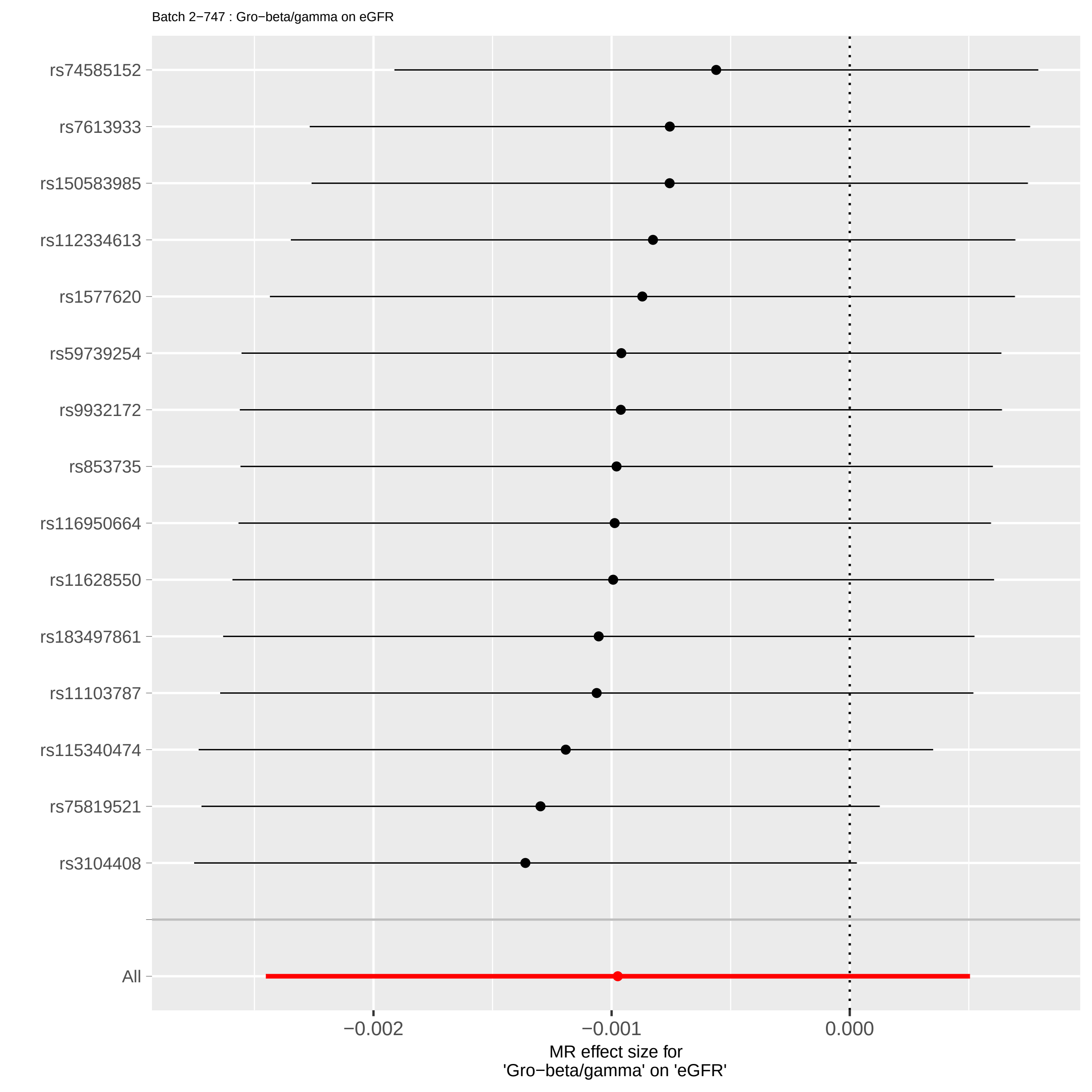

Batch 2−747 : Gro−beta/gamma on eGFR
rs74585152
rs7613933
rs150583985
rs112334613
rs1577620
rs59739254
rs9932172
rs853735
rs116950664
rs11628550
rs183497861
rs11103787
rs115340474
rs75819521
rs3104408
All
−0.002
−0.001
0.000
MR effect size for
'Gro−beta/gamma' on 'eGFR'

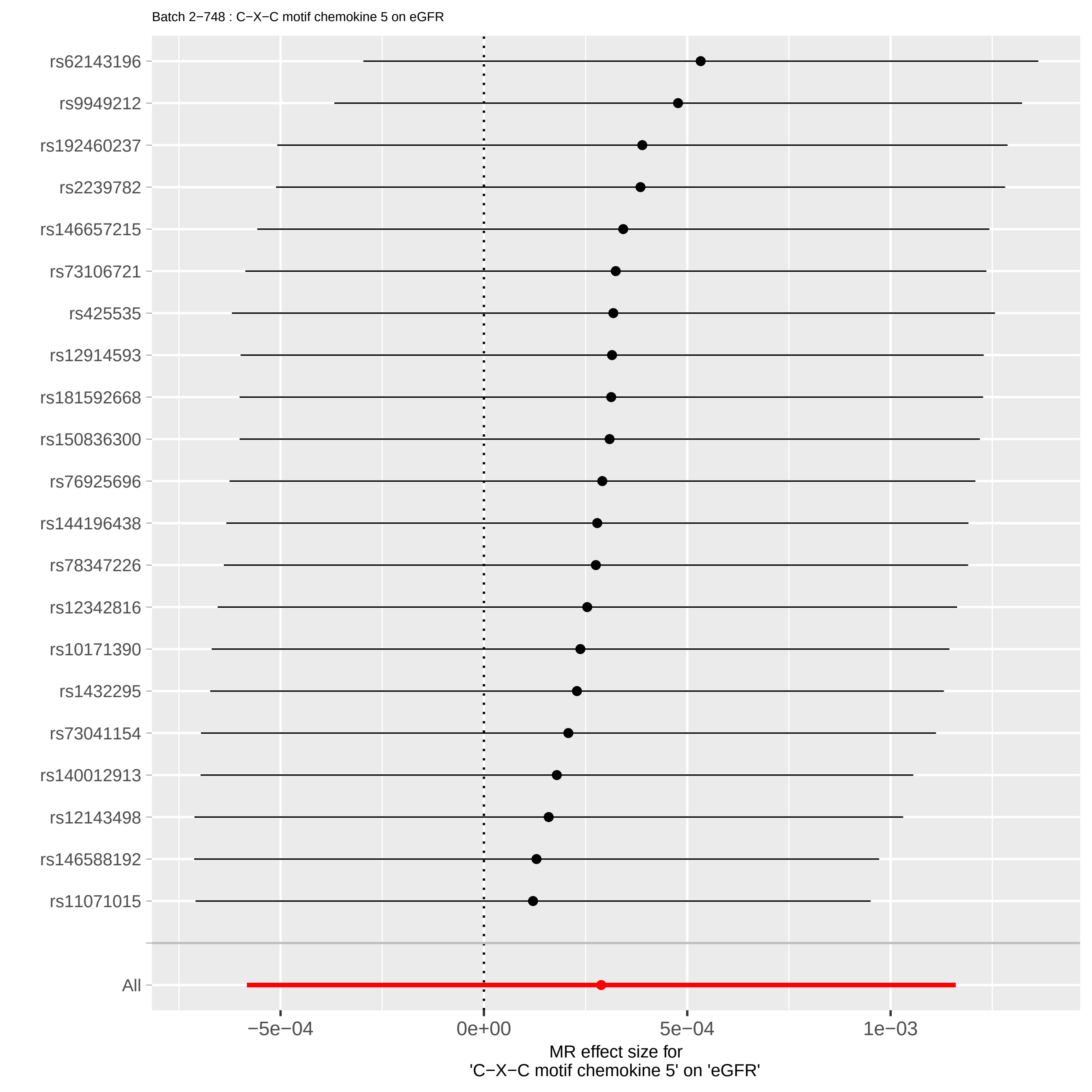
Batch 2−748 : C−X−C motif chemokine 5 on eGFR
rs62143196
rs9949212
rs192460237
rs2239782
rs146657215
rs73106721
rs425535
rs12914593
rs181592668
rs150836300
rs76925696
rs144196438
rs78347226
rs12342816
rs10171390
rs1432295
rs73041154
rs140012913
rs12143498
rs146588192
rs11071015
All
−5e−04
0e+00
5e−04
1e−03
MR effect size for
'C−X−C motif chemokine 5' on 'eGFR'

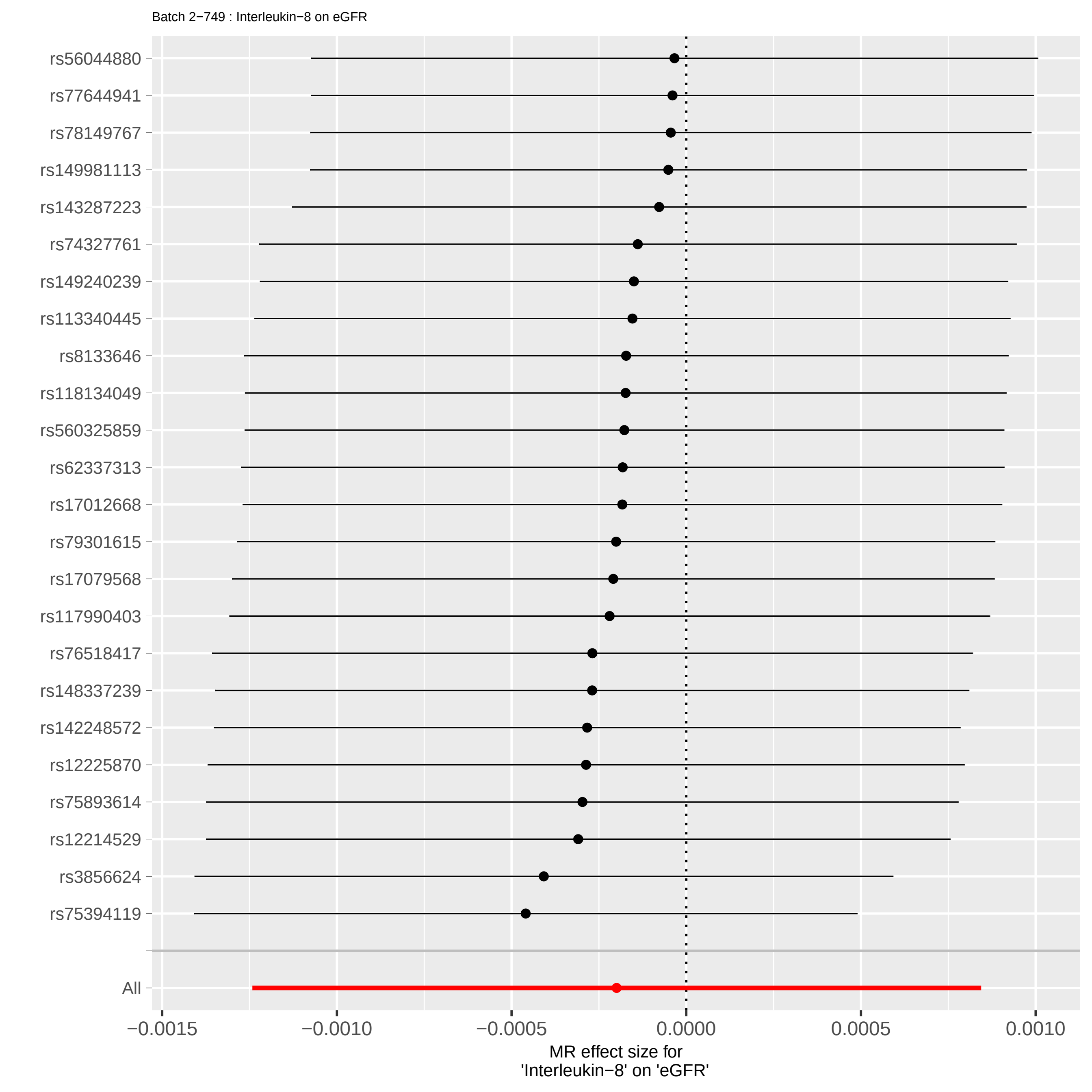

Batch 2−749 : Interleukin−8 on eGFR
rs56044880
rs77644941
rs78149767
rs149981113
rs143287223
rs74327761
rs149240239
rs113340445
rs8133646
rs118134049
rs560325859
rs62337313
rs17012668
rs79301615
rs17079568
rs117990403
rs76518417
rs148337239
rs142248572
rs12225870
rs75893614
rs12214529
rs3856624
rs75394119
All
−0.0015
−0.0010
−0.0005
0.0000
0.0005
0.0010
MR effect size for
'Interleukin−8' on 'eGFR'

Batch 2−750 : C−X−C motif chemokine 9 on eGFR

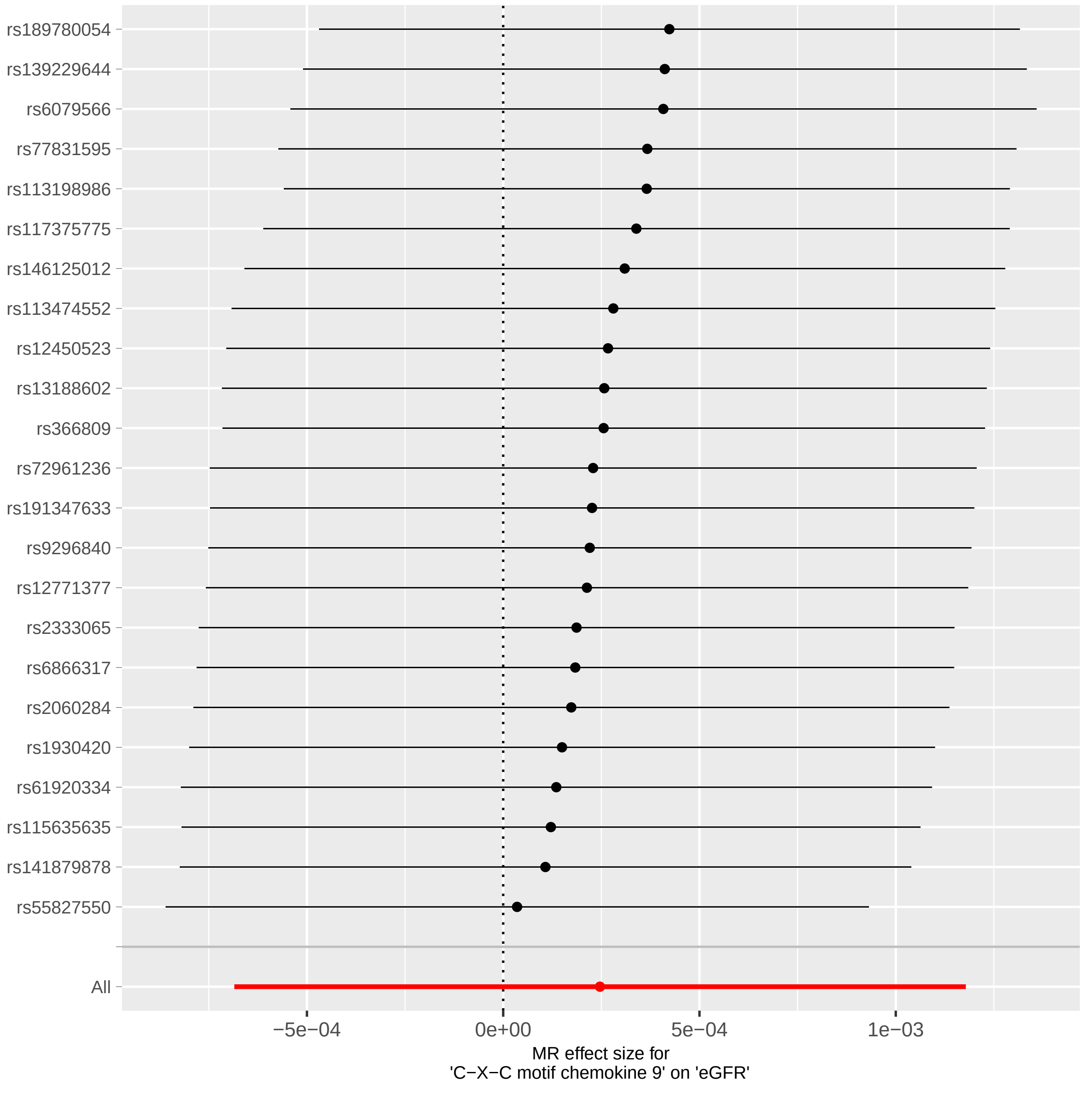

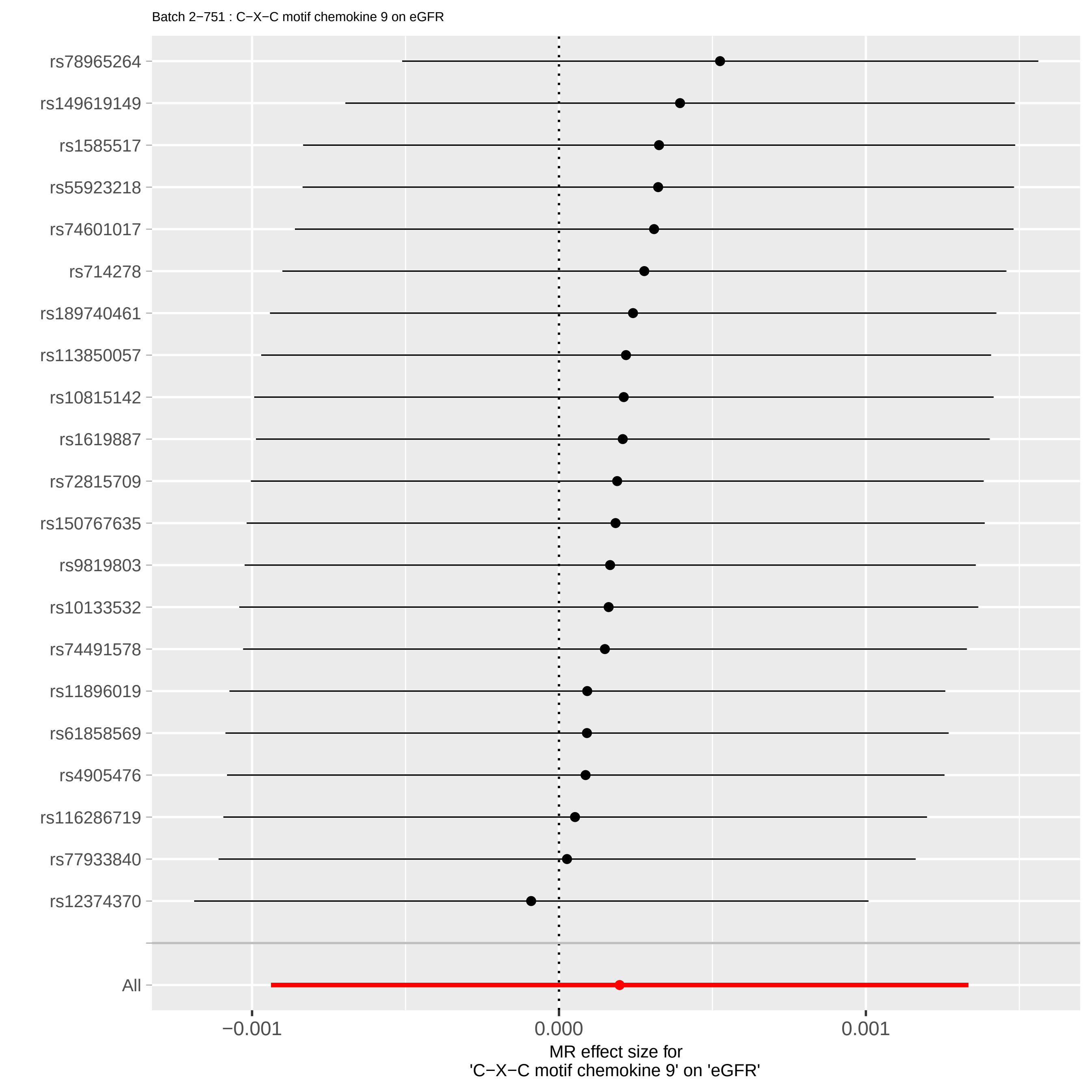
Batch 2−751 : C−X−C motif chemokine 9 on eGFR
rs78965264
rs149619149
rs1585517
rs55923218
rs74601017
rs714278
rs189740461
rs113850057
rs10815142
rs1619887
rs72815709
rs150767635
rs9819803
rs10133532
rs74491578
rs11896019
rs61858569
rs4905476
rs116286719
rs77933840
rs12374370
All
−0.001
0.000
0.001
MR effect size for
'C−X−C motif chemokine 9' on 'eGFR'

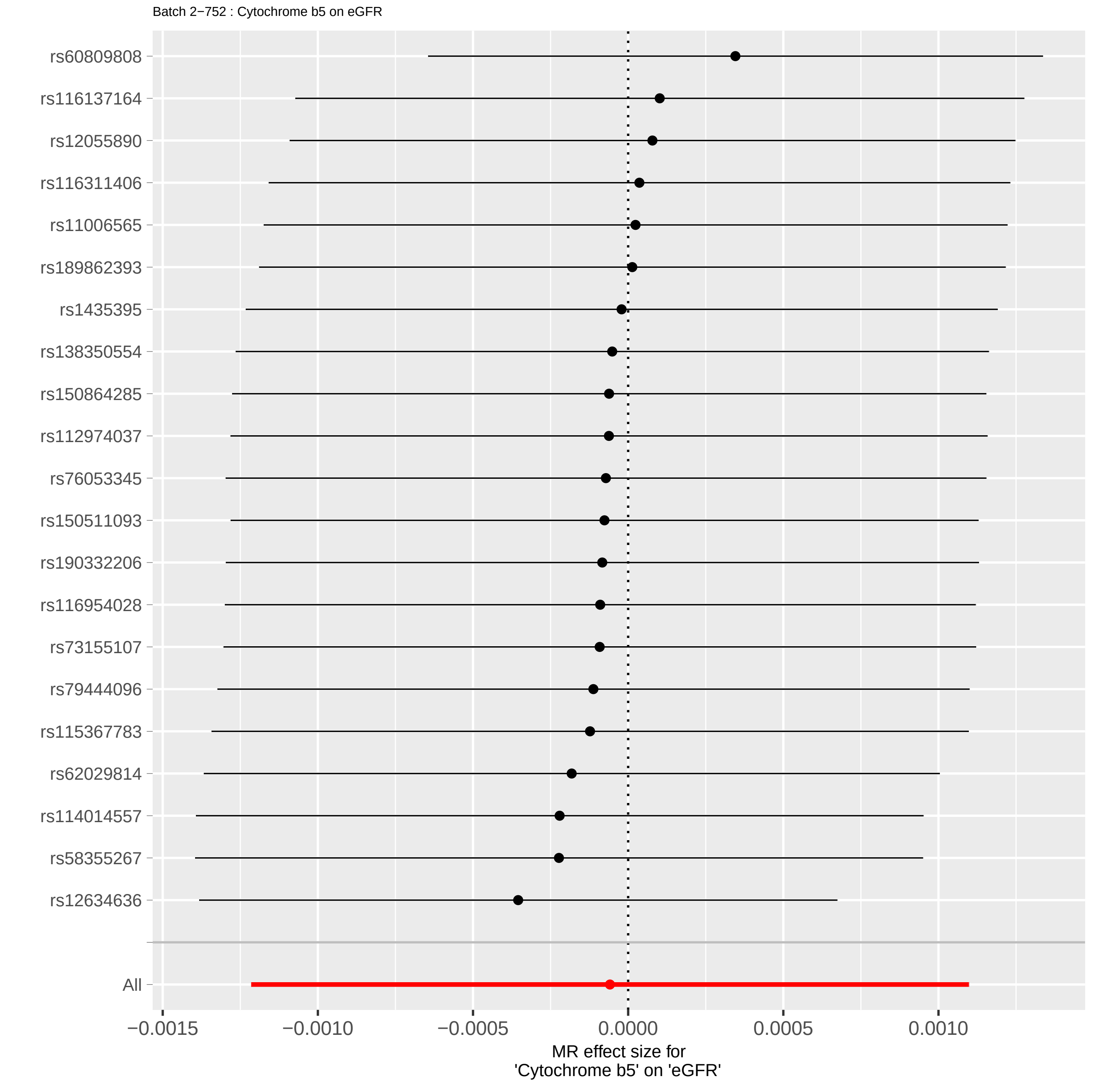

Batch 2−752 : Cytochrome b5 on eGFR
rs60809808
rs116137164
rs12055890
rs116311406
rs11006565
rs189862393
rs1435395
rs138350554
rs150864285
rs112974037
rs76053345
rs150511093
rs190332206
rs116954028
rs73155107
rs79444096
rs115367783
rs62029814
rs114014557
rs58355267
rs12634636
All
−0.0015
−0.0010
−0.0005
0.0000
0.0005
0.0010
MR effect size for
'Cytochrome b5' on 'eGFR'

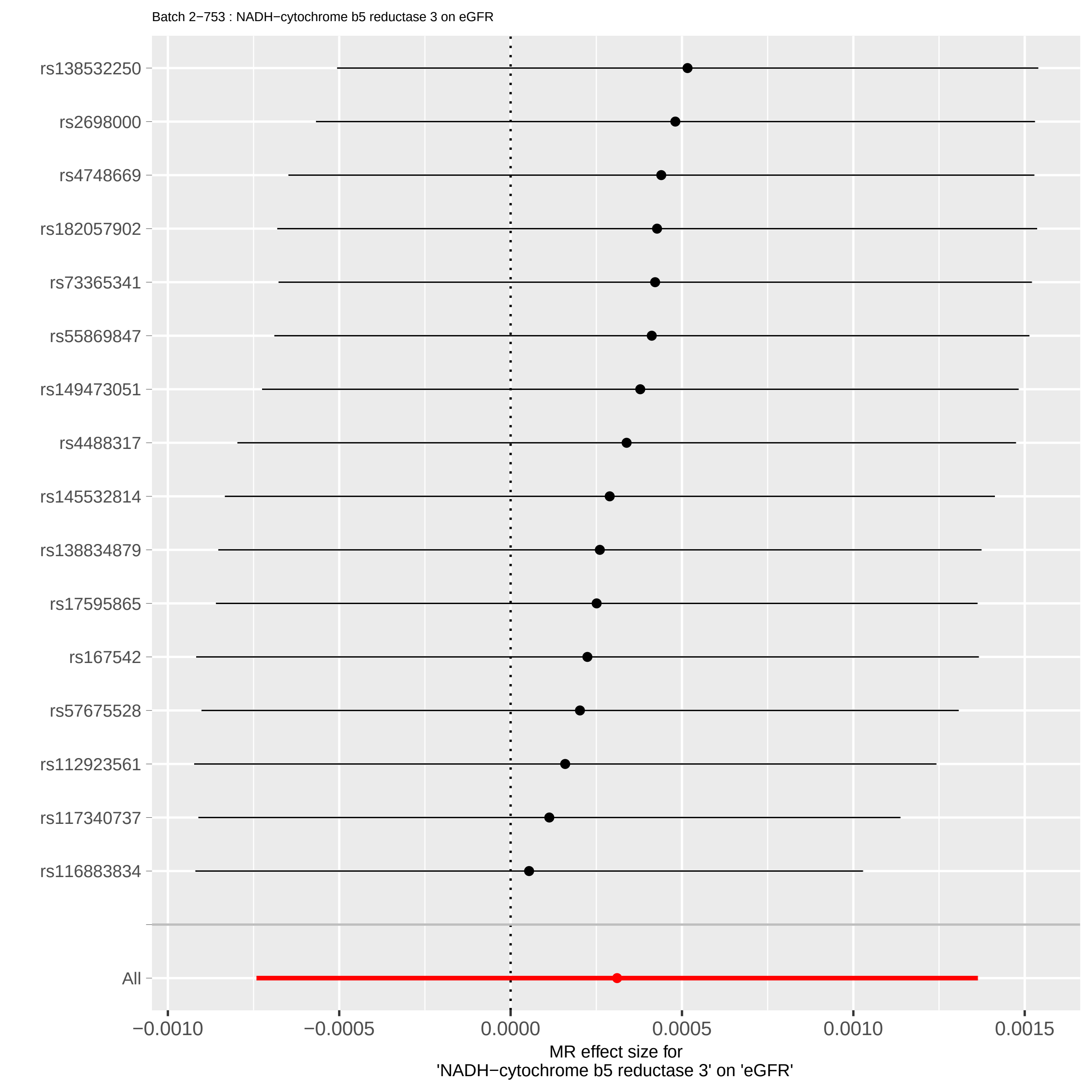

Batch 2−753 : NADH−cytochrome b5 reductase 3 on eGFR
rs138532250
rs2698000
rs4748669
rs182057902
rs73365341
rs55869847
rs149473051
rs4488317
rs145532814
rs138834879
rs17595865
rs167542
rs57675528
rs112923561
rs117340737
rs116883834
All
−0.0010
−0.0005
0.0000
0.0005
0.0010
0.0015
MR effect size for
'NADH−cytochrome b5 reductase 3' on 'eGFR'

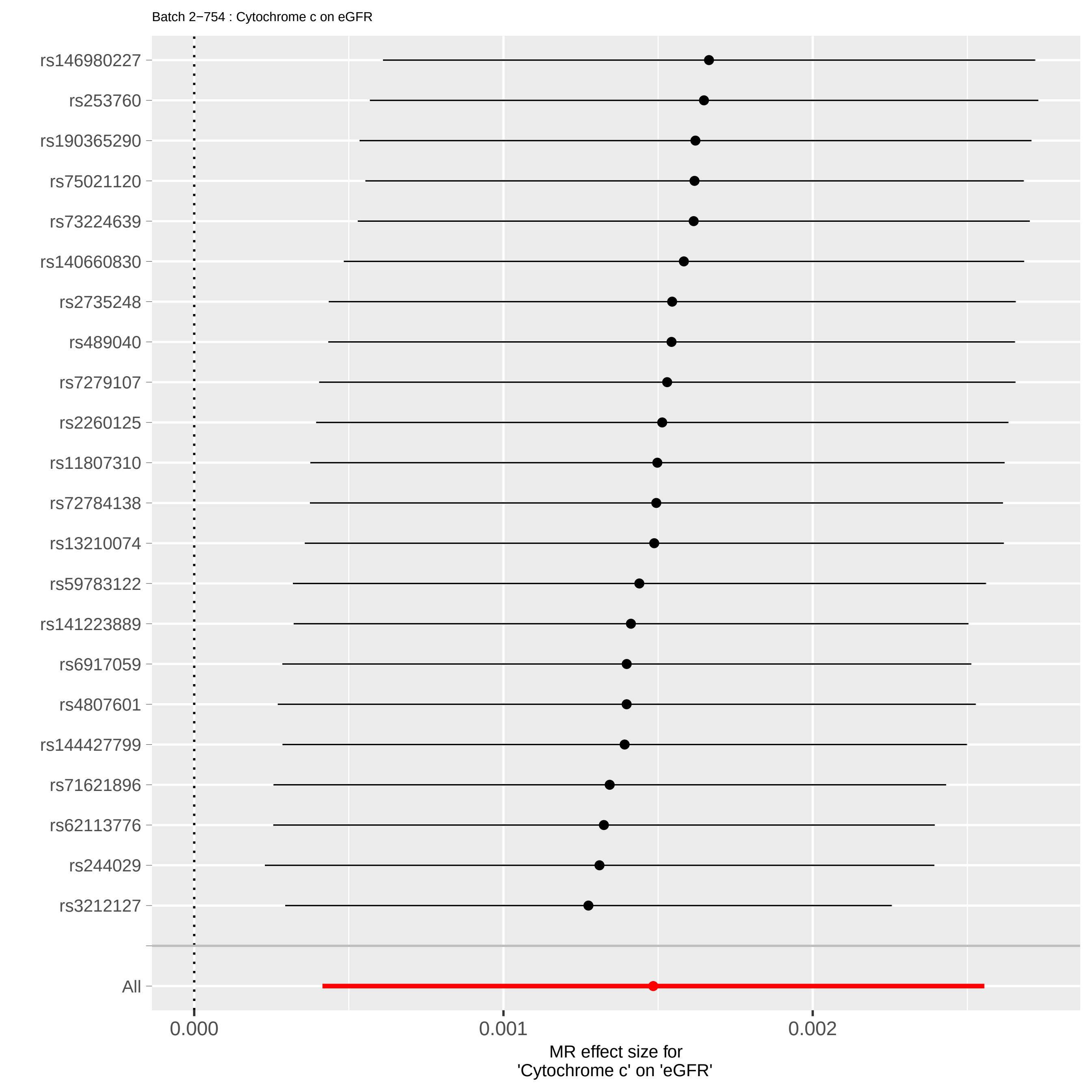
Batch 2−754 : Cytochrome c on eGFR
rs146980227
rs253760
rs190365290
rs75021120
rs73224639
rs140660830
rs2735248
rs489040
rs7279107
rs2260125
rs11807310
rs72784138
rs13210074
rs59783122
rs141223889
rs6917059
rs4807601
rs144427799
rs71621896
rs62113776
rs244029
rs3212127
All
0.000
0.001
0.002
MR effect size for
'Cytochrome c' on 'eGFR'

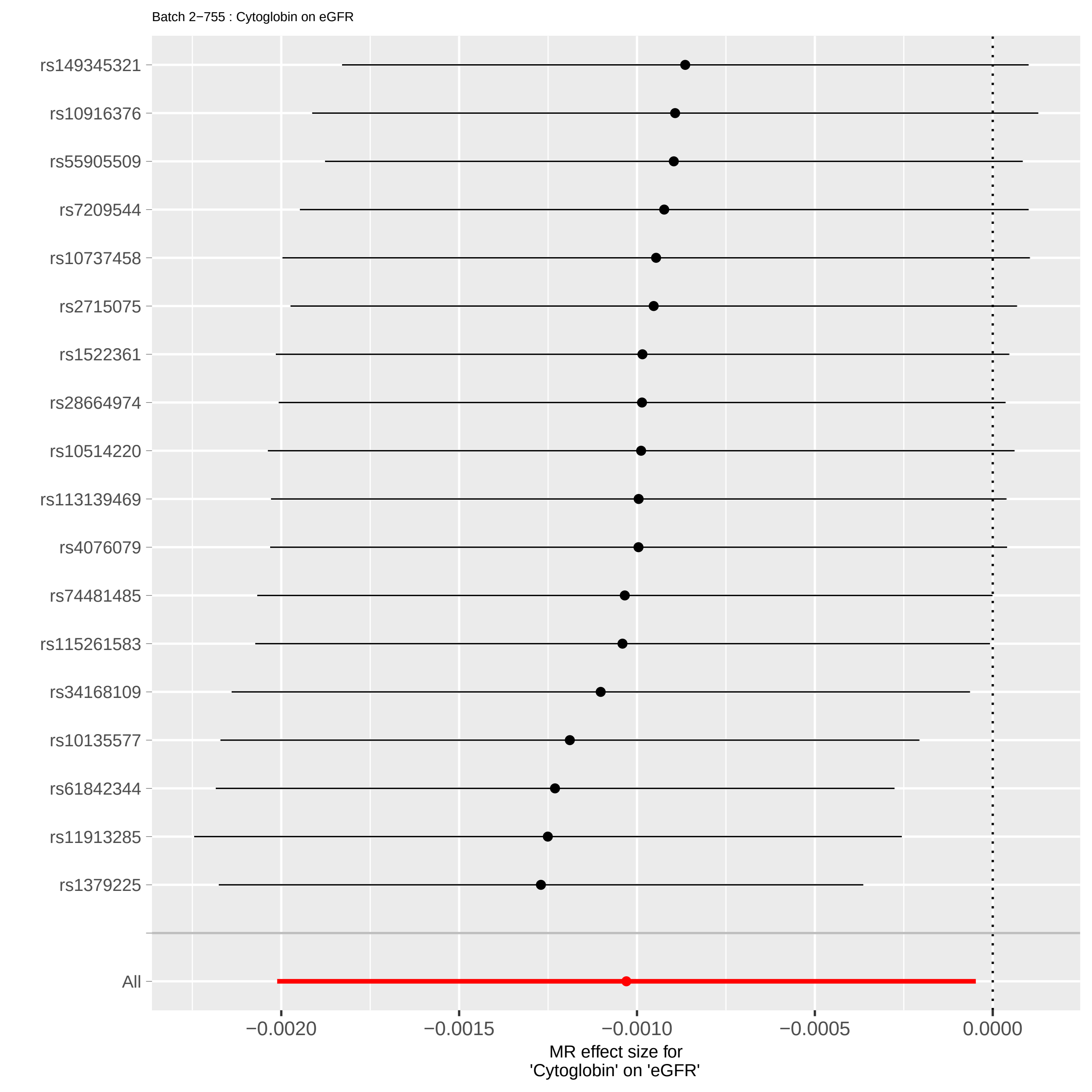
Batch 2−755 : Cytoglobin on eGFR
rs149345321
rs10916376
rs55905509
rs7209544
rs10737458
rs2715075
rs1522361
rs28664974
rs10514220
rs113139469
rs4076079
rs74481485
rs115261583
rs34168109
rs10135577
rs61842344
rs11913285
rs1379225
All
−0.0020
−0.0015
−0.0010
−0.0005
0.0000
MR effect size for
'Cytoglobin' on 'eGFR'

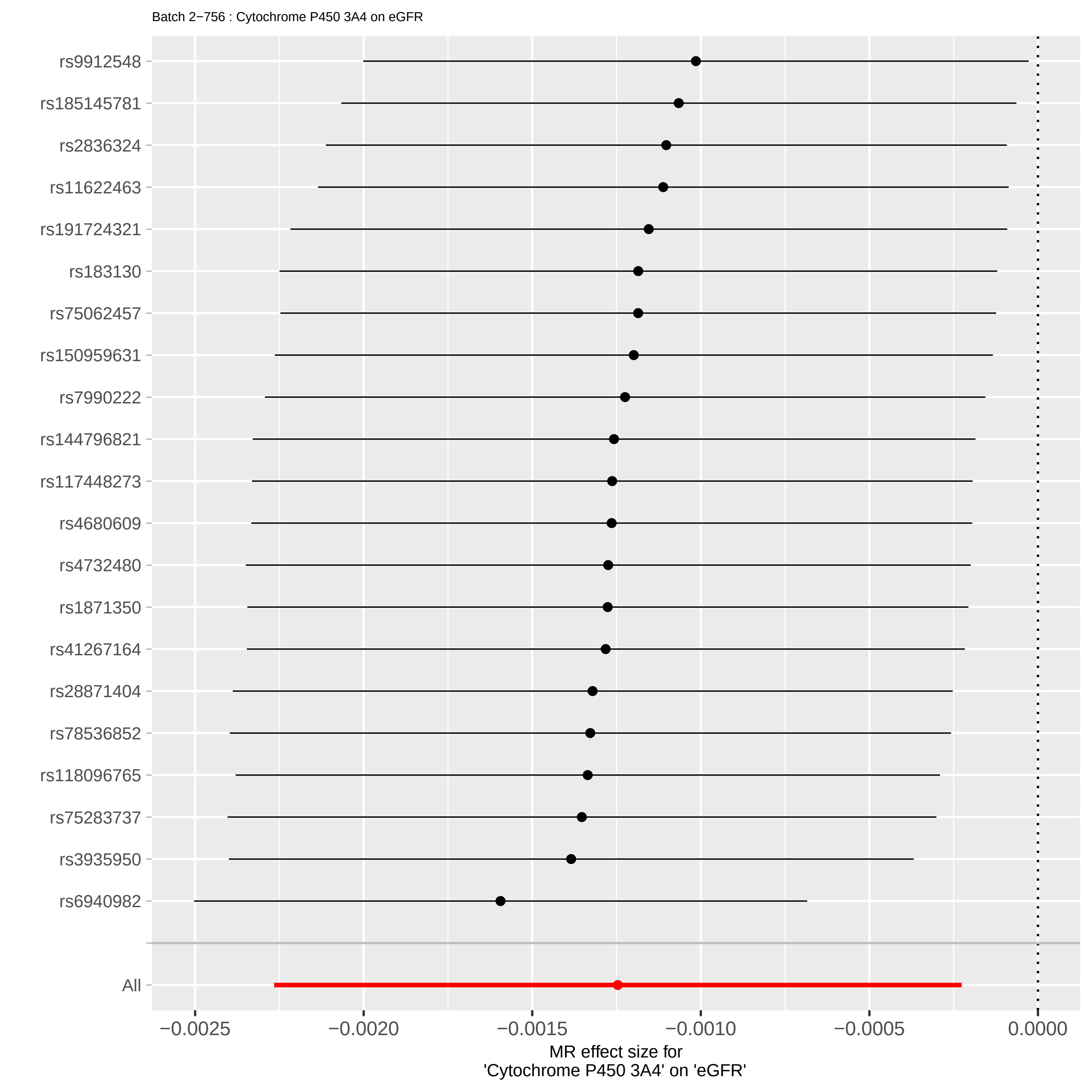

Batch 2−756 : Cytochrome P450 3A4 on eGFR
rs9912548
rs185145781
rs2836324
rs11622463
rs191724321
rs183130
rs75062457
rs150959631
rs7990222
rs144796821
rs117448273
rs4680609
rs4732480
rs1871350
rs41267164
rs28871404
rs78536852
rs118096765
rs75283737
rs3935950
rs6940982
All
−0.0025
−0.0020
−0.0015
−0.0010
−0.0005
0.0000
MR effect size for
'Cytochrome P450 3A4' on 'eGFR'

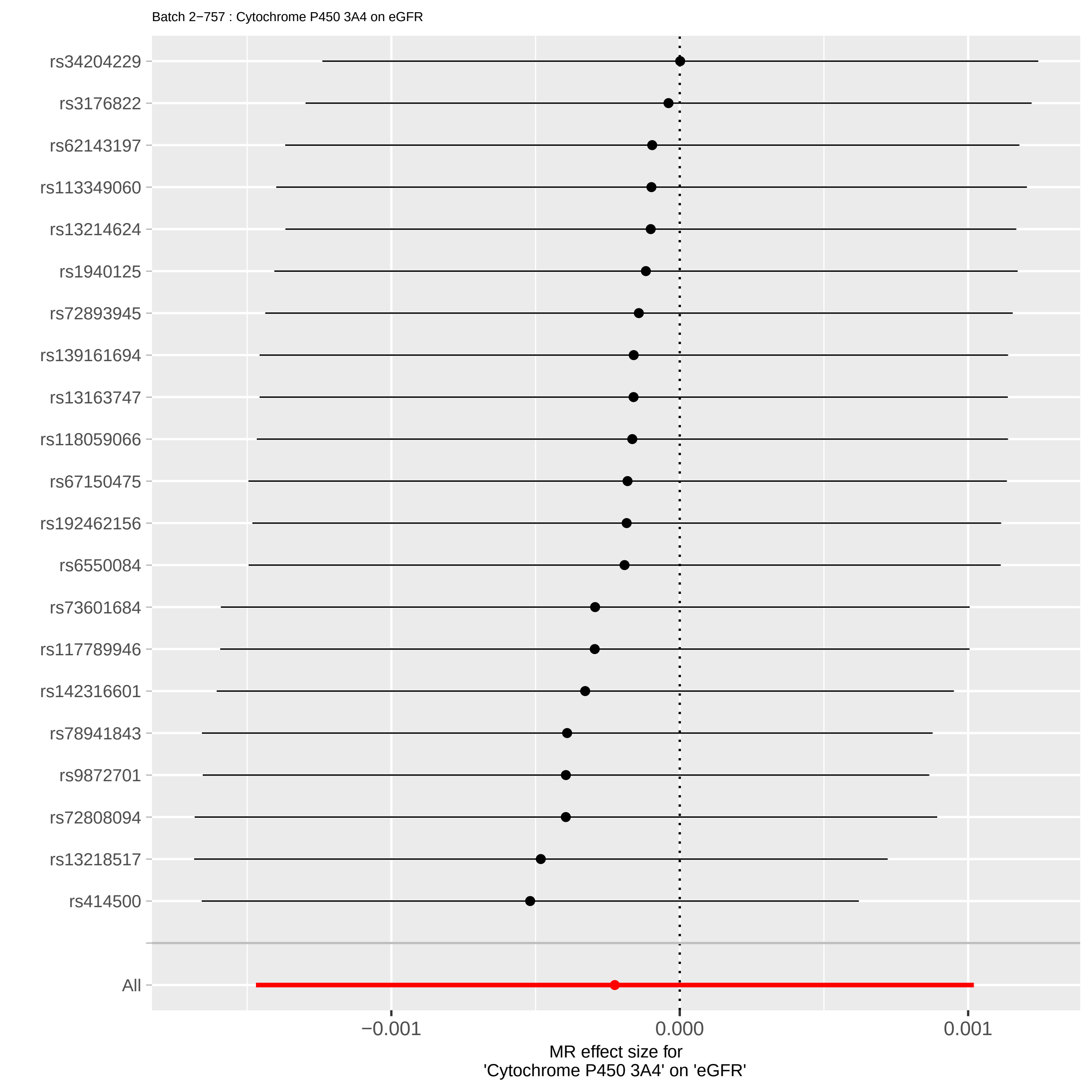

Batch 2−757 : Cytochrome P450 3A4 on eGFR
rs34204229
rs3176822
rs62143197
rs113349060
rs13214624
rs1940125
rs72893945
rs139161694
rs13163747
rs118059066
rs67150475
rs192462156
rs6550084
rs73601684
rs117789946
rs142316601
rs78941843
rs9872701
rs72808094
rs13218517
rs414500
All
−0.001
0.000
0.001
MR effect size for
'Cytochrome P450 3A4' on 'eGFR'

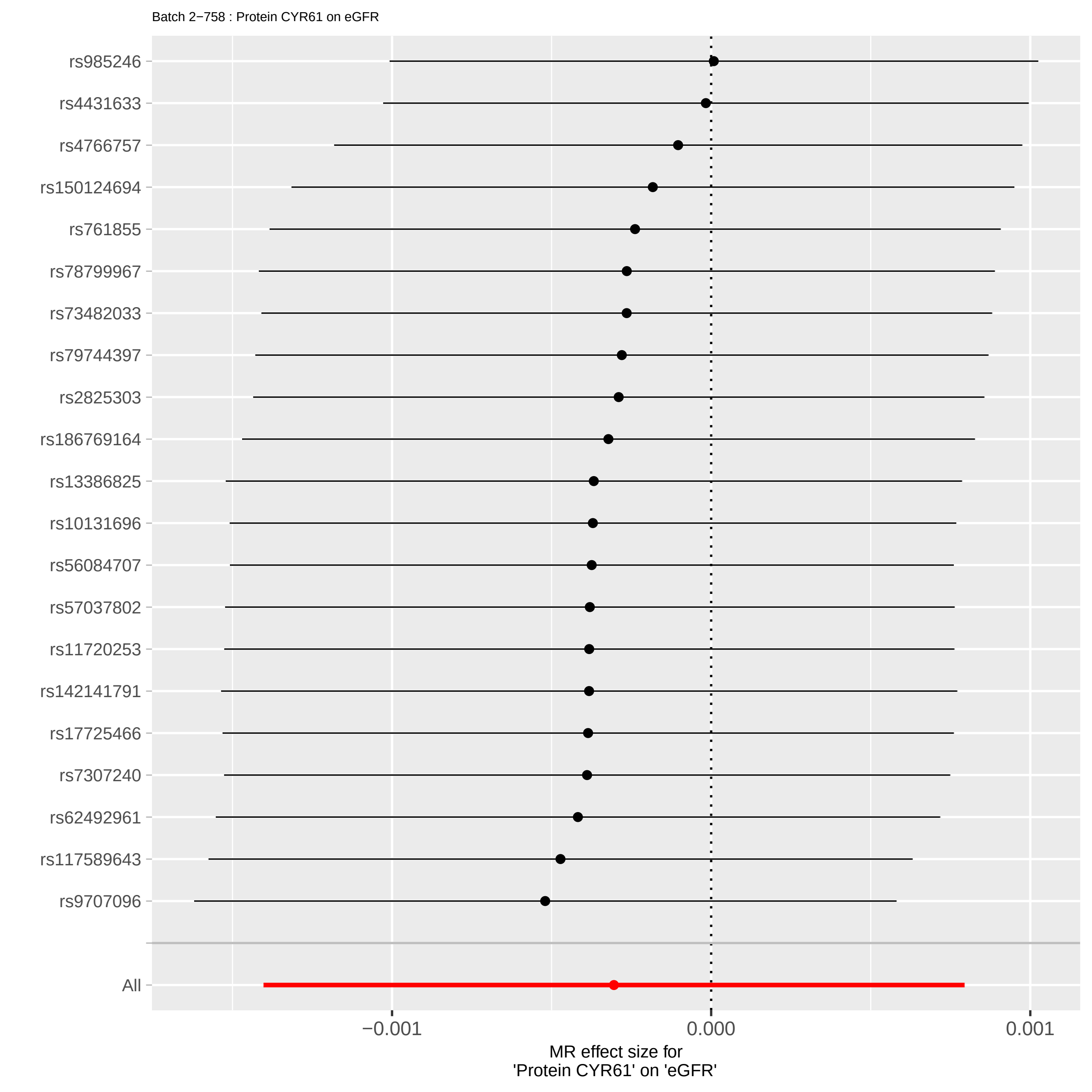

Batch 2−758 : Protein CYR61 on eGFR
rs985246
rs4431633
rs4766757
rs150124694
rs761855
rs78799967
rs73482033
rs79744397
rs2825303
rs186769164
rs13386825
rs10131696
rs56084707
rs57037802
rs11720253
rs142141791
rs17725466
rs7307240
rs62492961
rs117589643
rs9707096
All
−0.001
0.000
0.001
MR effect size for
'Protein CYR61' on 'eGFR'

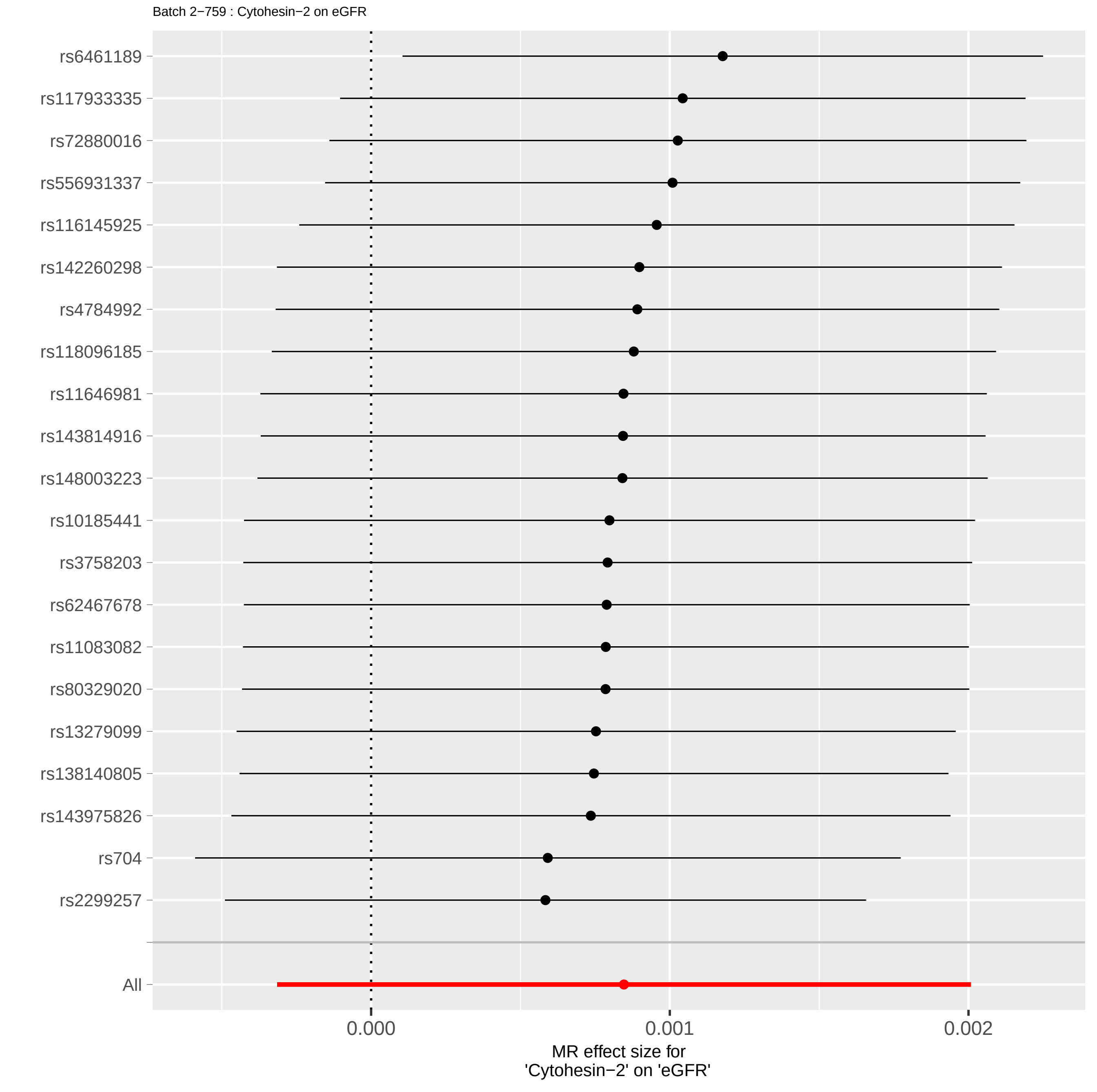

Batch 2−759 : Cytohesin−2 on eGFR
rs6461189
rs117933335
rs72880016
rs556931337
rs116145925
rs142260298
rs4784992
rs118096185
rs11646981
rs143814916
rs148003223
rs10185441
rs3758203
rs62467678
rs11083082
rs80329020
rs13279099
rs138140805
rs143975826
rs704
rs2299257
All
0.000
0.001
0.002
MR effect size for
'Cytohesin−2' on 'eGFR'

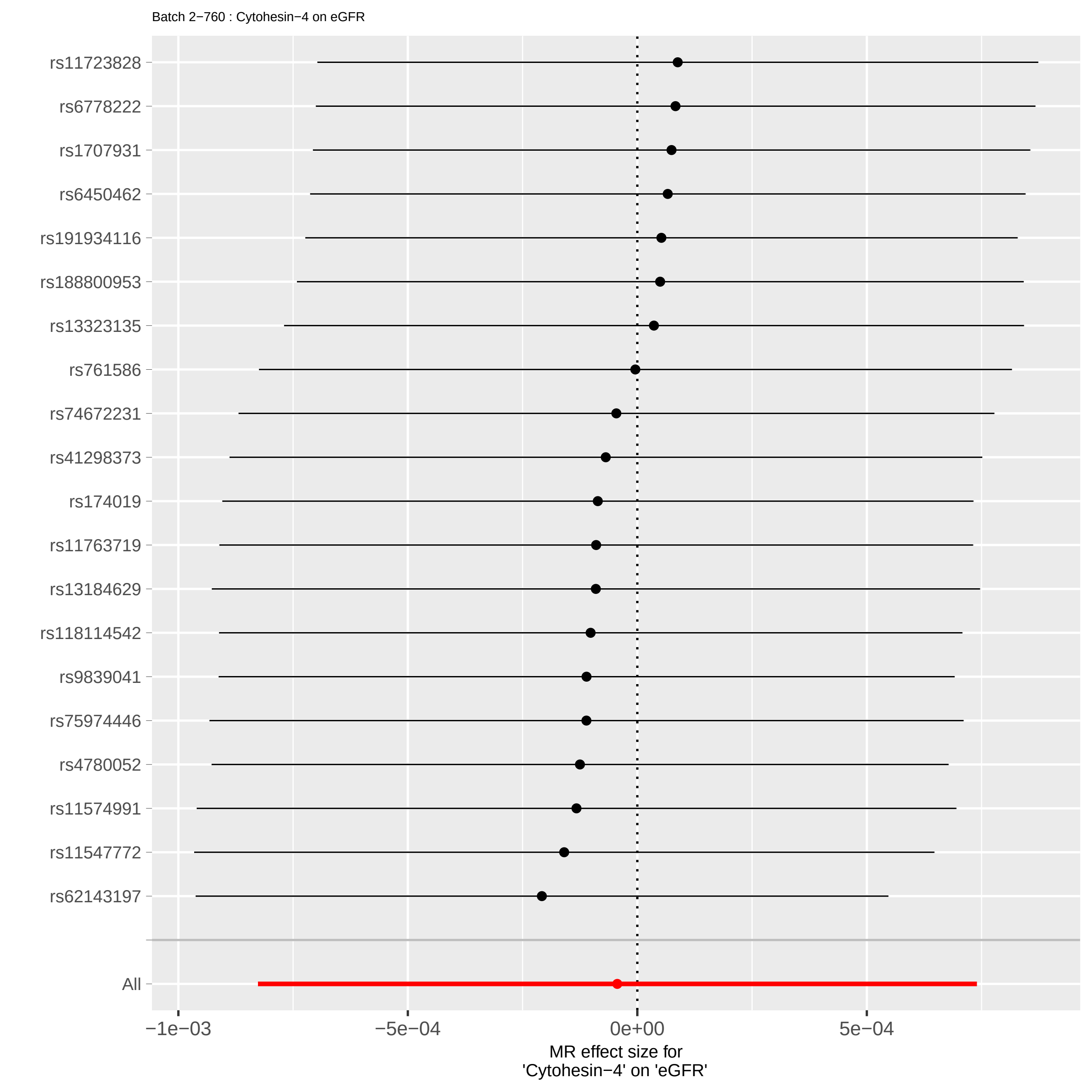
Batch 2−760 : Cytohesin−4 on eGFR
rs11723828
rs6778222
rs1707931
rs6450462
rs191934116
rs188800953
rs13323135
rs761586
rs74672231
rs41298373
rs174019
rs11763719
rs13184629
rs118114542
rs9839041
rs75974446
rs4780052
rs11574991
rs11547772
rs62143197
All
−1e−03
−5e−04
0e+00
5e−04
MR effect size for
'Cytohesin−4' on 'eGFR'

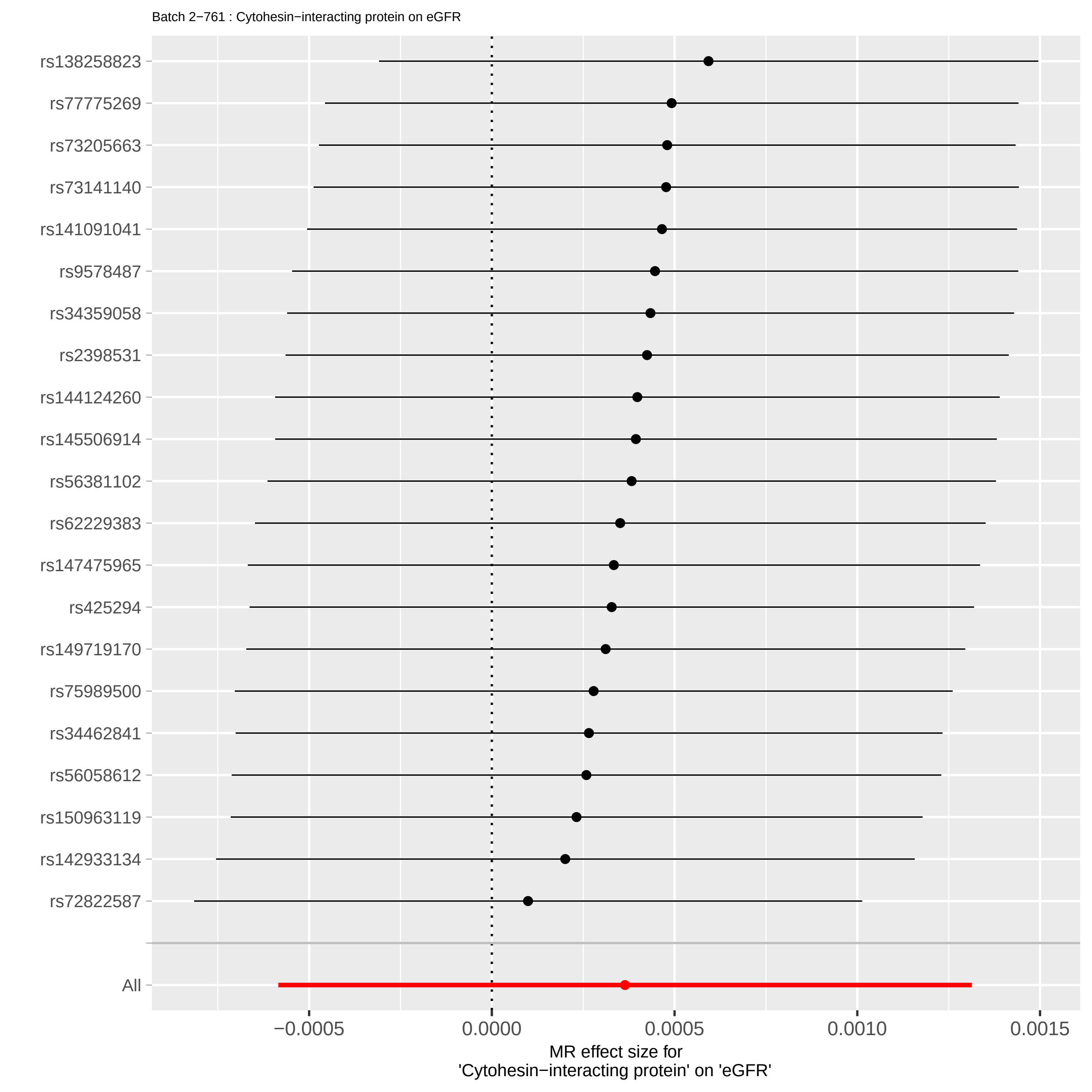

Batch 2−761 : Cytohesin−interacting protein on eGFR
rs138258823
rs77775269
rs73205663
rs73141140
rs141091041
rs9578487
rs34359058
rs2398531
rs144124260
rs145506914
rs56381102
rs62229383
rs147475965
rs425294
rs149719170
rs75989500
rs34462841
rs56058612
rs150963119
rs142933134
rs72822587
All
−0.0005
0.0000
0.0005
0.0010
0.0015
MR effect size for
'Cytohesin−interacting protein' on 'eGFR'

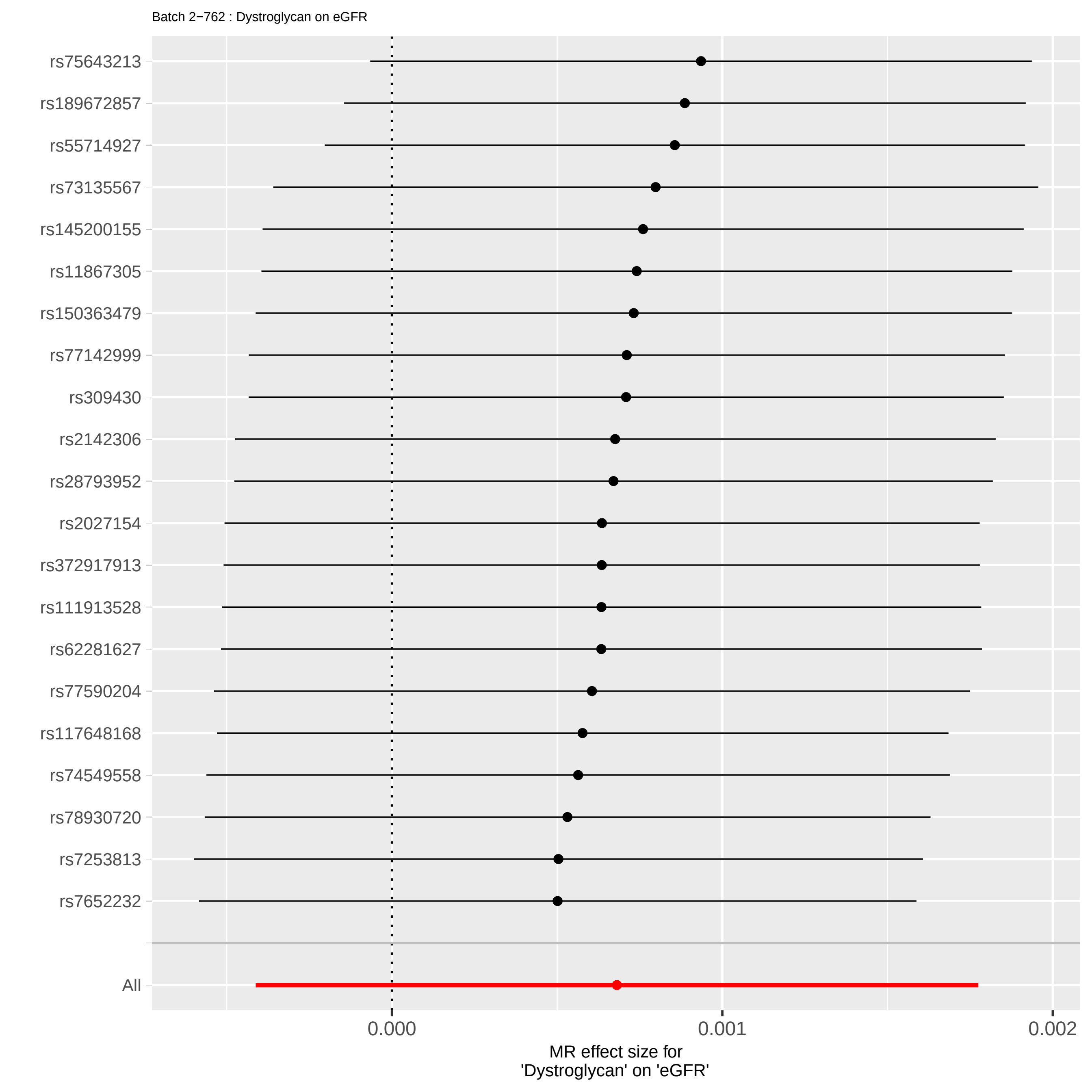
Batch 2−762 : Dystroglycan on eGFR
rs75643213
rs189672857
rs55714927
rs73135567
rs145200155
rs11867305
rs150363479
rs77142999
rs309430
rs2142306
rs28793952
rs2027154
rs372917913
rs111913528
rs62281627
rs77590204
rs117648168
rs74549558
rs78930720
rs7253813
rs7652232
All
0.000
0.001
0.002
MR effect size for
'Dystroglycan' on 'eGFR'

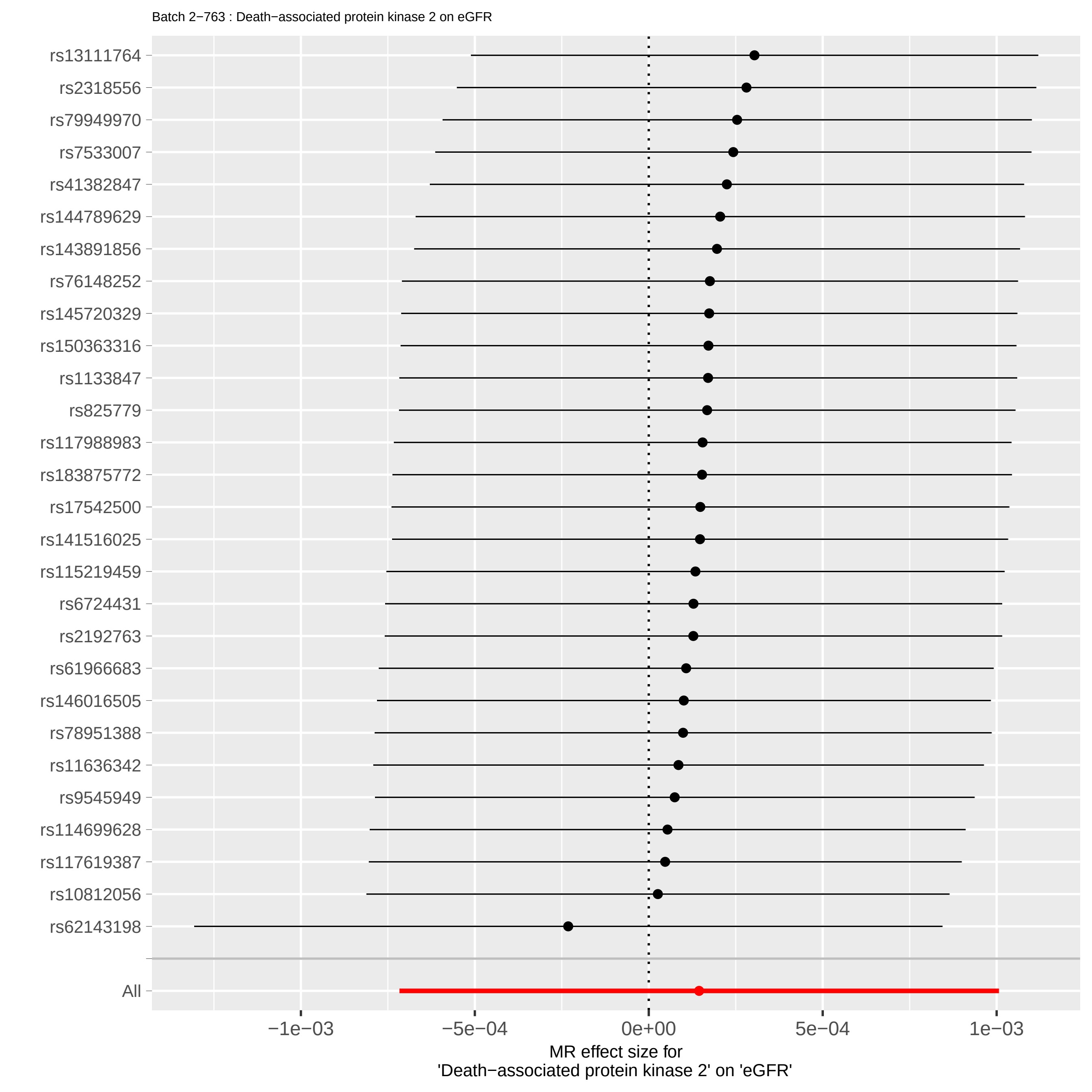
Batch 2−763 : Death−associated protein kinase 2 on eGFR
rs13111764
rs2318556
rs79949970
rs7533007
rs41382847
rs144789629
rs143891856
rs76148252
rs145720329
rs150363316
rs1133847
rs825779
rs117988983
rs183875772
rs17542500
rs141516025
rs115219459
rs6724431
rs2192763
rs61966683
rs146016505
rs78951388
rs11636342
rs9545949
rs114699628
rs117619387
rs10812056
rs62143198
All
−1e−03
−5e−04
0e+00
5e−04
1e−03
MR effect size for
'Death−associated protein kinase 2' on 'eGFR'

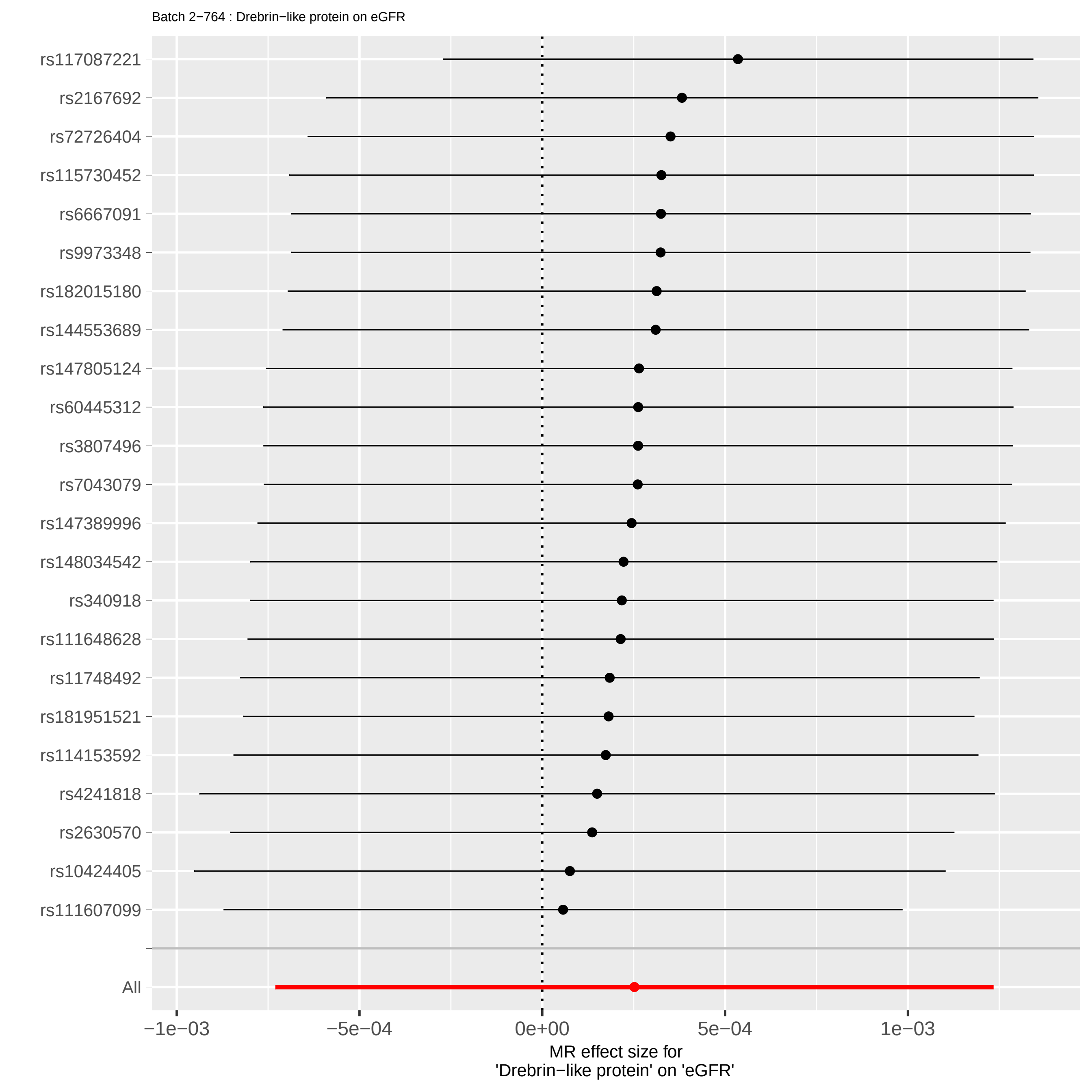
Batch 2−764 : Drebrin−like protein on eGFR
rs117087221
rs2167692
rs72726404
rs115730452
rs6667091
rs9973348
rs182015180
rs144553689
rs147805124
rs60445312
rs3807496
rs7043079
rs147389996
rs148034542
rs340918
rs111648628
rs11748492
rs181951521
rs114153592
rs4241818
rs2630570
rs10424405
rs111607099
All
−1e−03
−5e−04
0e+00
5e−04
1e−03
MR effect size for
'Drebrin−like protein' on 'eGFR'

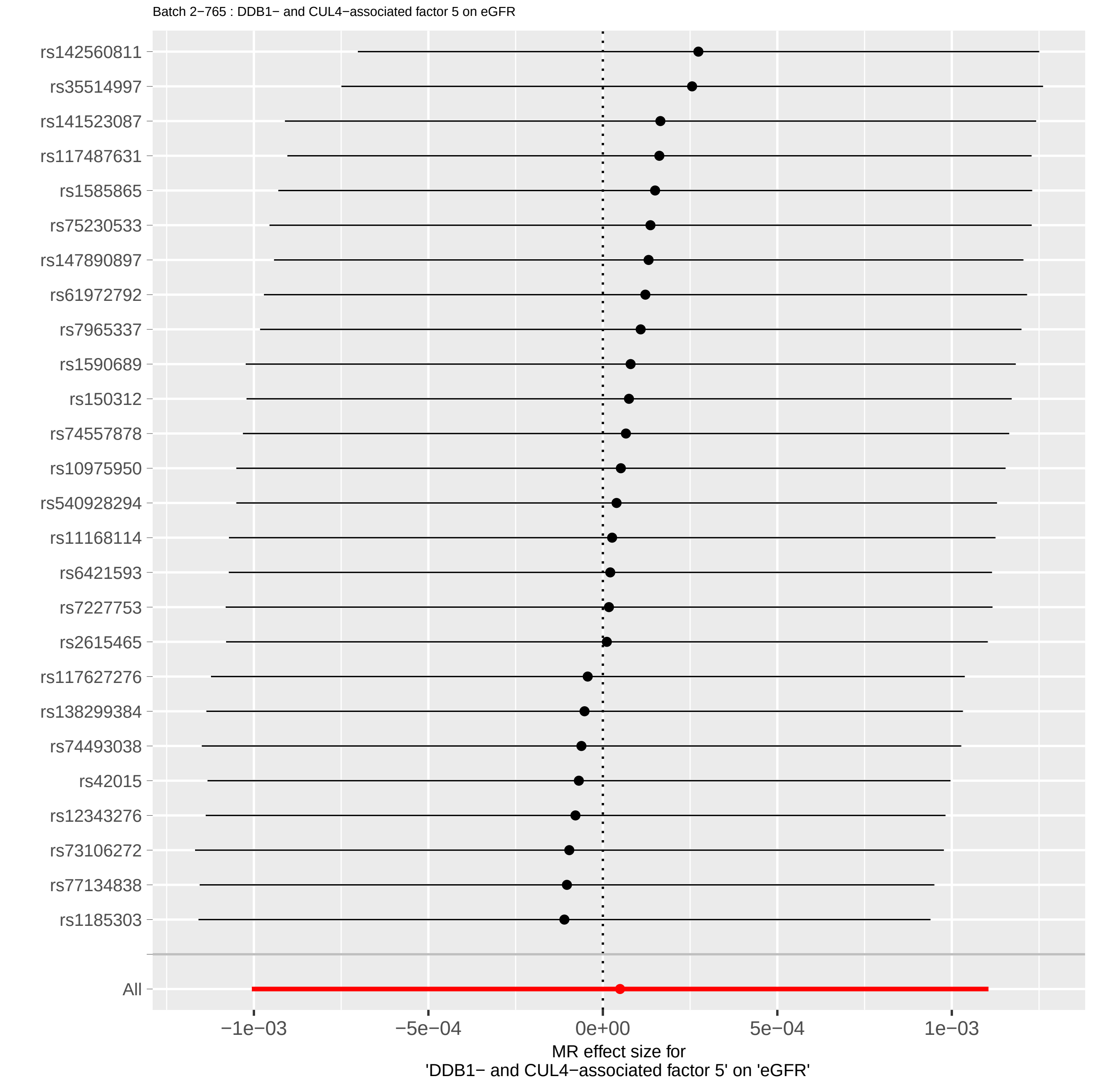

Batch 2−765 : DDB1− and CUL4−associated factor 5 on eGFR
rs142560811
rs35514997
rs141523087
rs117487631
rs1585865
rs75230533
rs147890897
rs61972792
rs7965337
rs1590689
rs150312
rs74557878
rs10975950
rs540928294
rs11168114
rs6421593
rs7227753
rs2615465
rs117627276
rs138299384
rs74493038
rs42015
rs12343276
rs73106272
rs77134838
rs1185303
All
−1e−03
−5e−04
0e+00
5e−04
1e−03
MR effect size for
'DDB1− and CUL4−associated factor 5' on 'eGFR'

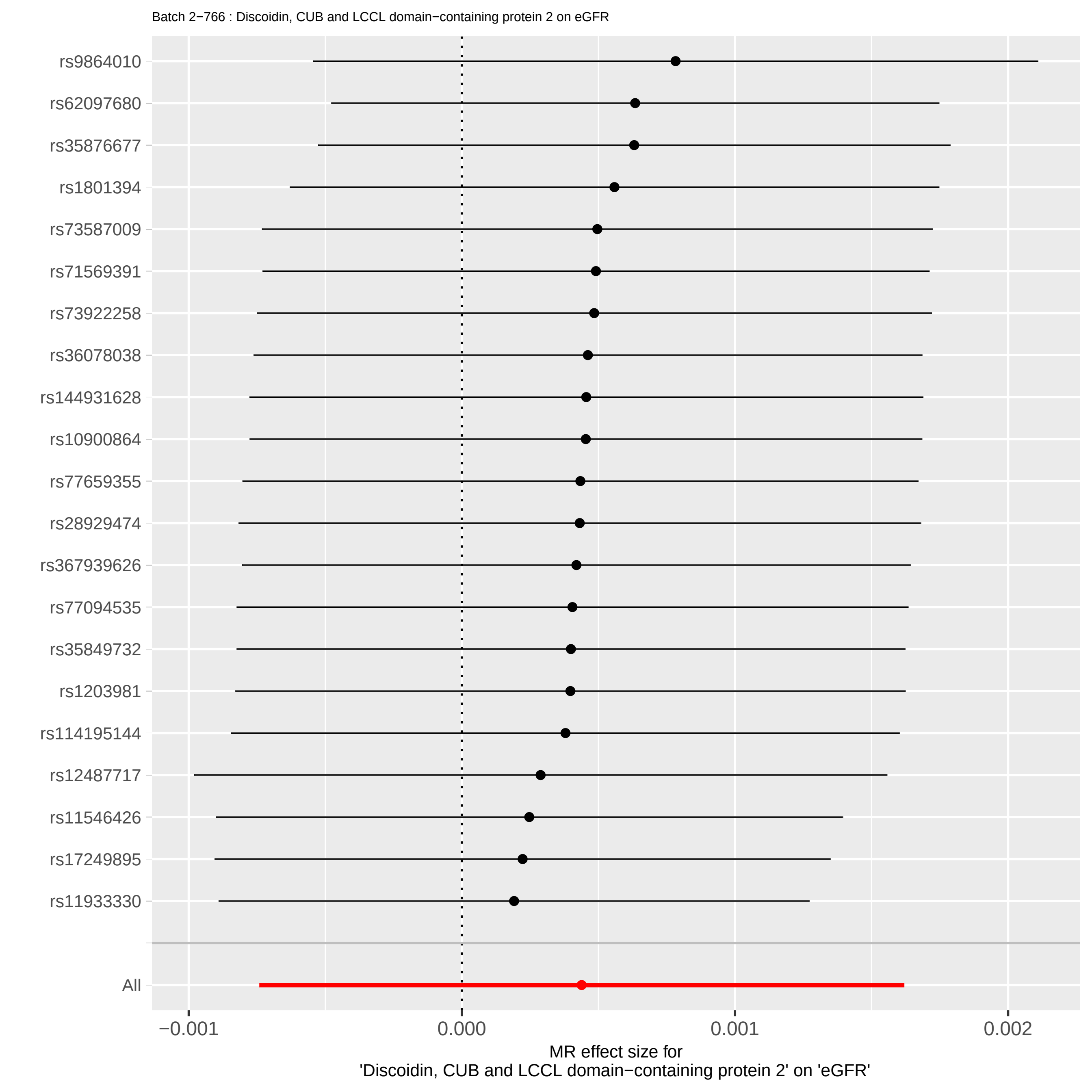

Batch 2−766 : Discoidin, CUB and LCCL domain−containing protein 2 on eGFR
rs9864010
rs62097680
rs35876677
rs1801394
rs73587009
rs71569391
rs73922258
rs36078038
rs144931628
rs10900864
rs77659355
rs28929474
rs367939626
rs77094535
rs35849732
rs1203981
rs114195144
rs12487717
rs11546426
rs17249895
rs11933330
All
−0.001
0.000
0.001
0.002
MR effect size for
'Discoidin, CUB and LCCL domain−containing protein 2' on 'eGFR'

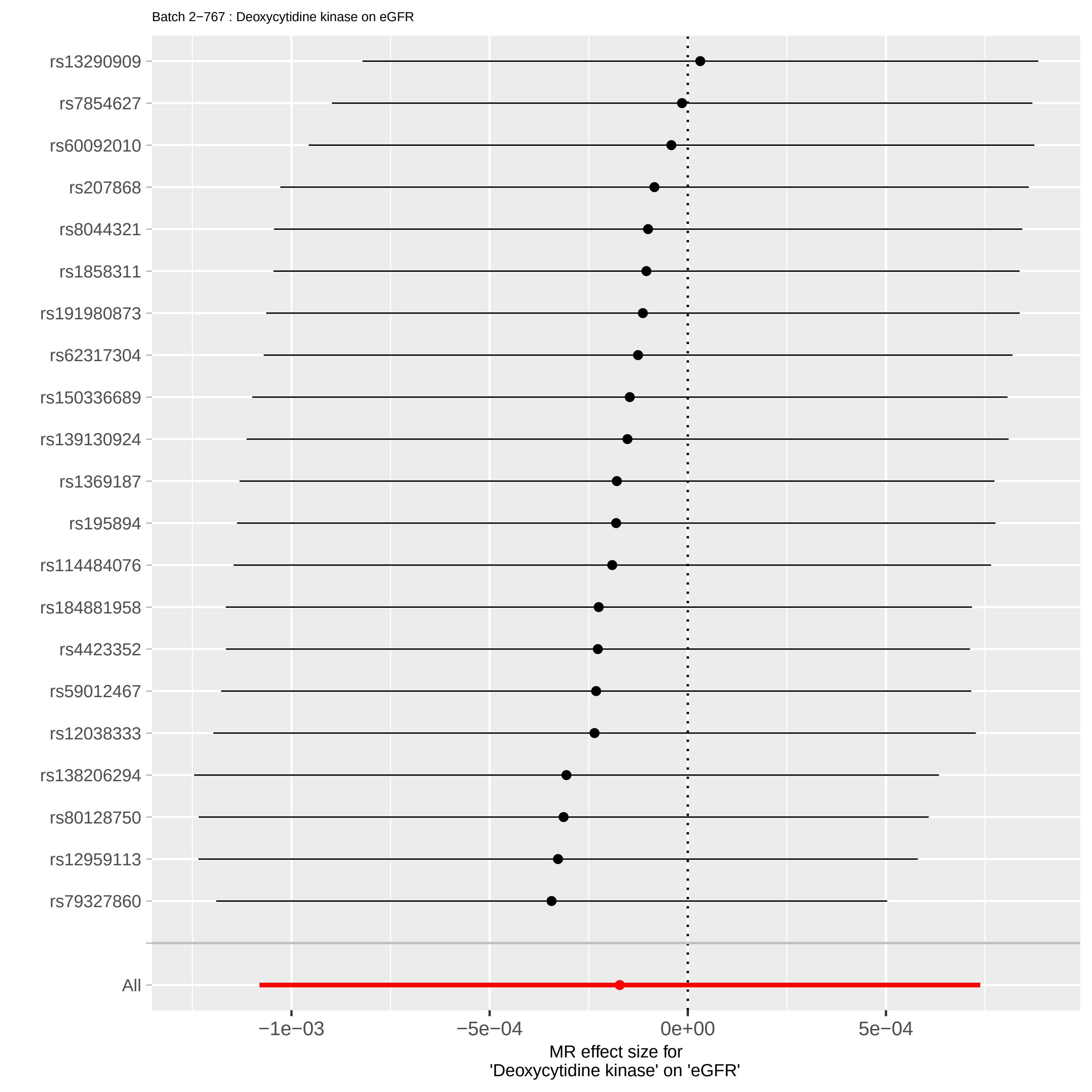

Batch 2−767 : Deoxycytidine kinase on eGFR
rs13290909
rs7854627
rs60092010
rs207868
rs8044321
rs1858311
rs191980873
rs62317304
rs150336689
rs139130924
rs1369187
rs195894
rs114484076
rs184881958
rs4423352
rs59012467
rs12038333
rs138206294
rs80128750
rs12959113
rs79327860
All
−1e−03
−5e−04
0e+00
5e−04
MR effect size for
'Deoxycytidine kinase' on 'eGFR'

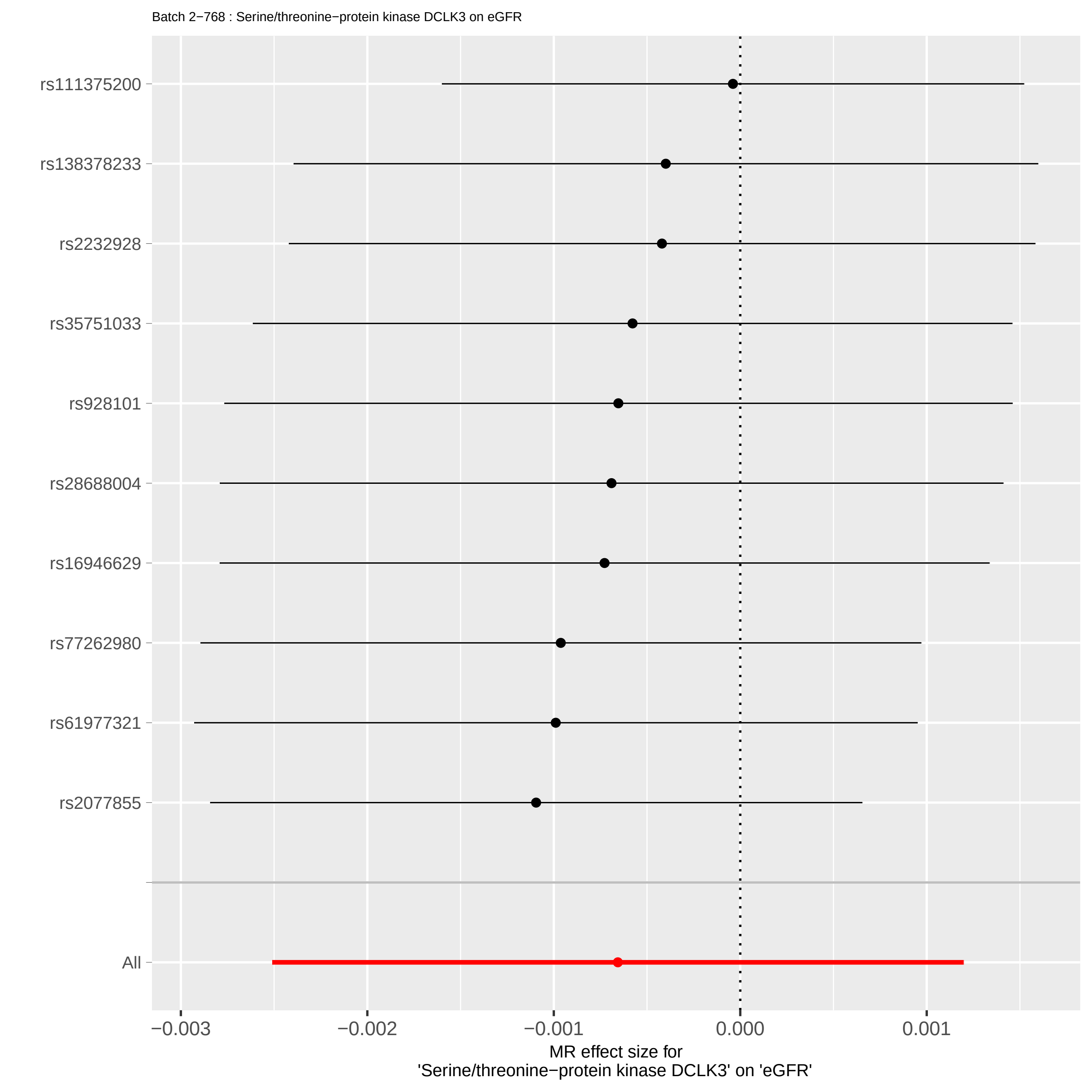
Batch 2−768 : Serine/threonine−protein kinase DCLK3 on eGFR
rs111375200
rs138378233
rs2232928
rs35751033
rs928101
rs28688004
rs16946629
rs77262980
rs61977321
rs2077855
All
−0.003
−0.002
−0.001
0.000
0.001
MR effect size for
'Serine/threonine−protein kinase DCLK3' on 'eGFR'

Batch 2−769 : Decorin on eGFR

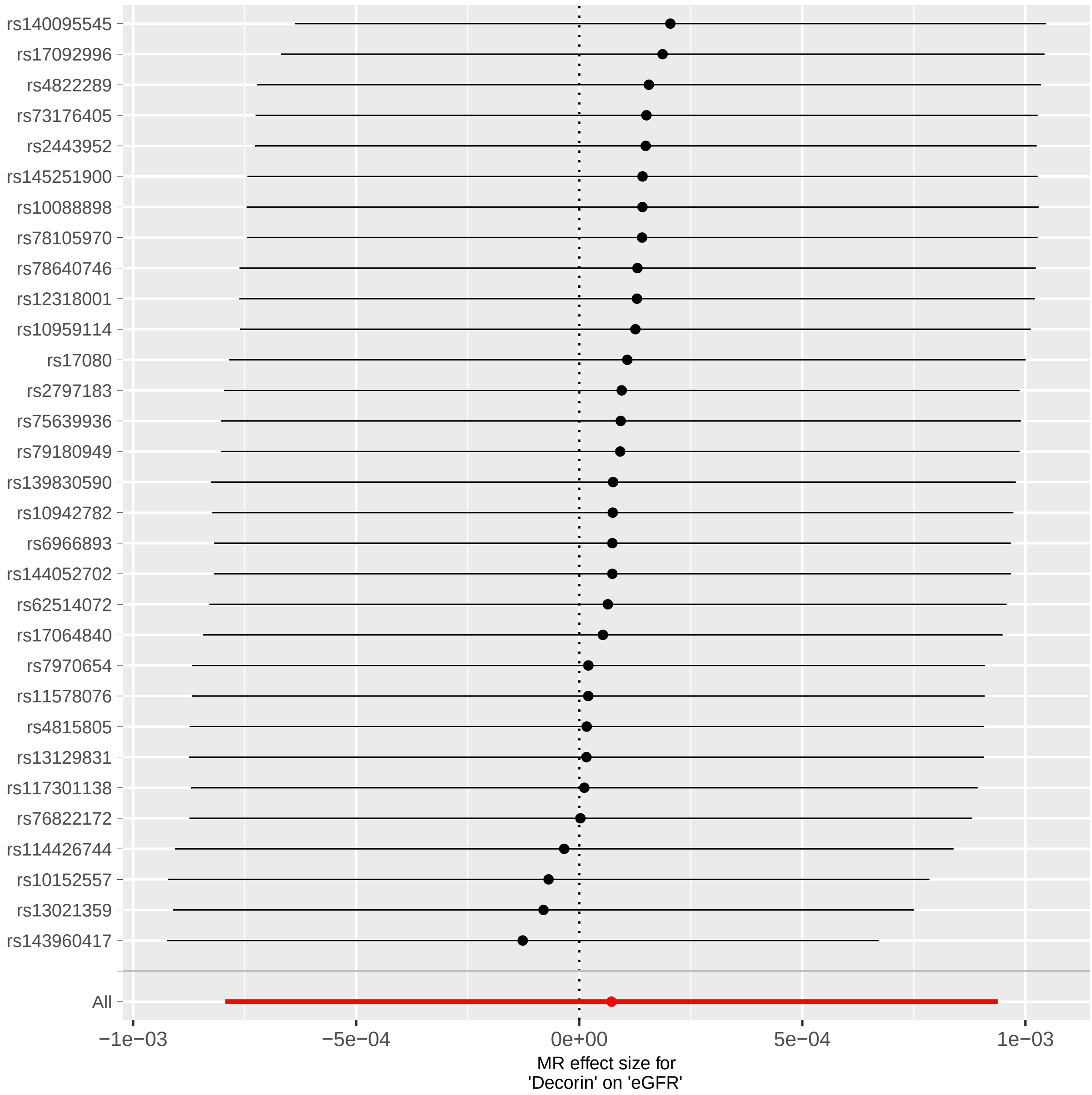

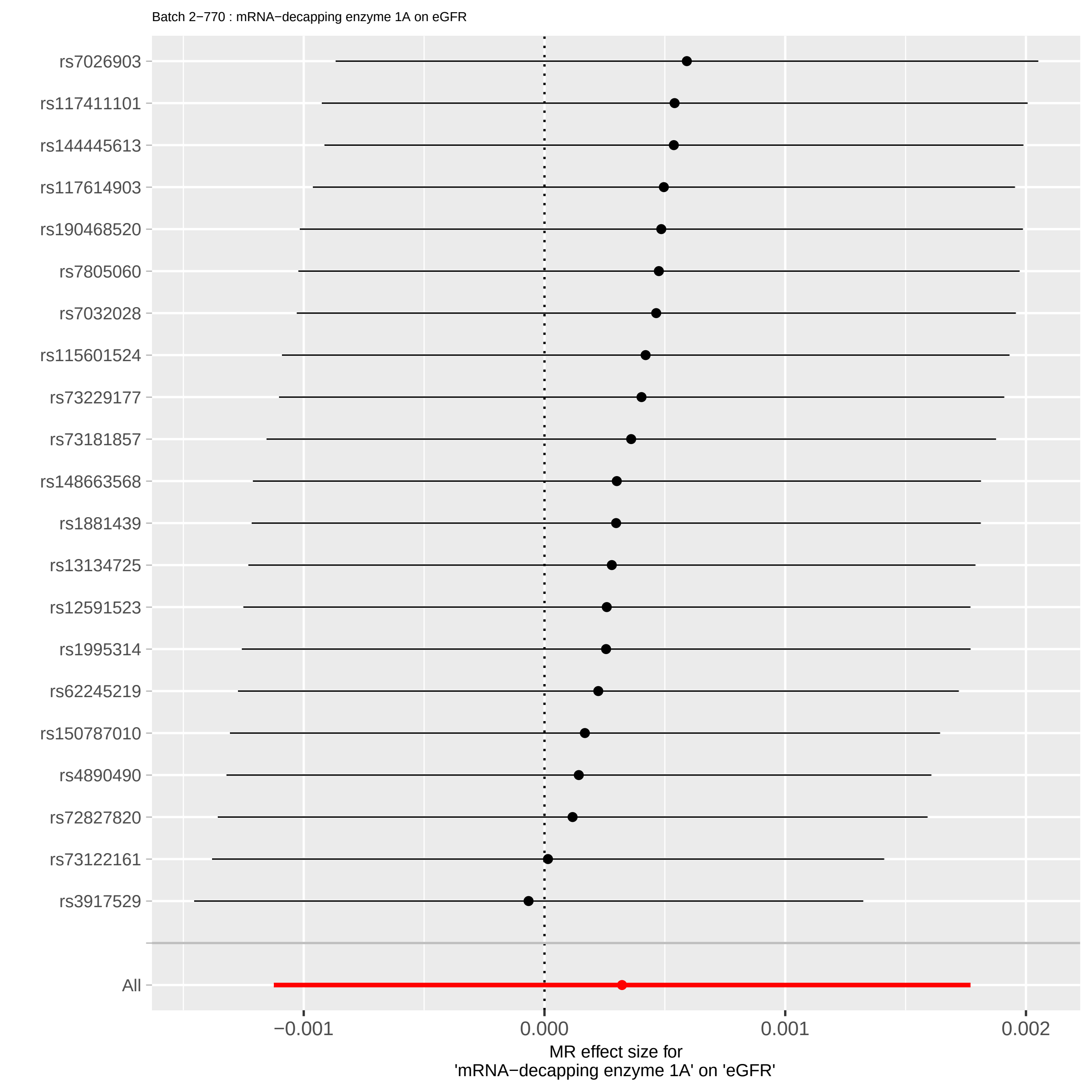

Batch 2−770 : mRNA−decapping enzyme 1A on eGFR
rs7026903
rs117411101
rs144445613
rs117614903
rs190468520
rs7805060
rs7032028
rs115601524
rs73229177
rs73181857
rs148663568
rs1881439
rs13134725
rs12591523
rs1995314
rs62245219
rs150787010
rs4890490
rs72827820
rs73122161
rs3917529
All
−0.001
0.000
0.001
0.002
MR effect size for
'mRNA−decapping enzyme 1A' on 'eGFR'

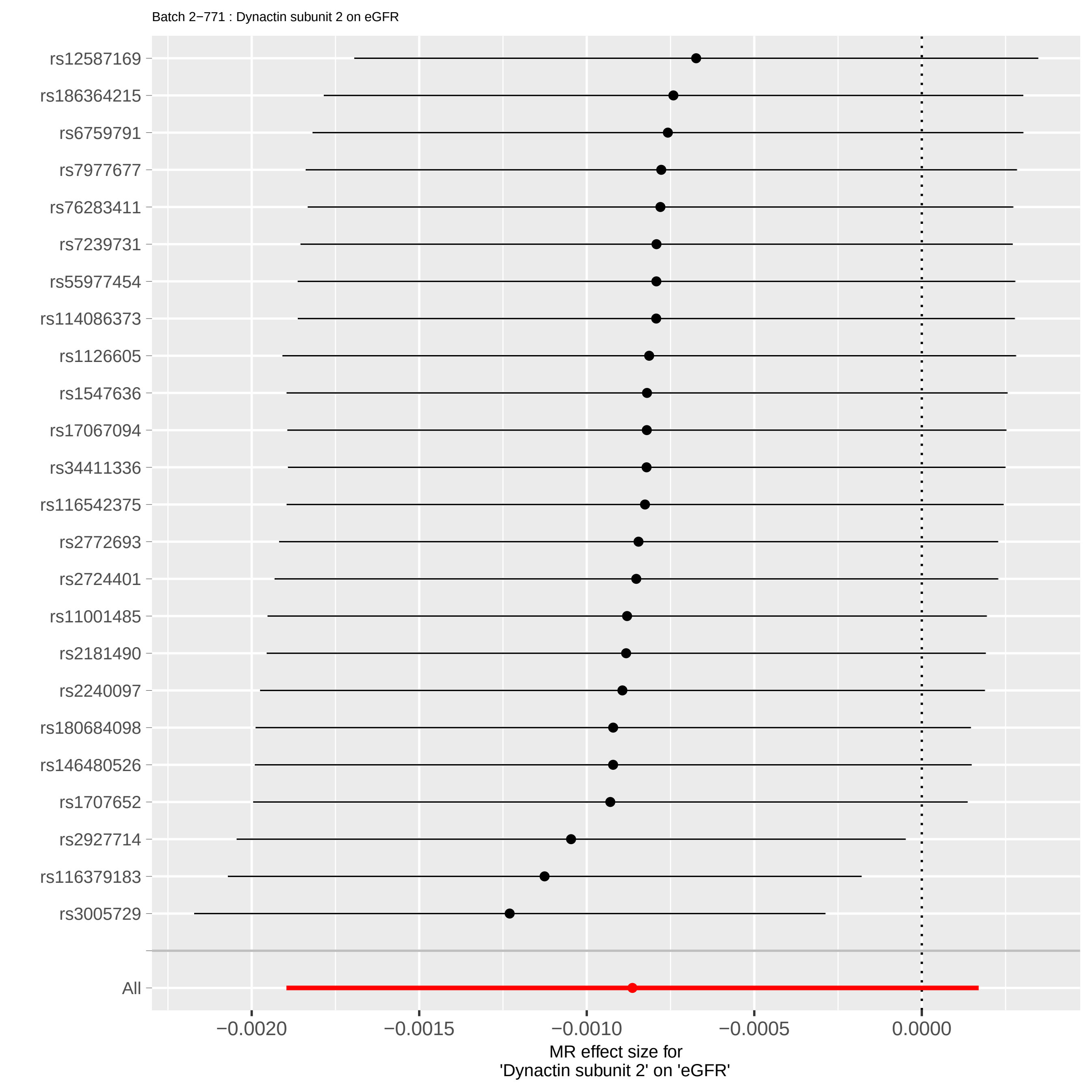
Batch 2−771 : Dynactin subunit 2 on eGFR
rs12587169
rs186364215
rs6759791
rs7977677
rs76283411
rs7239731
rs55977454
rs114086373
rs1126605
rs1547636
rs17067094
rs34411336
rs116542375
rs2772693
rs2724401
rs11001485
rs2181490
rs2240097
rs180684098
rs146480526
rs1707652
rs2927714
rs116379183
rs3005729
All
−0.0020
−0.0015
−0.0010
−0.0005
0.0000
MR effect size for
'Dynactin subunit 2' on 'eGFR'

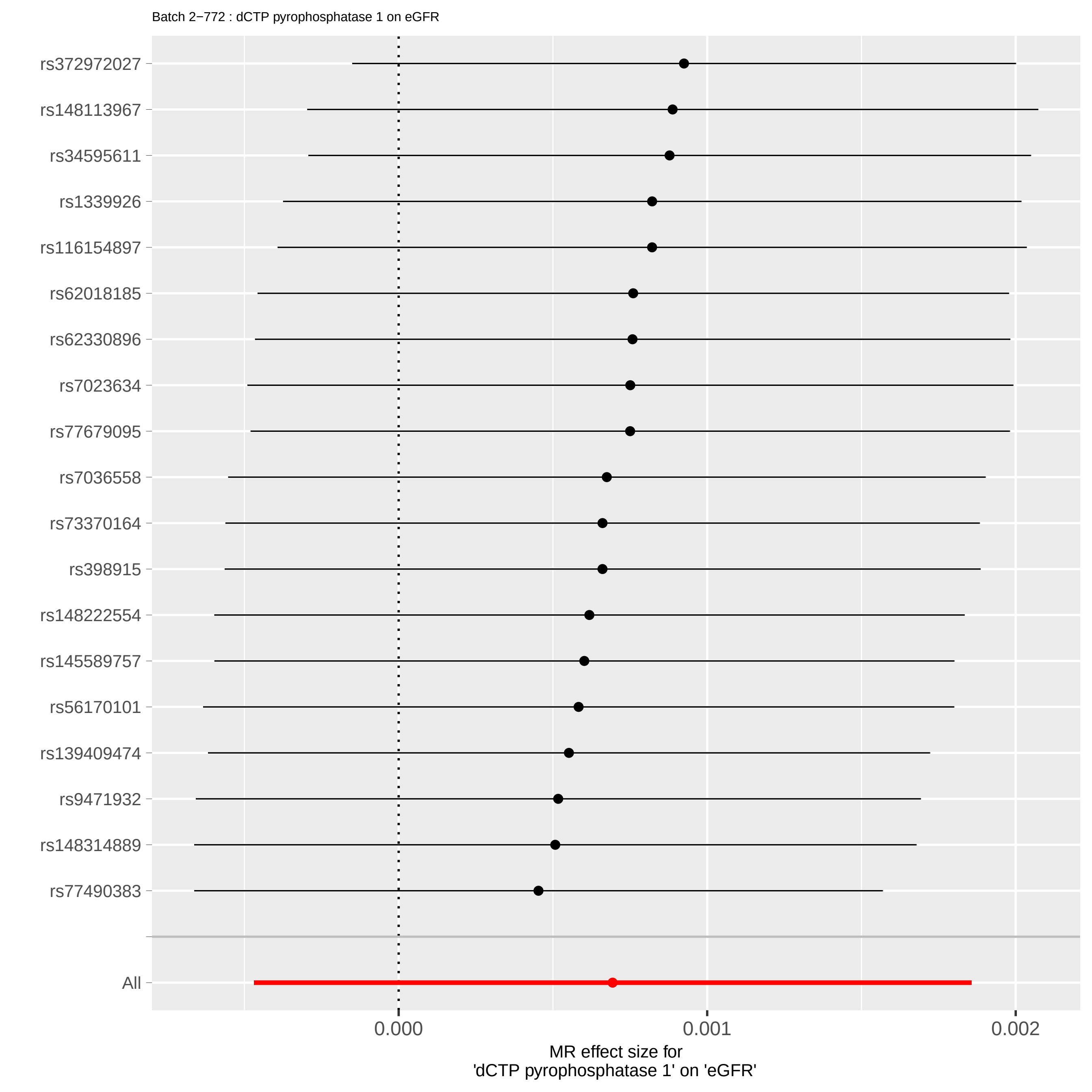

Batch 2−772 : dCTP pyrophosphatase 1 on eGFR
rs372972027
rs148113967
rs34595611
rs1339926
rs116154897
rs62018185
rs62330896
rs7023634
rs77679095
rs7036558
rs73370164
rs398915
rs148222554
rs145589757
rs56170101
rs139409474
rs9471932
rs148314889
rs77490383
All
0.000
0.001
0.002
MR effect size for
'dCTP pyrophosphatase 1' on 'eGFR'

Batch 2−773 : DCN1−like protein 3 on eGFR

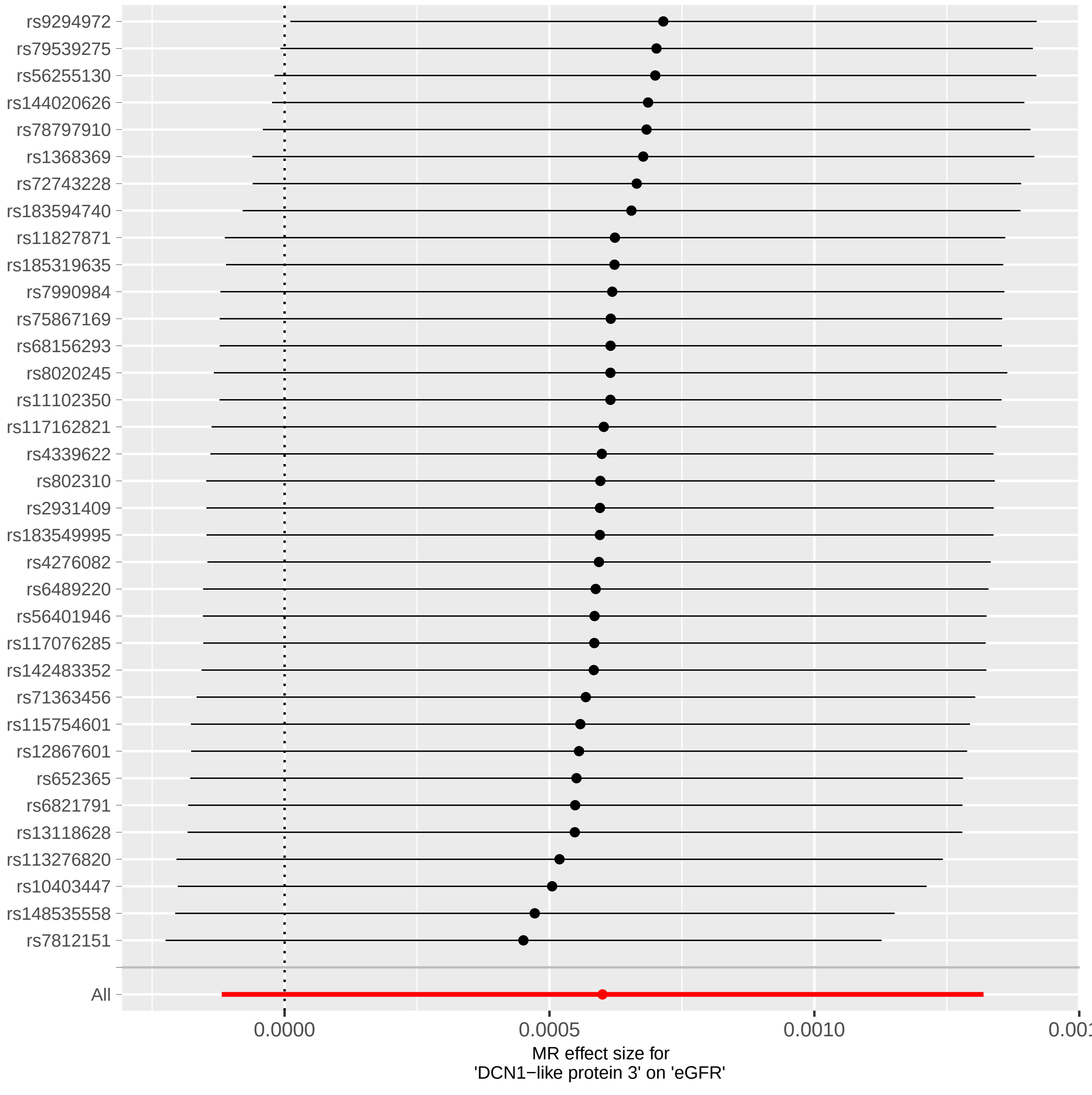

Batch 2−774 : DCN1−like protein 5 on eGFR

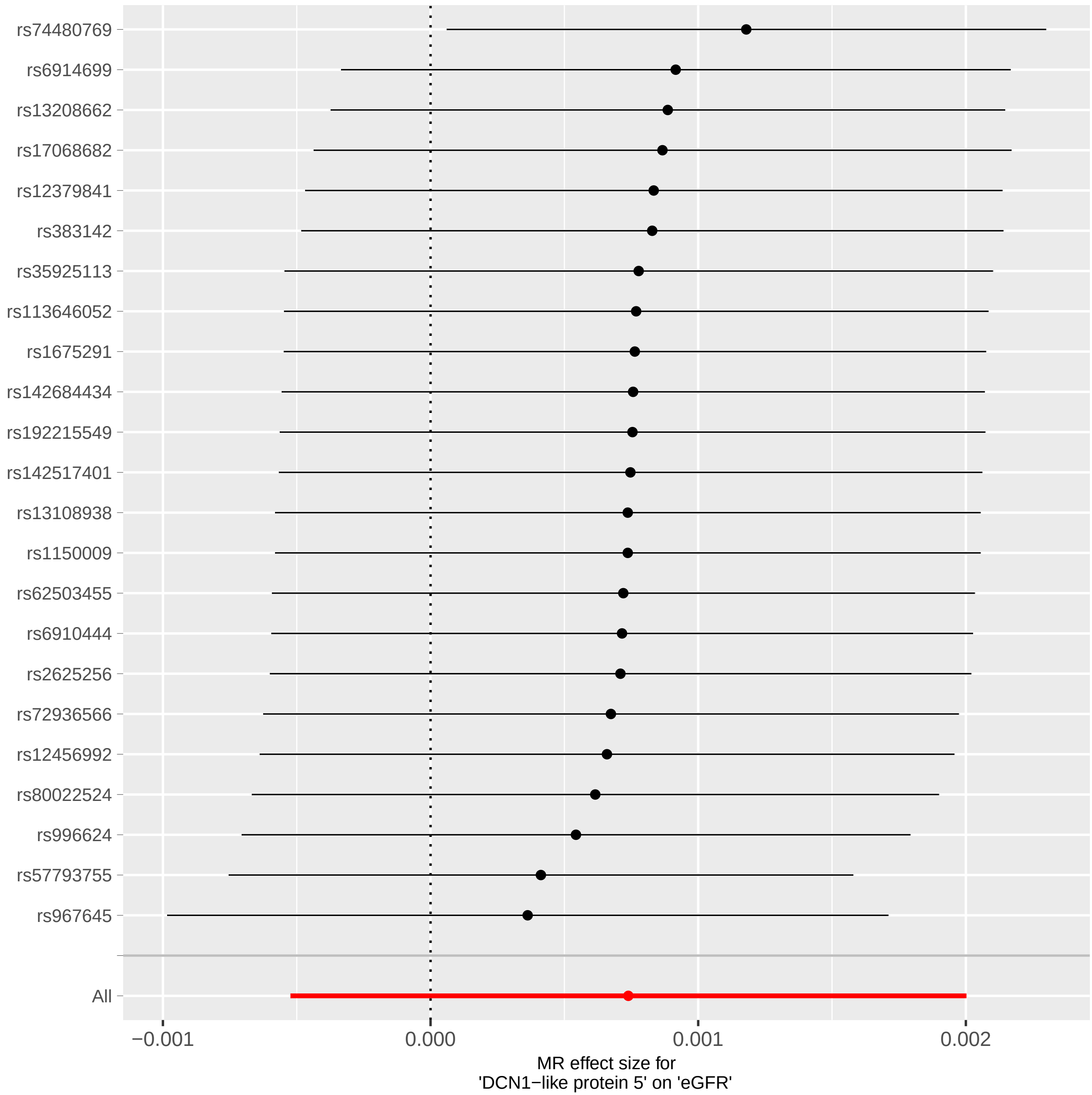

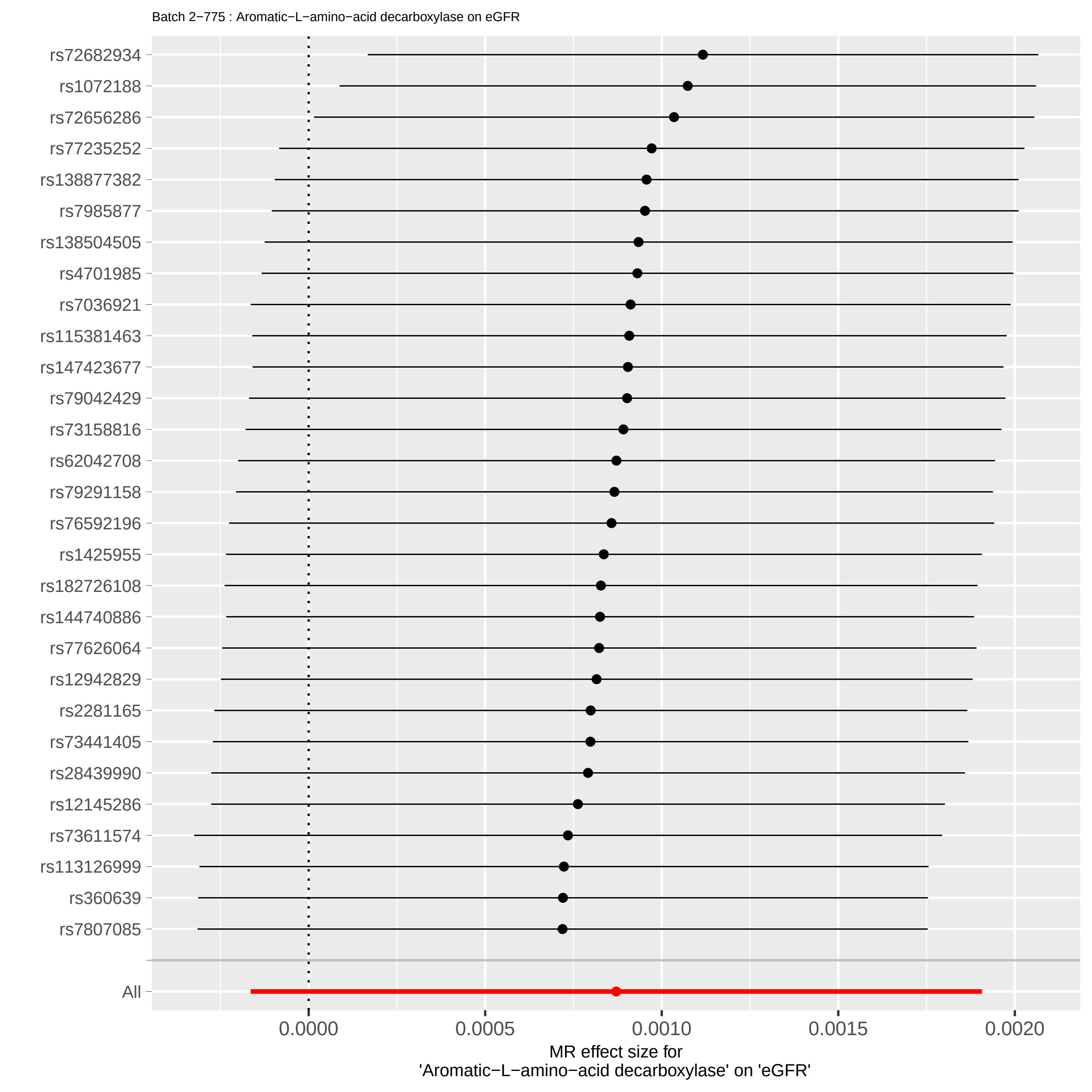

Batch 2−775 : Aromatic−L−amino−acid decarboxylase on eGFR
rs72682934
rs1072188
rs72656286
rs77235252
rs138877382
rs7985877
rs138504505
rs4701985
rs7036921
rs115381463
rs147423677
rs79042429
rs73158816
rs62042708
rs79291158
rs76592196
rs1425955
rs182726108
rs144740886
rs77626064
rs12942829
rs2281165
rs73441405
rs28439990
rs12145286
rs73611574
rs113126999
rs360639
rs7807085
All
0.0000
0.0005
0.0010
0.0015
0.0020
MR effect size for
'Aromatic−L−amino−acid decarboxylase' on 'eGFR'

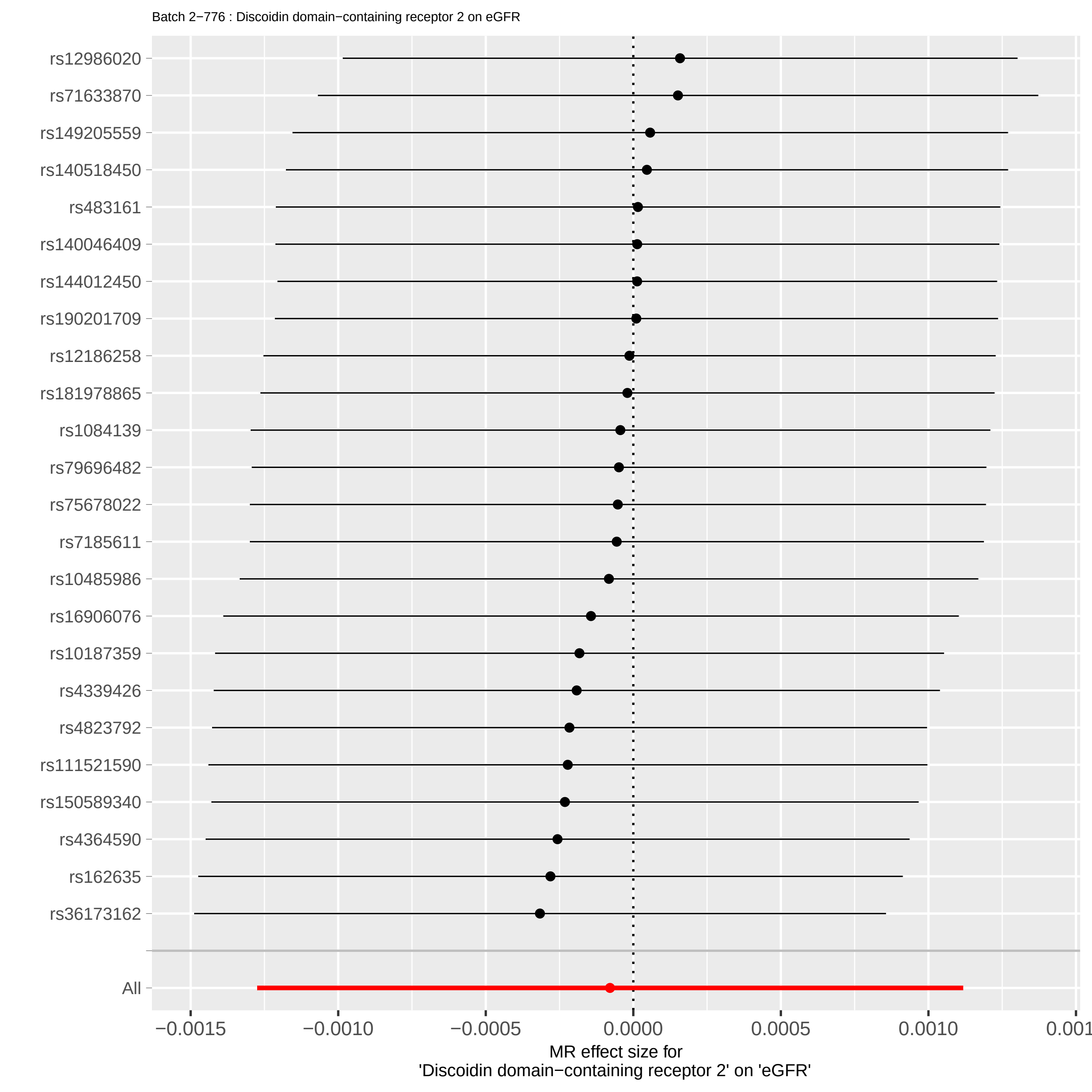

Batch 2−776 : Discoidin domain−containing receptor 2 on eGFR
rs12986020
rs71633870
rs149205559
rs140518450
rs483161
rs140046409
rs144012450
rs190201709
rs12186258
rs181978865
rs1084139
rs79696482
rs75678022
rs7185611
rs10485986
rs16906076
rs10187359
rs4339426
rs4823792
rs111521590
rs150589340
rs4364590
rs162635
rs36173162
All
−0.0015
−0.0010
−0.0005
0.0000
0.0005
0.0010
MR effect size for
'Discoidin domain−containing receptor 2' on 'eGFR'

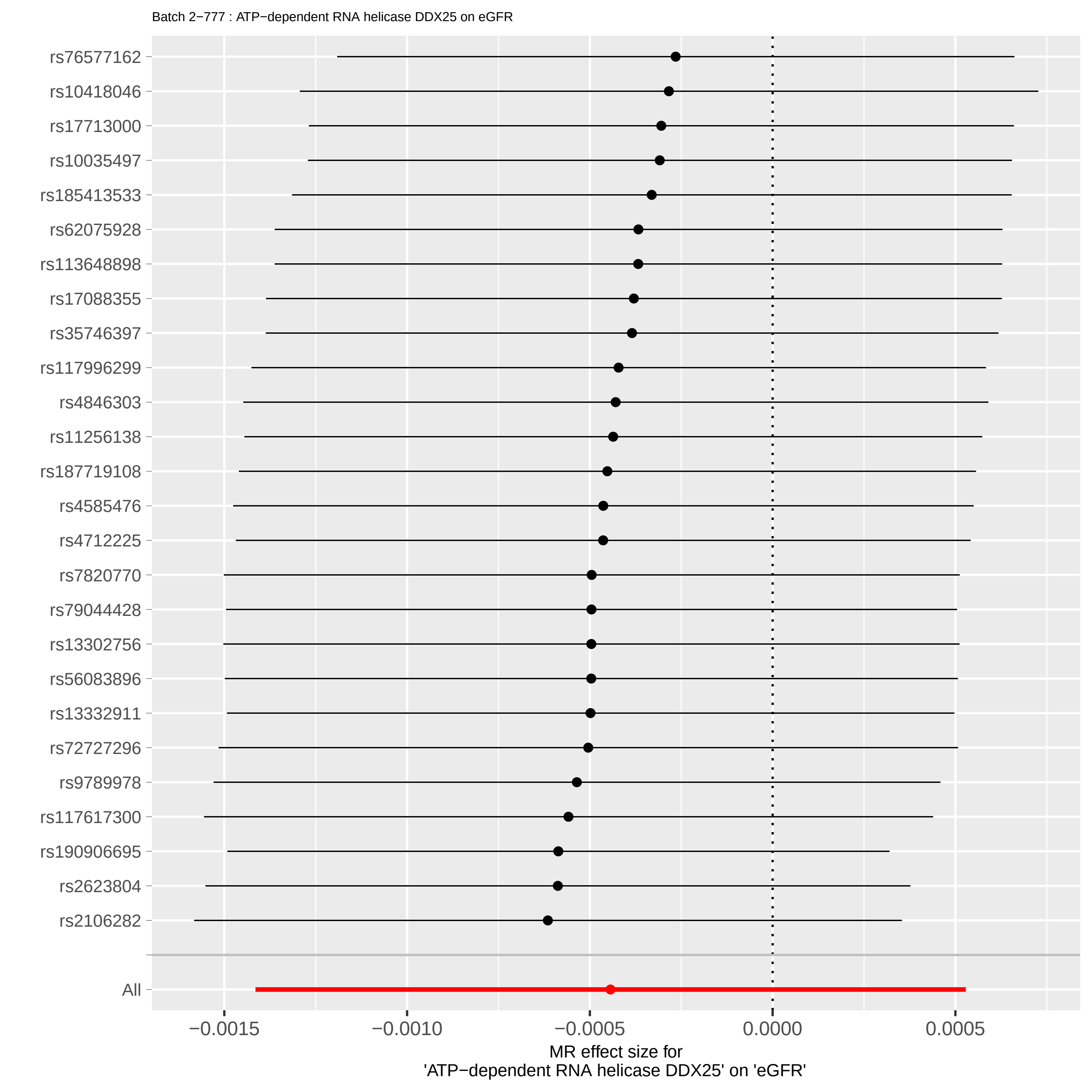

Batch 2−777 : ATP−dependent RNA helicase DDX25 on eGFR
rs76577162
rs10418046
rs17713000
rs10035497
rs185413533
rs62075928
rs113648898
rs17088355
rs35746397
rs117996299
rs4846303
rs11256138
rs187719108
rs4585476
rs4712225
rs7820770
rs79044428
rs13302756
rs56083896
rs13332911
rs72727296
rs9789978
rs117617300
rs190906695
rs2623804
rs2106282
All
−0.0015
−0.0010
−0.0005
0.0000
0.0005
MR effect size for
'ATP−dependent RNA helicase DDX25' on 'eGFR'

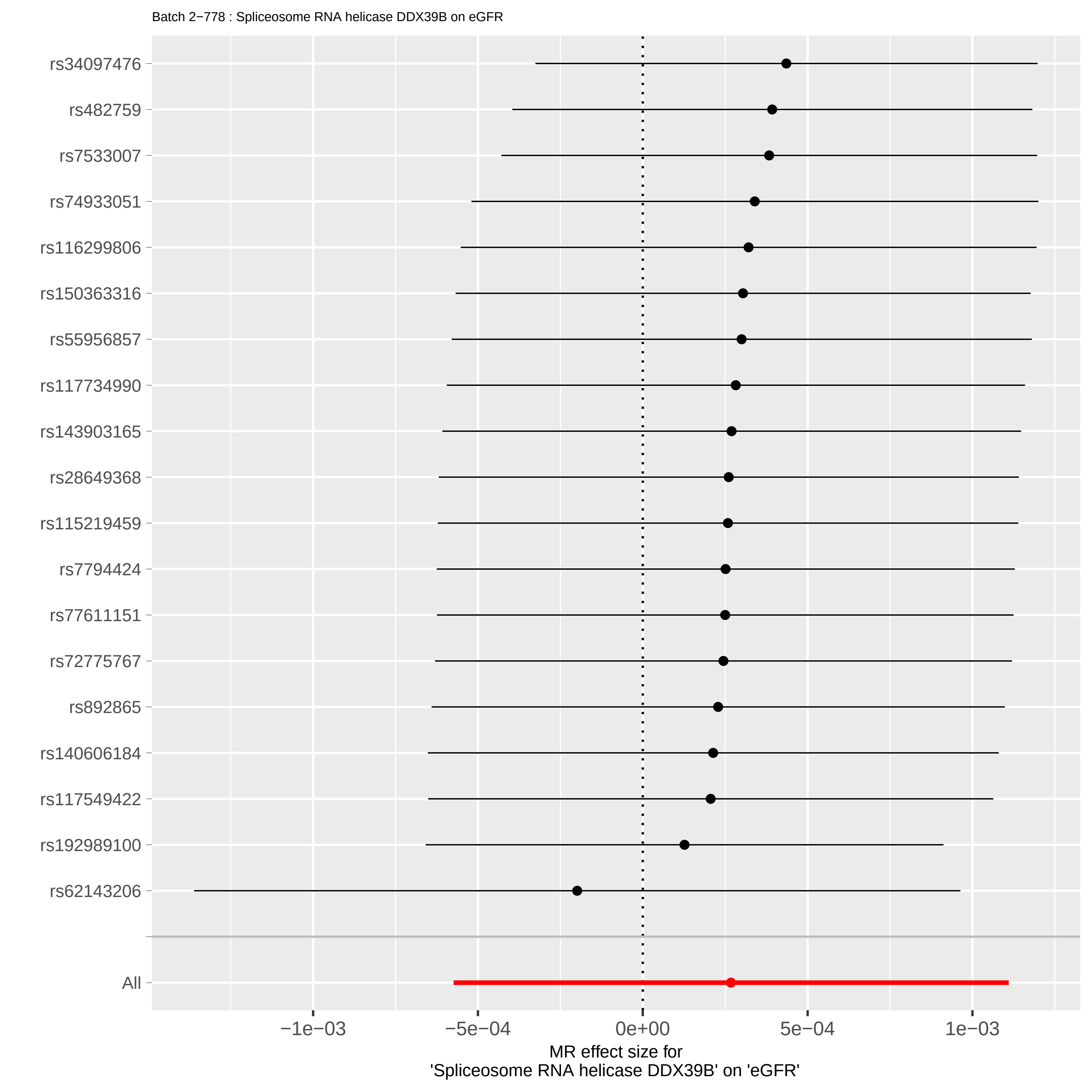

Batch 2−778 : Spliceosome RNA helicase DDX39B on eGFR
rs34097476
rs482759
rs7533007
rs74933051
rs116299806
rs150363316
rs55956857
rs117734990
rs143903165
rs28649368
rs115219459
rs7794424
rs77611151
rs72775767
rs892865
rs140606184
rs117549422
rs192989100
rs62143206
All
−1e−03
−5e−04
0e+00
5e−04
1e−03
MR effect size for
'Spliceosome RNA helicase DDX39B' on 'eGFR'

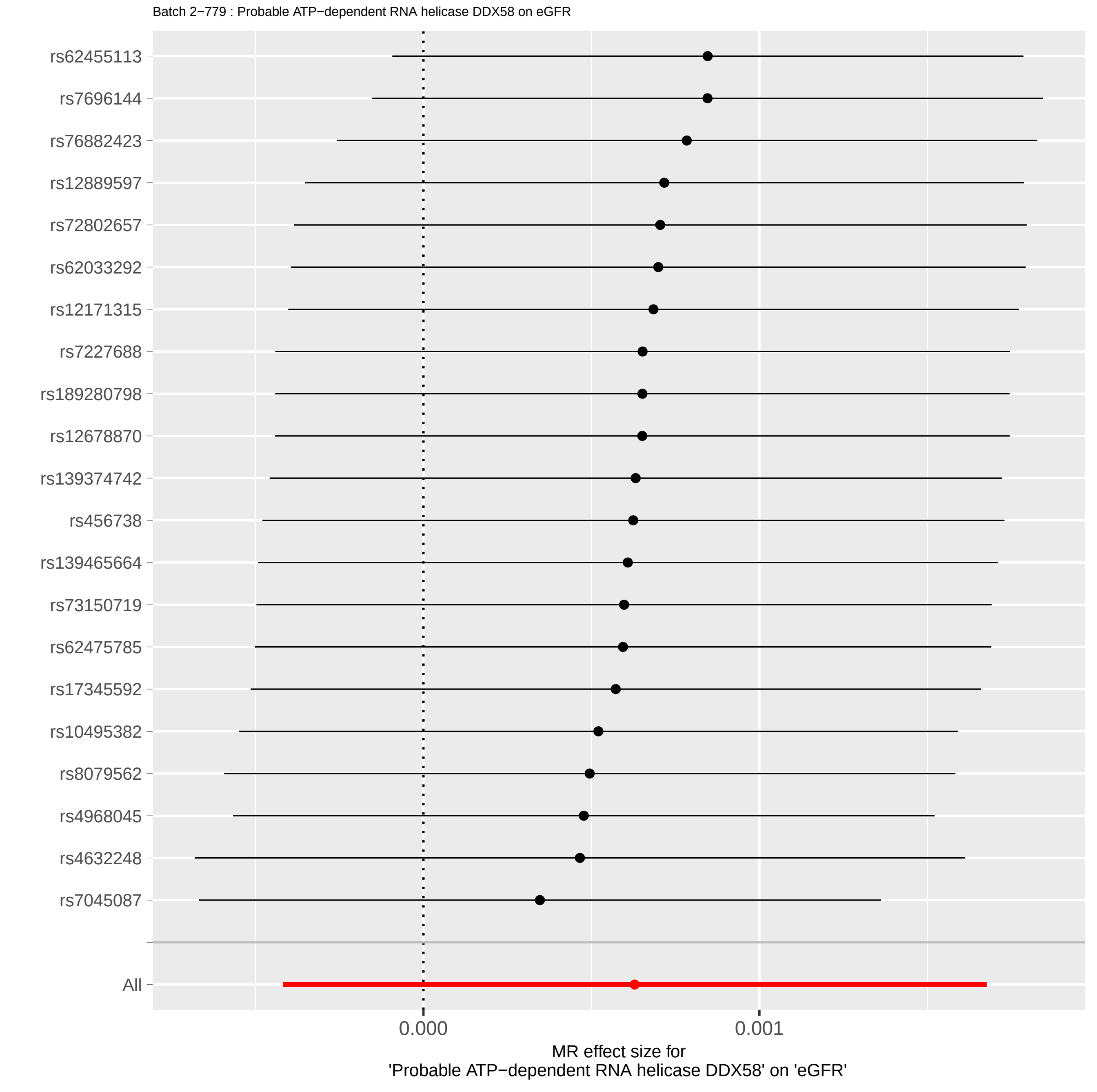

Batch 2−779 : Probable ATP−dependent RNA helicase DDX58 on eGFR
rs62455113
rs7696144
rs76882423
rs12889597
rs72802657
rs62033292
rs12171315
rs7227688
rs189280798
rs12678870
rs139374742
rs456738
rs139465664
rs73150719
rs62475785
rs17345592
rs10495382
rs8079562
rs4968045
rs4632248
rs7045087
All
0.000
0.001
MR effect size for
'Probable ATP−dependent RNA helicase DDX58' on 'eGFR'

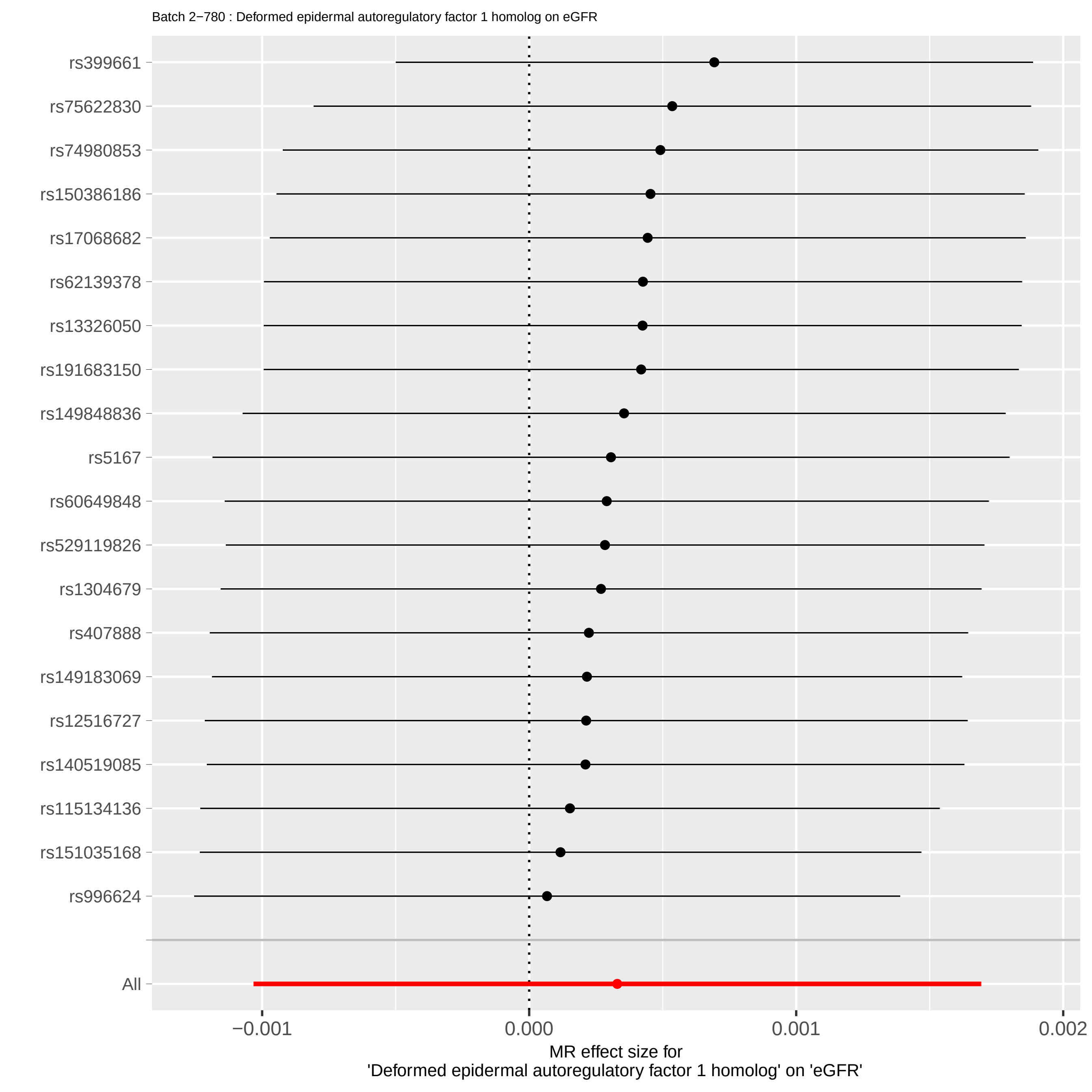
Batch 2−780 : Deformed epidermal autoregulatory factor 1 homolog on eGFR
rs399661
rs75622830
rs74980853
rs150386186
rs17068682
rs62139378
rs13326050
rs191683150
rs149848836
rs5167
rs60649848
rs529119826
rs1304679
rs407888
rs149183069
rs12516727
rs140519085
rs115134136
rs151035168
rs996624
All
−0.001
0.000
0.001
0.002
MR effect size for
'Deformed epidermal autoregulatory factor 1 homolog' on 'eGFR'

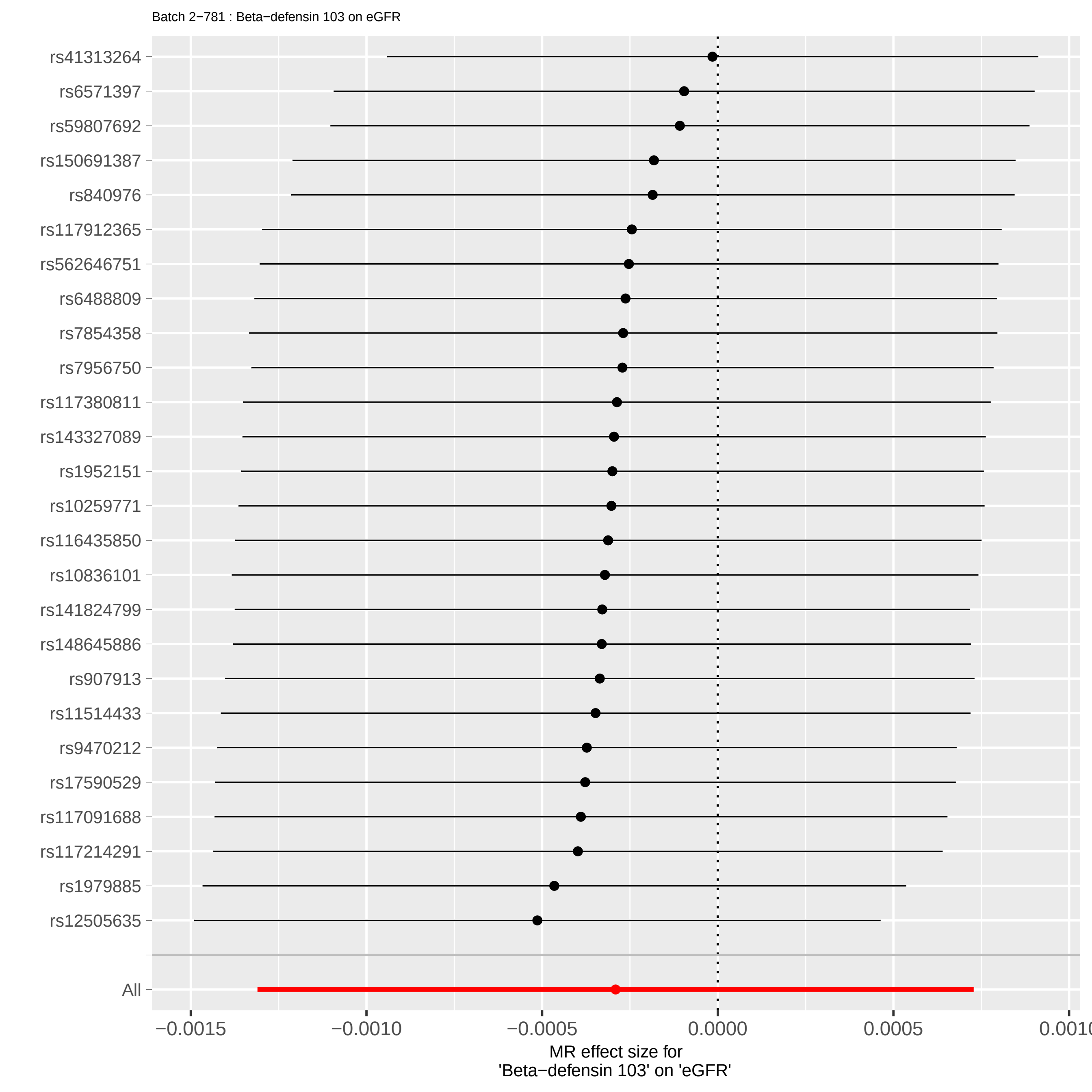

Batch 2−781 : Beta−defensin 103 on eGFR
rs41313264
rs6571397
rs59807692
rs150691387
rs840976
rs117912365
rs562646751
rs6488809
rs7854358
rs7956750
rs117380811
rs143327089
rs1952151
rs10259771
rs116435850
rs10836101
rs141824799
rs148645886
rs907913
rs11514433
rs9470212
rs17590529
rs117091688
rs117214291
rs1979885
rs12505635
All
−0.0015
−0.0010
−0.0005
0.0000
0.0005
MR effect size for
'Beta−defensin 103' on 'eGFR'

Batch 2−782 : Beta−defensin 104 on eGFR

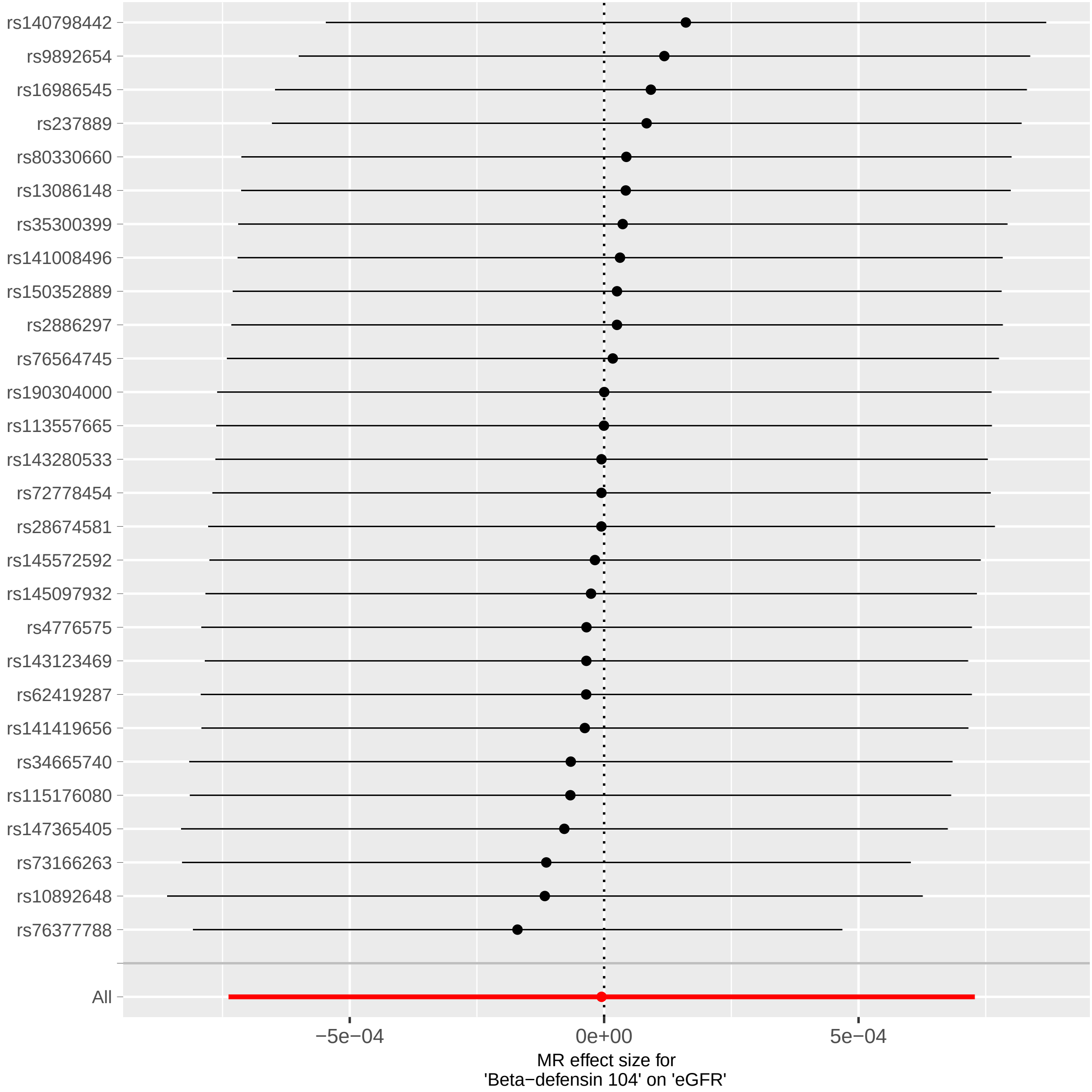

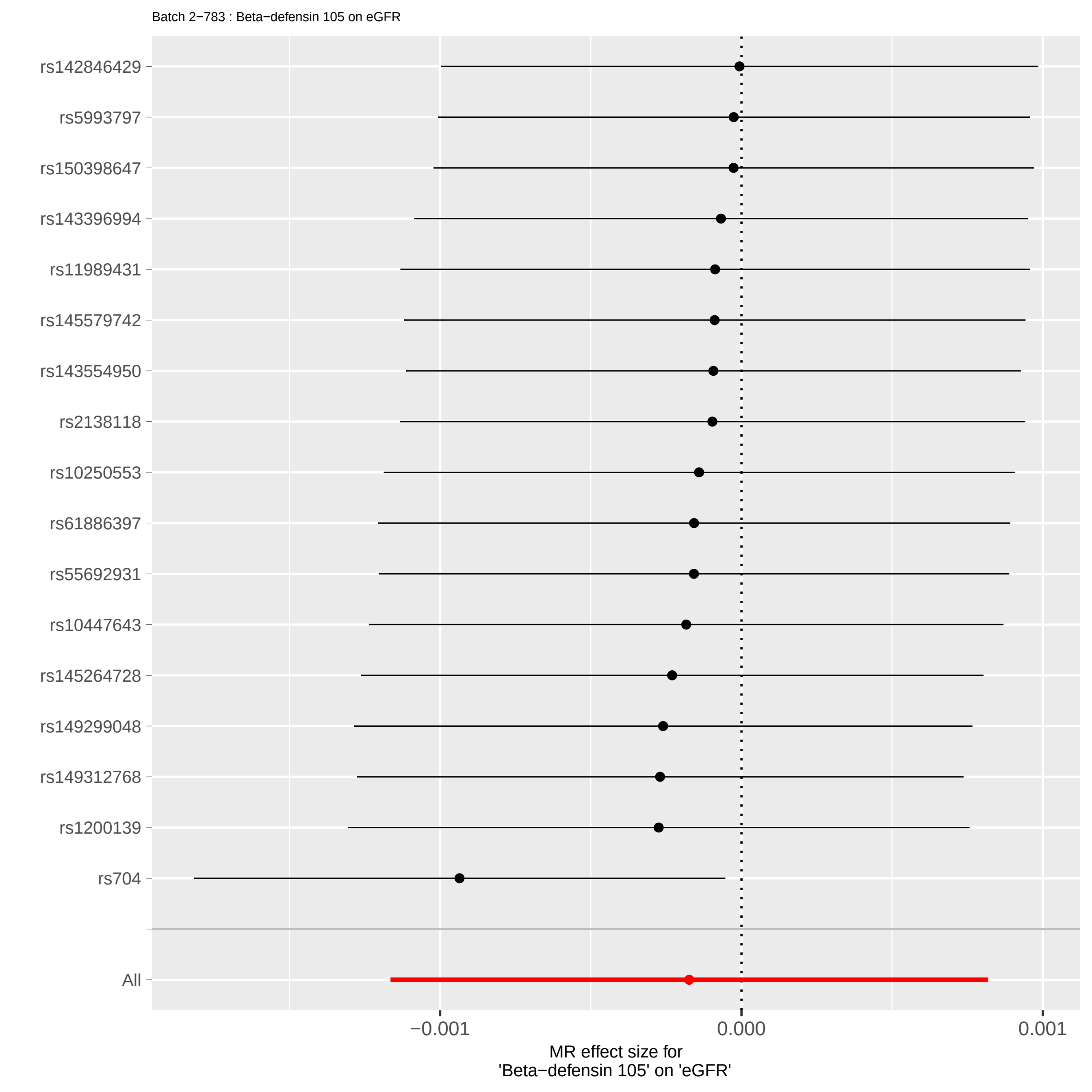

Batch 2−783 : Beta−defensin 105 on eGFR
rs142846429
rs5993797
rs150398647
rs143396994
rs11989431
rs145579742
rs143554950
rs2138118
rs10250553
rs61886397
rs55692931
rs10447643
rs145264728
rs149299048
rs149312768
rs1200139
rs704
All
−0.001
0.000
0.001
MR effect size for
'Beta−defensin 105' on 'eGFR'

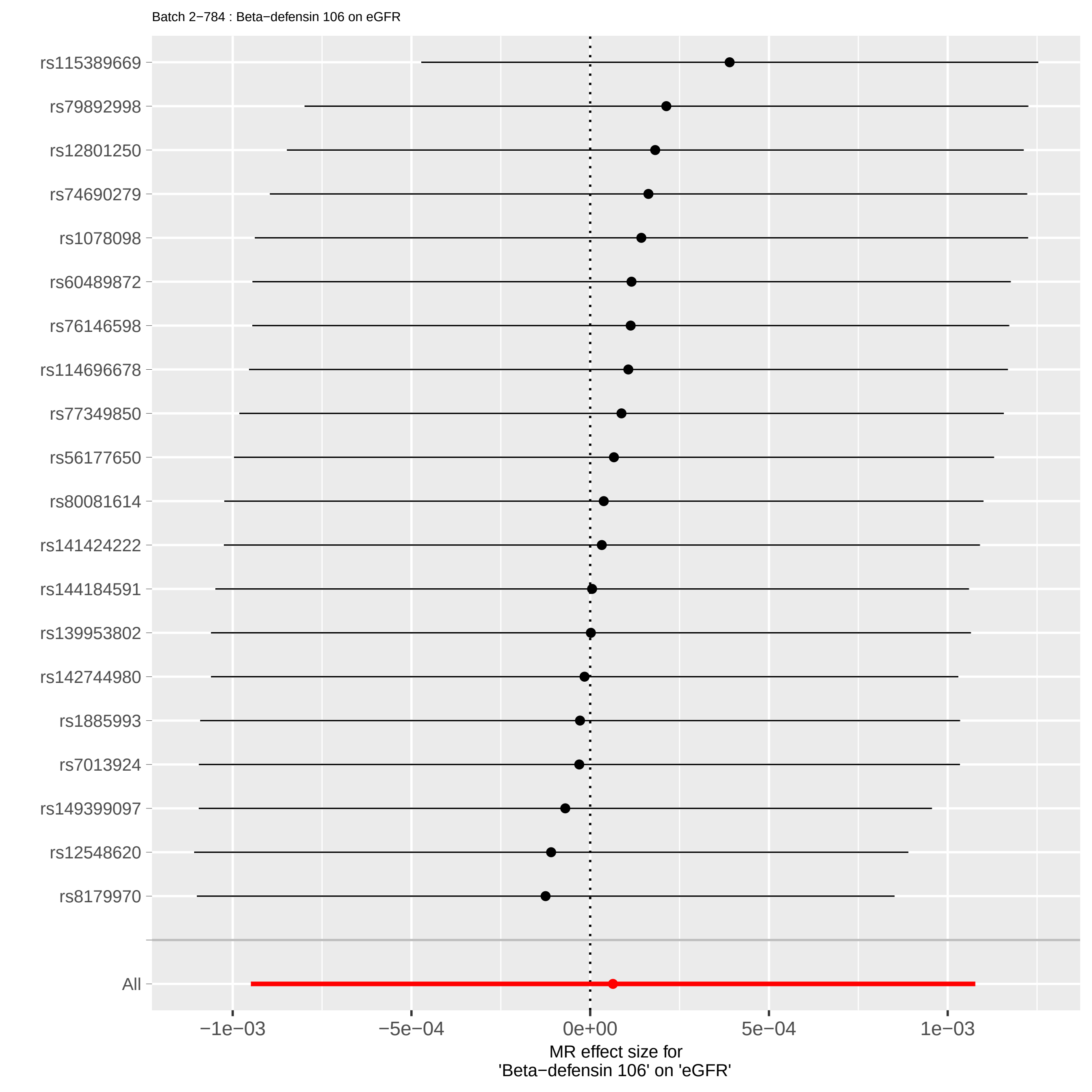

Batch 2−784 : Beta−defensin 106 on eGFR
rs115389669
rs79892998
rs12801250
rs74690279
rs1078098
rs60489872
rs76146598
rs114696678
rs77349850
rs56177650
rs80081614
rs141424222
rs144184591
rs139953802
rs142744980
rs1885993
rs7013924
rs149399097
rs12548620
rs8179970
All
−1e−03
−5e−04
0e+00
5e−04
1e−03
MR effect size for
'Beta−defensin 106' on 'eGFR'

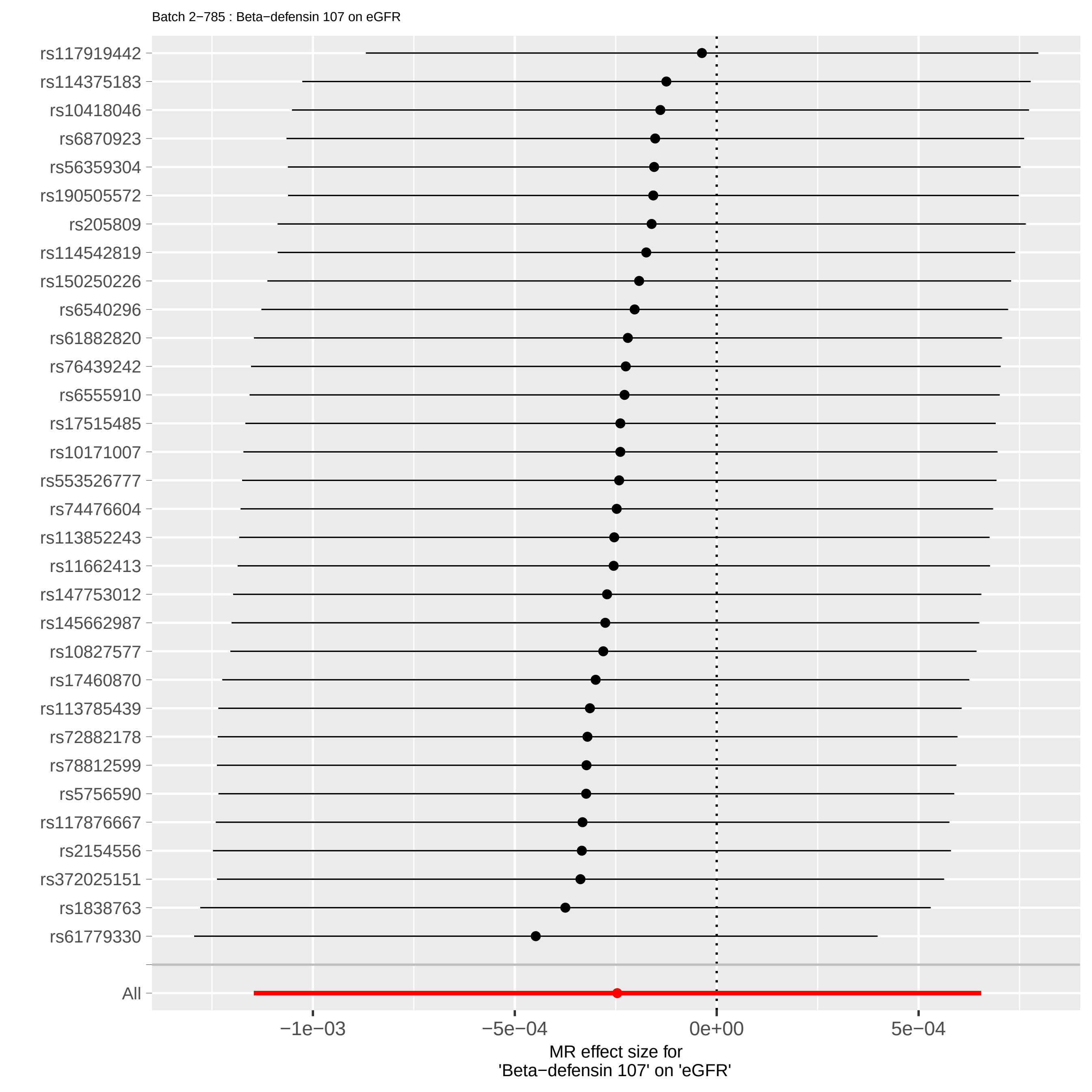

Batch 2−785 : Beta−defensin 107 on eGFR
rs117919442
rs114375183
rs10418046
rs6870923
rs56359304
rs190505572
rs205809
rs114542819
rs150250226
rs6540296
rs61882820
rs76439242
rs6555910
rs17515485
rs10171007
rs553526777
rs74476604
rs113852243
rs11662413
rs147753012
rs145662987
rs10827577
rs17460870
rs113785439
rs72882178
rs78812599
rs5756590
rs117876667
rs2154556
rs372025151
rs1838763
rs61779330
All
−1e−03
−5e−04
0e+00
5e−04
MR effect size for
'Beta−defensin 107' on 'eGFR'

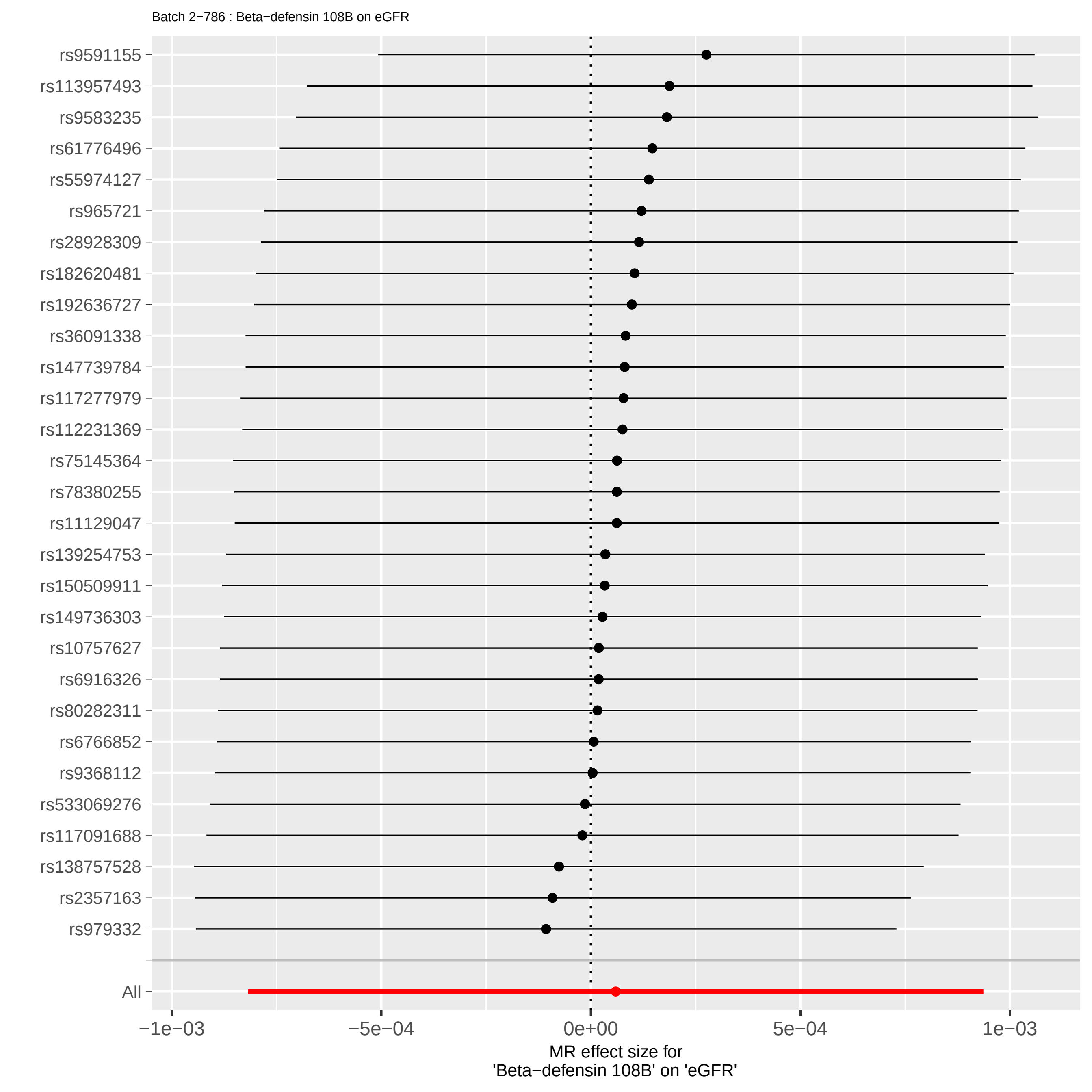

Batch 2−786 : Beta−defensin 108B on eGFR
rs9591155
rs113957493
rs9583235
rs61776496
rs55974127
rs965721
rs28928309
rs182620481
rs192636727
rs36091338
rs147739784
rs117277979
rs112231369
rs75145364
rs78380255
rs11129047
rs139254753
rs150509911
rs149736303
rs10757627
rs6916326
rs80282311
rs6766852
rs9368112
rs533069276
rs117091688
rs138757528
rs2357163
rs979332
All
−1e−03
−5e−04
0e+00
5e−04
1e−03
MR effect size for
'Beta−defensin 108B' on 'eGFR'

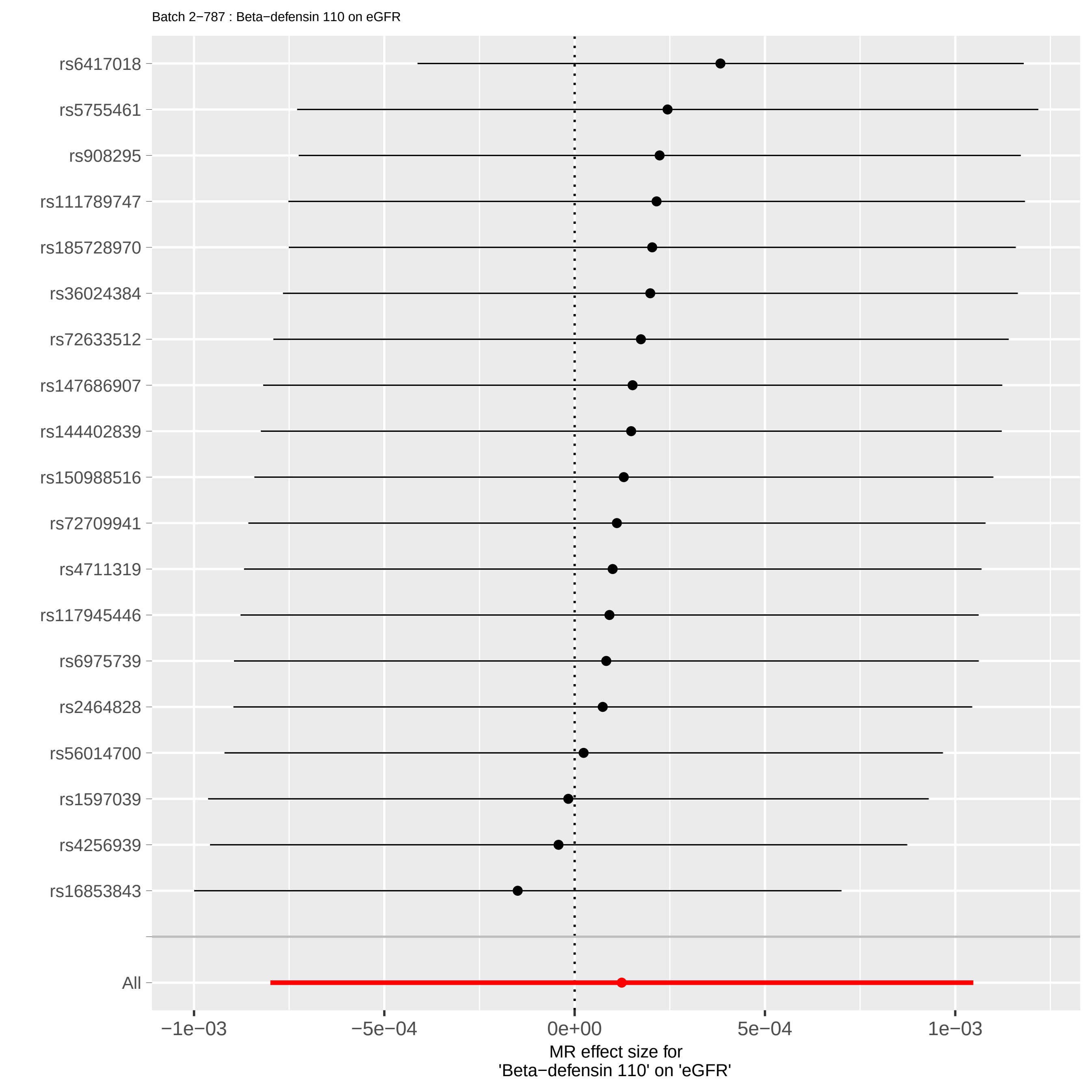

Batch 2−787 : Beta−defensin 110 on eGFR
rs6417018
rs5755461
rs908295
rs111789747
rs185728970
rs36024384
rs72633512
rs147686907
rs144402839
rs150988516
rs72709941
rs4711319
rs117945446
rs6975739
rs2464828
rs56014700
rs1597039
rs4256939
rs16853843
All
−1e−03
−5e−04
0e+00
5e−04
1e−03
MR effect size for
'Beta−defensin 110' on 'eGFR'

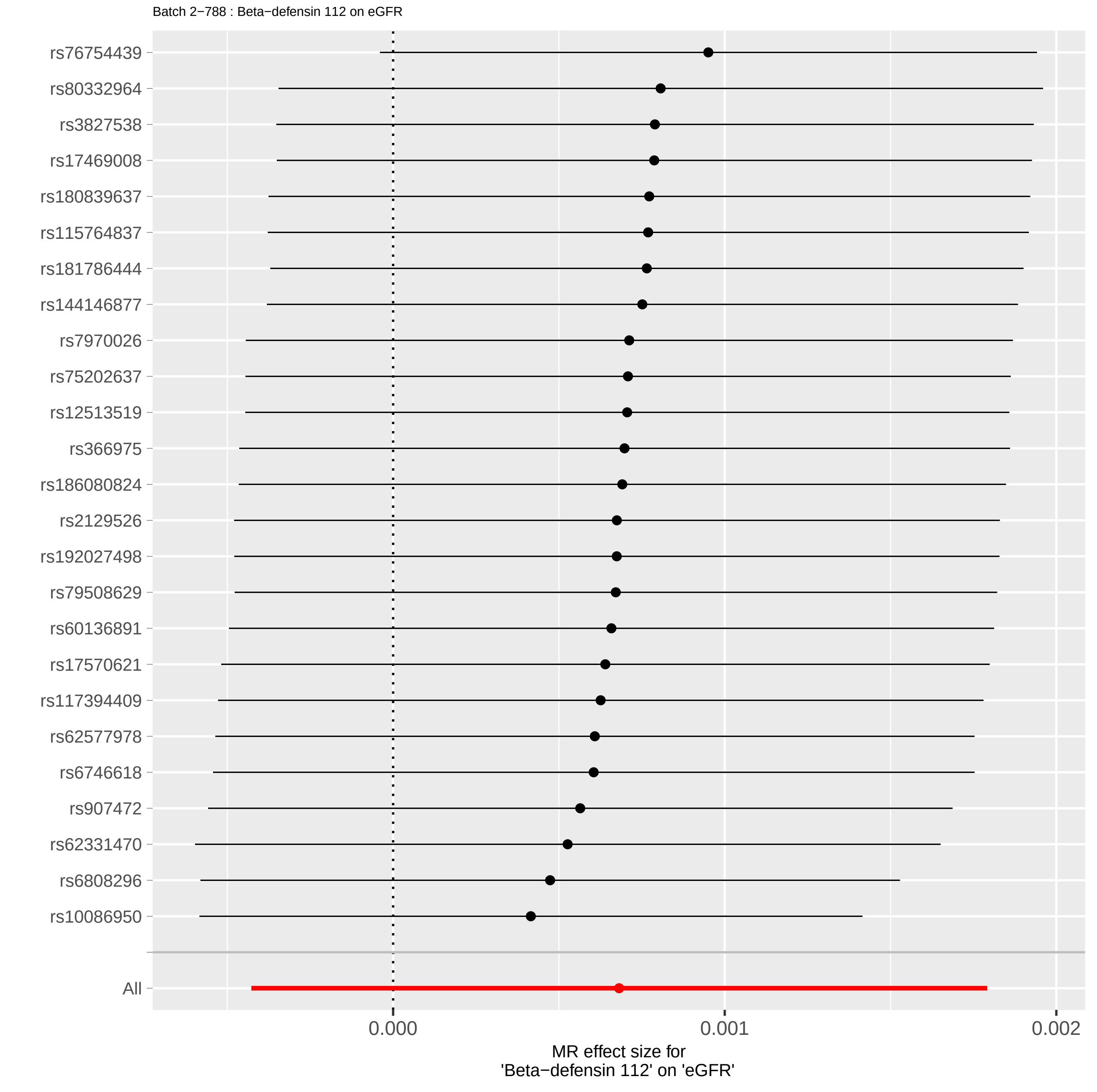

Batch 2−788 : Beta−defensin 112 on eGFR
rs76754439
rs80332964
rs3827538
rs17469008
rs180839637
rs115764837
rs181786444
rs144146877
rs7970026
rs75202637
rs12513519
rs366975
rs186080824
rs2129526
rs192027498
rs79508629
rs60136891
rs17570621
rs117394409
rs62577978
rs6746618
rs907472
rs62331470
rs6808296
rs10086950
All
0.000
0.001
0.002
MR effect size for
'Beta−defensin 112' on 'eGFR'

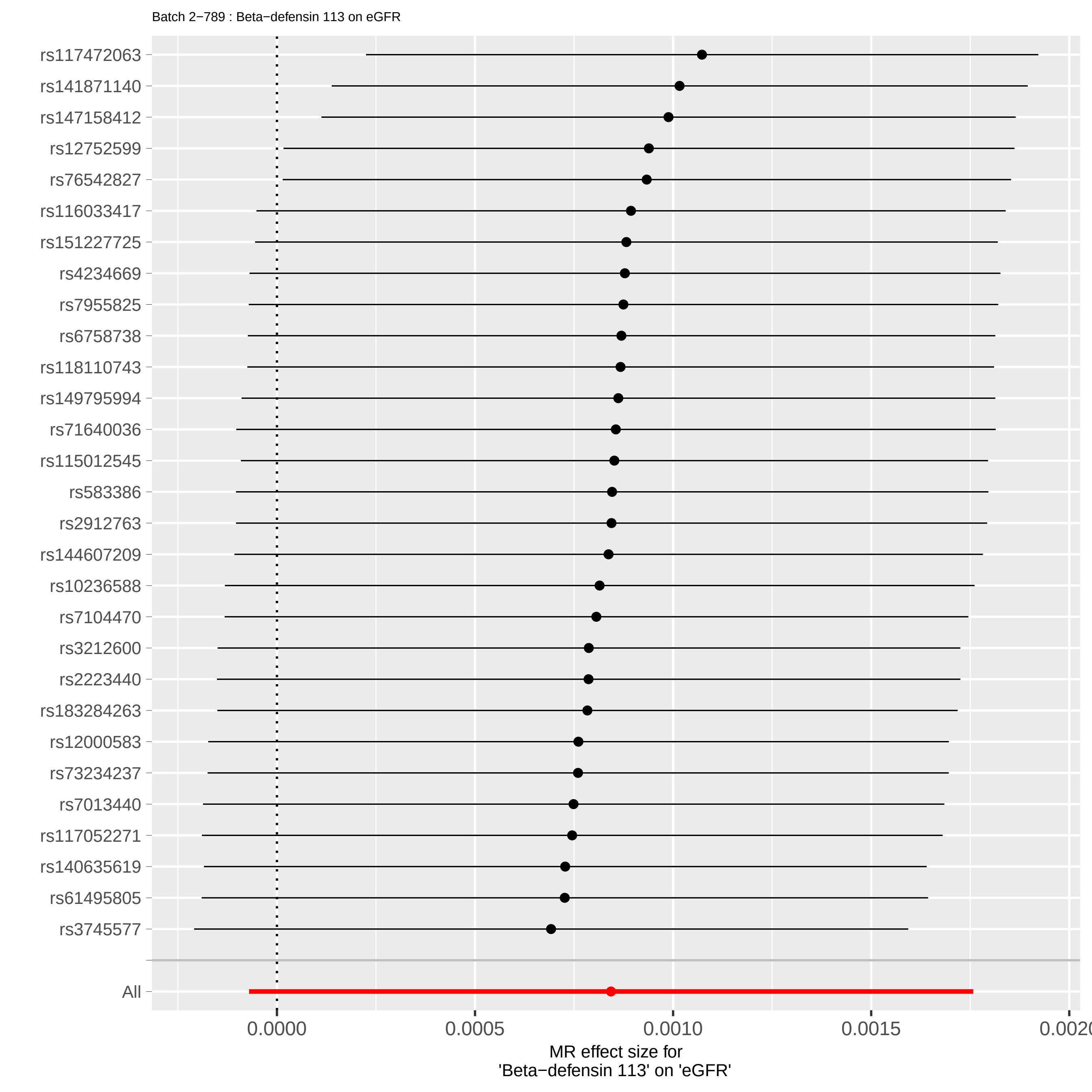
Batch 2−789 : Beta−defensin 113 on eGFR
rs117472063
rs141871140
rs147158412
rs12752599
rs76542827
rs116033417
rs151227725
rs4234669
rs7955825
rs6758738
rs118110743
rs149795994
rs71640036
rs115012545
rs583386
rs2912763
rs144607209
rs10236588
rs7104470
rs3212600
rs2223440
rs183284263
rs12000583
rs73234237
rs7013440
rs117052271
rs140635619
rs61495805
rs3745577
All
0.0000
0.0005
0.0010
0.0015
MR effect size for
'Beta−defensin 113' on 'eGFR'

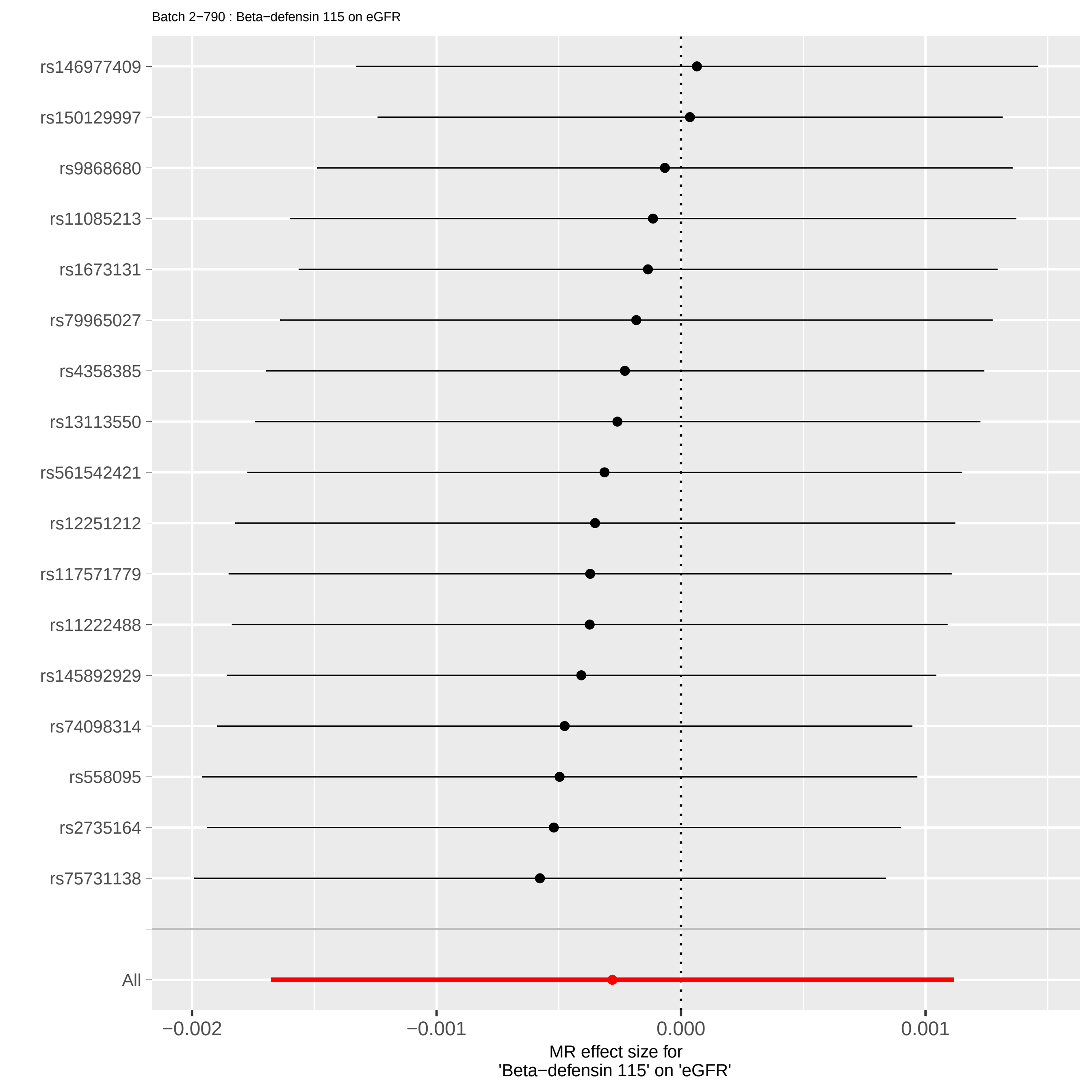
Batch 2−790 : Beta−defensin 115 on eGFR
rs146977409
rs150129997
rs9868680
rs11085213
rs1673131
rs79965027
rs4358385
rs13113550
rs561542421
rs12251212
rs117571779
rs11222488
rs145892929
rs74098314
rs558095
rs2735164
rs75731138
All
−0.002
−0.001
0.000
0.001
MR effect size for
'Beta−defensin 115' on 'eGFR'

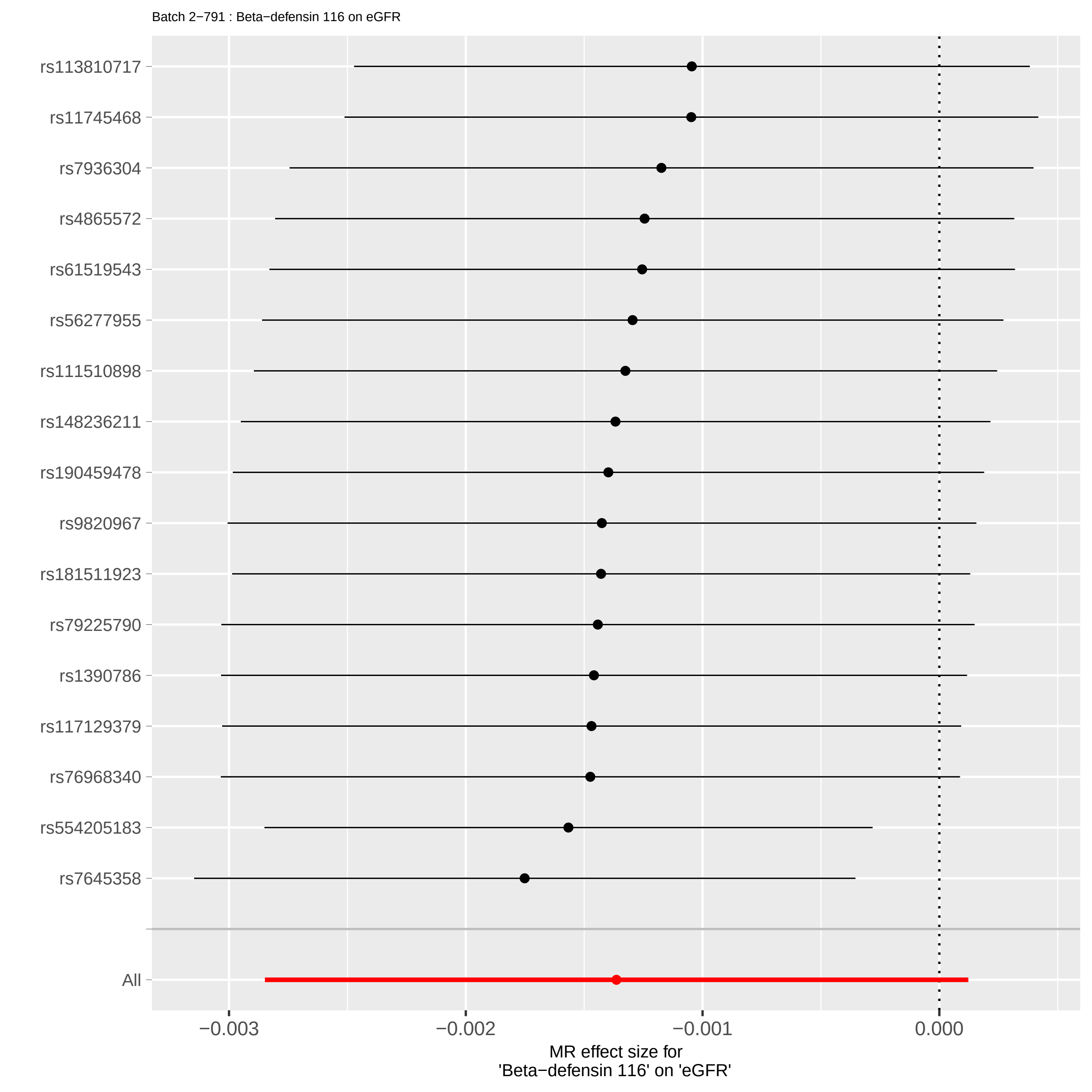

Batch 2−791 : Beta−defensin 116 on eGFR
rs113810717
rs11745468
rs7936304
rs4865572
rs61519543
rs56277955
rs111510898
rs148236211
rs190459478
rs9820967
rs181511923
rs79225790
rs1390786
rs117129379
rs76968340
rs554205183
rs7645358
All
−0.003
−0.002
−0.001
0.000
MR effect size for
'Beta−defensin 116' on 'eGFR'

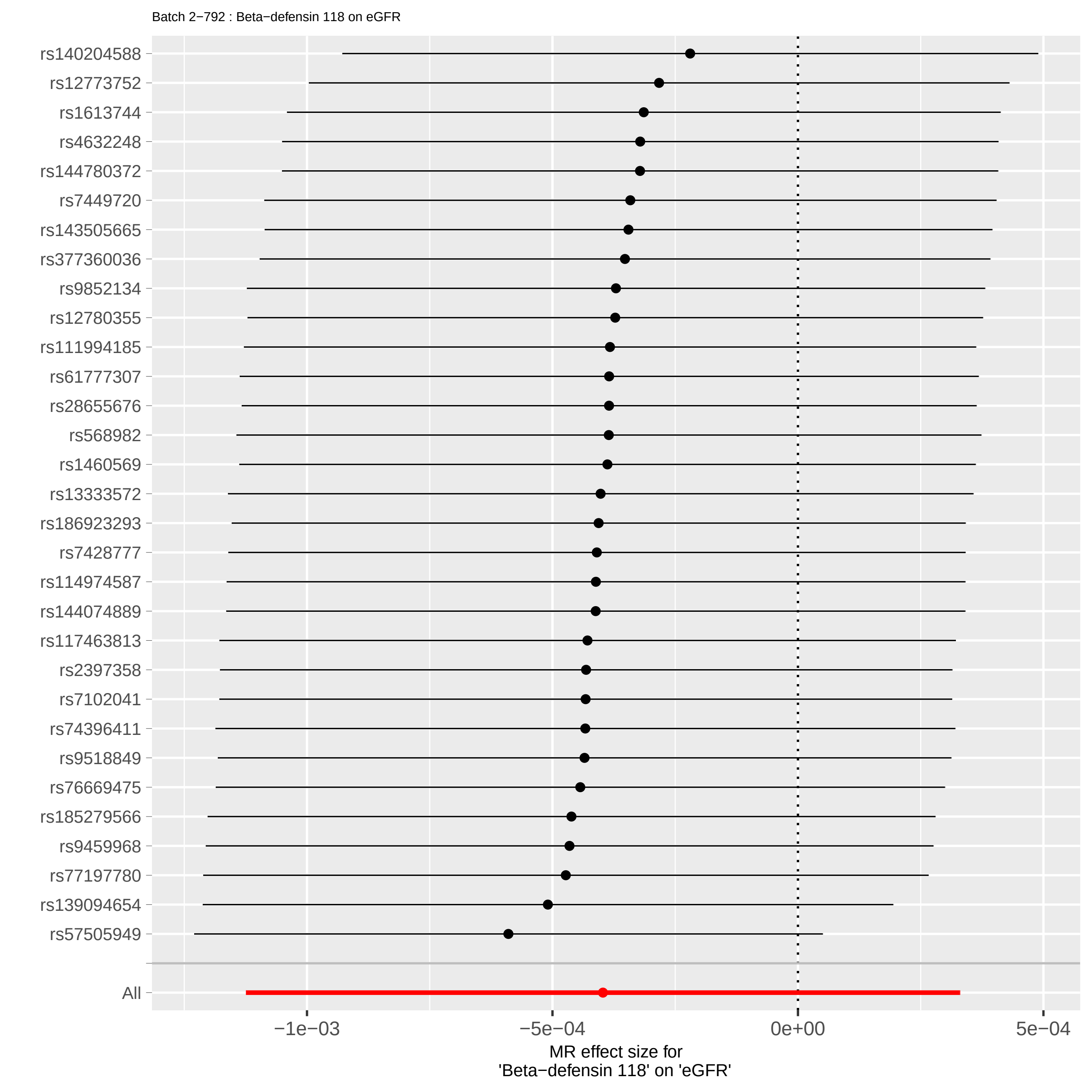

Batch 2−792 : Beta−defensin 118 on eGFR
rs140204588
rs12773752
rs1613744
rs4632248
rs144780372
rs7449720
rs143505665
rs377360036
rs9852134
rs12780355
rs111994185
rs61777307
rs28655676
rs568982
rs1460569
rs13333572
rs186923293
rs7428777
rs114974587
rs144074889
rs117463813
rs2397358
rs7102041
rs74396411
rs9518849
rs76669475
rs185279566
rs9459968
rs77197780
rs139094654
rs57505949
All
−1e−03
−5e−04
0e+00
5e−04
MR effect size for
'Beta−defensin 118' on 'eGFR'

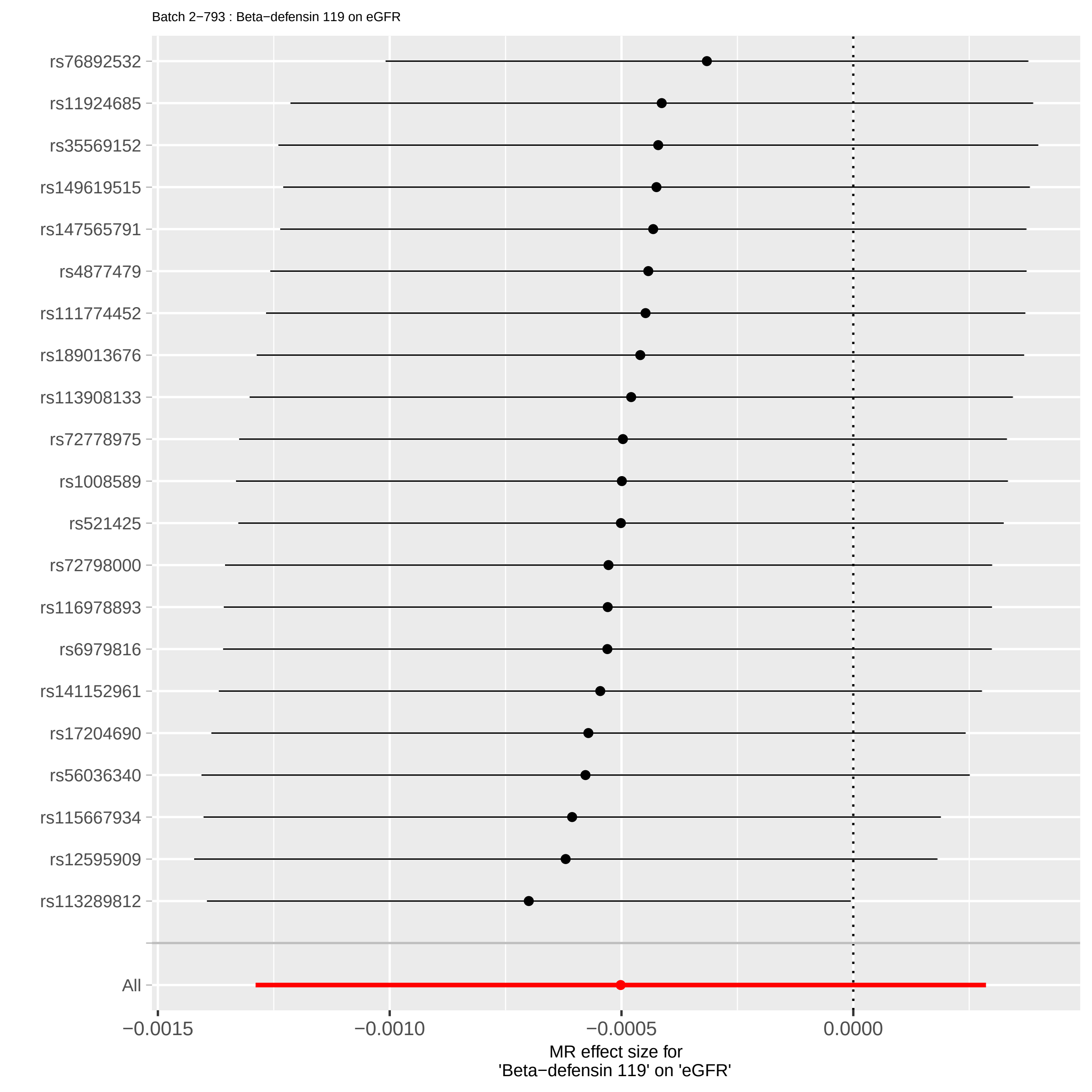

Batch 2−793 : Beta−defensin 119 on eGFR
rs76892532
rs11924685
rs35569152
rs149619515
rs147565791
rs4877479
rs111774452
rs189013676
rs113908133
rs72778975
rs1008589
rs521425
rs72798000
rs116978893
rs6979816
rs141152961
rs17204690
rs56036340
rs115667934
rs12595909
rs113289812
All
−0.0015
−0.0010
−0.0005
0.0000
MR effect size for
'Beta−defensin 119' on 'eGFR'

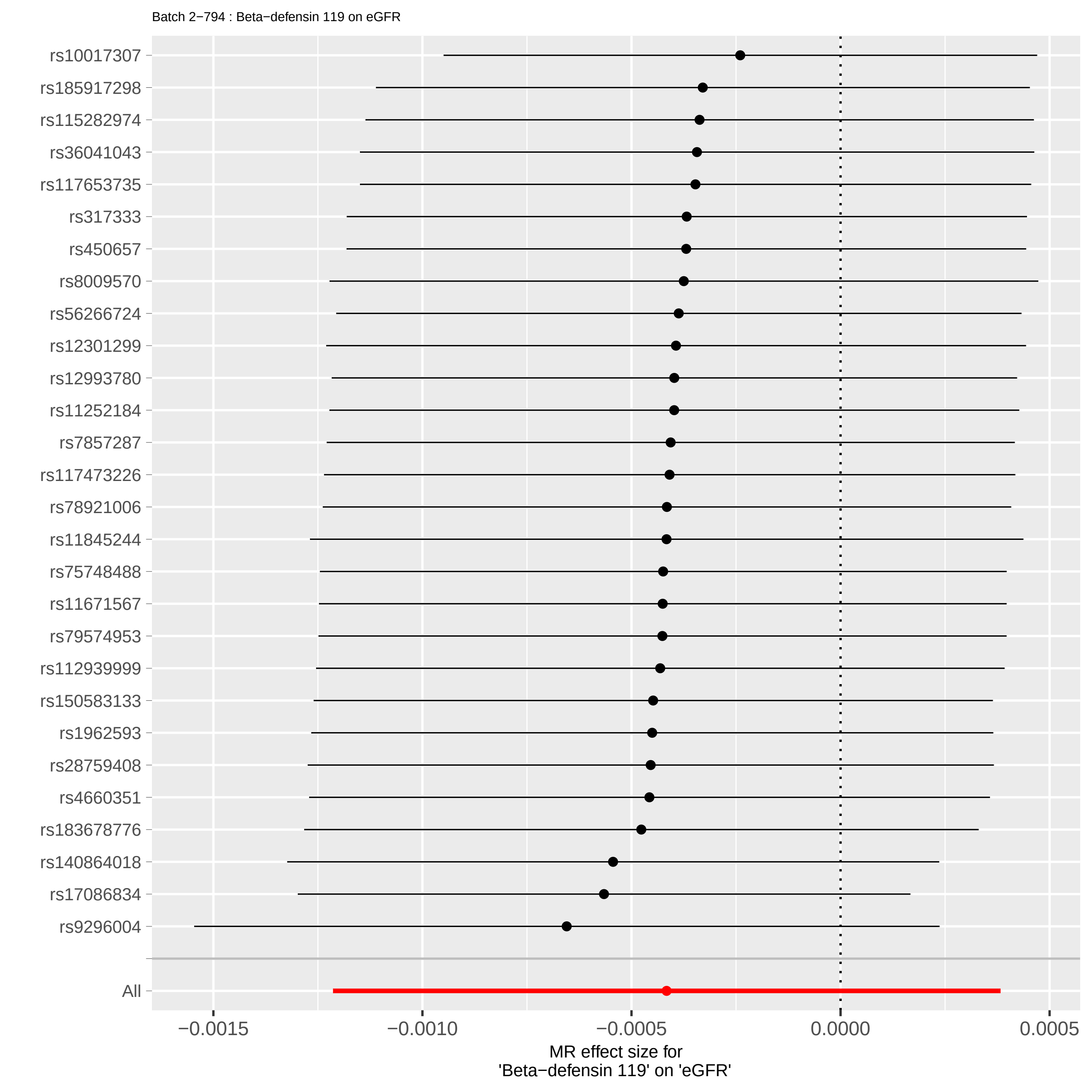

Batch 2−794 : Beta−defensin 119 on eGFR
rs10017307
rs185917298
rs115282974
rs36041043
rs117653735
rs317333
rs450657
rs8009570
rs56266724
rs12301299
rs12993780
rs11252184
rs7857287
rs117473226
rs78921006
rs11845244
rs75748488
rs11671567
rs79574953
rs112939999
rs150583133
rs1962593
rs28759408
rs4660351
rs183678776
rs140864018
rs17086834
rs9296004
All
−0.0015
−0.0010
−0.0005
0.0000
0.0005
MR effect size for
'Beta−defensin 119' on 'eGFR'

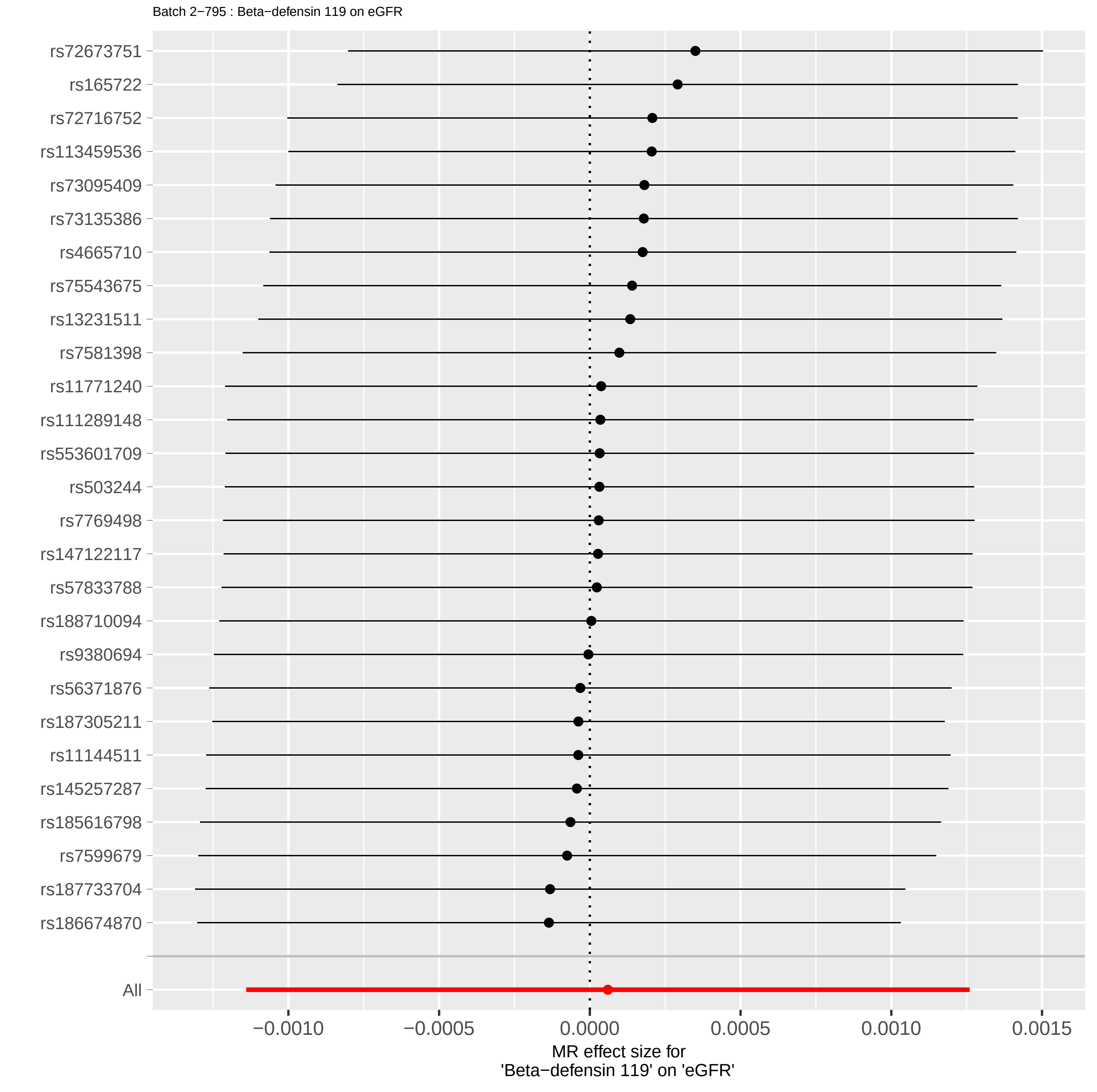

Batch 2−795 : Beta−defensin 119 on eGFR
rs72673751
rs165722
rs72716752
rs113459536
rs73095409
rs73135386
rs4665710
rs75543675
rs13231511
rs7581398
rs11771240
rs111289148
rs553601709
rs503244
rs7769498
rs147122117
rs57833788
rs188710094
rs9380694
rs56371876
rs187305211
rs11144511
rs145257287
rs185616798
rs7599679
rs187733704
rs186674870
All
−0.0010
−0.0005
0.0000
0.0005
0.0010
0.0015
MR effect size for
'Beta−defensin 119' on 'eGFR'

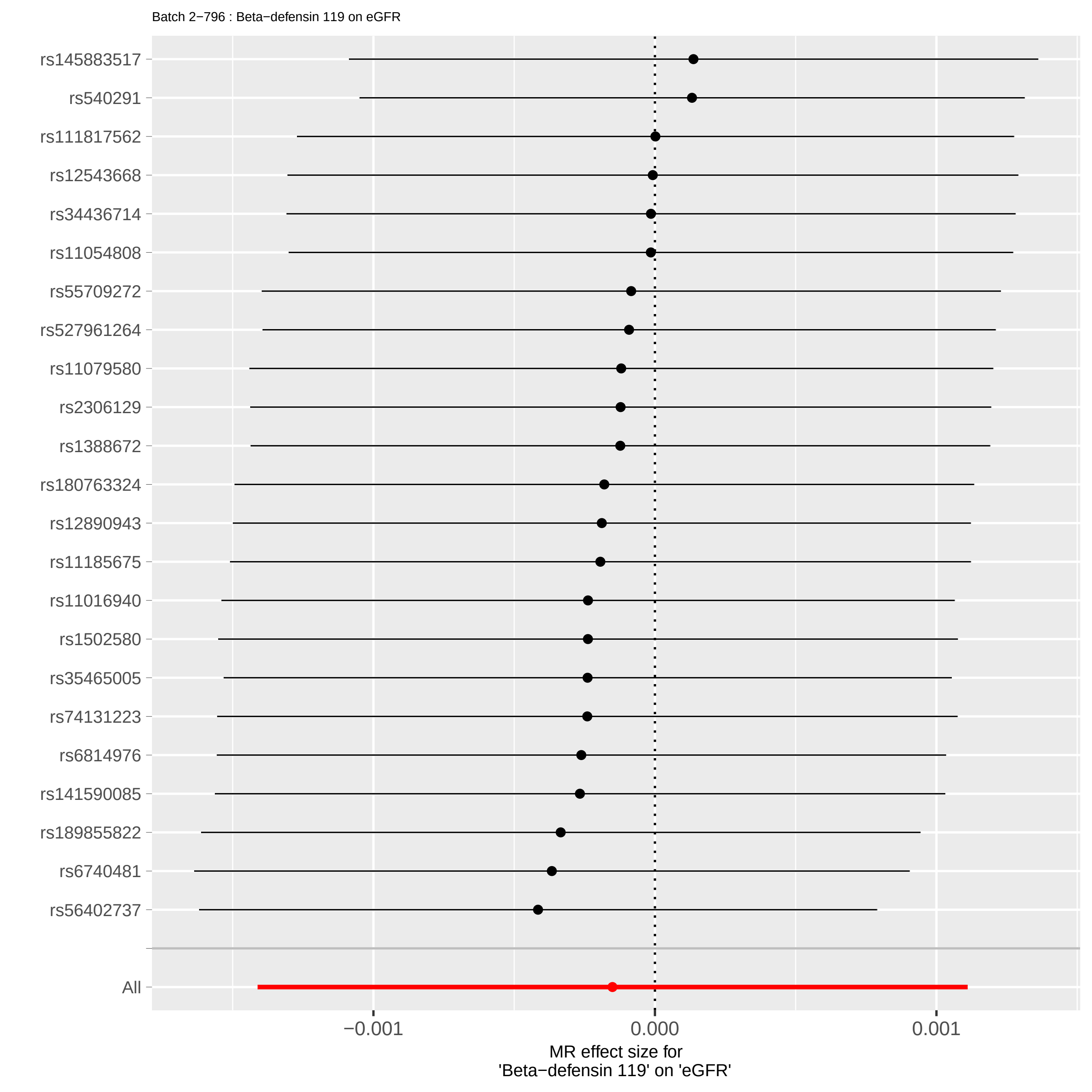

Batch 2−796 : Beta−defensin 119 on eGFR
rs145883517
rs540291
rs111817562
rs12543668
rs34436714
rs11054808
rs55709272
rs527961264
rs11079580
rs2306129
rs1388672
rs180763324
rs12890943
rs11185675
rs11016940
rs1502580
rs35465005
rs74131223
rs6814976
rs141590085
rs189855822
rs6740481
rs56402737
All
−0.001
0.000
0.001
MR effect size for
'Beta−defensin 119' on 'eGFR'

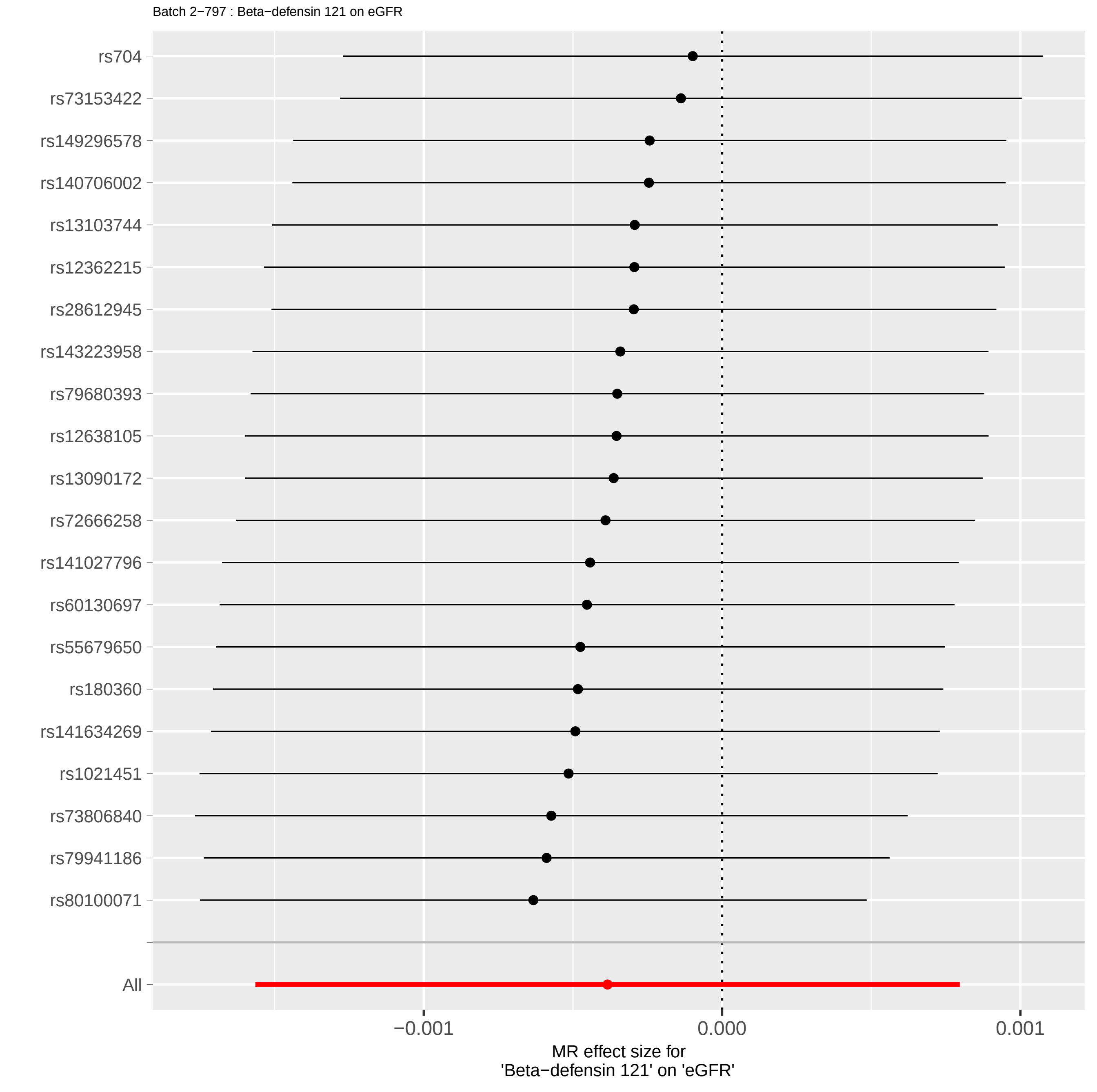

Batch 2−797 : Beta−defensin 121 on eGFR
rs704
rs73153422
rs149296578
rs140706002
rs13103744
rs12362215
rs28612945
rs143223958
rs79680393
rs12638105
rs13090172
rs72666258
rs141027796
rs60130697
rs55679650
rs180360
rs141634269
rs1021451
rs73806840
rs79941186
rs80100071
All
−0.001
0.000
0.001
MR effect size for
'Beta−defensin 121' on 'eGFR'

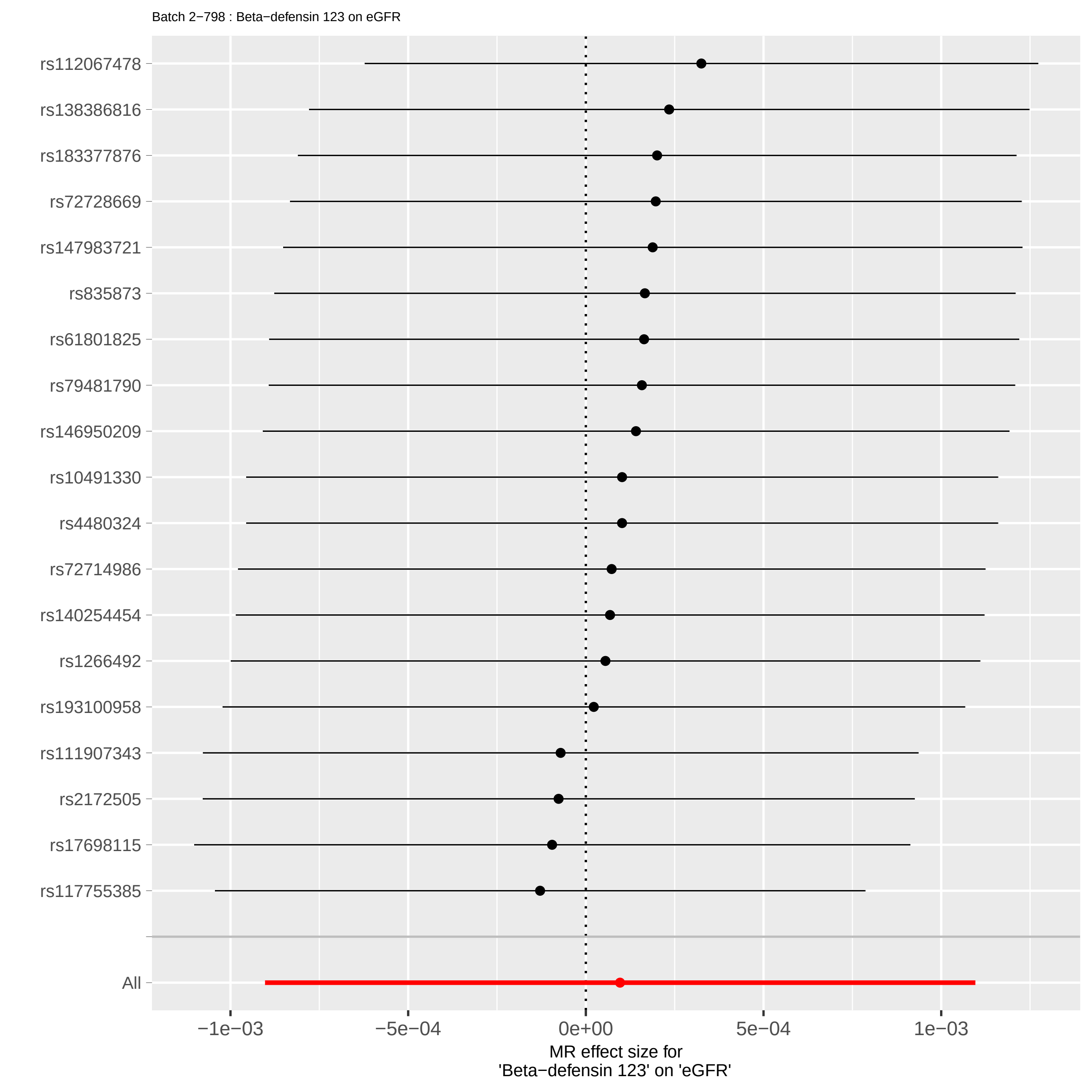

Batch 2−798 : Beta−defensin 123 on eGFR
rs112067478
rs138386816
rs183377876
rs72728669
rs147983721
rs835873
rs61801825
rs79481790
rs146950209
rs10491330
rs4480324
rs72714986
rs140254454
rs1266492
rs193100958
rs111907343
rs2172505
rs17698115
rs117755385
All
−1e−03
−5e−04
0e+00
5e−04
1e−03
MR effect size for
'Beta−defensin 123' on 'eGFR'

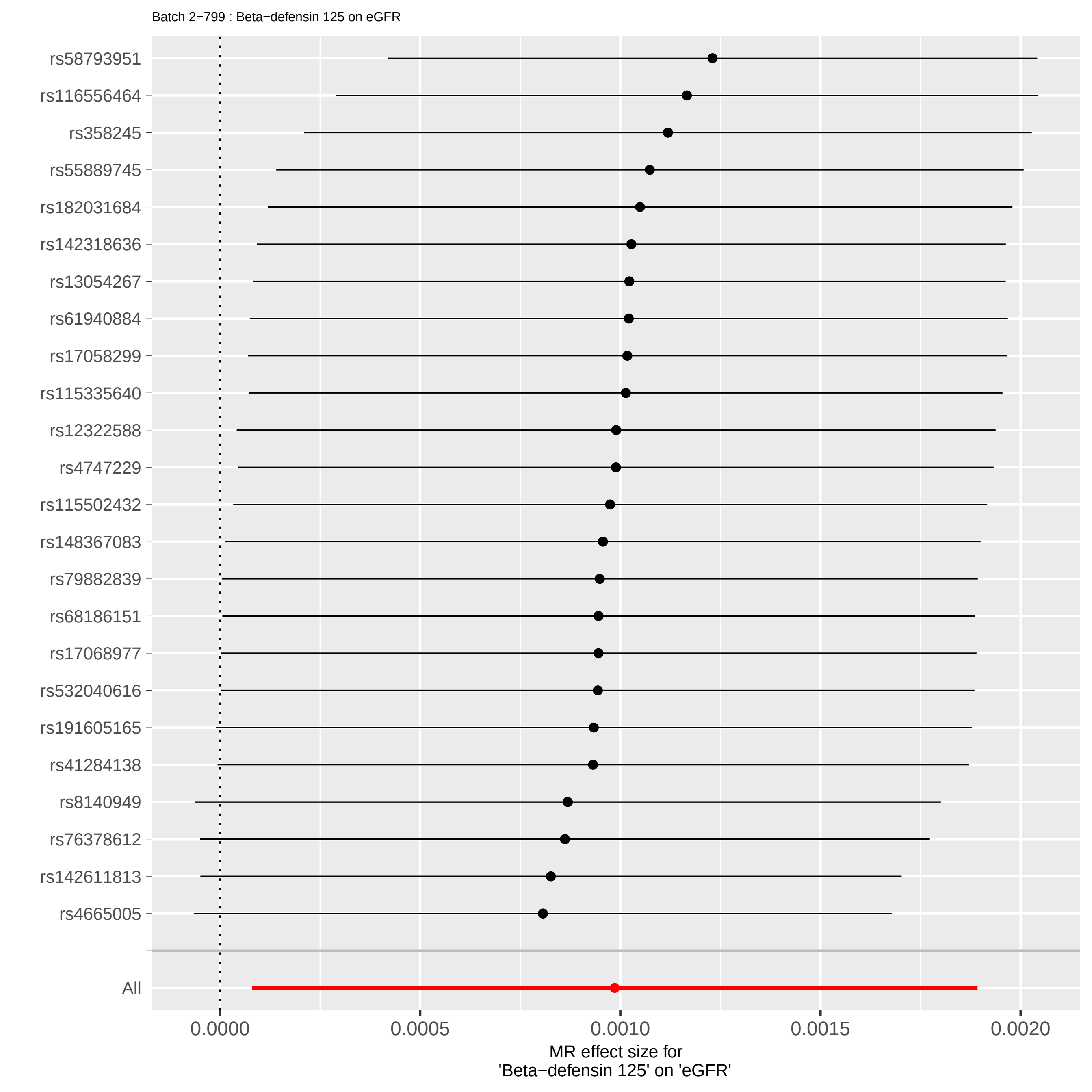
Batch 2−799 : Beta−defensin 125 on eGFR
rs58793951
rs116556464
rs358245
rs55889745
rs182031684
rs142318636
rs13054267
rs61940884
rs17058299
rs115335640
rs12322588
rs4747229
rs115502432
rs148367083
rs79882839
rs68186151
rs17068977
rs532040616
rs191605165
rs41284138
rs8140949
rs76378612
rs142611813
rs4665005
All
0.0000
0.0005
0.0010
0.0015
0.0020
MR effect size for
'Beta−defensin 125' on 'eGFR'

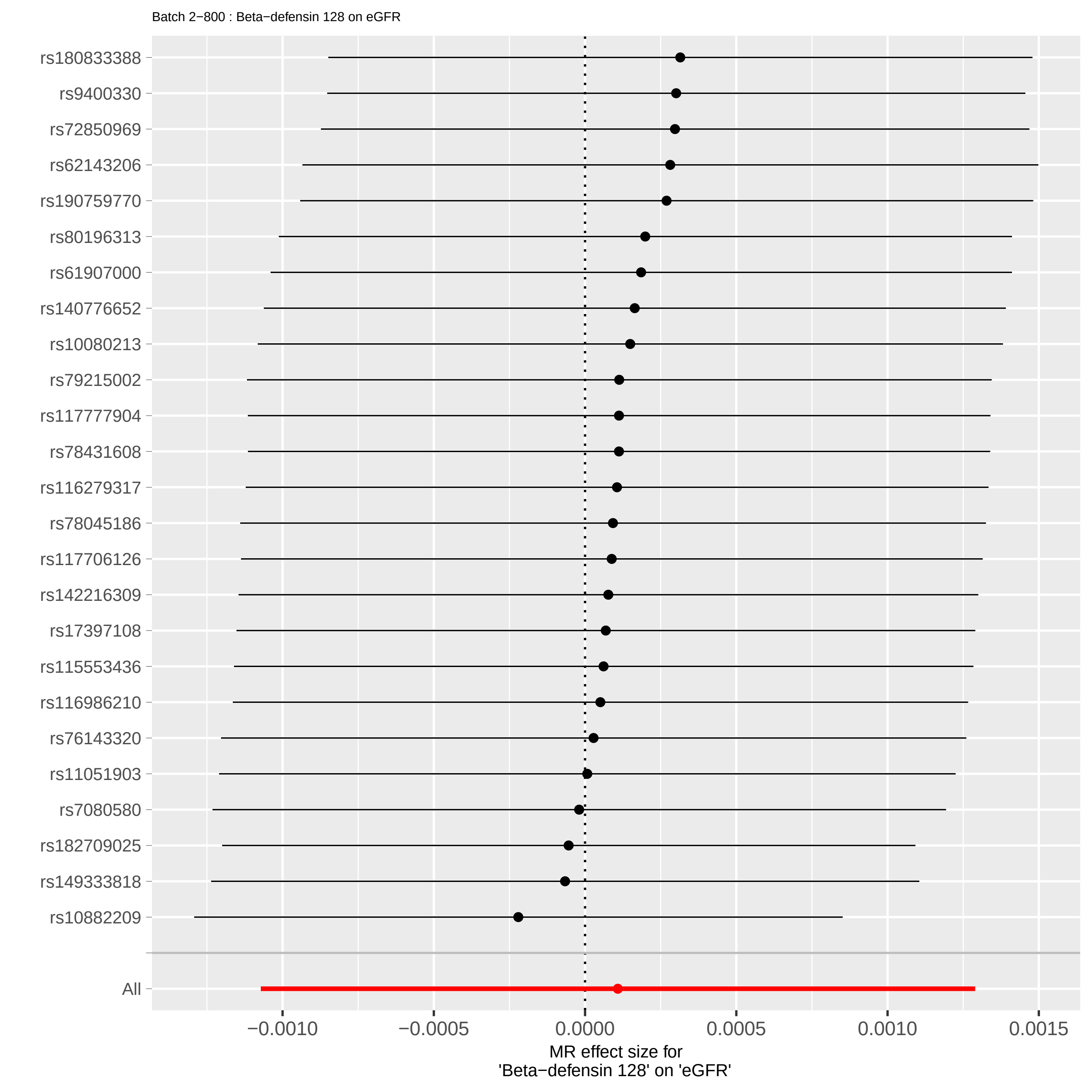

Batch 2−800 : Beta−defensin 128 on eGFR
rs180833388
rs9400330
rs72850969
rs62143206
rs190759770
rs80196313
rs61907000
rs140776652
rs10080213
rs79215002
rs117777904
rs78431608
rs116279317
rs78045186
rs117706126
rs142216309
rs17397108
rs115553436
rs116986210
rs76143320
rs11051903
rs7080580
rs182709025
rs149333818
rs10882209
All
−0.0010
−0.0005
0.0000
0.0005
0.0010
0.0015
MR effect size for
'Beta−defensin 128' on 'eGFR'

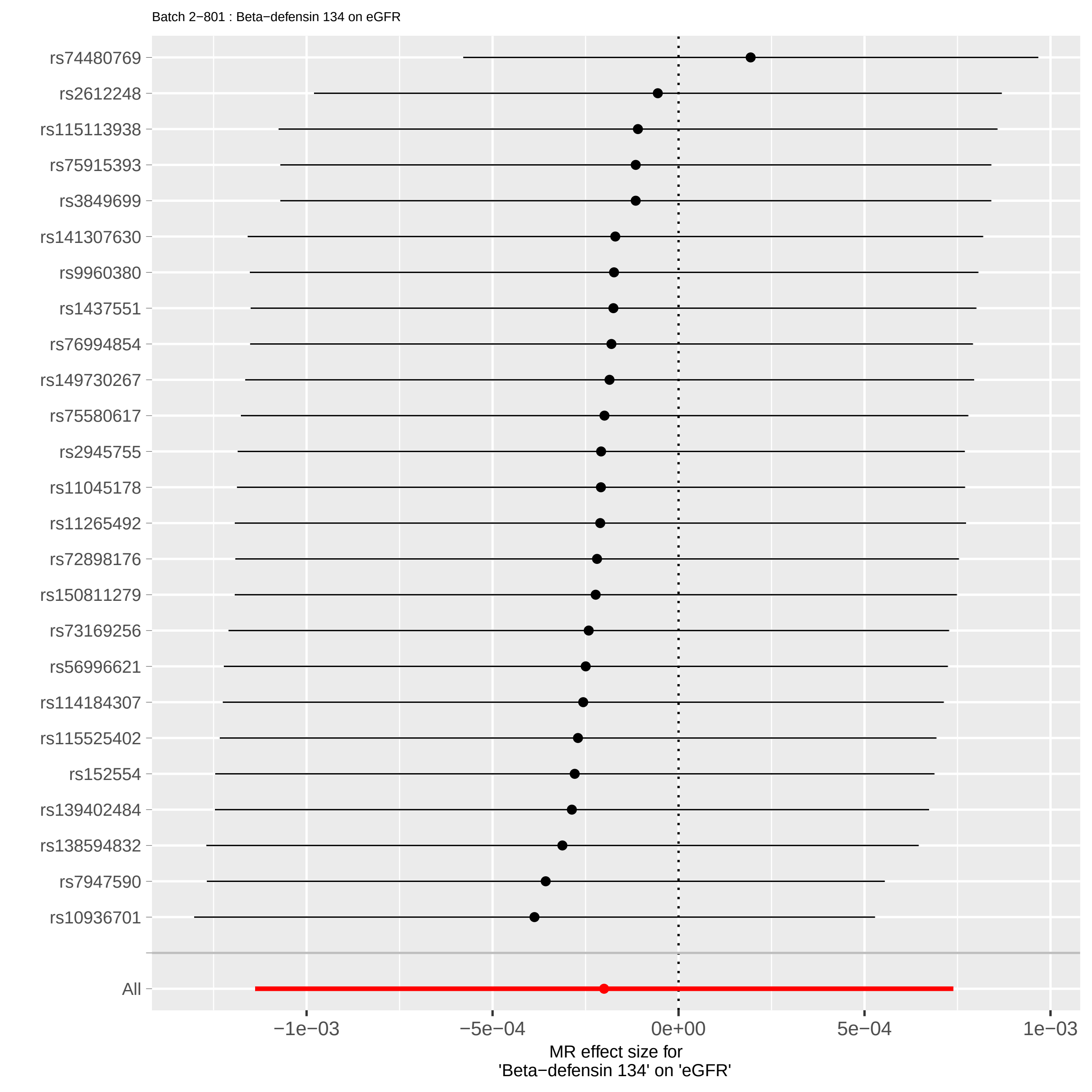

Batch 2−801 : Beta−defensin 134 on eGFR
rs74480769
rs2612248
rs115113938
rs75915393
rs3849699
rs141307630
rs9960380
rs1437551
rs76994854
rs149730267
rs75580617
rs2945755
rs11045178
rs11265492
rs72898176
rs150811279
rs73169256
rs56996621
rs114184307
rs115525402
rs152554
rs139402484
rs138594832
rs7947590
rs10936701
All
−1e−03
−5e−04
0e+00
5e−04
1e−03
MR effect size for
'Beta−defensin 134' on 'eGFR'

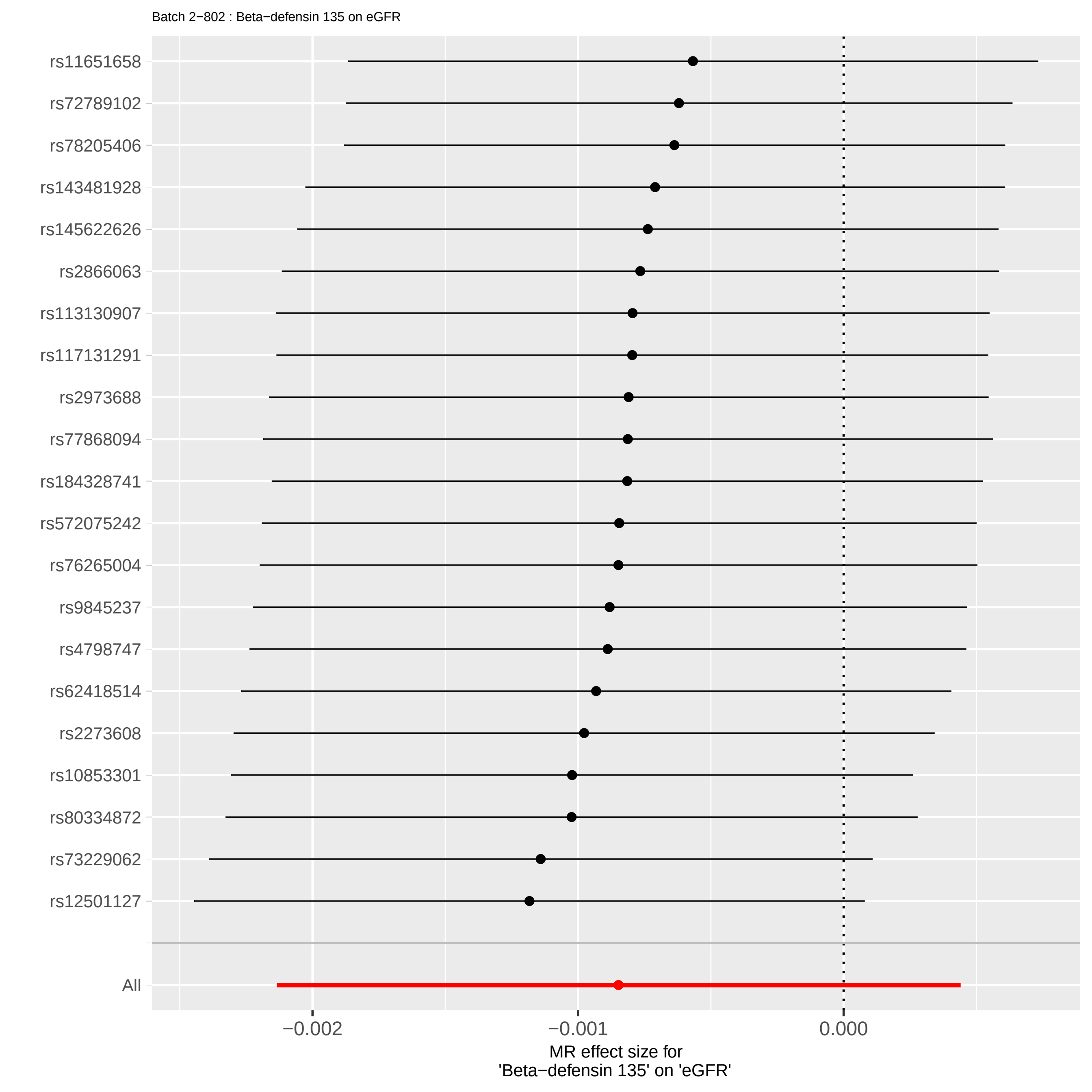
Batch 2−802 : Beta−defensin 135 on eGFR
rs11651658
rs72789102
rs78205406
rs143481928
rs145622626
rs2866063
rs113130907
rs117131291
rs2973688
rs77868094
rs184328741
rs572075242
rs76265004
rs9845237
rs4798747
rs62418514
rs2273608
rs10853301
rs80334872
rs73229062
rs12501127
All
−0.002
−0.001
0.000
MR effect size for
'Beta−defensin 135' on 'eGFR'

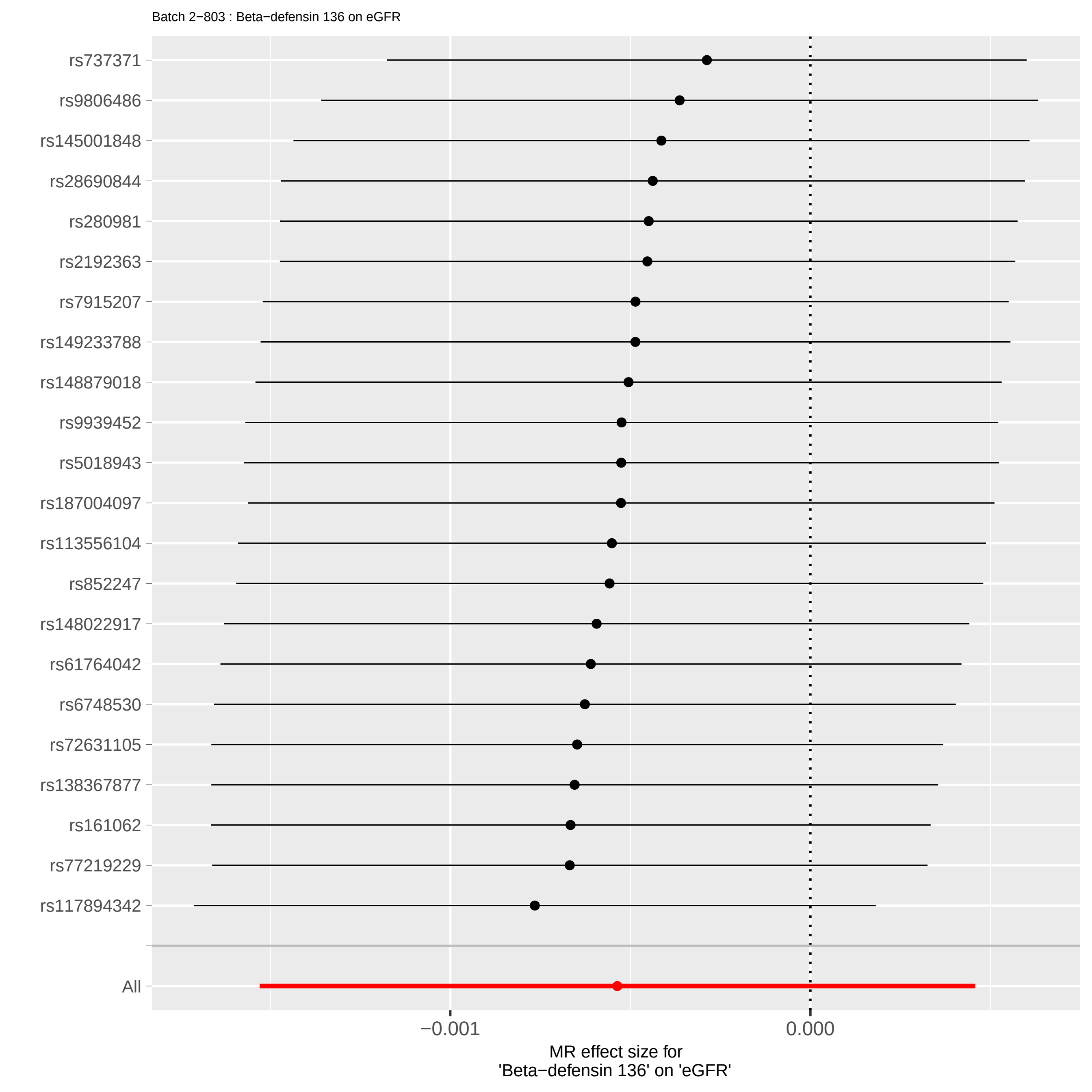

Batch 2−803 : Beta−defensin 136 on eGFR
rs737371
rs9806486
rs145001848
rs28690844
rs280981
rs2192363
rs7915207
rs149233788
rs148879018
rs9939452
rs5018943
rs187004097
rs113556104
rs852247
rs148022917
rs61764042
rs6748530
rs72631105
rs138367877
rs161062
rs77219229
rs117894342
All
−0.001
0.000
MR effect size for
'Beta−defensin 136' on 'eGFR'

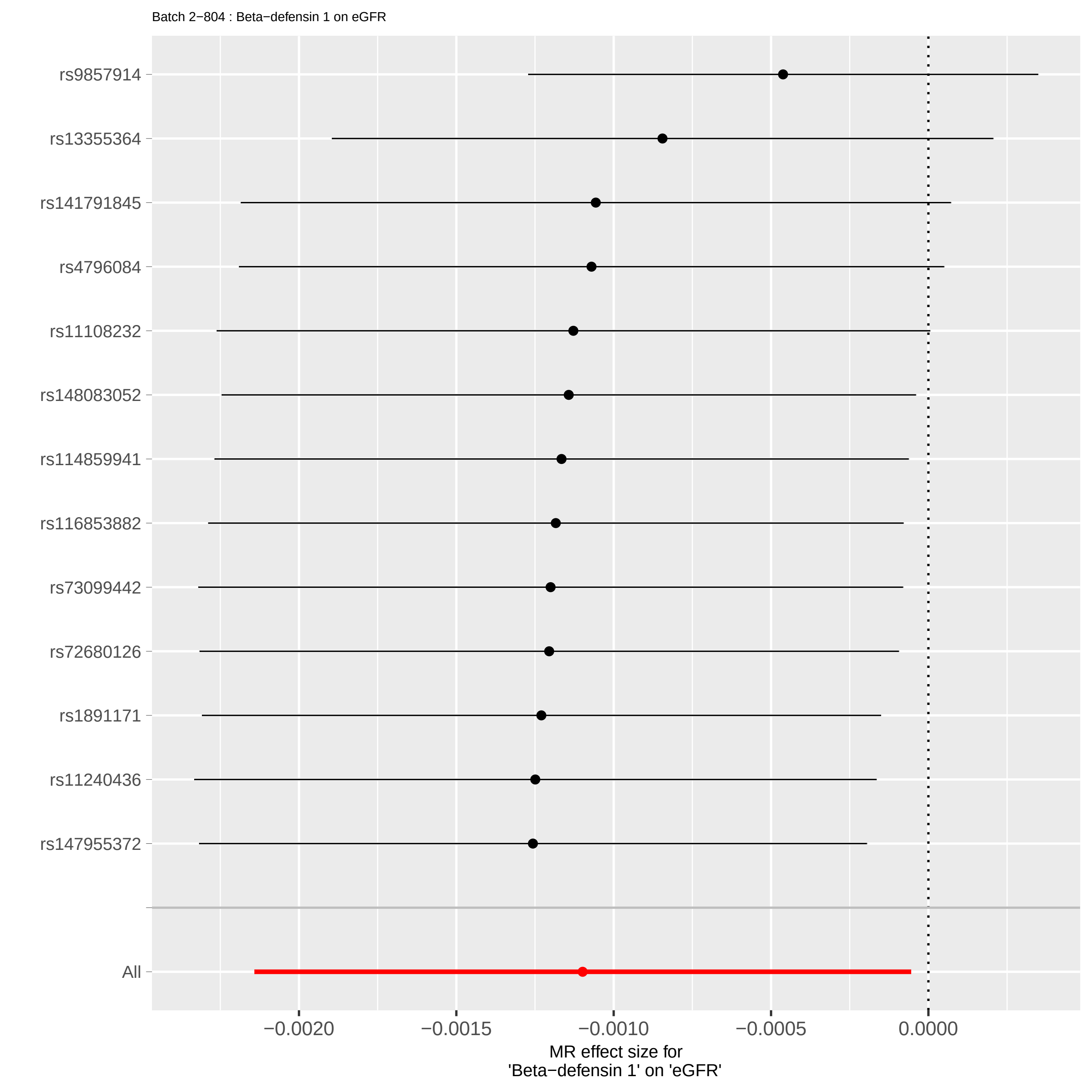

Batch 2−804 : Beta−defensin 1 on eGFR
rs9857914
rs13355364
rs141791845
rs4796084
rs11108232
rs148083052
rs114859941
rs116853882
rs73099442
rs72680126
rs1891171
rs11240436
rs147955372
All
−0.0020
−0.0015
−0.0010
−0.0005
0.0000
MR effect size for
'Beta−defensin 1' on 'eGFR'

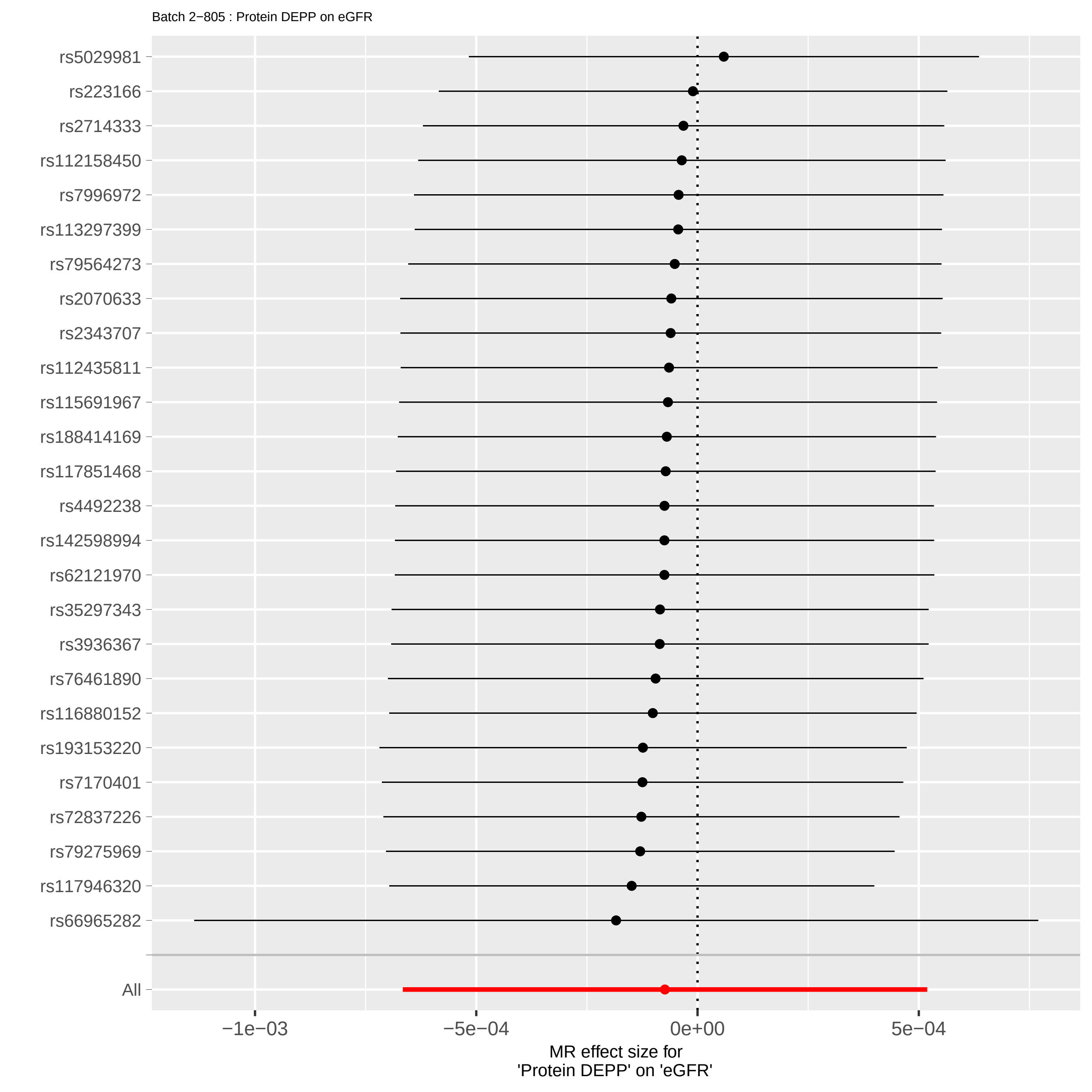

Batch 2−805 : Protein DEPP on eGFR
rs5029981
rs223166
rs2714333
rs112158450
rs7996972
rs113297399
rs79564273
rs2070633
rs2343707
rs112435811
rs115691967
rs188414169
rs117851468
rs4492238
rs142598994
rs62121970
rs35297343
rs3936367
rs76461890
rs116880152
rs193153220
rs7170401
rs72837226
rs79275969
rs117946320
rs66965282
All
−1e−03
−5e−04
0e+00
5e−04
MR effect size for
'Protein DEPP' on 'eGFR'

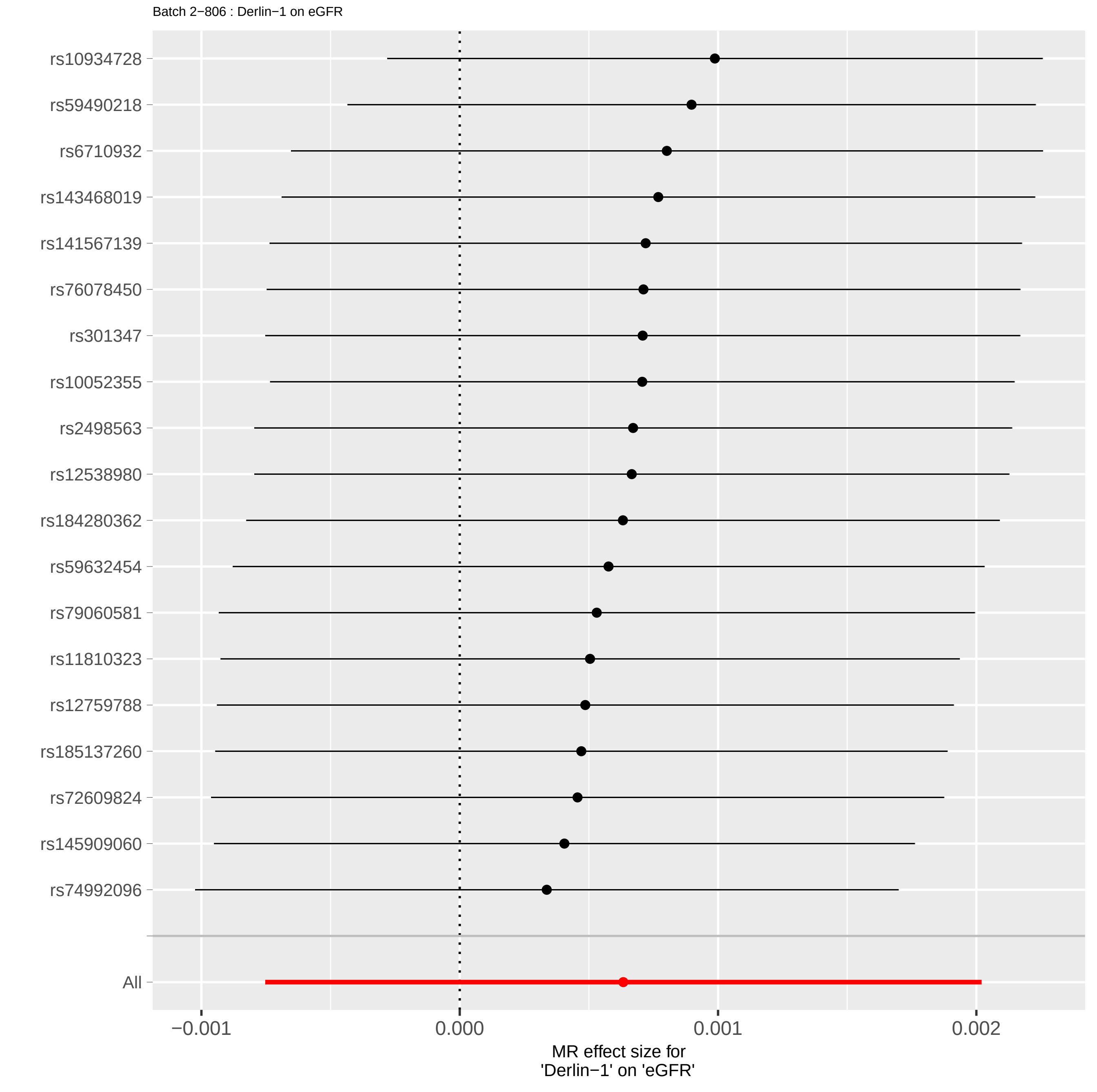
Batch 2−806 : Derlin−1 on eGFR
rs10934728
rs59490218
rs6710932
rs143468019
rs141567139
rs76078450
rs301347
rs10052355
rs2498563
rs12538980
rs184280362
rs59632454
rs79060581
rs11810323
rs12759788
rs185137260
rs72609824
rs145909060
rs74992096
All
−0.001
0.000
0.001
0.002
MR effect size for
'Derlin−1' on 'eGFR'

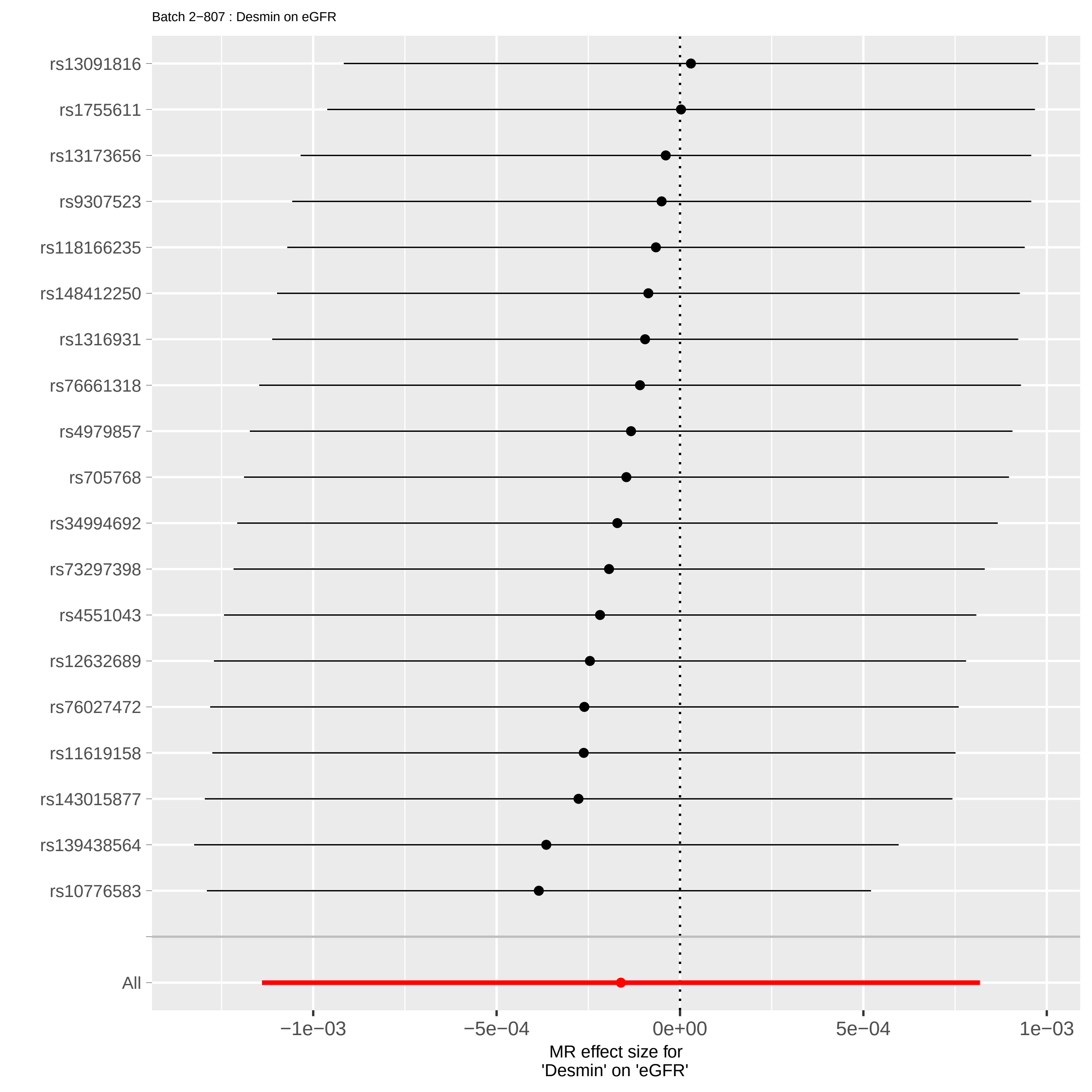
Batch 2−807 : Desmin on eGFR
rs13091816
rs1755611
rs13173656
rs9307523
rs118166235
rs148412250
rs1316931
rs76661318
rs4979857
rs705768
rs34994692
rs73297398
rs4551043
rs12632689
rs76027472
rs11619158
rs143015877
rs139438564
rs10776583
All
−1e−03
−5e−04
0e+00
5e−04
1e−03
MR effect size for
'Desmin' on 'eGFR'

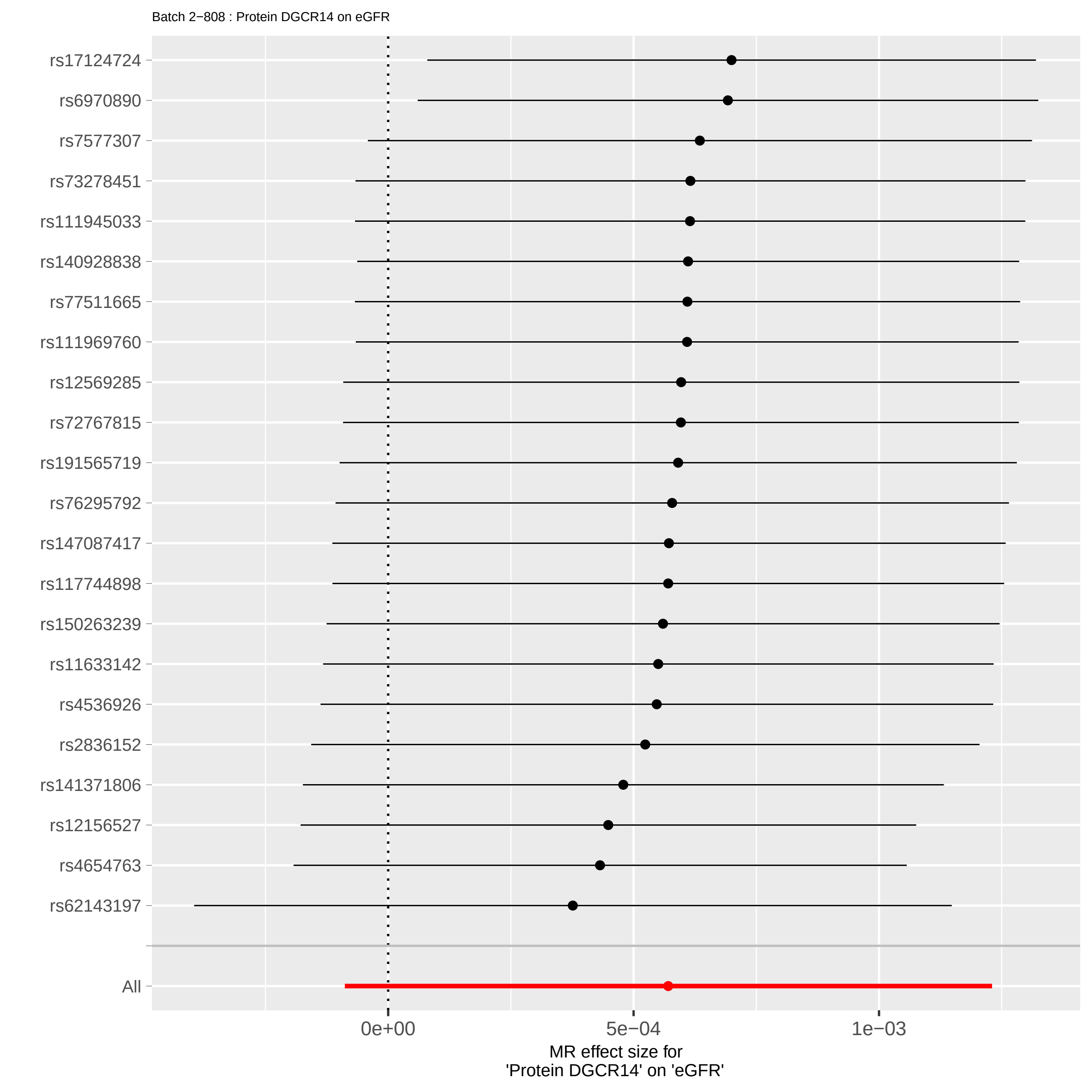
Batch 2−808 : Protein DGCR14 on eGFR
rs17124724
rs6970890
rs7577307
rs73278451
rs111945033
rs140928838
rs77511665
rs111969760
rs12569285
rs72767815
rs191565719
rs76295792
rs147087417
rs117744898
rs150263239
rs11633142
rs4536926
rs2836152
rs141371806
rs12156527
rs4654763
rs62143197
All
0e+00
5e−04
1e−03
MR effect size for
'Protein DGCR14' on 'eGFR'

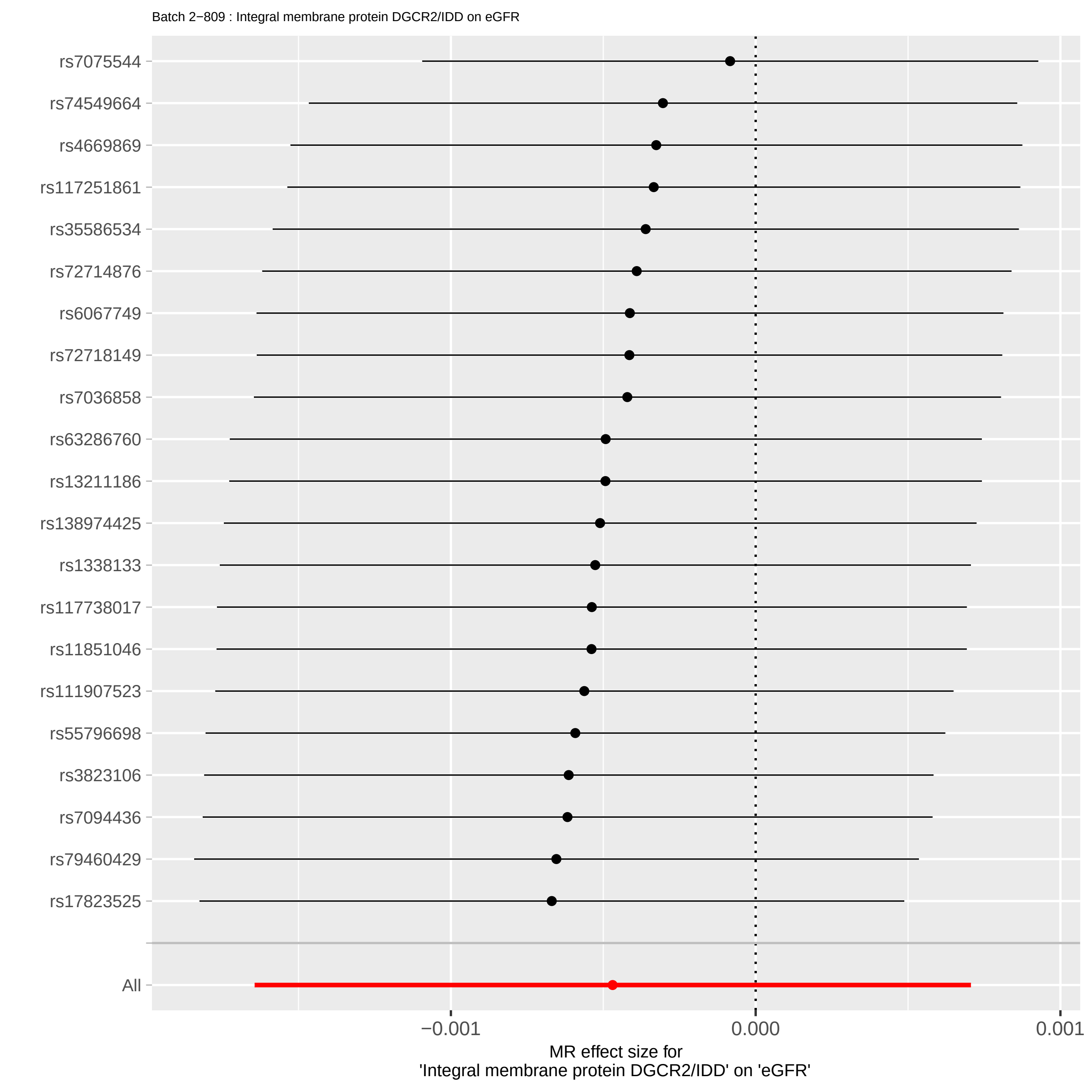

Batch 2−809 : Integral membrane protein DGCR2/IDD on eGFR
rs7075544
rs74549664
rs4669869
rs117251861
rs35586534
rs72714876
rs6067749
rs72718149
rs7036858
rs63286760
rs13211186
rs138974425
rs1338133
rs117738017
rs11851046
rs111907523
rs55796698
rs3823106
rs7094436
rs79460429
rs17823525
All
−0.001
0.000
0.001
MR effect size for
'Integral membrane protein DGCR2/IDD' on 'eGFR'

Batch 2−810 : Protein DGCR6 on eGFR

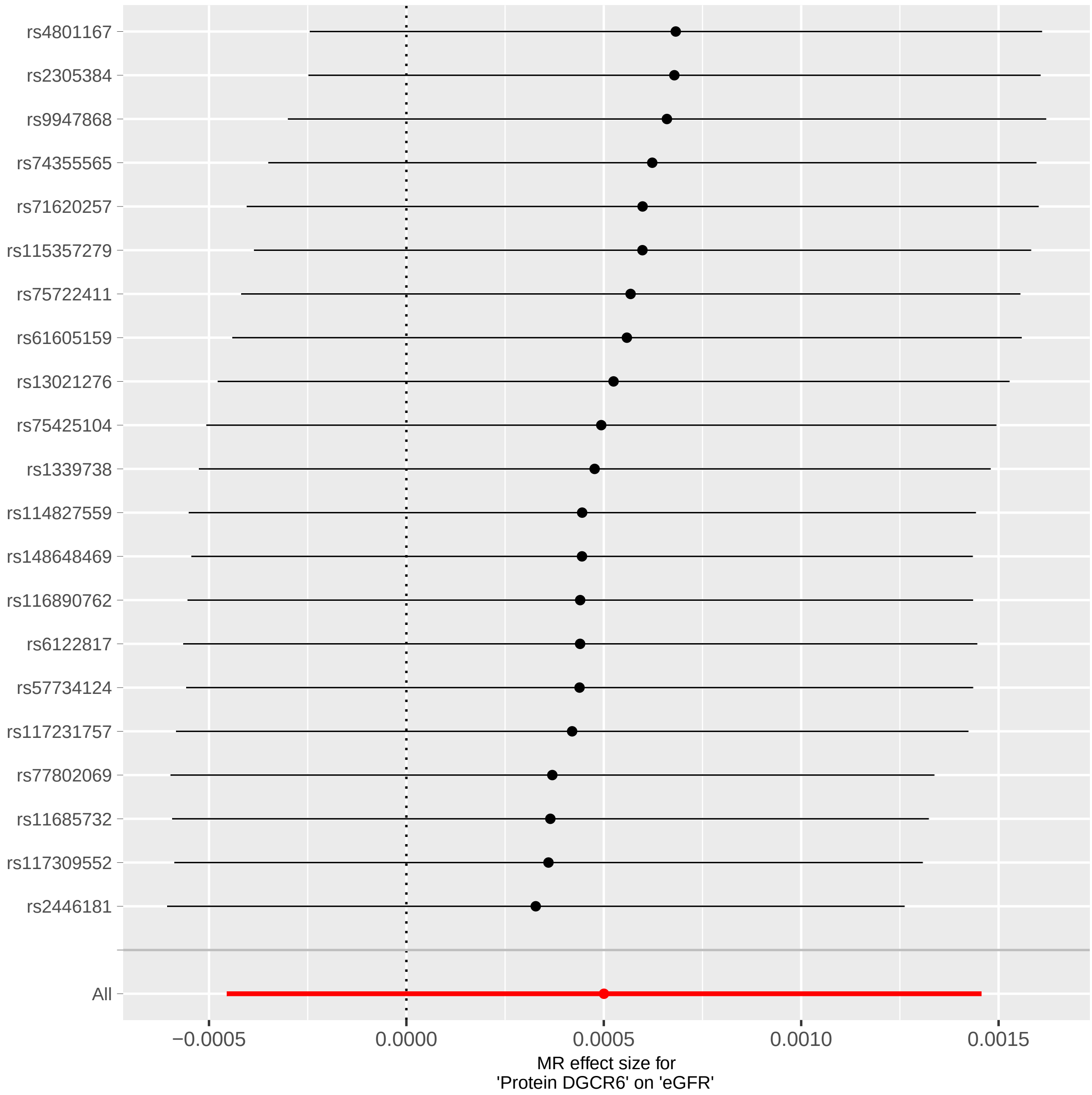

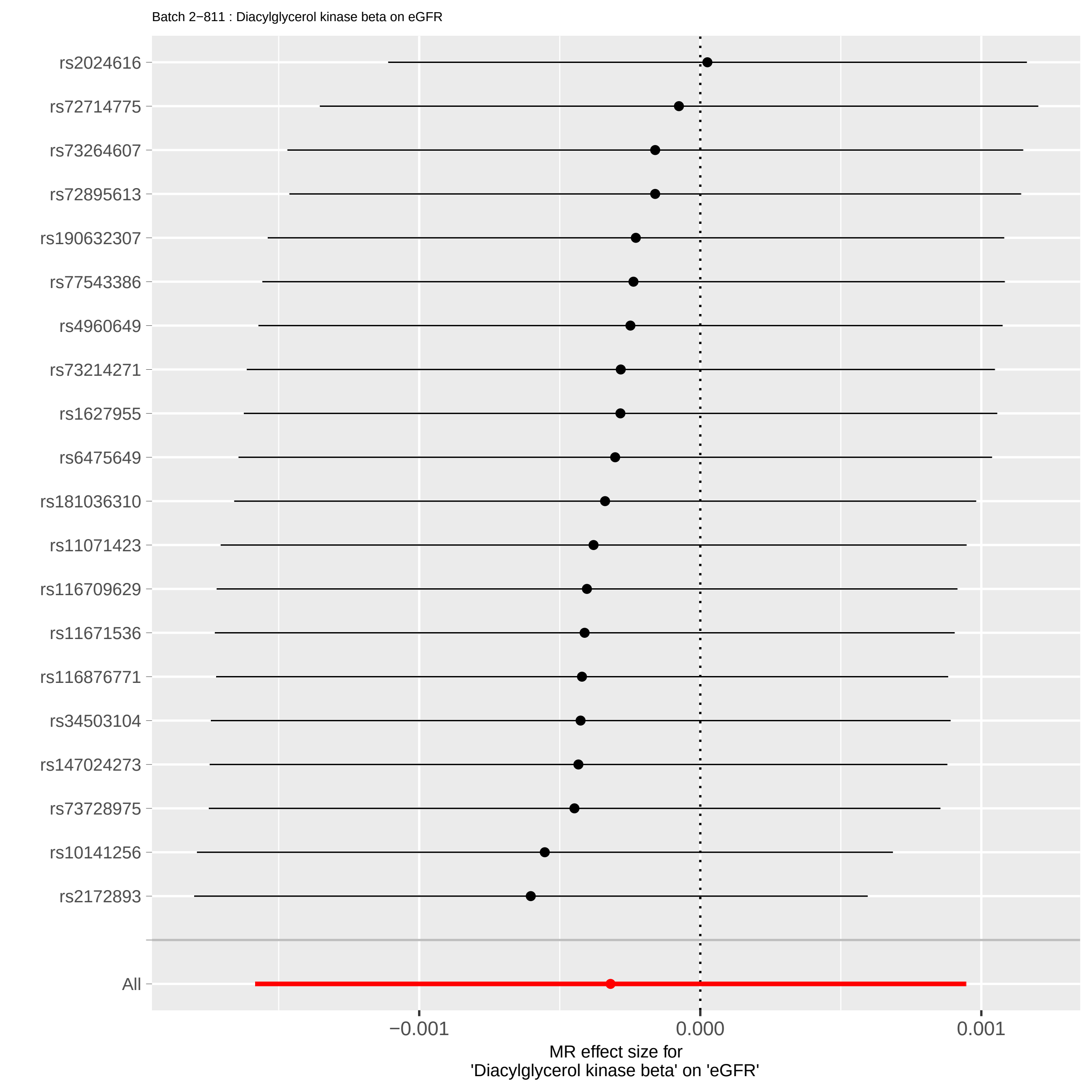

Batch 2−811 : Diacylglycerol kinase beta on eGFR
rs2024616
rs72714775
rs73264607
rs72895613
rs190632307
rs77543386
rs4960649
rs73214271
rs1627955
rs6475649
rs181036310
rs11071423
rs116709629
rs11671536
rs116876771
rs34503104
rs147024273
rs73728975
rs10141256
rs2172893
All
−0.001
0.000
0.001
MR effect size for
'Diacylglycerol kinase beta' on 'eGFR'

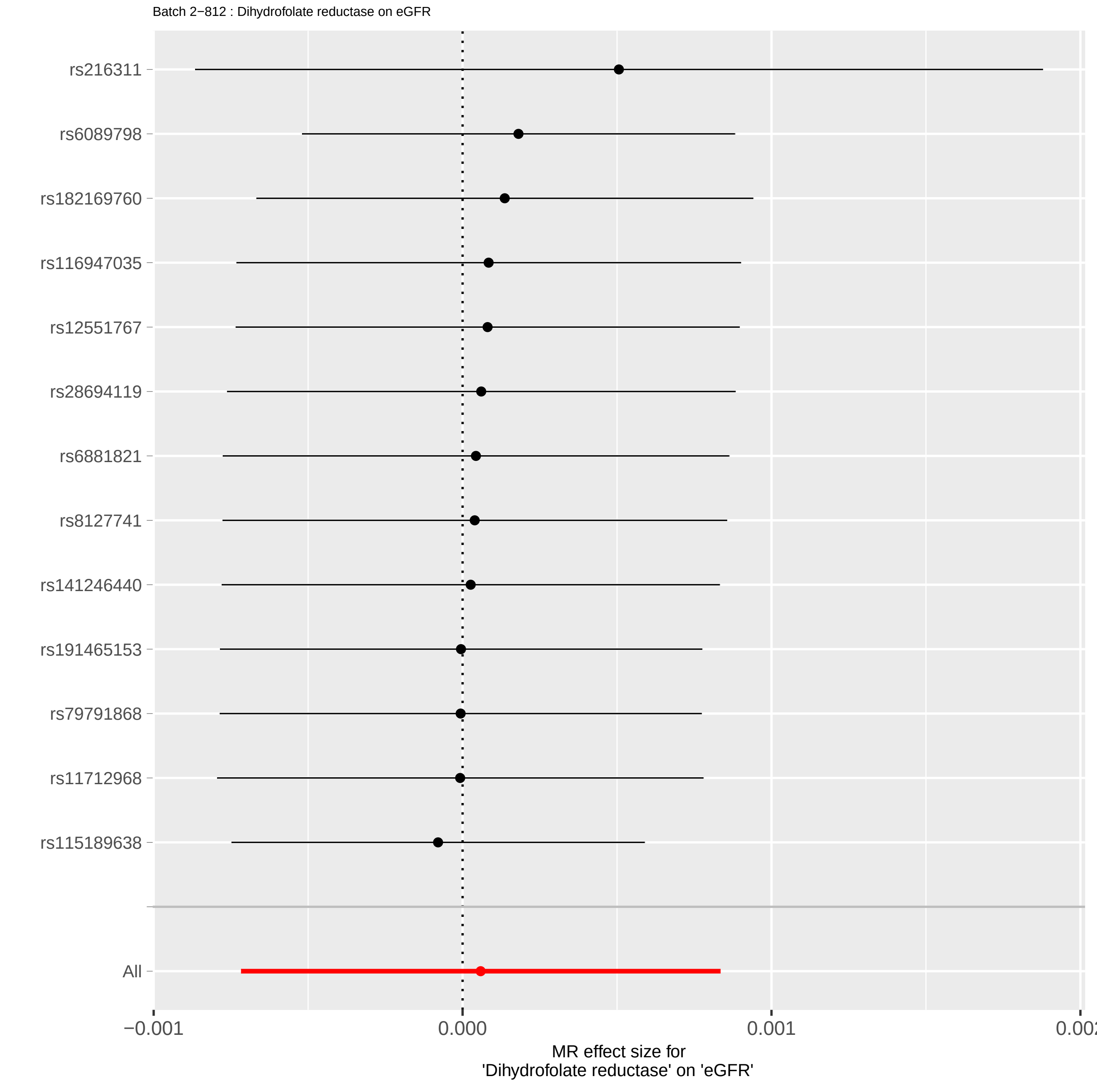
Batch 2−812 : Dihydrofolate reductase on eGFR
rs216311
rs6089798
rs182169760
rs116947035
rs12551767
rs28694119
rs6881821
rs8127741
rs141246440
rs191465153
rs79791868
rs11712968
rs115189638
All
−0.001
0.000
0.001
0.002
MR effect size for
'Dihydrofolate reductase' on 'eGFR'

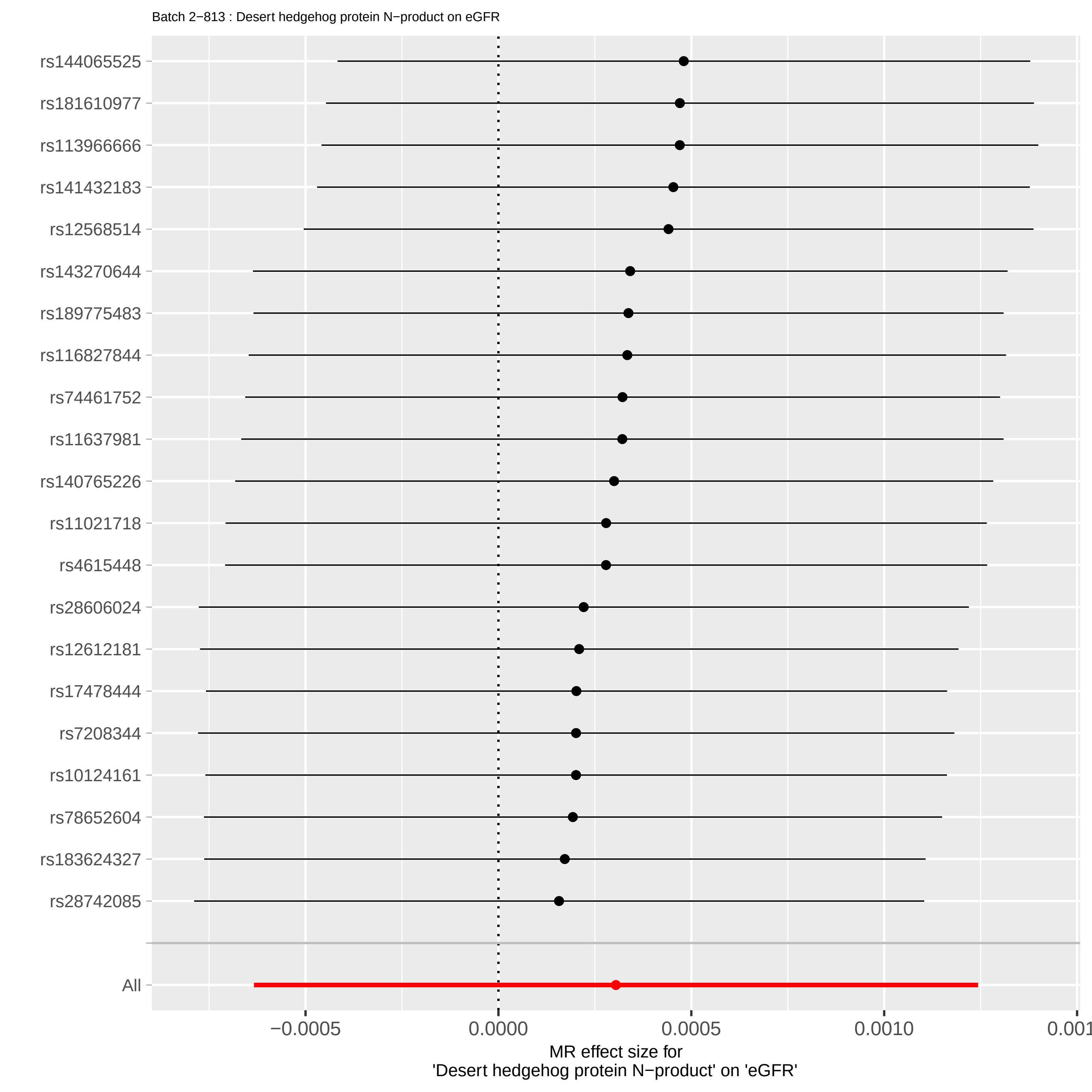

Batch 2−813 : Desert hedgehog protein N−product on eGFR
rs144065525
rs181610977
rs113966666
rs141432183
rs12568514
rs143270644
rs189775483
rs116827844
rs74461752
rs11637981
rs140765226
rs11021718
rs4615448
rs28606024
rs12612181
rs17478444
rs7208344
rs10124161
rs78652604
rs183624327
rs28742085
All
−0.0005
0.0000
0.0005
0.0010
MR effect size for
'Desert hedgehog protein N−product' on 'eGFR'

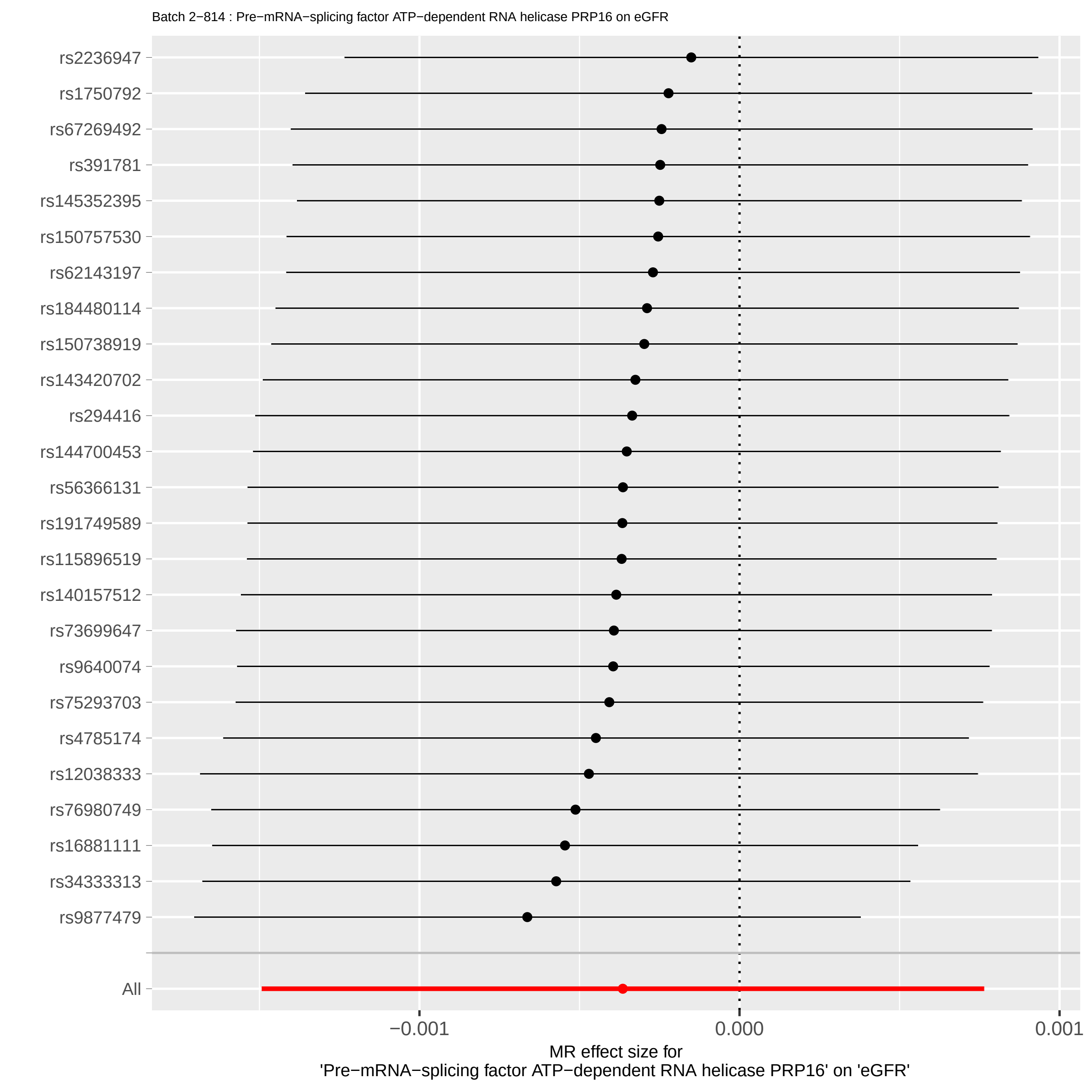

Batch 2−814 : Pre−mRNA−splicing factor ATP−dependent RNA helicase PRP16 on eGFR
rs2236947
rs1750792
rs67269492
rs391781
rs145352395
rs150757530
rs62143197
rs184480114
rs150738919
rs143420702
rs294416
rs144700453
rs56366131
rs191749589
rs115896519
rs140157512
rs73699647
rs9640074
rs75293703
rs4785174
rs12038333
rs76980749
rs16881111
rs34333313
rs9877479
All
−0.001
0.000
0.001
MR effect size for
'Pre−mRNA−splicing factor ATP−dependent RNA helicase PRP16' on 'eGFR'

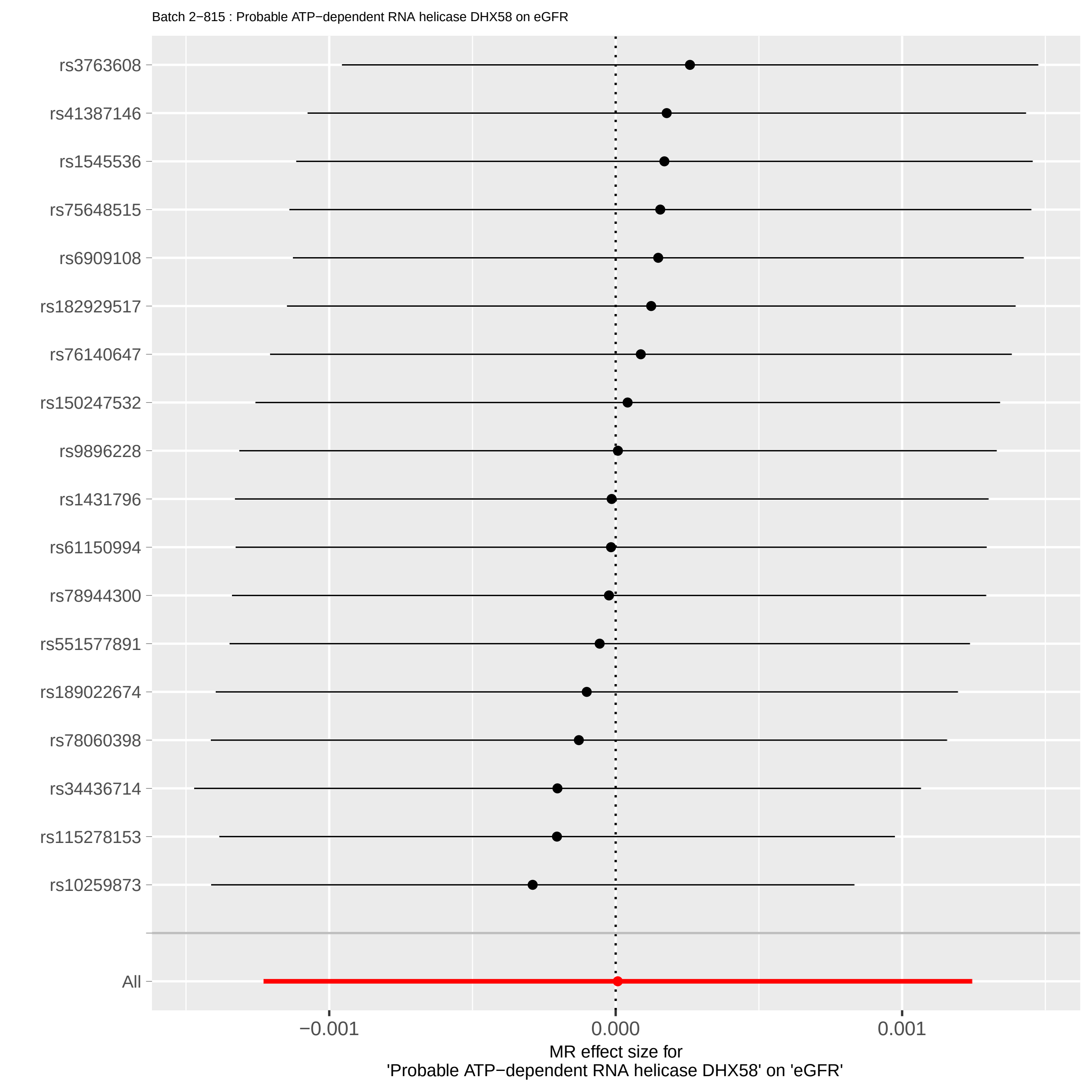

Batch 2−815 : Probable ATP−dependent RNA helicase DHX58 on eGFR
rs3763608
rs41387146
rs1545536
rs75648515
rs6909108
rs182929517
rs76140647
rs150247532
rs9896228
rs1431796
rs61150994
rs78944300
rs551577891
rs189022674
rs78060398
rs34436714
rs115278153
rs10259873
All
−0.001
0.000
0.001
MR effect size for
'Probable ATP−dependent RNA helicase DHX58' on 'eGFR'

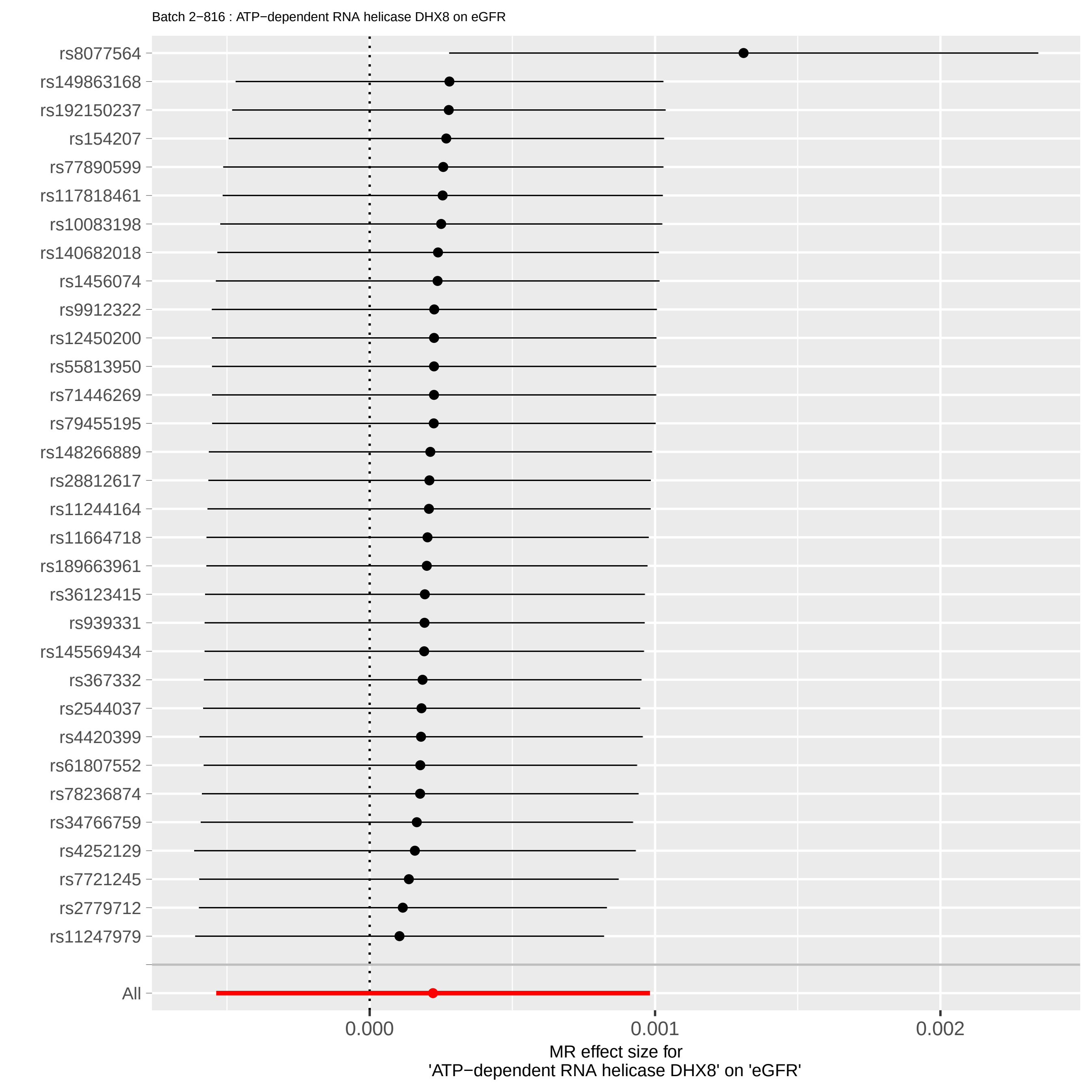
Batch 2−816 : ATP−dependent RNA helicase DHX8 on eGFR
rs8077564
rs149863168
rs192150237
rs154207
rs77890599
rs117818461
rs10083198
rs140682018
rs1456074
rs9912322
rs12450200
rs55813950
rs71446269
rs79455195
rs148266889
rs28812617
rs11244164
rs11664718
rs189663961
rs36123415
rs939331
rs145569434
rs367332
rs2544037
rs4420399
rs61807552
rs78236874
rs34766759
rs4252129
rs7721245
rs2779712
rs11247979
All
0.000
0.001
0.002
MR effect size for
'ATP−dependent RNA helicase DHX8' on 'eGFR'

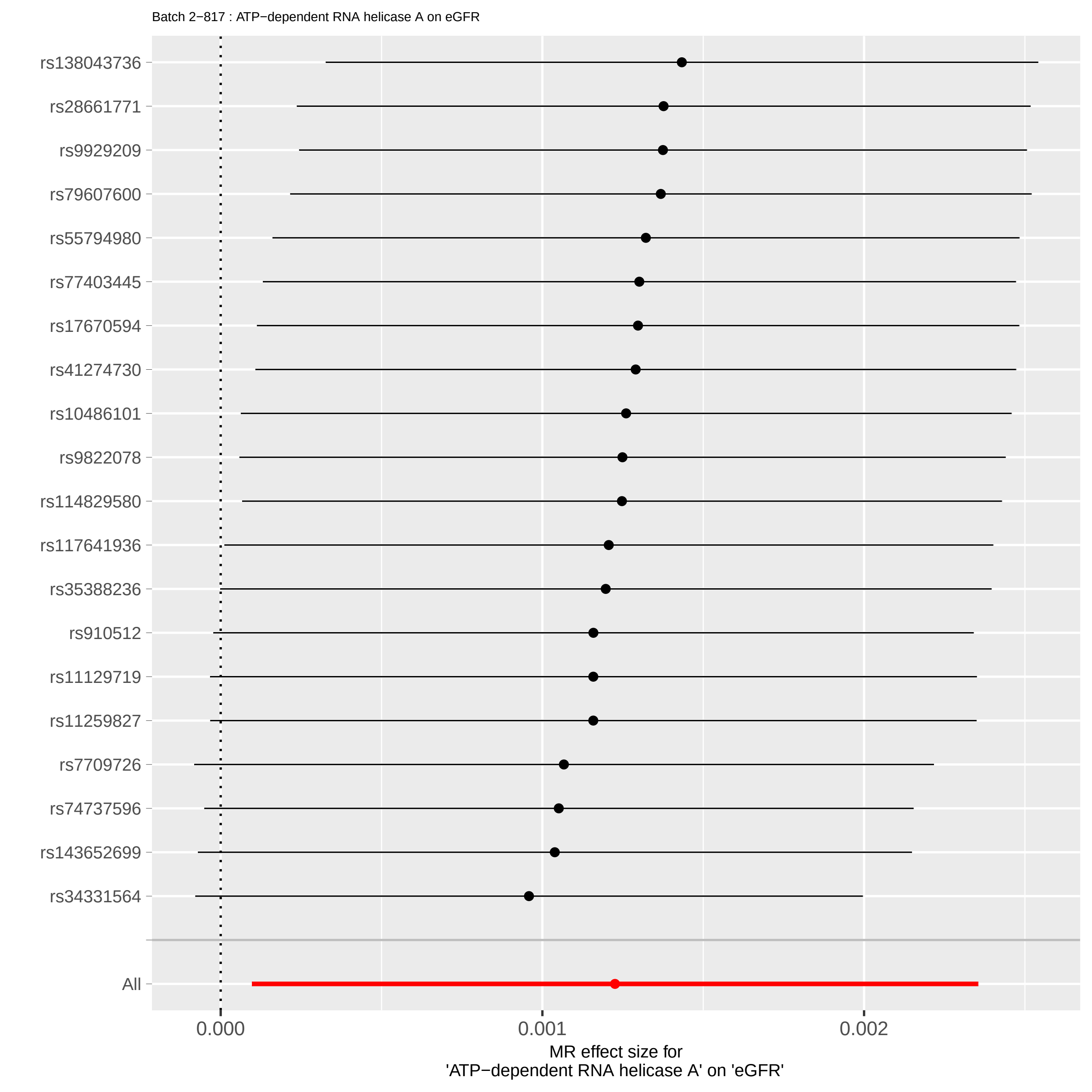
Batch 2−817 : ATP−dependent RNA helicase A on eGFR
rs138043736
rs28661771
rs9929209
rs79607600
rs55794980
rs77403445
rs17670594
rs41274730
rs10486101
rs9822078
rs114829580
rs117641936
rs35388236
rs910512
rs11129719
rs11259827
rs7709726
rs74737596
rs143652699
rs34331564
All
0.000
0.001
0.002
MR effect size for
'ATP−dependent RNA helicase A' on 'eGFR'

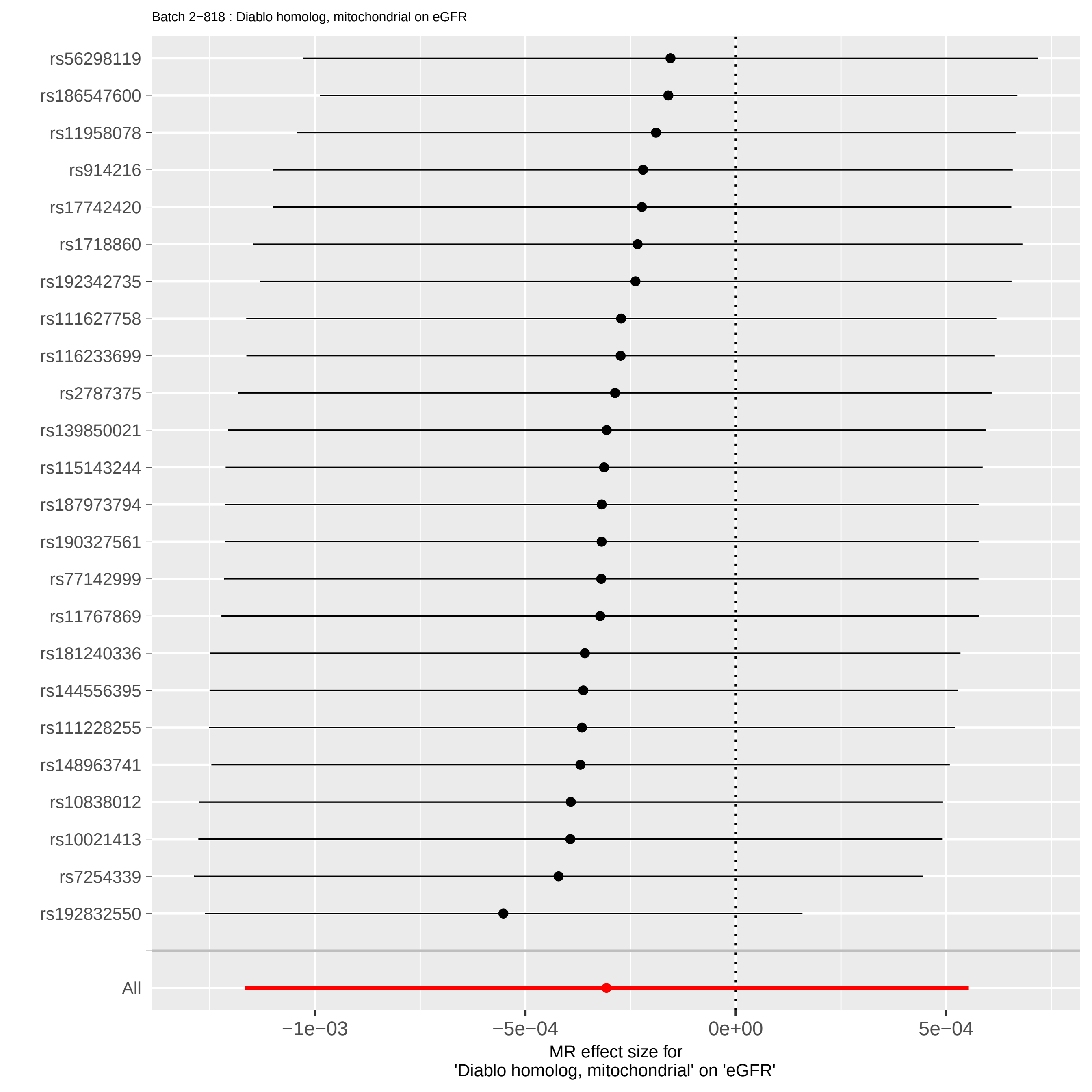

Batch 2−818 : Diablo homolog, mitochondrial on eGFR
rs56298119
rs186547600
rs11958078
rs914216
rs17742420
rs1718860
rs192342735
rs111627758
rs116233699
rs2787375
rs139850021
rs115143244
rs187973794
rs190327561
rs77142999
rs11767869
rs181240336
rs144556395
rs111228255
rs148963741
rs10838012
rs10021413
rs7254339
rs192832550
All
−1e−03
−5e−04
0e+00
5e−04
MR effect size for
'Diablo homolog, mitochondrial' on 'eGFR'

Batch 2−819 : Probable dimethyladenosine transferase on eGFR

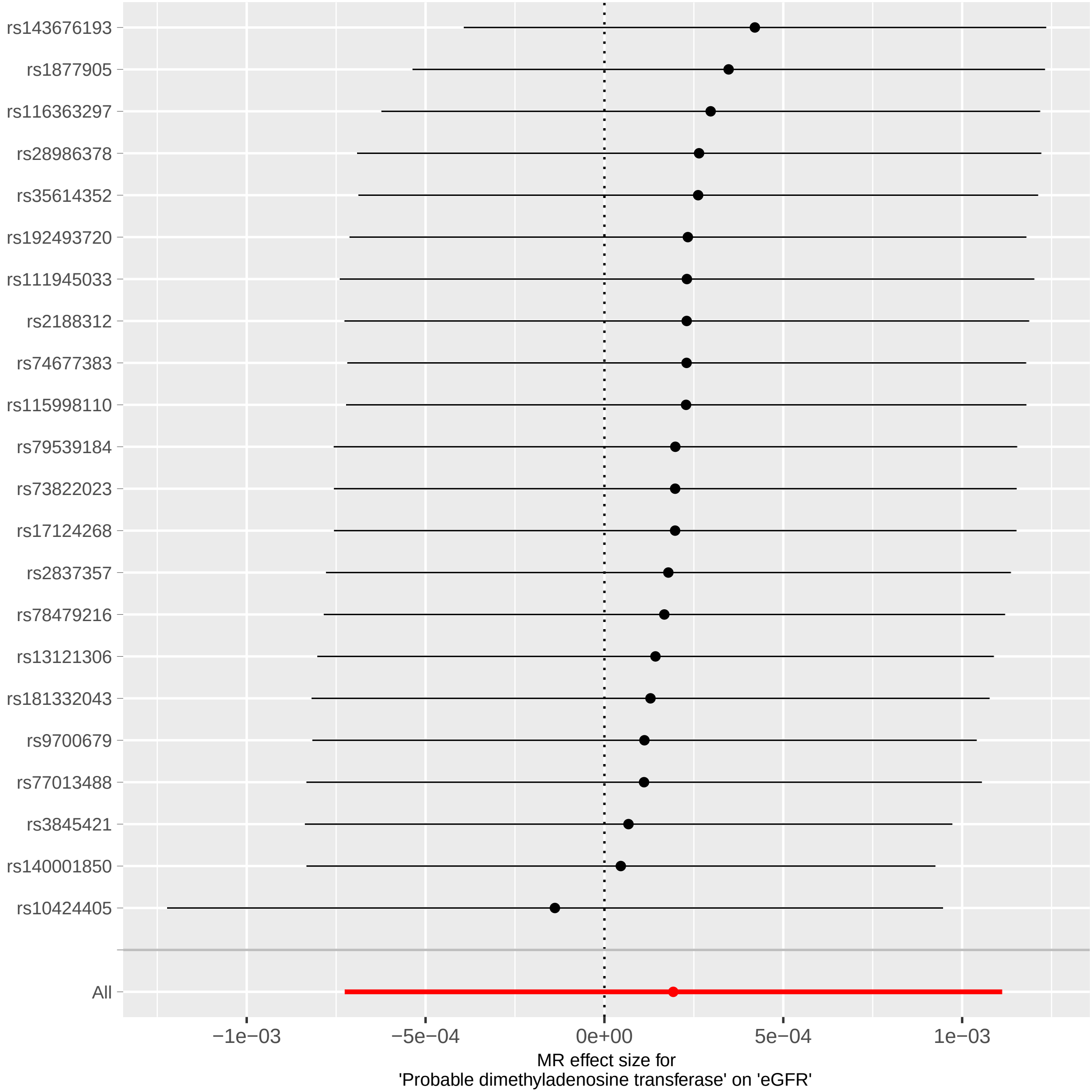

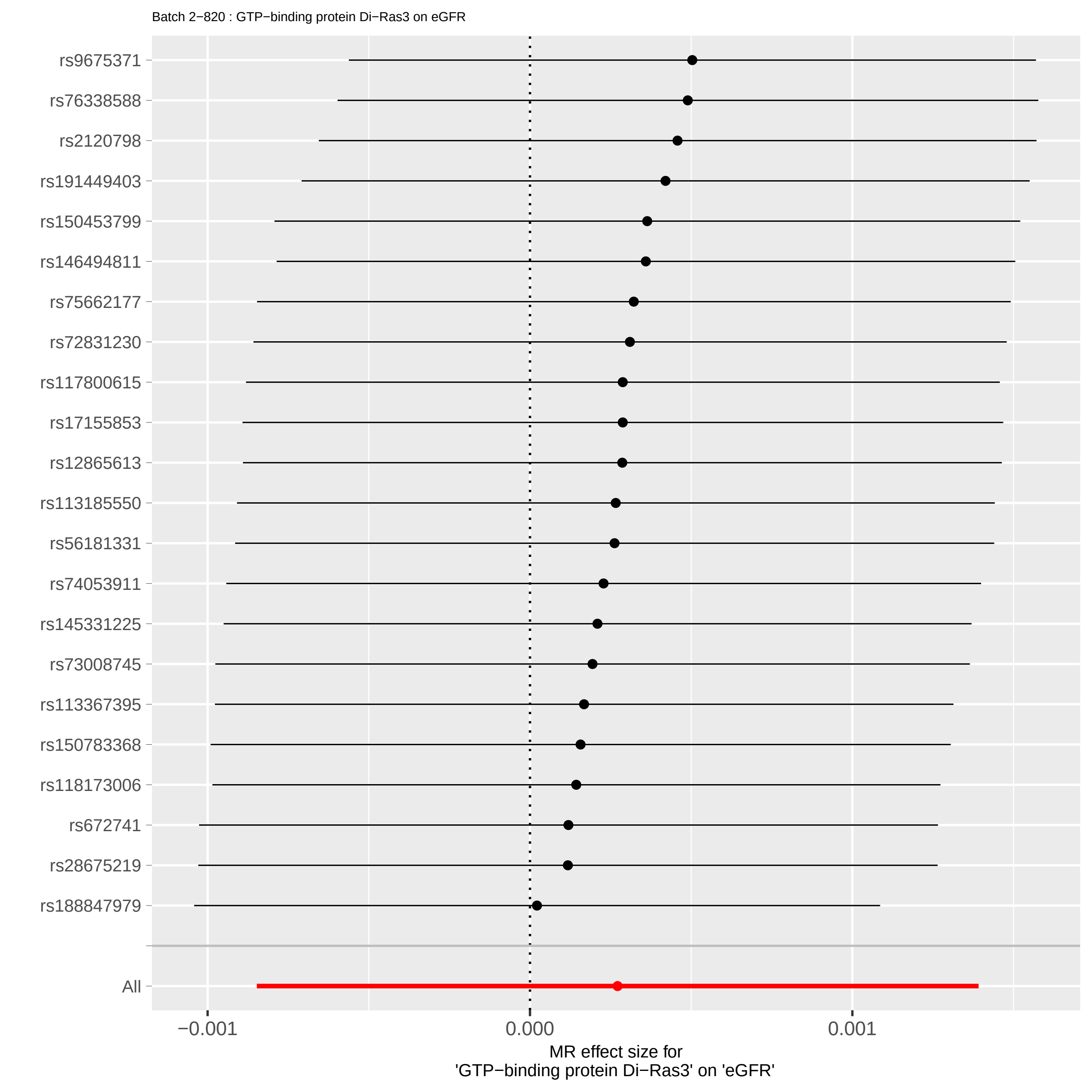

Batch 2−820 : GTP−binding protein Di−Ras3 on eGFR
rs9675371
rs76338588
rs2120798
rs191449403
rs150453799
rs146494811
rs75662177
rs72831230
rs117800615
rs17155853
rs12865613
rs113185550
rs56181331
rs74053911
rs145331225
rs73008745
rs113367395
rs150783368
rs118173006
rs672741
rs28675219
rs188847979
All
−0.001
0.000
0.001
MR effect size for
'GTP−binding protein Di−Ras3' on 'eGFR'

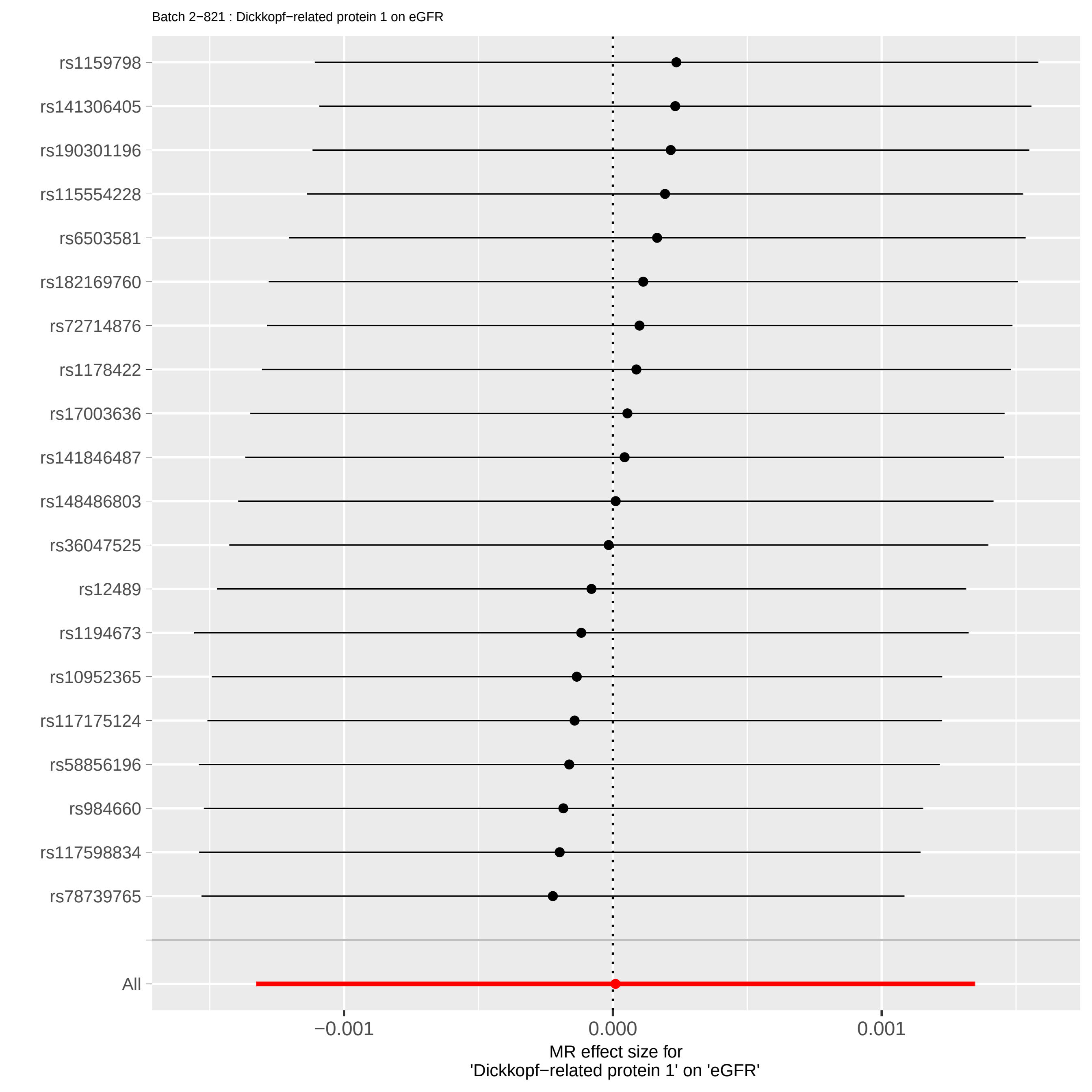

Batch 2−821 : Dickkopf−related protein 1 on eGFR
rs1159798
rs141306405
rs190301196
rs115554228
rs6503581
rs182169760
rs72714876
rs1178422
rs17003636
rs141846487
rs148486803
rs36047525
rs12489
rs1194673
rs10952365
rs117175124
rs58856196
rs984660
rs117598834
rs78739765
All
−0.001
0.000
0.001
MR effect size for
'Dickkopf−related protein 1' on 'eGFR'

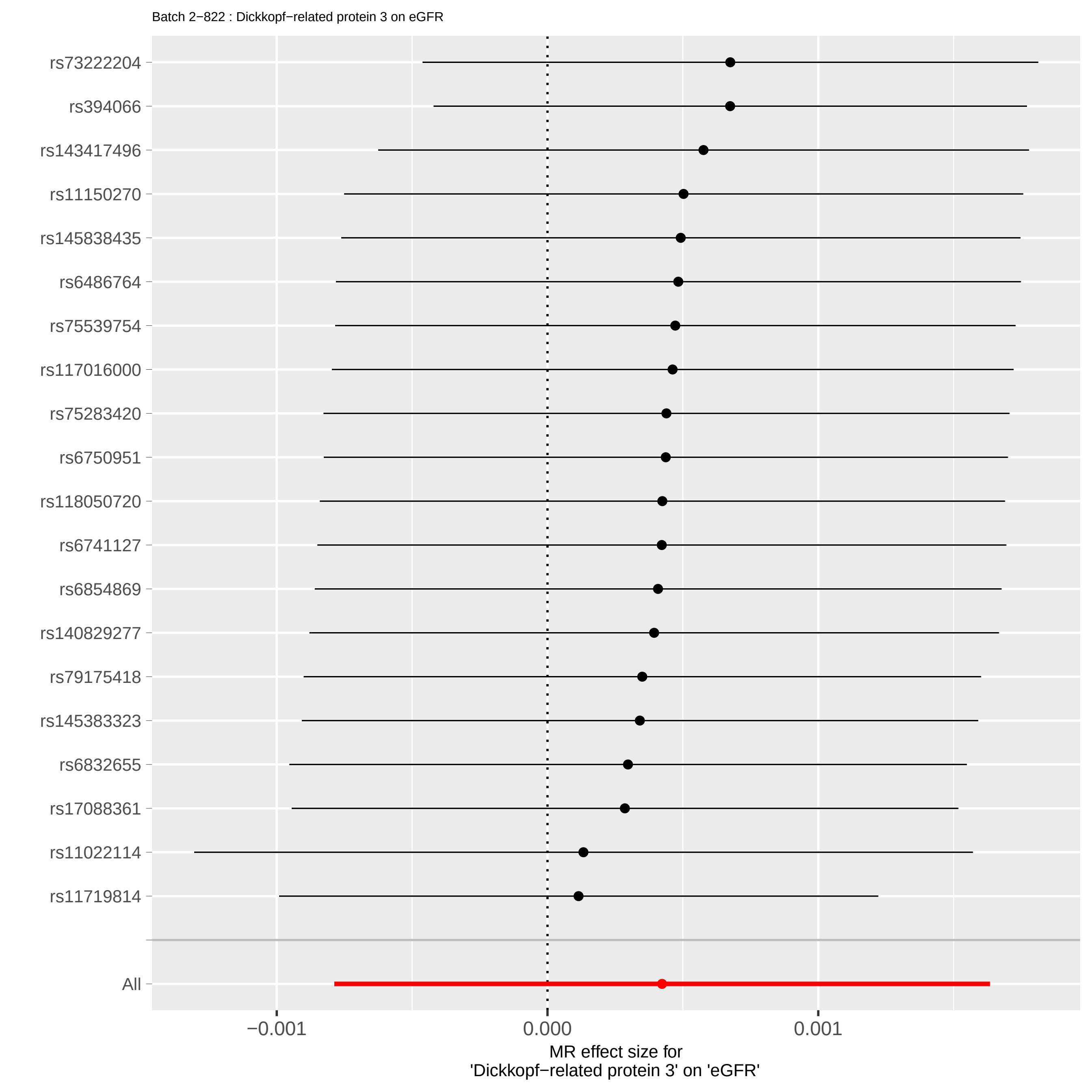

Batch 2−822 : Dickkopf−related protein 3 on eGFR
rs73222204
rs394066
rs143417496
rs11150270
rs145838435
rs6486764
rs75539754
rs117016000
rs75283420
rs6750951
rs118050720
rs6741127
rs6854869
rs140829277
rs79175418
rs145383323
rs6832655
rs17088361
rs11022114
rs11719814
All
−0.001
0.000
0.001
MR effect size for
'Dickkopf−related protein 3' on 'eGFR'

Batch 2−823 : Dickkopf−related protein 4 on eGFR

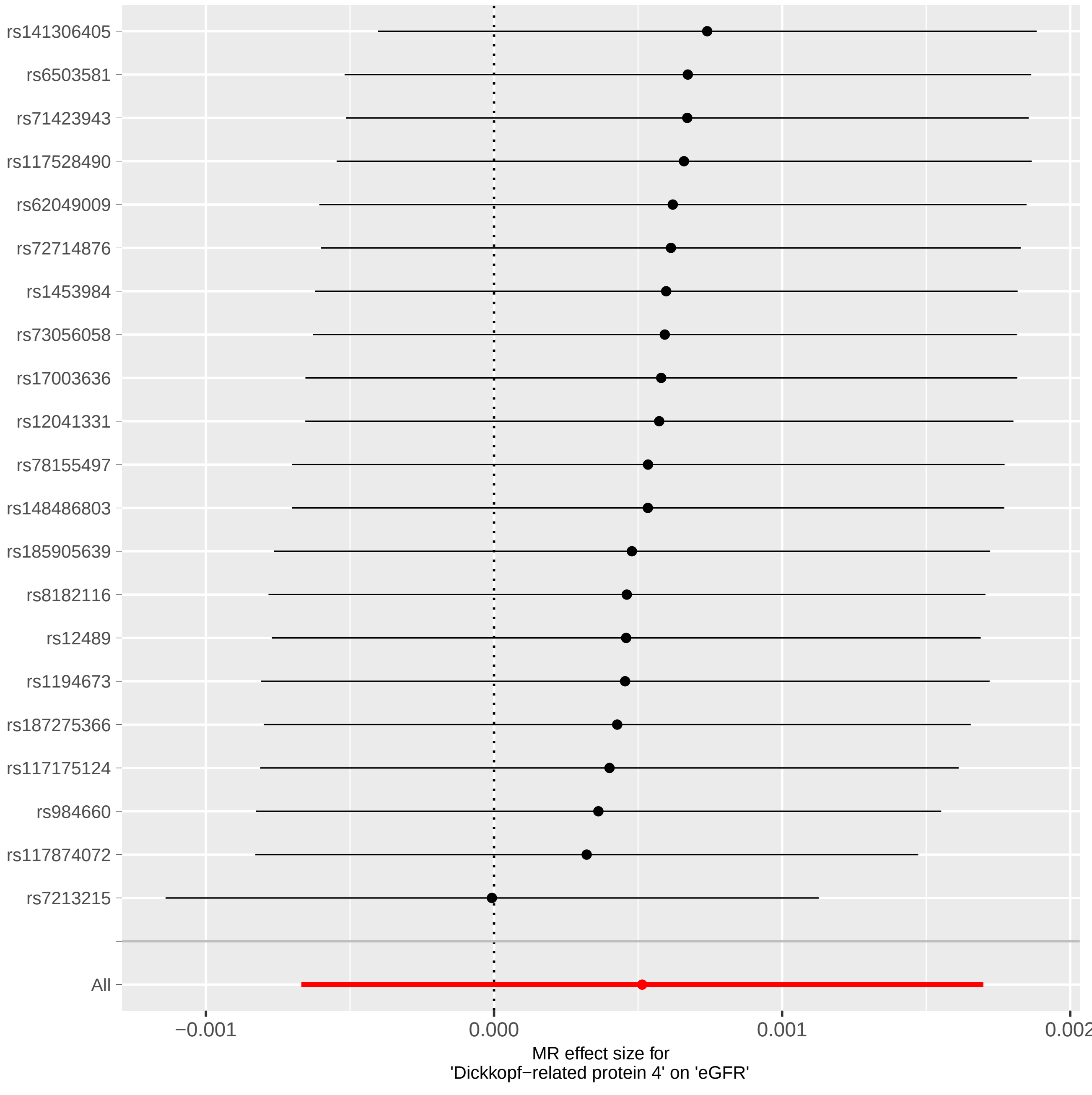

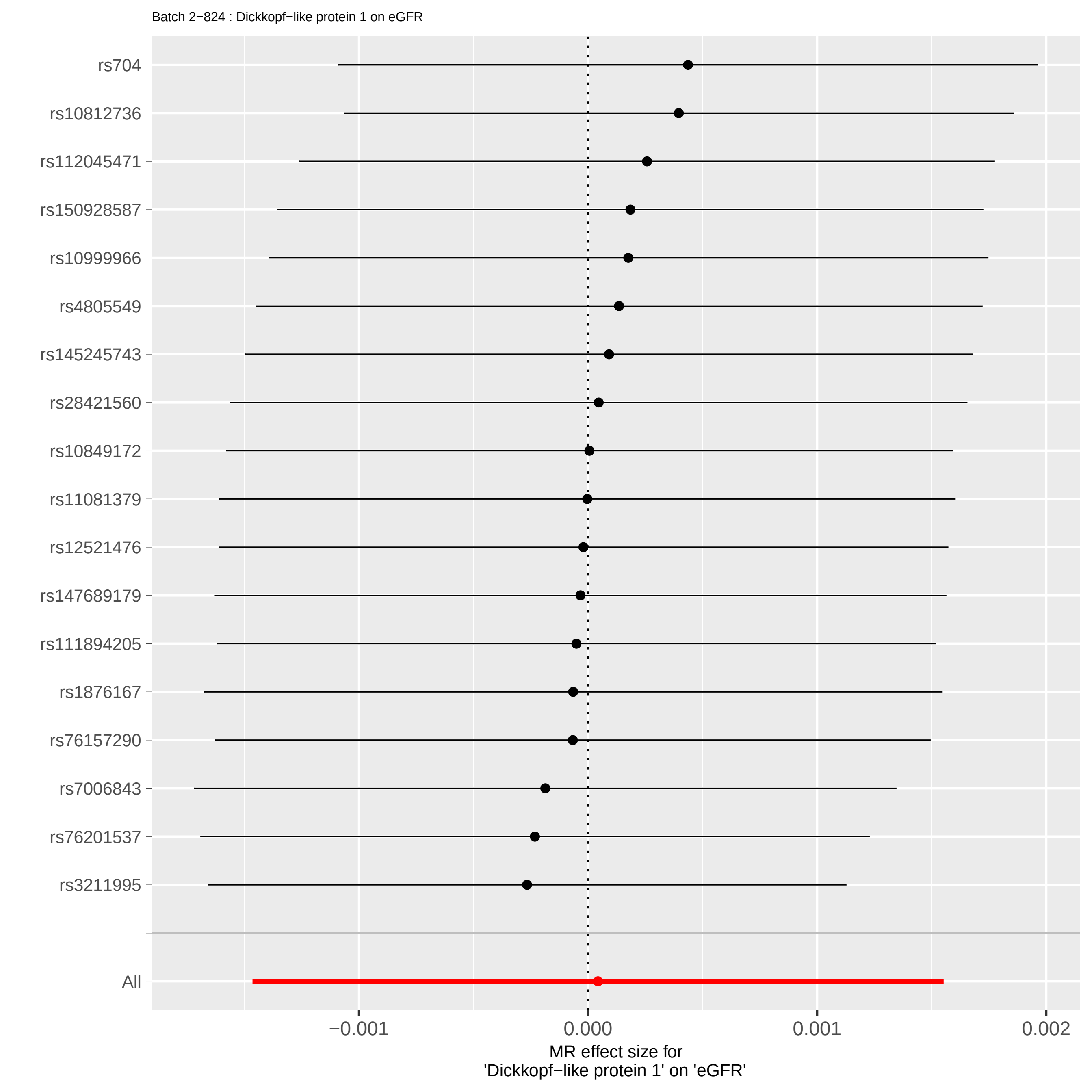

Batch 2−824 : Dickkopf−like protein 1 on eGFR
rs704
rs10812736
rs112045471
rs150928587
rs10999966
rs4805549
rs145245743
rs28421560
rs10849172
rs11081379
rs12521476
rs147689179
rs111894205
rs1876167
rs76157290
rs7006843
rs76201537
rs3211995
All
−0.001
0.000
0.001
0.002
MR effect size for
'Dickkopf−like protein 1' on 'eGFR'

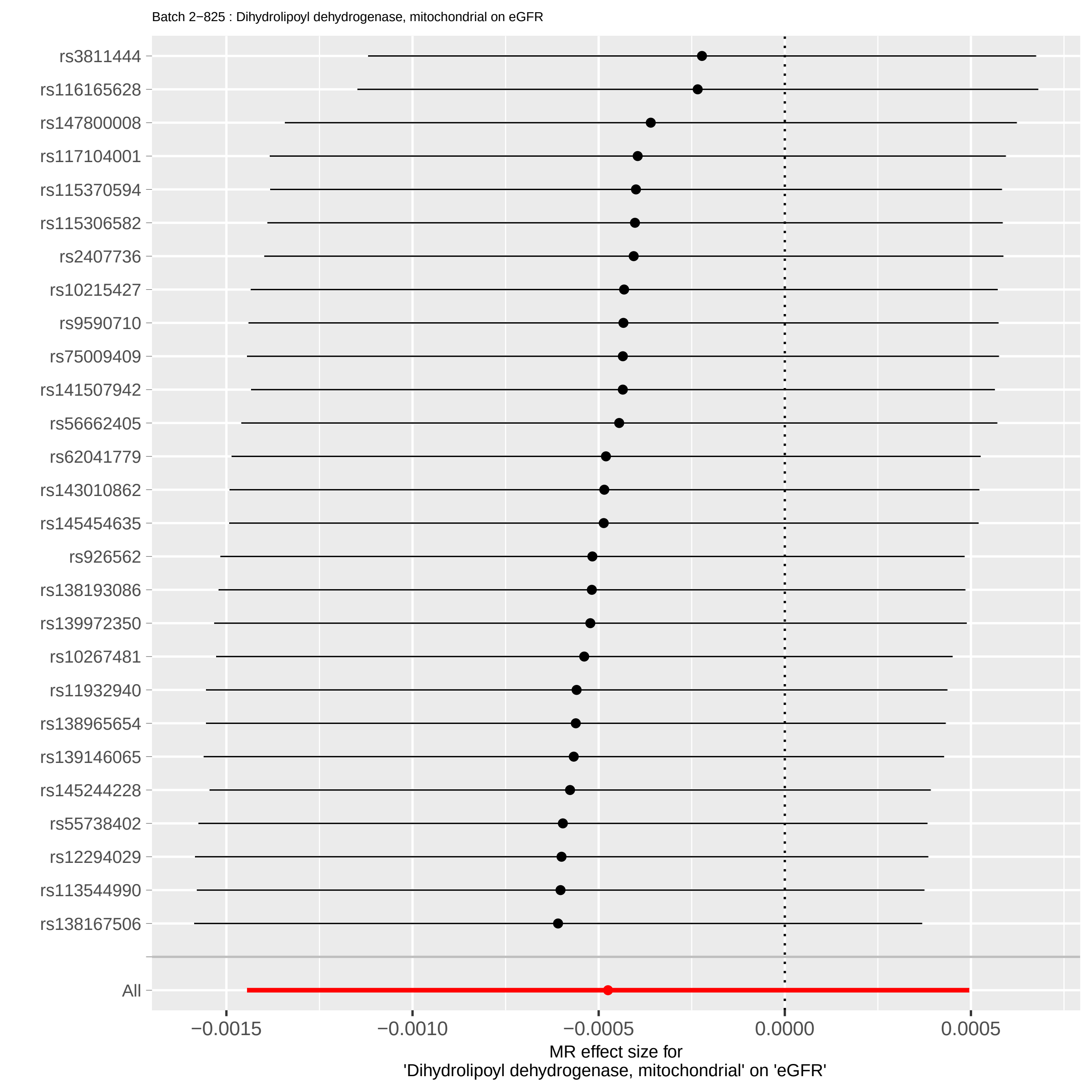

Batch 2−825 : Dihydrolipoyl dehydrogenase, mitochondrial on eGFR
rs3811444
rs116165628
rs147800008
rs117104001
rs115370594
rs115306582
rs2407736
rs10215427
rs9590710
rs75009409
rs141507942
rs56662405
rs62041779
rs143010862
rs145454635
rs926562
rs138193086
rs139972350
rs10267481
rs11932940
rs138965654
rs139146065
rs145244228
rs55738402
rs12294029
rs113544990
rs138167506
All
−0.0015
−0.0010
−0.0005
0.0000
0.0005
MR effect size for
'Dihydrolipoyl dehydrogenase, mitochondrial' on 'eGFR'

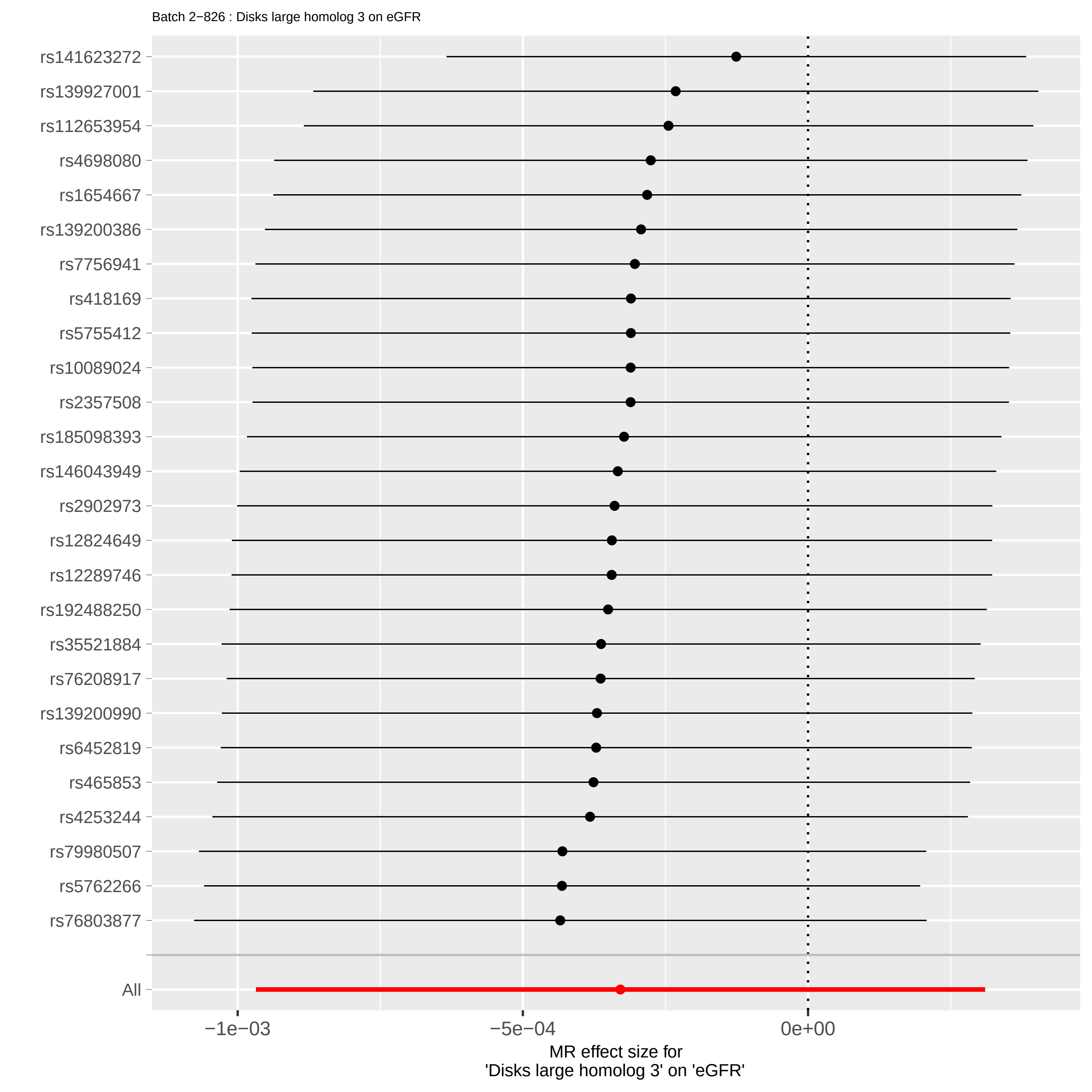

Batch 2−826 : Disks large homolog 3 on eGFR
rs141623272
rs139927001
rs112653954
rs4698080
rs1654667
rs139200386
rs7756941
rs418169
rs5755412
rs10089024
rs2357508
rs185098393
rs146043949
rs2902973
rs12824649
rs12289746
rs192488250
rs35521884
rs76208917
rs139200990
rs6452819
rs465853
rs4253244
rs79980507
rs5762266
rs76803877
All
−1e−03
−5e−04
0e+00
MR effect size for
'Disks large homolog 3' on 'eGFR'

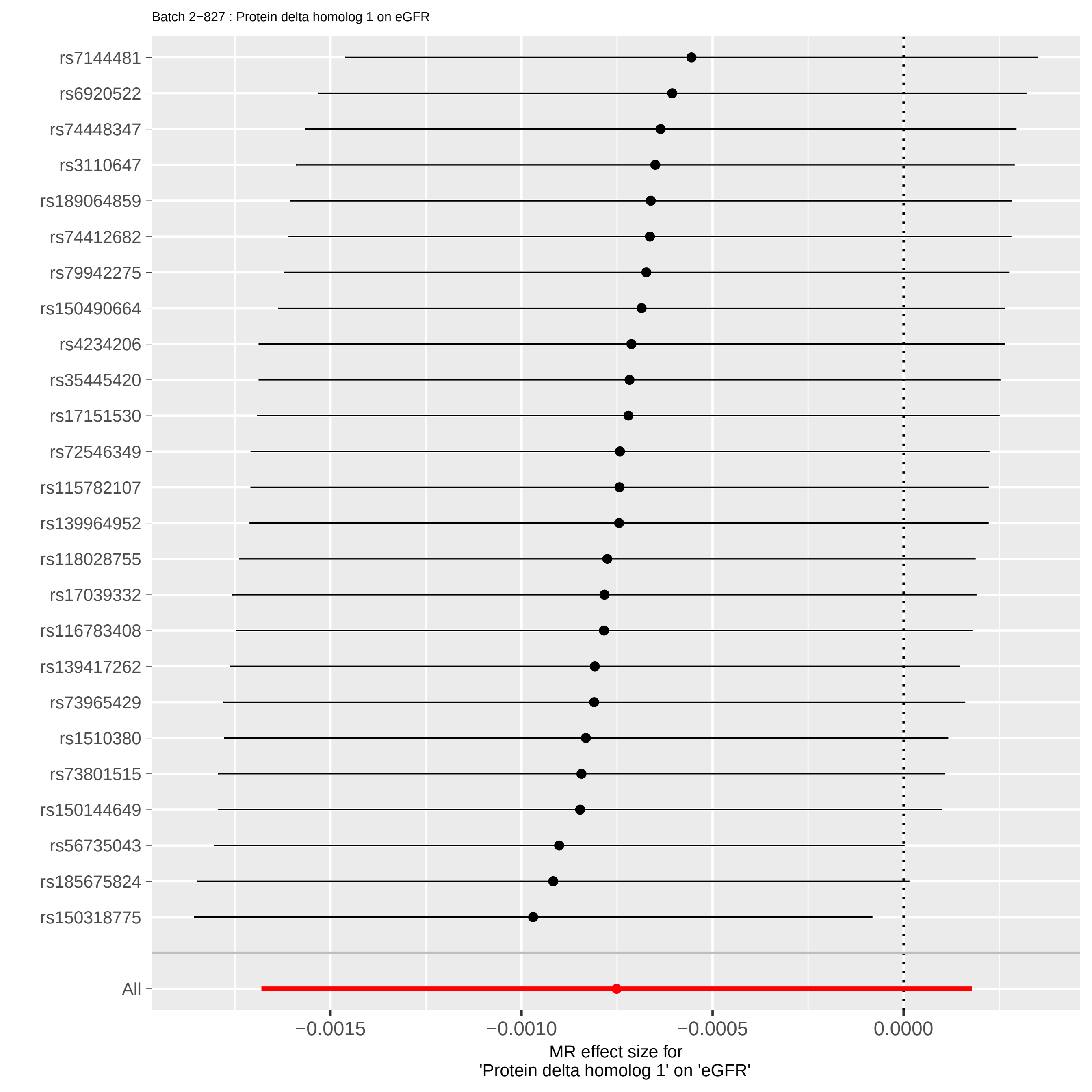
Batch 2−827 : Protein delta homolog 1 on eGFR
rs7144481
rs6920522
rs74448347
rs3110647
rs189064859
rs74412682
rs79942275
rs150490664
rs4234206
rs35445420
rs17151530
rs72546349
rs115782107
rs139964952
rs118028755
rs17039332
rs116783408
rs139417262
rs73965429
rs1510380
rs73801515
rs150144649
rs56735043
rs185675824
rs150318775
All
−0.0015
−0.0010
−0.0005
0.0000
MR effect size for
'Protein delta homolog 1' on 'eGFR'

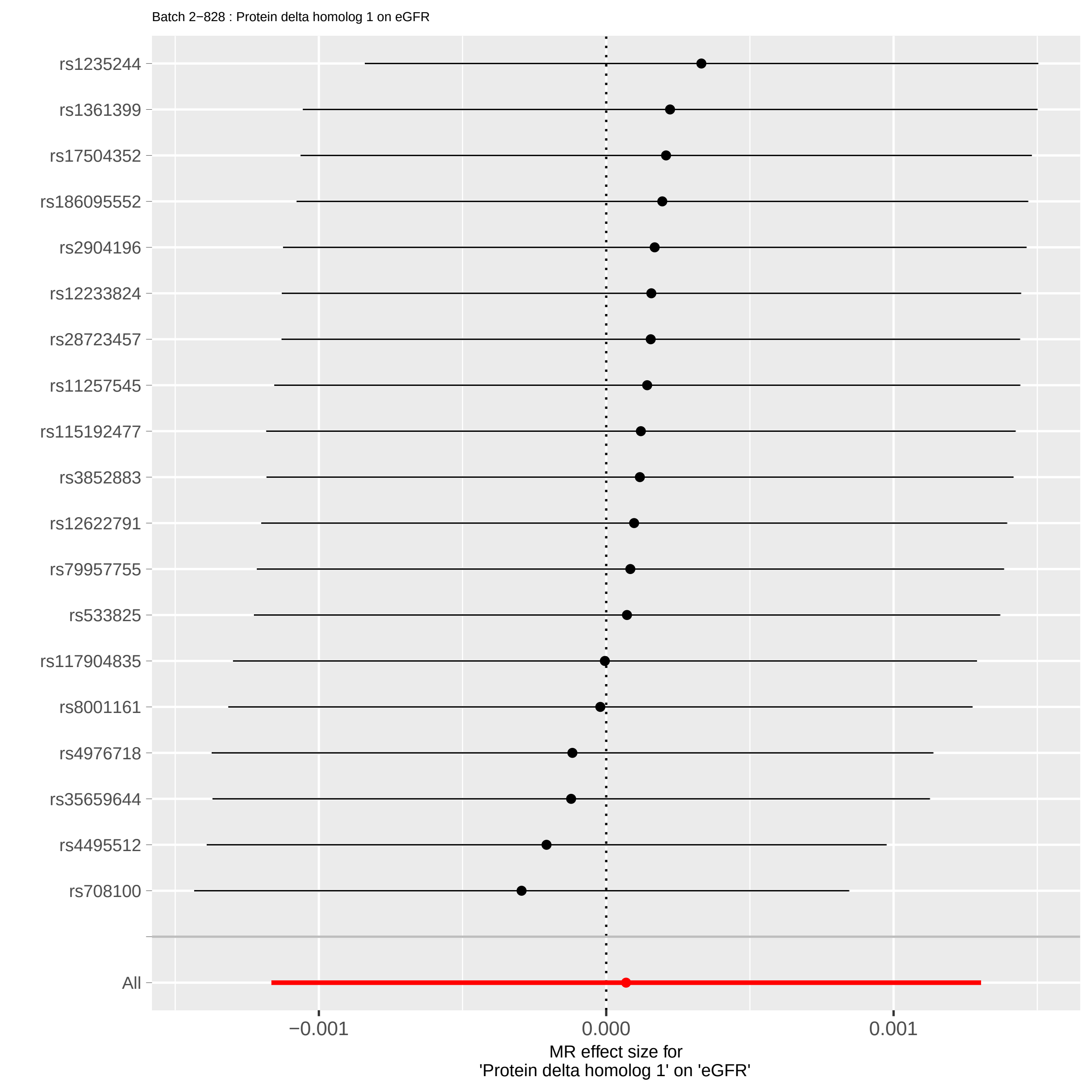

Batch 2−828 : Protein delta homolog 1 on eGFR
rs1235244
rs1361399
rs17504352
rs186095552
rs2904196
rs12233824
rs28723457
rs11257545
rs115192477
rs3852883
rs12622791
rs79957755
rs533825
rs117904835
rs8001161
rs4976718
rs35659644
rs4495512
rs708100
All
−0.001
0.000
0.001
MR effect size for
'Protein delta homolog 1' on 'eGFR'

Batch 2−829 : Protein delta homolog 2 on eGFR

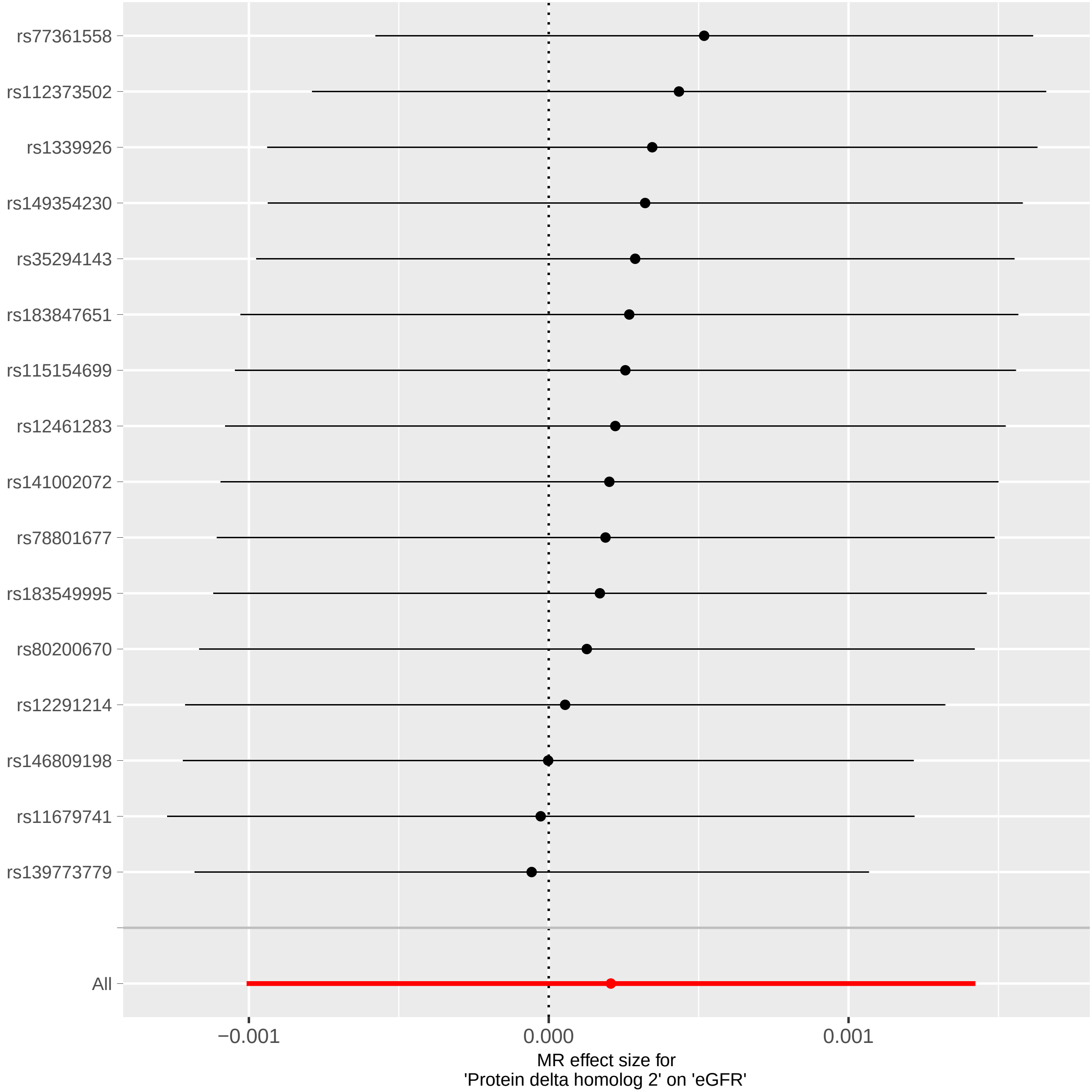

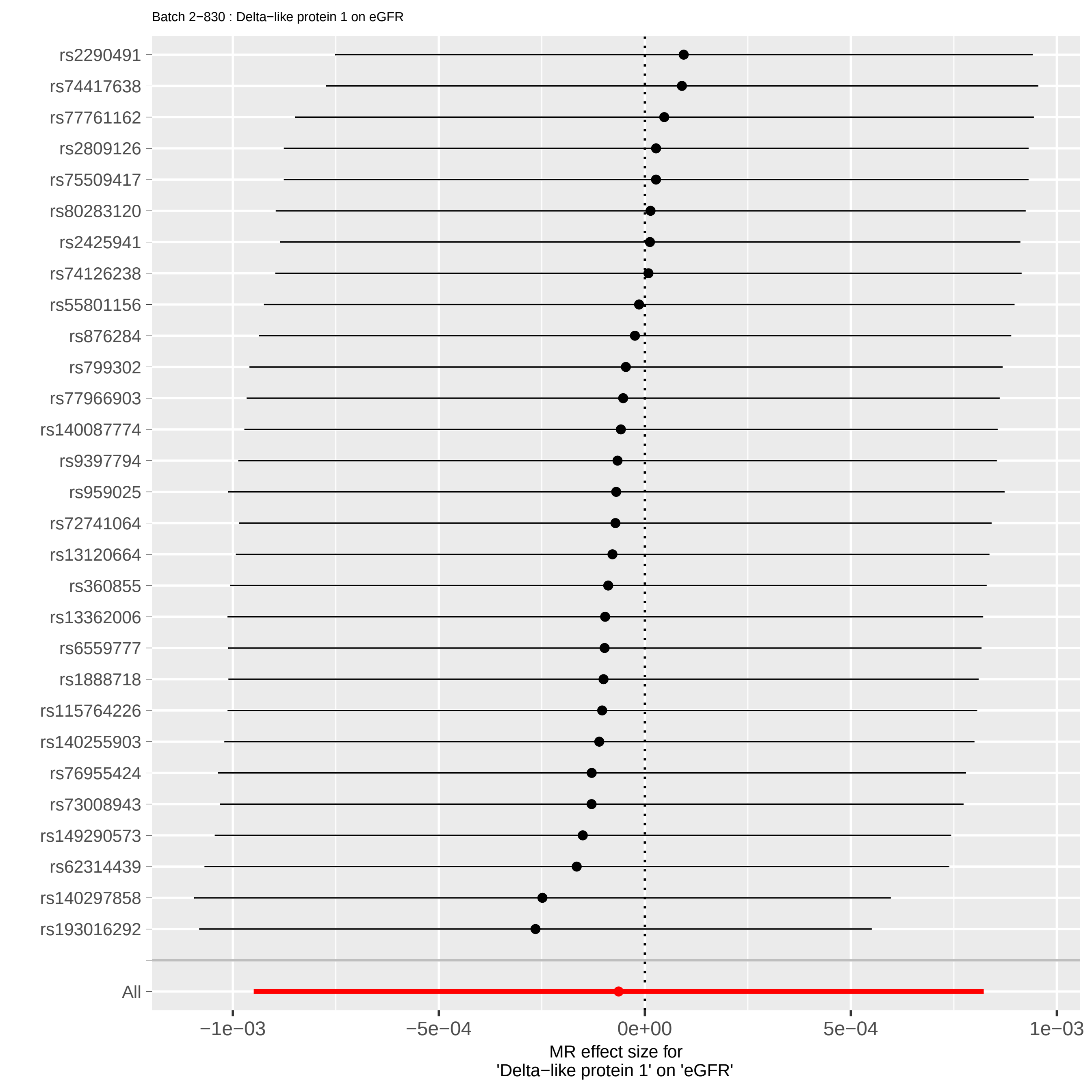

Batch 2−830 : Delta−like protein 1 on eGFR
rs2290491
rs74417638
rs77761162
rs2809126
rs75509417
rs80283120
rs2425941
rs74126238
rs55801156
rs876284
rs799302
rs77966903
rs140087774
rs9397794
rs959025
rs72741064
rs13120664
rs360855
rs13362006
rs6559777
rs1888718
rs115764226
rs140255903
rs76955424
rs73008943
rs149290573
rs62314439
rs140297858
rs193016292
All
−1e−03
−5e−04
0e+00
5e−04
1e−03
MR effect size for
'Delta−like protein 1' on 'eGFR'

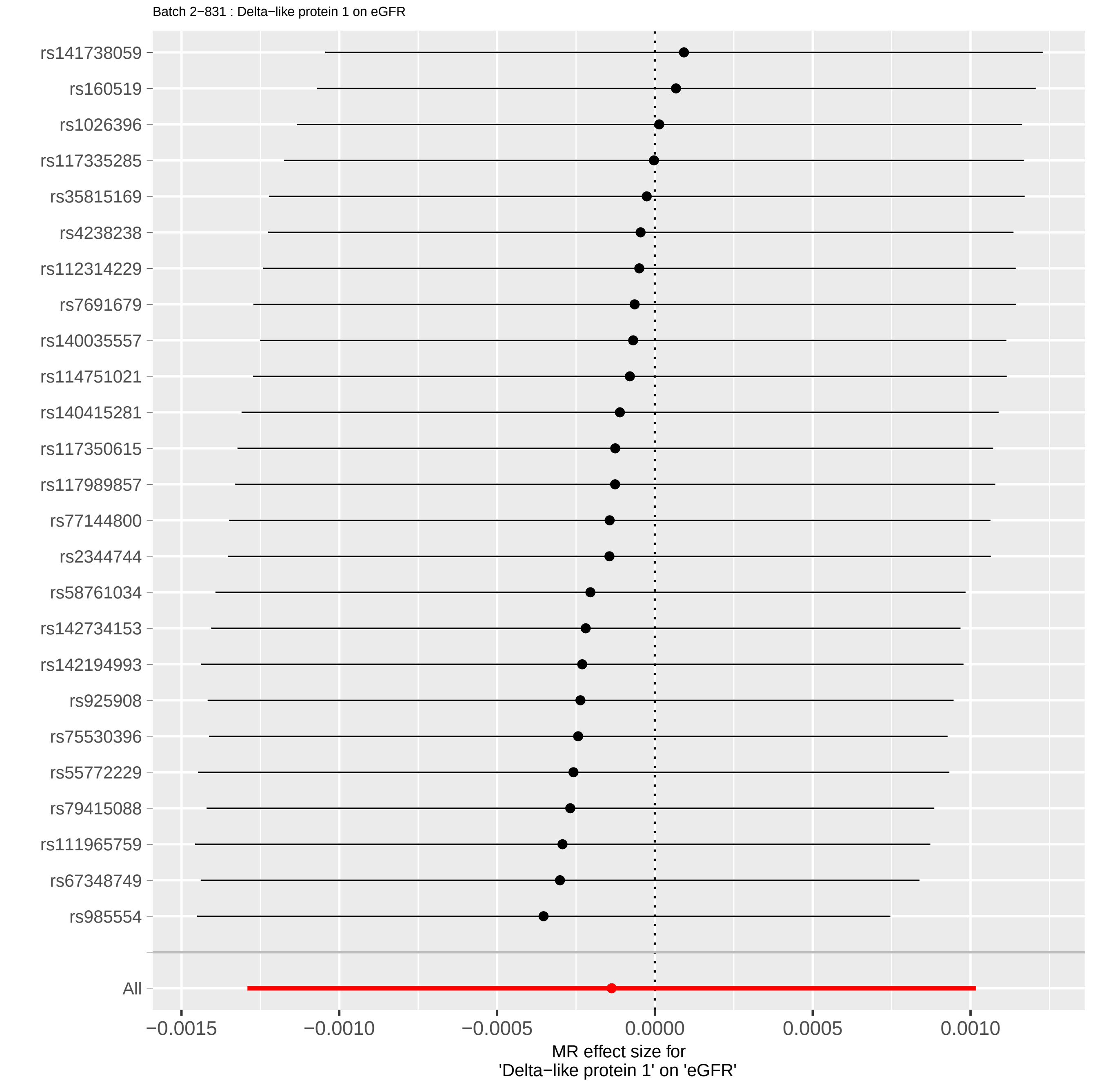

Batch 2−831 : Delta−like protein 1 on eGFR
rs141738059
rs160519
rs1026396
rs117335285
rs35815169
rs4238238
rs112314229
rs7691679
rs140035557
rs114751021
rs140415281
rs117350615
rs117989857
rs77144800
rs2344744
rs58761034
rs142734153
rs142194993
rs925908
rs75530396
rs55772229
rs79415088
rs111965759
rs67348749
rs985554
All
−0.0015
−0.0010
−0.0005
0.0000
0.0005
0.0010
MR effect size for
'Delta−like protein 1' on 'eGFR'

Batch 2−832 : Delta−like protein 4 on eGFR

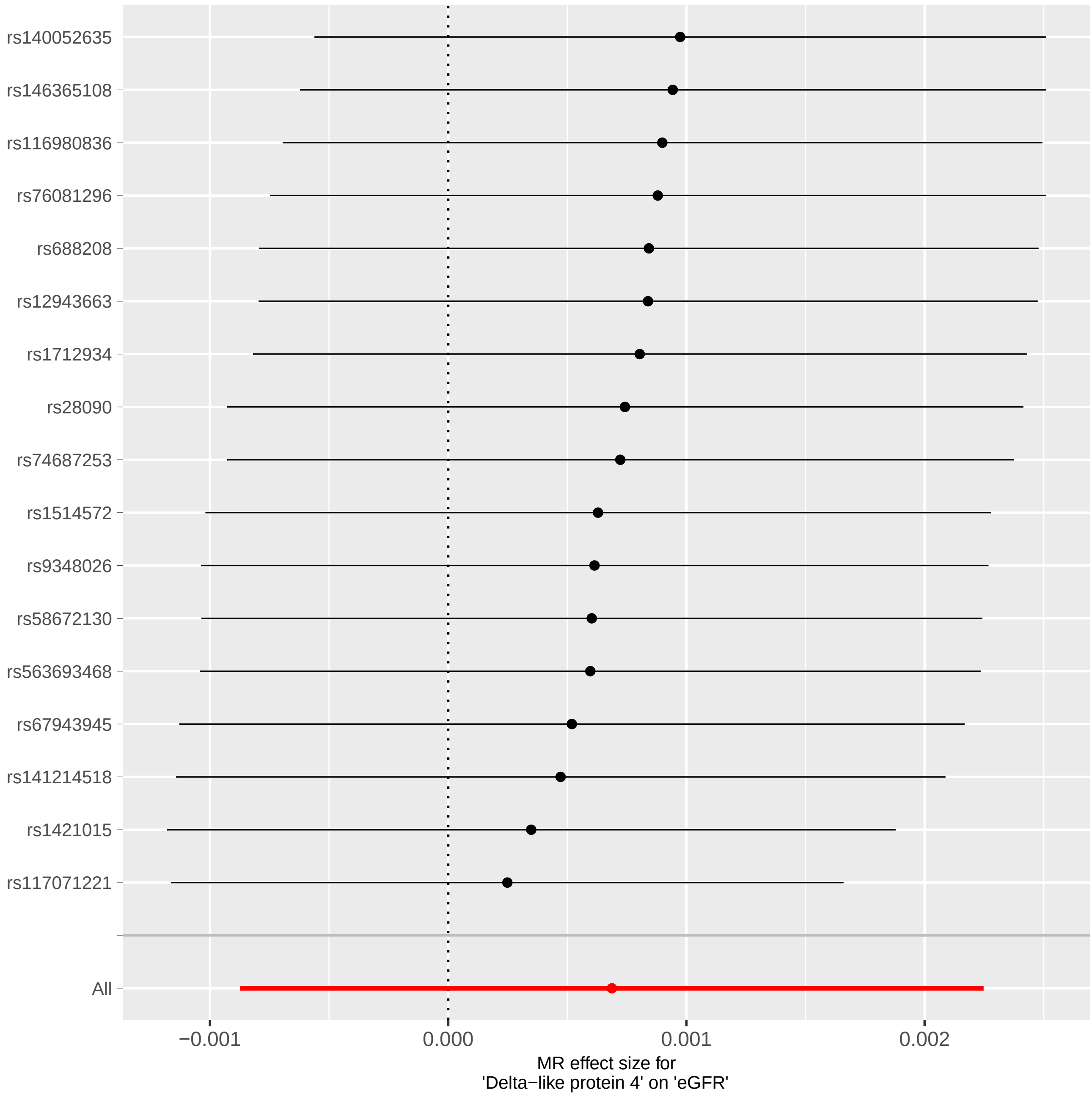

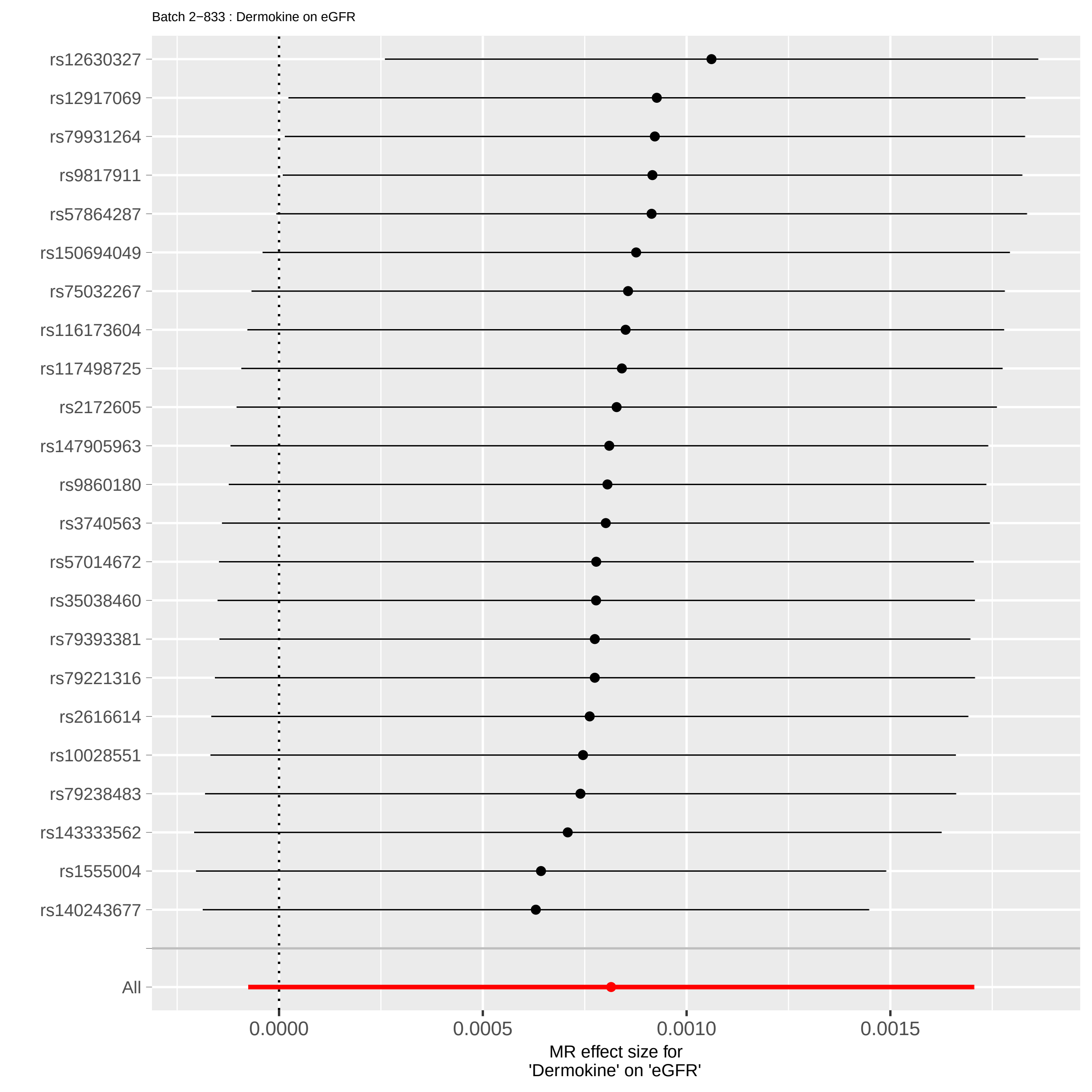
Batch 2−833 : Dermokine on eGFR
rs12630327
rs12917069
rs79931264
rs9817911
rs57864287
rs150694049
rs75032267
rs116173604
rs117498725
rs2172605
rs147905963
rs9860180
rs3740563
rs57014672
rs35038460
rs79393381
rs79221316
rs2616614
rs10028551
rs79238483
rs143333562
rs1555004
rs140243677
All
0.0000
0.0005
0.0010
0.0015
MR effect size for
'Dermokine' on 'eGFR'

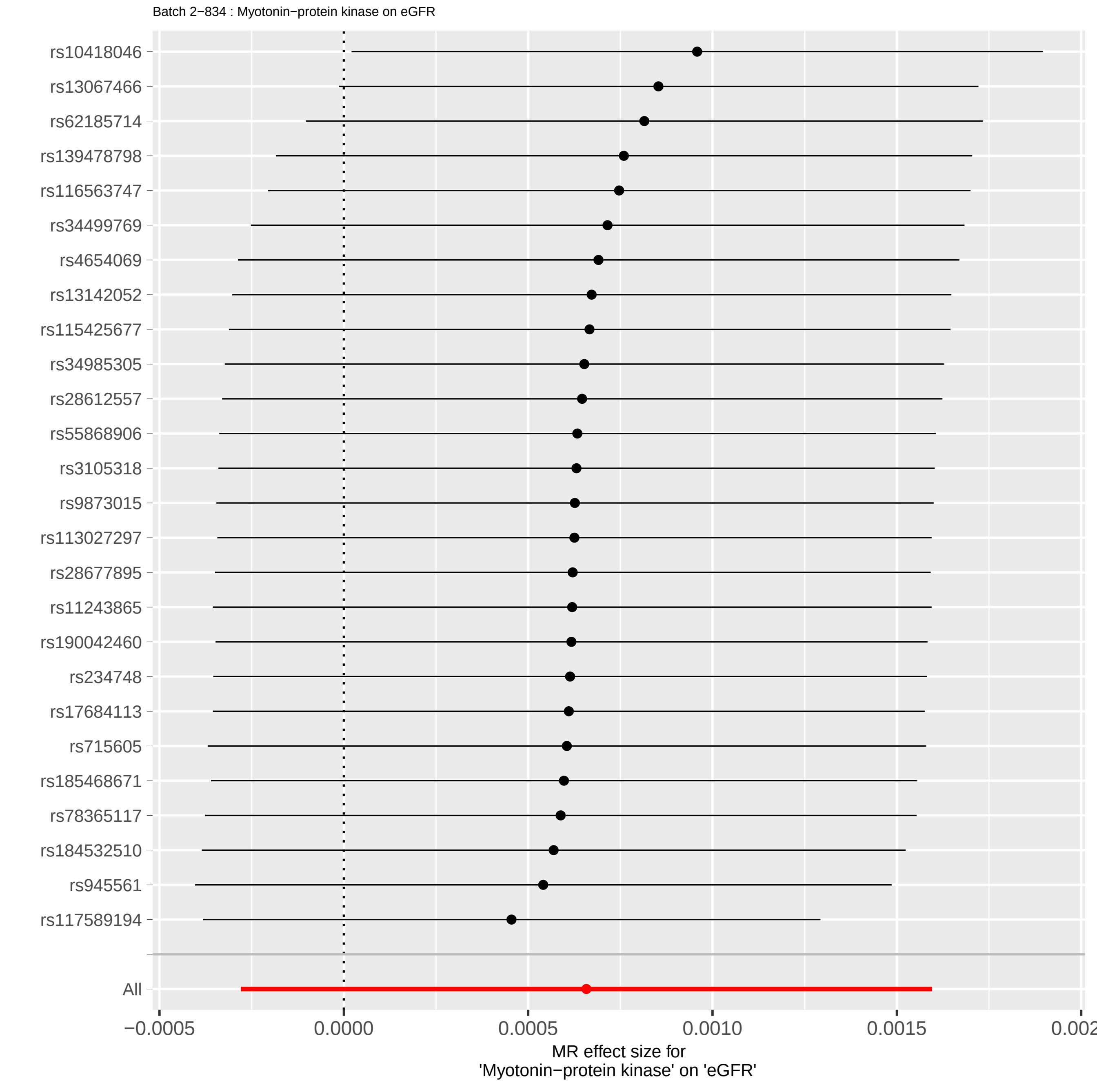

Batch 2−834 : Myotonin−protein kinase on eGFR
rs10418046
rs13067466
rs62185714
rs139478798
rs116563747
rs34499769
rs4654069
rs13142052
rs115425677
rs34985305
rs28612557
rs55868906
rs3105318
rs9873015
rs113027297
rs28677895
rs11243865
rs190042460
rs234748
rs17684113
rs715605
rs185468671
rs78365117
rs184532510
rs945561
rs117589194
All
−0.0005
0.0000
0.0005
0.0010
0.0015
0.002
MR effect size for
'Myotonin−protein kinase' on 'eGFR'

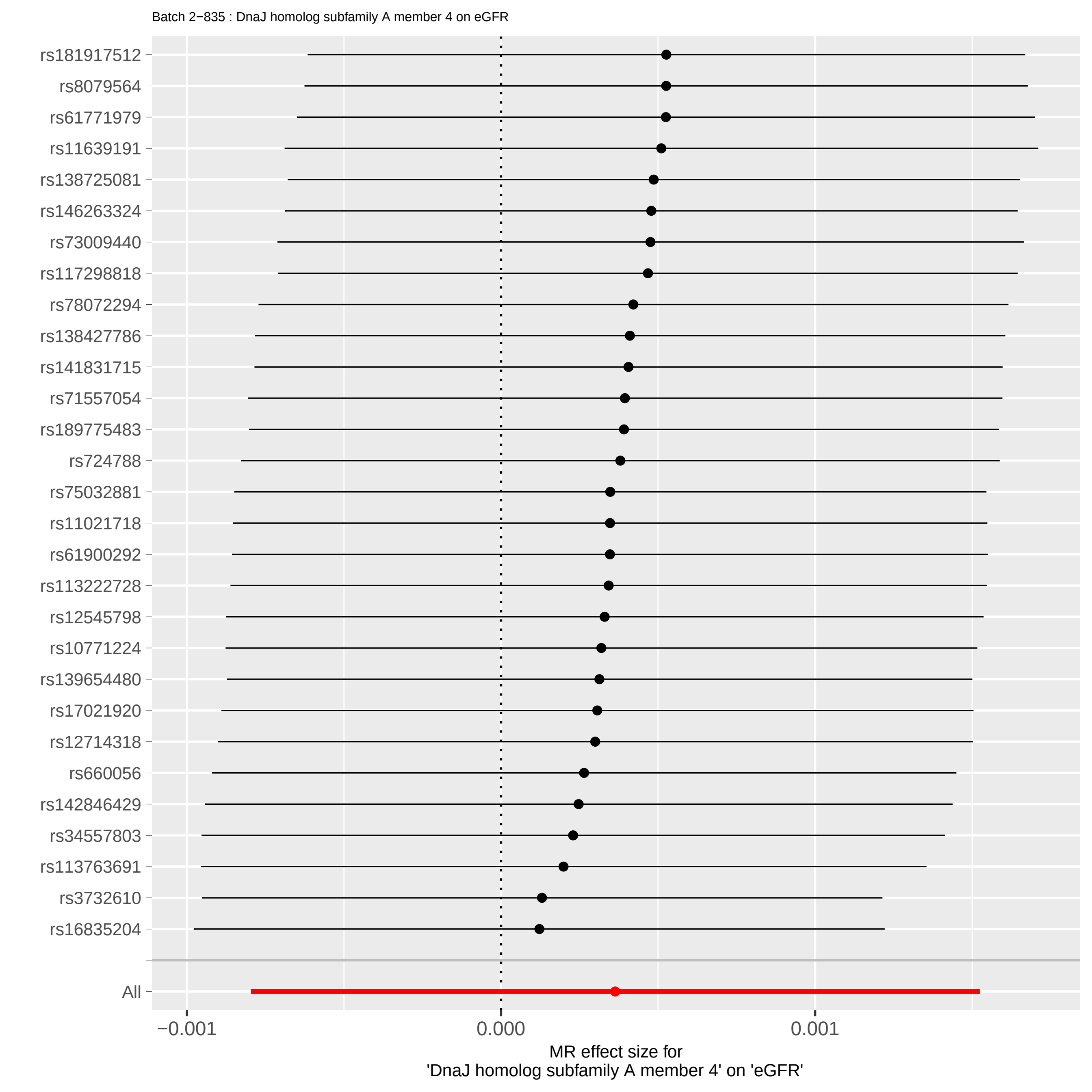

Batch 2−835 : DnaJ homolog subfamily A member 4 on eGFR
rs181917512
rs8079564
rs61771979
rs11639191
rs138725081
rs146263324
rs73009440
rs117298818
rs78072294
rs138427786
rs141831715
rs71557054
rs189775483
rs724788
rs75032881
rs11021718
rs61900292
rs113222728
rs12545798
rs10771224
rs139654480
rs17021920
rs12714318
rs660056
rs142846429
rs34557803
rs113763691
rs3732610
rs16835204
All
−0.001
0.000
0.001
MR effect size for
'DnaJ homolog subfamily A member 4' on 'eGFR'

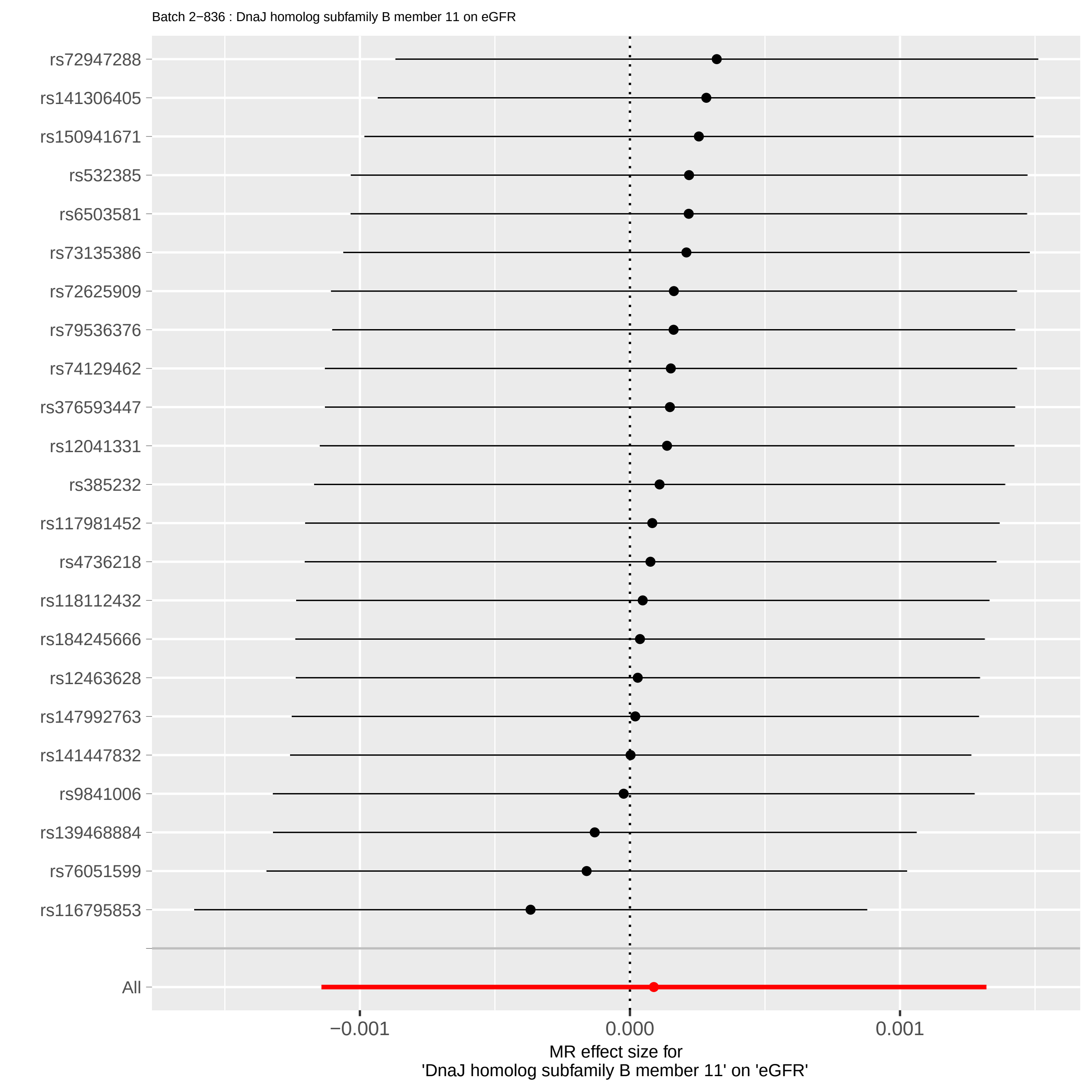
Batch 2−836 : DnaJ homolog subfamily B member 11 on eGFR
rs72947288
rs141306405
rs150941671
rs532385
rs6503581
rs73135386
rs72625909
rs79536376
rs74129462
rs376593447
rs12041331
rs385232
rs117981452
rs4736218
rs118112432
rs184245666
rs12463628
rs147992763
rs141447832
rs9841006
rs139468884
rs76051599
rs116795853
All
−0.001
0.000
0.001
MR effect size for
'DnaJ homolog subfamily B member 11' on 'eGFR'

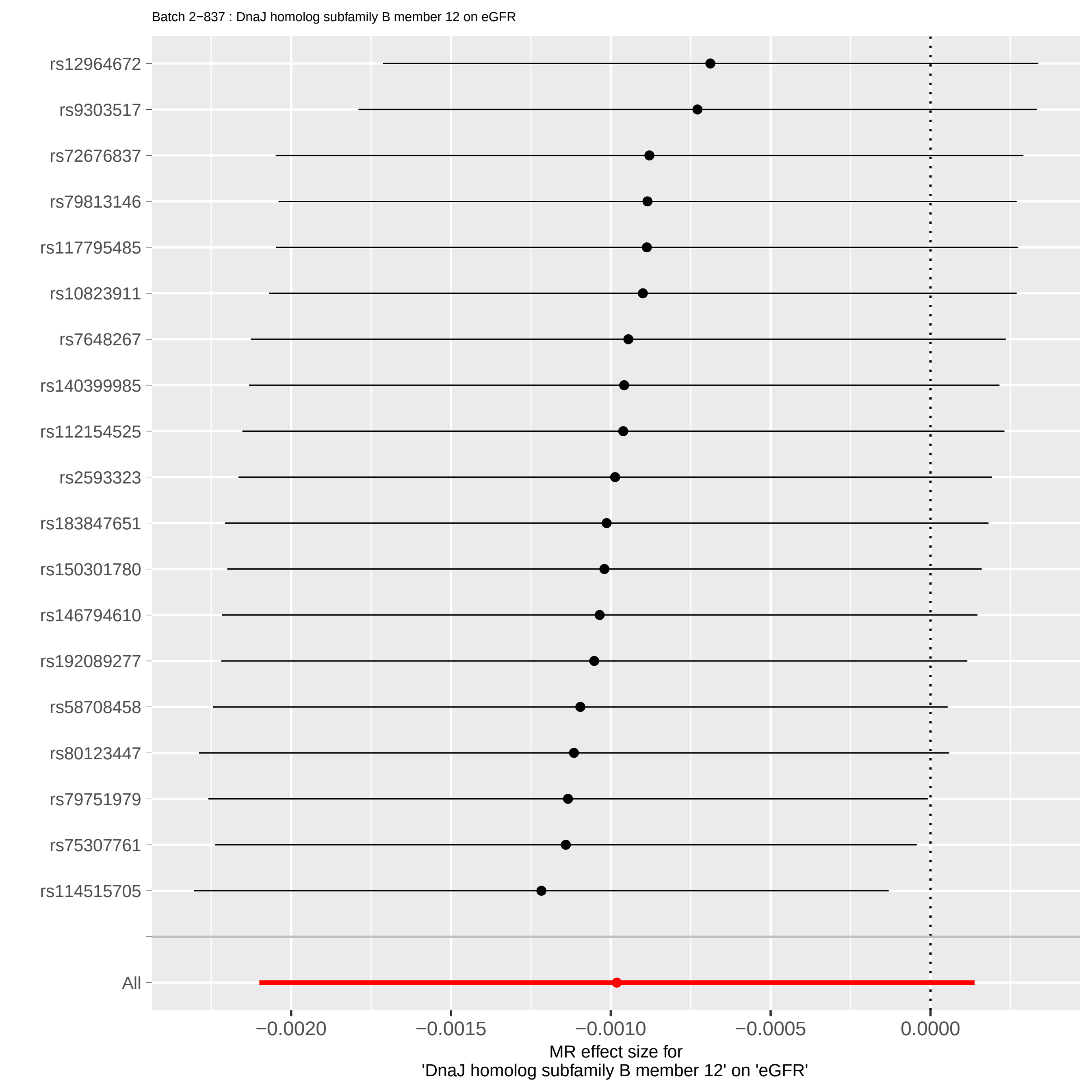
Batch 2−837 : DnaJ homolog subfamily B member 12 on eGFR
rs12964672
rs9303517
rs72676837
rs79813146
rs117795485
rs10823911
rs7648267
rs140399985
rs112154525
rs2593323
rs183847651
rs150301780
rs146794610
rs192089277
rs58708458
rs80123447
rs79751979
rs75307761
rs114515705
All
−0.0020
−0.0015
−0.0010
−0.0005
0.0000
MR effect size for
'DnaJ homolog subfamily B member 12' on 'eGFR'

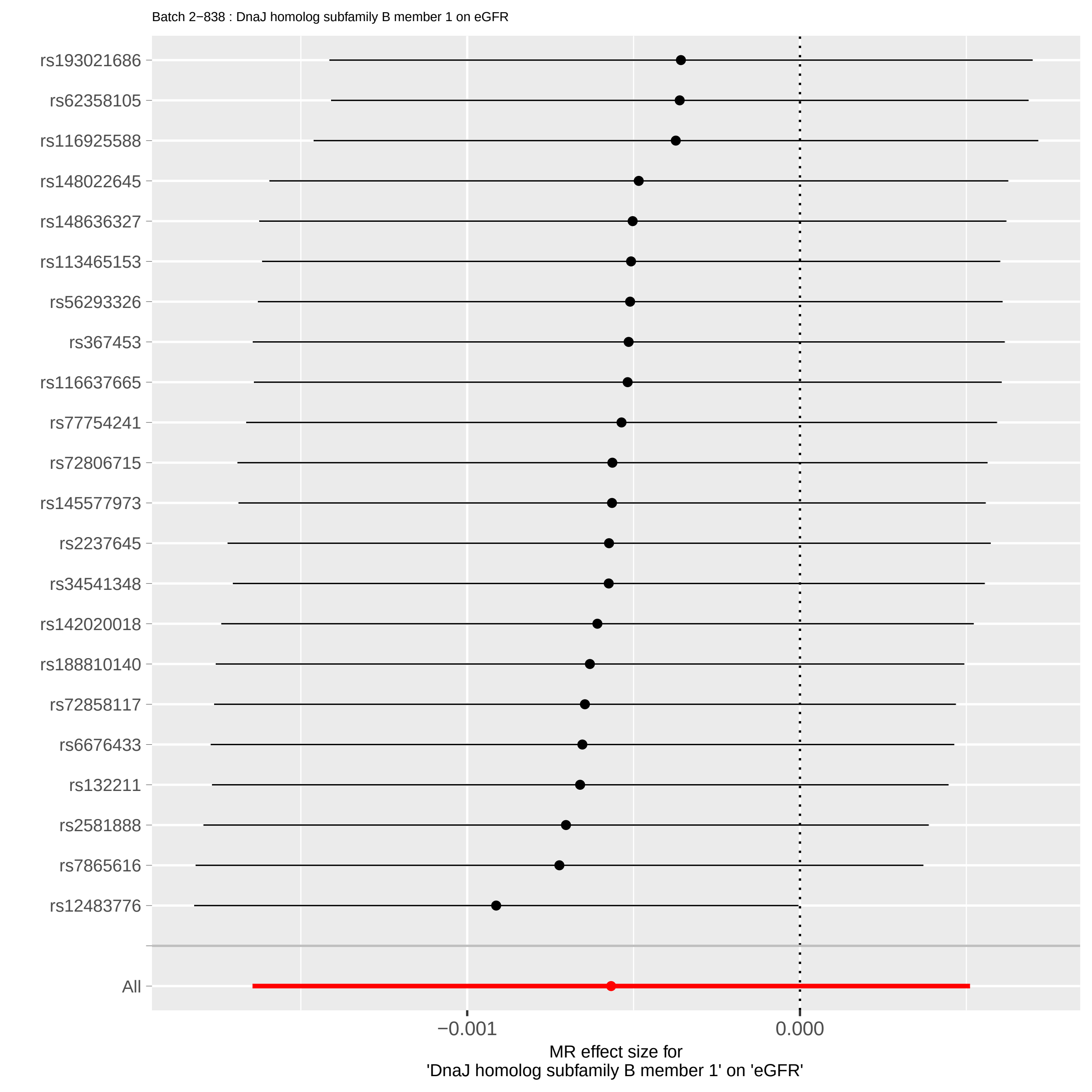
Batch 2−838 : DnaJ homolog subfamily B member 1 on eGFR
rs193021686
rs62358105
rs116925588
rs148022645
rs148636327
rs113465153
rs56293326
rs367453
rs116637665
rs77754241
rs72806715
rs145577973
rs2237645
rs34541348
rs142020018
rs188810140
rs72858117
rs6676433
rs132211
rs2581888
rs7865616
rs12483776
All
−0.001
0.000
MR effect size for
'DnaJ homolog subfamily B member 1' on 'eGFR'

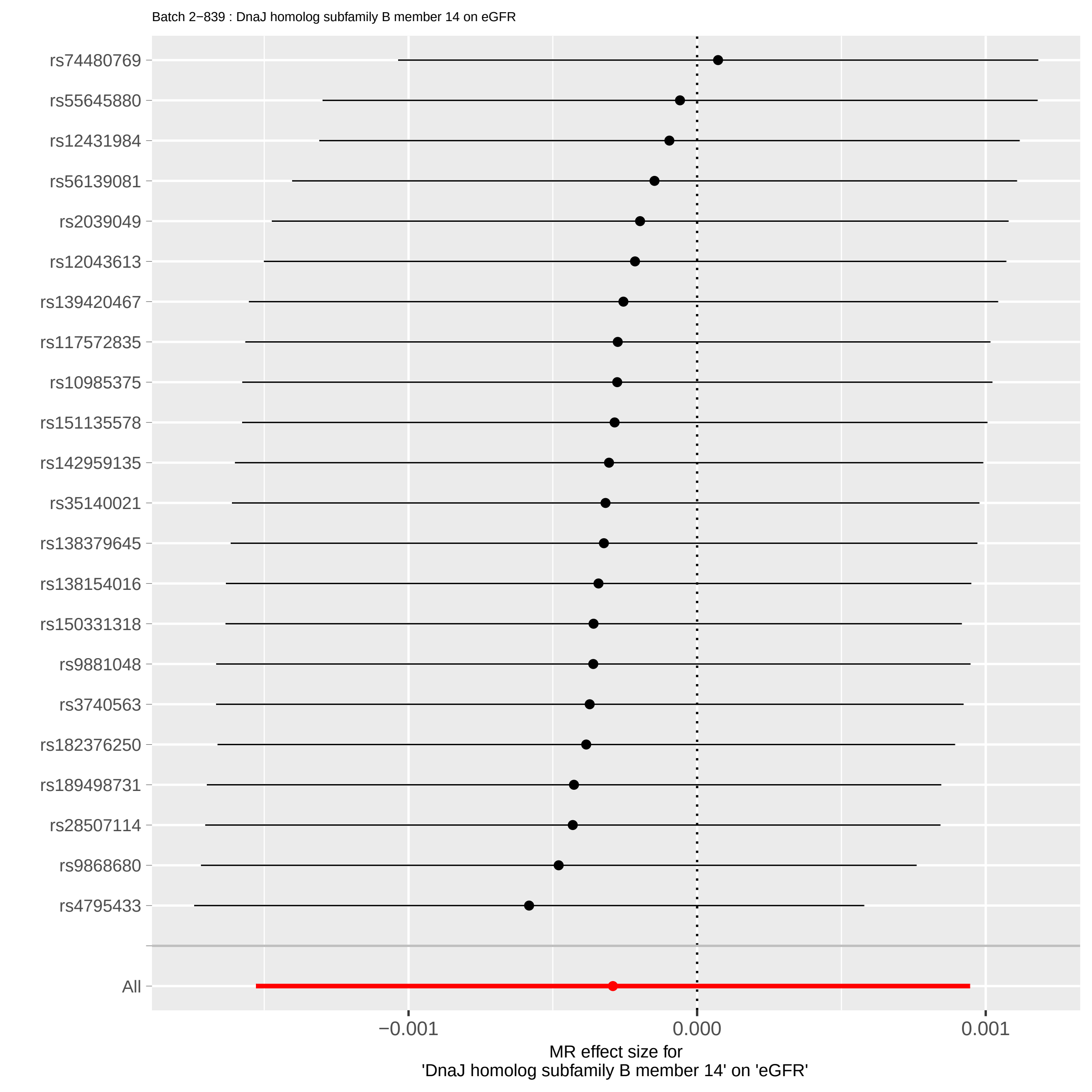
Batch 2−839 : DnaJ homolog subfamily B member 14 on eGFR
rs74480769
rs55645880
rs12431984
rs56139081
rs2039049
rs12043613
rs139420467
rs117572835
rs10985375
rs151135578
rs142959135
rs35140021
rs138379645
rs138154016
rs150331318
rs9881048
rs3740563
rs182376250
rs189498731
rs28507114
rs9868680
rs4795433
All
−0.001
0.000
0.001
MR effect size for
'DnaJ homolog subfamily B member 14' on 'eGFR'

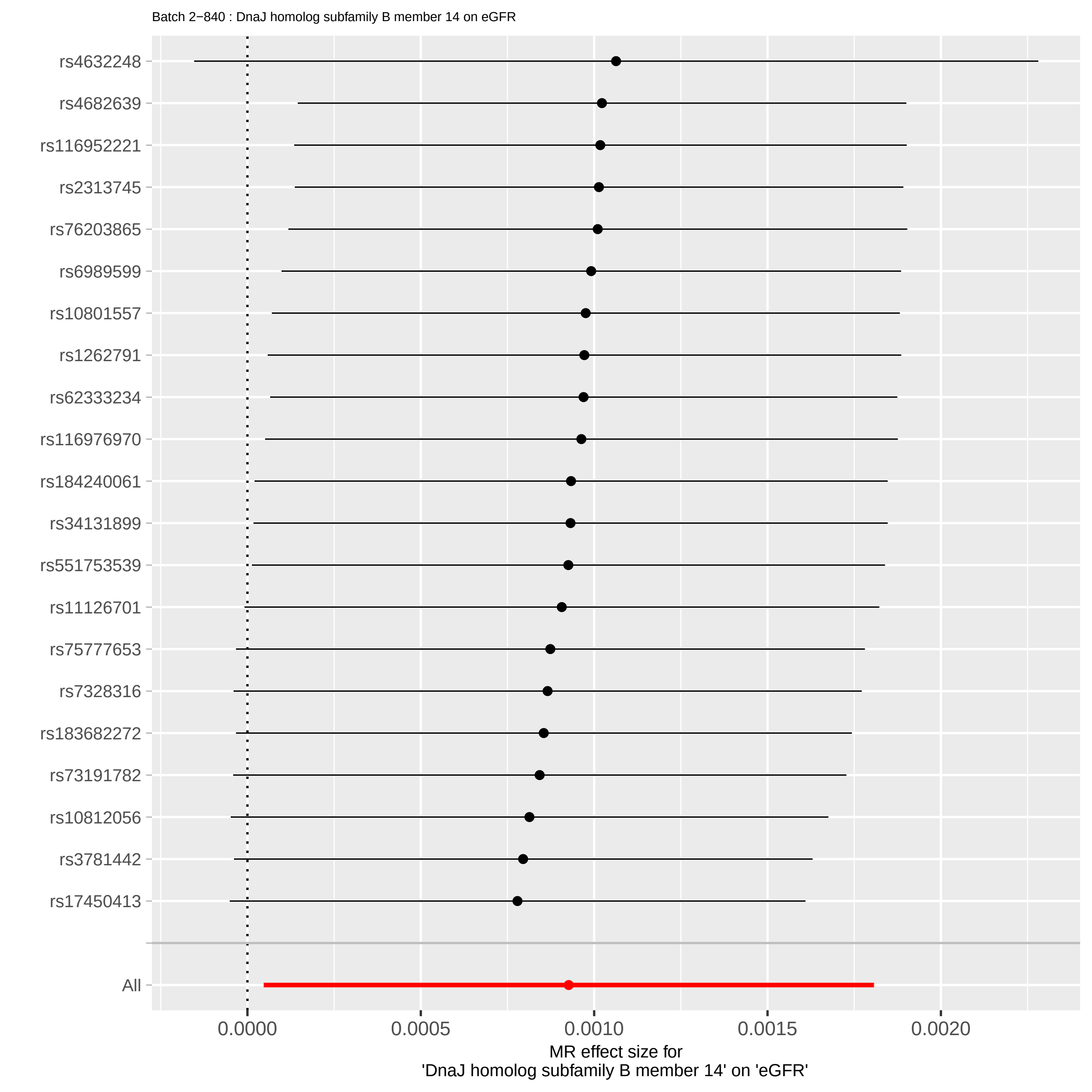
Batch 2−840 : DnaJ homolog subfamily B member 14 on eGFR
rs4632248
rs4682639
rs116952221
rs2313745
rs76203865
rs6989599
rs10801557
rs1262791
rs62333234
rs116976970
rs184240061
rs34131899
rs551753539
rs11126701
rs75777653
rs7328316
rs183682272
rs73191782
rs10812056
rs3781442
rs17450413
All
0.0000
0.0005
0.0010
0.0015
0.0020
MR effect size for
'DnaJ homolog subfamily B member 14' on 'eGFR'

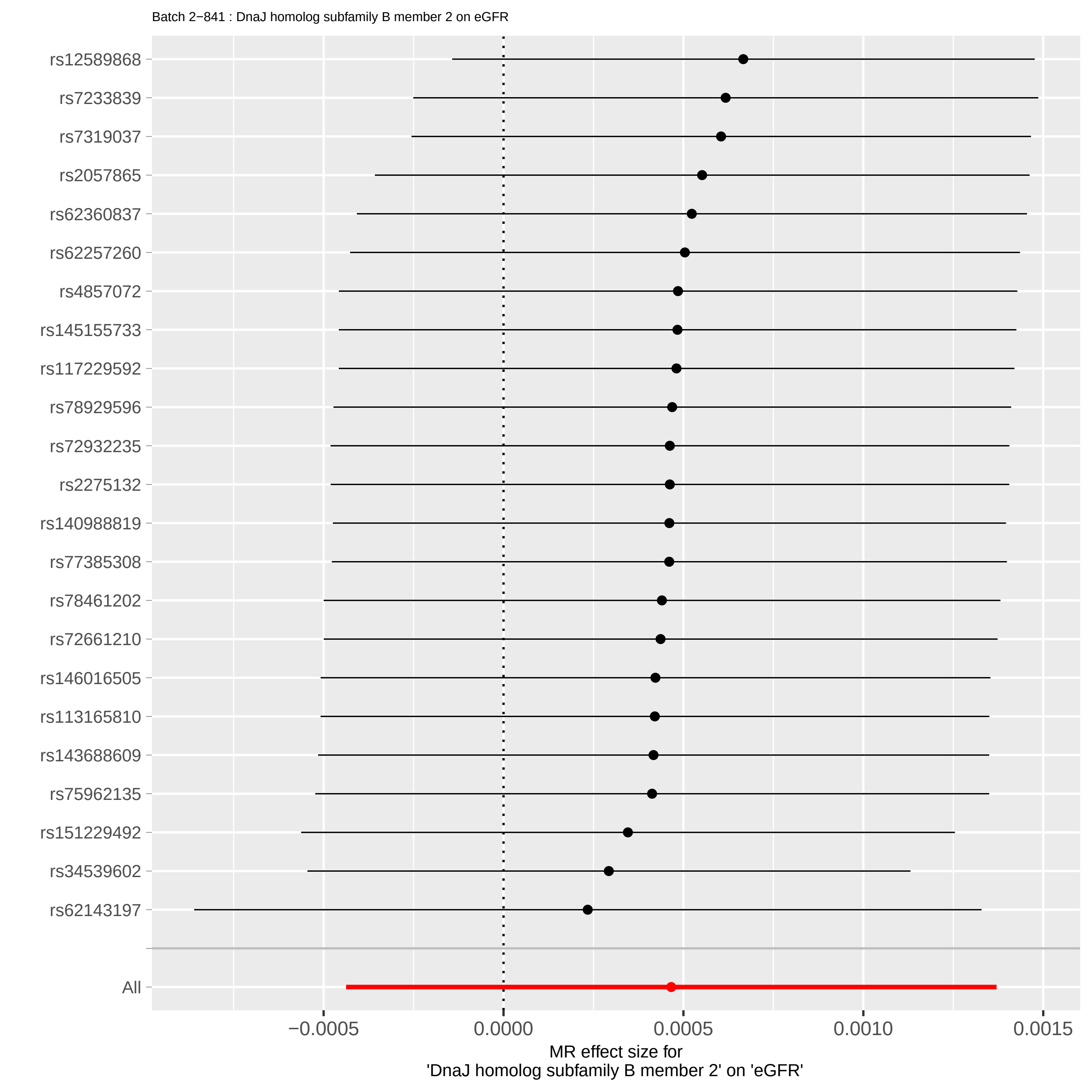

Batch 2−841 : DnaJ homolog subfamily B member 2 on eGFR
rs12589868
rs7233839
rs7319037
rs2057865
rs62360837
rs62257260
rs4857072
rs145155733
rs117229592
rs78929596
rs72932235
rs2275132
rs140988819
rs77385308
rs78461202
rs72661210
rs146016505
rs113165810
rs143688609
rs75962135
rs151229492
rs34539602
rs62143197
All
−0.0005
0.0000
0.0005
0.0010
0.0015
MR effect size for
'DnaJ homolog subfamily B member 2' on 'eGFR'

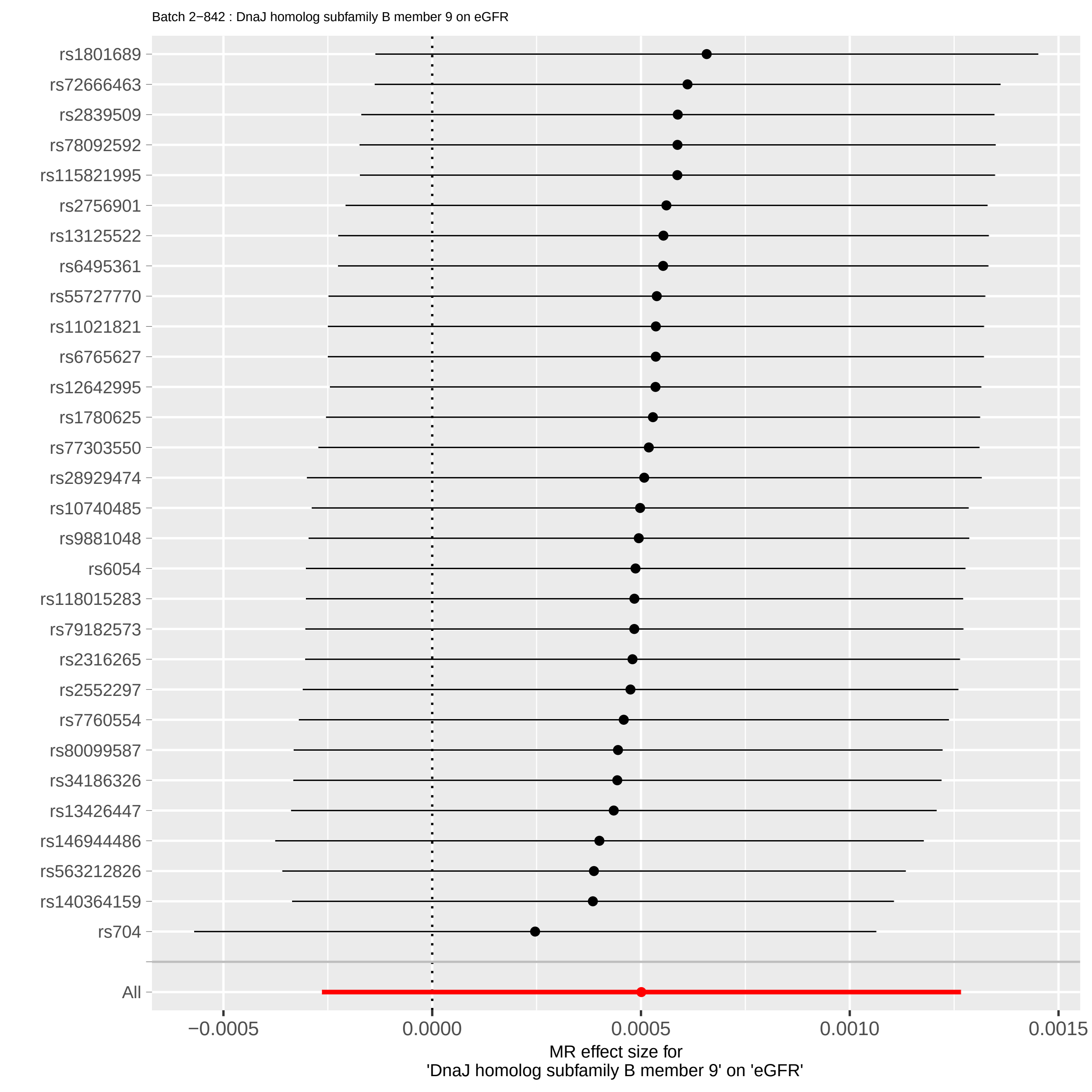

Batch 2−842 : DnaJ homolog subfamily B member 9 on eGFR
rs1801689
rs72666463
rs2839509
rs78092592
rs115821995
rs2756901
rs13125522
rs6495361
rs55727770
rs11021821
rs6765627
rs12642995
rs1780625
rs77303550
rs28929474
rs10740485
rs9881048
rs6054
rs118015283
rs79182573
rs2316265
rs2552297
rs7760554
rs80099587
rs34186326
rs13426447
rs146944486
rs563212826
rs140364159
rs704
All
−0.0005
0.0000
0.0005
0.0010
0.0015
MR effect size for
'DnaJ homolog subfamily B member 9' on 'eGFR'

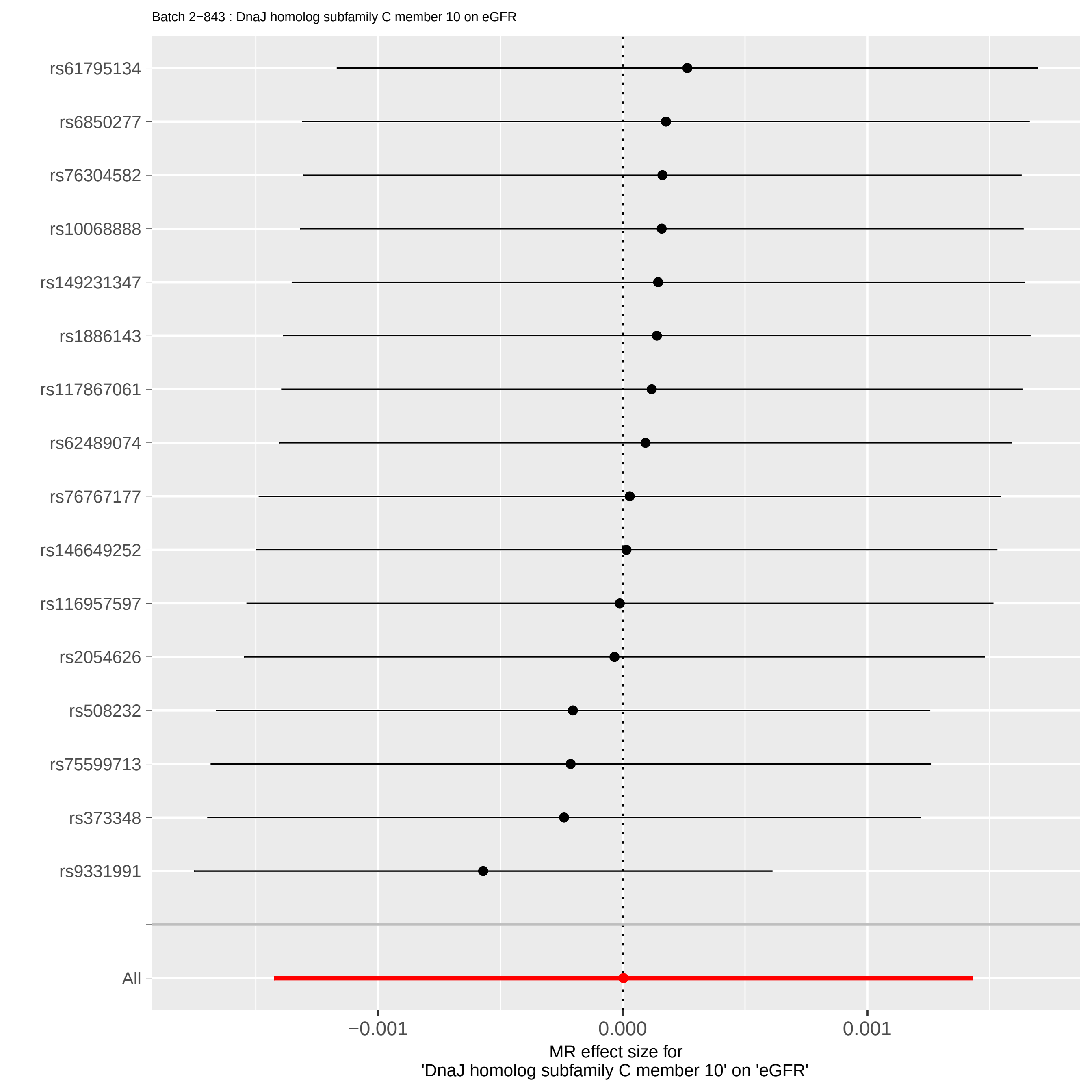

Batch 2−843 : DnaJ homolog subfamily C member 10 on eGFR
rs61795134
rs6850277
rs76304582
rs10068888
rs149231347
rs1886143
rs117867061
rs62489074
rs76767177
rs146649252
rs116957597
rs2054626
rs508232
rs75599713
rs373348
rs9331991
All
−0.001
0.000
0.001
MR effect size for
'DnaJ homolog subfamily C member 10' on 'eGFR'

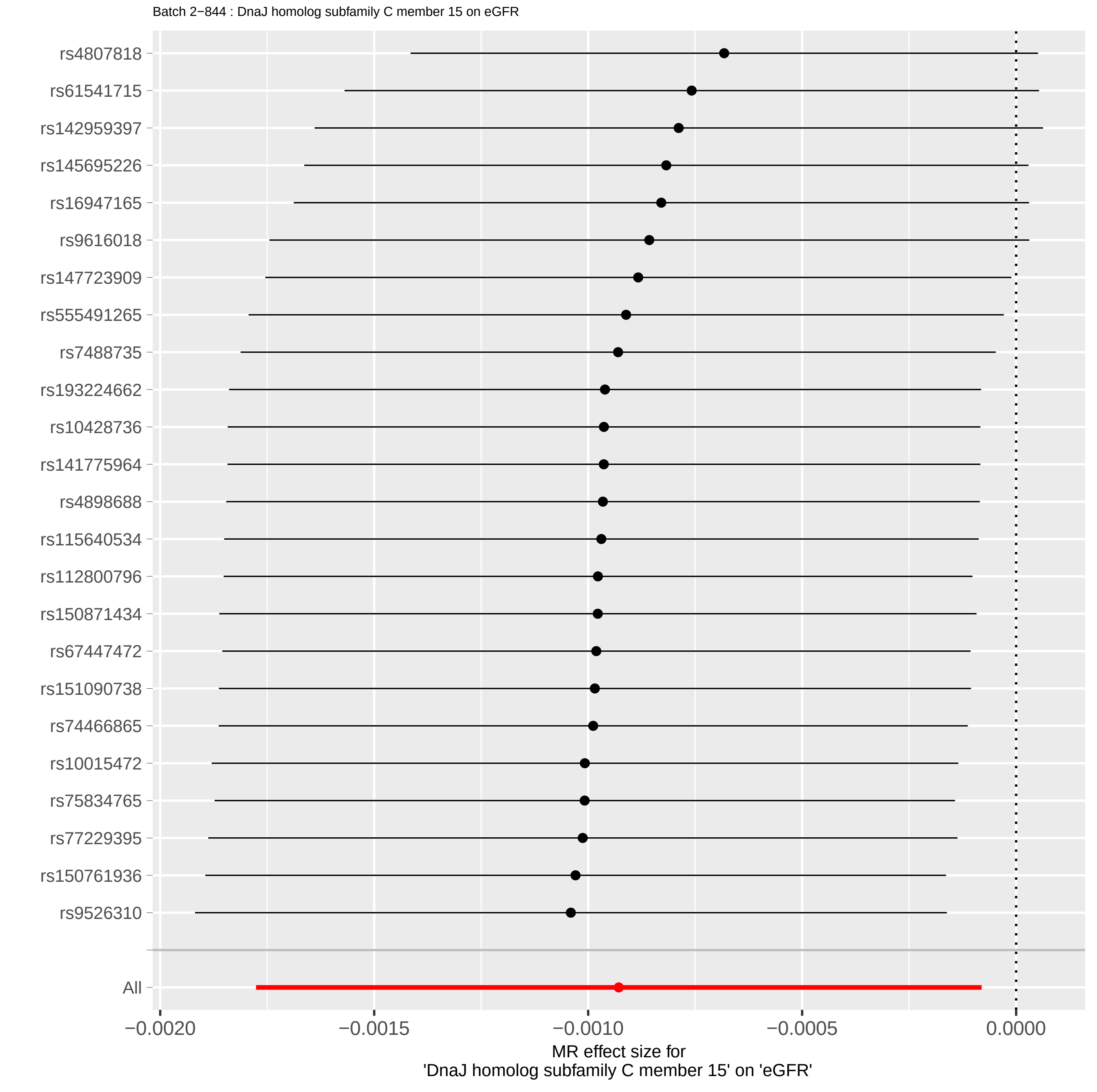

Batch 2−844 : DnaJ homolog subfamily C member 15 on eGFR
rs4807818
rs61541715
rs142959397
rs145695226
rs16947165
rs9616018
rs147723909
rs555491265
rs7488735
rs193224662
rs10428736
rs141775964
rs4898688
rs115640534
rs112800796
rs150871434
rs67447472
rs151090738
rs74466865
rs10015472
rs75834765
rs77229395
rs150761936
rs9526310
All
−0.0020
−0.0015
−0.0010
−0.0005
0.0000
MR effect size for
'DnaJ homolog subfamily C member 15' on 'eGFR'

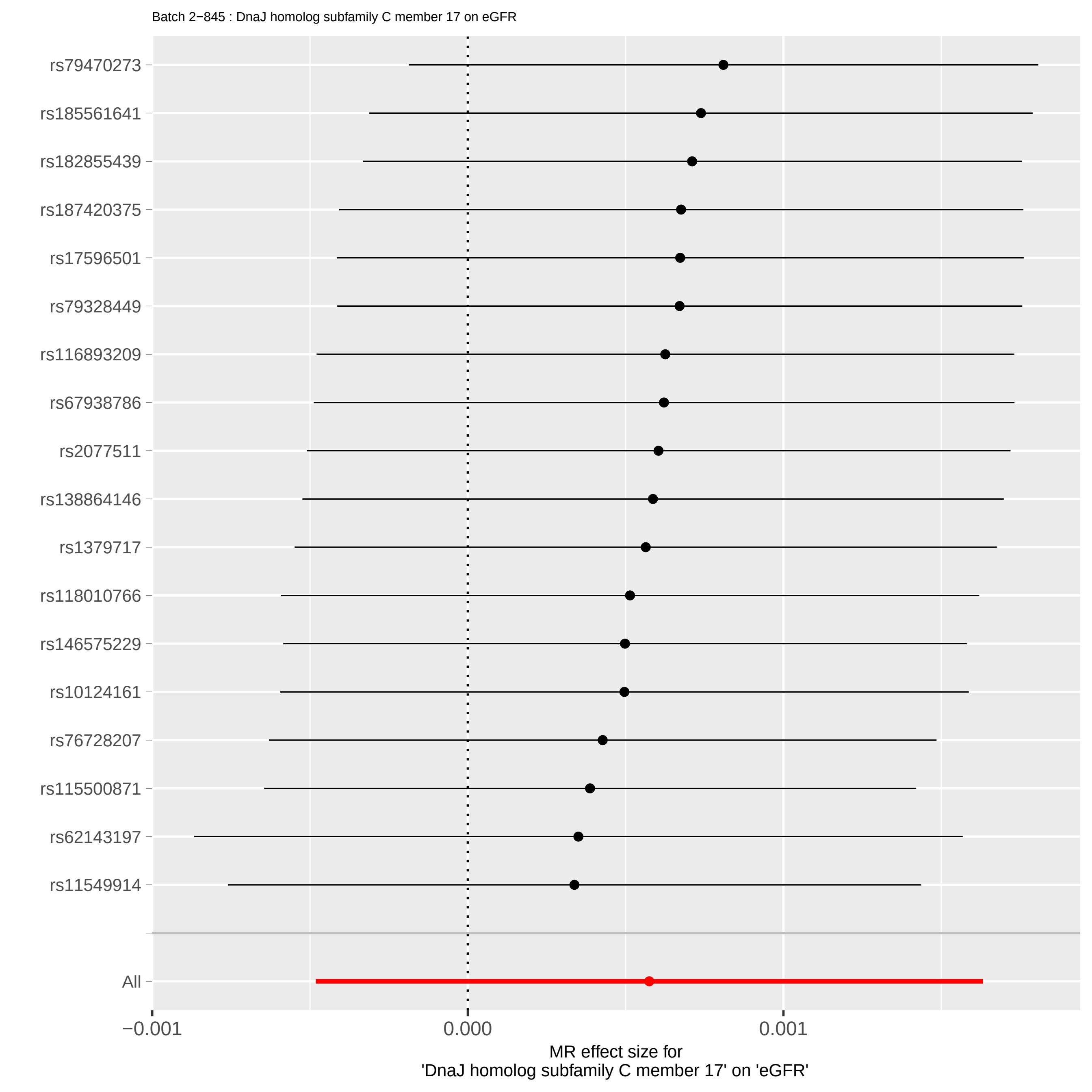

Batch 2−845 : DnaJ homolog subfamily C member 17 on eGFR
rs79470273
rs185561641
rs182855439
rs187420375
rs17596501
rs79328449
rs116893209
rs67938786
rs2077511
rs138864146
rs1379717
rs118010766
rs146575229
rs10124161
rs76728207
rs115500871
rs62143197
rs11549914
All
−0.001
0.000
0.001
MR effect size for
'DnaJ homolog subfamily C member 17' on 'eGFR'

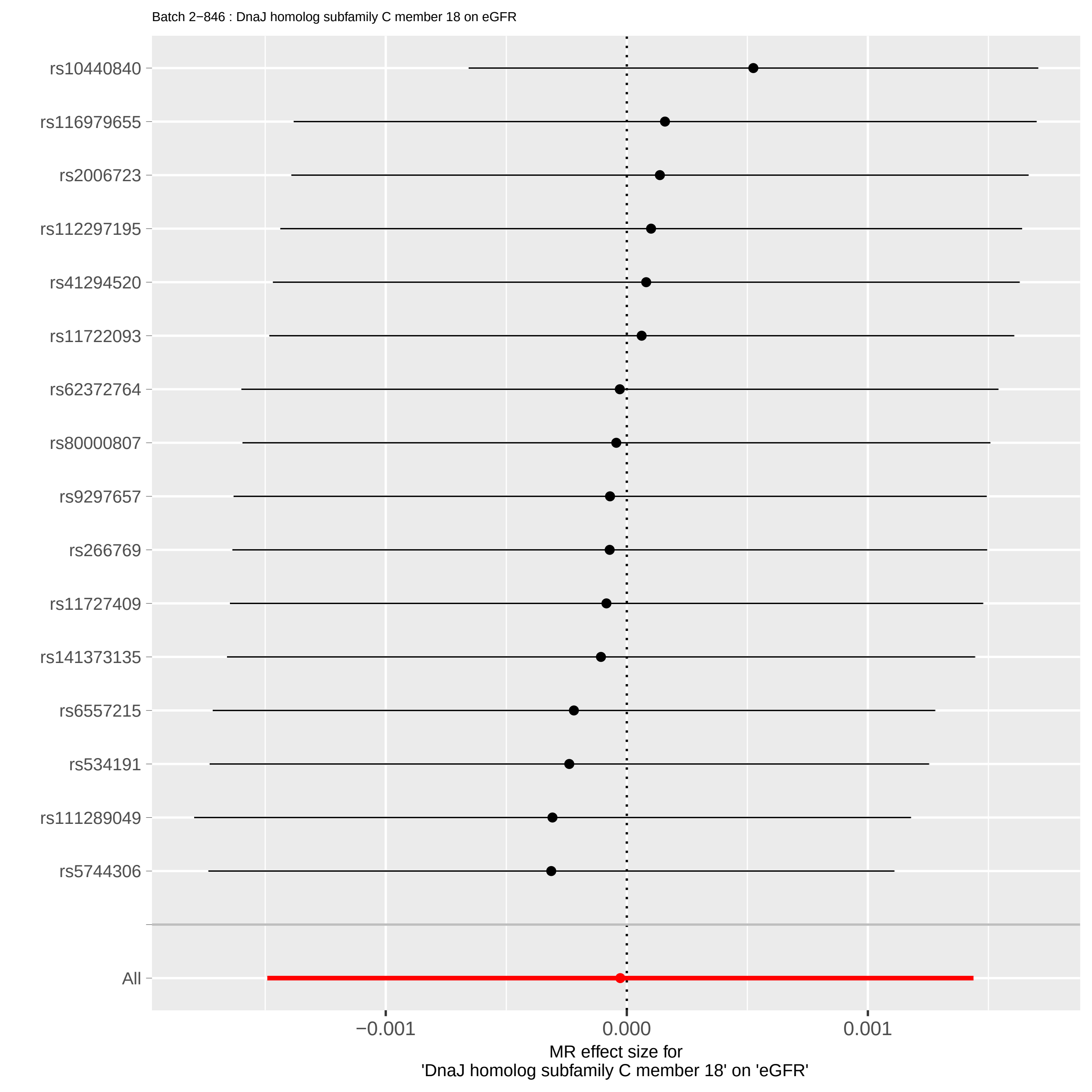
Batch 2−846 : DnaJ homolog subfamily C member 18 on eGFR
rs10440840
rs116979655
rs2006723
rs112297195
rs41294520
rs11722093
rs62372764
rs80000807
rs9297657
rs266769
rs11727409
rs141373135
rs6557215
rs534191
rs111289049
rs5744306
All
−0.001
0.000
0.001
MR effect size for
'DnaJ homolog subfamily C member 18' on 'eGFR'

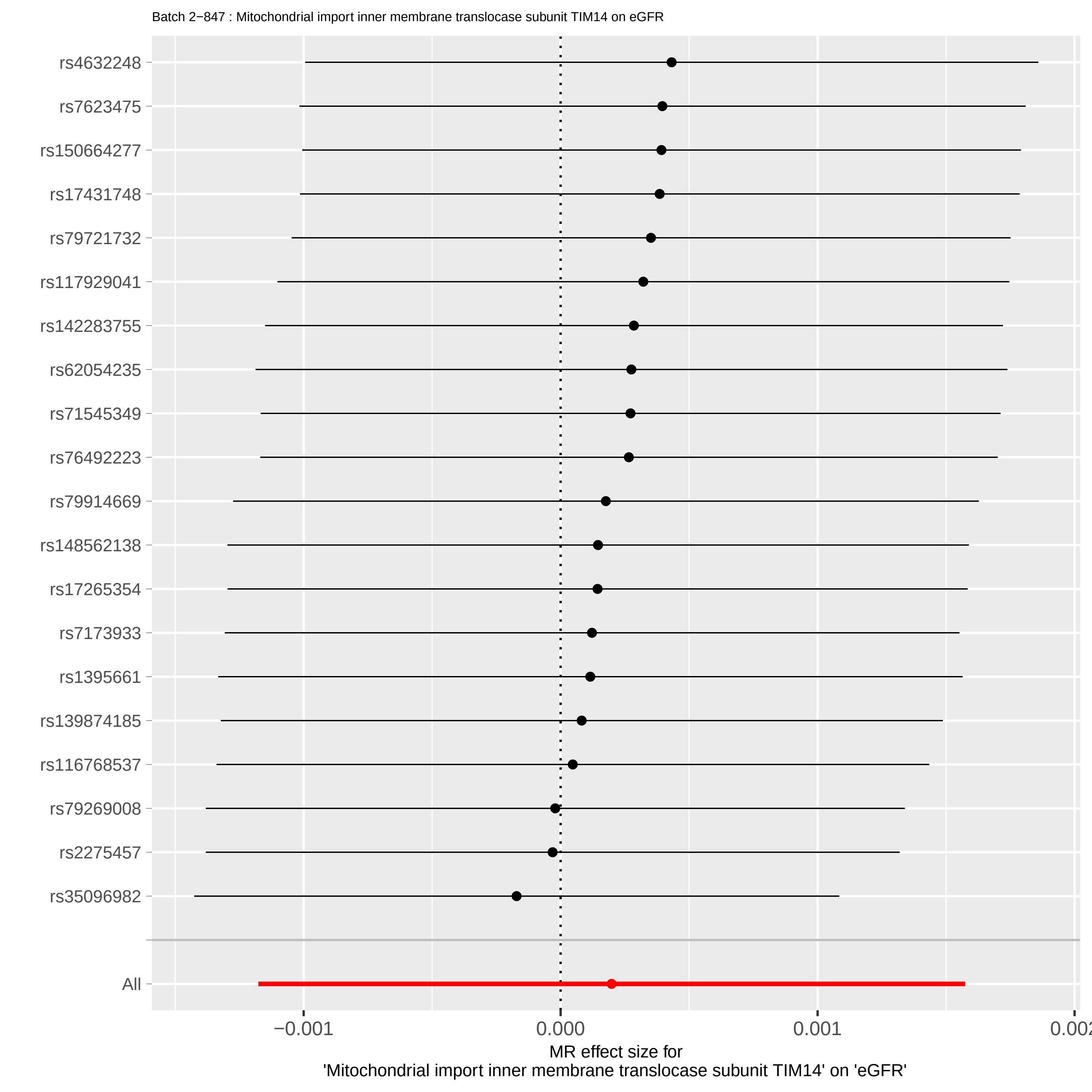

Batch 2−847 : Mitochondrial import inner membrane translocase subunit TIM14 on eGFR
rs4632248
rs7623475
rs150664277
rs17431748
rs79721732
rs117929041
rs142283755
rs62054235
rs71545349
rs76492223
rs79914669
rs148562138
rs17265354
rs7173933
rs1395661
rs139874185
rs116768537
rs79269008
rs2275457
rs35096982
All
−0.001
0.000
0.001
MR effect size for
'Mitochondrial import inner membrane translocase subunit TIM14' on 'eGFR'

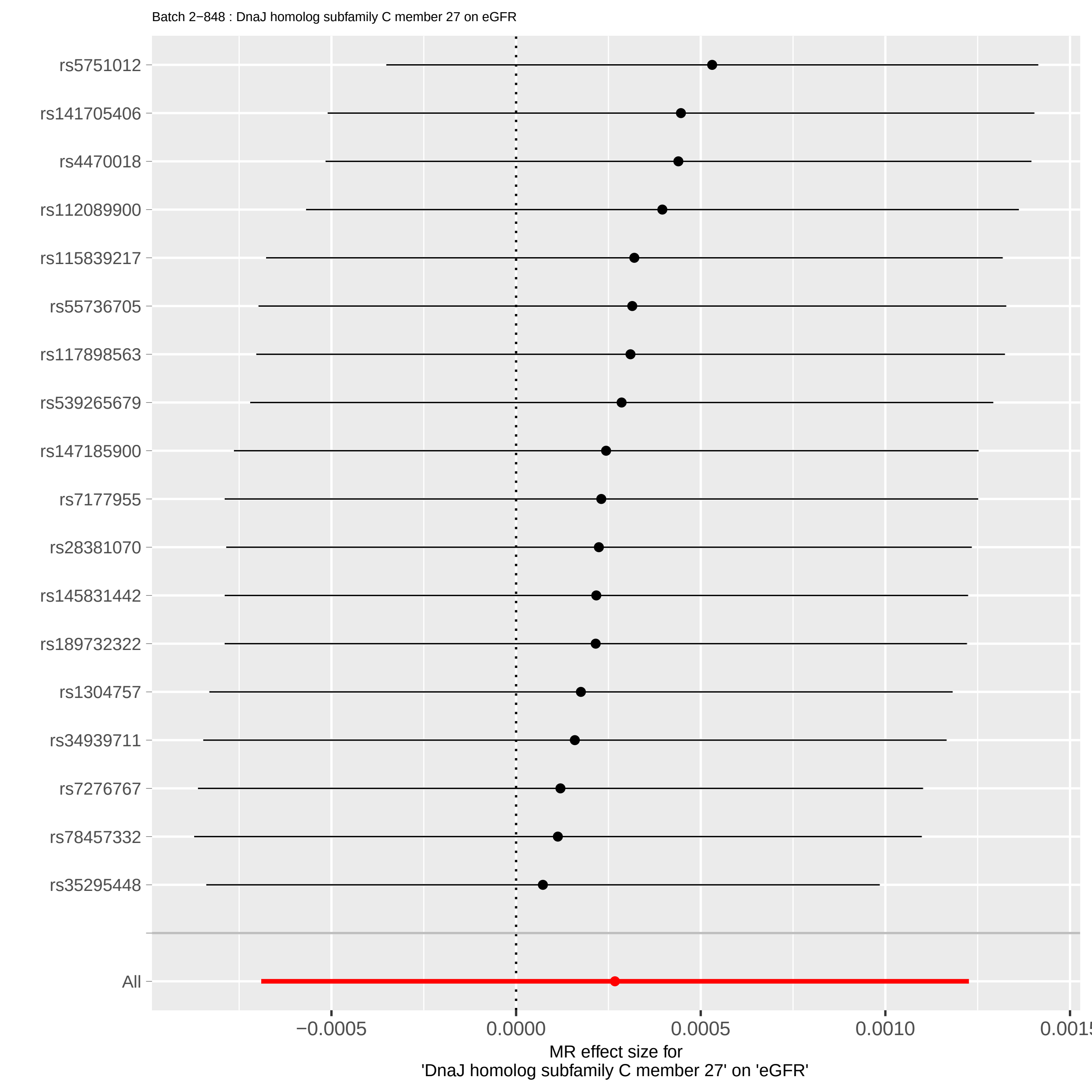

Batch 2−848 : DnaJ homolog subfamily C member 27 on eGFR
rs5751012
rs141705406
rs4470018
rs112089900
rs115839217
rs55736705
rs117898563
rs539265679
rs147185900
rs7177955
rs28381070
rs145831442
rs189732322
rs1304757
rs34939711
rs7276767
rs78457332
rs35295448
All
−0.0005
0.0000
0.0005
0.0010
MR effect size for
'DnaJ homolog subfamily C member 27' on 'eGFR'

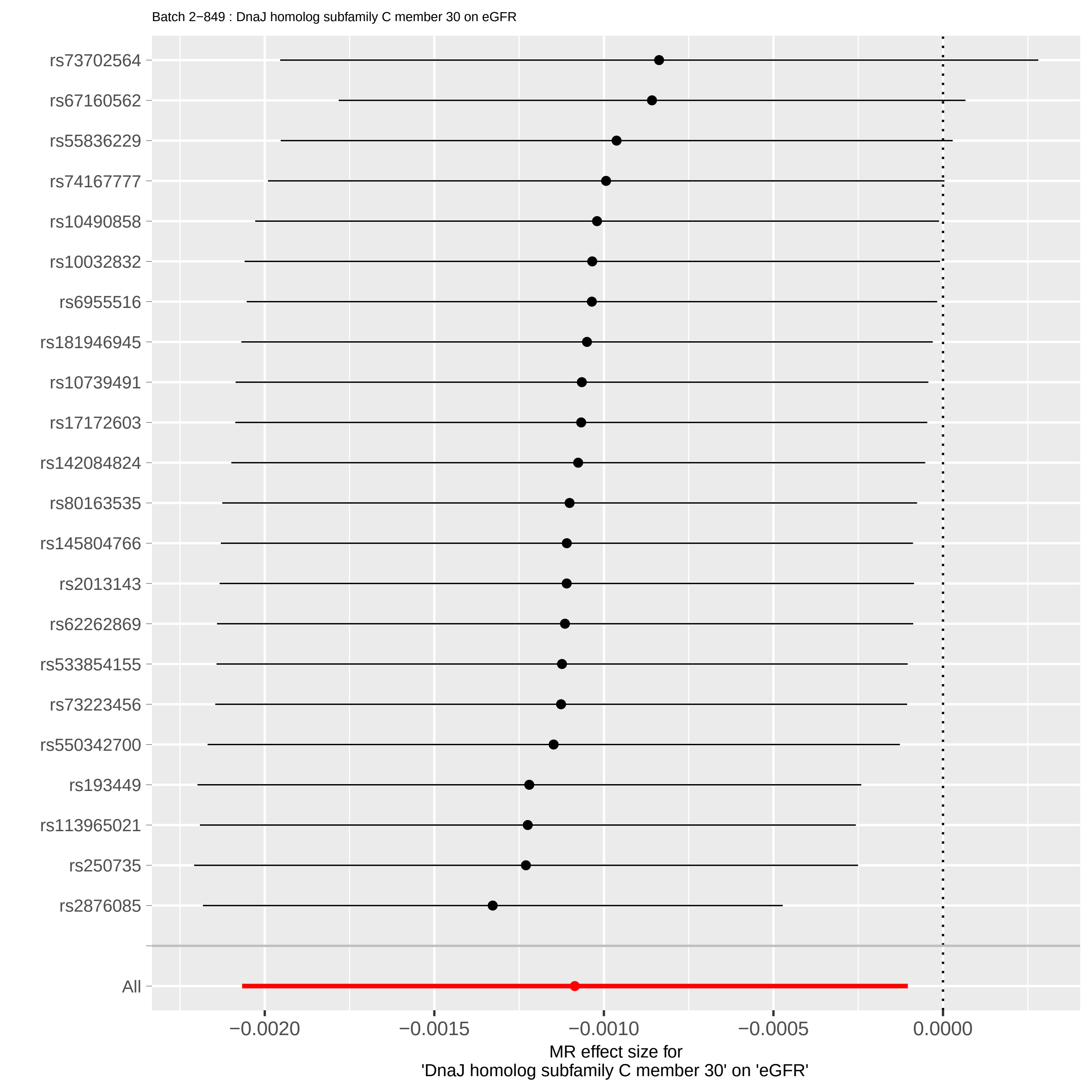

Batch 2−849 : DnaJ homolog subfamily C member 30 on eGFR
rs73702564
rs67160562
rs55836229
rs74167777
rs10490858
rs10032832
rs6955516
rs181946945
rs10739491
rs17172603
rs142084824
rs80163535
rs145804766
rs2013143
rs62262869
rs533854155
rs73223456
rs550342700
rs193449
rs113965021
rs250735
rs2876085
All
−0.0020
−0.0015
−0.0010
−0.0005
0.0000
MR effect size for
'DnaJ homolog subfamily C member 30' on 'eGFR'

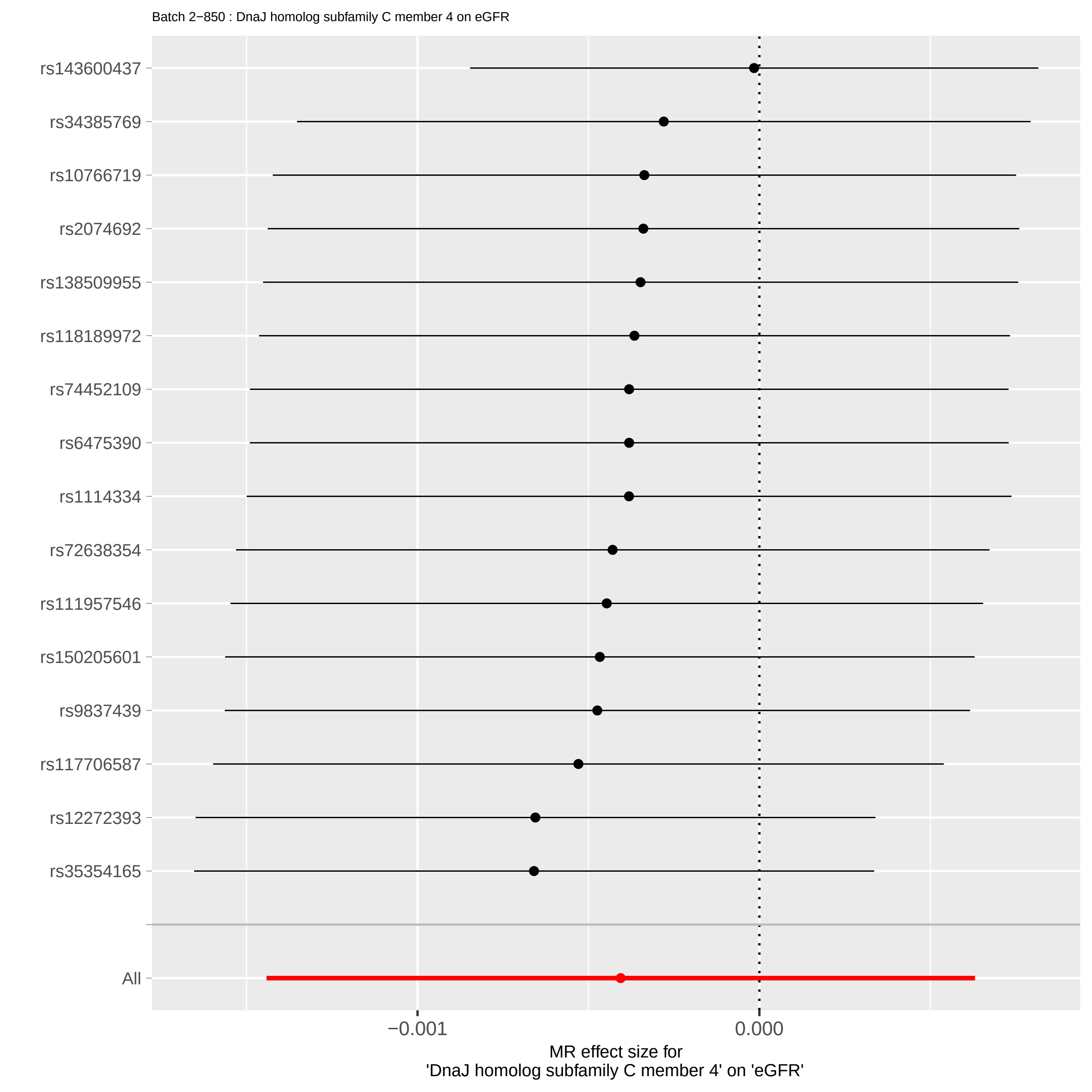

Batch 2−850 : DnaJ homolog subfamily C member 4 on eGFR
rs143600437
rs34385769
rs10766719
rs2074692
rs138509955
rs118189972
rs74452109
rs6475390
rs1114334
rs72638354
rs111957546
rs150205601
rs9837439
rs117706587
rs12272393
rs35354165
All
−0.001
0.000
MR effect size for
'DnaJ homolog subfamily C member 4' on 'eGFR'

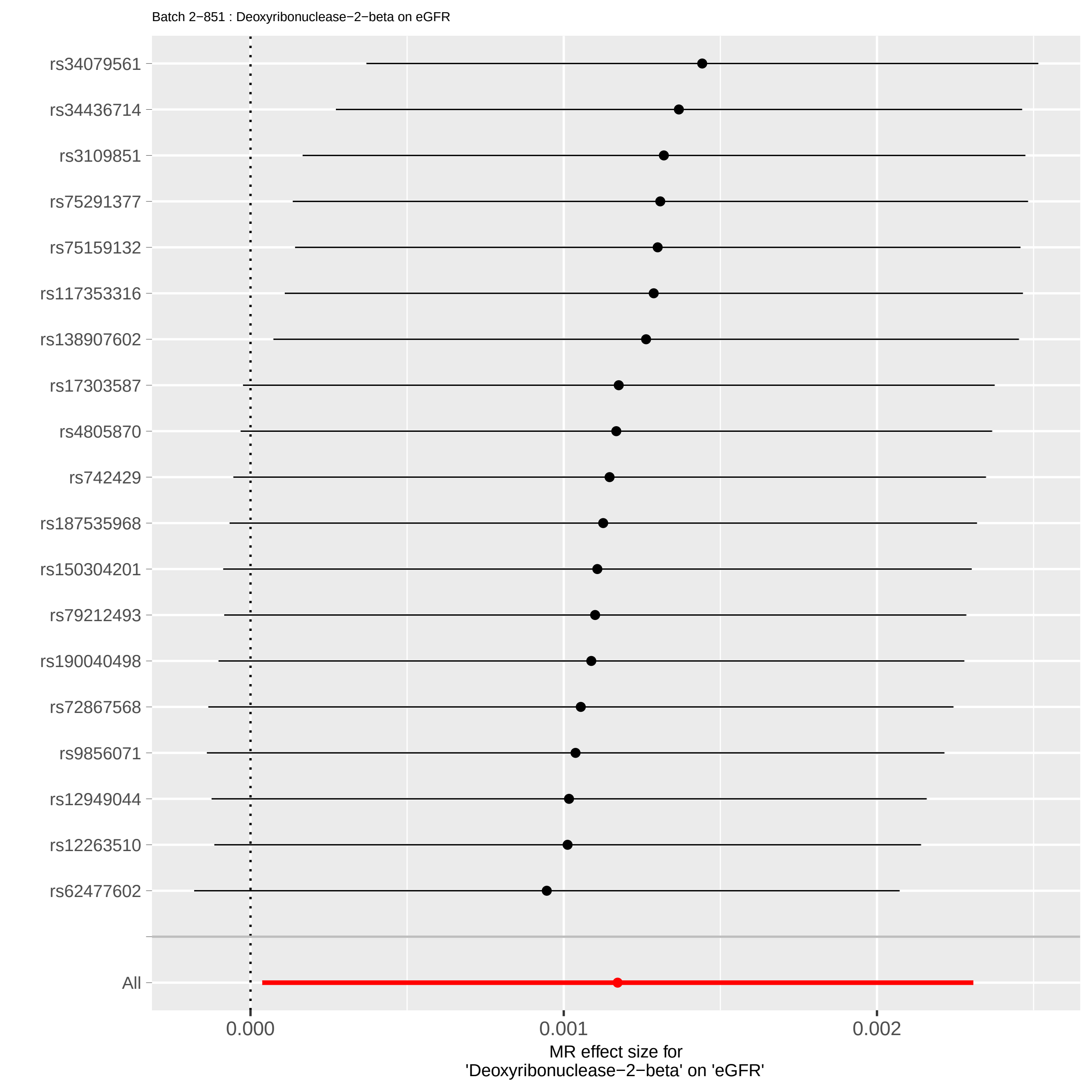

Batch 2−851 : Deoxyribonuclease−2−beta on eGFR
rs34079561
rs34436714
rs3109851
rs75291377
rs75159132
rs117353316
rs138907602
rs17303587
rs4805870
rs742429
rs187535968
rs150304201
rs79212493
rs190040498
rs72867568
rs9856071
rs12949044
rs12263510
rs62477602
All
0.000
0.001
0.002
MR effect size for
'Deoxyribonuclease−2−beta' on 'eGFR'

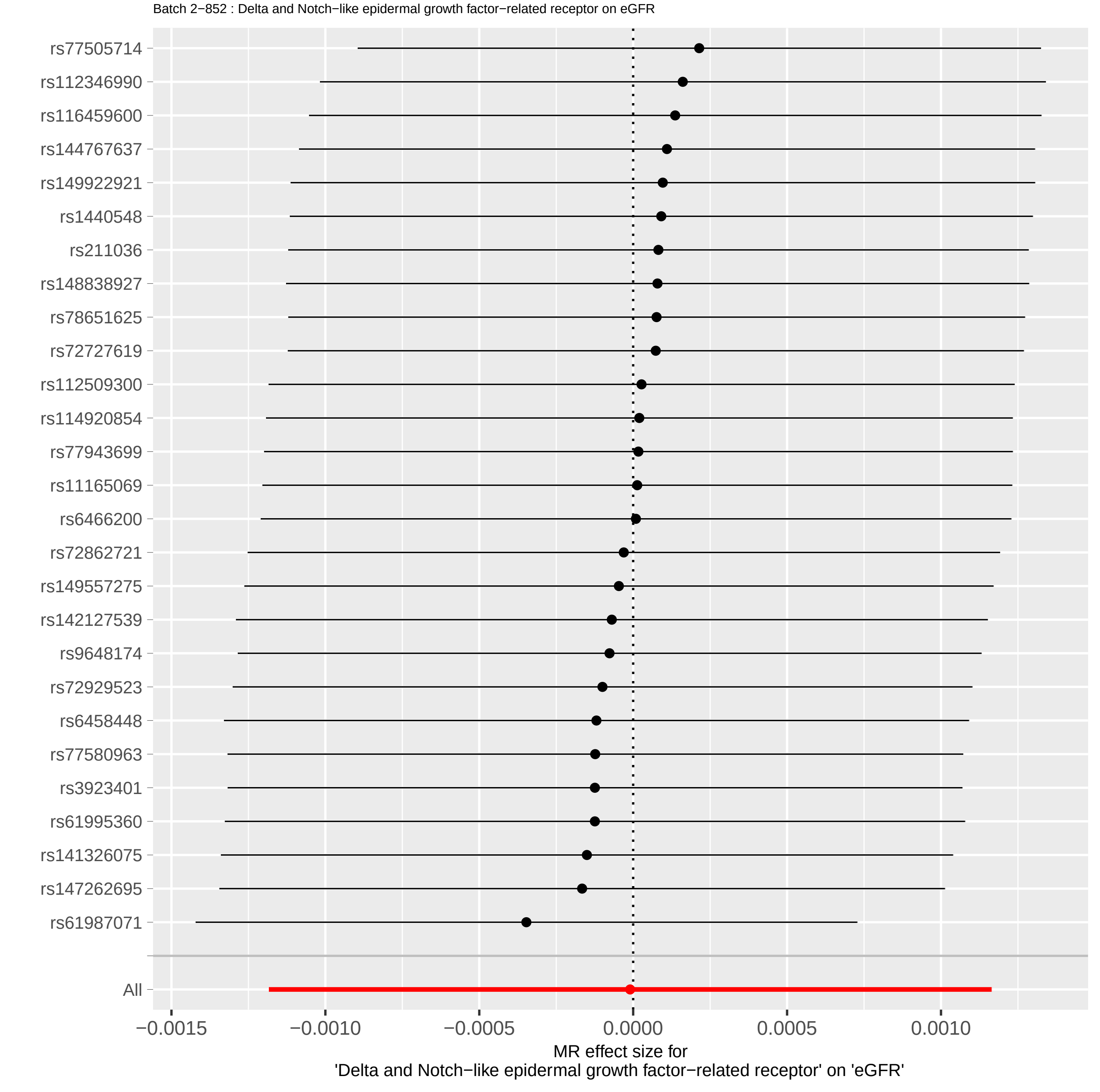

Batch 2−852 : Delta and Notch−like epidermal growth factor−related receptor on eGFR
rs77505714
rs112346990
rs116459600
rs144767637
rs149922921
rs1440548
rs211036
rs148838927
rs78651625
rs72727619
rs112509300
rs114920854
rs77943699
rs11165069
rs6466200
rs72862721
rs149557275
rs142127539
rs9648174
rs72929523
rs6458448
rs77580963
rs3923401
rs61995360
rs141326075
rs147262695
rs61987071
All
−0.0015
−0.0010
−0.0005
0.0000
0.0005
0.0010
MR effect size for
'Delta and Notch−like epidermal growth factor−related receptor' on 'eGFR'

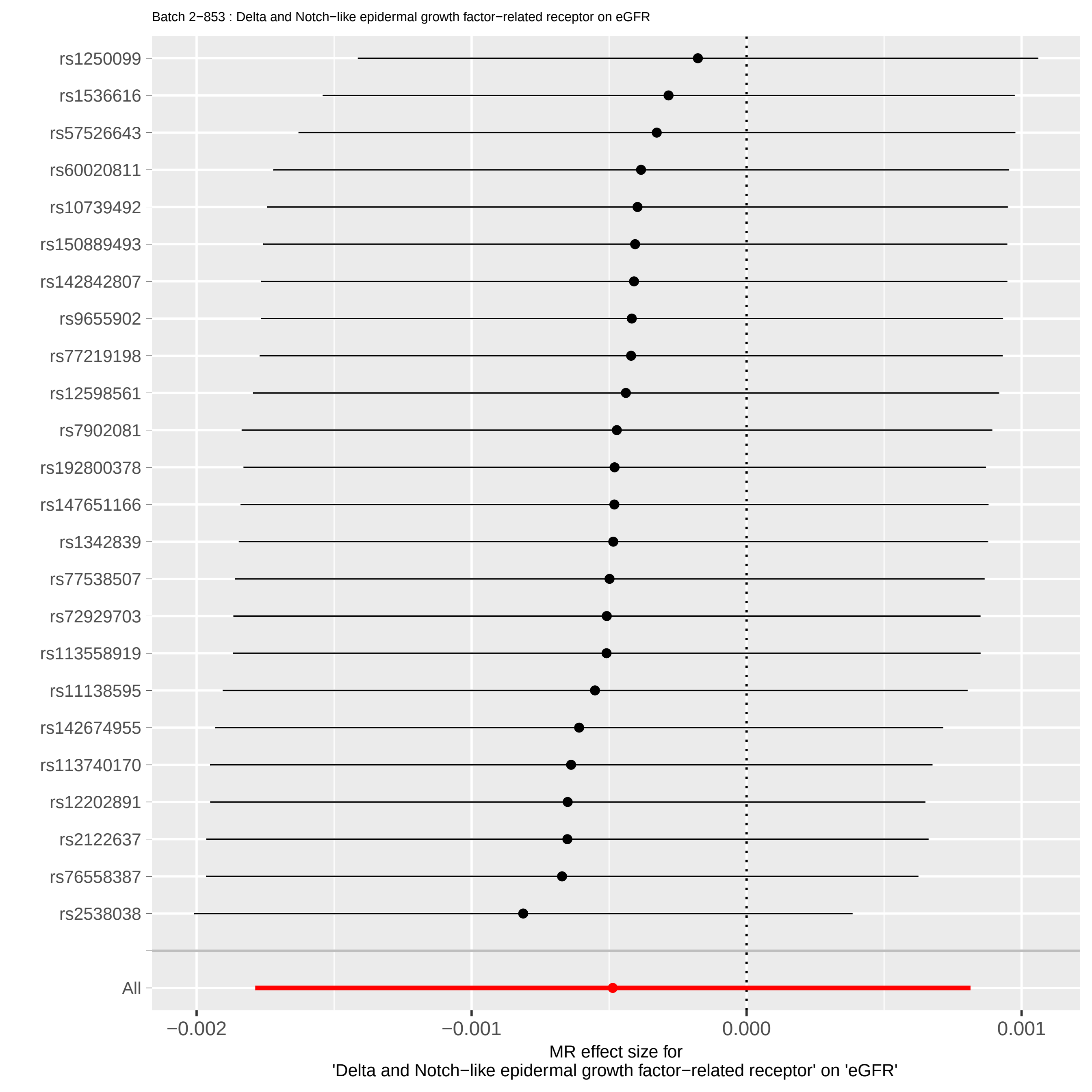

Batch 2−853 : Delta and Notch−like epidermal growth factor−related receptor on eGFR
rs1250099
rs1536616
rs57526643
rs60020811
rs10739492
rs150889493
rs142842807
rs9655902
rs77219198
rs12598561
rs7902081
rs192800378
rs147651166
rs1342839
rs77538507
rs72929703
rs113558919
rs11138595
rs142674955
rs113740170
rs12202891
rs2122637
rs76558387
rs2538038
All
−0.002
−0.001
0.000
0.001
MR effect size for
'Delta and Notch−like epidermal growth factor−related receptor' on 'eGFR'

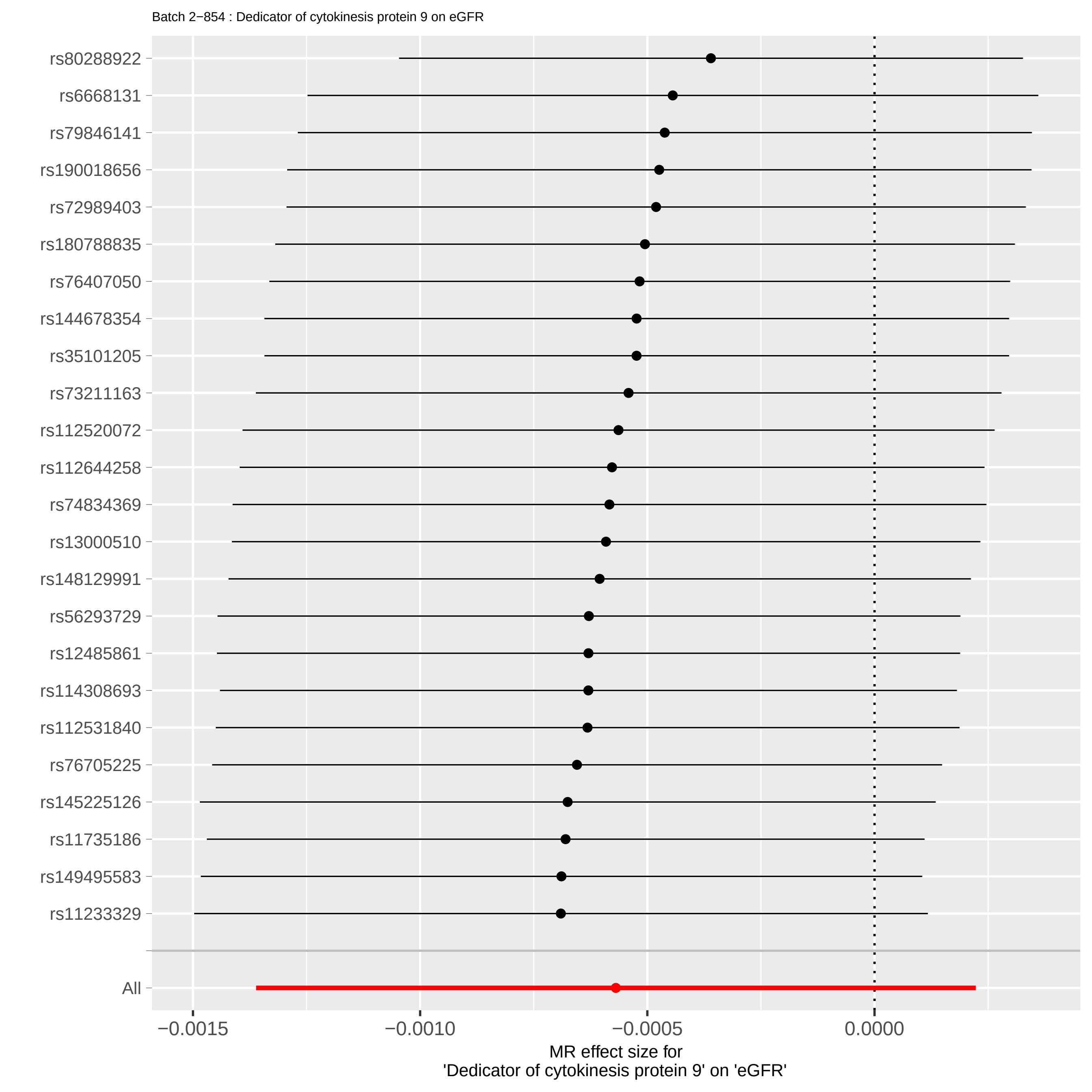

Batch 2−854 : Dedicator of cytokinesis protein 9 on eGFR
rs80288922
rs6668131
rs79846141
rs190018656
rs72989403
rs180788835
rs76407050
rs144678354
rs35101205
rs73211163
rs112520072
rs112644258
rs74834369
rs13000510
rs148129991
rs56293729
rs12485861
rs114308693
rs112531840
rs76705225
rs145225126
rs11735186
rs149495583
rs11233329
All
−0.0015
−0.0010
−0.0005
0.0000
MR effect size for
'Dedicator of cytokinesis protein 9' on 'eGFR'

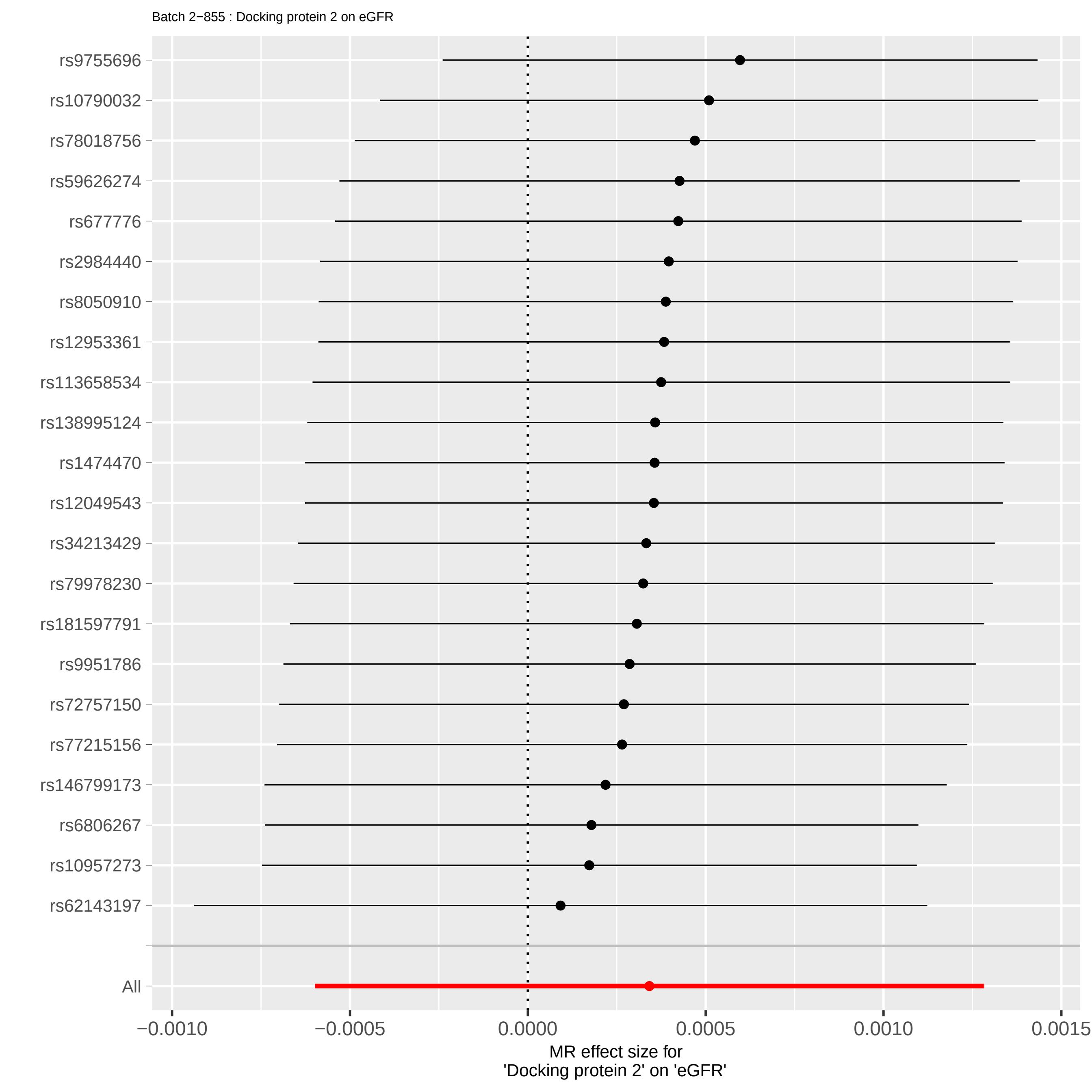

Batch 2−855 : Docking protein 2 on eGFR
rs9755696
rs10790032
rs78018756
rs59626274
rs677776
rs2984440
rs8050910
rs12953361
rs113658534
rs138995124
rs1474470
rs12049543
rs34213429
rs79978230
rs181597791
rs9951786
rs72757150
rs77215156
rs146799173
rs6806267
rs10957273
rs62143197
All
−0.0010
−0.0005
0.0000
0.0005
0.0010
0.0015
MR effect size for
'Docking protein 2' on 'eGFR'

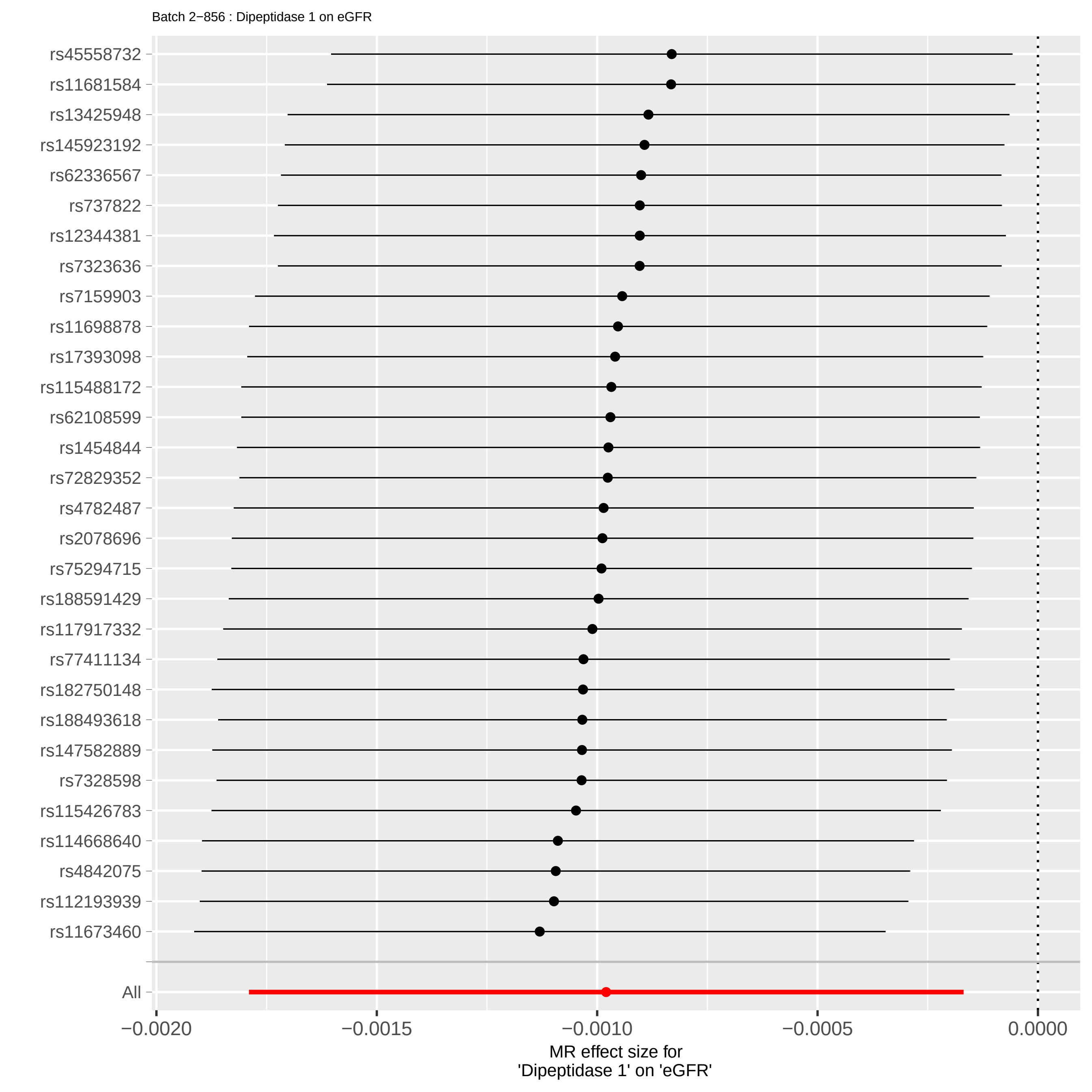

Batch 2−856 : Dipeptidase 1 on eGFR
rs45558732
rs11681584
rs13425948
rs145923192
rs62336567
rs737822
rs12344381
rs7323636
rs7159903
rs11698878
rs17393098
rs115488172
rs62108599
rs1454844
rs72829352
rs4782487
rs2078696
rs75294715
rs188591429
rs117917332
rs77411134
rs182750148
rs188493618
rs147582889
rs7328598
rs115426783
rs114668640
rs4842075
rs112193939
rs11673460
All
−0.0020
−0.0015
−0.0010
−0.0005
0.0000
MR effect size for
'Dipeptidase 1' on 'eGFR'

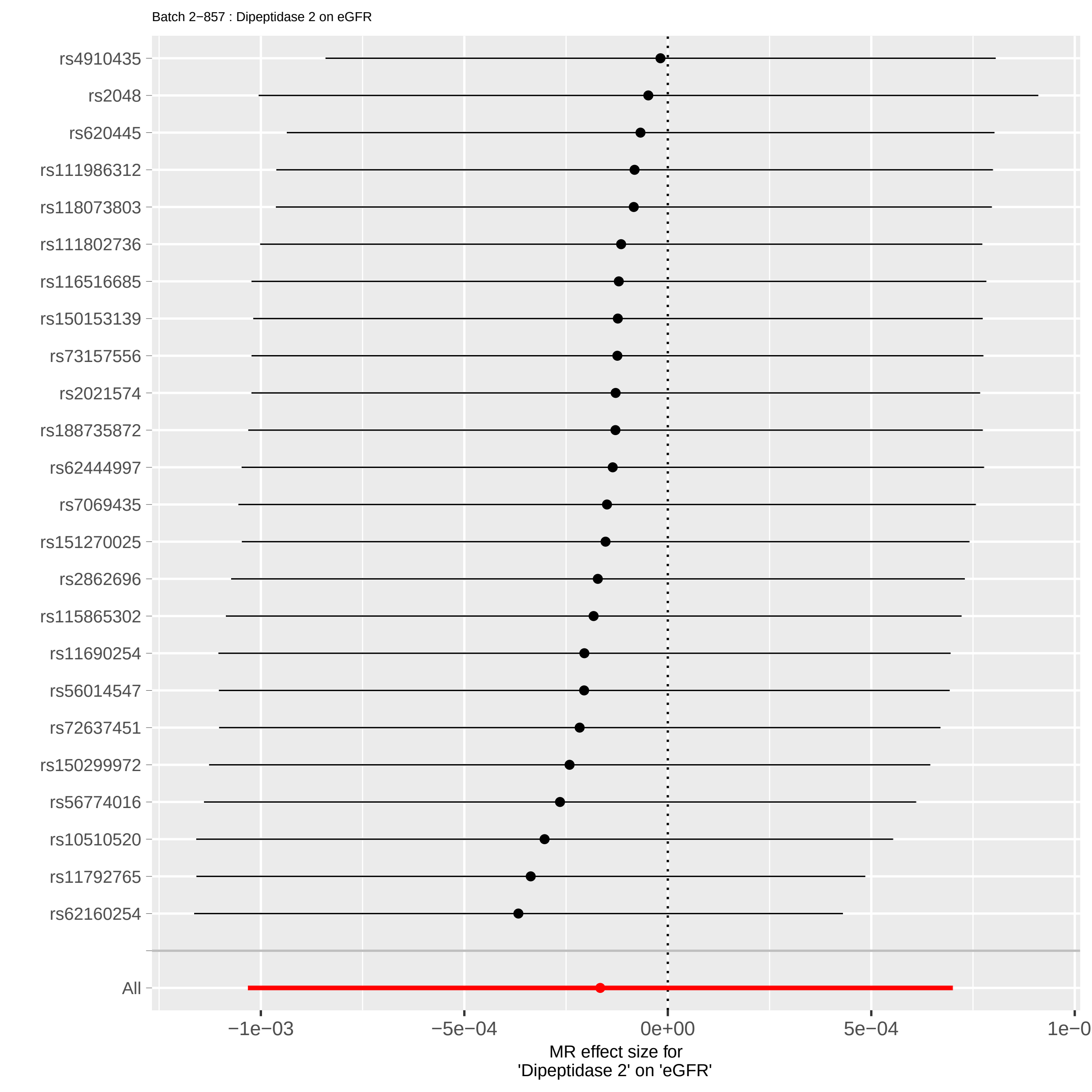

Batch 2−857 : Dipeptidase 2 on eGFR
rs4910435
rs2048
rs620445
rs111986312
rs118073803
rs111802736
rs116516685
rs150153139
rs73157556
rs2021574
rs188735872
rs62444997
rs7069435
rs151270025
rs2862696
rs115865302
rs11690254
rs56014547
rs72637451
rs150299972
rs56774016
rs10510520
rs11792765
rs62160254
All
−1e−03
−5e−04
0e+00
5e−04
MR effect size for
'Dipeptidase 2' on 'eGFR'

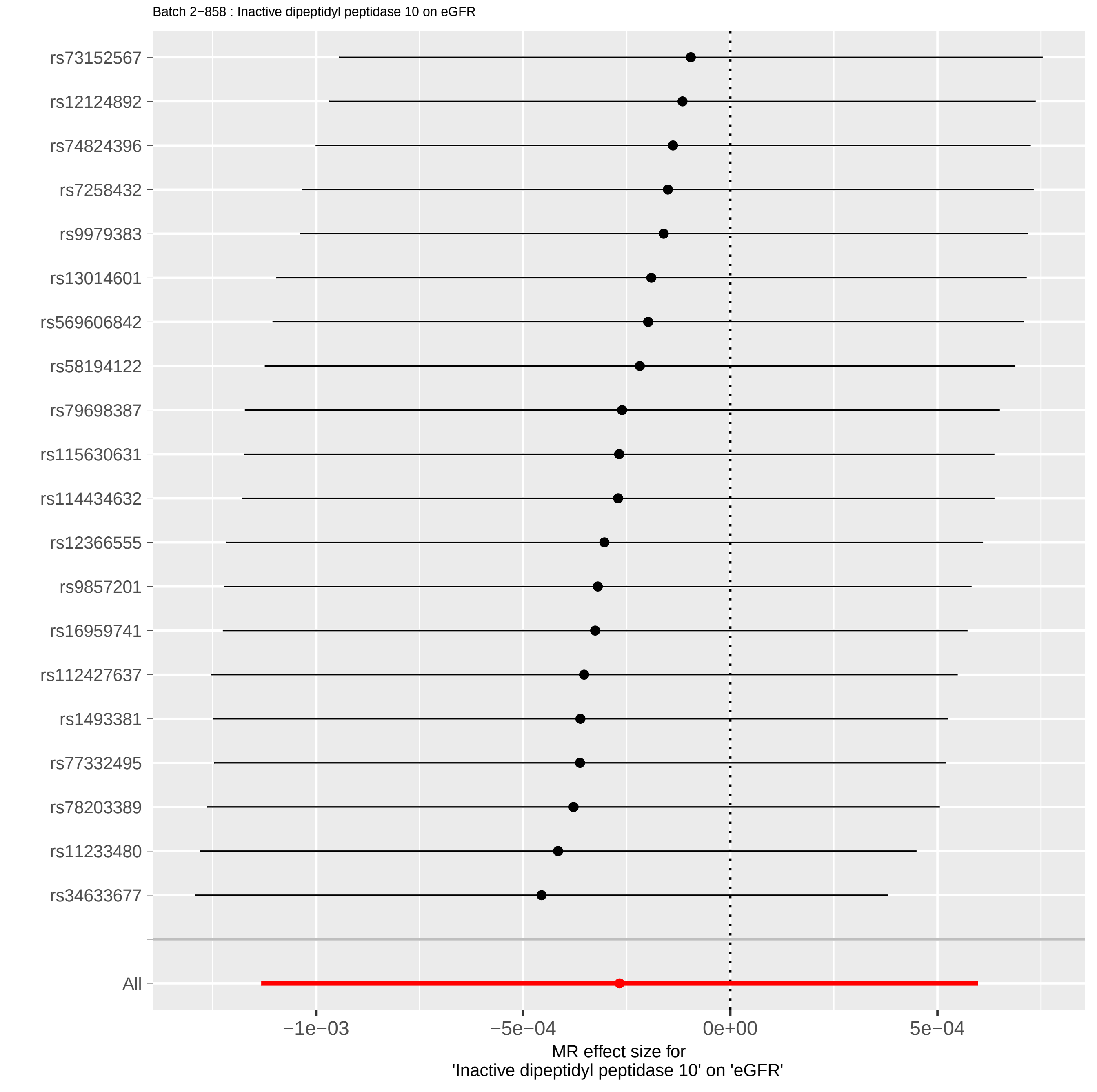

Batch 2−858 : Inactive dipeptidyl peptidase 10 on eGFR
rs73152567
rs12124892
rs74824396
rs7258432
rs9979383
rs13014601
rs569606842
rs58194122
rs79698387
rs115630631
rs114434632
rs12366555
rs9857201
rs16959741
rs112427637
rs1493381
rs77332495
rs78203389
rs11233480
rs34633677
All
−1e−03
−5e−04
0e+00
5e−04
MR effect size for
'Inactive dipeptidyl peptidase 10' on 'eGFR'

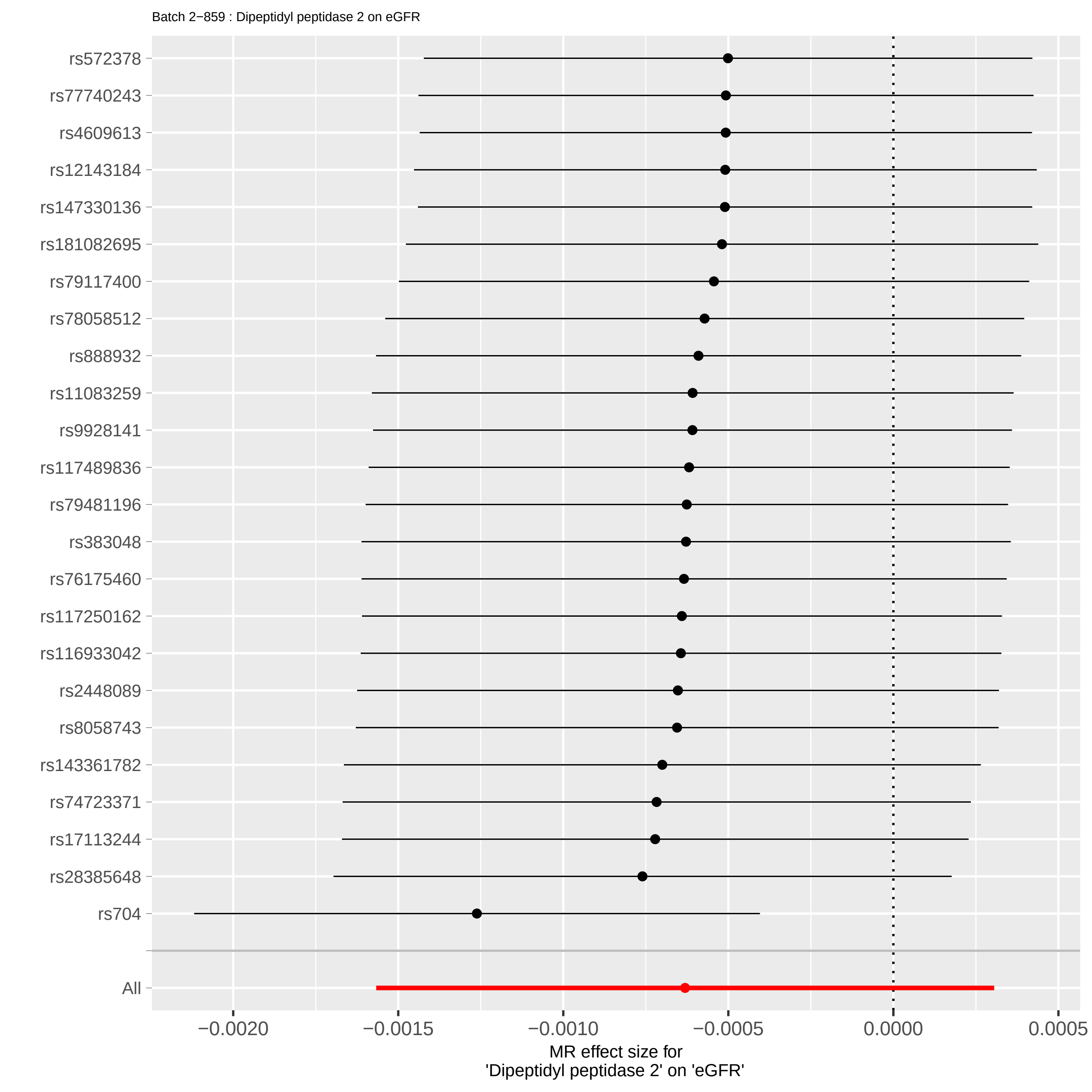
Batch 2−859 : Dipeptidyl peptidase 2 on eGFR
rs572378
rs77740243
rs4609613
rs12143184
rs147330136
rs181082695
rs79117400
rs78058512
rs888932
rs11083259
rs9928141
rs117489836
rs79481196
rs383048
rs76175460
rs117250162
rs116933042
rs2448089
rs8058743
rs143361782
rs74723371
rs17113244
rs28385648
rs704
All
−0.0020
−0.0015
−0.0010
−0.0005
0.0000
0.0005
MR effect size for
'Dipeptidyl peptidase 2' on 'eGFR'

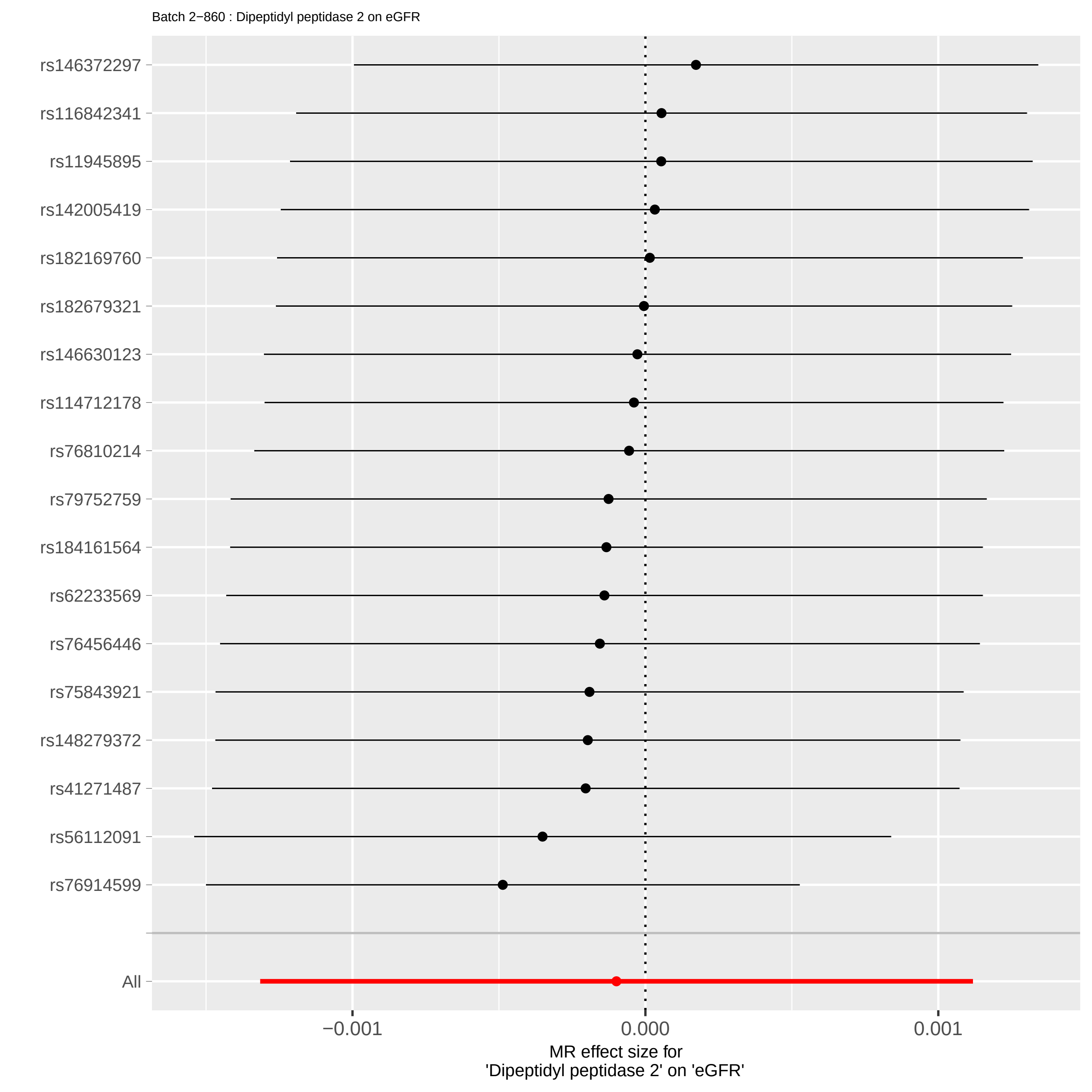
Batch 2−860 : Dipeptidyl peptidase 2 on eGFR
rs146372297
rs116842341
rs11945895
rs142005419
rs182169760
rs182679321
rs146630123
rs114712178
rs76810214
rs79752759
rs184161564
rs62233569
rs76456446
rs75843921
rs148279372
rs41271487
rs56112091
rs76914599
All
−0.001
0.000
0.001
MR effect size for
'Dipeptidyl peptidase 2' on 'eGFR'

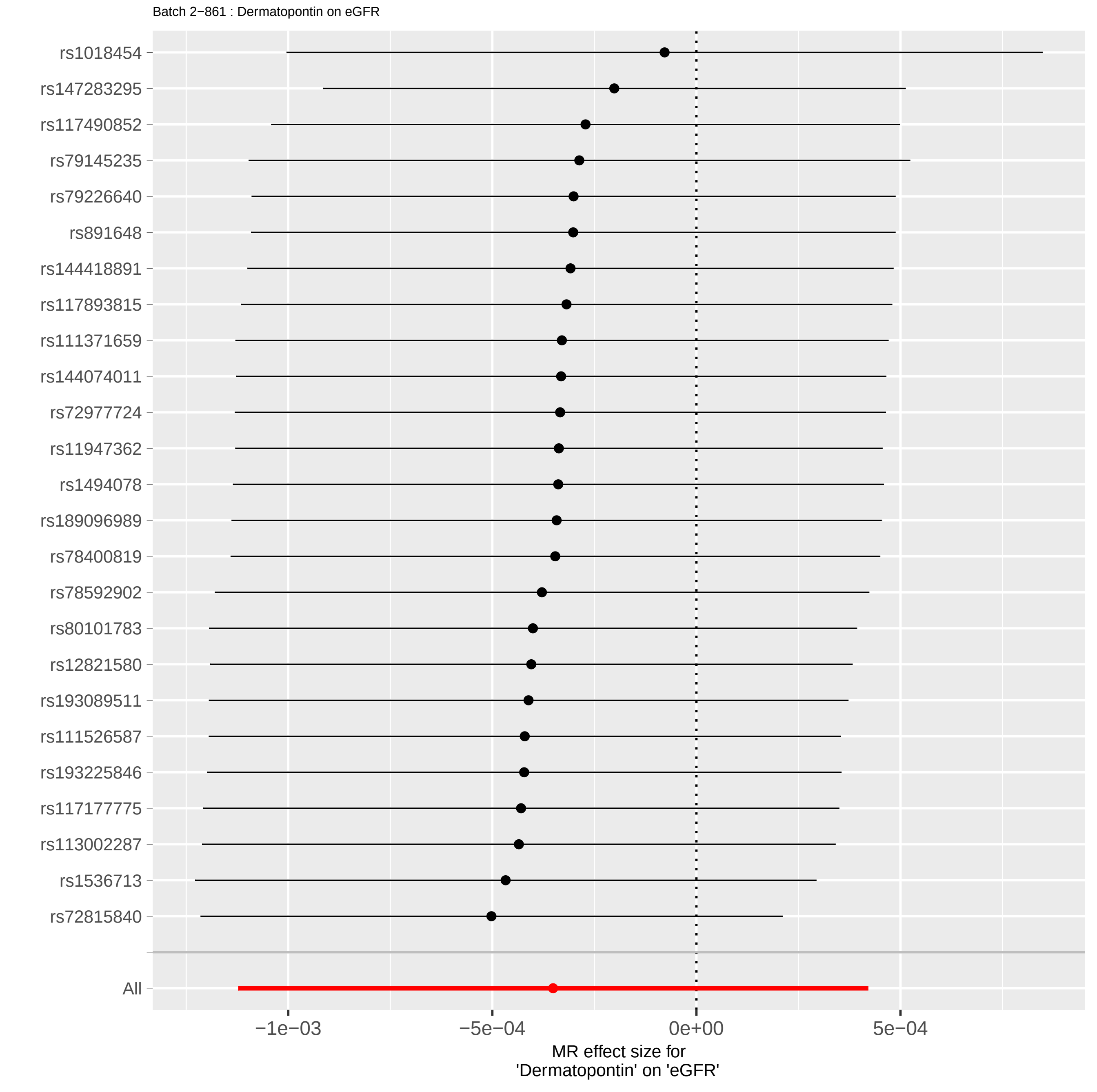
Batch 2−861 : Dermatopontin on eGFR
rs1018454
rs147283295
rs117490852
rs79145235
rs79226640
rs891648
rs144418891
rs117893815
rs111371659
rs144074011
rs72977724
rs11947362
rs1494078
rs189096989
rs78400819
rs78592902
rs80101783
rs12821580
rs193089511
rs111526587
rs193225846
rs117177775
rs113002287
rs1536713
rs72815840
All
−1e−03
−5e−04
0e+00
5e−04
MR effect size for
'Dermatopontin' on 'eGFR'

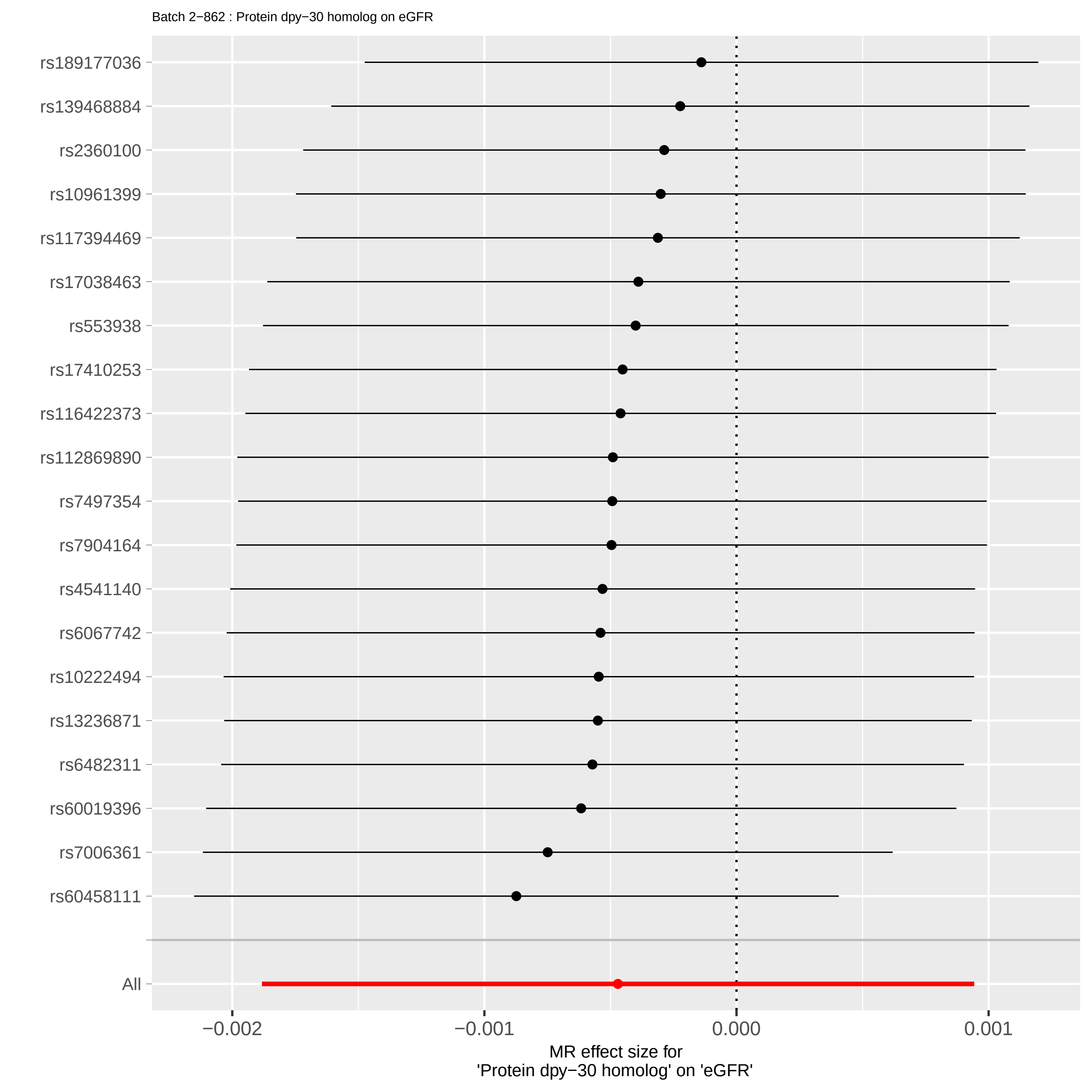
Batch 2−862 : Protein dpy−30 homolog on eGFR
rs189177036
rs139468884
rs2360100
rs10961399
rs117394469
rs17038463
rs553938
rs17410253
rs116422373
rs112869890
rs7497354
rs7904164
rs4541140
rs6067742
rs10222494
rs13236871
rs6482311
rs60019396
rs7006361
rs60458111
All
−0.002
−0.001
0.000
0.001
MR effect size for
'Protein dpy−30 homolog' on 'eGFR'

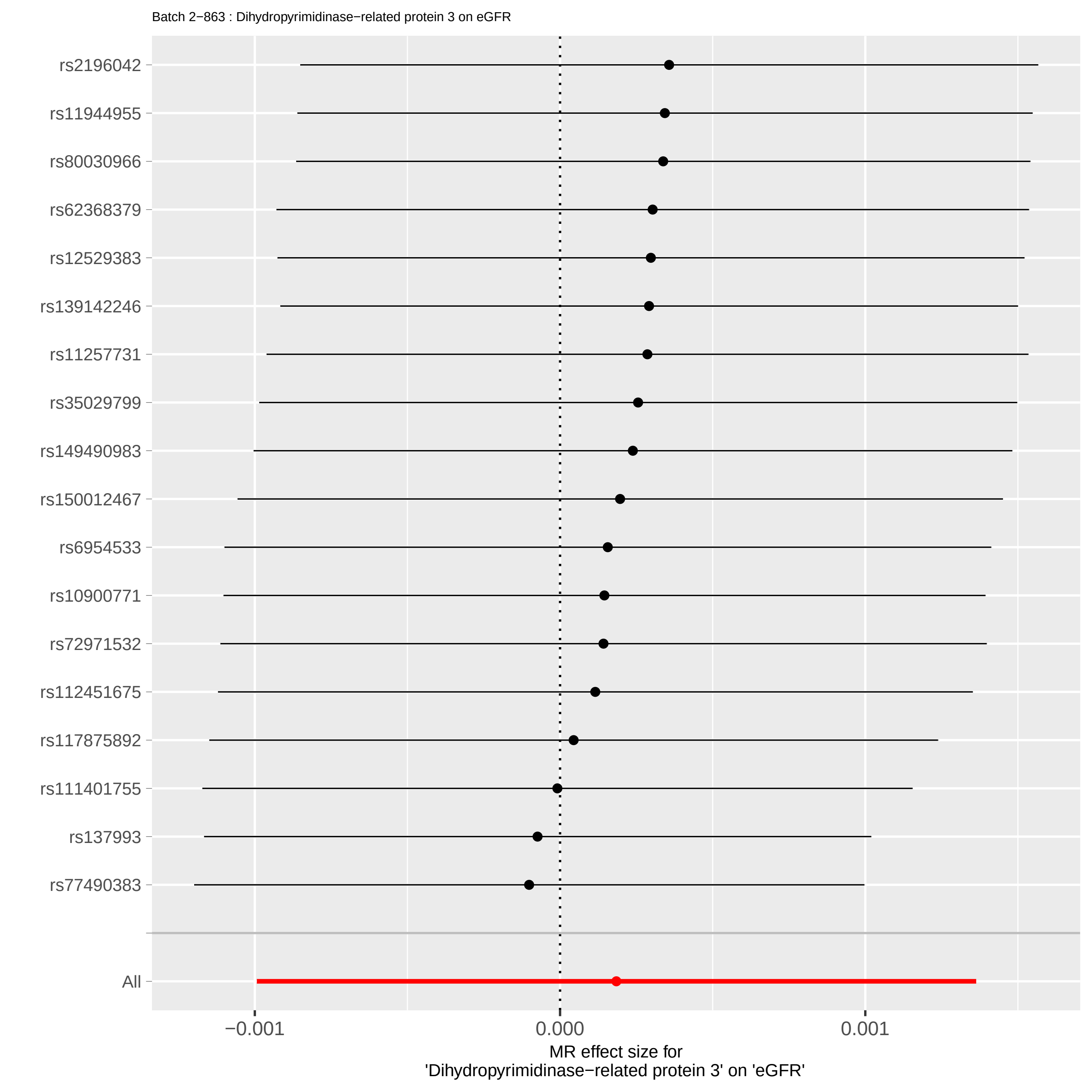

Batch 2−863 : Dihydropyrimidinase−related protein 3 on eGFR
rs2196042
rs11944955
rs80030966
rs62368379
rs12529383
rs139142246
rs11257731
rs35029799
rs149490983
rs150012467
rs6954533
rs10900771
rs72971532
rs112451675
rs117875892
rs111401755
rs137993
rs77490383
All
−0.001
0.000
0.001
MR effect size for
'Dihydropyrimidinase−related protein 3' on 'eGFR'

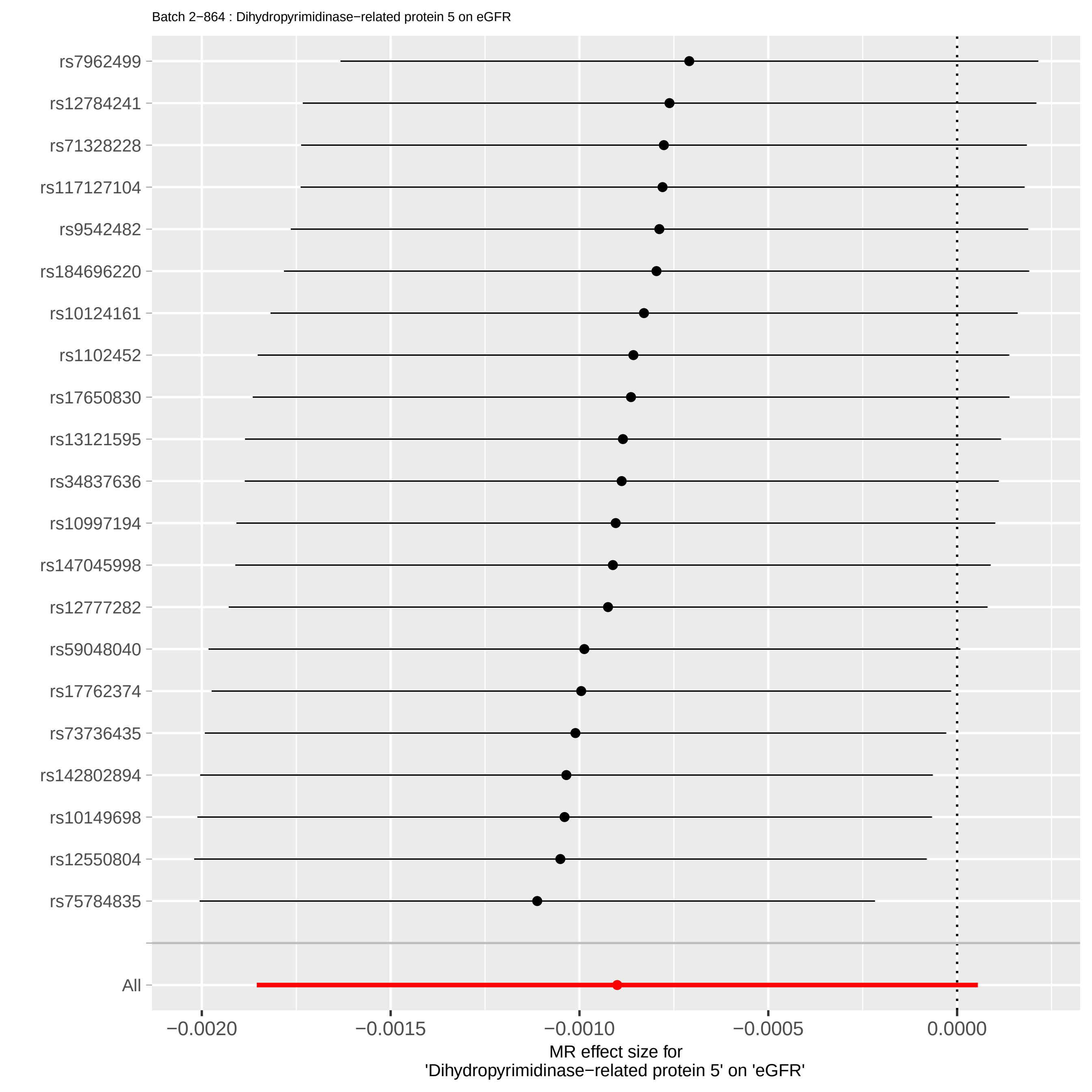

Batch 2−864 : Dihydropyrimidinase−related protein 5 on eGFR
rs7962499
rs12784241
rs71328228
rs117127104
rs9542482
rs184696220
rs10124161
rs1102452
rs17650830
rs13121595
rs34837636
rs10997194
rs147045998
rs12777282
rs59048040
rs17762374
rs73736435
rs142802894
rs10149698
rs12550804
rs75784835
All
−0.0020
−0.0015
−0.0010
−0.0005
0.0000
MR effect size for
'Dihydropyrimidinase−related protein 5' on 'eGFR'

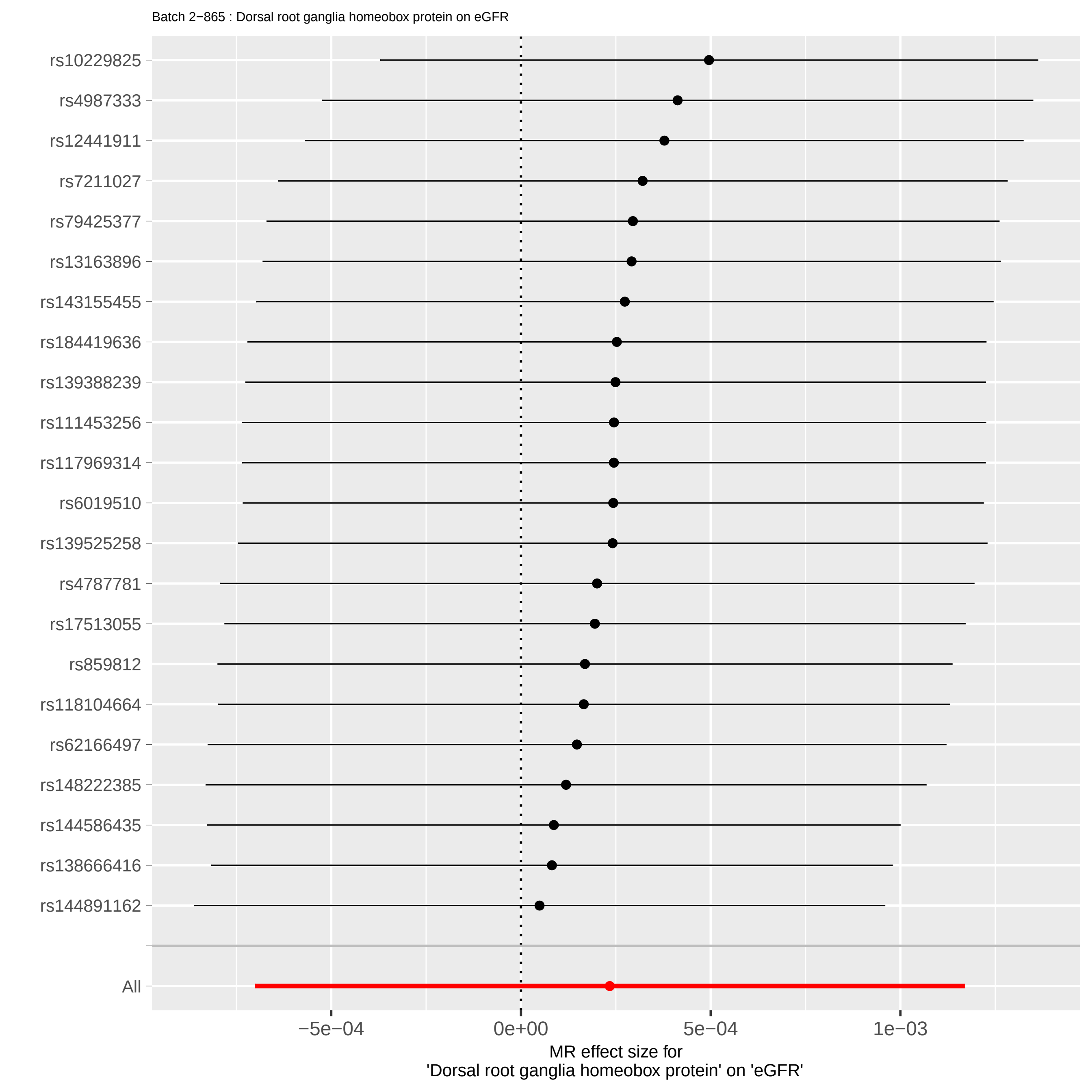

Batch 2−865 : Dorsal root ganglia homeobox protein on eGFR
rs10229825
rs4987333
rs12441911
rs7211027
rs79425377
rs13163896
rs143155455
rs184419636
rs139388239
rs111453256
rs117969314
rs6019510
rs139525258
rs4787781
rs17513055
rs859812
rs118104664
rs62166497
rs148222385
rs144586435
rs138666416
rs144891162
All
−5e−04
0e+00
5e−04
1e−03
MR effect size for
'Dorsal root ganglia homeobox protein' on 'eGFR'

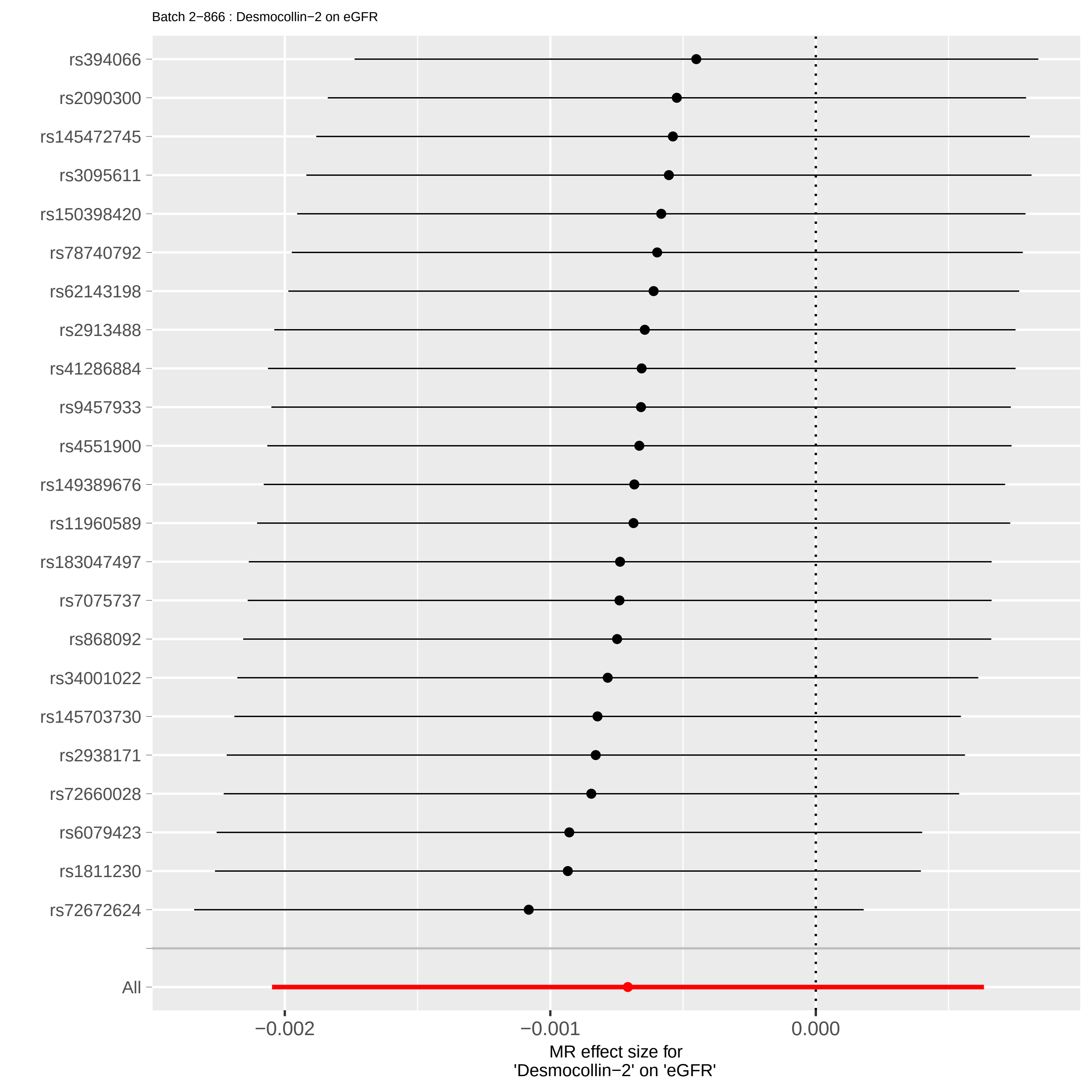

Batch 2−866 : Desmocollin−2 on eGFR
rs394066
rs2090300
rs145472745
rs3095611
rs150398420
rs78740792
rs62143198
rs2913488
rs41286884
rs9457933
rs4551900
rs149389676
rs11960589
rs183047497
rs7075737
rs868092
rs34001022
rs145703730
rs2938171
rs72660028
rs6079423
rs1811230
rs72672624
All
−0.002
−0.001
0.000
MR effect size for
'Desmocollin−2' on 'eGFR'

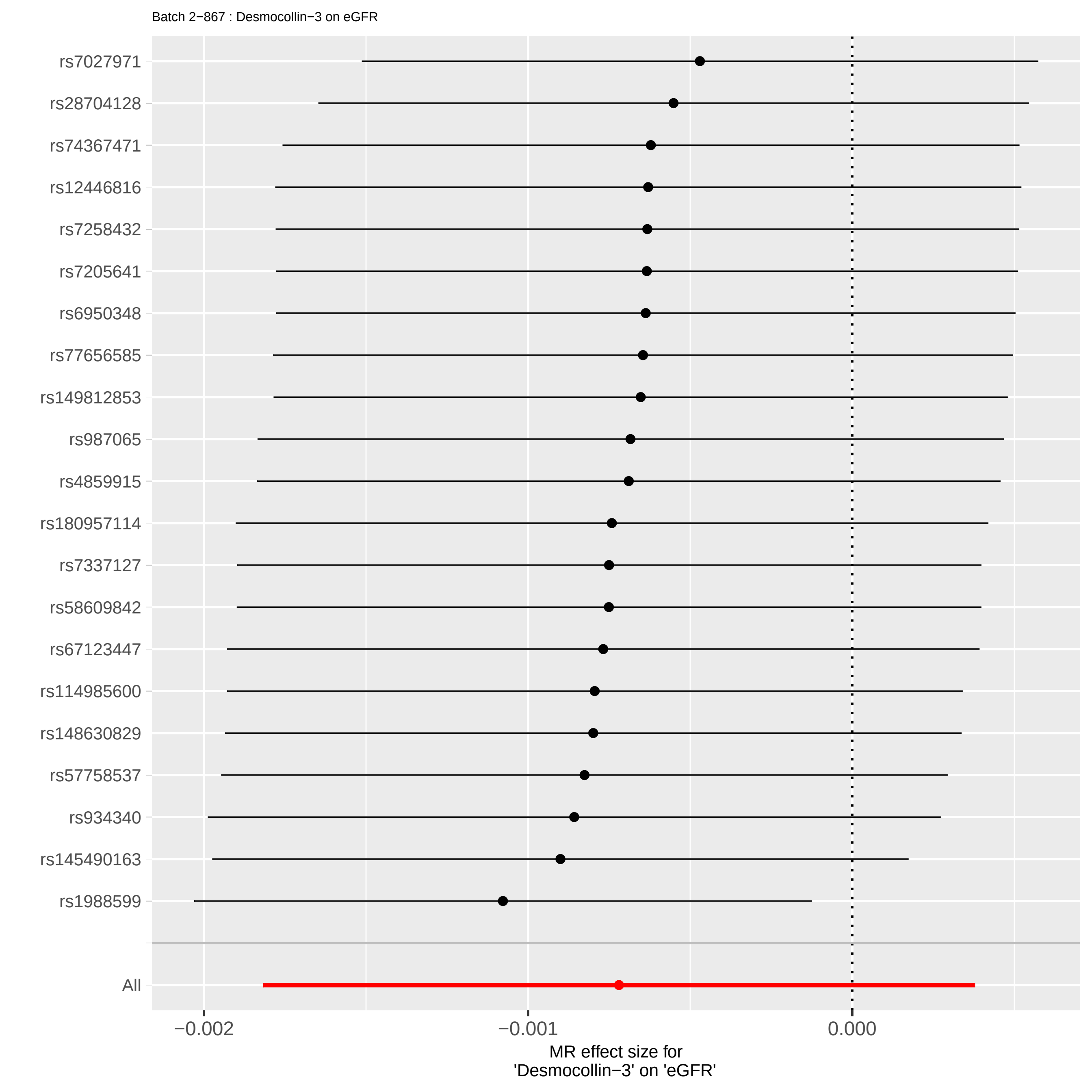

Batch 2−867 : Desmocollin−3 on eGFR
rs7027971
rs28704128
rs74367471
rs12446816
rs7258432
rs7205641
rs6950348
rs77656585
rs149812853
rs987065
rs4859915
rs180957114
rs7337127
rs58609842
rs67123447
rs114985600
rs148630829
rs57758537
rs934340
rs145490163
rs1988599
All
−0.002
−0.001
0.000
MR effect size for
'Desmocollin−3' on 'eGFR'

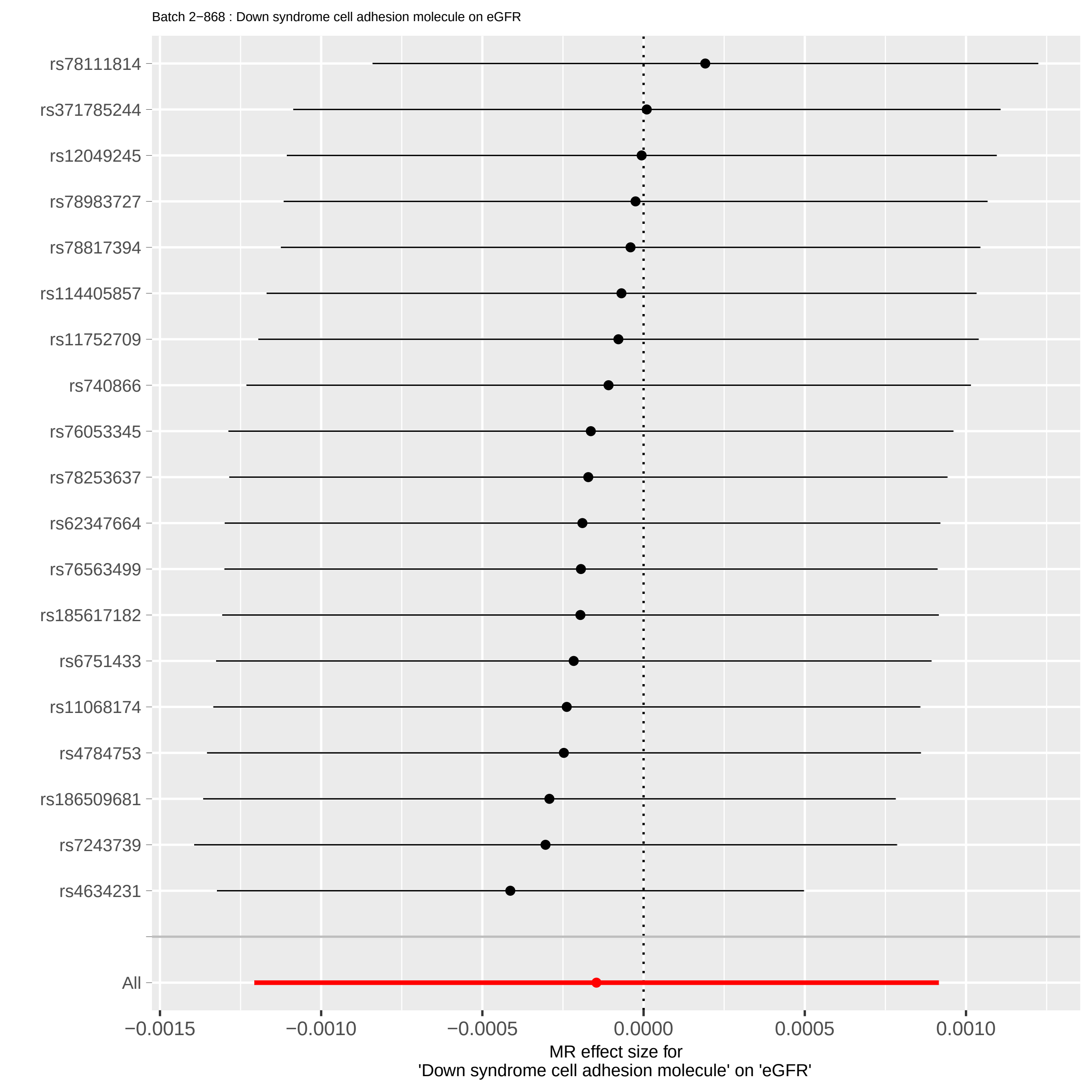
Batch 2−868 : Down syndrome cell adhesion molecule on eGFR
rs78111814
rs371785244
rs12049245
rs78983727
rs78817394
rs114405857
rs11752709
rs740866
rs76053345
rs78253637
rs62347664
rs76563499
rs185617182
rs6751433
rs11068174
rs4784753
rs186509681
rs7243739
rs4634231
All
−0.0015
−0.0010
−0.0005
0.0000
0.0005
0.0010
MR effect size for
'Down syndrome cell adhesion molecule' on 'eGFR'

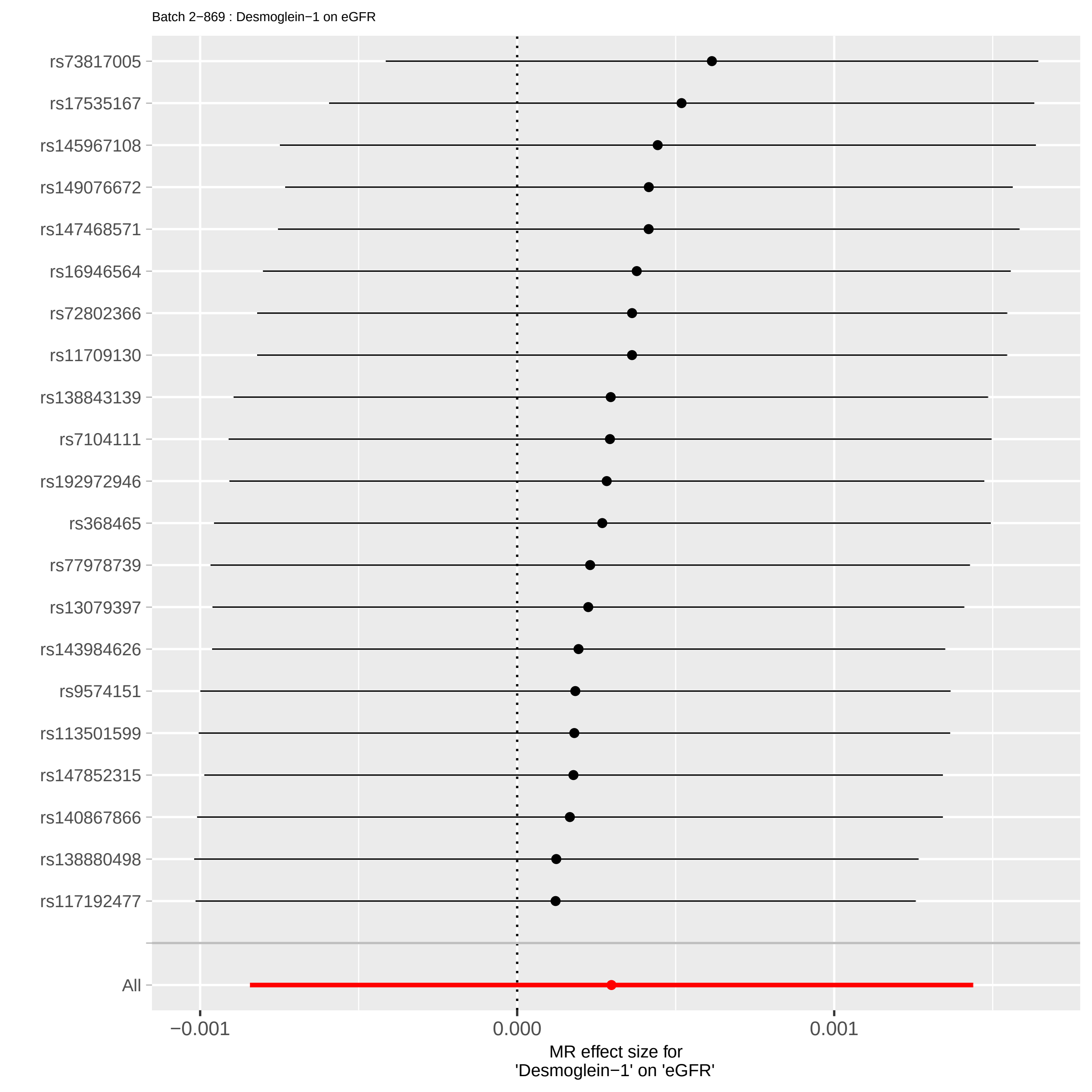

Batch 2−869 : Desmoglein−1 on eGFR
rs73817005
rs17535167
rs145967108
rs149076672
rs147468571
rs16946564
rs72802366
rs11709130
rs138843139
rs7104111
rs192972946
rs368465
rs77978739
rs13079397
rs143984626
rs9574151
rs113501599
rs147852315
rs140867866
rs138880498
rs117192477
All
−0.001
0.000
0.001
MR effect size for
'Desmoglein−1' on 'eGFR'

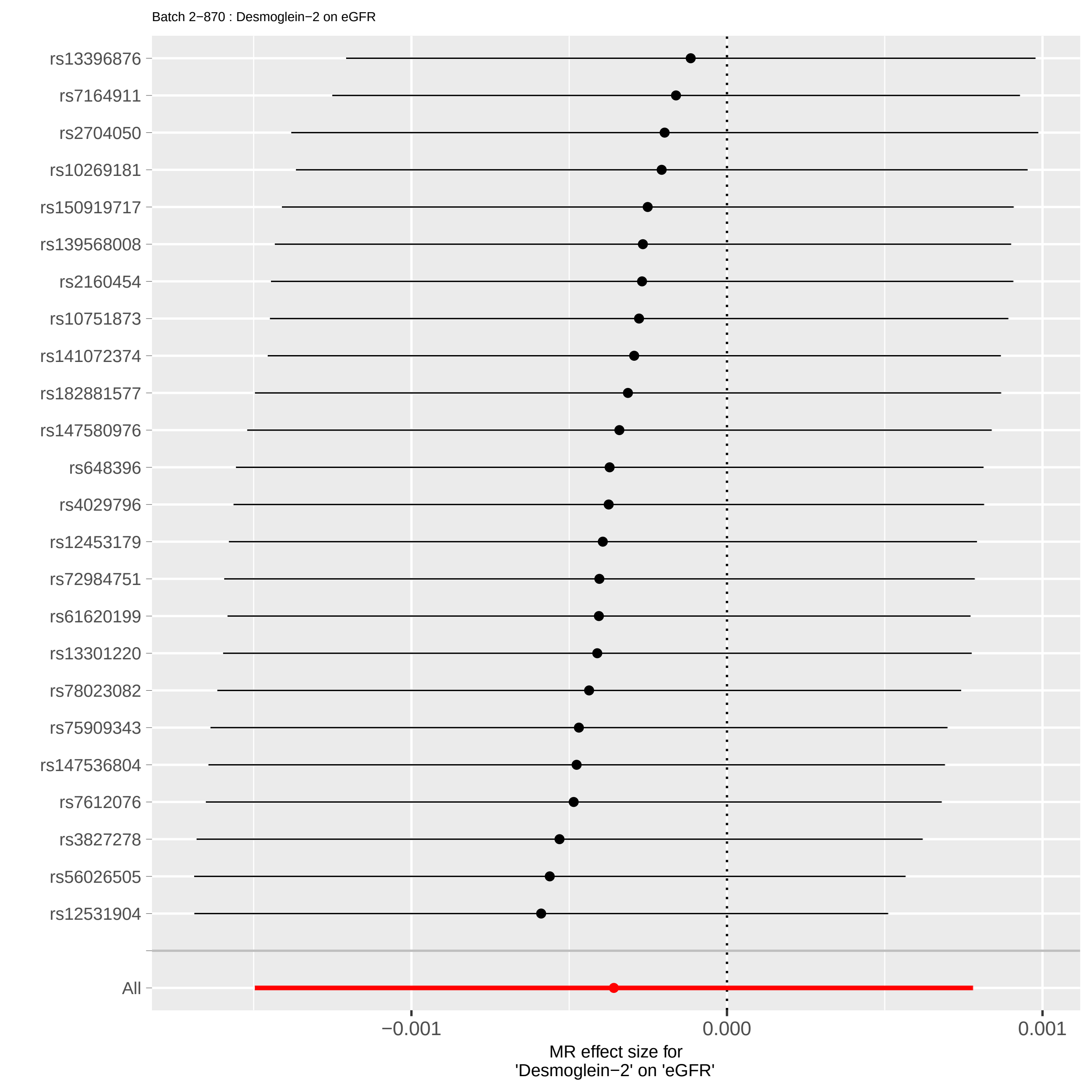

Batch 2−870 : Desmoglein−2 on eGFR
rs13396876
rs7164911
rs2704050
rs10269181
rs150919717
rs139568008
rs2160454
rs10751873
rs141072374
rs182881577
rs147580976
rs648396
rs4029796
rs12453179
rs72984751
rs61620199
rs13301220
rs78023082
rs75909343
rs147536804
rs7612076
rs3827278
rs56026505
rs12531904
All
−0.001
0.000
0.001
MR effect size for
'Desmoglein−2' on 'eGFR'

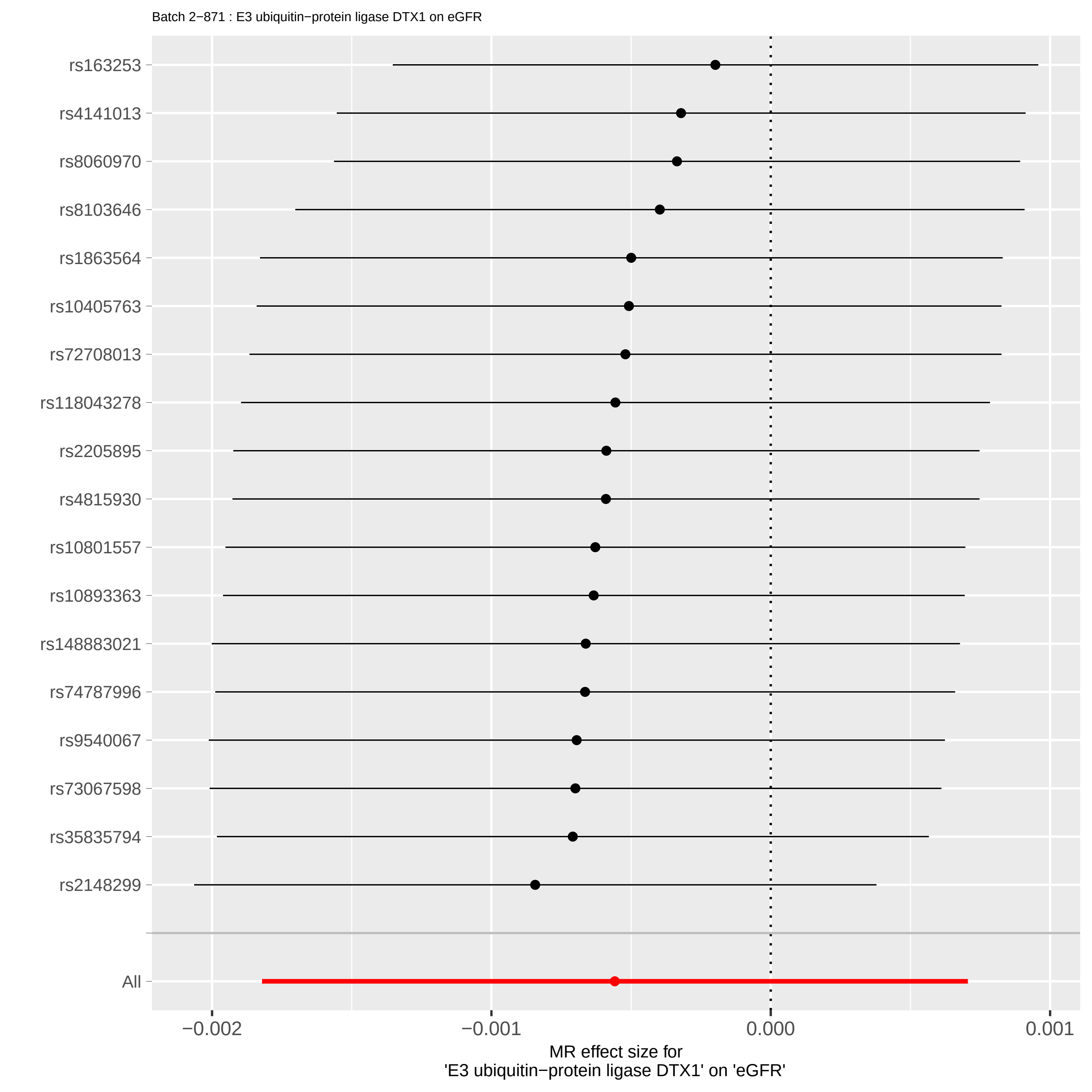

Batch 2−871 : E3 ubiquitin−protein ligase DTX1 on eGFR
rs163253
rs4141013
rs8060970
rs8103646
rs1863564
rs10405763
rs72708013
rs118043278
rs2205895
rs4815930
rs10801557
rs10893363
rs148883021
rs74787996
rs9540067
rs73067598
rs35835794
rs2148299
All
−0.002
−0.001
0.000
0.001
MR effect size for
'E3 ubiquitin−protein ligase DTX1' on 'eGFR'

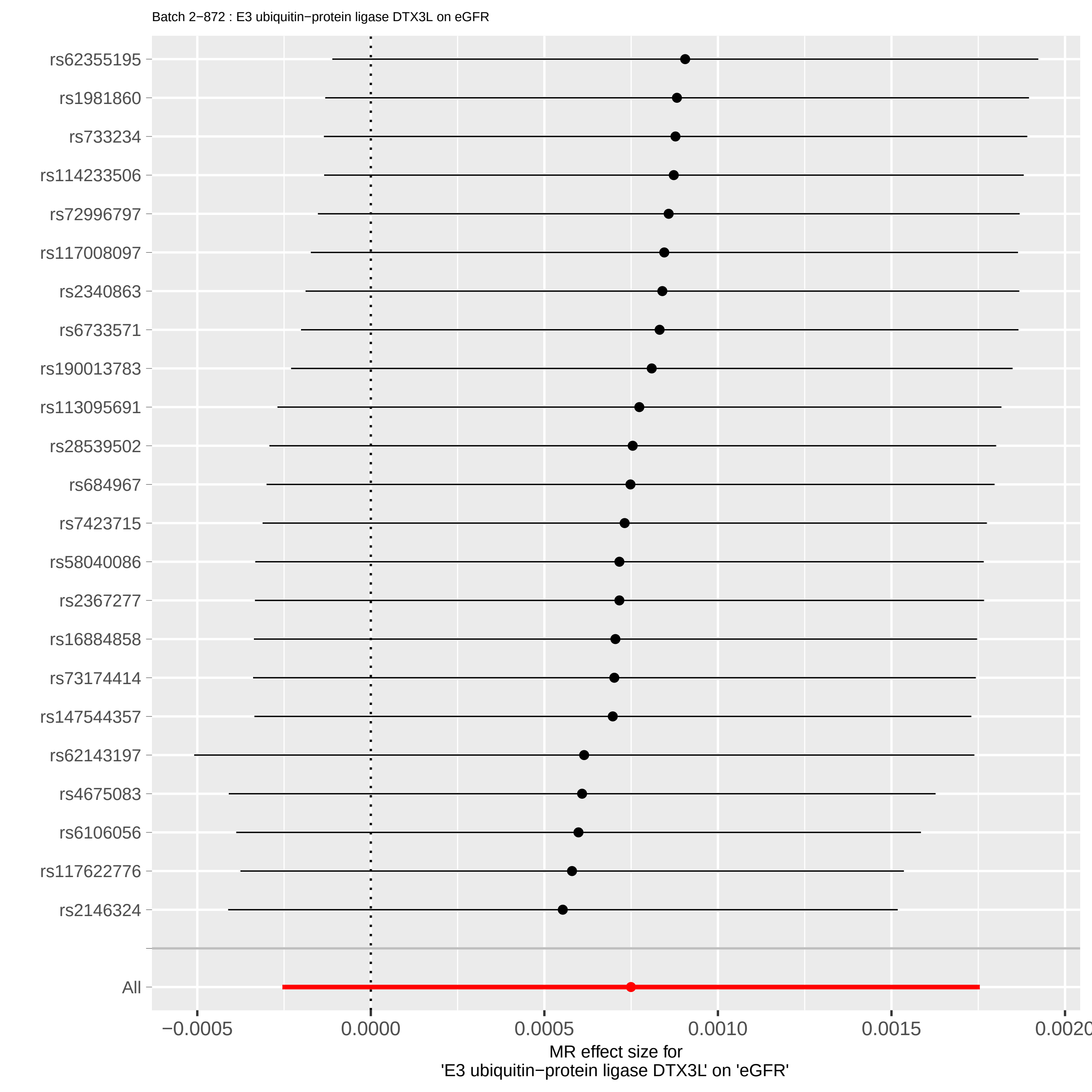

Batch 2−872 : E3 ubiquitin−protein ligase DTX3L on eGFR
rs62355195
rs1981860
rs733234
rs114233506
rs72996797
rs117008097
rs2340863
rs6733571
rs190013783
rs113095691
rs28539502
rs684967
rs7423715
rs58040086
rs2367277
rs16884858
rs73174414
rs147544357
rs62143197
rs4675083
rs6106056
rs117622776
rs2146324
All
−0.0005
0.0000
0.0005
0.0010
0.0015
MR effect size for
'E3 ubiquitin−protein ligase DTX3L' on 'eGFR'

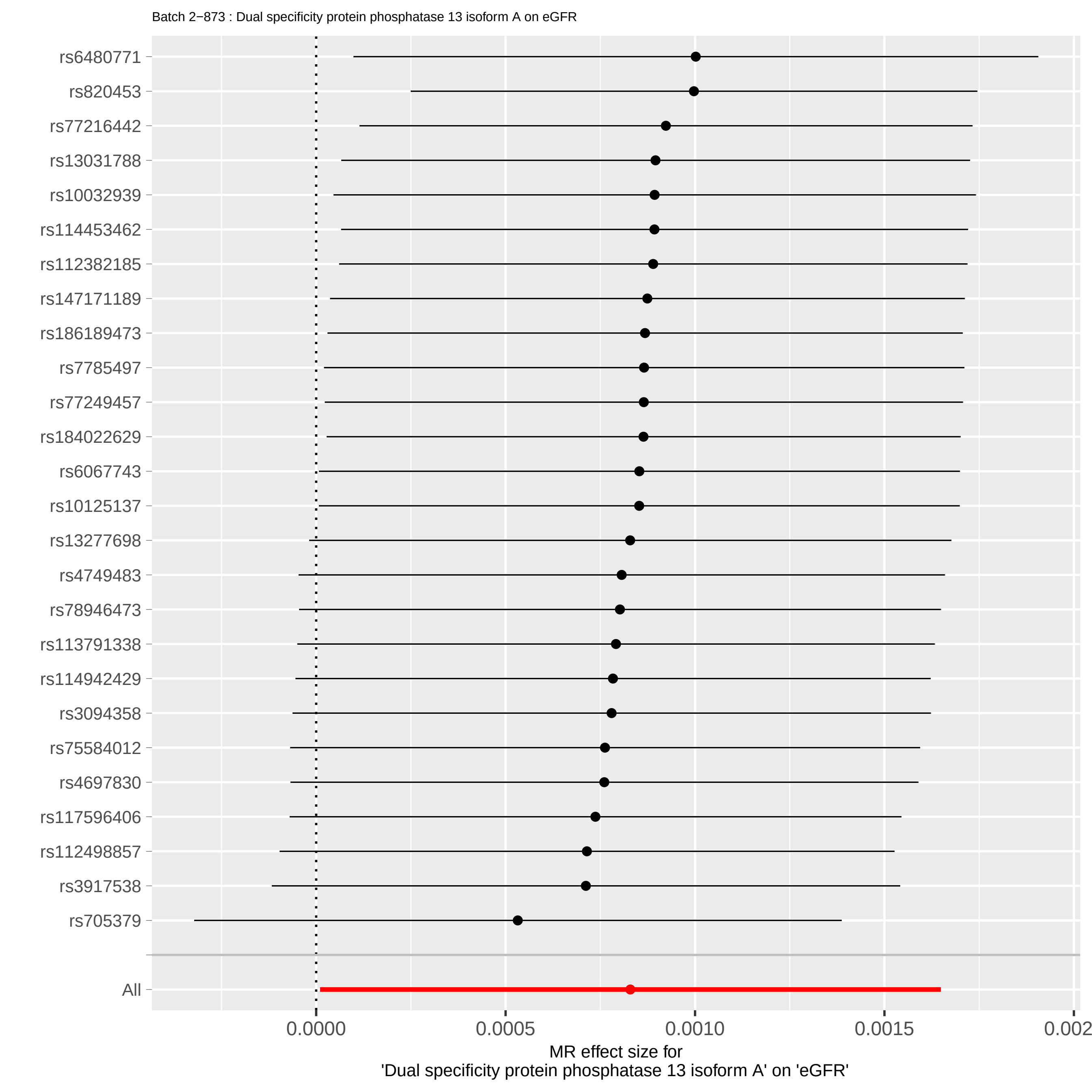

Batch 2−873 : Dual specificity protein phosphatase 13 isoform A on eGFR
rs6480771
rs820453
rs77216442
rs13031788
rs10032939
rs114453462
rs112382185
rs147171189
rs186189473
rs7785497
rs77249457
rs184022629
rs6067743
rs10125137
rs13277698
rs4749483
rs78946473
rs113791338
rs114942429
rs3094358
rs75584012
rs4697830
rs117596406
rs112498857
rs3917538
rs705379
All
0.0000
0.0005
0.0010
0.0015
0.002
MR effect size for
'Dual specificity protein phosphatase 13 isoform A' on 'eGFR'

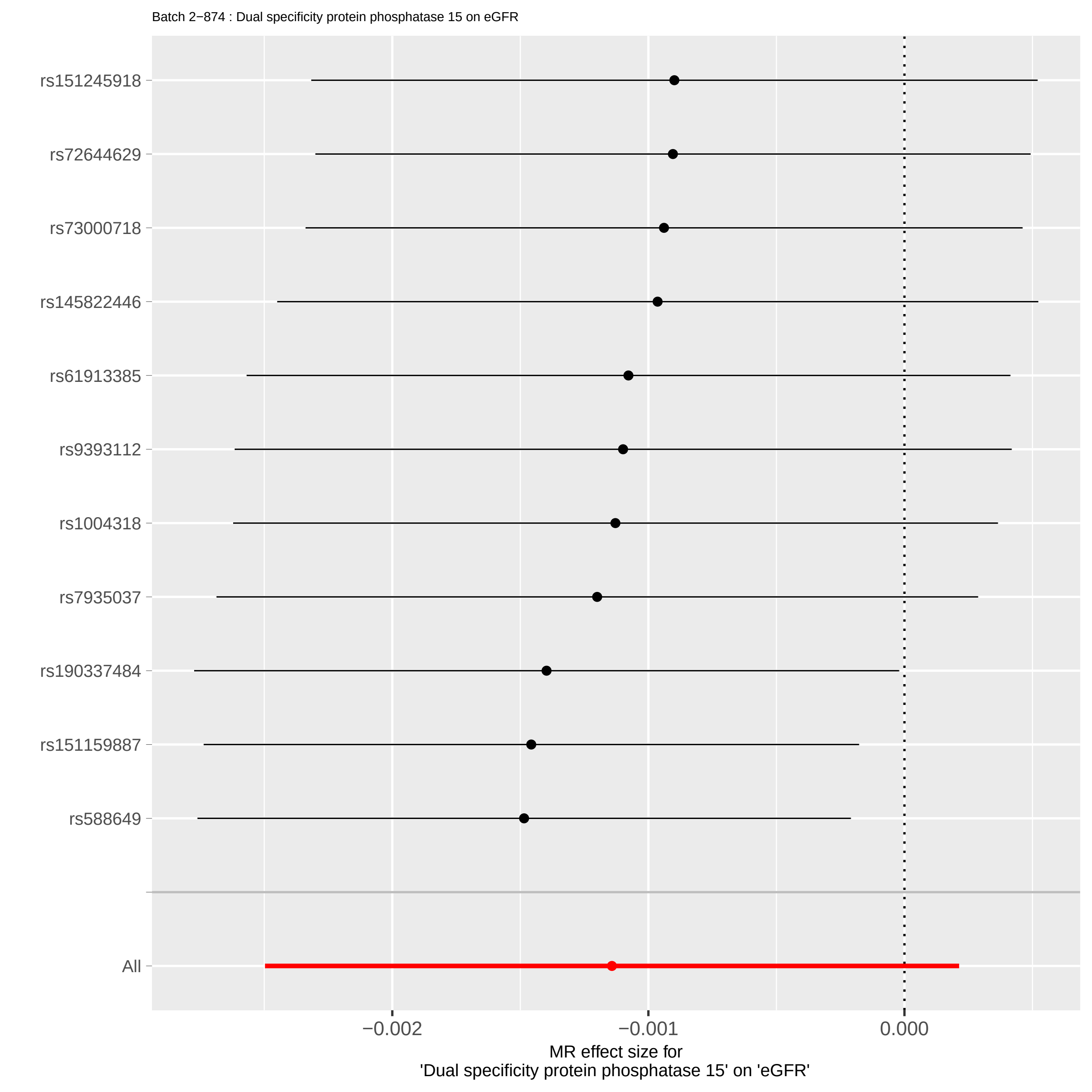

Batch 2−874 : Dual specificity protein phosphatase 15 on eGFR
rs151245918
rs72644629
rs73000718
rs145822446
rs61913385
rs9393112
rs1004318
rs7935037
rs190337484
rs151159887
rs588649
All
−0.002
−0.001
0.000
MR effect size for
'Dual specificity protein phosphatase 15' on 'eGFR'

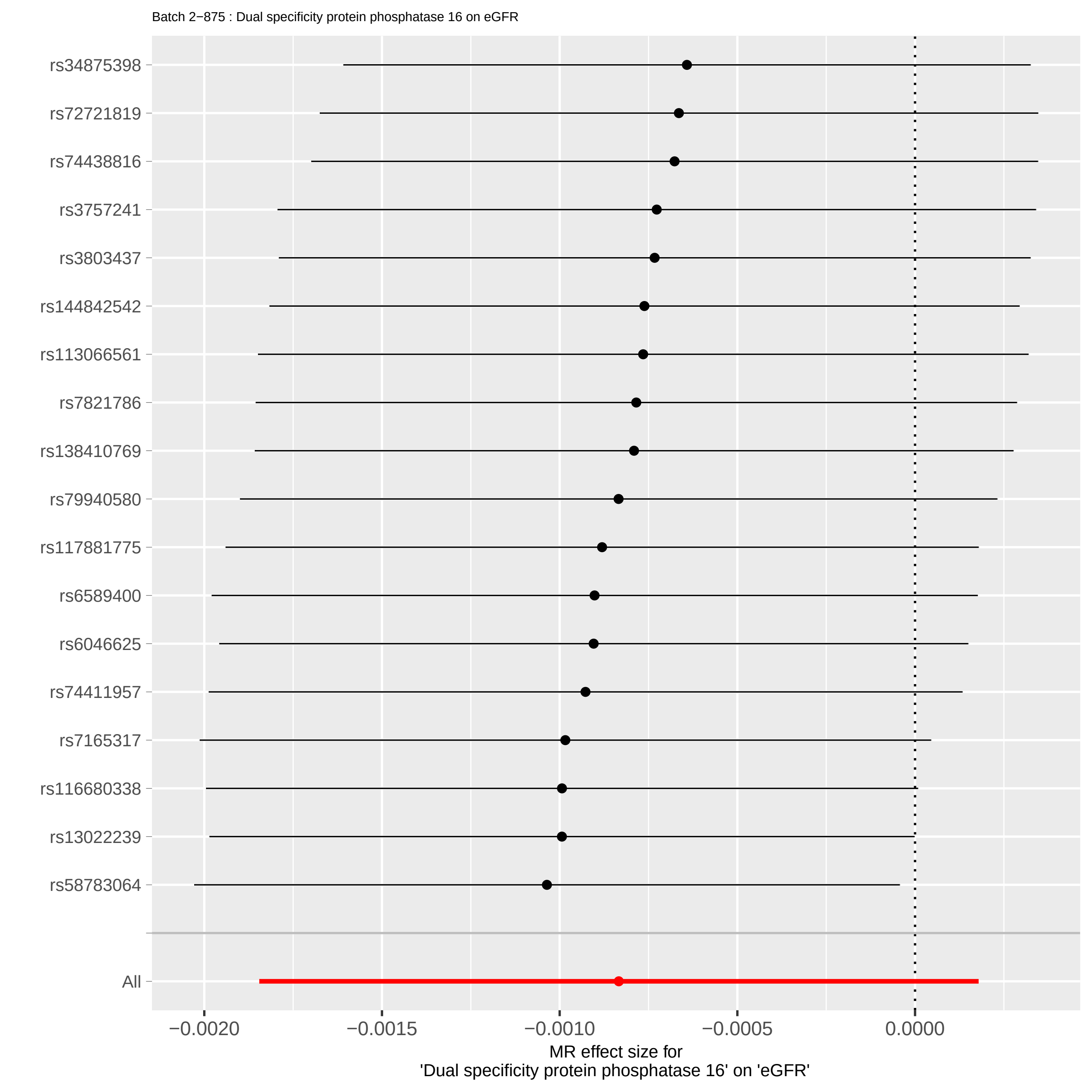

Batch 2−875 : Dual specificity protein phosphatase 16 on eGFR
rs34875398
rs72721819
rs74438816
rs3757241
rs3803437
rs144842542
rs113066561
rs7821786
rs138410769
rs79940580
rs117881775
rs6589400
rs6046625
rs74411957
rs7165317
rs116680338
rs13022239
rs58783064
All
−0.0020
−0.0015
−0.0010
−0.0005
0.0000
MR effect size for
'Dual specificity protein phosphatase 16' on 'eGFR'

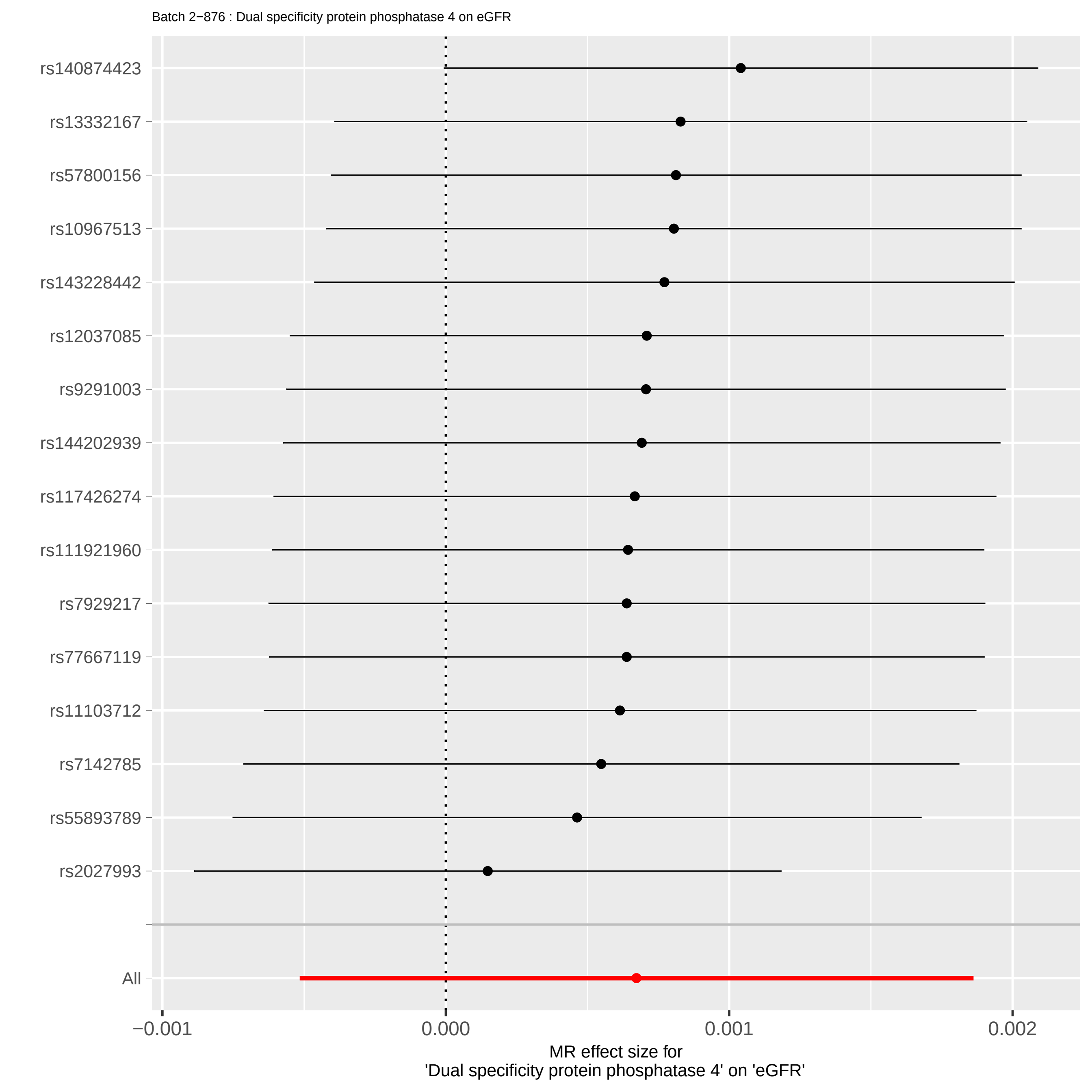
Batch 2−876 : Dual specificity protein phosphatase 4 on eGFR
rs140874423
rs13332167
rs57800156
rs10967513
rs143228442
rs12037085
rs9291003
rs144202939
rs117426274
rs111921960
rs7929217
rs77667119
rs11103712
rs7142785
rs55893789
rs2027993
All
−0.001
0.000
0.001
0.002
MR effect size for
'Dual specificity protein phosphatase 4' on 'eGFR'

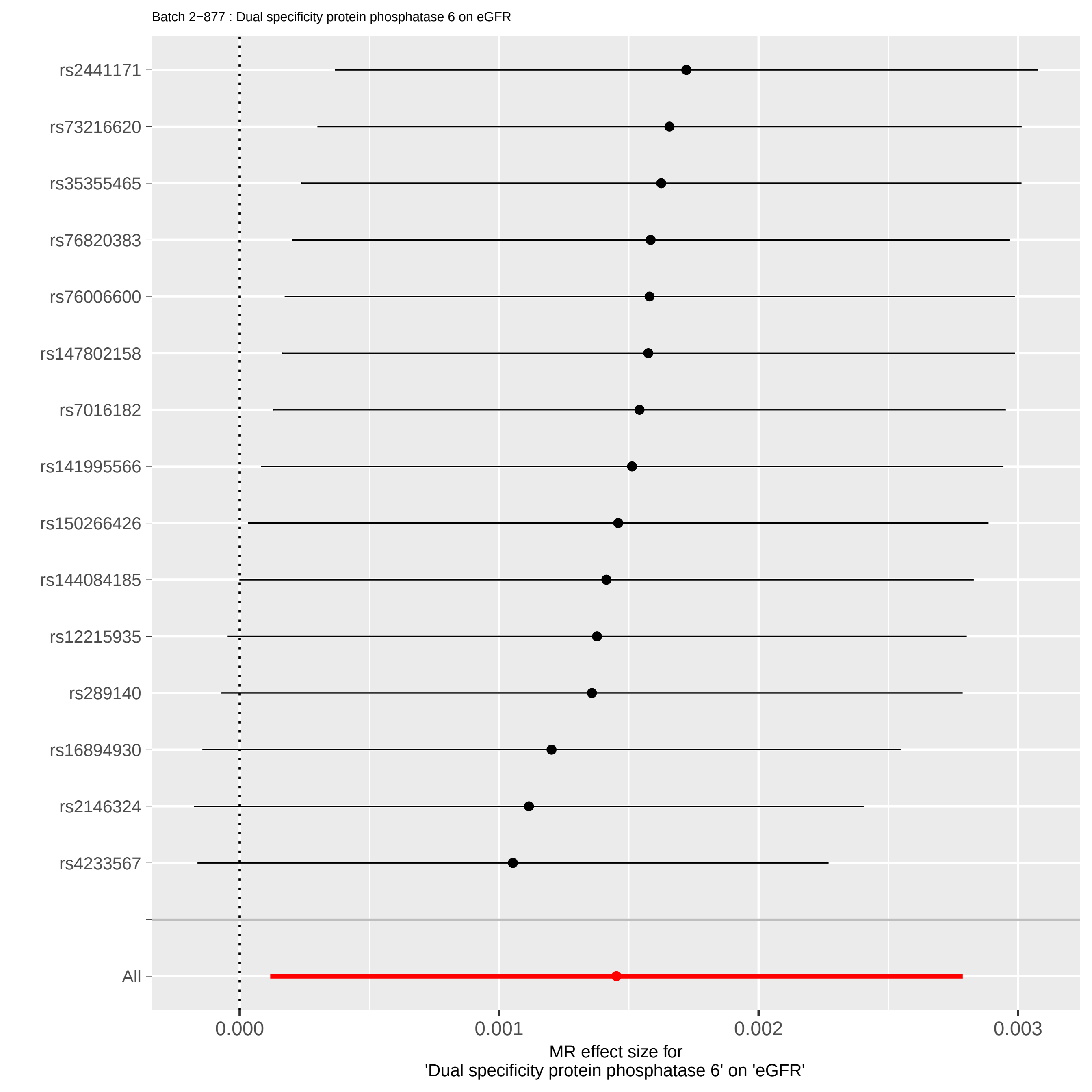

Batch 2−877 : Dual specificity protein phosphatase 6 on eGFR
rs2441171
rs73216620
rs35355465
rs76820383
rs76006600
rs147802158
rs7016182
rs141995566
rs150266426
rs144084185
rs12215935
rs289140
rs16894930
rs2146324
rs4233567
All
0.000
0.001
0.002
0.003
MR effect size for
'Dual specificity protein phosphatase 6' on 'eGFR'

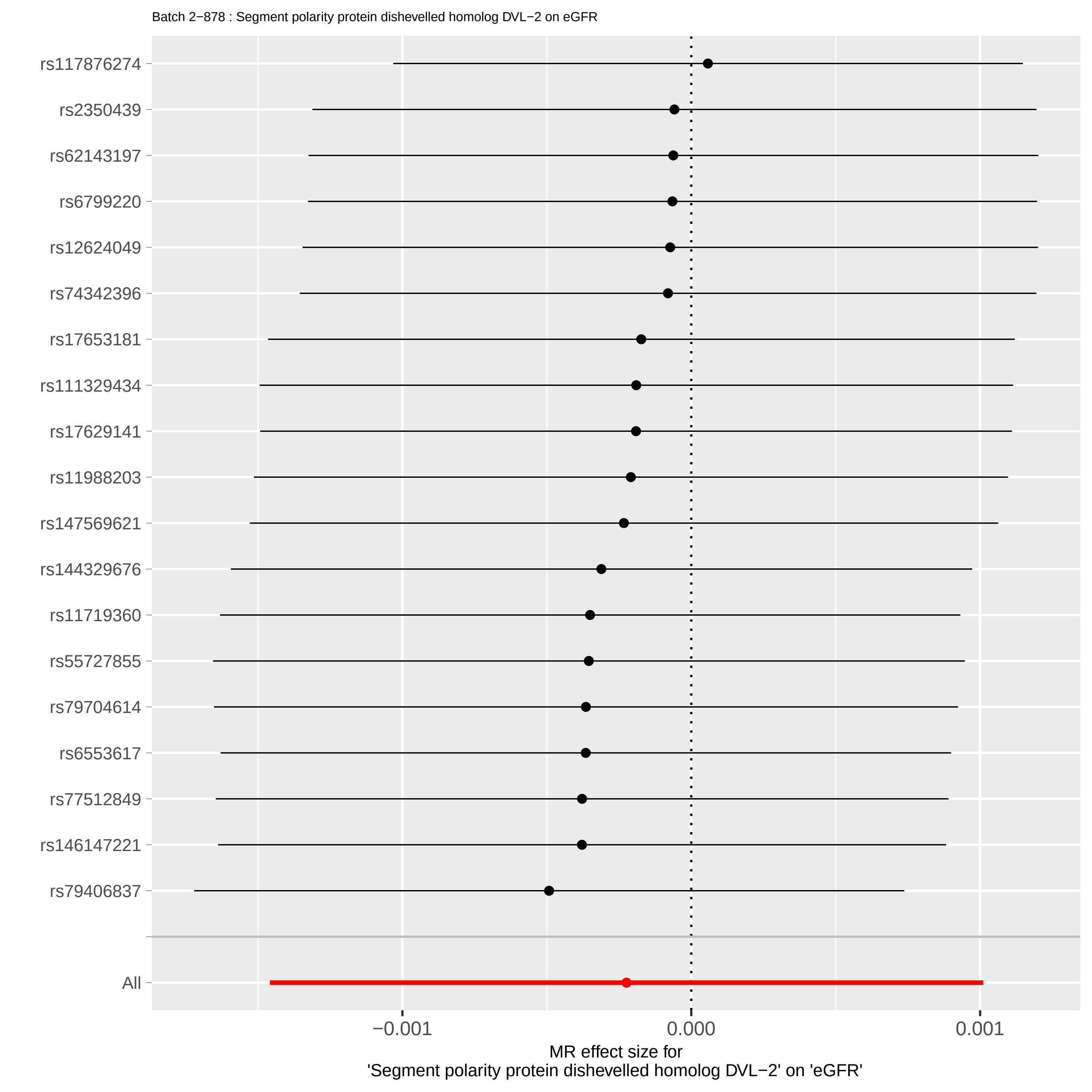

Batch 2−878 : Segment polarity protein dishevelled homolog DVL−2 on eGFR
rs117876274
rs2350439
rs62143197
rs6799220
rs12624049
rs74342396
rs17653181
rs111329434
rs17629141
rs11988203
rs147569621
rs144329676
rs11719360
rs55727855
rs79704614
rs6553617
rs77512849
rs146147221
rs79406837
All
−0.001
0.000
0.001
MR effect size for
'Segment polarity protein dishevelled homolog DVL−2' on 'eGFR'

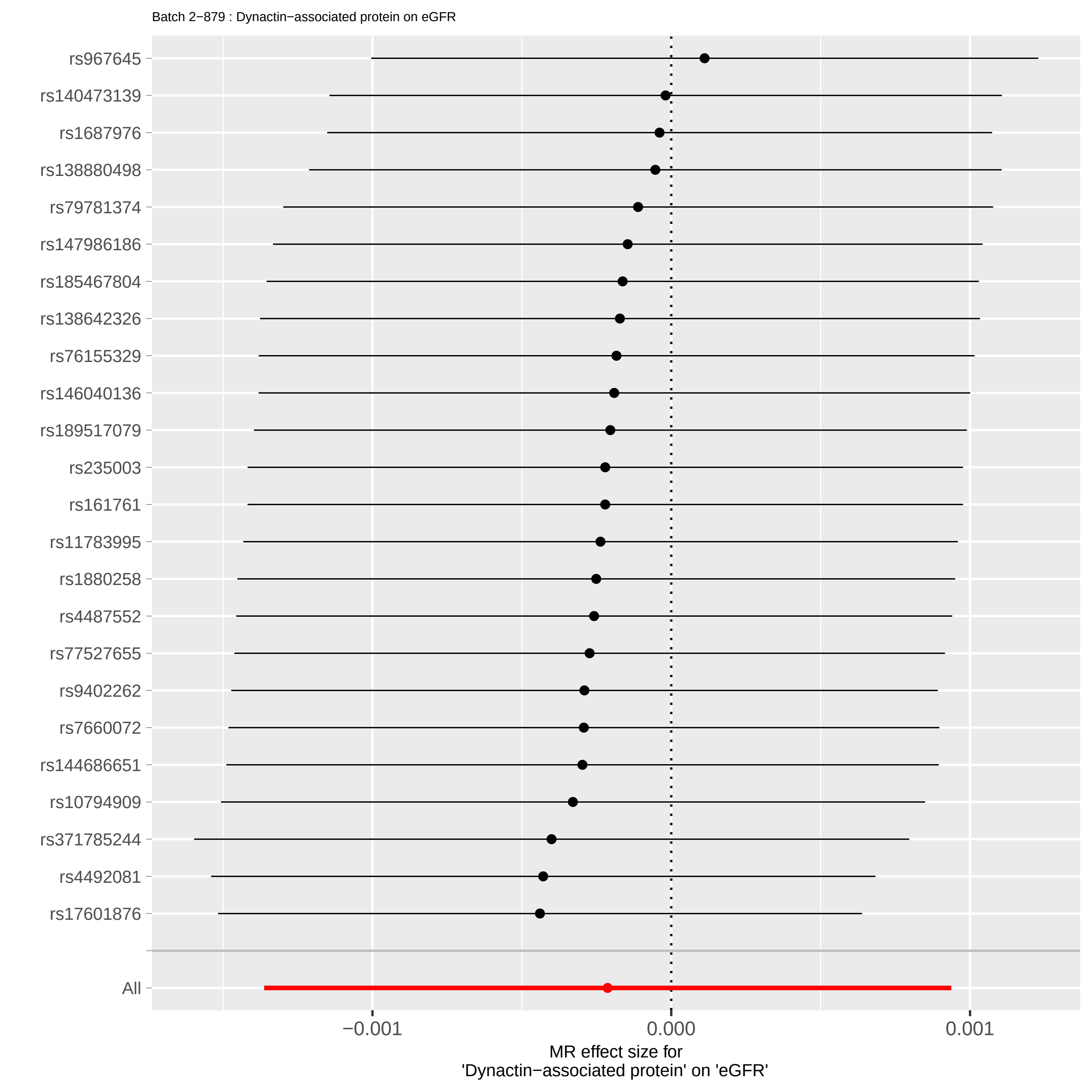

Batch 2−879 : Dynactin−associated protein on eGFR
rs967645
rs140473139
rs1687976
rs138880498
rs79781374
rs147986186
rs185467804
rs138642326
rs76155329
rs146040136
rs189517079
rs235003
rs161761
rs11783995
rs1880258
rs4487552
rs77527655
rs9402262
rs7660072
rs144686651
rs10794909
rs371785244
rs4492081
rs17601876
All
−0.001
0.000
0.001
MR effect size for
'Dynactin−associated protein' on 'eGFR'

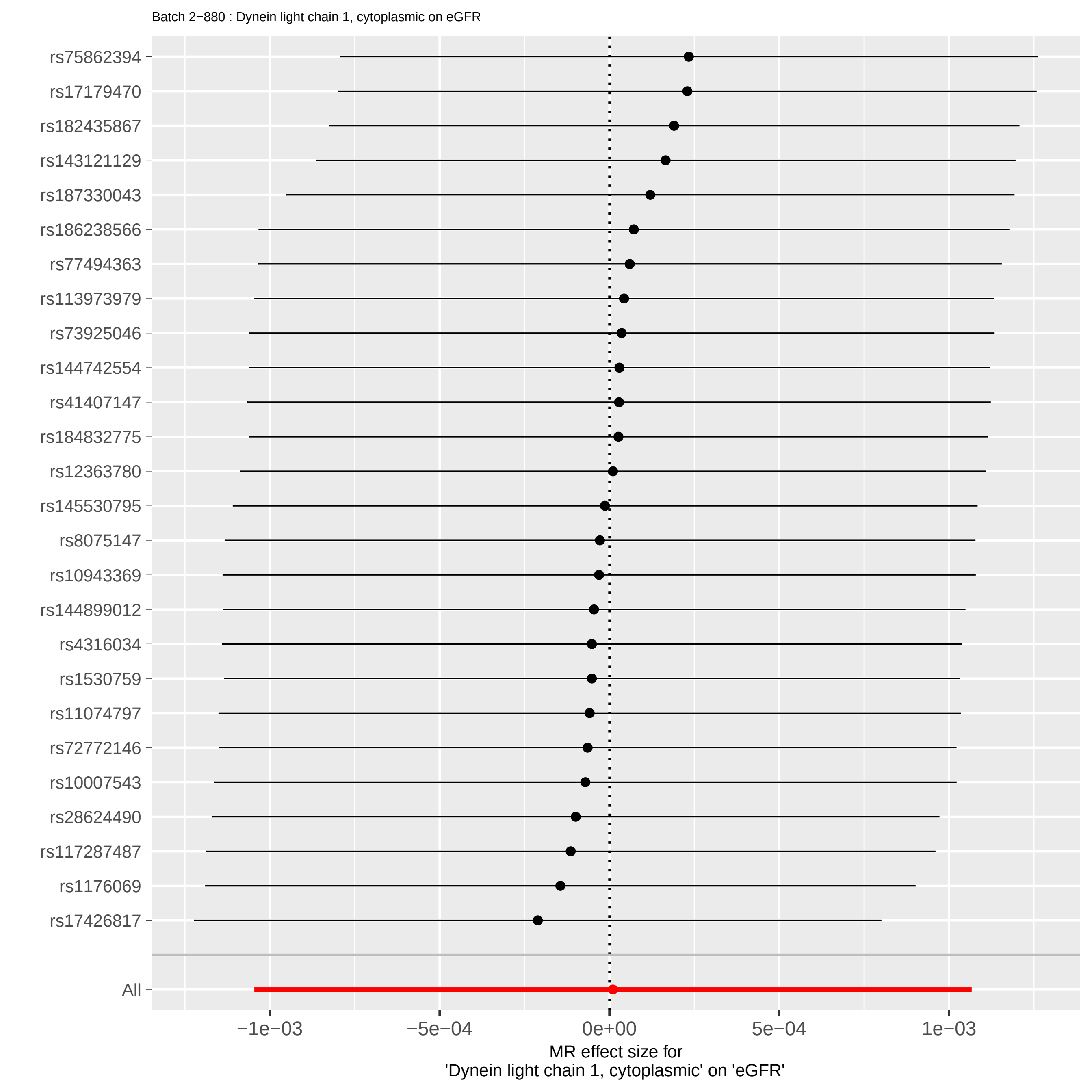

Batch 2−880 : Dynein light chain 1, cytoplasmic on eGFR
rs75862394
rs17179470
rs182435867
rs143121129
rs187330043
rs186238566
rs77494363
rs113973979
rs73925046
rs144742554
rs41407147
rs184832775
rs12363780
rs145530795
rs8075147
rs10943369
rs144899012
rs4316034
rs1530759
rs11074797
rs72772146
rs10007543
rs28624490
rs117287487
rs1176069
rs17426817
All
−1e−03
−5e−04
0e+00
5e−04
1e−03
MR effect size for
'Dynein light chain 1, cytoplasmic' on 'eGFR'

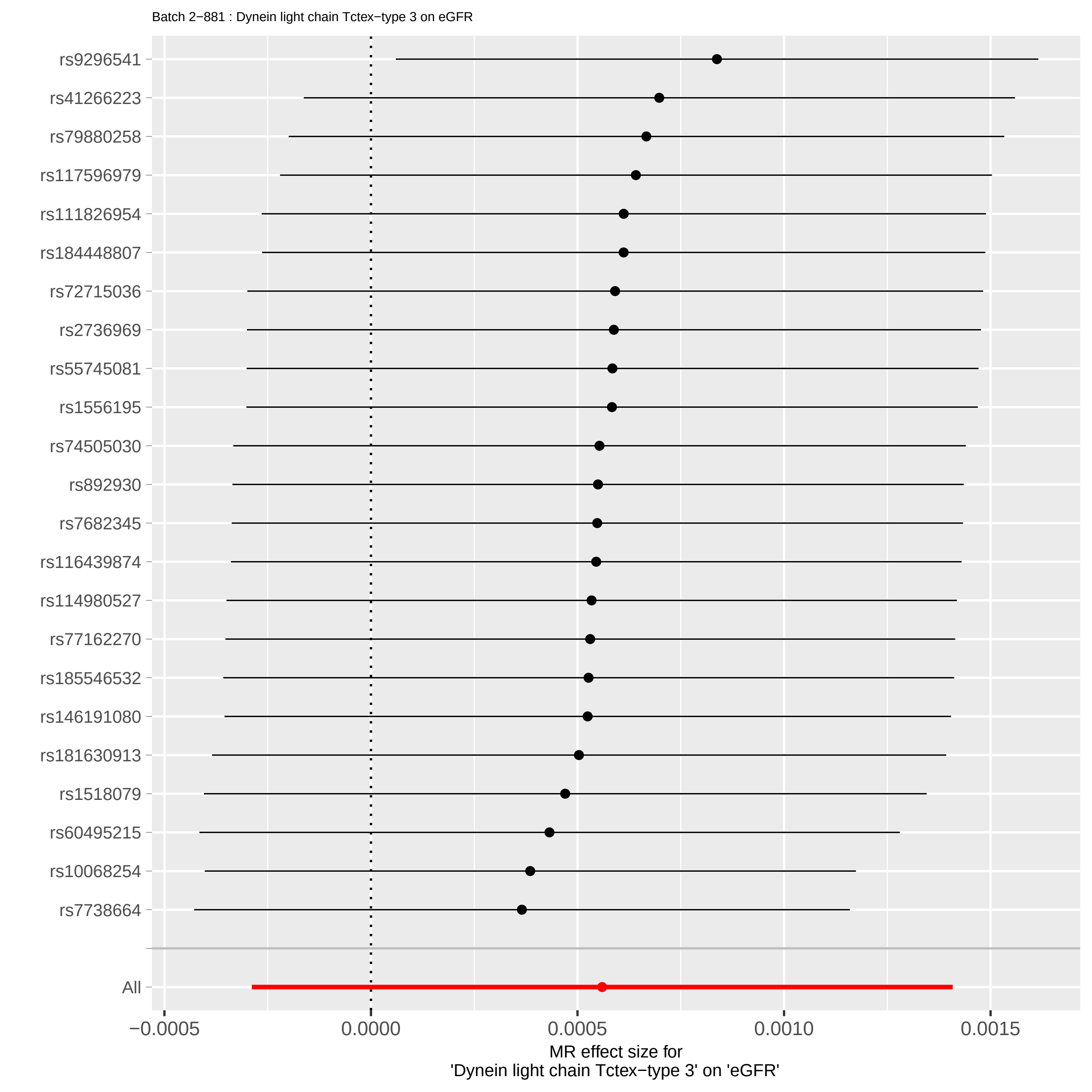

Batch 2−881 : Dynein light chain Tctex−type 3 on eGFR
rs9296541
rs41266223
rs79880258
rs117596979
rs111826954
rs184448807
rs72715036
rs2736969
rs55745081
rs1556195
rs74505030
rs892930
rs7682345
rs116439874
rs114980527
rs77162270
rs185546532
rs146191080
rs181630913
rs1518079
rs60495215
rs10068254
rs7738664
All
−0.0005
0.0000
0.0005
0.0010
0.0015
MR effect size for
'Dynein light chain Tctex−type 3' on 'eGFR'

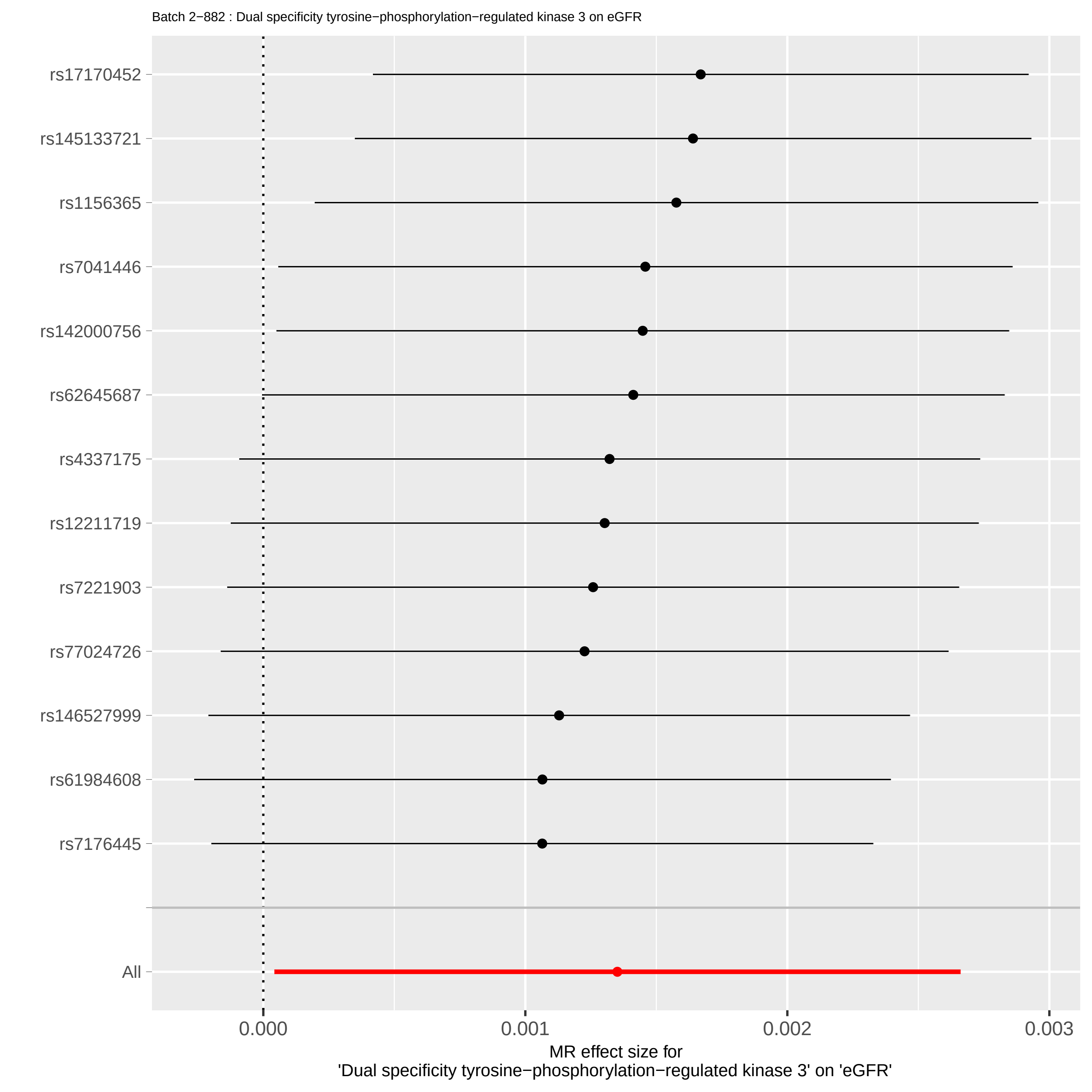
Batch 2−882 : Dual specificity tyrosine−phosphorylation−regulated kinase 3 on eGFR
rs17170452
rs145133721
rs1156365
rs7041446
rs142000756
rs62645687
rs4337175
rs12211719
rs7221903
rs77024726
rs146527999
rs61984608
rs7176445
All
0.000
0.001
0.002
0.003
MR effect size for
'Dual specificity tyrosine−phosphorylation−regulated kinase 3' on 'eGFR'

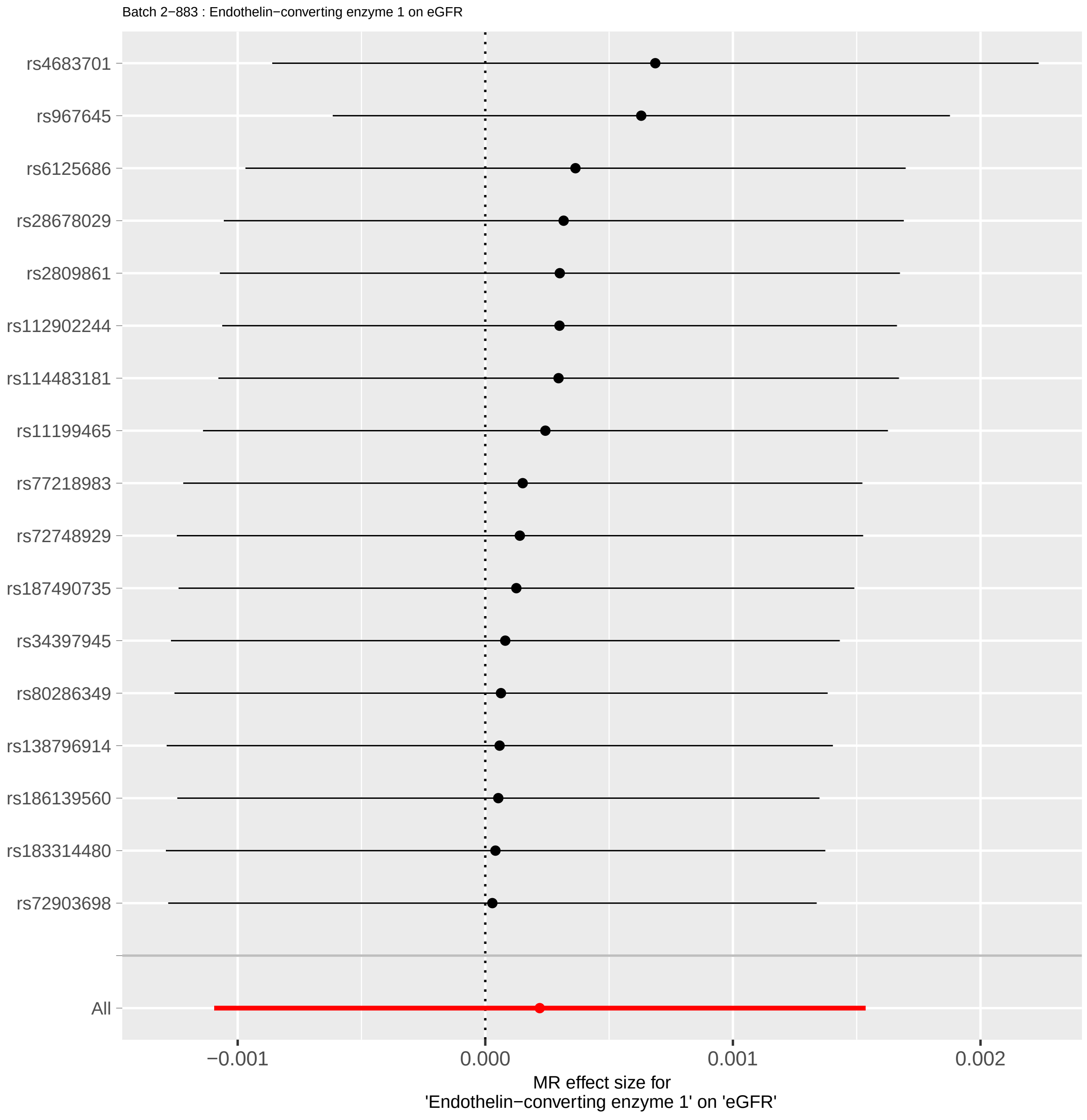

Batch 2−883 : Endothelin−converting enzyme 1 on eGFR
rs4683701
rs967645
rs6125686
rs28678029
rs2809861
rs112902244
rs114483181
rs11199465
rs77218983
rs72748929
rs187490735
rs34397945
rs80286349
rs138796914
rs186139560
rs183314480
rs72903698
All
−0.001
0.000
0.001
0.002
MR effect size for
'Endothelin−converting enzyme 1' on 'eGFR'

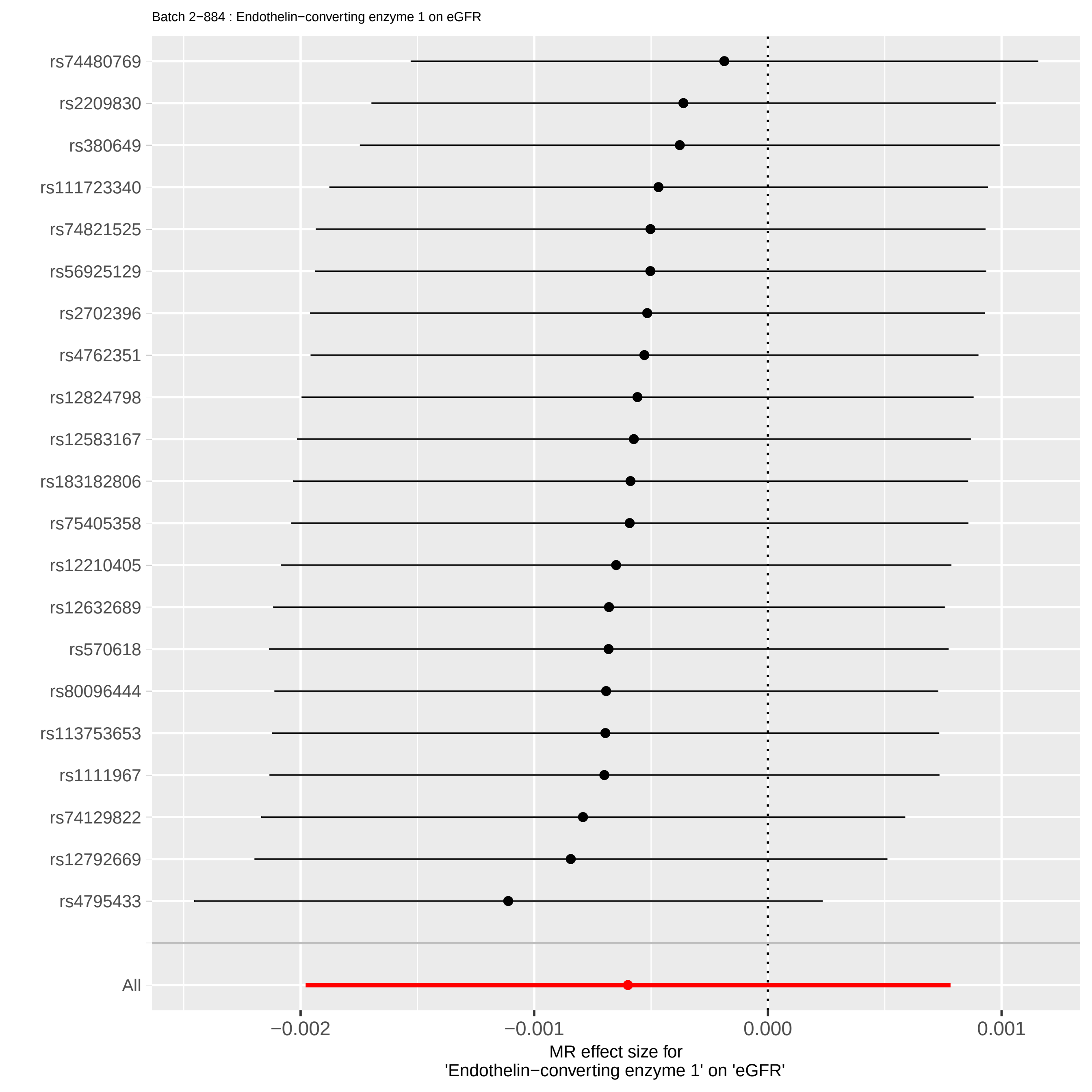

Batch 2−884 : Endothelin−converting enzyme 1 on eGFR
rs74480769
rs2209830
rs380649
rs111723340
rs74821525
rs56925129
rs2702396
rs4762351
rs12824798
rs12583167
rs183182806
rs75405358
rs12210405
rs12632689
rs570618
rs80096444
rs113753653
rs1111967
rs74129822
rs12792669
rs4795433
All
−0.002
−0.001
0.000
0.001
MR effect size for
'Endothelin−converting enzyme 1' on 'eGFR'

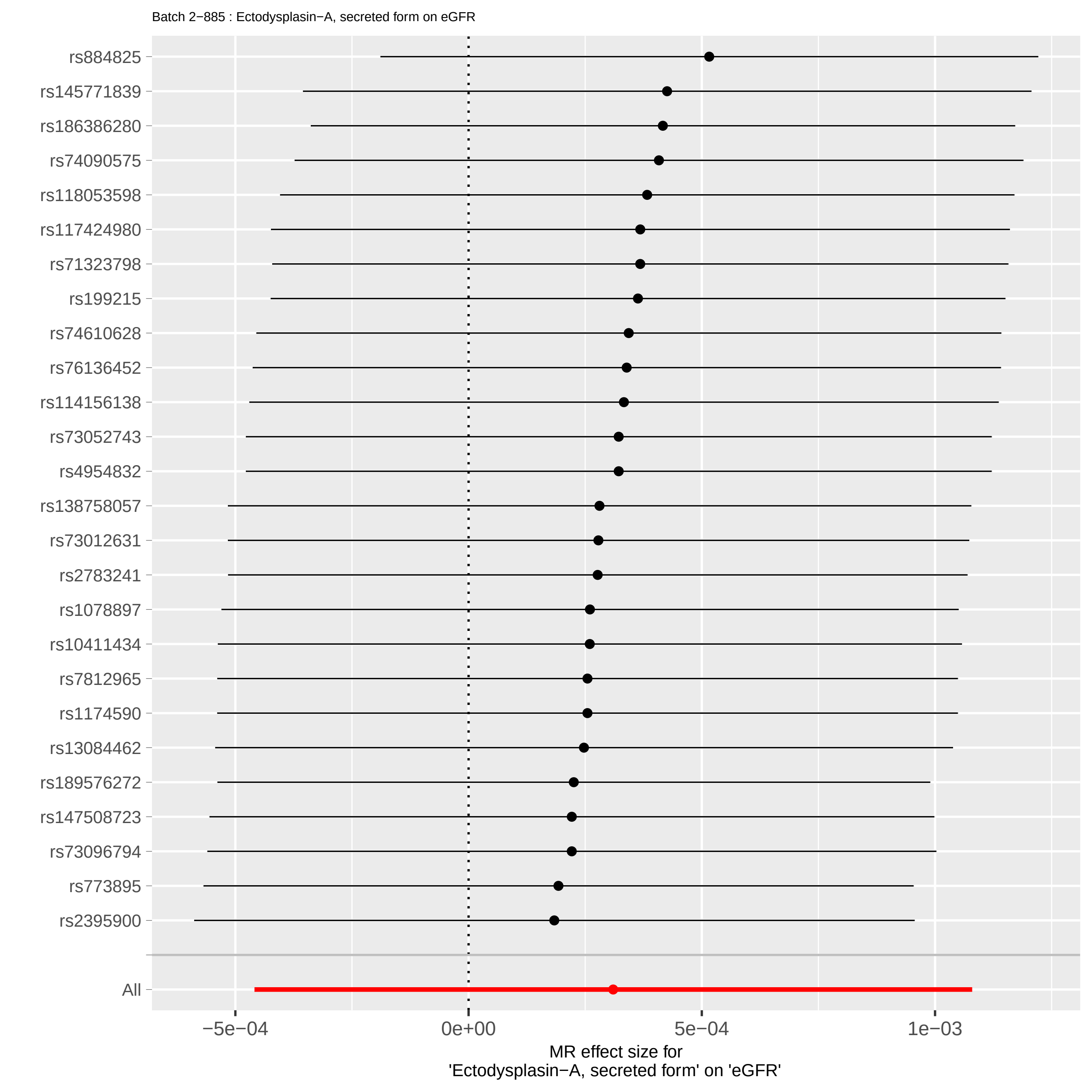
Batch 2−885 : Ectodysplasin−A, secreted form on eGFR
rs884825
rs145771839
rs186386280
rs74090575
rs118053598
rs117424980
rs71323798
rs199215
rs74610628
rs76136452
rs114156138
rs73052743
rs4954832
rs138758057
rs73012631
rs2783241
rs1078897
rs10411434
rs7812965
rs1174590
rs13084462
rs189576272
rs147508723
rs73096794
rs773895
rs2395900
All
−5e−04
0e+00
5e−04
1e−03
MR effect size for
'Ectodysplasin−A, secreted form' on 'eGFR'

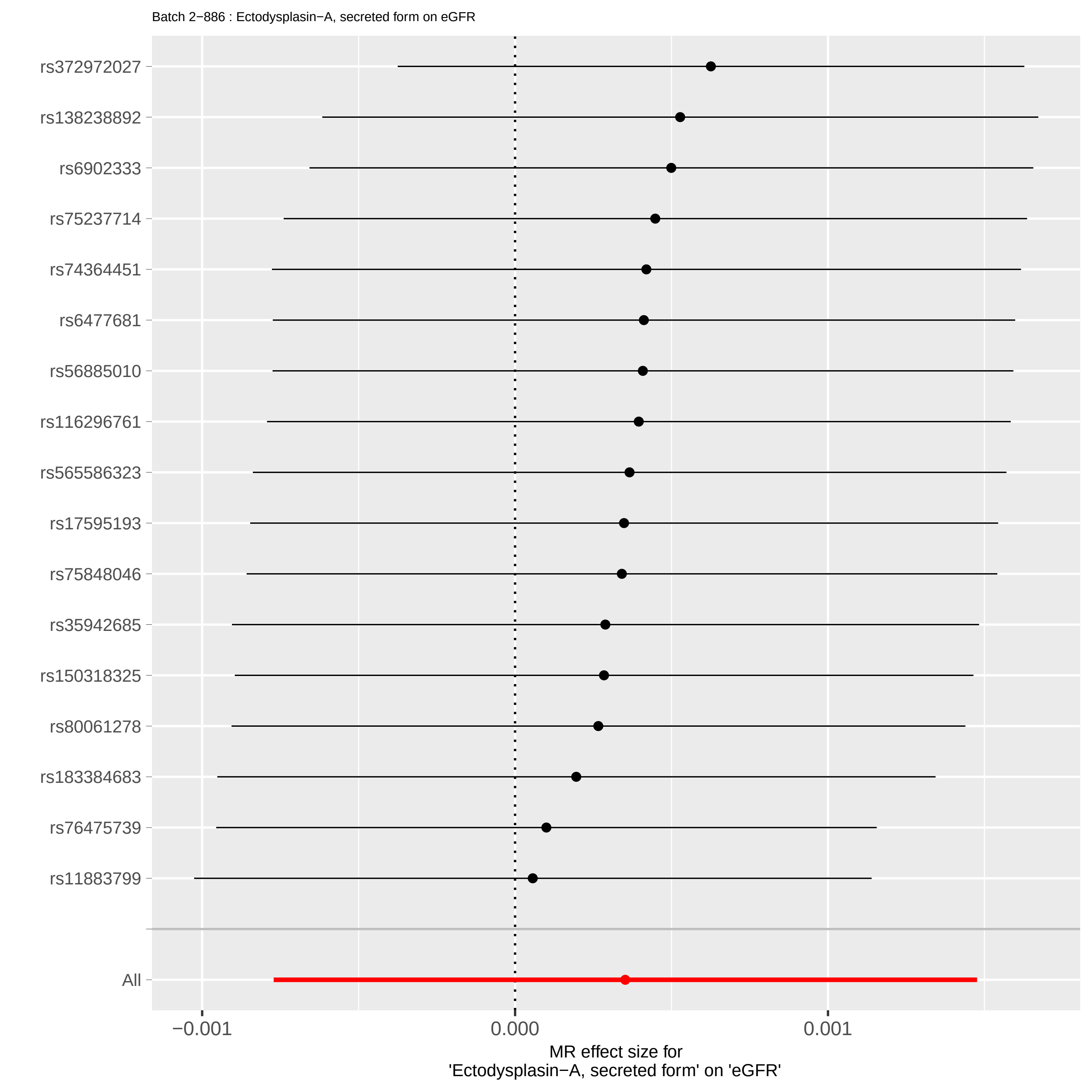

Batch 2−886 : Ectodysplasin−A, secreted form on eGFR
rs372972027
rs138238892
rs6902333
rs75237714
rs74364451
rs6477681
rs56885010
rs116296761
rs565586323
rs17595193
rs75848046
rs35942685
rs150318325
rs80061278
rs183384683
rs76475739
rs11883799
All
−0.001
0.000
0.001
MR effect size for
'Ectodysplasin−A, secreted form' on 'eGFR'

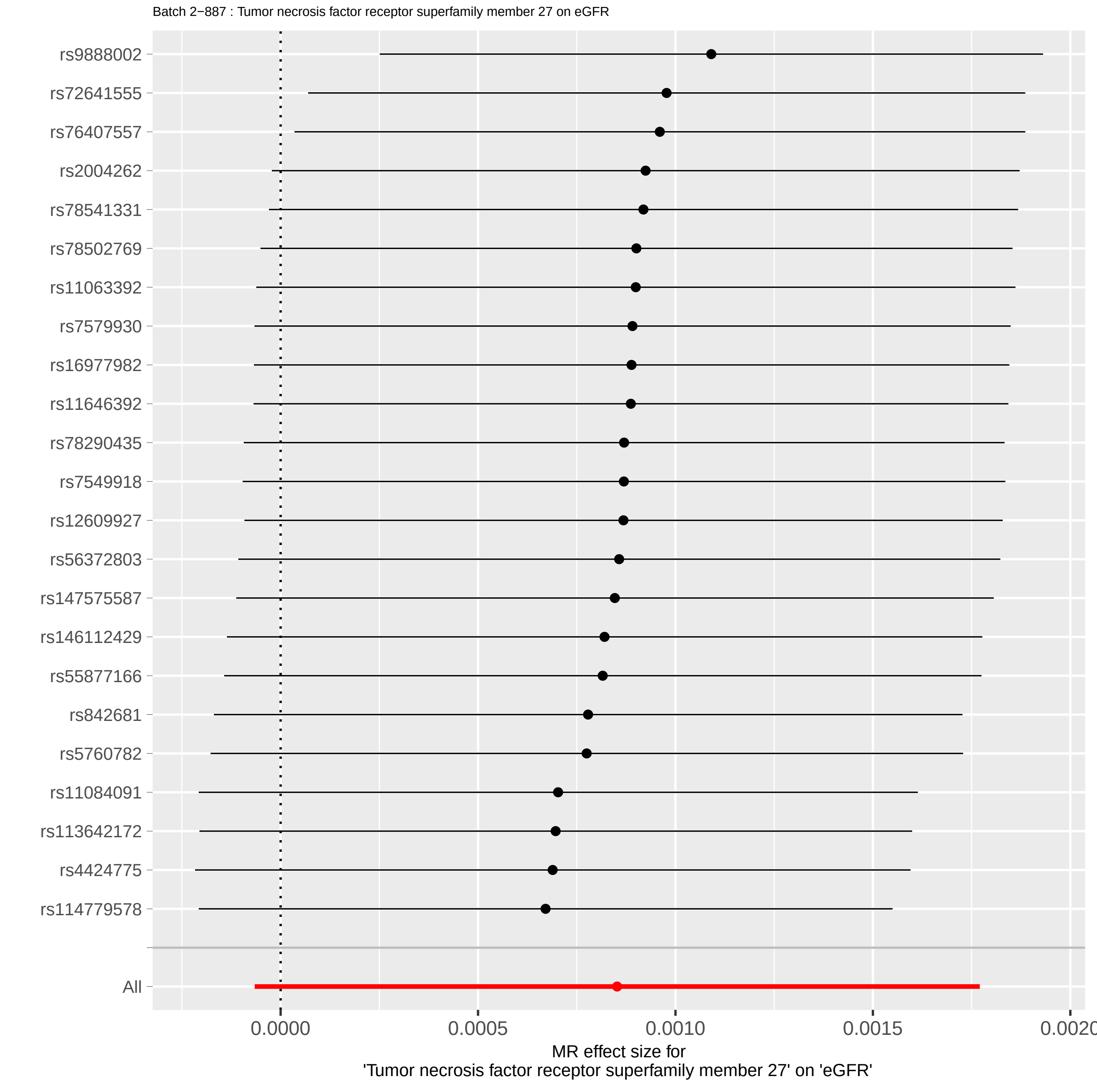
Batch 2−887 : Tumor necrosis factor receptor superfamily member 27 on eGFR
rs9888002
rs72641555
rs76407557
rs2004262
rs78541331
rs78502769
rs11063392
rs7579930
rs16977982
rs11646392
rs78290435
rs7549918
rs12609927
rs56372803
rs147575587
rs146112429
rs55877166
rs842681
rs5760782
rs11084091
rs113642172
rs4424775
rs114779578
All
0.0000
0.0005
0.0010
0.0015
0.0020
MR effect size for
'Tumor necrosis factor receptor superfamily member 27' on 'eGFR'

Batch 2−888 : Tumor necrosis factor receptor superfamily member EDAR on eGFR

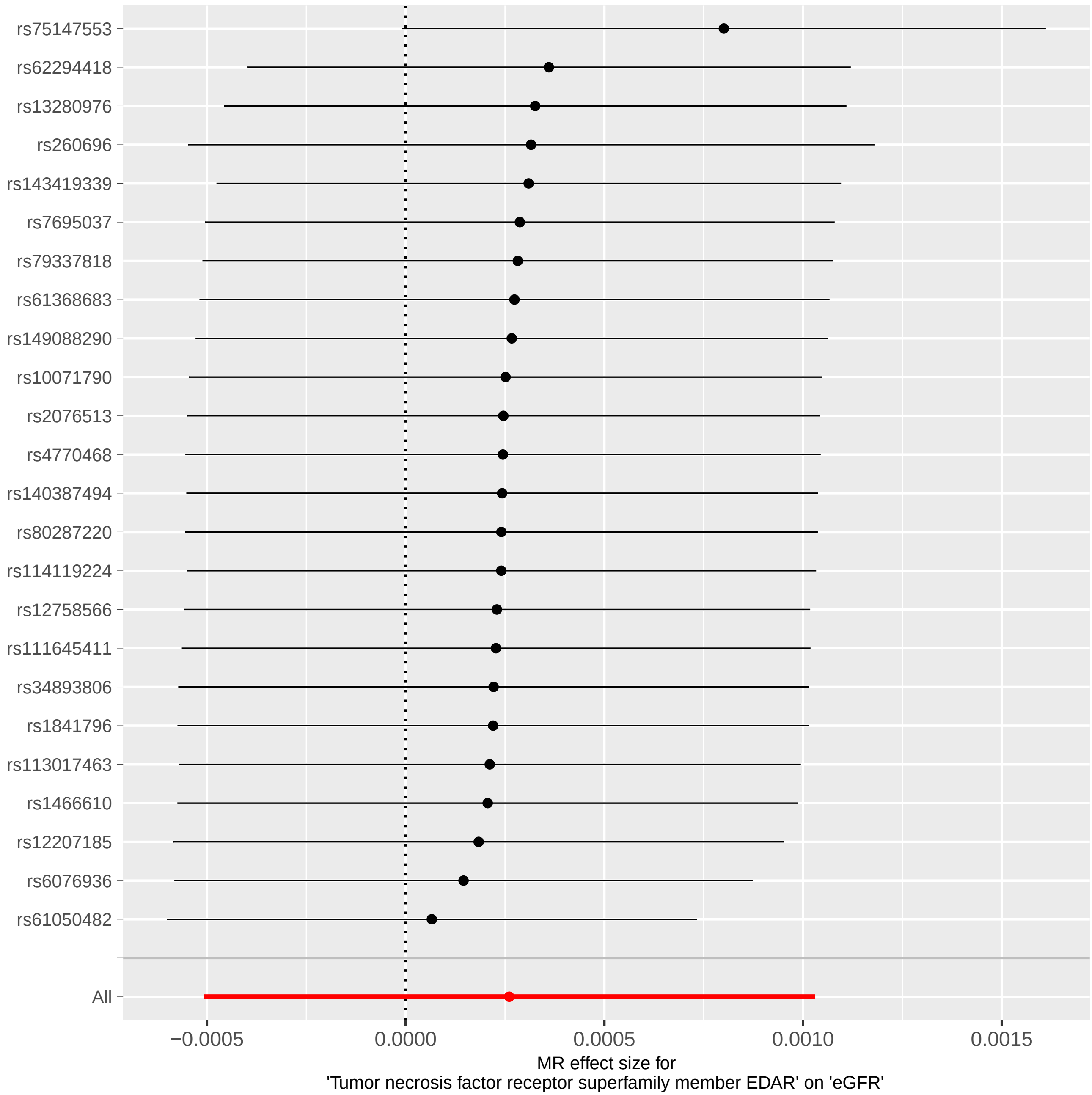

Batch 2−889 : Enhancer of mRNA−decapping protein 4 on eGFR

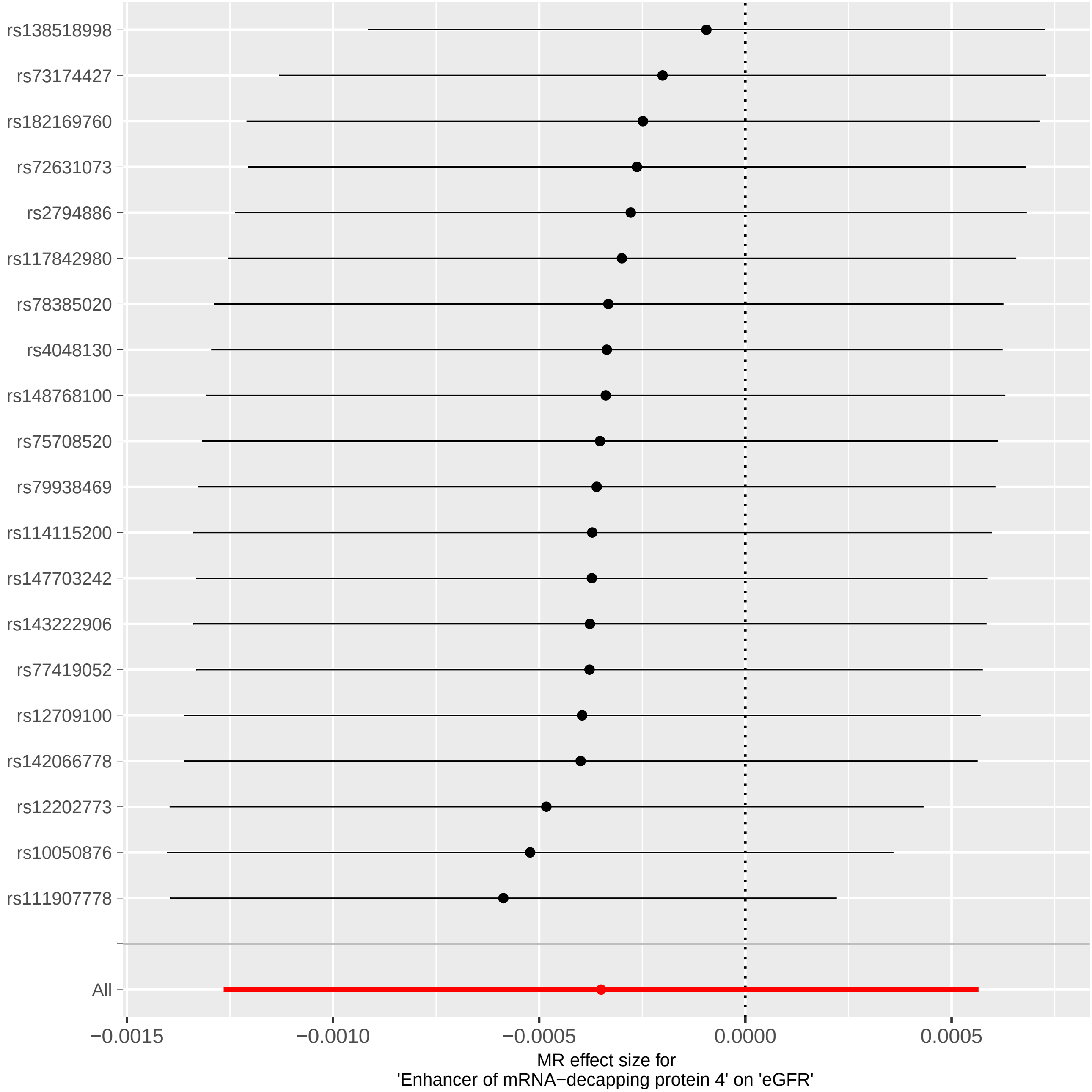

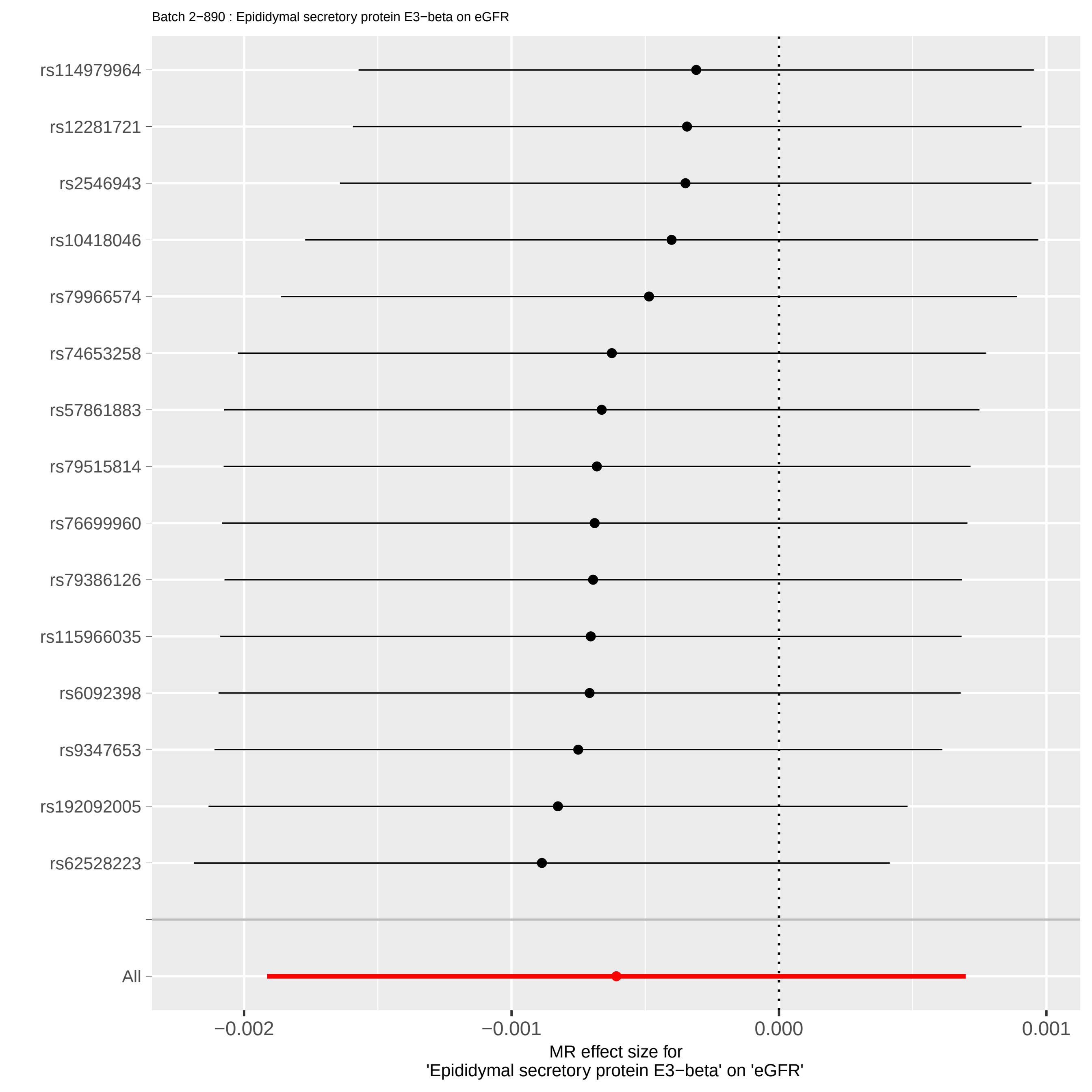

Batch 2−890 : Epididymal secretory protein E3−beta on eGFR
rs114979964
rs12281721
rs2546943
rs10418046
rs79966574
rs74653258
rs57861883
rs79515814
rs76699960
rs79386126
rs115966035
rs6092398
rs9347653
rs192092005
rs62528223
All
−0.002
−0.001
0.000
0.001
MR effect size for
'Epididymal secretory protein E3−beta' on 'eGFR'

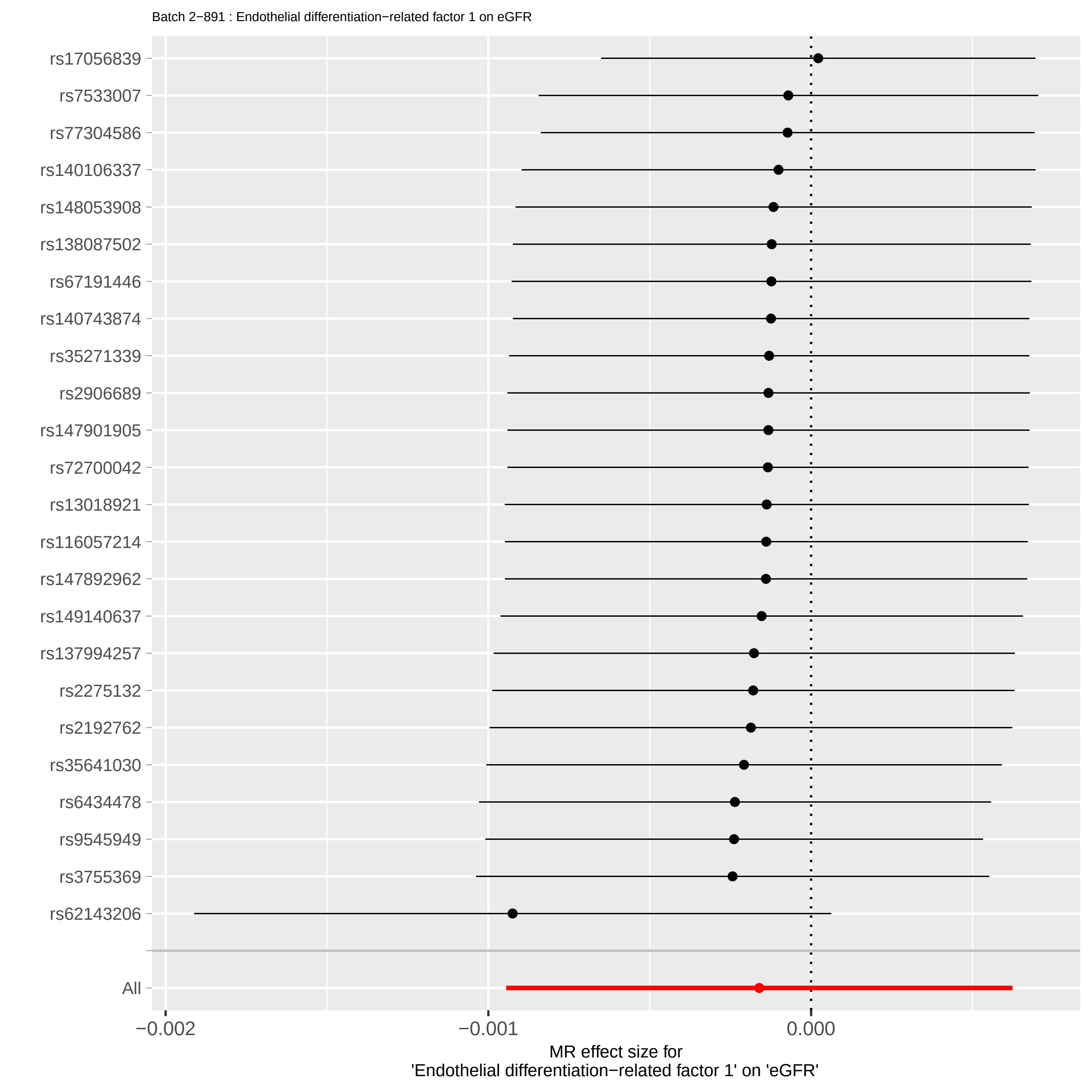
Batch 2−891 : Endothelial differentiation−related factor 1 on eGFR
rs17056839
rs7533007
rs77304586
rs140106337
rs148053908
rs138087502
rs67191446
rs140743874
rs35271339
rs2906689
rs147901905
rs72700042
rs13018921
rs116057214
rs147892962
rs149140637
rs137994257
rs2275132
rs2192762
rs35641030
rs6434478
rs9545949
rs3755369
rs62143206
All
−0.002
−0.001
0.000
MR effect size for
'Endothelial differentiation−related factor 1' on 'eGFR'

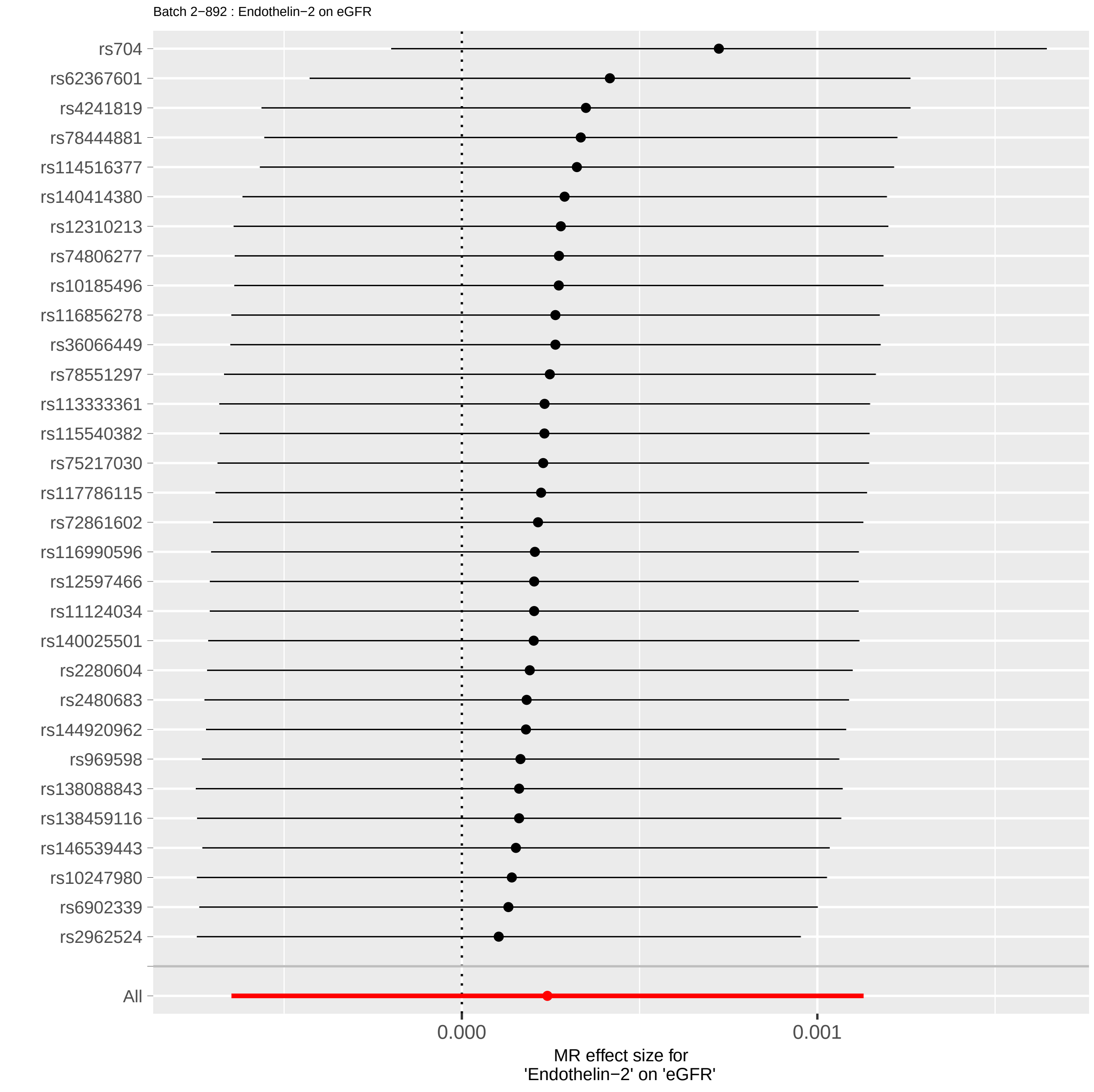

Batch 2−892 : Endothelin−2 on eGFR
rs704
rs62367601
rs4241819
rs78444881
rs114516377
rs140414380
rs12310213
rs74806277
rs10185496
rs116856278
rs36066449
rs78551297
rs113333361
rs115540382
rs75217030
rs117786115
rs72861602
rs116990596
rs12597466
rs11124034
rs140025501
rs2280604
rs2480683
rs144920962
rs969598
rs138088843
rs138459116
rs146539443
rs10247980
rs6902339
rs2962524
All
0.000
0.001
MR effect size for
'Endothelin−2' on 'eGFR'

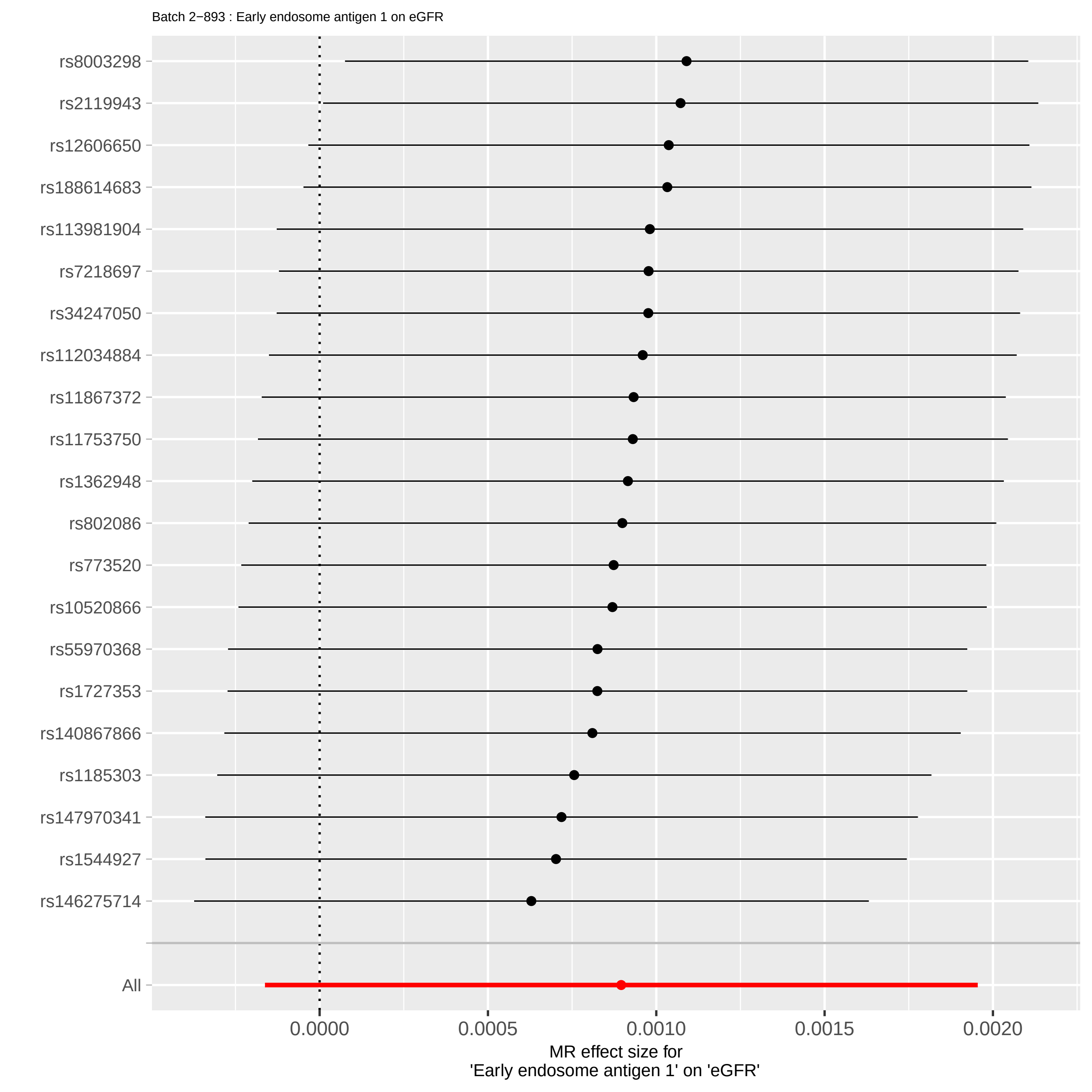
Batch 2−893 : Early endosome antigen 1 on eGFR
rs8003298
rs2119943
rs12606650
rs188614683
rs113981904
rs7218697
rs34247050
rs112034884
rs11867372
rs11753750
rs1362948
rs802086
rs773520
rs10520866
rs55970368
rs1727353
rs140867866
rs1185303
rs147970341
rs1544927
rs146275714
All
0.0000
0.0005
0.0010
0.0015
0.0020
MR effect size for
'Early endosome antigen 1' on 'eGFR'

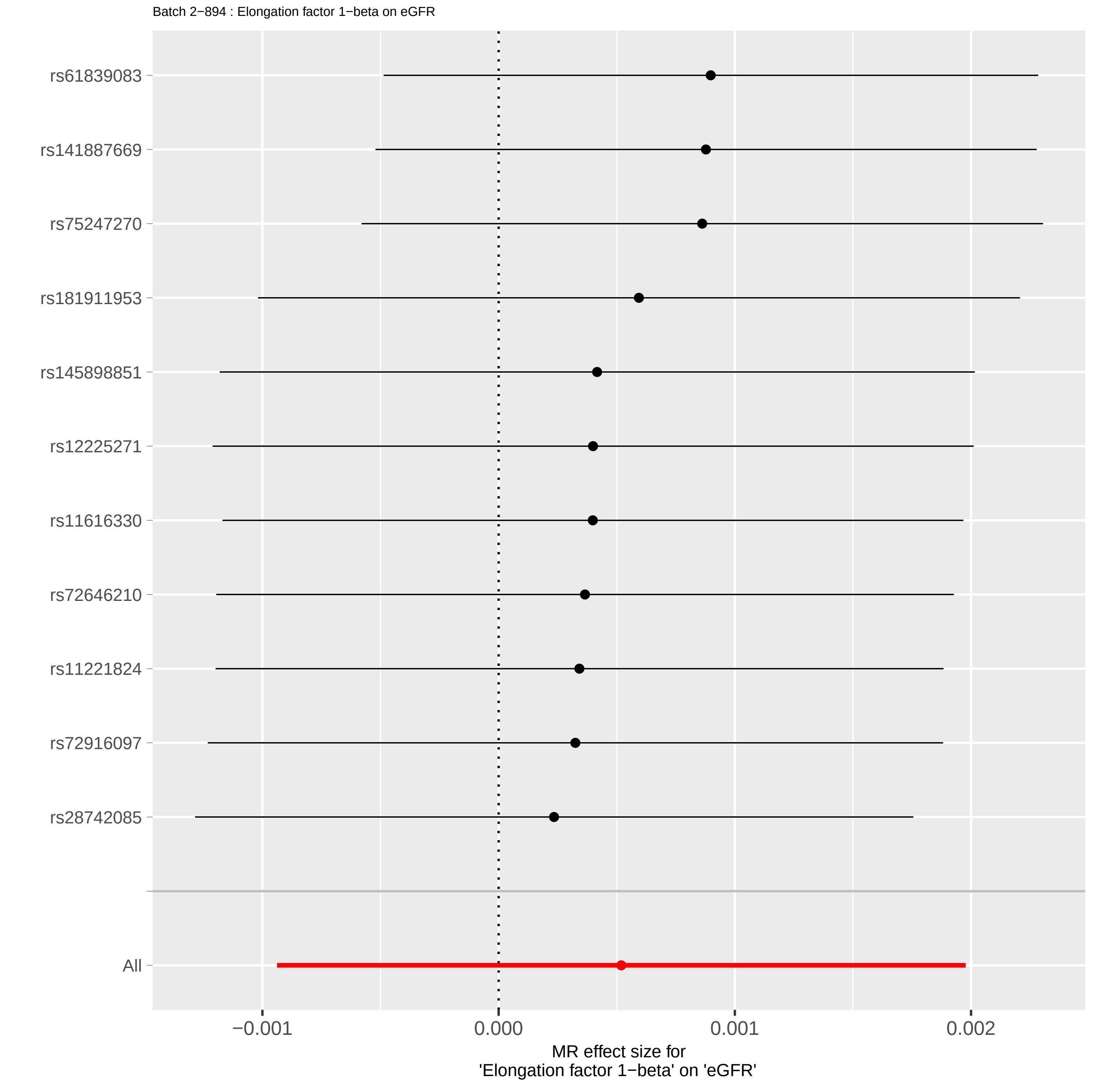

Batch 2−894 : Elongation factor 1−beta on eGFR
rs61839083
rs141887669
rs75247270
rs181911953
rs145898851
rs12225271
rs11616330
rs72646210
rs11221824
rs72916097
rs28742085
All
−0.001
0.000
0.001
0.002
MR effect size for
'Elongation factor 1−beta' on 'eGFR'

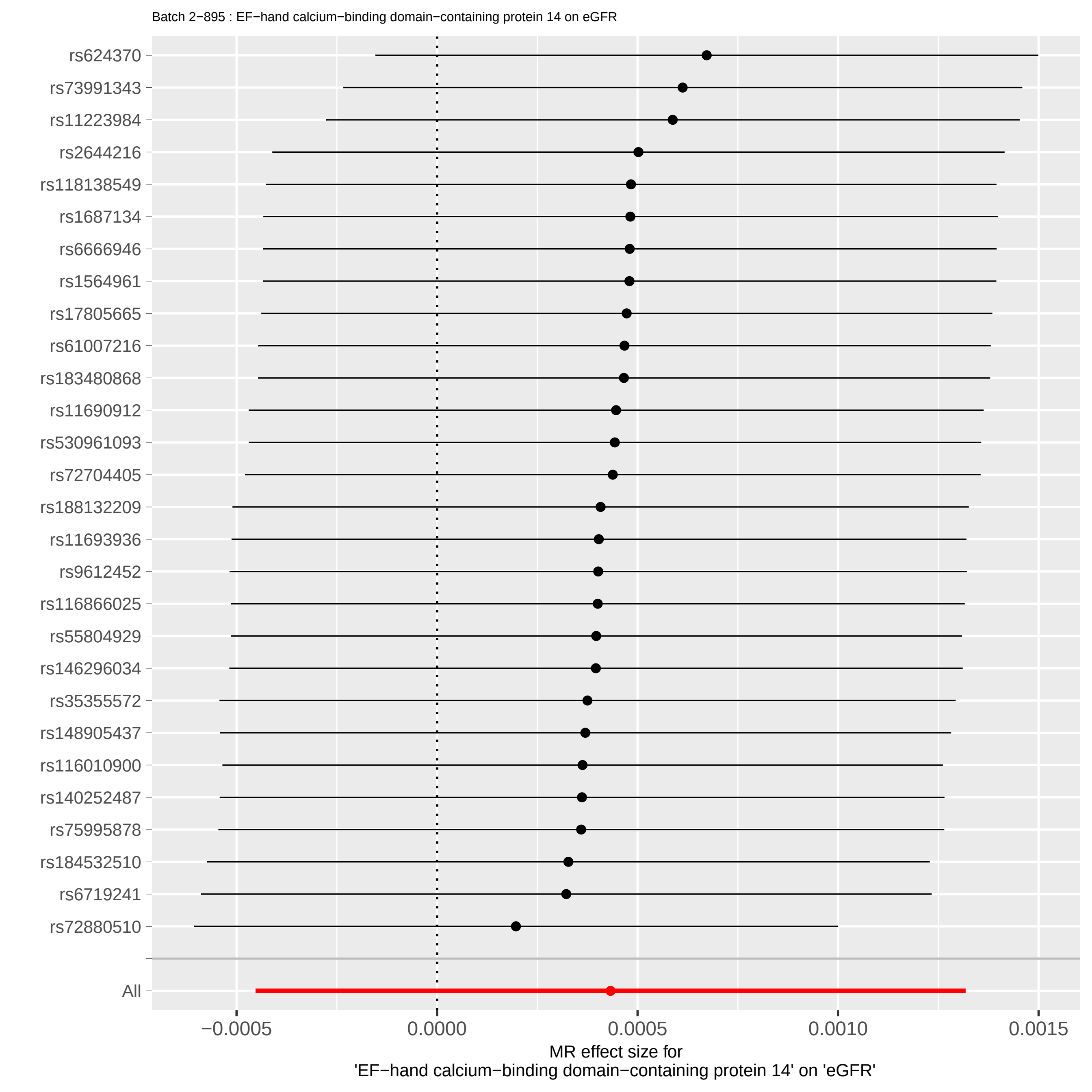

Batch 2−895 : EF−hand calcium−binding domain−containing protein 14 on eGFR
rs624370
rs73991343
rs11223984
rs2644216
rs118138549
rs1687134
rs6666946
rs1564961
rs17805665
rs61007216
rs183480868
rs11690912
rs530961093
rs72704405
rs188132209
rs11693936
rs9612452
rs116866025
rs55804929
rs146296034
rs35355572
rs148905437
rs116010900
rs140252487
rs75995878
rs184532510
rs6719241
rs72880510
All
−0.0005
0.0000
0.0005
0.0010
0.0015
MR effect size for
'EF−hand calcium−binding domain−containing protein 14' on 'eGFR'

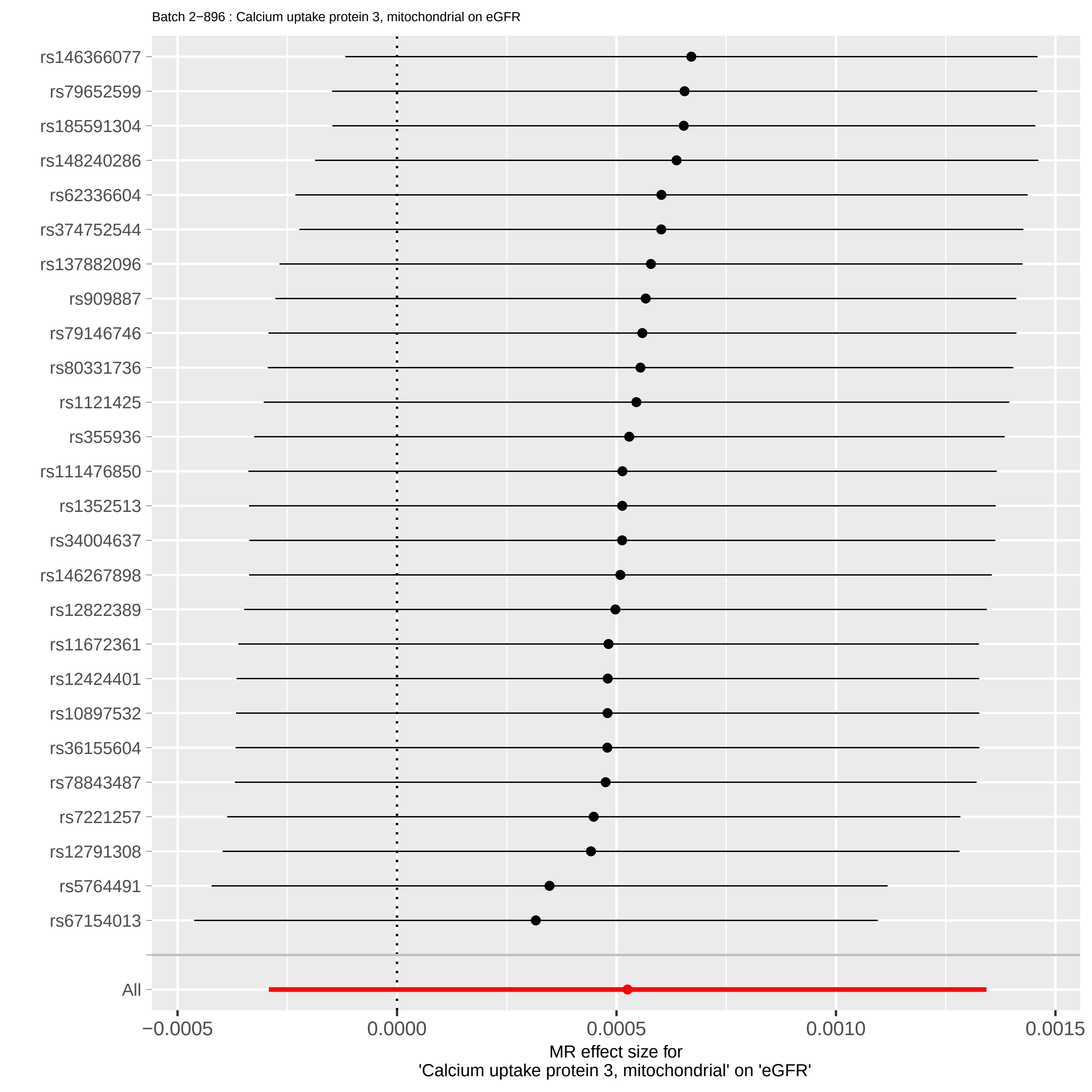
Batch 2−896 : Calcium uptake protein 3, mitochondrial on eGFR
rs146366077
rs79652599
rs185591304
rs148240286
rs62336604
rs374752544
rs137882096
rs909887
rs79146746
rs80331736
rs1121425
rs355936
rs111476850
rs1352513
rs34004637
rs146267898
rs12822389
rs11672361
rs12424401
rs10897532
rs36155604
rs78843487
rs7221257
rs12791308
rs5764491
rs67154013
All
−0.0005
0.0000
0.0005
0.0010
0.0015
MR effect size for
'Calcium uptake protein 3, mitochondrial' on 'eGFR'

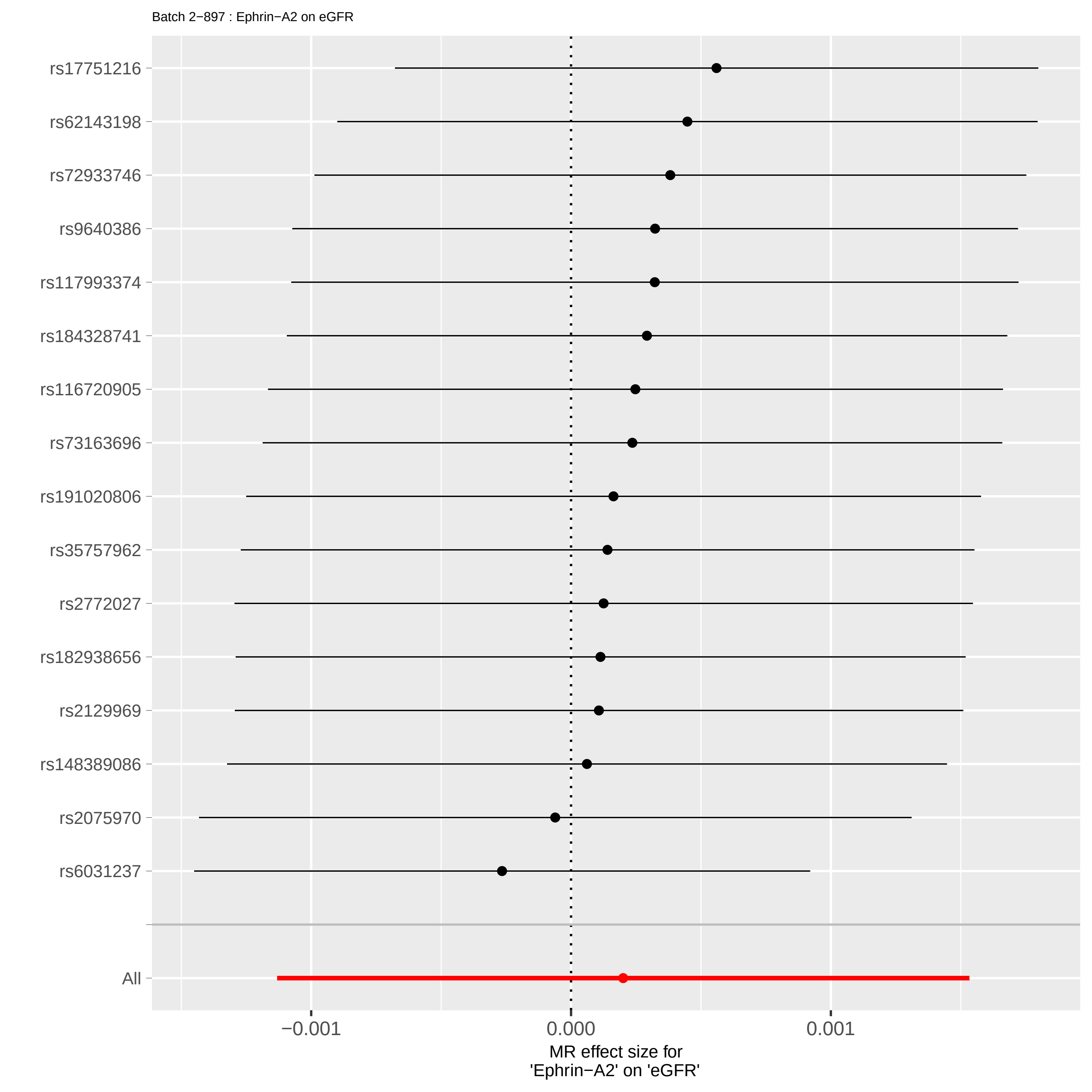

Batch 2−897 : Ephrin−A2 on eGFR
rs17751216
rs62143198
rs72933746
rs9640386
rs117993374
rs184328741
rs116720905
rs73163696
rs191020806
rs35757962
rs2772027
rs182938656
rs2129969
rs148389086
rs2075970
rs6031237
All
−0.001
0.000
0.001
MR effect size for
'Ephrin−A2' on 'eGFR'

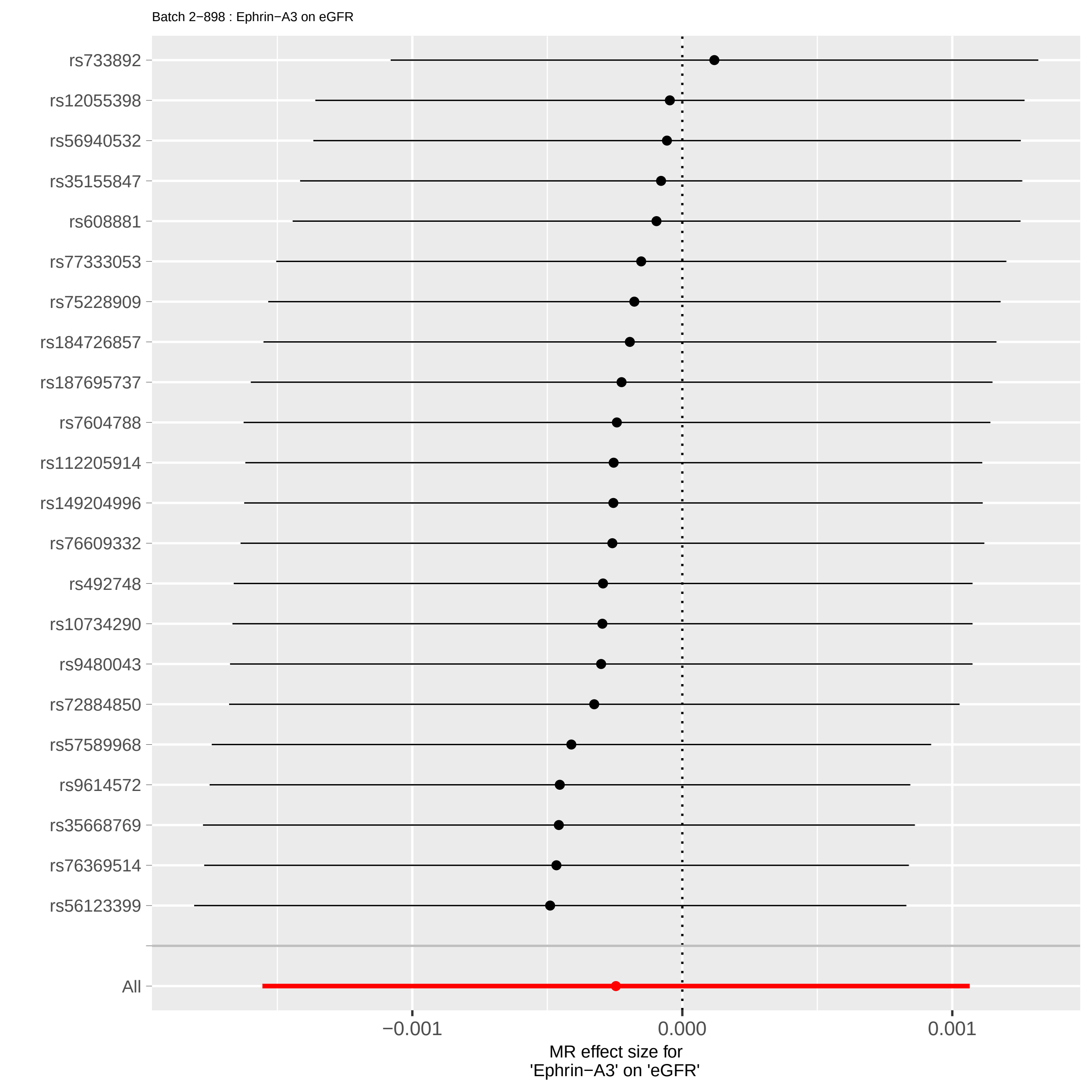

Batch 2−898 : Ephrin−A3 on eGFR
rs733892
rs12055398
rs56940532
rs35155847
rs608881
rs77333053
rs75228909
rs184726857
rs187695737
rs7604788
rs112205914
rs149204996
rs76609332
rs492748
rs10734290
rs9480043
rs72884850
rs57589968
rs9614572
rs35668769
rs76369514
rs56123399
All
−0.001
0.000
0.001
MR effect size for
'Ephrin−A3' on 'eGFR'

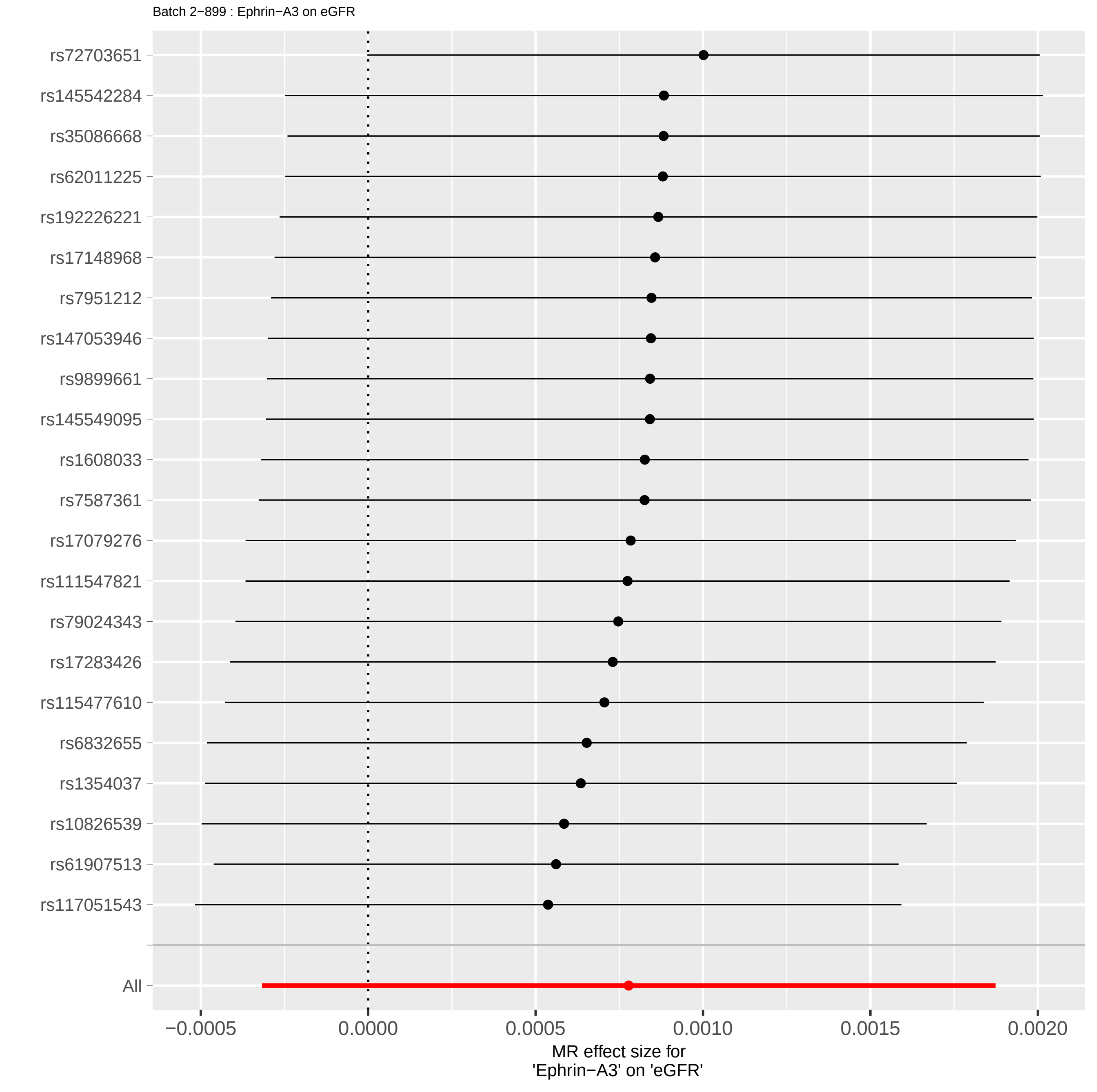

Batch 2−899 : Ephrin−A3 on eGFR
rs72703651
rs145542284
rs35086668
rs62011225
rs192226221
rs17148968
rs7951212
rs147053946
rs9899661
rs145549095
rs1608033
rs7587361
rs17079276
rs111547821
rs79024343
rs17283426
rs115477610
rs6832655
rs1354037
rs10826539
rs61907513
rs117051543
All
−0.0005
0.0000
0.0005
0.0010
0.0015
0.0020
MR effect size for
'Ephrin−A3' on 'eGFR'

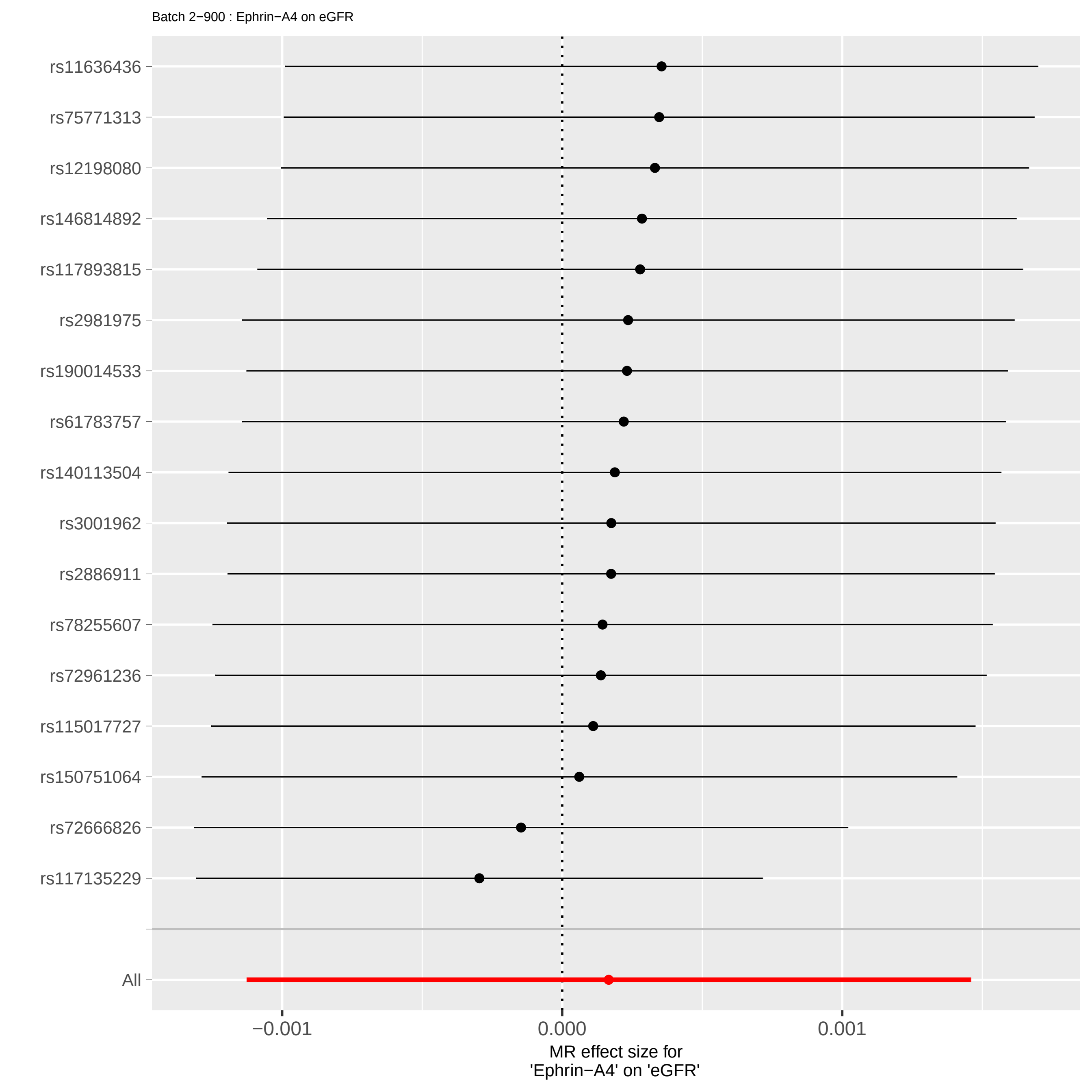

Batch 2−900 : Ephrin−A4 on eGFR
rs11636436
rs75771313
rs12198080
rs146814892
rs117893815
rs2981975
rs190014533
rs61783757
rs140113504
rs3001962
rs2886911
rs78255607
rs72961236
rs115017727
rs150751064
rs72666826
rs117135229
All
−0.001
0.000
0.001
MR effect size for
'Ephrin−A4' on 'eGFR'

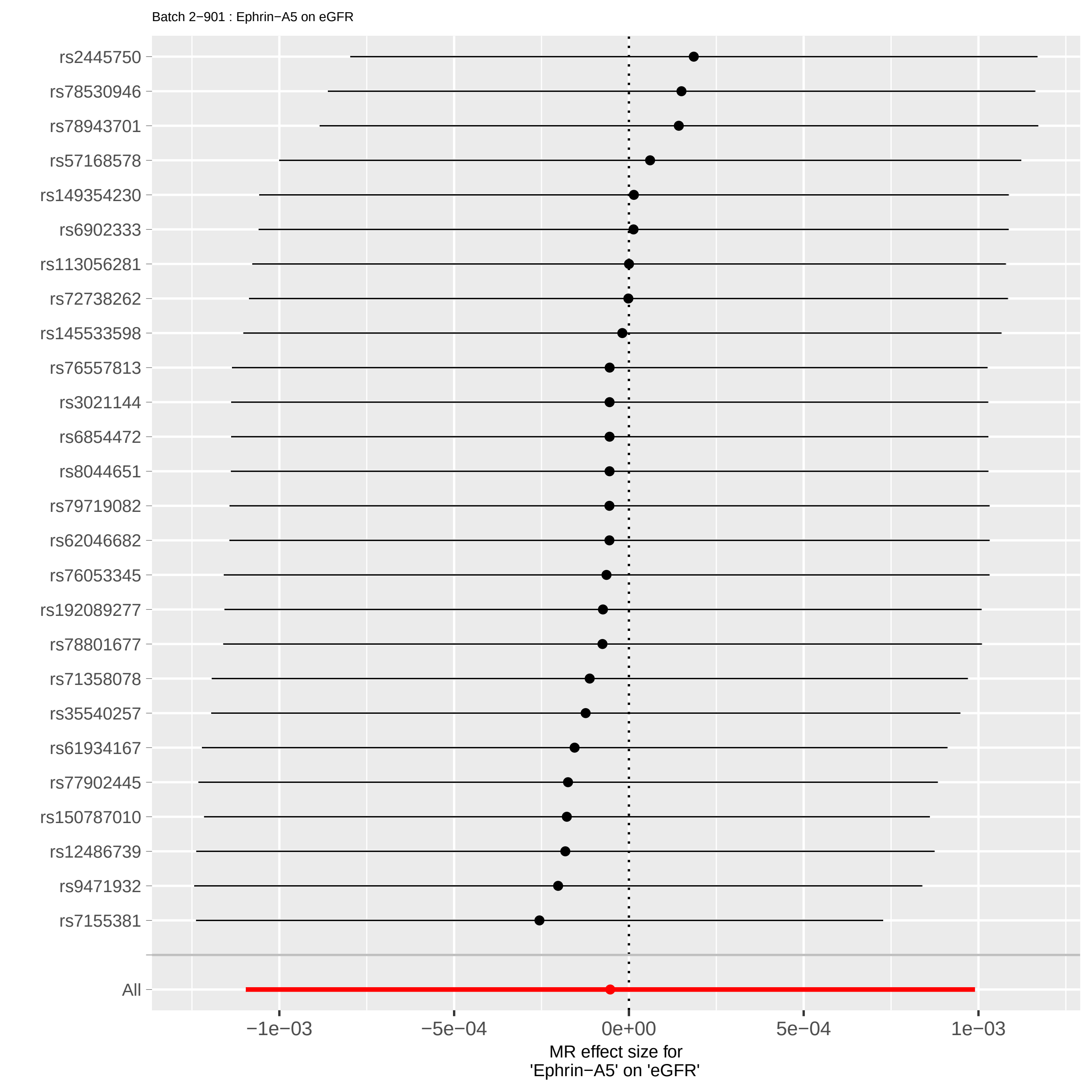
Batch 2−901 : Ephrin−A5 on eGFR
rs2445750
rs78530946
rs78943701
rs57168578
rs149354230
rs6902333
rs113056281
rs72738262
rs145533598
rs76557813
rs3021144
rs6854472
rs8044651
rs79719082
rs62046682
rs76053345
rs192089277
rs78801677
rs71358078
rs35540257
rs61934167
rs77902445
rs150787010
rs12486739
rs9471932
rs7155381
All
−1e−03
−5e−04
0e+00
5e−04
1e−03
MR effect size for
'Ephrin−A5' on 'eGFR'

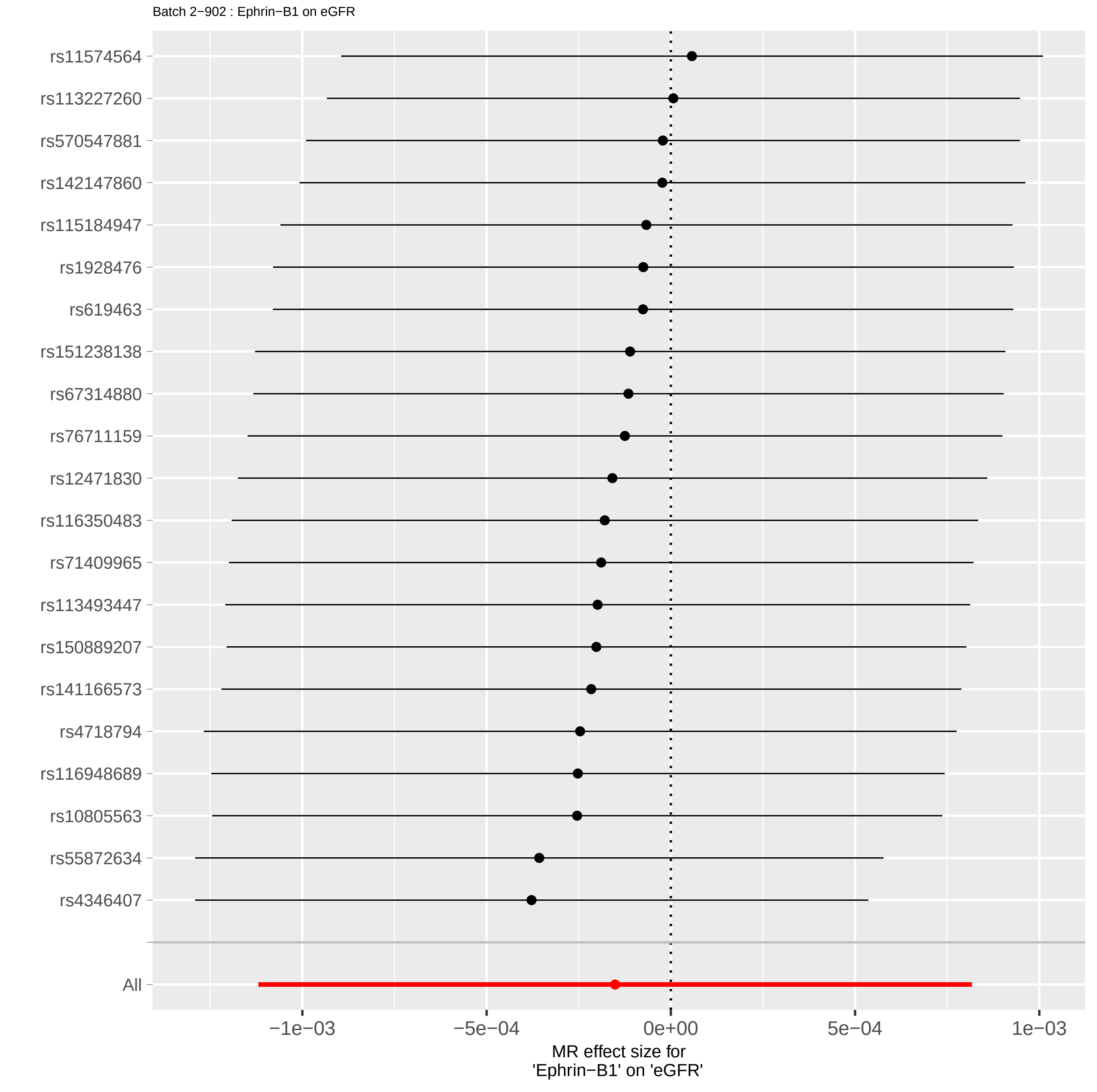
Batch 2−902 : Ephrin−B1 on eGFR
rs11574564
rs113227260
rs570547881
rs142147860
rs115184947
rs1928476
rs619463
rs151238138
rs67314880
rs76711159
rs12471830
rs116350483
rs71409965
rs113493447
rs150889207
rs141166573
rs4718794
rs116948689
rs10805563
rs55872634
rs4346407
All
−1e−03
−5e−04
0e+00
5e−04
1e−03
MR effect size for
'Ephrin−B1' on 'eGFR'

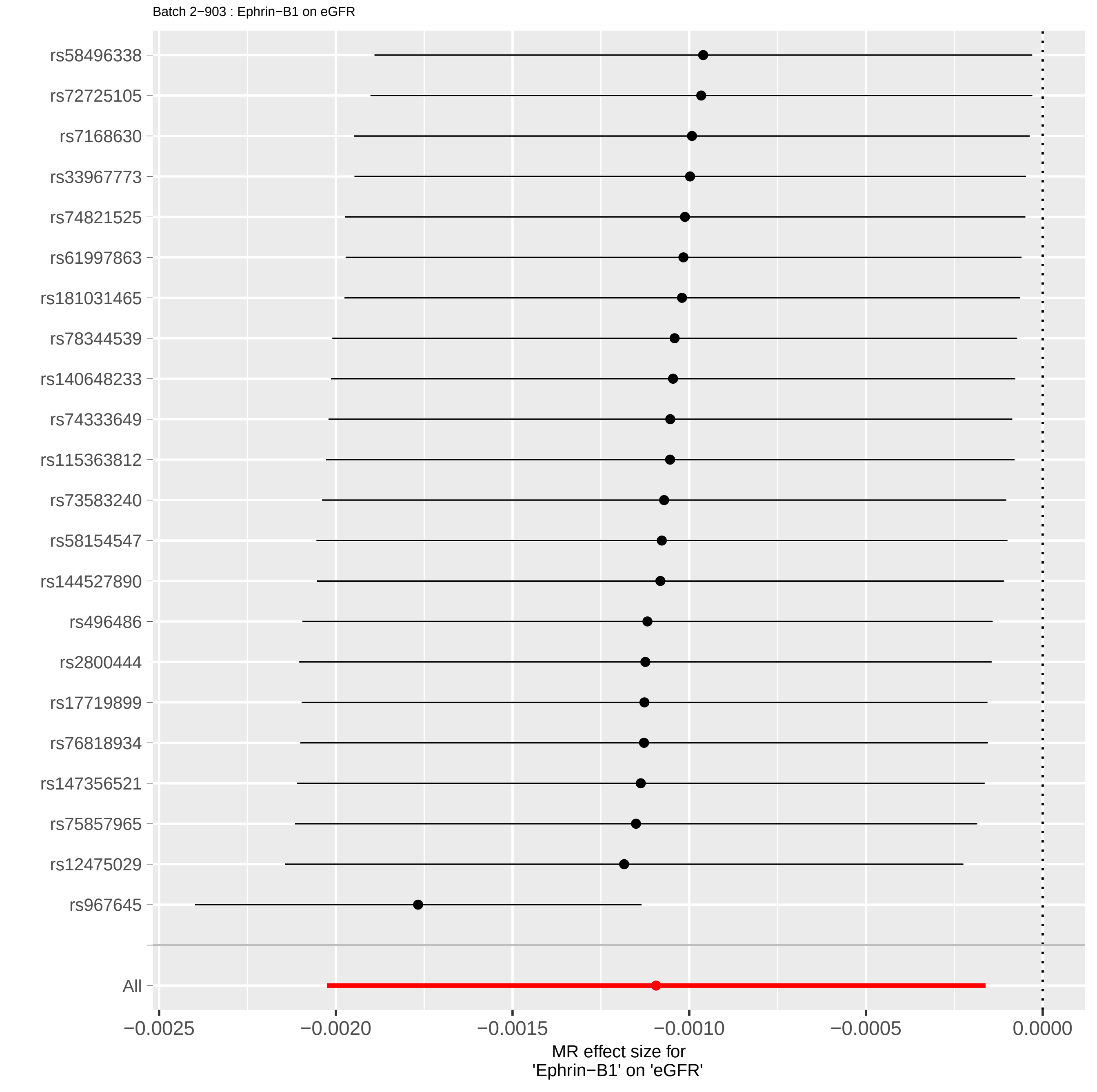
Batch 2−903 : Ephrin−B1 on eGFR
rs58496338
rs72725105
rs7168630
rs33967773
rs74821525
rs61997863
rs181031465
rs78344539
rs140648233
rs74333649
rs115363812
rs73583240
rs58154547
rs144527890
rs496486
rs2800444
rs17719899
rs76818934
rs147356521
rs75857965
rs12475029
rs967645
All
−0.0025
−0.0020
−0.0015
−0.0010
−0.0005
0.0000
MR effect size for
'Ephrin−B1' on 'eGFR'

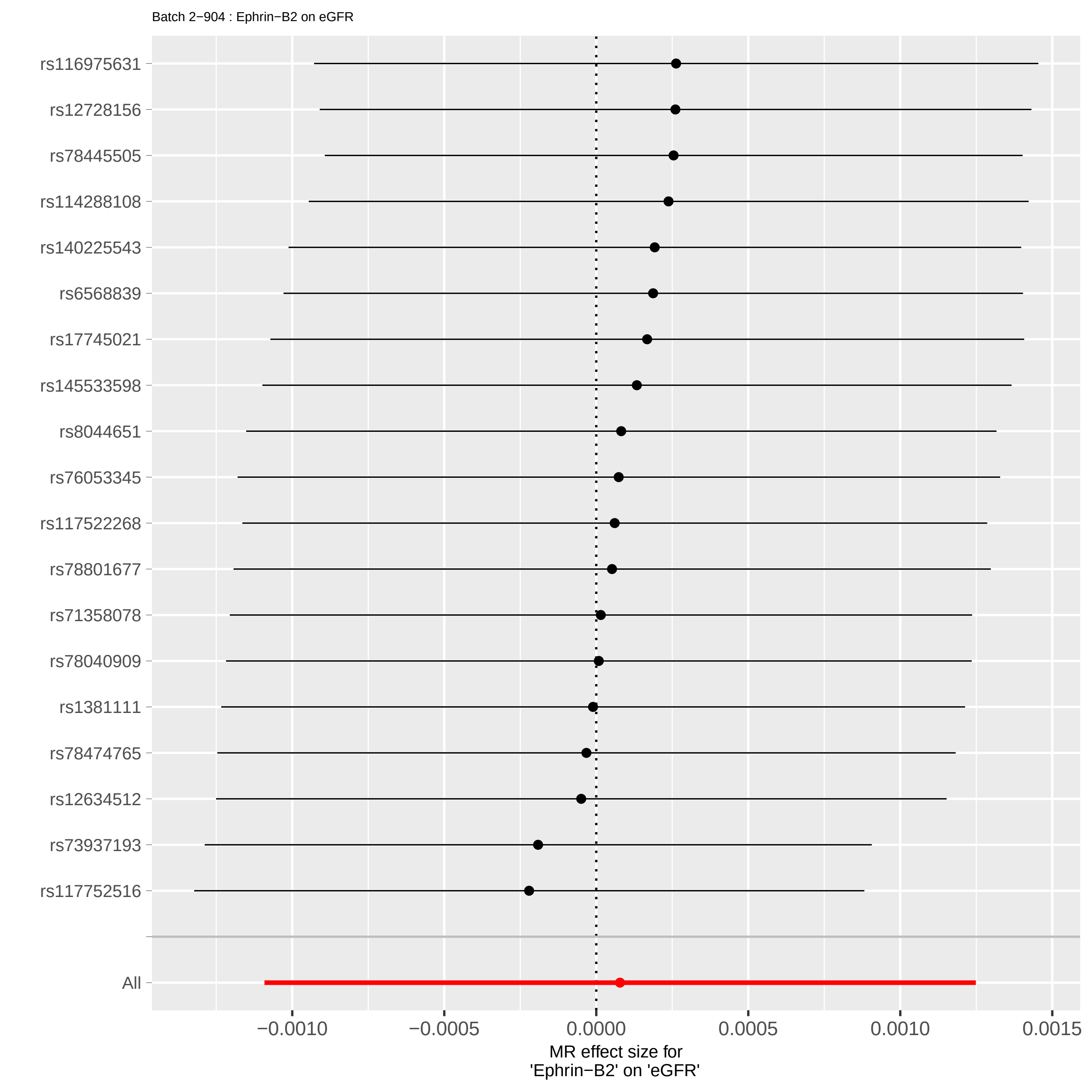
Batch 2−904 : Ephrin−B2 on eGFR
rs116975631
rs12728156
rs78445505
rs114288108
rs140225543
rs6568839
rs17745021
rs145533598
rs8044651
rs76053345
rs117522268
rs78801677
rs71358078
rs78040909
rs1381111
rs78474765
rs12634512
rs73937193
rs117752516
All
−0.0010
−0.0005
0.0000
0.0005
0.0010
0.0015
MR effect size for
'Ephrin−B2' on 'eGFR'

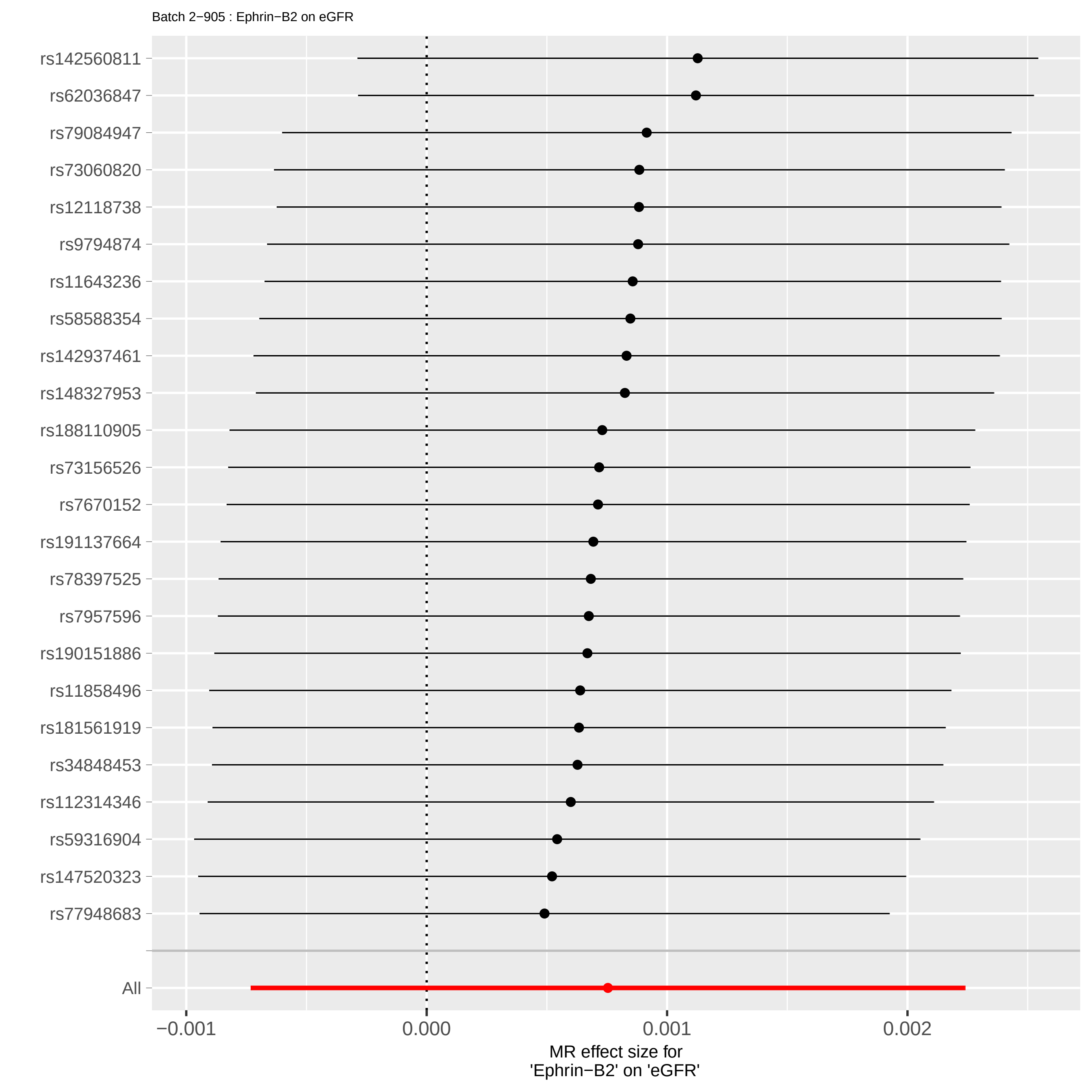

Batch 2−905 : Ephrin−B2 on eGFR
rs142560811
rs62036847
rs79084947
rs73060820
rs12118738
rs9794874
rs11643236
rs58588354
rs142937461
rs148327953
rs188110905
rs73156526
rs7670152
rs191137664
rs78397525
rs7957596
rs190151886
rs11858496
rs181561919
rs34848453
rs112314346
rs59316904
rs147520323
rs77948683
All
−0.001
0.000
0.001
0.002
MR effect size for
'Ephrin−B2' on 'eGFR'

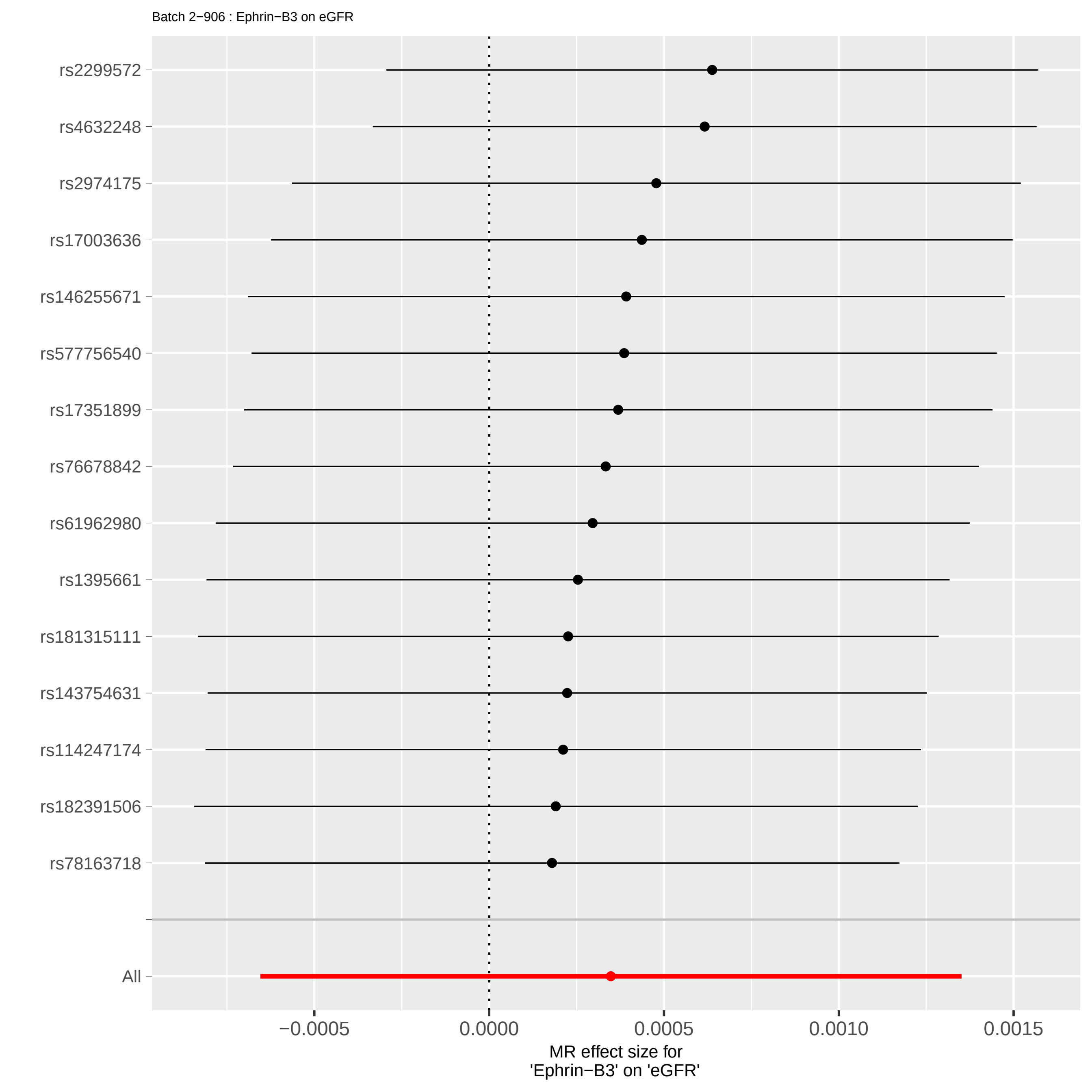

Batch 2−906 : Ephrin−B3 on eGFR
rs2299572
rs4632248
rs2974175
rs17003636
rs146255671
rs577756540
rs17351899
rs76678842
rs61962980
rs1395661
rs181315111
rs143754631
rs114247174
rs182391506
rs78163718
All
−0.0005
0.0000
0.0005
0.0010
0.0015
MR effect size for
'Ephrin−B3' on 'eGFR'

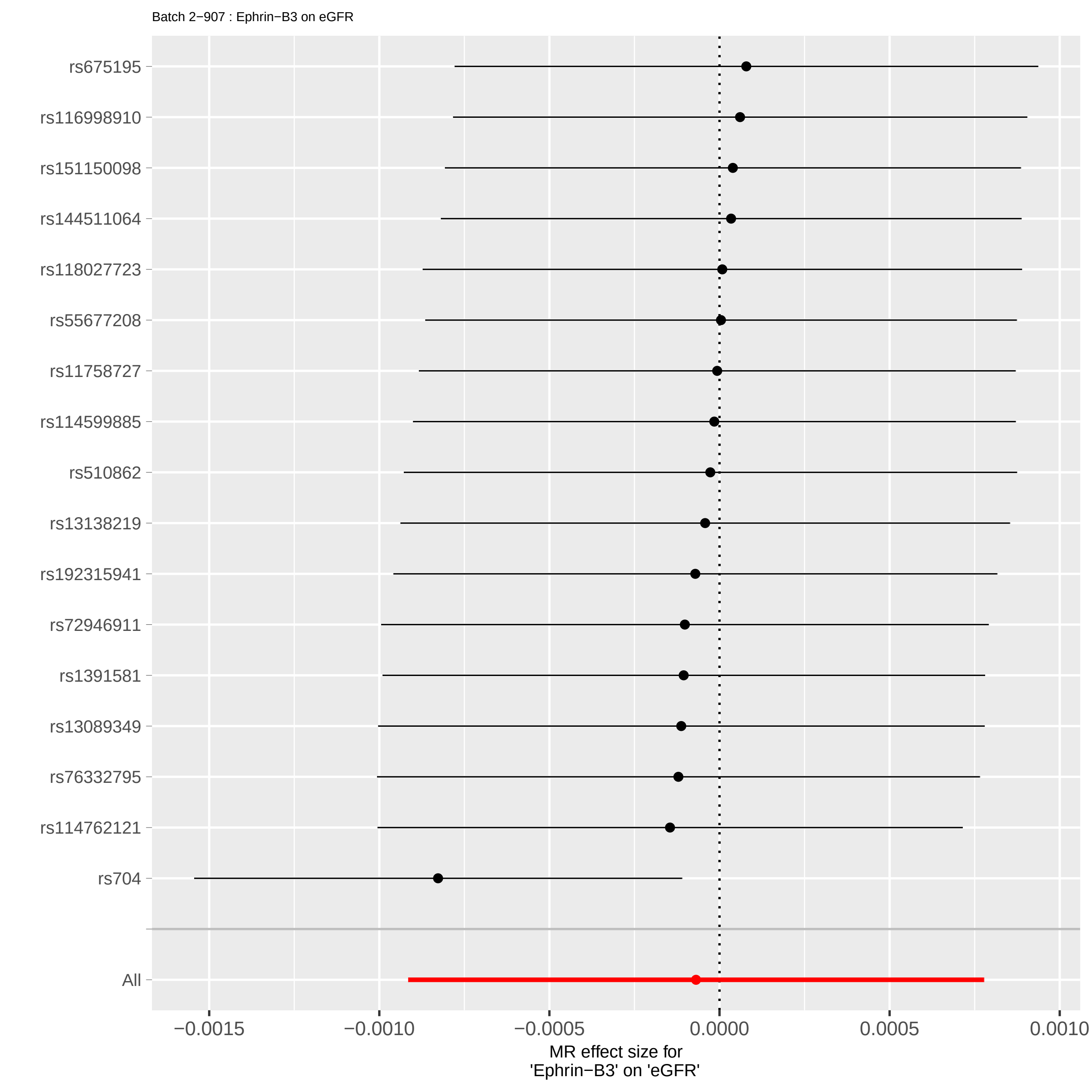

Batch 2−907 : Ephrin−B3 on eGFR
rs675195
rs116998910
rs151150098
rs144511064
rs118027723
rs55677208
rs11758727
rs114599885
rs510862
rs13138219
rs192315941
rs72946911
rs1391581
rs13089349
rs76332795
rs114762121
rs704
All
−0.0015
−0.0010
−0.0005
0.0000
0.0005
0.0010
MR effect size for
'Ephrin−B3' on 'eGFR'

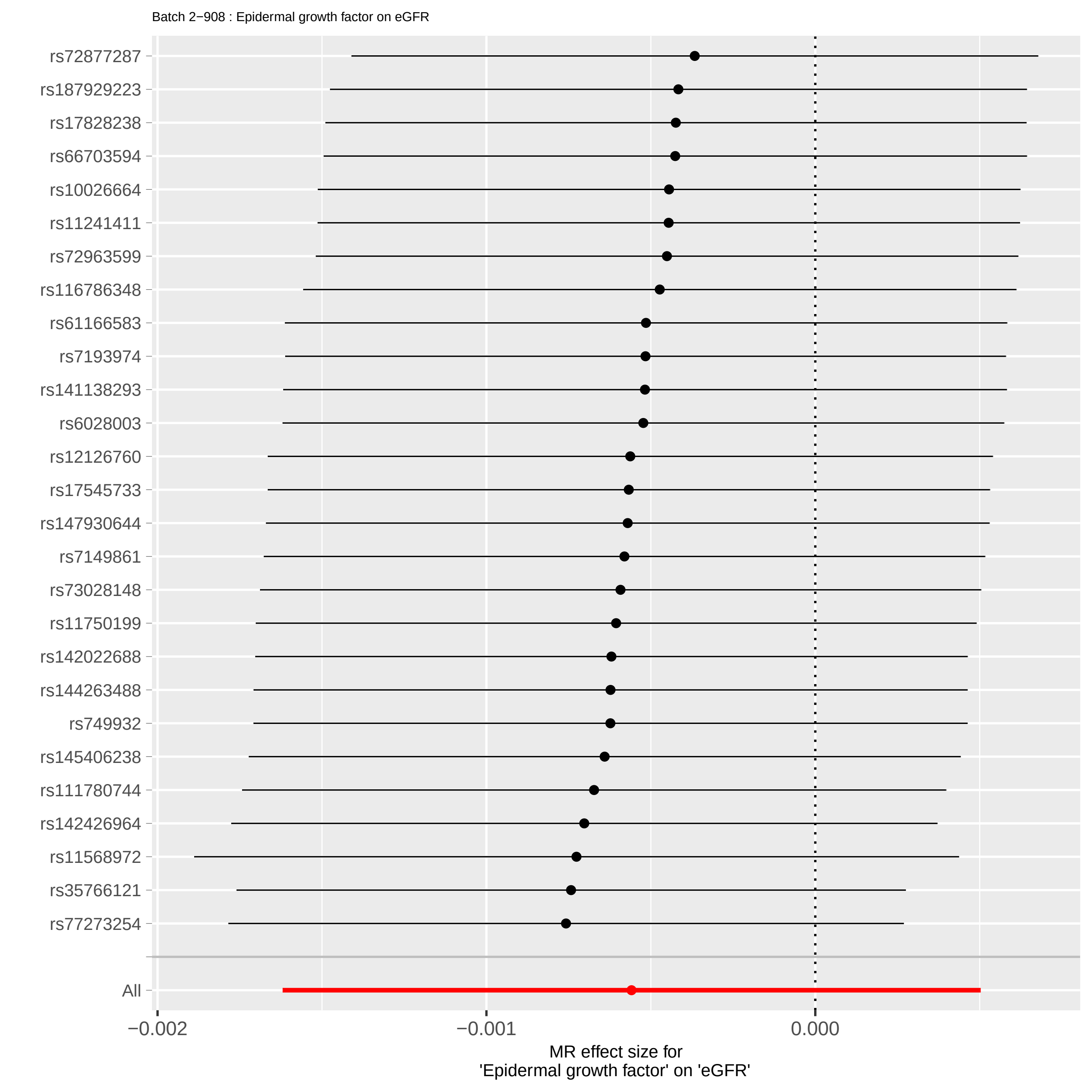
Batch 2−908 : Epidermal growth factor on eGFR
rs72877287
rs187929223
rs17828238
rs66703594
rs10026664
rs11241411
rs72963599
rs116786348
rs61166583
rs7193974
rs141138293
rs6028003
rs12126760
rs17545733
rs147930644
rs7149861
rs73028148
rs11750199
rs142022688
rs144263488
rs749932
rs145406238
rs111780744
rs142426964
rs11568972
rs35766121
rs77273254
All
−0.002
−0.001
0.000
MR effect size for
'Epidermal growth factor' on 'eGFR'

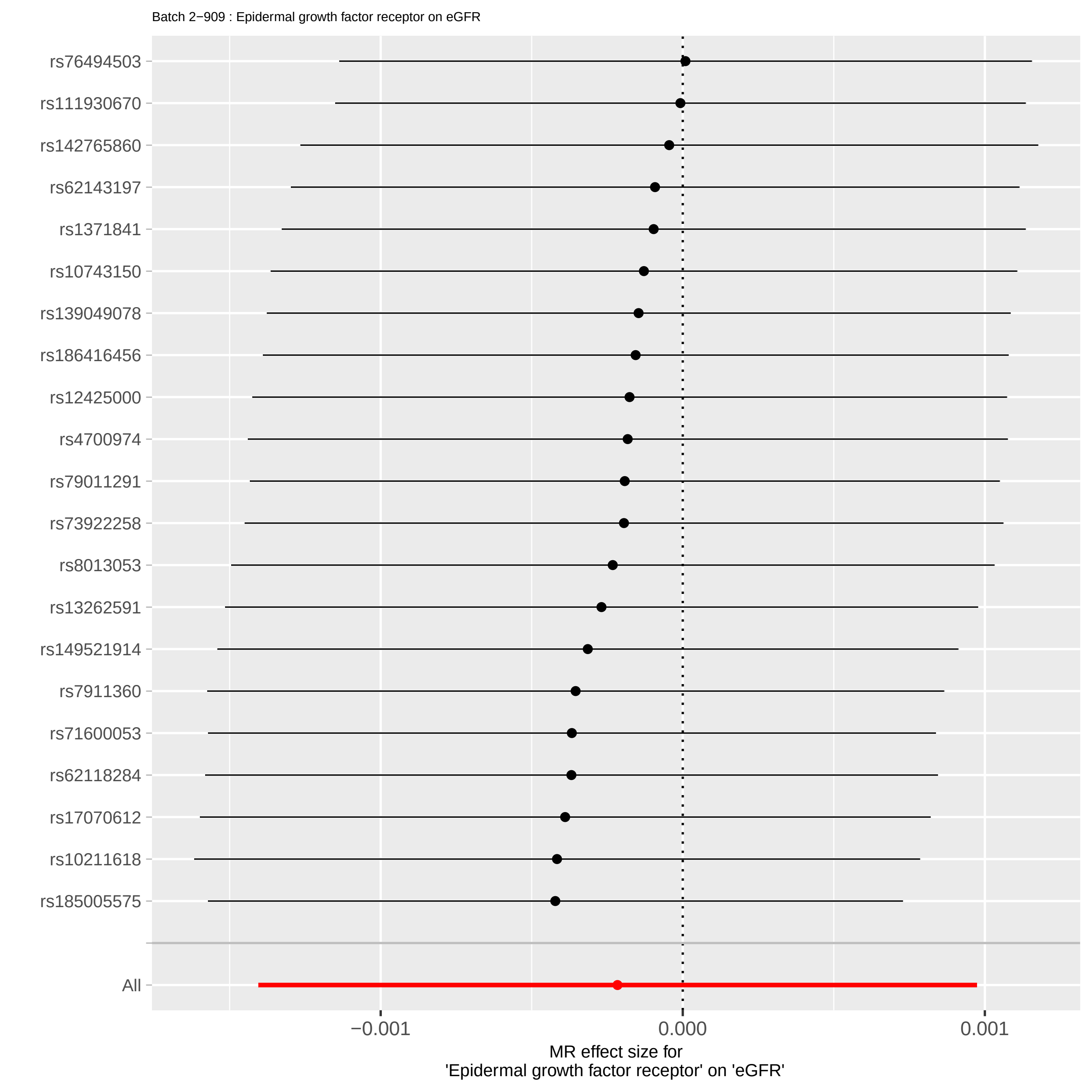

Batch 2−909 : Epidermal growth factor receptor on eGFR
rs76494503
rs111930670
rs142765860
rs62143197
rs1371841
rs10743150
rs139049078
rs186416456
rs12425000
rs4700974
rs79011291
rs73922258
rs8013053
rs13262591
rs149521914
rs7911360
rs71600053
rs62118284
rs17070612
rs10211618
rs185005575
All
−0.001
0.000
0.001
MR effect size for
'Epidermal growth factor receptor' on 'eGFR'

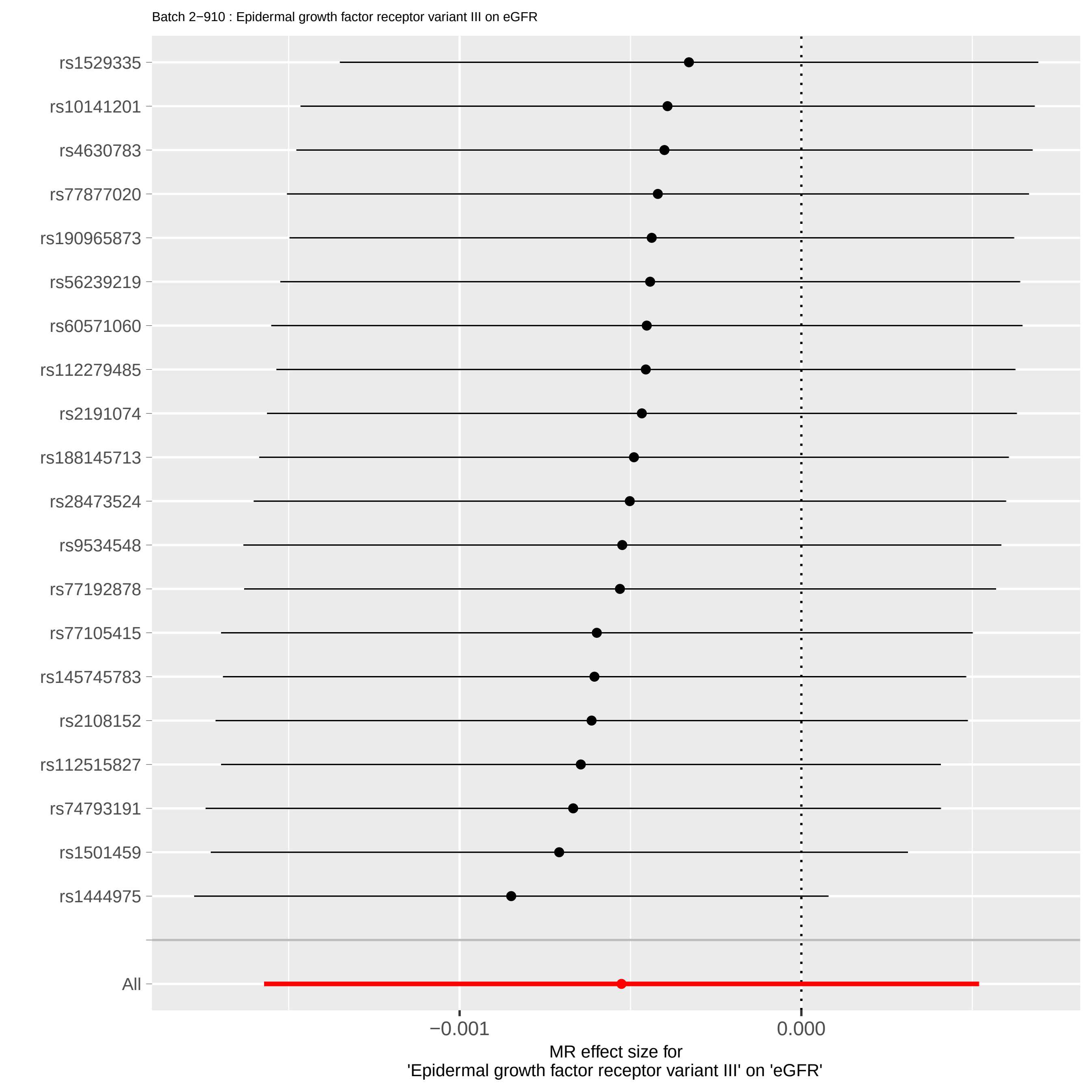

Batch 2−910 : Epidermal growth factor receptor variant III on eGFR
rs1529335
rs10141201
rs4630783
rs77877020
rs190965873
rs56239219
rs60571060
rs112279485
rs2191074
rs188145713
rs28473524
rs9534548
rs77192878
rs77105415
rs145745783
rs2108152
rs112515827
rs74793191
rs1501459
rs1444975
All
−0.001
0.000
MR effect size for
'Epidermal growth factor receptor variant III' on 'eGFR'

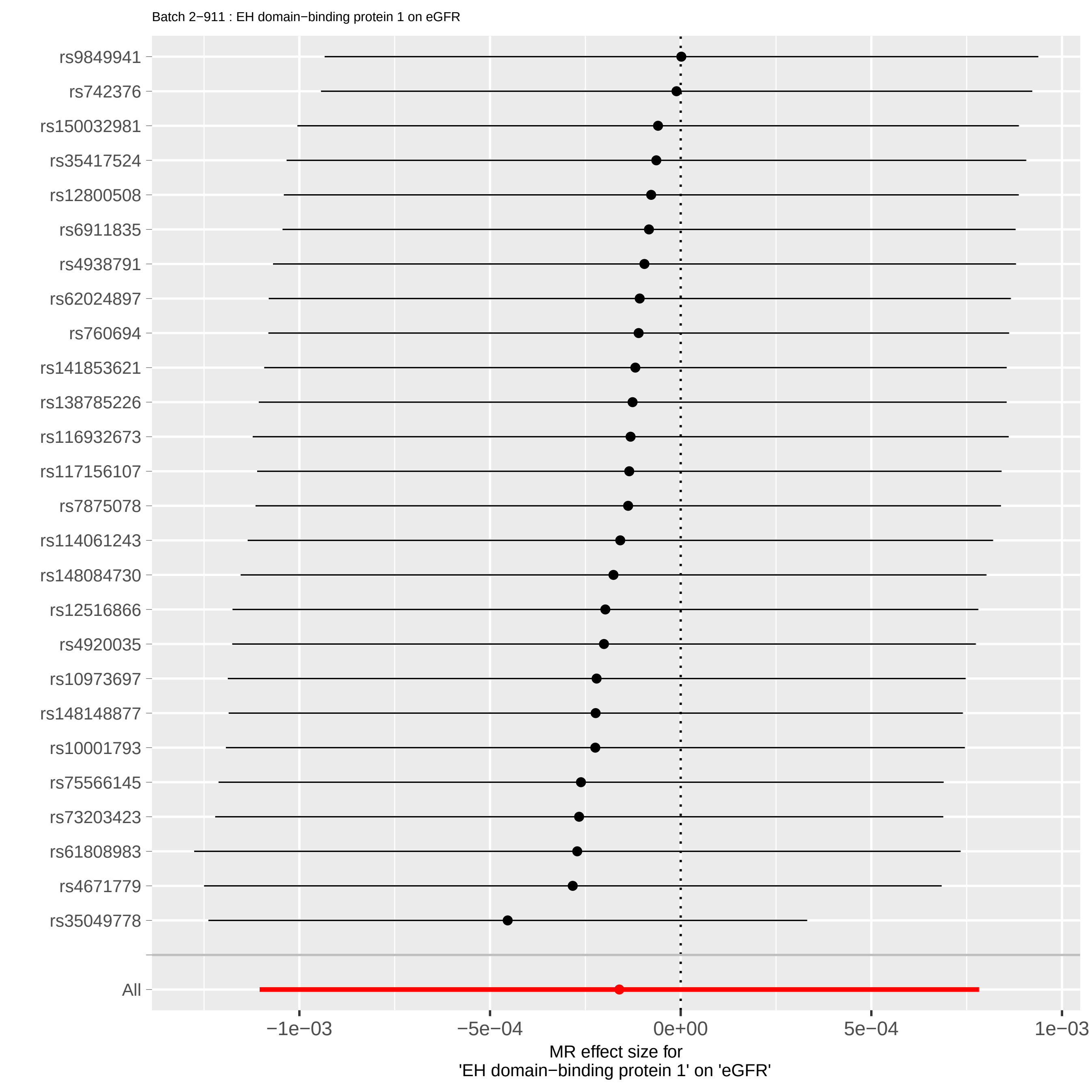

Batch 2−911 : EH domain−binding protein 1 on eGFR
rs9849941
rs742376
rs150032981
rs35417524
rs12800508
rs6911835
rs4938791
rs62024897
rs760694
rs141853621
rs138785226
rs116932673
rs117156107
rs7875078
rs114061243
rs148084730
rs12516866
rs4920035
rs10973697
rs148148877
rs10001793
rs75566145
rs73203423
rs61808983
rs4671779
rs35049778
All
−1e−03
−5e−04
0e+00
5e−04
1e−03
MR effect size for
'EH domain−binding protein 1' on 'eGFR'

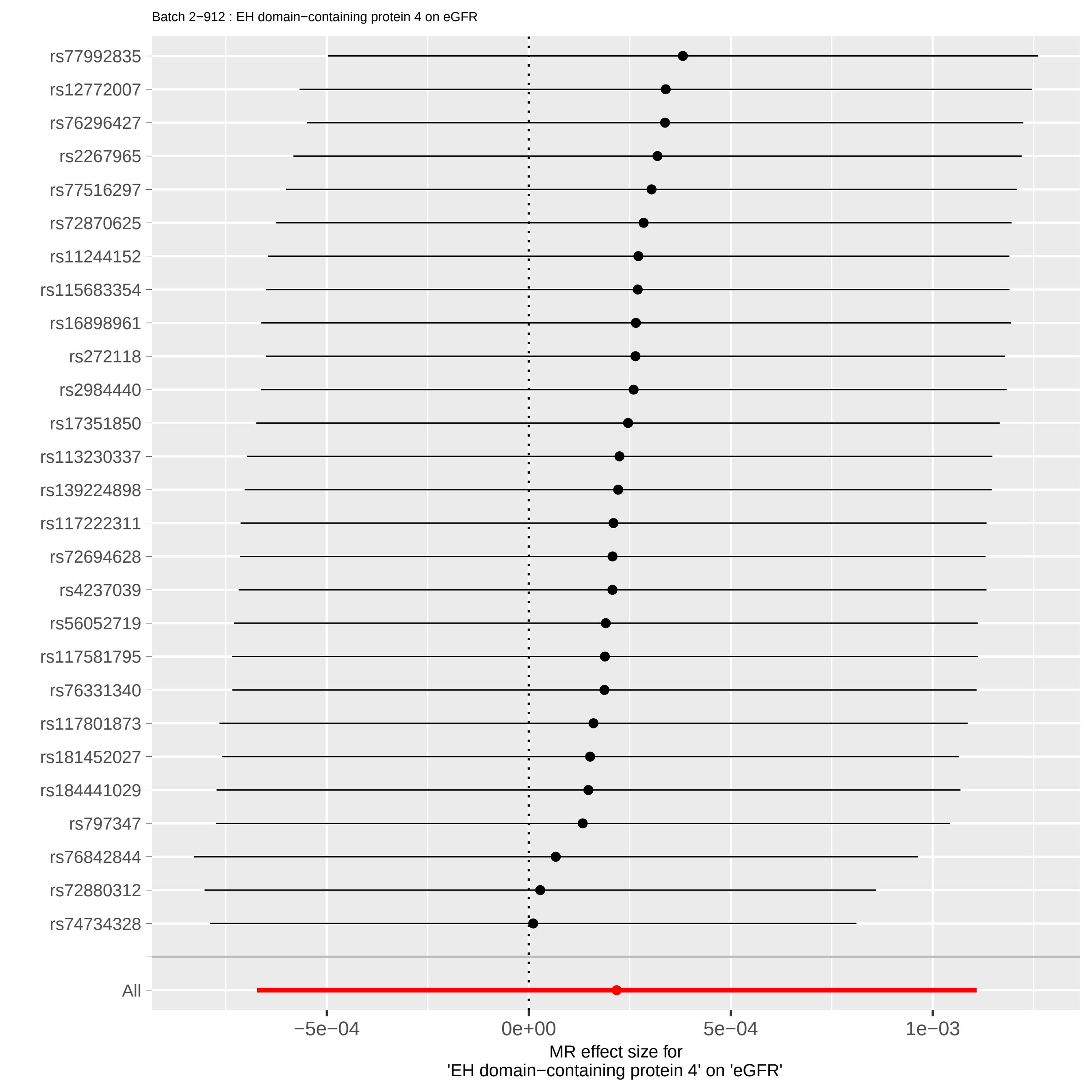

Batch 2−912 : EH domain−containing protein 4 on eGFR
rs77992835
rs12772007
rs76296427
rs2267965
rs77516297
rs72870625
rs11244152
rs115683354
rs16898961
rs272118
rs2984440
rs17351850
rs113230337
rs139224898
rs117222311
rs72694628
rs4237039
rs56052719
rs117581795
rs76331340
rs117801873
rs181452027
rs184441029
rs797347
rs76842844
rs72880312
rs74734328
All
−5e−04
0e+00
5e−04
1e−03
MR effect size for
'EH domain−containing protein 4' on 'eGFR'

Batch 2−913 : ETS homologous factor on eGFR

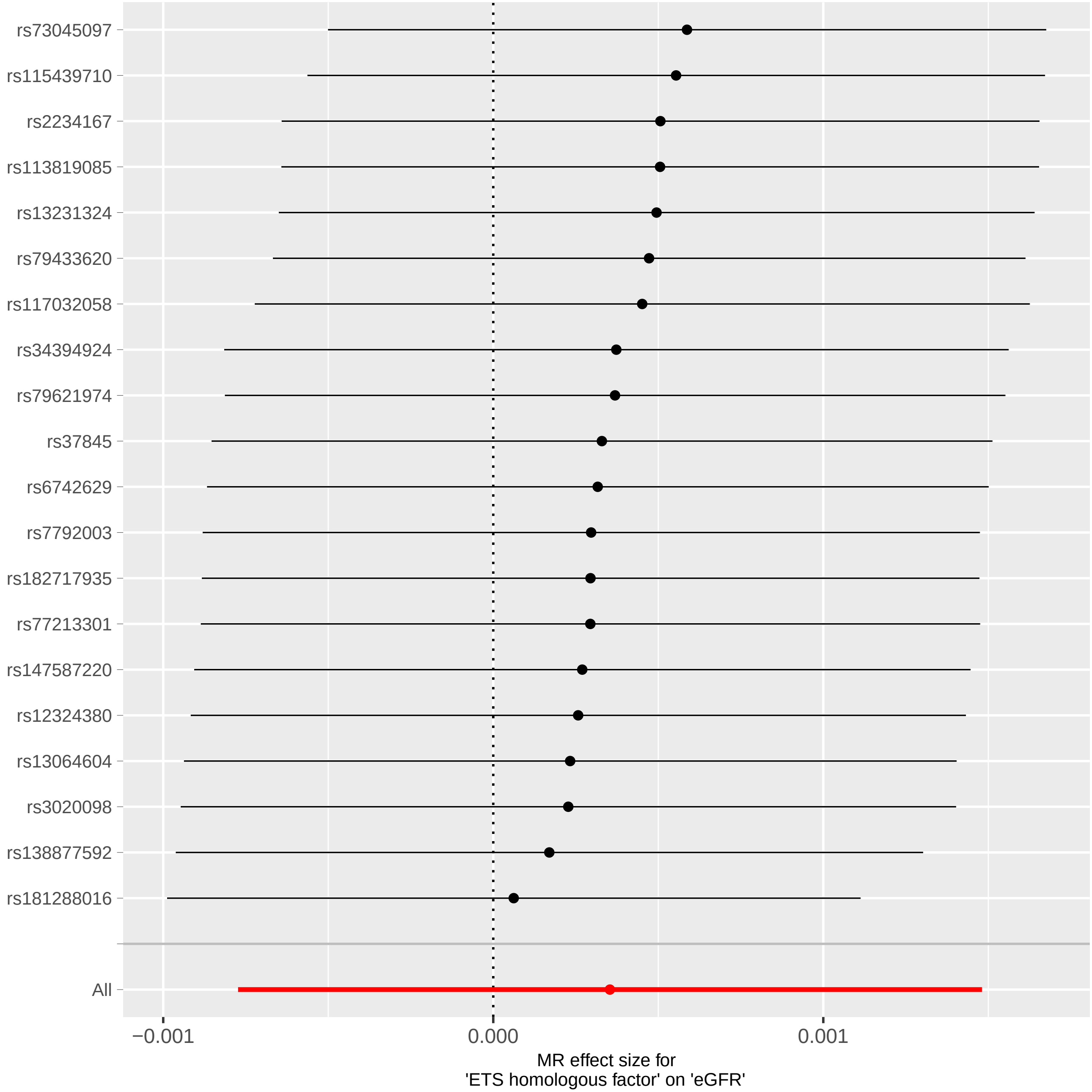

Batch 2−914 : Histone−lysine N−methyltransferase EHMT2 on eGFR

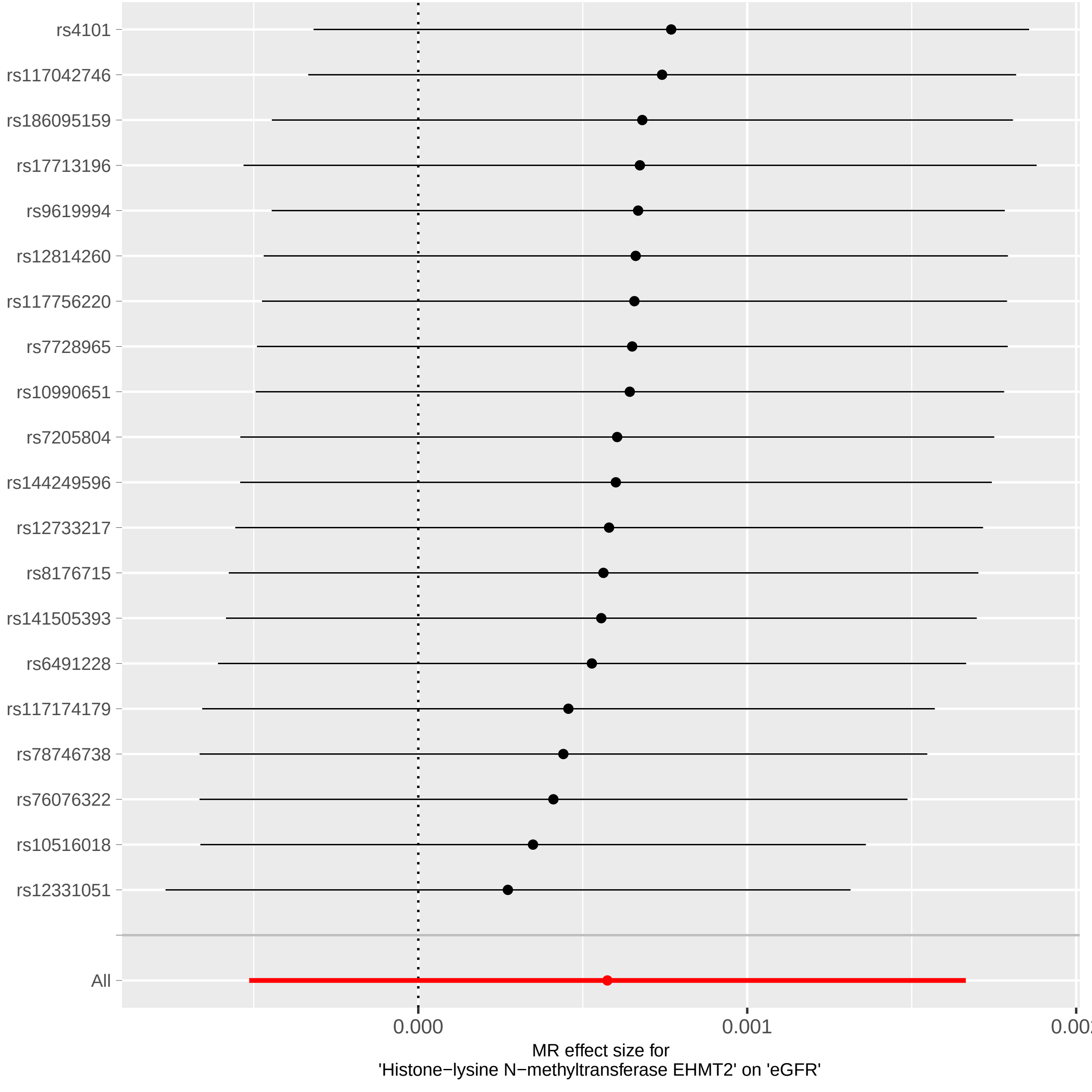

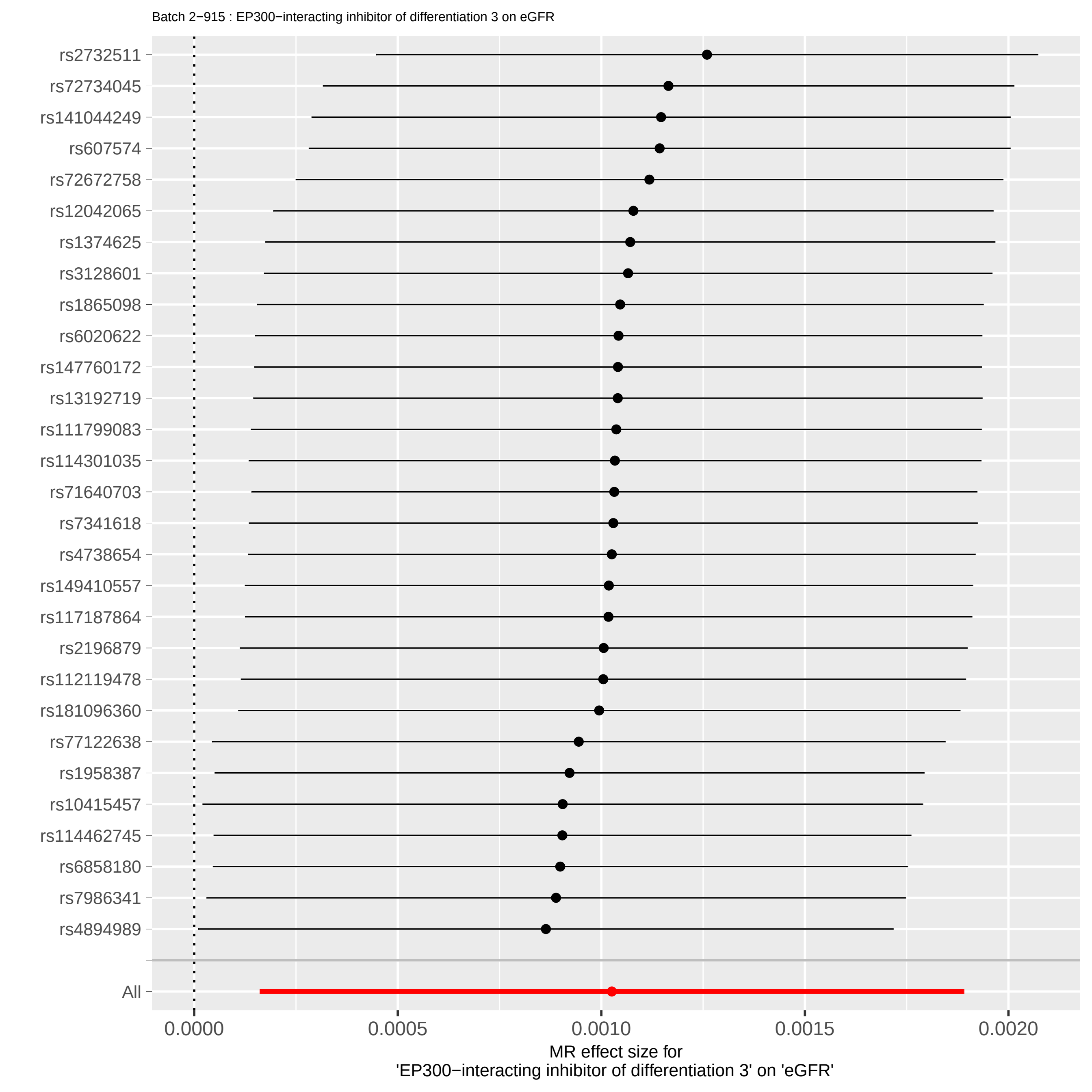
Batch 2−915 : EP300−interacting inhibitor of differentiation 3 on eGFR
rs2732511
rs72734045
rs141044249
rs607574
rs72672758
rs12042065
rs1374625
rs3128601
rs1865098
rs6020622
rs147760172
rs13192719
rs111799083
rs114301035
rs71640703
rs7341618
rs4738654
rs149410557
rs117187864
rs2196879
rs112119478
rs181096360
rs77122638
rs1958387
rs10415457
rs114462745
rs6858180
rs7986341
rs4894989
All
0.0000
0.0005
0.0010
0.0015
0.0020
MR effect size for
'EP300−interacting inhibitor of differentiation 3' on 'eGFR'

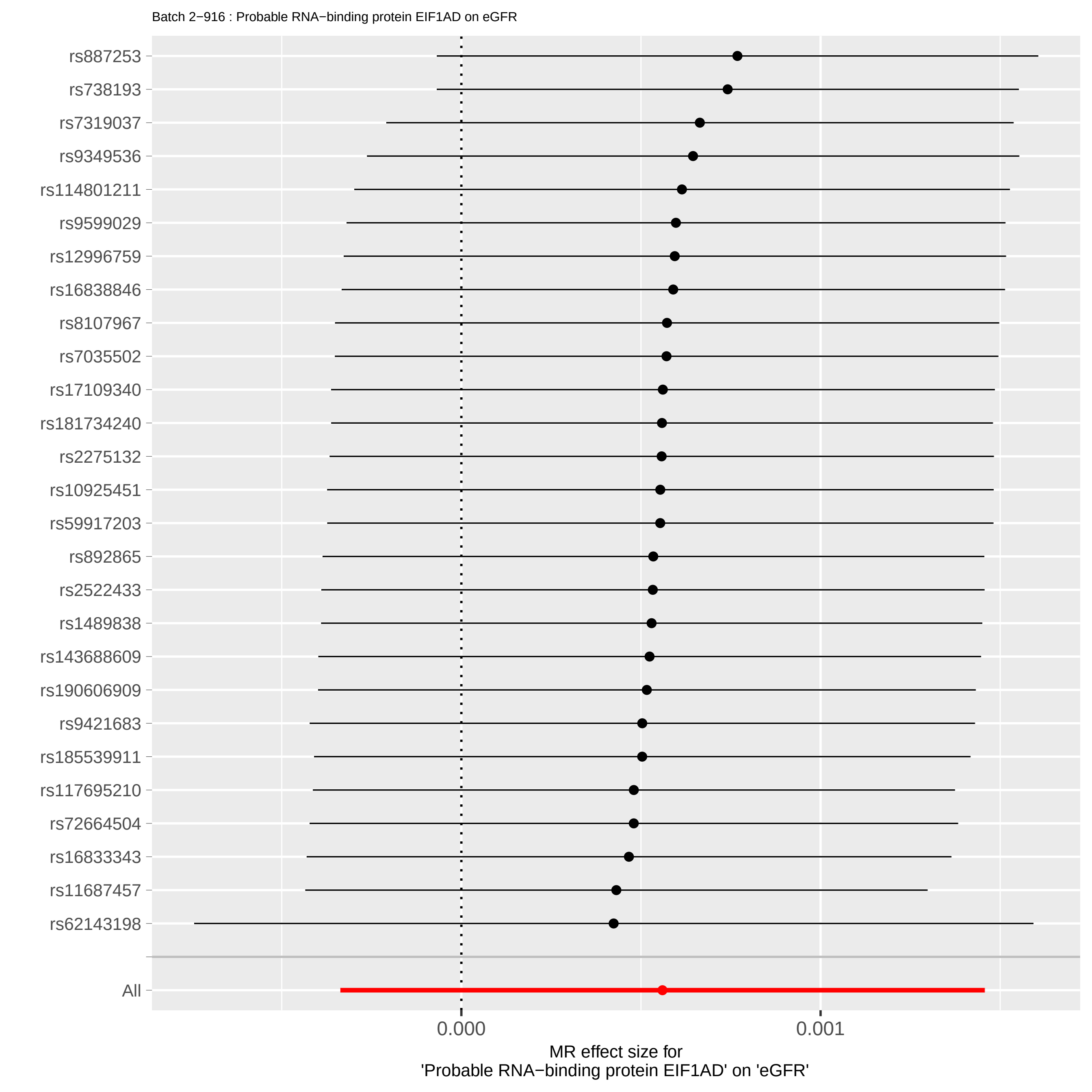

Batch 2−916 : Probable RNA−binding protein EIF1AD on eGFR
rs887253
rs738193
rs7319037
rs9349536
rs114801211
rs9599029
rs12996759
rs16838846
rs8107967
rs7035502
rs17109340
rs181734240
rs2275132
rs10925451
rs59917203
rs892865
rs2522433
rs1489838
rs143688609
rs190606909
rs9421683
rs185539911
rs117695210
rs72664504
rs16833343
rs11687457
rs62143198
All
0.000
0.001
MR effect size for
'Probable RNA−binding protein EIF1AD' on 'eGFR'

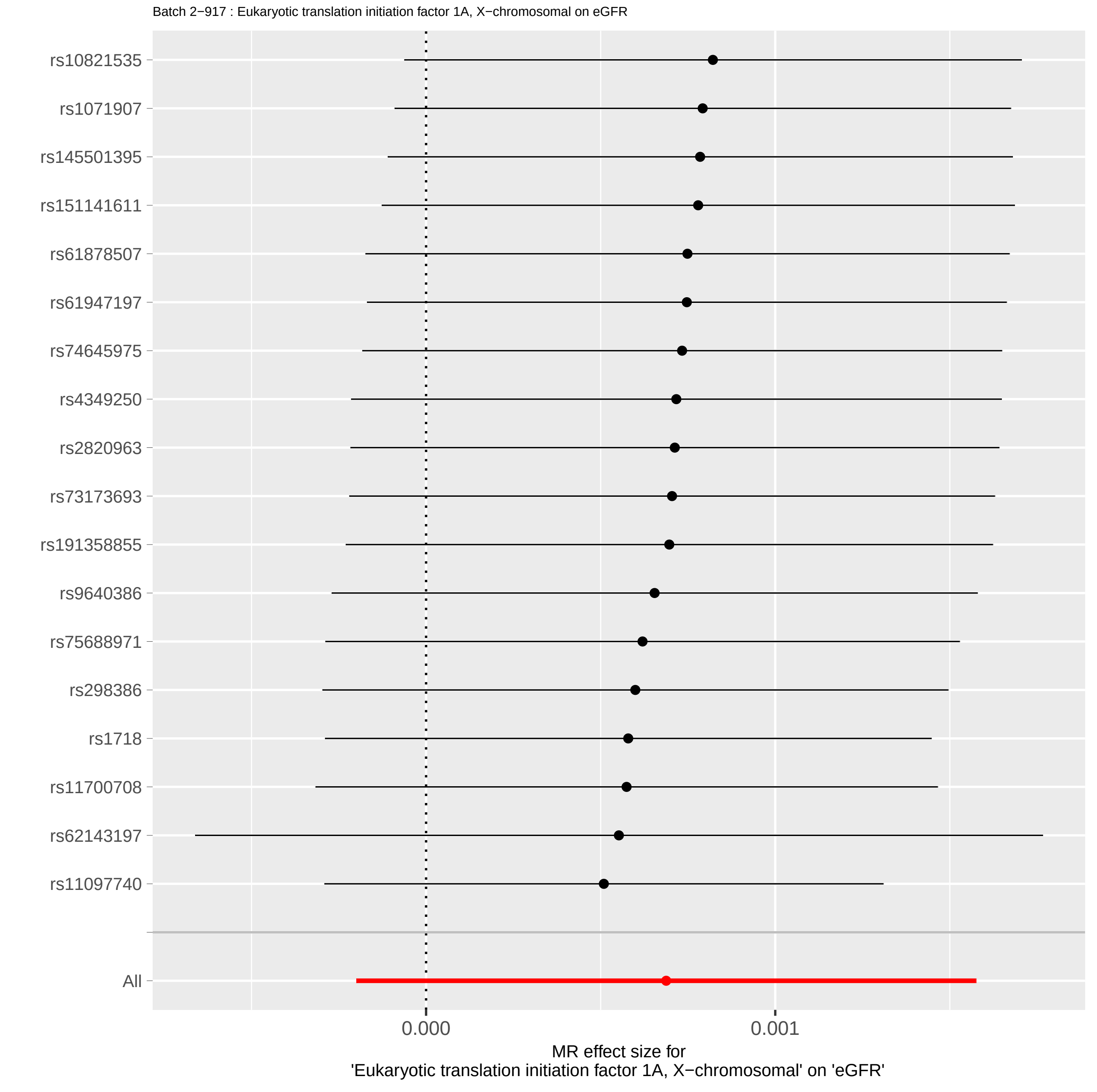
Batch 2−917 : Eukaryotic translation initiation factor 1A, X−chromosomal on eGFR
rs10821535
rs1071907
rs145501395
rs151141611
rs61878507
rs61947197
rs74645975
rs4349250
rs2820963
rs73173693
rs191358855
rs9640386
rs75688971
rs298386
rs1718
rs11700708
rs62143197
rs11097740
All
0.000
0.001
MR effect size for
'Eukaryotic translation initiation factor 1A, X−chromosomal' on 'eGFR'

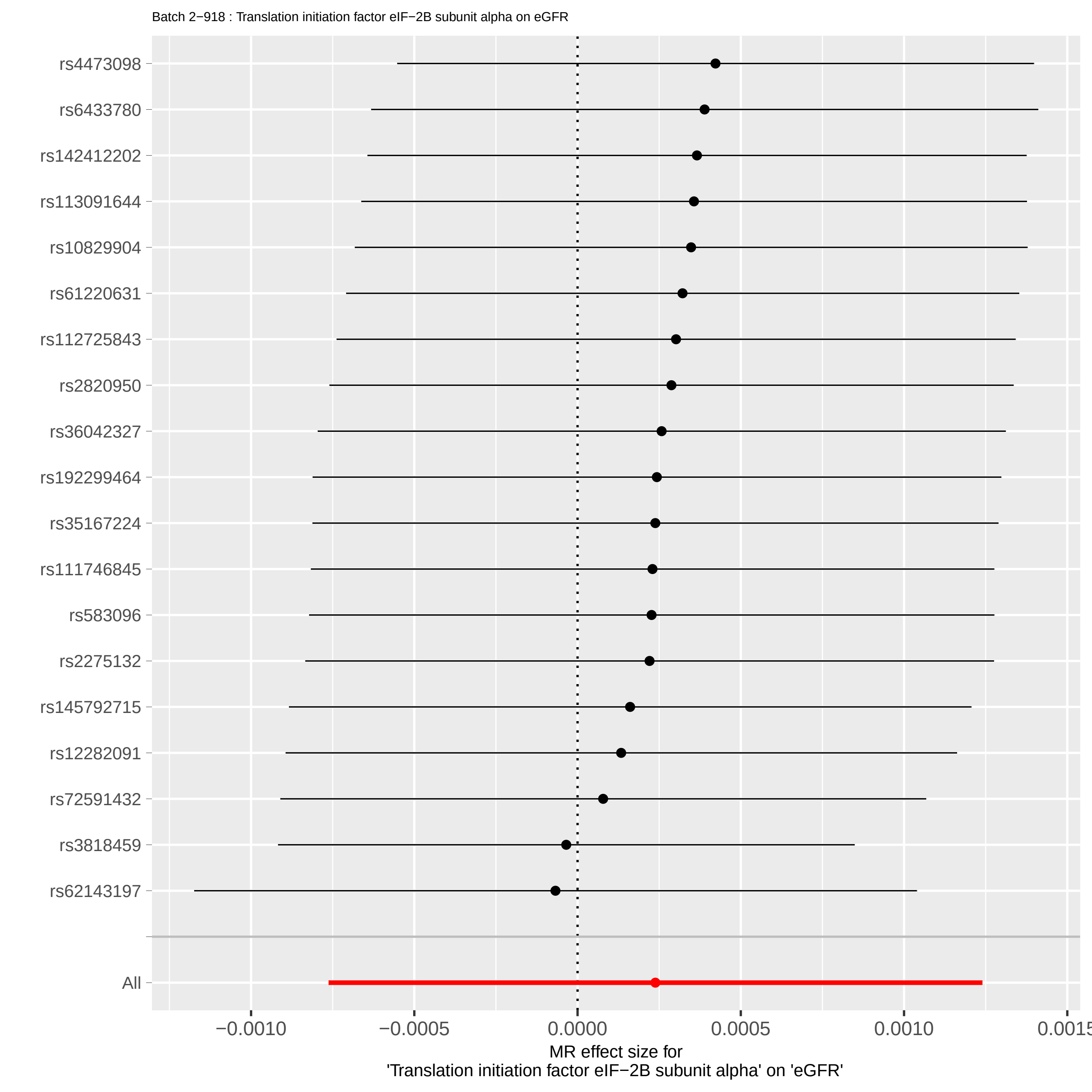

Batch 2−918 : Translation initiation factor eIF−2B subunit alpha on eGFR
rs4473098
rs6433780
rs142412202
rs113091644
rs10829904
rs61220631
rs112725843
rs2820950
rs36042327
rs192299464
rs35167224
rs111746845
rs583096
rs2275132
rs145792715
rs12282091
rs72591432
rs3818459
rs62143197
All
−0.0010
−0.0005
0.0000
0.0005
0.0010
0.0015
MR effect size for
'Translation initiation factor eIF−2B subunit alpha' on 'eGFR'

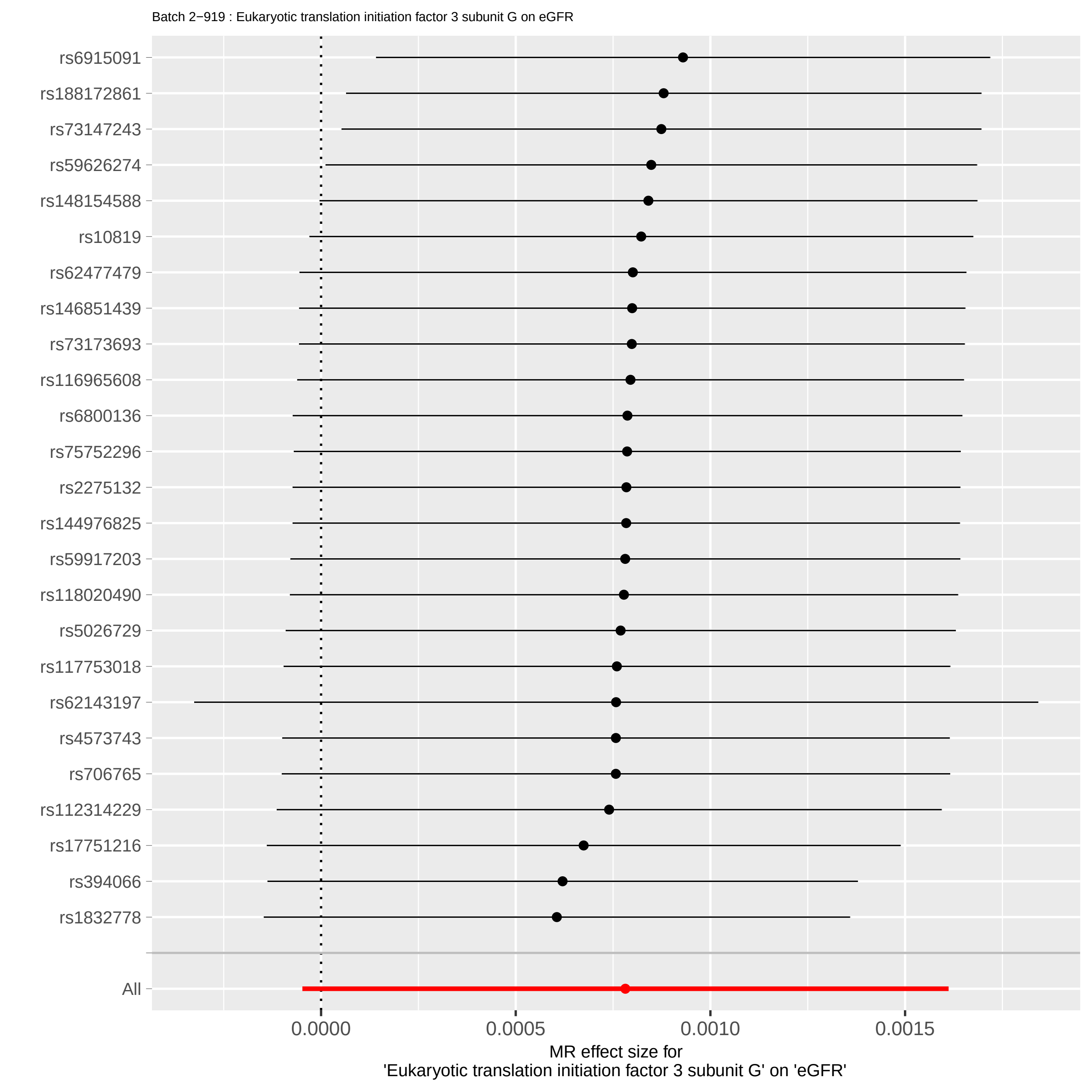

Batch 2−919 : Eukaryotic translation initiation factor 3 subunit G on eGFR
rs6915091
rs188172861
rs73147243
rs59626274
rs148154588
rs10819
rs62477479
rs146851439
rs73173693
rs116965608
rs6800136
rs75752296
rs2275132
rs144976825
rs59917203
rs118020490
rs5026729
rs117753018
rs62143197
rs4573743
rs706765
rs112314229
rs17751216
rs394066
rs1832778
All
0.0000
0.0005
0.0010
0.0015
MR effect size for
'Eukaryotic translation initiation factor 3 subunit G' on 'eGFR'

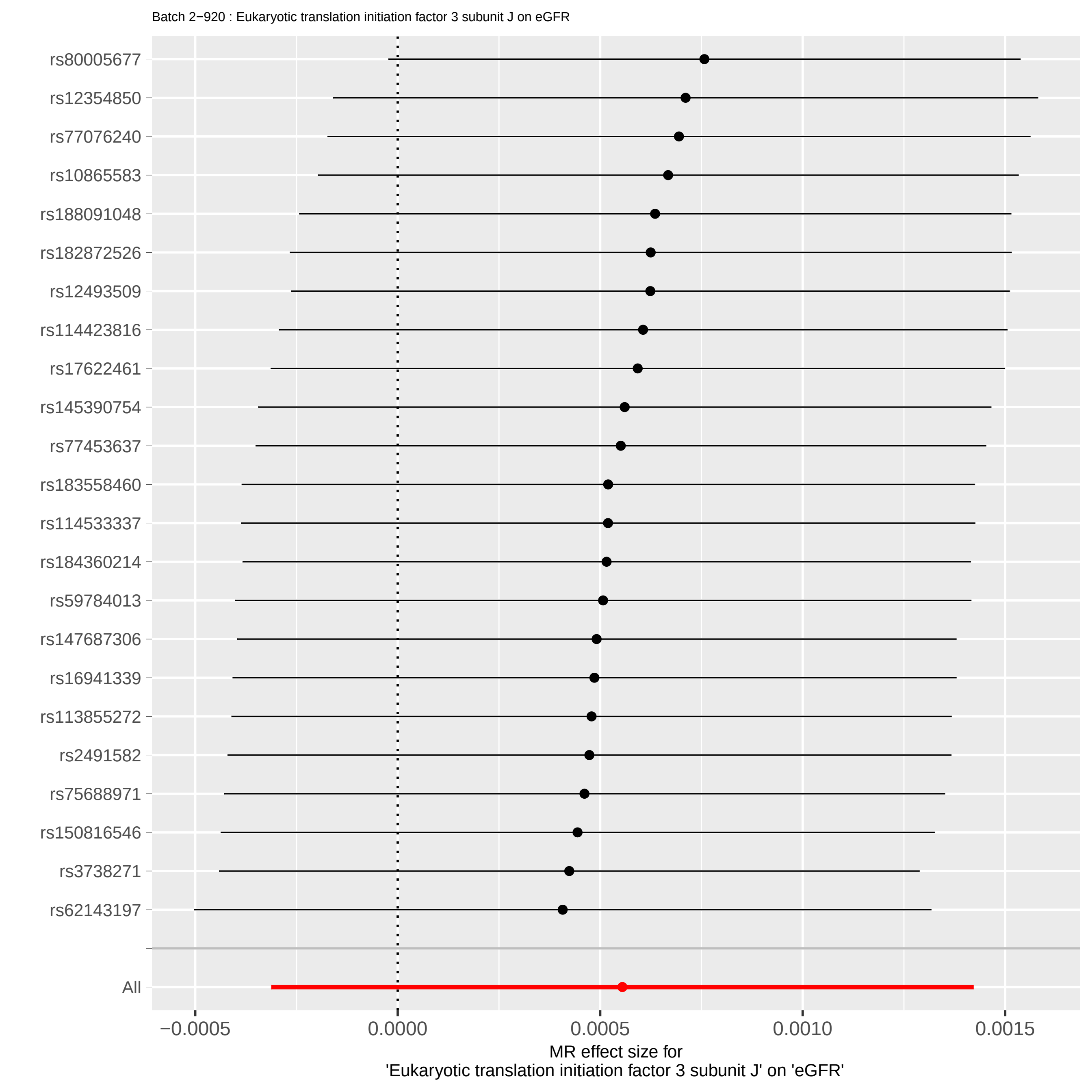

Batch 2−920 : Eukaryotic translation initiation factor 3 subunit J on eGFR
rs80005677
rs12354850
rs77076240
rs10865583
rs188091048
rs182872526
rs12493509
rs114423816
rs17622461
rs145390754
rs77453637
rs183558460
rs114533337
rs184360214
rs59784013
rs147687306
rs16941339
rs113855272
rs2491582
rs75688971
rs150816546
rs3738271
rs62143197
All
−0.0005
0.0000
0.0005
0.0010
0.0015
MR effect size for
'Eukaryotic translation initiation factor 3 subunit J' on 'eGFR'

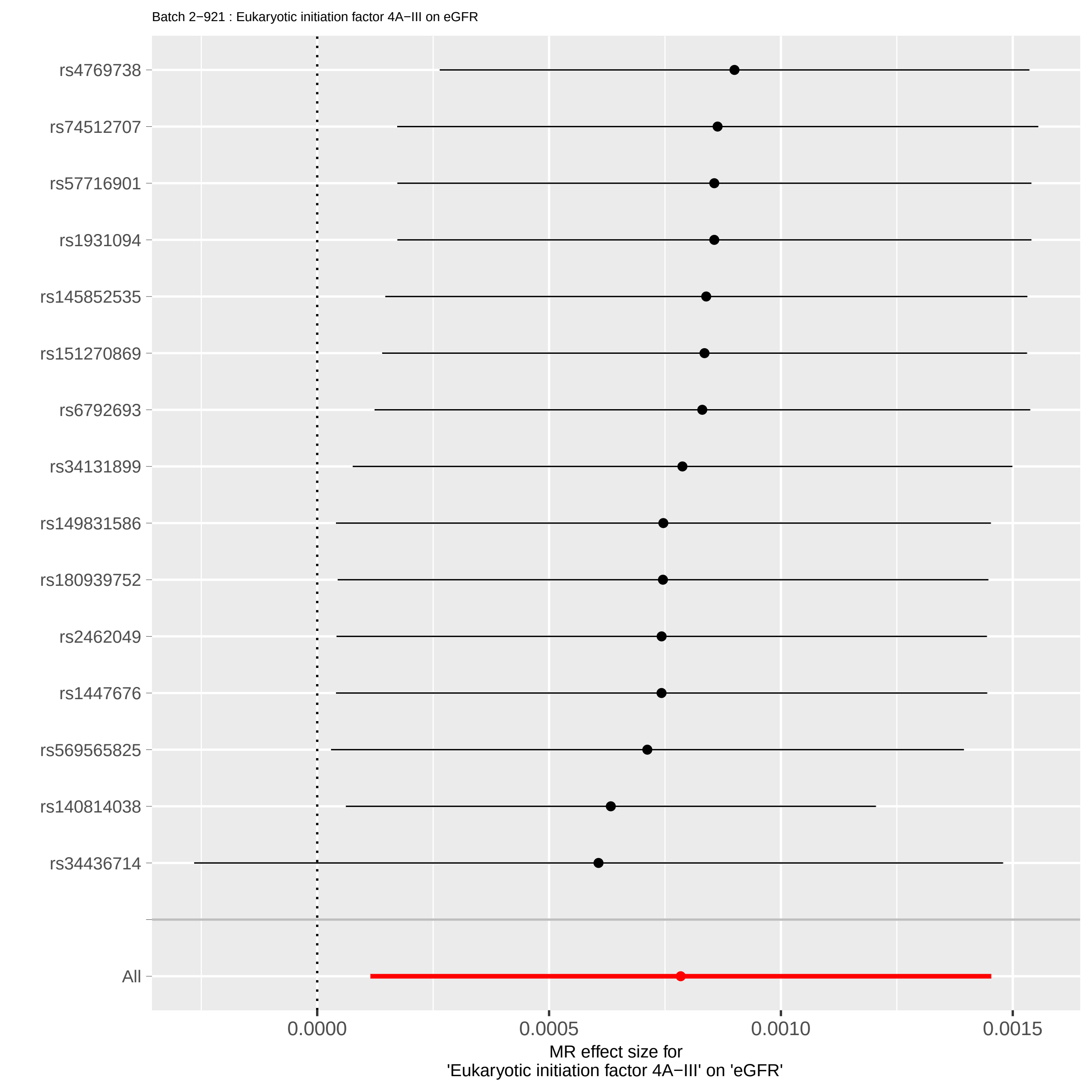

Batch 2−921 : Eukaryotic initiation factor 4A−III on eGFR
rs4769738
rs74512707
rs57716901
rs1931094
rs145852535
rs151270869
rs6792693
rs34131899
rs149831586
rs180939752
rs2462049
rs1447676
rs569565825
rs140814038
rs34436714
All
0.0000
0.0005
0.0010
0.0015
MR effect size for
'Eukaryotic initiation factor 4A−III' on 'eGFR'

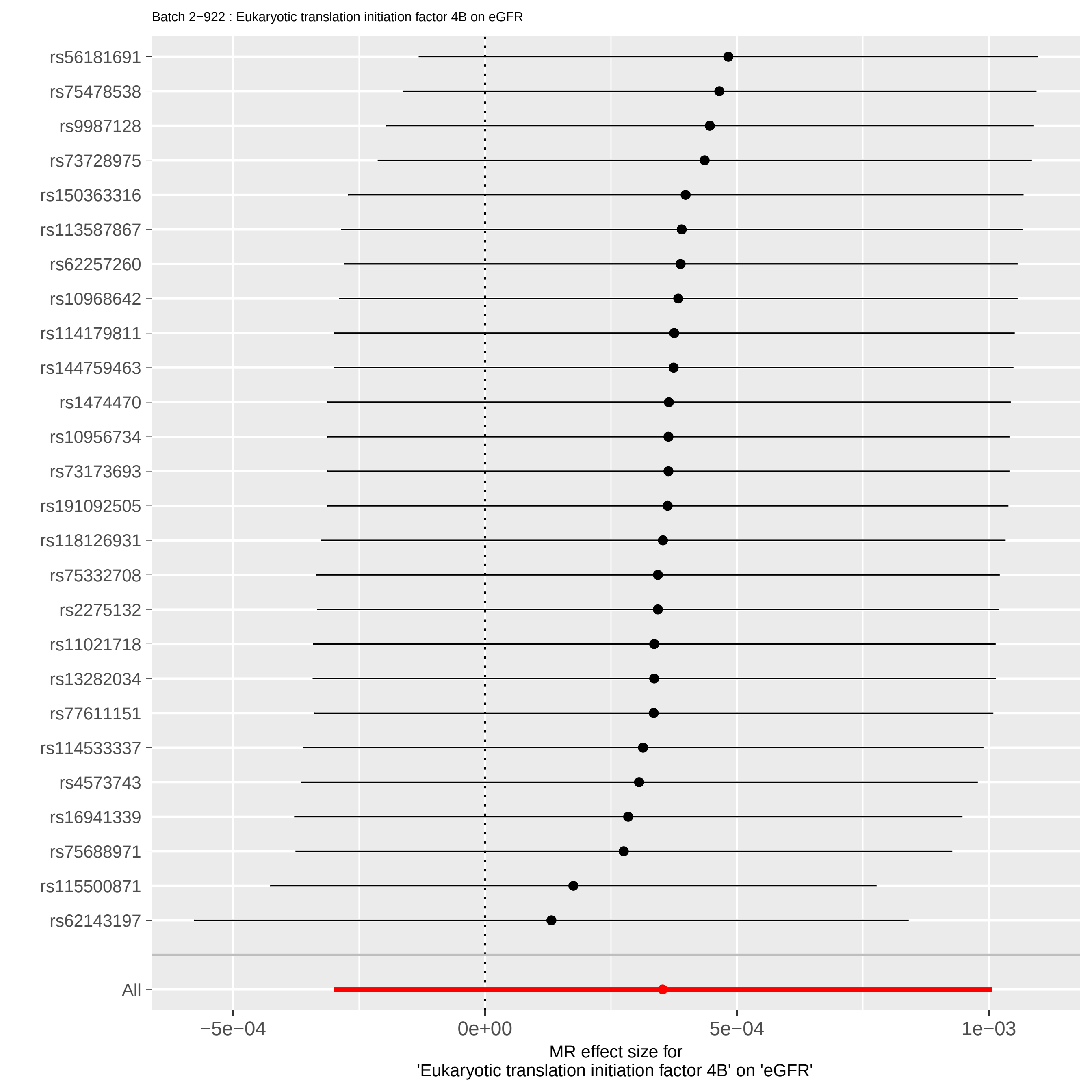
Batch 2−922 : Eukaryotic translation initiation factor 4B on eGFR
rs56181691
rs75478538
rs9987128
rs73728975
rs150363316
rs113587867
rs62257260
rs10968642
rs114179811
rs144759463
rs1474470
rs10956734
rs73173693
rs191092505
rs118126931
rs75332708
rs2275132
rs11021718
rs13282034
rs77611151
rs114533337
rs4573743
rs16941339
rs75688971
rs115500871
rs62143197
All
−5e−04
0e+00
5e−04
1e−03
MR effect size for
'Eukaryotic translation initiation factor 4B' on 'eGFR'

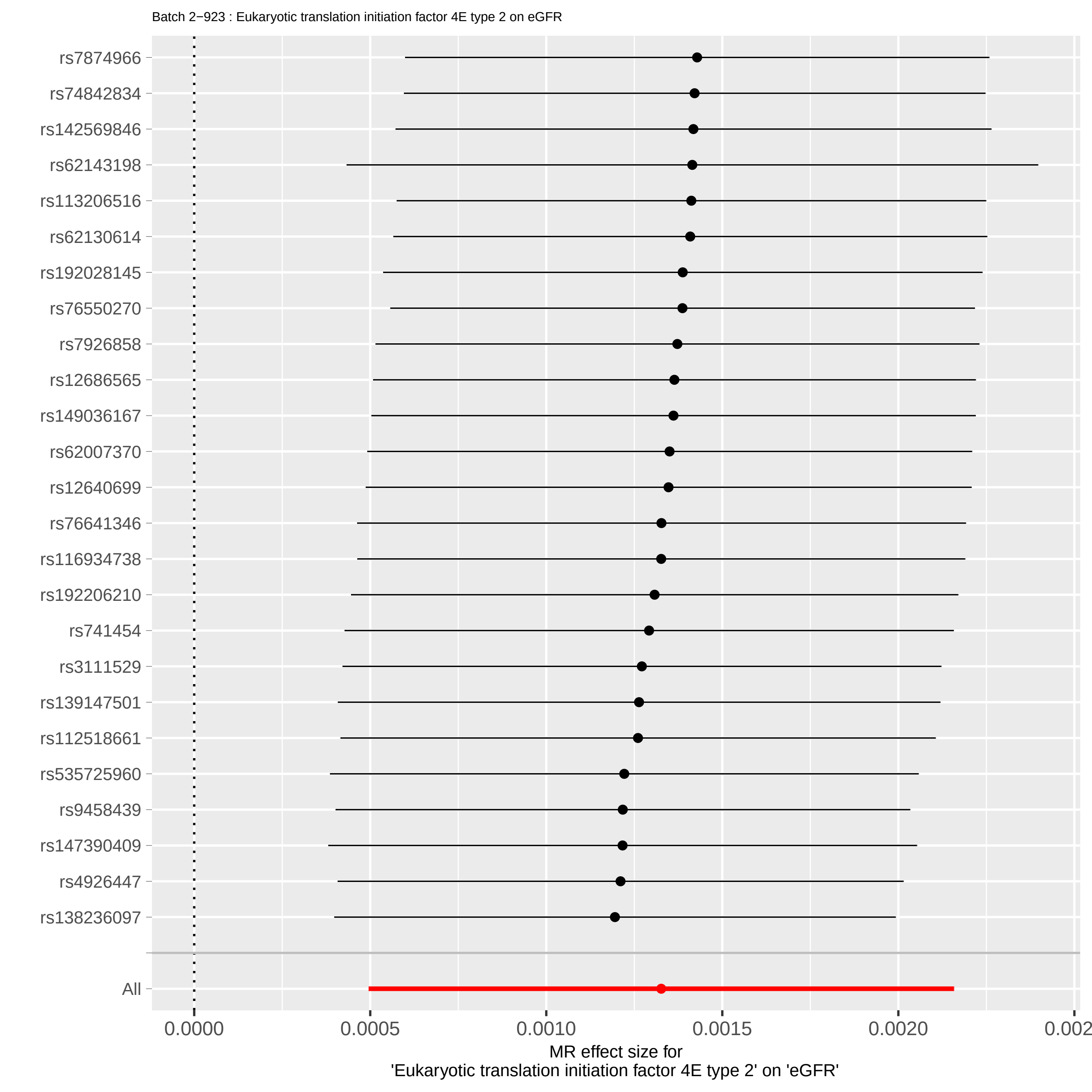

Batch 2−923 : Eukaryotic translation initiation factor 4E type 2 on eGFR
rs7874966
rs74842834
rs142569846
rs62143198
rs113206516
rs62130614
rs192028145
rs76550270
rs7926858
rs12686565
rs149036167
rs62007370
rs12640699
rs76641346
rs116934738
rs192206210
rs741454
rs3111529
rs139147501
rs112518661
rs535725960
rs9458439
rs147390409
rs4926447
rs138236097
All
0.0000
0.0005
0.0010
0.0015
0.0020
0.002
MR effect size for
'Eukaryotic translation initiation factor 4E type 2' on 'eGFR'

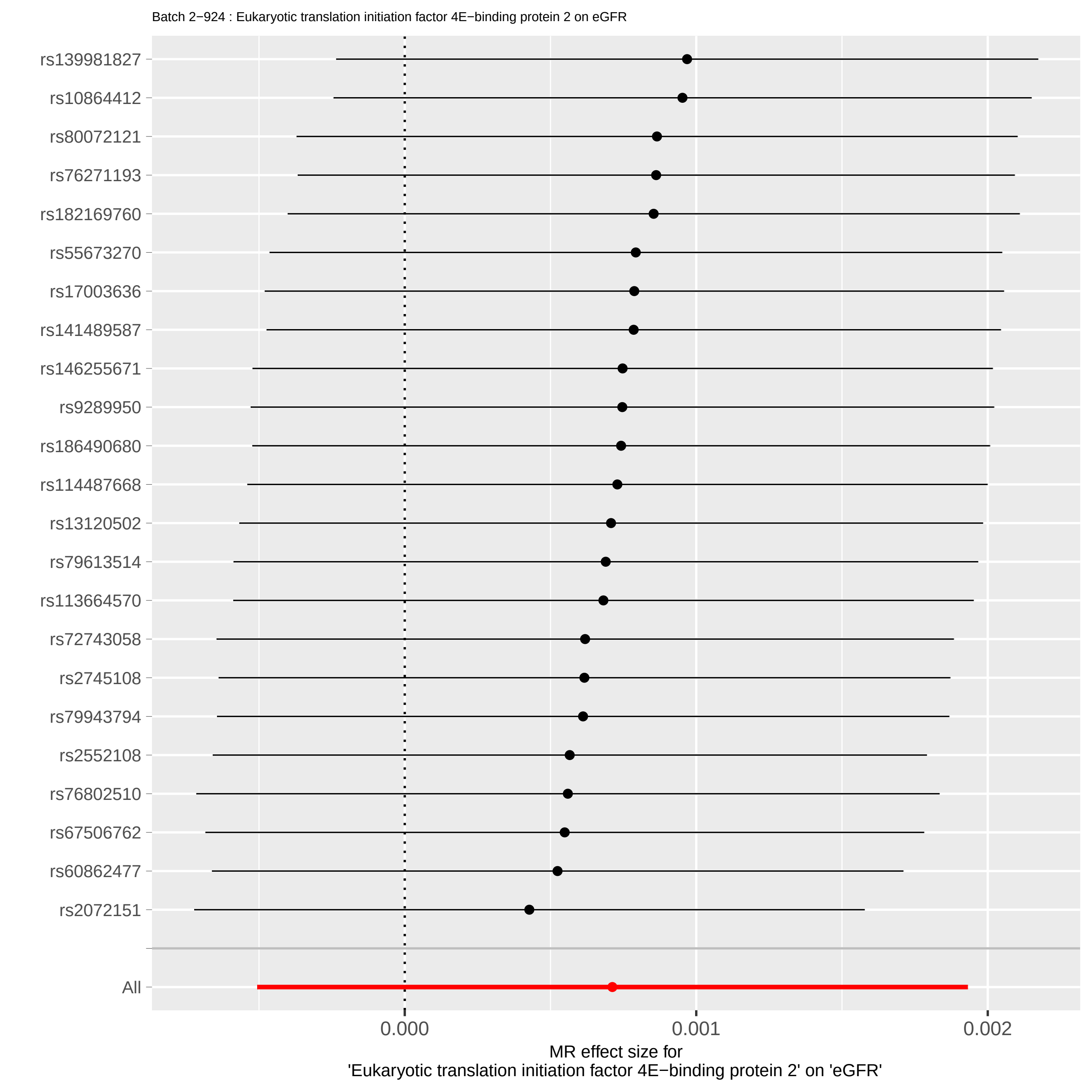
Batch 2−924 : Eukaryotic translation initiation factor 4E−binding protein 2 on eGFR
rs139981827
rs10864412
rs80072121
rs76271193
rs182169760
rs55673270
rs17003636
rs141489587
rs146255671
rs9289950
rs186490680
rs114487668
rs13120502
rs79613514
rs113664570
rs72743058
rs2745108
rs79943794
rs2552108
rs76802510
rs67506762
rs60862477
rs2072151
All
0.000
0.001
0.002
MR effect size for
'Eukaryotic translation initiation factor 4E−binding protein 2' on 'eGFR'

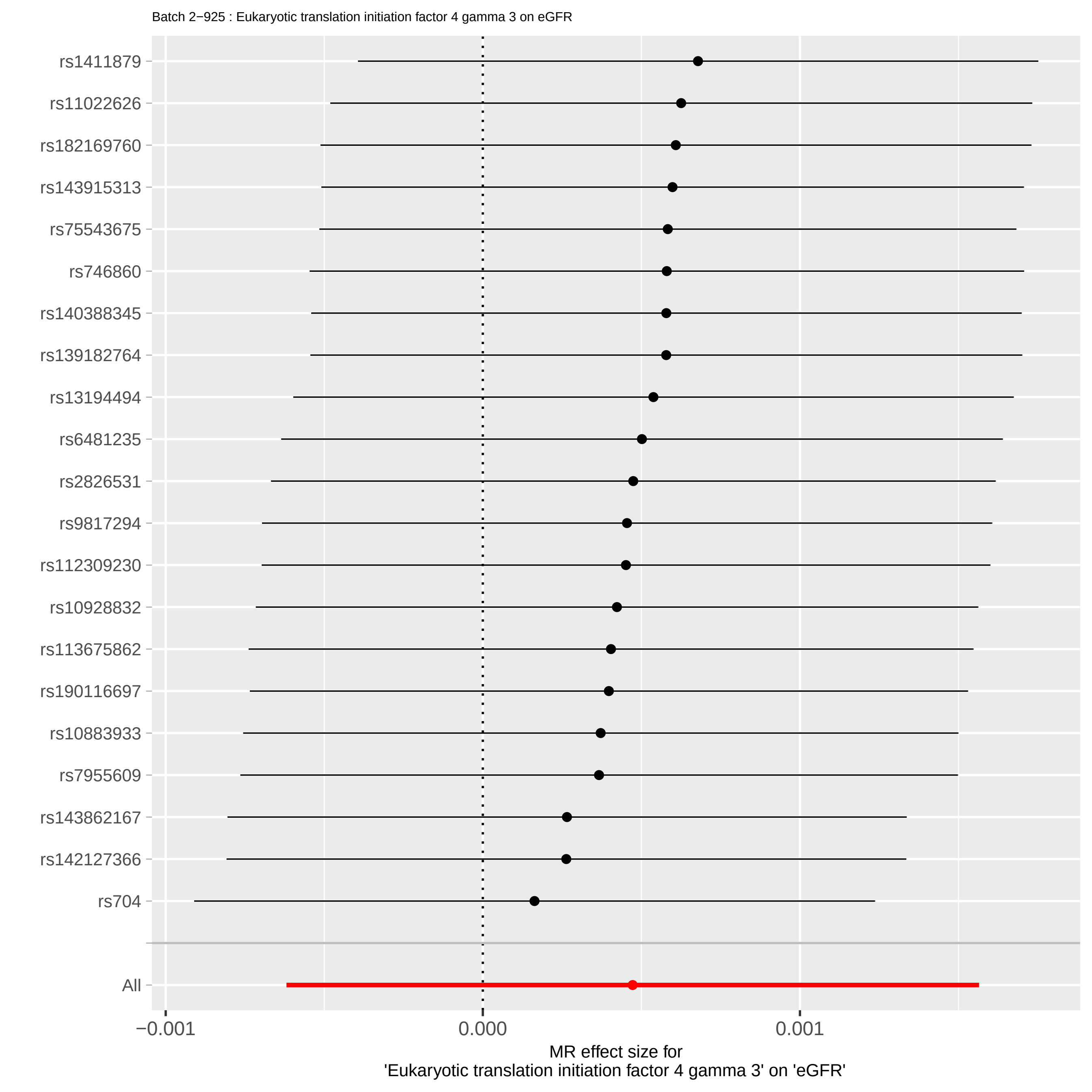

Batch 2−925 : Eukaryotic translation initiation factor 4 gamma 3 on eGFR
rs1411879
rs11022626
rs182169760
rs143915313
rs75543675
rs746860
rs140388345
rs139182764
rs13194494
rs6481235
rs2826531
rs9817294
rs112309230
rs10928832
rs113675862
rs190116697
rs10883933
rs7955609
rs143862167
rs142127366
rs704
All
−0.001
0.000
0.001
MR effect size for
'Eukaryotic translation initiation factor 4 gamma 3' on 'eGFR'

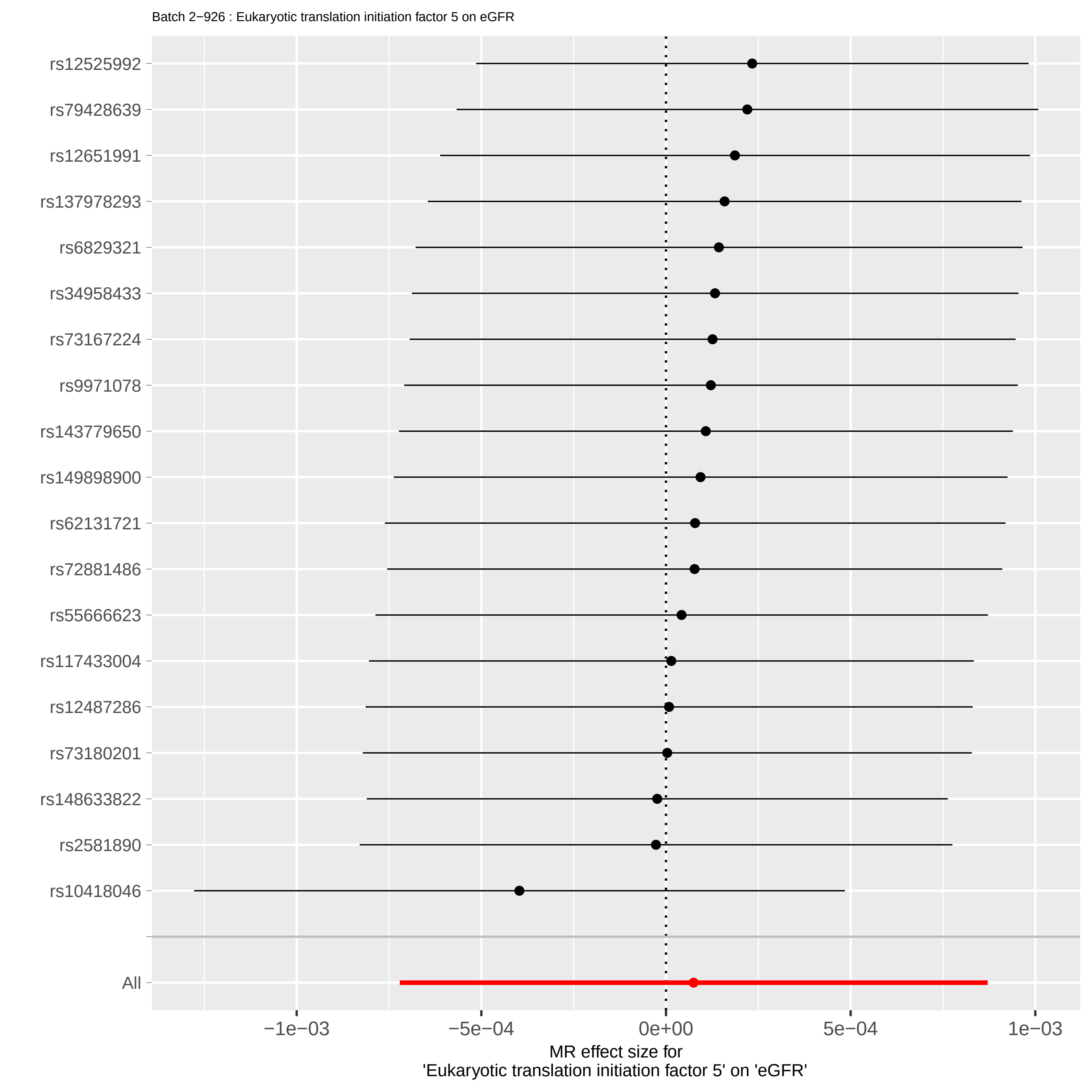

Batch 2−926 : Eukaryotic translation initiation factor 5 on eGFR
rs12525992
rs79428639
rs12651991
rs137978293
rs6829321
rs34958433
rs73167224
rs9971078
rs143779650
rs149898900
rs62131721
rs72881486
rs55666623
rs117433004
rs12487286
rs73180201
rs148633822
rs2581890
rs10418046
All
−1e−03
−5e−04
0e+00
5e−04
1e−03
MR effect size for
'Eukaryotic translation initiation factor 5' on 'eGFR'

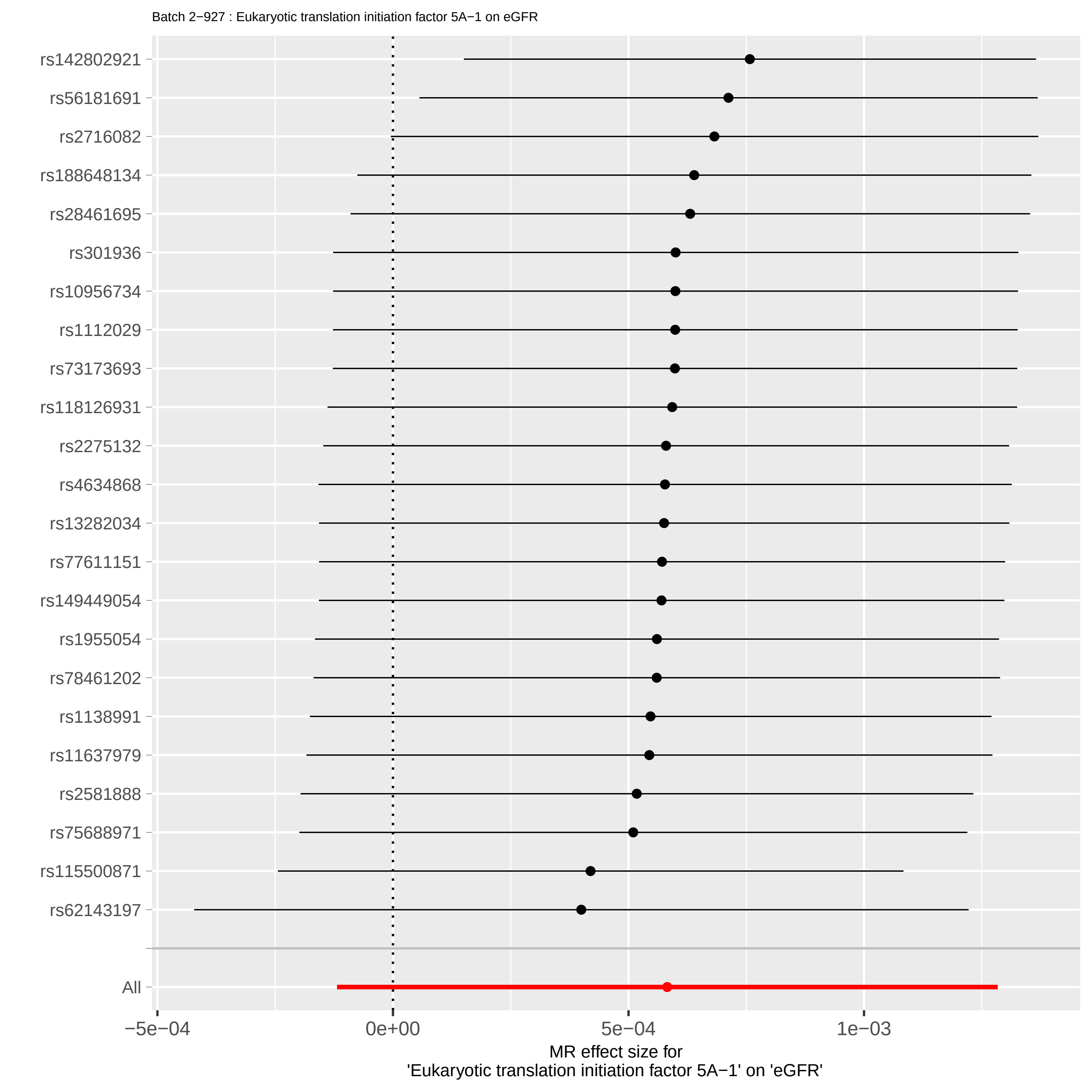

Batch 2−927 : Eukaryotic translation initiation factor 5A−1 on eGFR
rs142802921
rs56181691
rs2716082
rs188648134
rs28461695
rs301936
rs10956734
rs1112029
rs73173693
rs118126931
rs2275132
rs4634868
rs13282034
rs77611151
rs149449054
rs1955054
rs78461202
rs1138991
rs11637979
rs2581888
rs75688971
rs115500871
rs62143197
All
−5e−04
0e+00
5e−04
1e−03
MR effect size for
'Eukaryotic translation initiation factor 5A−1' on 'eGFR'

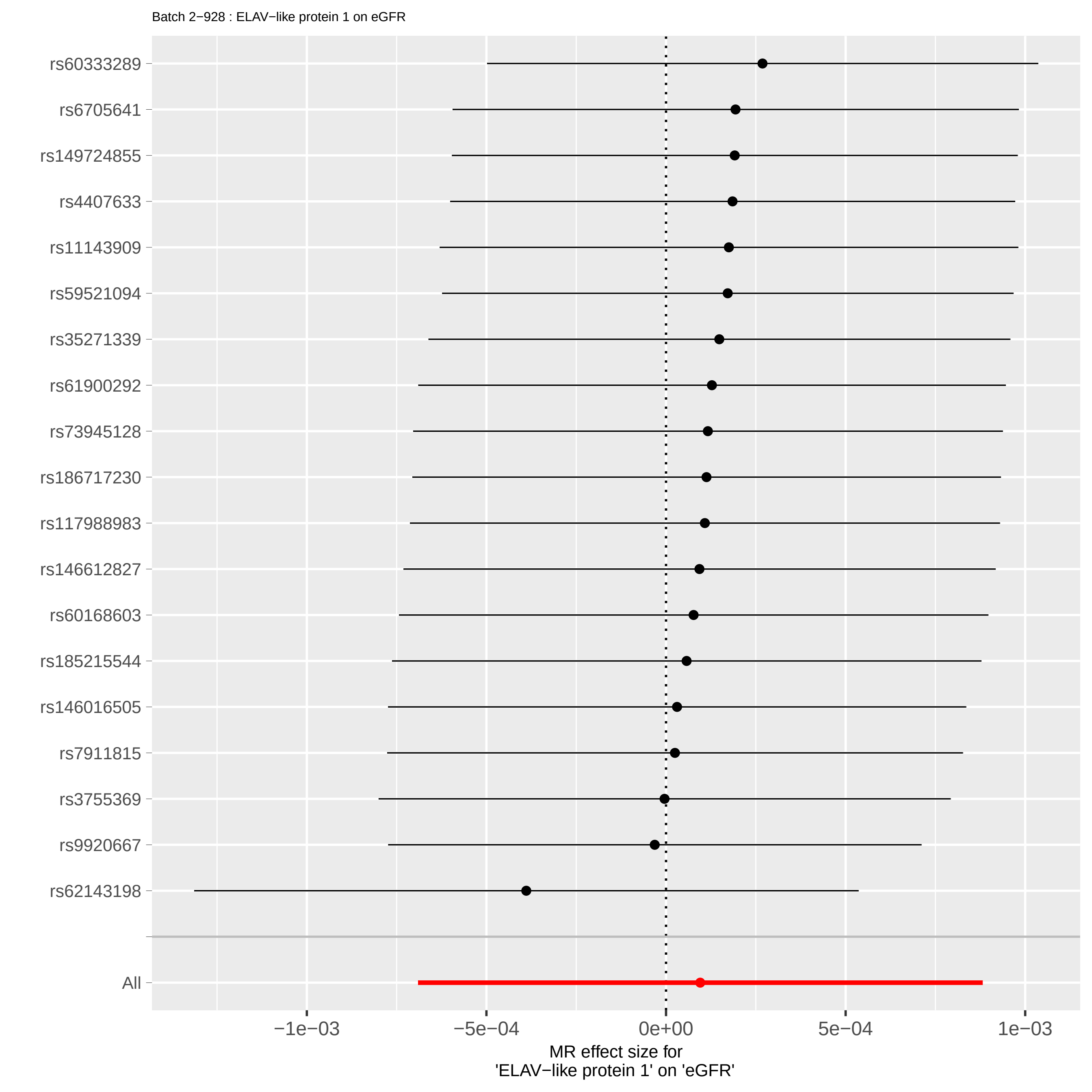
Batch 2−928 : ELAV−like protein 1 on eGFR
rs60333289
rs6705641
rs149724855
rs4407633
rs11143909
rs59521094
rs35271339
rs61900292
rs73945128
rs186717230
rs117988983
rs146612827
rs60168603
rs185215544
rs146016505
rs7911815
rs3755369
rs9920667
rs62143198
All
−1e−03
−5e−04
0e+00
5e−04
1e−03
MR effect size for
'ELAV−like protein 1' on 'eGFR'

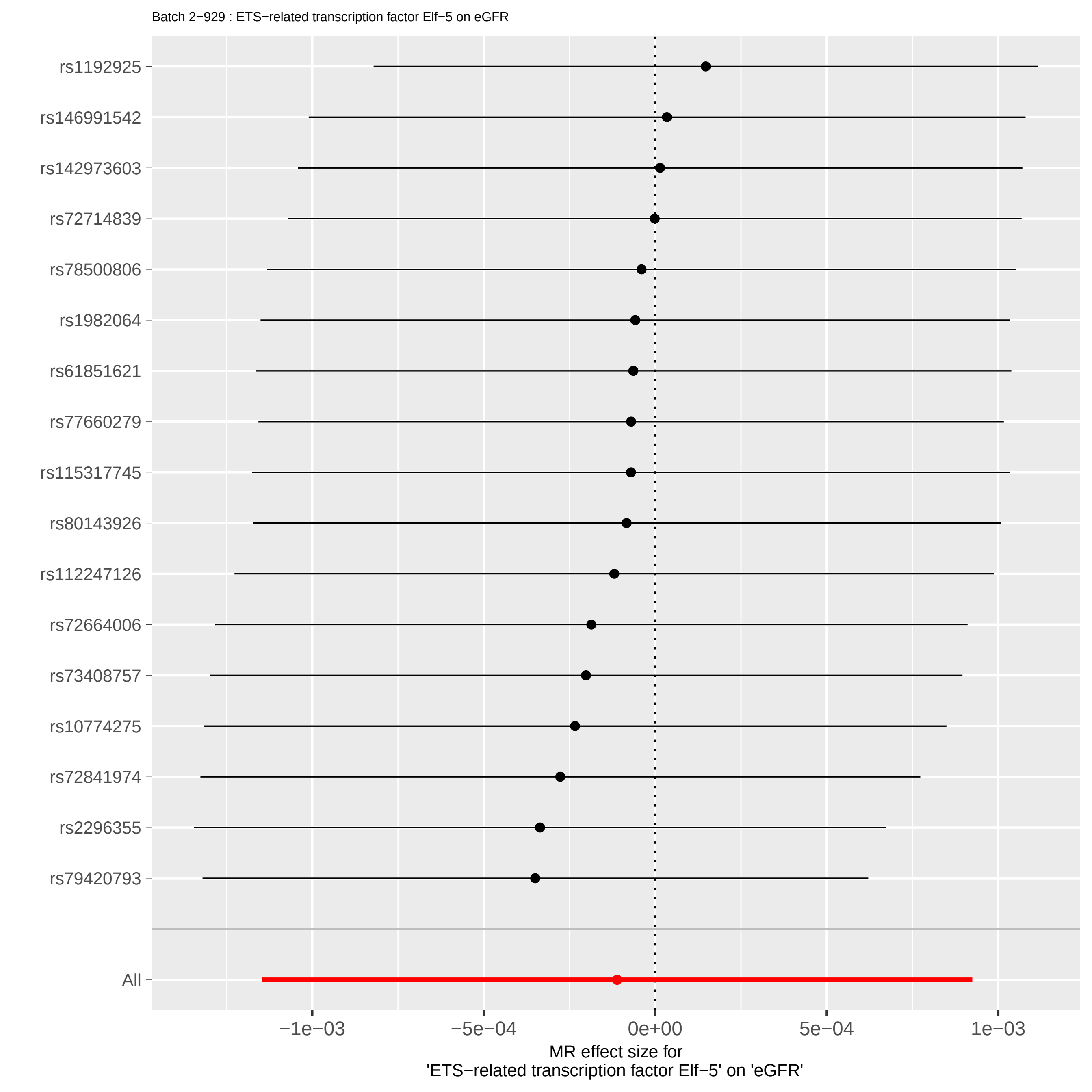
Batch 2−929 : ETS−related transcription factor Elf−5 on eGFR
rs1192925
rs146991542
rs142973603
rs72714839
rs78500806
rs1982064
rs61851621
rs77660279
rs115317745
rs80143926
rs112247126
rs72664006
rs73408757
rs10774275
rs72841974
rs2296355
rs79420793
All
−1e−03
−5e−04
0e+00
5e−04
1e−03
MR effect size for
'ETS−related transcription factor Elf−5' on 'eGFR'

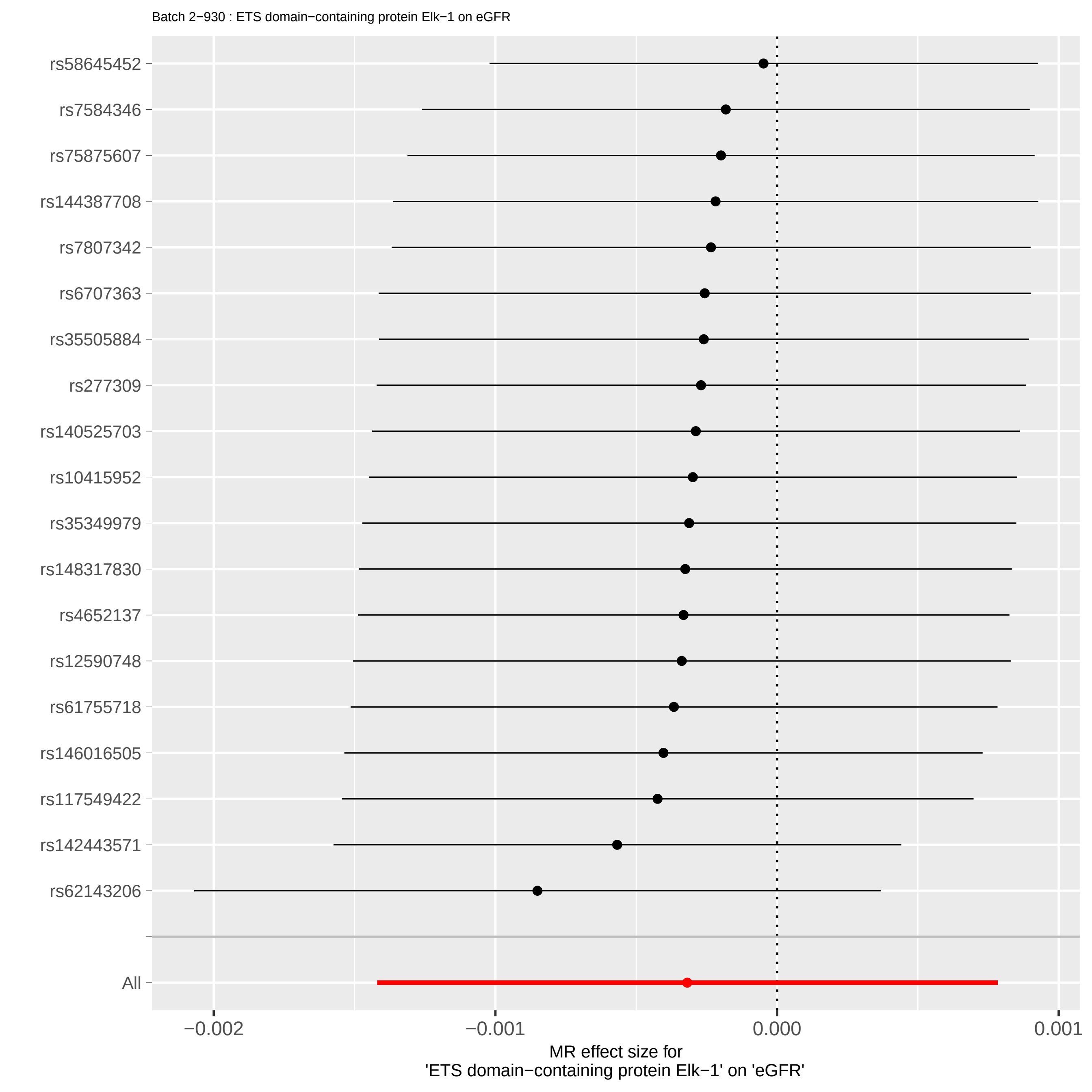

Batch 2−930 : ETS domain−containing protein Elk−1 on eGFR
rs58645452
rs7584346
rs75875607
rs144387708
rs7807342
rs6707363
rs35505884
rs277309
rs140525703
rs10415952
rs35349979
rs148317830
rs4652137
rs12590748
rs61755718
rs146016505
rs117549422
rs142443571
rs62143206
All
−0.002
−0.001
0.000
0.001
MR effect size for
'ETS domain−containing protein Elk−1' on 'eGFR'

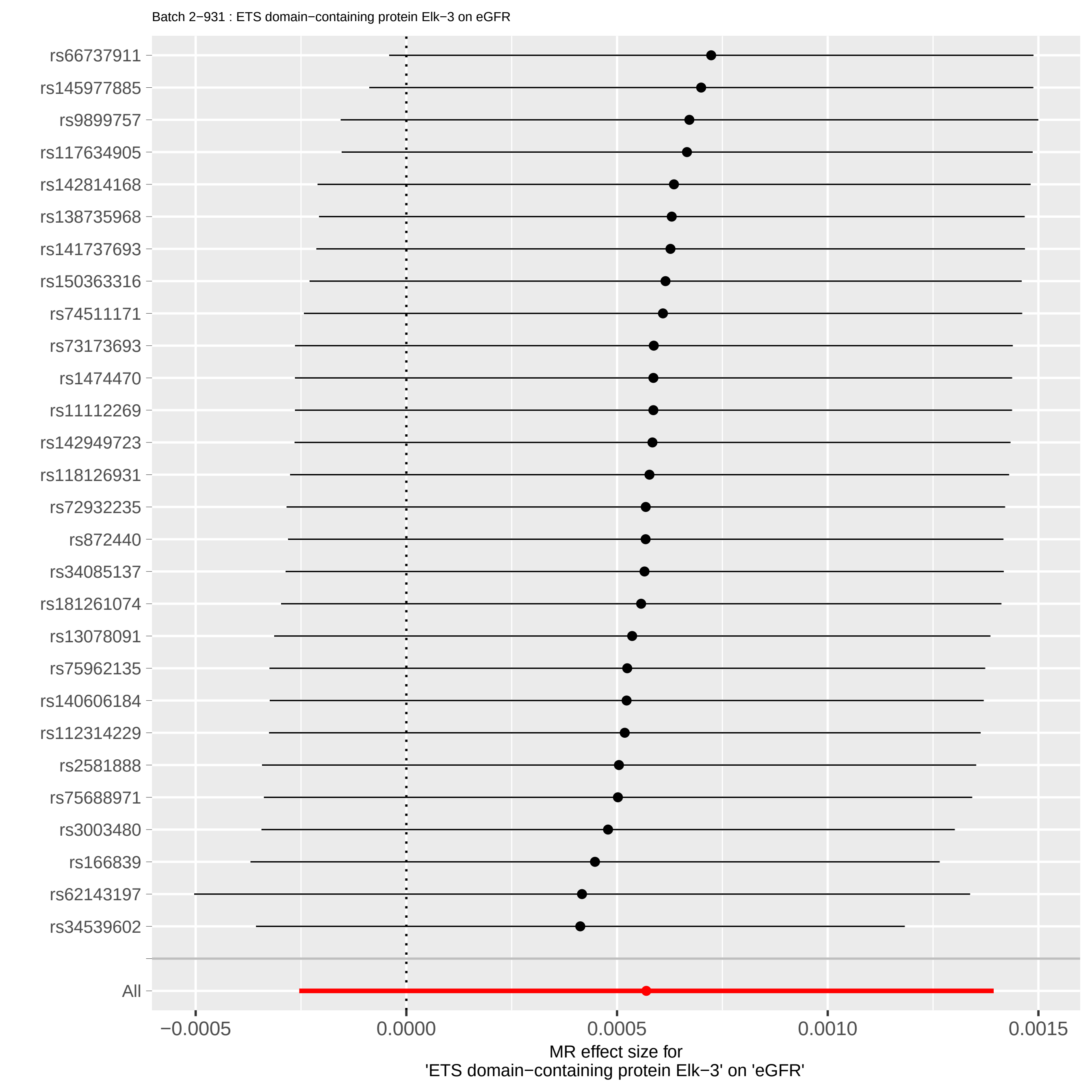

Batch 2−931 : ETS domain−containing protein Elk−3 on eGFR
rs66737911
rs145977885
rs9899757
rs117634905
rs142814168
rs138735968
rs141737693
rs150363316
rs74511171
rs73173693
rs1474470
rs11112269
rs142949723
rs118126931
rs72932235
rs872440
rs34085137
rs181261074
rs13078091
rs75962135
rs140606184
rs112314229
rs2581888
rs75688971
rs3003480
rs166839
rs62143197
rs34539602
All
−0.0005
0.0000
0.0005
0.0010
0.0015
MR effect size for
'ETS domain−containing protein Elk−3' on 'eGFR'

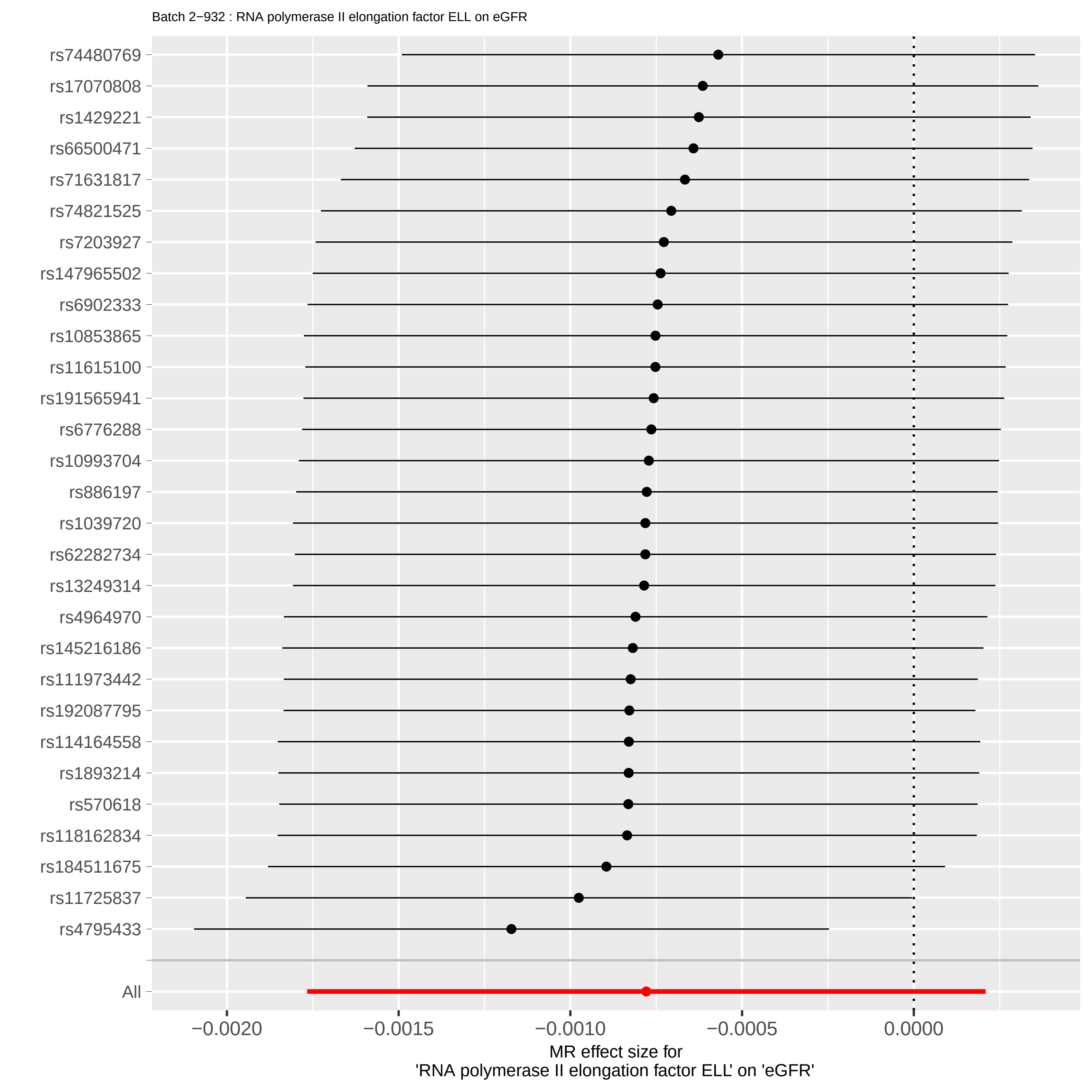

Batch 2−932 : RNA polymerase II elongation factor ELL on eGFR
rs74480769
rs17070808
rs1429221
rs66500471
rs71631817
rs74821525
rs7203927
rs147965502
rs6902333
rs10853865
rs11615100
rs191565941
rs6776288
rs10993704
rs886197
rs1039720
rs62282734
rs13249314
rs4964970
rs145216186
rs111973442
rs192087795
rs114164558
rs1893214
rs570618
rs118162834
rs184511675
rs11725837
rs4795433
All
−0.0020
−0.0015
−0.0010
−0.0005
0.0000
MR effect size for
'RNA polymerase II elongation factor ELL' on 'eGFR'

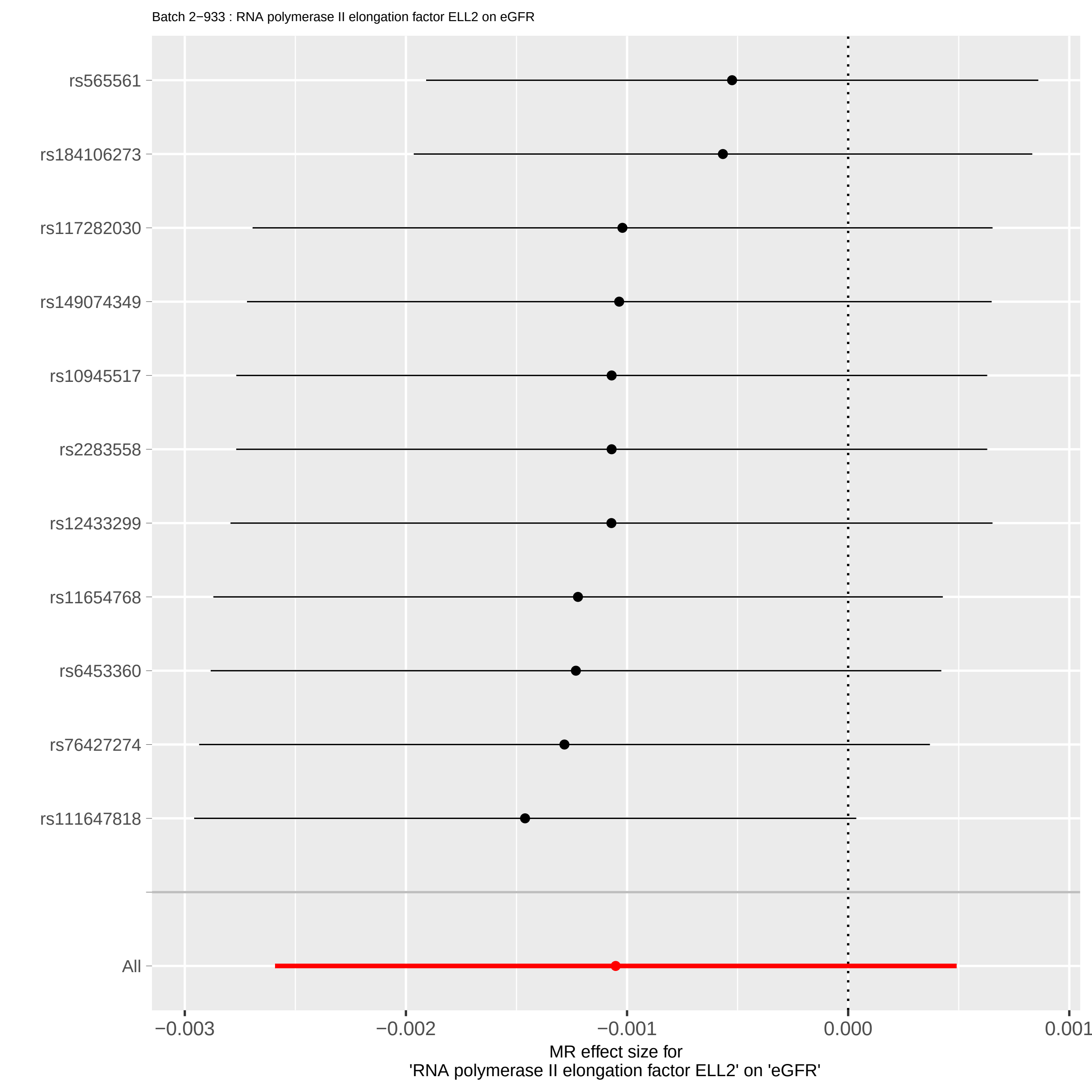

Batch 2−933 : RNA polymerase II elongation factor ELL2 on eGFR
rs565561
rs184106273
rs117282030
rs149074349
rs10945517
rs2283558
rs12433299
rs11654768
rs6453360
rs76427274
rs111647818
All
−0.003
−0.002
−0.001
0.000
0.001
MR effect size for
'RNA polymerase II elongation factor ELL2' on 'eGFR'

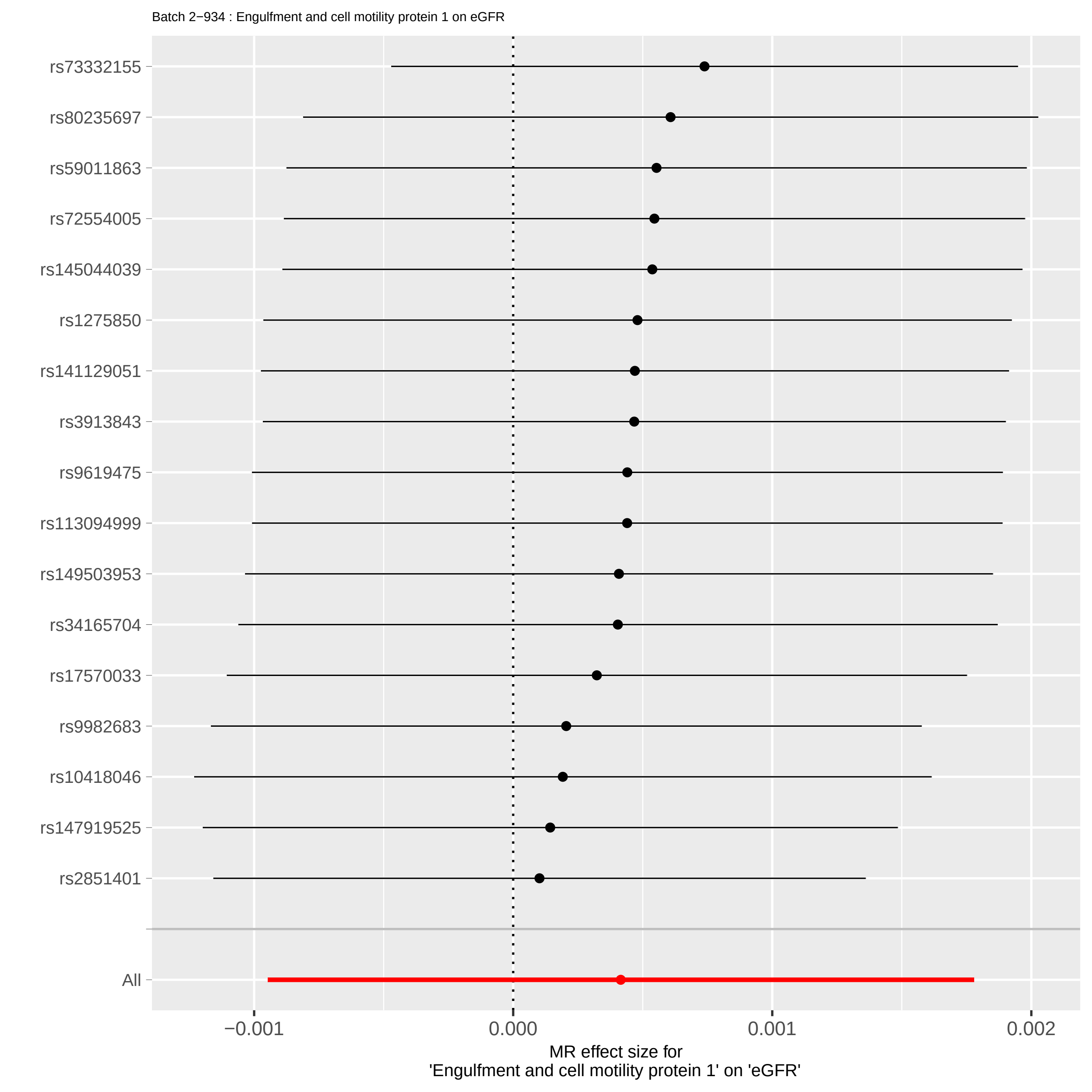

Batch 2−934 : Engulfment and cell motility protein 1 on eGFR
rs73332155
rs80235697
rs59011863
rs72554005
rs145044039
rs1275850
rs141129051
rs3913843
rs9619475
rs113094999
rs149503953
rs34165704
rs17570033
rs9982683
rs10418046
rs147919525
rs2851401
All
−0.001
0.000
0.001
0.002
MR effect size for
'Engulfment and cell motility protein 1' on 'eGFR'

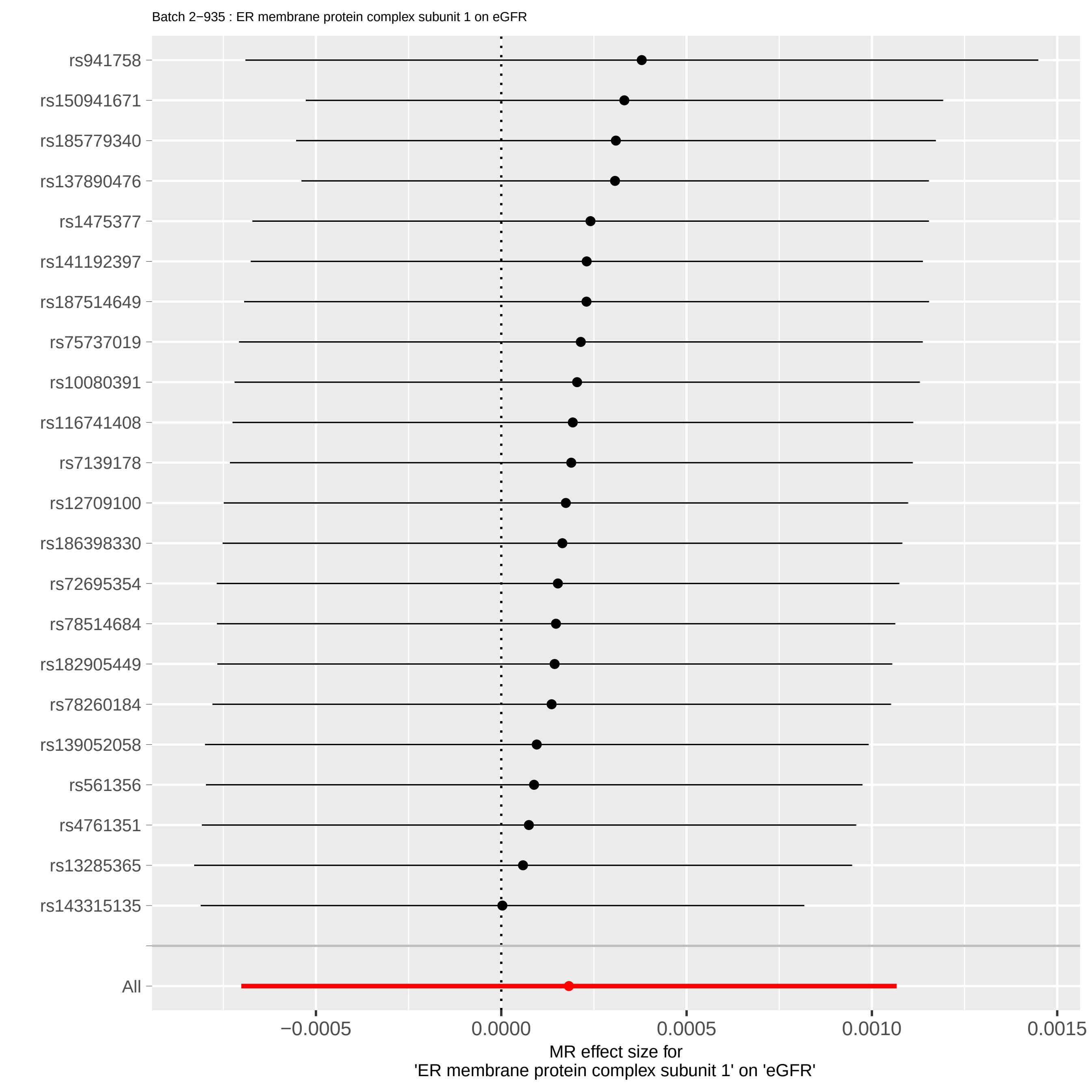
Batch 2−935 : ER membrane protein complex subunit 1 on eGFR
rs941758
rs150941671
rs185779340
rs137890476
rs1475377
rs141192397
rs187514649
rs75737019
rs10080391
rs116741408
rs7139178
rs12709100
rs186398330
rs72695354
rs78514684
rs182905449
rs78260184
rs139052058
rs561356
rs4761351
rs13285365
rs143315135
All
−0.0005
0.0000
0.0005
0.0010
0.0015
MR effect size for
'ER membrane protein complex subunit 1' on 'eGFR'

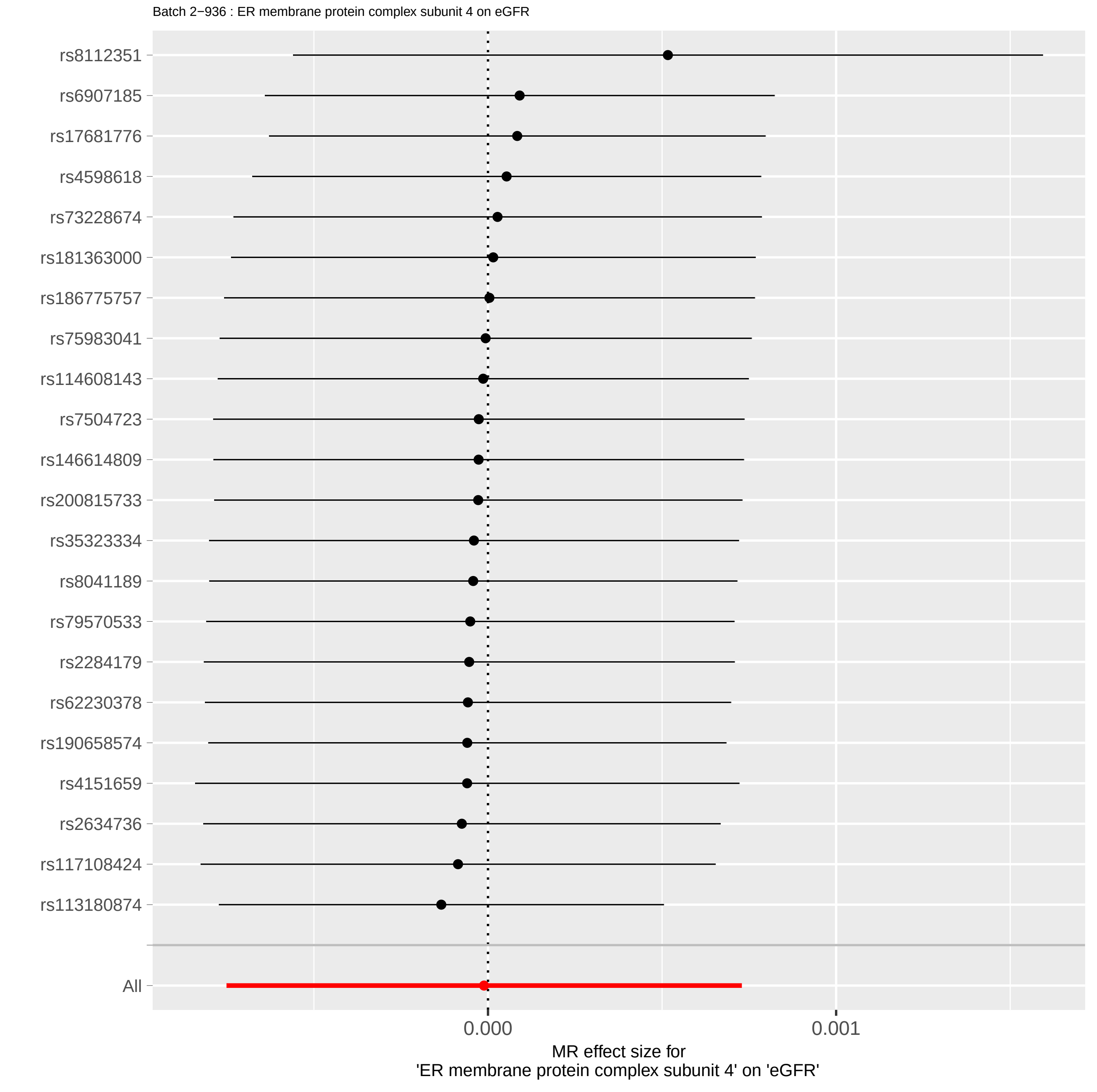

Batch 2−936 : ER membrane protein complex subunit 4 on eGFR
rs8112351
rs6907185
rs17681776
rs4598618
rs73228674
rs181363000
rs186775757
rs75983041
rs114608143
rs7504723
rs146614809
rs200815733
rs35323334
rs8041189
rs79570533
rs2284179
rs62230378
rs190658574
rs4151659
rs2634736
rs117108424
rs113180874
All
0.000
0.001
MR effect size for
'ER membrane protein complex subunit 4' on 'eGFR'

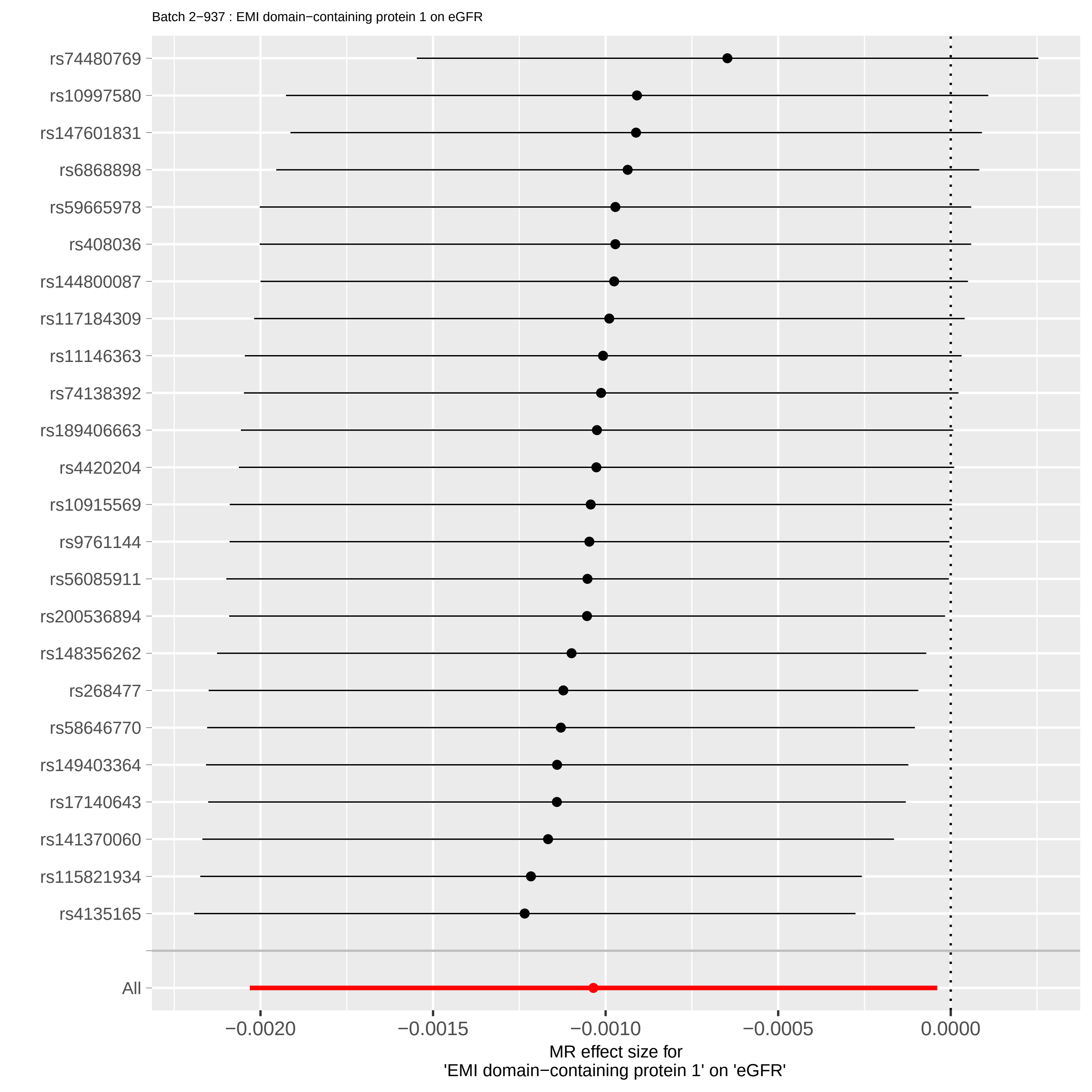

Batch 2−937 : EMI domain−containing protein 1 on eGFR
rs74480769
rs10997580
rs147601831
rs6868898
rs59665978
rs408036
rs144800087
rs117184309
rs11146363
rs74138392
rs189406663
rs4420204
rs10915569
rs9761144
rs56085911
rs200536894
rs148356262
rs268477
rs58646770
rs149403364
rs17140643
rs141370060
rs115821934
rs4135165
All
−0.0020
−0.0015
−0.0010
−0.0005
0.0000
MR effect size for
'EMI domain−containing protein 1' on 'eGFR'

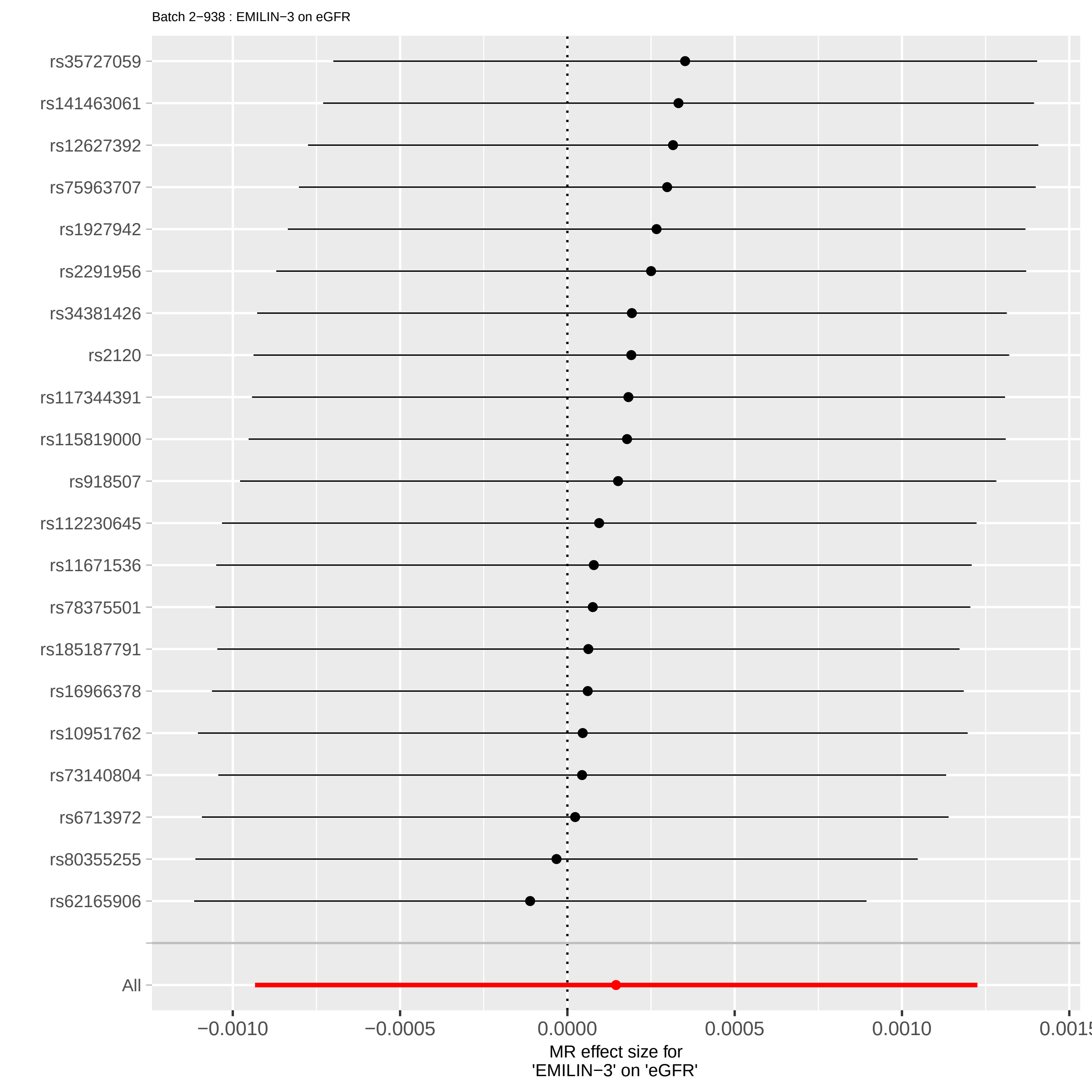

Batch 2−938 : EMILIN−3 on eGFR
rs35727059
rs141463061
rs12627392
rs75963707
rs1927942
rs2291956
rs34381426
rs2120
rs117344391
rs115819000
rs918507
rs112230645
rs11671536
rs78375501
rs185187791
rs16966378
rs10951762
rs73140804
rs6713972
rs80355255
rs62165906
All
−0.0010
−0.0005
0.0000
0.0005
0.0010
0.0015
MR effect size for
'EMILIN−3' on 'eGFR'

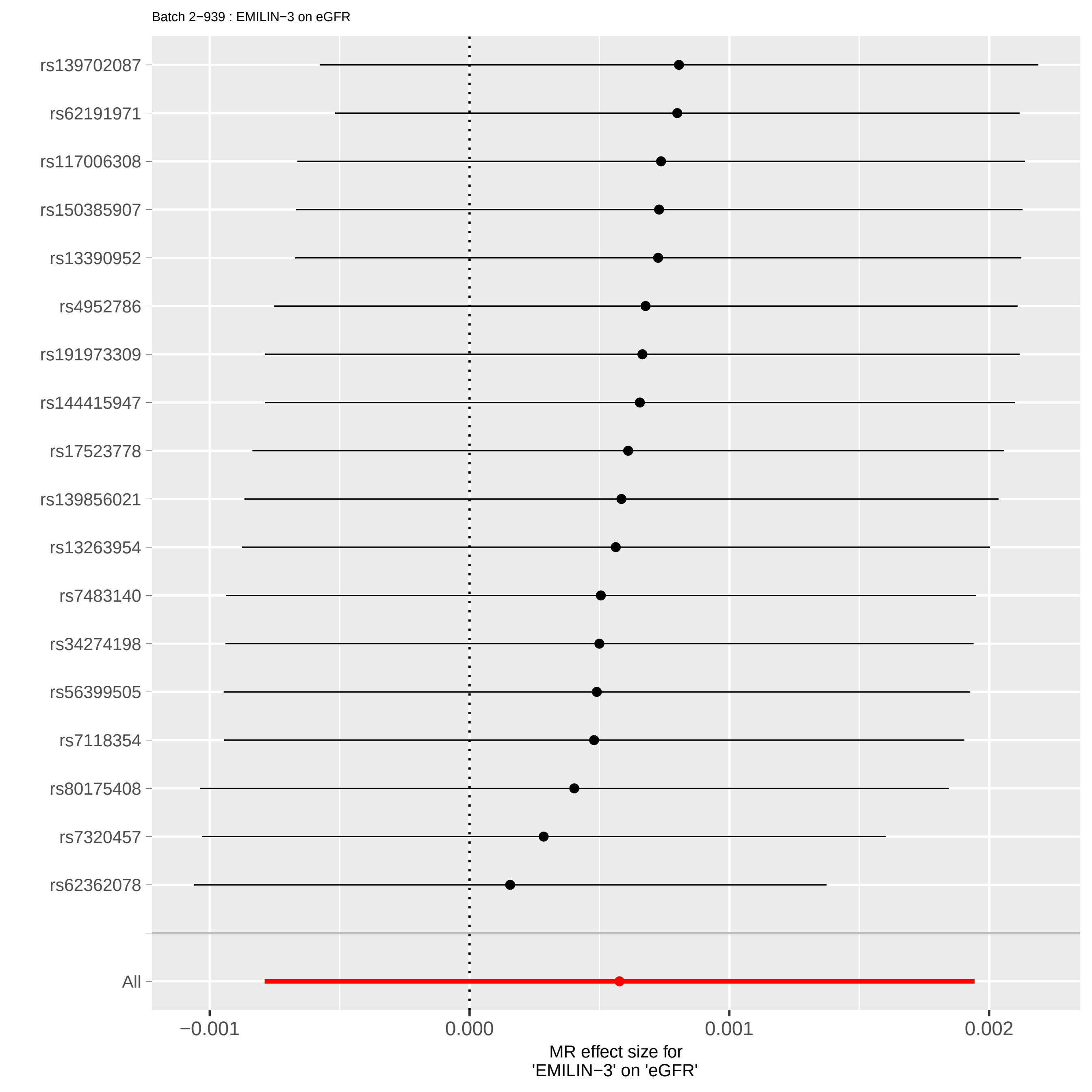

Batch 2−939 : EMILIN−3 on eGFR
rs139702087
rs62191971
rs117006308
rs150385907
rs13390952
rs4952786
rs191973309
rs144415947
rs17523778
rs139856021
rs13263954
rs7483140
rs34274198
rs56399505
rs7118354
rs80175408
rs7320457
rs62362078
All
−0.001
0.000
0.001
0.002
MR effect size for
'EMILIN−3' on 'eGFR'

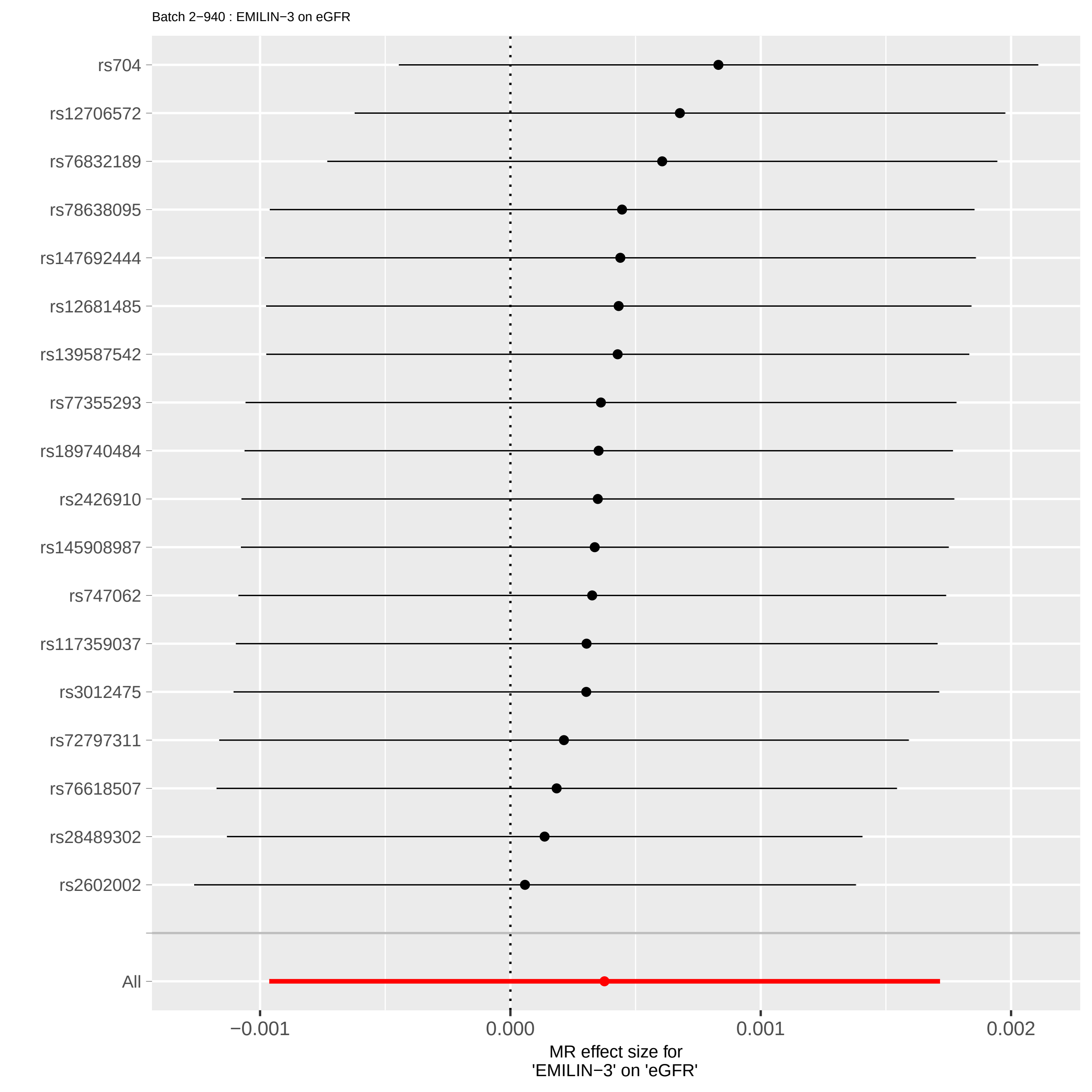

Batch 2−940 : EMILIN−3 on eGFR
rs704
rs12706572
rs76832189
rs78638095
rs147692444
rs12681485
rs139587542
rs77355293
rs189740484
rs2426910
rs145908987
rs747062
rs117359037
rs3012475
rs72797311
rs76618507
rs28489302
rs2602002
All
−0.001
0.000
0.001
0.002
MR effect size for
'EMILIN−3' on 'eGFR'

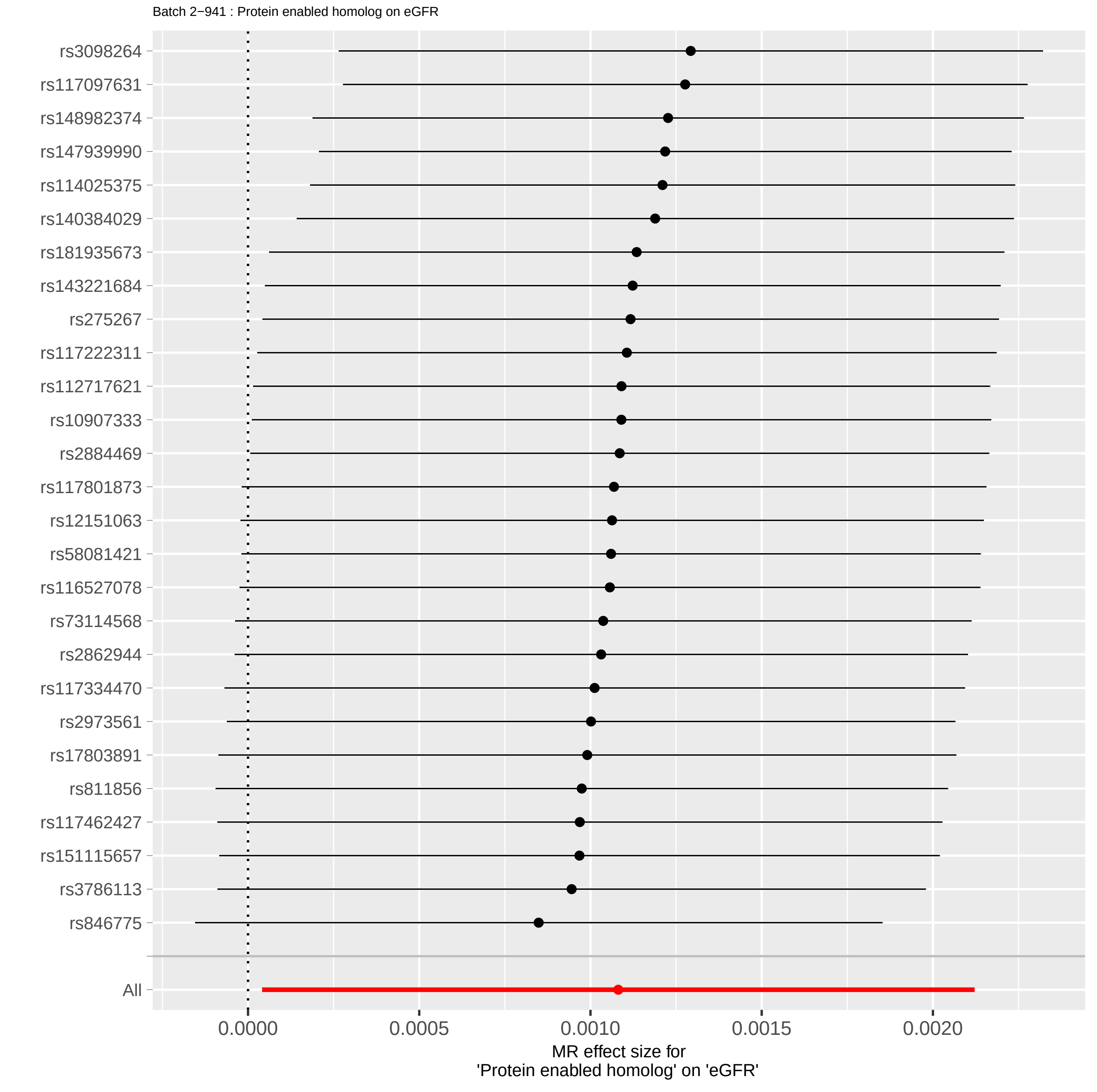

Batch 2−941 : Protein enabled homolog on eGFR
rs3098264
rs117097631
rs148982374
rs147939990
rs114025375
rs140384029
rs181935673
rs143221684
rs275267
rs117222311
rs112717621
rs10907333
rs2884469
rs117801873
rs12151063
rs58081421
rs116527078
rs73114568
rs2862944
rs117334470
rs2973561
rs17803891
rs811856
rs117462427
rs151115657
rs3786113
rs846775
All
0.0000
0.0005
0.0010
0.0015
0.0020
MR effect size for
'Protein enabled homolog' on 'eGFR'

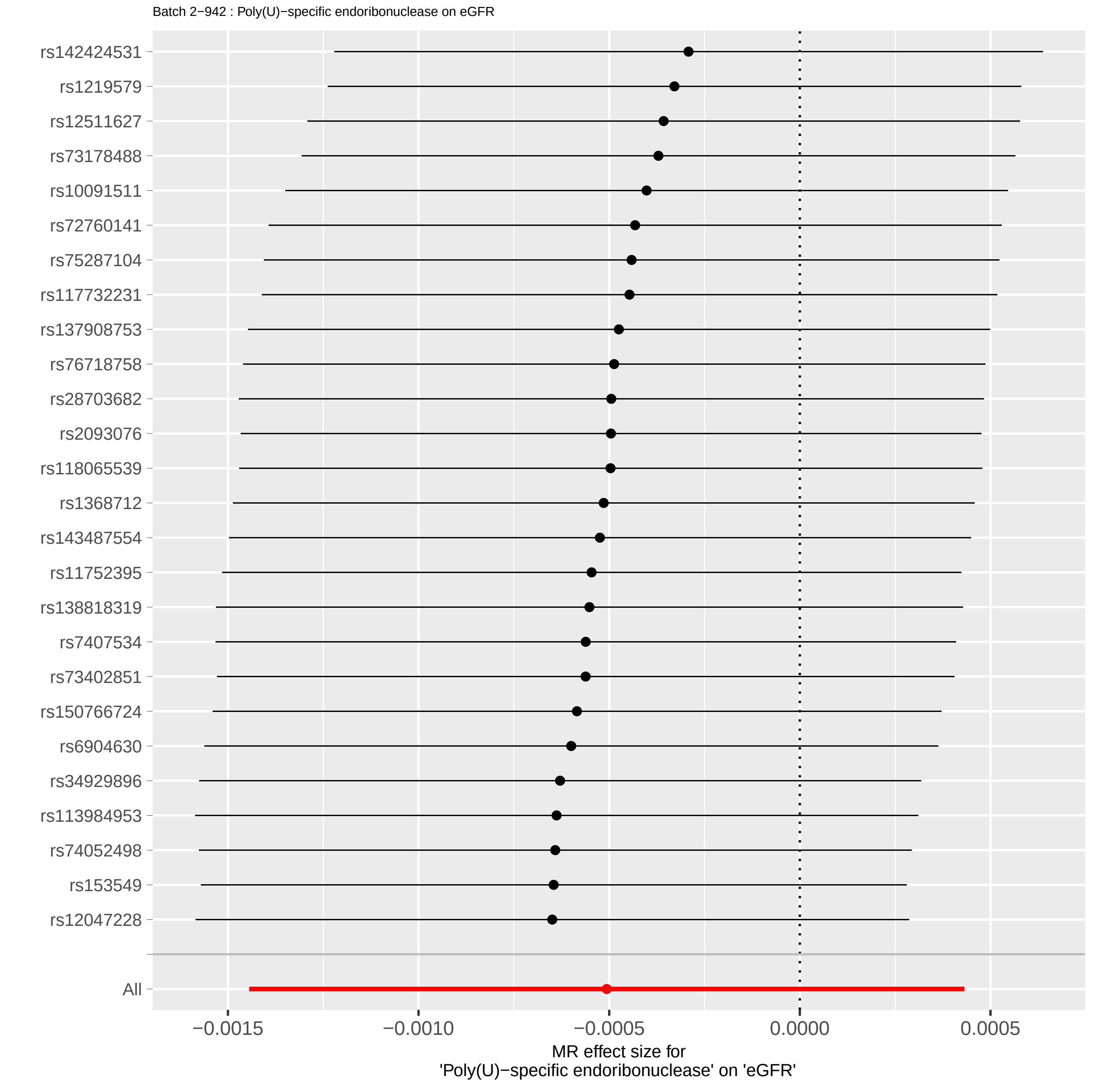
Batch 2−942 : Poly(U)−specific endoribonuclease on eGFR
rs142424531
rs1219579
rs12511627
rs73178488
rs10091511
rs72760141
rs75287104
rs117732231
rs137908753
rs76718758
rs28703682
rs2093076
rs118065539
rs1368712
rs143487554
rs11752395
rs138818319
rs7407534
rs73402851
rs150766724
rs6904630
rs34929896
rs113984953
rs74052498
rs153549
rs12047228
All
−0.0015
−0.0010
−0.0005
0.0000
0.0005
MR effect size for
'Poly(U)−specific endoribonuclease' on 'eGFR'

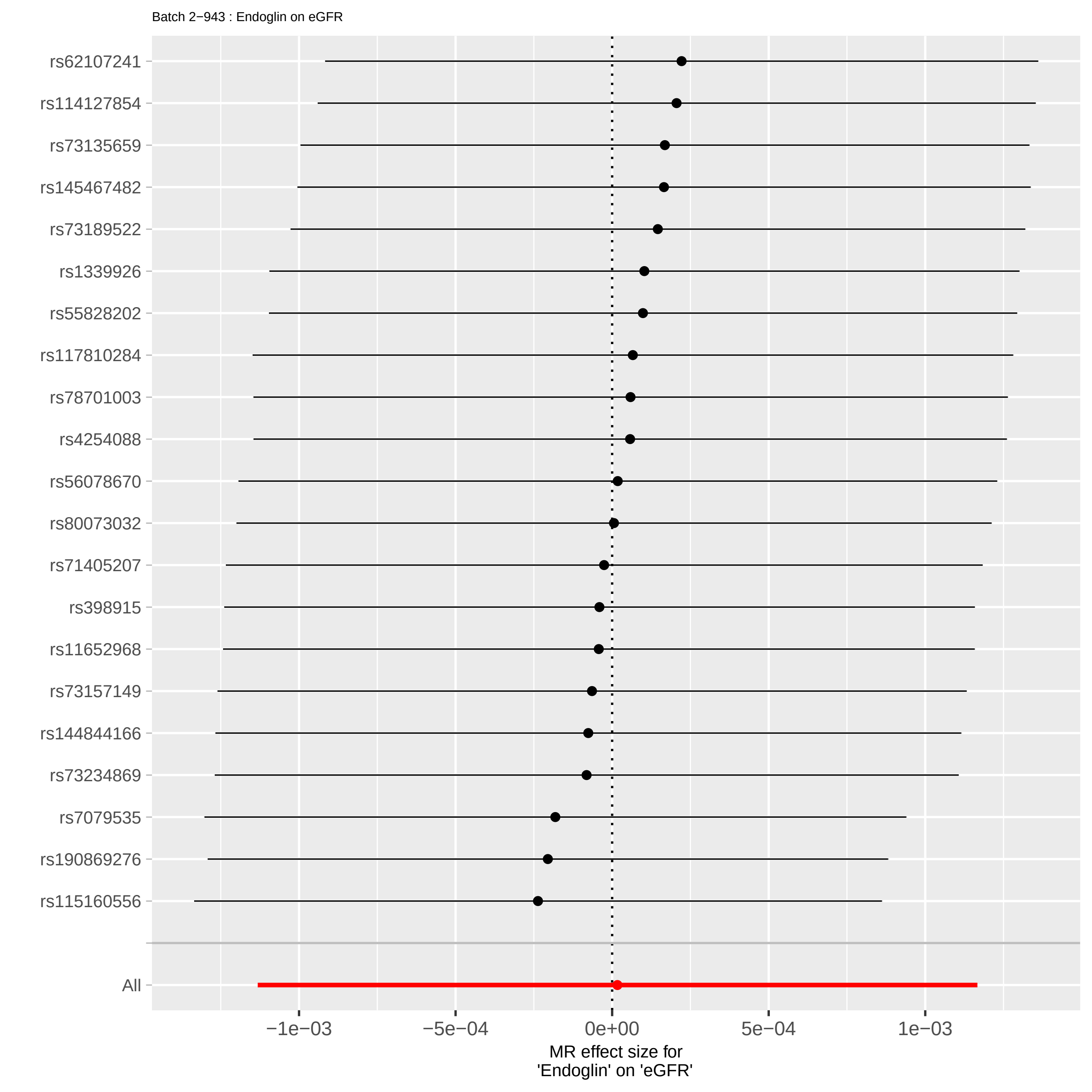
Batch 2−943 : Endoglin on eGFR
rs62107241
rs114127854
rs73135659
rs145467482
rs73189522
rs1339926
rs55828202
rs117810284
rs78701003
rs4254088
rs56078670
rs80073032
rs71405207
rs398915
rs11652968
rs73157149
rs144844166
rs73234869
rs7079535
rs190869276
rs115160556
All
−1e−03
−5e−04
0e+00
5e−04
1e−03
MR effect size for
'Endoglin' on 'eGFR'

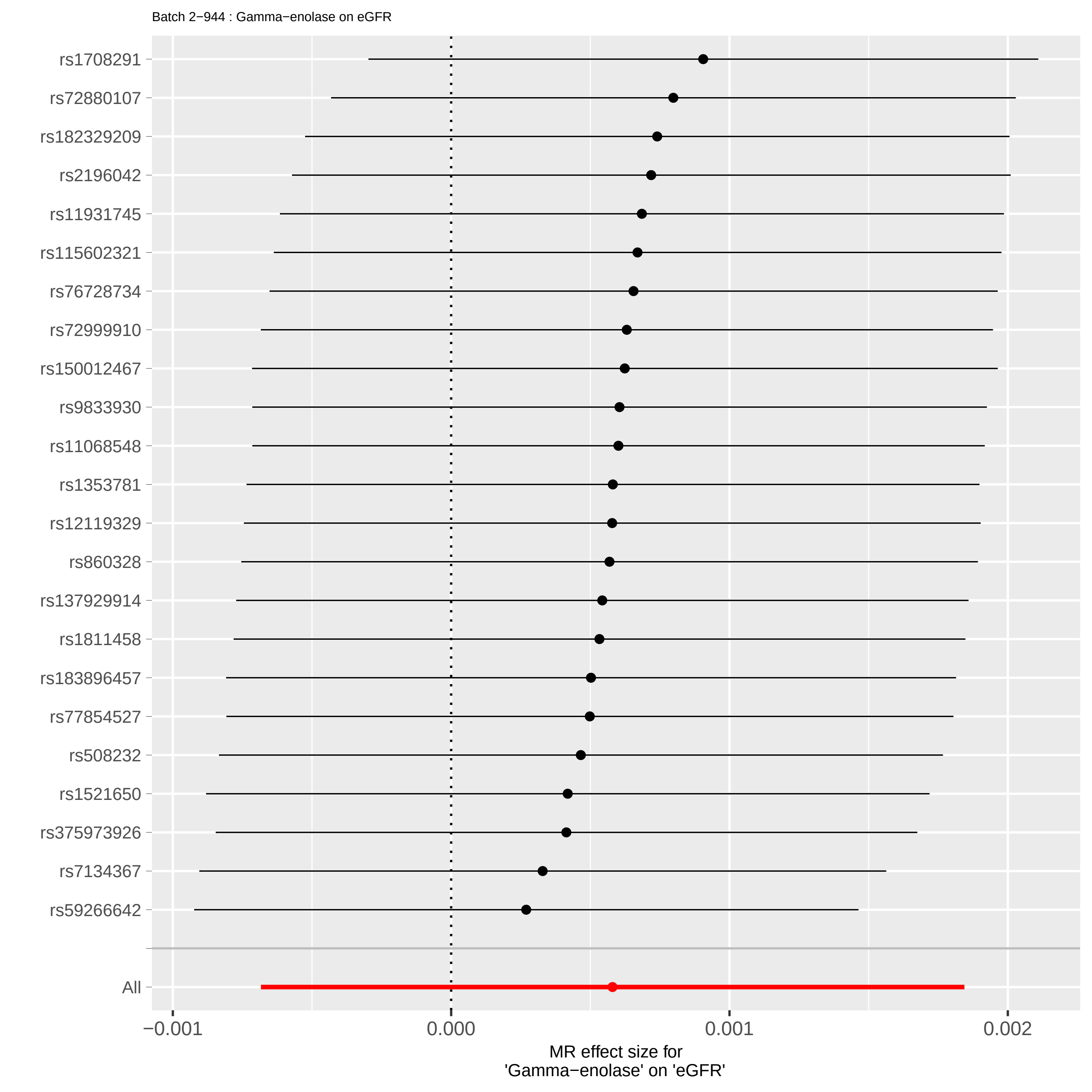

Batch 2−944 : Gamma−enolase on eGFR
rs1708291
rs72880107
rs182329209
rs2196042
rs11931745
rs115602321
rs76728734
rs72999910
rs150012467
rs9833930
rs11068548
rs1353781
rs12119329
rs860328
rs137929914
rs1811458
rs183896457
rs77854527
rs508232
rs1521650
rs375973926
rs7134367
rs59266642
All
−0.001
0.000
0.001
0.002
MR effect size for
'Gamma−enolase' on 'eGFR'

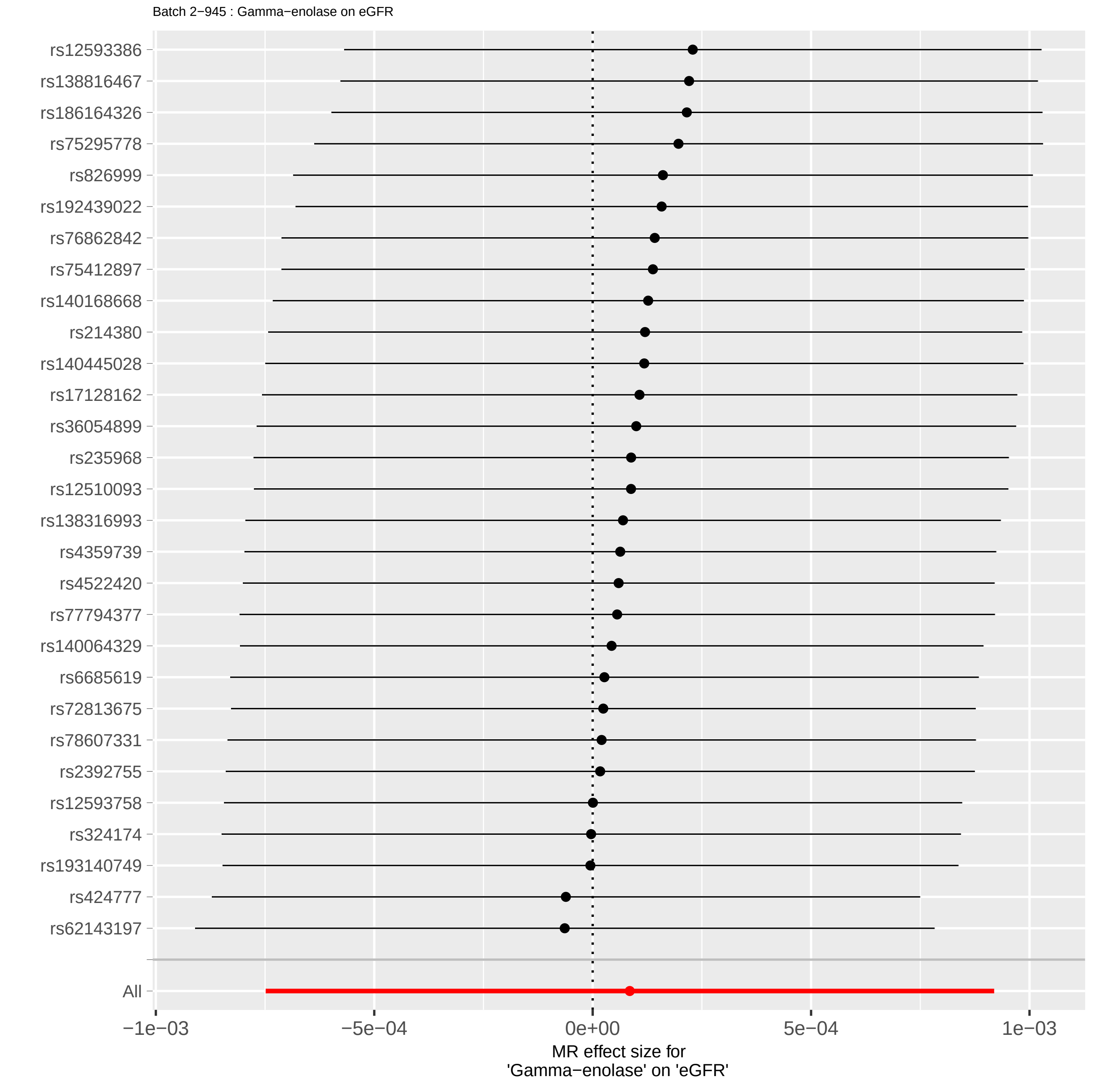

Batch 2−945 : Gamma−enolase on eGFR
rs12593386
rs138816467
rs186164326
rs75295778
rs826999
rs192439022
rs76862842
rs75412897
rs140168668
rs214380
rs140445028
rs17128162
rs36054899
rs235968
rs12510093
rs138316993
rs4359739
rs4522420
rs77794377
rs140064329
rs6685619
rs72813675
rs78607331
rs2392755
rs12593758
rs324174
rs193140749
rs424777
rs62143197
All
−1e−03
−5e−04
0e+00
5e−04
1e−03
MR effect size for
'Gamma−enolase' on 'eGFR'

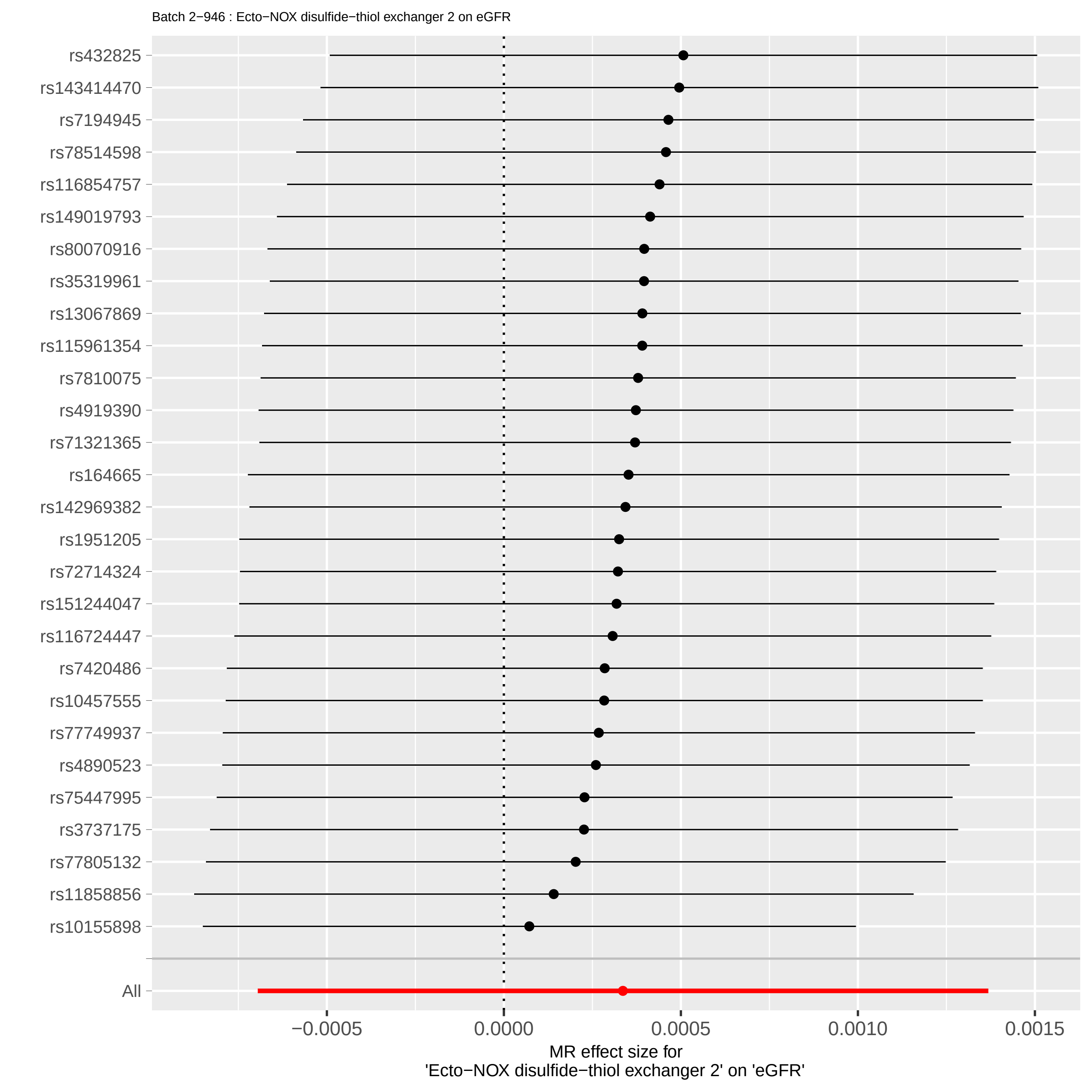
Batch 2−946 : Ecto−NOX disulfide−thiol exchanger 2 on eGFR
rs432825
rs143414470
rs7194945
rs78514598
rs116854757
rs149019793
rs80070916
rs35319961
rs13067869
rs115961354
rs7810075
rs4919390
rs71321365
rs164665
rs142969382
rs1951205
rs72714324
rs151244047
rs116724447
rs7420486
rs10457555
rs77749937
rs4890523
rs75447995
rs3737175
rs77805132
rs11858856
rs10155898
All
−0.0005
0.0000
0.0005
0.0010
0.0015
MR effect size for
'Ecto−NOX disulfide−thiol exchanger 2' on 'eGFR'

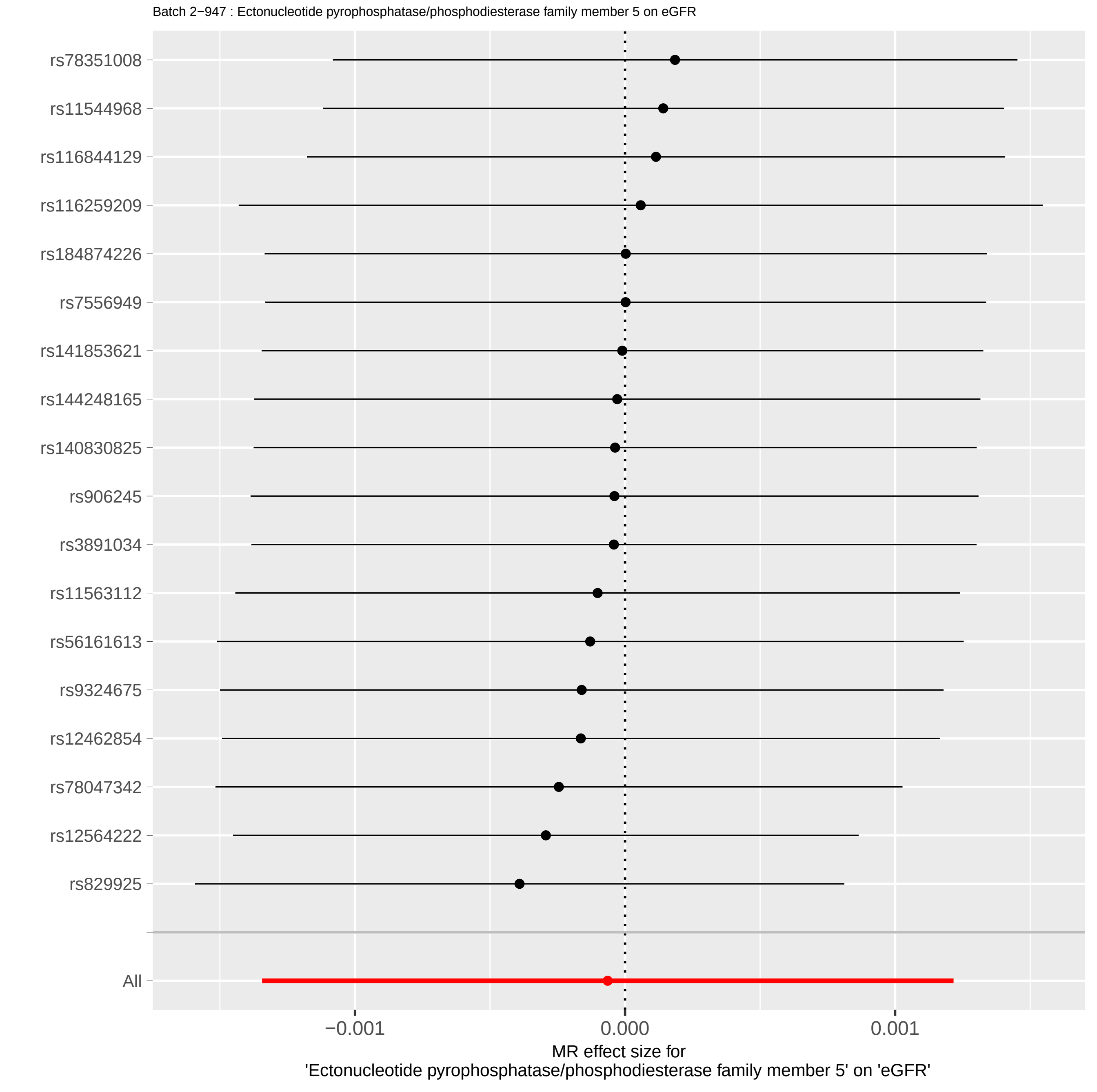
Batch 2−947 : Ectonucleotide pyrophosphatase/phosphodiesterase family member 5 on eGFR
rs78351008
rs11544968
rs116844129
rs116259209
rs184874226
rs7556949
rs141853621
rs144248165
rs140830825
rs906245
rs3891034
rs11563112
rs56161613
rs9324675
rs12462854
rs78047342
rs12564222
rs829925
All
−0.001
0.000
0.001
MR effect size for
'Ectonucleotide pyrophosphatase/phosphodiesterase family member 5' on 'eGFR'

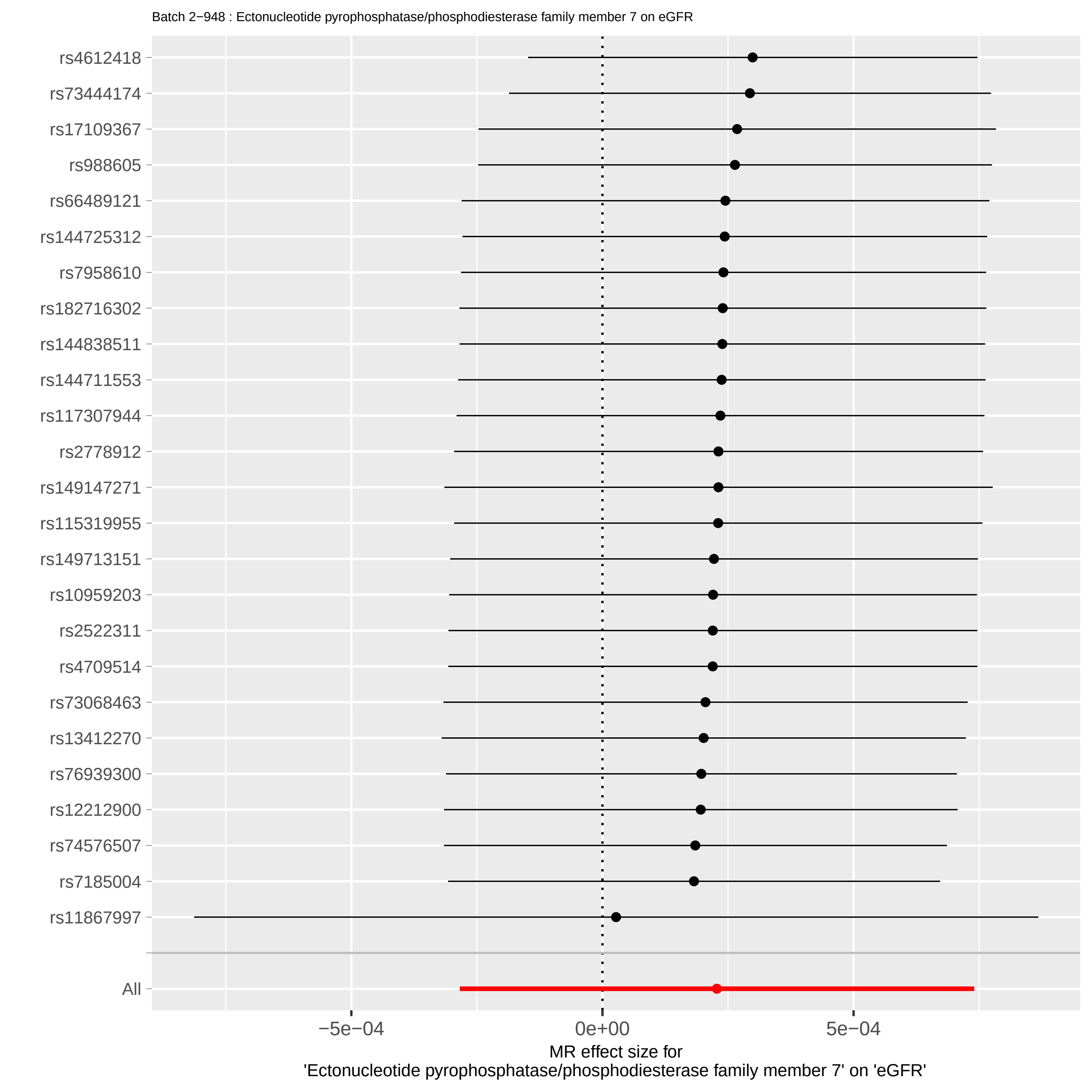

Batch 2−948 : Ectonucleotide pyrophosphatase/phosphodiesterase family member 7 on eGFR
rs4612418
rs73444174
rs17109367
rs988605
rs66489121
rs144725312
rs7958610
rs182716302
rs144838511
rs144711553
rs117307944
rs2778912
rs149147271
rs115319955
rs149713151
rs10959203
rs2522311
rs4709514
rs73068463
rs13412270
rs76939300
rs12212900
rs74576507
rs7185004
rs11867997
All
−5e−04
0e+00
5e−04
MR effect size for
'Ectonucleotide pyrophosphatase/phosphodiesterase family member 7' on 'eGFR'

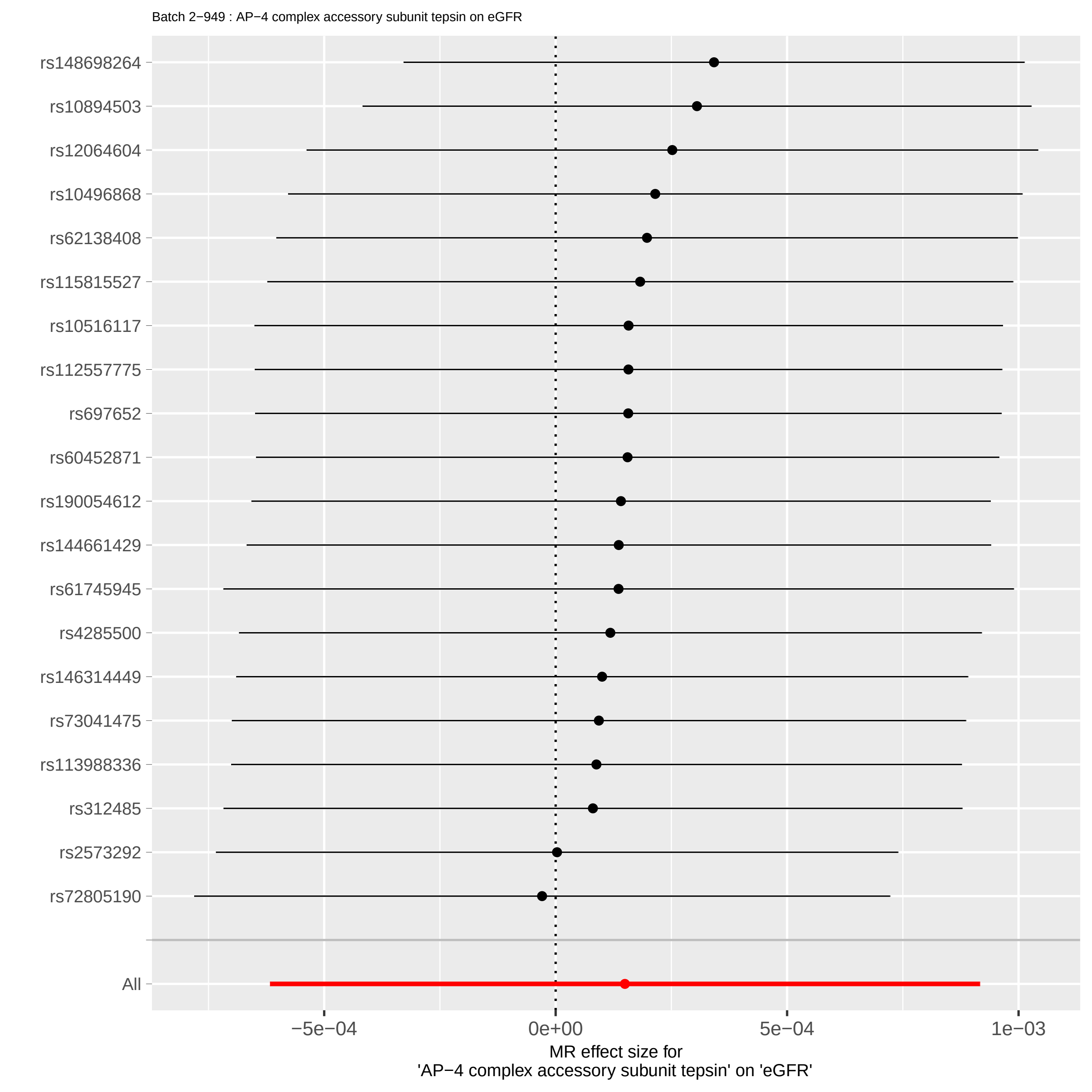

Batch 2−949 : AP−4 complex accessory subunit tepsin on eGFR
rs148698264
rs10894503
rs12064604
rs10496868
rs62138408
rs115815527
rs10516117
rs112557775
rs697652
rs60452871
rs190054612
rs144661429
rs61745945
rs4285500
rs146314449
rs73041475
rs113988336
rs312485
rs2573292
rs72805190
All
−5e−04
0e+00
5e−04
1e−03
MR effect size for
'AP−4 complex accessory subunit tepsin' on 'eGFR'

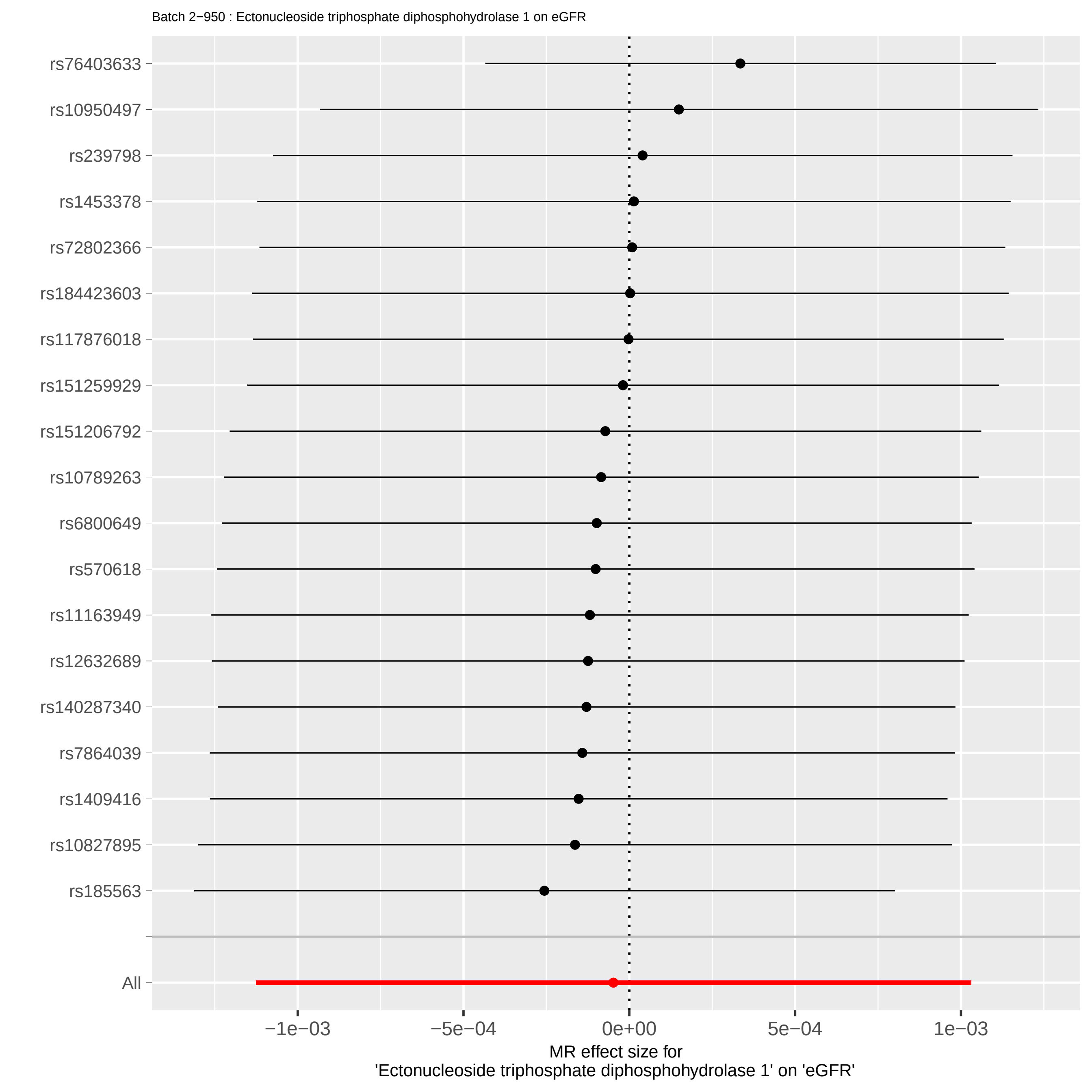

Batch 2−950 : Ectonucleoside triphosphate diphosphohydrolase 1 on eGFR
rs76403633
rs10950497
rs239798
rs1453378
rs72802366
rs184423603
rs117876018
rs151259929
rs151206792
rs10789263
rs6800649
rs570618
rs11163949
rs12632689
rs140287340
rs7864039
rs1409416
rs10827895
rs185563
All
−1e−03
−5e−04
0e+00
5e−04
1e−03
MR effect size for
'Ectonucleoside triphosphate diphosphohydrolase 1' on 'eGFR'

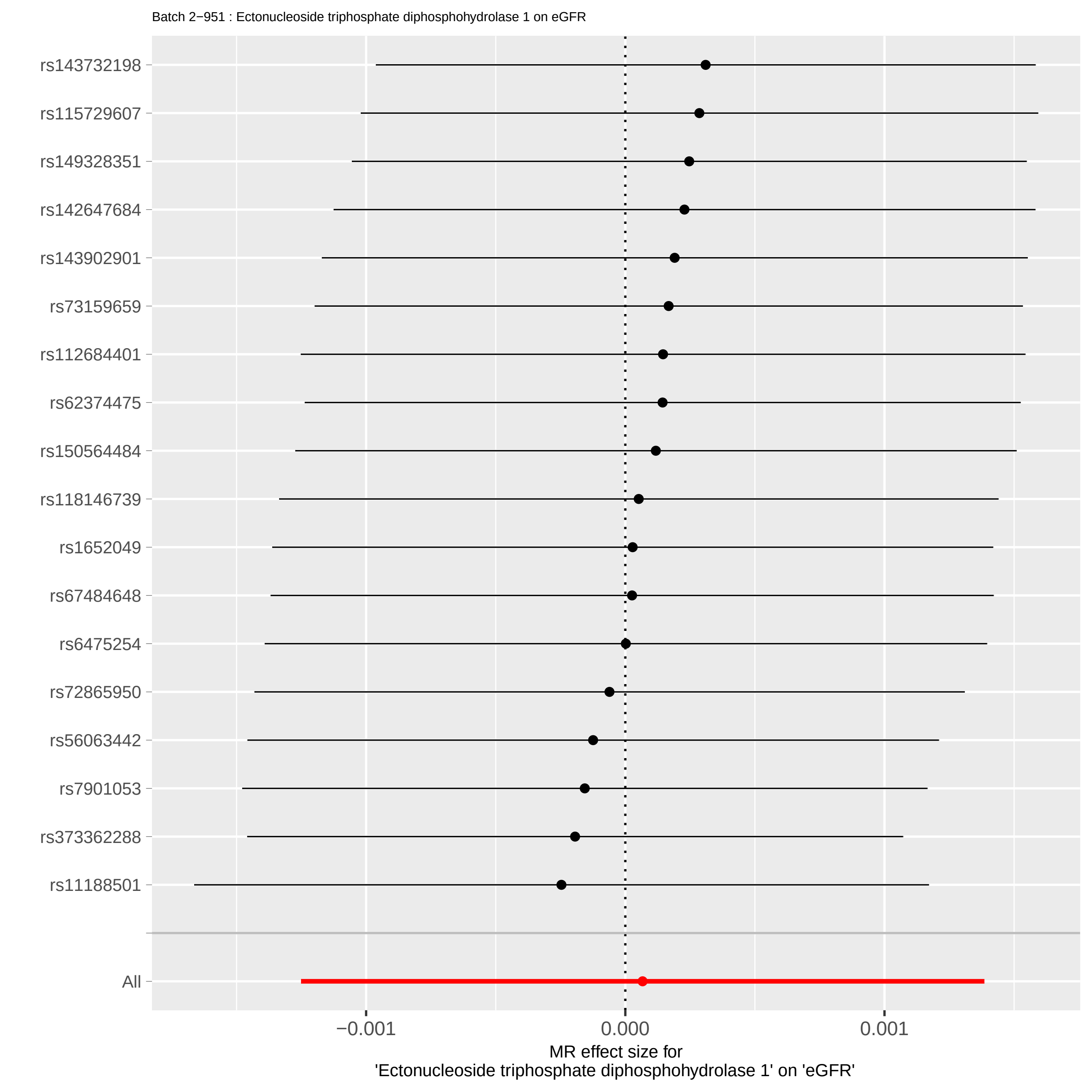

Batch 2−951 : Ectonucleoside triphosphate diphosphohydrolase 1 on eGFR
rs143732198
rs115729607
rs149328351
rs142647684
rs143902901
rs73159659
rs112684401
rs62374475
rs150564484
rs118146739
rs1652049
rs67484648
rs6475254
rs72865950
rs56063442
rs7901053
rs373362288
rs11188501
All
−0.001
0.000
0.001
MR effect size for
'Ectonucleoside triphosphate diphosphohydrolase 1' on 'eGFR'

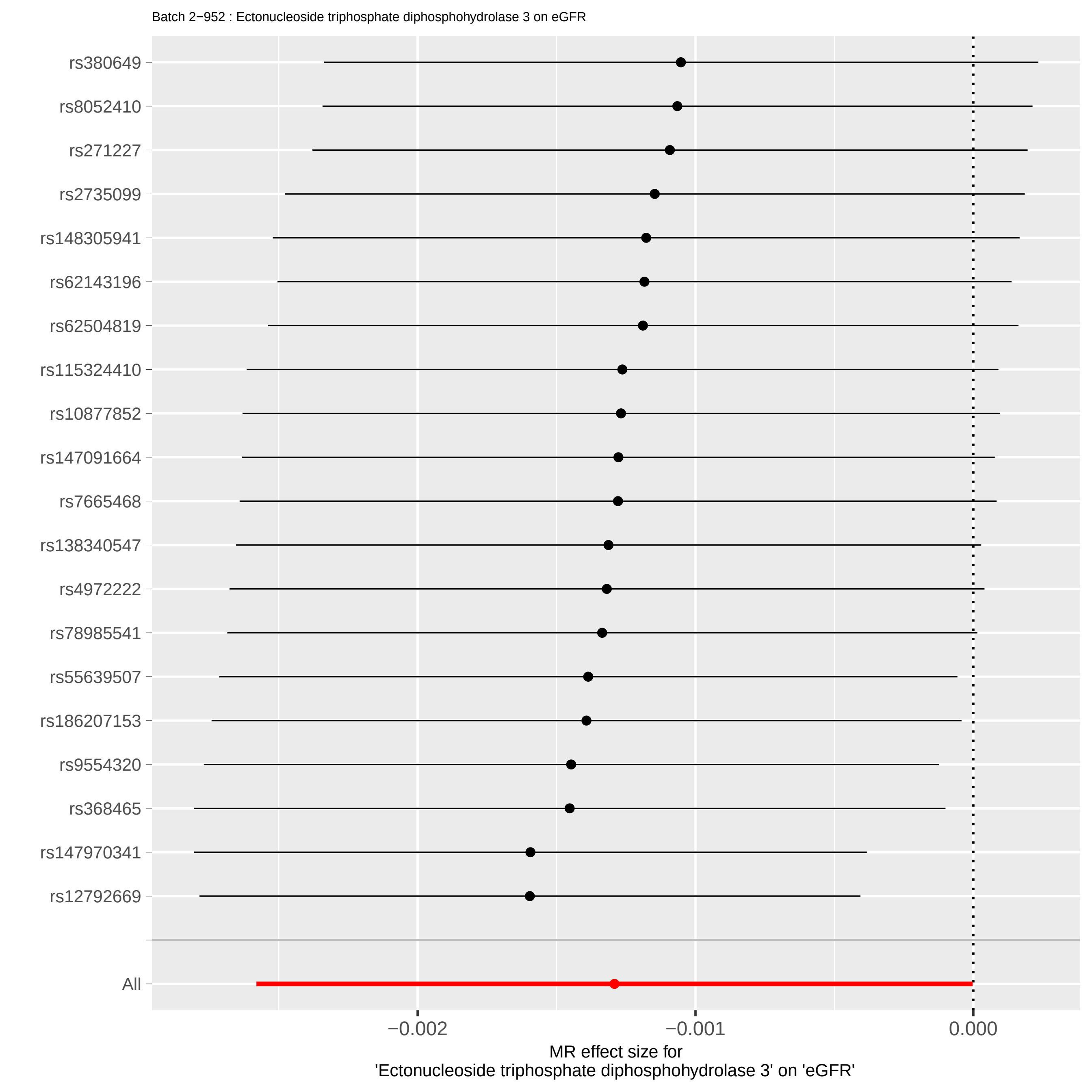
Batch 2−952 : Ectonucleoside triphosphate diphosphohydrolase 3 on eGFR
rs380649
rs8052410
rs271227
rs2735099
rs148305941
rs62143196
rs62504819
rs115324410
rs10877852
rs147091664
rs7665468
rs138340547
rs4972222
rs78985541
rs55639507
rs186207153
rs9554320
rs368465
rs147970341
rs12792669
All
−0.002
−0.001
0.000
MR effect size for
'Ectonucleoside triphosphate diphosphohydrolase 3' on 'eGFR'

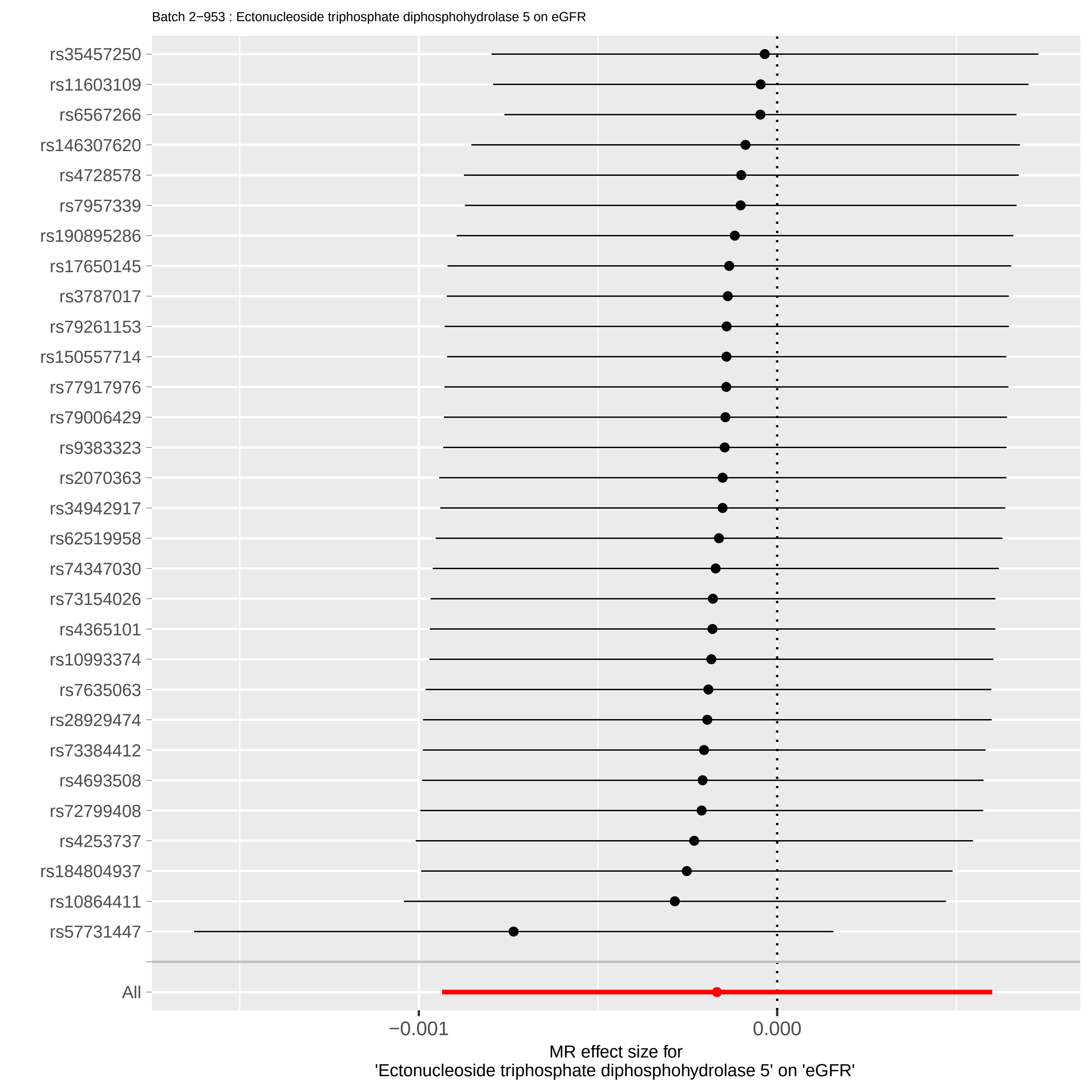

Batch 2−953 : Ectonucleoside triphosphate diphosphohydrolase 5 on eGFR
rs35457250
rs11603109
rs6567266
rs146307620
rs4728578
rs7957339
rs190895286
rs17650145
rs3787017
rs79261153
rs150557714
rs77917976
rs79006429
rs9383323
rs2070363
rs34942917
rs62519958
rs74347030
rs73154026
rs4365101
rs10993374
rs7635063
rs28929474
rs73384412
rs4693508
rs72799408
rs4253737
rs184804937
rs10864411
rs57731447
All
−0.001
0.000
MR effect size for
'Ectonucleoside triphosphate diphosphohydrolase 5' on 'eGFR'

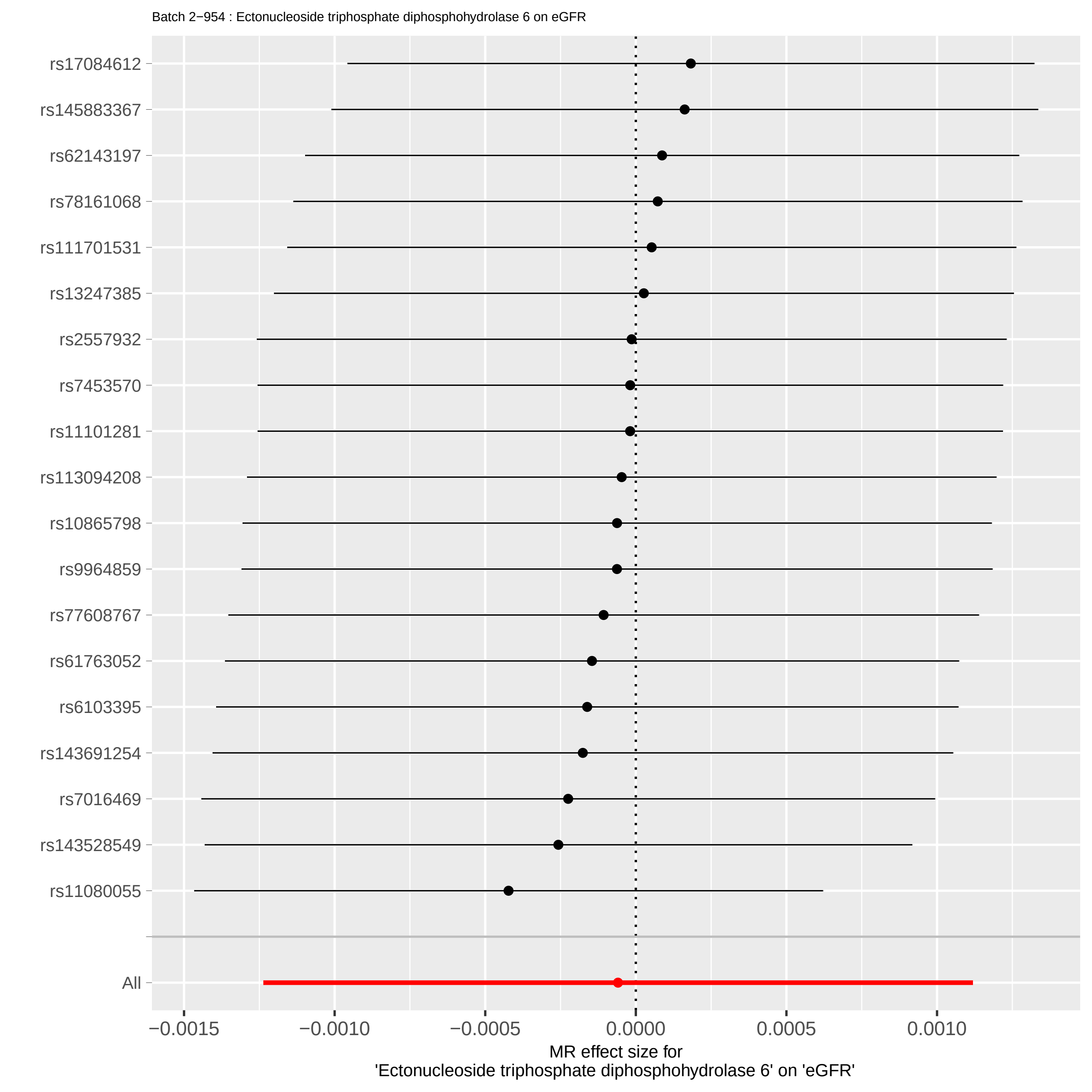

Batch 2−954 : Ectonucleoside triphosphate diphosphohydrolase 6 on eGFR
rs17084612
rs145883367
rs62143197
rs78161068
rs111701531
rs13247385
rs2557932
rs7453570
rs11101281
rs113094208
rs10865798
rs9964859
rs77608767
rs61763052
rs6103395
rs143691254
rs7016469
rs143528549
rs11080055
All
−0.0015
−0.0010
−0.0005
0.0000
0.0005
0.0010
MR effect size for
'Ectonucleoside triphosphate diphosphohydrolase 6' on 'eGFR'

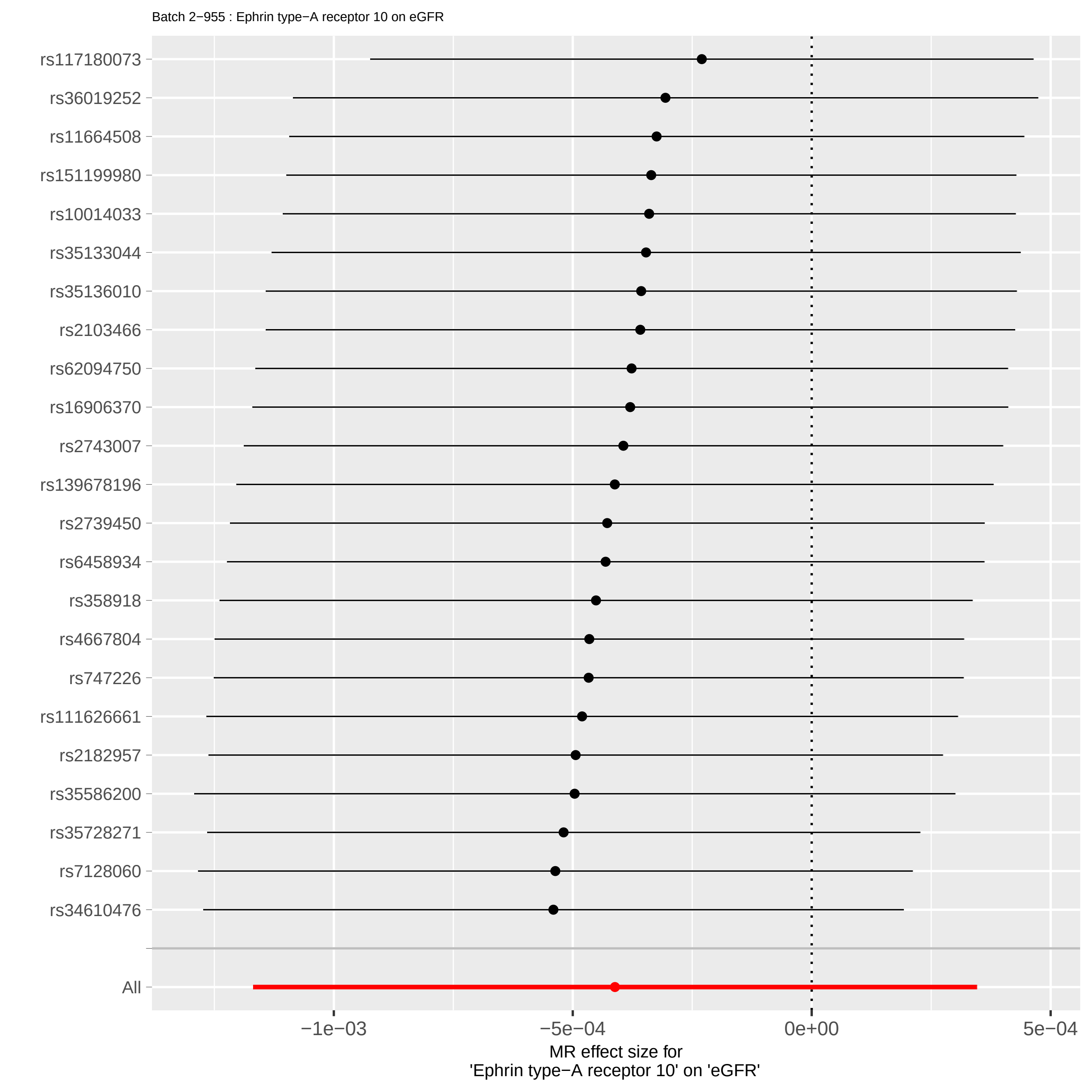

Batch 2−955 : Ephrin type−A receptor 10 on eGFR
rs117180073
rs36019252
rs11664508
rs151199980
rs10014033
rs35133044
rs35136010
rs2103466
rs62094750
rs16906370
rs2743007
rs139678196
rs2739450
rs6458934
rs358918
rs4667804
rs747226
rs111626661
rs2182957
rs35586200
rs35728271
rs7128060
rs34610476
All
−1e−03
−5e−04
0e+00
5e−04
MR effect size for
'Ephrin type−A receptor 10' on 'eGFR'

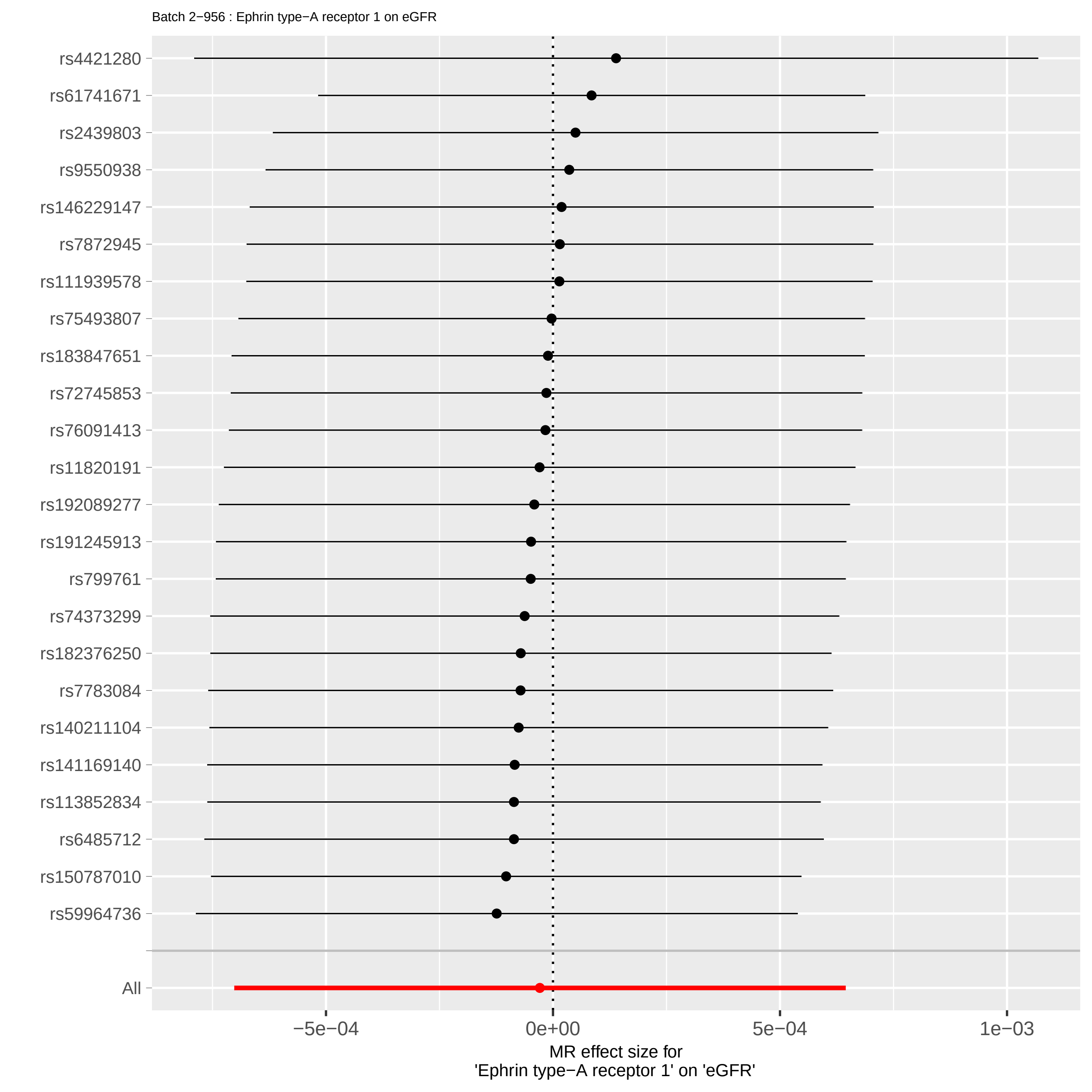

Batch 2−956 : Ephrin type−A receptor 1 on eGFR
rs4421280
rs61741671
rs2439803
rs9550938
rs146229147
rs7872945
rs111939578
rs75493807
rs183847651
rs72745853
rs76091413
rs11820191
rs192089277
rs191245913
rs799761
rs74373299
rs182376250
rs7783084
rs140211104
rs141169140
rs113852834
rs6485712
rs150787010
rs59964736
All
−5e−04
0e+00
5e−04
1e−03
MR effect size for
'Ephrin type−A receptor 1' on 'eGFR'

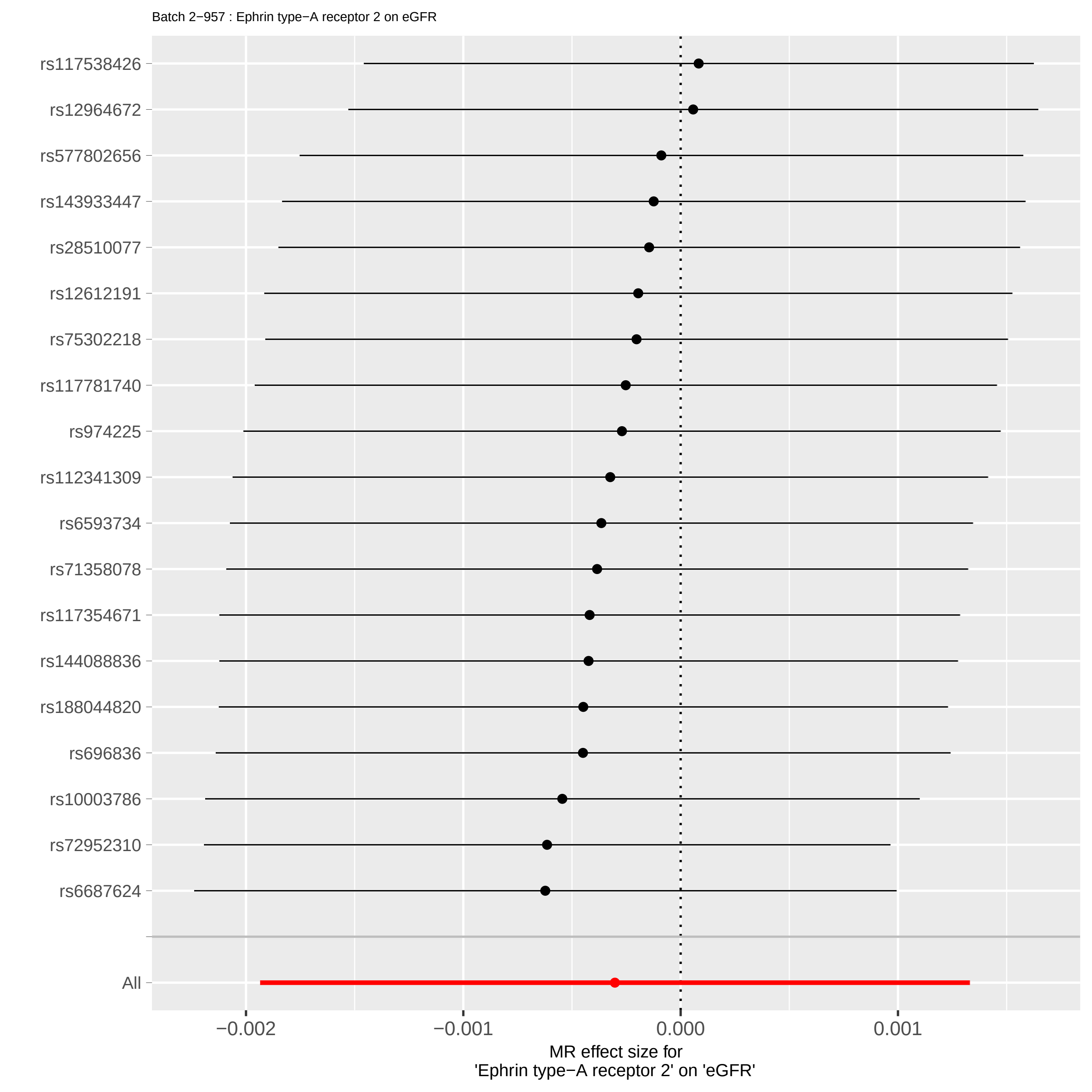

Batch 2−957 : Ephrin type−A receptor 2 on eGFR
rs117538426
rs12964672
rs577802656
rs143933447
rs28510077
rs12612191
rs75302218
rs117781740
rs974225
rs112341309
rs6593734
rs71358078
rs117354671
rs144088836
rs188044820
rs696836
rs10003786
rs72952310
rs6687624
All
−0.002
−0.001
0.000
0.001
MR effect size for
'Ephrin type−A receptor 2' on 'eGFR'

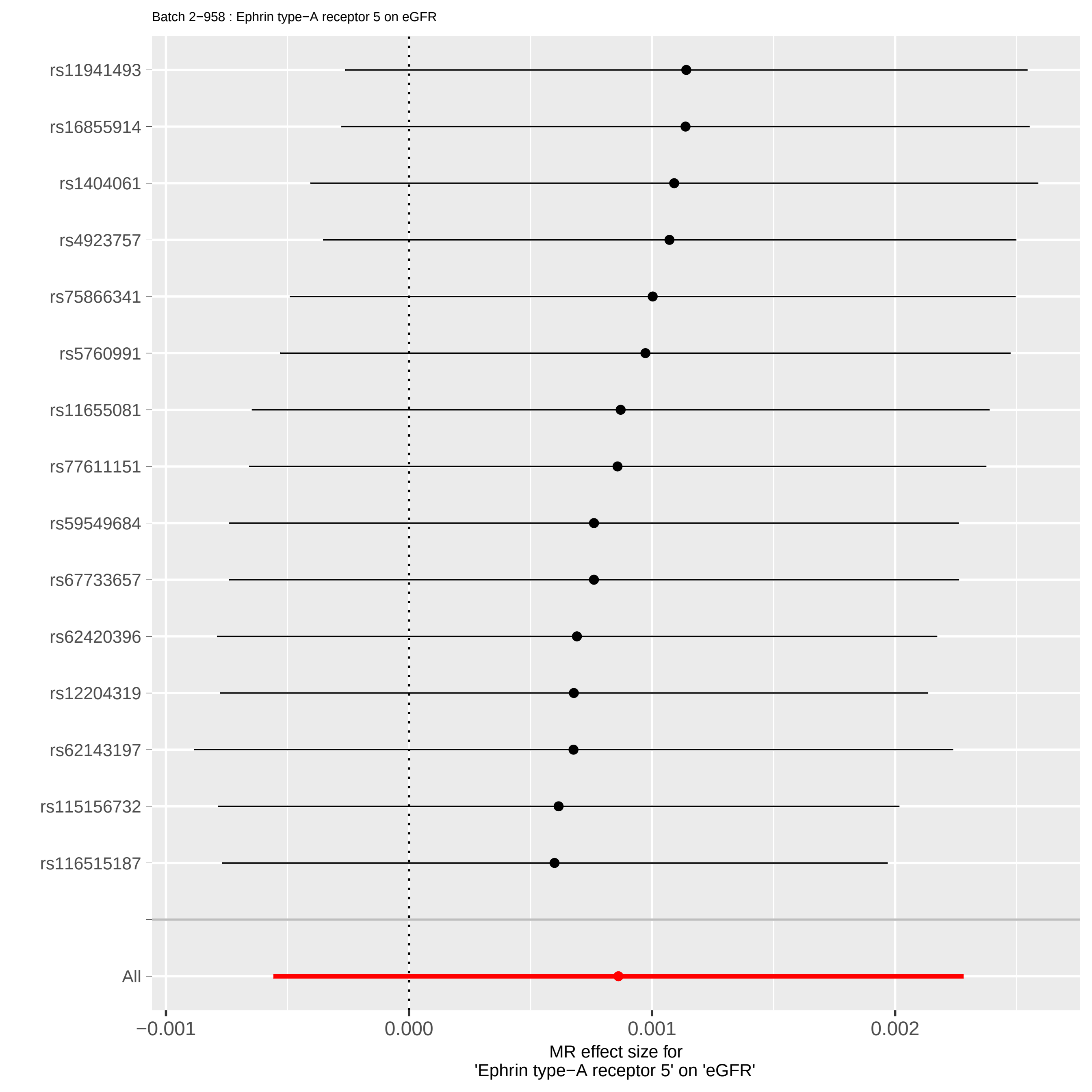

Batch 2−958 : Ephrin type−A receptor 5 on eGFR
rs11941493
rs16855914
rs1404061
rs4923757
rs75866341
rs5760991
rs11655081
rs77611151
rs59549684
rs67733657
rs62420396
rs12204319
rs62143197
rs115156732
rs116515187
All
−0.001
0.000
0.001
0.002
MR effect size for
'Ephrin type−A receptor 5' on 'eGFR'

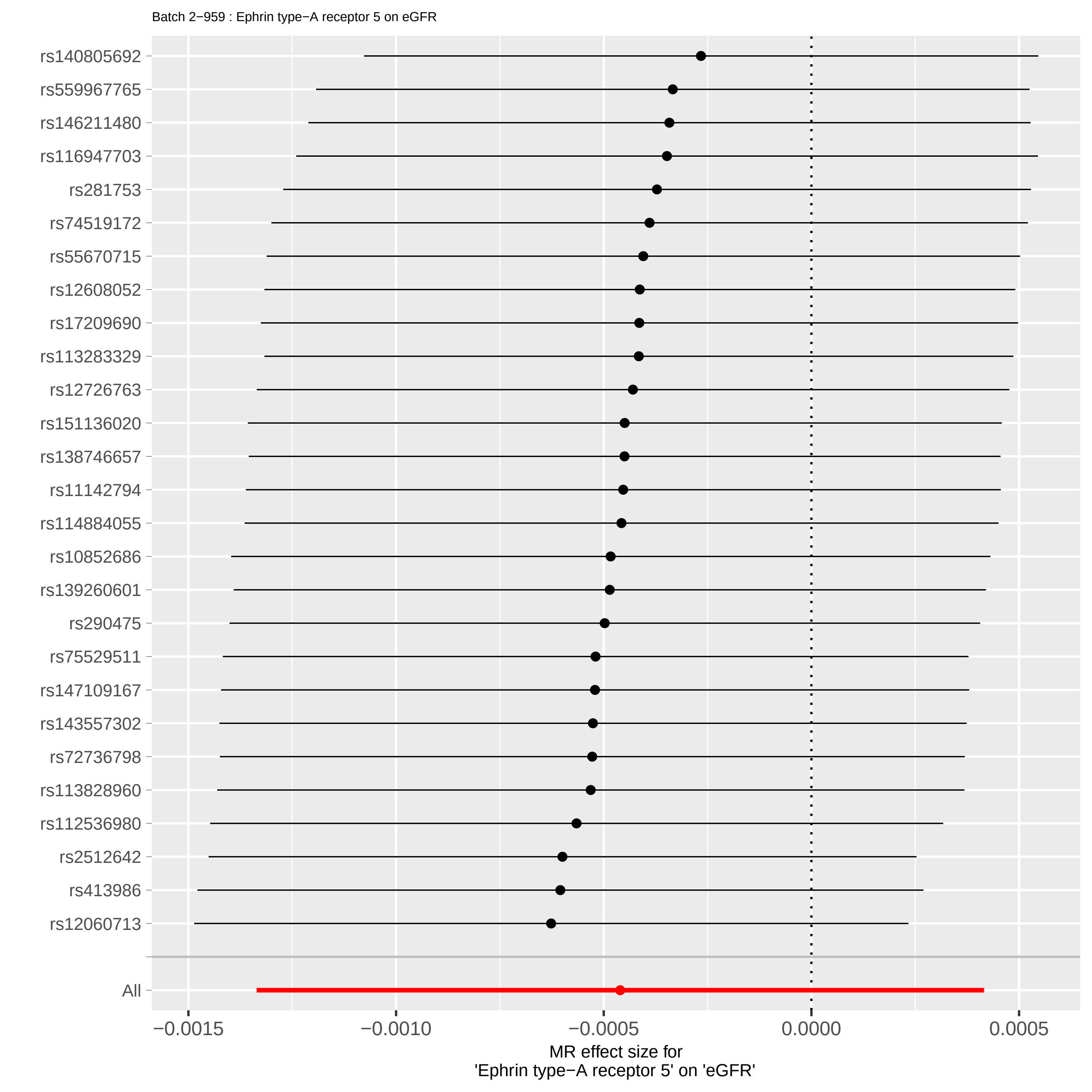

Batch 2−959 : Ephrin type−A receptor 5 on eGFR
rs140805692
rs559967765
rs146211480
rs116947703
rs281753
rs74519172
rs55670715
rs12608052
rs17209690
rs113283329
rs12726763
rs151136020
rs138746657
rs11142794
rs114884055
rs10852686
rs139260601
rs290475
rs75529511
rs147109167
rs143557302
rs72736798
rs113828960
rs112536980
rs2512642
rs413986
rs12060713
All
−0.0015
−0.0010
−0.0005
0.0000
0.0005
MR effect size for
'Ephrin type−A receptor 5' on 'eGFR'

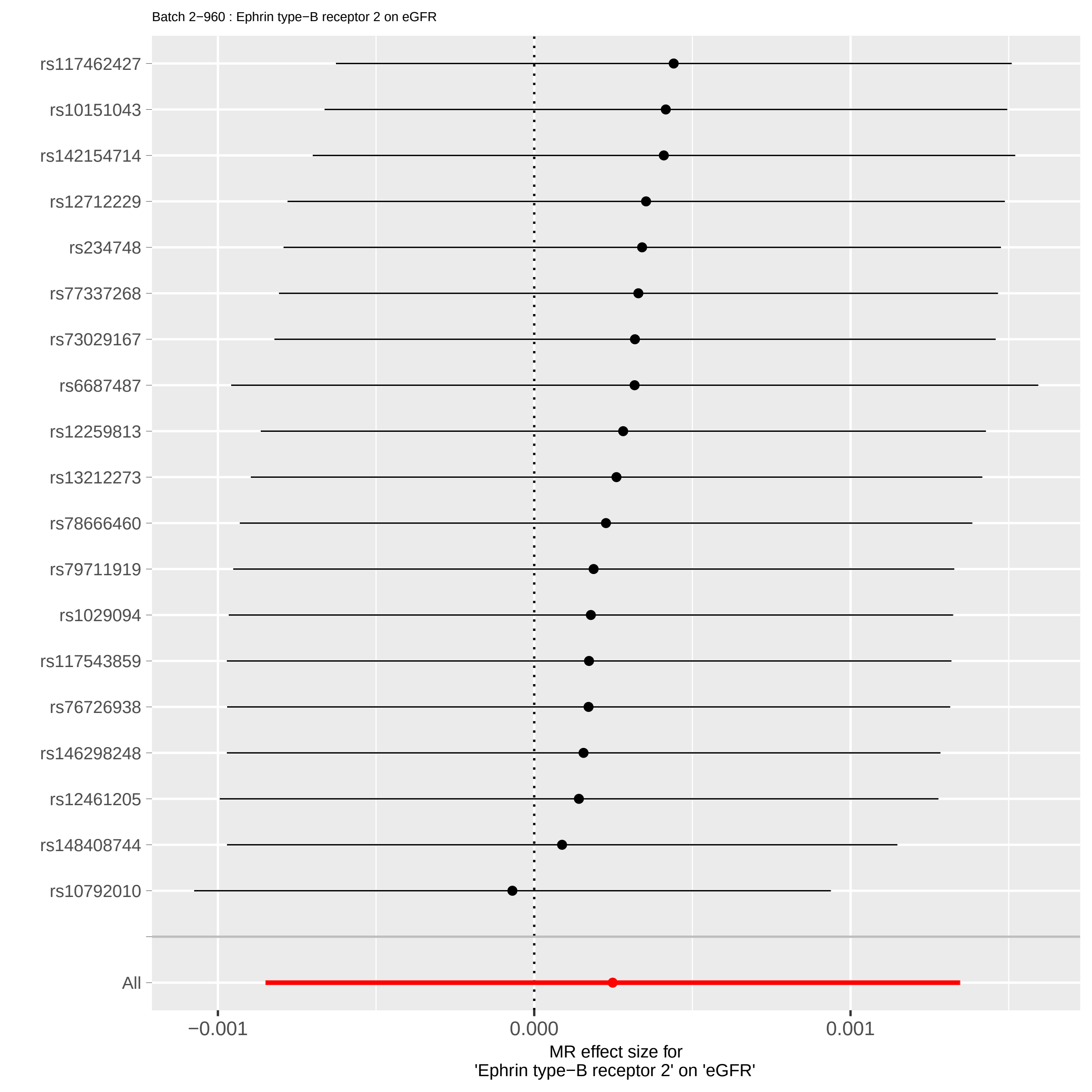

Batch 2−960 : Ephrin type−B receptor 2 on eGFR
rs117462427
rs10151043
rs142154714
rs12712229
rs234748
rs77337268
rs73029167
rs6687487
rs12259813
rs13212273
rs78666460
rs79711919
rs1029094
rs117543859
rs76726938
rs146298248
rs12461205
rs148408744
rs10792010
All
−0.001
0.000
0.001
MR effect size for
'Ephrin type−B receptor 2' on 'eGFR'

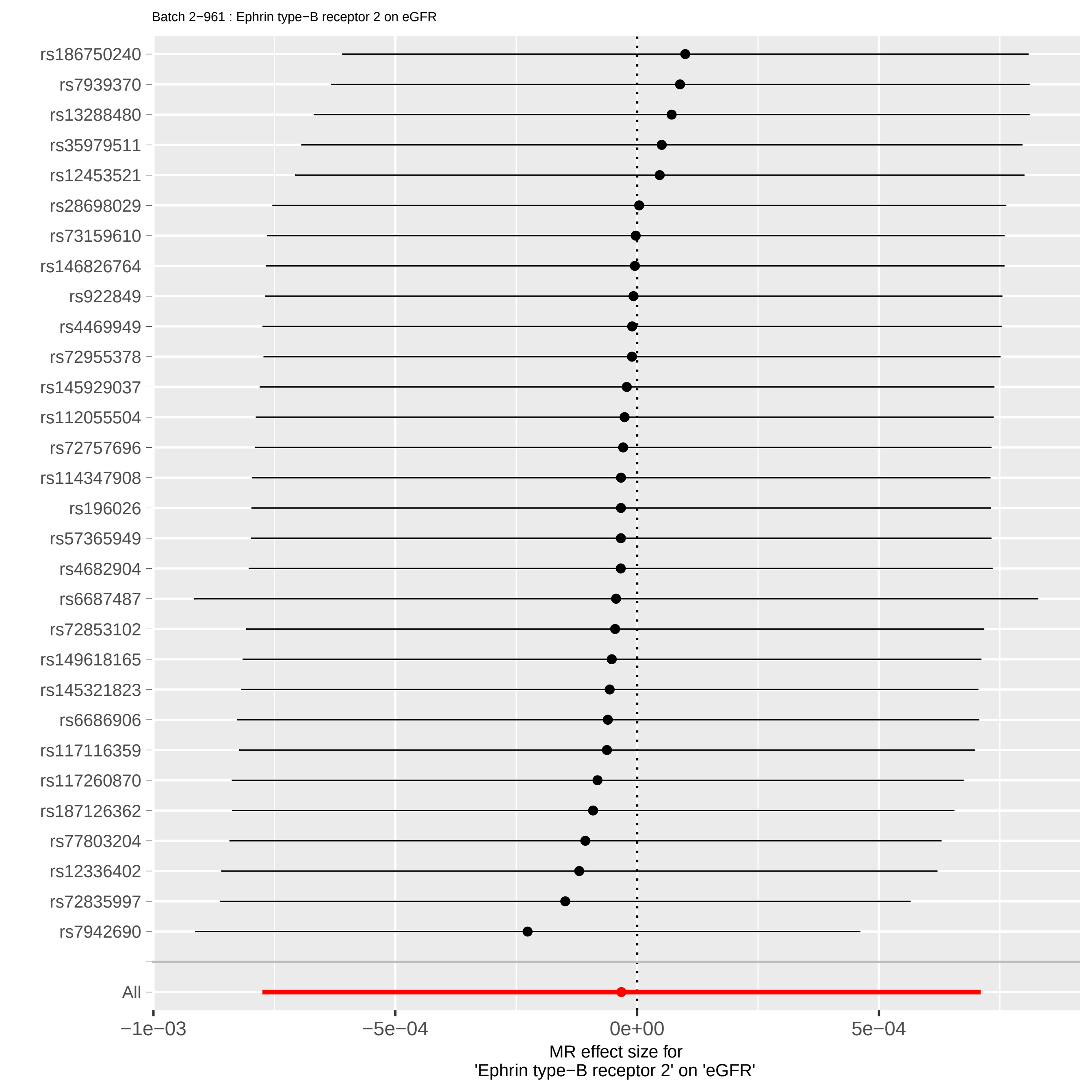
Batch 2−961 : Ephrin type−B receptor 2 on eGFR
rs186750240
rs7939370
rs13288480
rs35979511
rs12453521
rs28698029
rs73159610
rs146826764
rs922849
rs4469949
rs72955378
rs145929037
rs112055504
rs72757696
rs114347908
rs196026
rs57365949
rs4682904
rs6687487
rs72853102
rs149618165
rs145321823
rs6686906
rs117116359
rs117260870
rs187126362
rs77803204
rs12336402
rs72835997
rs7942690
All
−1e−03
−5e−04
0e+00
5e−04
MR effect size for
'Ephrin type−B receptor 2' on 'eGFR'

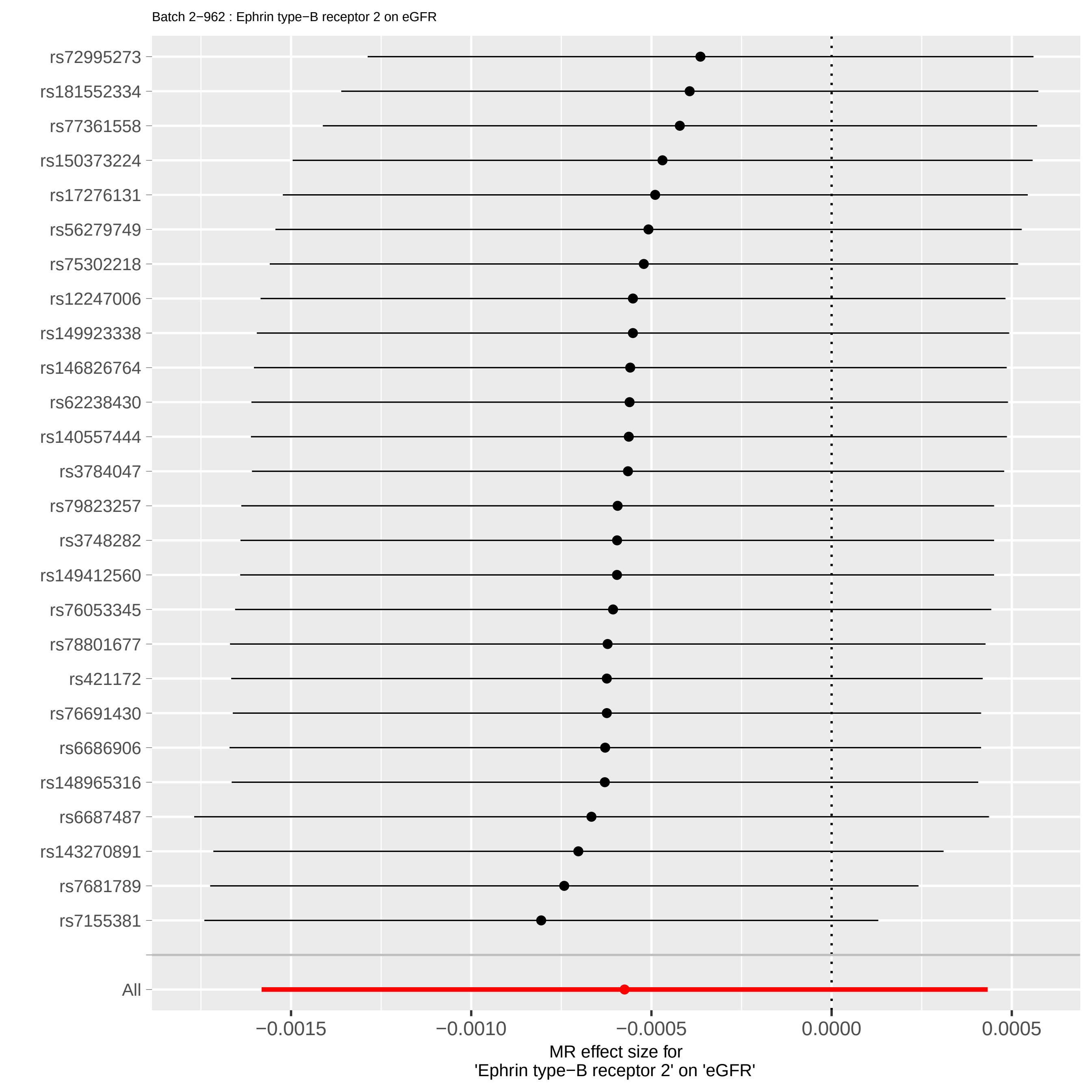
Batch 2−962 : Ephrin type−B receptor 2 on eGFR
rs72995273
rs181552334
rs77361558
rs150373224
rs17276131
rs56279749
rs75302218
rs12247006
rs149923338
rs146826764
rs62238430
rs140557444
rs3784047
rs79823257
rs3748282
rs149412560
rs76053345
rs78801677
rs421172
rs76691430
rs6686906
rs148965316
rs6687487
rs143270891
rs7681789
rs7155381
All
−0.0015
−0.0010
−0.0005
0.0000
0.0005
MR effect size for
'Ephrin type−B receptor 2' on 'eGFR'

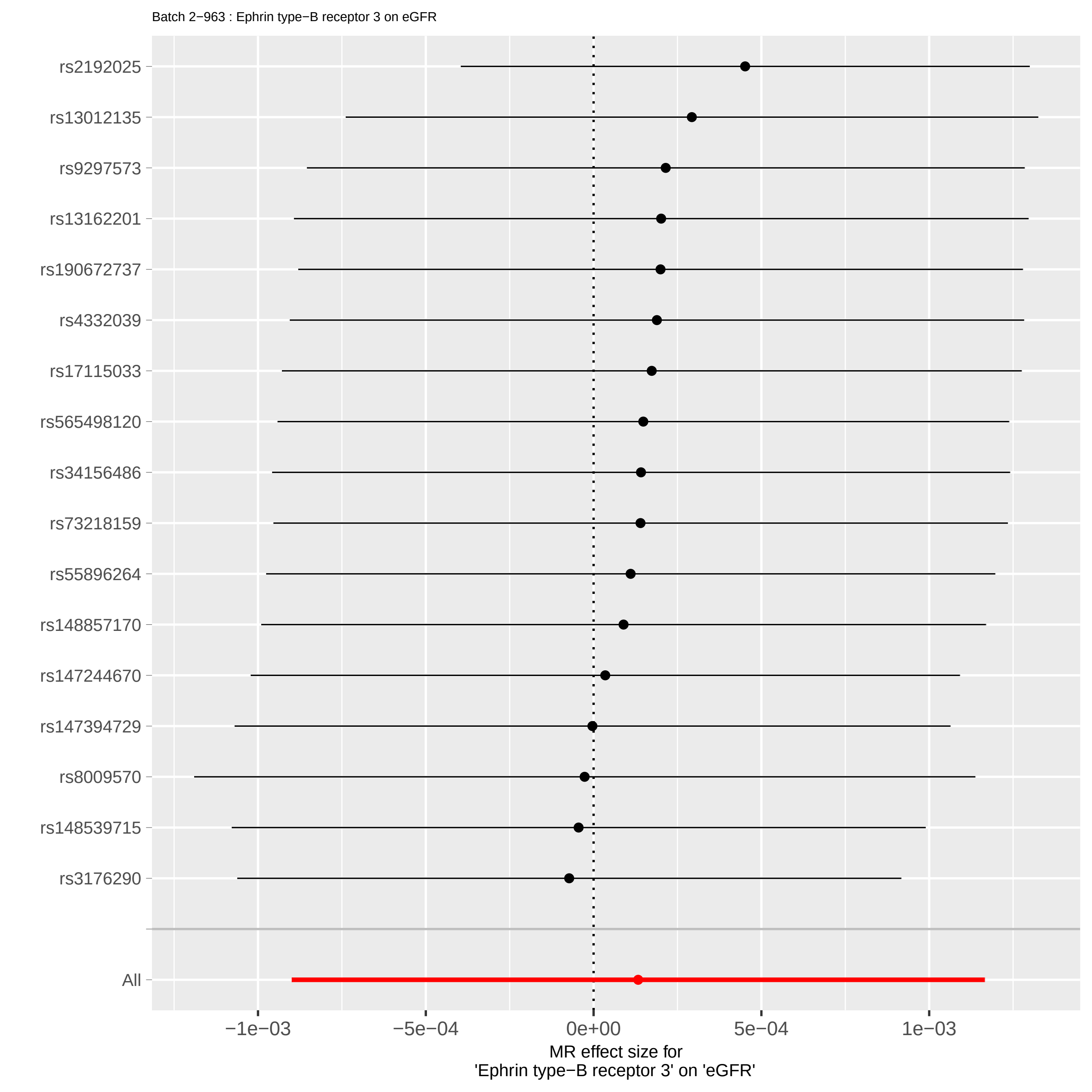

Batch 2−963 : Ephrin type−B receptor 3 on eGFR
rs2192025
rs13012135
rs9297573
rs13162201
rs190672737
rs4332039
rs17115033
rs565498120
rs34156486
rs73218159
rs55896264
rs148857170
rs147244670
rs147394729
rs8009570
rs148539715
rs3176290
All
−1e−03
−5e−04
0e+00
5e−04
1e−03
MR effect size for
'Ephrin type−B receptor 3' on 'eGFR'

Batch 2−964 : Ephrin type−B receptor 4 on eGFR

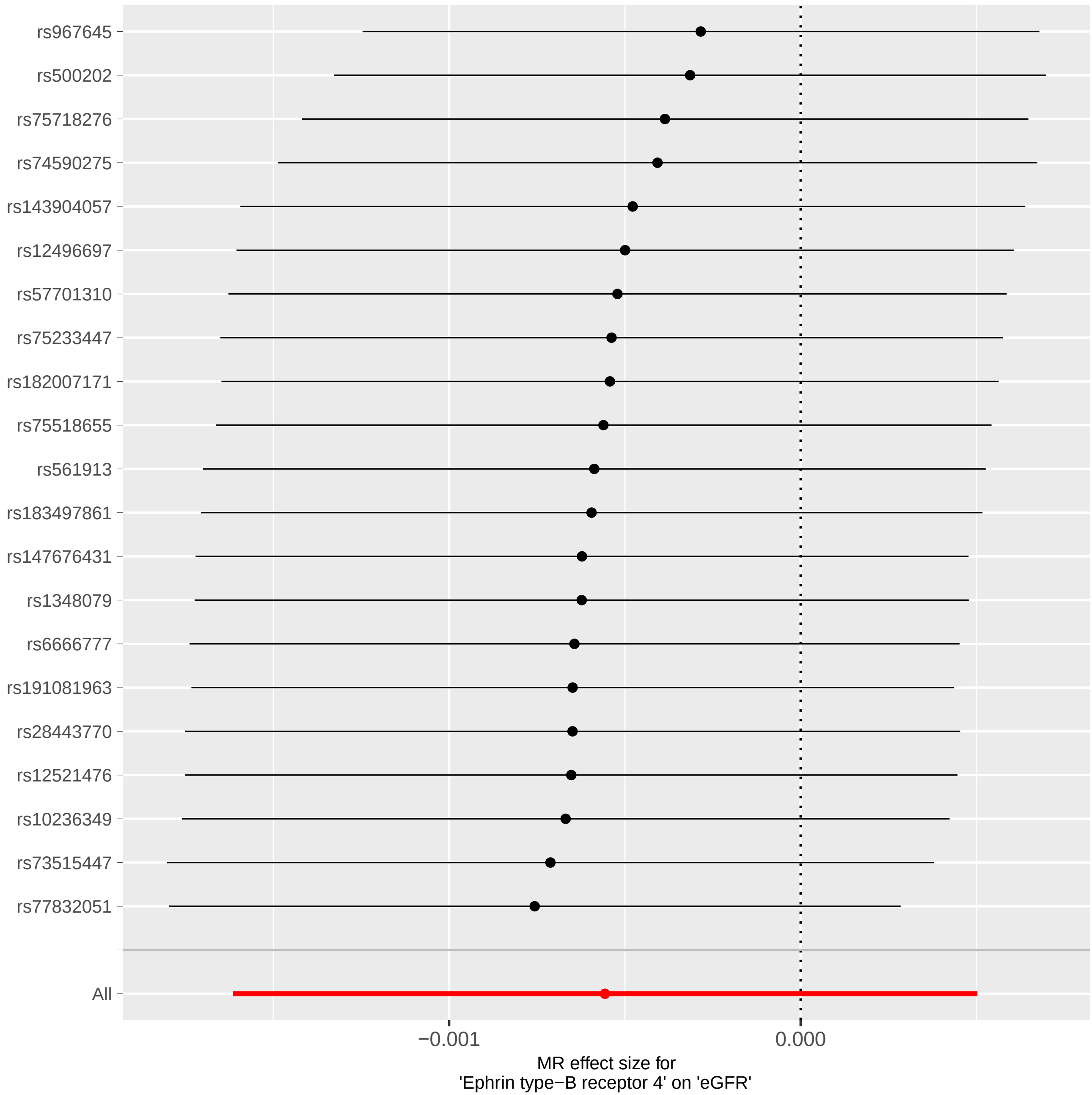

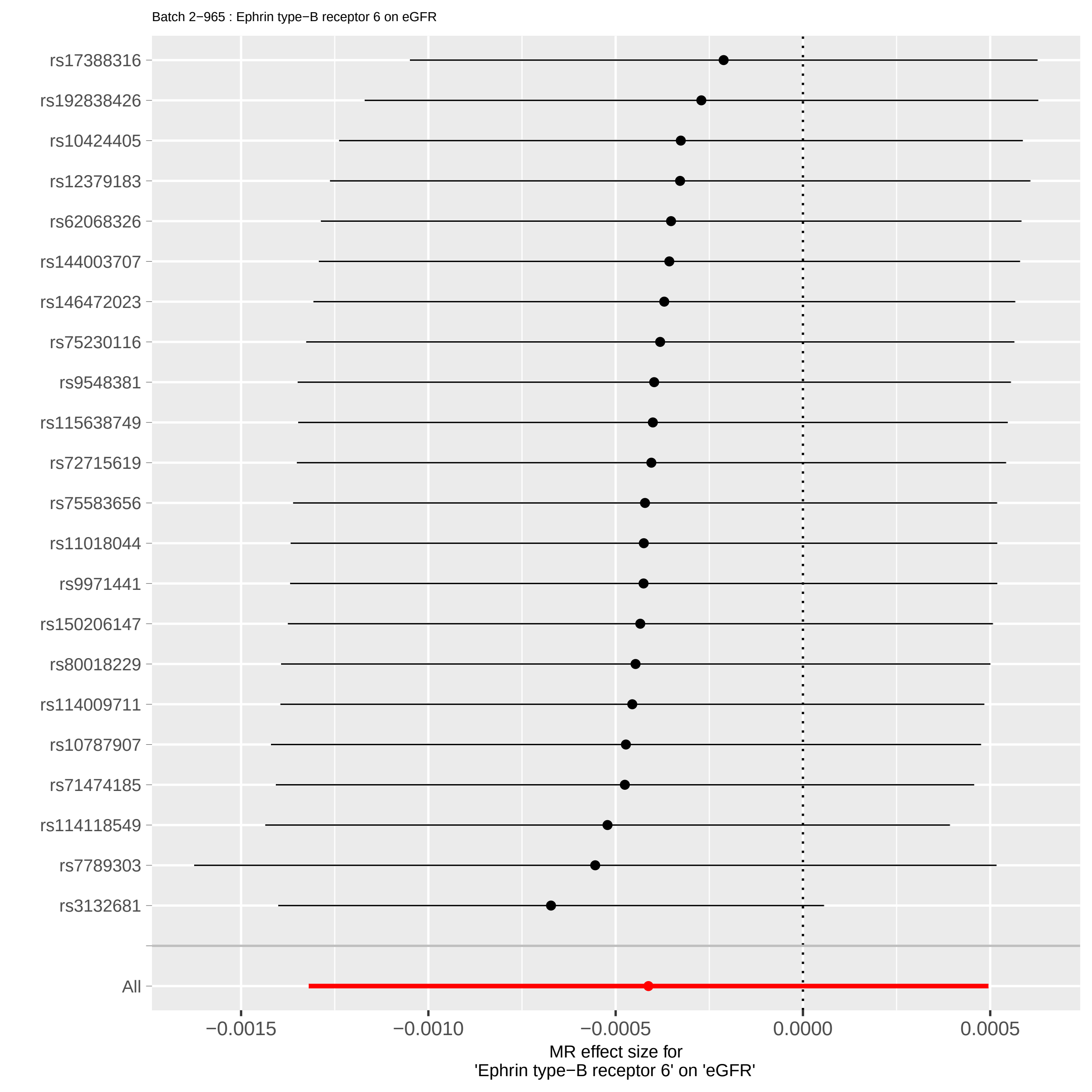

Batch 2−965 : Ephrin type−B receptor 6 on eGFR
rs17388316
rs192838426
rs10424405
rs12379183
rs62068326
rs144003707
rs146472023
rs75230116
rs9548381
rs115638749
rs72715619
rs75583656
rs11018044
rs9971441
rs150206147
rs80018229
rs114009711
rs10787907
rs71474185
rs114118549
rs7789303
rs3132681
All
−0.0015
−0.0010
−0.0005
0.0000
0.0005
MR effect size for
'Ephrin type−B receptor 6' on 'eGFR'

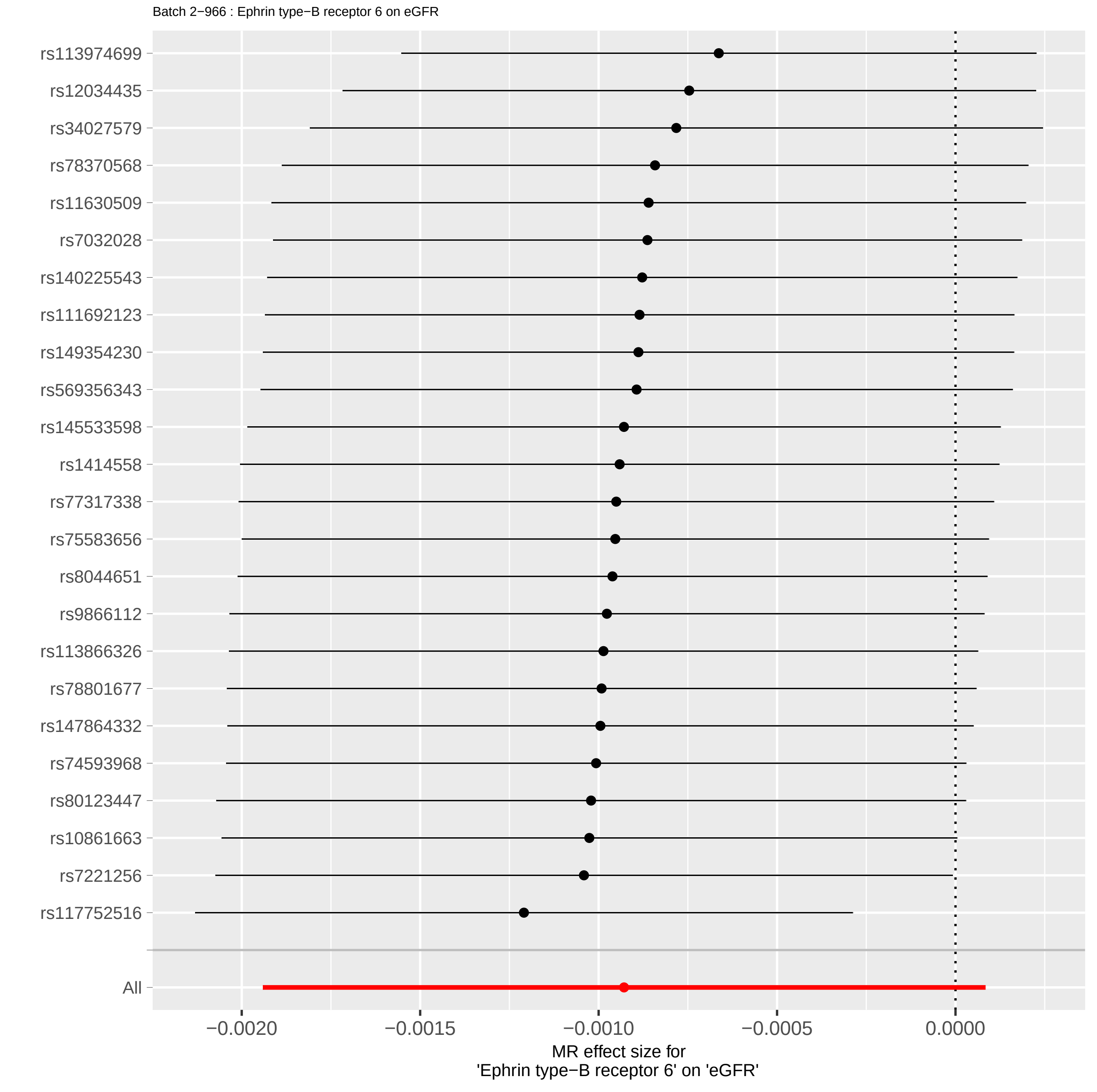

Batch 2−966 : Ephrin type−B receptor 6 on eGFR
rs113974699
rs12034435
rs34027579
rs78370568
rs11630509
rs7032028
rs140225543
rs111692123
rs149354230
rs569356343
rs145533598
rs1414558
rs77317338
rs75583656
rs8044651
rs9866112
rs113866326
rs78801677
rs147864332
rs74593968
rs80123447
rs10861663
rs7221256
rs117752516
All
−0.0020
−0.0015
−0.0010
−0.0005
0.0000
MR effect size for
'Ephrin type−B receptor 6' on 'eGFR'

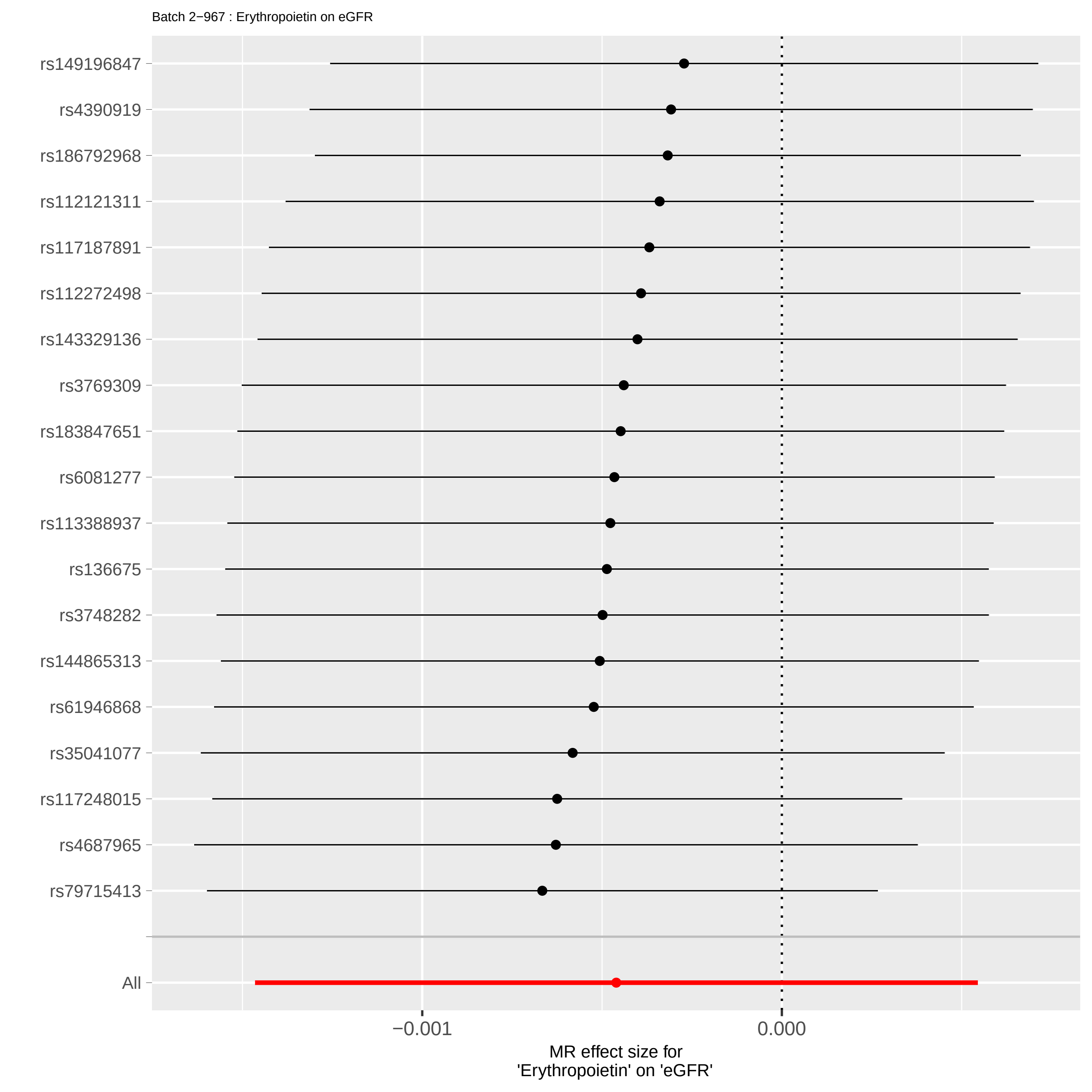
Batch 2−967 : Erythropoietin on eGFR
rs149196847
rs4390919
rs186792968
rs112121311
rs117187891
rs112272498
rs143329136
rs3769309
rs183847651
rs6081277
rs113388937
rs136675
rs3748282
rs144865313
rs61946868
rs35041077
rs117248015
rs4687965
rs79715413
All
−0.001
0.000
MR effect size for
'Erythropoietin' on 'eGFR'

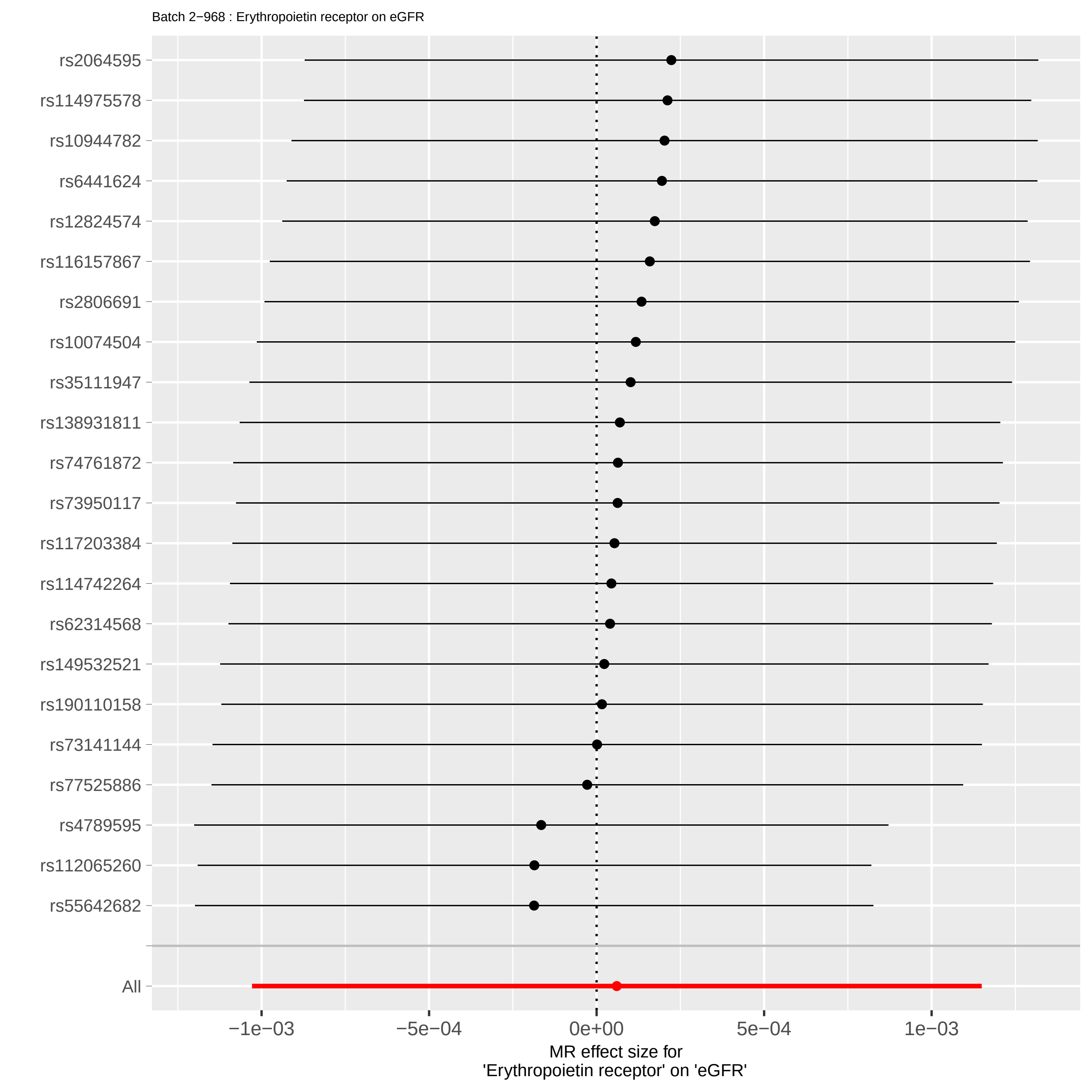

Batch 2−968 : Erythropoietin receptor on eGFR
rs2064595
rs114975578
rs10944782
rs6441624
rs12824574
rs116157867
rs2806691
rs10074504
rs35111947
rs138931811
rs74761872
rs73950117
rs117203384
rs114742264
rs62314568
rs149532521
rs190110158
rs73141144
rs77525886
rs4789595
rs112065260
rs55642682
All
−1e−03
−5e−04
0e+00
5e−04
1e−03
MR effect size for
'Erythropoietin receptor' on 'eGFR'

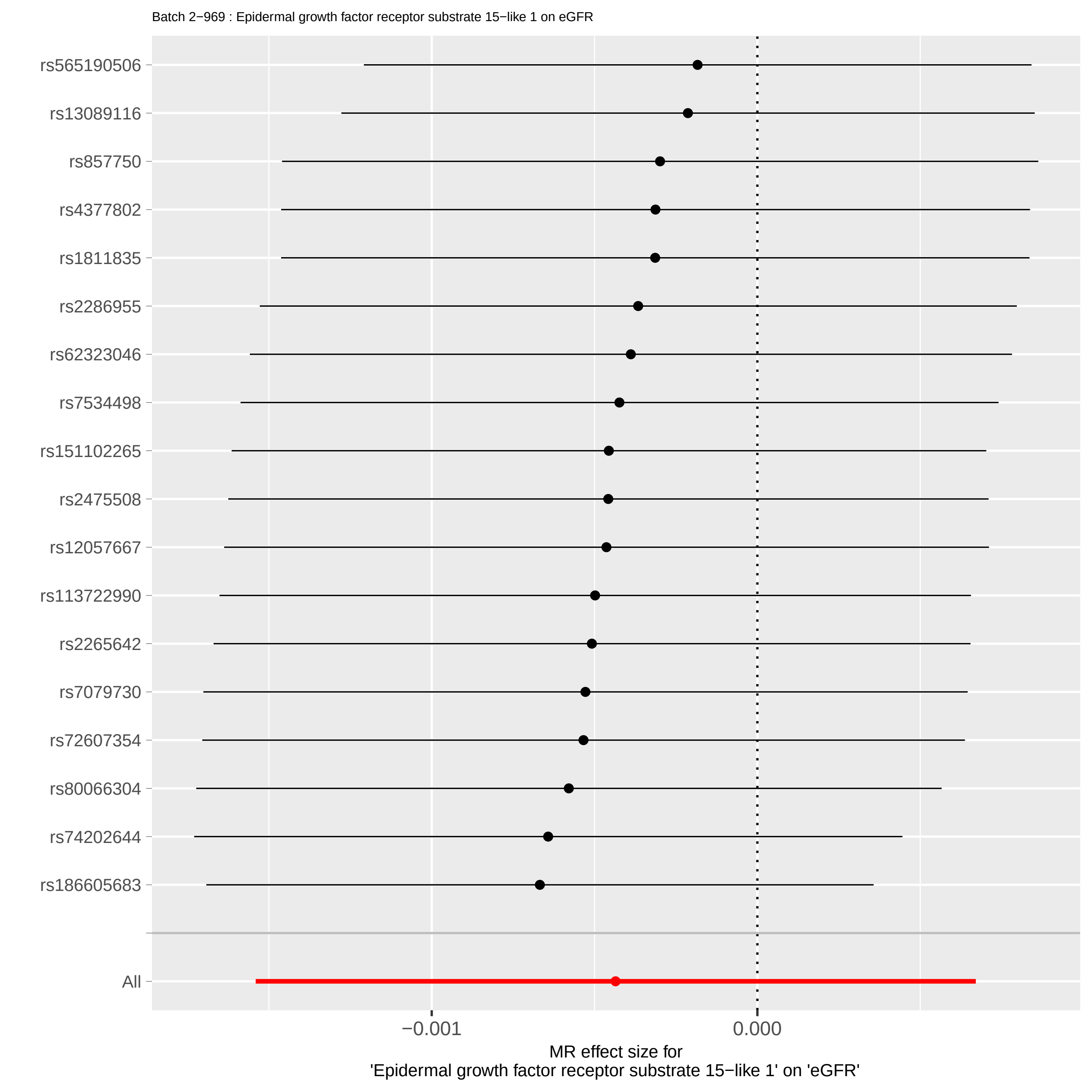
Batch 2−969 : Epidermal growth factor receptor substrate 15−like 1 on eGFR
rs565190506
rs13089116
rs857750
rs4377802
rs1811835
rs2286955
rs62323046
rs7534498
rs151102265
rs2475508
rs12057667
rs113722990
rs2265642
rs7079730
rs72607354
rs80066304
rs74202644
rs186605683
All
−0.001
0.000
MR effect size for
'Epidermal growth factor receptor substrate 15−like 1' on 'eGFR'

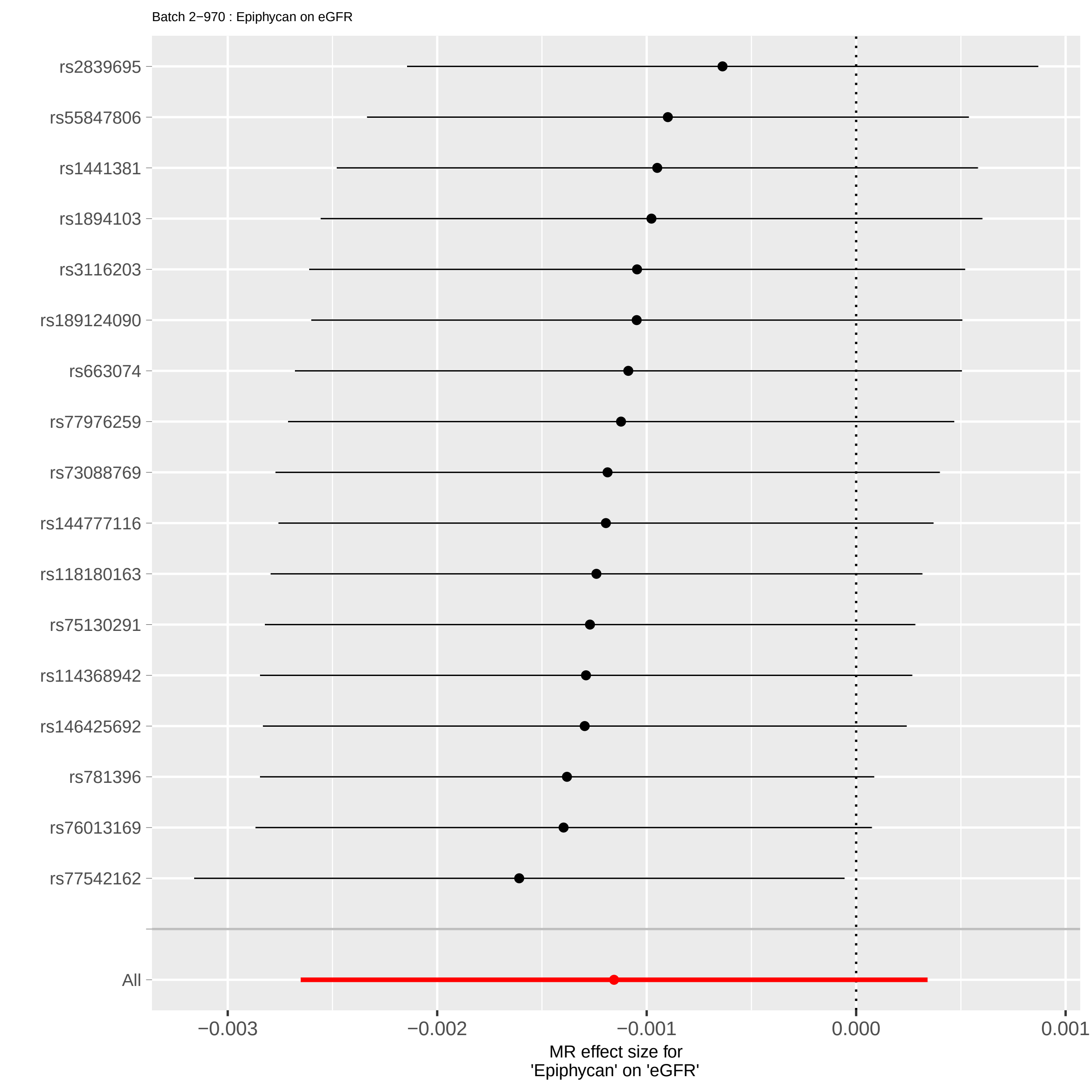

Batch 2−970 : Epiphycan on eGFR
rs2839695
rs55847806
rs1441381
rs1894103
rs3116203
rs189124090
rs663074
rs77976259
rs73088769
rs144777116
rs118180163
rs75130291
rs114368942
rs146425692
rs781396
rs76013169
rs77542162
All
−0.003
−0.002
−0.001
0.000
0.001
MR effect size for
'Epiphycan' on 'eGFR'

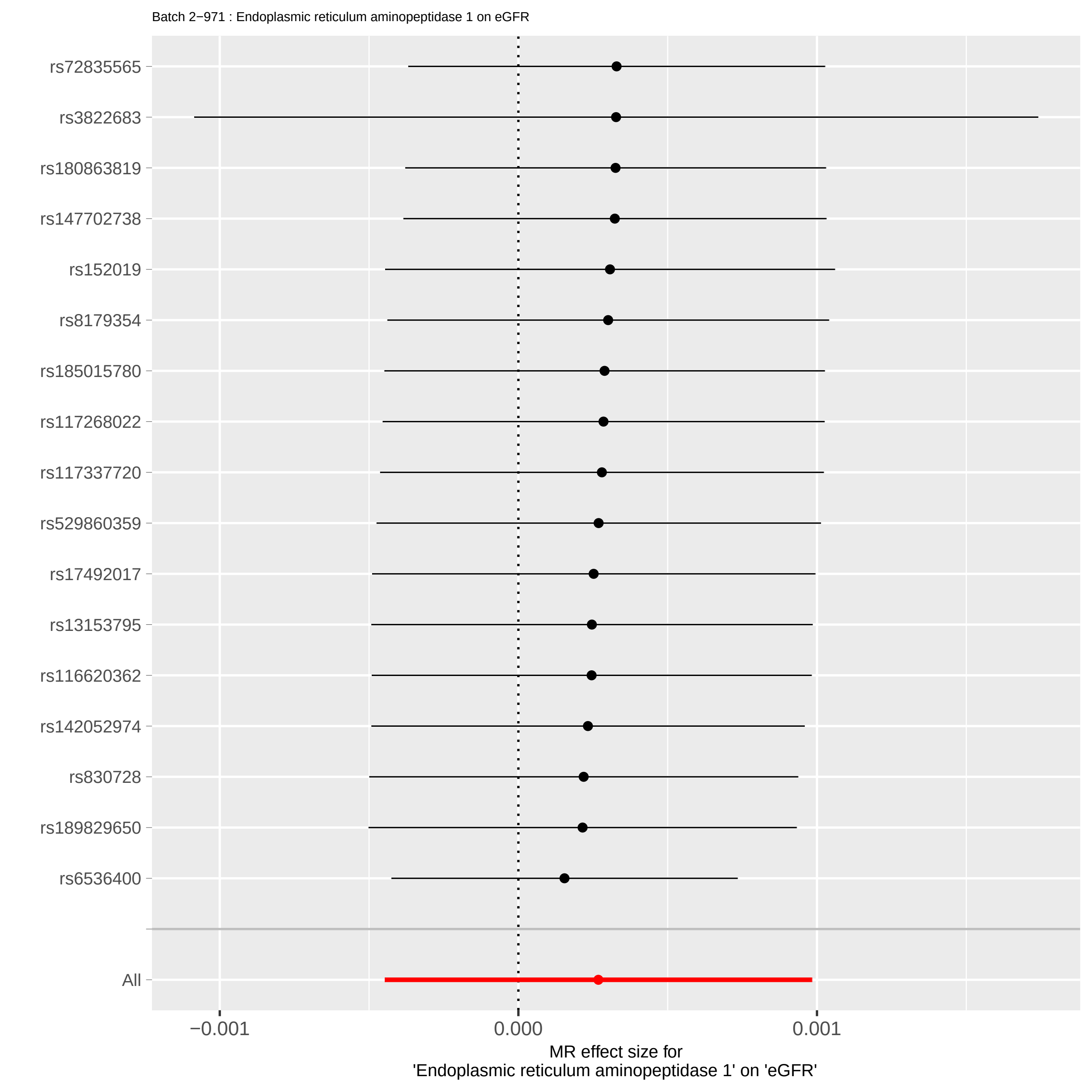

Batch 2−971 : Endoplasmic reticulum aminopeptidase 1 on eGFR
rs72835565
rs3822683
rs180863819
rs147702738
rs152019
rs8179354
rs185015780
rs117268022
rs117337720
rs529860359
rs17492017
rs13153795
rs116620362
rs142052974
rs830728
rs189829650
rs6536400
All
−0.001
0.000
0.001
MR effect size for
'Endoplasmic reticulum aminopeptidase 1' on 'eGFR'

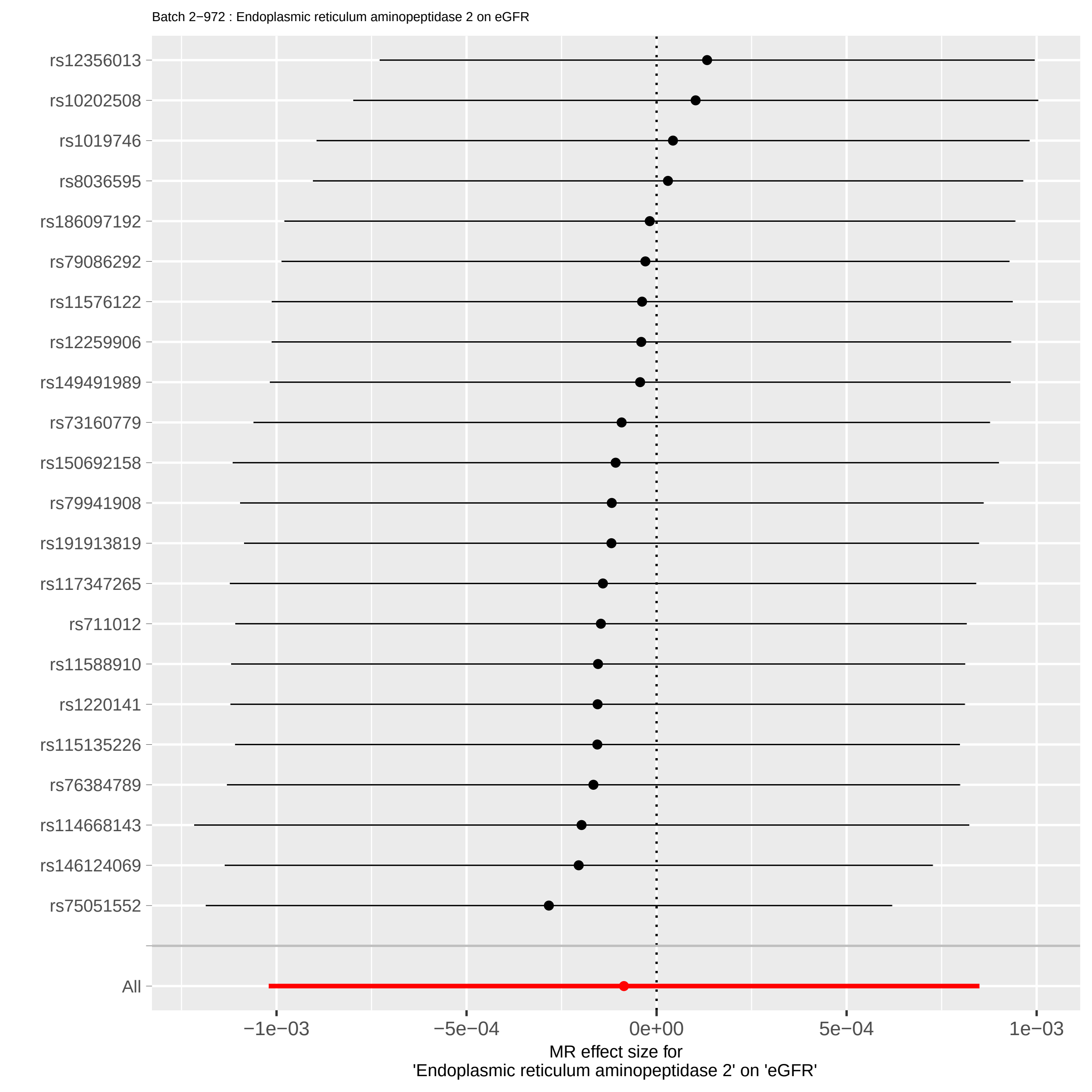

Batch 2−972 : Endoplasmic reticulum aminopeptidase 2 on eGFR
rs12356013
rs10202508
rs1019746
rs8036595
rs186097192
rs79086292
rs11576122
rs12259906
rs149491989
rs73160779
rs150692158
rs79941908
rs191913819
rs117347265
rs711012
rs11588910
rs1220141
rs115135226
rs76384789
rs114668143
rs146124069
rs75051552
All
−1e−03
−5e−04
0e+00
5e−04
1e−03
MR effect size for
'Endoplasmic reticulum aminopeptidase 2' on 'eGFR'

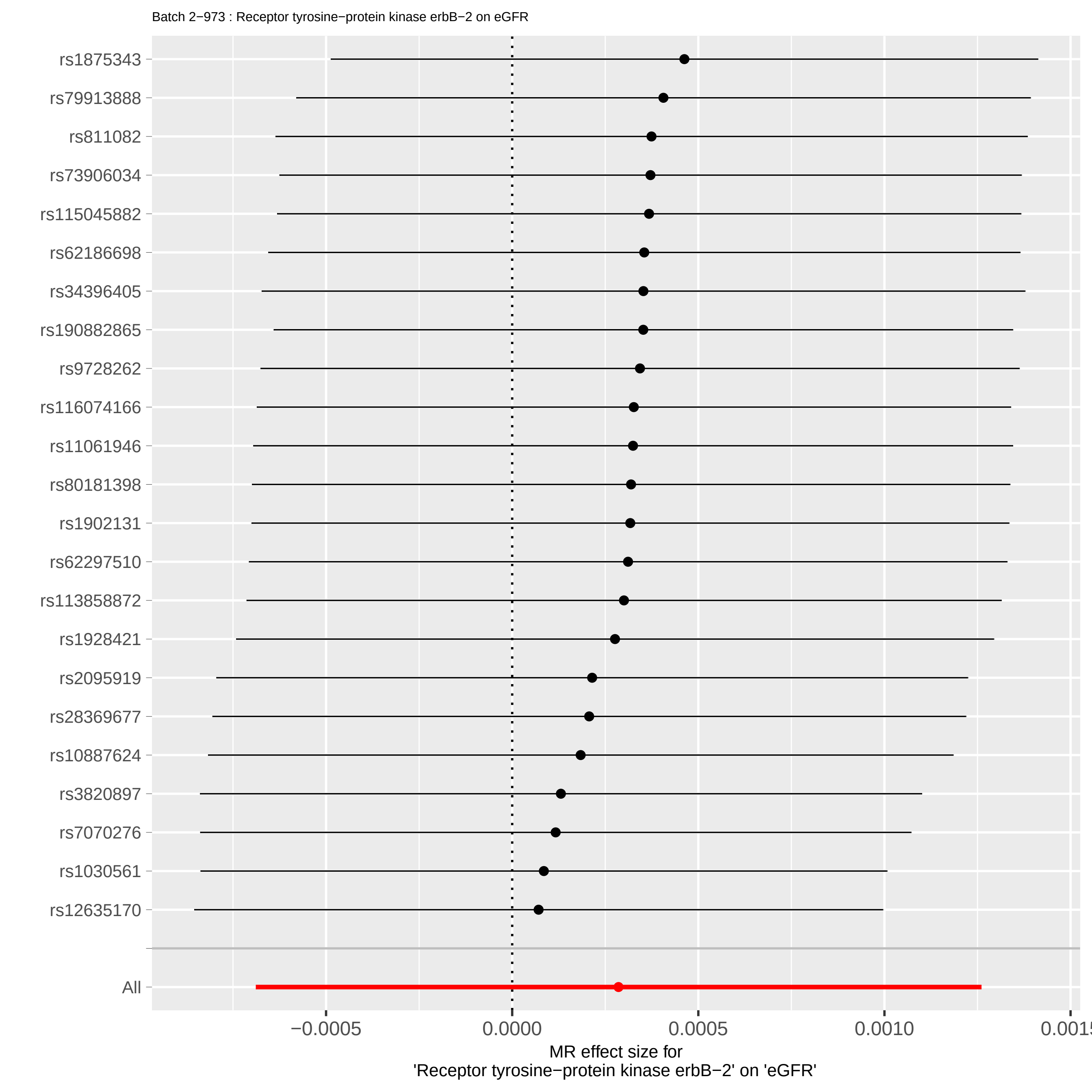

Batch 2−973 : Receptor tyrosine−protein kinase erbB−2 on eGFR
rs1875343
rs79913888
rs811082
rs73906034
rs115045882
rs62186698
rs34396405
rs190882865
rs9728262
rs116074166
rs11061946
rs80181398
rs1902131
rs62297510
rs113858872
rs1928421
rs2095919
rs28369677
rs10887624
rs3820897
rs7070276
rs1030561
rs12635170
All
−0.0005
0.0000
0.0005
0.0010
MR effect size for
'Receptor tyrosine−protein kinase erbB−2' on 'eGFR'

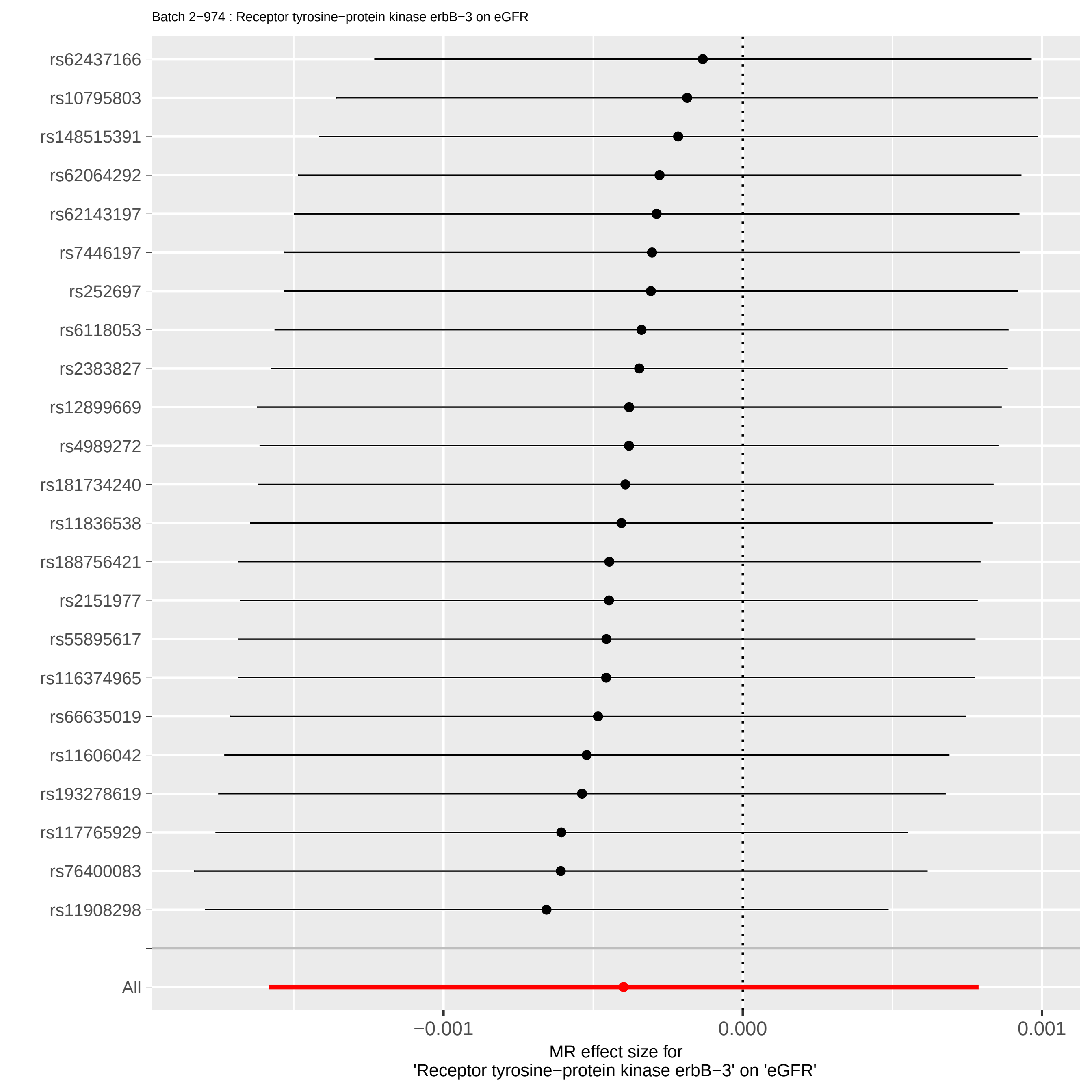

Batch 2−974 : Receptor tyrosine−protein kinase erbB−3 on eGFR
rs62437166
rs10795803
rs148515391
rs62064292
rs62143197
rs7446197
rs252697
rs6118053
rs2383827
rs12899669
rs4989272
rs181734240
rs11836538
rs188756421
rs2151977
rs55895617
rs116374965
rs66635019
rs11606042
rs193278619
rs117765929
rs76400083
rs11908298
All
−0.001
0.000
0.001
MR effect size for
'Receptor tyrosine−protein kinase erbB−3' on 'eGFR'

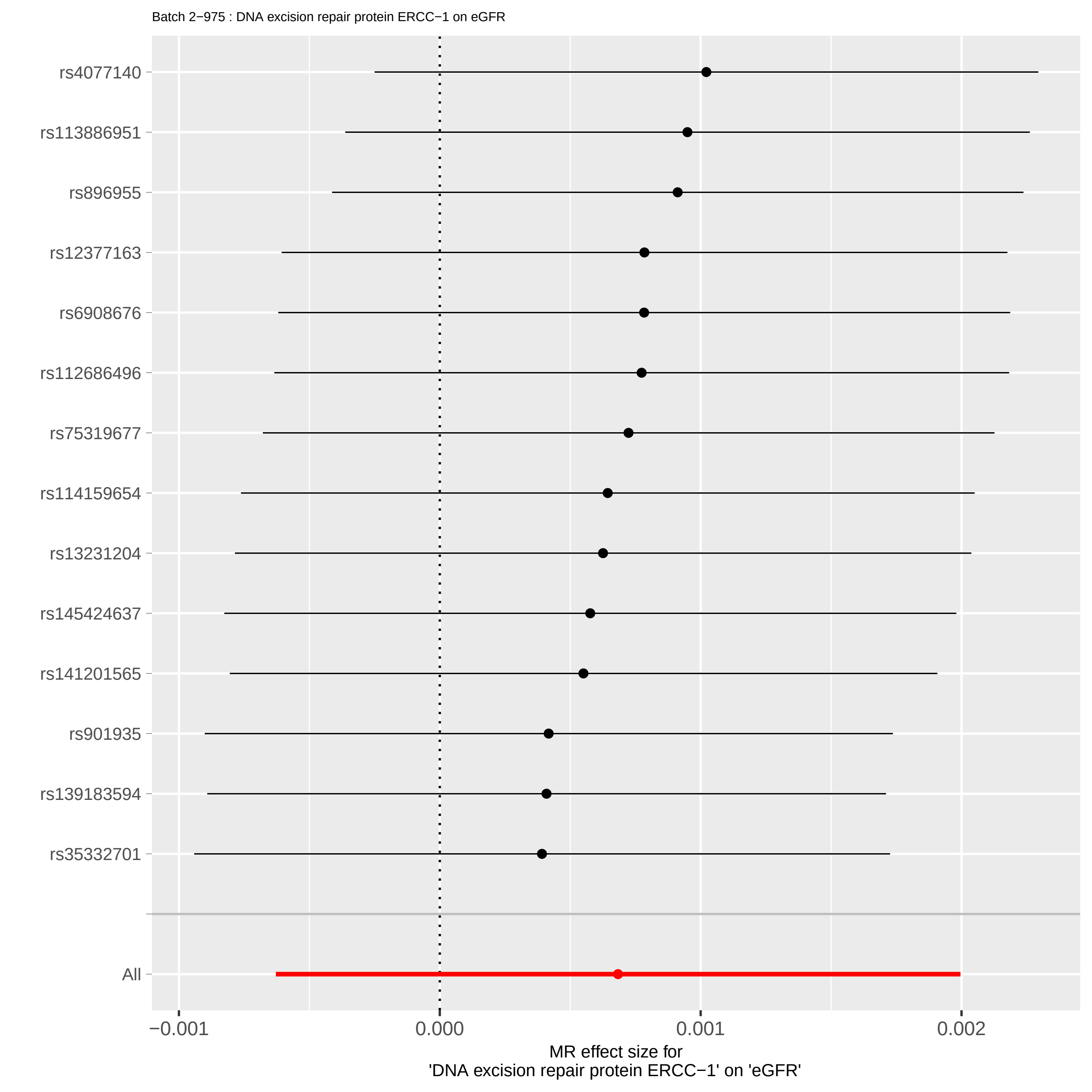

Batch 2−975 : DNA excision repair protein ERCC−1 on eGFR
rs4077140
rs113886951
rs896955
rs12377163
rs6908676
rs112686496
rs75319677
rs114159654
rs13231204
rs145424637
rs141201565
rs901935
rs139183594
rs35332701
All
−0.001
0.000
0.001
0.002
MR effect size for
'DNA excision repair protein ERCC−1' on 'eGFR'

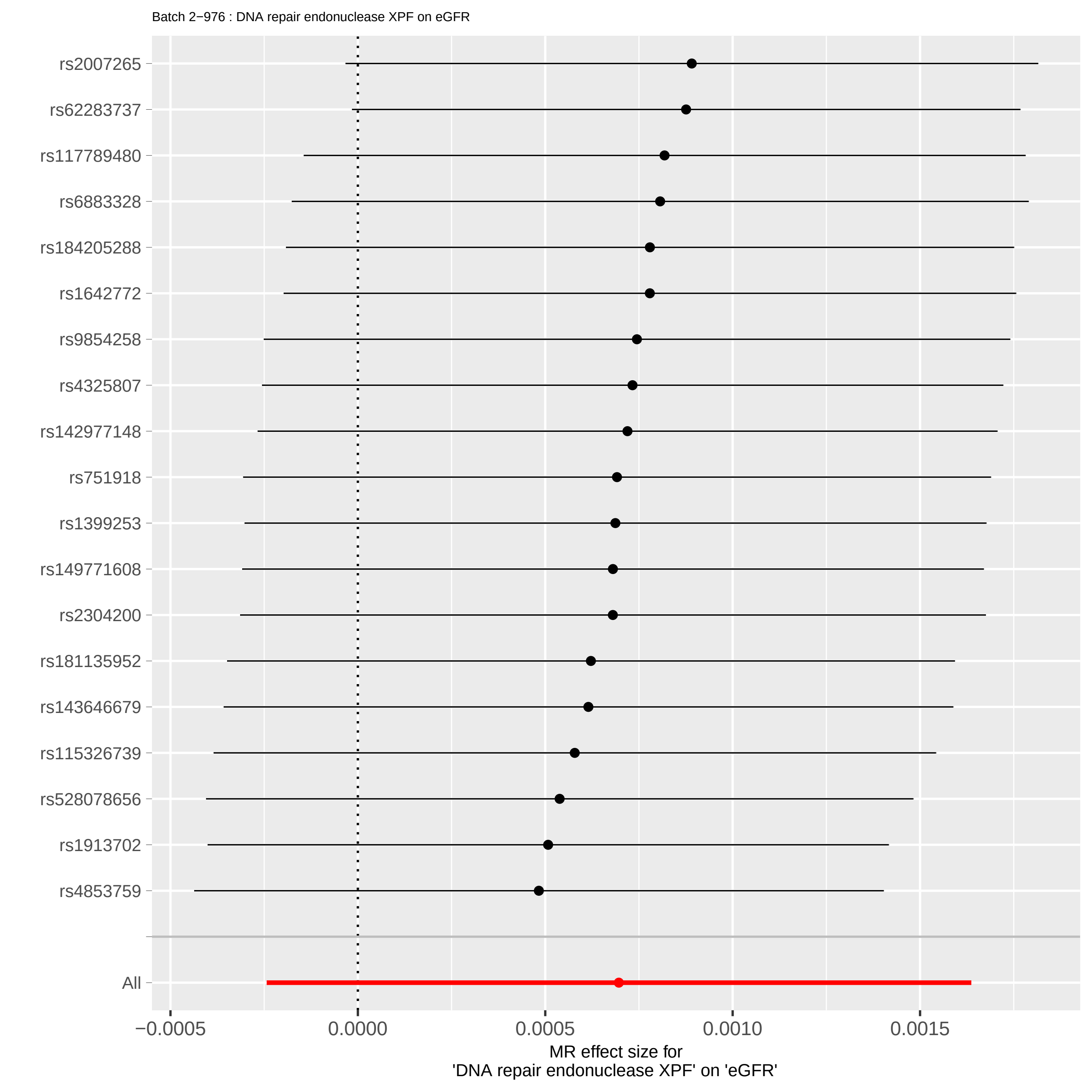

Batch 2−976 : DNA repair endonuclease XPF on eGFR
rs2007265
rs62283737
rs117789480
rs6883328
rs184205288
rs1642772
rs9854258
rs4325807
rs142977148
rs751918
rs1399253
rs149771608
rs2304200
rs181135952
rs143646679
rs115326739
rs528078656
rs1913702
rs4853759
All
−0.0005
0.0000
0.0005
0.0010
0.0015
MR effect size for
'DNA repair endonuclease XPF' on 'eGFR'

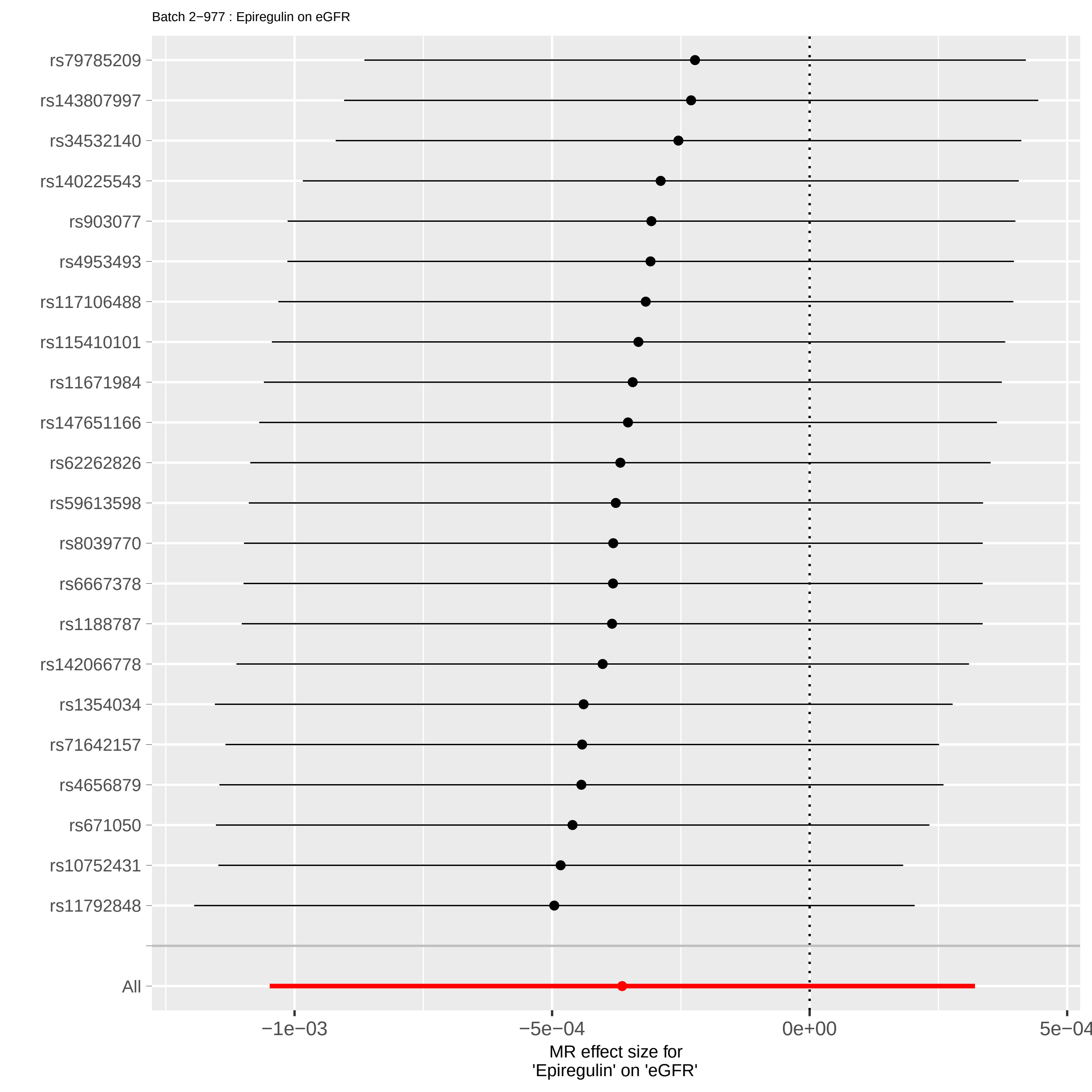

Batch 2−977 : Epiregulin on eGFR
rs79785209
rs143807997
rs34532140
rs140225543
rs903077
rs4953493
rs117106488
rs115410101
rs11671984
rs147651166
rs62262826
rs59613598
rs8039770
rs6667378
rs1188787
rs142066778
rs1354034
rs71642157
rs4656879
rs671050
rs10752431
rs11792848
All
−1e−03
−5e−04
0e+00
5e−04
MR effect size for
'Epiregulin' on 'eGFR'

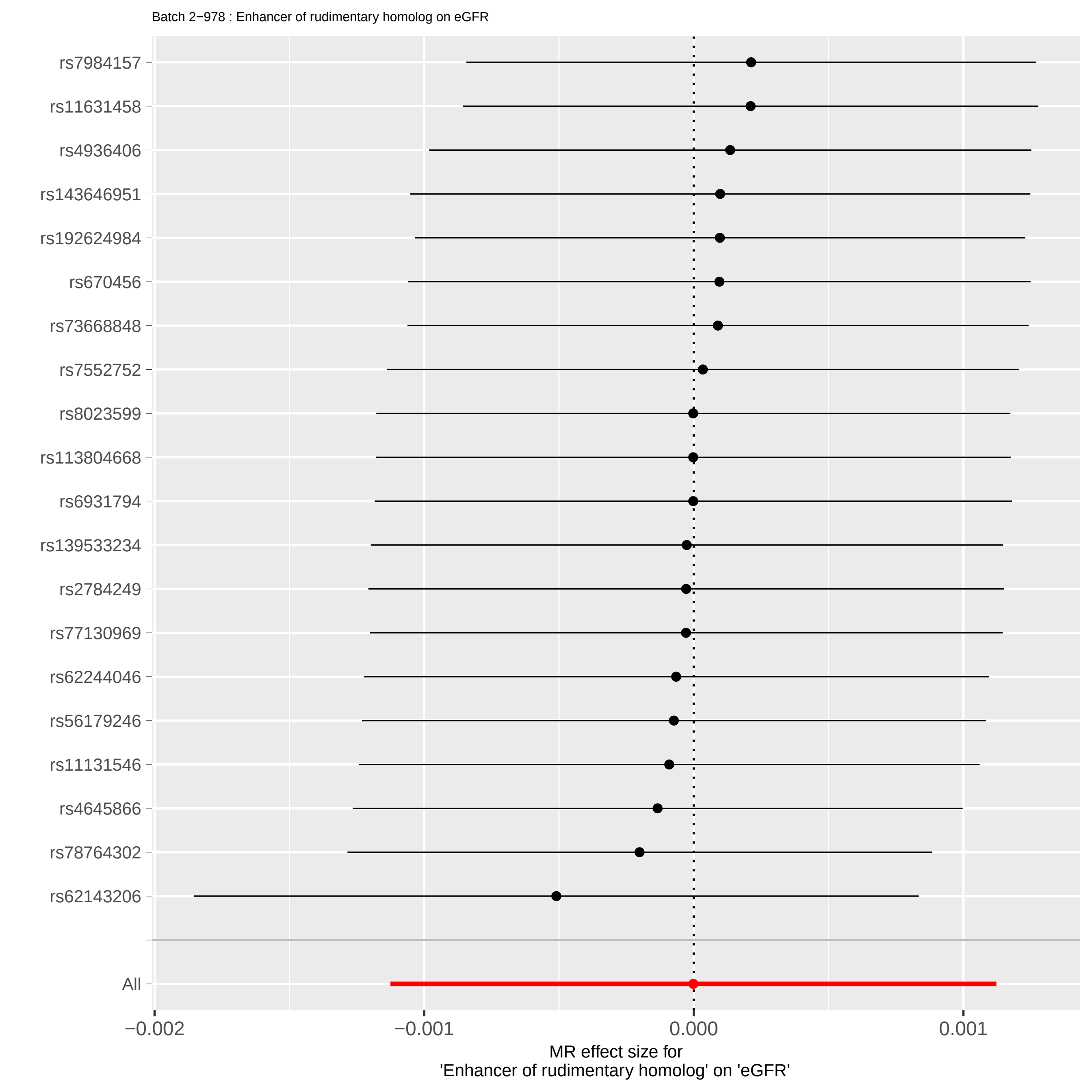

Batch 2−978 : Enhancer of rudimentary homolog on eGFR
rs7984157
rs11631458
rs4936406
rs143646951
rs192624984
rs670456
rs73668848
rs7552752
rs8023599
rs113804668
rs6931794
rs139533234
rs2784249
rs77130969
rs62244046
rs56179246
rs11131546
rs4645866
rs78764302
rs62143206
All
−0.002
−0.001
0.000
0.001
MR effect size for
'Enhancer of rudimentary homolog' on 'eGFR'

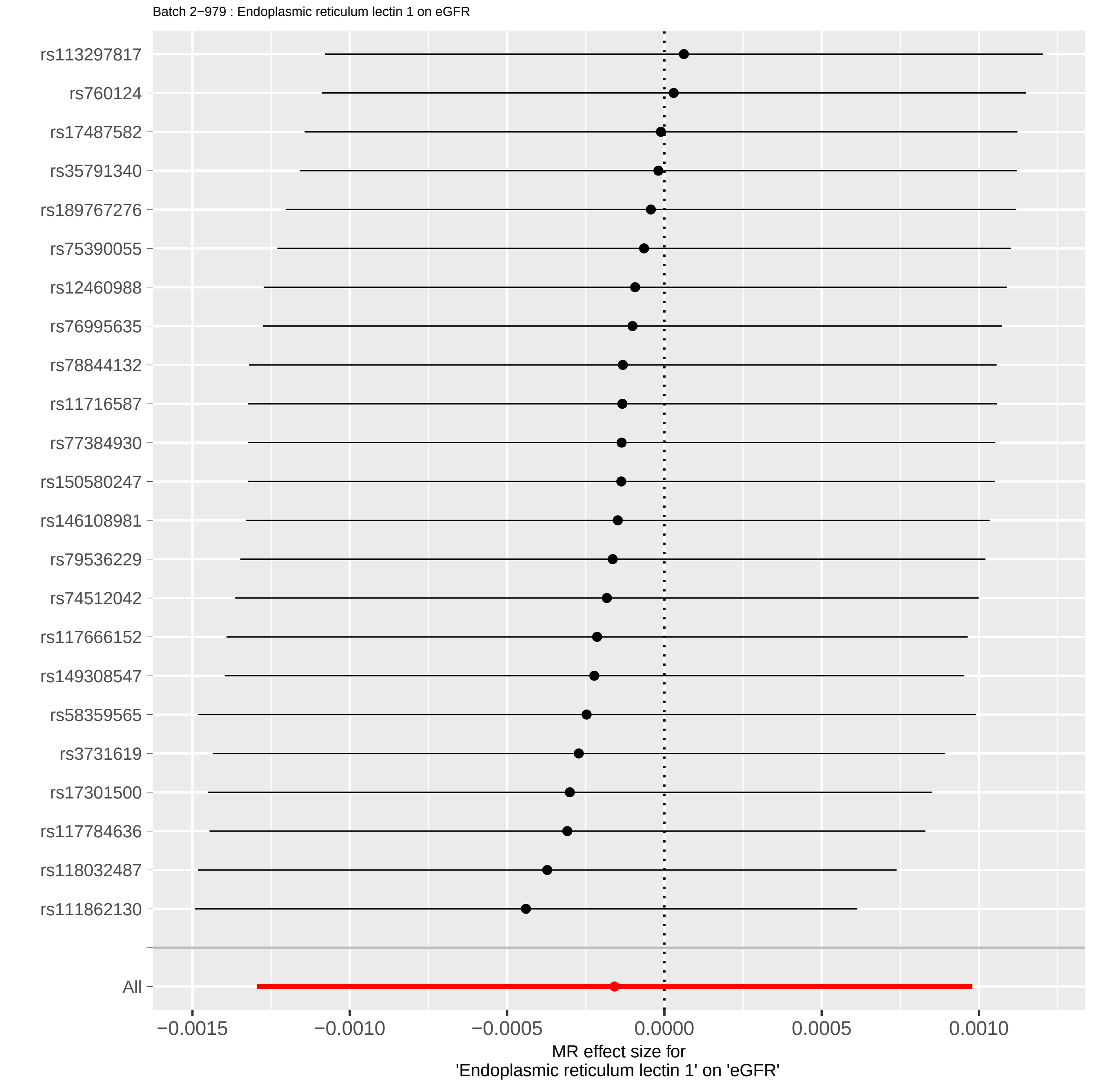

Batch 2−979 : Endoplasmic reticulum lectin 1 on eGFR
rs113297817
rs760124
rs17487582
rs35791340
rs189767276
rs75390055
rs12460988
rs76995635
rs78844132
rs11716587
rs77384930
rs150580247
rs146108981
rs79536229
rs74512042
rs117666152
rs149308547
rs58359565
rs3731619
rs17301500
rs117784636
rs118032487
rs111862130
All
−0.0015
−0.0010
−0.0005
0.0000
0.0005
0.0010
MR effect size for
'Endoplasmic reticulum lectin 1' on 'eGFR'

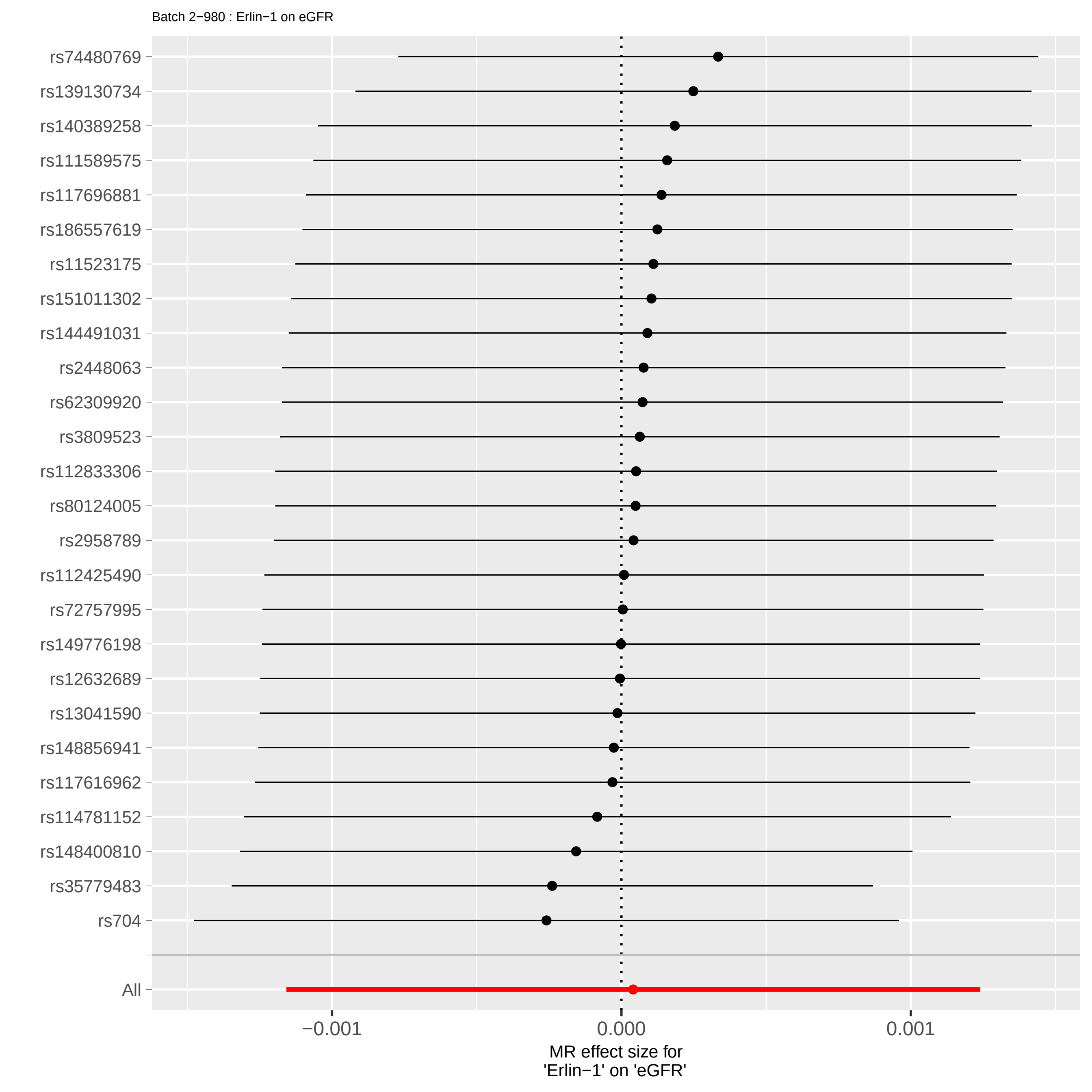

Batch 2−980 : Erlin−1 on eGFR
rs74480769
rs139130734
rs140389258
rs111589575
rs117696881
rs186557619
rs11523175
rs151011302
rs144491031
rs2448063
rs62309920
rs3809523
rs112833306
rs80124005
rs2958789
rs112425490
rs72757995
rs149776198
rs12632689
rs13041590
rs148856941
rs117616962
rs114781152
rs148400810
rs35779483
rs704
All
−0.001
0.000
0.001
MR effect size for
'Erlin−1' on 'eGFR'

Batch 2−981 : Erythroid membrane−associated protein on eGFR

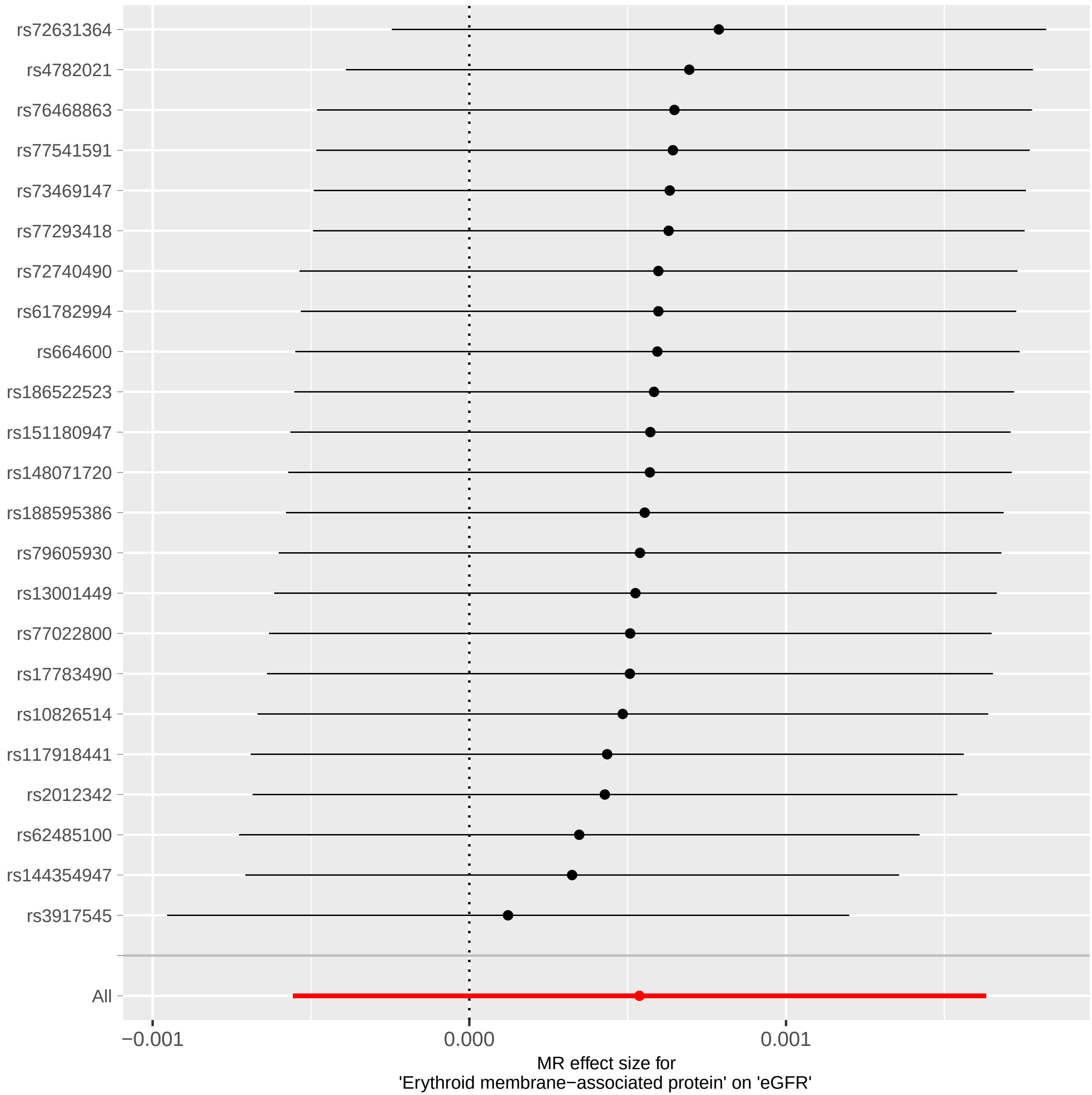

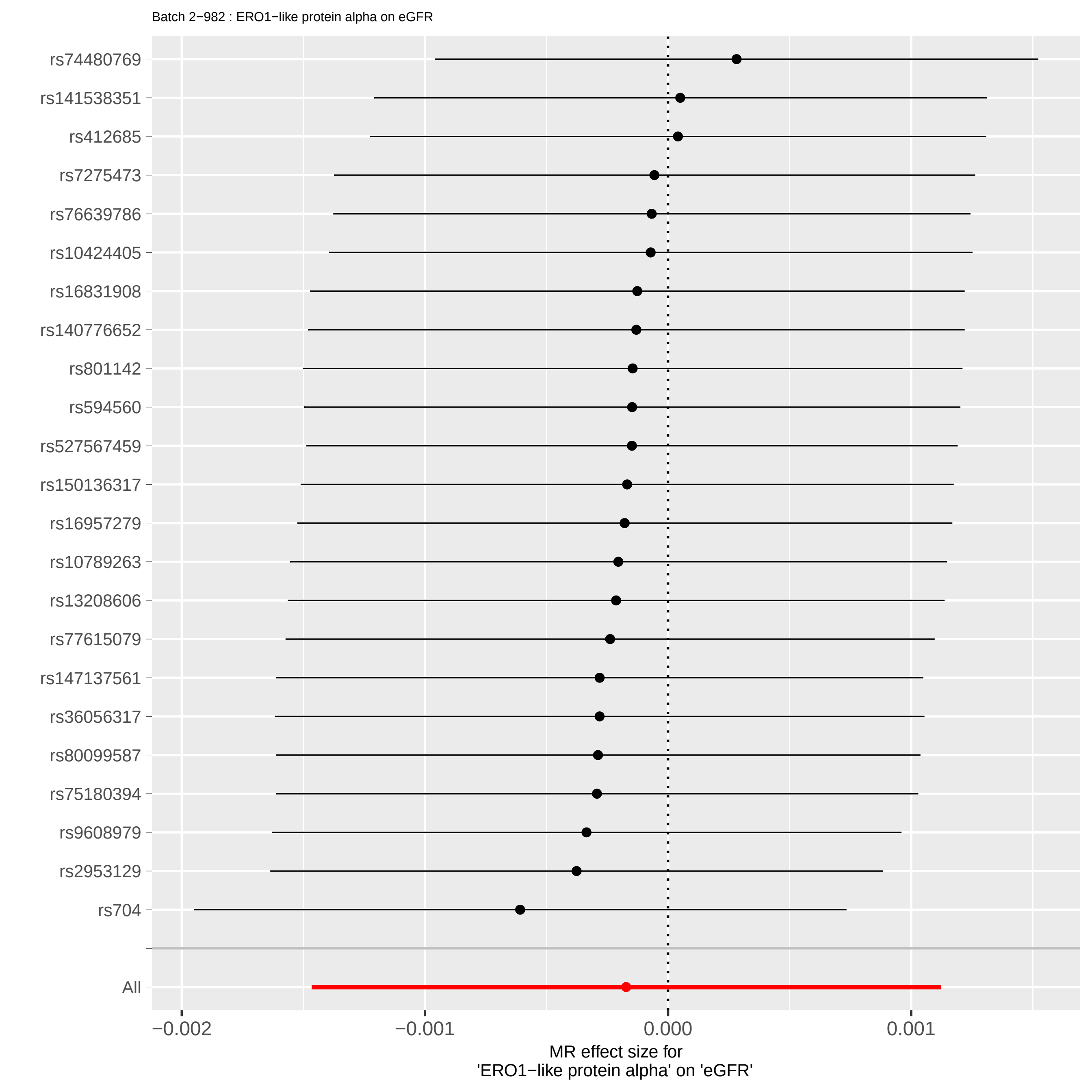

Batch 2−982 : ERO1−like protein alpha on eGFR
rs74480769
rs141538351
rs412685
rs7275473
rs76639786
rs10424405
rs16831908
rs140776652
rs801142
rs594560
rs527567459
rs150136317
rs16957279
rs10789263
rs13208606
rs77615079
rs147137561
rs36056317
rs80099587
rs75180394
rs9608979
rs2953129
rs704
All
−0.002
−0.001
0.000
0.001
MR effect size for
'ERO1−like protein alpha' on 'eGFR'

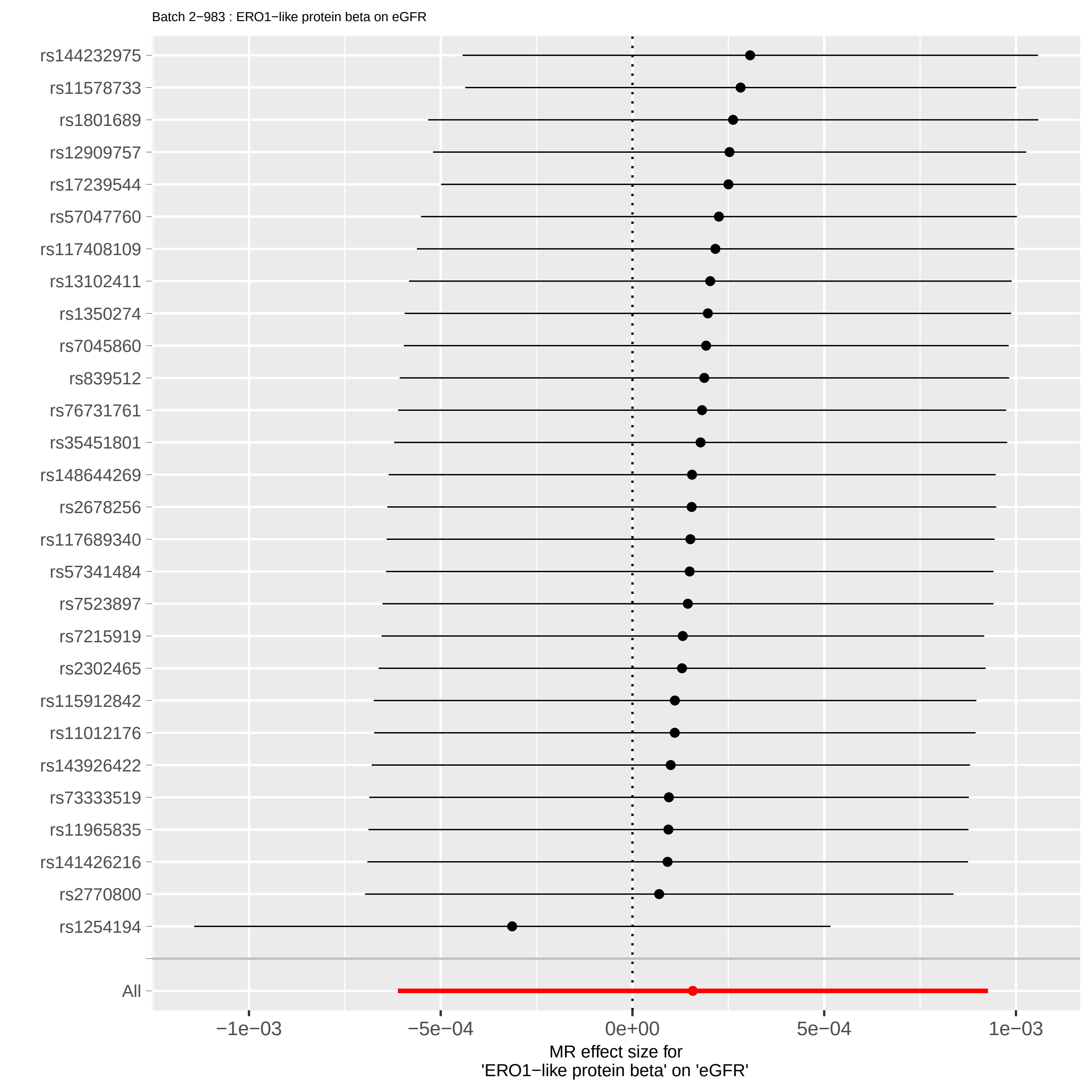

Batch 2−983 : ERO1−like protein beta on eGFR
rs144232975
rs11578733
rs1801689
rs12909757
rs17239544
rs57047760
rs117408109
rs13102411
rs1350274
rs7045860
rs839512
rs76731761
rs35451801
rs148644269
rs2678256
rs117689340
rs57341484
rs7523897
rs7215919
rs2302465
rs115912842
rs11012176
rs143926422
rs73333519
rs11965835
rs141426216
rs2770800
rs1254194
All
−1e−03
−5e−04
0e+00
5e−04
1e−03
MR effect size for
'ERO1−like protein beta' on 'eGFR'

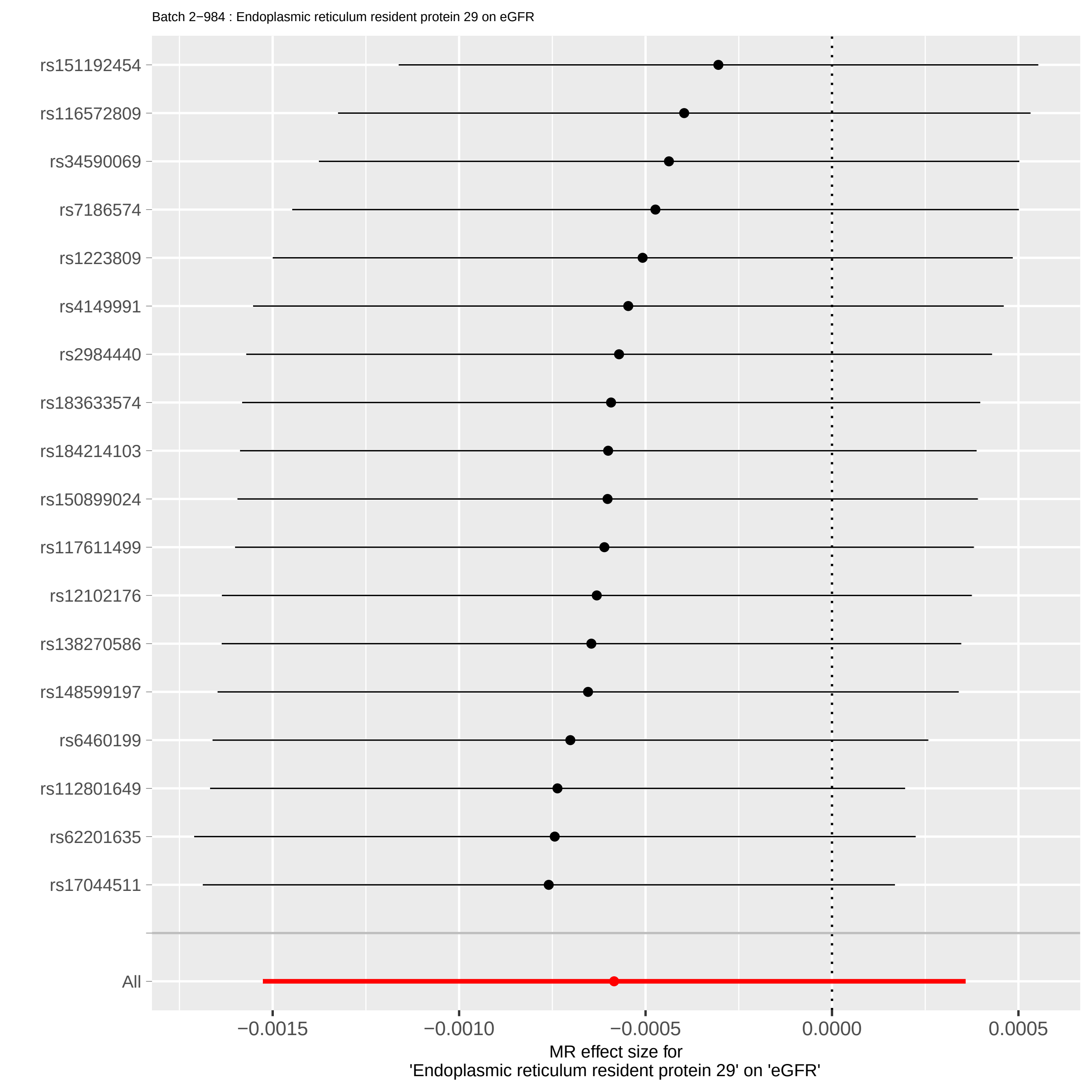

Batch 2−984 : Endoplasmic reticulum resident protein 29 on eGFR
rs151192454
rs116572809
rs34590069
rs7186574
rs1223809
rs4149991
rs2984440
rs183633574
rs184214103
rs150899024
rs117611499
rs12102176
rs138270586
rs148599197
rs6460199
rs112801649
rs62201635
rs17044511
All
−0.0015
−0.0010
−0.0005
0.0000
0.0005
MR effect size for
'Endoplasmic reticulum resident protein 29' on 'eGFR'

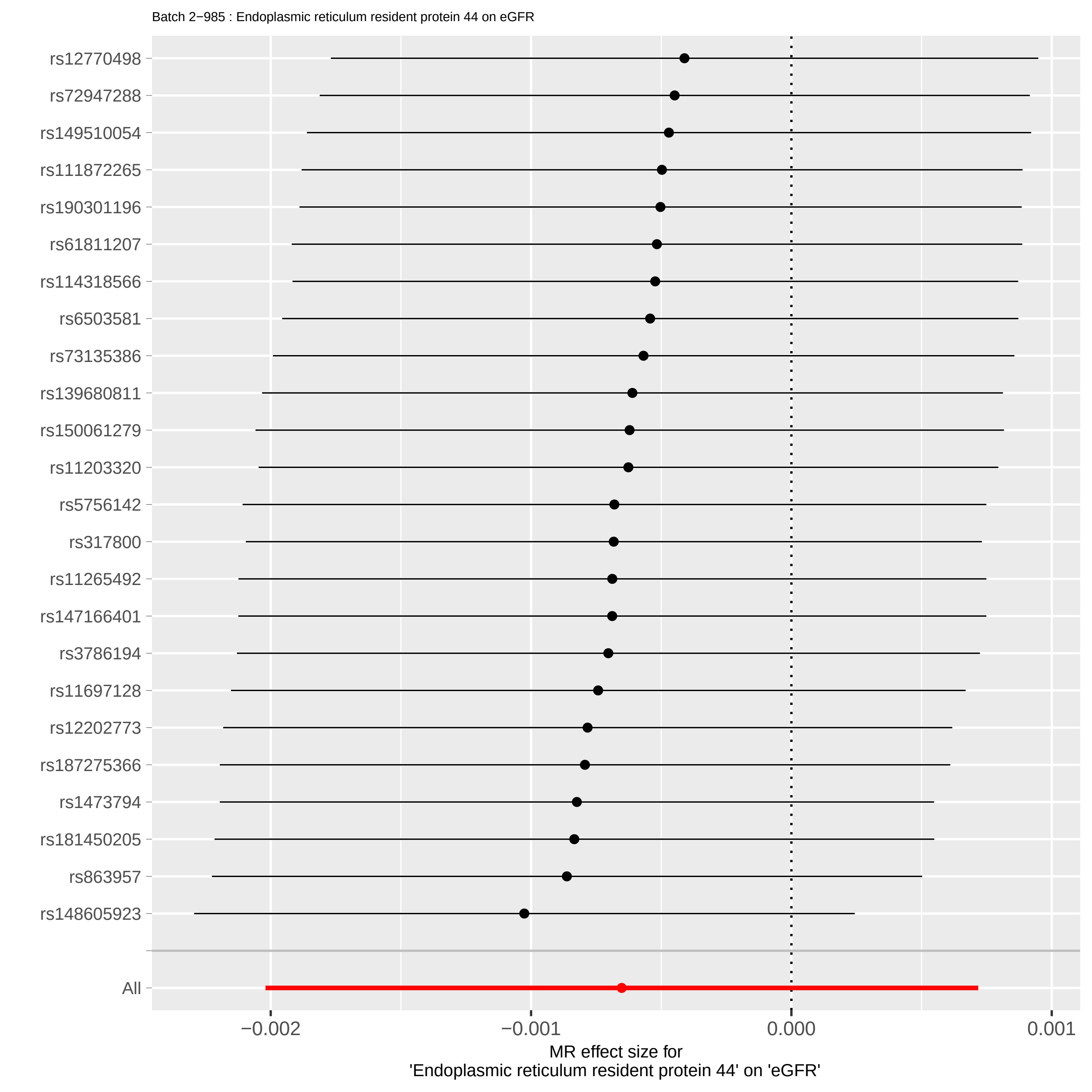

Batch 2−985 : Endoplasmic reticulum resident protein 44 on eGFR
rs12770498
rs72947288
rs149510054
rs111872265
rs190301196
rs61811207
rs114318566
rs6503581
rs73135386
rs139680811
rs150061279
rs11203320
rs5756142
rs317800
rs11265492
rs147166401
rs3786194
rs11697128
rs12202773
rs187275366
rs1473794
rs181450205
rs863957
rs148605923
All
−0.002
−0.001
0.000
0.001
MR effect size for
'Endoplasmic reticulum resident protein 44' on 'eGFR'

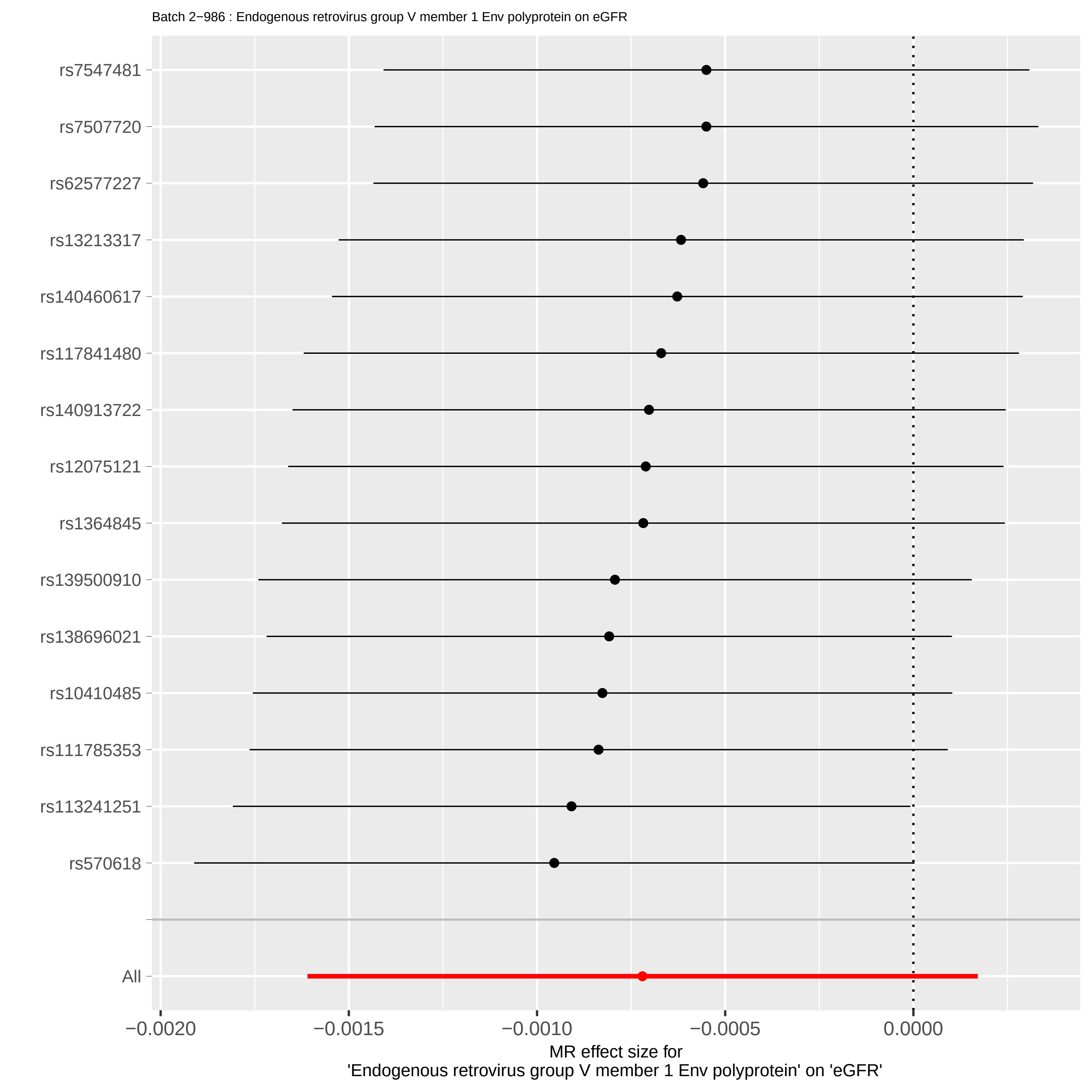

Batch 2−986 : Endogenous retrovirus group V member 1 Env polyprotein on eGFR
rs7547481
rs7507720
rs62577227
rs13213317
rs140460617
rs117841480
rs140913722
rs12075121
rs1364845
rs139500910
rs138696021
rs10410485
rs111785353
rs113241251
rs570618
All
−0.0020
−0.0015
−0.0010
−0.0005
0.0000
MR effect size for
'Endogenous retrovirus group V member 1 Env polyprotein' on 'eGFR'

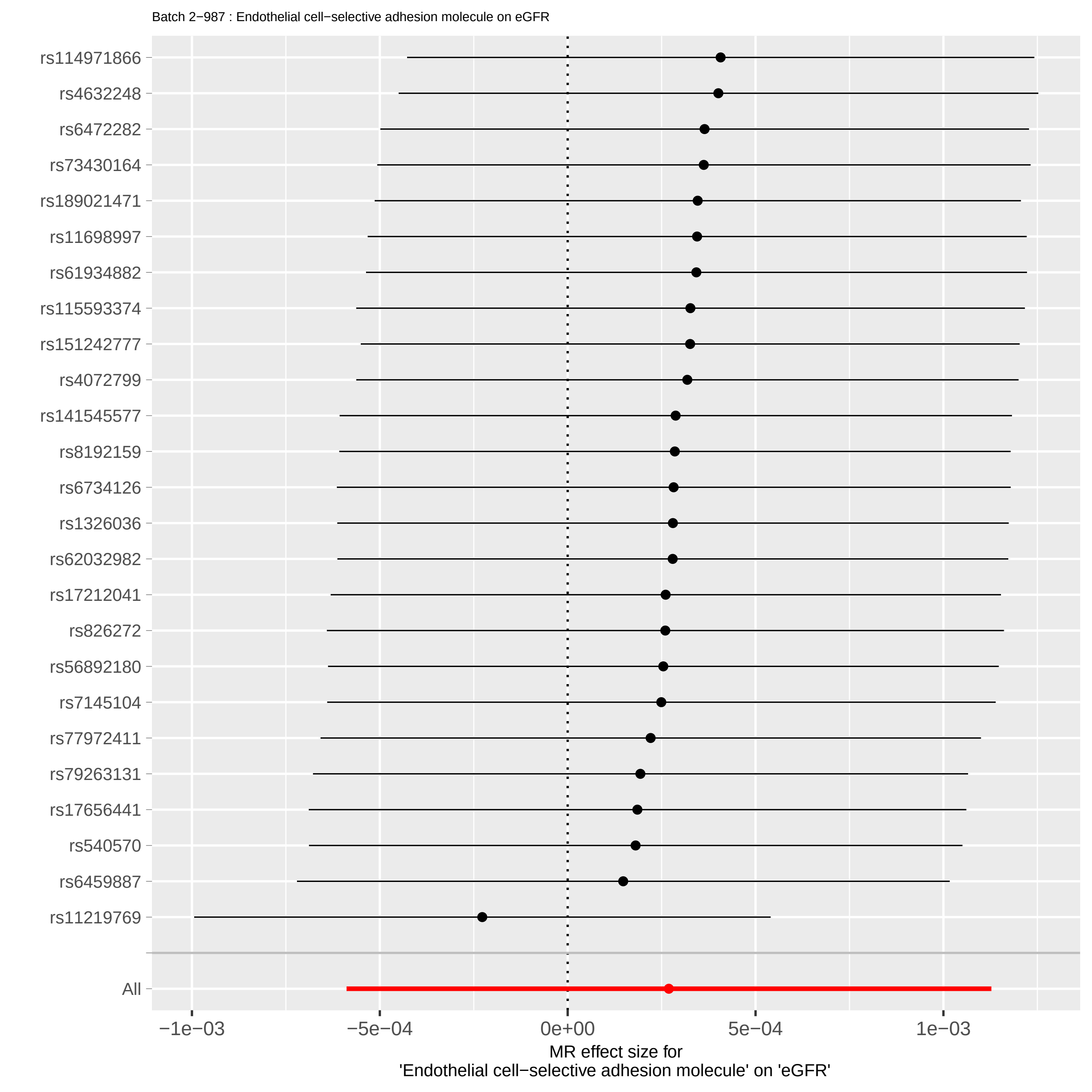
Batch 2−987 : Endothelial cell−selective adhesion molecule on eGFR
rs114971866
rs4632248
rs6472282
rs73430164
rs189021471
rs11698997
rs61934882
rs115593374
rs151242777
rs4072799
rs141545577
rs8192159
rs6734126
rs1326036
rs62032982
rs17212041
rs826272
rs56892180
rs7145104
rs77972411
rs79263131
rs17656441
rs540570
rs6459887
rs11219769
All
−1e−03
−5e−04
0e+00
5e−04
1e−03
MR effect size for
'Endothelial cell−selective adhesion molecule' on 'eGFR'

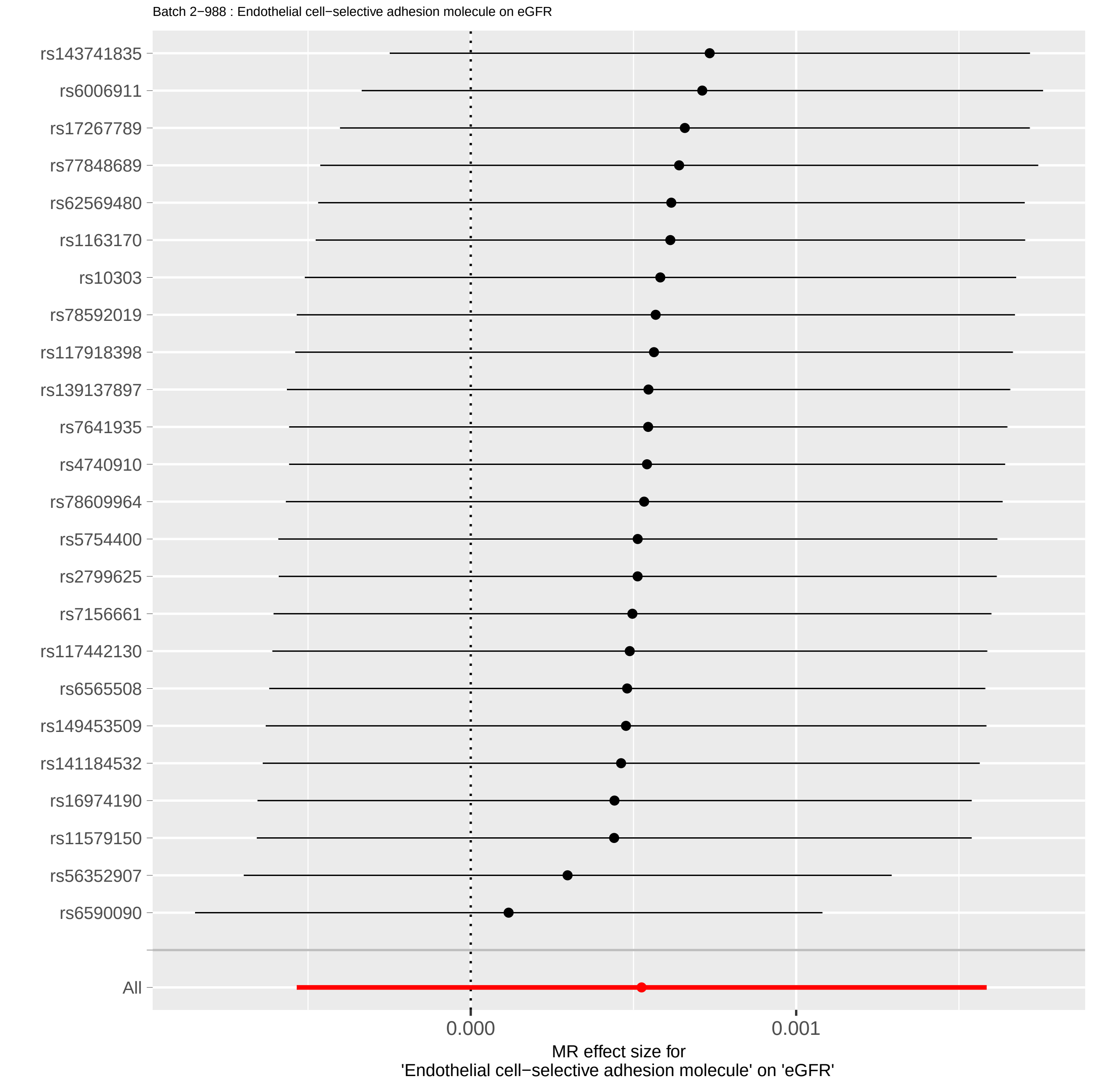

Batch 2−988 : Endothelial cell−selective adhesion molecule on eGFR
rs143741835
rs6006911
rs17267789
rs77848689
rs62569480
rs1163170
rs10303
rs78592019
rs117918398
rs139137897
rs7641935
rs4740910
rs78609964
rs5754400
rs2799625
rs7156661
rs117442130
rs6565508
rs149453509
rs141184532
rs16974190
rs11579150
rs56352907
rs6590090
All
0.000
0.001
MR effect size for
'Endothelial cell−selective adhesion molecule' on 'eGFR'

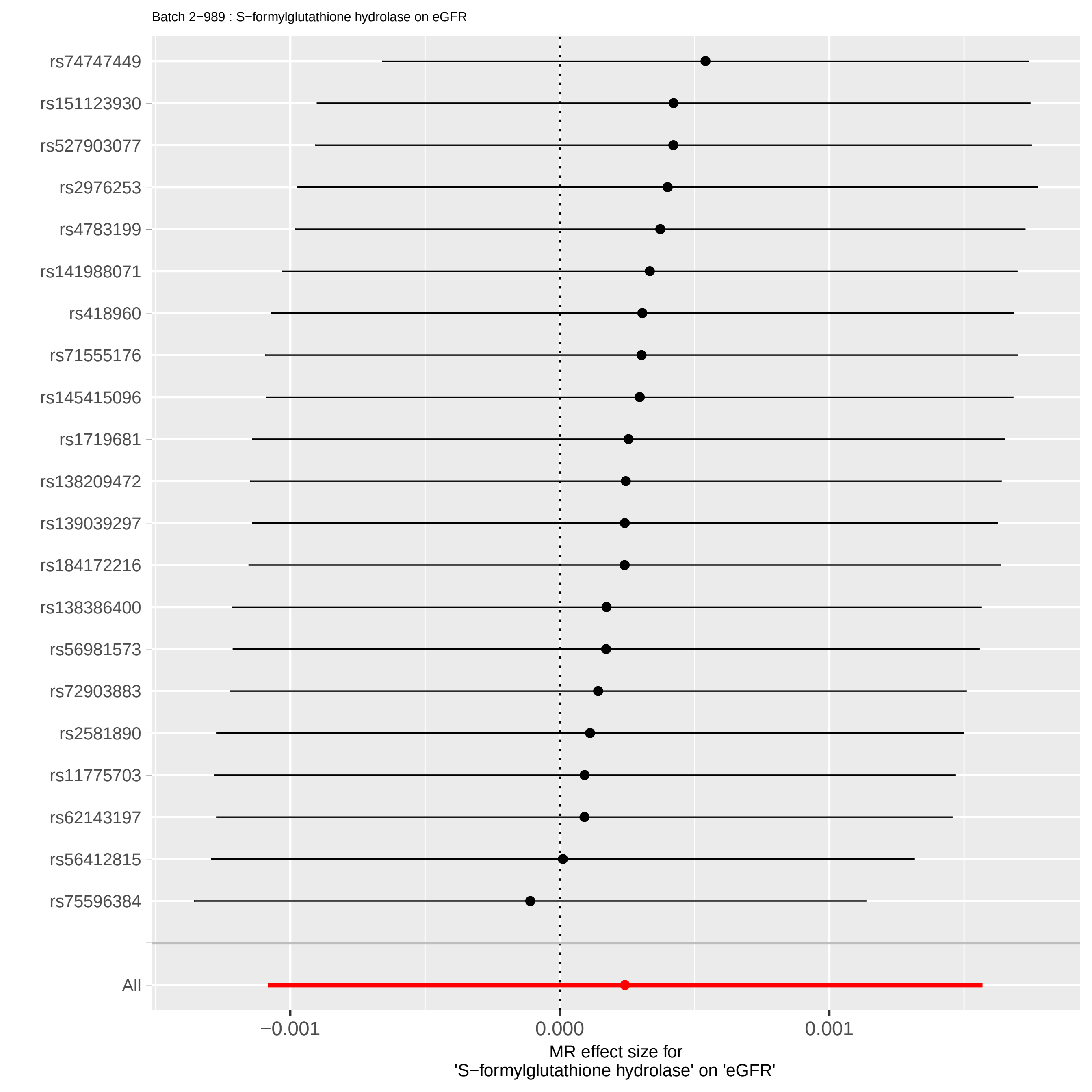

Batch 2−989 : S−formylglutathione hydrolase on eGFR
rs74747449
rs151123930
rs527903077
rs2976253
rs4783199
rs141988071
rs418960
rs71555176
rs145415096
rs1719681
rs138209472
rs139039297
rs184172216
rs138386400
rs56981573
rs72903883
rs2581890
rs11775703
rs62143197
rs56412815
rs75596384
All
−0.001
0.000
0.001
MR effect size for
'S−formylglutathione hydrolase' on 'eGFR'

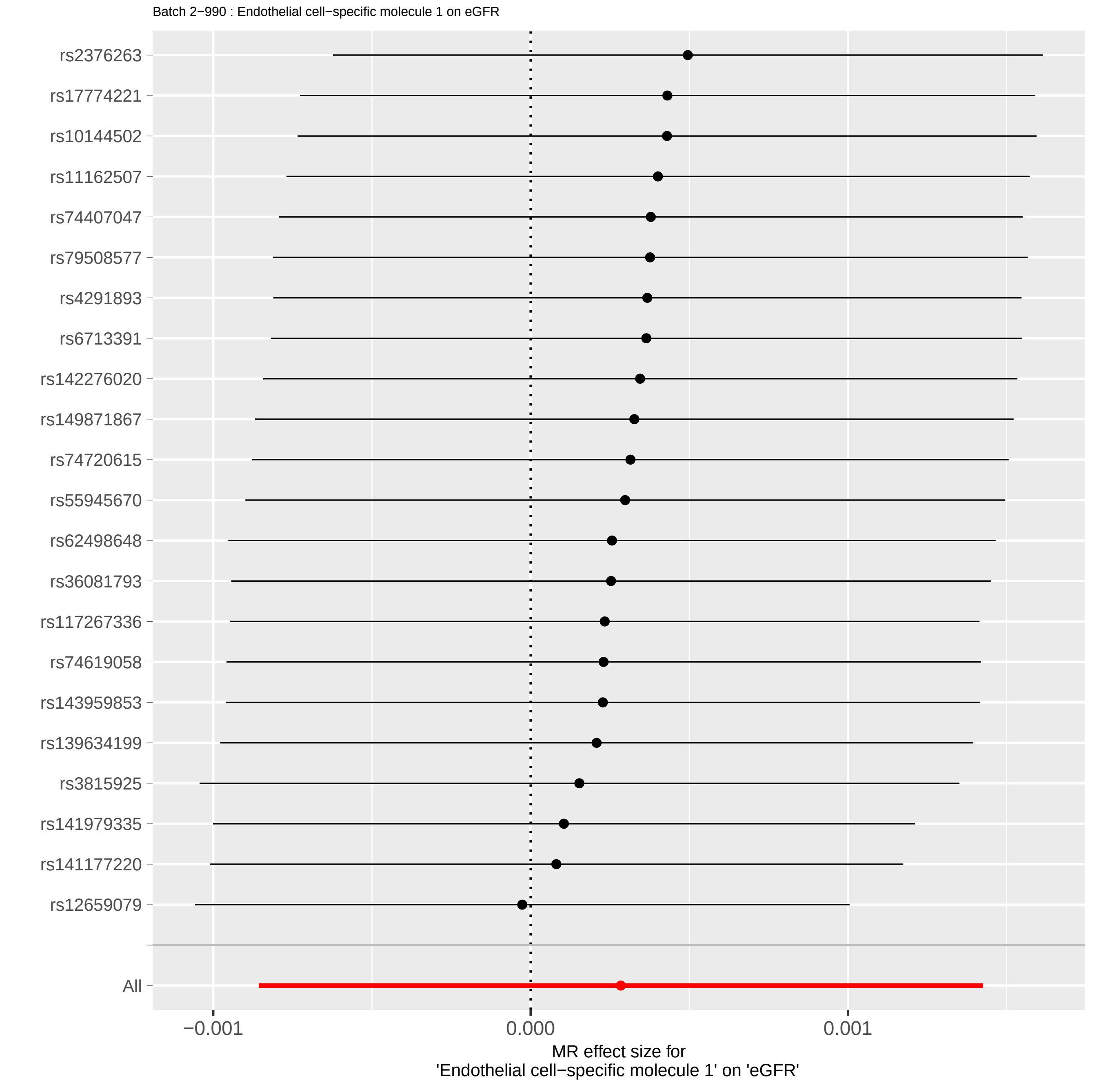

Batch 2−990 : Endothelial cell−specific molecule 1 on eGFR
rs2376263
rs17774221
rs10144502
rs11162507
rs74407047
rs79508577
rs4291893
rs6713391
rs142276020
rs149871867
rs74720615
rs55945670
rs62498648
rs36081793
rs117267336
rs74619058
rs143959853
rs139634199
rs3815925
rs141979335
rs141177220
rs12659079
All
−0.001
0.000
0.001
MR effect size for
'Endothelial cell−specific molecule 1' on 'eGFR'

Batch 2−991 : Estrogen receptor on eGFR

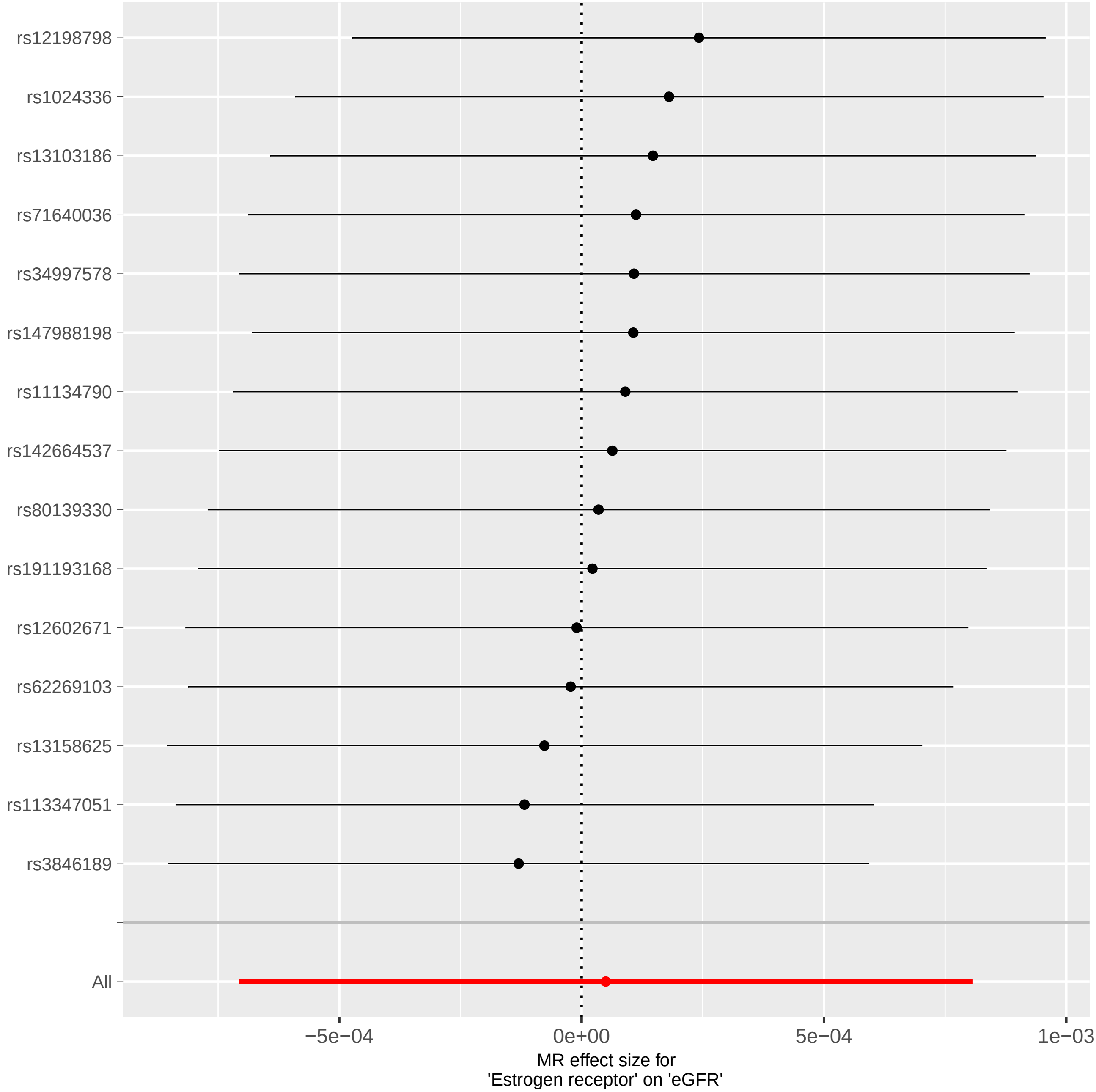

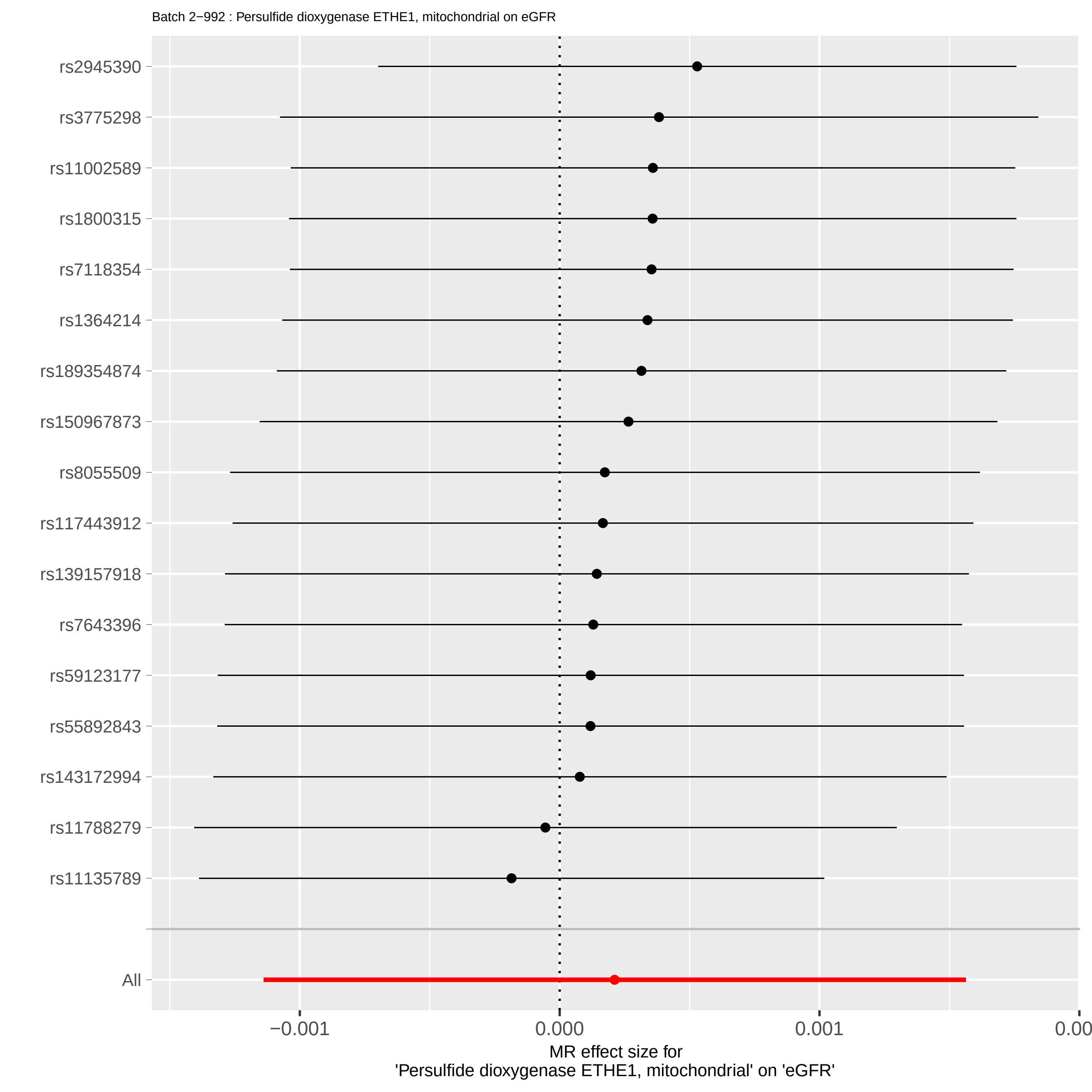
Batch 2−992 : Persulfide dioxygenase ETHE1, mitochondrial on eGFR
rs2945390
rs3775298
rs11002589
rs1800315
rs7118354
rs1364214
rs189354874
rs150967873
rs8055509
rs117443912
rs139157918
rs7643396
rs59123177
rs55892843
rs143172994
rs11788279
rs11135789
All
−0.001
0.000
0.001
0.00
MR effect size for
'Persulfide dioxygenase ETHE1, mitochondrial' on 'eGFR'

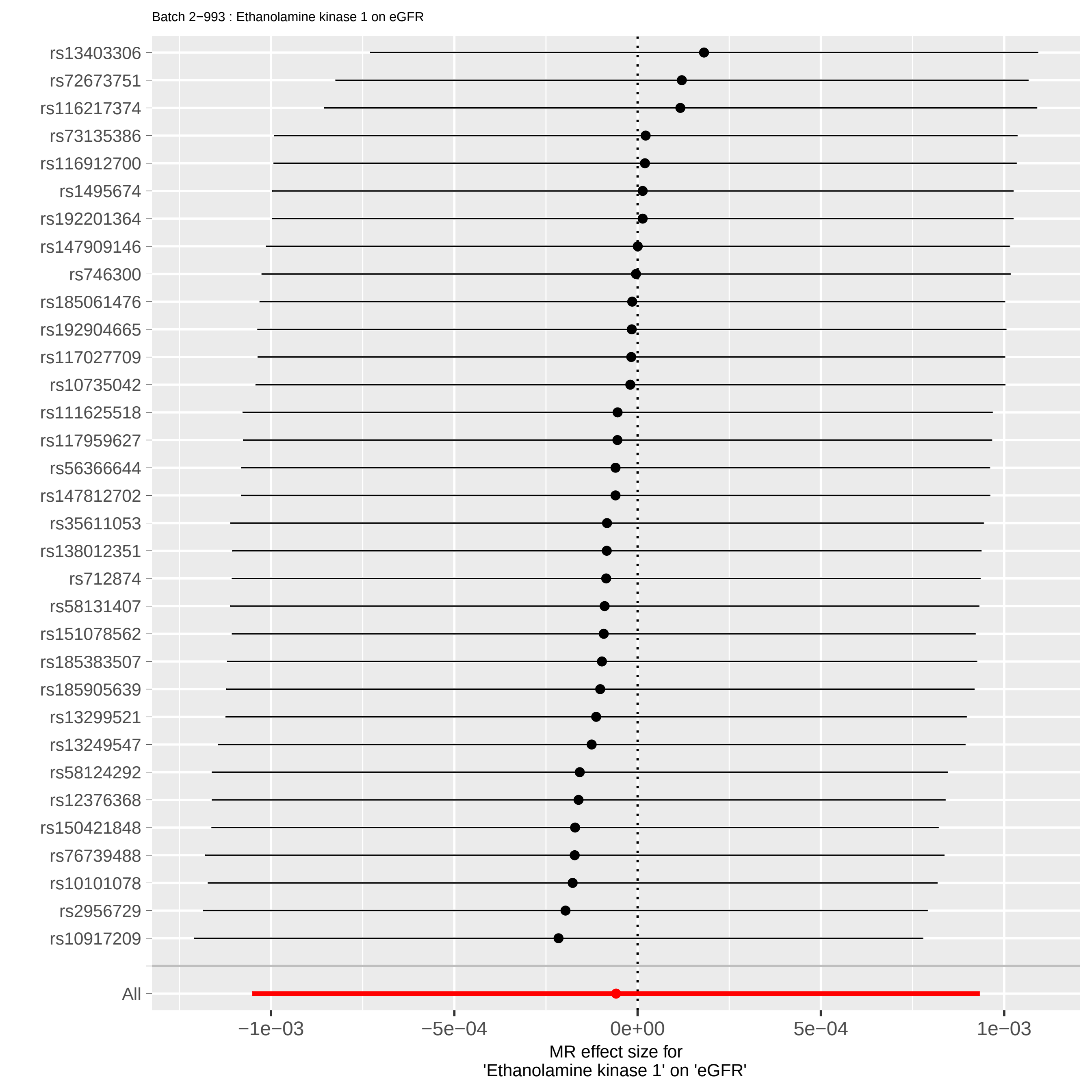

Batch 2−993 : Ethanolamine kinase 1 on eGFR
rs13403306
rs72673751
rs116217374
rs73135386
rs116912700
rs1495674
rs192201364
rs147909146
rs746300
rs185061476
rs192904665
rs117027709
rs10735042
rs111625518
rs117959627
rs56366644
rs147812702
rs35611053
rs138012351
rs712874
rs58131407
rs151078562
rs185383507
rs185905639
rs13299521
rs13249547
rs58124292
rs12376368
rs150421848
rs76739488
rs10101078
rs2956729
rs10917209
All
−1e−03
−5e−04
0e+00
5e−04
1e−03
MR effect size for
'Ethanolamine kinase 1' on 'eGFR'

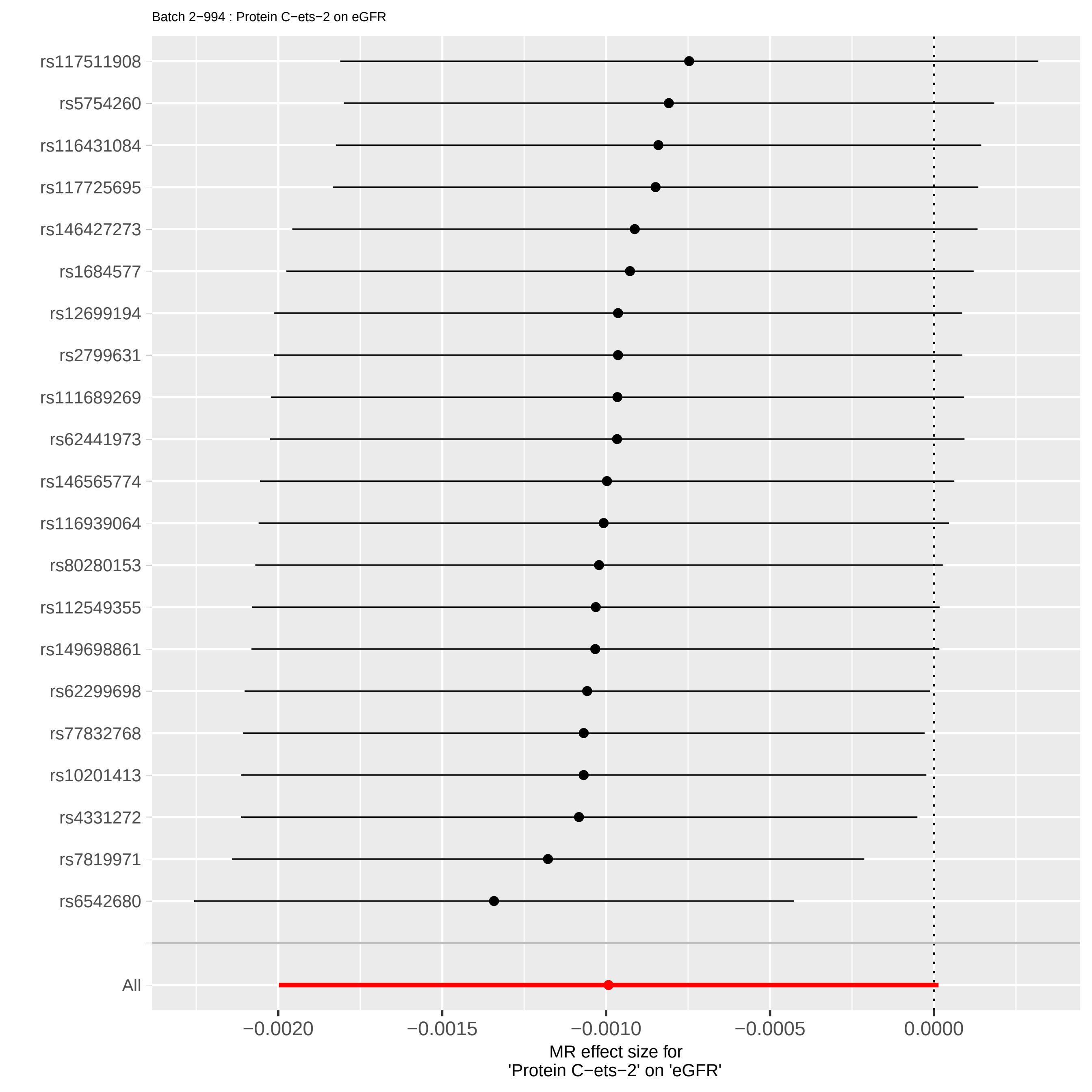

Batch 2−994 : Protein C−ets−2 on eGFR
rs117511908
rs5754260
rs116431084
rs117725695
rs146427273
rs1684577
rs12699194
rs2799631
rs111689269
rs62441973
rs146565774
rs116939064
rs80280153
rs112549355
rs149698861
rs62299698
rs77832768
rs10201413
rs4331272
rs7819971
rs6542680
All
−0.0020
−0.0015
−0.0010
−0.0005
0.0000
MR effect size for
'Protein C−ets−2' on 'eGFR'

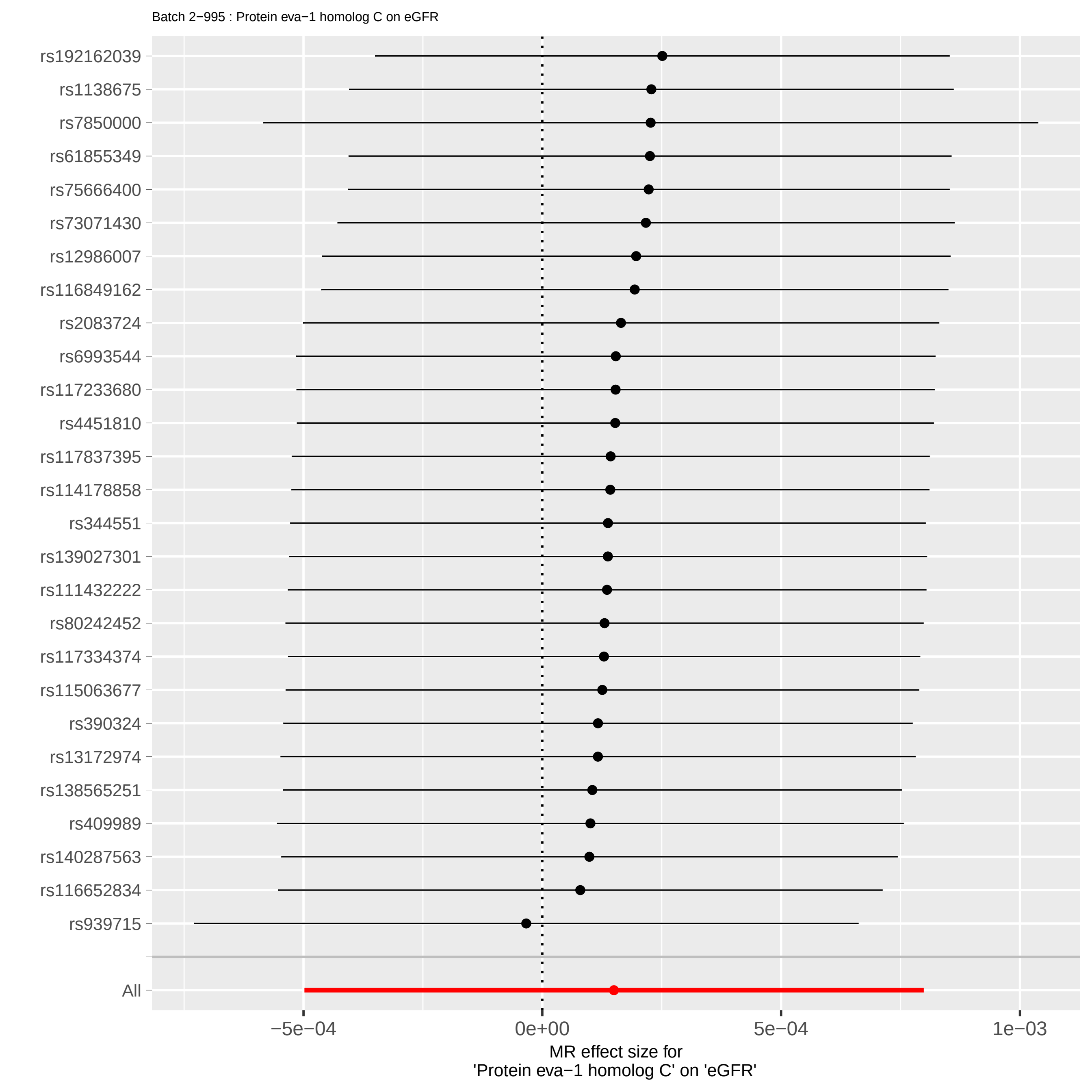

Batch 2−995 : Protein eva−1 homolog C on eGFR
rs192162039
rs1138675
rs7850000
rs61855349
rs75666400
rs73071430
rs12986007
rs116849162
rs2083724
rs6993544
rs117233680
rs4451810
rs117837395
rs114178858
rs344551
rs139027301
rs111432222
rs80242452
rs117334374
rs115063677
rs390324
rs13172974
rs138565251
rs409989
rs140287563
rs116652834
rs939715
All
−5e−04
0e+00
5e−04
1e−03
MR effect size for
'Protein eva−1 homolog C' on 'eGFR'

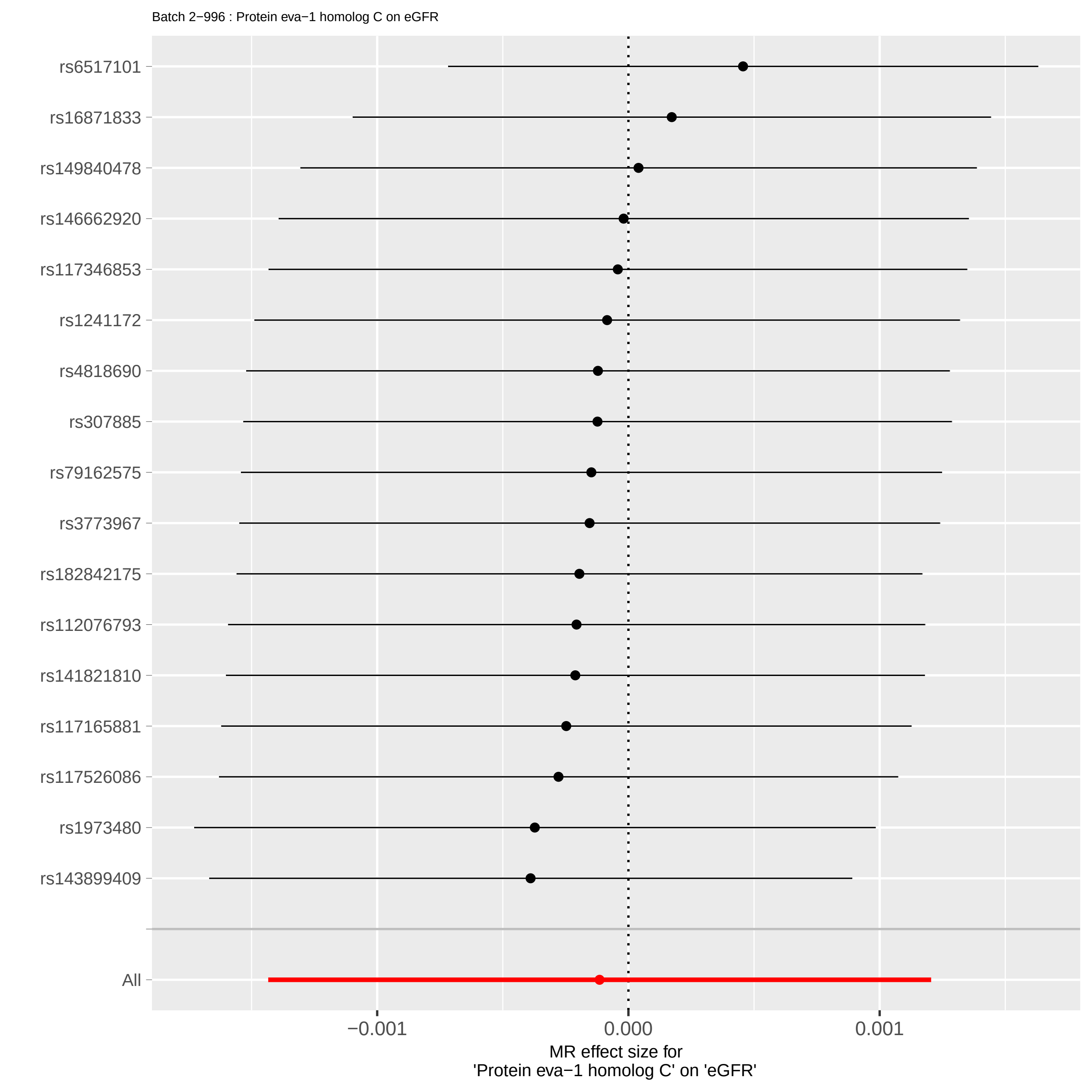

Batch 2−996 : Protein eva−1 homolog C on eGFR
rs6517101
rs16871833
rs149840478
rs146662920
rs117346853
rs1241172
rs4818690
rs307885
rs79162575
rs3773967
rs182842175
rs112076793
rs141821810
rs117165881
rs117526086
rs1973480
rs143899409
All
−0.001
0.000
0.001
MR effect size for
'Protein eva−1 homolog C' on 'eGFR'

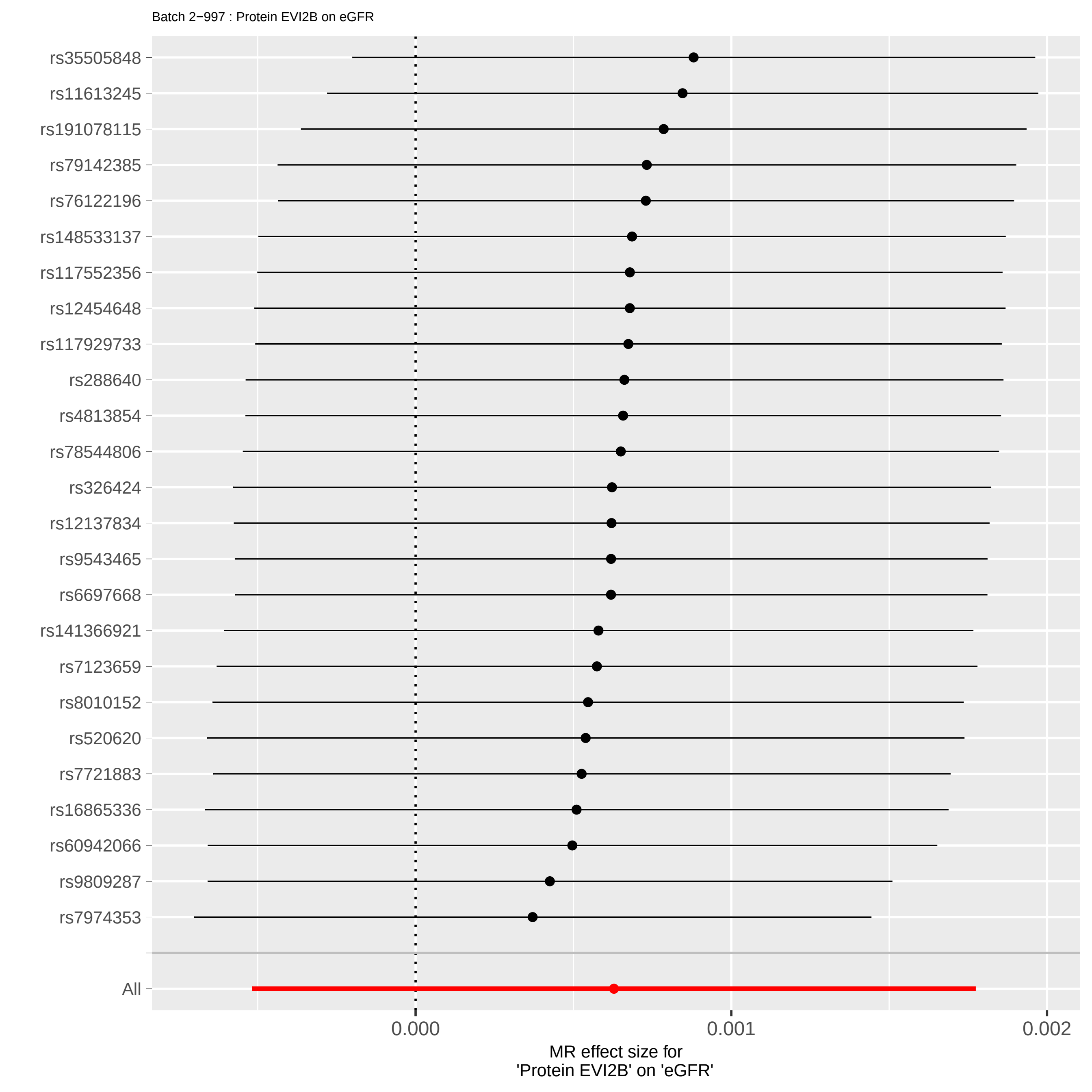
Batch 2−997 : Protein EVI2B on eGFR
rs35505848
rs11613245
rs191078115
rs79142385
rs76122196
rs148533137
rs117552356
rs12454648
rs117929733
rs288640
rs4813854
rs78544806
rs326424
rs12137834
rs9543465
rs6697668
rs141366921
rs7123659
rs8010152
rs520620
rs7721883
rs16865336
rs60942066
rs9809287
rs7974353
All
0.000
0.001
0.002
MR effect size for
'Protein EVI2B' on 'eGFR'

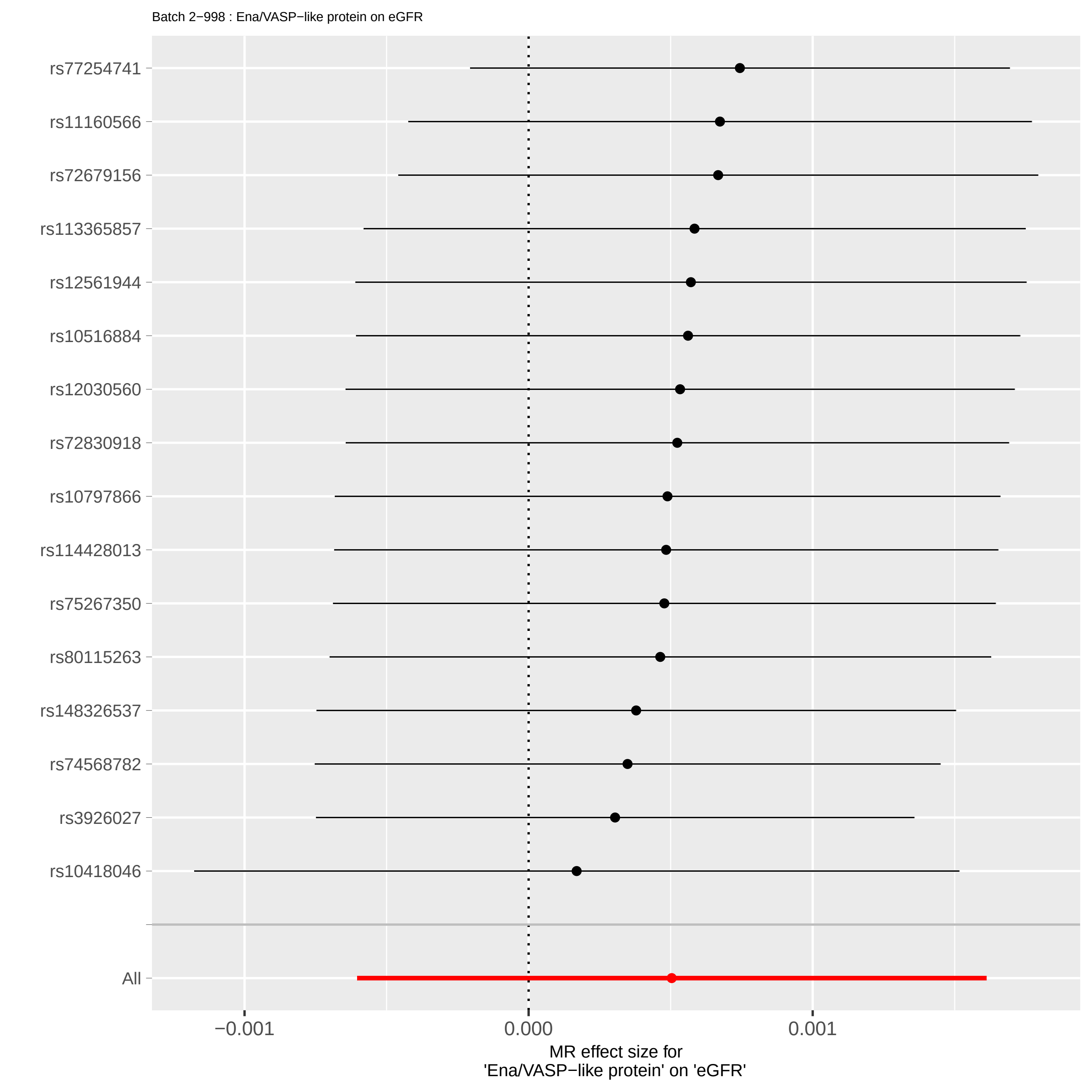

Batch 2−998 : Ena/VASP−like protein on eGFR
rs77254741
rs11160566
rs72679156
rs113365857
rs12561944
rs10516884
rs12030560
rs72830918
rs10797866
rs114428013
rs75267350
rs80115263
rs148326537
rs74568782
rs3926027
rs10418046
All
−0.001
0.000
0.001
MR effect size for
'Ena/VASP−like protein' on 'eGFR'

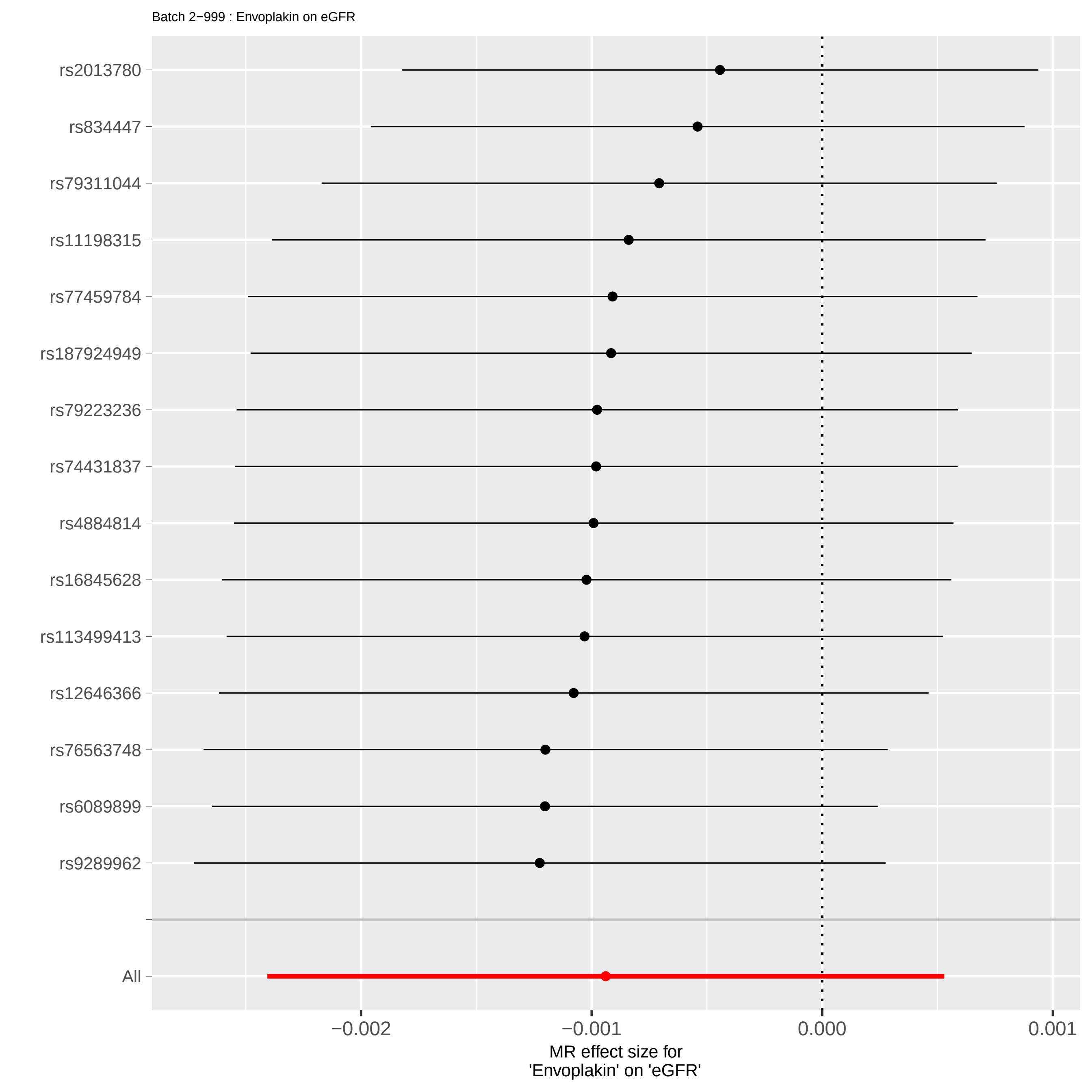

Batch 2−999 : Envoplakin on eGFR
rs2013780
rs834447
rs79311044
rs11198315
rs77459784
rs187924949
rs79223236
rs74431837
rs4884814
rs16845628
rs113499413
rs12646366
rs76563748
rs6089899
rs9289962
All
−0.002
−0.001
0.000
0.001
MR effect size for
'Envoplakin' on 'eGFR'

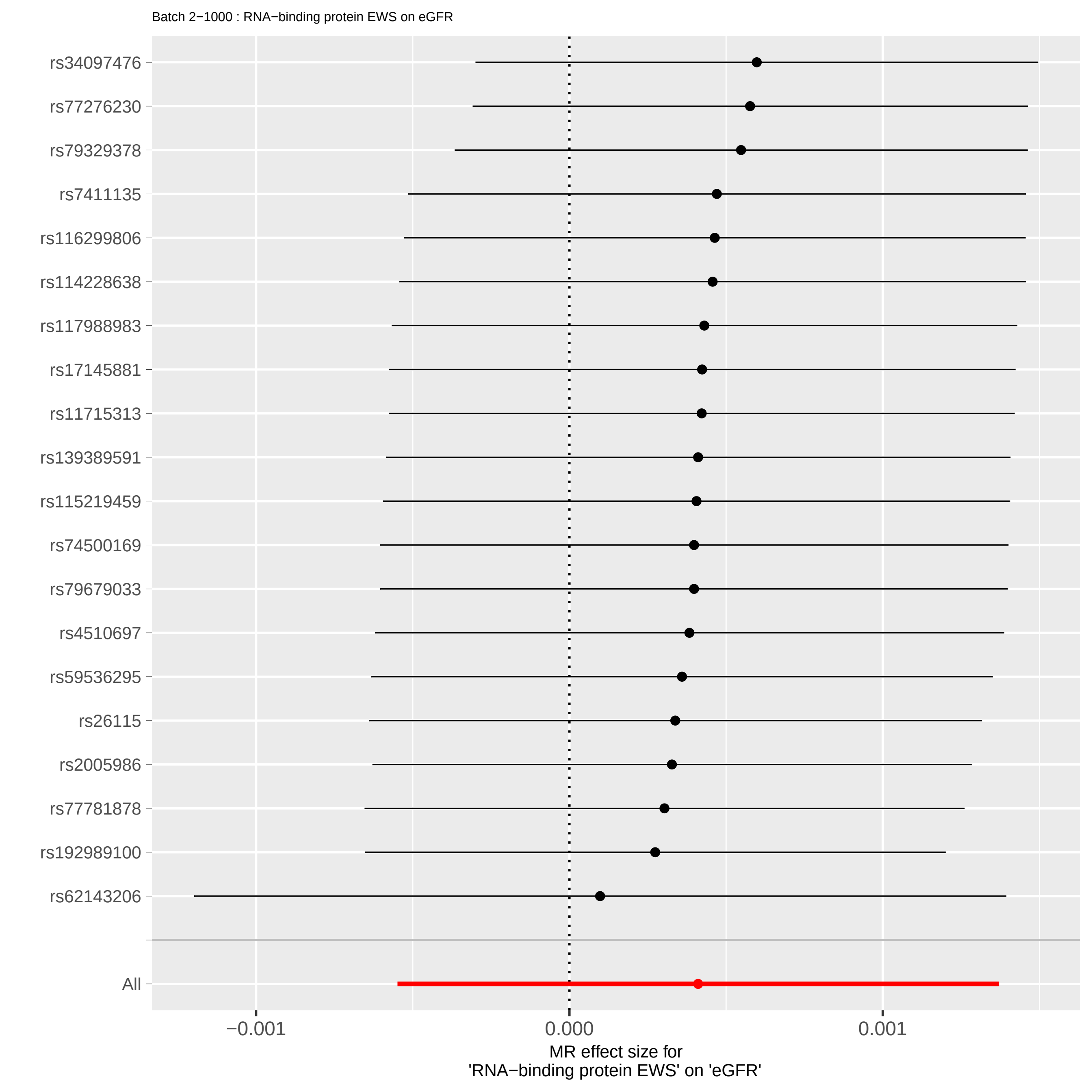

Batch 2−1000 : RNA−binding protein EWS on eGFR
rs34097476
rs77276230
rs79329378
rs7411135
rs116299806
rs114228638
rs117988983
rs17145881
rs11715313
rs139389591
rs115219459
rs74500169
rs79679033
rs4510697
rs59536295
rs26115
rs2005986
rs77781878
rs192989100
rs62143206
All
−0.001
0.000
0.001
MR effect size for
'RNA−binding protein EWS' on 'eGFR'

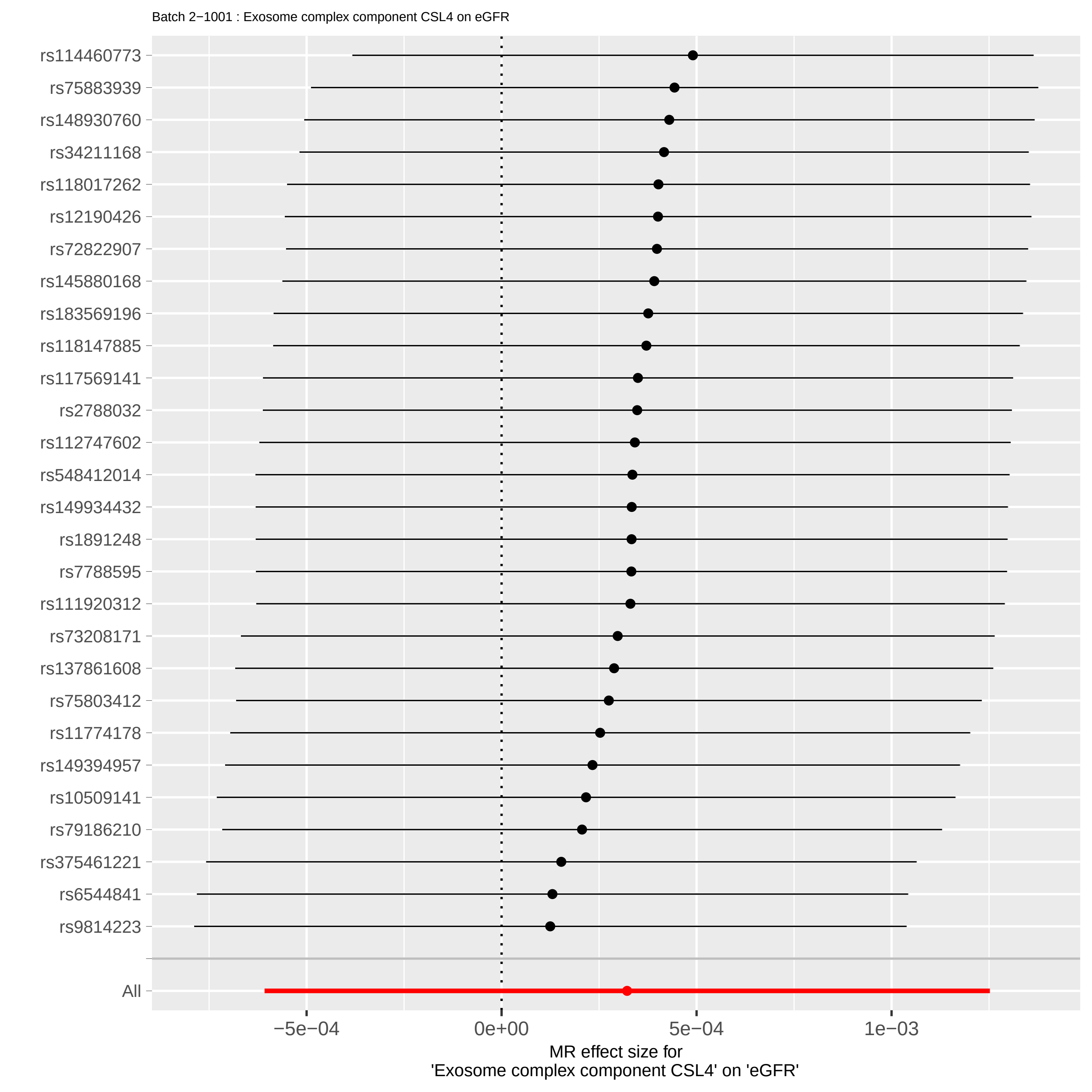

Batch 2−1001 : Exosome complex component CSL4 on eGFR
rs114460773
rs75883939
rs148930760
rs34211168
rs118017262
rs12190426
rs72822907
rs145880168
rs183569196
rs118147885
rs117569141
rs2788032
rs112747602
rs548412014
rs149934432
rs1891248
rs7788595
rs111920312
rs73208171
rs137861608
rs75803412
rs11774178
rs149394957
rs10509141
rs79186210
rs375461221
rs6544841
rs9814223
All
−5e−04
0e+00
5e−04
1e−03
MR effect size for
'Exosome complex component CSL4' on 'eGFR'

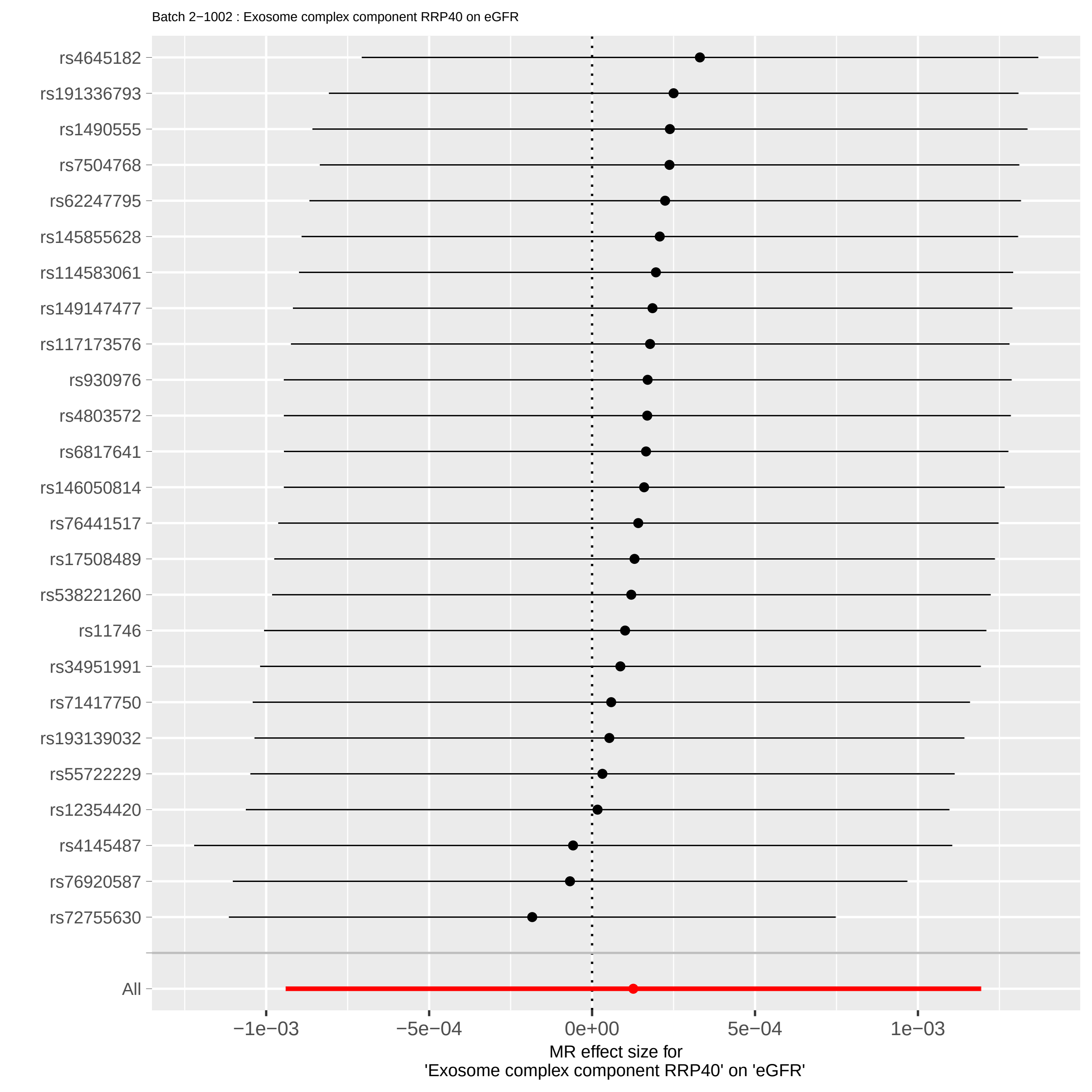

Batch 2−1002 : Exosome complex component RRP40 on eGFR
rs4645182
rs191336793
rs1490555
rs7504768
rs62247795
rs145855628
rs114583061
rs149147477
rs117173576
rs930976
rs4803572
rs6817641
rs146050814
rs76441517
rs17508489
rs538221260
rs11746
rs34951991
rs71417750
rs193139032
rs55722229
rs12354420
rs4145487
rs76920587
rs72755630
All
−1e−03
−5e−04
0e+00
5e−04
1e−03
MR effect size for
'Exosome complex component RRP40' on 'eGFR'

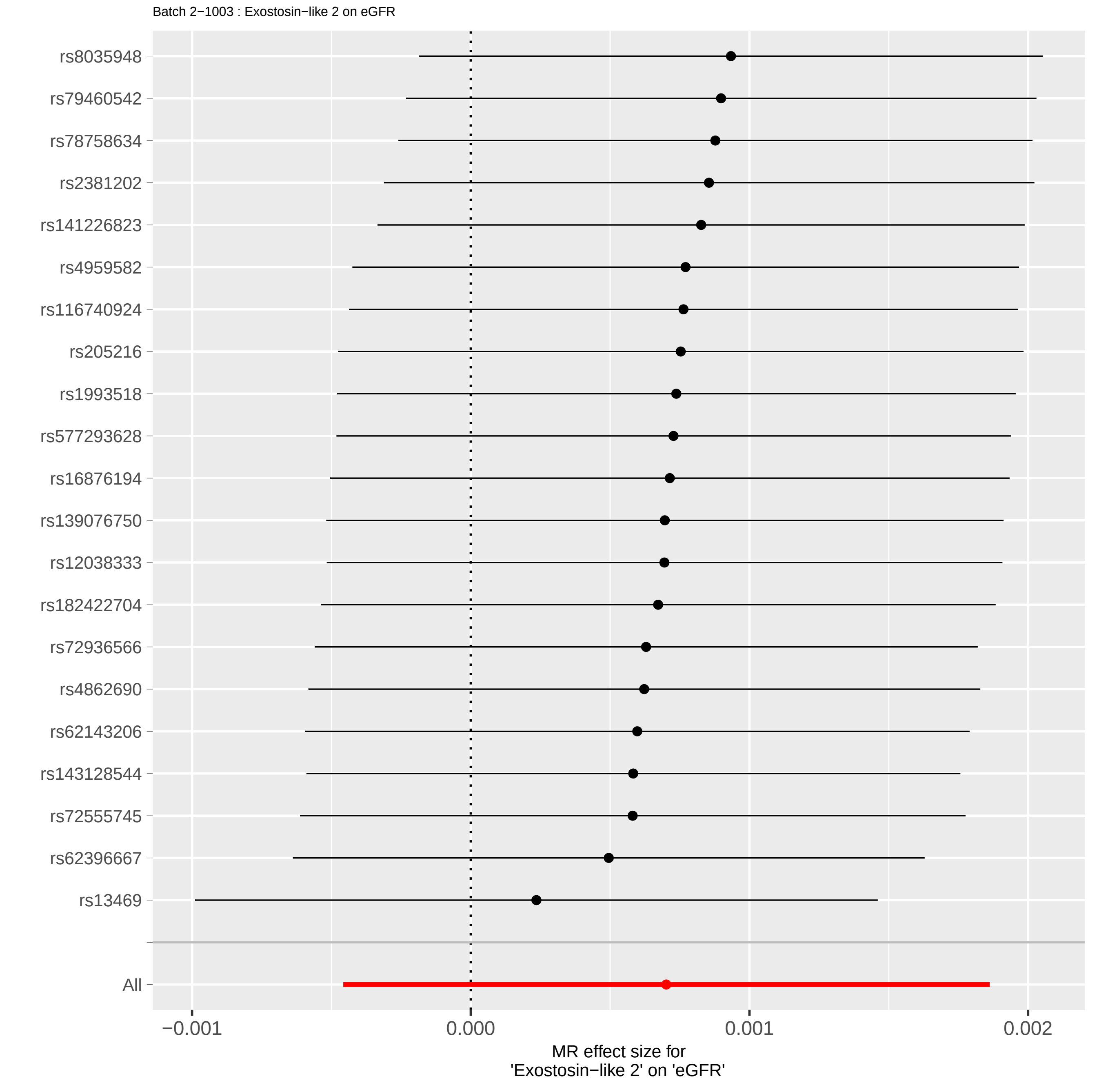

Batch 2−1003 : Exostosin−like 2 on eGFR
rs8035948
rs79460542
rs78758634
rs2381202
rs141226823
rs4959582
rs116740924
rs205216
rs1993518
rs577293628
rs16876194
rs139076750
rs12038333
rs182422704
rs72936566
rs4862690
rs62143206
rs143128544
rs72555745
rs62396667
rs13469
All
−0.001
0.000
0.001
0.002
MR effect size for
'Exostosin−like 2' on 'eGFR'

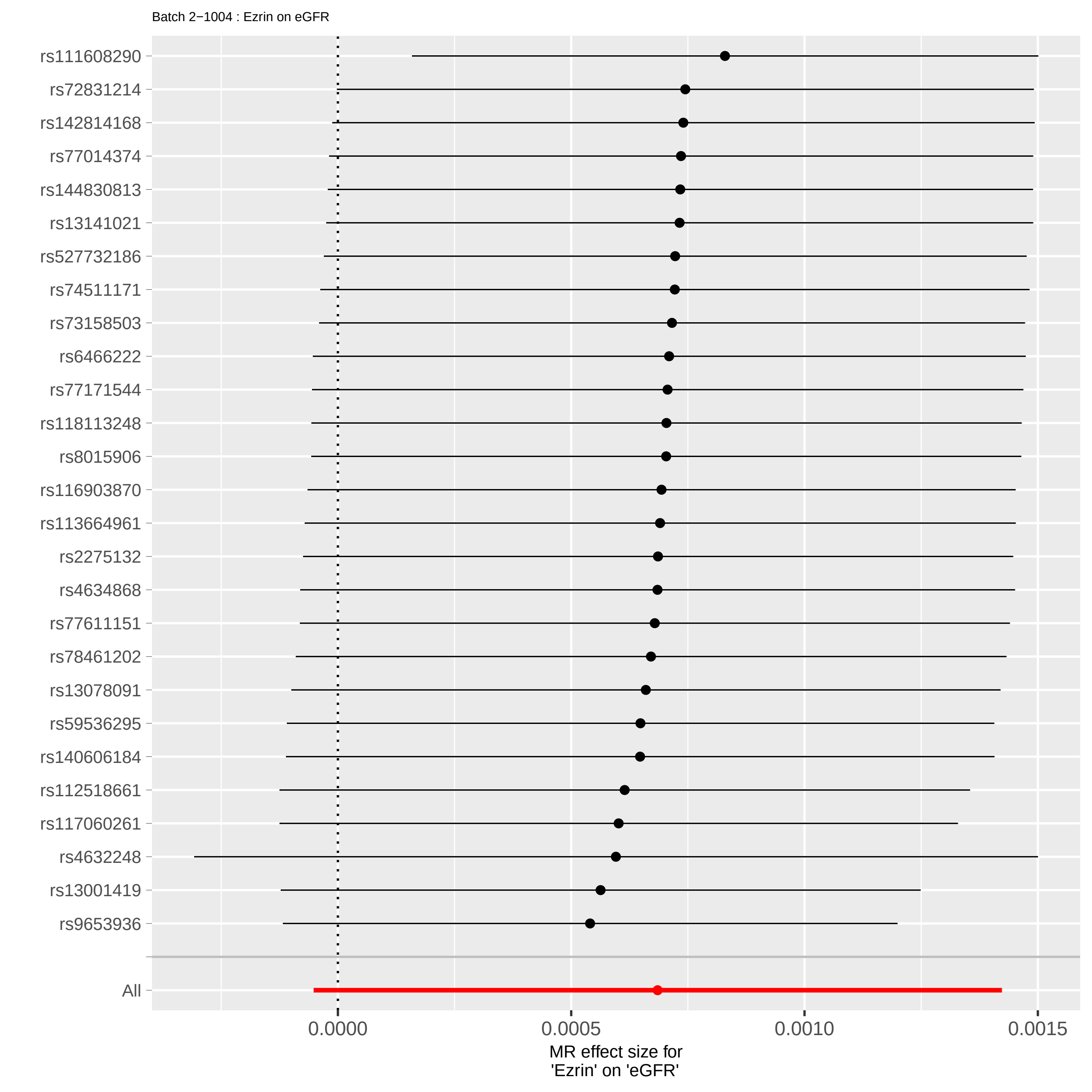

Batch 2−1004 : Ezrin on eGFR
rs111608290
rs72831214
rs142814168
rs77014374
rs144830813
rs13141021
rs527732186
rs74511171
rs73158503
rs6466222
rs77171544
rs118113248
rs8015906
rs116903870
rs113664961
rs2275132
rs4634868
rs77611151
rs78461202
rs13078091
rs59536295
rs140606184
rs112518661
rs117060261
rs4632248
rs13001419
rs9653936
All
0.0000
0.0005
0.0010
0.0015
MR effect size for
'Ezrin' on 'eGFR'

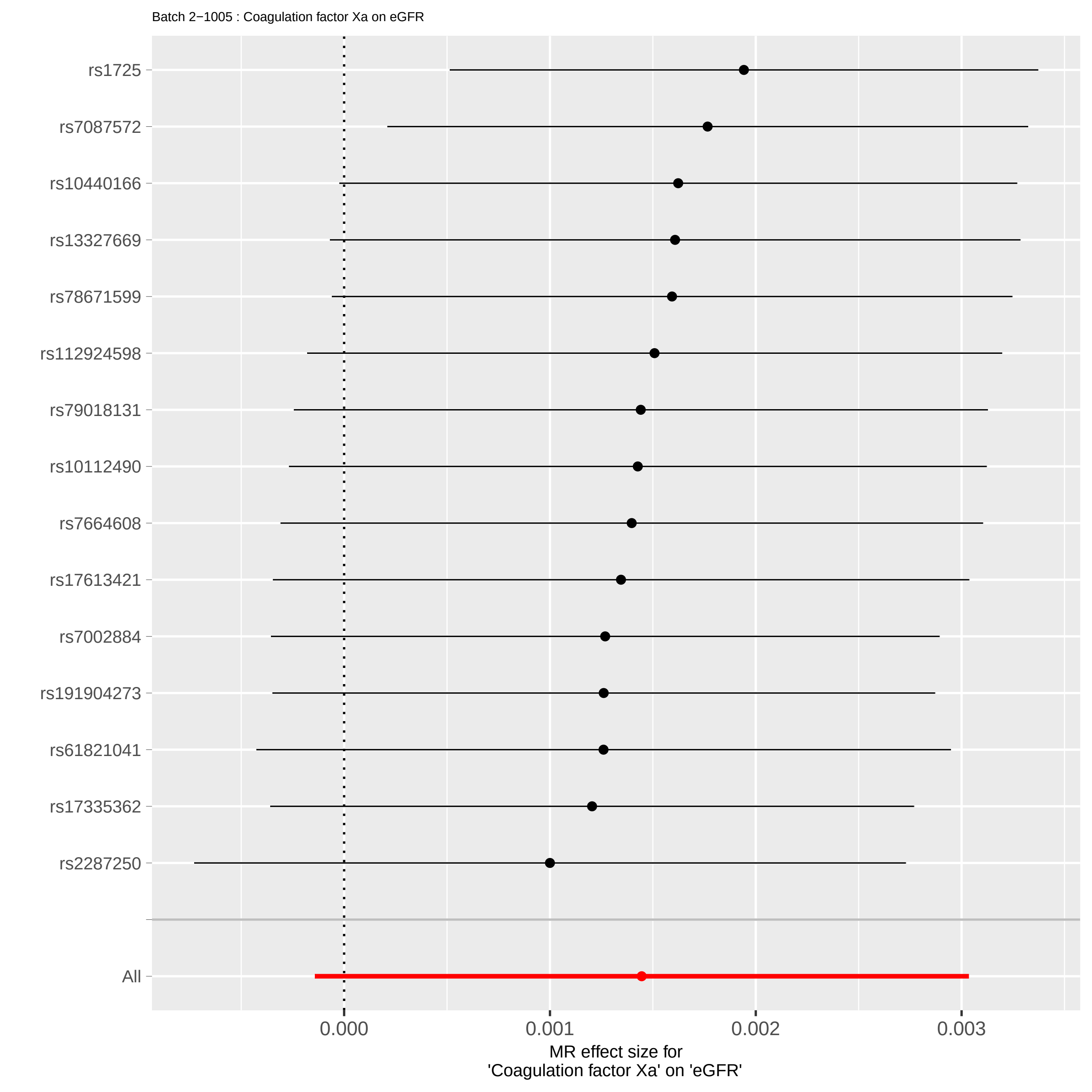

Batch 2−1005 : Coagulation factor Xa on eGFR
rs1725
rs7087572
rs10440166
rs13327669
rs78671599
rs112924598
rs79018131
rs10112490
rs7664608
rs17613421
rs7002884
rs191904273
rs61821041
rs17335362
rs2287250
All
0.000
0.001
0.002
0.003
MR effect size for
'Coagulation factor Xa' on 'eGFR'

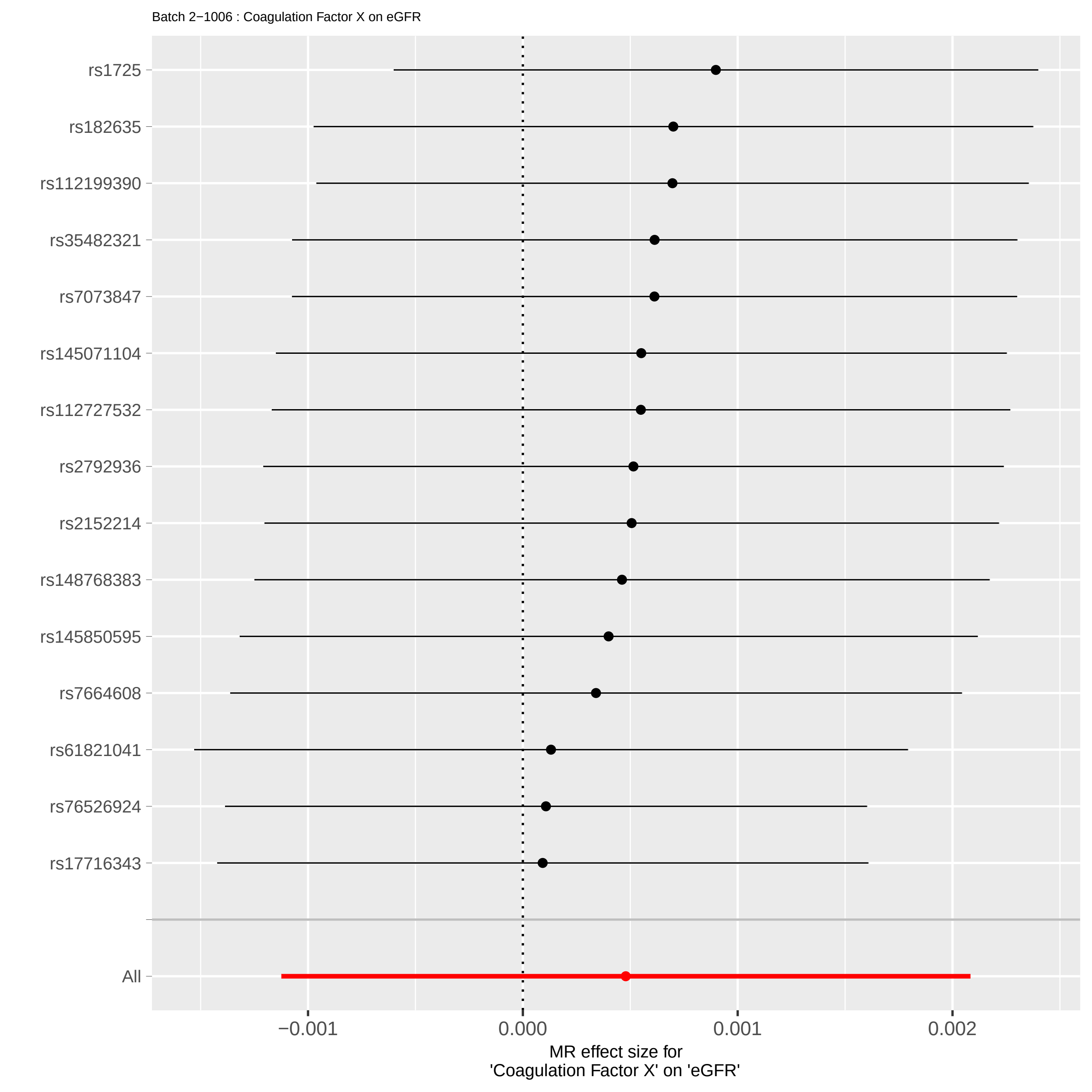

Batch 2−1006 : Coagulation Factor X on eGFR
rs1725
rs182635
rs112199390
rs35482321
rs7073847
rs145071104
rs112727532
rs2792936
rs2152214
rs148768383
rs145850595
rs7664608
rs61821041
rs76526924
rs17716343
All
−0.001
0.000
0.001
0.002
MR effect size for
'Coagulation Factor X' on 'eGFR'

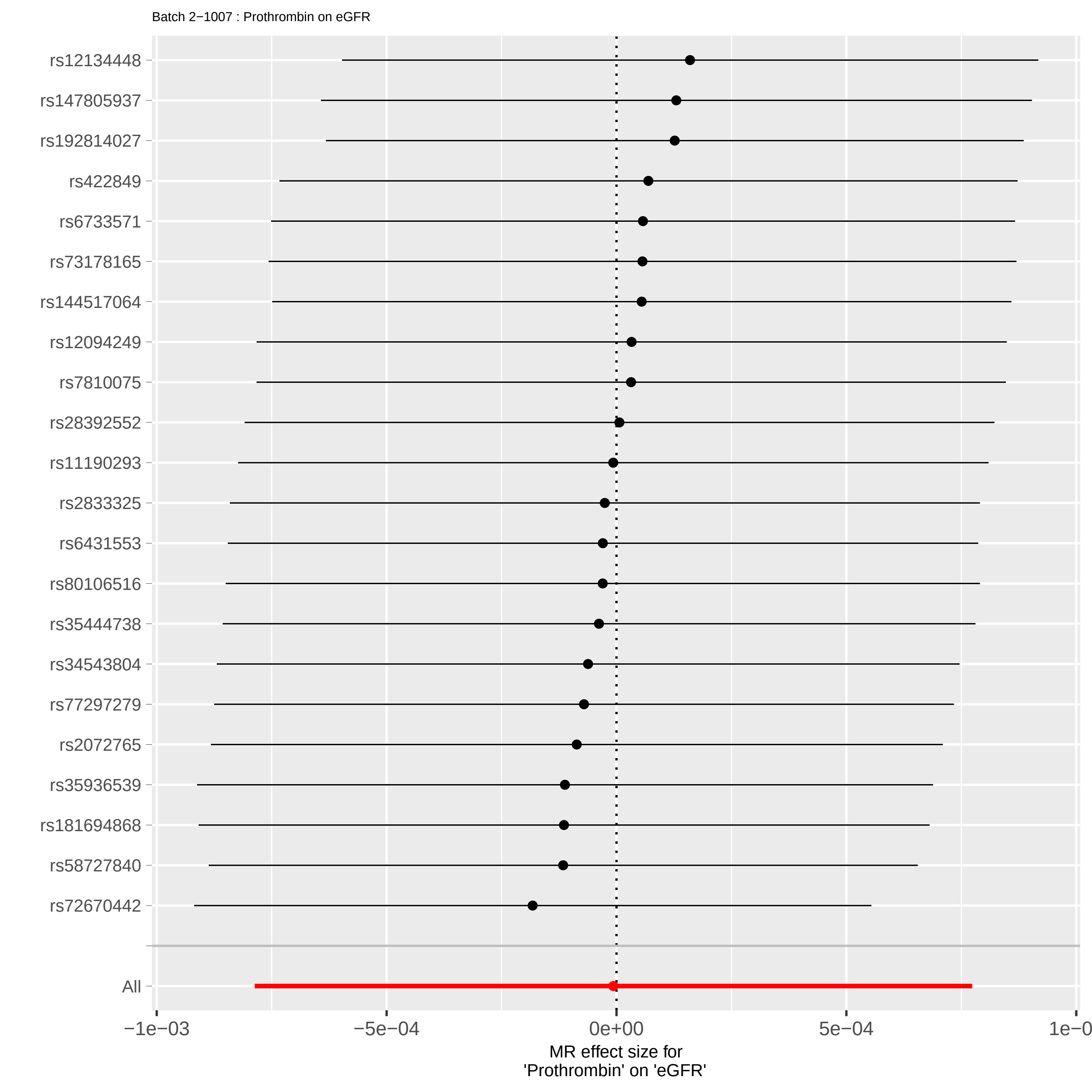

Batch 2−1007 : Prothrombin on eGFR
rs12134448
rs147805937
rs192814027
rs422849
rs6733571
rs73178165
rs144517064
rs12094249
rs7810075
rs28392552
rs11190293
rs2833325
rs6431553
rs80106516
rs35444738
rs34543804
rs77297279
rs2072765
rs35936539
rs181694868
rs58727840
rs72670442
All
−1e−03
−5e−04
0e+00
5e−04
MR effect size for
'Prothrombin' on 'eGFR'

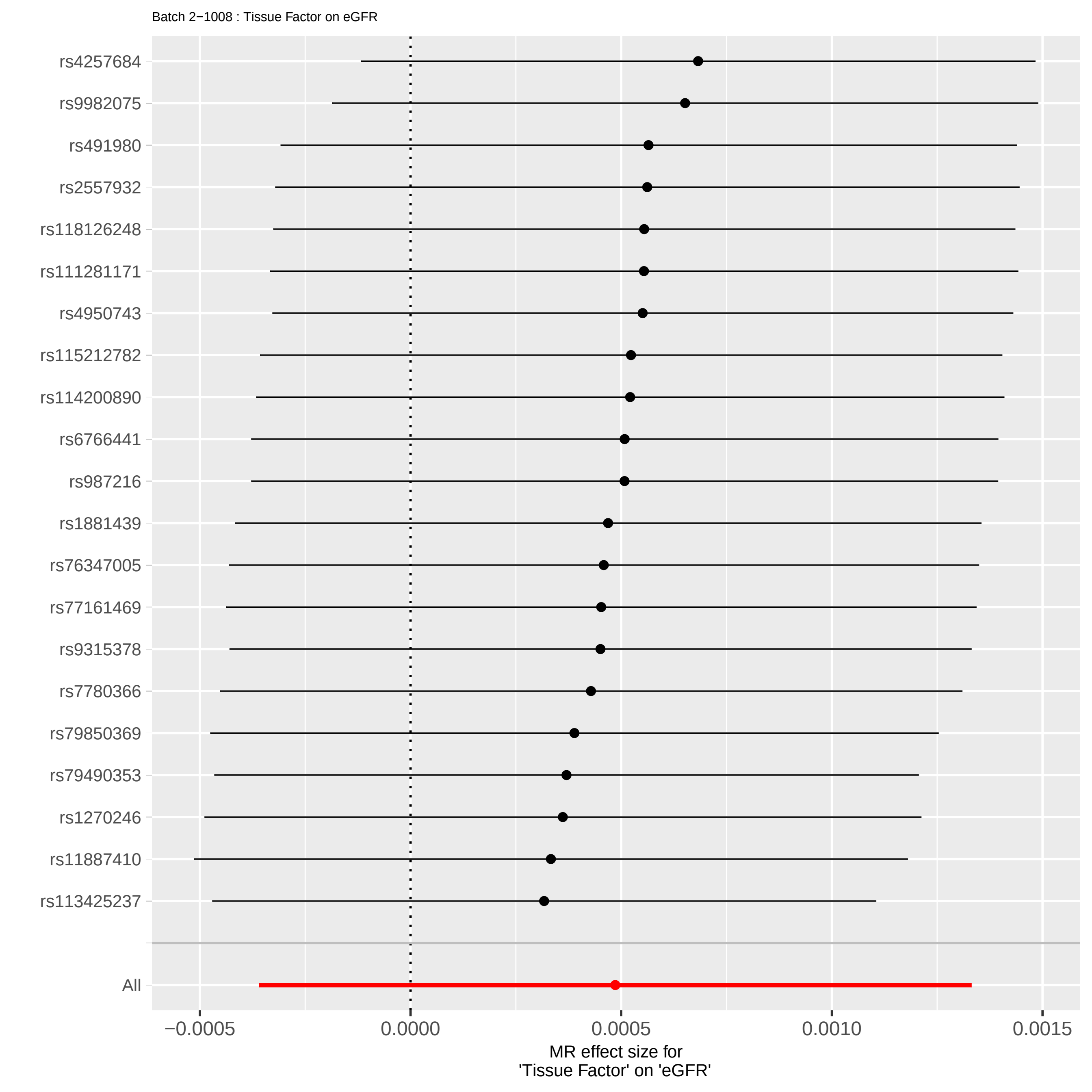

Batch 2−1008 : Tissue Factor on eGFR
rs4257684
rs9982075
rs491980
rs2557932
rs118126248
rs111281171
rs4950743
rs115212782
rs114200890
rs6766441
rs987216
rs1881439
rs76347005
rs77161469
rs9315378
rs7780366
rs79850369
rs79490353
rs1270246
rs11887410
rs113425237
All
−0.0005
0.0000
0.0005
0.0010
0.0015
MR effect size for
'Tissue Factor' on 'eGFR'

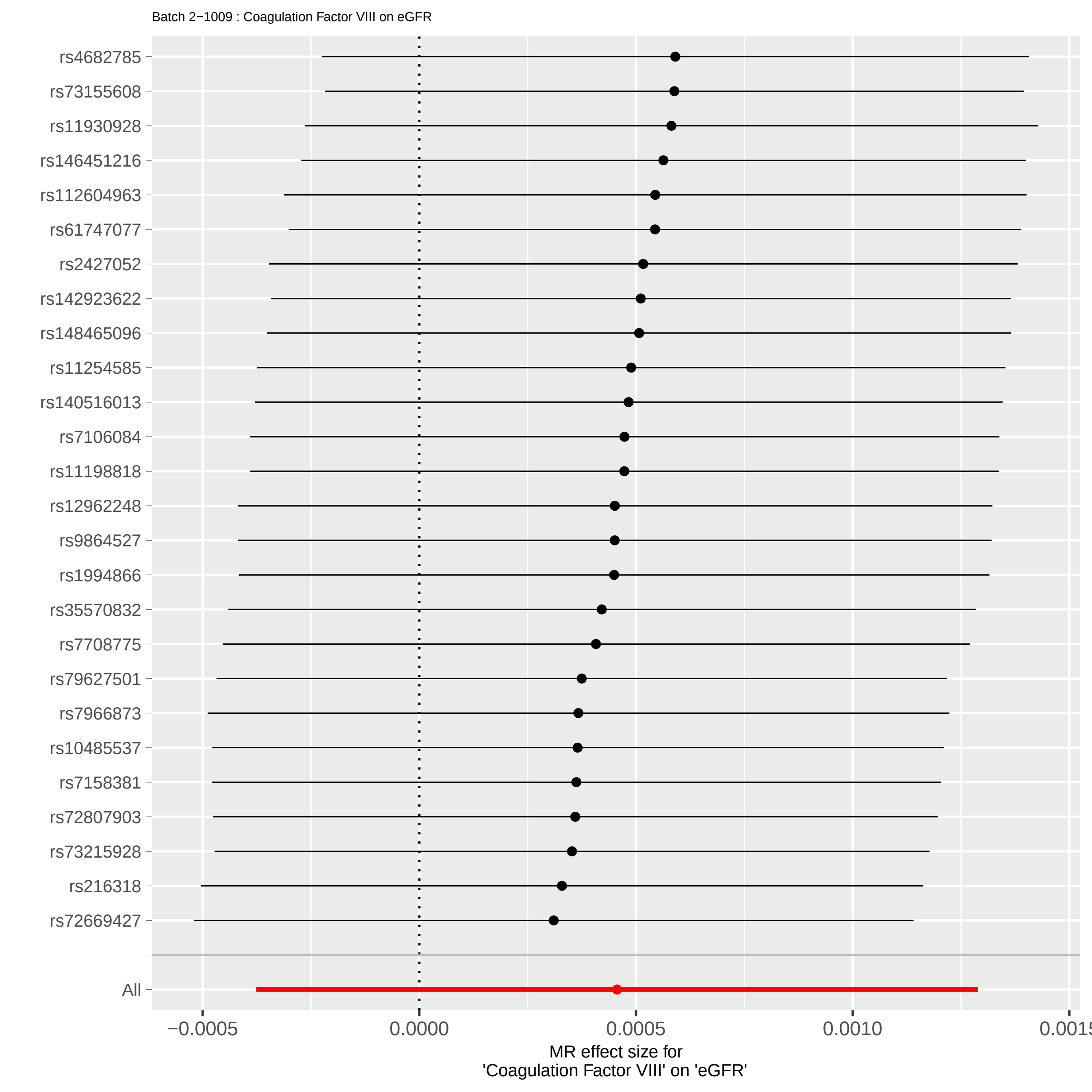

Batch 2−1009 : Coagulation Factor VIII on eGFR
rs4682785
rs73155608
rs11930928
rs146451216
rs112604963
rs61747077
rs2427052
rs142923622
rs148465096
rs11254585
rs140516013
rs7106084
rs11198818
rs12962248
rs9864527
rs1994866
rs35570832
rs7708775
rs79627501
rs7966873
rs10485537
rs7158381
rs72807903
rs73215928
rs216318
rs72669427
All
−0.0005
0.0000
0.0005
0.0010
MR effect size for
'Coagulation Factor VIII' on 'eGFR'

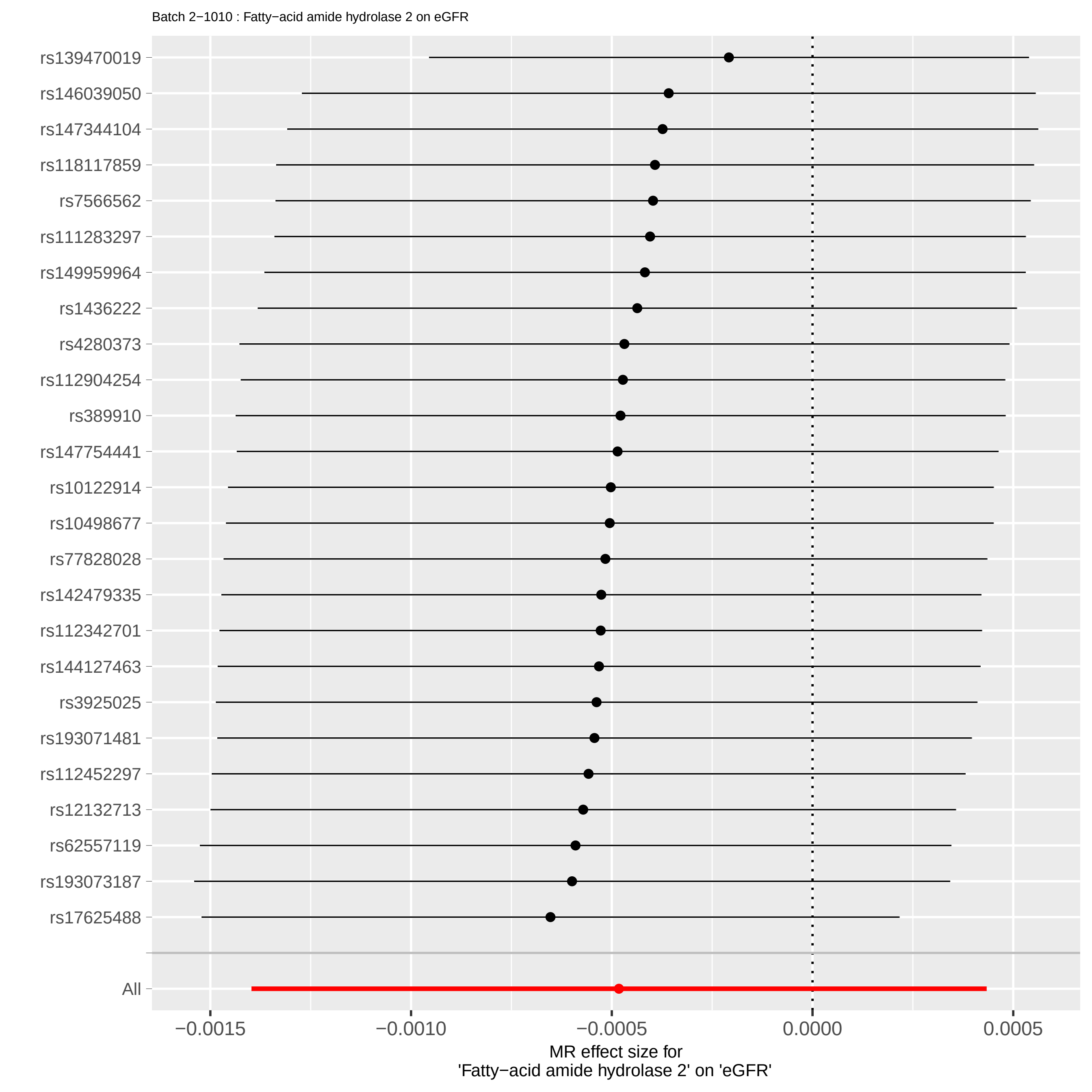

Batch 2−1010 : Fatty−acid amide hydrolase 2 on eGFR
rs139470019
rs146039050
rs147344104
rs118117859
rs7566562
rs111283297
rs149959964
rs1436222
rs4280373
rs112904254
rs389910
rs147754441
rs10122914
rs10498677
rs77828028
rs142479335
rs112342701
rs144127463
rs3925025
rs193071481
rs112452297
rs12132713
rs62557119
rs193073187
rs17625488
All
−0.0015
−0.0010
−0.0005
0.0000
0.0005
MR effect size for
'Fatty−acid amide hydrolase 2' on 'eGFR'

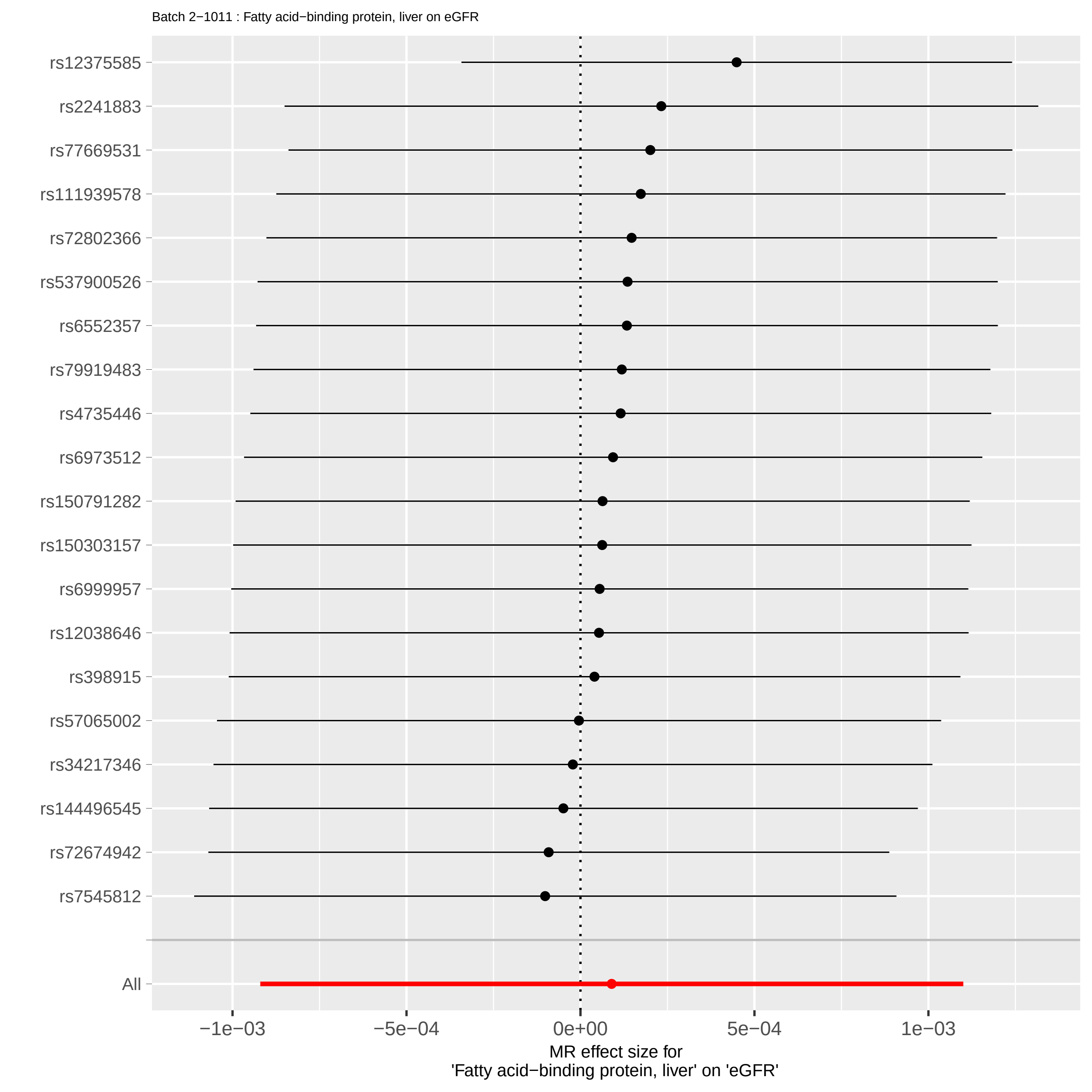

Batch 2−1011 : Fatty acid−binding protein, liver on eGFR
rs12375585
rs2241883
rs77669531
rs111939578
rs72802366
rs537900526
rs6552357
rs79919483
rs4735446
rs6973512
rs150791282
rs150303157
rs6999957
rs12038646
rs398915
rs57065002
rs34217346
rs144496545
rs72674942
rs7545812
All
−1e−03
−5e−04
0e+00
5e−04
1e−03
MR effect size for
'Fatty acid−binding protein, liver' on 'eGFR'

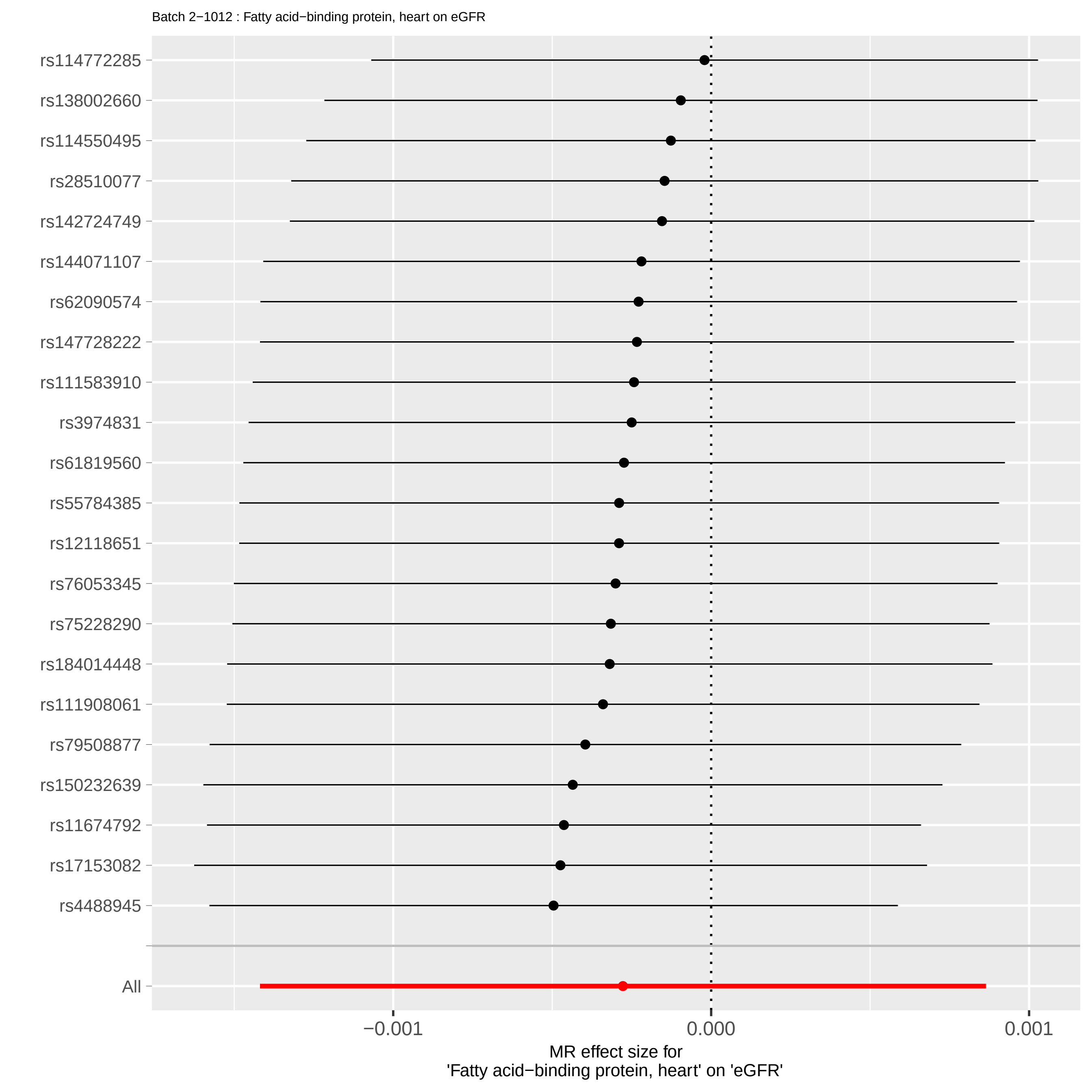

Batch 2−1012 : Fatty acid−binding protein, heart on eGFR
rs114772285
rs138002660
rs114550495
rs28510077
rs142724749
rs144071107
rs62090574
rs147728222
rs111583910
rs3974831
rs61819560
rs55784385
rs12118651
rs76053345
rs75228290
rs184014448
rs111908061
rs79508877
rs150232639
rs11674792
rs17153082
rs4488945
All
−0.001
0.000
0.001
MR effect size for
'Fatty acid−binding protein, heart' on 'eGFR'

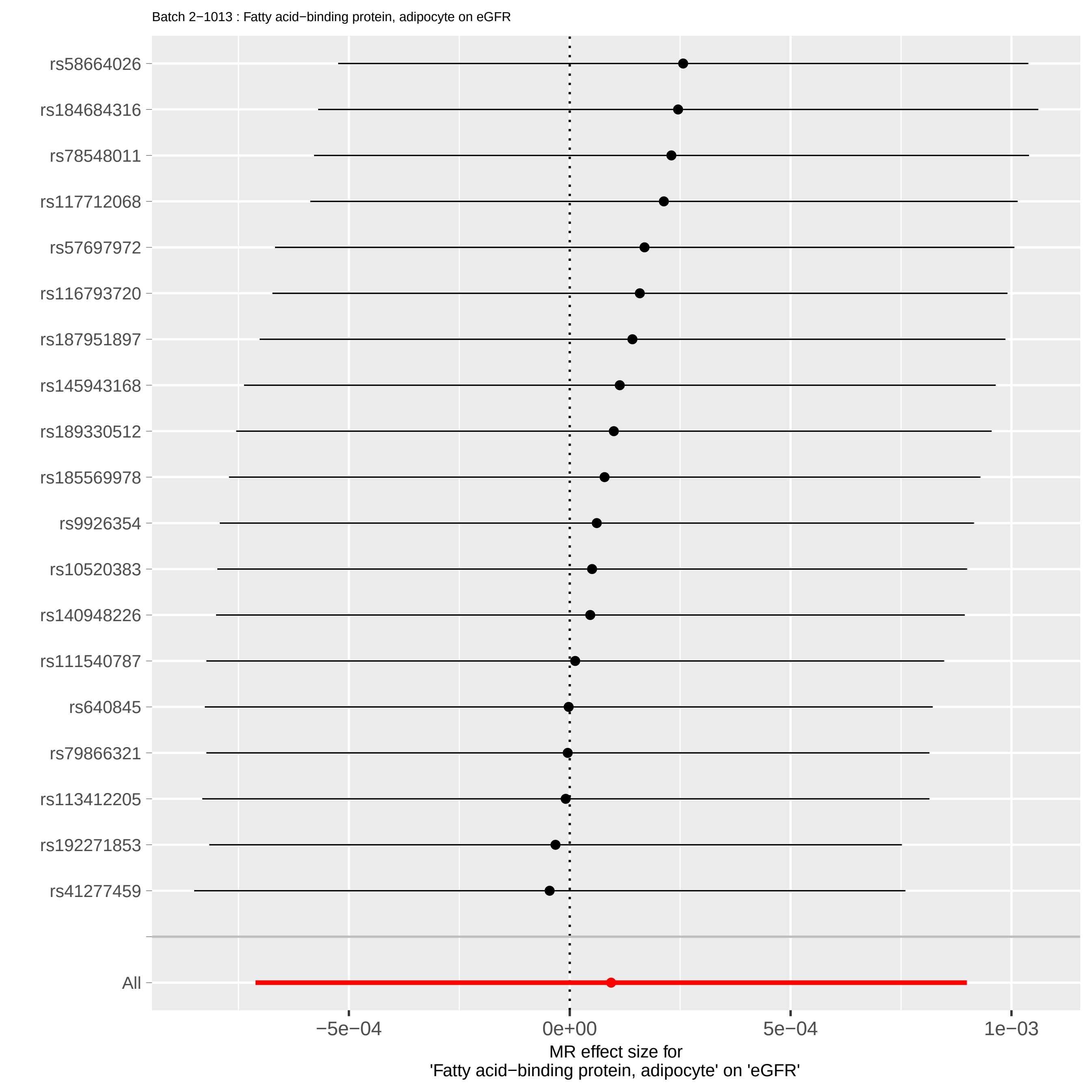

Batch 2−1013 : Fatty acid−binding protein, adipocyte on eGFR
rs58664026
rs184684316
rs78548011
rs117712068
rs57697972
rs116793720
rs187951897
rs145943168
rs189330512
rs185569978
rs9926354
rs10520383
rs140948226
rs111540787
rs640845
rs79866321
rs113412205
rs192271853
rs41277459
All
−5e−04
0e+00
5e−04
1e−03
MR effect size for
'Fatty acid−binding protein, adipocyte' on 'eGFR'

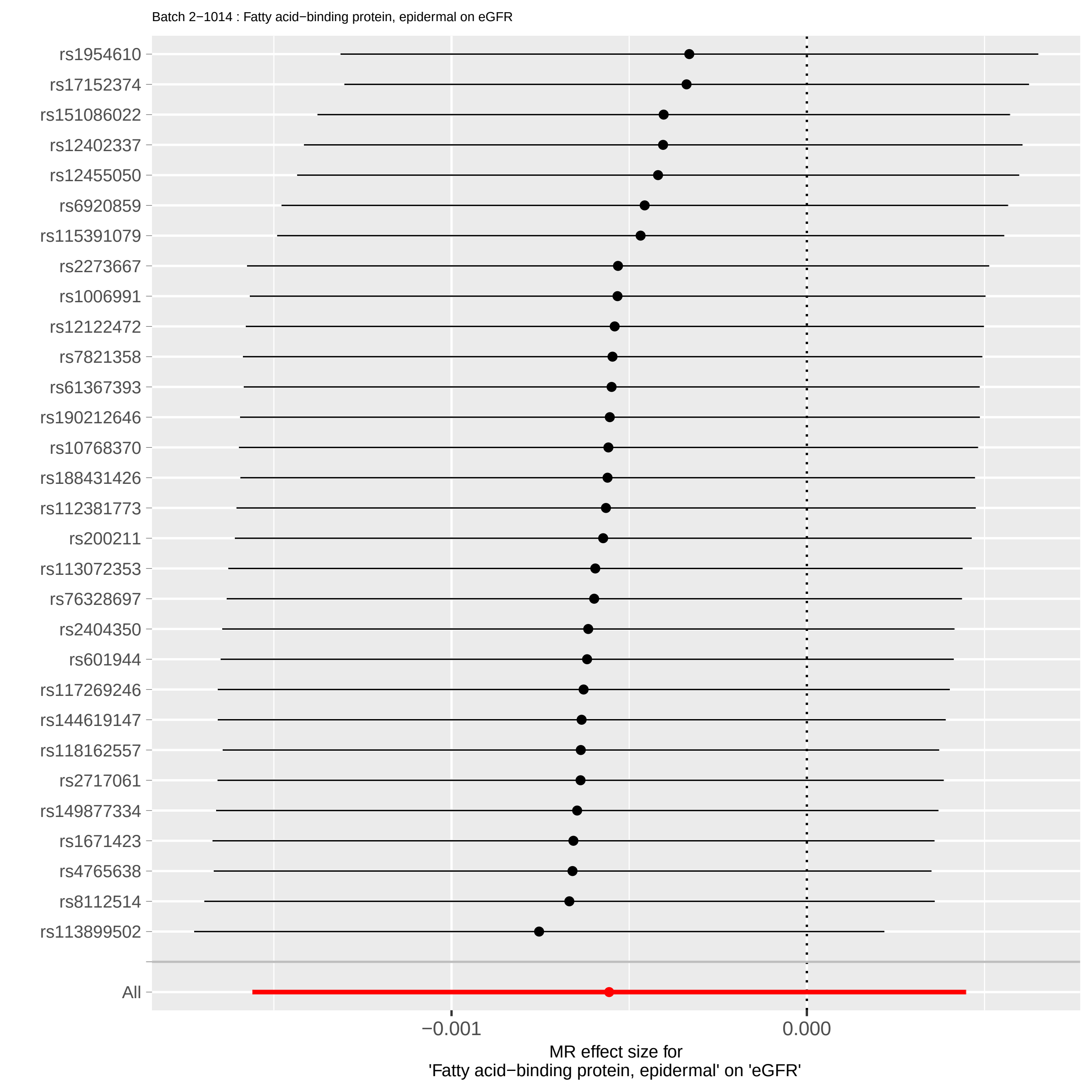
Batch 2−1014 : Fatty acid−binding protein, epidermal on eGFR
rs1954610
rs17152374
rs151086022
rs12402337
rs12455050
rs6920859
rs115391079
rs2273667
rs1006991
rs12122472
rs7821358
rs61367393
rs190212646
rs10768370
rs188431426
rs112381773
rs200211
rs113072353
rs76328697
rs2404350
rs601944
rs117269246
rs144619147
rs118162557
rs2717061
rs149877334
rs1671423
rs4765638
rs8112514
rs113899502
All
−0.001
0.000
MR effect size for
'Fatty acid−binding protein, epidermal' on 'eGFR'

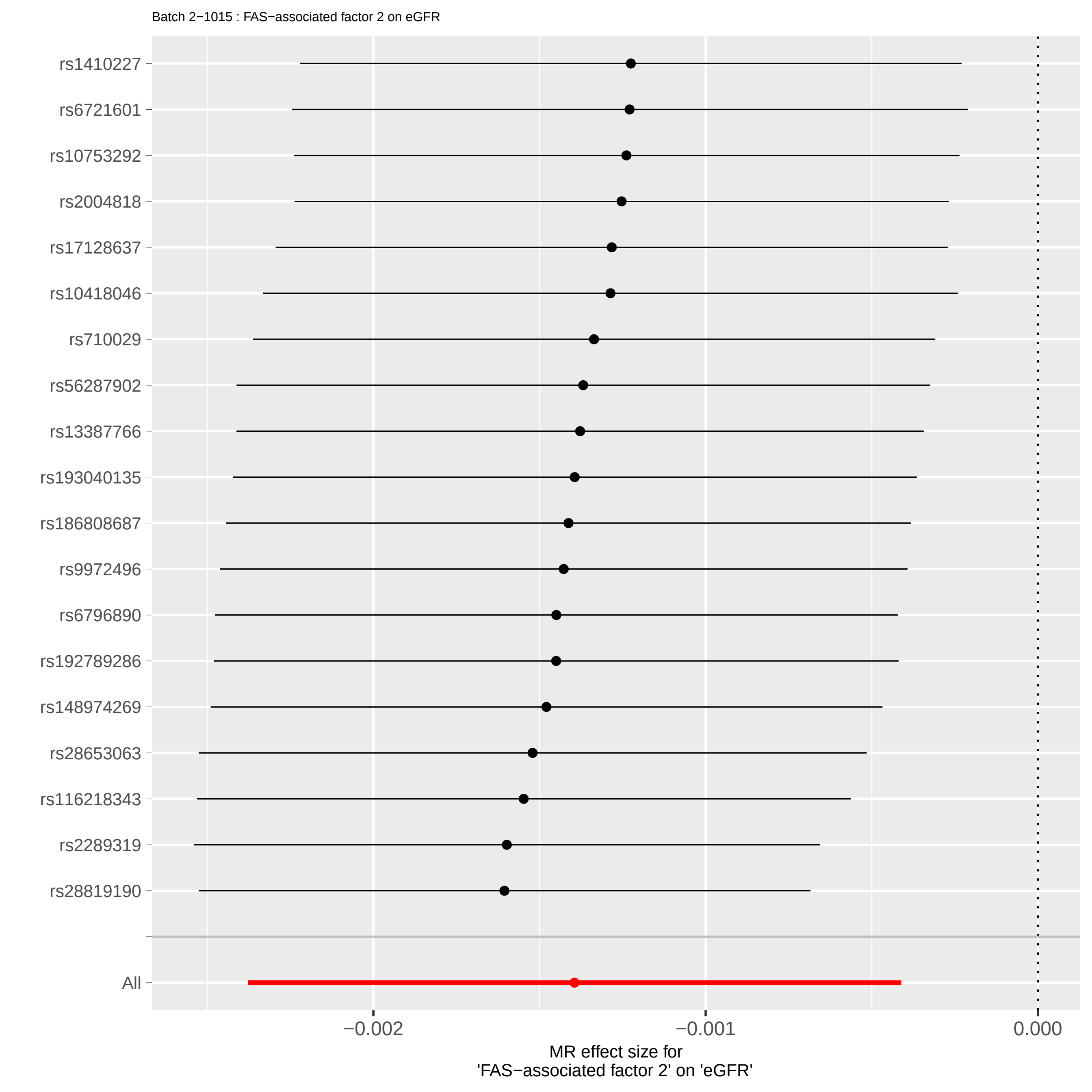

Batch 2−1015 : FAS−associated factor 2 on eGFR
rs1410227
rs6721601
rs10753292
rs2004818
rs17128637
rs10418046
rs710029
rs56287902
rs13387766
rs193040135
rs186808687
rs9972496
rs6796890
rs192789286
rs148974269
rs28653063
rs116218343
rs2289319
rs28819190
All
−0.002
−0.001
0.000
MR effect size for
'FAS−associated factor 2' on 'eGFR'

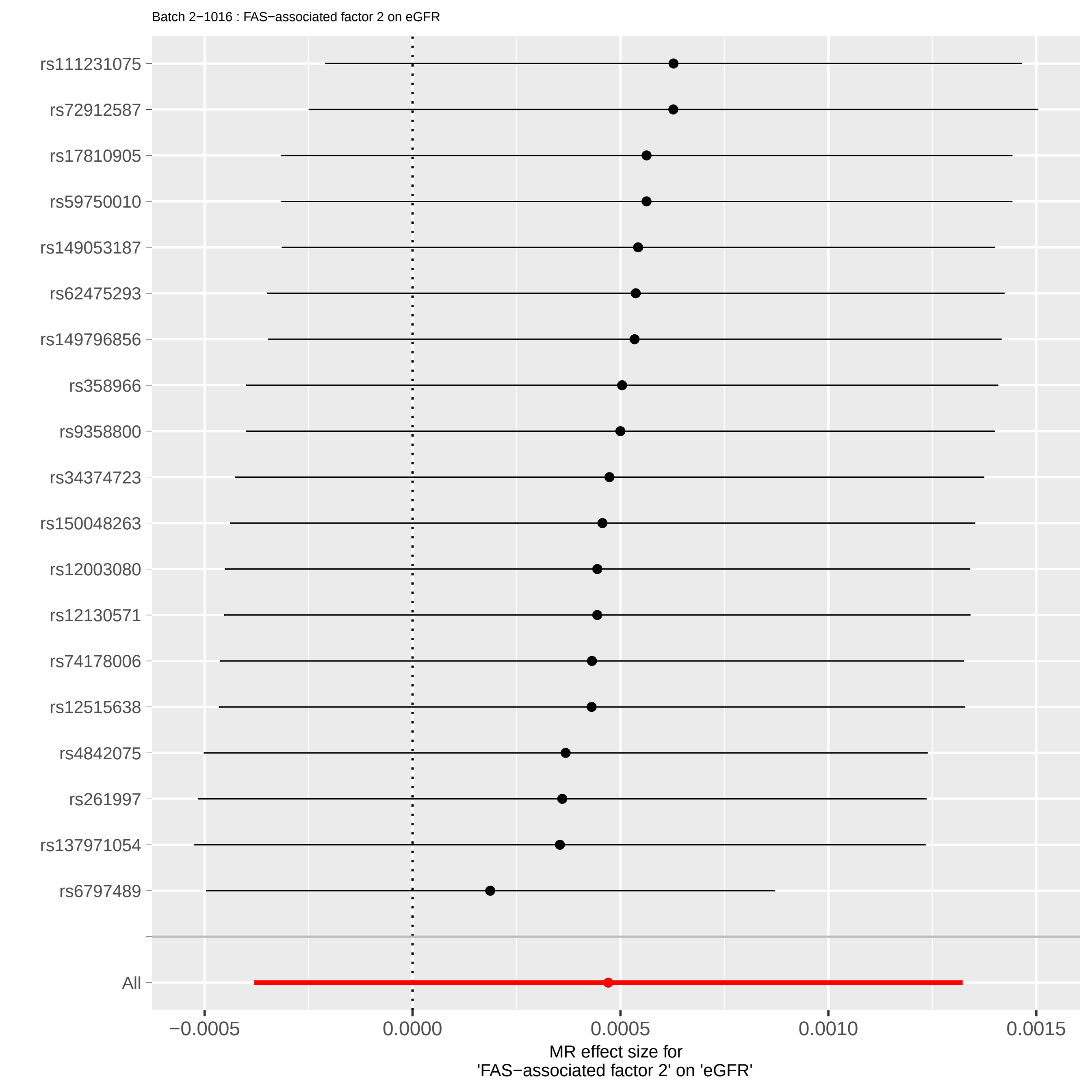

Batch 2−1016 : FAS−associated factor 2 on eGFR
rs111231075
rs72912587
rs17810905
rs59750010
rs149053187
rs62475293
rs149796856
rs358966
rs9358800
rs34374723
rs150048263
rs12003080
rs12130571
rs74178006
rs12515638
rs4842075
rs261997
rs137971054
rs6797489
All
−0.0005
0.0000
0.0005
0.0010
0.0015
MR effect size for
'FAS−associated factor 2' on 'eGFR'

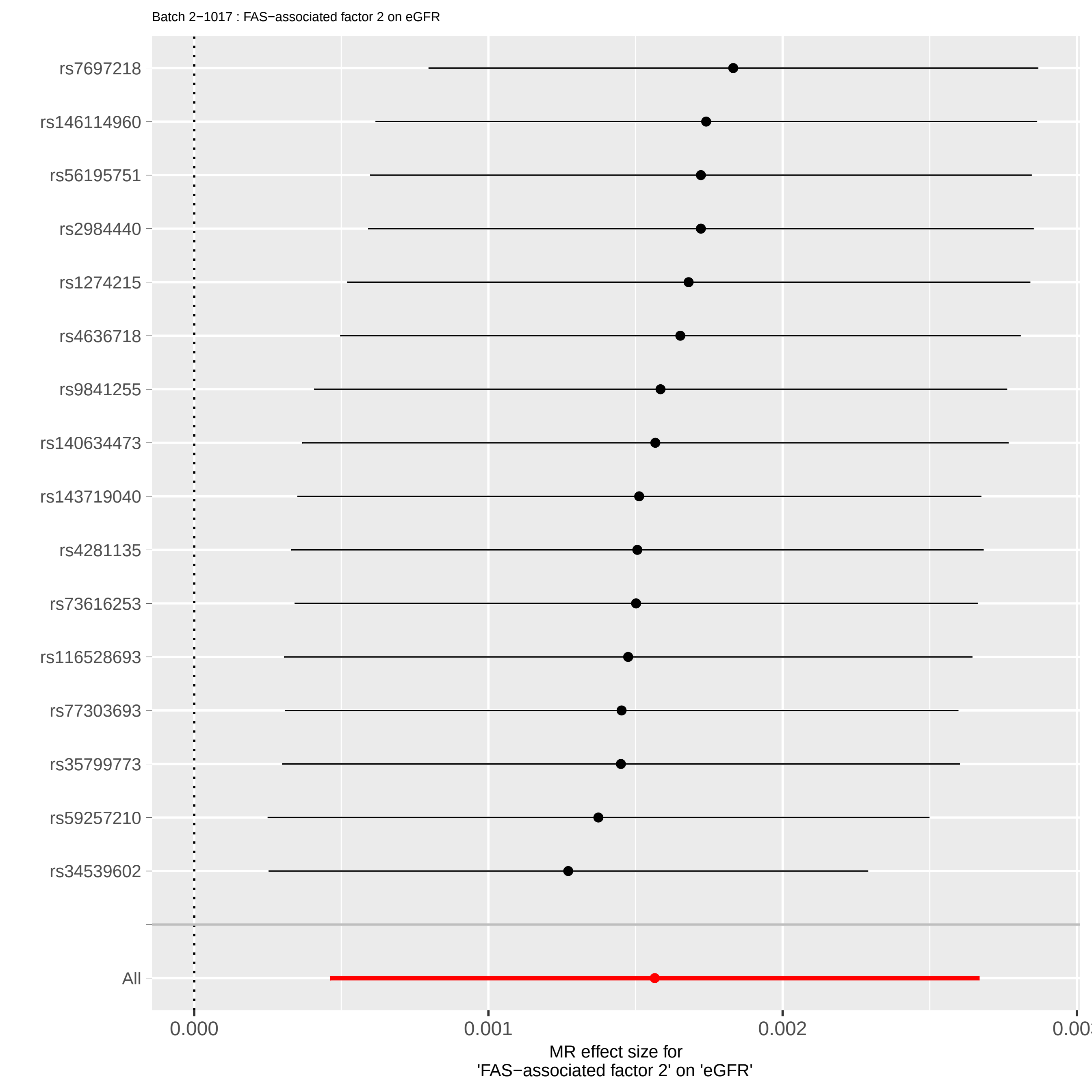

Batch 2−1017 : FAS−associated factor 2 on eGFR
rs7697218
rs146114960
rs56195751
rs2984440
rs1274215
rs4636718
rs9841255
rs140634473
rs143719040
rs4281135
rs73616253
rs116528693
rs77303693
rs35799773
rs59257210
rs34539602
All
0.000
0.001
0.002
MR effect size for
'FAS−associated factor 2' on 'eGFR'

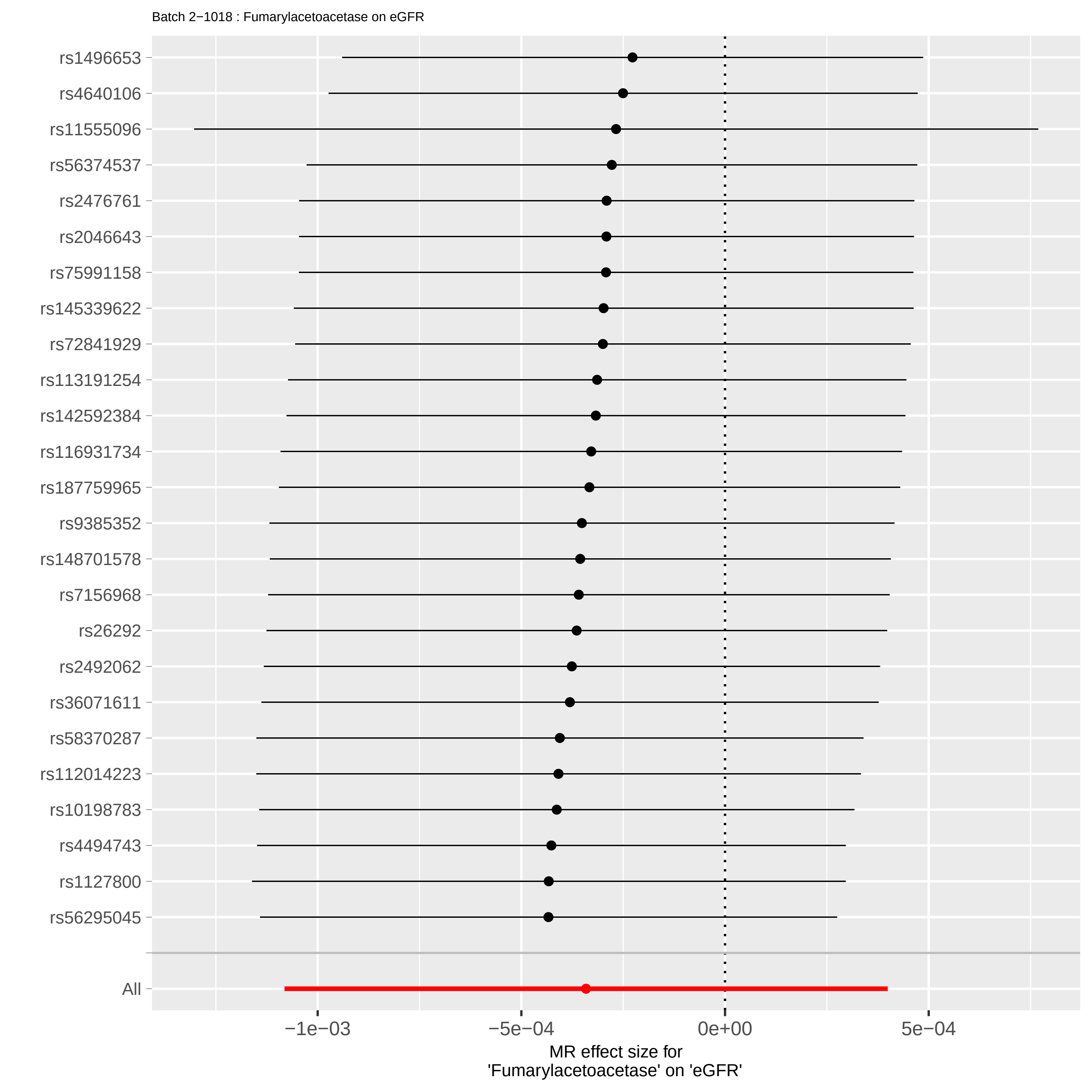
Batch 2−1018 : Fumarylacetoacetase on eGFR
rs1496653
rs4640106
rs11555096
rs56374537
rs2476761
rs2046643
rs75991158
rs145339622
rs72841929
rs113191254
rs142592384
rs116931734
rs187759965
rs9385352
rs148701578
rs7156968
rs26292
rs2492062
rs36071611
rs58370287
rs112014223
rs10198783
rs4494743
rs1127800
rs56295045
All
−1e−03
−5e−04
0e+00
5e−04
MR effect size for
'Fumarylacetoacetase' on 'eGFR'

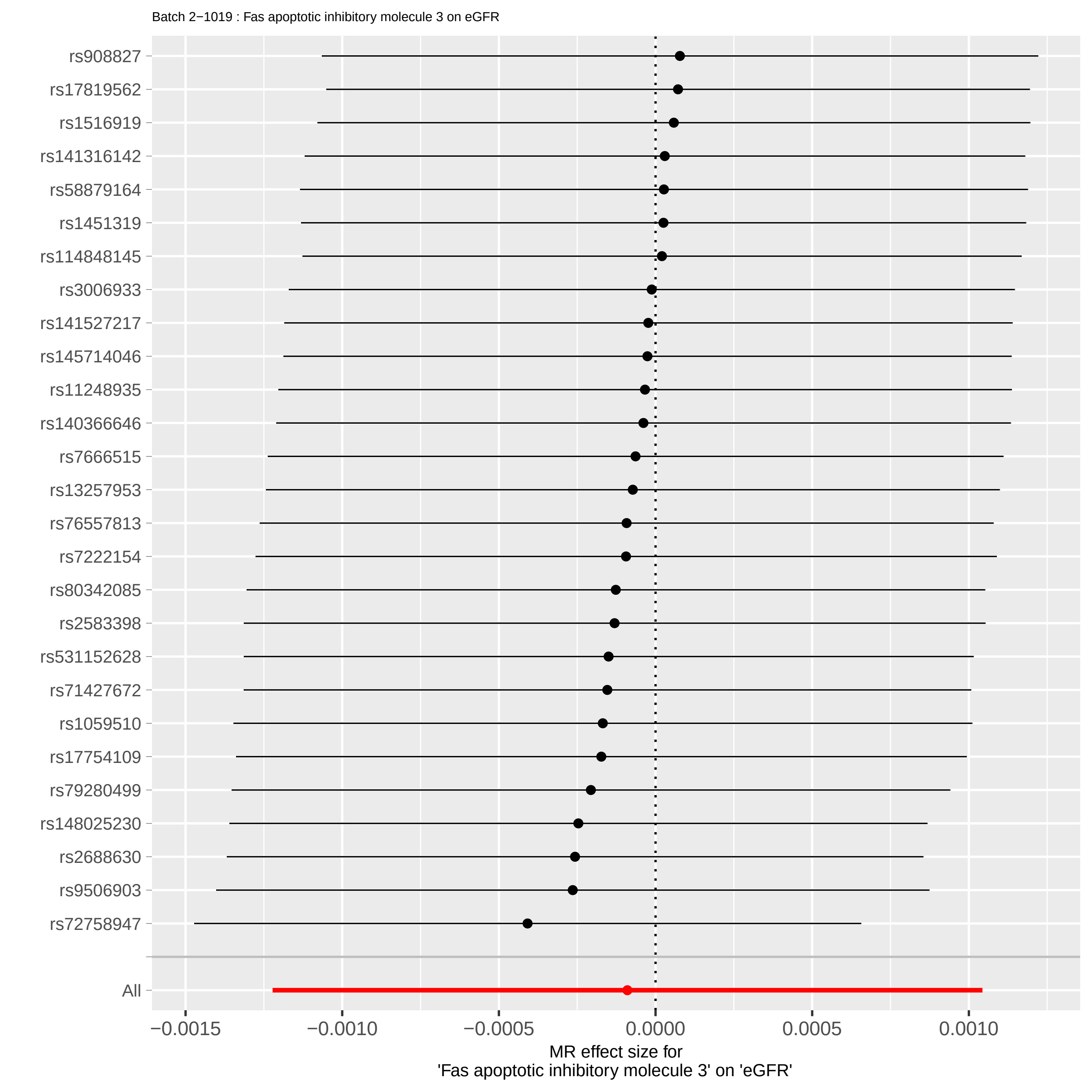

Batch 2−1019 : Fas apoptotic inhibitory molecule 3 on eGFR
rs908827
rs17819562
rs1516919
rs141316142
rs58879164
rs1451319
rs114848145
rs3006933
rs141527217
rs145714046
rs11248935
rs140366646
rs7666515
rs13257953
rs76557813
rs7222154
rs80342085
rs2583398
rs531152628
rs71427672
rs1059510
rs17754109
rs79280499
rs148025230
rs2688630
rs9506903
rs72758947
All
−0.0015
−0.0010
−0.0005
0.0000
0.0005
0.0010
MR effect size for
'Fas apoptotic inhibitory molecule 3' on 'eGFR'

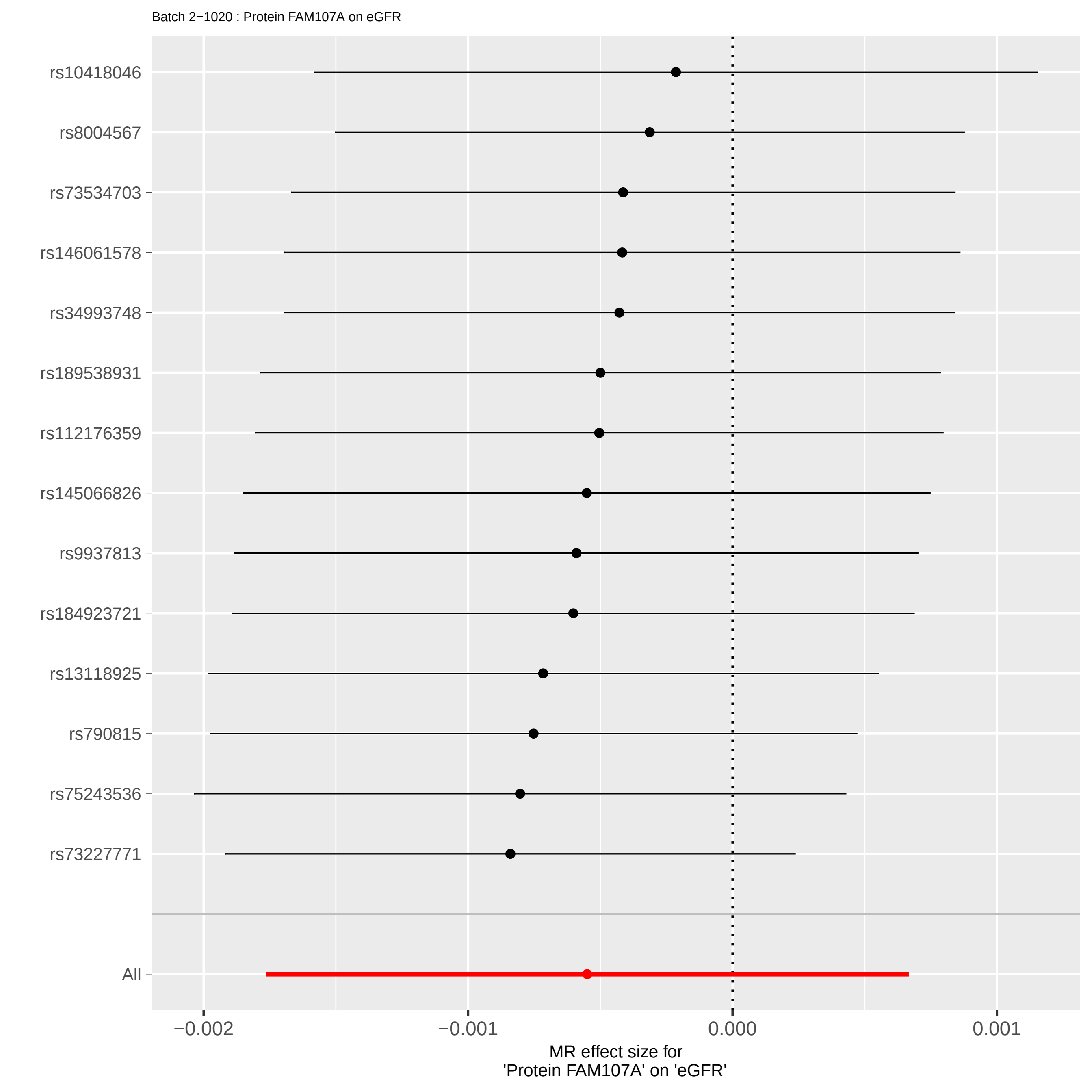
Batch 2−1020 : Protein FAM107A on eGFR
rs10418046
rs8004567
rs73534703
rs146061578
rs34993748
rs189538931
rs112176359
rs145066826
rs9937813
rs184923721
rs13118925
rs790815
rs75243536
rs73227771
All
−0.002
−0.001
0.000
0.001
MR effect size for
'Protein FAM107A' on 'eGFR'

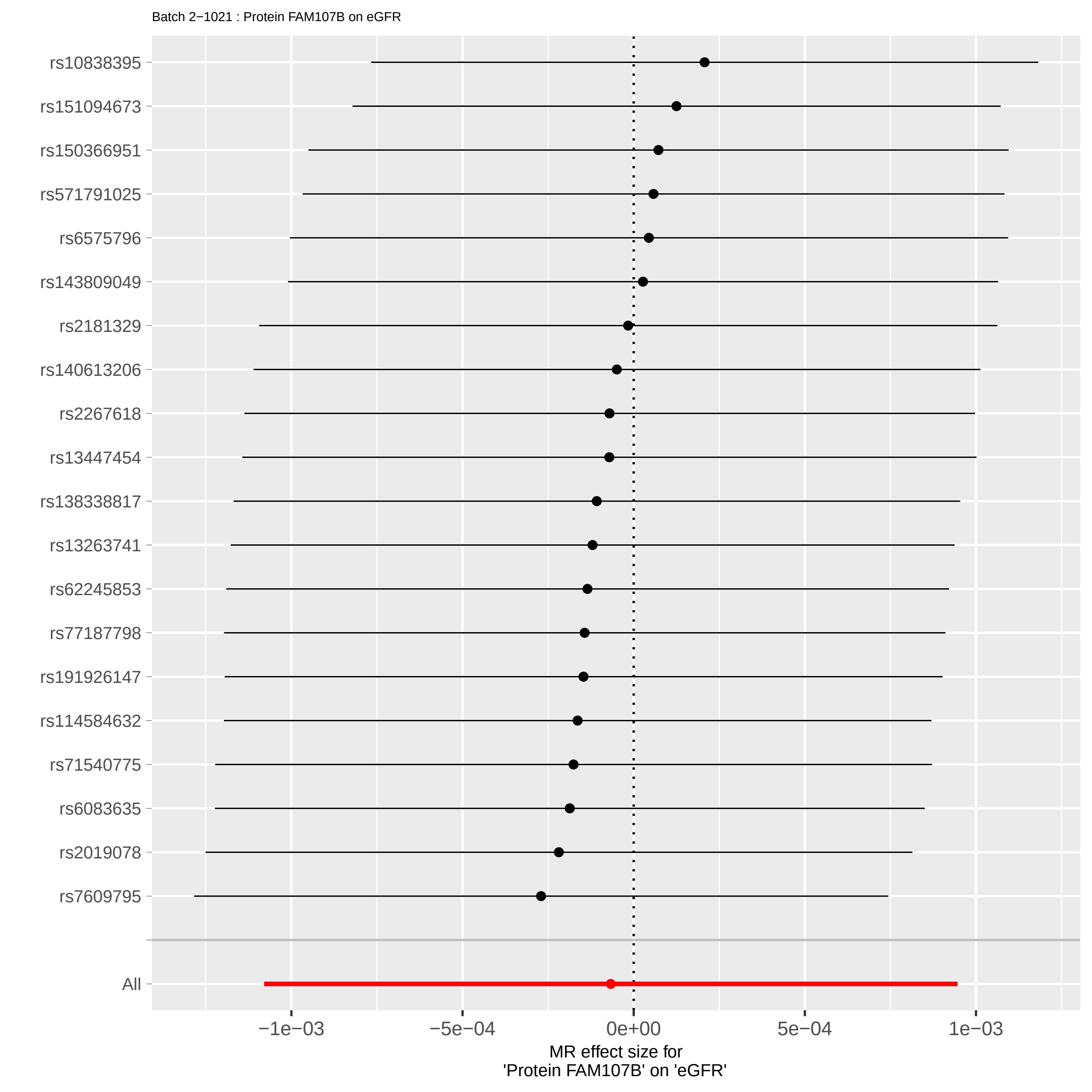

Batch 2−1021 : Protein FAM107B on eGFR
rs10838395
rs151094673
rs150366951
rs571791025
rs6575796
rs143809049
rs2181329
rs140613206
rs2267618
rs13447454
rs138338817
rs13263741
rs62245853
rs77187798
rs191926147
rs114584632
rs71540775
rs6083635
rs2019078
rs7609795
All
−1e−03
−5e−04
0e+00
5e−04
1e−03
MR effect size for
'Protein FAM107B' on 'eGFR'

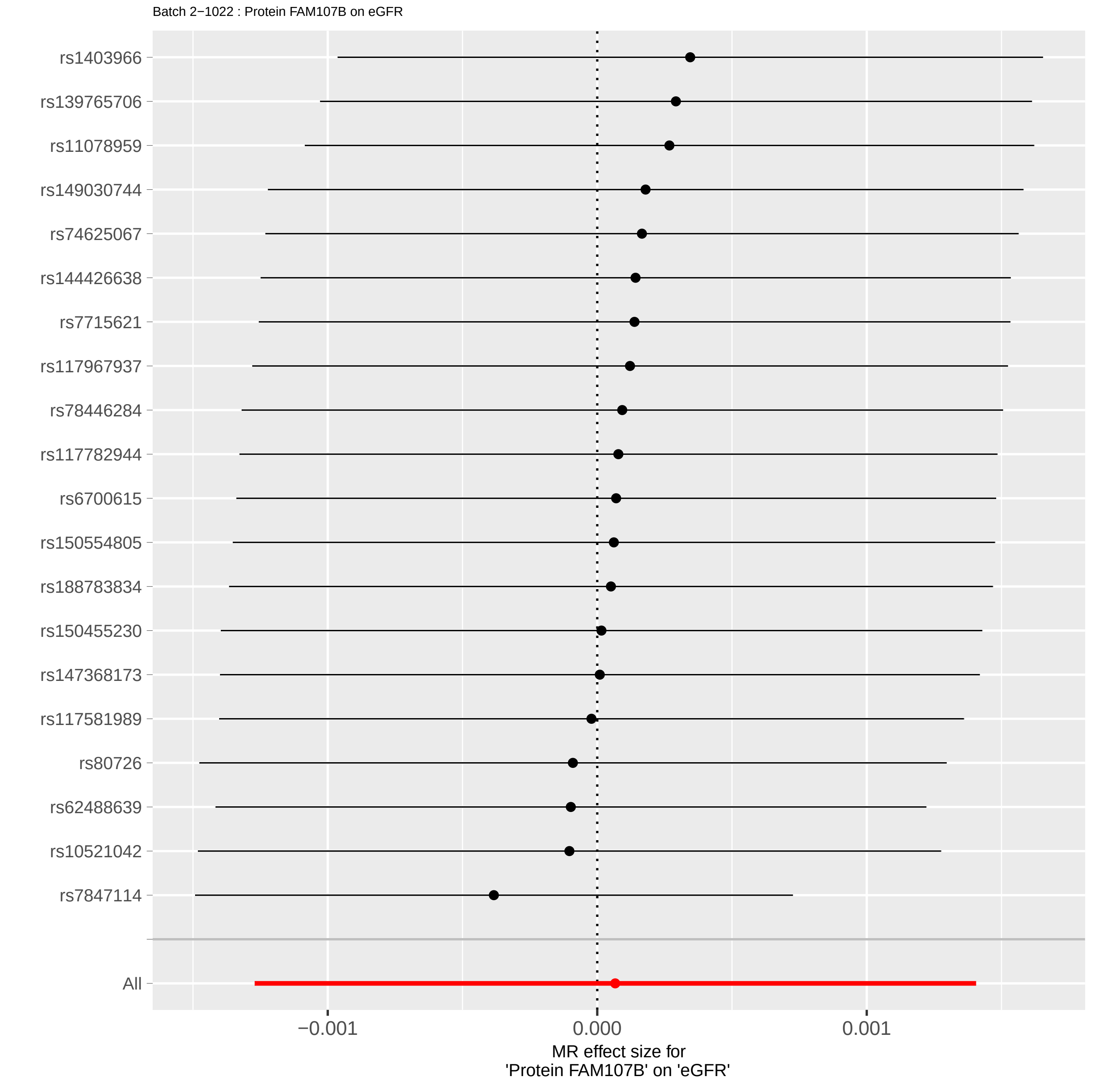
Batch 2−1022 : Protein FAM107B on eGFR
rs1403966
rs139765706
rs11078959
rs149030744
rs74625067
rs144426638
rs7715621
rs117967937
rs78446284
rs117782944
rs6700615
rs150554805
rs188783834
rs150455230
rs147368173
rs117581989
rs80726
rs62488639
rs10521042
rs7847114
All
−0.001
0.000
0.001
MR effect size for
'Protein FAM107B' on 'eGFR'

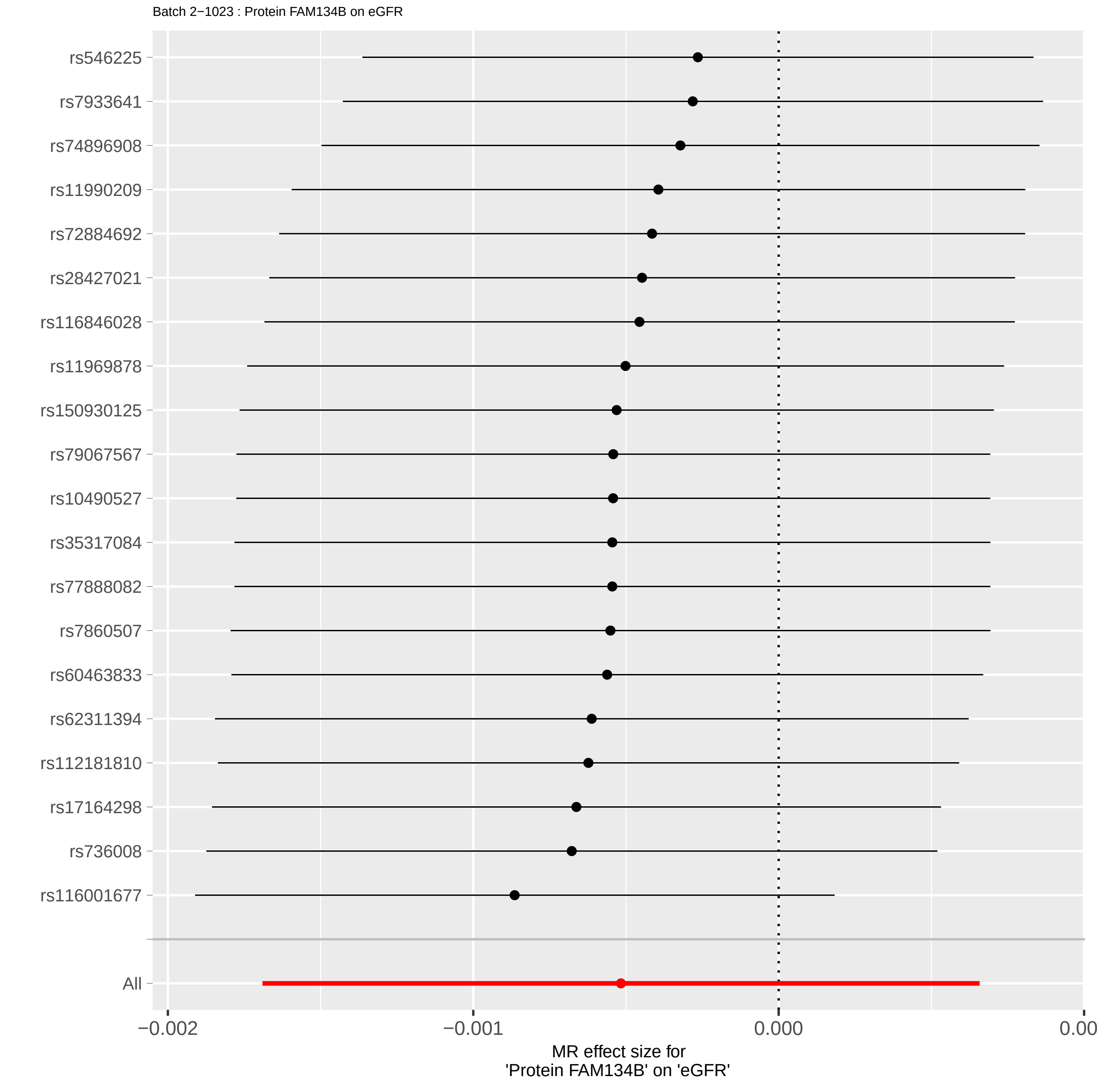

Batch 2−1023 : Protein FAM134B on eGFR
rs546225
rs7933641
rs74896908
rs11990209
rs72884692
rs28427021
rs116846028
rs11969878
rs150930125
rs79067567
rs10490527
rs35317084
rs77888082
rs7860507
rs60463833
rs62311394
rs112181810
rs17164298
rs736008
rs116001677
All
−0.002
−0.001
0.000
0.00
MR effect size for
'Protein FAM134B' on 'eGFR'

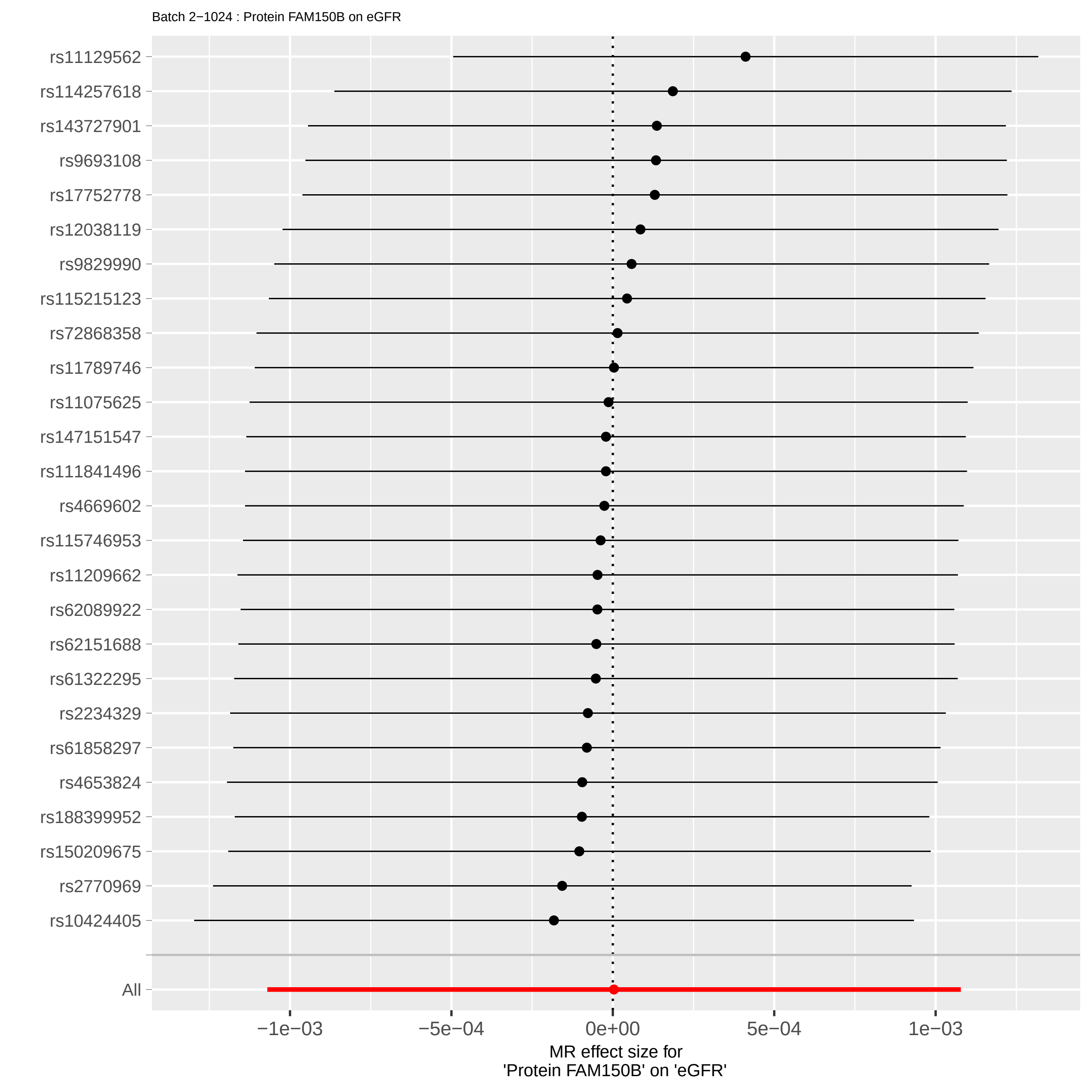

Batch 2−1024 : Protein FAM150B on eGFR
rs11129562
rs114257618
rs143727901
rs9693108
rs17752778
rs12038119
rs9829990
rs115215123
rs72868358
rs11789746
rs11075625
rs147151547
rs111841496
rs4669602
rs115746953
rs11209662
rs62089922
rs62151688
rs61322295
rs2234329
rs61858297
rs4653824
rs188399952
rs150209675
rs2770969
rs10424405
All
−1e−03
−5e−04
0e+00
5e−04
1e−03
MR effect size for
'Protein FAM150B' on 'eGFR'

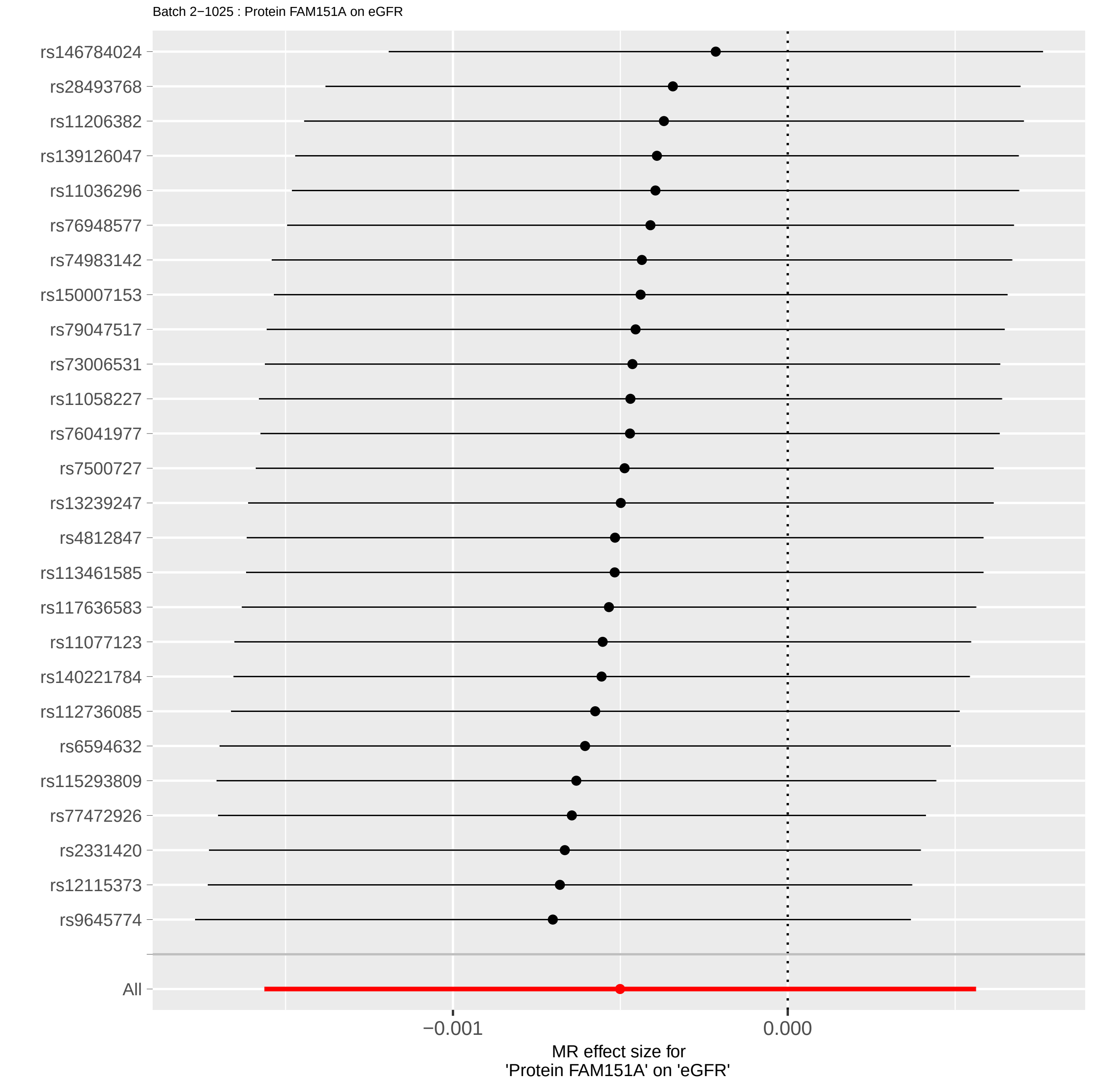

Batch 2−1025 : Protein FAM151A on eGFR
rs146784024
rs28493768
rs11206382
rs139126047
rs11036296
rs76948577
rs74983142
rs150007153
rs79047517
rs73006531
rs11058227
rs76041977
rs7500727
rs13239247
rs4812847
rs113461585
rs117636583
rs11077123
rs140221784
rs112736085
rs6594632
rs115293809
rs77472926
rs2331420
rs12115373
rs9645774
All
−0.001
0.000
MR effect size for
'Protein FAM151A' on 'eGFR'

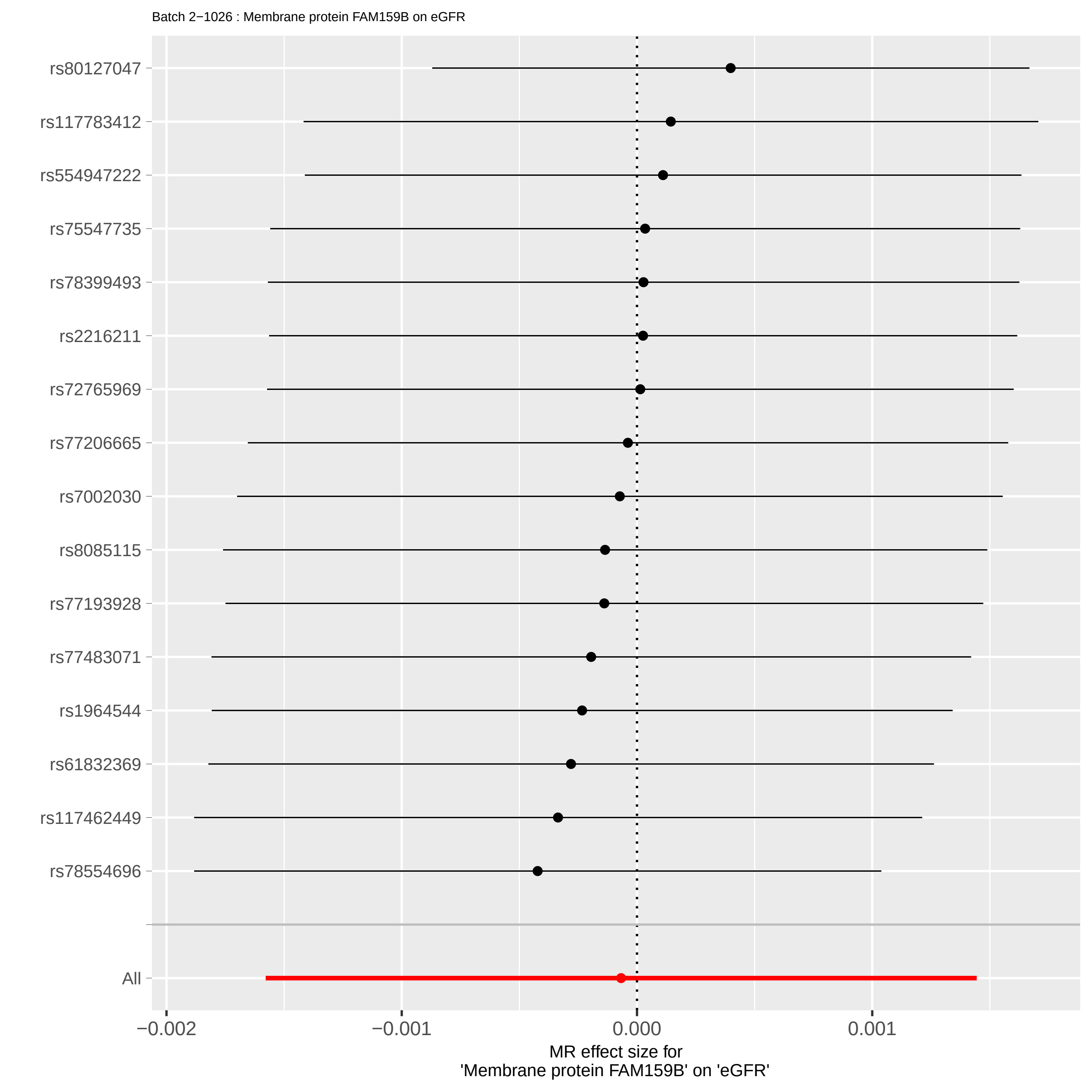

Batch 2−1026 : Membrane protein FAM159B on eGFR
rs80127047
rs117783412
rs554947222
rs75547735
rs78399493
rs2216211
rs72765969
rs77206665
rs7002030
rs8085115
rs77193928
rs77483071
rs1964544
rs61832369
rs117462449
rs78554696
All
−0.002
−0.001
0.000
0.001
MR effect size for
'Membrane protein FAM159B' on 'eGFR'

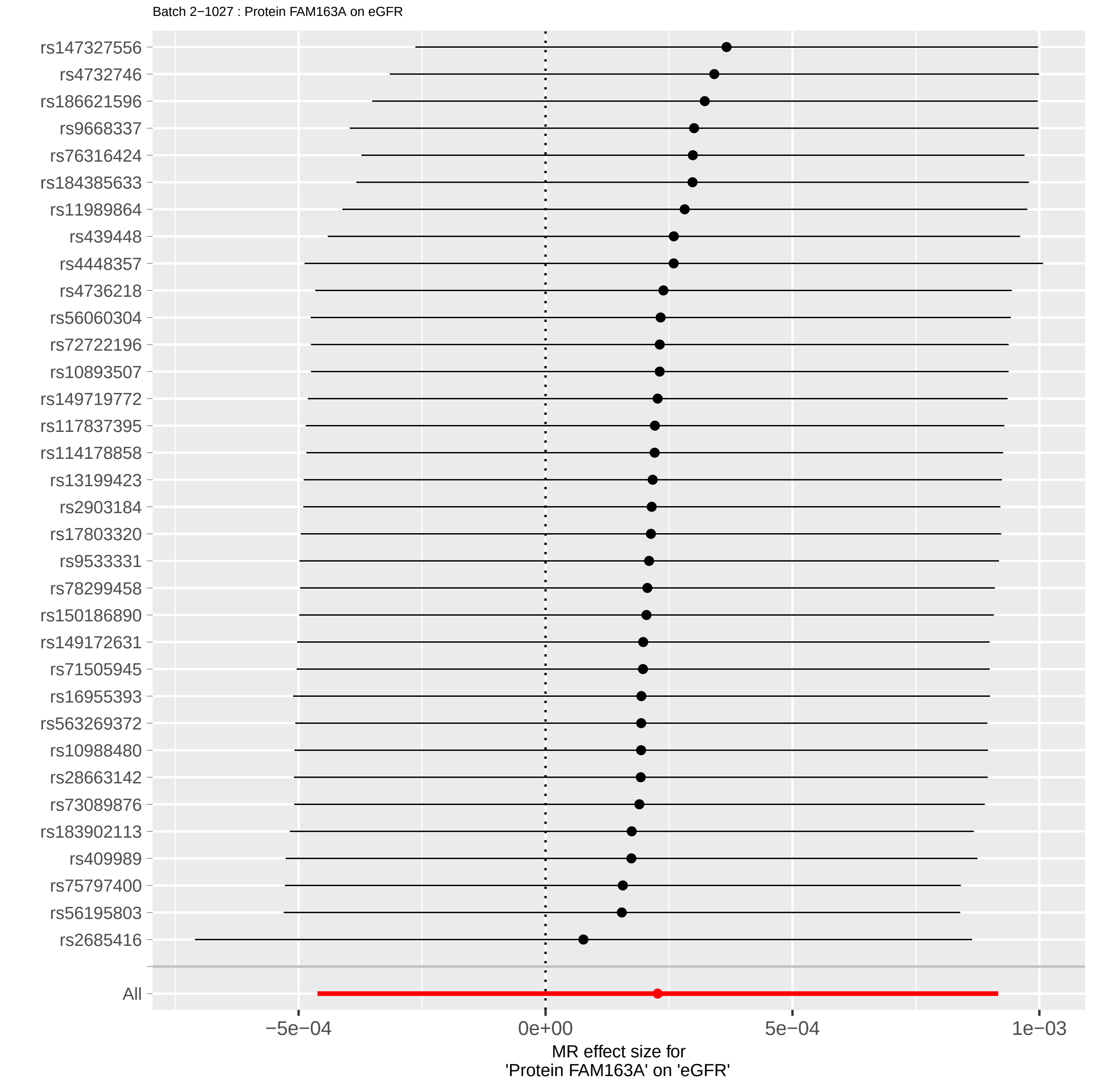

Batch 2−1027 : Protein FAM163A on eGFR
rs147327556
rs4732746
rs186621596
rs9668337
rs76316424
rs184385633
rs11989864
rs439448
rs4448357
rs4736218
rs56060304
rs72722196
rs10893507
rs149719772
rs117837395
rs114178858
rs13199423
rs2903184
rs17803320
rs9533331
rs78299458
rs150186890
rs149172631
rs71505945
rs16955393
rs563269372
rs10988480
rs28663142
rs73089876
rs183902113
rs409989
rs75797400
rs56195803
rs2685416
All
−5e−04
0e+00
5e−04
1e−03
MR effect size for
'Protein FAM163A' on 'eGFR'

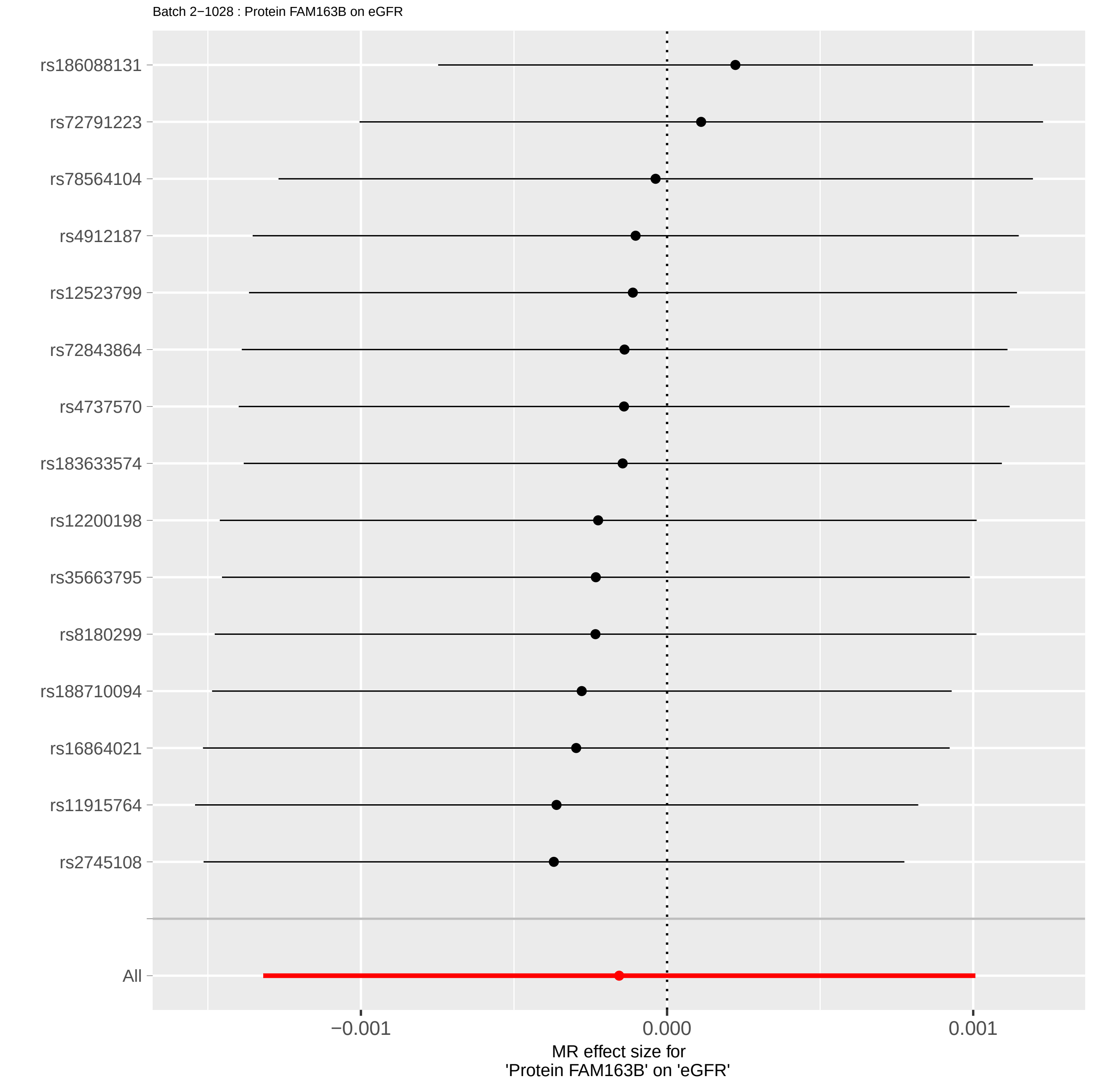

Batch 2−1028 : Protein FAM163B on eGFR
rs186088131
rs72791223
rs78564104
rs4912187
rs12523799
rs72843864
rs4737570
rs183633574
rs12200198
rs35663795
rs8180299
rs188710094
rs16864021
rs11915764
rs2745108
All
−0.001
0.000
0.001
MR effect size for
'Protein FAM163B' on 'eGFR'

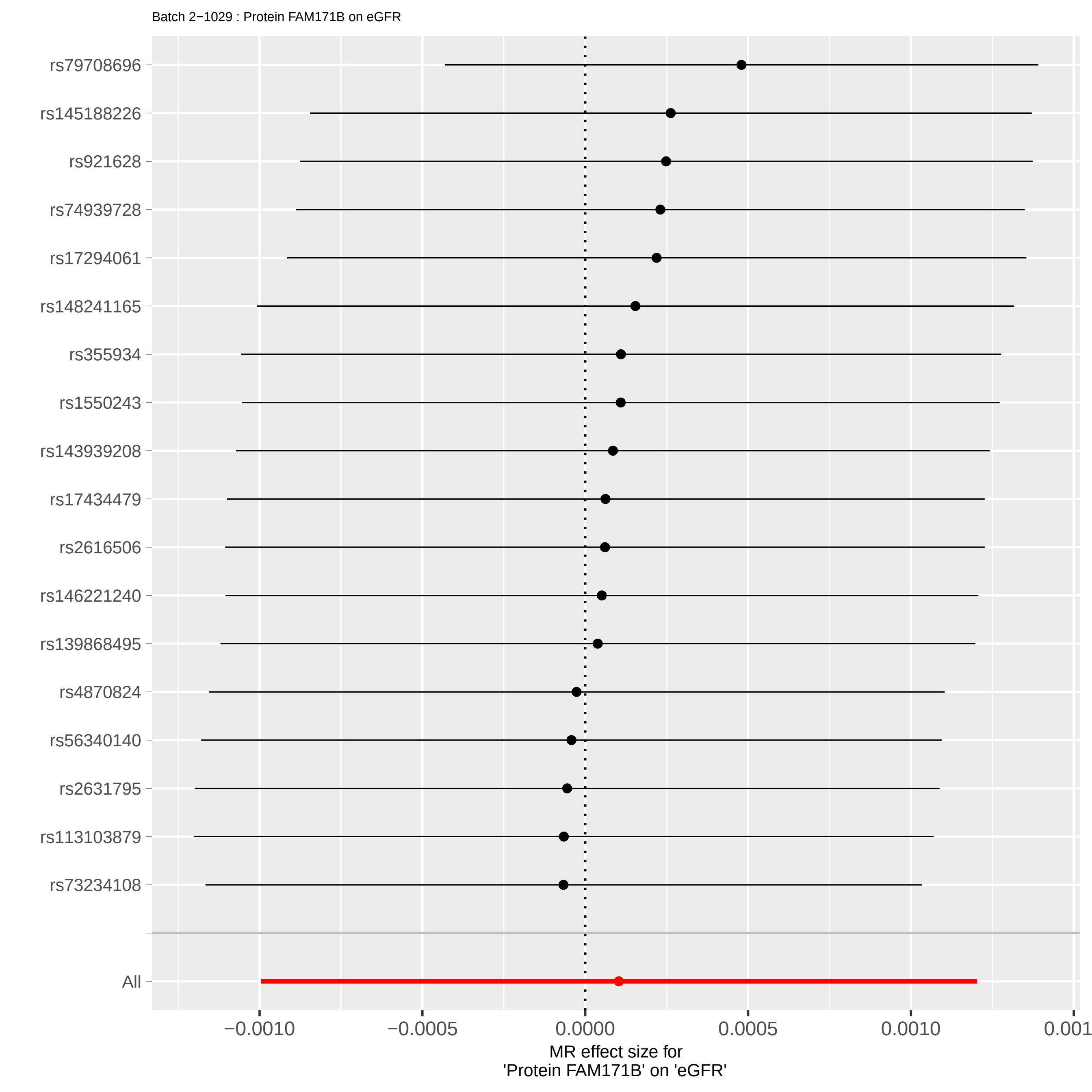

Batch 2−1029 : Protein FAM171B on eGFR
rs79708696
rs145188226
rs921628
rs74939728
rs17294061
rs148241165
rs355934
rs1550243
rs143939208
rs17434479
rs2616506
rs146221240
rs139868495
rs4870824
rs56340140
rs2631795
rs113103879
rs73234108
All
−0.0010
−0.0005
0.0000
0.0005
0.0010
0.001
MR effect size for
'Protein FAM171B' on 'eGFR'

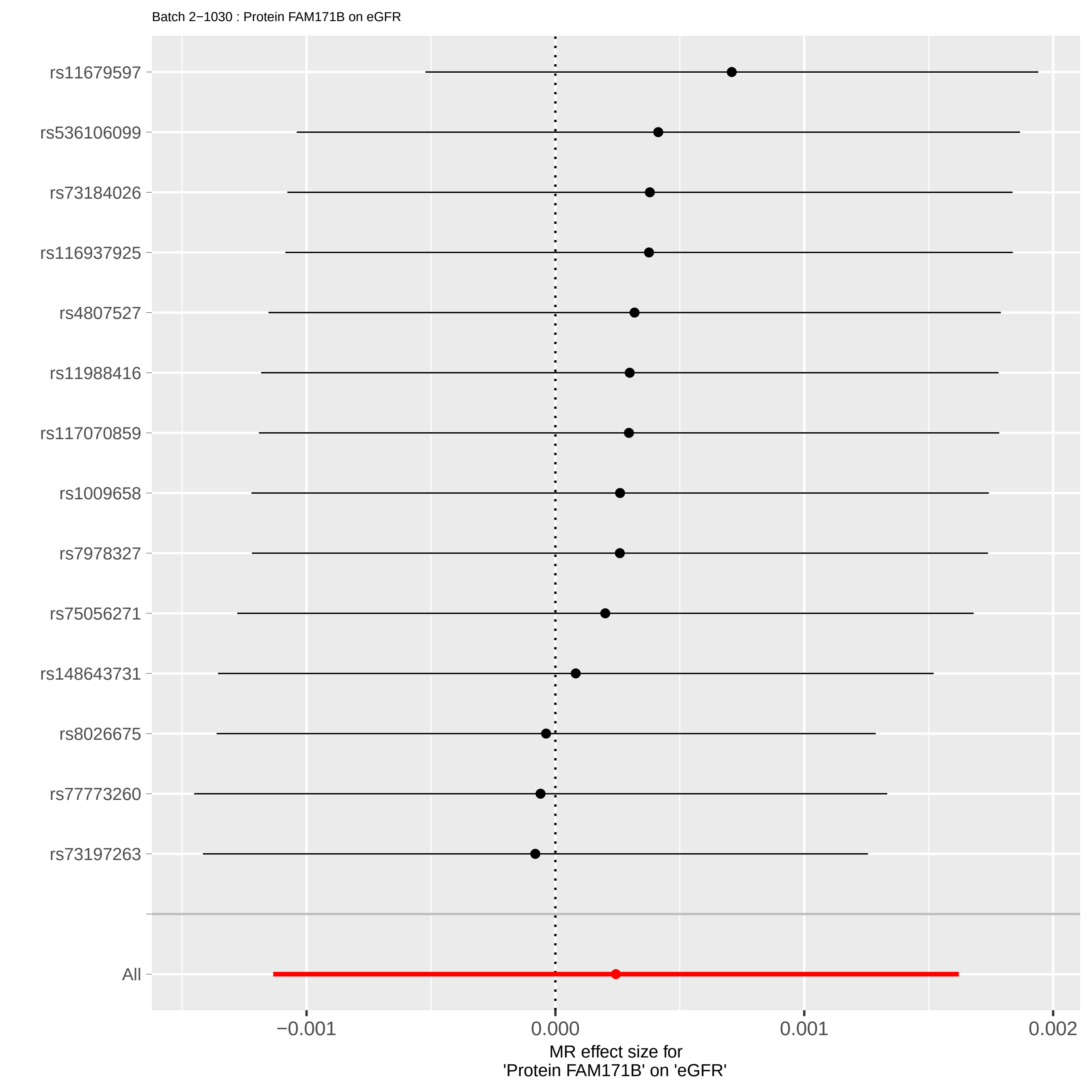

Batch 2−1030 : Protein FAM171B on eGFR
rs11679597
rs536106099
rs73184026
rs116937925
rs4807527
rs11988416
rs117070859
rs1009658
rs7978327
rs75056271
rs148643731
rs8026675
rs77773260
rs73197263
All
−0.001
0.000
0.001
0.002
MR effect size for
'Protein FAM171B' on 'eGFR'

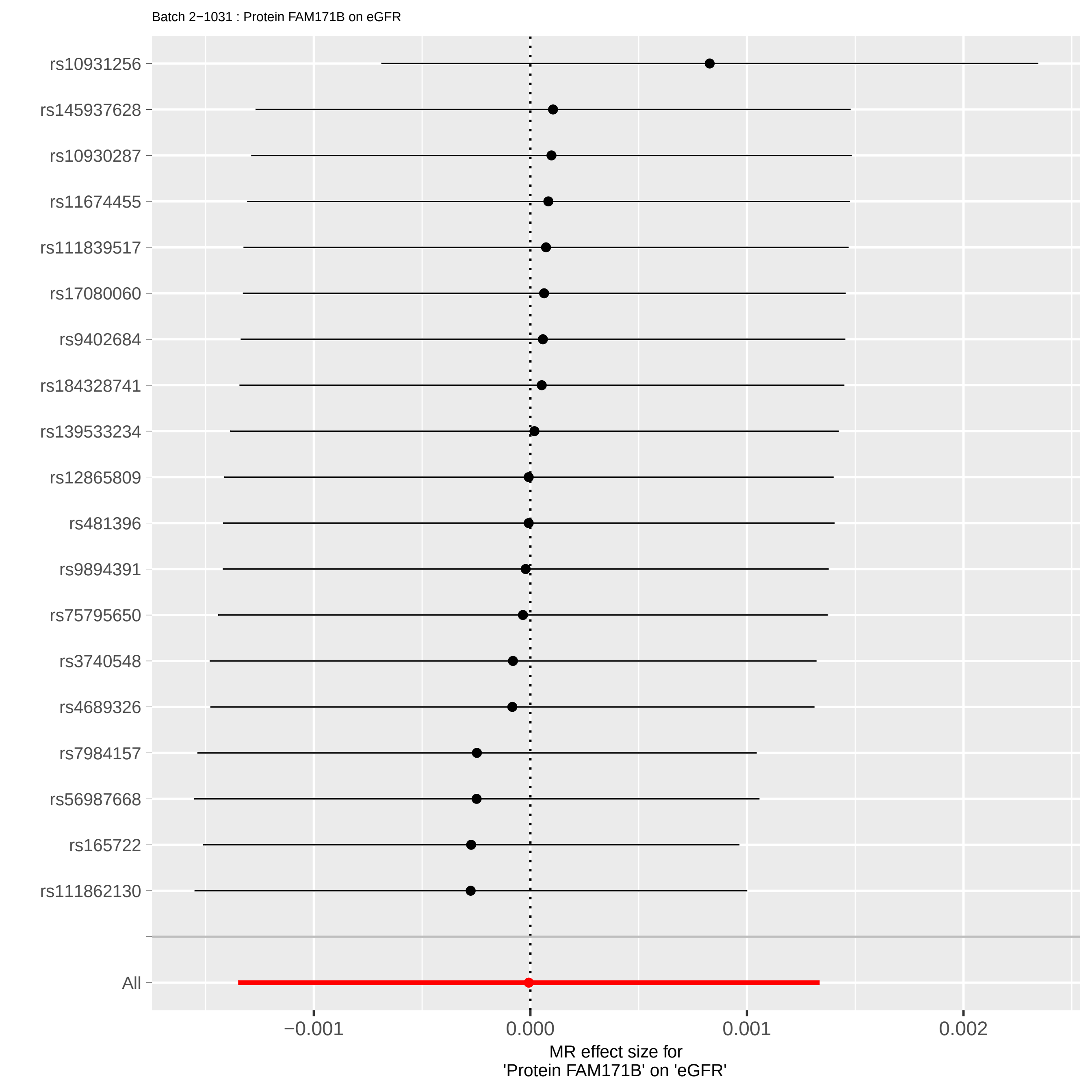

Batch 2−1031 : Protein FAM171B on eGFR
rs10931256
rs145937628
rs10930287
rs11674455
rs111839517
rs17080060
rs9402684
rs184328741
rs139533234
rs12865809
rs481396
rs9894391
rs75795650
rs3740548
rs4689326
rs7984157
rs56987668
rs165722
rs111862130
All
−0.001
0.000
0.001
0.002
MR effect size for
'Protein FAM171B' on 'eGFR'

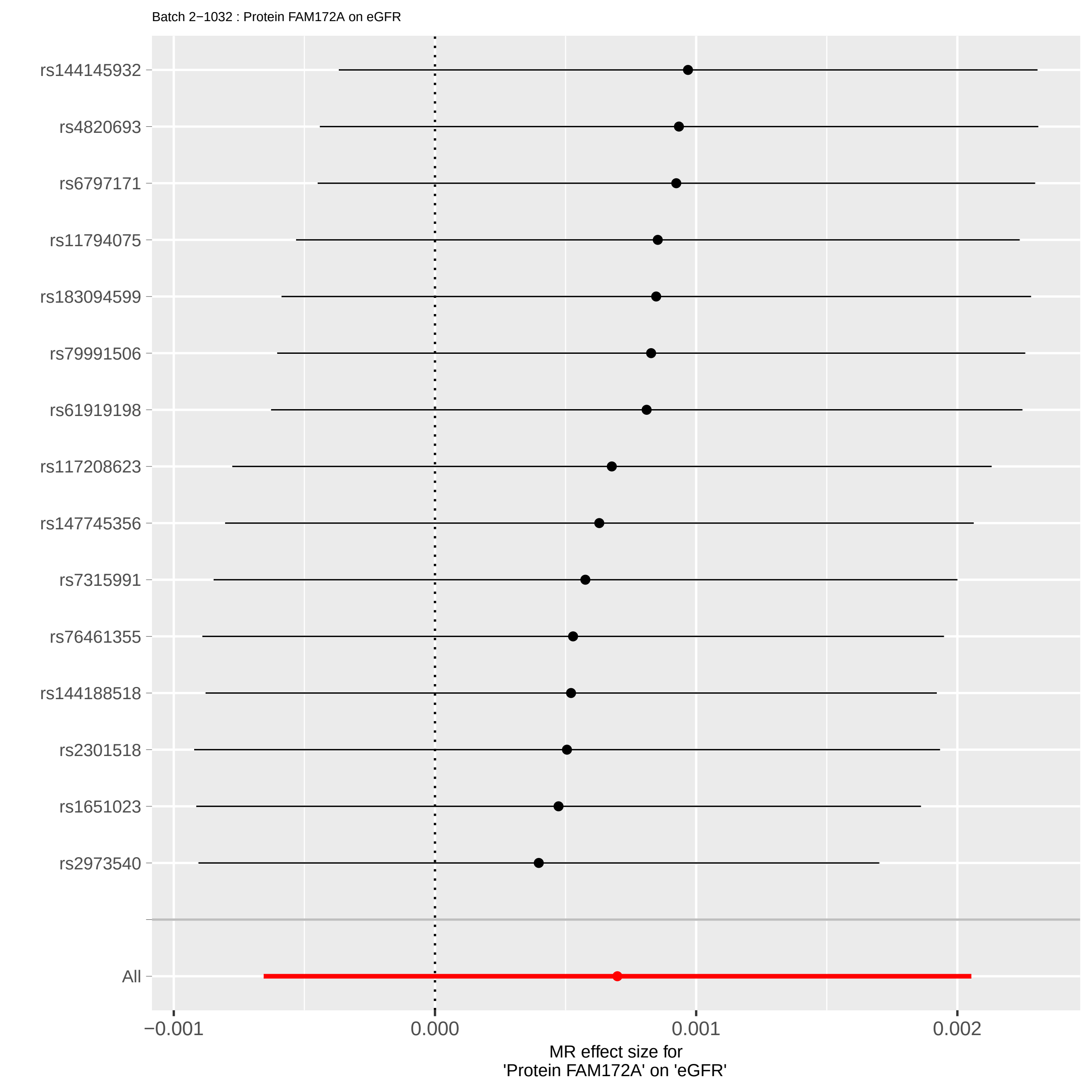

Batch 2−1032 : Protein FAM172A on eGFR
rs144145932
rs4820693
rs6797171
rs11794075
rs183094599
rs79991506
rs61919198
rs117208623
rs147745356
rs7315991
rs76461355
rs144188518
rs2301518
rs1651023
rs2973540
All
−0.001
0.000
0.001
0.002
MR effect size for
'Protein FAM172A' on 'eGFR'

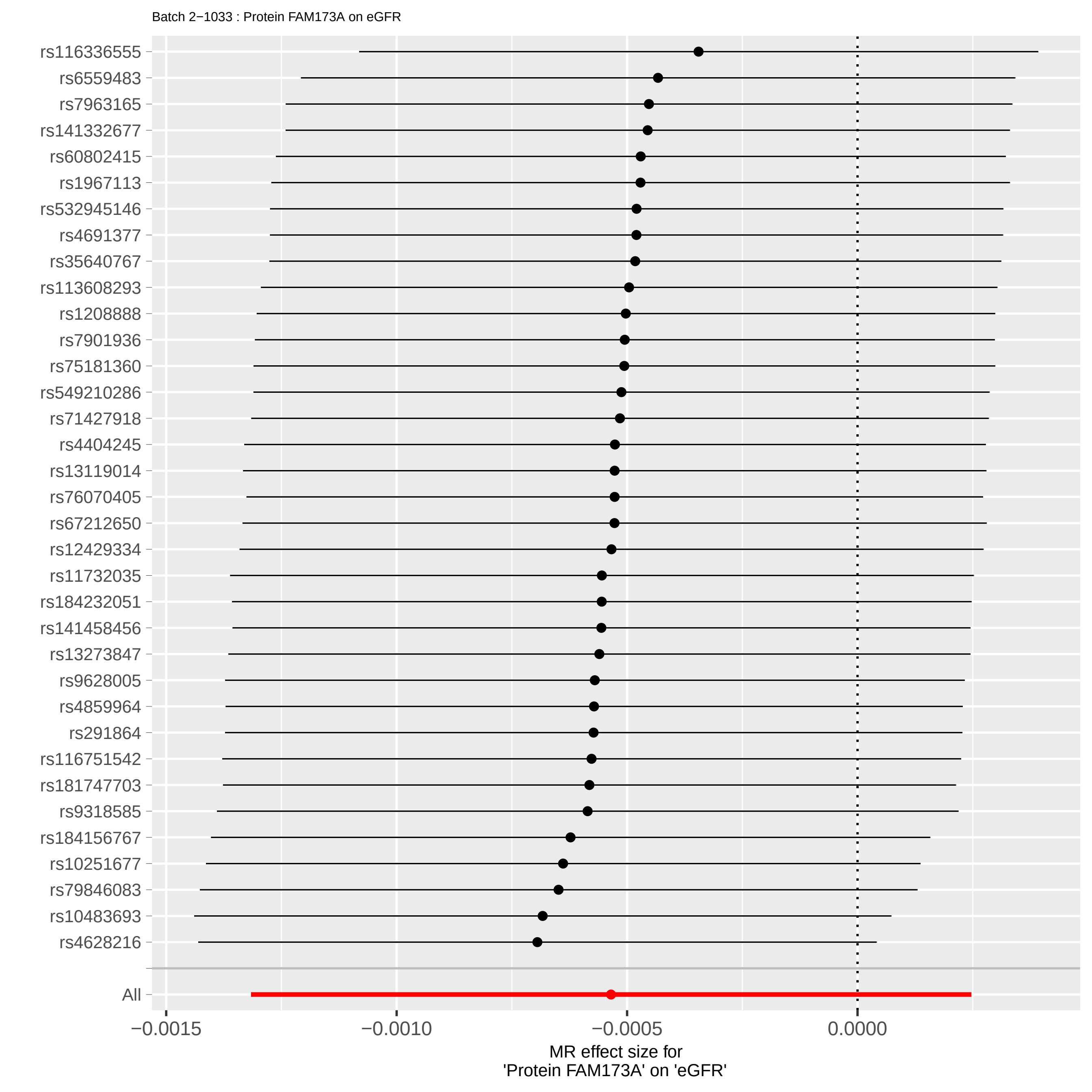

Batch 2−1033 : Protein FAM173A on eGFR
rs116336555
rs6559483
rs7963165
rs141332677
rs60802415
rs1967113
rs532945146
rs4691377
rs35640767
rs113608293
rs1208888
rs7901936
rs75181360
rs549210286
rs71427918
rs4404245
rs13119014
rs76070405
rs67212650
rs12429334
rs11732035
rs184232051
rs141458456
rs13273847
rs9628005
rs4859964
rs291864
rs116751542
rs181747703
rs9318585
rs184156767
rs10251677
rs79846083
rs10483693
rs4628216
All
−0.0015
−0.0010
−0.0005
0.0000
MR effect size for
'Protein FAM173A' on 'eGFR'

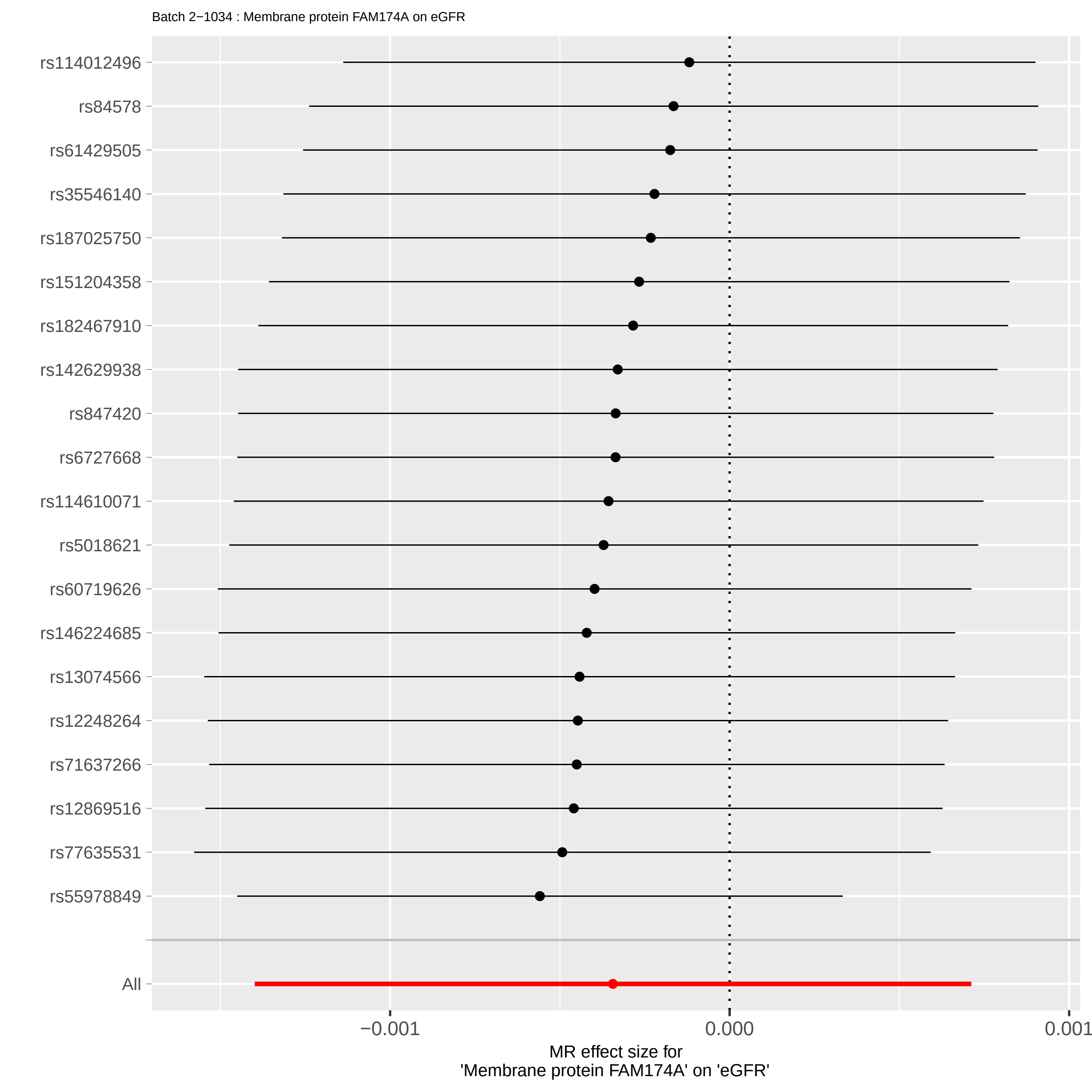
Batch 2−1034 : Membrane protein FAM174A on eGFR
rs114012496
rs84578
rs61429505
rs35546140
rs187025750
rs151204358
rs182467910
rs142629938
rs847420
rs6727668
rs114610071
rs5018621
rs60719626
rs146224685
rs13074566
rs12248264
rs71637266
rs12869516
rs77635531
rs55978849
All
−0.001
0.000
0.001
MR effect size for
'Membrane protein FAM174A' on 'eGFR'

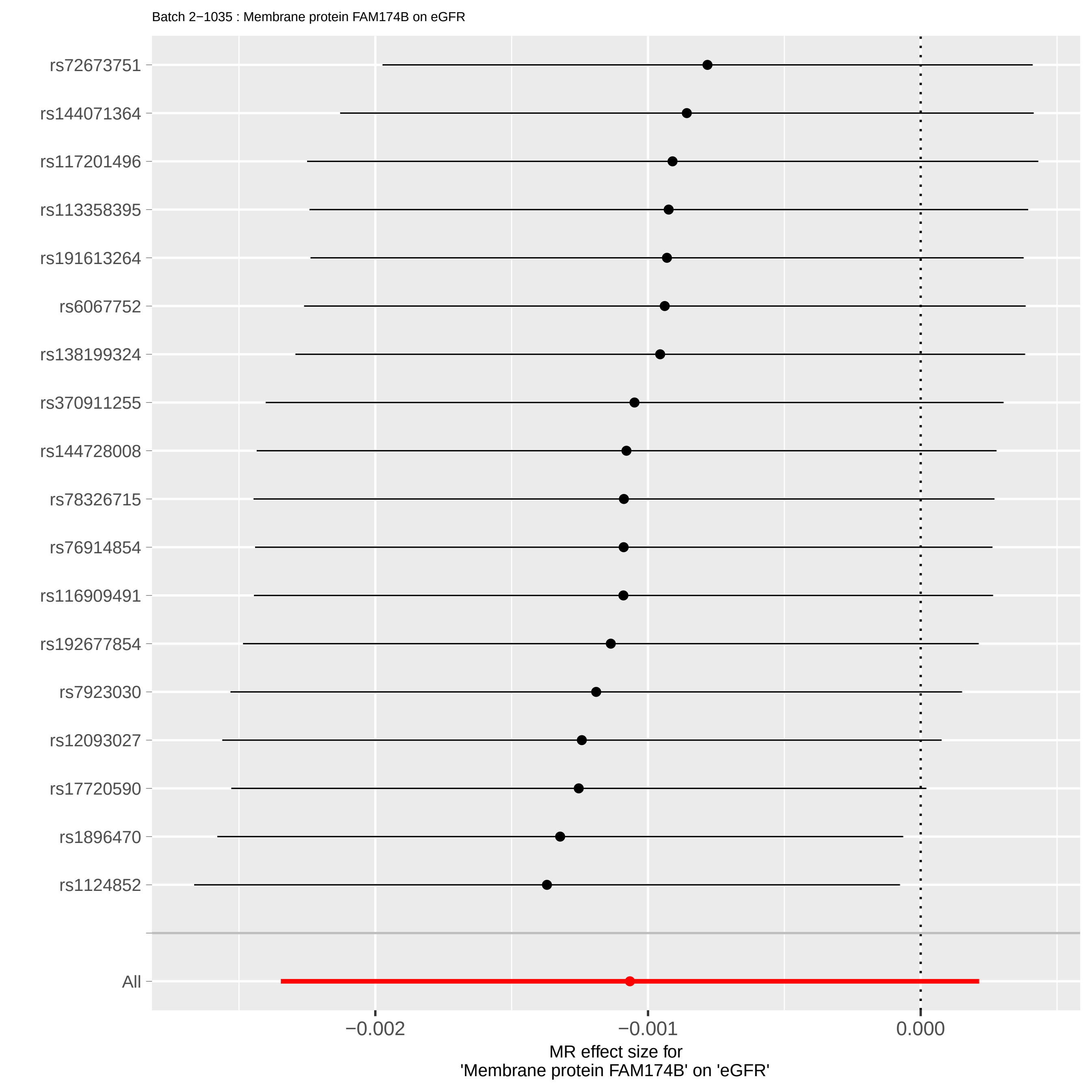

Batch 2−1035 : Membrane protein FAM174B on eGFR
rs72673751
rs144071364
rs117201496
rs113358395
rs191613264
rs6067752
rs138199324
rs370911255
rs144728008
rs78326715
rs76914854
rs116909491
rs192677854
rs7923030
rs12093027
rs17720590
rs1896470
rs1124852
All
−0.002
−0.001
0.000
MR effect size for
'Membrane protein FAM174B' on 'eGFR'

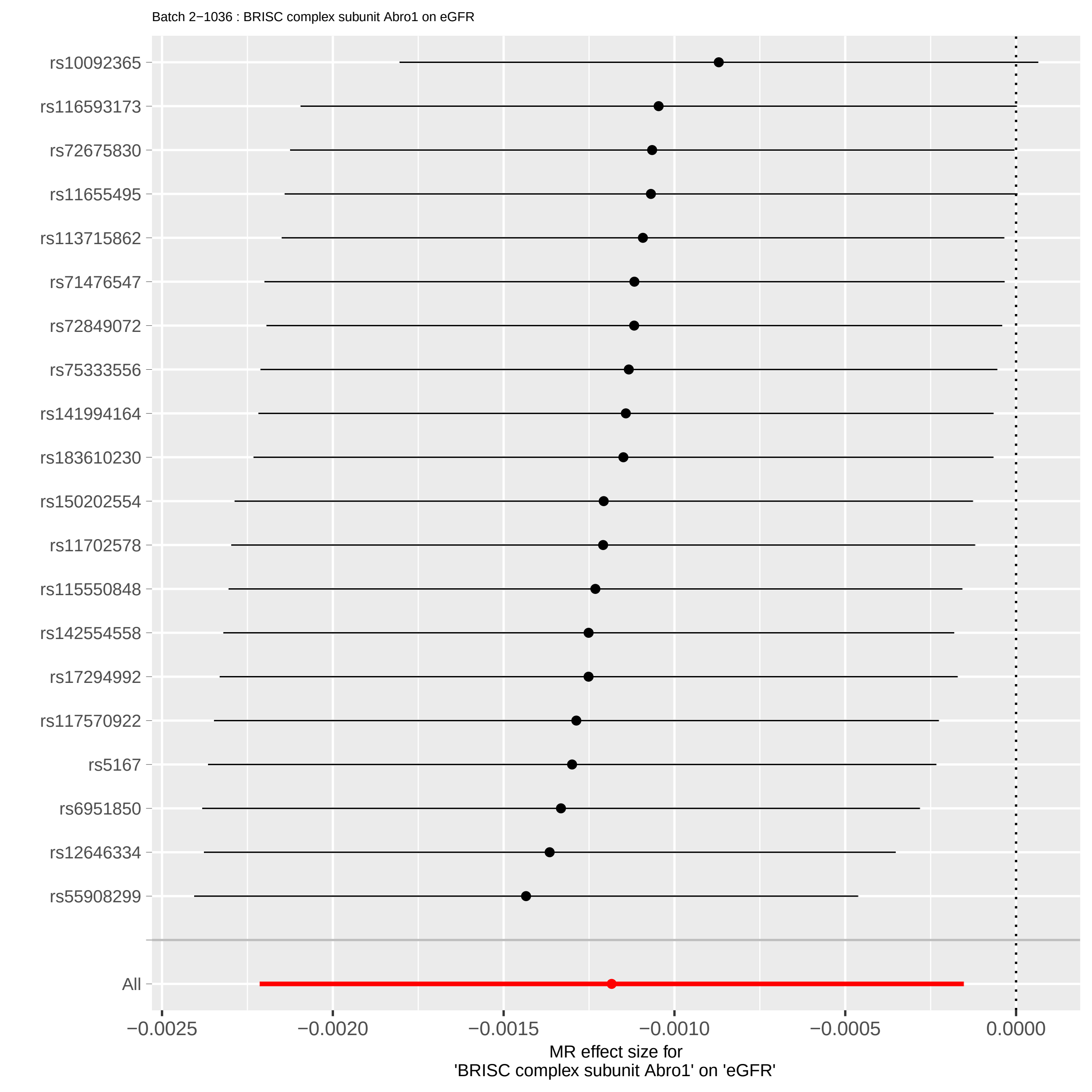

Batch 2−1036 : BRISC complex subunit Abro1 on eGFR
rs10092365
rs116593173
rs72675830
rs11655495
rs113715862
rs71476547
rs72849072
rs75333556
rs141994164
rs183610230
rs150202554
rs11702578
rs115550848
rs142554558
rs17294992
rs117570922
rs5167
rs6951850
rs12646334
rs55908299
All
−0.0025
−0.0020
−0.0015
−0.0010
−0.0005
0.0000
MR effect size for
'BRISC complex subunit Abro1' on 'eGFR'

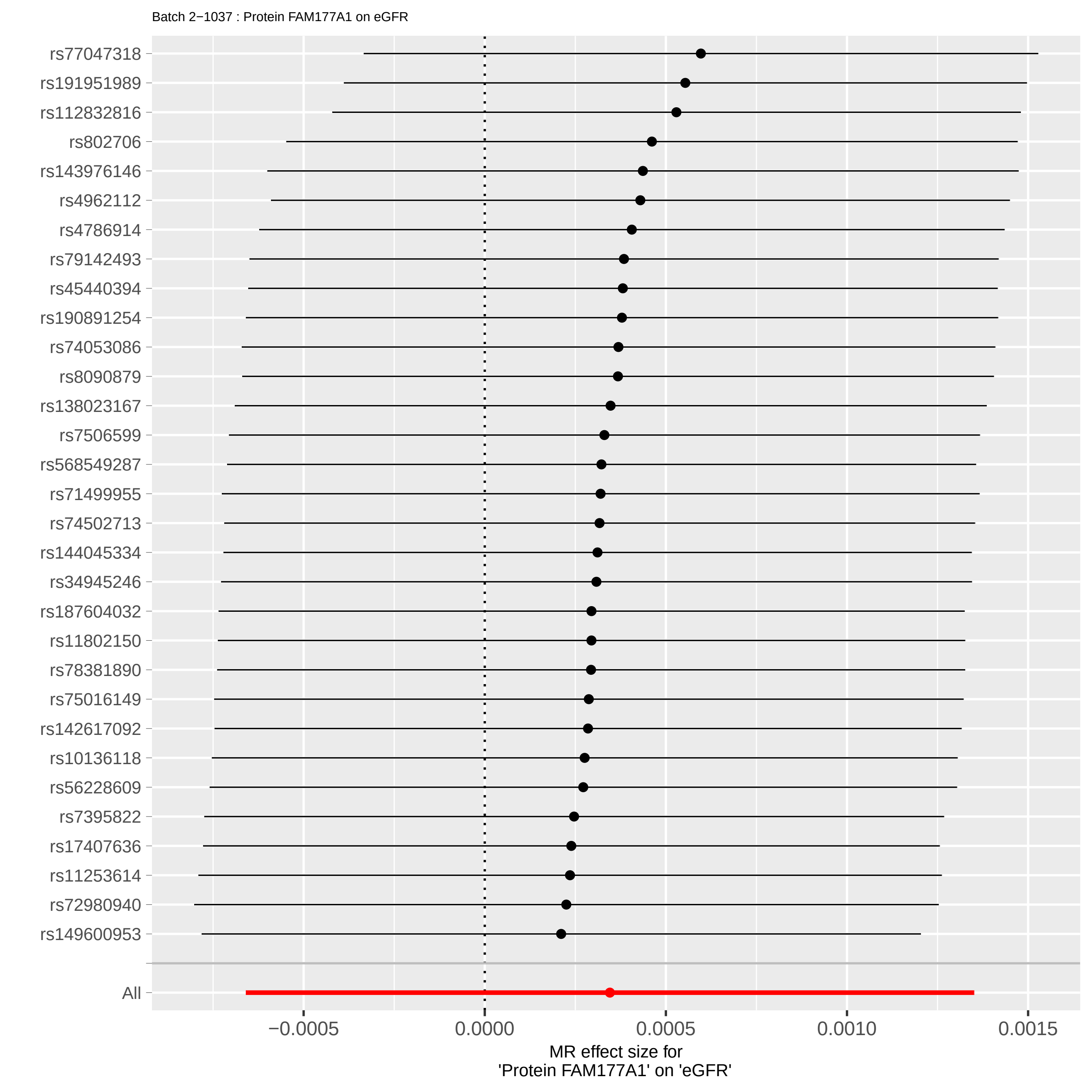
Batch 2−1037 : Protein FAM177A1 on eGFR
rs77047318
rs191951989
rs112832816
rs802706
rs143976146
rs4962112
rs4786914
rs79142493
rs45440394
rs190891254
rs74053086
rs8090879
rs138023167
rs7506599
rs568549287
rs71499955
rs74502713
rs144045334
rs34945246
rs187604032
rs11802150
rs78381890
rs75016149
rs142617092
rs10136118
rs56228609
rs7395822
rs17407636
rs11253614
rs72980940
rs149600953
All
−0.0005
0.0000
0.0005
0.0010
0.0015
MR effect size for
'Protein FAM177A1' on 'eGFR'

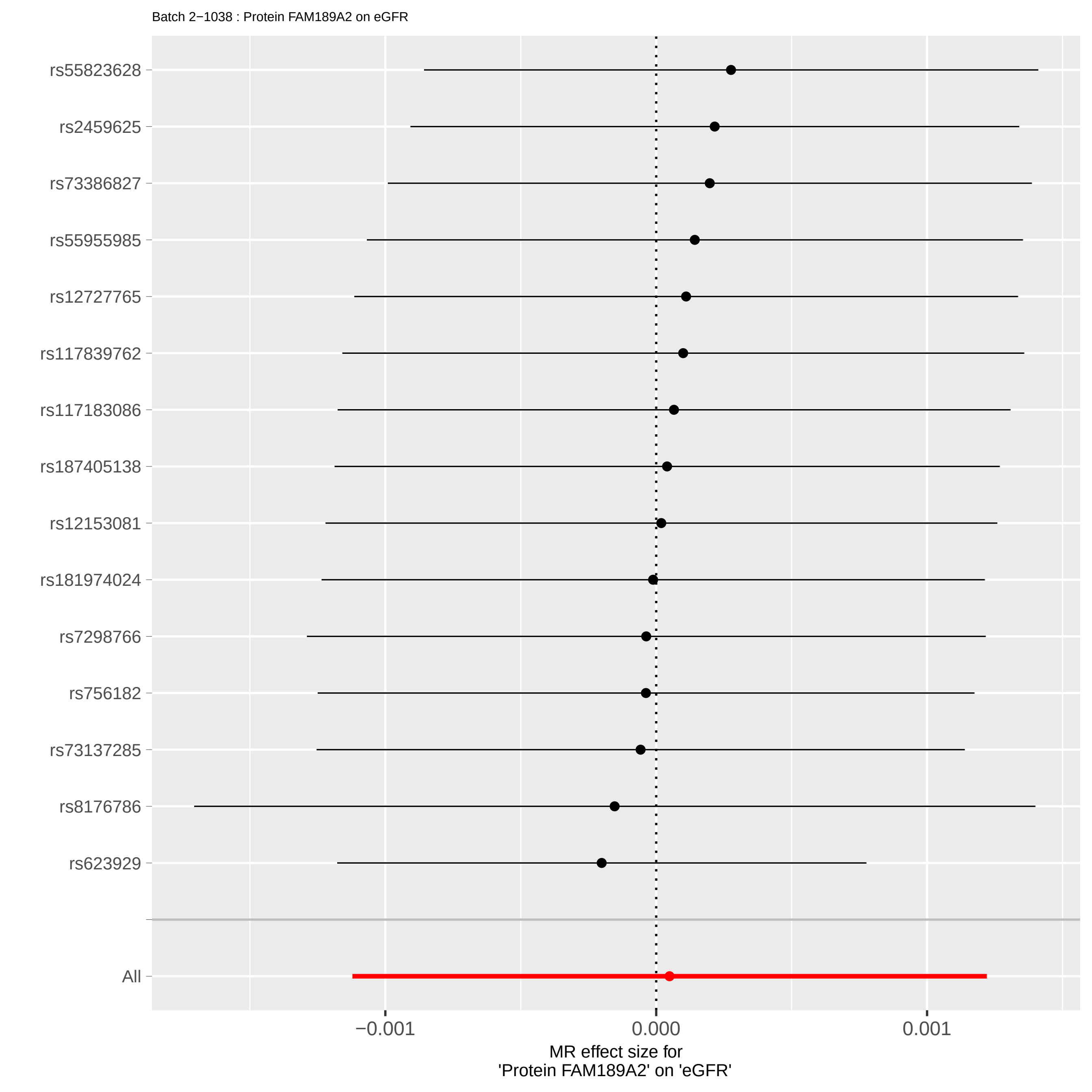

Batch 2−1038 : Protein FAM189A2 on eGFR
rs55823628
rs2459625
rs73386827
rs55955985
rs12727765
rs117839762
rs117183086
rs187405138
rs12153081
rs181974024
rs7298766
rs756182
rs73137285
rs8176786
rs623929
All
−0.001
0.000
0.001
MR effect size for
'Protein FAM189A2' on 'eGFR'

Batch 2−1039 : Protein FAM189A2 on eGFR

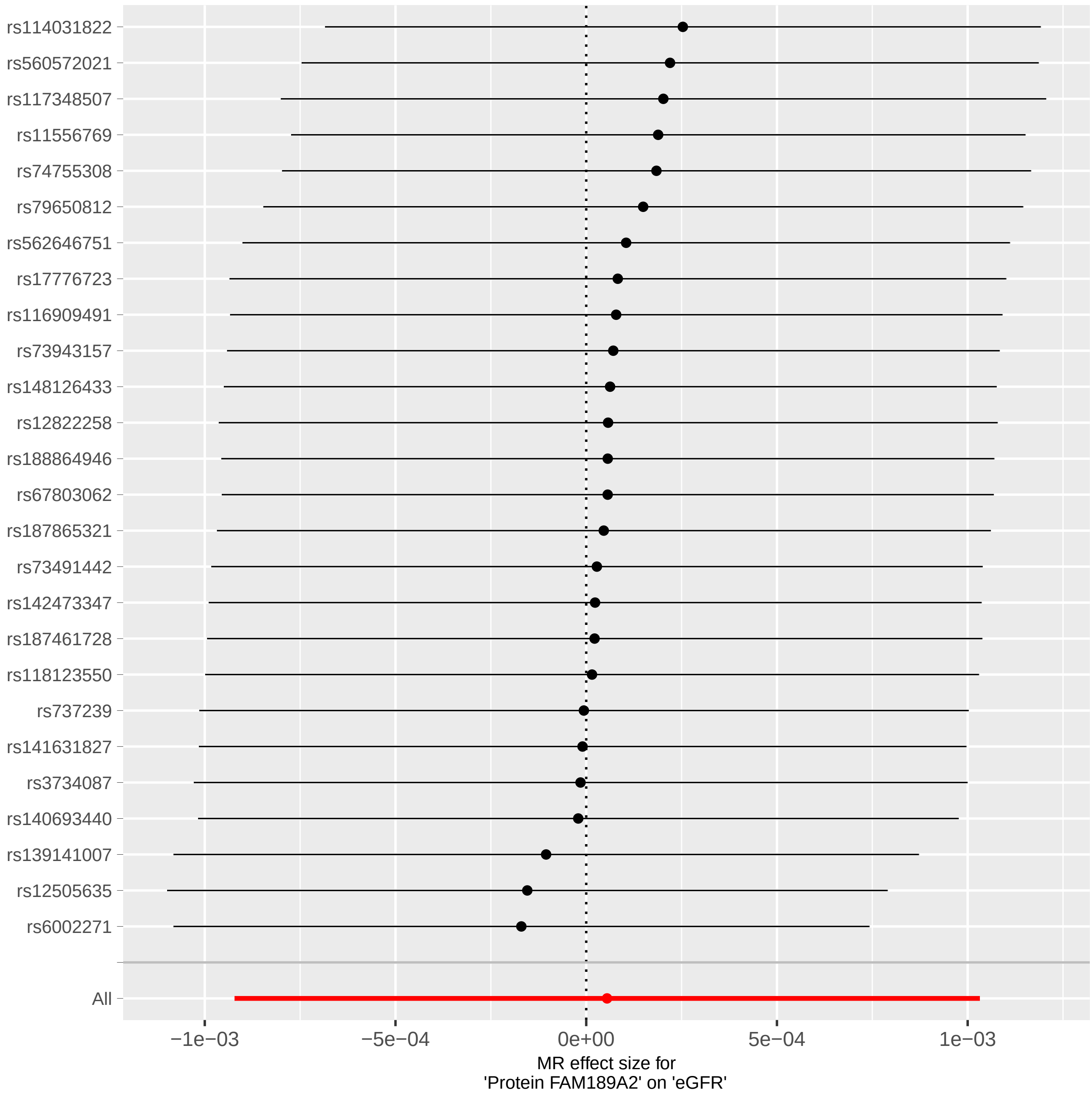

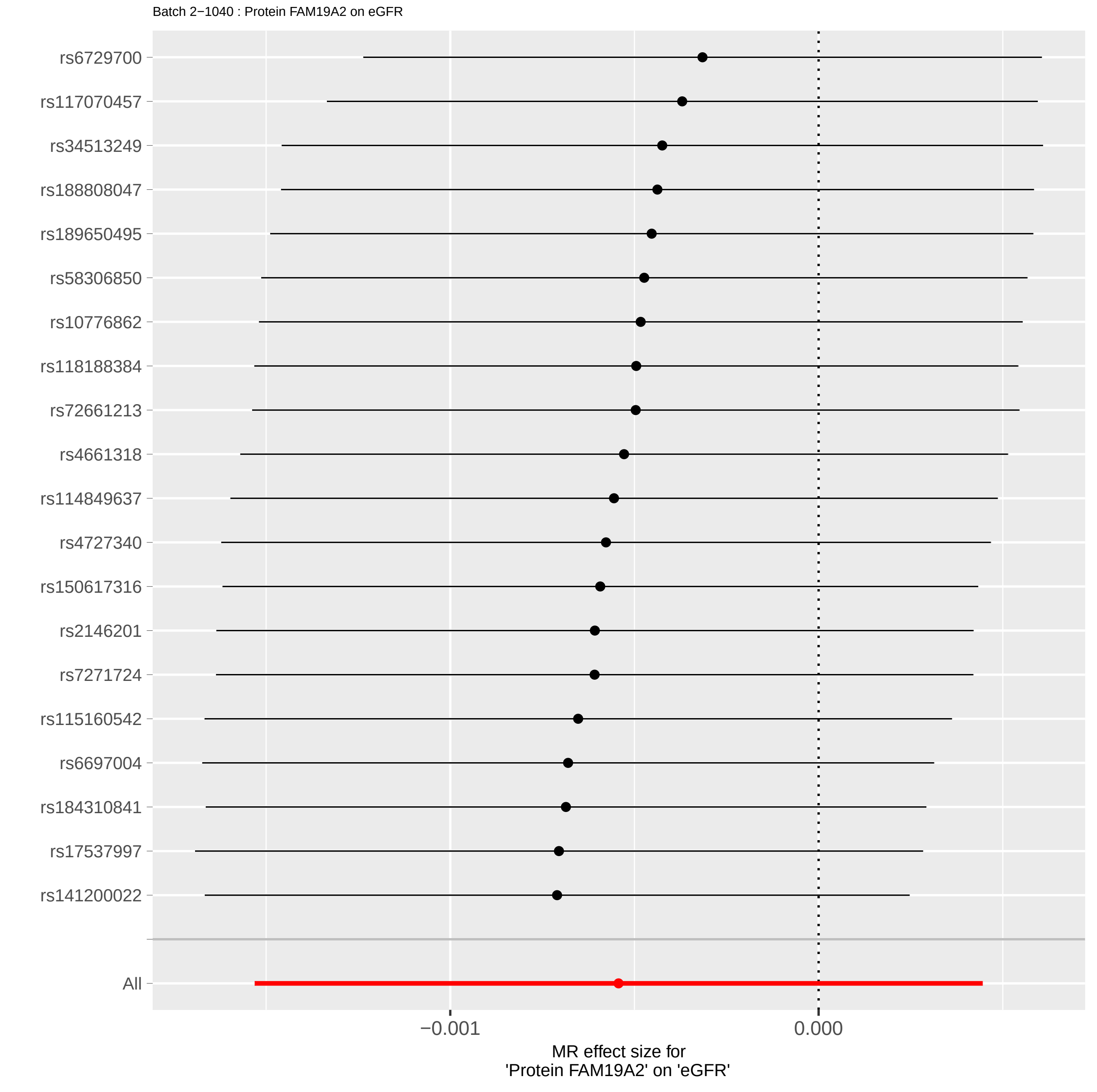

Batch 2−1040 : Protein FAM19A2 on eGFR
rs6729700
rs117070457
rs34513249
rs188808047
rs189650495
rs58306850
rs10776862
rs118188384
rs72661213
rs4661318
rs114849637
rs4727340
rs150617316
rs2146201
rs7271724
rs115160542
rs6697004
rs184310841
rs17537997
rs141200022
All
−0.001
0.000
MR effect size for
'Protein FAM19A2' on 'eGFR'

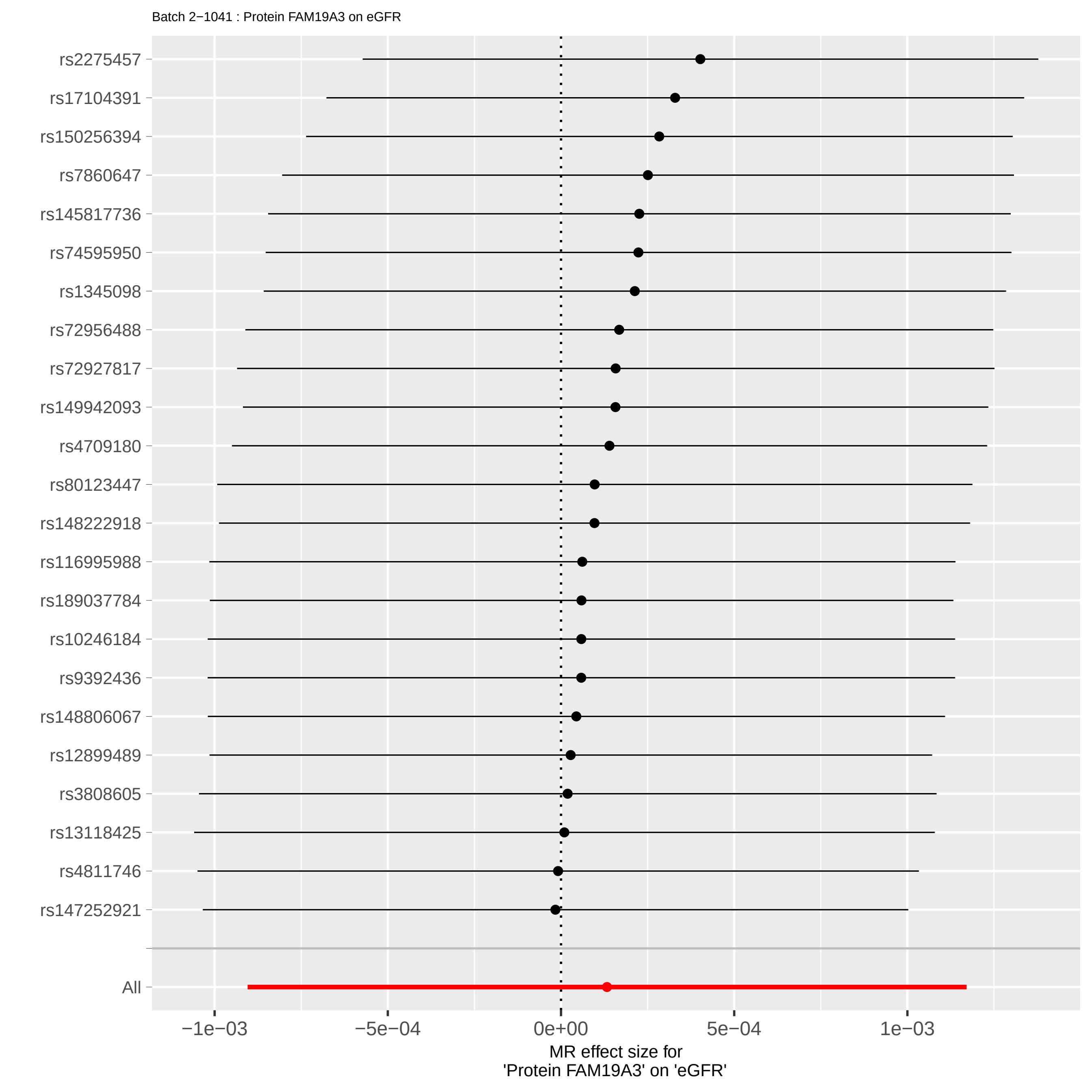

Batch 2−1041 : Protein FAM19A3 on eGFR
rs2275457
rs17104391
rs150256394
rs7860647
rs145817736
rs74595950
rs1345098
rs72956488
rs72927817
rs149942093
rs4709180
rs80123447
rs148222918
rs116995988
rs189037784
rs10246184
rs9392436
rs148806067
rs12899489
rs3808605
rs13118425
rs4811746
rs147252921
All
−1e−03
−5e−04
0e+00
5e−04
1e−03
MR effect size for
'Protein FAM19A3' on 'eGFR'

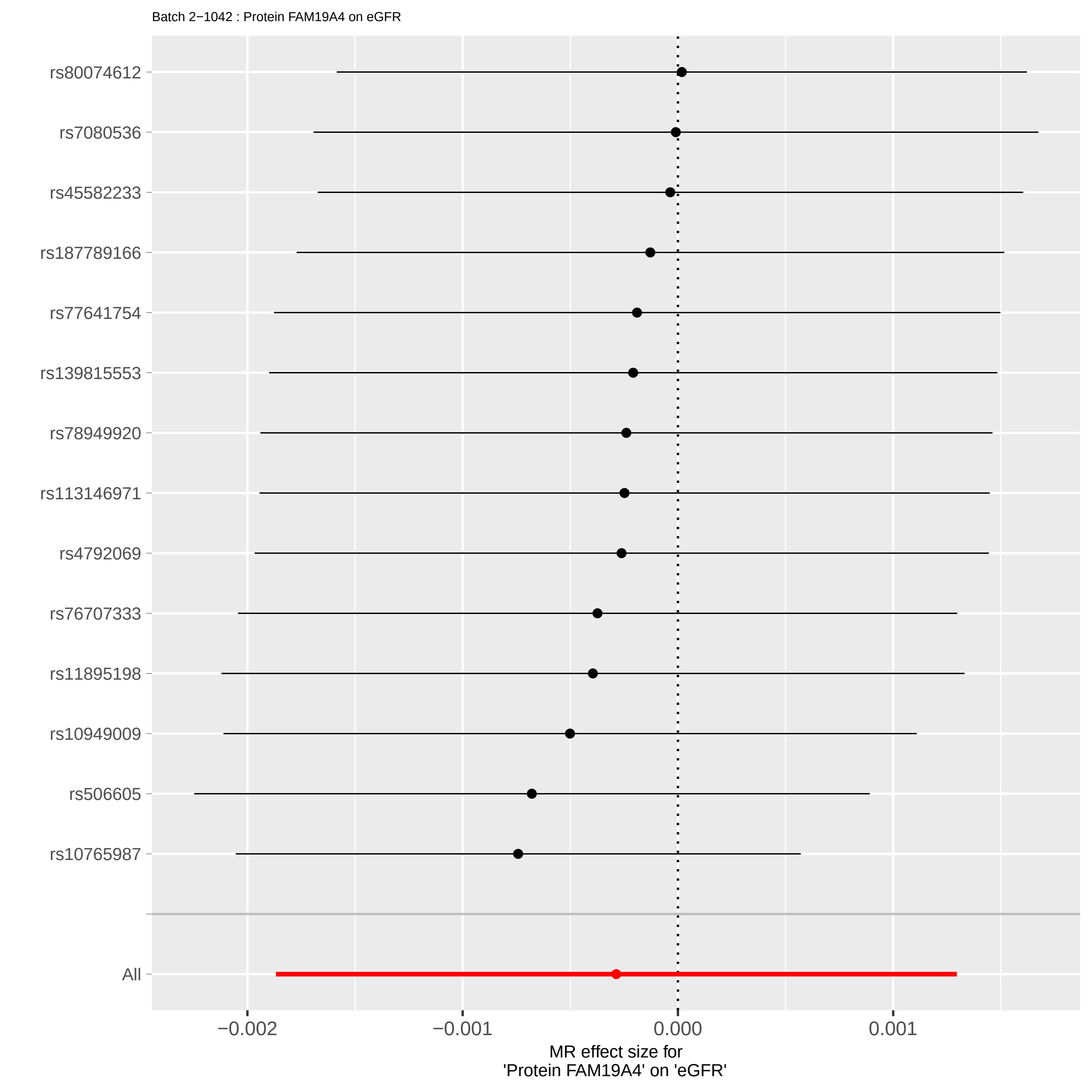
Batch 2−1042 : Protein FAM19A4 on eGFR
rs80074612
rs7080536
rs45582233
rs187789166
rs77641754
rs139815553
rs78949920
rs113146971
rs4792069
rs76707333
rs11895198
rs10949009
rs506605
rs10765987
All
−0.002
−0.001
0.000
0.001
MR effect size for
'Protein FAM19A4' on 'eGFR'

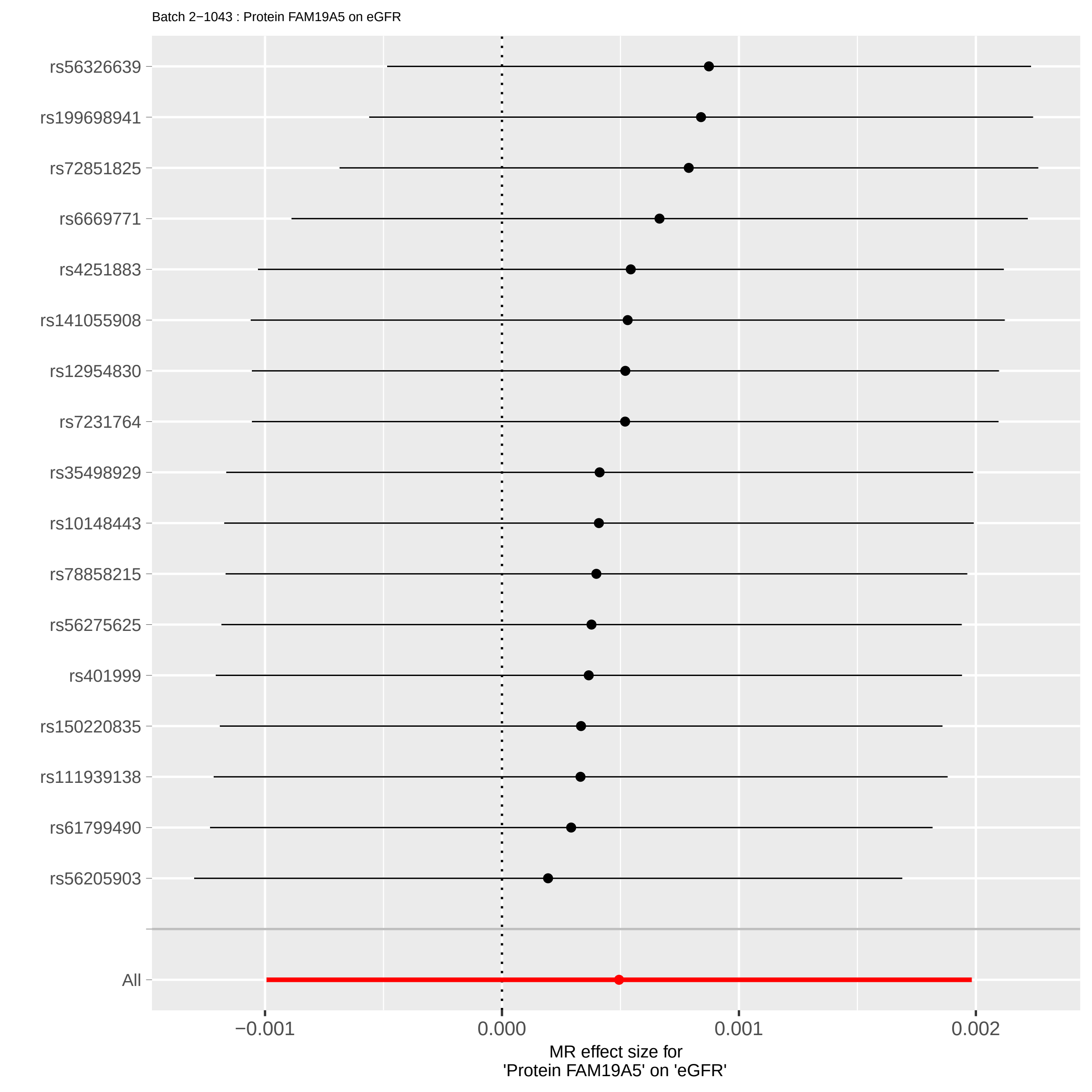

Batch 2−1043 : Protein FAM19A5 on eGFR
rs56326639
rs199698941
rs72851825
rs6669771
rs4251883
rs141055908
rs12954830
rs7231764
rs35498929
rs10148443
rs78858215
rs56275625
rs401999
rs150220835
rs111939138
rs61799490
rs56205903
All
−0.001
0.000
0.001
0.002
MR effect size for
'Protein FAM19A5' on 'eGFR'

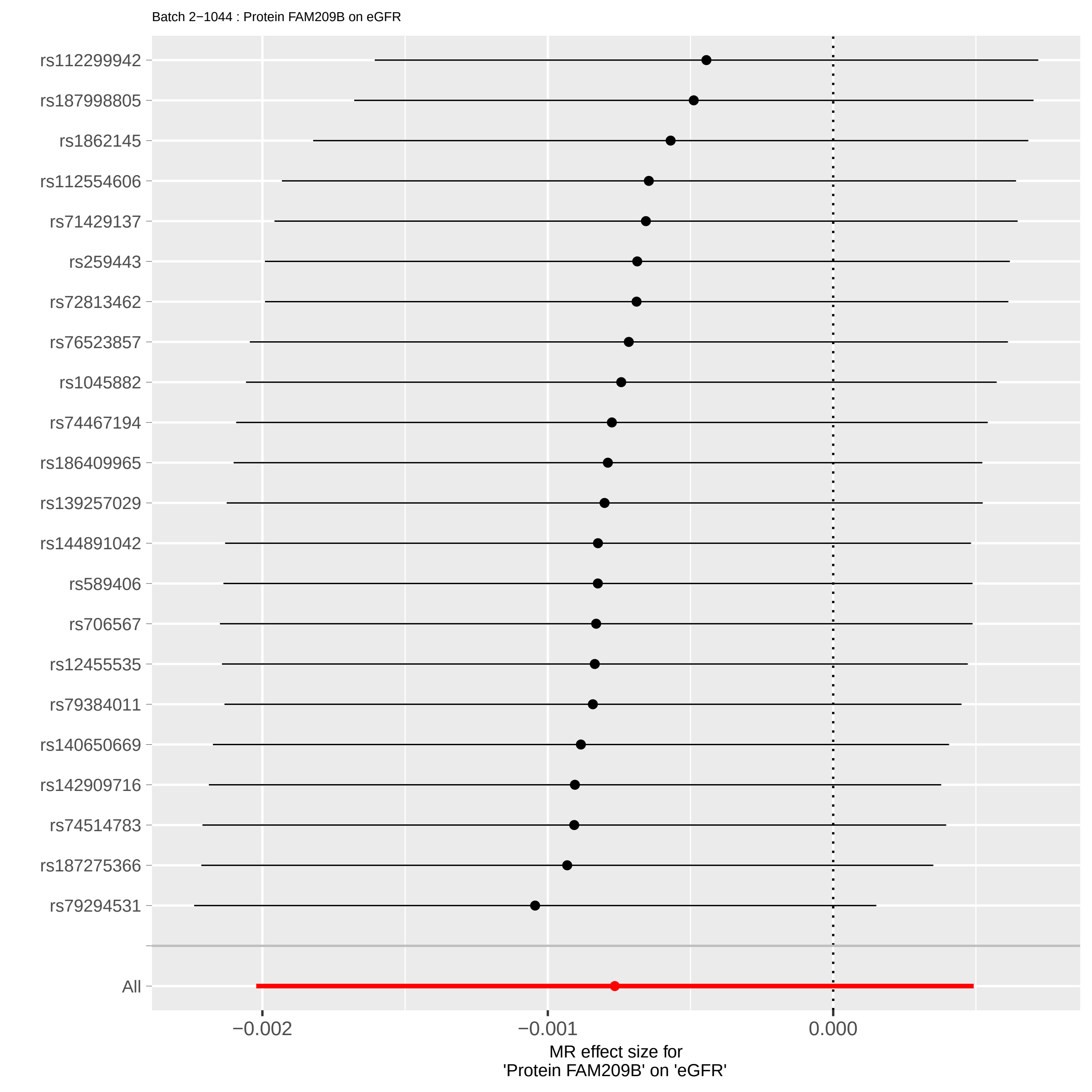
Batch 2−1044 : Protein FAM209B on eGFR
rs112299942
rs187998805
rs1862145
rs112554606
rs71429137
rs259443
rs72813462
rs76523857
rs1045882
rs74467194
rs186409965
rs139257029
rs144891042
rs589406
rs706567
rs12455535
rs79384011
rs140650669
rs142909716
rs74514783
rs187275366
rs79294531
All
−0.002
−0.001
0.000
MR effect size for
'Protein FAM209B' on 'eGFR'

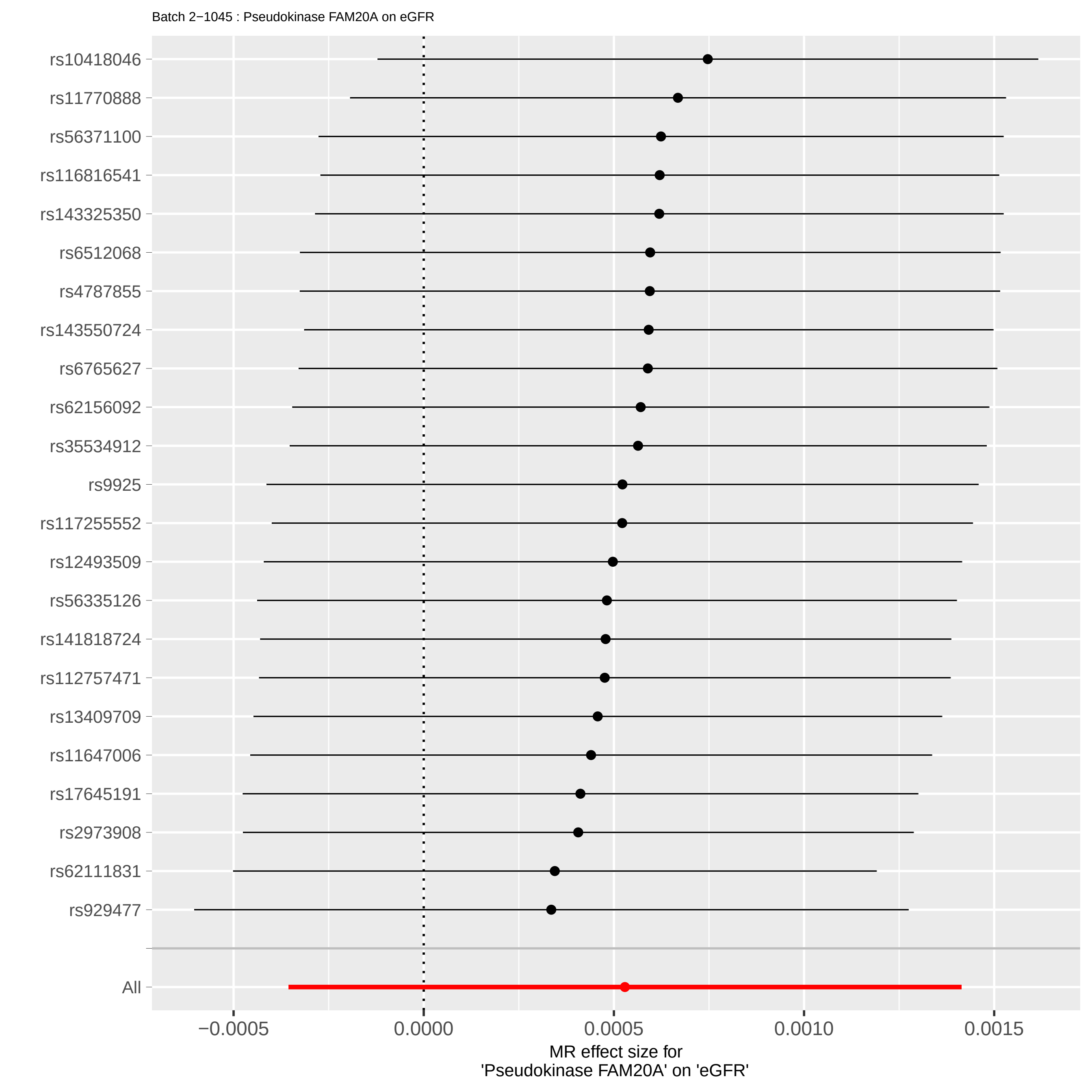
Batch 2−1045 : Pseudokinase FAM20A on eGFR
rs10418046
rs11770888
rs56371100
rs116816541
rs143325350
rs6512068
rs4787855
rs143550724
rs6765627
rs62156092
rs35534912
rs9925
rs117255552
rs12493509
rs56335126
rs141818724
rs112757471
rs13409709
rs11647006
rs17645191
rs2973908
rs62111831
rs929477
All
−0.0005
0.0000
0.0005
0.0010
0.0015
MR effect size for
'Pseudokinase FAM20A' on 'eGFR'

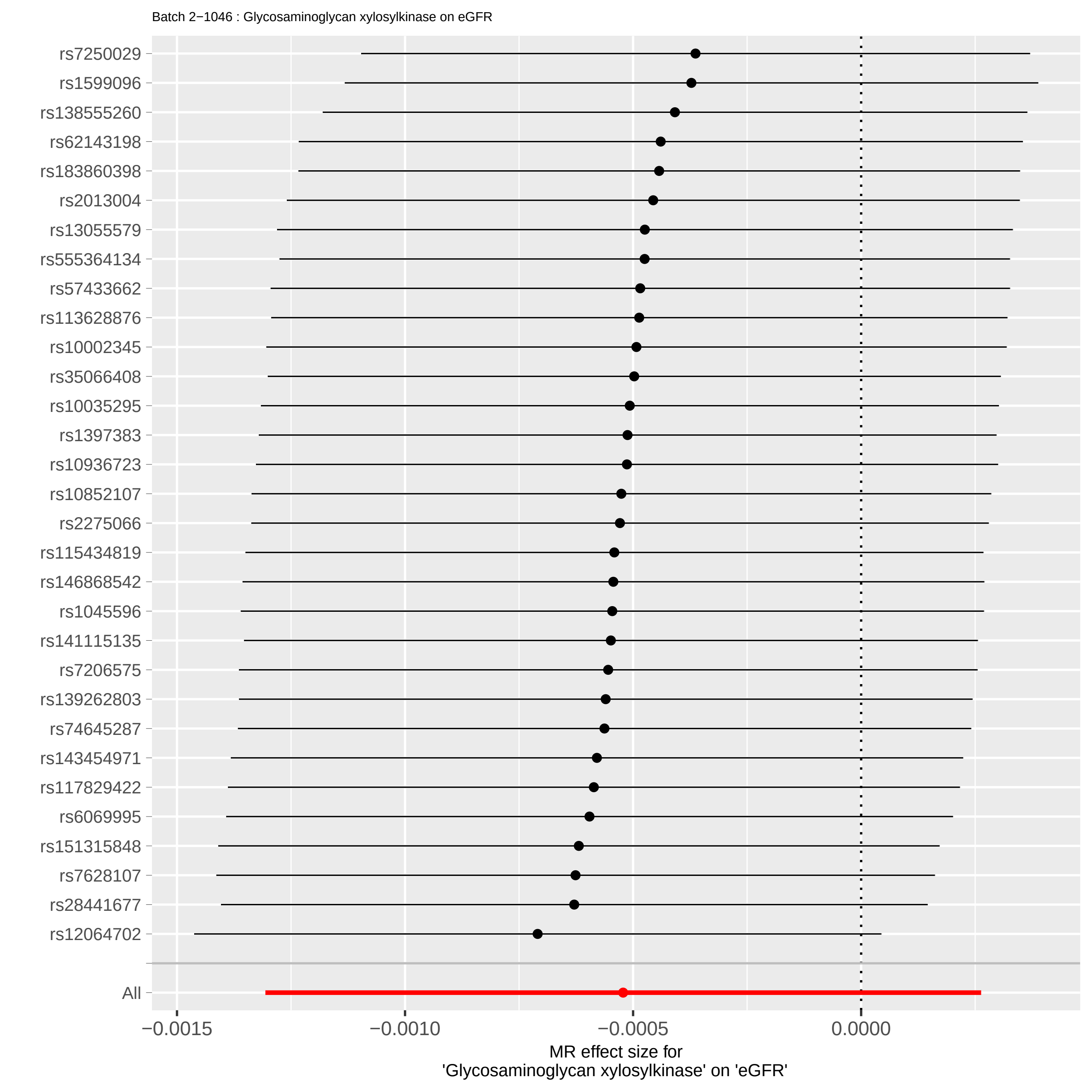

Batch 2−1046 : Glycosaminoglycan xylosylkinase on eGFR
rs7250029
rs1599096
rs138555260
rs62143198
rs183860398
rs2013004
rs13055579
rs555364134
rs57433662
rs113628876
rs10002345
rs35066408
rs10035295
rs1397383
rs10936723
rs10852107
rs2275066
rs115434819
rs146868542
rs1045596
rs141115135
rs7206575
rs139262803
rs74645287
rs143454971
rs117829422
rs6069995
rs151315848
rs7628107
rs28441677
rs12064702
All
−0.0015
−0.0010
−0.0005
0.0000
MR effect size for
'Glycosaminoglycan xylosylkinase' on 'eGFR'

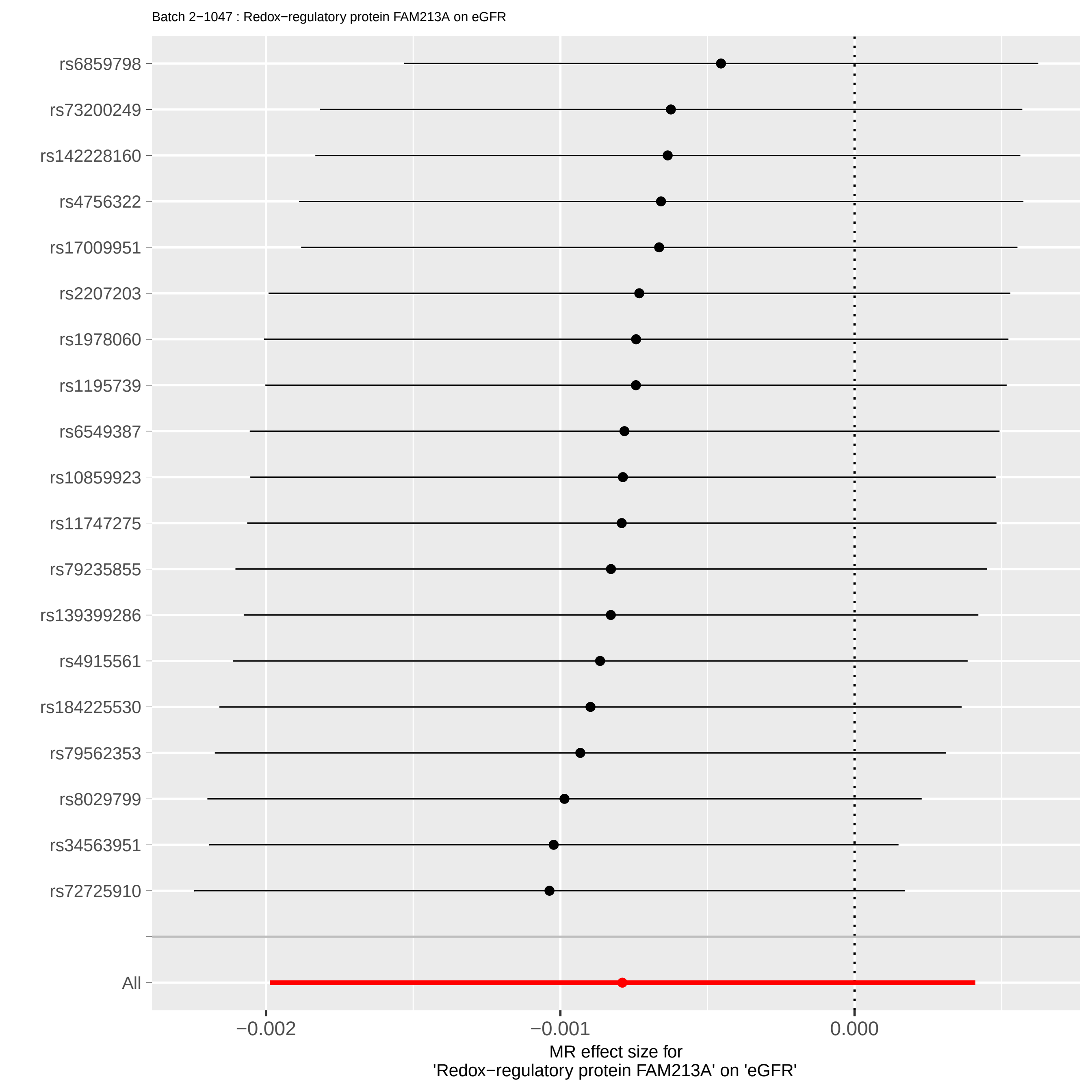

Batch 2−1047 : Redox−regulatory protein FAM213A on eGFR
rs6859798
rs73200249
rs142228160
rs4756322
rs17009951
rs2207203
rs1978060
rs1195739
rs6549387
rs10859923
rs11747275
rs79235855
rs139399286
rs4915561
rs184225530
rs79562353
rs8029799
rs34563951
rs72725910
All
−0.002
−0.001
0.000
MR effect size for
'Redox−regulatory protein FAM213A' on 'eGFR'

Batch 2−1048 : Protein FAM24B on eGFR

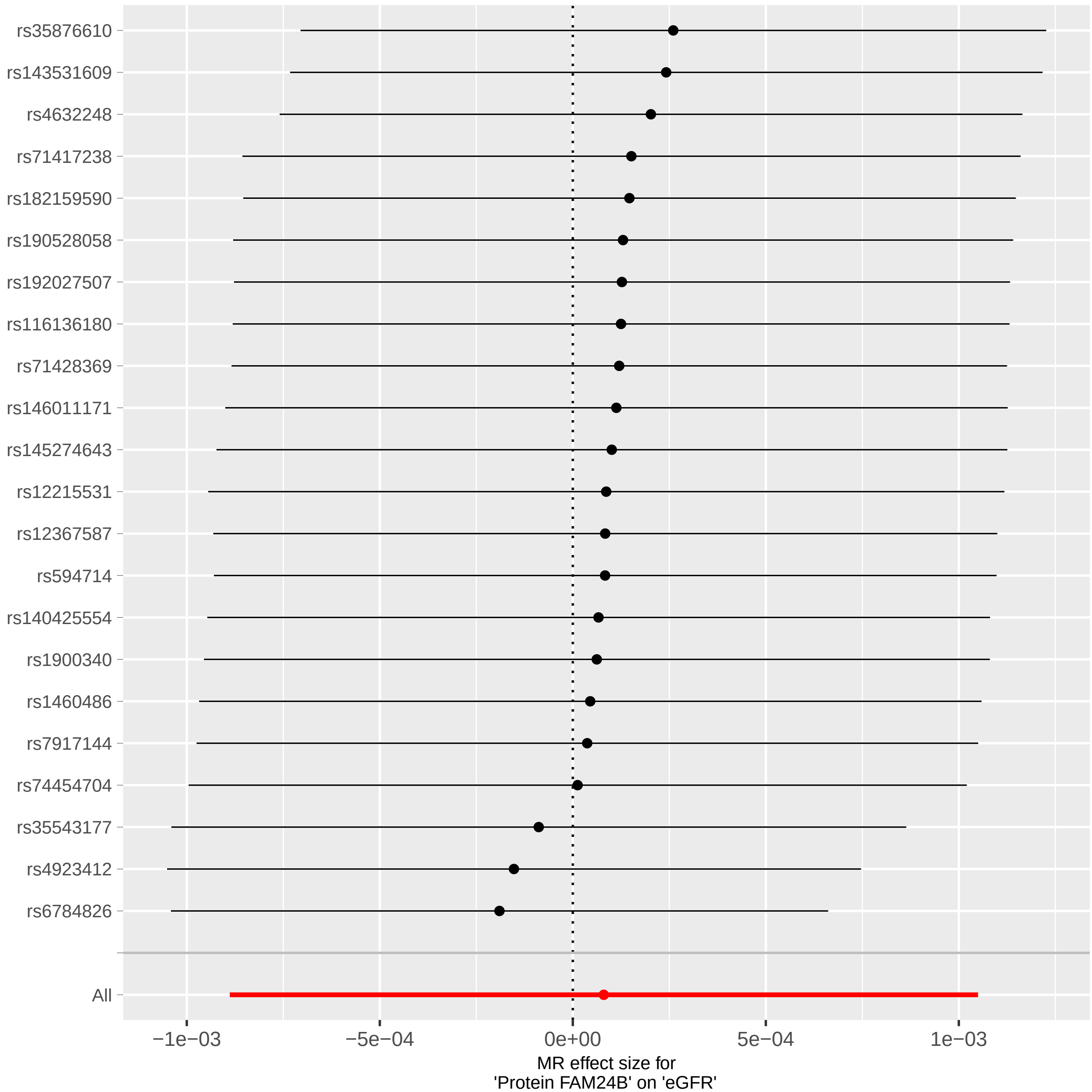

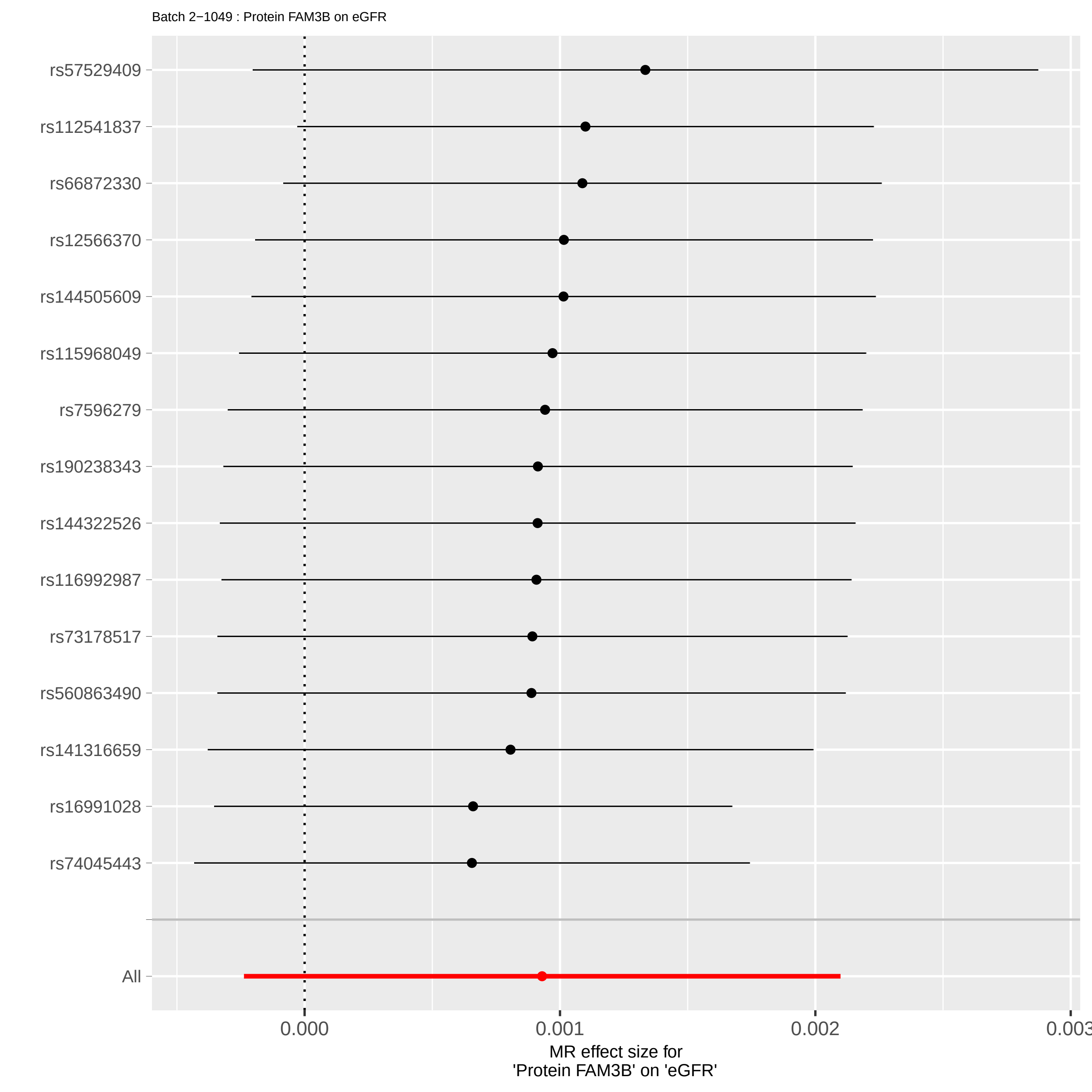

Batch 2−1049 : Protein FAM3B on eGFR
rs57529409
rs112541837
rs66872330
rs12566370
rs144505609
rs115968049
rs7596279
rs190238343
rs144322526
rs116992987
rs73178517
rs560863490
rs141316659
rs16991028
rs74045443
All
0.000
0.001
0.002
0.003
MR effect size for
'Protein FAM3B' on 'eGFR'

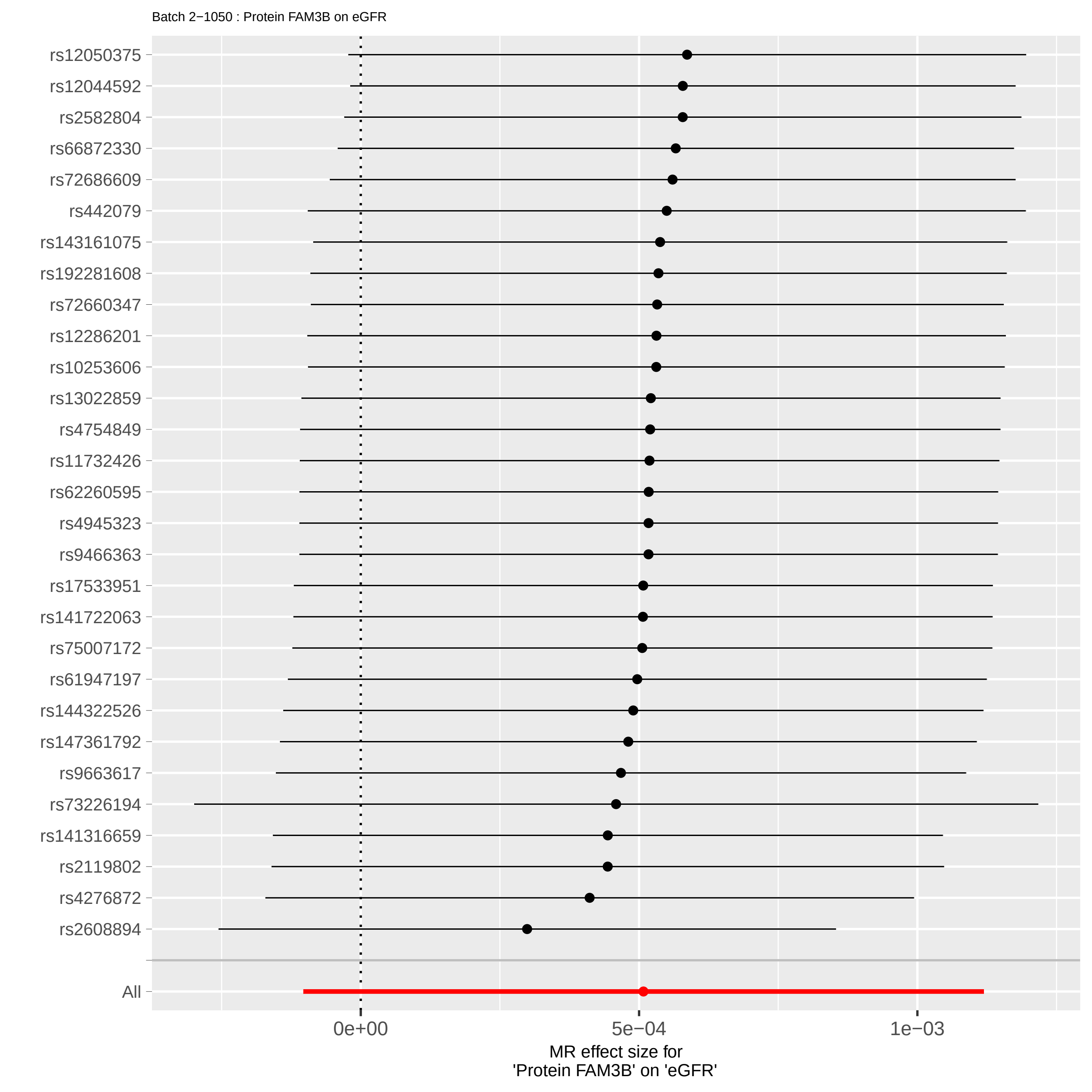

Batch 2−1050 : Protein FAM3B on eGFR
rs12050375
rs12044592
rs2582804
rs66872330
rs72686609
rs442079
rs143161075
rs192281608
rs72660347
rs12286201
rs10253606
rs13022859
rs4754849
rs11732426
rs62260595
rs4945323
rs9466363
rs17533951
rs141722063
rs75007172
rs61947197
rs144322526
rs147361792
rs9663617
rs73226194
rs141316659
rs2119802
rs4276872
rs2608894
All
0e+00
5e−04
1e−03
MR effect size for
'Protein FAM3B' on 'eGFR'

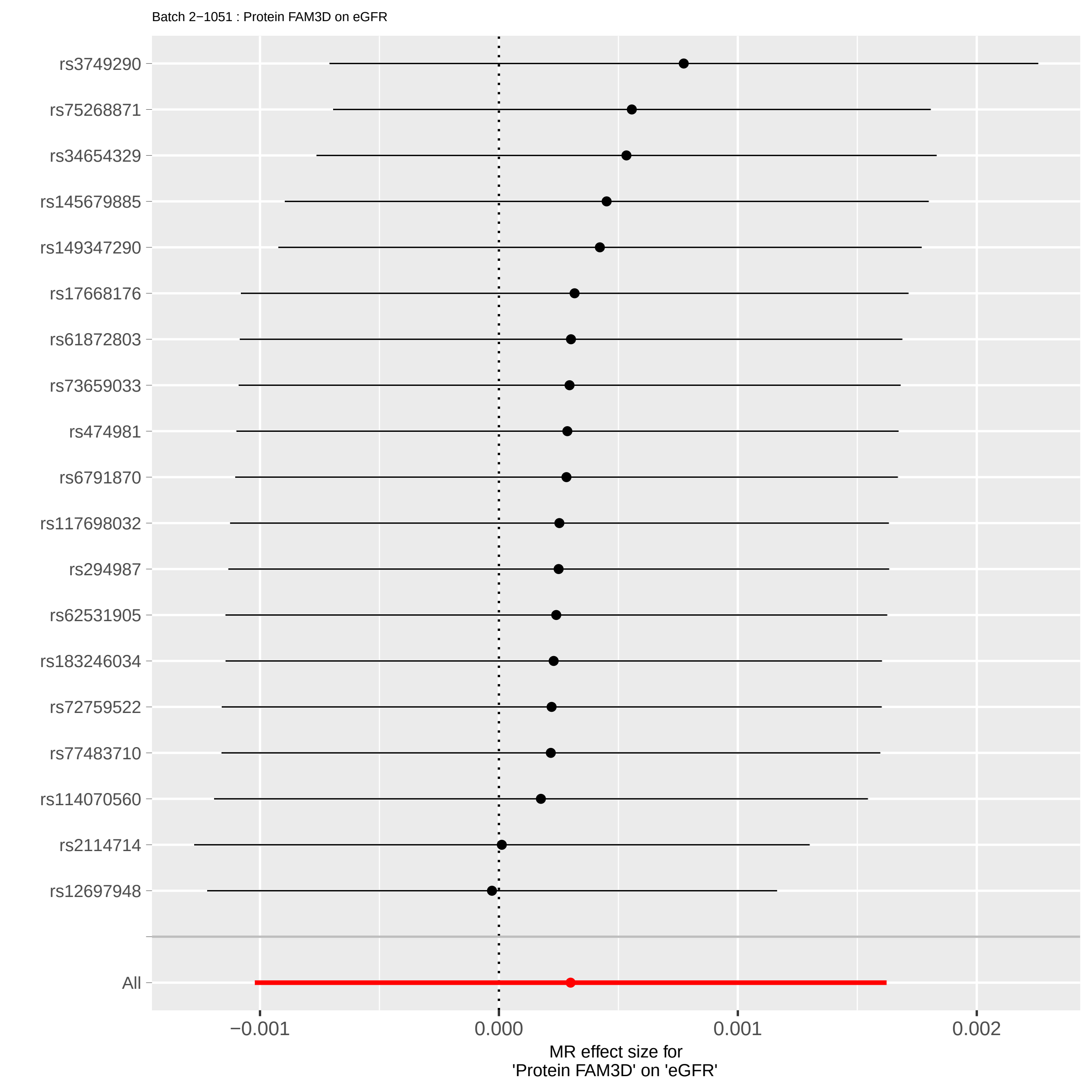

Batch 2−1051 : Protein FAM3D on eGFR
rs3749290
rs75268871
rs34654329
rs145679885
rs149347290
rs17668176
rs61872803
rs73659033
rs474981
rs6791870
rs117698032
rs294987
rs62531905
rs183246034
rs72759522
rs77483710
rs114070560
rs2114714
rs12697948
All
−0.001
0.000
0.001
0.002
MR effect size for
'Protein FAM3D' on 'eGFR'

Batch 2−1052 : MIP18 family protein FAM96A on eGFR

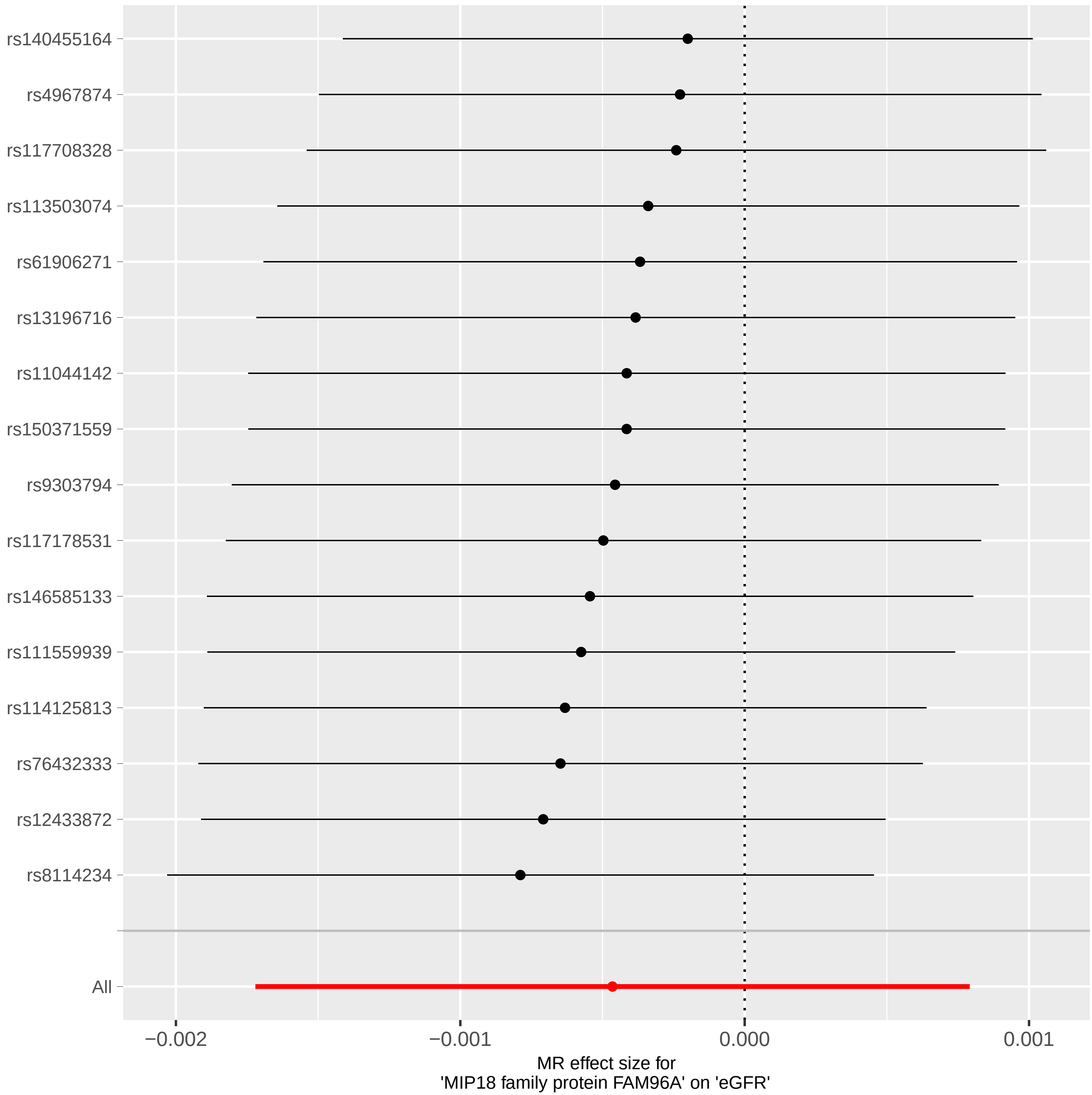

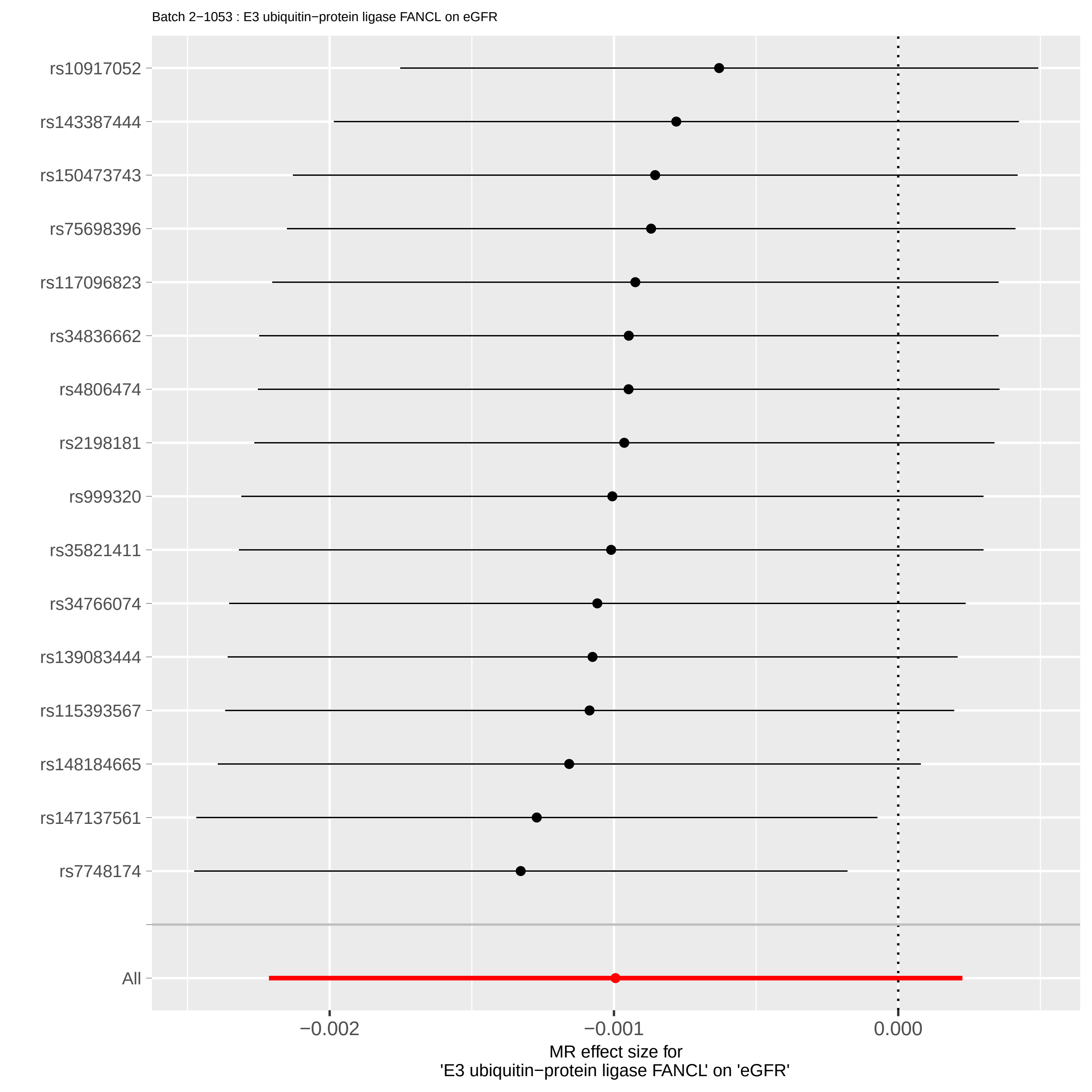

Batch 2−1053 : E3 ubiquitin−protein ligase FANCL on eGFR
rs10917052
rs143387444
rs150473743
rs75698396
rs117096823
rs34836662
rs4806474
rs2198181
rs999320
rs35821411
rs34766074
rs139083444
rs115393567
rs148184665
rs147137561
rs7748174
All
−0.002
−0.001
0.000
MR effect size for
'E3 ubiquitin−protein ligase FANCL' on 'eGFR'

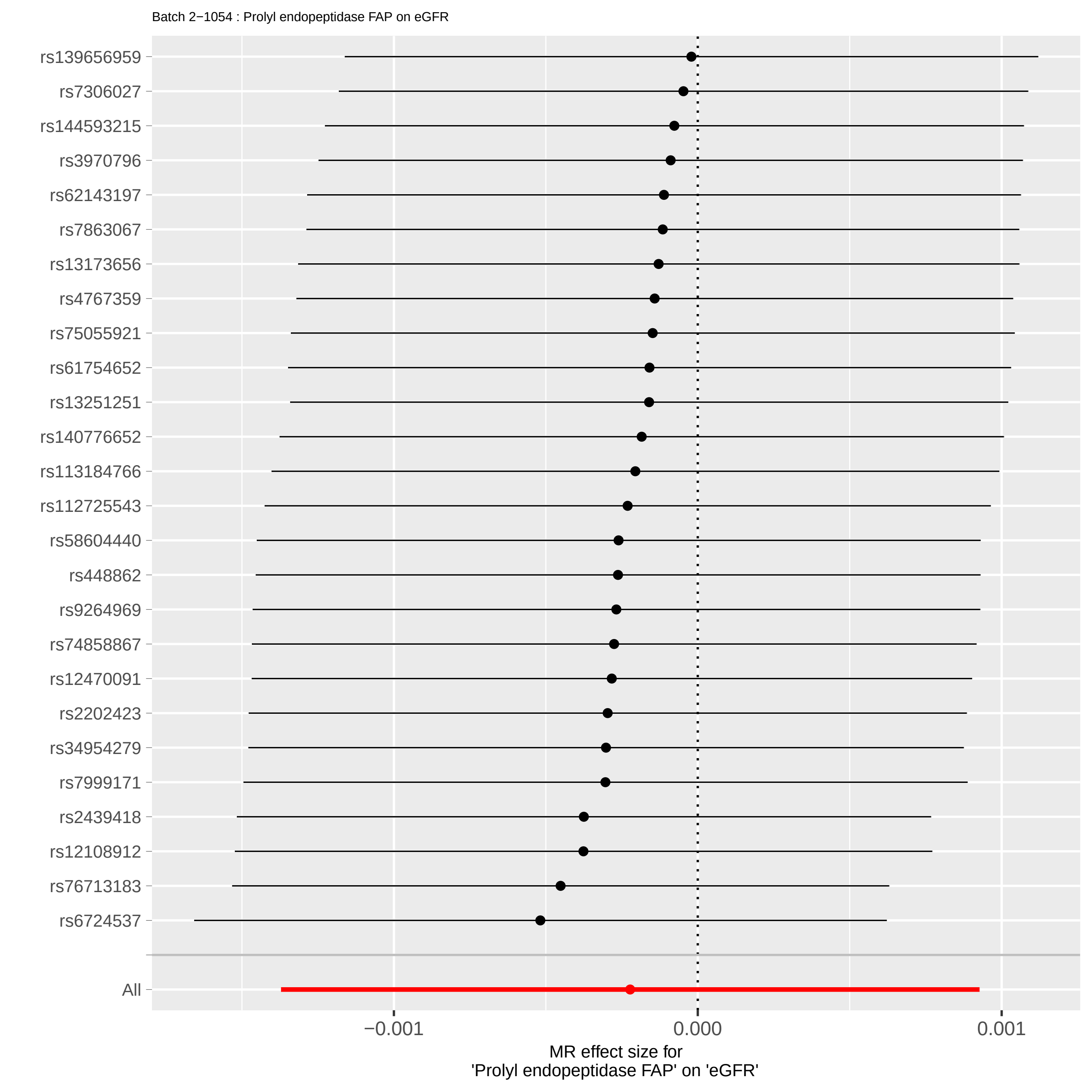
Batch 2−1054 : Prolyl endopeptidase FAP on eGFR
rs139656959
rs7306027
rs144593215
rs3970796
rs62143197
rs7863067
rs13173656
rs4767359
rs75055921
rs61754652
rs13251251
rs140776652
rs113184766
rs112725543
rs58604440
rs448862
rs9264969
rs74858867
rs12470091
rs2202423
rs34954279
rs7999171
rs2439418
rs12108912
rs76713183
rs6724537
All
−0.001
0.000
0.001
MR effect size for
'Prolyl endopeptidase FAP' on 'eGFR'

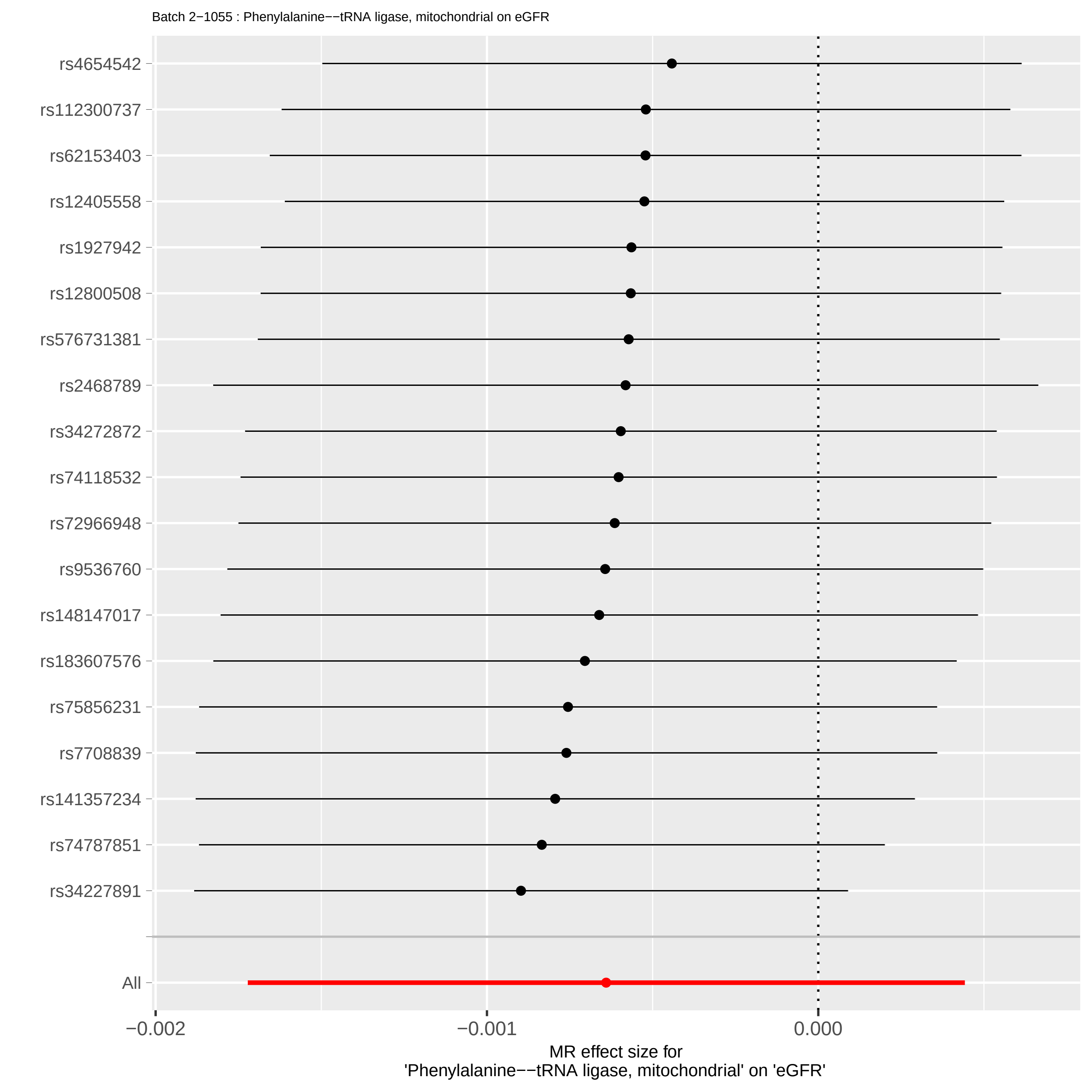
Batch 2−1055 : Phenylalanine−−tRNA ligase, mitochondrial on eGFR
rs4654542
rs112300737
rs62153403
rs12405558
rs1927942
rs12800508
rs576731381
rs2468789
rs34272872
rs74118532
rs72966948
rs9536760
rs148147017
rs183607576
rs75856231
rs7708839
rs141357234
rs74787851
rs34227891
All
−0.002
−0.001
0.000
MR effect size for
'Phenylalanine−−tRNA ligase, mitochondrial' on 'eGFR'

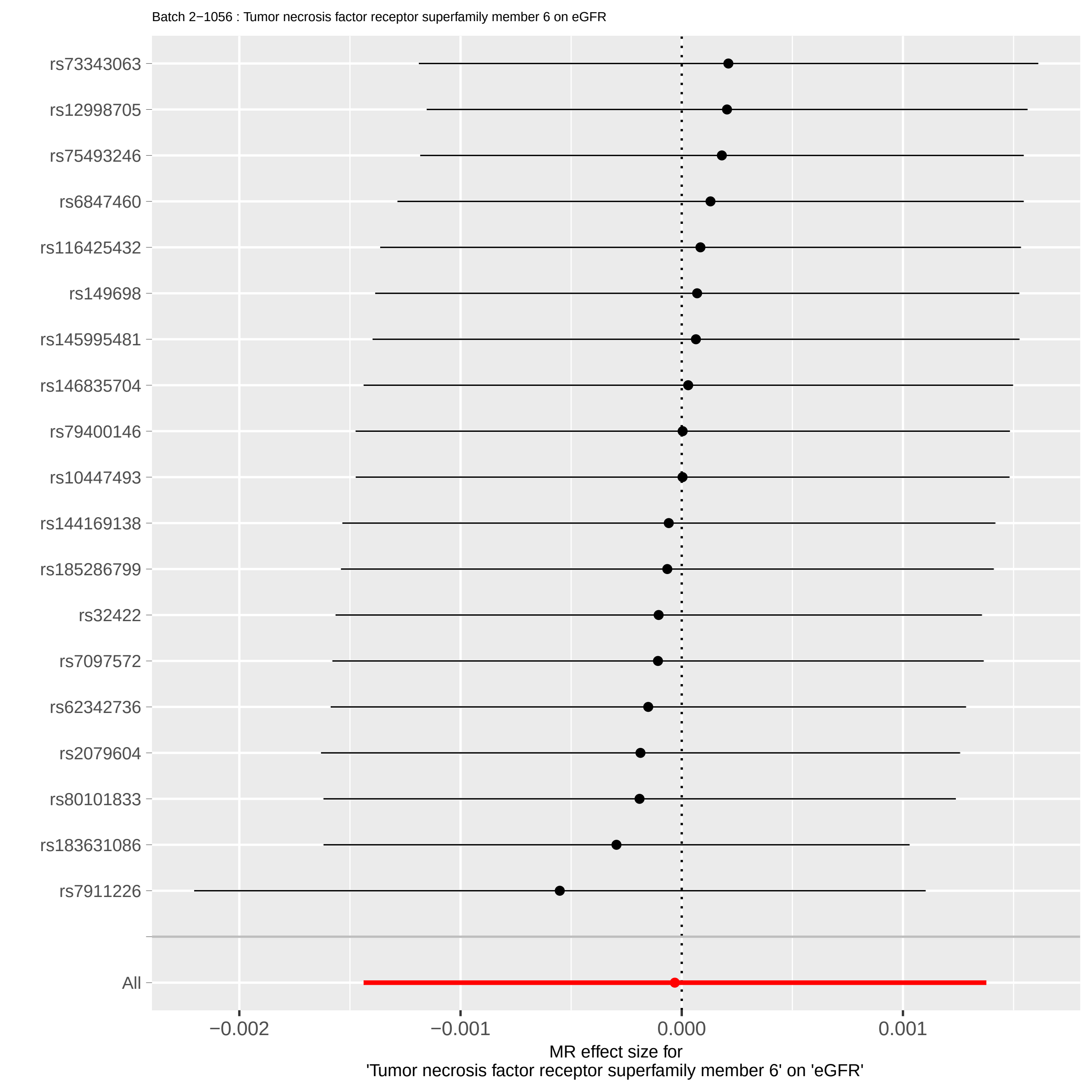
Batch 2−1056 : Tumor necrosis factor receptor superfamily member 6 on eGFR
rs73343063
rs12998705
rs75493246
rs6847460
rs116425432
rs149698
rs145995481
rs146835704
rs79400146
rs10447493
rs144169138
rs185286799
rs32422
rs7097572
rs62342736
rs2079604
rs80101833
rs183631086
rs7911226
All
−0.002
−0.001
0.000
0.001
MR effect size for
'Tumor necrosis factor receptor superfamily member 6' on 'eGFR'

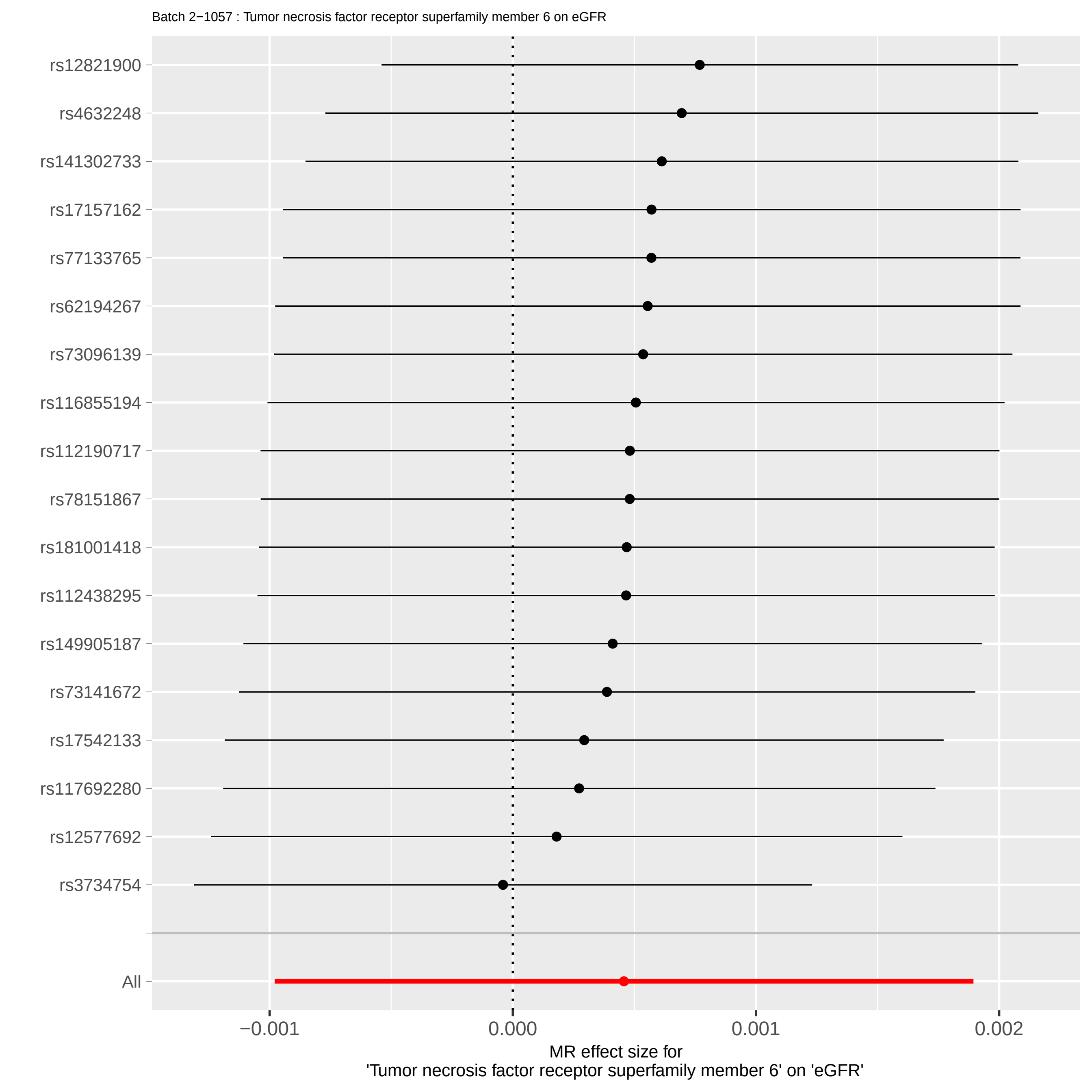

Batch 2−1057 : Tumor necrosis factor receptor superfamily member 6 on eGFR
rs12821900
rs4632248
rs141302733
rs17157162
rs77133765
rs62194267
rs73096139
rs116855194
rs112190717
rs78151867
rs181001418
rs112438295
rs149905187
rs73141672
rs17542133
rs117692280
rs12577692
rs3734754
All
−0.001
0.000
0.001
0.002
MR effect size for
'Tumor necrosis factor receptor superfamily member 6' on 'eGFR'

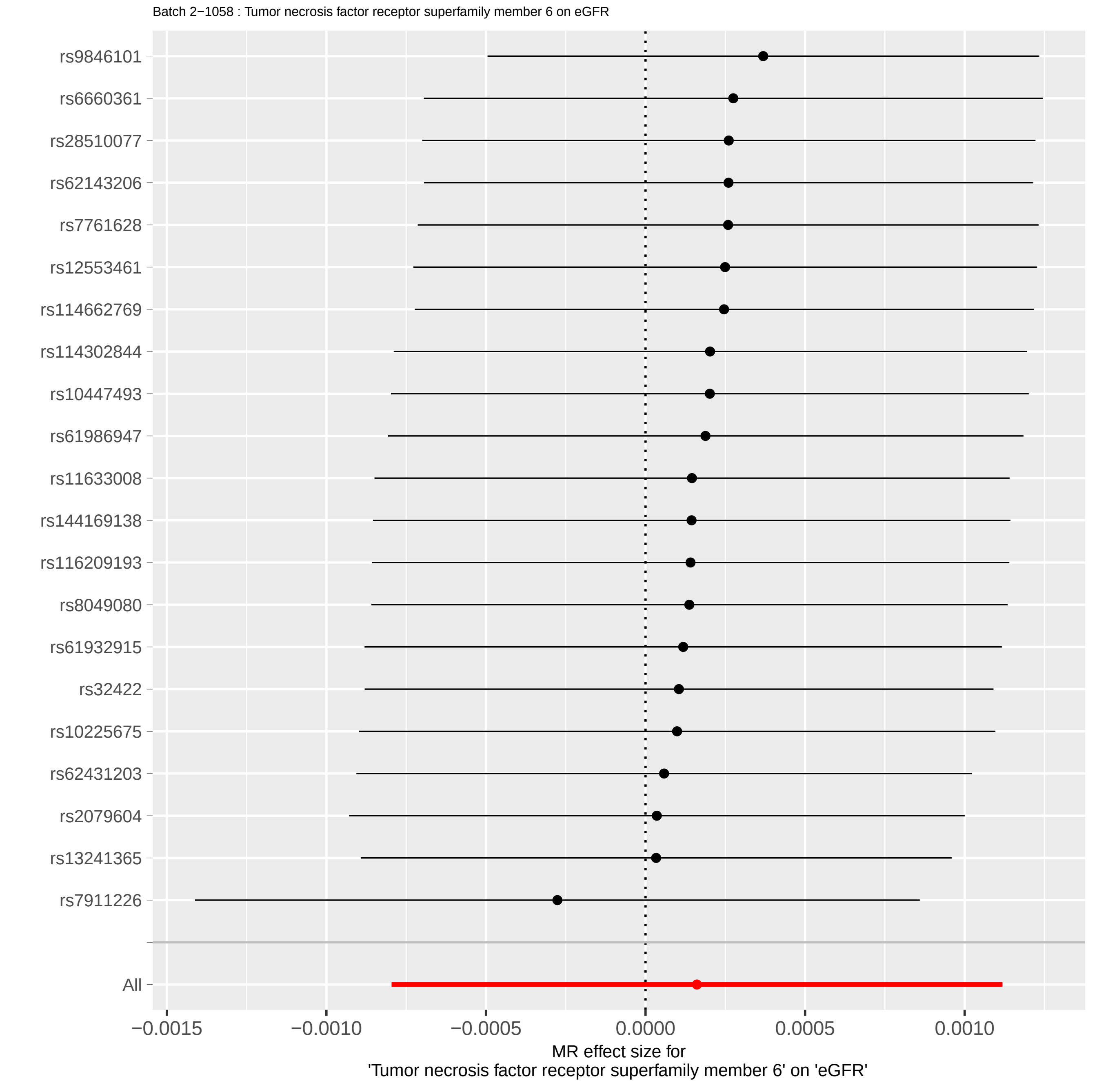

Batch 2−1058 : Tumor necrosis factor receptor superfamily member 6 on eGFR
rs9846101
rs6660361
rs28510077
rs62143206
rs7761628
rs12553461
rs114662769
rs114302844
rs10447493
rs61986947
rs11633008
rs144169138
rs116209193
rs8049080
rs61932915
rs32422
rs10225675
rs62431203
rs2079604
rs13241365
rs7911226
All
−0.0015
−0.0010
−0.0005
0.0000
0.0005
0.0010
MR effect size for
'Tumor necrosis factor receptor superfamily member 6' on 'eGFR'

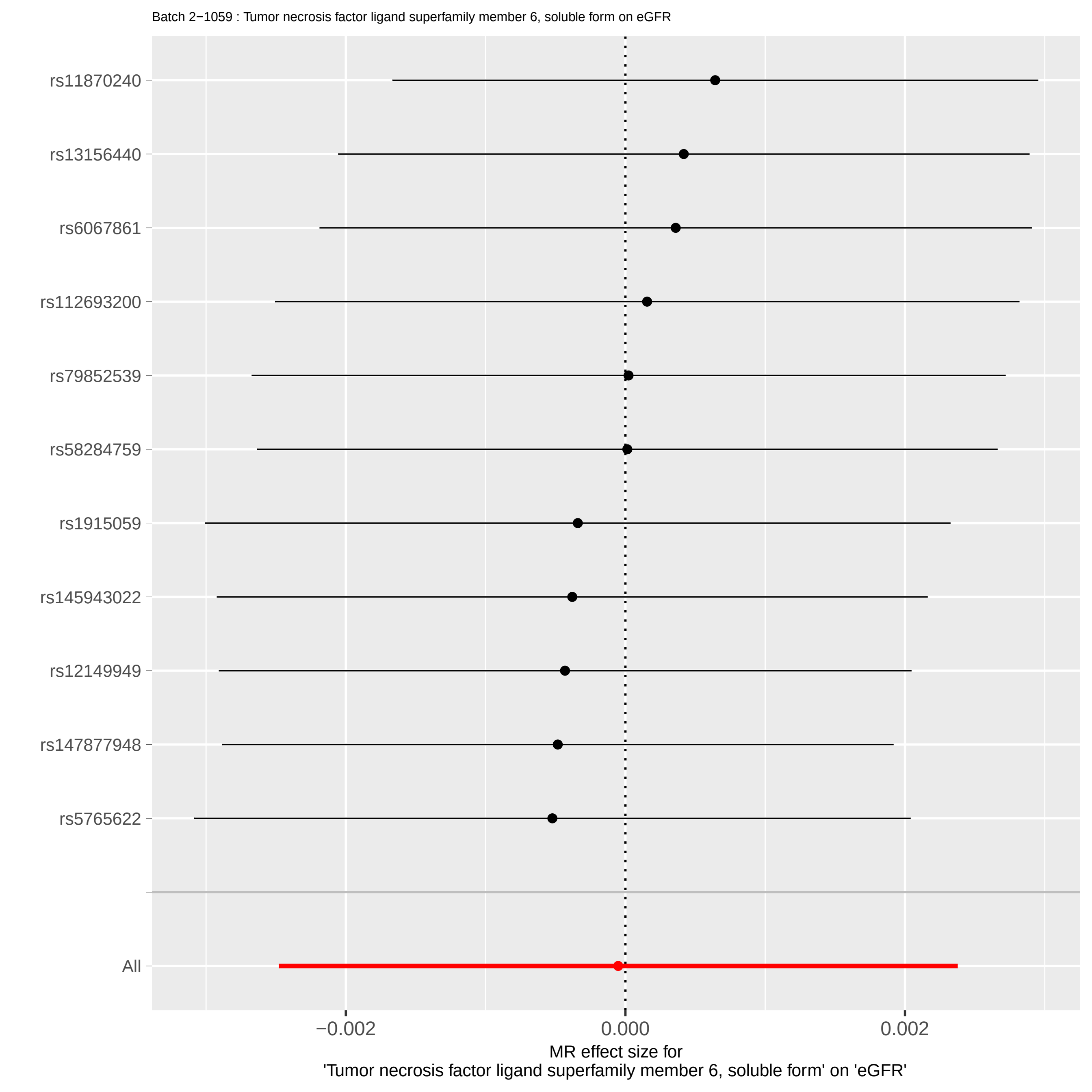

Batch 2−1059 : Tumor necrosis factor ligand superfamily member 6, soluble form on eGFR
rs11870240
rs13156440
rs6067861
rs112693200
rs79852539
rs58284759
rs1915059
rs145943022
rs12149949
rs147877948
rs5765622
All
−0.002
0.000
0.002
MR effect size for
'Tumor necrosis factor ligand superfamily member 6, soluble form' on 'eGFR'

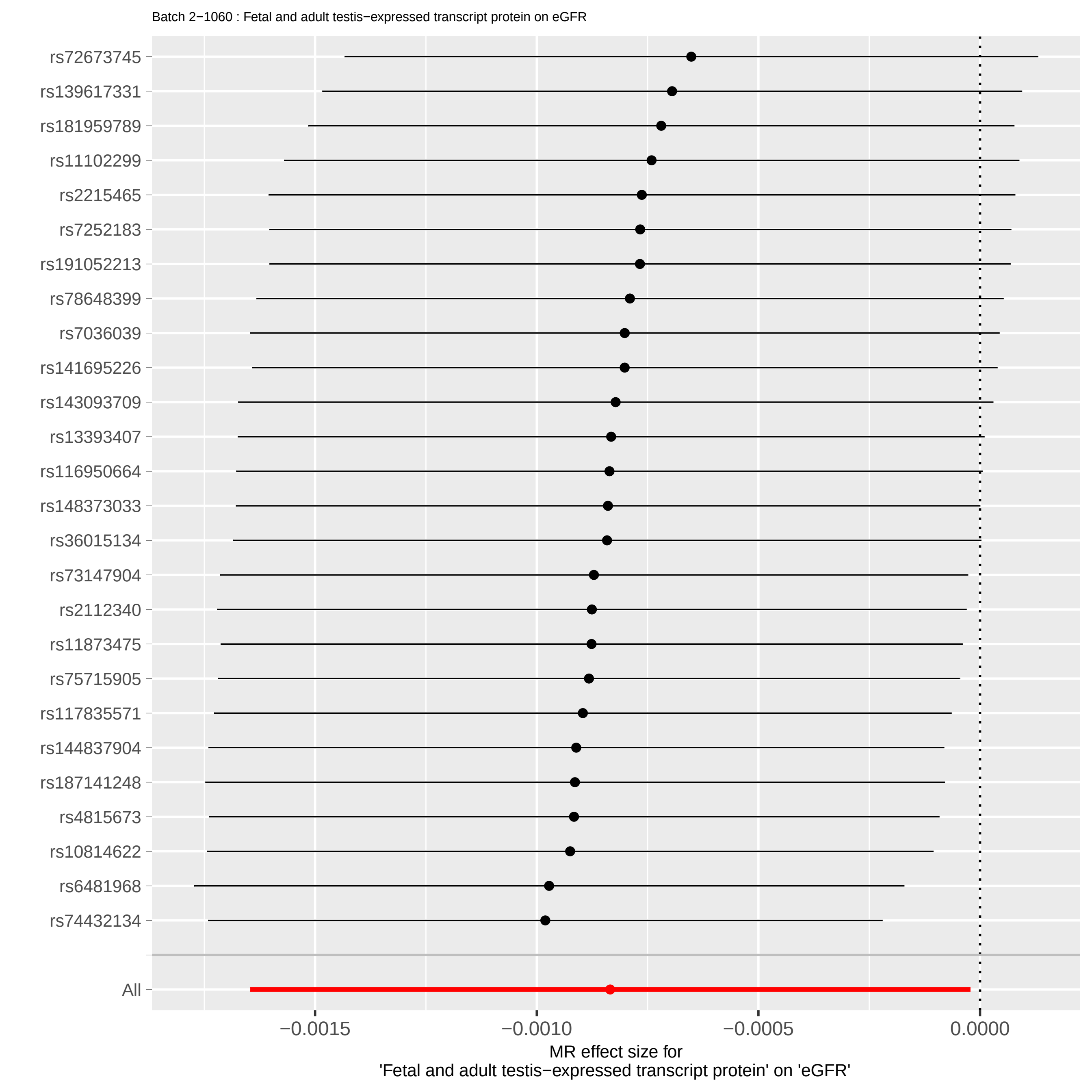
Batch 2−1060 : Fetal and adult testis−expressed transcript protein on eGFR
rs72673745
rs139617331
rs181959789
rs11102299
rs2215465
rs7252183
rs191052213
rs78648399
rs7036039
rs141695226
rs143093709
rs13393407
rs116950664
rs148373033
rs36015134
rs73147904
rs2112340
rs11873475
rs75715905
rs117835571
rs144837904
rs187141248
rs4815673
rs10814622
rs6481968
rs74432134
All
−0.0015
−0.0010
−0.0005
0.0000
MR effect size for
'Fetal and adult testis−expressed transcript protein' on 'eGFR'

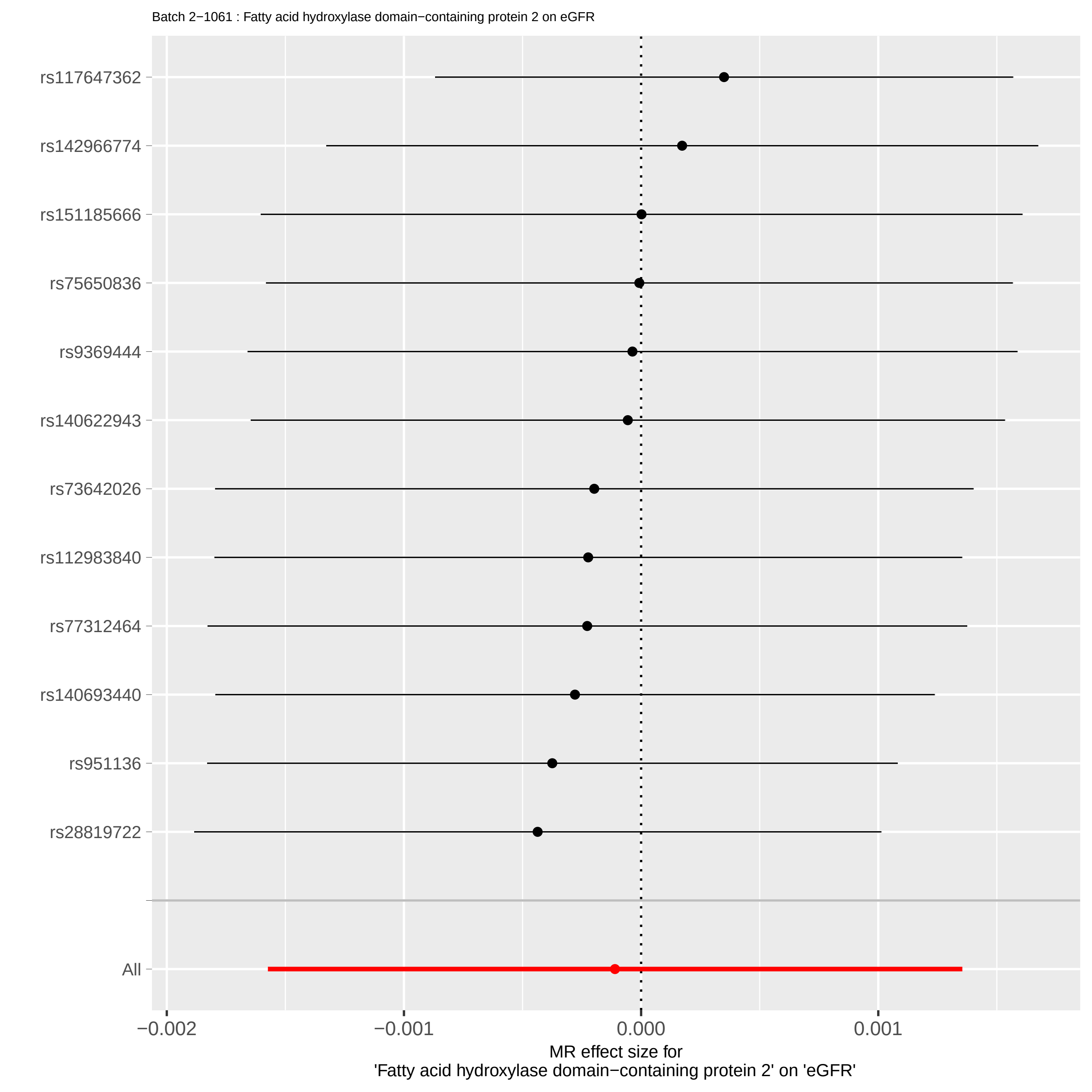

Batch 2−1061 : Fatty acid hydroxylase domain−containing protein 2 on eGFR
rs117647362
rs142966774
rs151185666
rs75650836
rs9369444
rs140622943
rs73642026
rs112983840
rs77312464
rs140693440
rs951136
rs28819722
All
−0.002
−0.001
0.000
0.001
MR effect size for
'Fatty acid hydroxylase domain−containing protein 2' on 'eGFR'

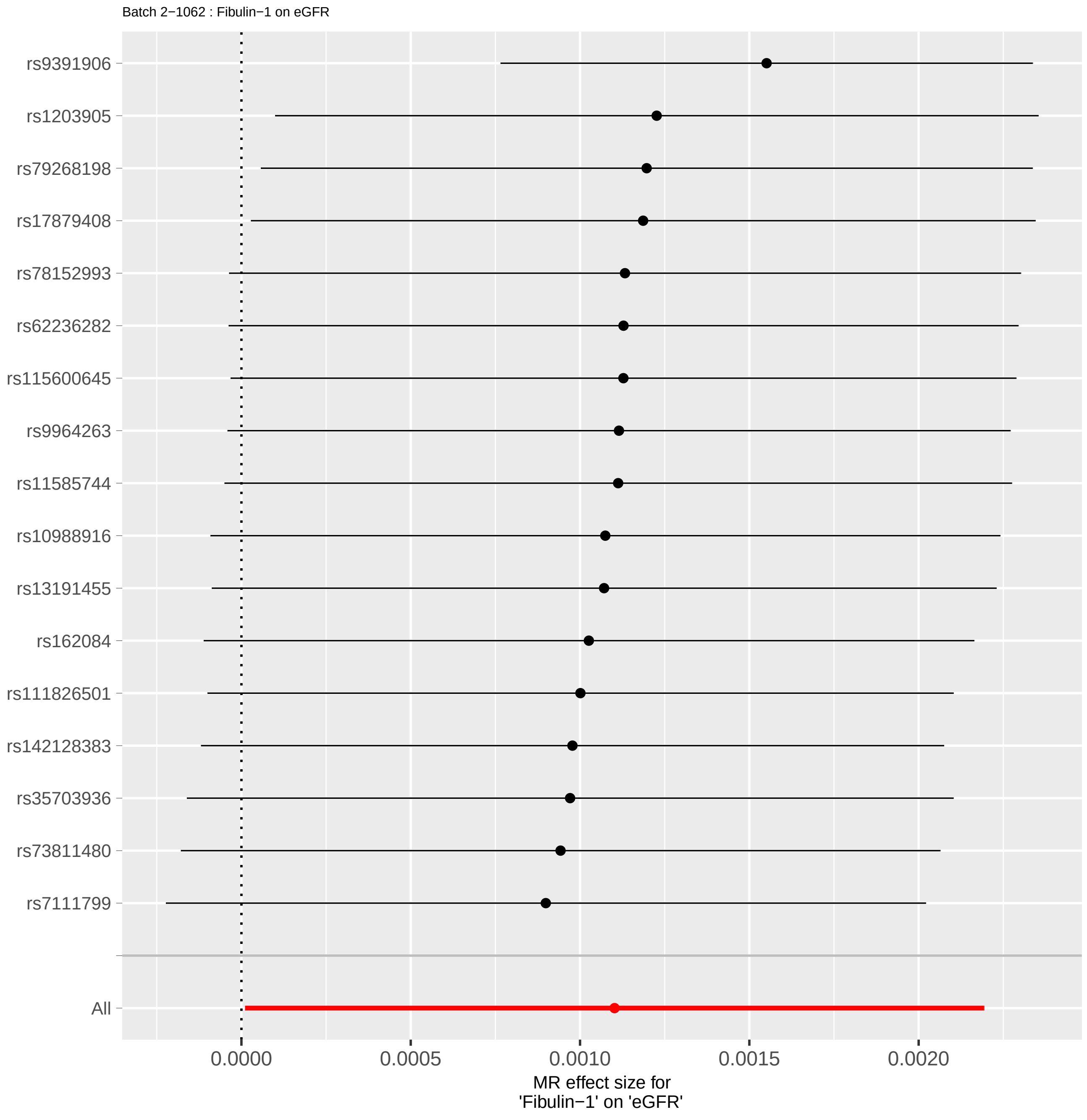

Batch 2−1062 : Fibulin−1 on eGFR
rs9391906
rs1203905
rs79268198
rs17879408
rs78152993
rs62236282
rs115600645
rs9964263
rs11585744
rs10988916
rs13191455
rs162084
rs111826501
rs142128383
rs35703936
rs73811480
rs7111799
All
0.0000
0.0005
0.0010
0.0015
0.0020
MR effect size for
'Fibulin−1' on 'eGFR'

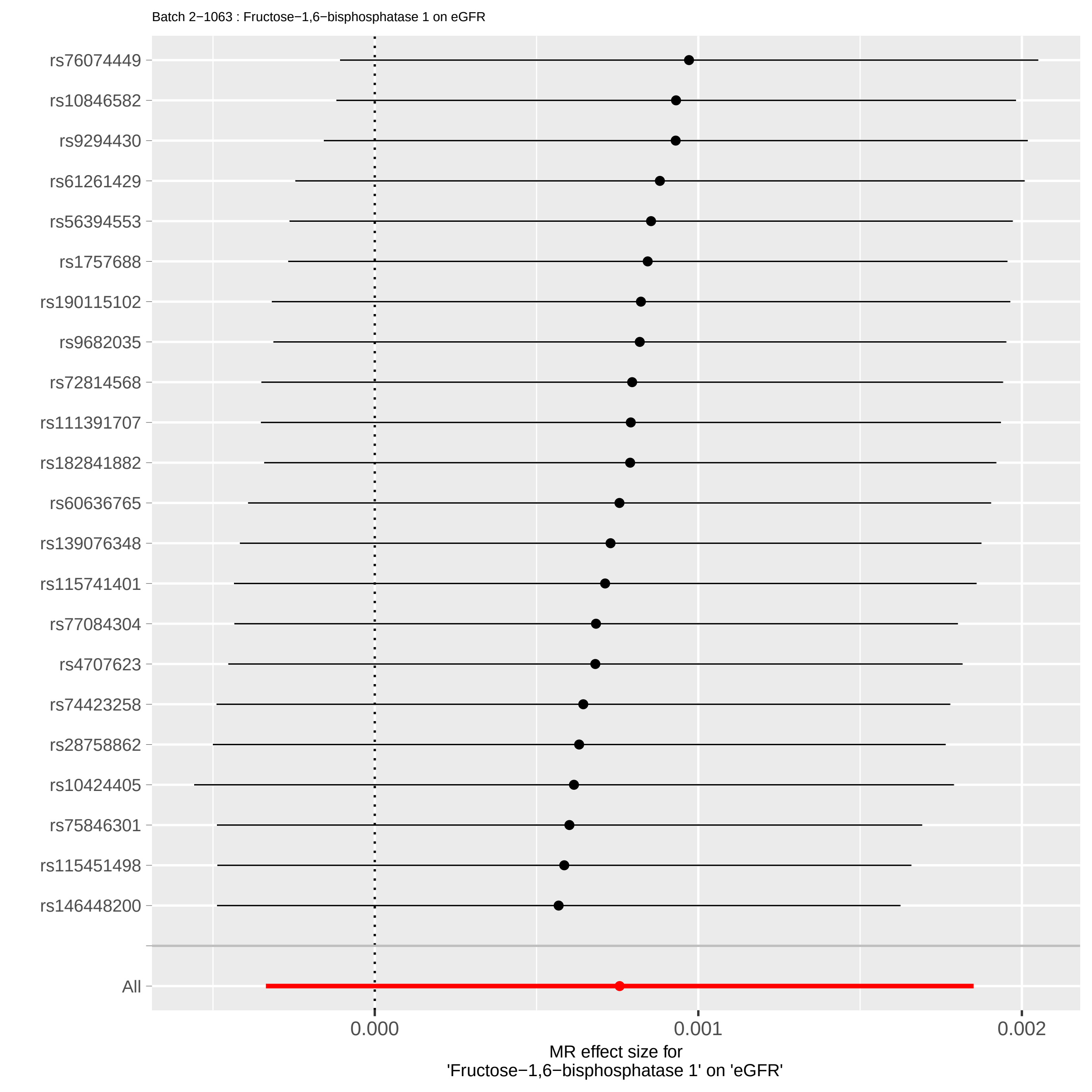

Batch 2−1063 : Fructose−1,6−bisphosphatase 1 on eGFR
rs76074449
rs10846582
rs9294430
rs61261429
rs56394553
rs1757688
rs190115102
rs9682035
rs72814568
rs111391707
rs182841882
rs60636765
rs139076348
rs115741401
rs77084304
rs4707623
rs74423258
rs28758862
rs10424405
rs75846301
rs115451498
rs146448200
All
0.000
0.001
0.002
MR effect size for
'Fructose−1,6−bisphosphatase 1' on 'eGFR'

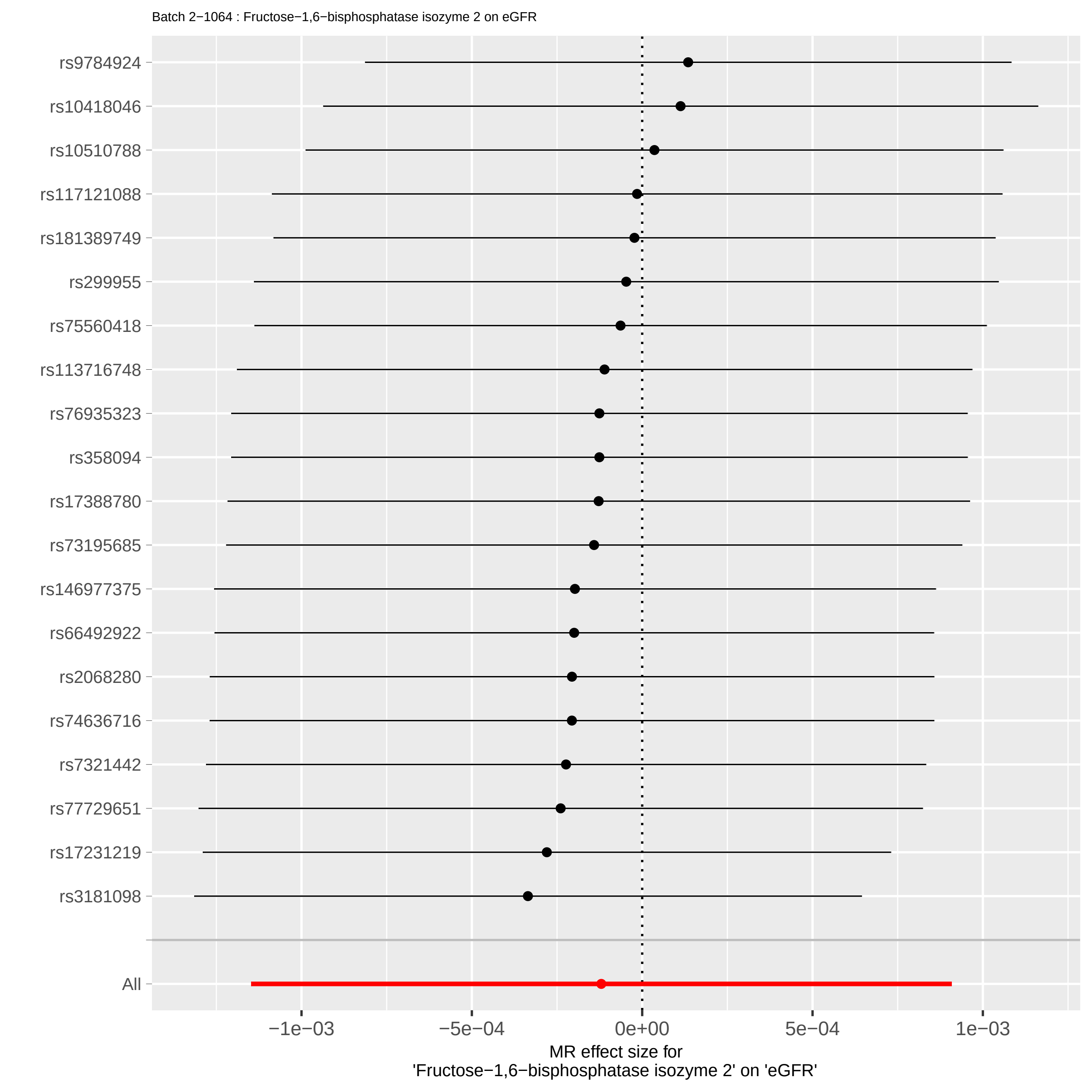

Batch 2−1064 : Fructose−1,6−bisphosphatase isozyme 2 on eGFR
rs9784924
rs10418046
rs10510788
rs117121088
rs181389749
rs299955
rs75560418
rs113716748
rs76935323
rs358094
rs17388780
rs73195685
rs146977375
rs66492922
rs2068280
rs74636716
rs7321442
rs77729651
rs17231219
rs3181098
All
−1e−03
−5e−04
0e+00
5e−04
1e−03
MR effect size for
'Fructose−1,6−bisphosphatase isozyme 2' on 'eGFR'

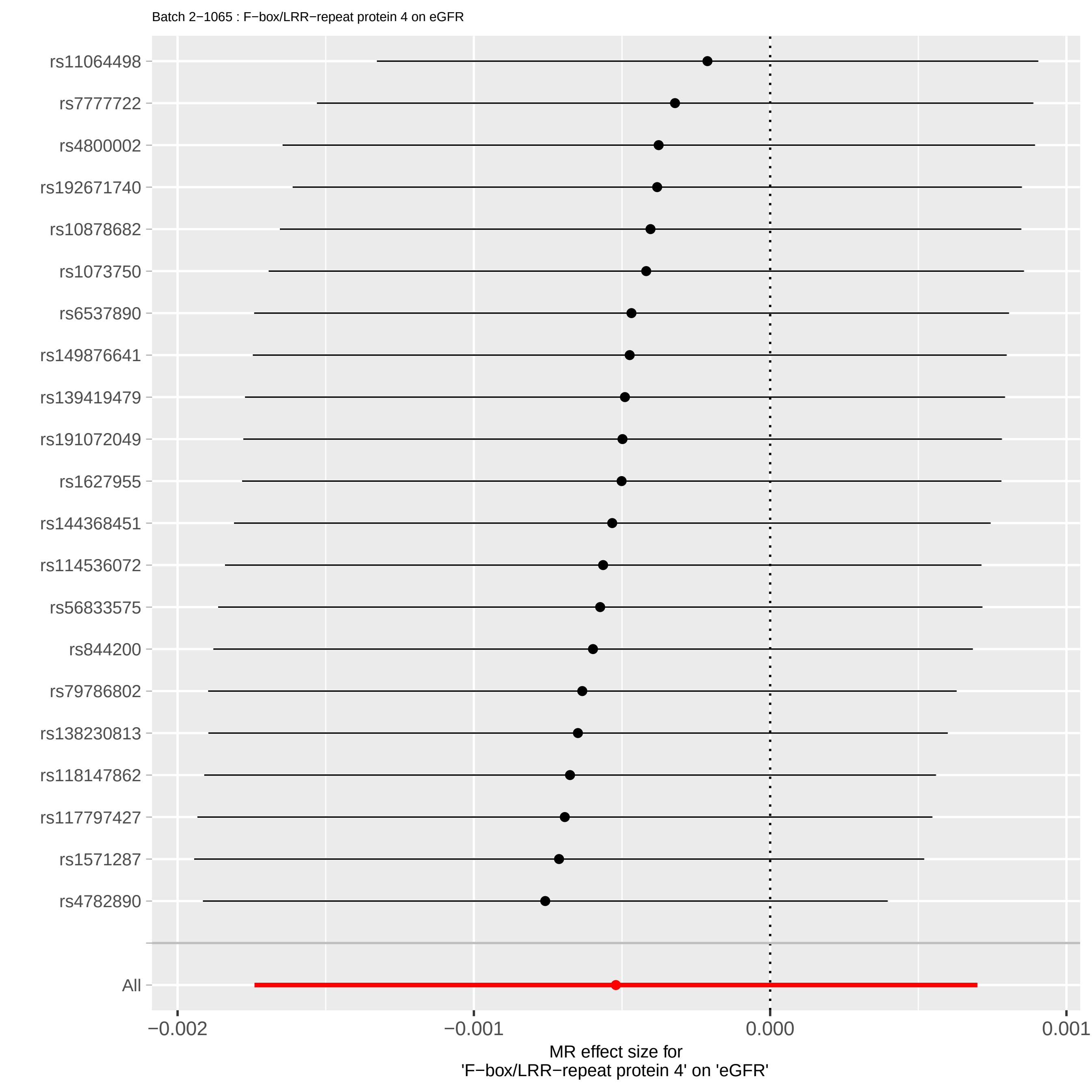

Batch 2−1065 : F−box/LRR−repeat protein 4 on eGFR
rs11064498
rs7777722
rs4800002
rs192671740
rs10878682
rs1073750
rs6537890
rs149876641
rs139419479
rs191072049
rs1627955
rs144368451
rs114536072
rs56833575
rs844200
rs79786802
rs138230813
rs118147862
rs117797427
rs1571287
rs4782890
All
−0.002
−0.001
0.000
0.001
MR effect size for
'F−box/LRR−repeat protein 4' on 'eGFR'

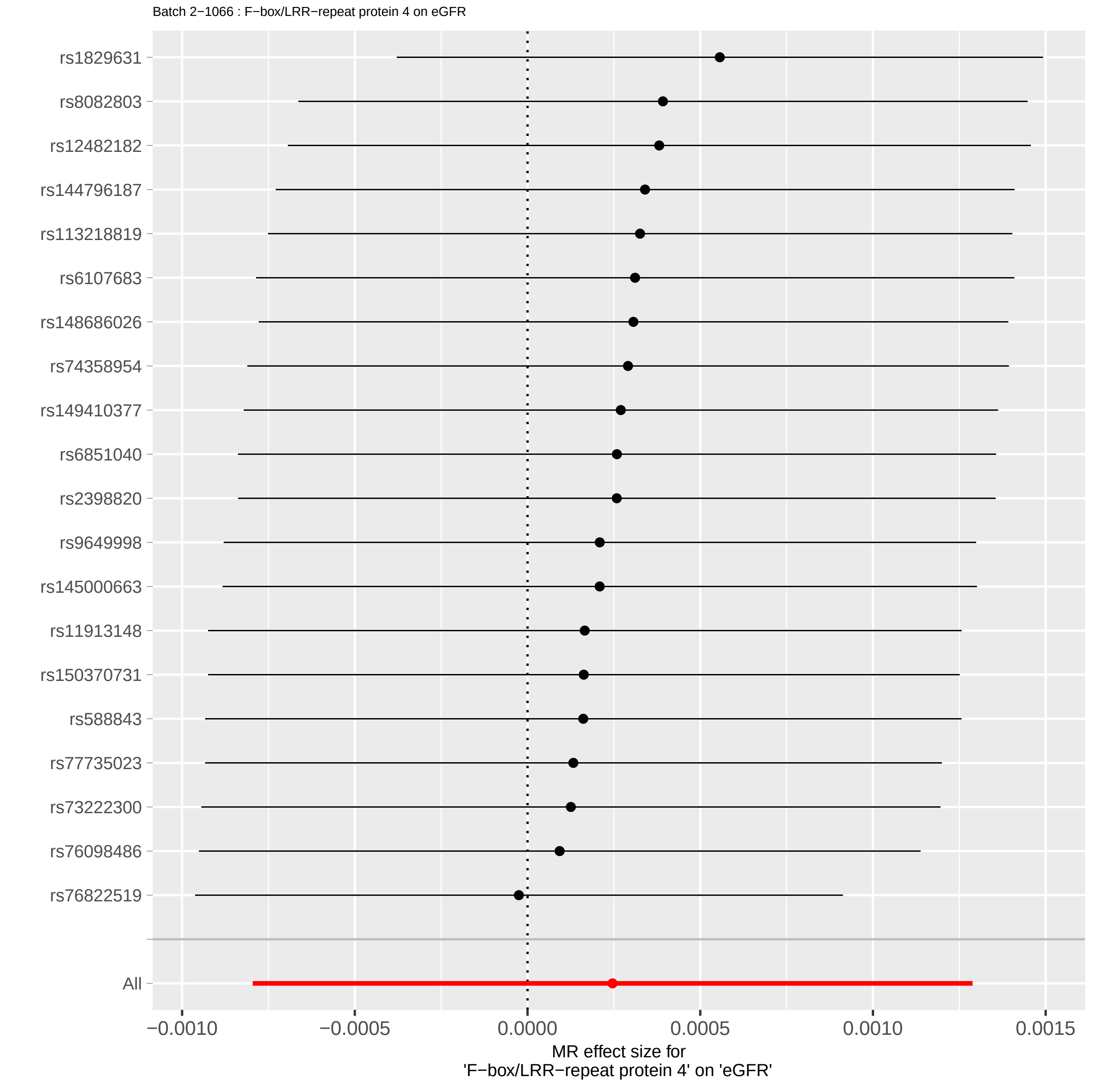
Batch 2−1066 : F−box/LRR−repeat protein 4 on eGFR
rs1829631
rs8082803
rs12482182
rs144796187
rs113218819
rs6107683
rs148686026
rs74358954
rs149410377
rs6851040
rs2398820
rs9649998
rs145000663
rs11913148
rs150370731
rs588843
rs77735023
rs73222300
rs76098486
rs76822519
All
−0.0010
−0.0005
0.0000
0.0005
0.0010
0.0015
MR effect size for
'F−box/LRR−repeat protein 4' on 'eGFR'

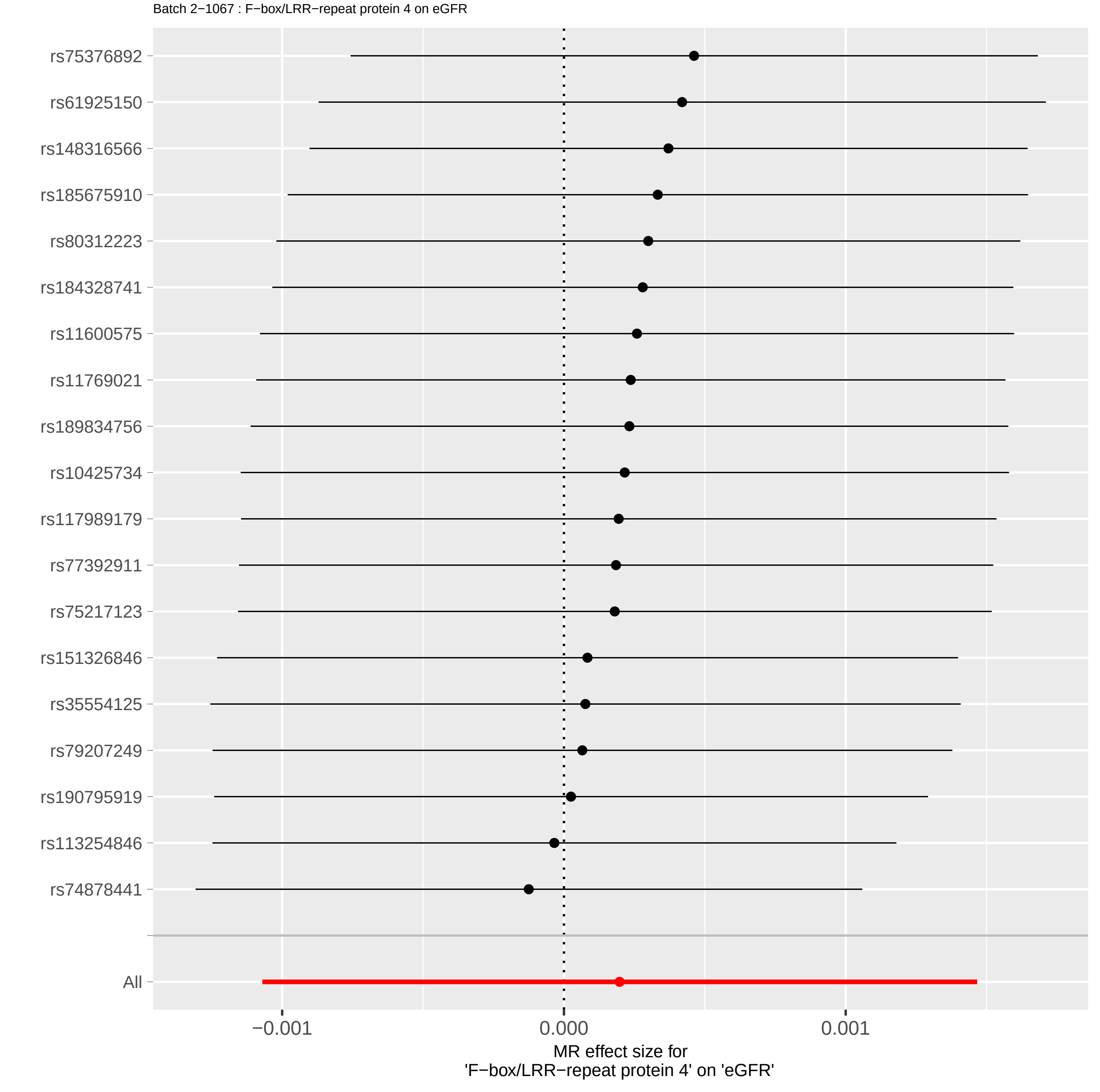
Batch 2−1067 : F−box/LRR−repeat protein 4 on eGFR
rs75376892
rs61925150
rs148316566
rs185675910
rs80312223
rs184328741
rs11600575
rs11769021
rs189834756
rs10425734
rs117989179
rs77392911
rs75217123
rs151326846
rs35554125
rs79207249
rs190795919
rs113254846
rs74878441
All
−0.001
0.000
0.001
MR effect size for
'F−box/LRR−repeat protein 4' on 'eGFR'

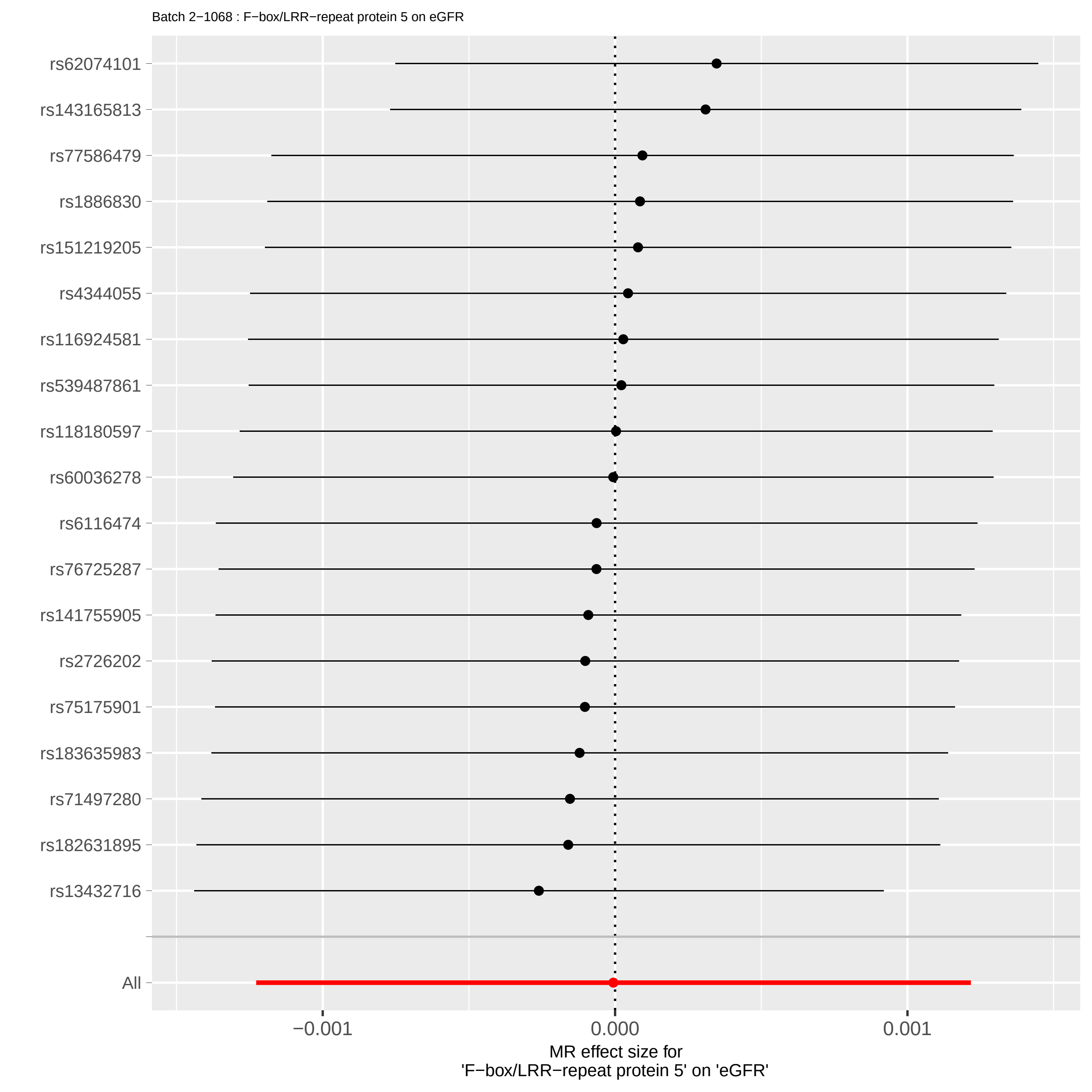

Batch 2−1068 : F−box/LRR−repeat protein 5 on eGFR
rs62074101
rs143165813
rs77586479
rs1886830
rs151219205
rs4344055
rs116924581
rs539487861
rs118180597
rs60036278
rs6116474
rs76725287
rs141755905
rs2726202
rs75175901
rs183635983
rs71497280
rs182631895
rs13432716
All
−0.001
0.000
0.001
MR effect size for
'F−box/LRR−repeat protein 5' on 'eGFR'

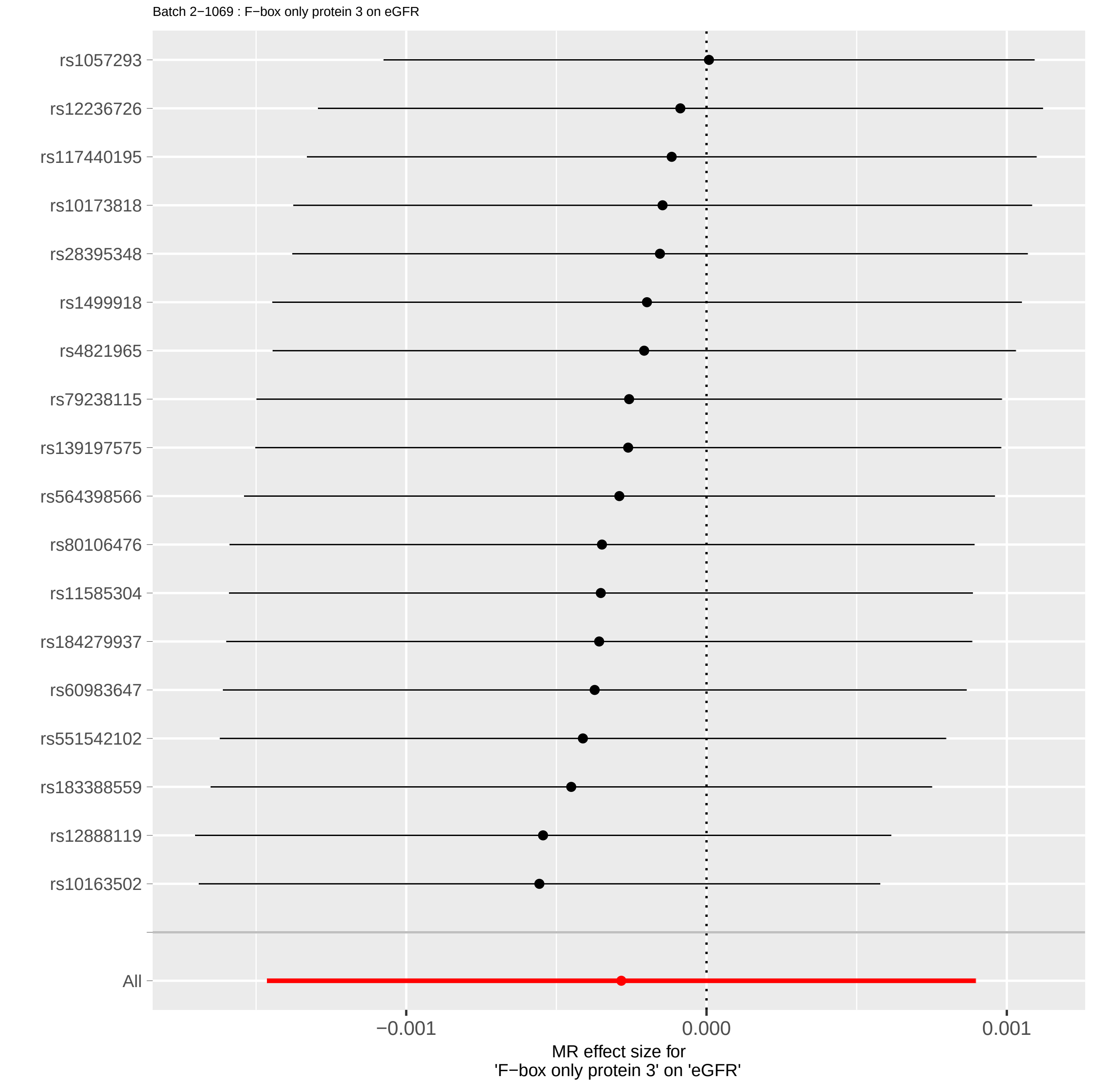
Batch 2−1069 : F−box only protein 3 on eGFR
rs1057293
rs12236726
rs117440195
rs10173818
rs28395348
rs1499918
rs4821965
rs79238115
rs139197575
rs564398566
rs80106476
rs11585304
rs184279937
rs60983647
rs551542102
rs183388559
rs12888119
rs10163502
All
−0.001
0.000
0.001
MR effect size for
'F−box only protein 3' on 'eGFR'

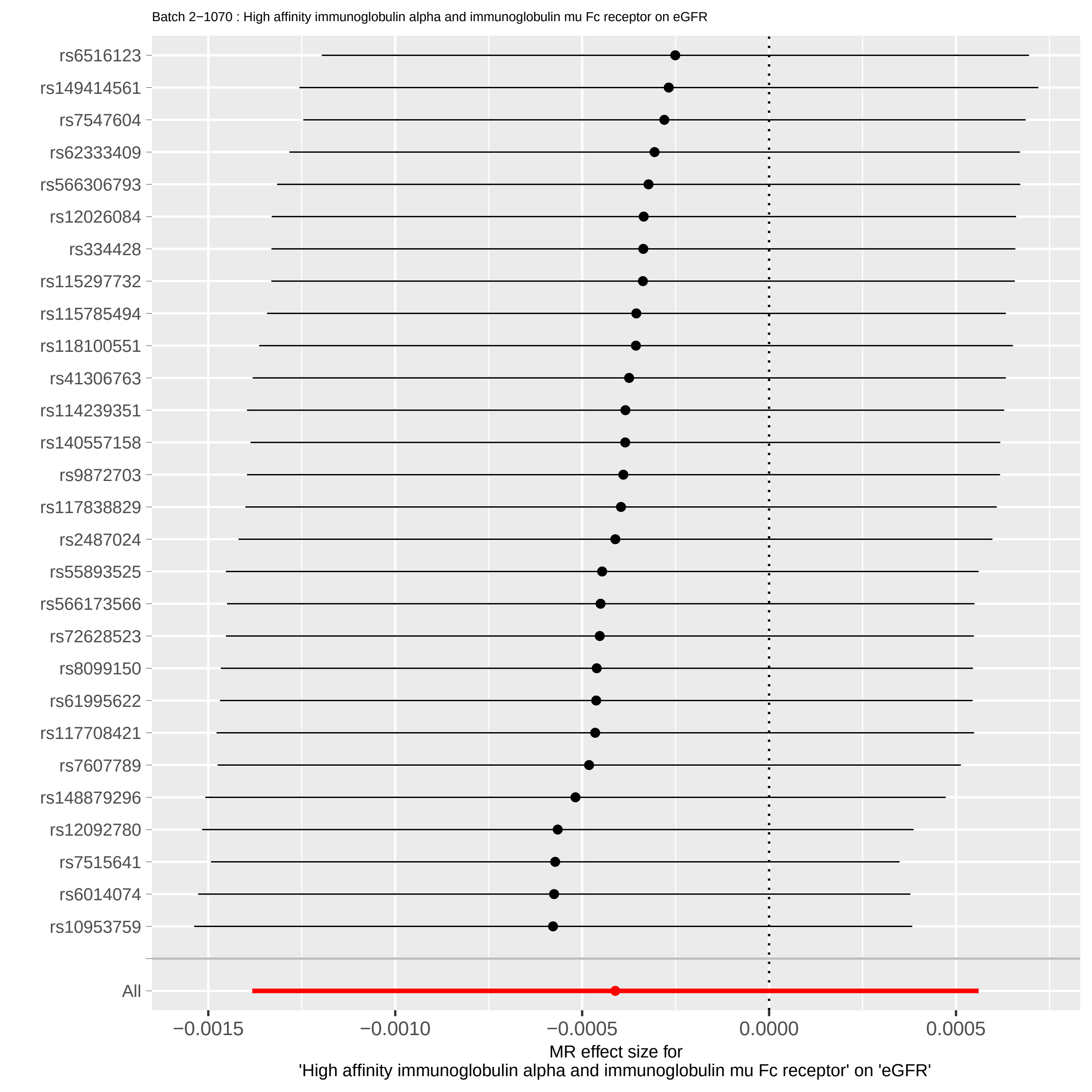
Batch 2−1070 : High affinity immunoglobulin alpha and immunoglobulin mu Fc receptor on eGFR
rs6516123
rs149414561
rs7547604
rs62333409
rs566306793
rs12026084
rs334428
rs115297732
rs115785494
rs118100551
rs41306763
rs114239351
rs140557158
rs9872703
rs117838829
rs2487024
rs55893525
rs566173566
rs72628523
rs8099150
rs61995622
rs117708421
rs7607789
rs148879296
rs12092780
rs7515641
rs6014074
rs10953759
All
−0.0015
−0.0010
−0.0005
0.0000
0.0005
MR effect size for
'High affinity immunoglobulin alpha and immunoglobulin mu Fc receptor' on 'eGFR'

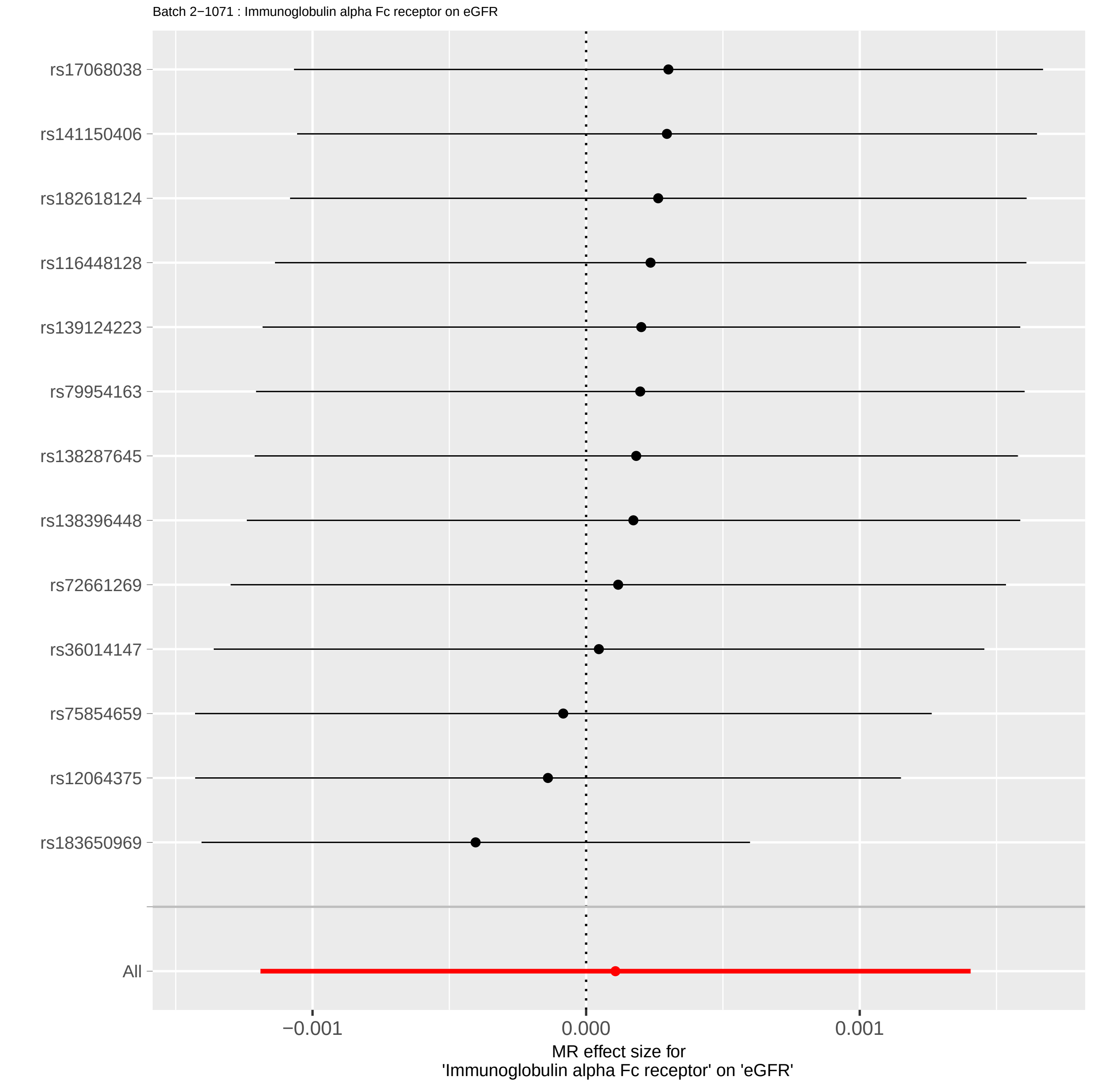

Batch 2−1071 : Immunoglobulin alpha Fc receptor on eGFR
rs17068038
rs141150406
rs182618124
rs116448128
rs139124223
rs79954163
rs138287645
rs138396448
rs72661269
rs36014147
rs75854659
rs12064375
rs183650969
All
−0.001
0.000
0.001
MR effect size for
'Immunoglobulin alpha Fc receptor' on 'eGFR'

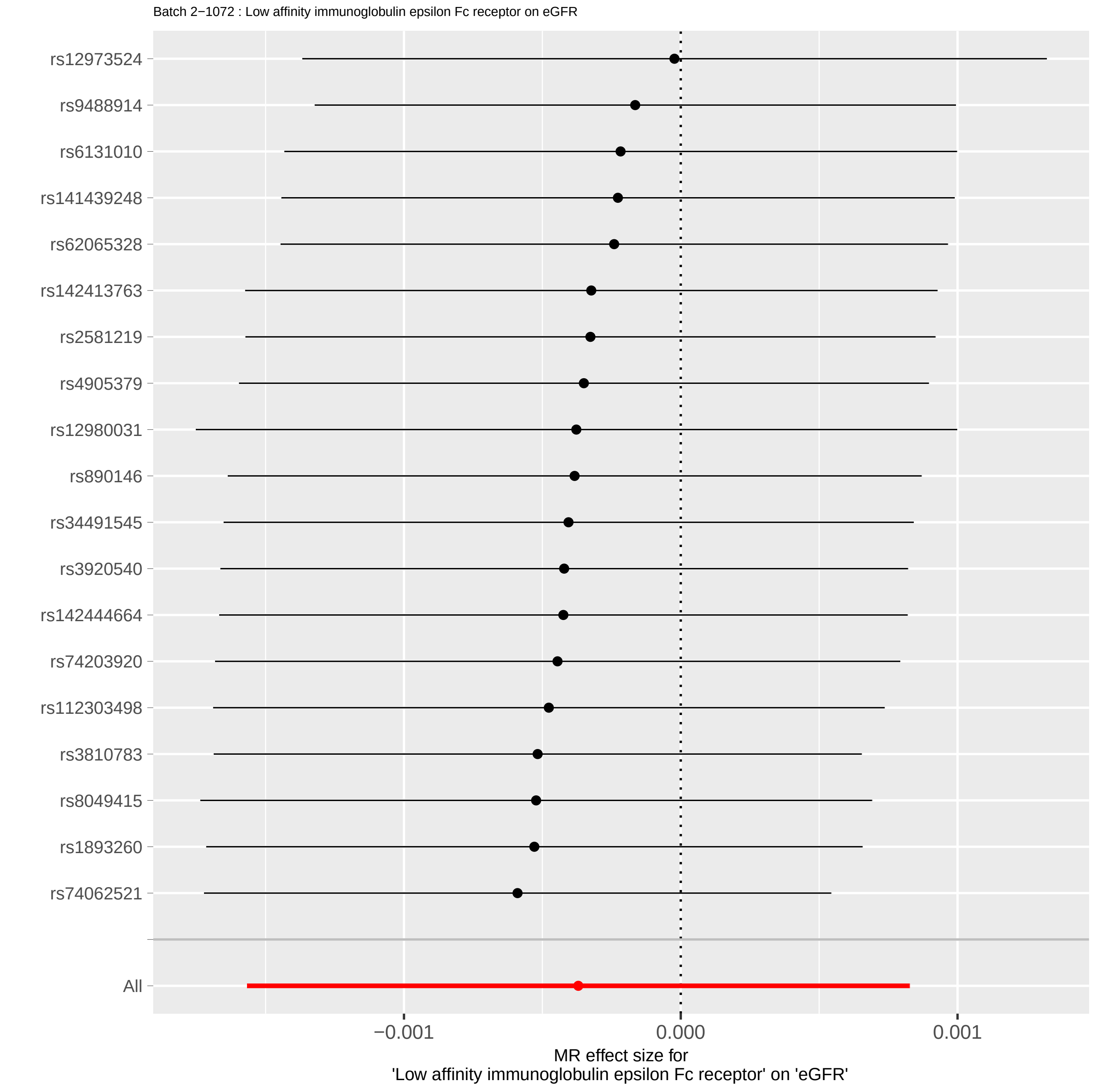

Batch 2−1072 : Low affinity immunoglobulin epsilon Fc receptor on eGFR
rs12973524
rs9488914
rs6131010
rs141439248
rs62065328
rs142413763
rs2581219
rs4905379
rs12980031
rs890146
rs34491545
rs3920540
rs142444664
rs74203920
rs112303498
rs3810783
rs8049415
rs1893260
rs74062521
All
−0.001
0.000
0.001
MR effect size for
'Low affinity immunoglobulin epsilon Fc receptor' on 'eGFR'

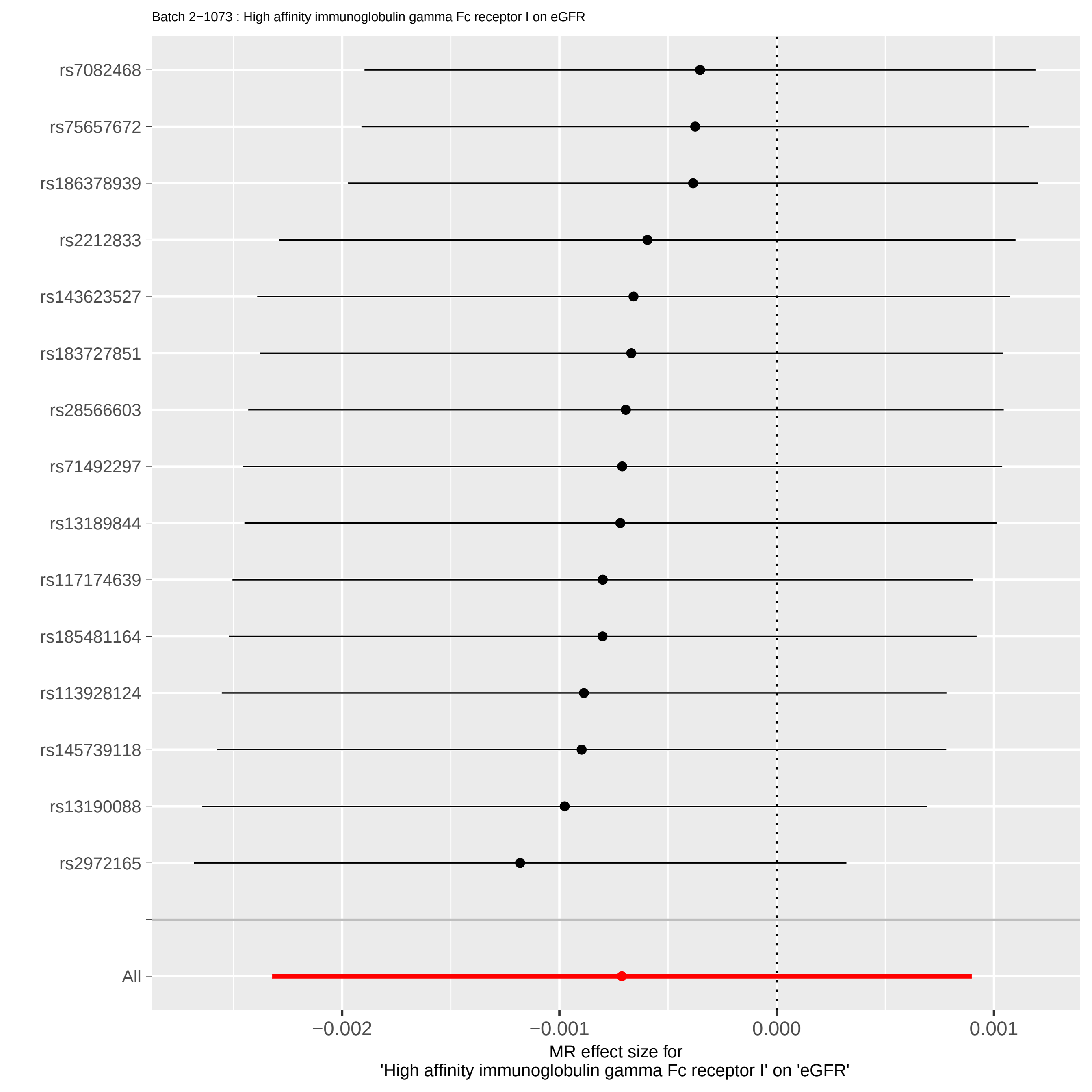

Batch 2−1073 : High affinity immunoglobulin gamma Fc receptor I on eGFR
rs7082468
rs75657672
rs186378939
rs2212833
rs143623527
rs183727851
rs28566603
rs71492297
rs13189844
rs117174639
rs185481164
rs113928124
rs145739118
rs13190088
rs2972165
All
−0.002
−0.001
0.000
0.001
MR effect size for
'High affinity immunoglobulin gamma Fc receptor I' on 'eGFR'

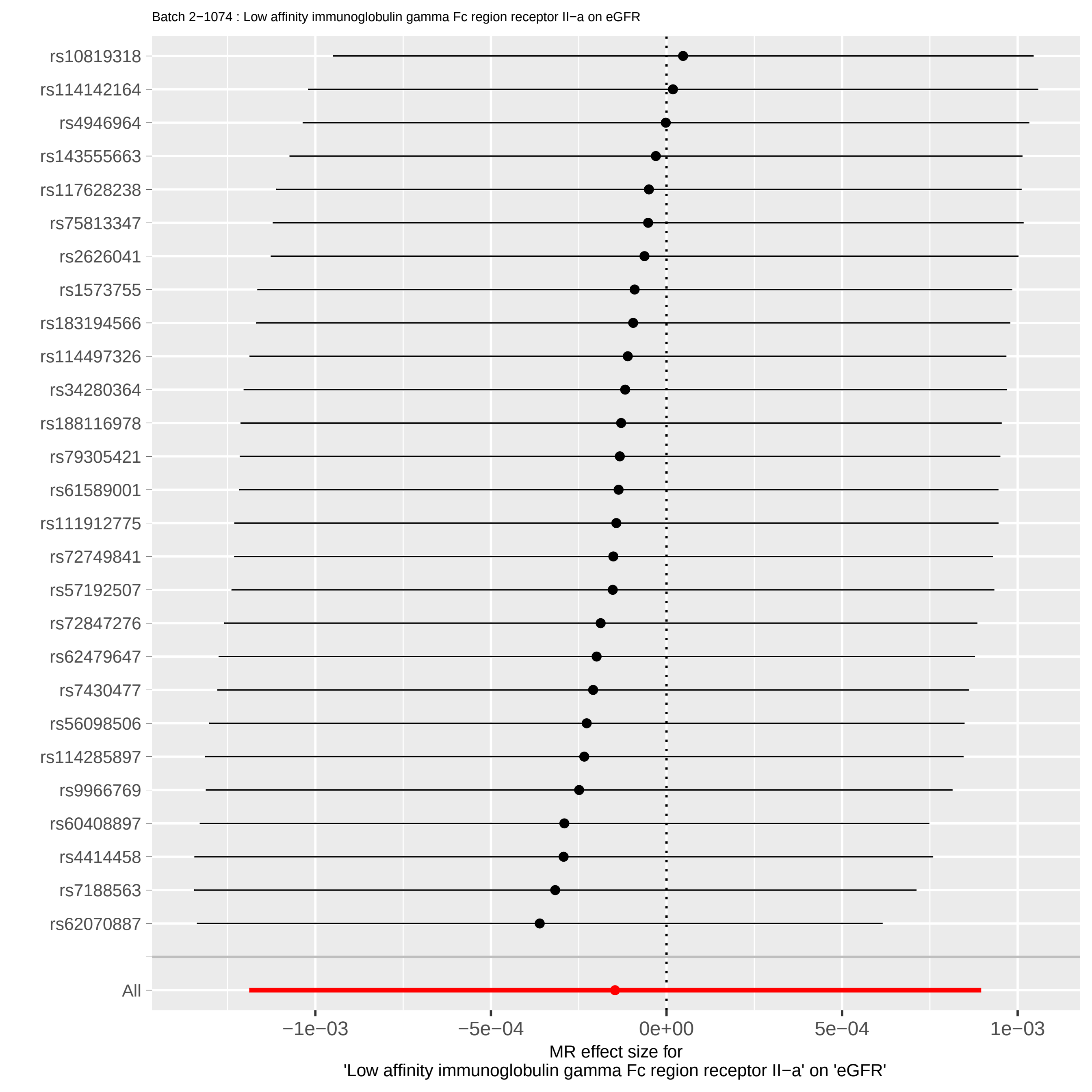

Batch 2−1074 : Low affinity immunoglobulin gamma Fc region receptor II−a on eGFR
rs10819318
rs114142164
rs4946964
rs143555663
rs117628238
rs75813347
rs2626041
rs1573755
rs183194566
rs114497326
rs34280364
rs188116978
rs79305421
rs61589001
rs111912775
rs72749841
rs57192507
rs72847276
rs62479647
rs7430477
rs56098506
rs114285897
rs9966769
rs60408897
rs4414458
rs7188563
rs62070887
All
−1e−03
−5e−04
0e+00
5e−04
1e−03
MR effect size for
'Low affinity immunoglobulin gamma Fc region receptor II−a' on 'eGFR'

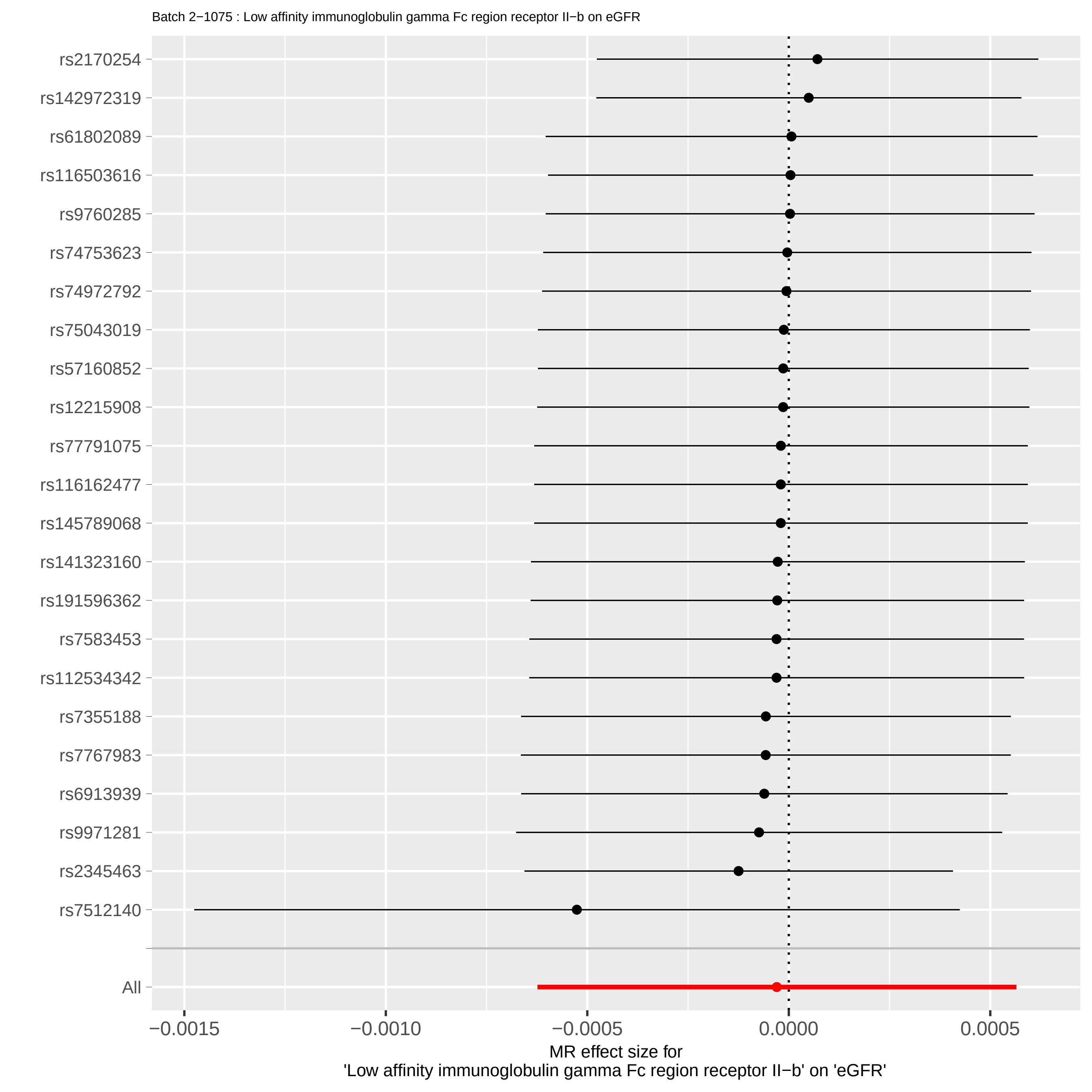

Batch 2−1075 : Low affinity immunoglobulin gamma Fc region receptor II−b on eGFR
rs2170254
rs142972319
rs61802089
rs116503616
rs9760285
rs74753623
rs74972792
rs75043019
rs57160852
rs12215908
rs77791075
rs116162477
rs145789068
rs141323160
rs191596362
rs7583453
rs112534342
rs7355188
rs7767983
rs6913939
rs9971281
rs2345463
rs7512140
All
−0.0015
−0.0010
−0.0005
0.0000
0.0005
MR effect size for
'Low affinity immunoglobulin gamma Fc region receptor II−b' on 'eGFR'

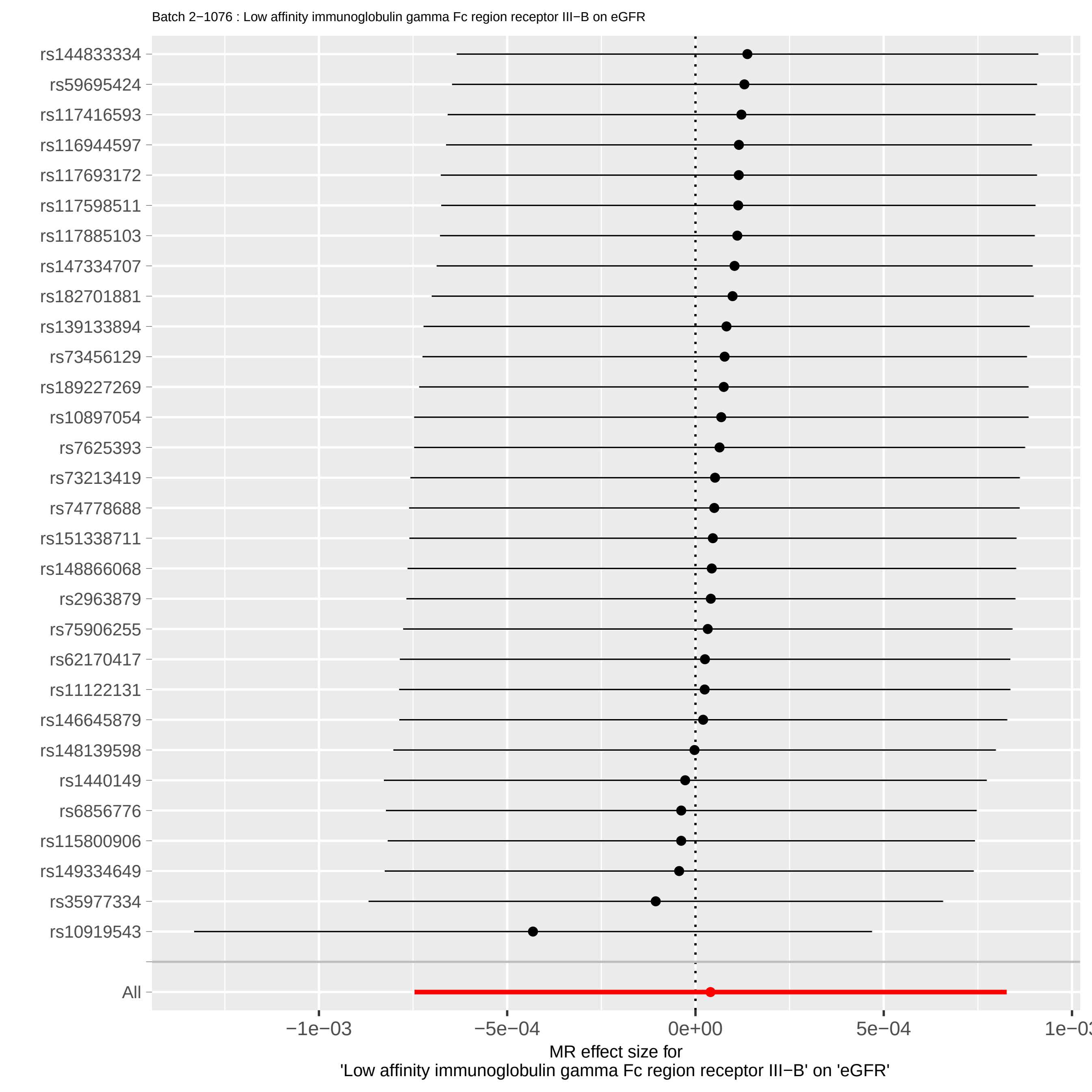
Batch 2−1076 : Low affinity immunoglobulin gamma Fc region receptor III−B on eGFR
rs144833334
rs59695424
rs117416593
rs116944597
rs117693172
rs117598511
rs117885103
rs147334707
rs182701881
rs139133894
rs73456129
rs189227269
rs10897054
rs7625393
rs73213419
rs74778688
rs151338711
rs148866068
rs2963879
rs75906255
rs62170417
rs11122131
rs146645879
rs148139598
rs1440149
rs6856776
rs115800906
rs149334649
rs35977334
rs10919543
All
−1e−03
−5e−04
0e+00
5e−04
1e−03
MR effect size for
'Low affinity immunoglobulin gamma Fc region receptor III−B' on 'eGFR'

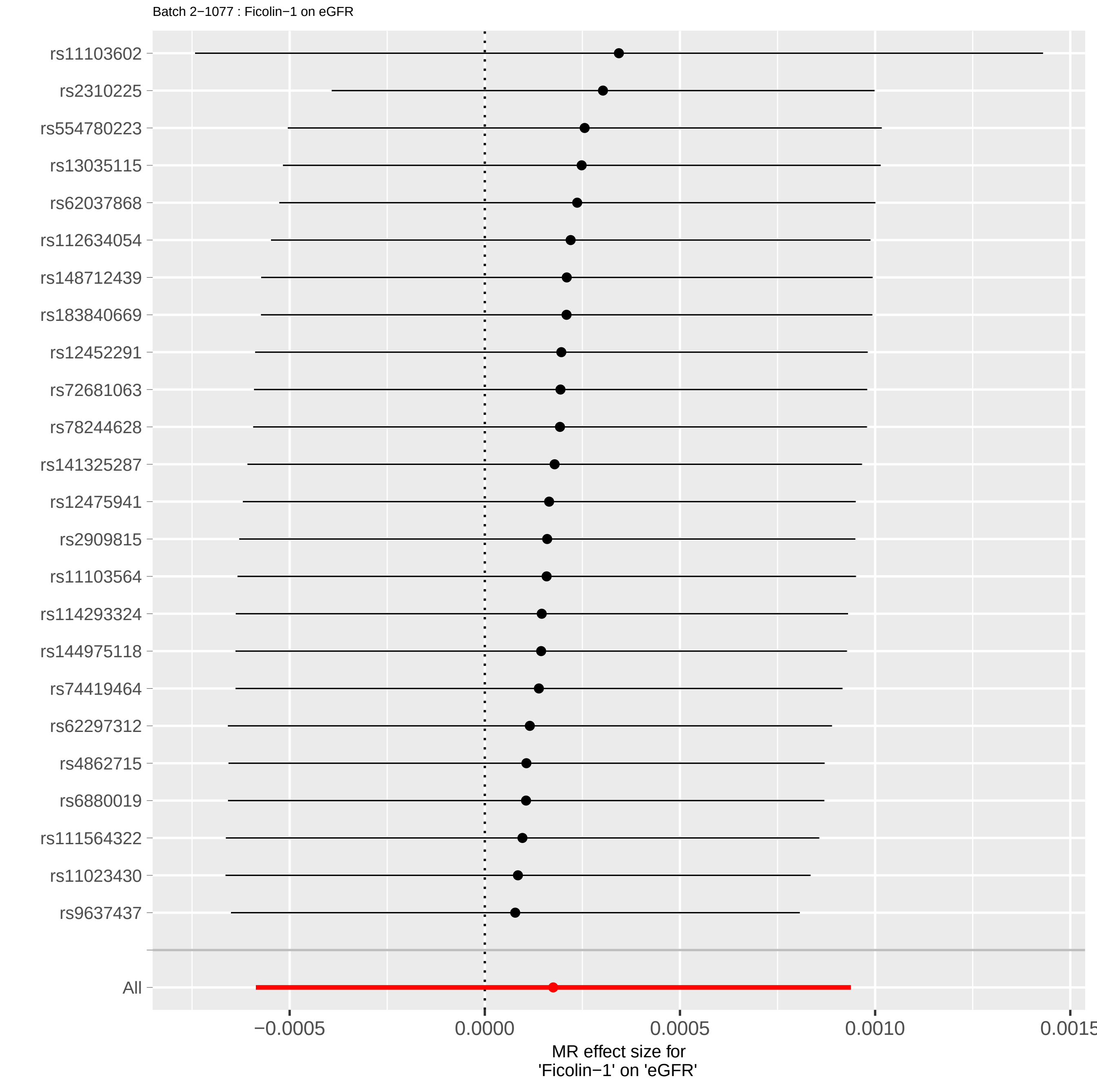

Batch 2−1077 : Ficolin−1 on eGFR
rs11103602
rs2310225
rs554780223
rs13035115
rs62037868
rs112634054
rs148712439
rs183840669
rs12452291
rs72681063
rs78244628
rs141325287
rs12475941
rs2909815
rs11103564
rs114293324
rs144975118
rs74419464
rs62297312
rs4862715
rs6880019
rs111564322
rs11023430
rs9637437
All
−0.0005
0.0000
0.0005
0.0010
0.0015
MR effect size for
'Ficolin−1' on 'eGFR'

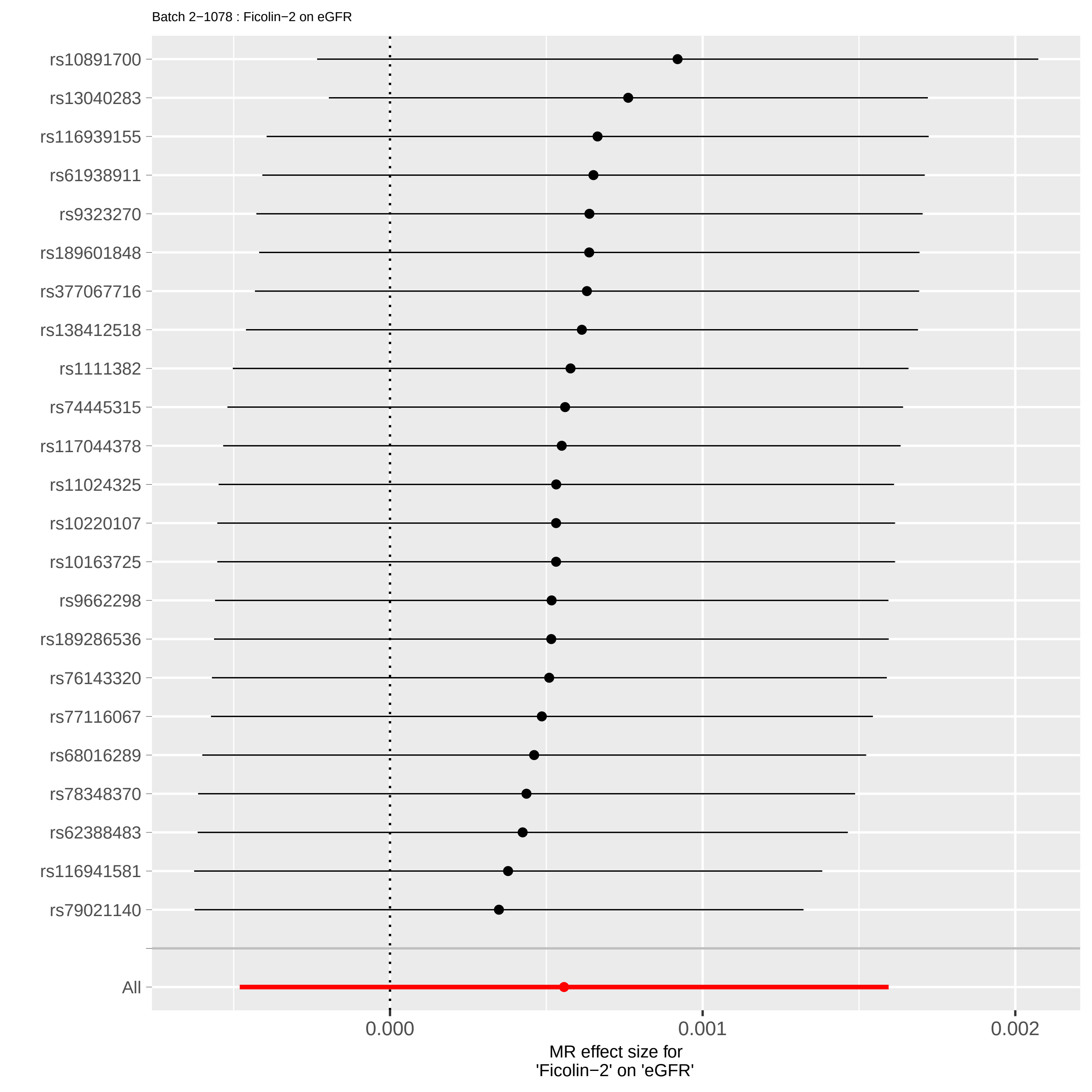
Batch 2−1078 : Ficolin−2 on eGFR
rs10891700
rs13040283
rs116939155
rs61938911
rs9323270
rs189601848
rs377067716
rs138412518
rs1111382
rs74445315
rs117044378
rs11024325
rs10220107
rs10163725
rs9662298
rs189286536
rs76143320
rs77116067
rs68016289
rs78348370
rs62388483
rs116941581
rs79021140
All
0.000
0.001
0.002
MR effect size for
'Ficolin−2' on 'eGFR'

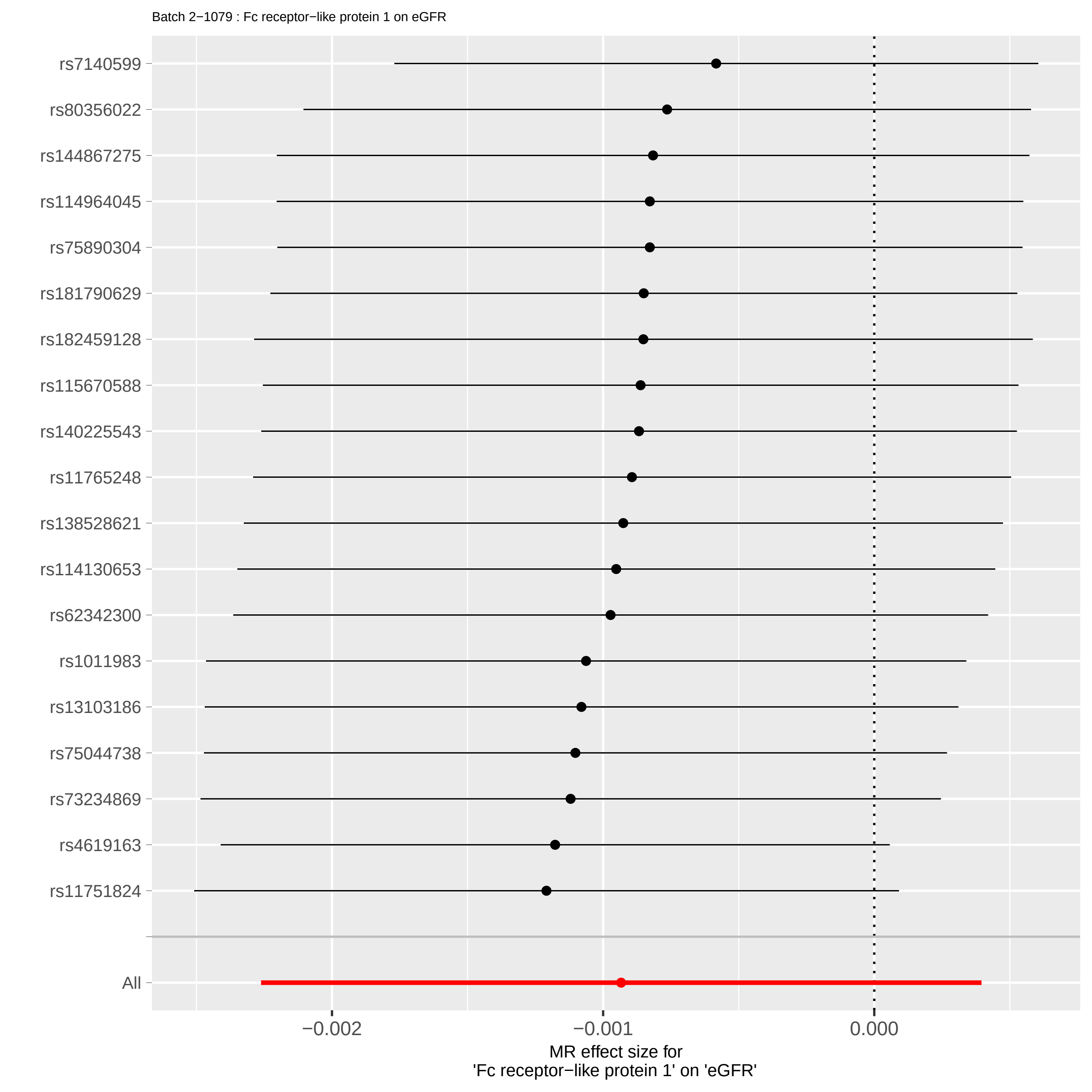

Batch 2−1079 : Fc receptor−like protein 1 on eGFR
rs7140599
rs80356022
rs144867275
rs114964045
rs75890304
rs181790629
rs182459128
rs115670588
rs140225543
rs11765248
rs138528621
rs114130653
rs62342300
rs1011983
rs13103186
rs75044738
rs73234869
rs4619163
rs11751824
All
−0.002
−0.001
0.000
MR effect size for
'Fc receptor−like protein 1' on 'eGFR'

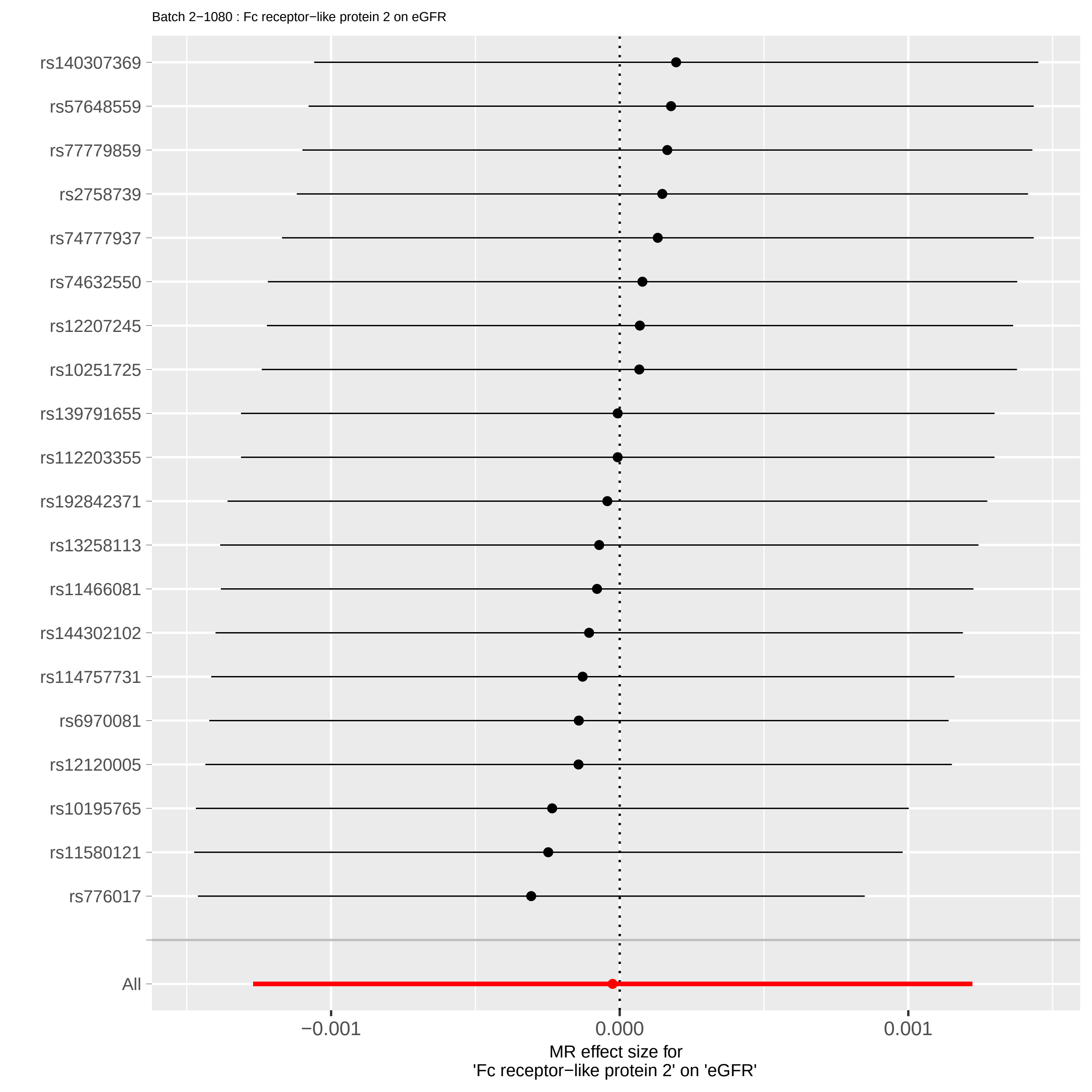

Batch 2−1080 : Fc receptor−like protein 2 on eGFR
rs140307369
rs57648559
rs77779859
rs2758739
rs74777937
rs74632550
rs12207245
rs10251725
rs139791655
rs112203355
rs192842371
rs13258113
rs11466081
rs144302102
rs114757731
rs6970081
rs12120005
rs10195765
rs11580121
rs776017
All
−0.001
0.000
0.001
MR effect size for
'Fc receptor−like protein 2' on 'eGFR'

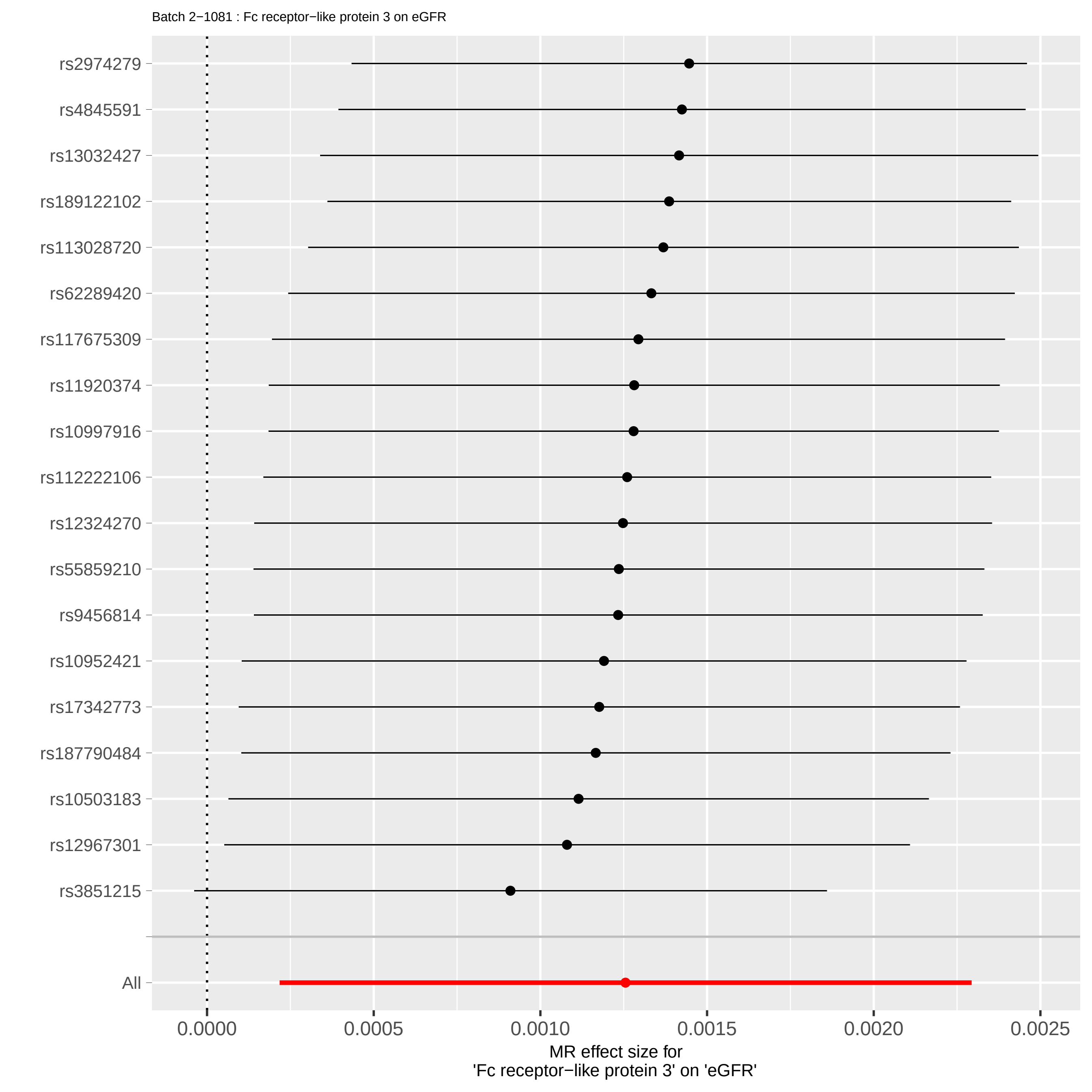
Batch 2−1081 : Fc receptor−like protein 3 on eGFR
rs2974279
rs4845591
rs13032427
rs189122102
rs113028720
rs62289420
rs117675309
rs11920374
rs10997916
rs112222106
rs12324270
rs55859210
rs9456814
rs10952421
rs17342773
rs187790484
rs10503183
rs12967301
rs3851215
All
0.0000
0.0005
0.0010
0.0015
0.0020
0.0025
MR effect size for
'Fc receptor−like protein 3' on 'eGFR'

Batch 2−1082 : Fc receptor−like protein 4 on eGFR

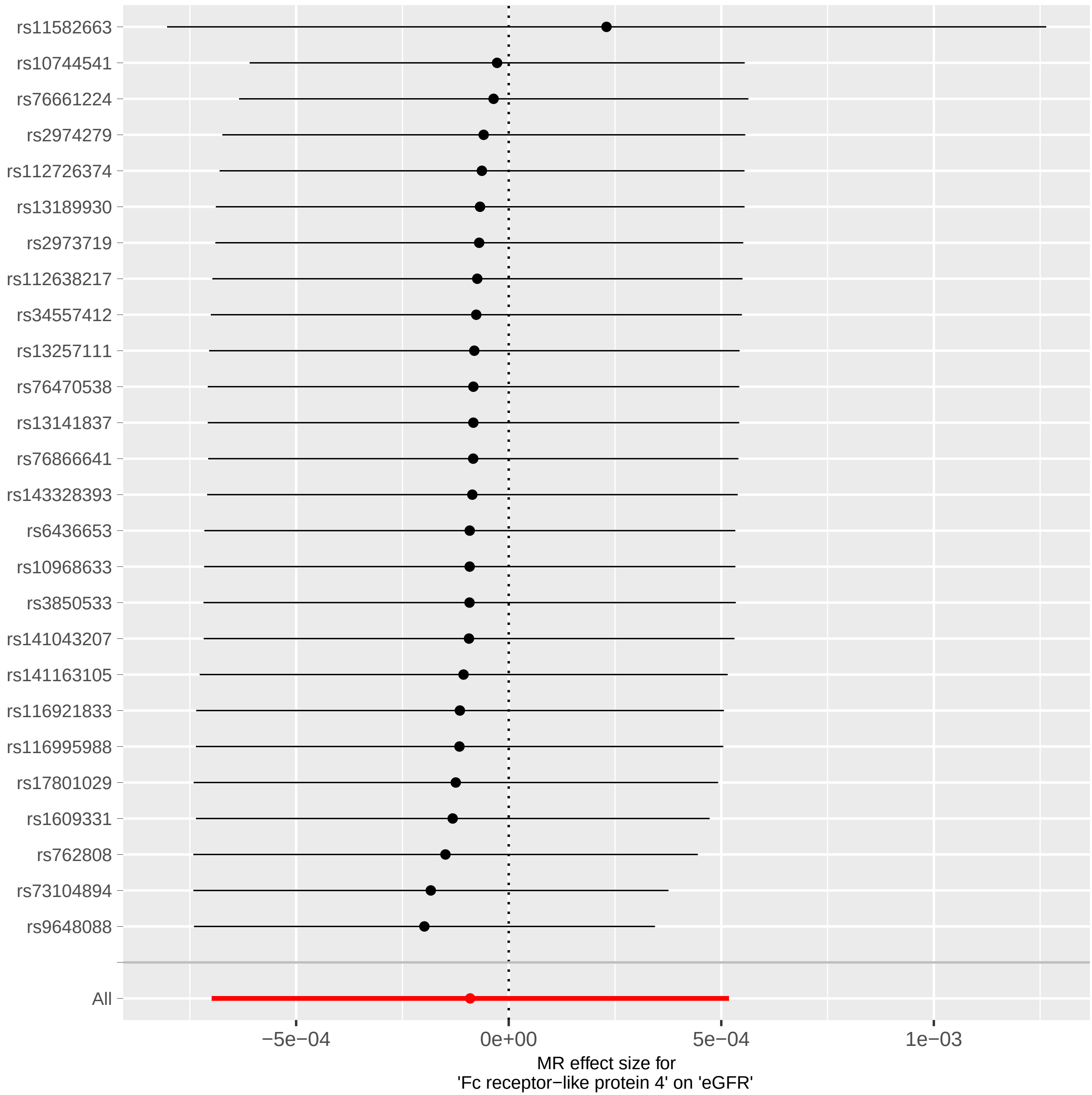

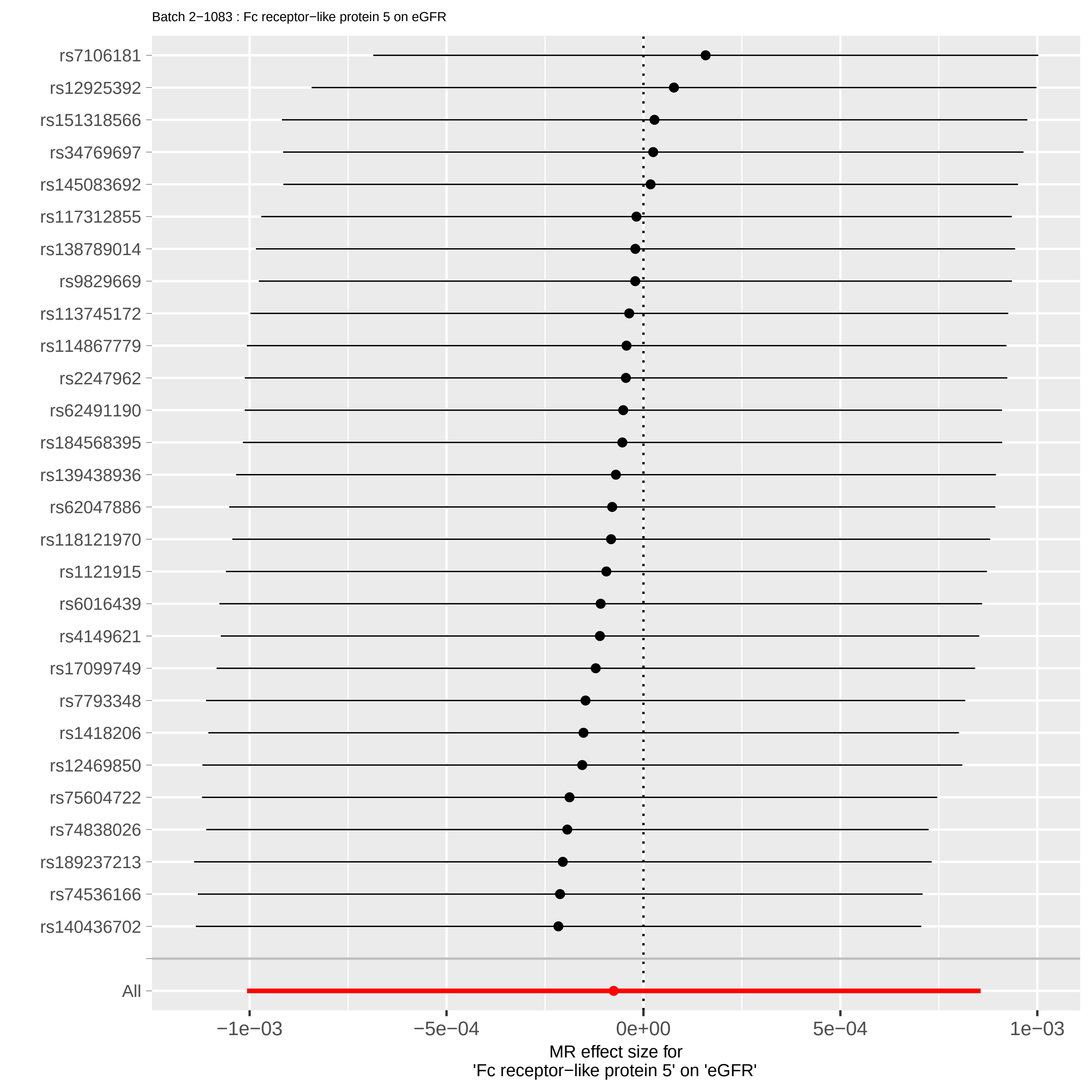

Batch 2−1083 : Fc receptor−like protein 5 on eGFR
rs7106181
rs12925392
rs151318566
rs34769697
rs145083692
rs117312855
rs138789014
rs9829669
rs113745172
rs114867779
rs2247962
rs62491190
rs184568395
rs139438936
rs62047886
rs118121970
rs1121915
rs6016439
rs4149621
rs17099749
rs7793348
rs1418206
rs12469850
rs75604722
rs74838026
rs189237213
rs74536166
rs140436702
All
−1e−03
−5e−04
0e+00
5e−04
1e−03
MR effect size for
'Fc receptor−like protein 5' on 'eGFR'

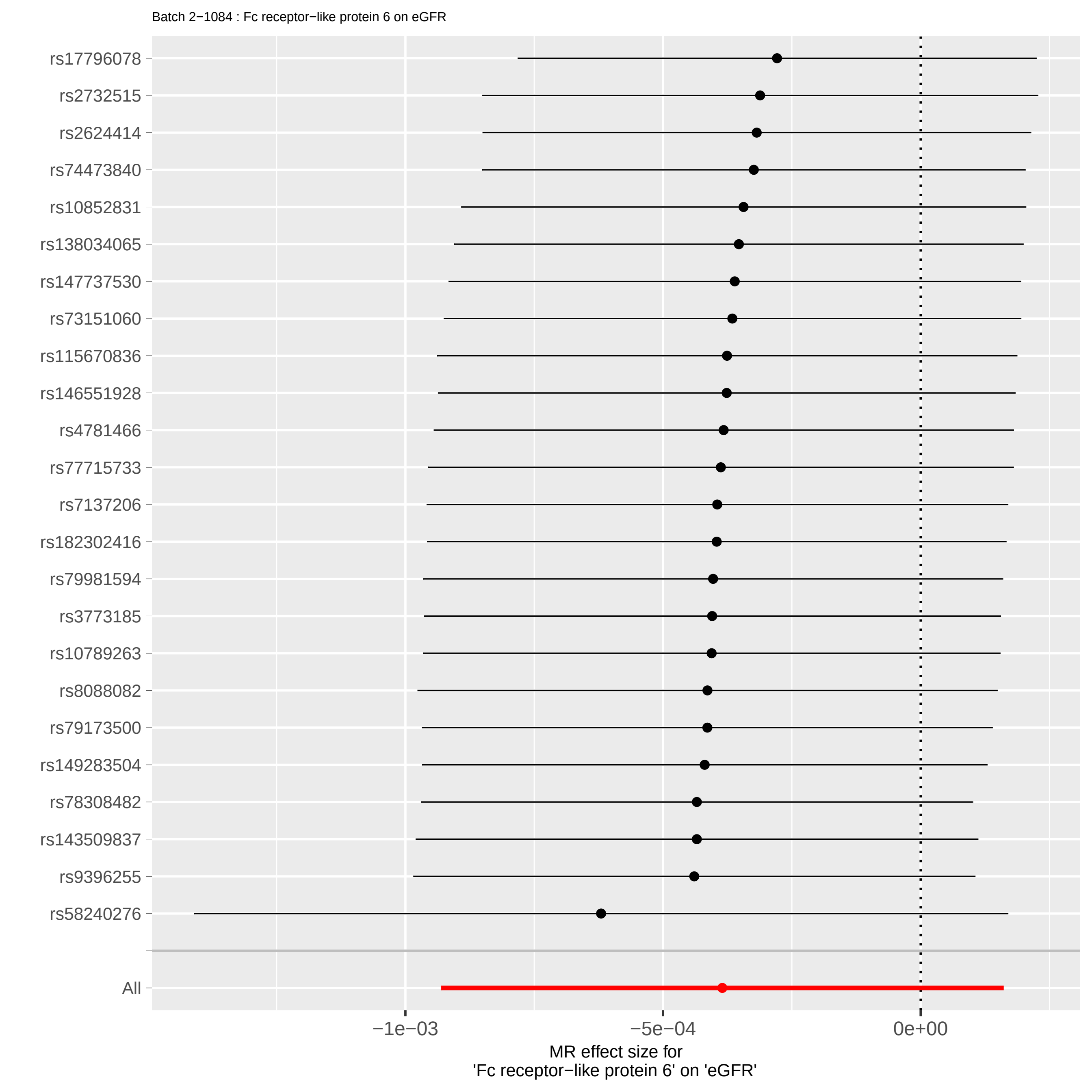

Batch 2−1084 : Fc receptor−like protein 6 on eGFR
rs17796078
rs2732515
rs2624414
rs74473840
rs10852831
rs138034065
rs147737530
rs73151060
rs115670836
rs146551928
rs4781466
rs77715733
rs7137206
rs182302416
rs79981594
rs3773185
rs10789263
rs8088082
rs79173500
rs149283504
rs78308482
rs143509837
rs9396255
rs58240276
All
−1e−03
−5e−04
0e+00
MR effect size for
'Fc receptor−like protein 6' on 'eGFR'

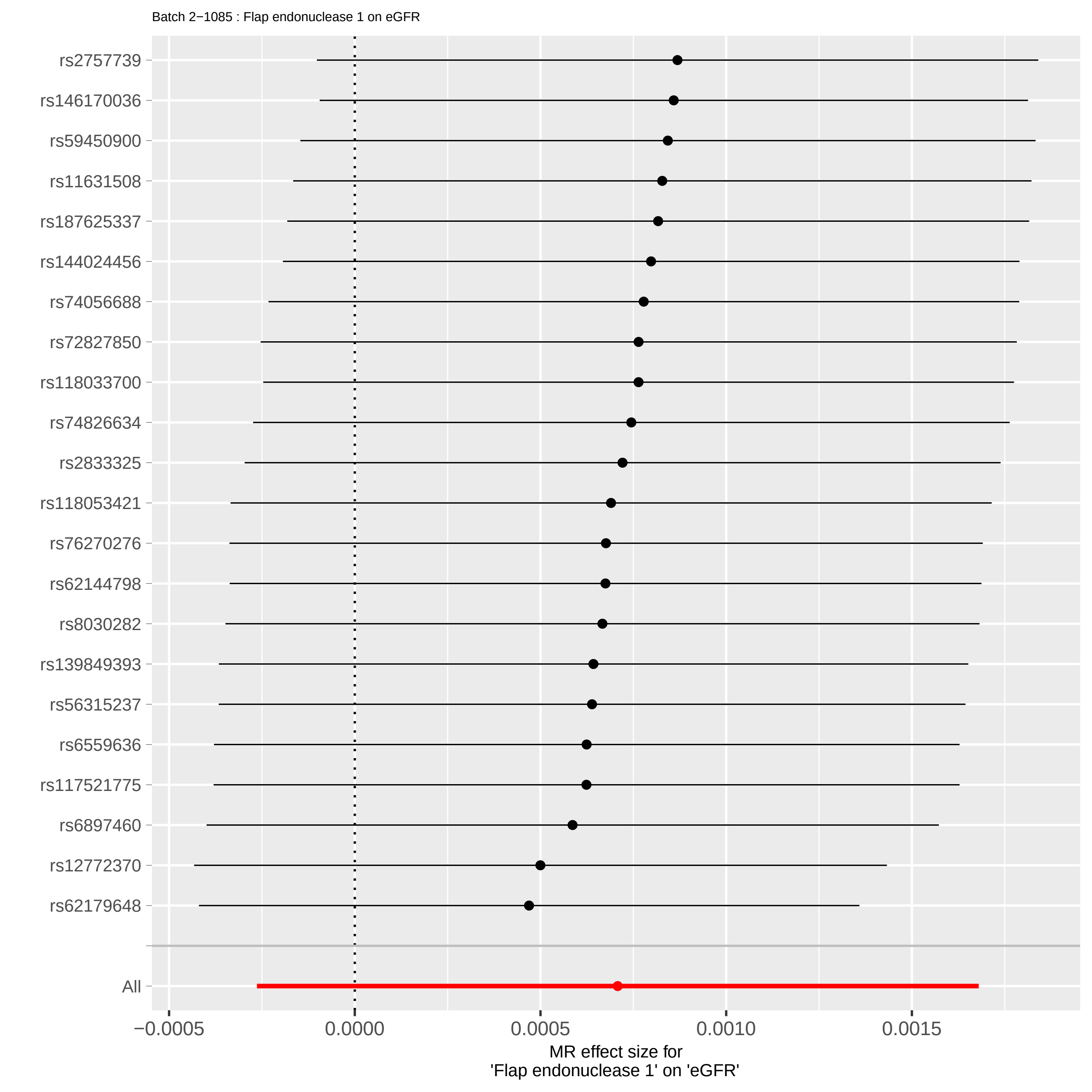

Batch 2−1085 : Flap endonuclease 1 on eGFR
rs2757739
rs146170036
rs59450900
rs11631508
rs187625337
rs144024456
rs74056688
rs72827850
rs118033700
rs74826634
rs2833325
rs118053421
rs76270276
rs62144798
rs8030282
rs139849393
rs56315237
rs6559636
rs117521775
rs6897460
rs12772370
rs62179648
All
−0.0005
0.0000
0.0005
0.0010
0.0015
MR effect size for
'Flap endonuclease 1' on 'eGFR'

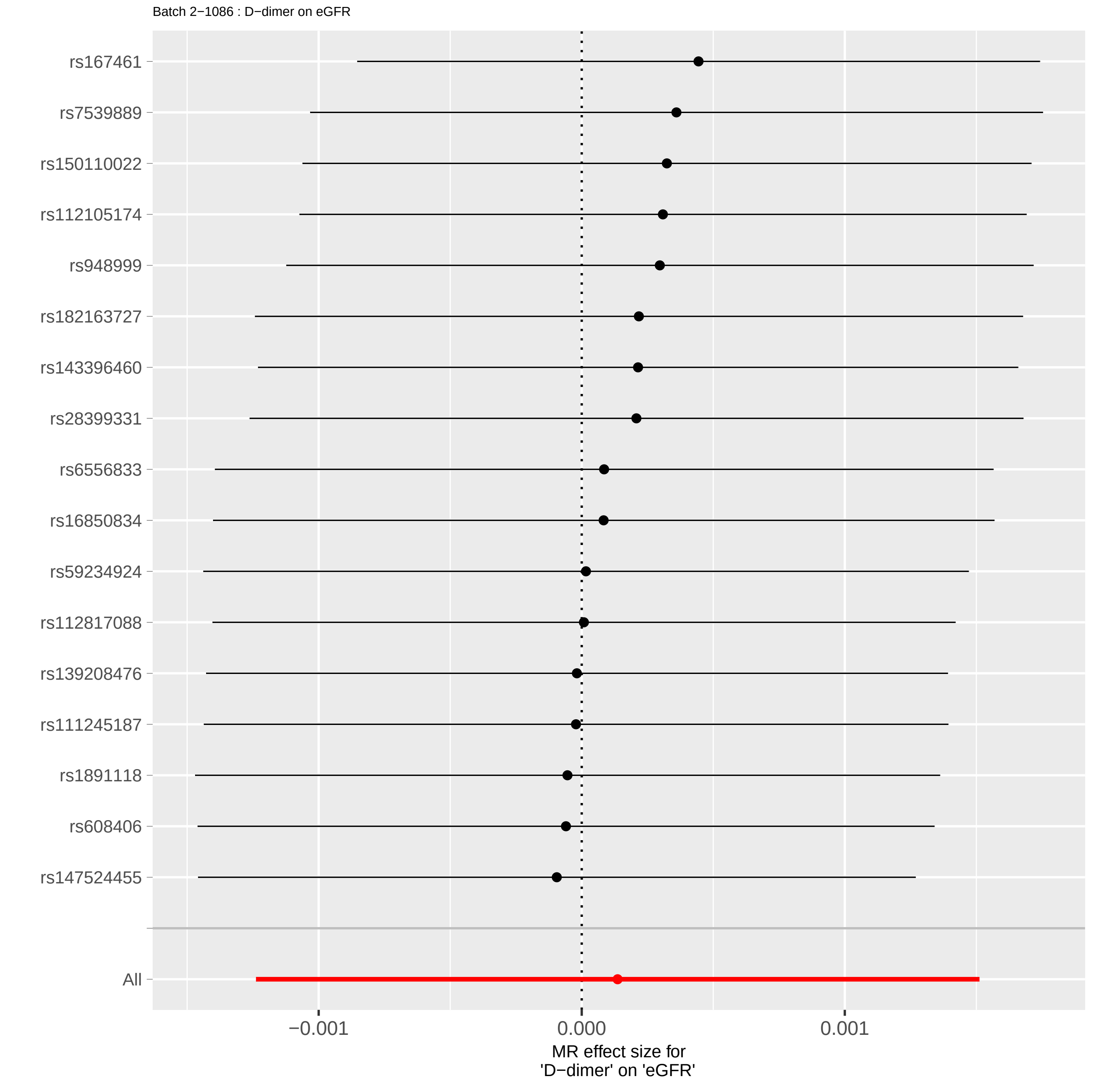

Batch 2−1086 : D−dimer on eGFR
rs167461
rs7539889
rs150110022
rs112105174
rs948999
rs182163727
rs143396460
rs28399331
rs6556833
rs16850834
rs59234924
rs112817088
rs139208476
rs111245187
rs1891118
rs608406
rs147524455
All
−0.001
0.000
0.001
MR effect size for
'D−dimer' on 'eGFR'

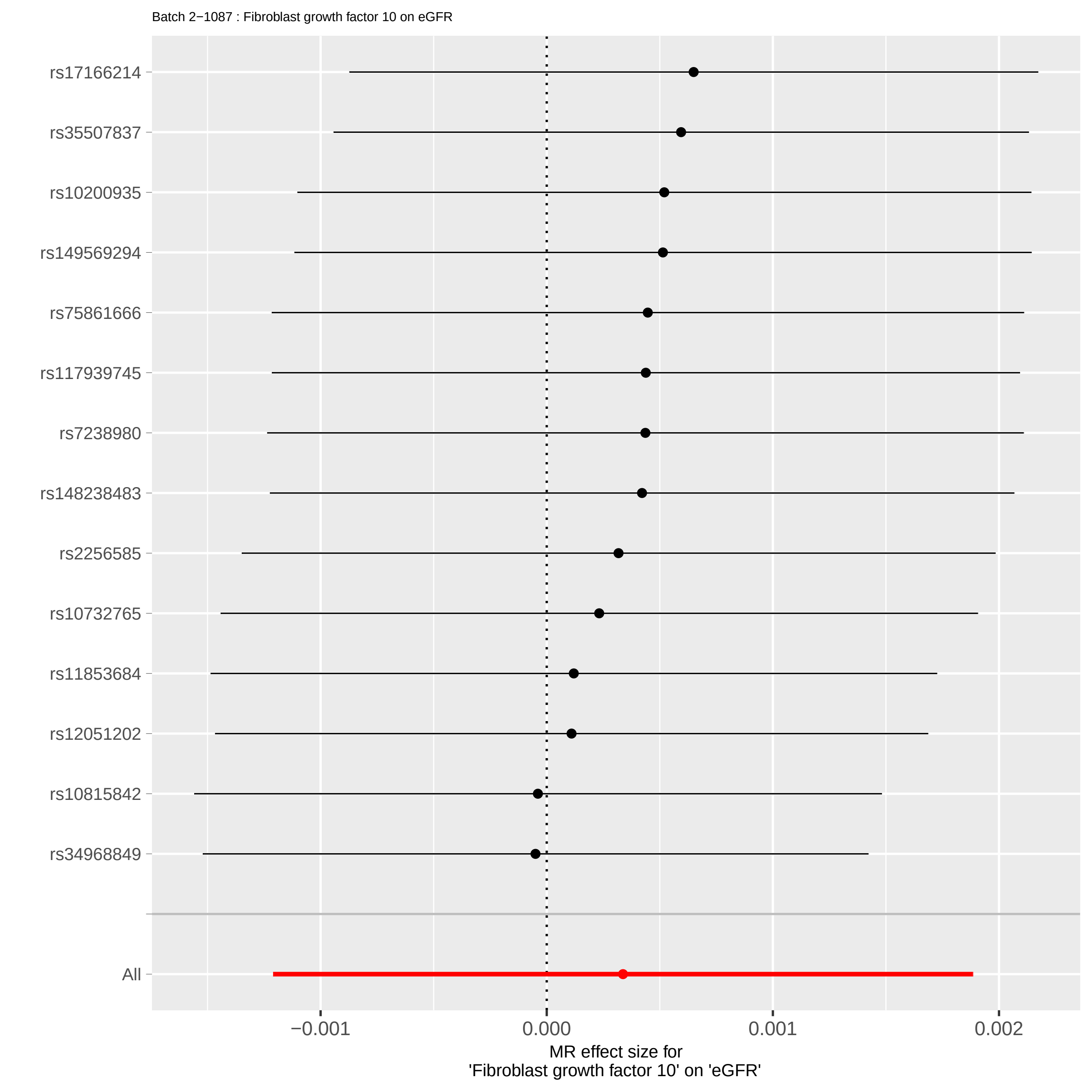

Batch 2−1087 : Fibroblast growth factor 10 on eGFR
rs17166214
rs35507837
rs10200935
rs149569294
rs75861666
rs117939745
rs7238980
rs148238483
rs2256585
rs10732765
rs11853684
rs12051202
rs10815842
rs34968849
All
−0.001
0.000
0.001
0.002
MR effect size for
'Fibroblast growth factor 10' on 'eGFR'

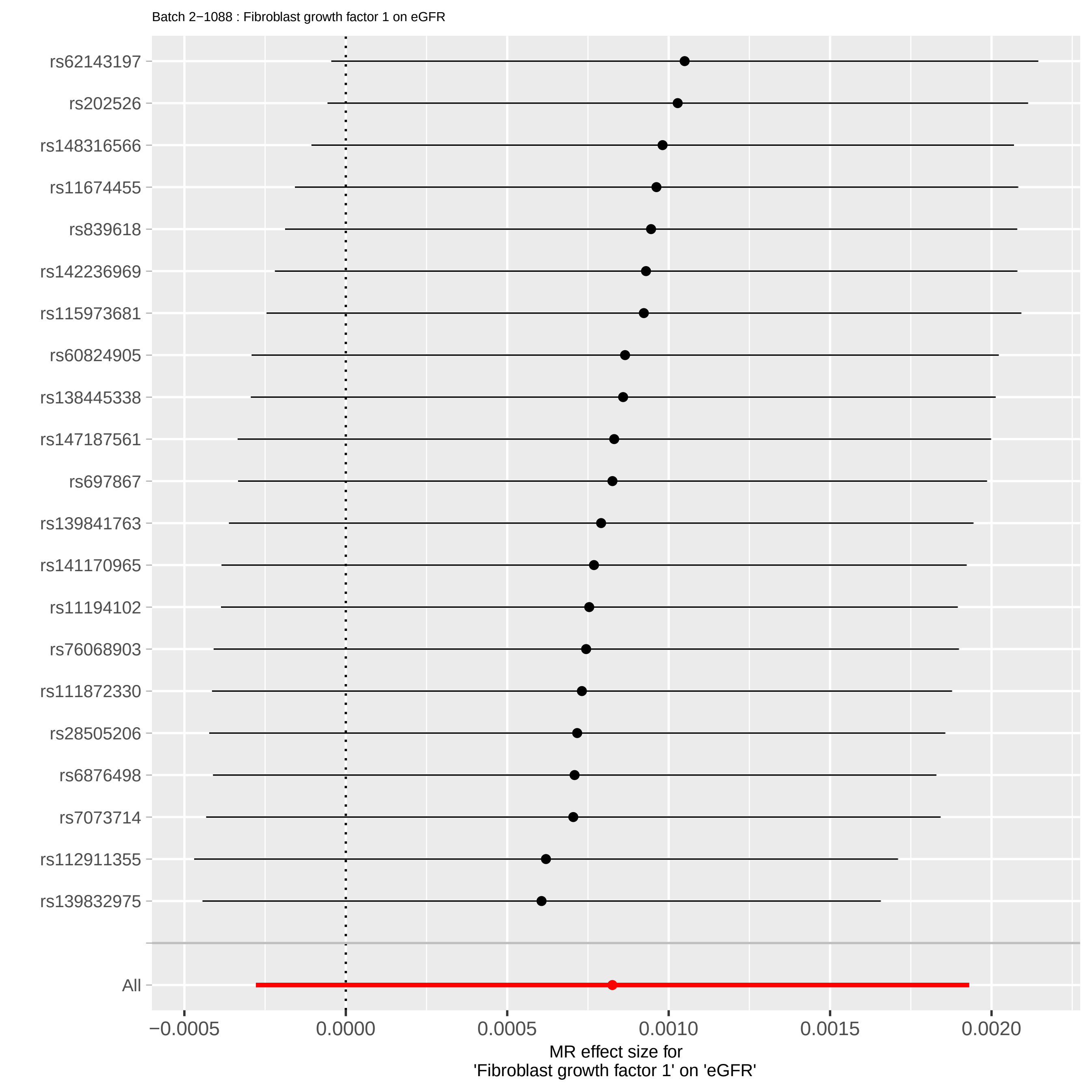

Batch 2−1088 : Fibroblast growth factor 1 on eGFR
rs62143197
rs202526
rs148316566
rs11674455
rs839618
rs142236969
rs115973681
rs60824905
rs138445338
rs147187561
rs697867
rs139841763
rs141170965
rs11194102
rs76068903
rs111872330
rs28505206
rs6876498
rs7073714
rs112911355
rs139832975
All
−0.0005
0.0000
0.0005
0.0010
0.0015
0.0020
MR effect size for
'Fibroblast growth factor 1' on 'eGFR'

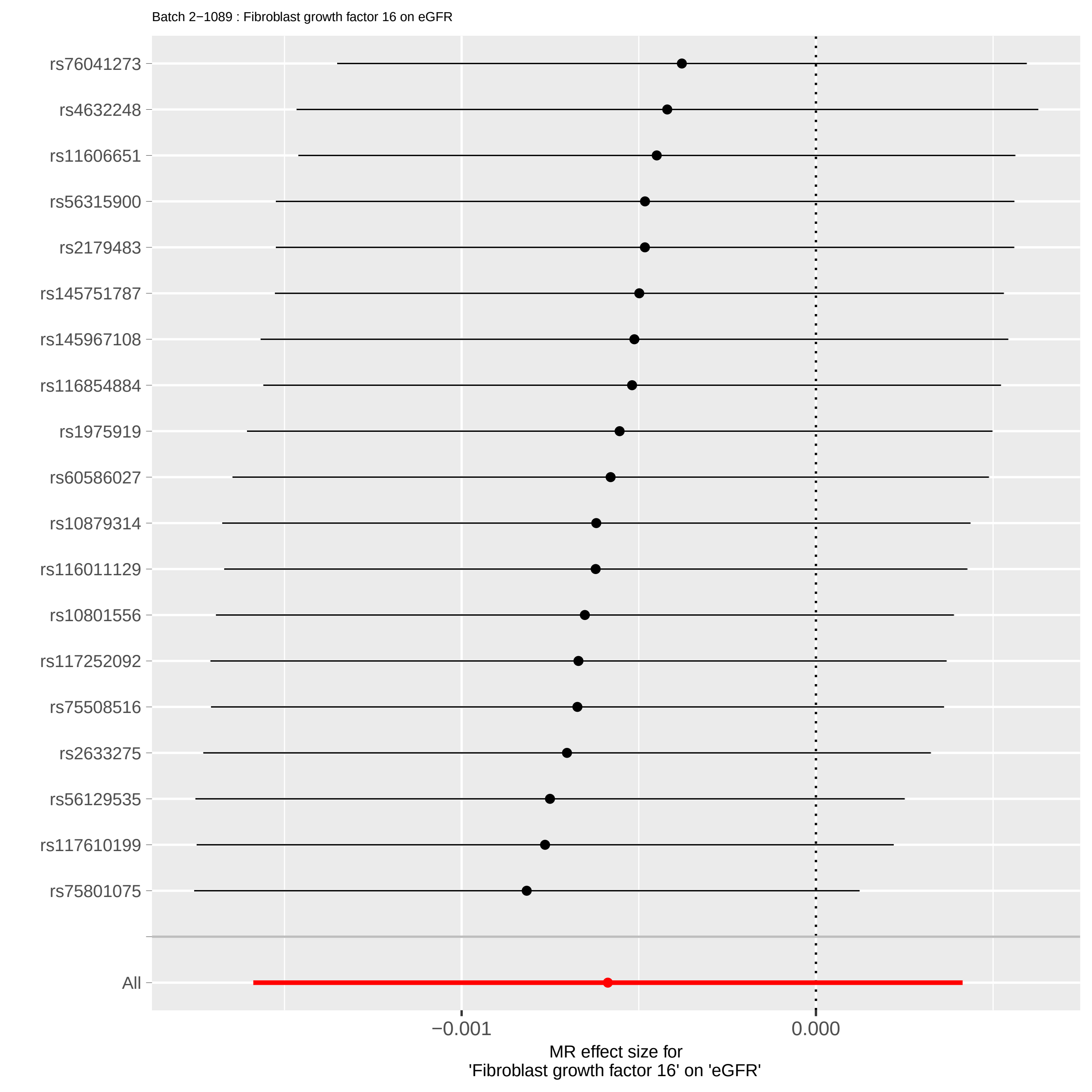

Batch 2−1089 : Fibroblast growth factor 16 on eGFR
rs76041273
rs4632248
rs11606651
rs56315900
rs2179483
rs145751787
rs145967108
rs116854884
rs1975919
rs60586027
rs10879314
rs116011129
rs10801556
rs117252092
rs75508516
rs2633275
rs56129535
rs117610199
rs75801075
All
−0.001
0.000
MR effect size for
'Fibroblast growth factor 16' on 'eGFR'

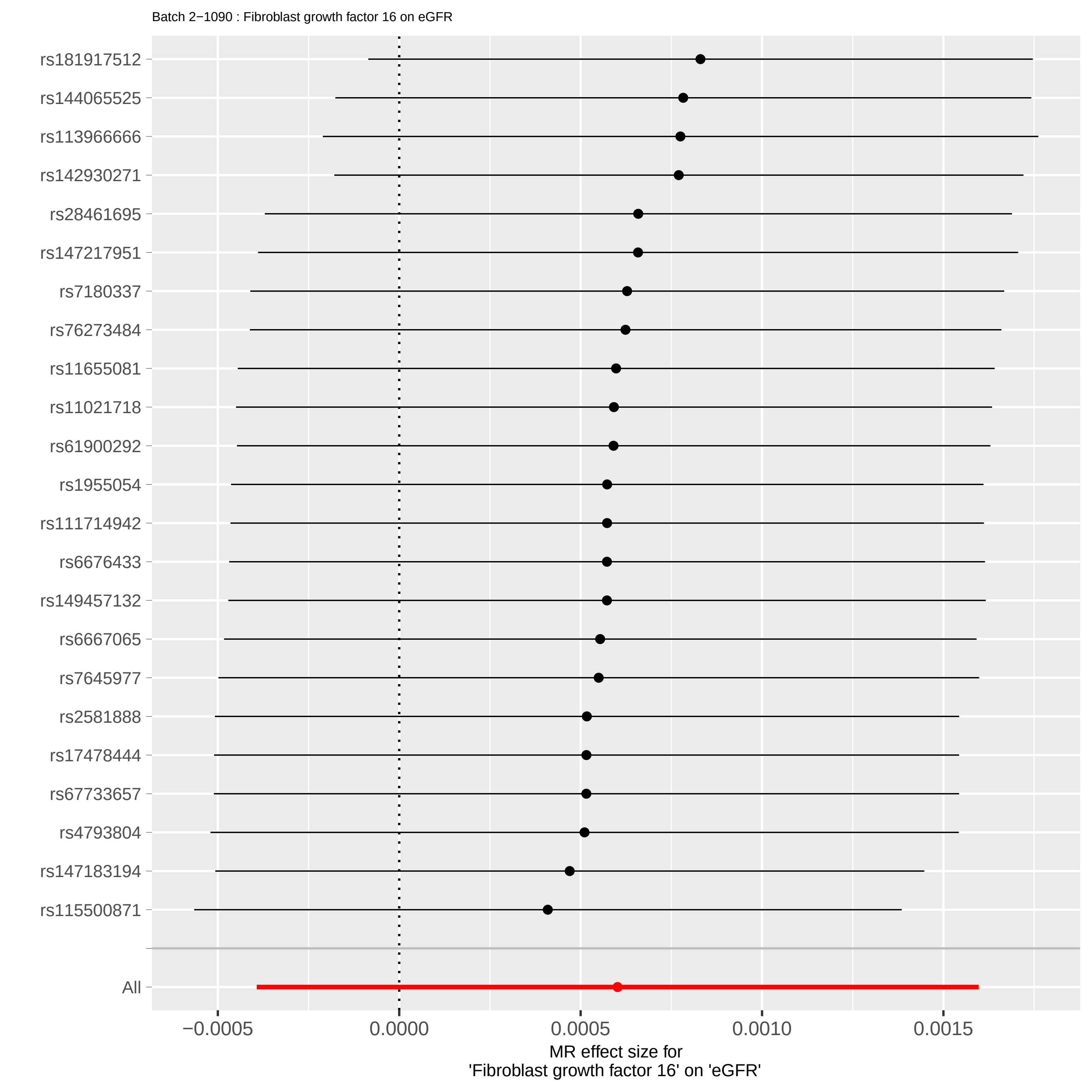
Batch 2−1090 : Fibroblast growth factor 16 on eGFR
rs181917512
rs144065525
rs113966666
rs142930271
rs28461695
rs147217951
rs7180337
rs76273484
rs11655081
rs11021718
rs61900292
rs1955054
rs111714942
rs6676433
rs149457132
rs6667065
rs7645977
rs2581888
rs17478444
rs67733657
rs4793804
rs147183194
rs115500871
All
−0.0005
0.0000
0.0005
0.0010
0.0015
MR effect size for
'Fibroblast growth factor 16' on 'eGFR'

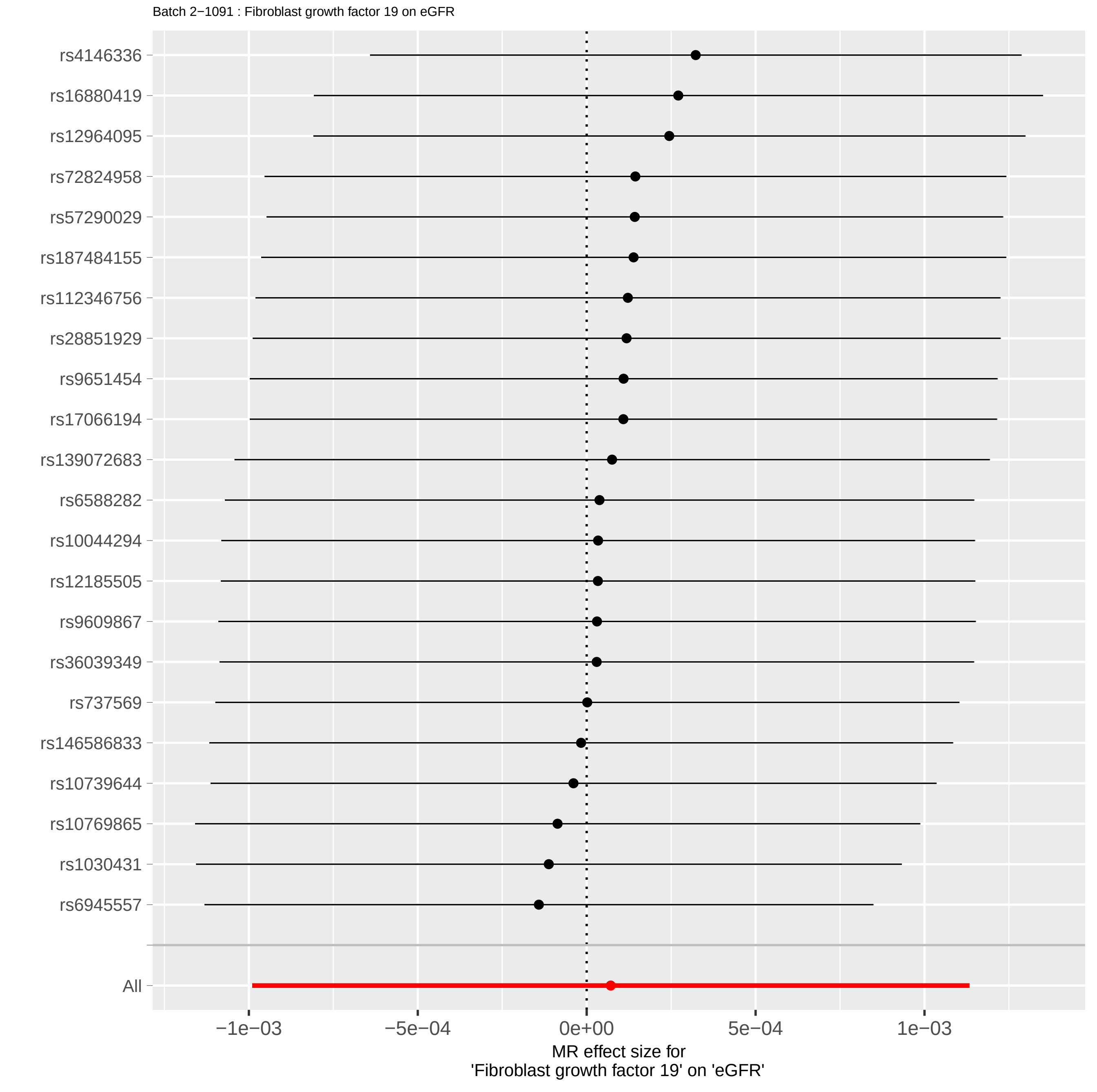

Batch 2−1091 : Fibroblast growth factor 19 on eGFR
rs4146336
rs16880419
rs12964095
rs72824958
rs57290029
rs187484155
rs112346756
rs28851929
rs9651454
rs17066194
rs139072683
rs6588282
rs10044294
rs12185505
rs9609867
rs36039349
rs737569
rs146586833
rs10739644
rs10769865
rs1030431
rs6945557
All
−1e−03
−5e−04
0e+00
5e−04
1e−03
MR effect size for
'Fibroblast growth factor 19' on 'eGFR'

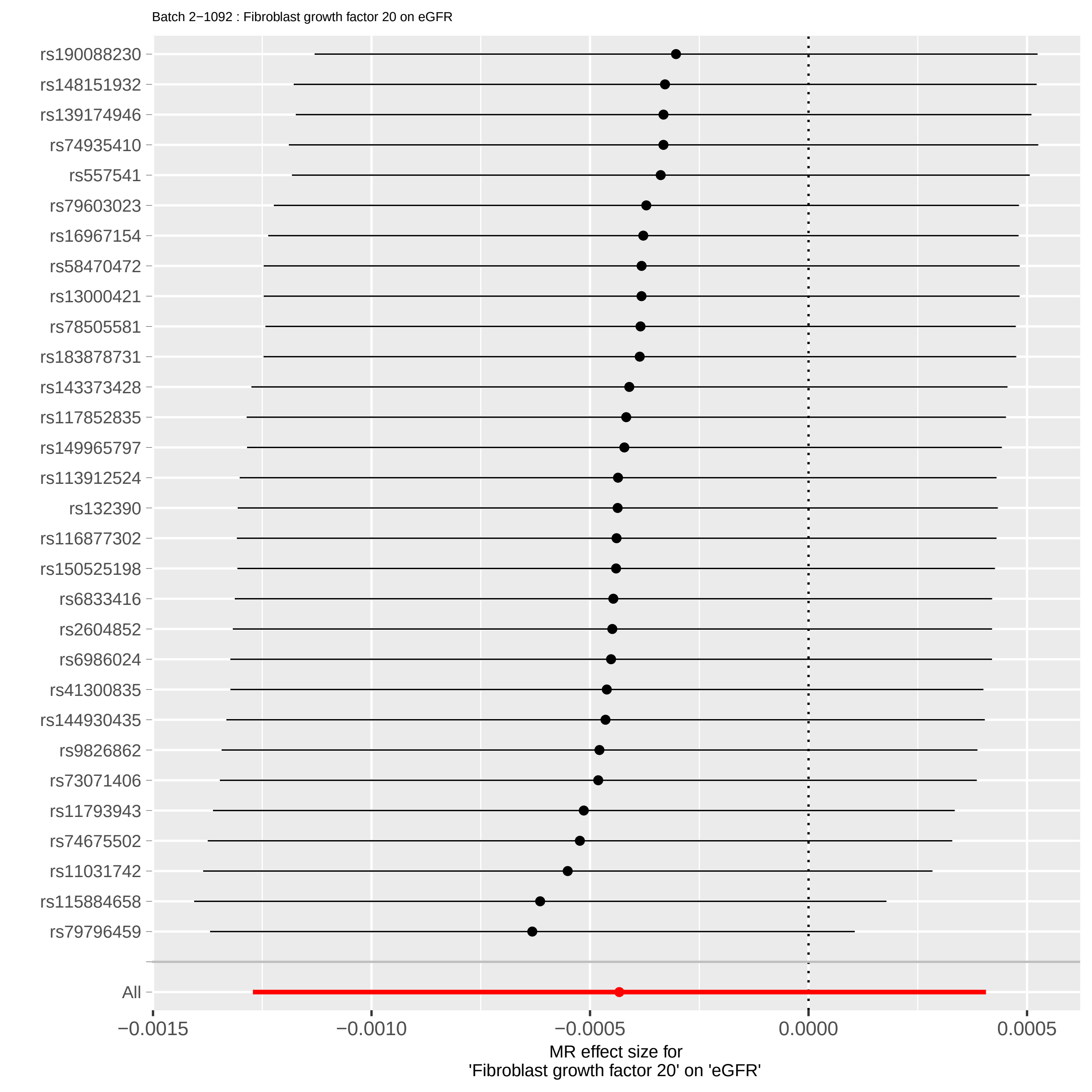

Batch 2−1092 : Fibroblast growth factor 20 on eGFR
rs190088230
rs148151932
rs139174946
rs74935410
rs557541
rs79603023
rs16967154
rs58470472
rs13000421
rs78505581
rs183878731
rs143373428
rs117852835
rs149965797
rs113912524
rs132390
rs116877302
rs150525198
rs6833416
rs2604852
rs6986024
rs41300835
rs144930435
rs9826862
rs73071406
rs11793943
rs74675502
rs11031742
rs115884658
rs79796459
All
−0.0015
−0.0010
−0.0005
0.0000
0.0005
MR effect size for
'Fibroblast growth factor 20' on 'eGFR'

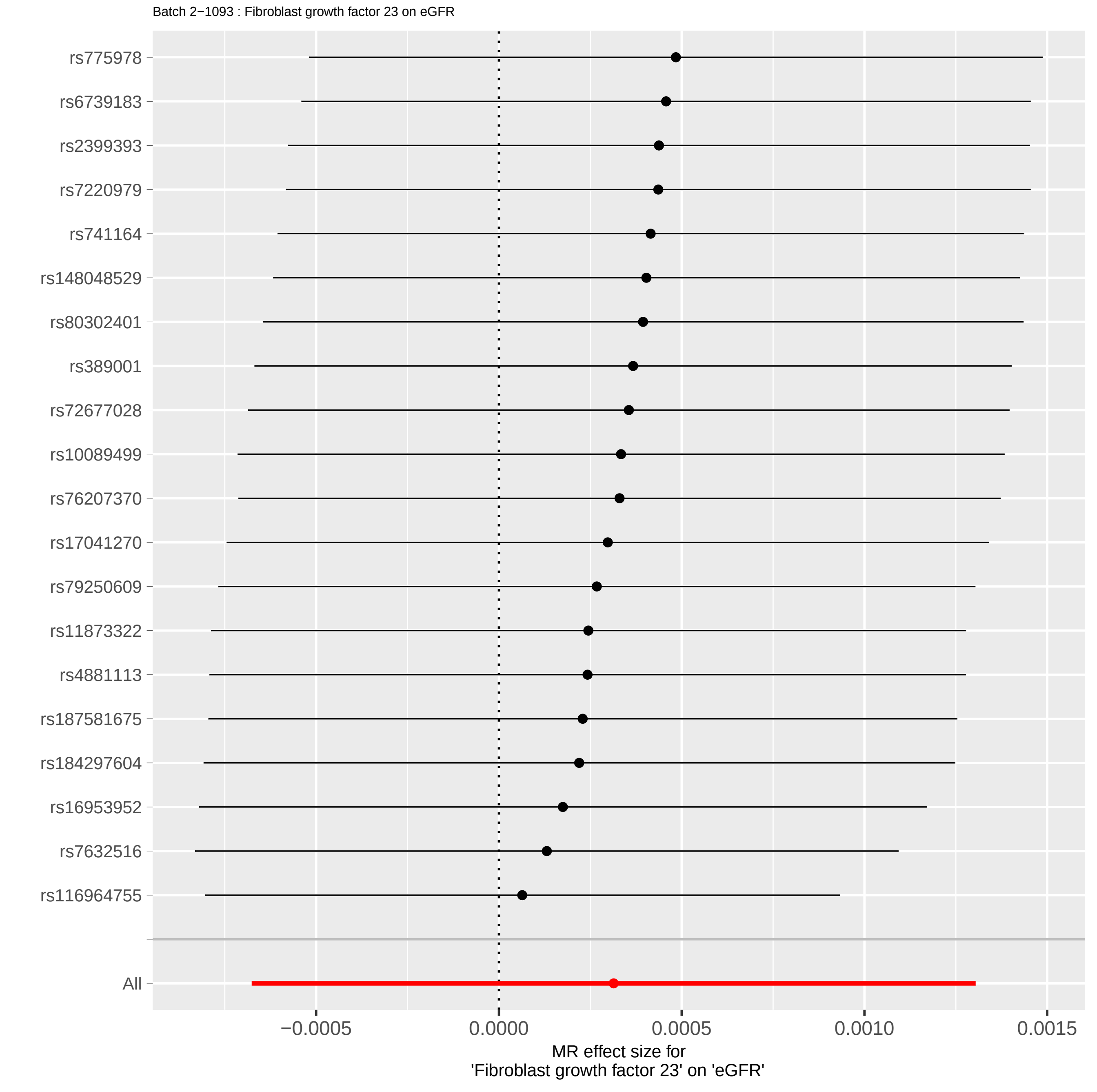

Batch 2−1093 : Fibroblast growth factor 23 on eGFR
rs775978
rs6739183
rs2399393
rs7220979
rs741164
rs148048529
rs80302401
rs389001
rs72677028
rs10089499
rs76207370
rs17041270
rs79250609
rs11873322
rs4881113
rs187581675
rs184297604
rs16953952
rs7632516
rs116964755
All
−0.0005
0.0000
0.0005
0.0010
0.0015
MR effect size for
'Fibroblast growth factor 23' on 'eGFR'

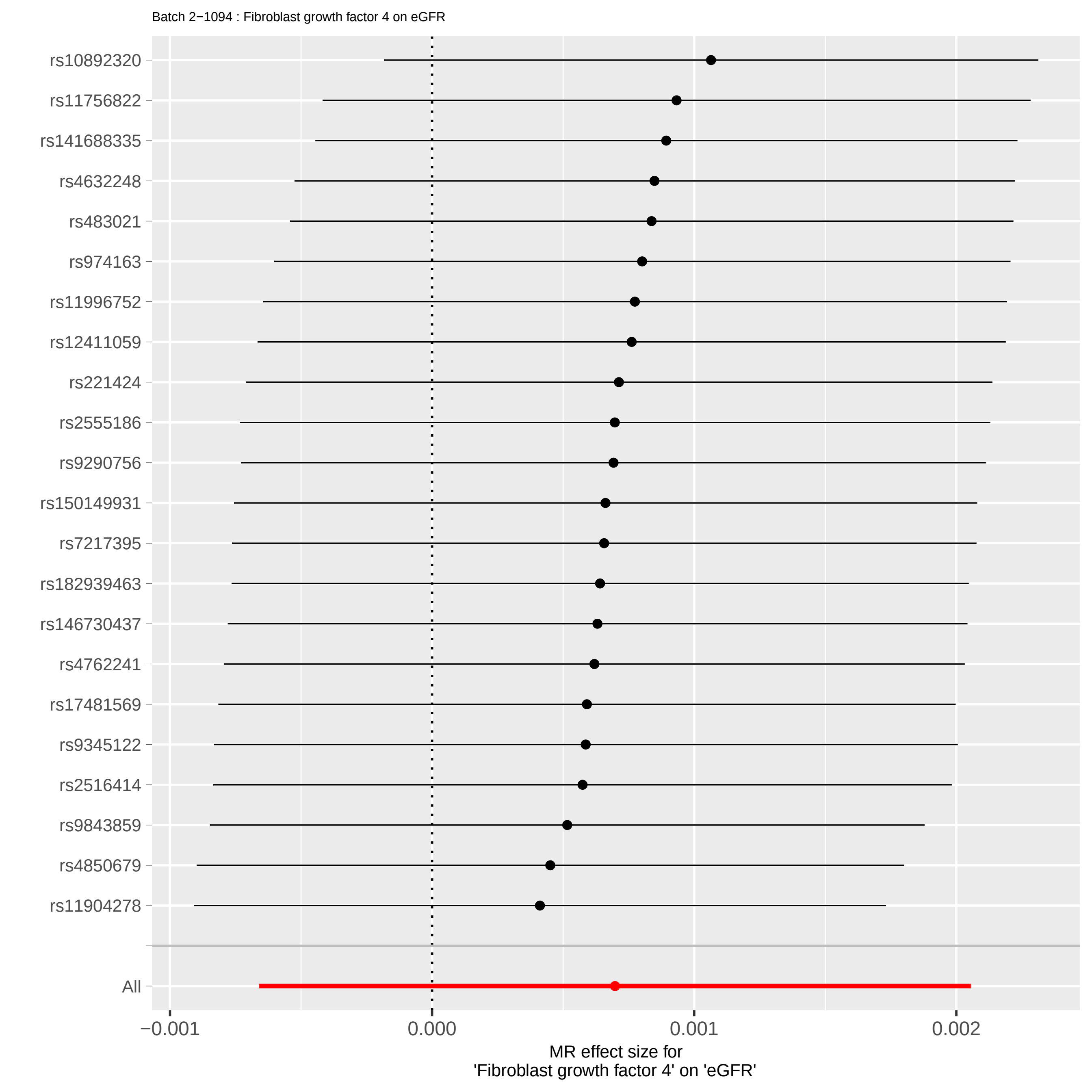

Batch 2−1094 : Fibroblast growth factor 4 on eGFR
rs10892320
rs11756822
rs141688335
rs4632248
rs483021
rs974163
rs11996752
rs12411059
rs221424
rs2555186
rs9290756
rs150149931
rs7217395
rs182939463
rs146730437
rs4762241
rs17481569
rs9345122
rs2516414
rs9843859
rs4850679
rs11904278
All
−0.001
0.000
0.001
0.002
MR effect size for
'Fibroblast growth factor 4' on 'eGFR'

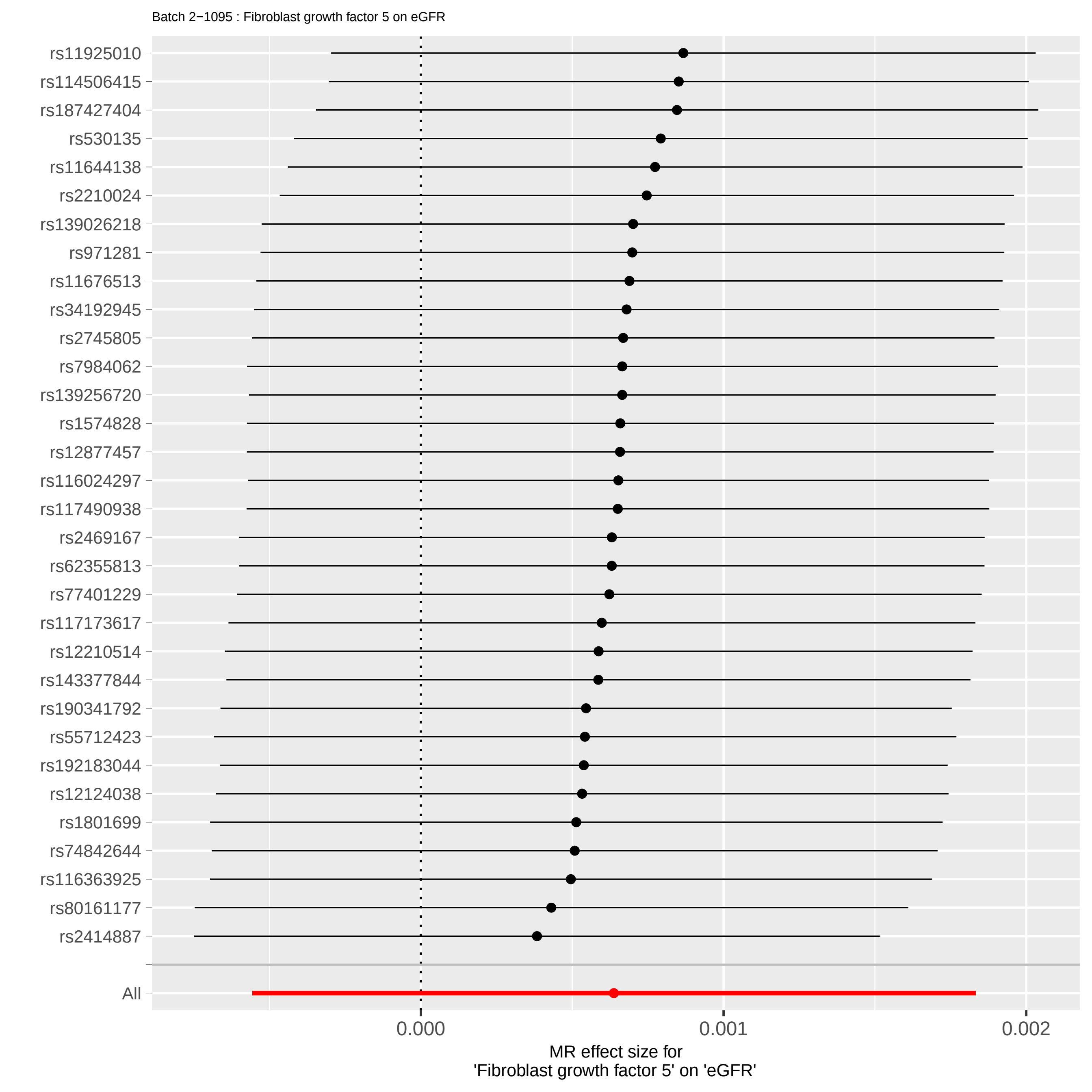

Batch 2−1095 : Fibroblast growth factor 5 on eGFR
rs11925010
rs114506415
rs187427404
rs530135
rs11644138
rs2210024
rs139026218
rs971281
rs11676513
rs34192945
rs2745805
rs7984062
rs139256720
rs1574828
rs12877457
rs116024297
rs117490938
rs2469167
rs62355813
rs77401229
rs117173617
rs12210514
rs143377844
rs190341792
rs55712423
rs192183044
rs12124038
rs1801699
rs74842644
rs116363925
rs80161177
rs2414887
All
0.000
0.001
0.002
MR effect size for
'Fibroblast growth factor 5' on 'eGFR'

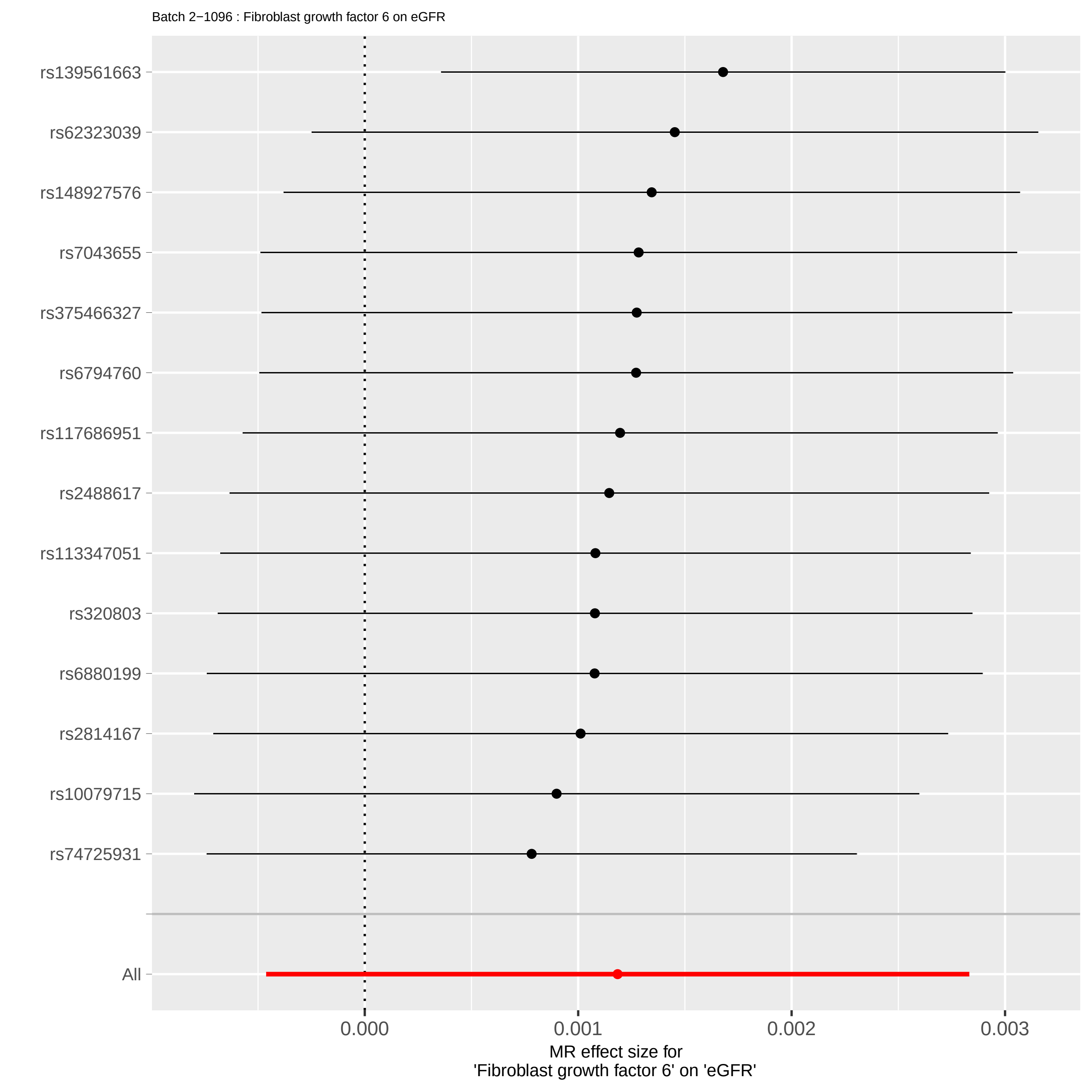

Batch 2−1096 : Fibroblast growth factor 6 on eGFR
rs139561663
rs62323039
rs148927576
rs7043655
rs375466327
rs6794760
rs117686951
rs2488617
rs113347051
rs320803
rs6880199
rs2814167
rs10079715
rs74725931
All
0.000
0.001
0.002
0.003
MR effect size for
'Fibroblast growth factor 6' on 'eGFR'

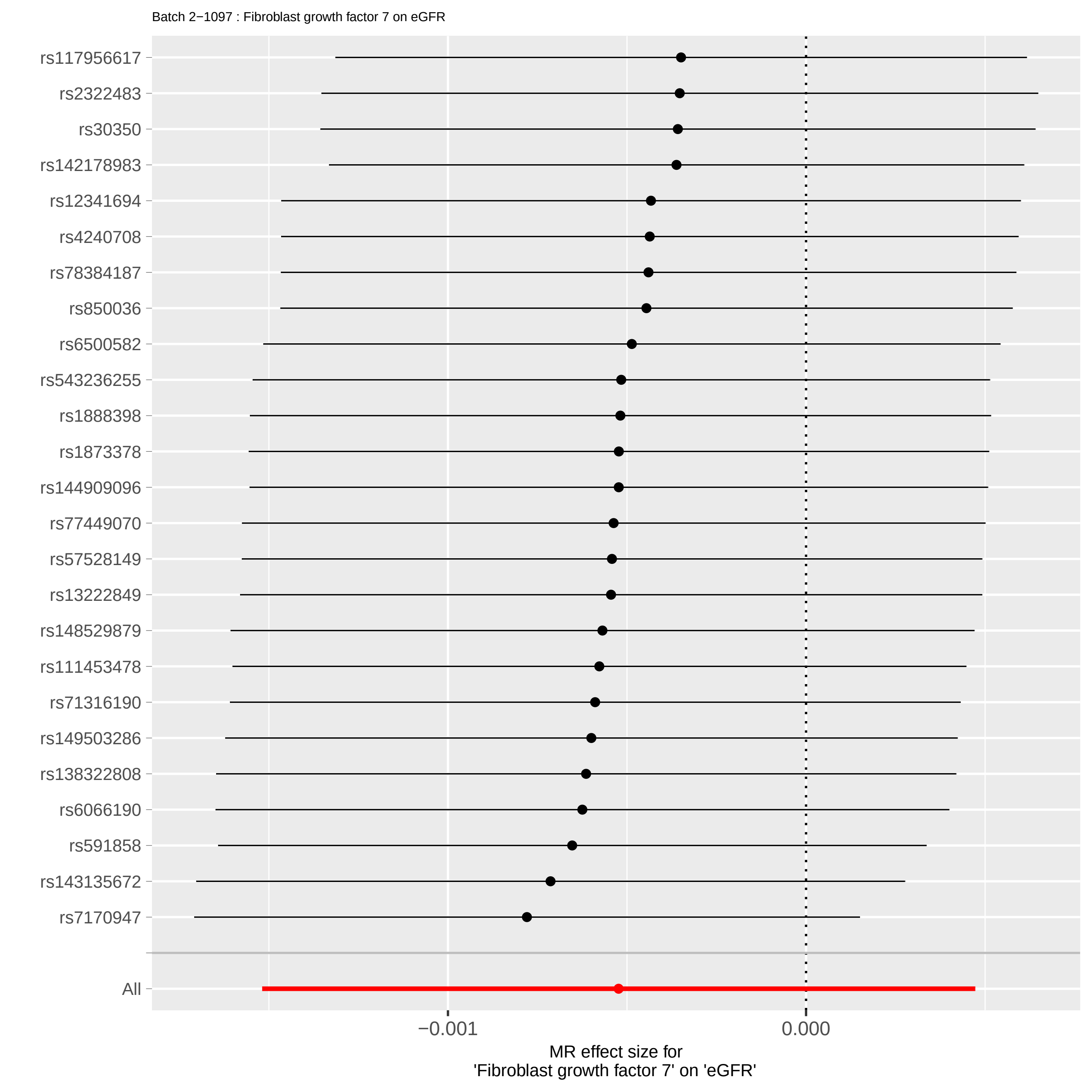
Batch 2−1097 : Fibroblast growth factor 7 on eGFR
rs117956617
rs2322483
rs30350
rs142178983
rs12341694
rs4240708
rs78384187
rs850036
rs6500582
rs543236255
rs1888398
rs1873378
rs144909096
rs77449070
rs57528149
rs13222849
rs148529879
rs111453478
rs71316190
rs149503286
rs138322808
rs6066190
rs591858
rs143135672
rs7170947
All
−0.001
0.000
MR effect size for
'Fibroblast growth factor 7' on 'eGFR'

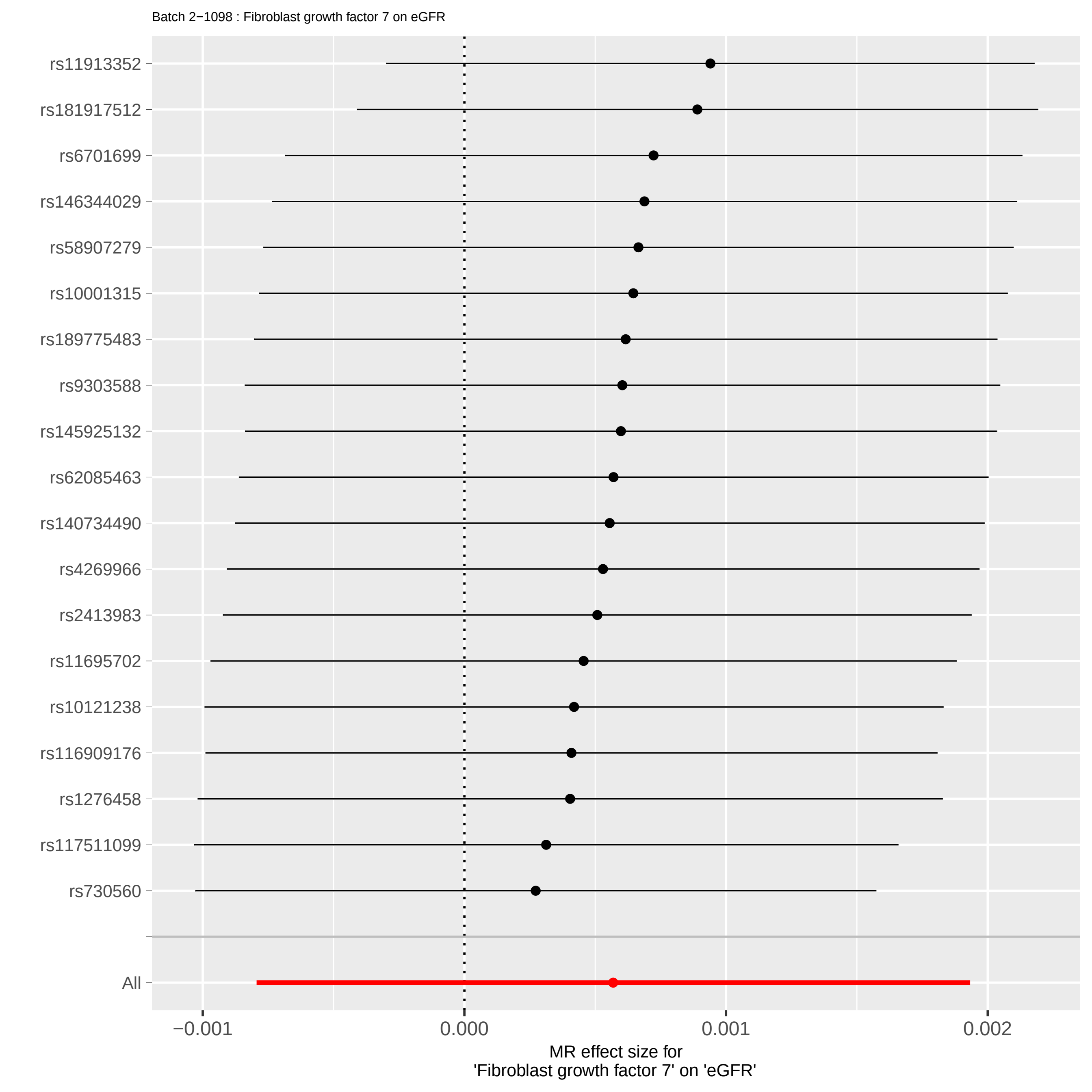

Batch 2−1098 : Fibroblast growth factor 7 on eGFR
rs11913352
rs181917512
rs6701699
rs146344029
rs58907279
rs10001315
rs189775483
rs9303588
rs145925132
rs62085463
rs140734490
rs4269966
rs2413983
rs11695702
rs10121238
rs116909176
rs1276458
rs117511099
rs730560
All
−0.001
0.000
0.001
0.002
MR effect size for
'Fibroblast growth factor 7' on 'eGFR'

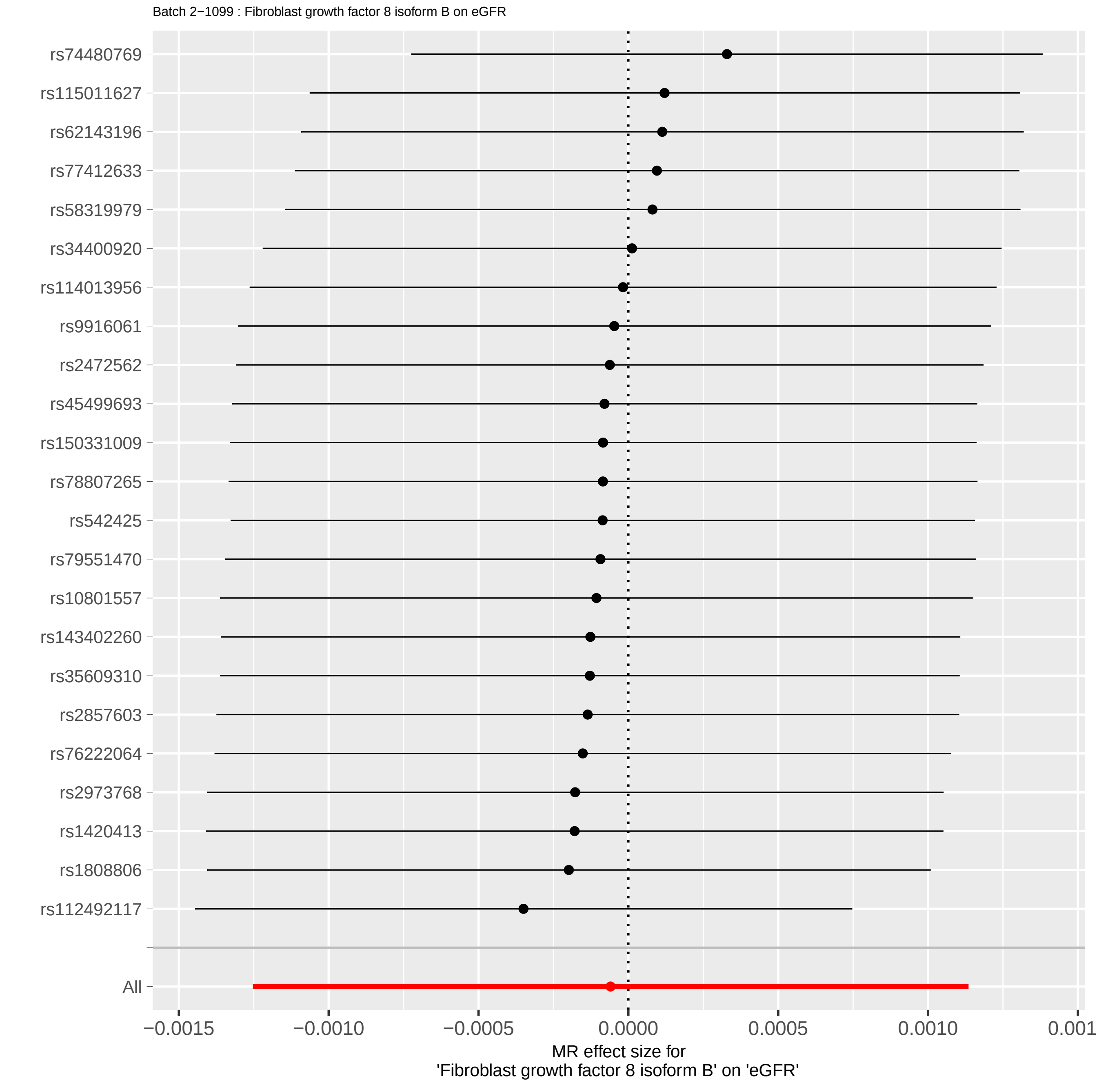

Batch 2−1099 : Fibroblast growth factor 8 isoform B on eGFR
rs74480769
rs115011627
rs62143196
rs77412633
rs58319979
rs34400920
rs114013956
rs9916061
rs2472562
rs45499693
rs150331009
rs78807265
rs542425
rs79551470
rs10801557
rs143402260
rs35609310
rs2857603
rs76222064
rs2973768
rs1420413
rs1808806
rs112492117
All
−0.0015
−0.0010
−0.0005
0.0000
0.0005
0.0010
0.001
MR effect size for
'Fibroblast growth factor 8 isoform B' on 'eGFR'

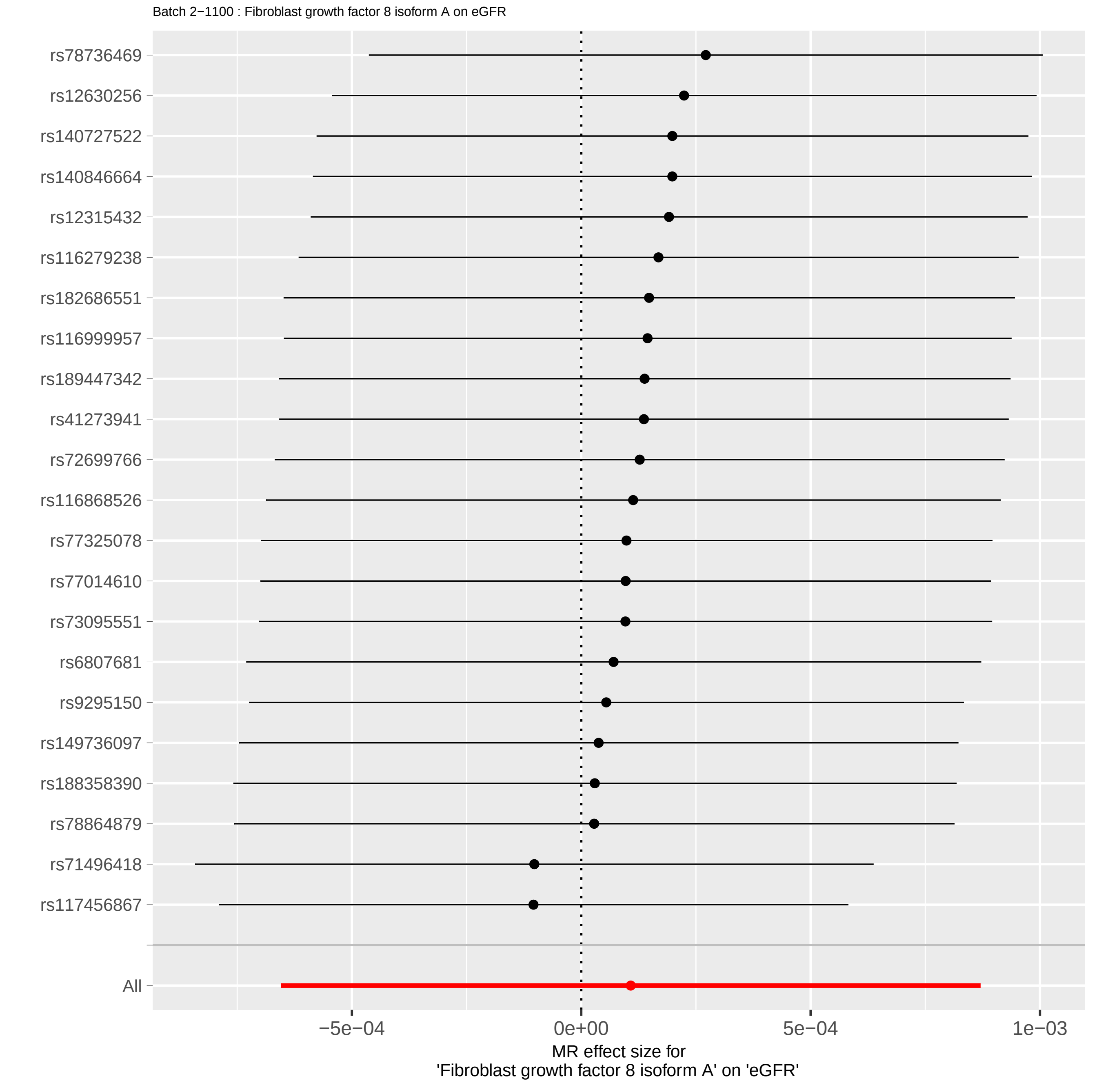

Batch 2−1100 : Fibroblast growth factor 8 isoform A on eGFR
rs78736469
rs12630256
rs140727522
rs140846664
rs12315432
rs116279238
rs182686551
rs116999957
rs189447342
rs41273941
rs72699766
rs116868526
rs77325078
rs77014610
rs73095551
rs6807681
rs9295150
rs149736097
rs188358390
rs78864879
rs71496418
rs117456867
All
−5e−04
0e+00
5e−04
1e−03
MR effect size for
'Fibroblast growth factor 8 isoform A' on 'eGFR'

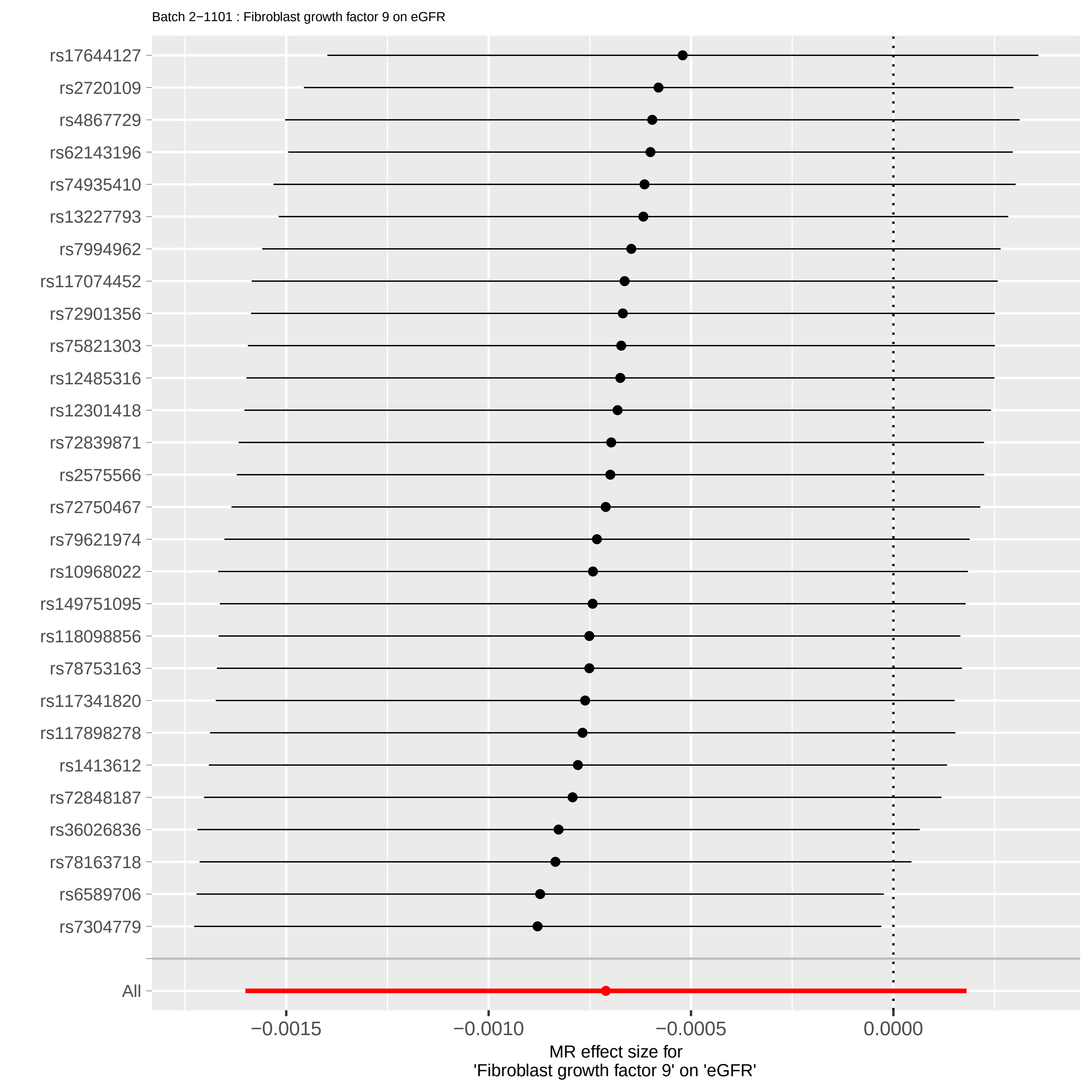

Batch 2−1101 : Fibroblast growth factor 9 on eGFR
rs17644127
rs2720109
rs4867729
rs62143196
rs74935410
rs13227793
rs7994962
rs117074452
rs72901356
rs75821303
rs12485316
rs12301418
rs72839871
rs2575566
rs72750467
rs79621974
rs10968022
rs149751095
rs118098856
rs78753163
rs117341820
rs117898278
rs1413612
rs72848187
rs36026836
rs78163718
rs6589706
rs7304779
All
−0.0015
−0.0010
−0.0005
0.0000
MR effect size for
'Fibroblast growth factor 9' on 'eGFR'

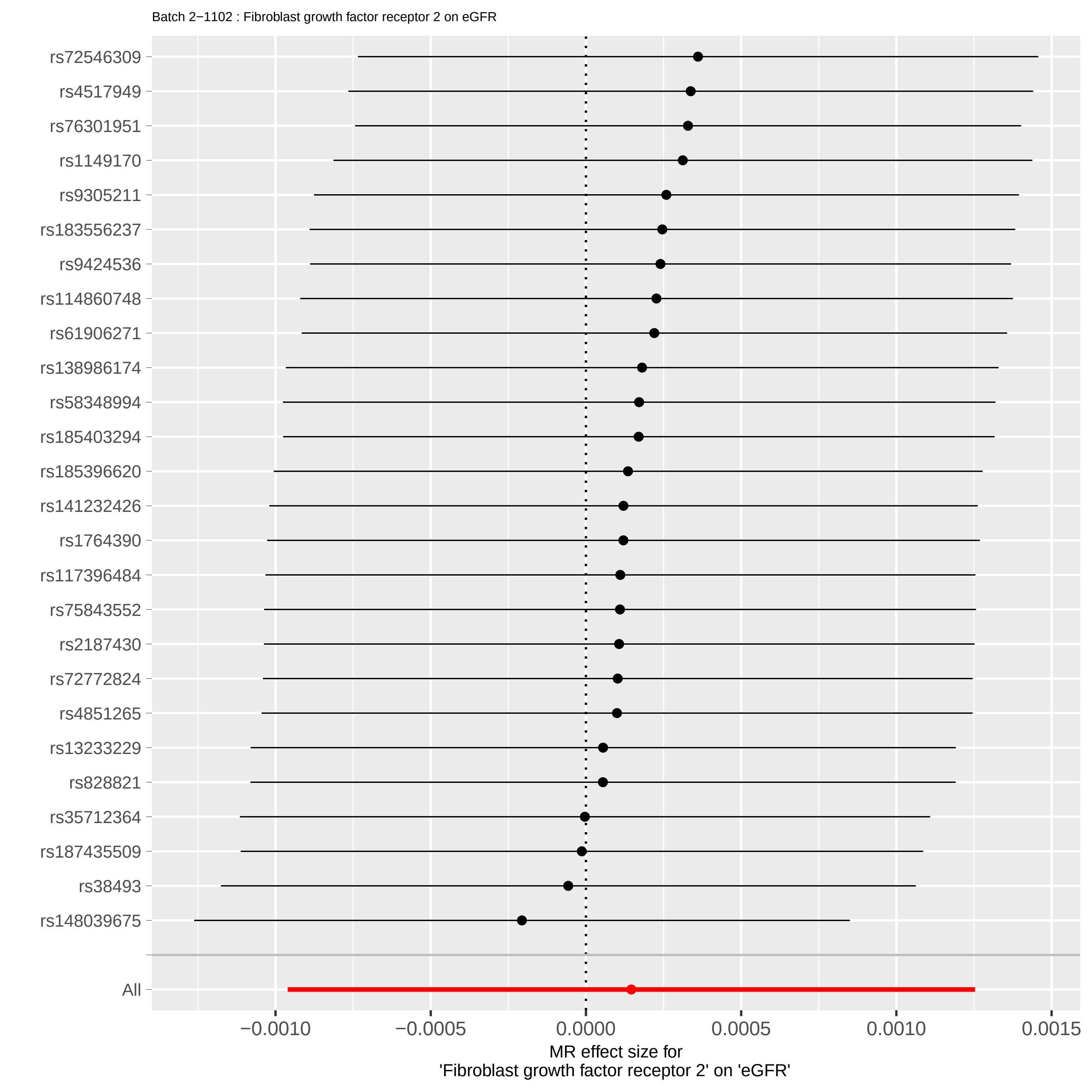

Batch 2−1102 : Fibroblast growth factor receptor 2 on eGFR
rs72546309
rs4517949
rs76301951
rs1149170
rs9305211
rs183556237
rs9424536
rs114860748
rs61906271
rs138986174
rs58348994
rs185403294
rs185396620
rs141232426
rs1764390
rs117396484
rs75843552
rs2187430
rs72772824
rs4851265
rs13233229
rs828821
rs35712364
rs187435509
rs38493
rs148039675
All
−0.0010
−0.0005
0.0000
0.0005
0.0010
0.0015
MR effect size for
'Fibroblast growth factor receptor 2' on 'eGFR'

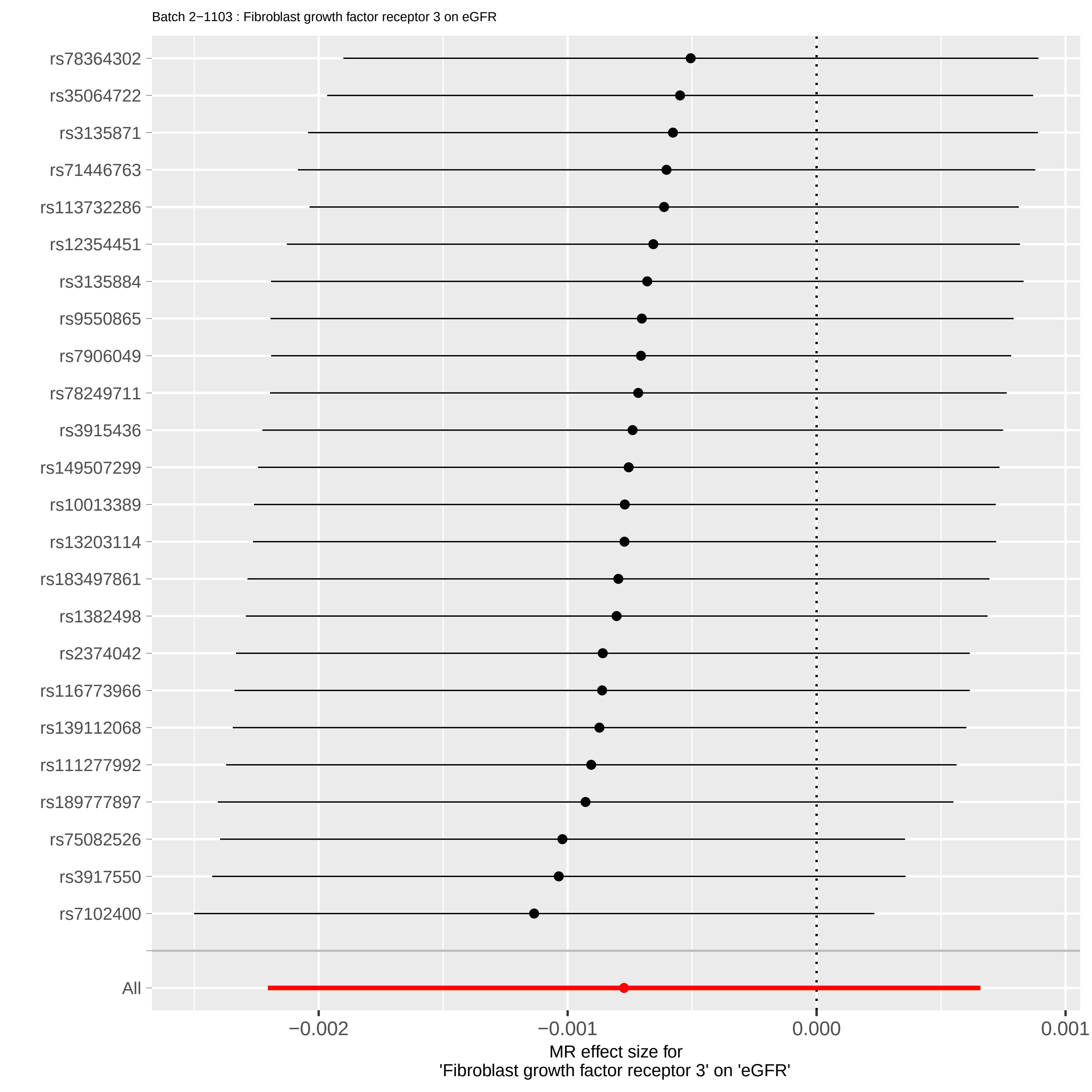

Batch 2−1103 : Fibroblast growth factor receptor 3 on eGFR
rs78364302
rs35064722
rs3135871
rs71446763
rs113732286
rs12354451
rs3135884
rs9550865
rs7906049
rs78249711
rs3915436
rs149507299
rs10013389
rs13203114
rs183497861
rs1382498
rs2374042
rs116773966
rs139112068
rs111277992
rs189777897
rs75082526
rs3917550
rs7102400
All
−0.002
−0.001
0.000
0.001
MR effect size for
'Fibroblast growth factor receptor 3' on 'eGFR'

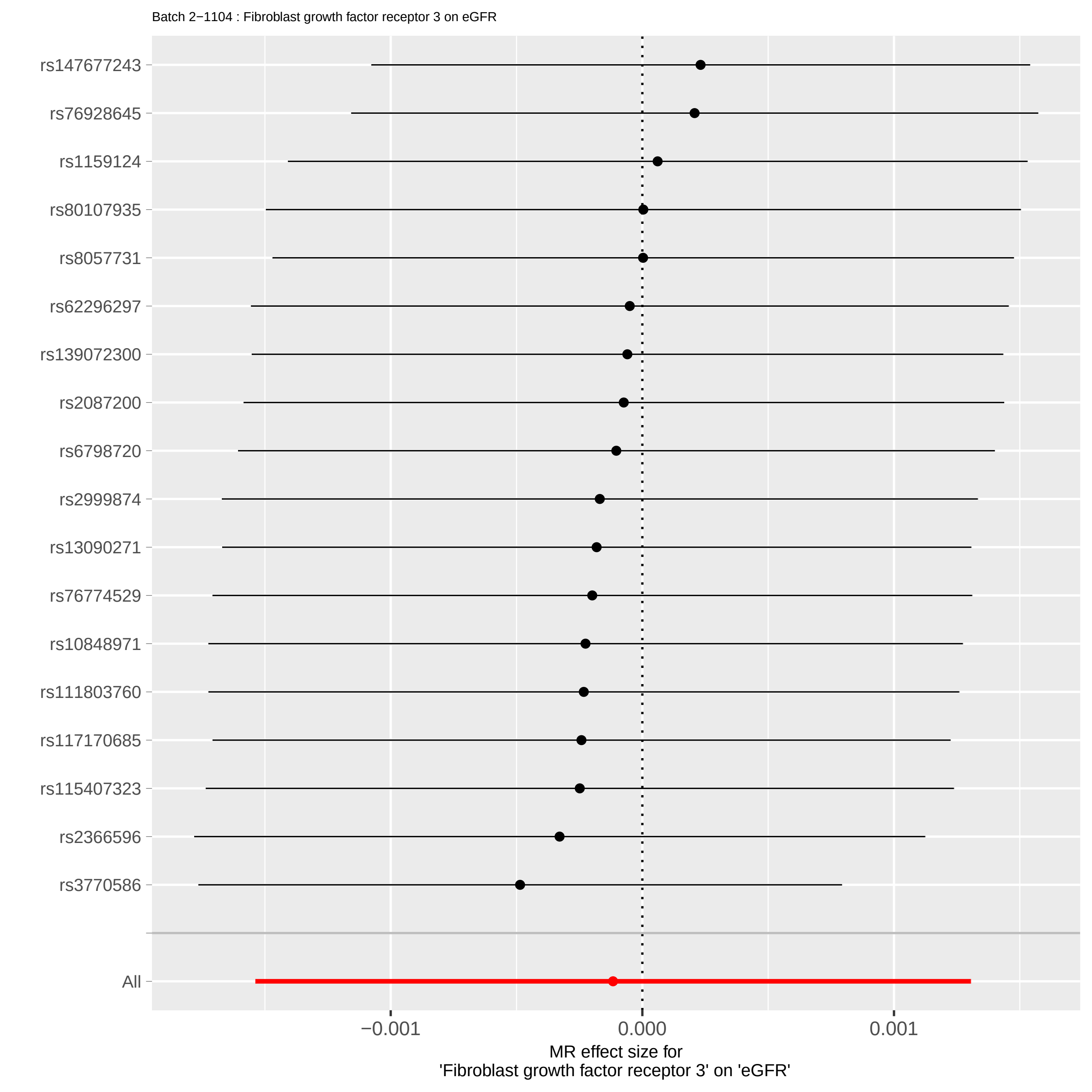

Batch 2−1104 : Fibroblast growth factor receptor 3 on eGFR
rs147677243
rs76928645
rs1159124
rs80107935
rs8057731
rs62296297
rs139072300
rs2087200
rs6798720
rs2999874
rs13090271
rs76774529
rs10848971
rs111803760
rs117170685
rs115407323
rs2366596
rs3770586
All
−0.001
0.000
0.001
MR effect size for
'Fibroblast growth factor receptor 3' on 'eGFR'

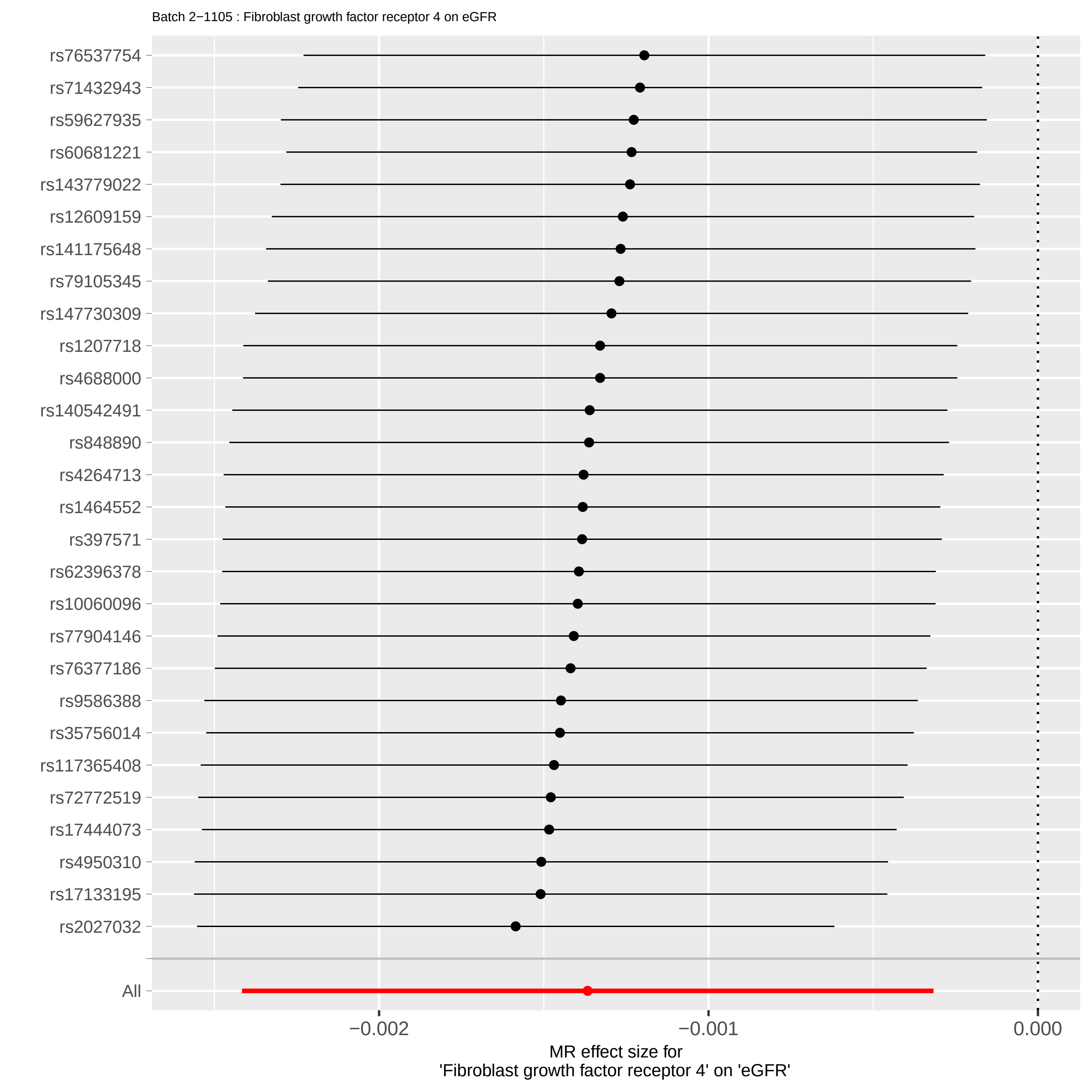

Batch 2−1105 : Fibroblast growth factor receptor 4 on eGFR
rs76537754
rs71432943
rs59627935
rs60681221
rs143779022
rs12609159
rs141175648
rs79105345
rs147730309
rs1207718
rs4688000
rs140542491
rs848890
rs4264713
rs1464552
rs397571
rs62396378
rs10060096
rs77904146
rs76377186
rs9586388
rs35756014
rs117365408
rs72772519
rs17444073
rs4950310
rs17133195
rs2027032
All
−0.002
−0.001
0.000
MR effect size for
'Fibroblast growth factor receptor 4' on 'eGFR'

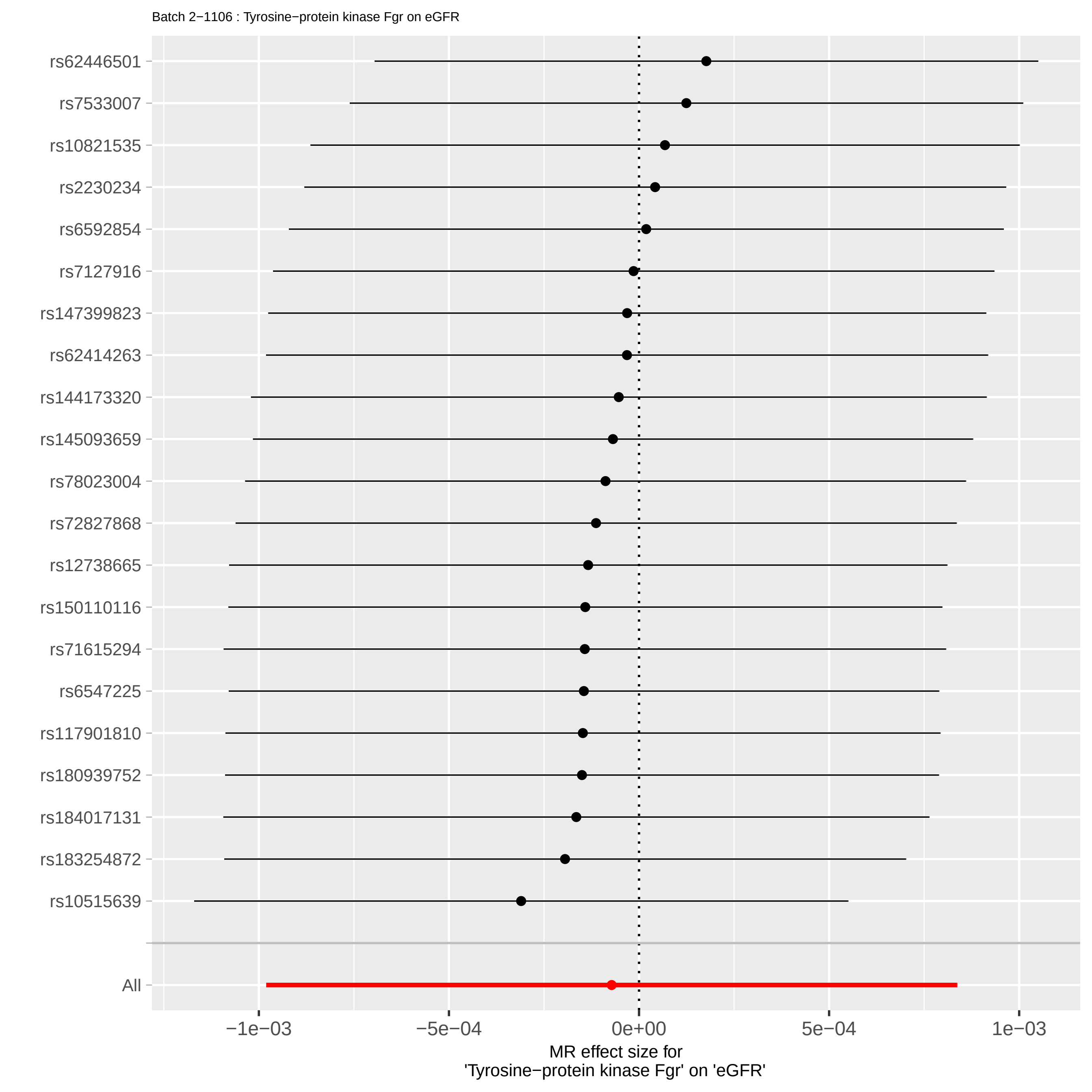

Batch 2−1106 : Tyrosine−protein kinase Fgr on eGFR
rs62446501
rs7533007
rs10821535
rs2230234
rs6592854
rs7127916
rs147399823
rs62414263
rs144173320
rs145093659
rs78023004
rs72827868
rs12738665
rs150110116
rs71615294
rs6547225
rs117901810
rs180939752
rs184017131
rs183254872
rs10515639
All
−1e−03
−5e−04
0e+00
5e−04
1e−03
MR effect size for
'Tyrosine−protein kinase Fgr' on 'eGFR'

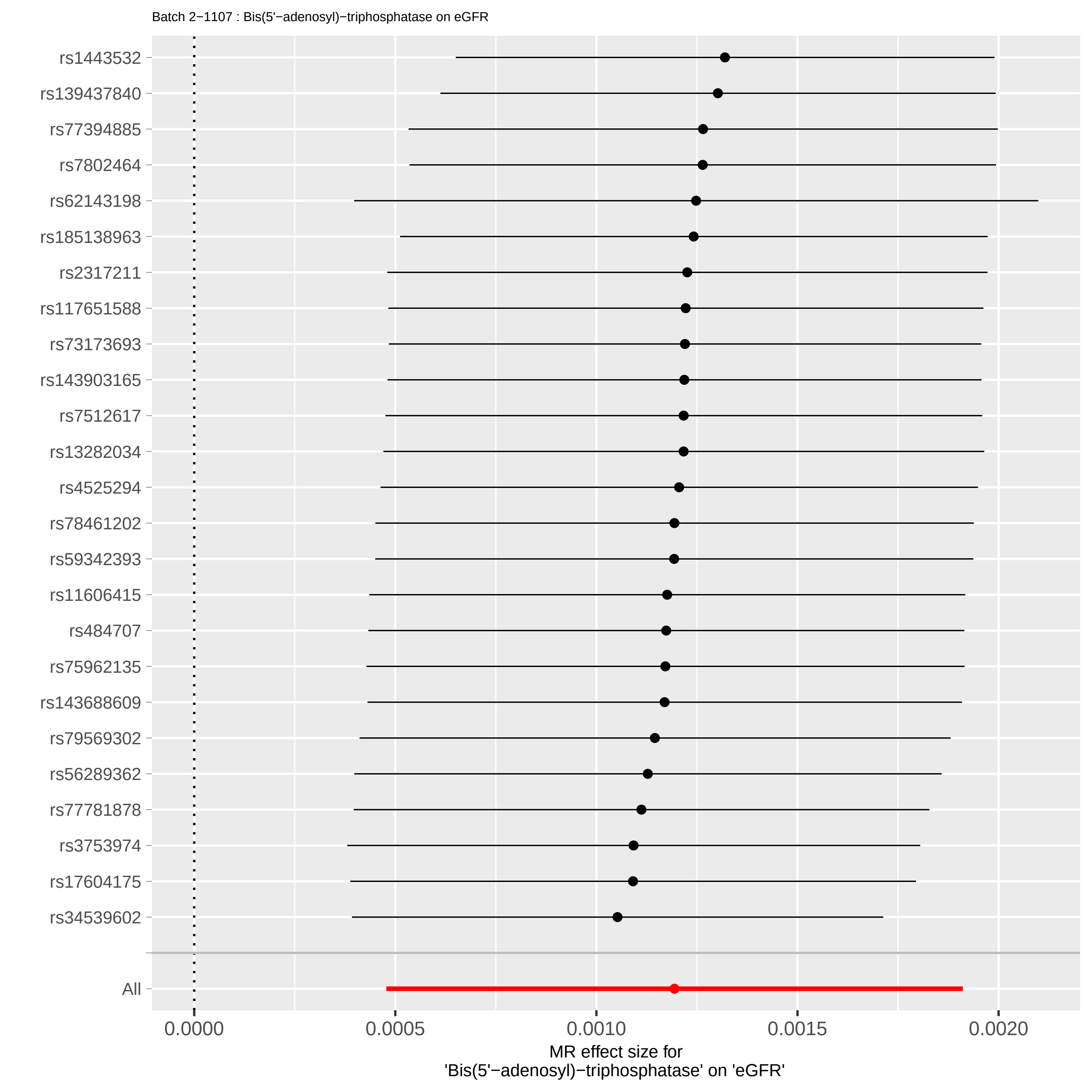
Batch 2−1107 : Bis(5'−adenosyl)−triphosphatase on eGFR
rs1443532
rs139437840
rs77394885
rs7802464
rs62143198
rs185138963
rs2317211
rs117651588
rs73173693
rs143903165
rs7512617
rs13282034
rs4525294
rs78461202
rs59342393
rs11606415
rs484707
rs75962135
rs143688609
rs79569302
rs56289362
rs77781878
rs3753974
rs17604175
rs34539602
All
0.0000
0.0005
0.0010
0.0015
0.0020
MR effect size for
'Bis(5'−adenosyl)−triphosphatase' on 'eGFR'

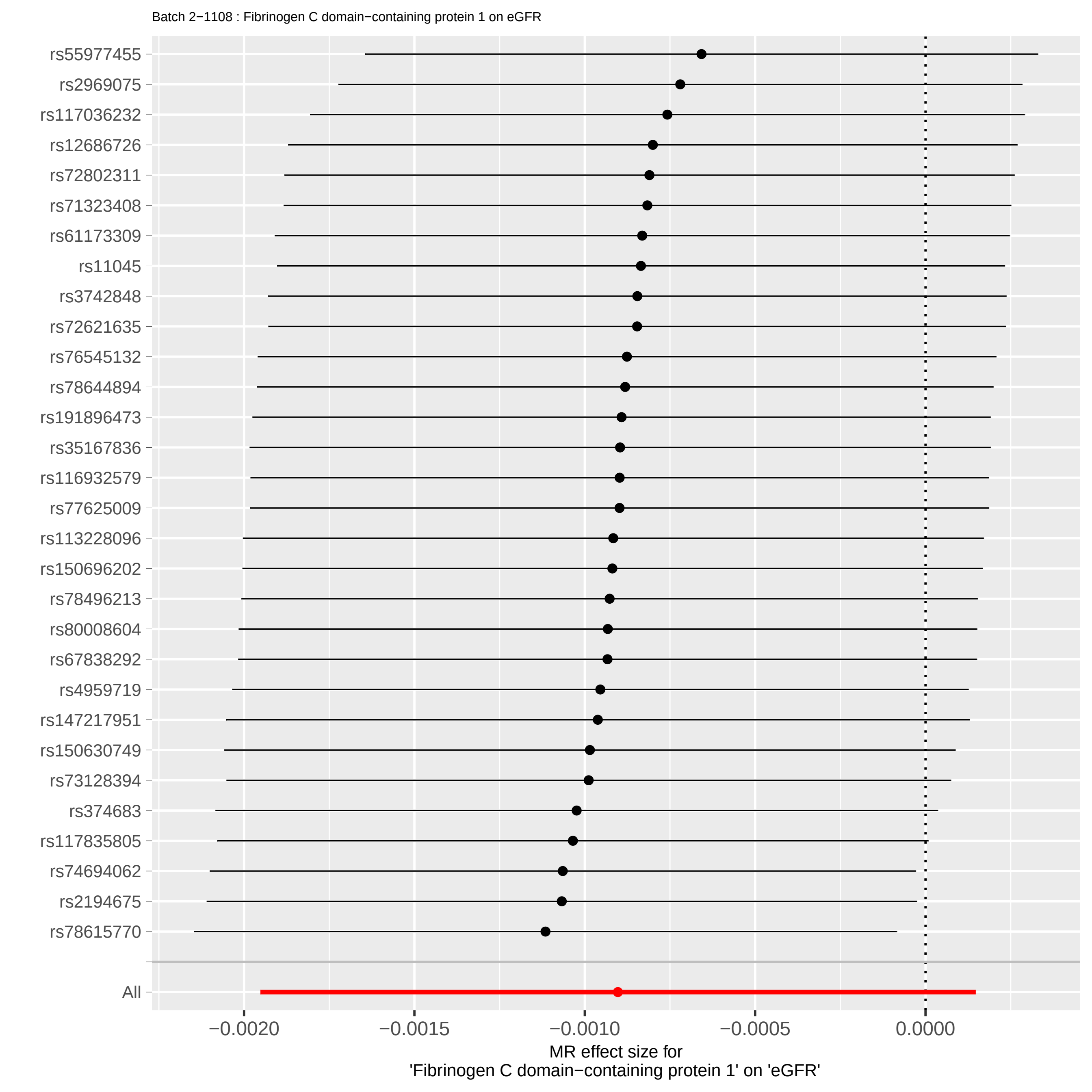

Batch 2−1108 : Fibrinogen C domain−containing protein 1 on eGFR
rs55977455
rs2969075
rs117036232
rs12686726
rs72802311
rs71323408
rs61173309
rs11045
rs3742848
rs72621635
rs76545132
rs78644894
rs191896473
rs35167836
rs116932579
rs77625009
rs113228096
rs150696202
rs78496213
rs80008604
rs67838292
rs4959719
rs147217951
rs150630749
rs73128394
rs374683
rs117835805
rs74694062
rs2194675
rs78615770
All
−0.0020
−0.0015
−0.0010
−0.0005
0.0000
MR effect size for
'Fibrinogen C domain−containing protein 1' on 'eGFR'

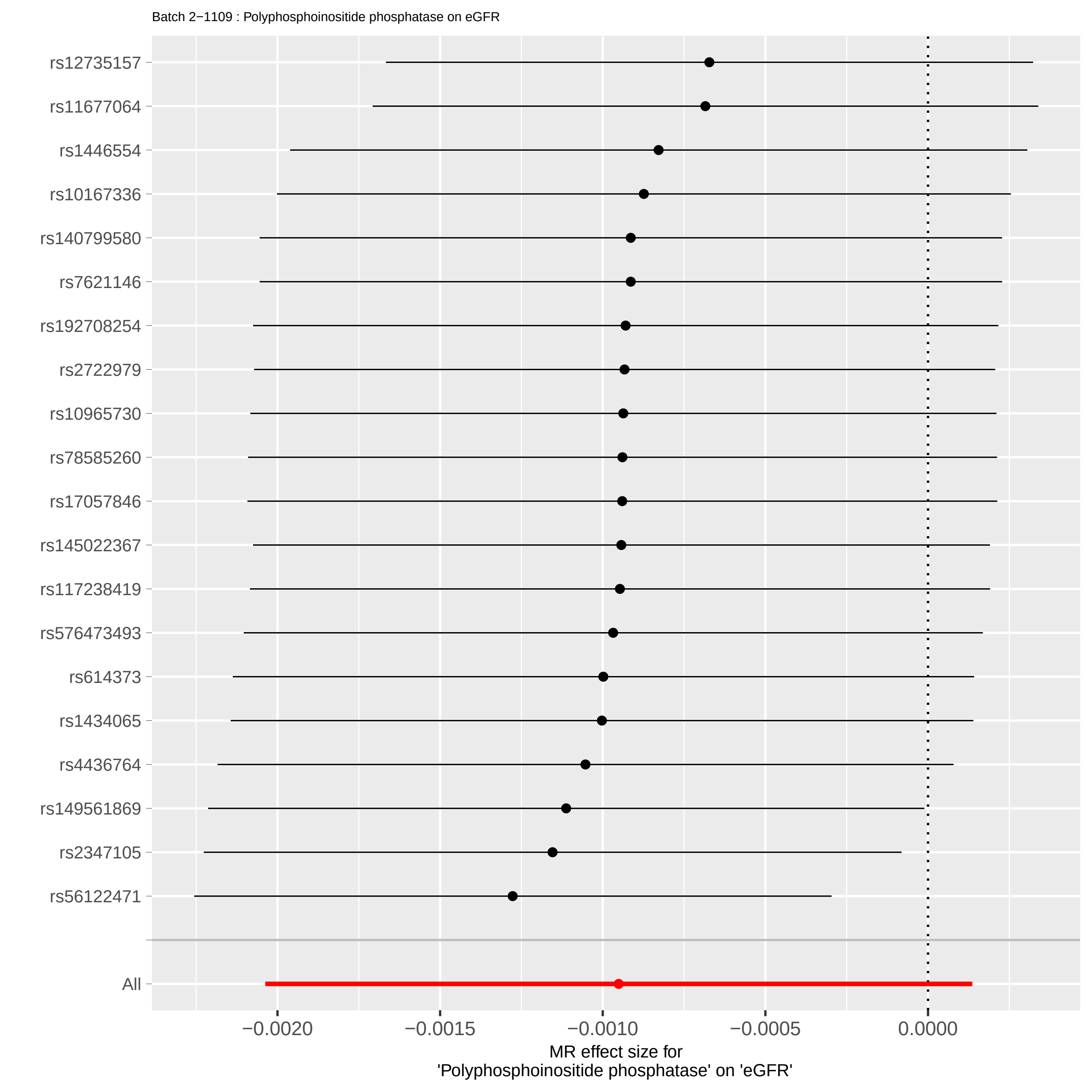
Batch 2−1109 : Polyphosphoinositide phosphatase on eGFR
rs12735157
rs11677064
rs1446554
rs10167336
rs140799580
rs7621146
rs192708254
rs2722979
rs10965730
rs78585260
rs17057846
rs145022367
rs117238419
rs576473493
rs614373
rs1434065
rs4436764
rs149561869
rs2347105
rs56122471
All
−0.0020
−0.0015
−0.0010
−0.0005
0.0000
MR effect size for
'Polyphosphoinositide phosphatase' on 'eGFR'

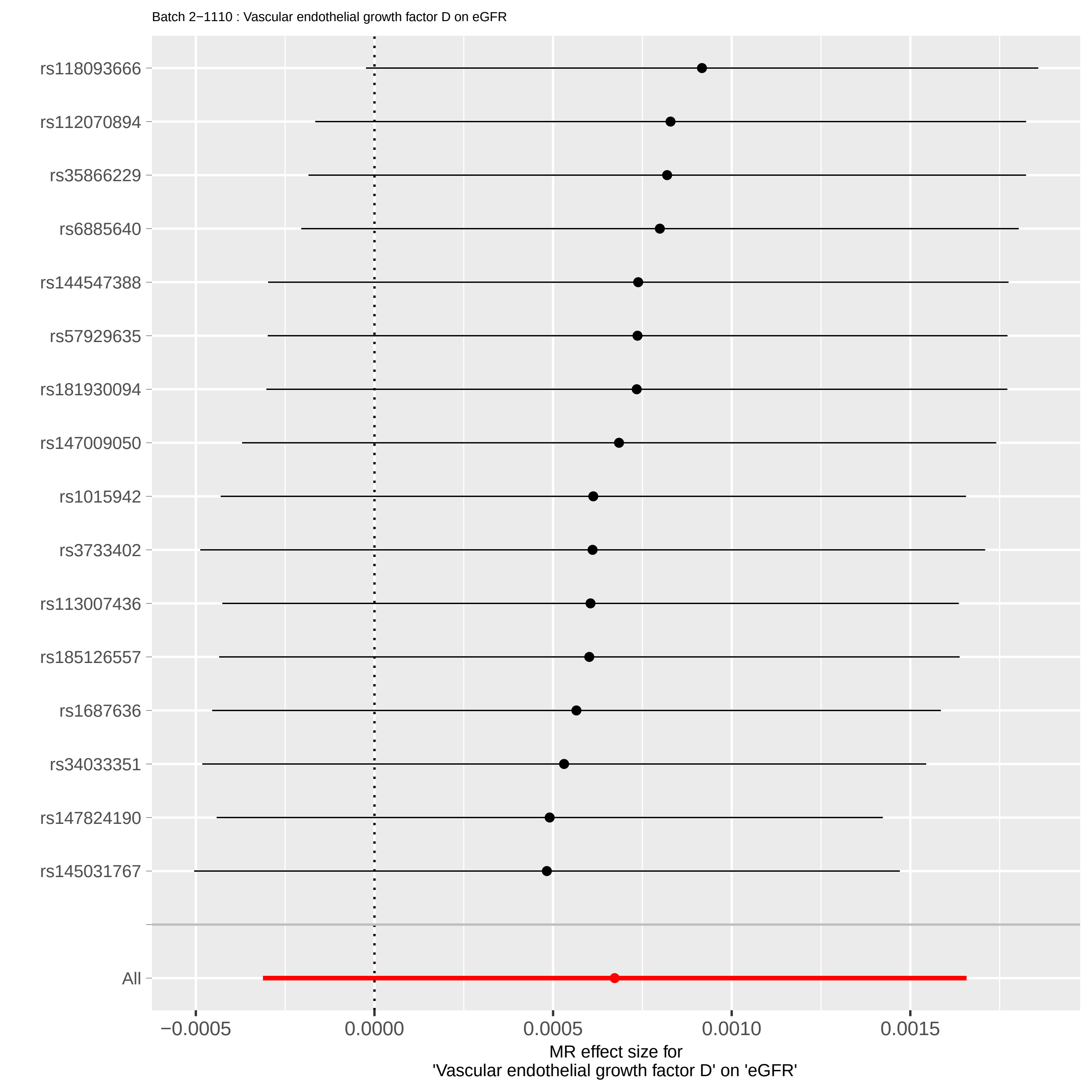

Batch 2−1110 : Vascular endothelial growth factor D on eGFR
rs118093666
rs112070894
rs35866229
rs6885640
rs144547388
rs57929635
rs181930094
rs147009050
rs1015942
rs3733402
rs113007436
rs185126557
rs1687636
rs34033351
rs147824190
rs145031767
All
−0.0005
0.0000
0.0005
0.0010
0.0015
MR effect size for
'Vascular endothelial growth factor D' on 'eGFR'

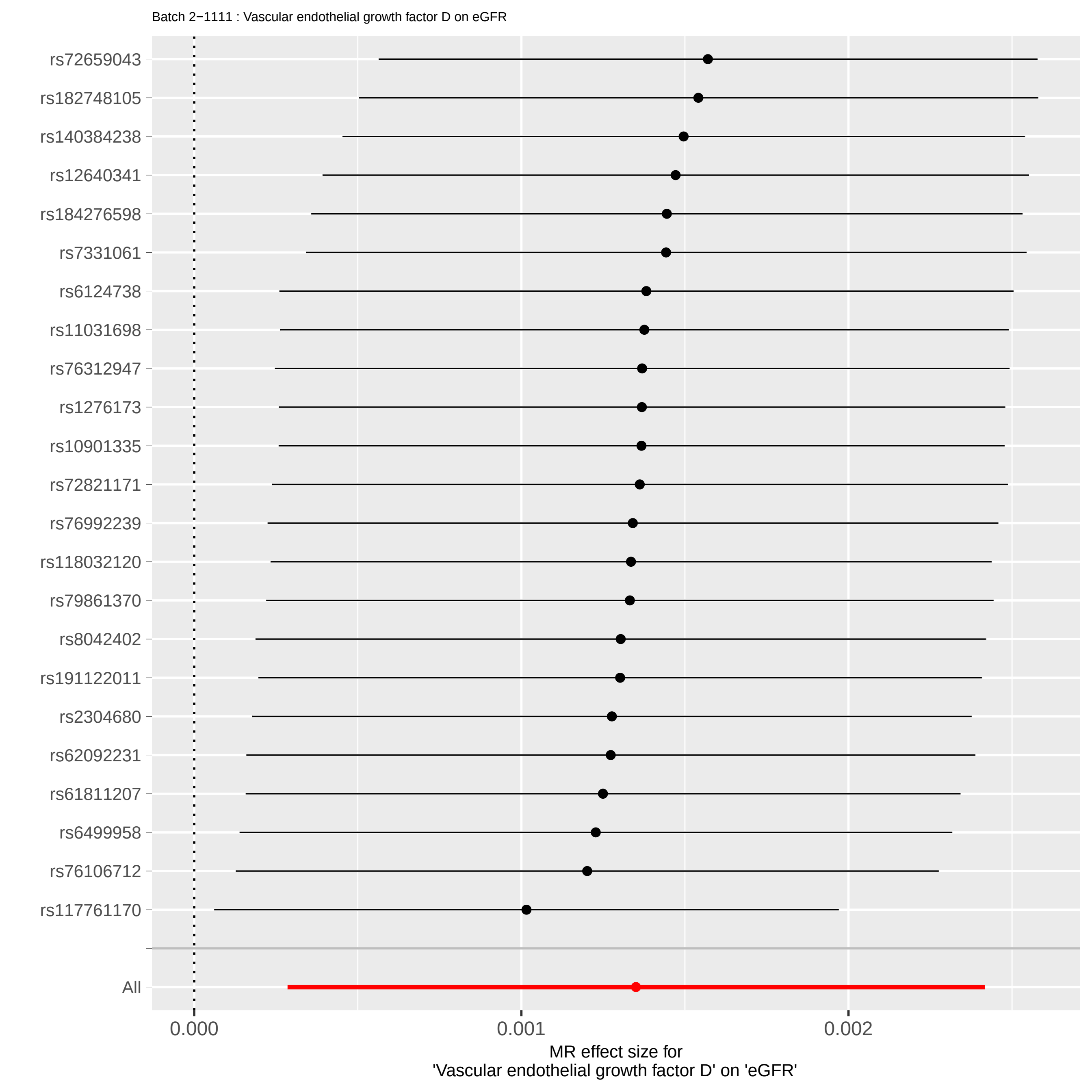

Batch 2−1111 : Vascular endothelial growth factor D on eGFR
rs72659043
rs182748105
rs140384238
rs12640341
rs184276598
rs7331061
rs6124738
rs11031698
rs76312947
rs1276173
rs10901335
rs72821171
rs76992239
rs118032120
rs79861370
rs8042402
rs191122011
rs2304680
rs62092231
rs61811207
rs6499958
rs76106712
rs117761170
All
0.000
0.001
0.002
MR effect size for
'Vascular endothelial growth factor D' on 'eGFR'

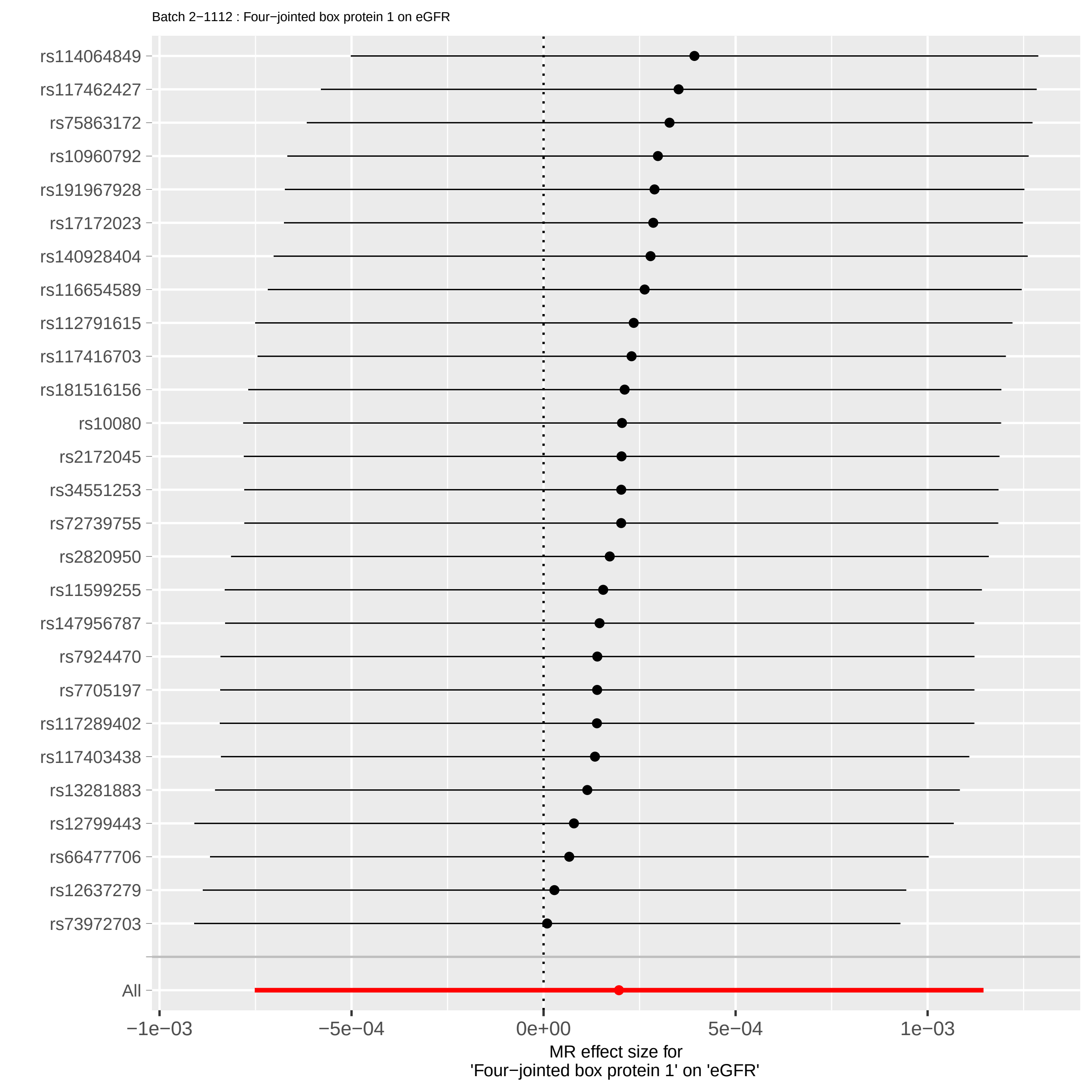

Batch 2−1112 : Four−jointed box protein 1 on eGFR
rs114064849
rs117462427
rs75863172
rs10960792
rs191967928
rs17172023
rs140928404
rs116654589
rs112791615
rs117416703
rs181516156
rs10080
rs2172045
rs34551253
rs72739755
rs2820950
rs11599255
rs147956787
rs7924470
rs7705197
rs117289402
rs117403438
rs13281883
rs12799443
rs66477706
rs12637279
rs73972703
All
−1e−03
−5e−04
0e+00
5e−04
1e−03
MR effect size for
'Four−jointed box protein 1' on 'eGFR'

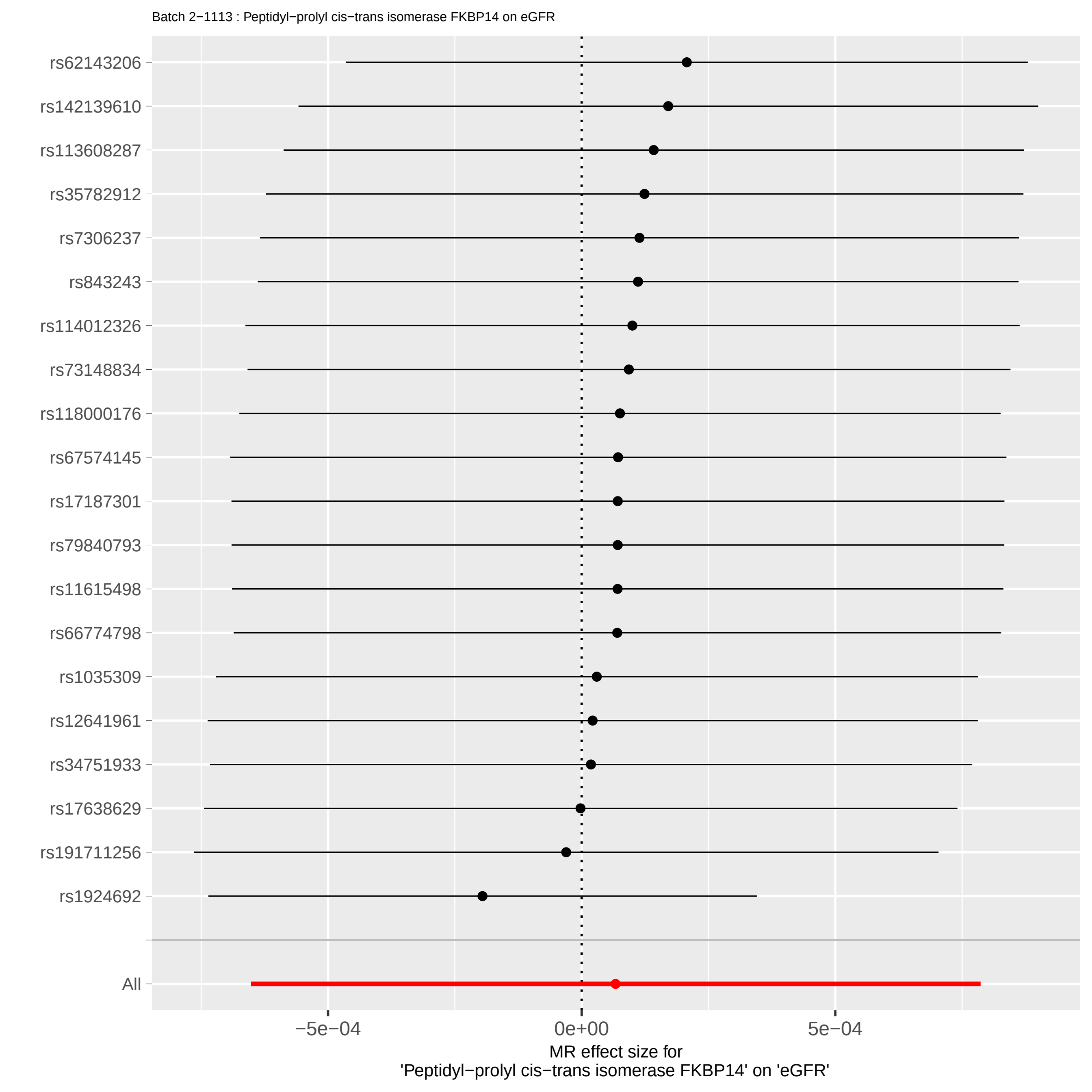

Batch 2−1113 : Peptidyl−prolyl cis−trans isomerase FKBP14 on eGFR
rs62143206
rs142139610
rs113608287
rs35782912
rs7306237
rs843243
rs114012326
rs73148834
rs118000176
rs67574145
rs17187301
rs79840793
rs11615498
rs66774798
rs1035309
rs12641961
rs34751933
rs17638629
rs191711256
rs1924692
All
−5e−04
0e+00
5e−04
MR effect size for
'Peptidyl−prolyl cis−trans isomerase FKBP14' on 'eGFR'

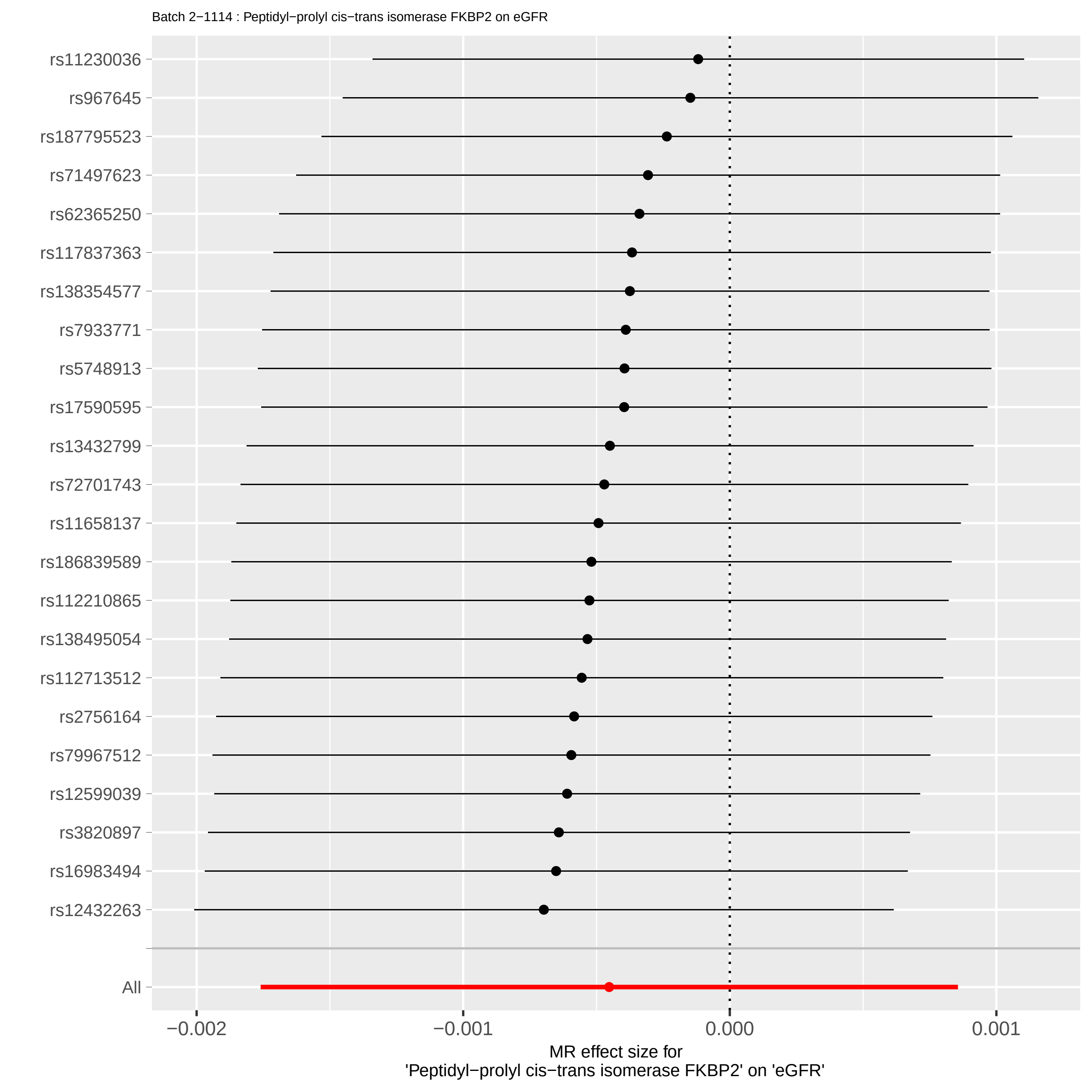
Batch 2−1114 : Peptidyl−prolyl cis−trans isomerase FKBP2 on eGFR
rs11230036
rs967645
rs187795523
rs71497623
rs62365250
rs117837363
rs138354577
rs7933771
rs5748913
rs17590595
rs13432799
rs72701743
rs11658137
rs186839589
rs112210865
rs138495054
rs112713512
rs2756164
rs79967512
rs12599039
rs3820897
rs16983494
rs12432263
All
−0.002
−0.001
0.000
0.001
MR effect size for
'Peptidyl−prolyl cis−trans isomerase FKBP2' on 'eGFR'

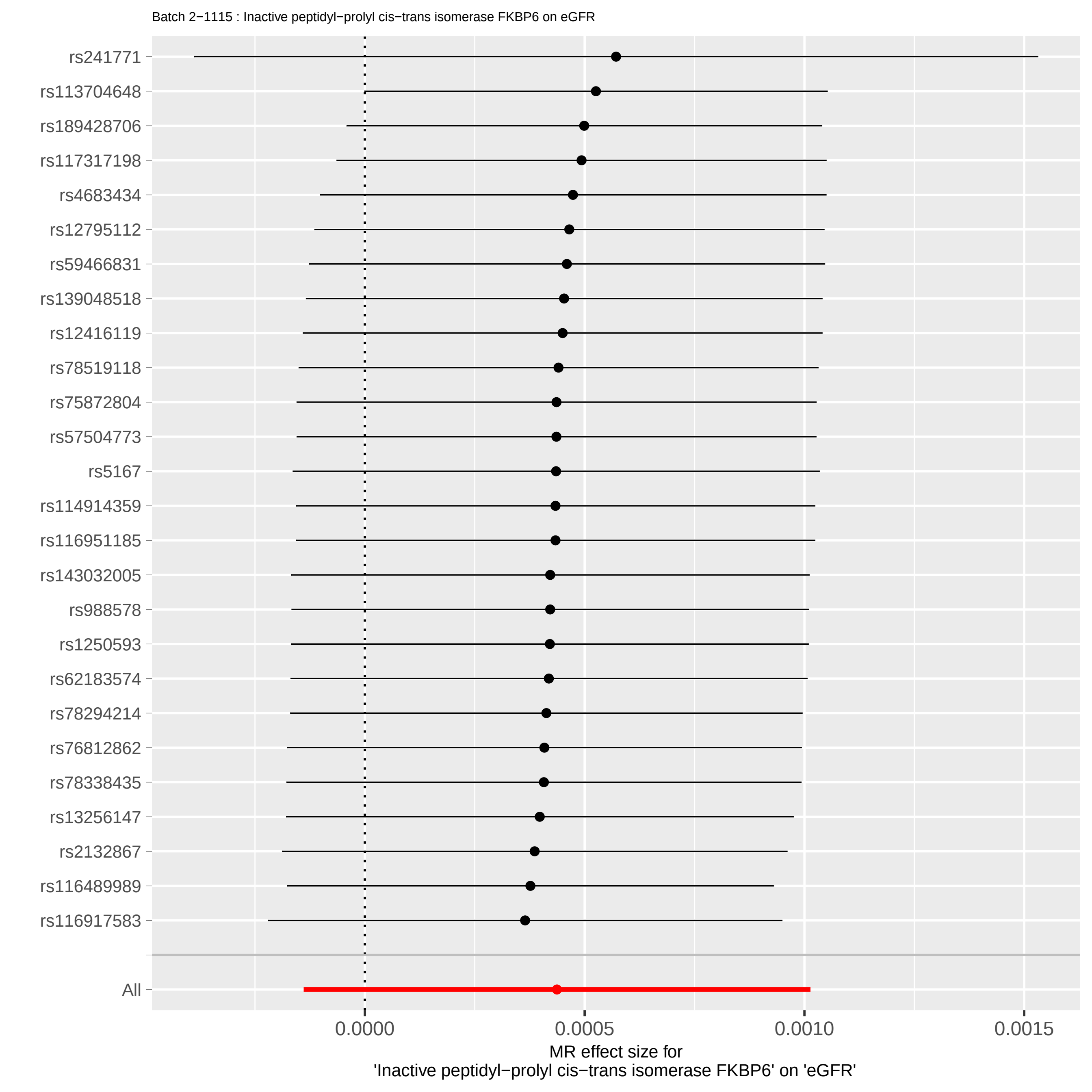

Batch 2−1115 : Inactive peptidyl−prolyl cis−trans isomerase FKBP6 on eGFR
rs241771
rs113704648
rs189428706
rs117317198
rs4683434
rs12795112
rs59466831
rs139048518
rs12416119
rs78519118
rs75872804
rs57504773
rs5167
rs114914359
rs116951185
rs143032005
rs988578
rs1250593
rs62183574
rs78294214
rs76812862
rs78338435
rs13256147
rs2132867
rs116489989
rs116917583
All
0.0000
0.0005
0.0010
0.0015
MR effect size for
'Inactive peptidyl−prolyl cis−trans isomerase FKBP6' on 'eGFR'

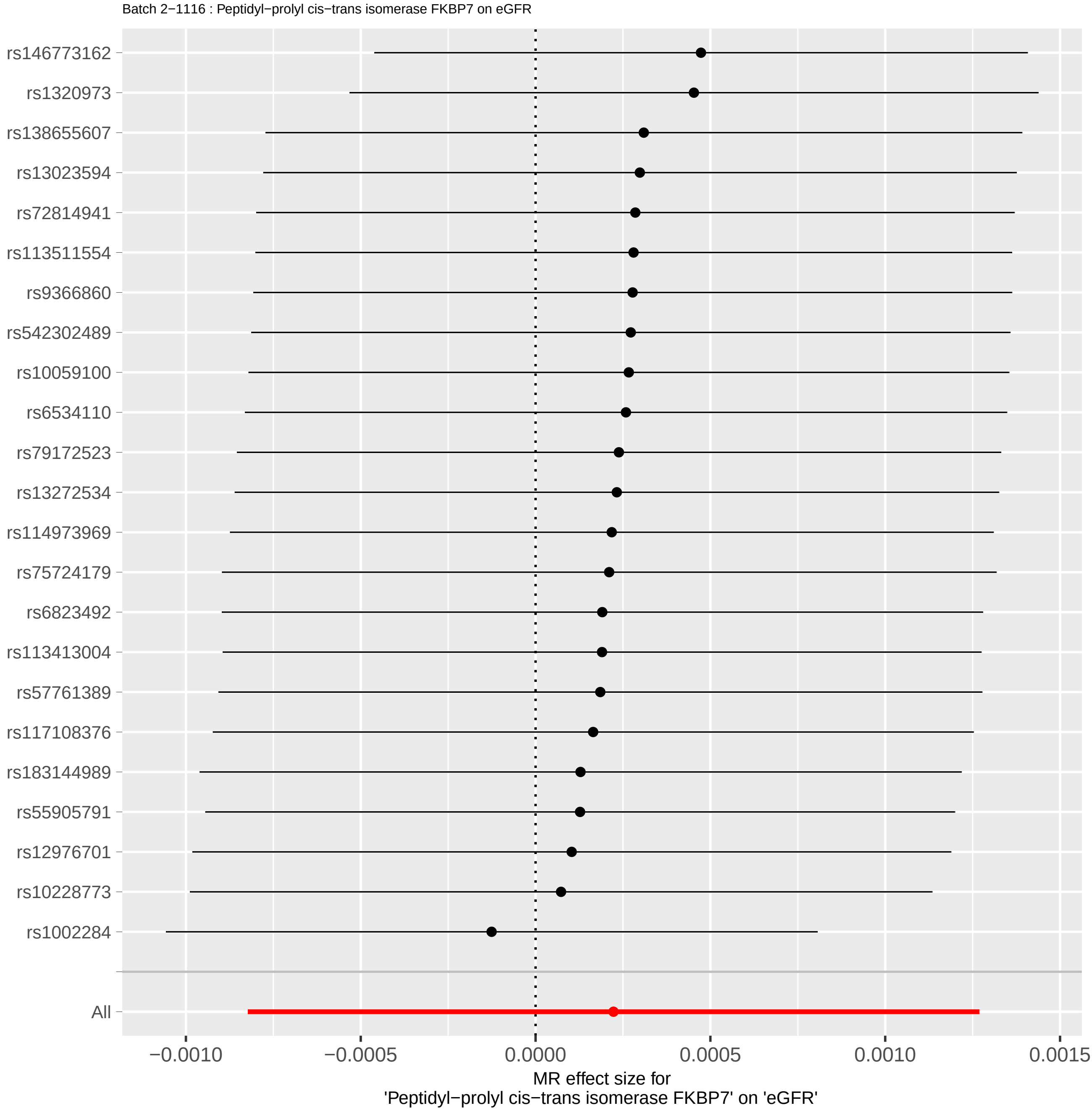

Batch 2−1116 : Peptidyl−prolyl cis−trans isomerase FKBP7 on eGFR
rs146773162
rs1320973
rs138655607
rs13023594
rs72814941
rs113511554
rs9366860
rs542302489
rs10059100
rs6534110
rs79172523
rs13272534
rs114973969
rs75724179
rs6823492
rs113413004
rs57761389
rs117108376
rs183144989
rs55905791
rs12976701
rs10228773
rs1002284
All
−0.0010
−0.0005
0.0000
0.0005
0.0010
0.0015
MR effect size for
'Peptidyl−prolyl cis−trans isomerase FKBP7' on 'eGFR'

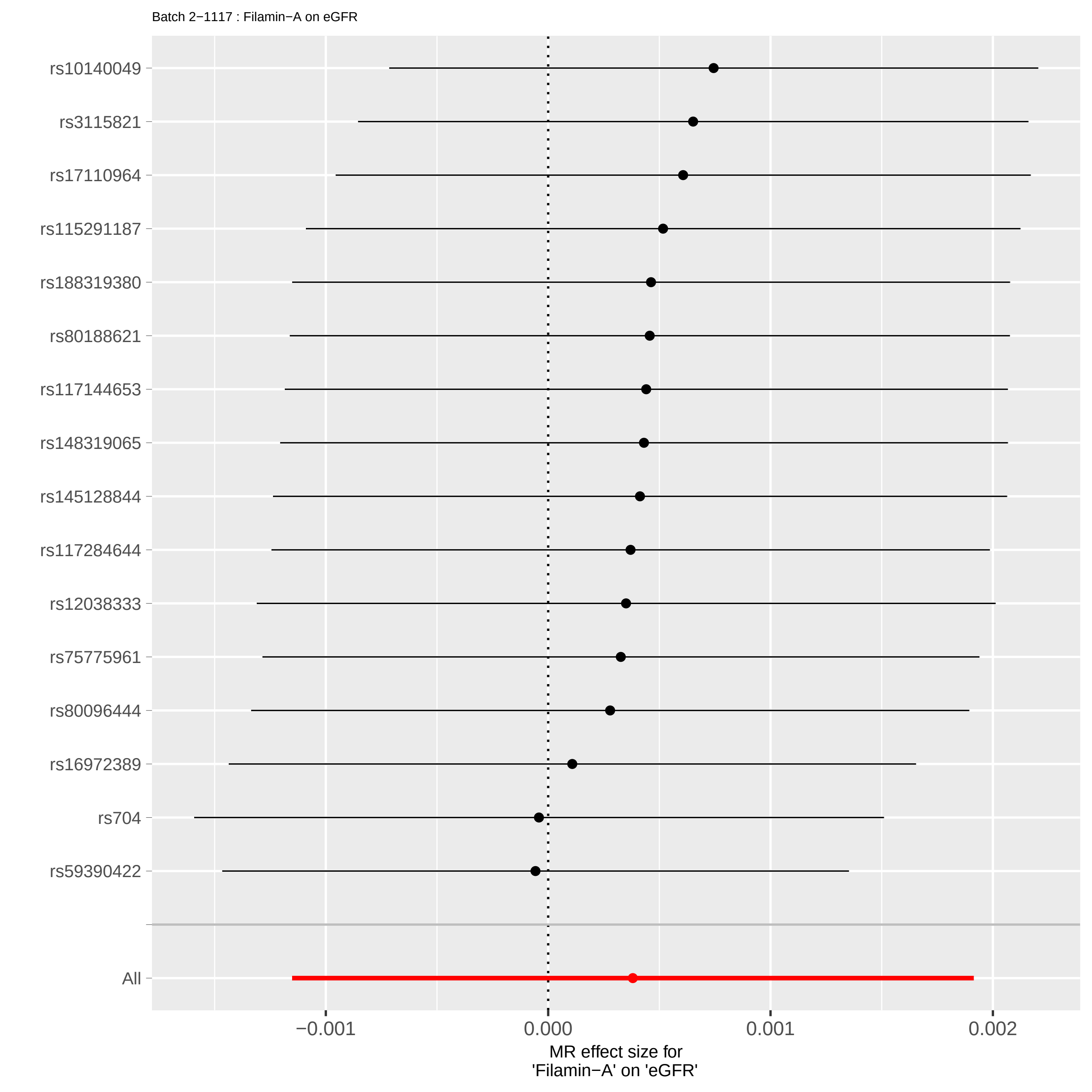

Batch 2−1117 : Filamin−A on eGFR
rs10140049
rs3115821
rs17110964
rs115291187
rs188319380
rs80188621
rs117144653
rs148319065
rs145128844
rs117284644
rs12038333
rs75775961
rs80096444
rs16972389
rs704
rs59390422
All
−0.001
0.000
0.001
0.002
MR effect size for
'Filamin−A' on 'eGFR'

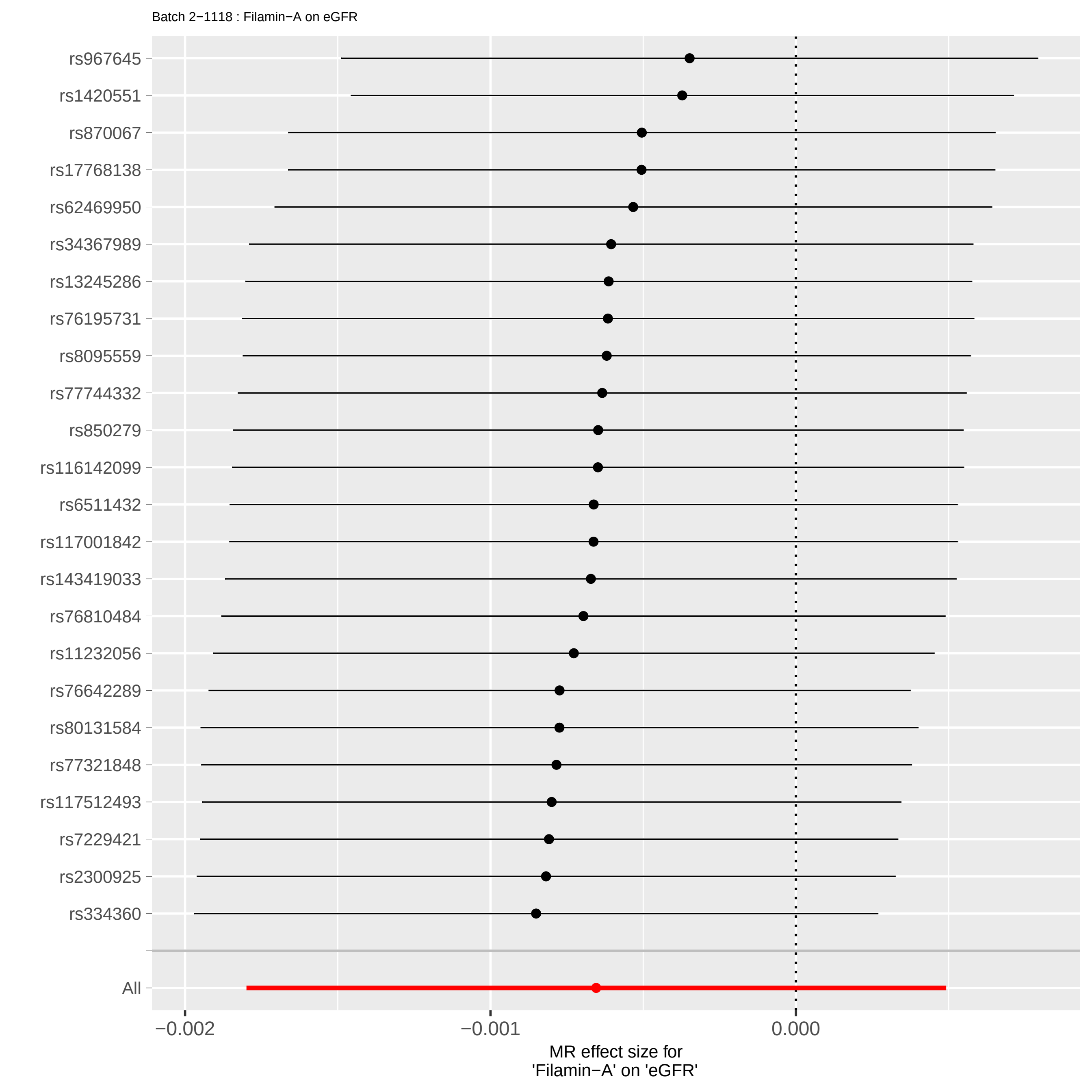
Batch 2−1118 : Filamin−A on eGFR
rs967645
rs1420551
rs870067
rs17768138
rs62469950
rs34367989
rs13245286
rs76195731
rs8095559
rs77744332
rs850279
rs116142099
rs6511432
rs117001842
rs143419033
rs76810484
rs11232056
rs76642289
rs80131584
rs77321848
rs117512493
rs7229421
rs2300925
rs334360
All
−0.002
−0.001
0.000
MR effect size for
'Filamin−A' on 'eGFR'

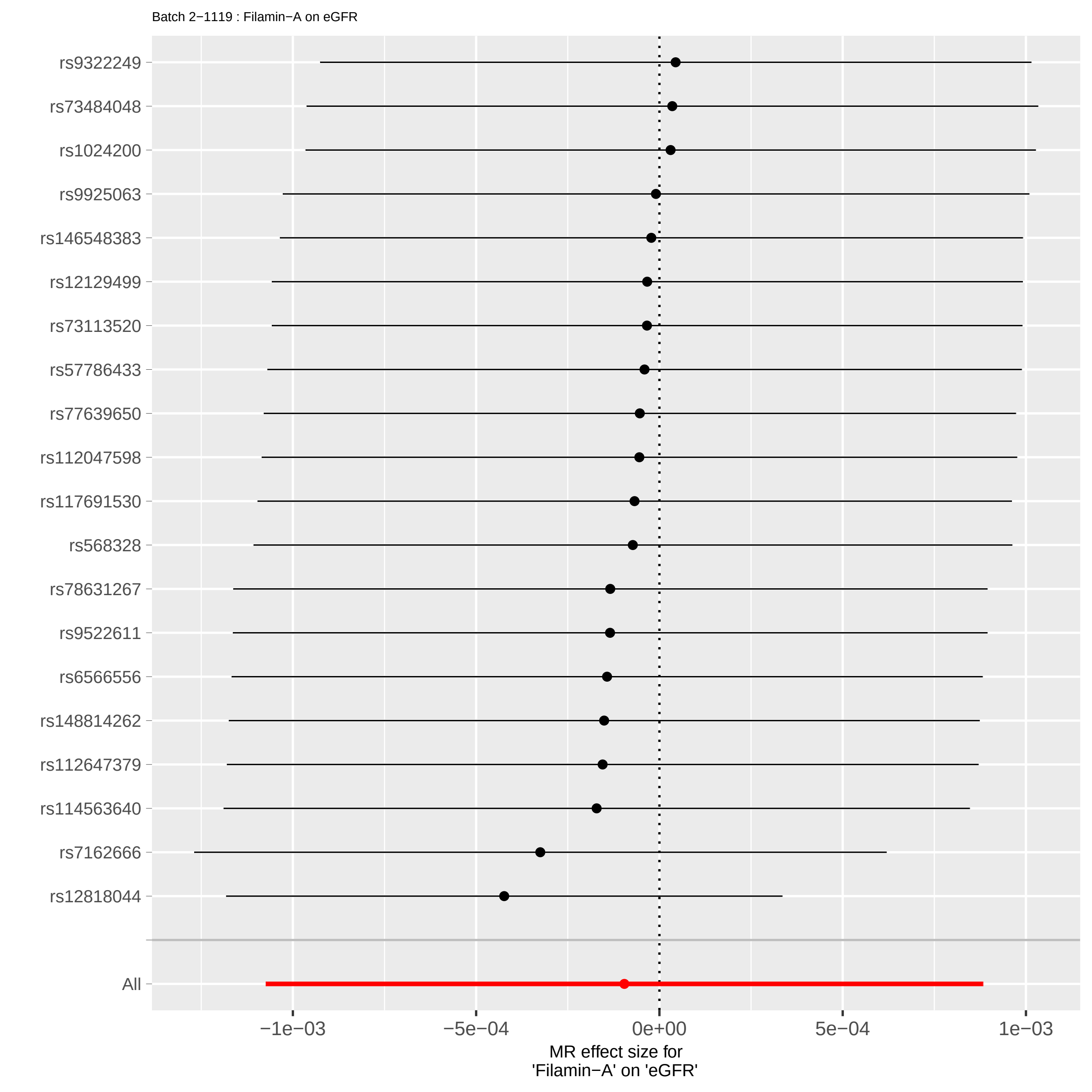

Batch 2−1119 : Filamin−A on eGFR
rs9322249
rs73484048
rs1024200
rs9925063
rs146548383
rs12129499
rs73113520
rs57786433
rs77639650
rs112047598
rs117691530
rs568328
rs78631267
rs9522611
rs6566556
rs148814262
rs112647379
rs114563640
rs7162666
rs12818044
All
−1e−03
−5e−04
0e+00
5e−04
1e−03
MR effect size for
'Filamin−A' on 'eGFR'

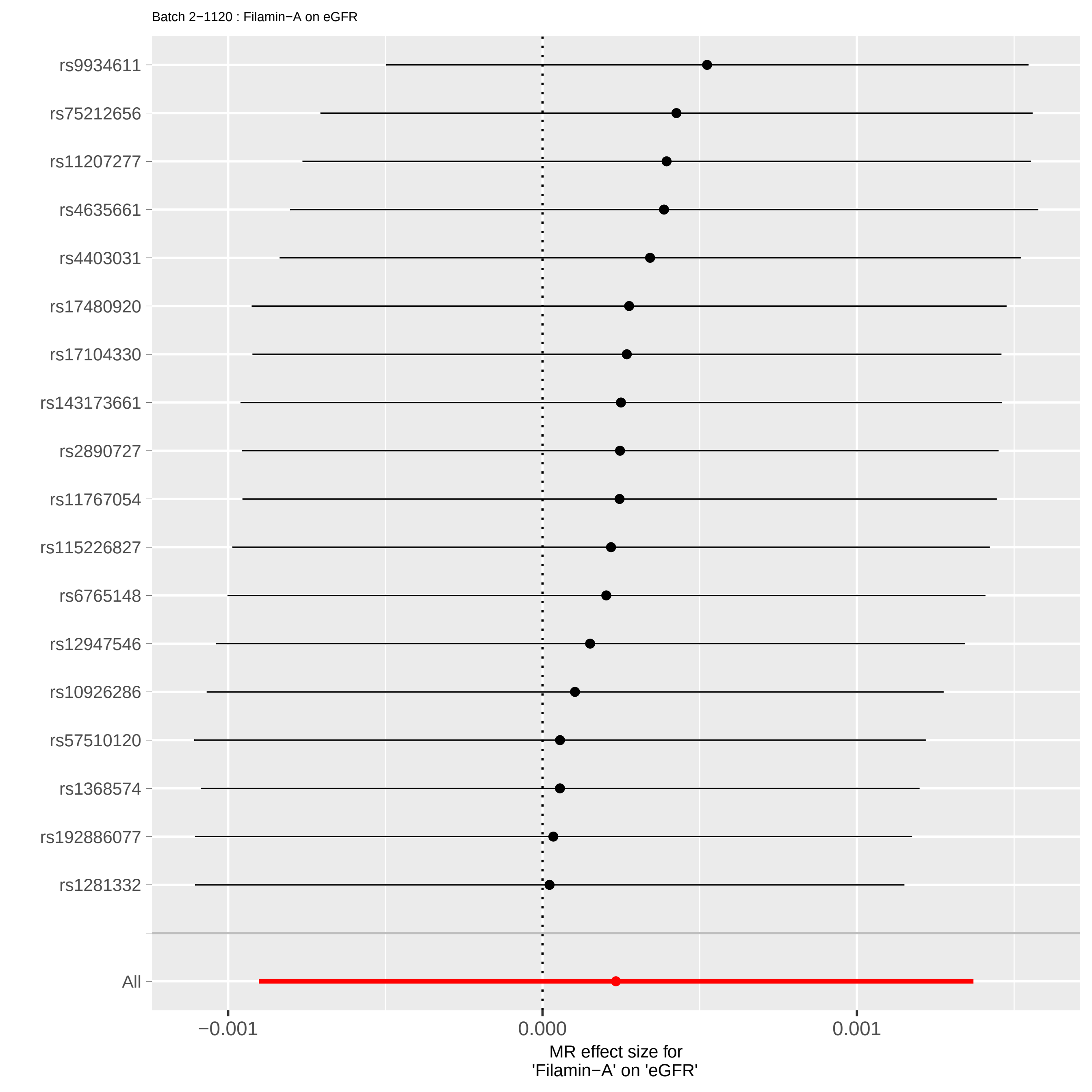

Batch 2−1120 : Filamin−A on eGFR
rs9934611
rs75212656
rs11207277
rs4635661
rs4403031
rs17480920
rs17104330
rs143173661
rs2890727
rs11767054
rs115226827
rs6765148
rs12947546
rs10926286
rs57510120
rs1368574
rs192886077
rs1281332
All
−0.001
0.000
0.001
MR effect size for
'Filamin−A' on 'eGFR'

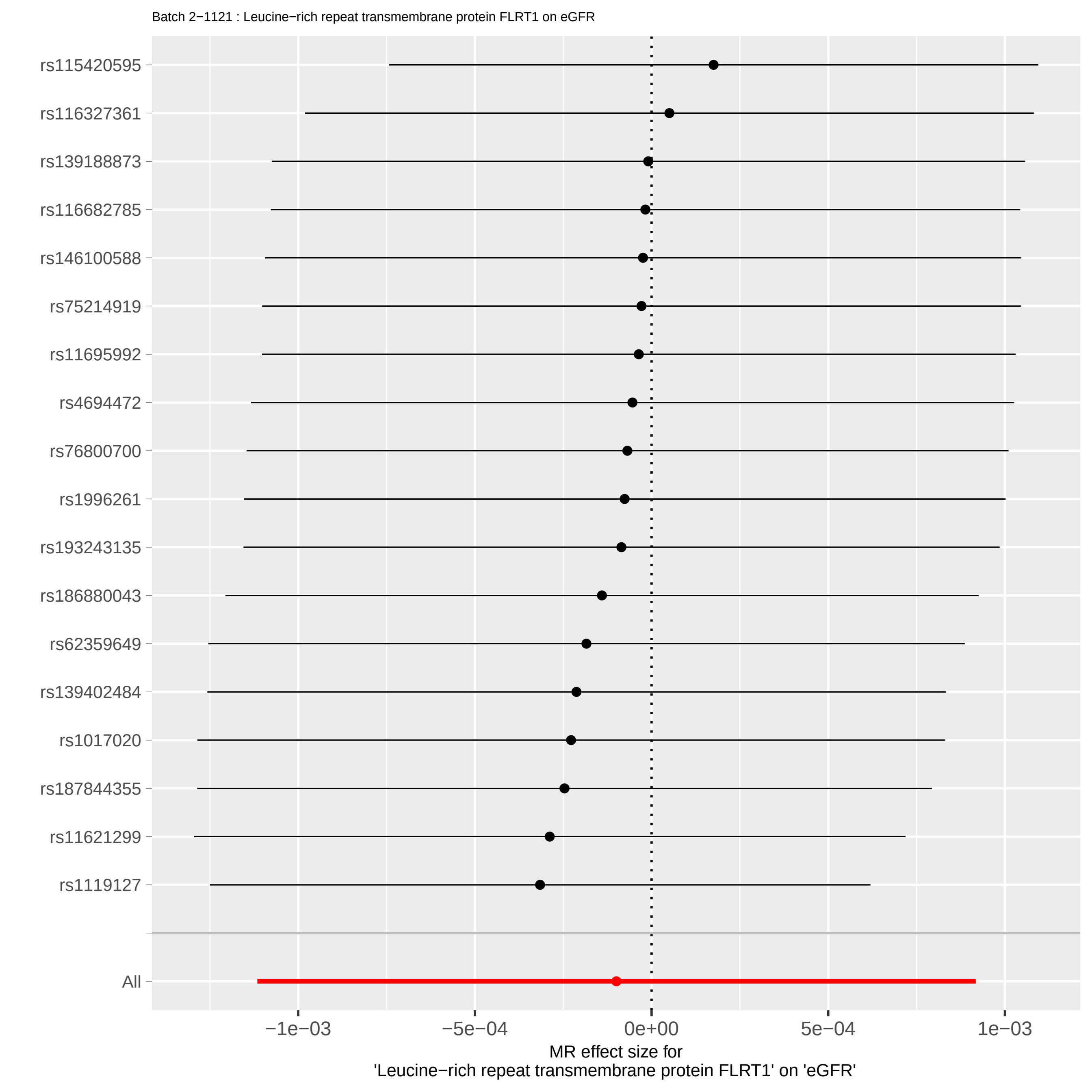

Batch 2−1121 : Leucine−rich repeat transmembrane protein FLRT1 on eGFR
rs115420595
rs116327361
rs139188873
rs116682785
rs146100588
rs75214919
rs11695992
rs4694472
rs76800700
rs1996261
rs193243135
rs186880043
rs62359649
rs139402484
rs1017020
rs187844355
rs11621299
rs1119127
All
−1e−03
−5e−04
0e+00
5e−04
1e−03
MR effect size for
'Leucine−rich repeat transmembrane protein FLRT1' on 'eGFR'

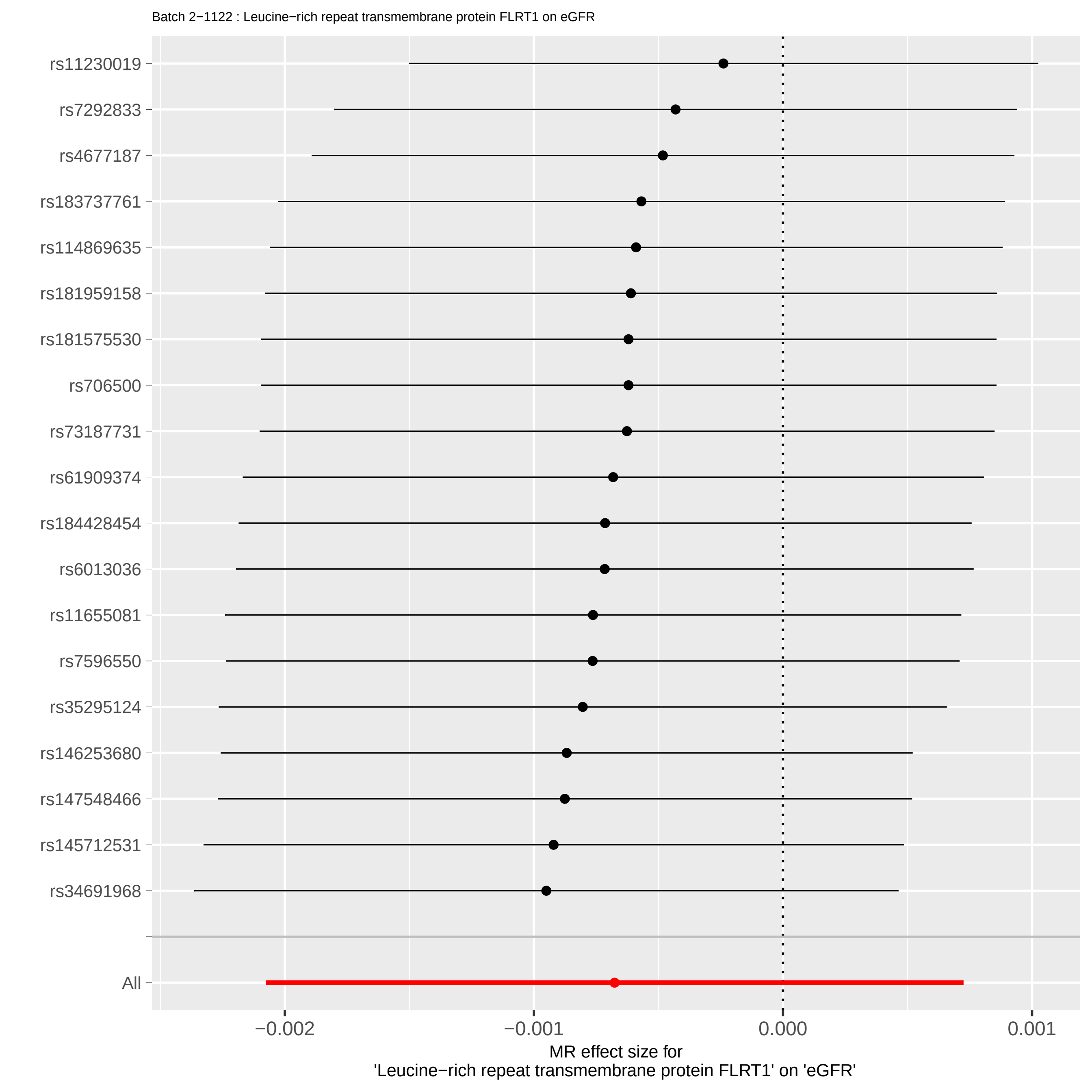

Batch 2−1122 : Leucine−rich repeat transmembrane protein FLRT1 on eGFR
rs11230019
rs7292833
rs4677187
rs183737761
rs114869635
rs181959158
rs181575530
rs706500
rs73187731
rs61909374
rs184428454
rs6013036
rs11655081
rs7596550
rs35295124
rs146253680
rs147548466
rs145712531
rs34691968
All
−0.002
−0.001
0.000
0.001
MR effect size for
'Leucine−rich repeat transmembrane protein FLRT1' on 'eGFR'

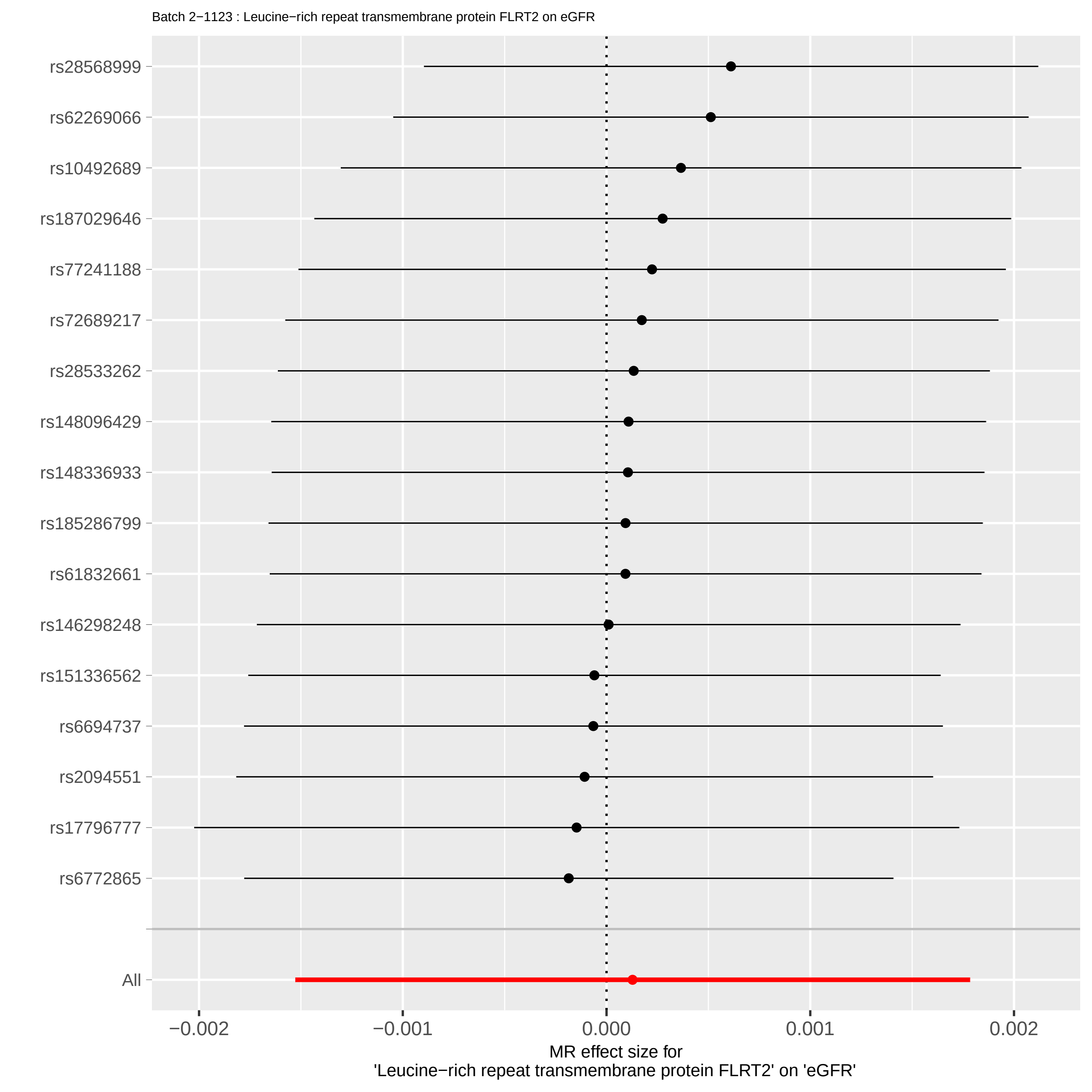

Batch 2−1123 : Leucine−rich repeat transmembrane protein FLRT2 on eGFR
rs28568999
rs62269066
rs10492689
rs187029646
rs77241188
rs72689217
rs28533262
rs148096429
rs148336933
rs185286799
rs61832661
rs146298248
rs151336562
rs6694737
rs2094551
rs17796777
rs6772865
All
−0.002
−0.001
0.000
0.001
0.002
MR effect size for
'Leucine−rich repeat transmembrane protein FLRT2' on 'eGFR'

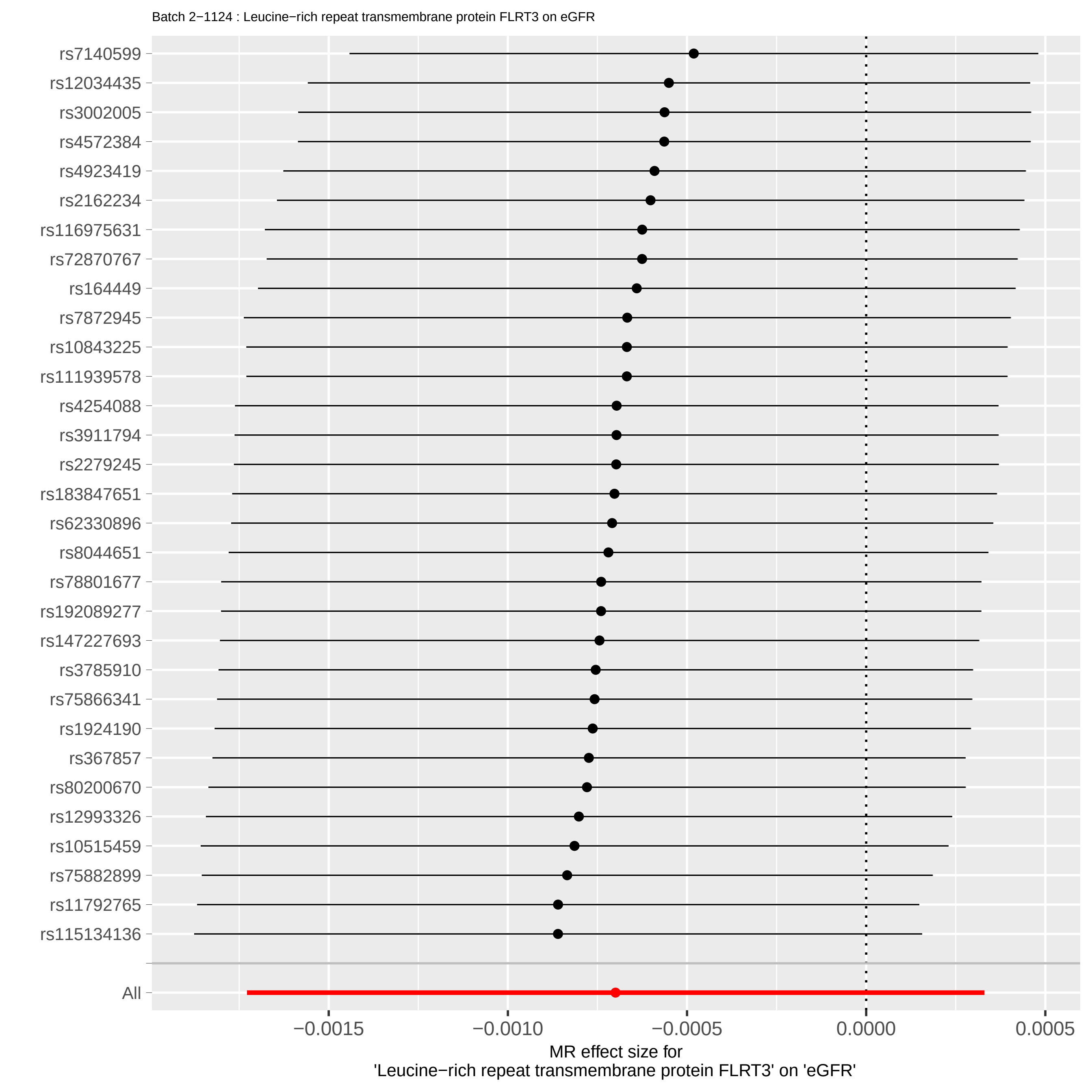
Batch 2−1124 : Leucine−rich repeat transmembrane protein FLRT3 on eGFR
rs7140599
rs12034435
rs3002005
rs4572384
rs4923419
rs2162234
rs116975631
rs72870767
rs164449
rs7872945
rs10843225
rs111939578
rs4254088
rs3911794
rs2279245
rs183847651
rs62330896
rs8044651
rs78801677
rs192089277
rs147227693
rs3785910
rs75866341
rs1924190
rs367857
rs80200670
rs12993326
rs10515459
rs75882899
rs11792765
rs115134136
All
−0.0015
−0.0010
−0.0005
0.0000
0.0005
MR effect size for
'Leucine−rich repeat transmembrane protein FLRT3' on 'eGFR'

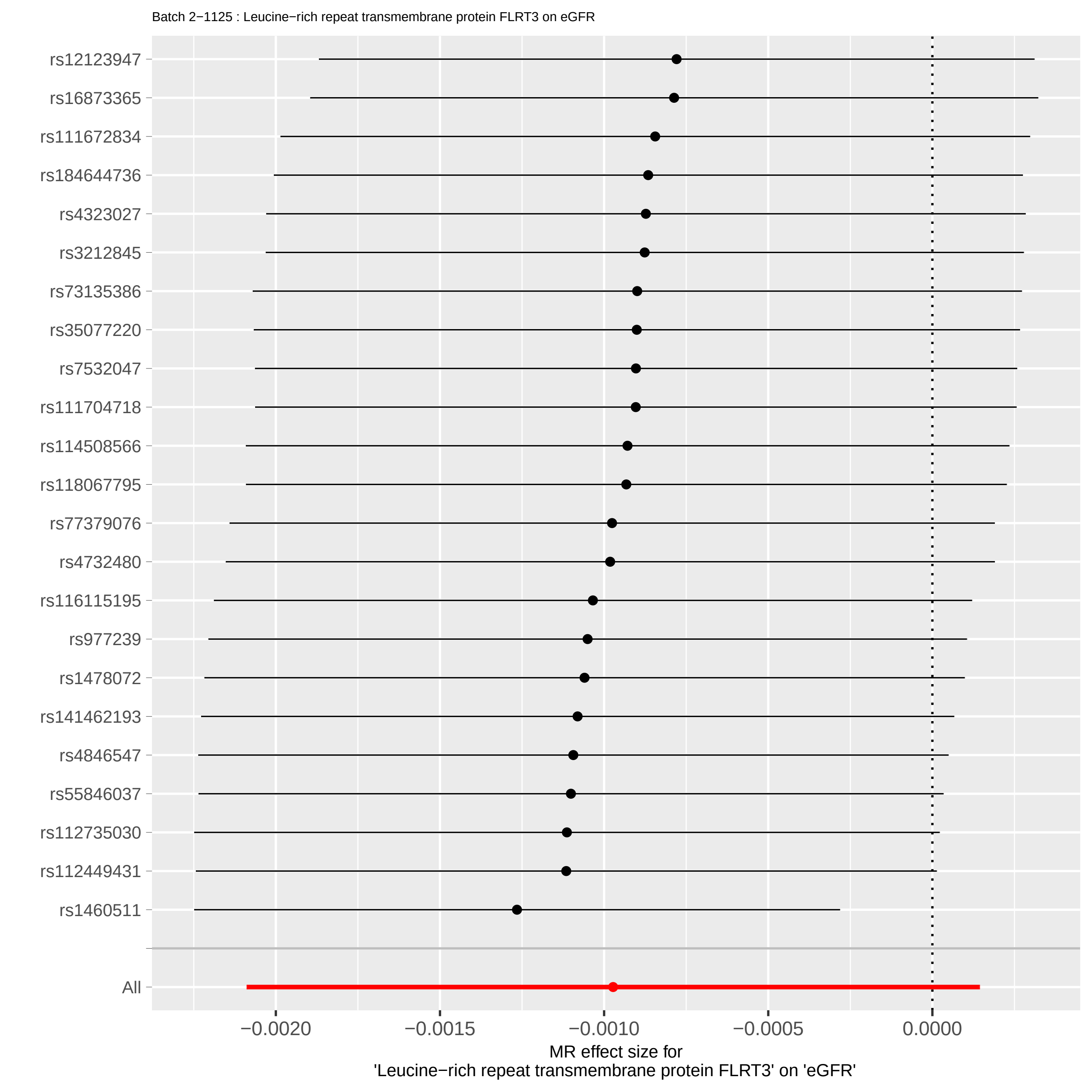

Batch 2−1125 : Leucine−rich repeat transmembrane protein FLRT3 on eGFR
rs12123947
rs16873365
rs111672834
rs184644736
rs4323027
rs3212845
rs73135386
rs35077220
rs7532047
rs111704718
rs114508566
rs118067795
rs77379076
rs4732480
rs116115195
rs977239
rs1478072
rs141462193
rs4846547
rs55846037
rs112735030
rs112449431
rs1460511
All
−0.0020
−0.0015
−0.0010
−0.0005
0.0000
MR effect size for
'Leucine−rich repeat transmembrane protein FLRT3' on 'eGFR'

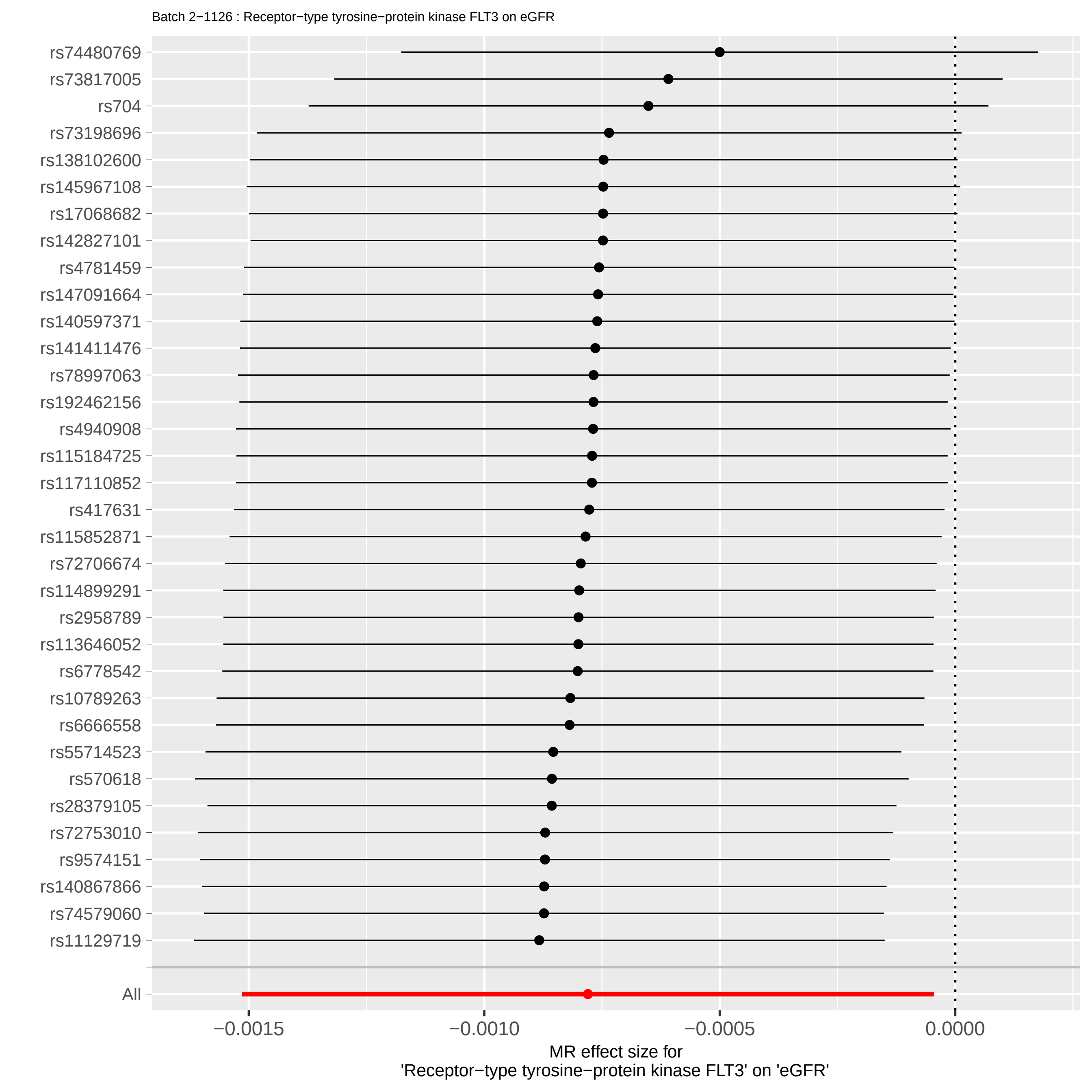
Batch 2−1126 : Receptor−type tyrosine−protein kinase FLT3 on eGFR
rs74480769
rs73817005
rs704
rs73198696
rs138102600
rs145967108
rs17068682
rs142827101
rs4781459
rs147091664
rs140597371
rs141411476
rs78997063
rs192462156
rs4940908
rs115184725
rs117110852
rs417631
rs115852871
rs72706674
rs114899291
rs2958789
rs113646052
rs6778542
rs10789263
rs6666558
rs55714523
rs570618
rs28379105
rs72753010
rs9574151
rs140867866
rs74579060
rs11129719
All
−0.0015
−0.0010
−0.0005
0.0000
MR effect size for
'Receptor−type tyrosine−protein kinase FLT3' on 'eGFR'

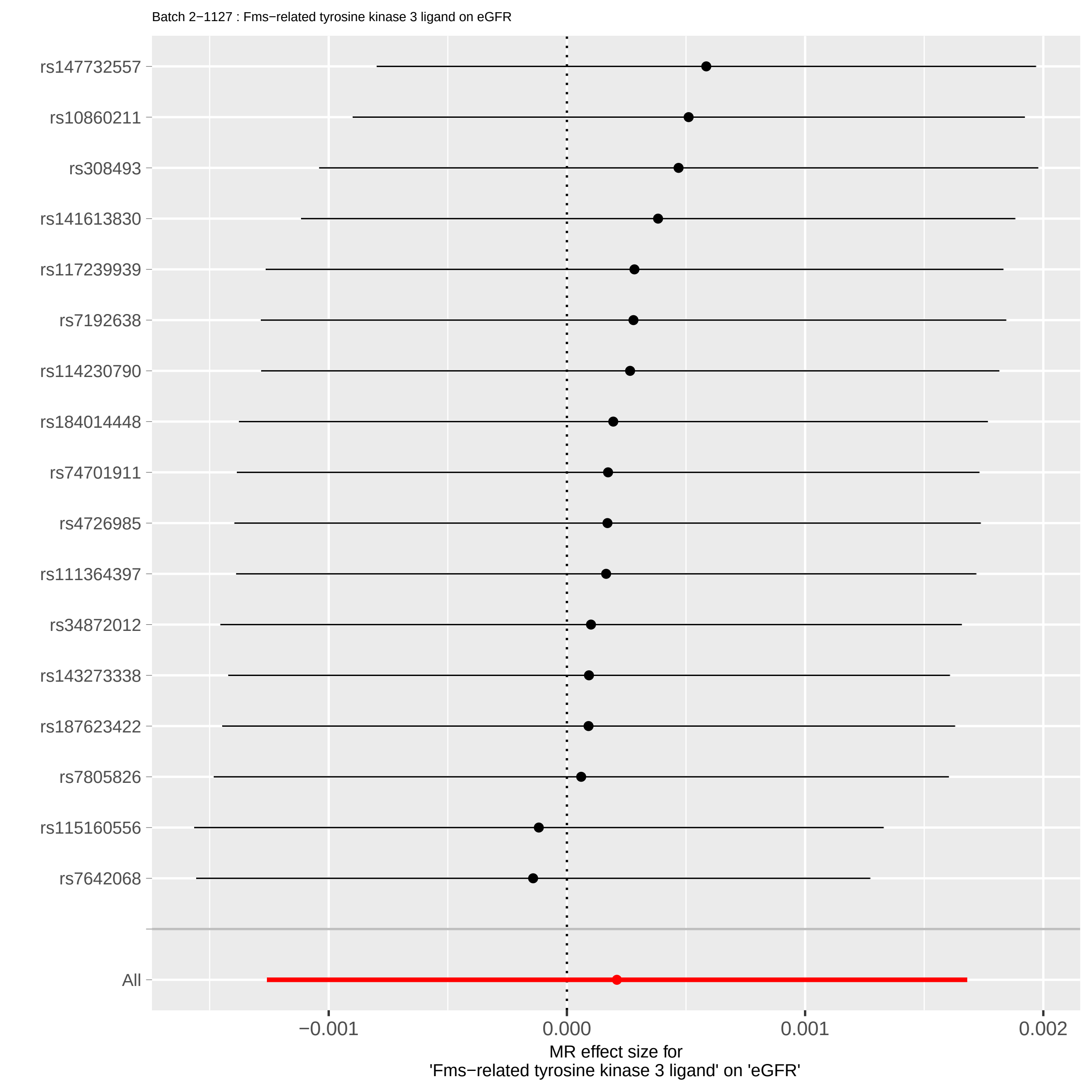
Batch 2−1127 : Fms−related tyrosine kinase 3 ligand on eGFR
rs147732557
rs10860211
rs308493
rs141613830
rs117239939
rs7192638
rs114230790
rs184014448
rs74701911
rs4726985
rs111364397
rs34872012
rs143273338
rs187623422
rs7805826
rs115160556
rs7642068
All
−0.001
0.000
0.001
0.002
MR effect size for
'Fms−related tyrosine kinase 3 ligand' on 'eGFR'

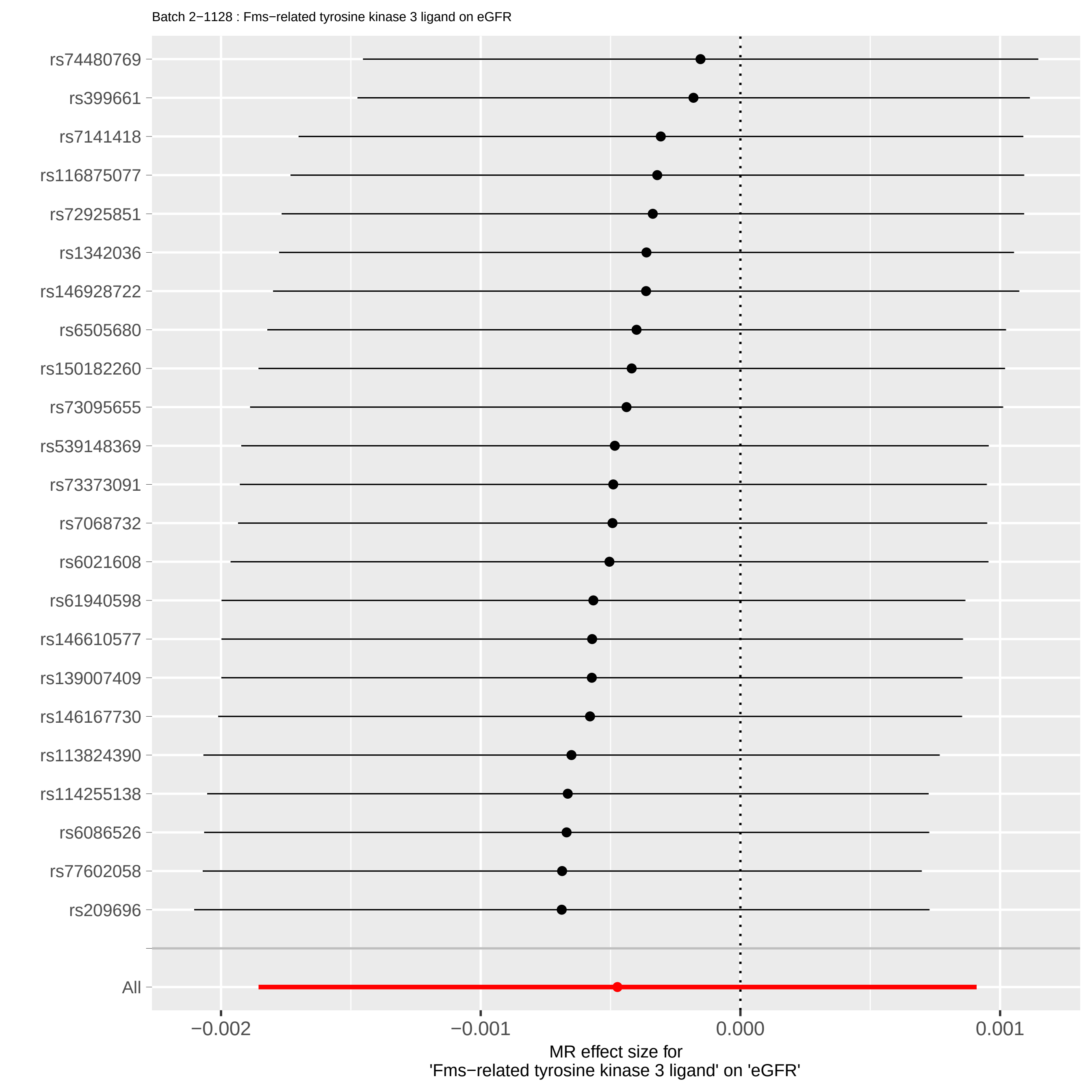

Batch 2−1128 : Fms−related tyrosine kinase 3 ligand on eGFR
rs74480769
rs399661
rs7141418
rs116875077
rs72925851
rs1342036
rs146928722
rs6505680
rs150182260
rs73095655
rs539148369
rs73373091
rs7068732
rs6021608
rs61940598
rs146610577
rs139007409
rs146167730
rs113824390
rs114255138
rs6086526
rs77602058
rs209696
All
−0.002
−0.001
0.000
0.001
MR effect size for
'Fms−related tyrosine kinase 3 ligand' on 'eGFR'

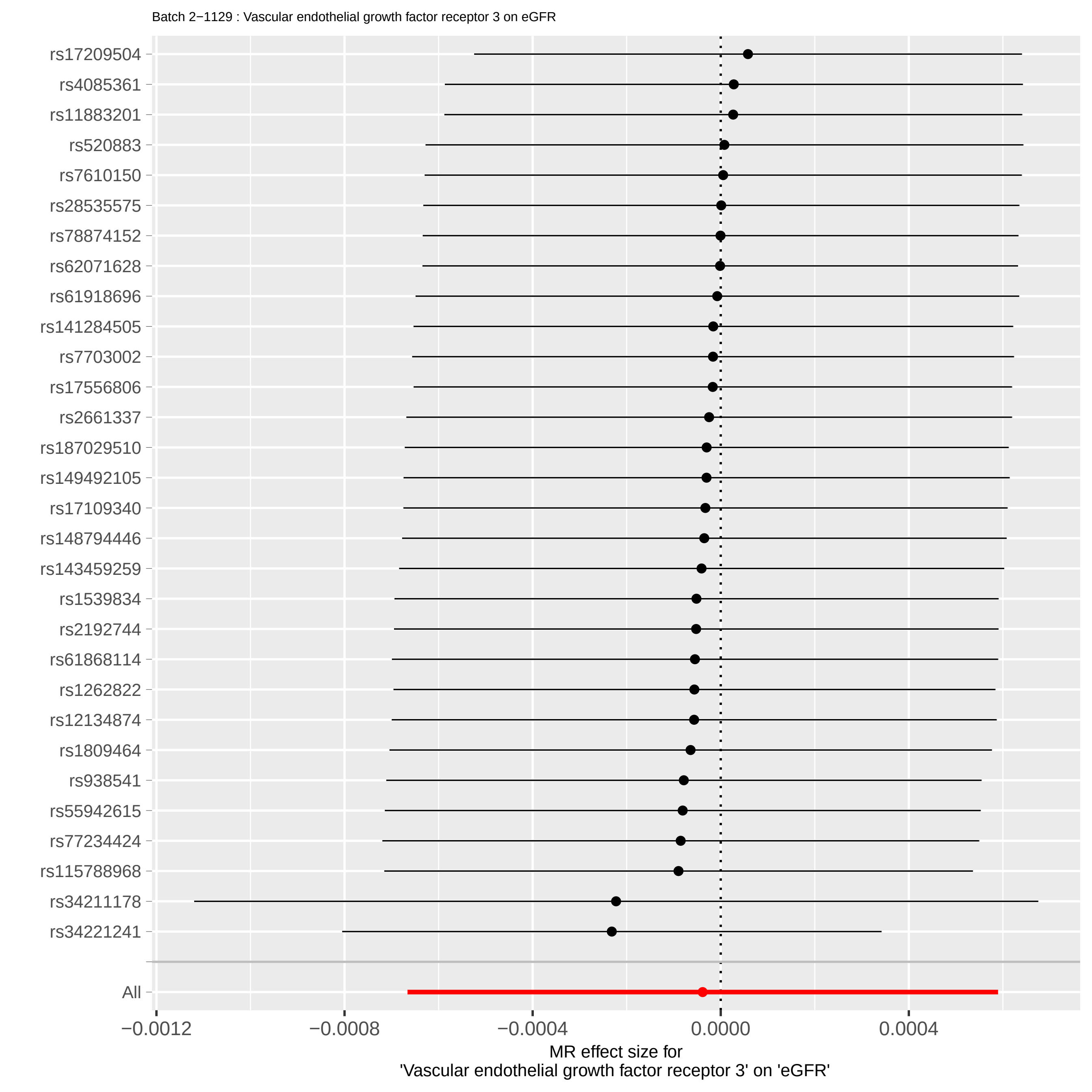

Batch 2−1129 : Vascular endothelial growth factor receptor 3 on eGFR
rs17209504
rs4085361
rs11883201
rs520883
rs7610150
rs28535575
rs78874152
rs62071628
rs61918696
rs141284505
rs7703002
rs17556806
rs2661337
rs187029510
rs149492105
rs17109340
rs148794446
rs143459259
rs1539834
rs2192744
rs61868114
rs1262822
rs12134874
rs1809464
rs938541
rs55942615
rs77234424
rs115788968
rs34211178
rs34221241
All
−0.0012
−0.0008
−0.0004
0.0000
0.0004
MR effect size for
'Vascular endothelial growth factor receptor 3' on 'eGFR'

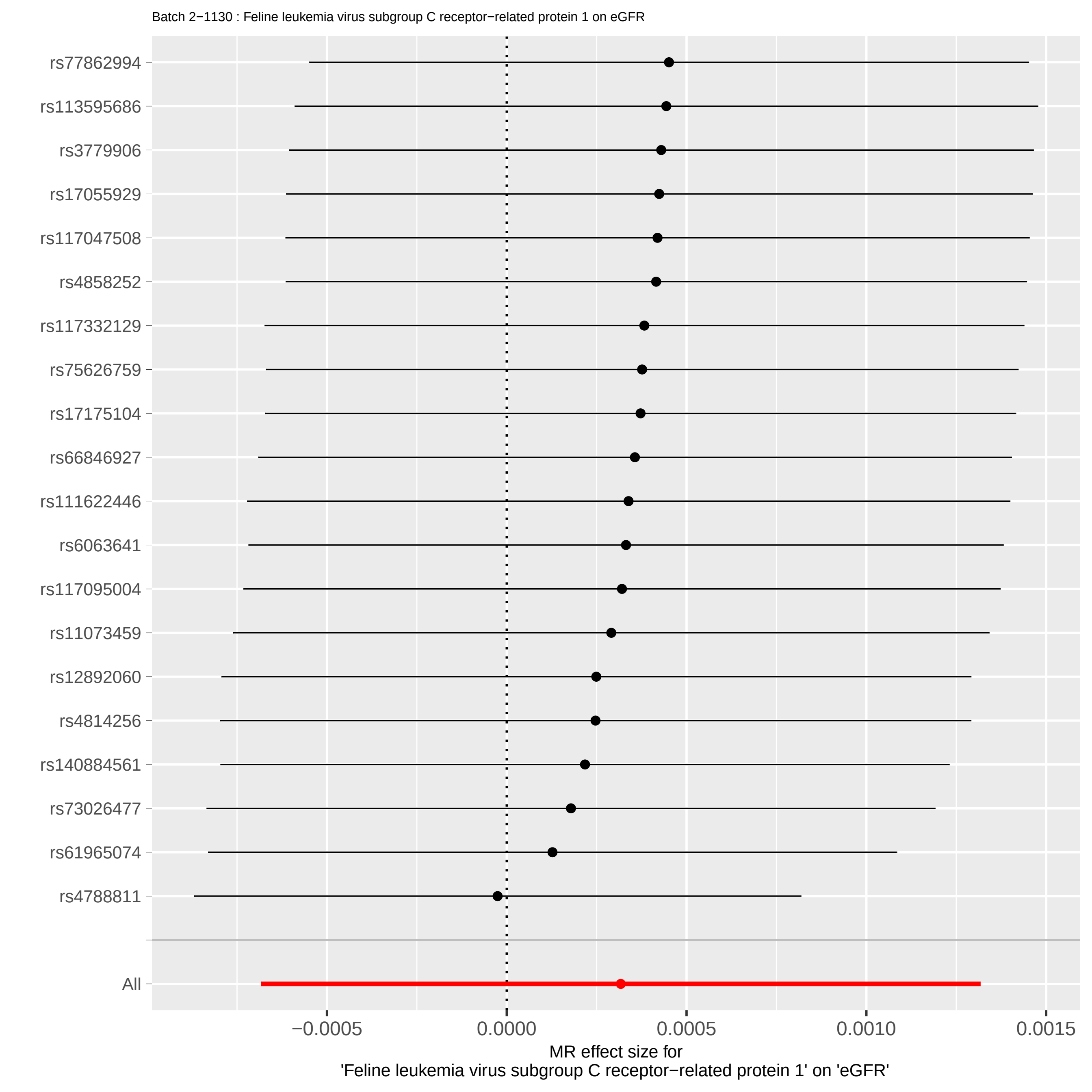
Batch 2−1130 : Feline leukemia virus subgroup C receptor−related protein 1 on eGFR
rs77862994
rs113595686
rs3779906
rs17055929
rs117047508
rs4858252
rs117332129
rs75626759
rs17175104
rs66846927
rs111622446
rs6063641
rs117095004
rs11073459
rs12892060
rs4814256
rs140884561
rs73026477
rs61965074
rs4788811
All
−0.0005
0.0000
0.0005
0.0010
0.0015
MR effect size for
'Feline leukemia virus subgroup C receptor−related protein 1' on 'eGFR'

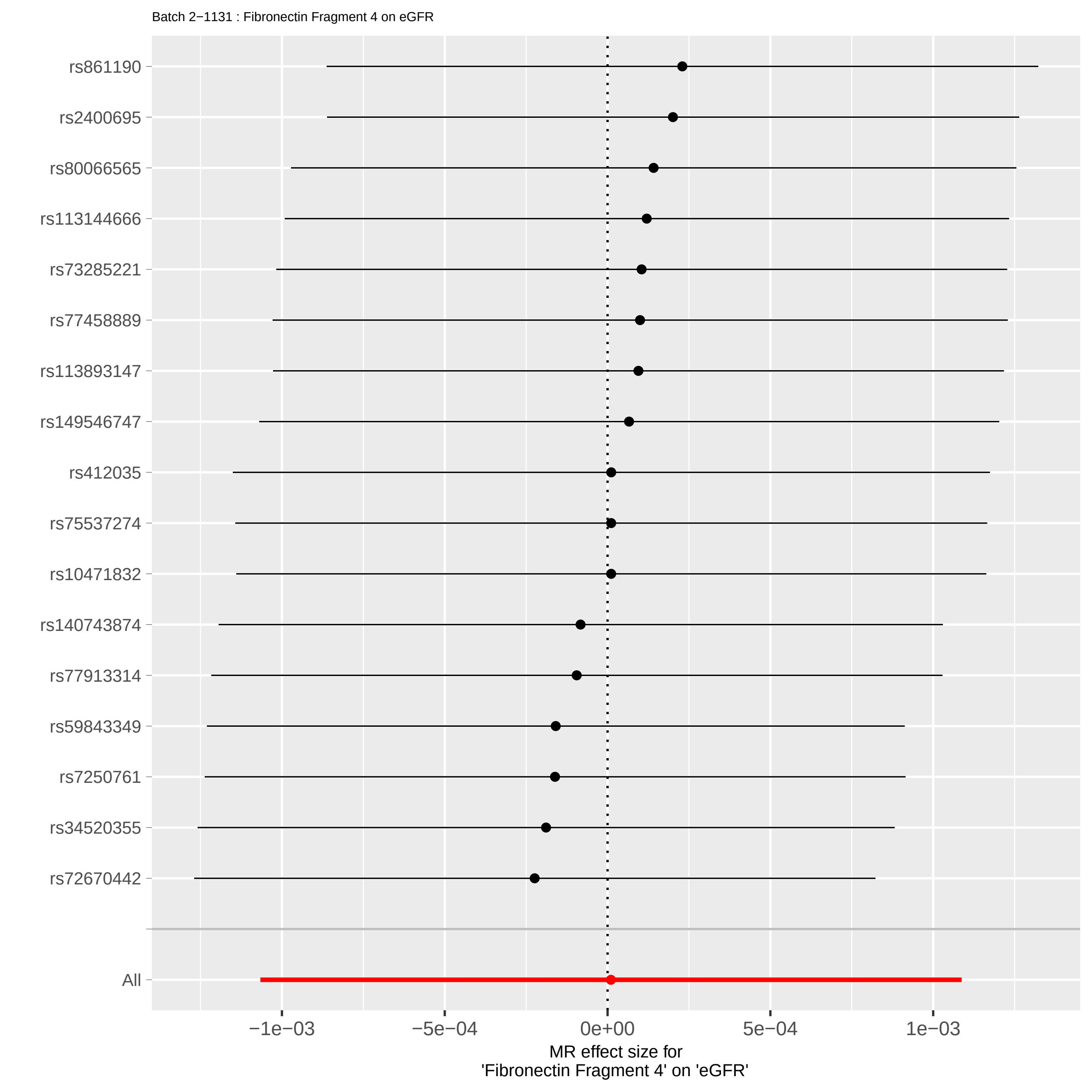

Batch 2−1131 : Fibronectin Fragment 4 on eGFR
rs861190
rs2400695
rs80066565
rs113144666
rs73285221
rs77458889
rs113893147
rs149546747
rs412035
rs75537274
rs10471832
rs140743874
rs77913314
rs59843349
rs7250761
rs34520355
rs72670442
All
−1e−03
−5e−04
0e+00
5e−04
1e−03
MR effect size for
'Fibronectin Fragment 4' on 'eGFR'

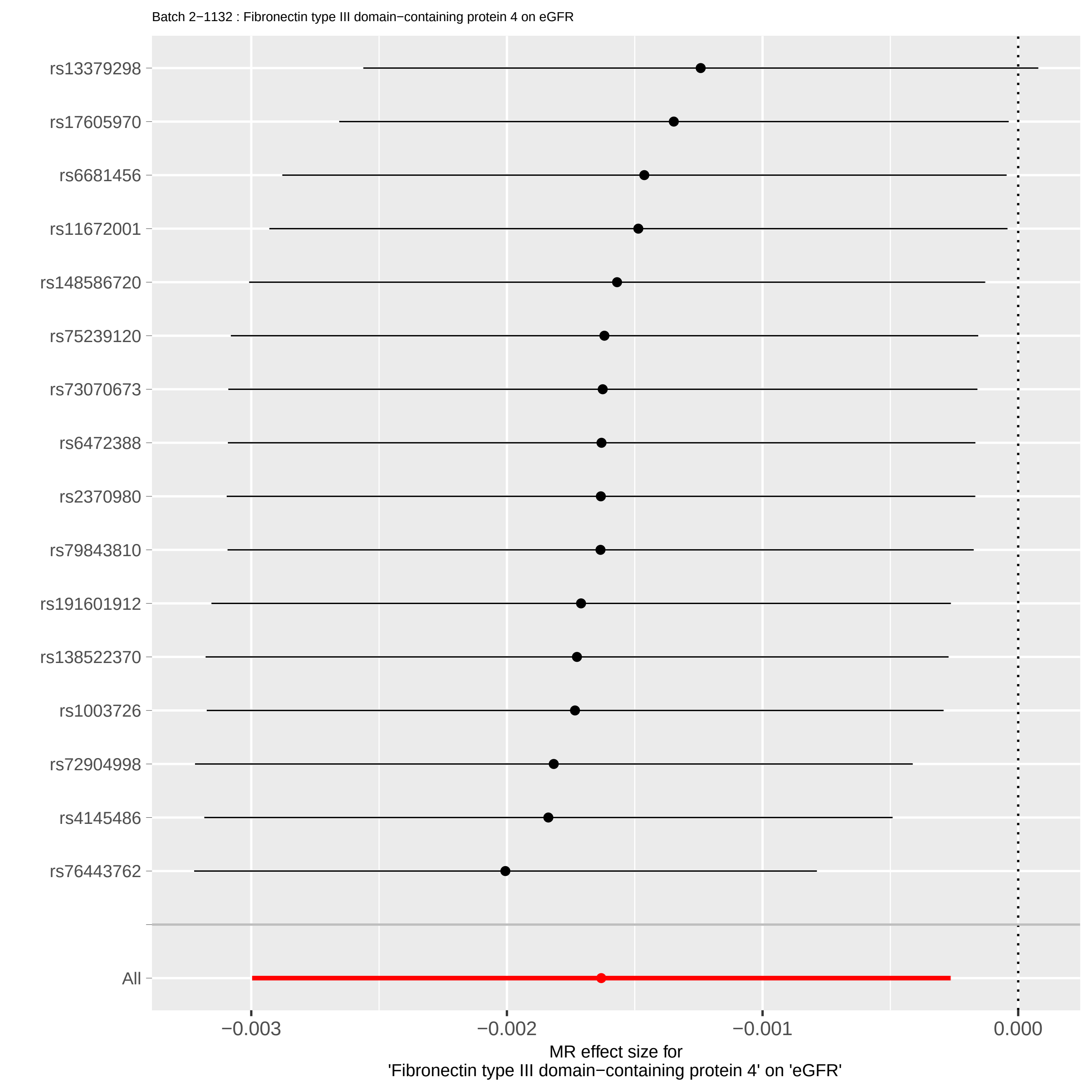

Batch 2−1132 : Fibronectin type III domain−containing protein 4 on eGFR
rs13379298
rs17605970
rs6681456
rs11672001
rs148586720
rs75239120
rs73070673
rs6472388
rs2370980
rs79843810
rs191601912
rs138522370
rs1003726
rs72904998
rs4145486
rs76443762
All
−0.003
−0.002
−0.001
0.000
MR effect size for
'Fibronectin type III domain−containing protein 4' on 'eGFR'

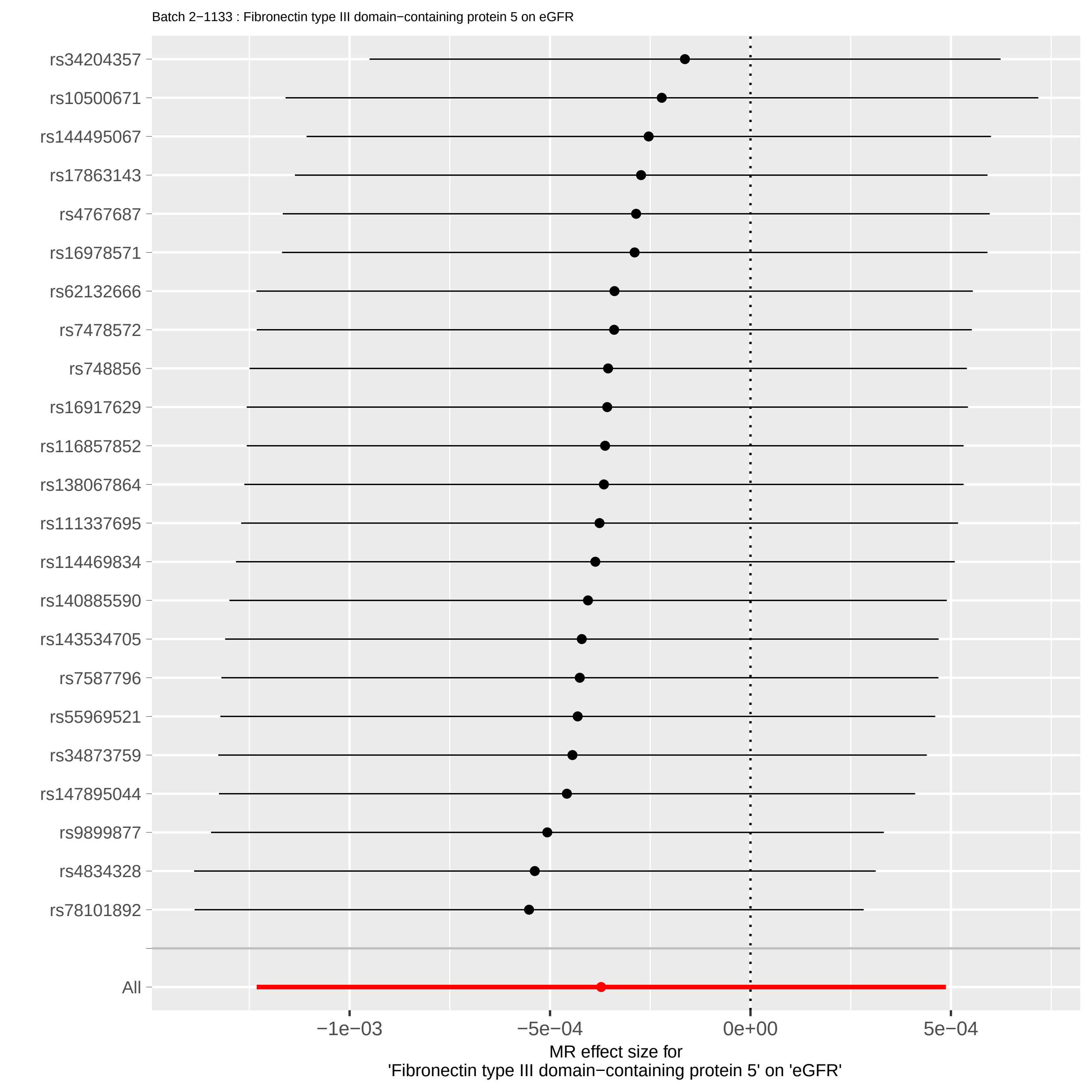

Batch 2−1133 : Fibronectin type III domain−containing protein 5 on eGFR
rs34204357
rs10500671
rs144495067
rs17863143
rs4767687
rs16978571
rs62132666
rs7478572
rs748856
rs16917629
rs116857852
rs138067864
rs111337695
rs114469834
rs140885590
rs143534705
rs7587796
rs55969521
rs34873759
rs147895044
rs9899877
rs4834328
rs78101892
All
−1e−03
−5e−04
0e+00
5e−04
MR effect size for
'Fibronectin type III domain−containing protein 5' on 'eGFR'

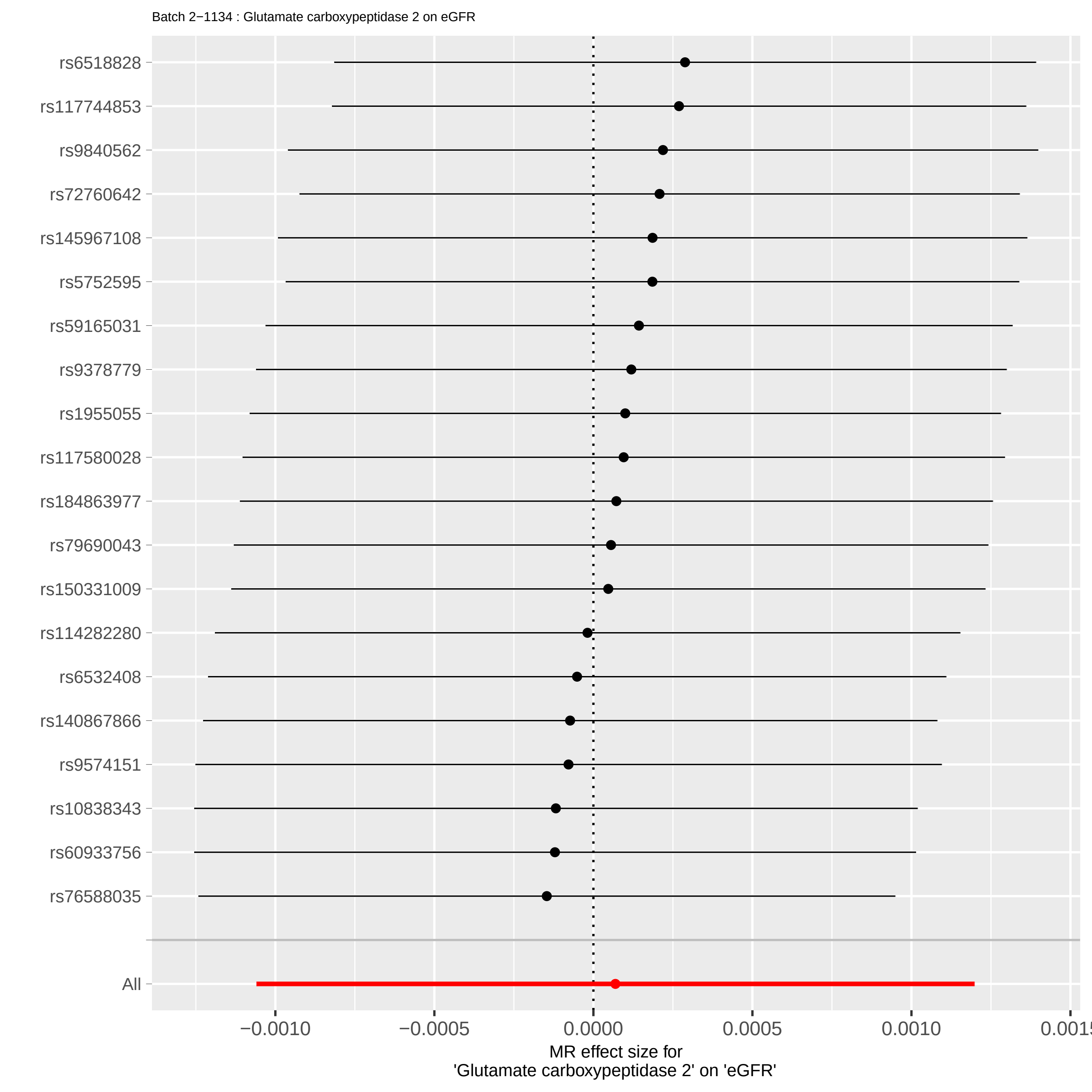
Batch 2−1134 : Glutamate carboxypeptidase 2 on eGFR
rs6518828
rs117744853
rs9840562
rs72760642
rs145967108
rs5752595
rs59165031
rs9378779
rs1955055
rs117580028
rs184863977
rs79690043
rs150331009
rs114282280
rs6532408
rs140867866
rs9574151
rs10838343
rs60933756
rs76588035
All
−0.0010
−0.0005
0.0000
0.0005
0.0010
MR effect size for
'Glutamate carboxypeptidase 2' on 'eGFR'

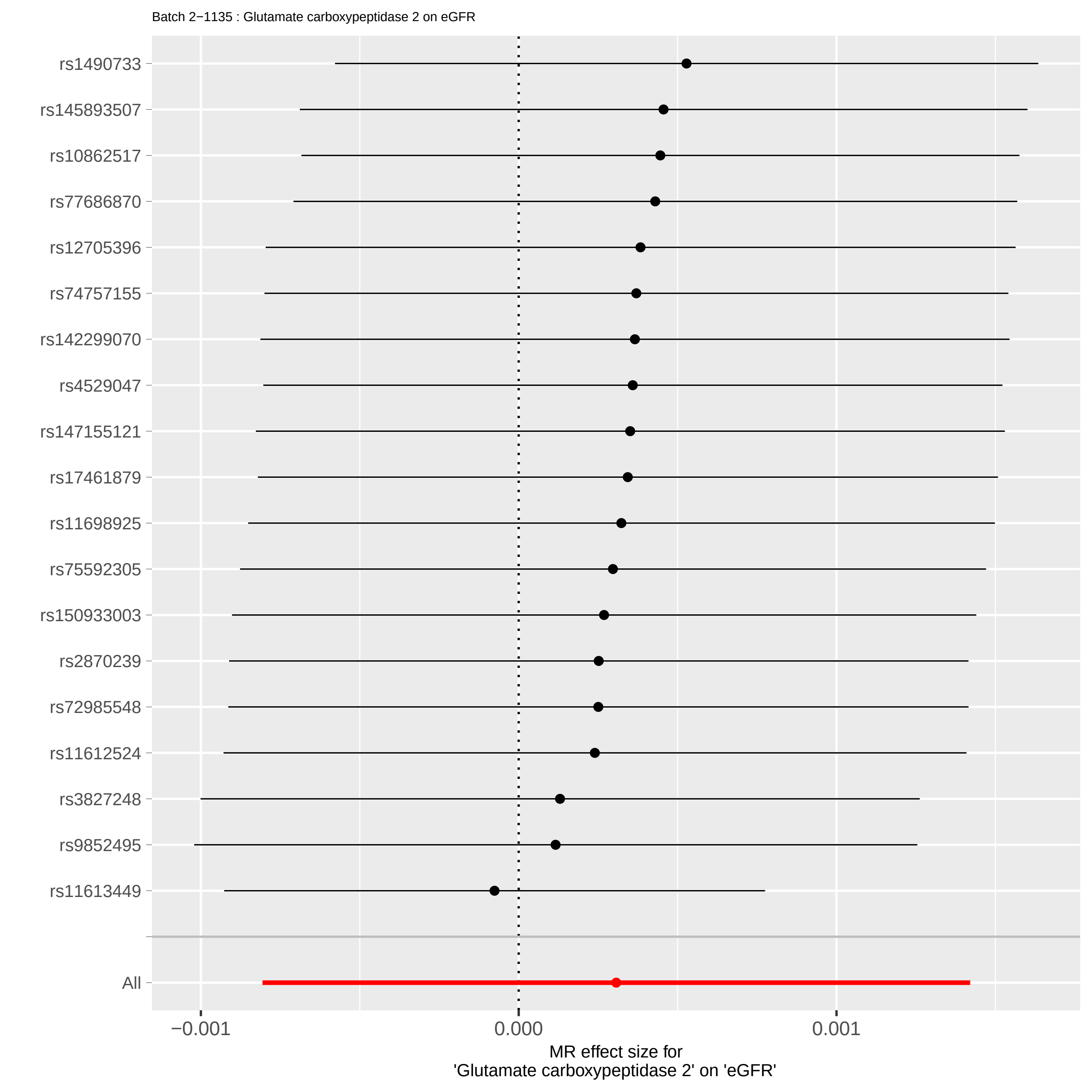
Batch 2−1135 : Glutamate carboxypeptidase 2 on eGFR
rs1490733
rs145893507
rs10862517
rs77686870
rs12705396
rs74757155
rs142299070
rs4529047
rs147155121
rs17461879
rs11698925
rs75592305
rs150933003
rs2870239
rs72985548
rs11612524
rs3827248
rs9852495
rs11613449
All
−0.001
0.000
0.001
MR effect size for
'Glutamate carboxypeptidase 2' on 'eGFR'

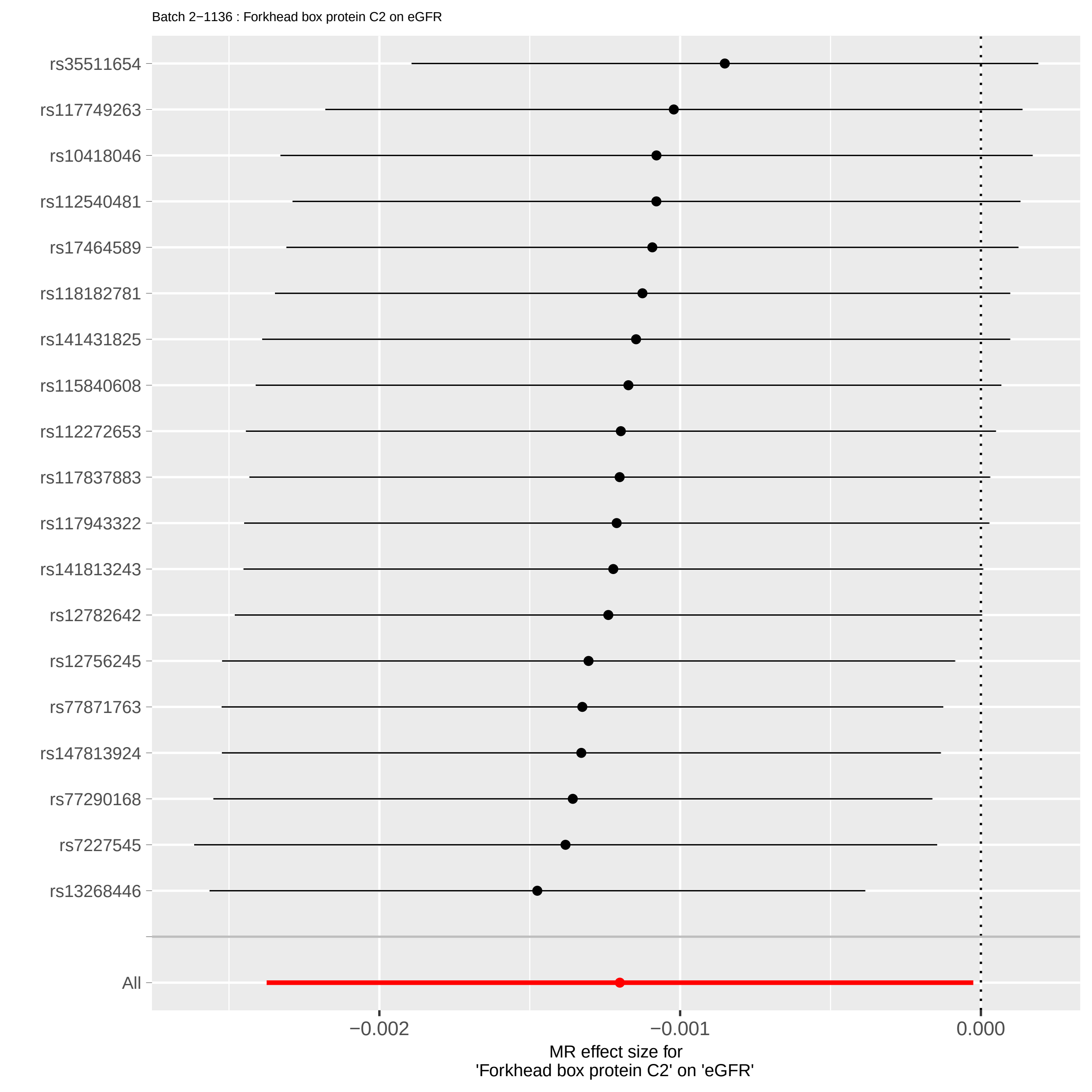

Batch 2−1136 : Forkhead box protein C2 on eGFR
rs35511654
rs117749263
rs10418046
rs112540481
rs17464589
rs118182781
rs141431825
rs115840608
rs112272653
rs117837883
rs117943322
rs141813243
rs12782642
rs12756245
rs77871763
rs147813924
rs77290168
rs7227545
rs13268446
All
−0.002
−0.001
0.000
MR effect size for
'Forkhead box protein C2' on 'eGFR'

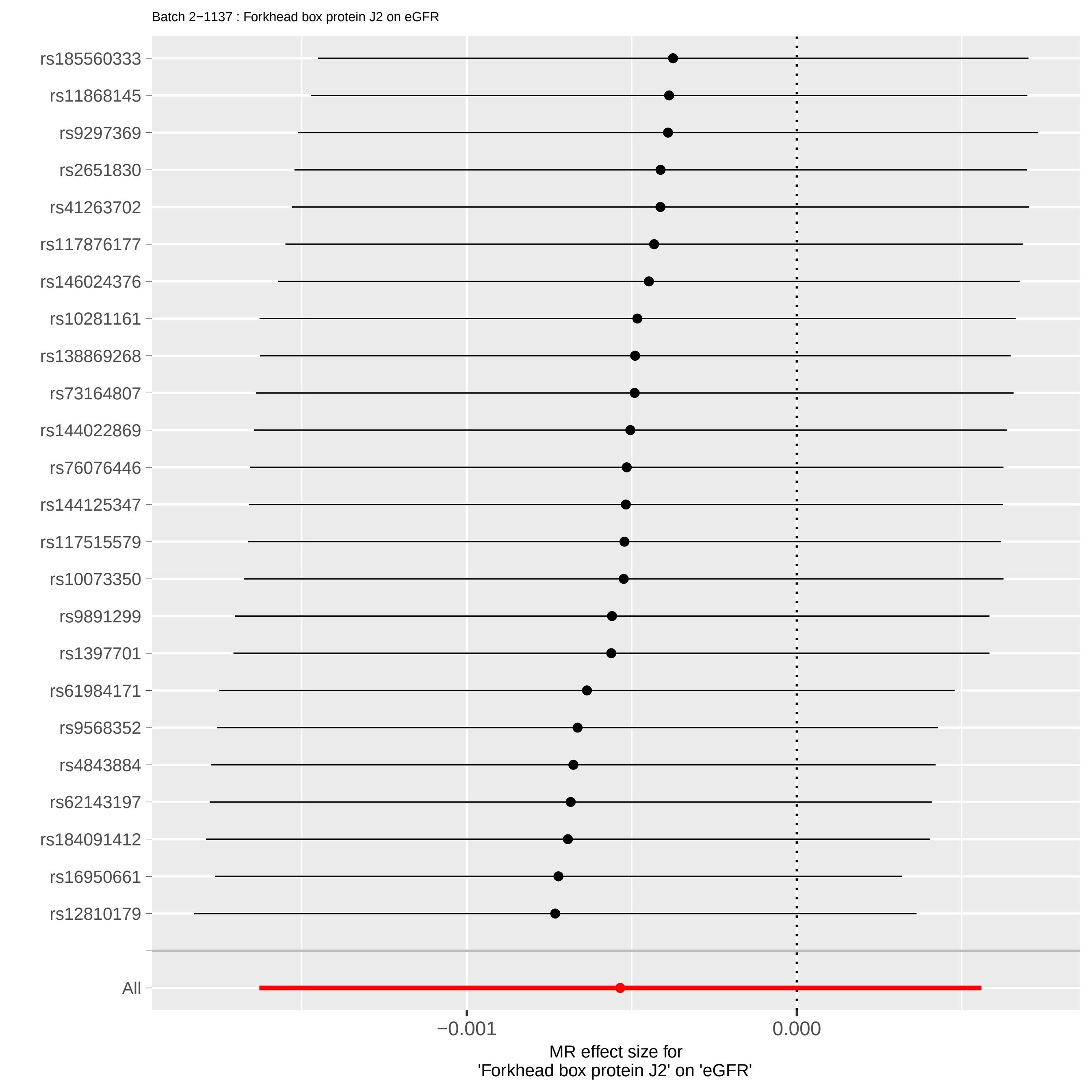

Batch 2−1137 : Forkhead box protein J2 on eGFR
rs185560333
rs11868145
rs9297369
rs2651830
rs41263702
rs117876177
rs146024376
rs10281161
rs138869268
rs73164807
rs144022869
rs76076446
rs144125347
rs117515579
rs10073350
rs9891299
rs1397701
rs61984171
rs9568352
rs4843884
rs62143197
rs184091412
rs16950661
rs12810179
All
−0.001
0.000
MR effect size for
'Forkhead box protein J2' on 'eGFR'

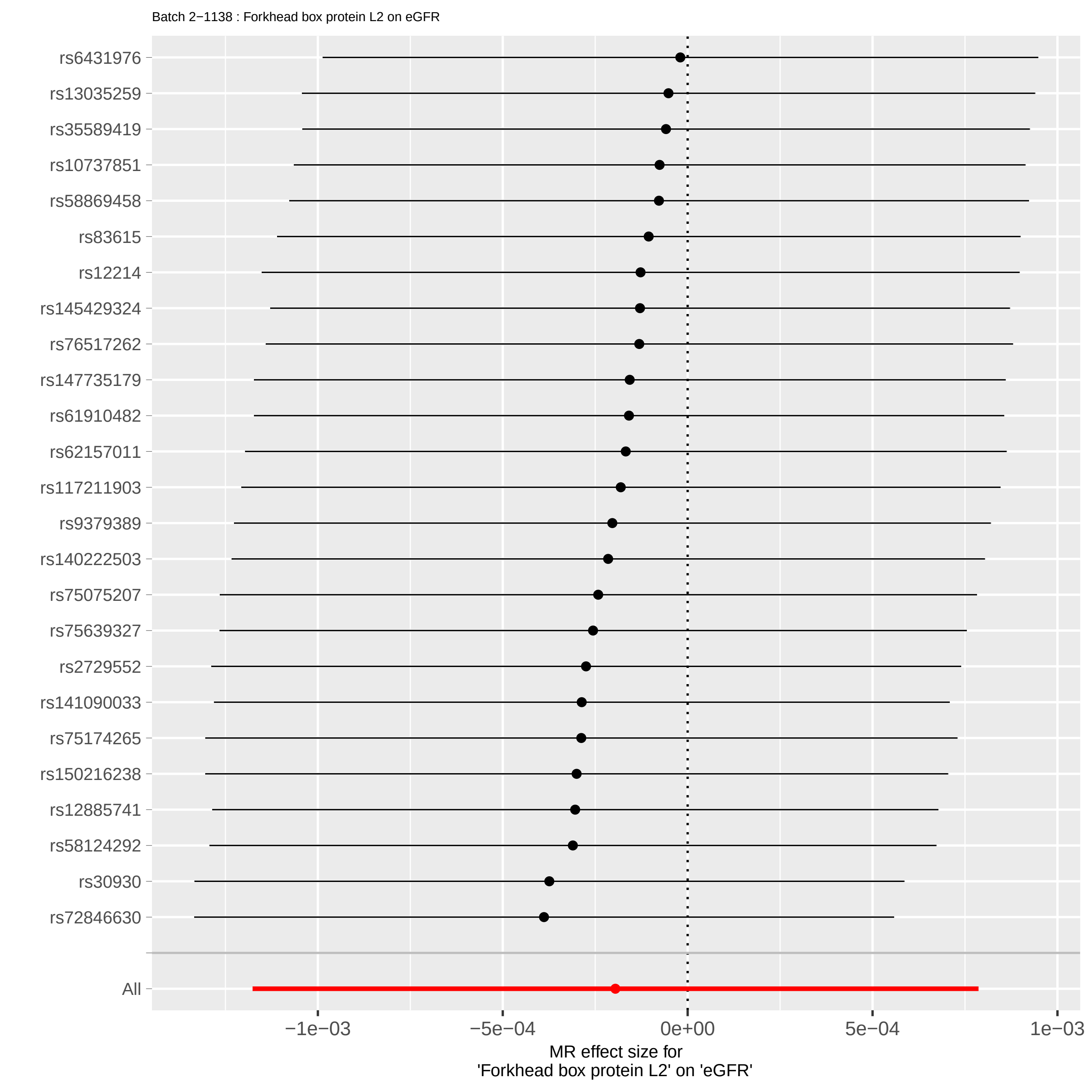

Batch 2−1138 : Forkhead box protein L2 on eGFR
rs6431976
rs13035259
rs35589419
rs10737851
rs58869458
rs83615
rs12214
rs145429324
rs76517262
rs147735179
rs61910482
rs62157011
rs117211903
rs9379389
rs140222503
rs75075207
rs75639327
rs2729552
rs141090033
rs75174265
rs150216238
rs12885741
rs58124292
rs30930
rs72846630
All
−1e−03
−5e−04
0e+00
5e−04
1e−03
MR effect size for
'Forkhead box protein L2' on 'eGFR'

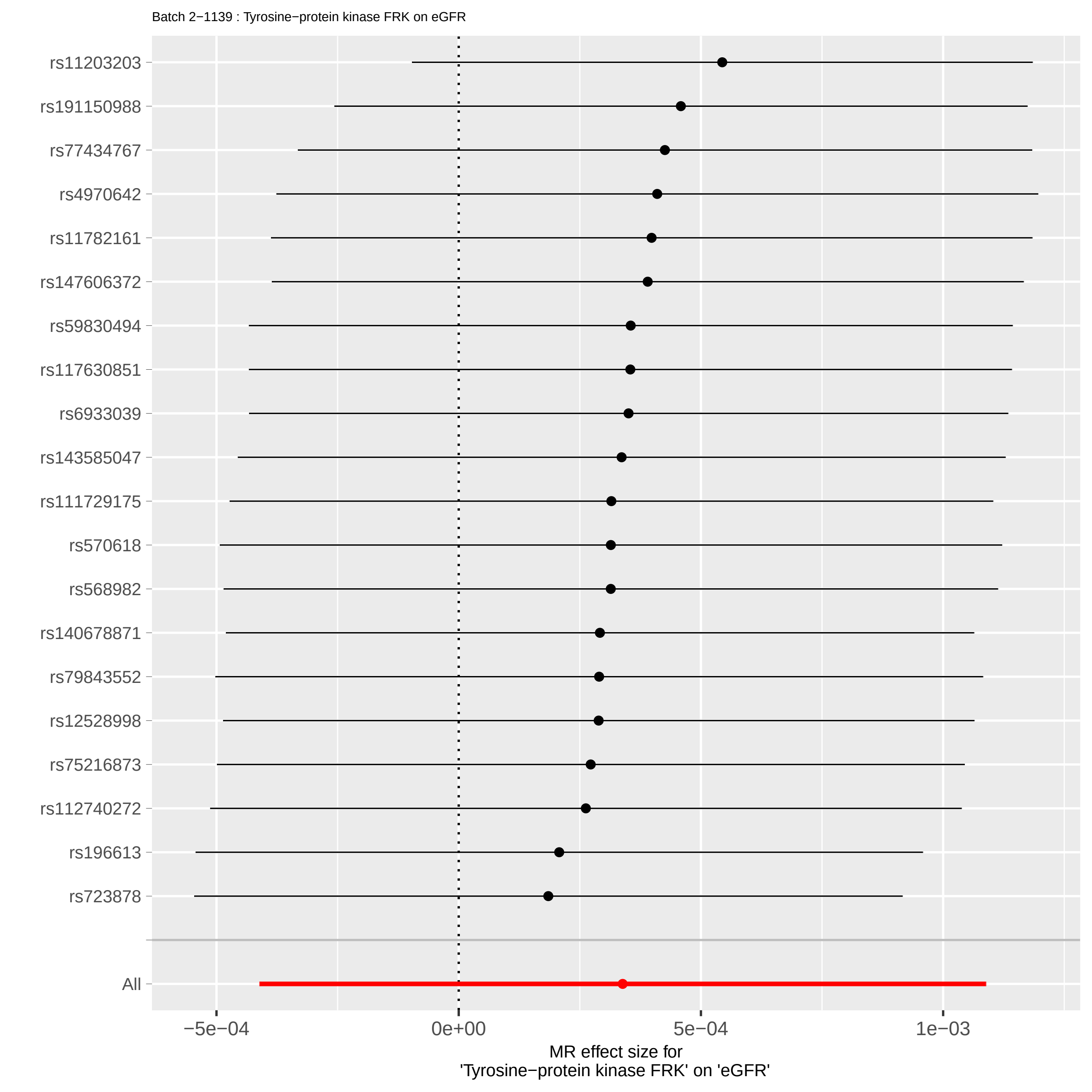

Batch 2−1139 : Tyrosine−protein kinase FRK on eGFR
rs11203203
rs191150988
rs77434767
rs4970642
rs11782161
rs147606372
rs59830494
rs117630851
rs6933039
rs143585047
rs111729175
rs570618
rs568982
rs140678871
rs79843552
rs12528998
rs75216873
rs112740272
rs196613
rs723878
All
−5e−04
0e+00
5e−04
1e−03
MR effect size for
'Tyrosine−protein kinase FRK' on 'eGFR'

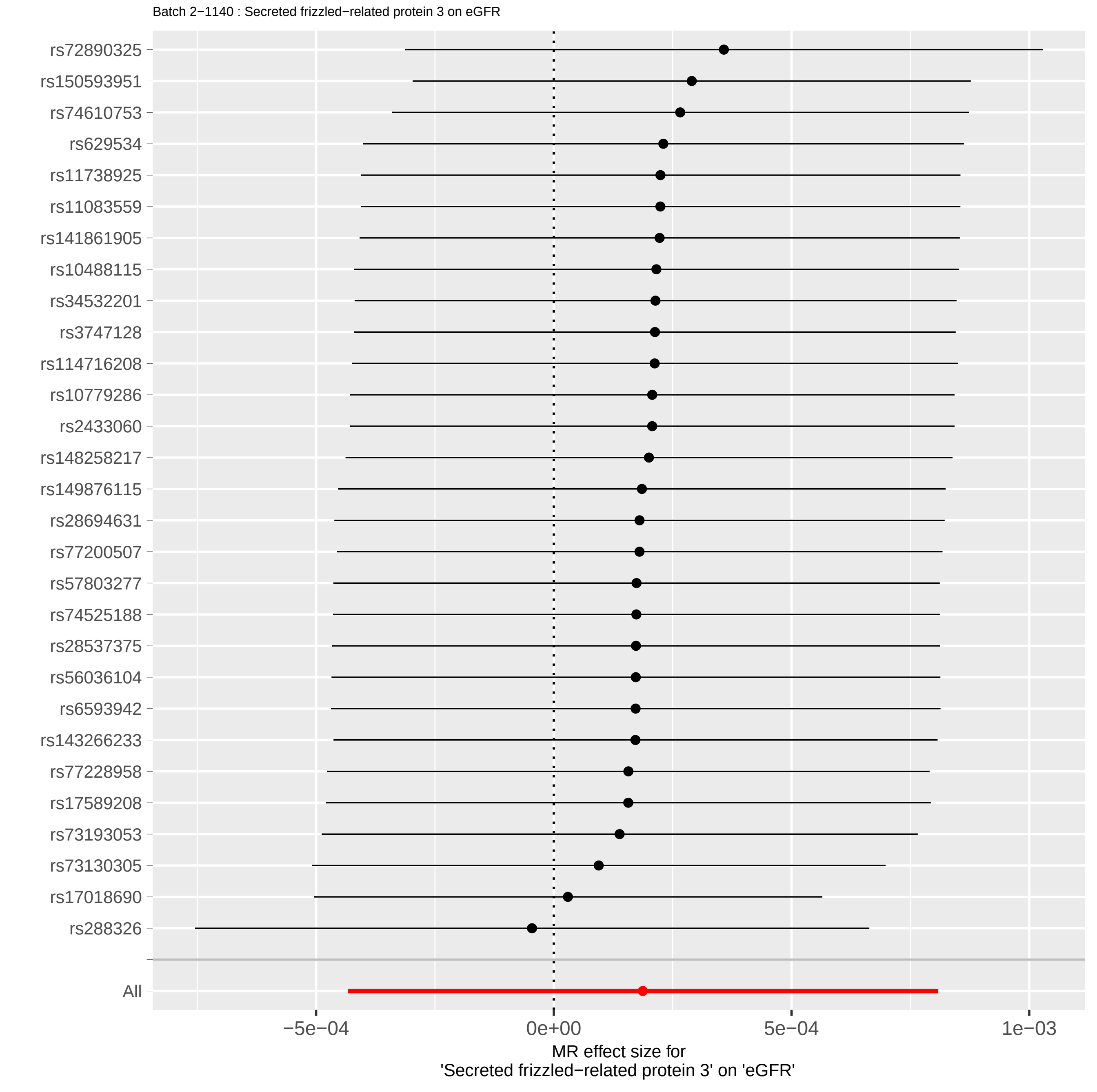
Batch 2−1140 : Secreted frizzled−related protein 3 on eGFR
rs72890325
rs150593951
rs74610753
rs629534
rs11738925
rs11083559
rs141861905
rs10488115
rs34532201
rs3747128
rs114716208
rs10779286
rs2433060
rs148258217
rs149876115
rs28694631
rs77200507
rs57803277
rs74525188
rs28537375
rs56036104
rs6593942
rs143266233
rs77228958
rs17589208
rs73193053
rs73130305
rs17018690
rs288326
All
−5e−04
0e+00
5e−04
1e−03
MR effect size for
'Secreted frizzled−related protein 3' on 'eGFR'

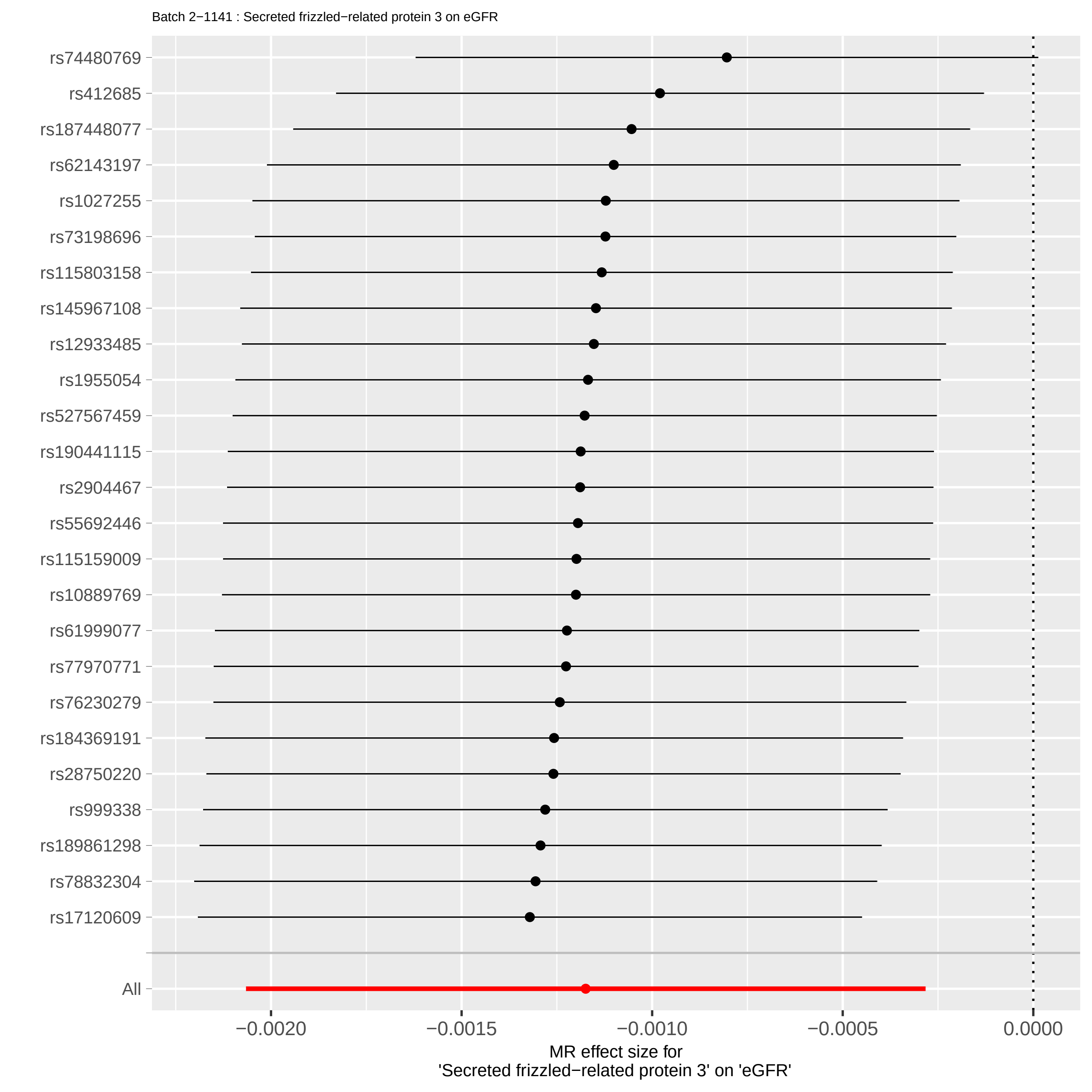

Batch 2−1141 : Secreted frizzled−related protein 3 on eGFR
rs74480769
rs412685
rs187448077
rs62143197
rs1027255
rs73198696
rs115803158
rs145967108
rs12933485
rs1955054
rs527567459
rs190441115
rs2904467
rs55692446
rs115159009
rs10889769
rs61999077
rs77970771
rs76230279
rs184369191
rs28750220
rs999338
rs189861298
rs78832304
rs17120609
All
−0.0020
−0.0015
−0.0010
−0.0005
0.0000
MR effect size for
'Secreted frizzled−related protein 3' on 'eGFR'

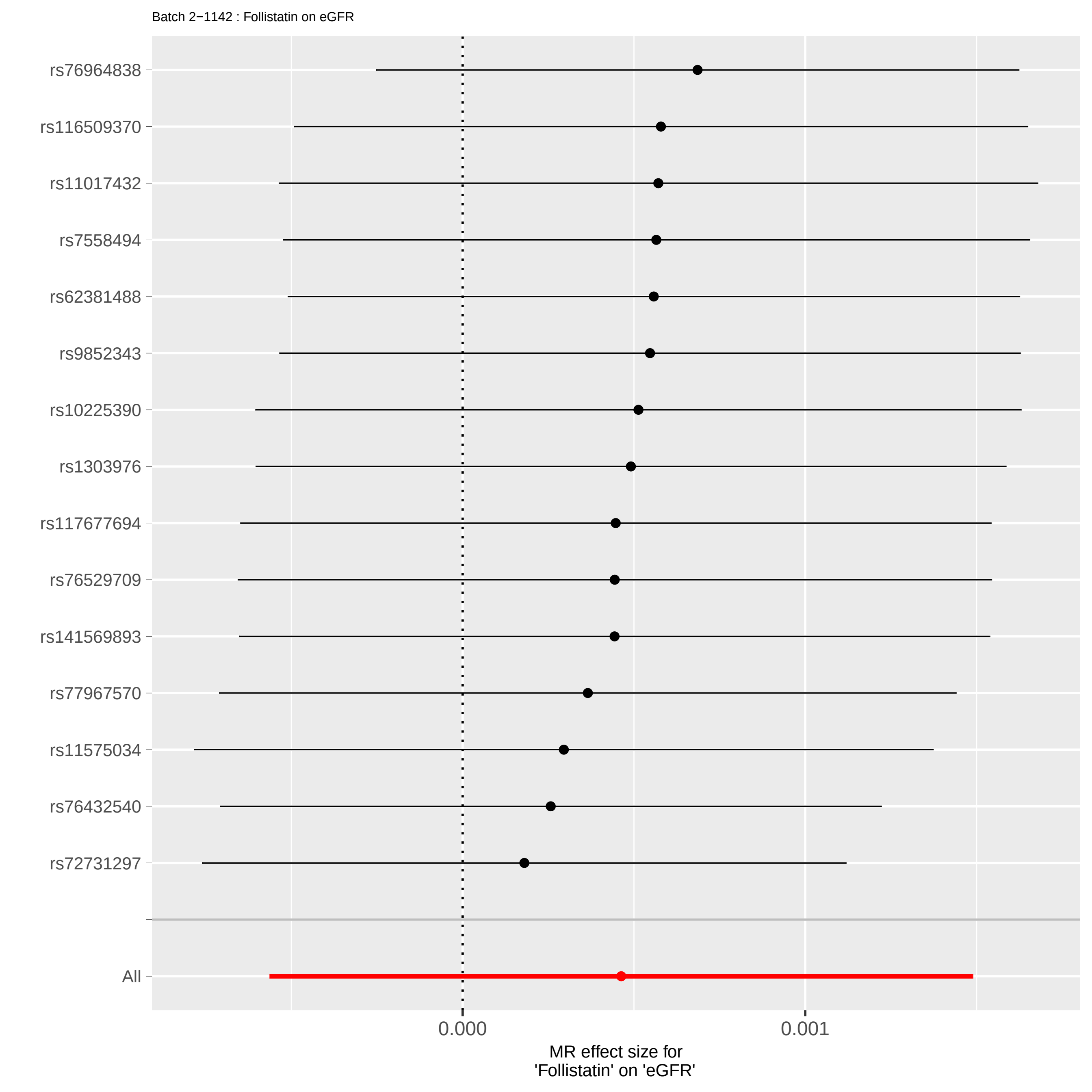
Batch 2−1142 : Follistatin on eGFR
rs76964838
rs116509370
rs11017432
rs7558494
rs62381488
rs9852343
rs10225390
rs1303976
rs117677694
rs76529709
rs141569893
rs77967570
rs11575034
rs76432540
rs72731297
All
0.000
0.001
MR effect size for
'Follistatin' on 'eGFR'

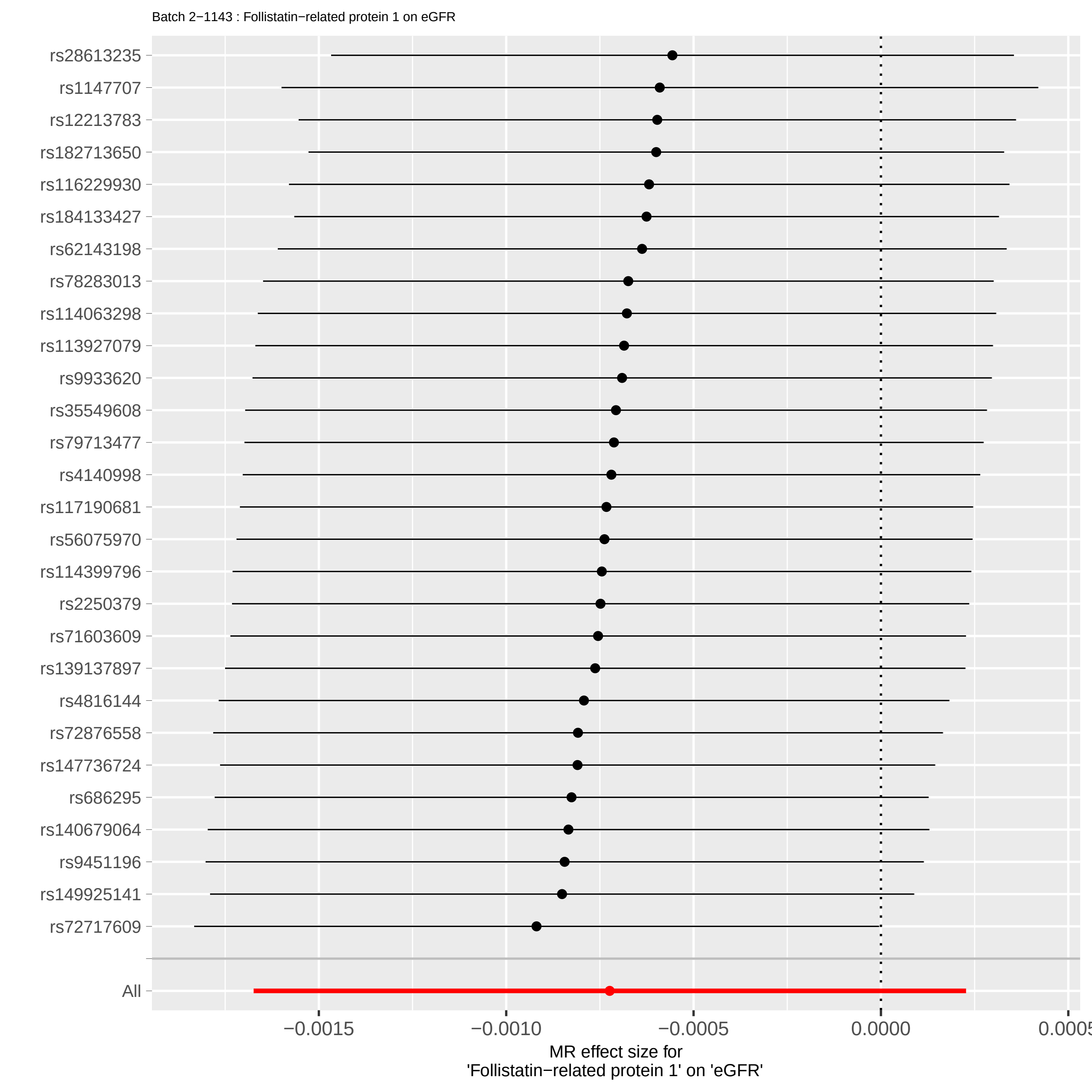

Batch 2−1143 : Follistatin−related protein 1 on eGFR
rs28613235
rs1147707
rs12213783
rs182713650
rs116229930
rs184133427
rs62143198
rs78283013
rs114063298
rs113927079
rs9933620
rs35549608
rs79713477
rs4140998
rs117190681
rs56075970
rs114399796
rs2250379
rs71603609
rs139137897
rs4816144
rs72876558
rs147736724
rs686295
rs140679064
rs9451196
rs149925141
rs72717609
All
−0.0015
−0.0010
−0.0005
0.0000
MR effect size for
'Follistatin−related protein 1' on 'eGFR'

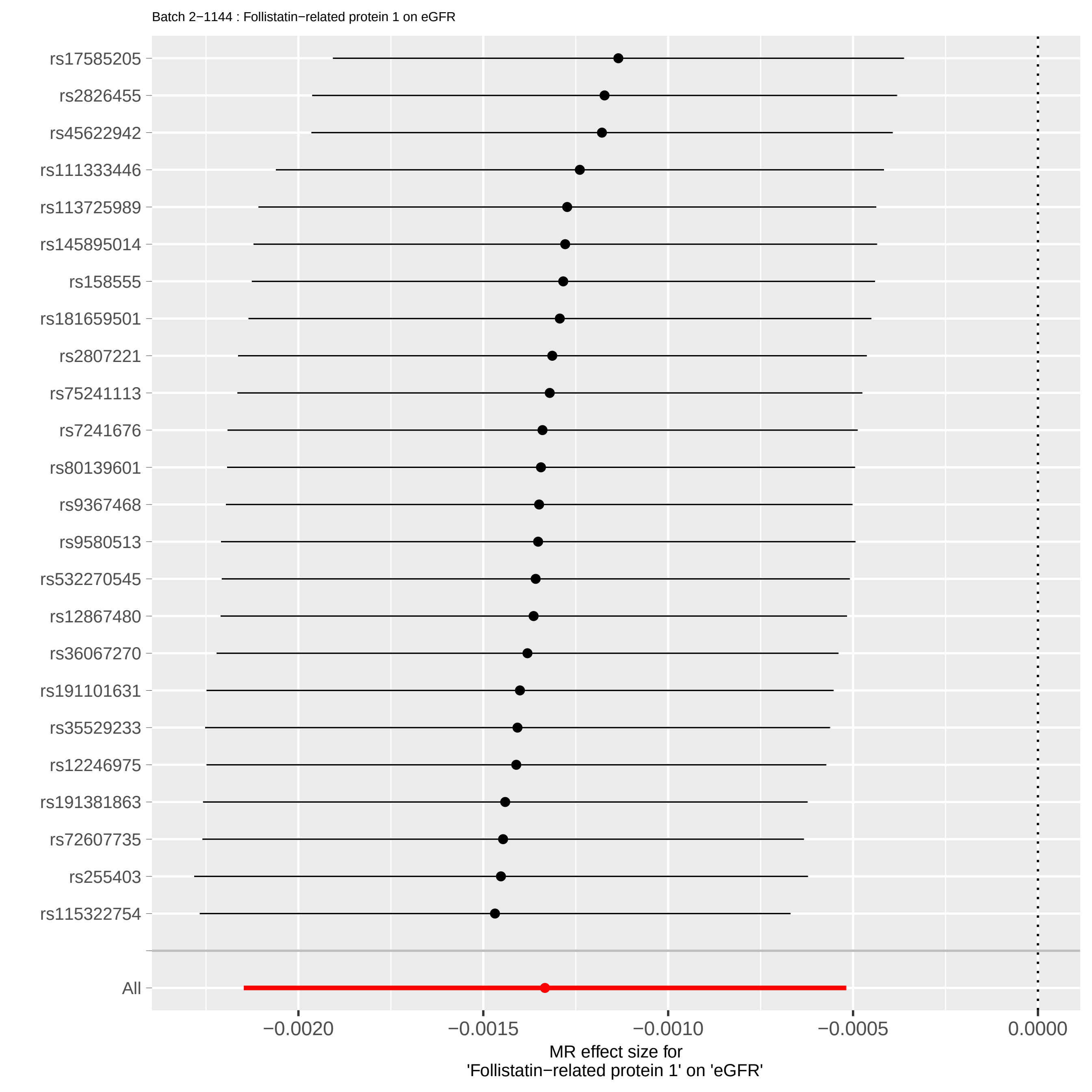

Batch 2−1144 : Follistatin−related protein 1 on eGFR
rs17585205
rs2826455
rs45622942
rs111333446
rs113725989
rs145895014
rs158555
rs181659501
rs2807221
rs75241113
rs7241676
rs80139601
rs9367468
rs9580513
rs532270545
rs12867480
rs36067270
rs191101631
rs35529233
rs12246975
rs191381863
rs72607735
rs255403
rs115322754
All
−0.0020
−0.0015
−0.0010
−0.0005
0.0000
MR effect size for
'Follistatin−related protein 1' on 'eGFR'

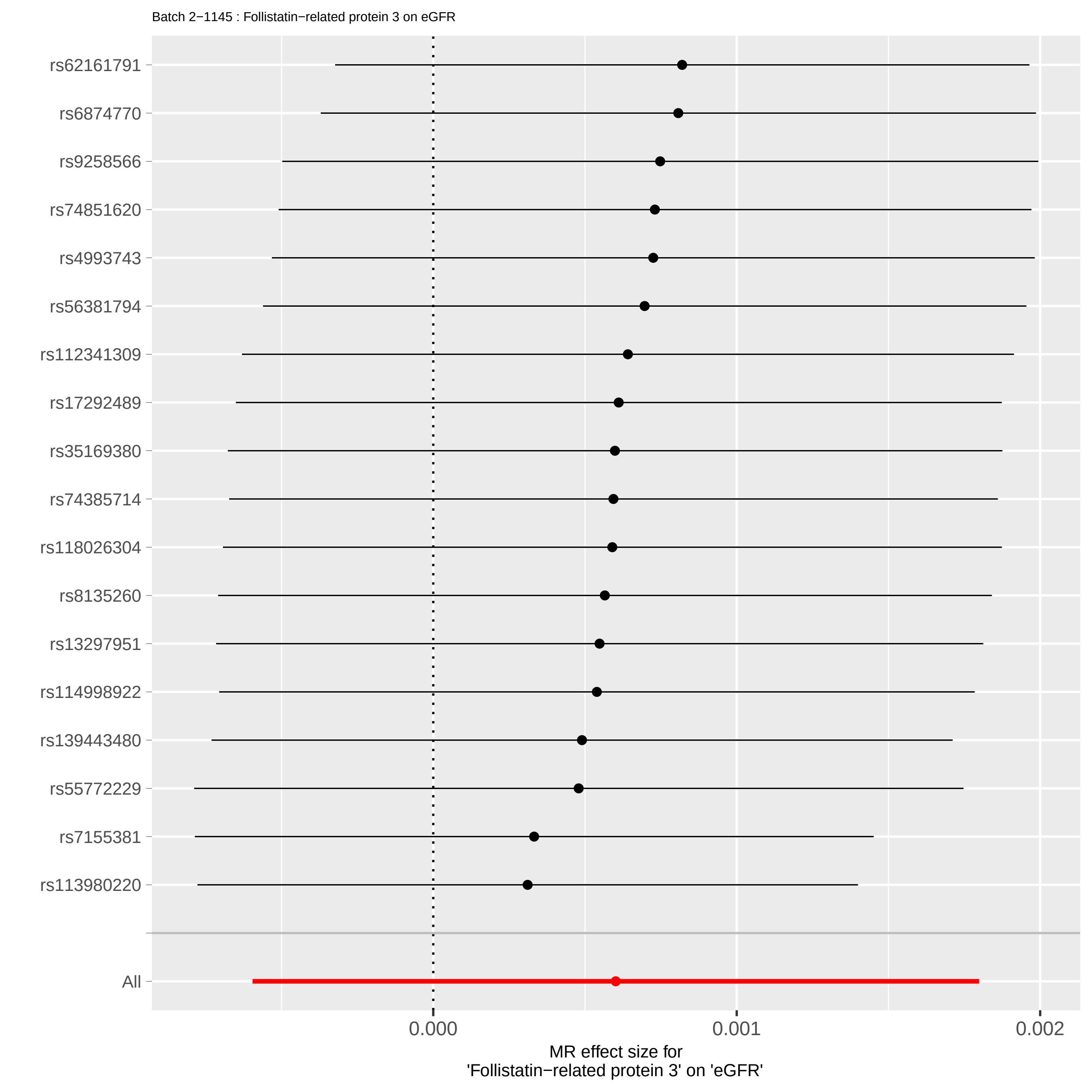
Batch 2−1145 : Follistatin−related protein 3 on eGFR
rs62161791
rs6874770
rs9258566
rs74851620
rs4993743
rs56381794
rs112341309
rs17292489
rs35169380
rs74385714
rs118026304
rs8135260
rs13297951
rs114998922
rs139443480
rs55772229
rs7155381
rs113980220
All
0.000
0.001
0.002
MR effect size for
'Follistatin−related protein 3' on 'eGFR'

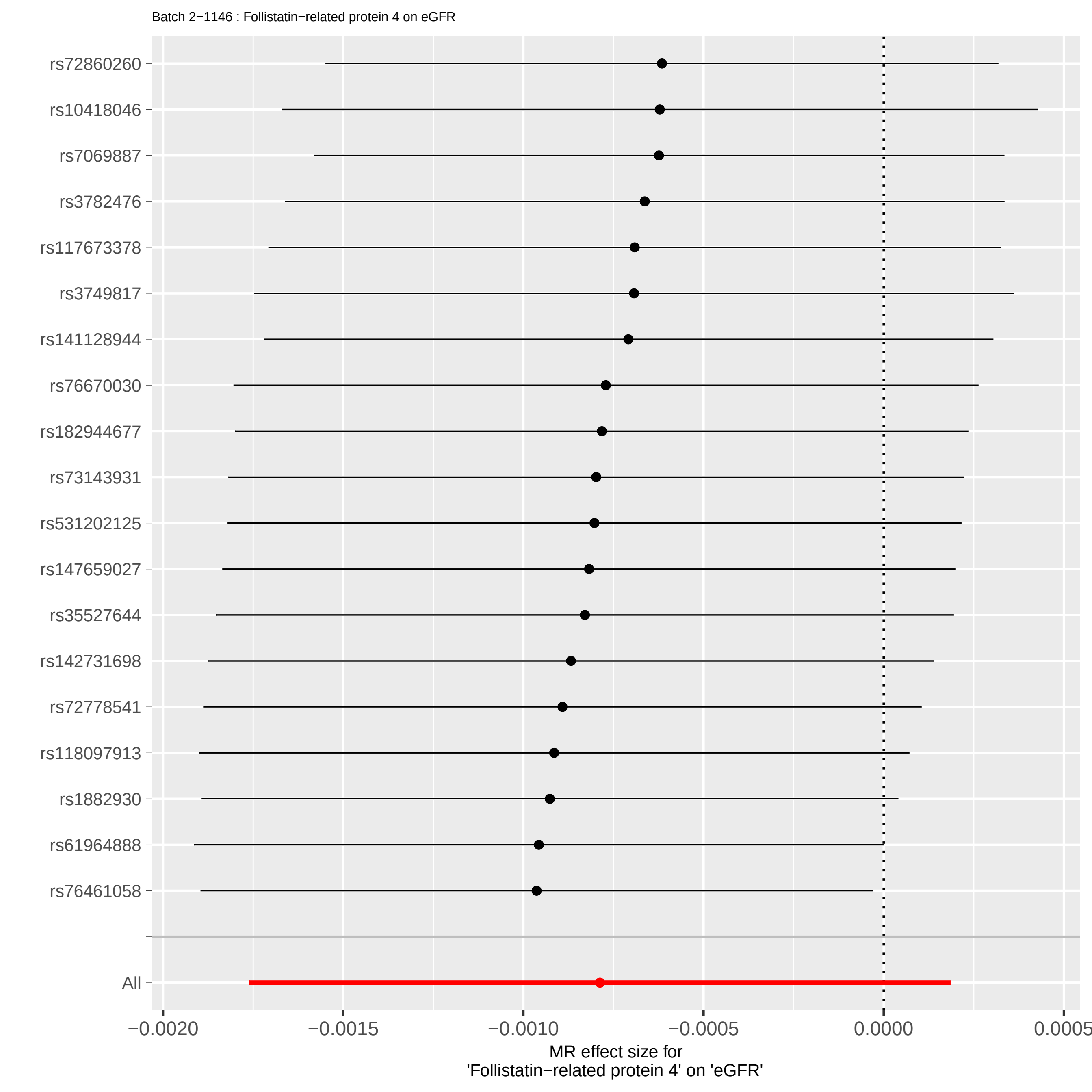

Batch 2−1146 : Follistatin−related protein 4 on eGFR
rs72860260
rs10418046
rs7069887
rs3782476
rs117673378
rs3749817
rs141128944
rs76670030
rs182944677
rs73143931
rs531202125
rs147659027
rs35527644
rs142731698
rs72778541
rs118097913
rs1882930
rs61964888
rs76461058
All
−0.0020
−0.0015
−0.0010
−0.0005
0.0000
0.0005
MR effect size for
'Follistatin−related protein 4' on 'eGFR'

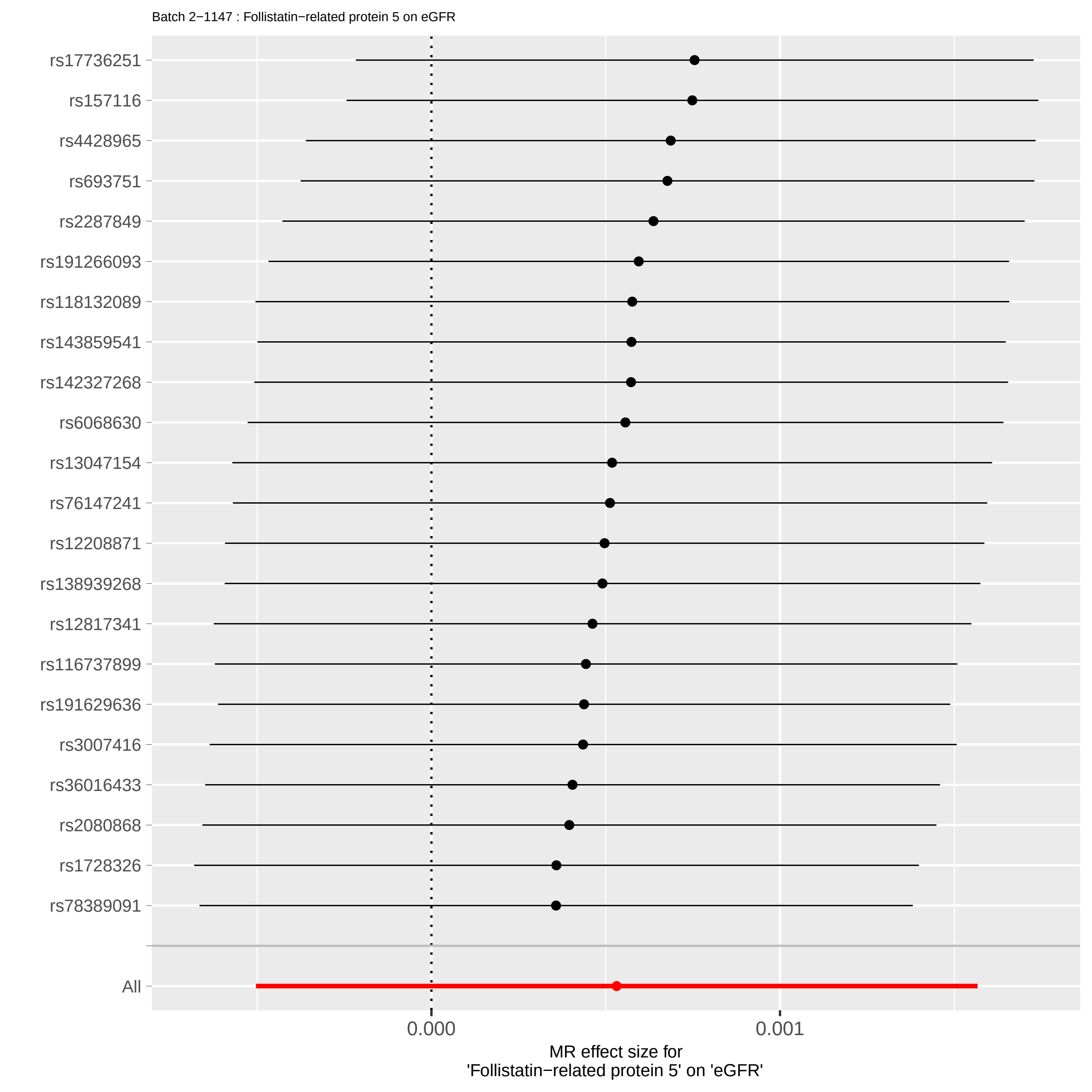

Batch 2−1147 : Follistatin−related protein 5 on eGFR
rs17736251
rs157116
rs4428965
rs693751
rs2287849
rs191266093
rs118132089
rs143859541
rs142327268
rs6068630
rs13047154
rs76147241
rs12208871
rs138939268
rs12817341
rs116737899
rs191629636
rs3007416
rs36016433
rs2080868
rs1728326
rs78389091
All
0.000
0.001
MR effect size for
'Follistatin−related protein 5' on 'eGFR'

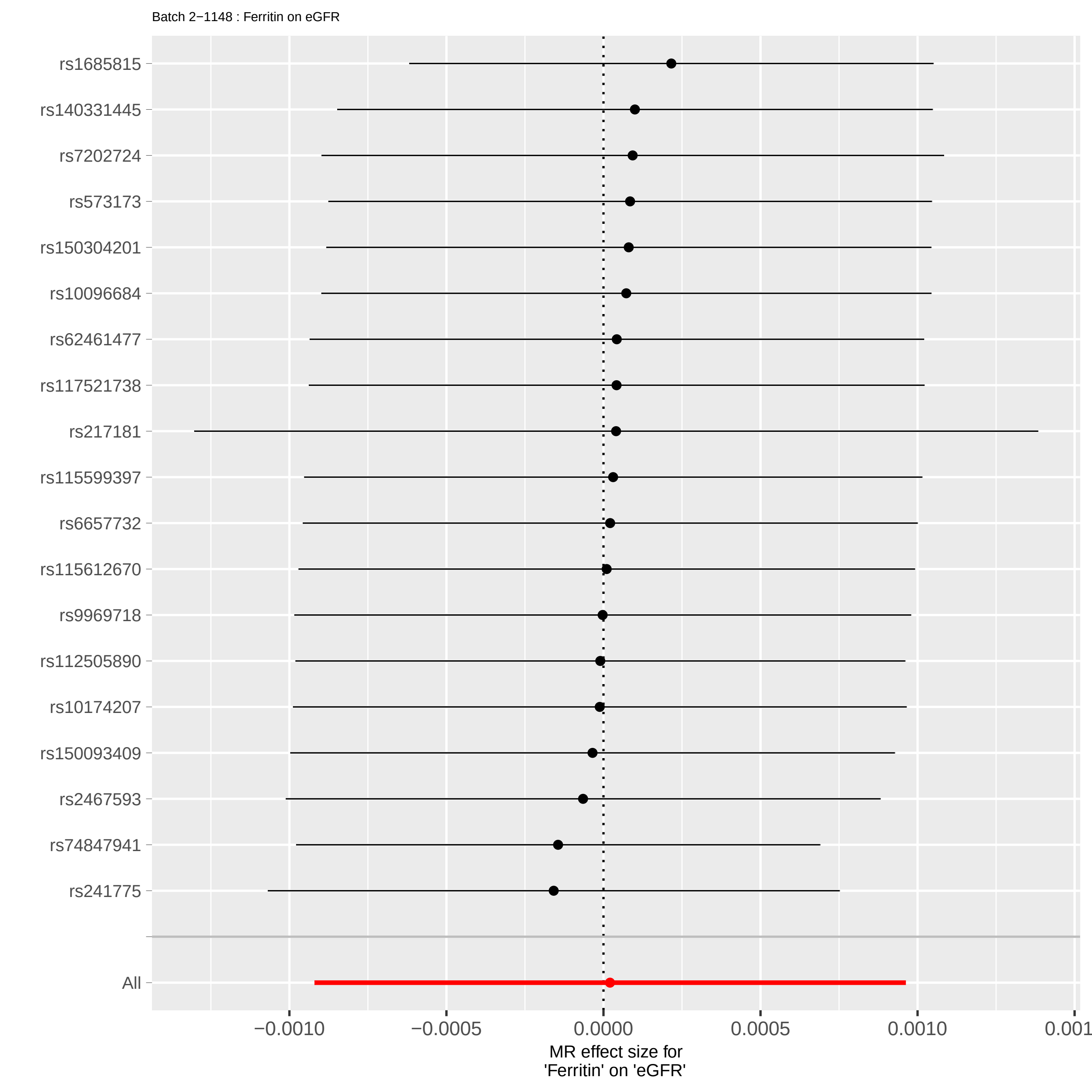

Batch 2−1148 : Ferritin on eGFR
rs1685815
rs140331445
rs7202724
rs573173
rs150304201
rs10096684
rs62461477
rs117521738
rs217181
rs115599397
rs6657732
rs115612670
rs9969718
rs112505890
rs10174207
rs150093409
rs2467593
rs74847941
rs241775
All
−0.0010
−0.0005
0.0000
0.0005
0.0010
0.001
MR effect size for
'Ferritin' on 'eGFR'

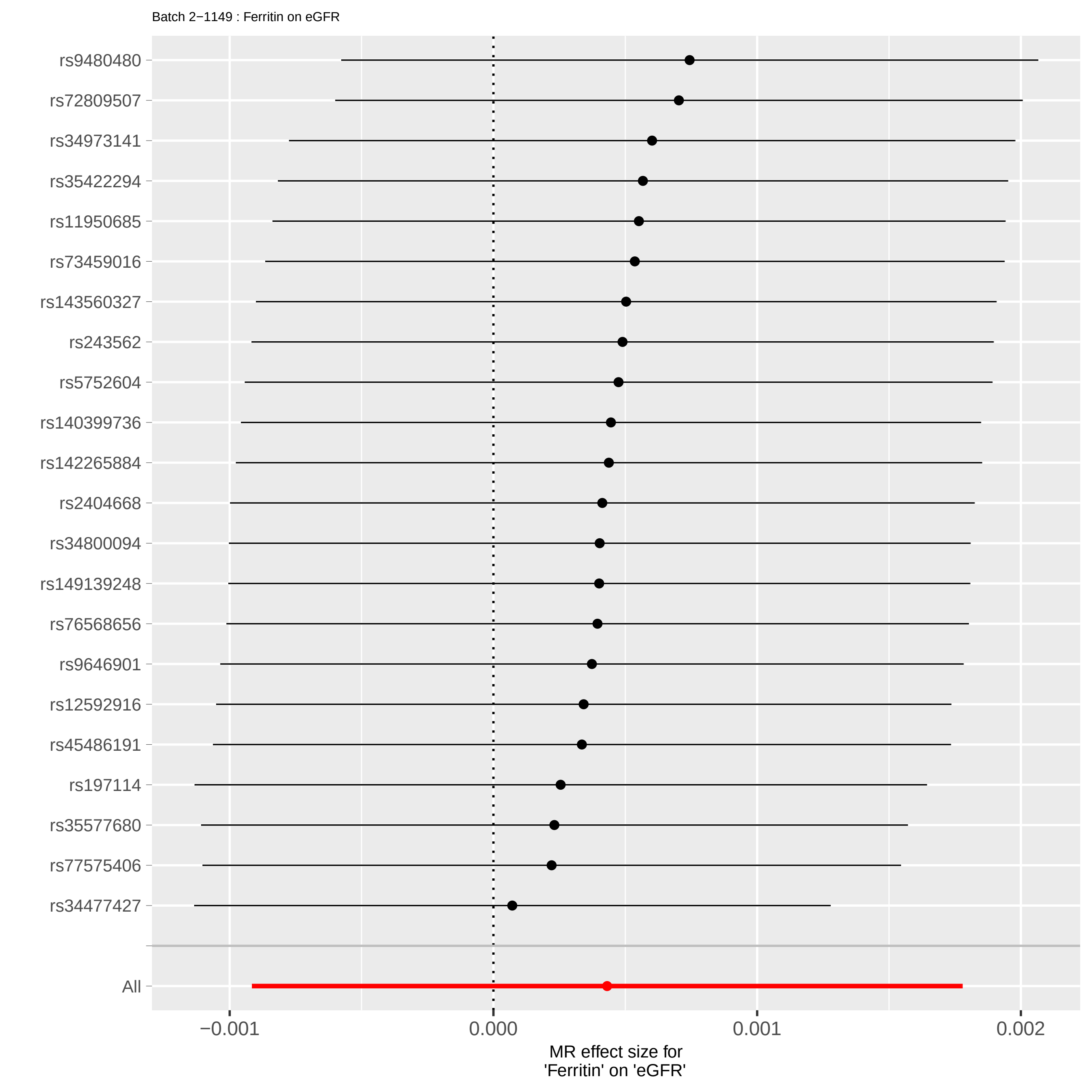

Batch 2−1149 : Ferritin on eGFR
rs9480480
rs72809507
rs34973141
rs35422294
rs11950685
rs73459016
rs143560327
rs243562
rs5752604
rs140399736
rs142265884
rs2404668
rs34800094
rs149139248
rs76568656
rs9646901
rs12592916
rs45486191
rs197114
rs35577680
rs77575406
rs34477427
All
−0.001
0.000
0.001
0.002
MR effect size for
'Ferritin' on 'eGFR'

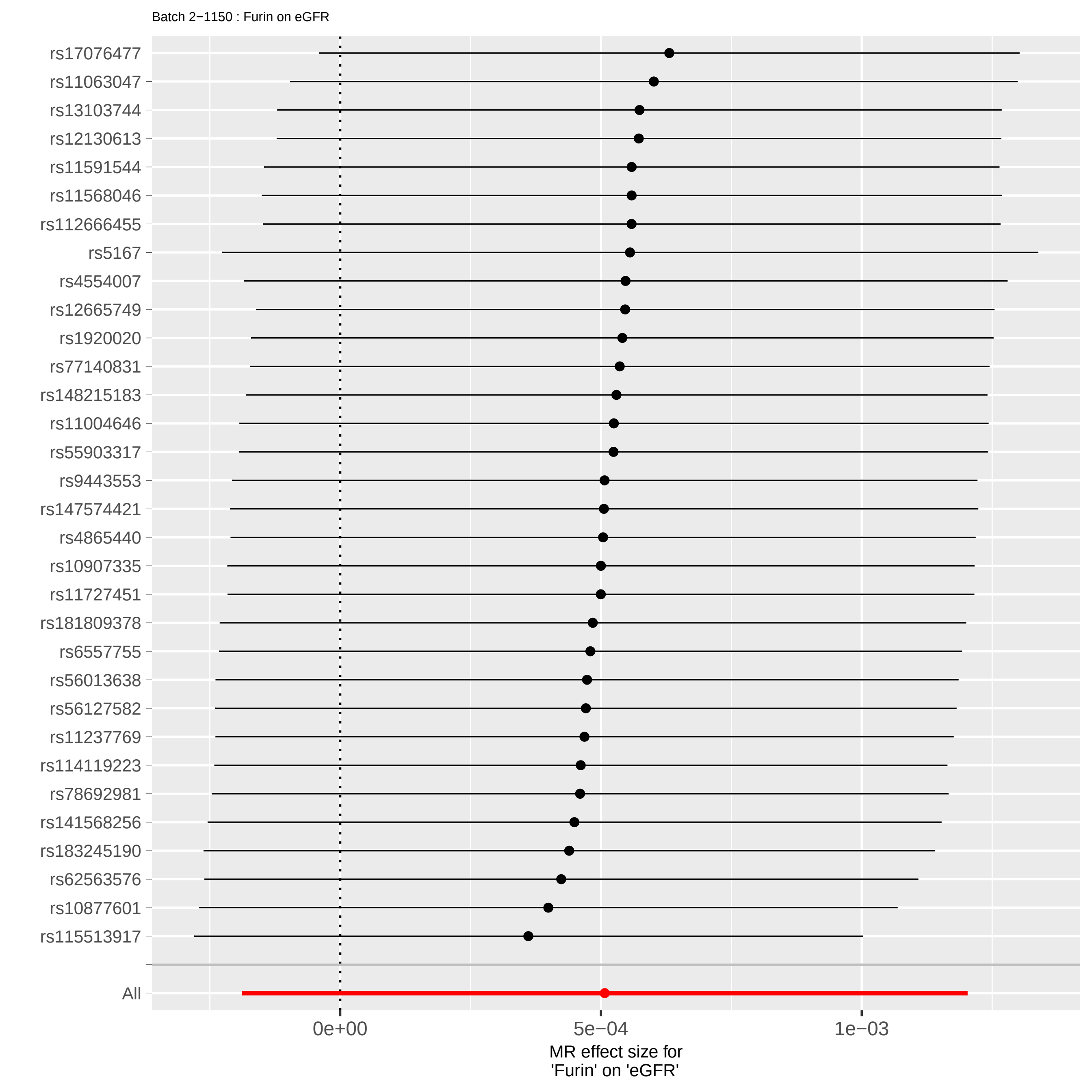

Batch 2−1150 : Furin on eGFR
rs17076477
rs11063047
rs13103744
rs12130613
rs11591544
rs11568046
rs112666455
rs5167
rs4554007
rs12665749
rs1920020
rs77140831
rs148215183
rs11004646
rs55903317
rs9443553
rs147574421
rs4865440
rs10907335
rs11727451
rs181809378
rs6557755
rs56013638
rs56127582
rs11237769
rs114119223
rs78692981
rs141568256
rs183245190
rs62563576
rs10877601
rs115513917
All
0e+00
5e−04
1e−03
MR effect size for
'Furin' on 'eGFR'

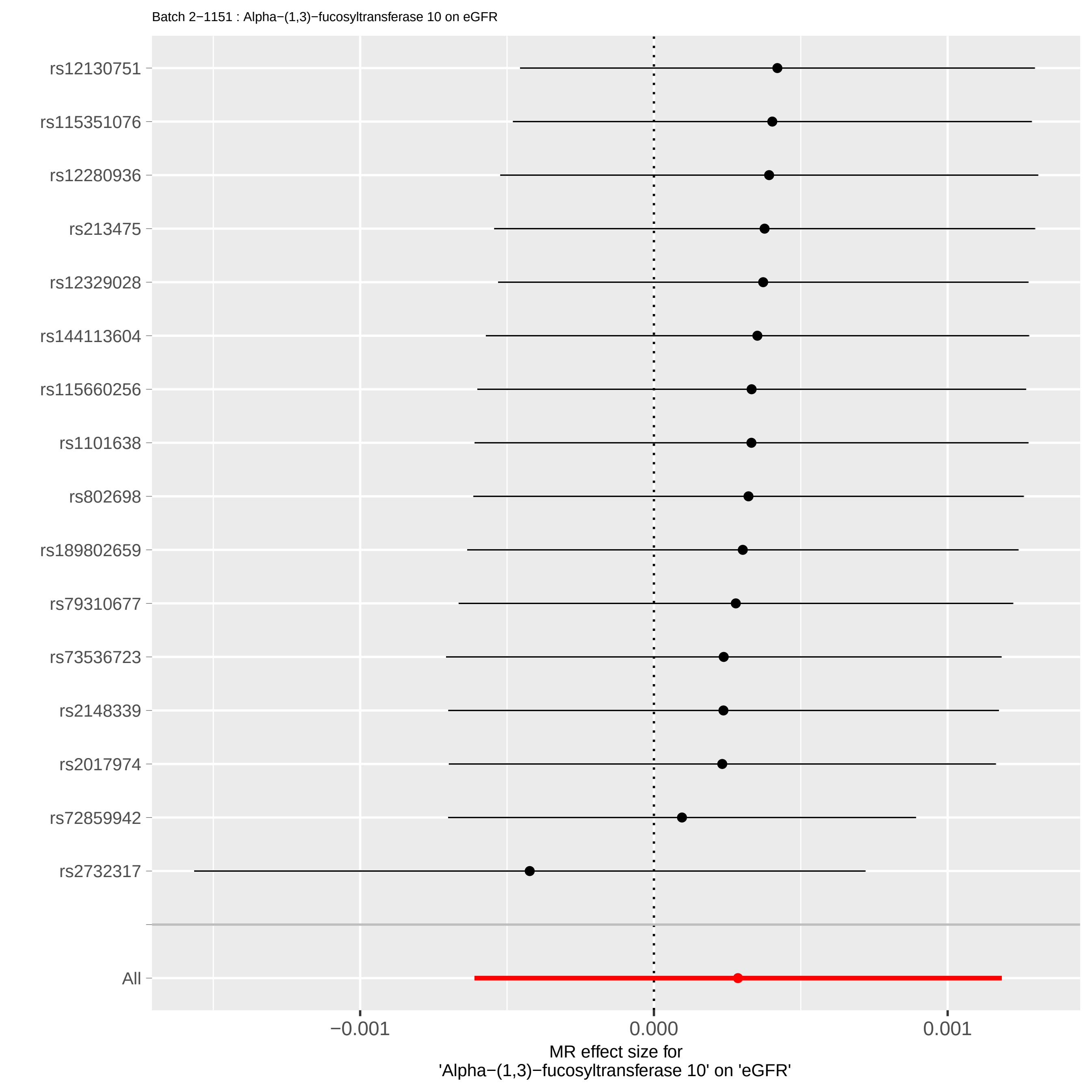
Batch 2−1151 : Alpha−(1,3)−fucosyltransferase 10 on eGFR
rs12130751
rs115351076
rs12280936
rs213475
rs12329028
rs144113604
rs115660256
rs1101638
rs802698
rs189802659
rs79310677
rs73536723
rs2148339
rs2017974
rs72859942
rs2732317
All
−0.001
0.000
0.001
MR effect size for
'Alpha−(1,3)−fucosyltransferase 10' on 'eGFR'

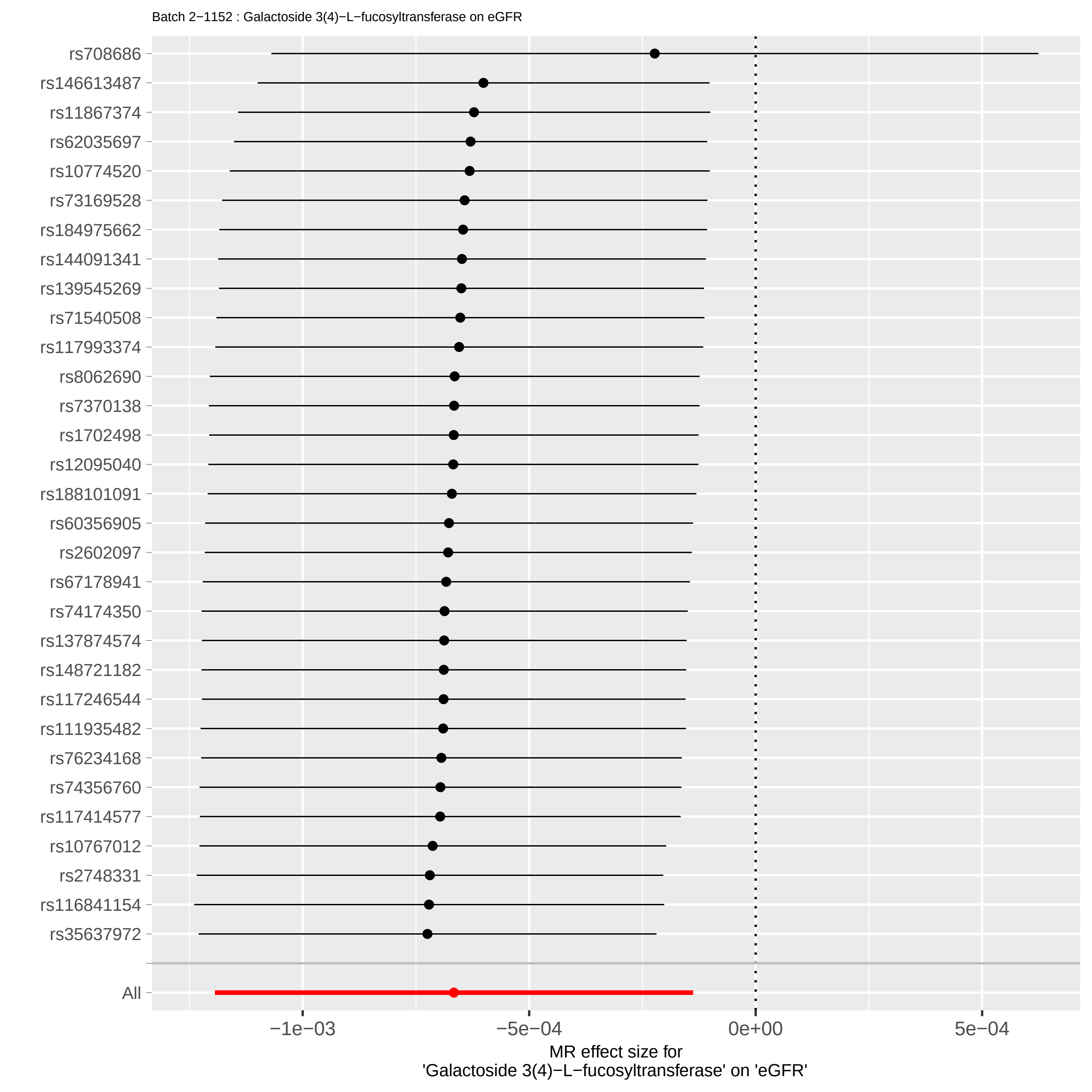
Batch 2−1152 : Galactoside 3(4)−L−fucosyltransferase on eGFR
rs708686
rs146613487
rs11867374
rs62035697
rs10774520
rs73169528
rs184975662
rs144091341
rs139545269
rs71540508
rs117993374
rs8062690
rs7370138
rs1702498
rs12095040
rs188101091
rs60356905
rs2602097
rs67178941
rs74174350
rs137874574
rs148721182
rs117246544
rs111935482
rs76234168
rs74356760
rs117414577
rs10767012
rs2748331
rs116841154
rs35637972
All
−1e−03
−5e−04
0e+00
5e−04
MR effect size for
'Galactoside 3(4)−L−fucosyltransferase' on 'eGFR'

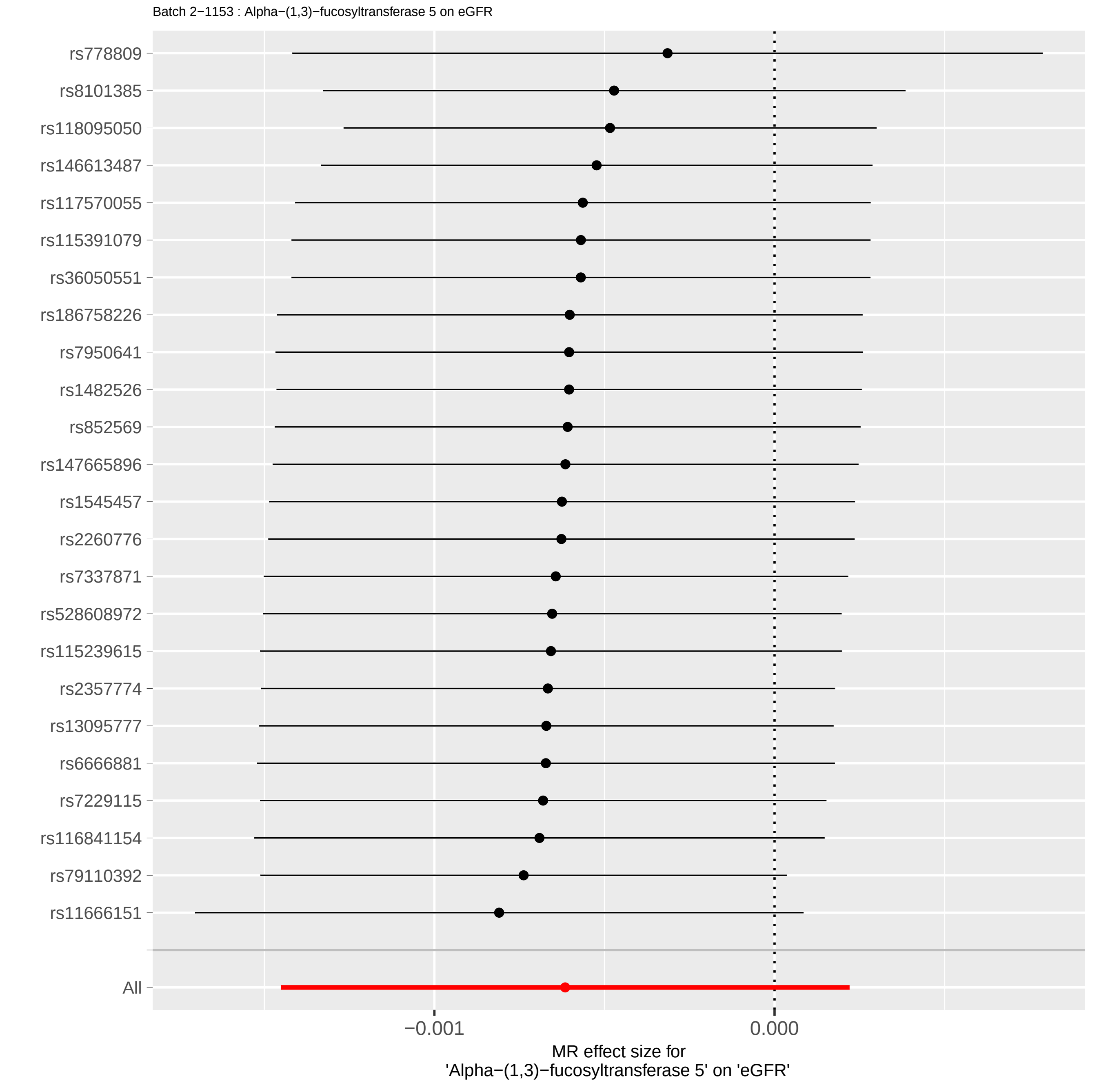

Batch 2−1153 : Alpha−(1,3)−fucosyltransferase 5 on eGFR
rs778809
rs8101385
rs118095050
rs146613487
rs117570055
rs115391079
rs36050551
rs186758226
rs7950641
rs1482526
rs852569
rs147665896
rs1545457
rs2260776
rs7337871
rs528608972
rs115239615
rs2357774
rs13095777
rs6666881
rs7229115
rs116841154
rs79110392
rs11666151
All
−0.001
0.000
MR effect size for
'Alpha−(1,3)−fucosyltransferase 5' on 'eGFR'

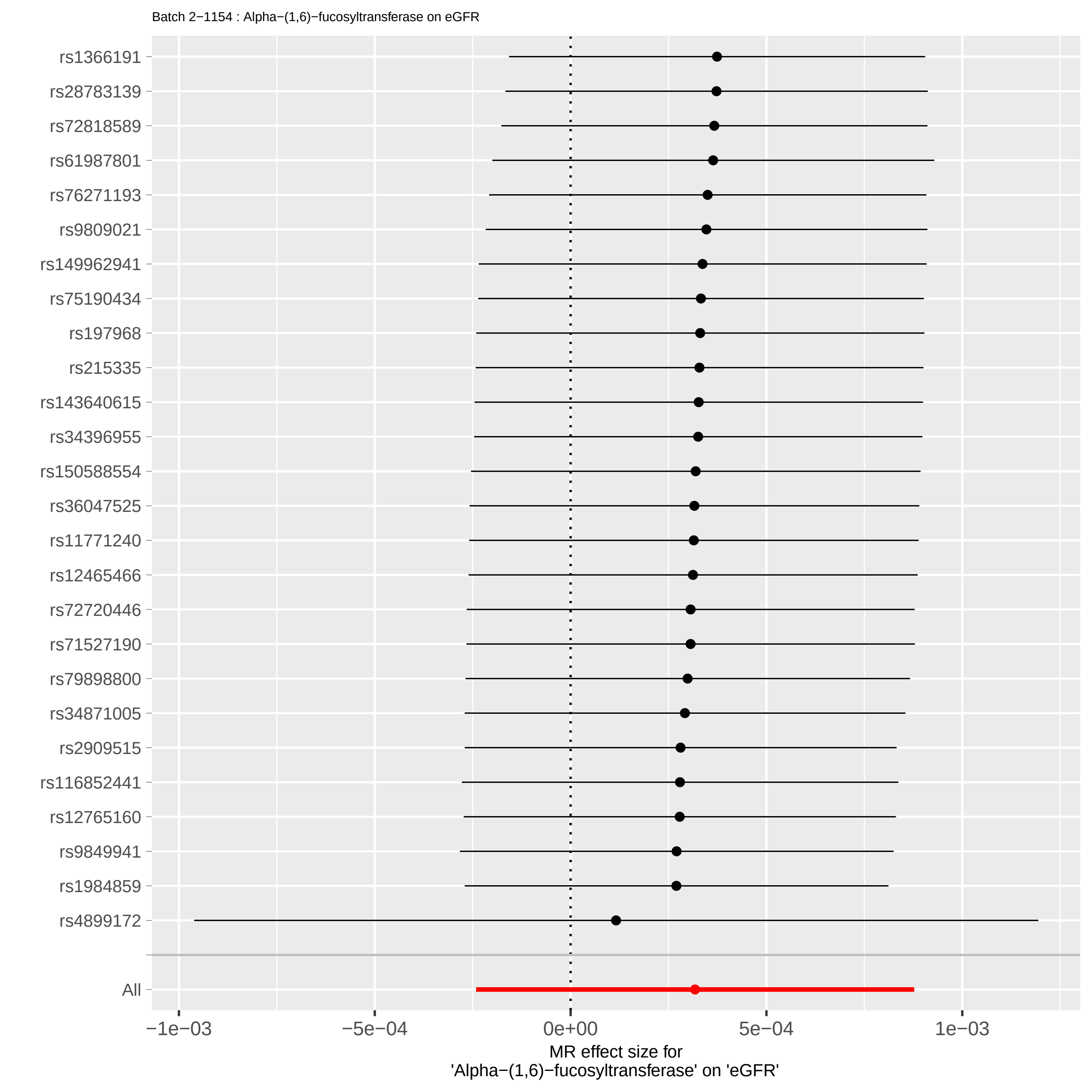

Batch 2−1154 : Alpha−(1,6)−fucosyltransferase on eGFR
rs1366191
rs28783139
rs72818589
rs61987801
rs76271193
rs9809021
rs149962941
rs75190434
rs197968
rs215335
rs143640615
rs34396955
rs150588554
rs36047525
rs11771240
rs12465466
rs72720446
rs71527190
rs79898800
rs34871005
rs2909515
rs116852441
rs12765160
rs9849941
rs1984859
rs4899172
All
−1e−03
−5e−04
0e+00
5e−04
1e−03
MR effect size for
'Alpha−(1,6)−fucosyltransferase' on 'eGFR'

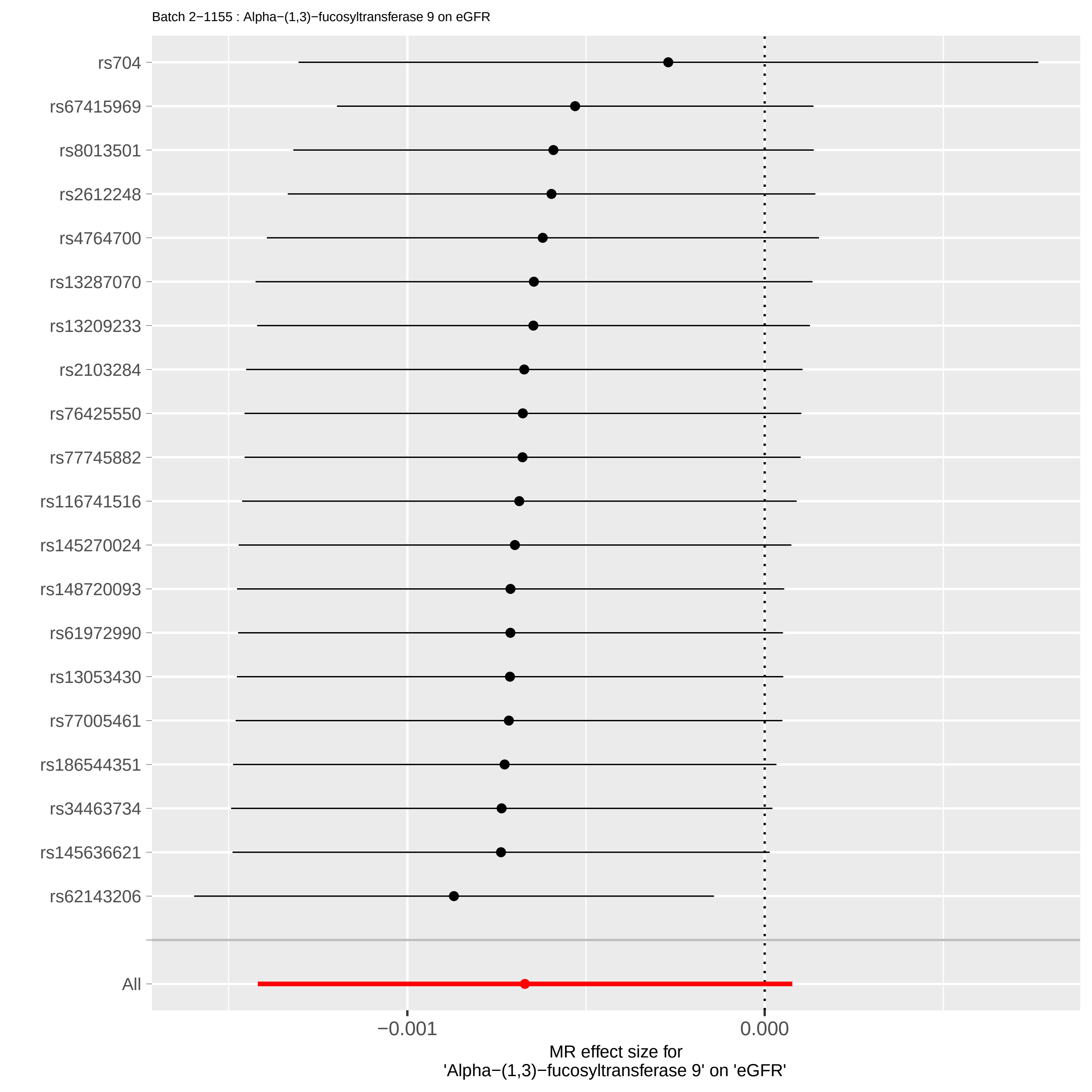

Batch 2−1155 : Alpha−(1,3)−fucosyltransferase 9 on eGFR
rs704
rs67415969
rs8013501
rs2612248
rs4764700
rs13287070
rs13209233
rs2103284
rs76425550
rs77745882
rs116741516
rs145270024
rs148720093
rs61972990
rs13053430
rs77005461
rs186544351
rs34463734
rs145636621
rs62143206
All
−0.001
0.000
MR effect size for
'Alpha−(1,3)−fucosyltransferase 9' on 'eGFR'

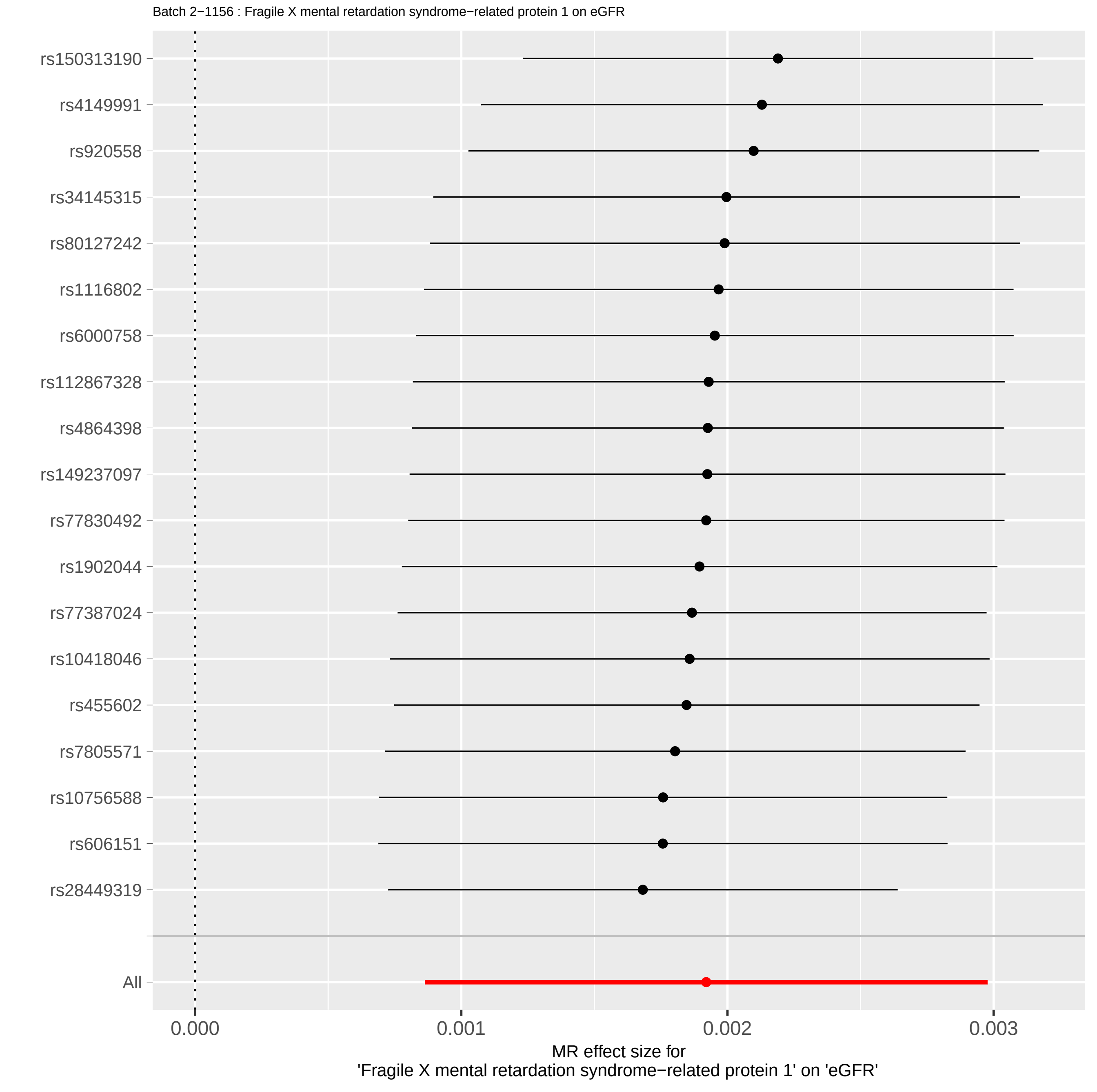
Batch 2−1156 : Fragile X mental retardation syndrome−related protein 1 on eGFR
rs150313190
rs4149991
rs920558
rs34145315
rs80127242
rs1116802
rs6000758
rs112867328
rs4864398
rs149237097
rs77830492
rs1902044
rs77387024
rs10418046
rs455602
rs7805571
rs10756588
rs606151
rs28449319
All
0.000
0.001
0.002
0.003
MR effect size for
'Fragile X mental retardation syndrome−related protein 1' on 'eGFR'

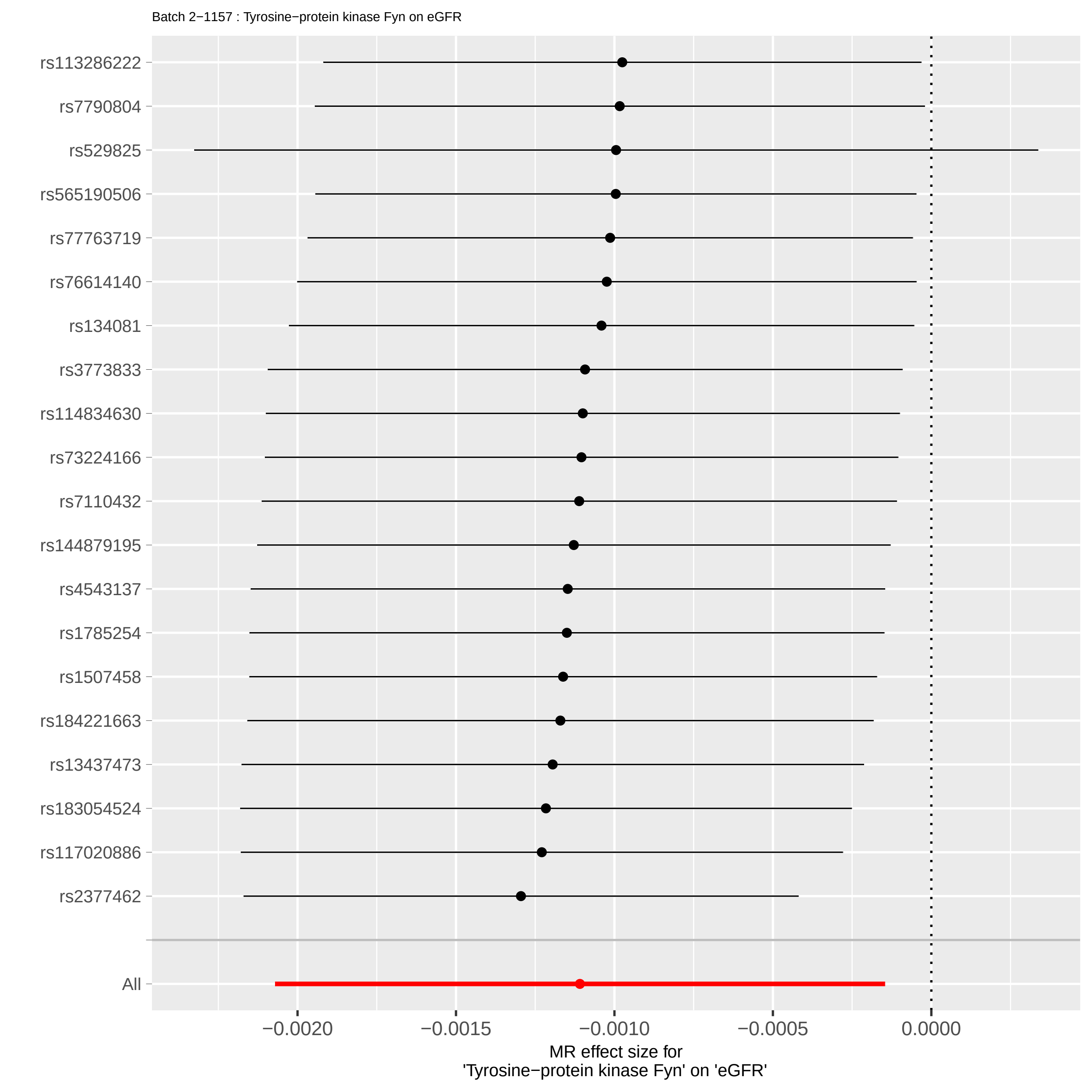

Batch 2−1157 : Tyrosine−protein kinase Fyn on eGFR
rs113286222
rs7790804
rs529825
rs565190506
rs77763719
rs76614140
rs134081
rs3773833
rs114834630
rs73224166
rs7110432
rs144879195
rs4543137
rs1785254
rs1507458
rs184221663
rs13437473
rs183054524
rs117020886
rs2377462
All
−0.0020
−0.0015
−0.0010
−0.0005
0.0000
MR effect size for
'Tyrosine−protein kinase Fyn' on 'eGFR'

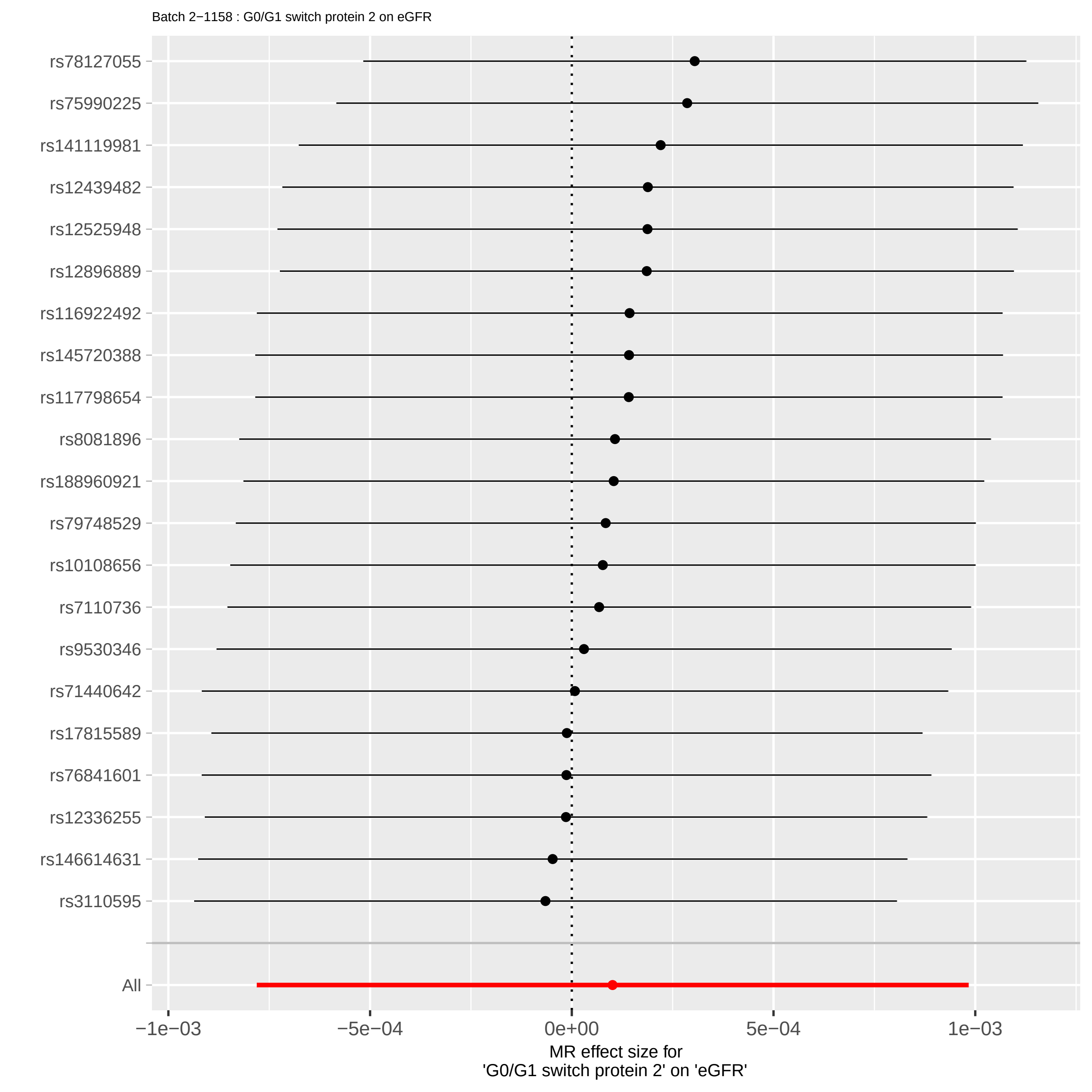

Batch 2−1158 : G0/G1 switch protein 2 on eGFR
rs78127055
rs75990225
rs141119981
rs12439482
rs12525948
rs12896889
rs116922492
rs145720388
rs117798654
rs8081896
rs188960921
rs79748529
rs10108656
rs7110736
rs9530346
rs71440642
rs17815589
rs76841601
rs12336255
rs146614631
rs3110595
All
−1e−03
−5e−04
0e+00
5e−04
1e−03
MR effect size for
'G0/G1 switch protein 2' on 'eGFR'

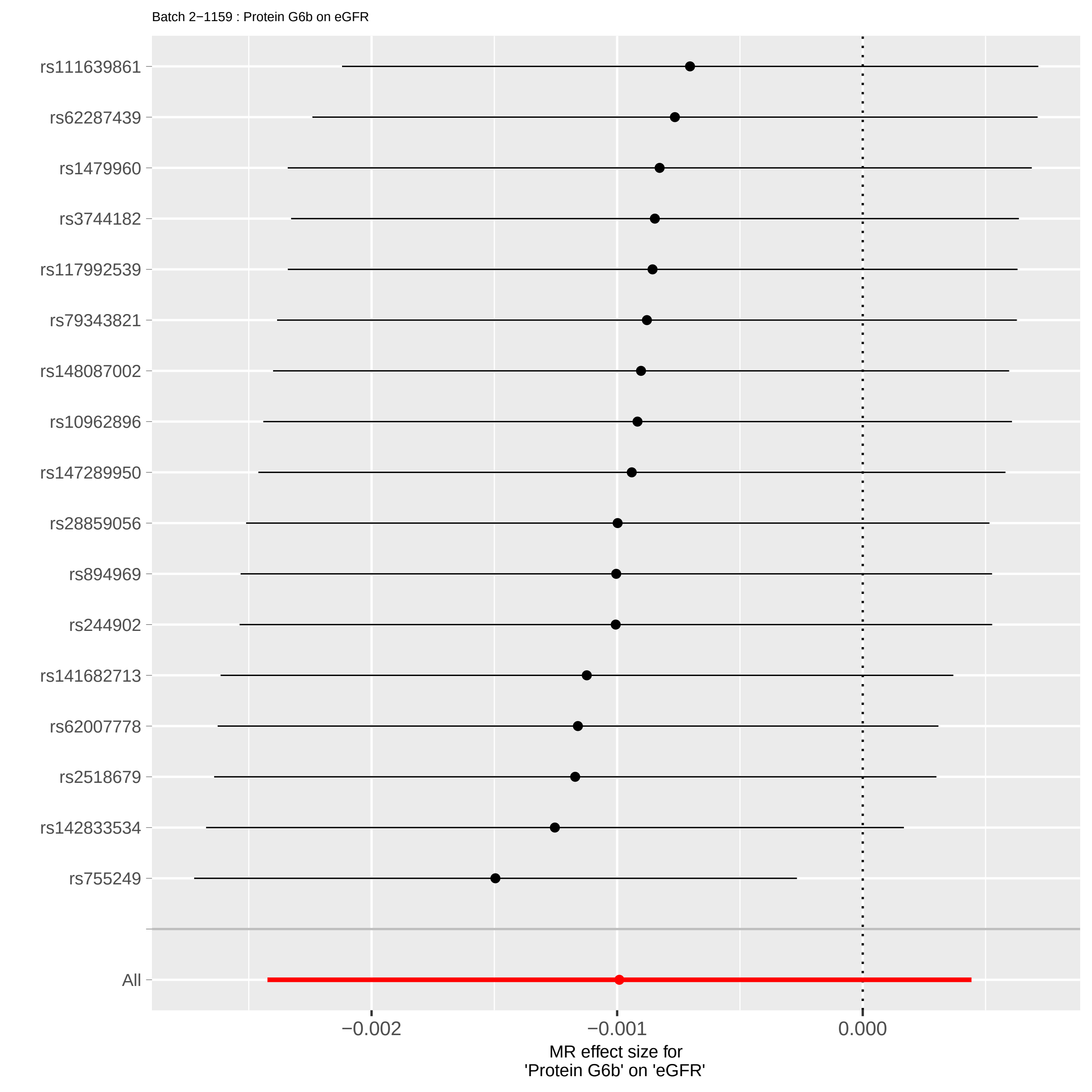
Batch 2−1159 : Protein G6b on eGFR
rs111639861
rs62287439
rs1479960
rs3744182
rs117992539
rs79343821
rs148087002
rs10962896
rs147289950
rs28859056
rs894969
rs244902
rs141682713
rs62007778
rs2518679
rs142833534
rs755249
All
−0.002
−0.001
0.000
MR effect size for
'Protein G6b' on 'eGFR'

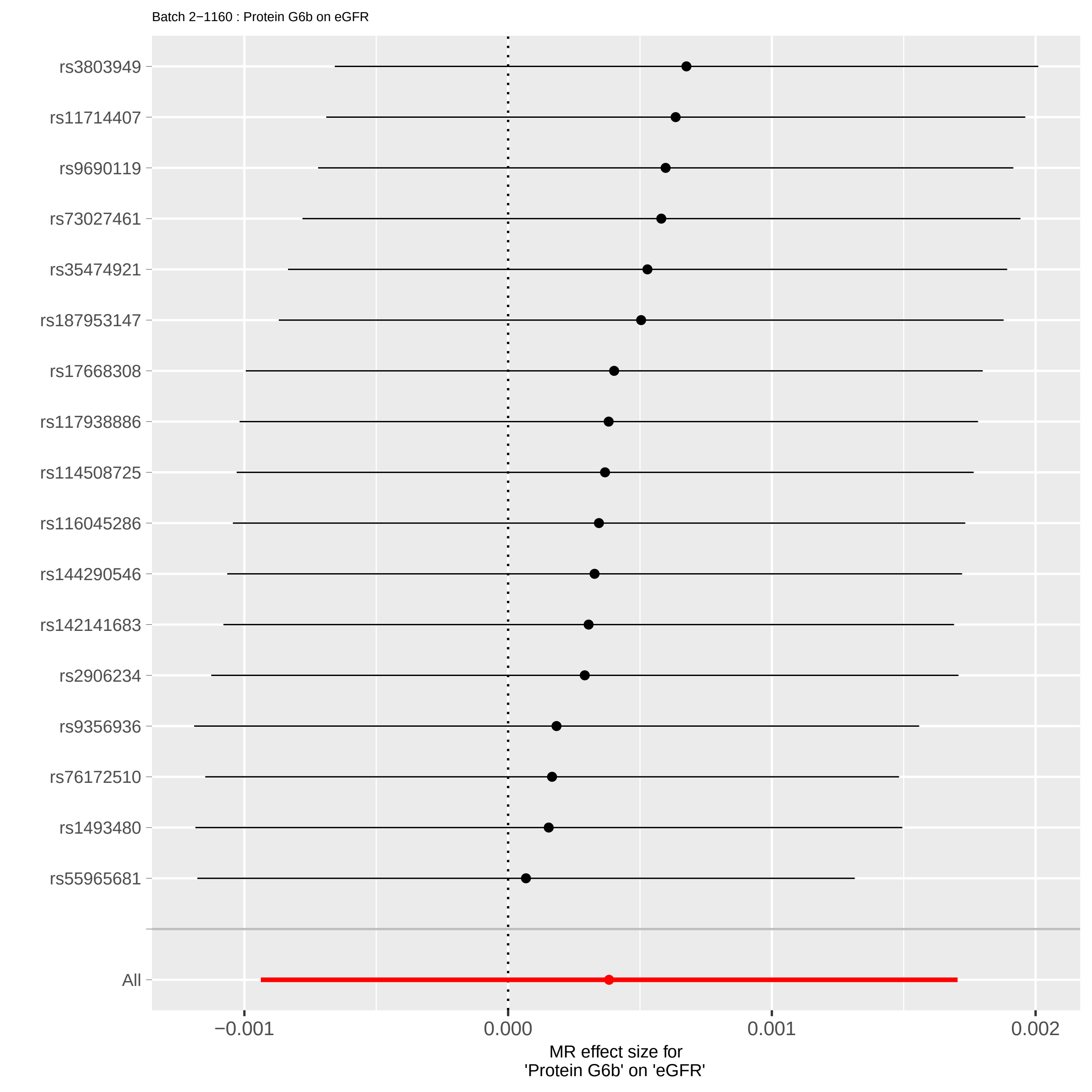
Batch 2−1160 : Protein G6b on eGFR
rs3803949
rs11714407
rs9690119
rs73027461
rs35474921
rs187953147
rs17668308
rs117938886
rs114508725
rs116045286
rs144290546
rs142141683
rs2906234
rs9356936
rs76172510
rs1493480
rs55965681
All
−0.001
0.000
0.001
0.002
MR effect size for
'Protein G6b' on 'eGFR'

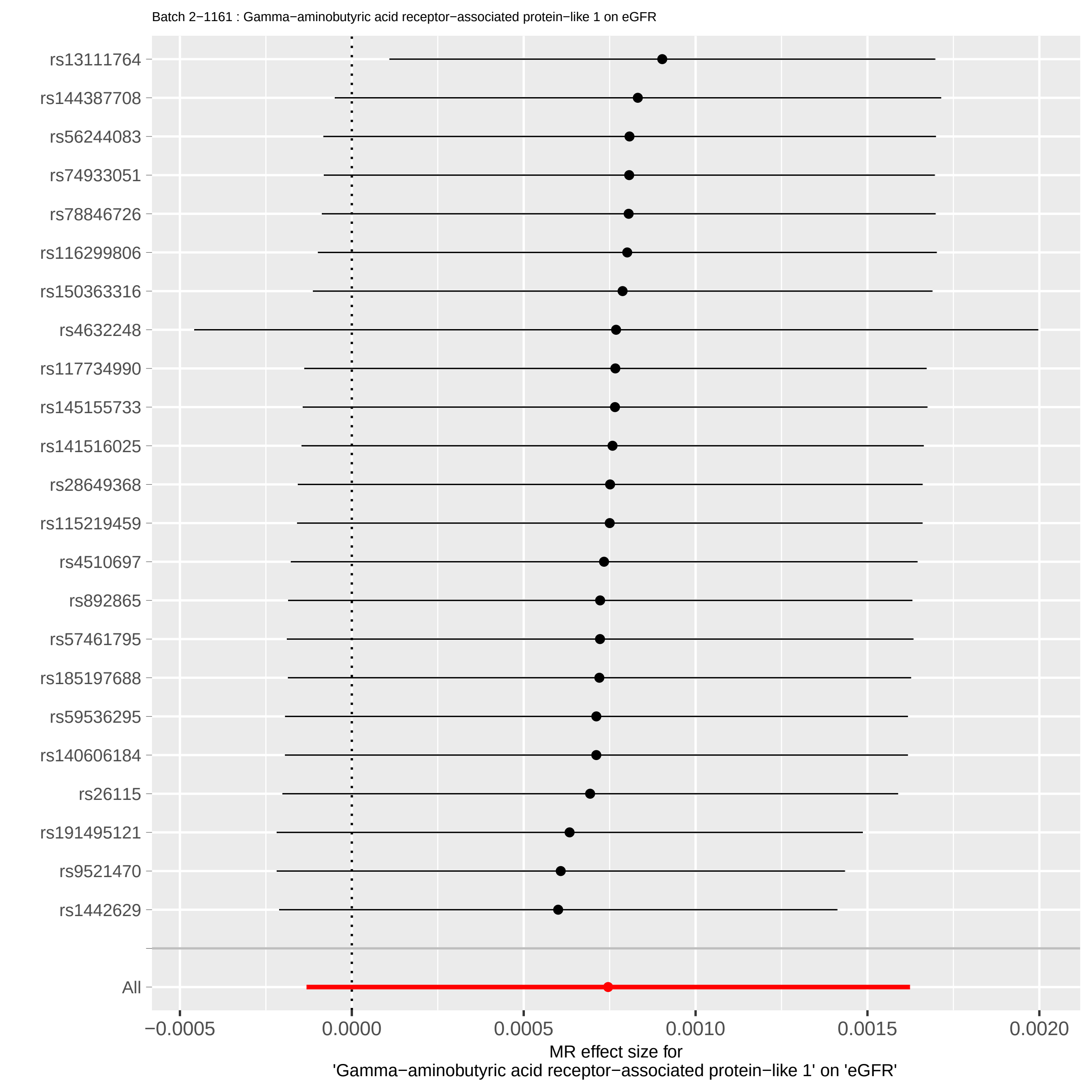

Batch 2−1161 : Gamma−aminobutyric acid receptor−associated protein−like 1 on eGFR
rs13111764
rs144387708
rs56244083
rs74933051
rs78846726
rs116299806
rs150363316
rs4632248
rs117734990
rs145155733
rs141516025
rs28649368
rs115219459
rs4510697
rs892865
rs57461795
rs185197688
rs59536295
rs140606184
rs26115
rs191495121
rs9521470
rs1442629
All
−0.0005
0.0000
0.0005
0.0010
0.0015
0.0020
MR effect size for
'Gamma−aminobutyric acid receptor−associated protein−like 1' on 'eGFR'

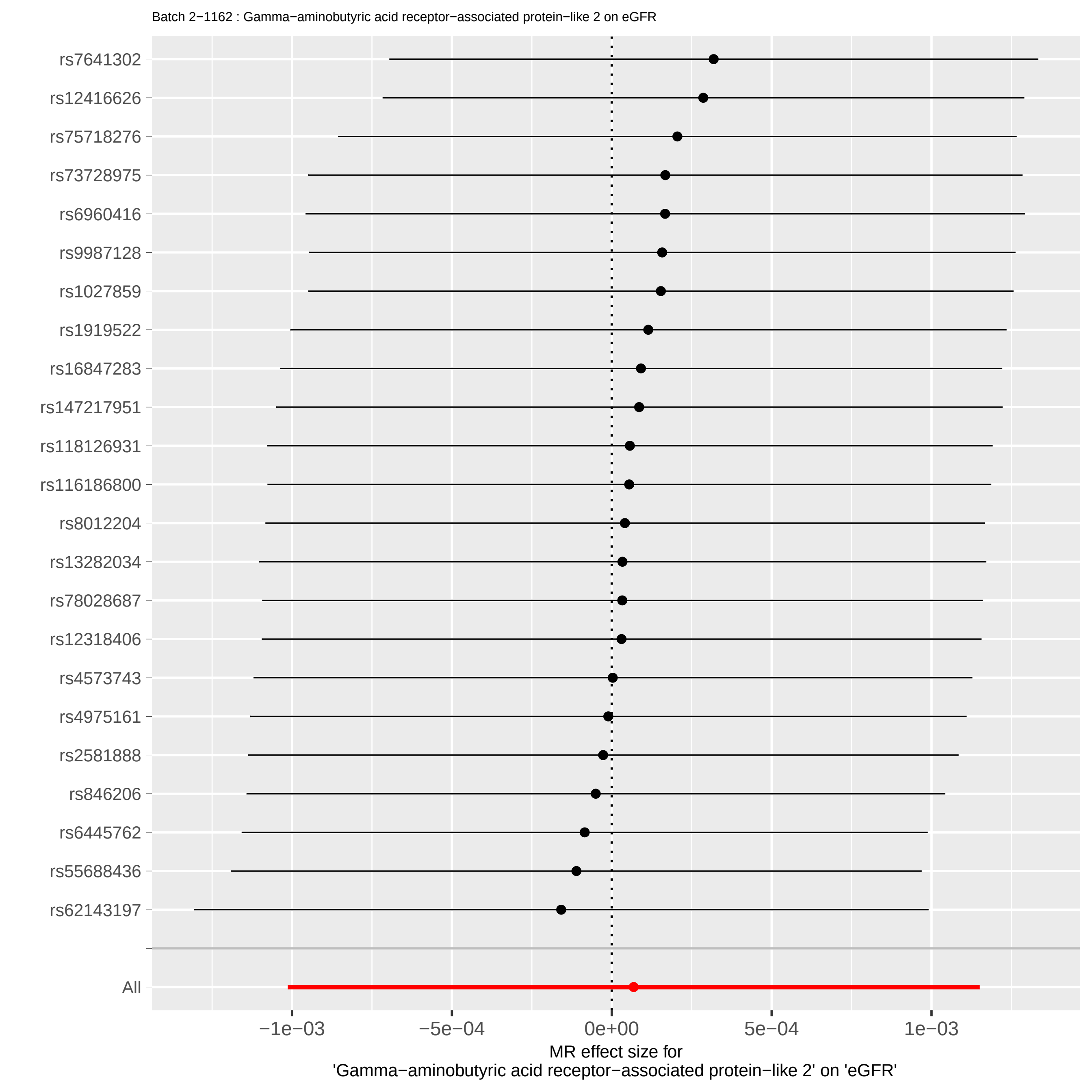
Batch 2−1162 : Gamma−aminobutyric acid receptor−associated protein−like 2 on eGFR
rs7641302
rs12416626
rs75718276
rs73728975
rs6960416
rs9987128
rs1027859
rs1919522
rs16847283
rs147217951
rs118126931
rs116186800
rs8012204
rs13282034
rs78028687
rs12318406
rs4573743
rs4975161
rs2581888
rs846206
rs6445762
rs55688436
rs62143197
All
−1e−03
−5e−04
0e+00
5e−04
1e−03
MR effect size for
'Gamma−aminobutyric acid receptor−associated protein−like 2' on 'eGFR'

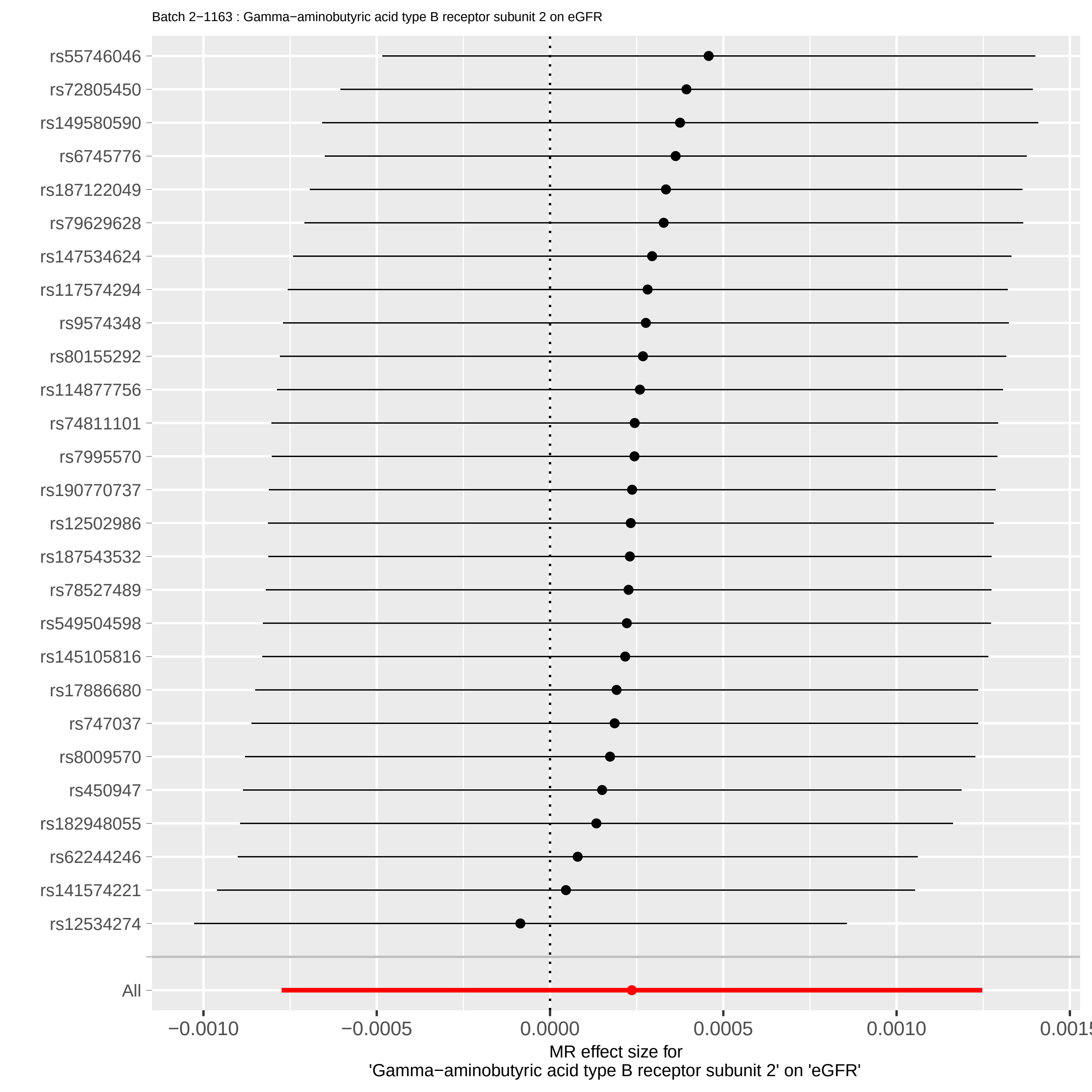

Batch 2−1163 : Gamma−aminobutyric acid type B receptor subunit 2 on eGFR
rs55746046
rs72805450
rs149580590
rs6745776
rs187122049
rs79629628
rs147534624
rs117574294
rs9574348
rs80155292
rs114877756
rs74811101
rs7995570
rs190770737
rs12502986
rs187543532
rs78527489
rs549504598
rs145105816
rs17886680
rs747037
rs8009570
rs450947
rs182948055
rs62244246
rs141574221
rs12534274
All
−0.0010
−0.0005
0.0000
0.0005
0.0010
0.0015
MR effect size for
'Gamma−aminobutyric acid type B receptor subunit 2' on 'eGFR'

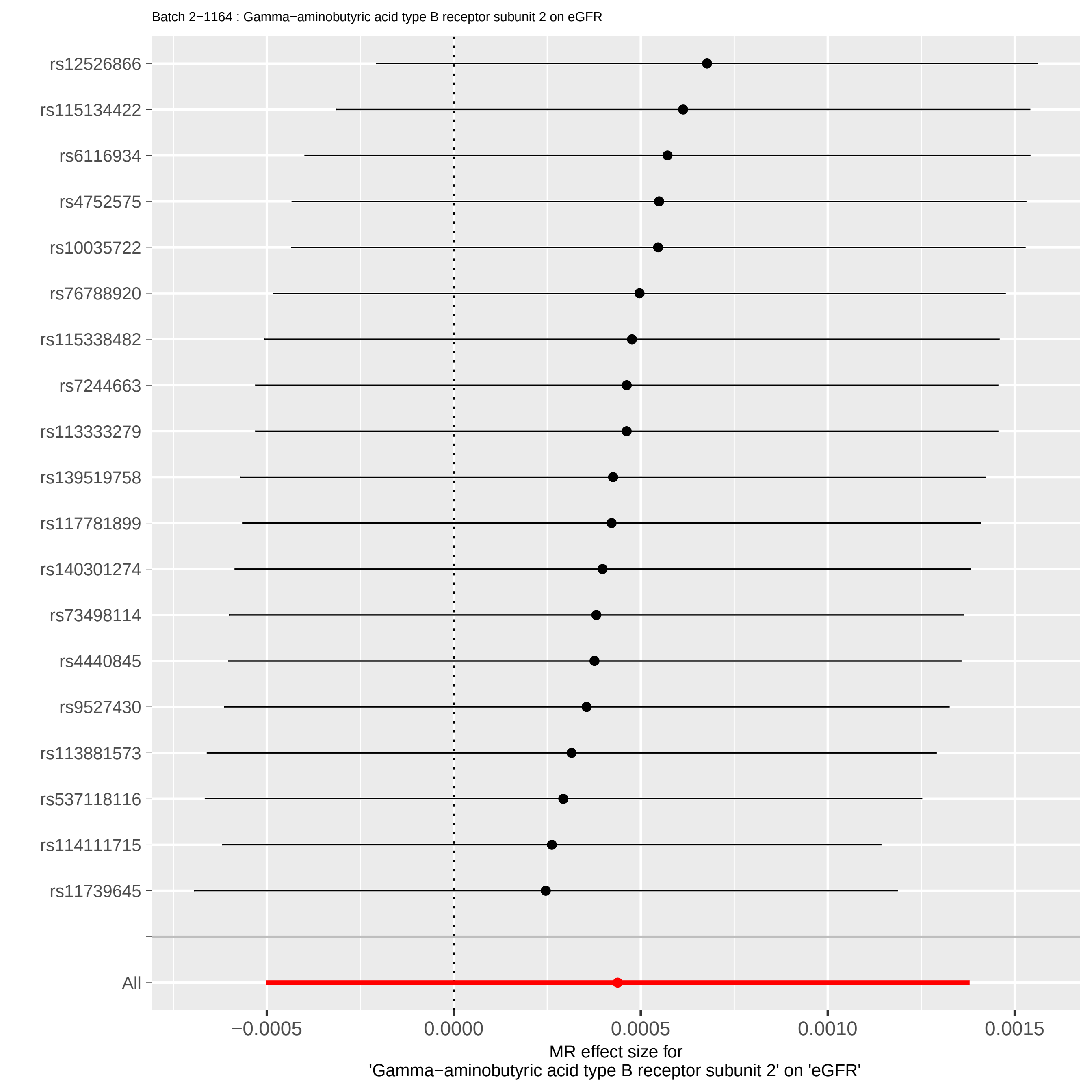

Batch 2−1164 : Gamma−aminobutyric acid type B receptor subunit 2 on eGFR
rs12526866
rs115134422
rs6116934
rs4752575
rs10035722
rs76788920
rs115338482
rs7244663
rs113333279
rs139519758
rs117781899
rs140301274
rs73498114
rs4440845
rs9527430
rs113881573
rs537118116
rs114111715
rs11739645
All
−0.0005
0.0000
0.0005
0.0010
0.0015
MR effect size for
'Gamma−aminobutyric acid type B receptor subunit 2' on 'eGFR'

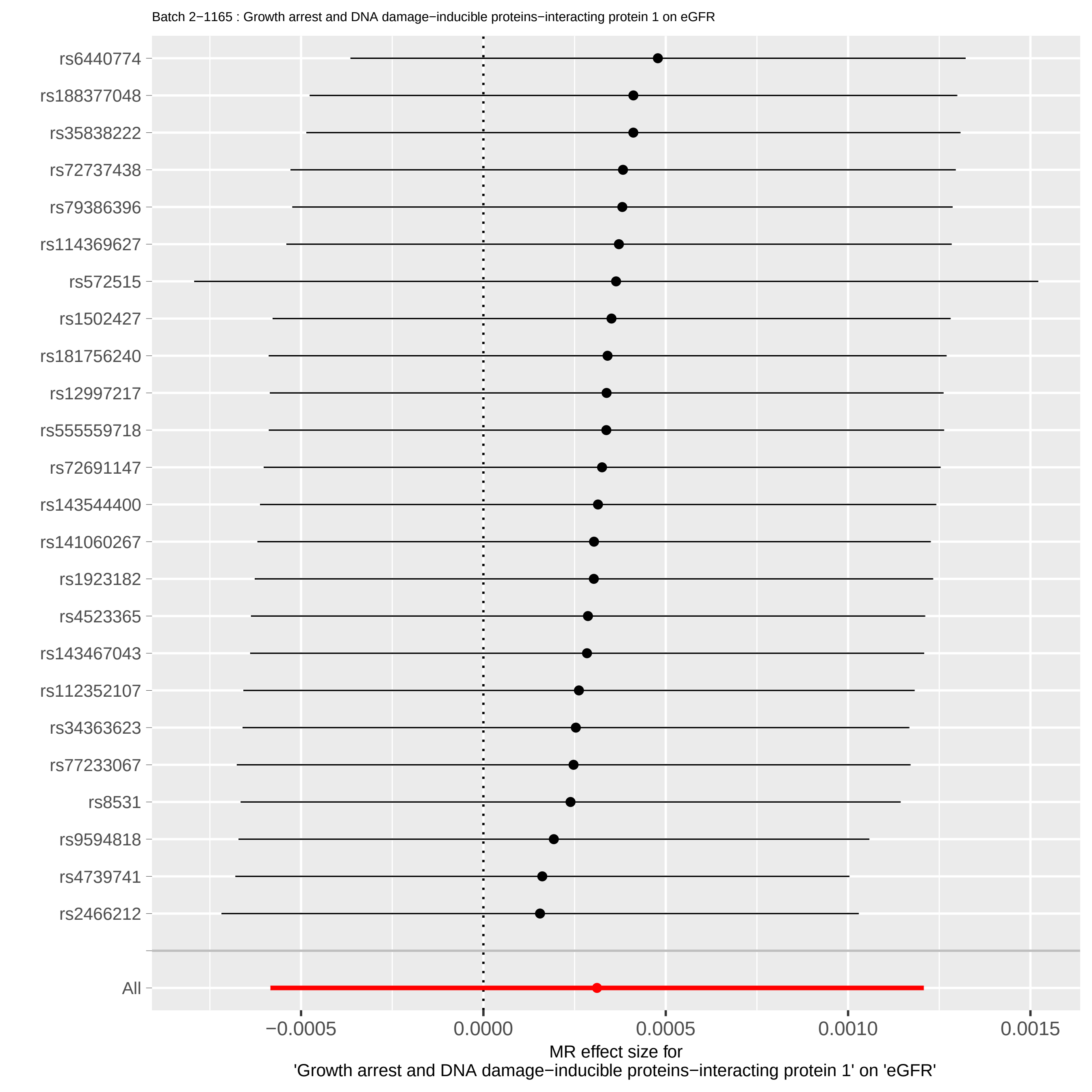
Batch 2−1165 : Growth arrest and DNA damage−inducible proteins−interacting protein 1 on eGFR
rs6440774
rs188377048
rs35838222
rs72737438
rs79386396
rs114369627
rs572515
rs1502427
rs181756240
rs12997217
rs555559718
rs72691147
rs143544400
rs141060267
rs1923182
rs4523365
rs143467043
rs112352107
rs34363623
rs77233067
rs8531
rs9594818
rs4739741
rs2466212
All
−0.0005
0.0000
0.0005
0.0010
0.0015
MR effect size for
'Growth arrest and DNA damage−inducible proteins−interacting protein 1' on 'eGFR'

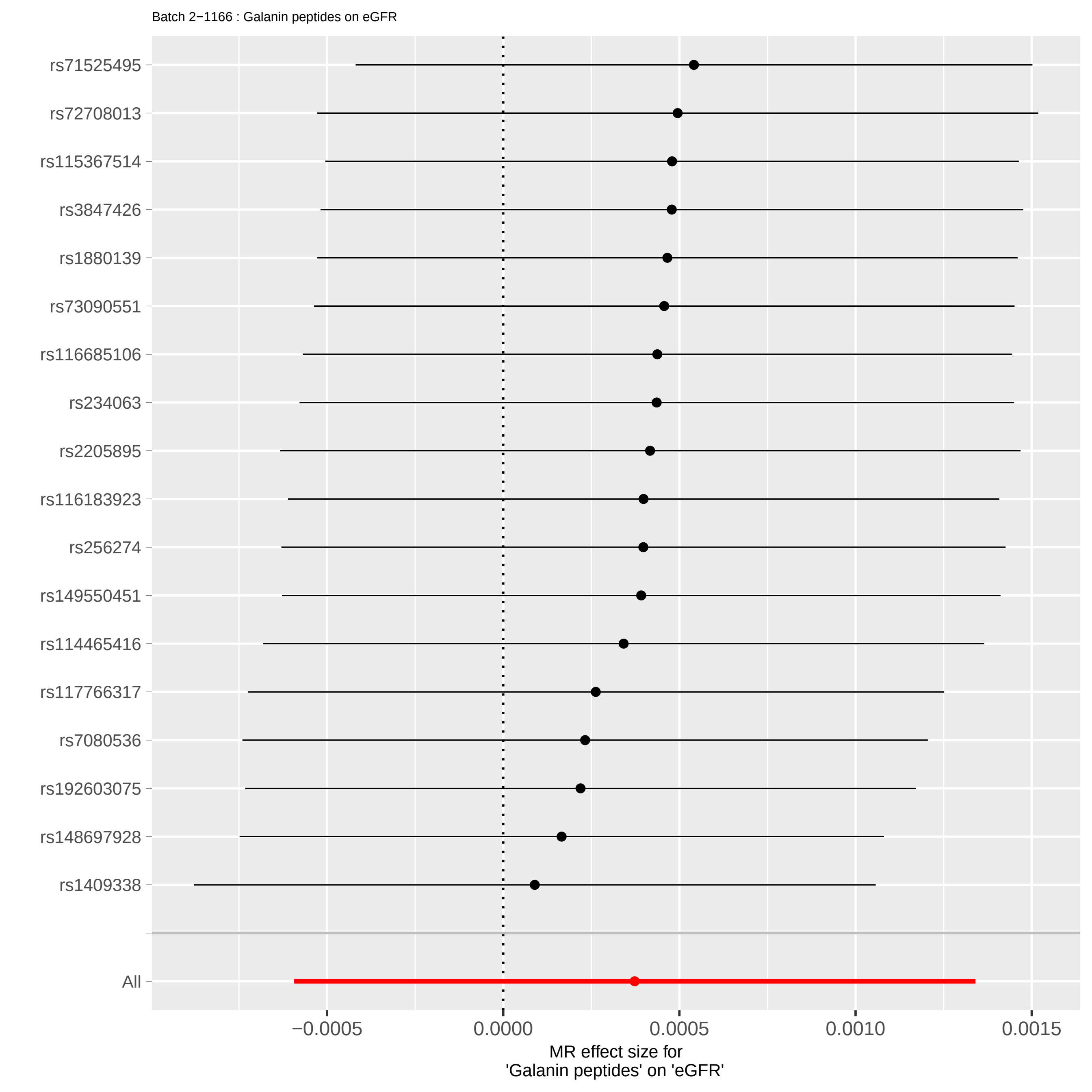

Batch 2−1166 : Galanin peptides on eGFR
rs71525495
rs72708013
rs115367514
rs3847426
rs1880139
rs73090551
rs116685106
rs234063
rs2205895
rs116183923
rs256274
rs149550451
rs114465416
rs117766317
rs7080536
rs192603075
rs148697928
rs1409338
All
−0.0005
0.0000
0.0005
0.0010
0.0015
MR effect size for
'Galanin peptides' on 'eGFR'

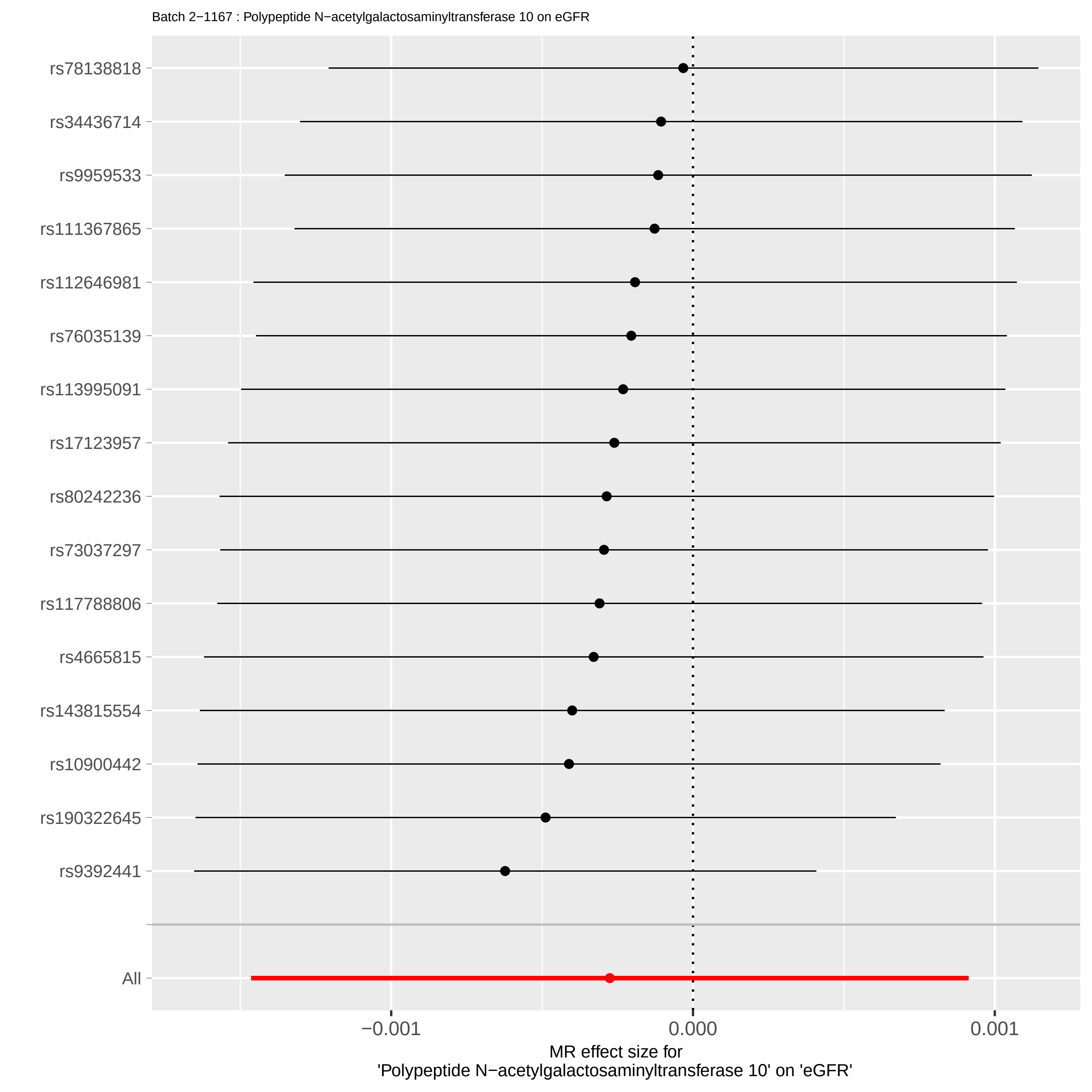
Batch 2−1167 : Polypeptide N−acetylgalactosaminyltransferase 10 on eGFR
rs78138818
rs34436714
rs9959533
rs111367865
rs112646981
rs76035139
rs113995091
rs17123957
rs80242236
rs73037297
rs117788806
rs4665815
rs143815554
rs10900442
rs190322645
rs9392441
All
−0.001
0.000
0.001
MR effect size for
'Polypeptide N−acetylgalactosaminyltransferase 10' on 'eGFR'

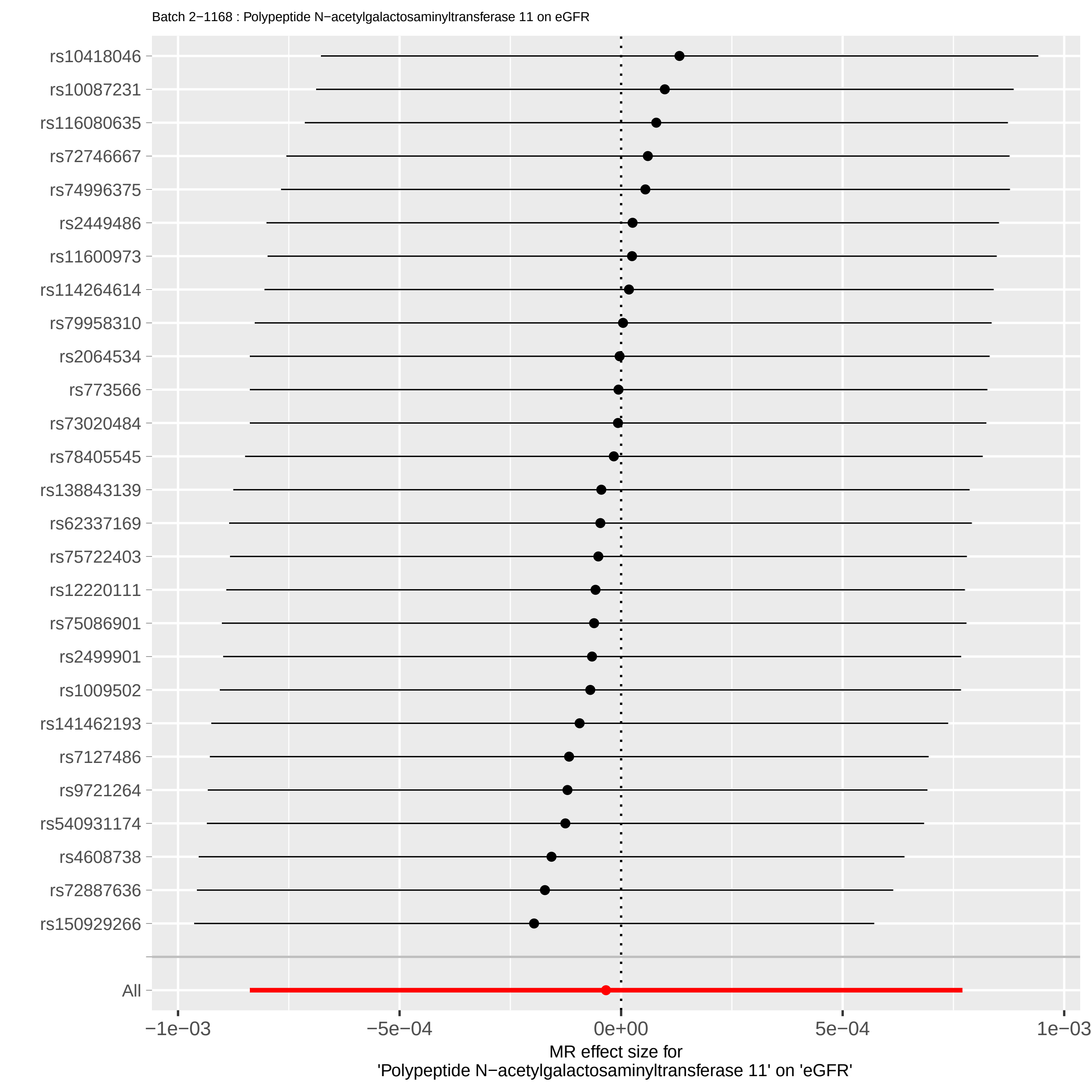

Batch 2−1168 : Polypeptide N−acetylgalactosaminyltransferase 11 on eGFR
rs10418046
rs10087231
rs116080635
rs72746667
rs74996375
rs2449486
rs11600973
rs114264614
rs79958310
rs2064534
rs773566
rs73020484
rs78405545
rs138843139
rs62337169
rs75722403
rs12220111
rs75086901
rs2499901
rs1009502
rs141462193
rs7127486
rs9721264
rs540931174
rs4608738
rs72887636
rs150929266
All
−1e−03
−5e−04
0e+00
5e−04
1e−03
MR effect size for
'Polypeptide N−acetylgalactosaminyltransferase 11' on 'eGFR'

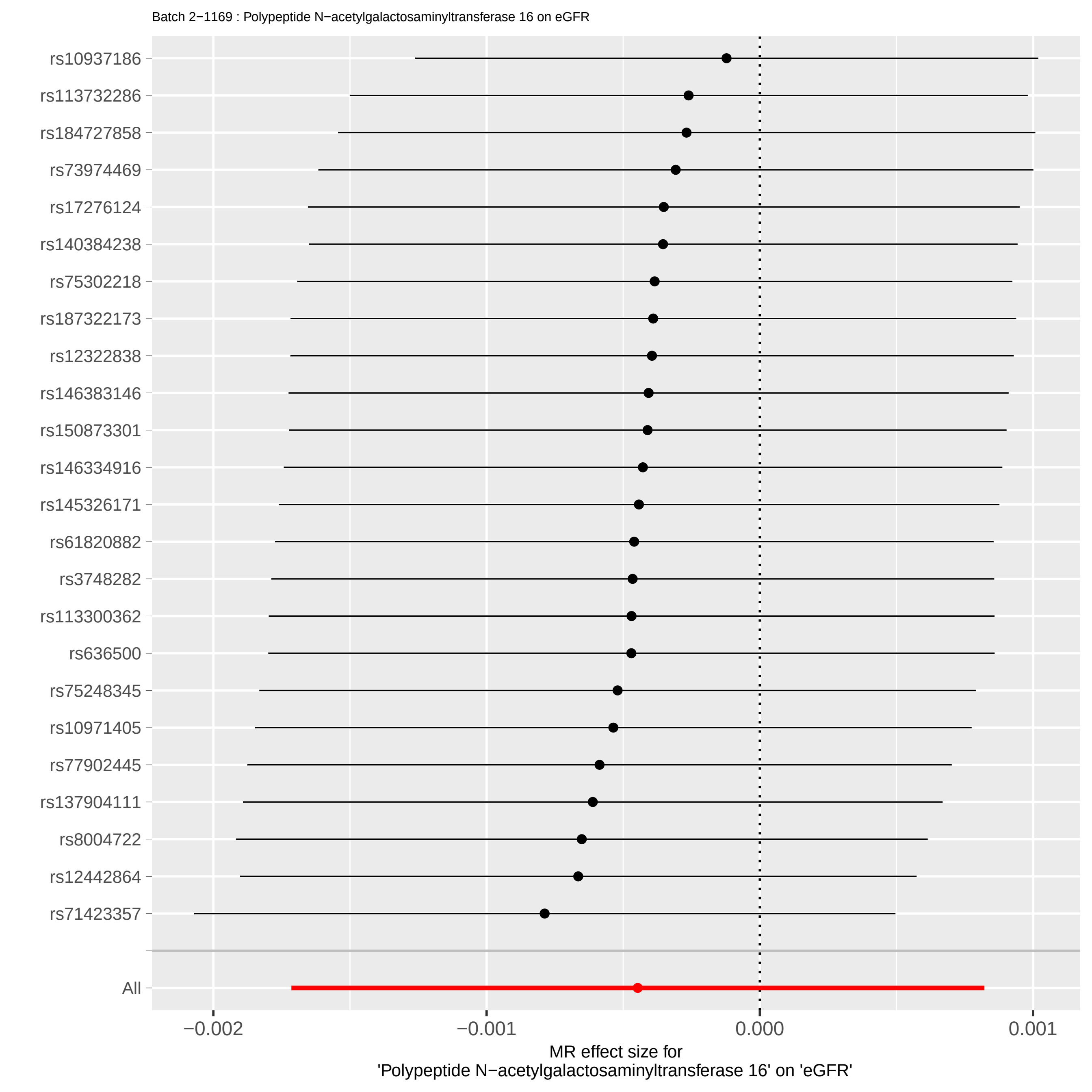

Batch 2−1169 : Polypeptide N−acetylgalactosaminyltransferase 16 on eGFR
rs10937186
rs113732286
rs184727858
rs73974469
rs17276124
rs140384238
rs75302218
rs187322173
rs12322838
rs146383146
rs150873301
rs146334916
rs145326171
rs61820882
rs3748282
rs113300362
rs636500
rs75248345
rs10971405
rs77902445
rs137904111
rs8004722
rs12442864
rs71423357
All
−0.002
−0.001
0.000
0.001
MR effect size for
'Polypeptide N−acetylgalactosaminyltransferase 16' on 'eGFR'

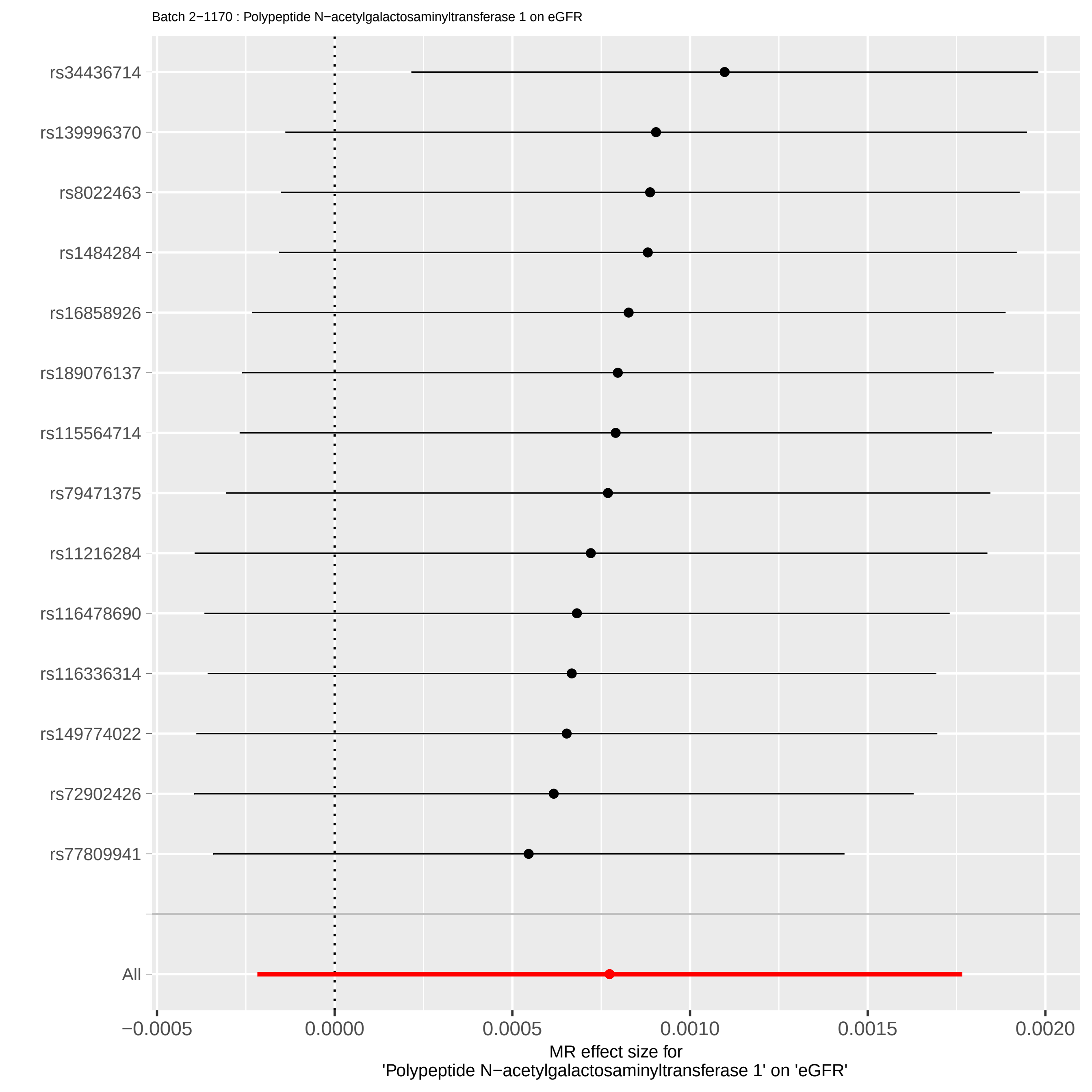

Batch 2−1170 : Polypeptide N−acetylgalactosaminyltransferase 1 on eGFR
rs34436714
rs139996370
rs8022463
rs1484284
rs16858926
rs189076137
rs115564714
rs79471375
rs11216284
rs116478690
rs116336314
rs149774022
rs72902426
rs77809941
All
−0.0005
0.0000
0.0005
0.0010
0.0015
0.0020
MR effect size for
'Polypeptide N−acetylgalactosaminyltransferase 1' on 'eGFR'

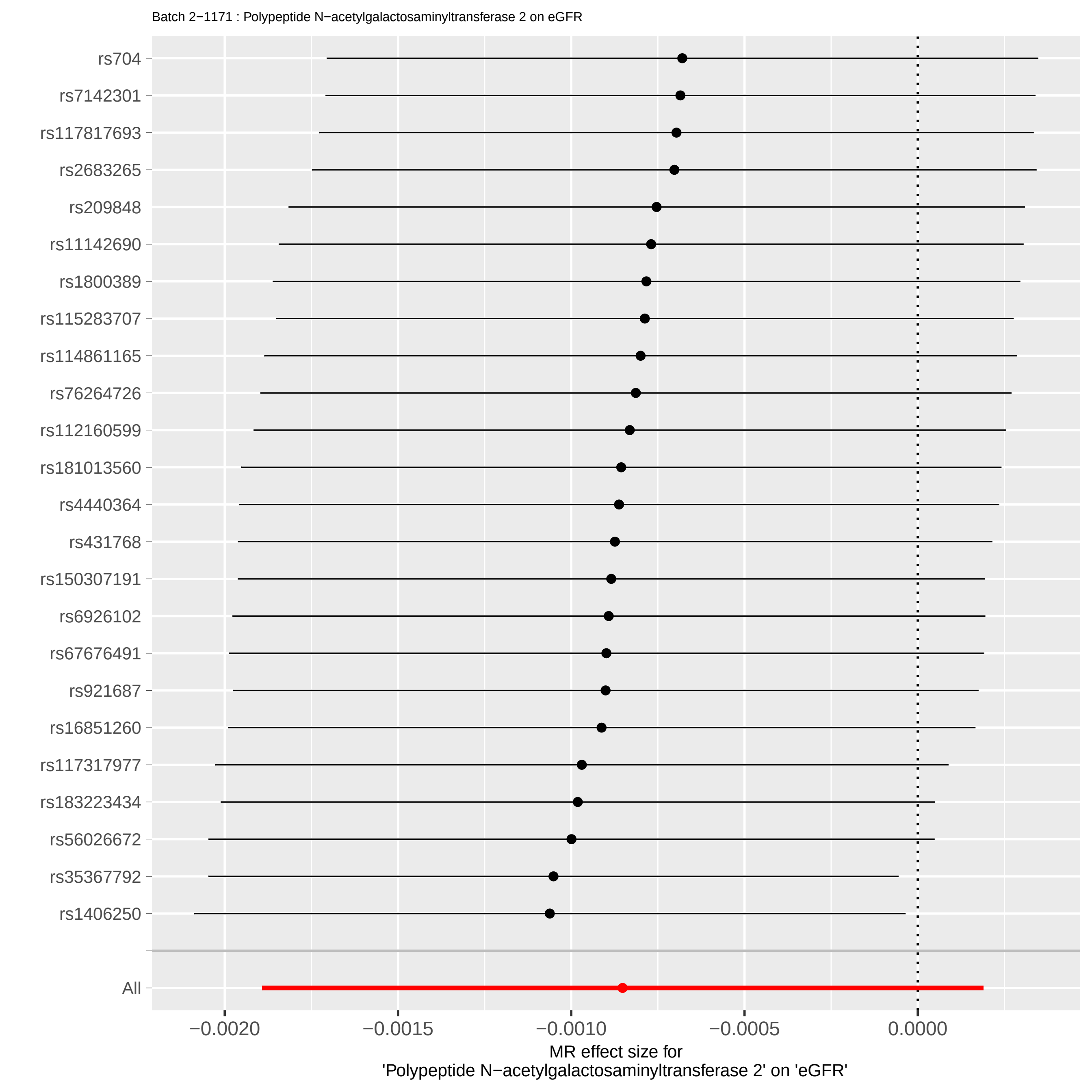

Batch 2−1171 : Polypeptide N−acetylgalactosaminyltransferase 2 on eGFR
rs704
rs7142301
rs117817693
rs2683265
rs209848
rs11142690
rs1800389
rs115283707
rs114861165
rs76264726
rs112160599
rs181013560
rs4440364
rs431768
rs150307191
rs6926102
rs67676491
rs921687
rs16851260
rs117317977
rs183223434
rs56026672
rs35367792
rs1406250
All
−0.0020
−0.0015
−0.0010
−0.0005
0.0000
MR effect size for
'Polypeptide N−acetylgalactosaminyltransferase 2' on 'eGFR'

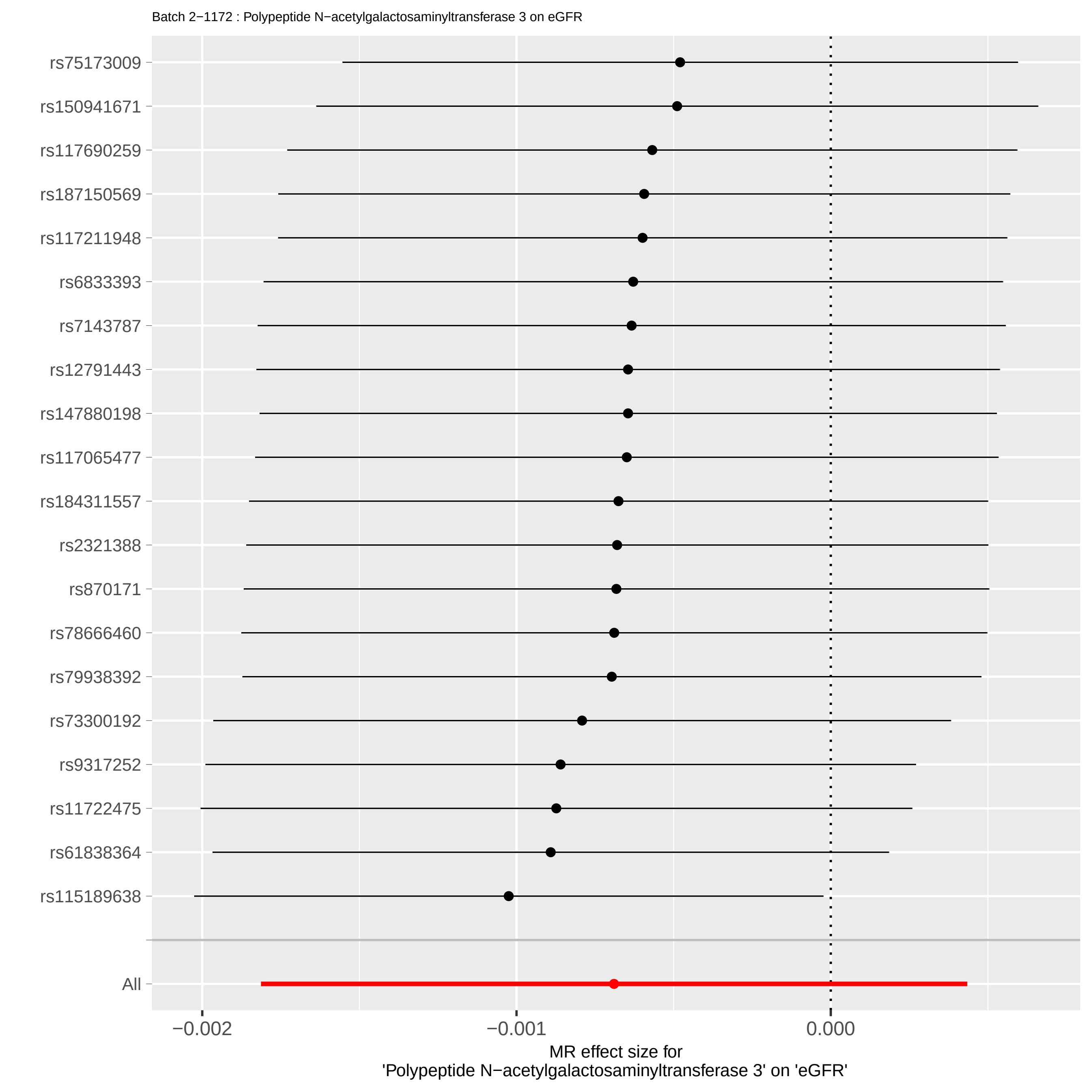

Batch 2−1172 : Polypeptide N−acetylgalactosaminyltransferase 3 on eGFR
rs75173009
rs150941671
rs117690259
rs187150569
rs117211948
rs6833393
rs7143787
rs12791443
rs147880198
rs117065477
rs184311557
rs2321388
rs870171
rs78666460
rs79938392
rs73300192
rs9317252
rs11722475
rs61838364
rs115189638
All
−0.002
−0.001
0.000
MR effect size for
'Polypeptide N−acetylgalactosaminyltransferase 3' on 'eGFR'

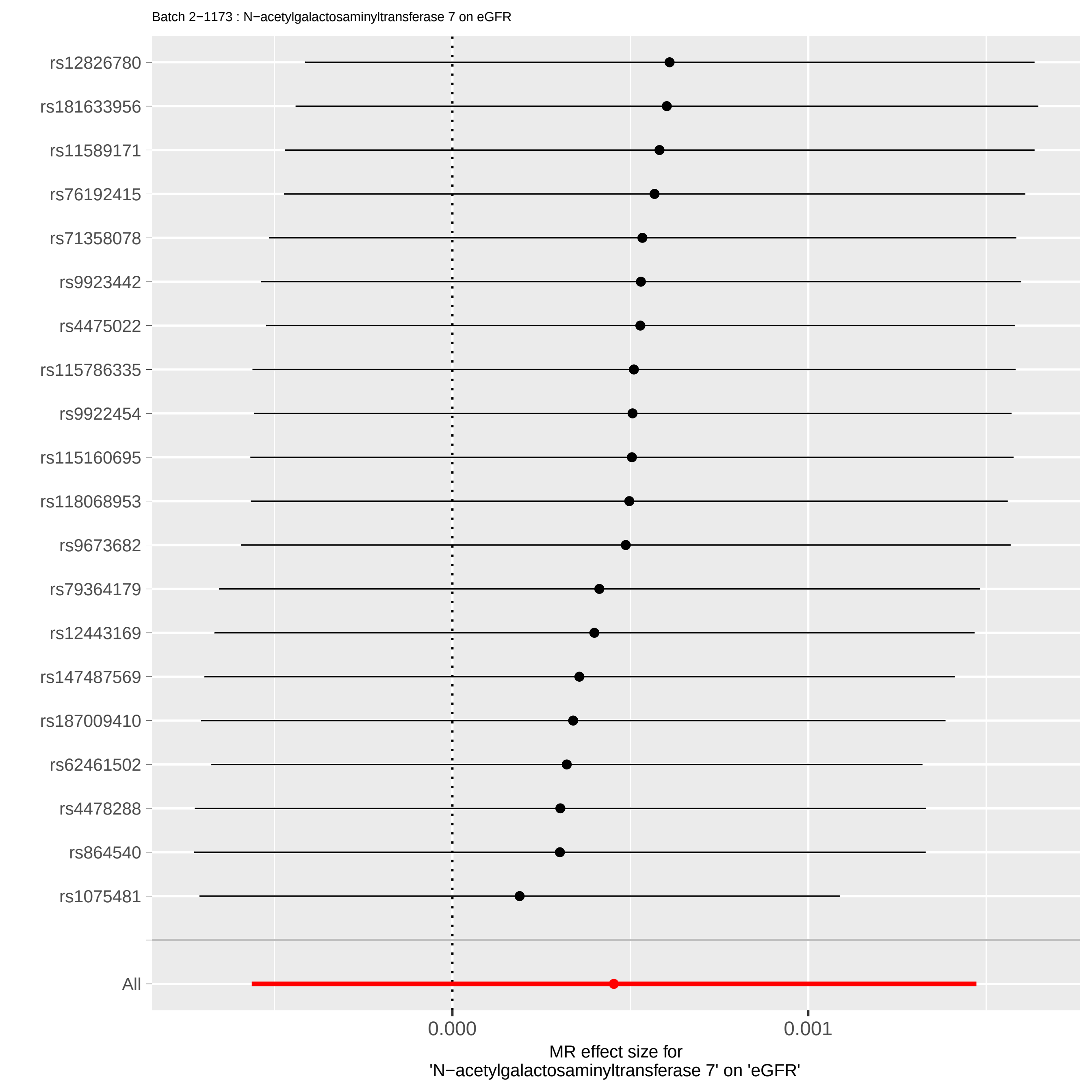

Batch 2−1173 : N−acetylgalactosaminyltransferase 7 on eGFR
rs12826780
rs181633956
rs11589171
rs76192415
rs71358078
rs9923442
rs4475022
rs115786335
rs9922454
rs115160695
rs118068953
rs9673682
rs79364179
rs12443169
rs147487569
rs187009410
rs62461502
rs4478288
rs864540
rs1075481
All
0.000
0.001
MR effect size for
'N−acetylgalactosaminyltransferase 7' on 'eGFR'

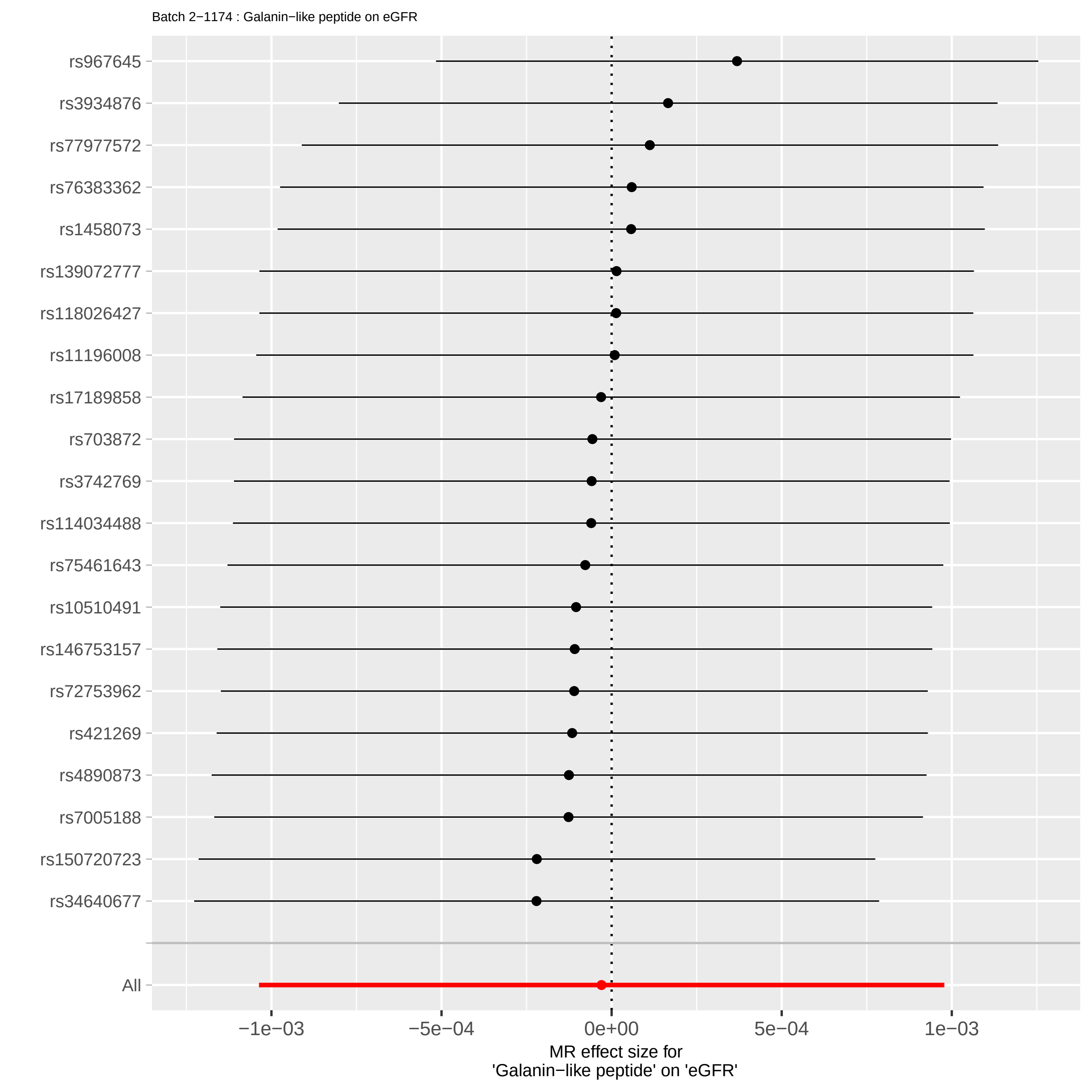

Batch 2−1174 : Galanin−like peptide on eGFR
rs967645
rs3934876
rs77977572
rs76383362
rs1458073
rs139072777
rs118026427
rs11196008
rs17189858
rs703872
rs3742769
rs114034488
rs75461643
rs10510491
rs146753157
rs72753962
rs421269
rs4890873
rs7005188
rs150720723
rs34640677
All
−1e−03
−5e−04
0e+00
5e−04
1e−03
MR effect size for
'Galanin−like peptide' on 'eGFR'

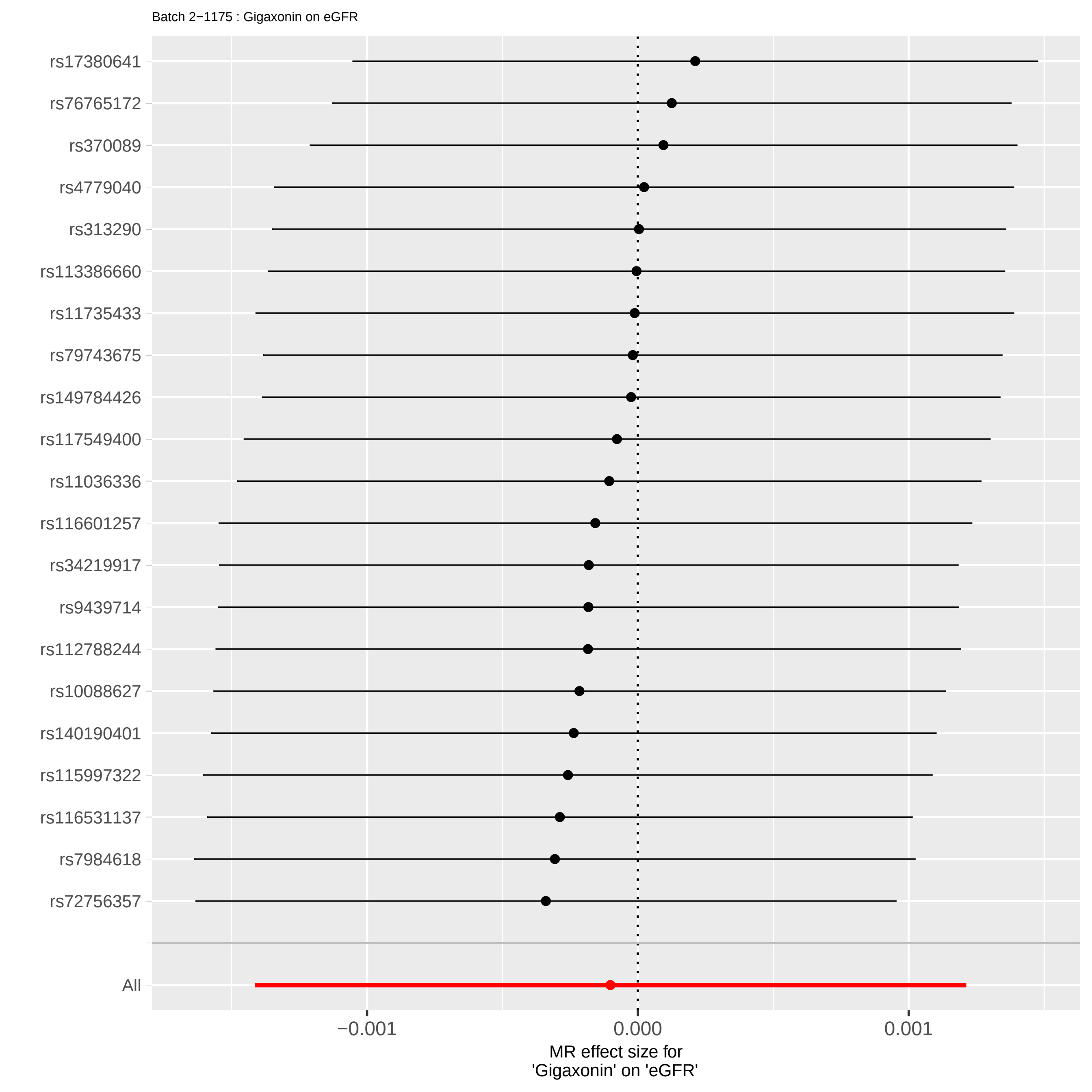

Batch 2−1175 : Gigaxonin on eGFR
rs17380641
rs76765172
rs370089
rs4779040
rs313290
rs113386660
rs11735433
rs79743675
rs149784426
rs117549400
rs11036336
rs116601257
rs34219917
rs9439714
rs112788244
rs10088627
rs140190401
rs115997322
rs116531137
rs7984618
rs72756357
All
−0.001
0.000
0.001
MR effect size for
'Gigaxonin' on 'eGFR'

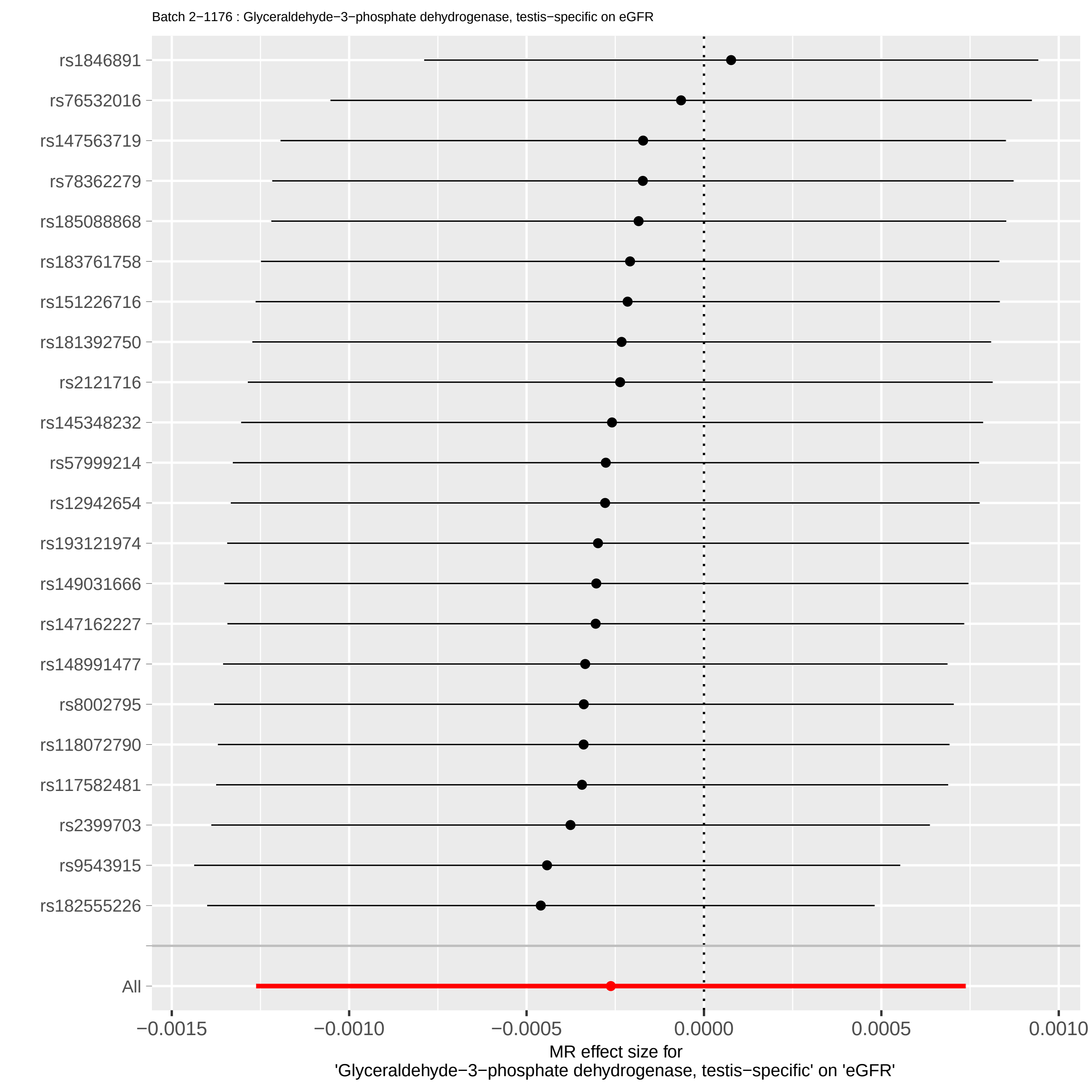
Batch 2−1176 : Glyceraldehyde−3−phosphate dehydrogenase, testis−specific on eGFR
rs1846891
rs76532016
rs147563719
rs78362279
rs185088868
rs183761758
rs151226716
rs181392750
rs2121716
rs145348232
rs57999214
rs12942654
rs193121974
rs149031666
rs147162227
rs148991477
rs8002795
rs118072790
rs117582481
rs2399703
rs9543915
rs182555226
All
−0.0015
−0.0010
−0.0005
0.0000
0.0005
0.0010
MR effect size for
'Glyceraldehyde−3−phosphate dehydrogenase, testis−specific' on 'eGFR'

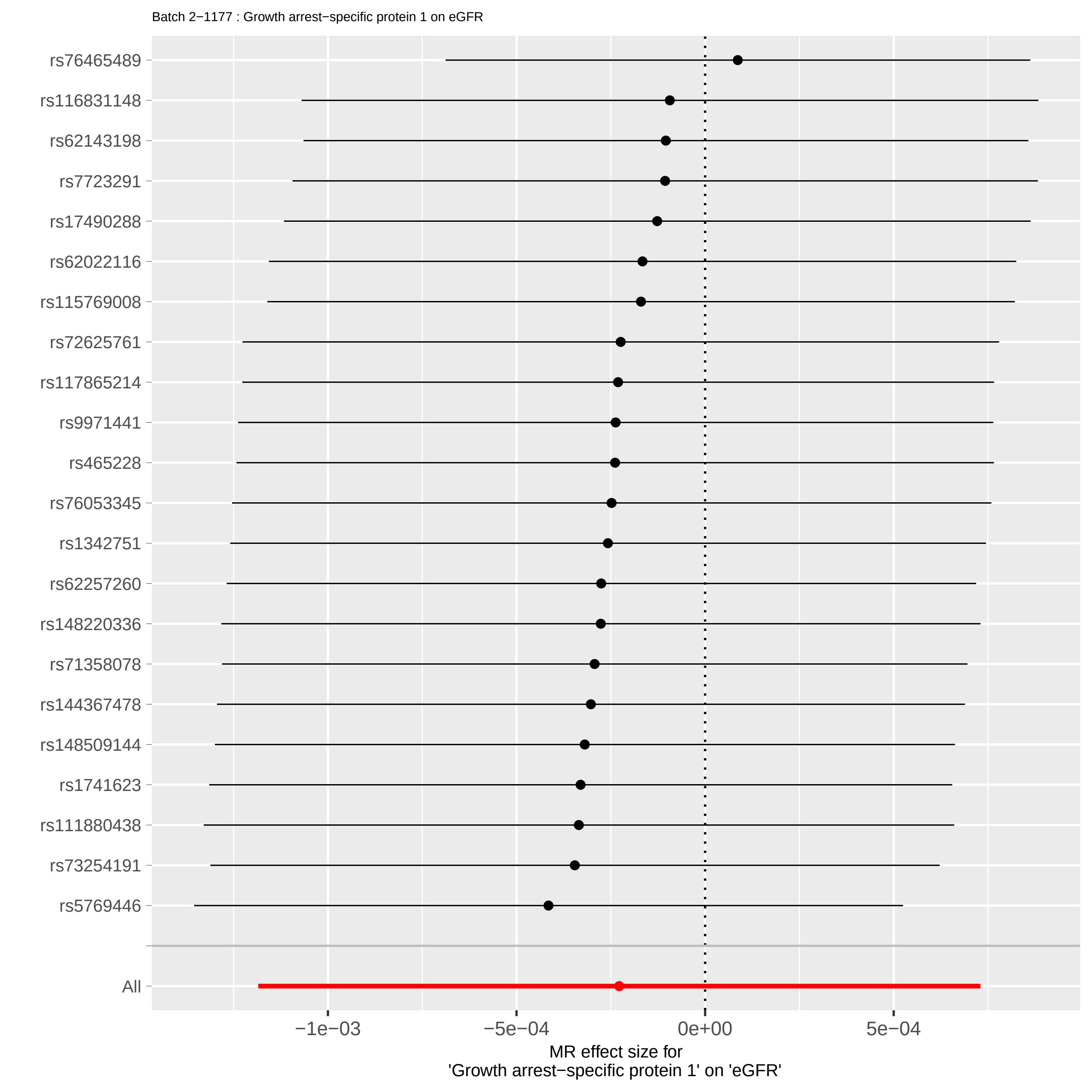

Batch 2−1177 : Growth arrest−specific protein 1 on eGFR
rs76465489
rs116831148
rs62143198
rs7723291
rs17490288
rs62022116
rs115769008
rs72625761
rs117865214
rs9971441
rs465228
rs76053345
rs1342751
rs62257260
rs148220336
rs71358078
rs144367478
rs148509144
rs1741623
rs111880438
rs73254191
rs5769446
All
−1e−03
−5e−04
0e+00
5e−04
MR effect size for
'Growth arrest−specific protein 1' on 'eGFR'

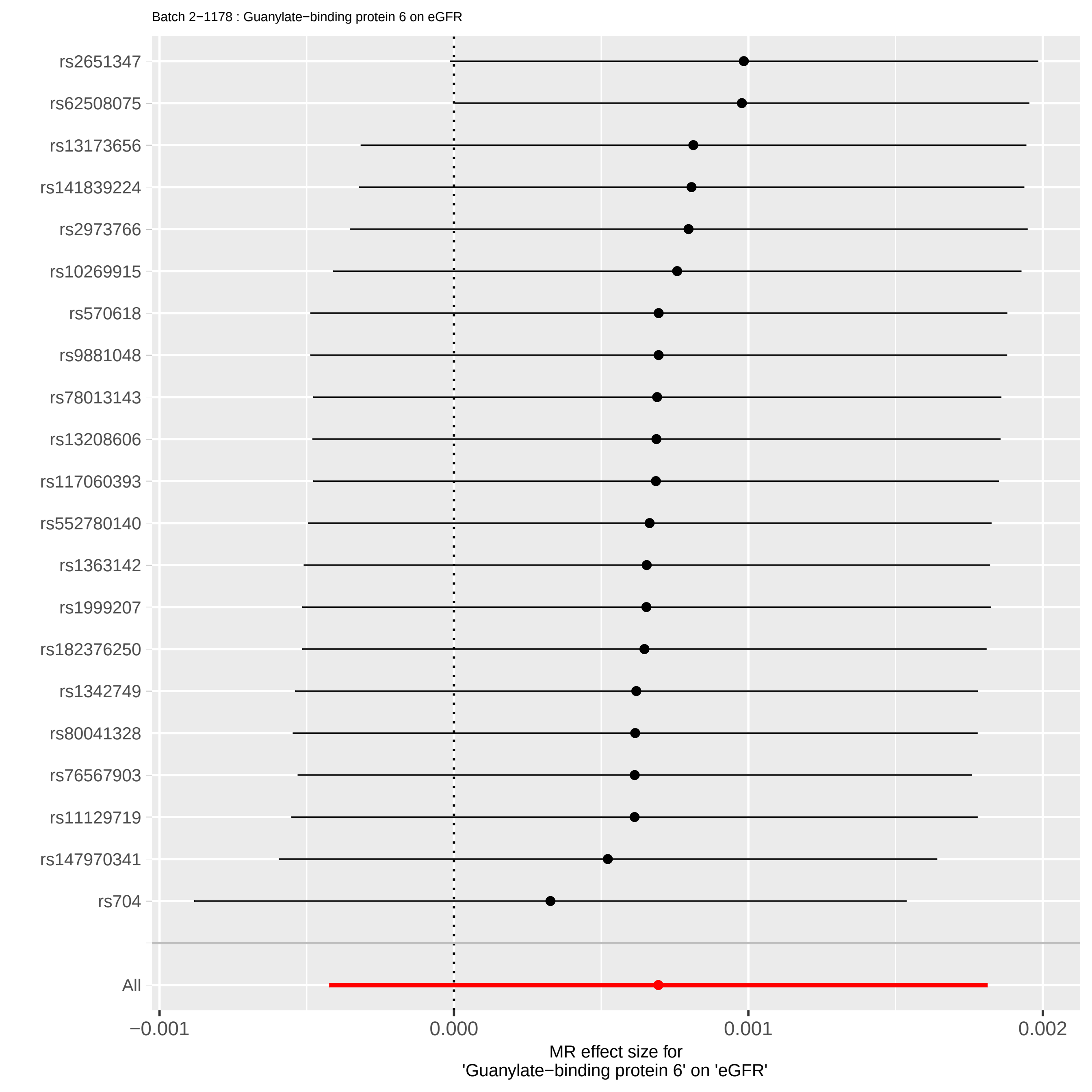

Batch 2−1178 : Guanylate−binding protein 6 on eGFR
rs2651347
rs62508075
rs13173656
rs141839224
rs2973766
rs10269915
rs570618
rs9881048
rs78013143
rs13208606
rs117060393
rs552780140
rs1363142
rs1999207
rs182376250
rs1342749
rs80041328
rs76567903
rs11129719
rs147970341
rs704
All
−0.001
0.000
0.001
0.002
MR effect size for
'Guanylate−binding protein 6' on 'eGFR'

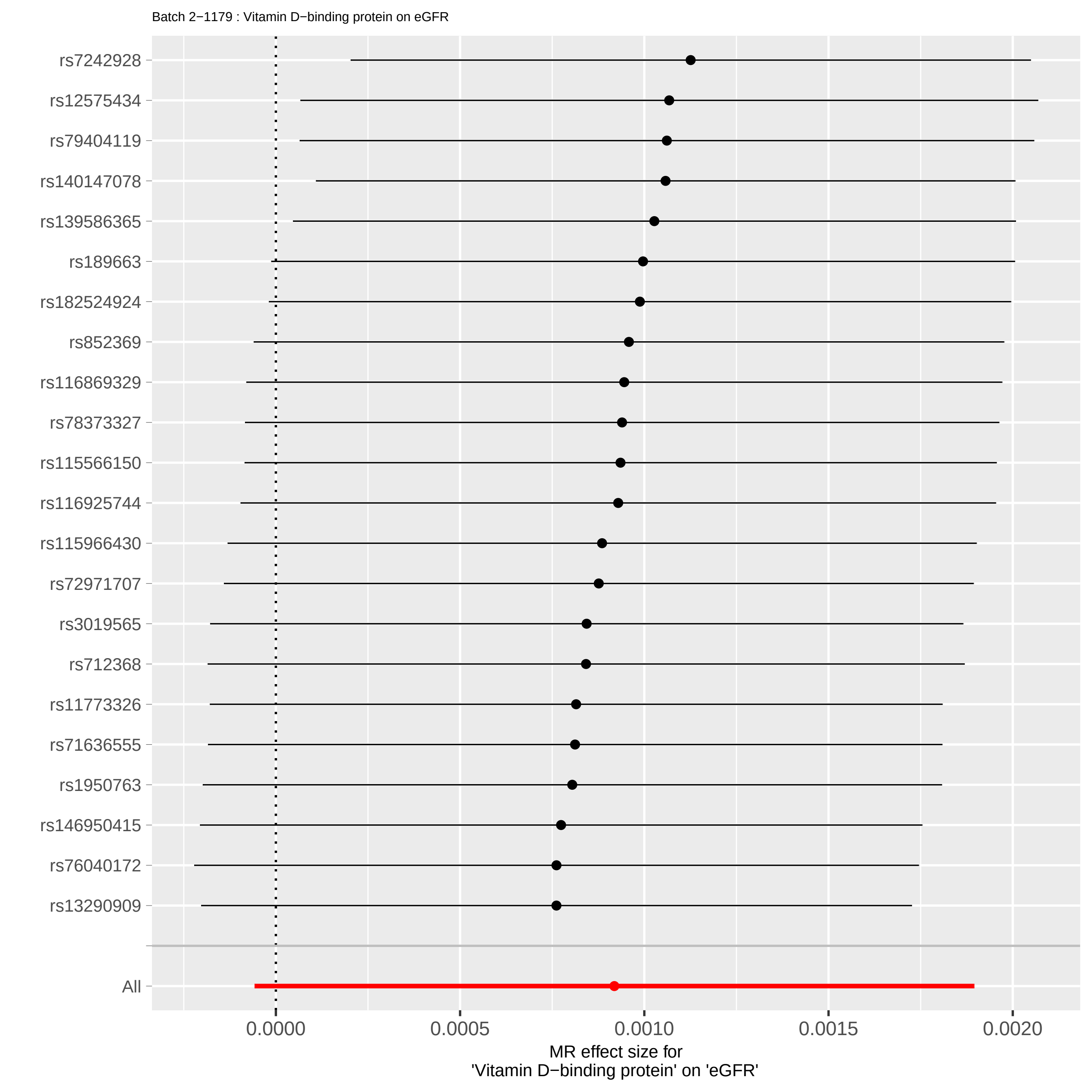

Batch 2−1179 : Vitamin D−binding protein on eGFR
rs7242928
rs12575434
rs79404119
rs140147078
rs139586365
rs189663
rs182524924
rs852369
rs116869329
rs78373327
rs115566150
rs116925744
rs115966430
rs72971707
rs3019565
rs712368
rs11773326
rs71636555
rs1950763
rs146950415
rs76040172
rs13290909
All
0.0000
0.0005
0.0010
0.0015
0.0020
MR effect size for
'Vitamin D−binding protein' on 'eGFR'

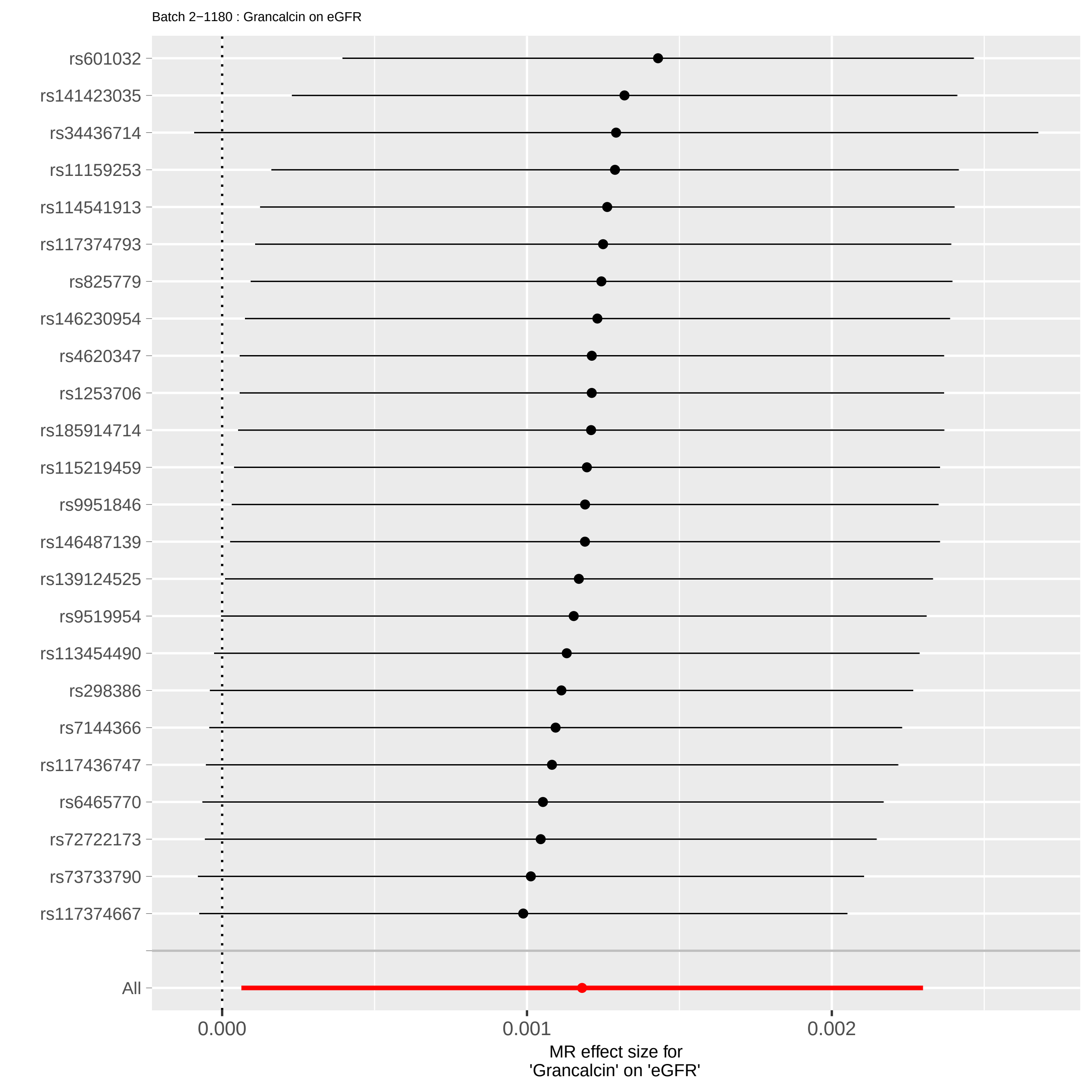

Batch 2−1180 : Grancalcin on eGFR
rs601032
rs141423035
rs34436714
rs11159253
rs114541913
rs117374793
rs825779
rs146230954
rs4620347
rs1253706
rs185914714
rs115219459
rs9951846
rs146487139
rs139124525
rs9519954
rs113454490
rs298386
rs7144366
rs117436747
rs6465770
rs72722173
rs73733790
rs117374667
All
0.000
0.001
0.002
MR effect size for
'Grancalcin' on 'eGFR'

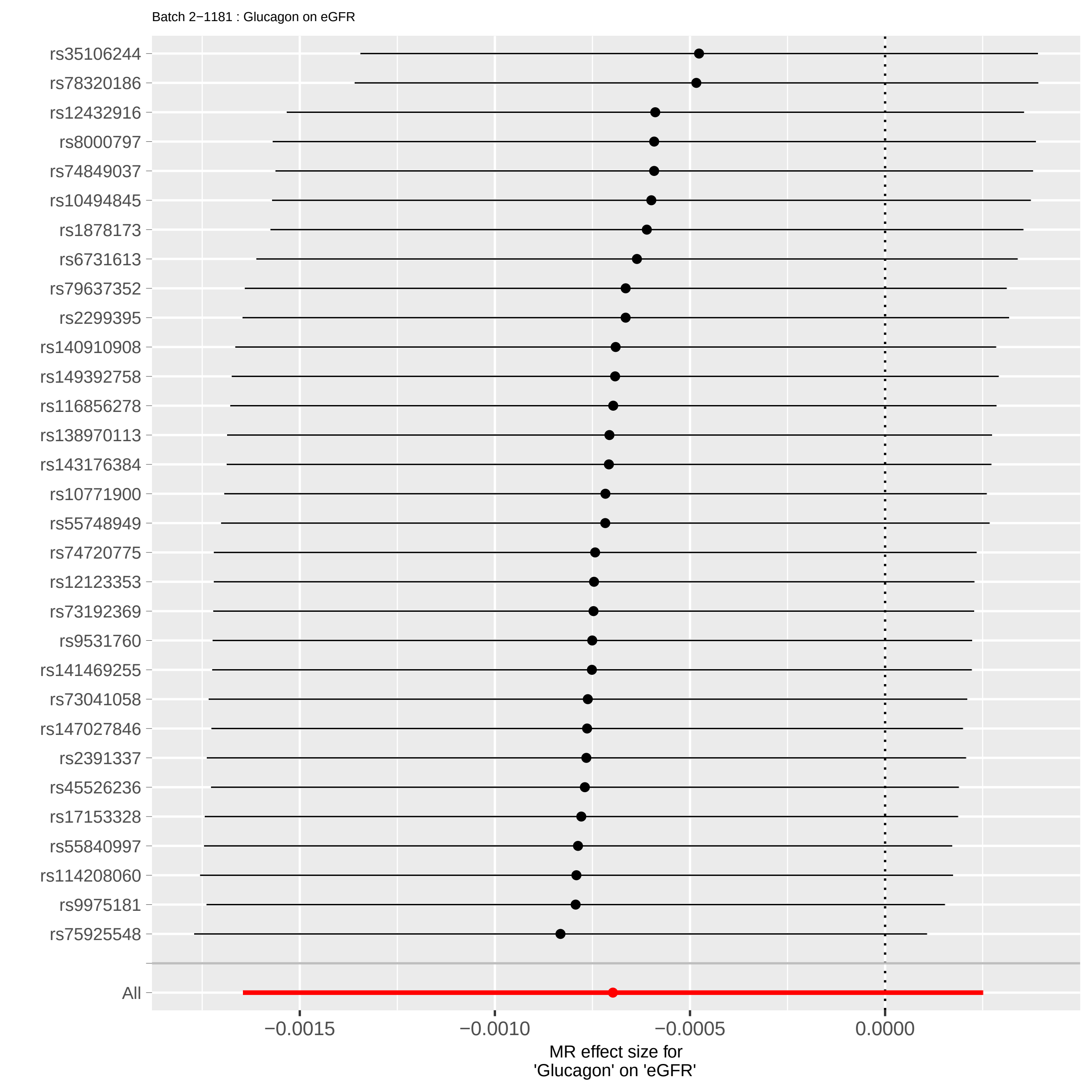
Batch 2−1181 : Glucagon on eGFR
rs35106244
rs78320186
rs12432916
rs8000797
rs74849037
rs10494845
rs1878173
rs6731613
rs79637352
rs2299395
rs140910908
rs149392758
rs116856278
rs138970113
rs143176384
rs10771900
rs55748949
rs74720775
rs12123353
rs73192369
rs9531760
rs141469255
rs73041058
rs147027846
rs2391337
rs45526236
rs17153328
rs55840997
rs114208060
rs9975181
rs75925548
All
−0.0015
−0.0010
−0.0005
0.0000
MR effect size for
'Glucagon' on 'eGFR'

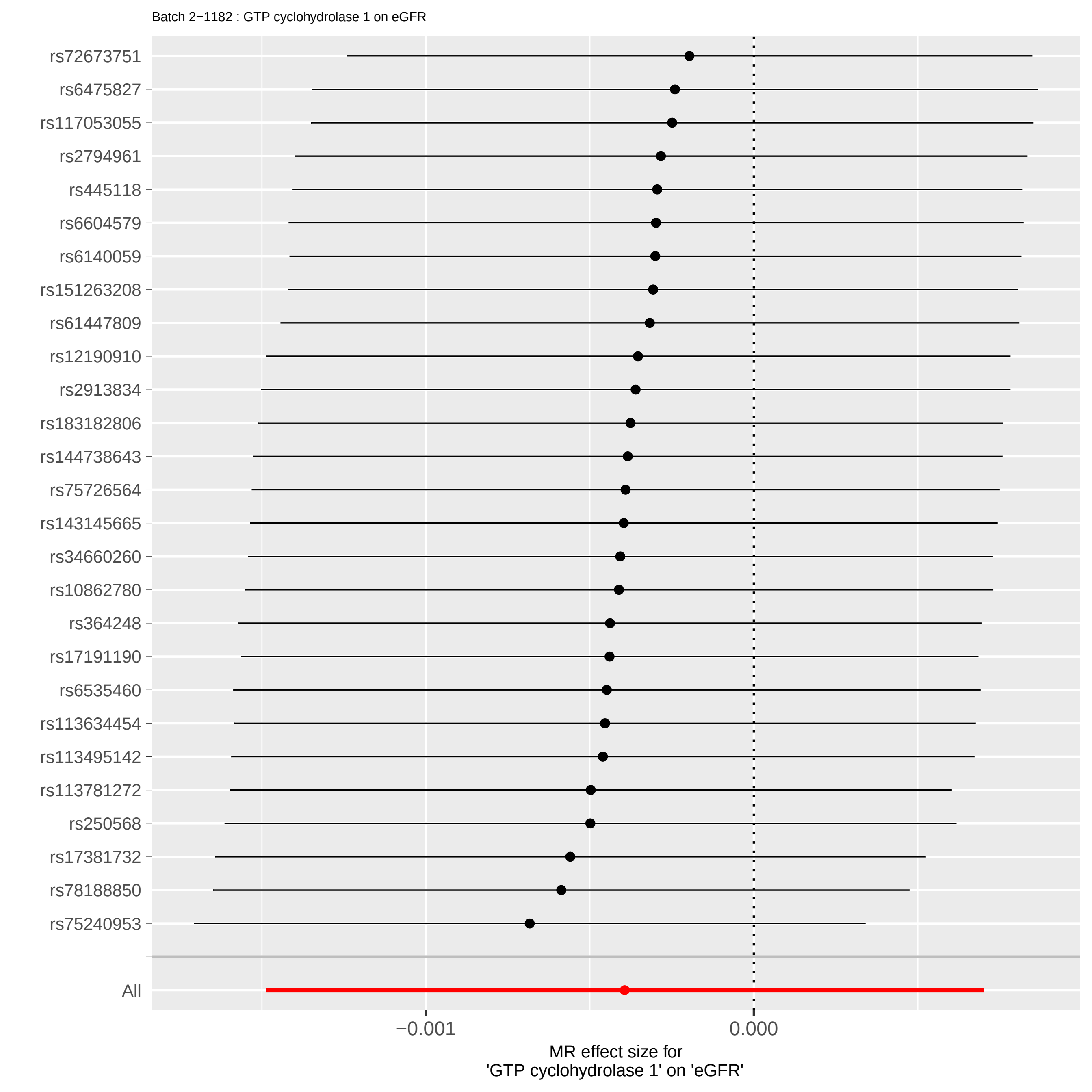

Batch 2−1182 : GTP cyclohydrolase 1 on eGFR
rs72673751
rs6475827
rs117053055
rs2794961
rs445118
rs6604579
rs6140059
rs151263208
rs61447809
rs12190910
rs2913834
rs183182806
rs144738643
rs75726564
rs143145665
rs34660260
rs10862780
rs364248
rs17191190
rs6535460
rs113634454
rs113495142
rs113781272
rs250568
rs17381732
rs78188850
rs75240953
All
−0.001
0.000
MR effect size for
'GTP cyclohydrolase 1' on 'eGFR'

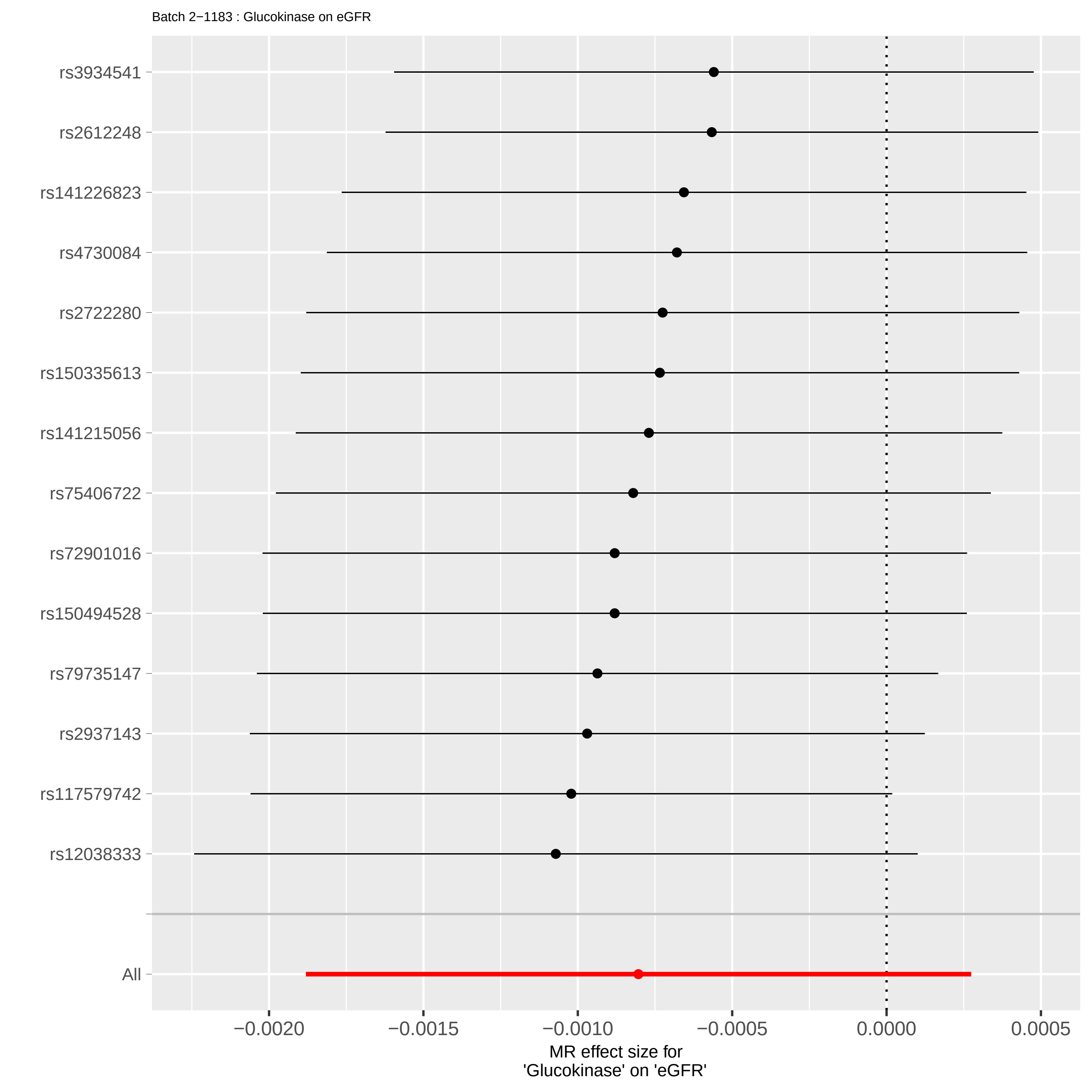

Batch 2−1183 : Glucokinase on eGFR
rs3934541
rs2612248
rs141226823
rs4730084
rs2722280
rs150335613
rs141215056
rs75406722
rs72901016
rs150494528
rs79735147
rs2937143
rs117579742
rs12038333
All
−0.0020
−0.0015
−0.0010
−0.0005
0.0000
0.0005
MR effect size for
'Glucokinase' on 'eGFR'

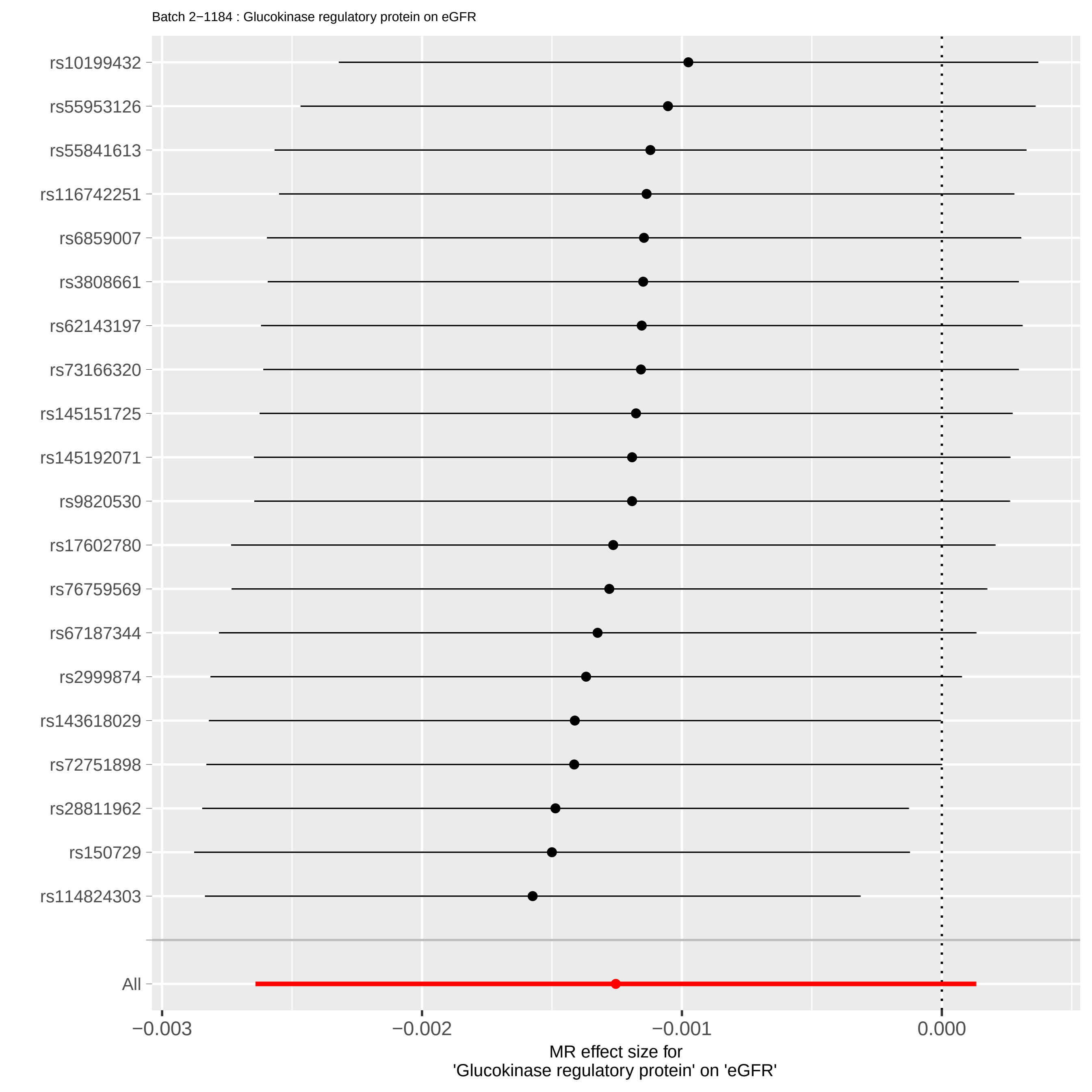

Batch 2−1184 : Glucokinase regulatory protein on eGFR
rs10199432
rs55953126
rs55841613
rs116742251
rs6859007
rs3808661
rs62143197
rs73166320
rs145151725
rs145192071
rs9820530
rs17602780
rs76759569
rs67187344
rs2999874
rs143618029
rs72751898
rs28811962
rs150729
rs114824303
All
−0.003
−0.002
−0.001
0.000
MR effect size for
'Glucokinase regulatory protein' on 'eGFR'

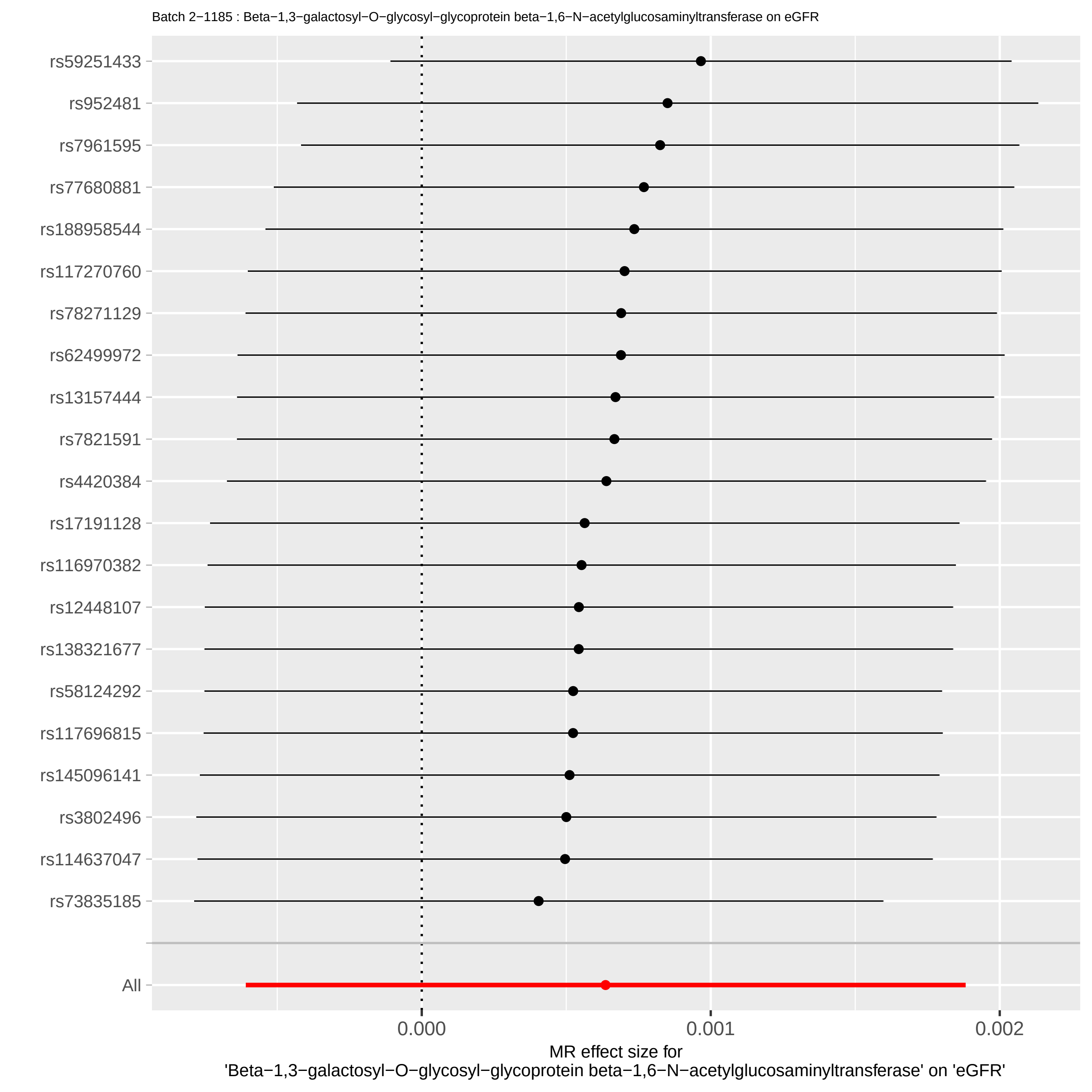

Batch 2−1185 : Beta−1,3−galactosyl−O−glycosyl−glycoprotein beta−1,6−N−acetylglucosaminyltransferase on eGFR
rs59251433
rs952481
rs7961595
rs77680881
rs188958544
rs117270760
rs78271129
rs62499972
rs13157444
rs7821591
rs4420384
rs17191128
rs116970382
rs12448107
rs138321677
rs58124292
rs117696815
rs145096141
rs3802496
rs114637047
rs73835185
All
0.000
0.001
0.002
MR effect size for
'Beta−1,3−galactosyl−O−glycosyl−glycoprotein beta−1,6−N−acetylglucosaminyltransferase' on 'eGFR'

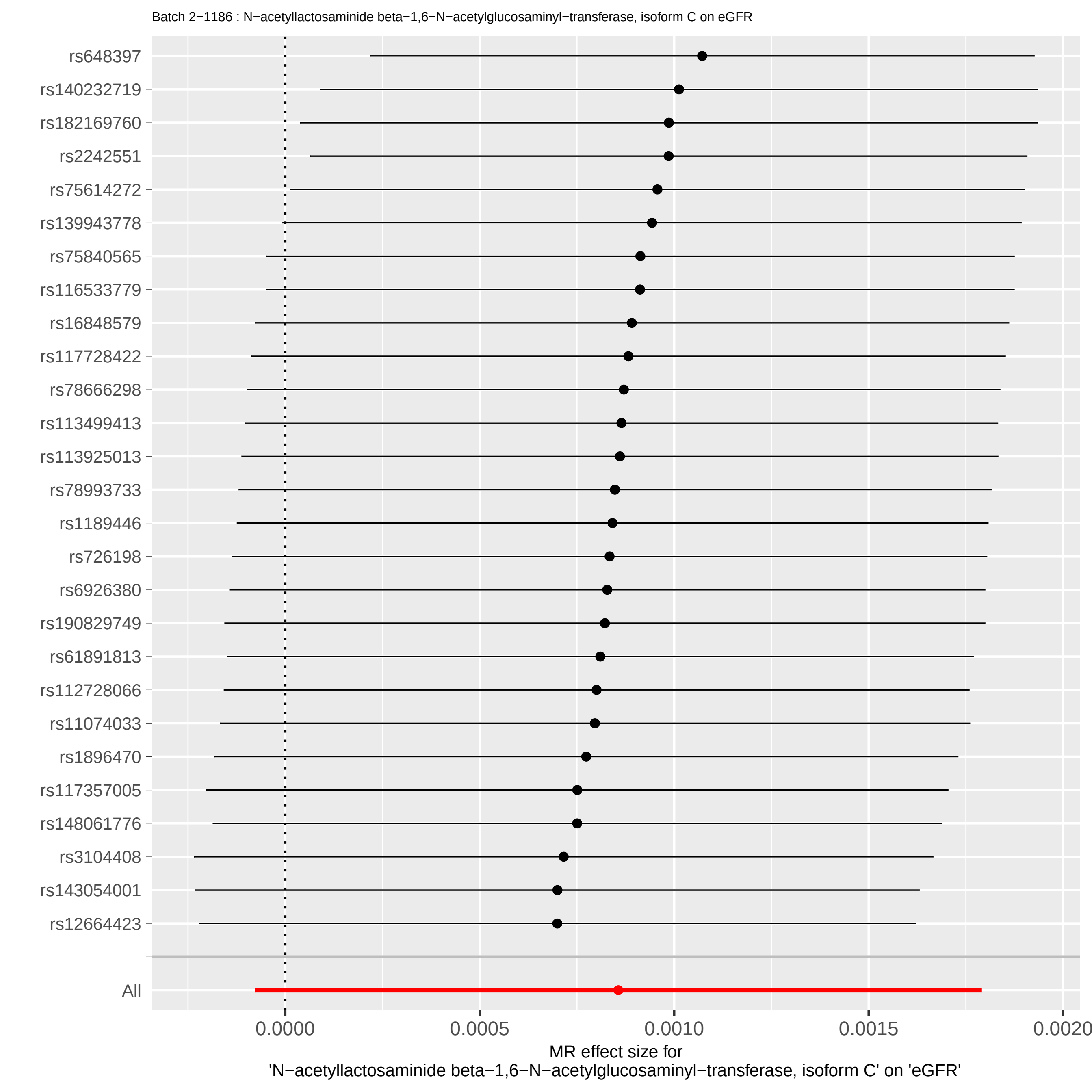
Batch 2−1186 : N−acetyllactosaminide beta−1,6−N−acetylglucosaminyl−transferase, isoform C on eGFR
rs648397
rs140232719
rs182169760
rs2242551
rs75614272
rs139943778
rs75840565
rs116533779
rs16848579
rs117728422
rs78666298
rs113499413
rs113925013
rs78993733
rs1189446
rs726198
rs6926380
rs190829749
rs61891813
rs112728066
rs11074033
rs1896470
rs117357005
rs148061776
rs3104408
rs143054001
rs12664423
All
0.0000
0.0005
0.0010
0.0015
0.0020
MR effect size for
'N−acetyllactosaminide beta−1,6−N−acetylglucosaminyl−transferase, isoform C' on 'eGFR'

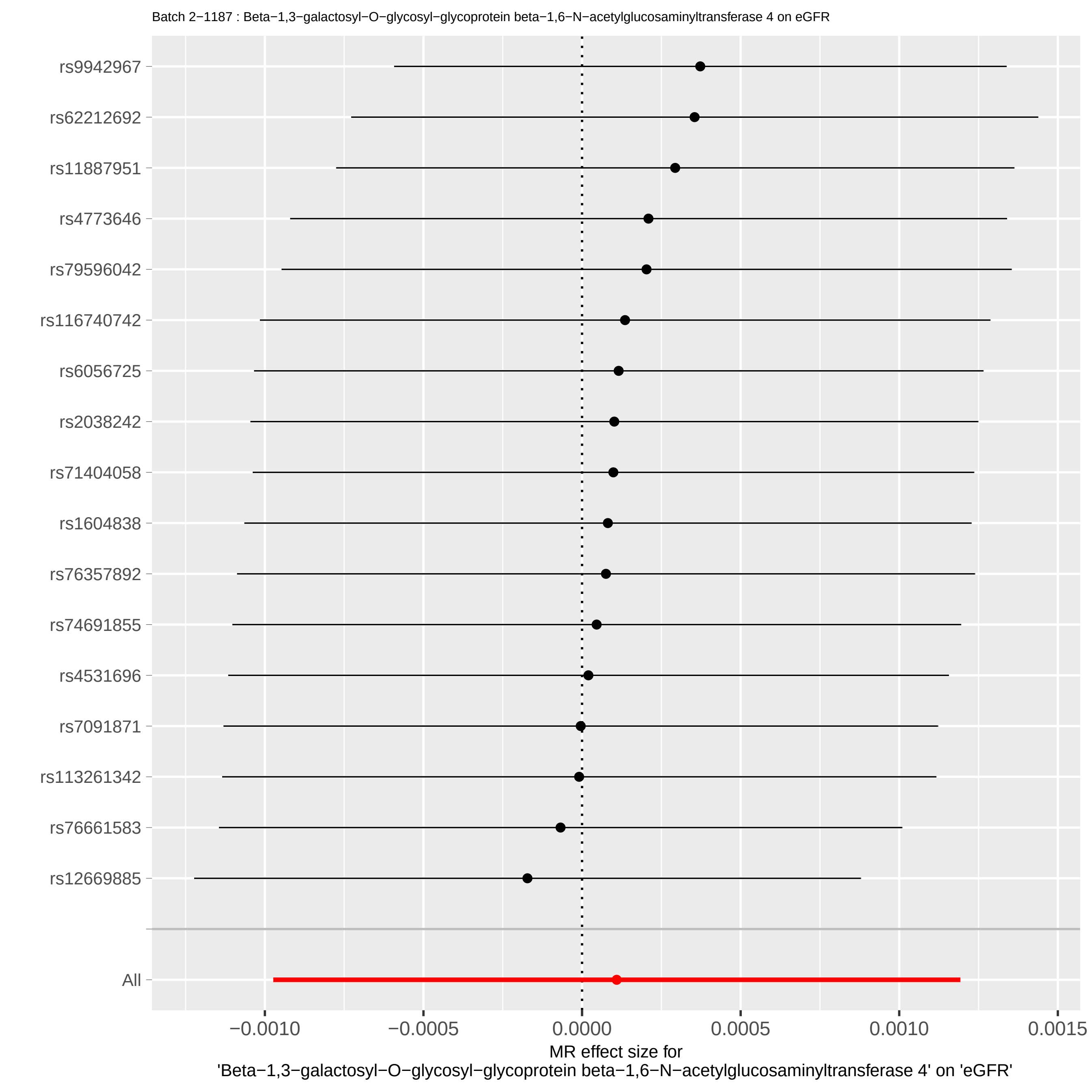
Batch 2−1187 : Beta−1,3−galactosyl−O−glycosyl−glycoprotein beta−1,6−N−acetylglucosaminyltransferase 4 on eGFR
rs9942967
rs62212692
rs11887951
rs4773646
rs79596042
rs116740742
rs6056725
rs2038242
rs71404058
rs1604838
rs76357892
rs74691855
rs4531696
rs7091871
rs113261342
rs76661583
rs12669885
All
−0.0010
−0.0005
0.0000
0.0005
0.0010
0.0015
MR effect size for
'Beta−1,3−galactosyl−O−glycosyl−glycoprotein beta−1,6−N−acetylglucosaminyltransferase 4' on 'eGFR'

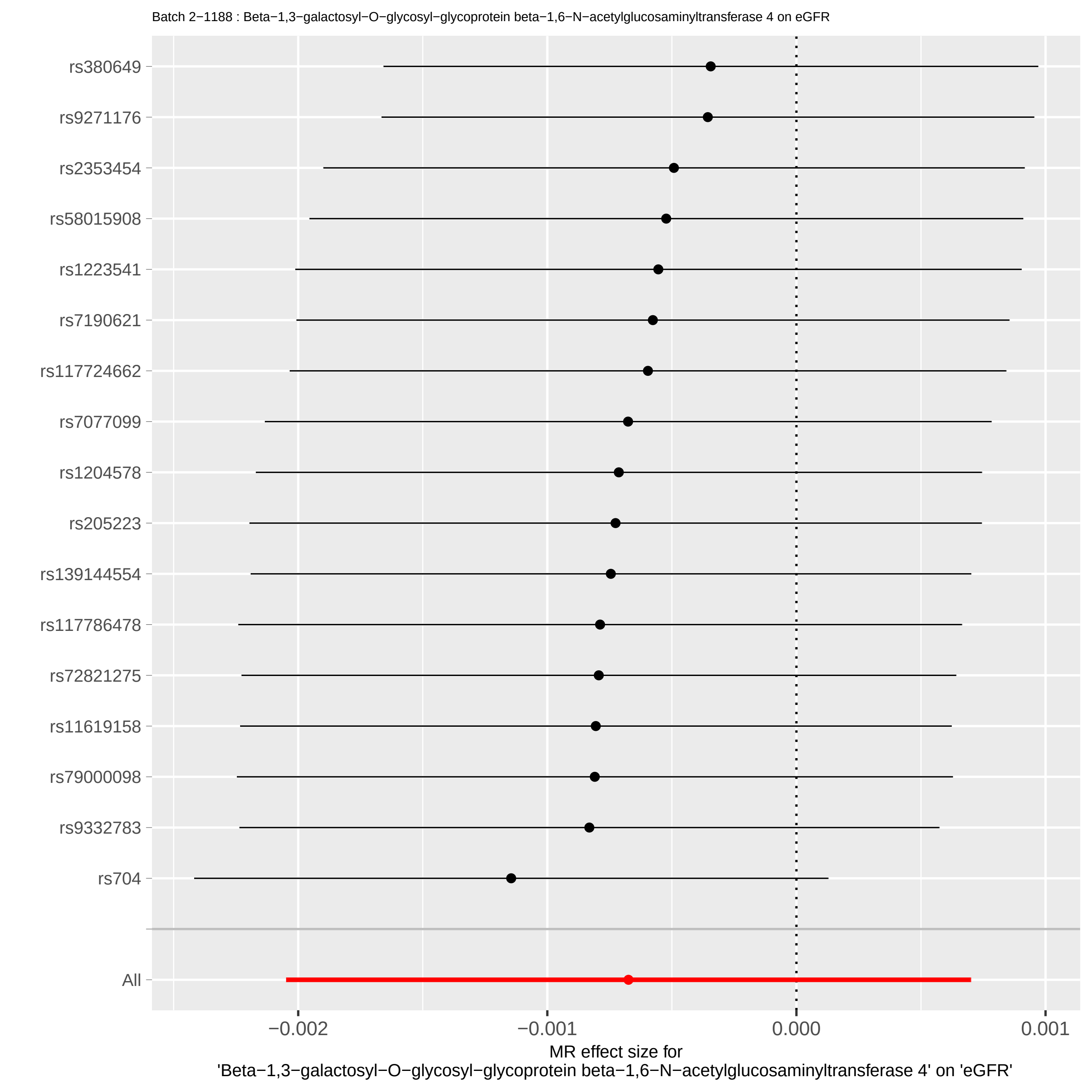

Batch 2−1188 : Beta−1,3−galactosyl−O−glycosyl−glycoprotein beta−1,6−N−acetylglucosaminyltransferase 4 on eGFR
rs380649
rs9271176
rs2353454
rs58015908
rs1223541
rs7190621
rs117724662
rs7077099
rs1204578
rs205223
rs139144554
rs117786478
rs72821275
rs11619158
rs79000098
rs9332783
rs704
All
−0.002
−0.001
0.000
0.001
MR effect size for
'Beta−1,3−galactosyl−O−glycosyl−glycoprotein beta−1,6−N−acetylglucosaminyltransferase 4' on 'eGFR'

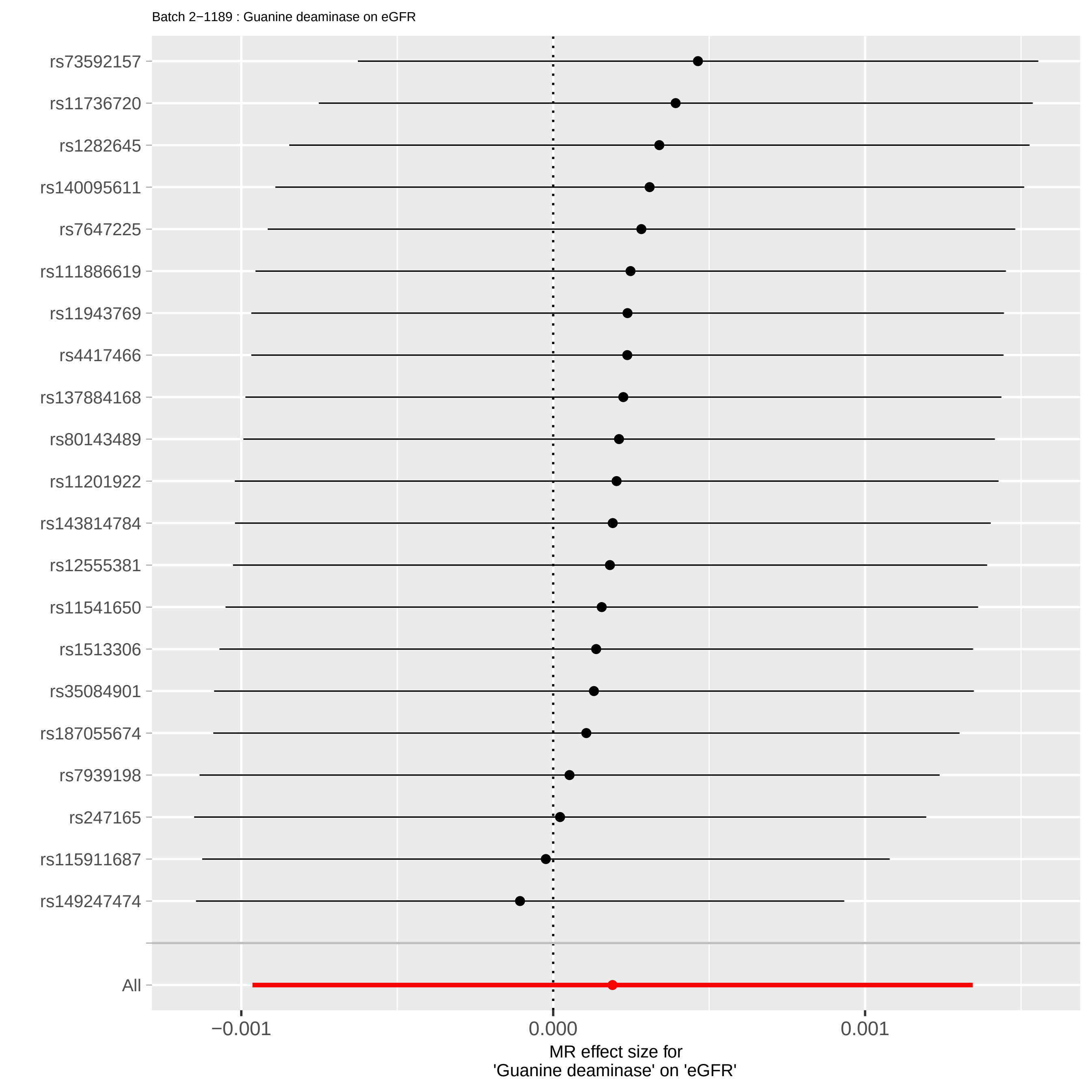

Batch 2−1189 : Guanine deaminase on eGFR
rs73592157
rs11736720
rs1282645
rs140095611
rs7647225
rs111886619
rs11943769
rs4417466
rs137884168
rs80143489
rs11201922
rs143814784
rs12555381
rs11541650
rs1513306
rs35084901
rs187055674
rs7939198
rs247165
rs115911687
rs149247474
All
−0.001
0.000
0.001
MR effect size for
'Guanine deaminase' on 'eGFR'

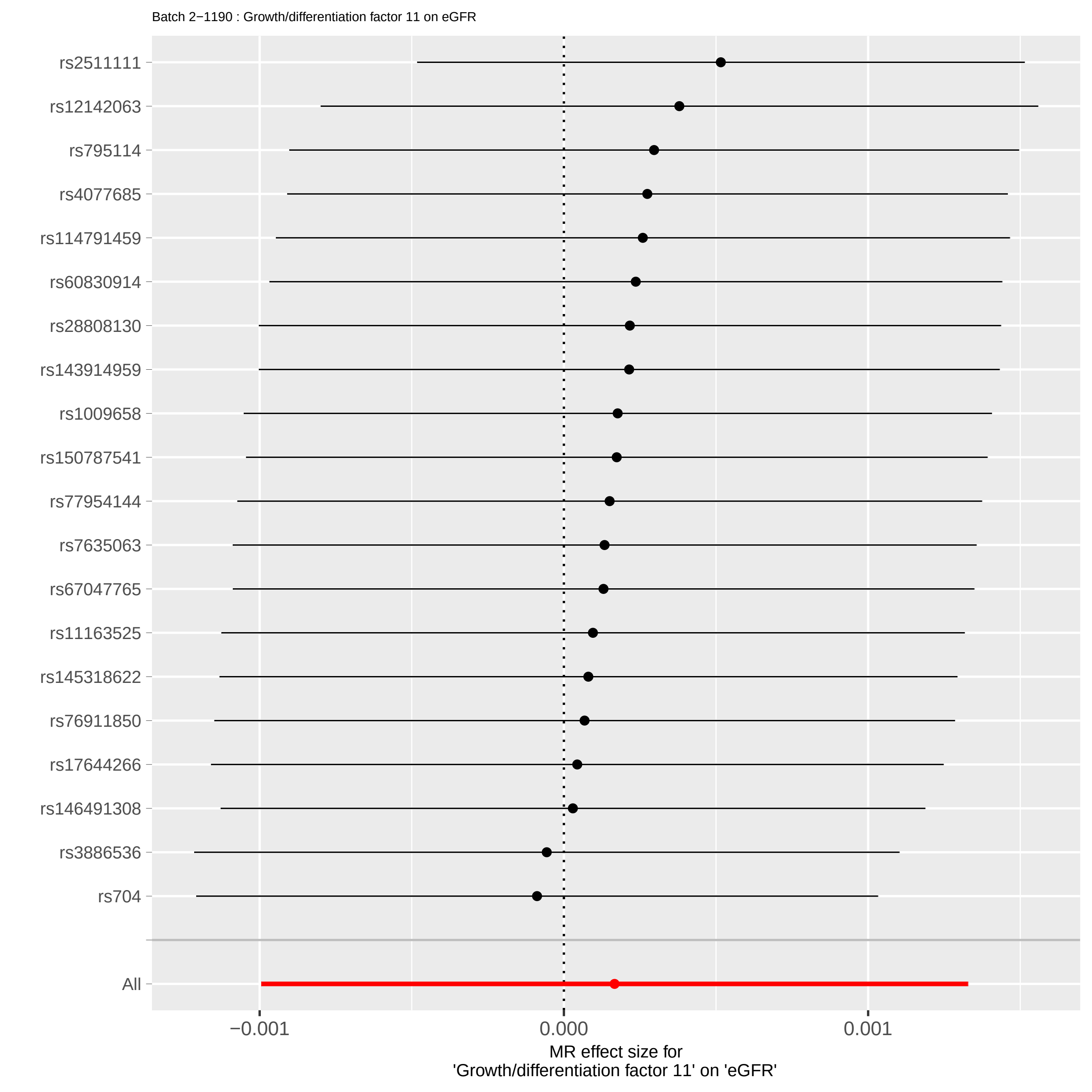

Batch 2−1190 : Growth/differentiation factor 11 on eGFR
rs2511111
rs12142063
rs795114
rs4077685
rs114791459
rs60830914
rs28808130
rs143914959
rs1009658
rs150787541
rs77954144
rs7635063
rs67047765
rs11163525
rs145318622
rs76911850
rs17644266
rs146491308
rs3886536
rs704
All
−0.001
0.000
0.001
MR effect size for
'Growth/differentiation factor 11' on 'eGFR'

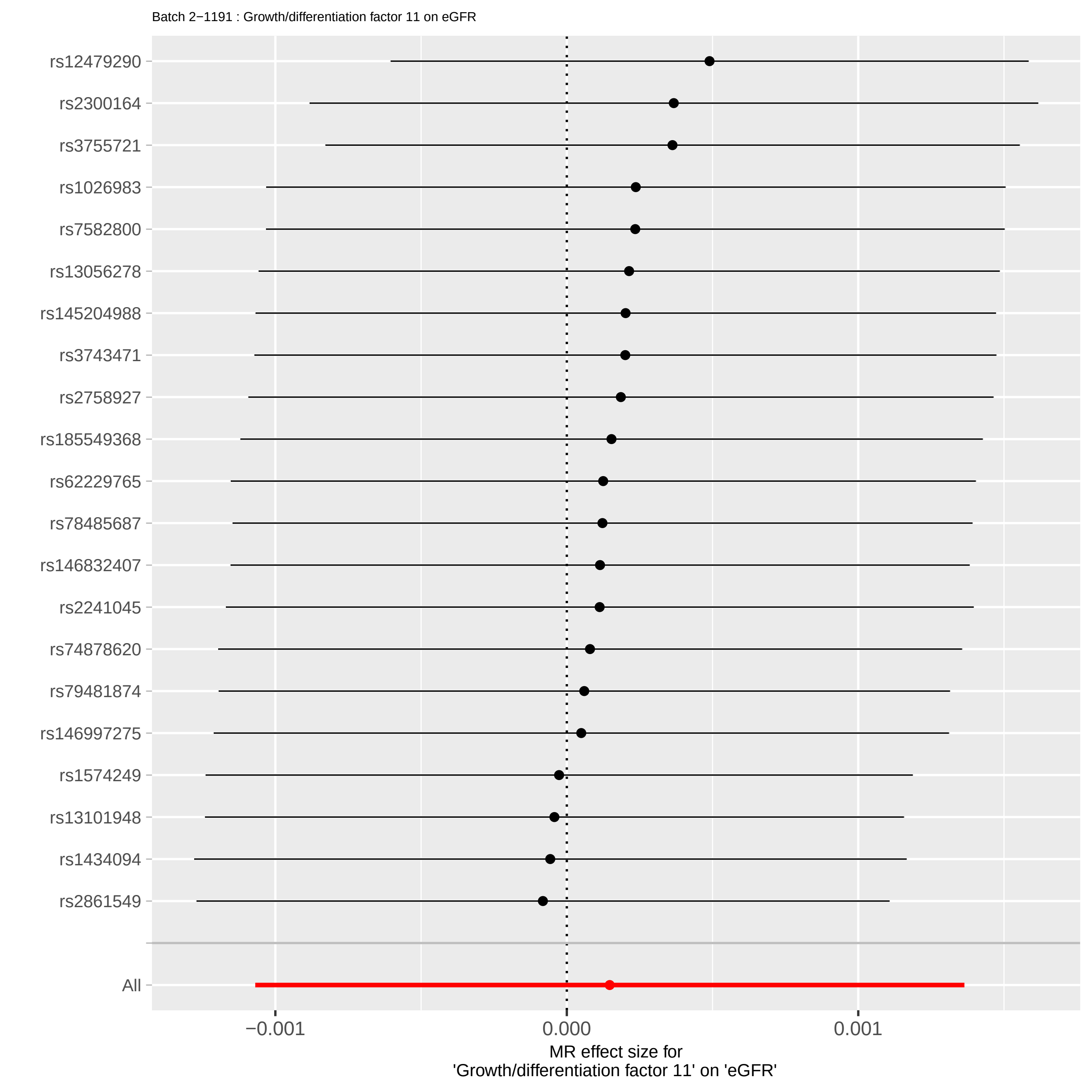

Batch 2−1191 : Growth/differentiation factor 11 on eGFR
rs12479290
rs2300164
rs3755721
rs1026983
rs7582800
rs13056278
rs145204988
rs3743471
rs2758927
rs185549368
rs62229765
rs78485687
rs146832407
rs2241045
rs74878620
rs79481874
rs146997275
rs1574249
rs13101948
rs1434094
rs2861549
All
−0.001
0.000
0.001
MR effect size for
'Growth/differentiation factor 11' on 'eGFR'

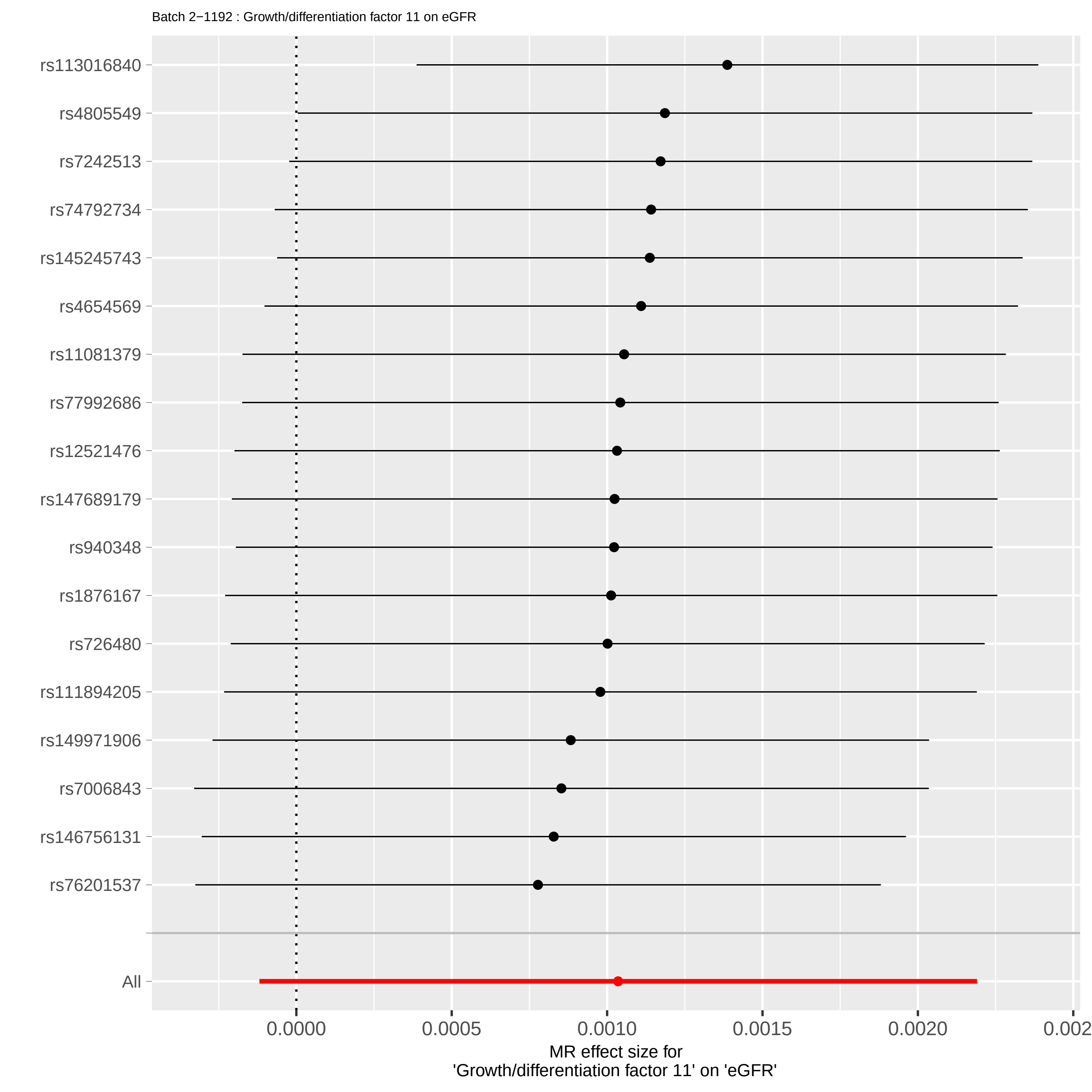

Batch 2−1192 : Growth/differentiation factor 11 on eGFR
rs113016840
rs4805549
rs7242513
rs74792734
rs145245743
rs4654569
rs11081379
rs77992686
rs12521476
rs147689179
rs940348
rs1876167
rs726480
rs111894205
rs149971906
rs7006843
rs146756131
rs76201537
All
0.0000
0.0005
0.0010
0.0015
0.0020
MR effect size for
'Growth/differentiation factor 11' on 'eGFR'

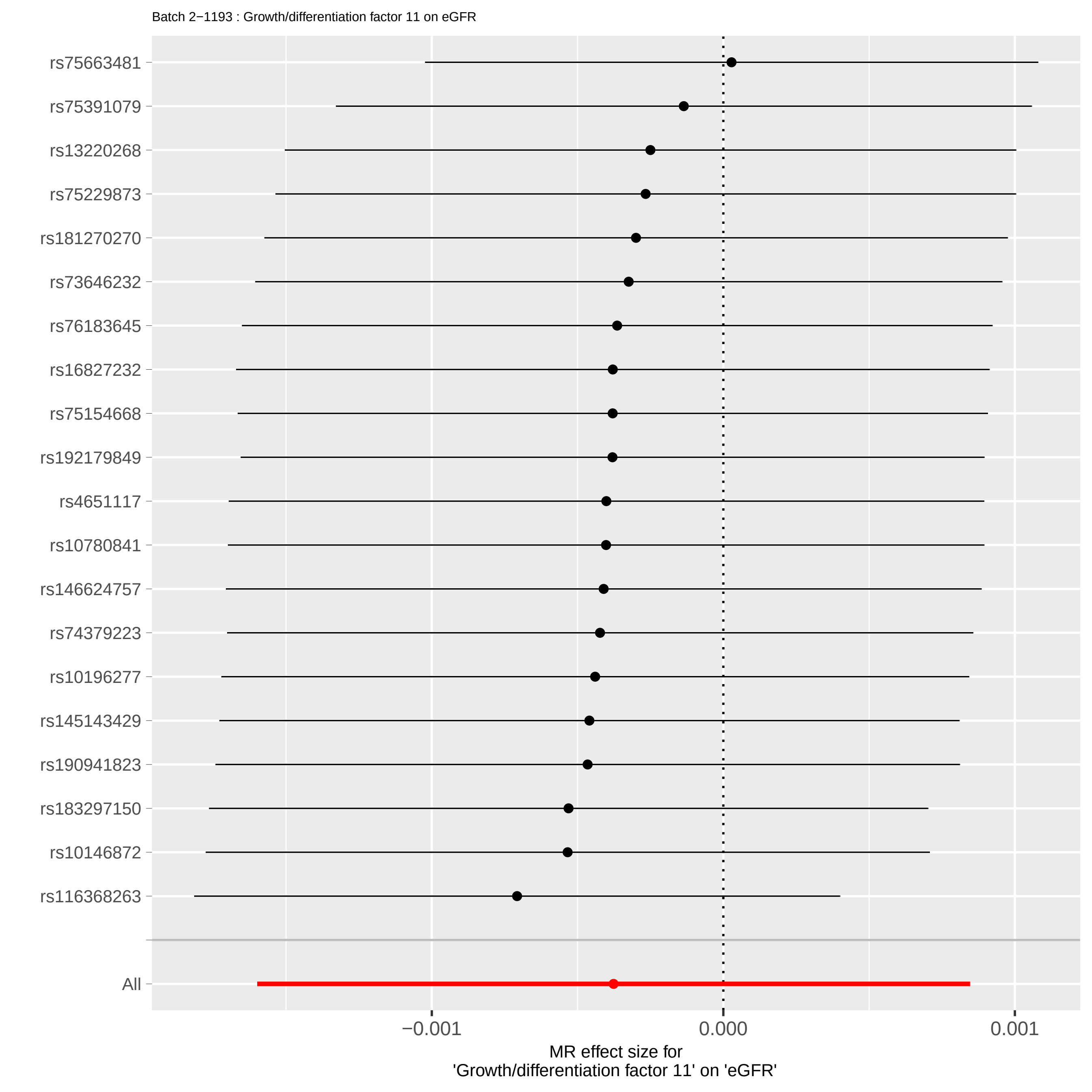
Batch 2−1193 : Growth/differentiation factor 11 on eGFR
rs75663481
rs75391079
rs13220268
rs75229873
rs181270270
rs73646232
rs76183645
rs16827232
rs75154668
rs192179849
rs4651117
rs10780841
rs146624757
rs74379223
rs10196277
rs145143429
rs190941823
rs183297150
rs10146872
rs116368263
All
−0.001
0.000
0.001
MR effect size for
'Growth/differentiation factor 11' on 'eGFR'

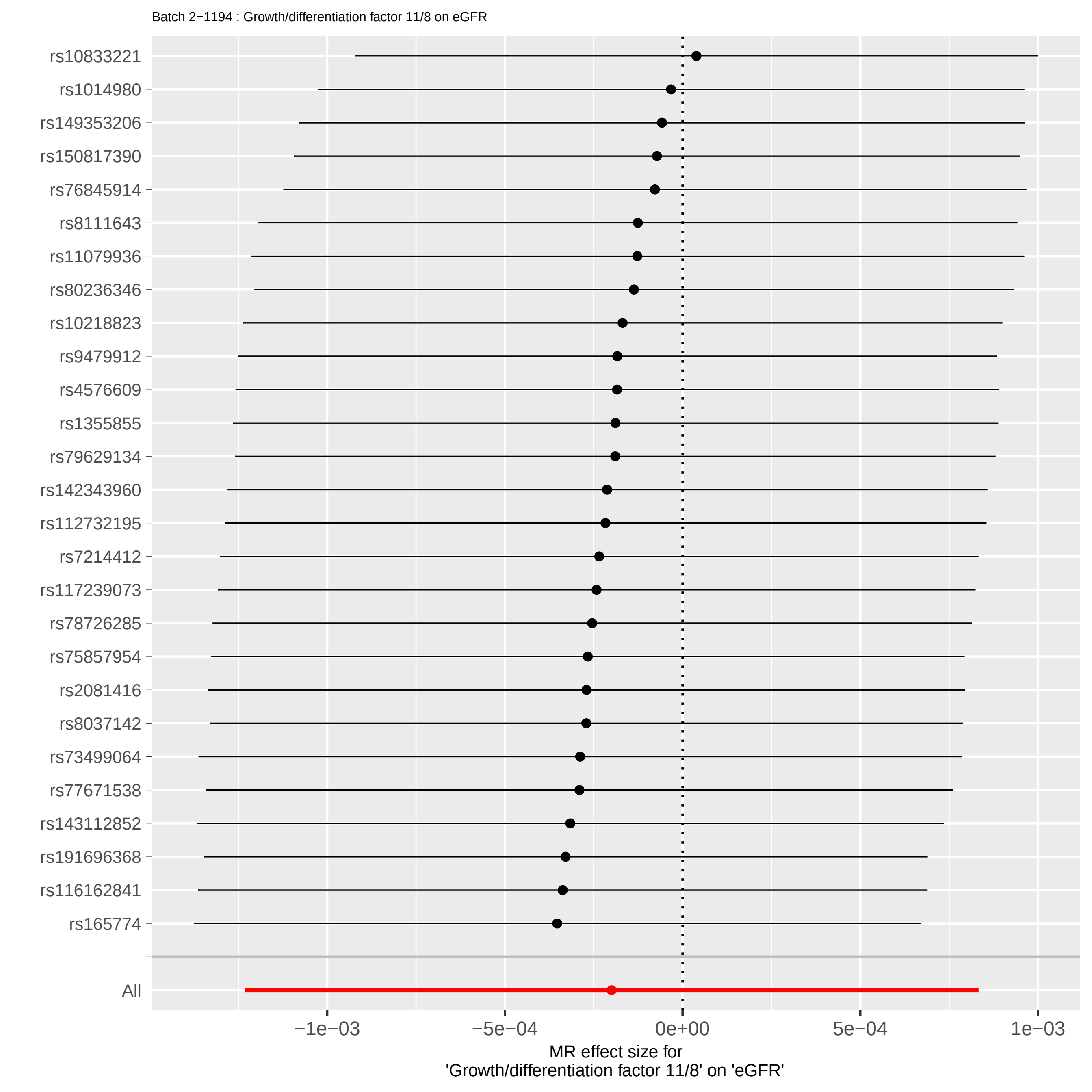

Batch 2−1194 : Growth/differentiation factor 11/8 on eGFR
rs10833221
rs1014980
rs149353206
rs150817390
rs76845914
rs8111643
rs11079936
rs80236346
rs10218823
rs9479912
rs4576609
rs1355855
rs79629134
rs142343960
rs112732195
rs7214412
rs117239073
rs78726285
rs75857954
rs2081416
rs8037142
rs73499064
rs77671538
rs143112852
rs191696368
rs116162841
rs165774
All
−1e−03
−5e−04
0e+00
5e−04
1e−03
MR effect size for
'Growth/differentiation factor 11/8' on 'eGFR'

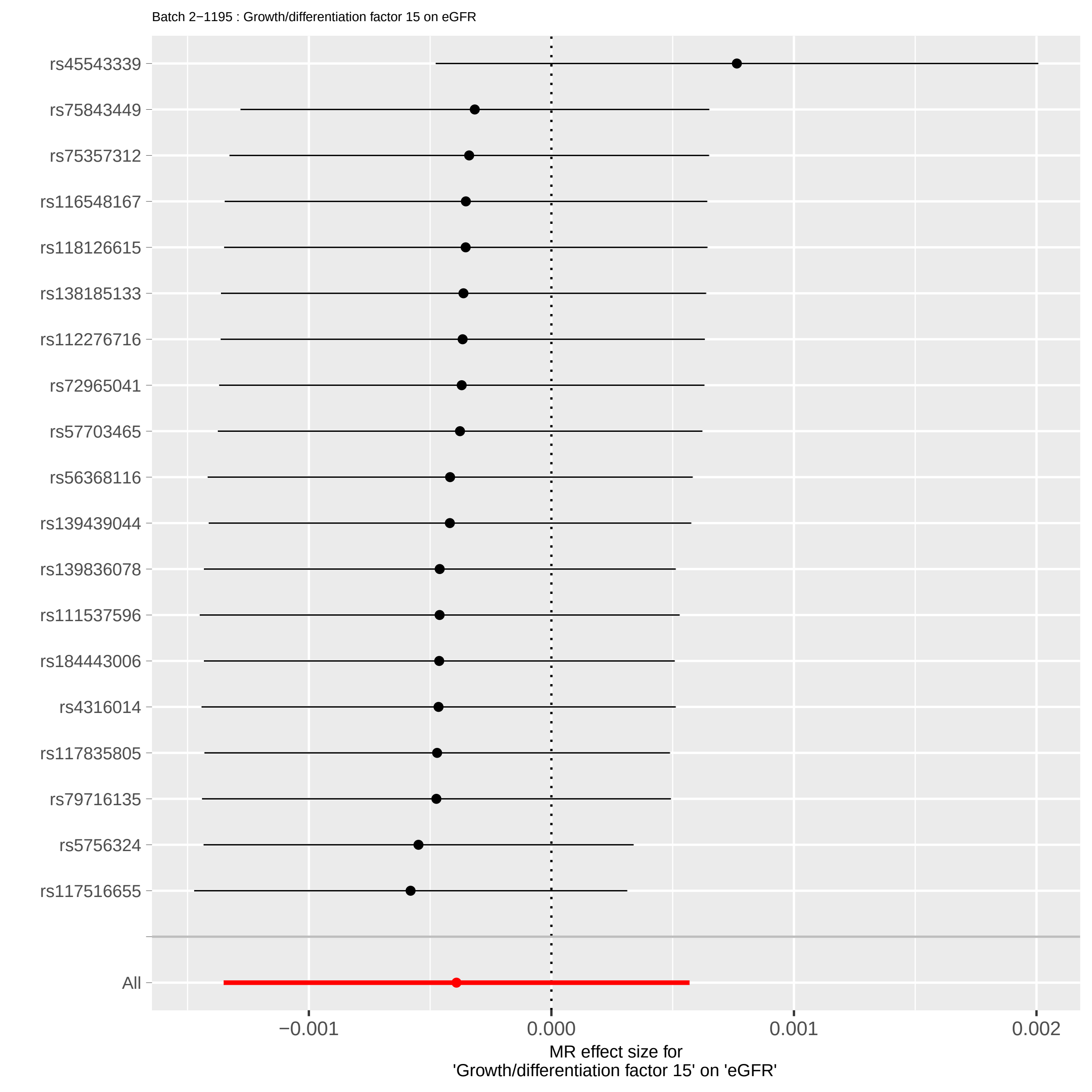

Batch 2−1195 : Growth/differentiation factor 15 on eGFR
rs45543339
rs75843449
rs75357312
rs116548167
rs118126615
rs138185133
rs112276716
rs72965041
rs57703465
rs56368116
rs139439044
rs139836078
rs111537596
rs184443006
rs4316014
rs117835805
rs79716135
rs5756324
rs117516655
All
−0.001
0.000
0.001
0.002
MR effect size for
'Growth/differentiation factor 15' on 'eGFR'

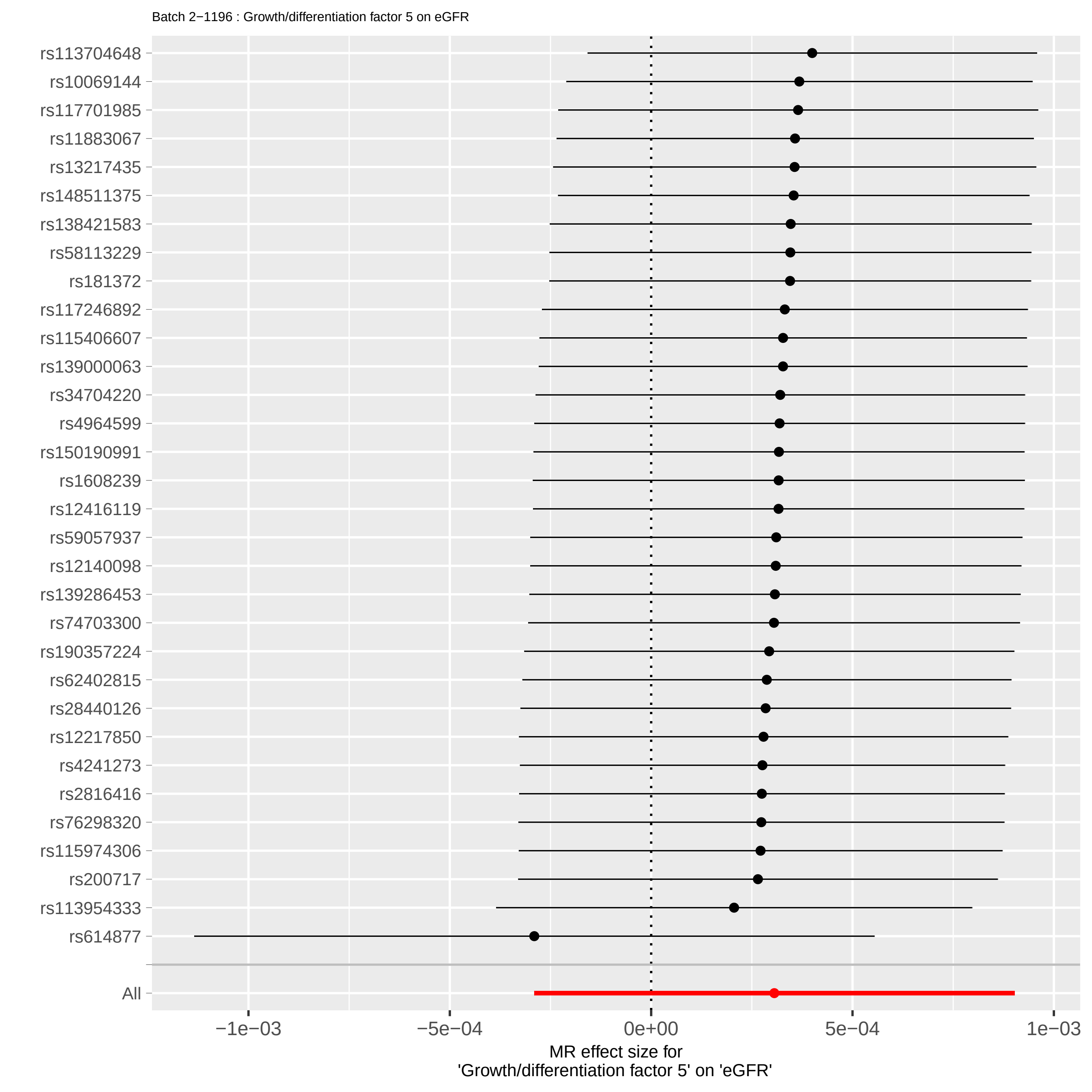
Batch 2−1196 : Growth/differentiation factor 5 on eGFR
rs113704648
rs10069144
rs117701985
rs11883067
rs13217435
rs148511375
rs138421583
rs58113229
rs181372
rs117246892
rs115406607
rs139000063
rs34704220
rs4964599
rs150190991
rs1608239
rs12416119
rs59057937
rs12140098
rs139286453
rs74703300
rs190357224
rs62402815
rs28440126
rs12217850
rs4241273
rs2816416
rs76298320
rs115974306
rs200717
rs113954333
rs614877
All
−1e−03
−5e−04
0e+00
5e−04
1e−03
MR effect size for
'Growth/differentiation factor 5' on 'eGFR'

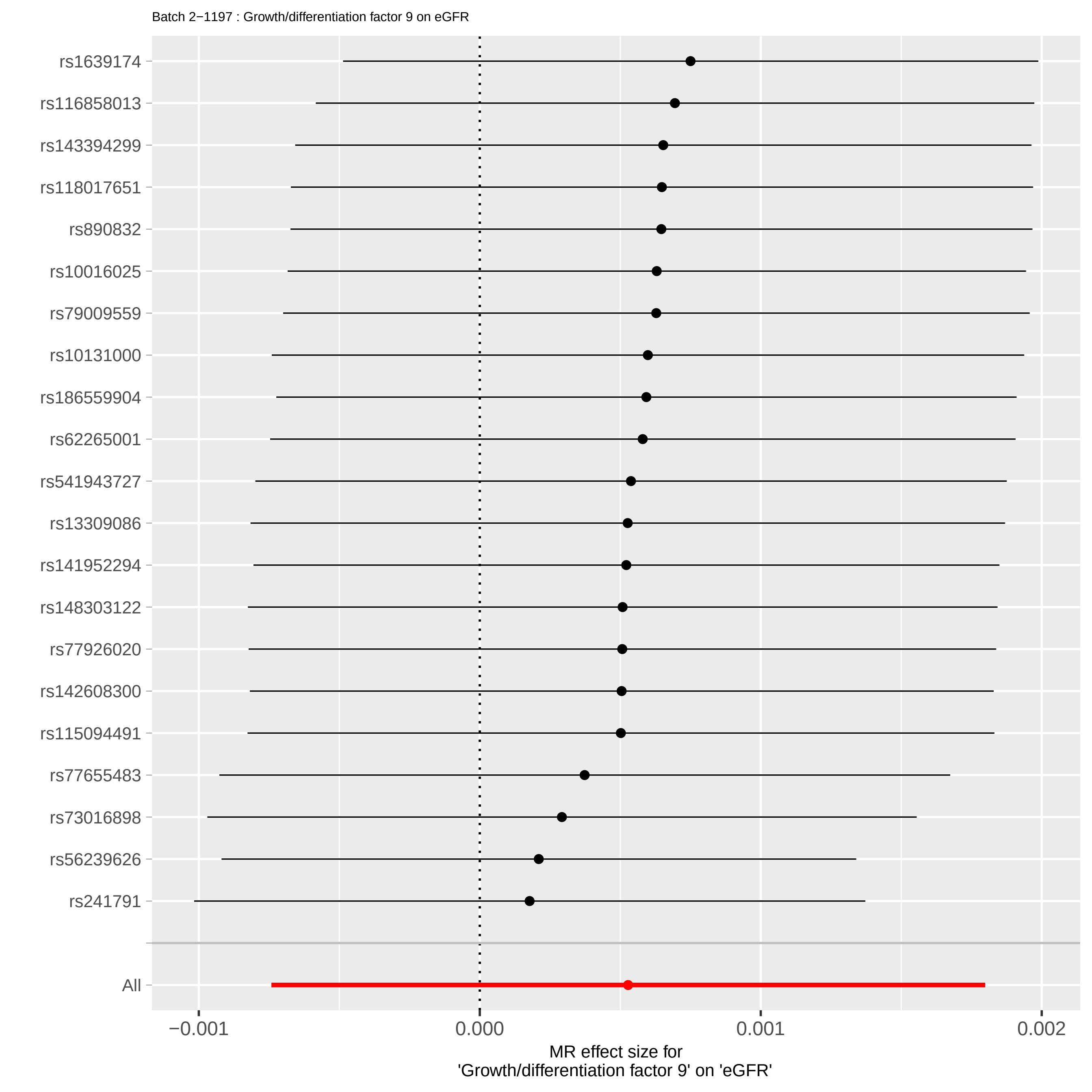
Batch 2−1197 : Growth/differentiation factor 9 on eGFR
rs1639174
rs116858013
rs143394299
rs118017651
rs890832
rs10016025
rs79009559
rs10131000
rs186559904
rs62265001
rs541943727
rs13309086
rs141952294
rs148303122
rs77926020
rs142608300
rs115094491
rs77655483
rs73016898
rs56239626
rs241791
All
−0.001
0.000
0.001
0.002
MR effect size for
'Growth/differentiation factor 9' on 'eGFR'

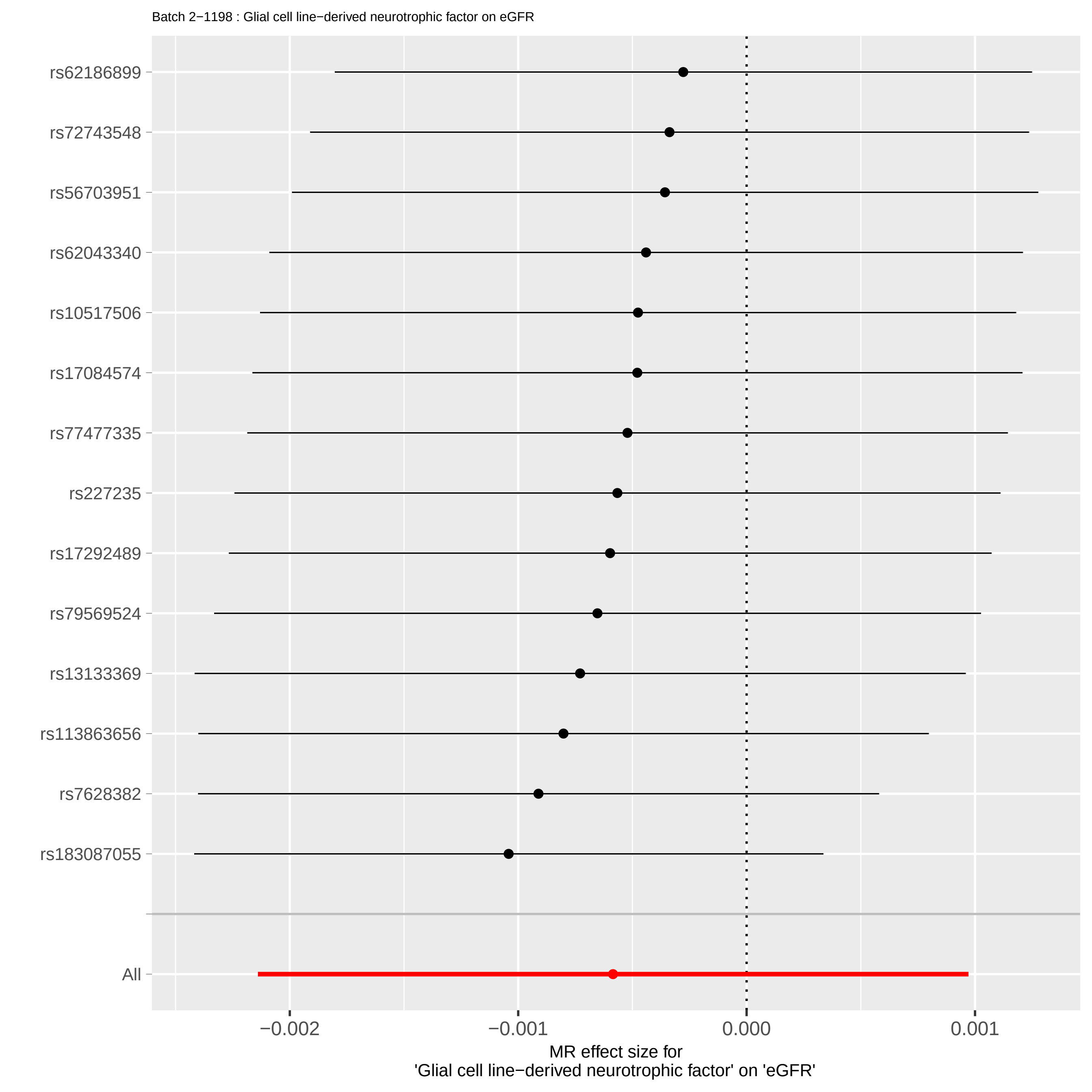
Batch 2−1198 : Glial cell line−derived neurotrophic factor on eGFR
rs62186899
rs72743548
rs56703951
rs62043340
rs10517506
rs17084574
rs77477335
rs227235
rs17292489
rs79569524
rs13133369
rs113863656
rs7628382
rs183087055
All
−0.002
−0.001
0.000
0.001
MR effect size for
'Glial cell line−derived neurotrophic factor' on 'eGFR'

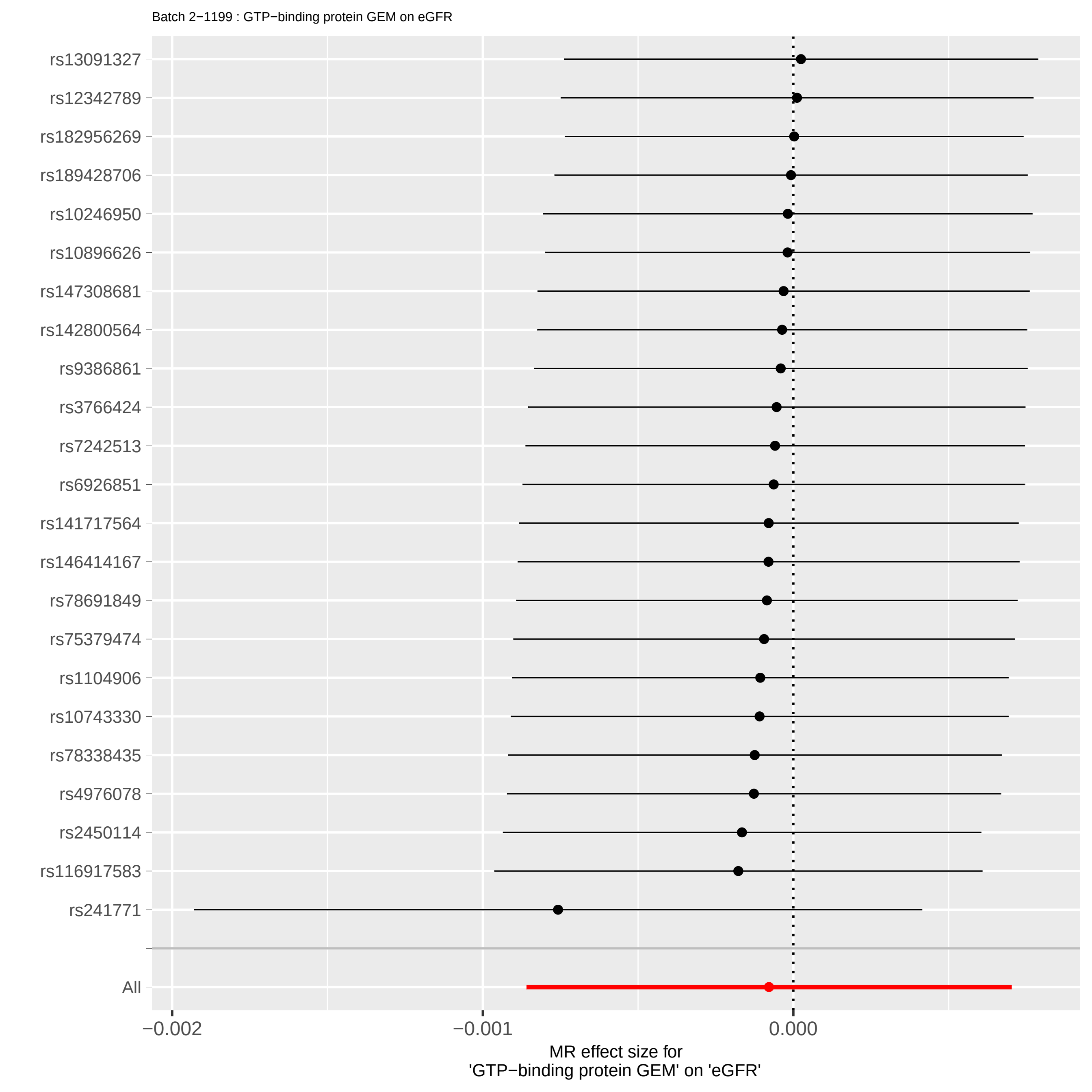
Batch 2−1199 : GTP−binding protein GEM on eGFR
rs13091327
rs12342789
rs182956269
rs189428706
rs10246950
rs10896626
rs147308681
rs142800564
rs9386861
rs3766424
rs7242513
rs6926851
rs141717564
rs146414167
rs78691849
rs75379474
rs1104906
rs10743330
rs78338435
rs4976078
rs2450114
rs116917583
rs241771
All
−0.002
−0.001
0.000
MR effect size for
'GTP−binding protein GEM' on 'eGFR'

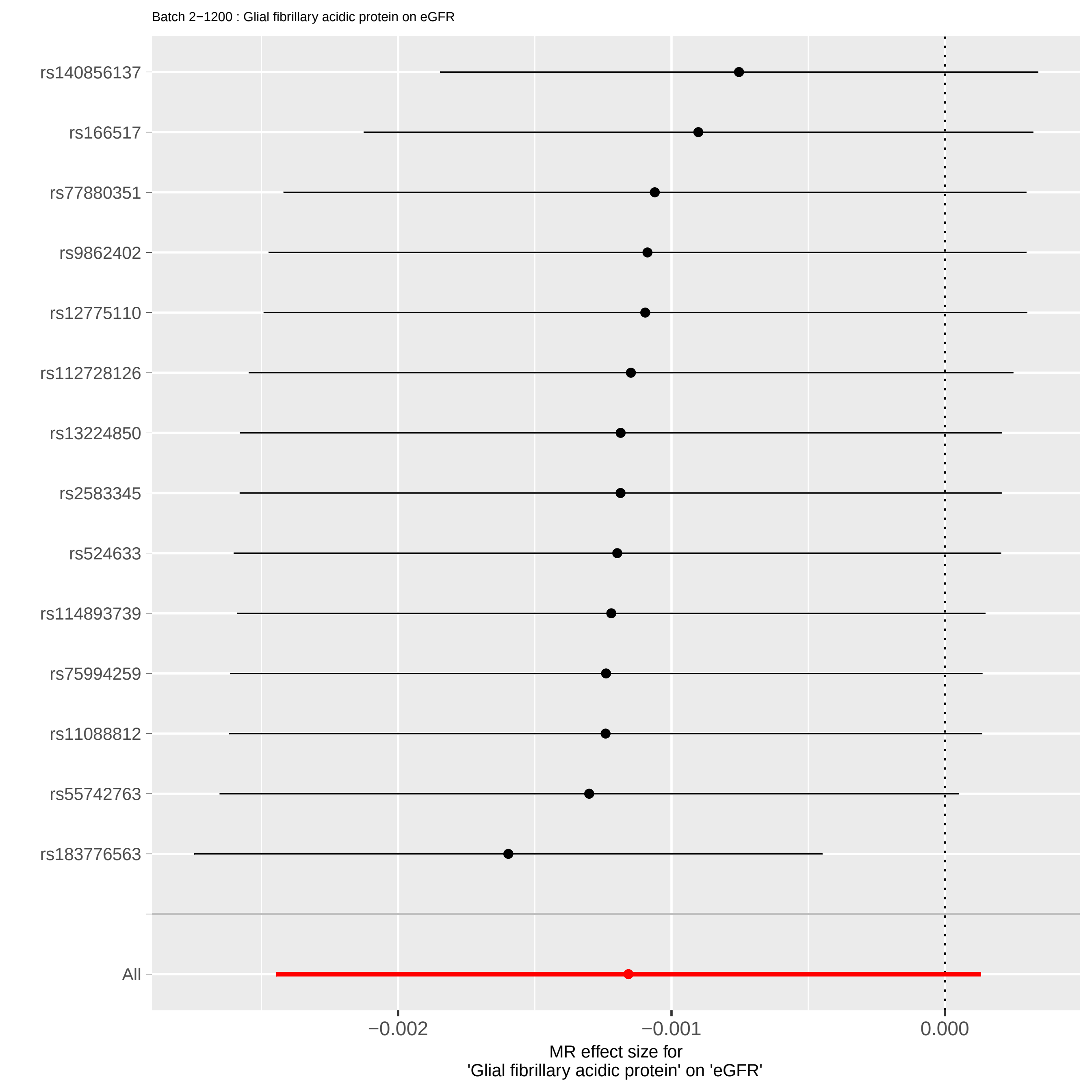

Batch 2−1200 : Glial fibrillary acidic protein on eGFR
rs140856137
rs166517
rs77880351
rs9862402
rs12775110
rs112728126
rs13224850
rs2583345
rs524633
rs114893739
rs75994259
rs11088812
rs55742763
rs183776563
All
−0.002
−0.001
0.000
MR effect size for
'Glial fibrillary acidic protein' on 'eGFR'

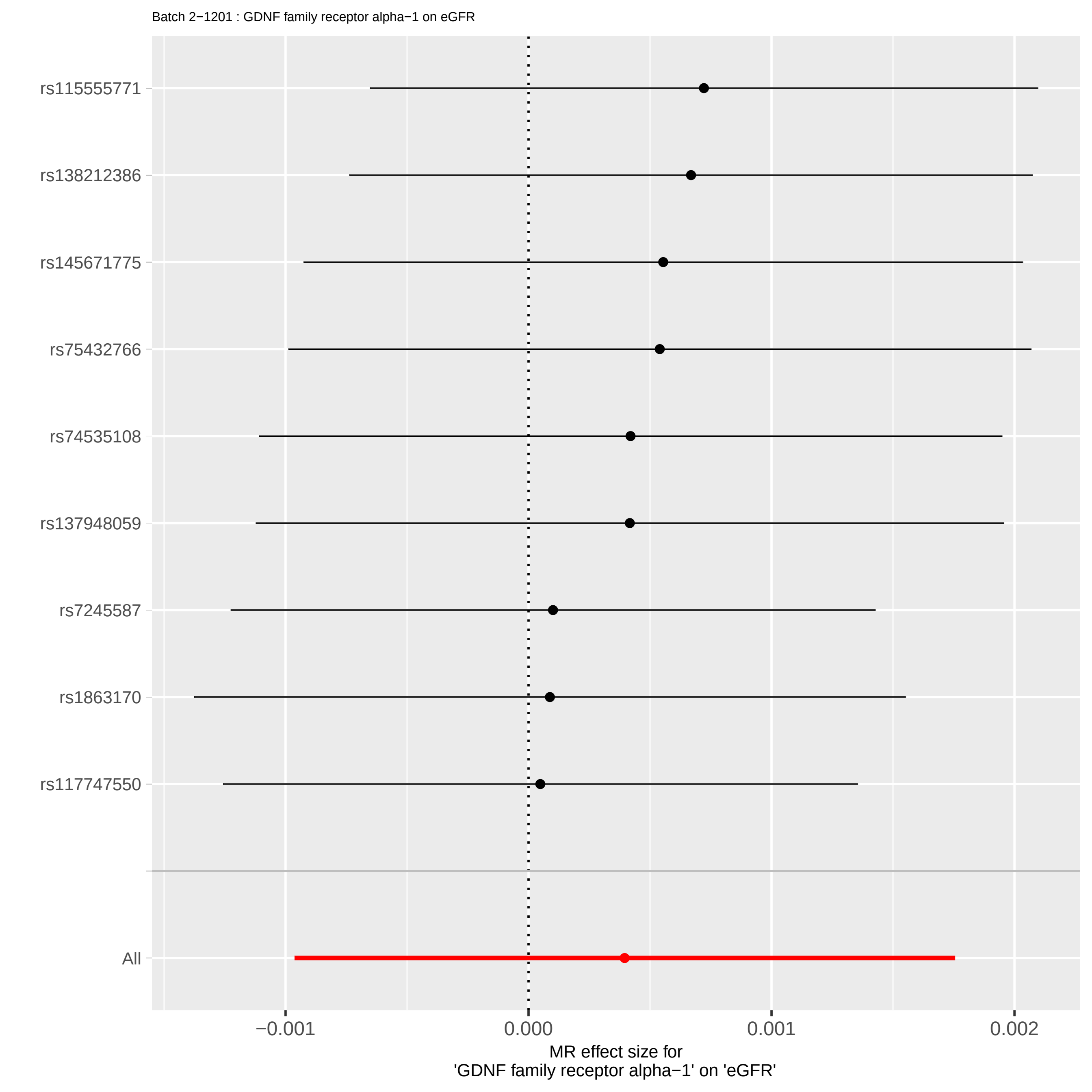

Batch 2−1201 : GDNF family receptor alpha−1 on eGFR
rs115555771
rs138212386
rs145671775
rs75432766
rs74535108
rs137948059
rs7245587
rs1863170
rs117747550
All
−0.001
0.000
0.001
0.002
MR effect size for
'GDNF family receptor alpha−1' on 'eGFR'

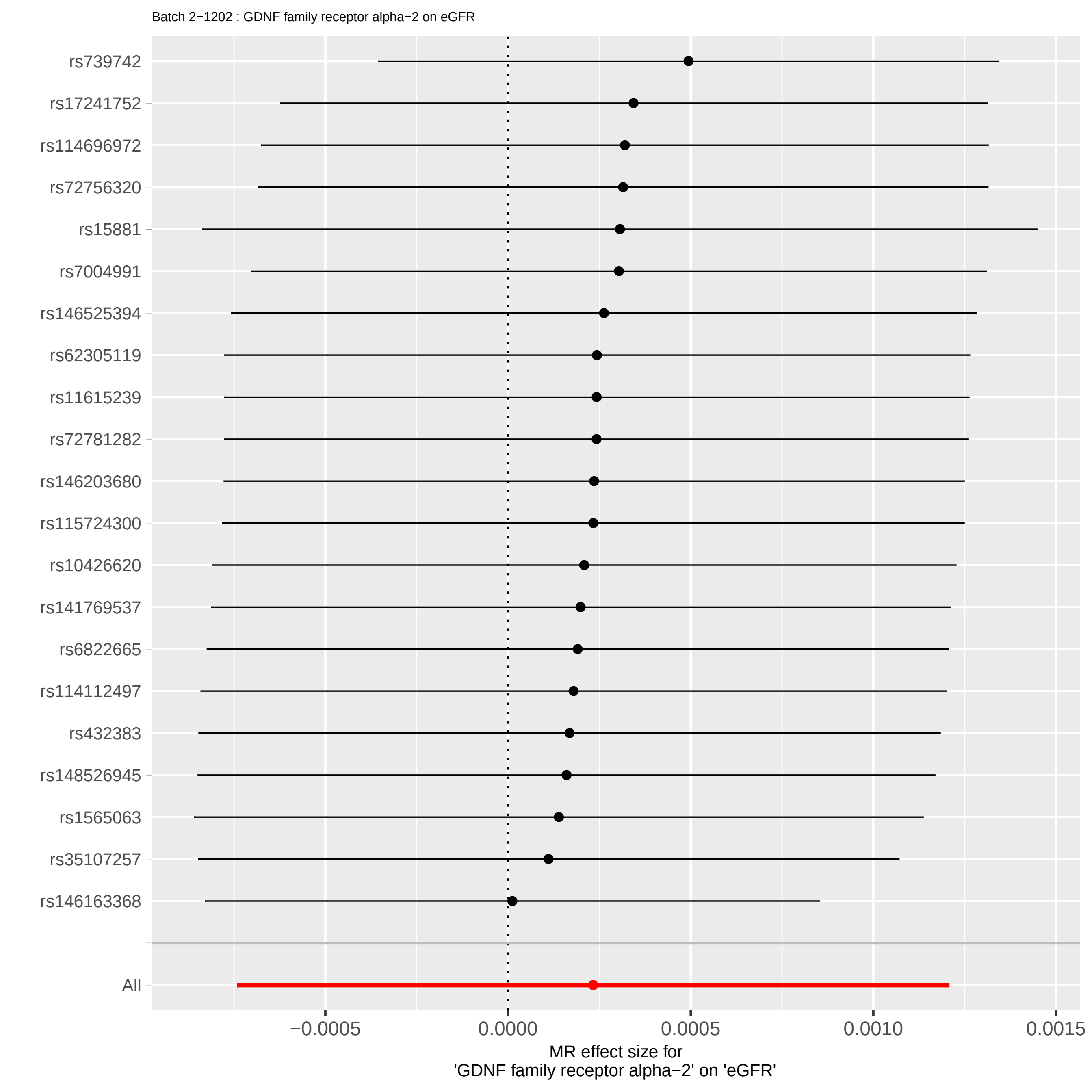

Batch 2−1202 : GDNF family receptor alpha−2 on eGFR
rs739742
rs17241752
rs114696972
rs72756320
rs15881
rs7004991
rs146525394
rs62305119
rs11615239
rs72781282
rs146203680
rs115724300
rs10426620
rs141769537
rs6822665
rs114112497
rs432383
rs148526945
rs1565063
rs35107257
rs146163368
All
−0.0005
0.0000
0.0005
0.0010
0.0015
MR effect size for
'GDNF family receptor alpha−2' on 'eGFR'

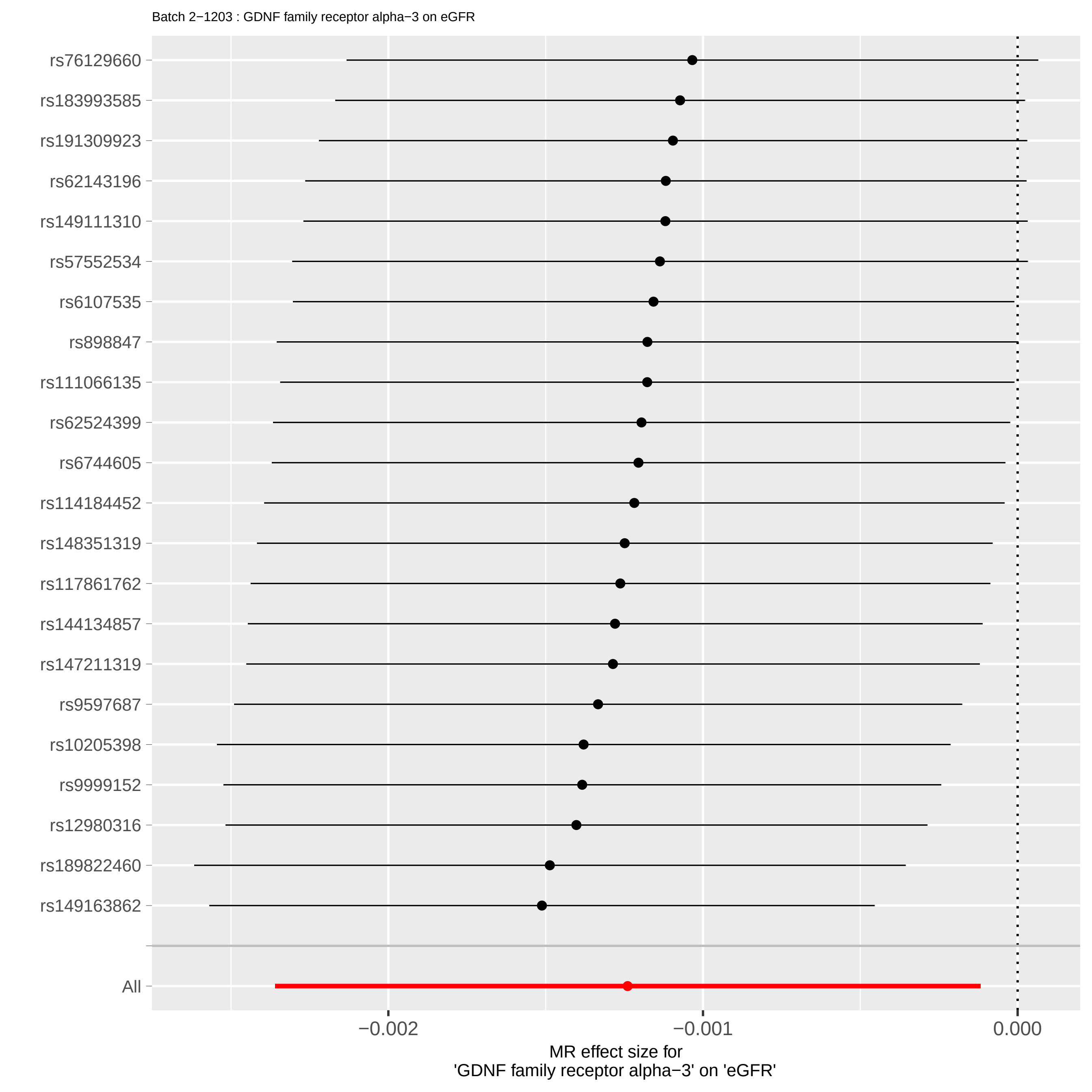

Batch 2−1203 : GDNF family receptor alpha−3 on eGFR
rs76129660
rs183993585
rs191309923
rs62143196
rs149111310
rs57552534
rs6107535
rs898847
rs111066135
rs62524399
rs6744605
rs114184452
rs148351319
rs117861762
rs144134857
rs147211319
rs9597687
rs10205398
rs9999152
rs12980316
rs189822460
rs149163862
All
−0.002
−0.001
0.000
MR effect size for
'GDNF family receptor alpha−3' on 'eGFR'

Batch 2−1204 : GDNF family receptor alpha−like on eGFR

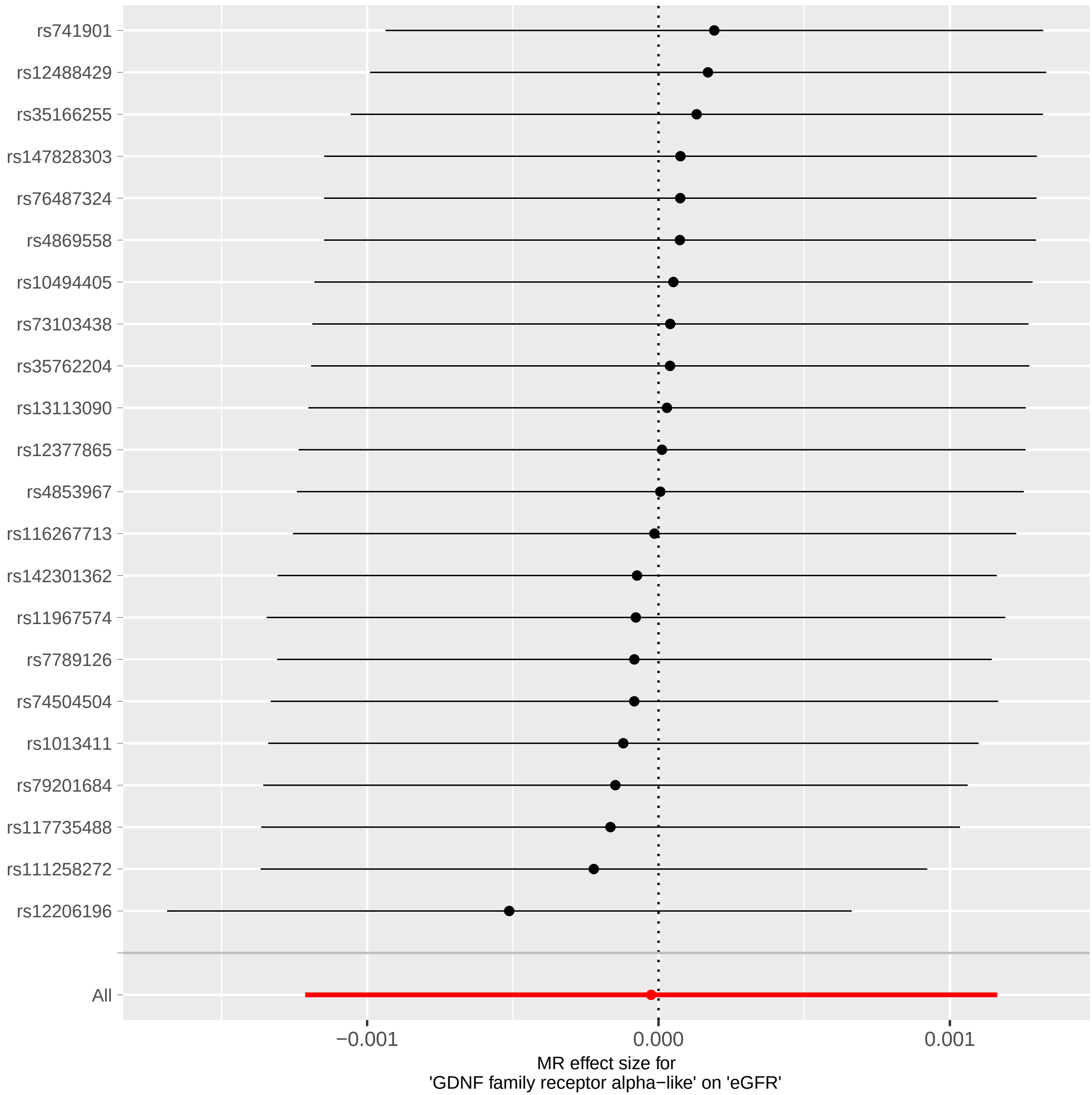

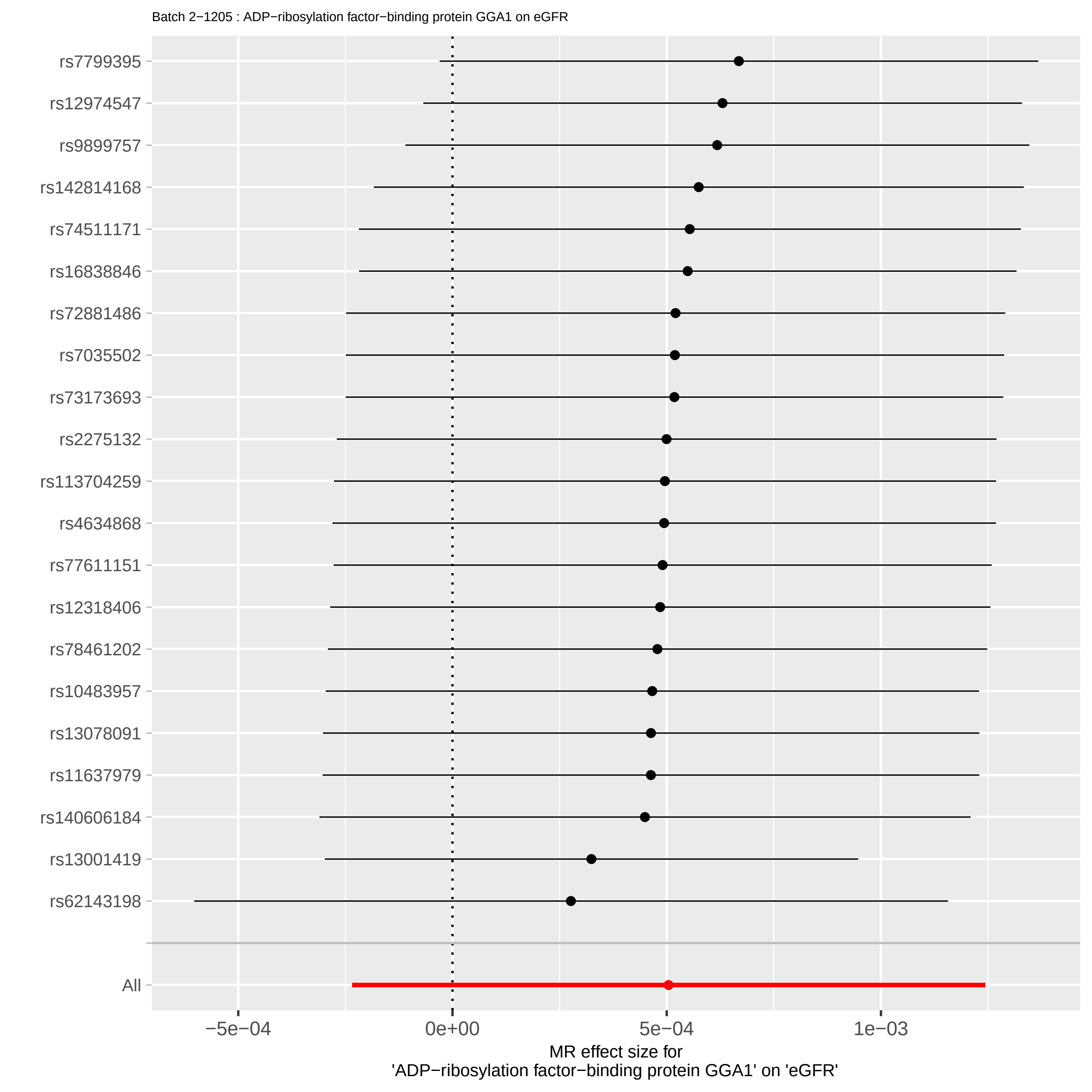

Batch 2−1205 : ADP−ribosylation factor−binding protein GGA1 on eGFR
rs7799395
rs12974547
rs9899757
rs142814168
rs74511171
rs16838846
rs72881486
rs7035502
rs73173693
rs2275132
rs113704259
rs4634868
rs77611151
rs12318406
rs78461202
rs10483957
rs13078091
rs11637979
rs140606184
rs13001419
rs62143198
All
−5e−04
0e+00
5e−04
1e−03
MR effect size for
'ADP−ribosylation factor−binding protein GGA1' on 'eGFR'

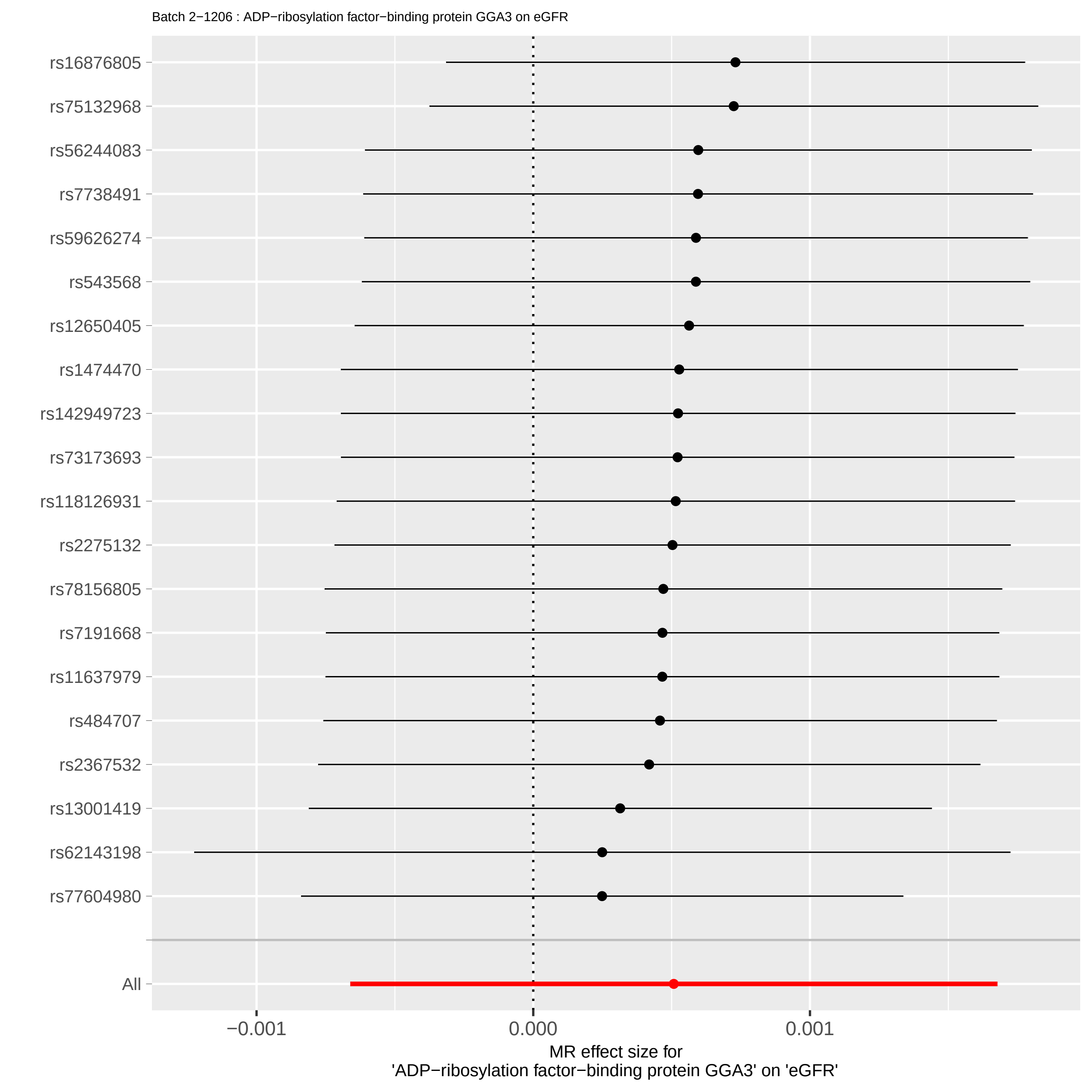
Batch 2−1206 : ADP−ribosylation factor−binding protein GGA3 on eGFR
rs16876805
rs75132968
rs56244083
rs7738491
rs59626274
rs543568
rs12650405
rs1474470
rs142949723
rs73173693
rs118126931
rs2275132
rs78156805
rs7191668
rs11637979
rs484707
rs2367532
rs13001419
rs62143198
rs77604980
All
−0.001
0.000
0.001
MR effect size for
'ADP−ribosylation factor−binding protein GGA3' on 'eGFR'

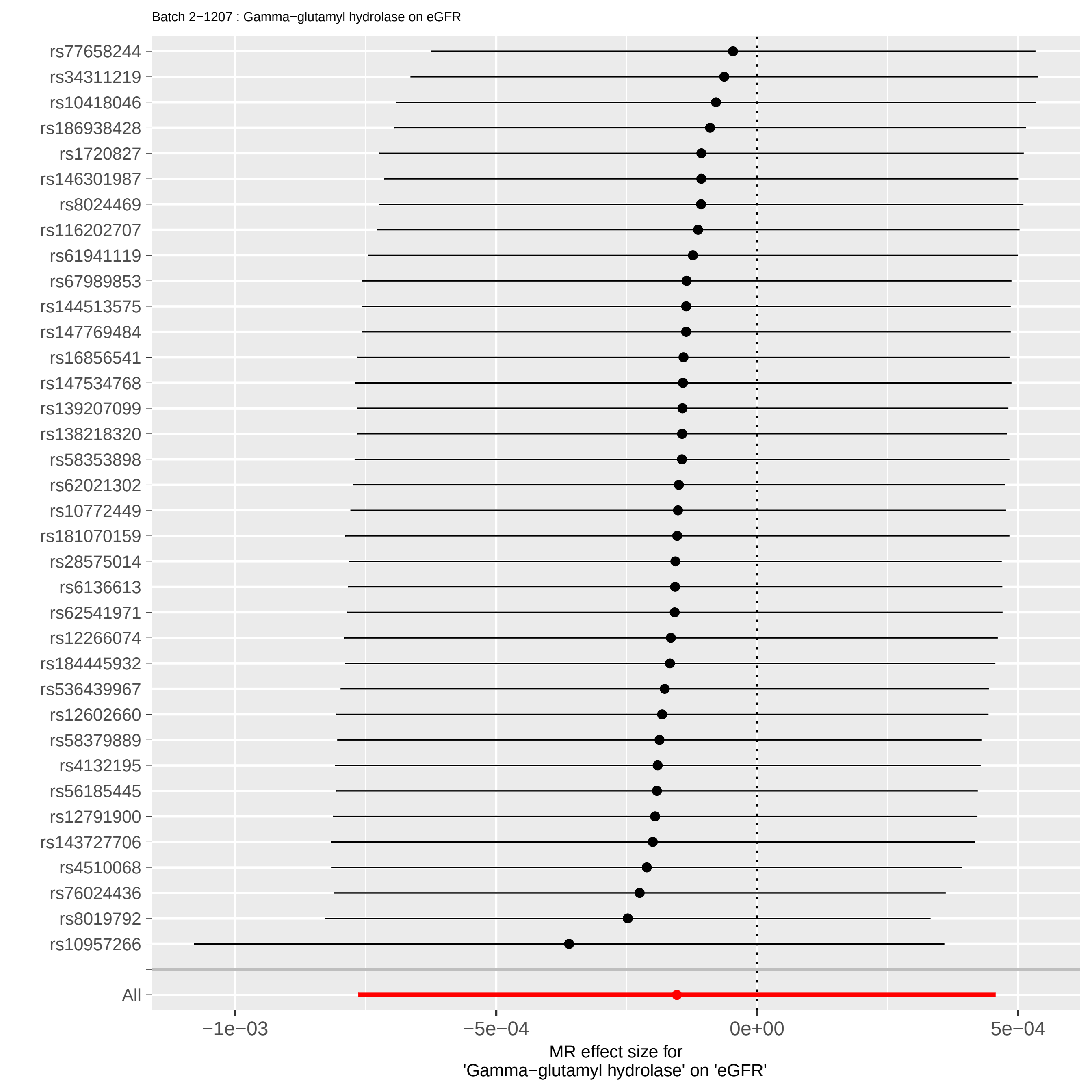

Batch 2−1207 : Gamma−glutamyl hydrolase on eGFR
rs77658244
rs34311219
rs10418046
rs186938428
rs1720827
rs146301987
rs8024469
rs116202707
rs61941119
rs67989853
rs144513575
rs147769484
rs16856541
rs147534768
rs139207099
rs138218320
rs58353898
rs62021302
rs10772449
rs181070159
rs28575014
rs6136613
rs62541971
rs12266074
rs184445932
rs536439967
rs12602660
rs58379889
rs4132195
rs56185445
rs12791900
rs143727706
rs4510068
rs76024436
rs8019792
rs10957266
All
−1e−03
−5e−04
0e+00
5e−04
MR effect size for
'Gamma−glutamyl hydrolase' on 'eGFR'

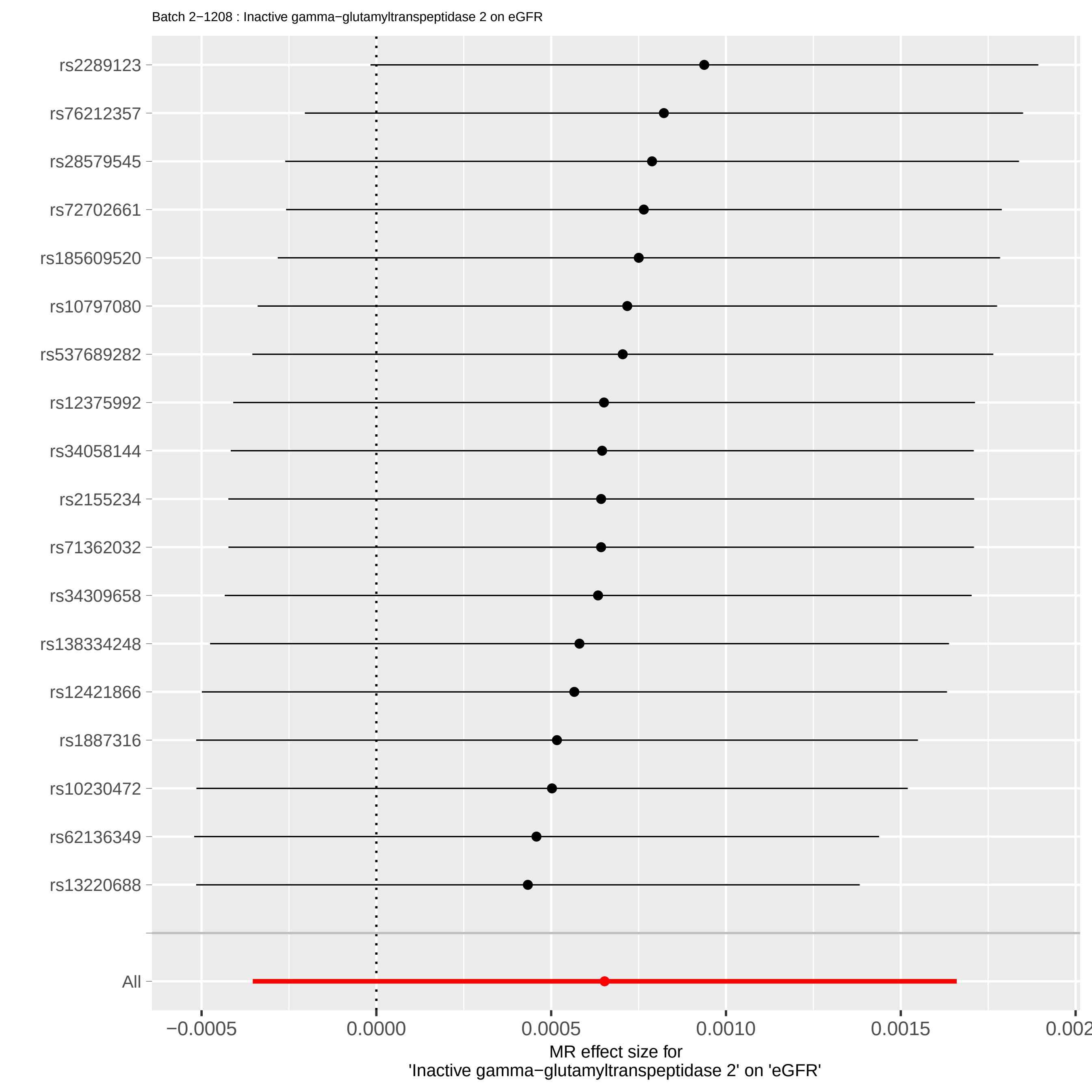
Batch 2−1208 : Inactive gamma−glutamyltranspeptidase 2 on eGFR
rs2289123
rs76212357
rs28579545
rs72702661
rs185609520
rs10797080
rs537689282
rs12375992
rs34058144
rs2155234
rs71362032
rs34309658
rs138334248
rs12421866
rs1887316
rs10230472
rs62136349
rs13220688
All
−0.0005
0.0000
0.0005
0.0010
0.0015
MR effect size for
'Inactive gamma−glutamyltranspeptidase 2' on 'eGFR'

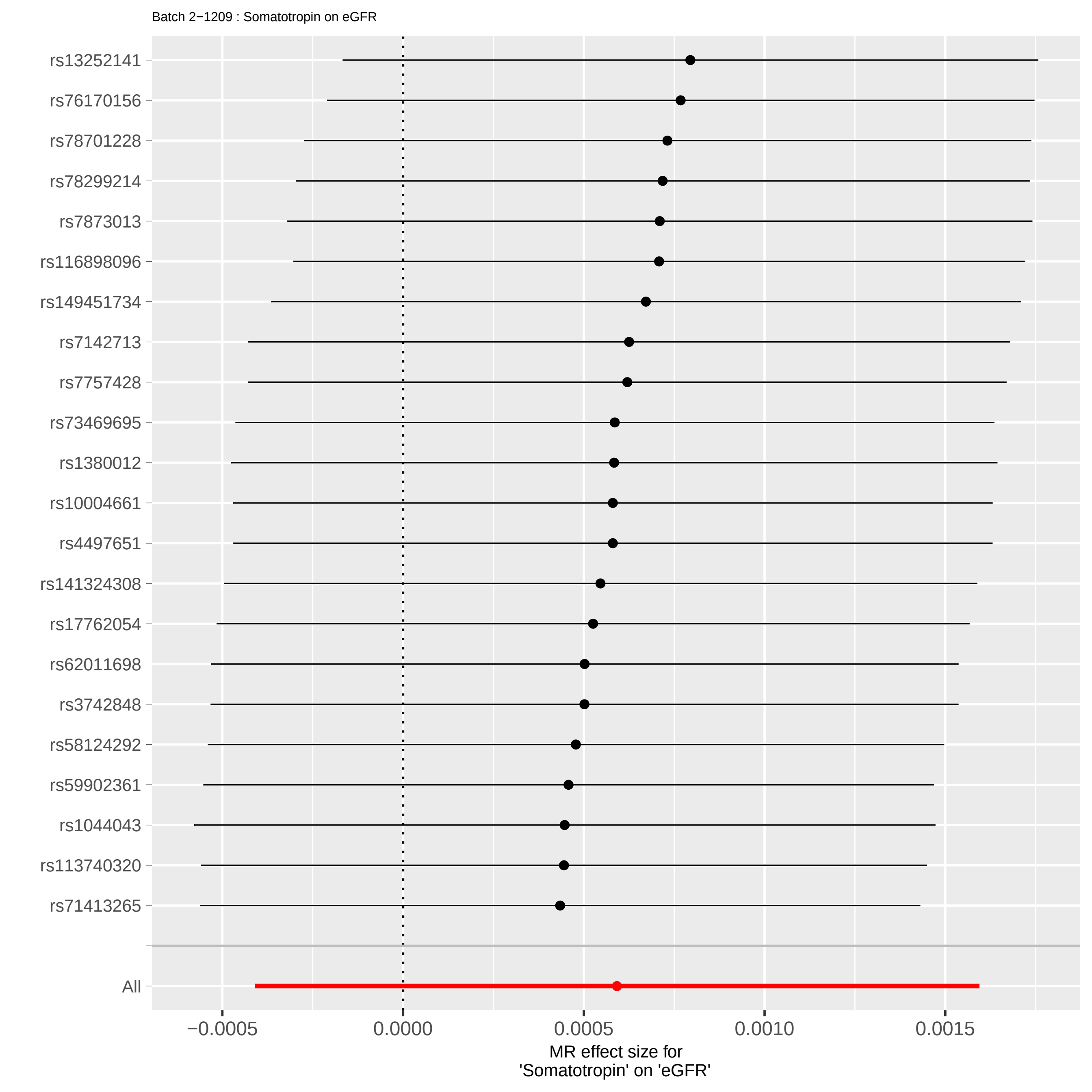
Batch 2−1209 : Somatotropin on eGFR
rs13252141
rs76170156
rs78701228
rs78299214
rs7873013
rs116898096
rs149451734
rs7142713
rs7757428
rs73469695
rs1380012
rs10004661
rs4497651
rs141324308
rs17762054
rs62011698
rs3742848
rs58124292
rs59902361
rs1044043
rs113740320
rs71413265
All
−0.0005
0.0000
0.0005
0.0010
0.0015
MR effect size for
'Somatotropin' on 'eGFR'

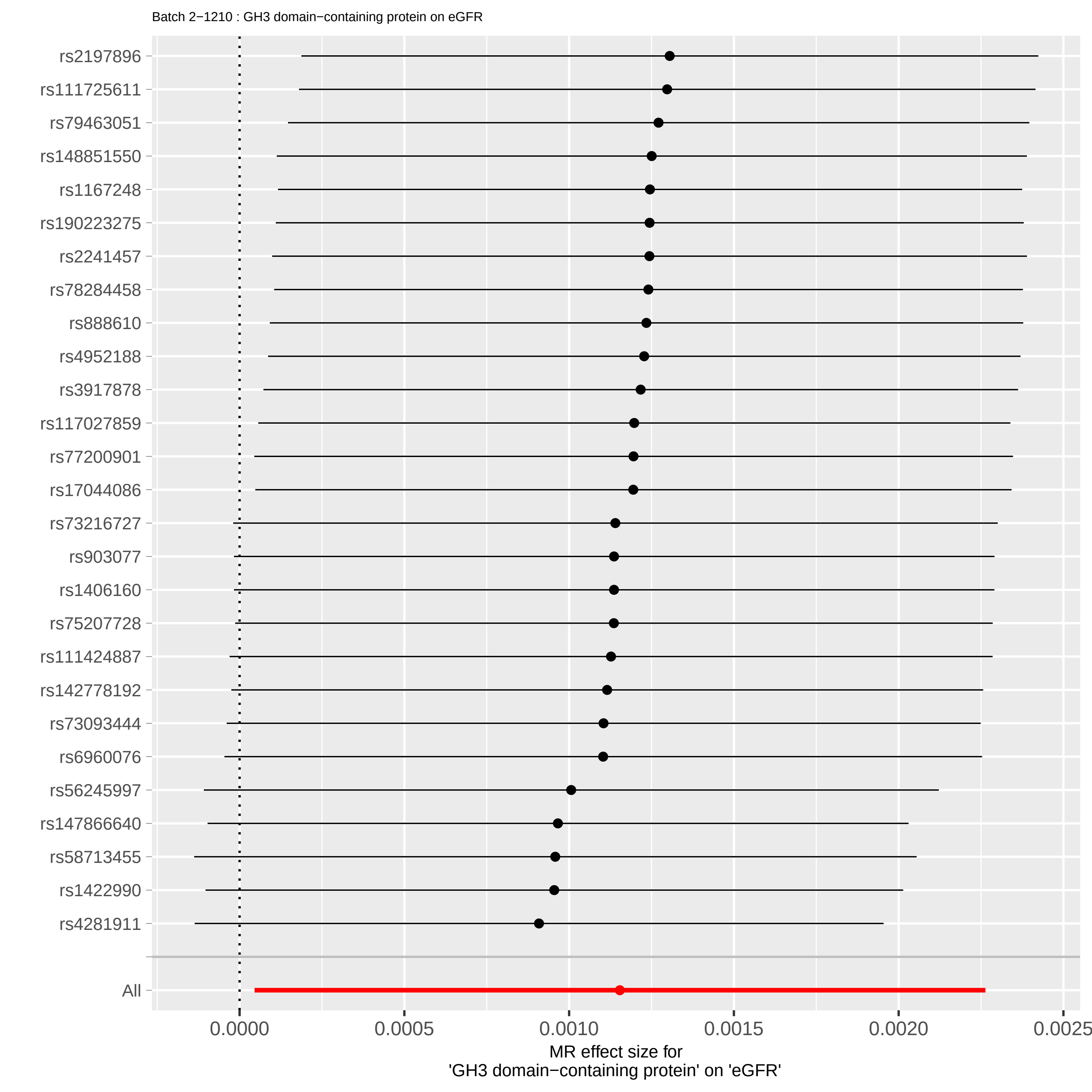

Batch 2−1210 : GH3 domain−containing protein on eGFR
rs2197896
rs111725611
rs79463051
rs148851550
rs1167248
rs190223275
rs2241457
rs78284458
rs888610
rs4952188
rs3917878
rs117027859
rs77200901
rs17044086
rs73216727
rs903077
rs1406160
rs75207728
rs111424887
rs142778192
rs73093444
rs6960076
rs56245997
rs147866640
rs58713455
rs1422990
rs4281911
All
0.0000
0.0005
0.0010
0.0015
0.0020
0.0025
MR effect size for
'GH3 domain−containing protein' on 'eGFR'

Batch 2−1211 : Growth hormone receptor on eGFR

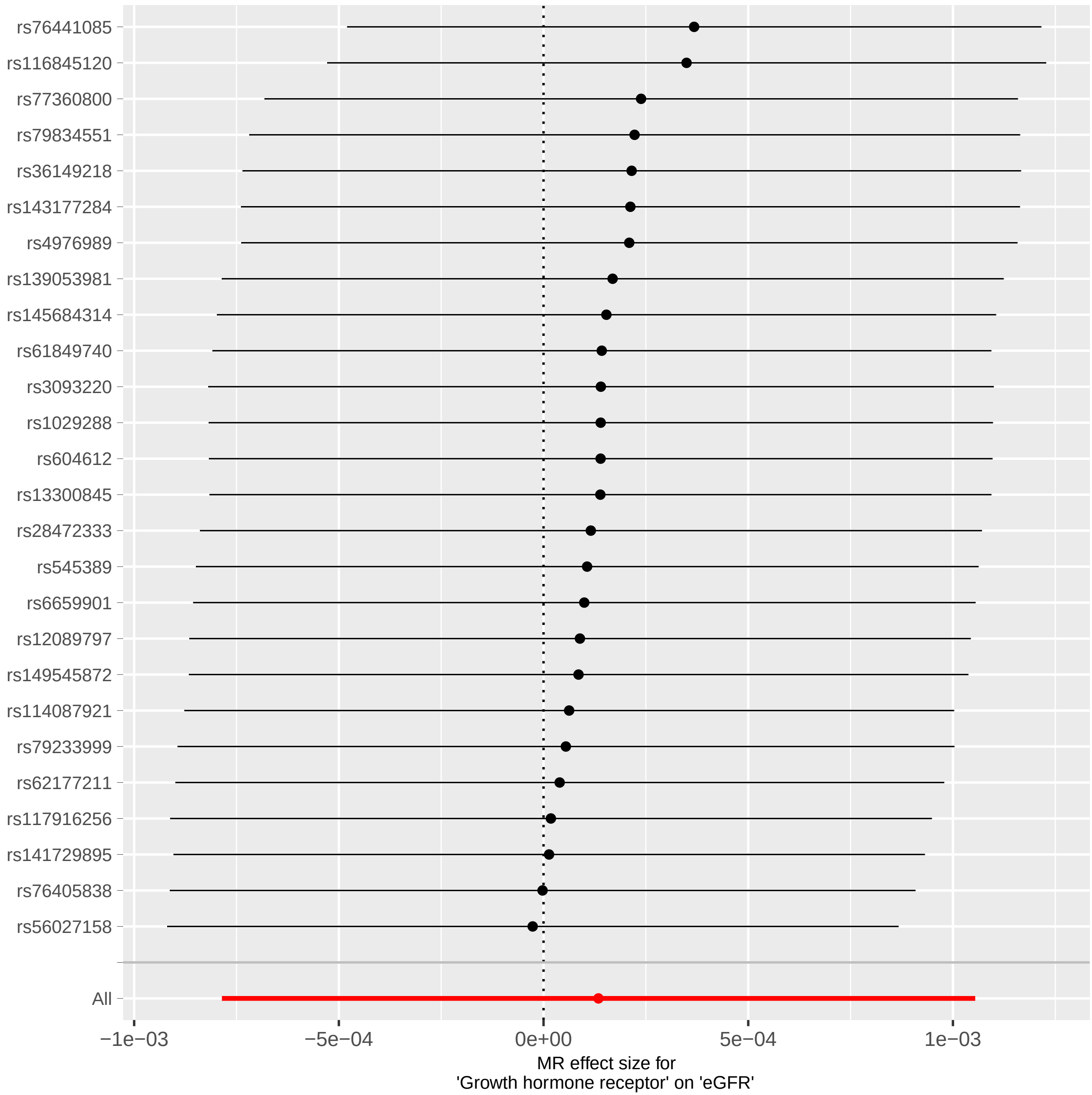

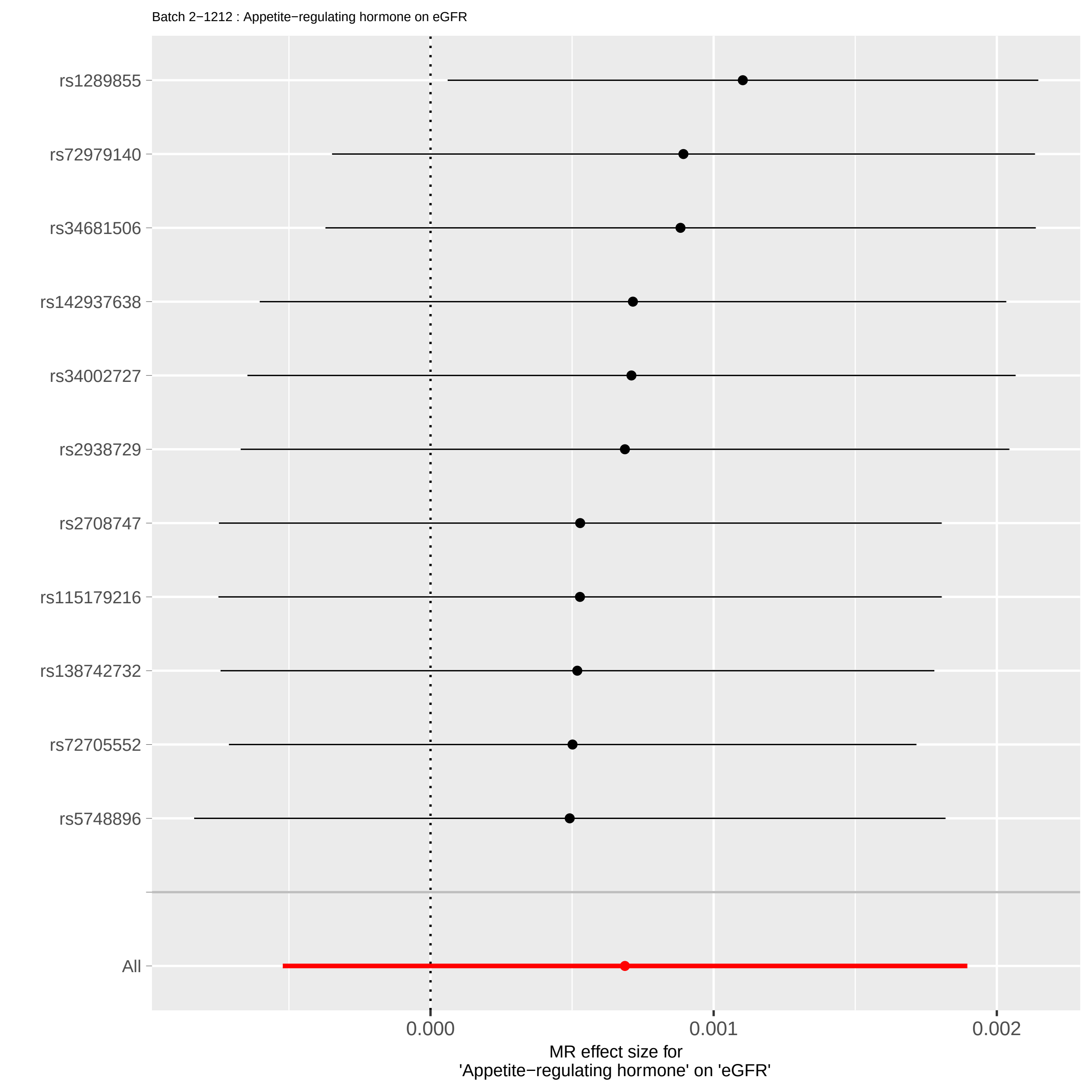

Batch 2−1212 : Appetite−regulating hormone on eGFR
rs1289855
rs72979140
rs34681506
rs142937638
rs34002727
rs2938729
rs2708747
rs115179216
rs138742732
rs72705552
rs5748896
All
0.000
0.001
0.002
MR effect size for
'Appetite−regulating hormone' on 'eGFR'

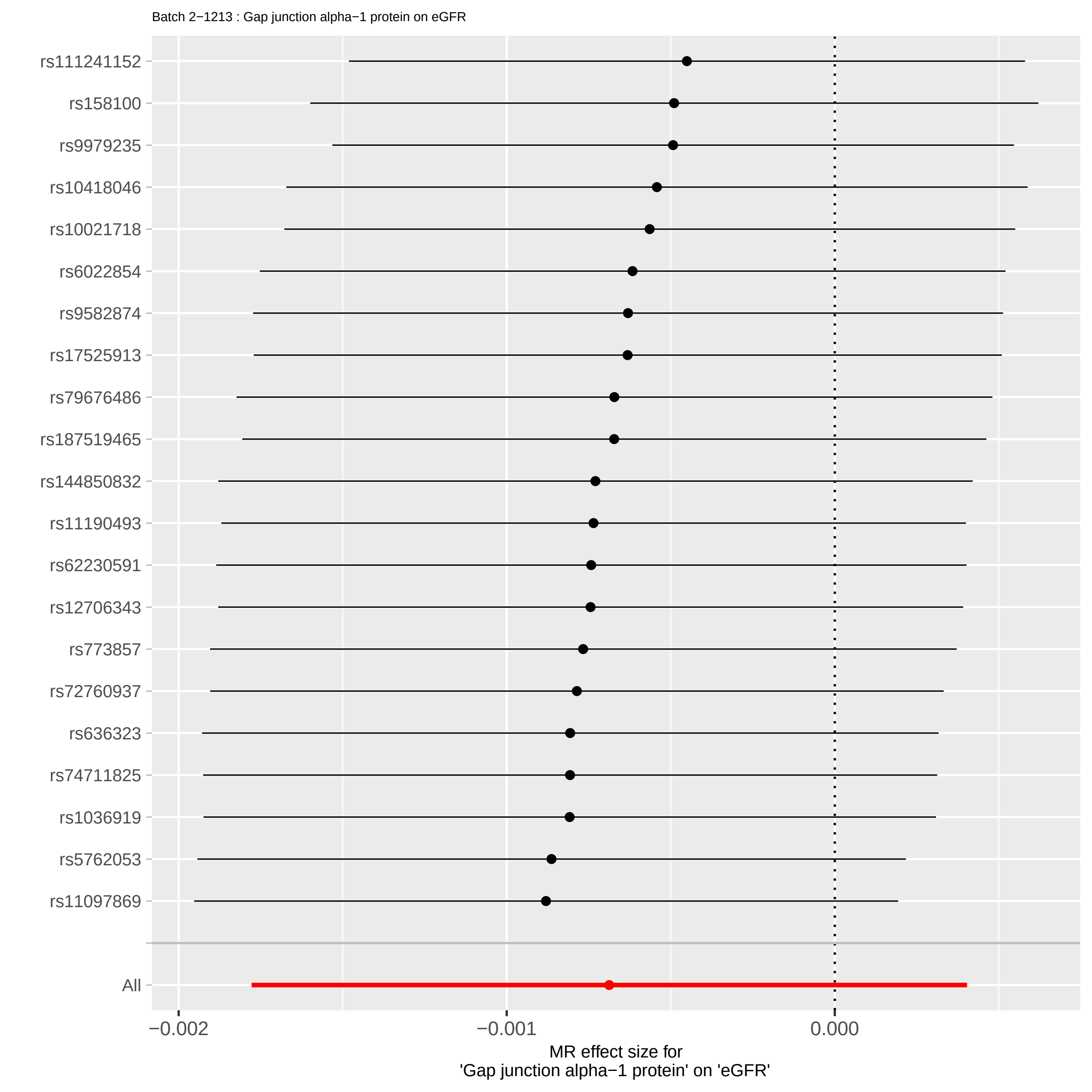

Batch 2−1213 : Gap junction alpha−1 protein on eGFR
rs111241152
rs158100
rs9979235
rs10418046
rs10021718
rs6022854
rs9582874
rs17525913
rs79676486
rs187519465
rs144850832
rs11190493
rs62230591
rs12706343
rs773857
rs72760937
rs636323
rs74711825
rs1036919
rs5762053
rs11097869
All
−0.002
−0.001
0.000
MR effect size for
'Gap junction alpha−1 protein' on 'eGFR'

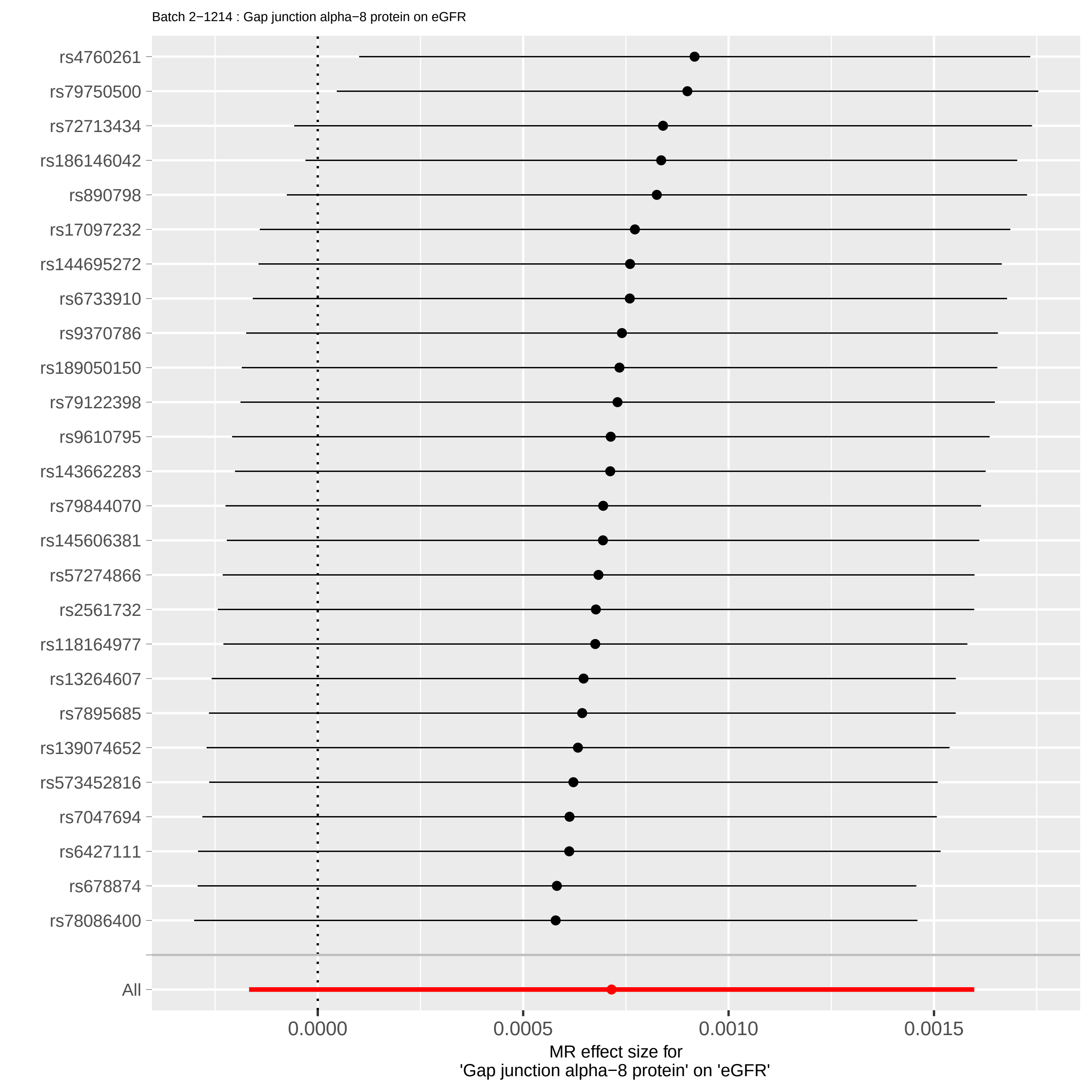

Batch 2−1214 : Gap junction alpha−8 protein on eGFR
rs4760261
rs79750500
rs72713434
rs186146042
rs890798
rs17097232
rs144695272
rs6733910
rs9370786
rs189050150
rs79122398
rs9610795
rs143662283
rs79844070
rs145606381
rs57274866
rs2561732
rs118164977
rs13264607
rs7895685
rs139074652
rs573452816
rs7047694
rs6427111
rs678874
rs78086400
All
0.0000
0.0005
0.0010
0.0015
MR effect size for
'Gap junction alpha−8 protein' on 'eGFR'

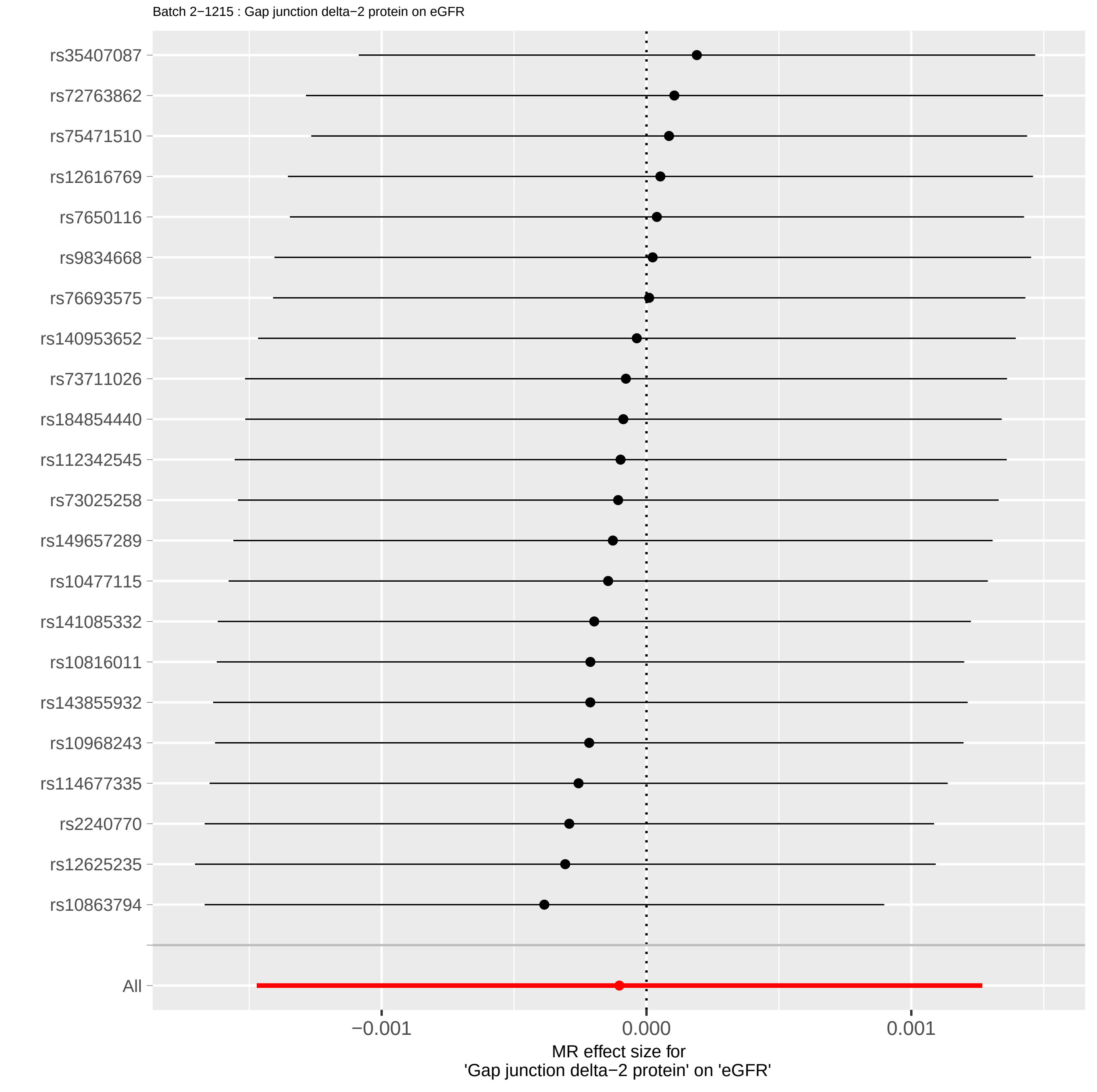

Batch 2−1215 : Gap junction delta−2 protein on eGFR
rs35407087
rs72763862
rs75471510
rs12616769
rs7650116
rs9834668
rs76693575
rs140953652
rs73711026
rs184854440
rs112342545
rs73025258
rs149657289
rs10477115
rs141085332
rs10816011
rs143855932
rs10968243
rs114677335
rs2240770
rs12625235
rs10863794
All
−0.001
0.000
0.001
MR effect size for
'Gap junction delta−2 protein' on 'eGFR'

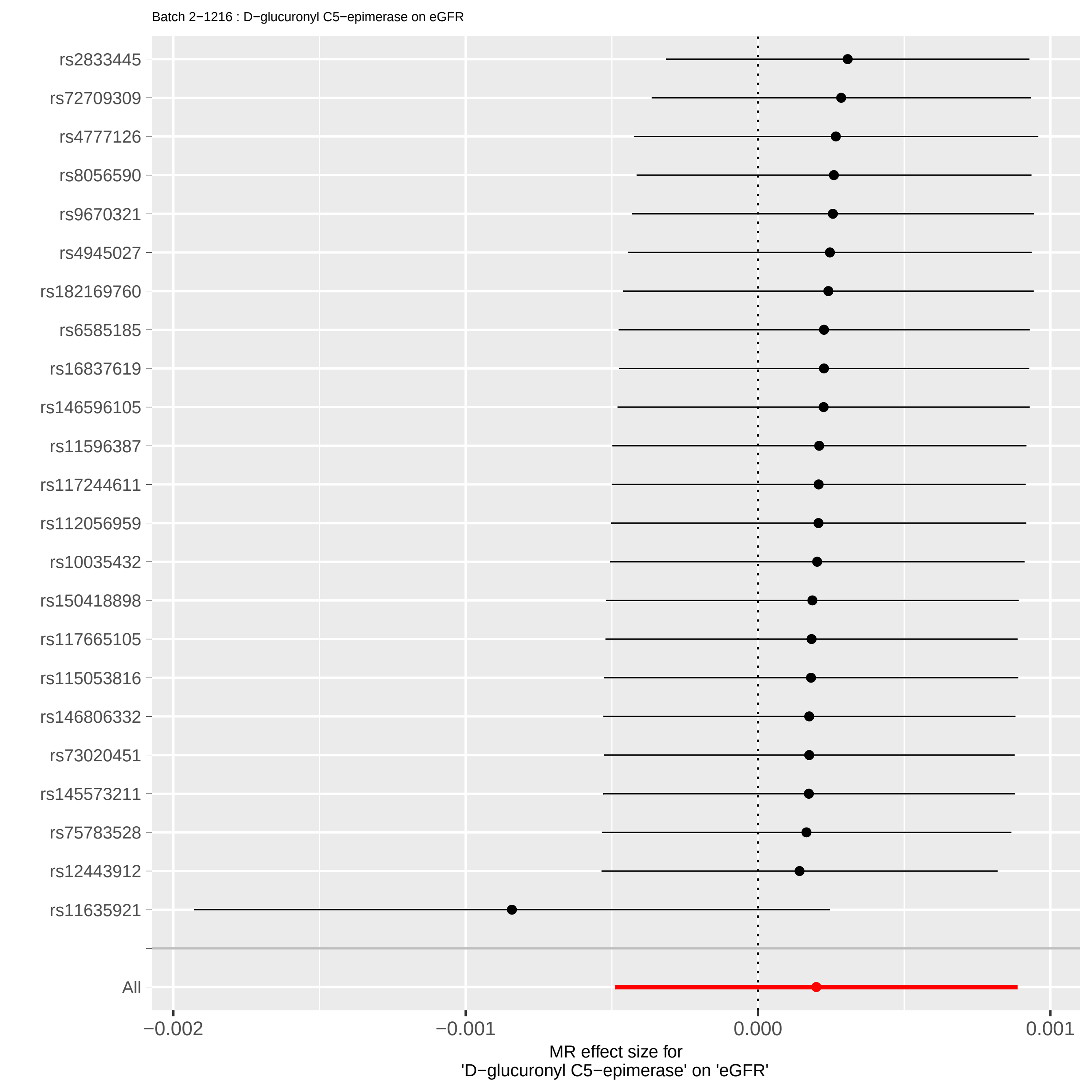

Batch 2−1216 : D−glucuronyl C5−epimerase on eGFR
rs2833445
rs72709309
rs4777126
rs8056590
rs9670321
rs4945027
rs182169760
rs6585185
rs16837619
rs146596105
rs11596387
rs117244611
rs112056959
rs10035432
rs150418898
rs117665105
rs115053816
rs146806332
rs73020451
rs145573211
rs75783528
rs12443912
rs11635921
All
−0.002
−0.001
0.000
0.001
MR effect size for
'D−glucuronyl C5−epimerase' on 'eGFR'

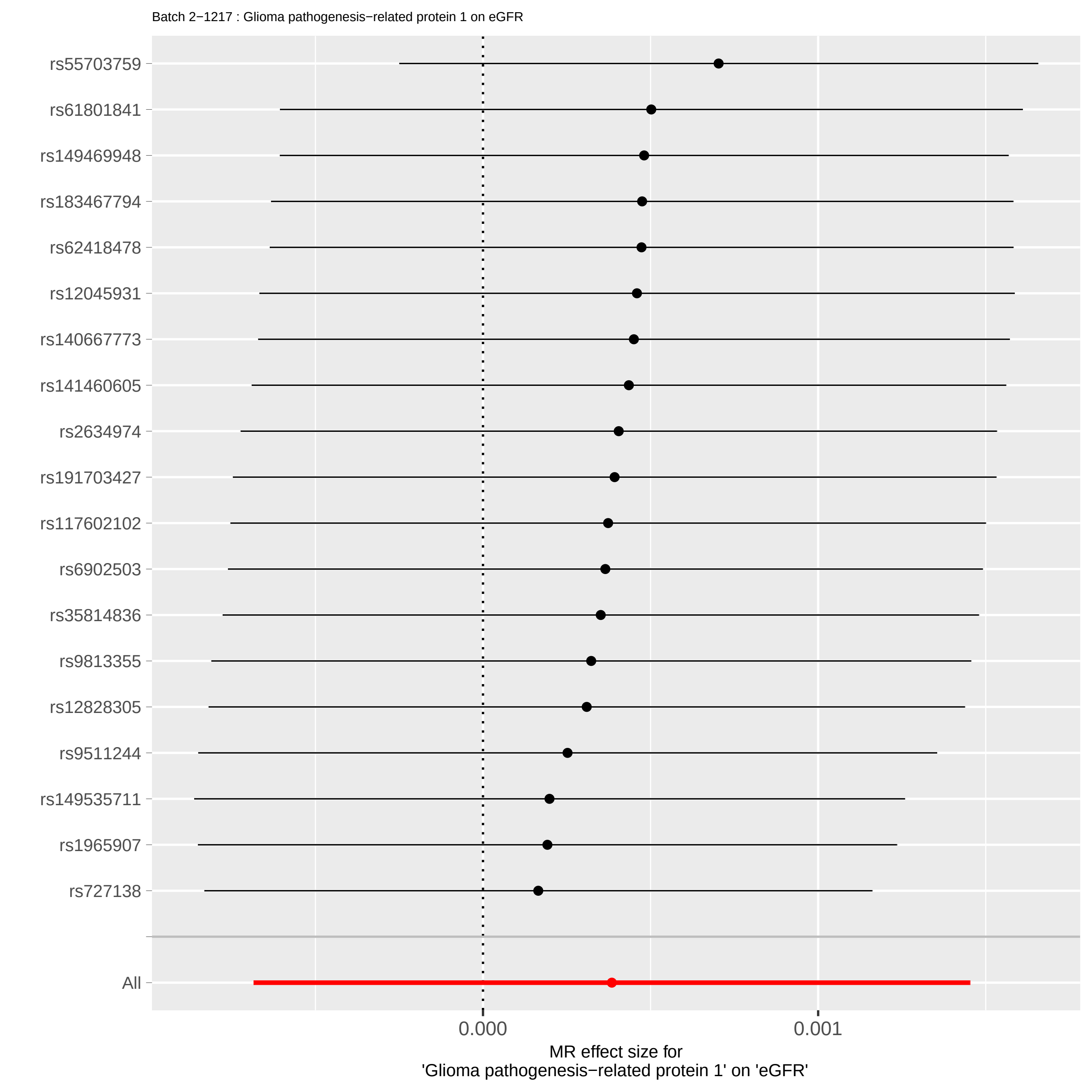

Batch 2−1217 : Glioma pathogenesis−related protein 1 on eGFR
rs55703759
rs61801841
rs149469948
rs183467794
rs62418478
rs12045931
rs140667773
rs141460605
rs2634974
rs191703427
rs117602102
rs6902503
rs35814836
rs9813355
rs12828305
rs9511244
rs149535711
rs1965907
rs727138
All
0.000
0.001
MR effect size for
'Glioma pathogenesis−related protein 1' on 'eGFR'

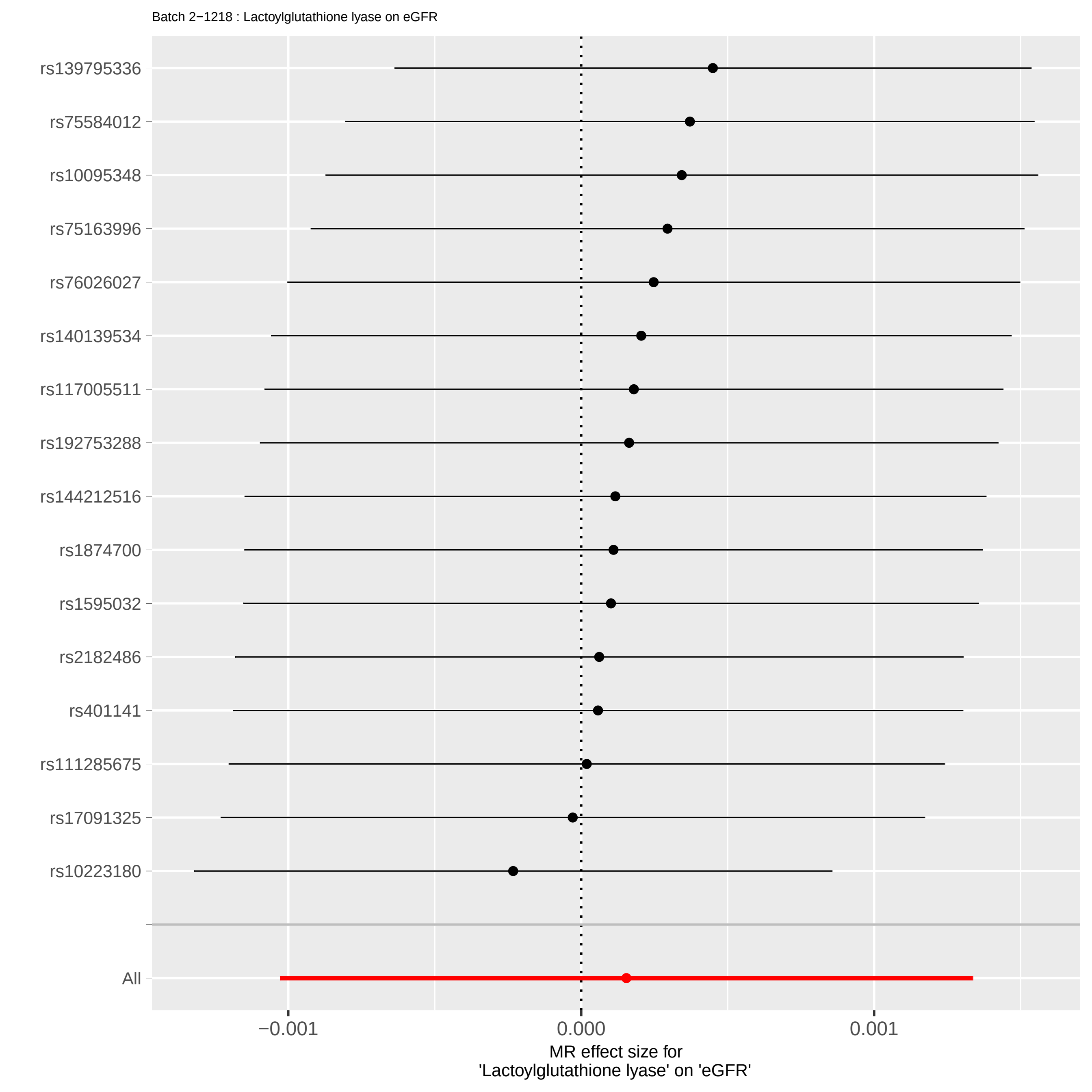
Batch 2−1218 : Lactoylglutathione lyase on eGFR
rs139795336
rs75584012
rs10095348
rs75163996
rs76026027
rs140139534
rs117005511
rs192753288
rs144212516
rs1874700
rs1595032
rs2182486
rs401141
rs111285675
rs17091325
rs10223180
All
−0.001
0.000
0.001
MR effect size for
'Lactoylglutathione lyase' on 'eGFR'

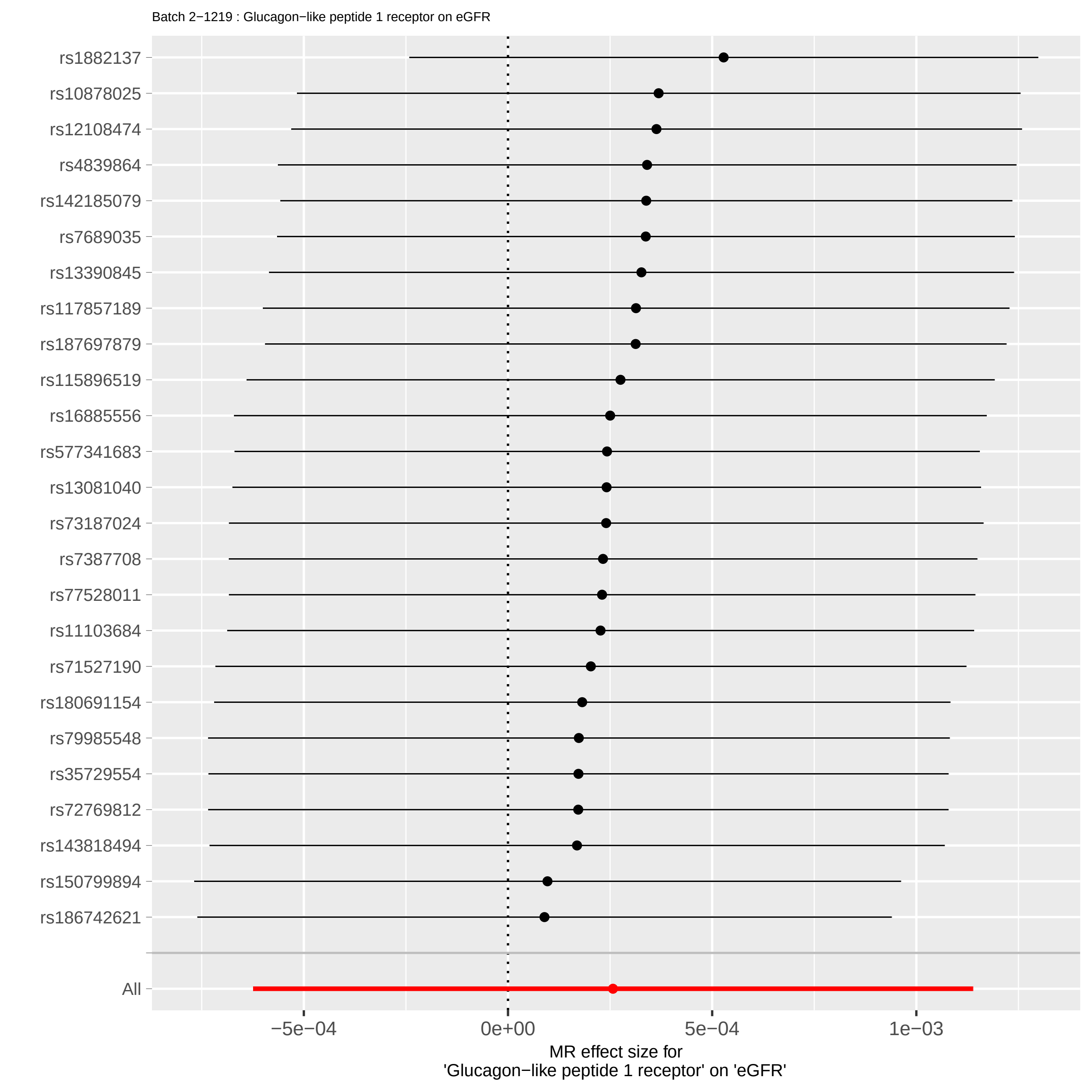

Batch 2−1219 : Glucagon−like peptide 1 receptor on eGFR
rs1882137
rs10878025
rs12108474
rs4839864
rs142185079
rs7689035
rs13390845
rs117857189
rs187697879
rs115896519
rs16885556
rs577341683
rs13081040
rs73187024
rs7387708
rs77528011
rs11103684
rs71527190
rs180691154
rs79985548
rs35729554
rs72769812
rs143818494
rs150799894
rs186742621
All
−5e−04
0e+00
5e−04
1e−03
MR effect size for
'Glucagon−like peptide 1 receptor' on 'eGFR'

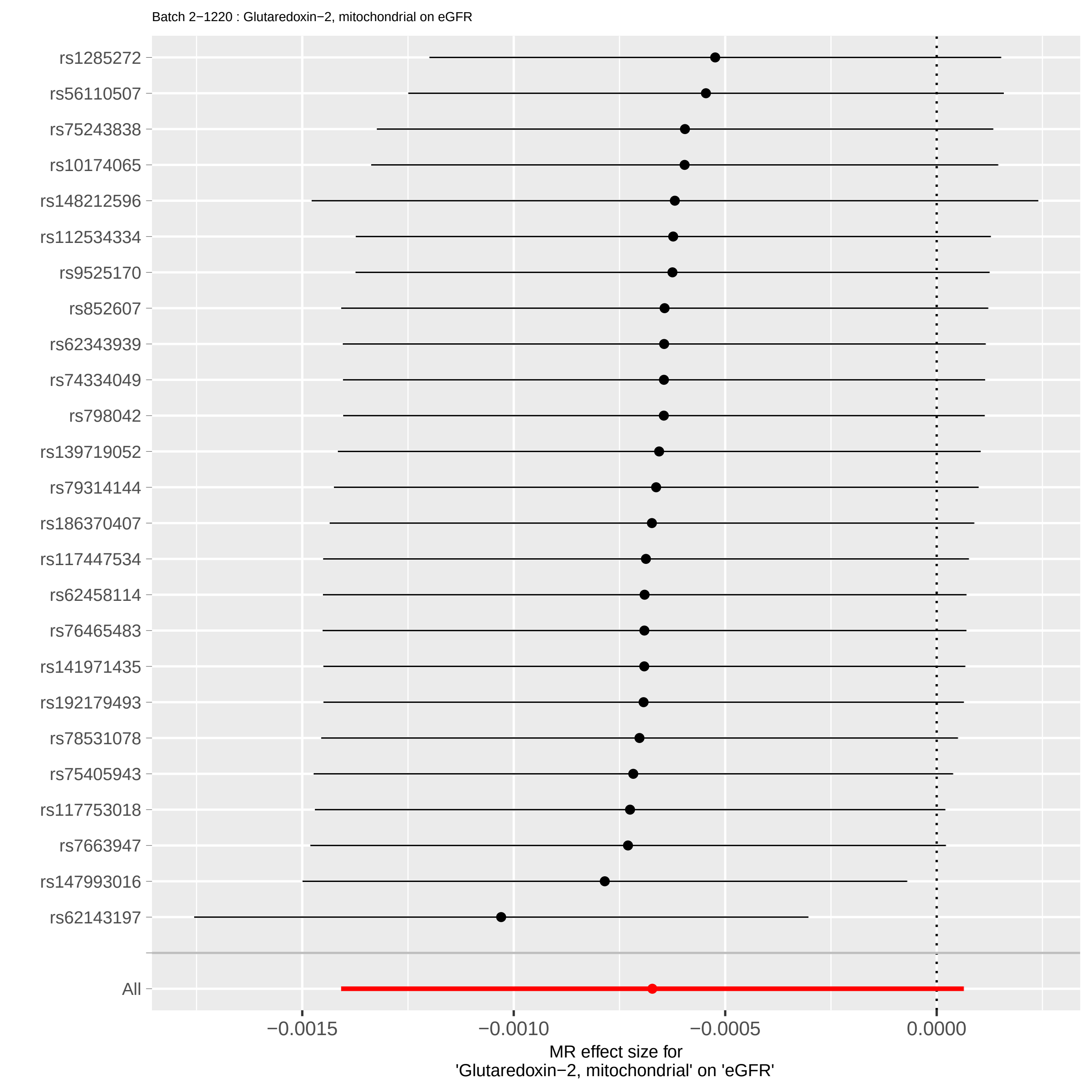

Batch 2−1220 : Glutaredoxin−2, mitochondrial on eGFR
rs1285272
rs56110507
rs75243838
rs10174065
rs148212596
rs112534334
rs9525170
rs852607
rs62343939
rs74334049
rs798042
rs139719052
rs79314144
rs186370407
rs117447534
rs62458114
rs76465483
rs141971435
rs192179493
rs78531078
rs75405943
rs117753018
rs7663947
rs147993016
rs62143197
All
−0.0015
−0.0010
−0.0005
0.0000
MR effect size for
'Glutaredoxin−2, mitochondrial' on 'eGFR'

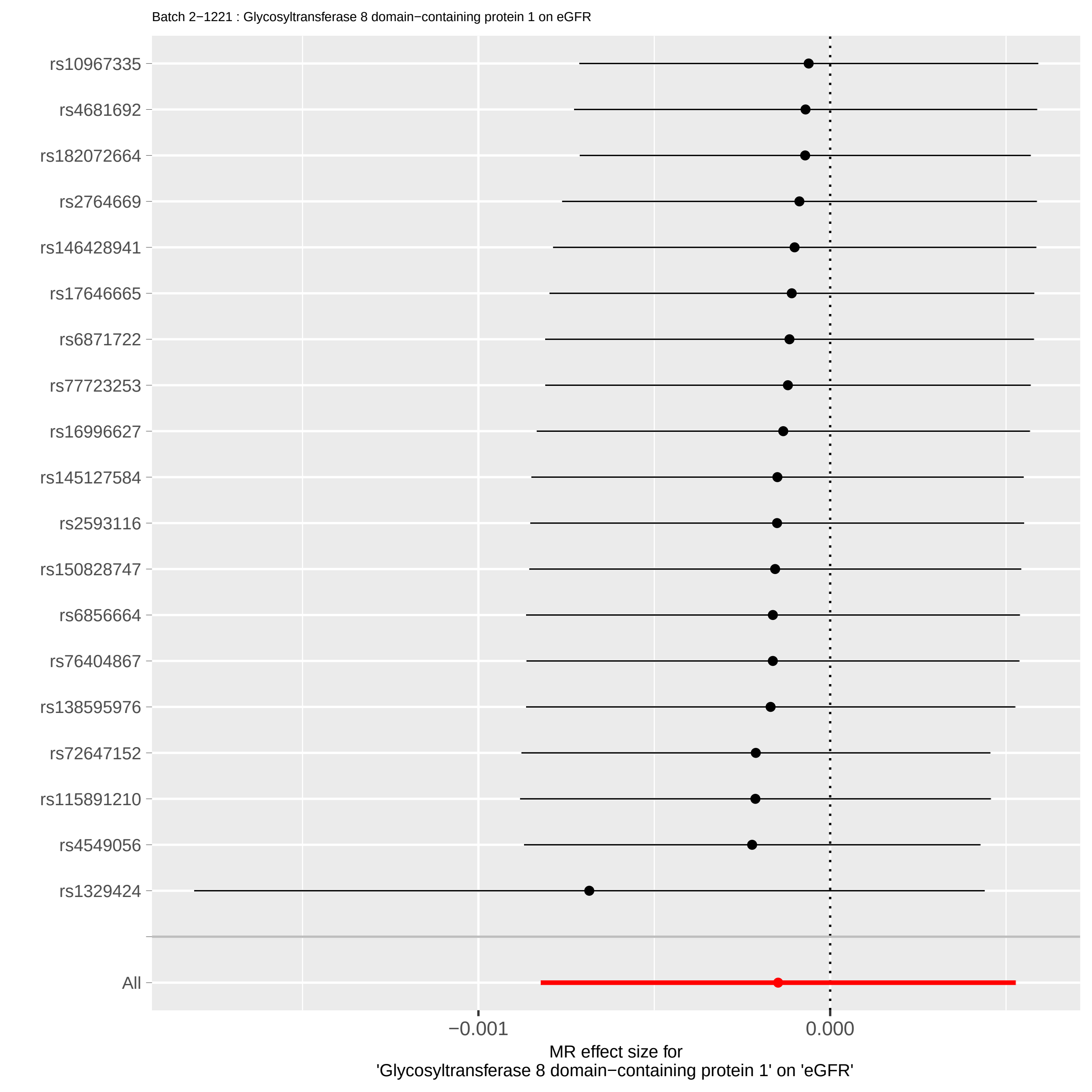

Batch 2−1221 : Glycosyltransferase 8 domain−containing protein 1 on eGFR
rs10967335
rs4681692
rs182072664
rs2764669
rs146428941
rs17646665
rs6871722
rs77723253
rs16996627
rs145127584
rs2593116
rs150828747
rs6856664
rs76404867
rs138595976
rs72647152
rs115891210
rs4549056
rs1329424
All
−0.001
0.000
MR effect size for
'Glycosyltransferase 8 domain−containing protein 1' on 'eGFR'

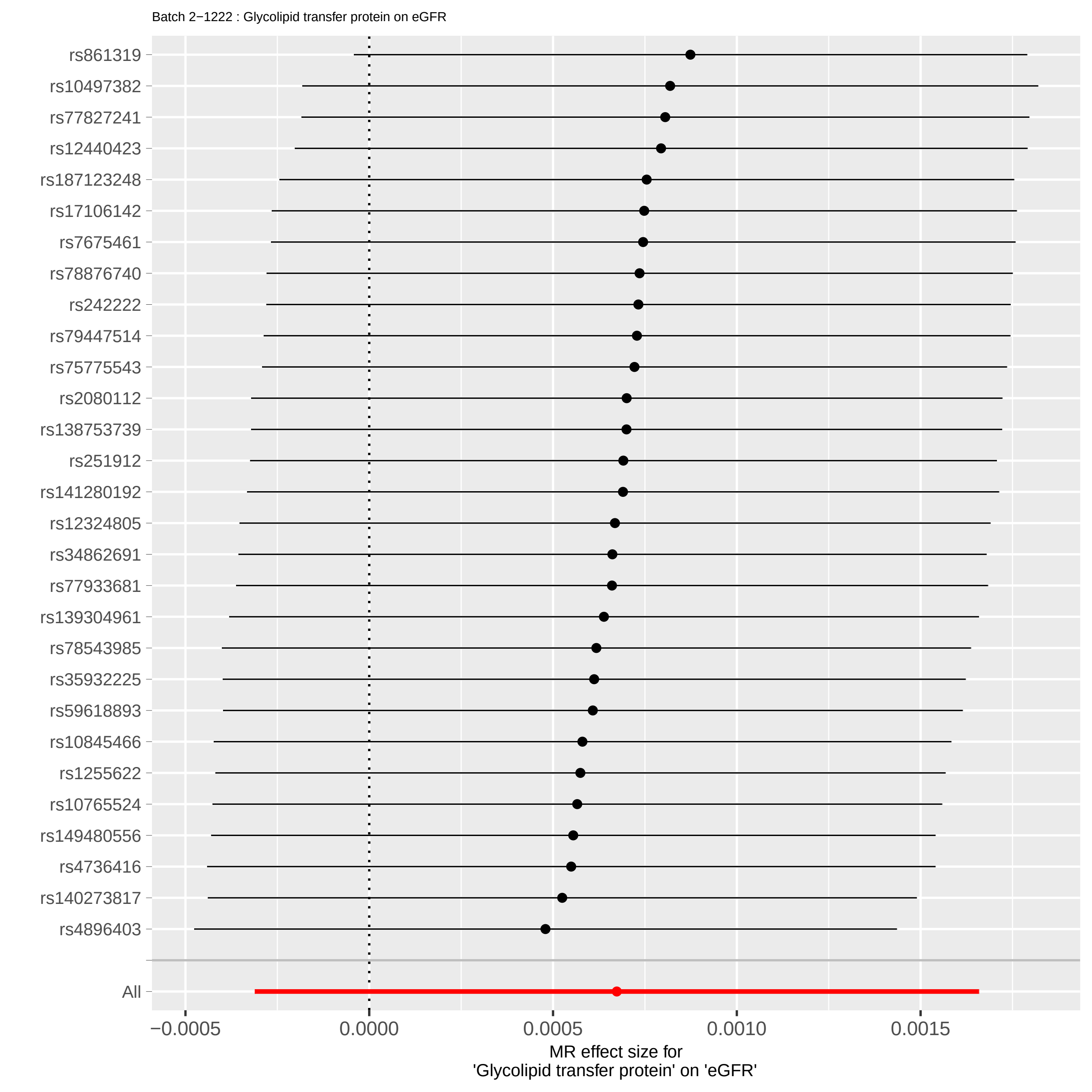
Batch 2−1222 : Glycolipid transfer protein on eGFR
rs861319
rs10497382
rs77827241
rs12440423
rs187123248
rs17106142
rs7675461
rs78876740
rs242222
rs79447514
rs75775543
rs2080112
rs138753739
rs251912
rs141280192
rs12324805
rs34862691
rs77933681
rs139304961
rs78543985
rs35932225
rs59618893
rs10845466
rs1255622
rs10765524
rs149480556
rs4736416
rs140273817
rs4896403
All
−0.0005
0.0000
0.0005
0.0010
0.0015
MR effect size for
'Glycolipid transfer protein' on 'eGFR'

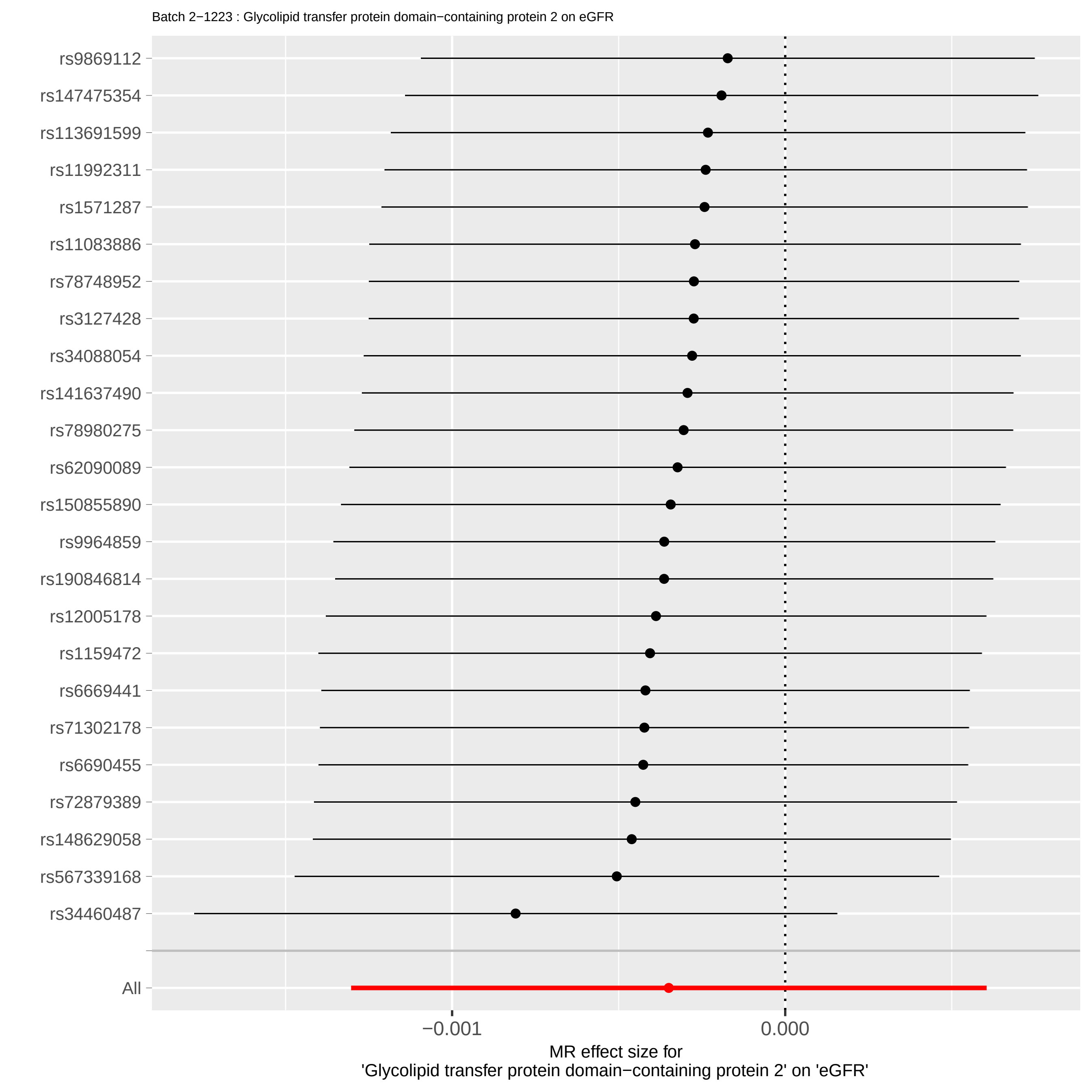

Batch 2−1223 : Glycolipid transfer protein domain−containing protein 2 on eGFR
rs9869112
rs147475354
rs113691599
rs11992311
rs1571287
rs11083886
rs78748952
rs3127428
rs34088054
rs141637490
rs78980275
rs62090089
rs150855890
rs9964859
rs190846814
rs12005178
rs1159472
rs6669441
rs71302178
rs6690455
rs72879389
rs148629058
rs567339168
rs34460487
All
−0.001
0.000
MR effect size for
'Glycolipid transfer protein domain−containing protein 2' on 'eGFR'

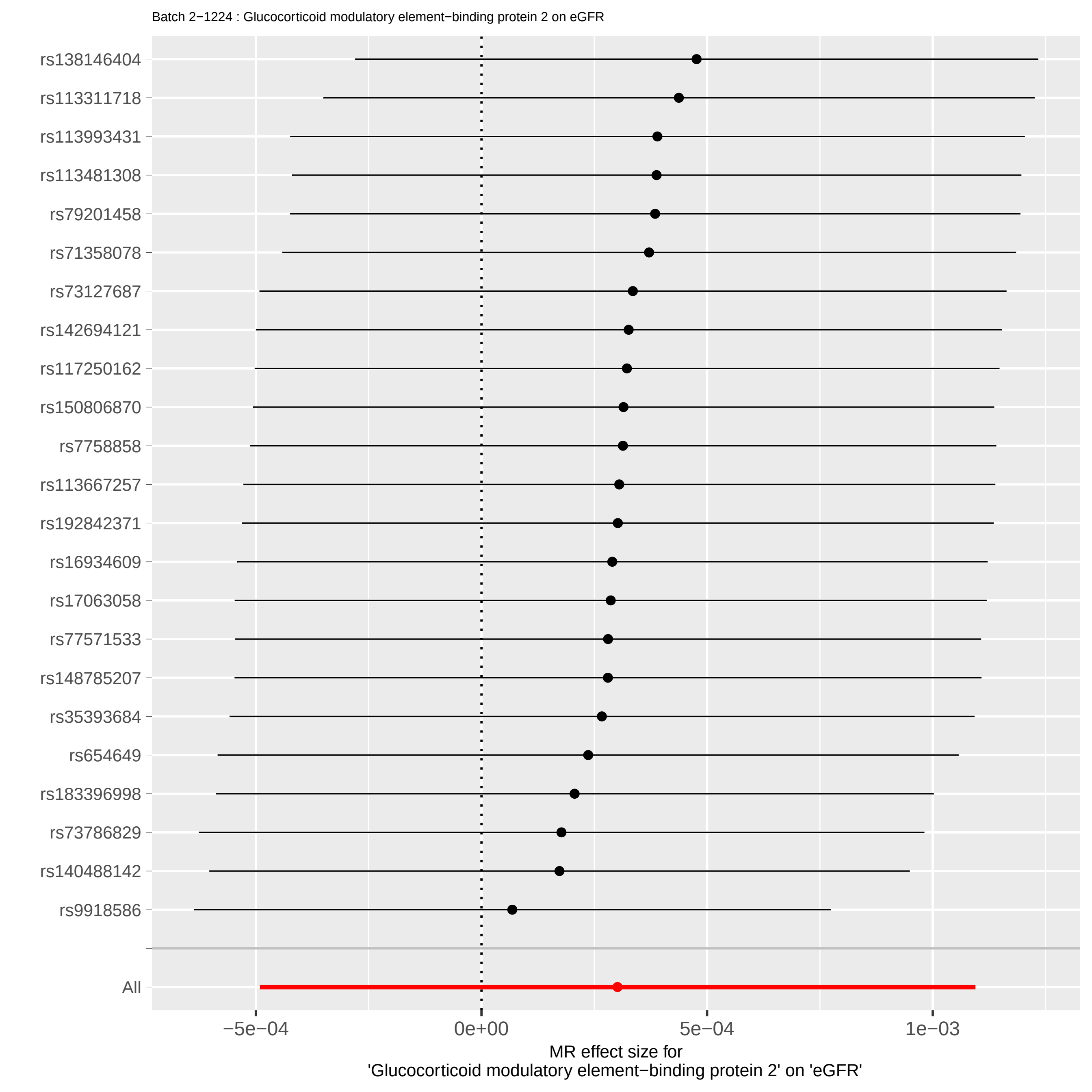
Batch 2−1224 : Glucocorticoid modulatory element−binding protein 2 on eGFR
rs138146404
rs113311718
rs113993431
rs113481308
rs79201458
rs71358078
rs73127687
rs142694121
rs117250162
rs150806870
rs7758858
rs113667257
rs192842371
rs16934609
rs17063058
rs77571533
rs148785207
rs35393684
rs654649
rs183396998
rs73786829
rs140488142
rs9918586
All
−5e−04
0e+00
5e−04
1e−03
MR effect size for
'Glucocorticoid modulatory element−binding protein 2' on 'eGFR'

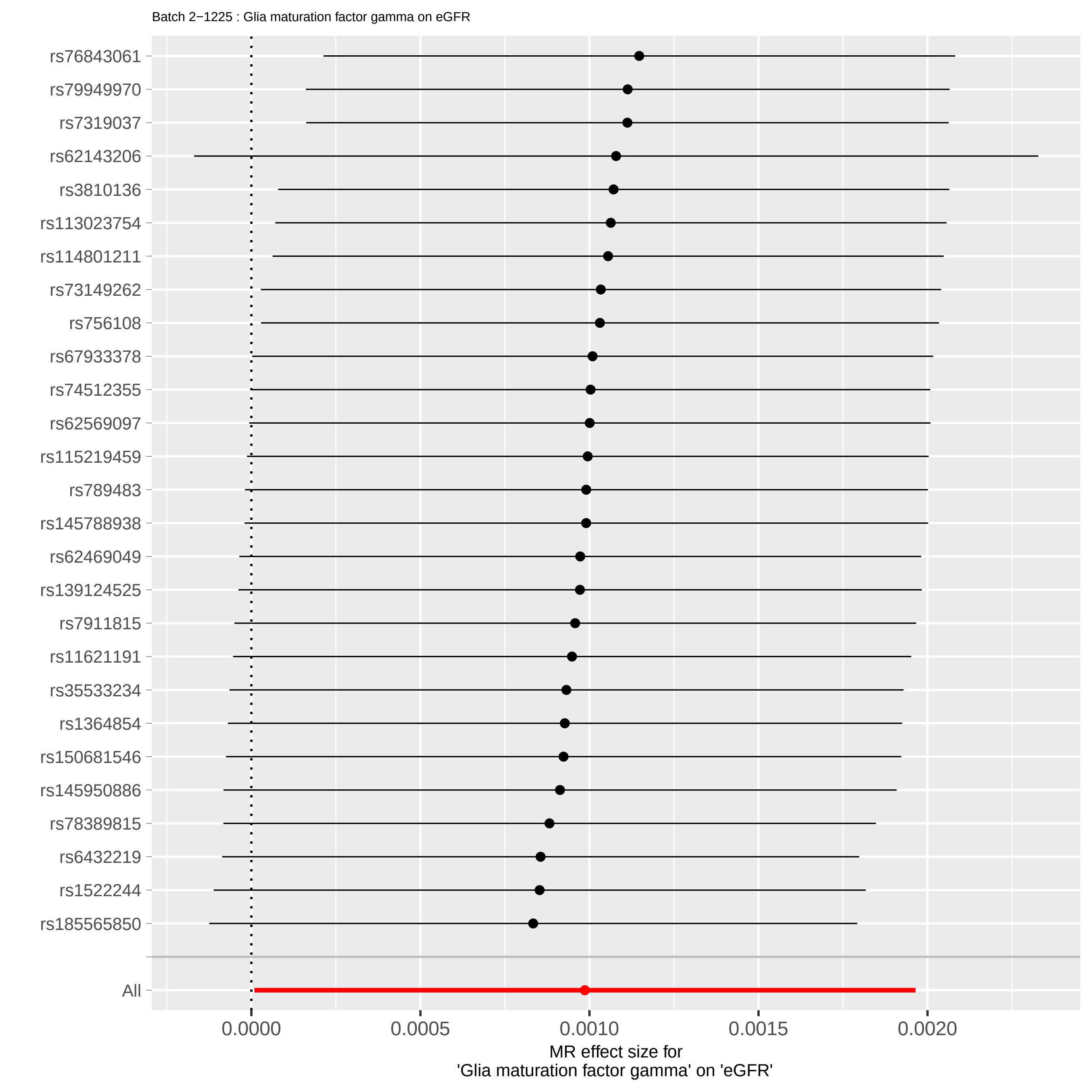

Batch 2−1225 : Glia maturation factor gamma on eGFR
rs76843061
rs79949970
rs7319037
rs62143206
rs3810136
rs113023754
rs114801211
rs73149262
rs756108
rs67933378
rs74512355
rs62569097
rs115219459
rs789483
rs145788938
rs62469049
rs139124525
rs7911815
rs11621191
rs35533234
rs1364854
rs150681546
rs145950886
rs78389815
rs6432219
rs1522244
rs185565850
All
0.0000
0.0005
0.0010
0.0015
0.0020
MR effect size for
'Glia maturation factor gamma' on 'eGFR'

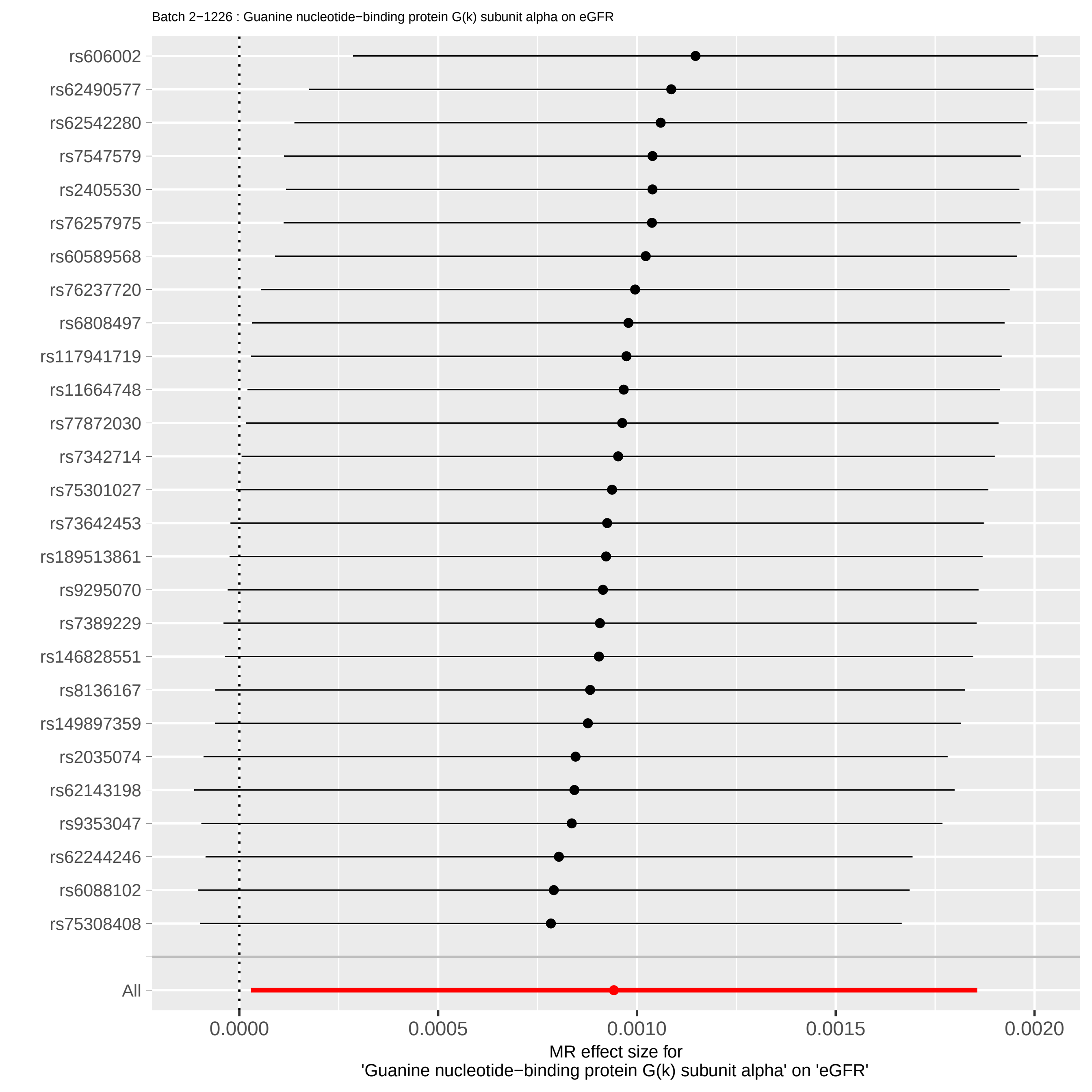

Batch 2−1226 : Guanine nucleotide−binding protein G(k) subunit alpha on eGFR
rs606002
rs62490577
rs62542280
rs7547579
rs2405530
rs76257975
rs60589568
rs76237720
rs6808497
rs117941719
rs11664748
rs77872030
rs7342714
rs75301027
rs73642453
rs189513861
rs9295070
rs7389229
rs146828551
rs8136167
rs149897359
rs2035074
rs62143198
rs9353047
rs62244246
rs6088102
rs75308408
All
0.0000
0.0005
0.0010
0.0015
0.0020
MR effect size for
'Guanine nucleotide−binding protein G(k) subunit alpha' on 'eGFR'

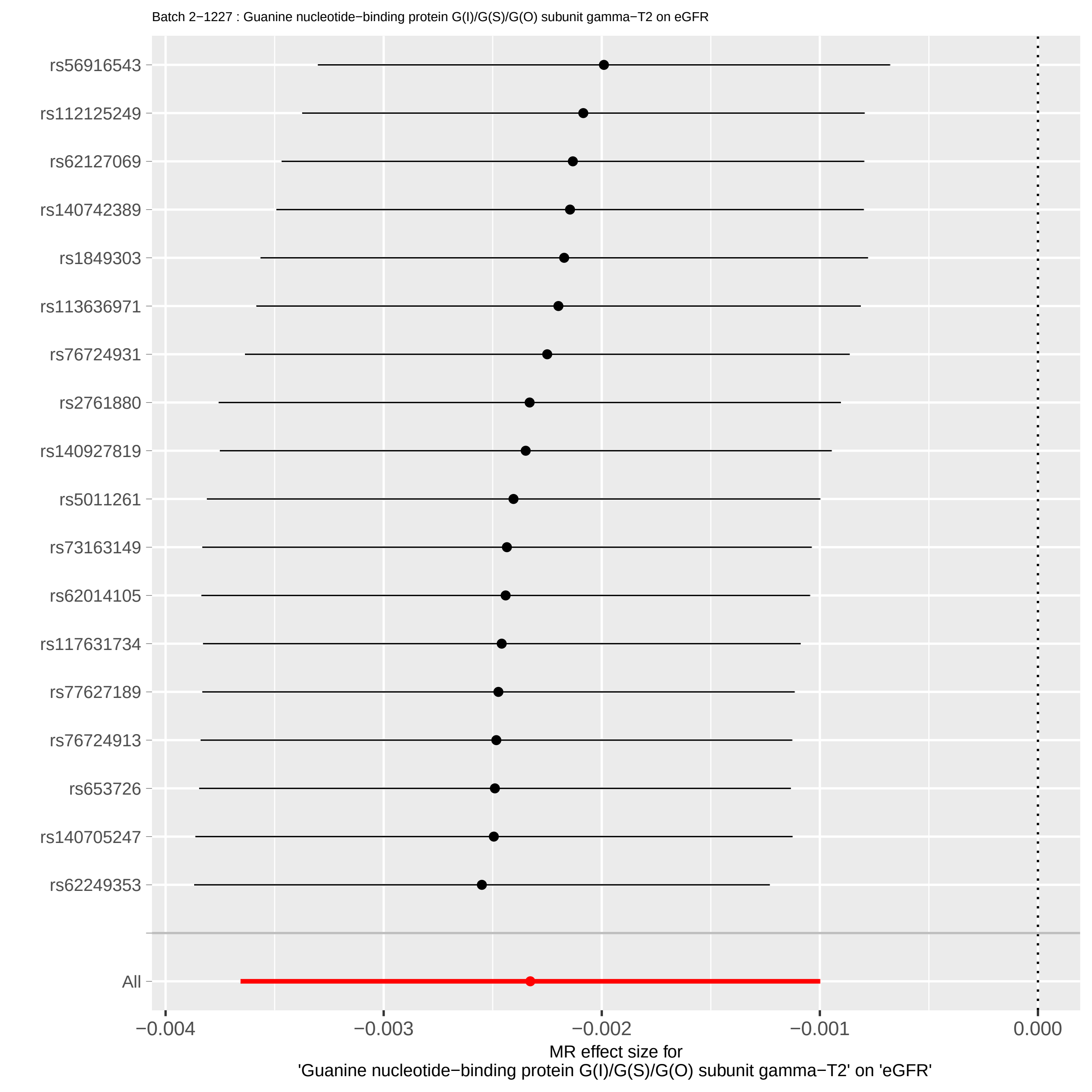

Batch 2−1227 : Guanine nucleotide−binding protein G(I)/G(S)/G(O) subunit gamma−T2 on eGFR
rs56916543
rs112125249
rs62127069
rs140742389
rs1849303
rs113636971
rs76724931
rs2761880
rs140927819
rs5011261
rs73163149
rs62014105
rs117631734
rs77627189
rs76724913
rs653726
rs140705247
rs62249353
All
−0.004
−0.003
−0.002
−0.001
0.000
MR effect size for
'Guanine nucleotide−binding protein G(I)/G(S)/G(O) subunit gamma−T2' on 'eGFR'

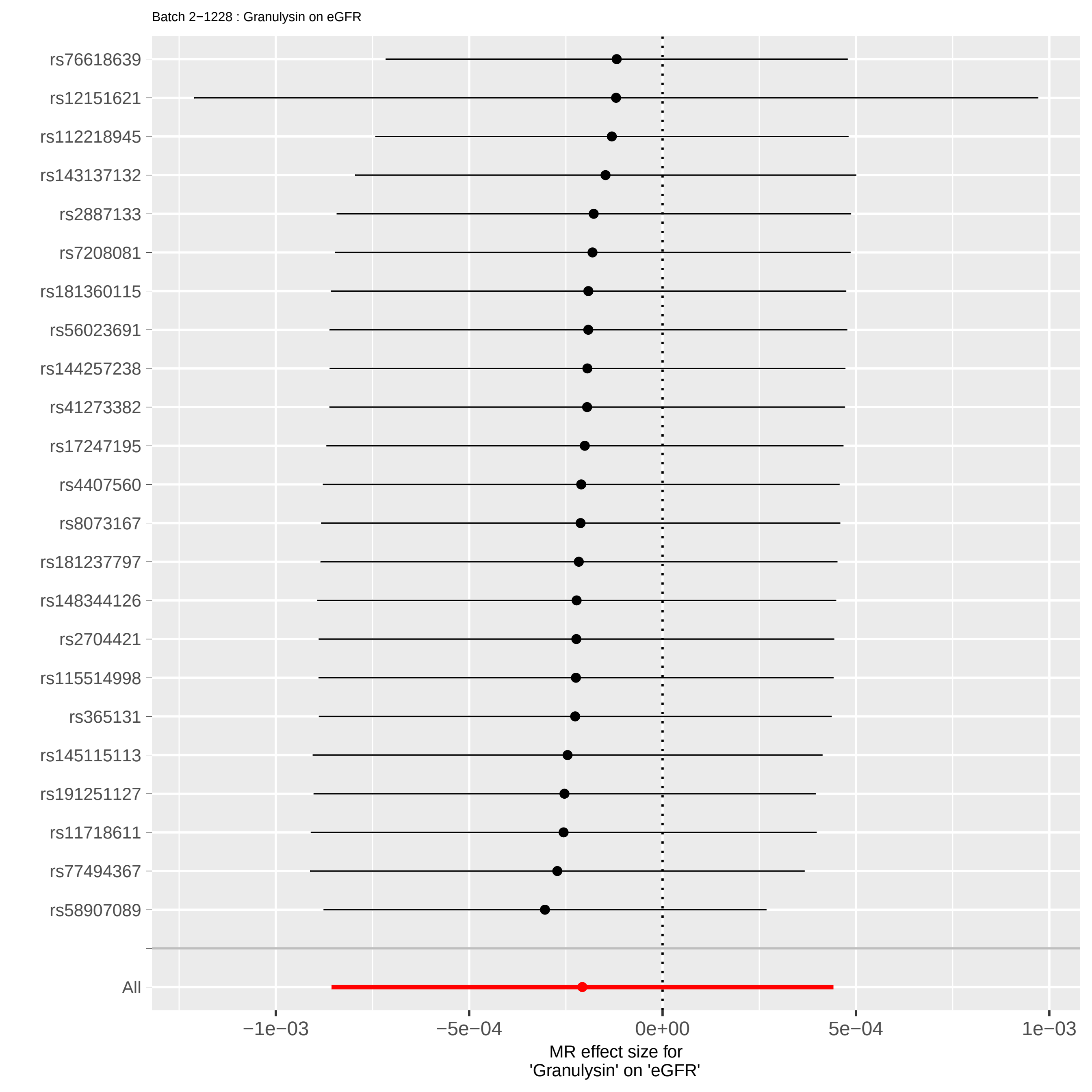

Batch 2−1228 : Granulysin on eGFR
rs76618639
rs12151621
rs112218945
rs143137132
rs2887133
rs7208081
rs181360115
rs56023691
rs144257238
rs41273382
rs17247195
rs4407560
rs8073167
rs181237797
rs148344126
rs2704421
rs115514998
rs365131
rs145115113
rs191251127
rs11718611
rs77494367
rs58907089
All
−1e−03
−5e−04
0e+00
5e−04
1e−03
MR effect size for
'Granulysin' on 'eGFR'

Batch 2−1229 : Glycine N−methyltransferase on eGFR

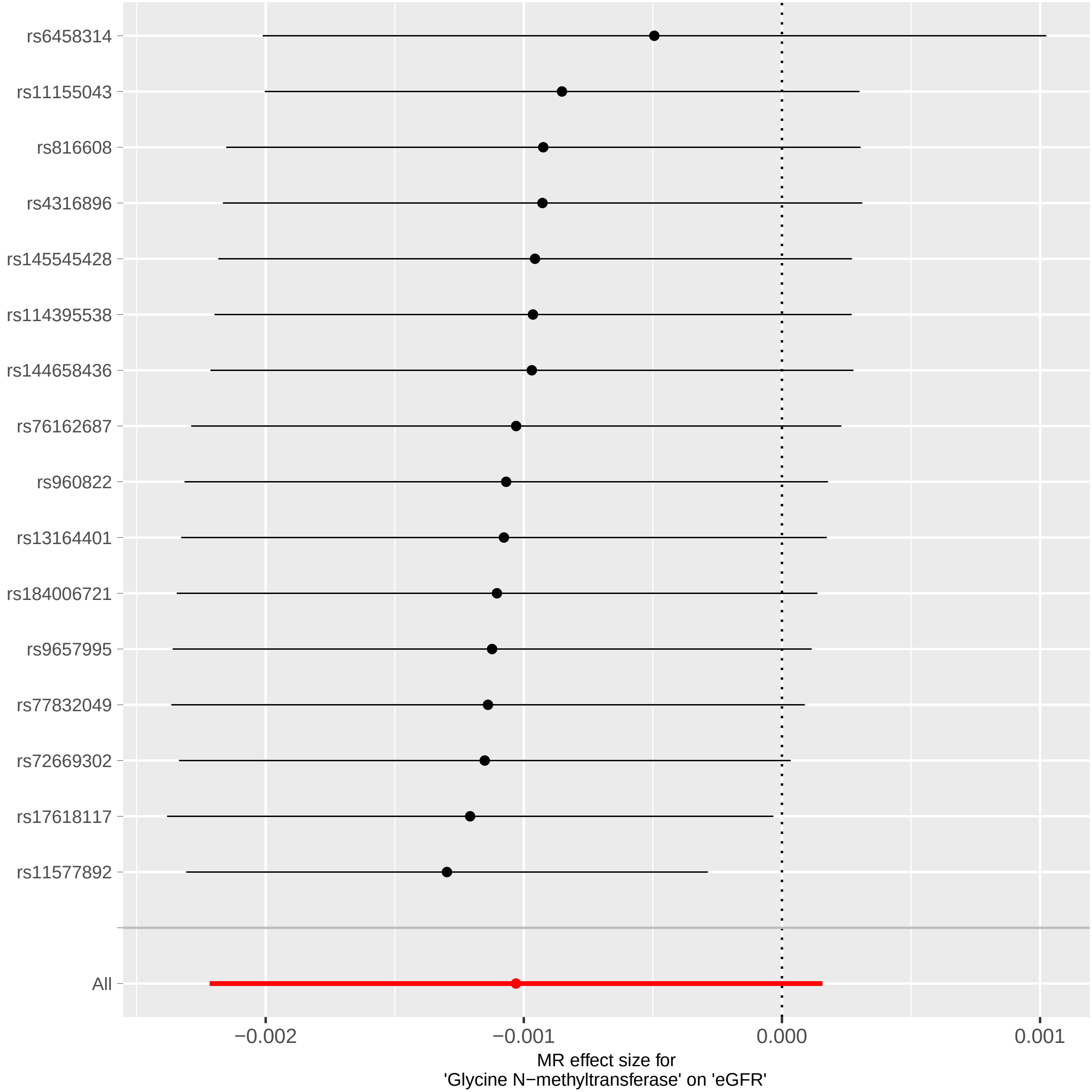

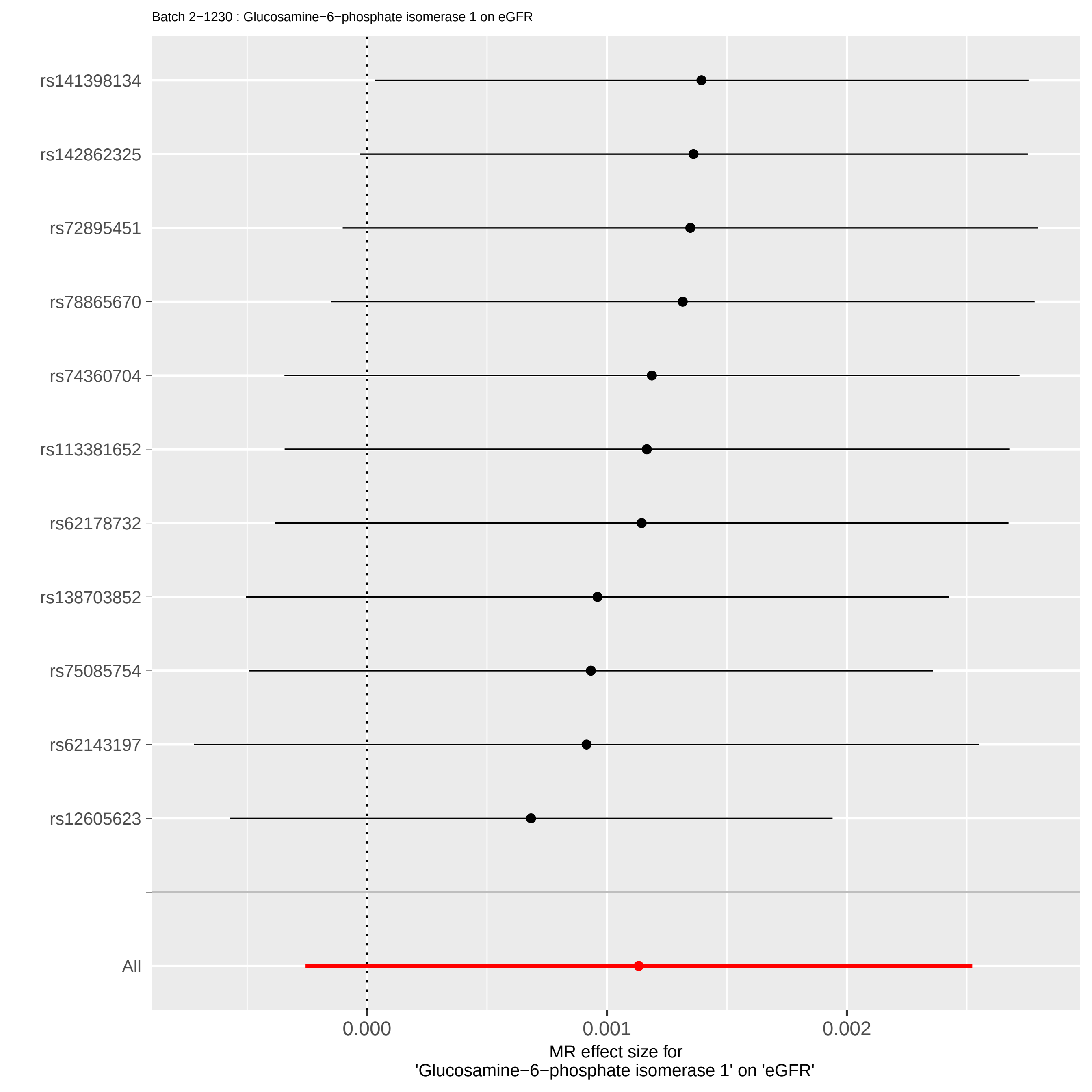

Batch 2−1230 : Glucosamine−6−phosphate isomerase 1 on eGFR
rs141398134
rs142862325
rs72895451
rs78865670
rs74360704
rs113381652
rs62178732
rs138703852
rs75085754
rs62143197
rs12605623
All
0.000
0.001
0.002
MR effect size for
'Glucosamine−6−phosphate isomerase 1' on 'eGFR'

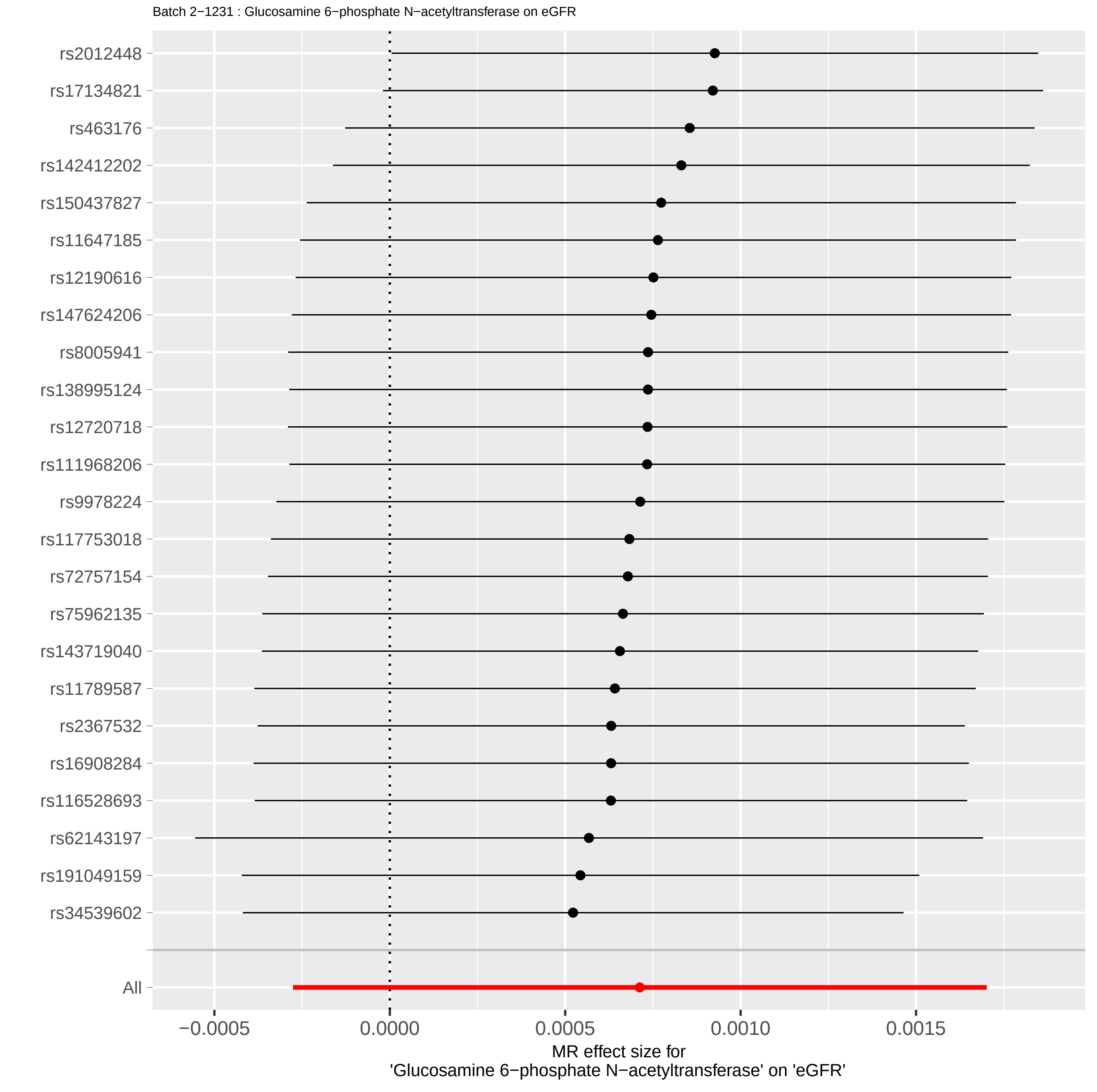

Batch 2−1231 : Glucosamine 6−phosphate N−acetyltransferase on eGFR
rs2012448
rs17134821
rs463176
rs142412202
rs150437827
rs11647185
rs12190616
rs147624206
rs8005941
rs138995124
rs12720718
rs111968206
rs9978224
rs117753018
rs72757154
rs75962135
rs143719040
rs11789587
rs2367532
rs16908284
rs116528693
rs62143197
rs191049159
rs34539602
All
−0.0005
0.0000
0.0005
0.0010
0.0015
MR effect size for
'Glucosamine 6−phosphate N−acetyltransferase' on 'eGFR'

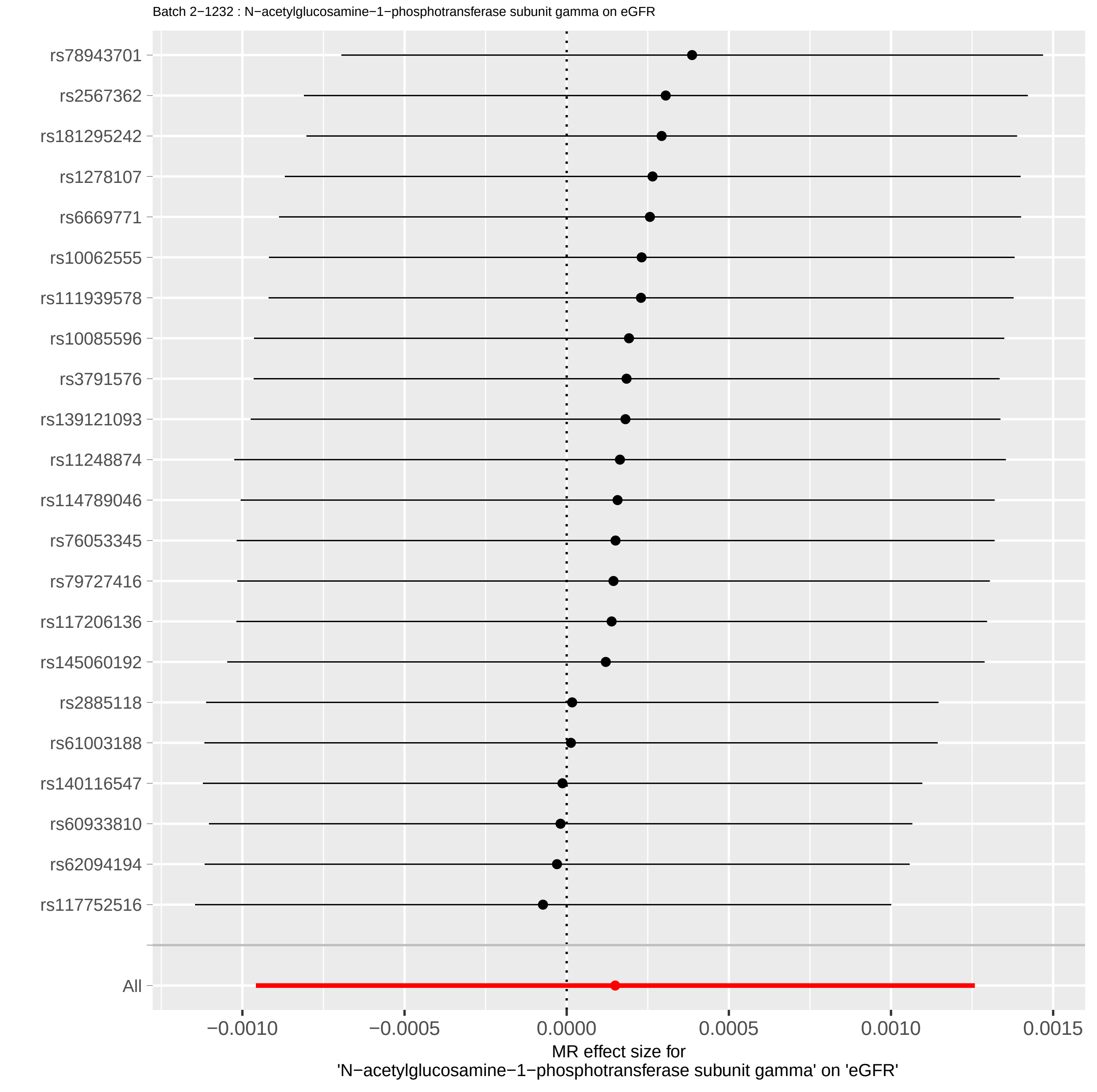

Batch 2−1232 : N−acetylglucosamine−1−phosphotransferase subunit gamma on eGFR
rs78943701
rs2567362
rs181295242
rs1278107
rs6669771
rs10062555
rs111939578
rs10085596
rs3791576
rs139121093
rs11248874
rs114789046
rs76053345
rs79727416
rs117206136
rs145060192
rs2885118
rs61003188
rs140116547
rs60933810
rs62094194
rs117752516
All
−0.0010
−0.0005
0.0000
0.0005
0.0010
0.0015
MR effect size for
'N−acetylglucosamine−1−phosphotransferase subunit gamma' on 'eGFR'

Batch 2−1233 : Progonadoliberin−1 on eGFR

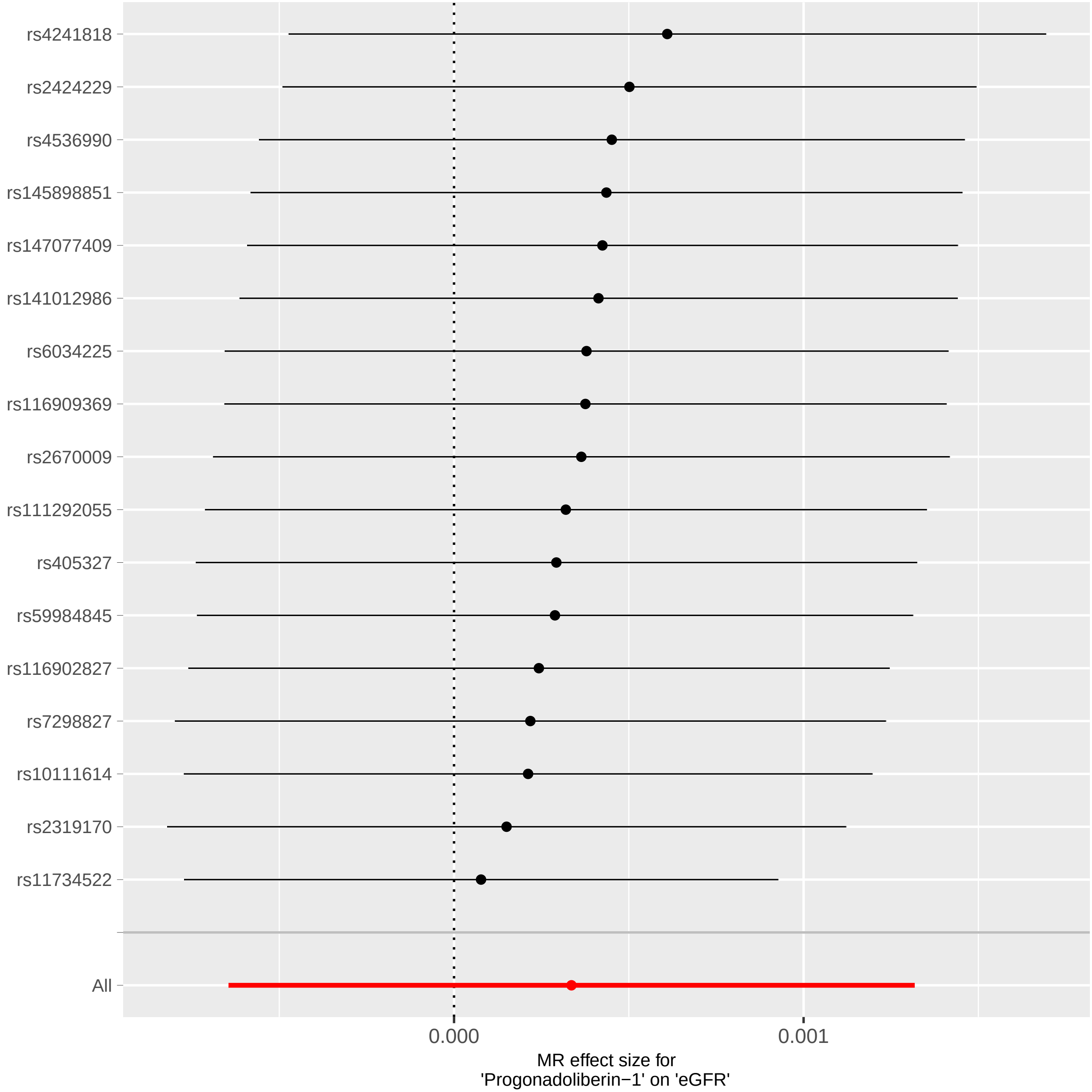

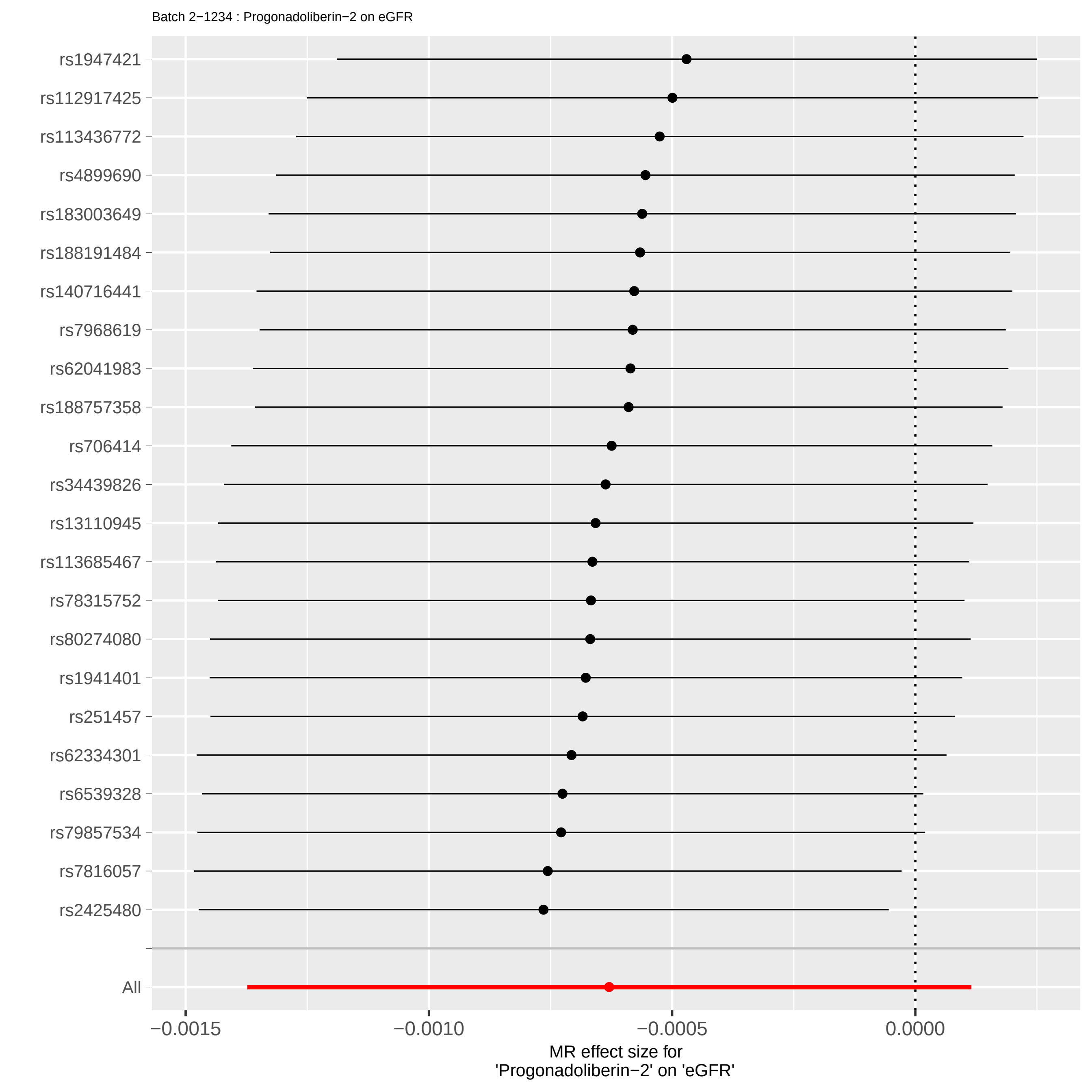
Batch 2−1234 : Progonadoliberin−2 on eGFR
rs1947421
rs112917425
rs113436772
rs4899690
rs183003649
rs188191484
rs140716441
rs7968619
rs62041983
rs188757358
rs706414
rs34439826
rs13110945
rs113685467
rs78315752
rs80274080
rs1941401
rs251457
rs62334301
rs6539328
rs79857534
rs7816057
rs2425480
All
−0.0015
−0.0010
−0.0005
0.0000
MR effect size for
'Progonadoliberin−2' on 'eGFR'

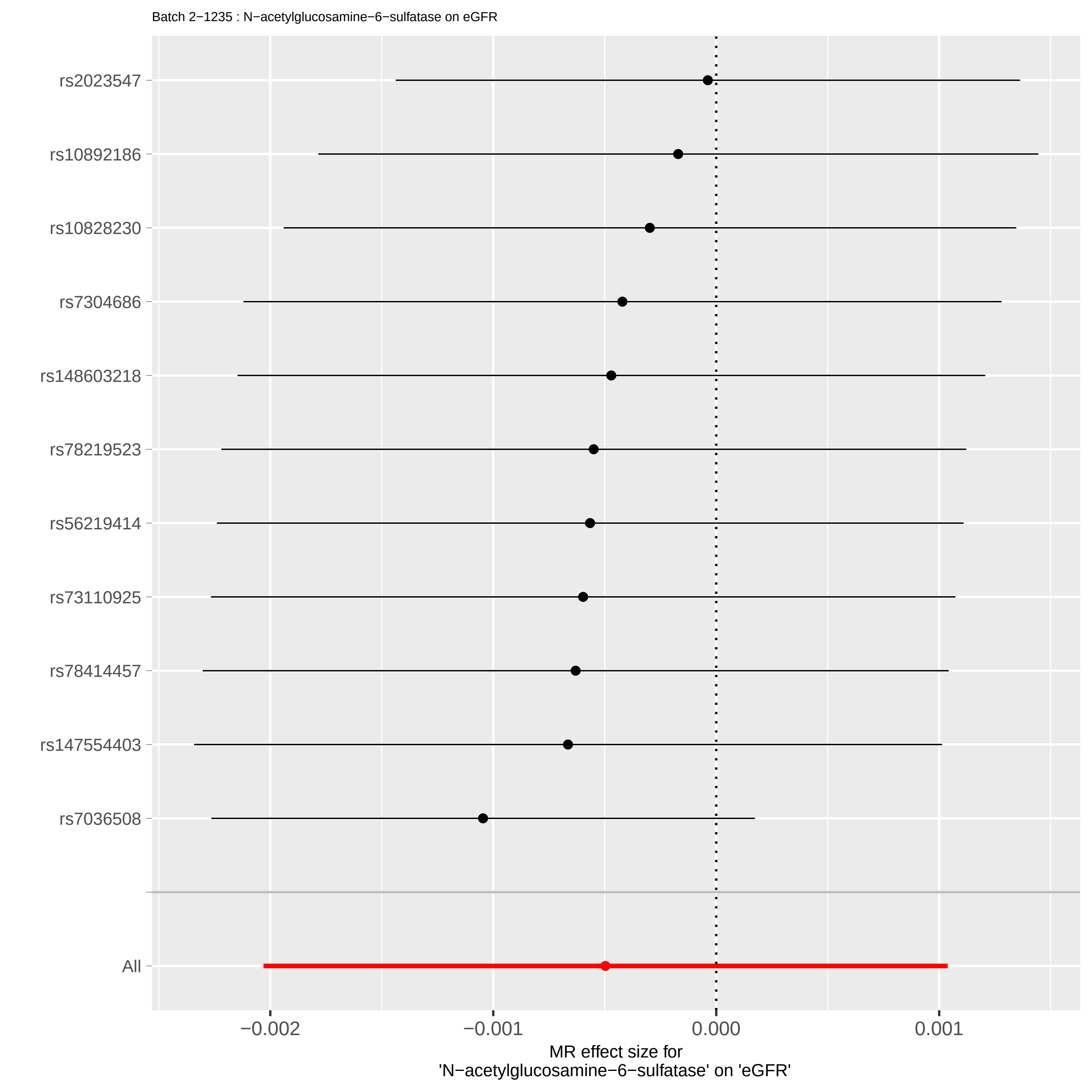

Batch 2−1235 : N−acetylglucosamine−6−sulfatase on eGFR
rs2023547
rs10892186
rs10828230
rs7304686
rs148603218
rs78219523
rs56219414
rs73110925
rs78414457
rs147554403
rs7036508
All
−0.002
−0.001
0.000
0.001
MR effect size for
'N−acetylglucosamine−6−sulfatase' on 'eGFR'

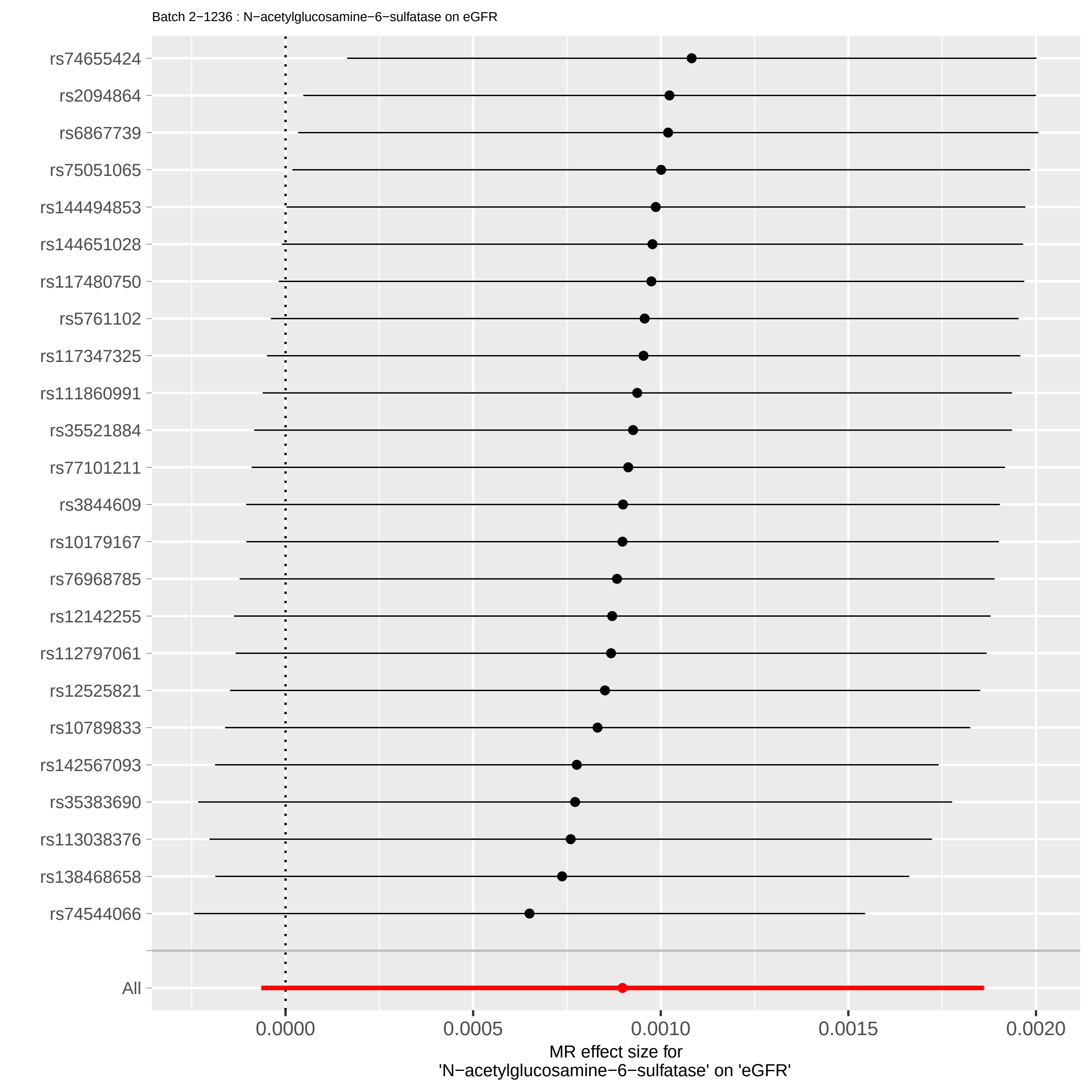

Batch 2−1236 : N−acetylglucosamine−6−sulfatase on eGFR
rs74655424
rs2094864
rs6867739
rs75051065
rs144494853
rs144651028
rs117480750
rs5761102
rs117347325
rs111860991
rs35521884
rs77101211
rs3844609
rs10179167
rs76968785
rs12142255
rs112797061
rs12525821
rs10789833
rs142567093
rs35383690
rs113038376
rs138468658
rs74544066
All
0.0000
0.0005
0.0010
0.0015
0.0020
MR effect size for
'N−acetylglucosamine−6−sulfatase' on 'eGFR'

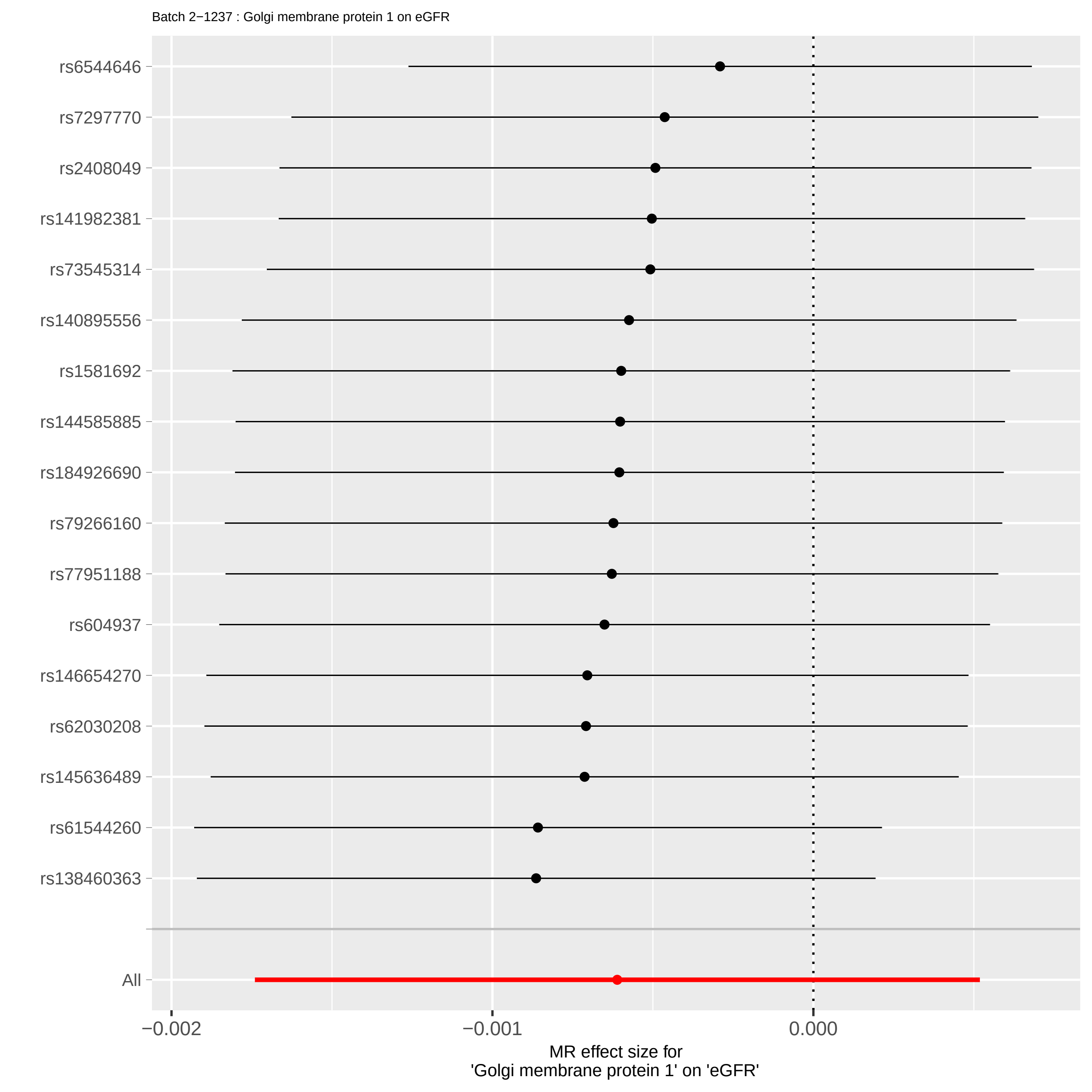

Batch 2−1237 : Golgi membrane protein 1 on eGFR
rs6544646
rs7297770
rs2408049
rs141982381
rs73545314
rs140895556
rs1581692
rs144585885
rs184926690
rs79266160
rs77951188
rs604937
rs146654270
rs62030208
rs145636489
rs61544260
rs138460363
All
−0.002
−0.001
0.000
MR effect size for
'Golgi membrane protein 1' on 'eGFR'

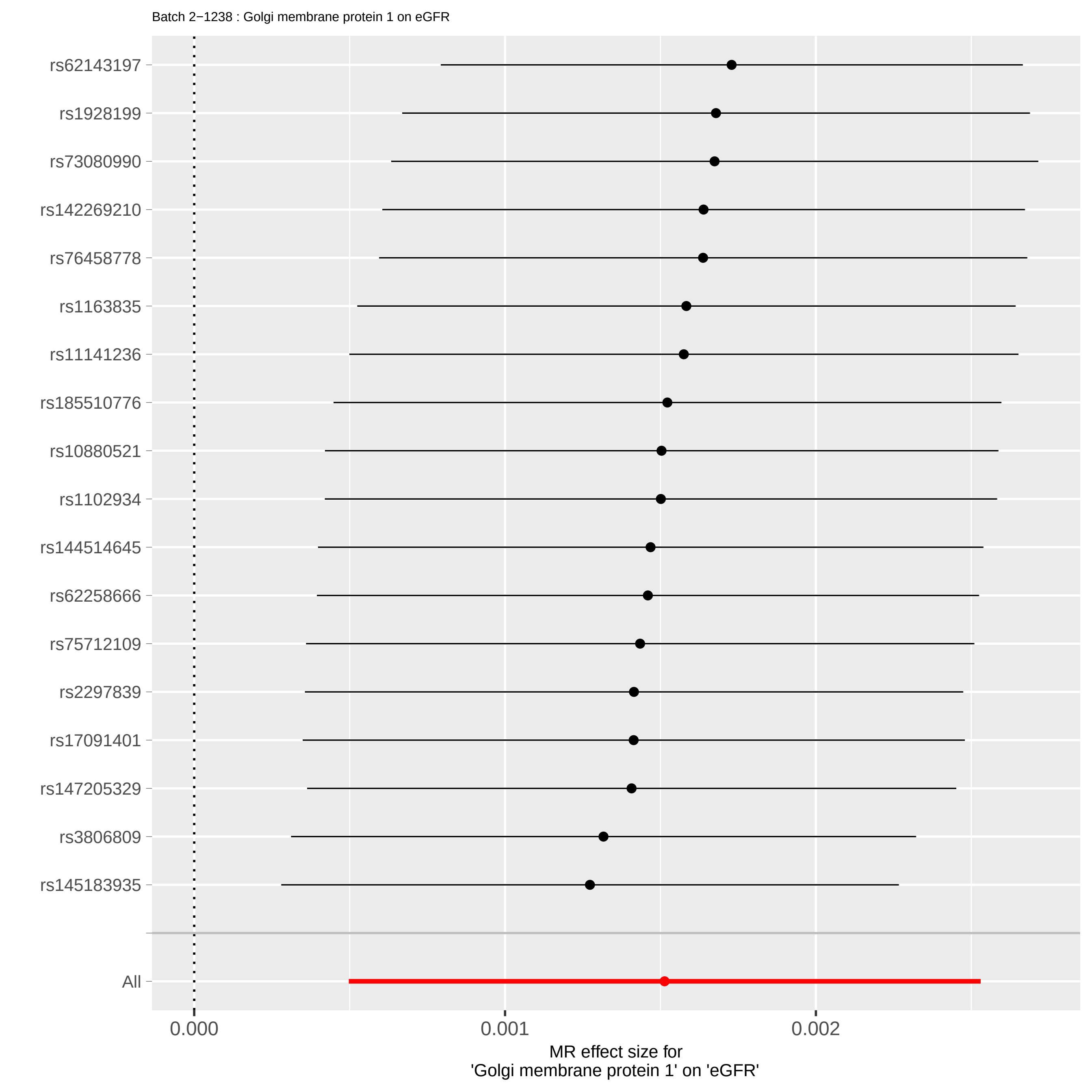

Batch 2−1238 : Golgi membrane protein 1 on eGFR
rs62143197
rs1928199
rs73080990
rs142269210
rs76458778
rs1163835
rs11141236
rs185510776
rs10880521
rs1102934
rs144514645
rs62258666
rs75712109
rs2297839
rs17091401
rs147205329
rs3806809
rs145183935
All
0.000
0.001
0.002
MR effect size for
'Golgi membrane protein 1' on 'eGFR'

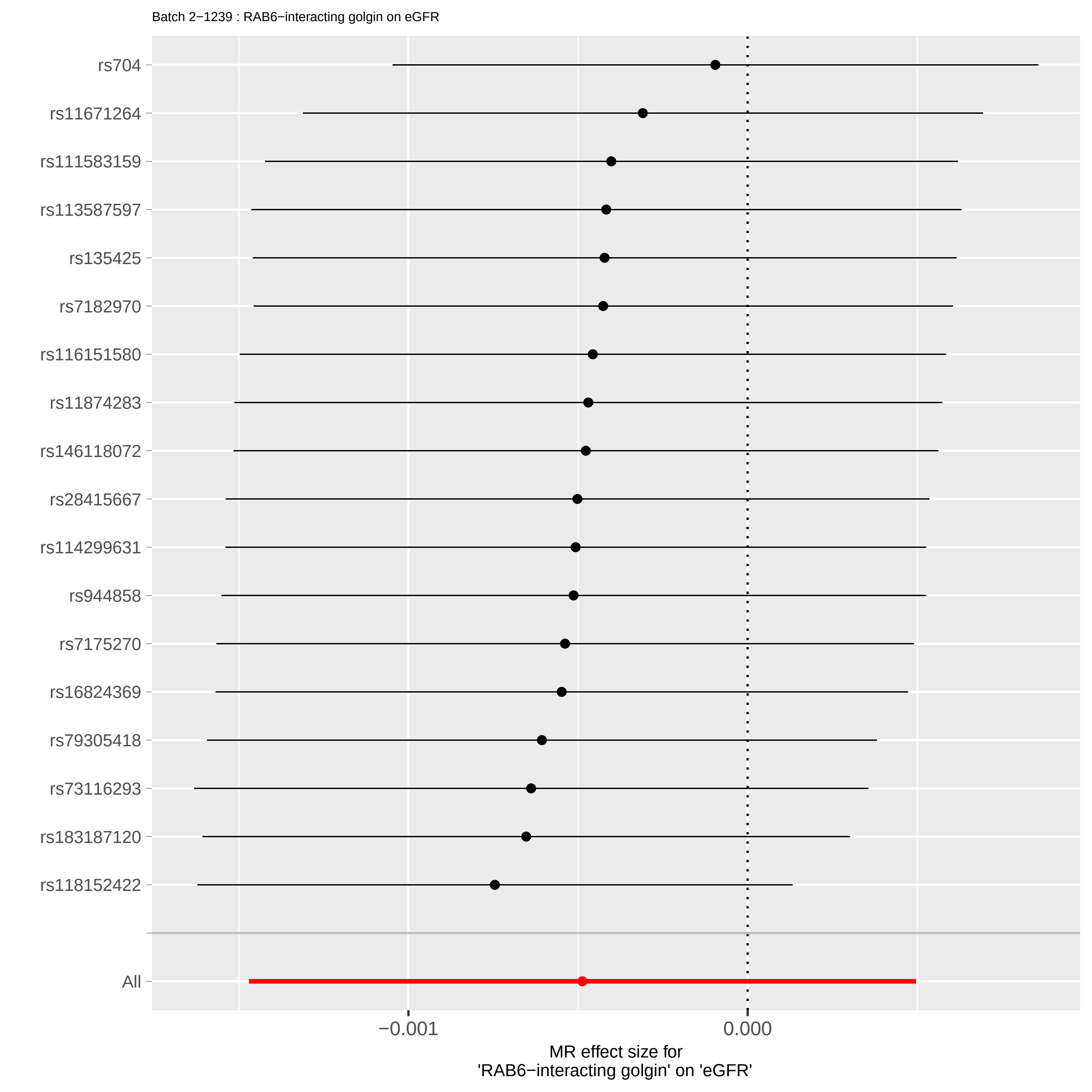

Batch 2−1239 : RAB6−interacting golgin on eGFR
rs704
rs11671264
rs111583159
rs113587597
rs135425
rs7182970
rs116151580
rs11874283
rs146118072
rs28415667
rs114299631
rs944858
rs7175270
rs16824369
rs79305418
rs73116293
rs183187120
rs118152422
All
−0.001
0.000
MR effect size for
'RAB6−interacting golgin' on 'eGFR'

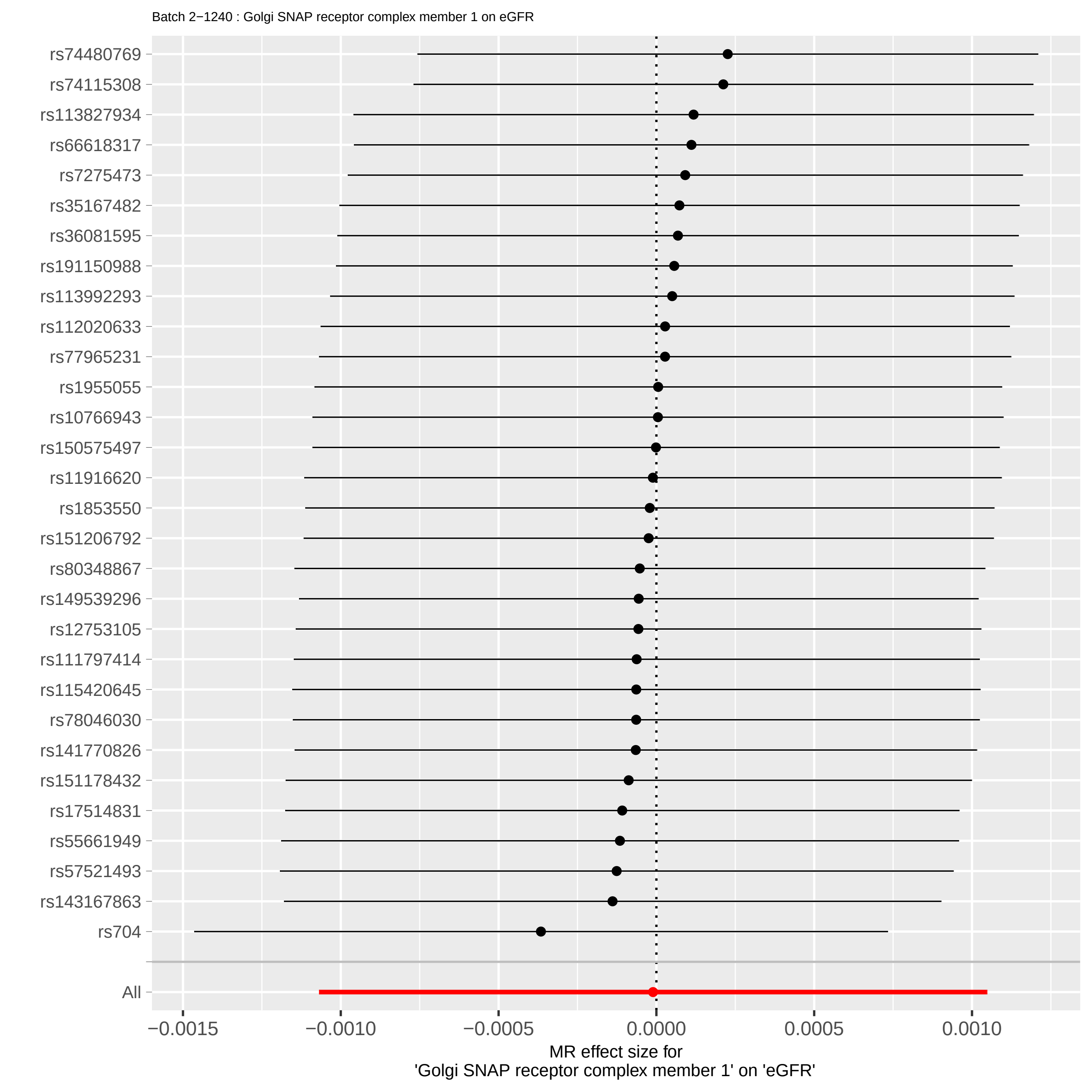

Batch 2−1240 : Golgi SNAP receptor complex member 1 on eGFR
rs74480769
rs74115308
rs113827934
rs66618317
rs7275473
rs35167482
rs36081595
rs191150988
rs113992293
rs112020633
rs77965231
rs1955055
rs10766943
rs150575497
rs11916620
rs1853550
rs151206792
rs80348867
rs149539296
rs12753105
rs111797414
rs115420645
rs78046030
rs141770826
rs151178432
rs17514831
rs55661949
rs57521493
rs143167863
rs704
All
−0.0015
−0.0010
−0.0005
0.0000
0.0005
0.0010
MR effect size for
'Golgi SNAP receptor complex member 1' on 'eGFR'

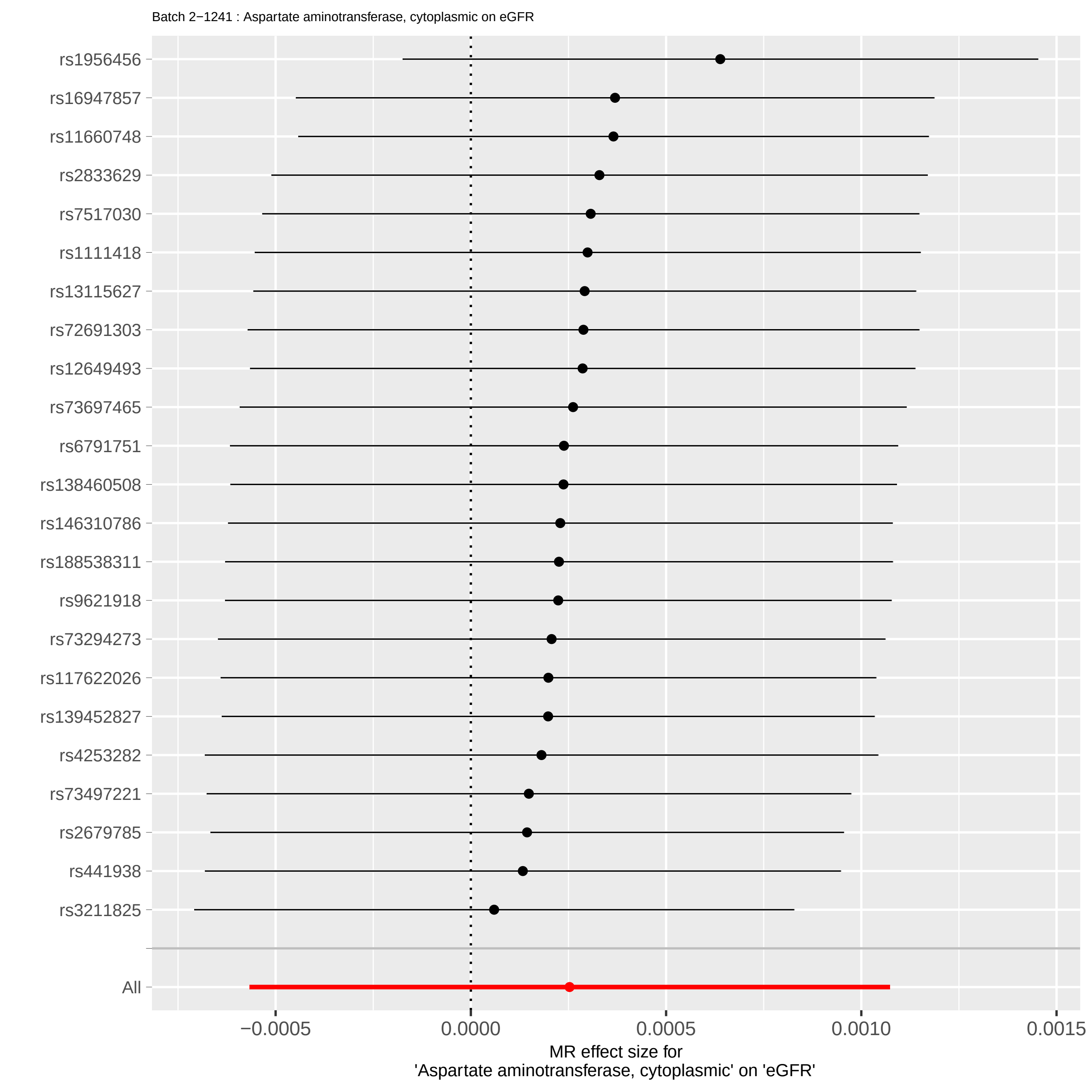

Batch 2−1241 : Aspartate aminotransferase, cytoplasmic on eGFR
rs1956456
rs16947857
rs11660748
rs2833629
rs7517030
rs1111418
rs13115627
rs72691303
rs12649493
rs73697465
rs6791751
rs138460508
rs146310786
rs188538311
rs9621918
rs73294273
rs117622026
rs139452827
rs4253282
rs73497221
rs2679785
rs441938
rs3211825
All
−0.0005
0.0000
0.0005
0.0010
0.0015
MR effect size for
'Aspartate aminotransferase, cytoplasmic' on 'eGFR'

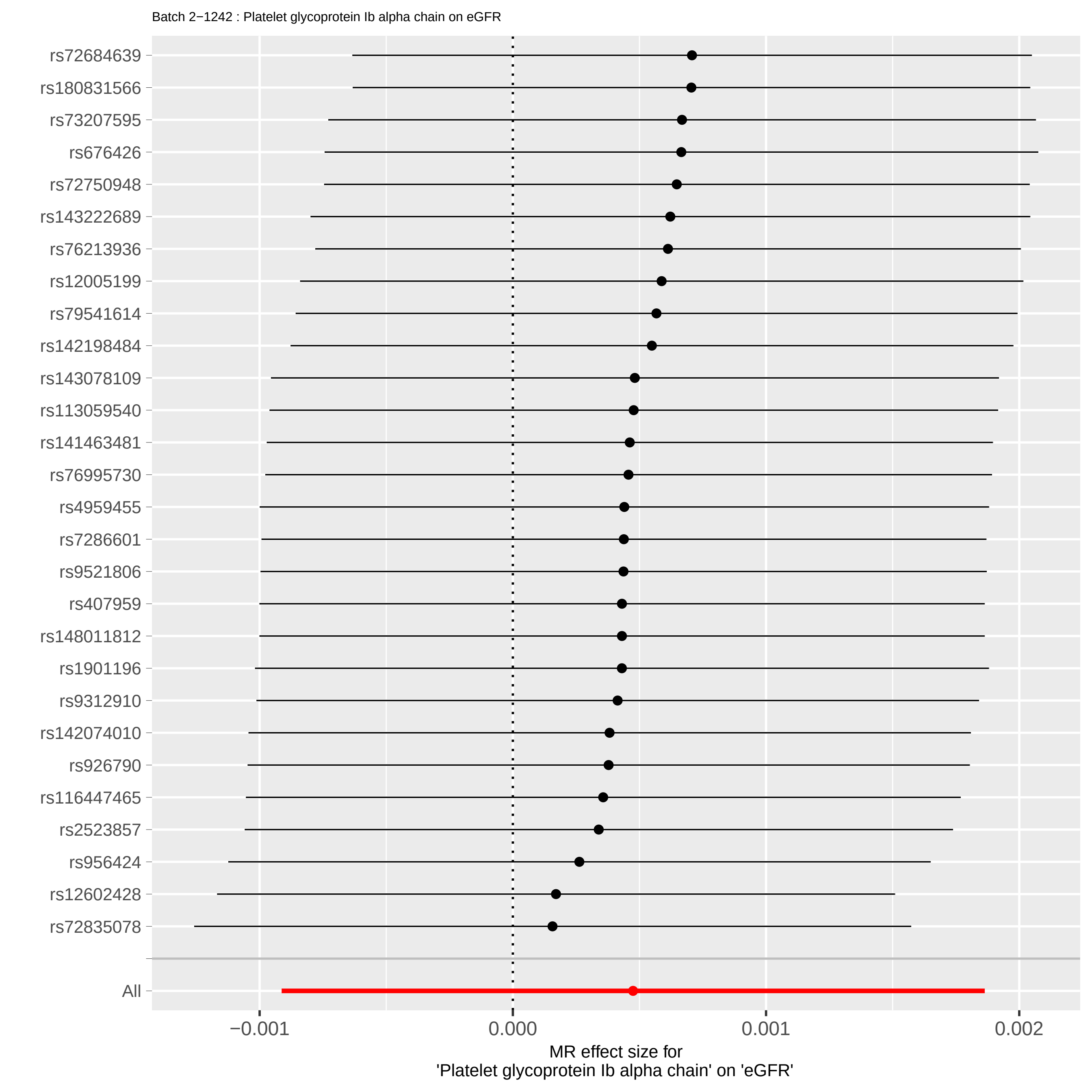

Batch 2−1242 : Platelet glycoprotein Ib alpha chain on eGFR
rs72684639
rs180831566
rs73207595
rs676426
rs72750948
rs143222689
rs76213936
rs12005199
rs79541614
rs142198484
rs143078109
rs113059540
rs141463481
rs76995730
rs4959455
rs7286601
rs9521806
rs407959
rs148011812
rs1901196
rs9312910
rs142074010
rs926790
rs116447465
rs2523857
rs956424
rs12602428
rs72835078
All
−0.001
0.000
0.001
0.002
MR effect size for
'Platelet glycoprotein Ib alpha chain' on 'eGFR'

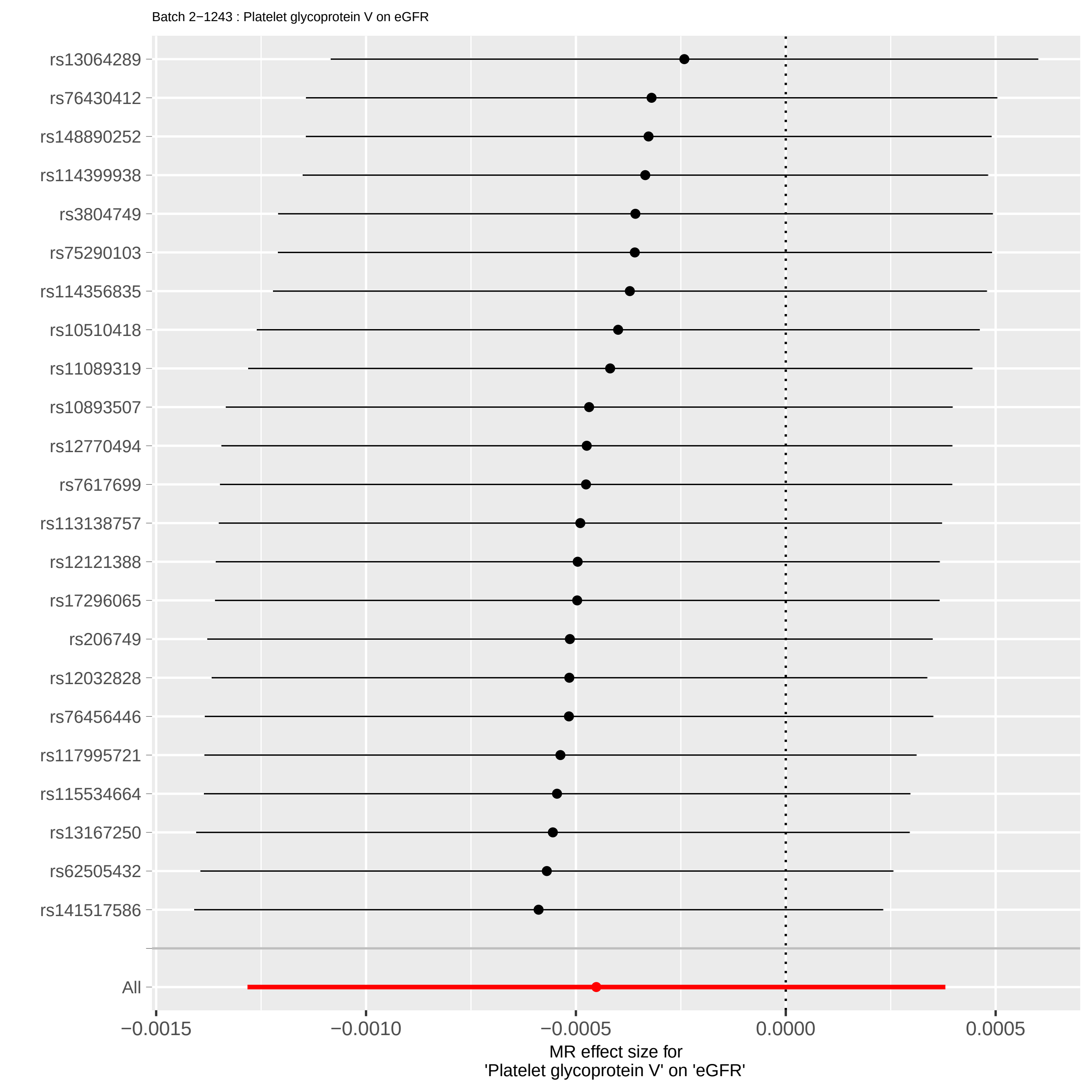

Batch 2−1243 : Platelet glycoprotein V on eGFR
rs13064289
rs76430412
rs148890252
rs114399938
rs3804749
rs75290103
rs114356835
rs10510418
rs11089319
rs10893507
rs12770494
rs7617699
rs113138757
rs12121388
rs17296065
rs206749
rs12032828
rs76456446
rs117995721
rs115534664
rs13167250
rs62505432
rs141517586
All
−0.0015
−0.0010
−0.0005
0.0000
0.0005
MR effect size for
'Platelet glycoprotein V' on 'eGFR'

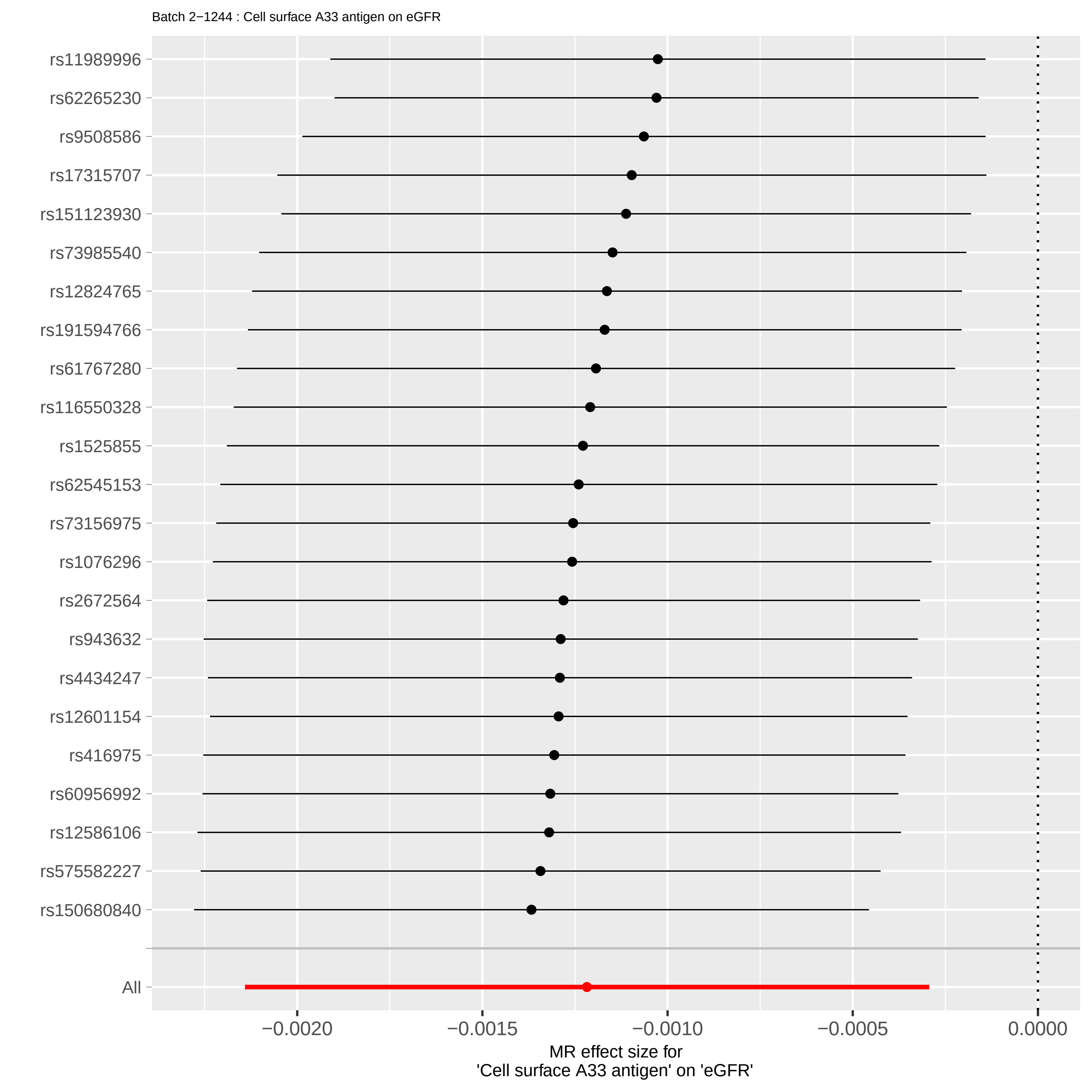
Batch 2−1244 : Cell surface A33 antigen on eGFR
rs11989996
rs62265230
rs9508586
rs17315707
rs151123930
rs73985540
rs12824765
rs191594766
rs61767280
rs116550328
rs1525855
rs62545153
rs73156975
rs1076296
rs2672564
rs943632
rs4434247
rs12601154
rs416975
rs60956992
rs12586106
rs575582227
rs150680840
All
−0.0020
−0.0015
−0.0010
−0.0005
0.0000
MR effect size for
'Cell surface A33 antigen' on 'eGFR'

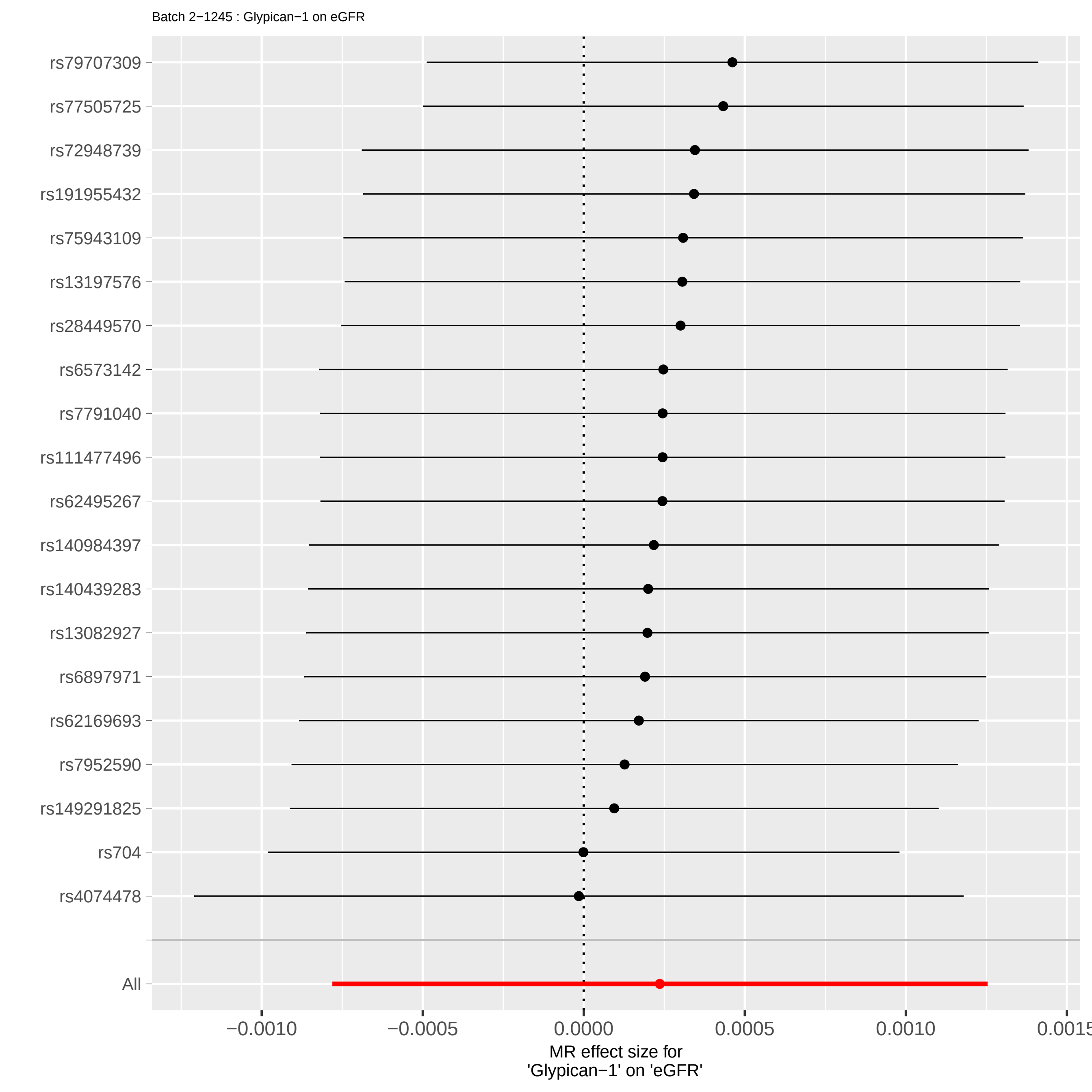

Batch 2−1245 : Glypican−1 on eGFR
rs79707309
rs77505725
rs72948739
rs191955432
rs75943109
rs13197576
rs28449570
rs6573142
rs7791040
rs111477496
rs62495267
rs140984397
rs140439283
rs13082927
rs6897971
rs62169693
rs7952590
rs149291825
rs704
rs4074478
All
−0.0010
−0.0005
0.0000
0.0005
0.0010
0.0015
MR effect size for
'Glypican−1' on 'eGFR'

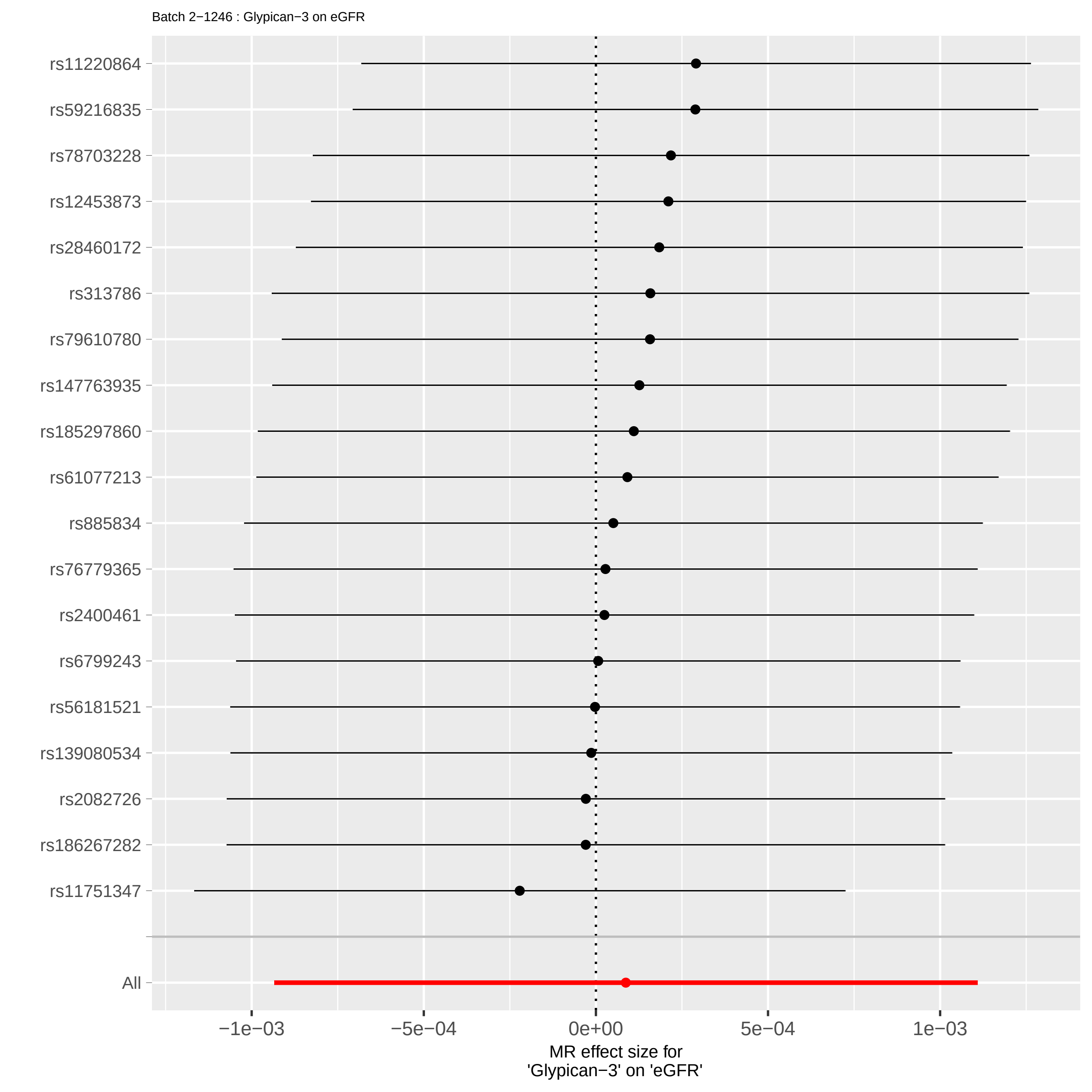

Batch 2−1246 : Glypican−3 on eGFR
rs11220864
rs59216835
rs78703228
rs12453873
rs28460172
rs313786
rs79610780
rs147763935
rs185297860
rs61077213
rs885834
rs76779365
rs2400461
rs6799243
rs56181521
rs139080534
rs2082726
rs186267282
rs11751347
All
−1e−03
−5e−04
0e+00
5e−04
1e−03
MR effect size for
'Glypican−3' on 'eGFR'

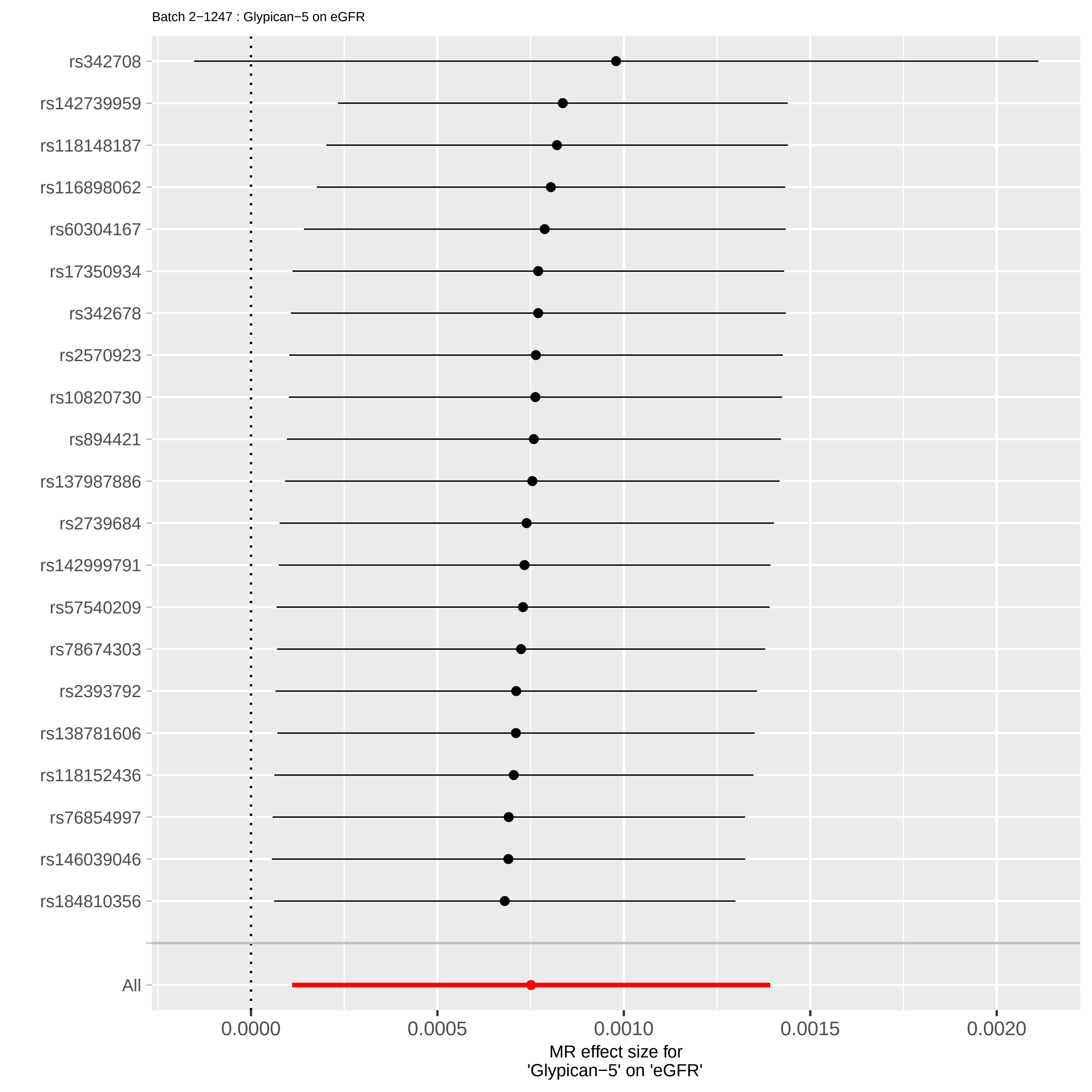

Batch 2−1247 : Glypican−5 on eGFR
rs342708
rs142739959
rs118148187
rs116898062
rs60304167
rs17350934
rs342678
rs2570923
rs10820730
rs894421
rs137987886
rs2739684
rs142999791
rs57540209
rs78674303
rs2393792
rs138781606
rs118152436
rs76854997
rs146039046
rs184810356
All
0.0000
0.0005
0.0010
0.0015
0.0020
MR effect size for
'Glypican−5' on 'eGFR'

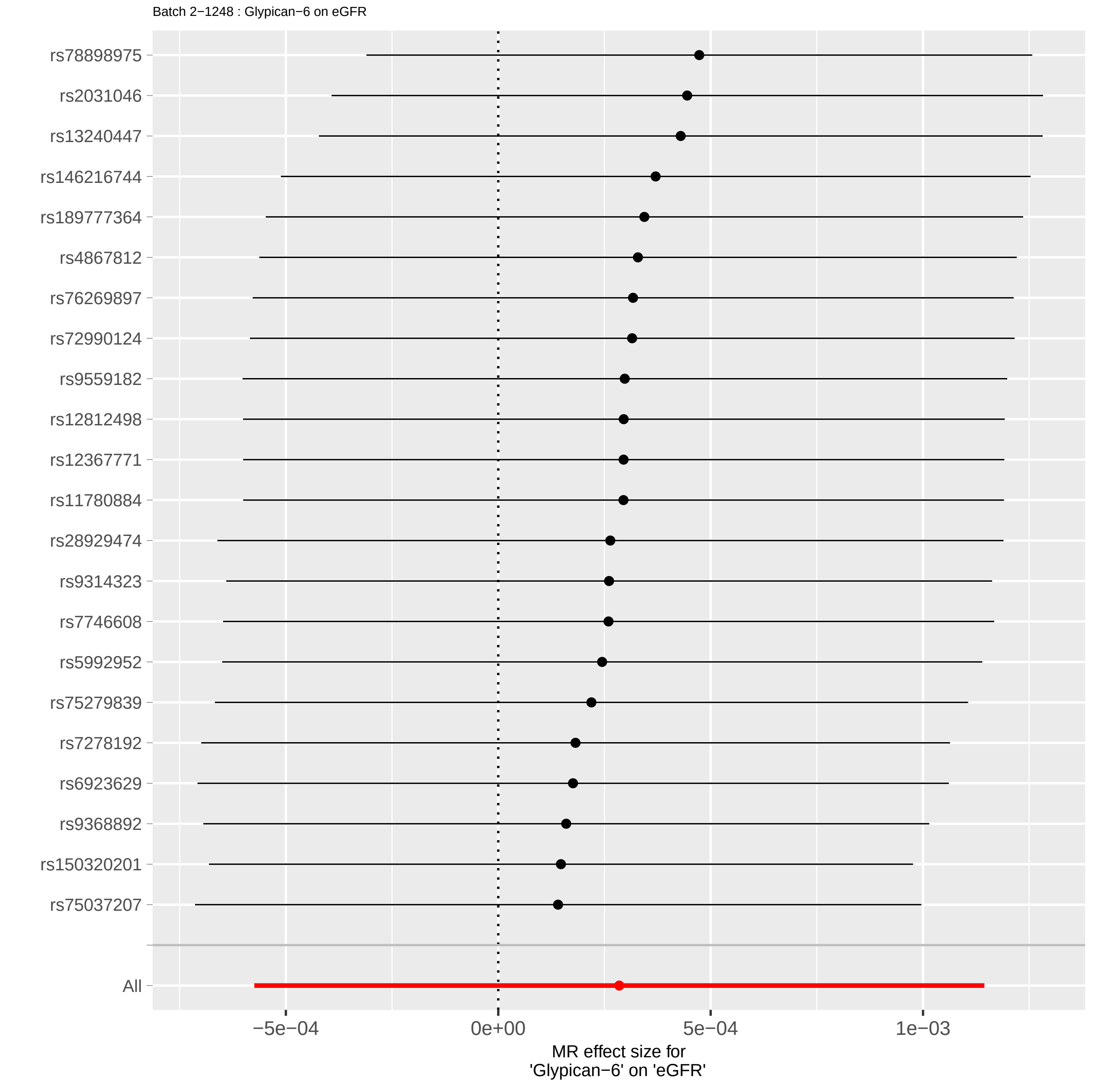

Batch 2−1248 : Glypican−6 on eGFR
rs78898975
rs2031046
rs13240447
rs146216744
rs189777364
rs4867812
rs76269897
rs72990124
rs9559182
rs12812498
rs12367771
rs11780884
rs28929474
rs9314323
rs7746608
rs5992952
rs75279839
rs7278192
rs6923629
rs9368892
rs150320201
rs75037207
All
−5e−04
0e+00
5e−04
1e−03
MR effect size for
'Glypican−6' on 'eGFR'

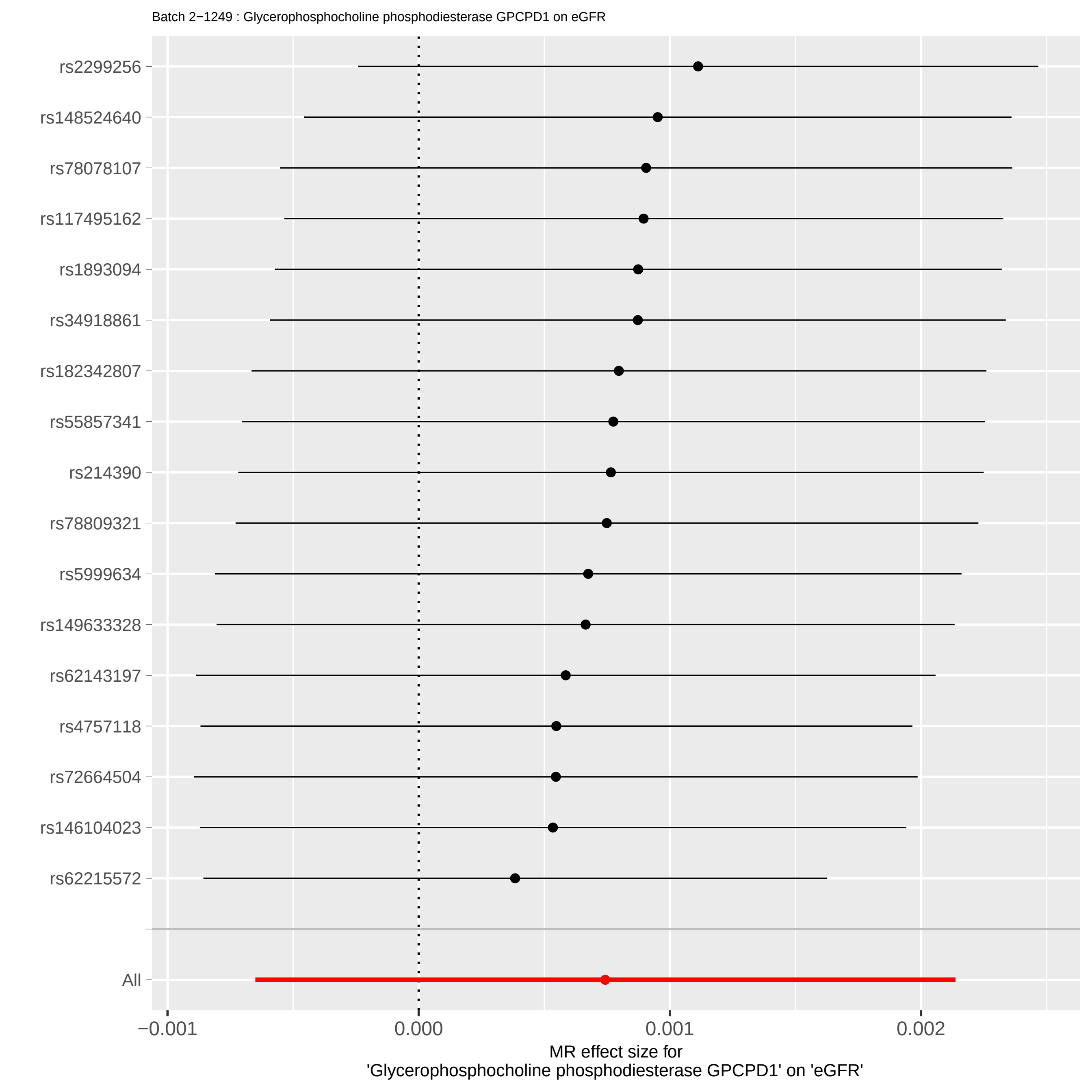

Batch 2−1249 : Glycerophosphocholine phosphodiesterase GPCPD1 on eGFR
rs2299256
rs148524640
rs78078107
rs117495162
rs1893094
rs34918861
rs182342807
rs55857341
rs214390
rs78809321
rs5999634
rs149633328
rs62143197
rs4757118
rs72664504
rs146104023
rs62215572
All
−0.001
0.000
0.001
0.002
MR effect size for
'Glycerophosphocholine phosphodiesterase GPCPD1' on 'eGFR'

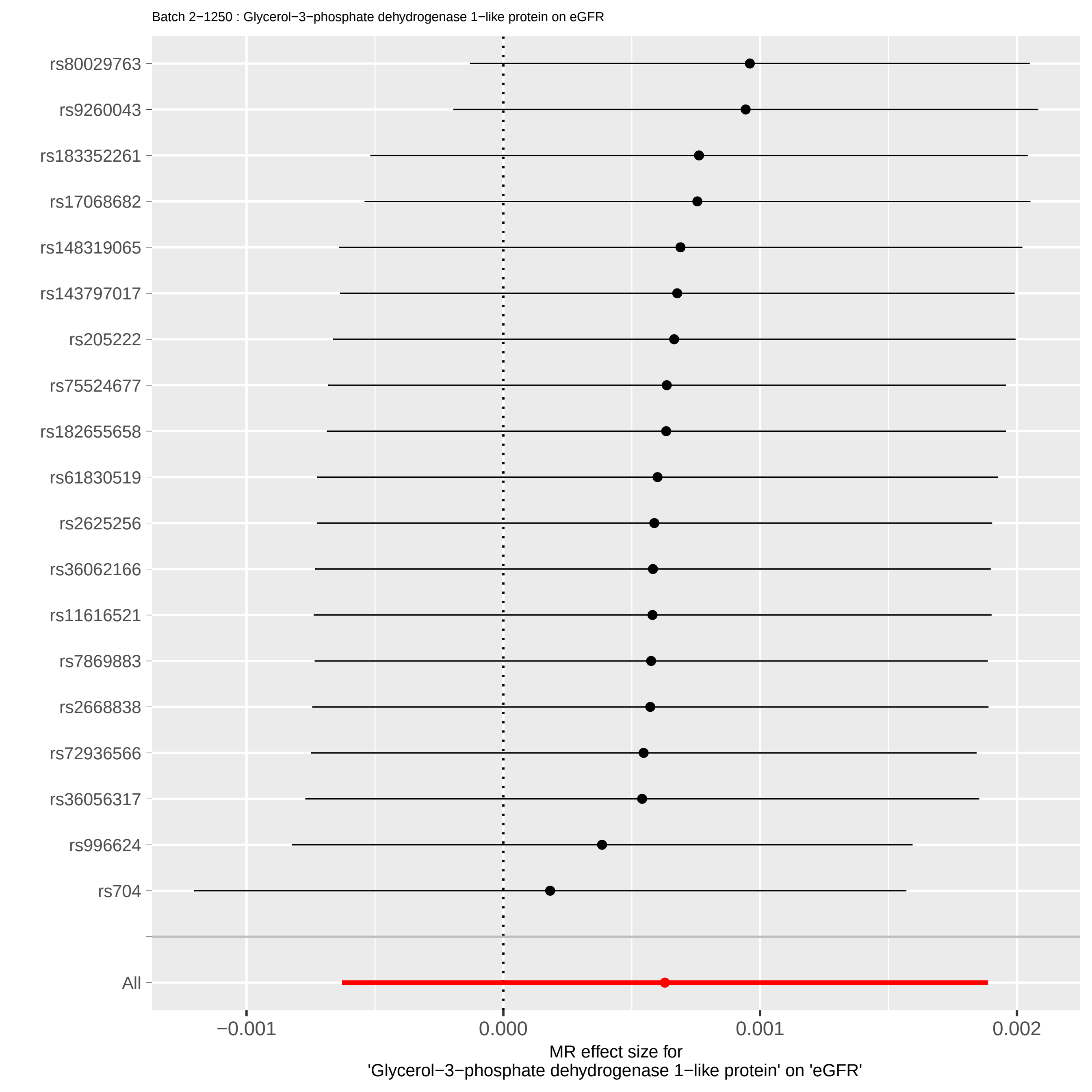
Batch 2−1250 : Glycerol−3−phosphate dehydrogenase 1−like protein on eGFR
rs80029763
rs9260043
rs183352261
rs17068682
rs148319065
rs143797017
rs205222
rs75524677
rs182655658
rs61830519
rs2625256
rs36062166
rs11616521
rs7869883
rs2668838
rs72936566
rs36056317
rs996624
rs704
All
−0.001
0.000
0.001
0.002
MR effect size for
'Glycerol−3−phosphate dehydrogenase 1−like protein' on 'eGFR'

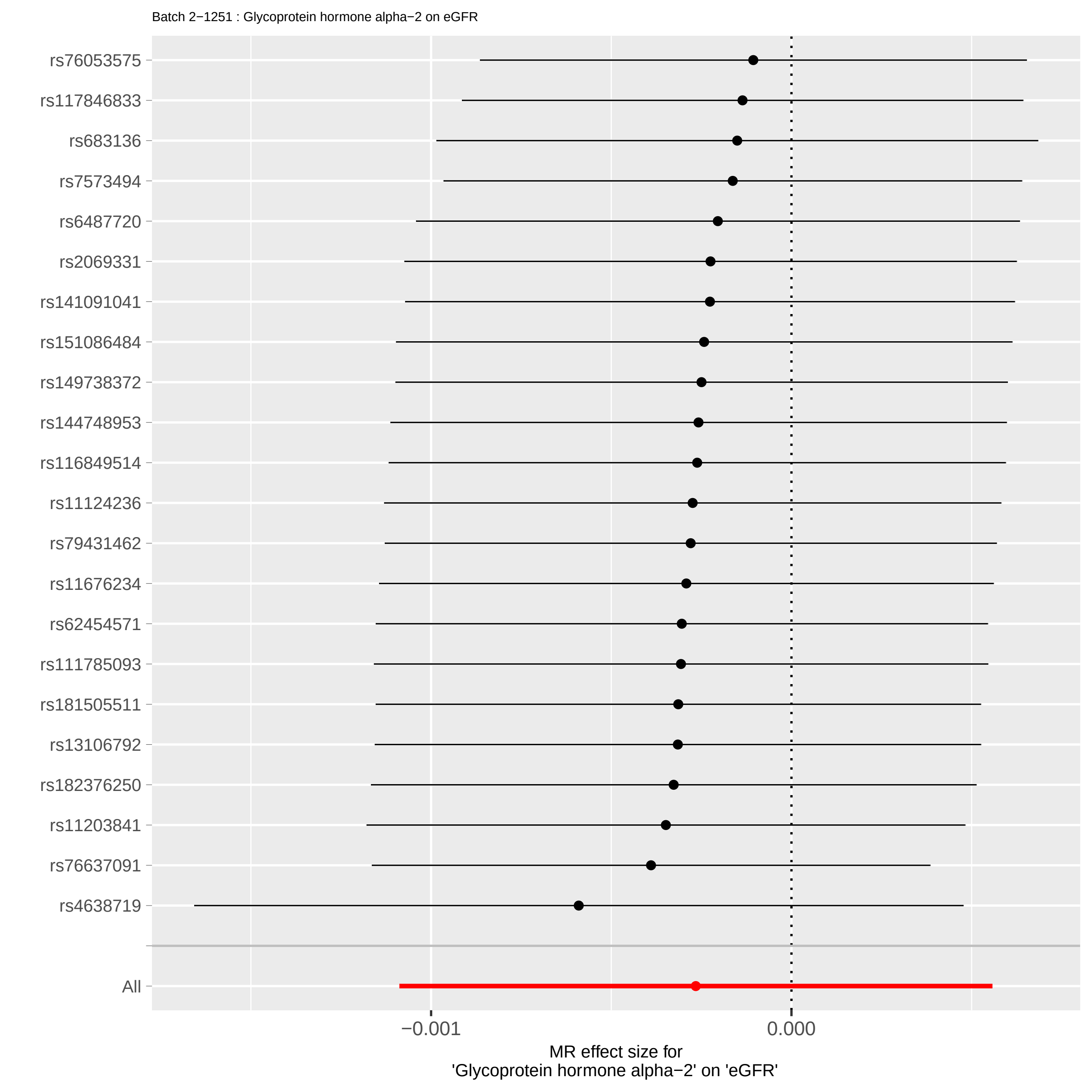

Batch 2−1251 : Glycoprotein hormone alpha−2 on eGFR
rs76053575
rs117846833
rs683136
rs7573494
rs6487720
rs2069331
rs141091041
rs151086484
rs149738372
rs144748953
rs116849514
rs11124236
rs79431462
rs11676234
rs62454571
rs111785093
rs181505511
rs13106792
rs182376250
rs11203841
rs76637091
rs4638719
All
−0.001
0.000
MR effect size for
'Glycoprotein hormone alpha−2' on 'eGFR'

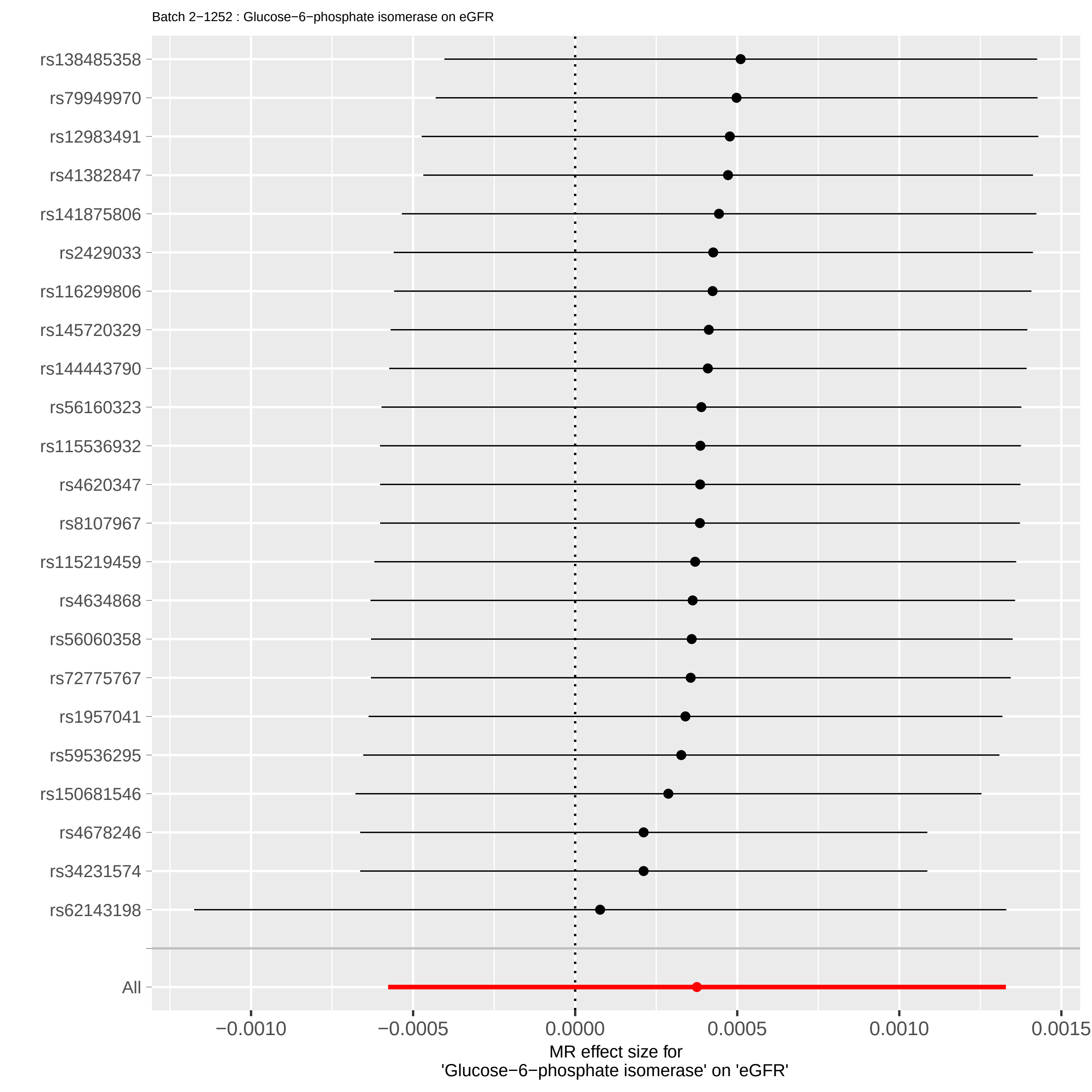

Batch 2−1252 : Glucose−6−phosphate isomerase on eGFR
rs138485358
rs79949970
rs12983491
rs41382847
rs141875806
rs2429033
rs116299806
rs145720329
rs144443790
rs56160323
rs115536932
rs4620347
rs8107967
rs115219459
rs4634868
rs56060358
rs72775767
rs1957041
rs59536295
rs150681546
rs4678246
rs34231574
rs62143198
All
−0.0010
−0.0005
0.0000
0.0005
0.0010
0.0015
MR effect size for
'Glucose−6−phosphate isomerase' on 'eGFR'

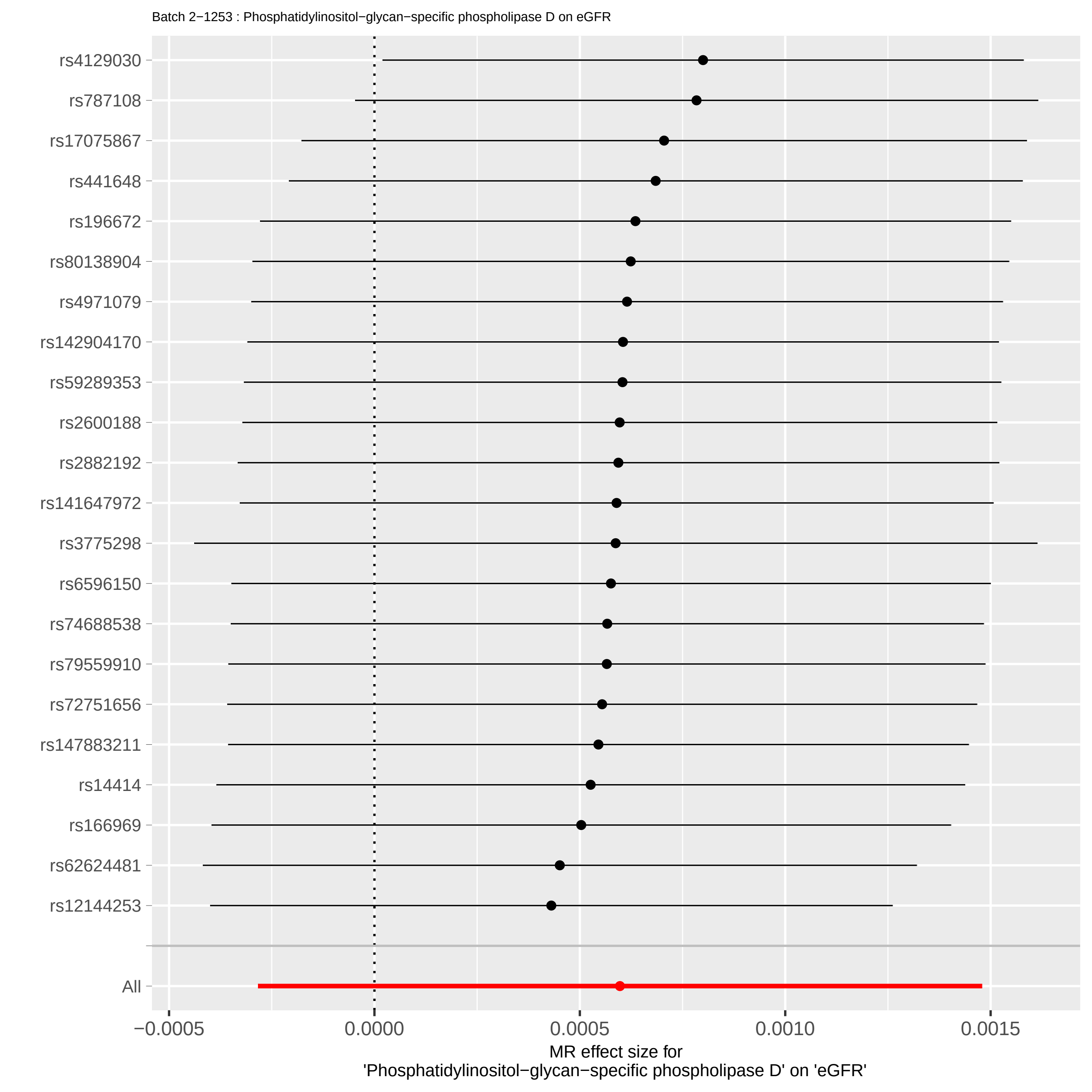

Batch 2−1253 : Phosphatidylinositol−glycan−specific phospholipase D on eGFR
rs4129030
rs787108
rs17075867
rs441648
rs196672
rs80138904
rs4971079
rs142904170
rs59289353
rs2600188
rs2882192
rs141647972
rs3775298
rs6596150
rs74688538
rs79559910
rs72751656
rs147883211
rs14414
rs166969
rs62624481
rs12144253
All
−0.0005
0.0000
0.0005
0.0010
0.0015
MR effect size for
'Phosphatidylinositol−glycan−specific phospholipase D' on 'eGFR'

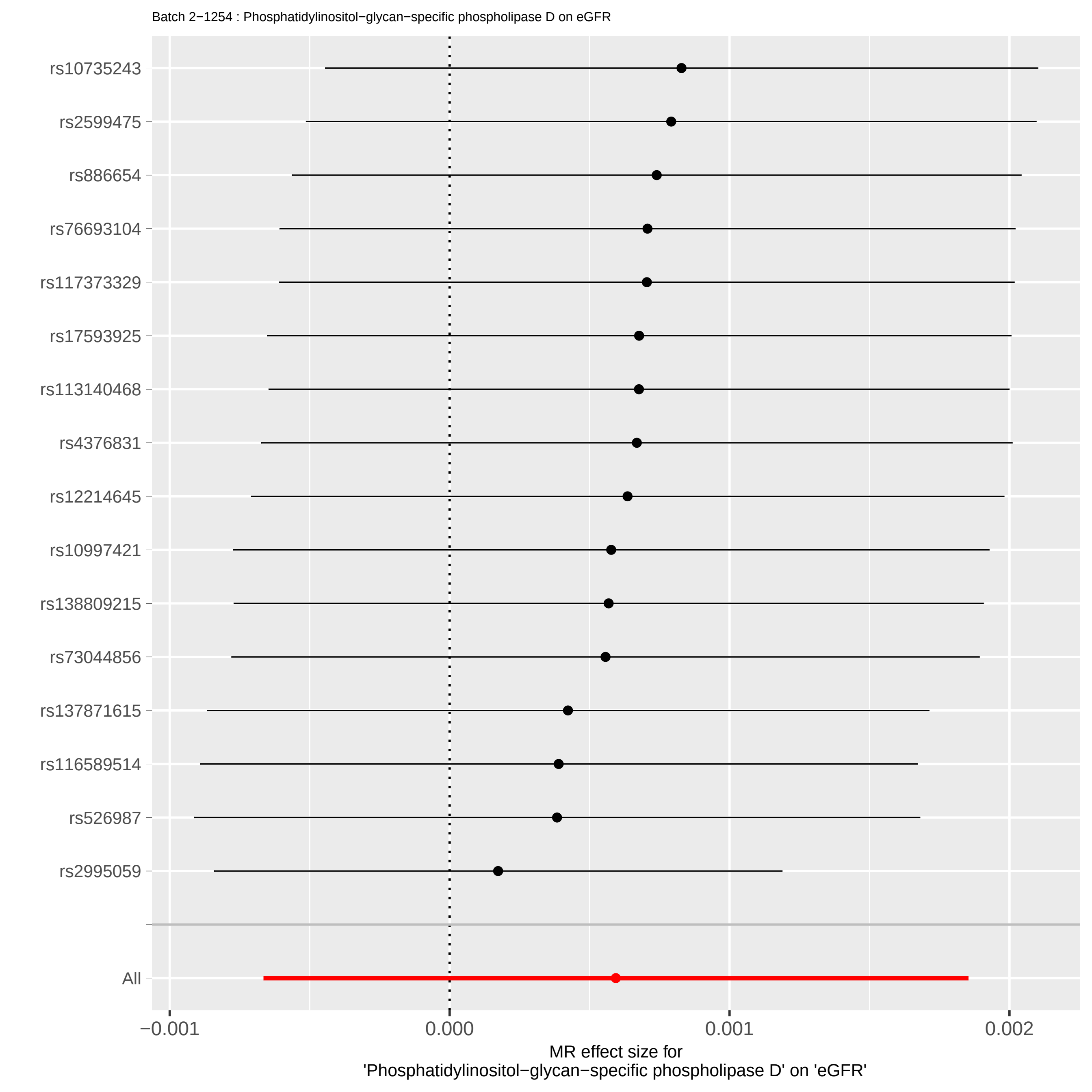
Batch 2−1254 : Phosphatidylinositol−glycan−specific phospholipase D on eGFR
rs10735243
rs2599475
rs886654
rs76693104
rs117373329
rs17593925
rs113140468
rs4376831
rs12214645
rs10997421
rs138809215
rs73044856
rs137871615
rs116589514
rs526987
rs2995059
All
−0.001
0.000
0.001
0.002
MR effect size for
'Phosphatidylinositol−glycan−specific phospholipase D' on 'eGFR'

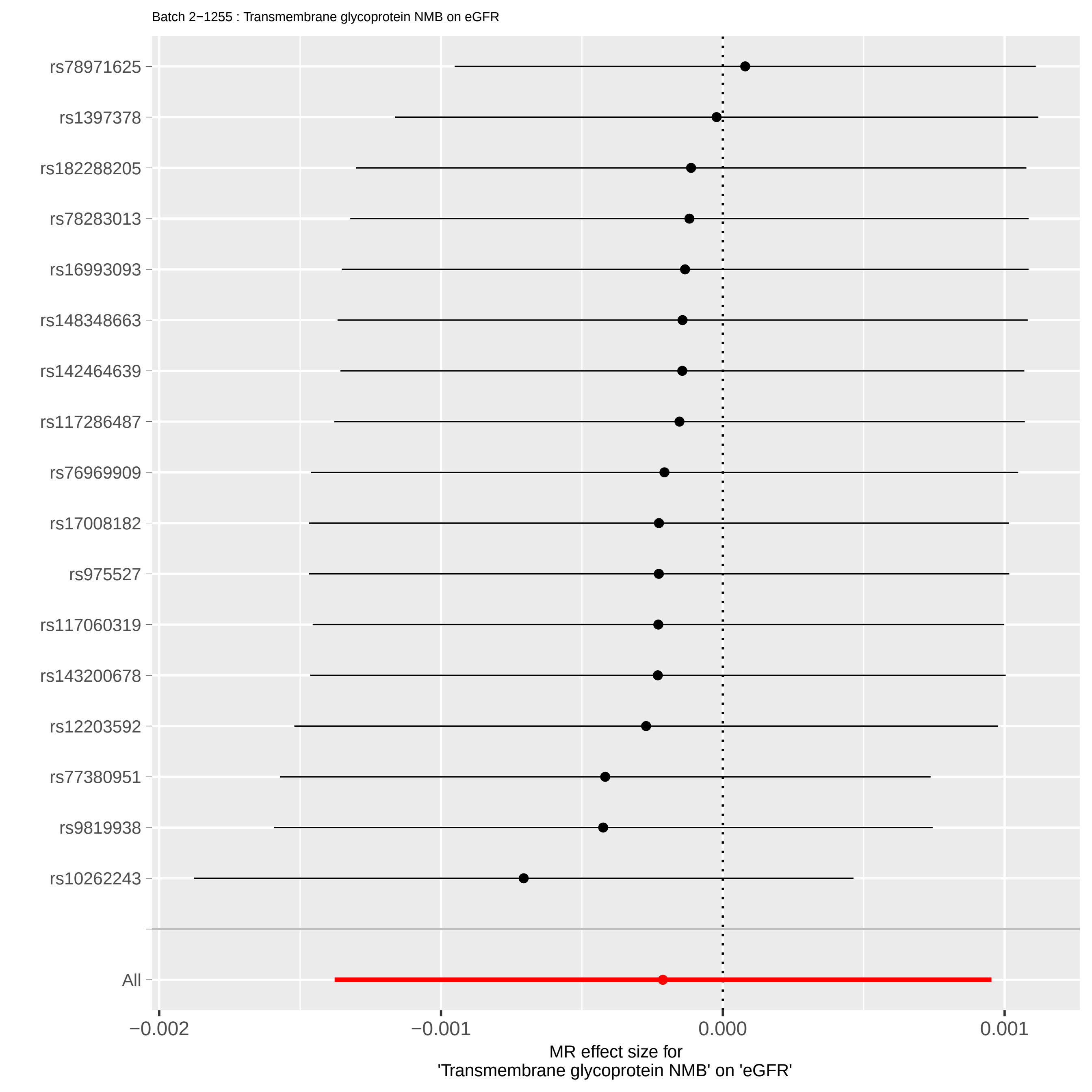
Batch 2−1255 : Transmembrane glycoprotein NMB on eGFR
rs78971625
rs1397378
rs182288205
rs78283013
rs16993093
rs148348663
rs142464639
rs117286487
rs76969909
rs17008182
rs975527
rs117060319
rs143200678
rs12203592
rs77380951
rs9819938
rs10262243
All
−0.002
−0.001
0.000
0.001
MR effect size for
'Transmembrane glycoprotein NMB' on 'eGFR'

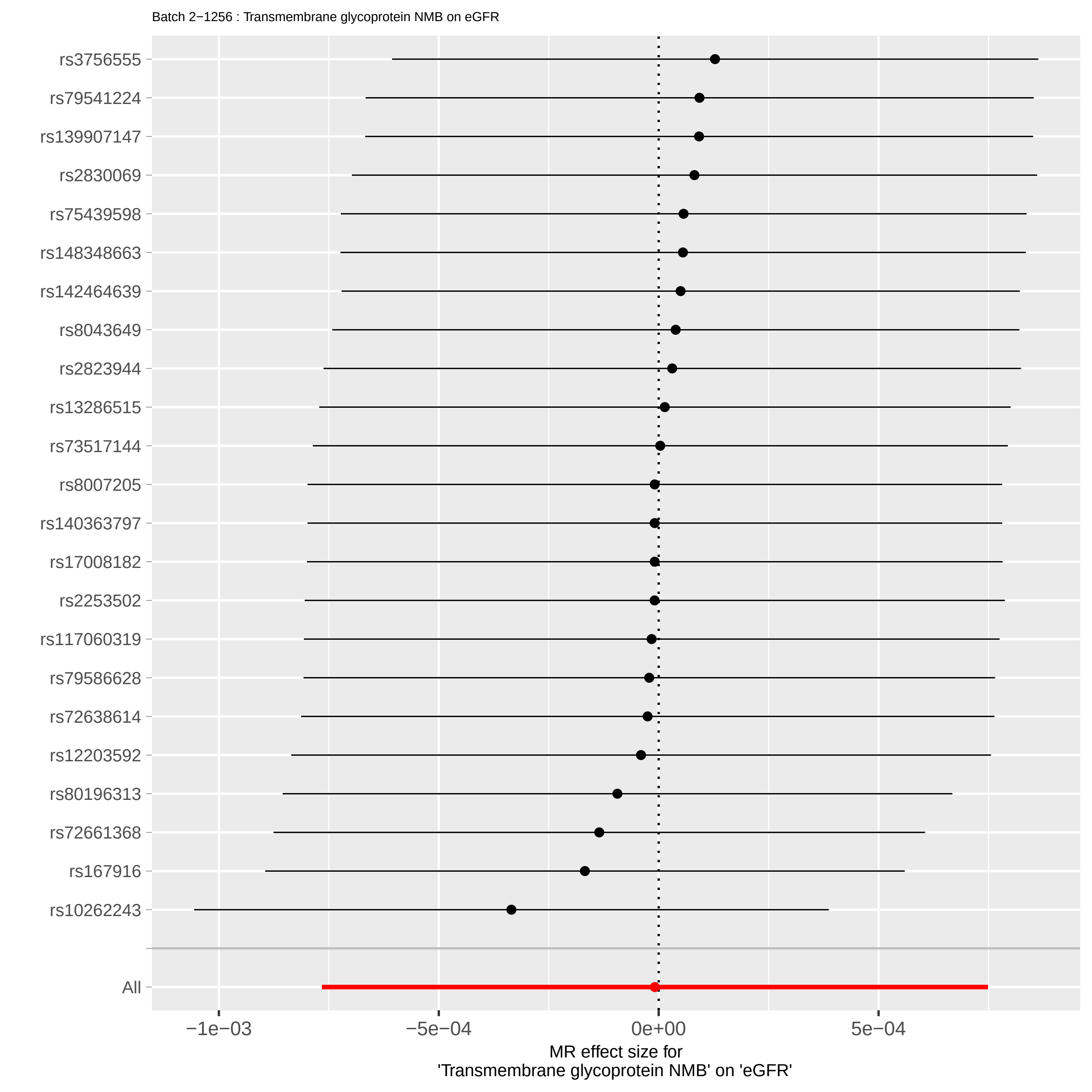
Batch 2−1256 : Transmembrane glycoprotein NMB on eGFR
rs3756555
rs79541224
rs139907147
rs2830069
rs75439598
rs148348663
rs142464639
rs8043649
rs2823944
rs13286515
rs73517144
rs8007205
rs140363797
rs17008182
rs2253502
rs117060319
rs79586628
rs72638614
rs12203592
rs80196313
rs72661368
rs167916
rs10262243
All
−1e−03
−5e−04
0e+00
5e−04
MR effect size for
'Transmembrane glycoprotein NMB' on 'eGFR'

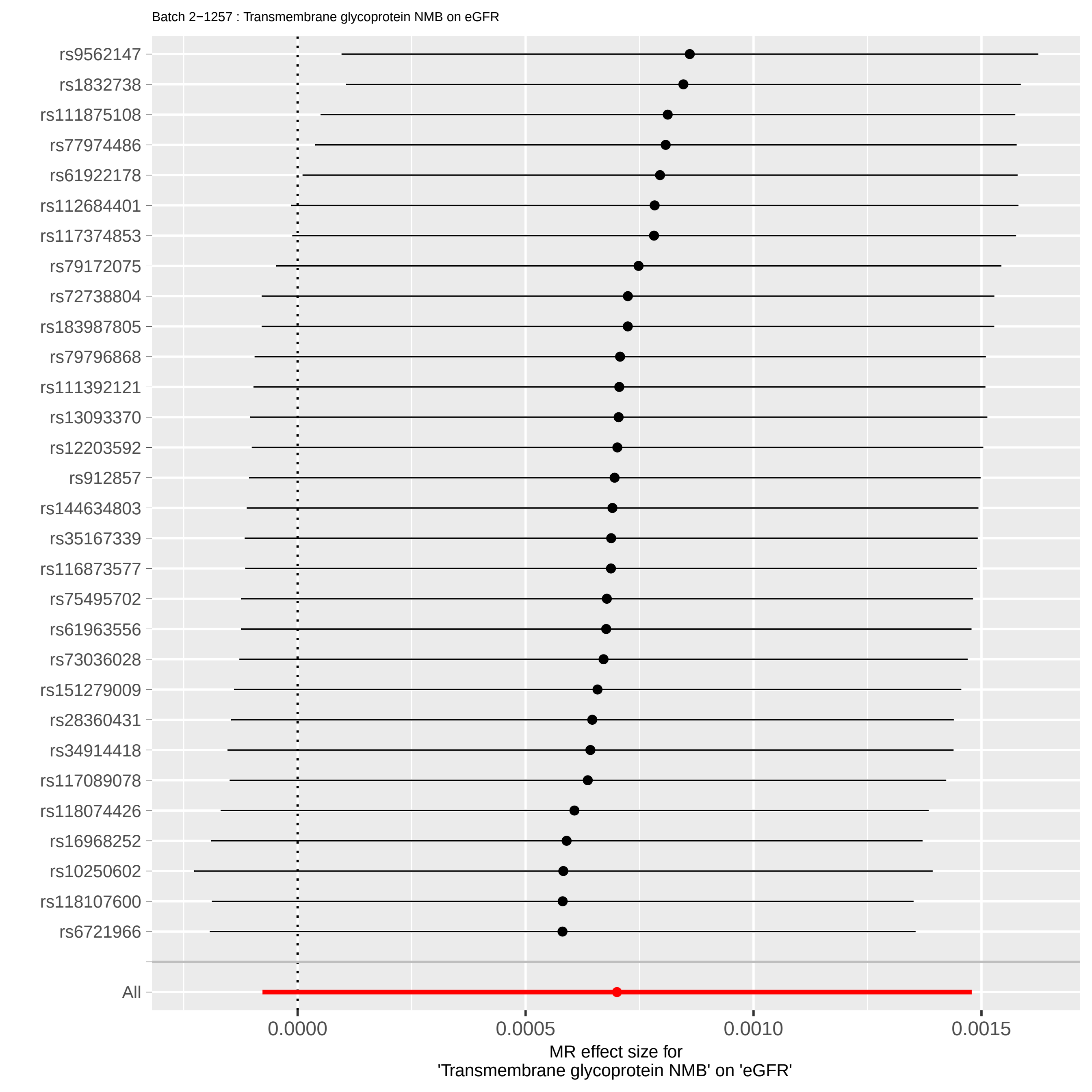
Batch 2−1257 : Transmembrane glycoprotein NMB on eGFR
rs9562147
rs1832738
rs111875108
rs77974486
rs61922178
rs112684401
rs117374853
rs79172075
rs72738804
rs183987805
rs79796868
rs111392121
rs13093370
rs12203592
rs912857
rs144634803
rs35167339
rs116873577
rs75495702
rs61963556
rs73036028
rs151279009
rs28360431
rs34914418
rs117089078
rs118074426
rs16968252
rs10250602
rs118107600
rs6721966
All
0.0000
0.0005
0.0010
0.0015
MR effect size for
'Transmembrane glycoprotein NMB' on 'eGFR'

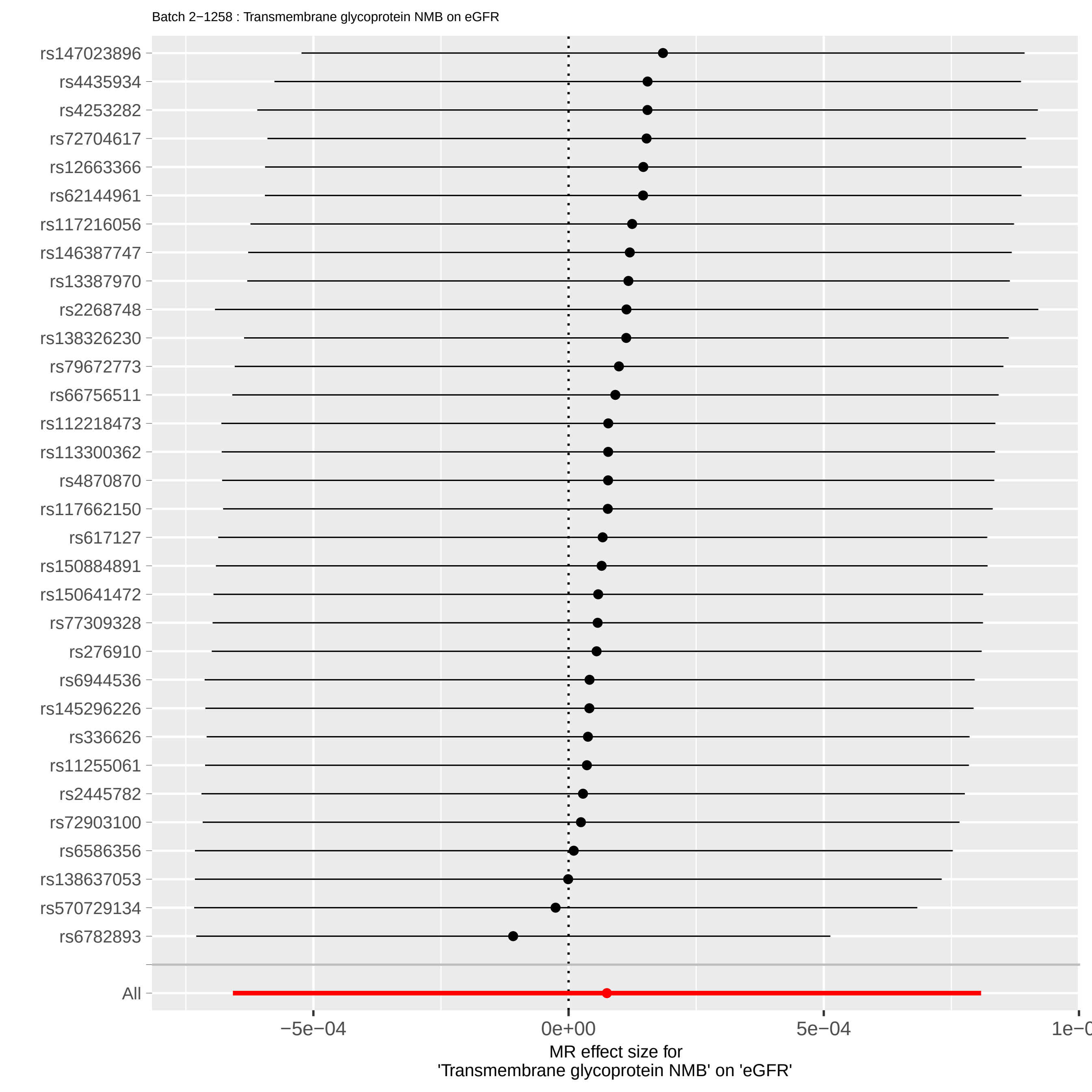

Batch 2−1258 : Transmembrane glycoprotein NMB on eGFR
rs147023896
rs4435934
rs4253282
rs72704617
rs12663366
rs62144961
rs117216056
rs146387747
rs13387970
rs2268748
rs138326230
rs79672773
rs66756511
rs112218473
rs113300362
rs4870870
rs117662150
rs617127
rs150884891
rs150641472
rs77309328
rs276910
rs6944536
rs145296226
rs336626
rs11255061
rs2445782
rs72903100
rs6586356
rs138637053
rs570729134
rs6782893
All
−5e−04
0e+00
5e−04
MR effect size for
'Transmembrane glycoprotein NMB' on 'eGFR'

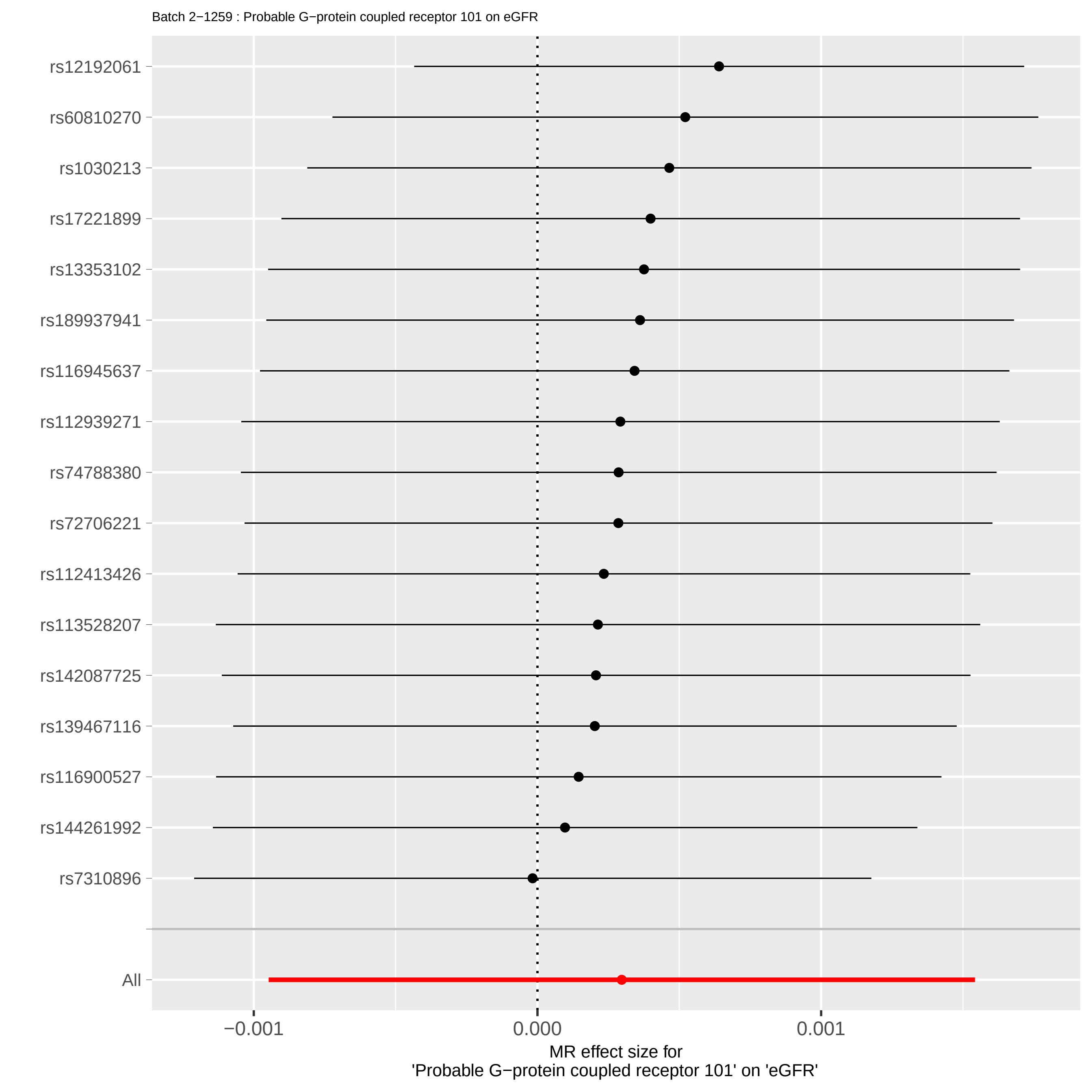
Batch 2−1259 : Probable G−protein coupled receptor 101 on eGFR
rs12192061
rs60810270
rs1030213
rs17221899
rs13353102
rs189937941
rs116945637
rs112939271
rs74788380
rs72706221
rs112413426
rs113528207
rs142087725
rs139467116
rs116900527
rs144261992
rs7310896
All
−0.001
0.000
0.001
MR effect size for
'Probable G−protein coupled receptor 101' on 'eGFR'

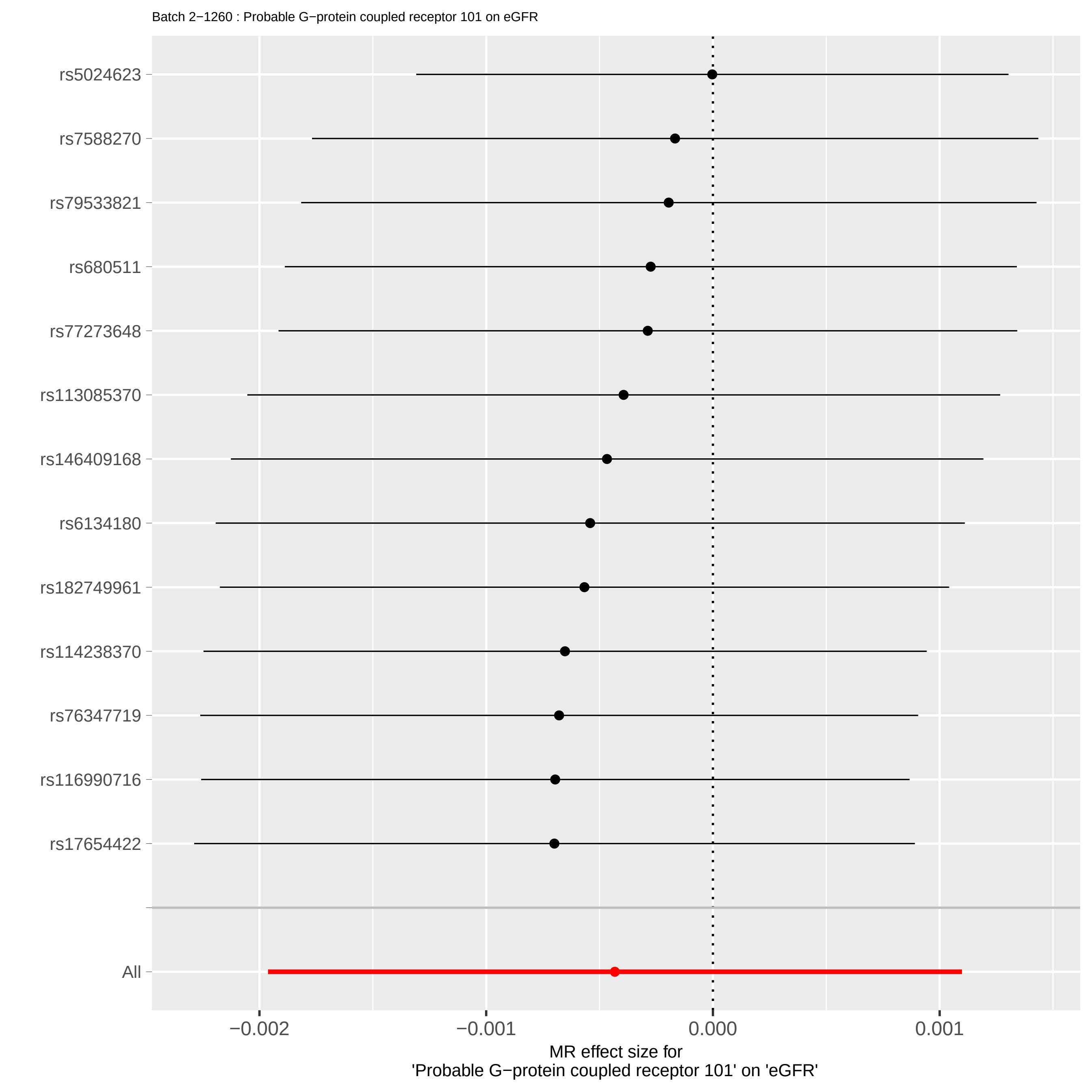

Batch 2−1260 : Probable G−protein coupled receptor 101 on eGFR
rs5024623
rs7588270
rs79533821
rs680511
rs77273648
rs113085370
rs146409168
rs6134180
rs182749961
rs114238370
rs76347719
rs116990716
rs17654422
All
−0.002
−0.001
0.000
0.001
MR effect size for
'Probable G−protein coupled receptor 101' on 'eGFR'

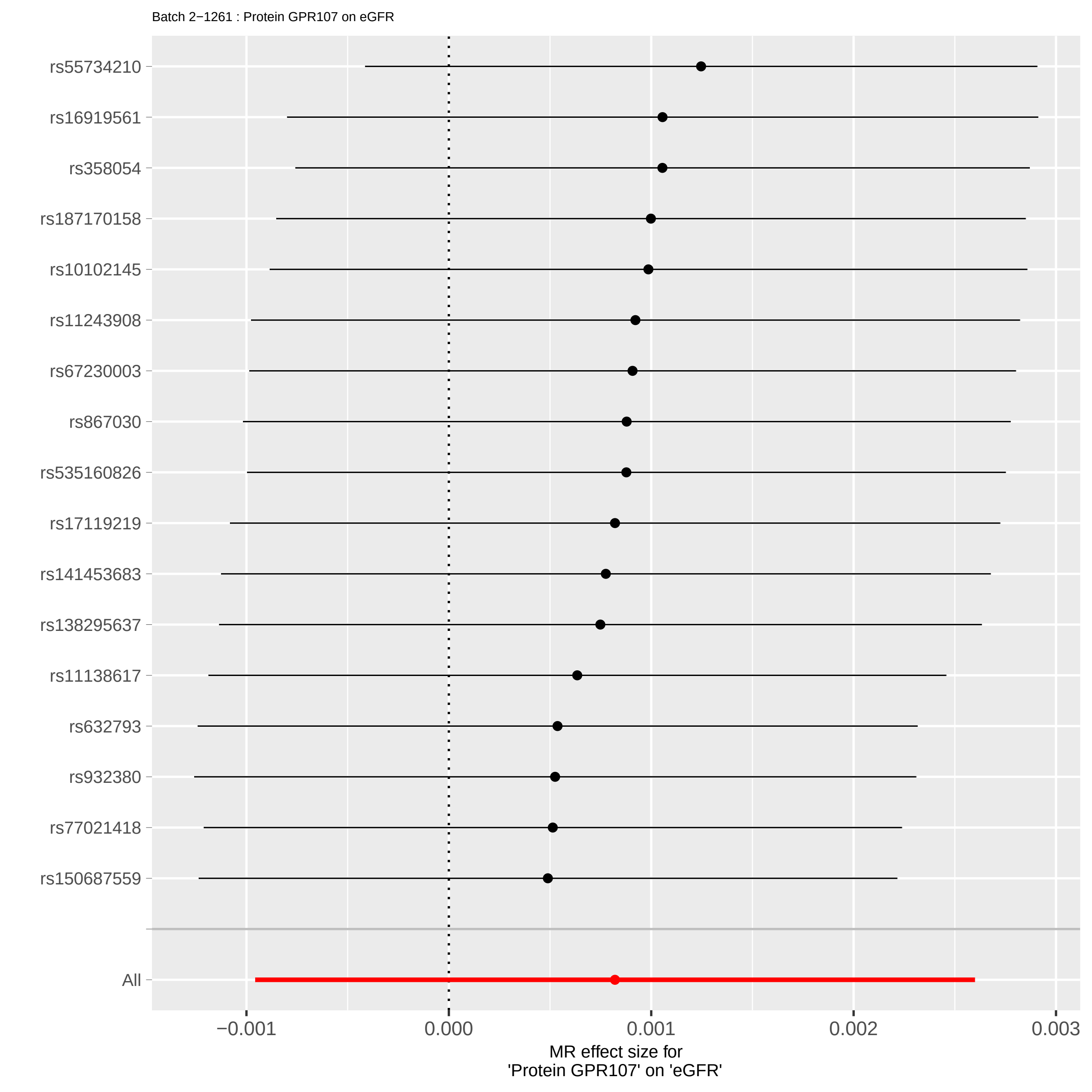
Batch 2−1261 : Protein GPR107 on eGFR
rs55734210
rs16919561
rs358054
rs187170158
rs10102145
rs11243908
rs67230003
rs867030
rs535160826
rs17119219
rs141453683
rs138295637
rs11138617
rs632793
rs932380
rs77021418
rs150687559
All
−0.001
0.000
0.001
0.002
0.003
MR effect size for
'Protein GPR107' on 'eGFR'

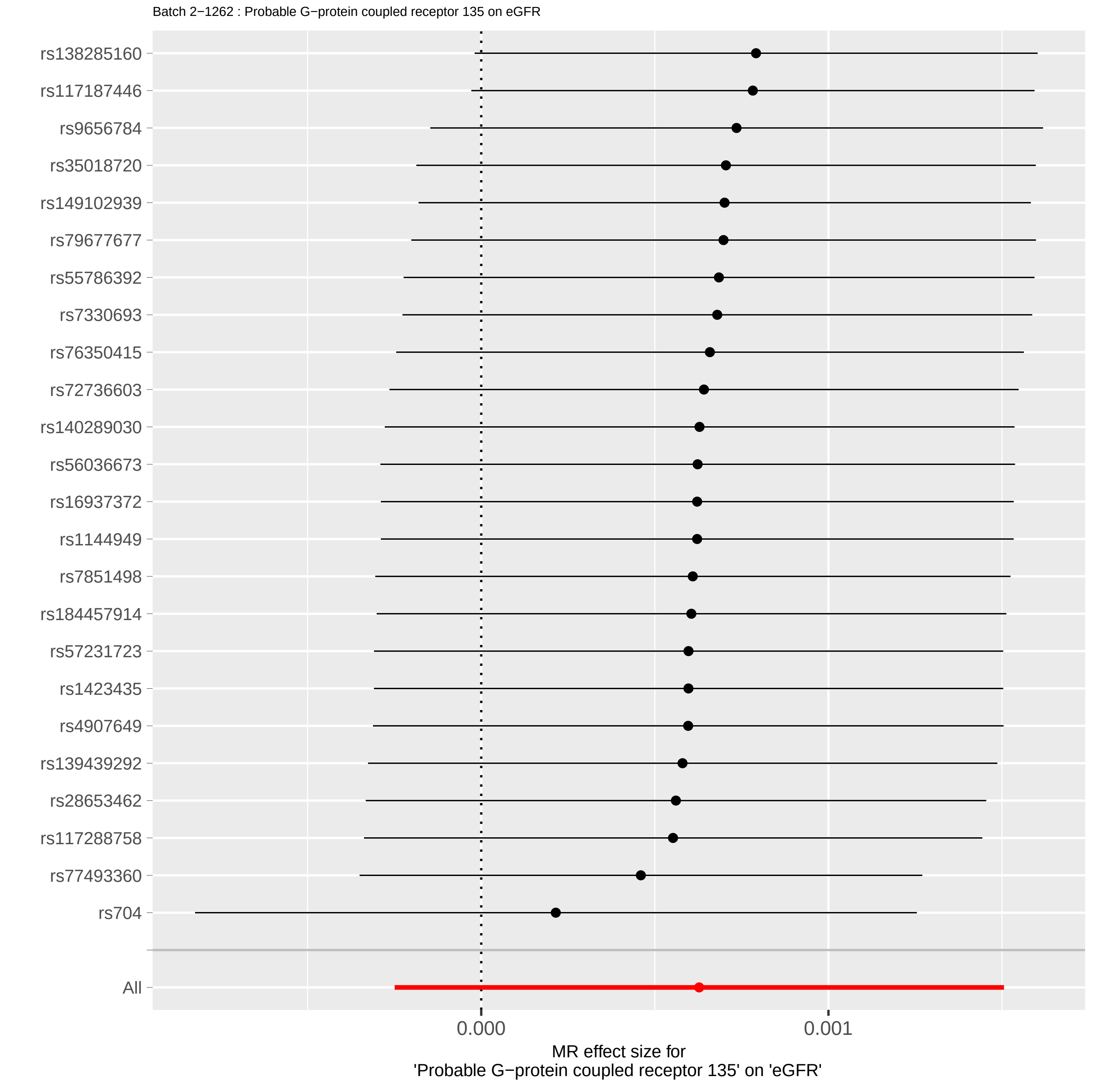

Batch 2−1262 : Probable G−protein coupled receptor 135 on eGFR
rs138285160
rs117187446
rs9656784
rs35018720
rs149102939
rs79677677
rs55786392
rs7330693
rs76350415
rs72736603
rs140289030
rs56036673
rs16937372
rs1144949
rs7851498
rs184457914
rs57231723
rs1423435
rs4907649
rs139439292
rs28653462
rs117288758
rs77493360
rs704
All
0.000
0.001
MR effect size for
'Probable G−protein coupled receptor 135' on 'eGFR'

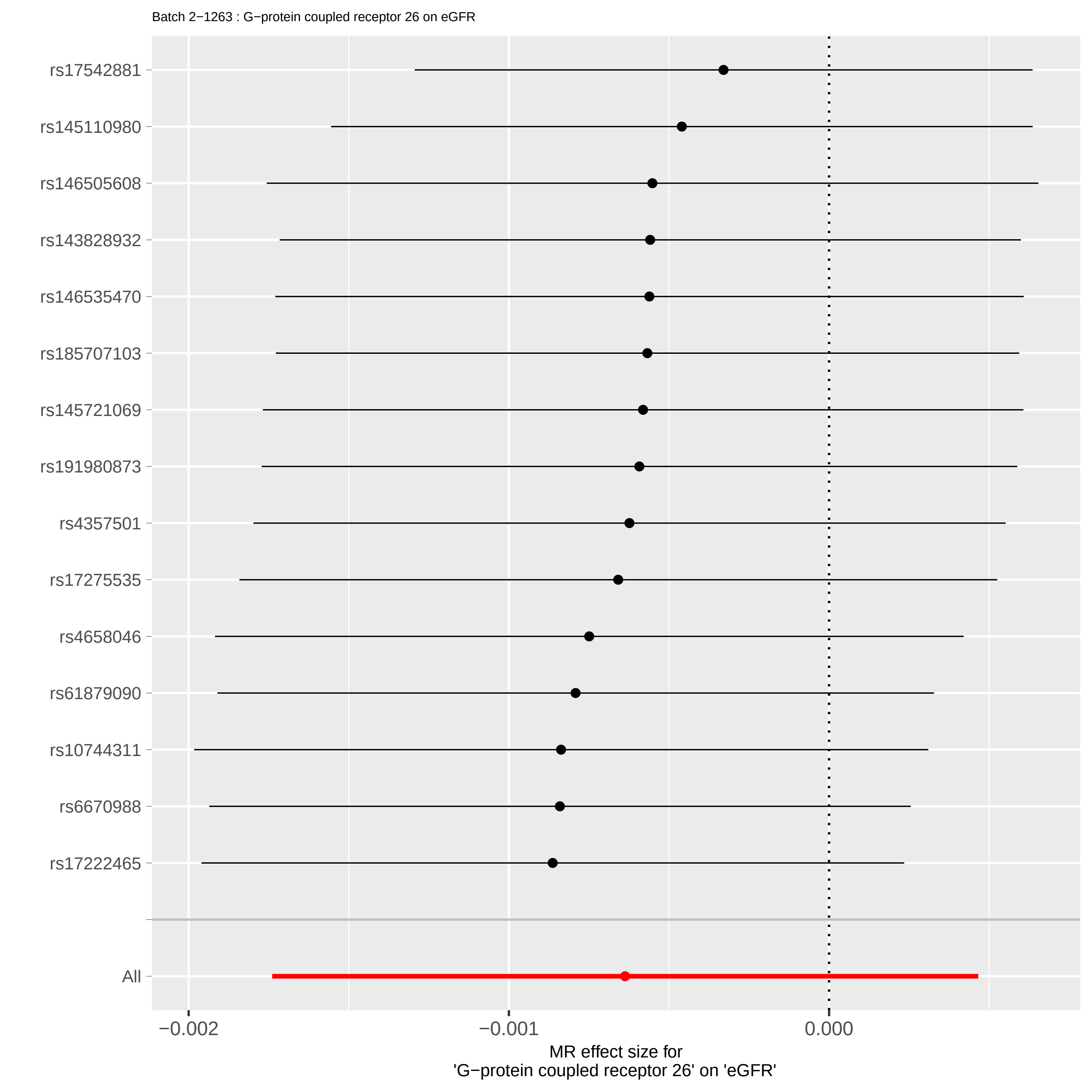

Batch 2−1263 : G−protein coupled receptor 26 on eGFR
rs17542881
rs145110980
rs146505608
rs143828932
rs146535470
rs185707103
rs145721069
rs191980873
rs4357501
rs17275535
rs4658046
rs61879090
rs10744311
rs6670988
rs17222465
All
−0.002
−0.001
0.000
MR effect size for
'G−protein coupled receptor 26' on 'eGFR'

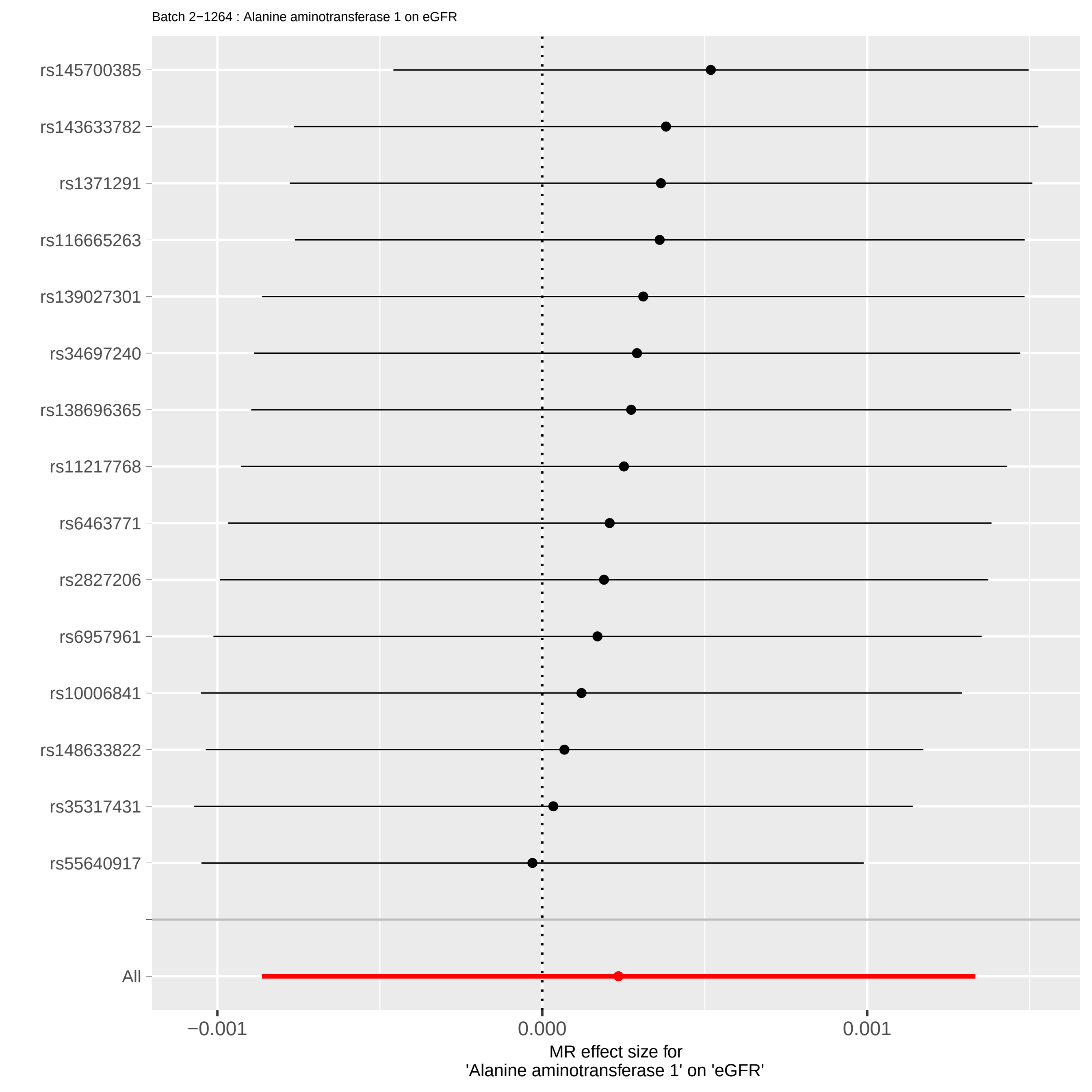
Batch 2−1264 : Alanine aminotransferase 1 on eGFR
rs145700385
rs143633782
rs1371291
rs116665263
rs139027301
rs34697240
rs138696365
rs11217768
rs6463771
rs2827206
rs6957961
rs10006841
rs148633822
rs35317431
rs55640917
All
−0.001
0.000
0.001
MR effect size for
'Alanine aminotransferase 1' on 'eGFR'

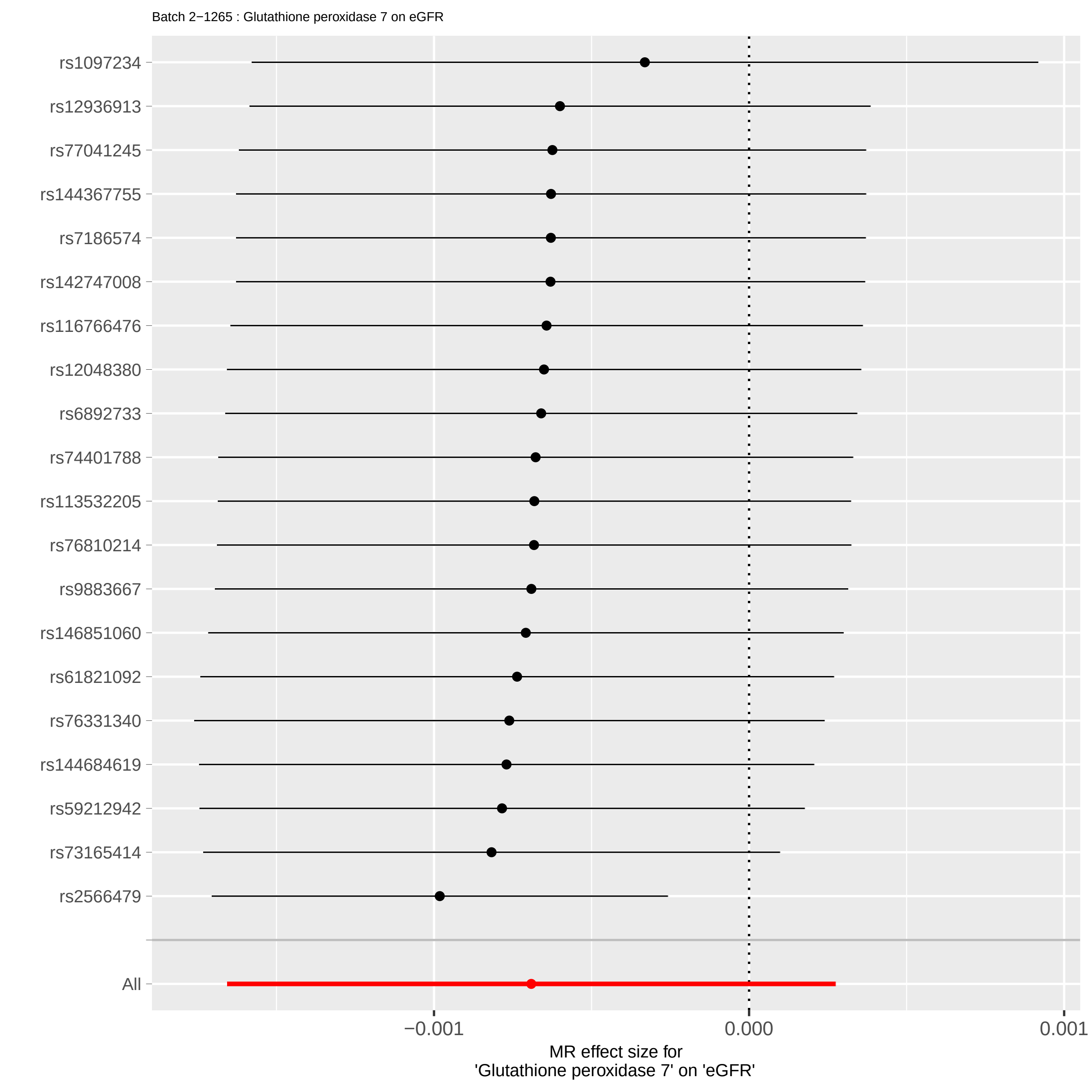

Batch 2−1265 : Glutathione peroxidase 7 on eGFR
rs1097234
rs12936913
rs77041245
rs144367755
rs7186574
rs142747008
rs116766476
rs12048380
rs6892733
rs74401788
rs113532205
rs76810214
rs9883667
rs146851060
rs61821092
rs76331340
rs144684619
rs59212942
rs73165414
rs2566479
All
−0.001
0.000
0.001
MR effect size for
'Glutathione peroxidase 7' on 'eGFR'

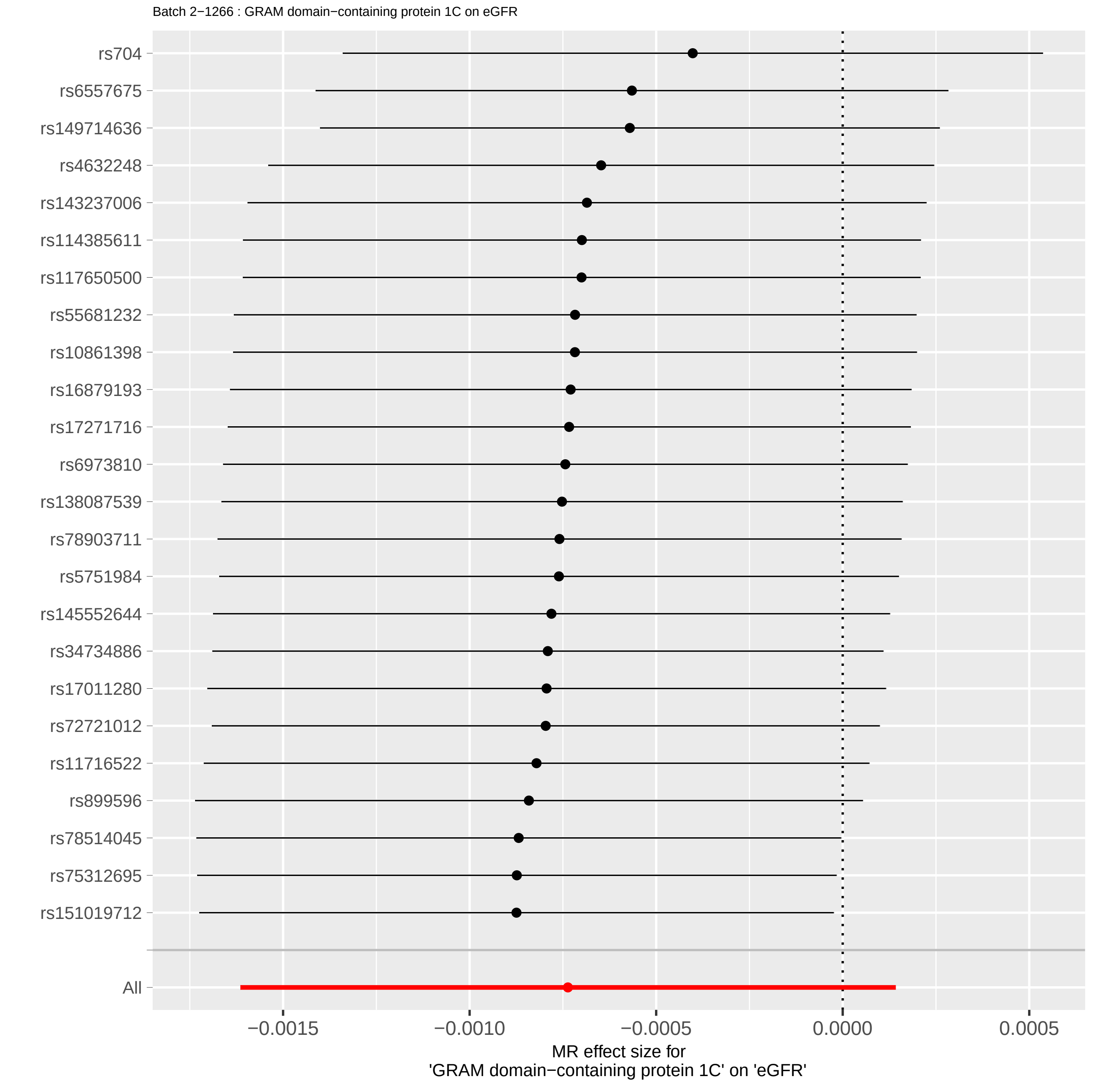

Batch 2−1266 : GRAM domain−containing protein 1C on eGFR
rs704
rs6557675
rs149714636
rs4632248
rs143237006
rs114385611
rs117650500
rs55681232
rs10861398
rs16879193
rs17271716
rs6973810
rs138087539
rs78903711
rs5751984
rs145552644
rs34734886
rs17011280
rs72721012
rs11716522
rs899596
rs78514045
rs75312695
rs151019712
All
−0.0015
−0.0010
−0.0005
0.0000
0.0005
MR effect size for
'GRAM domain−containing protein 1C' on 'eGFR'

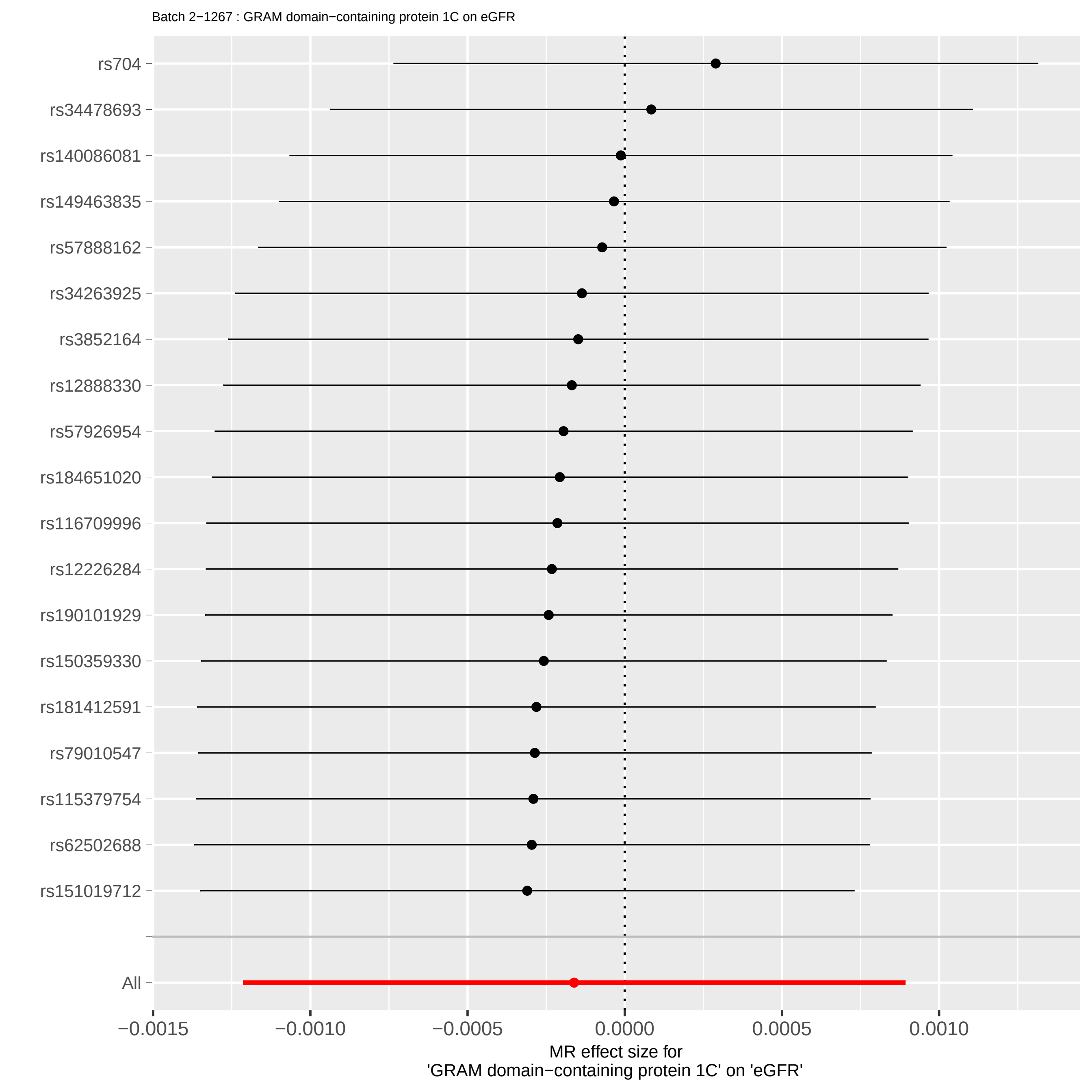

Batch 2−1267 : GRAM domain−containing protein 1C on eGFR
rs704
rs34478693
rs140086081
rs149463835
rs57888162
rs34263925
rs3852164
rs12888330
rs57926954
rs184651020
rs116709996
rs12226284
rs190101929
rs150359330
rs181412591
rs79010547
rs115379754
rs62502688
rs151019712
All
−0.0015
−0.0010
−0.0005
0.0000
0.0005
0.0010
MR effect size for
'GRAM domain−containing protein 1C' on 'eGFR'

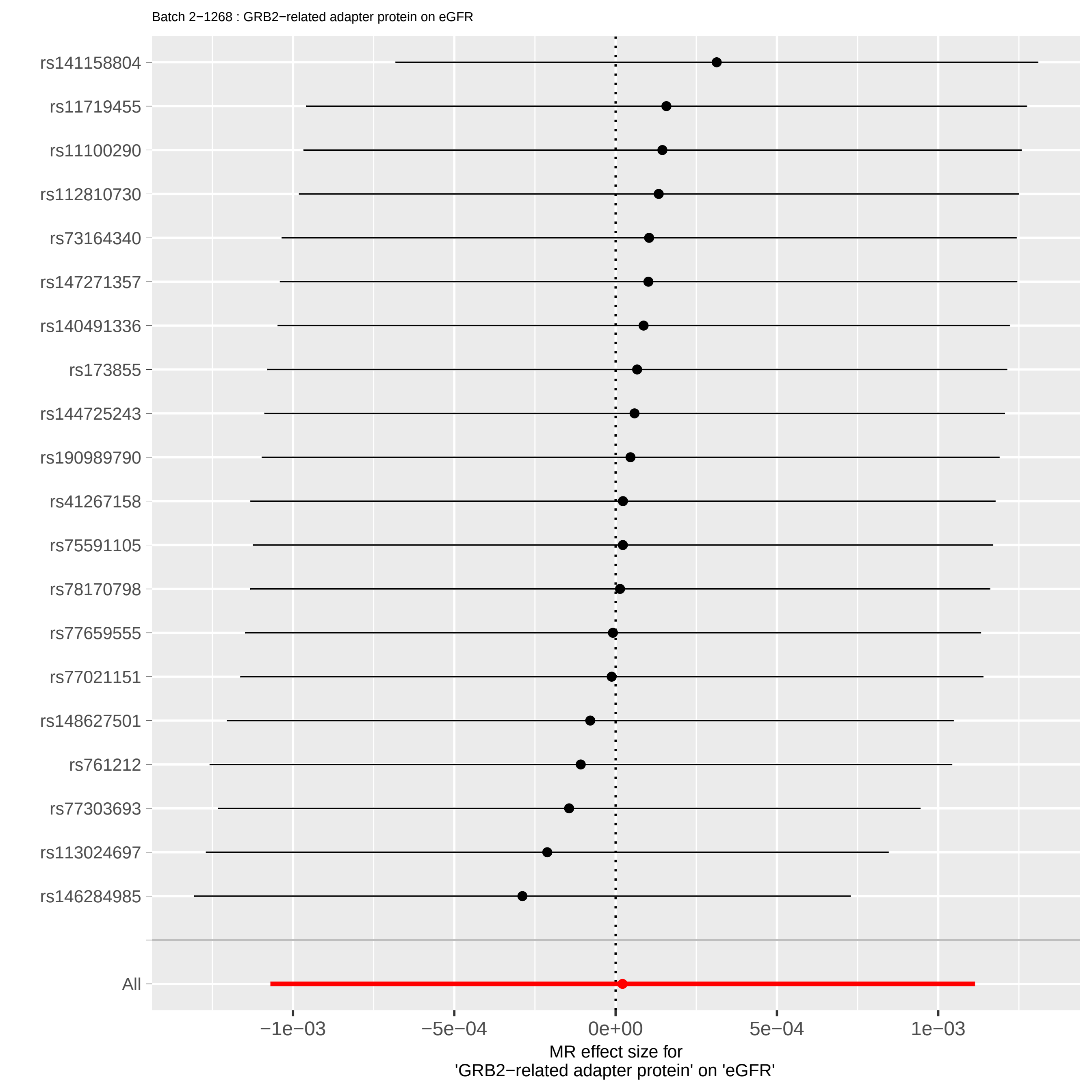

Batch 2−1268 : GRB2−related adapter protein on eGFR
rs141158804
rs11719455
rs11100290
rs112810730
rs73164340
rs147271357
rs140491336
rs173855
rs144725243
rs190989790
rs41267158
rs75591105
rs78170798
rs77659555
rs77021151
rs148627501
rs761212
rs77303693
rs113024697
rs146284985
All
−1e−03
−5e−04
0e+00
5e−04
1e−03
MR effect size for
'GRB2−related adapter protein' on 'eGFR'

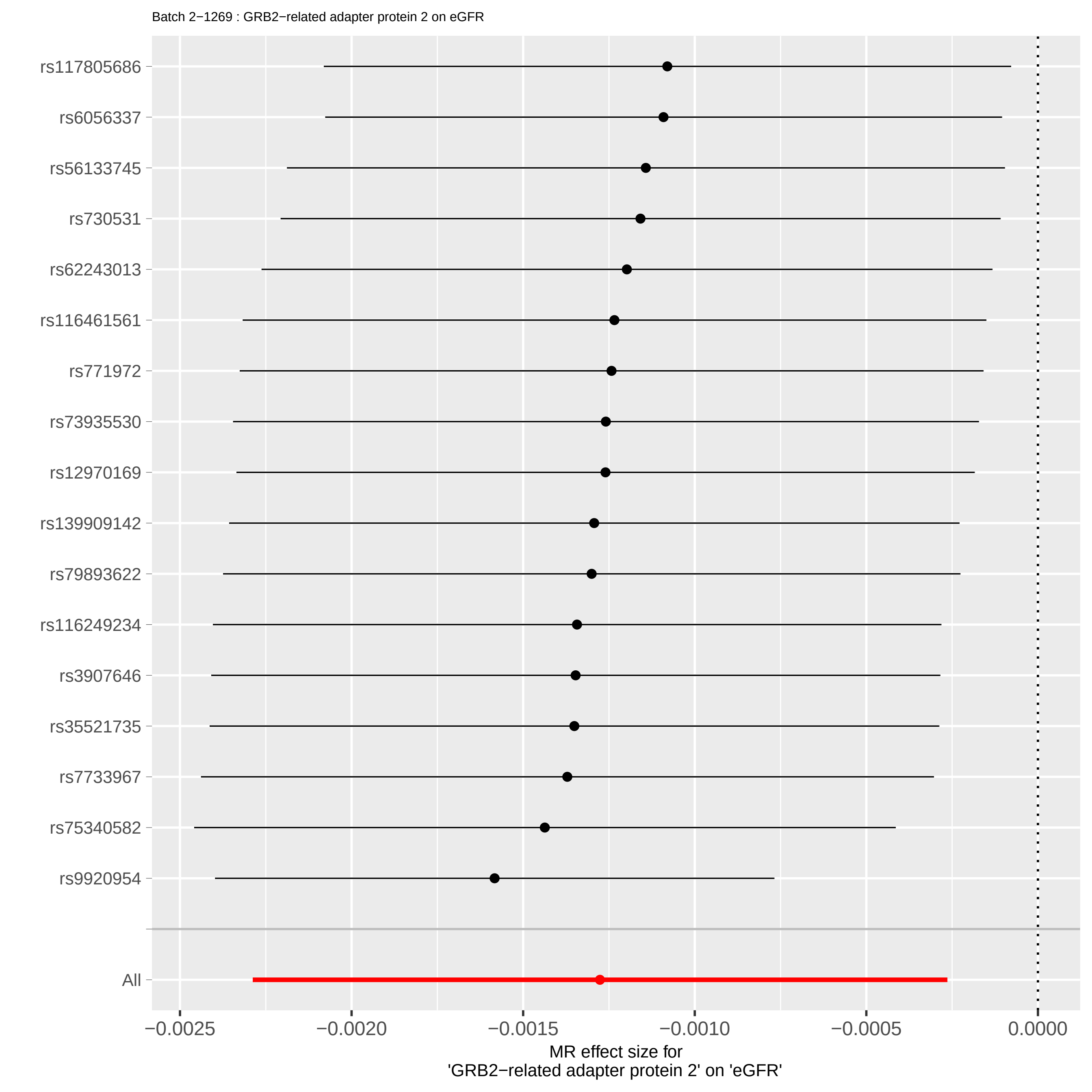

Batch 2−1269 : GRB2−related adapter protein 2 on eGFR
rs117805686
rs6056337
rs56133745
rs730531
rs62243013
rs116461561
rs771972
rs73935530
rs12970169
rs139909142
rs79893622
rs116249234
rs3907646
rs35521735
rs7733967
rs75340582
rs9920954
All
−0.0025
−0.0020
−0.0015
−0.0010
−0.0005
0.0000
MR effect size for
'GRB2−related adapter protein 2' on 'eGFR'

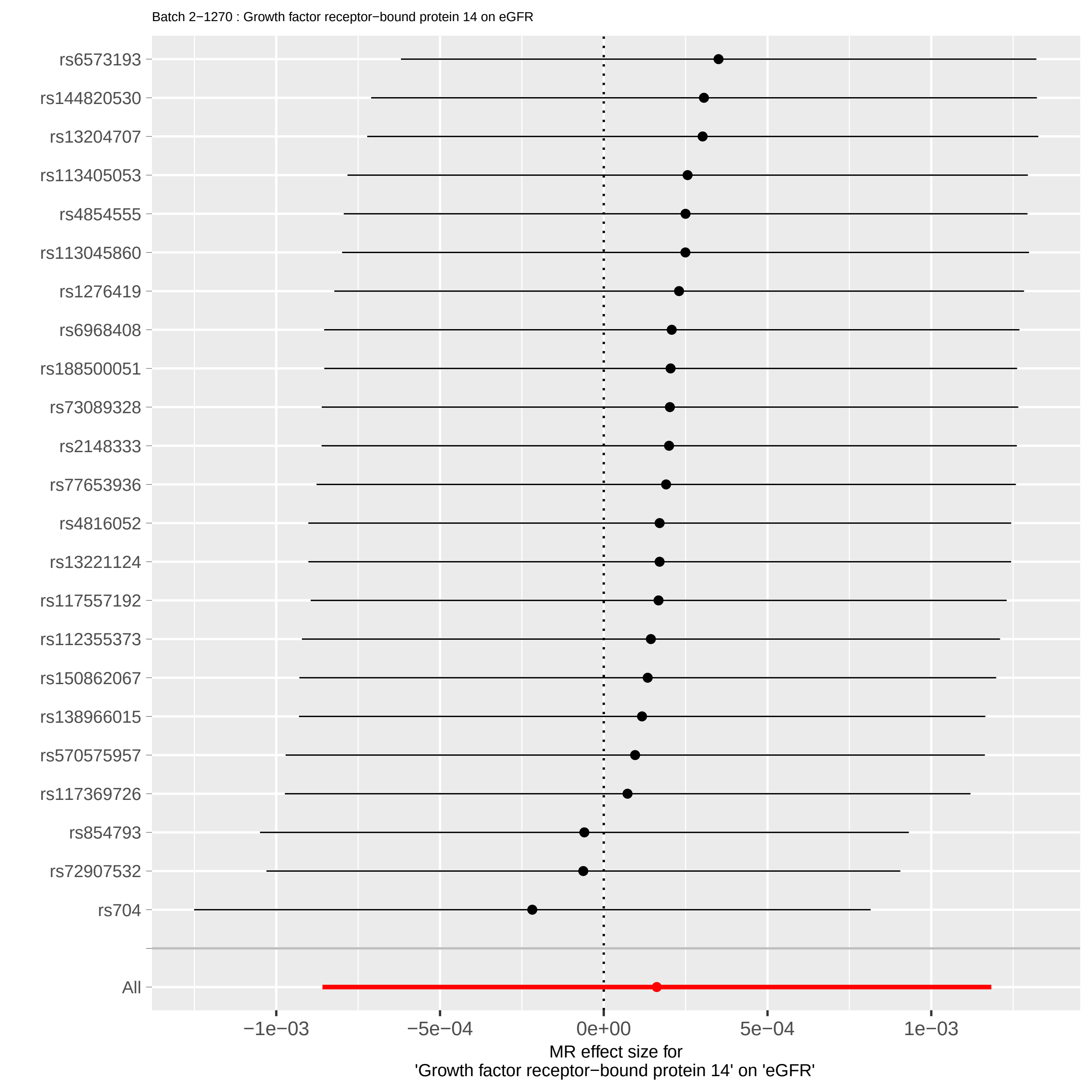

Batch 2−1270 : Growth factor receptor−bound protein 14 on eGFR
rs6573193
rs144820530
rs13204707
rs113405053
rs4854555
rs113045860
rs1276419
rs6968408
rs188500051
rs73089328
rs2148333
rs77653936
rs4816052
rs13221124
rs117557192
rs112355373
rs150862067
rs138966015
rs570575957
rs117369726
rs854793
rs72907532
rs704
All
−1e−03
−5e−04
0e+00
5e−04
1e−03
MR effect size for
'Growth factor receptor−bound protein 14' on 'eGFR'

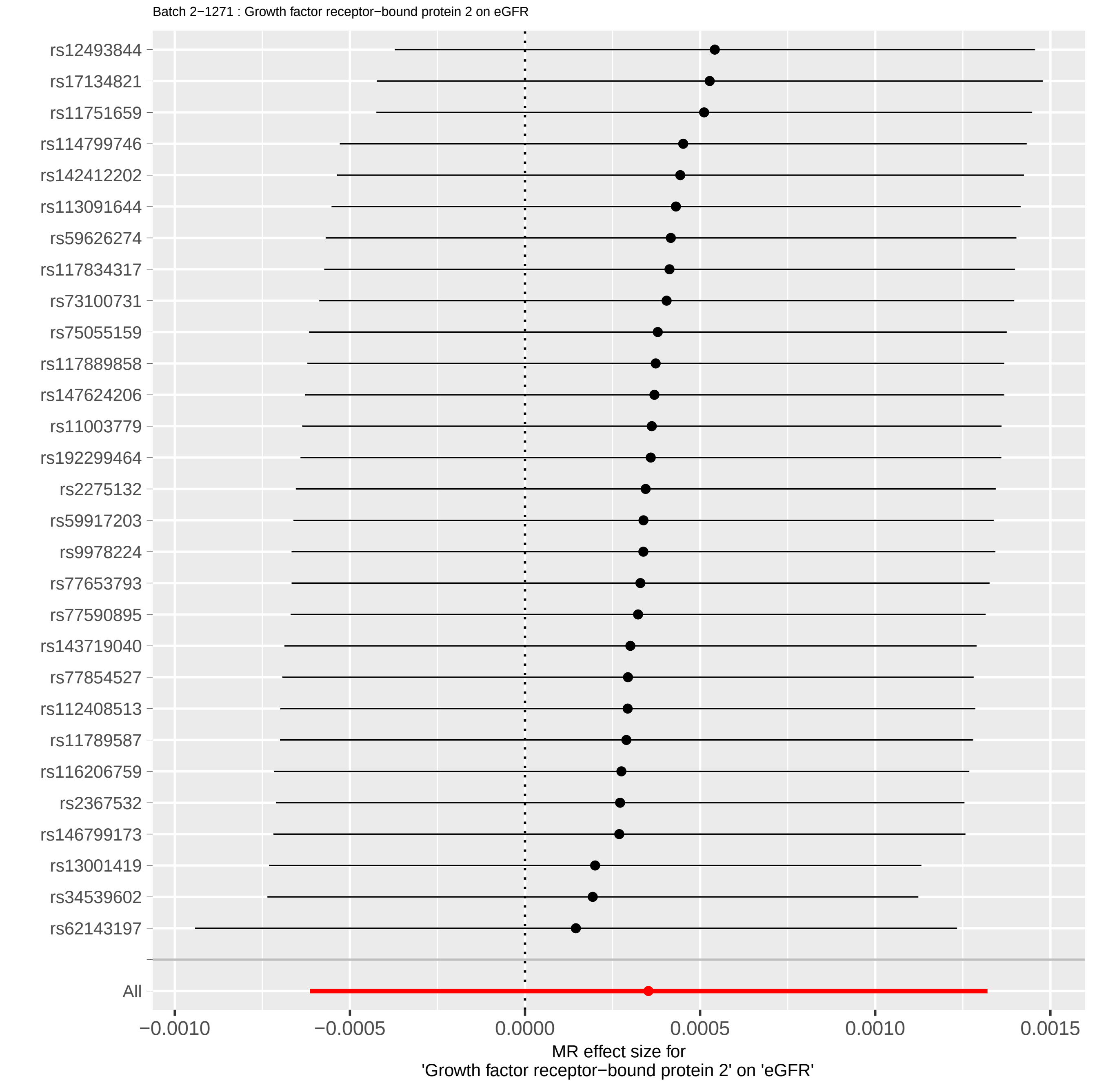

Batch 2−1271 : Growth factor receptor−bound protein 2 on eGFR
rs12493844
rs17134821
rs11751659
rs114799746
rs142412202
rs113091644
rs59626274
rs117834317
rs73100731
rs75055159
rs117889858
rs147624206
rs11003779
rs192299464
rs2275132
rs59917203
rs9978224
rs77653793
rs77590895
rs143719040
rs77854527
rs112408513
rs11789587
rs116206759
rs2367532
rs146799173
rs13001419
rs34539602
rs62143197
All
−0.0010
−0.0005
0.0000
0.0005
0.0010
0.0015
MR effect size for
'Growth factor receptor−bound protein 2' on 'eGFR'

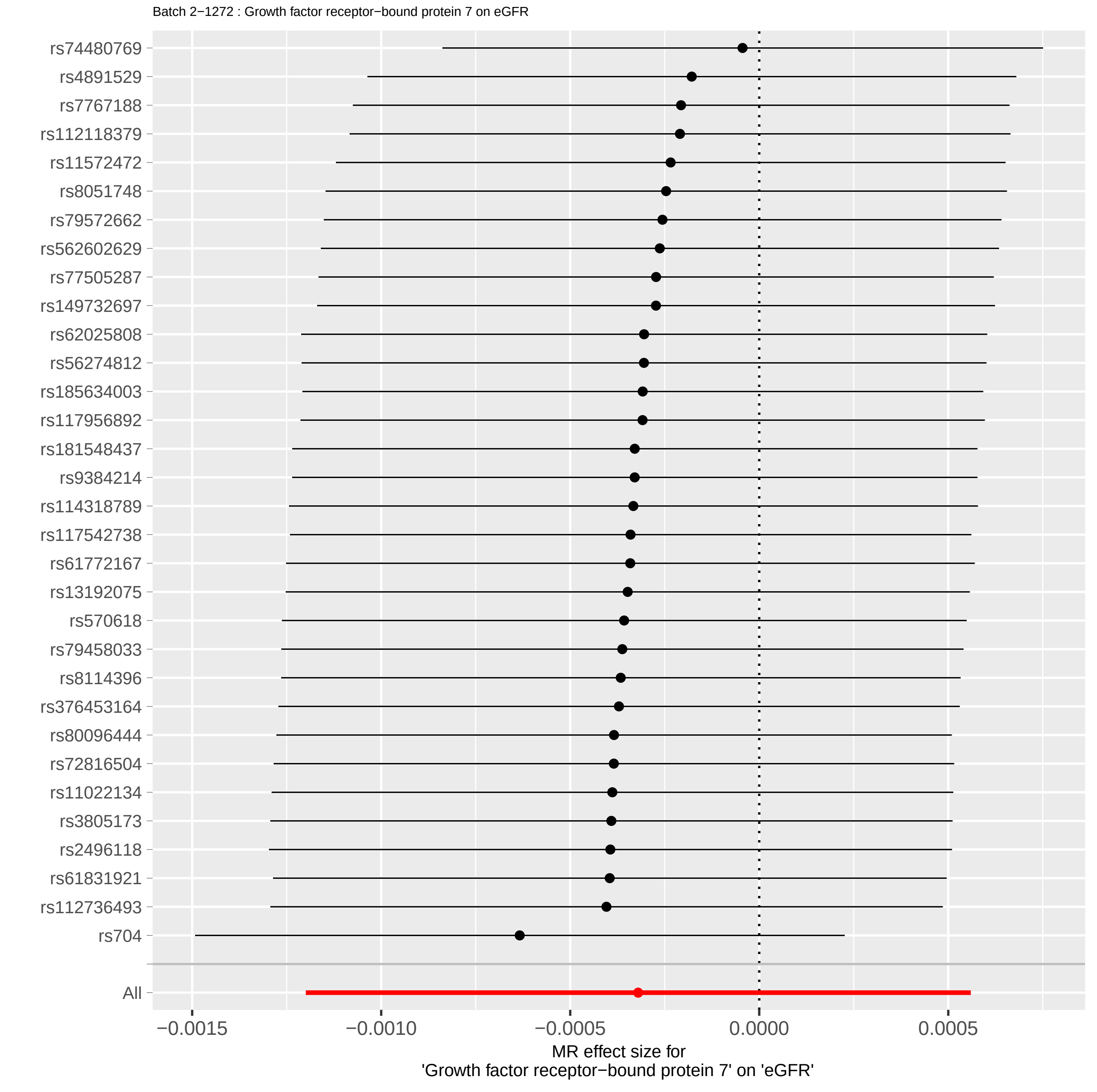

Batch 2−1272 : Growth factor receptor−bound protein 7 on eGFR
rs74480769
rs4891529
rs7767188
rs112118379
rs11572472
rs8051748
rs79572662
rs562602629
rs77505287
rs149732697
rs62025808
rs56274812
rs185634003
rs117956892
rs181548437
rs9384214
rs114318789
rs117542738
rs61772167
rs13192075
rs570618
rs79458033
rs8114396
rs376453164
rs80096444
rs72816504
rs11022134
rs3805173
rs2496118
rs61831921
rs112736493
rs704
All
−0.0015
−0.0010
−0.0005
0.0000
0.0005
MR effect size for
'Growth factor receptor−bound protein 7' on 'eGFR'

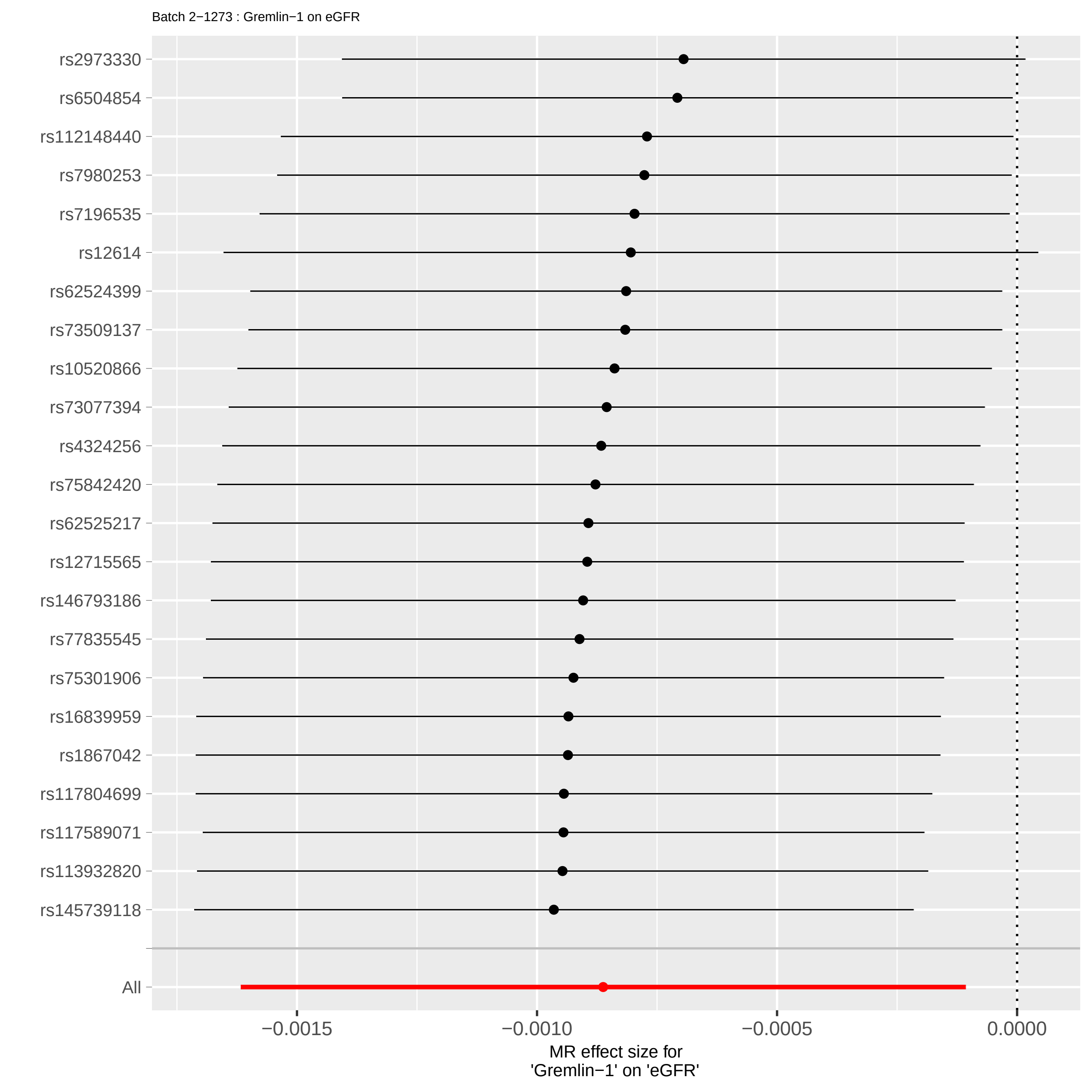
Batch 2−1273 : Gremlin−1 on eGFR
rs2973330
rs6504854
rs112148440
rs7980253
rs7196535
rs12614
rs62524399
rs73509137
rs10520866
rs73077394
rs4324256
rs75842420
rs62525217
rs12715565
rs146793186
rs77835545
rs75301906
rs16839959
rs1867042
rs117804699
rs117589071
rs113932820
rs145739118
All
−0.0015
−0.0010
−0.0005
0.0000
MR effect size for
'Gremlin−1' on 'eGFR'

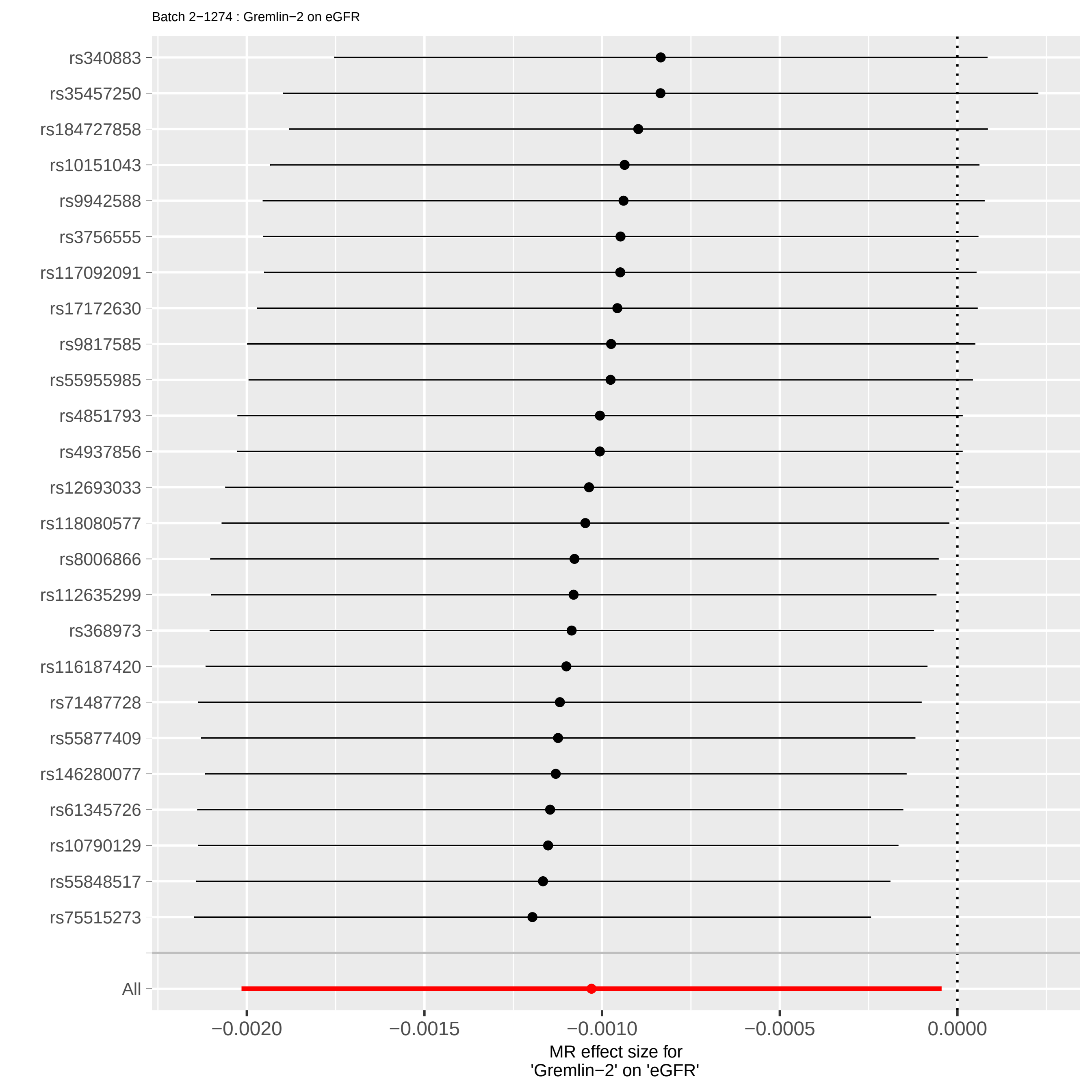
Batch 2−1274 : Gremlin−2 on eGFR
rs340883
rs35457250
rs184727858
rs10151043
rs9942588
rs3756555
rs117092091
rs17172630
rs9817585
rs55955985
rs4851793
rs4937856
rs12693033
rs118080577
rs8006866
rs112635299
rs368973
rs116187420
rs71487728
rs55877409
rs146280077
rs61345726
rs10790129
rs55848517
rs75515273
All
−0.0020
−0.0015
−0.0010
−0.0005
0.0000
MR effect size for
'Gremlin−2' on 'eGFR'

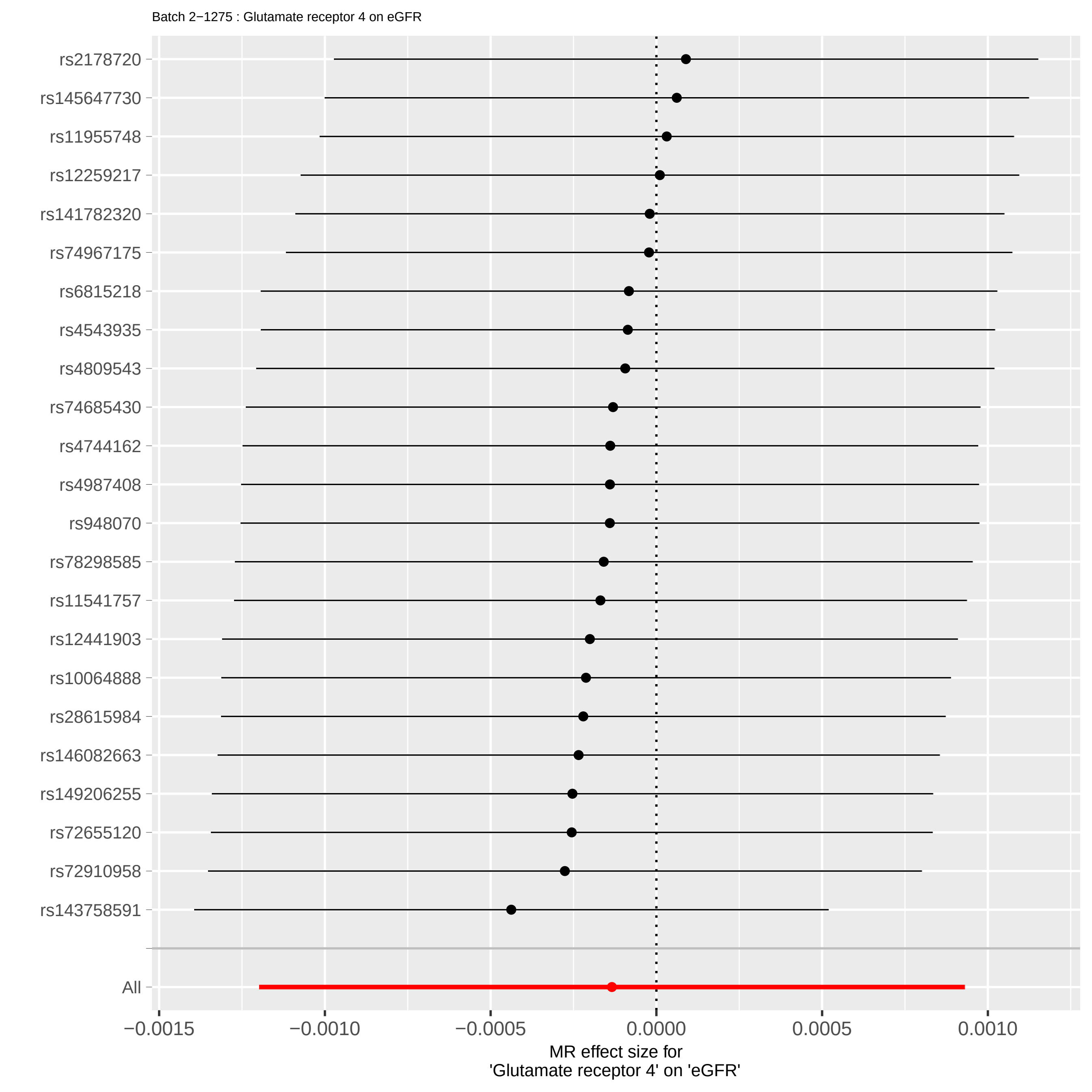

Batch 2−1275 : Glutamate receptor 4 on eGFR
rs2178720
rs145647730
rs11955748
rs12259217
rs141782320
rs74967175
rs6815218
rs4543935
rs4809543
rs74685430
rs4744162
rs4987408
rs948070
rs78298585
rs11541757
rs12441903
rs10064888
rs28615984
rs146082663
rs149206255
rs72655120
rs72910958
rs143758591
All
−0.0015
−0.0010
−0.0005
0.0000
0.0005
0.0010
MR effect size for
'Glutamate receptor 4' on 'eGFR'

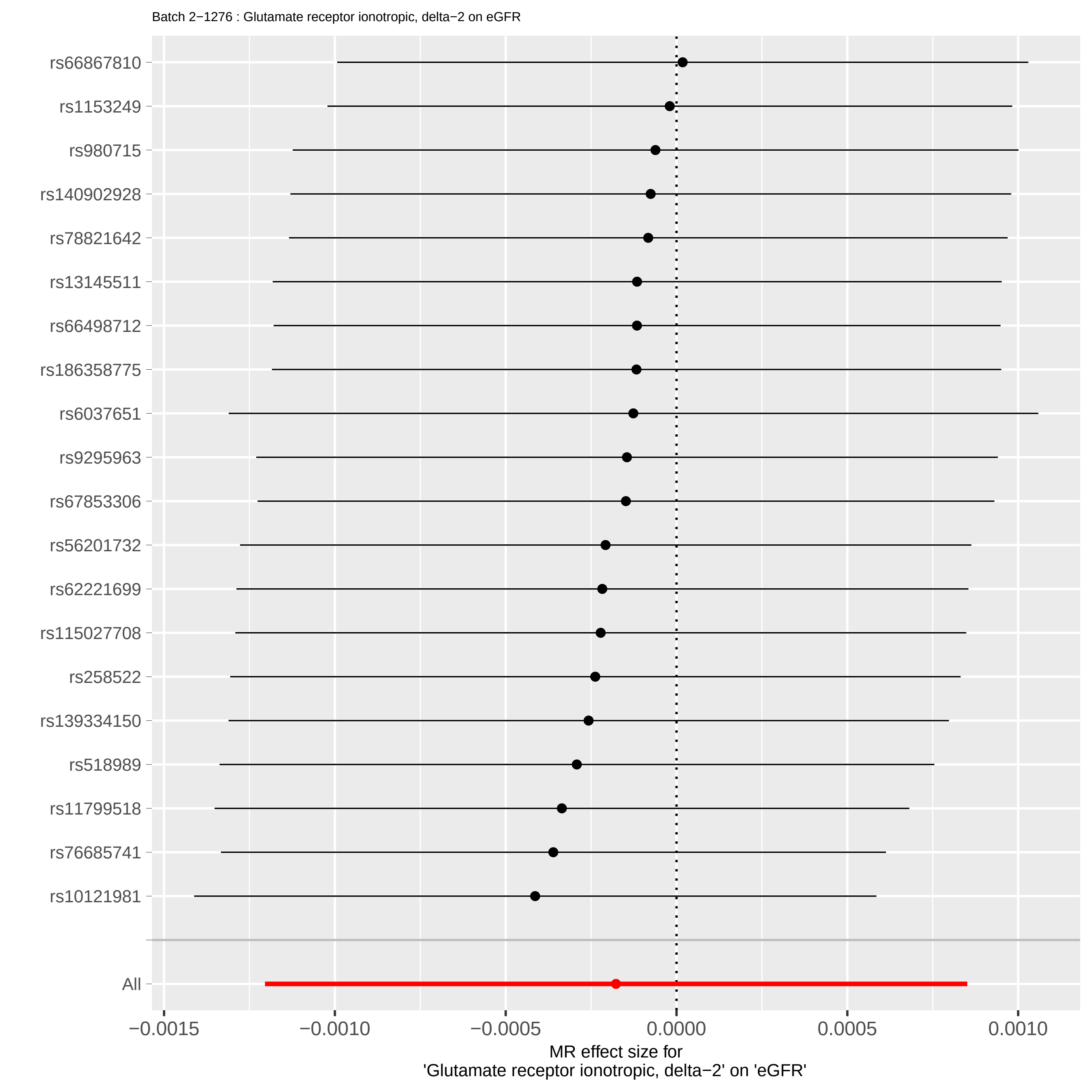

Batch 2−1276 : Glutamate receptor ionotropic, delta−2 on eGFR
rs66867810
rs1153249
rs980715
rs140902928
rs78821642
rs13145511
rs66498712
rs186358775
rs6037651
rs9295963
rs67853306
rs56201732
rs62221699
rs115027708
rs258522
rs139334150
rs518989
rs11799518
rs76685741
rs10121981
All
−0.0015
−0.0010
−0.0005
0.0000
0.0005
0.0010
MR effect size for
'Glutamate receptor ionotropic, delta−2' on 'eGFR'

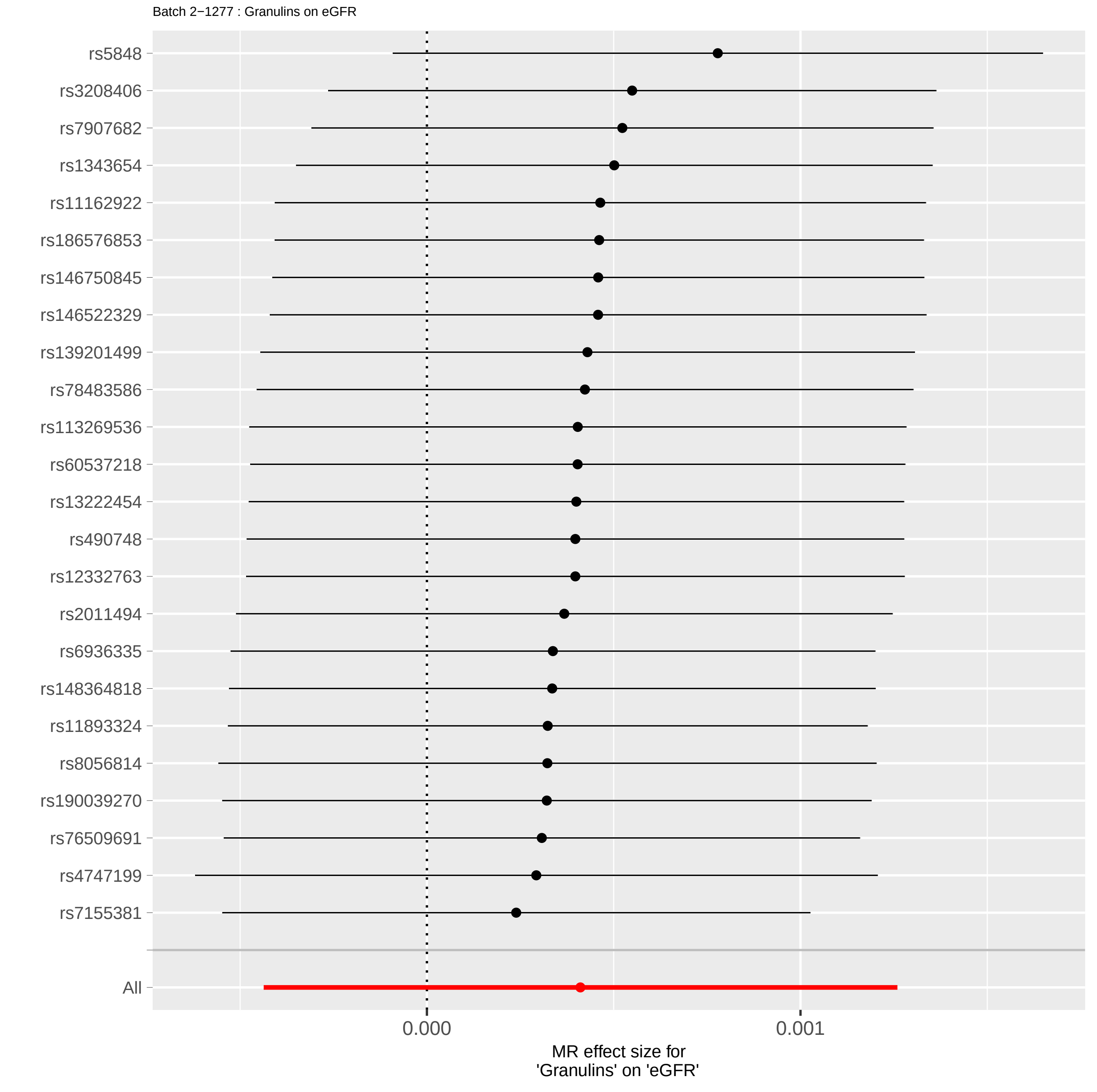

Batch 2−1277 : Granulins on eGFR
rs5848
rs3208406
rs7907682
rs1343654
rs11162922
rs186576853
rs146750845
rs146522329
rs139201499
rs78483586
rs113269536
rs60537218
rs13222454
rs490748
rs12332763
rs2011494
rs6936335
rs148364818
rs11893324
rs8056814
rs190039270
rs76509691
rs4747199
rs7155381
All
0.000
0.001
MR effect size for
'Granulins' on 'eGFR'

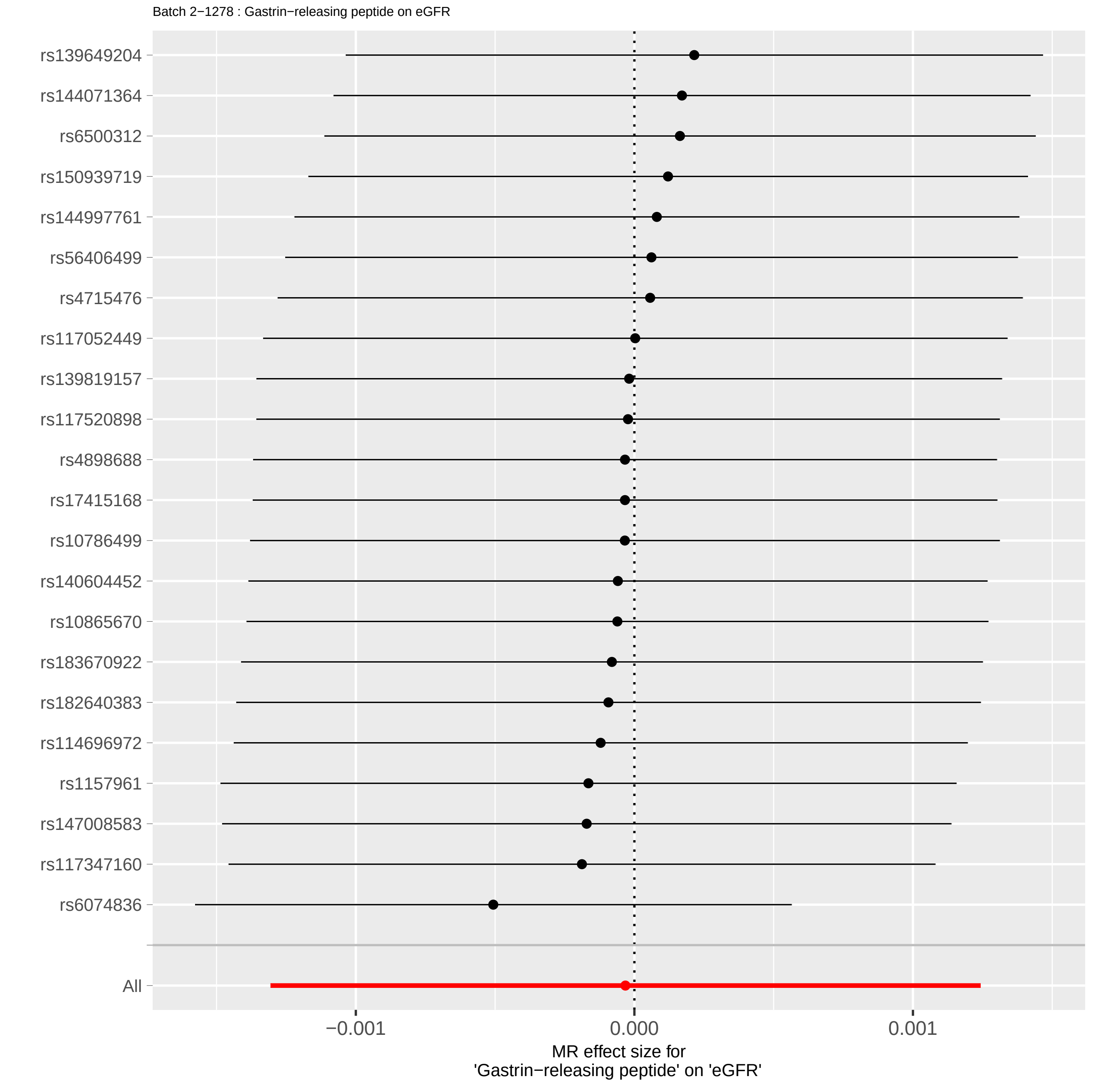

Batch 2−1278 : Gastrin−releasing peptide on eGFR
rs139649204
rs144071364
rs6500312
rs150939719
rs144997761
rs56406499
rs4715476
rs117052449
rs139819157
rs117520898
rs4898688
rs17415168
rs10786499
rs140604452
rs10865670
rs183670922
rs182640383
rs114696972
rs1157961
rs147008583
rs117347160
rs6074836
All
−0.001
0.000
0.001
MR effect size for
'Gastrin−releasing peptide' on 'eGFR'

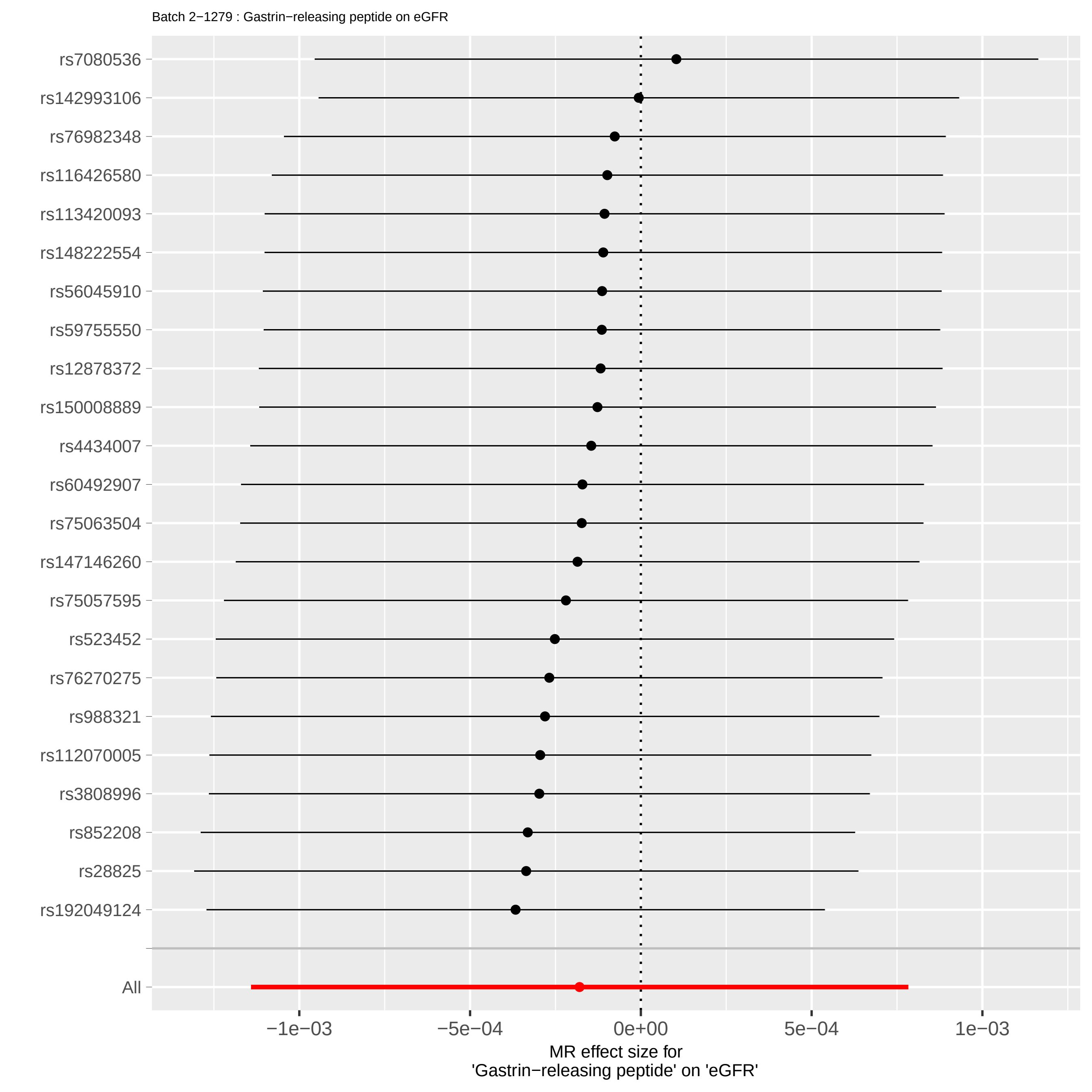

Batch 2−1279 : Gastrin−releasing peptide on eGFR
rs7080536
rs142993106
rs76982348
rs116426580
rs113420093
rs148222554
rs56045910
rs59755550
rs12878372
rs150008889
rs4434007
rs60492907
rs75063504
rs147146260
rs75057595
rs523452
rs76270275
rs988321
rs112070005
rs3808996
rs852208
rs28825
rs192049124
All
−1e−03
−5e−04
0e+00
5e−04
1e−03
MR effect size for
'Gastrin−releasing peptide' on 'eGFR'

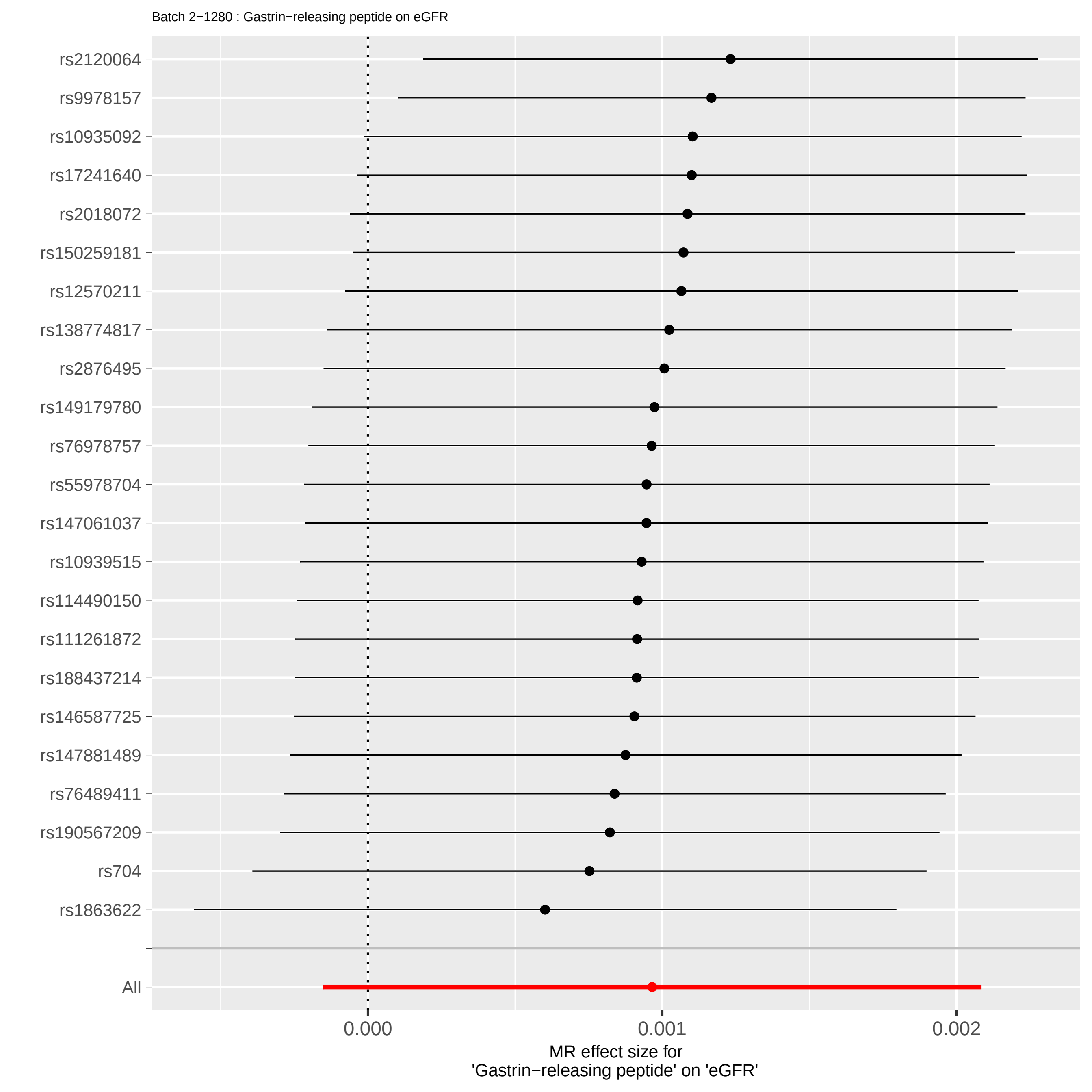
Batch 2−1280 : Gastrin−releasing peptide on eGFR
rs2120064
rs9978157
rs10935092
rs17241640
rs2018072
rs150259181
rs12570211
rs138774817
rs2876495
rs149179780
rs76978757
rs55978704
rs147061037
rs10939515
rs114490150
rs111261872
rs188437214
rs146587725
rs147881489
rs76489411
rs190567209
rs704
rs1863622
All
0.000
0.001
0.002
MR effect size for
'Gastrin−releasing peptide' on 'eGFR'

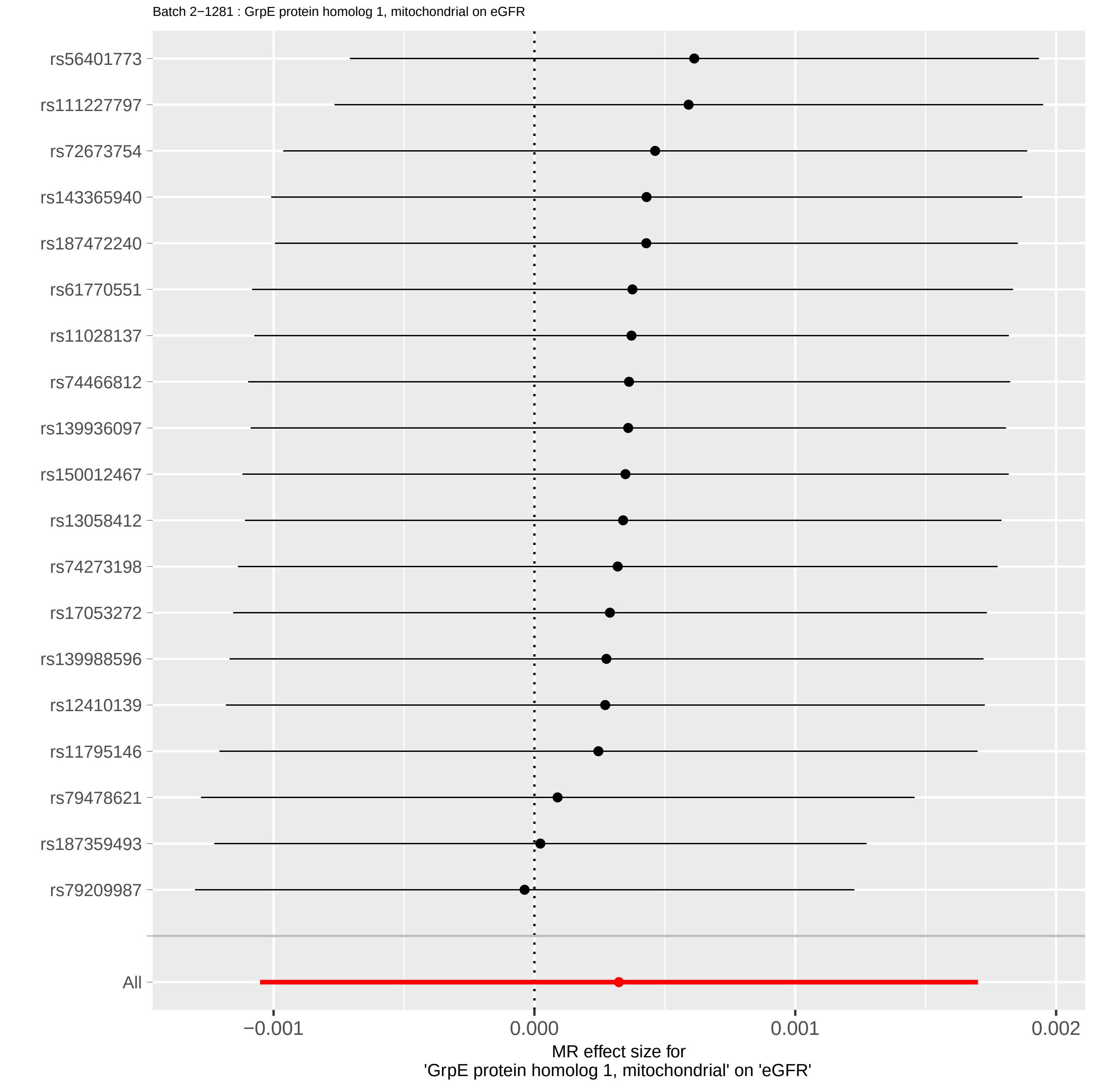

Batch 2−1281 : GrpE protein homolog 1, mitochondrial on eGFR
rs56401773
rs111227797
rs72673754
rs143365940
rs187472240
rs61770551
rs11028137
rs74466812
rs139936097
rs150012467
rs13058412
rs74273198
rs17053272
rs139988596
rs12410139
rs11795146
rs79478621
rs187359493
rs79209987
All
−0.001
0.000
0.001
0.002
MR effect size for
'GrpE protein homolog 1, mitochondrial' on 'eGFR'

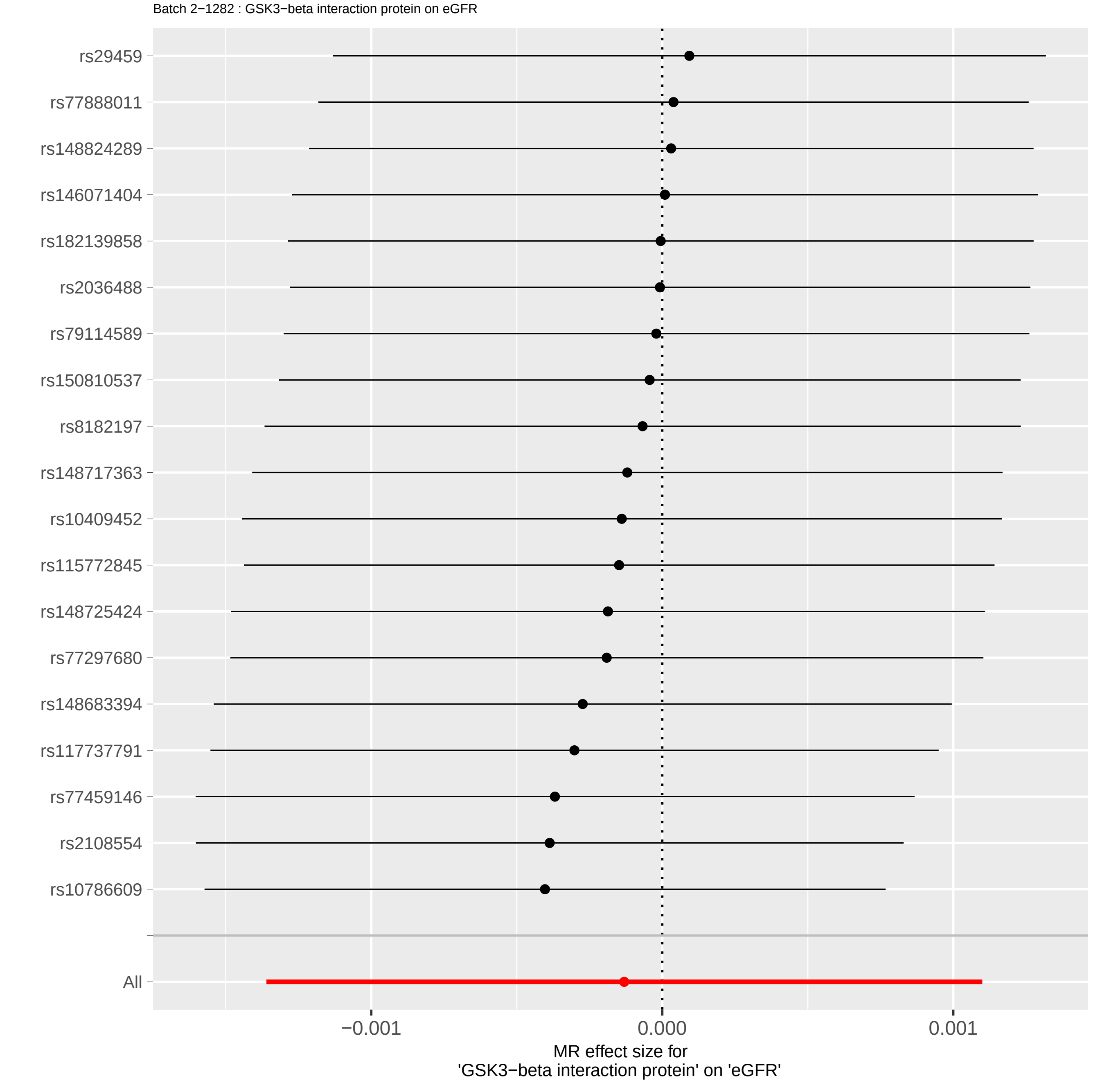
Batch 2−1282 : GSK3−beta interaction protein on eGFR
rs29459
rs77888011
rs148824289
rs146071404
rs182139858
rs2036488
rs79114589
rs150810537
rs8182197
rs148717363
rs10409452
rs115772845
rs148725424
rs77297680
rs148683394
rs117737791
rs77459146
rs2108554
rs10786609
All
−0.001
0.000
0.001
MR effect size for
'GSK3−beta interaction protein' on 'eGFR'

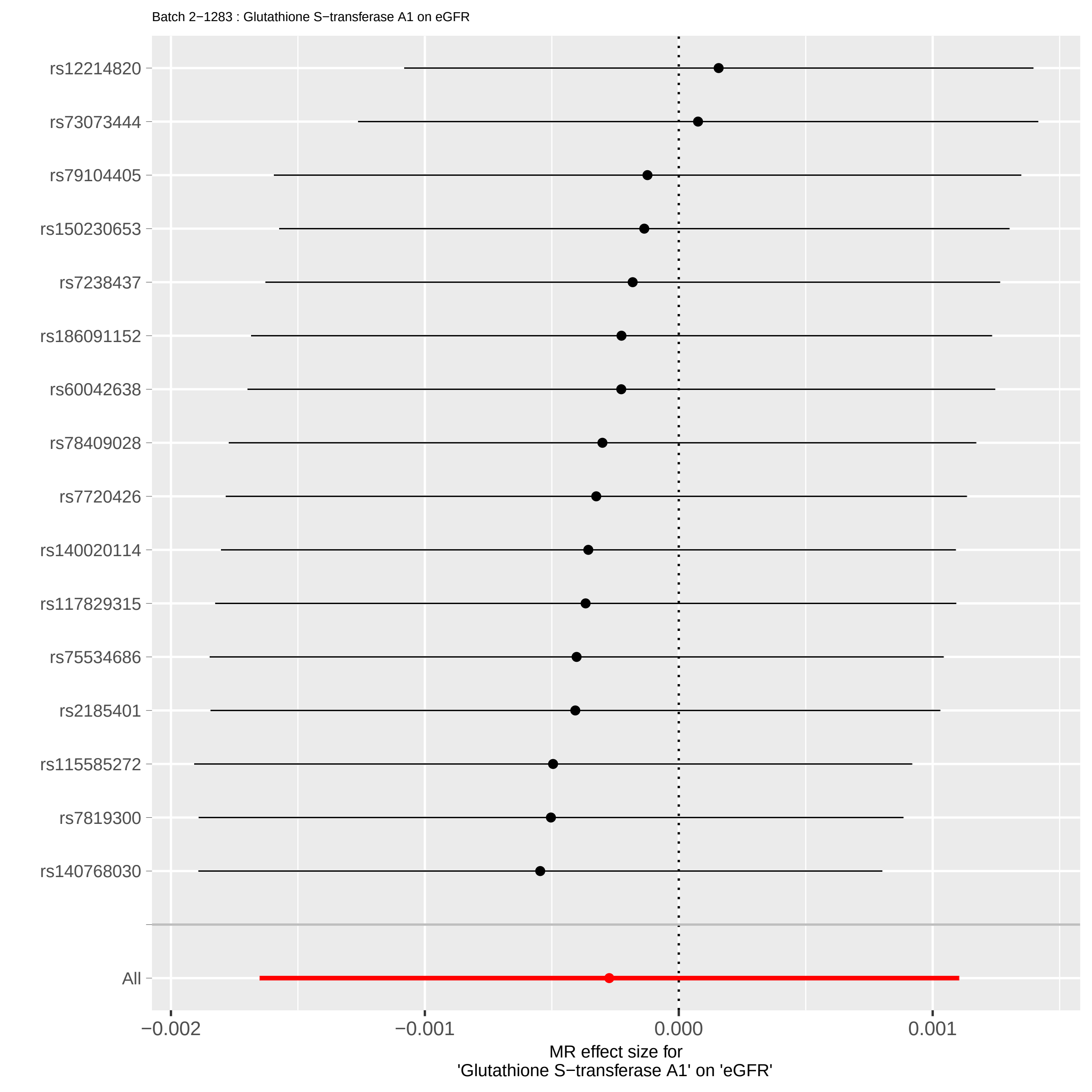

Batch 2−1283 : Glutathione S−transferase A1 on eGFR
rs12214820
rs73073444
rs79104405
rs150230653
rs7238437
rs186091152
rs60042638
rs78409028
rs7720426
rs140020114
rs117829315
rs75534686
rs2185401
rs115585272
rs7819300
rs140768030
All
−0.002
−0.001
0.000
0.001
MR effect size for
'Glutathione S−transferase A1' on 'eGFR'

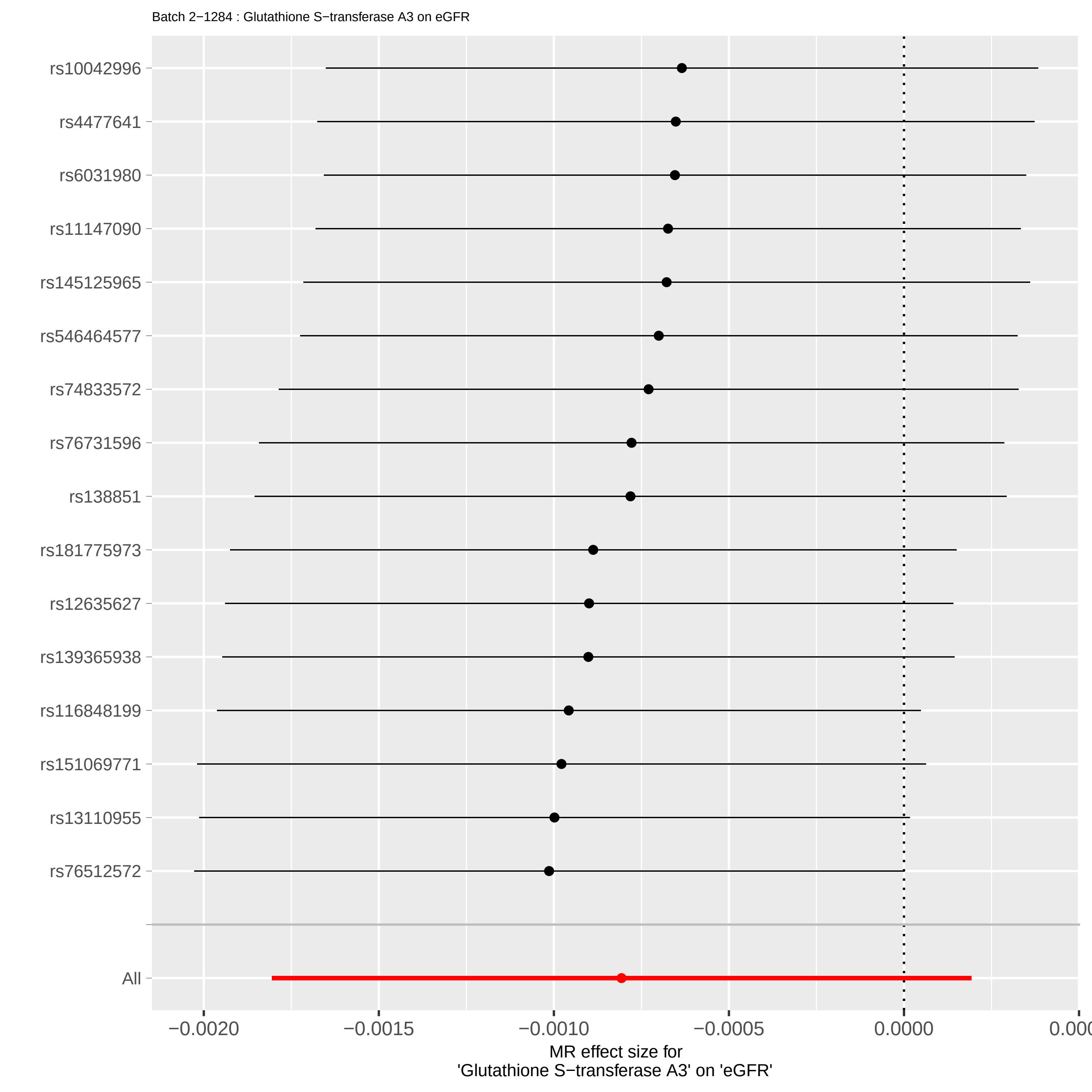

Batch 2−1284 : Glutathione S−transferase A3 on eGFR
rs10042996
rs4477641
rs6031980
rs11147090
rs145125965
rs546464577
rs74833572
rs76731596
rs138851
rs181775973
rs12635627
rs139365938
rs116848199
rs151069771
rs13110955
rs76512572
All
−0.0020
−0.0015
−0.0010
−0.0005
0.0000
MR effect size for
'Glutathione S−transferase A3' on 'eGFR'

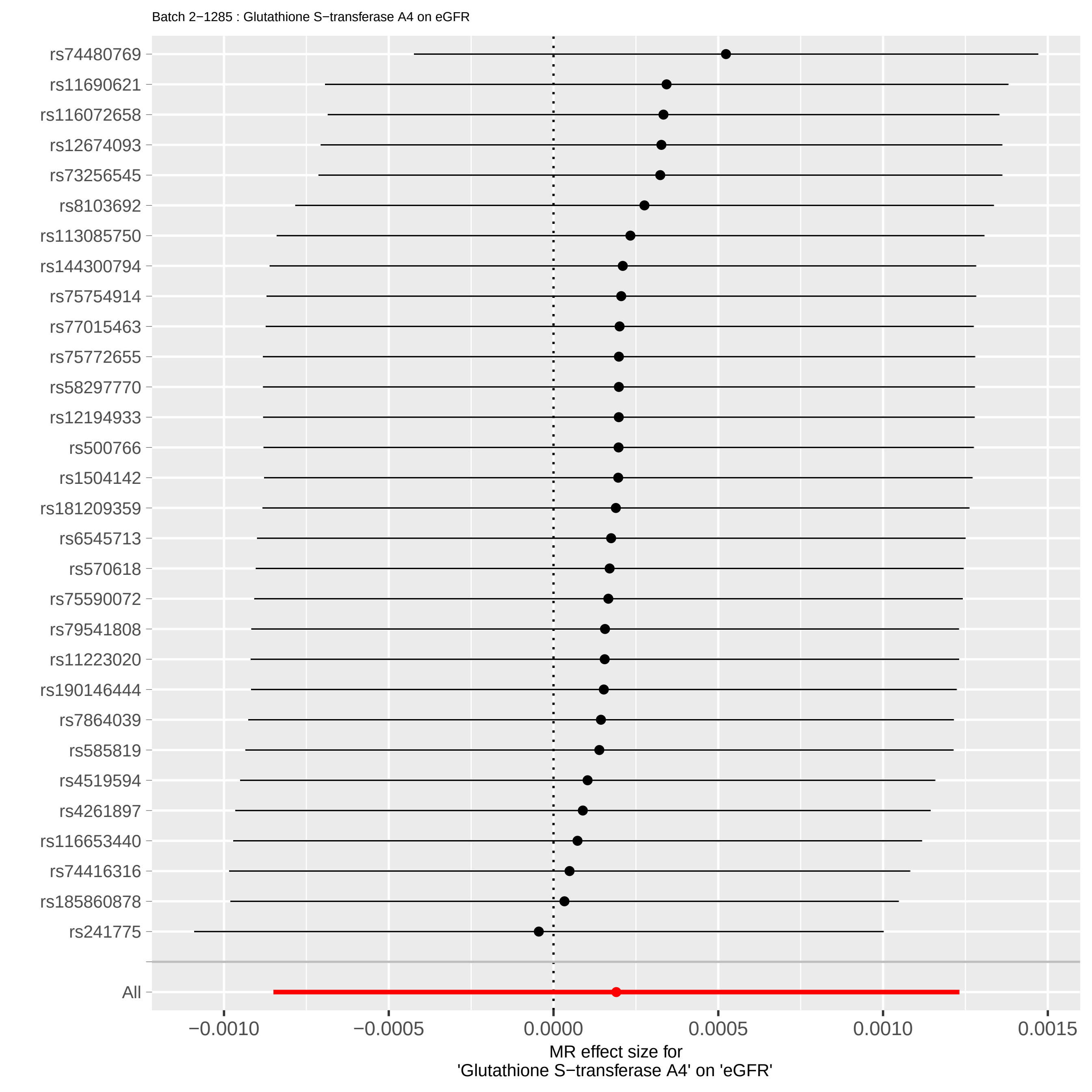

Batch 2−1285 : Glutathione S−transferase A4 on eGFR
rs74480769
rs11690621
rs116072658
rs12674093
rs73256545
rs8103692
rs113085750
rs144300794
rs75754914
rs77015463
rs75772655
rs58297770
rs12194933
rs500766
rs1504142
rs181209359
rs6545713
rs570618
rs75590072
rs79541808
rs11223020
rs190146444
rs7864039
rs585819
rs4519594
rs4261897
rs116653440
rs74416316
rs185860878
rs241775
All
−0.0010
−0.0005
0.0000
0.0005
0.0010
0.0015
MR effect size for
'Glutathione S−transferase A4' on 'eGFR'

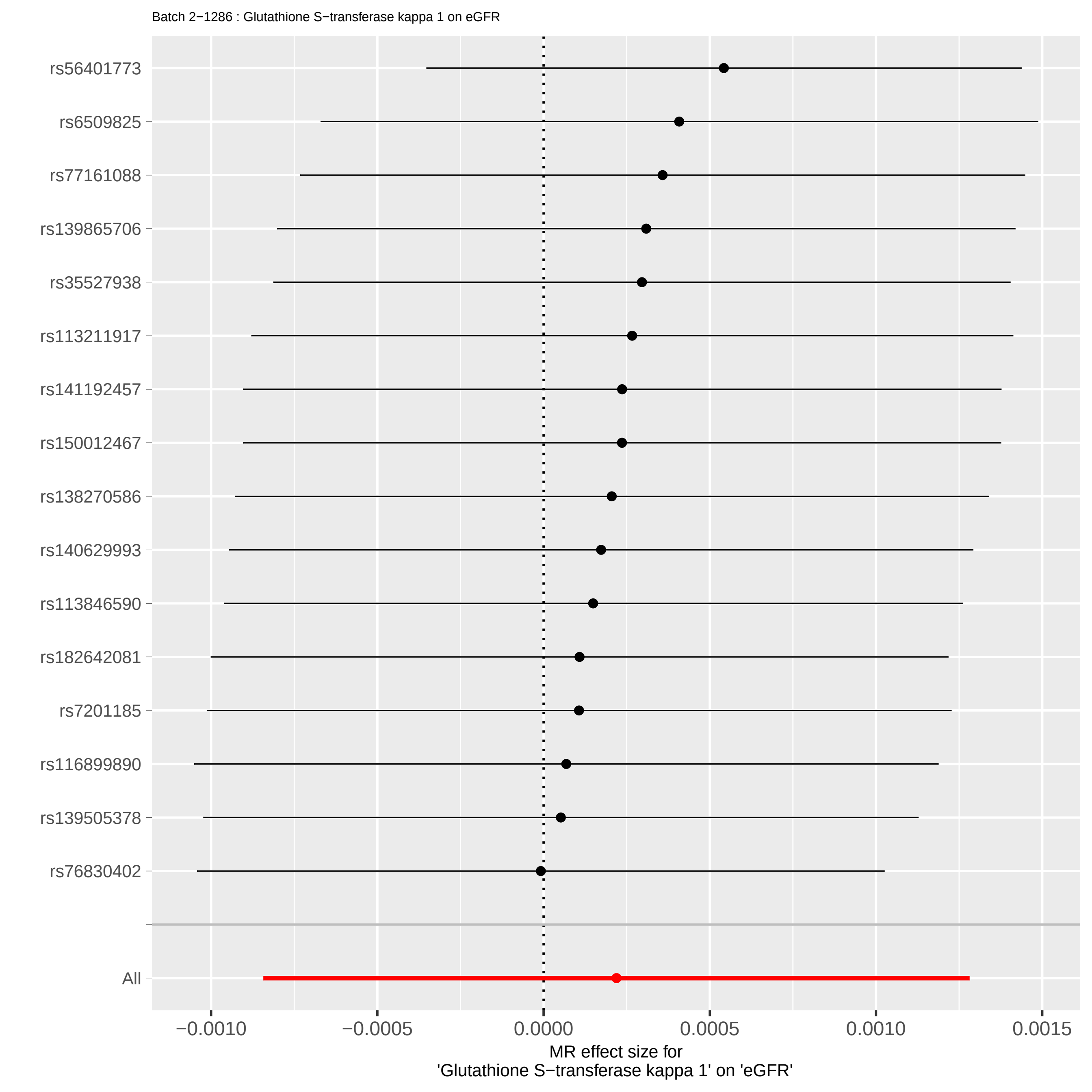

Batch 2−1286 : Glutathione S−transferase kappa 1 on eGFR
rs56401773
rs6509825
rs77161088
rs139865706
rs35527938
rs113211917
rs141192457
rs150012467
rs138270586
rs140629993
rs113846590
rs182642081
rs7201185
rs116899890
rs139505378
rs76830402
All
−0.0010
−0.0005
0.0000
0.0005
0.0010
0.0015
MR effect size for
'Glutathione S−transferase kappa 1' on 'eGFR'

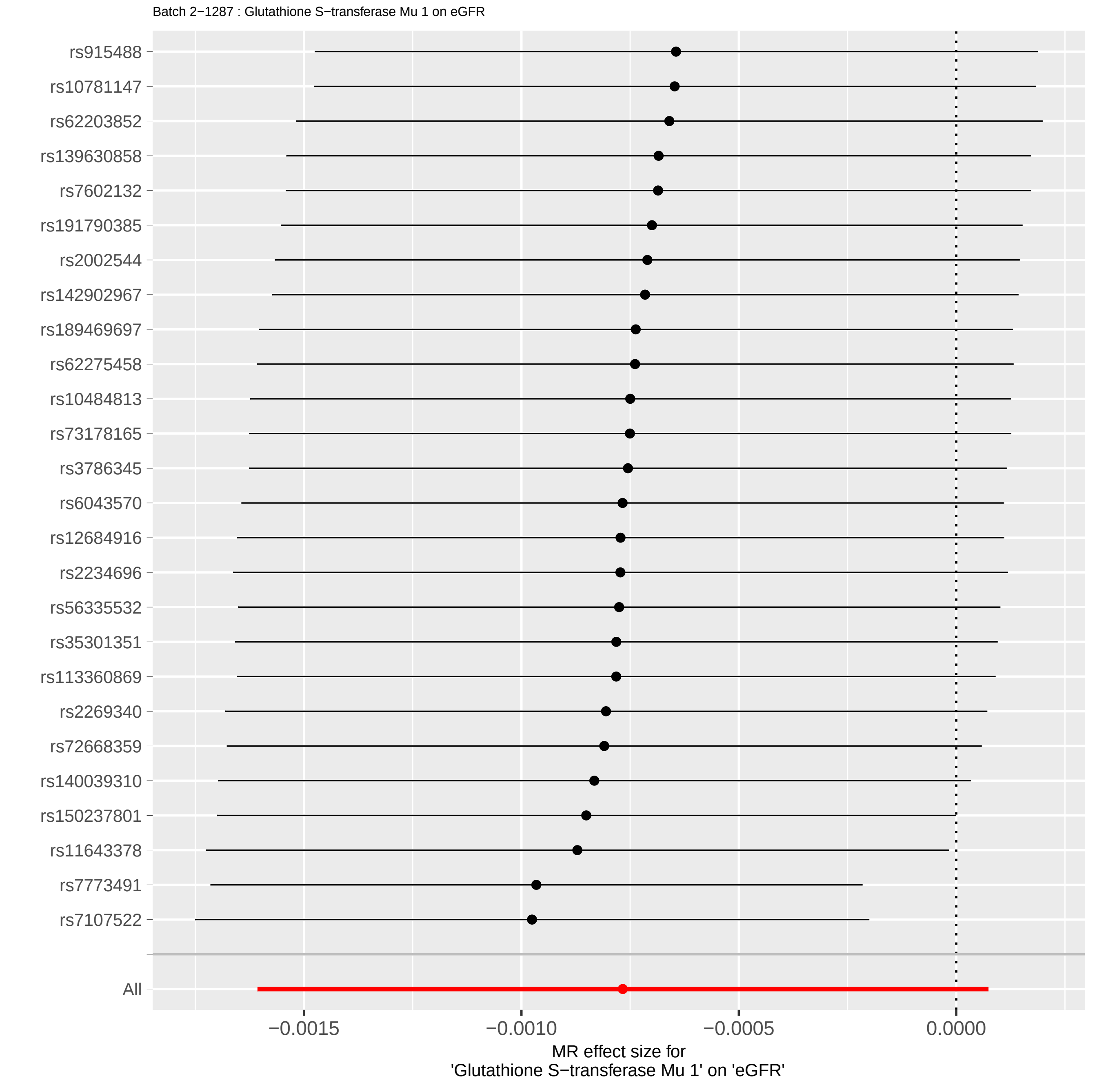

Batch 2−1287 : Glutathione S−transferase Mu 1 on eGFR
rs915488
rs10781147
rs62203852
rs139630858
rs7602132
rs191790385
rs2002544
rs142902967
rs189469697
rs62275458
rs10484813
rs73178165
rs3786345
rs6043570
rs12684916
rs2234696
rs56335532
rs35301351
rs113360869
rs2269340
rs72668359
rs140039310
rs150237801
rs11643378
rs7773491
rs7107522
All
−0.0015
−0.0010
−0.0005
0.0000
MR effect size for
'Glutathione S−transferase Mu 1' on 'eGFR'

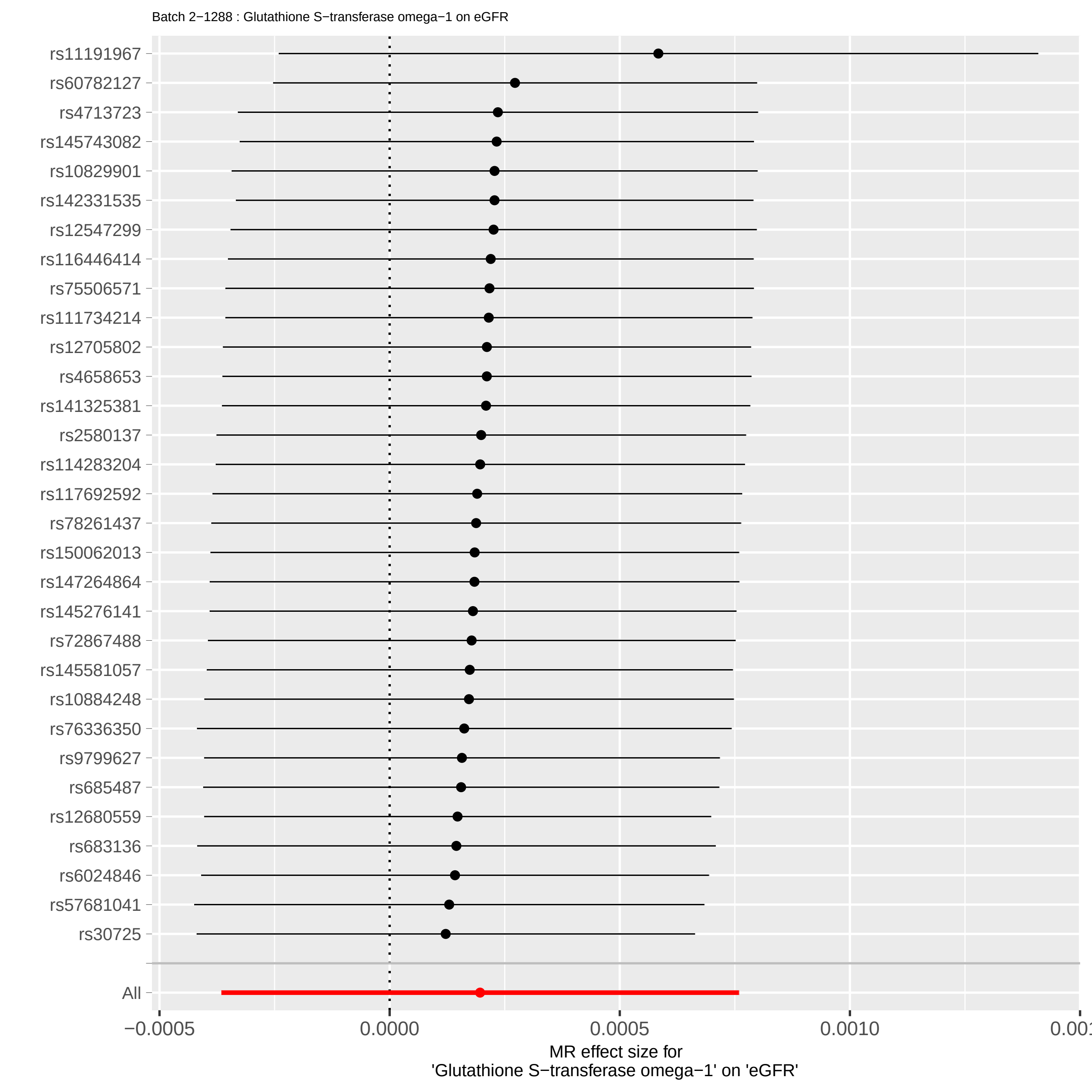

Batch 2−1288 : Glutathione S−transferase omega−1 on eGFR
rs11191967
rs60782127
rs4713723
rs145743082
rs10829901
rs142331535
rs12547299
rs116446414
rs75506571
rs111734214
rs12705802
rs4658653
rs141325381
rs2580137
rs114283204
rs117692592
rs78261437
rs150062013
rs147264864
rs145276141
rs72867488
rs145581057
rs10884248
rs76336350
rs9799627
rs685487
rs12680559
rs683136
rs6024846
rs57681041
rs30725
All
−0.0005
0.0000
0.0005
0.0010
MR effect size for
'Glutathione S−transferase omega−1' on 'eGFR'

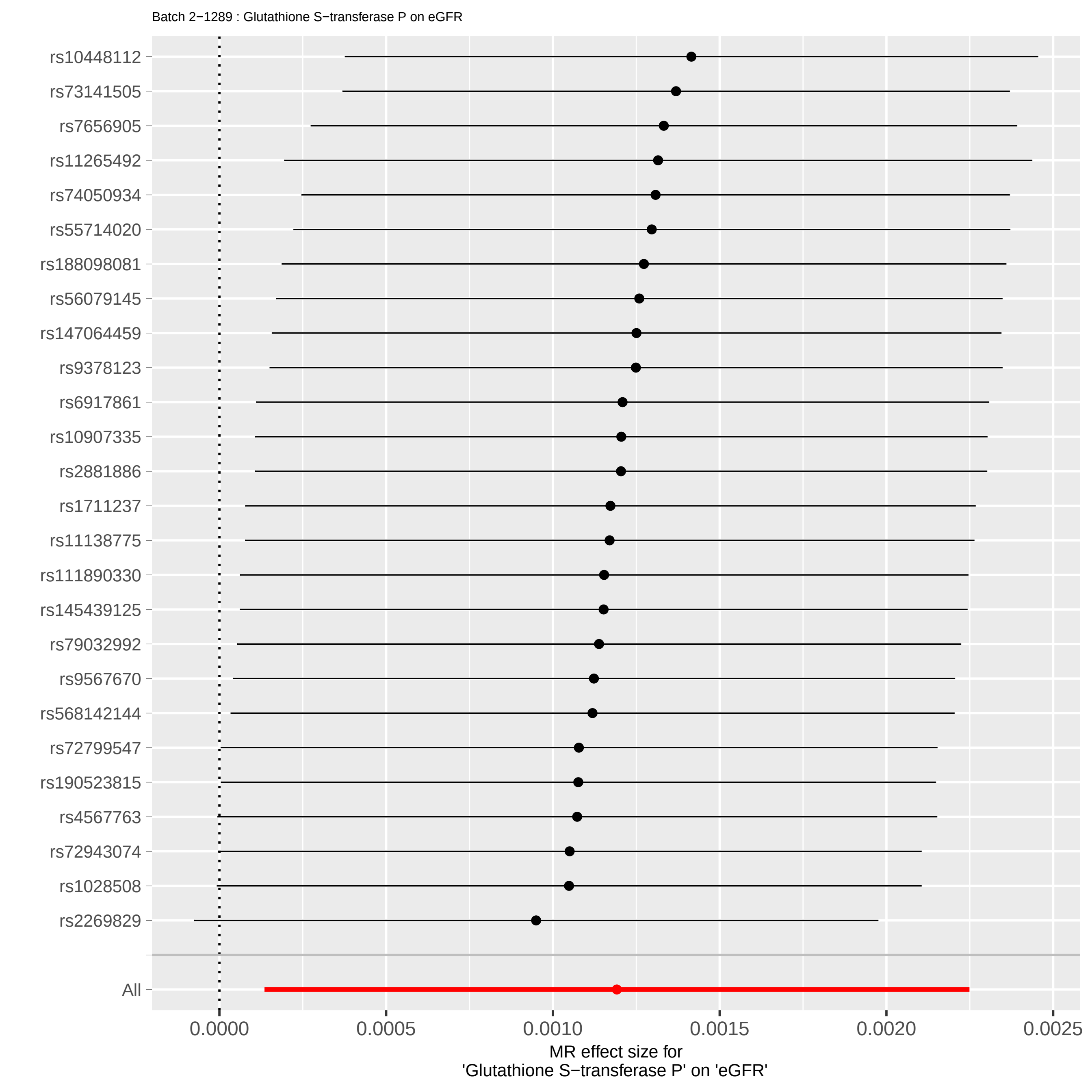

Batch 2−1289 : Glutathione S−transferase P on eGFR
rs10448112
rs73141505
rs7656905
rs11265492
rs74050934
rs55714020
rs188098081
rs56079145
rs147064459
rs9378123
rs6917861
rs10907335
rs2881886
rs1711237
rs11138775
rs111890330
rs145439125
rs79032992
rs9567670
rs568142144
rs72799547
rs190523815
rs4567763
rs72943074
rs1028508
rs2269829
All
0.0000
0.0005
0.0010
0.0015
0.0020
0.0025
MR effect size for
'Glutathione S−transferase P' on 'eGFR'

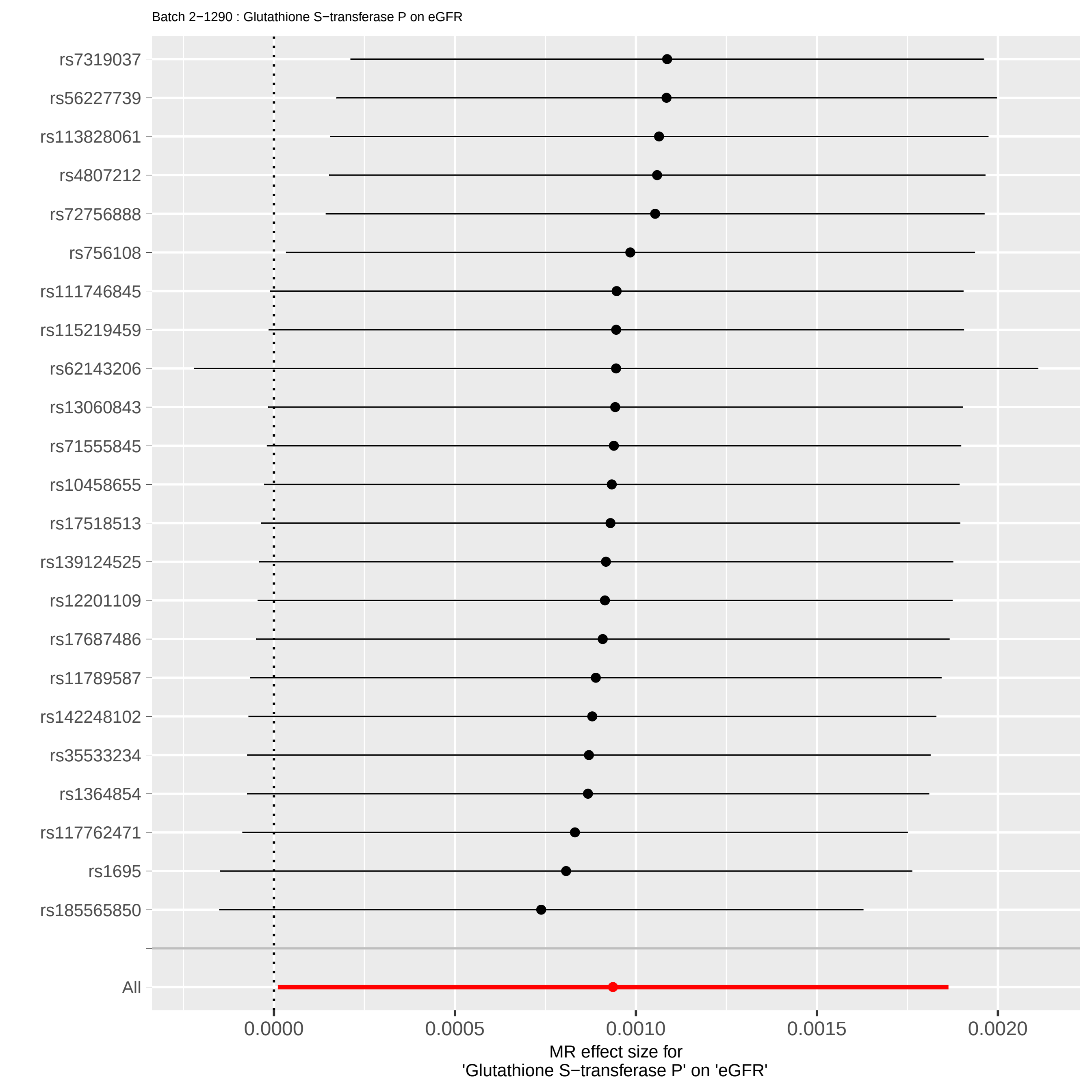
Batch 2−1290 : Glutathione S−transferase P on eGFR
rs7319037
rs56227739
rs113828061
rs4807212
rs72756888
rs756108
rs111746845
rs115219459
rs62143206
rs13060843
rs71555845
rs10458655
rs17518513
rs139124525
rs12201109
rs17687486
rs11789587
rs142248102
rs35533234
rs1364854
rs117762471
rs1695
rs185565850
All
0.0000
0.0005
0.0010
0.0015
0.0020
MR effect size for
'Glutathione S−transferase P' on 'eGFR'

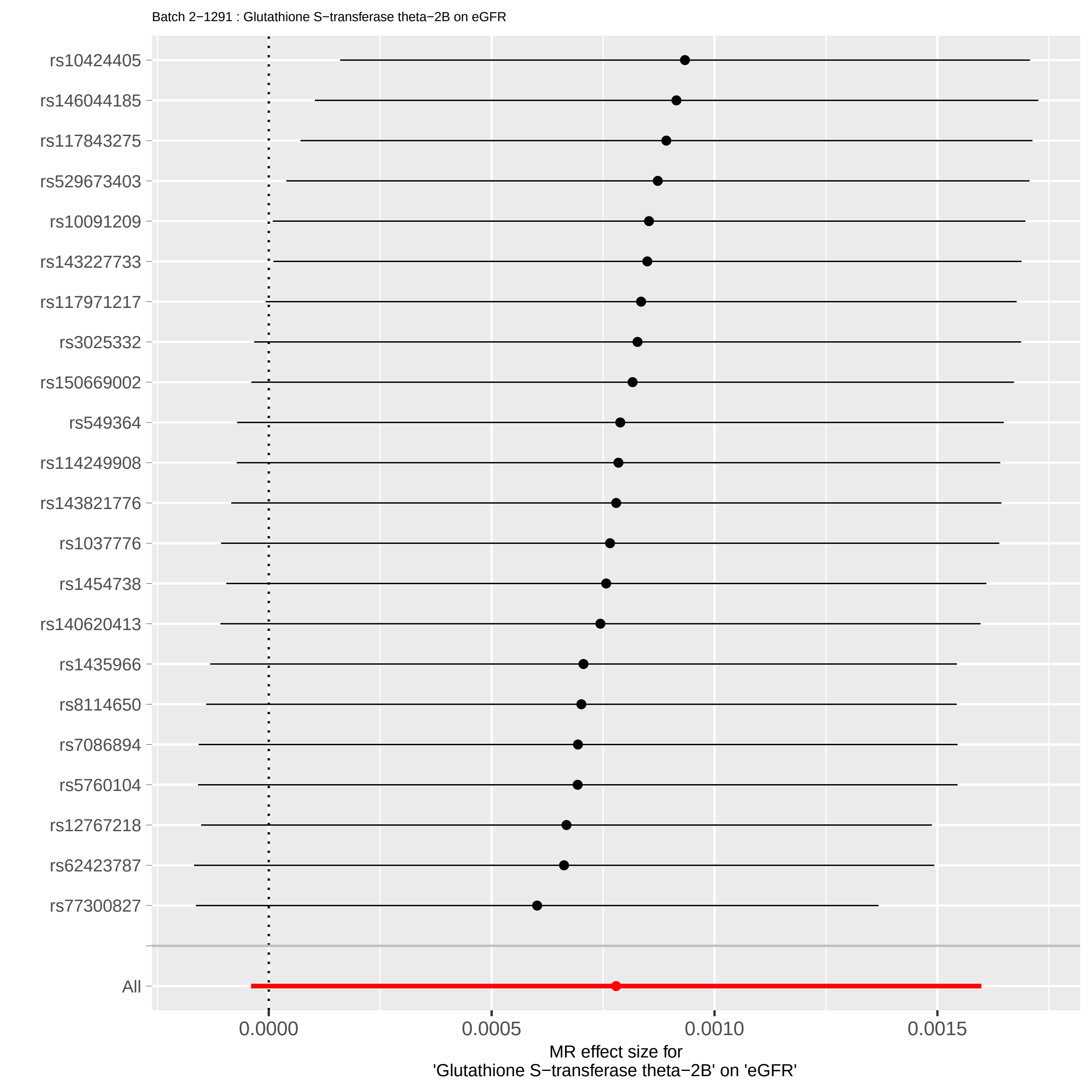

Batch 2−1291 : Glutathione S−transferase theta−2B on eGFR
rs10424405
rs146044185
rs117843275
rs529673403
rs10091209
rs143227733
rs117971217
rs3025332
rs150669002
rs549364
rs114249908
rs143821776
rs1037776
rs1454738
rs140620413
rs1435966
rs8114650
rs7086894
rs5760104
rs12767218
rs62423787
rs77300827
All
0.0000
0.0005
0.0010
0.0015
MR effect size for
'Glutathione S−transferase theta−2B' on 'eGFR'

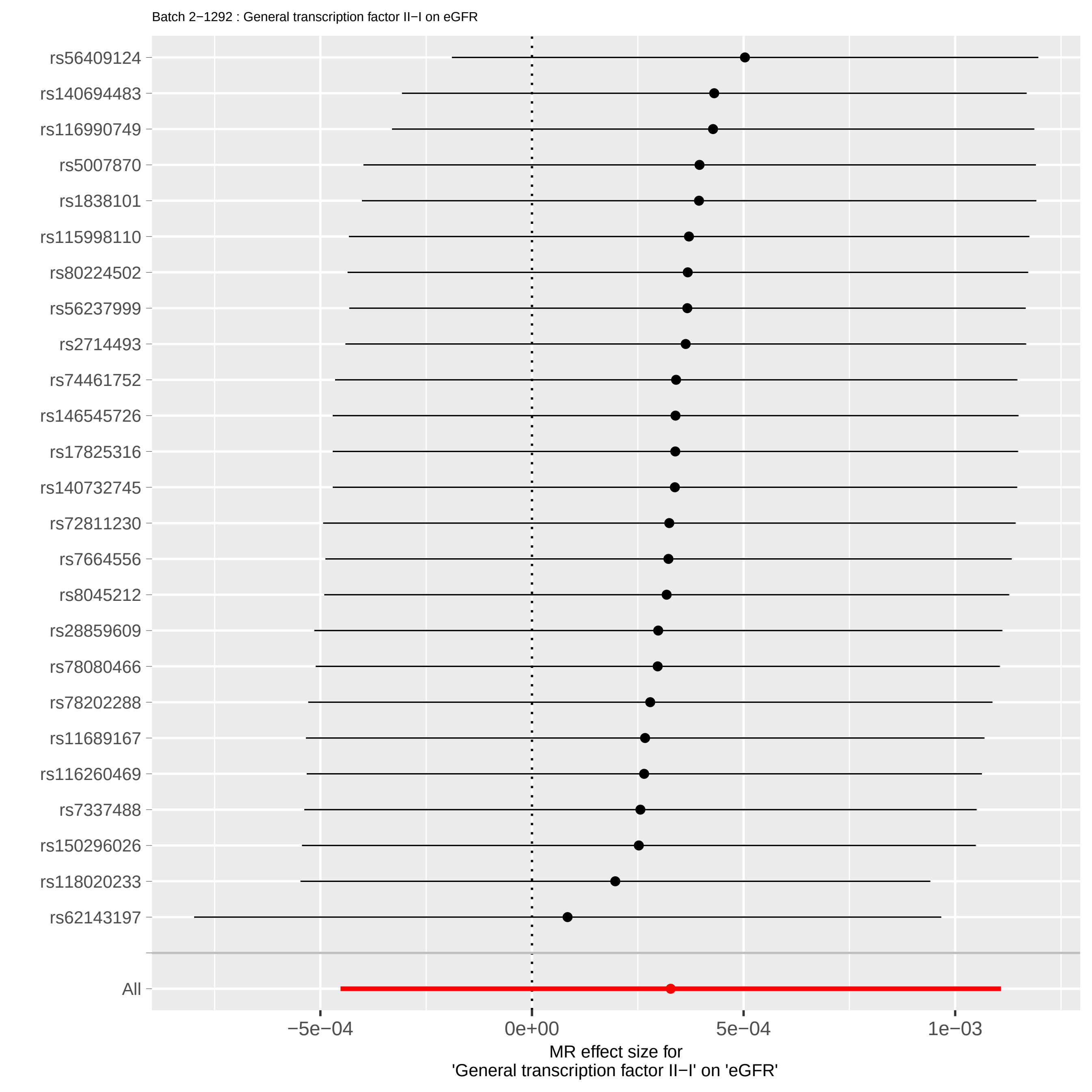

Batch 2−1292 : General transcription factor II−I on eGFR
rs56409124
rs140694483
rs116990749
rs5007870
rs1838101
rs115998110
rs80224502
rs56237999
rs2714493
rs74461752
rs146545726
rs17825316
rs140732745
rs72811230
rs7664556
rs8045212
rs28859609
rs78080466
rs78202288
rs11689167
rs116260469
rs7337488
rs150296026
rs118020233
rs62143197
All
−5e−04
0e+00
5e−04
1e−03
MR effect size for
'General transcription factor II−I' on 'eGFR'

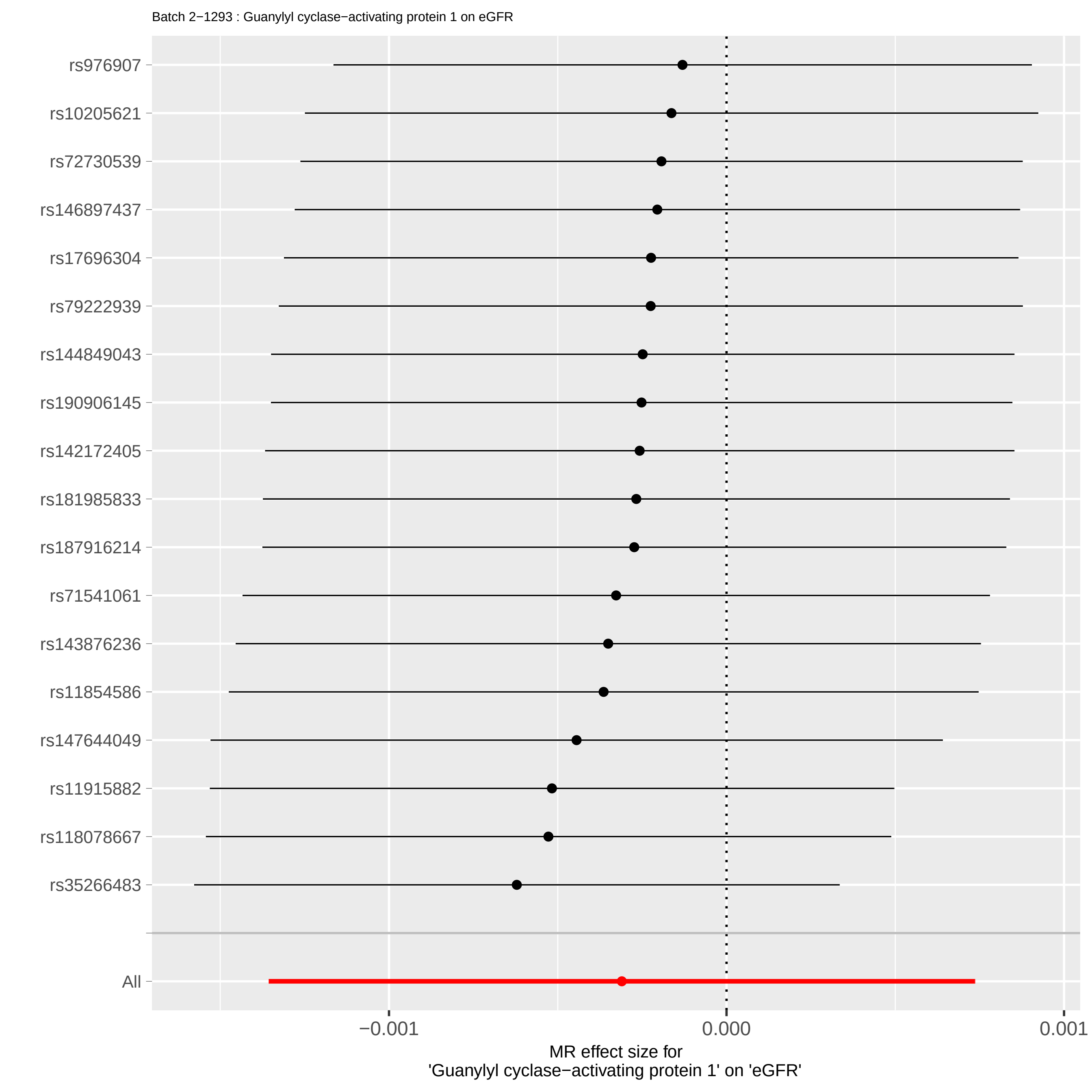

Batch 2−1293 : Guanylyl cyclase−activating protein 1 on eGFR
rs976907
rs10205621
rs72730539
rs146897437
rs17696304
rs79222939
rs144849043
rs190906145
rs142172405
rs181985833
rs187916214
rs71541061
rs143876236
rs11854586
rs147644049
rs11915882
rs118078667
rs35266483
All
−0.001
0.000
0.001
MR effect size for
'Guanylyl cyclase−activating protein 1' on 'eGFR'

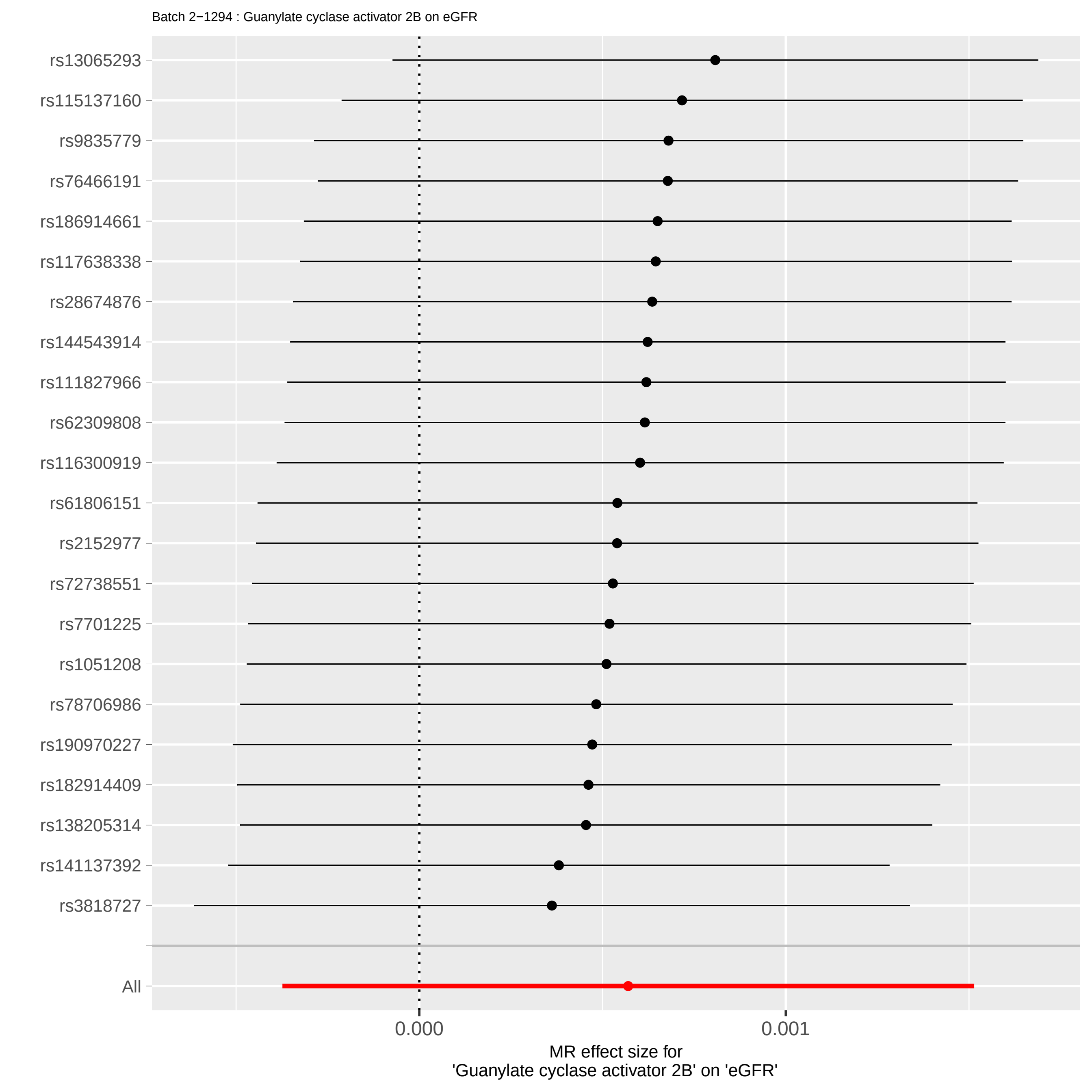
Batch 2−1294 : Guanylate cyclase activator 2B on eGFR
rs13065293
rs115137160
rs9835779
rs76466191
rs186914661
rs117638338
rs28674876
rs144543914
rs111827966
rs62309808
rs116300919
rs61806151
rs2152977
rs72738551
rs7701225
rs1051208
rs78706986
rs190970227
rs182914409
rs138205314
rs141137392
rs3818727
All
0.000
0.001
MR effect size for
'Guanylate cyclase activator 2B' on 'eGFR'

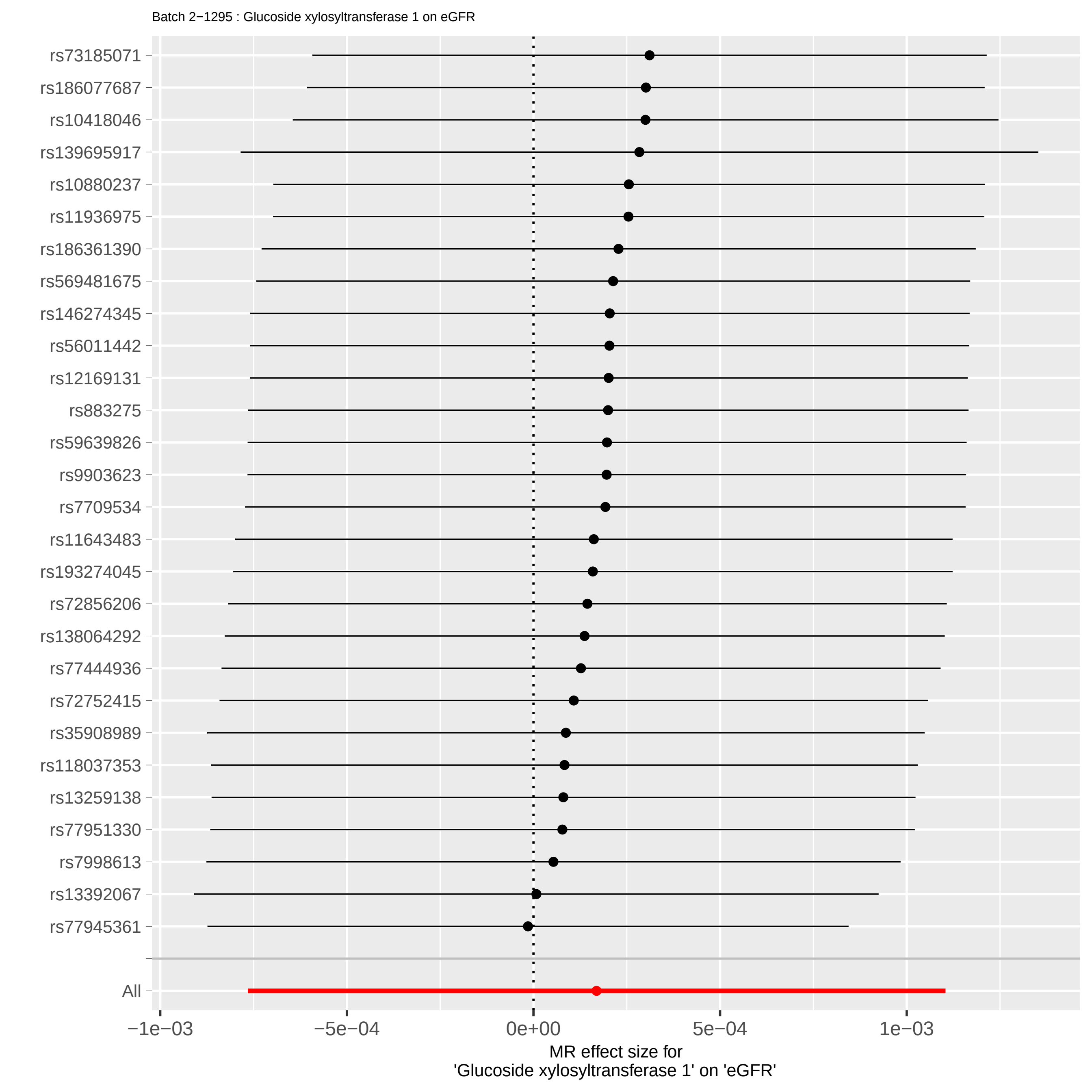

Batch 2−1295 : Glucoside xylosyltransferase 1 on eGFR
rs73185071
rs186077687
rs10418046
rs139695917
rs10880237
rs11936975
rs186361390
rs569481675
rs146274345
rs56011442
rs12169131
rs883275
rs59639826
rs9903623
rs7709534
rs11643483
rs193274045
rs72856206
rs138064292
rs77444936
rs72752415
rs35908989
rs118037353
rs13259138
rs77951330
rs7998613
rs13392067
rs77945361
All
−1e−03
−5e−04
0e+00
5e−04
1e−03
MR effect size for
'Glucoside xylosyltransferase 1' on 'eGFR'

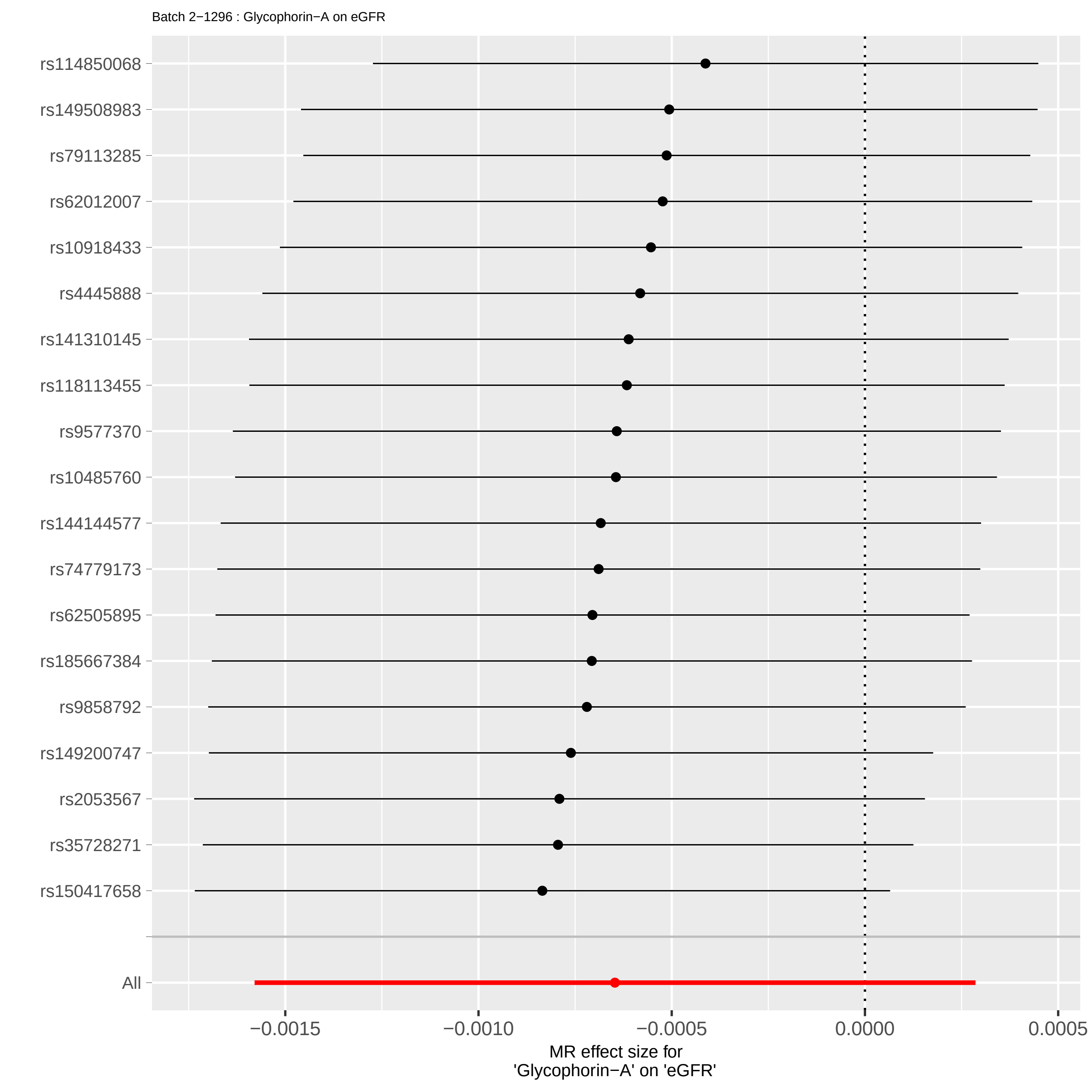

Batch 2−1296 : Glycophorin−A on eGFR
rs114850068
rs149508983
rs79113285
rs62012007
rs10918433
rs4445888
rs141310145
rs118113455
rs9577370
rs10485760
rs144144577
rs74779173
rs62505895
rs185667384
rs9858792
rs149200747
rs2053567
rs35728271
rs150417658
All
−0.0015
−0.0010
−0.0005
0.0000
0.0005
MR effect size for
'Glycophorin−A' on 'eGFR'

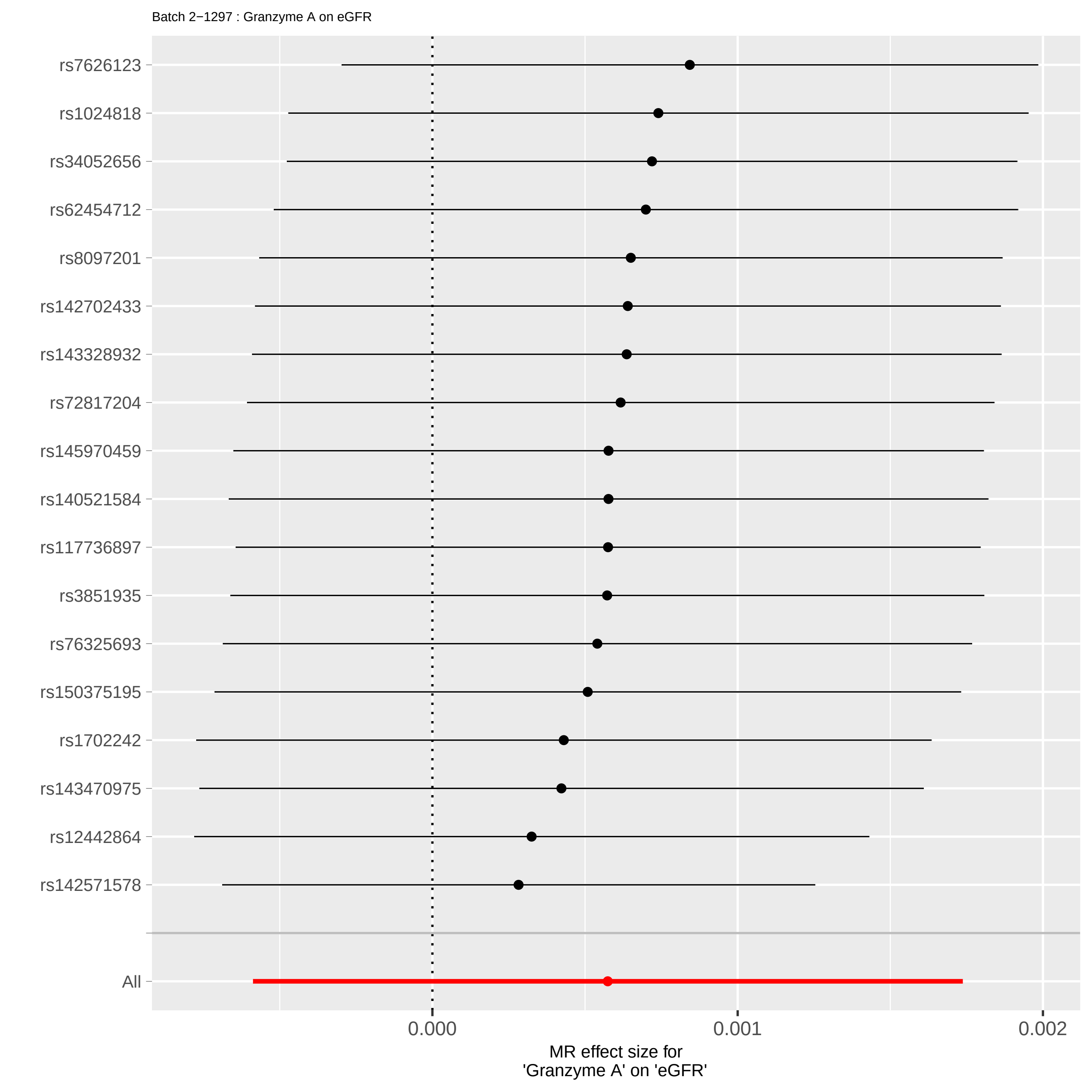

Batch 2−1297 : Granzyme A on eGFR
rs7626123
rs1024818
rs34052656
rs62454712
rs8097201
rs142702433
rs143328932
rs72817204
rs145970459
rs140521584
rs117736897
rs3851935
rs76325693
rs150375195
rs1702242
rs143470975
rs12442864
rs142571578
All
0.000
0.001
0.002
MR effect size for
'Granzyme A' on 'eGFR'

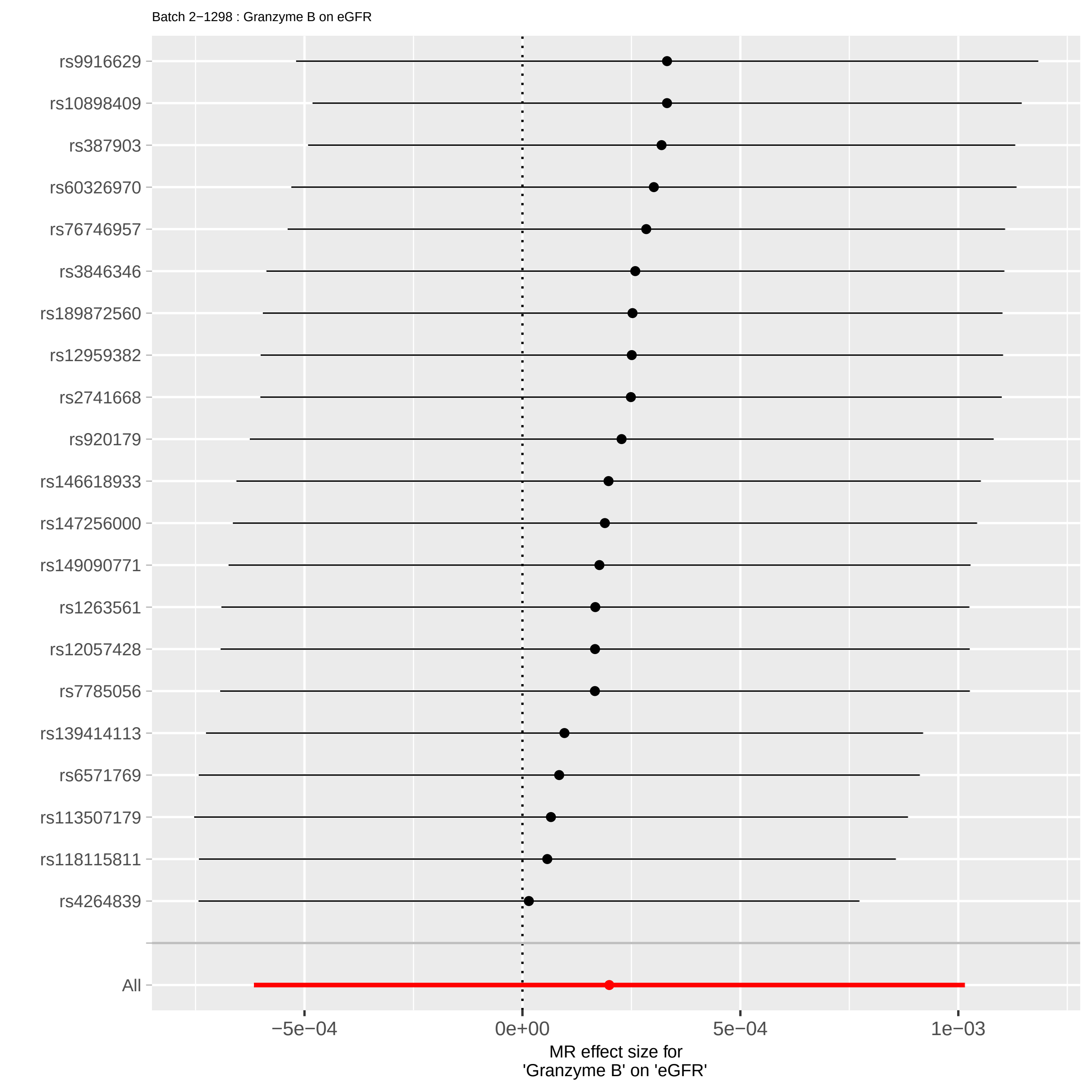

Batch 2−1298 : Granzyme B on eGFR
rs9916629
rs10898409
rs387903
rs60326970
rs76746957
rs3846346
rs189872560
rs12959382
rs2741668
rs920179
rs146618933
rs147256000
rs149090771
rs1263561
rs12057428
rs7785056
rs139414113
rs6571769
rs113507179
rs118115811
rs4264839
All
−5e−04
0e+00
5e−04
1e−03
MR effect size for
'Granzyme B' on 'eGFR'

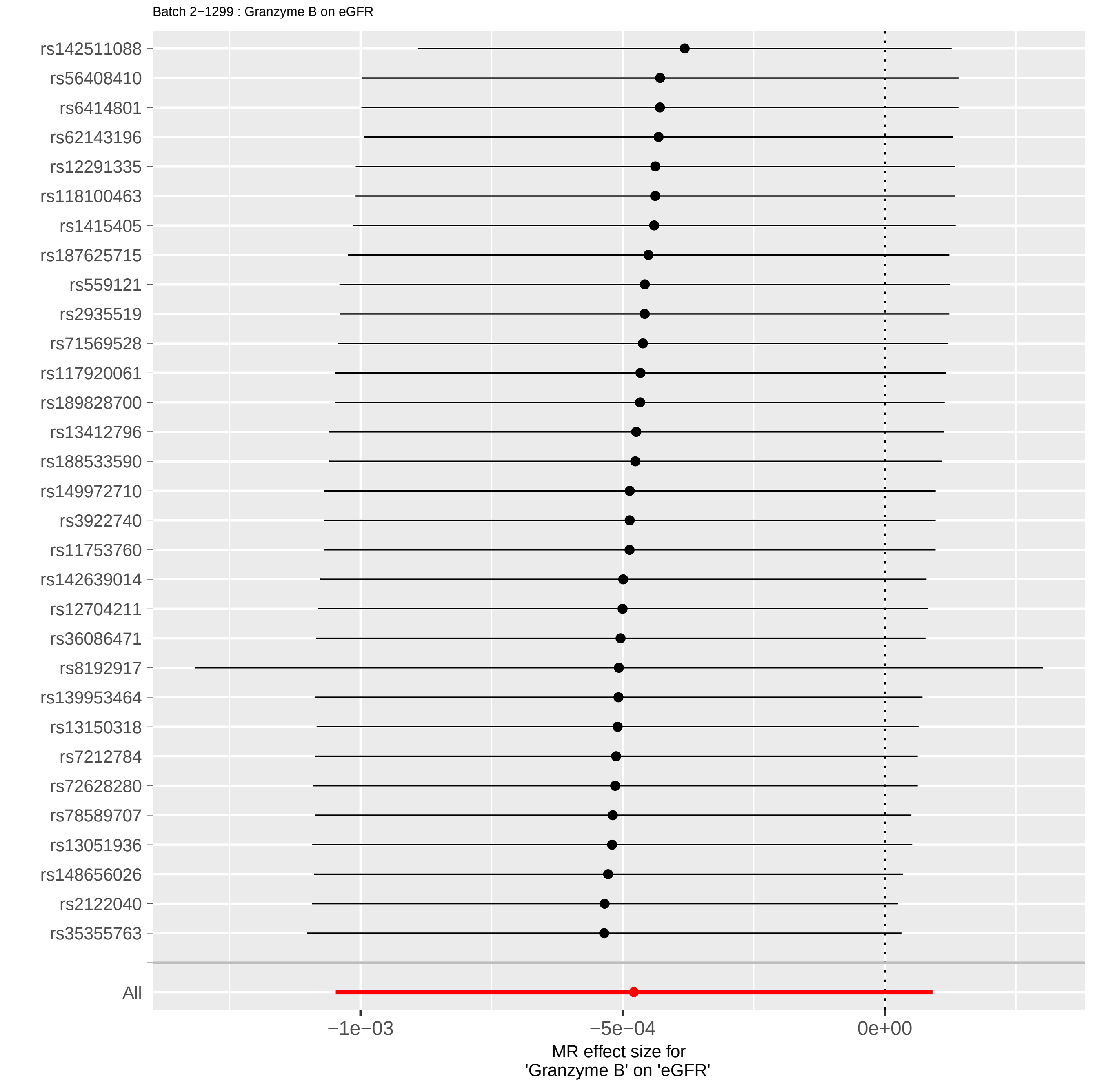

Batch 2−1299 : Granzyme B on eGFR
rs142511088
rs56408410
rs6414801
rs62143196
rs12291335
rs118100463
rs1415405
rs187625715
rs559121
rs2935519
rs71569528
rs117920061
rs189828700
rs13412796
rs188533590
rs149972710
rs3922740
rs11753760
rs142639014
rs12704211
rs36086471
rs8192917
rs139953464
rs13150318
rs7212784
rs72628280
rs78589707
rs13051936
rs148656026
rs2122040
rs35355763
All
−1e−03
−5e−04
0e+00
MR effect size for
'Granzyme B' on 'eGFR'

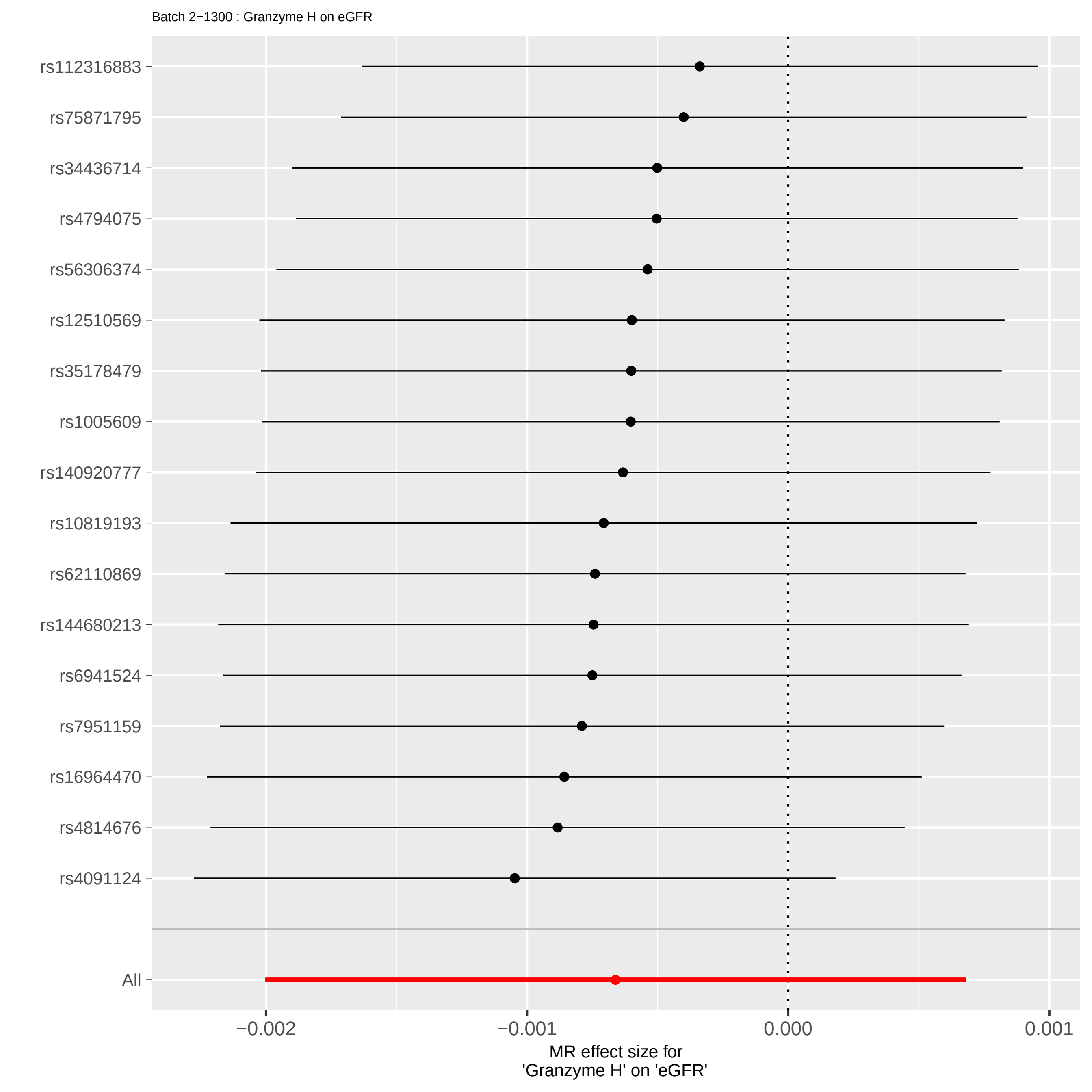

Batch 2−1300 : Granzyme H on eGFR
rs112316883
rs75871795
rs34436714
rs4794075
rs56306374
rs12510569
rs35178479
rs1005609
rs140920777
rs10819193
rs62110869
rs144680213
rs6941524
rs7951159
rs16964470
rs4814676
rs4091124
All
−0.002
−0.001
0.000
0.001
MR effect size for
'Granzyme H' on 'eGFR'

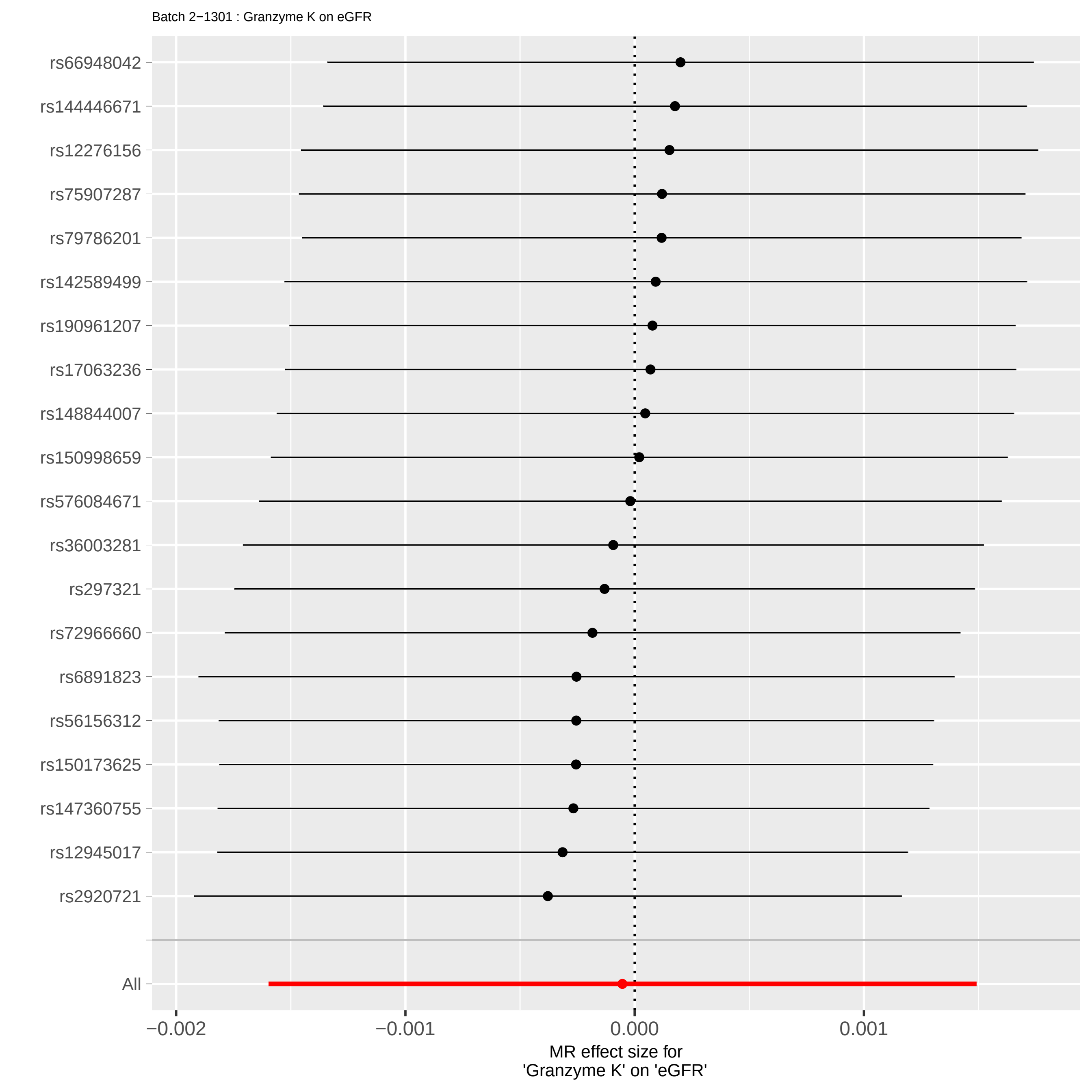

Batch 2−1301 : Granzyme K on eGFR
rs66948042
rs144446671
rs12276156
rs75907287
rs79786201
rs142589499
rs190961207
rs17063236
rs148844007
rs150998659
rs576084671
rs36003281
rs297321
rs72966660
rs6891823
rs56156312
rs150173625
rs147360755
rs12945017
rs2920721
All
−0.002
−0.001
0.000
0.001
MR effect size for
'Granzyme K' on 'eGFR'

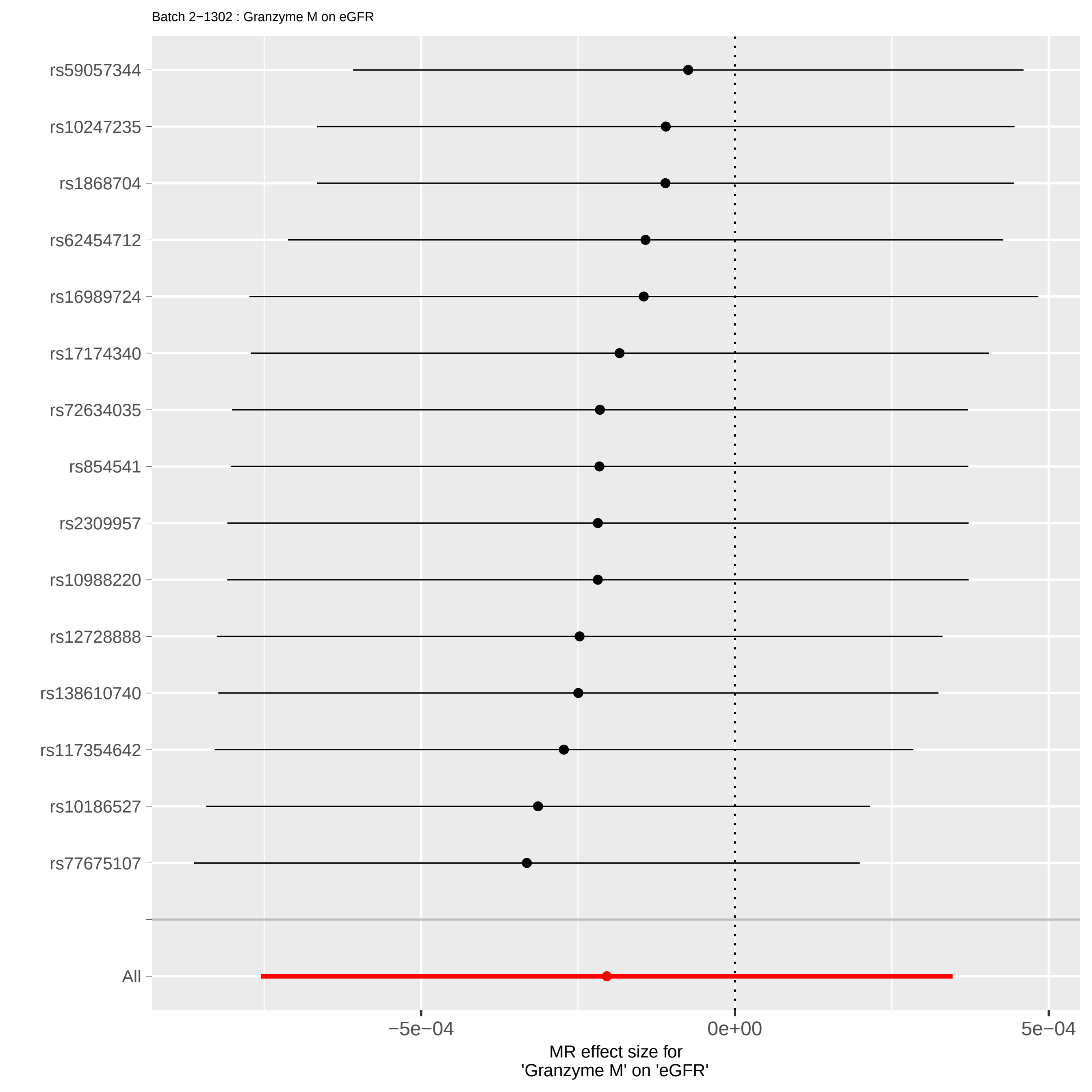
Batch 2−1302 : Granzyme M on eGFR
rs59057344
rs10247235
rs1868704
rs62454712
rs16989724
rs17174340
rs72634035
rs854541
rs2309957
rs10988220
rs12728888
rs138610740
rs117354642
rs10186527
rs77675107
All
−5e−04
0e+00
5e−04
MR effect size for
'Granzyme M' on 'eGFR'

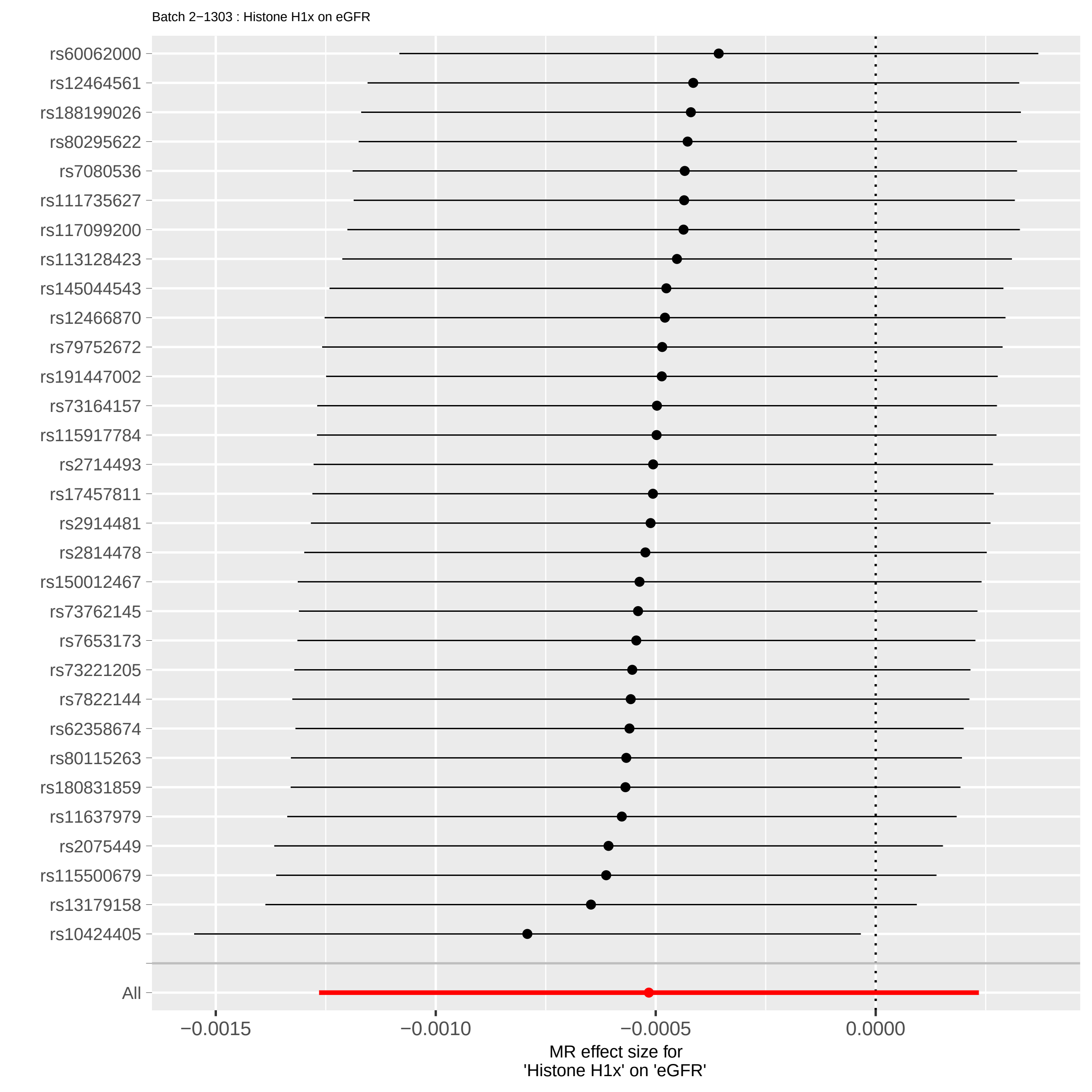
Batch 2−1303 : Histone H1x on eGFR
rs60062000
rs12464561
rs188199026
rs80295622
rs7080536
rs111735627
rs117099200
rs113128423
rs145044543
rs12466870
rs79752672
rs191447002
rs73164157
rs115917784
rs2714493
rs17457811
rs2914481
rs2814478
rs150012467
rs73762145
rs7653173
rs73221205
rs7822144
rs62358674
rs80115263
rs180831859
rs11637979
rs2075449
rs115500679
rs13179158
rs10424405
All
−0.0015
−0.0010
−0.0005
0.0000
MR effect size for
'Histone H1x' on 'eGFR'

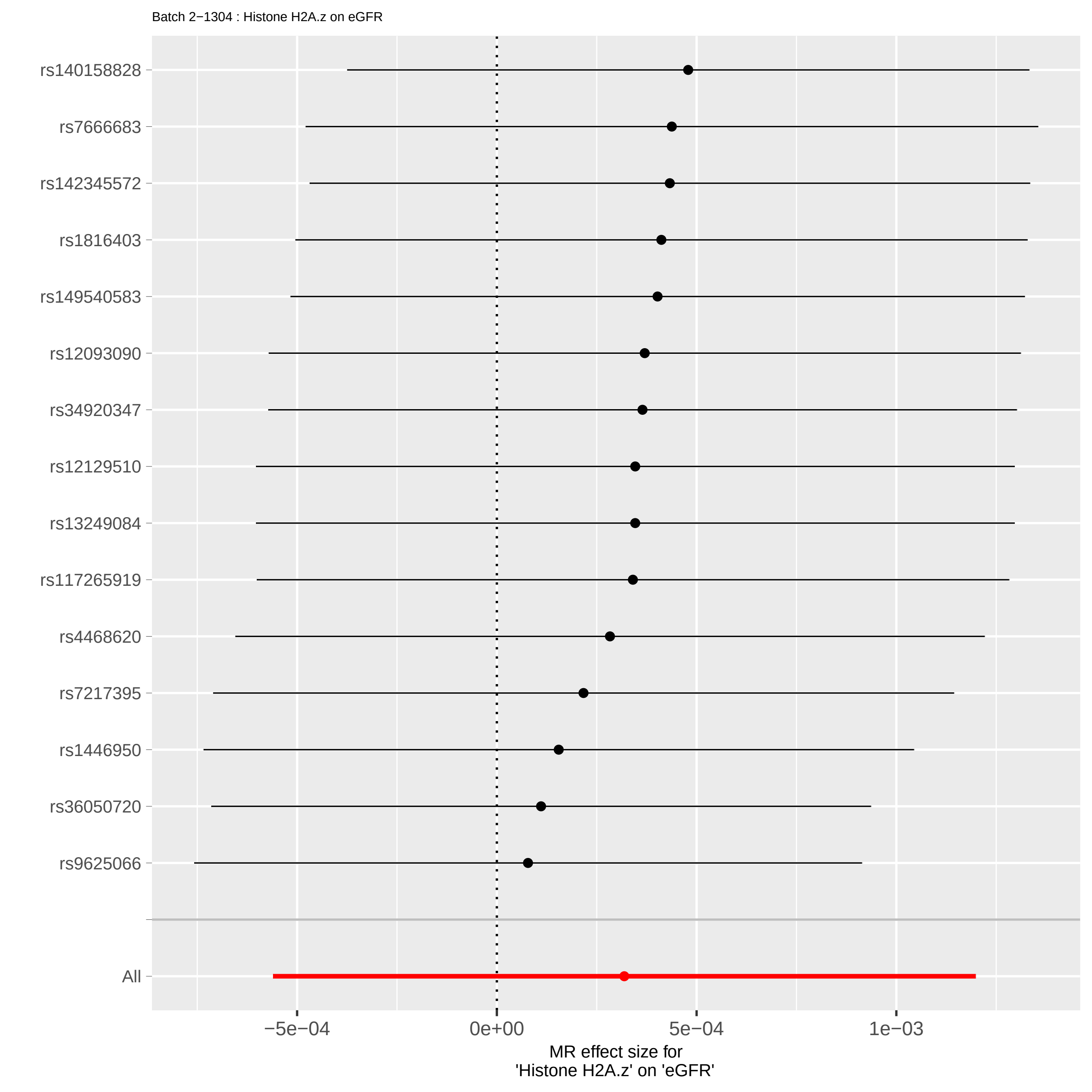

Batch 2−1304 : Histone H2A.z on eGFR
rs140158828
rs7666683
rs142345572
rs1816403
rs149540583
rs12093090
rs34920347
rs12129510
rs13249084
rs117265919
rs4468620
rs7217395
rs1446950
rs36050720
rs9625066
All
−5e−04
0e+00
5e−04
1e−03
MR effect size for
'Histone H2A.z' on 'eGFR'

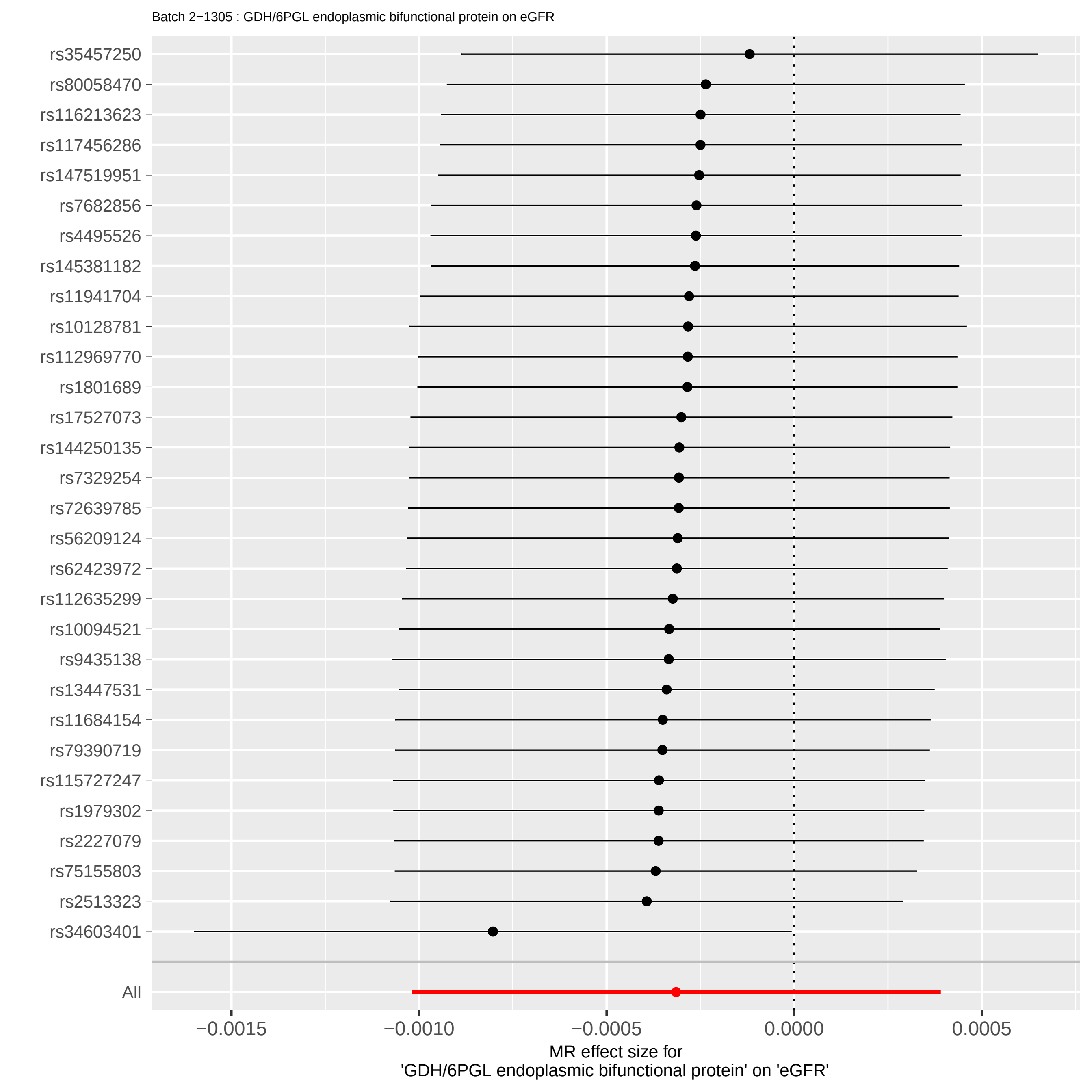

Batch 2−1305 : GDH/6PGL endoplasmic bifunctional protein on eGFR
rs35457250
rs80058470
rs116213623
rs117456286
rs147519951
rs7682856
rs4495526
rs145381182
rs11941704
rs10128781
rs112969770
rs1801689
rs17527073
rs144250135
rs7329254
rs72639785
rs56209124
rs62423972
rs112635299
rs10094521
rs9435138
rs13447531
rs11684154
rs79390719
rs115727247
rs1979302
rs2227079
rs75155803
rs2513323
rs34603401
All
−0.0015
−0.0010
−0.0005
0.0000
0.0005
MR effect size for
'GDH/6PGL endoplasmic bifunctional protein' on 'eGFR'

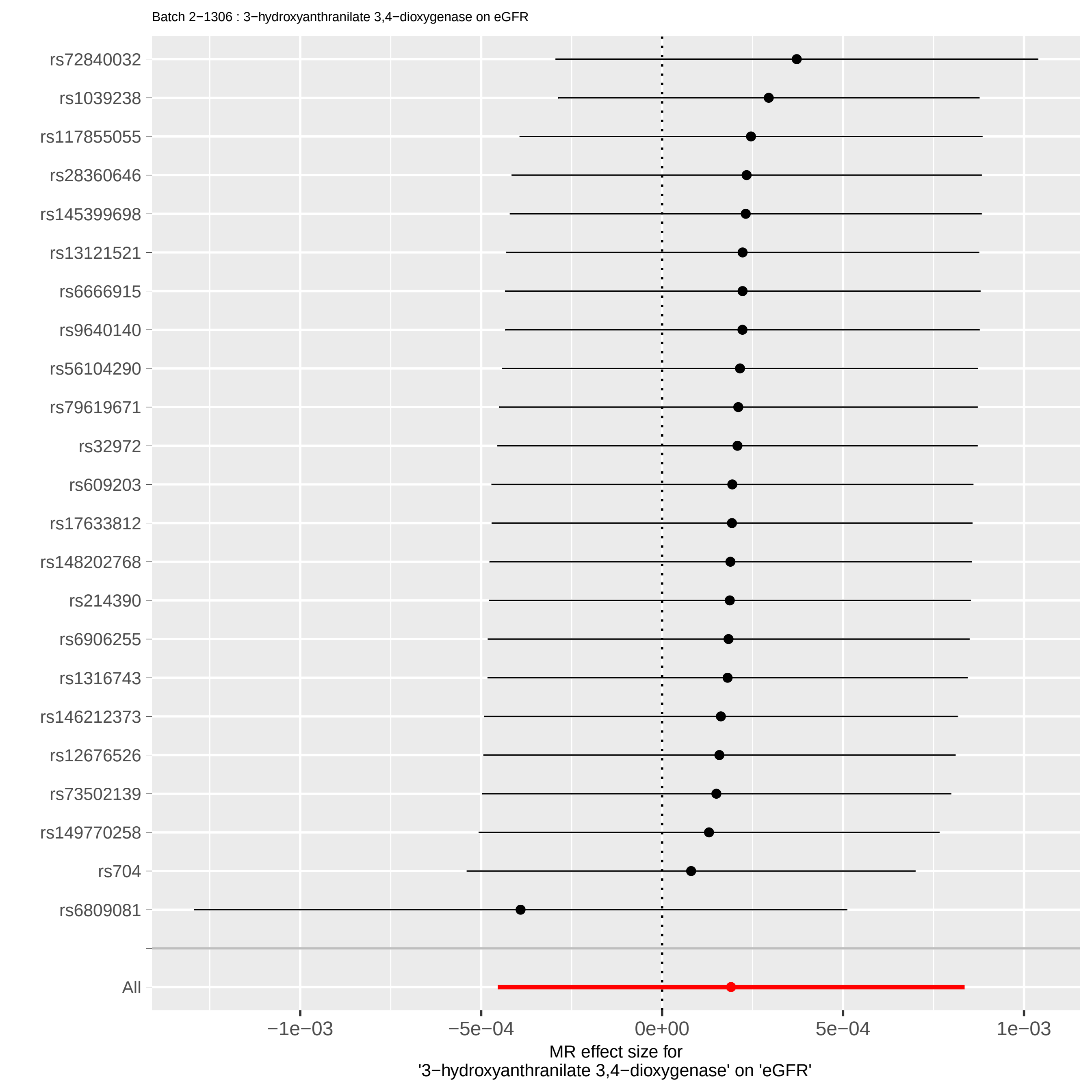

Batch 2−1306 : 3−hydroxyanthranilate 3,4−dioxygenase on eGFR
rs72840032
rs1039238
rs117855055
rs28360646
rs145399698
rs13121521
rs6666915
rs9640140
rs56104290
rs79619671
rs32972
rs609203
rs17633812
rs148202768
rs214390
rs6906255
rs1316743
rs146212373
rs12676526
rs73502139
rs149770258
rs704
rs6809081
All
−1e−03
−5e−04
0e+00
5e−04
1e−03
MR effect size for
'3−hydroxyanthranilate 3,4−dioxygenase' on 'eGFR'

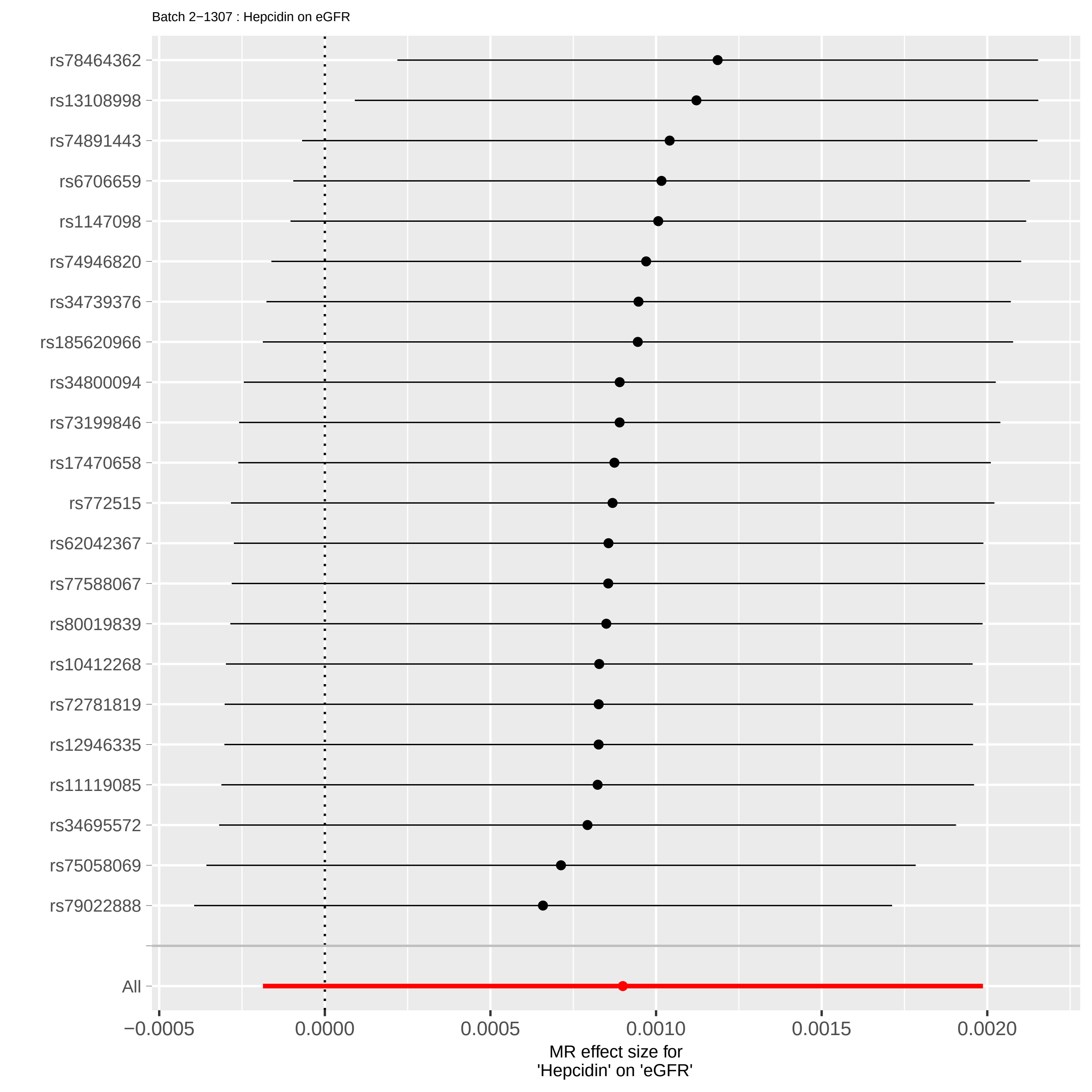
Batch 2−1307 : Hepcidin on eGFR
rs78464362
rs13108998
rs74891443
rs6706659
rs1147098
rs74946820
rs34739376
rs185620966
rs34800094
rs73199846
rs17470658
rs772515
rs62042367
rs77588067
rs80019839
rs10412268
rs72781819
rs12946335
rs11119085
rs34695572
rs75058069
rs79022888
All
−0.0005
0.0000
0.0005
0.0010
0.0015
0.0020
MR effect size for
'Hepcidin' on 'eGFR'

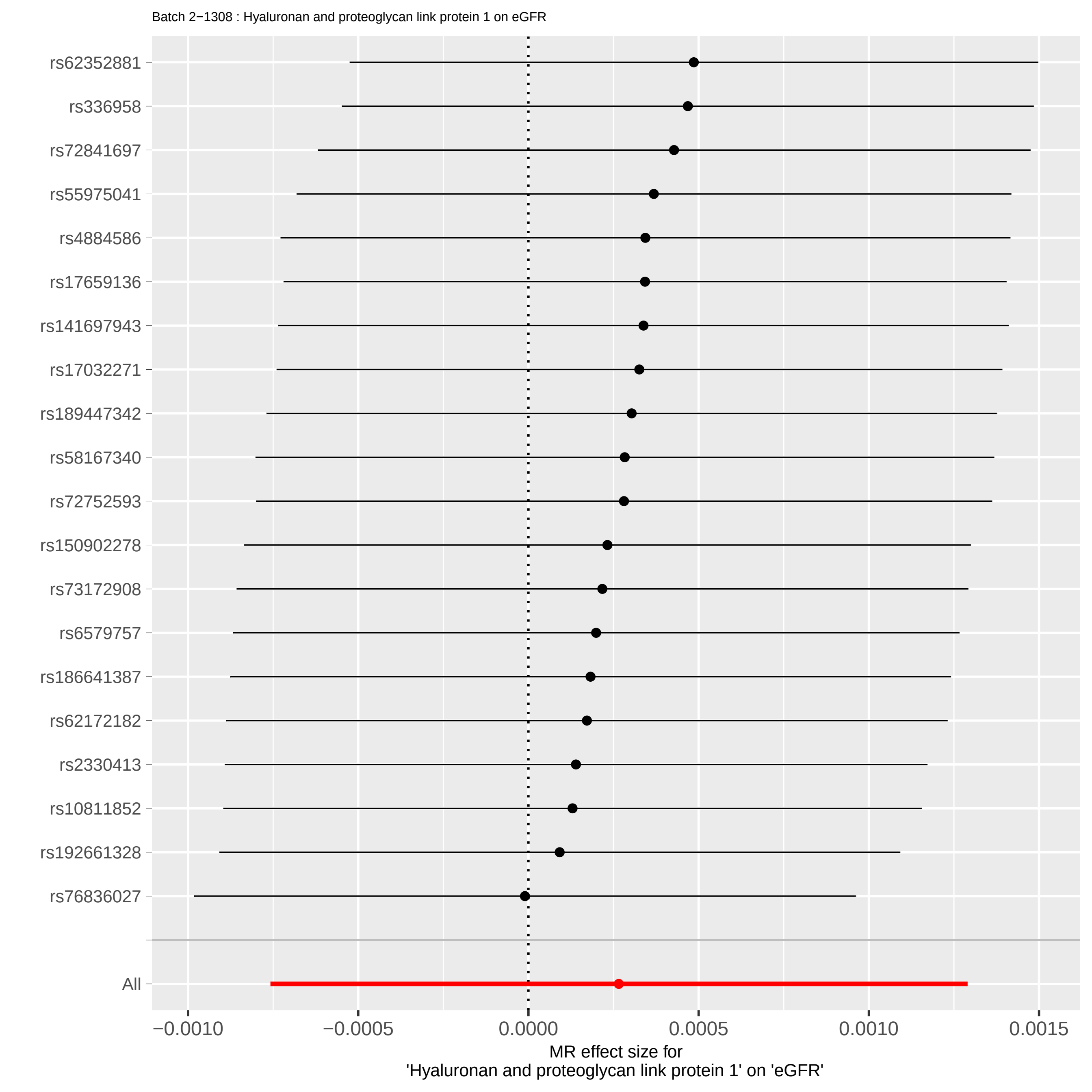
Batch 2−1308 : Hyaluronan and proteoglycan link protein 1 on eGFR
rs62352881
rs336958
rs72841697
rs55975041
rs4884586
rs17659136
rs141697943
rs17032271
rs189447342
rs58167340
rs72752593
rs150902278
rs73172908
rs6579757
rs186641387
rs62172182
rs2330413
rs10811852
rs192661328
rs76836027
All
−0.0010
−0.0005
0.0000
0.0005
0.0010
0.0015
MR effect size for
'Hyaluronan and proteoglycan link protein 1' on 'eGFR'

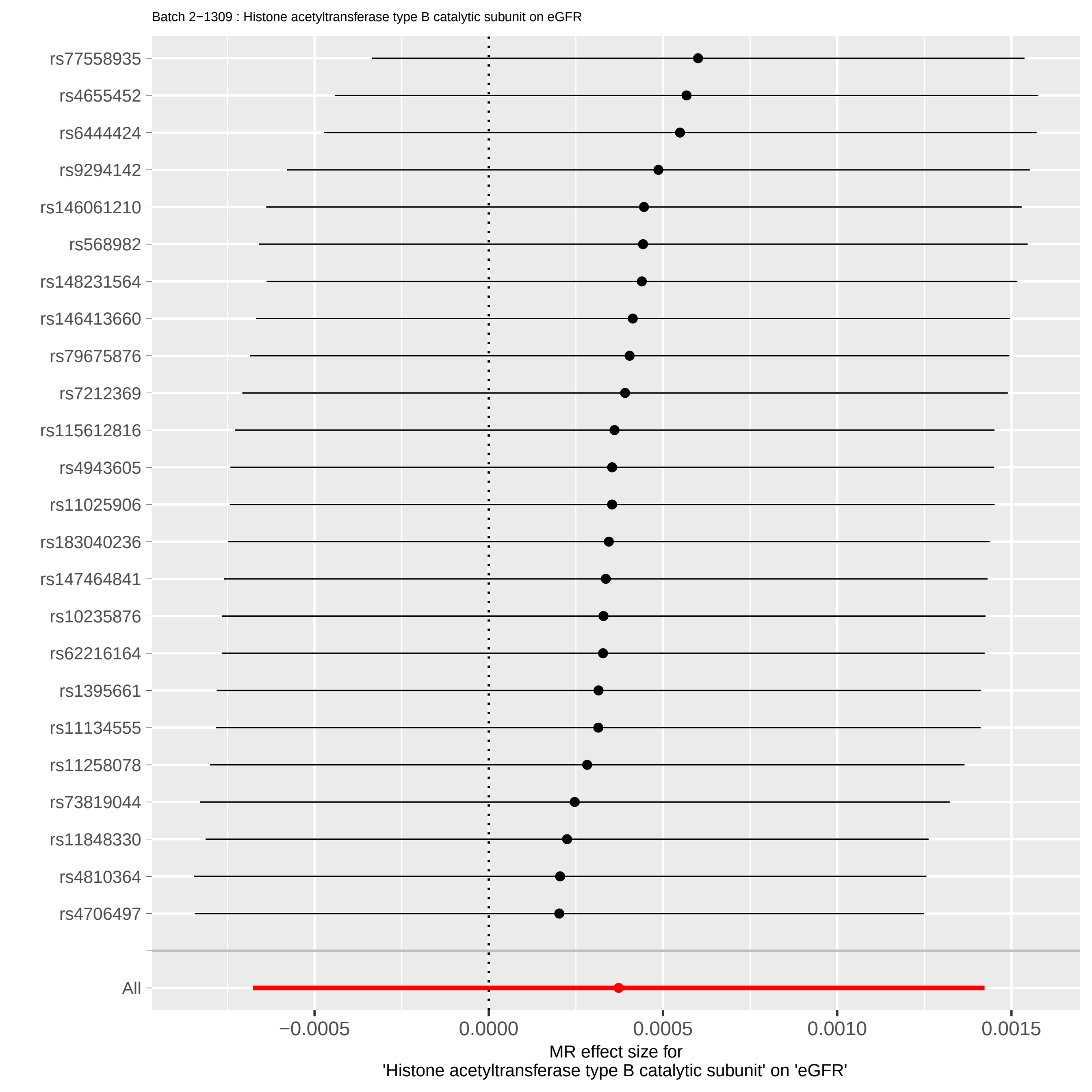
Batch 2−1309 : Histone acetyltransferase type B catalytic subunit on eGFR
rs77558935
rs4655452
rs6444424
rs9294142
rs146061210
rs568982
rs148231564
rs146413660
rs79675876
rs7212369
rs115612816
rs4943605
rs11025906
rs183040236
rs147464841
rs10235876
rs62216164
rs1395661
rs11134555
rs11258078
rs73819044
rs11848330
rs4810364
rs4706497
All
−0.0005
0.0000
0.0005
0.0010
0.0015
MR effect size for
'Histone acetyltransferase type B catalytic subunit' on 'eGFR'

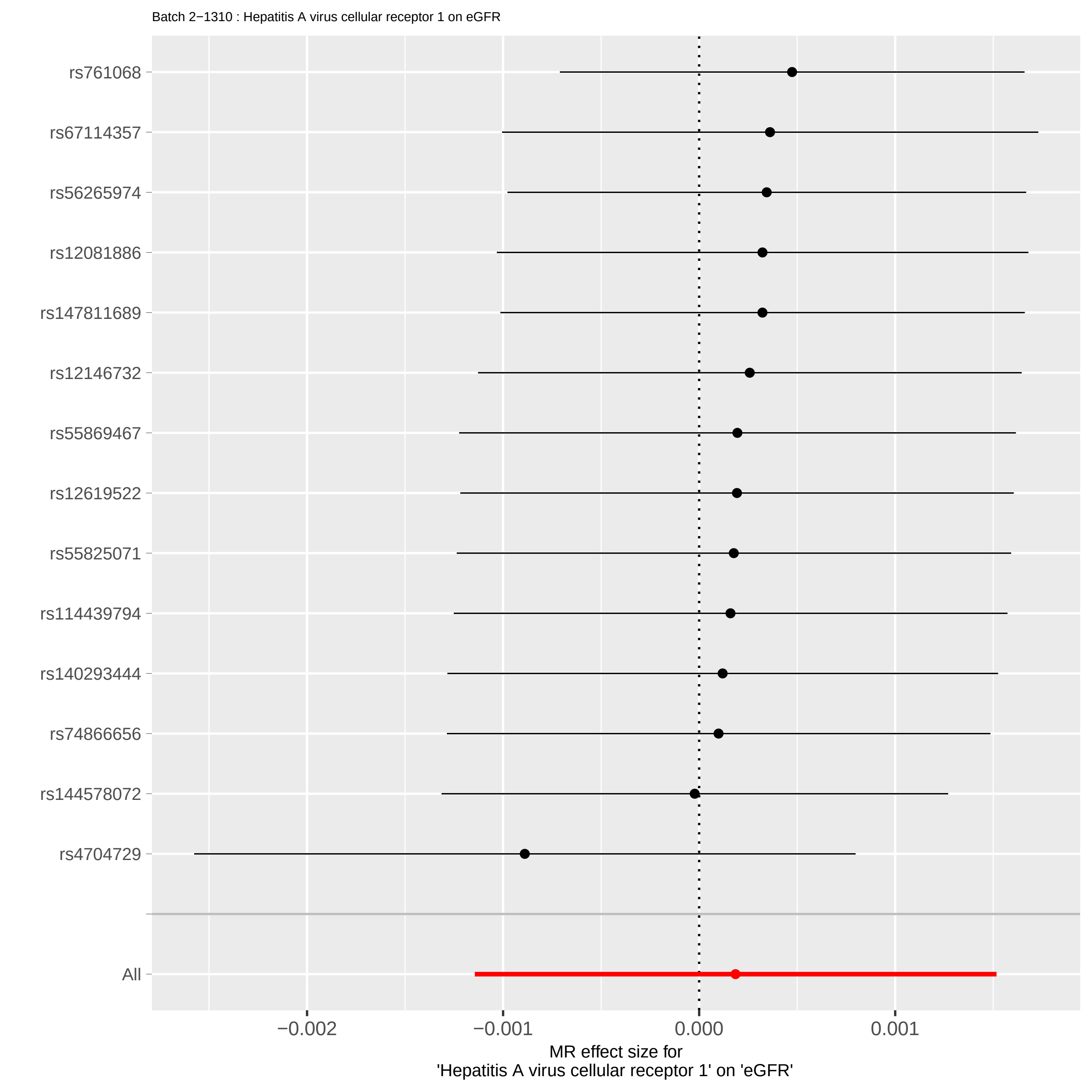
Batch 2−1310 : Hepatitis A virus cellular receptor 1 on eGFR
rs761068
rs67114357
rs56265974
rs12081886
rs147811689
rs12146732
rs55869467
rs12619522
rs55825071
rs114439794
rs140293444
rs74866656
rs144578072
rs4704729
All
−0.002
−0.001
0.000
0.001
MR effect size for
'Hepatitis A virus cellular receptor 1' on 'eGFR'

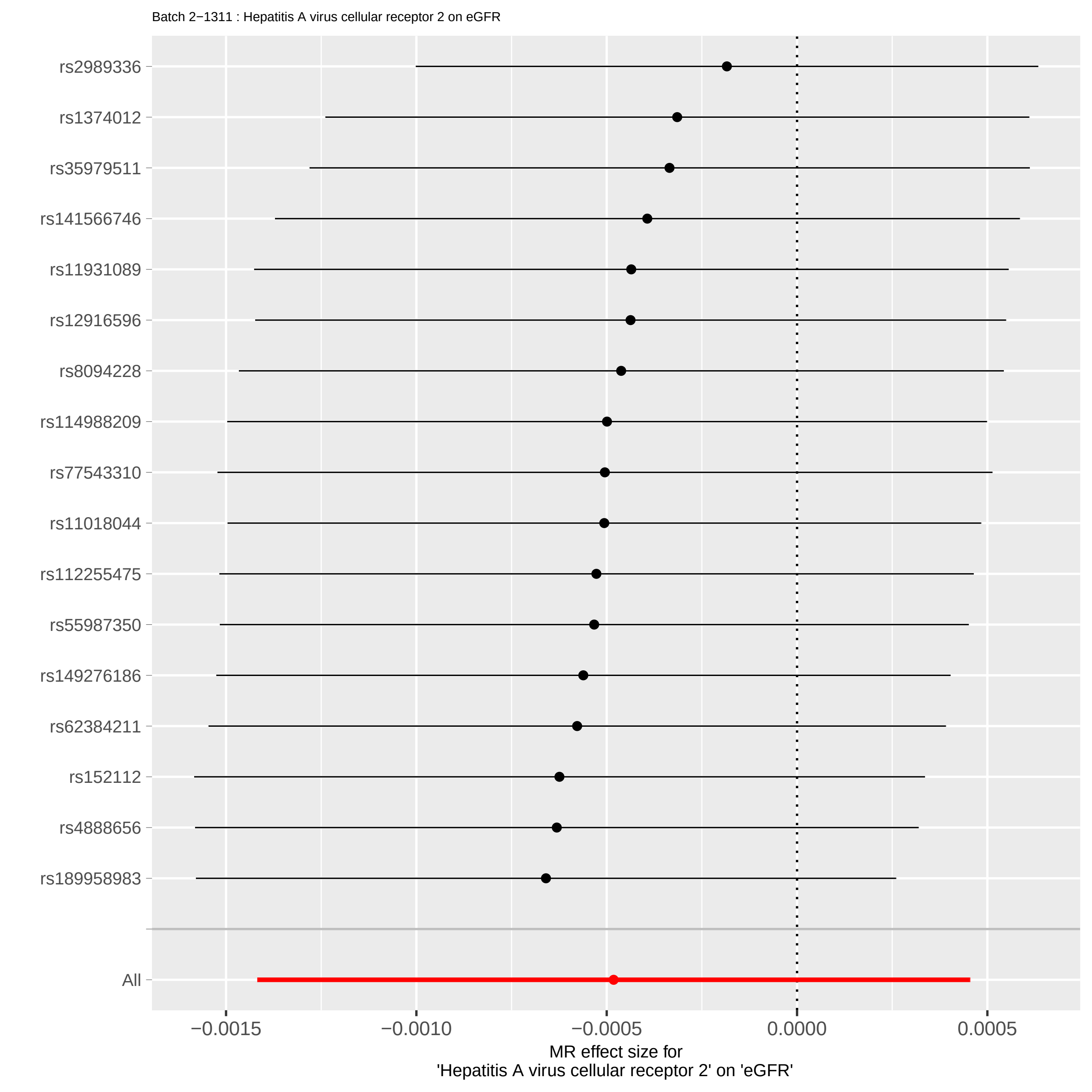
Batch 2−1311 : Hepatitis A virus cellular receptor 2 on eGFR
rs2989336
rs1374012
rs35979511
rs141566746
rs11931089
rs12916596
rs8094228
rs114988209
rs77543310
rs11018044
rs112255475
rs55987350
rs149276186
rs62384211
rs152112
rs4888656
rs189958983
All
−0.0015
−0.0010
−0.0005
0.0000
0.0005
MR effect size for
'Hepatitis A virus cellular receptor 2' on 'eGFR'

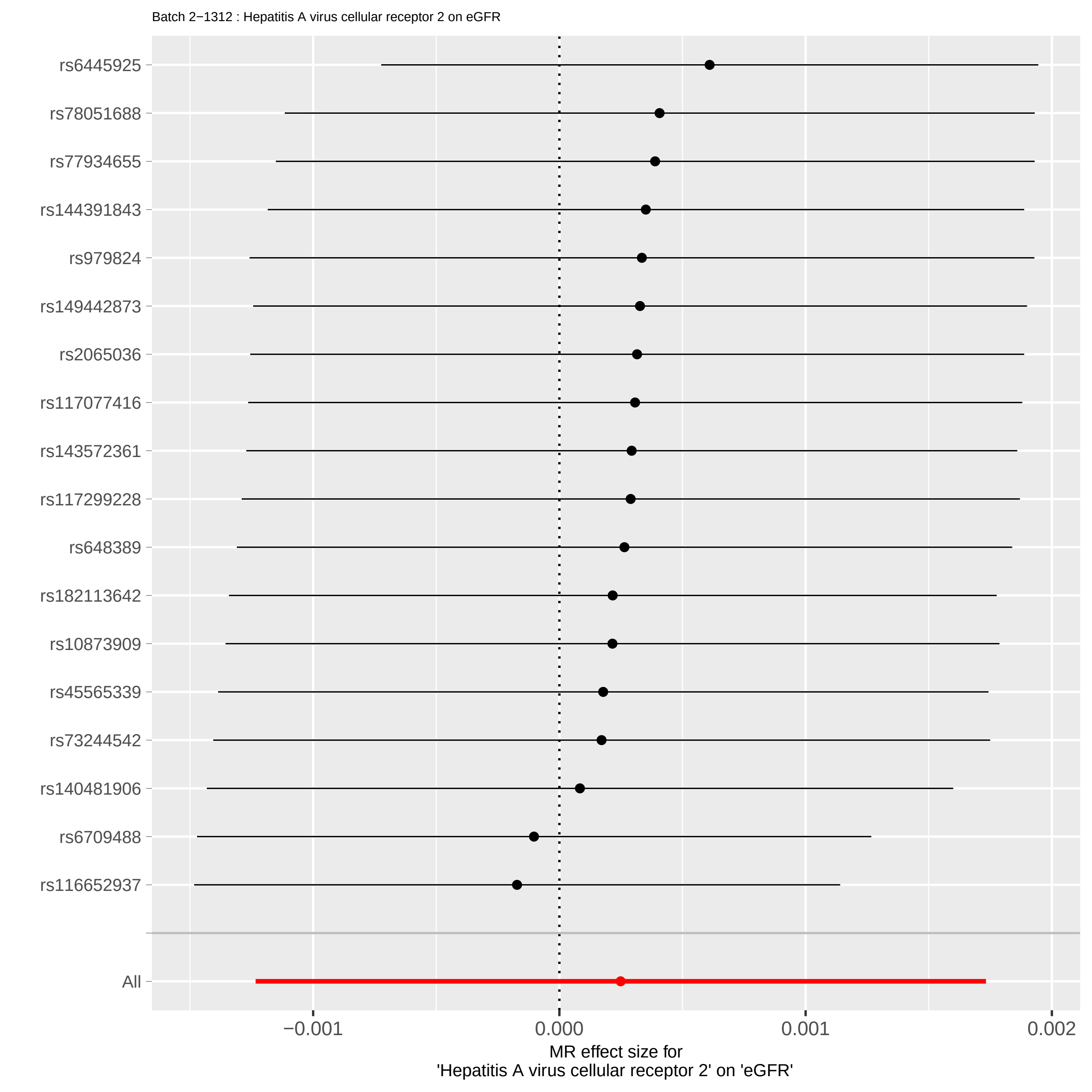

Batch 2−1312 : Hepatitis A virus cellular receptor 2 on eGFR
rs6445925
rs78051688
rs77934655
rs144391843
rs979824
rs149442873
rs2065036
rs117077416
rs143572361
rs117299228
rs648389
rs182113642
rs10873909
rs45565339
rs73244542
rs140481906
rs6709488
rs116652937
All
−0.001
0.000
0.001
0.002
MR effect size for
'Hepatitis A virus cellular receptor 2' on 'eGFR'

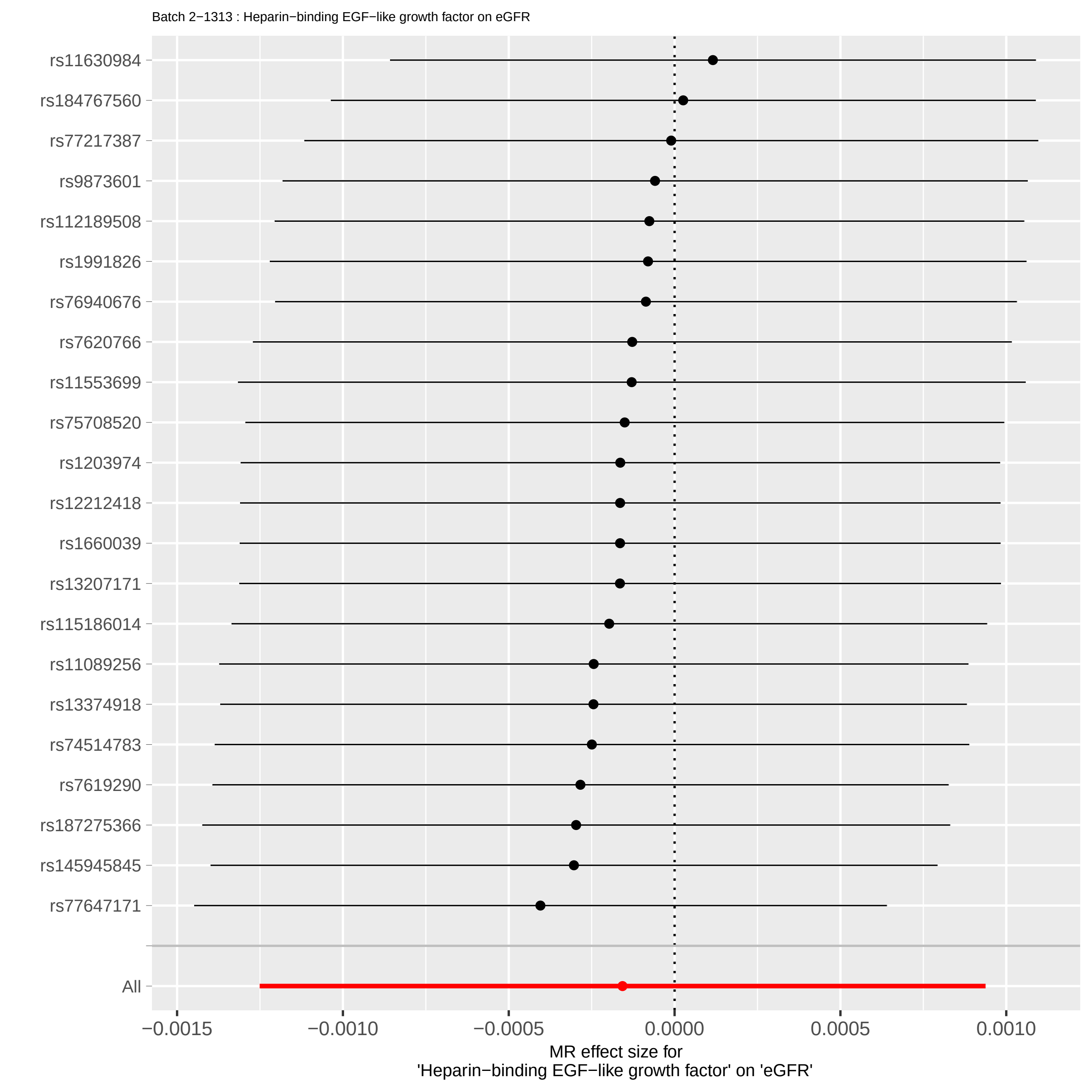

Batch 2−1313 : Heparin−binding EGF−like growth factor on eGFR
rs11630984
rs184767560
rs77217387
rs9873601
rs112189508
rs1991826
rs76940676
rs7620766
rs11553699
rs75708520
rs1203974
rs12212418
rs1660039
rs13207171
rs115186014
rs11089256
rs13374918
rs74514783
rs7619290
rs187275366
rs145945845
rs77647171
All
−0.0015
−0.0010
−0.0005
0.0000
0.0005
0.0010
MR effect size for
'Heparin−binding EGF−like growth factor' on 'eGFR'

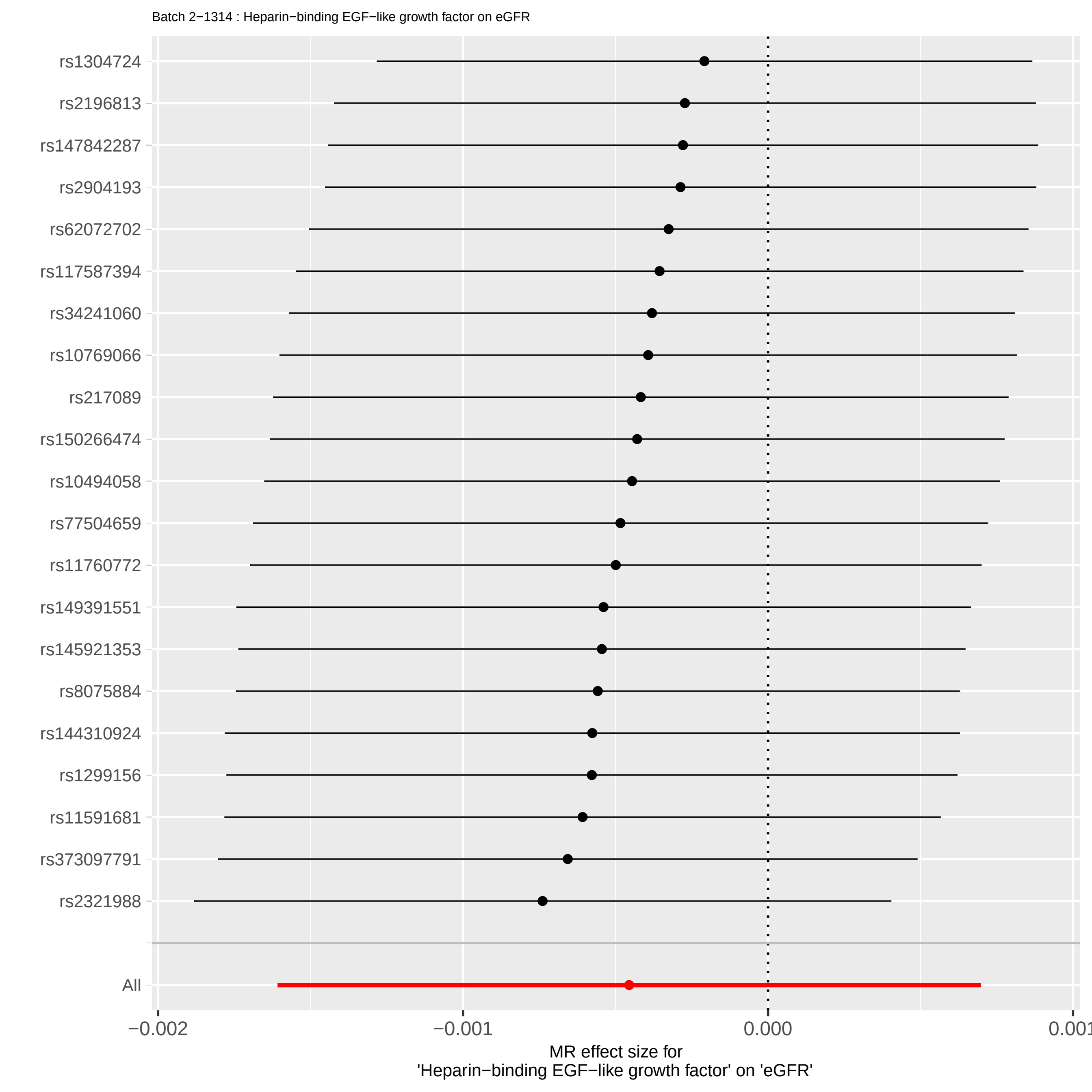

Batch 2−1314 : Heparin−binding EGF−like growth factor on eGFR
rs1304724
rs2196813
rs147842287
rs2904193
rs62072702
rs117587394
rs34241060
rs10769066
rs217089
rs150266474
rs10494058
rs77504659
rs11760772
rs149391551
rs145921353
rs8075884
rs144310924
rs1299156
rs11591681
rs373097791
rs2321988
All
−0.002
−0.001
0.000
0.001
MR effect size for
'Heparin−binding EGF−like growth factor' on 'eGFR'

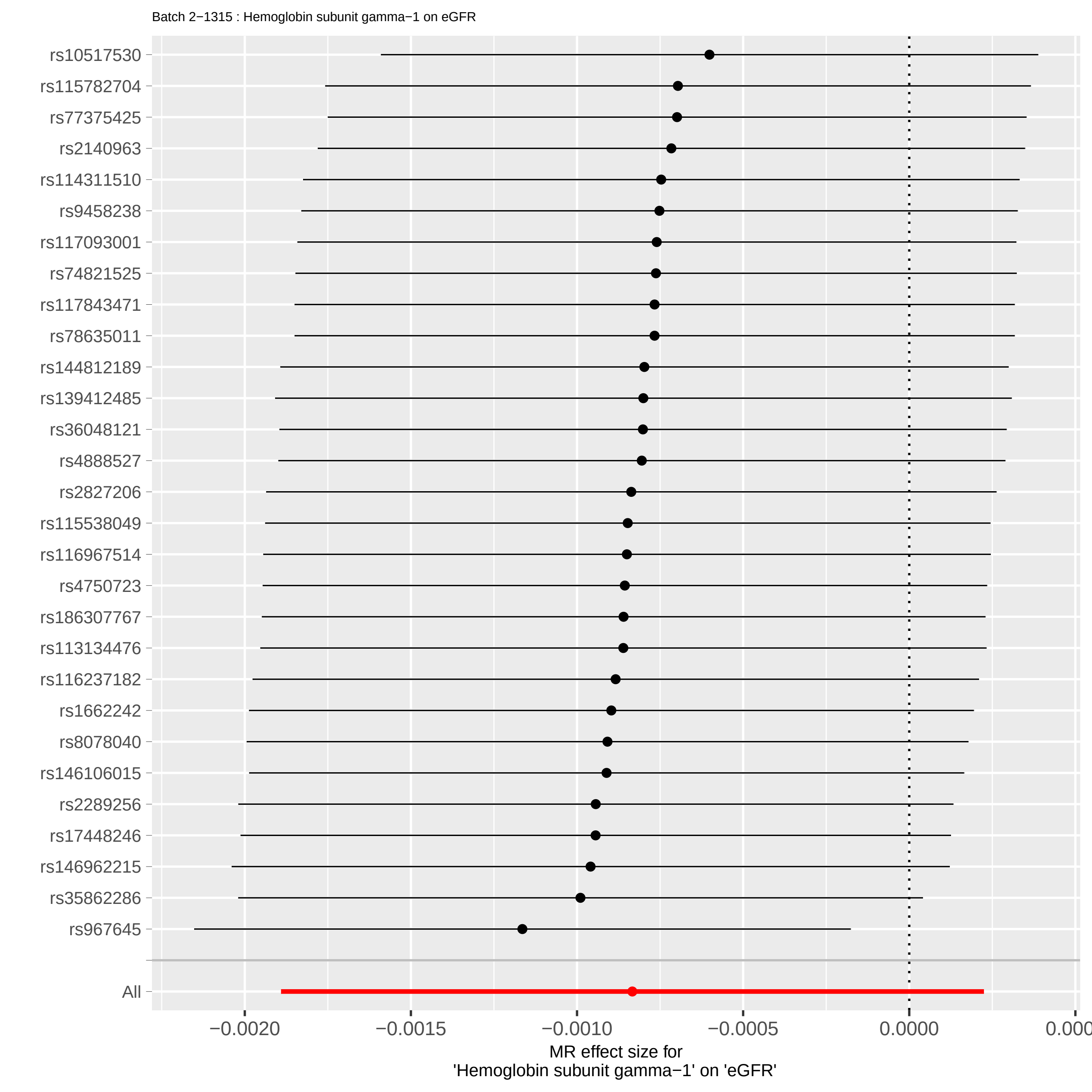

Batch 2−1315 : Hemoglobin subunit gamma−1 on eGFR
rs10517530
rs115782704
rs77375425
rs2140963
rs114311510
rs9458238
rs117093001
rs74821525
rs117843471
rs78635011
rs144812189
rs139412485
rs36048121
rs4888527
rs2827206
rs115538049
rs116967514
rs4750723
rs186307767
rs113134476
rs116237182
rs1662242
rs8078040
rs146106015
rs2289256
rs17448246
rs146962215
rs35862286
rs967645
All
−0.0020
−0.0015
−0.0010
−0.0005
0.0000
MR effect size for
'Hemoglobin subunit gamma−1' on 'eGFR'

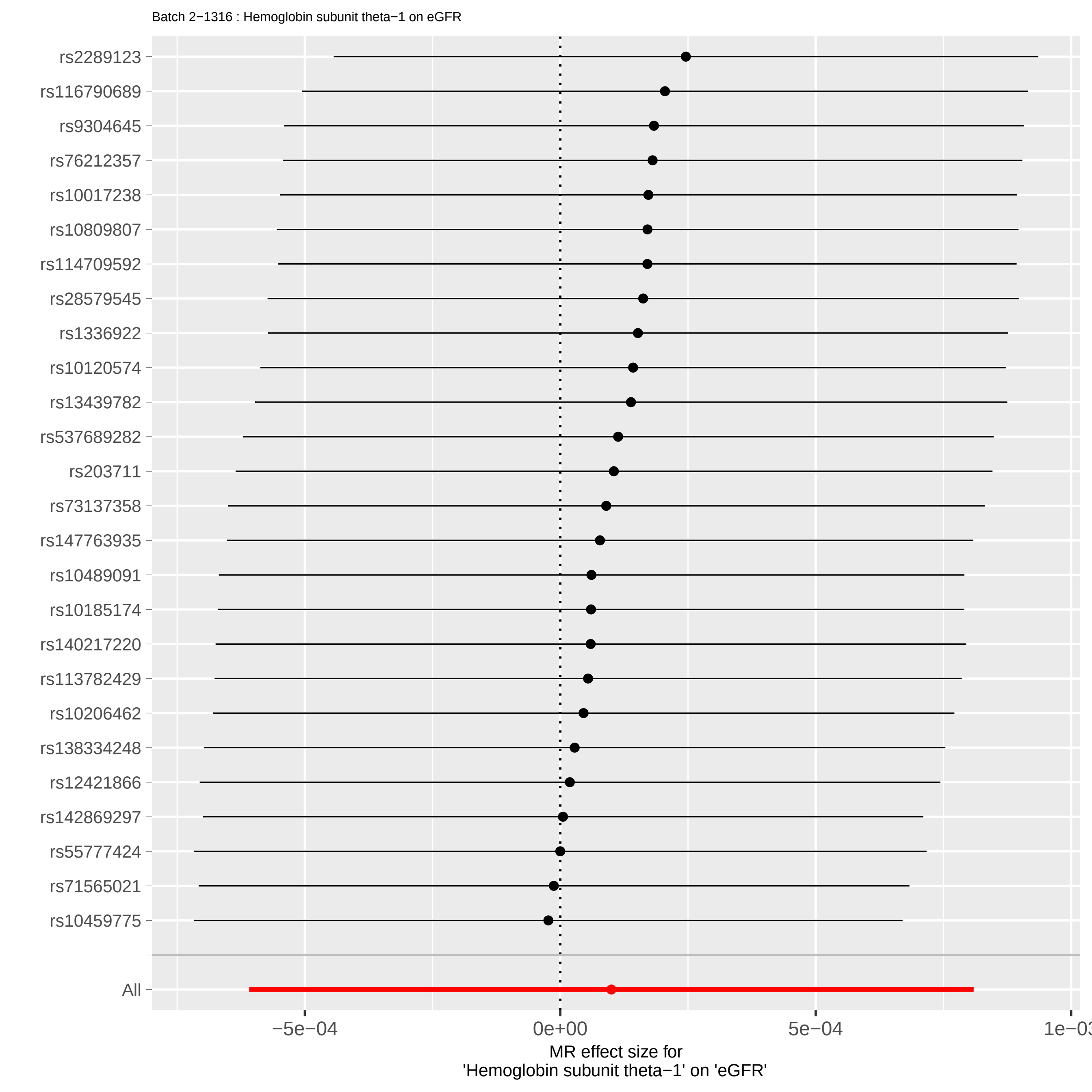
Batch 2−1316 : Hemoglobin subunit theta−1 on eGFR
rs2289123
rs116790689
rs9304645
rs76212357
rs10017238
rs10809807
rs114709592
rs28579545
rs1336922
rs10120574
rs13439782
rs537689282
rs203711
rs73137358
rs147763935
rs10489091
rs10185174
rs140217220
rs113782429
rs10206462
rs138334248
rs12421866
rs142869297
rs55777424
rs71565021
rs10459775
All
−5e−04
0e+00
5e−04
1e−03
MR effect size for
'Hemoglobin subunit theta−1' on 'eGFR'

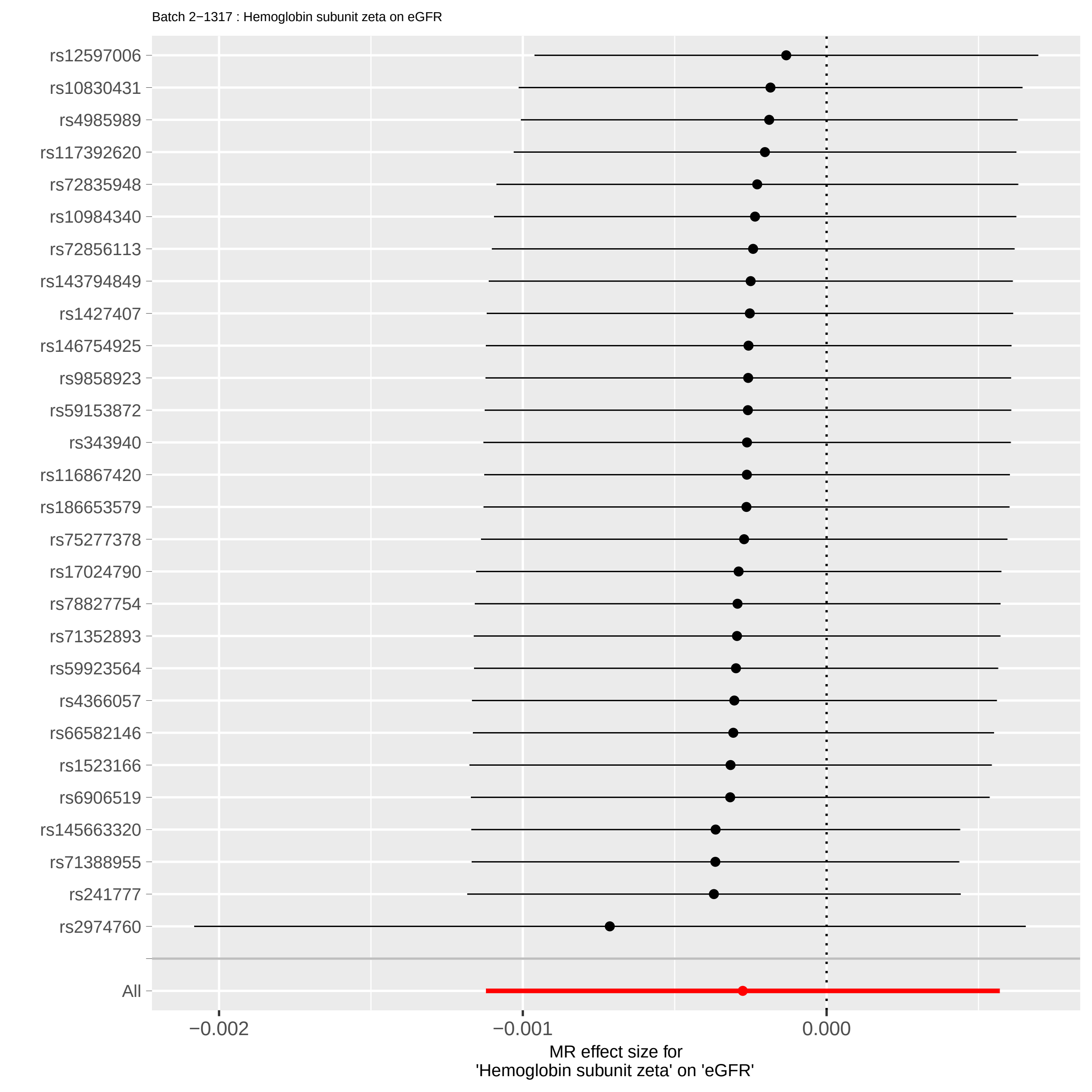

Batch 2−1317 : Hemoglobin subunit zeta on eGFR
rs12597006
rs10830431
rs4985989
rs117392620
rs72835948
rs10984340
rs72856113
rs143794849
rs1427407
rs146754925
rs9858923
rs59153872
rs343940
rs116867420
rs186653579
rs75277378
rs17024790
rs78827754
rs71352893
rs59923564
rs4366057
rs66582146
rs1523166
rs6906519
rs145663320
rs71388955
rs241777
rs2974760
All
−0.002
−0.001
0.000
MR effect size for
'Hemoglobin subunit zeta' on 'eGFR'

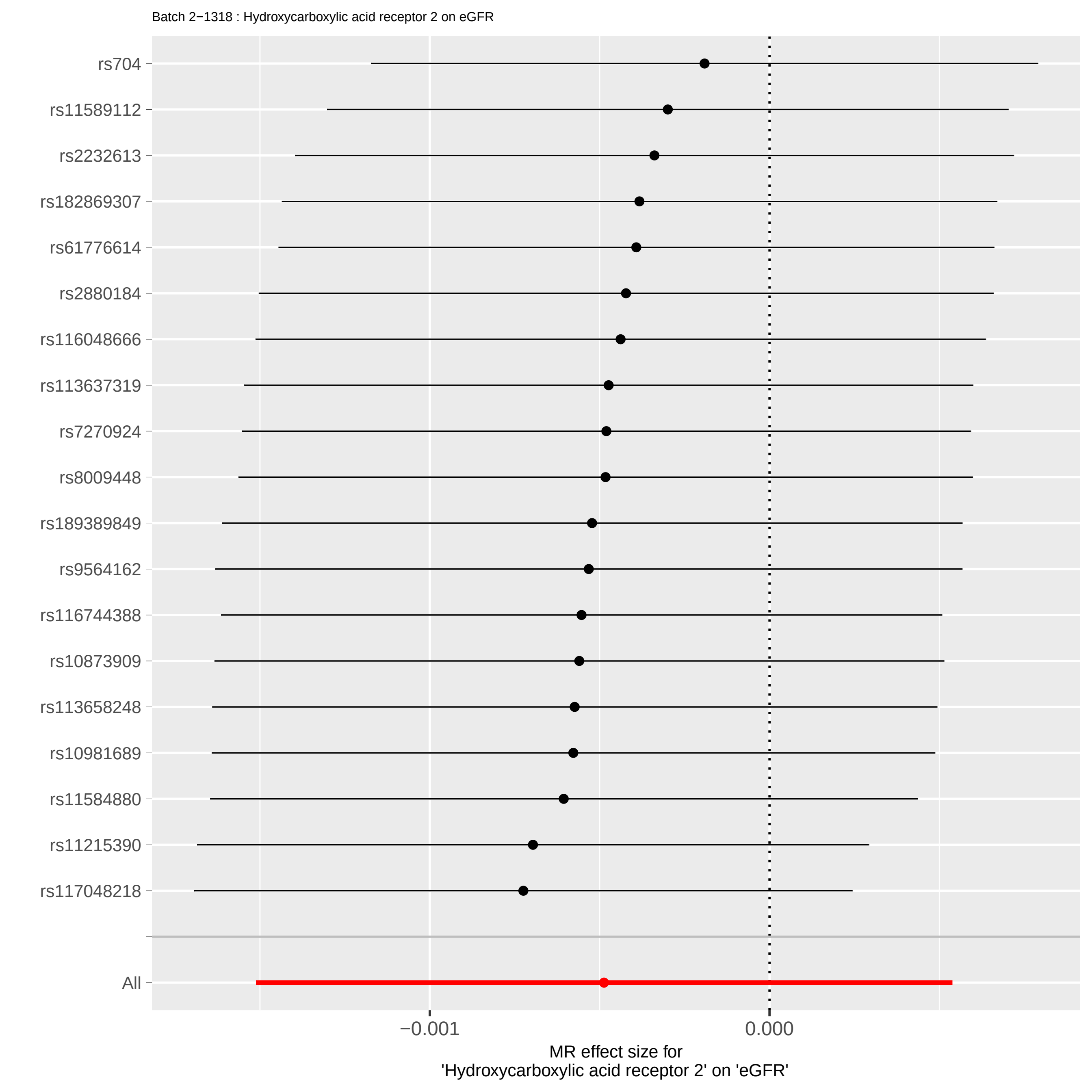
Batch 2−1318 : Hydroxycarboxylic acid receptor 2 on eGFR
rs704
rs11589112
rs2232613
rs182869307
rs61776614
rs2880184
rs116048666
rs113637319
rs7270924
rs8009448
rs189389849
rs9564162
rs116744388
rs10873909
rs113658248
rs10981689
rs11584880
rs11215390
rs117048218
All
−0.001
0.000
MR effect size for
'Hydroxycarboxylic acid receptor 2' on 'eGFR'

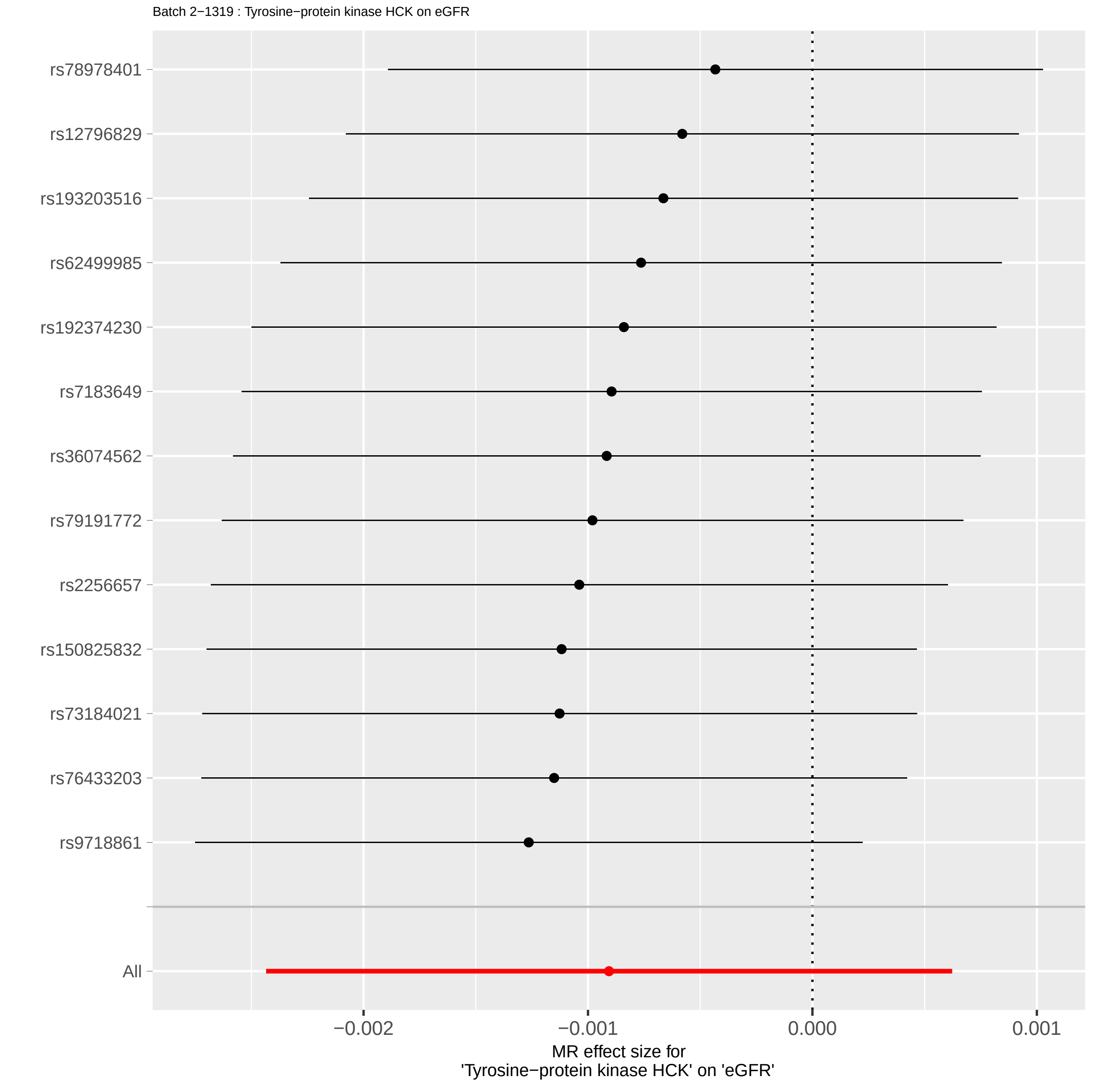

Batch 2−1319 : Tyrosine−protein kinase HCK on eGFR
rs78978401
rs12796829
rs193203516
rs62499985
rs192374230
rs7183649
rs36074562
rs79191772
rs2256657
rs150825832
rs73184021
rs76433203
rs9718861
All
−0.002
−0.001
0.000
0.001
MR effect size for
'Tyrosine−protein kinase HCK' on 'eGFR'

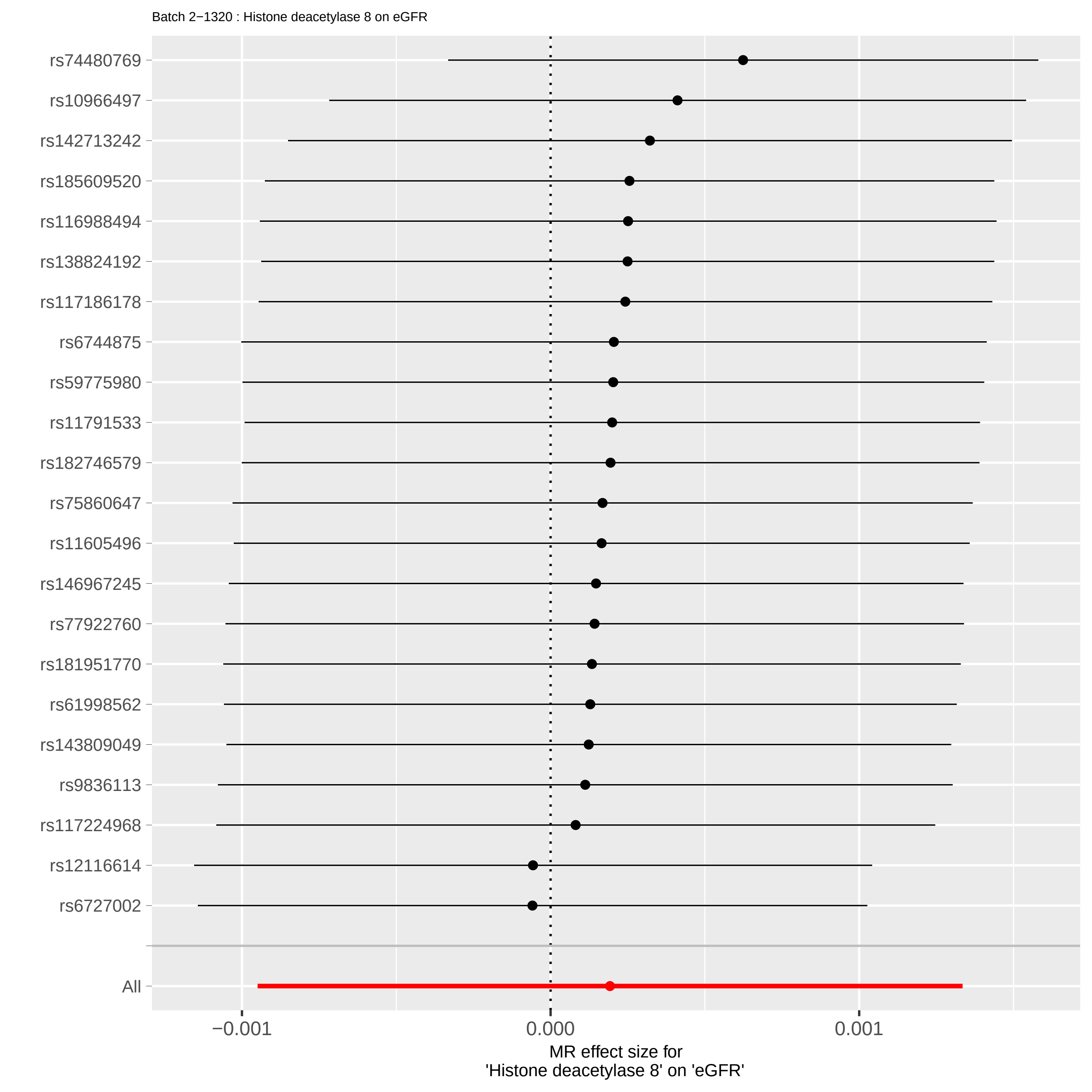

Batch 2−1320 : Histone deacetylase 8 on eGFR
rs74480769
rs10966497
rs142713242
rs185609520
rs116988494
rs138824192
rs117186178
rs6744875
rs59775980
rs11791533
rs182746579
rs75860647
rs11605496
rs146967245
rs77922760
rs181951770
rs61998562
rs143809049
rs9836113
rs117224968
rs12116614
rs6727002
All
−0.001
0.000
0.001
MR effect size for
'Histone deacetylase 8' on 'eGFR'

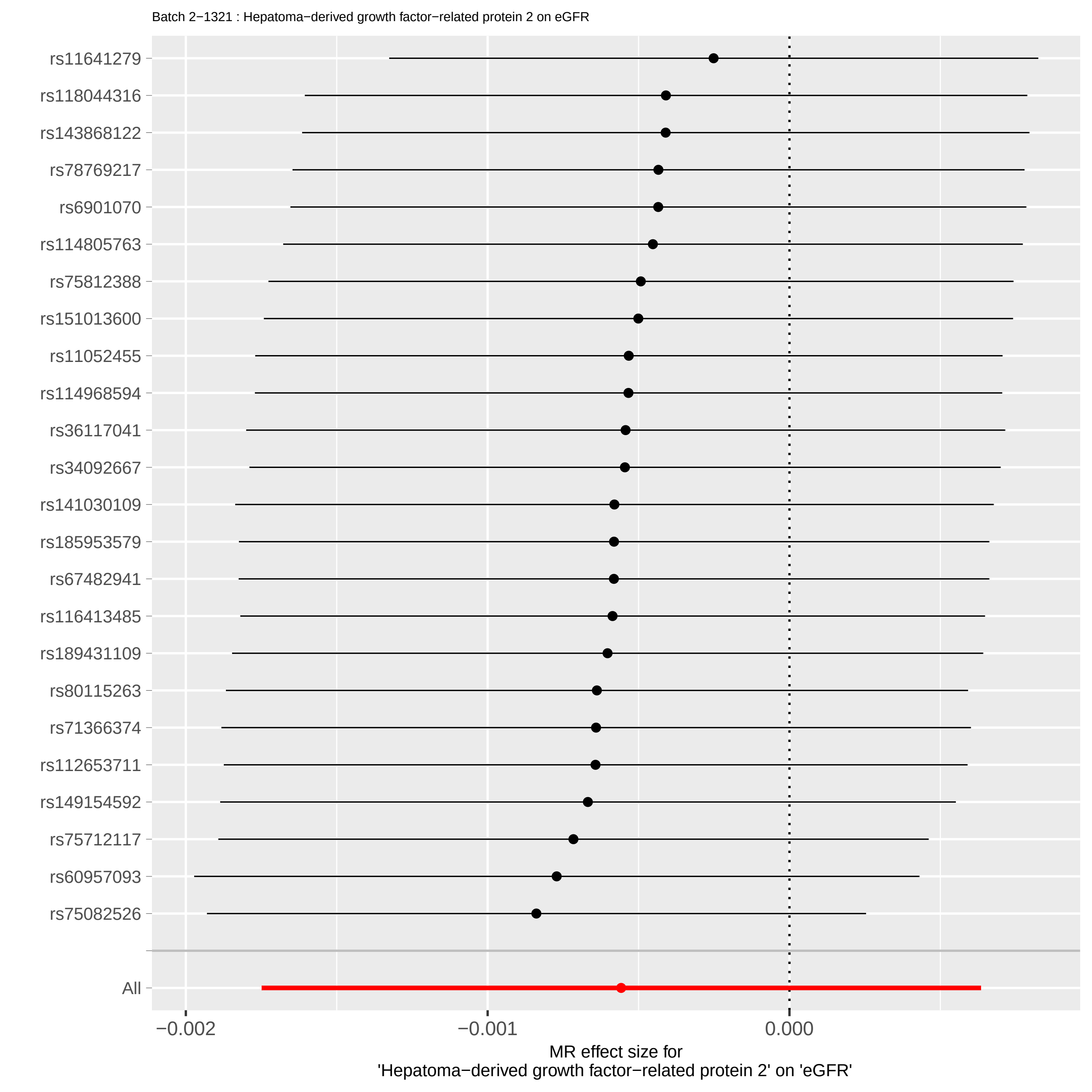

Batch 2−1321 : Hepatoma−derived growth factor−related protein 2 on eGFR
rs11641279
rs118044316
rs143868122
rs78769217
rs6901070
rs114805763
rs75812388
rs151013600
rs11052455
rs114968594
rs36117041
rs34092667
rs141030109
rs185953579
rs67482941
rs116413485
rs189431109
rs80115263
rs71366374
rs112653711
rs149154592
rs75712117
rs60957093
rs75082526
All
−0.002
−0.001
0.000
MR effect size for
'Hepatoma−derived growth factor−related protein 2' on 'eGFR'

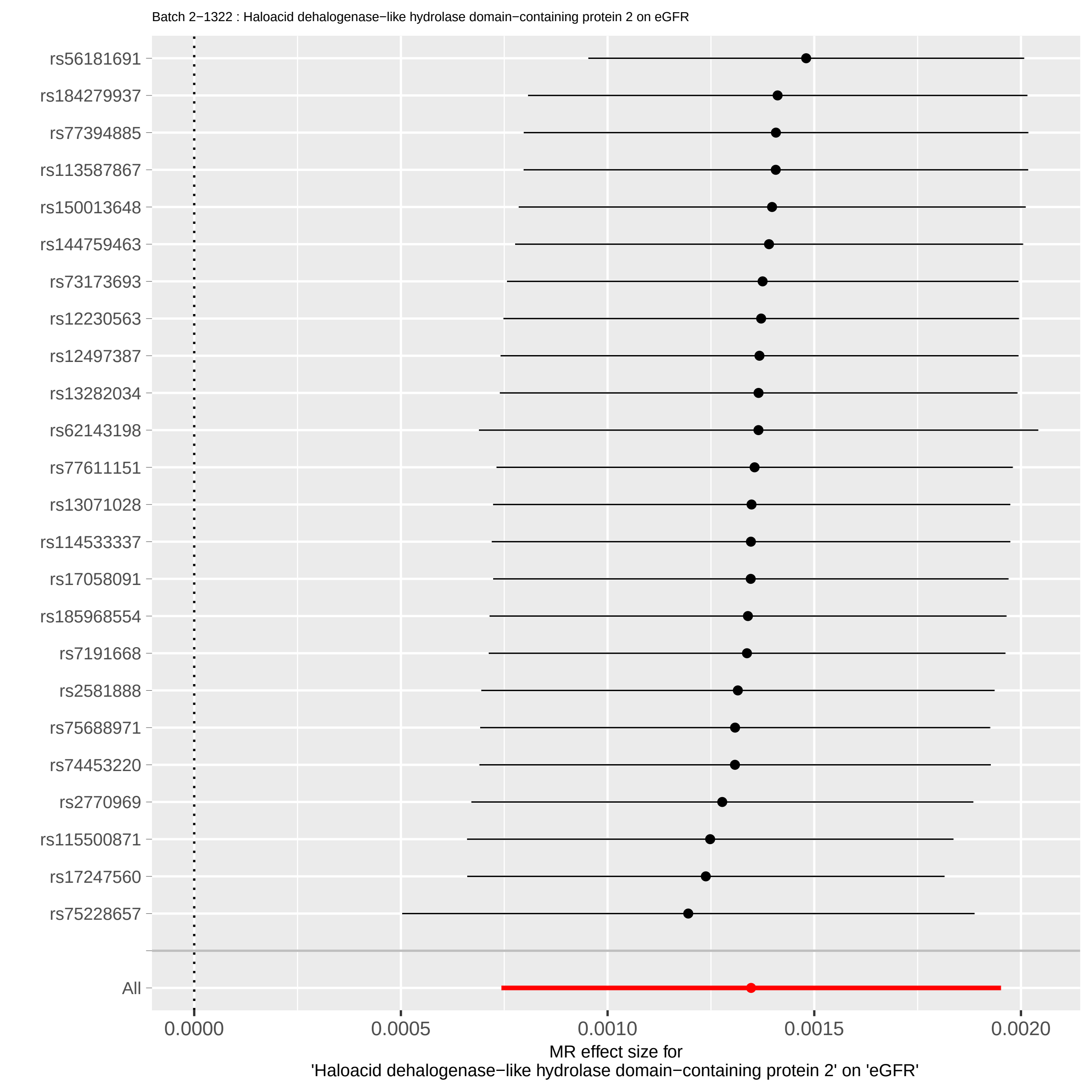
Batch 2−1322 : Haloacid dehalogenase−like hydrolase domain−containing protein 2 on eGFR
rs56181691
rs184279937
rs77394885
rs113587867
rs150013648
rs144759463
rs73173693
rs12230563
rs12497387
rs13282034
rs62143198
rs77611151
rs13071028
rs114533337
rs17058091
rs185968554
rs7191668
rs2581888
rs75688971
rs74453220
rs2770969
rs115500871
rs17247560
rs75228657
All
0.0000
0.0005
0.0010
0.0015
0.0020
MR effect size for
'Haloacid dehalogenase−like hydrolase domain−containing protein 2' on 'eGFR'

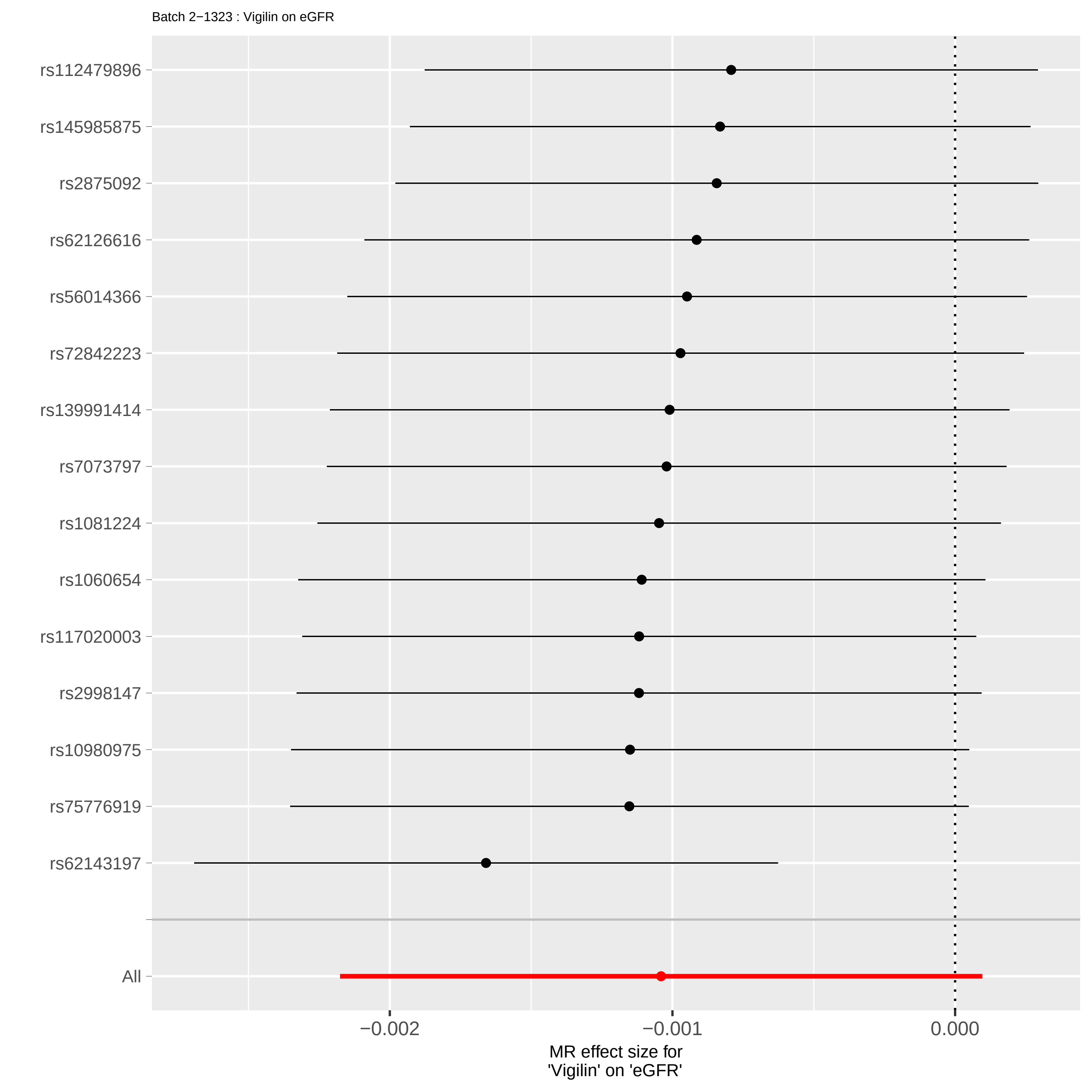

Batch 2−1323 : Vigilin on eGFR
rs112479896
rs145985875
rs2875092
rs62126616
rs56014366
rs72842223
rs139991414
rs7073797
rs1081224
rs1060654
rs117020003
rs2998147
rs10980975
rs75776919
rs62143197
All
−0.002
−0.001
0.000
MR effect size for
'Vigilin' on 'eGFR'

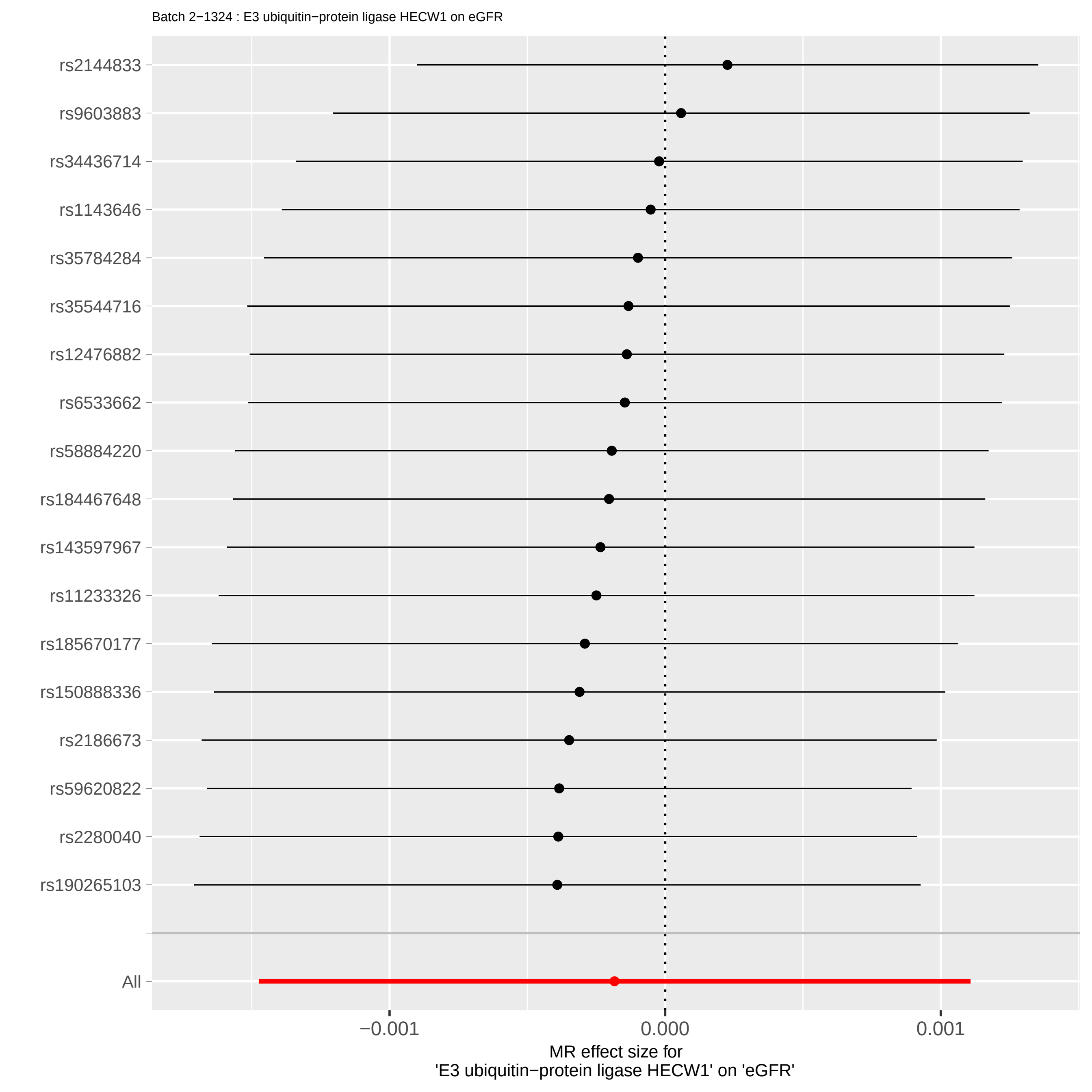

Batch 2−1324 : E3 ubiquitin−protein ligase HECW1 on eGFR
rs2144833
rs9603883
rs34436714
rs1143646
rs35784284
rs35544716
rs12476882
rs6533662
rs58884220
rs184467648
rs143597967
rs11233326
rs185670177
rs150888336
rs2186673
rs59620822
rs2280040
rs190265103
All
−0.001
0.000
0.001
MR effect size for
'E3 ubiquitin−protein ligase HECW1' on 'eGFR'

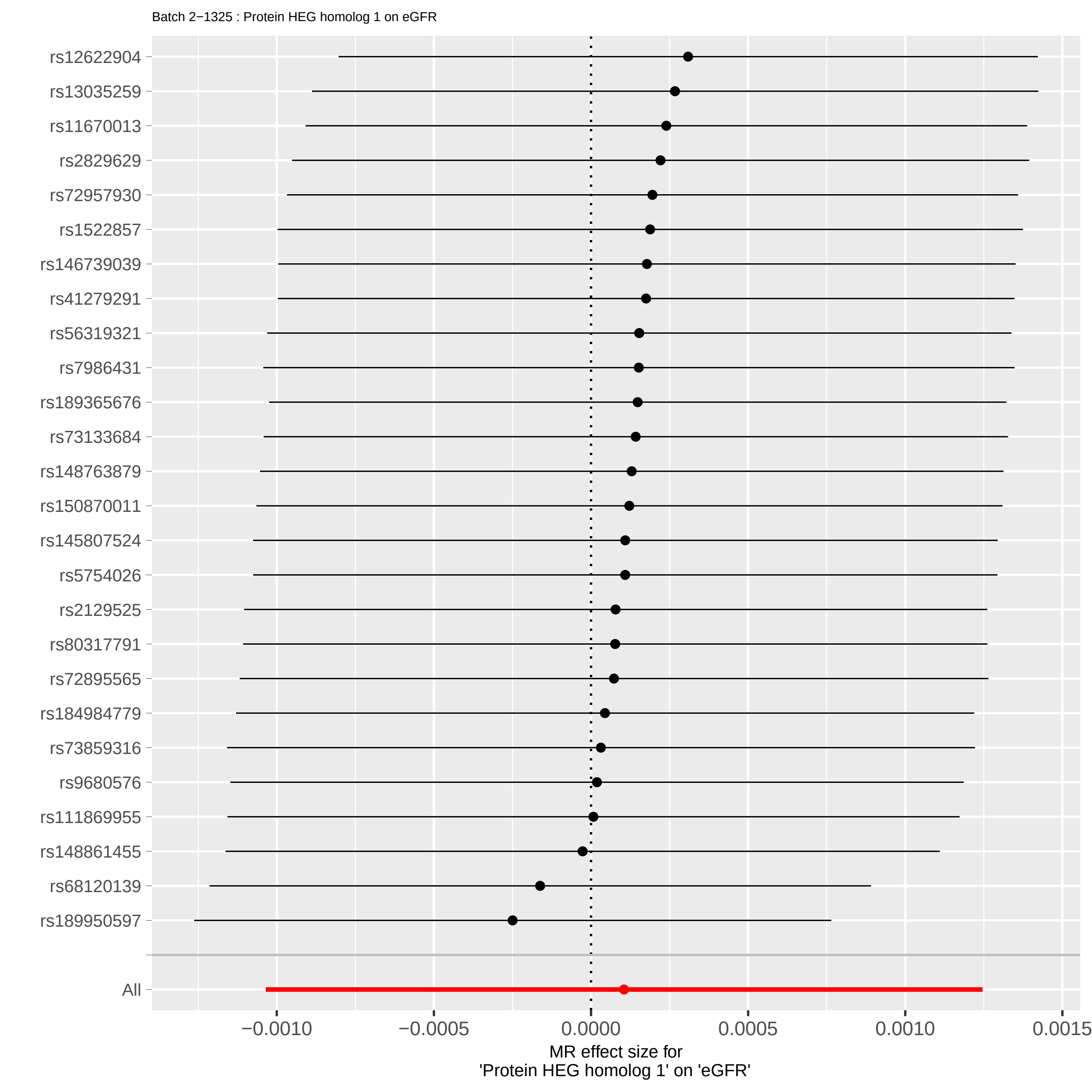
Batch 2−1325 : Protein HEG homolog 1 on eGFR
rs12622904
rs13035259
rs11670013
rs2829629
rs72957930
rs1522857
rs146739039
rs41279291
rs56319321
rs7986431
rs189365676
rs73133684
rs148763879
rs150870011
rs145807524
rs5754026
rs2129525
rs80317791
rs72895565
rs184984779
rs73859316
rs9680576
rs111869955
rs148861455
rs68120139
rs189950597
All
−0.0010
−0.0005
0.0000
0.0005
0.0010
0.0015
MR effect size for
'Protein HEG homolog 1' on 'eGFR'

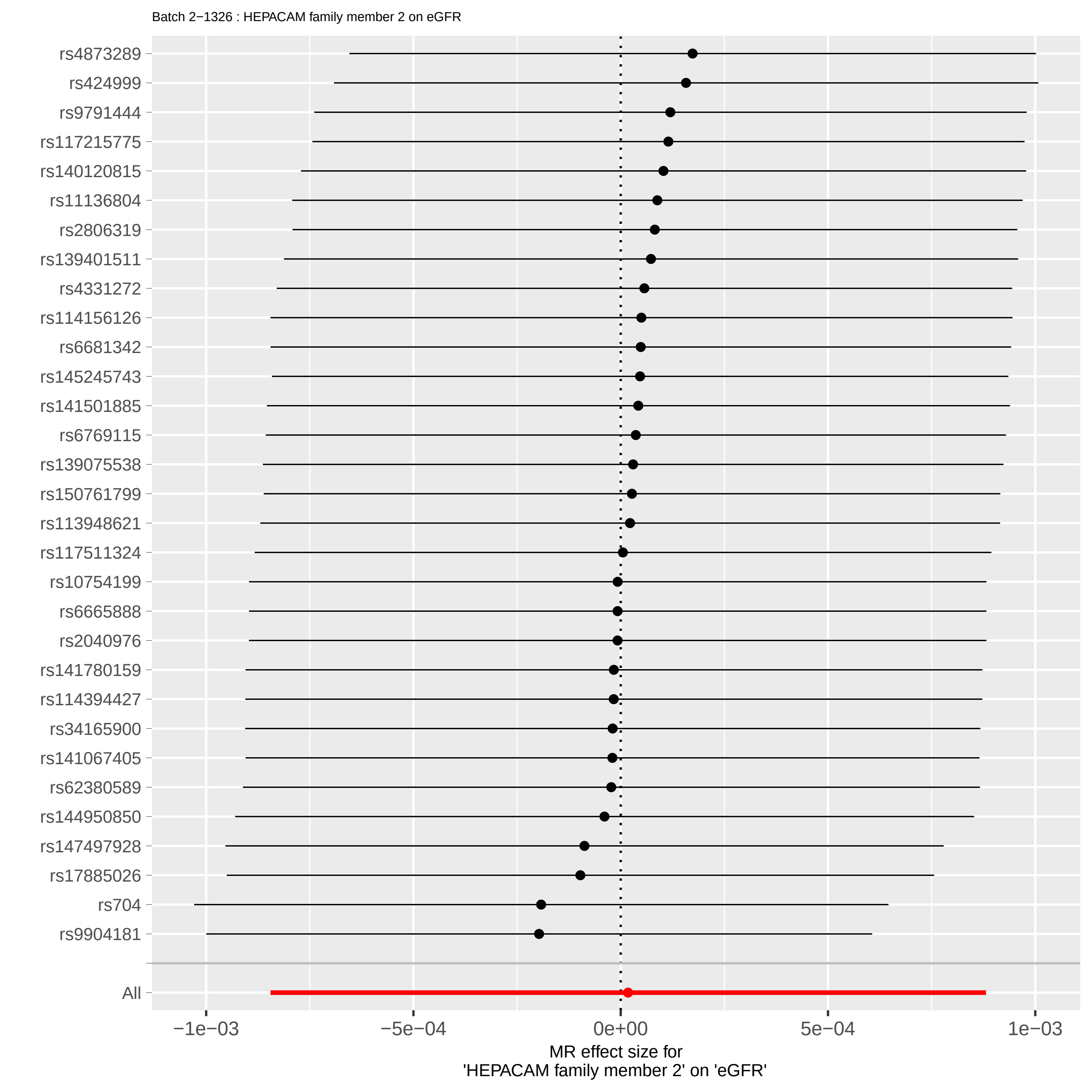

Batch 2−1326 : HEPACAM family member 2 on eGFR
rs4873289
rs424999
rs9791444
rs117215775
rs140120815
rs11136804
rs2806319
rs139401511
rs4331272
rs114156126
rs6681342
rs145245743
rs141501885
rs6769115
rs139075538
rs150761799
rs113948621
rs117511324
rs10754199
rs6665888
rs2040976
rs141780159
rs114394427
rs34165900
rs141067405
rs62380589
rs144950850
rs147497928
rs17885026
rs704
rs9904181
All
−1e−03
−5e−04
0e+00
5e−04
1e−03
MR effect size for
'HEPACAM family member 2' on 'eGFR'

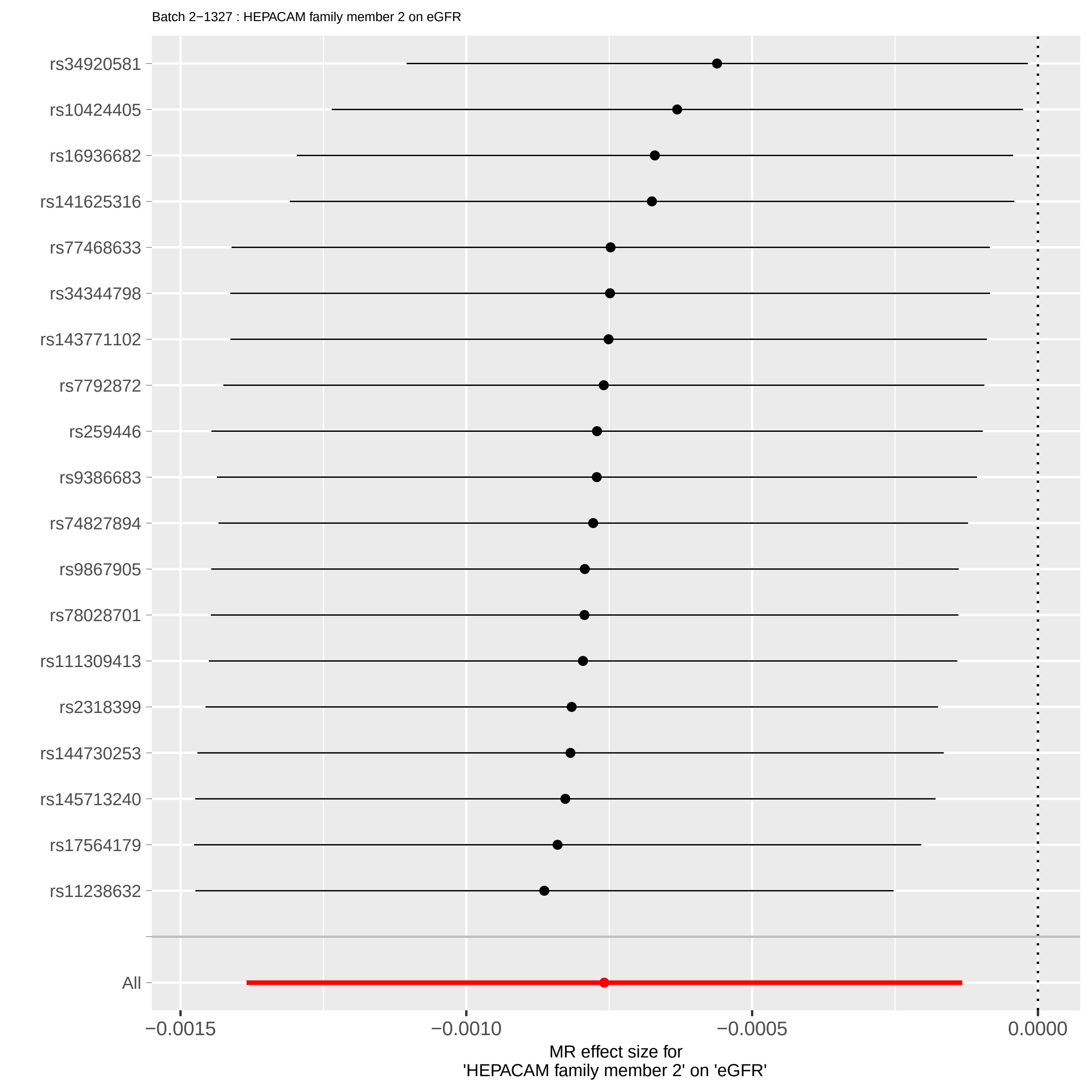

Batch 2−1327 : HEPACAM family member 2 on eGFR
rs34920581
rs10424405
rs16936682
rs141625316
rs77468633
rs34344798
rs143771102
rs7792872
rs259446
rs9386683
rs74827894
rs9867905
rs78028701
rs111309413
rs2318399
rs144730253
rs145713240
rs17564179
rs11238632
All
−0.0015
−0.0010
−0.0005
0.0000
MR effect size for
'HEPACAM family member 2' on 'eGFR'

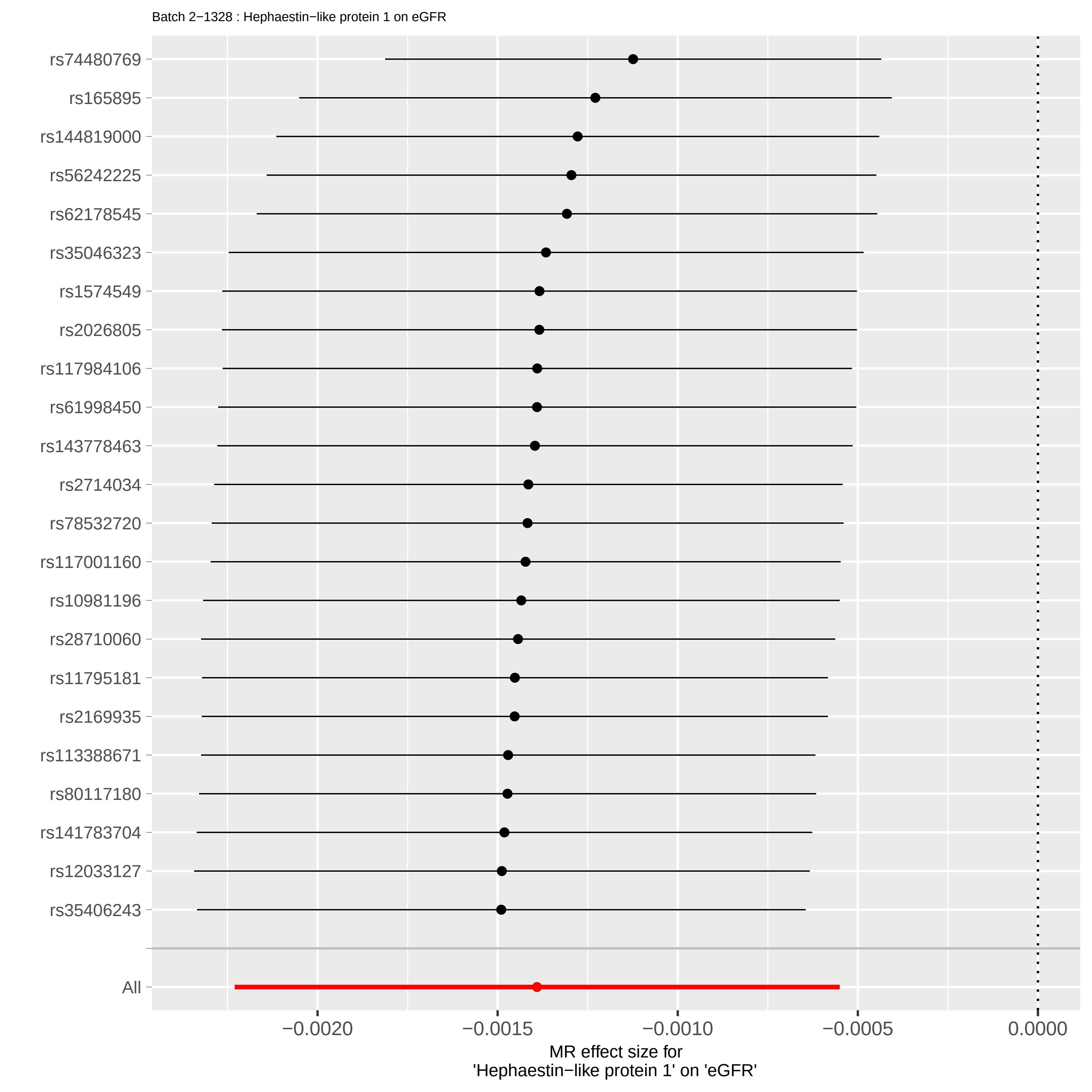

Batch 2−1328 : Hephaestin−like protein 1 on eGFR
rs74480769
rs165895
rs144819000
rs56242225
rs62178545
rs35046323
rs1574549
rs2026805
rs117984106
rs61998450
rs143778463
rs2714034
rs78532720
rs117001160
rs10981196
rs28710060
rs11795181
rs2169935
rs113388671
rs80117180
rs141783704
rs12033127
rs35406243
All
−0.0020
−0.0015
−0.0010
−0.0005
0.0000
MR effect size for
'Hephaestin−like protein 1' on 'eGFR'

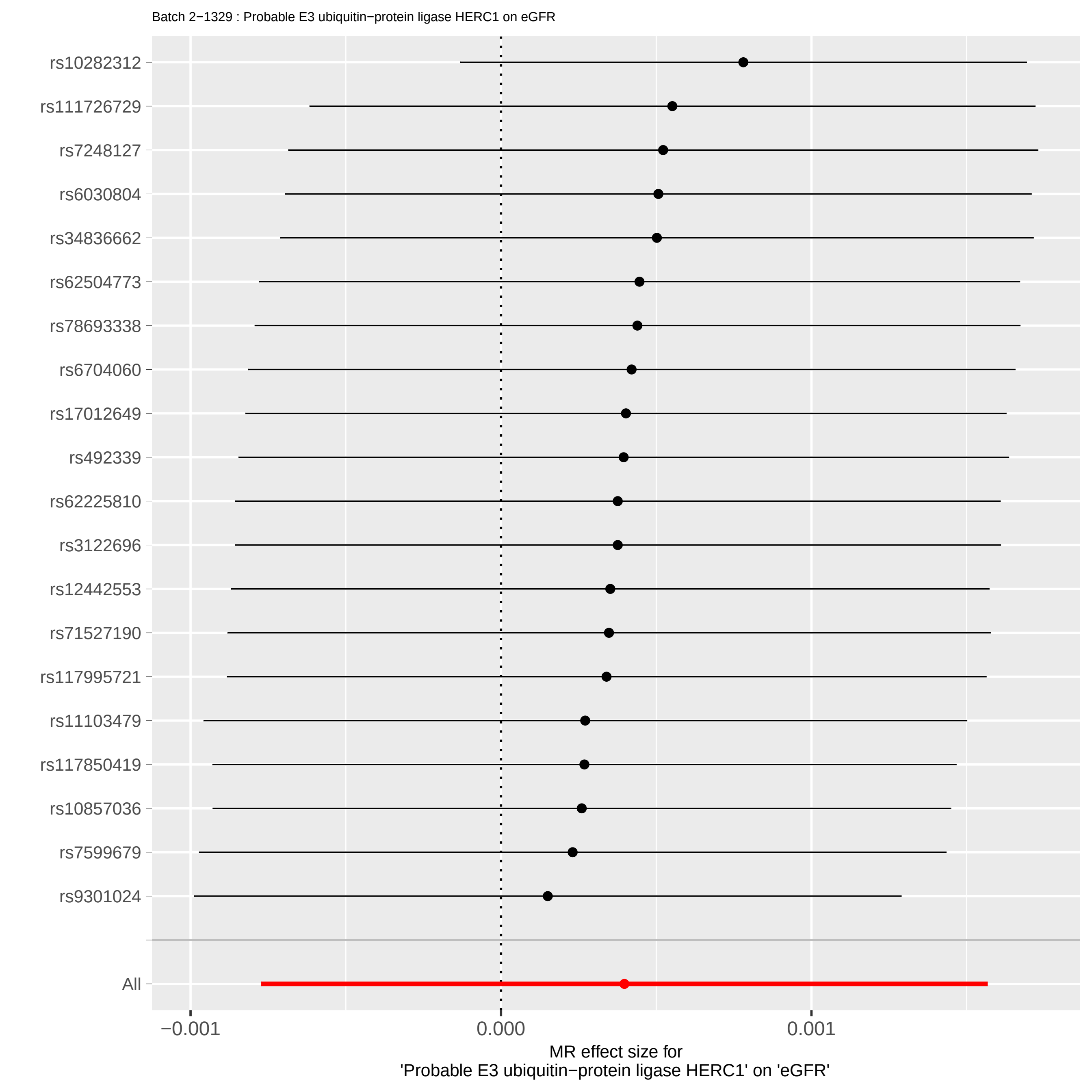
Batch 2−1329 : Probable E3 ubiquitin−protein ligase HERC1 on eGFR
rs10282312
rs111726729
rs7248127
rs6030804
rs34836662
rs62504773
rs78693338
rs6704060
rs17012649
rs492339
rs62225810
rs3122696
rs12442553
rs71527190
rs117995721
rs11103479
rs117850419
rs10857036
rs7599679
rs9301024
All
−0.001
0.000
0.001
MR effect size for
'Probable E3 ubiquitin−protein ligase HERC1' on 'eGFR'

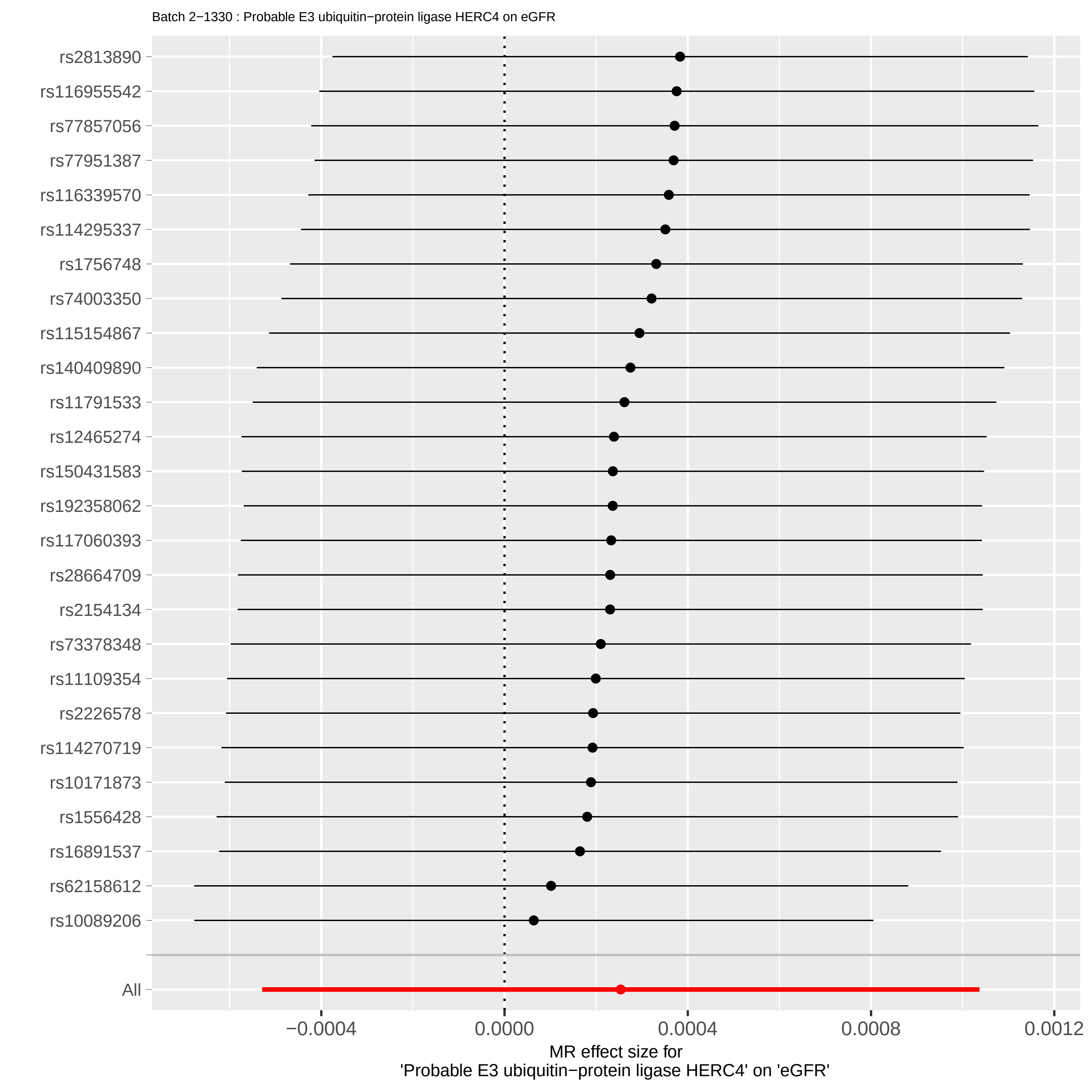
Batch 2−1330 : Probable E3 ubiquitin−protein ligase HERC4 on eGFR
rs2813890
rs116955542
rs77857056
rs77951387
rs116339570
rs114295337
rs1756748
rs74003350
rs115154867
rs140409890
rs11791533
rs12465274
rs150431583
rs192358062
rs117060393
rs28664709
rs2154134
rs73378348
rs11109354
rs2226578
rs114270719
rs10171873
rs1556428
rs16891537
rs62158612
rs10089206
All
−0.0004
0.0000
0.0004
0.0008
0.0012
MR effect size for
'Probable E3 ubiquitin−protein ligase HERC4' on 'eGFR'

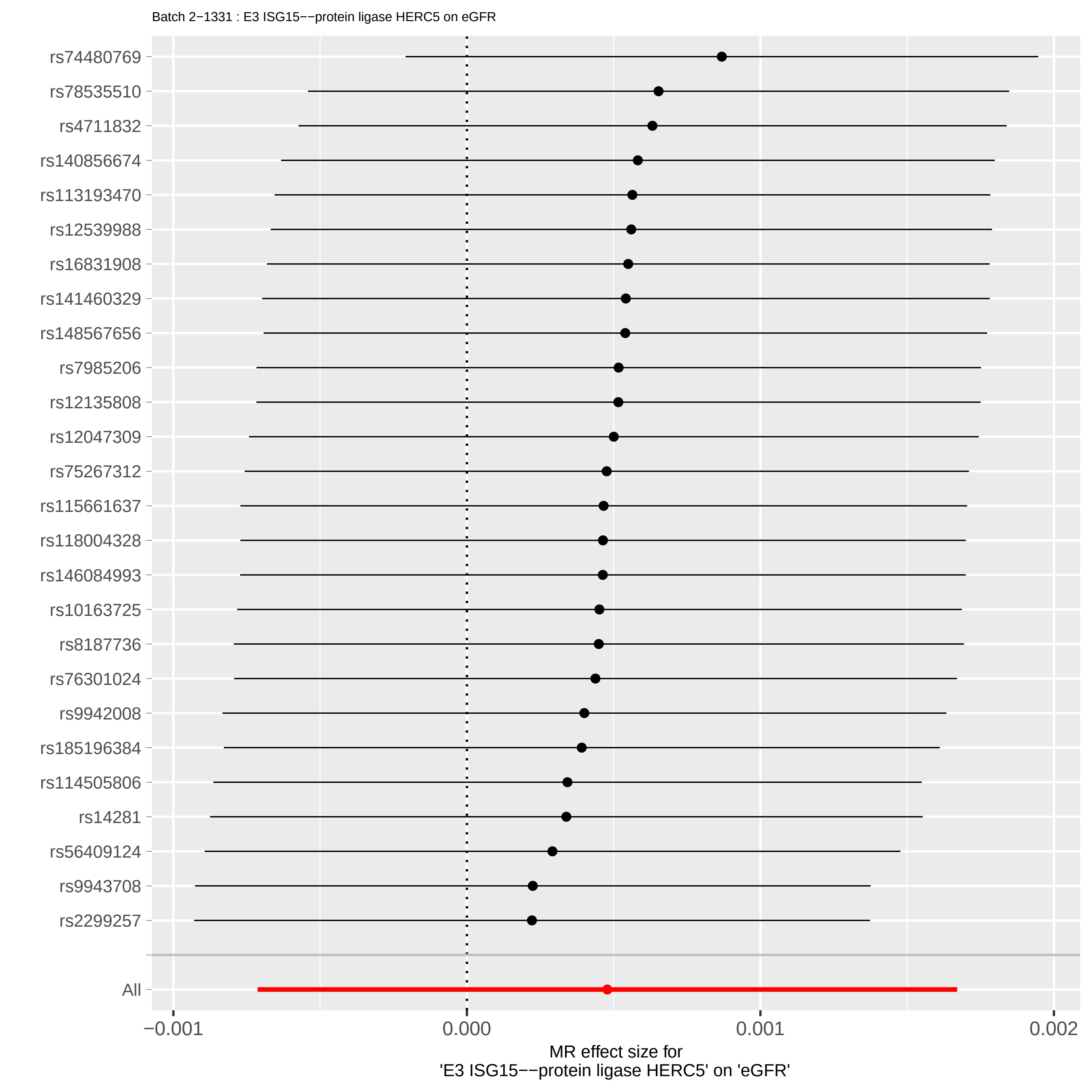
Batch 2−1331 : E3 ISG15−−protein ligase HERC5 on eGFR
rs74480769
rs78535510
rs4711832
rs140856674
rs113193470
rs12539988
rs16831908
rs141460329
rs148567656
rs7985206
rs12135808
rs12047309
rs75267312
rs115661637
rs118004328
rs146084993
rs10163725
rs8187736
rs76301024
rs9942008
rs185196384
rs114505806
rs14281
rs56409124
rs9943708
rs2299257
All
−0.001
0.000
0.001
0.002
MR effect size for
'E3 ISG15−−protein ligase HERC5' on 'eGFR'

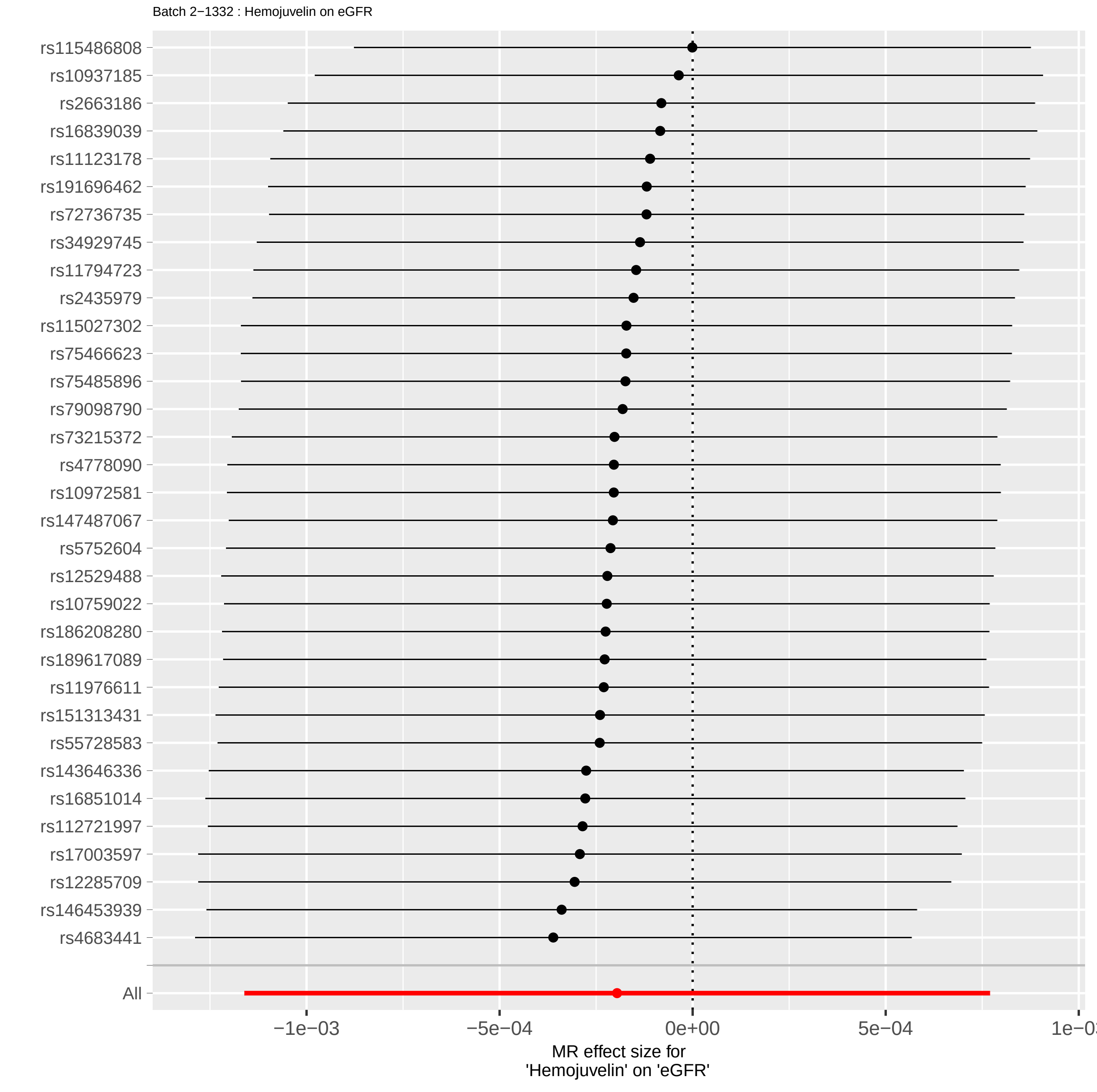
Batch 2−1332 : Hemojuvelin on eGFR
rs115486808
rs10937185
rs2663186
rs16839039
rs11123178
rs191696462
rs72736735
rs34929745
rs11794723
rs2435979
rs115027302
rs75466623
rs75485896
rs79098790
rs73215372
rs4778090
rs10972581
rs147487067
rs5752604
rs12529488
rs10759022
rs186208280
rs189617089
rs11976611
rs151313431
rs55728583
rs143646336
rs16851014
rs112721997
rs17003597
rs12285709
rs146453939
rs4683441
All
−1e−03
−5e−04
0e+00
5e−04
MR effect size for
'Hemojuvelin' on 'eGFR'

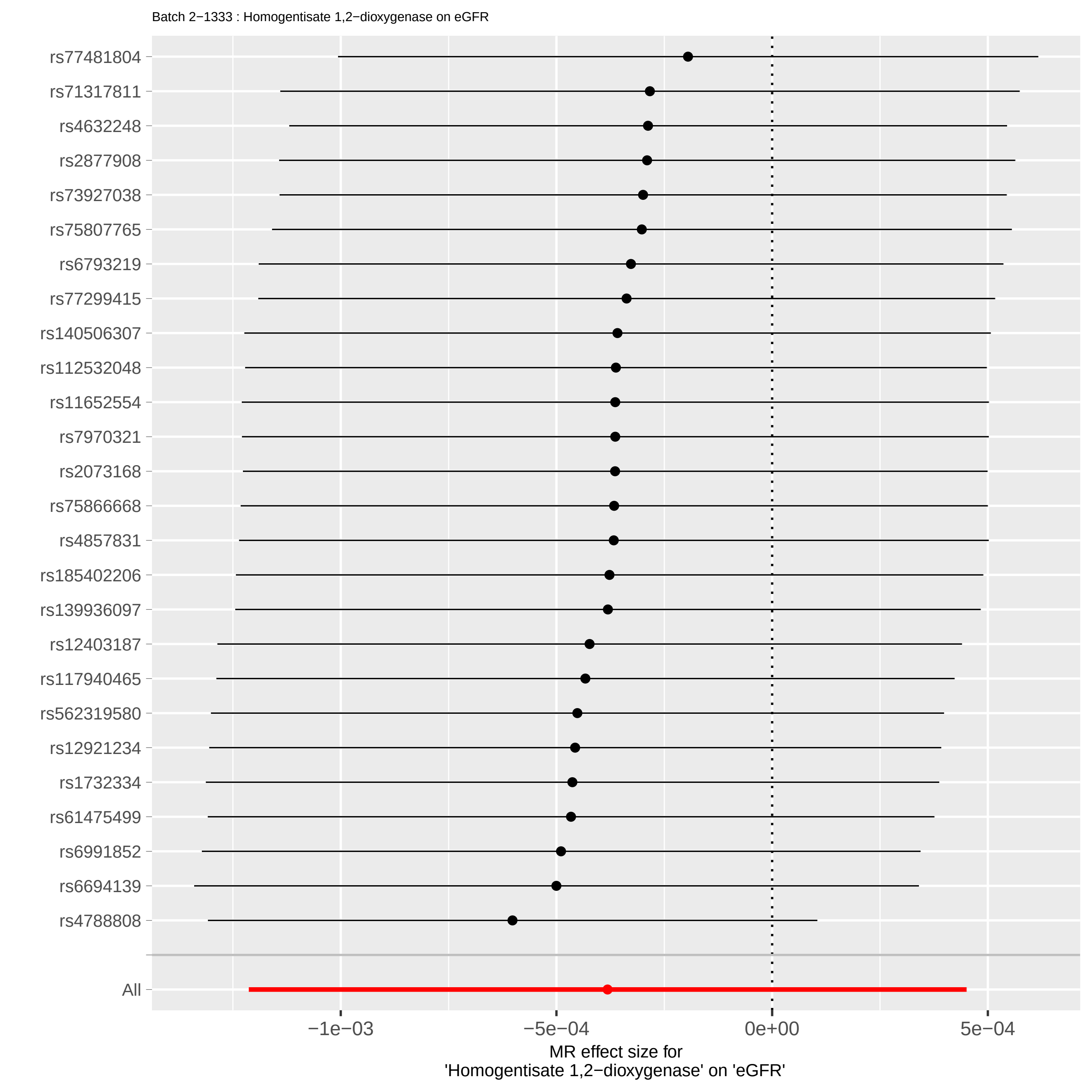

Batch 2−1333 : Homogentisate 1,2−dioxygenase on eGFR
rs77481804
rs71317811
rs4632248
rs2877908
rs73927038
rs75807765
rs6793219
rs77299415
rs140506307
rs112532048
rs11652554
rs7970321
rs2073168
rs75866668
rs4857831
rs185402206
rs139936097
rs12403187
rs117940465
rs562319580
rs12921234
rs1732334
rs61475499
rs6991852
rs6694139
rs4788808
All
−1e−03
−5e−04
0e+00
5e−04
MR effect size for
'Homogentisate 1,2−dioxygenase' on 'eGFR'

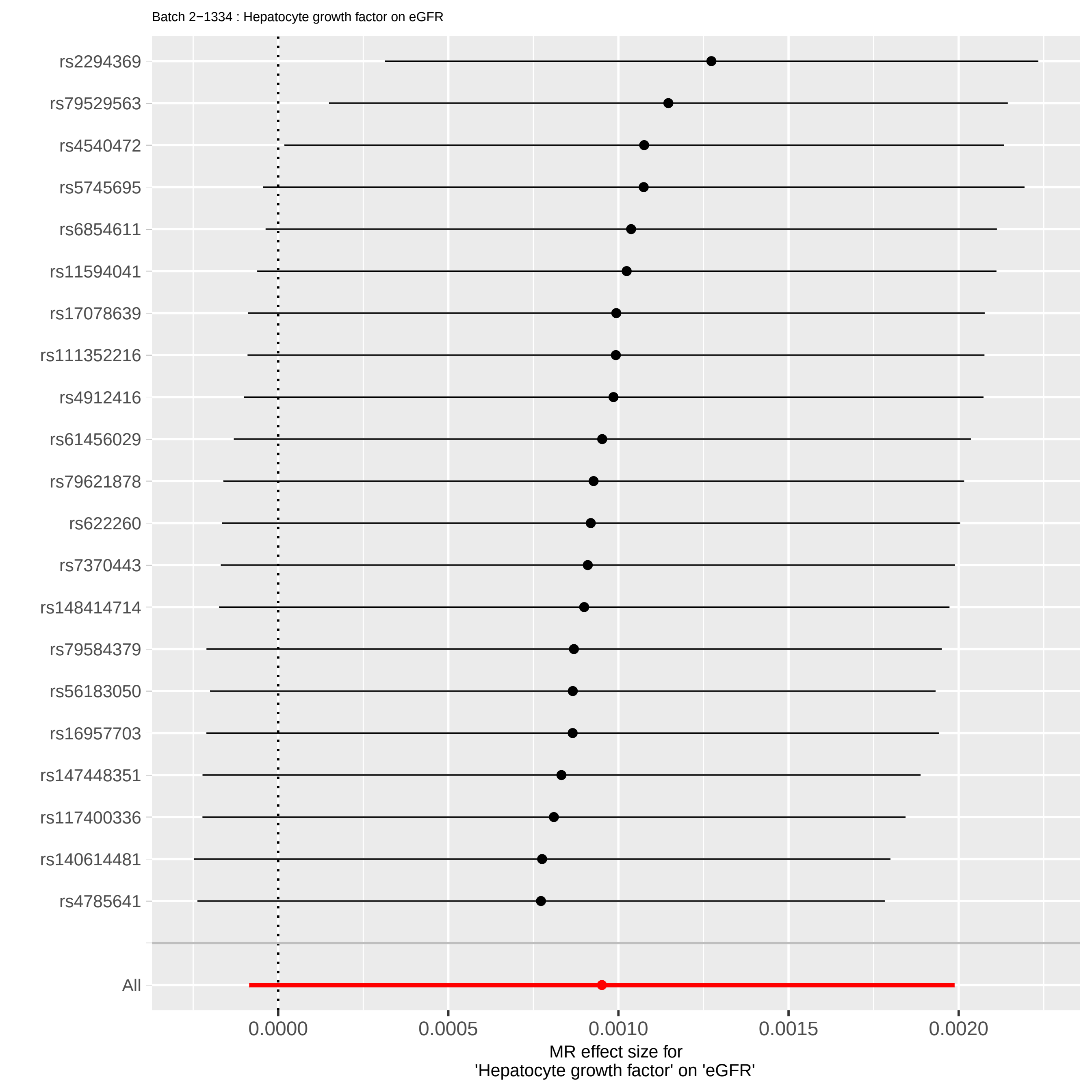
Batch 2−1334 : Hepatocyte growth factor on eGFR
rs2294369
rs79529563
rs4540472
rs5745695
rs6854611
rs11594041
rs17078639
rs111352216
rs4912416
rs61456029
rs79621878
rs622260
rs7370443
rs148414714
rs79584379
rs56183050
rs16957703
rs147448351
rs117400336
rs140614481
rs4785641
All
0.0000
0.0005
0.0010
0.0015
0.0020
MR effect size for
'Hepatocyte growth factor' on 'eGFR'

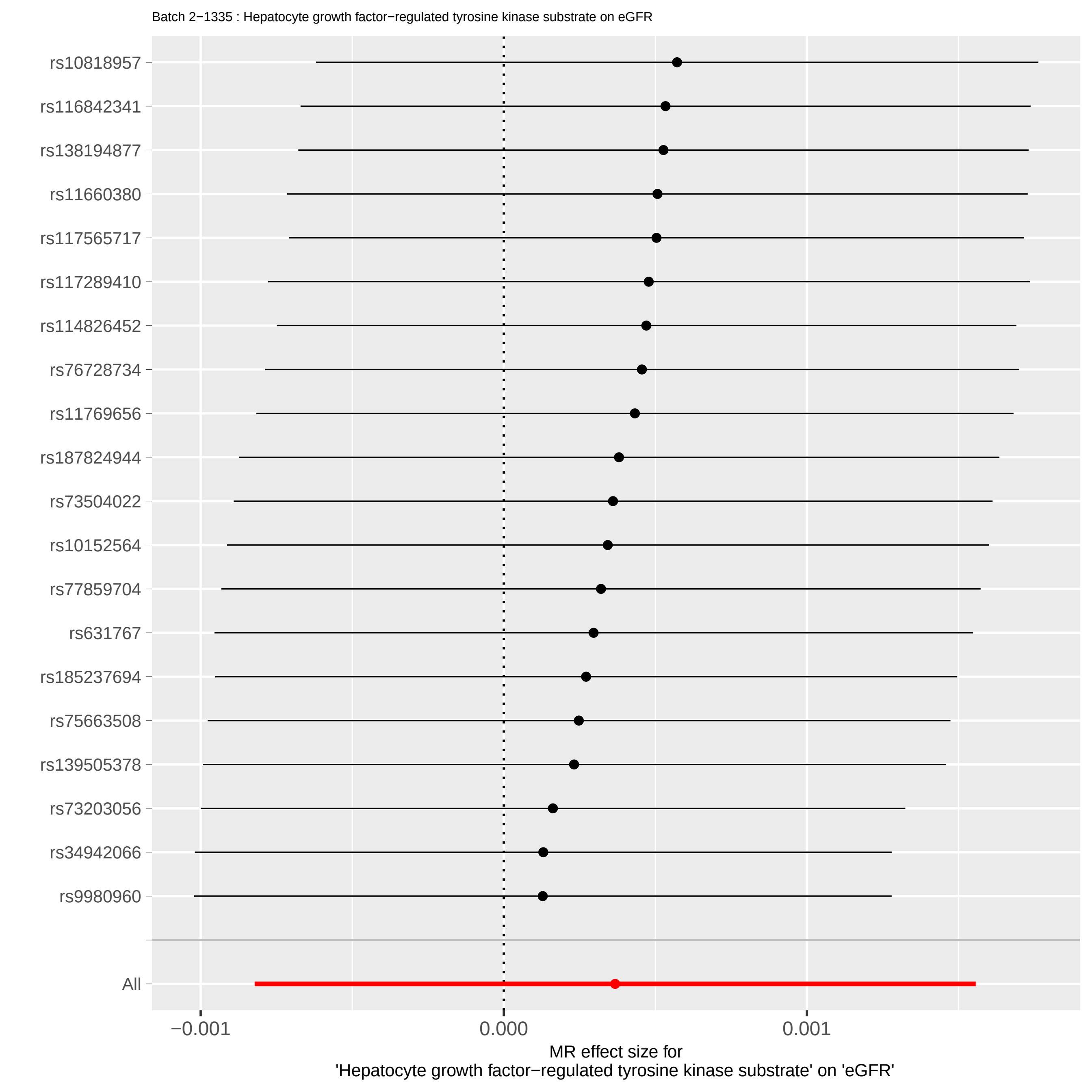

Batch 2−1335 : Hepatocyte growth factor−regulated tyrosine kinase substrate on eGFR
rs10818957
rs116842341
rs138194877
rs11660380
rs117565717
rs117289410
rs114826452
rs76728734
rs11769656
rs187824944
rs73504022
rs10152564
rs77859704
rs631767
rs185237694
rs75663508
rs139505378
rs73203056
rs34942066
rs9980960
All
−0.001
0.000
0.001
MR effect size for
'Hepatocyte growth factor−regulated tyrosine kinase substrate' on 'eGFR'

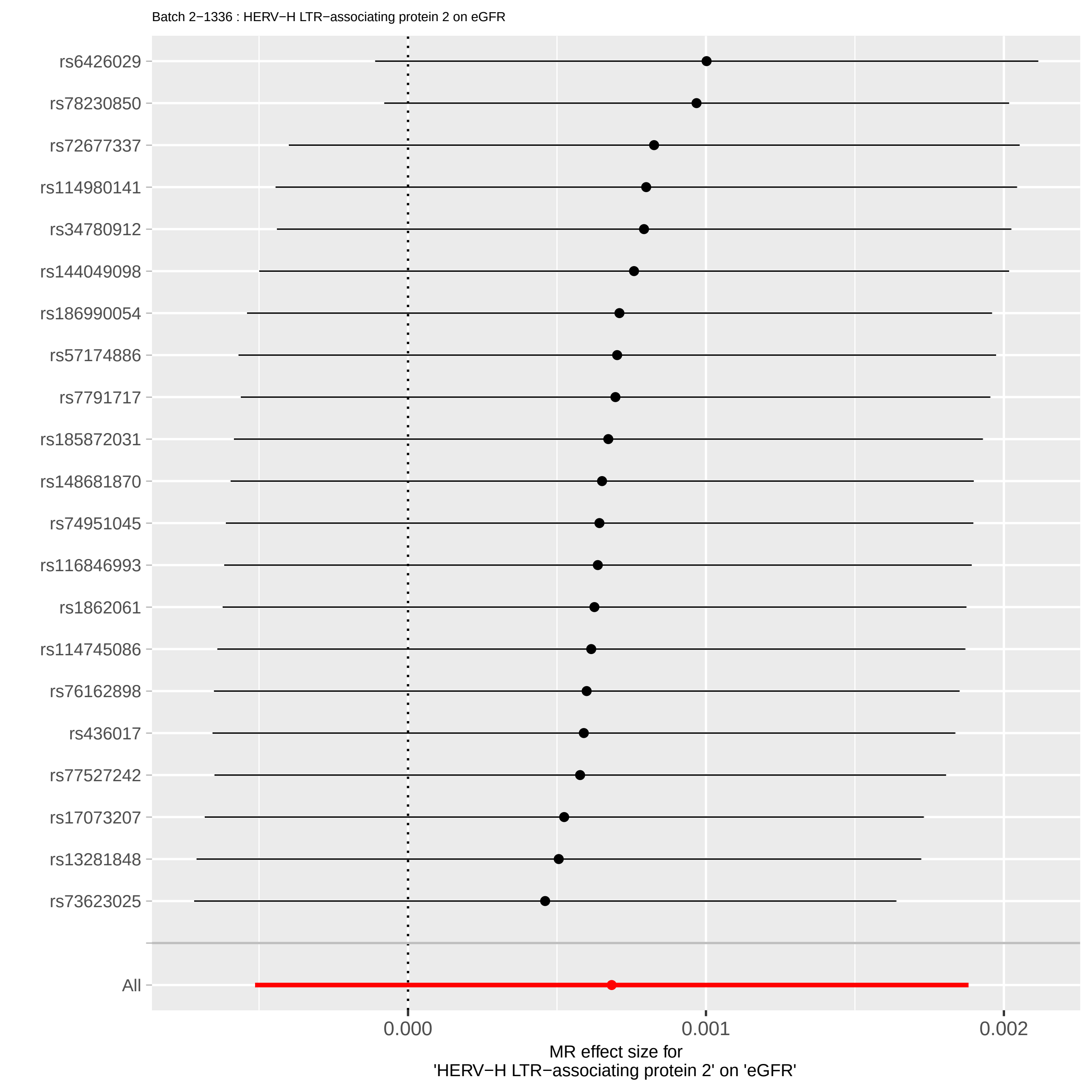

Batch 2−1336 : HERV−H LTR−associating protein 2 on eGFR
rs6426029
rs78230850
rs72677337
rs114980141
rs34780912
rs144049098
rs186990054
rs57174886
rs7791717
rs185872031
rs148681870
rs74951045
rs116846993
rs1862061
rs114745086
rs76162898
rs436017
rs77527242
rs17073207
rs13281848
rs73623025
All
0.000
0.001
0.002
MR effect size for
'HERV−H LTR−associating protein 2' on 'eGFR'

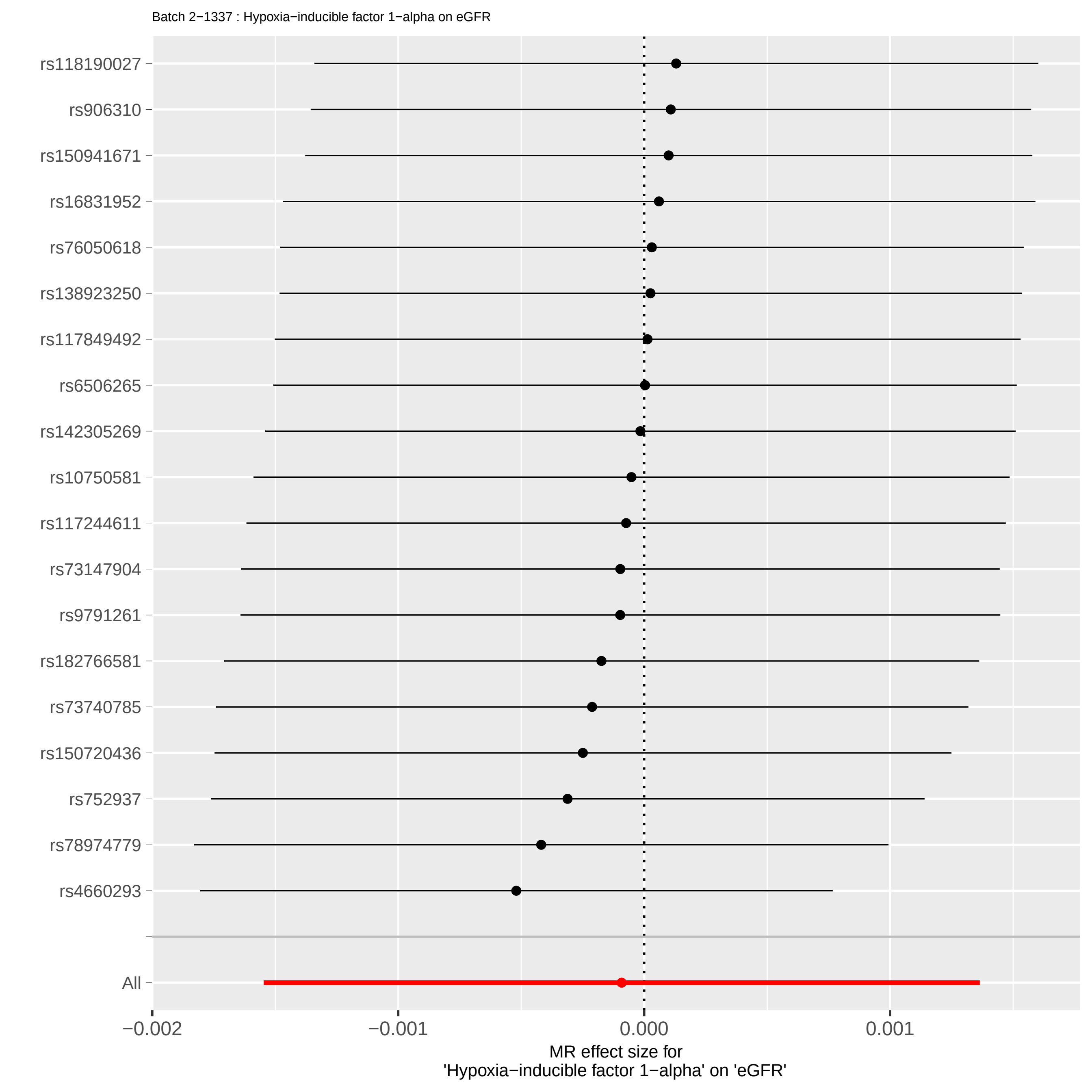

Batch 2−1337 : Hypoxia−inducible factor 1−alpha on eGFR
rs118190027
rs906310
rs150941671
rs16831952
rs76050618
rs138923250
rs117849492
rs6506265
rs142305269
rs10750581
rs117244611
rs73147904
rs9791261
rs182766581
rs73740785
rs150720436
rs752937
rs78974779
rs4660293
All
−0.002
−0.001
0.000
0.001
MR effect size for
'Hypoxia−inducible factor 1−alpha' on 'eGFR'

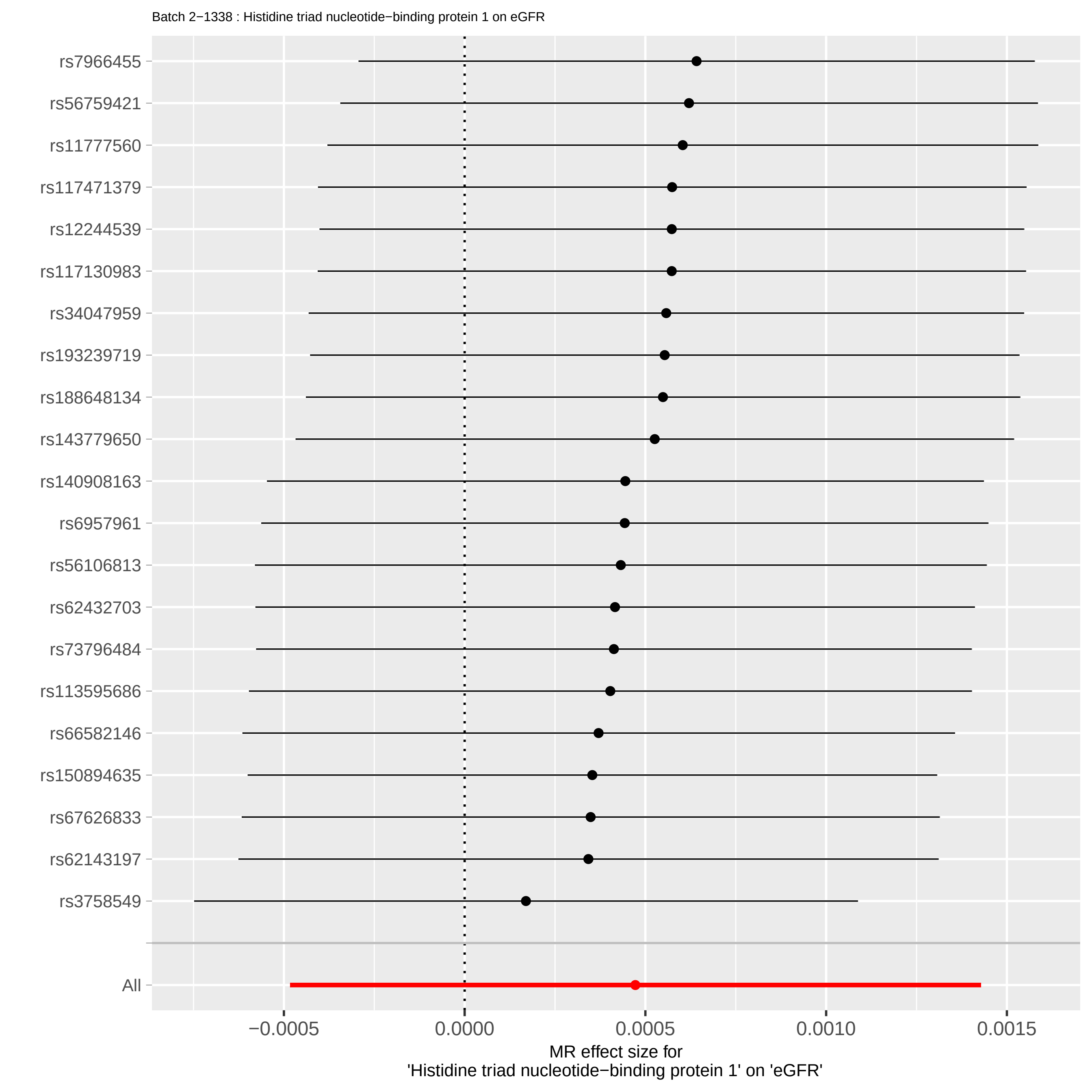

Batch 2−1338 : Histidine triad nucleotide−binding protein 1 on eGFR
rs7966455
rs56759421
rs11777560
rs117471379
rs12244539
rs117130983
rs34047959
rs193239719
rs188648134
rs143779650
rs140908163
rs6957961
rs56106813
rs62432703
rs73796484
rs113595686
rs66582146
rs150894635
rs67626833
rs62143197
rs3758549
All
−0.0005
0.0000
0.0005
0.0010
0.0015
MR effect size for
'Histidine triad nucleotide−binding protein 1' on 'eGFR'

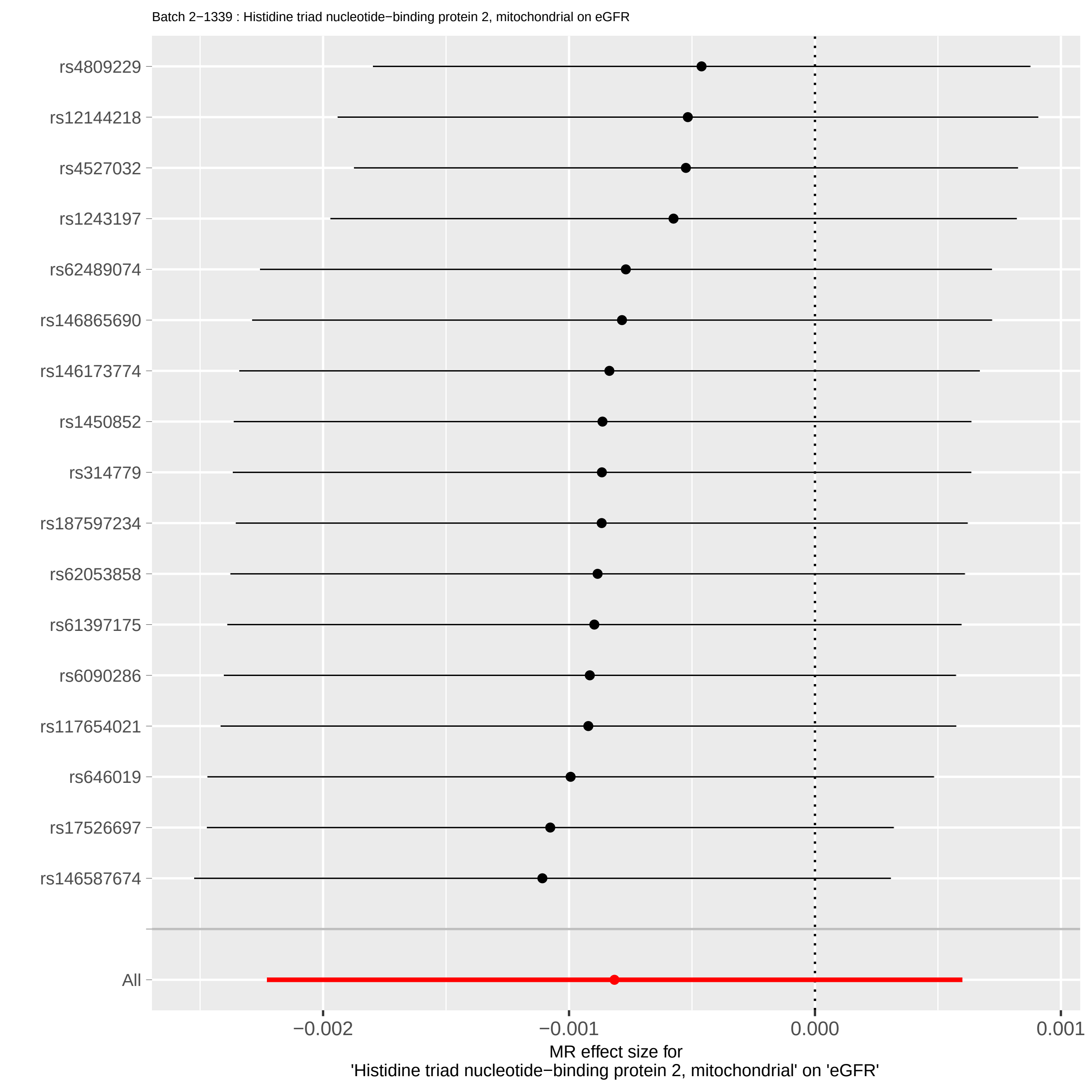

Batch 2−1339 : Histidine triad nucleotide−binding protein 2, mitochondrial on eGFR
rs4809229
rs12144218
rs4527032
rs1243197
rs62489074
rs146865690
rs146173774
rs1450852
rs314779
rs187597234
rs62053858
rs61397175
rs6090286
rs117654021
rs646019
rs17526697
rs146587674
All
−0.002
−0.001
0.000
0.001
MR effect size for
'Histidine triad nucleotide−binding protein 2, mitochondrial' on 'eGFR'

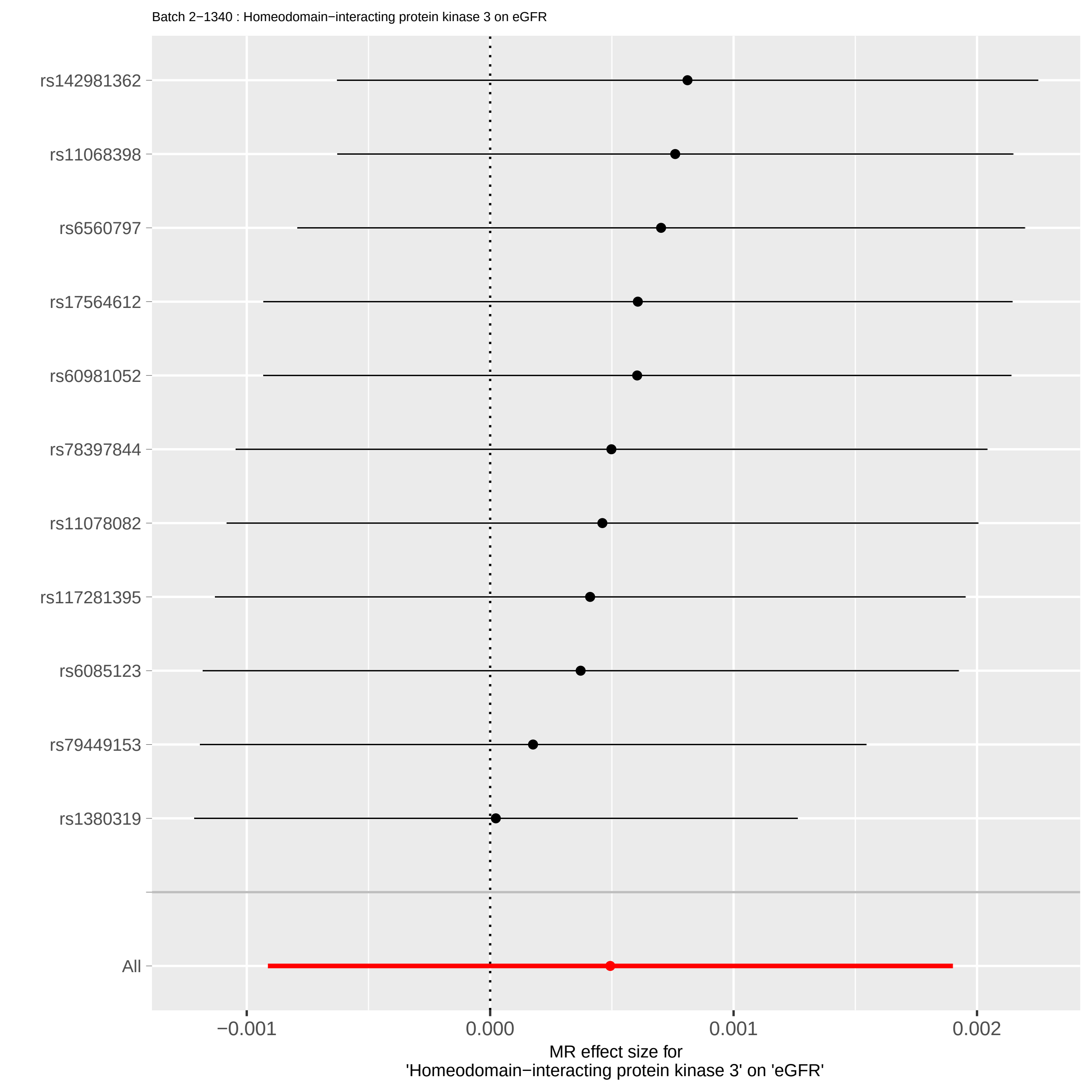

Batch 2−1340 : Homeodomain−interacting protein kinase 3 on eGFR
rs142981362
rs11068398
rs6560797
rs17564612
rs60981052
rs78397844
rs11078082
rs117281395
rs6085123
rs79449153
rs1380319
All
−0.001
0.000
0.001
0.002
MR effect size for
'Homeodomain−interacting protein kinase 3' on 'eGFR'

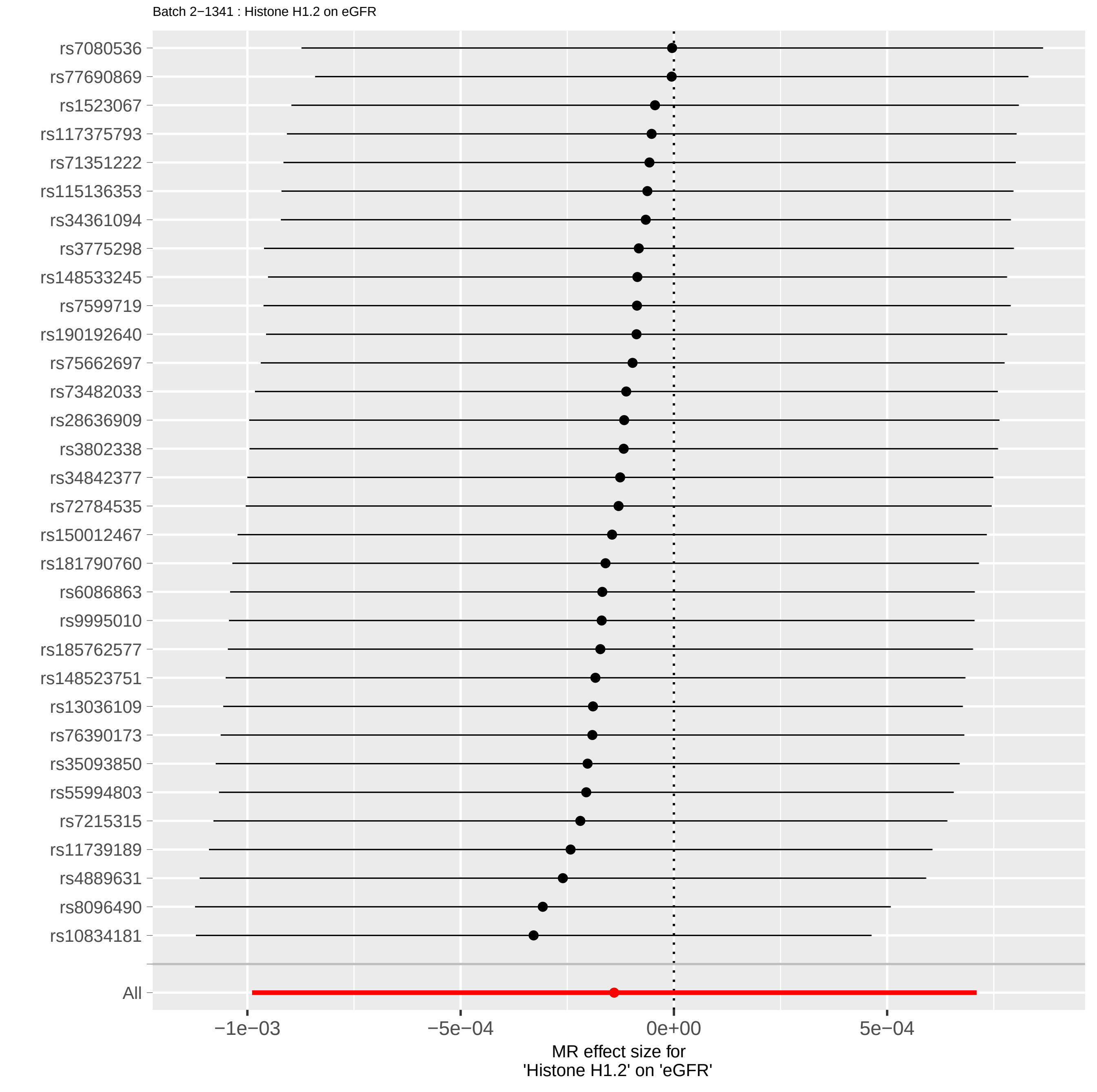

Batch 2−1341 : Histone H1.2 on eGFR
rs7080536
rs77690869
rs1523067
rs117375793
rs71351222
rs115136353
rs34361094
rs3775298
rs148533245
rs7599719
rs190192640
rs75662697
rs73482033
rs28636909
rs3802338
rs34842377
rs72784535
rs150012467
rs181790760
rs6086863
rs9995010
rs185762577
rs148523751
rs13036109
rs76390173
rs35093850
rs55994803
rs7215315
rs11739189
rs4889631
rs8096490
rs10834181
All
−1e−03
−5e−04
0e+00
5e−04
MR effect size for
'Histone H1.2' on 'eGFR'

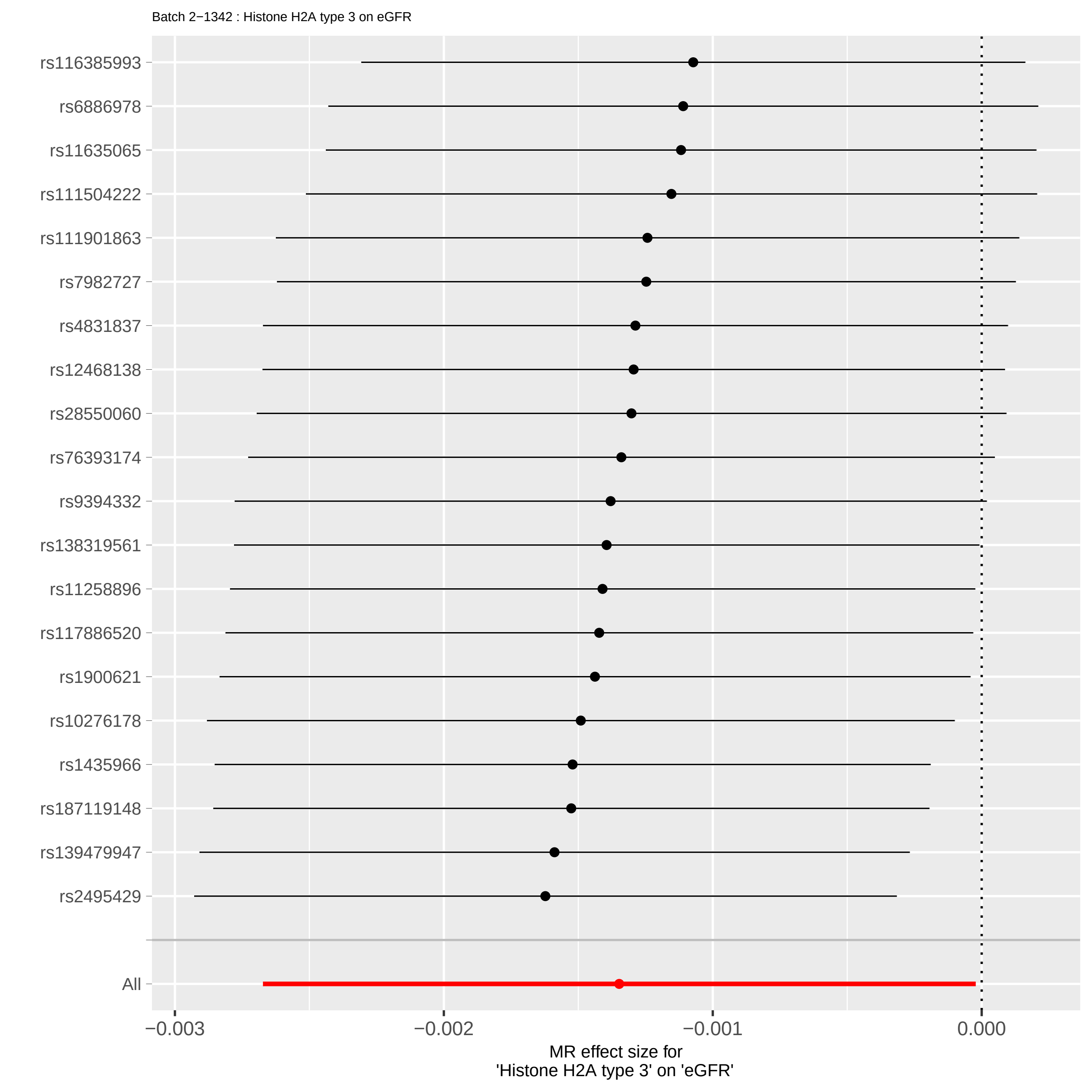

Batch 2−1342 : Histone H2A type 3 on eGFR
rs116385993
rs6886978
rs11635065
rs111504222
rs111901863
rs7982727
rs4831837
rs12468138
rs28550060
rs76393174
rs9394332
rs138319561
rs11258896
rs117886520
rs1900621
rs10276178
rs1435966
rs187119148
rs139479947
rs2495429
All
−0.003
−0.002
−0.001
0.000
MR effect size for
'Histone H2A type 3' on 'eGFR'

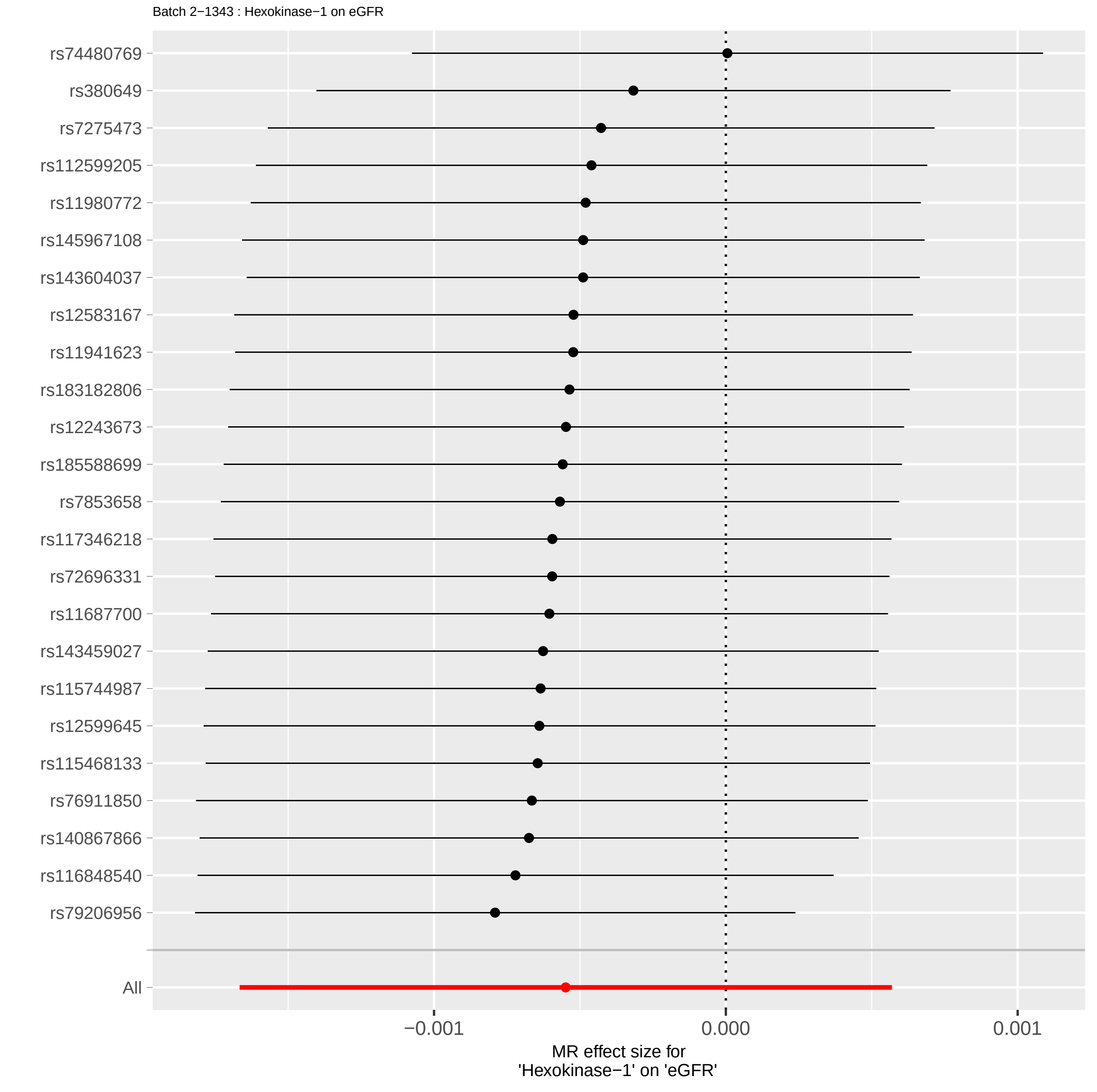

Batch 2−1343 : Hexokinase−1 on eGFR
rs74480769
rs380649
rs7275473
rs112599205
rs11980772
rs145967108
rs143604037
rs12583167
rs11941623
rs183182806
rs12243673
rs185588699
rs7853658
rs117346218
rs72696331
rs11687700
rs143459027
rs115744987
rs12599645
rs115468133
rs76911850
rs140867866
rs116848540
rs79206956
All
−0.001
0.000
0.001
MR effect size for
'Hexokinase−1' on 'eGFR'

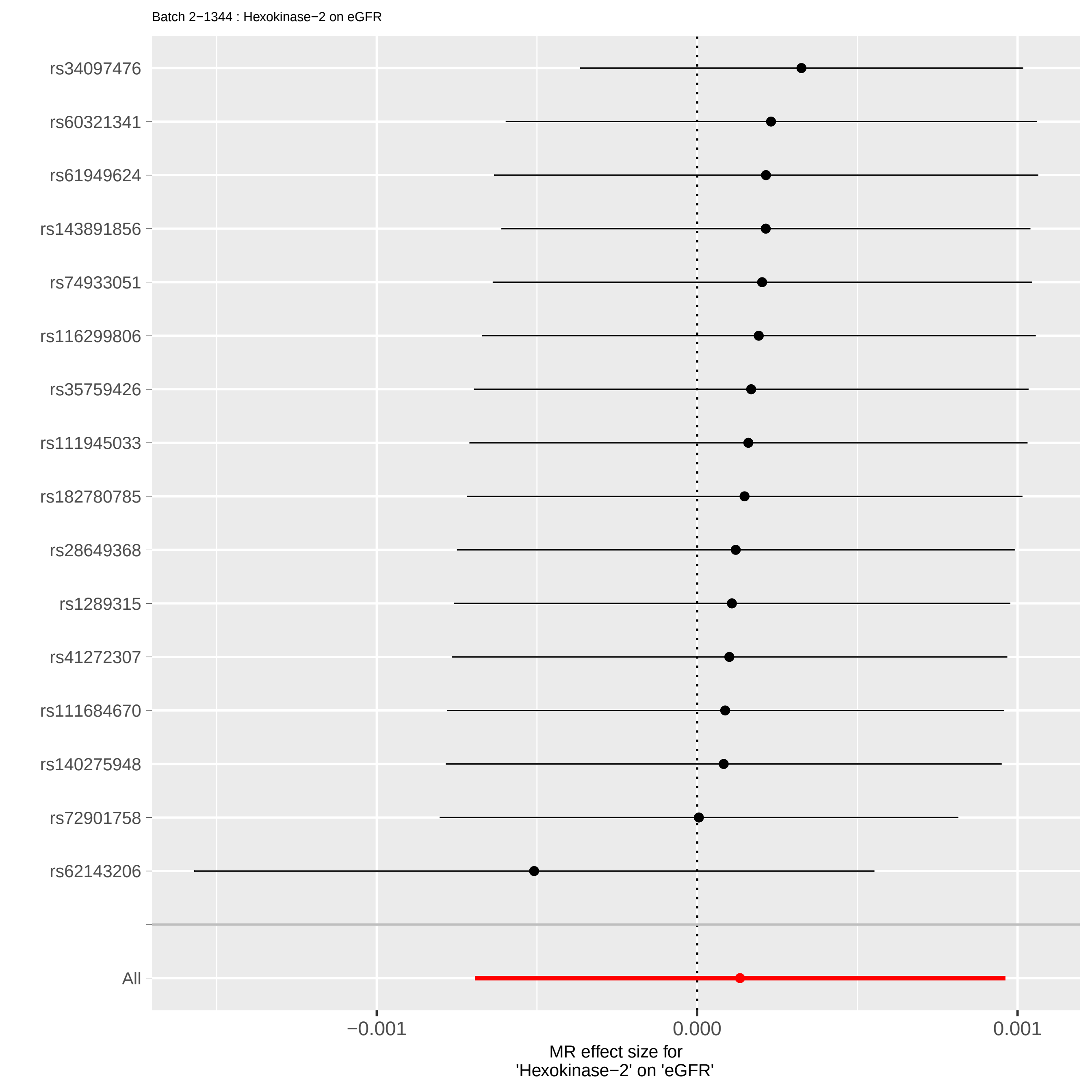

Batch 2−1344 : Hexokinase−2 on eGFR
rs34097476
rs60321341
rs61949624
rs143891856
rs74933051
rs116299806
rs35759426
rs111945033
rs182780785
rs28649368
rs1289315
rs41272307
rs111684670
rs140275948
rs72901758
rs62143206
All
−0.001
0.000
0.001
MR effect size for
'Hexokinase−2' on 'eGFR'

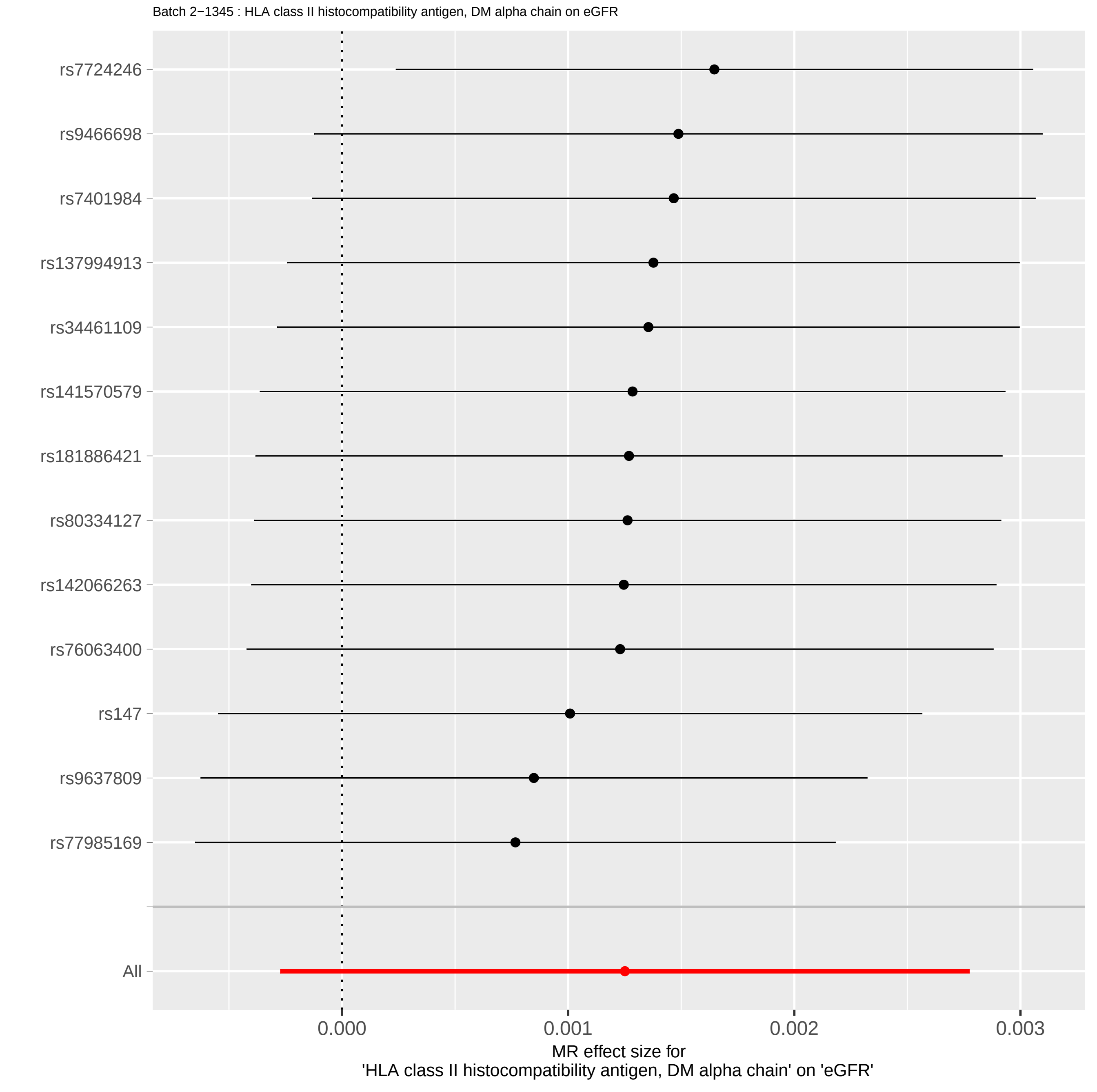

Batch 2−1345 : HLA class II histocompatibility antigen, DM alpha chain on eGFR
rs7724246
rs9466698
rs7401984
rs137994913
rs34461109
rs141570579
rs181886421
rs80334127
rs142066263
rs76063400
rs147
rs9637809
rs77985169
All
0.000
0.001
0.002
0.003
MR effect size for
'HLA class II histocompatibility antigen, DM alpha chain' on 'eGFR'

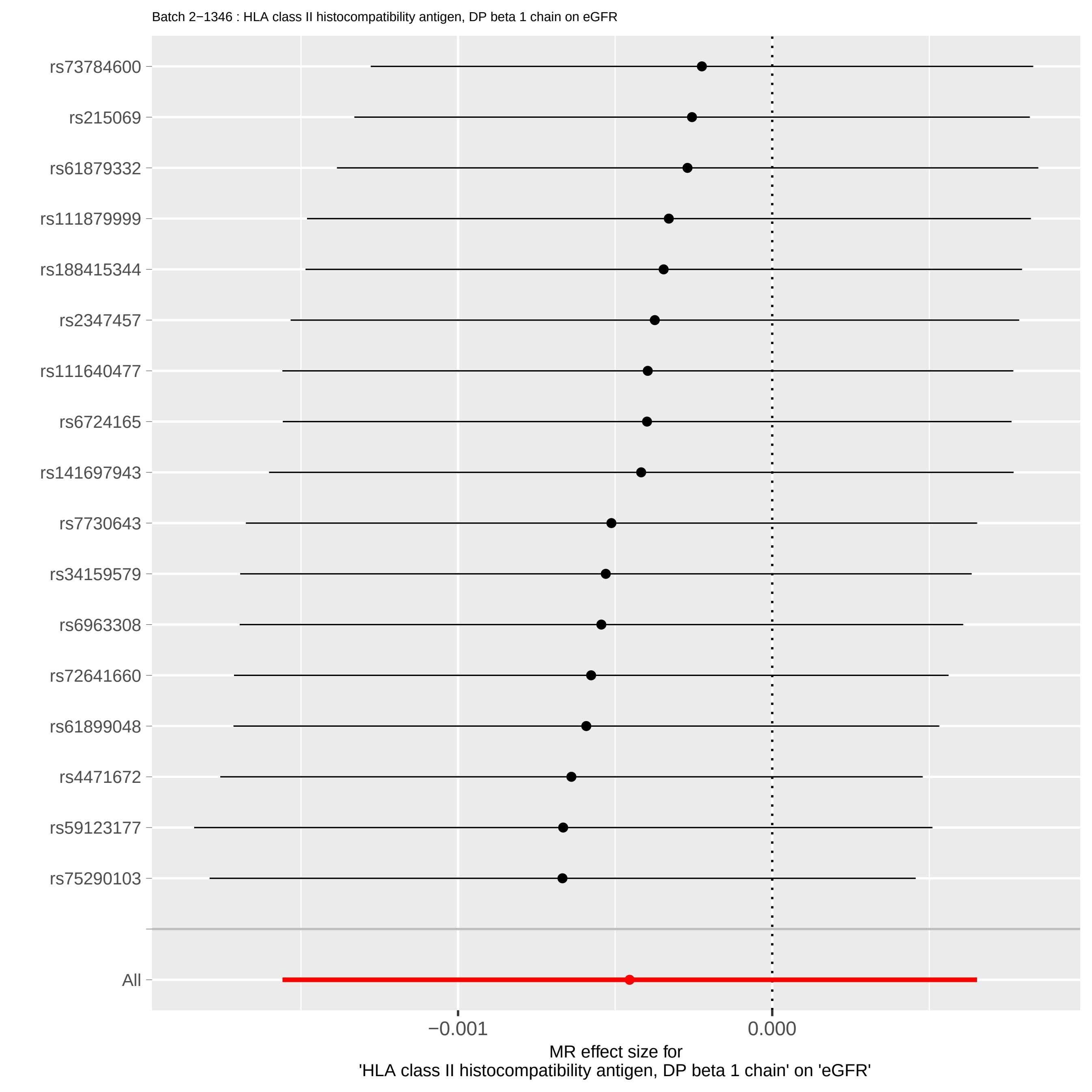

Batch 2−1346 : HLA class II histocompatibility antigen, DP beta 1 chain on eGFR
rs73784600
rs215069
rs61879332
rs111879999
rs188415344
rs2347457
rs111640477
rs6724165
rs141697943
rs7730643
rs34159579
rs6963308
rs72641660
rs61899048
rs4471672
rs59123177
rs75290103
All
−0.001
0.000
MR effect size for
'HLA class II histocompatibility antigen, DP beta 1 chain' on 'eGFR'

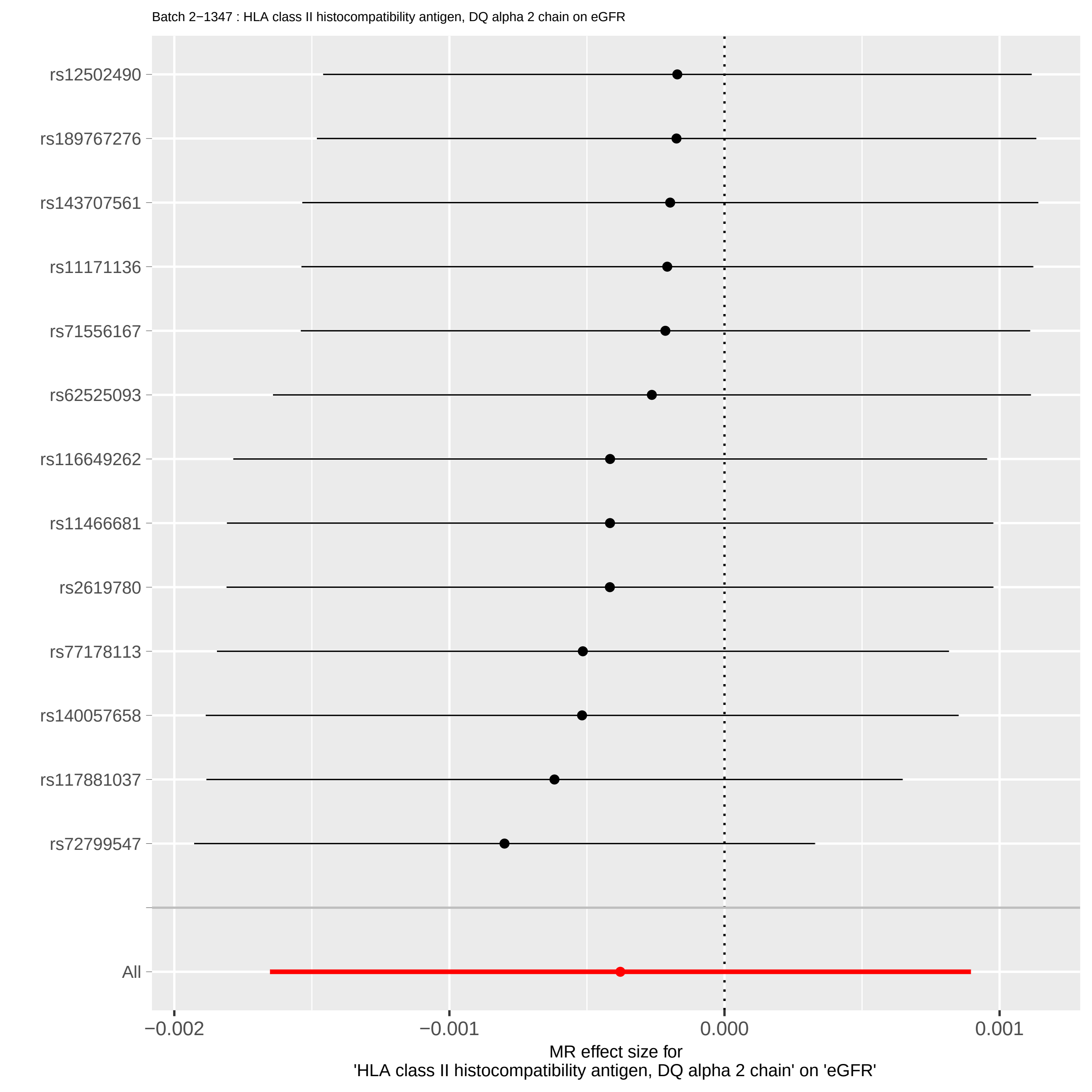

Batch 2−1347 : HLA class II histocompatibility antigen, DQ alpha 2 chain on eGFR
rs12502490
rs189767276
rs143707561
rs11171136
rs71556167
rs62525093
rs116649262
rs11466681
rs2619780
rs77178113
rs140057658
rs117881037
rs72799547
All
−0.002
−0.001
0.000
0.001
MR effect size for
'HLA class II histocompatibility antigen, DQ alpha 2 chain' on 'eGFR'

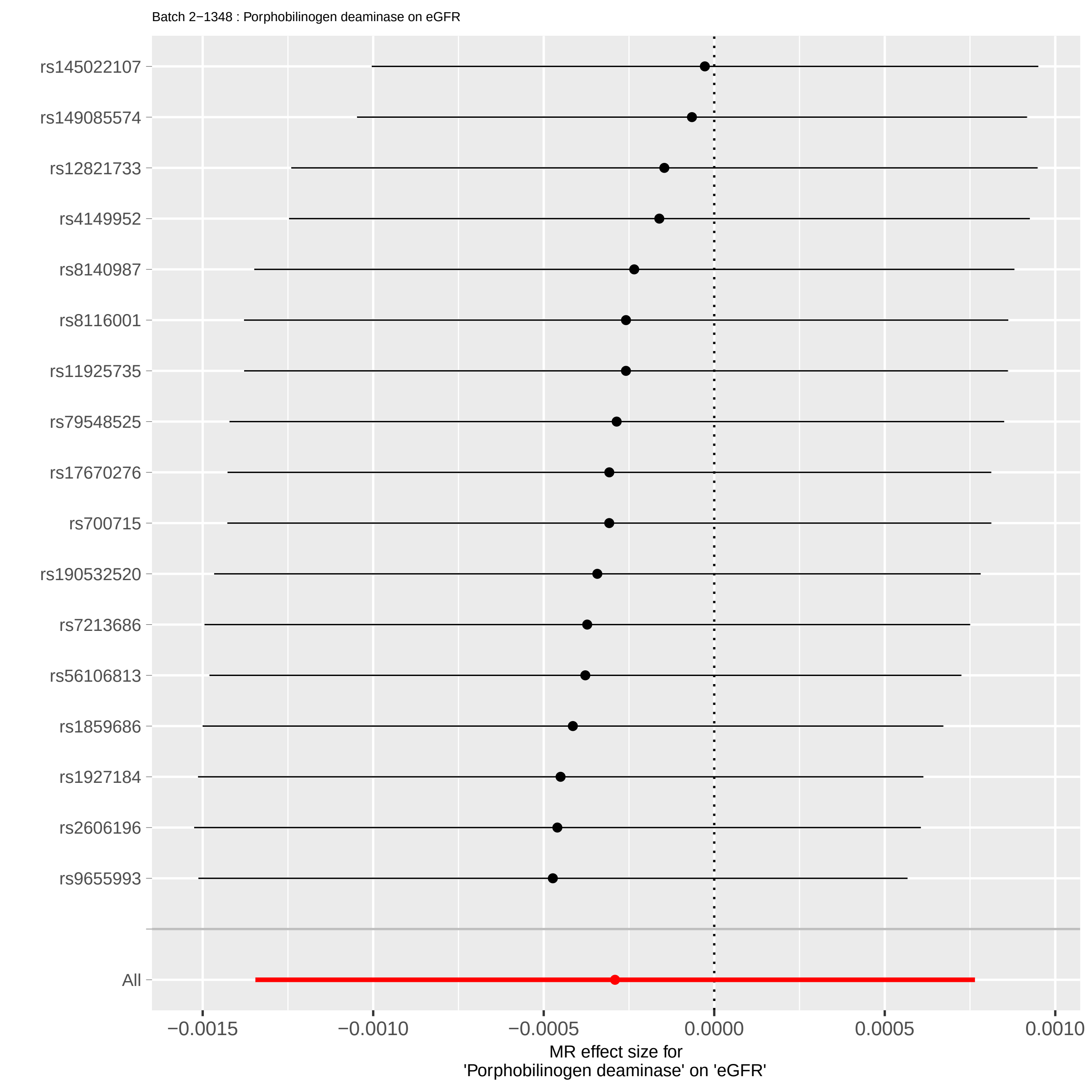

Batch 2−1348 : Porphobilinogen deaminase on eGFR
rs145022107
rs149085574
rs12821733
rs4149952
rs8140987
rs8116001
rs11925735
rs79548525
rs17670276
rs700715
rs190532520
rs7213686
rs56106813
rs1859686
rs1927184
rs2606196
rs9655993
All
−0.0015
−0.0010
−0.0005
0.0000
0.0005
0.0010
MR effect size for
'Porphobilinogen deaminase' on 'eGFR'

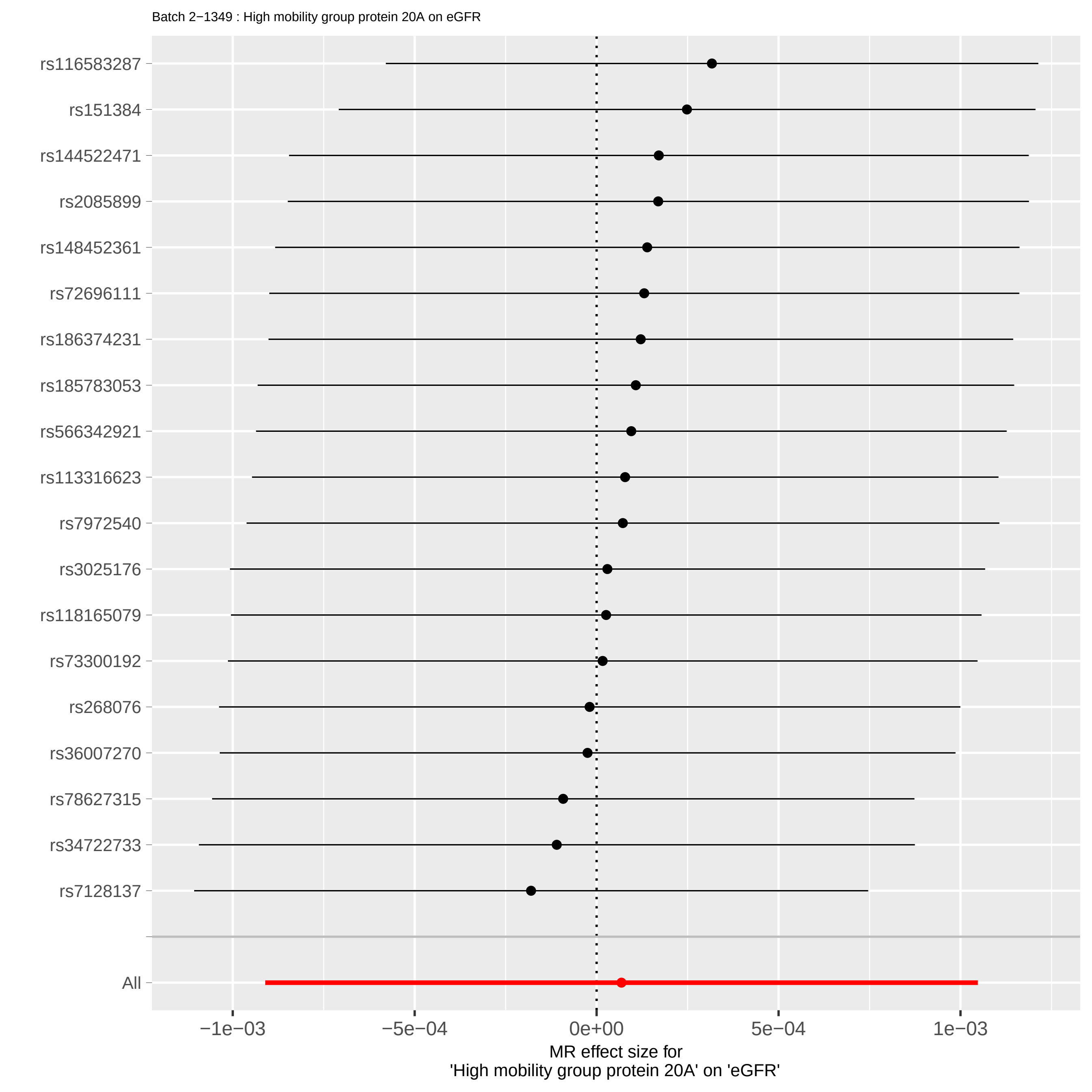

Batch 2−1349 : High mobility group protein 20A on eGFR
rs116583287
rs151384
rs144522471
rs2085899
rs148452361
rs72696111
rs186374231
rs185783053
rs566342921
rs113316623
rs7972540
rs3025176
rs118165079
rs73300192
rs268076
rs36007270
rs78627315
rs34722733
rs7128137
All
−1e−03
−5e−04
0e+00
5e−04
1e−03
MR effect size for
'High mobility group protein 20A' on 'eGFR'

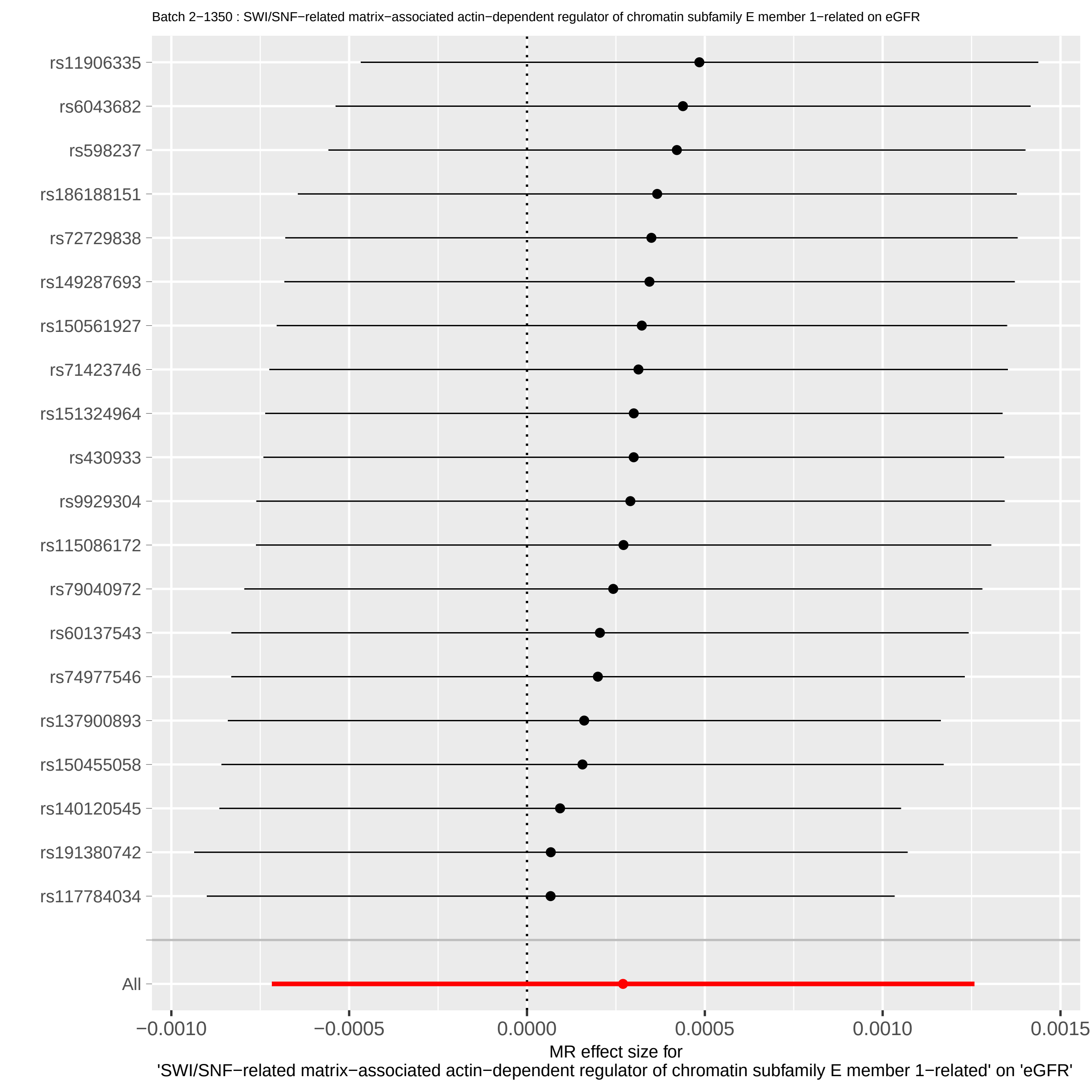

Batch 2−1350 : SWI/SNF−related matrix−associated actin−dependent regulator of chromatin subfamily E member 1−related on eGFR
rs11906335
rs6043682
rs598237
rs186188151
rs72729838
rs149287693
rs150561927
rs71423746
rs151324964
rs430933
rs9929304
rs115086172
rs79040972
rs60137543
rs74977546
rs137900893
rs150455058
rs140120545
rs191380742
rs117784034
All
−0.0010
−0.0005
0.0000
0.0005
0.0010
0.0015
MR effect size for
'SWI/SNF−related matrix−associated actin−dependent regulator of chromatin subfamily E member 1−related' on 'eGFR'

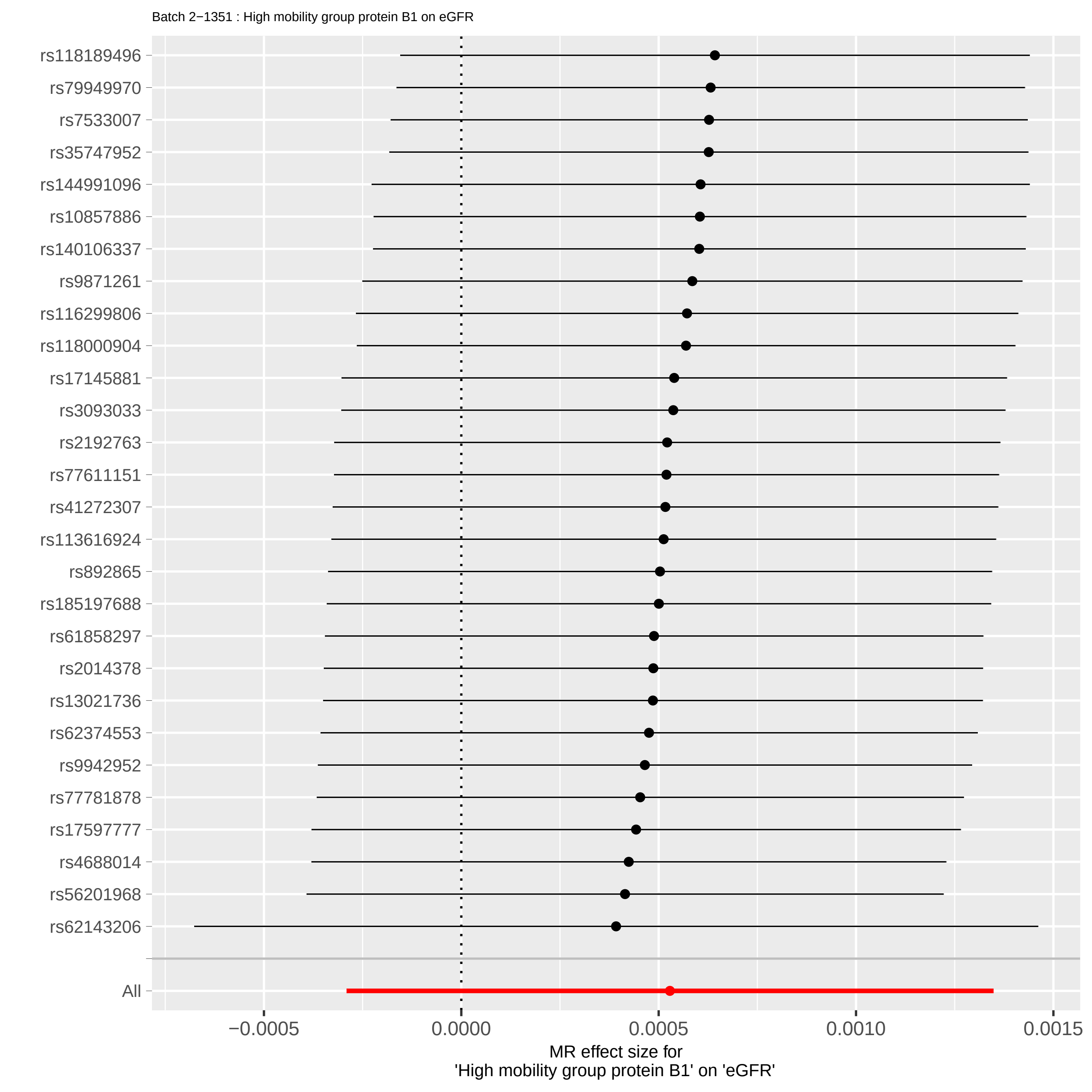

Batch 2−1351 : High mobility group protein B1 on eGFR
rs118189496
rs79949970
rs7533007
rs35747952
rs144991096
rs10857886
rs140106337
rs9871261
rs116299806
rs118000904
rs17145881
rs3093033
rs2192763
rs77611151
rs41272307
rs113616924
rs892865
rs185197688
rs61858297
rs2014378
rs13021736
rs62374553
rs9942952
rs77781878
rs17597777
rs4688014
rs56201968
rs62143206
All
−0.0005
0.0000
0.0005
0.0010
0.0015
MR effect size for
'High mobility group protein B1' on 'eGFR'

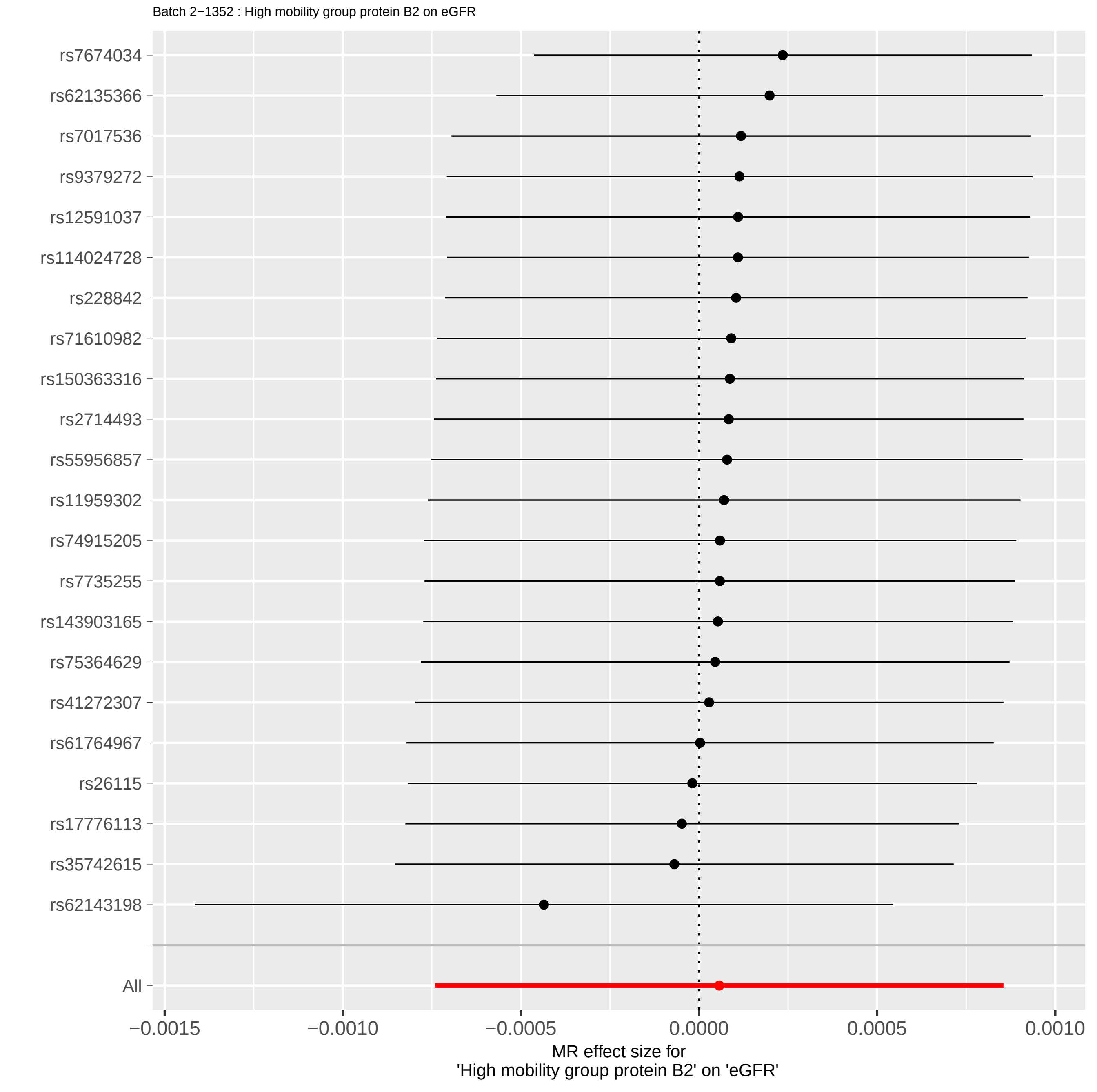

Batch 2−1352 : High mobility group protein B2 on eGFR
rs7674034
rs62135366
rs7017536
rs9379272
rs12591037
rs114024728
rs228842
rs71610982
rs150363316
rs2714493
rs55956857
rs11959302
rs74915205
rs7735255
rs143903165
rs75364629
rs41272307
rs61764967
rs26115
rs17776113
rs35742615
rs62143198
All
−0.0015
−0.0010
−0.0005
0.0000
0.0005
0.0010
MR effect size for
'High mobility group protein B2' on 'eGFR'

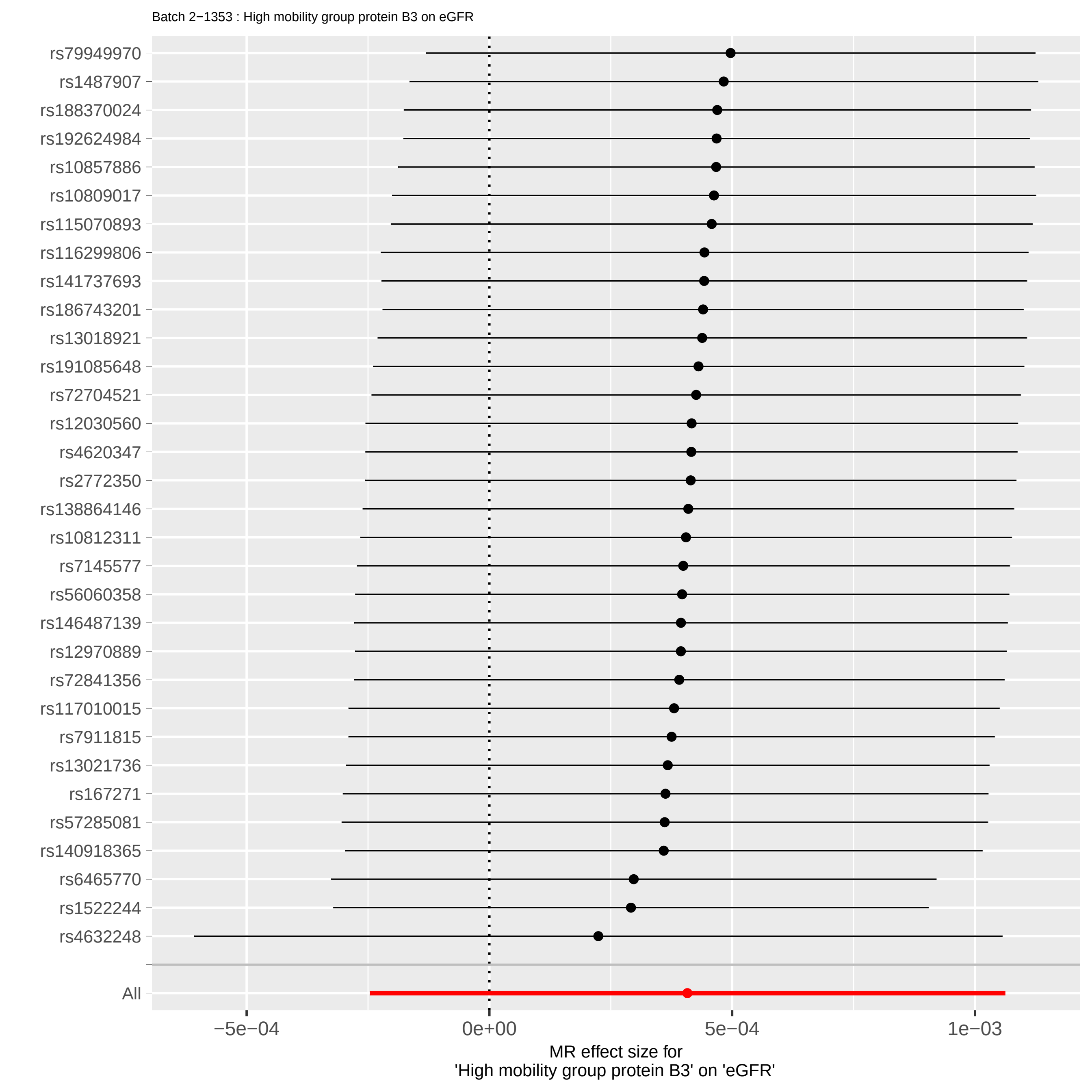

Batch 2−1353 : High mobility group protein B3 on eGFR
rs79949970
rs1487907
rs188370024
rs192624984
rs10857886
rs10809017
rs115070893
rs116299806
rs141737693
rs186743201
rs13018921
rs191085648
rs72704521
rs12030560
rs4620347
rs2772350
rs138864146
rs10812311
rs7145577
rs56060358
rs146487139
rs12970889
rs72841356
rs117010015
rs7911815
rs13021736
rs167271
rs57285081
rs140918365
rs6465770
rs1522244
rs4632248
All
−5e−04
0e+00
5e−04
1e−03
MR effect size for
'High mobility group protein B3' on 'eGFR'

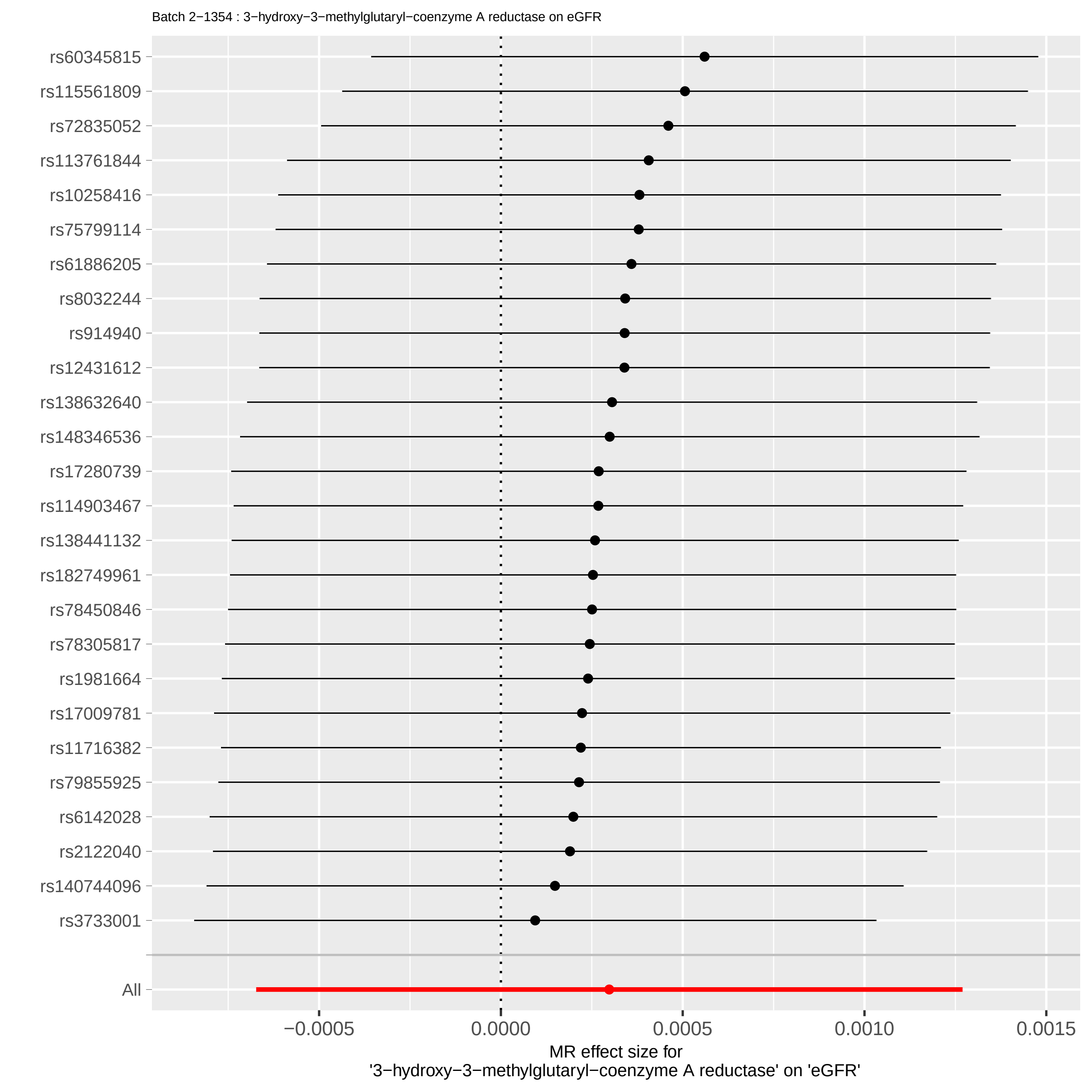
Batch 2−1354 : 3−hydroxy−3−methylglutaryl−coenzyme A reductase on eGFR
rs60345815
rs115561809
rs72835052
rs113761844
rs10258416
rs75799114
rs61886205
rs8032244
rs914940
rs12431612
rs138632640
rs148346536
rs17280739
rs114903467
rs138441132
rs182749961
rs78450846
rs78305817
rs1981664
rs17009781
rs11716382
rs79855925
rs6142028
rs2122040
rs140744096
rs3733001
All
−0.0005
0.0000
0.0005
0.0010
0.0015
MR effect size for
'3−hydroxy−3−methylglutaryl−coenzyme A reductase' on 'eGFR'

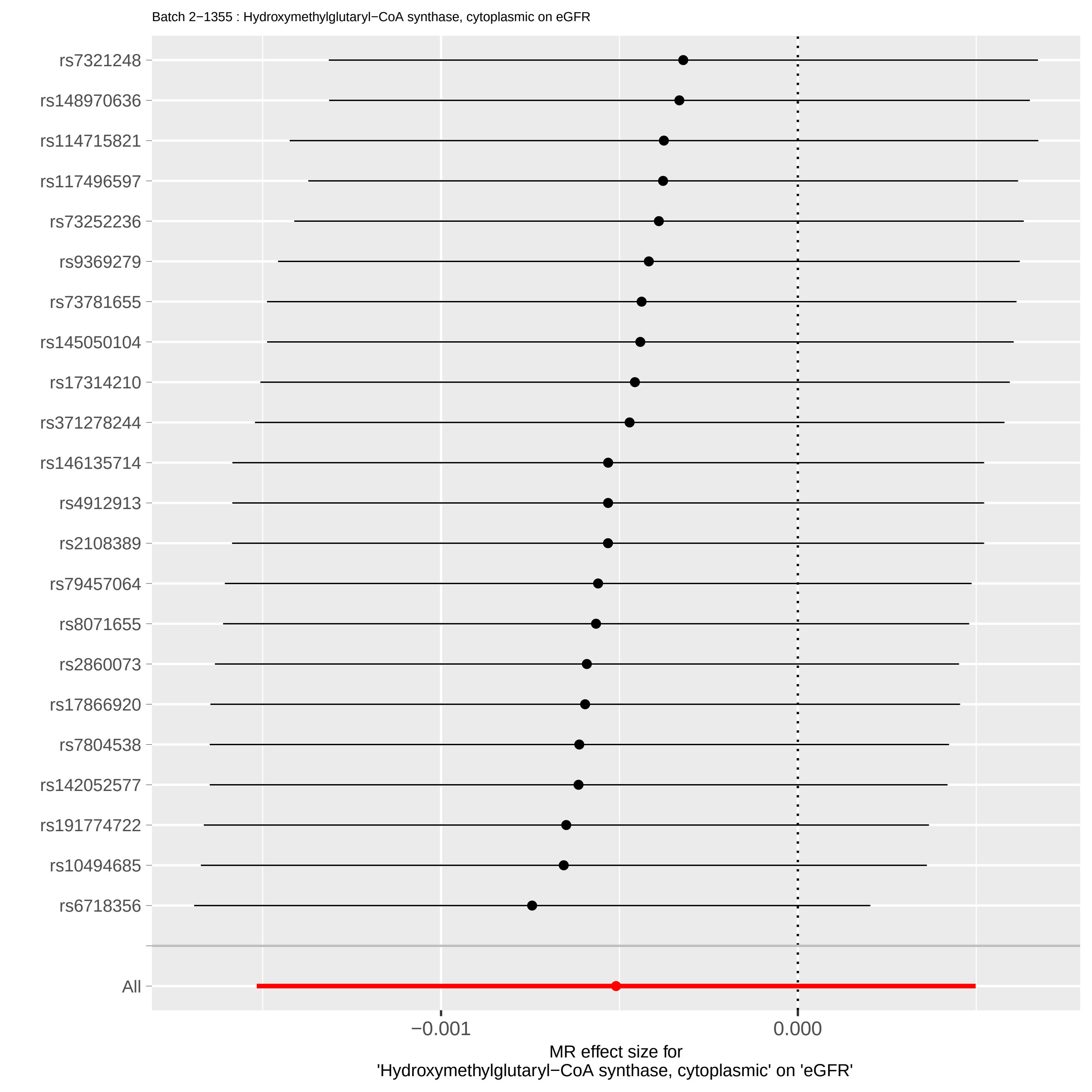

Batch 2−1355 : Hydroxymethylglutaryl−CoA synthase, cytoplasmic on eGFR
rs7321248
rs148970636
rs114715821
rs117496597
rs73252236
rs9369279
rs73781655
rs145050104
rs17314210
rs371278244
rs146135714
rs4912913
rs2108389
rs79457064
rs8071655
rs2860073
rs17866920
rs7804538
rs142052577
rs191774722
rs10494685
rs6718356
All
−0.001
0.000
MR effect size for
'Hydroxymethylglutaryl−CoA synthase, cytoplasmic' on 'eGFR'

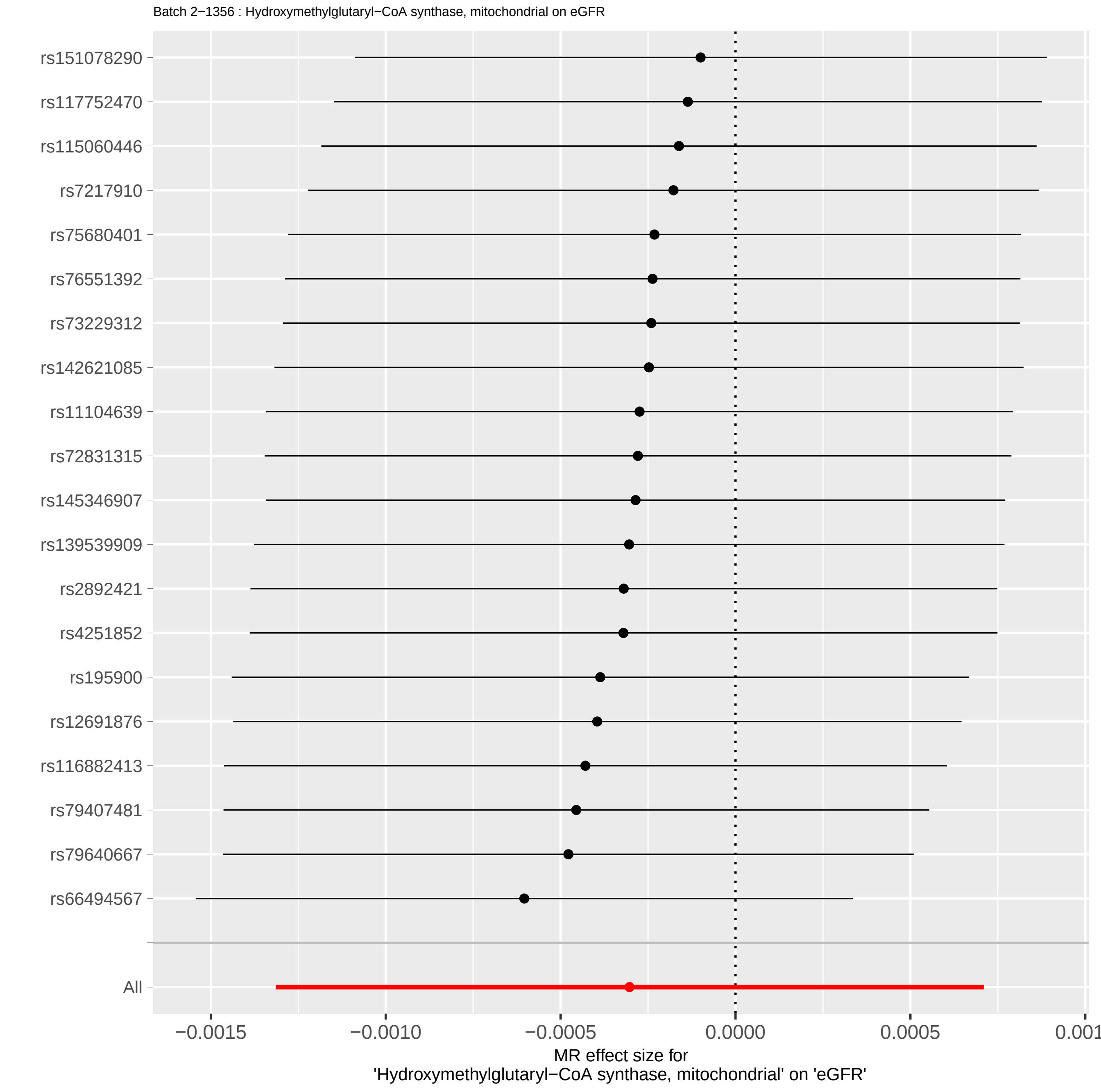

Batch 2−1356 : Hydroxymethylglutaryl−CoA synthase, mitochondrial on eGFR
rs151078290
rs117752470
rs115060446
rs7217910
rs75680401
rs76551392
rs73229312
rs142621085
rs11104639
rs72831315
rs145346907
rs139539909
rs2892421
rs4251852
rs195900
rs12691876
rs116882413
rs79407481
rs79640667
rs66494567
All
−0.0015
−0.0010
−0.0005
0.0000
0.0005
0.001
MR effect size for
'Hydroxymethylglutaryl−CoA synthase, mitochondrial' on 'eGFR'

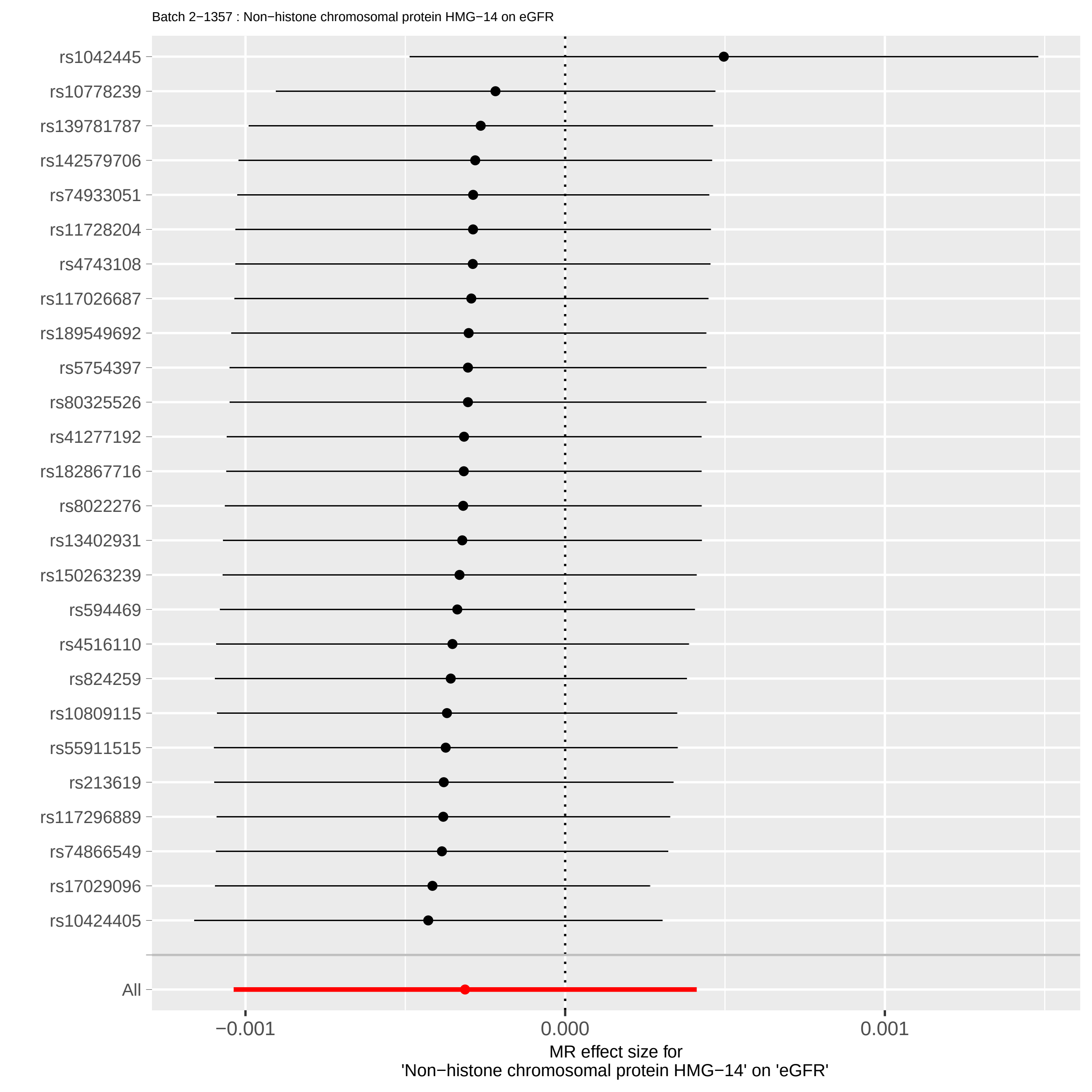

Batch 2−1357 : Non−histone chromosomal protein HMG−14 on eGFR
rs1042445
rs10778239
rs139781787
rs142579706
rs74933051
rs11728204
rs4743108
rs117026687
rs189549692
rs5754397
rs80325526
rs41277192
rs182867716
rs8022276
rs13402931
rs150263239
rs594469
rs4516110
rs824259
rs10809115
rs55911515
rs213619
rs117296889
rs74866549
rs17029096
rs10424405
All
−0.001
0.000
0.001
MR effect size for
'Non−histone chromosomal protein HMG−14' on 'eGFR'

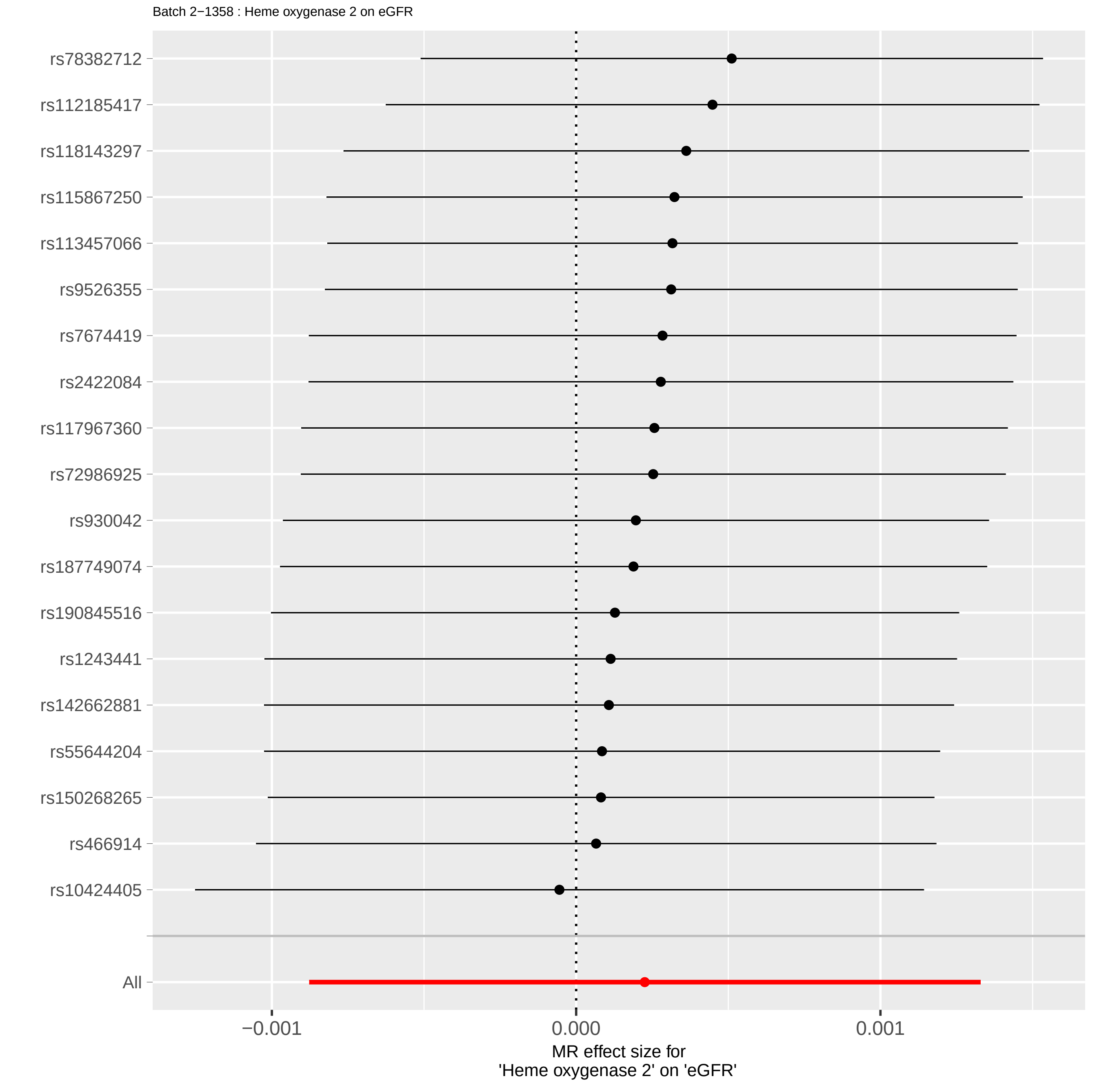

Batch 2−1358 : Heme oxygenase 2 on eGFR
rs78382712
rs112185417
rs118143297
rs115867250
rs113457066
rs9526355
rs7674419
rs2422084
rs117967360
rs72986925
rs930042
rs187749074
rs190845516
rs1243441
rs142662881
rs55644204
rs150268265
rs466914
rs10424405
All
−0.001
0.000
0.001
MR effect size for
'Heme oxygenase 2' on 'eGFR'

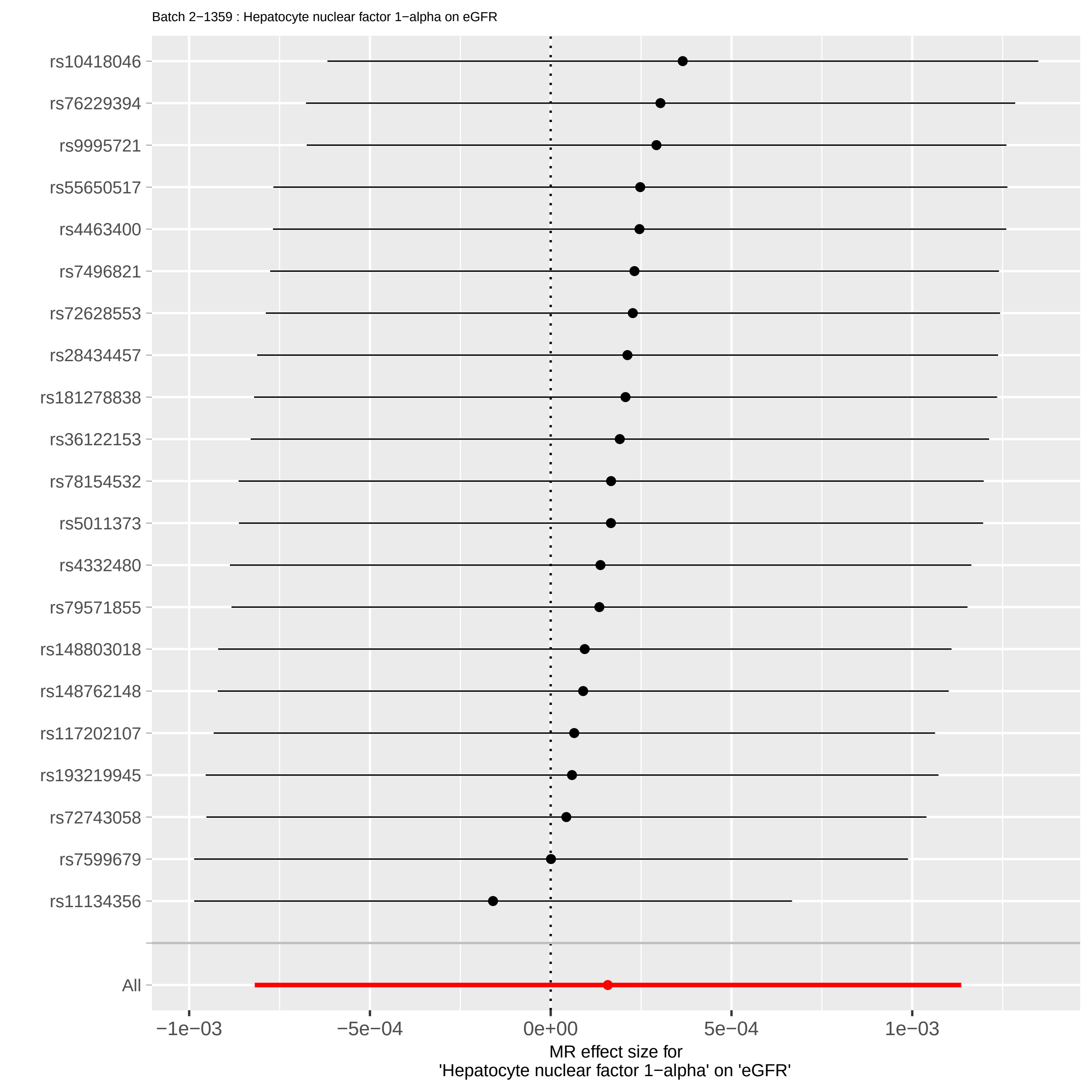
Batch 2−1359 : Hepatocyte nuclear factor 1−alpha on eGFR
rs10418046
rs76229394
rs9995721
rs55650517
rs4463400
rs7496821
rs72628553
rs28434457
rs181278838
rs36122153
rs78154532
rs5011373
rs4332480
rs79571855
rs148803018
rs148762148
rs117202107
rs193219945
rs72743058
rs7599679
rs11134356
All
−1e−03
−5e−04
0e+00
5e−04
1e−03
MR effect size for
'Hepatocyte nuclear factor 1−alpha' on 'eGFR'

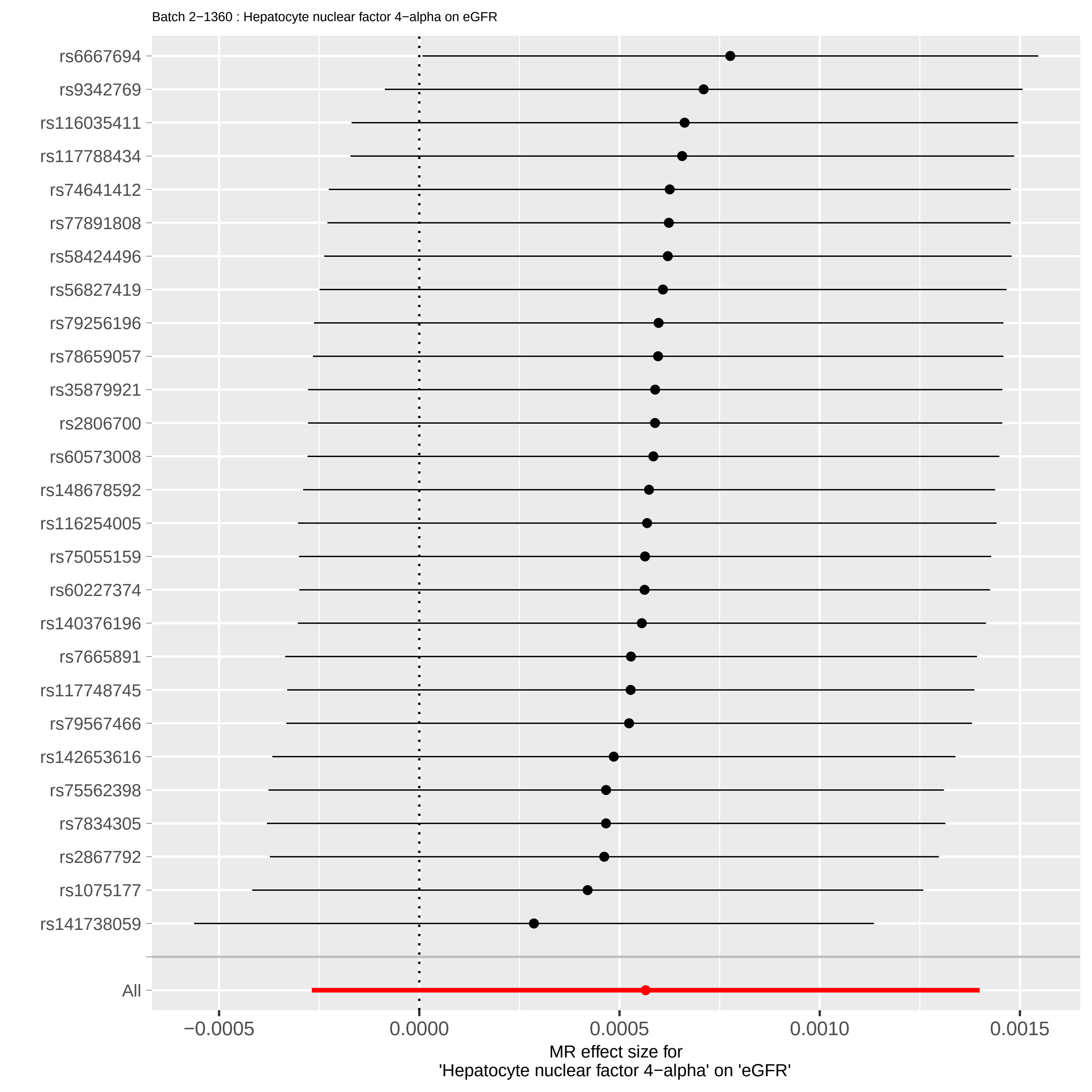

Batch 2−1360 : Hepatocyte nuclear factor 4−alpha on eGFR
rs6667694
rs9342769
rs116035411
rs117788434
rs74641412
rs77891808
rs58424496
rs56827419
rs79256196
rs78659057
rs35879921
rs2806700
rs60573008
rs148678592
rs116254005
rs75055159
rs60227374
rs140376196
rs7665891
rs117748745
rs79567466
rs142653616
rs75562398
rs7834305
rs2867792
rs1075177
rs141738059
All
−0.0005
0.0000
0.0005
0.0010
0.0015
MR effect size for
'Hepatocyte nuclear factor 4−alpha' on 'eGFR'

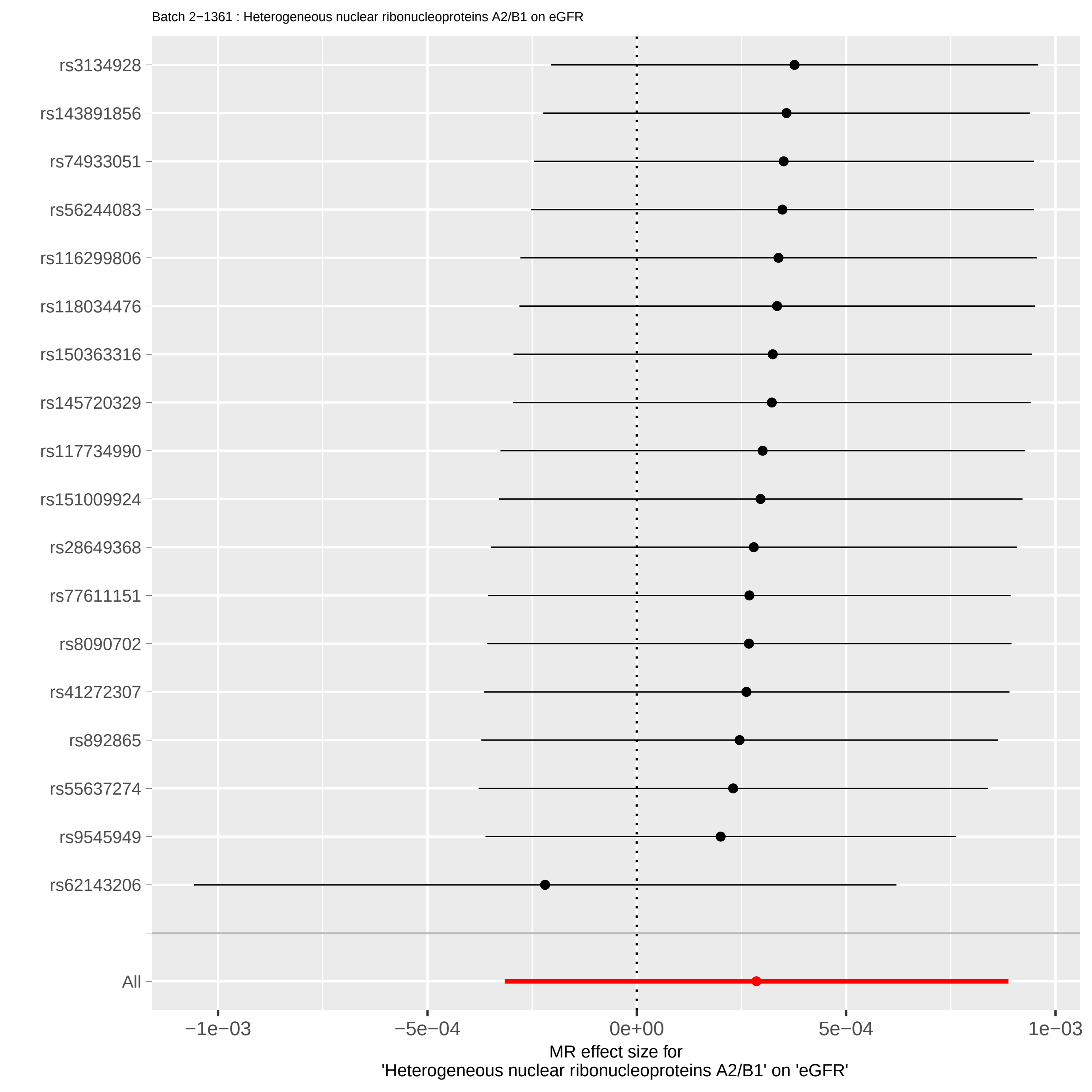

Batch 2−1361 : Heterogeneous nuclear ribonucleoproteins A2/B1 on eGFR
rs3134928
rs143891856
rs74933051
rs56244083
rs116299806
rs118034476
rs150363316
rs145720329
rs117734990
rs151009924
rs28649368
rs77611151
rs8090702
rs41272307
rs892865
rs55637274
rs9545949
rs62143206
All
−1e−03
−5e−04
0e+00
5e−04
1e−03
MR effect size for
'Heterogeneous nuclear ribonucleoproteins A2/B1' on 'eGFR'

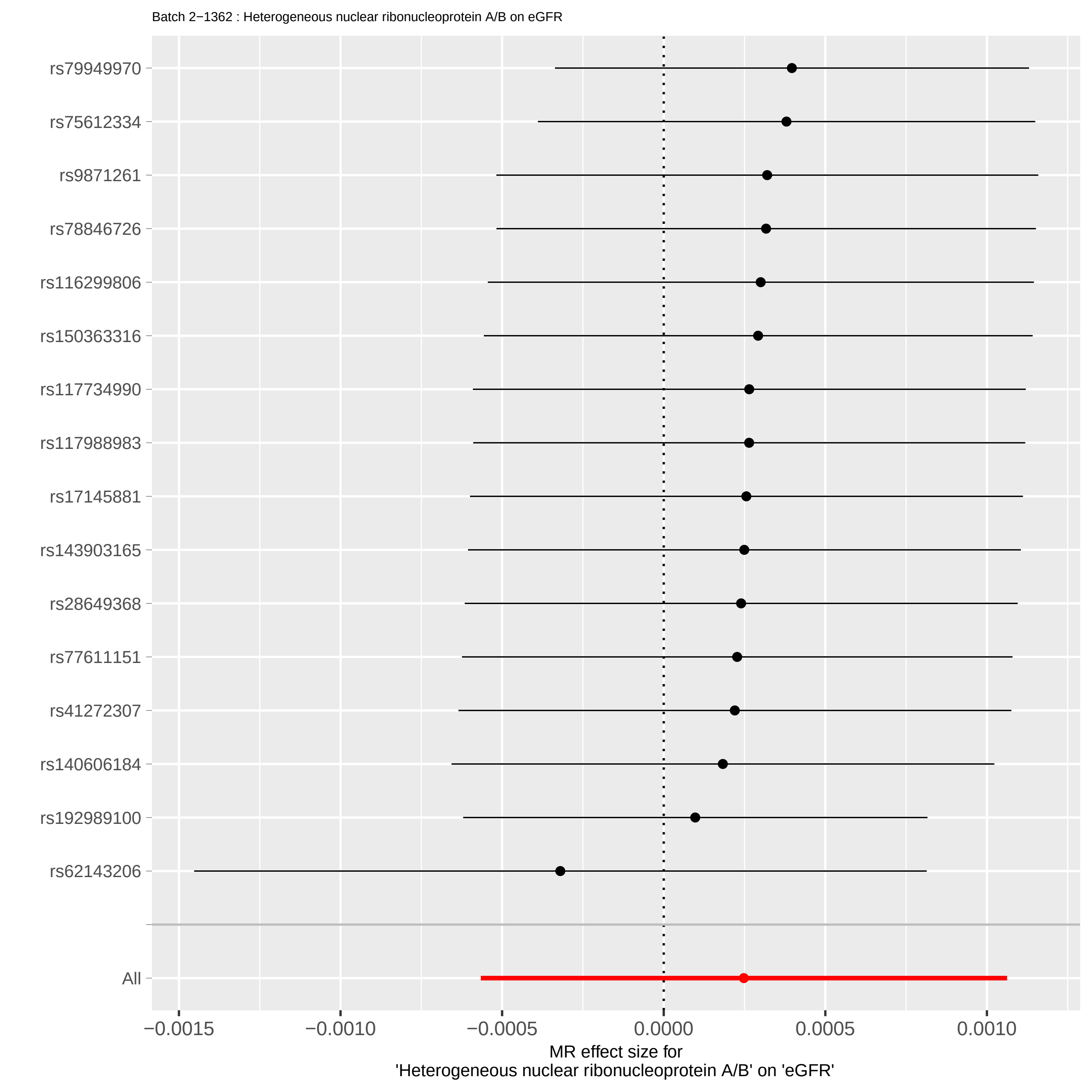

Batch 2−1362 : Heterogeneous nuclear ribonucleoprotein A/B on eGFR
rs79949970
rs75612334
rs9871261
rs78846726
rs116299806
rs150363316
rs117734990
rs117988983
rs17145881
rs143903165
rs28649368
rs77611151
rs41272307
rs140606184
rs192989100
rs62143206
All
−0.0015
−0.0010
−0.0005
0.0000
0.0005
0.0010
MR effect size for
'Heterogeneous nuclear ribonucleoprotein A/B' on 'eGFR'

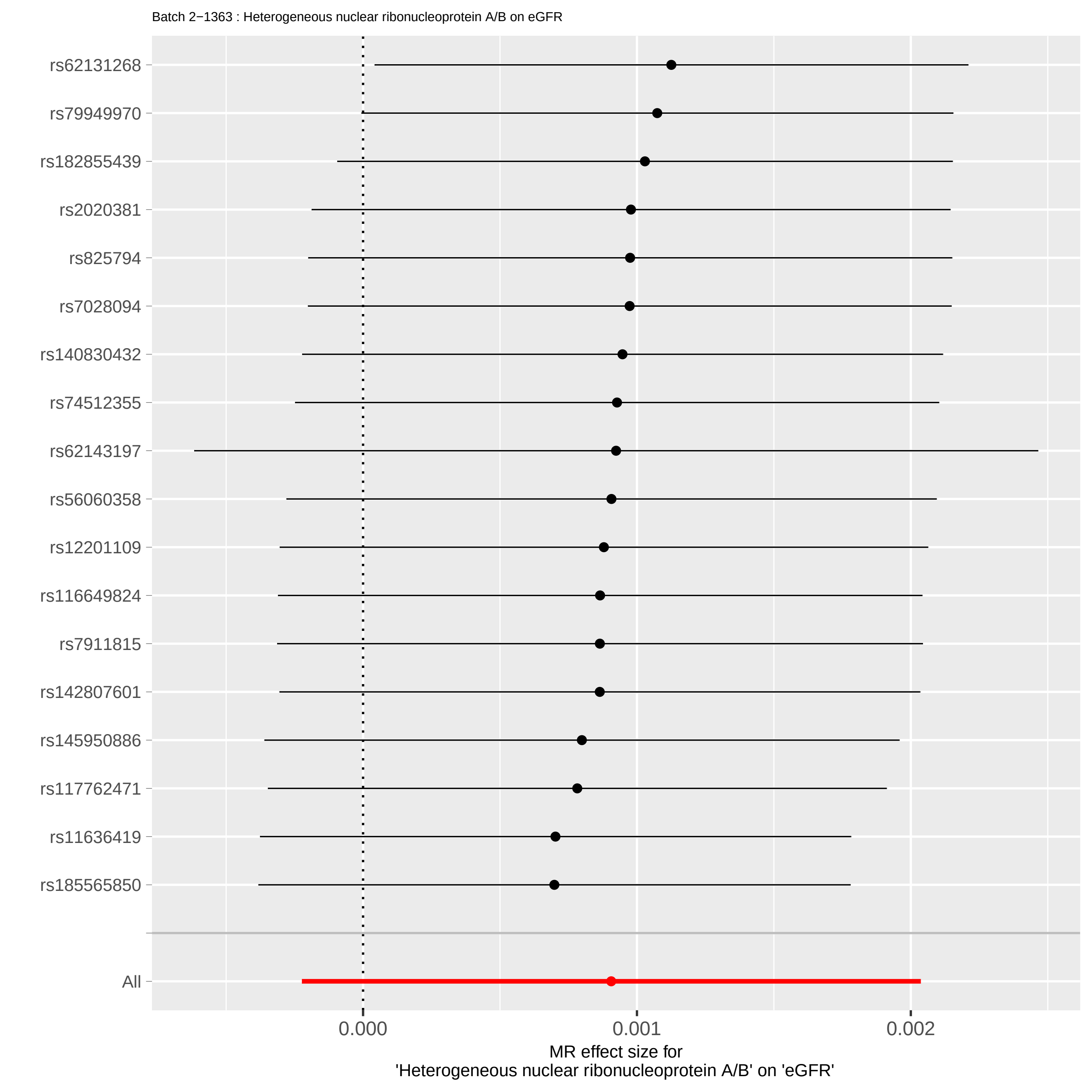

Batch 2−1363 : Heterogeneous nuclear ribonucleoprotein A/B on eGFR
rs62131268
rs79949970
rs182855439
rs2020381
rs825794
rs7028094
rs140830432
rs74512355
rs62143197
rs56060358
rs12201109
rs116649824
rs7911815
rs142807601
rs145950886
rs117762471
rs11636419
rs185565850
All
0.000
0.001
0.002
MR effect size for
'Heterogeneous nuclear ribonucleoprotein A/B' on 'eGFR'

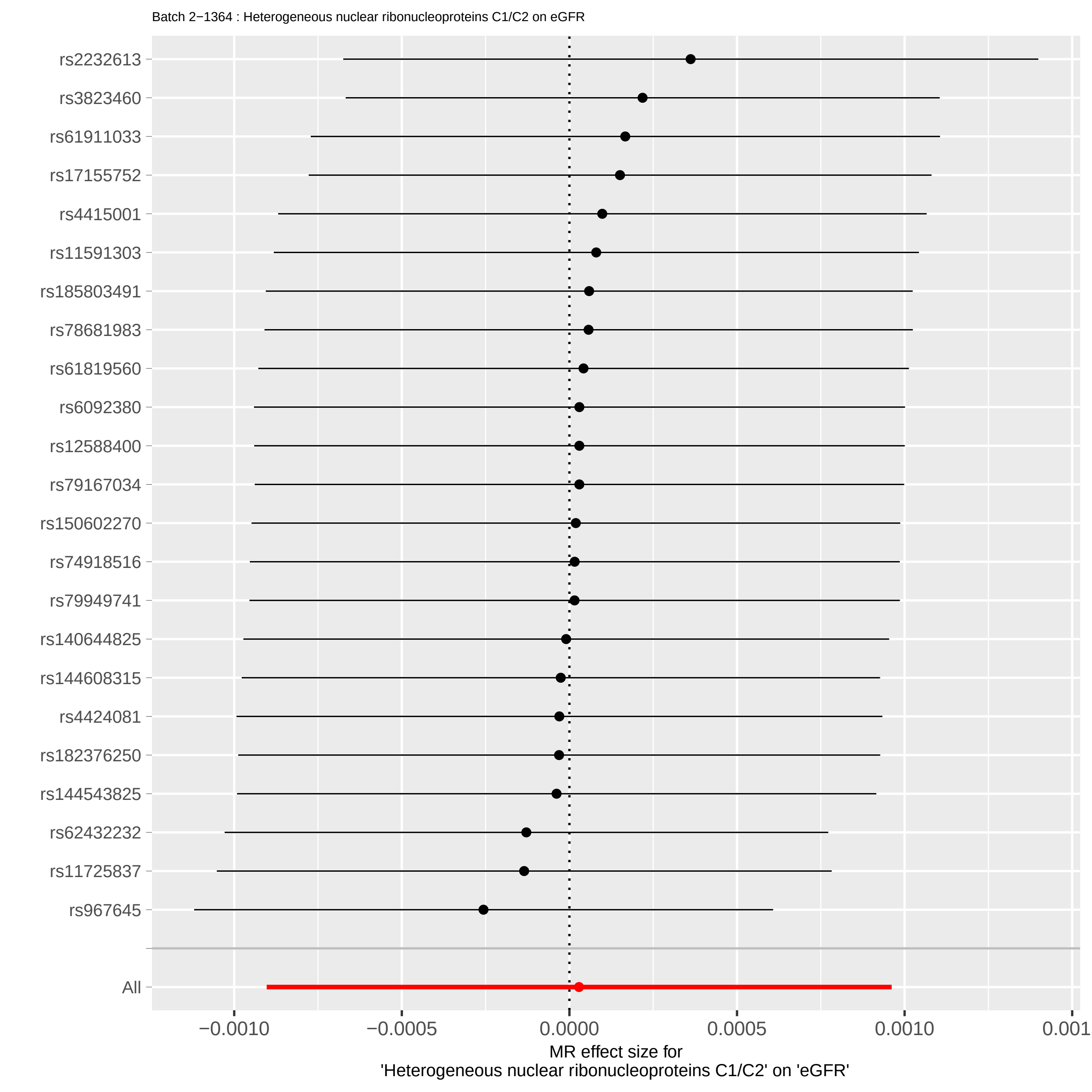

Batch 2−1364 : Heterogeneous nuclear ribonucleoproteins C1/C2 on eGFR
rs2232613
rs3823460
rs61911033
rs17155752
rs4415001
rs11591303
rs185803491
rs78681983
rs61819560
rs6092380
rs12588400
rs79167034
rs150602270
rs74918516
rs79949741
rs140644825
rs144608315
rs4424081
rs182376250
rs144543825
rs62432232
rs11725837
rs967645
All
−0.0010
−0.0005
0.0000
0.0005
0.0010
0.001
MR effect size for
'Heterogeneous nuclear ribonucleoproteins C1/C2' on 'eGFR'

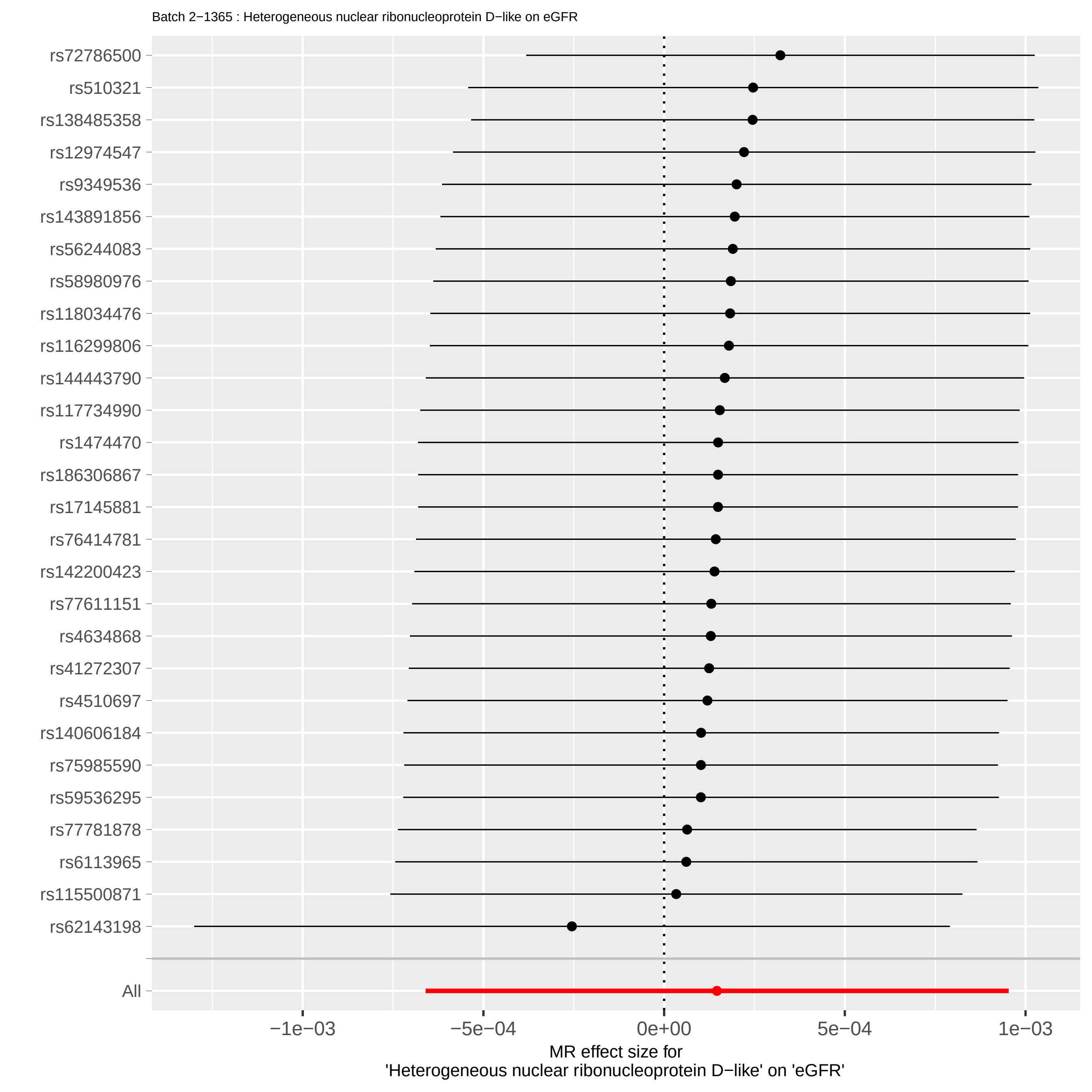

Batch 2−1365 : Heterogeneous nuclear ribonucleoprotein D−like on eGFR
rs72786500
rs510321
rs138485358
rs12974547
rs9349536
rs143891856
rs56244083
rs58980976
rs118034476
rs116299806
rs144443790
rs117734990
rs1474470
rs186306867
rs17145881
rs76414781
rs142200423
rs77611151
rs4634868
rs41272307
rs4510697
rs140606184
rs75985590
rs59536295
rs77781878
rs6113965
rs115500871
rs62143198
All
−1e−03
−5e−04
0e+00
5e−04
1e−03
MR effect size for
'Heterogeneous nuclear ribonucleoprotein D−like' on 'eGFR'

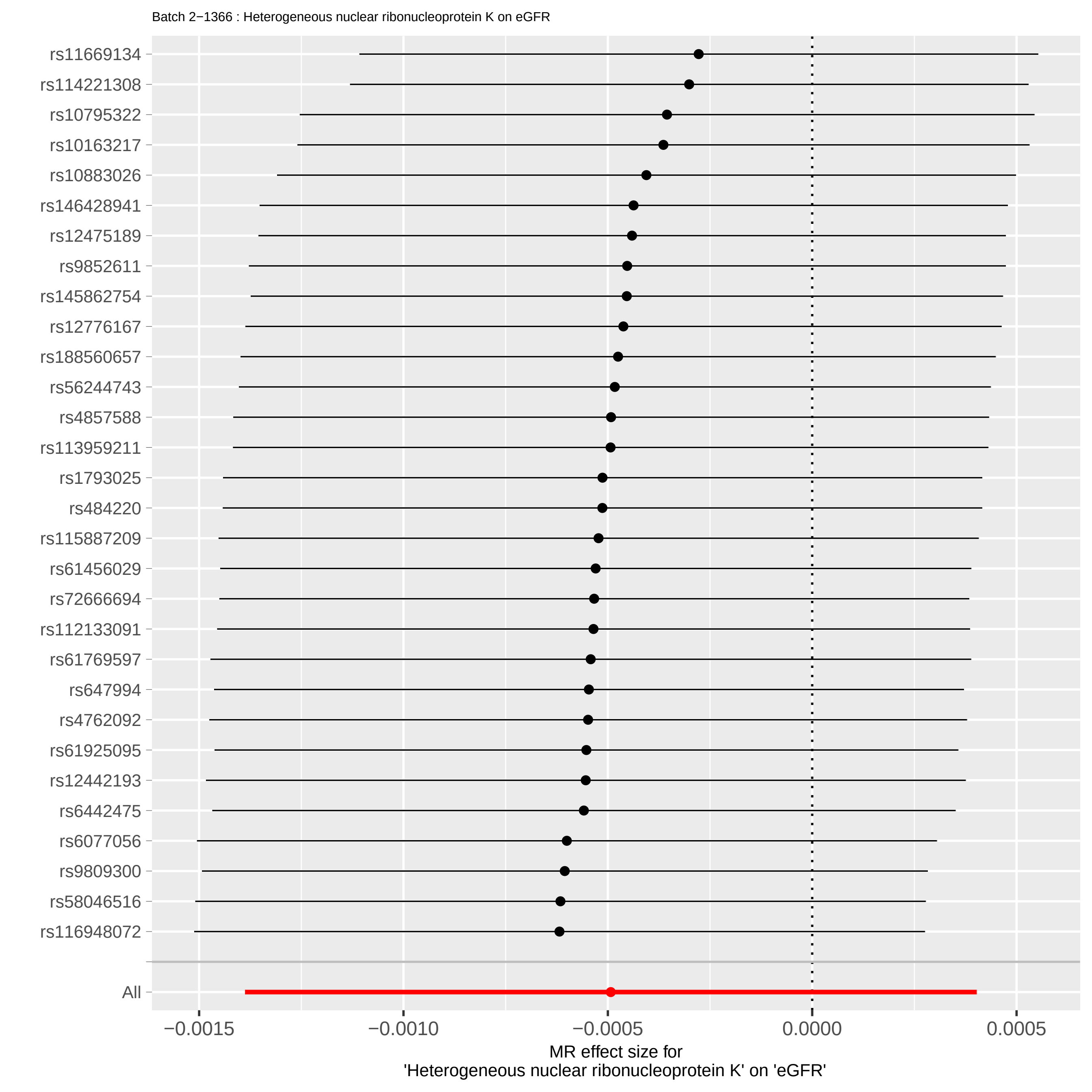

Batch 2−1366 : Heterogeneous nuclear ribonucleoprotein K on eGFR
rs11669134
rs114221308
rs10795322
rs10163217
rs10883026
rs146428941
rs12475189
rs9852611
rs145862754
rs12776167
rs188560657
rs56244743
rs4857588
rs113959211
rs1793025
rs484220
rs115887209
rs61456029
rs72666694
rs112133091
rs61769597
rs647994
rs4762092
rs61925095
rs12442193
rs6442475
rs6077056
rs9809300
rs58046516
rs116948072
All
−0.0015
−0.0010
−0.0005
0.0000
0.0005
MR effect size for
'Heterogeneous nuclear ribonucleoprotein K' on 'eGFR'

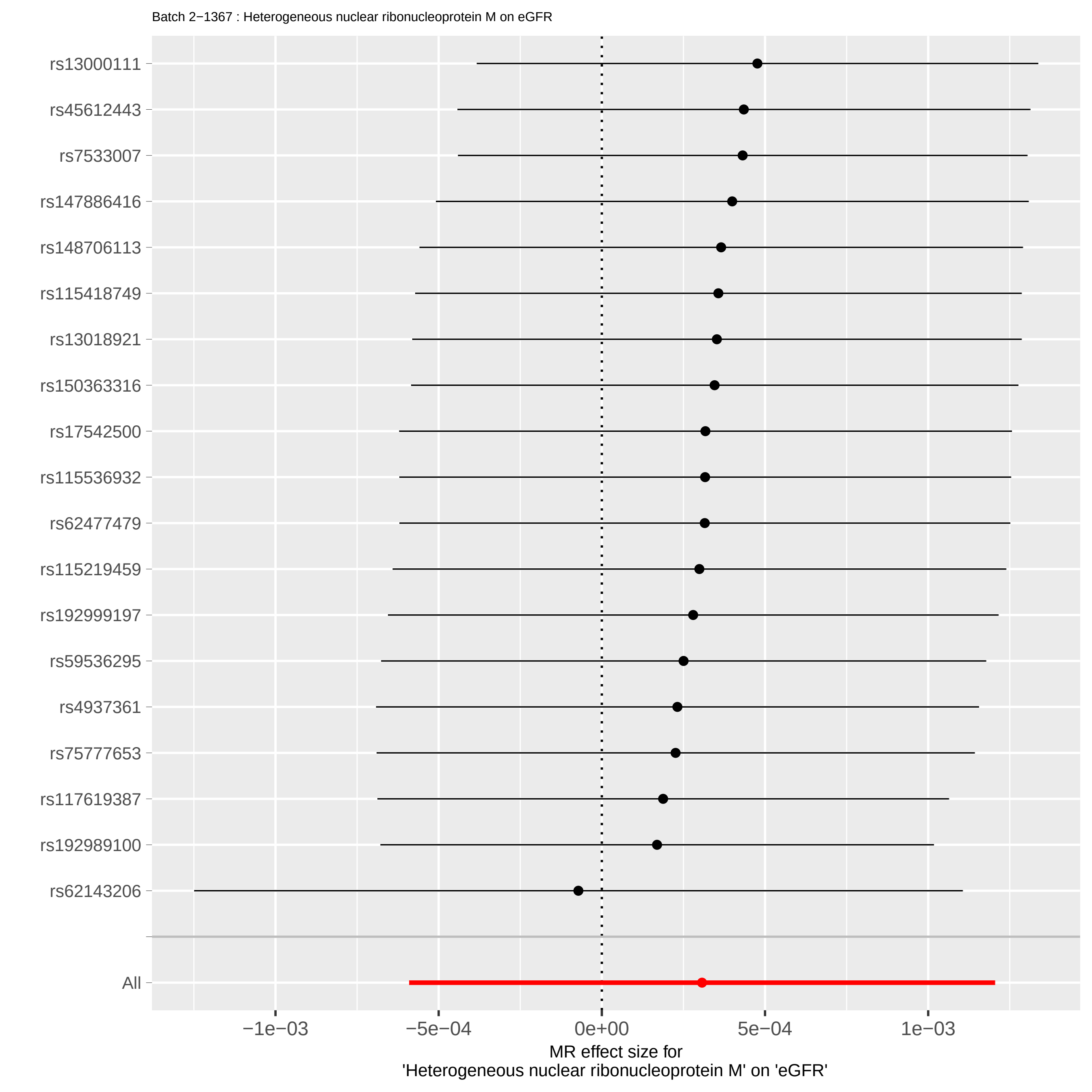

Batch 2−1367 : Heterogeneous nuclear ribonucleoprotein M on eGFR
rs13000111
rs45612443
rs7533007
rs147886416
rs148706113
rs115418749
rs13018921
rs150363316
rs17542500
rs115536932
rs62477479
rs115219459
rs192999197
rs59536295
rs4937361
rs75777653
rs117619387
rs192989100
rs62143206
All
−1e−03
−5e−04
0e+00
5e−04
1e−03
MR effect size for
'Heterogeneous nuclear ribonucleoprotein M' on 'eGFR'

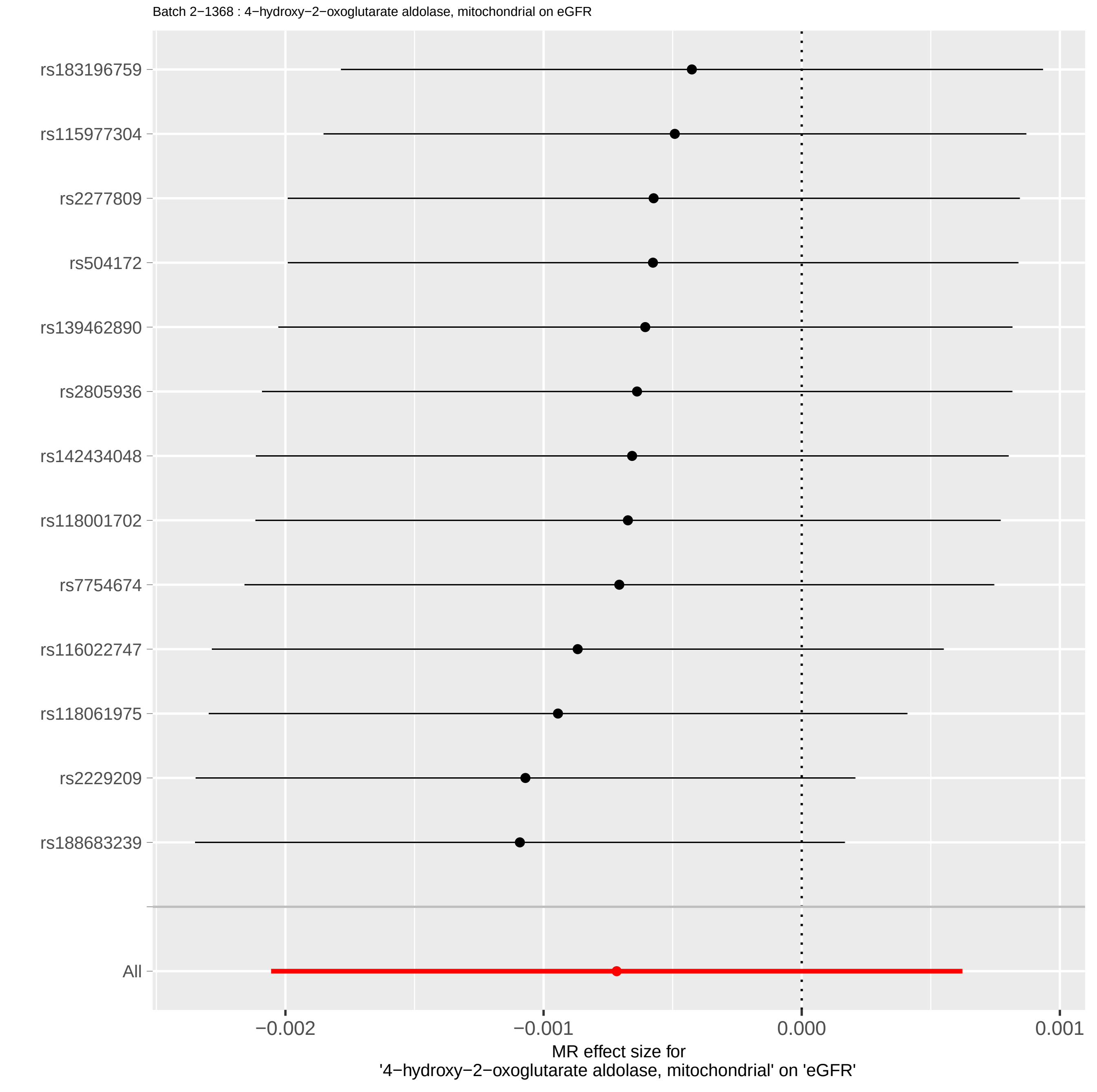
Batch 2−1368 : 4−hydroxy−2−oxoglutarate aldolase, mitochondrial on eGFR
rs183196759
rs115977304
rs2277809
rs504172
rs139462890
rs2805936
rs142434048
rs118001702
rs7754674
rs116022747
rs118061975
rs2229209
rs188683239
All
−0.002
−0.001
0.000
0.001
MR effect size for
'4−hydroxy−2−oxoglutarate aldolase, mitochondrial' on 'eGFR'

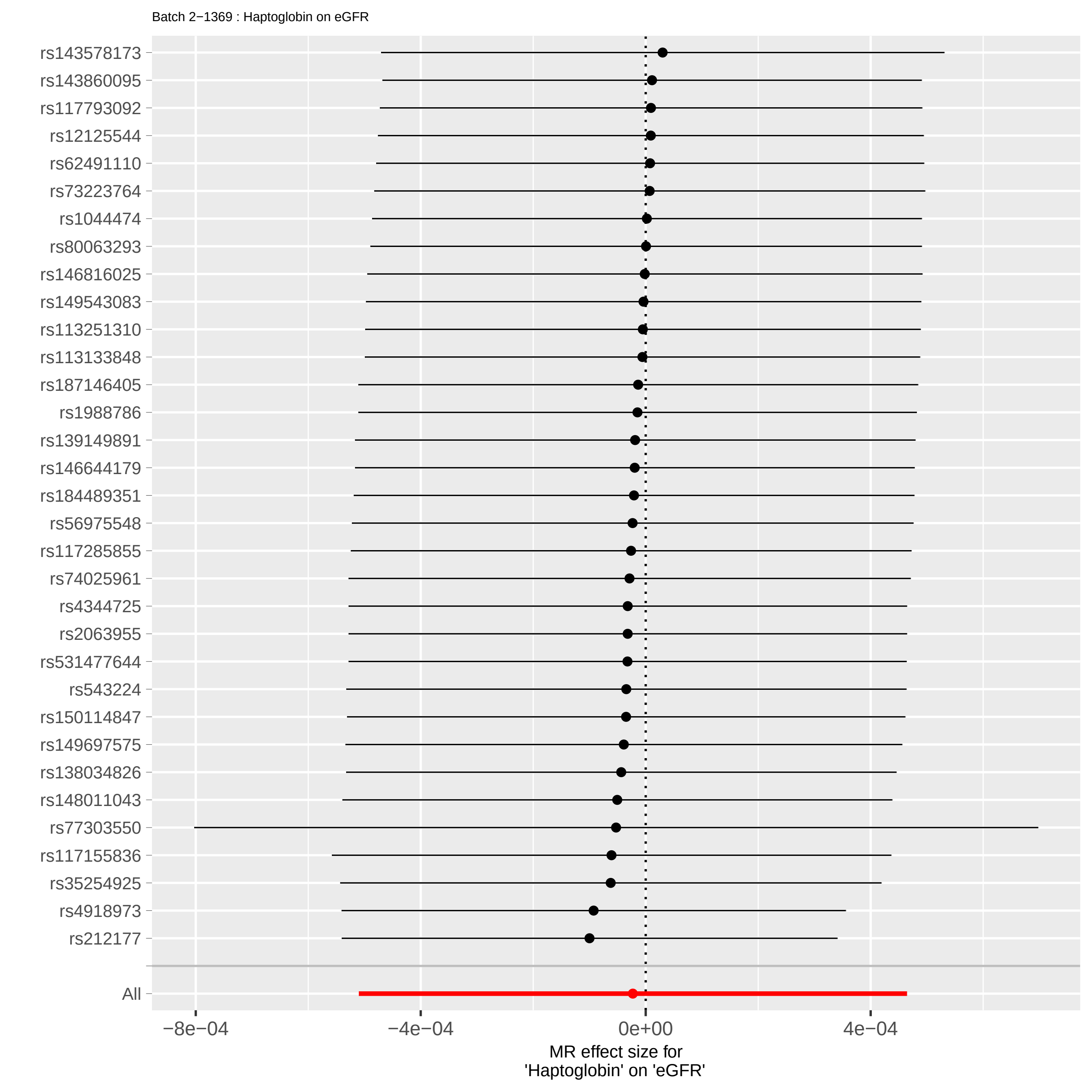

Batch 2−1369 : Haptoglobin on eGFR
rs143578173
rs143860095
rs117793092
rs12125544
rs62491110
rs73223764
rs1044474
rs80063293
rs146816025
rs149543083
rs113251310
rs113133848
rs187146405
rs1988786
rs139149891
rs146644179
rs184489351
rs56975548
rs117285855
rs74025961
rs4344725
rs2063955
rs531477644
rs543224
rs150114847
rs149697575
rs138034826
rs148011043
rs77303550
rs117155836
rs35254925
rs4918973
rs212177
All
−8e−04
−4e−04
0e+00
4e−04
MR effect size for
'Haptoglobin' on 'eGFR'

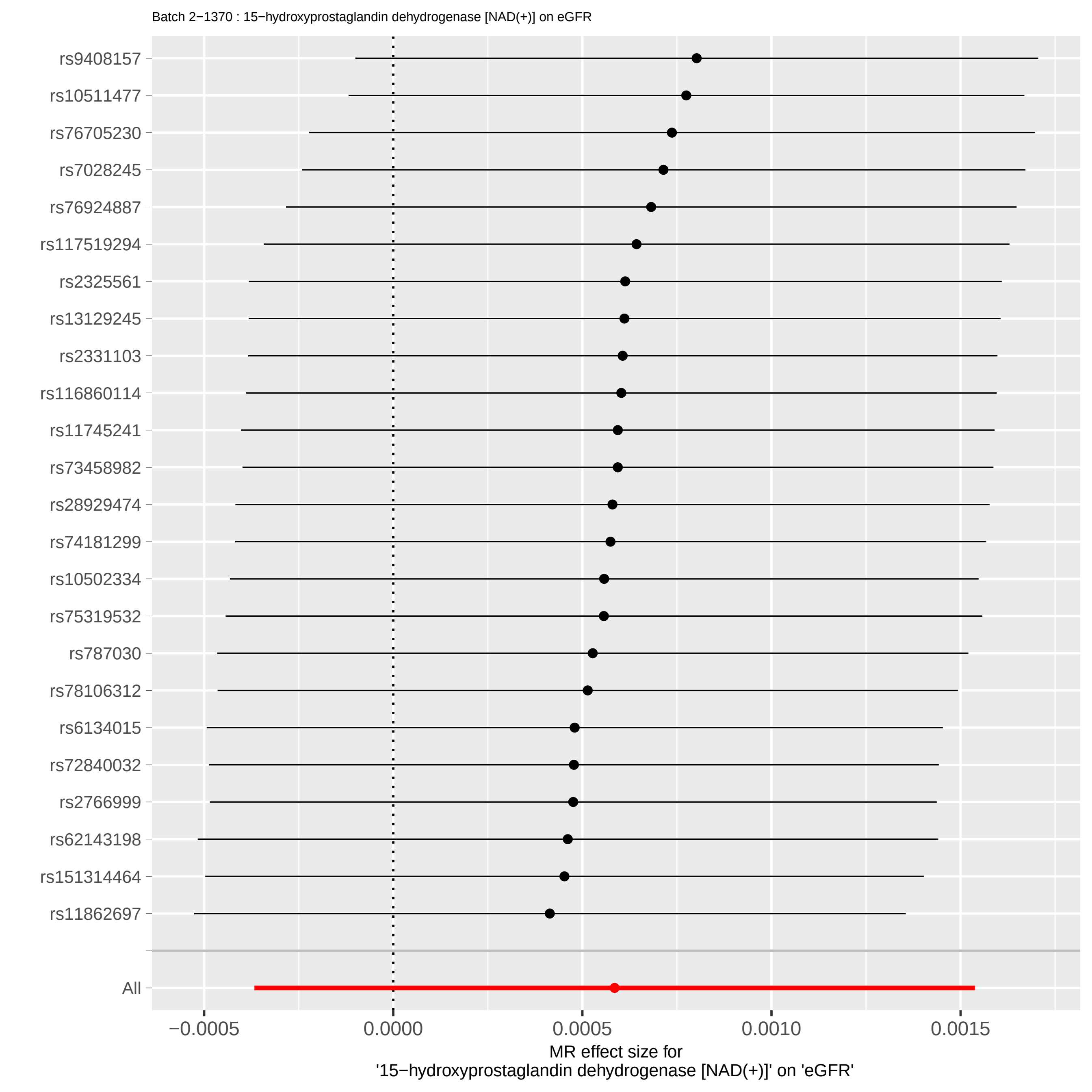

Batch 2−1370 : 15−hydroxyprostaglandin dehydrogenase [NAD(+)] on eGFR
rs9408157
rs10511477
rs76705230
rs7028245
rs76924887
rs117519294
rs2325561
rs13129245
rs2331103
rs116860114
rs11745241
rs73458982
rs28929474
rs74181299
rs10502334
rs75319532
rs787030
rs78106312
rs6134015
rs72840032
rs2766999
rs62143198
rs151314464
rs11862697
All
−0.0005
0.0000
0.0005
0.0010
0.0015
MR effect size for
'15−hydroxyprostaglandin dehydrogenase [NAD(+)]' on 'eGFR'

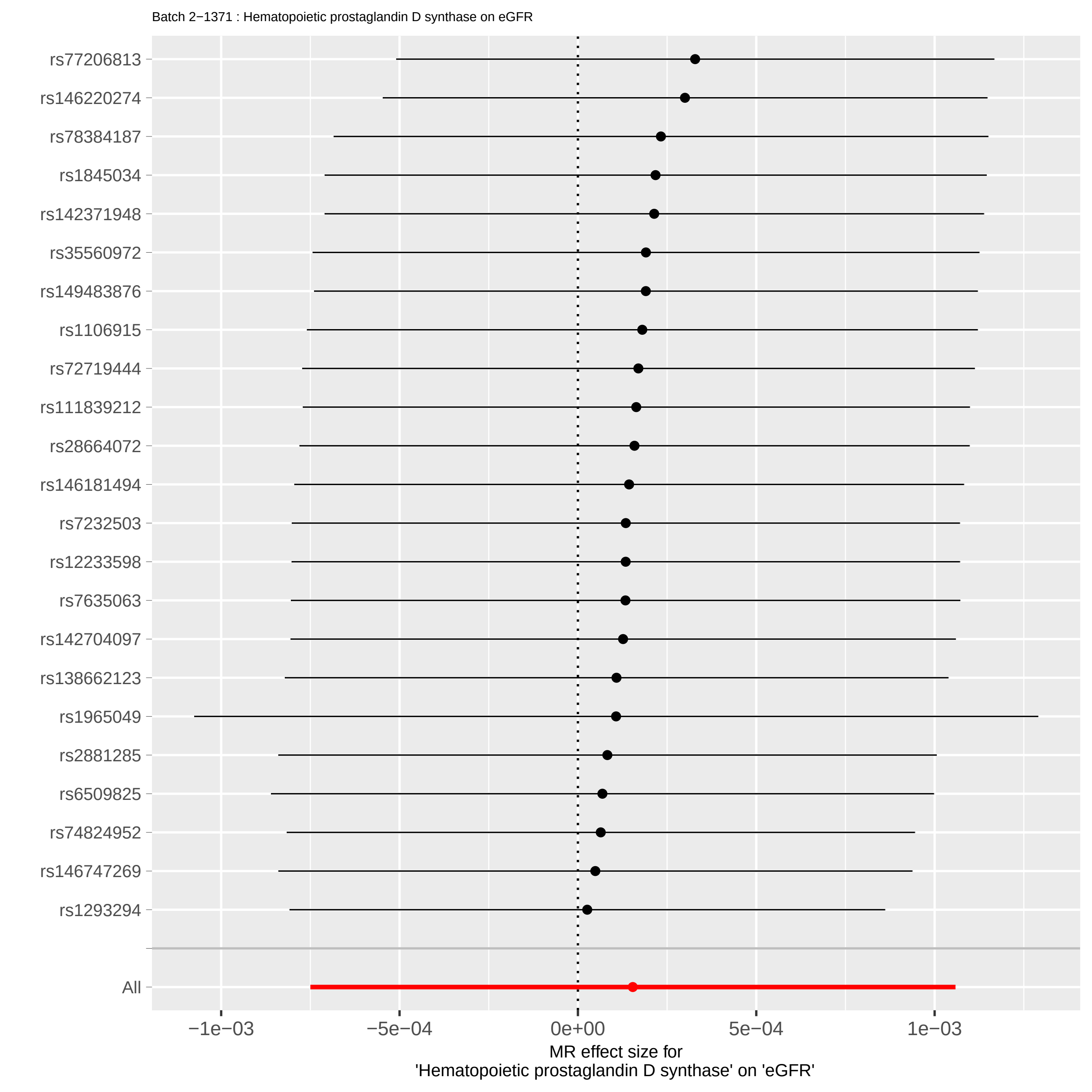

Batch 2−1371 : Hematopoietic prostaglandin D synthase on eGFR
rs77206813
rs146220274
rs78384187
rs1845034
rs142371948
rs35560972
rs149483876
rs1106915
rs72719444
rs111839212
rs28664072
rs146181494
rs7232503
rs12233598
rs7635063
rs142704097
rs138662123
rs1965049
rs2881285
rs6509825
rs74824952
rs146747269
rs1293294
All
−1e−03
−5e−04
0e+00
5e−04
1e−03
MR effect size for
'Hematopoietic prostaglandin D synthase' on 'eGFR'

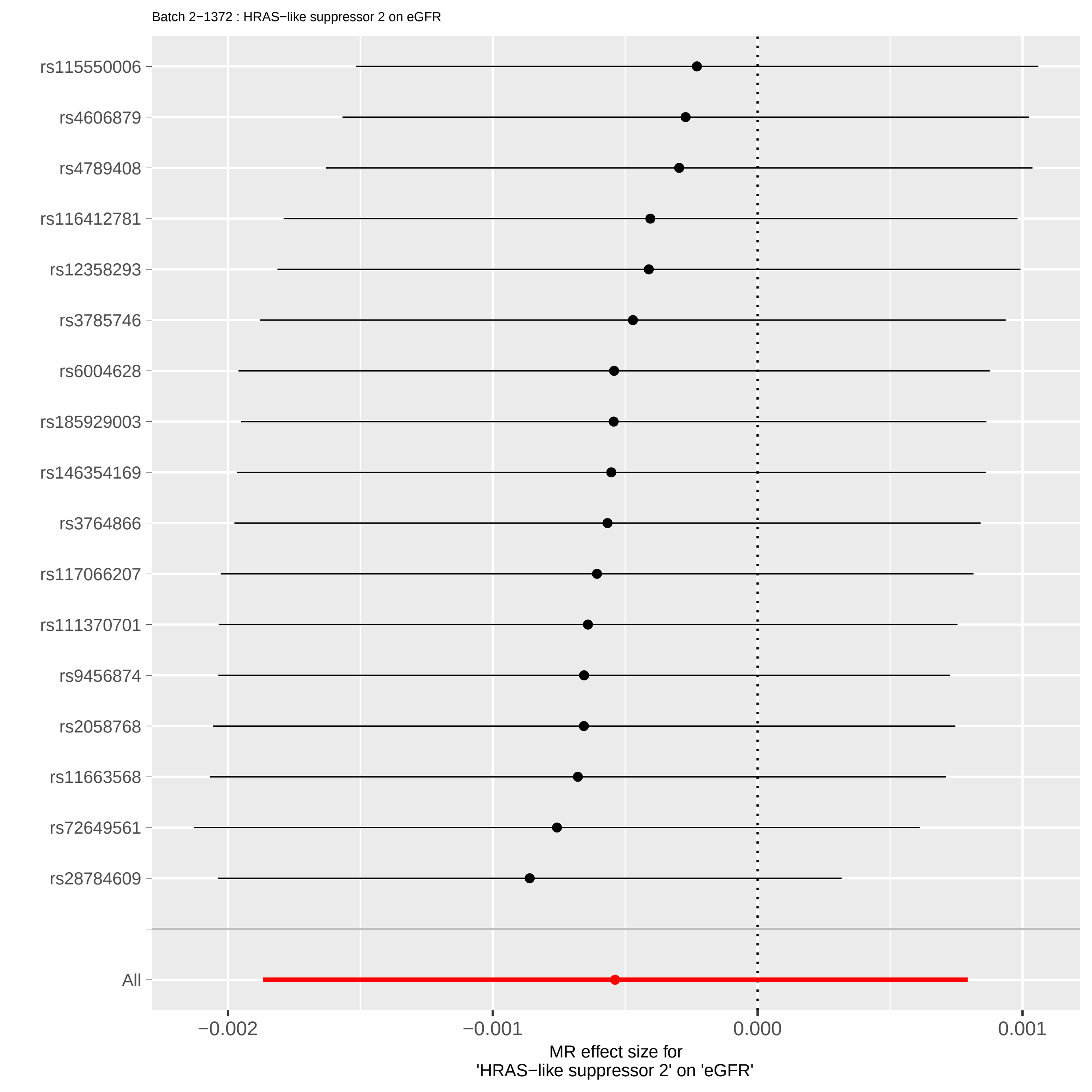

Batch 2−1372 : HRAS−like suppressor 2 on eGFR
rs115550006
rs4606879
rs4789408
rs116412781
rs12358293
rs3785746
rs6004628
rs185929003
rs146354169
rs3764866
rs117066207
rs111370701
rs9456874
rs2058768
rs11663568
rs72649561
rs28784609
All
−0.002
−0.001
0.000
0.001
MR effect size for
'HRAS−like suppressor 2' on 'eGFR'

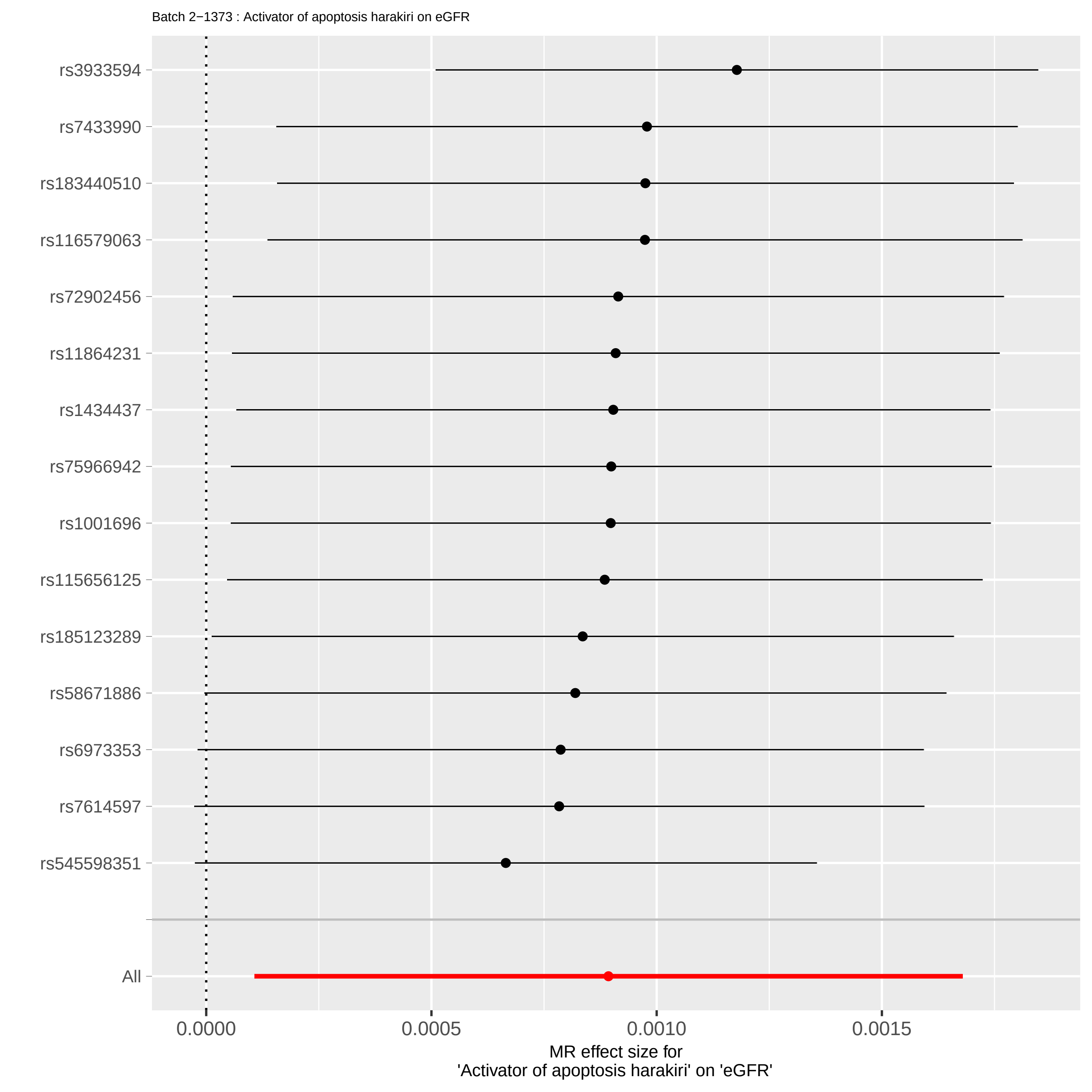
Batch 2−1373 : Activator of apoptosis harakiri on eGFR
rs3933594
rs7433990
rs183440510
rs116579063
rs72902456
rs11864231
rs1434437
rs75966942
rs1001696
rs115656125
rs185123289
rs58671886
rs6973353
rs7614597
rs545598351
All
0.0000
0.0005
0.0010
0.0015
MR effect size for
'Activator of apoptosis harakiri' on 'eGFR'

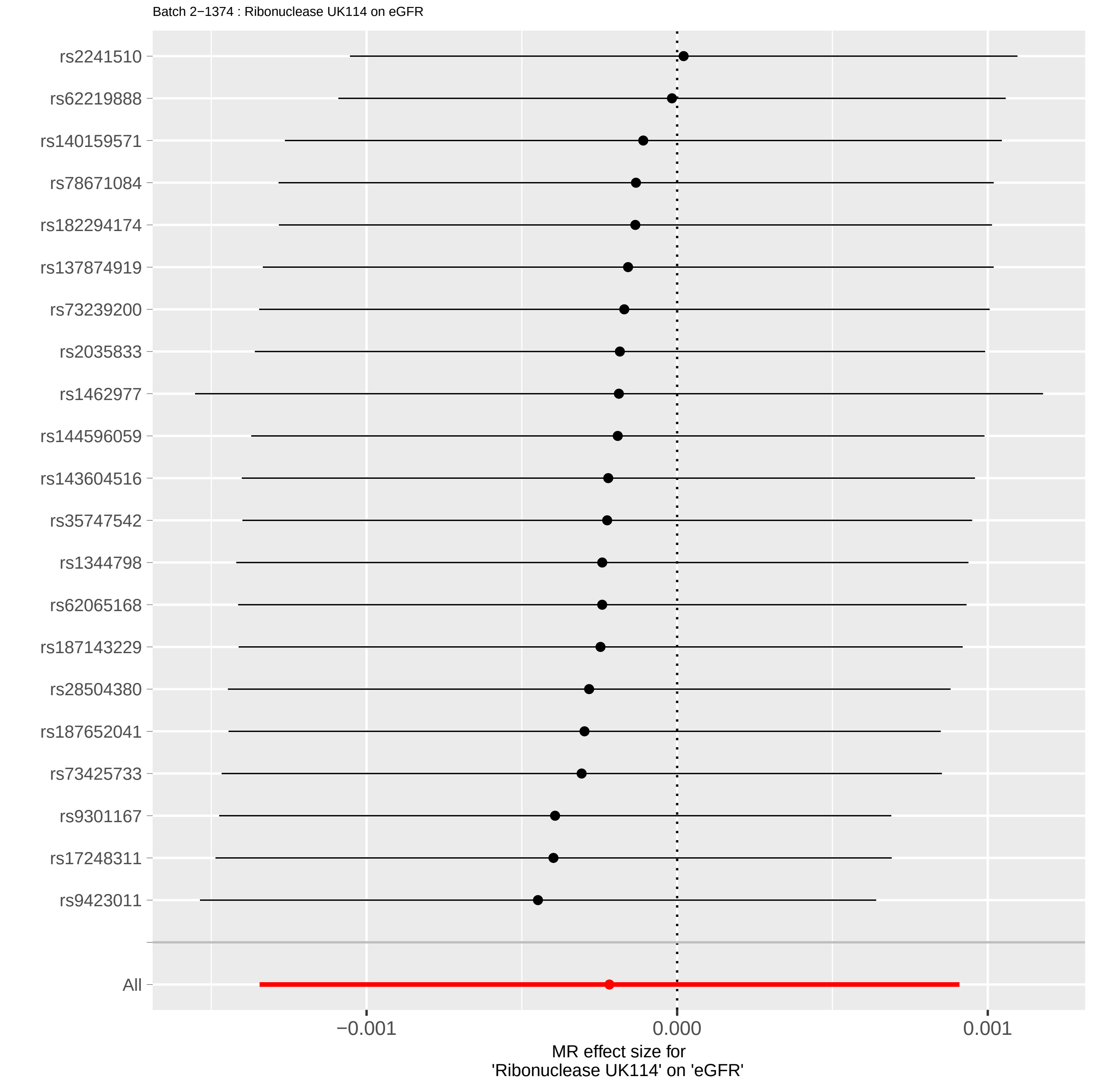

Batch 2−1374 : Ribonuclease UK114 on eGFR
rs2241510
rs62219888
rs140159571
rs78671084
rs182294174
rs137874919
rs73239200
rs2035833
rs1462977
rs144596059
rs143604516
rs35747542
rs1344798
rs62065168
rs187143229
rs28504380
rs187652041
rs73425733
rs9301167
rs17248311
rs9423011
All
−0.001
0.000
0.001
MR effect size for
'Ribonuclease UK114' on 'eGFR'

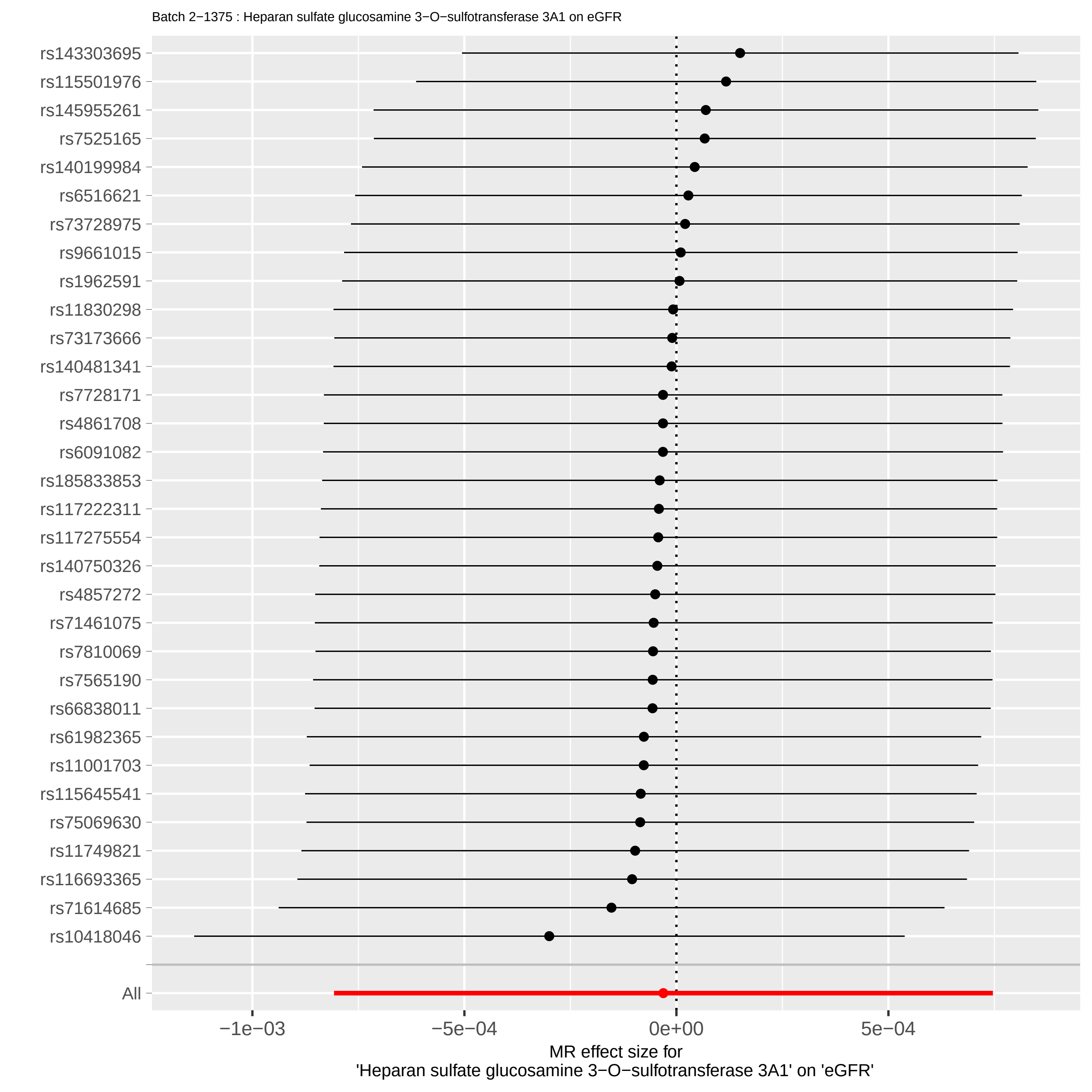
Batch 2−1375 : Heparan sulfate glucosamine 3−O−sulfotransferase 3A1 on eGFR
rs143303695
rs115501976
rs145955261
rs7525165
rs140199984
rs6516621
rs73728975
rs9661015
rs1962591
rs11830298
rs73173666
rs140481341
rs7728171
rs4861708
rs6091082
rs185833853
rs117222311
rs117275554
rs140750326
rs4857272
rs71461075
rs7810069
rs7565190
rs66838011
rs61982365
rs11001703
rs115645541
rs75069630
rs11749821
rs116693365
rs71614685
rs10418046
All
−1e−03
−5e−04
0e+00
5e−04
MR effect size for
'Heparan sulfate glucosamine 3−O−sulfotransferase 3A1' on 'eGFR'

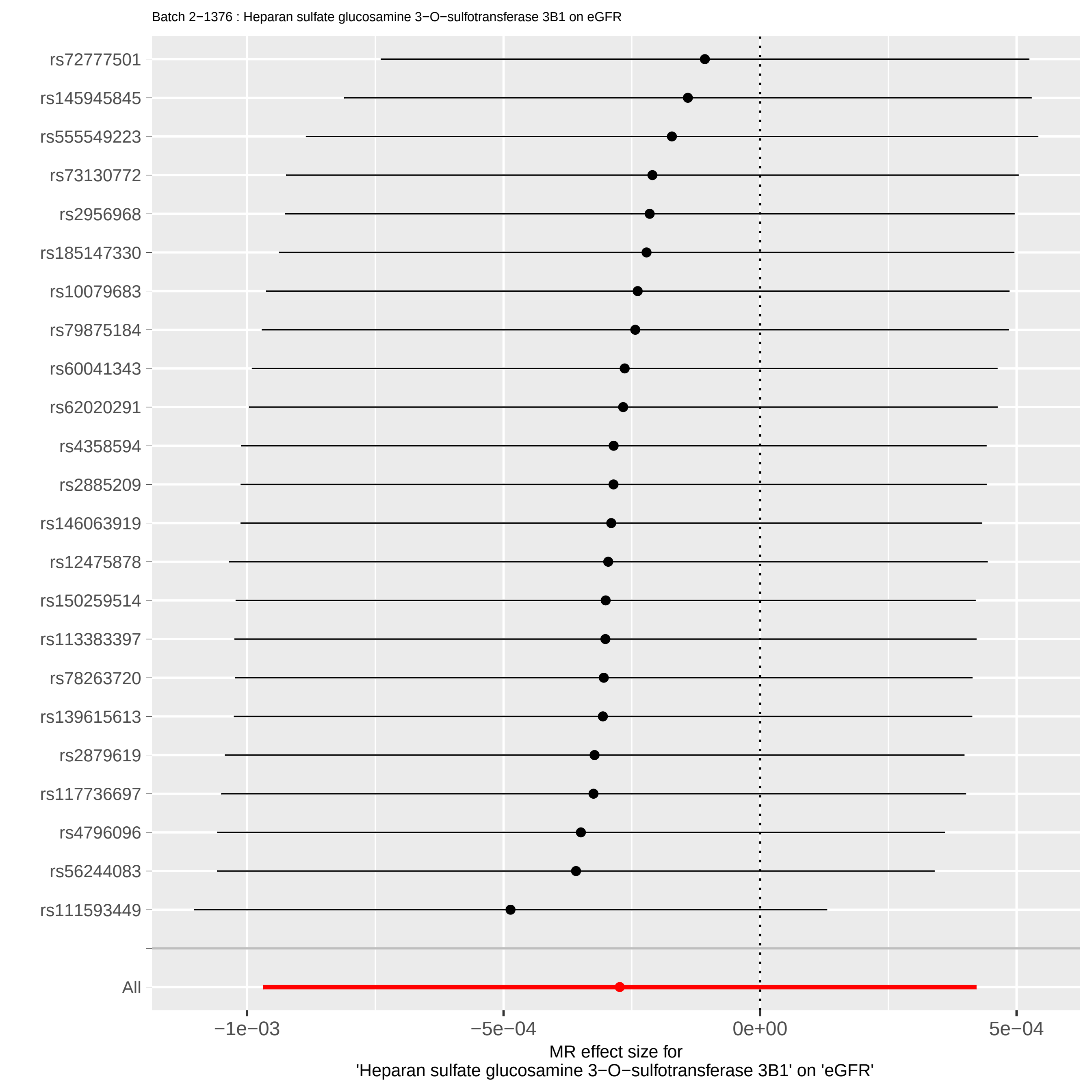

Batch 2−1376 : Heparan sulfate glucosamine 3−O−sulfotransferase 3B1 on eGFR
rs72777501
rs145945845
rs555549223
rs73130772
rs2956968
rs185147330
rs10079683
rs79875184
rs60041343
rs62020291
rs4358594
rs2885209
rs146063919
rs12475878
rs150259514
rs113383397
rs78263720
rs139615613
rs2879619
rs117736697
rs4796096
rs56244083
rs111593449
All
−1e−03
−5e−04
0e+00
5e−04
MR effect size for
'Heparan sulfate glucosamine 3−O−sulfotransferase 3B1' on 'eGFR'

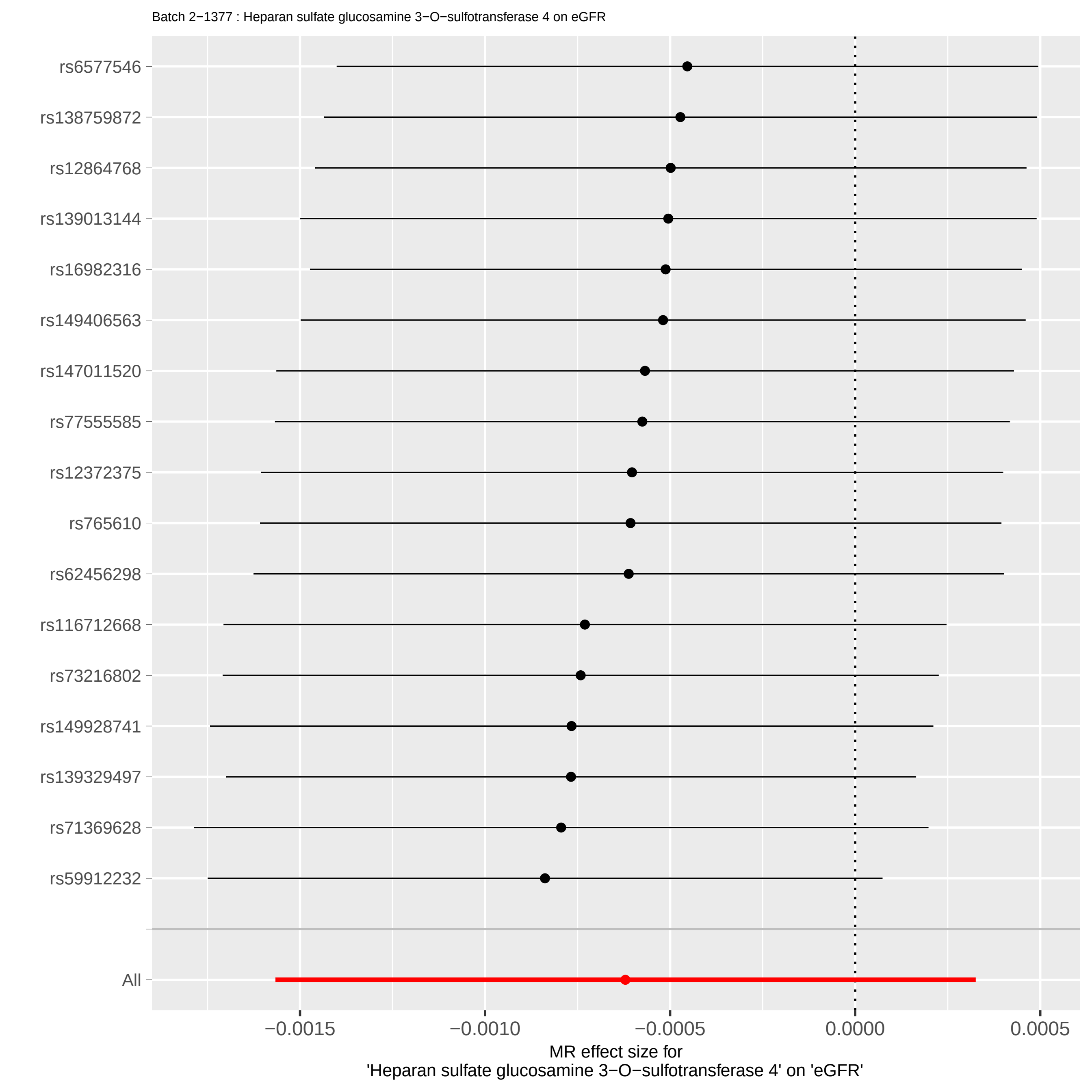

Batch 2−1377 : Heparan sulfate glucosamine 3−O−sulfotransferase 4 on eGFR
rs6577546
rs138759872
rs12864768
rs139013144
rs16982316
rs149406563
rs147011520
rs77555585
rs12372375
rs765610
rs62456298
rs116712668
rs73216802
rs149928741
rs139329497
rs71369628
rs59912232
All
−0.0015
−0.0010
−0.0005
0.0000
0.0005
MR effect size for
'Heparan sulfate glucosamine 3−O−sulfotransferase 4' on 'eGFR'

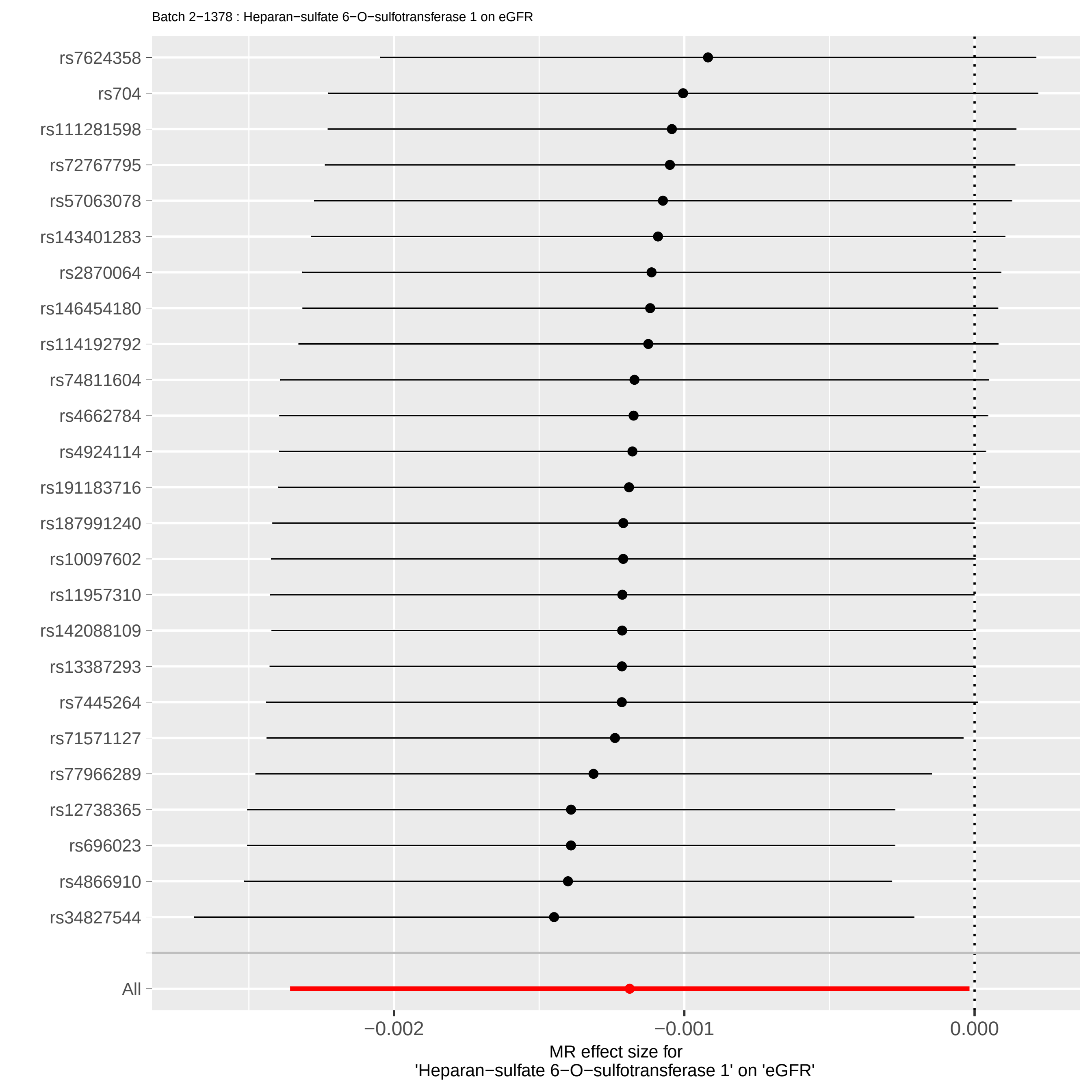

Batch 2−1378 : Heparan−sulfate 6−O−sulfotransferase 1 on eGFR
rs7624358
rs704
rs111281598
rs72767795
rs57063078
rs143401283
rs2870064
rs146454180
rs114192792
rs74811604
rs4662784
rs4924114
rs191183716
rs187991240
rs10097602
rs11957310
rs142088109
rs13387293
rs7445264
rs71571127
rs77966289
rs12738365
rs696023
rs4866910
rs34827544
All
−0.002
−0.001
0.000
MR effect size for
'Heparan−sulfate 6−O−sulfotransferase 1' on 'eGFR'

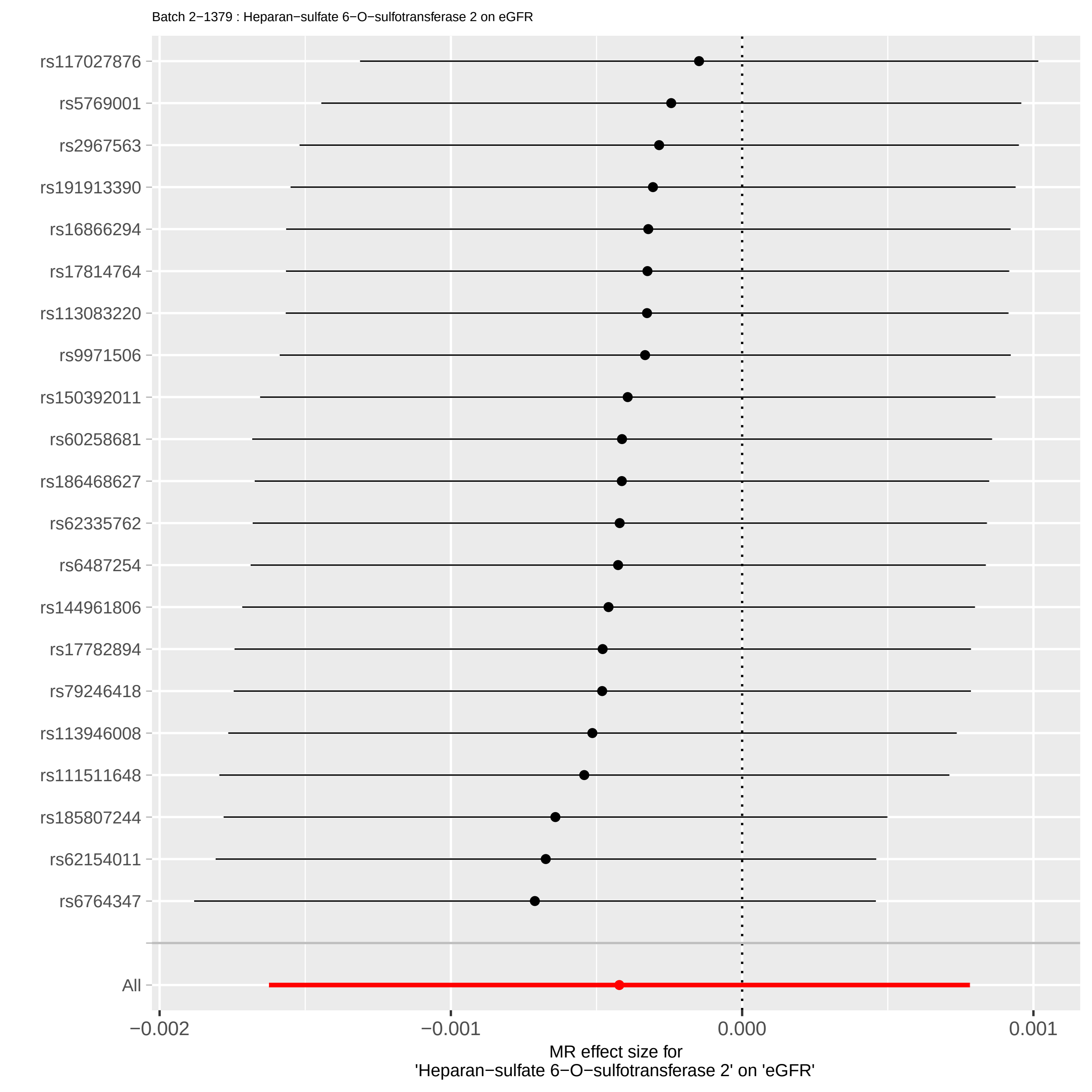
Batch 2−1379 : Heparan−sulfate 6−O−sulfotransferase 2 on eGFR
rs117027876
rs5769001
rs2967563
rs191913390
rs16866294
rs17814764
rs113083220
rs9971506
rs150392011
rs60258681
rs186468627
rs62335762
rs6487254
rs144961806
rs17782894
rs79246418
rs113946008
rs111511648
rs185807244
rs62154011
rs6764347
All
−0.002
−0.001
0.000
0.001
MR effect size for
'Heparan−sulfate 6−O−sulfotransferase 2' on 'eGFR'

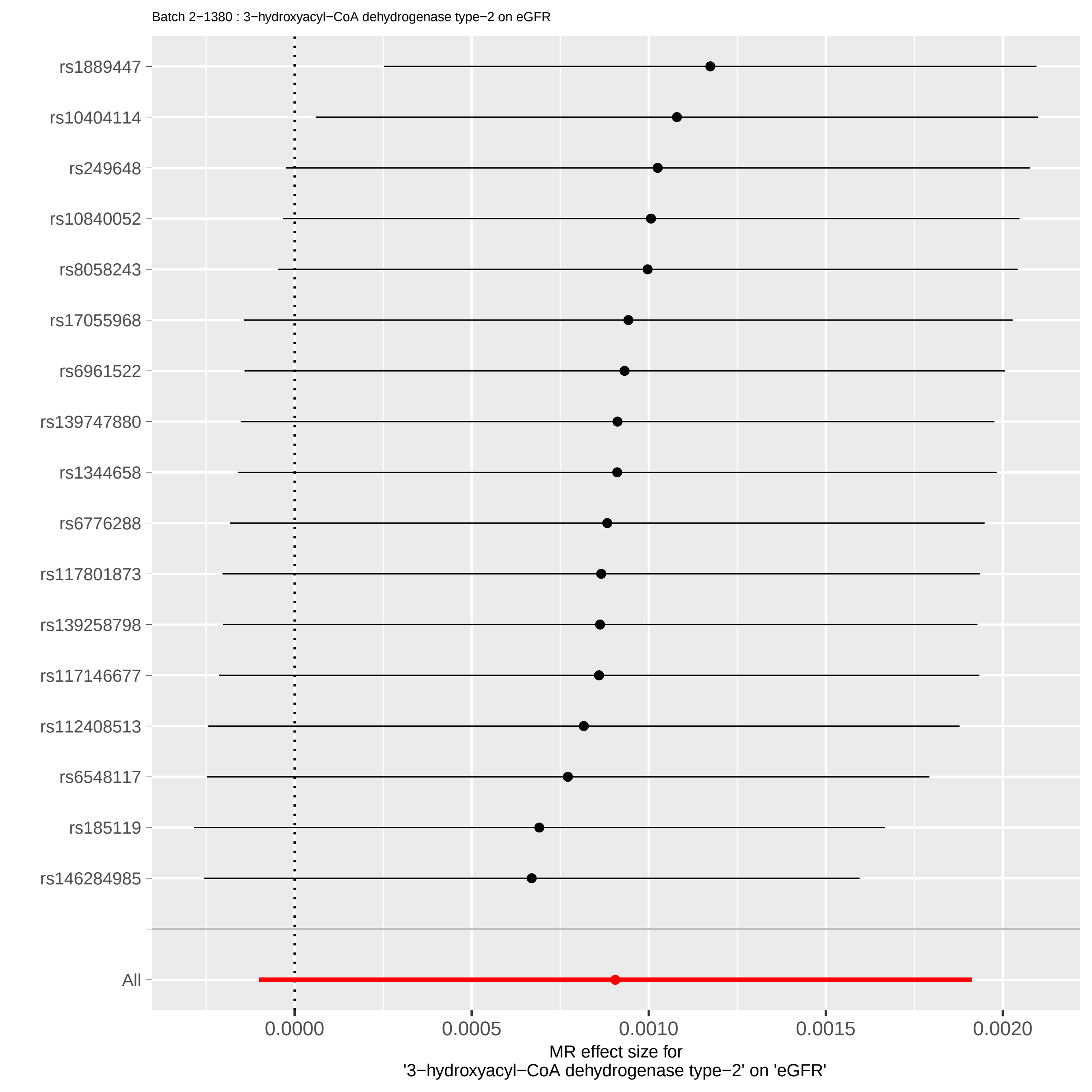

Batch 2−1380 : 3−hydroxyacyl−CoA dehydrogenase type−2 on eGFR
rs1889447
rs10404114
rs249648
rs10840052
rs8058243
rs17055968
rs6961522
rs139747880
rs1344658
rs6776288
rs117801873
rs139258798
rs117146677
rs112408513
rs6548117
rs185119
rs146284985
All
0.0000
0.0005
0.0010
0.0015
0.0020
MR effect size for
'3−hydroxyacyl−CoA dehydrogenase type−2' on 'eGFR'

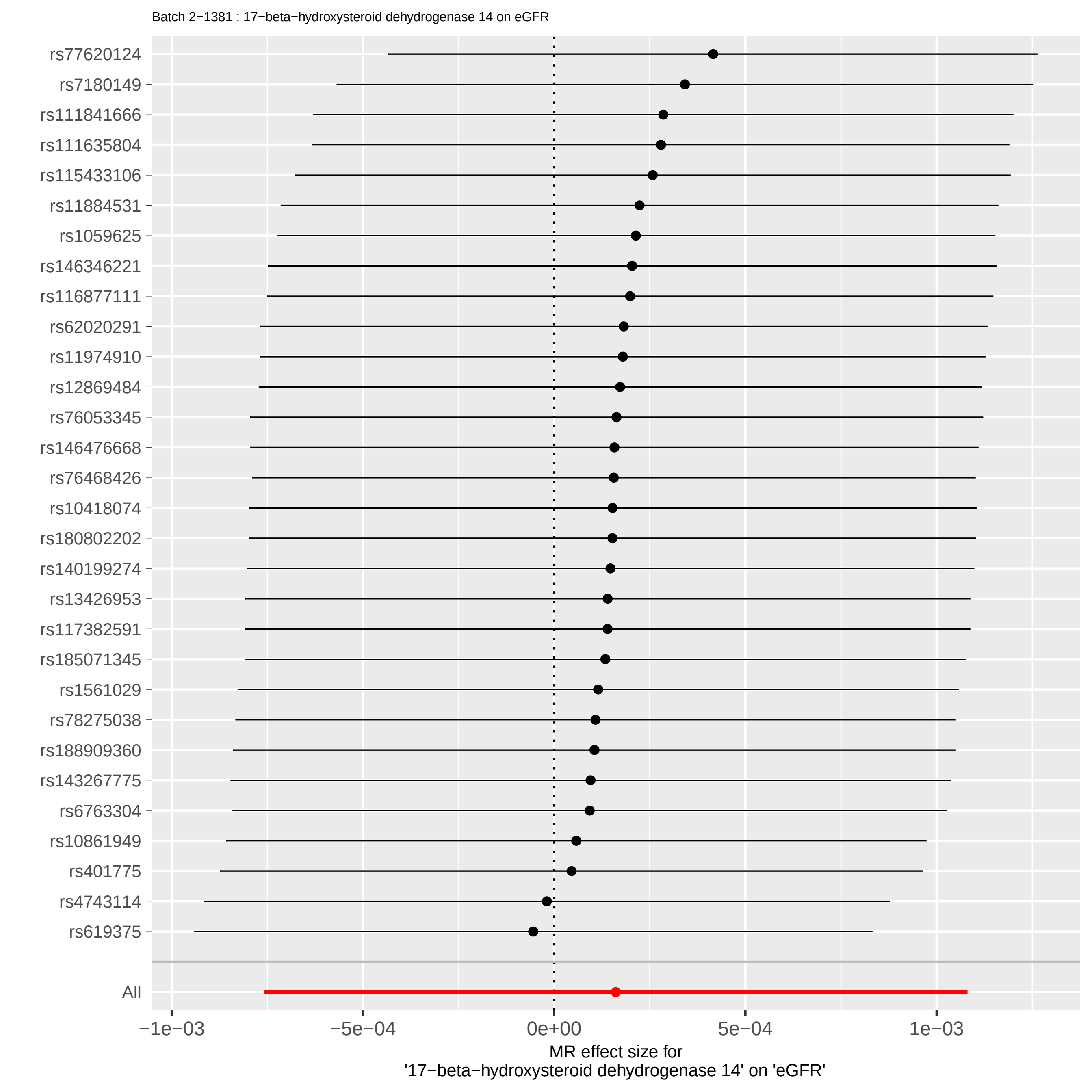

Batch 2−1381 : 17−beta−hydroxysteroid dehydrogenase 14 on eGFR
rs77620124
rs7180149
rs111841666
rs111635804
rs115433106
rs11884531
rs1059625
rs146346221
rs116877111
rs62020291
rs11974910
rs12869484
rs76053345
rs146476668
rs76468426
rs10418074
rs180802202
rs140199274
rs13426953
rs117382591
rs185071345
rs1561029
rs78275038
rs188909360
rs143267775
rs6763304
rs10861949
rs401775
rs4743114
rs619375
All
−1e−03
−5e−04
0e+00
5e−04
1e−03
MR effect size for
'17−beta−hydroxysteroid dehydrogenase 14' on 'eGFR'

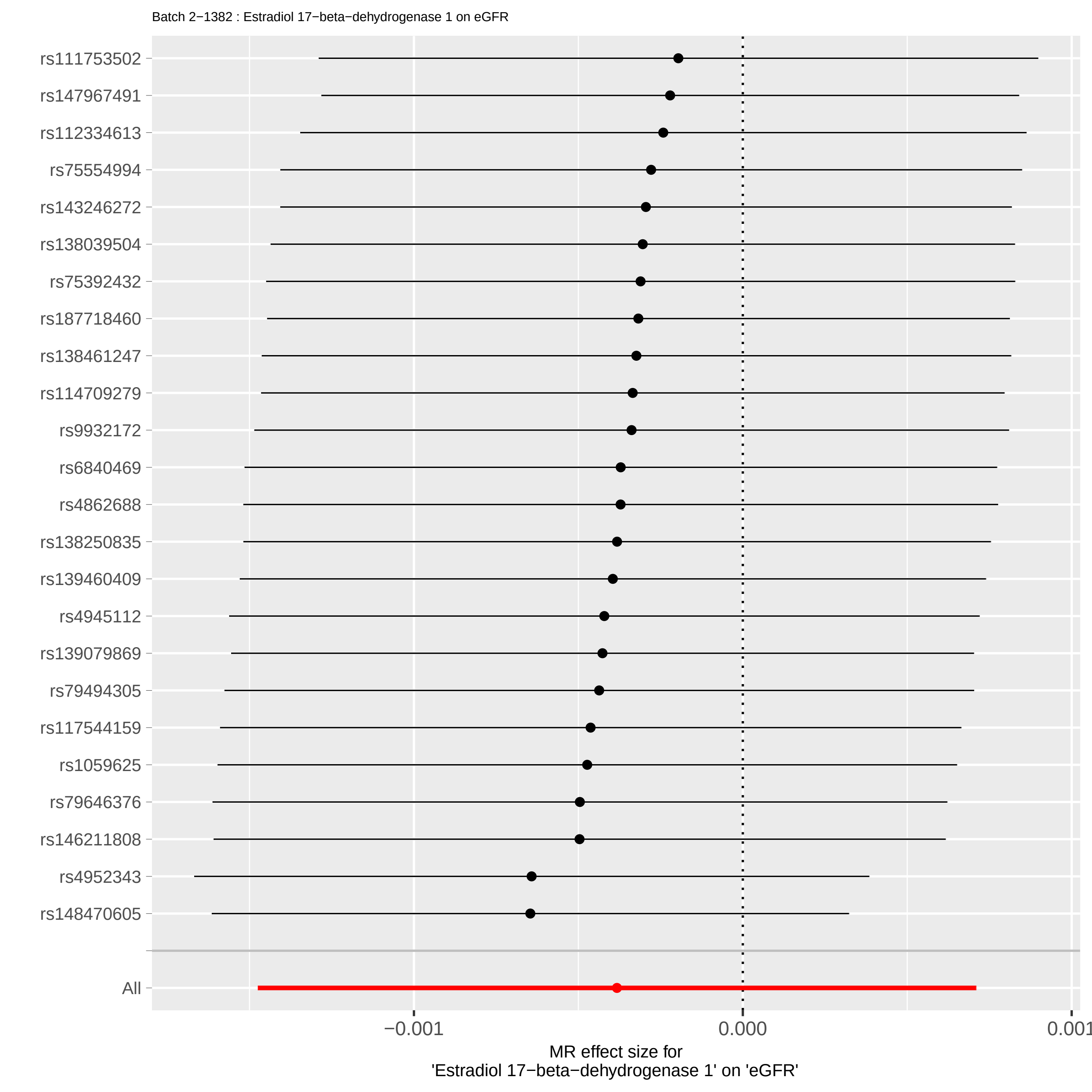
Batch 2−1382 : Estradiol 17−beta−dehydrogenase 1 on eGFR
rs111753502
rs147967491
rs112334613
rs75554994
rs143246272
rs138039504
rs75392432
rs187718460
rs138461247
rs114709279
rs9932172
rs6840469
rs4862688
rs138250835
rs139460409
rs4945112
rs139079869
rs79494305
rs117544159
rs1059625
rs79646376
rs146211808
rs4952343
rs148470605
All
−0.001
0.000
0.001
MR effect size for
'Estradiol 17−beta−dehydrogenase 1' on 'eGFR'

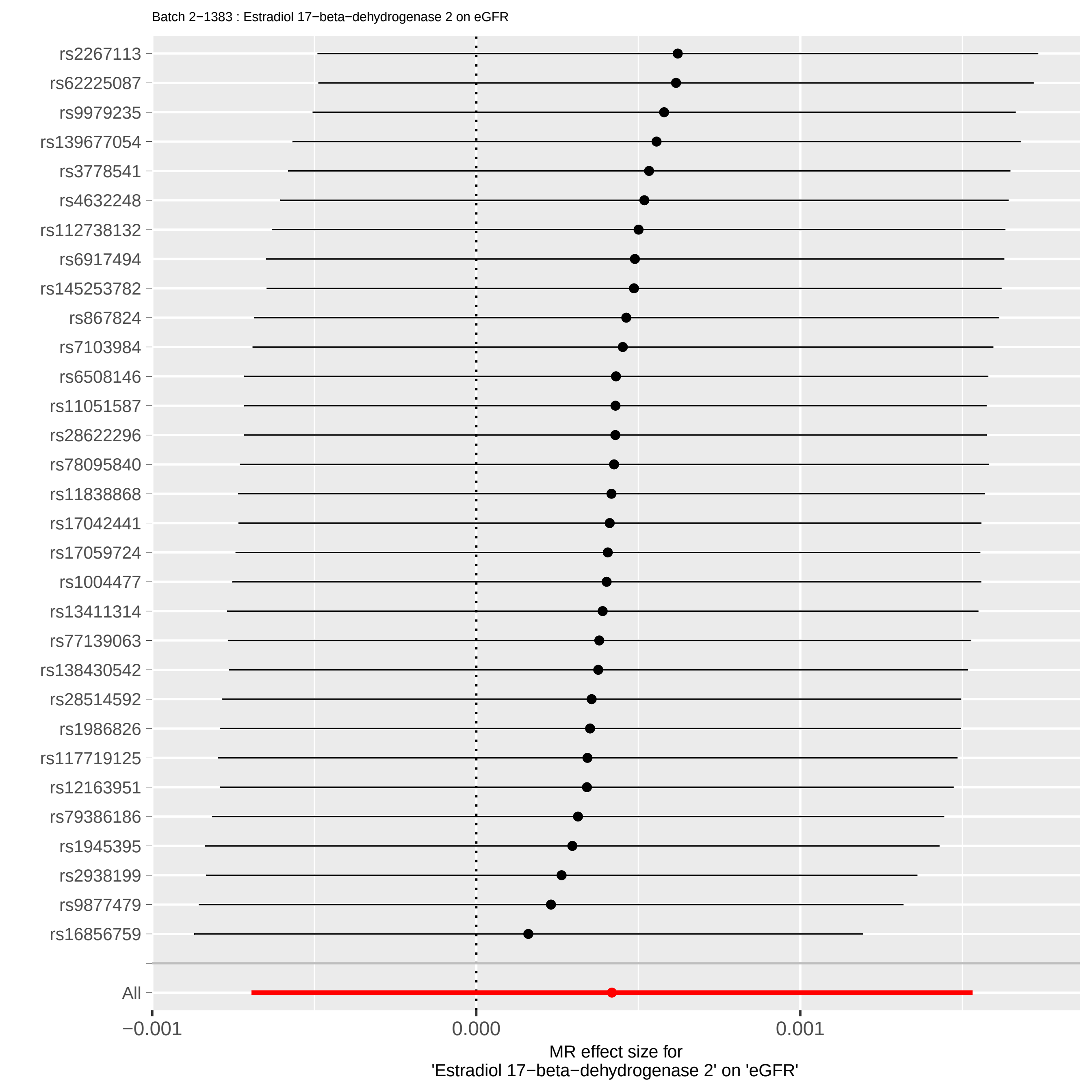
Batch 2−1383 : Estradiol 17−beta−dehydrogenase 2 on eGFR
rs2267113
rs62225087
rs9979235
rs139677054
rs3778541
rs4632248
rs112738132
rs6917494
rs145253782
rs867824
rs7103984
rs6508146
rs11051587
rs28622296
rs78095840
rs11838868
rs17042441
rs17059724
rs1004477
rs13411314
rs77139063
rs138430542
rs28514592
rs1986826
rs117719125
rs12163951
rs79386186
rs1945395
rs2938199
rs9877479
rs16856759
All
−0.001
0.000
0.001
MR effect size for
'Estradiol 17−beta−dehydrogenase 2' on 'eGFR'

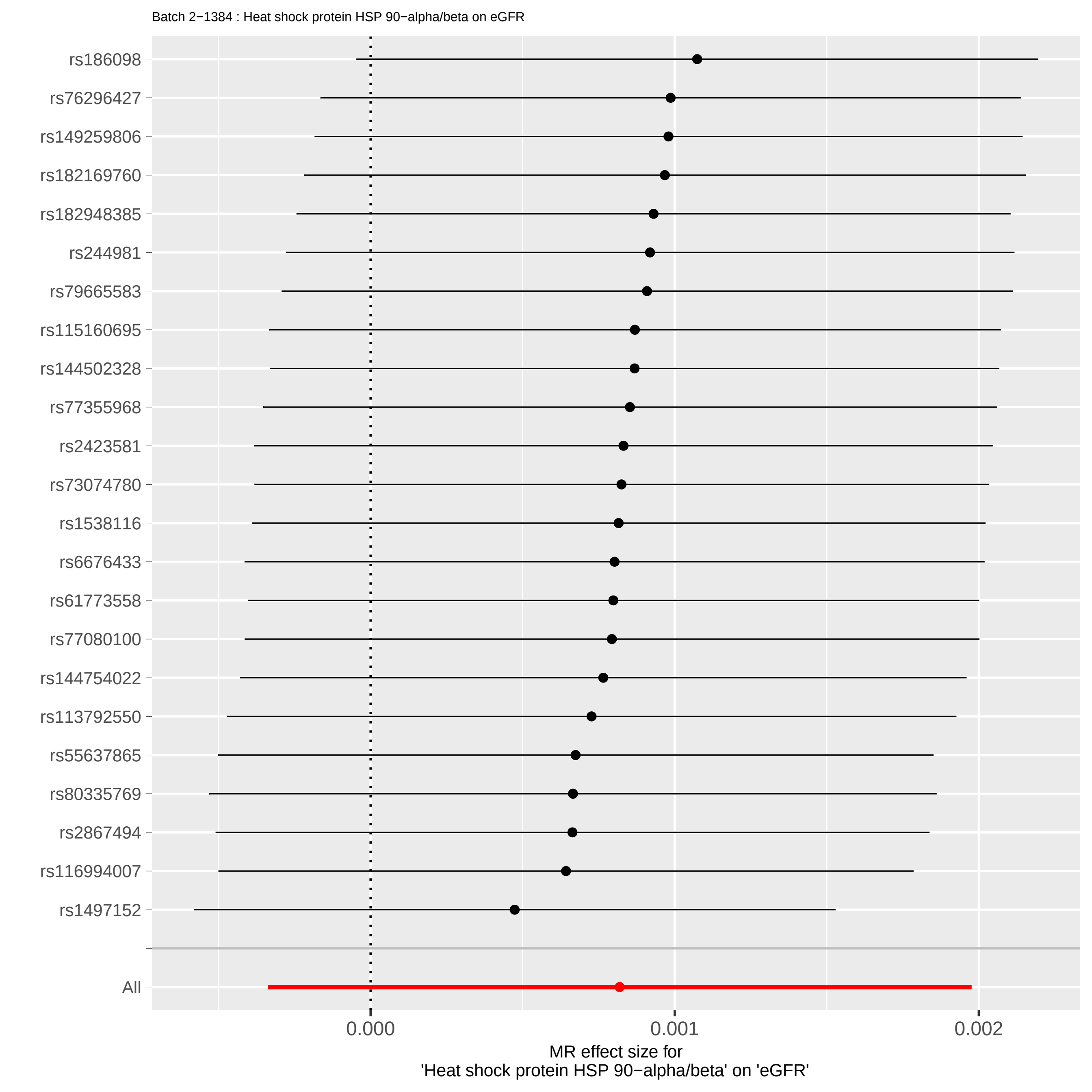

Batch 2−1384 : Heat shock protein HSP 90−alpha/beta on eGFR
rs186098
rs76296427
rs149259806
rs182169760
rs182948385
rs244981
rs79665583
rs115160695
rs144502328
rs77355968
rs2423581
rs73074780
rs1538116
rs6676433
rs61773558
rs77080100
rs144754022
rs113792550
rs55637865
rs80335769
rs2867494
rs116994007
rs1497152
All
0.000
0.001
0.002
MR effect size for
'Heat shock protein HSP 90−alpha/beta' on 'eGFR'

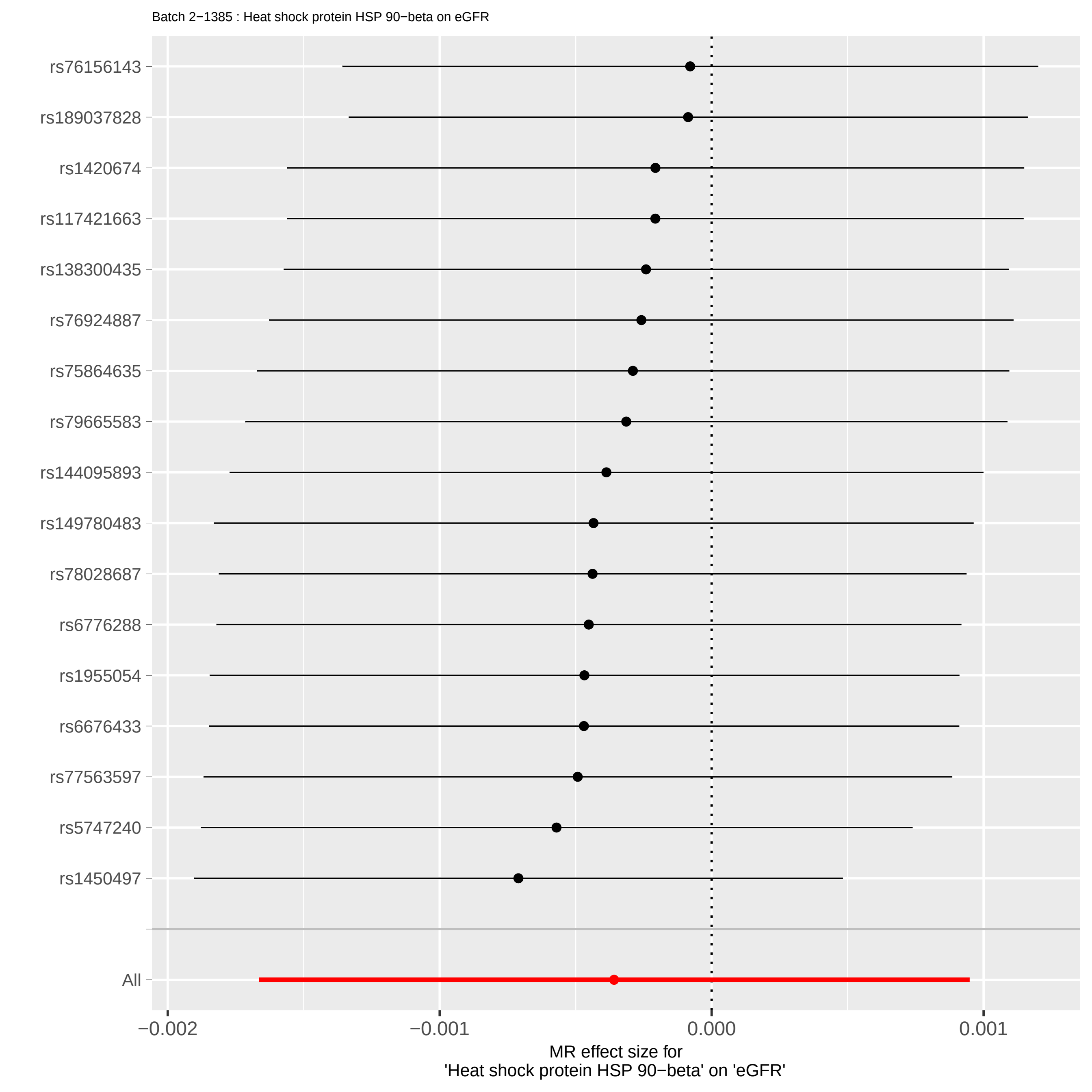

Batch 2−1385 : Heat shock protein HSP 90−beta on eGFR
rs76156143
rs189037828
rs1420674
rs117421663
rs138300435
rs76924887
rs75864635
rs79665583
rs144095893
rs149780483
rs78028687
rs6776288
rs1955054
rs6676433
rs77563597
rs5747240
rs1450497
All
−0.002
−0.001
0.000
0.001
MR effect size for
'Heat shock protein HSP 90−beta' on 'eGFR'

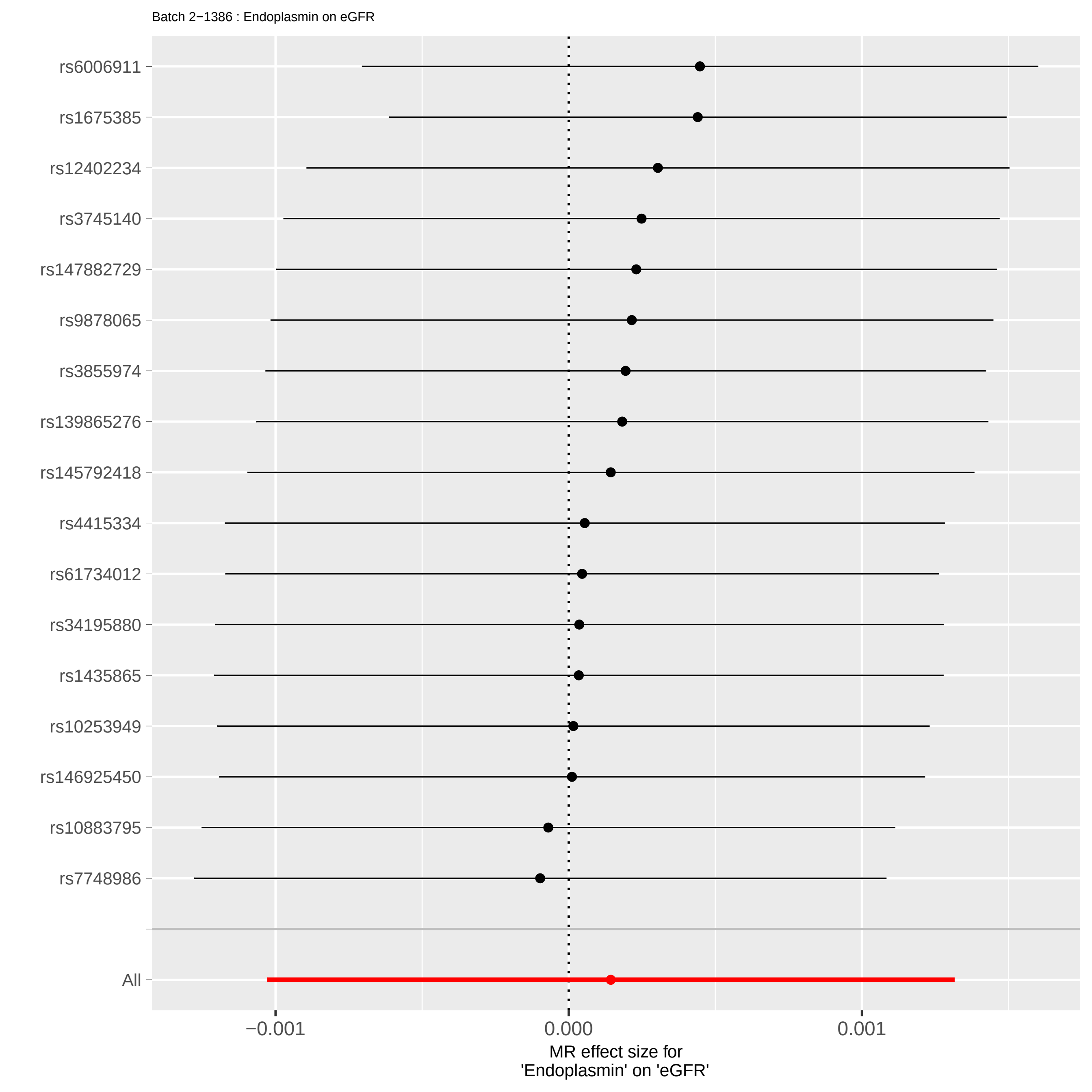

Batch 2−1386 : Endoplasmin on eGFR
rs6006911
rs1675385
rs12402234
rs3745140
rs147882729
rs9878065
rs3855974
rs139865276
rs145792418
rs4415334
rs61734012
rs34195880
rs1435865
rs10253949
rs146925450
rs10883795
rs7748986
All
−0.001
0.000
0.001
MR effect size for
'Endoplasmin' on 'eGFR'

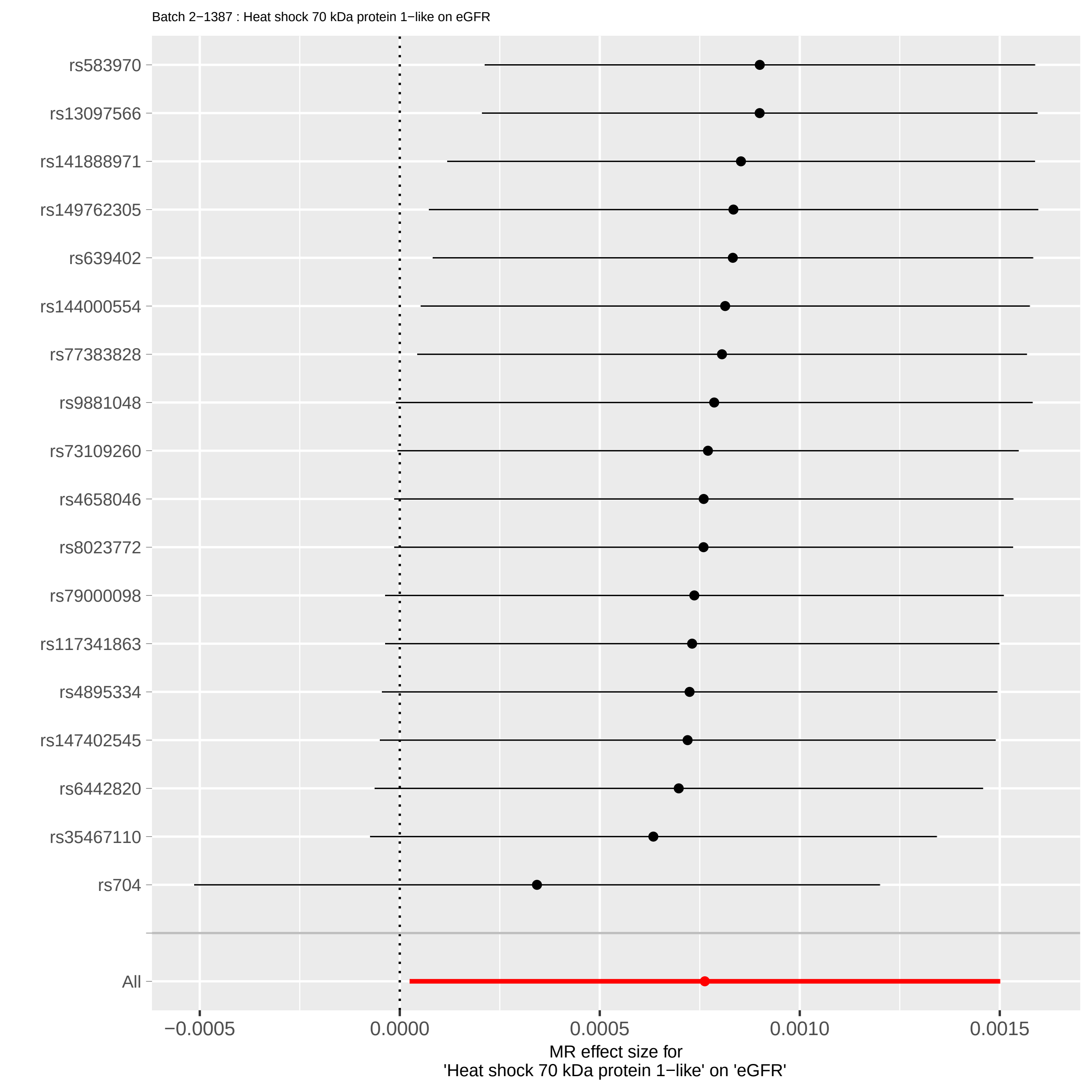

Batch 2−1387 : Heat shock 70 kDa protein 1−like on eGFR
rs583970
rs13097566
rs141888971
rs149762305
rs639402
rs144000554
rs77383828
rs9881048
rs73109260
rs4658046
rs8023772
rs79000098
rs117341863
rs4895334
rs147402545
rs6442820
rs35467110
rs704
All
−0.0005
0.0000
0.0005
0.0010
0.0015
MR effect size for
'Heat shock 70 kDa protein 1−like' on 'eGFR'

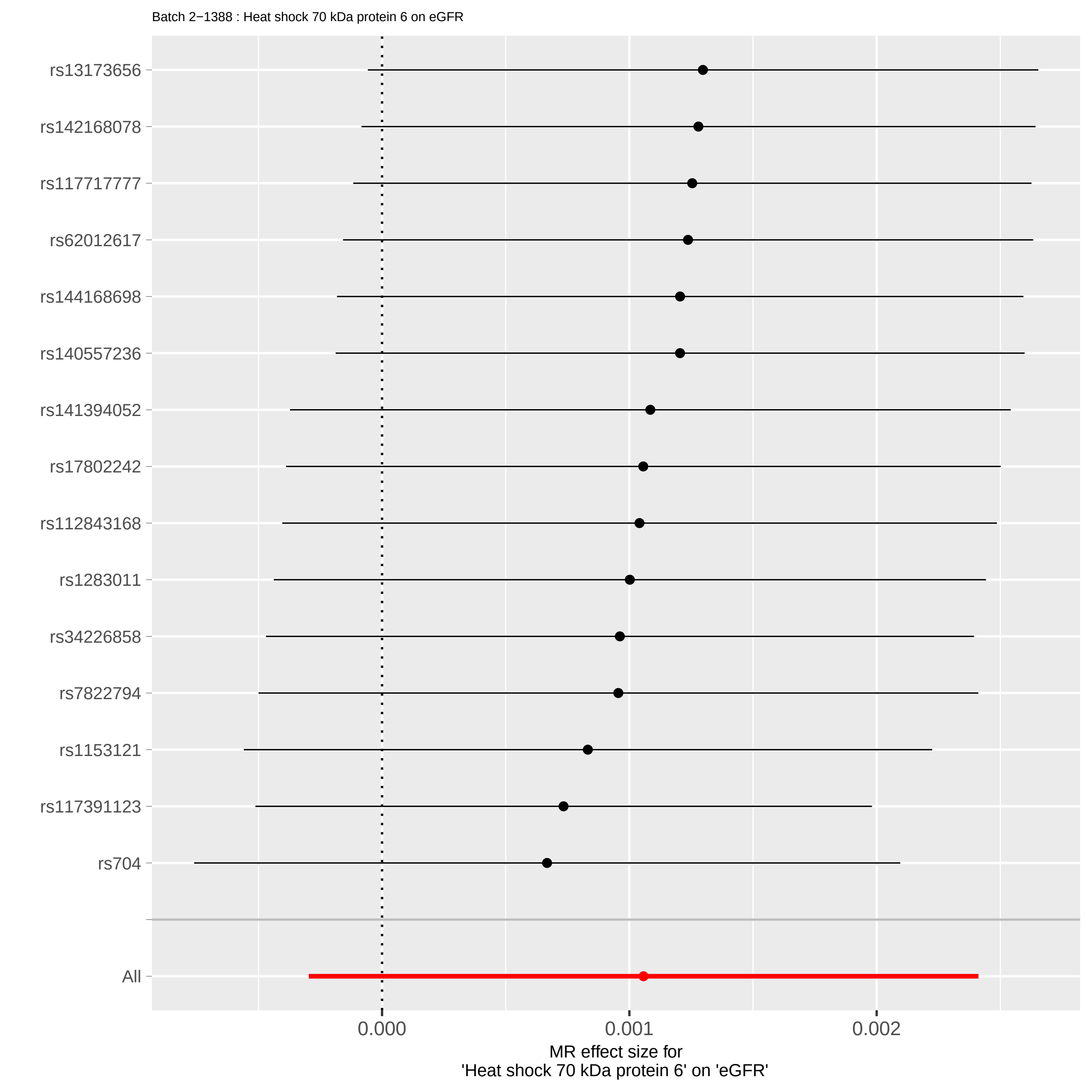

Batch 2−1388 : Heat shock 70 kDa protein 6 on eGFR
rs13173656
rs142168078
rs117717777
rs62012617
rs144168698
rs140557236
rs141394052
rs17802242
rs112843168
rs1283011
rs34226858
rs7822794
rs1153121
rs117391123
rs704
All
0.000
0.001
0.002
MR effect size for
'Heat shock 70 kDa protein 6' on 'eGFR'

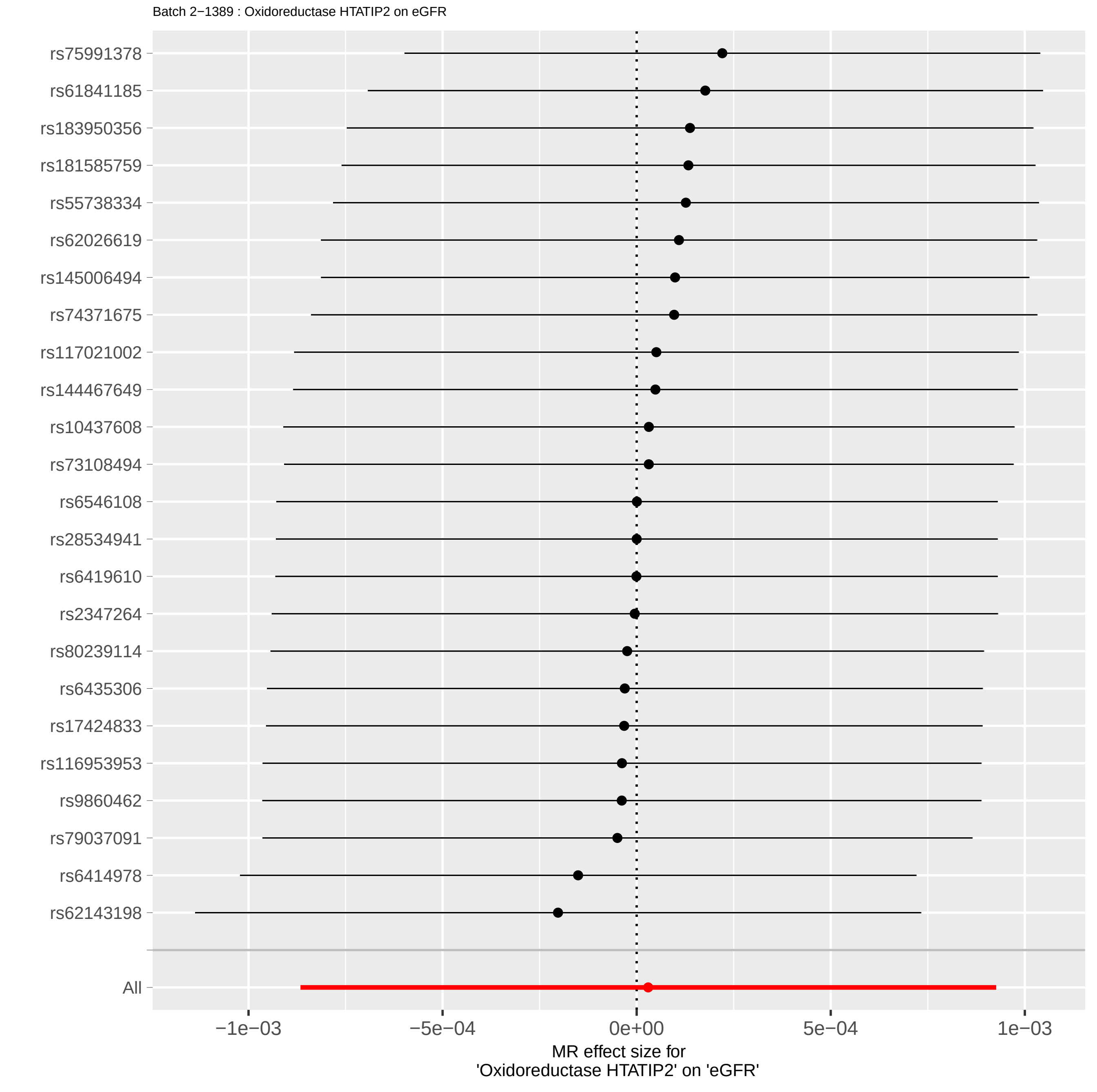

Batch 2−1389 : Oxidoreductase HTATIP2 on eGFR
rs75991378
rs61841185
rs183950356
rs181585759
rs55738334
rs62026619
rs145006494
rs74371675
rs117021002
rs144467649
rs10437608
rs73108494
rs6546108
rs28534941
rs6419610
rs2347264
rs80239114
rs6435306
rs17424833
rs116953953
rs9860462
rs79037091
rs6414978
rs62143198
All
−1e−03
−5e−04
0e+00
5e−04
1e−03
MR effect size for
'Oxidoreductase HTATIP2' on 'eGFR'

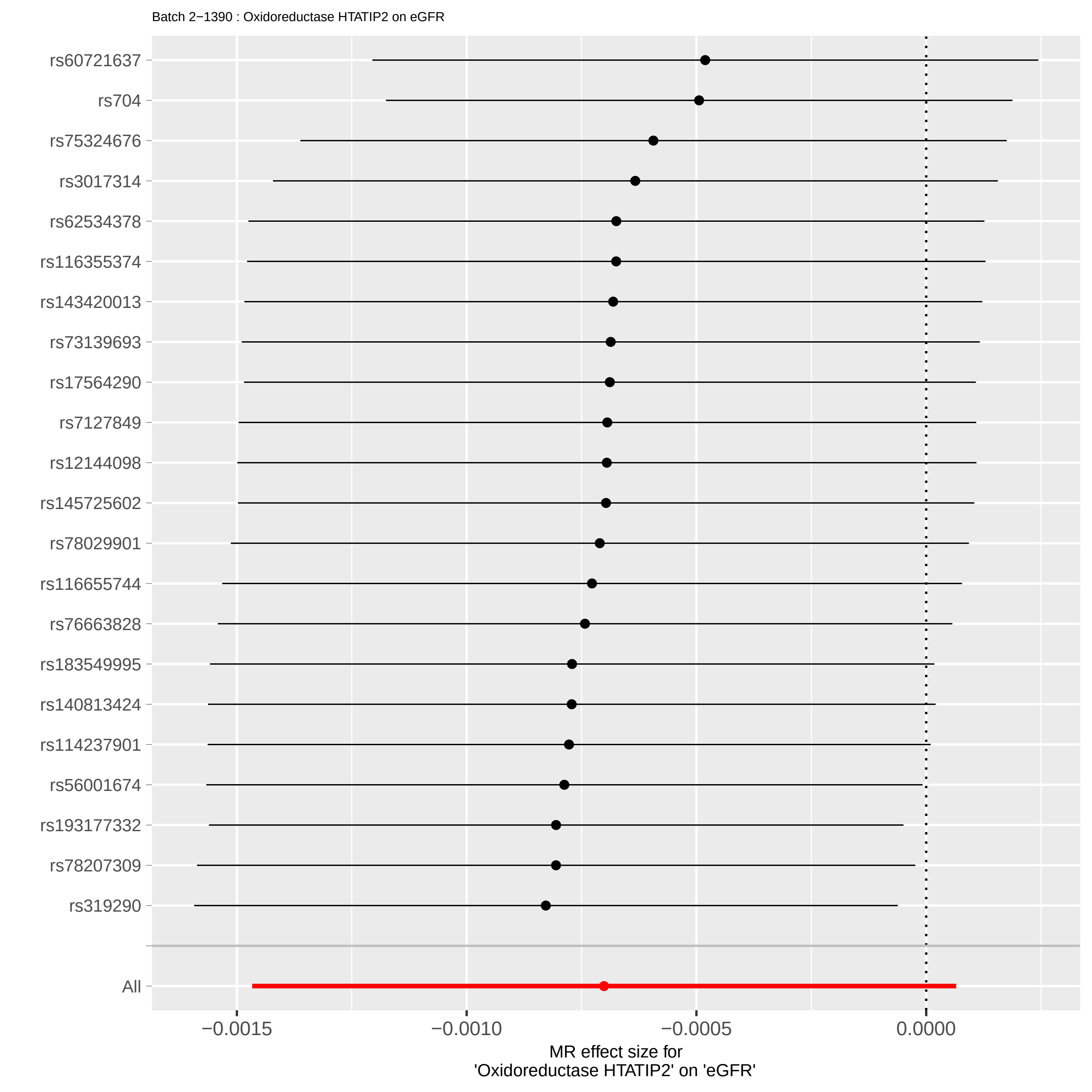

Batch 2−1390 : Oxidoreductase HTATIP2 on eGFR
rs60721637
rs704
rs75324676
rs3017314
rs62534378
rs116355374
rs143420013
rs73139693
rs17564290
rs7127849
rs12144098
rs145725602
rs78029901
rs116655744
rs76663828
rs183549995
rs140813424
rs114237901
rs56001674
rs193177332
rs78207309
rs319290
All
−0.0015
−0.0010
−0.0005
0.0000
MR effect size for
'Oxidoreductase HTATIP2' on 'eGFR'

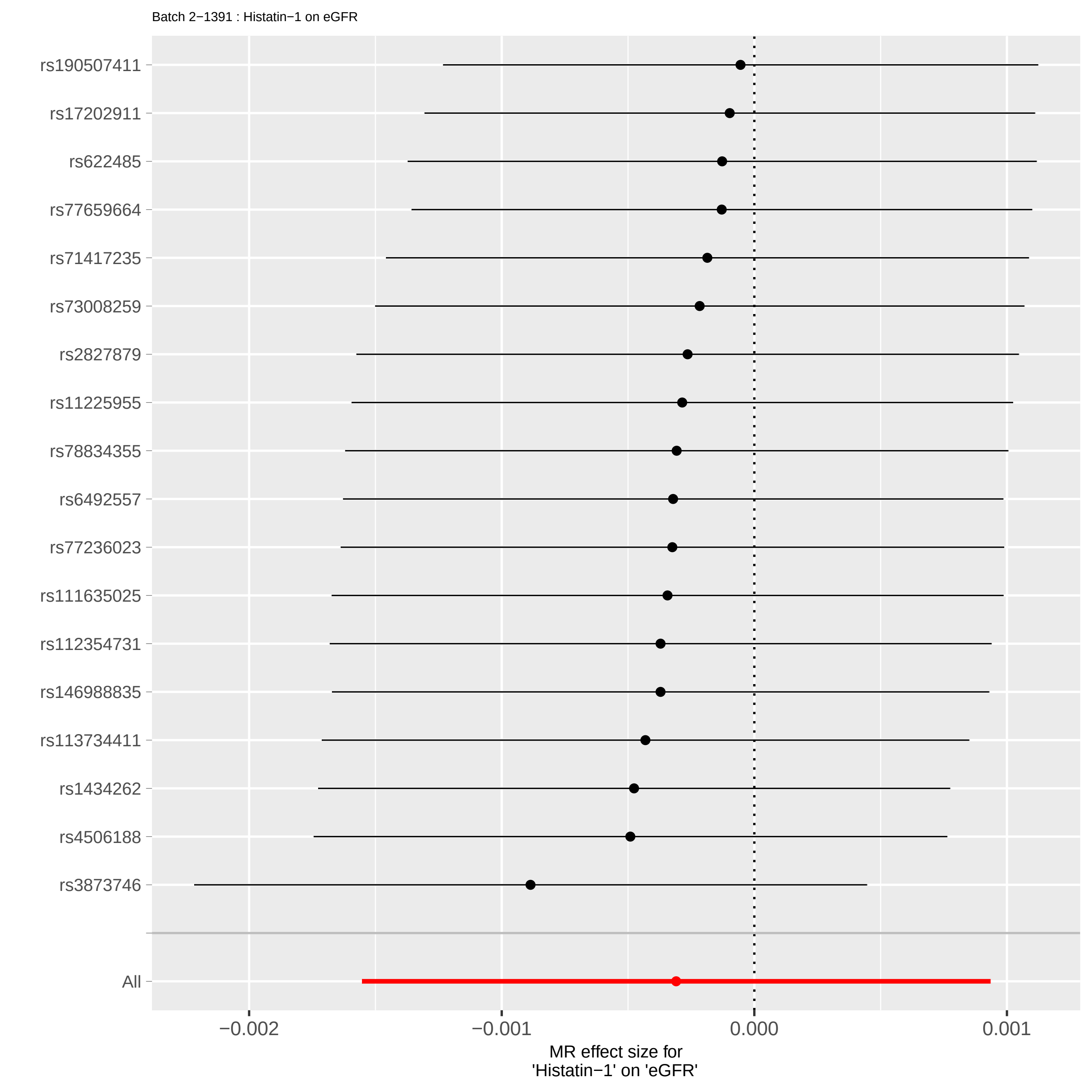
Batch 2−1391 : Histatin−1 on eGFR
rs190507411
rs17202911
rs622485
rs77659664
rs71417235
rs73008259
rs2827879
rs11225955
rs78834355
rs6492557
rs77236023
rs111635025
rs112354731
rs146988835
rs113734411
rs1434262
rs4506188
rs3873746
All
−0.002
−0.001
0.000
0.001
MR effect size for
'Histatin−1' on 'eGFR'

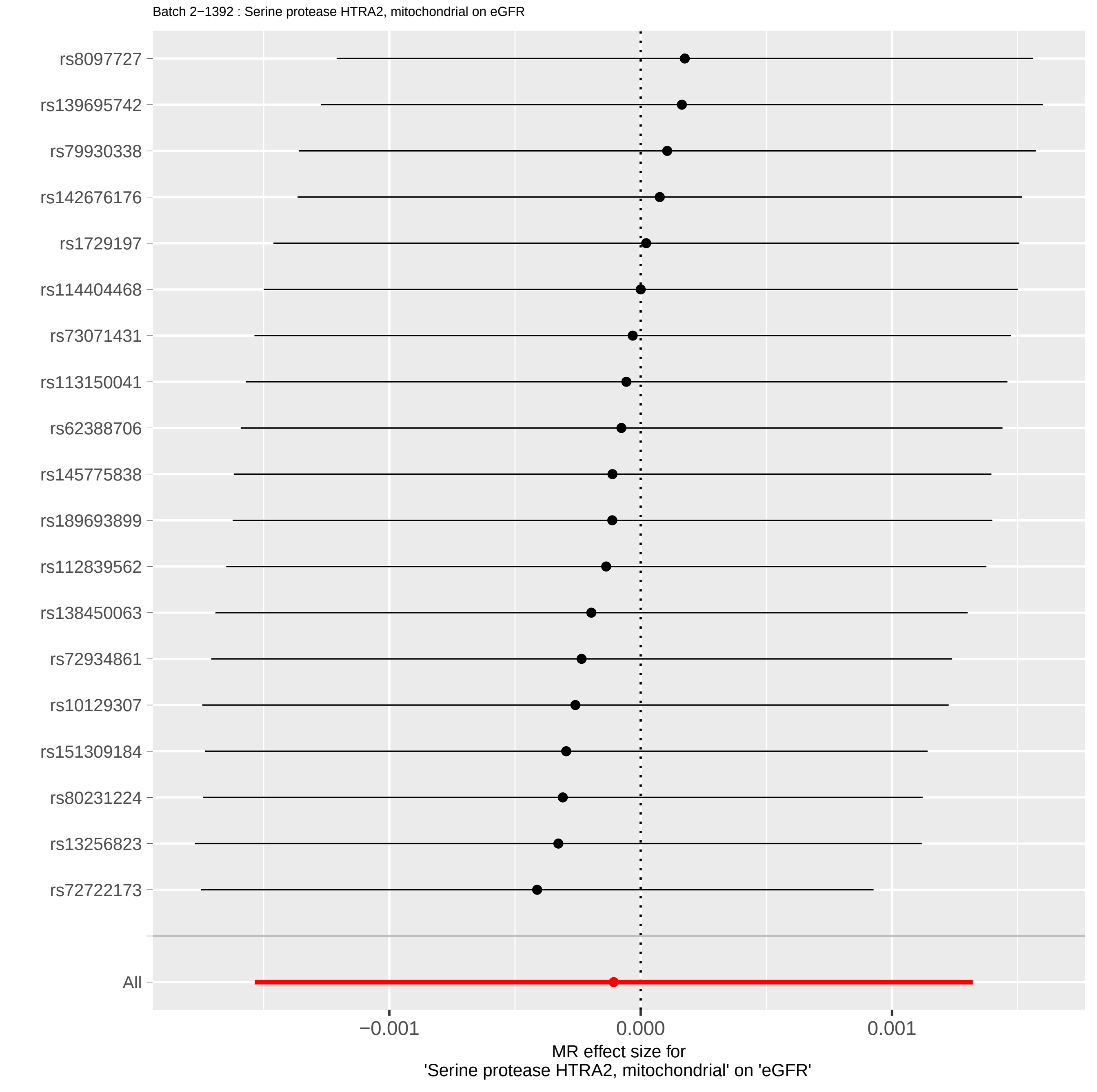

Batch 2−1392 : Serine protease HTRA2, mitochondrial on eGFR
rs8097727
rs139695742
rs79930338
rs142676176
rs1729197
rs114404468
rs73071431
rs113150041
rs62388706
rs145775838
rs189693899
rs112839562
rs138450063
rs72934861
rs10129307
rs151309184
rs80231224
rs13256823
rs72722173
All
−0.001
0.000
0.001
MR effect size for
'Serine protease HTRA2, mitochondrial' on 'eGFR'

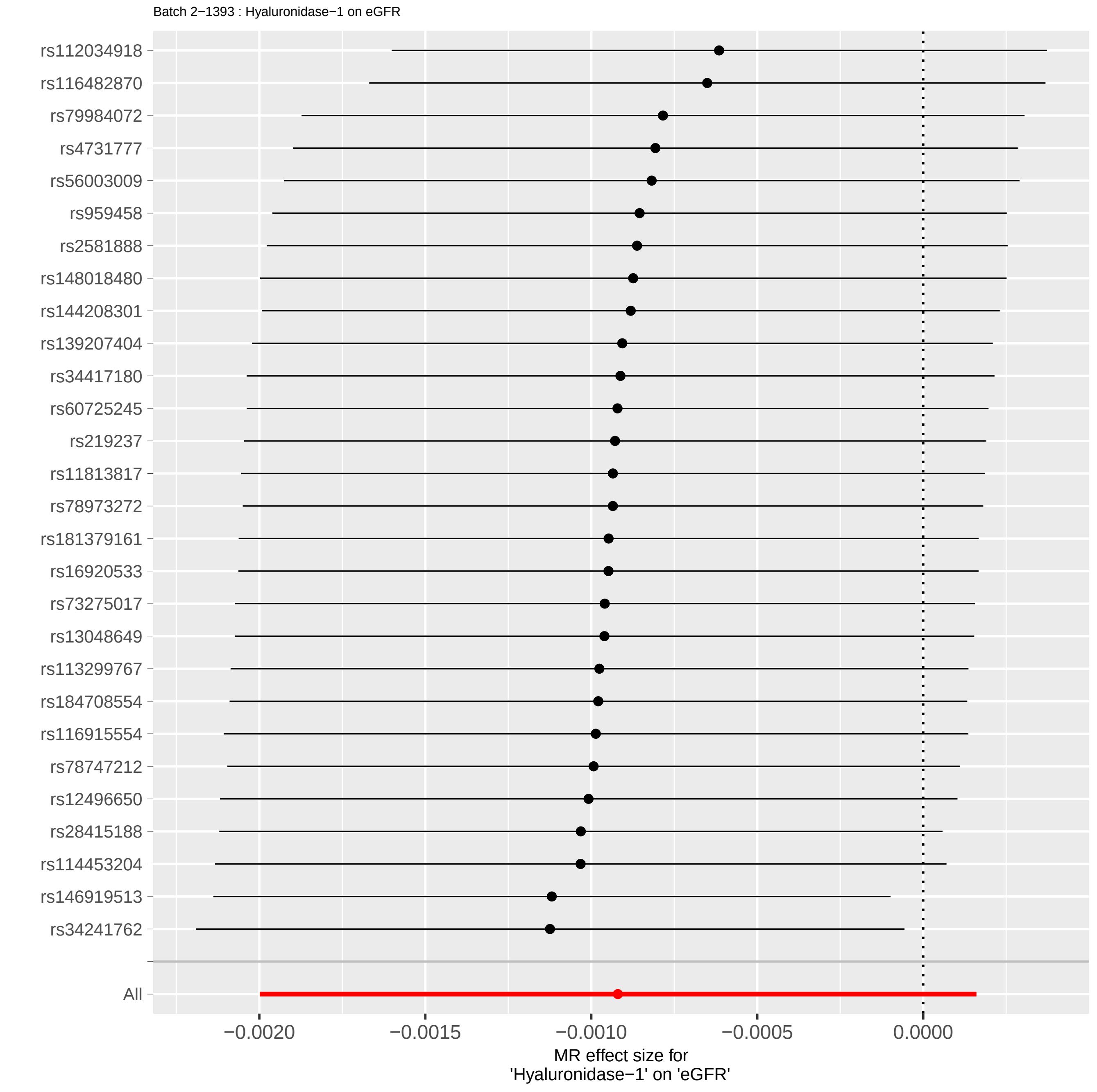

Batch 2−1393 : Hyaluronidase−1 on eGFR
rs112034918
rs116482870
rs79984072
rs4731777
rs56003009
rs959458
rs2581888
rs148018480
rs144208301
rs139207404
rs34417180
rs60725245
rs219237
rs11813817
rs78973272
rs181379161
rs16920533
rs73275017
rs13048649
rs113299767
rs184708554
rs116915554
rs78747212
rs12496650
rs28415188
rs114453204
rs146919513
rs34241762
All
−0.0020
−0.0015
−0.0010
−0.0005
0.0000
MR effect size for
'Hyaluronidase−1' on 'eGFR'

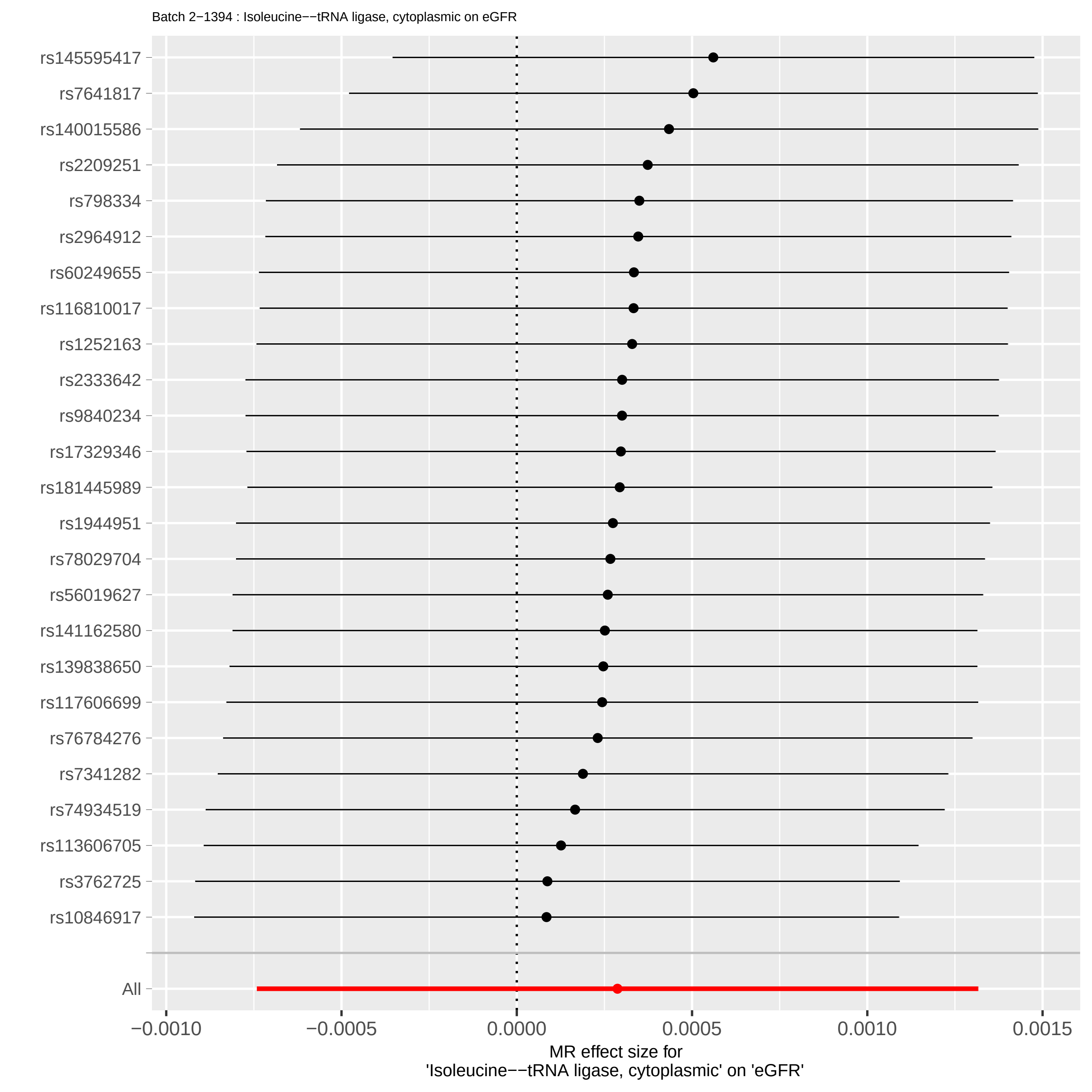

Batch 2−1394 : Isoleucine−−tRNA ligase, cytoplasmic on eGFR
rs145595417
rs7641817
rs140015586
rs2209251
rs798334
rs2964912
rs60249655
rs116810017
rs1252163
rs2333642
rs9840234
rs17329346
rs181445989
rs1944951
rs78029704
rs56019627
rs141162580
rs139838650
rs117606699
rs76784276
rs7341282
rs74934519
rs113606705
rs3762725
rs10846917
All
−0.0010
−0.0005
0.0000
0.0005
0.0010
0.0015
MR effect size for
'Isoleucine−−tRNA ligase, cytoplasmic' on 'eGFR'

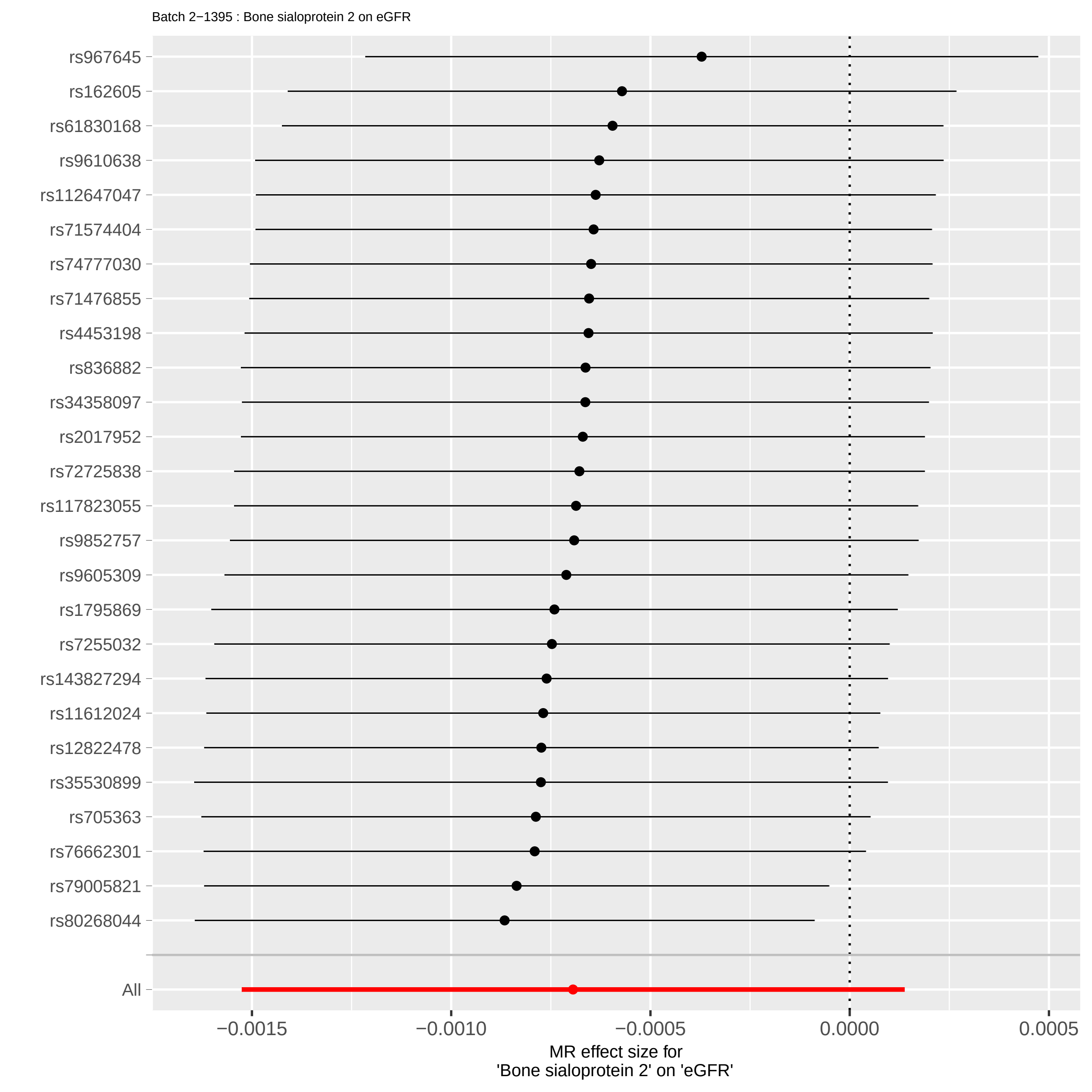

Batch 2−1395 : Bone sialoprotein 2 on eGFR
rs967645
rs162605
rs61830168
rs9610638
rs112647047
rs71574404
rs74777030
rs71476855
rs4453198
rs836882
rs34358097
rs2017952
rs72725838
rs117823055
rs9852757
rs9605309
rs1795869
rs7255032
rs143827294
rs11612024
rs12822478
rs35530899
rs705363
rs76662301
rs79005821
rs80268044
All
−0.0015
−0.0010
−0.0005
0.0000
0.0005
MR effect size for
'Bone sialoprotein 2' on 'eGFR'

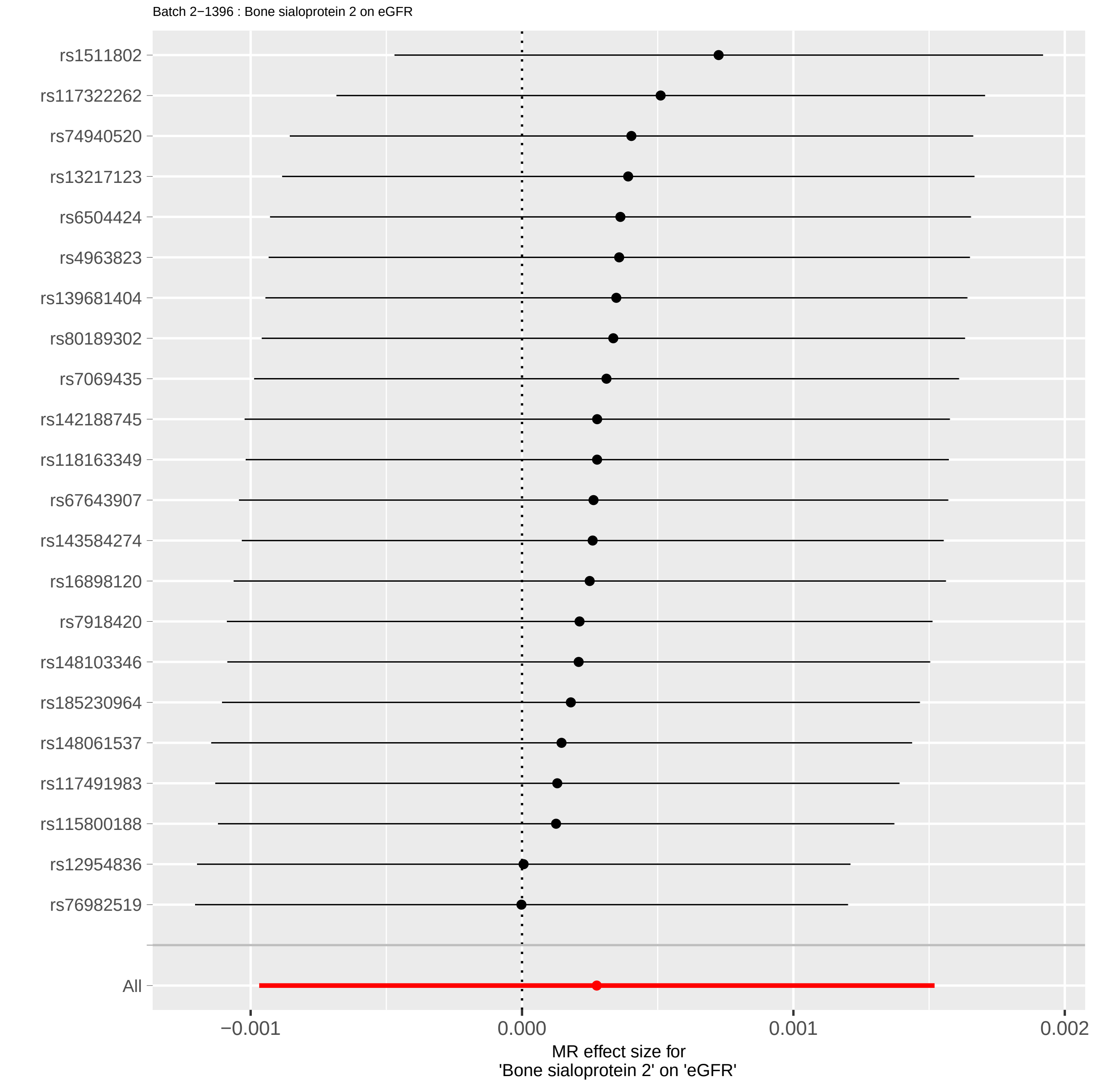
Batch 2−1396 : Bone sialoprotein 2 on eGFR
rs1511802
rs117322262
rs74940520
rs13217123
rs6504424
rs4963823
rs139681404
rs80189302
rs7069435
rs142188745
rs118163349
rs67643907
rs143584274
rs16898120
rs7918420
rs148103346
rs185230964
rs148061537
rs117491983
rs115800188
rs12954836
rs76982519
All
−0.001
0.000
0.001
0.002
MR effect size for
'Bone sialoprotein 2' on 'eGFR'

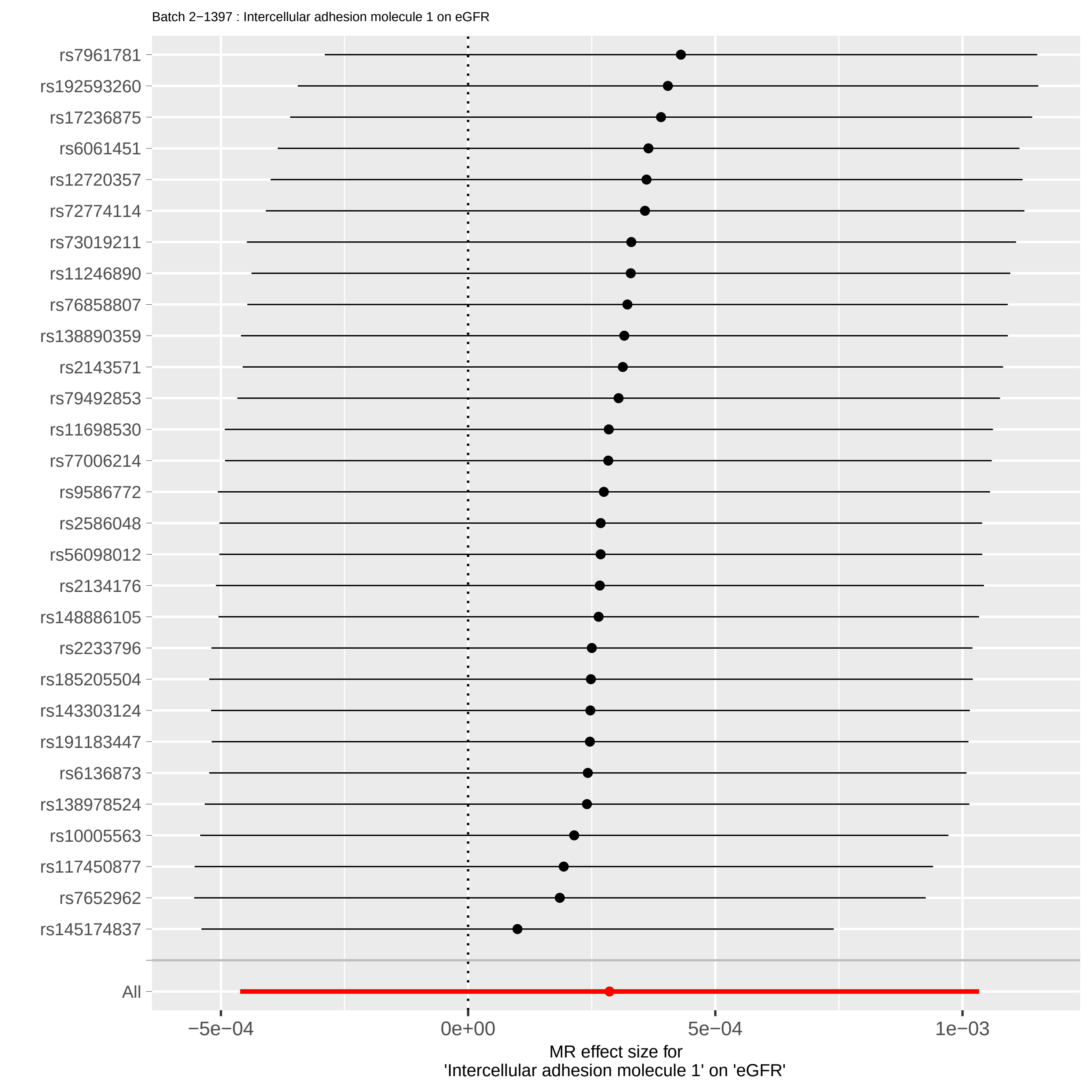

Batch 2−1397 : Intercellular adhesion molecule 1 on eGFR
rs7961781
rs192593260
rs17236875
rs6061451
rs12720357
rs72774114
rs73019211
rs11246890
rs76858807
rs138890359
rs2143571
rs79492853
rs11698530
rs77006214
rs9586772
rs2586048
rs56098012
rs2134176
rs148886105
rs2233796
rs185205504
rs143303124
rs191183447
rs6136873
rs138978524
rs10005563
rs117450877
rs7652962
rs145174837
All
−5e−04
0e+00
5e−04
1e−03
MR effect size for
'Intercellular adhesion molecule 1' on 'eGFR'

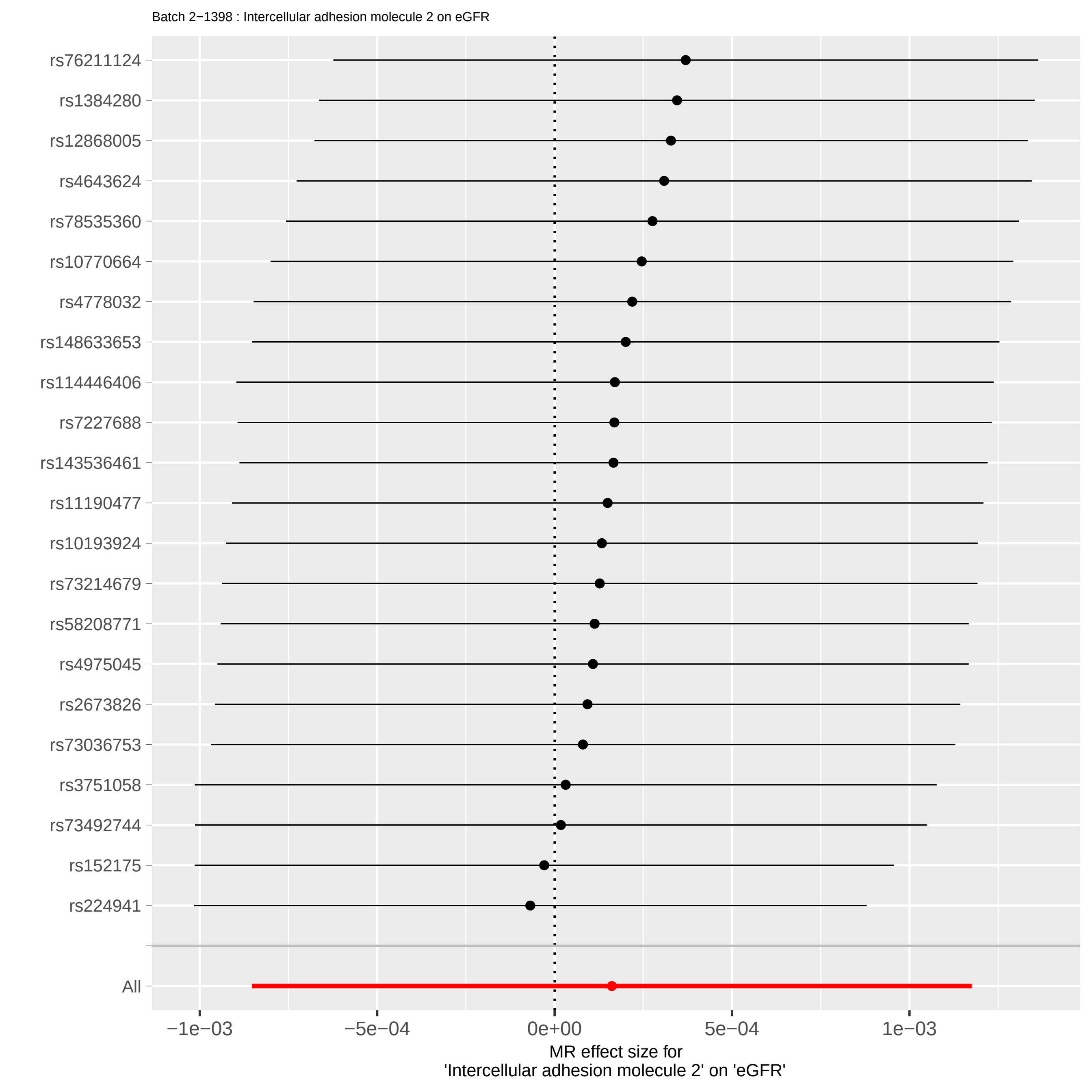

Batch 2−1398 : Intercellular adhesion molecule 2 on eGFR
rs76211124
rs1384280
rs12868005
rs4643624
rs78535360
rs10770664
rs4778032
rs148633653
rs114446406
rs7227688
rs143536461
rs11190477
rs10193924
rs73214679
rs58208771
rs4975045
rs2673826
rs73036753
rs3751058
rs73492744
rs152175
rs224941
All
−1e−03
−5e−04
0e+00
5e−04
1e−03
MR effect size for
'Intercellular adhesion molecule 2' on 'eGFR'

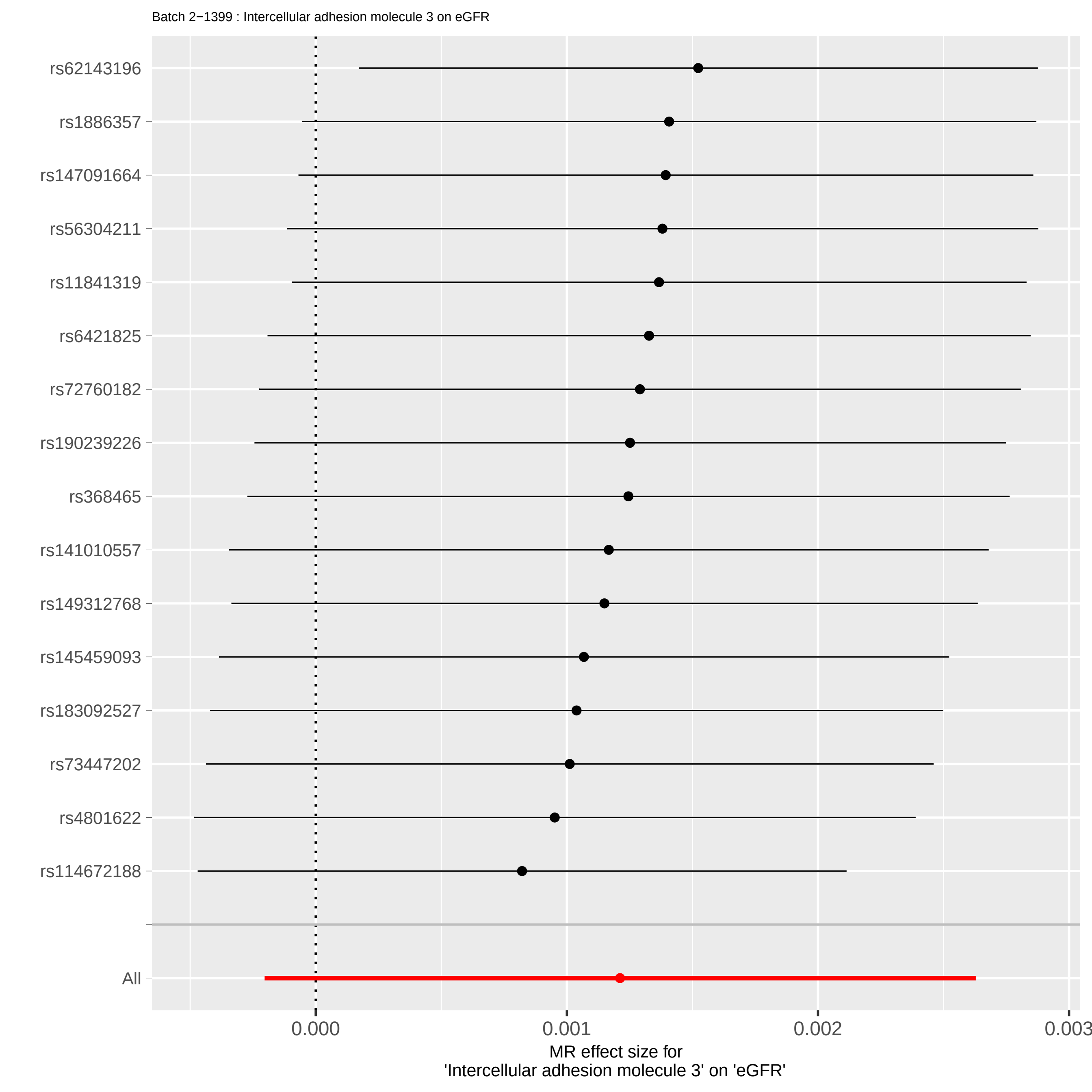

Batch 2−1399 : Intercellular adhesion molecule 3 on eGFR
rs62143196
rs1886357
rs147091664
rs56304211
rs11841319
rs6421825
rs72760182
rs190239226
rs368465
rs141010557
rs149312768
rs145459093
rs183092527
rs73447202
rs4801622
rs114672188
All
0.000
0.001
0.002
0.003
MR effect size for
'Intercellular adhesion molecule 3' on 'eGFR'

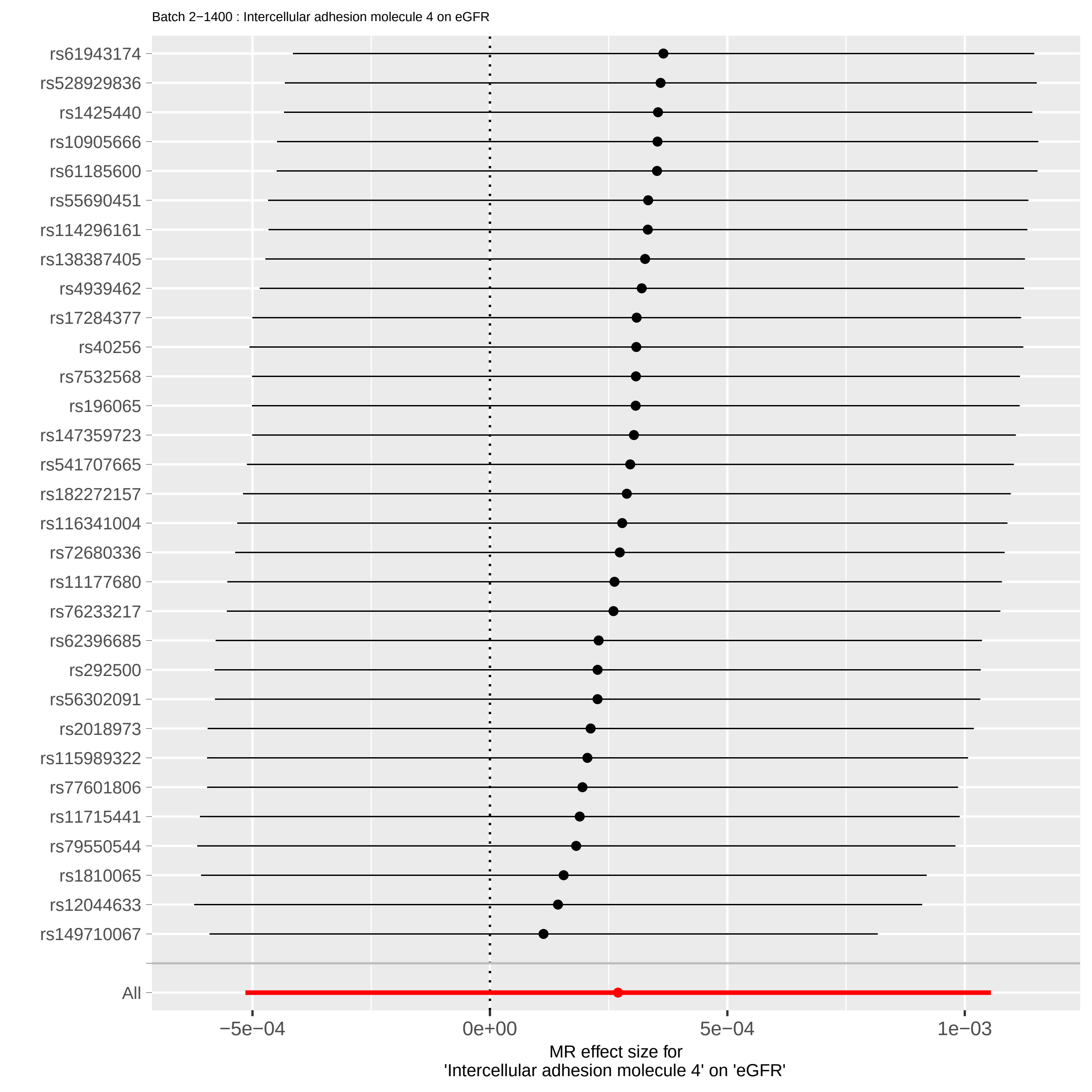

Batch 2−1400 : Intercellular adhesion molecule 4 on eGFR
rs61943174
rs528929836
rs1425440
rs10905666
rs61185600
rs55690451
rs114296161
rs138387405
rs4939462
rs17284377
rs40256
rs7532568
rs196065
rs147359723
rs541707665
rs182272157
rs116341004
rs72680336
rs11177680
rs76233217
rs62396685
rs292500
rs56302091
rs2018973
rs115989322
rs77601806
rs11715441
rs79550544
rs1810065
rs12044633
rs149710067
All
−5e−04
0e+00
5e−04
1e−03
MR effect size for
'Intercellular adhesion molecule 4' on 'eGFR'

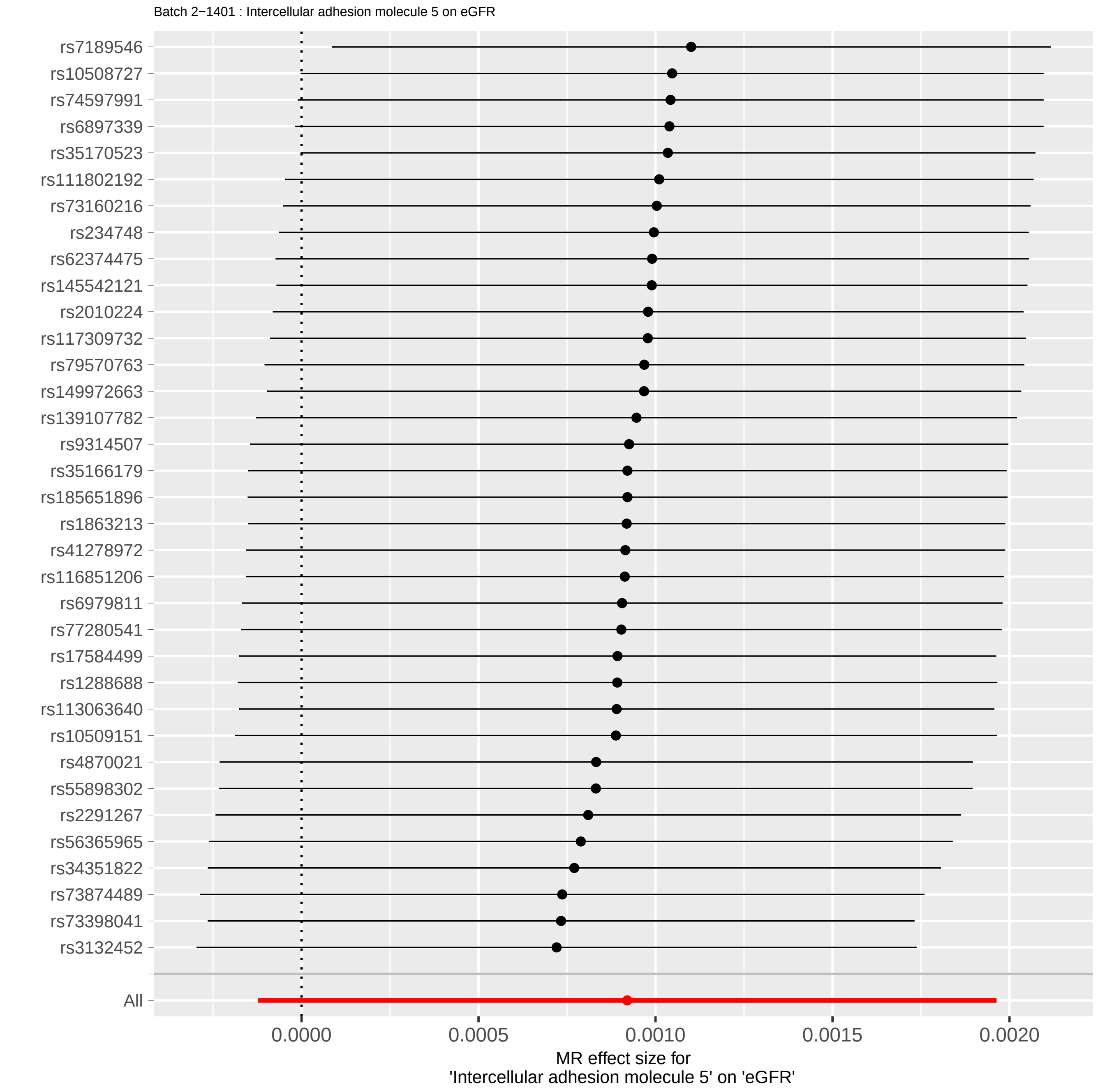

Batch 2−1401 : Intercellular adhesion molecule 5 on eGFR
rs7189546
rs10508727
rs74597991
rs6897339
rs35170523
rs111802192
rs73160216
rs234748
rs62374475
rs145542121
rs2010224
rs117309732
rs79570763
rs149972663
rs139107782
rs9314507
rs35166179
rs185651896
rs1863213
rs41278972
rs116851206
rs6979811
rs77280541
rs17584499
rs1288688
rs113063640
rs10509151
rs4870021
rs55898302
rs2291267
rs56365965
rs34351822
rs73874489
rs73398041
rs3132452
All
0.0000
0.0005
0.0010
0.0015
0.0020
MR effect size for
'Intercellular adhesion molecule 5' on 'eGFR'

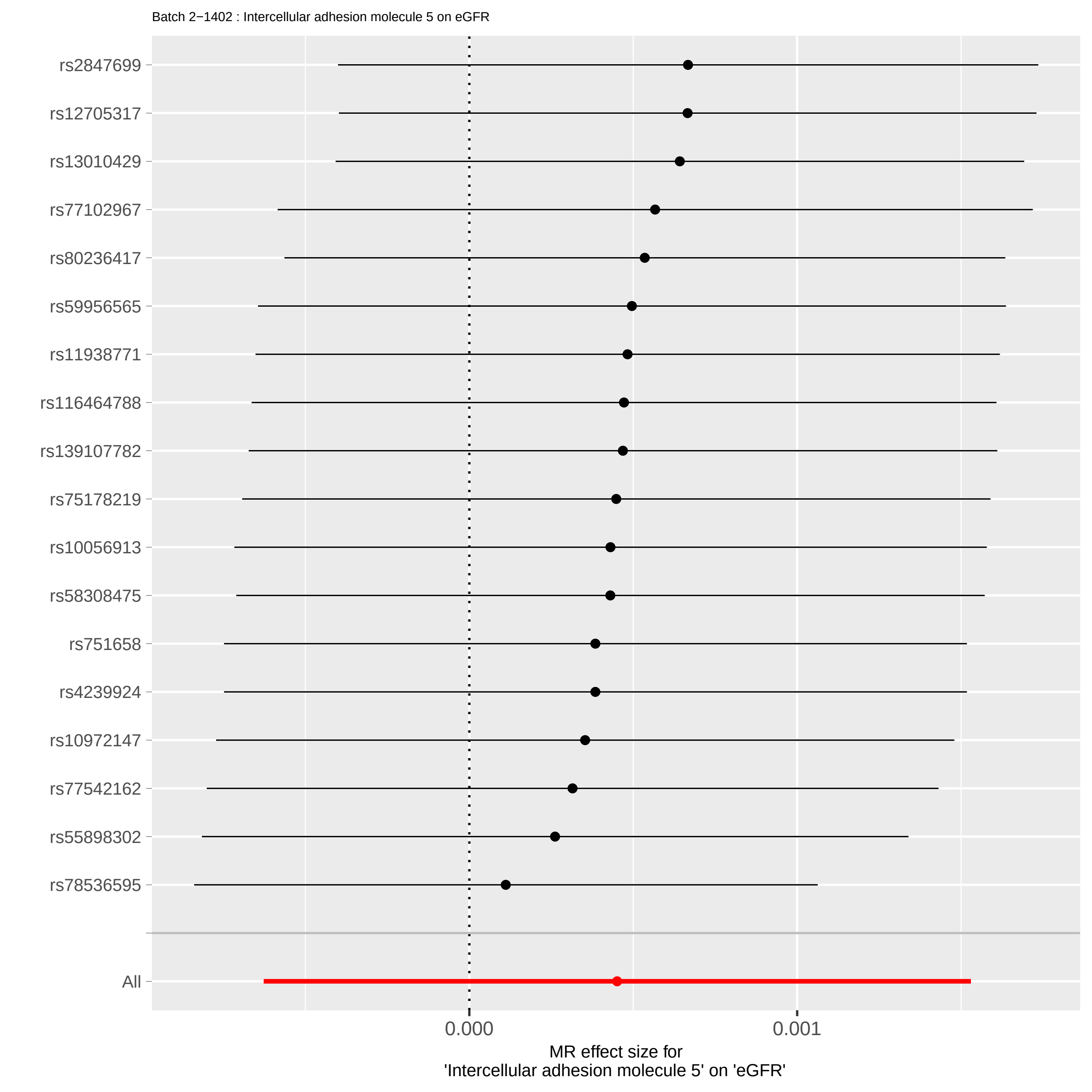

Batch 2−1402 : Intercellular adhesion molecule 5 on eGFR
rs2847699
rs12705317
rs13010429
rs77102967
rs80236417
rs59956565
rs11938771
rs116464788
rs139107782
rs75178219
rs10056913
rs58308475
rs751658
rs4239924
rs10972147
rs77542162
rs55898302
rs78536595
All
0.000
0.001
MR effect size for
'Intercellular adhesion molecule 5' on 'eGFR'

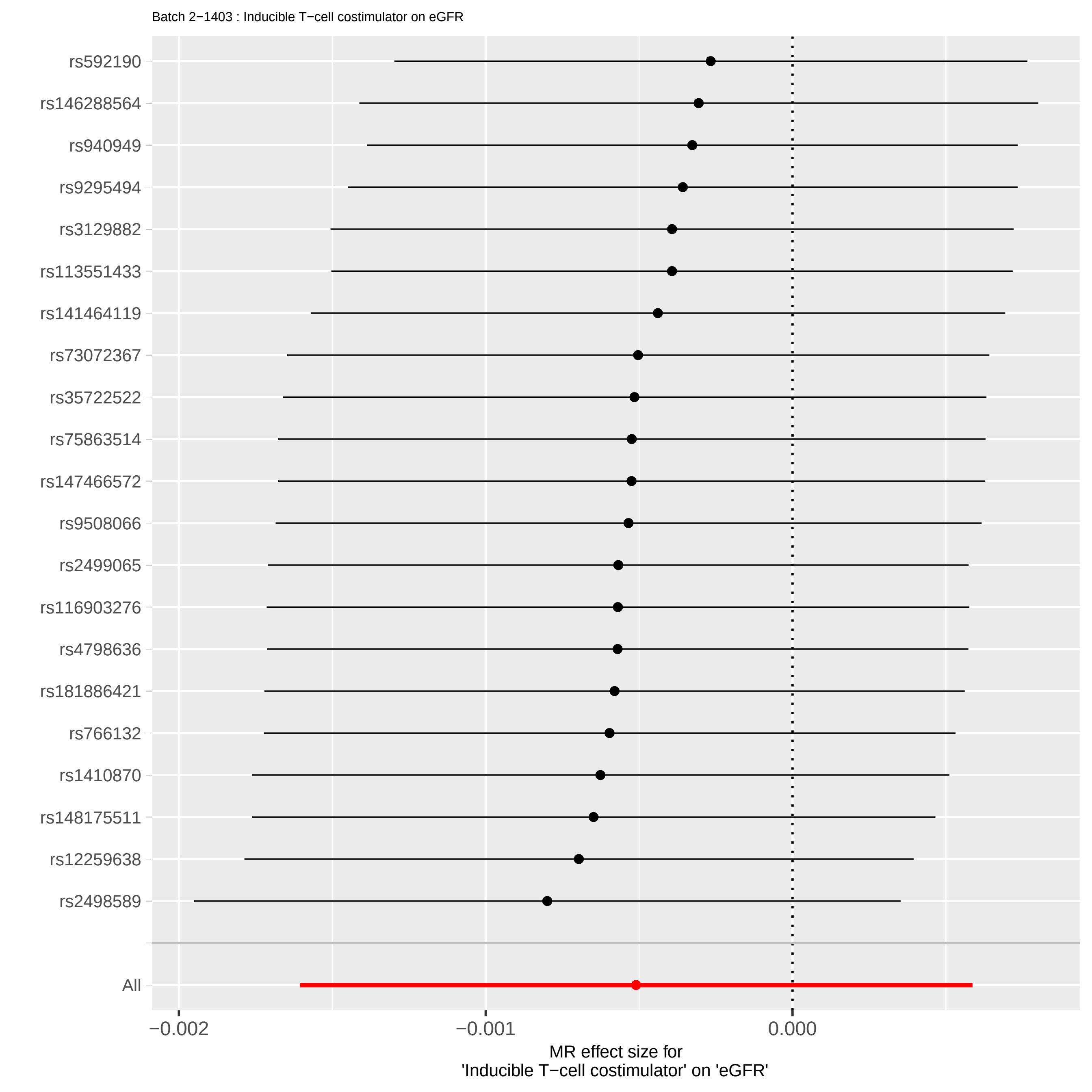
Batch 2−1403 : Inducible T−cell costimulator on eGFR
rs592190
rs146288564
rs940949
rs9295494
rs3129882
rs113551433
rs141464119
rs73072367
rs35722522
rs75863514
rs147466572
rs9508066
rs2499065
rs116903276
rs4798636
rs181886421
rs766132
rs1410870
rs148175511
rs12259638
rs2498589
All
−0.002
−0.001
0.000
MR effect size for
'Inducible T−cell costimulator' on 'eGFR'

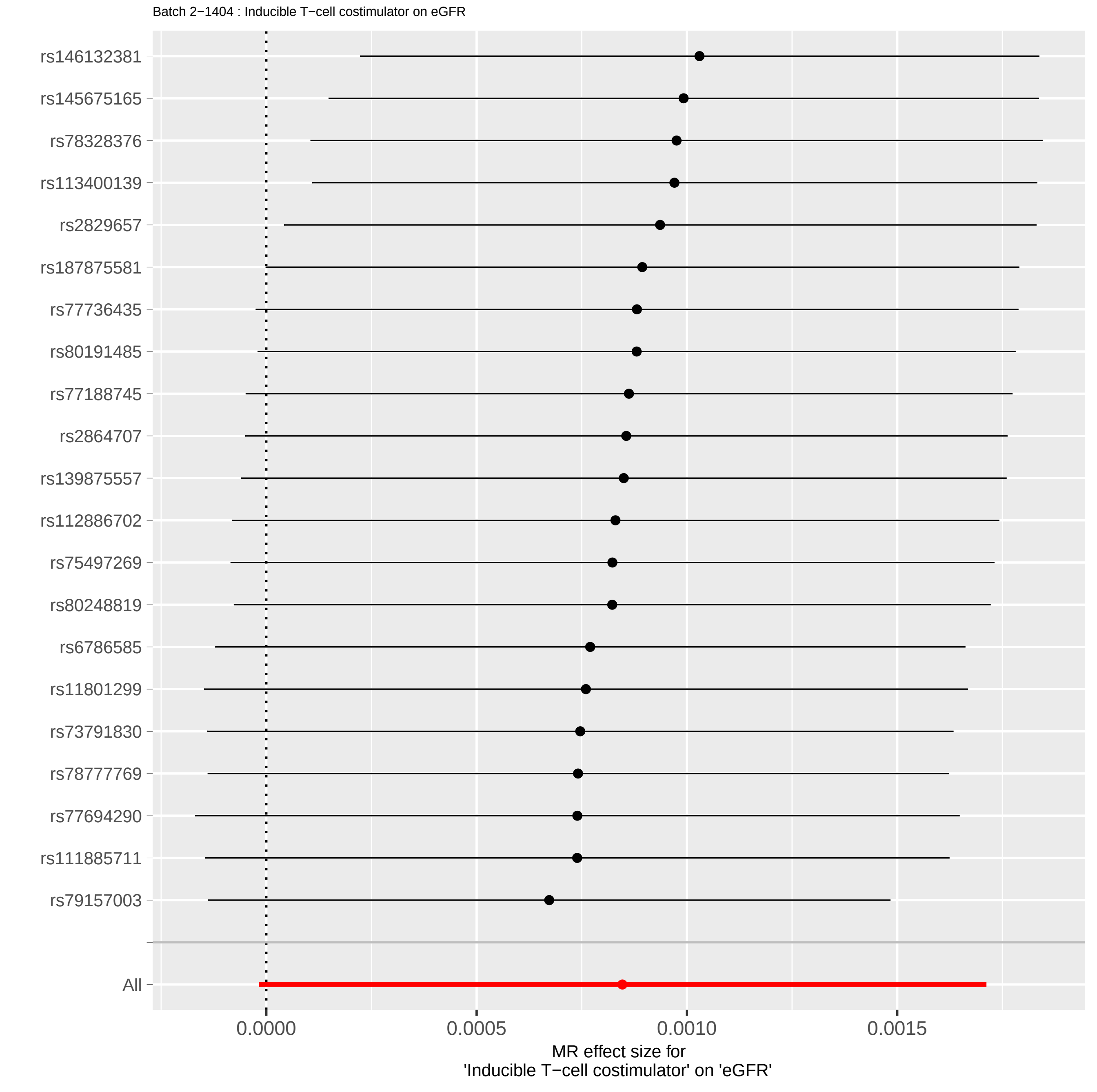

Batch 2−1404 : Inducible T−cell costimulator on eGFR
rs146132381
rs145675165
rs78328376
rs113400139
rs2829657
rs187875581
rs77736435
rs80191485
rs77188745
rs2864707
rs139875557
rs112886702
rs75497269
rs80248819
rs6786585
rs11801299
rs73791830
rs78777769
rs77694290
rs111885711
rs79157003
All
0.0000
0.0005
0.0010
0.0015
MR effect size for
'Inducible T−cell costimulator' on 'eGFR'

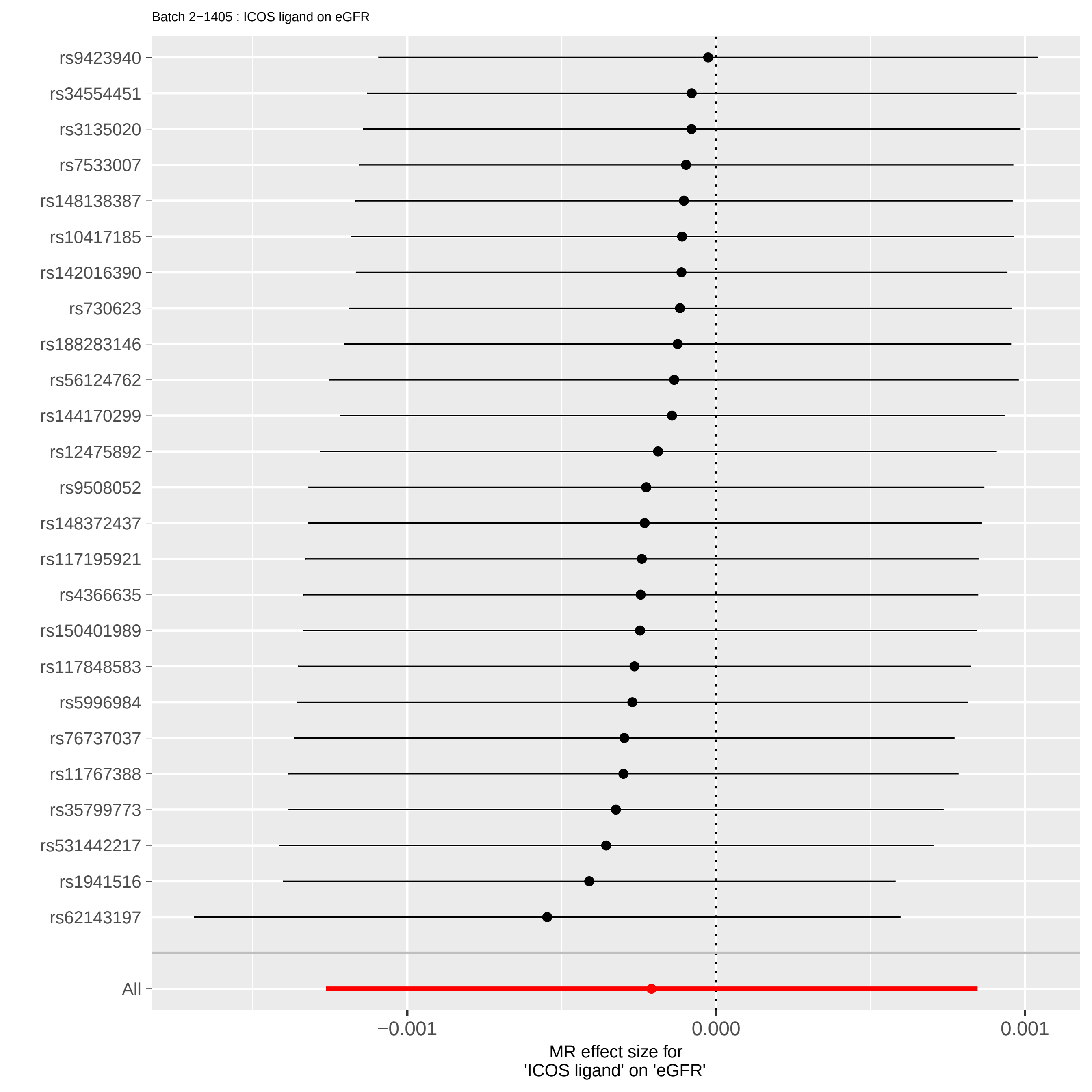

Batch 2−1405 : ICOS ligand on eGFR
rs9423940
rs34554451
rs3135020
rs7533007
rs148138387
rs10417185
rs142016390
rs730623
rs188283146
rs56124762
rs144170299
rs12475892
rs9508052
rs148372437
rs117195921
rs4366635
rs150401989
rs117848583
rs5996984
rs76737037
rs11767388
rs35799773
rs531442217
rs1941516
rs62143197
All
−0.001
0.000
0.001
MR effect size for
'ICOS ligand' on 'eGFR'

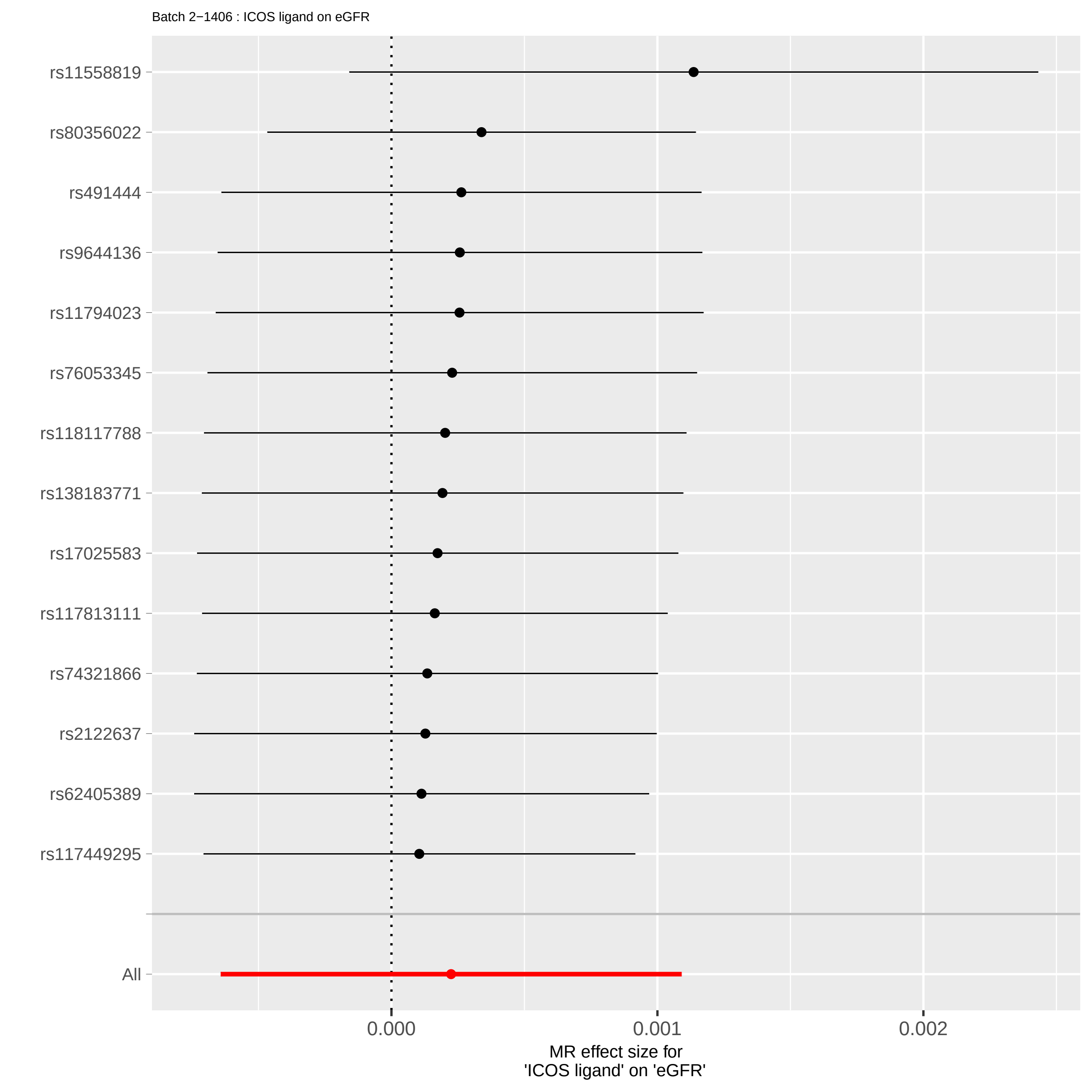
Batch 2−1406 : ICOS ligand on eGFR
rs11558819
rs80356022
rs491444
rs9644136
rs11794023
rs76053345
rs118117788
rs138183771
rs17025583
rs117813111
rs74321866
rs2122637
rs62405389
rs117449295
All
0.000
0.001
0.002
MR effect size for
'ICOS ligand' on 'eGFR'

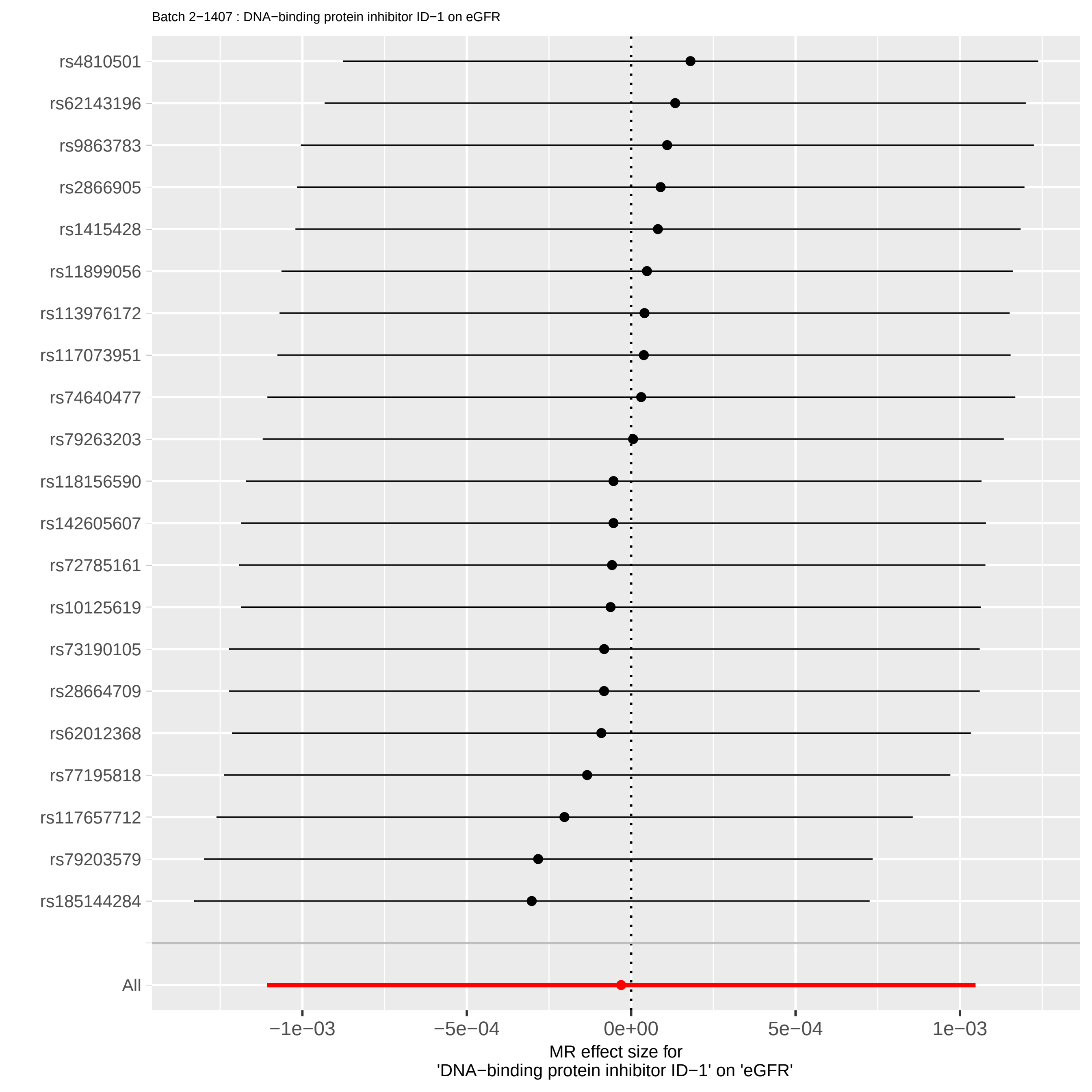

Batch 2−1407 : DNA−binding protein inhibitor ID−1 on eGFR
rs4810501
rs62143196
rs9863783
rs2866905
rs1415428
rs11899056
rs113976172
rs117073951
rs74640477
rs79263203
rs118156590
rs142605607
rs72785161
rs10125619
rs73190105
rs28664709
rs62012368
rs77195818
rs117657712
rs79203579
rs185144284
All
−1e−03
−5e−04
0e+00
5e−04
1e−03
MR effect size for
'DNA−binding protein inhibitor ID−1' on 'eGFR'

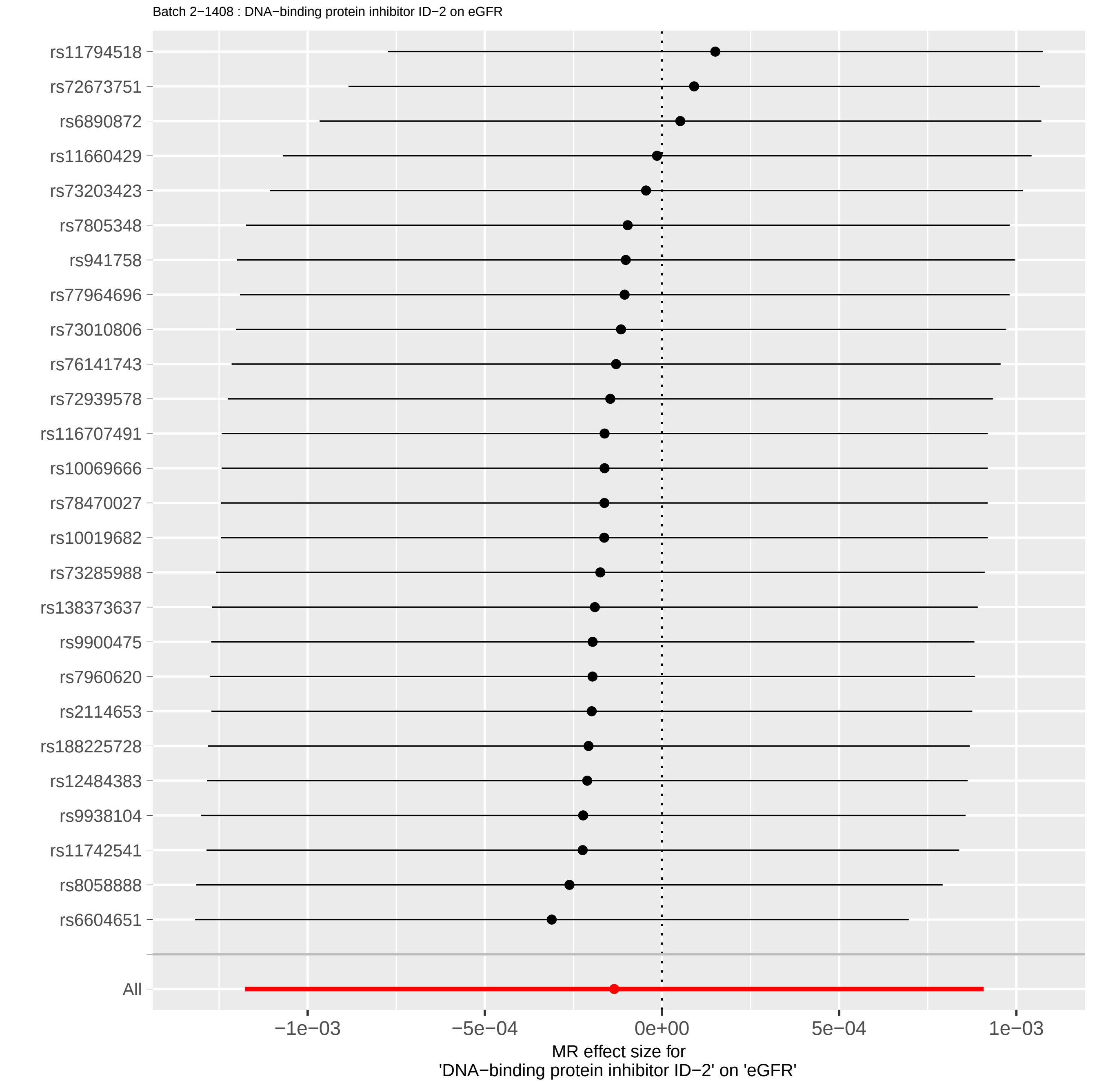

Batch 2−1408 : DNA−binding protein inhibitor ID−2 on eGFR
rs11794518
rs72673751
rs6890872
rs11660429
rs73203423
rs7805348
rs941758
rs77964696
rs73010806
rs76141743
rs72939578
rs116707491
rs10069666
rs78470027
rs10019682
rs73285988
rs138373637
rs9900475
rs7960620
rs2114653
rs188225728
rs12484383
rs9938104
rs11742541
rs8058888
rs6604651
All
−1e−03
−5e−04
0e+00
5e−04
1e−03
MR effect size for
'DNA−binding protein inhibitor ID−2' on 'eGFR'

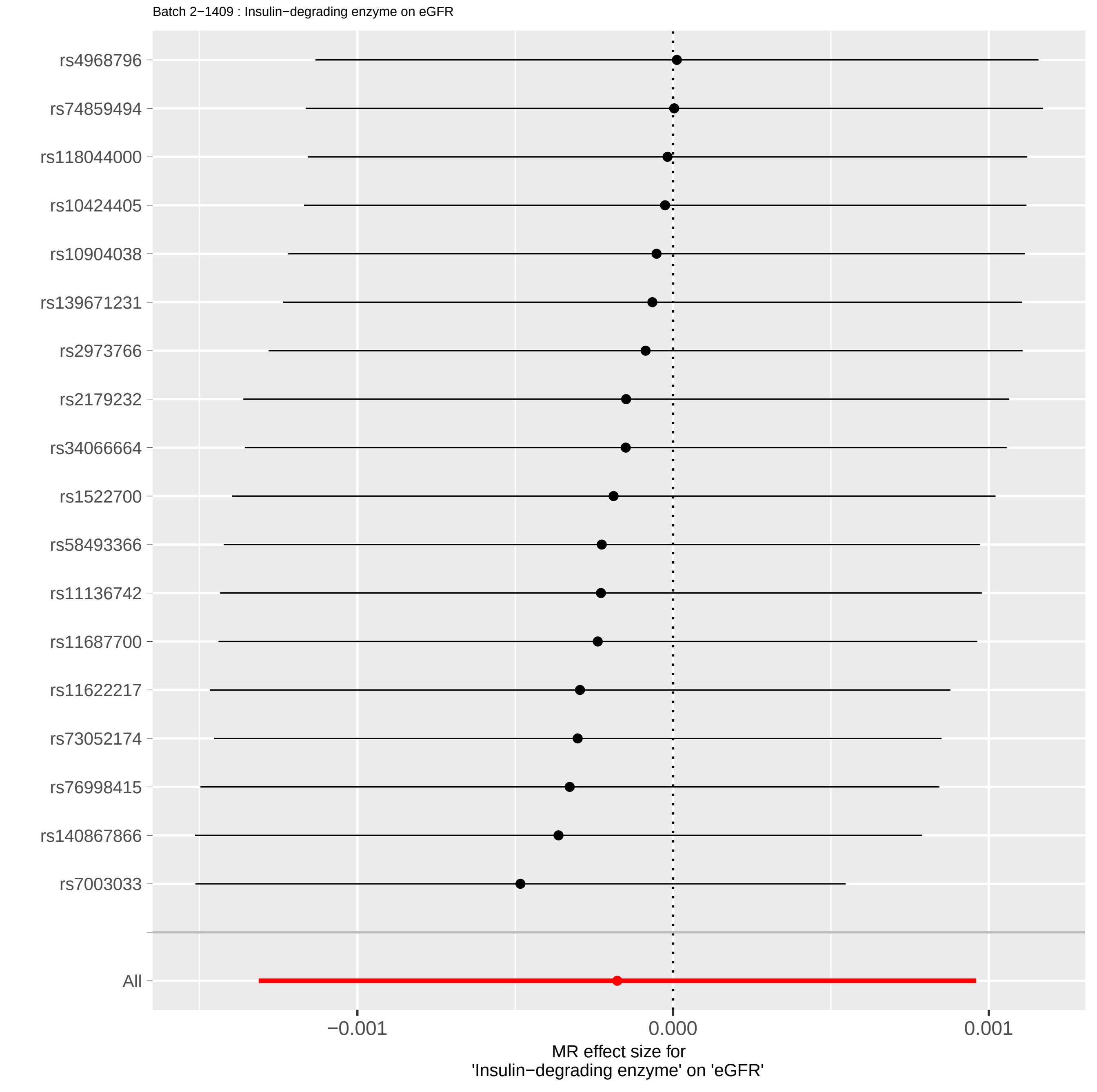

Batch 2−1409 : Insulin−degrading enzyme on eGFR
rs4968796
rs74859494
rs118044000
rs10424405
rs10904038
rs139671231
rs2973766
rs2179232
rs34066664
rs1522700
rs58493366
rs11136742
rs11687700
rs11622217
rs73052174
rs76998415
rs140867866
rs7003033
All
−0.001
0.000
0.001
MR effect size for
'Insulin−degrading enzyme' on 'eGFR'

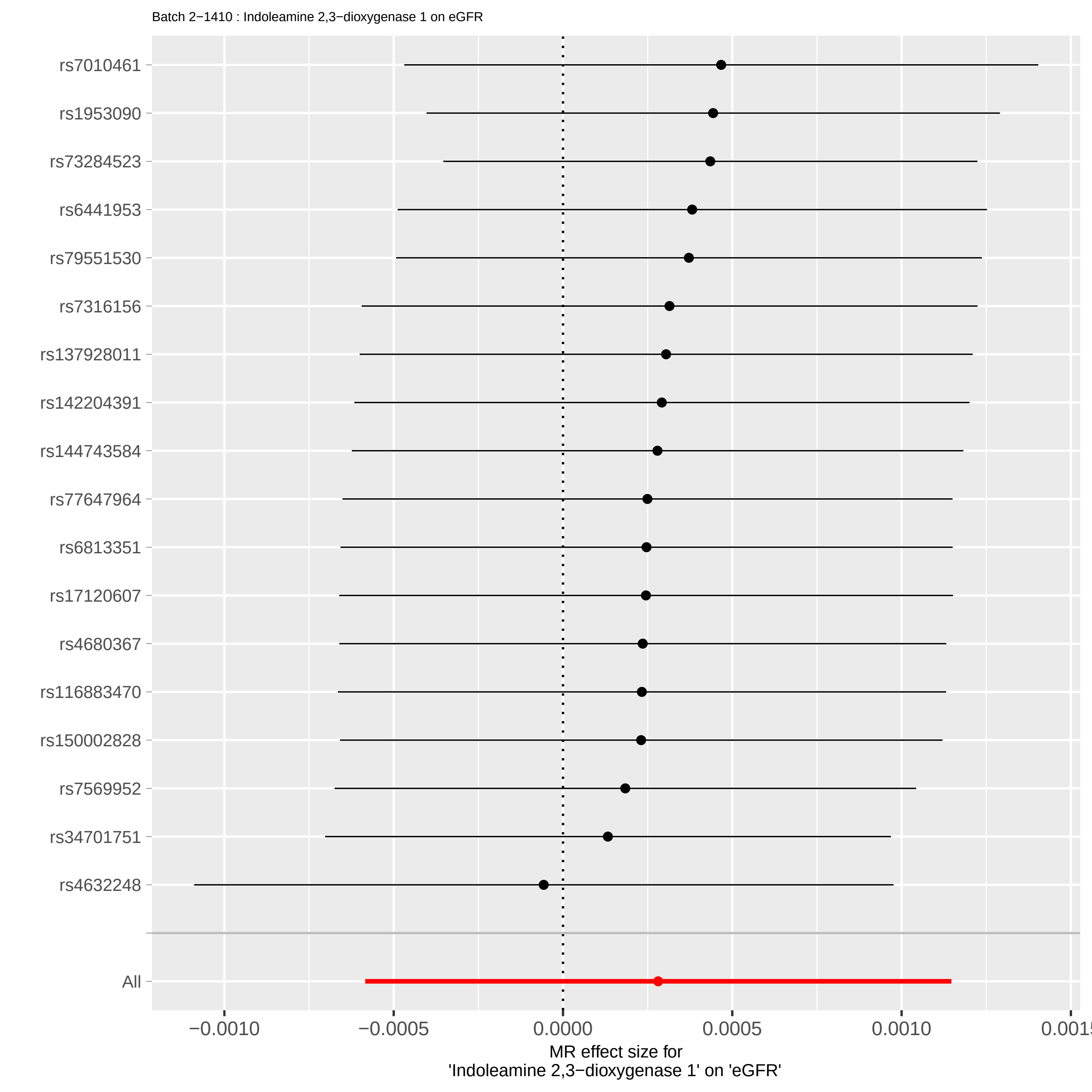

Batch 2−1410 : Indoleamine 2,3−dioxygenase 1 on eGFR
rs7010461
rs1953090
rs73284523
rs6441953
rs79551530
rs7316156
rs137928011
rs142204391
rs144743584
rs77647964
rs6813351
rs17120607
rs4680367
rs116883470
rs150002828
rs7569952
rs34701751
rs4632248
All
−0.0010
−0.0005
0.0000
0.0005
0.0010
MR effect size for
'Indoleamine 2,3−dioxygenase 1' on 'eGFR'

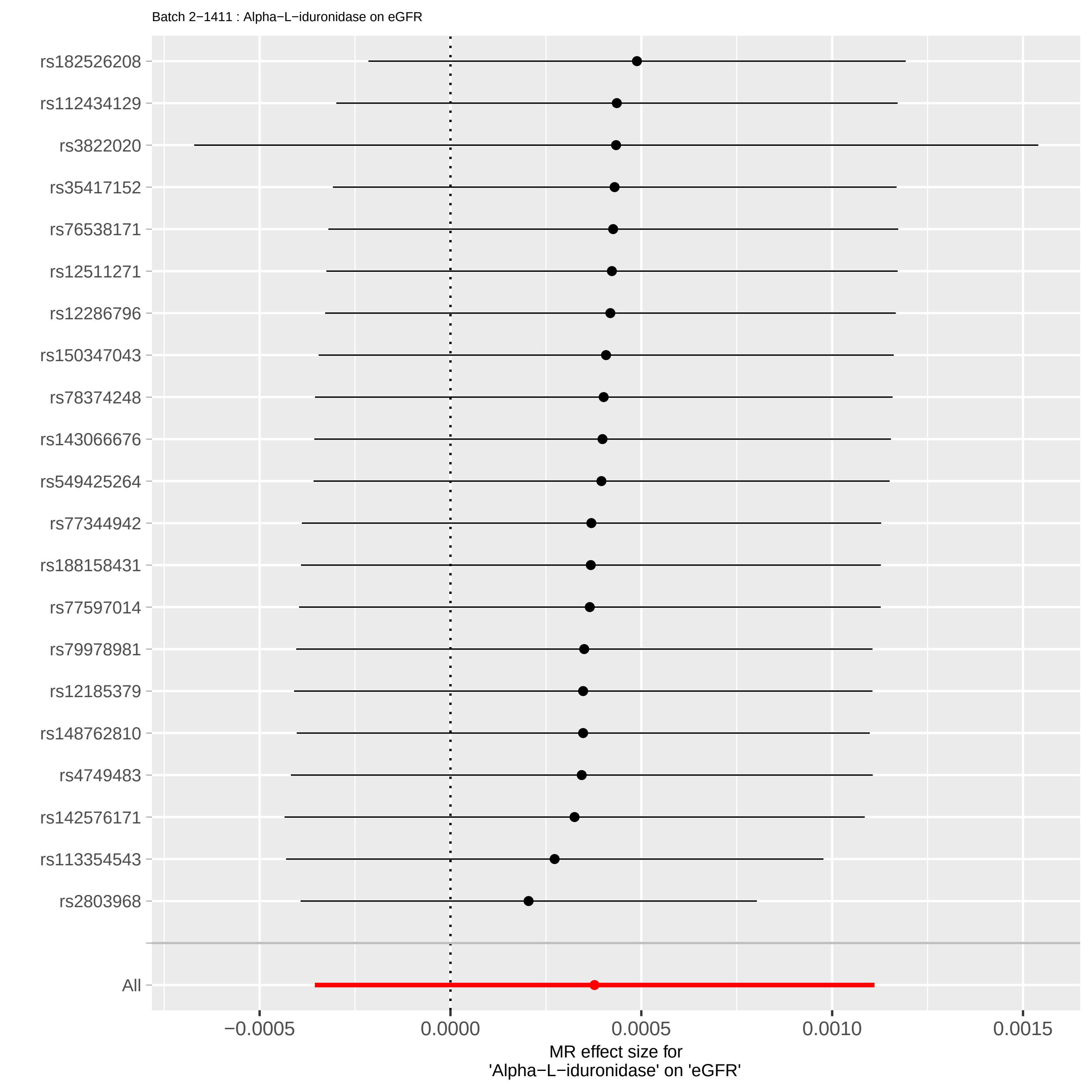

Batch 2−1411 : Alpha−L−iduronidase on eGFR
rs182526208
rs112434129
rs3822020
rs35417152
rs76538171
rs12511271
rs12286796
rs150347043
rs78374248
rs143066676
rs549425264
rs77344942
rs188158431
rs77597014
rs79978981
rs12185379
rs148762810
rs4749483
rs142576171
rs113354543
rs2803968
All
−0.0005
0.0000
0.0005
0.0010
0.0015
MR effect size for
'Alpha−L−iduronidase' on 'eGFR'

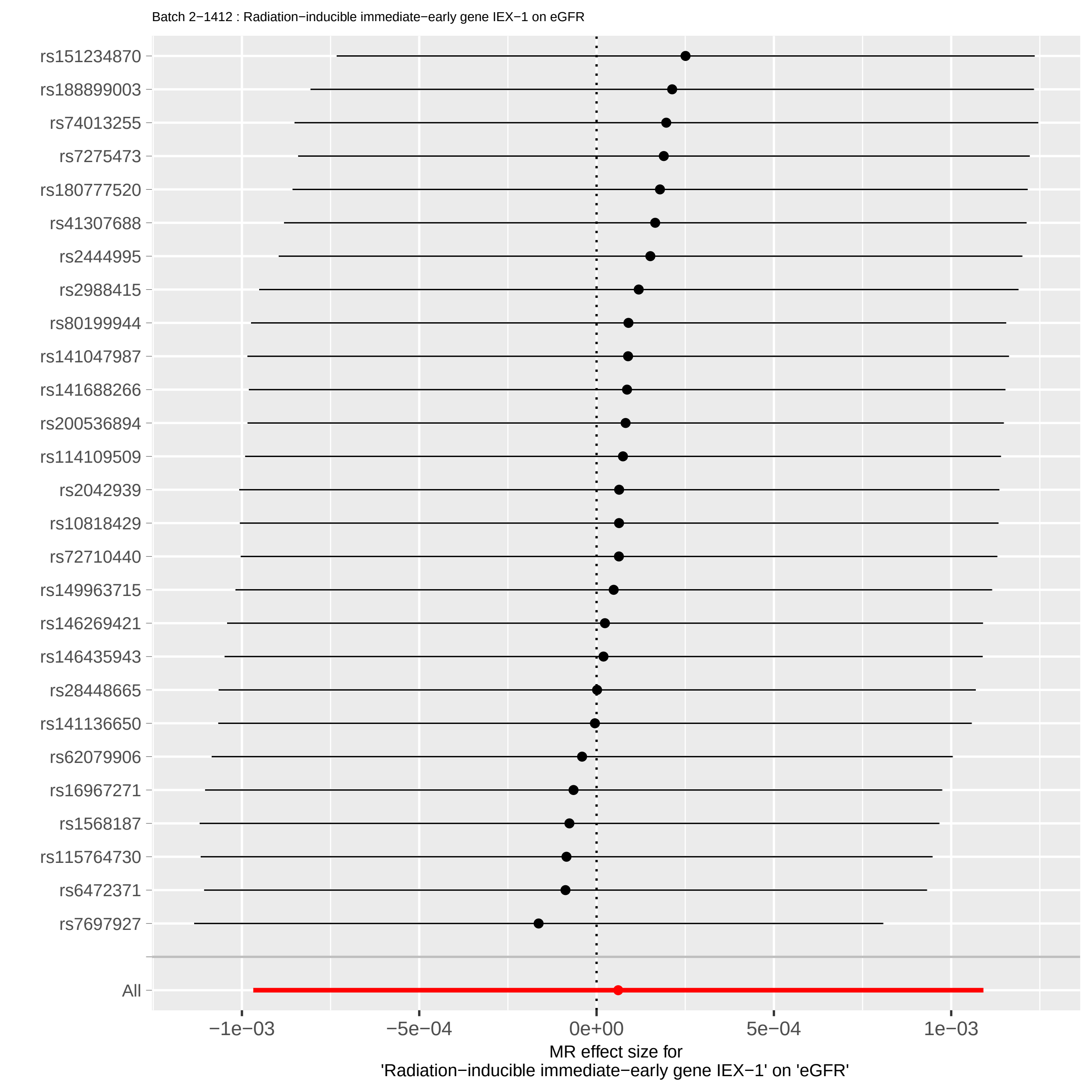

Batch 2−1412 : Radiation−inducible immediate−early gene IEX−1 on eGFR
rs151234870
rs188899003
rs74013255
rs7275473
rs180777520
rs41307688
rs2444995
rs2988415
rs80199944
rs141047987
rs141688266
rs200536894
rs114109509
rs2042939
rs10818429
rs72710440
rs149963715
rs146269421
rs146435943
rs28448665
rs141136650
rs62079906
rs16967271
rs1568187
rs115764730
rs6472371
rs7697927
All
−1e−03
−5e−04
0e+00
5e−04
1e−03
MR effect size for
'Radiation−inducible immediate−early gene IEX−1' on 'eGFR'

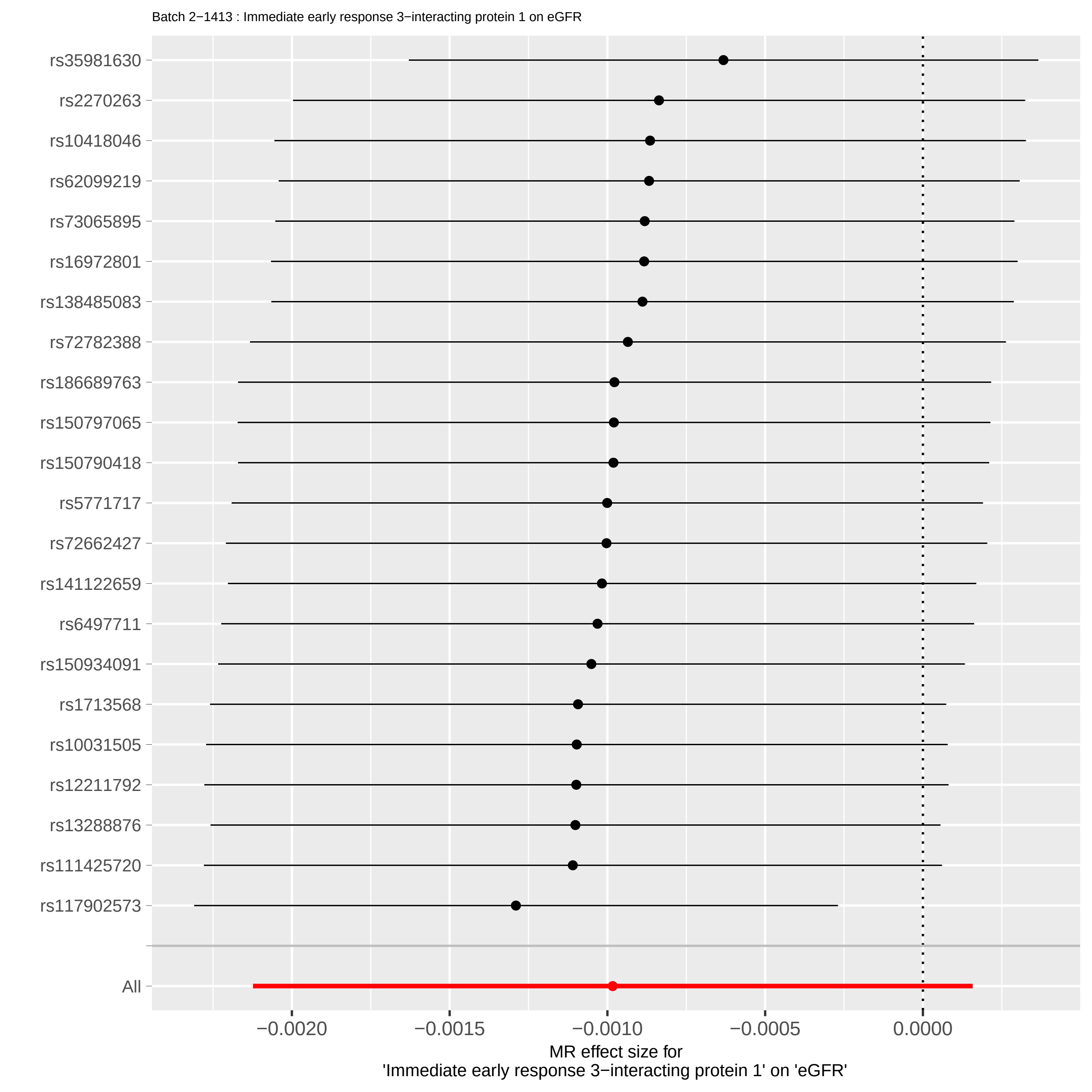
Batch 2−1413 : Immediate early response 3−interacting protein 1 on eGFR
rs35981630
rs2270263
rs10418046
rs62099219
rs73065895
rs16972801
rs138485083
rs72782388
rs186689763
rs150797065
rs150790418
rs5771717
rs72662427
rs141122659
rs6497711
rs150934091
rs1713568
rs10031505
rs12211792
rs13288876
rs111425720
rs117902573
All
−0.0020
−0.0015
−0.0010
−0.0005
0.0000
MR effect size for
'Immediate early response 3−interacting protein 1' on 'eGFR'

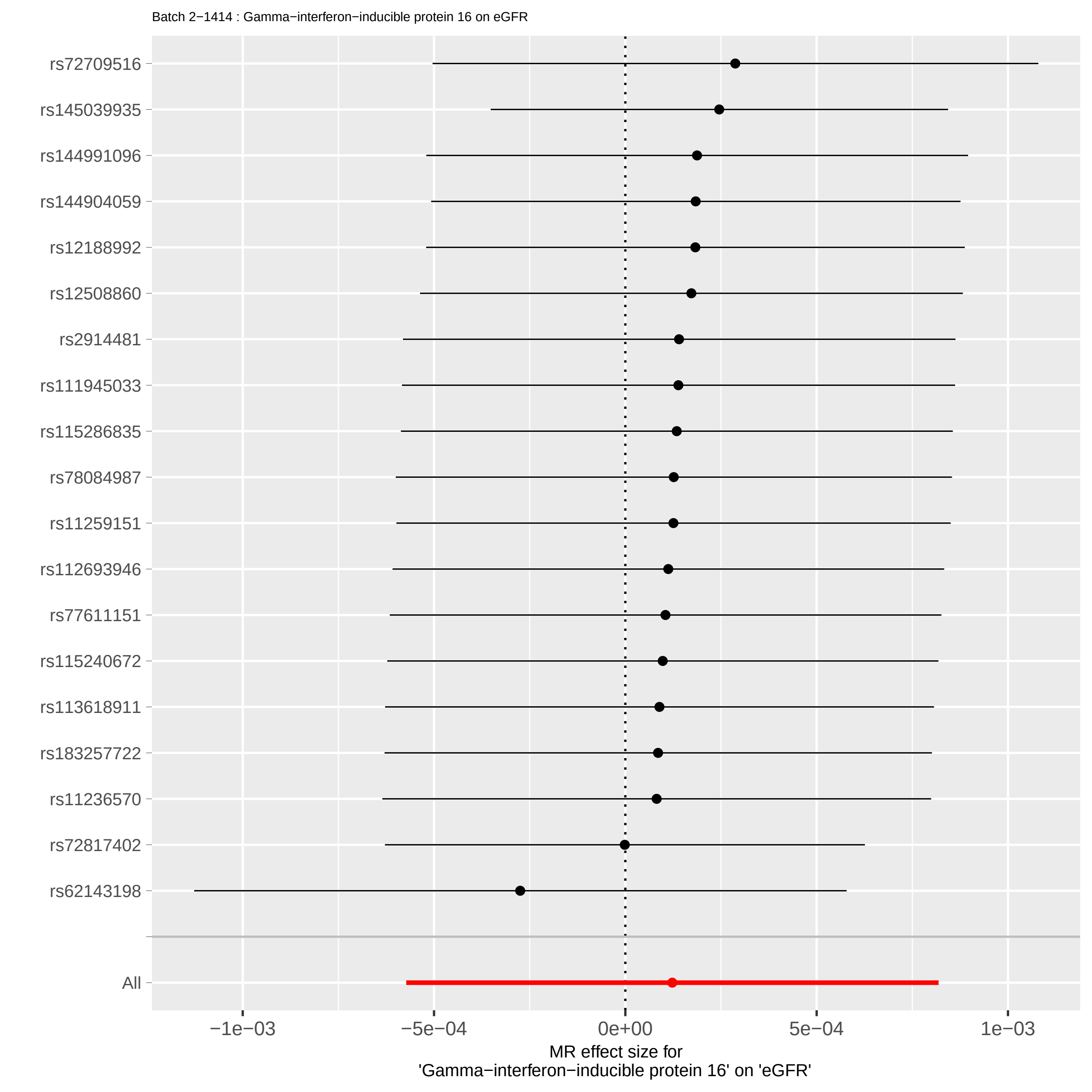

Batch 2−1414 : Gamma−interferon−inducible protein 16 on eGFR
rs72709516
rs145039935
rs144991096
rs144904059
rs12188992
rs12508860
rs2914481
rs111945033
rs115286835
rs78084987
rs11259151
rs112693946
rs77611151
rs115240672
rs113618911
rs183257722
rs11236570
rs72817402
rs62143198
All
−1e−03
−5e−04
0e+00
5e−04
1e−03
MR effect size for
'Gamma−interferon−inducible protein 16' on 'eGFR'

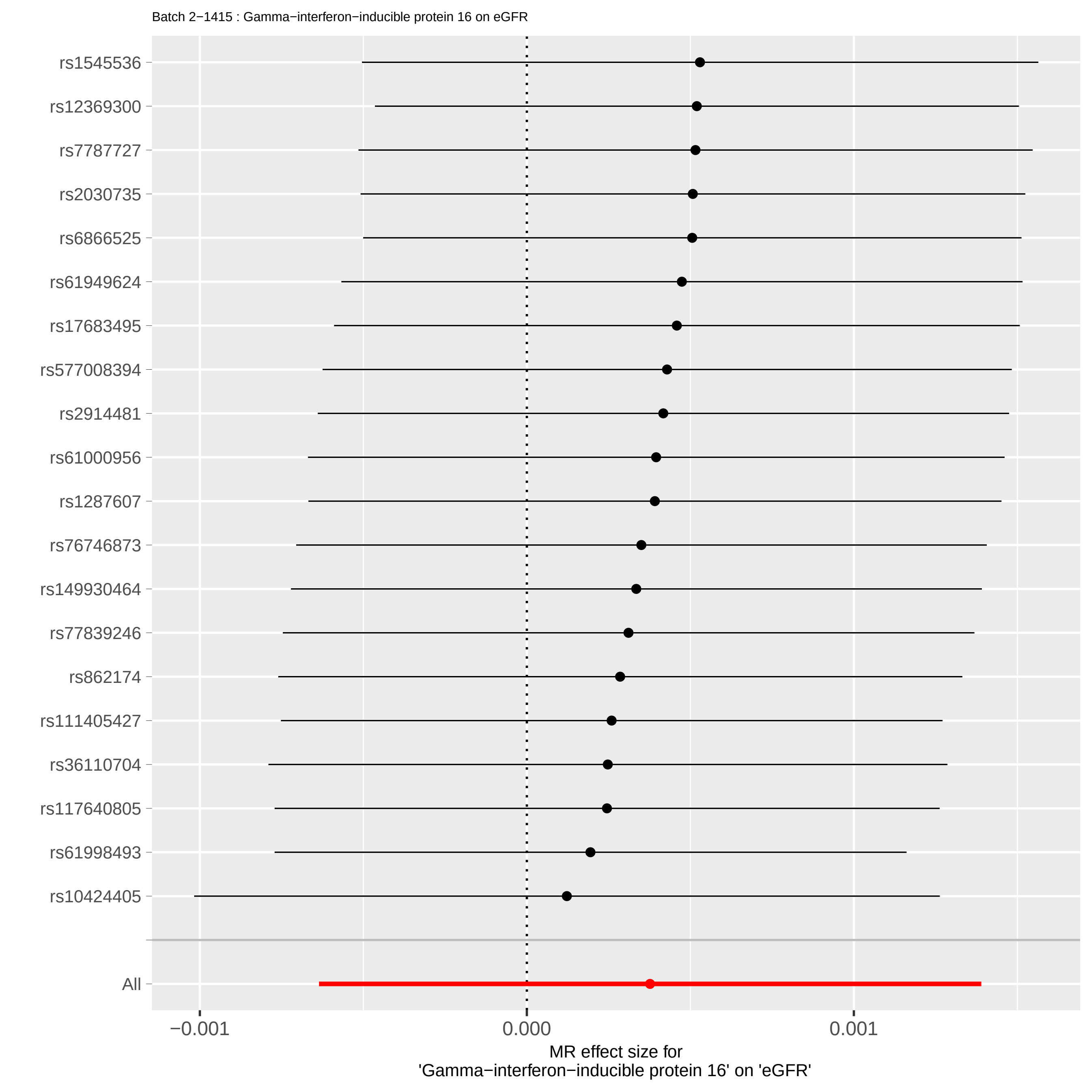
Batch 2−1415 : Gamma−interferon−inducible protein 16 on eGFR
rs1545536
rs12369300
rs7787727
rs2030735
rs6866525
rs61949624
rs17683495
rs577008394
rs2914481
rs61000956
rs1287607
rs76746873
rs149930464
rs77839246
rs862174
rs111405427
rs36110704
rs117640805
rs61998493
rs10424405
All
−0.001
0.000
0.001
MR effect size for
'Gamma−interferon−inducible protein 16' on 'eGFR'

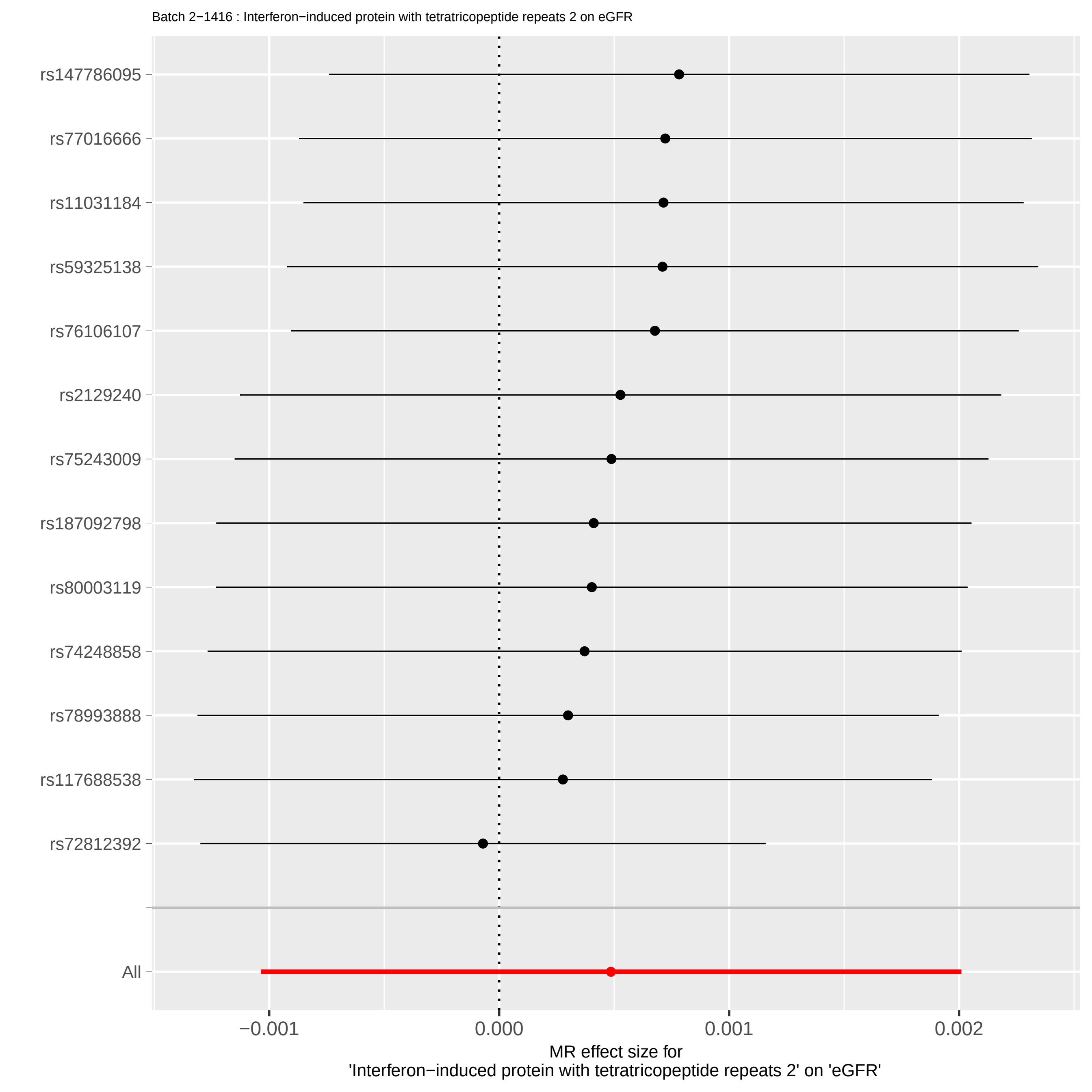

Batch 2−1416 : Interferon−induced protein with tetratricopeptide repeats 2 on eGFR
rs147786095
rs77016666
rs11031184
rs59325138
rs76106107
rs2129240
rs75243009
rs187092798
rs80003119
rs74248858
rs78993888
rs117688538
rs72812392
All
−0.001
0.000
0.001
0.002
MR effect size for
'Interferon−induced protein with tetratricopeptide repeats 2' on 'eGFR'

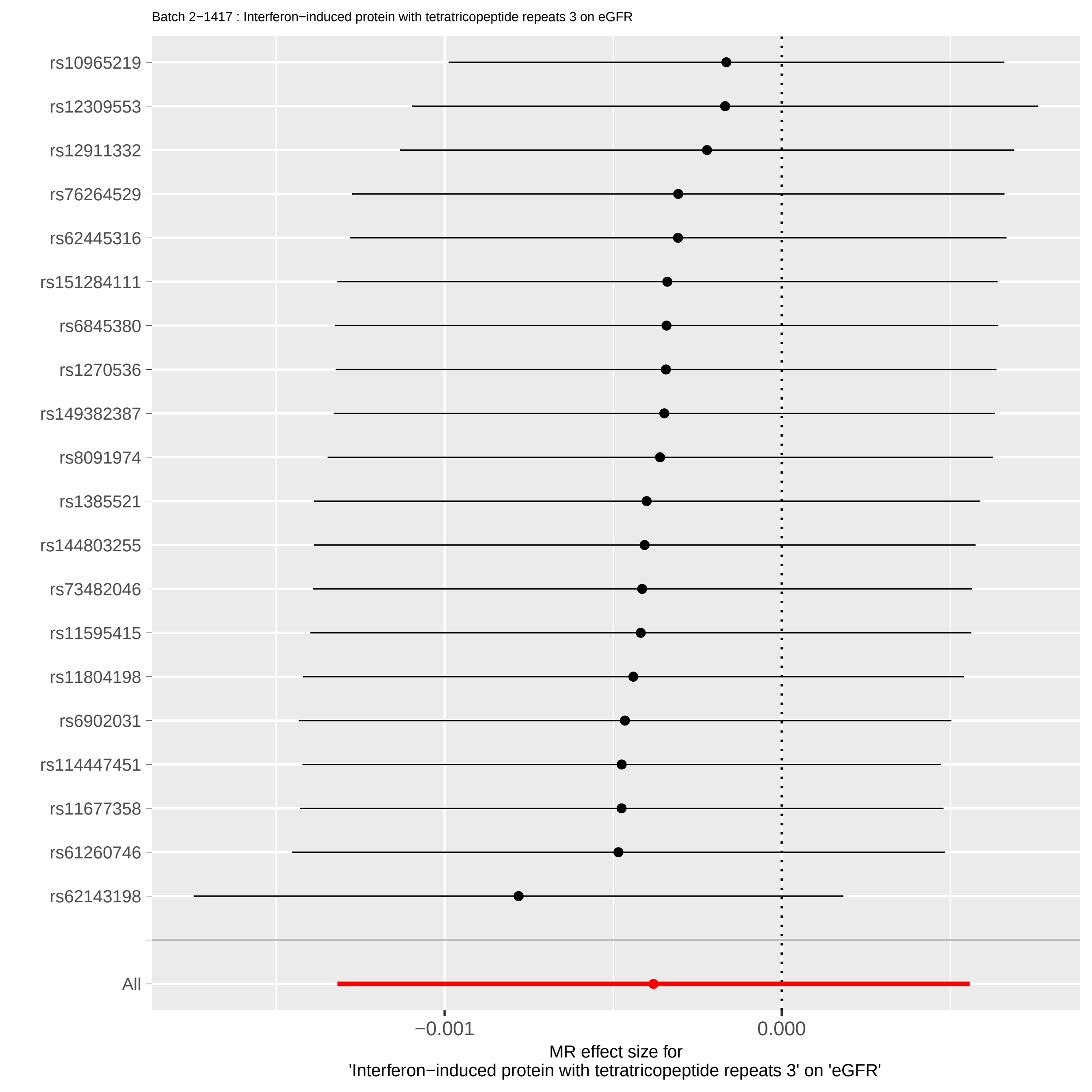
Batch 2−1417 : Interferon−induced protein with tetratricopeptide repeats 3 on eGFR
rs10965219
rs12309553
rs12911332
rs76264529
rs62445316
rs151284111
rs6845380
rs1270536
rs149382387
rs8091974
rs1385521
rs144803255
rs73482046
rs11595415
rs11804198
rs6902031
rs114447451
rs11677358
rs61260746
rs62143198
All
−0.001
0.000
MR effect size for
'Interferon−induced protein with tetratricopeptide repeats 3' on 'eGFR'

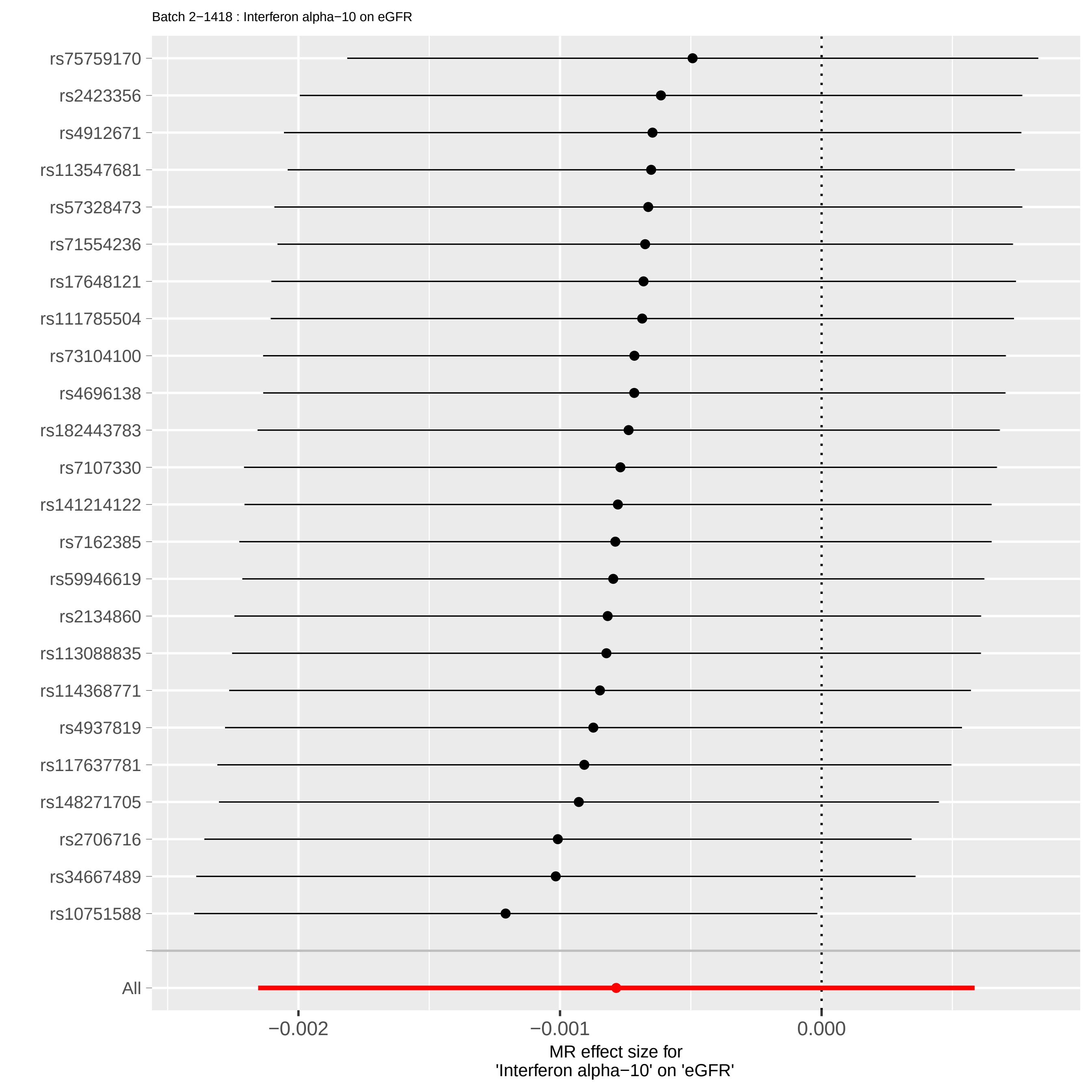

Batch 2−1418 : Interferon alpha−10 on eGFR
rs75759170
rs2423356
rs4912671
rs113547681
rs57328473
rs71554236
rs17648121
rs111785504
rs73104100
rs4696138
rs182443783
rs7107330
rs141214122
rs7162385
rs59946619
rs2134860
rs113088835
rs114368771
rs4937819
rs117637781
rs148271705
rs2706716
rs34667489
rs10751588
All
−0.002
−0.001
0.000
MR effect size for
'Interferon alpha−10' on 'eGFR'

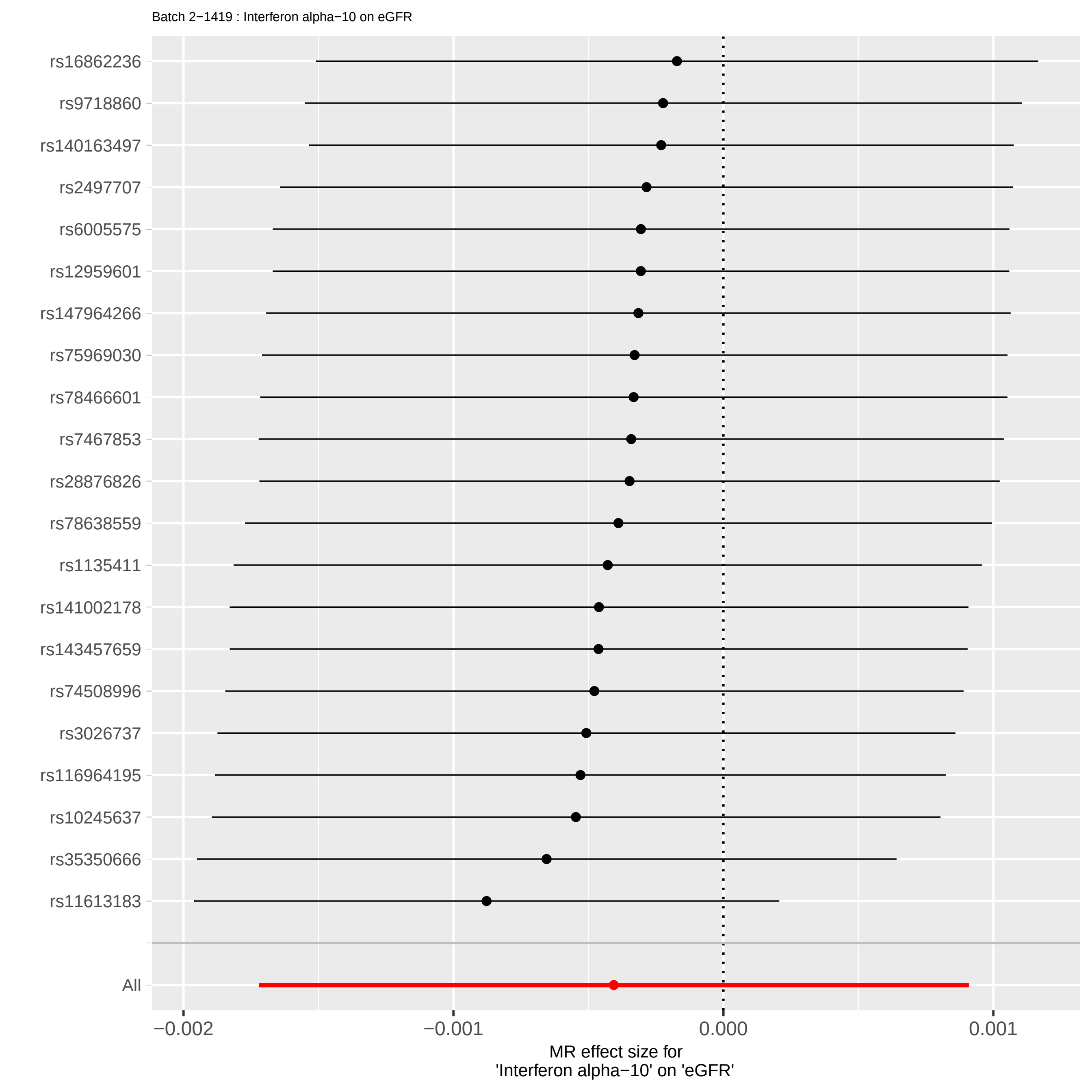

Batch 2−1419 : Interferon alpha−10 on eGFR
rs16862236
rs9718860
rs140163497
rs2497707
rs6005575
rs12959601
rs147964266
rs75969030
rs78466601
rs7467853
rs28876826
rs78638559
rs1135411
rs141002178
rs143457659
rs74508996
rs3026737
rs116964195
rs10245637
rs35350666
rs11613183
All
−0.002
−0.001
0.000
0.001
MR effect size for
'Interferon alpha−10' on 'eGFR'

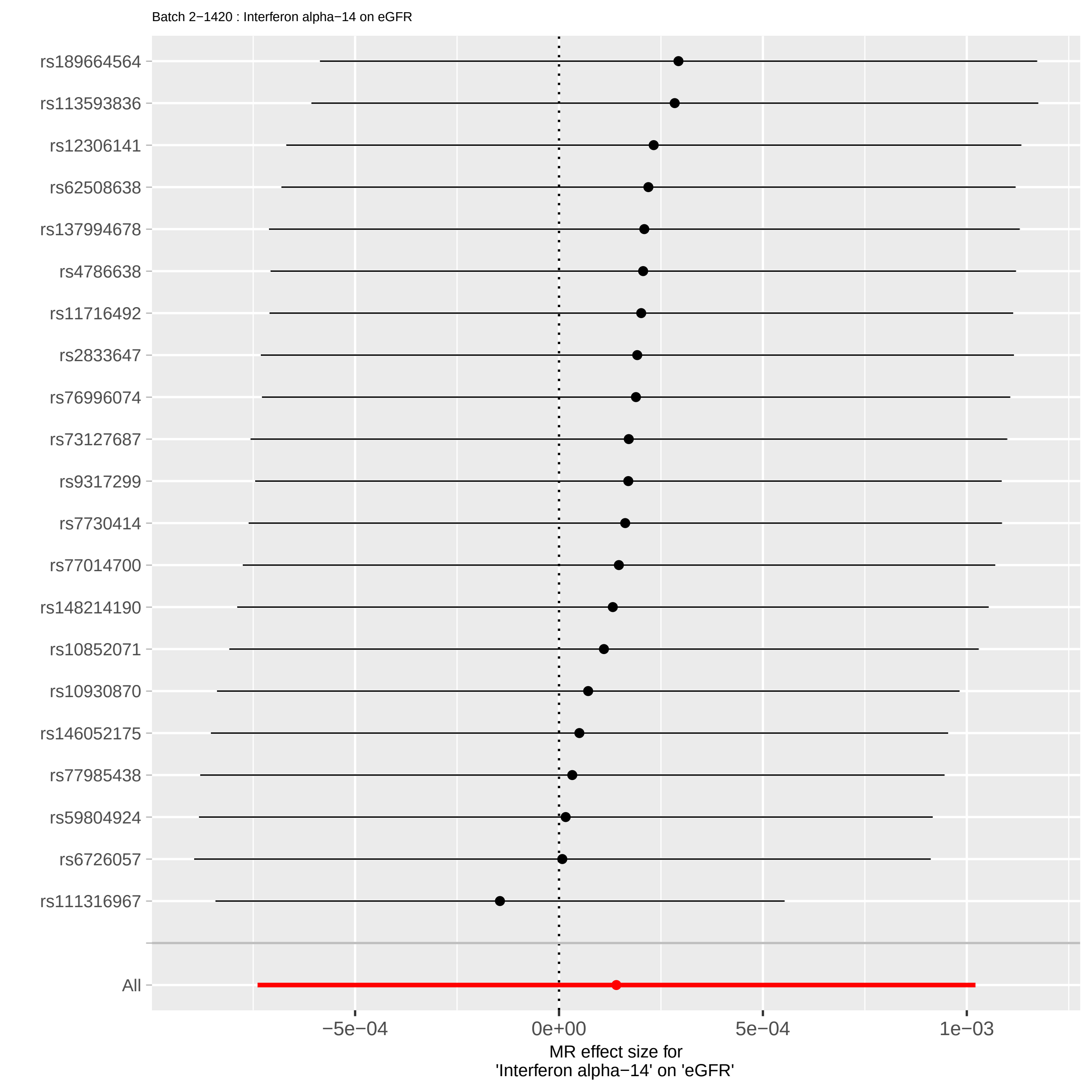

Batch 2−1420 : Interferon alpha−14 on eGFR
rs189664564
rs113593836
rs12306141
rs62508638
rs137994678
rs4786638
rs11716492
rs2833647
rs76996074
rs73127687
rs9317299
rs7730414
rs77014700
rs148214190
rs10852071
rs10930870
rs146052175
rs77985438
rs59804924
rs6726057
rs111316967
All
−5e−04
0e+00
5e−04
1e−03
MR effect size for
'Interferon alpha−14' on 'eGFR'

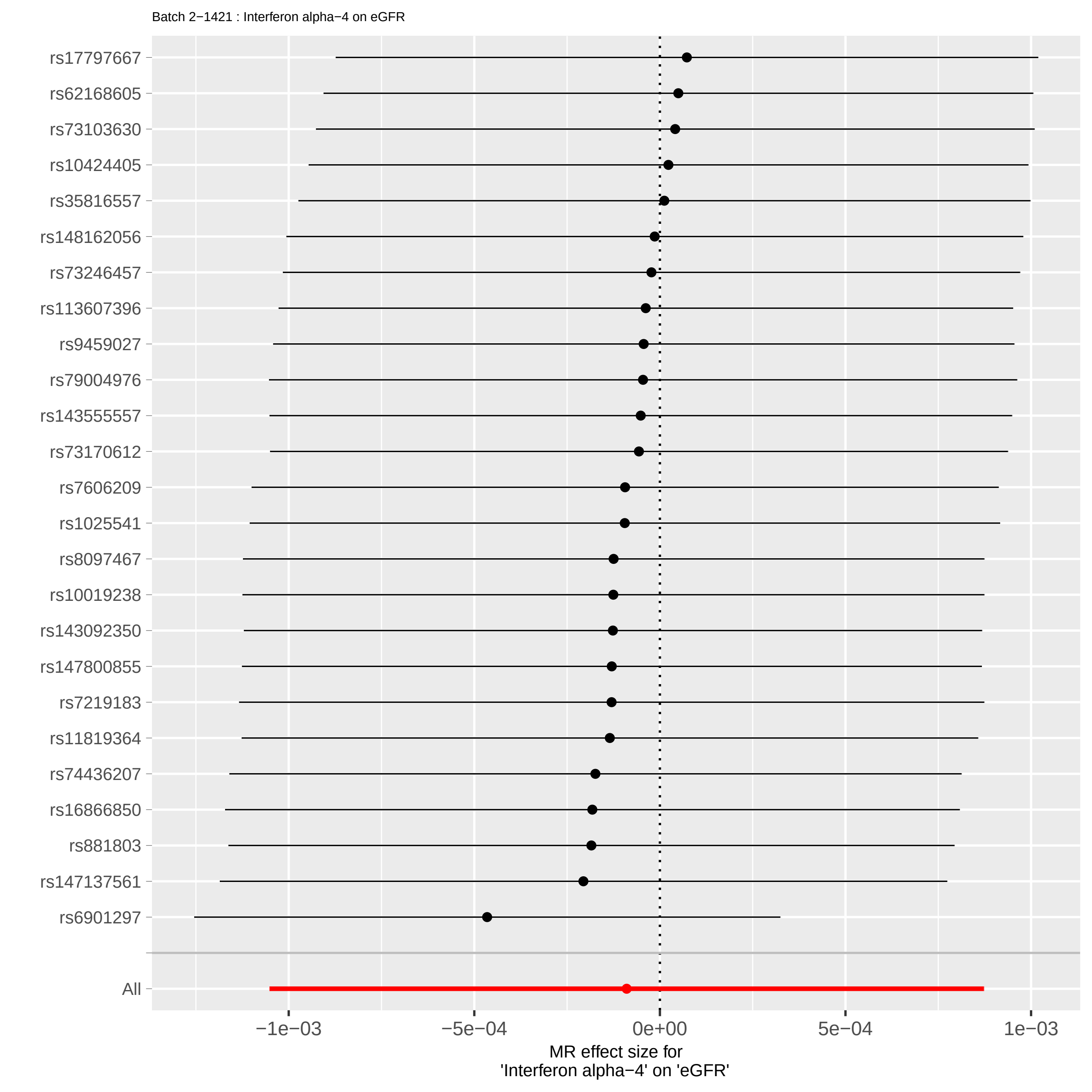

Batch 2−1421 : Interferon alpha−4 on eGFR
rs17797667
rs62168605
rs73103630
rs10424405
rs35816557
rs148162056
rs73246457
rs113607396
rs9459027
rs79004976
rs143555557
rs73170612
rs7606209
rs1025541
rs8097467
rs10019238
rs143092350
rs147800855
rs7219183
rs11819364
rs74436207
rs16866850
rs881803
rs147137561
rs6901297
All
−1e−03
−5e−04
0e+00
5e−04
1e−03
MR effect size for
'Interferon alpha−4' on 'eGFR'

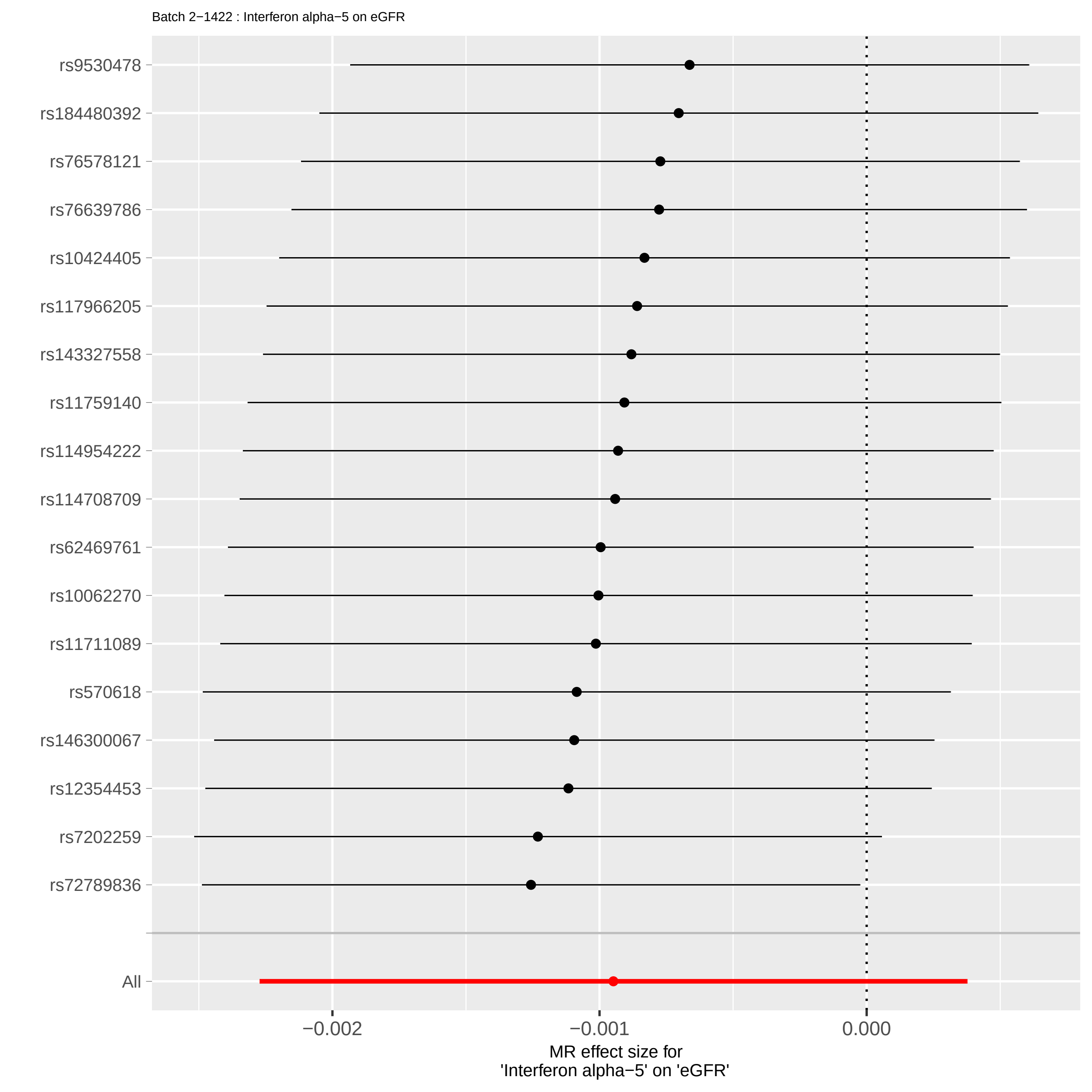

Batch 2−1422 : Interferon alpha−5 on eGFR
rs9530478
rs184480392
rs76578121
rs76639786
rs10424405
rs117966205
rs143327558
rs11759140
rs114954222
rs114708709
rs62469761
rs10062270
rs11711089
rs570618
rs146300067
rs12354453
rs7202259
rs72789836
All
−0.002
−0.001
0.000
MR effect size for
'Interferon alpha−5' on 'eGFR'

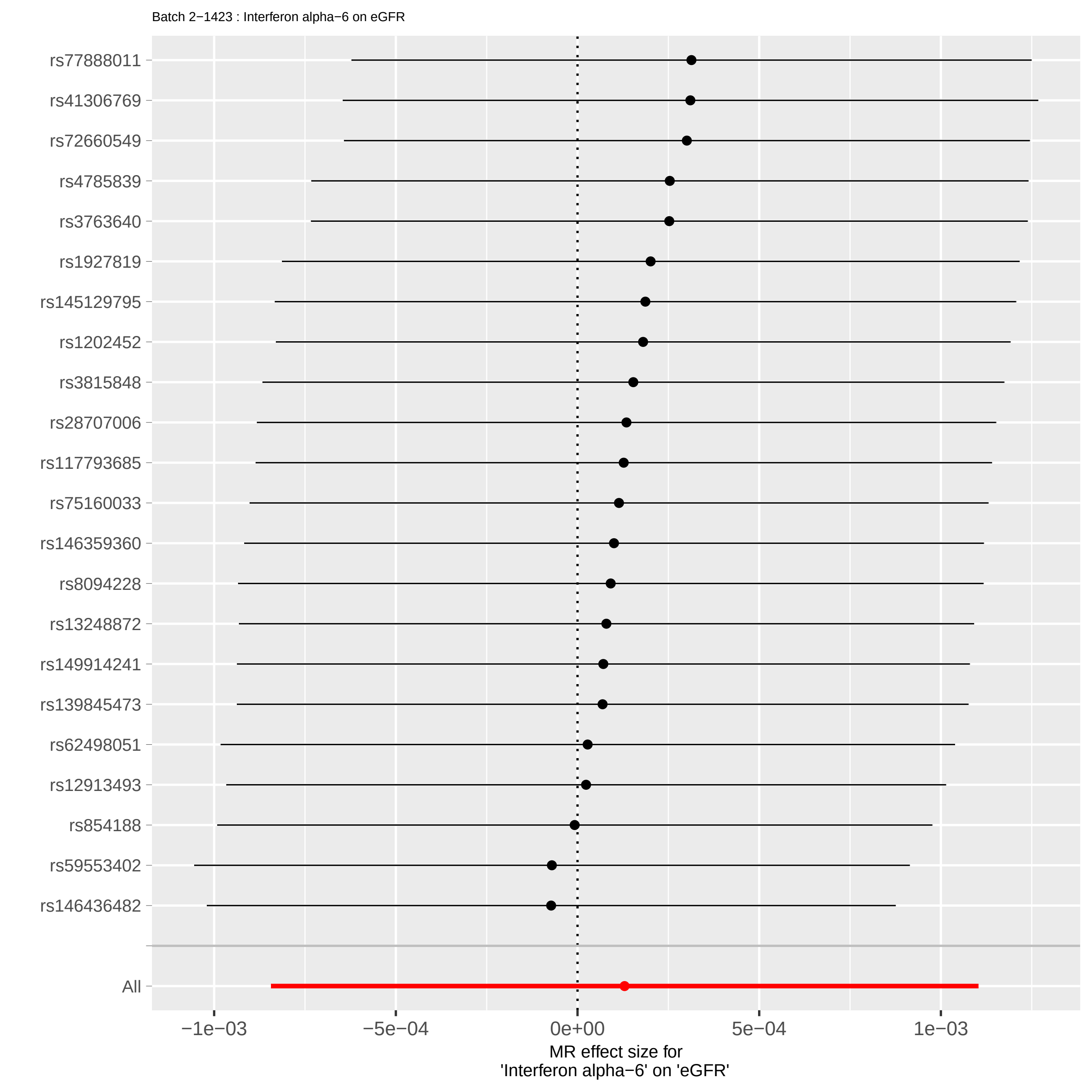

Batch 2−1423 : Interferon alpha−6 on eGFR
rs77888011
rs41306769
rs72660549
rs4785839
rs3763640
rs1927819
rs145129795
rs1202452
rs3815848
rs28707006
rs117793685
rs75160033
rs146359360
rs8094228
rs13248872
rs149914241
rs139845473
rs62498051
rs12913493
rs854188
rs59553402
rs146436482
All
−1e−03
−5e−04
0e+00
5e−04
1e−03
MR effect size for
'Interferon alpha−6' on 'eGFR'

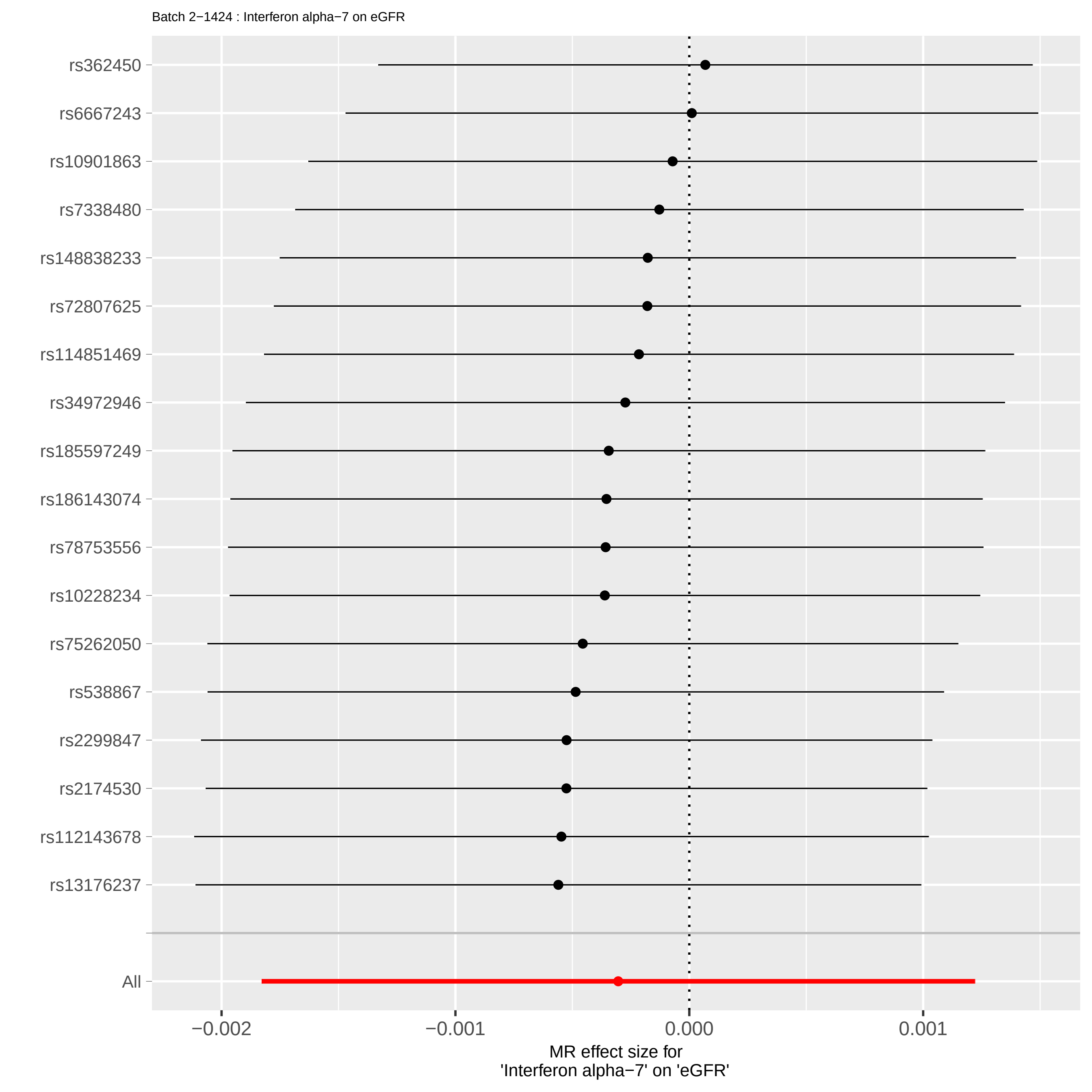

Batch 2−1424 : Interferon alpha−7 on eGFR
rs362450
rs6667243
rs10901863
rs7338480
rs148838233
rs72807625
rs114851469
rs34972946
rs185597249
rs186143074
rs78753556
rs10228234
rs75262050
rs538867
rs2299847
rs2174530
rs112143678
rs13176237
All
−0.002
−0.001
0.000
0.001
MR effect size for
'Interferon alpha−7' on 'eGFR'

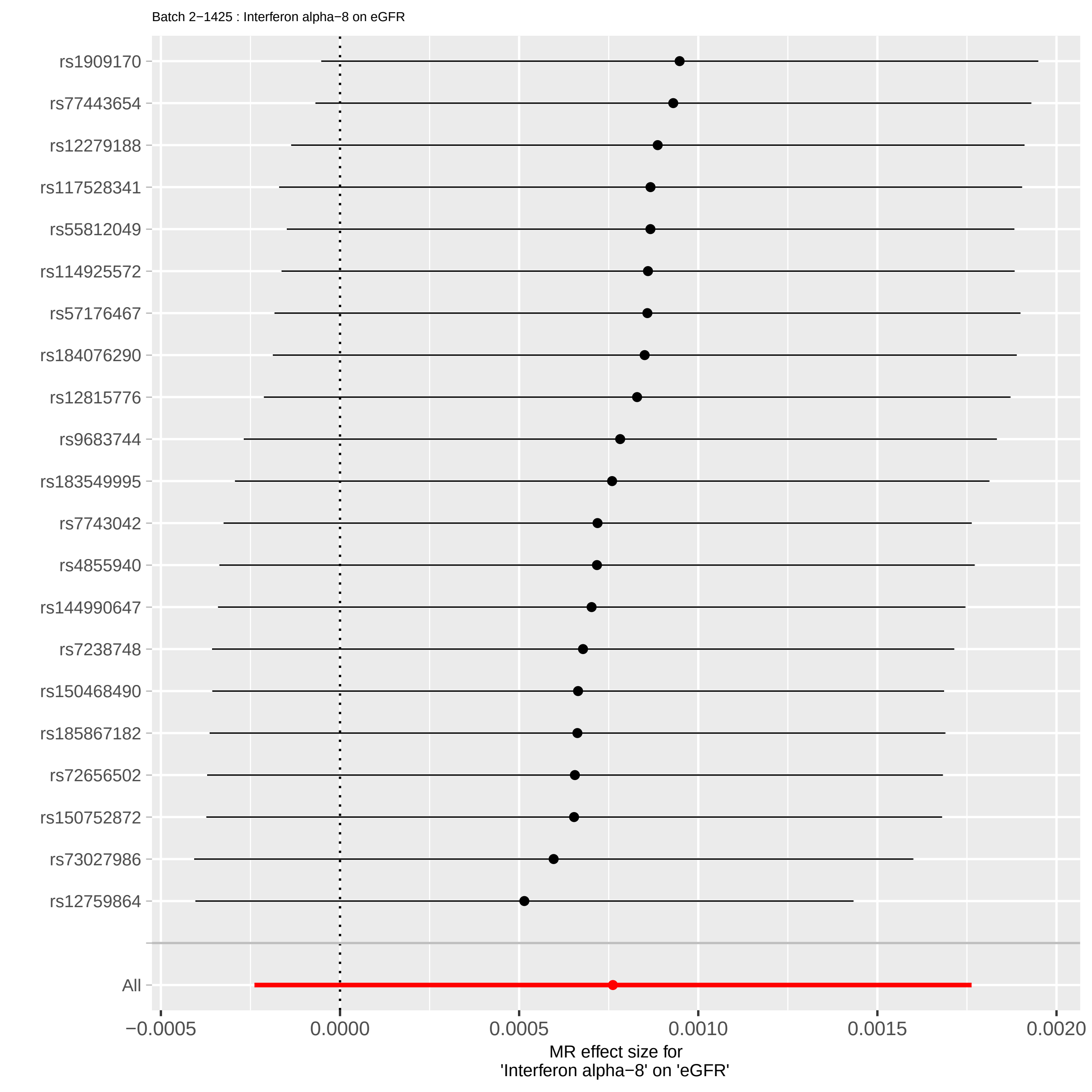

Batch 2−1425 : Interferon alpha−8 on eGFR
rs1909170
rs77443654
rs12279188
rs117528341
rs55812049
rs114925572
rs57176467
rs184076290
rs12815776
rs9683744
rs183549995
rs7743042
rs4855940
rs144990647
rs7238748
rs150468490
rs185867182
rs72656502
rs150752872
rs73027986
rs12759864
All
−0.0005
0.0000
0.0005
0.0010
0.0015
0.0020
MR effect size for
'Interferon alpha−8' on 'eGFR'

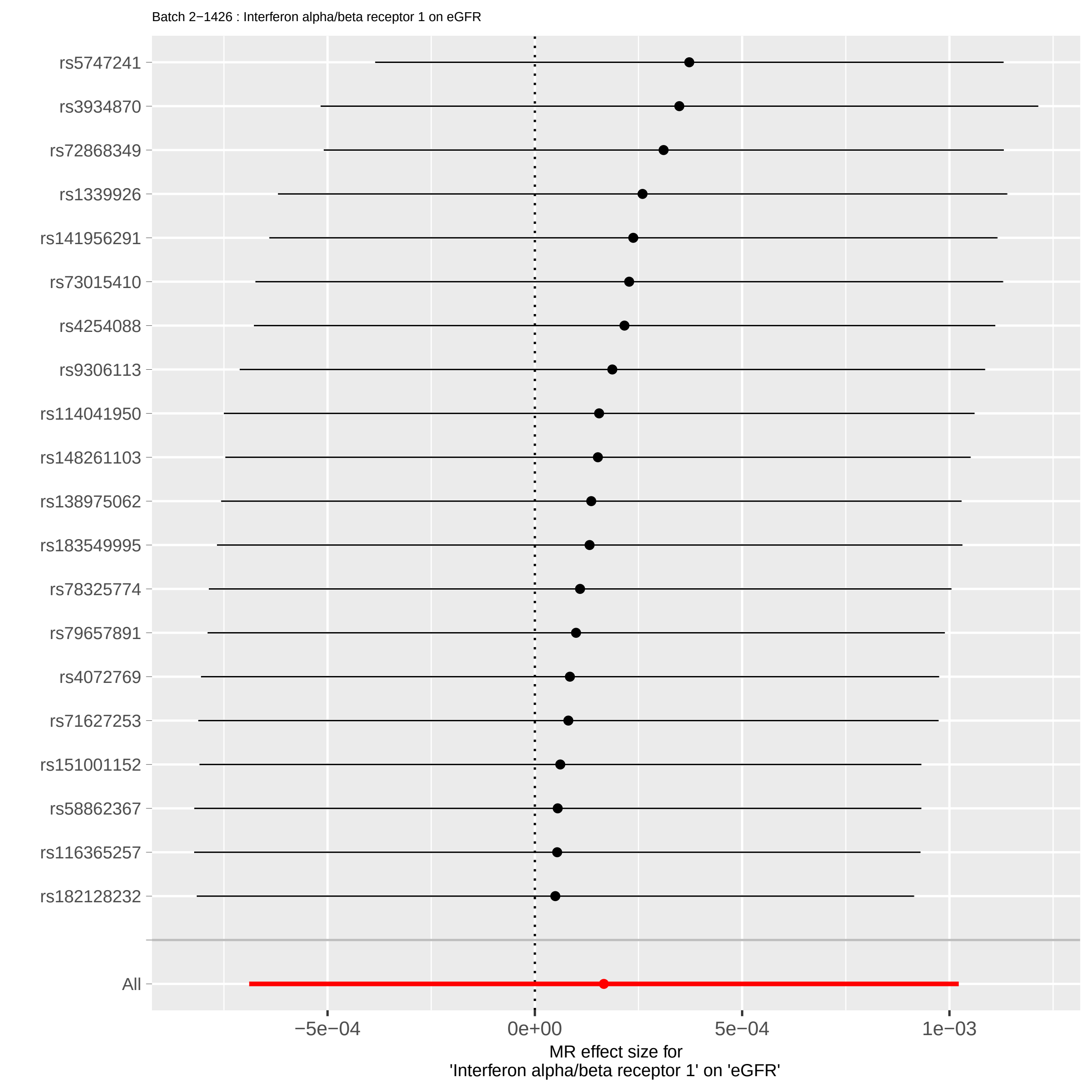

Batch 2−1426 : Interferon alpha/beta receptor 1 on eGFR
rs5747241
rs3934870
rs72868349
rs1339926
rs141956291
rs73015410
rs4254088
rs9306113
rs114041950
rs148261103
rs138975062
rs183549995
rs78325774
rs79657891
rs4072769
rs71627253
rs151001152
rs58862367
rs116365257
rs182128232
All
−5e−04
0e+00
5e−04
1e−03
MR effect size for
'Interferon alpha/beta receptor 1' on 'eGFR'

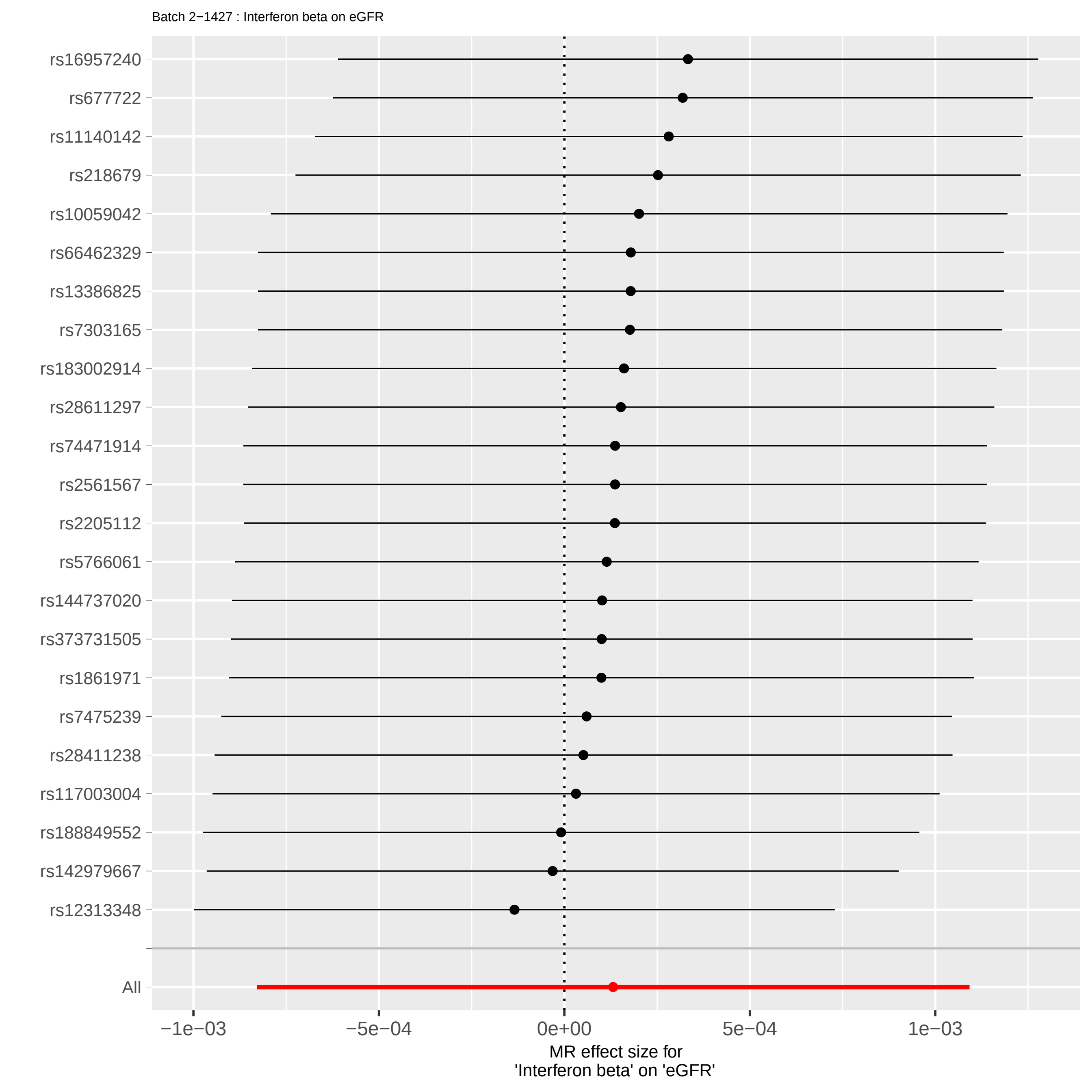

Batch 2−1427 : Interferon beta on eGFR
rs16957240
rs677722
rs11140142
rs218679
rs10059042
rs66462329
rs13386825
rs7303165
rs183002914
rs28611297
rs74471914
rs2561567
rs2205112
rs5766061
rs144737020
rs373731505
rs1861971
rs7475239
rs28411238
rs117003004
rs188849552
rs142979667
rs12313348
All
−1e−03
−5e−04
0e+00
5e−04
1e−03
MR effect size for
'Interferon beta' on 'eGFR'

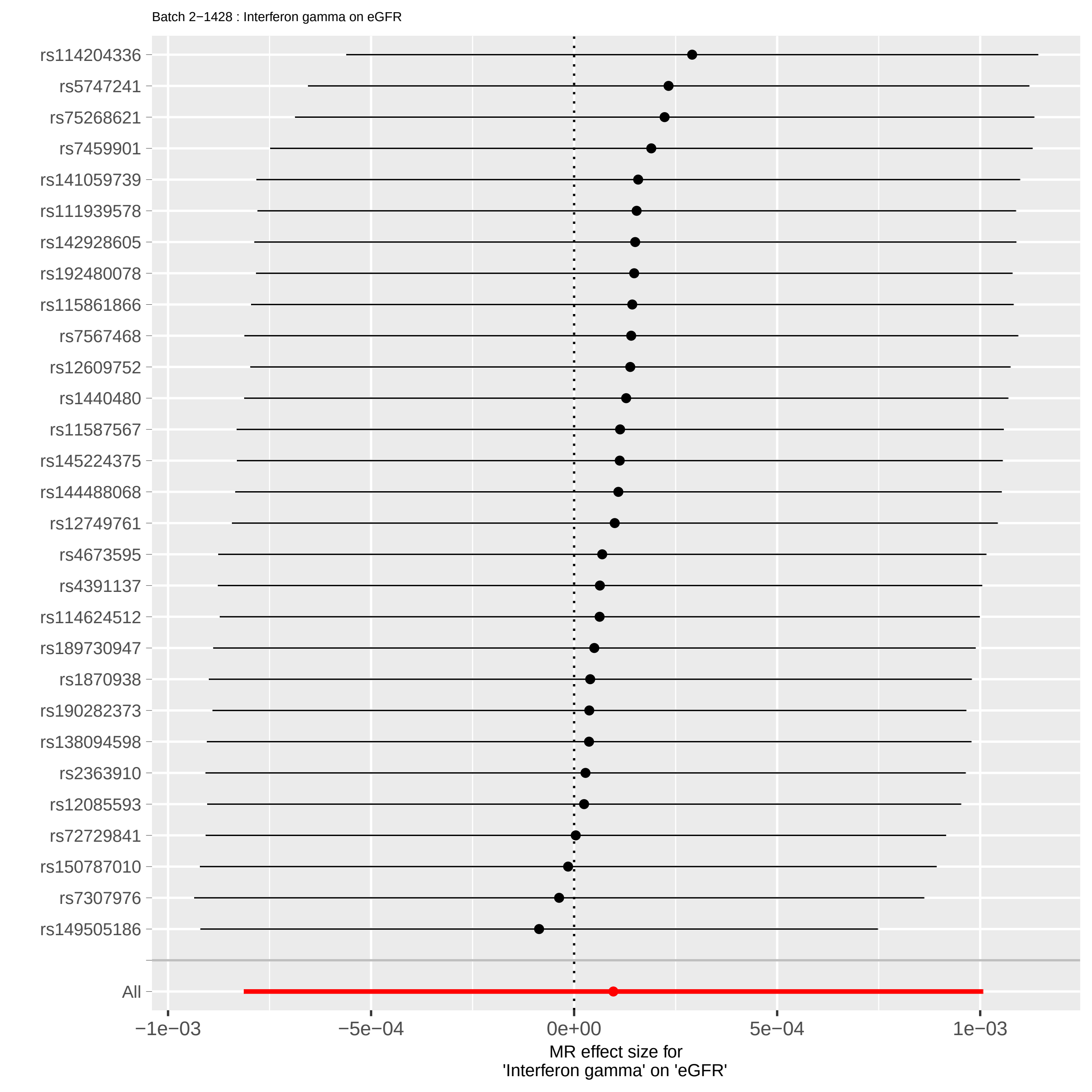

Batch 2−1428 : Interferon gamma on eGFR
rs114204336
rs5747241
rs75268621
rs7459901
rs141059739
rs111939578
rs142928605
rs192480078
rs115861866
rs7567468
rs12609752
rs1440480
rs11587567
rs145224375
rs144488068
rs12749761
rs4673595
rs4391137
rs114624512
rs189730947
rs1870938
rs190282373
rs138094598
rs2363910
rs12085593
rs72729841
rs150787010
rs7307976
rs149505186
All
−1e−03
−5e−04
0e+00
5e−04
1e−03
MR effect size for
'Interferon gamma' on 'eGFR'

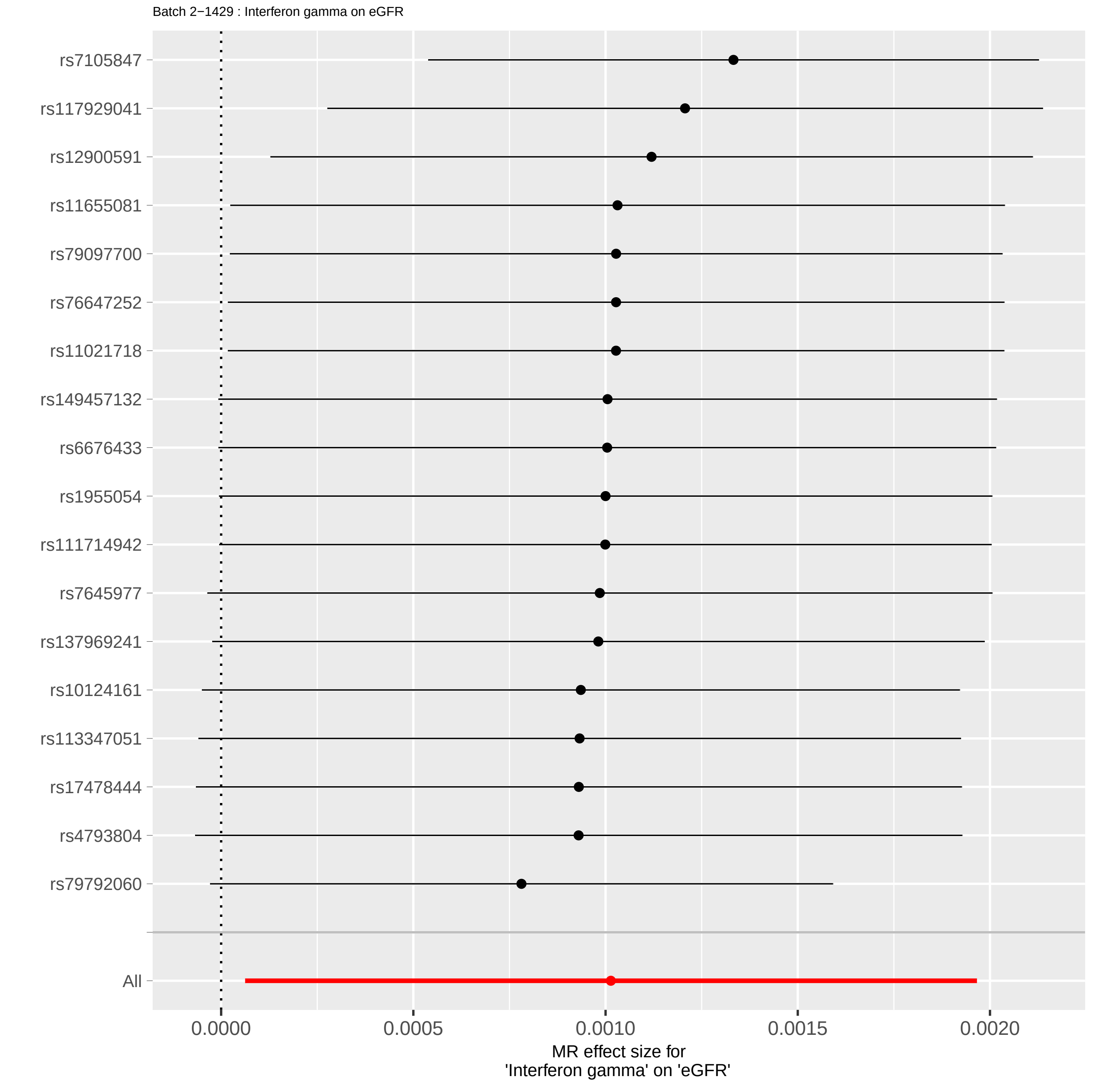

Batch 2−1429 : Interferon gamma on eGFR
rs7105847
rs117929041
rs12900591
rs11655081
rs79097700
rs76647252
rs11021718
rs149457132
rs6676433
rs1955054
rs111714942
rs7645977
rs137969241
rs10124161
rs113347051
rs17478444
rs4793804
rs79792060
All
0.0000
0.0005
0.0010
0.0015
0.0020
MR effect size for
'Interferon gamma' on 'eGFR'

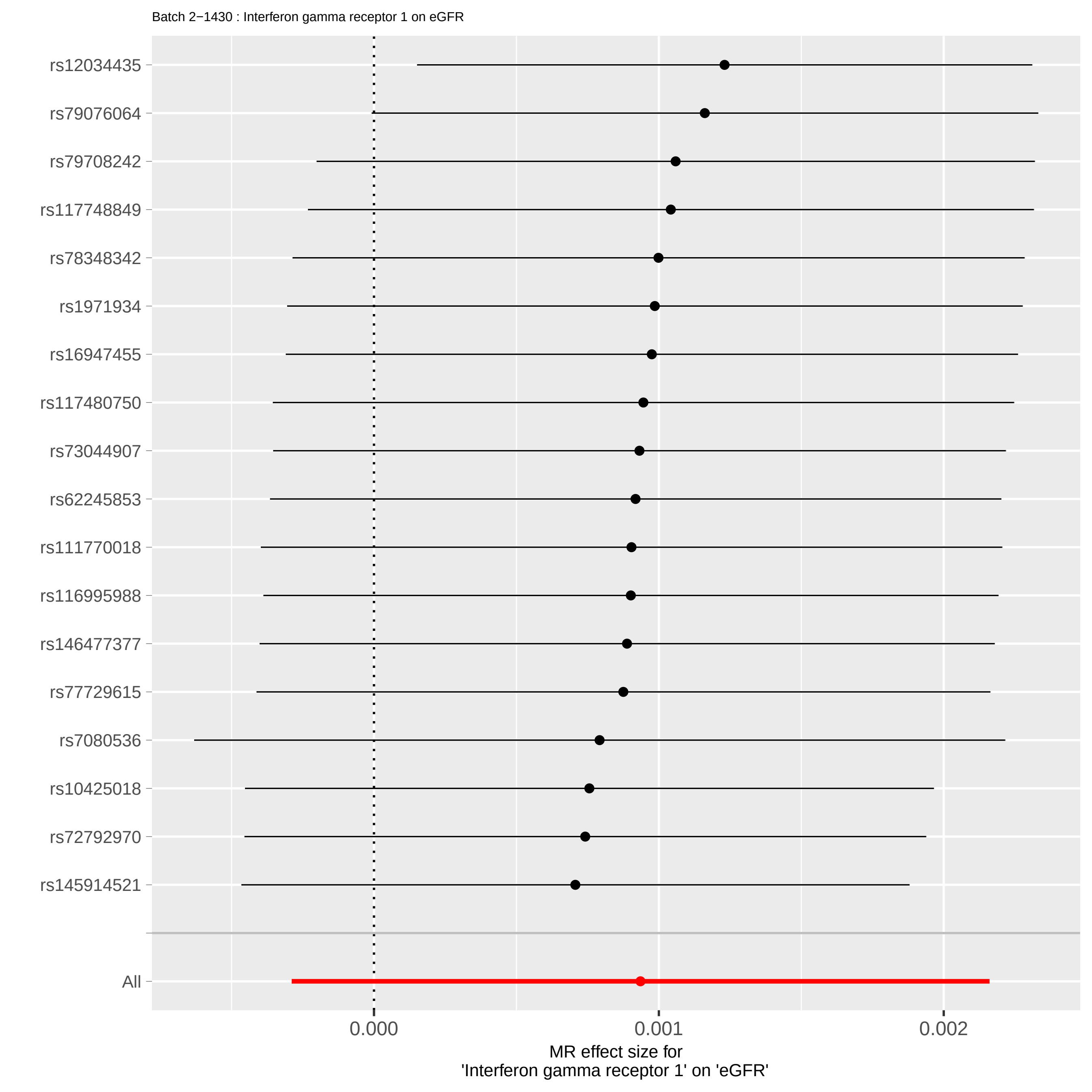

Batch 2−1430 : Interferon gamma receptor 1 on eGFR
rs12034435
rs79076064
rs79708242
rs117748849
rs78348342
rs1971934
rs16947455
rs117480750
rs73044907
rs62245853
rs111770018
rs116995988
rs146477377
rs77729615
rs7080536
rs10425018
rs72792970
rs145914521
All
0.000
0.001
0.002
MR effect size for
'Interferon gamma receptor 1' on 'eGFR'

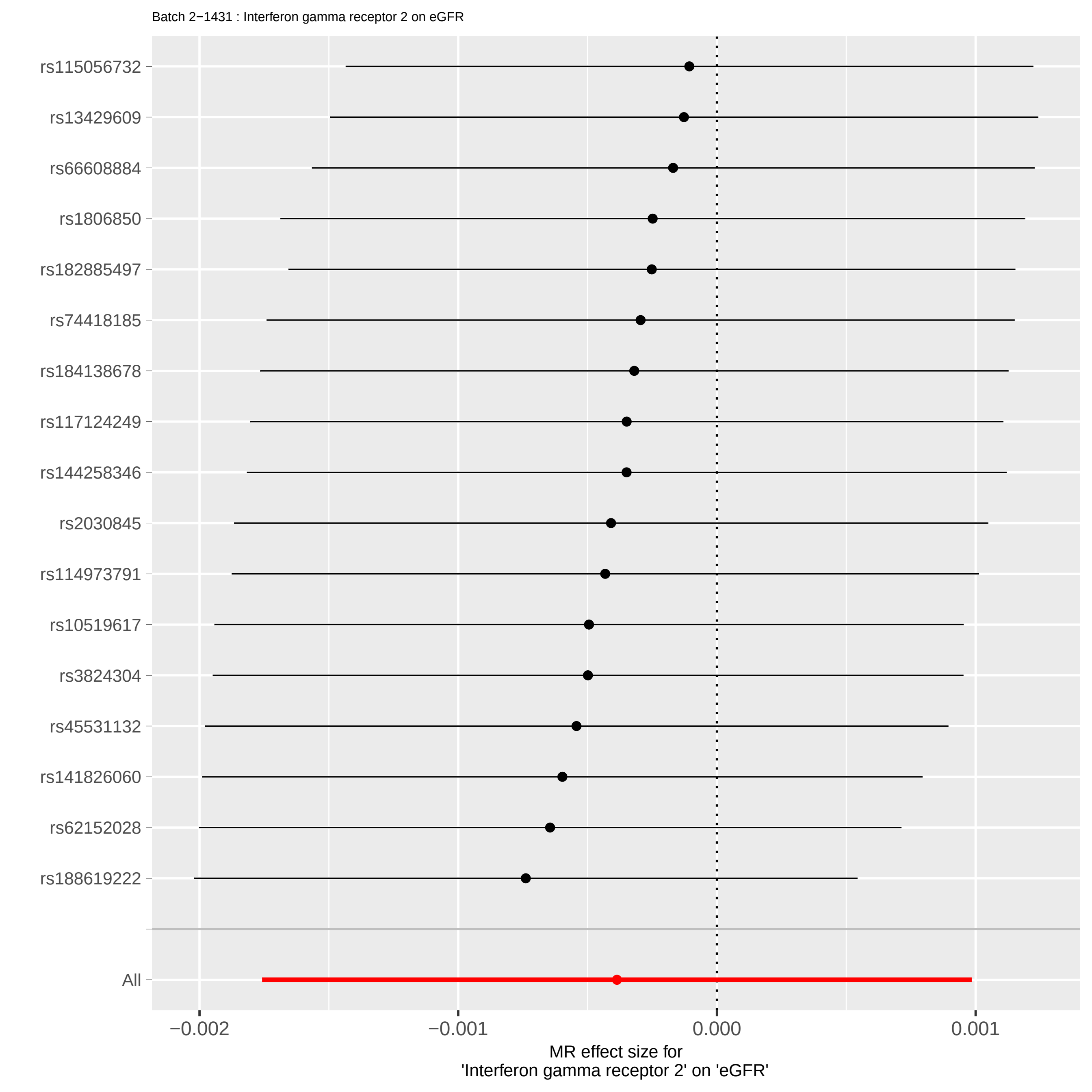

Batch 2−1431 : Interferon gamma receptor 2 on eGFR
rs115056732
rs13429609
rs66608884
rs1806850
rs182885497
rs74418185
rs184138678
rs117124249
rs144258346
rs2030845
rs114973791
rs10519617
rs3824304
rs45531132
rs141826060
rs62152028
rs188619222
All
−0.002
−0.001
0.000
0.001
MR effect size for
'Interferon gamma receptor 2' on 'eGFR'

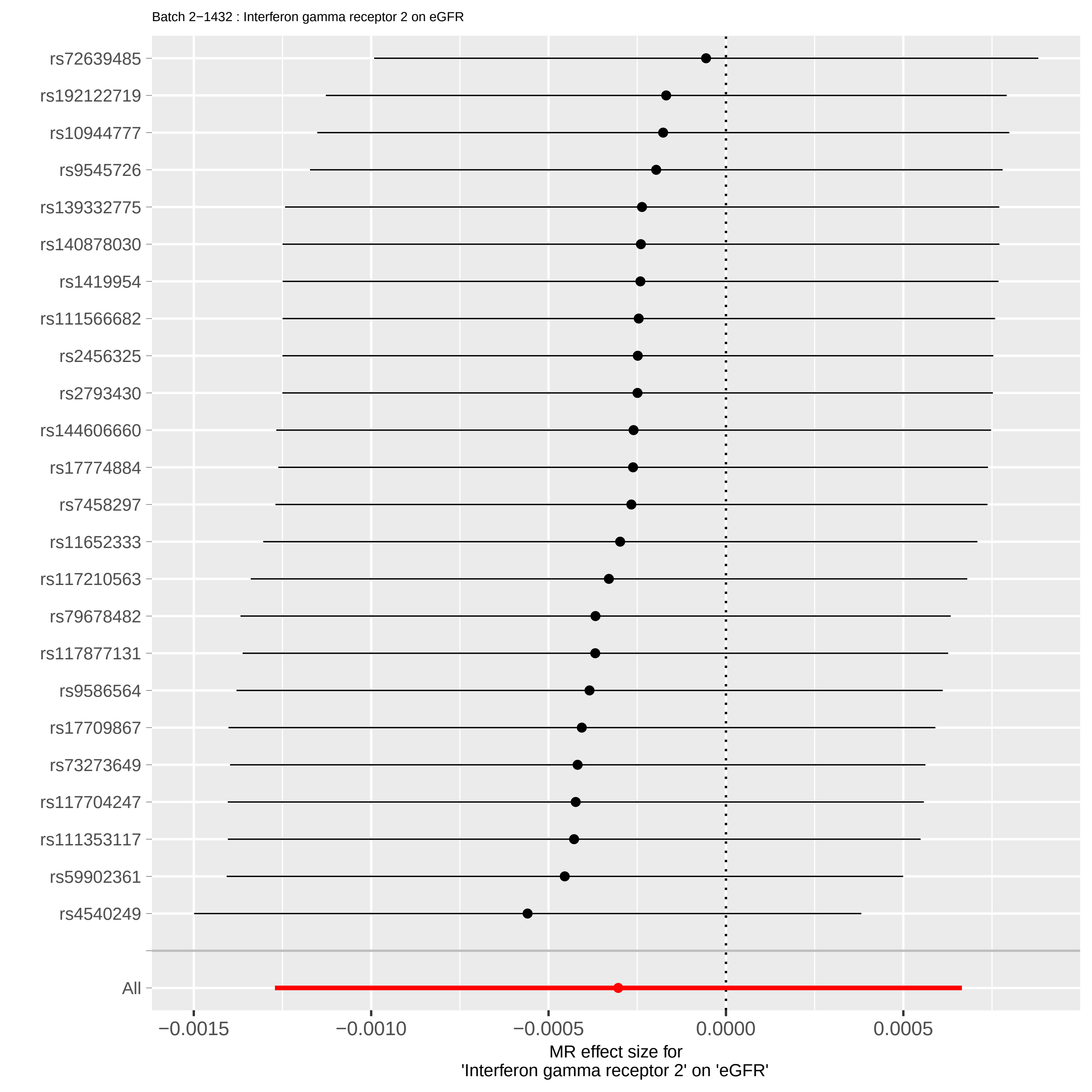

Batch 2−1432 : Interferon gamma receptor 2 on eGFR
rs72639485
rs192122719
rs10944777
rs9545726
rs139332775
rs140878030
rs1419954
rs111566682
rs2456325
rs2793430
rs144606660
rs17774884
rs7458297
rs11652333
rs117210563
rs79678482
rs117877131
rs9586564
rs17709867
rs73273649
rs117704247
rs111353117
rs59902361
rs4540249
All
−0.0015
−0.0010
−0.0005
0.0000
0.0005
MR effect size for
'Interferon gamma receptor 2' on 'eGFR'

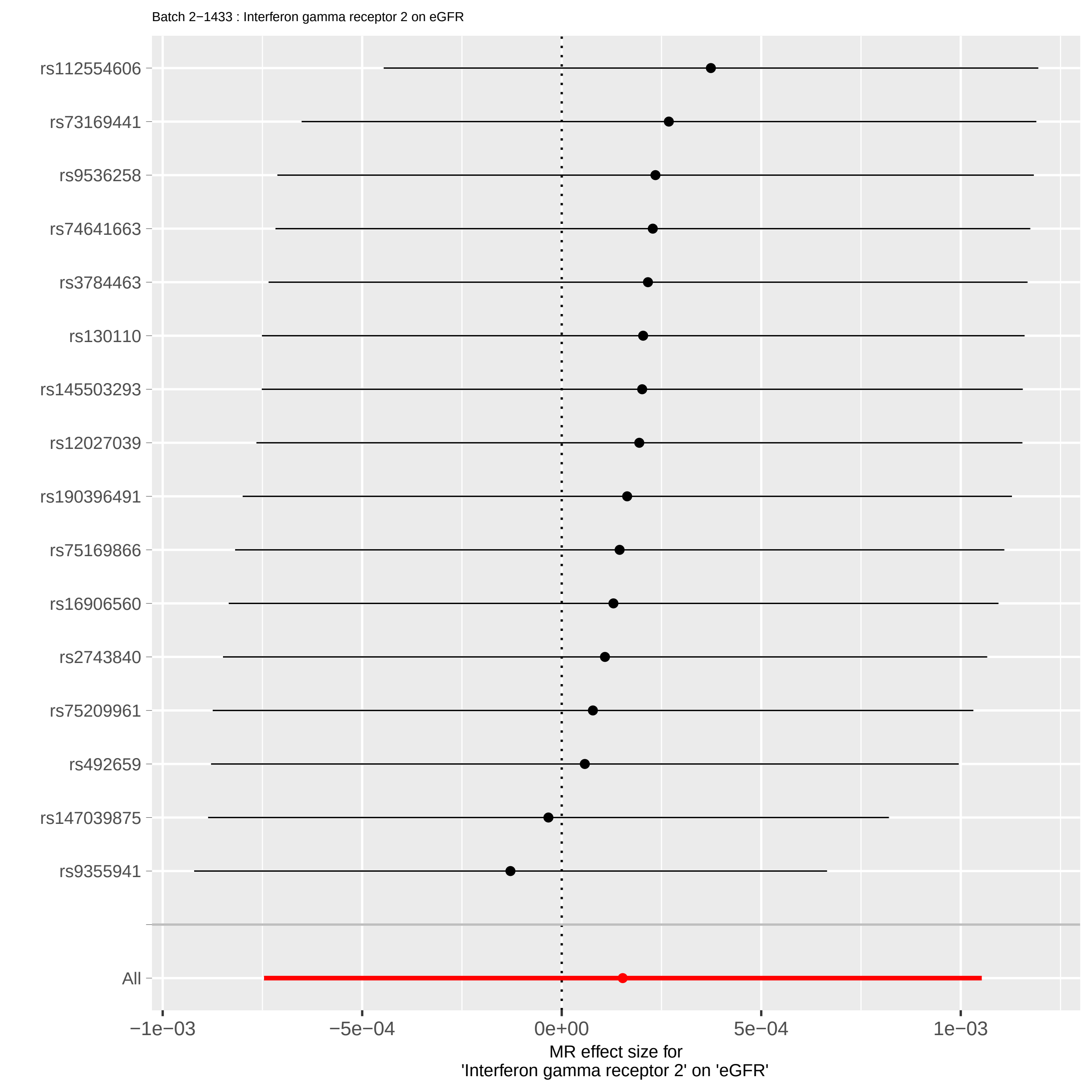

Batch 2−1433 : Interferon gamma receptor 2 on eGFR
rs112554606
rs73169441
rs9536258
rs74641663
rs3784463
rs130110
rs145503293
rs12027039
rs190396491
rs75169866
rs16906560
rs2743840
rs75209961
rs492659
rs147039875
rs9355941
All
−1e−03
−5e−04
0e+00
5e−04
1e−03
MR effect size for
'Interferon gamma receptor 2' on 'eGFR'

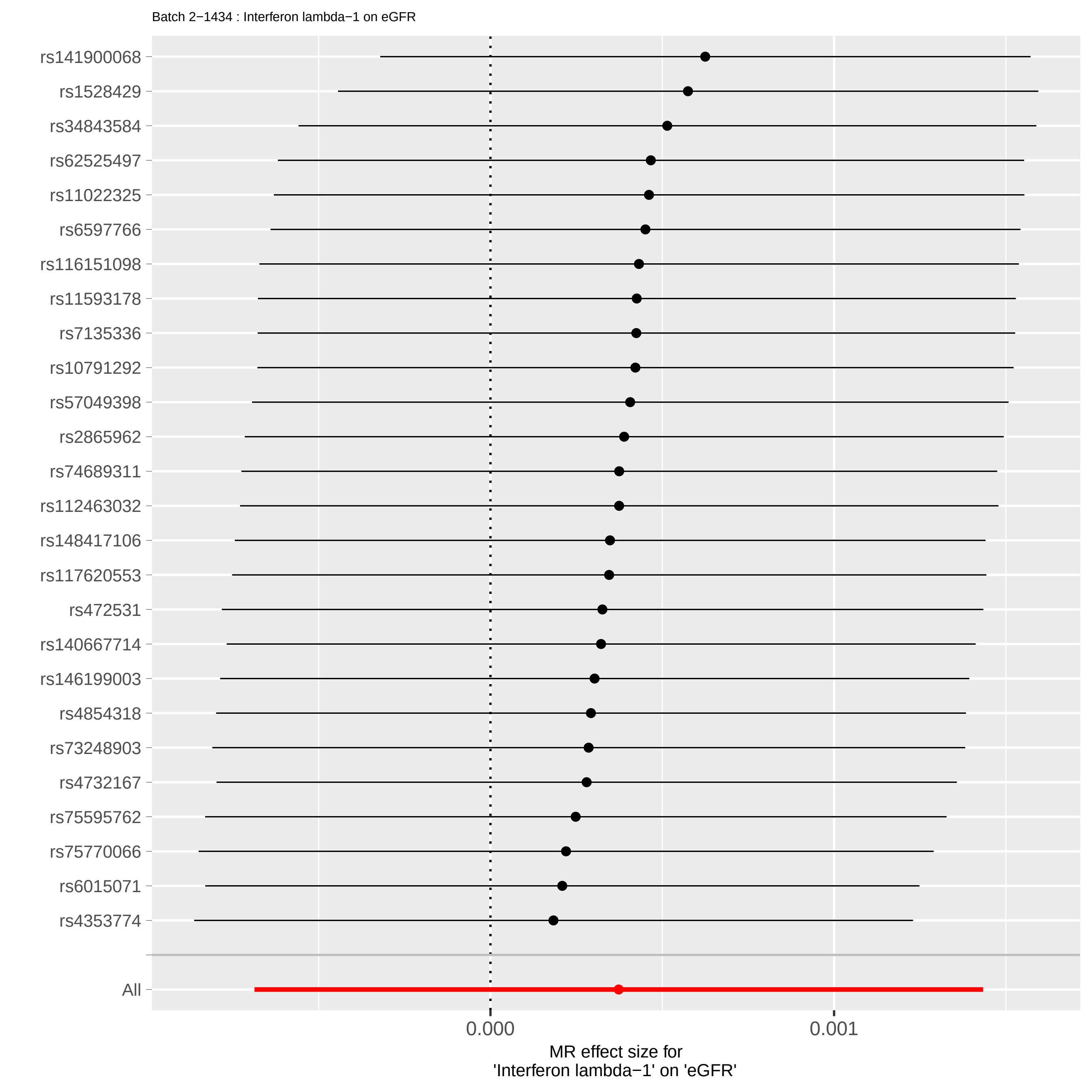
Batch 2−1434 : Interferon lambda−1 on eGFR
rs141900068
rs1528429
rs34843584
rs62525497
rs11022325
rs6597766
rs116151098
rs11593178
rs7135336
rs10791292
rs57049398
rs2865962
rs74689311
rs112463032
rs148417106
rs117620553
rs472531
rs140667714
rs146199003
rs4854318
rs73248903
rs4732167
rs75595762
rs75770066
rs6015071
rs4353774
All
0.000
0.001
MR effect size for
'Interferon lambda−1' on 'eGFR'

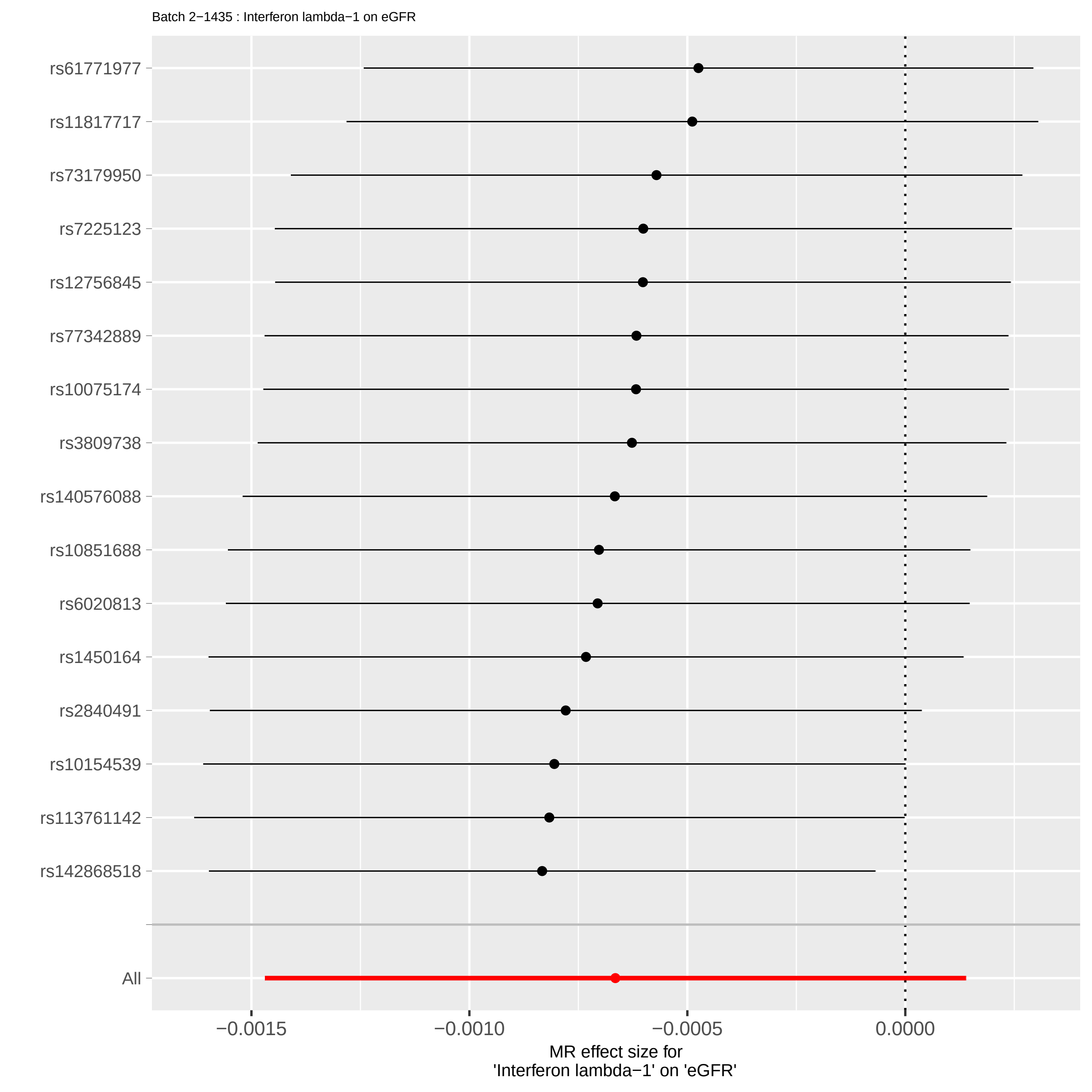
Batch 2−1435 : Interferon lambda−1 on eGFR
rs61771977
rs11817717
rs73179950
rs7225123
rs12756845
rs77342889
rs10075174
rs3809738
rs140576088
rs10851688
rs6020813
rs1450164
rs2840491
rs10154539
rs113761142
rs142868518
All
−0.0015
−0.0010
−0.0005
0.0000
MR effect size for
'Interferon lambda−1' on 'eGFR'

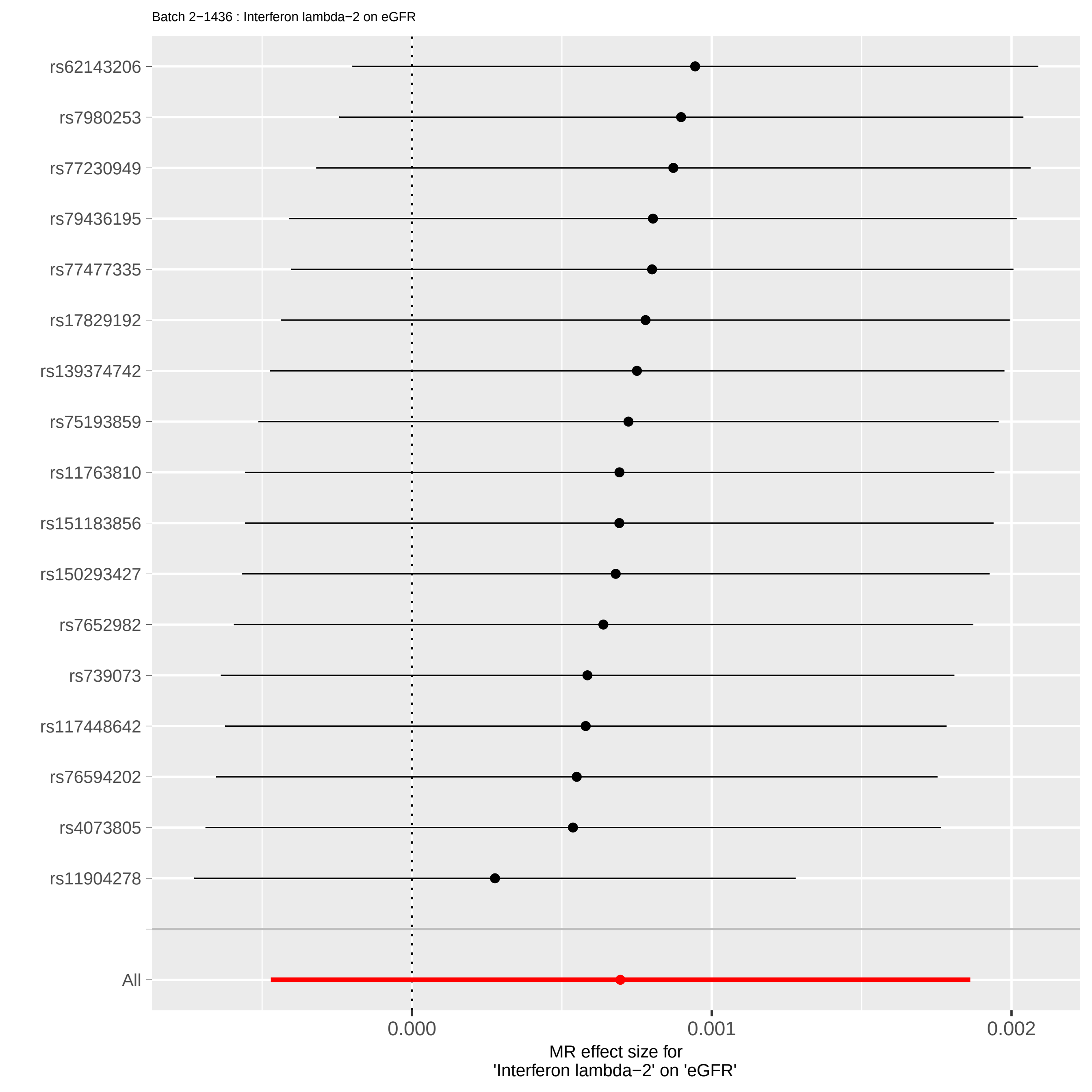

Batch 2−1436 : Interferon lambda−2 on eGFR
rs62143206
rs7980253
rs77230949
rs79436195
rs77477335
rs17829192
rs139374742
rs75193859
rs11763810
rs151183856
rs150293427
rs7652982
rs739073
rs117448642
rs76594202
rs4073805
rs11904278
All
0.000
0.001
0.002
MR effect size for
'Interferon lambda−2' on 'eGFR'

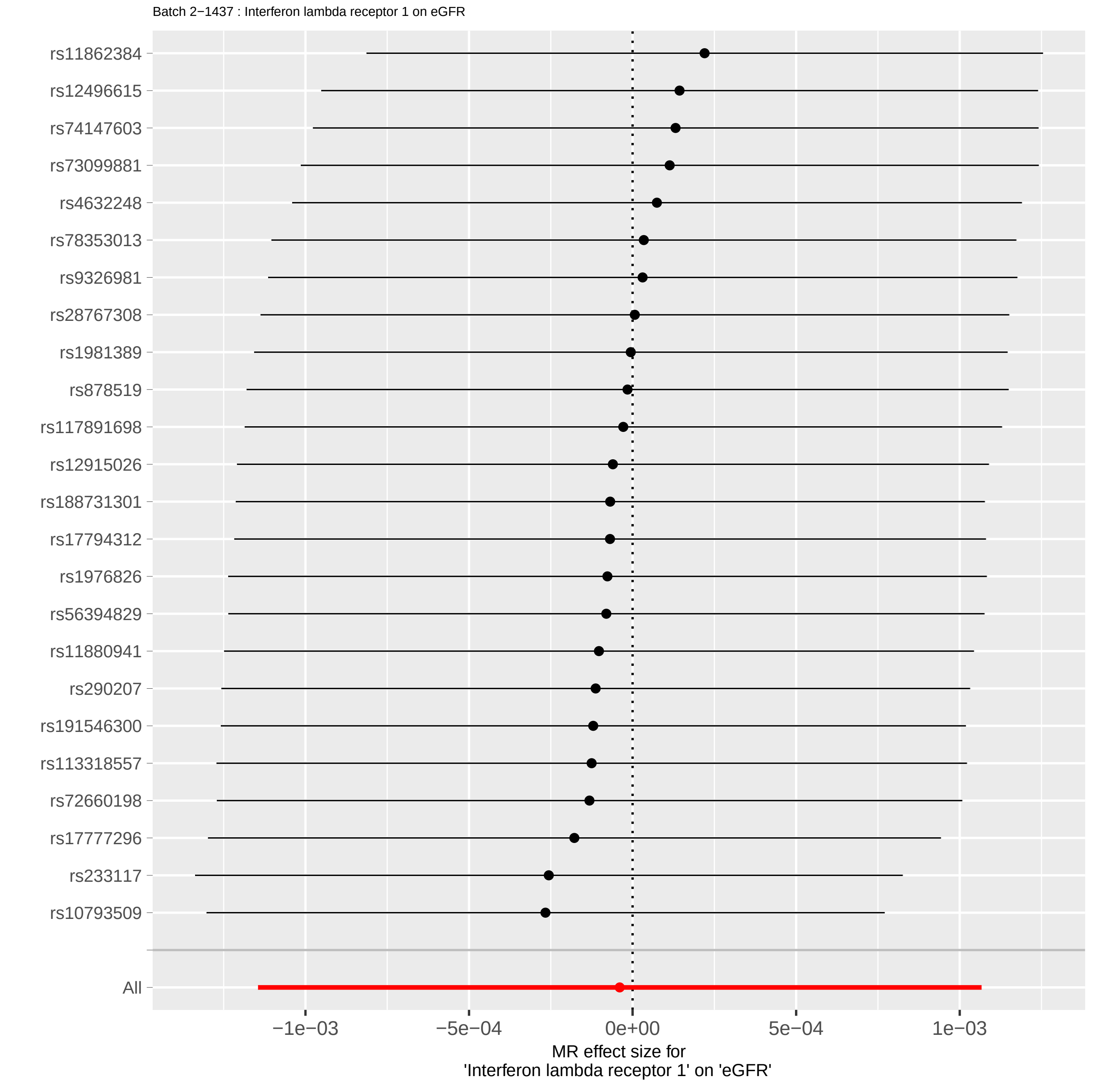

Batch 2−1437 : Interferon lambda receptor 1 on eGFR
rs11862384
rs12496615
rs74147603
rs73099881
rs4632248
rs78353013
rs9326981
rs28767308
rs1981389
rs878519
rs117891698
rs12915026
rs188731301
rs17794312
rs1976826
rs56394829
rs11880941
rs290207
rs191546300
rs113318557
rs72660198
rs17777296
rs233117
rs10793509
All
−1e−03
−5e−04
0e+00
5e−04
1e−03
MR effect size for
'Interferon lambda receptor 1' on 'eGFR'

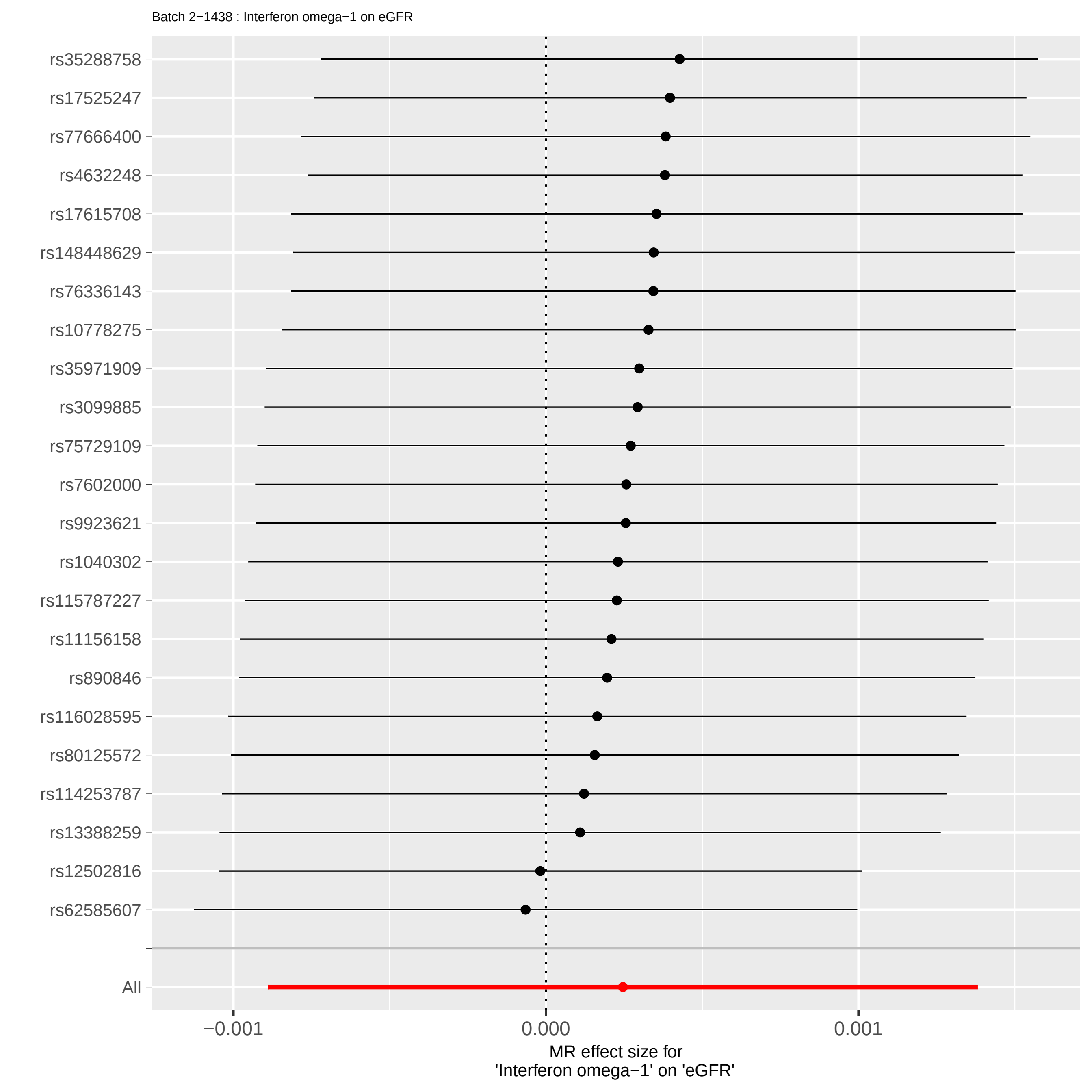
Batch 2−1438 : Interferon omega−1 on eGFR
rs35288758
rs17525247
rs77666400
rs4632248
rs17615708
rs148448629
rs76336143
rs10778275
rs35971909
rs3099885
rs75729109
rs7602000
rs9923621
rs1040302
rs115787227
rs11156158
rs890846
rs116028595
rs80125572
rs114253787
rs13388259
rs12502816
rs62585607
All
−0.001
0.000
0.001
MR effect size for
'Interferon omega−1' on 'eGFR'

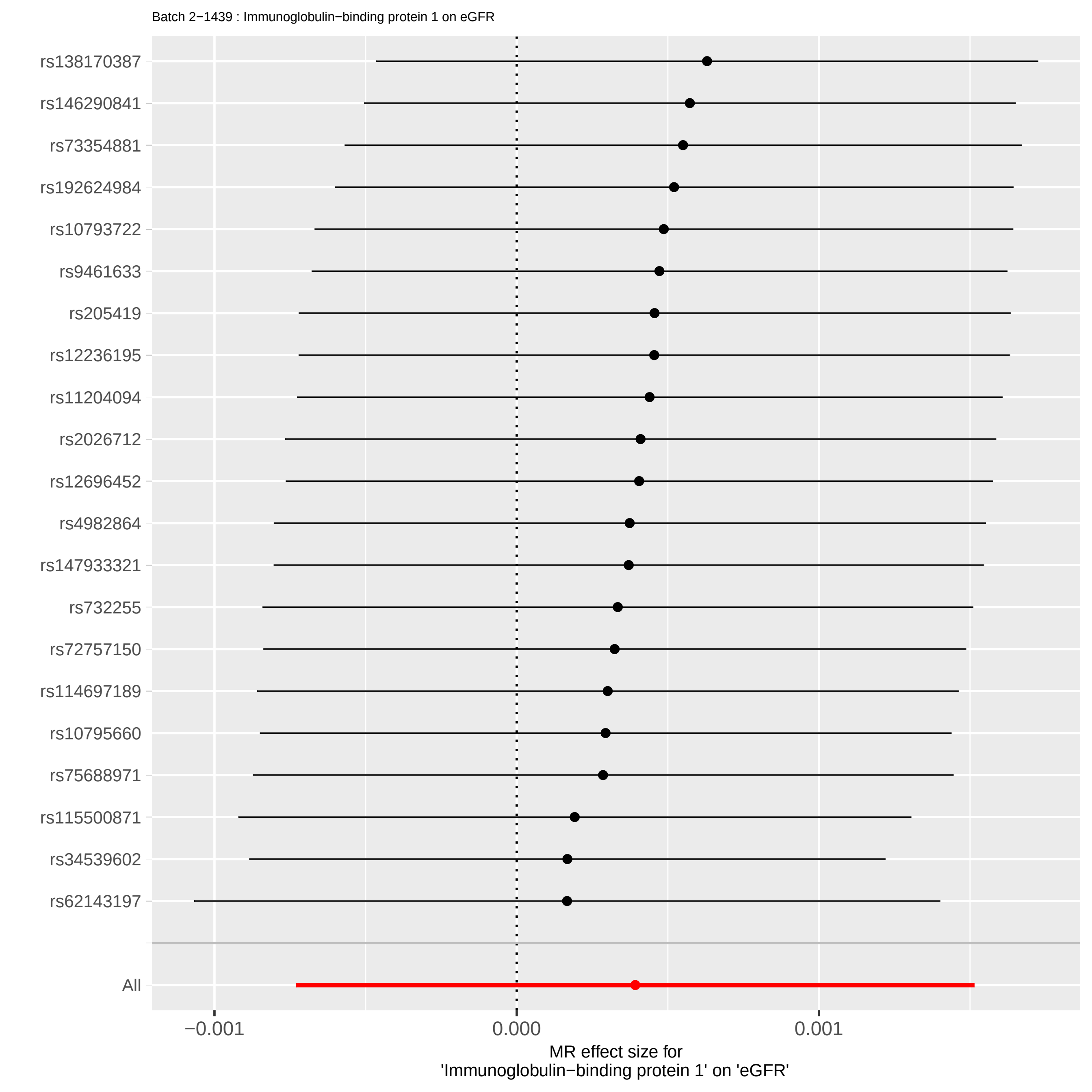

Batch 2−1439 : Immunoglobulin−binding protein 1 on eGFR
rs138170387
rs146290841
rs73354881
rs192624984
rs10793722
rs9461633
rs205419
rs12236195
rs11204094
rs2026712
rs12696452
rs4982864
rs147933321
rs732255
rs72757150
rs114697189
rs10795660
rs75688971
rs115500871
rs34539602
rs62143197
All
−0.001
0.000
0.001
MR effect size for
'Immunoglobulin−binding protein 1' on 'eGFR'

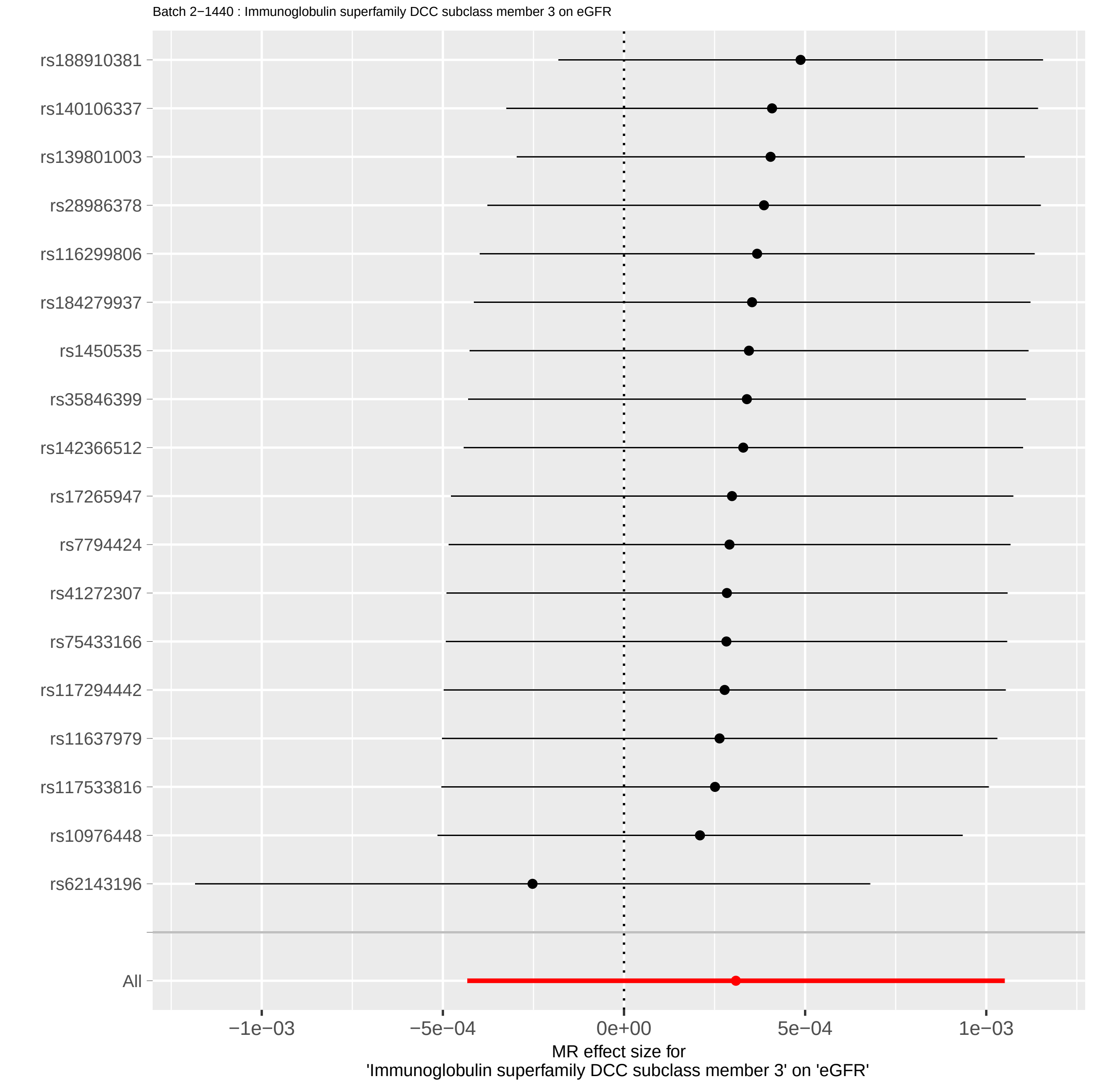

Batch 2−1440 : Immunoglobulin superfamily DCC subclass member 3 on eGFR
rs188910381
rs140106337
rs139801003
rs28986378
rs116299806
rs184279937
rs1450535
rs35846399
rs142366512
rs17265947
rs7794424
rs41272307
rs75433166
rs117294442
rs11637979
rs117533816
rs10976448
rs62143196
All
−1e−03
−5e−04
0e+00
5e−04
1e−03
MR effect size for
'Immunoglobulin superfamily DCC subclass member 3' on 'eGFR'

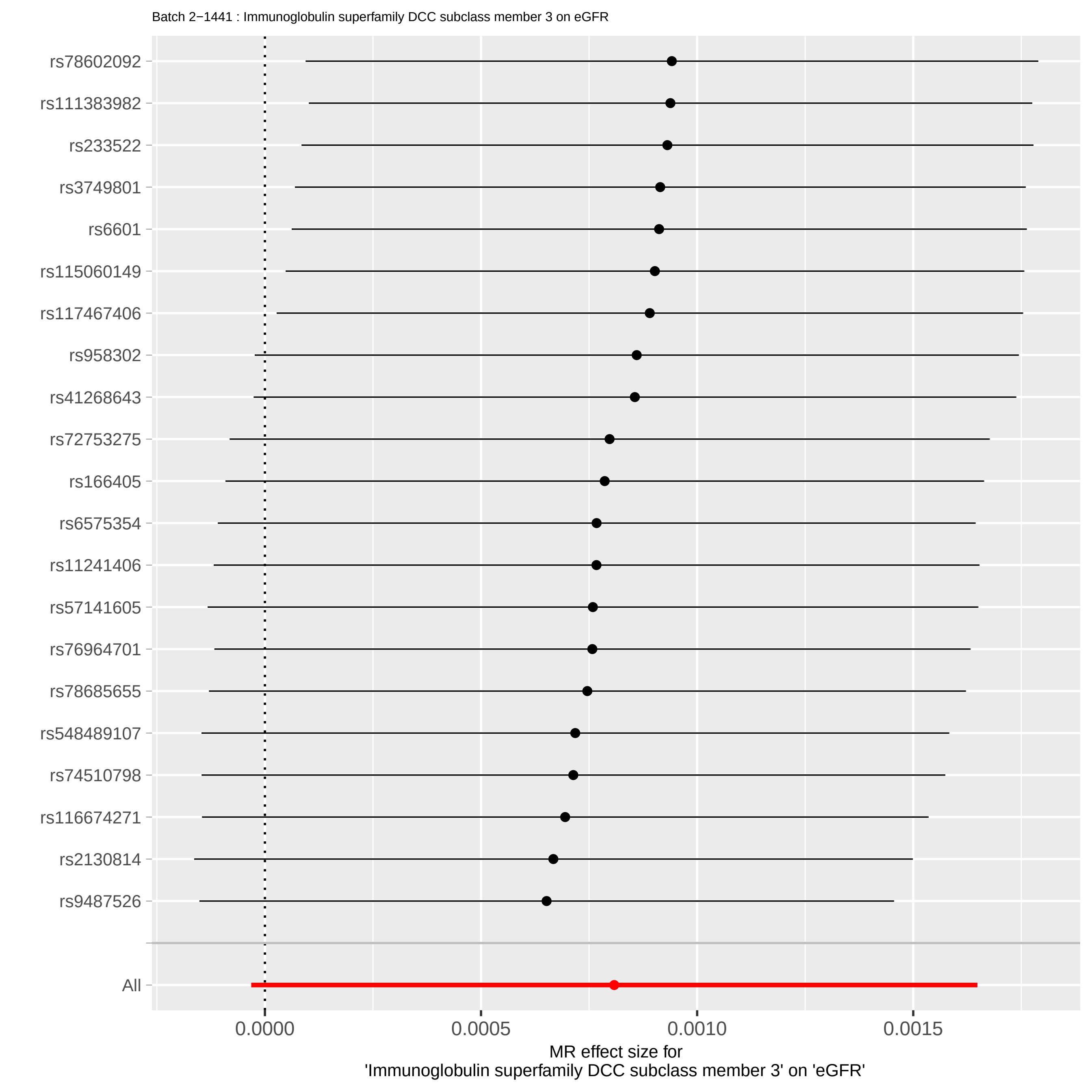

Batch 2−1441 : Immunoglobulin superfamily DCC subclass member 3 on eGFR
rs78602092
rs111383982
rs233522
rs3749801
rs6601
rs115060149
rs117467406
rs958302
rs41268643
rs72753275
rs166405
rs6575354
rs11241406
rs57141605
rs76964701
rs78685655
rs548489107
rs74510798
rs116674271
rs2130814
rs9487526
All
0.0000
0.0005
0.0010
0.0015
MR effect size for
'Immunoglobulin superfamily DCC subclass member 3' on 'eGFR'

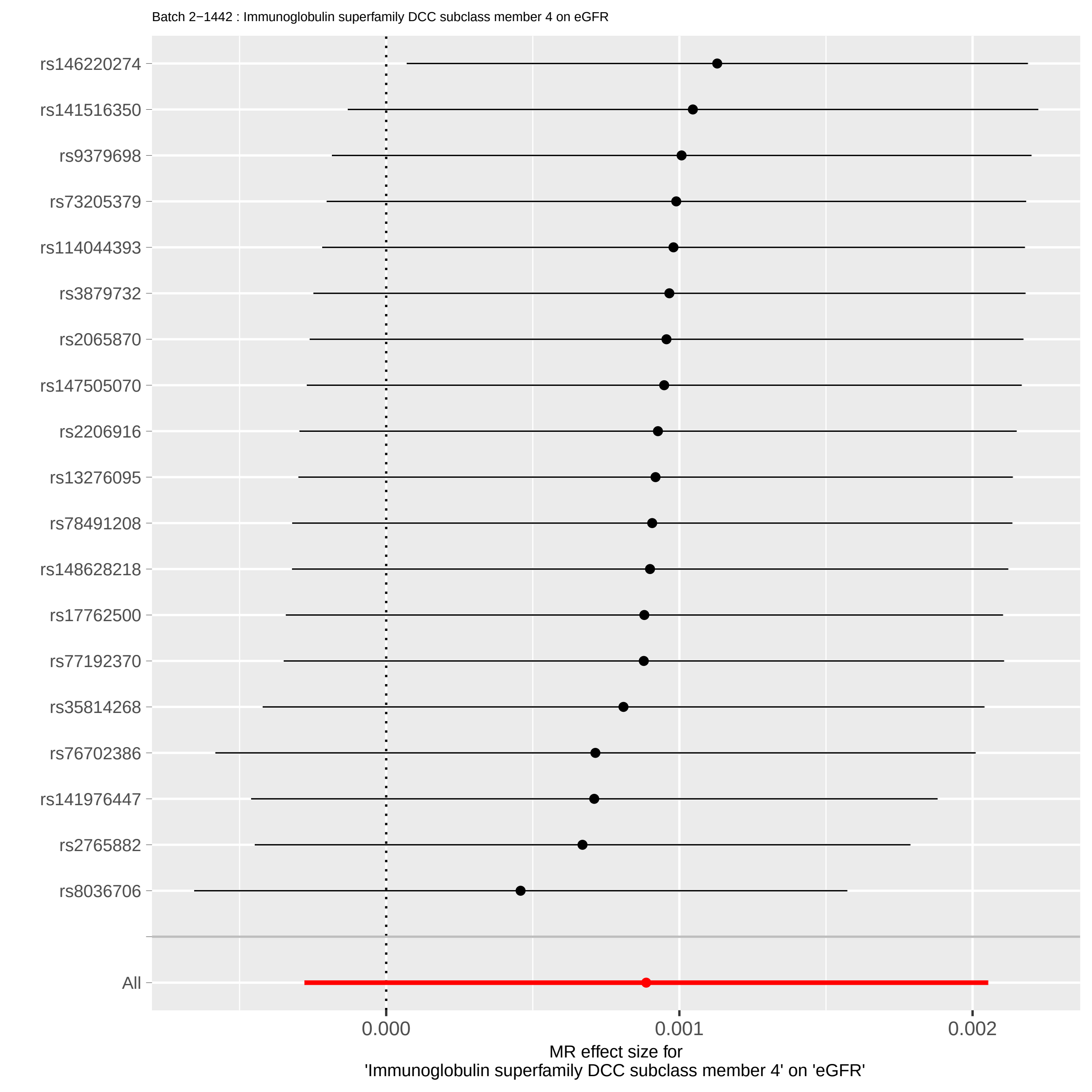

Batch 2−1442 : Immunoglobulin superfamily DCC subclass member 4 on eGFR
rs146220274
rs141516350
rs9379698
rs73205379
rs114044393
rs3879732
rs2065870
rs147505070
rs2206916
rs13276095
rs78491208
rs148628218
rs17762500
rs77192370
rs35814268
rs76702386
rs141976447
rs2765882
rs8036706
All
0.000
0.001
0.002
MR effect size for
'Immunoglobulin superfamily DCC subclass member 4' on 'eGFR'

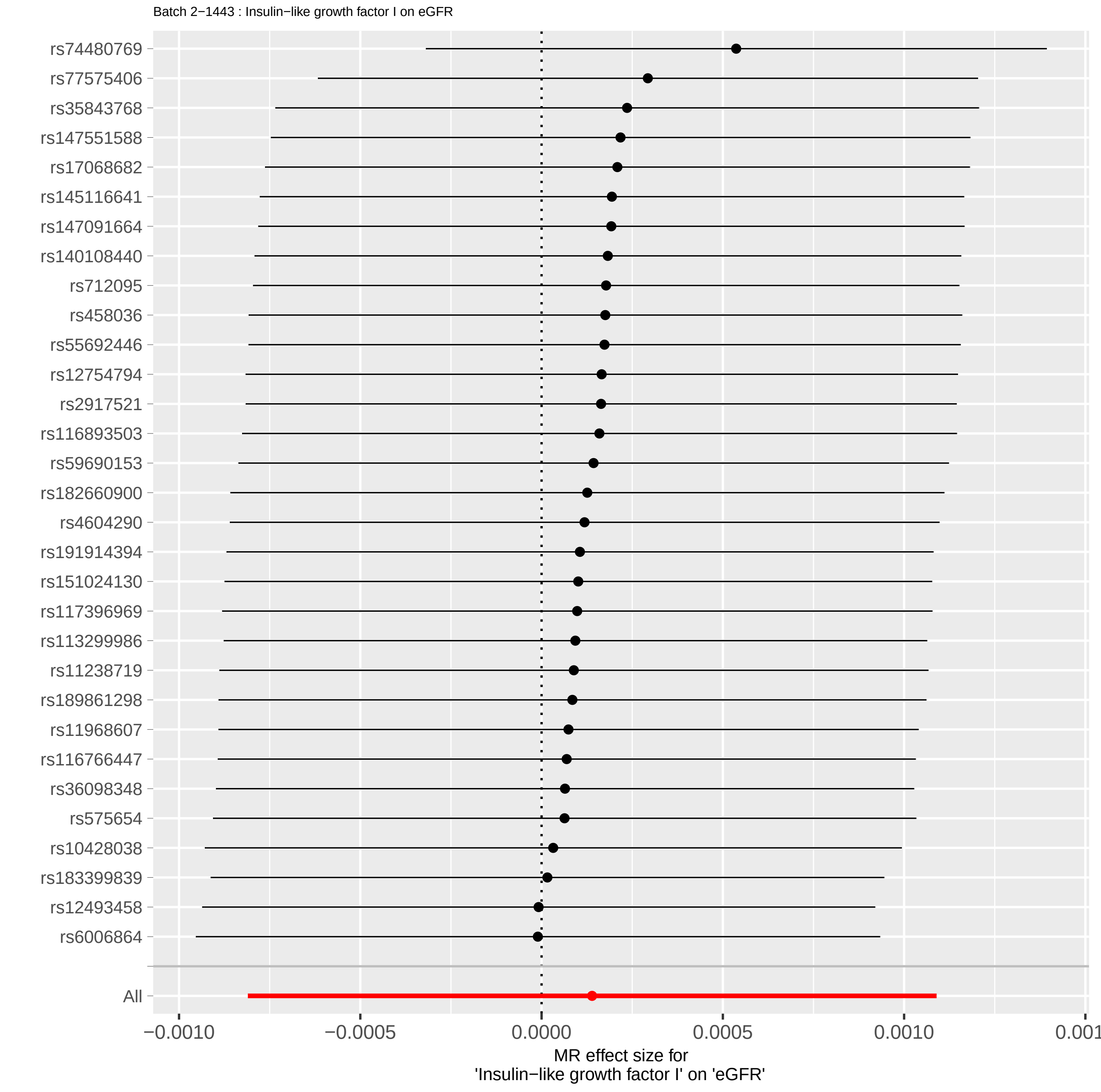

Batch 2−1443 : Insulin−like growth factor I on eGFR
rs74480769
rs77575406
rs35843768
rs147551588
rs17068682
rs145116641
rs147091664
rs140108440
rs712095
rs458036
rs55692446
rs12754794
rs2917521
rs116893503
rs59690153
rs182660900
rs4604290
rs191914394
rs151024130
rs117396969
rs113299986
rs11238719
rs189861298
rs11968607
rs116766447
rs36098348
rs575654
rs10428038
rs183399839
rs12493458
rs6006864
All
−0.0010
−0.0005
0.0000
0.0005
0.0010
MR effect size for
'Insulin−like growth factor I' on 'eGFR'

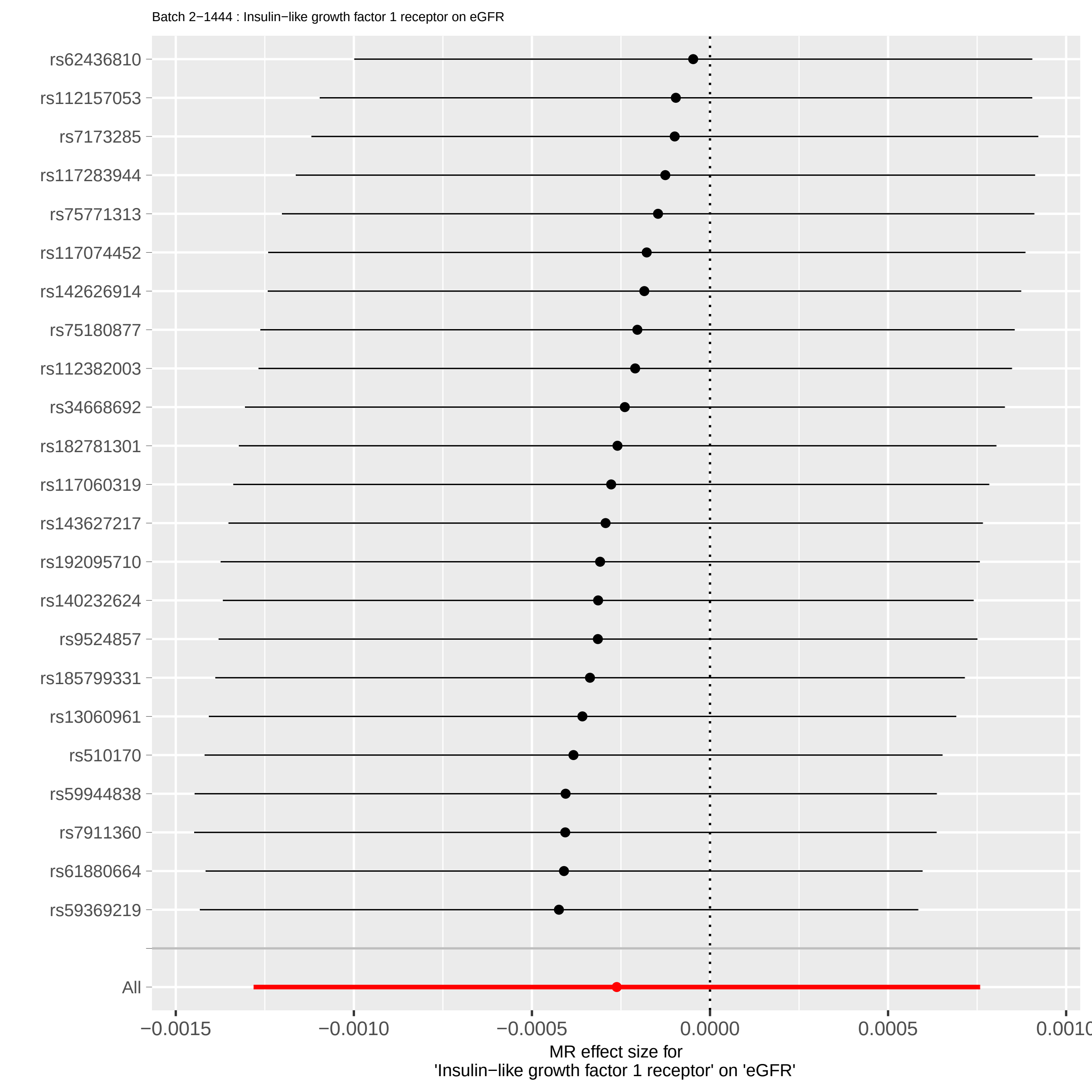

Batch 2−1444 : Insulin−like growth factor 1 receptor on eGFR
rs62436810
rs112157053
rs7173285
rs117283944
rs75771313
rs117074452
rs142626914
rs75180877
rs112382003
rs34668692
rs182781301
rs117060319
rs143627217
rs192095710
rs140232624
rs9524857
rs185799331
rs13060961
rs510170
rs59944838
rs7911360
rs61880664
rs59369219
All
−0.0015
−0.0010
−0.0005
0.0000
0.0005
0.0010
MR effect size for
'Insulin−like growth factor 1 receptor' on 'eGFR'

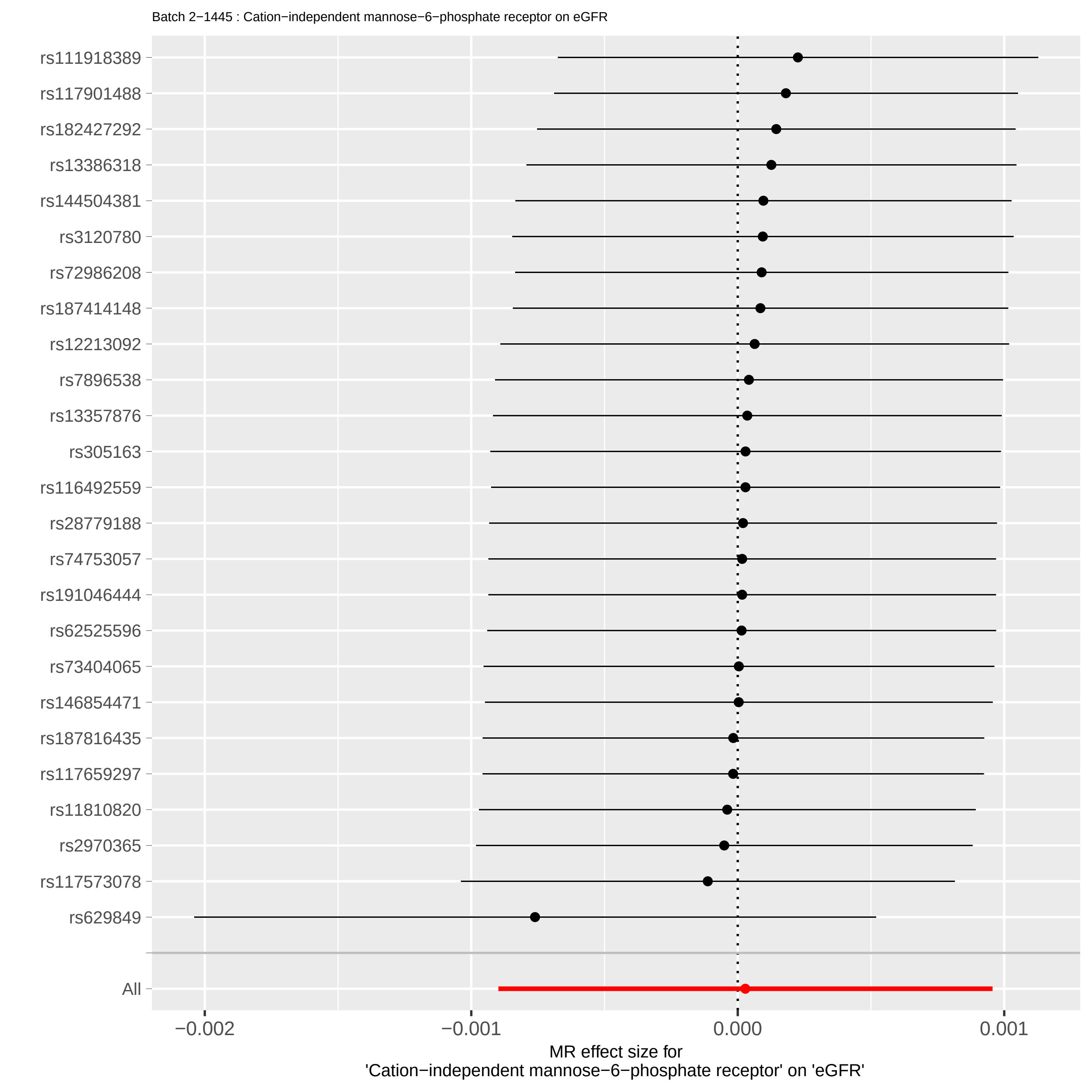

Batch 2−1445 : Cation−independent mannose−6−phosphate receptor on eGFR
rs111918389
rs117901488
rs182427292
rs13386318
rs144504381
rs3120780
rs72986208
rs187414148
rs12213092
rs7896538
rs13357876
rs305163
rs116492559
rs28779188
rs74753057
rs191046444
rs62525596
rs73404065
rs146854471
rs187816435
rs117659297
rs11810820
rs2970365
rs117573078
rs629849
All
−0.002
−0.001
0.000
0.001
MR effect size for
'Cation−independent mannose−6−phosphate receptor' on 'eGFR'

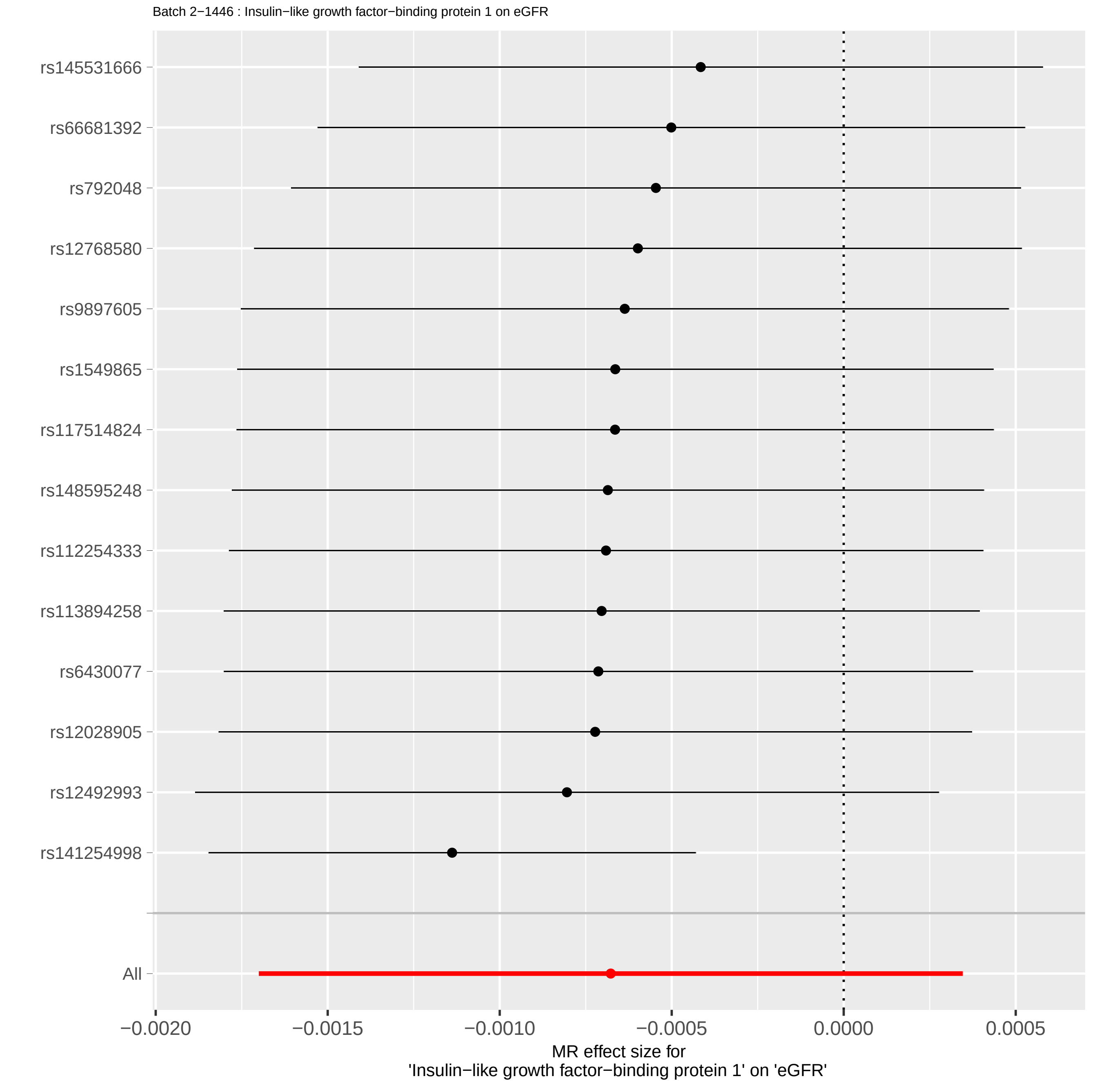

Batch 2−1446 : Insulin−like growth factor−binding protein 1 on eGFR
rs145531666
rs66681392
rs792048
rs12768580
rs9897605
rs1549865
rs117514824
rs148595248
rs112254333
rs113894258
rs6430077
rs12028905
rs12492993
rs141254998
All
−0.0020
−0.0015
−0.0010
−0.0005
0.0000
0.0005
MR effect size for
'Insulin−like growth factor−binding protein 1' on 'eGFR'

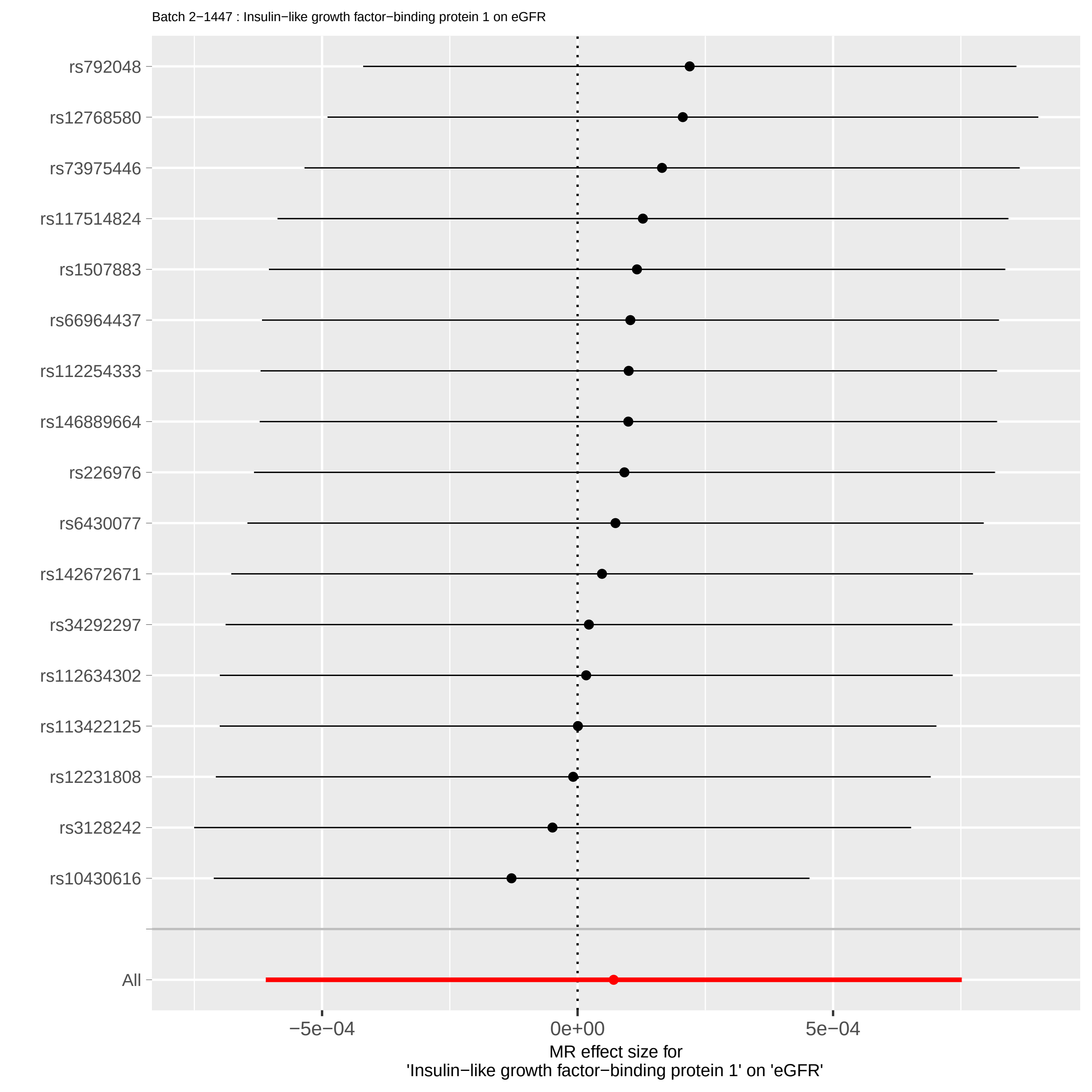
Batch 2−1447 : Insulin−like growth factor−binding protein 1 on eGFR
rs792048
rs12768580
rs73975446
rs117514824
rs1507883
rs66964437
rs112254333
rs146889664
rs226976
rs6430077
rs142672671
rs34292297
rs112634302
rs113422125
rs12231808
rs3128242
rs10430616
All
−5e−04
0e+00
5e−04
MR effect size for
'Insulin−like growth factor−binding protein 1' on 'eGFR'

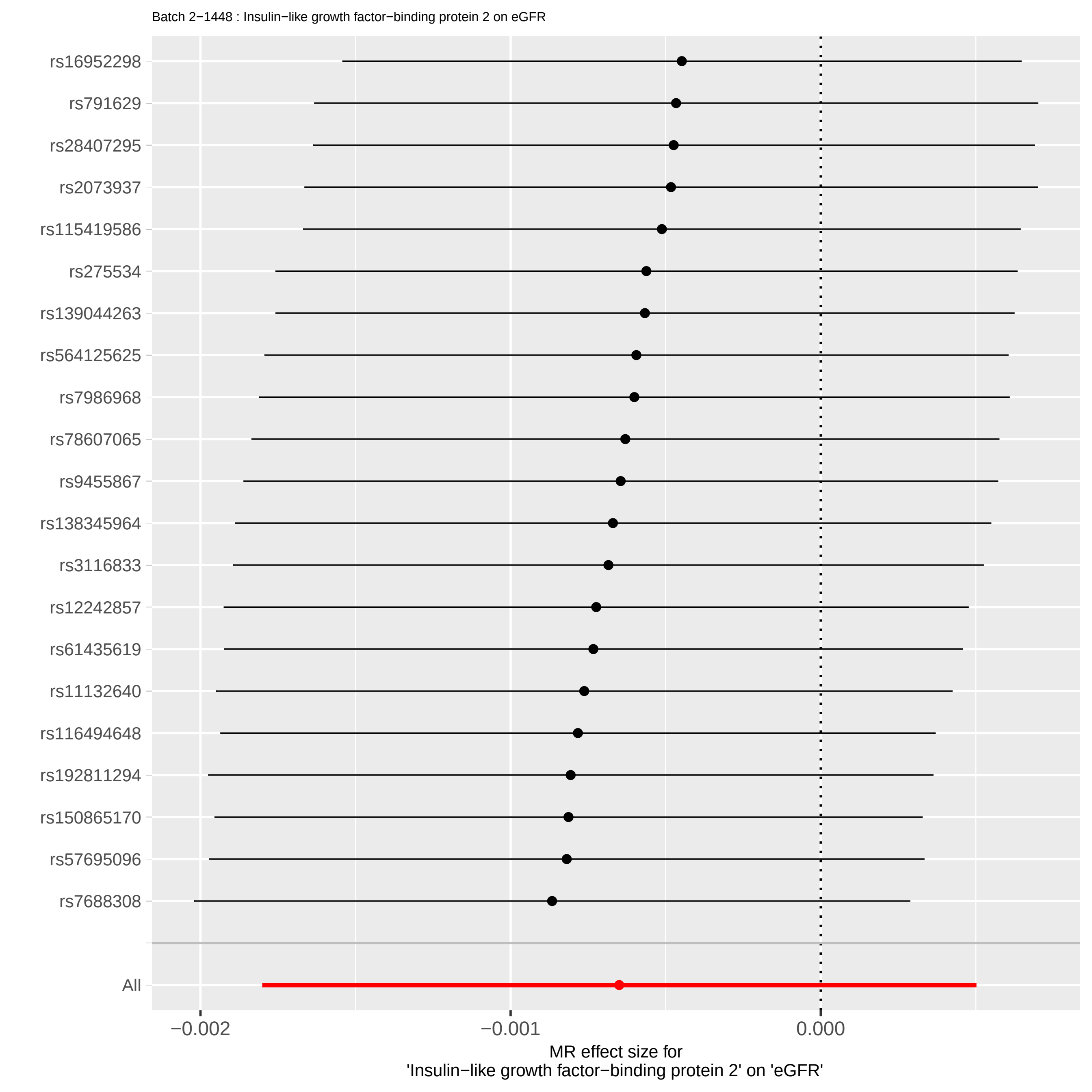

Batch 2−1448 : Insulin−like growth factor−binding protein 2 on eGFR
rs16952298
rs791629
rs28407295
rs2073937
rs115419586
rs275534
rs139044263
rs564125625
rs7986968
rs78607065
rs9455867
rs138345964
rs3116833
rs12242857
rs61435619
rs11132640
rs116494648
rs192811294
rs150865170
rs57695096
rs7688308
All
−0.002
−0.001
0.000
MR effect size for
'Insulin−like growth factor−binding protein 2' on 'eGFR'

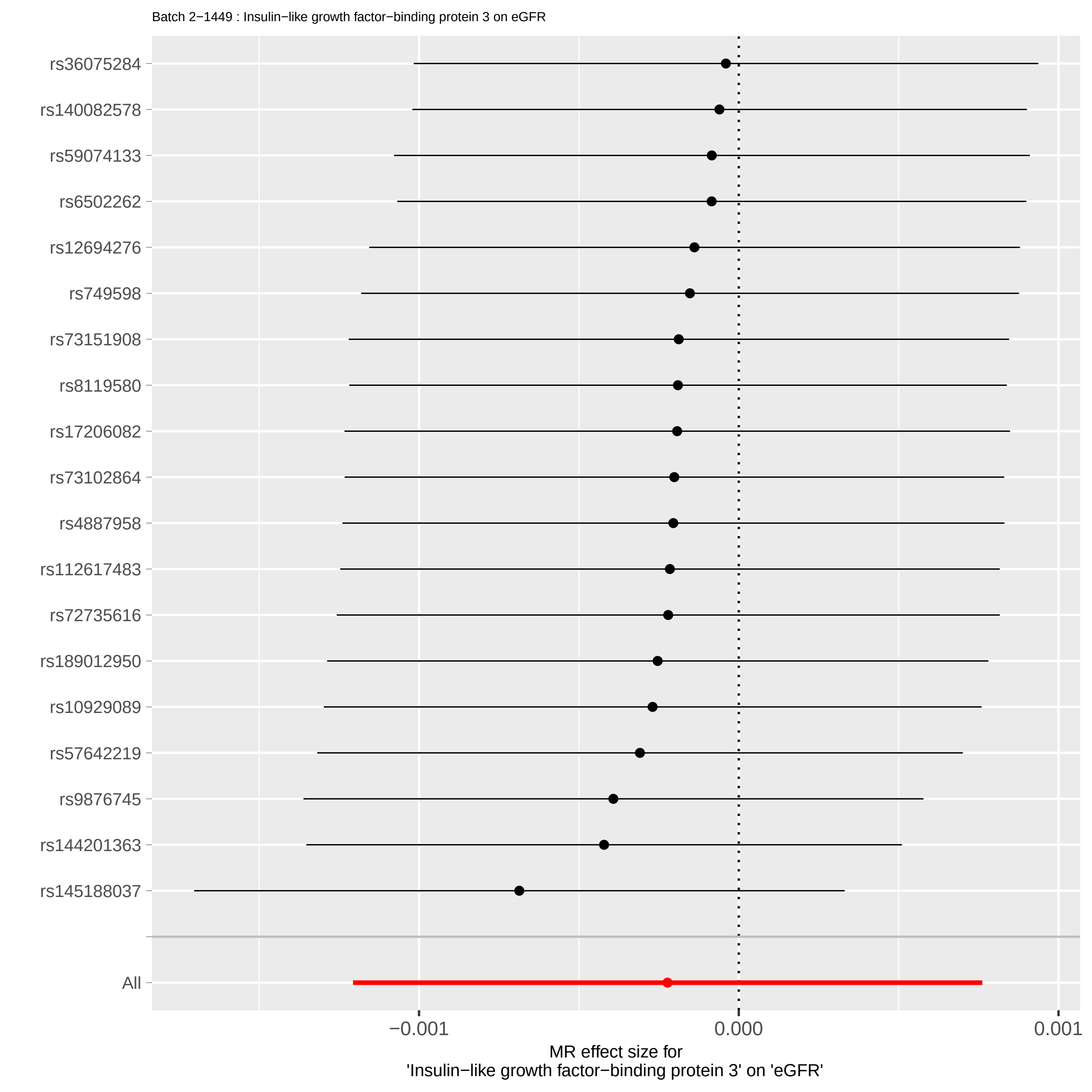

Batch 2−1449 : Insulin−like growth factor−binding protein 3 on eGFR
rs36075284
rs140082578
rs59074133
rs6502262
rs12694276
rs749598
rs73151908
rs8119580
rs17206082
rs73102864
rs4887958
rs112617483
rs72735616
rs189012950
rs10929089
rs57642219
rs9876745
rs144201363
rs145188037
All
−0.001
0.000
0.001
MR effect size for
'Insulin−like growth factor−binding protein 3' on 'eGFR'

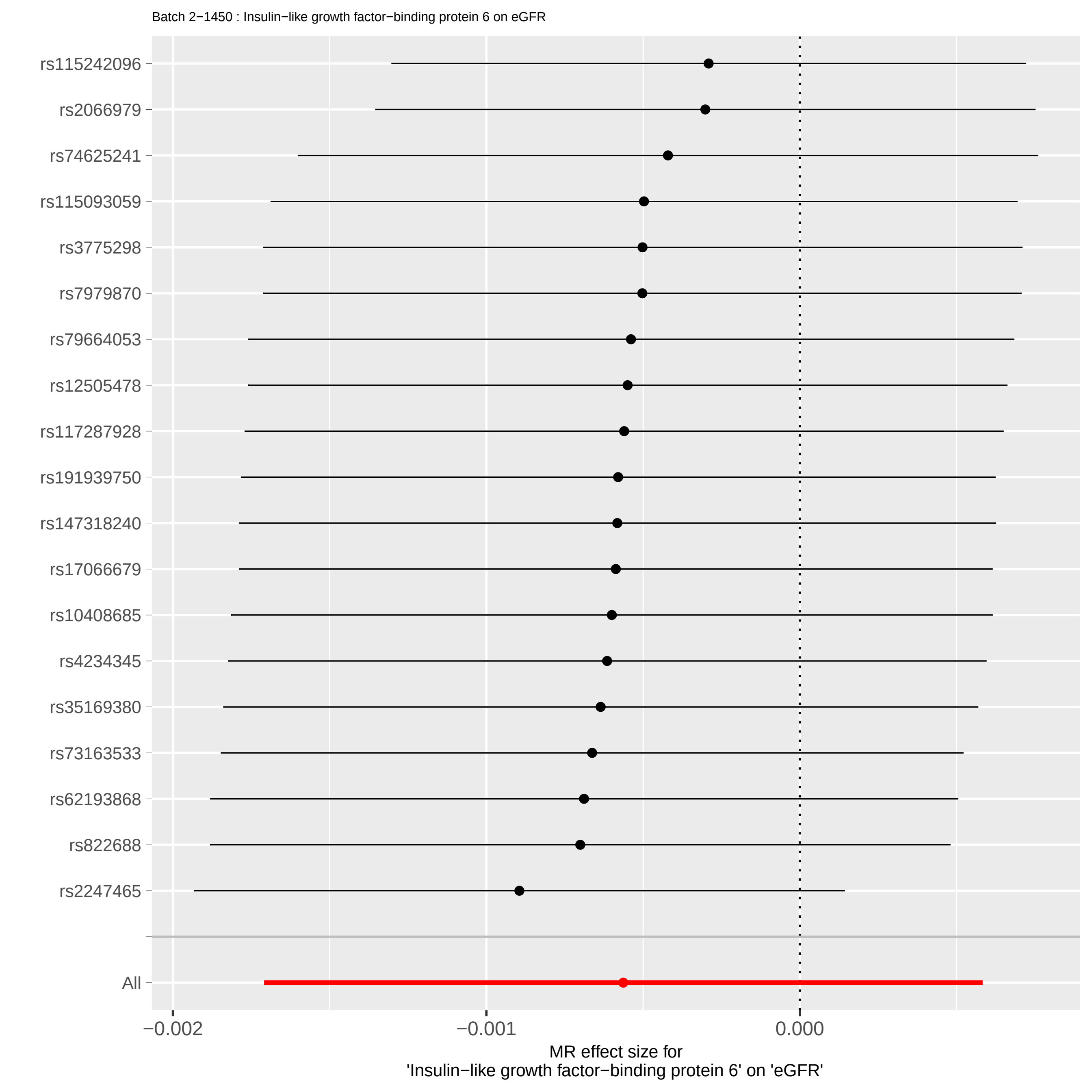

Batch 2−1450 : Insulin−like growth factor−binding protein 6 on eGFR
rs115242096
rs2066979
rs74625241
rs115093059
rs3775298
rs7979870
rs79664053
rs12505478
rs117287928
rs191939750
rs147318240
rs17066679
rs10408685
rs4234345
rs35169380
rs73163533
rs62193868
rs822688
rs2247465
All
−0.002
−0.001
0.000
MR effect size for
'Insulin−like growth factor−binding protein 6' on 'eGFR'

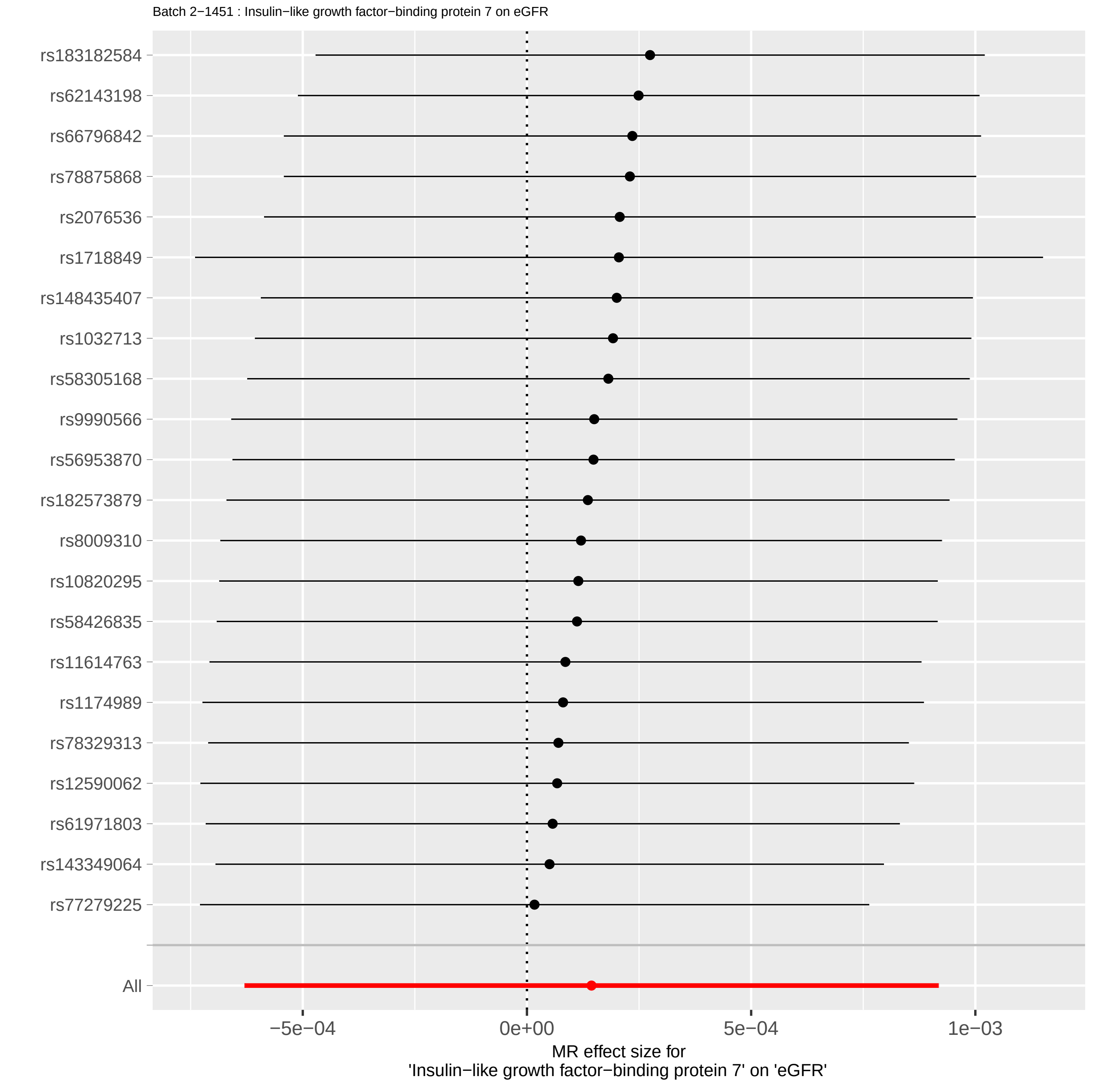
Batch 2−1451 : Insulin−like growth factor−binding protein 7 on eGFR
rs183182584
rs62143198
rs66796842
rs78875868
rs2076536
rs1718849
rs148435407
rs1032713
rs58305168
rs9990566
rs56953870
rs182573879
rs8009310
rs10820295
rs58426835
rs11614763
rs1174989
rs78329313
rs12590062
rs61971803
rs143349064
rs77279225
All
−5e−04
0e+00
5e−04
1e−03
MR effect size for
'Insulin−like growth factor−binding protein 7' on 'eGFR'

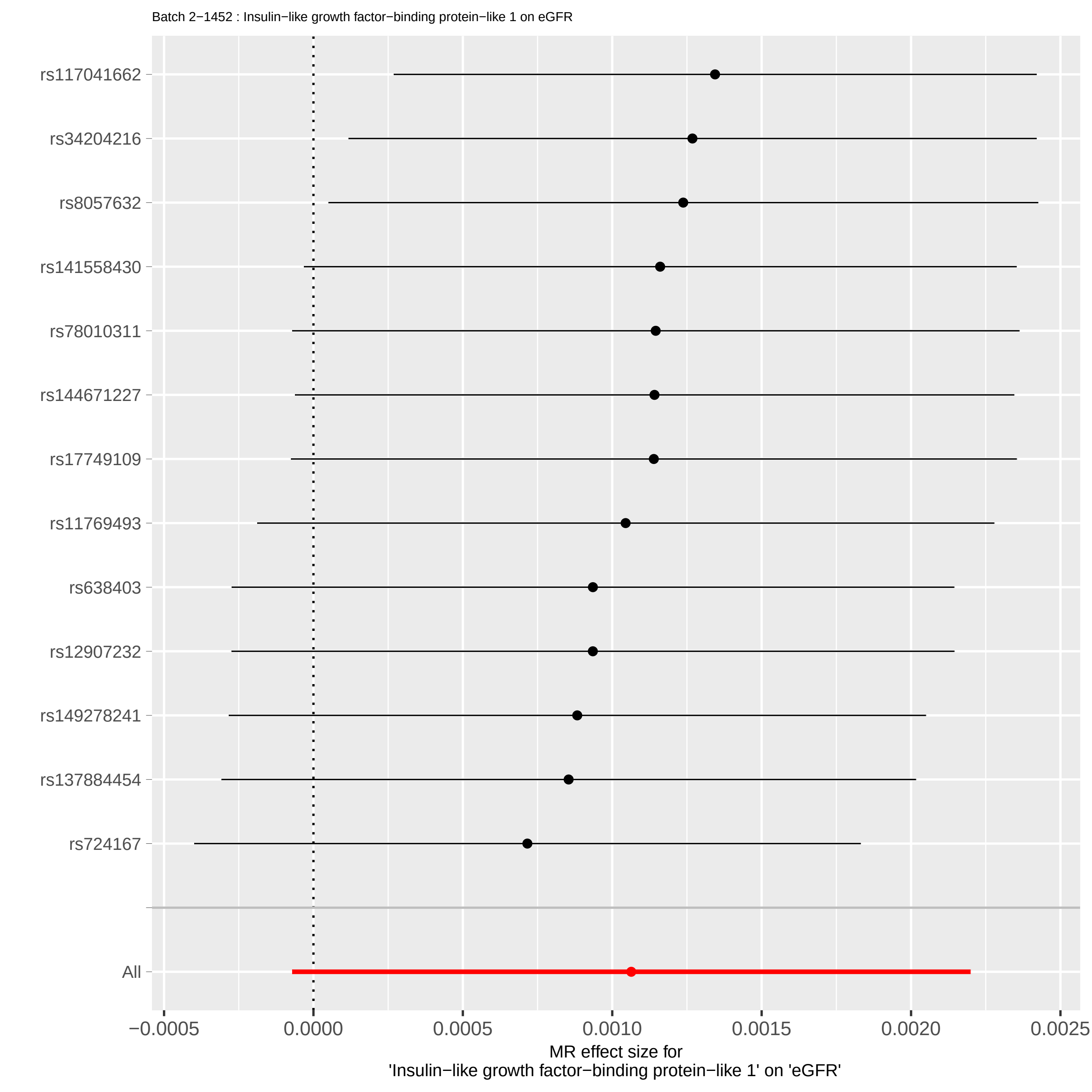

Batch 2−1452 : Insulin−like growth factor−binding protein−like 1 on eGFR
rs117041662
rs34204216
rs8057632
rs141558430
rs78010311
rs144671227
rs17749109
rs11769493
rs638403
rs12907232
rs149278241
rs137884454
rs724167
All
−0.0005
0.0000
0.0005
0.0010
0.0015
0.0020
0.0025
MR effect size for
'Insulin−like growth factor−binding protein−like 1' on 'eGFR'

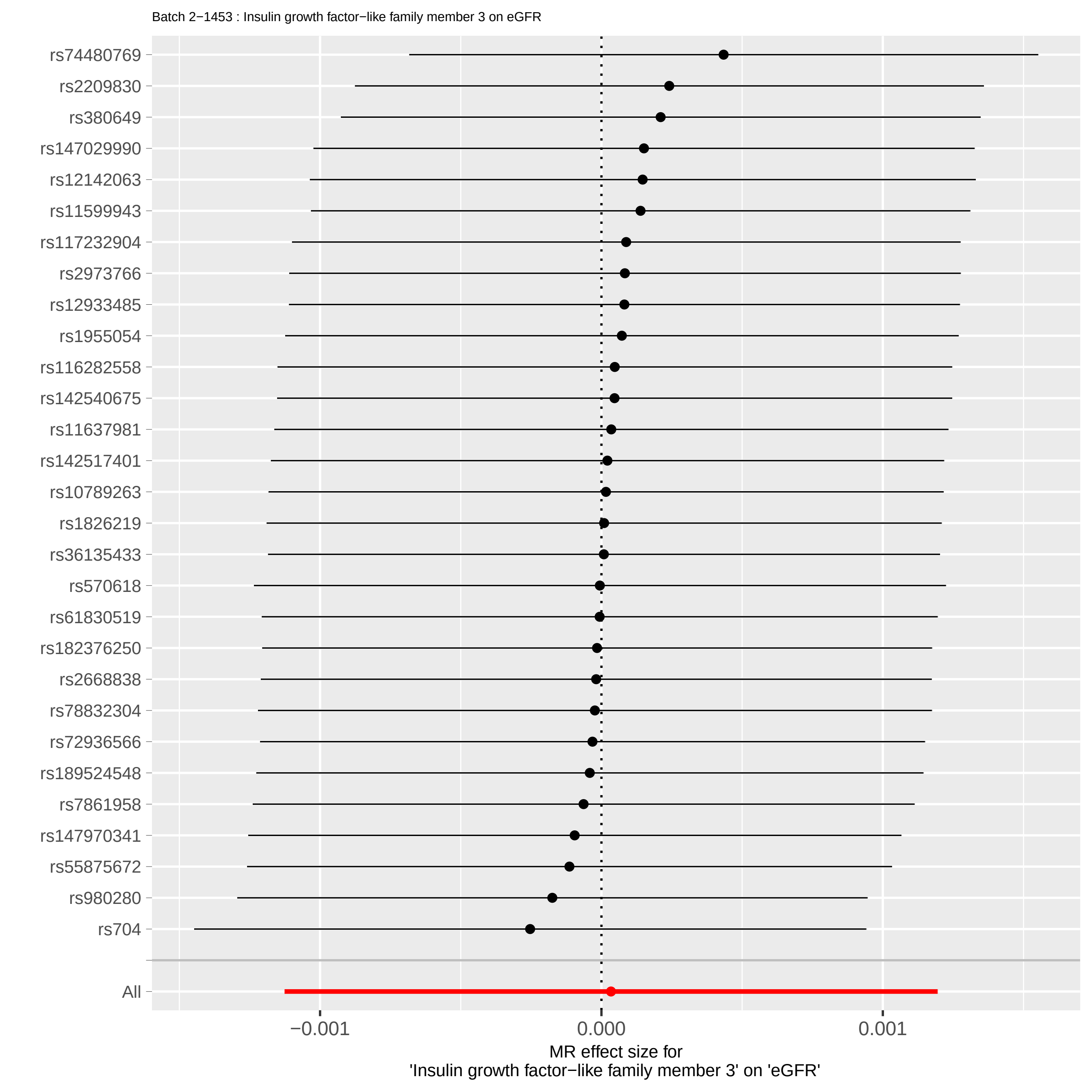
Batch 2−1453 : Insulin growth factor−like family member 3 on eGFR
rs74480769
rs2209830
rs380649
rs147029990
rs12142063
rs11599943
rs117232904
rs2973766
rs12933485
rs1955054
rs116282558
rs142540675
rs11637981
rs142517401
rs10789263
rs1826219
rs36135433
rs570618
rs61830519
rs182376250
rs2668838
rs78832304
rs72936566
rs189524548
rs7861958
rs147970341
rs55875672
rs980280
rs704
All
−0.001
0.000
0.001
MR effect size for
'Insulin growth factor−like family member 3' on 'eGFR'

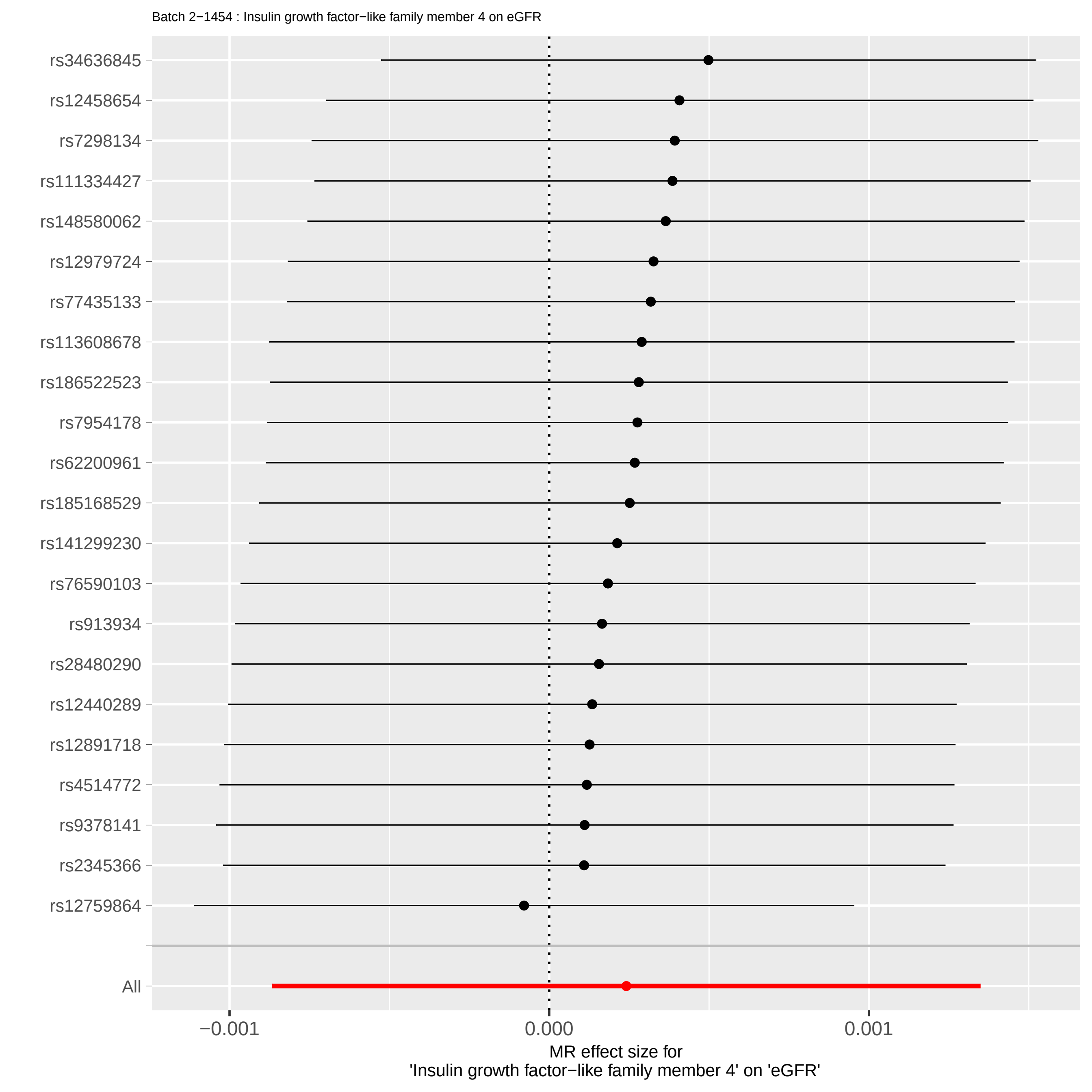
Batch 2−1454 : Insulin growth factor−like family member 4 on eGFR
rs34636845
rs12458654
rs7298134
rs111334427
rs148580062
rs12979724
rs77435133
rs113608678
rs186522523
rs7954178
rs62200961
rs185168529
rs141299230
rs76590103
rs913934
rs28480290
rs12440289
rs12891718
rs4514772
rs9378141
rs2345366
rs12759864
All
−0.001
0.000
0.001
MR effect size for
'Insulin growth factor−like family member 4' on 'eGFR'

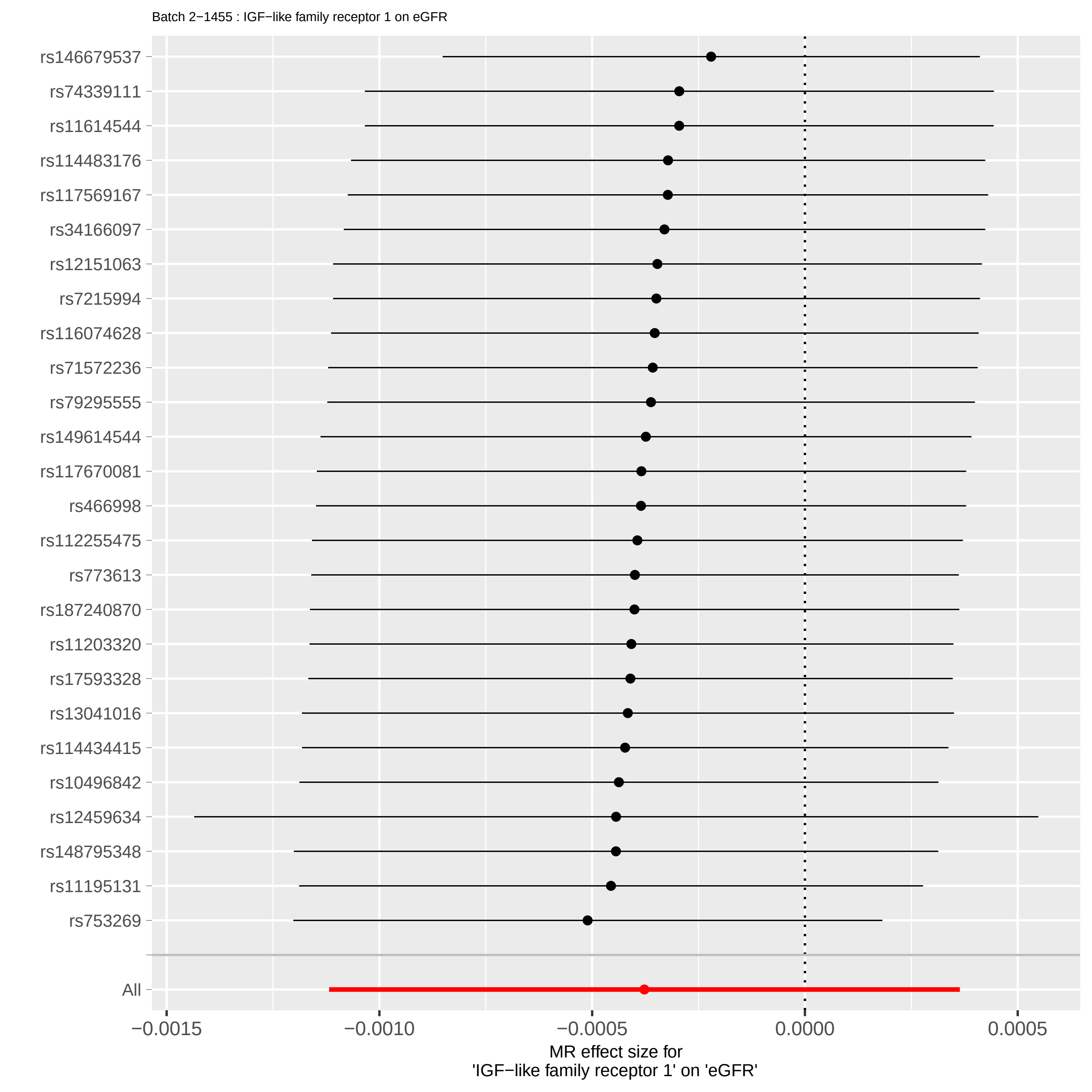

Batch 2−1455 : IGF−like family receptor 1 on eGFR
rs146679537
rs74339111
rs11614544
rs114483176
rs117569167
rs34166097
rs12151063
rs7215994
rs116074628
rs71572236
rs79295555
rs149614544
rs117670081
rs466998
rs112255475
rs773613
rs187240870
rs11203320
rs17593328
rs13041016
rs114434415
rs10496842
rs12459634
rs148795348
rs11195131
rs753269
All
−0.0015
−0.0010
−0.0005
0.0000
0.0005
MR effect size for
'IGF−like family receptor 1' on 'eGFR'

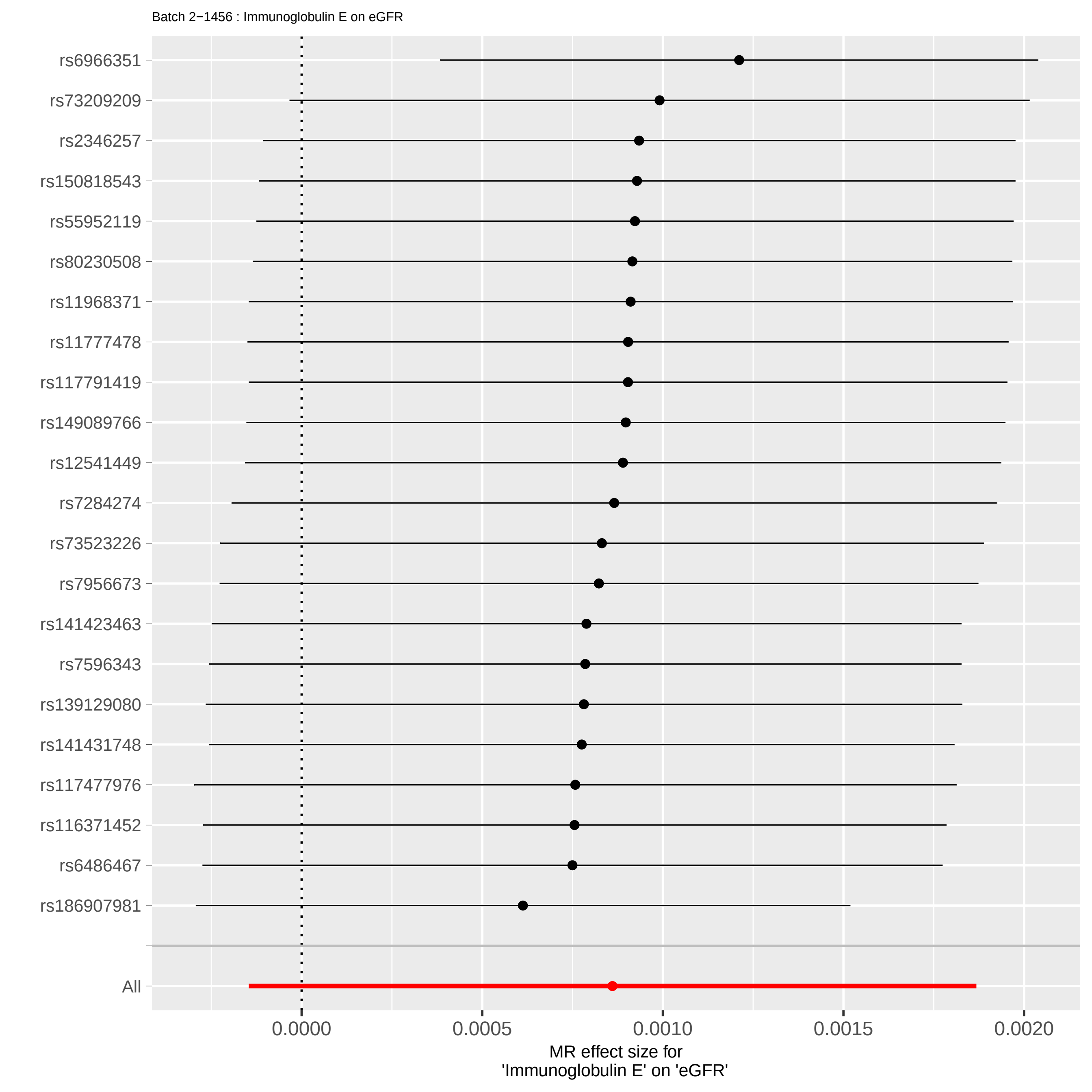

Batch 2−1456 : Immunoglobulin E on eGFR
rs6966351
rs73209209
rs2346257
rs150818543
rs55952119
rs80230508
rs11968371
rs11777478
rs117791419
rs149089766
rs12541449
rs7284274
rs73523226
rs7956673
rs141423463
rs7596343
rs139129080
rs141431748
rs117477976
rs116371452
rs6486467
rs186907981
All
0.0000
0.0005
0.0010
0.0015
0.0020
MR effect size for
'Immunoglobulin E' on 'eGFR'

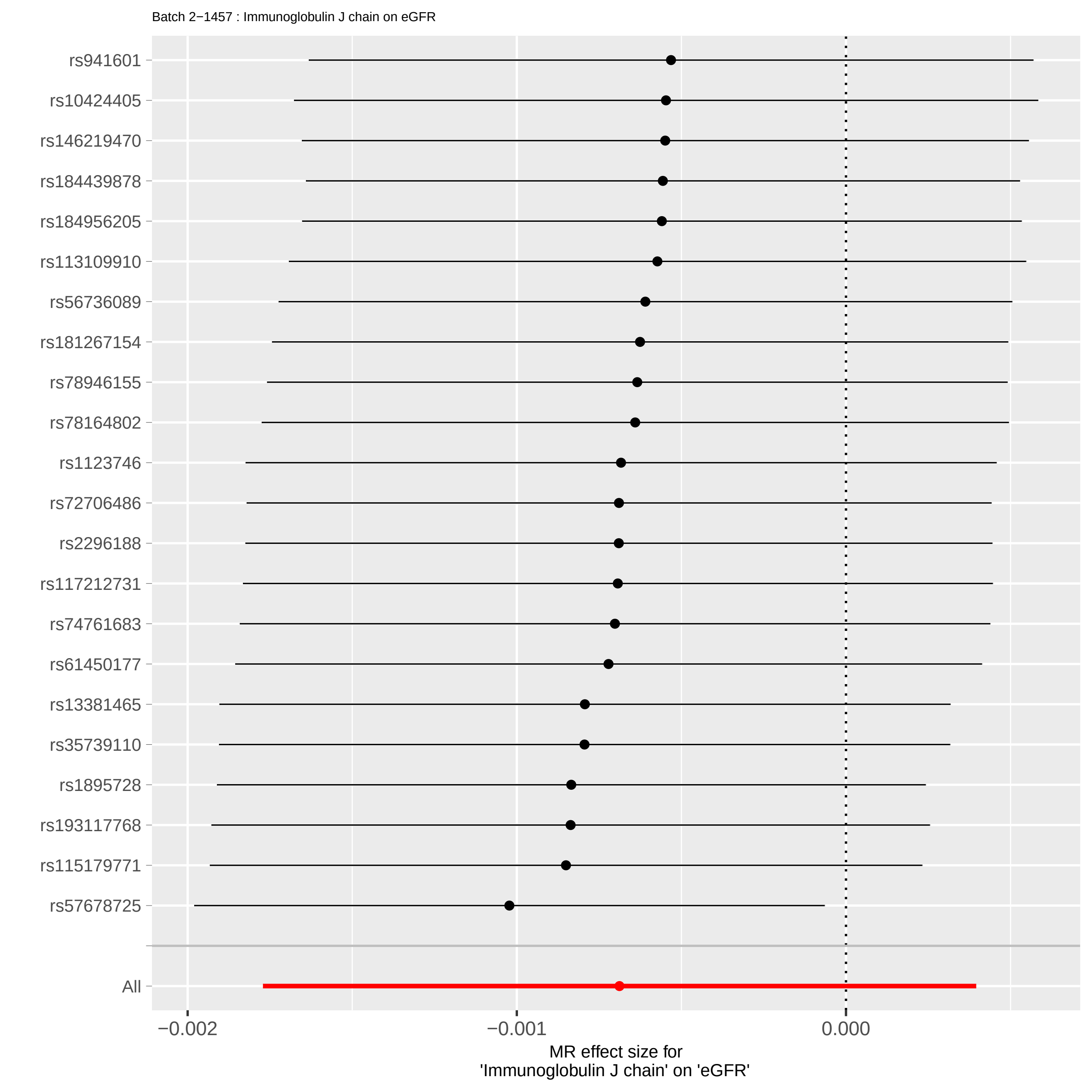

Batch 2−1457 : Immunoglobulin J chain on eGFR
rs941601
rs10424405
rs146219470
rs184439878
rs184956205
rs113109910
rs56736089
rs181267154
rs78946155
rs78164802
rs1123746
rs72706486
rs2296188
rs117212731
rs74761683
rs61450177
rs13381465
rs35739110
rs1895728
rs193117768
rs115179771
rs57678725
All
−0.002
−0.001
0.000
MR effect size for
'Immunoglobulin J chain' on 'eGFR'

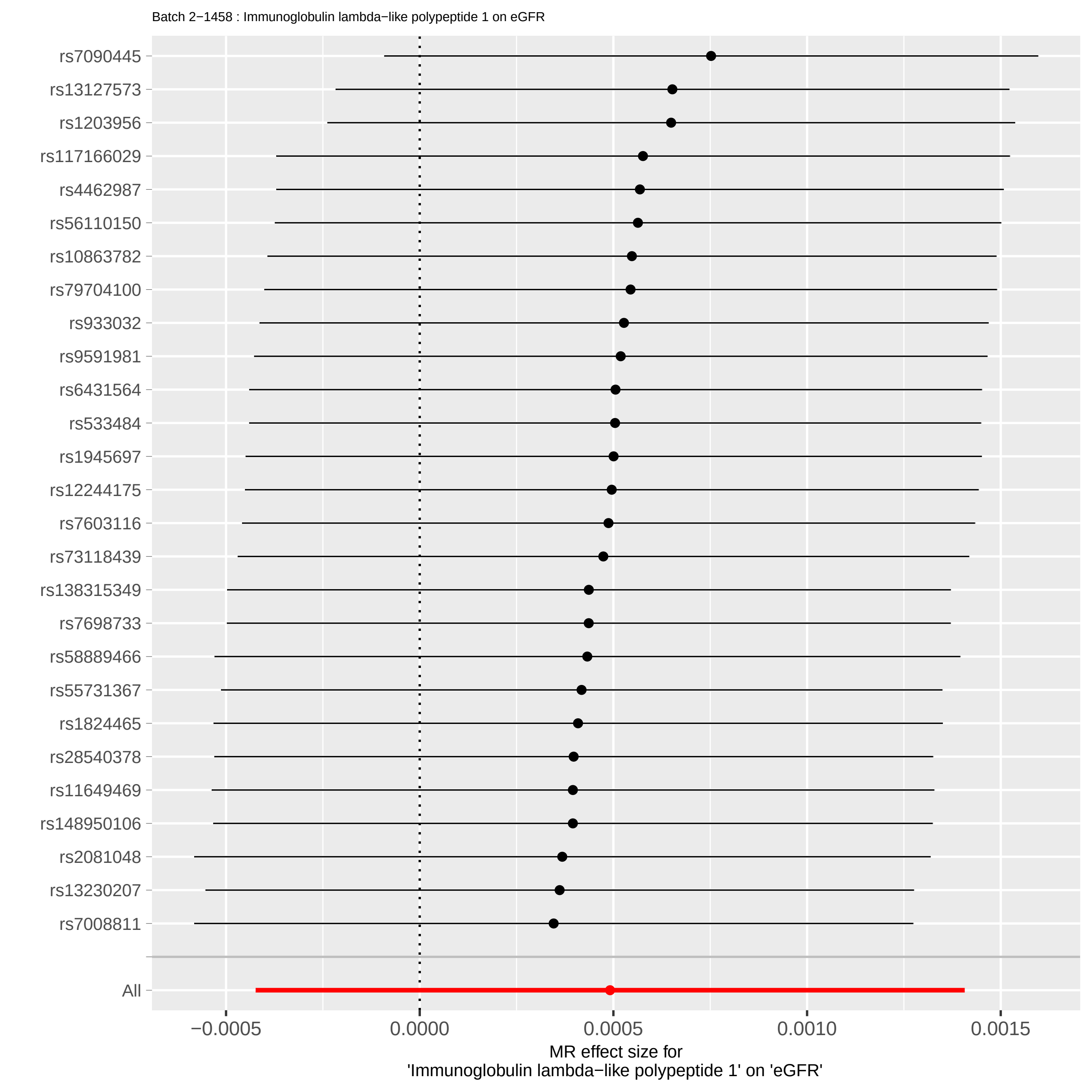

Batch 2−1458 : Immunoglobulin lambda−like polypeptide 1 on eGFR
rs7090445
rs13127573
rs1203956
rs117166029
rs4462987
rs56110150
rs10863782
rs79704100
rs933032
rs9591981
rs6431564
rs533484
rs1945697
rs12244175
rs7603116
rs73118439
rs138315349
rs7698733
rs58889466
rs55731367
rs1824465
rs28540378
rs11649469
rs148950106
rs2081048
rs13230207
rs7008811
All
−0.0005
0.0000
0.0005
0.0010
0.0015
MR effect size for
'Immunoglobulin lambda−like polypeptide 1' on 'eGFR'

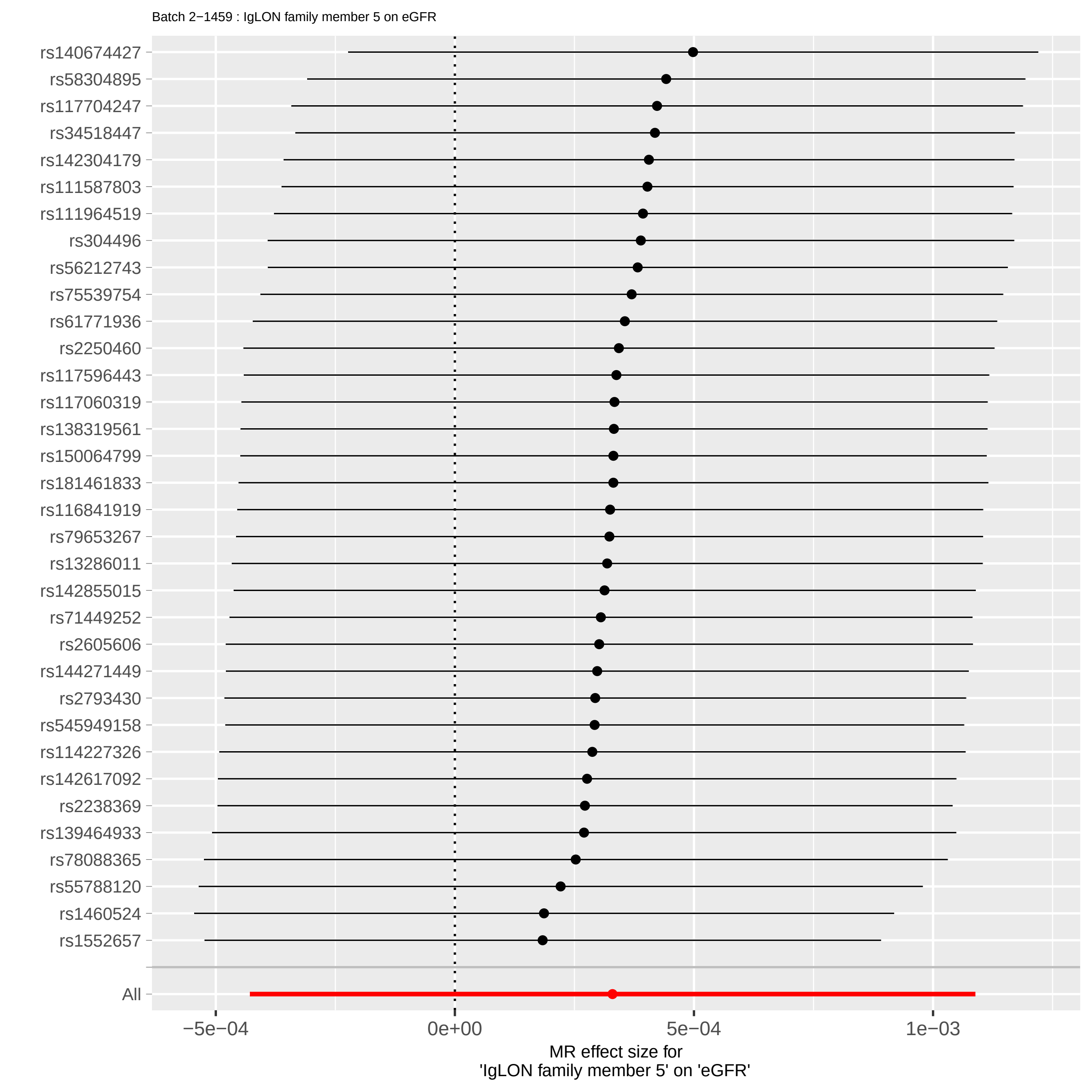
Batch 2−1459 : IgLON family member 5 on eGFR
rs140674427
rs58304895
rs117704247
rs34518447
rs142304179
rs111587803
rs111964519
rs304496
rs56212743
rs75539754
rs61771936
rs2250460
rs117596443
rs117060319
rs138319561
rs150064799
rs181461833
rs116841919
rs79653267
rs13286011
rs142855015
rs71449252
rs2605606
rs144271449
rs2793430
rs545949158
rs114227326
rs142617092
rs2238369
rs139464933
rs78088365
rs55788120
rs1460524
rs1552657
All
−5e−04
0e+00
5e−04
1e−03
MR effect size for
'IgLON family member 5' on 'eGFR'

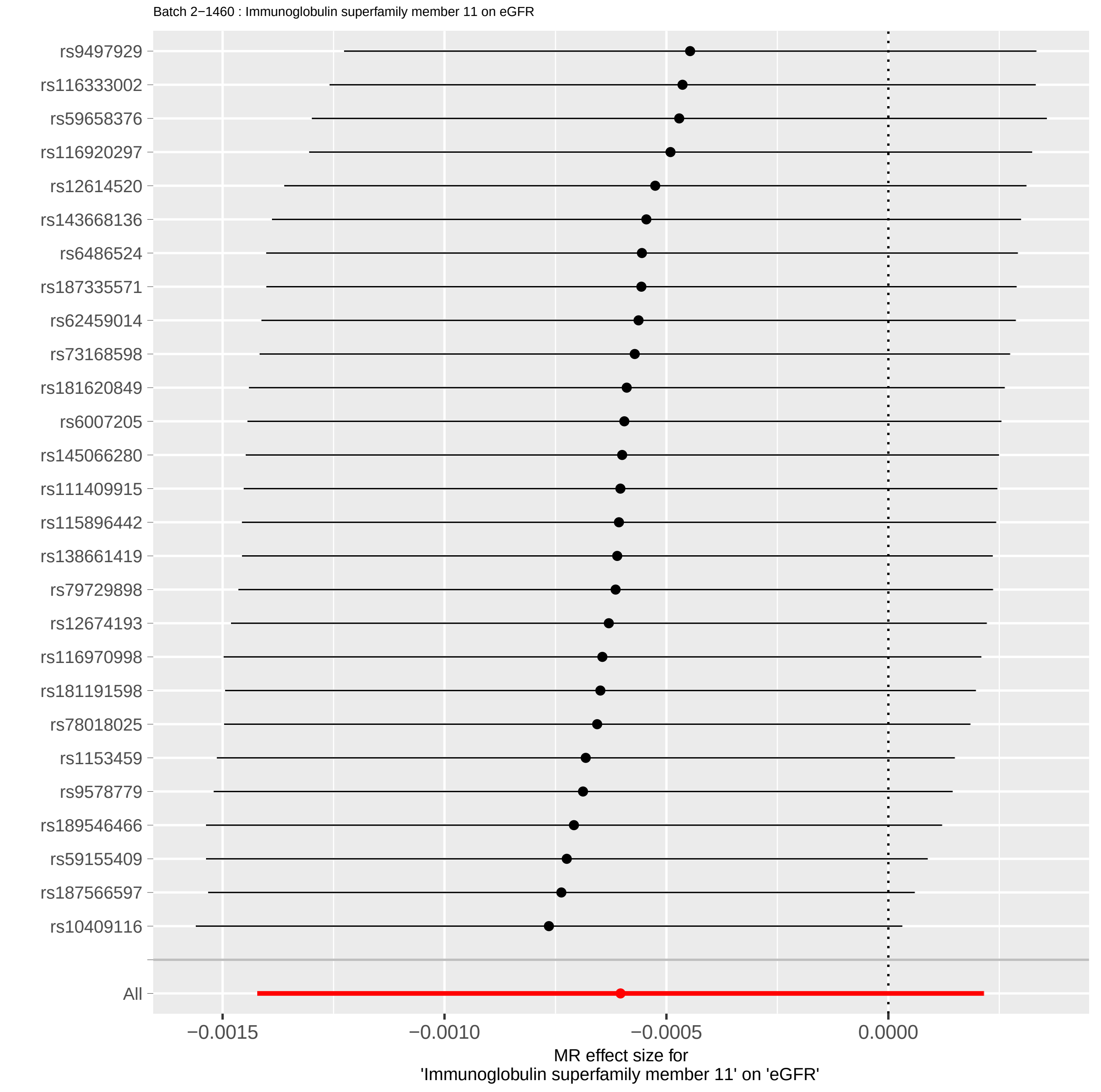

Batch 2−1460 : Immunoglobulin superfamily member 11 on eGFR
rs9497929
rs116333002
rs59658376
rs116920297
rs12614520
rs143668136
rs6486524
rs187335571
rs62459014
rs73168598
rs181620849
rs6007205
rs145066280
rs111409915
rs115896442
rs138661419
rs79729898
rs12674193
rs116970998
rs181191598
rs78018025
rs1153459
rs9578779
rs189546466
rs59155409
rs187566597
rs10409116
All
−0.0015
−0.0010
−0.0005
0.0000
MR effect size for
'Immunoglobulin superfamily member 11' on 'eGFR'

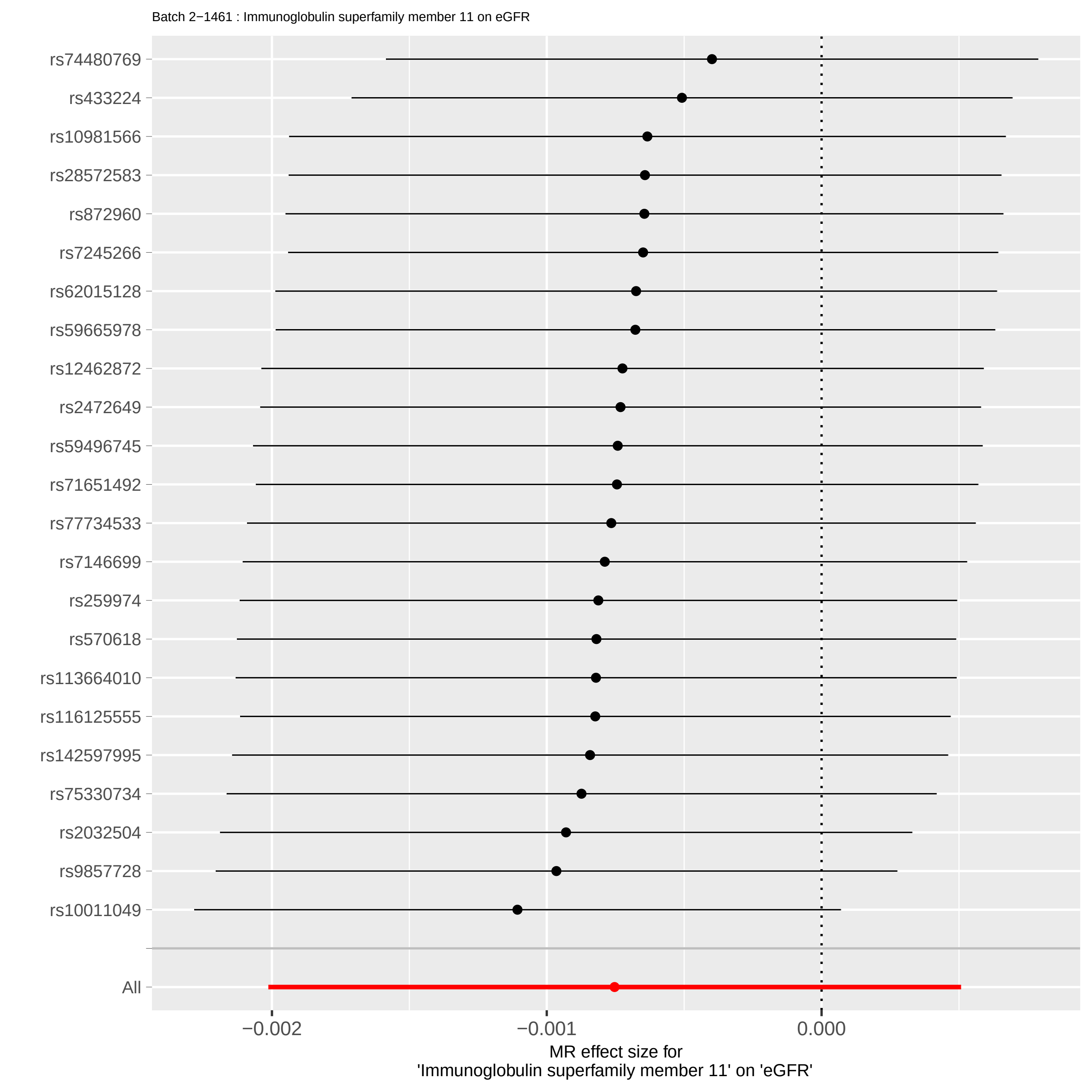

Batch 2−1461 : Immunoglobulin superfamily member 11 on eGFR
rs74480769
rs433224
rs10981566
rs28572583
rs872960
rs7245266
rs62015128
rs59665978
rs12462872
rs2472649
rs59496745
rs71651492
rs77734533
rs7146699
rs259974
rs570618
rs113664010
rs116125555
rs142597995
rs75330734
rs2032504
rs9857728
rs10011049
All
−0.002
−0.001
0.000
MR effect size for
'Immunoglobulin superfamily member 11' on 'eGFR'

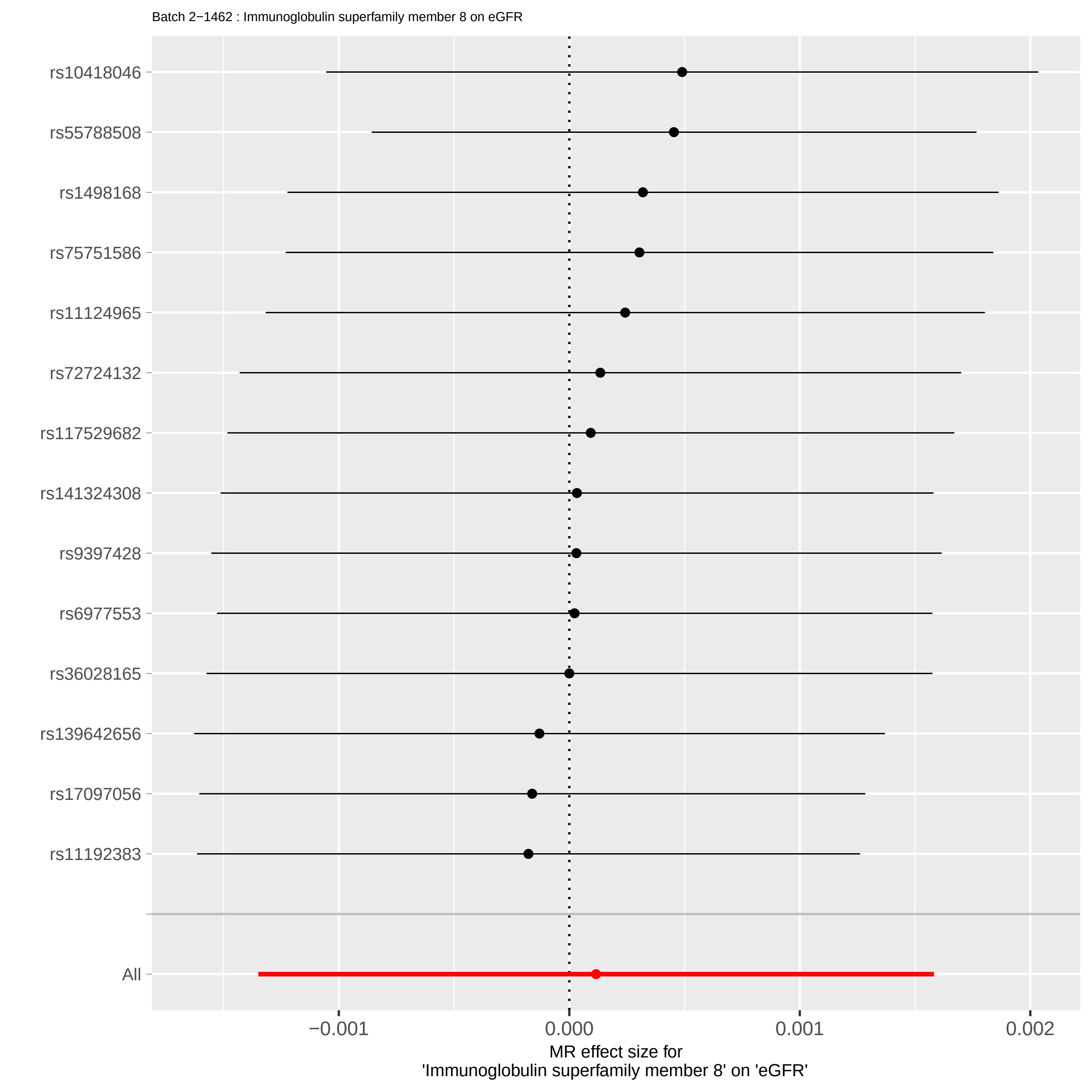

Batch 2−1462 : Immunoglobulin superfamily member 8 on eGFR
rs10418046
rs55788508
rs1498168
rs75751586
rs11124965
rs72724132
rs117529682
rs141324308
rs9397428
rs6977553
rs36028165
rs139642656
rs17097056
rs11192383
All
−0.001
0.000
0.001
0.002
MR effect size for
'Immunoglobulin superfamily member 8' on 'eGFR'

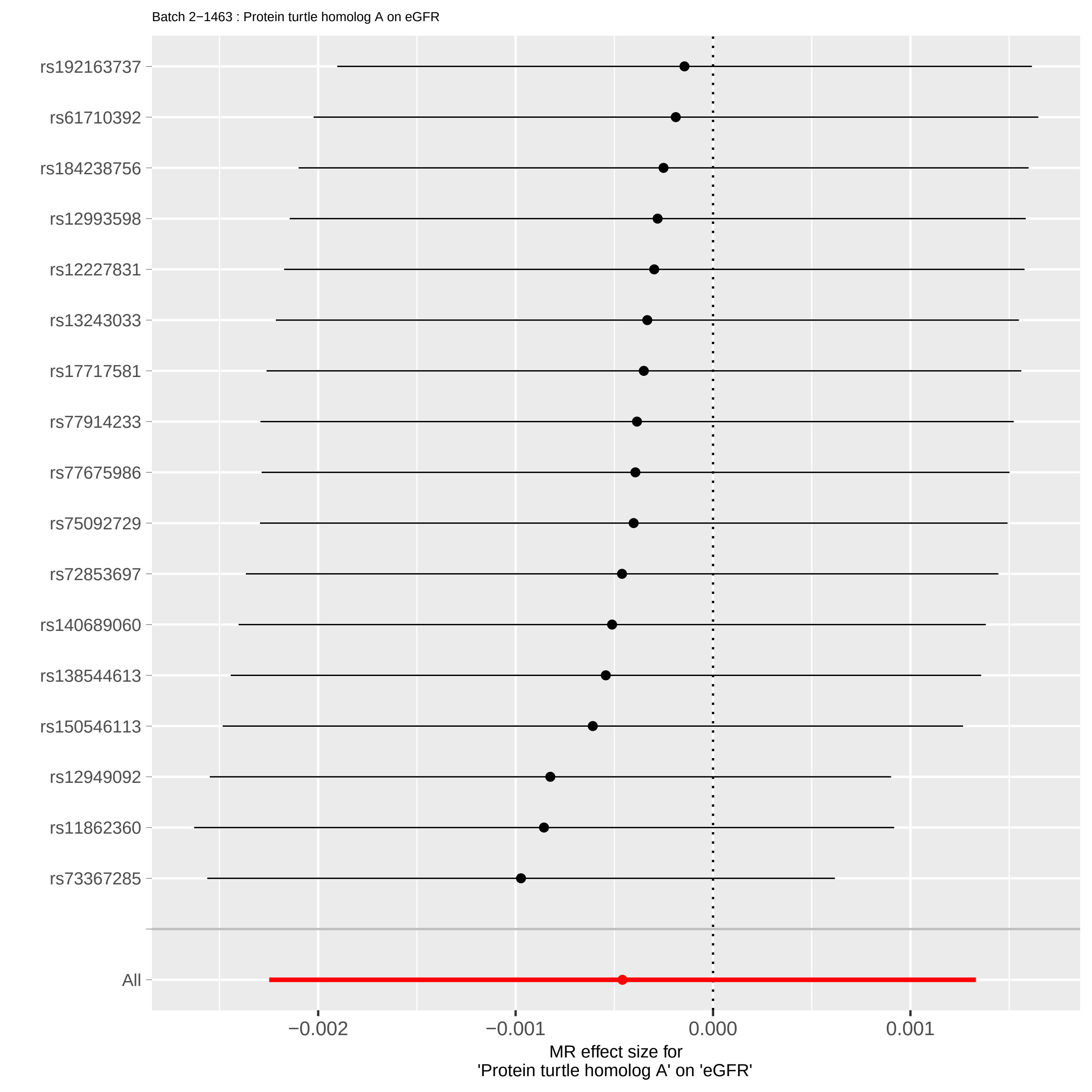

Batch 2−1463 : Protein turtle homolog A on eGFR
rs192163737
rs61710392
rs184238756
rs12993598
rs12227831
rs13243033
rs17717581
rs77914233
rs77675986
rs75092729
rs72853697
rs140689060
rs138544613
rs150546113
rs12949092
rs11862360
rs73367285
All
−0.002
−0.001
0.000
0.001
MR effect size for
'Protein turtle homolog A' on 'eGFR'

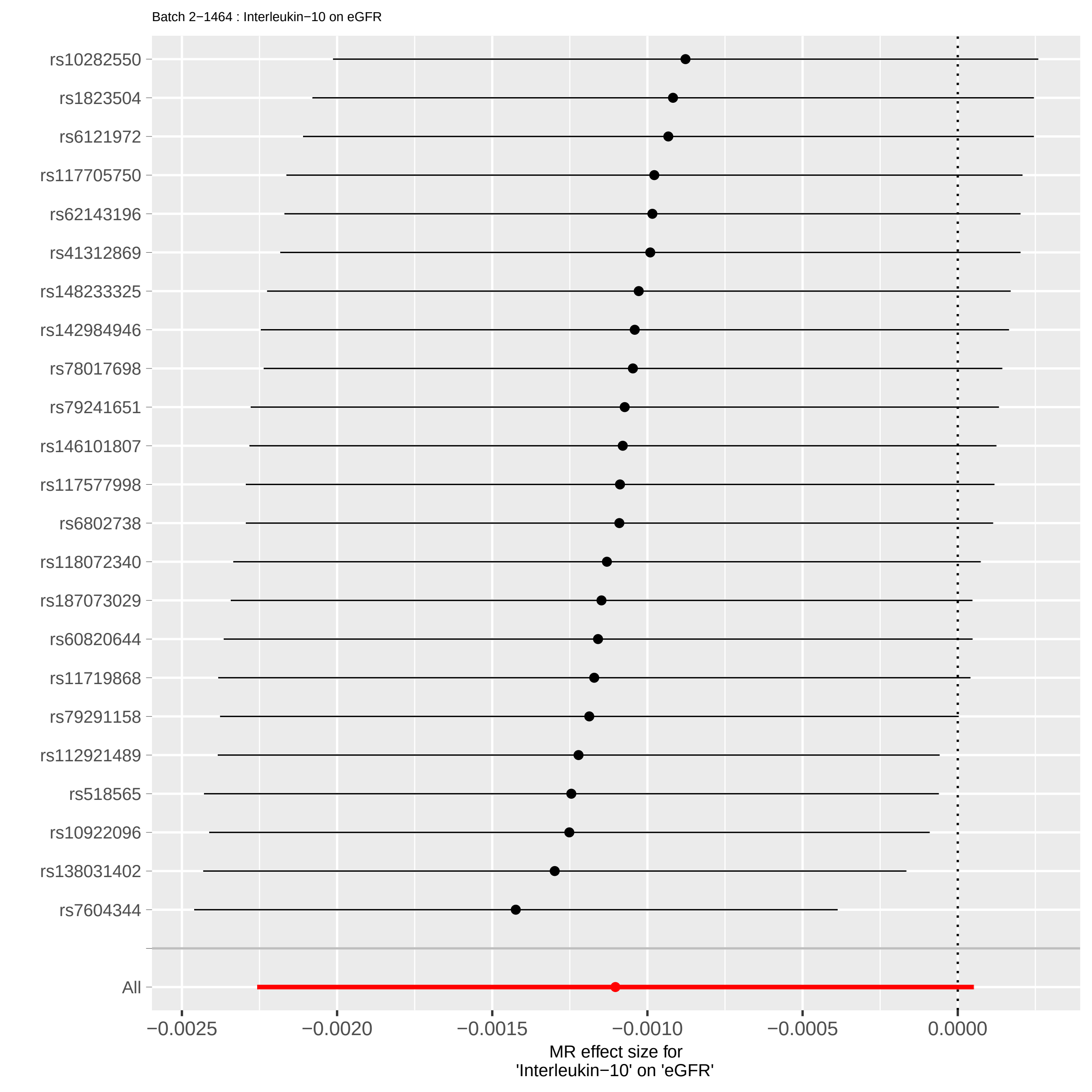

Batch 2−1464 : Interleukin−10 on eGFR
rs10282550
rs1823504
rs6121972
rs117705750
rs62143196
rs41312869
rs148233325
rs142984946
rs78017698
rs79241651
rs146101807
rs117577998
rs6802738
rs118072340
rs187073029
rs60820644
rs11719868
rs79291158
rs112921489
rs518565
rs10922096
rs138031402
rs7604344
All
−0.0025
−0.0020
−0.0015
−0.0010
−0.0005
0.0000
MR effect size for
'Interleukin−10' on 'eGFR'

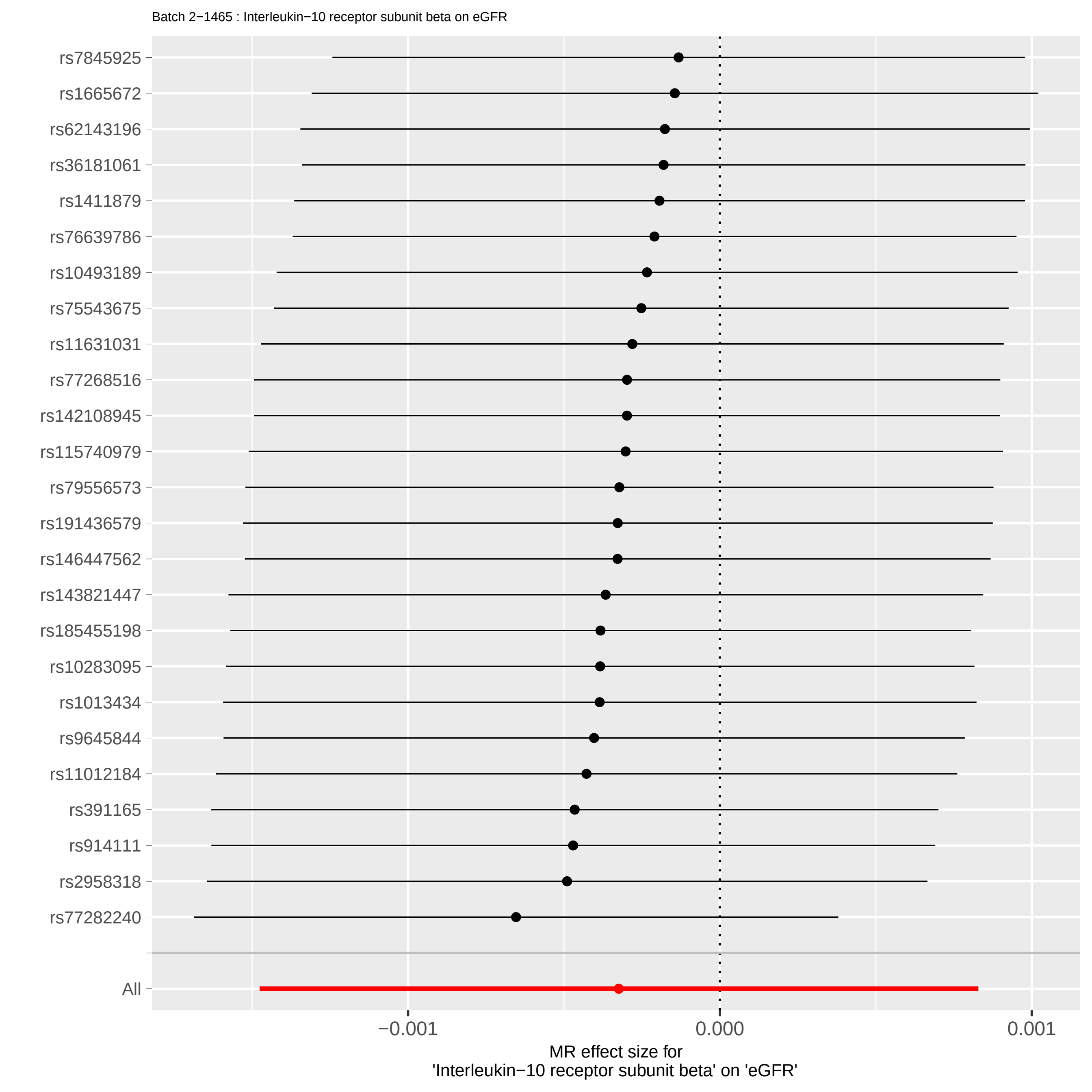

Batch 2−1465 : Interleukin−10 receptor subunit beta on eGFR
rs7845925
rs1665672
rs62143196
rs36181061
rs1411879
rs76639786
rs10493189
rs75543675
rs11631031
rs77268516
rs142108945
rs115740979
rs79556573
rs191436579
rs146447562
rs143821447
rs185455198
rs10283095
rs1013434
rs9645844
rs11012184
rs391165
rs914111
rs2958318
rs77282240
All
−0.001
0.000
0.001
MR effect size for
'Interleukin−10 receptor subunit beta' on 'eGFR'

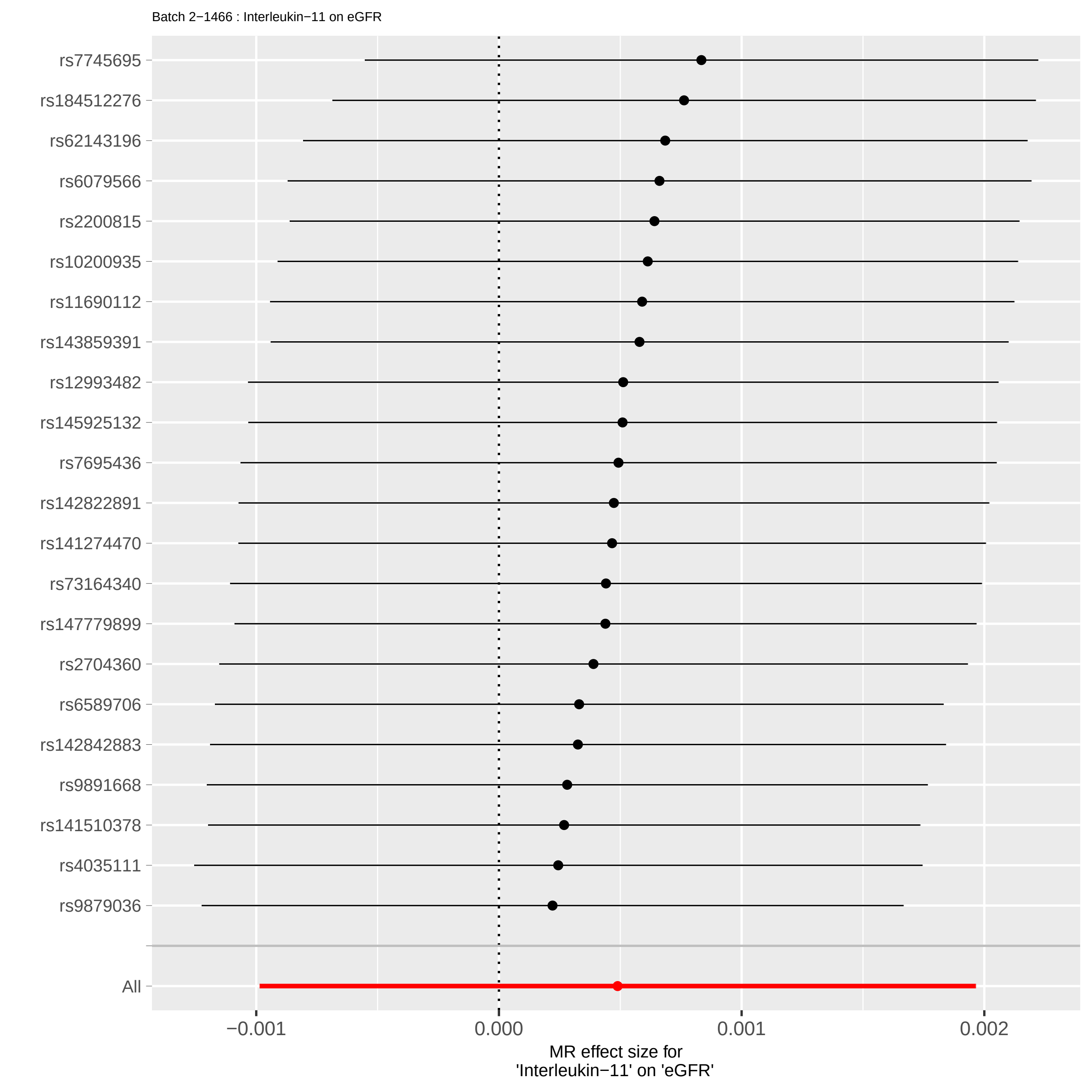
Batch 2−1466 : Interleukin−11 on eGFR
rs7745695
rs184512276
rs62143196
rs6079566
rs2200815
rs10200935
rs11690112
rs143859391
rs12993482
rs145925132
rs7695436
rs142822891
rs141274470
rs73164340
rs147779899
rs2704360
rs6589706
rs142842883
rs9891668
rs141510378
rs4035111
rs9879036
All
−0.001
0.000
0.001
0.002
MR effect size for
'Interleukin−11' on 'eGFR'

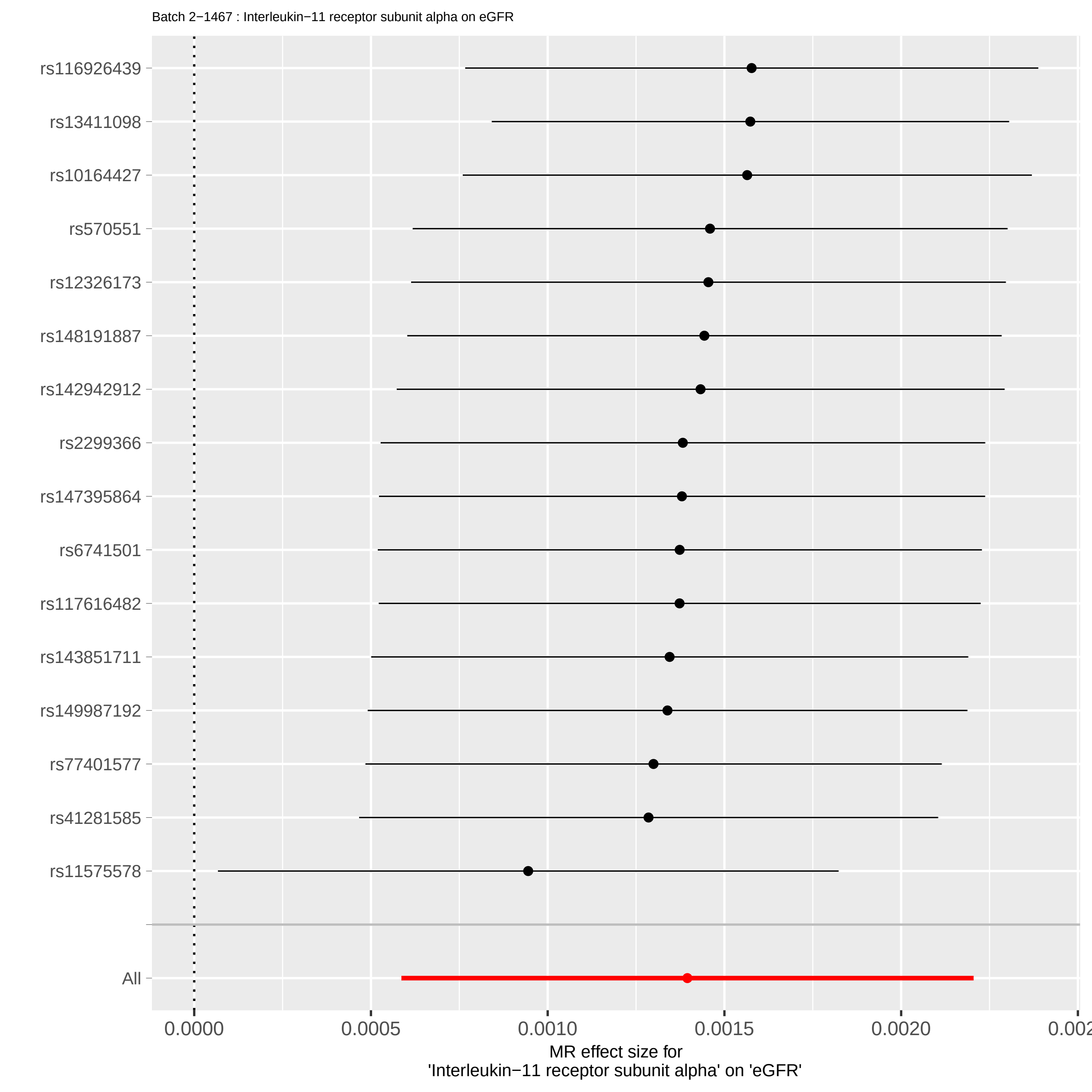

Batch 2−1467 : Interleukin−11 receptor subunit alpha on eGFR
rs116926439
rs13411098
rs10164427
rs570551
rs12326173
rs148191887
rs142942912
rs2299366
rs147395864
rs6741501
rs117616482
rs143851711
rs149987192
rs77401577
rs41281585
rs11575578
All
0.0000
0.0005
0.0010
0.0015
0.0020
MR effect size for
'Interleukin−11 receptor subunit alpha' on 'eGFR'

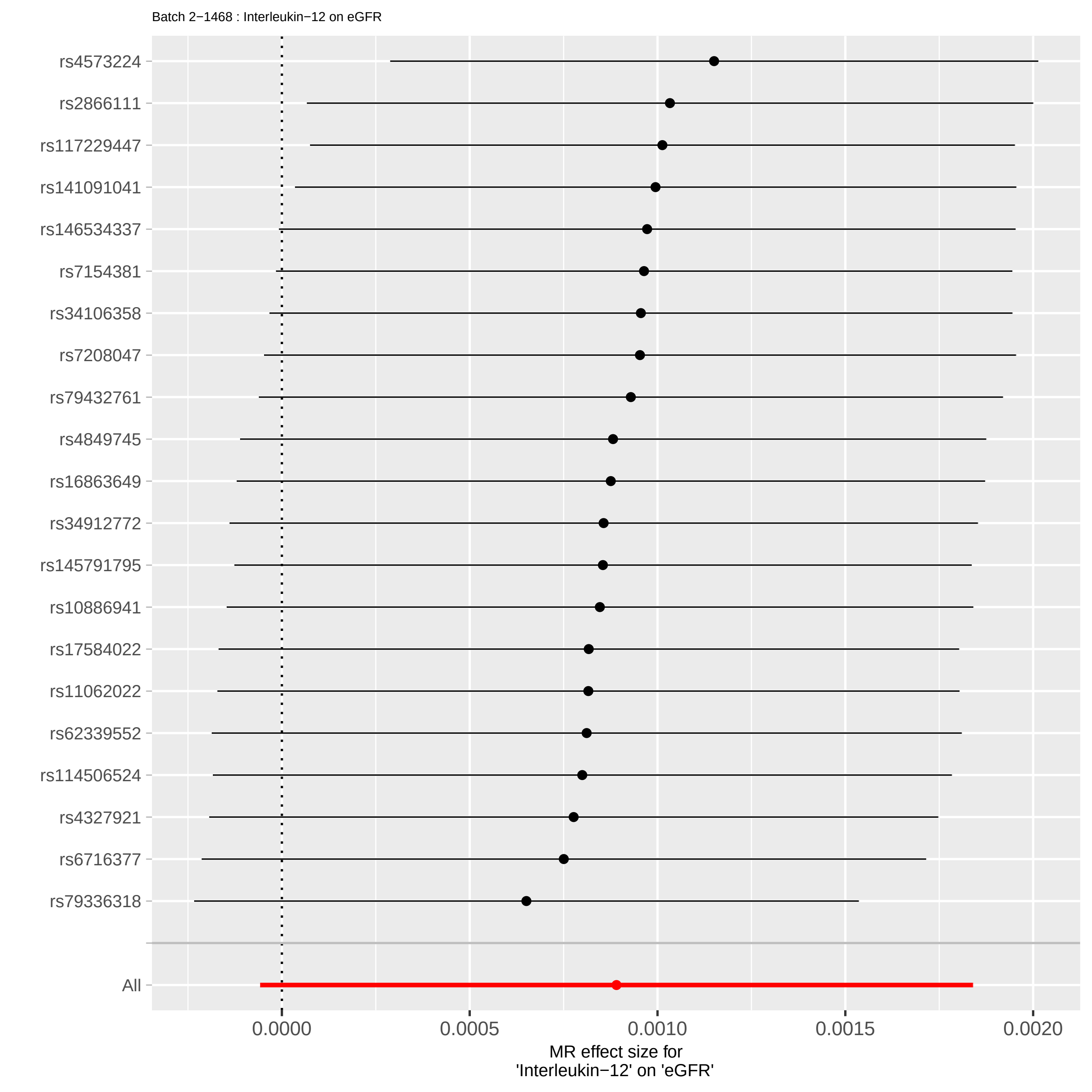
Batch 2−1468 : Interleukin−12 on eGFR
rs4573224
rs2866111
rs117229447
rs141091041
rs146534337
rs7154381
rs34106358
rs7208047
rs79432761
rs4849745
rs16863649
rs34912772
rs145791795
rs10886941
rs17584022
rs11062022
rs62339552
rs114506524
rs4327921
rs6716377
rs79336318
All
0.0000
0.0005
0.0010
0.0015
0.0020
MR effect size for
'Interleukin−12' on 'eGFR'

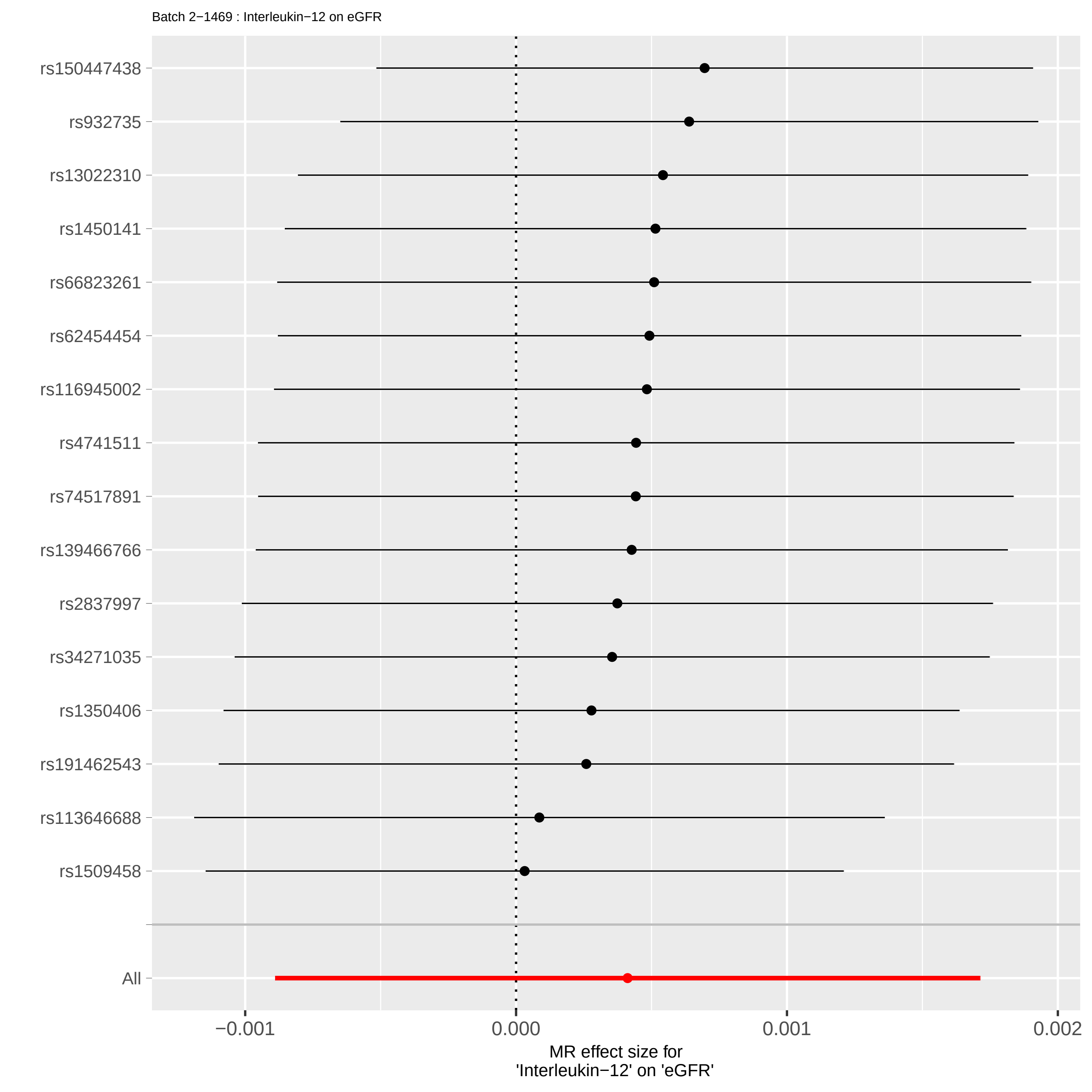

Batch 2−1469 : Interleukin−12 on eGFR
rs150447438
rs932735
rs13022310
rs1450141
rs66823261
rs62454454
rs116945002
rs4741511
rs74517891
rs139466766
rs2837997
rs34271035
rs1350406
rs191462543
rs113646688
rs1509458
All
−0.001
0.000
0.001
0.002
MR effect size for
'Interleukin−12' on 'eGFR'

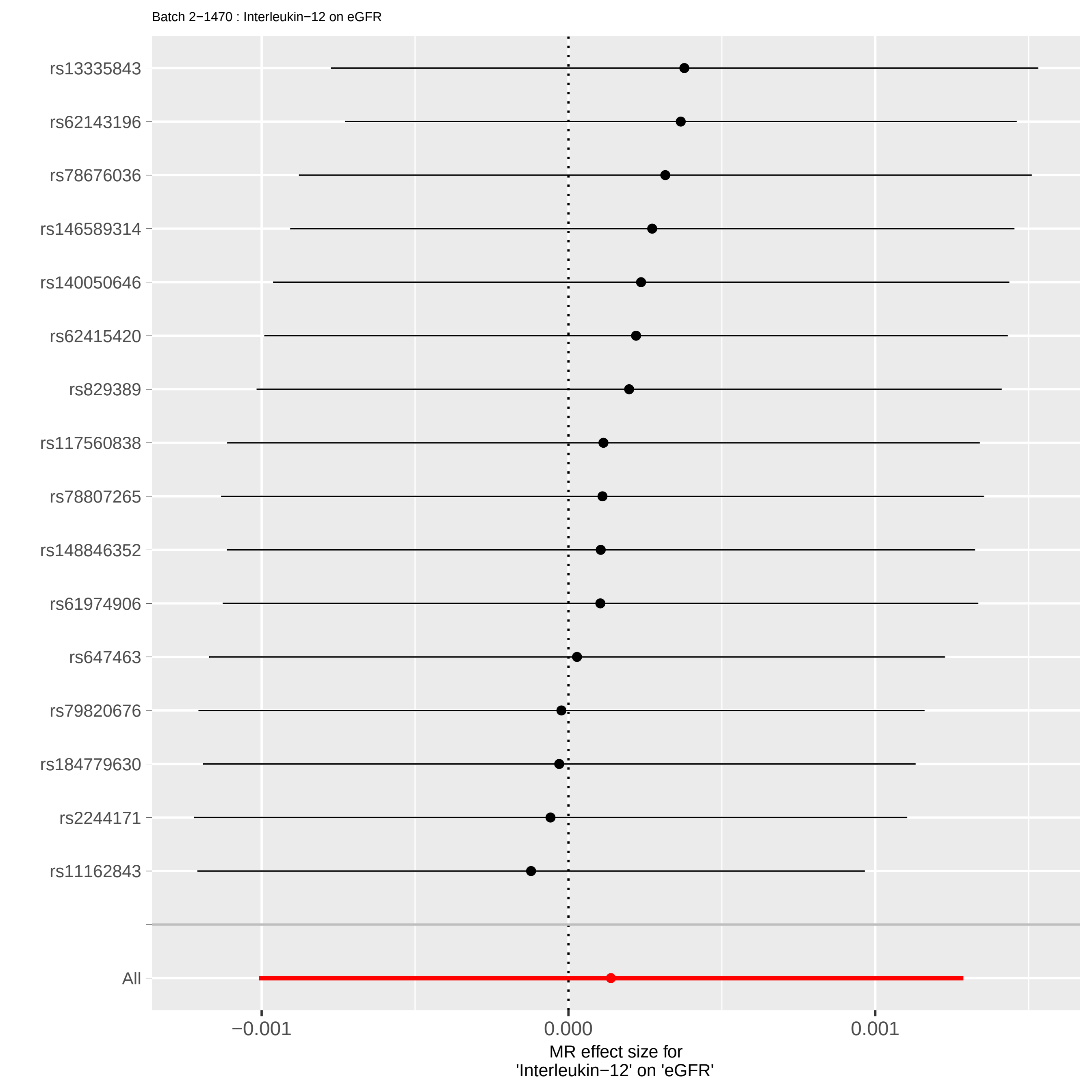

Batch 2−1470 : Interleukin−12 on eGFR
rs13335843
rs62143196
rs78676036
rs146589314
rs140050646
rs62415420
rs829389
rs117560838
rs78807265
rs148846352
rs61974906
rs647463
rs79820676
rs184779630
rs2244171
rs11162843
All
−0.001
0.000
0.001
MR effect size for
'Interleukin−12' on 'eGFR'

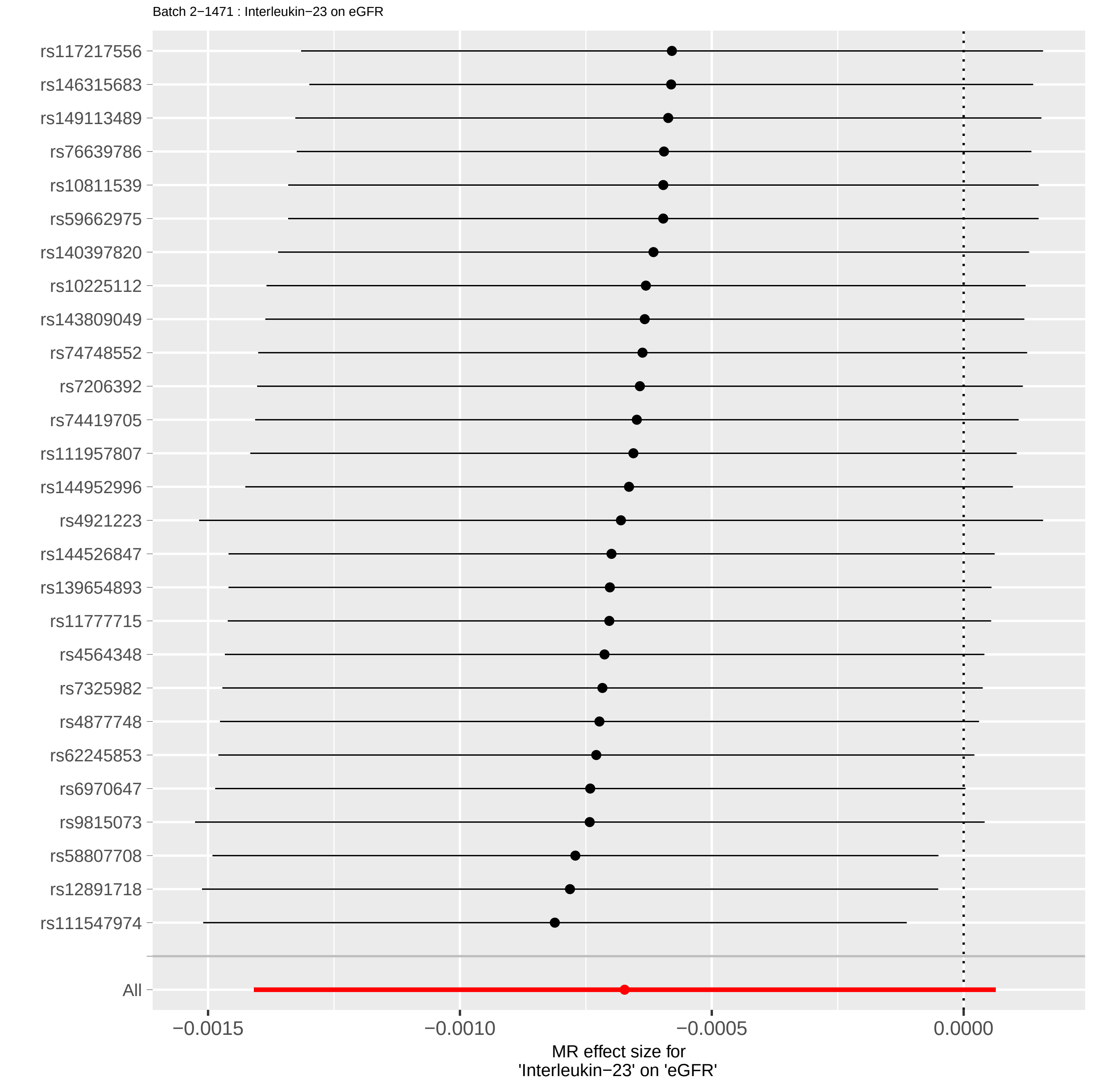

Batch 2−1471 : Interleukin−23 on eGFR
rs117217556
rs146315683
rs149113489
rs76639786
rs10811539
rs59662975
rs140397820
rs10225112
rs143809049
rs74748552
rs7206392
rs74419705
rs111957807
rs144952996
rs4921223
rs144526847
rs139654893
rs11777715
rs4564348
rs7325982
rs4877748
rs62245853
rs6970647
rs9815073
rs58807708
rs12891718
rs111547974
All
−0.0015
−0.0010
−0.0005
0.0000
MR effect size for
'Interleukin−23' on 'eGFR'

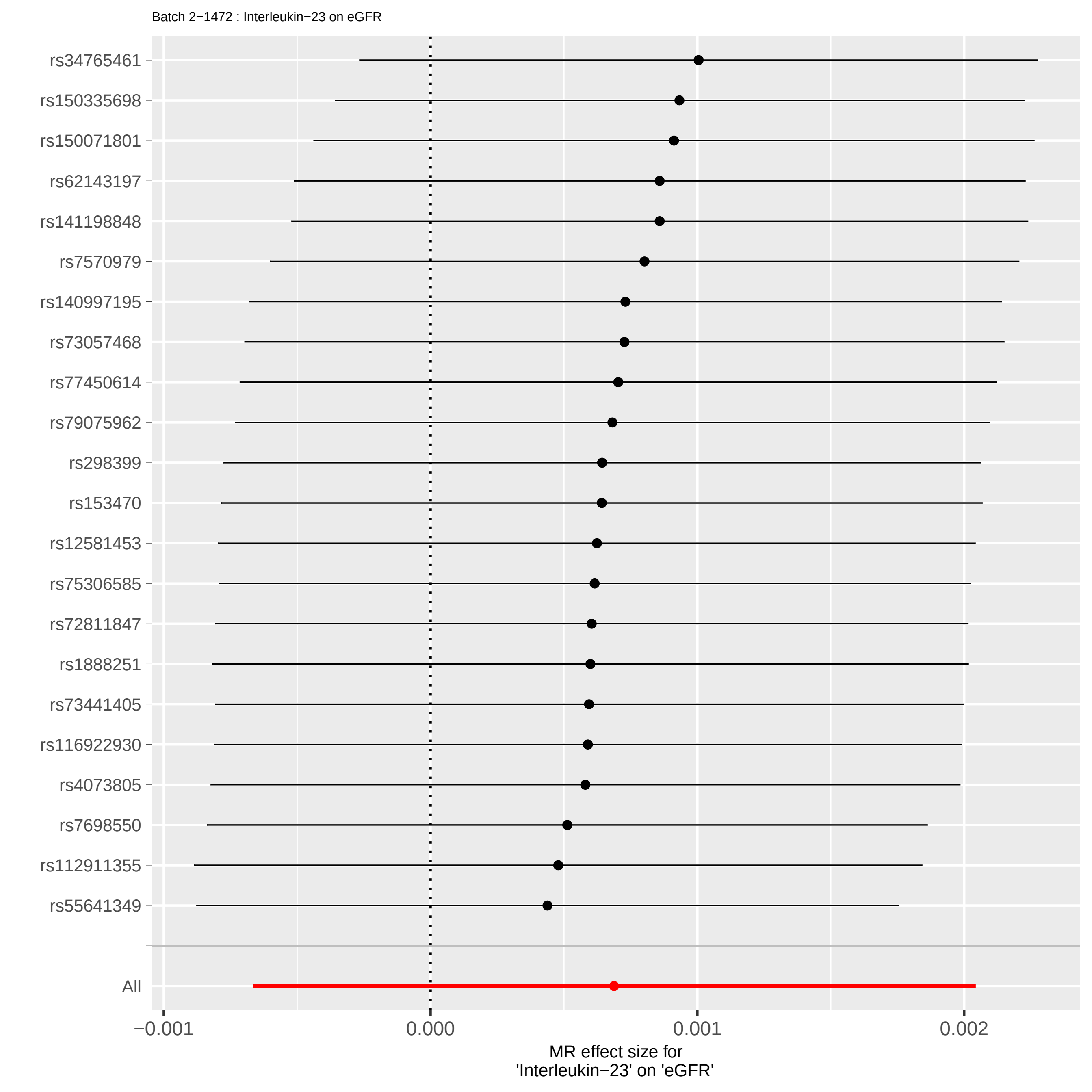

Batch 2−1472 : Interleukin−23 on eGFR
rs34765461
rs150335698
rs150071801
rs62143197
rs141198848
rs7570979
rs140997195
rs73057468
rs77450614
rs79075962
rs298399
rs153470
rs12581453
rs75306585
rs72811847
rs1888251
rs73441405
rs116922930
rs4073805
rs7698550
rs112911355
rs55641349
All
−0.001
0.000
0.001
0.002
MR effect size for
'Interleukin−23' on 'eGFR'

Batch 2−1473 : Interleukin−12 receptor subunit beta−1 on eGFR

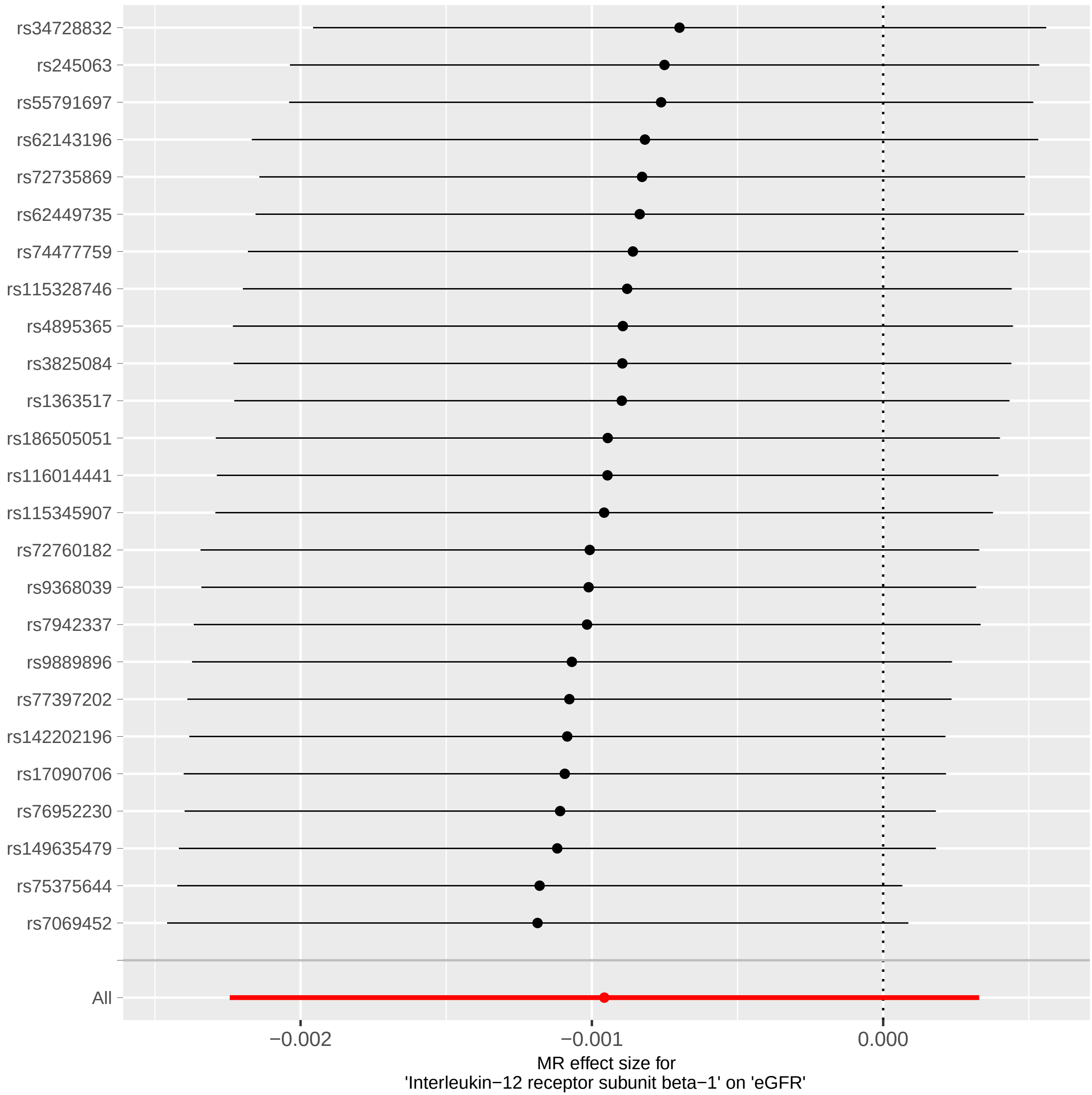

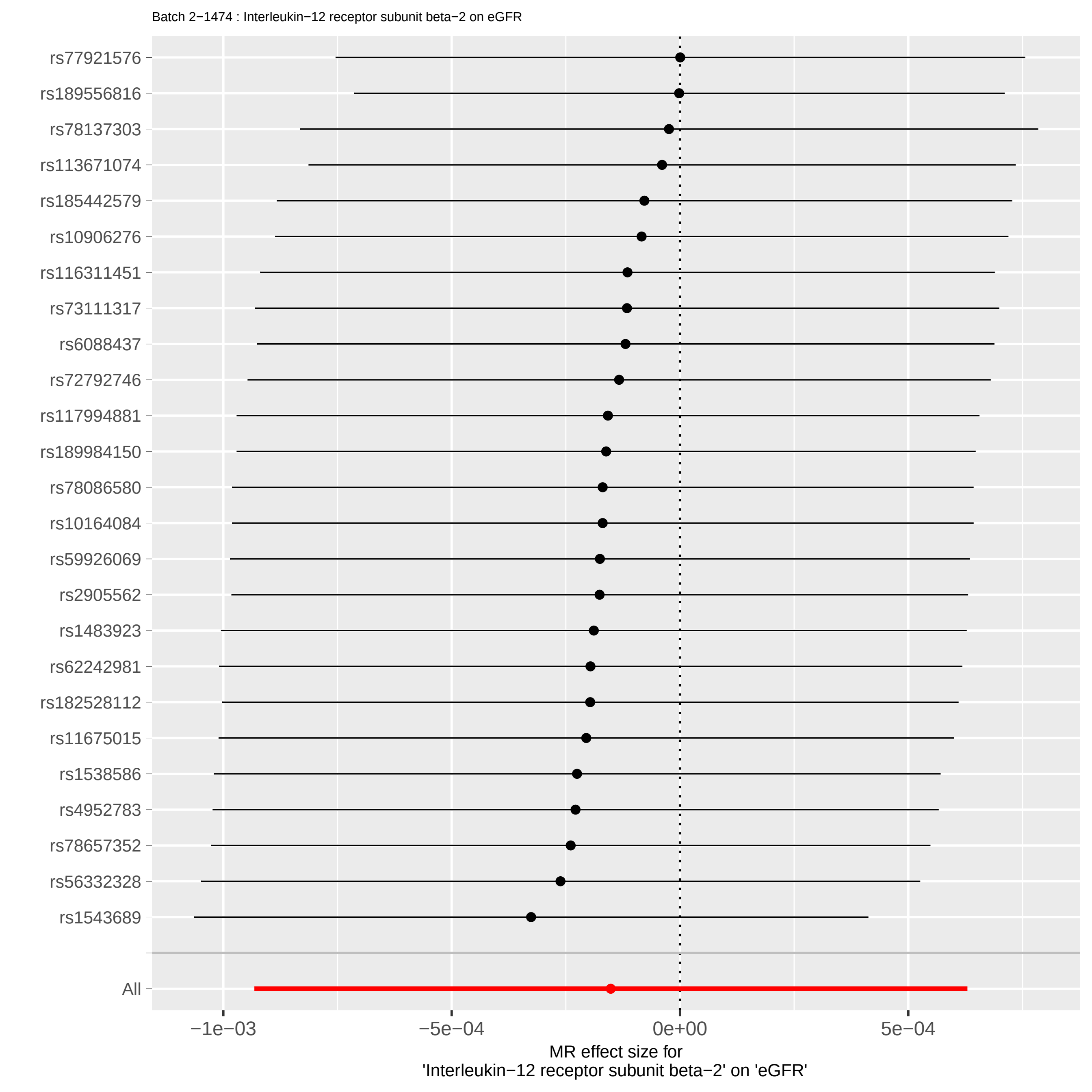

Batch 2−1474 : Interleukin−12 receptor subunit beta−2 on eGFR
rs77921576
rs189556816
rs78137303
rs113671074
rs185442579
rs10906276
rs116311451
rs73111317
rs6088437
rs72792746
rs117994881
rs189984150
rs78086580
rs10164084
rs59926069
rs2905562
rs1483923
rs62242981
rs182528112
rs11675015
rs1538586
rs4952783
rs78657352
rs56332328
rs1543689
All
−1e−03
−5e−04
0e+00
5e−04
MR effect size for
'Interleukin−12 receptor subunit beta−2' on 'eGFR'

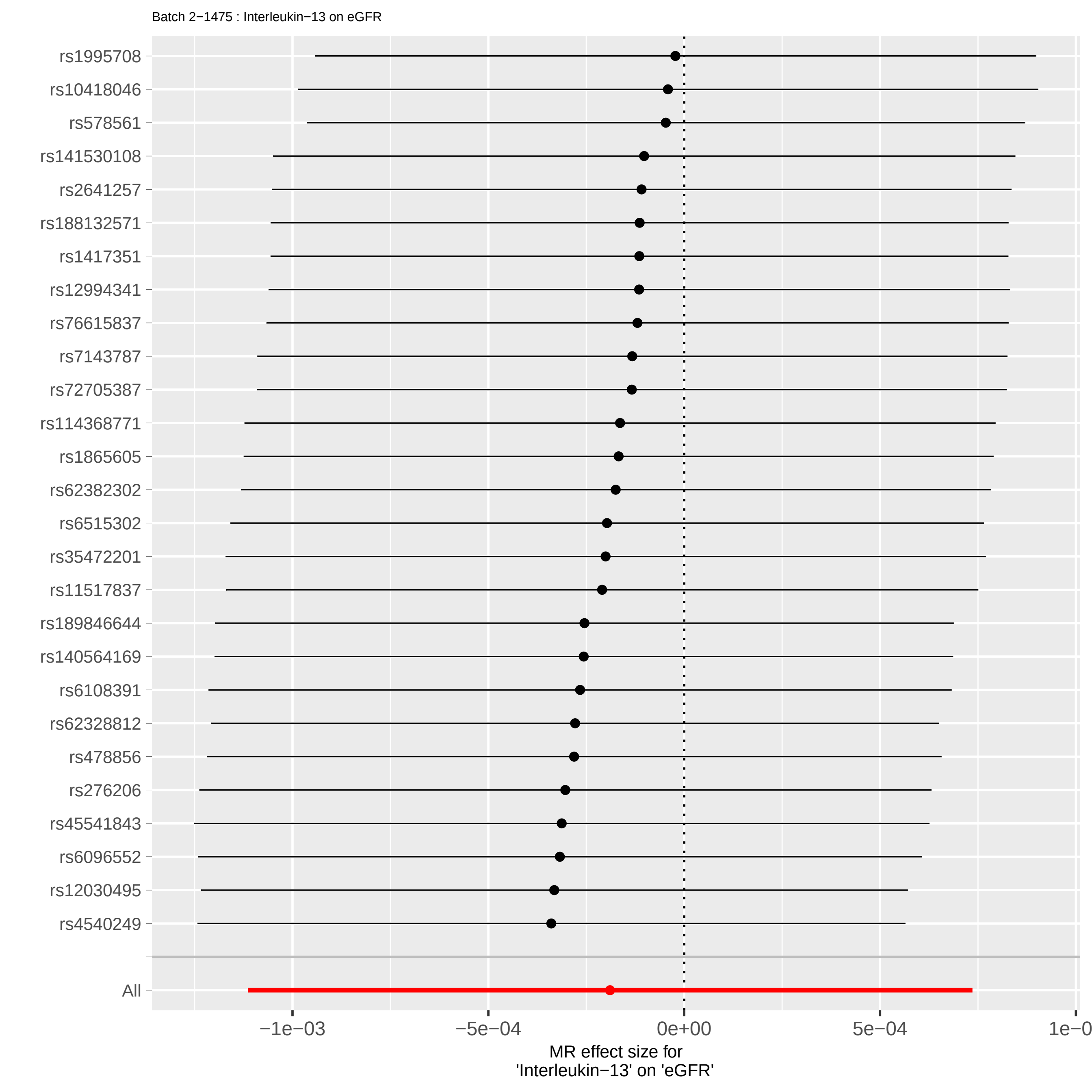

Batch 2−1475 : Interleukin−13 on eGFR
rs1995708
rs10418046
rs578561
rs141530108
rs2641257
rs188132571
rs1417351
rs12994341
rs76615837
rs7143787
rs72705387
rs114368771
rs1865605
rs62382302
rs6515302
rs35472201
rs11517837
rs189846644
rs140564169
rs6108391
rs62328812
rs478856
rs276206
rs45541843
rs6096552
rs12030495
rs4540249
All
−1e−03
−5e−04
0e+00
5e−04
1e−0
MR effect size for
'Interleukin−13' on 'eGFR'

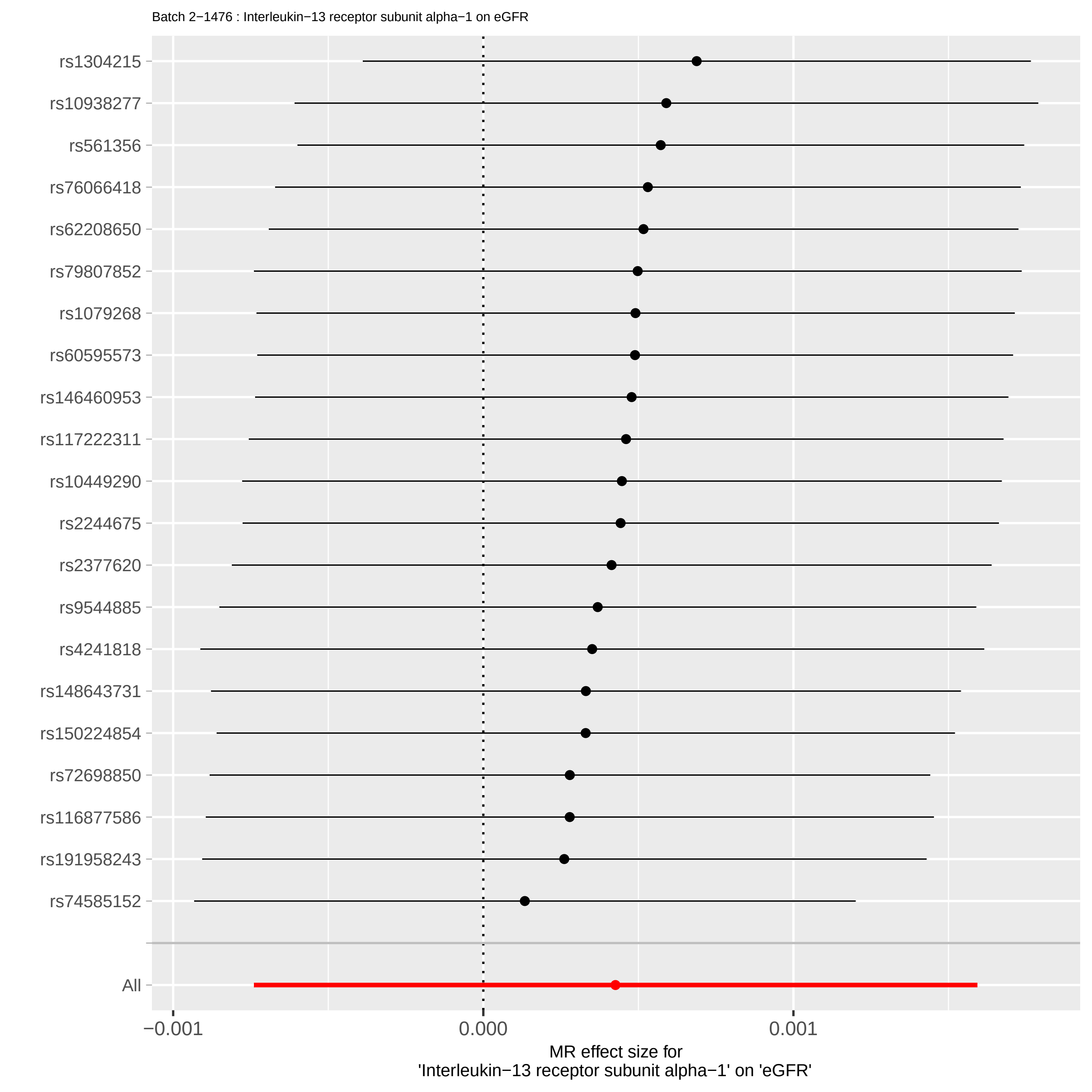

Batch 2−1476 : Interleukin−13 receptor subunit alpha−1 on eGFR
rs1304215
rs10938277
rs561356
rs76066418
rs62208650
rs79807852
rs1079268
rs60595573
rs146460953
rs117222311
rs10449290
rs2244675
rs2377620
rs9544885
rs4241818
rs148643731
rs150224854
rs72698850
rs116877586
rs191958243
rs74585152
All
−0.001
0.000
0.001
MR effect size for
'Interleukin−13 receptor subunit alpha−1' on 'eGFR'

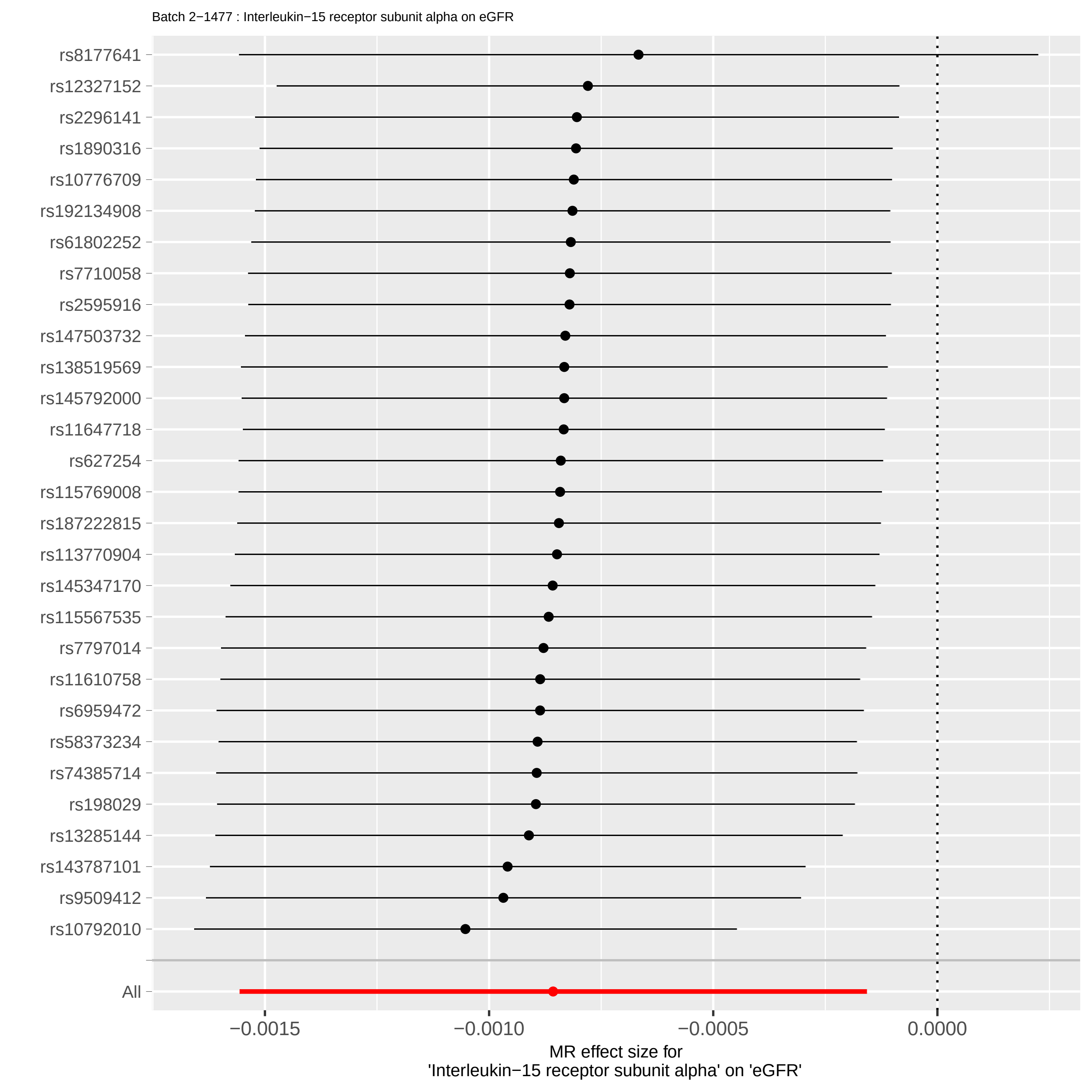

Batch 2−1477 : Interleukin−15 receptor subunit alpha on eGFR
rs8177641
rs12327152
rs2296141
rs1890316
rs10776709
rs192134908
rs61802252
rs7710058
rs2595916
rs147503732
rs138519569
rs145792000
rs11647718
rs627254
rs115769008
rs187222815
rs113770904
rs145347170
rs115567535
rs7797014
rs11610758
rs6959472
rs58373234
rs74385714
rs198029
rs13285144
rs143787101
rs9509412
rs10792010
All
−0.0015
−0.0010
−0.0005
0.0000
MR effect size for
'Interleukin−15 receptor subunit alpha' on 'eGFR'

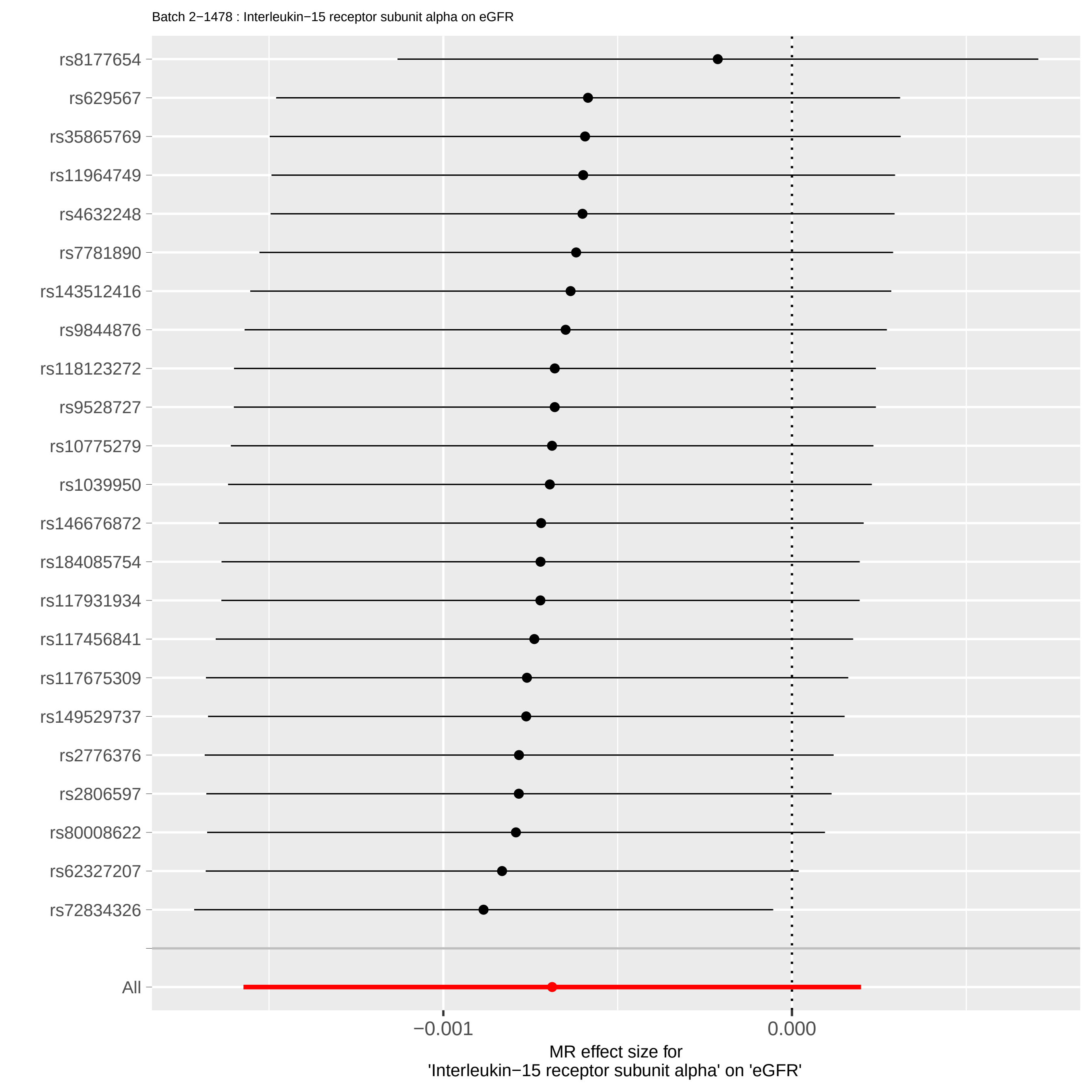
Batch 2−1478 : Interleukin−15 receptor subunit alpha on eGFR
rs8177654
rs629567
rs35865769
rs11964749
rs4632248
rs7781890
rs143512416
rs9844876
rs118123272
rs9528727
rs10775279
rs1039950
rs146676872
rs184085754
rs117931934
rs117456841
rs117675309
rs149529737
rs2776376
rs2806597
rs80008622
rs62327207
rs72834326
All
−0.001
0.000
MR effect size for
'Interleukin−15 receptor subunit alpha' on 'eGFR'

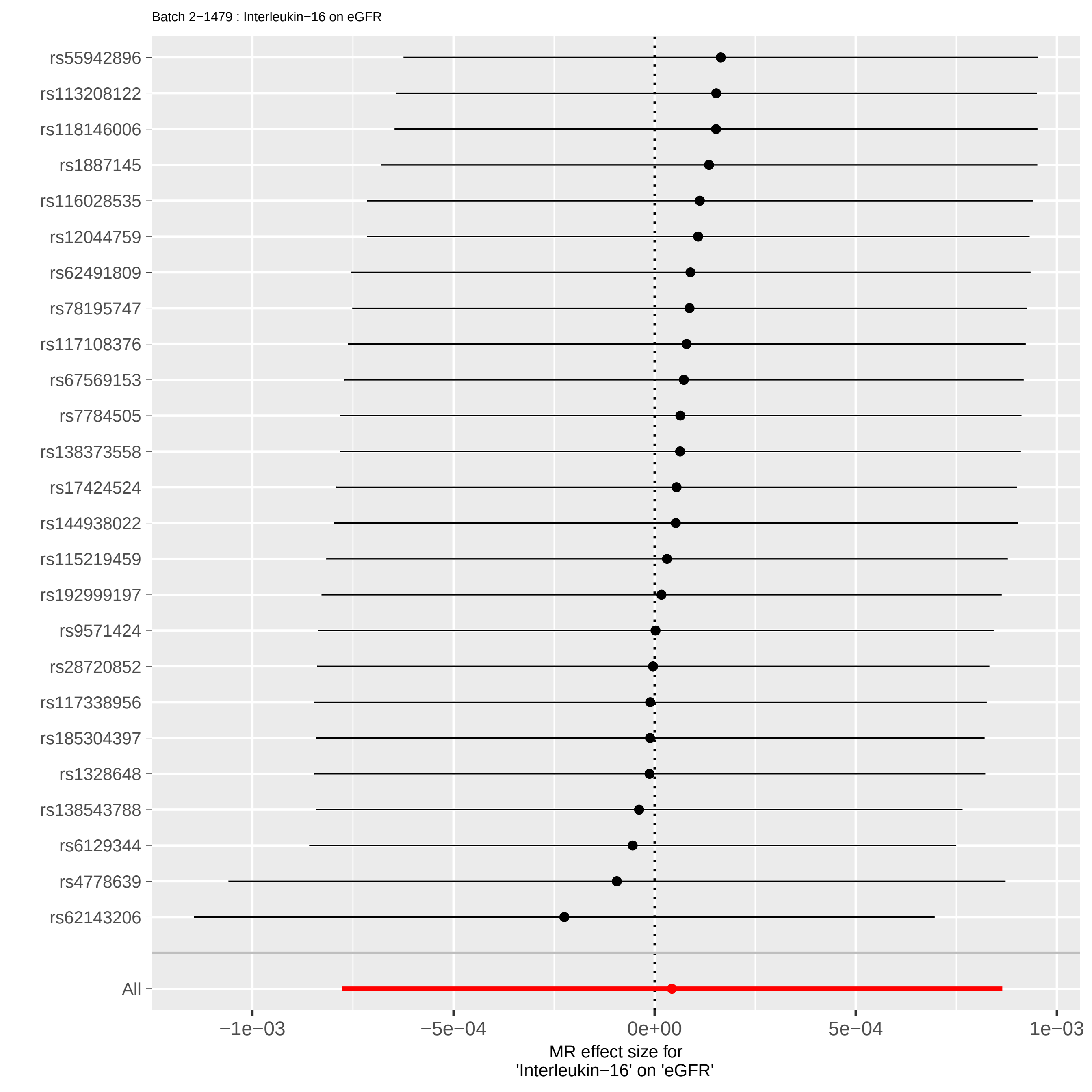

Batch 2−1479 : Interleukin−16 on eGFR
rs55942896
rs113208122
rs118146006
rs1887145
rs116028535
rs12044759
rs62491809
rs78195747
rs117108376
rs67569153
rs7784505
rs138373558
rs17424524
rs144938022
rs115219459
rs192999197
rs9571424
rs28720852
rs117338956
rs185304397
rs1328648
rs138543788
rs6129344
rs4778639
rs62143206
All
−1e−03
−5e−04
0e+00
5e−04
1e−03
MR effect size for
'Interleukin−16' on 'eGFR'

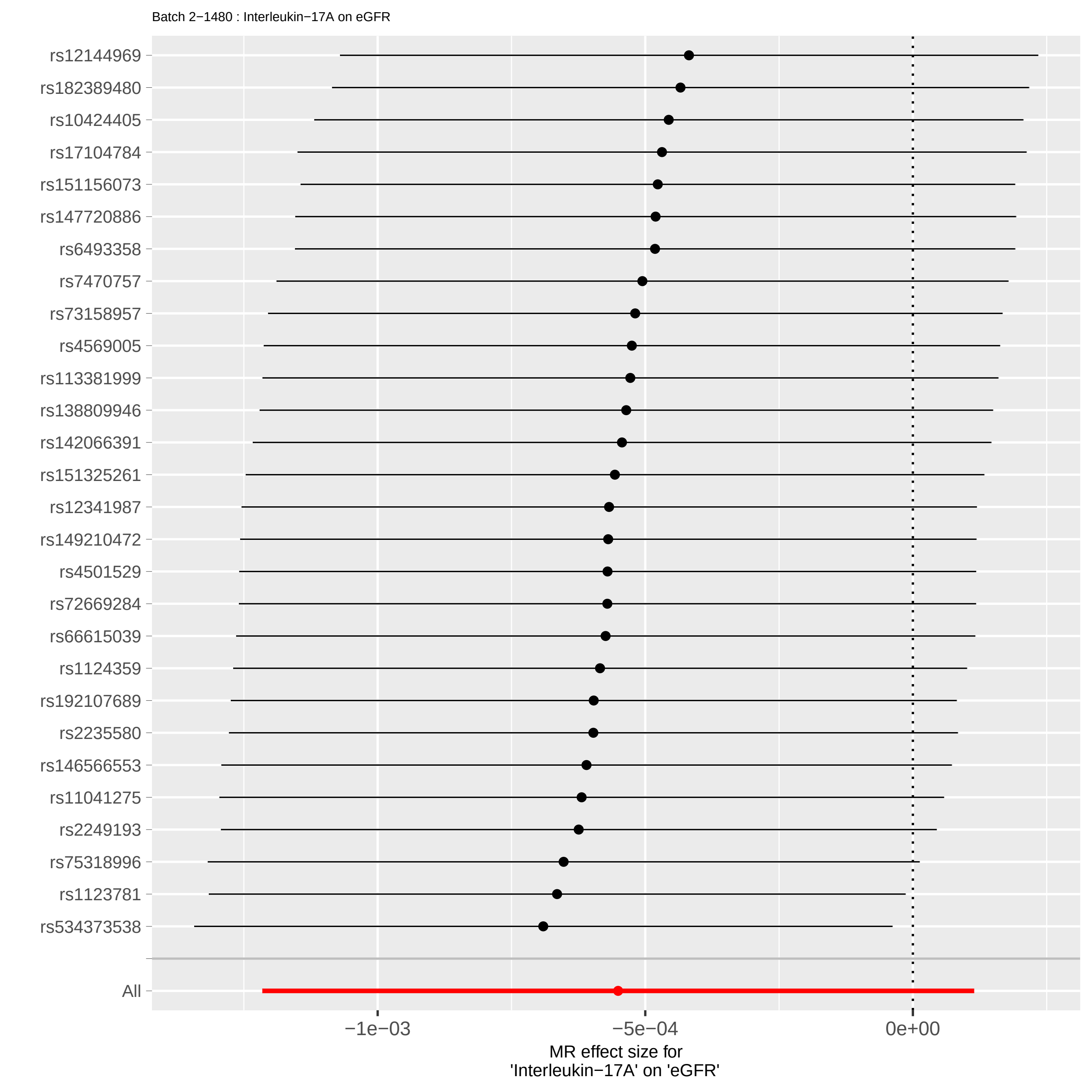

Batch 2−1480 : Interleukin−17A on eGFR
rs12144969
rs182389480
rs10424405
rs17104784
rs151156073
rs147720886
rs6493358
rs7470757
rs73158957
rs4569005
rs113381999
rs138809946
rs142066391
rs151325261
rs12341987
rs149210472
rs4501529
rs72669284
rs66615039
rs1124359
rs192107689
rs2235580
rs146566553
rs11041275
rs2249193
rs75318996
rs1123781
rs534373538
All
−1e−03
−5e−04
0e+00
MR effect size for
'Interleukin−17A' on 'eGFR'

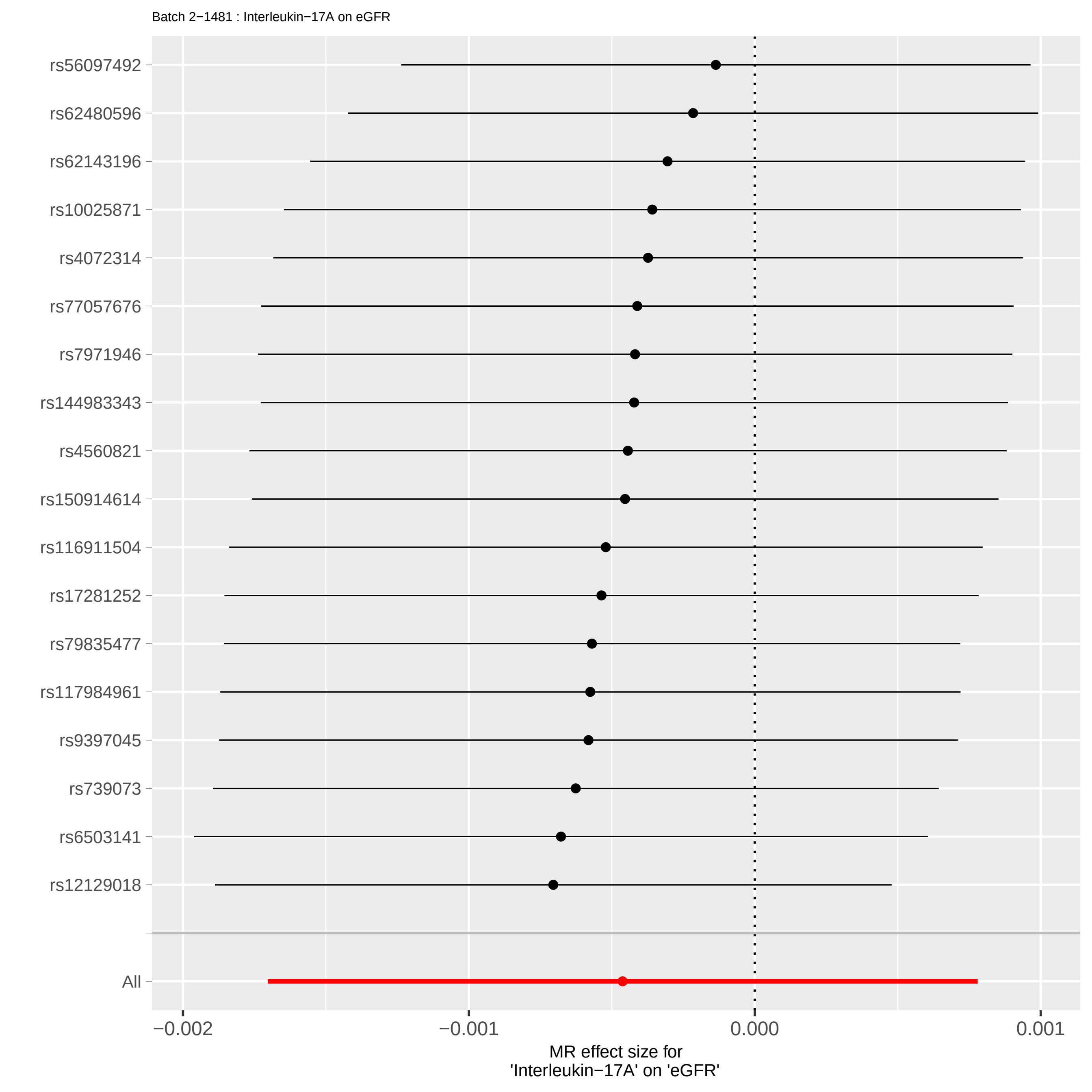

Batch 2−1481 : Interleukin−17A on eGFR
rs56097492
rs62480596
rs62143196
rs10025871
rs4072314
rs77057676
rs7971946
rs144983343
rs4560821
rs150914614
rs116911504
rs17281252
rs79835477
rs117984961
rs9397045
rs739073
rs6503141
rs12129018
All
−0.002
−0.001
0.000
0.001
MR effect size for
'Interleukin−17A' on 'eGFR'

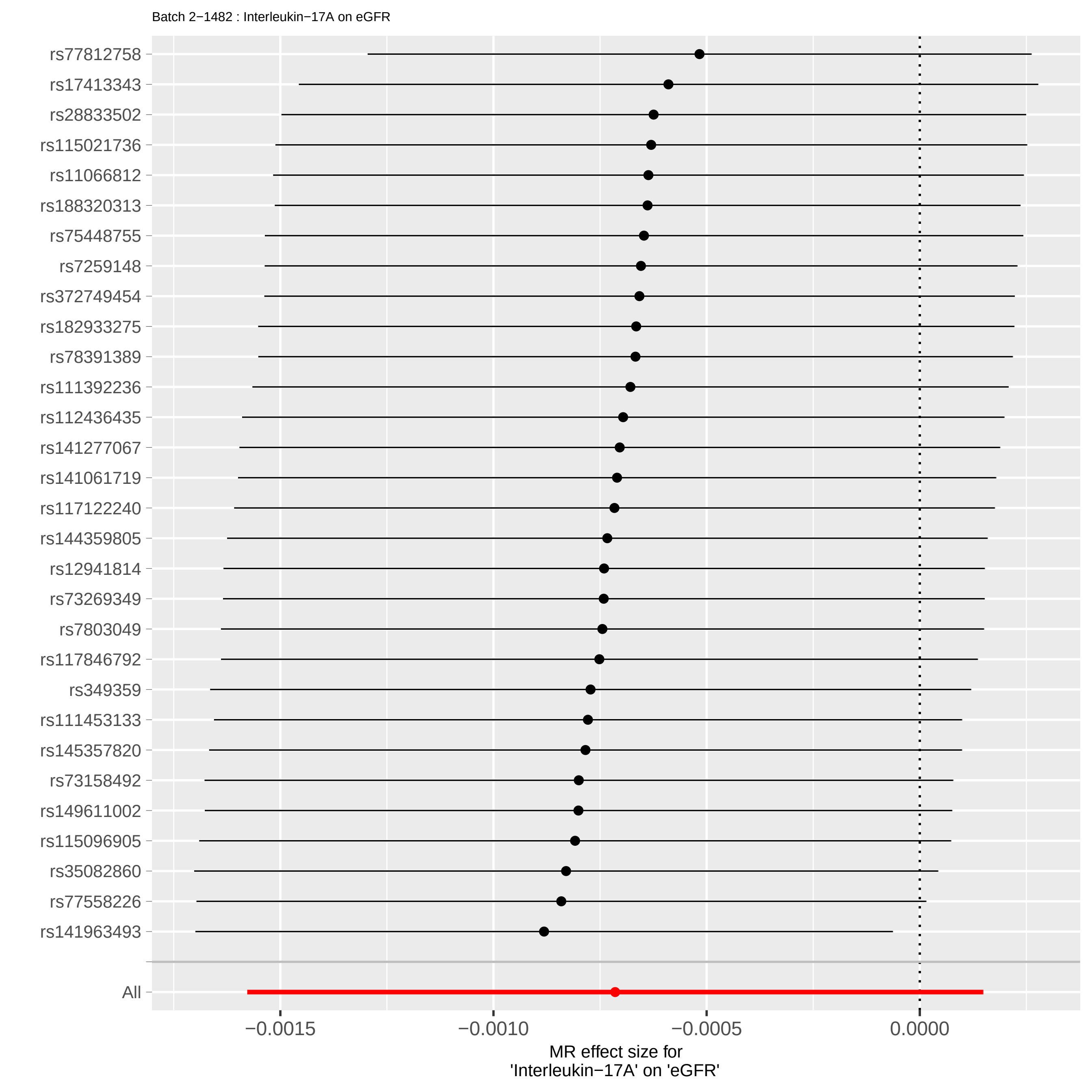

Batch 2−1482 : Interleukin−17A on eGFR
rs77812758
rs17413343
rs28833502
rs115021736
rs11066812
rs188320313
rs75448755
rs7259148
rs372749454
rs182933275
rs78391389
rs111392236
rs112436435
rs141277067
rs141061719
rs117122240
rs144359805
rs12941814
rs73269349
rs7803049
rs117846792
rs349359
rs111453133
rs145357820
rs73158492
rs149611002
rs115096905
rs35082860
rs77558226
rs141963493
All
−0.0015
−0.0010
−0.0005
0.0000
MR effect size for
'Interleukin−17A' on 'eGFR'

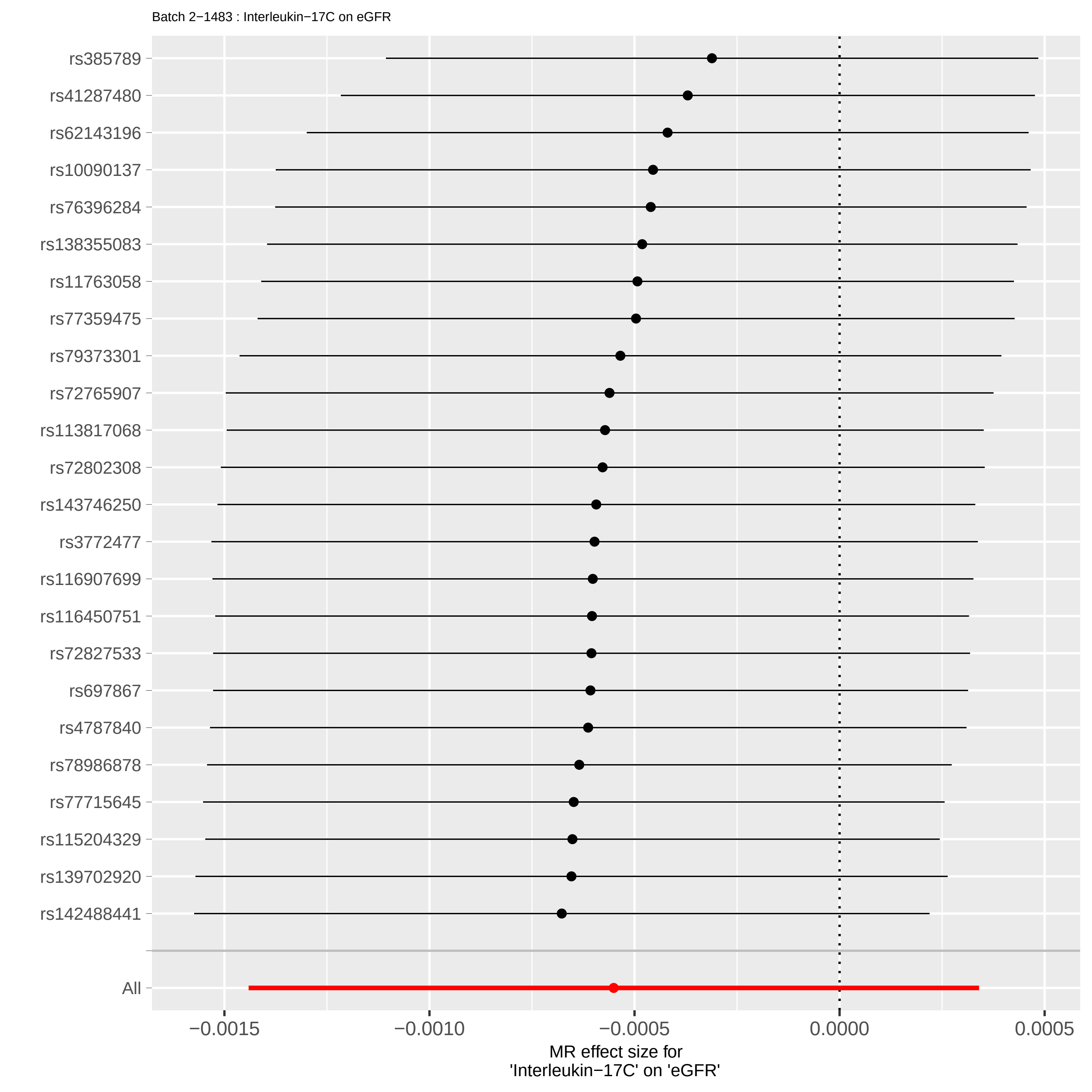

Batch 2−1483 : Interleukin−17C on eGFR
rs385789
rs41287480
rs62143196
rs10090137
rs76396284
rs138355083
rs11763058
rs77359475
rs79373301
rs72765907
rs113817068
rs72802308
rs143746250
rs3772477
rs116907699
rs116450751
rs72827533
rs697867
rs4787840
rs78986878
rs77715645
rs115204329
rs139702920
rs142488441
All
−0.0015
−0.0010
−0.0005
0.0000
0.0005
MR effect size for
'Interleukin−17C' on 'eGFR'

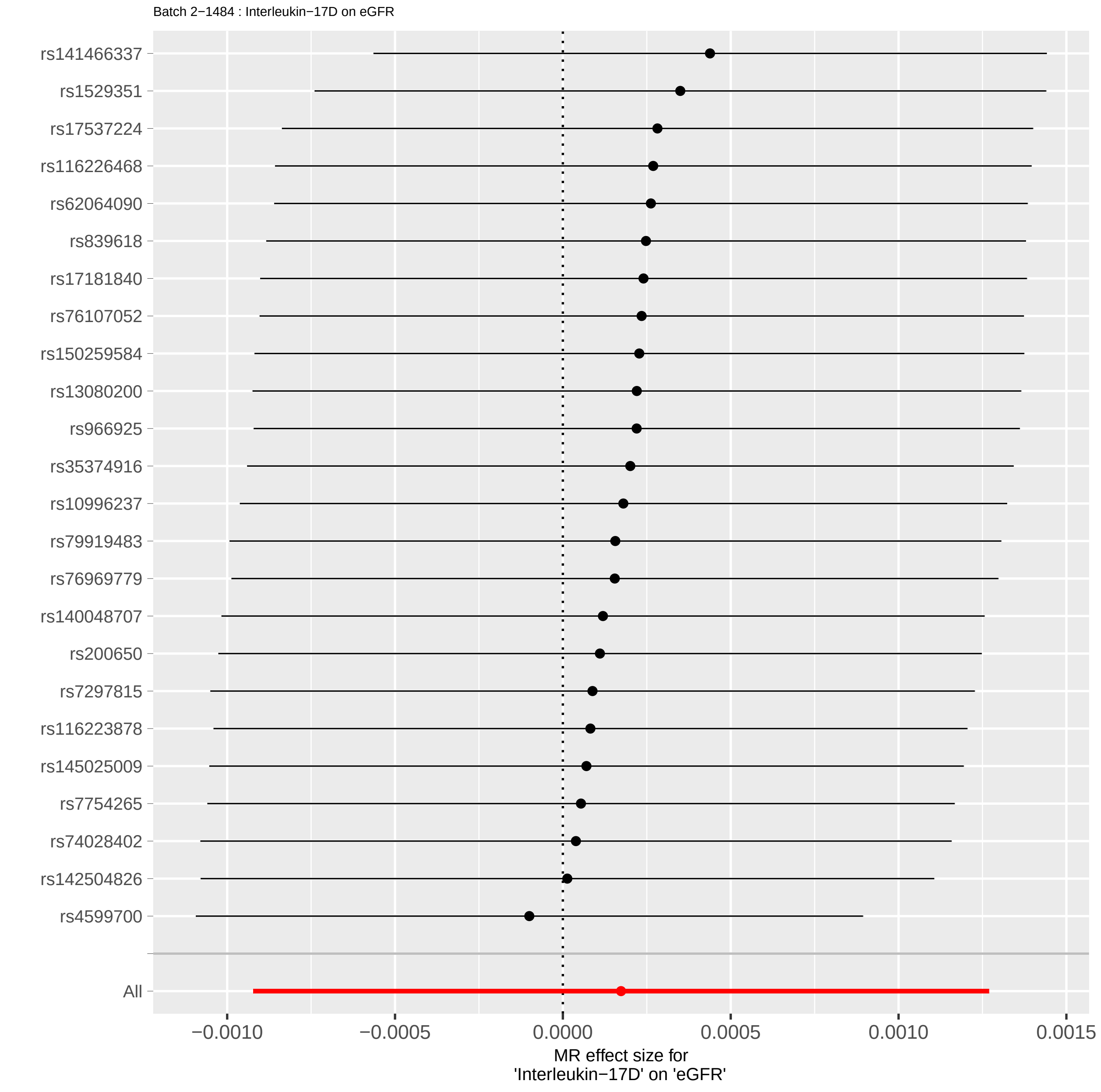
Batch 2−1484 : Interleukin−17D on eGFR
rs141466337
rs1529351
rs17537224
rs116226468
rs62064090
rs839618
rs17181840
rs76107052
rs150259584
rs13080200
rs966925
rs35374916
rs10996237
rs79919483
rs76969779
rs140048707
rs200650
rs7297815
rs116223878
rs145025009
rs7754265
rs74028402
rs142504826
rs4599700
All
−0.0010
−0.0005
0.0000
0.0005
0.0010
0.0015
MR effect size for
'Interleukin−17D' on 'eGFR'

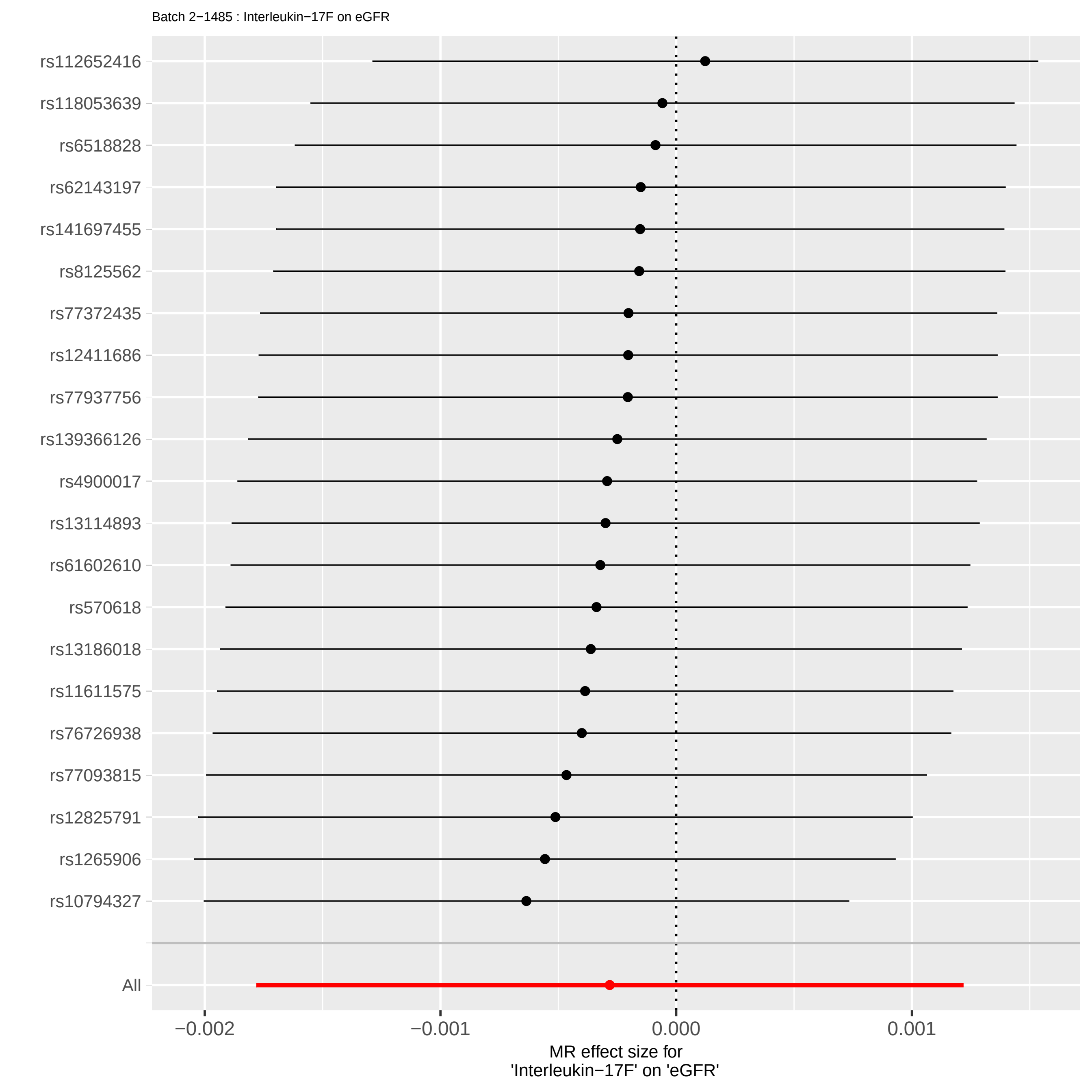

Batch 2−1485 : Interleukin−17F on eGFR
rs112652416
rs118053639
rs6518828
rs62143197
rs141697455
rs8125562
rs77372435
rs12411686
rs77937756
rs139366126
rs4900017
rs13114893
rs61602610
rs570618
rs13186018
rs11611575
rs76726938
rs77093815
rs12825791
rs1265906
rs10794327
All
−0.002
−0.001
0.000
0.001
MR effect size for
'Interleukin−17F' on 'eGFR'

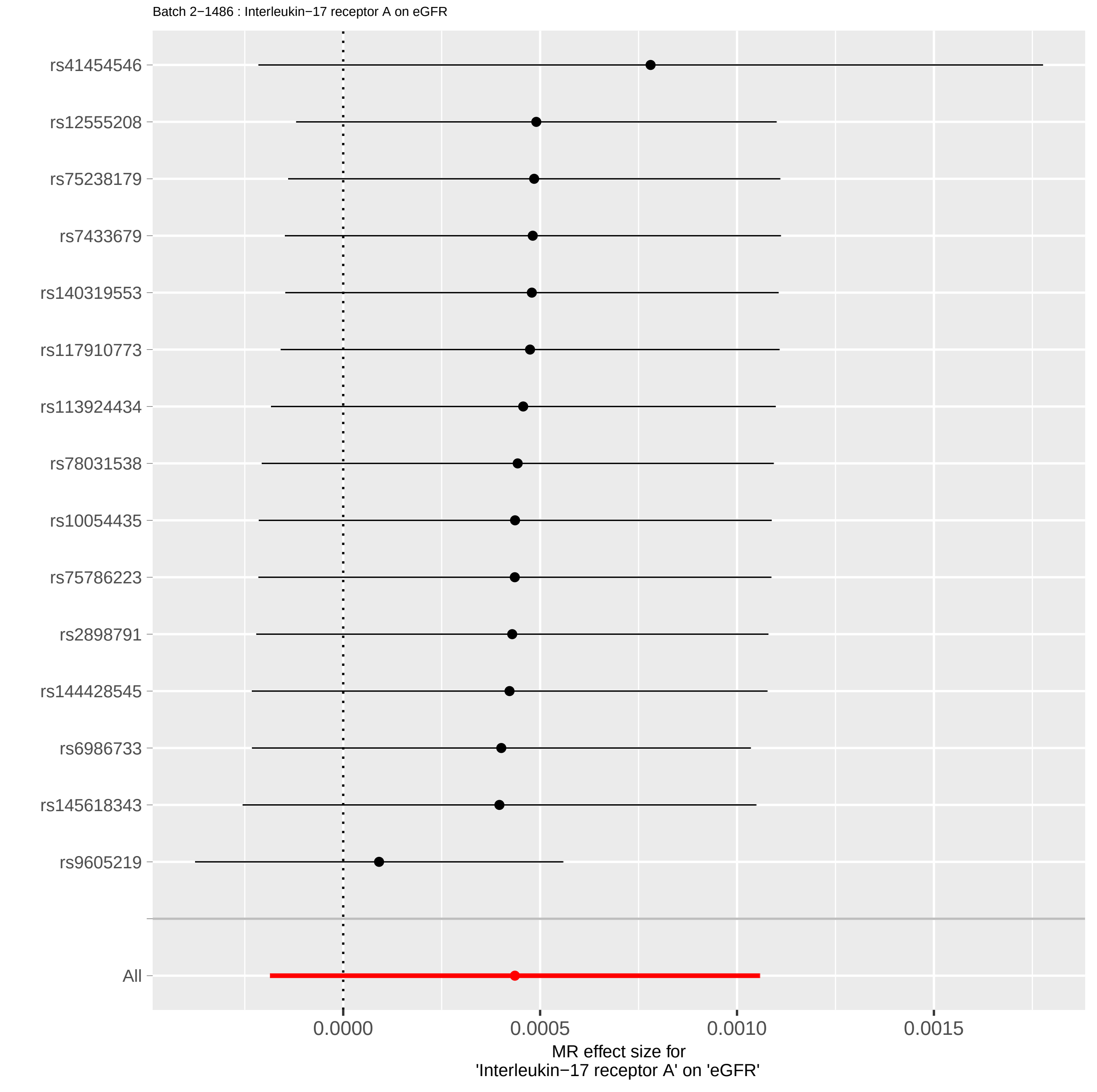
Batch 2−1486 : Interleukin−17 receptor A on eGFR
rs41454546
rs12555208
rs75238179
rs7433679
rs140319553
rs117910773
rs113924434
rs78031538
rs10054435
rs75786223
rs2898791
rs144428545
rs6986733
rs145618343
rs9605219
All
0.0000
0.0005
0.0010
0.0015
MR effect size for
'Interleukin−17 receptor A' on 'eGFR'

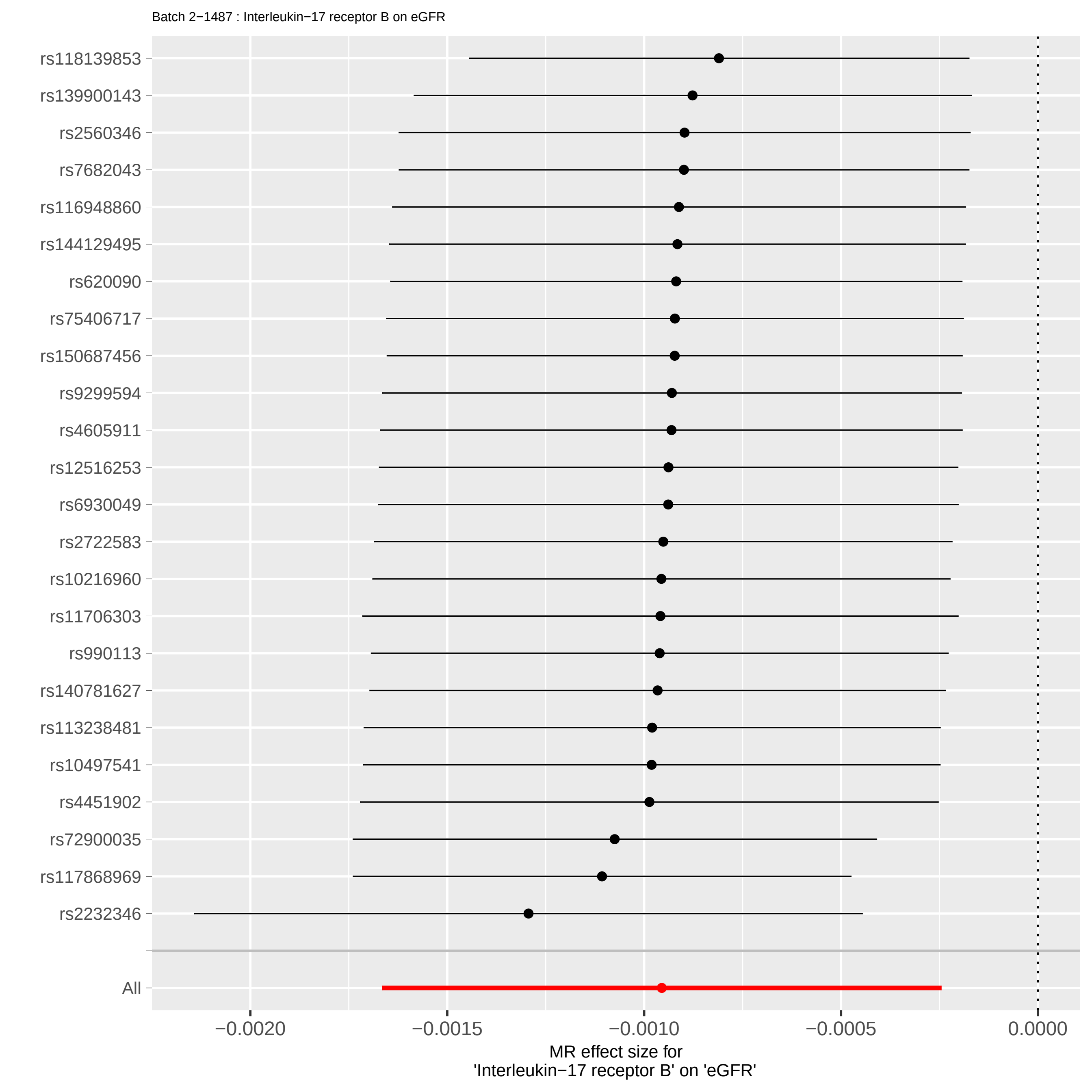

Batch 2−1487 : Interleukin−17 receptor B on eGFR
rs118139853
rs139900143
rs2560346
rs7682043
rs116948860
rs144129495
rs620090
rs75406717
rs150687456
rs9299594
rs4605911
rs12516253
rs6930049
rs2722583
rs10216960
rs11706303
rs990113
rs140781627
rs113238481
rs10497541
rs4451902
rs72900035
rs117868969
rs2232346
All
−0.0020
−0.0015
−0.0010
−0.0005
0.0000
MR effect size for
'Interleukin−17 receptor B' on 'eGFR'

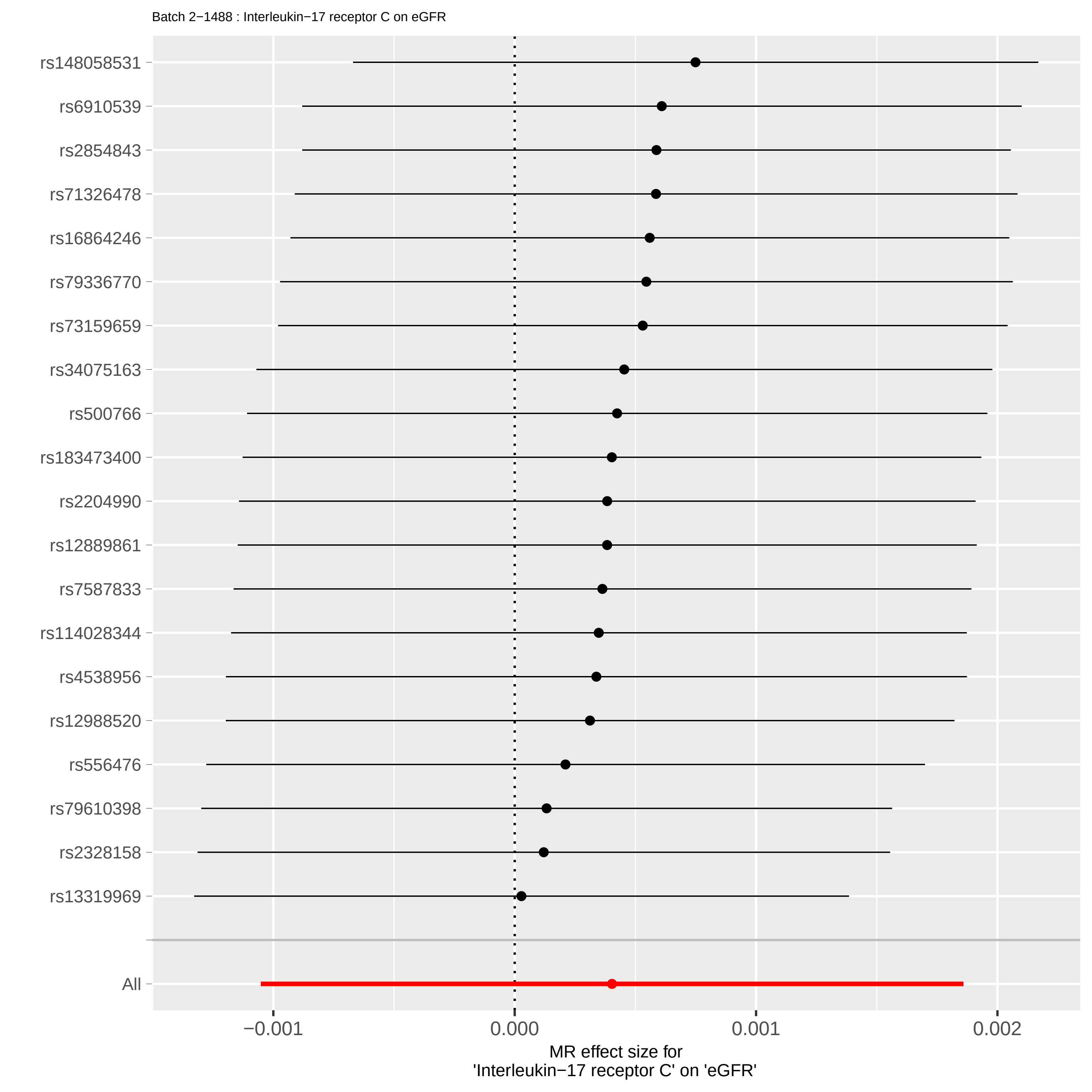

Batch 2−1488 : Interleukin−17 receptor C on eGFR
rs148058531
rs6910539
rs2854843
rs71326478
rs16864246
rs79336770
rs73159659
rs34075163
rs500766
rs183473400
rs2204990
rs12889861
rs7587833
rs114028344
rs4538956
rs12988520
rs556476
rs79610398
rs2328158
rs13319969
All
−0.001
0.000
0.001
0.002
MR effect size for
'Interleukin−17 receptor C' on 'eGFR'

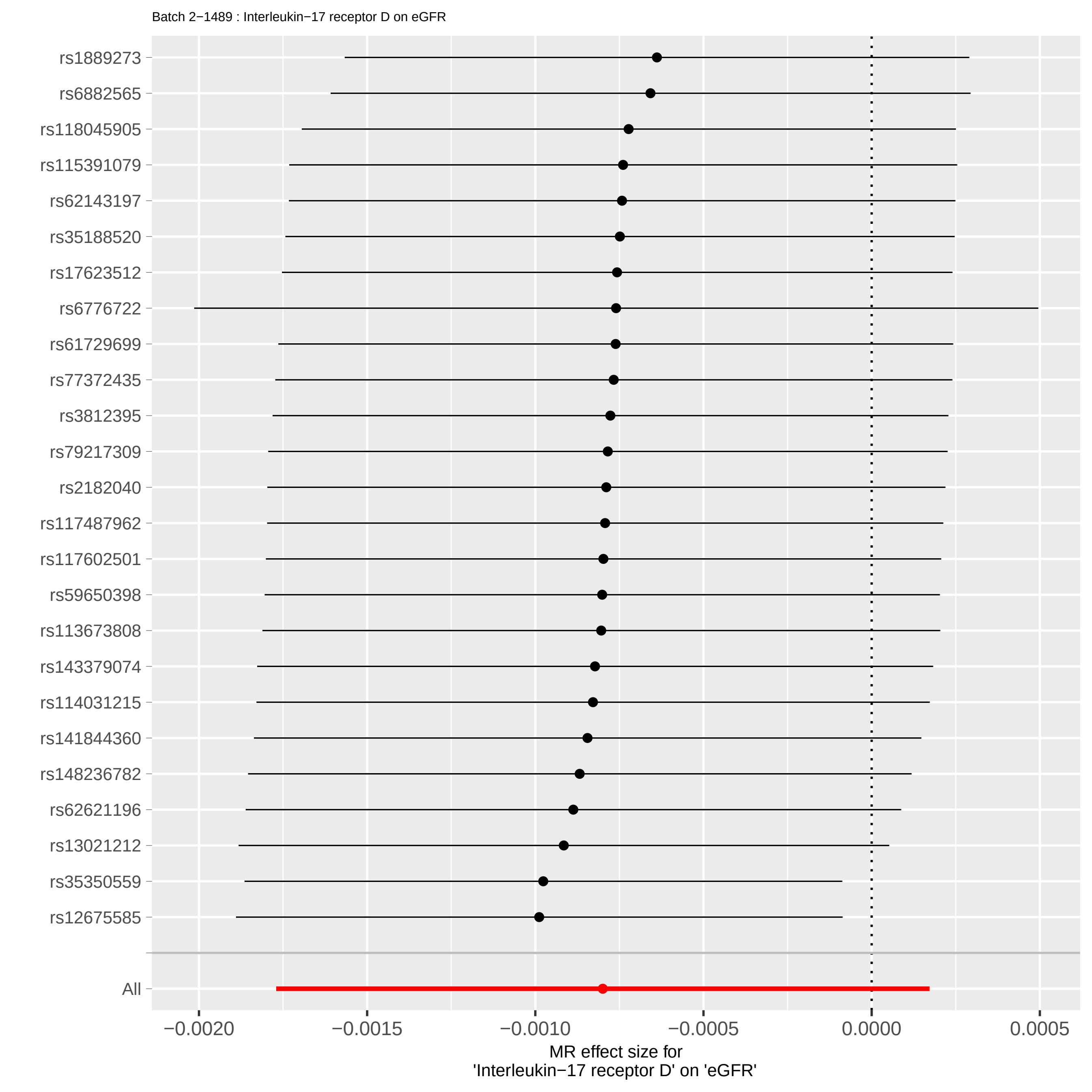
Batch 2−1489 : Interleukin−17 receptor D on eGFR
rs1889273
rs6882565
rs118045905
rs115391079
rs62143197
rs35188520
rs17623512
rs6776722
rs61729699
rs77372435
rs3812395
rs79217309
rs2182040
rs117487962
rs117602501
rs59650398
rs113673808
rs143379074
rs114031215
rs141844360
rs148236782
rs62621196
rs13021212
rs35350559
rs12675585
All
−0.0020
−0.0015
−0.0010
−0.0005
0.0000
0.0005
MR effect size for
'Interleukin−17 receptor D' on 'eGFR'

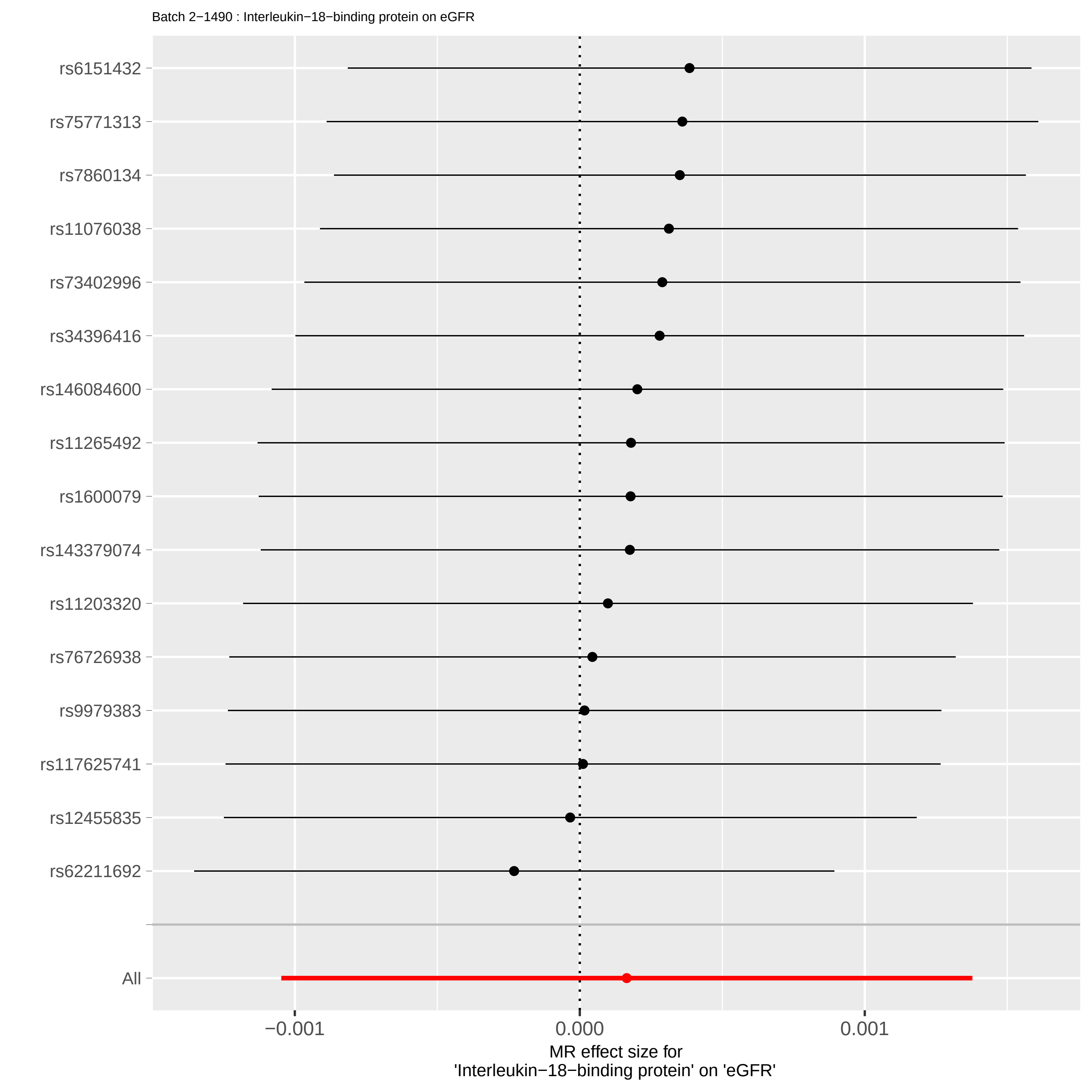
Batch 2−1490 : Interleukin−18−binding protein on eGFR
rs6151432
rs75771313
rs7860134
rs11076038
rs73402996
rs34396416
rs146084600
rs11265492
rs1600079
rs143379074
rs11203320
rs76726938
rs9979383
rs117625741
rs12455835
rs62211692
All
−0.001
0.000
0.001
MR effect size for
'Interleukin−18−binding protein' on 'eGFR'

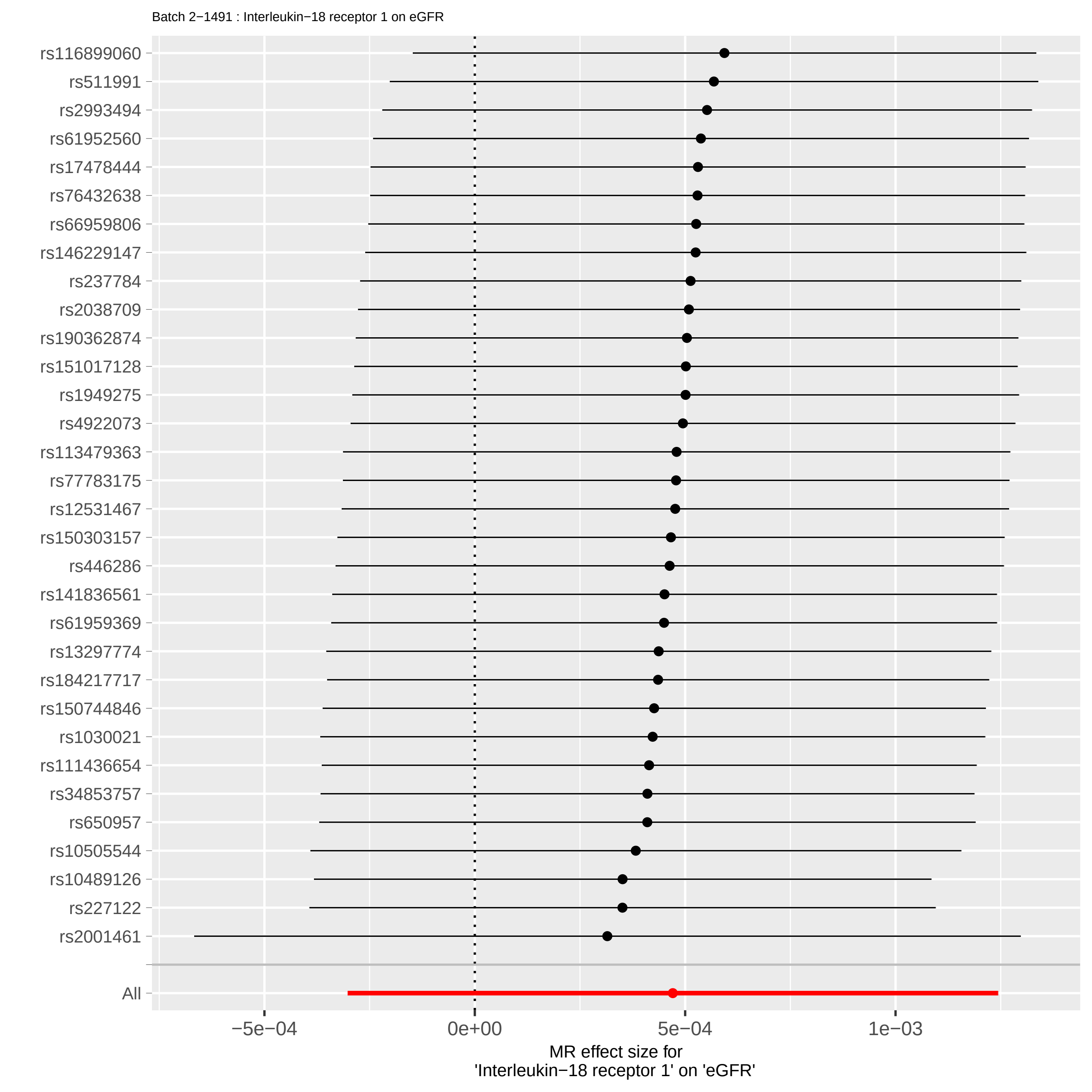
Batch 2−1491 : Interleukin−18 receptor 1 on eGFR
rs116899060
rs511991
rs2993494
rs61952560
rs17478444
rs76432638
rs66959806
rs146229147
rs237784
rs2038709
rs190362874
rs151017128
rs1949275
rs4922073
rs113479363
rs77783175
rs12531467
rs150303157
rs446286
rs141836561
rs61959369
rs13297774
rs184217717
rs150744846
rs1030021
rs111436654
rs34853757
rs650957
rs10505544
rs10489126
rs227122
rs2001461
All
−5e−04
0e+00
5e−04
1e−03
MR effect size for
'Interleukin−18 receptor 1' on 'eGFR'

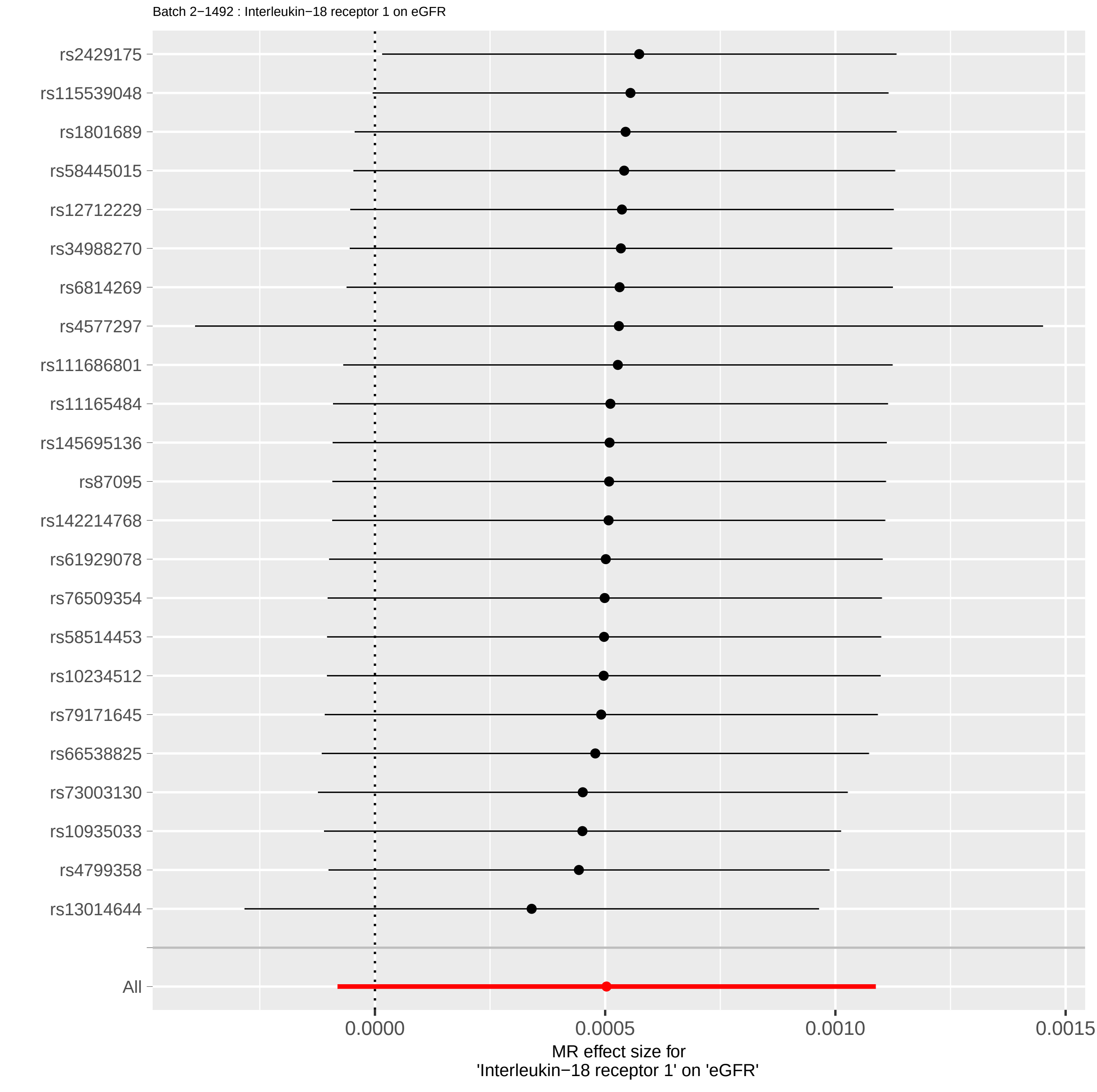

Batch 2−1492 : Interleukin−18 receptor 1 on eGFR
rs2429175
rs115539048
rs1801689
rs58445015
rs12712229
rs34988270
rs6814269
rs4577297
rs111686801
rs11165484
rs145695136
rs87095
rs142214768
rs61929078
rs76509354
rs58514453
rs10234512
rs79171645
rs66538825
rs73003130
rs10935033
rs4799358
rs13014644
All
0.0000
0.0005
0.0010
0.0015
MR effect size for
'Interleukin−18 receptor 1' on 'eGFR'

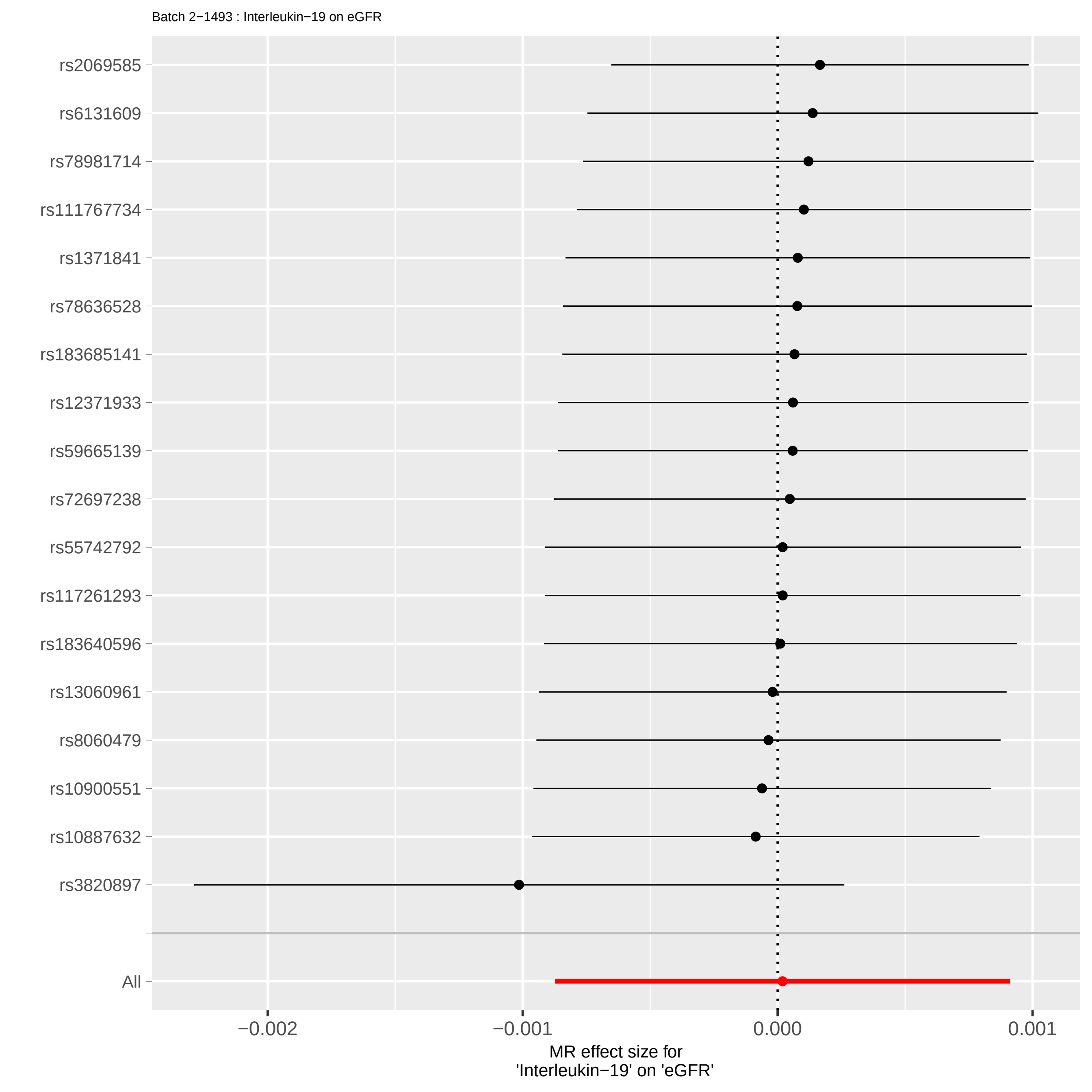

Batch 2−1493 : Interleukin−19 on eGFR
rs2069585
rs6131609
rs78981714
rs111767734
rs1371841
rs78636528
rs183685141
rs12371933
rs59665139
rs72697238
rs55742792
rs117261293
rs183640596
rs13060961
rs8060479
rs10900551
rs10887632
rs3820897
All
−0.002
−0.001
0.000
0.001
MR effect size for
'Interleukin−19' on 'eGFR'

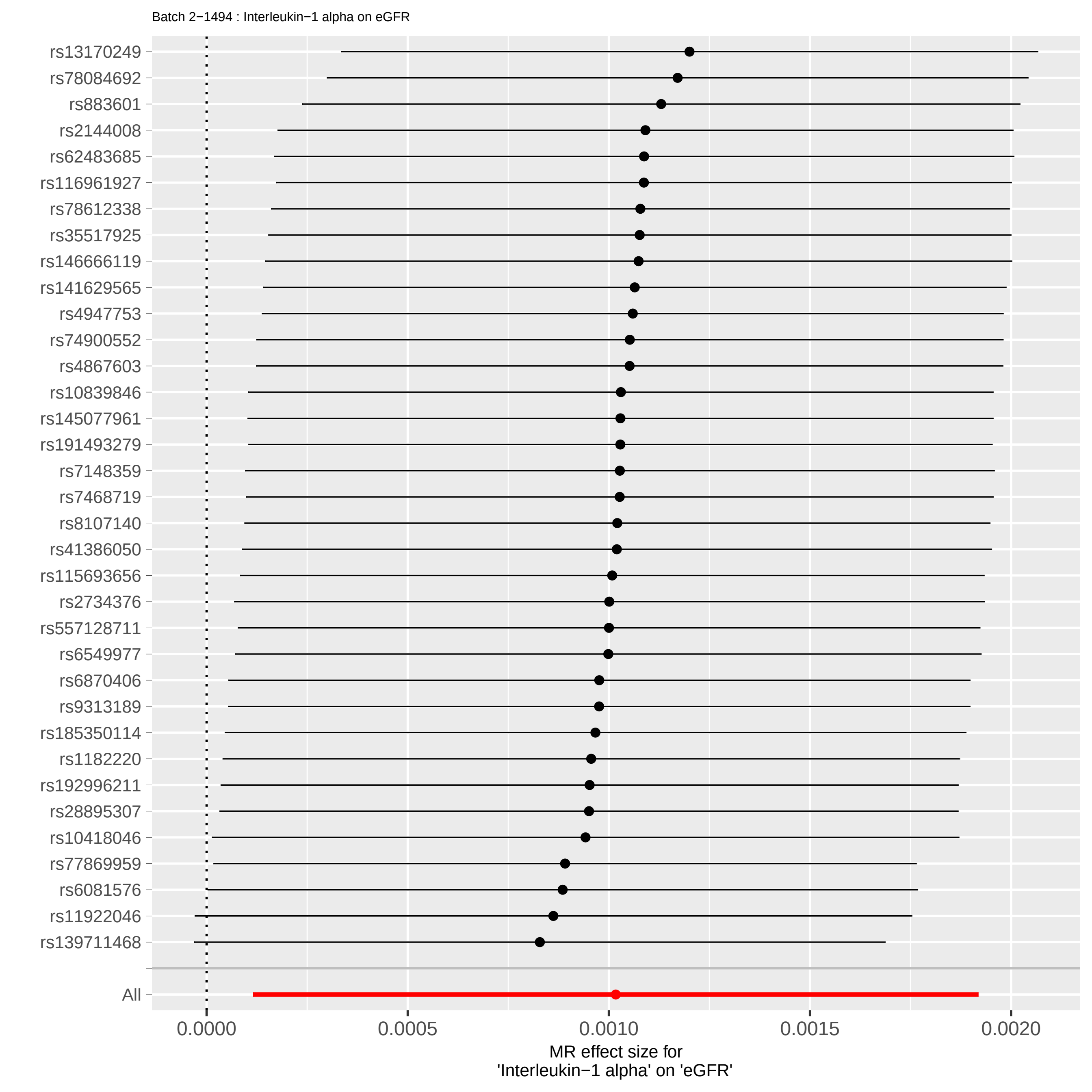

Batch 2−1494 : Interleukin−1 alpha on eGFR
rs13170249
rs78084692
rs883601
rs2144008
rs62483685
rs116961927
rs78612338
rs35517925
rs146666119
rs141629565
rs4947753
rs74900552
rs4867603
rs10839846
rs145077961
rs191493279
rs7148359
rs7468719
rs8107140
rs41386050
rs115693656
rs2734376
rs557128711
rs6549977
rs6870406
rs9313189
rs185350114
rs1182220
rs192996211
rs28895307
rs10418046
rs77869959
rs6081576
rs11922046
rs139711468
All
0.0000
0.0005
0.0010
0.0015
0.0020
MR effect size for
'Interleukin−1 alpha' on 'eGFR'

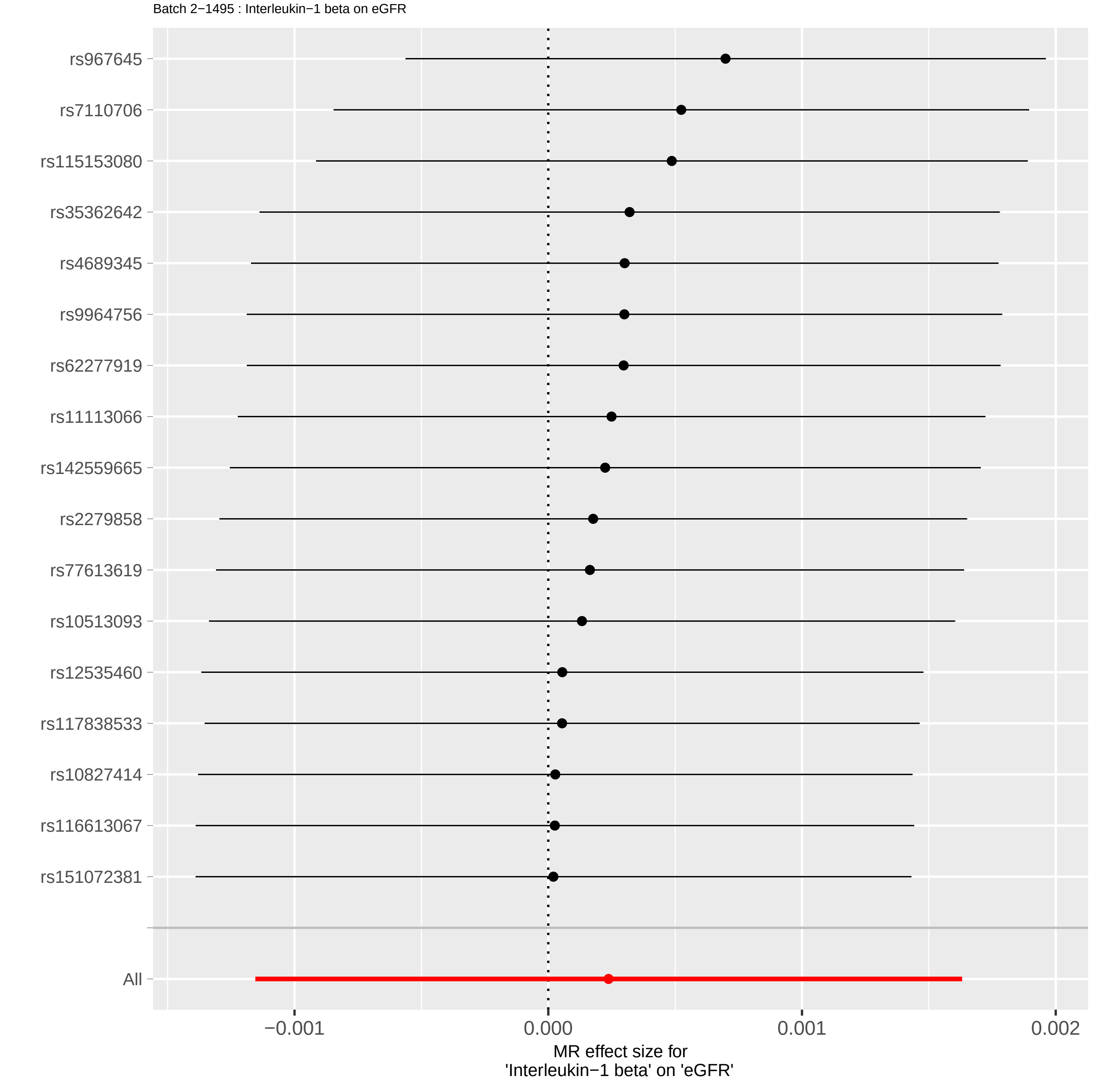

Batch 2−1495 : Interleukin−1 beta on eGFR
rs967645
rs7110706
rs115153080
rs35362642
rs4689345
rs9964756
rs62277919
rs11113066
rs142559665
rs2279858
rs77613619
rs10513093
rs12535460
rs117838533
rs10827414
rs116613067
rs151072381
All
−0.001
0.000
0.001
0.002
MR effect size for
'Interleukin−1 beta' on 'eGFR'

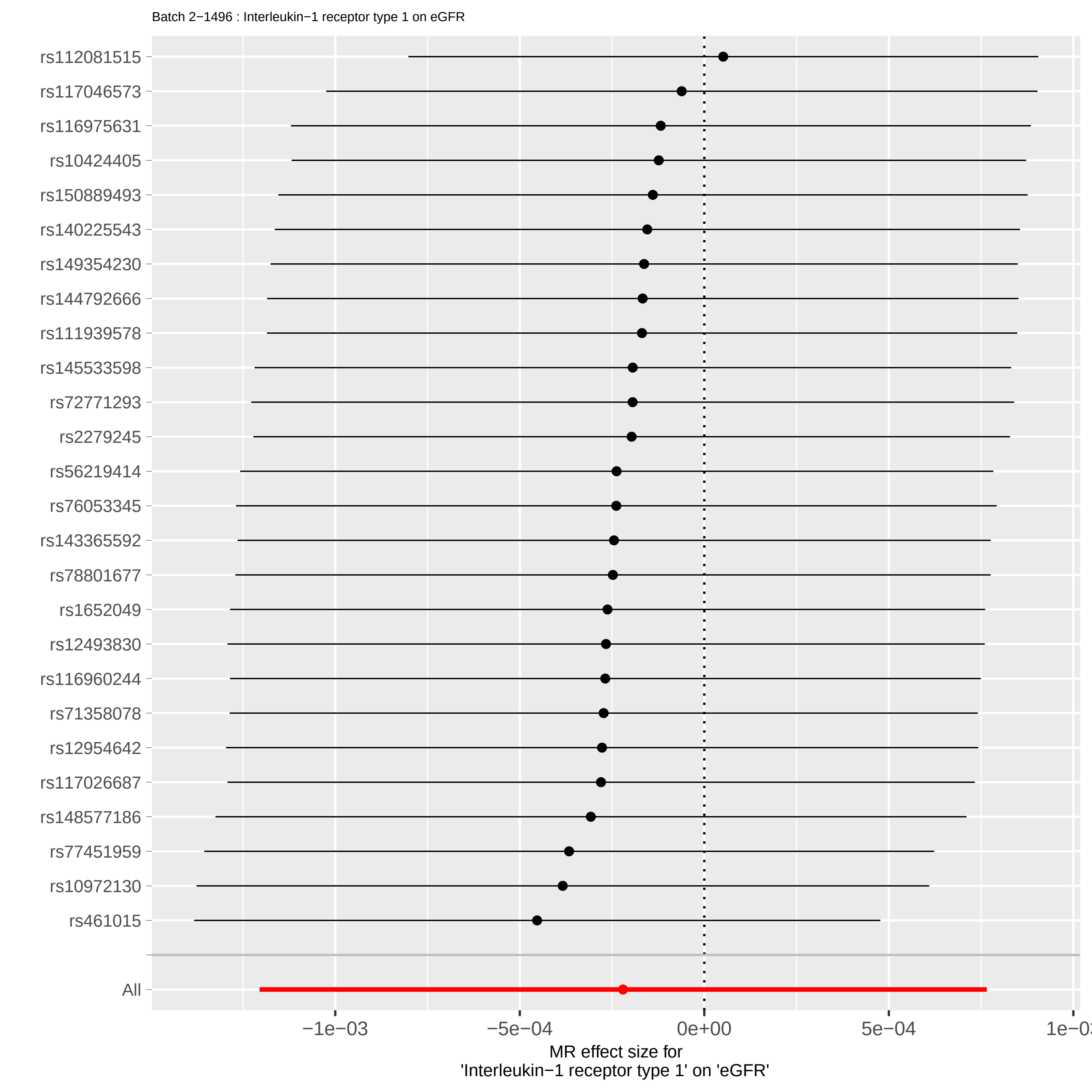

Batch 2−1496 : Interleukin−1 receptor type 1 on eGFR
rs112081515
rs117046573
rs116975631
rs10424405
rs150889493
rs140225543
rs149354230
rs144792666
rs111939578
rs145533598
rs72771293
rs2279245
rs56219414
rs76053345
rs143365592
rs78801677
rs1652049
rs12493830
rs116960244
rs71358078
rs12954642
rs117026687
rs148577186
rs77451959
rs10972130
rs461015
All
−1e−03
−5e−04
0e+00
5e−04
MR effect size for
'Interleukin−1 receptor type 1' on 'eGFR'

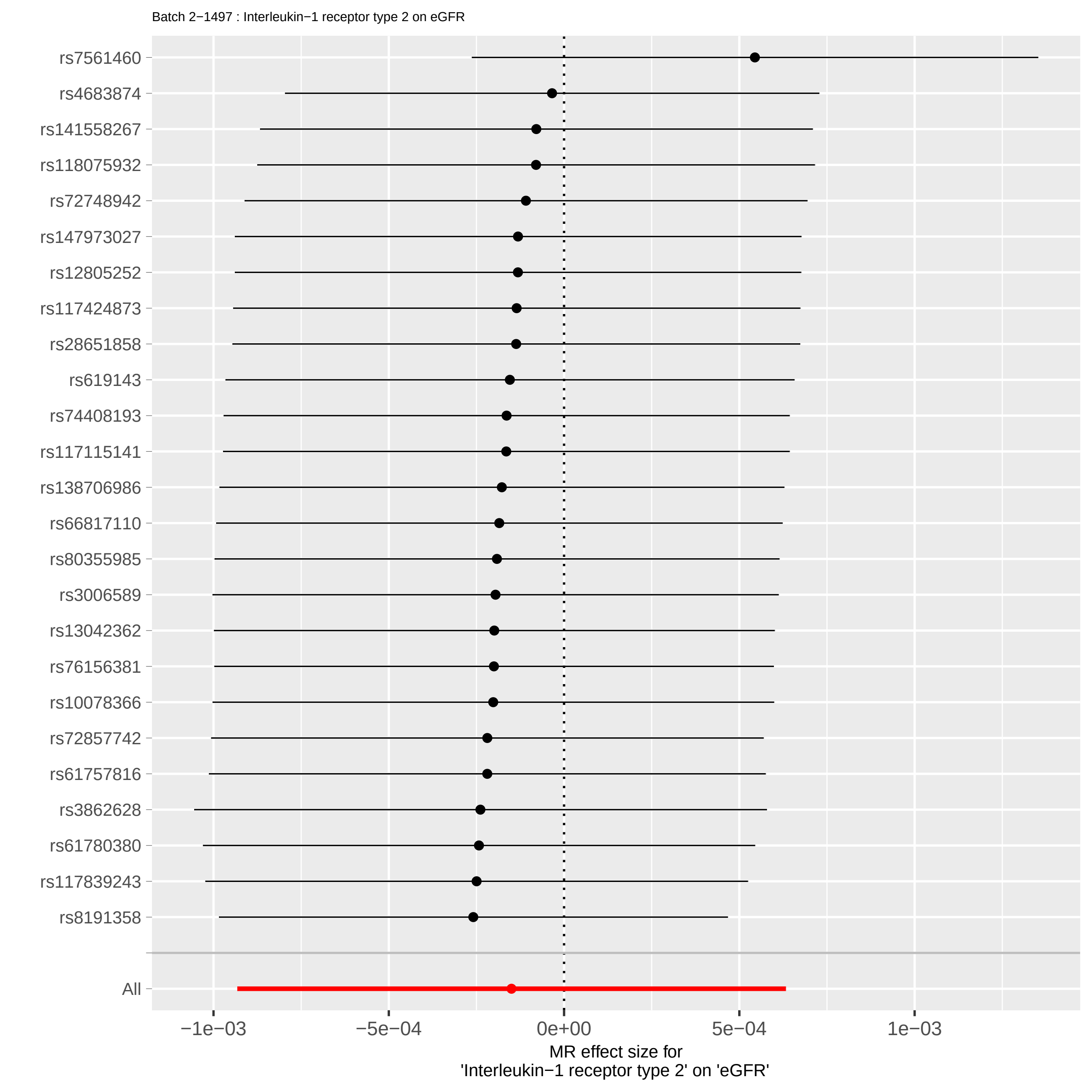

Batch 2−1497 : Interleukin−1 receptor type 2 on eGFR
rs7561460
rs4683874
rs141558267
rs118075932
rs72748942
rs147973027
rs12805252
rs117424873
rs28651858
rs619143
rs74408193
rs117115141
rs138706986
rs66817110
rs80355985
rs3006589
rs13042362
rs76156381
rs10078366
rs72857742
rs61757816
rs3862628
rs61780380
rs117839243
rs8191358
All
−1e−03
−5e−04
0e+00
5e−04
1e−03
MR effect size for
'Interleukin−1 receptor type 2' on 'eGFR'

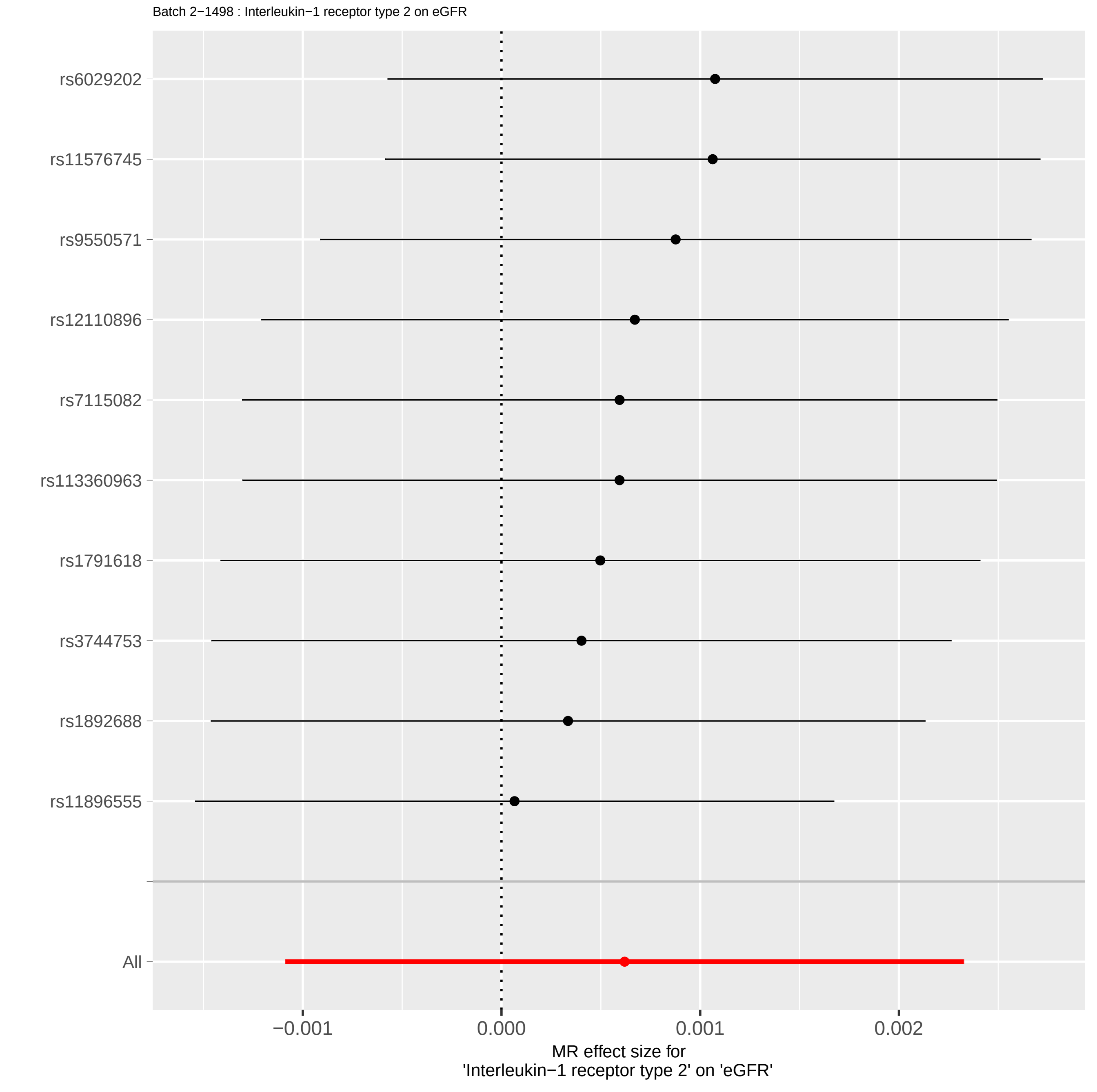

Batch 2−1498 : Interleukin−1 receptor type 2 on eGFR
rs6029202
rs11576745
rs9550571
rs12110896
rs7115082
rs113360963
rs1791618
rs3744753
rs1892688
rs11896555
All
−0.001
0.000
0.001
0.002
MR effect size for
'Interleukin−1 receptor type 2' on 'eGFR'

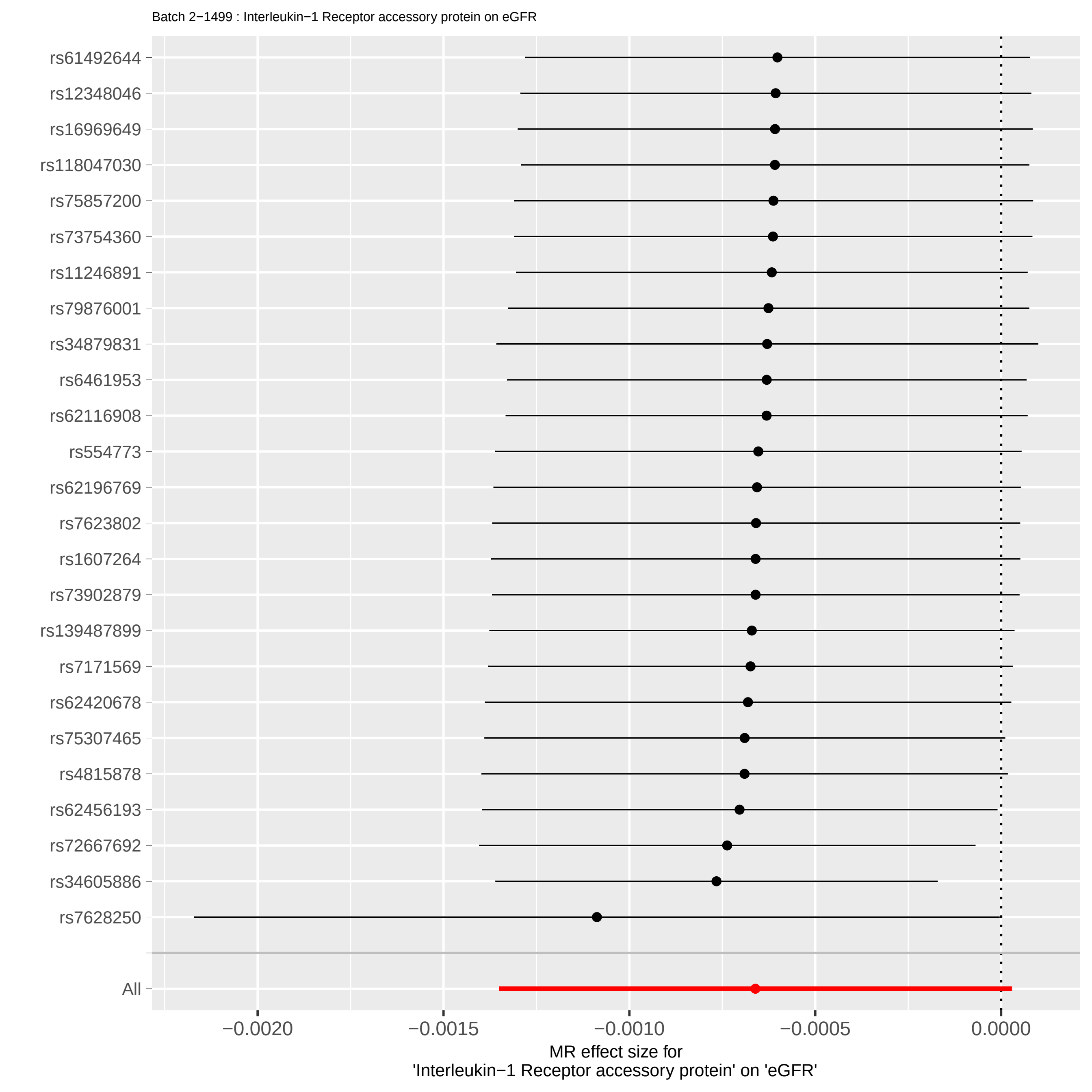

Batch 2−1499 : Interleukin−1 Receptor accessory protein on eGFR
rs61492644
rs12348046
rs16969649
rs118047030
rs75857200
rs73754360
rs11246891
rs79876001
rs34879831
rs6461953
rs62116908
rs554773
rs62196769
rs7623802
rs1607264
rs73902879
rs139487899
rs7171569
rs62420678
rs75307465
rs4815878
rs62456193
rs72667692
rs34605886
rs7628250
All
−0.0020
−0.0015
−0.0010
−0.0005
0.0000
MR effect size for
'Interleukin−1 Receptor accessory protein' on 'eGFR'

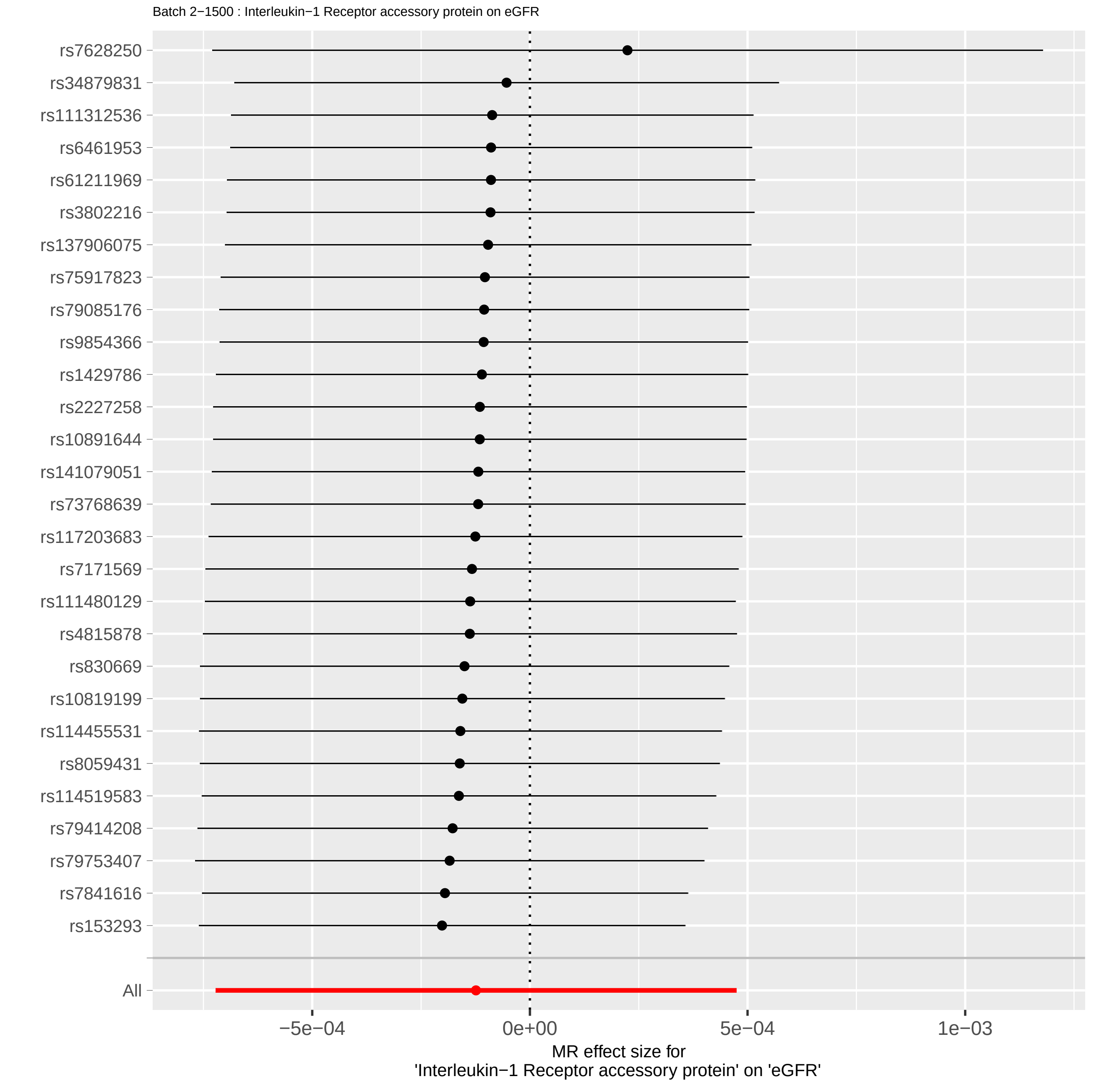

Batch 2−1500 : Interleukin−1 Receptor accessory protein on eGFR
rs7628250
rs34879831
rs111312536
rs6461953
rs61211969
rs3802216
rs137906075
rs75917823
rs79085176
rs9854366
rs1429786
rs2227258
rs10891644
rs141079051
rs73768639
rs117203683
rs7171569
rs111480129
rs4815878
rs830669
rs10819199
rs114455531
rs8059431
rs114519583
rs79414208
rs79753407
rs7841616
rs153293
All
−5e−04
0e+00
5e−04
1e−03
MR effect size for
'Interleukin−1 Receptor accessory protein' on 'eGFR'

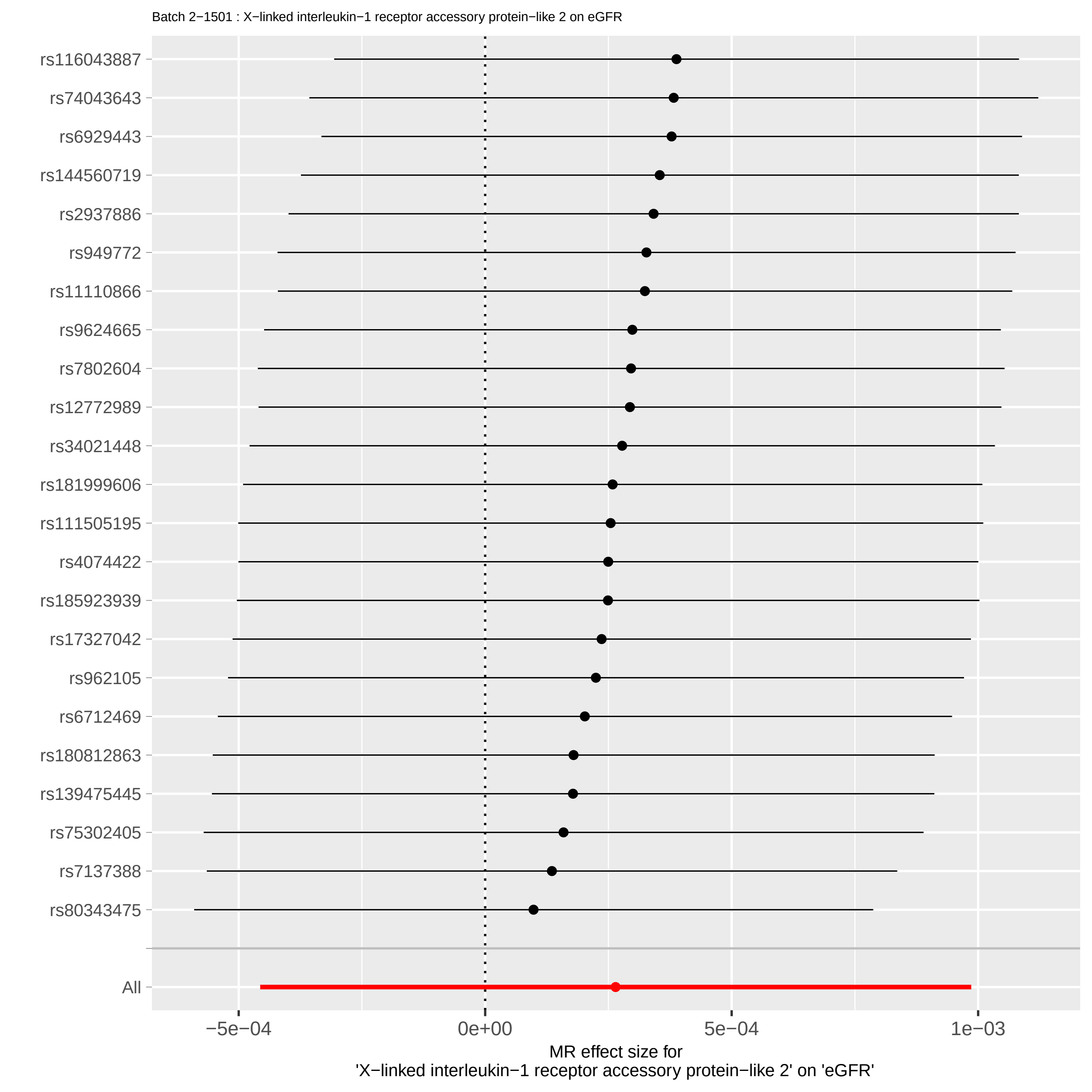
Batch 2−1501 : X−linked interleukin−1 receptor accessory protein−like 2 on eGFR
rs116043887
rs74043643
rs6929443
rs144560719
rs2937886
rs949772
rs11110866
rs9624665
rs7802604
rs12772989
rs34021448
rs181999606
rs111505195
rs4074422
rs185923939
rs17327042
rs962105
rs6712469
rs180812863
rs139475445
rs75302405
rs7137388
rs80343475
All
−5e−04
0e+00
5e−04
1e−03
MR effect size for
'X−linked interleukin−1 receptor accessory protein−like 2' on 'eGFR'

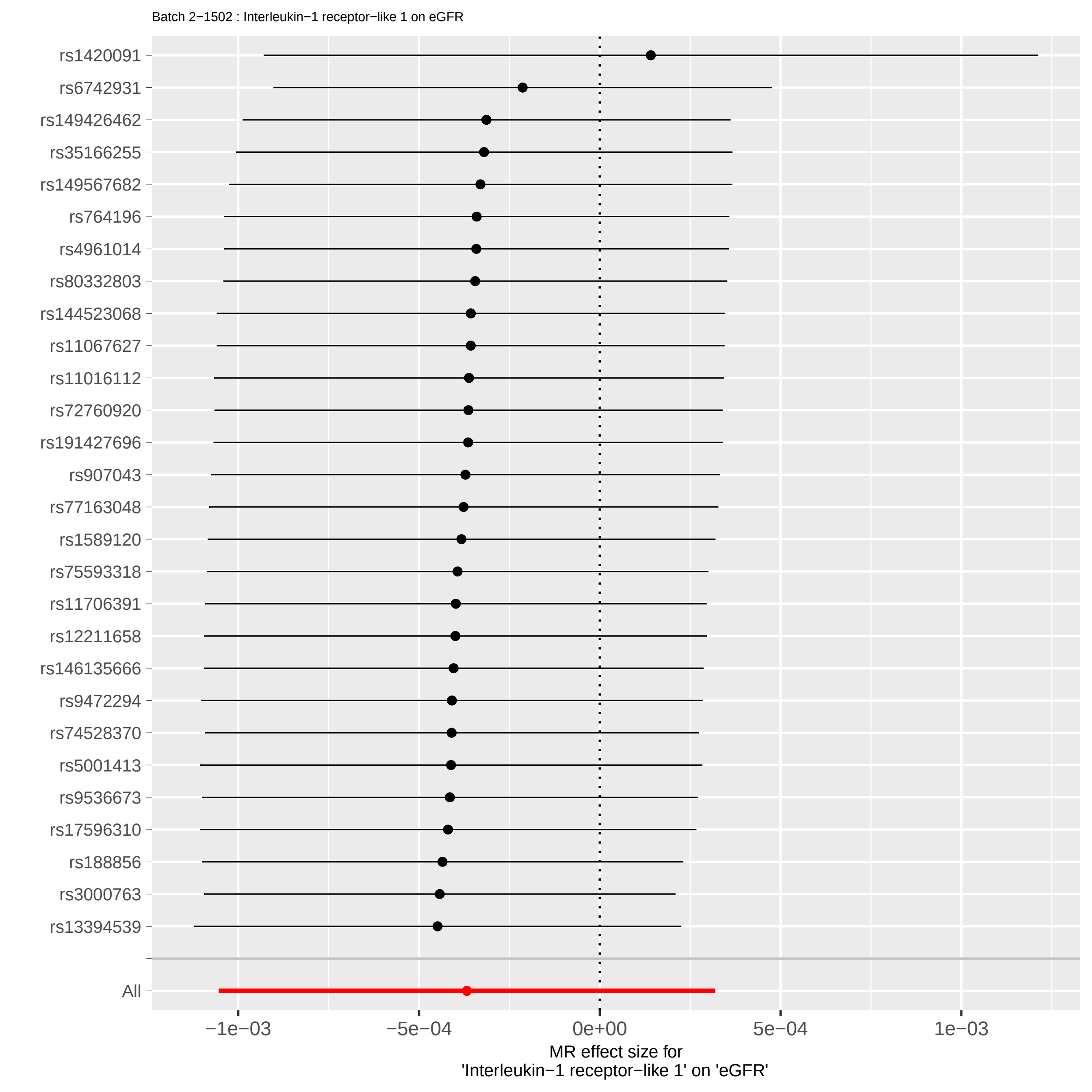

Batch 2−1502 : Interleukin−1 receptor−like 1 on eGFR
rs1420091
rs6742931
rs149426462
rs35166255
rs149567682
rs764196
rs4961014
rs80332803
rs144523068
rs11067627
rs11016112
rs72760920
rs191427696
rs907043
rs77163048
rs1589120
rs75593318
rs11706391
rs12211658
rs146135666
rs9472294
rs74528370
rs5001413
rs9536673
rs17596310
rs188856
rs3000763
rs13394539
All
−1e−03
−5e−04
0e+00
5e−04
1e−03
MR effect size for
'Interleukin−1 receptor−like 1' on 'eGFR'

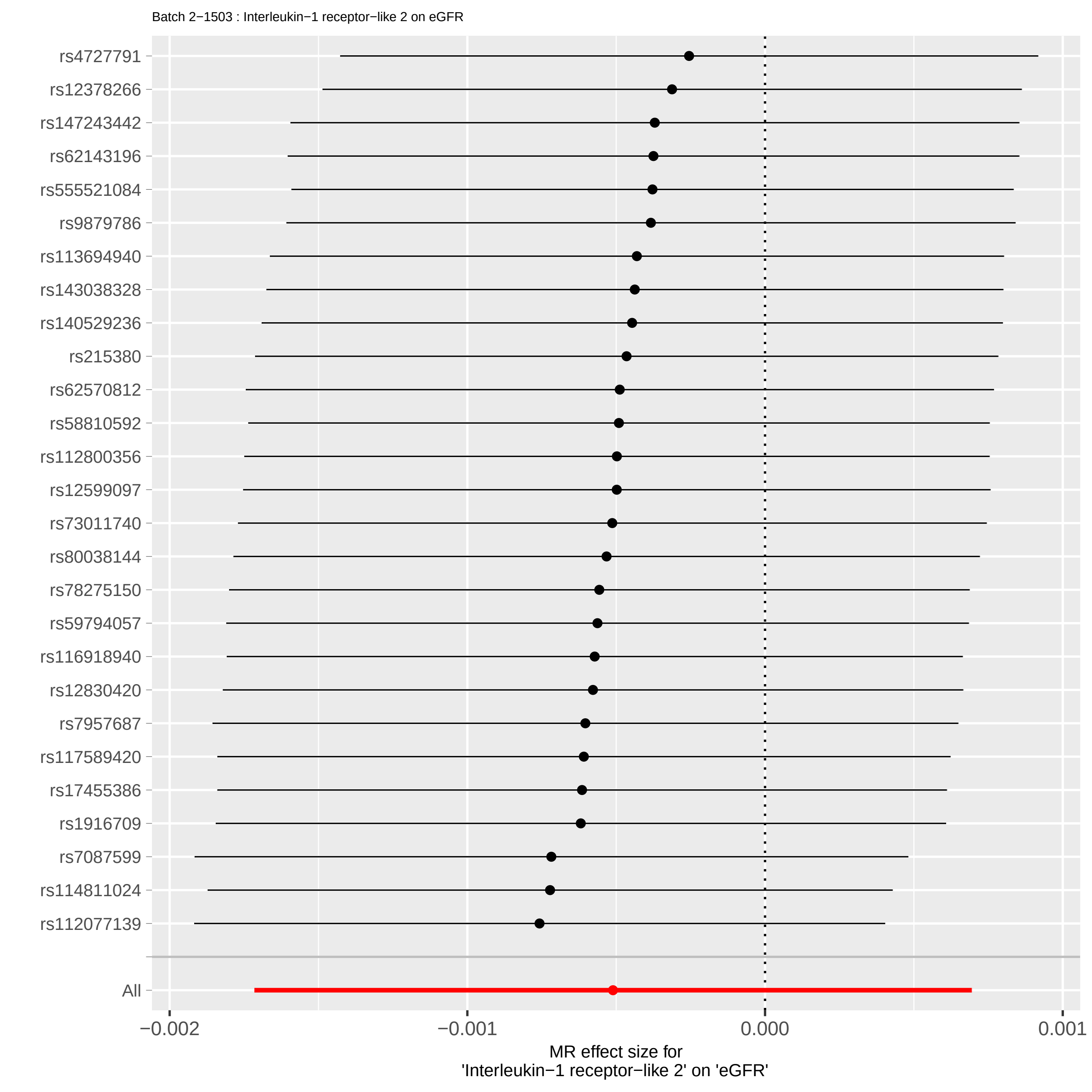

Batch 2−1503 : Interleukin−1 receptor−like 2 on eGFR
rs4727791
rs12378266
rs147243442
rs62143196
rs555521084
rs9879786
rs113694940
rs143038328
rs140529236
rs215380
rs62570812
rs58810592
rs112800356
rs12599097
rs73011740
rs80038144
rs78275150
rs59794057
rs116918940
rs12830420
rs7957687
rs117589420
rs17455386
rs1916709
rs7087599
rs114811024
rs112077139
All
−0.002
−0.001
0.000
0.001
MR effect size for
'Interleukin−1 receptor−like 2' on 'eGFR'

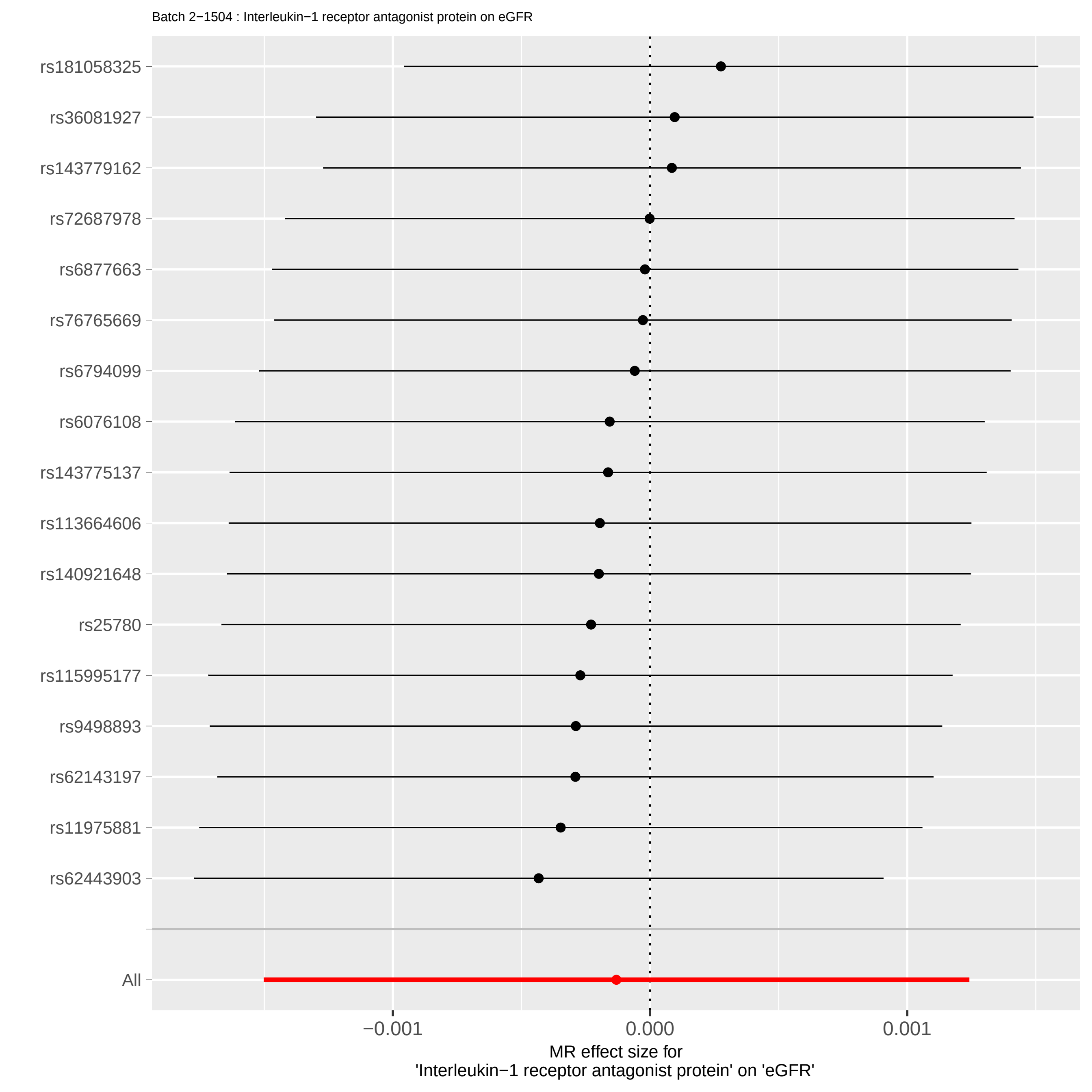

Batch 2−1504 : Interleukin−1 receptor antagonist protein on eGFR
rs181058325
rs36081927
rs143779162
rs72687978
rs6877663
rs76765669
rs6794099
rs6076108
rs143775137
rs113664606
rs140921648
rs25780
rs115995177
rs9498893
rs62143197
rs11975881
rs62443903
All
−0.001
0.000
0.001
MR effect size for
'Interleukin−1 receptor antagonist protein' on 'eGFR'

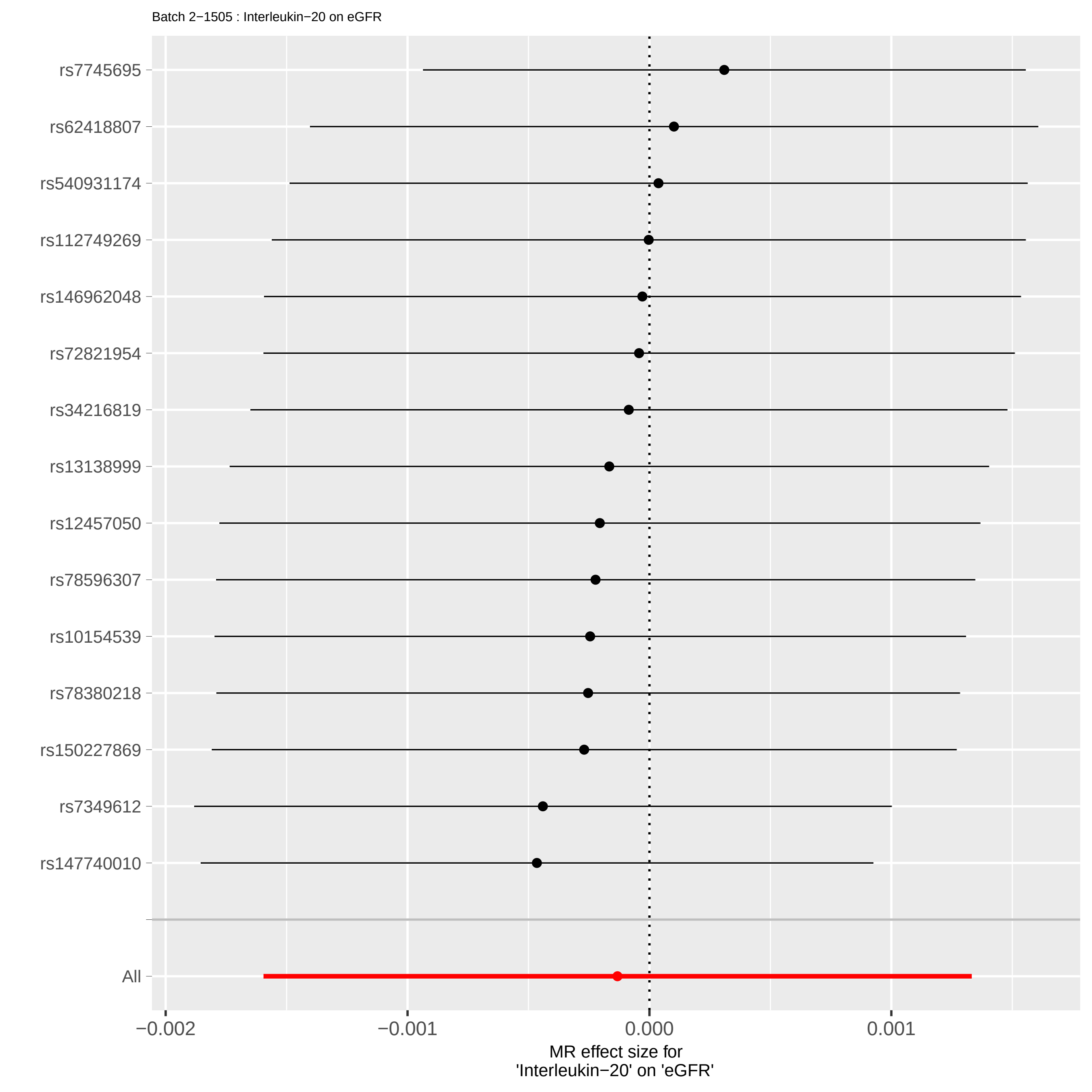
Batch 2−1505 : Interleukin−20 on eGFR
rs7745695
rs62418807
rs540931174
rs112749269
rs146962048
rs72821954
rs34216819
rs13138999
rs12457050
rs78596307
rs10154539
rs78380218
rs150227869
rs7349612
rs147740010
All
−0.002
−0.001
0.000
0.001
MR effect size for
'Interleukin−20' on 'eGFR'

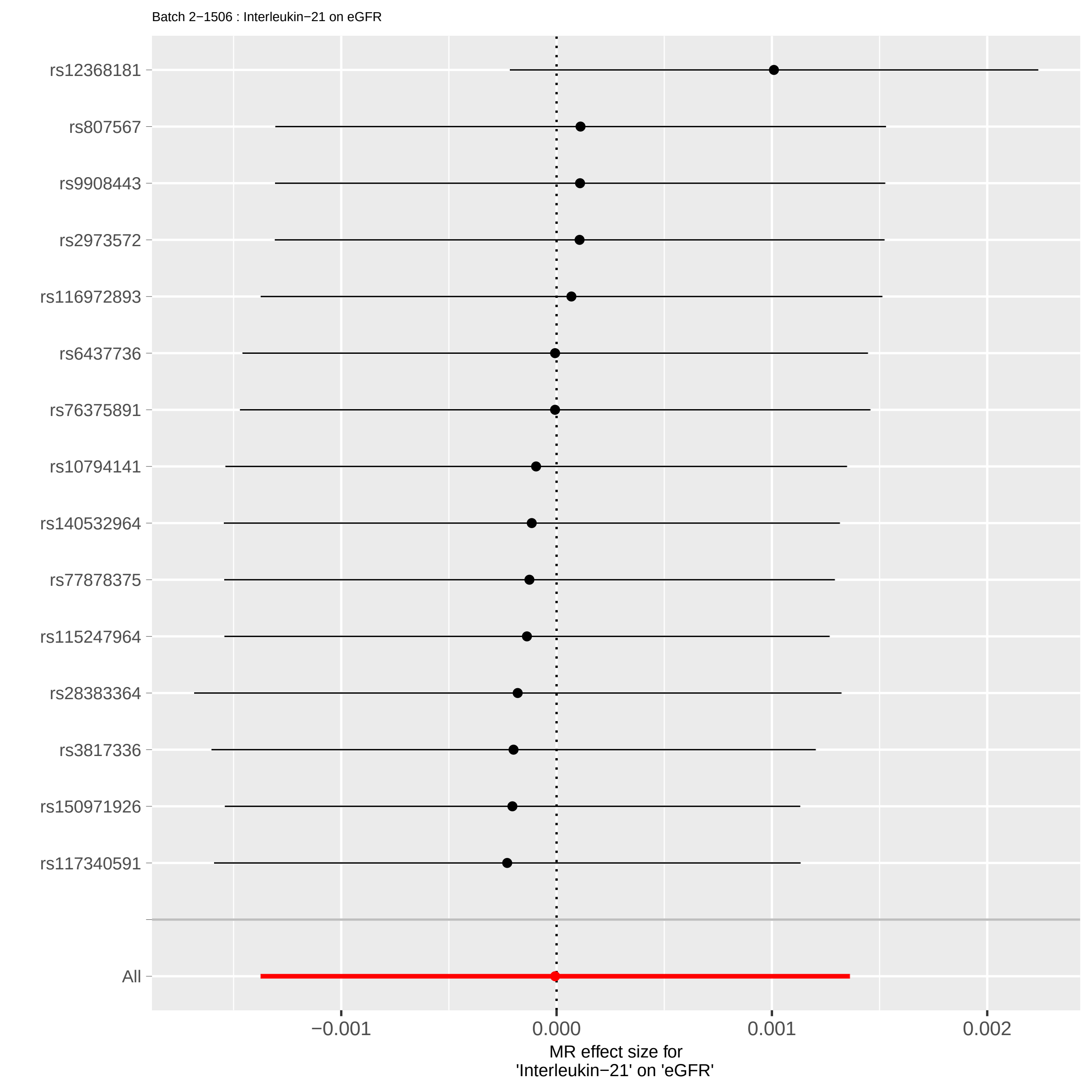

Batch 2−1506 : Interleukin−21 on eGFR
rs12368181
rs807567
rs9908443
rs2973572
rs116972893
rs6437736
rs76375891
rs10794141
rs140532964
rs77878375
rs115247964
rs28383364
rs3817336
rs150971926
rs117340591
All
−0.001
0.000
0.001
0.002
MR effect size for
'Interleukin−21' on 'eGFR'

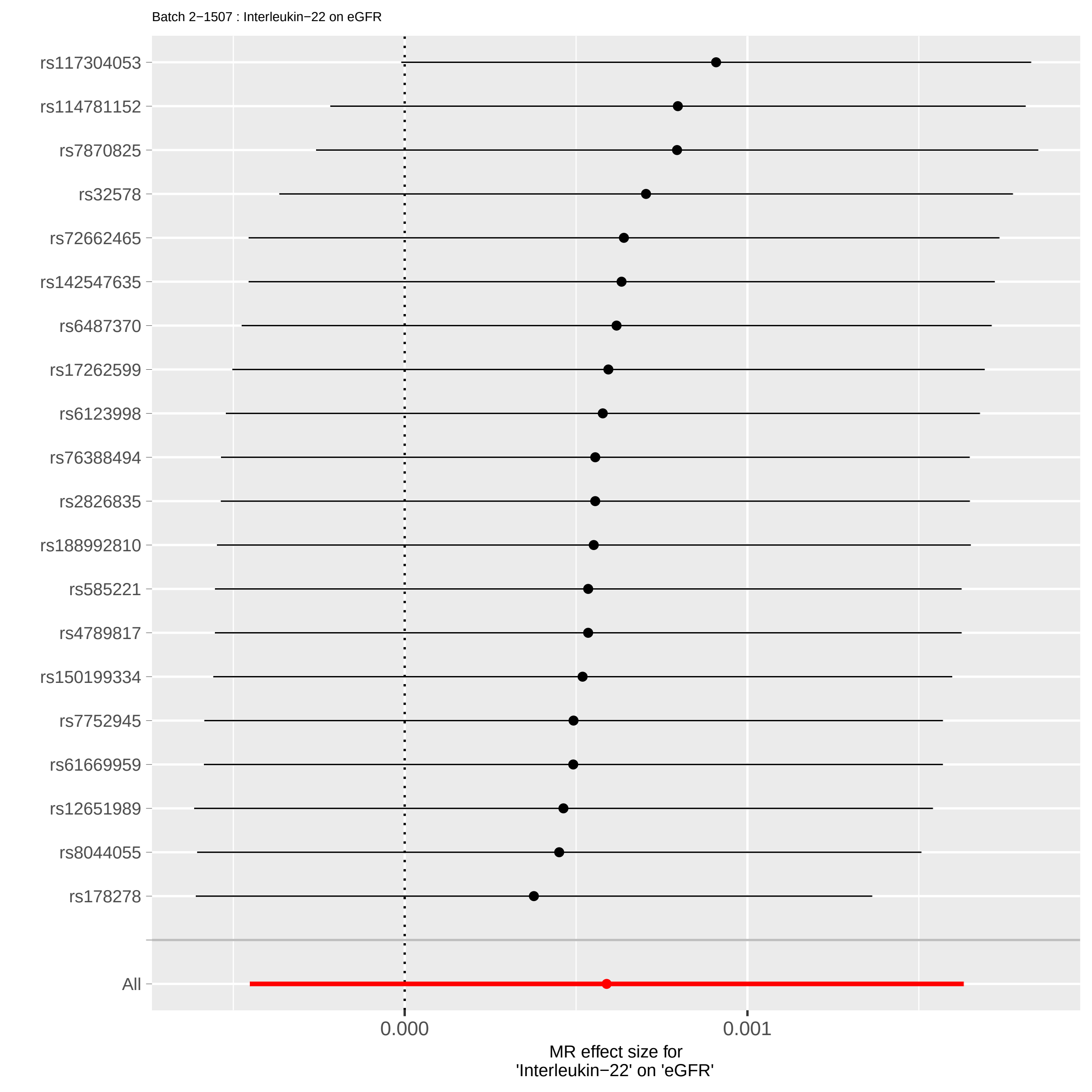

Batch 2−1507 : Interleukin−22 on eGFR
rs117304053
rs114781152
rs7870825
rs32578
rs72662465
rs142547635
rs6487370
rs17262599
rs6123998
rs76388494
rs2826835
rs188992810
rs585221
rs4789817
rs150199334
rs7752945
rs61669959
rs12651989
rs8044055
rs178278
All
0.000
0.001
MR effect size for
'Interleukin−22' on 'eGFR'

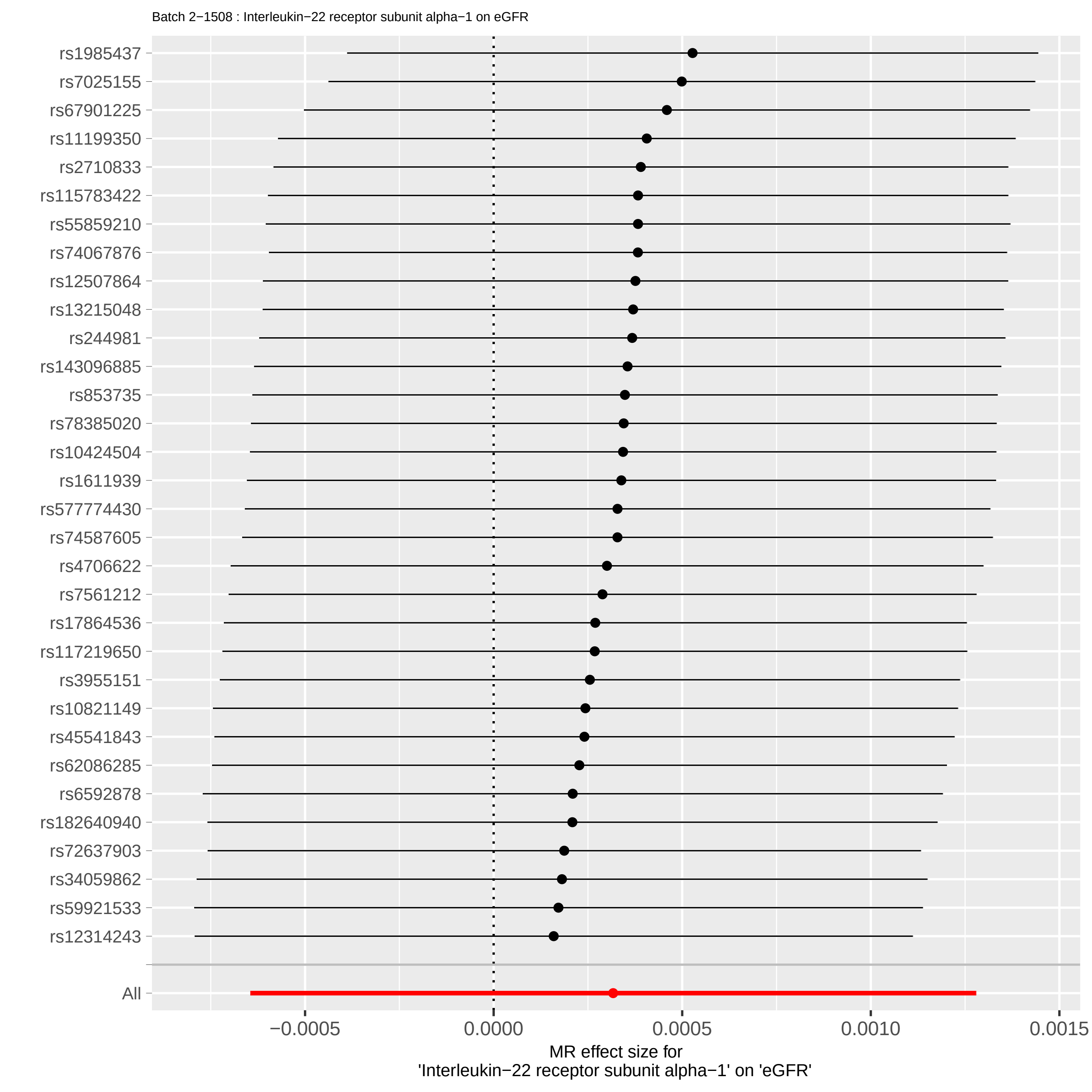

Batch 2−1508 : Interleukin−22 receptor subunit alpha−1 on eGFR
rs1985437
rs7025155
rs67901225
rs11199350
rs2710833
rs115783422
rs55859210
rs74067876
rs12507864
rs13215048
rs244981
rs143096885
rs853735
rs78385020
rs10424504
rs1611939
rs577774430
rs74587605
rs4706622
rs7561212
rs17864536
rs117219650
rs3955151
rs10821149
rs45541843
rs62086285
rs6592878
rs182640940
rs72637903
rs34059862
rs59921533
rs12314243
All
−0.0005
0.0000
0.0005
0.0010
0.0015
MR effect size for
'Interleukin−22 receptor subunit alpha−1' on 'eGFR'

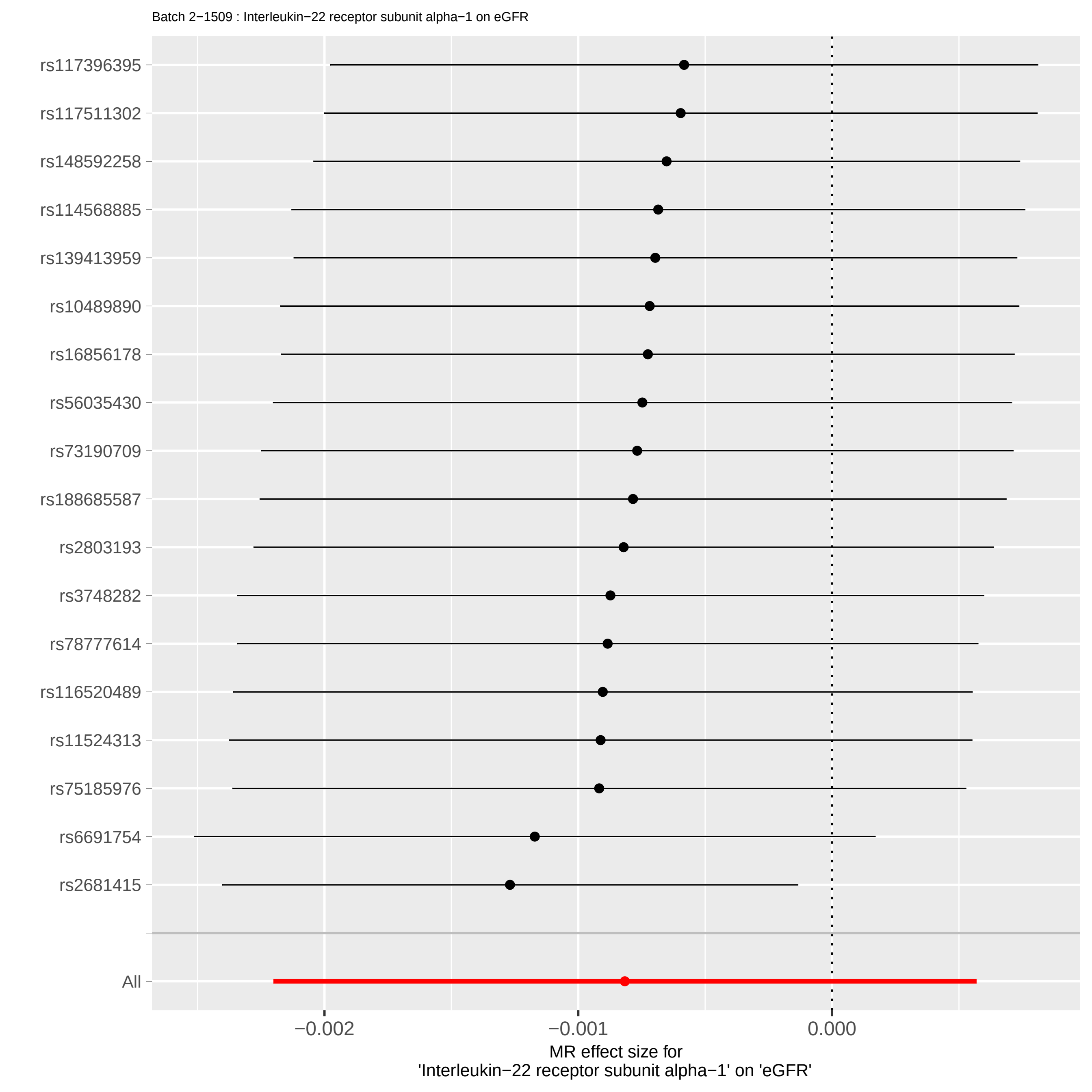

Batch 2−1509 : Interleukin−22 receptor subunit alpha−1 on eGFR
rs117396395
rs117511302
rs148592258
rs114568885
rs139413959
rs10489890
rs16856178
rs56035430
rs73190709
rs188685587
rs2803193
rs3748282
rs78777614
rs116520489
rs11524313
rs75185976
rs6691754
rs2681415
All
−0.002
−0.001
0.000
MR effect size for
'Interleukin−22 receptor subunit alpha−1' on 'eGFR'

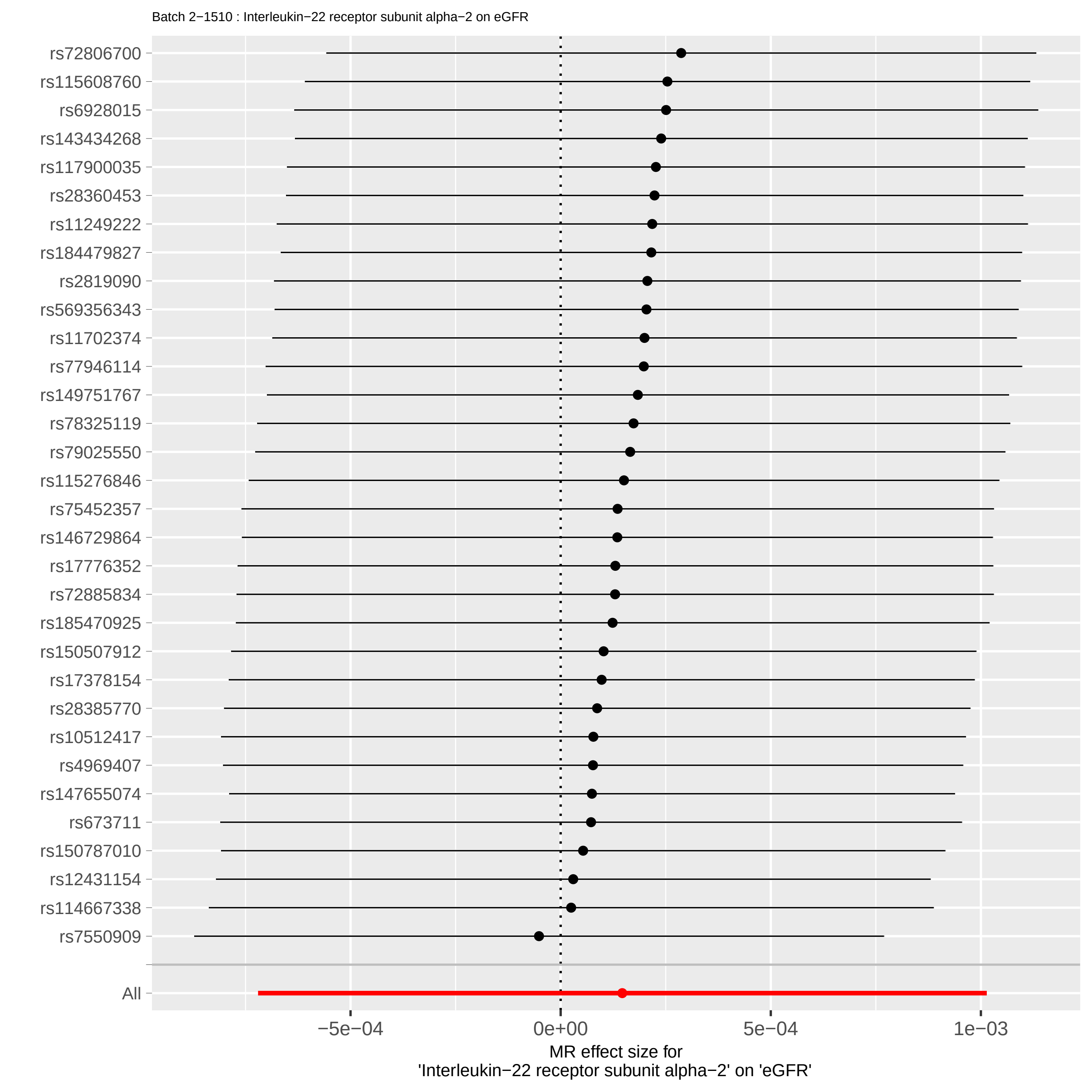

Batch 2−1510 : Interleukin−22 receptor subunit alpha−2 on eGFR
rs72806700
rs115608760
rs6928015
rs143434268
rs117900035
rs28360453
rs11249222
rs184479827
rs2819090
rs569356343
rs11702374
rs77946114
rs149751767
rs78325119
rs79025550
rs115276846
rs75452357
rs146729864
rs17776352
rs72885834
rs185470925
rs150507912
rs17378154
rs28385770
rs10512417
rs4969407
rs147655074
rs673711
rs150787010
rs12431154
rs114667338
rs7550909
All
−5e−04
0e+00
5e−04
1e−03
MR effect size for
'Interleukin−22 receptor subunit alpha−2' on 'eGFR'

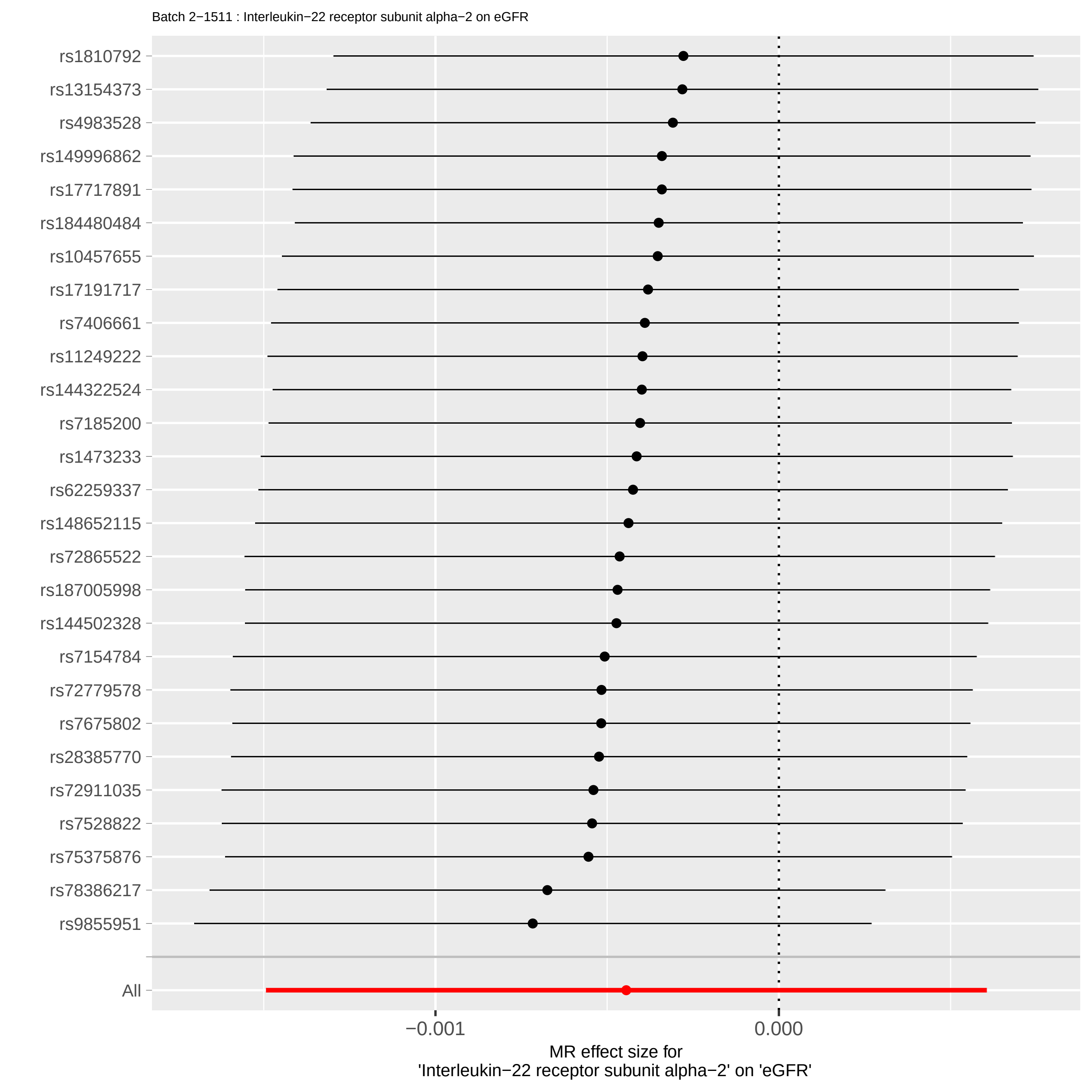
Batch 2−1511 : Interleukin−22 receptor subunit alpha−2 on eGFR
rs1810792
rs13154373
rs4983528
rs149996862
rs17717891
rs184480484
rs10457655
rs17191717
rs7406661
rs11249222
rs144322524
rs7185200
rs1473233
rs62259337
rs148652115
rs72865522
rs187005998
rs144502328
rs7154784
rs72779578
rs7675802
rs28385770
rs72911035
rs7528822
rs75375876
rs78386217
rs9855951
All
−0.001
0.000
MR effect size for
'Interleukin−22 receptor subunit alpha−2' on 'eGFR'

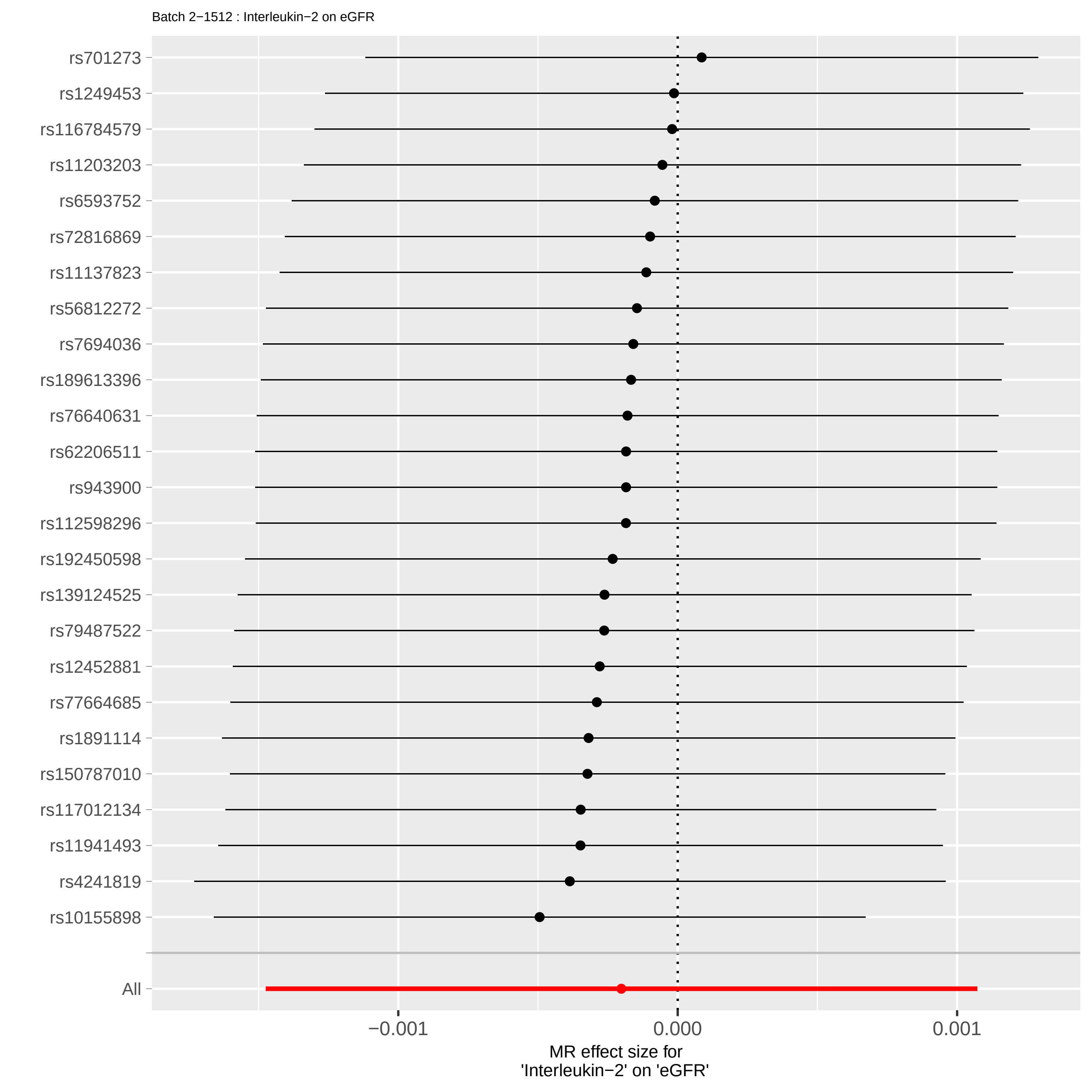
Batch 2−1512 : Interleukin−2 on eGFR
rs701273
rs1249453
rs116784579
rs11203203
rs6593752
rs72816869
rs11137823
rs56812272
rs7694036
rs189613396
rs76640631
rs62206511
rs943900
rs112598296
rs192450598
rs139124525
rs79487522
rs12452881
rs77664685
rs1891114
rs150787010
rs117012134
rs11941493
rs4241819
rs10155898
All
−0.001
0.000
0.001
MR effect size for
'Interleukin−2' on 'eGFR'

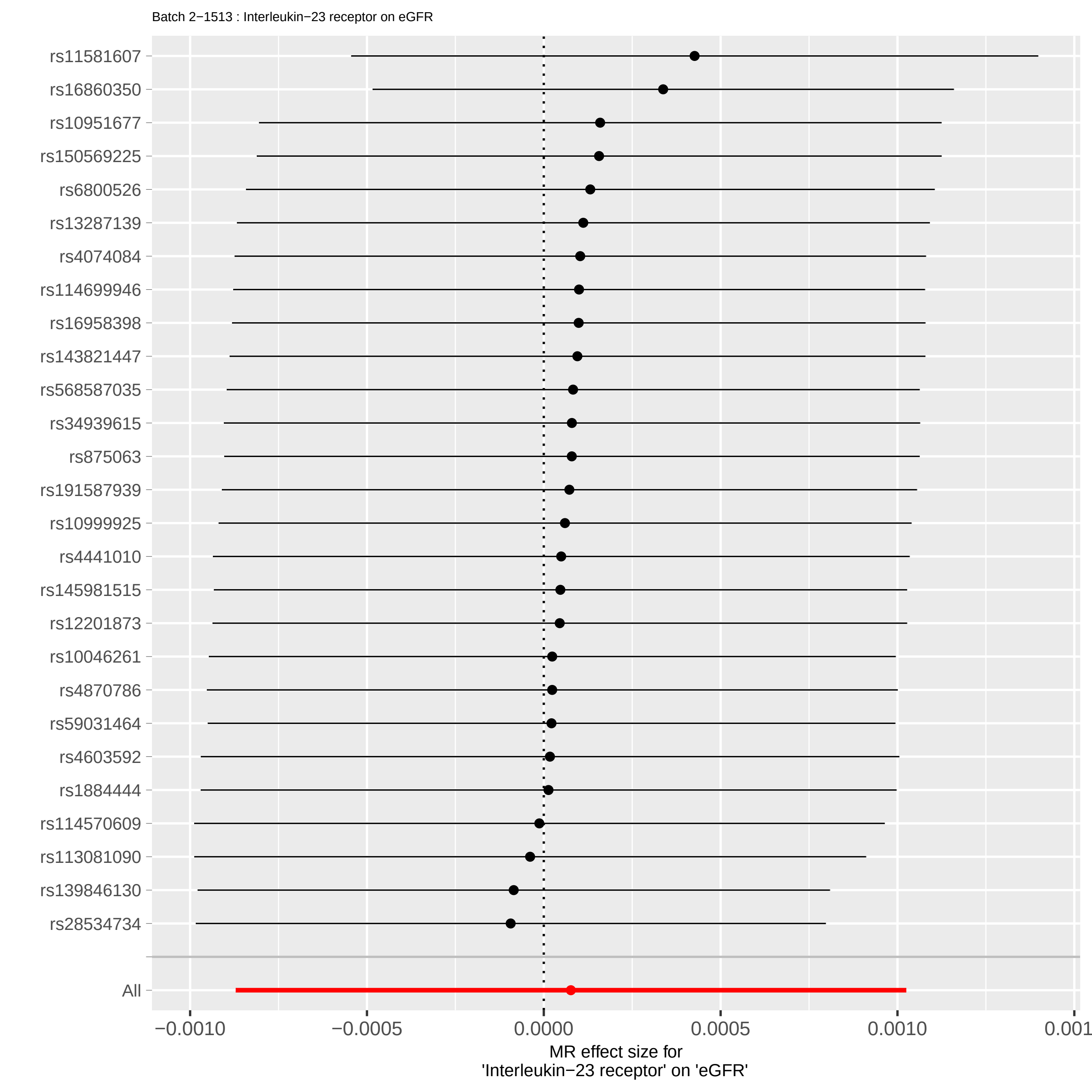
Batch 2−1513 : Interleukin−23 receptor on eGFR
rs11581607
rs16860350
rs10951677
rs150569225
rs6800526
rs13287139
rs4074084
rs114699946
rs16958398
rs143821447
rs568587035
rs34939615
rs875063
rs191587939
rs10999925
rs4441010
rs145981515
rs12201873
rs10046261
rs4870786
rs59031464
rs4603592
rs1884444
rs114570609
rs113081090
rs139846130
rs28534734
All
−0.0010
−0.0005
0.0000
0.0005
0.0010
MR effect size for
'Interleukin−23 receptor' on 'eGFR'

Batch 2−1514 : Interleukin−24 on eGFR

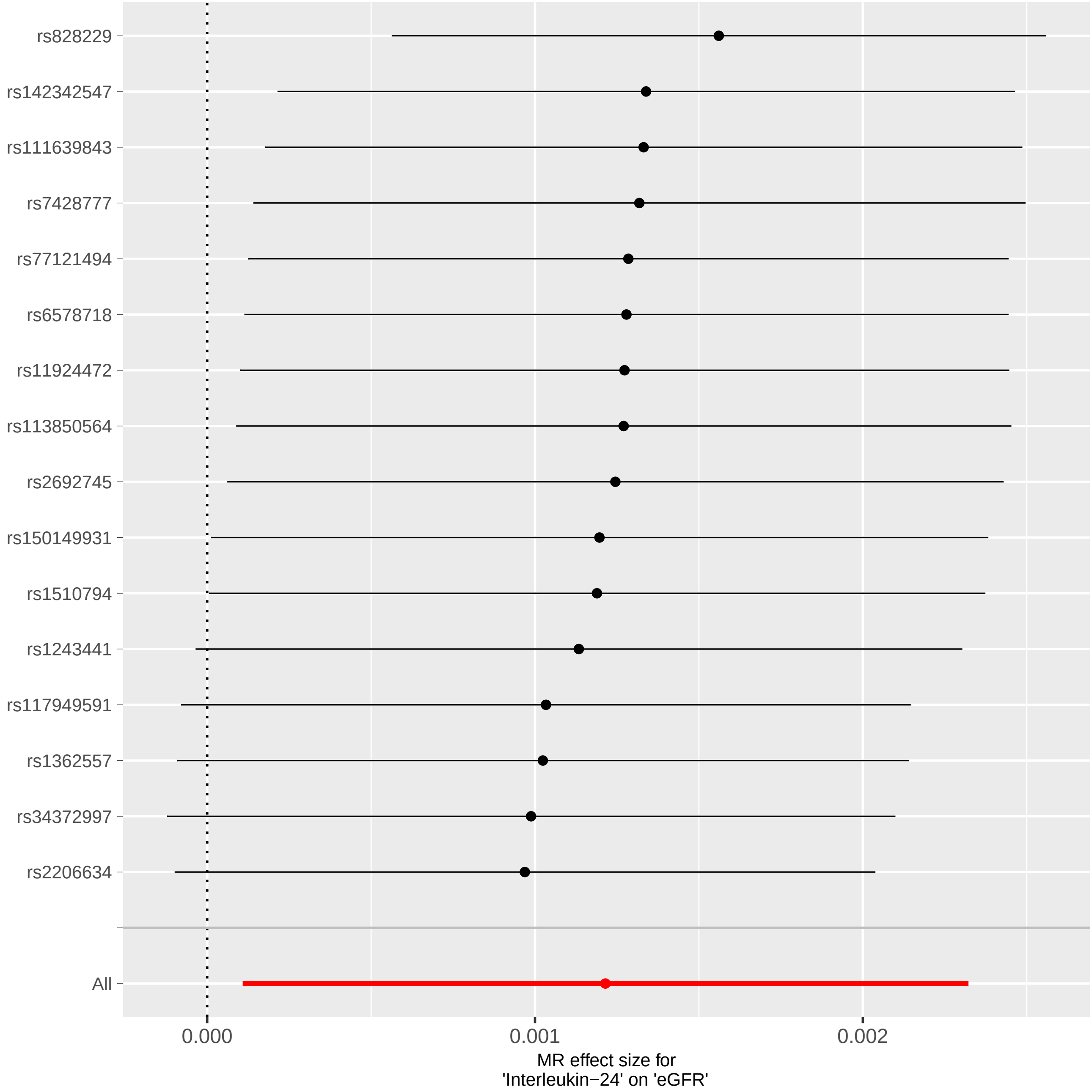

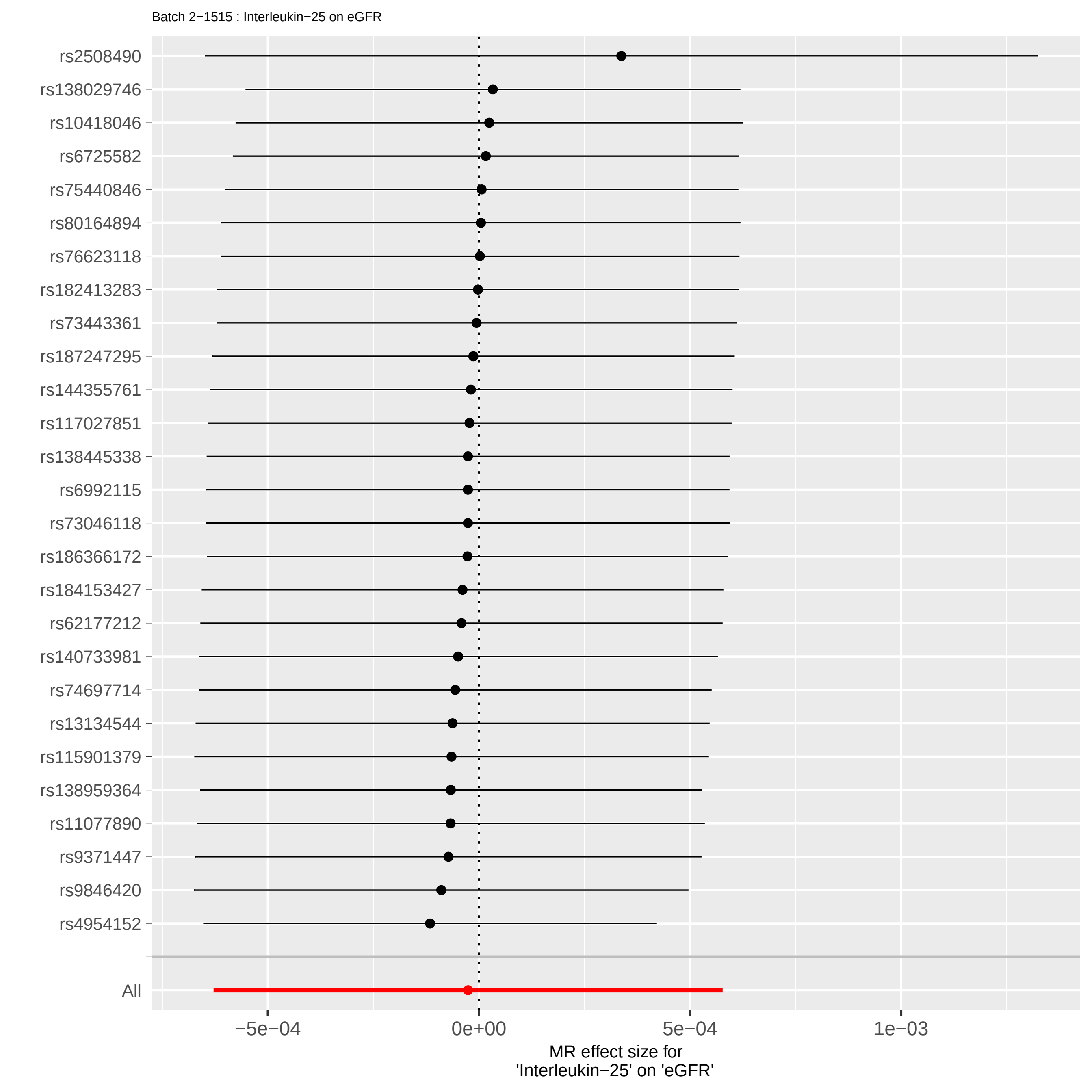
Batch 2−1515 : Interleukin−25 on eGFR
rs2508490
rs138029746
rs10418046
rs6725582
rs75440846
rs80164894
rs76623118
rs182413283
rs73443361
rs187247295
rs144355761
rs117027851
rs138445338
rs6992115
rs73046118
rs186366172
rs184153427
rs62177212
rs140733981
rs74697714
rs13134544
rs115901379
rs138959364
rs11077890
rs9371447
rs9846420
rs4954152
All
−5e−04
0e+00
5e−04
1e−03
MR effect size for
'Interleukin−25' on 'eGFR'

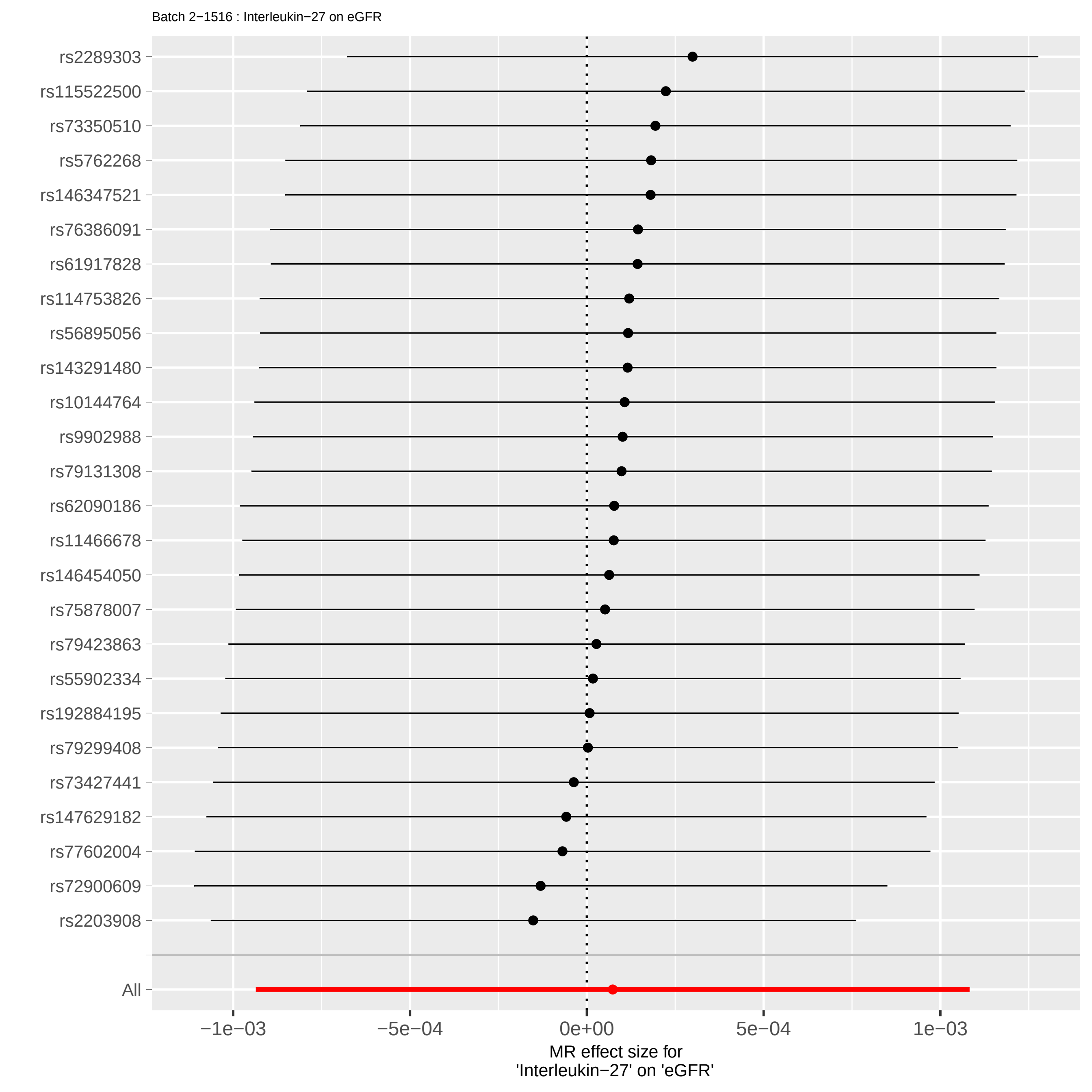

Batch 2−1516 : Interleukin−27 on eGFR
rs2289303
rs115522500
rs73350510
rs5762268
rs146347521
rs76386091
rs61917828
rs114753826
rs56895056
rs143291480
rs10144764
rs9902988
rs79131308
rs62090186
rs11466678
rs146454050
rs75878007
rs79423863
rs55902334
rs192884195
rs79299408
rs73427441
rs147629182
rs77602004
rs72900609
rs2203908
All
−1e−03
−5e−04
0e+00
5e−04
1e−03
MR effect size for
'Interleukin−27' on 'eGFR'

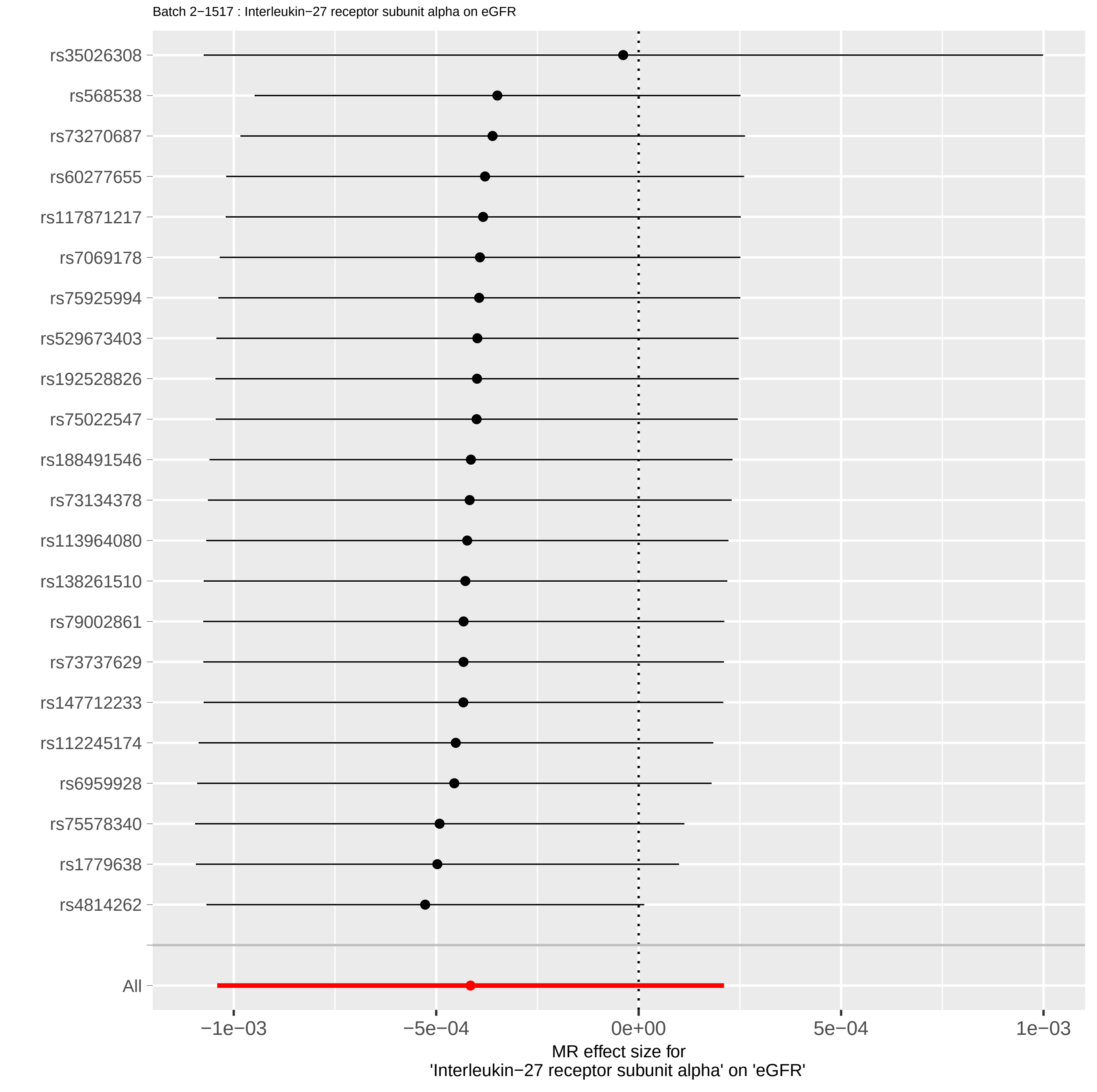
Batch 2−1517 : Interleukin−27 receptor subunit alpha on eGFR
rs35026308
rs568538
rs73270687
rs60277655
rs117871217
rs7069178
rs75925994
rs529673403
rs192528826
rs75022547
rs188491546
rs73134378
rs113964080
rs138261510
rs79002861
rs73737629
rs147712233
rs112245174
rs6959928
rs75578340
rs1779638
rs4814262
All
−1e−03
−5e−04
0e+00
5e−04
1e−03
MR effect size for
'Interleukin−27 receptor subunit alpha' on 'eGFR'

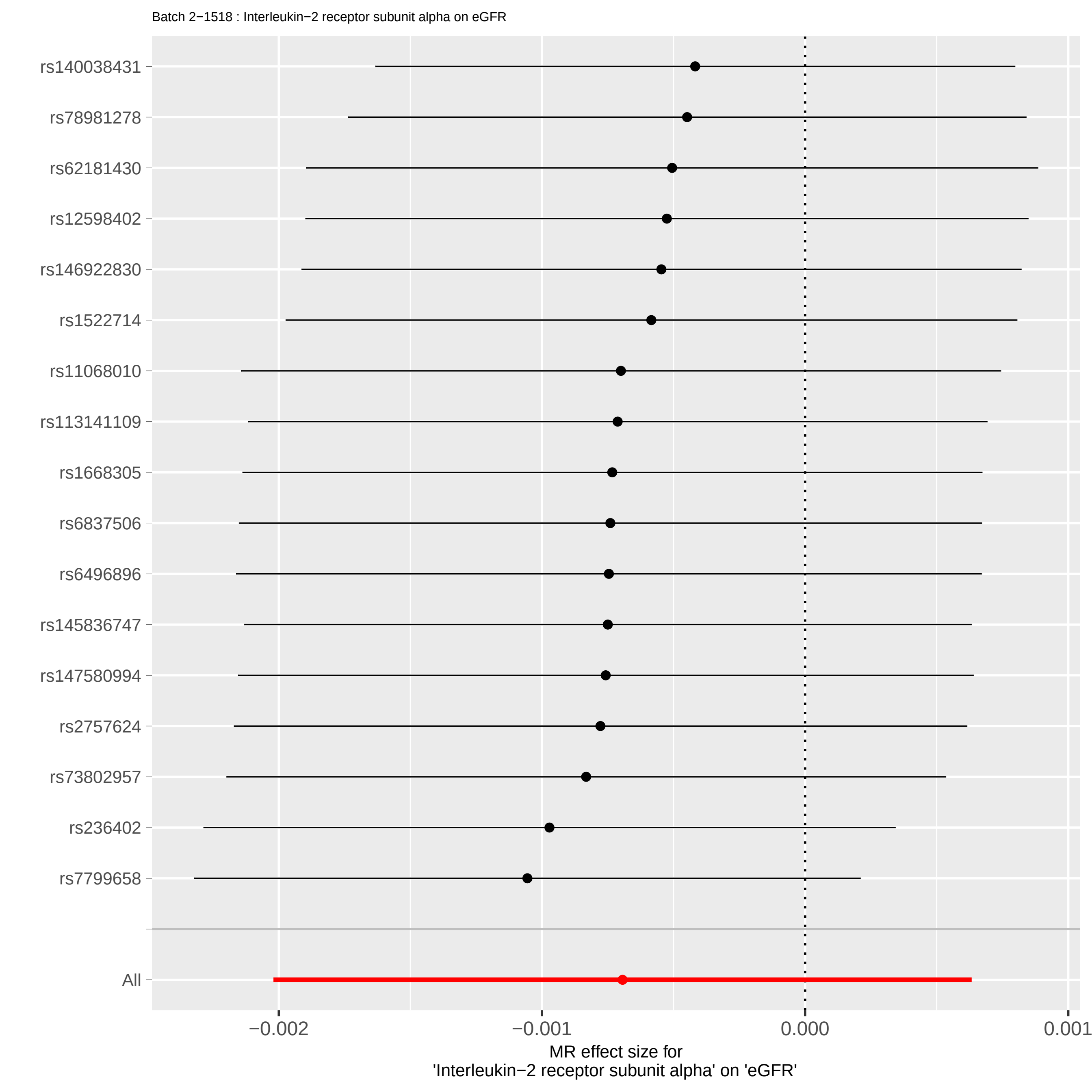

Batch 2−1518 : Interleukin−2 receptor subunit alpha on eGFR
rs140038431
rs78981278
rs62181430
rs12598402
rs146922830
rs1522714
rs11068010
rs113141109
rs1668305
rs6837506
rs6496896
rs145836747
rs147580994
rs2757624
rs73802957
rs236402
rs7799658
All
−0.002
−0.001
0.000
0.001
MR effect size for
'Interleukin−2 receptor subunit alpha' on 'eGFR'

Batch 2−1519 : Interleukin−2 receptor subunit beta on eGFR

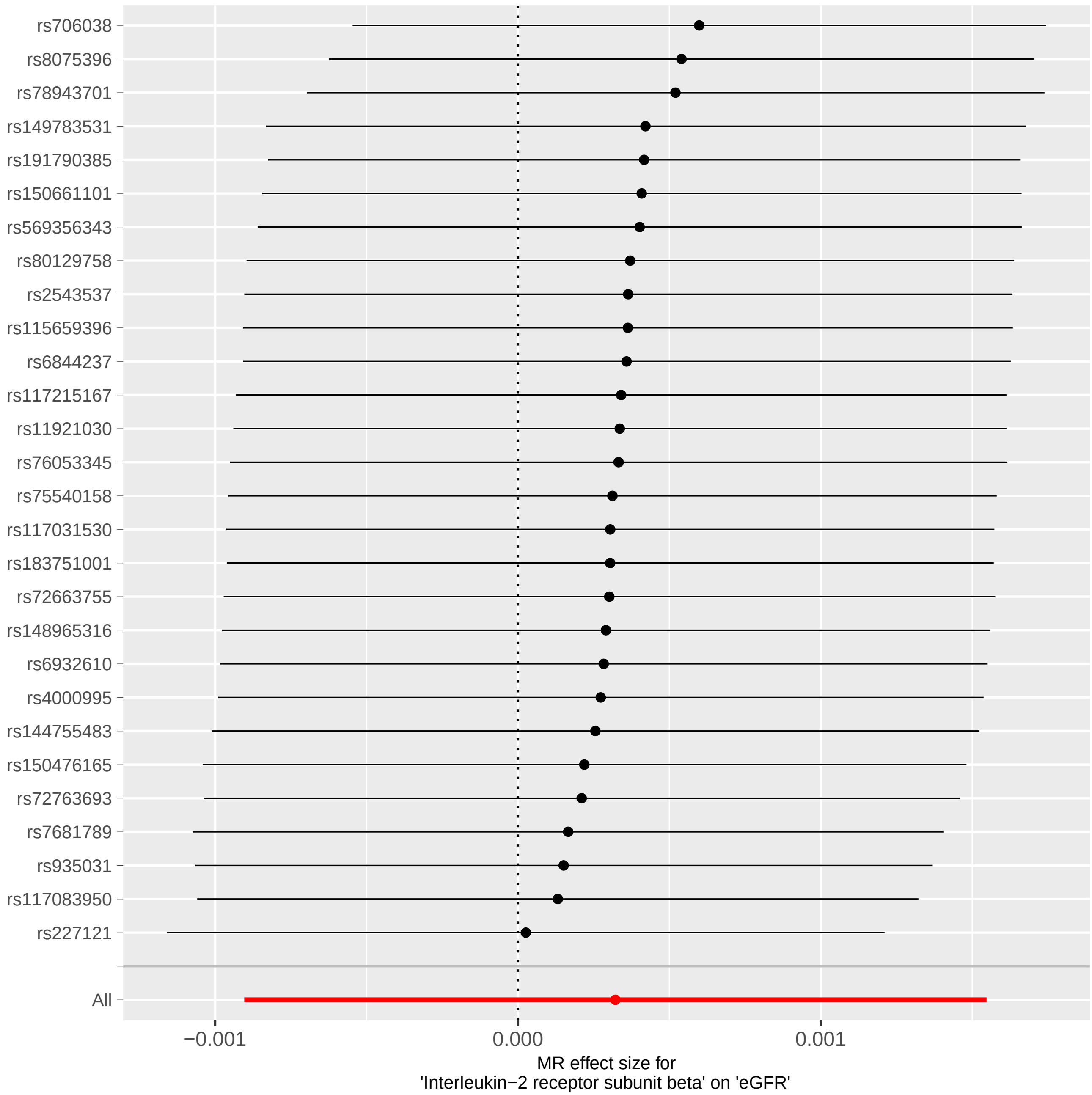

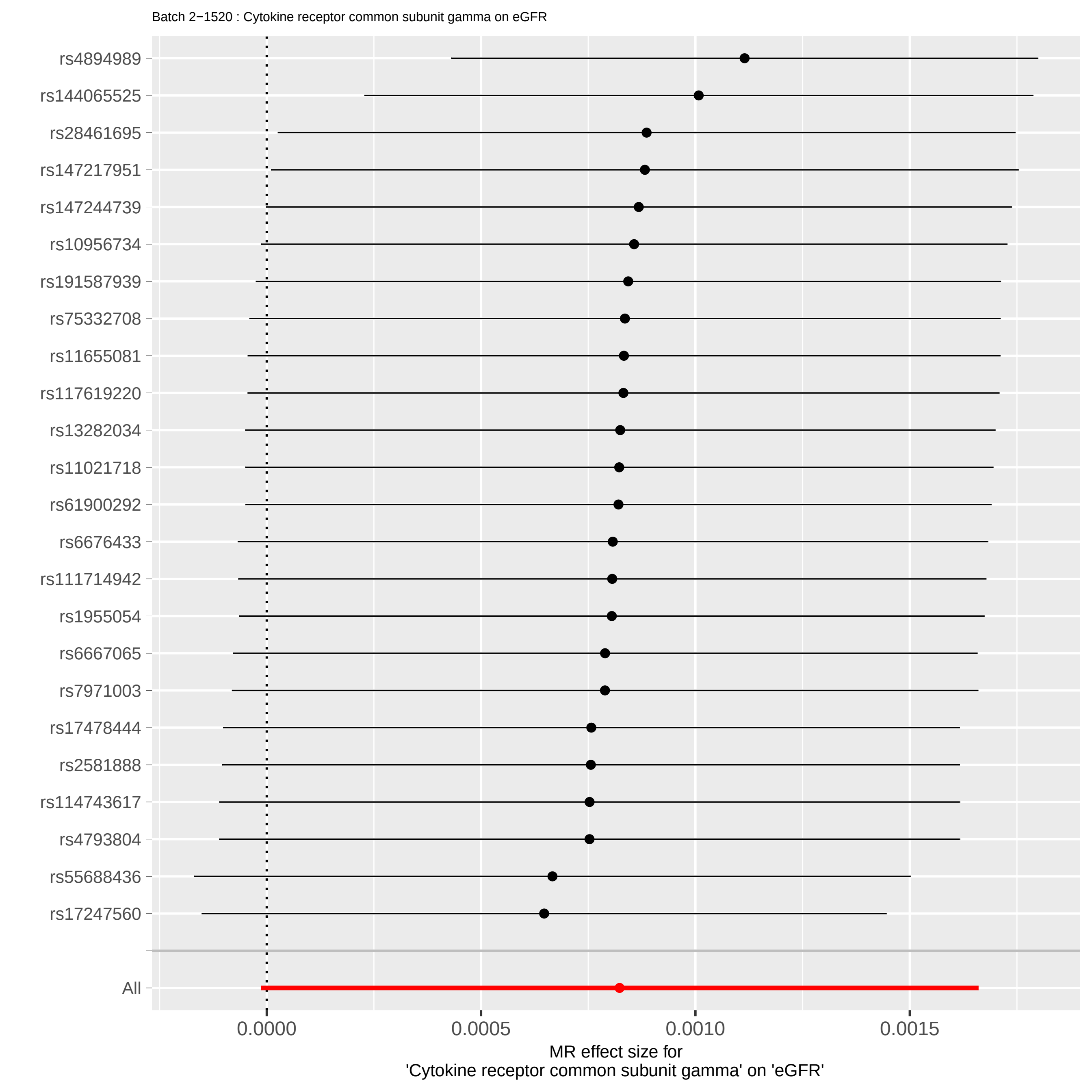
Batch 2−1520 : Cytokine receptor common subunit gamma on eGFR
rs4894989
rs144065525
rs28461695
rs147217951
rs147244739
rs10956734
rs191587939
rs75332708
rs11655081
rs117619220
rs13282034
rs11021718
rs61900292
rs6676433
rs111714942
rs1955054
rs6667065
rs7971003
rs17478444
rs2581888
rs114743617
rs4793804
rs55688436
rs17247560
All
0.0000
0.0005
0.0010
0.0015
MR effect size for
'Cytokine receptor common subunit gamma' on 'eGFR'

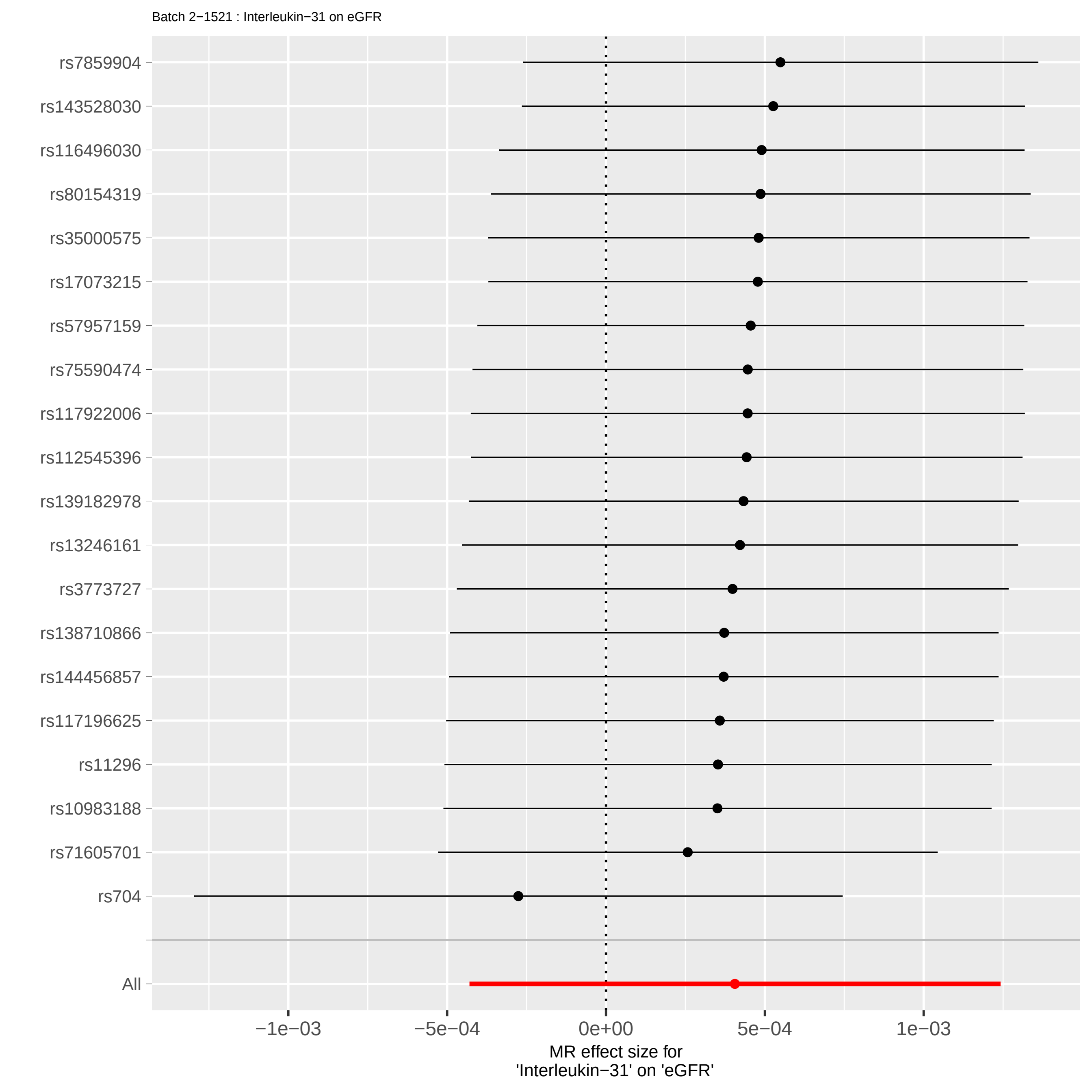

Batch 2−1521 : Interleukin−31 on eGFR
rs7859904
rs143528030
rs116496030
rs80154319
rs35000575
rs17073215
rs57957159
rs75590474
rs117922006
rs112545396
rs139182978
rs13246161
rs3773727
rs138710866
rs144456857
rs117196625
rs11296
rs10983188
rs71605701
rs704
All
−1e−03
−5e−04
0e+00
5e−04
1e−03
MR effect size for
'Interleukin−31' on 'eGFR'

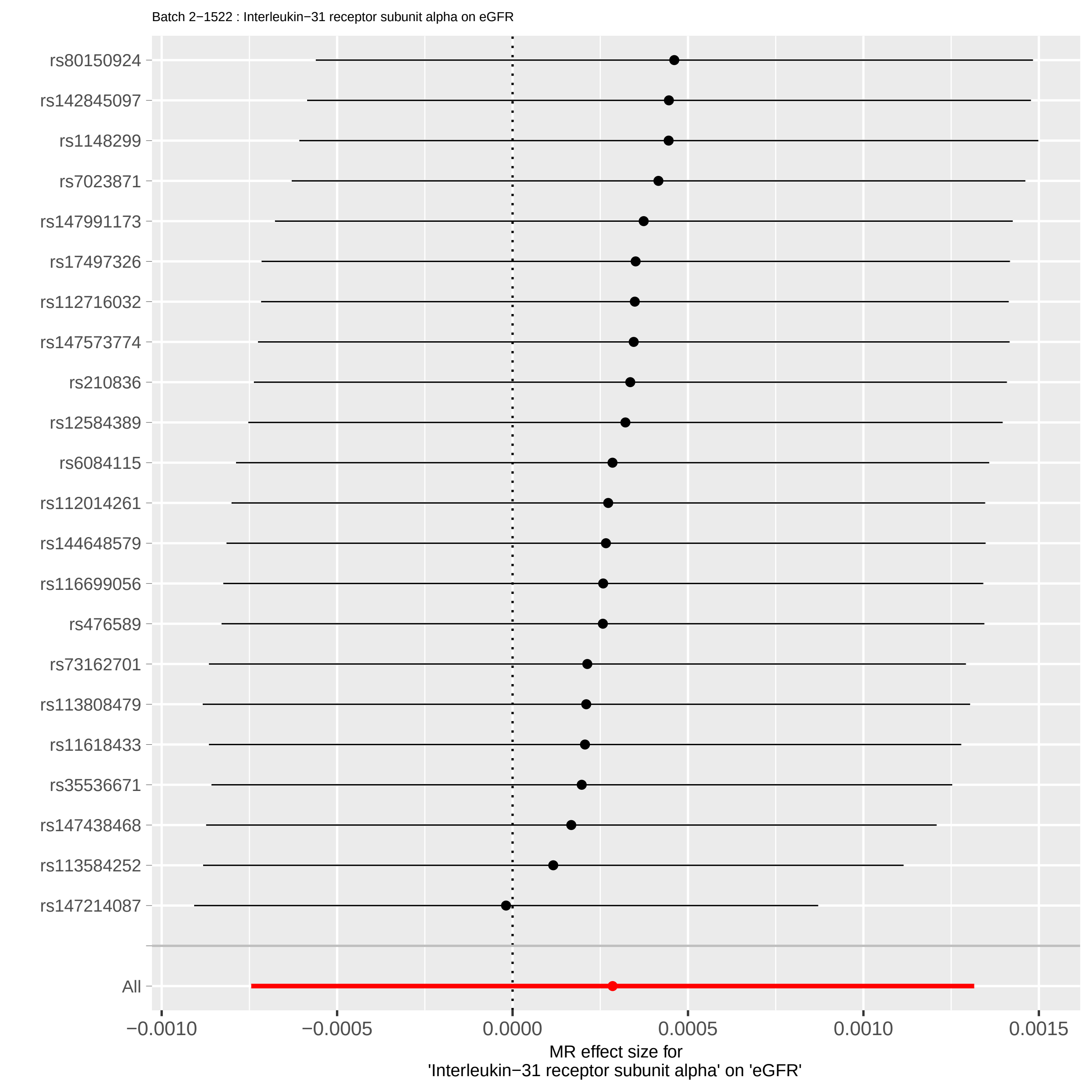

Batch 2−1522 : Interleukin−31 receptor subunit alpha on eGFR
rs80150924
rs142845097
rs1148299
rs7023871
rs147991173
rs17497326
rs112716032
rs147573774
rs210836
rs12584389
rs6084115
rs112014261
rs144648579
rs116699056
rs476589
rs73162701
rs113808479
rs11618433
rs35536671
rs147438468
rs113584252
rs147214087
All
−0.0010
−0.0005
0.0000
0.0005
0.0010
0.0015
MR effect size for
'Interleukin−31 receptor subunit alpha' on 'eGFR'

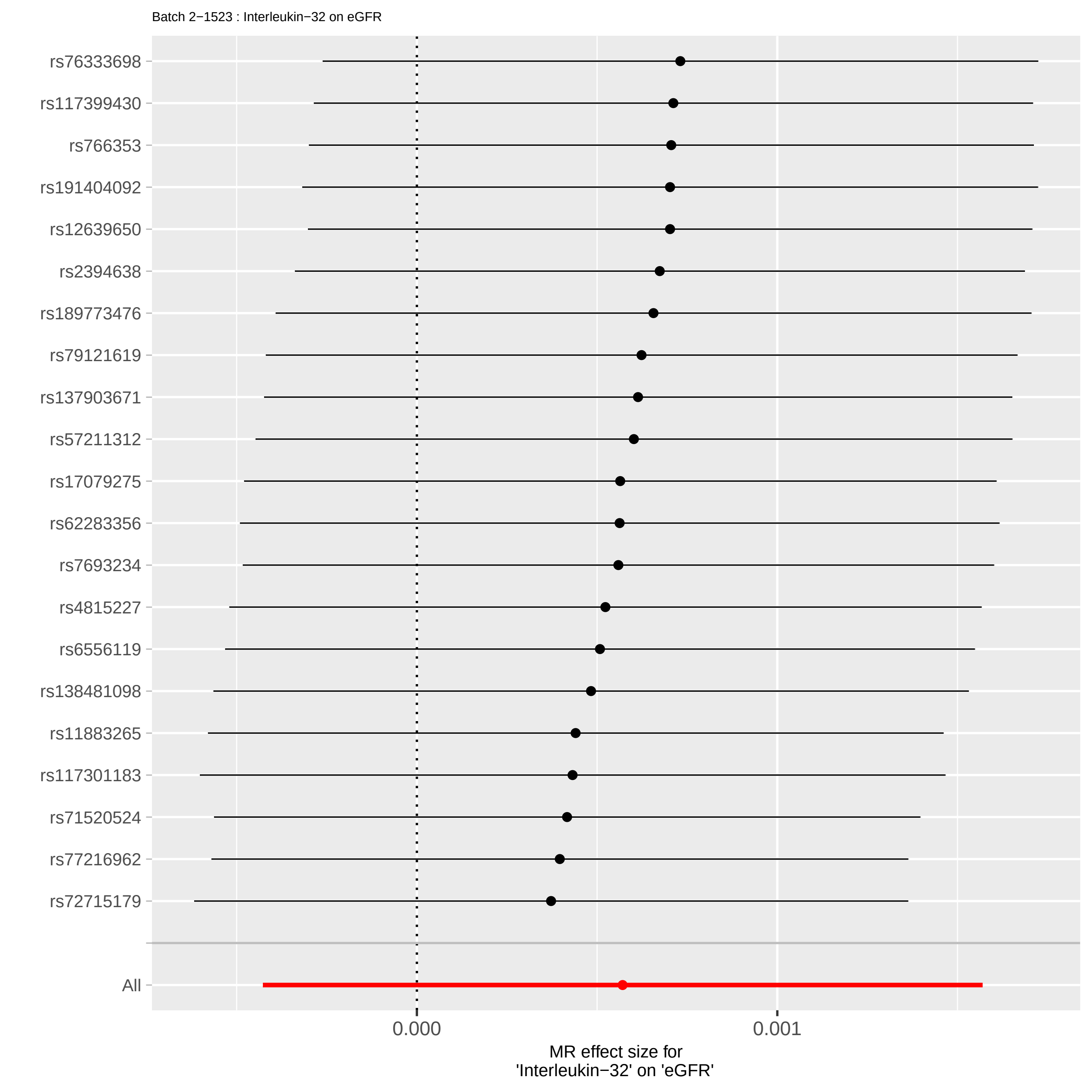
Batch 2−1523 : Interleukin−32 on eGFR
rs76333698
rs117399430
rs766353
rs191404092
rs12639650
rs2394638
rs189773476
rs79121619
rs137903671
rs57211312
rs17079275
rs62283356
rs7693234
rs4815227
rs6556119
rs138481098
rs11883265
rs117301183
rs71520524
rs77216962
rs72715179
All
0.000
0.001
MR effect size for
'Interleukin−32' on 'eGFR'

Batch 2−1524 : Interleukin−34 on eGFR

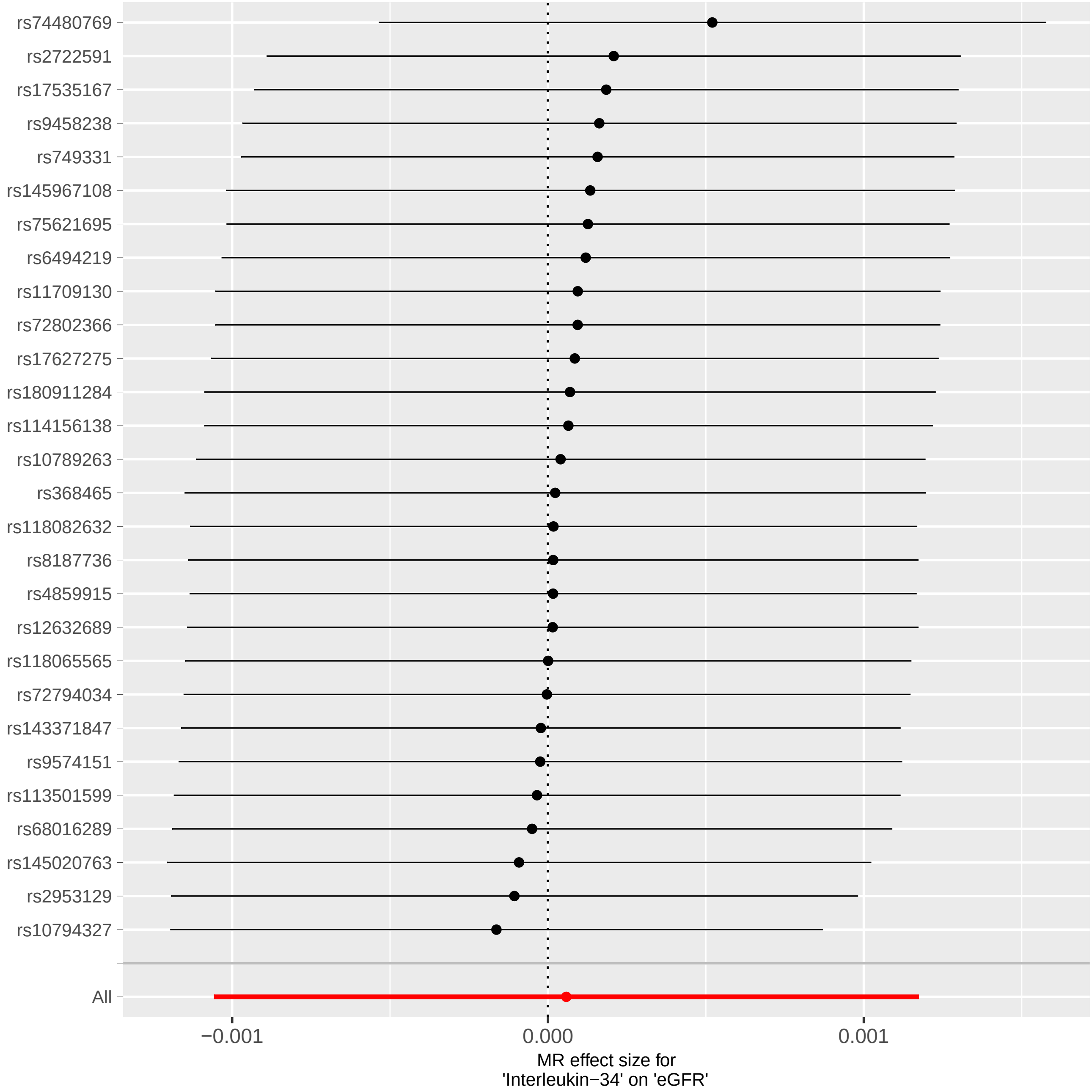

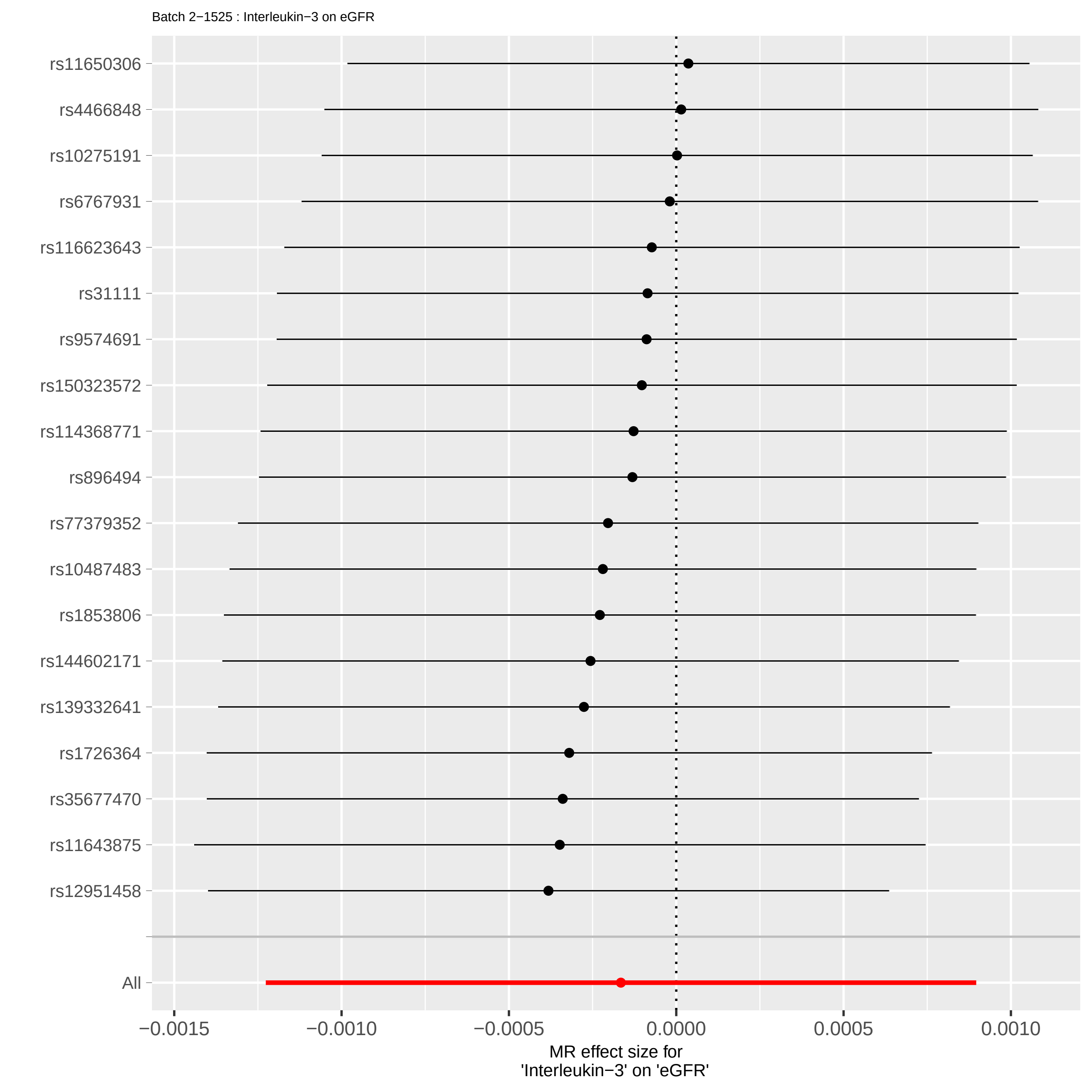

Batch 2−1525 : Interleukin−3 on eGFR
rs11650306
rs4466848
rs10275191
rs6767931
rs116623643
rs31111
rs9574691
rs150323572
rs114368771
rs896494
rs77379352
rs10487483
rs1853806
rs144602171
rs139332641
rs1726364
rs35677470
rs11643875
rs12951458
All
−0.0015
−0.0010
−0.0005
0.0000
0.0005
0.0010
MR effect size for
'Interleukin−3' on 'eGFR'

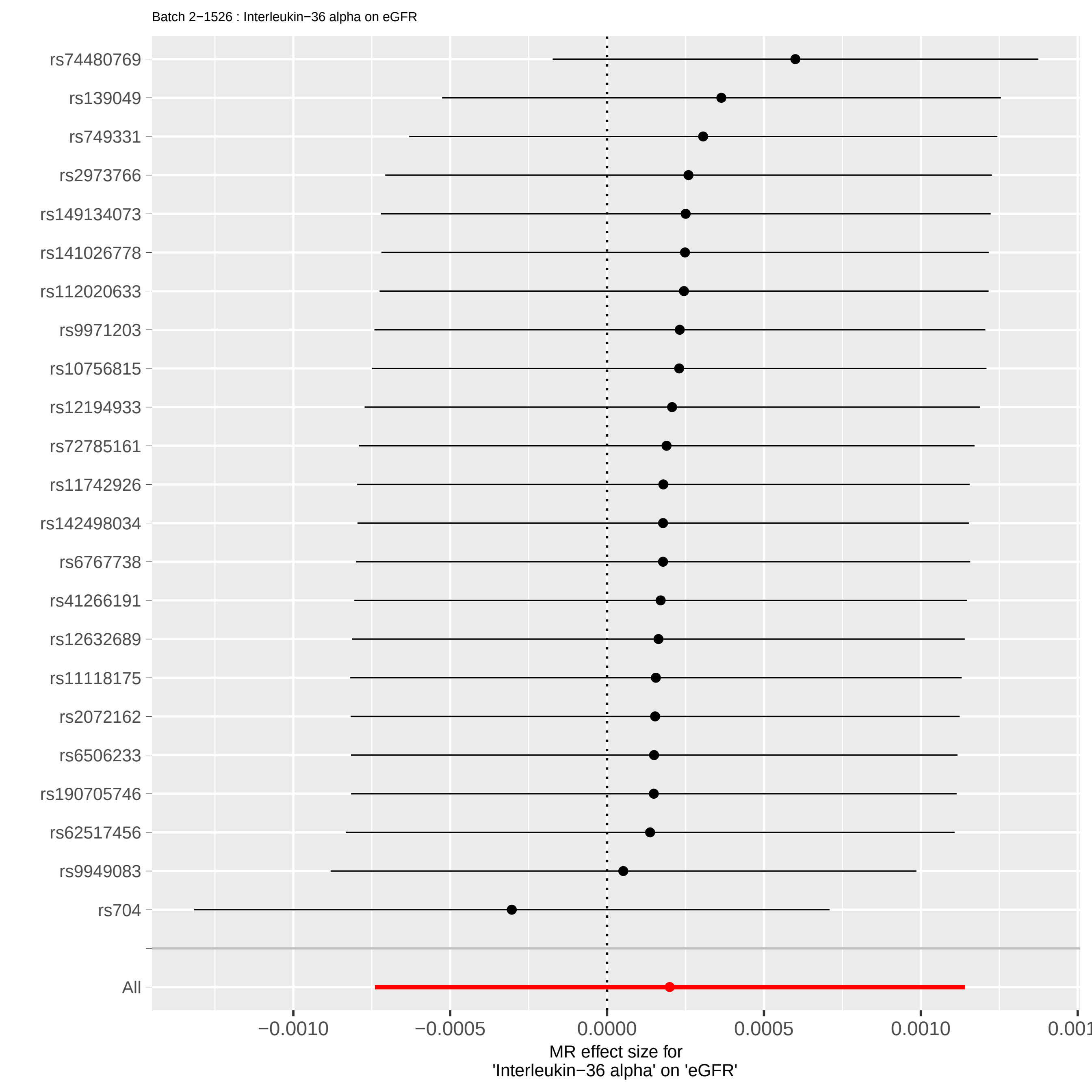

Batch 2−1526 : Interleukin−36 alpha on eGFR
rs74480769
rs139049
rs749331
rs2973766
rs149134073
rs141026778
rs112020633
rs9971203
rs10756815
rs12194933
rs72785161
rs11742926
rs142498034
rs6767738
rs41266191
rs12632689
rs11118175
rs2072162
rs6506233
rs190705746
rs62517456
rs9949083
rs704
All
−0.0010
−0.0005
0.0000
0.0005
0.0010
MR effect size for
'Interleukin−36 alpha' on 'eGFR'

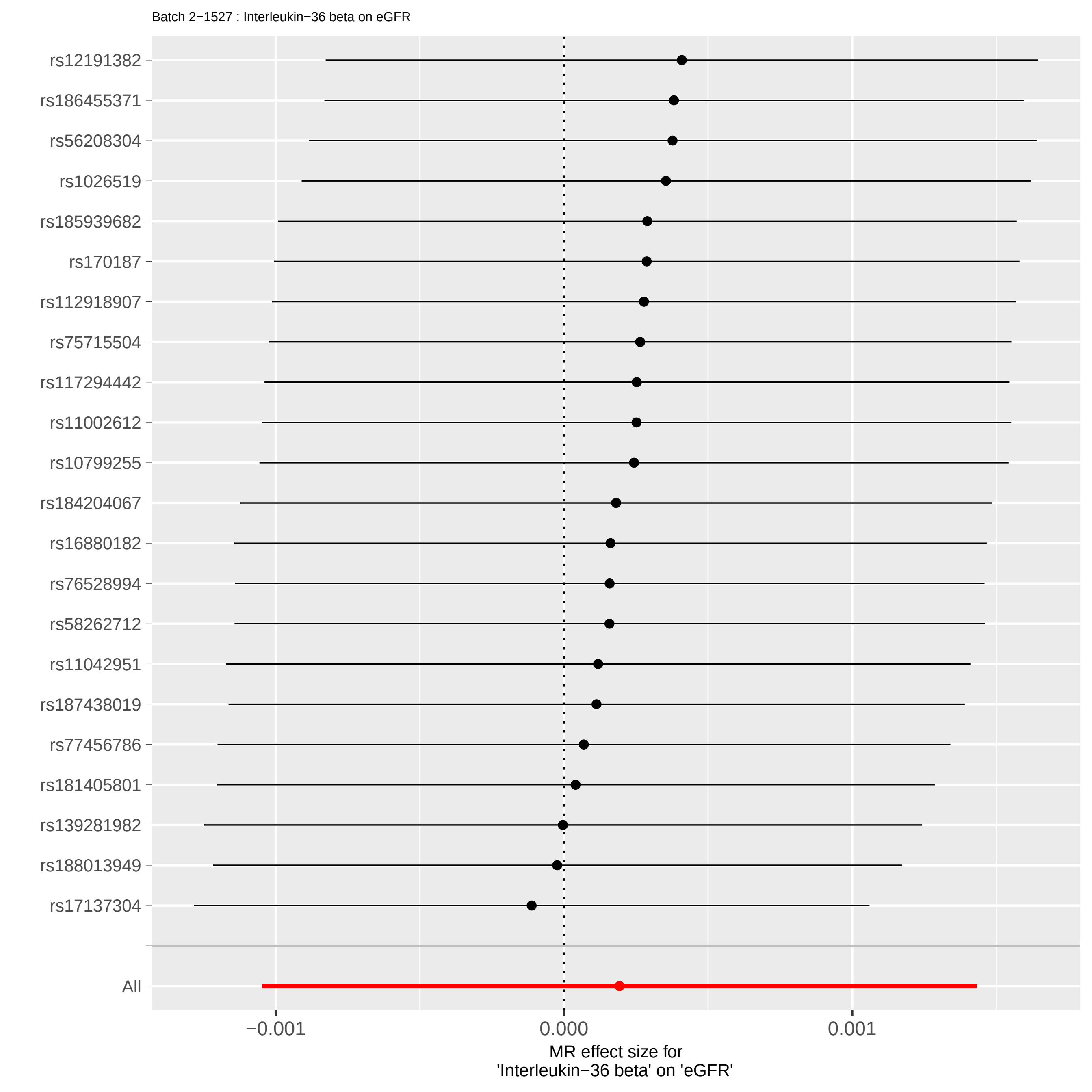

Batch 2−1527 : Interleukin−36 beta on eGFR
rs12191382
rs186455371
rs56208304
rs1026519
rs185939682
rs170187
rs112918907
rs75715504
rs117294442
rs11002612
rs10799255
rs184204067
rs16880182
rs76528994
rs58262712
rs11042951
rs187438019
rs77456786
rs181405801
rs139281982
rs188013949
rs17137304
All
−0.001
0.000
0.001
MR effect size for
'Interleukin−36 beta' on 'eGFR'

Batch 2−1528 : Interleukin−36 gamma on eGFR

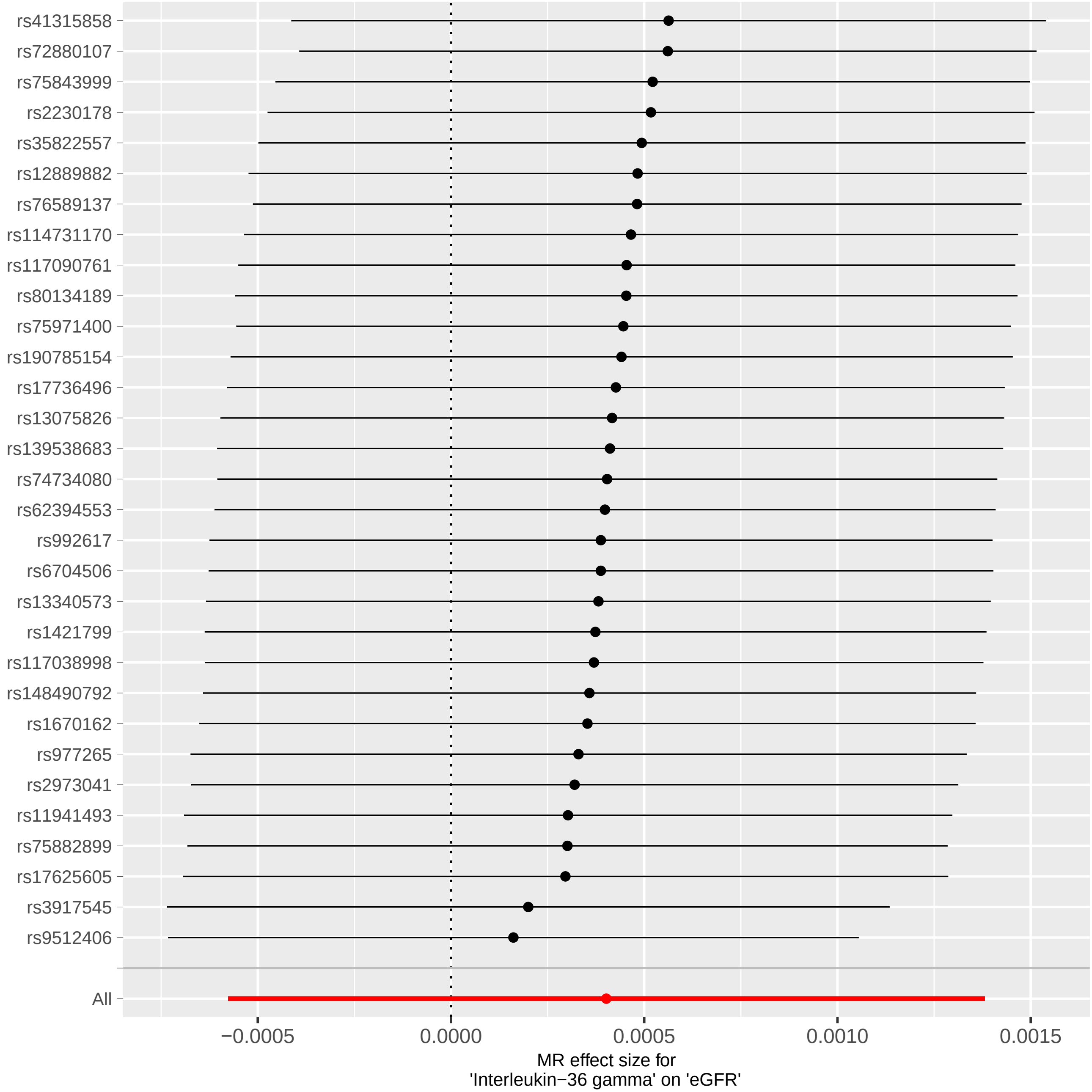

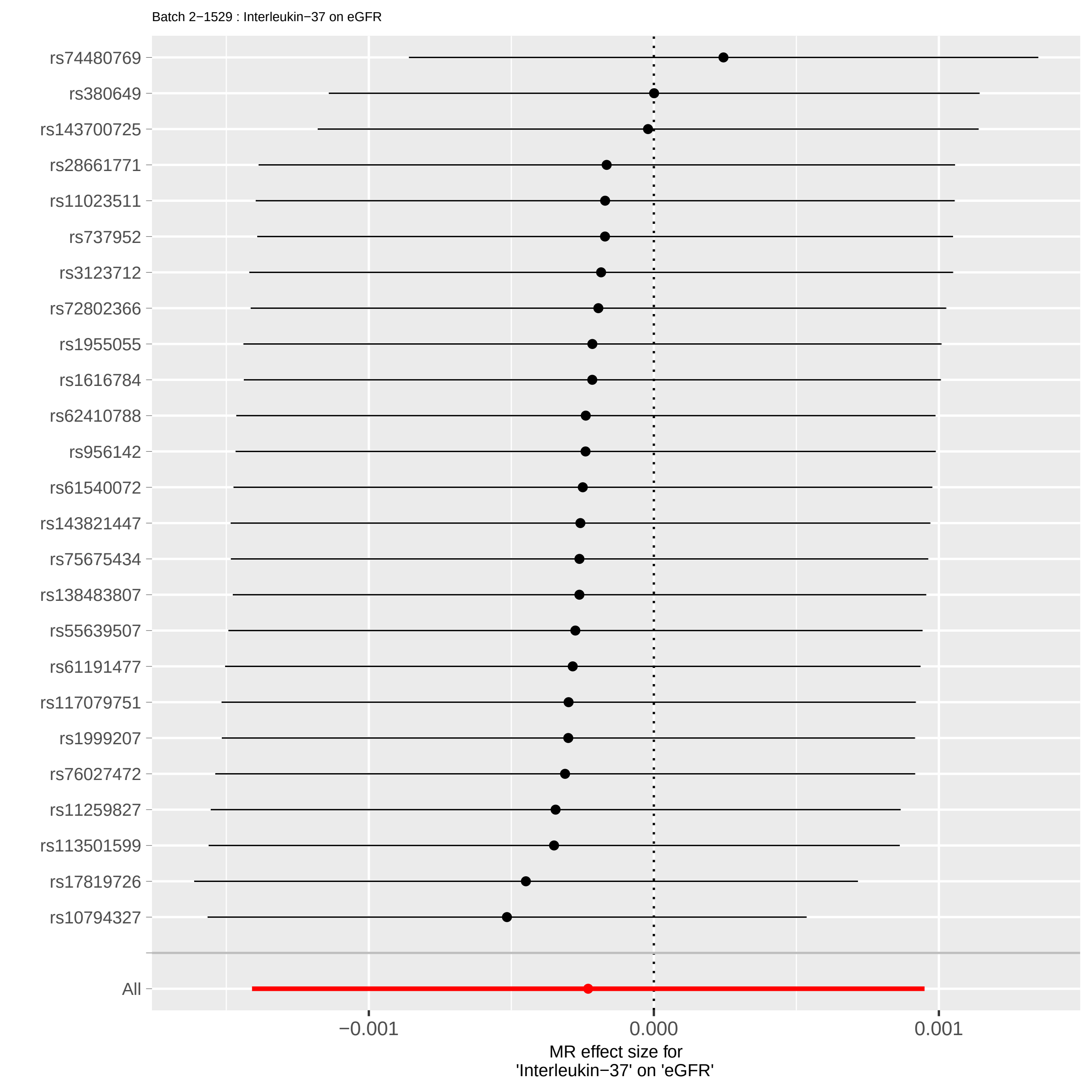

Batch 2−1529 : Interleukin−37 on eGFR
rs74480769
rs380649
rs143700725
rs28661771
rs11023511
rs737952
rs3123712
rs72802366
rs1955055
rs1616784
rs62410788
rs956142
rs61540072
rs143821447
rs75675434
rs138483807
rs55639507
rs61191477
rs117079751
rs1999207
rs76027472
rs11259827
rs113501599
rs17819726
rs10794327
All
−0.001
0.000
0.001
MR effect size for
'Interleukin−37' on 'eGFR'

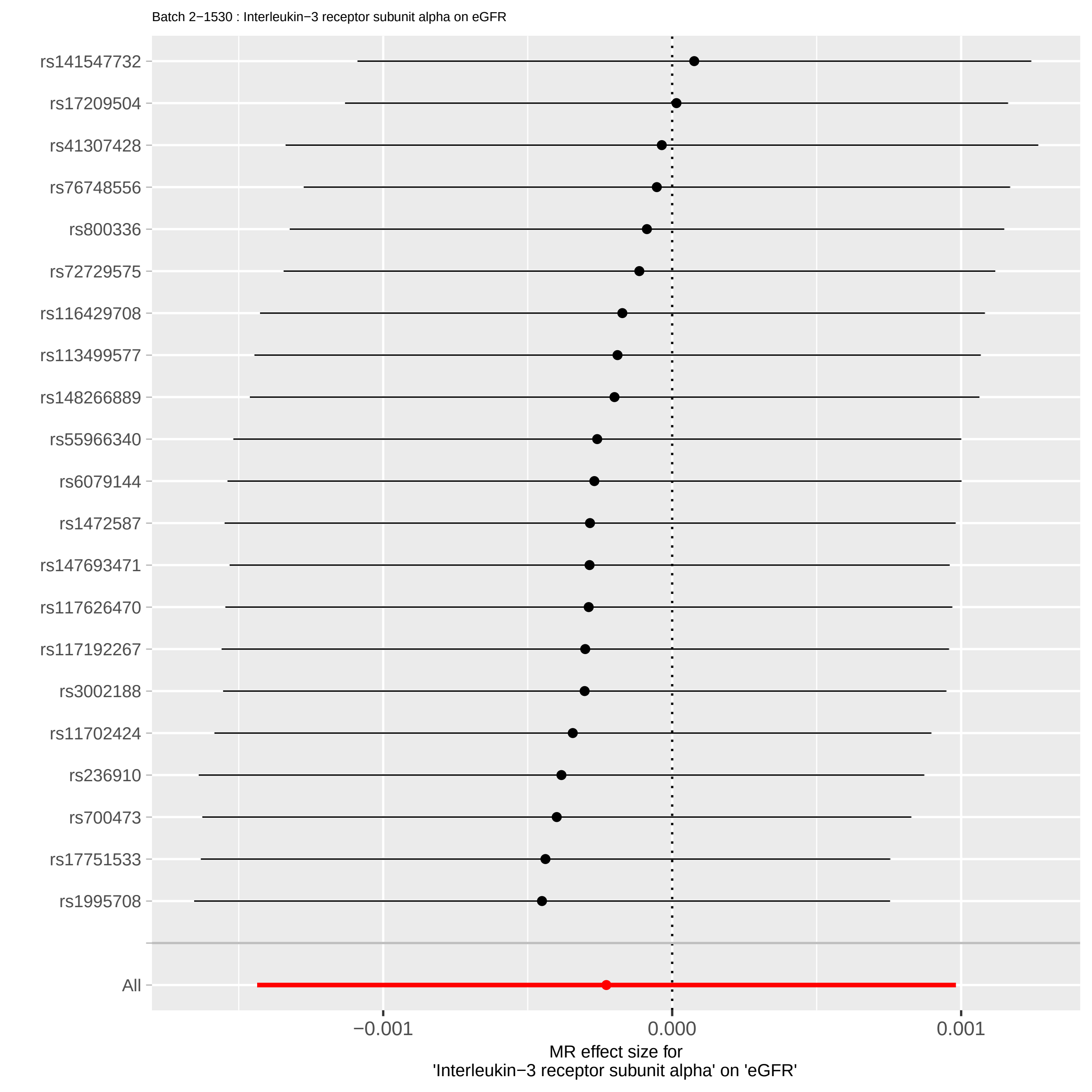

Batch 2−1530 : Interleukin−3 receptor subunit alpha on eGFR
rs141547732
rs17209504
rs41307428
rs76748556
rs800336
rs72729575
rs116429708
rs113499577
rs148266889
rs55966340
rs6079144
rs1472587
rs147693471
rs117626470
rs117192267
rs3002188
rs11702424
rs236910
rs700473
rs17751533
rs1995708
All
−0.001
0.000
0.001
MR effect size for
'Interleukin−3 receptor subunit alpha' on 'eGFR'

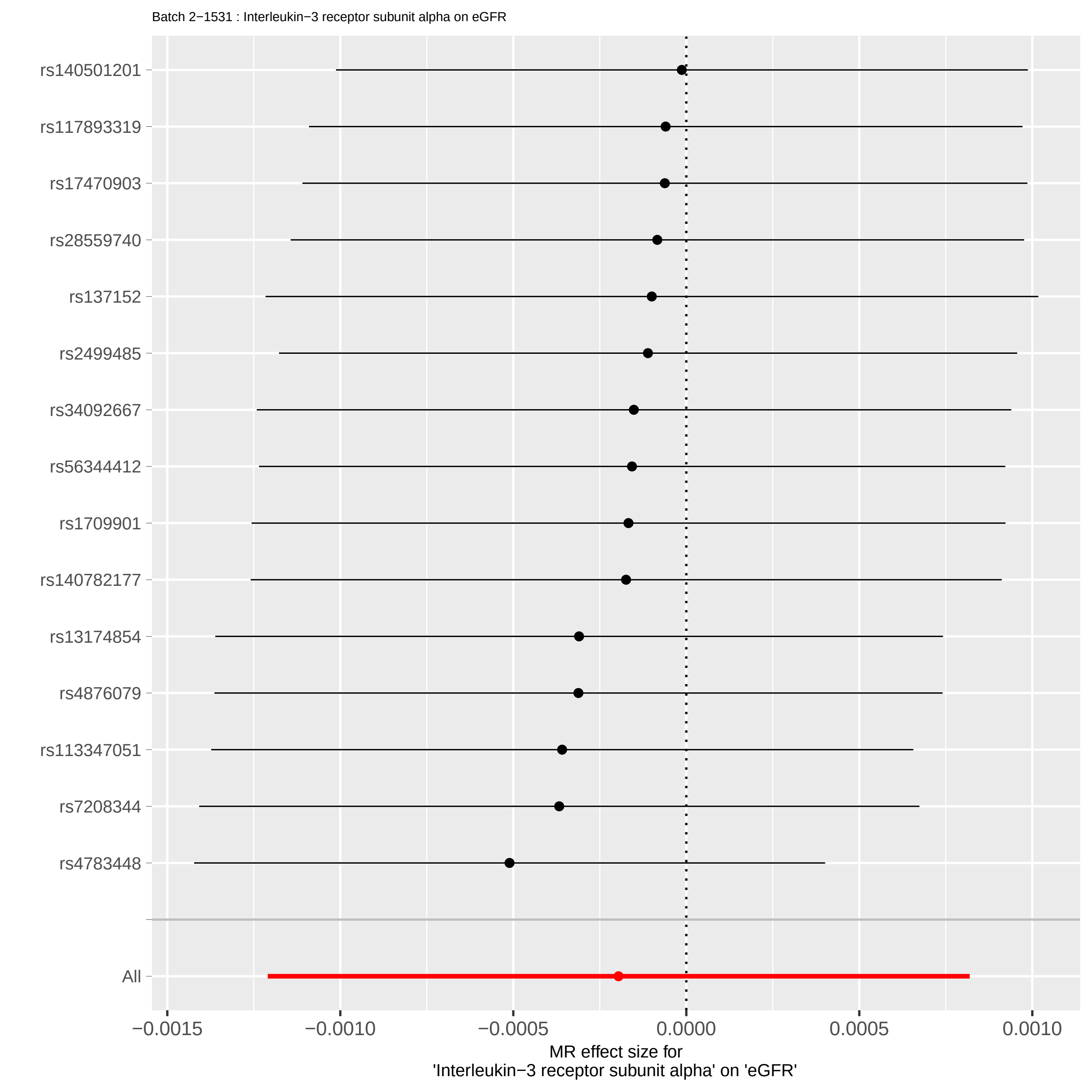
Batch 2−1531 : Interleukin−3 receptor subunit alpha on eGFR
rs140501201
rs117893319
rs17470903
rs28559740
rs137152
rs2499485
rs34092667
rs56344412
rs1709901
rs140782177
rs13174854
rs4876079
rs113347051
rs7208344
rs4783448
All
−0.0015
−0.0010
−0.0005
0.0000
0.0005
0.0010
MR effect size for
'Interleukin−3 receptor subunit alpha' on 'eGFR'

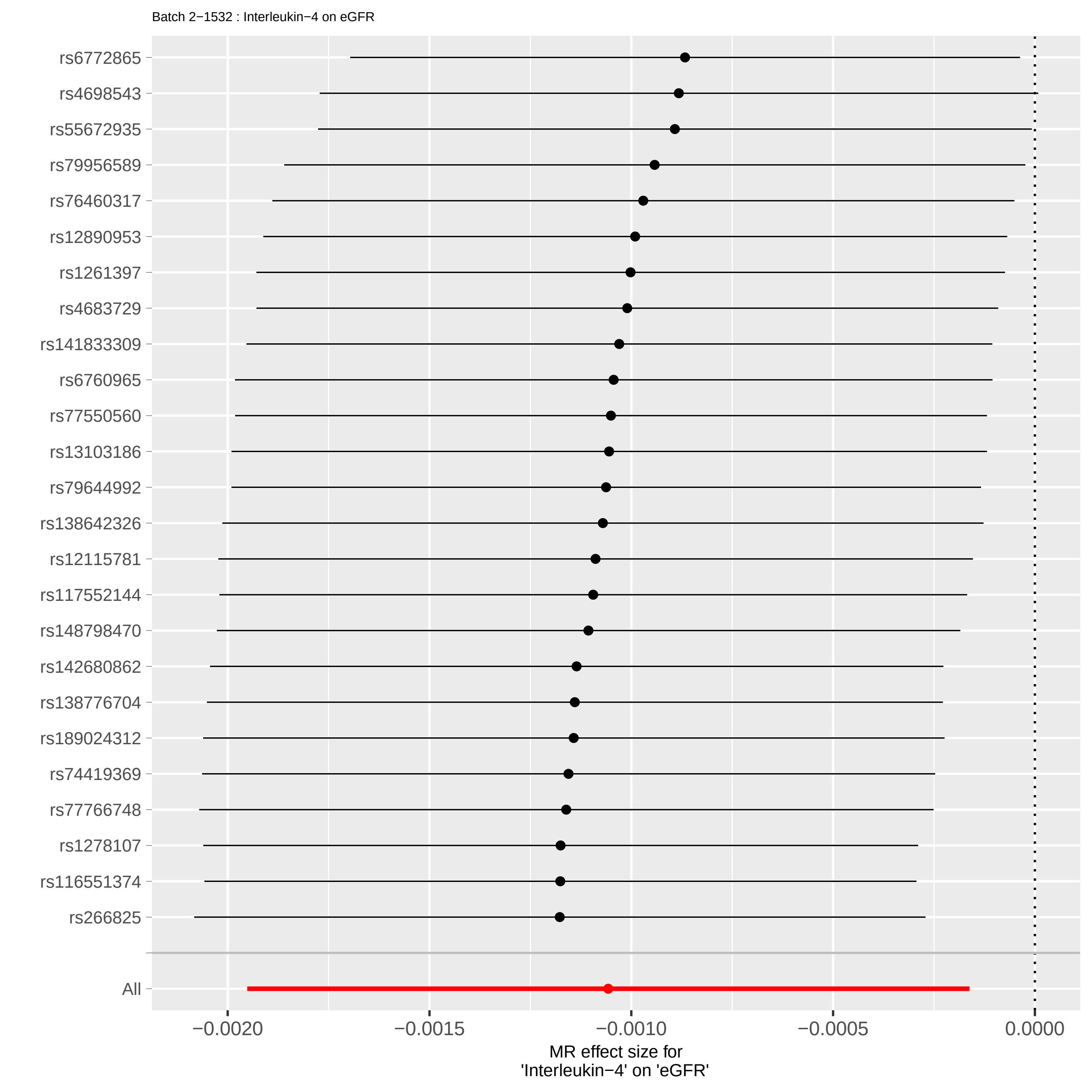

Batch 2−1532 : Interleukin−4 on eGFR
rs6772865
rs4698543
rs55672935
rs79956589
rs76460317
rs12890953
rs1261397
rs4683729
rs141833309
rs6760965
rs77550560
rs13103186
rs79644992
rs138642326
rs12115781
rs117552144
rs148798470
rs142680862
rs138776704
rs189024312
rs74419369
rs77766748
rs1278107
rs116551374
rs266825
All
−0.0020
−0.0015
−0.0010
−0.0005
0.0000
MR effect size for
'Interleukin−4' on 'eGFR'

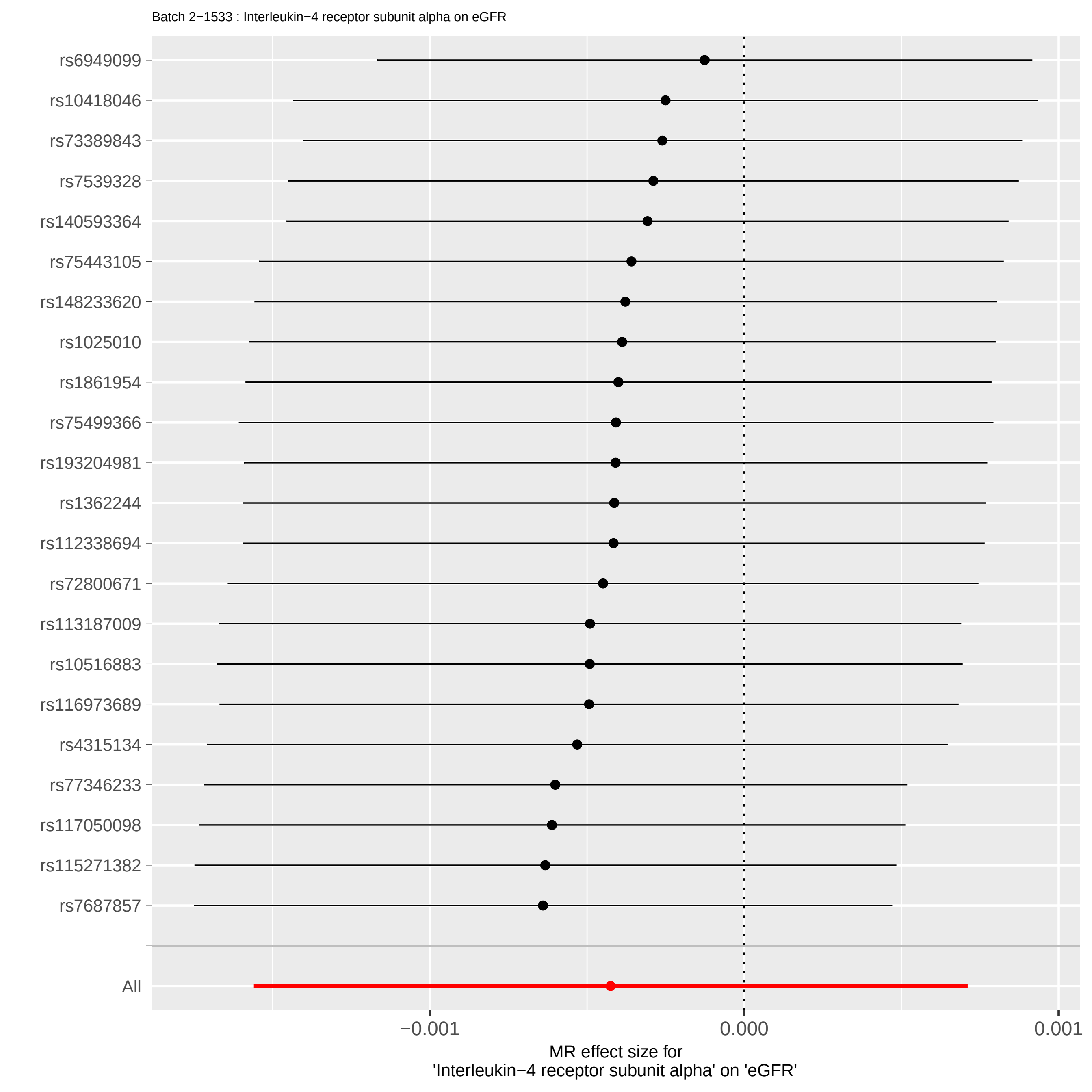
Batch 2−1533 : Interleukin−4 receptor subunit alpha on eGFR
rs6949099
rs10418046
rs73389843
rs7539328
rs140593364
rs75443105
rs148233620
rs1025010
rs1861954
rs75499366
rs193204981
rs1362244
rs112338694
rs72800671
rs113187009
rs10516883
rs116973689
rs4315134
rs77346233
rs117050098
rs115271382
rs7687857
All
−0.001
0.000
0.001
MR effect size for
'Interleukin−4 receptor subunit alpha' on 'eGFR'

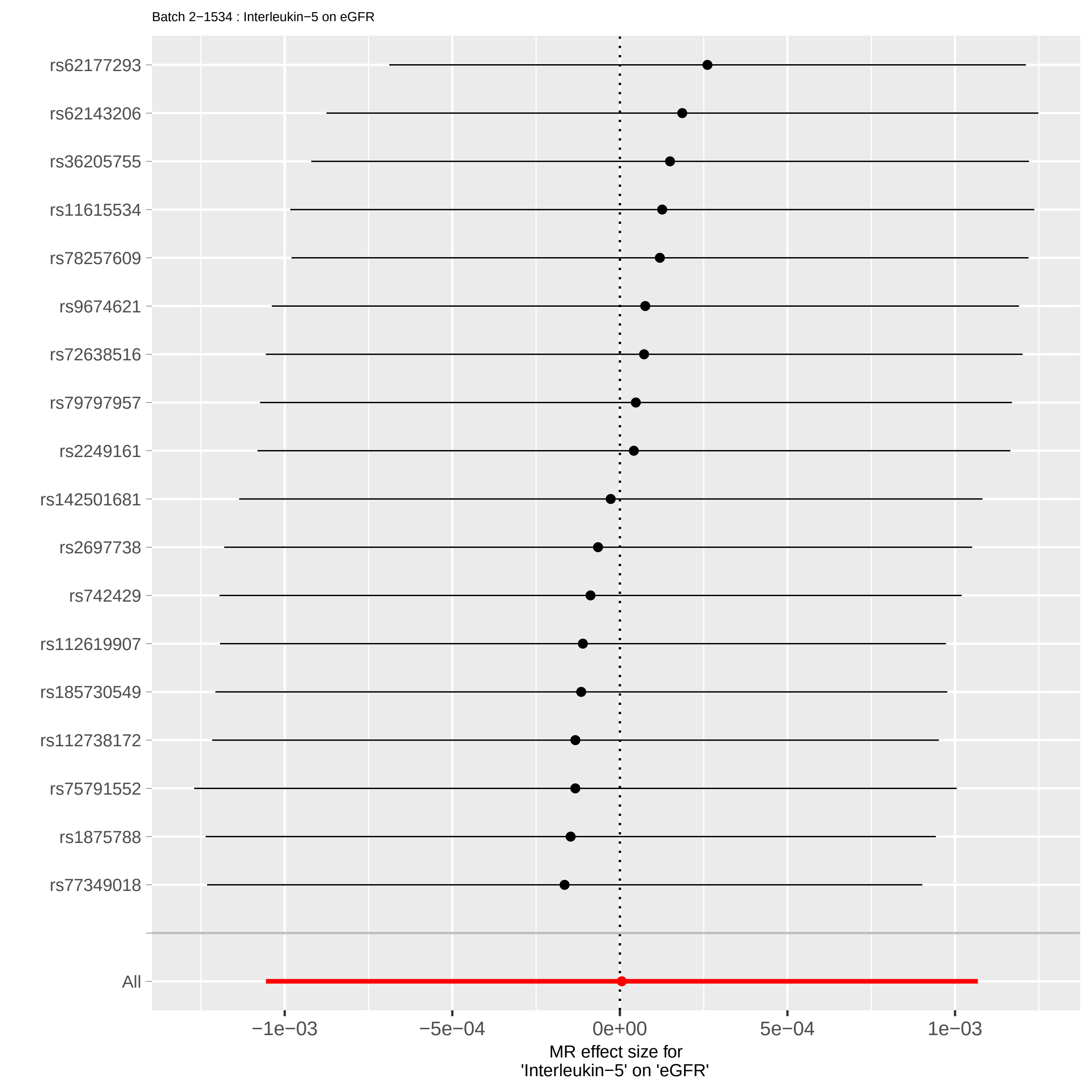

Batch 2−1534 : Interleukin−5 on eGFR
rs62177293
rs62143206
rs36205755
rs11615534
rs78257609
rs9674621
rs72638516
rs79797957
rs2249161
rs142501681
rs2697738
rs742429
rs112619907
rs185730549
rs112738172
rs75791552
rs1875788
rs77349018
All
−1e−03
−5e−04
0e+00
5e−04
1e−03
MR effect size for
'Interleukin−5' on 'eGFR'

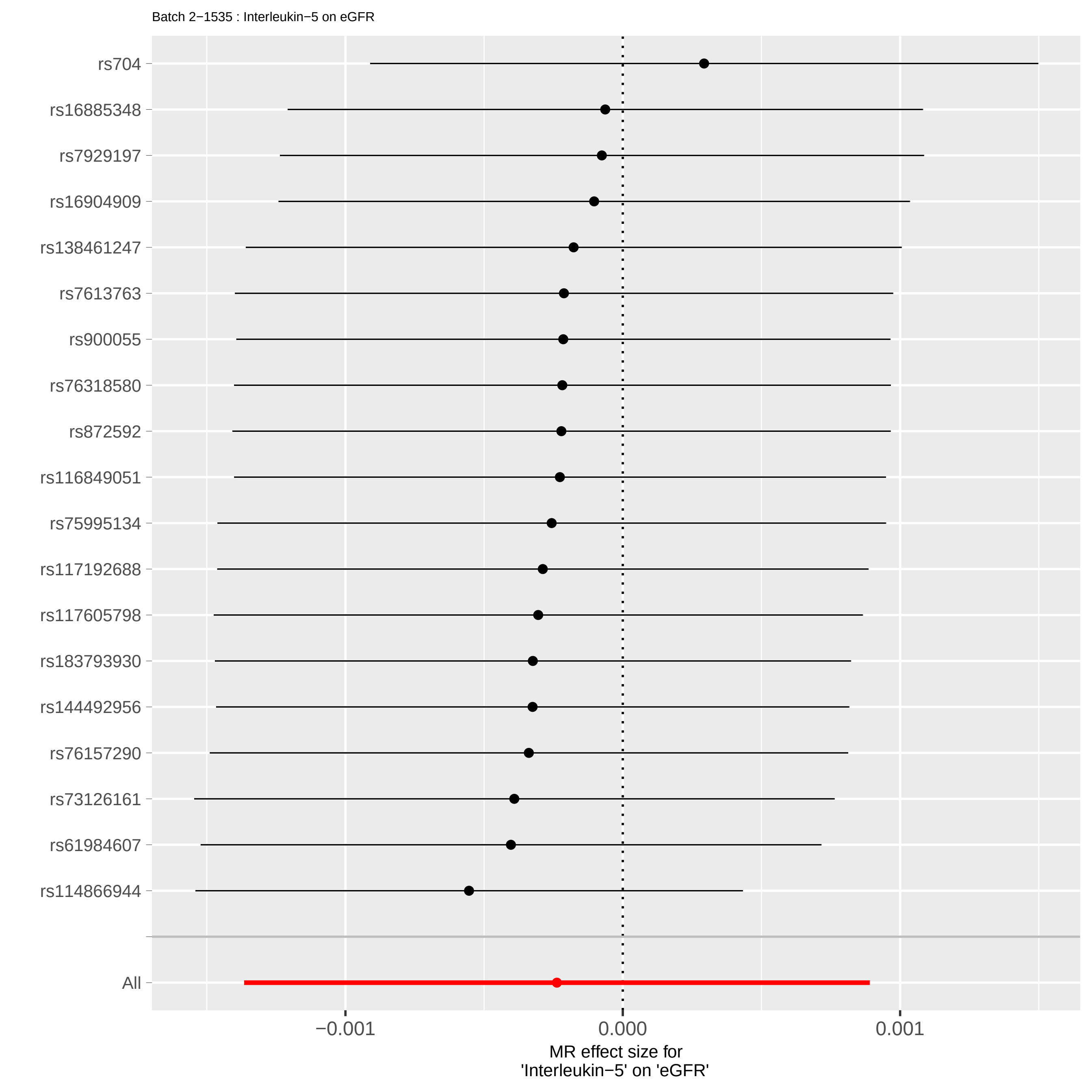

Batch 2−1535 : Interleukin−5 on eGFR
rs704
rs16885348
rs7929197
rs16904909
rs138461247
rs7613763
rs900055
rs76318580
rs872592
rs116849051
rs75995134
rs117192688
rs117605798
rs183793930
rs144492956
rs76157290
rs73126161
rs61984607
rs114866944
All
−0.001
0.000
0.001
MR effect size for
'Interleukin−5' on 'eGFR'

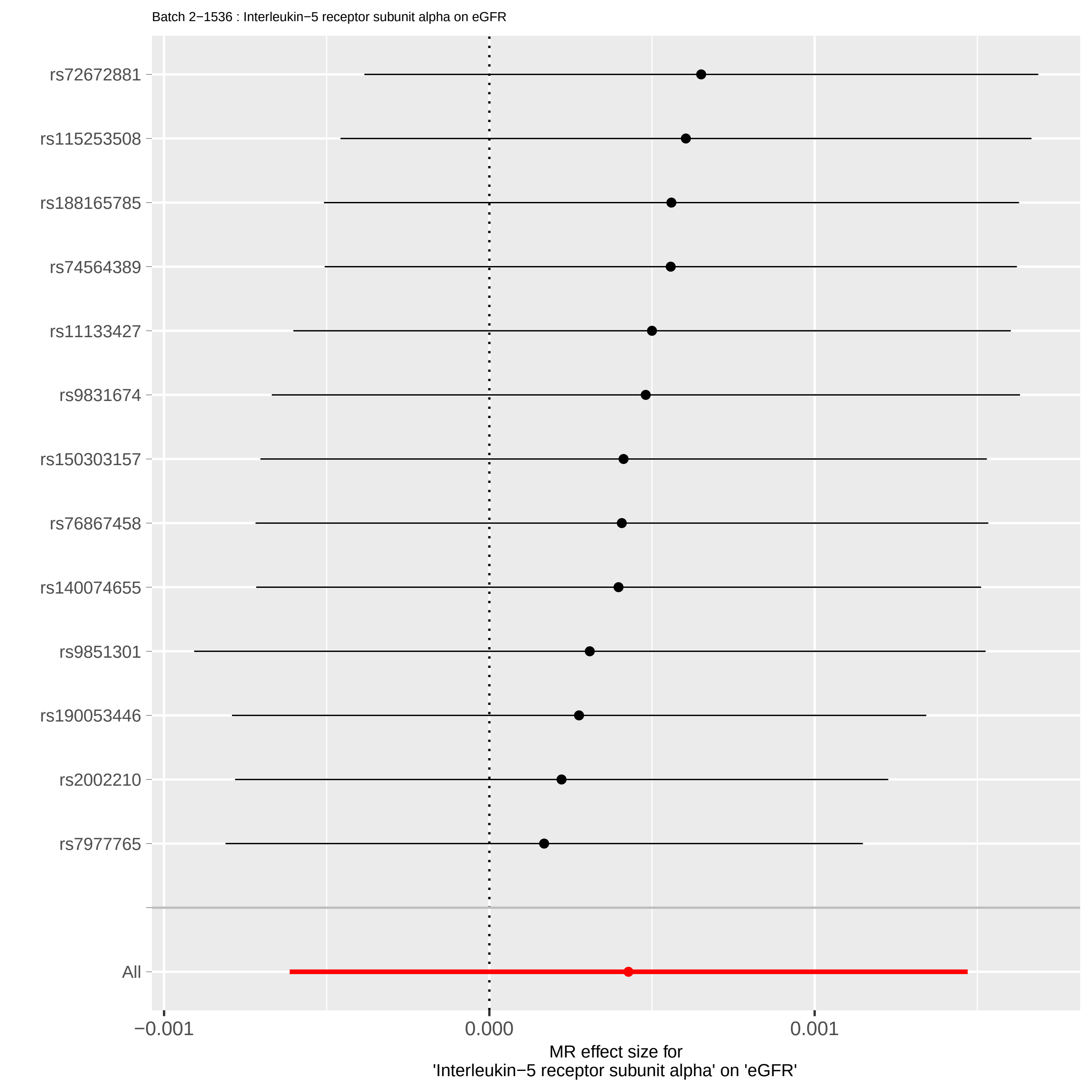

Batch 2−1536 : Interleukin−5 receptor subunit alpha on eGFR
rs72672881
rs115253508
rs188165785
rs74564389
rs11133427
rs9831674
rs150303157
rs76867458
rs140074655
rs9851301
rs190053446
rs2002210
rs7977765
All
−0.001
0.000
0.001
MR effect size for
'Interleukin−5 receptor subunit alpha' on 'eGFR'

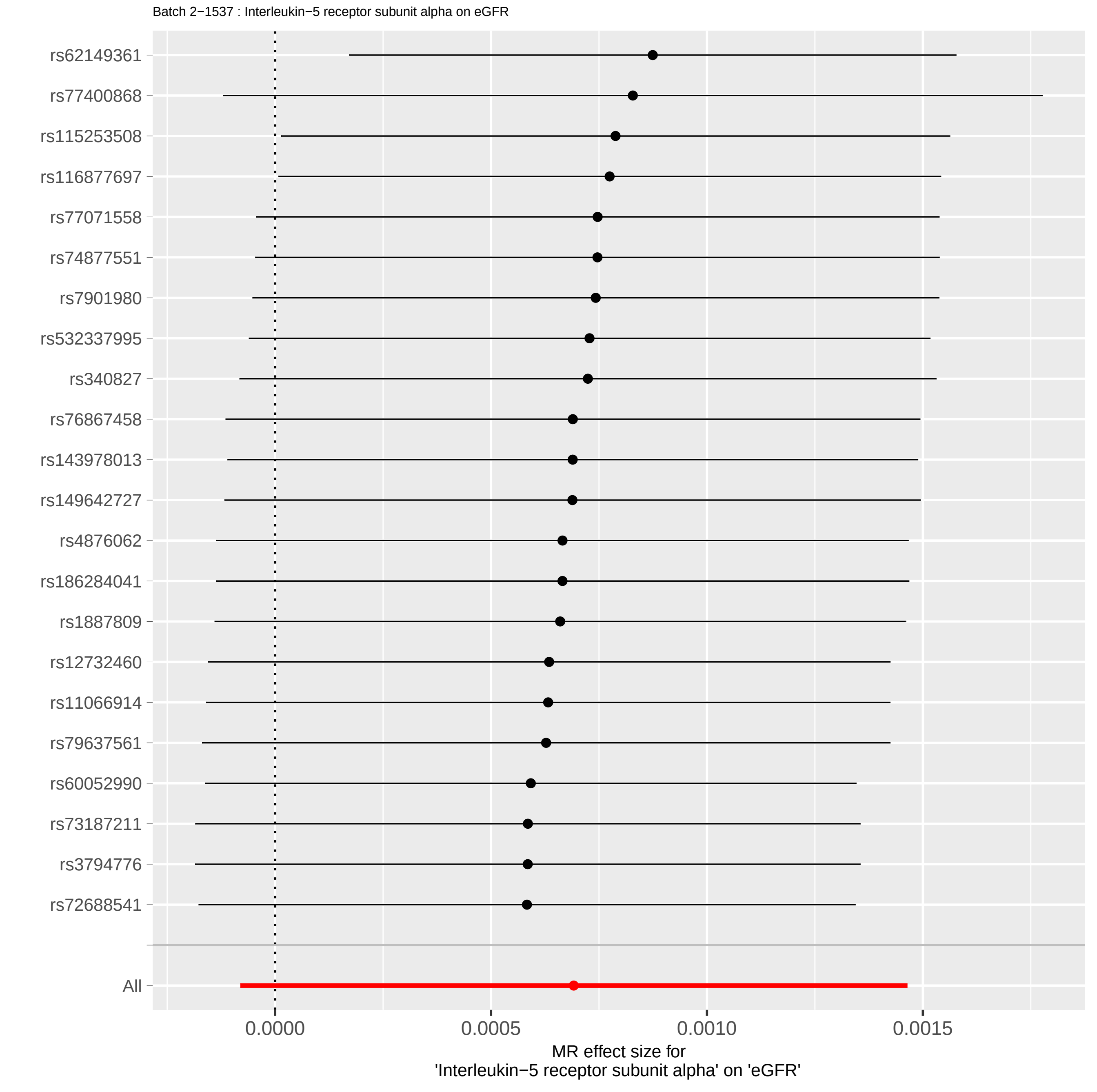

Batch 2−1537 : Interleukin−5 receptor subunit alpha on eGFR
rs62149361
rs77400868
rs115253508
rs116877697
rs77071558
rs74877551
rs7901980
rs532337995
rs340827
rs76867458
rs143978013
rs149642727
rs4876062
rs186284041
rs1887809
rs12732460
rs11066914
rs79637561
rs60052990
rs73187211
rs3794776
rs72688541
All
0.0000
0.0005
0.0010
0.0015
MR effect size for
'Interleukin−5 receptor subunit alpha' on 'eGFR'

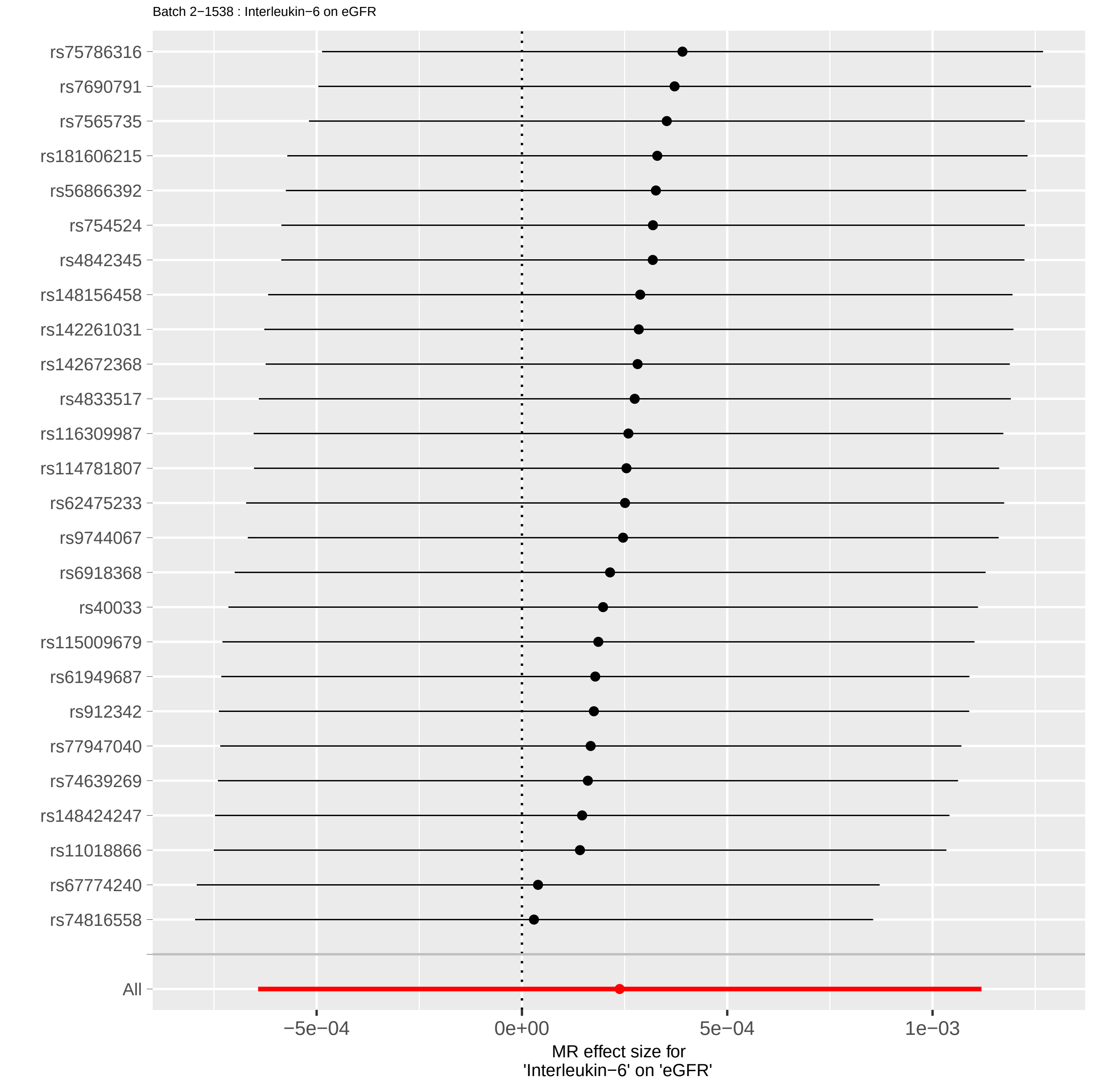

Batch 2−1538 : Interleukin−6 on eGFR
rs75786316
rs7690791
rs7565735
rs181606215
rs56866392
rs754524
rs4842345
rs148156458
rs142261031
rs142672368
rs4833517
rs116309987
rs114781807
rs62475233
rs9744067
rs6918368
rs40033
rs115009679
rs61949687
rs912342
rs77947040
rs74639269
rs148424247
rs11018866
rs67774240
rs74816558
All
−5e−04
0e+00
5e−04
1e−03
MR effect size for
'Interleukin−6' on 'eGFR'

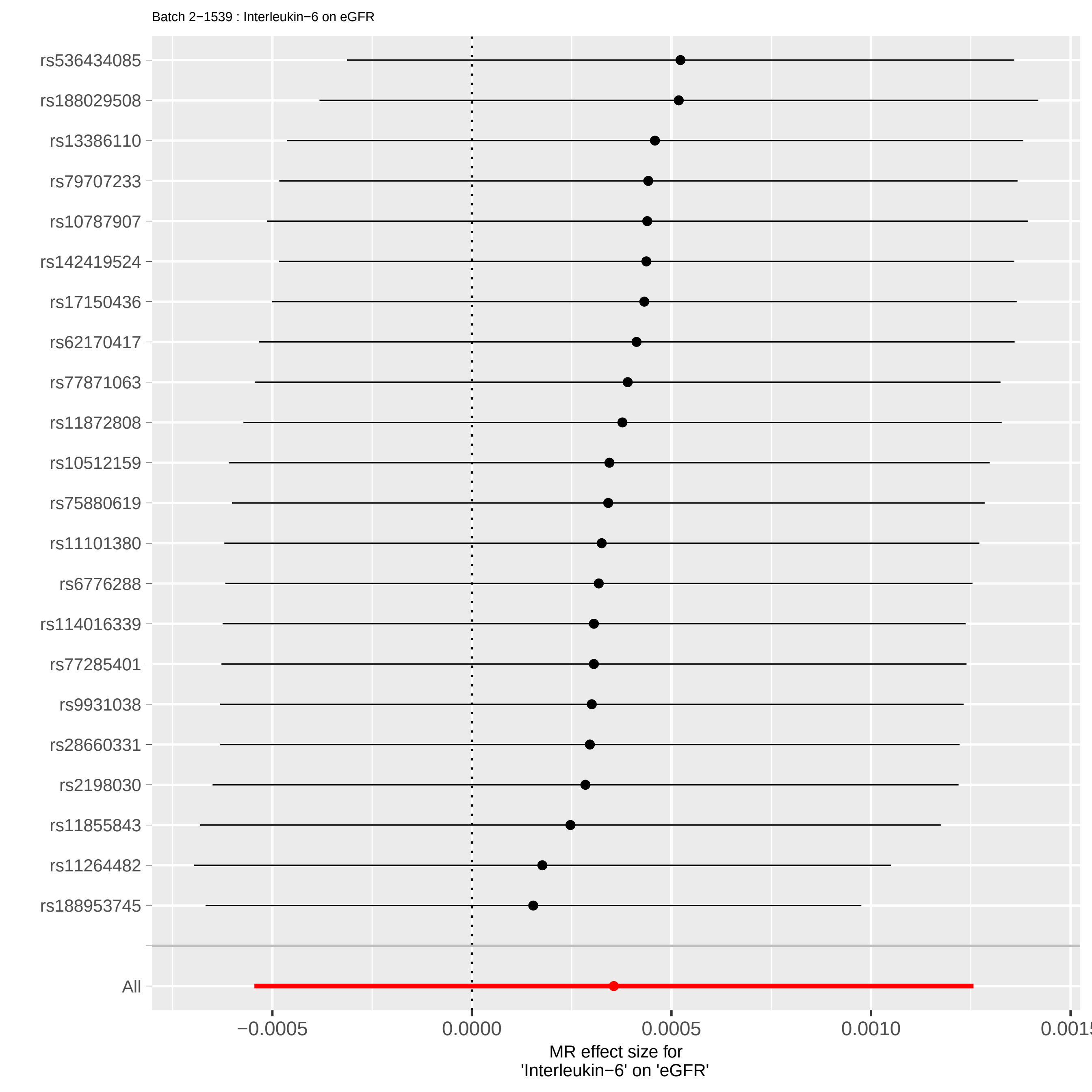
Batch 2−1539 : Interleukin−6 on eGFR
rs536434085
rs188029508
rs13386110
rs79707233
rs10787907
rs142419524
rs17150436
rs62170417
rs77871063
rs11872808
rs10512159
rs75880619
rs11101380
rs6776288
rs114016339
rs77285401
rs9931038
rs28660331
rs2198030
rs11855843
rs11264482
rs188953745
All
−0.0005
0.0000
0.0005
0.0010
MR effect size for
'Interleukin−6' on 'eGFR'

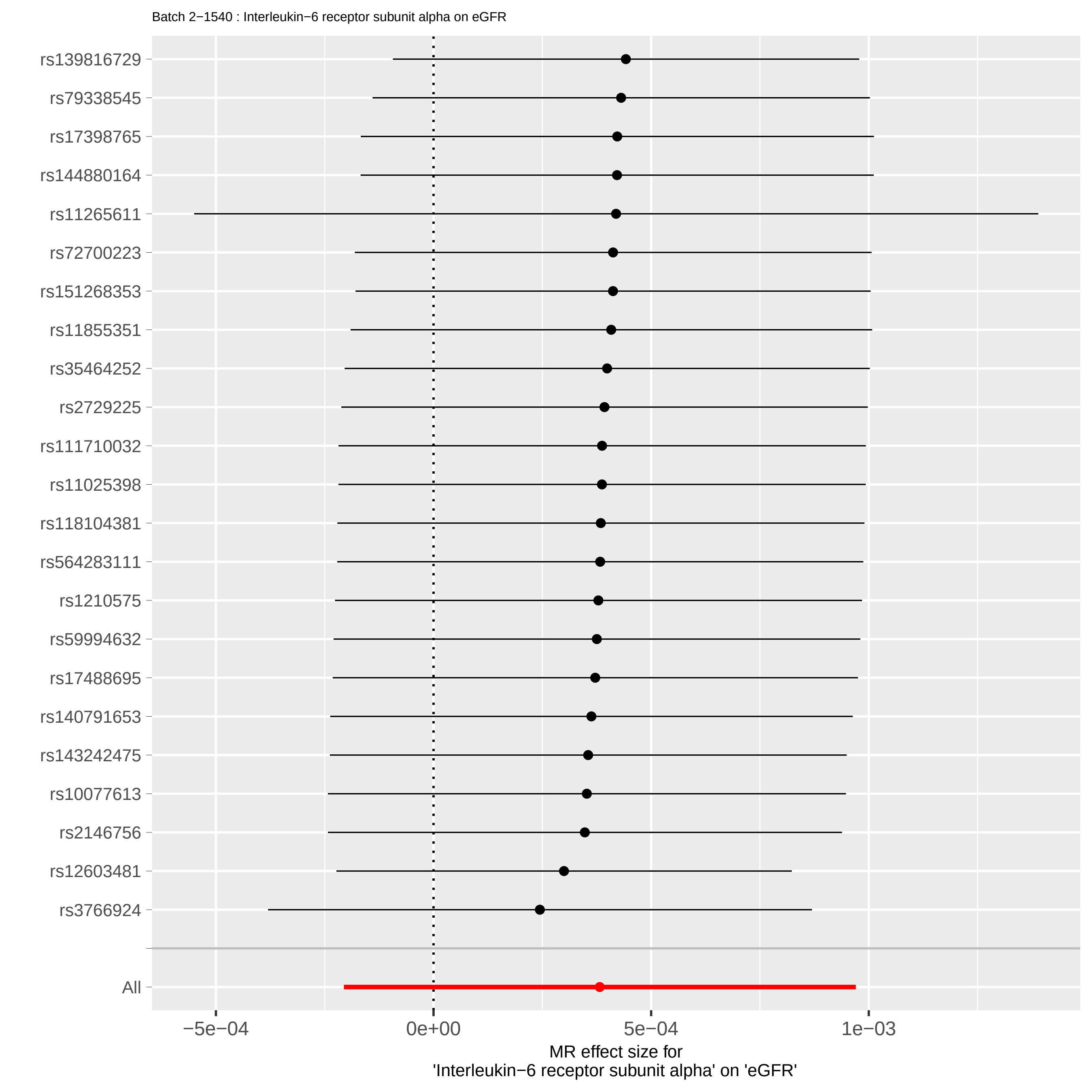

Batch 2−1540 : Interleukin−6 receptor subunit alpha on eGFR
rs139816729
rs79338545
rs17398765
rs144880164
rs11265611
rs72700223
rs151268353
rs11855351
rs35464252
rs2729225
rs111710032
rs11025398
rs118104381
rs564283111
rs1210575
rs59994632
rs17488695
rs140791653
rs143242475
rs10077613
rs2146756
rs12603481
rs3766924
All
−5e−04
0e+00
5e−04
1e−03
MR effect size for
'Interleukin−6 receptor subunit alpha' on 'eGFR'

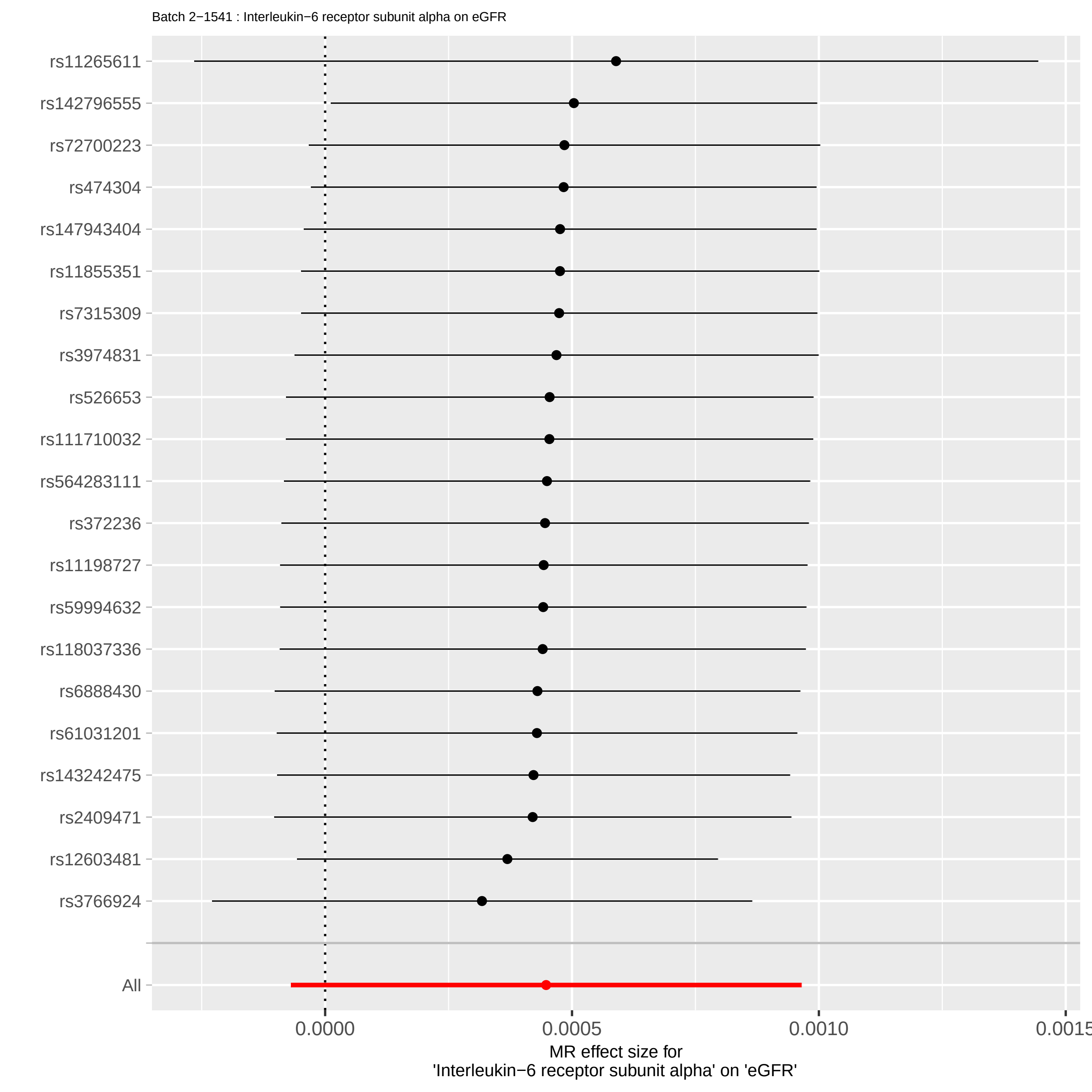

Batch 2−1541 : Interleukin−6 receptor subunit alpha on eGFR
rs11265611
rs142796555
rs72700223
rs474304
rs147943404
rs11855351
rs7315309
rs3974831
rs526653
rs111710032
rs564283111
rs372236
rs11198727
rs59994632
rs118037336
rs6888430
rs61031201
rs143242475
rs2409471
rs12603481
rs3766924
All
0.0000
0.0005
0.0010
0.0015
MR effect size for
'Interleukin−6 receptor subunit alpha' on 'eGFR'

Batch 2−1542 : Interleukin−6 receptor subunit beta on eGFR

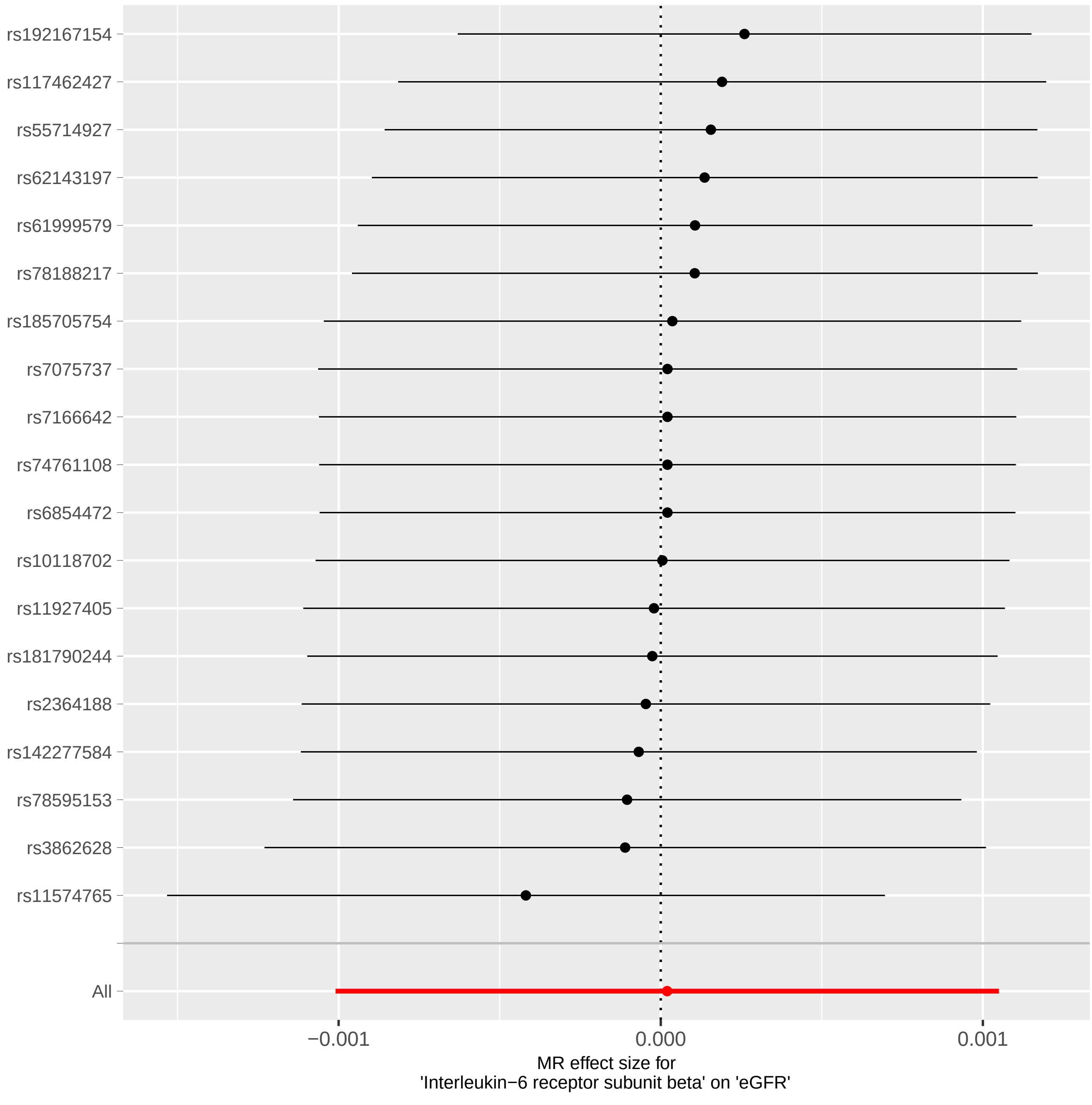

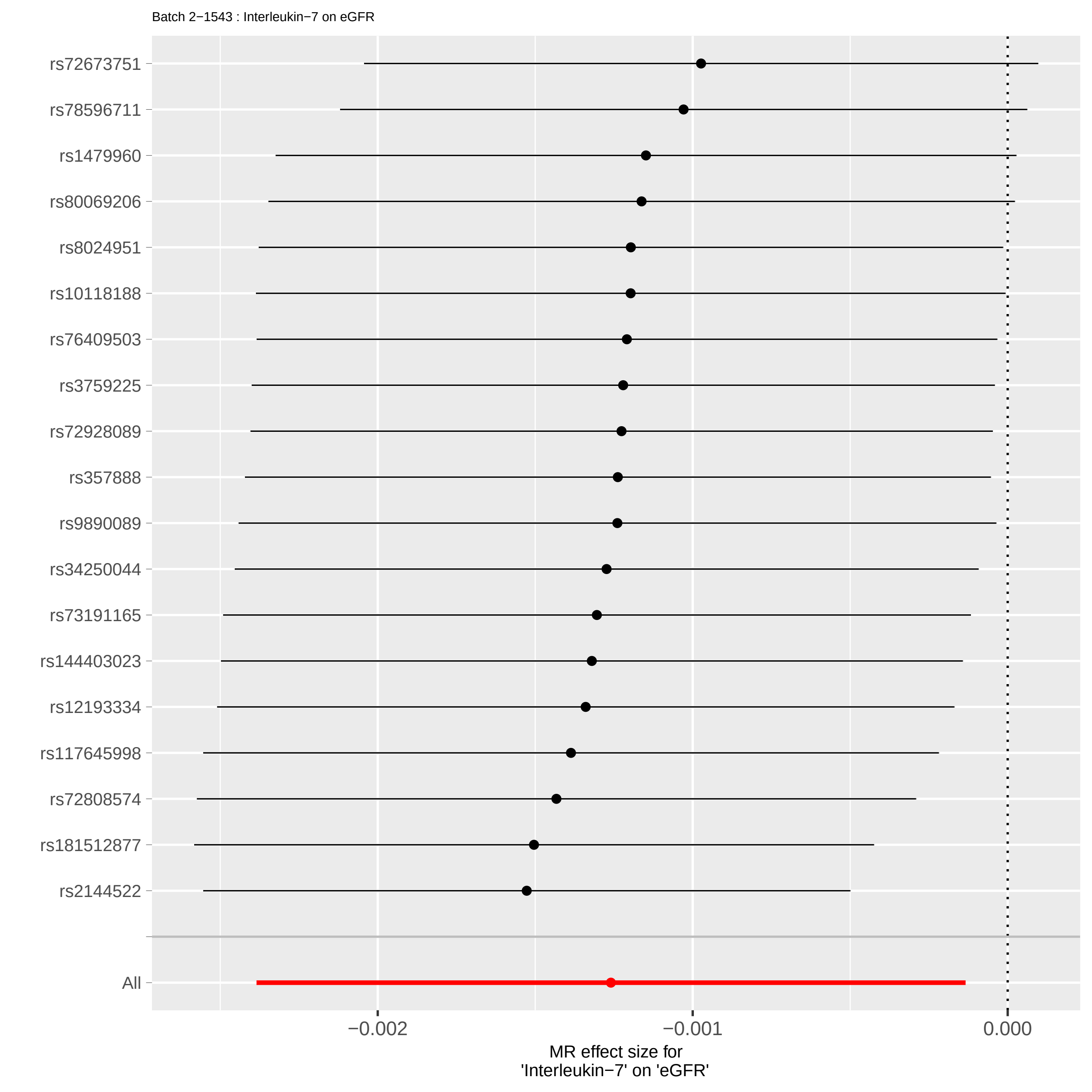

Batch 2−1543 : Interleukin−7 on eGFR
rs72673751
rs78596711
rs1479960
rs80069206
rs8024951
rs10118188
rs76409503
rs3759225
rs72928089
rs357888
rs9890089
rs34250044
rs73191165
rs144403023
rs12193334
rs117645998
rs72808574
rs181512877
rs2144522
All
−0.002
−0.001
0.000
MR effect size for
'Interleukin−7' on 'eGFR'

Batch 2−1544 : Interleukin−7 receptor subunit alpha on eGFR

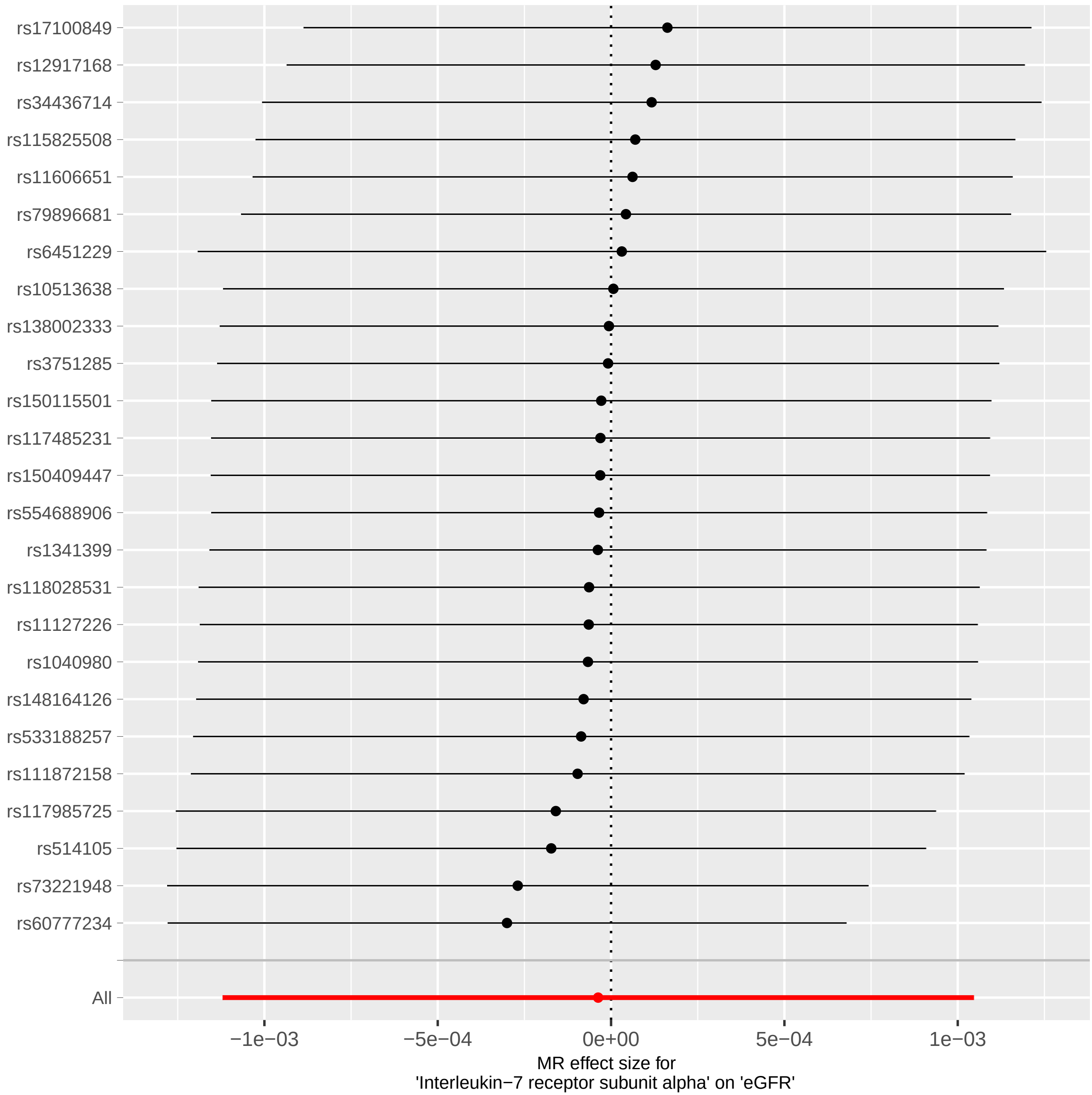

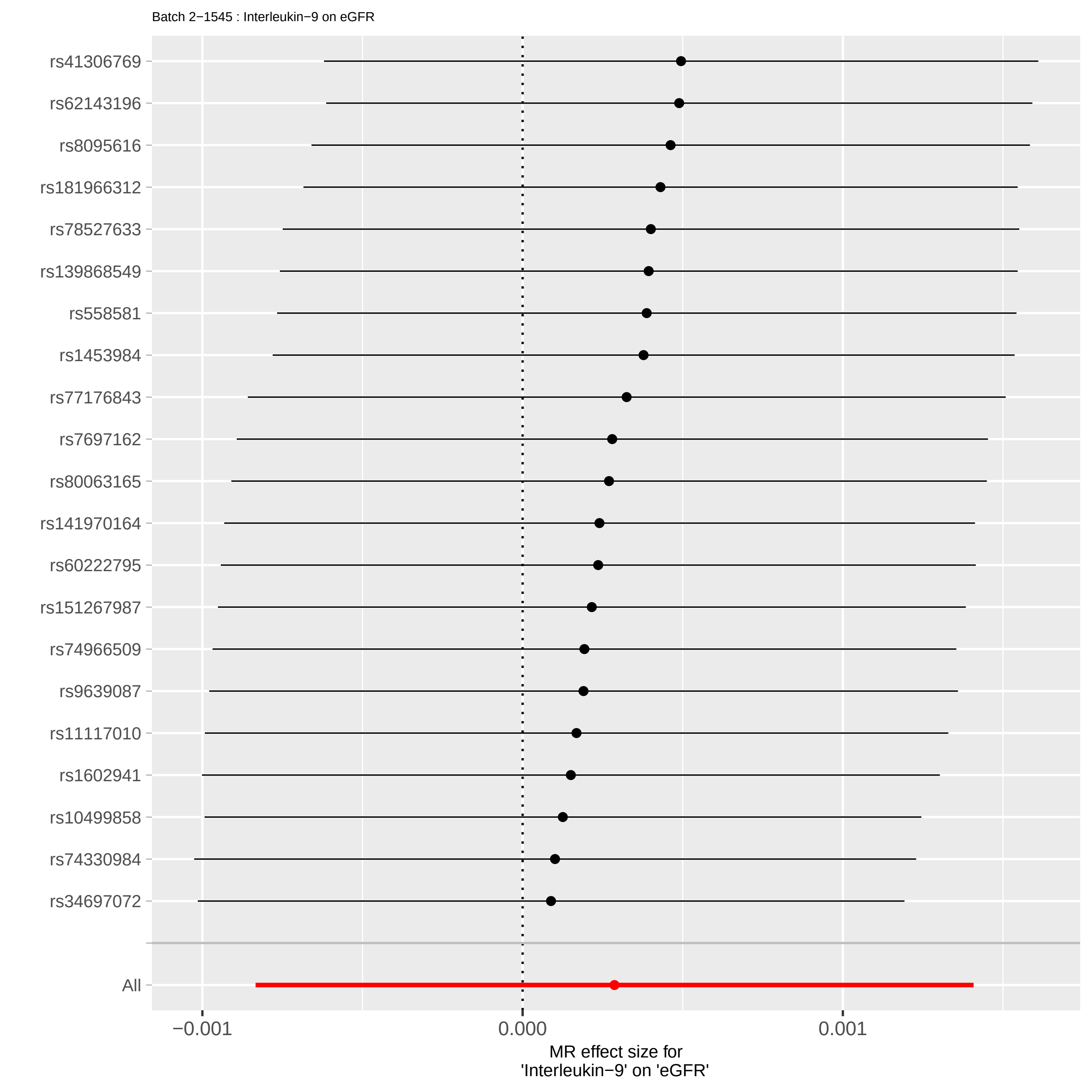

Batch 2−1545 : Interleukin−9 on eGFR
rs41306769
rs62143196
rs8095616
rs181966312
rs78527633
rs139868549
rs558581
rs1453984
rs77176843
rs7697162
rs80063165
rs141970164
rs60222795
rs151267987
rs74966509
rs9639087
rs11117010
rs1602941
rs10499858
rs74330984
rs34697072
All
−0.001
0.000
0.001
MR effect size for
'Interleukin−9' on 'eGFR'

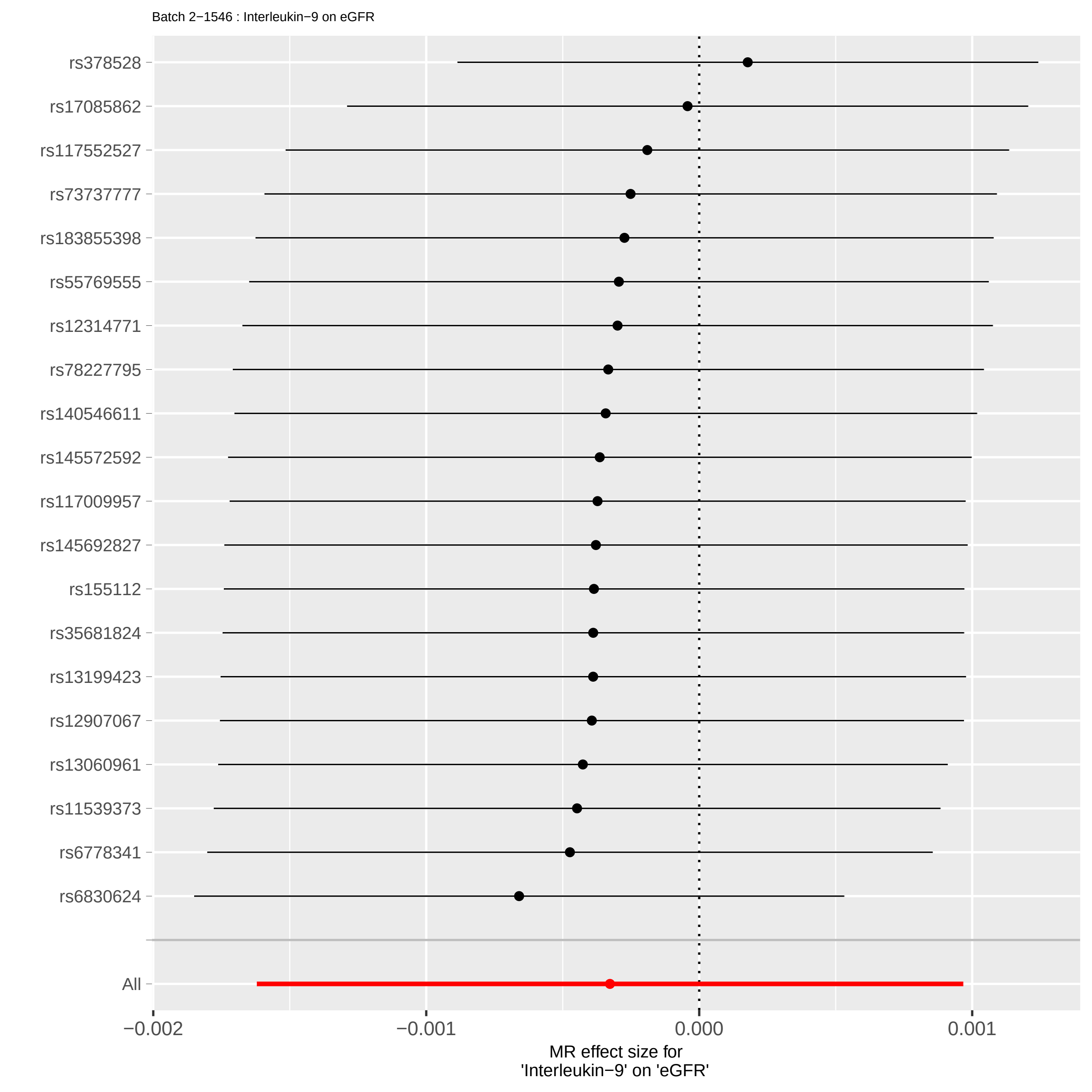

Batch 2−1546 : Interleukin−9 on eGFR
rs378528
rs17085862
rs117552527
rs73737777
rs183855398
rs55769555
rs12314771
rs78227795
rs140546611
rs145572592
rs117009957
rs145692827
rs155112
rs35681824
rs13199423
rs12907067
rs13060961
rs11539373
rs6778341
rs6830624
All
−0.002
−0.001
0.000
0.001
MR effect size for
'Interleukin−9' on 'eGFR'

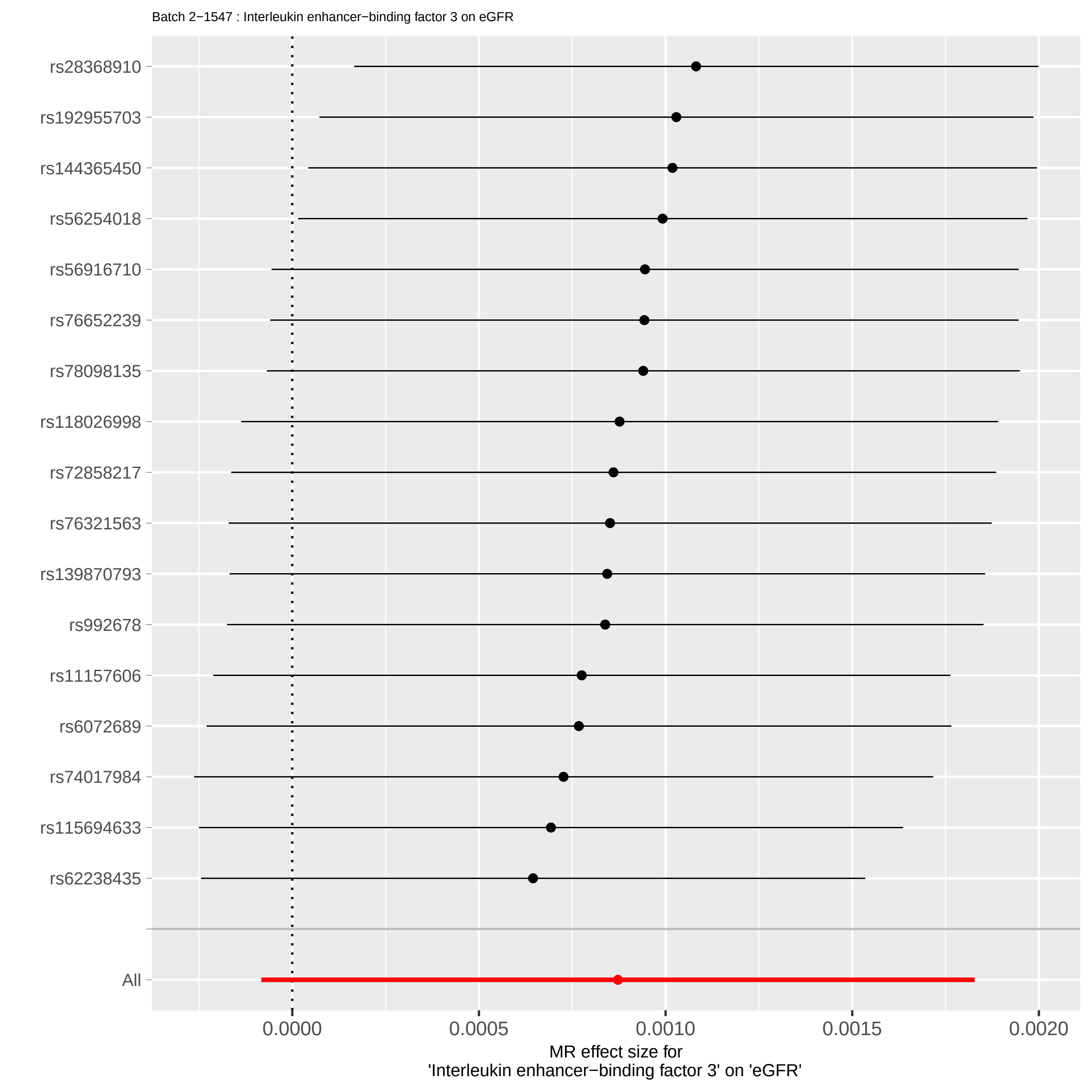

Batch 2−1547 : Interleukin enhancer−binding factor 3 on eGFR
rs28368910
rs192955703
rs144365450
rs56254018
rs56916710
rs76652239
rs78098135
rs118026998
rs72858217
rs76321563
rs139870793
rs992678
rs11157606
rs6072689
rs74017984
rs115694633
rs62238435
All
0.0000
0.0005
0.0010
0.0015
0.0020
MR effect size for
'Interleukin enhancer−binding factor 3' on 'eGFR'

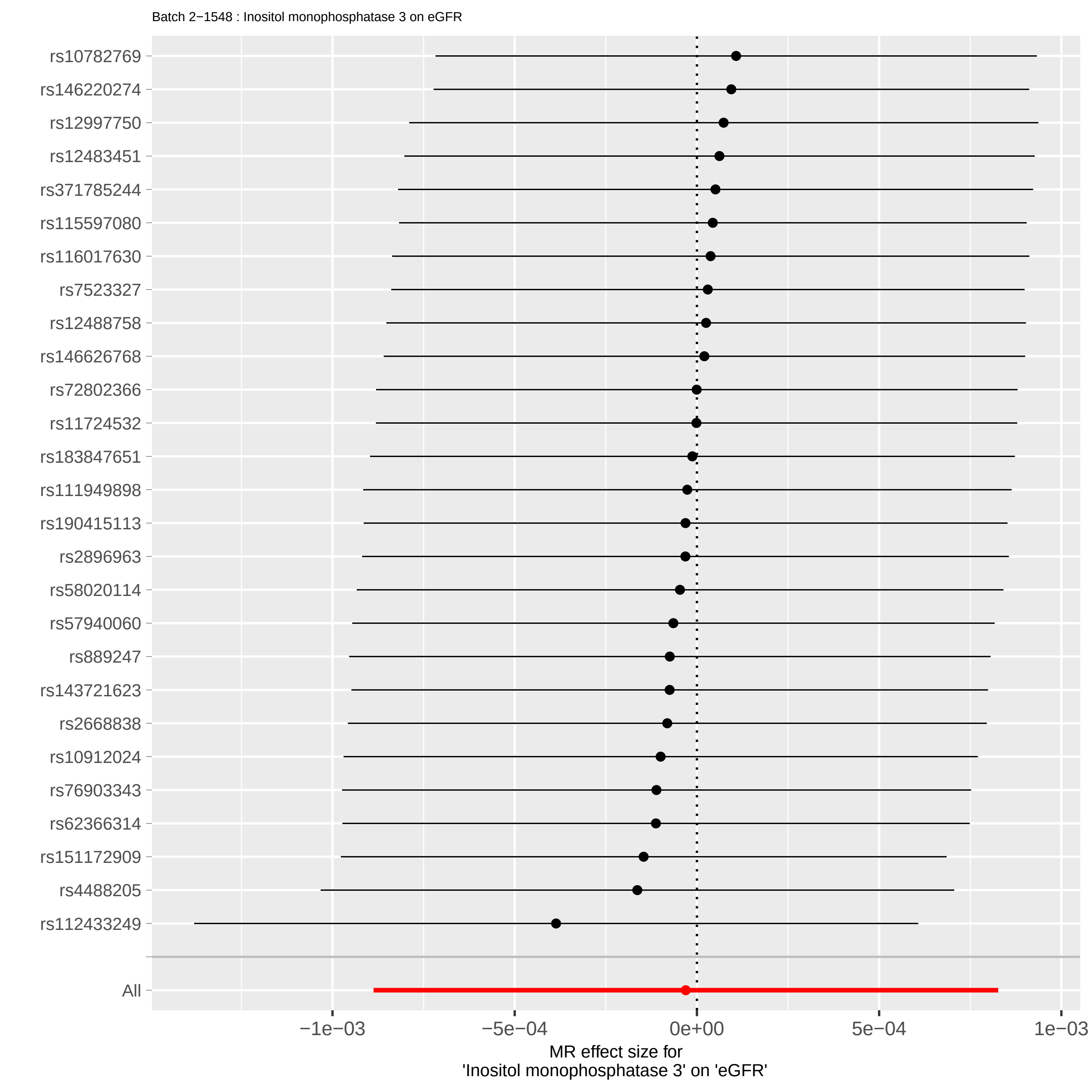

Batch 2−1548 : Inositol monophosphatase 3 on eGFR
rs10782769
rs146220274
rs12997750
rs12483451
rs371785244
rs115597080
rs116017630
rs7523327
rs12488758
rs146626768
rs72802366
rs11724532
rs183847651
rs111949898
rs190415113
rs2896963
rs58020114
rs57940060
rs889247
rs143721623
rs2668838
rs10912024
rs76903343
rs62366314
rs151172909
rs4488205
rs112433249
All
−1e−03
−5e−04
0e+00
5e−04
1e−03
MR effect size for
'Inositol monophosphatase 3' on 'eGFR'

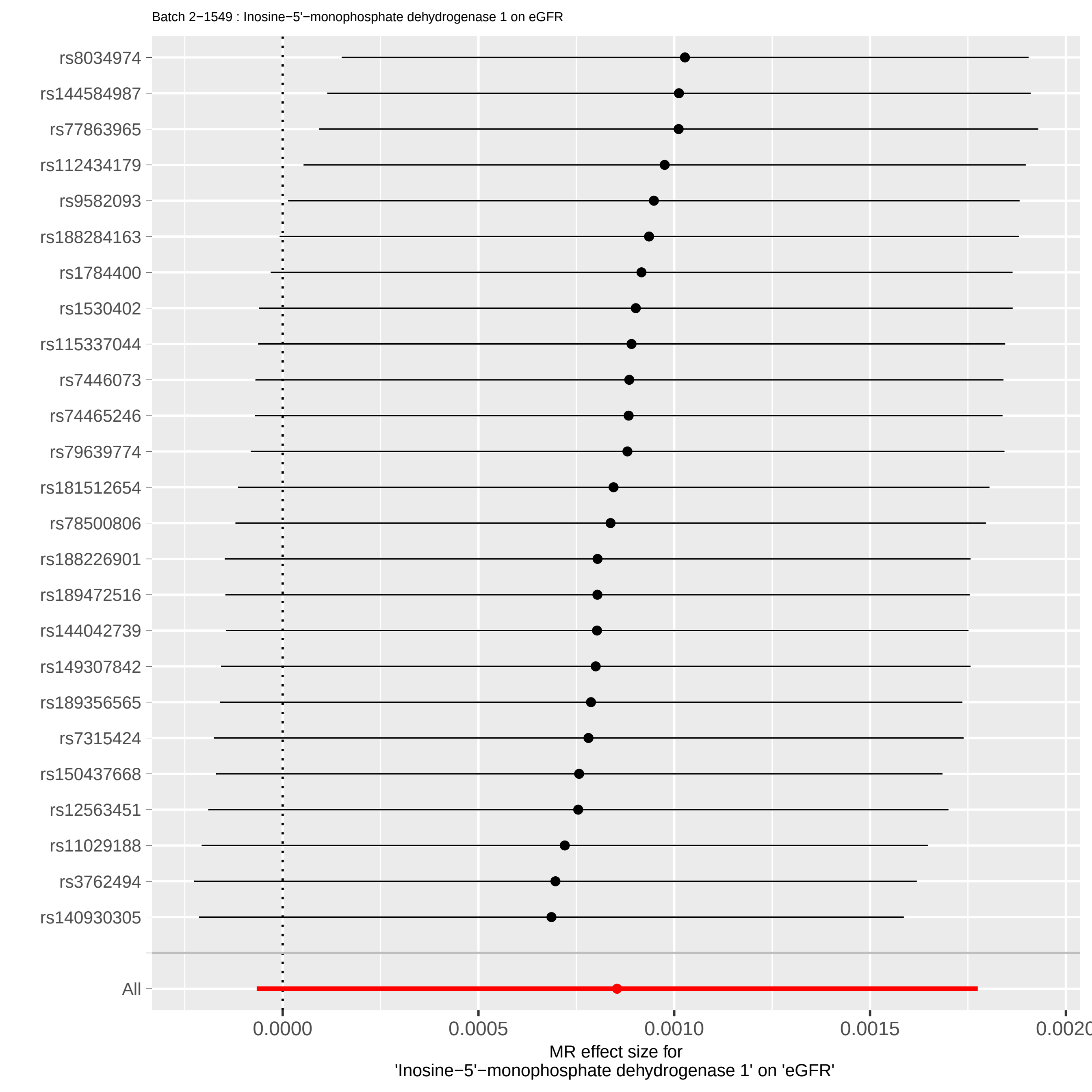

Batch 2−1549 : Inosine−5'−monophosphate dehydrogenase 1 on eGFR
rs8034974
rs144584987
rs77863965
rs112434179
rs9582093
rs188284163
rs1784400
rs1530402
rs115337044
rs7446073
rs74465246
rs79639774
rs181512654
rs78500806
rs188226901
rs189472516
rs144042739
rs149307842
rs189356565
rs7315424
rs150437668
rs12563451
rs11029188
rs3762494
rs140930305
All
0.0000
0.0005
0.0010
0.0015
0.0020
MR effect size for
'Inosine−5'−monophosphate dehydrogenase 1' on 'eGFR'

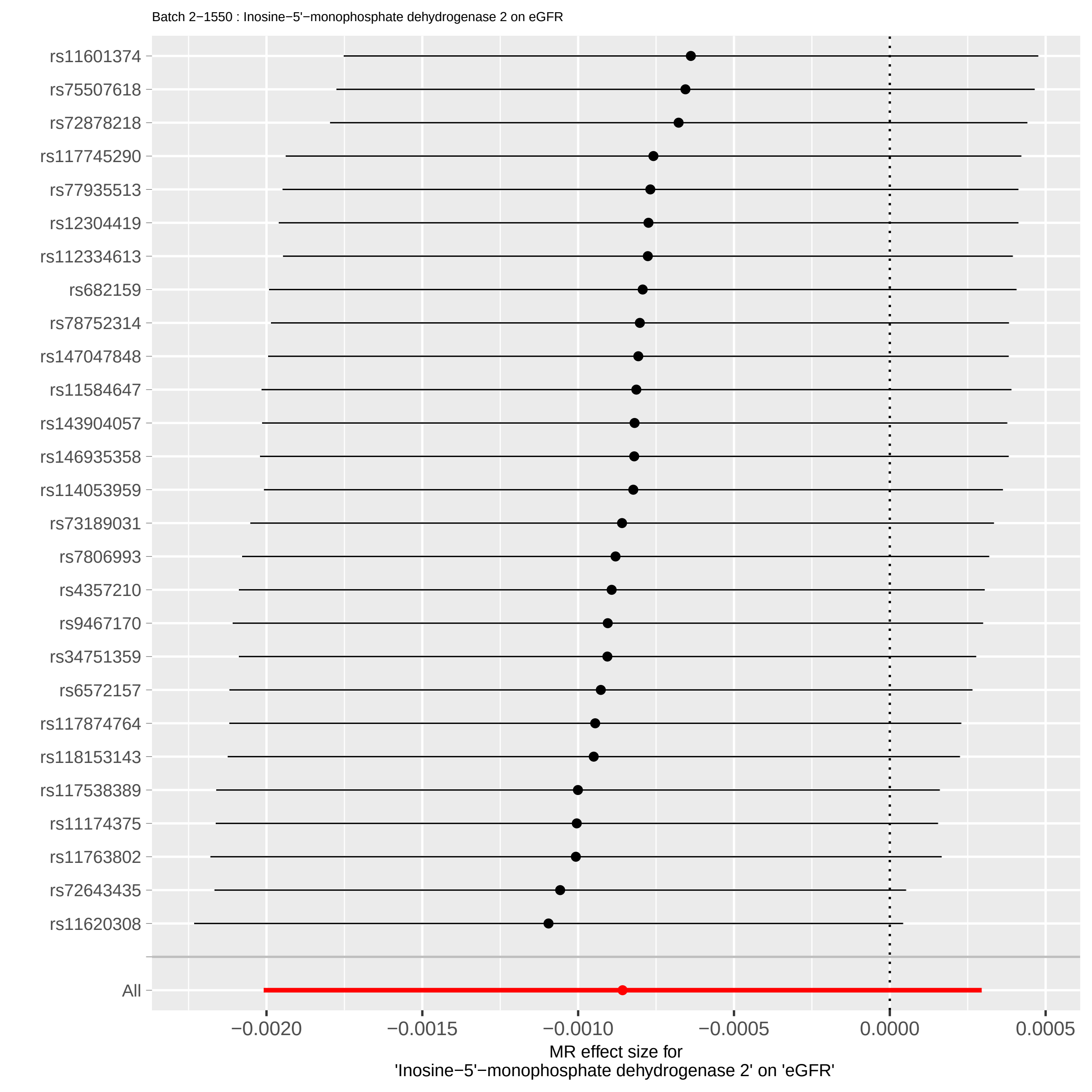

Batch 2−1550 : Inosine−5'−monophosphate dehydrogenase 2 on eGFR
rs11601374
rs75507618
rs72878218
rs117745290
rs77935513
rs12304419
rs112334613
rs682159
rs78752314
rs147047848
rs11584647
rs143904057
rs146935358
rs114053959
rs73189031
rs7806993
rs4357210
rs9467170
rs34751359
rs6572157
rs117874764
rs118153143
rs117538389
rs11174375
rs11763802
rs72643435
rs11620308
All
−0.0020
−0.0015
−0.0010
−0.0005
0.0000
0.0005
MR effect size for
'Inosine−5'−monophosphate dehydrogenase 2' on 'eGFR'

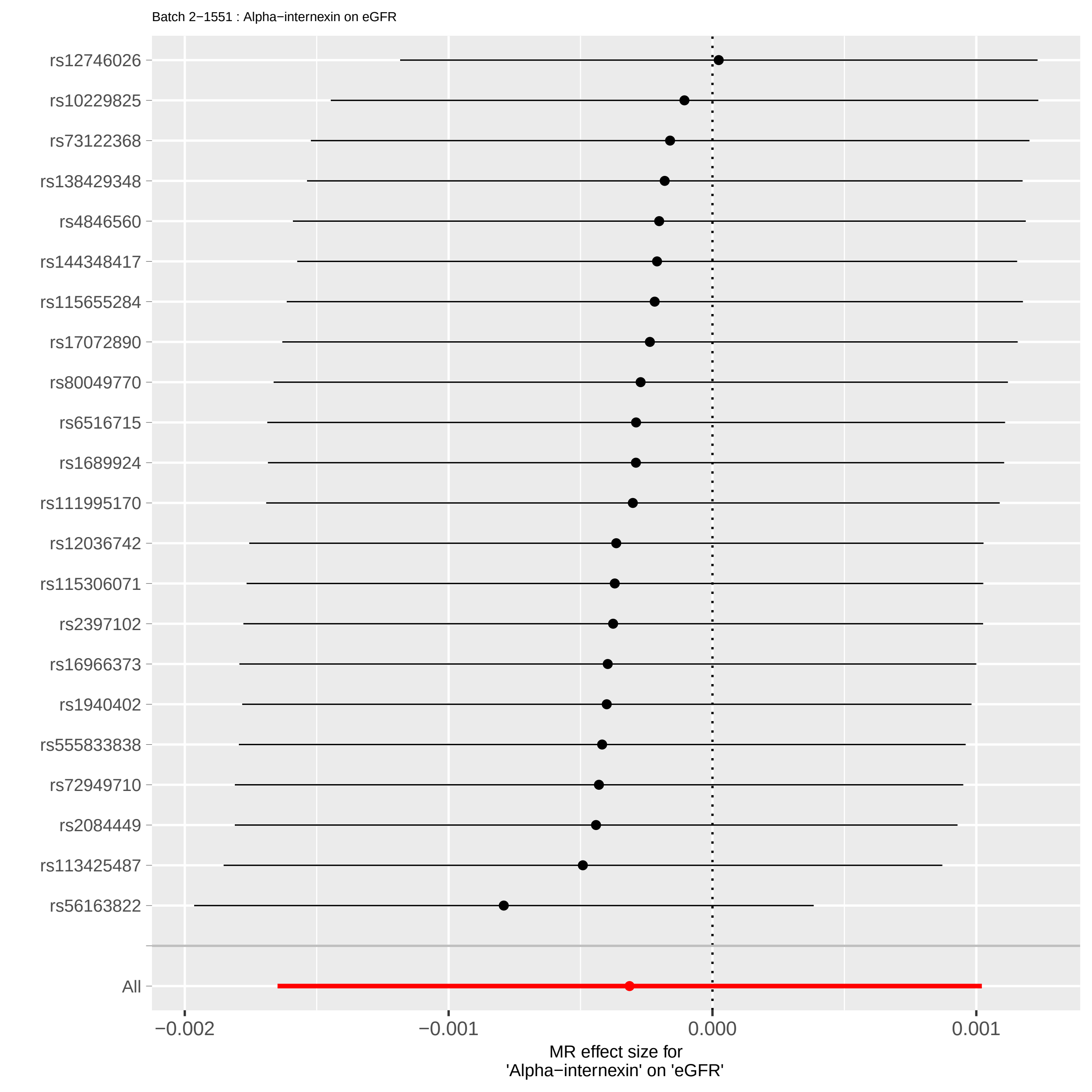

Batch 2−1551 : Alpha−internexin on eGFR
rs12746026
rs10229825
rs73122368
rs138429348
rs4846560
rs144348417
rs115655284
rs17072890
rs80049770
rs6516715
rs1689924
rs111995170
rs12036742
rs115306071
rs2397102
rs16966373
rs1940402
rs555833838
rs72949710
rs2084449
rs113425487
rs56163822
All
−0.002
−0.001
0.000
0.001
MR effect size for
'Alpha−internexin' on 'eGFR'

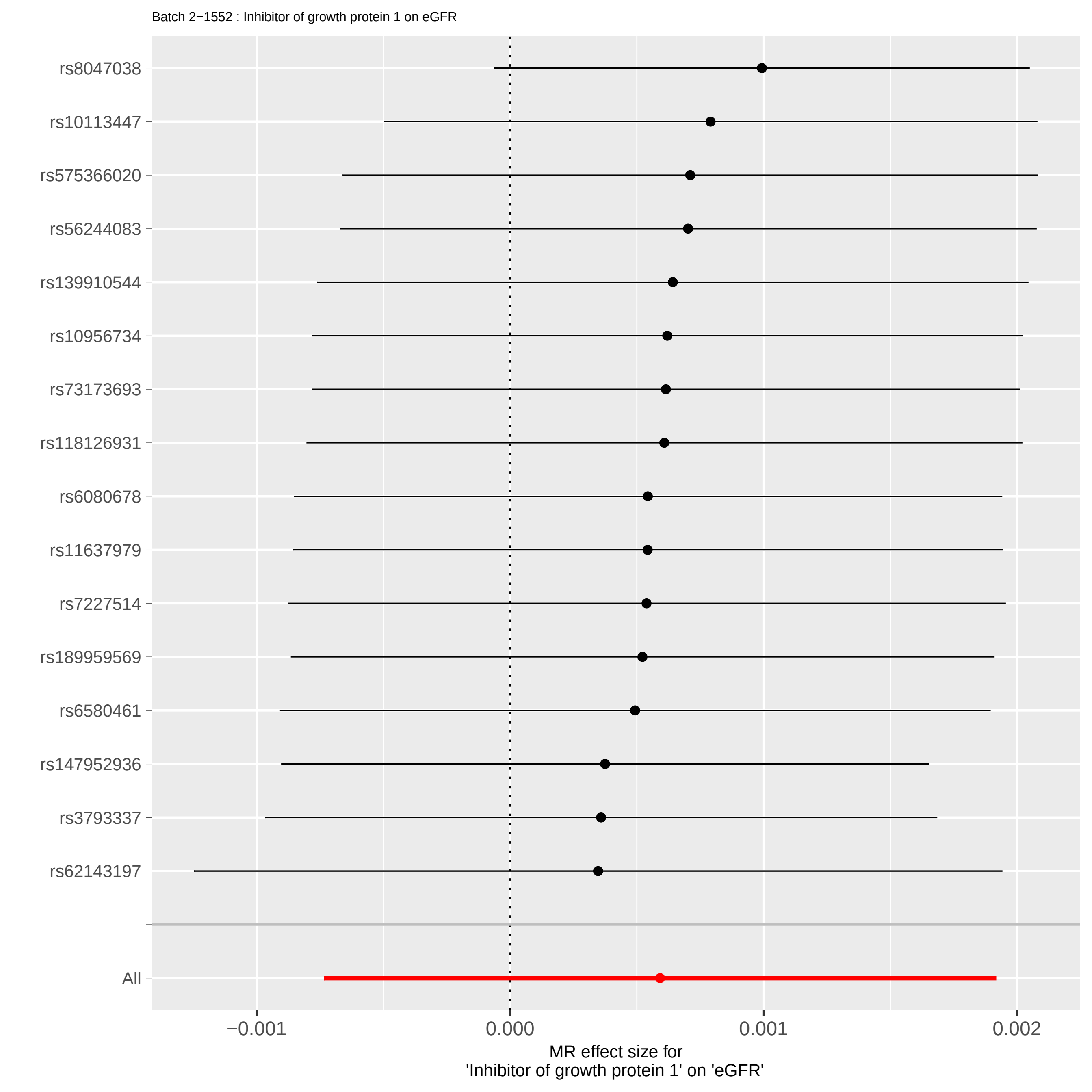

Batch 2−1552 : Inhibitor of growth protein 1 on eGFR
rs8047038
rs10113447
rs575366020
rs56244083
rs139910544
rs10956734
rs73173693
rs118126931
rs6080678
rs11637979
rs7227514
rs189959569
rs6580461
rs147952936
rs3793337
rs62143197
All
−0.001
0.000
0.001
0.002
MR effect size for
'Inhibitor of growth protein 1' on 'eGFR'

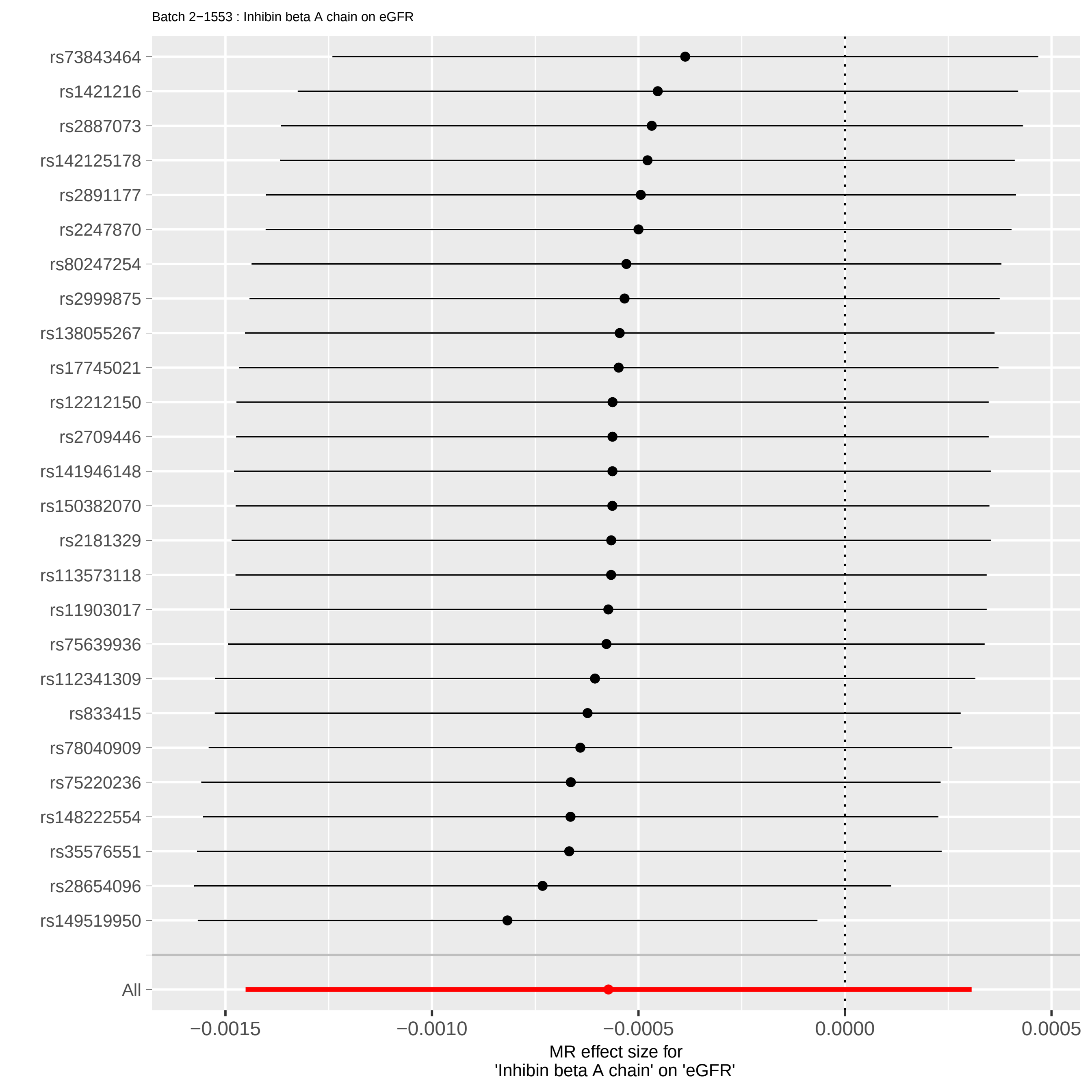
Batch 2−1553 : Inhibin beta A chain on eGFR
rs73843464
rs1421216
rs2887073
rs142125178
rs2891177
rs2247870
rs80247254
rs2999875
rs138055267
rs17745021
rs12212150
rs2709446
rs141946148
rs150382070
rs2181329
rs113573118
rs11903017
rs75639936
rs112341309
rs833415
rs78040909
rs75220236
rs148222554
rs35576551
rs28654096
rs149519950
All
−0.0015
−0.0010
−0.0005
0.0000
0.0005
MR effect size for
'Inhibin beta A chain' on 'eGFR'

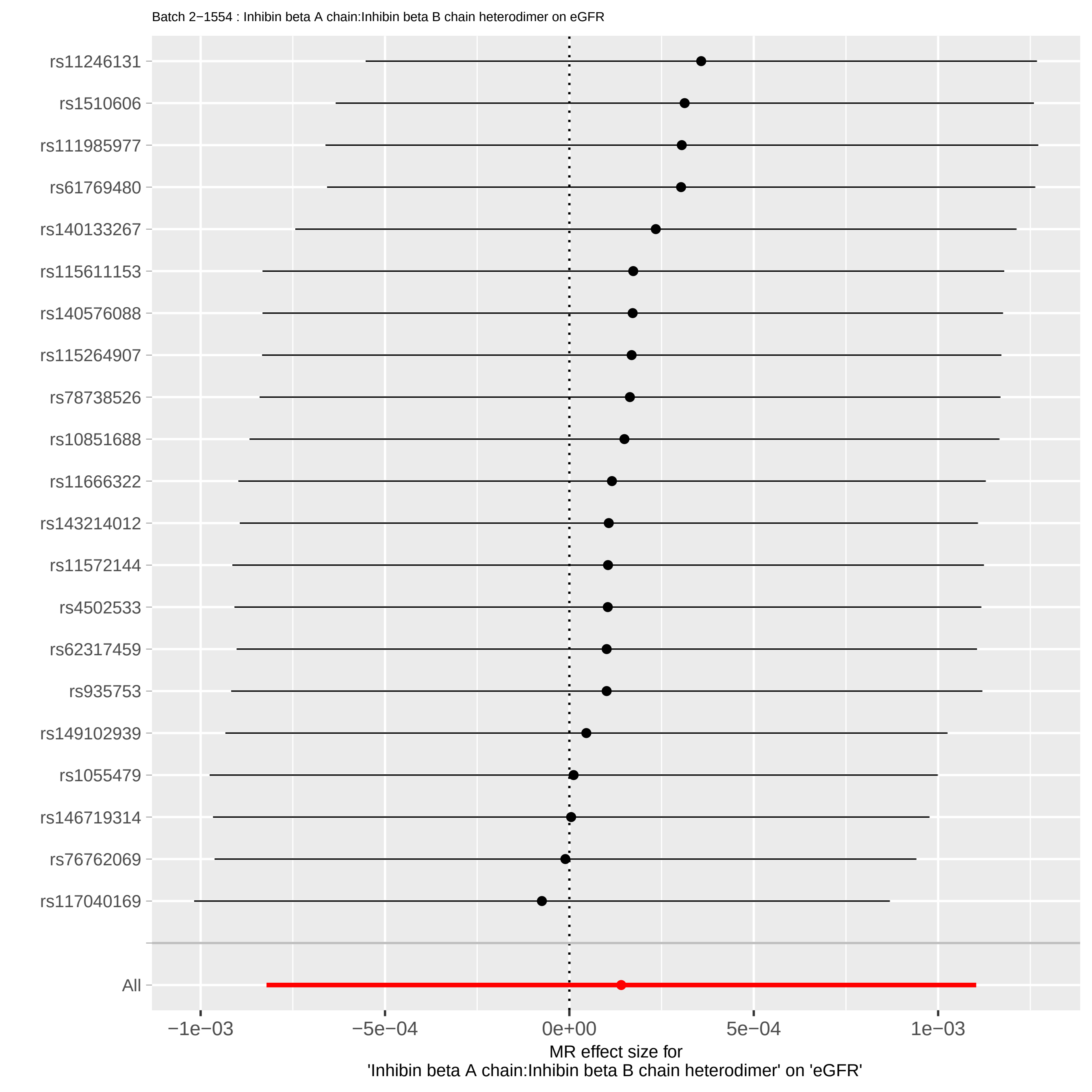

Batch 2−1554 : Inhibin beta A chain:Inhibin beta B chain heterodimer on eGFR
rs11246131
rs1510606
rs111985977
rs61769480
rs140133267
rs115611153
rs140576088
rs115264907
rs78738526
rs10851688
rs11666322
rs143214012
rs11572144
rs4502533
rs62317459
rs935753
rs149102939
rs1055479
rs146719314
rs76762069
rs117040169
All
−1e−03
−5e−04
0e+00
5e−04
1e−03
MR effect size for
'Inhibin beta A chain:Inhibin beta B chain heterodimer' on 'eGFR'

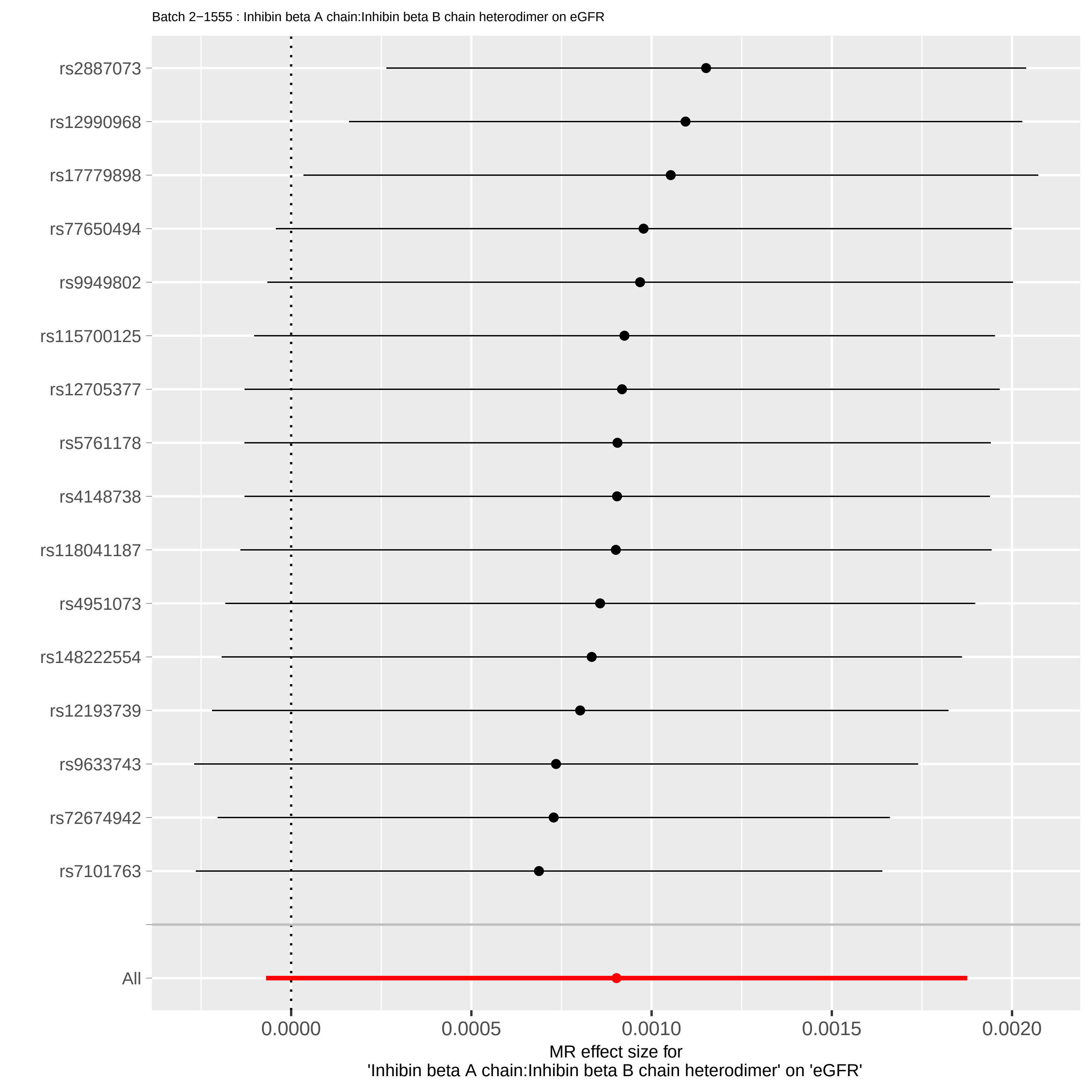

Batch 2−1555 : Inhibin beta A chain:Inhibin beta B chain heterodimer on eGFR
rs2887073
rs12990968
rs17779898
rs77650494
rs9949802
rs115700125
rs12705377
rs5761178
rs4148738
rs118041187
rs4951073
rs148222554
rs12193739
rs9633743
rs72674942
rs7101763
All
0.0000
0.0005
0.0010
0.0015
0.0020
MR effect size for
'Inhibin beta A chain:Inhibin beta B chain heterodimer' on 'eGFR'

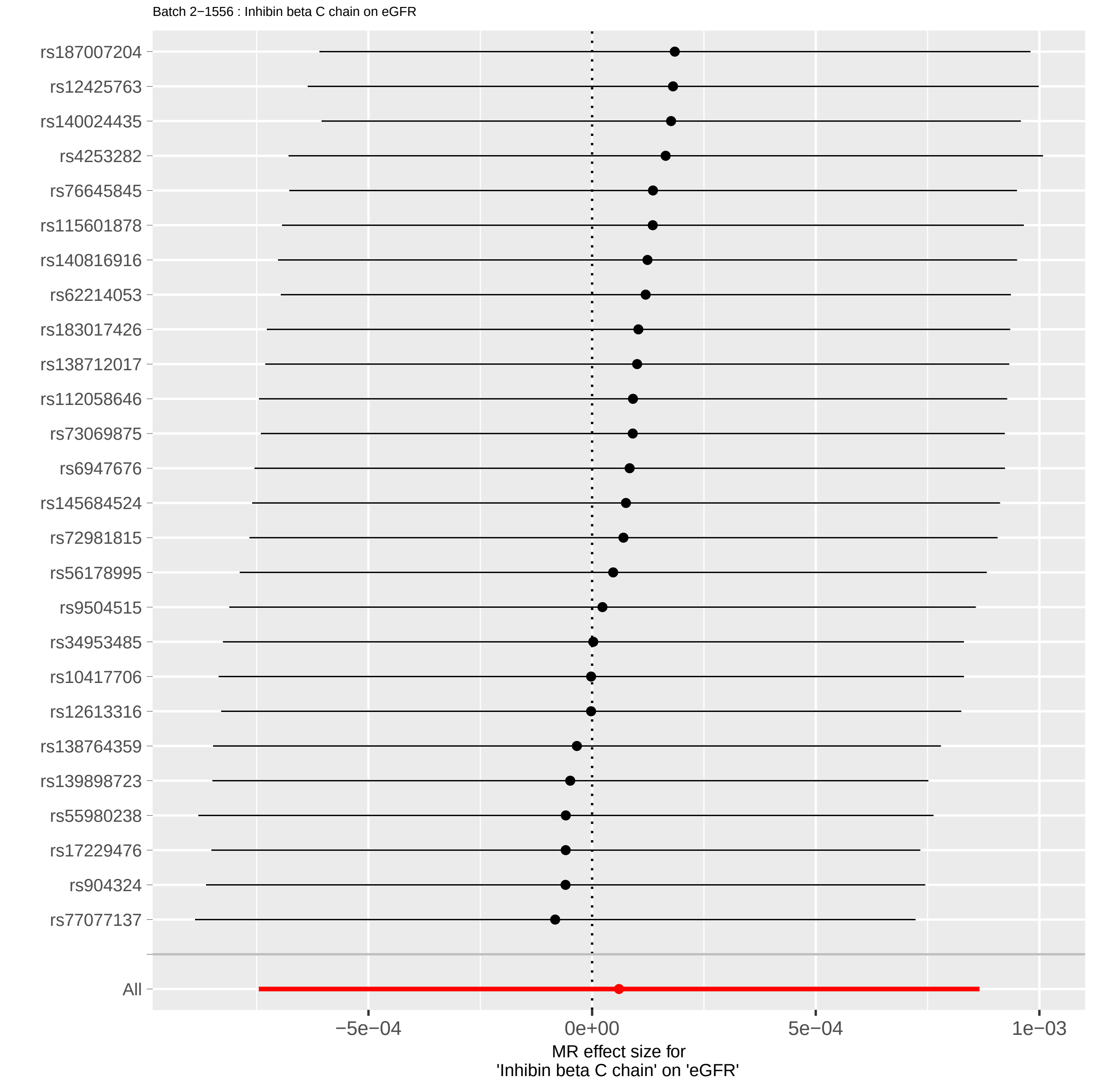

Batch 2−1556 : Inhibin beta C chain on eGFR
rs187007204
rs12425763
rs140024435
rs4253282
rs76645845
rs115601878
rs140816916
rs62214053
rs183017426
rs138712017
rs112058646
rs73069875
rs6947676
rs145684524
rs72981815
rs56178995
rs9504515
rs34953485
rs10417706
rs12613316
rs138764359
rs139898723
rs55980238
rs17229476
rs904324
rs77077137
All
−5e−04
0e+00
5e−04
1e−03
MR effect size for
'Inhibin beta C chain' on 'eGFR'

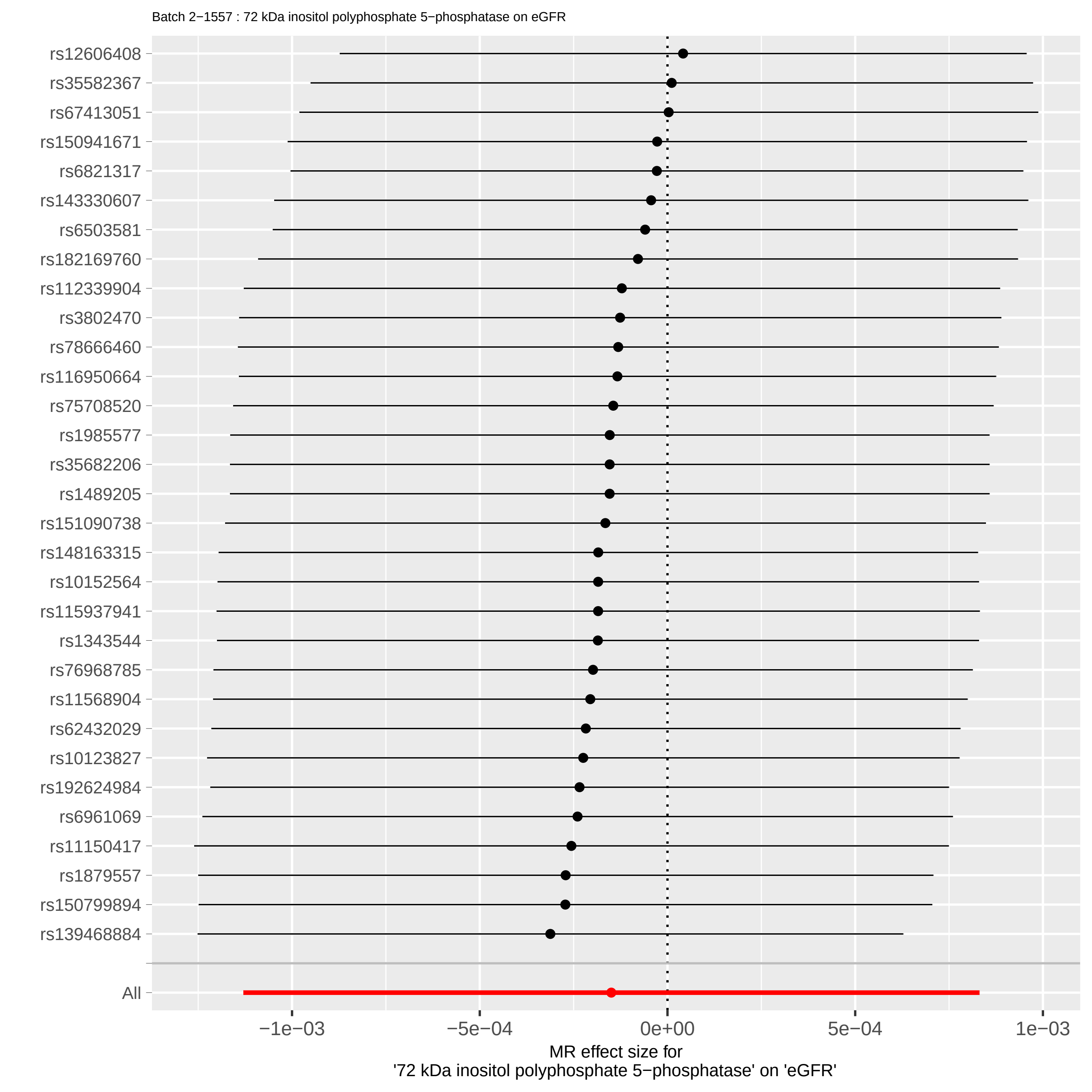

Batch 2−1557 : 72 kDa inositol polyphosphate 5−phosphatase on eGFR
rs12606408
rs35582367
rs67413051
rs150941671
rs6821317
rs143330607
rs6503581
rs182169760
rs112339904
rs3802470
rs78666460
rs116950664
rs75708520
rs1985577
rs35682206
rs1489205
rs151090738
rs148163315
rs10152564
rs115937941
rs1343544
rs76968785
rs11568904
rs62432029
rs10123827
rs192624984
rs6961069
rs11150417
rs1879557
rs150799894
rs139468884
All
−1e−03
−5e−04
0e+00
5e−04
1e−03
MR effect size for
'72 kDa inositol polyphosphate 5−phosphatase' on 'eGFR'

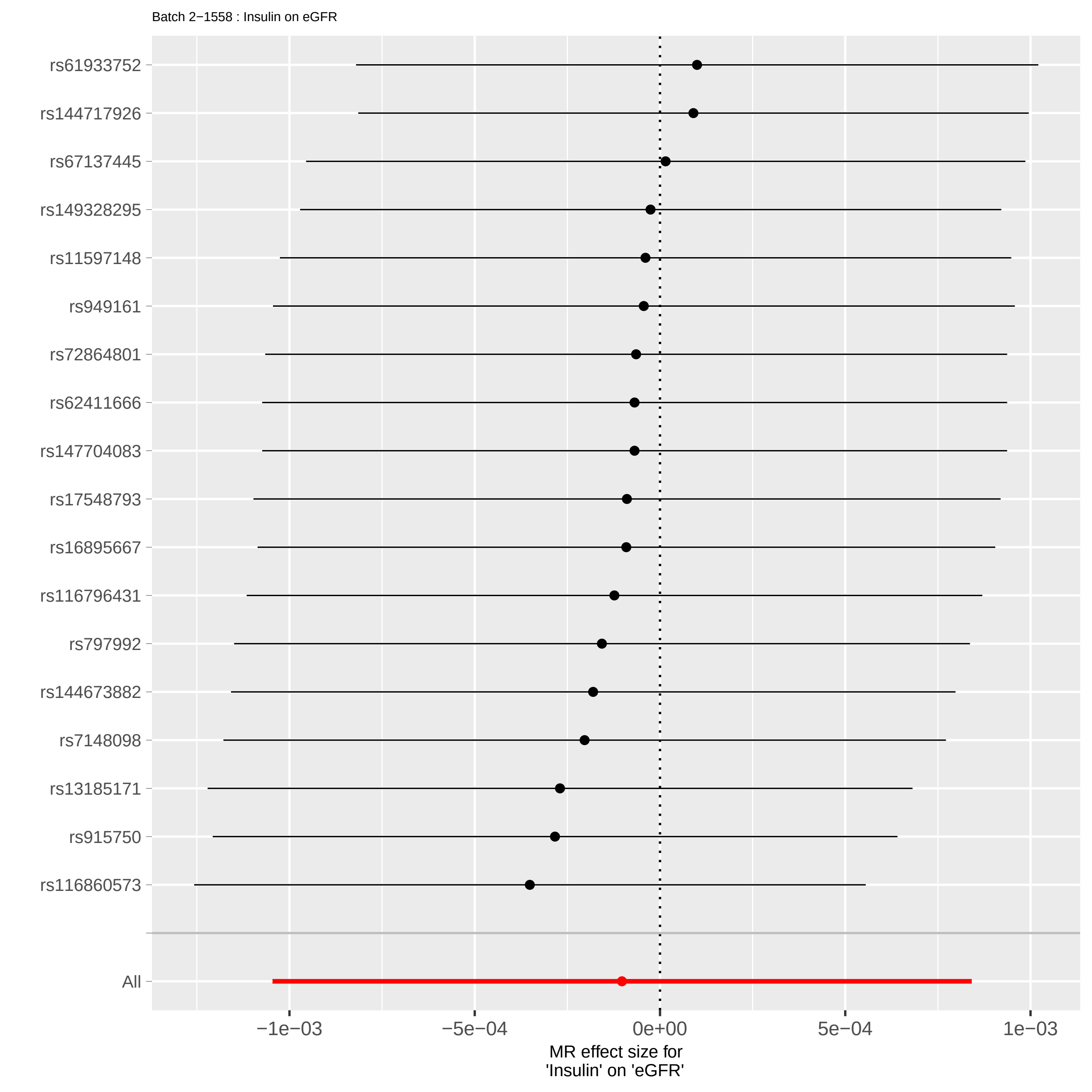
Batch 2−1558 : Insulin on eGFR
rs61933752
rs144717926
rs67137445
rs149328295
rs11597148
rs949161
rs72864801
rs62411666
rs147704083
rs17548793
rs16895667
rs116796431
rs797992
rs144673882
rs7148098
rs13185171
rs915750
rs116860573
All
−1e−03
−5e−04
0e+00
5e−04
1e−03
MR effect size for
'Insulin' on 'eGFR'

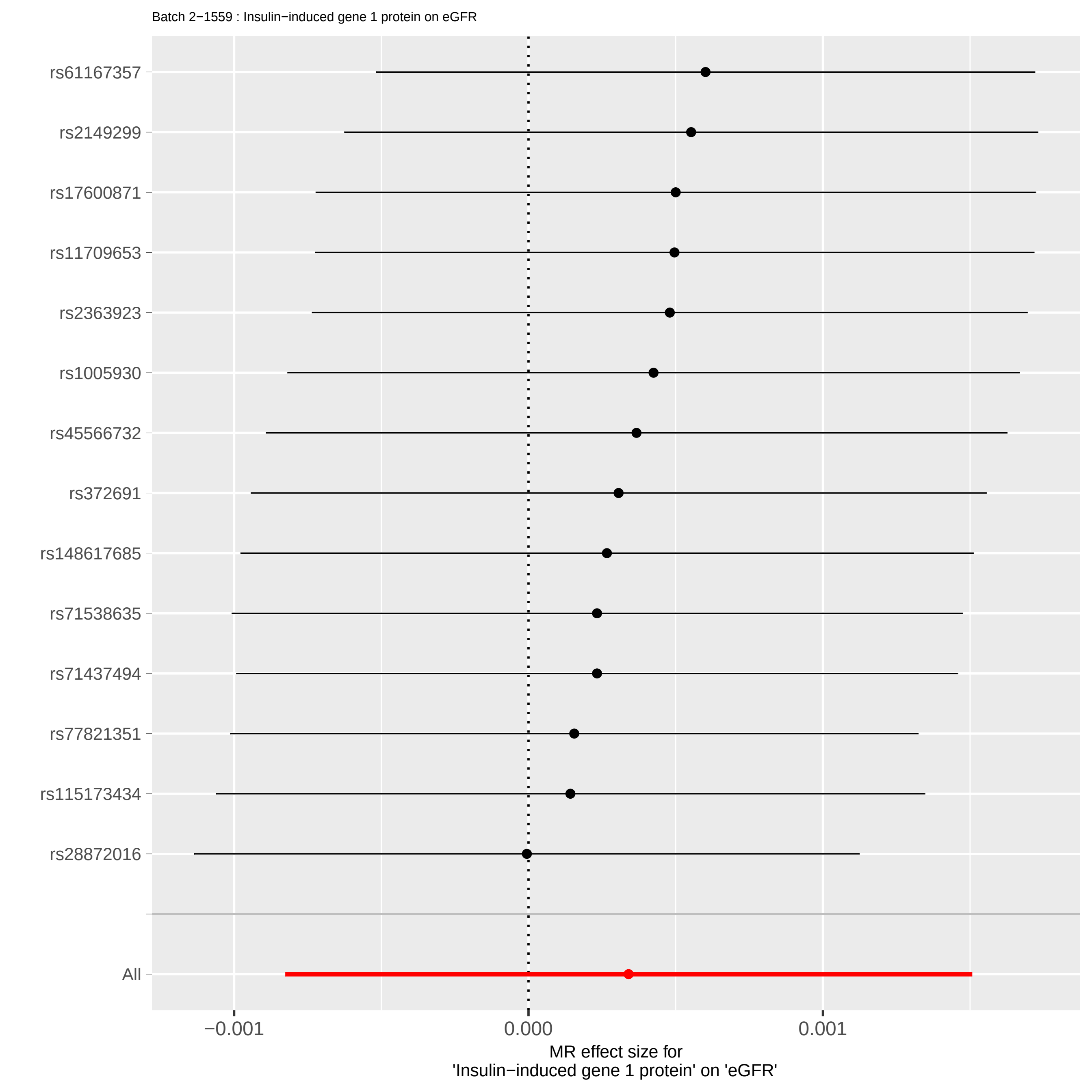

Batch 2−1559 : Insulin−induced gene 1 protein on eGFR
rs61167357
rs2149299
rs17600871
rs11709653
rs2363923
rs1005930
rs45566732
rs372691
rs148617685
rs71538635
rs71437494
rs77821351
rs115173434
rs28872016
All
−0.001
0.000
0.001
MR effect size for
'Insulin−induced gene 1 protein' on 'eGFR'

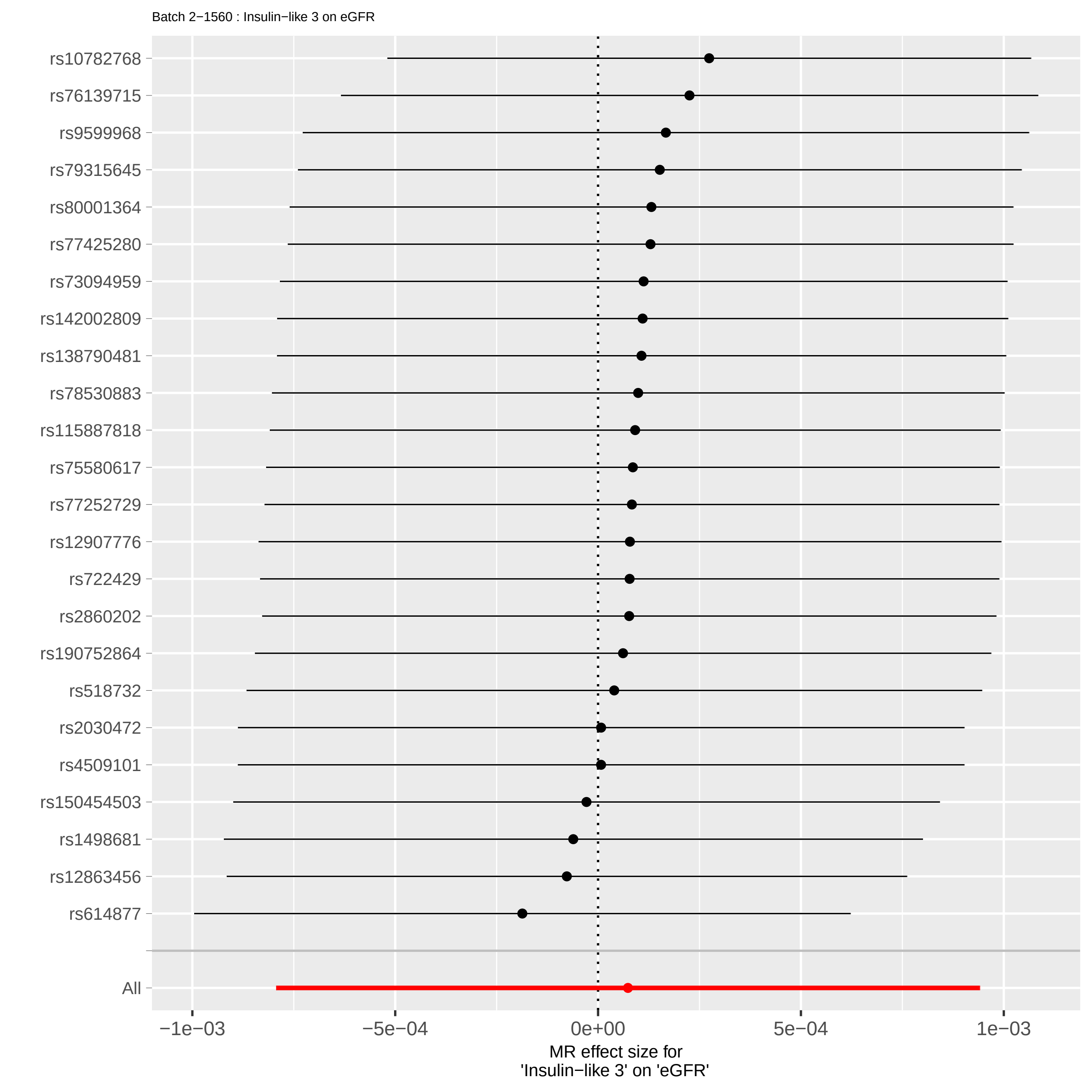
Batch 2−1560 : Insulin−like 3 on eGFR
rs10782768
rs76139715
rs9599968
rs79315645
rs80001364
rs77425280
rs73094959
rs142002809
rs138790481
rs78530883
rs115887818
rs75580617
rs77252729
rs12907776
rs722429
rs2860202
rs190752864
rs518732
rs2030472
rs4509101
rs150454503
rs1498681
rs12863456
rs614877
All
−1e−03
−5e−04
0e+00
5e−04
1e−03
MR effect size for
'Insulin−like 3' on 'eGFR'

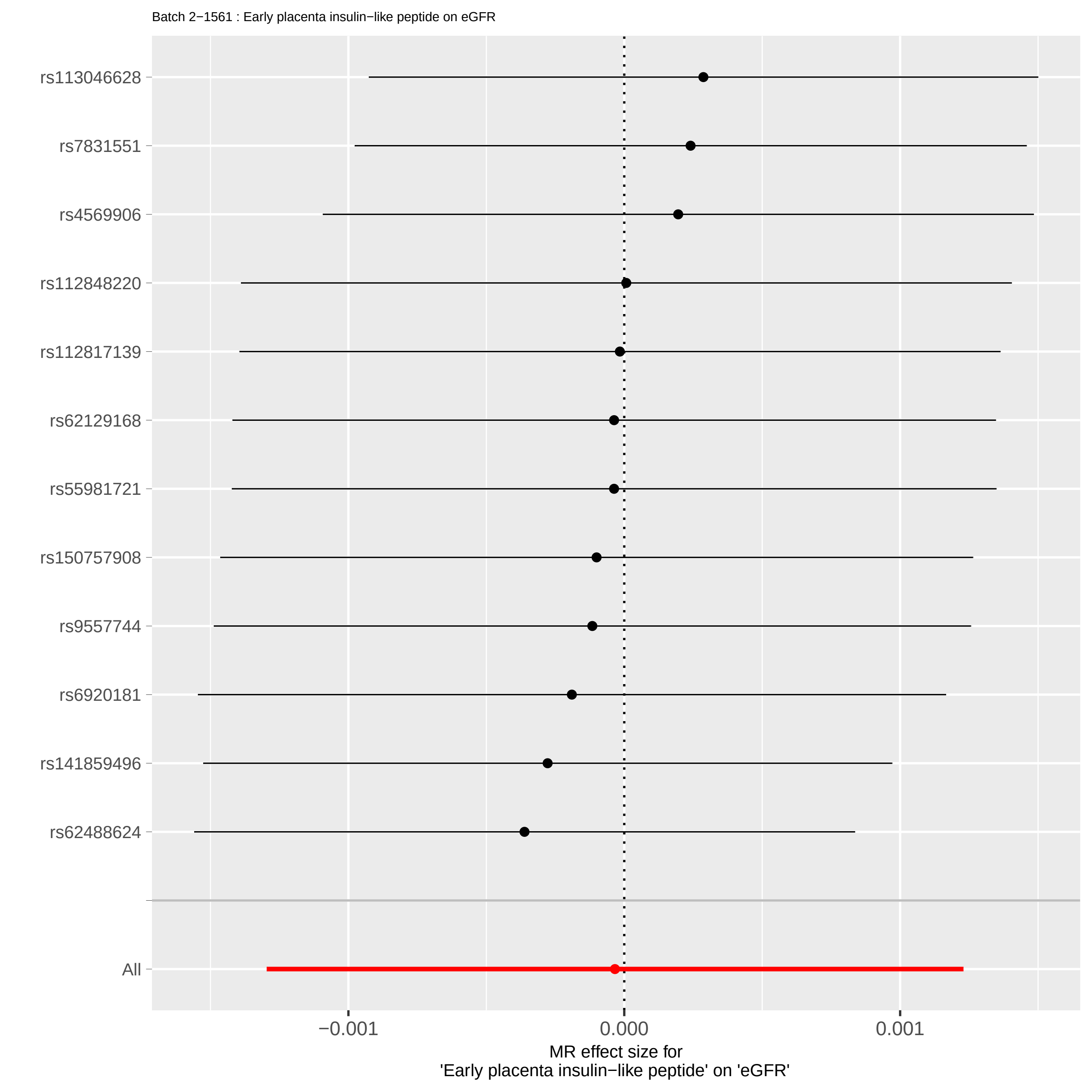

Batch 2−1561 : Early placenta insulin−like peptide on eGFR
rs113046628
rs7831551
rs4569906
rs112848220
rs112817139
rs62129168
rs55981721
rs150757908
rs9557744
rs6920181
rs141859496
rs62488624
All
−0.001
0.000
0.001
MR effect size for
'Early placenta insulin−like peptide' on 'eGFR'

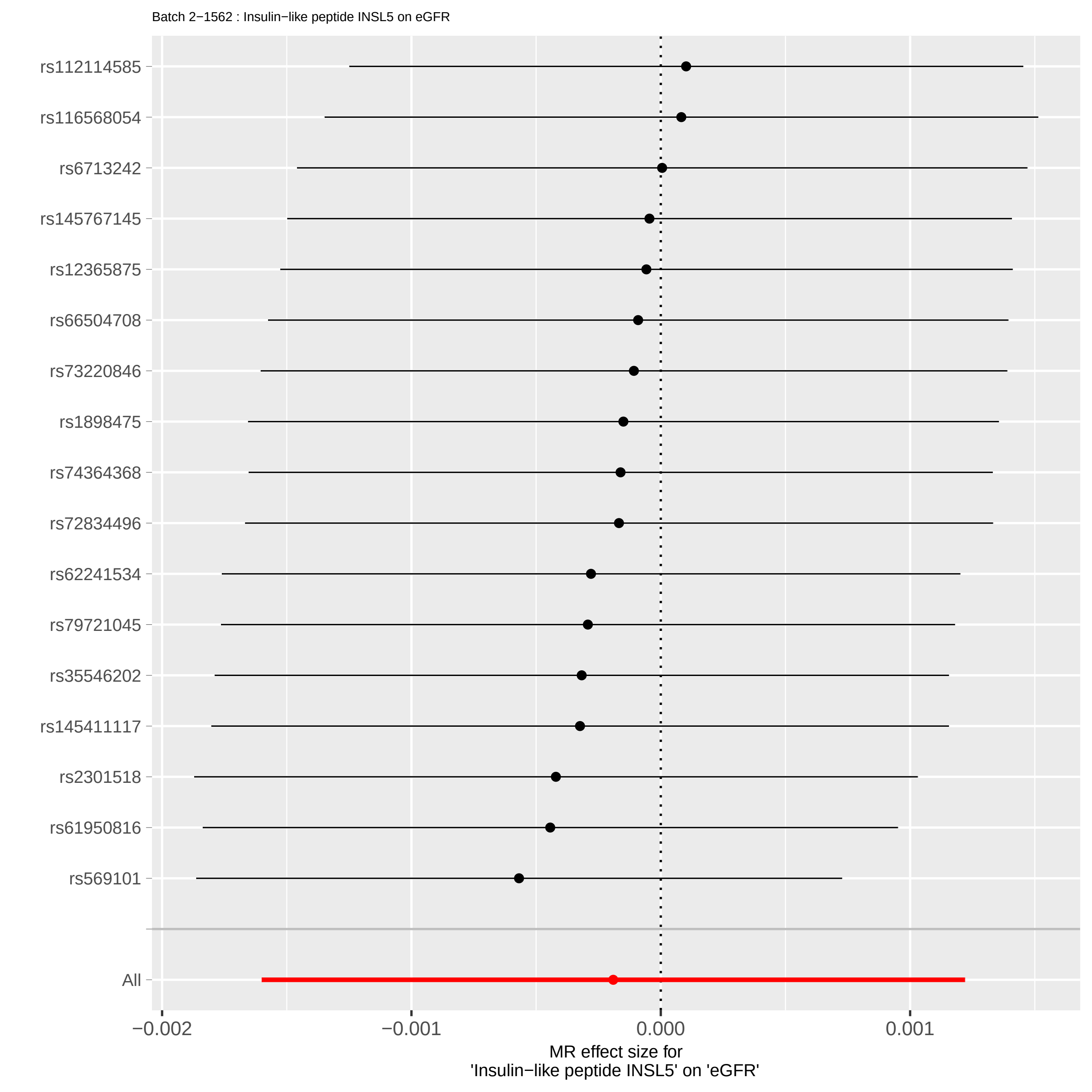

Batch 2−1562 : Insulin−like peptide INSL5 on eGFR
rs112114585
rs116568054
rs6713242
rs145767145
rs12365875
rs66504708
rs73220846
rs1898475
rs74364368
rs72834496
rs62241534
rs79721045
rs35546202
rs145411117
rs2301518
rs61950816
rs569101
All
−0.002
−0.001
0.000
0.001
MR effect size for
'Insulin−like peptide INSL5' on 'eGFR'

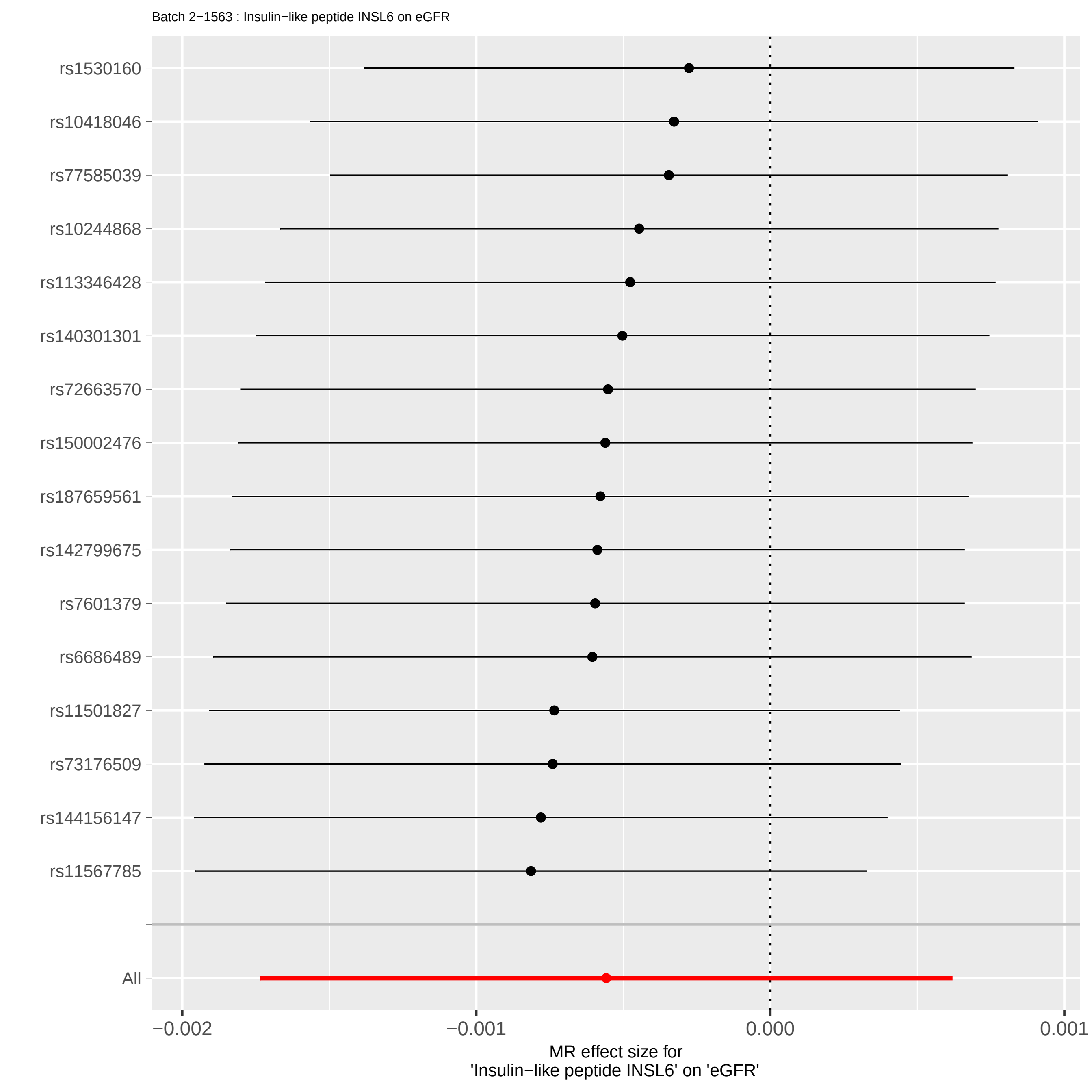
Batch 2−1563 : Insulin−like peptide INSL6 on eGFR
rs1530160
rs10418046
rs77585039
rs10244868
rs113346428
rs140301301
rs72663570
rs150002476
rs187659561
rs142799675
rs7601379
rs6686489
rs11501827
rs73176509
rs144156147
rs11567785
All
−0.002
−0.001
0.000
0.001
MR effect size for
'Insulin−like peptide INSL6' on 'eGFR'

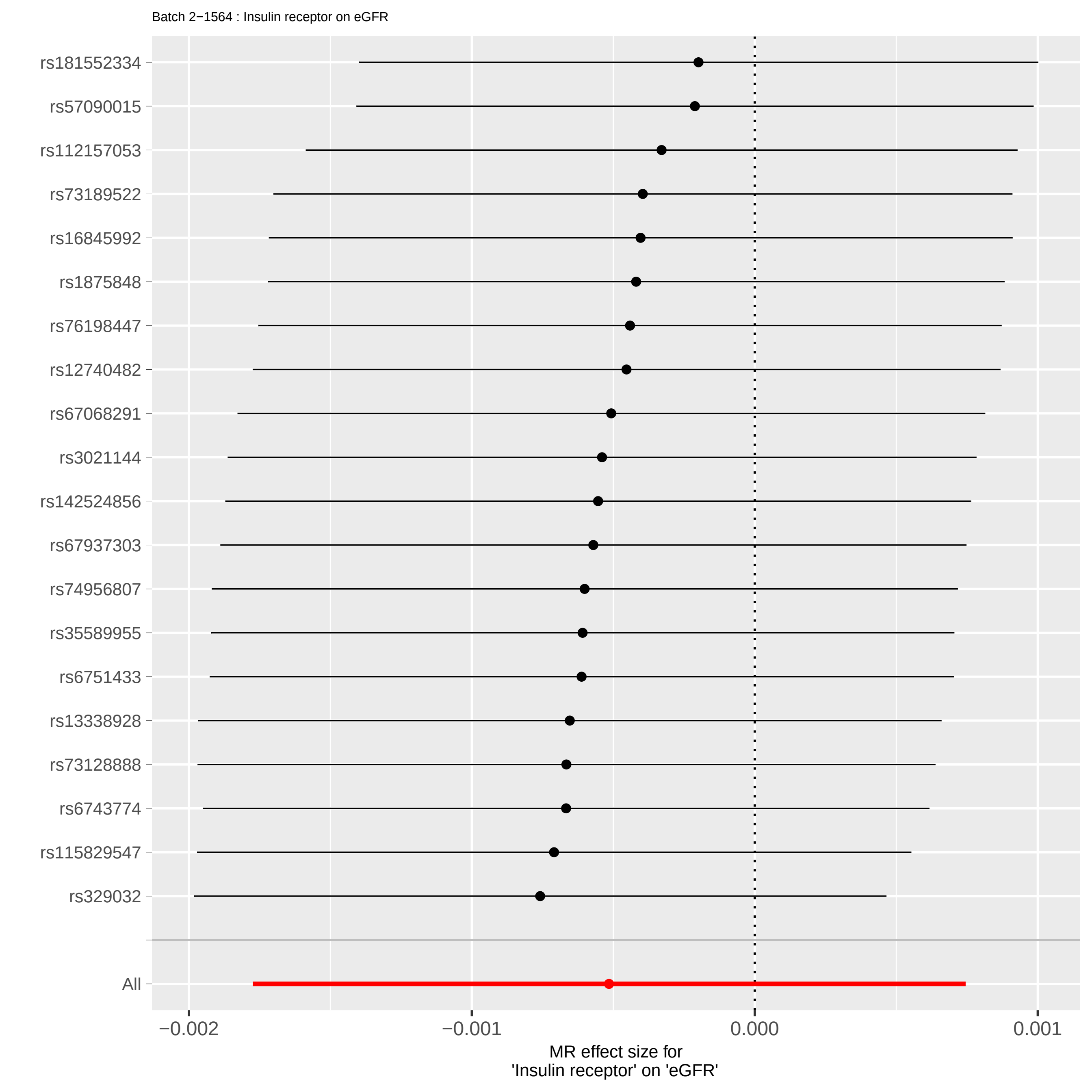

Batch 2−1564 : Insulin receptor on eGFR
rs181552334
rs57090015
rs112157053
rs73189522
rs16845992
rs1875848
rs76198447
rs12740482
rs67068291
rs3021144
rs142524856
rs67937303
rs74956807
rs35589955
rs6751433
rs13338928
rs73128888
rs6743774
rs115829547
rs329032
All
−0.002
−0.001
0.000
0.001
MR effect size for
'Insulin receptor' on 'eGFR'

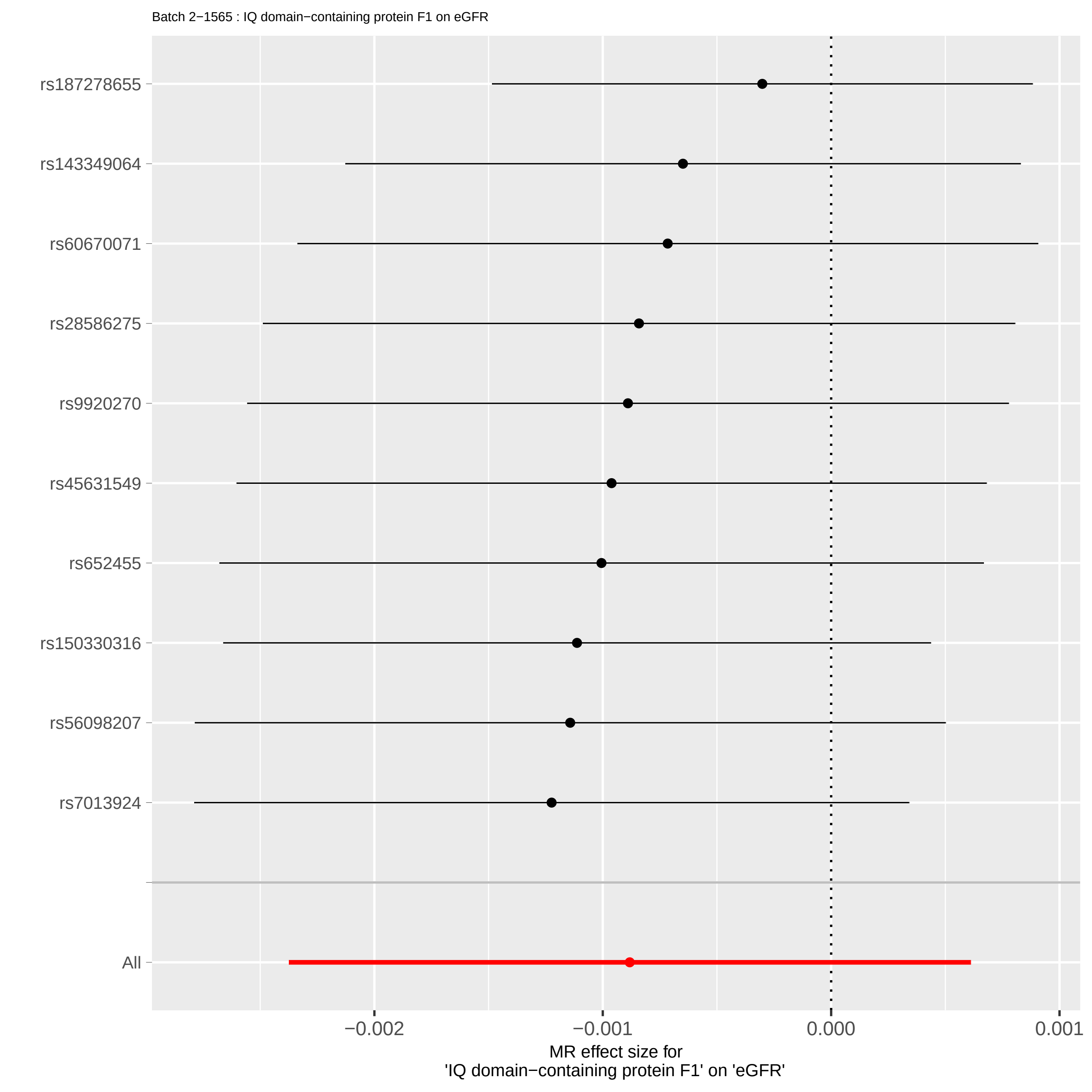

Batch 2−1565 : IQ domain−containing protein F1 on eGFR
rs187278655
rs143349064
rs60670071
rs28586275
rs9920270
rs45631549
rs652455
rs150330316
rs56098207
rs7013924
All
−0.002
−0.001
0.000
0.001
MR effect size for
'IQ domain−containing protein F1' on 'eGFR'

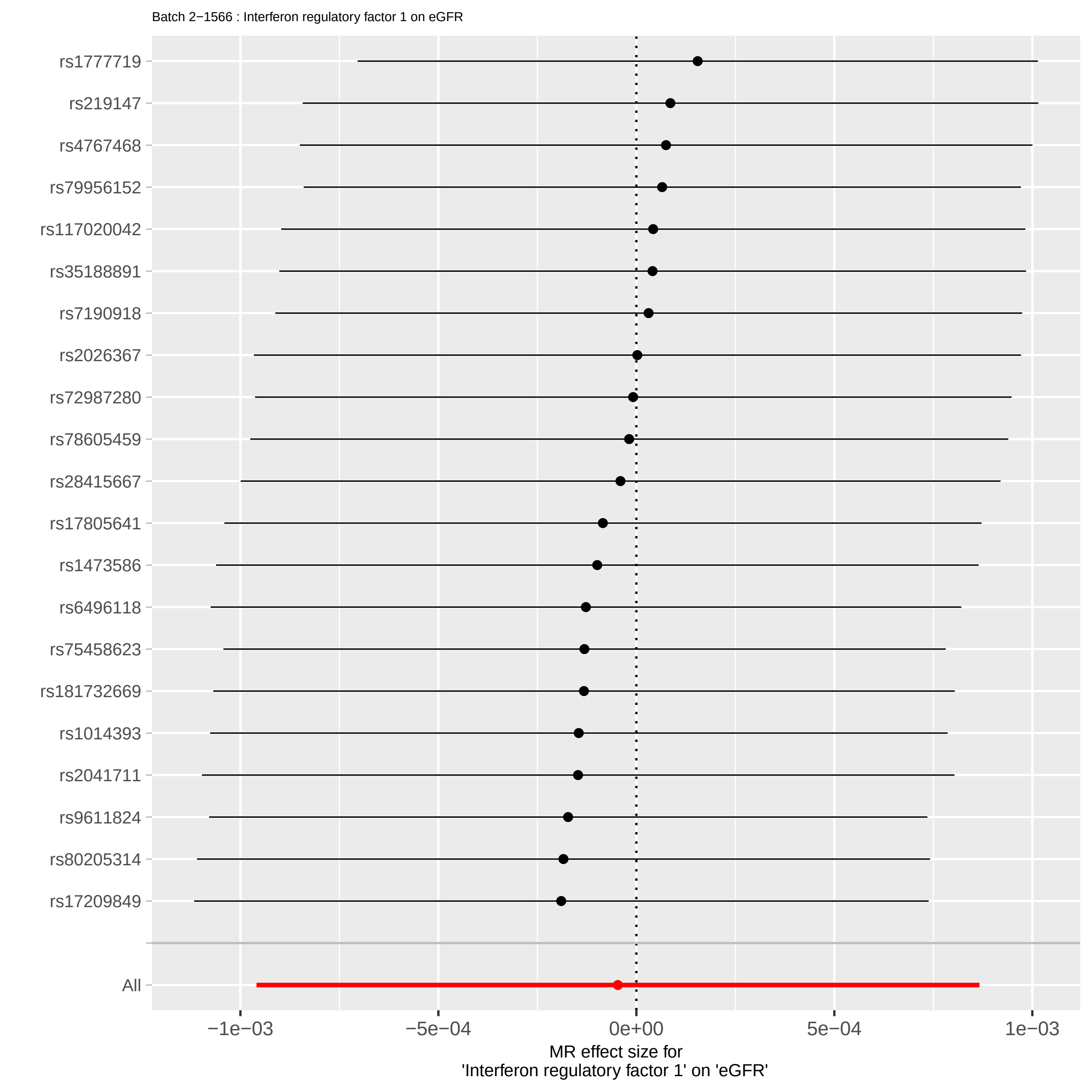

Batch 2−1566 : Interferon regulatory factor 1 on eGFR
rs1777719
rs219147
rs4767468
rs79956152
rs117020042
rs35188891
rs7190918
rs2026367
rs72987280
rs78605459
rs28415667
rs17805641
rs1473586
rs6496118
rs75458623
rs181732669
rs1014393
rs2041711
rs9611824
rs80205314
rs17209849
All
−1e−03
−5e−04
0e+00
5e−04
1e−03
MR effect size for
'Interferon regulatory factor 1' on 'eGFR'

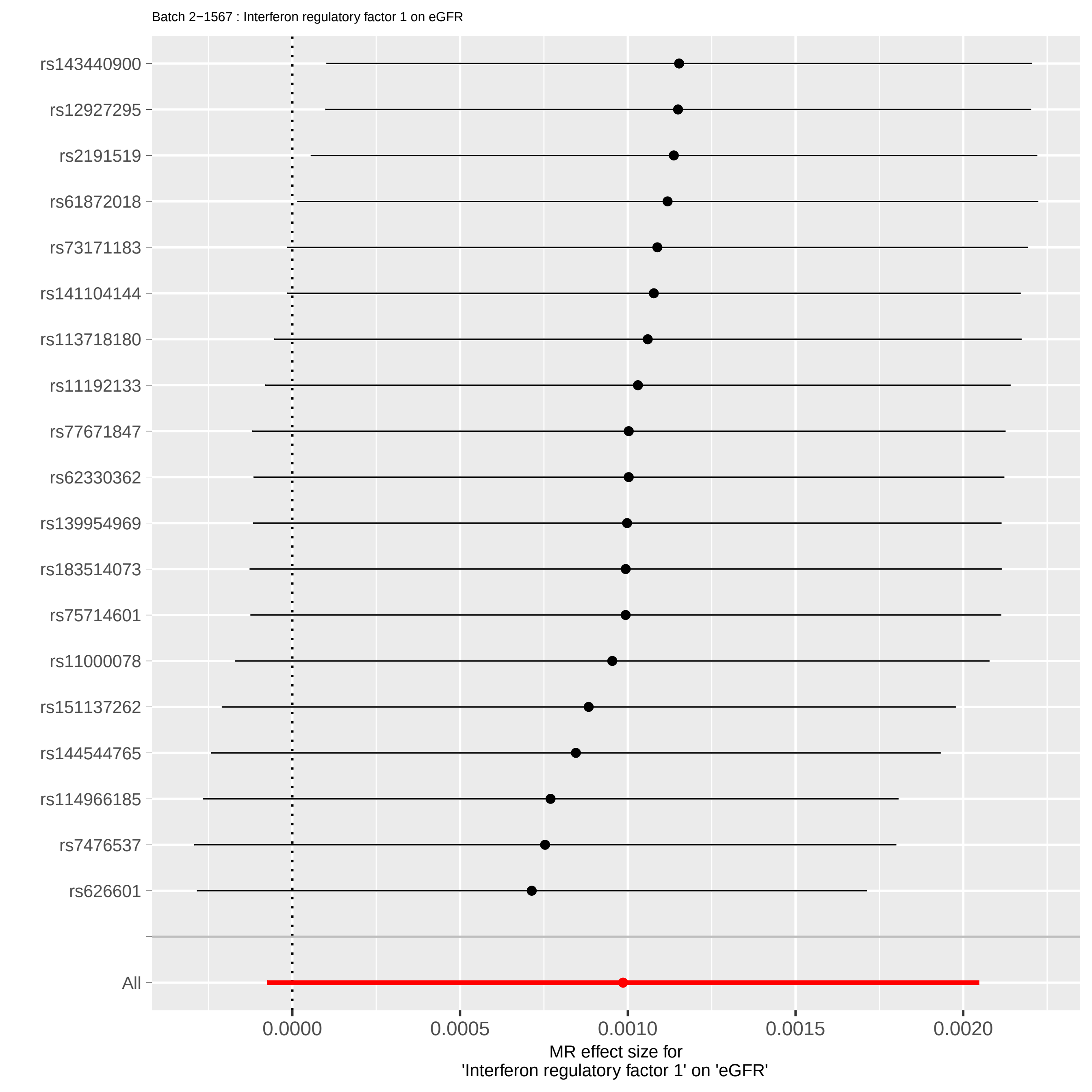
Batch 2−1567 : Interferon regulatory factor 1 on eGFR
rs143440900
rs12927295
rs2191519
rs61872018
rs73171183
rs141104144
rs113718180
rs11192133
rs77671847
rs62330362
rs139954969
rs183514073
rs75714601
rs11000078
rs151137262
rs144544765
rs114966185
rs7476537
rs626601
All
0.0000
0.0005
0.0010
0.0015
0.0020
MR effect size for
'Interferon regulatory factor 1' on 'eGFR'

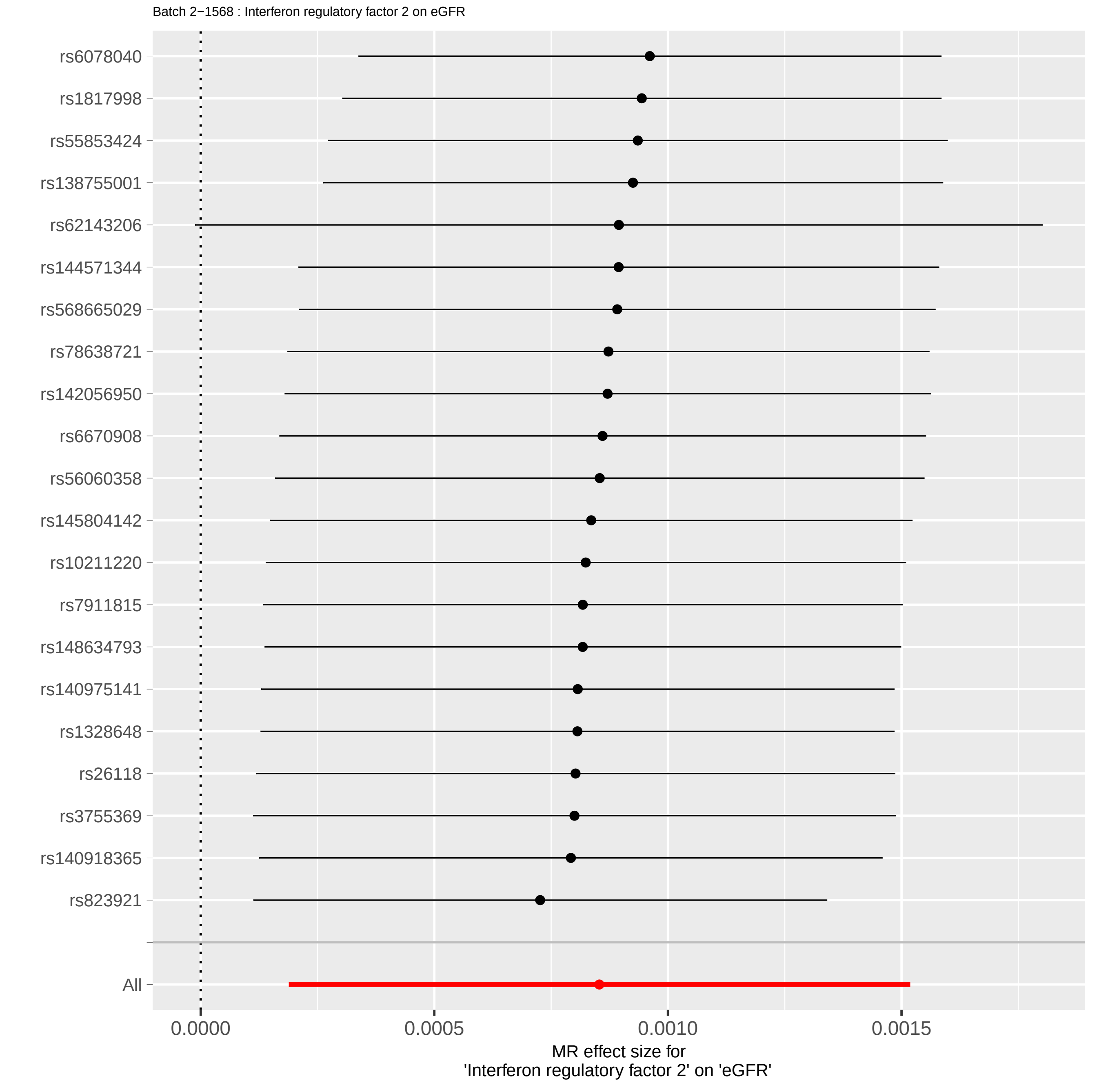

Batch 2−1568 : Interferon regulatory factor 2 on eGFR
rs6078040
rs1817998
rs55853424
rs138755001
rs62143206
rs144571344
rs568665029
rs78638721
rs142056950
rs6670908
rs56060358
rs145804142
rs10211220
rs7911815
rs148634793
rs140975141
rs1328648
rs26118
rs3755369
rs140918365
rs823921
All
0.0000
0.0005
0.0010
0.0015
MR effect size for
'Interferon regulatory factor 2' on 'eGFR'

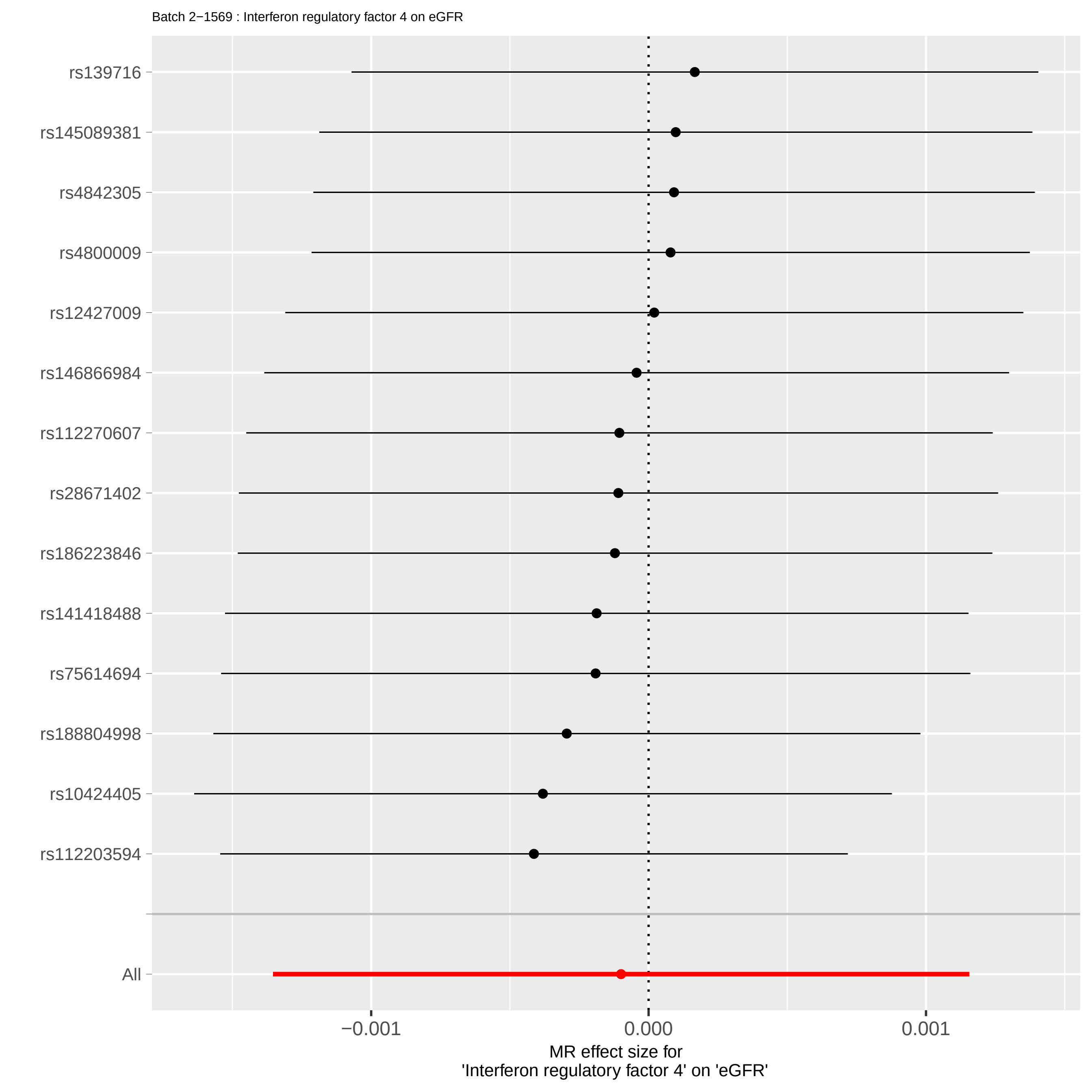

Batch 2−1569 : Interferon regulatory factor 4 on eGFR
rs139716
rs145089381
rs4842305
rs4800009
rs12427009
rs146866984
rs112270607
rs28671402
rs186223846
rs141418488
rs75614694
rs188804998
rs10424405
rs112203594
All
−0.001
0.000
0.001
MR effect size for
'Interferon regulatory factor 4' on 'eGFR'

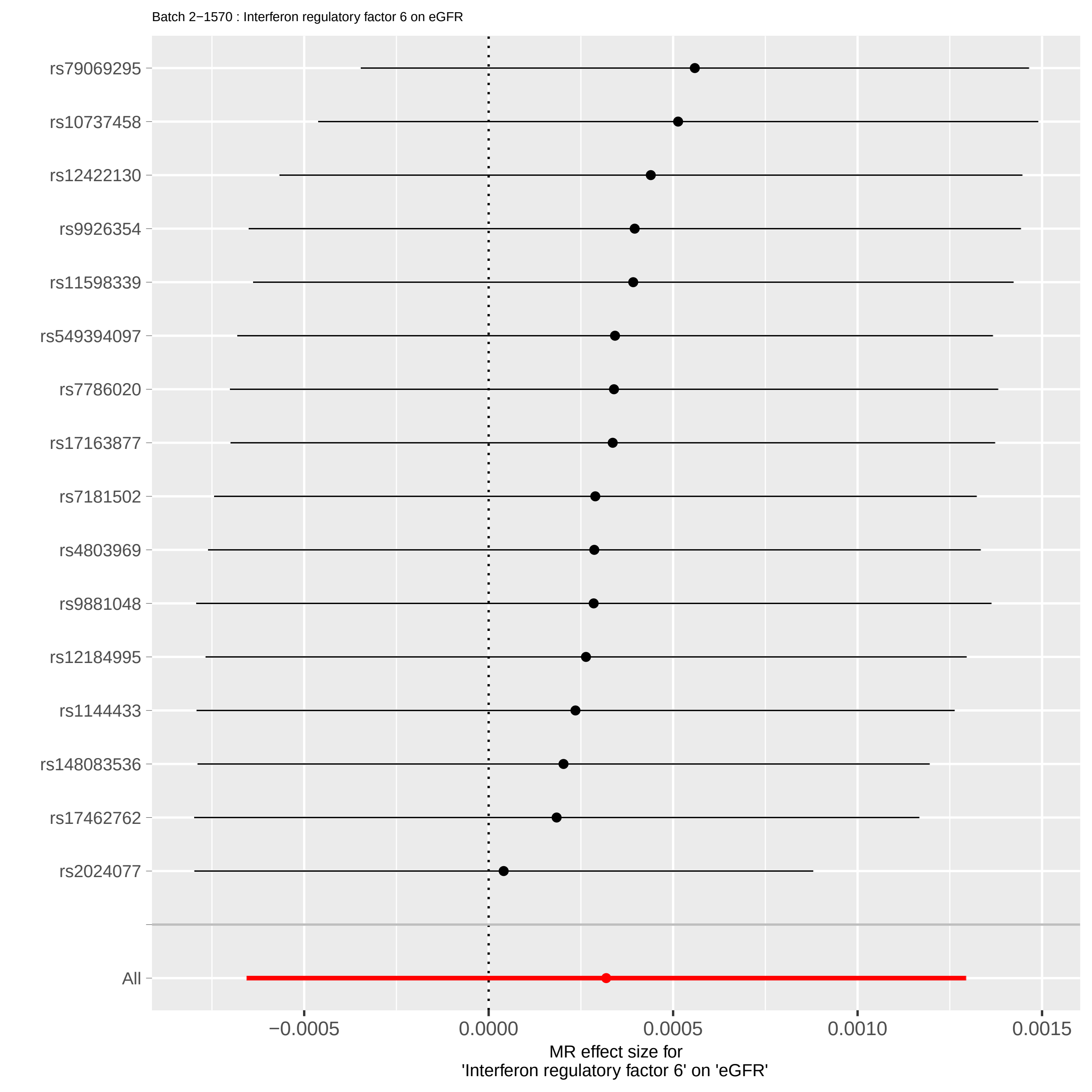

Batch 2−1570 : Interferon regulatory factor 6 on eGFR
rs79069295
rs10737458
rs12422130
rs9926354
rs11598339
rs549394097
rs7786020
rs17163877
rs7181502
rs4803969
rs9881048
rs12184995
rs1144433
rs148083536
rs17462762
rs2024077
All
−0.0005
0.0000
0.0005
0.0010
0.0015
MR effect size for
'Interferon regulatory factor 6' on 'eGFR'

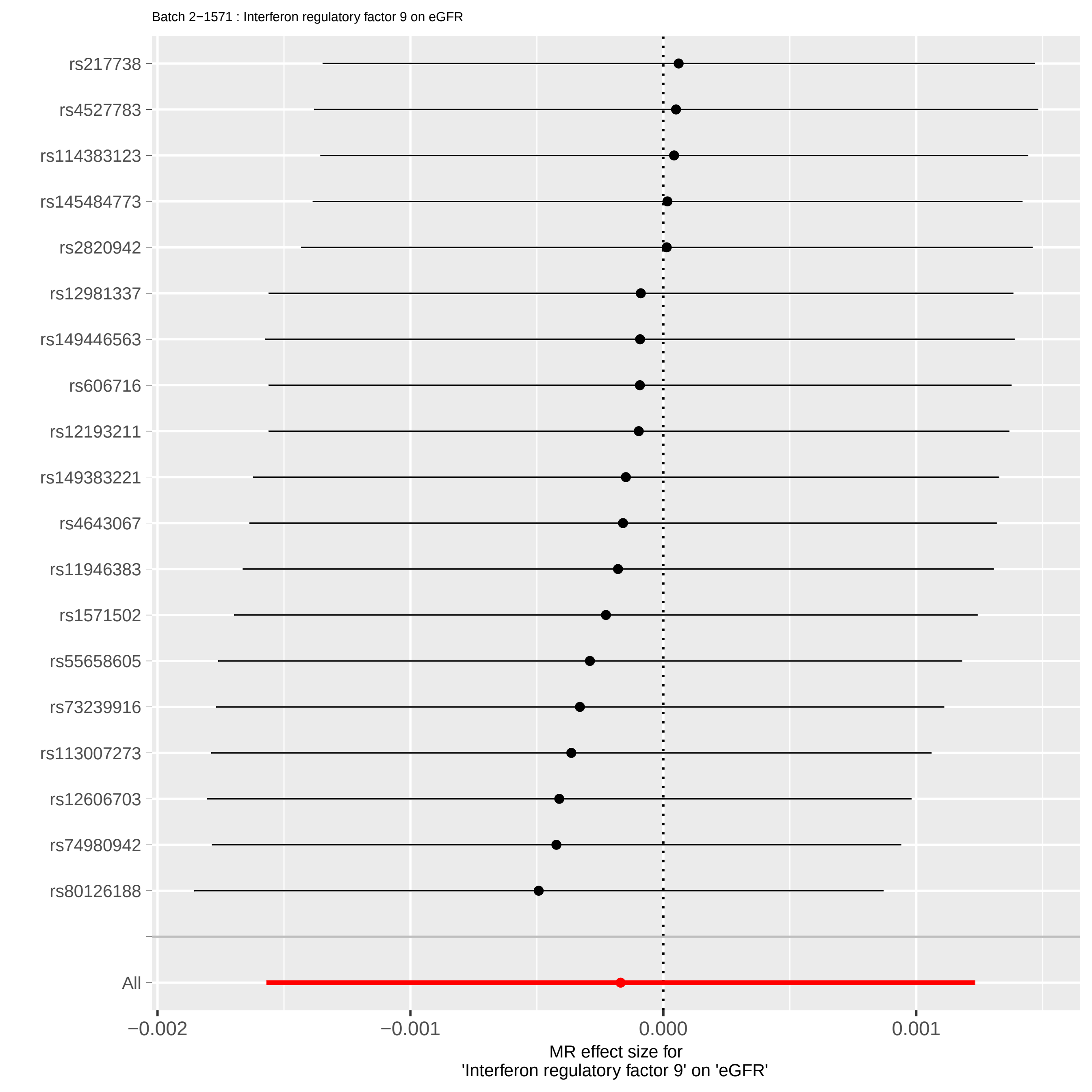

Batch 2−1571 : Interferon regulatory factor 9 on eGFR
rs217738
rs4527783
rs114383123
rs145484773
rs2820942
rs12981337
rs149446563
rs606716
rs12193211
rs149383221
rs4643067
rs11946383
rs1571502
rs55658605
rs73239916
rs113007273
rs12606703
rs74980942
rs80126188
All
−0.002
−0.001
0.000
0.001
MR effect size for
'Interferon regulatory factor 9' on 'eGFR'

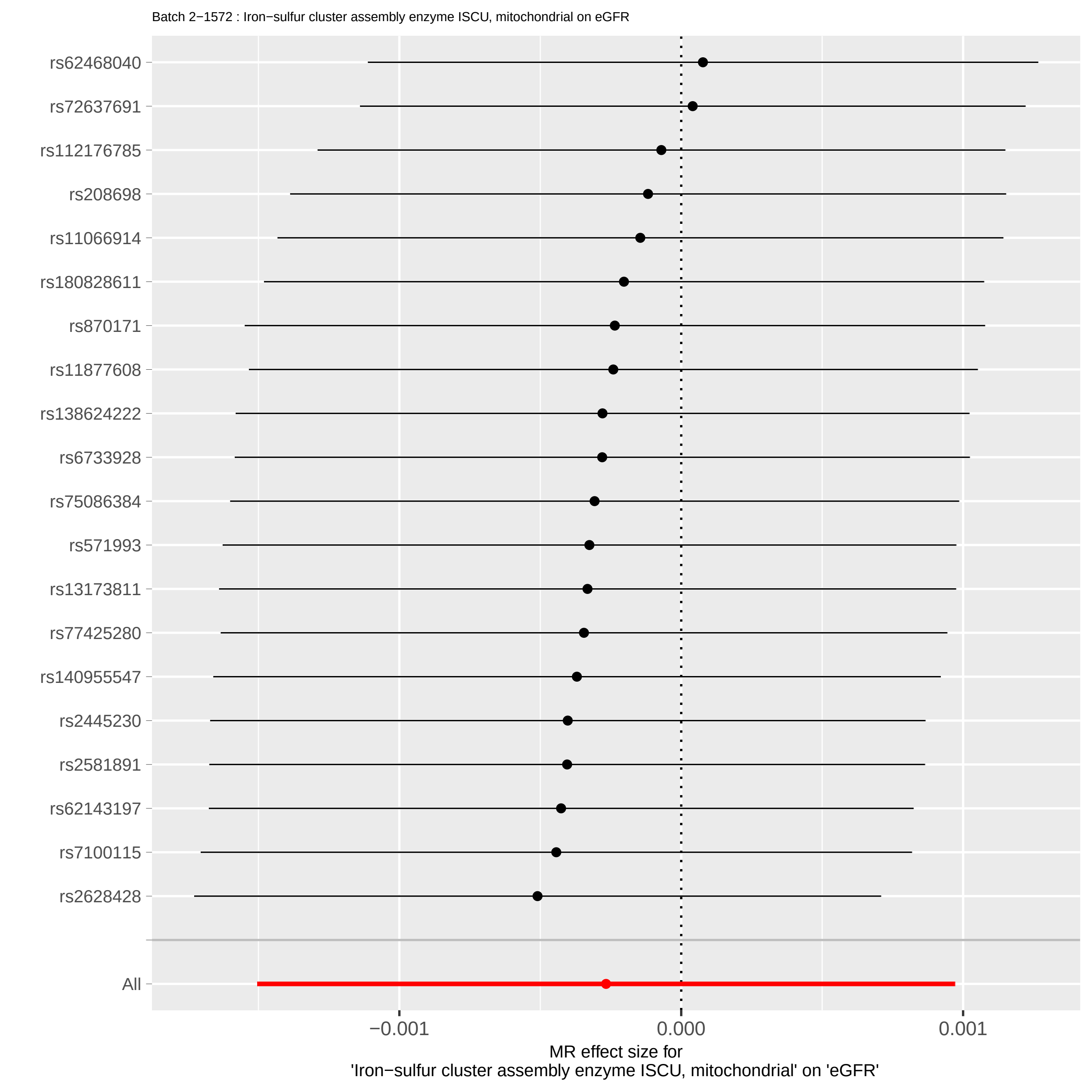

Batch 2−1572 : Iron−sulfur cluster assembly enzyme ISCU, mitochondrial on eGFR
rs62468040
rs72637691
rs112176785
rs208698
rs11066914
rs180828611
rs870171
rs11877608
rs138624222
rs6733928
rs75086384
rs571993
rs13173811
rs77425280
rs140955547
rs2445230
rs2581891
rs62143197
rs7100115
rs2628428
All
−0.001
0.000
0.001
MR effect size for
'Iron−sulfur cluster assembly enzyme ISCU, mitochondrial' on 'eGFR'

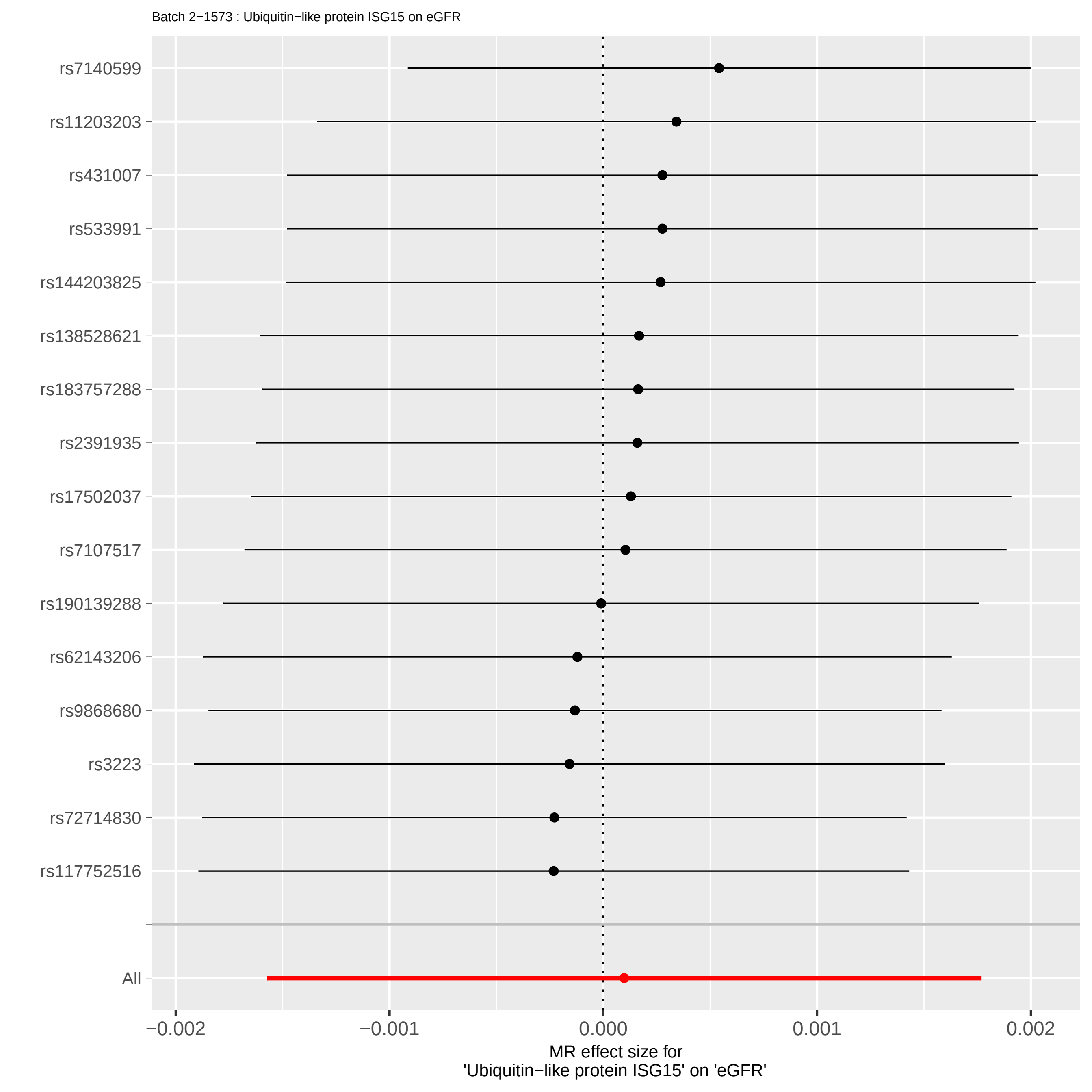

Batch 2−1573 : Ubiquitin−like protein ISG15 on eGFR
rs7140599
rs11203203
rs431007
rs533991
rs144203825
rs138528621
rs183757288
rs2391935
rs17502037
rs7107517
rs190139288
rs62143206
rs9868680
rs3223
rs72714830
rs117752516
All
−0.002
−0.001
0.000
0.001
0.002
MR effect size for
'Ubiquitin−like protein ISG15' on 'eGFR'

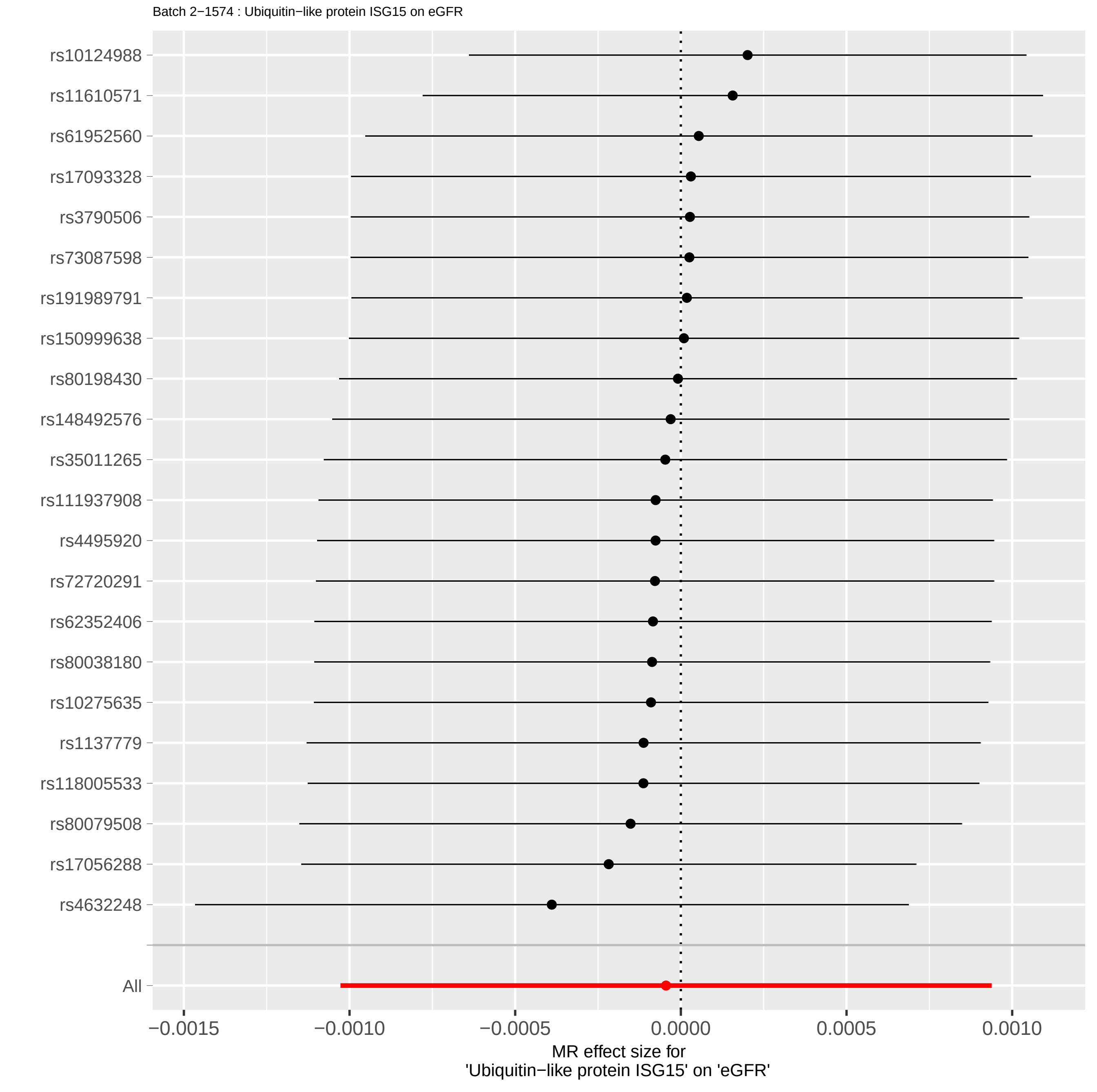

Batch 2−1574 : Ubiquitin−like protein ISG15 on eGFR
rs10124988
rs11610571
rs61952560
rs17093328
rs3790506
rs73087598
rs191989791
rs150999638
rs80198430
rs148492576
rs35011265
rs111937908
rs4495920
rs72720291
rs62352406
rs80038180
rs10275635
rs1137779
rs118005533
rs80079508
rs17056288
rs4632248
All
−0.0015
−0.0010
−0.0005
0.0000
0.0005
0.0010
MR effect size for
'Ubiquitin−like protein ISG15' on 'eGFR'

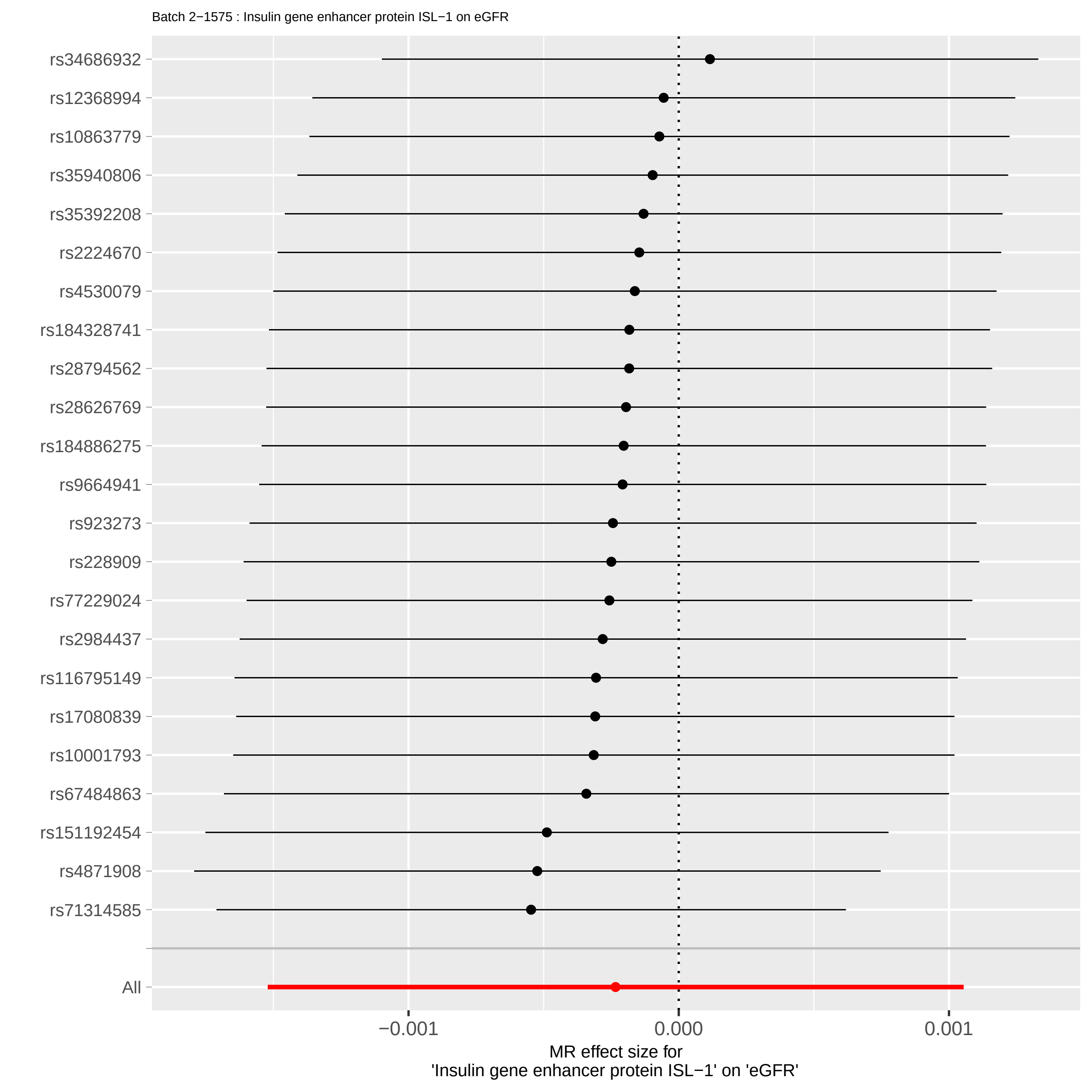

Batch 2−1575 : Insulin gene enhancer protein ISL−1 on eGFR
rs34686932
rs12368994
rs10863779
rs35940806
rs35392208
rs2224670
rs4530079
rs184328741
rs28794562
rs28626769
rs184886275
rs9664941
rs923273
rs228909
rs77229024
rs2984437
rs116795149
rs17080839
rs10001793
rs67484863
rs151192454
rs4871908
rs71314585
All
−0.001
0.000
0.001
MR effect size for
'Insulin gene enhancer protein ISL−1' on 'eGFR'

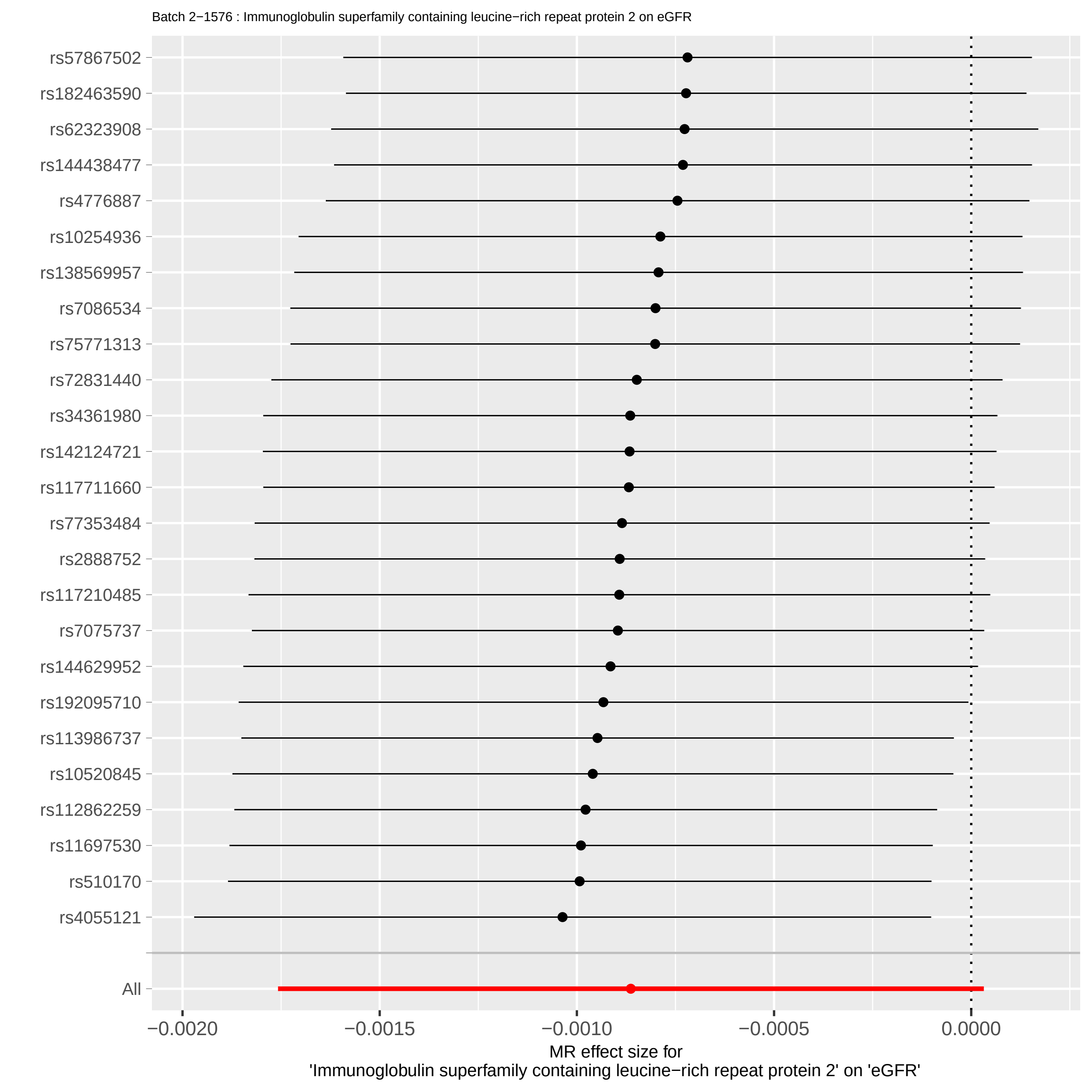
Batch 2−1576 : Immunoglobulin superfamily containing leucine−rich repeat protein 2 on eGFR
rs57867502
rs182463590
rs62323908
rs144438477
rs4776887
rs10254936
rs138569957
rs7086534
rs75771313
rs72831440
rs34361980
rs142124721
rs117711660
rs77353484
rs2888752
rs117210485
rs7075737
rs144629952
rs192095710
rs113986737
rs10520845
rs112862259
rs11697530
rs510170
rs4055121
All
−0.0020
−0.0015
−0.0010
−0.0005
0.0000
MR effect size for
'Immunoglobulin superfamily containing leucine−rich repeat protein 2' on 'eGFR'

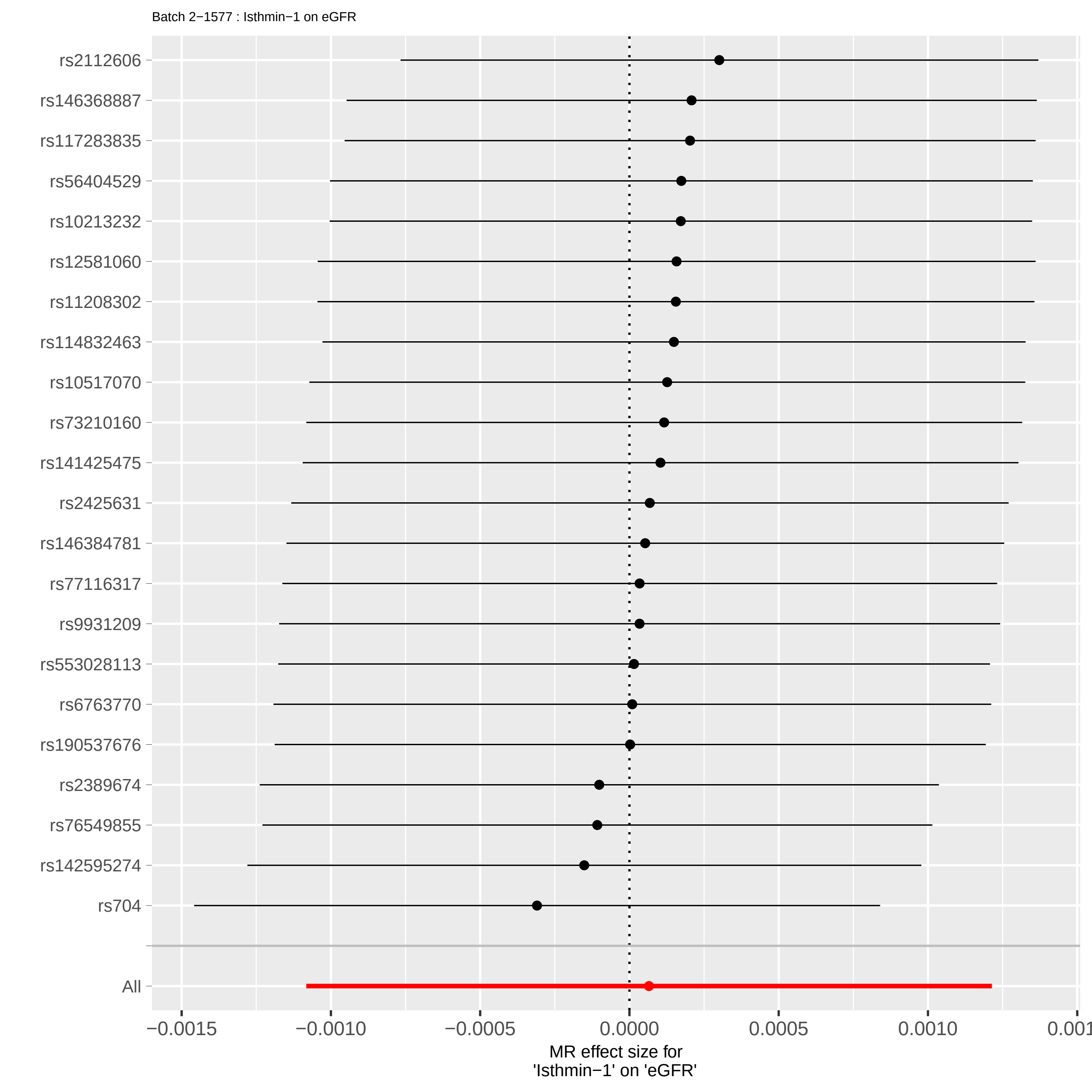

Batch 2−1577 : Isthmin−1 on eGFR
rs2112606
rs146368887
rs117283835
rs56404529
rs10213232
rs12581060
rs11208302
rs114832463
rs10517070
rs73210160
rs141425475
rs2425631
rs146384781
rs77116317
rs9931209
rs553028113
rs6763770
rs190537676
rs2389674
rs76549855
rs142595274
rs704
All
−0.0015
−0.0010
−0.0005
0.0000
0.0005
0.0010
MR effect size for
'Isthmin−1' on 'eGFR'

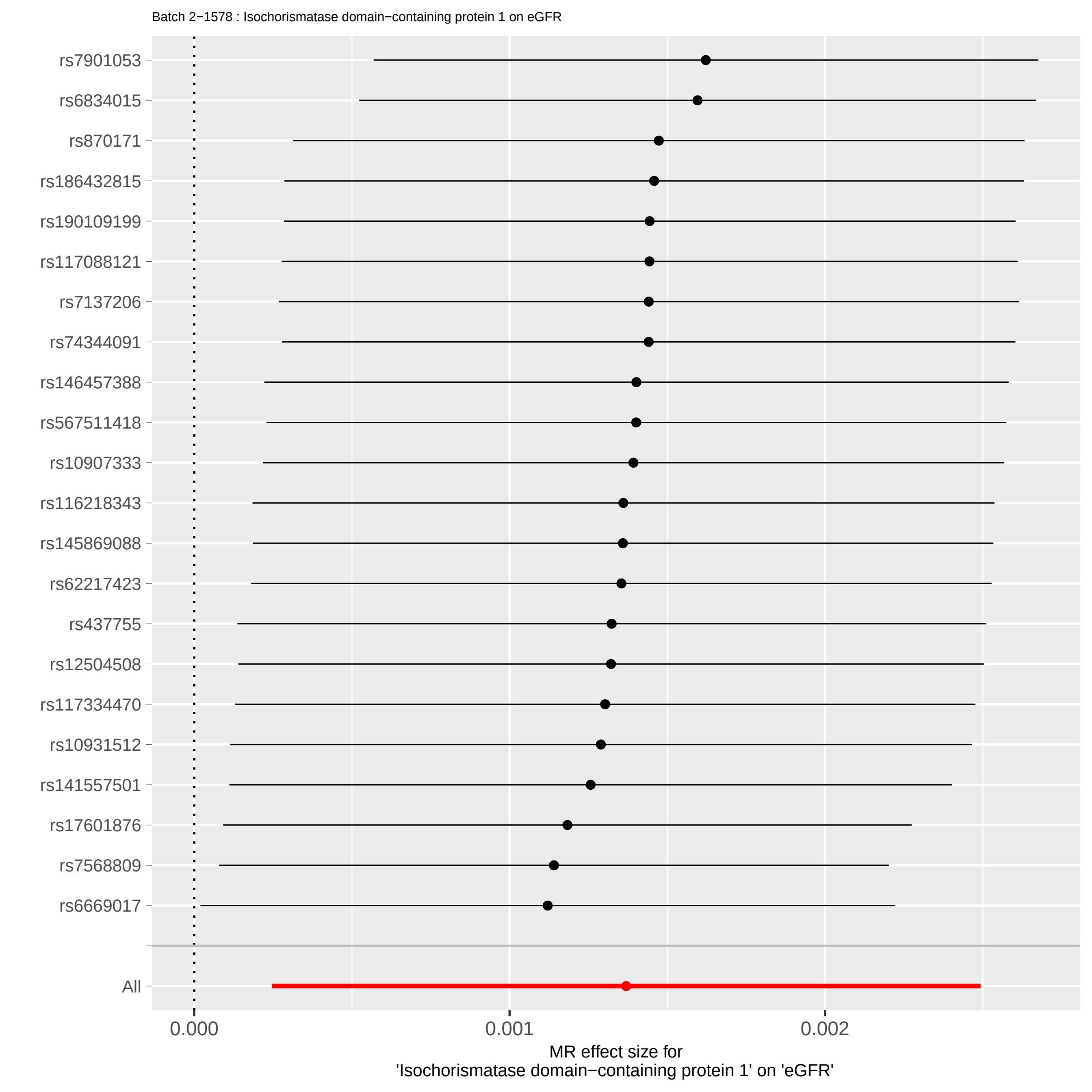
Batch 2−1578 : Isochorismatase domain−containing protein 1 on eGFR
rs7901053
rs6834015
rs870171
rs186432815
rs190109199
rs117088121
rs7137206
rs74344091
rs146457388
rs567511418
rs10907333
rs116218343
rs145869088
rs62217423
rs437755
rs12504508
rs117334470
rs10931512
rs141557501
rs17601876
rs7568809
rs6669017
All
0.000
0.001
0.002
MR effect size for
'Isochorismatase domain−containing protein 1' on 'eGFR'

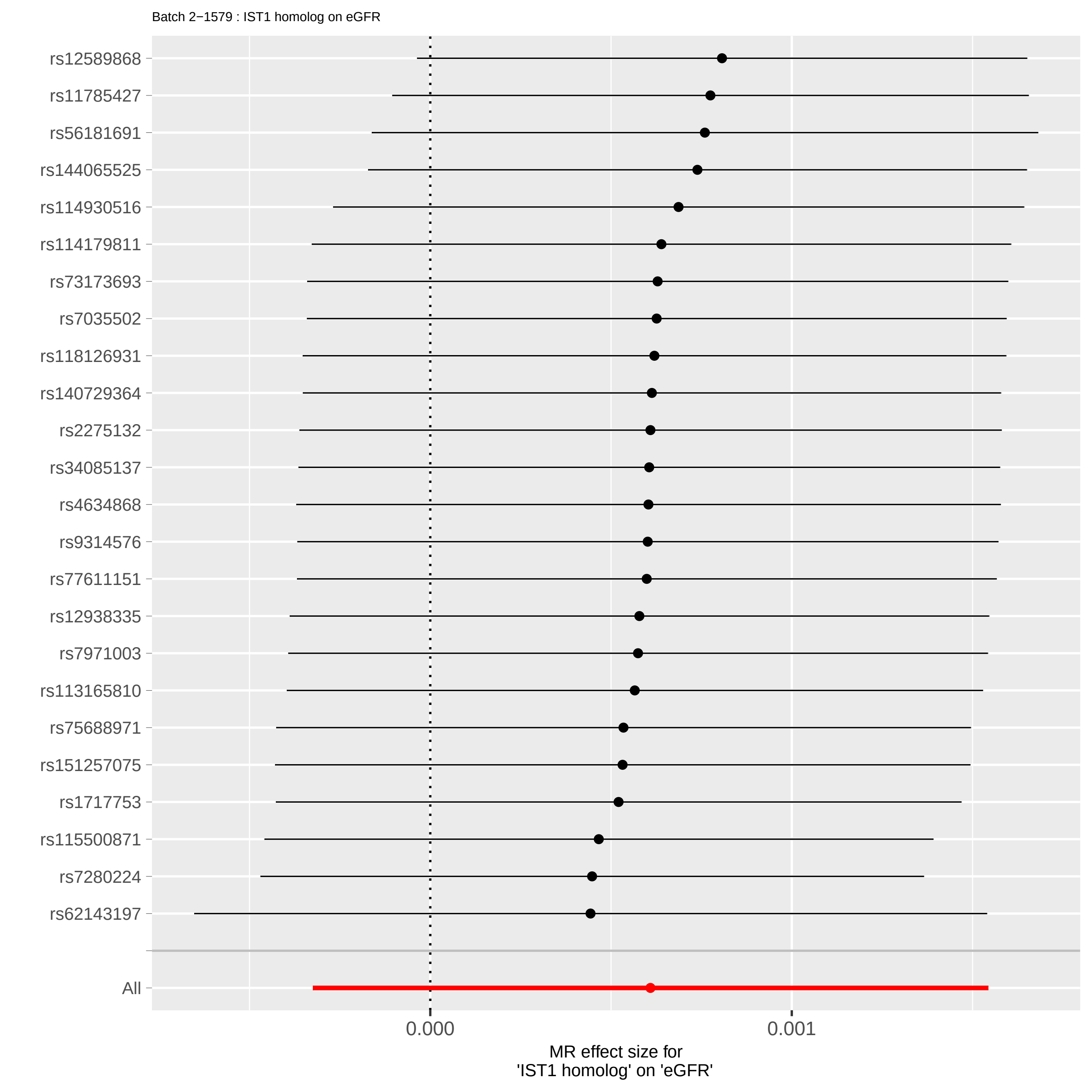

Batch 2−1579 : IST1 homolog on eGFR
rs12589868
rs11785427
rs56181691
rs144065525
rs114930516
rs114179811
rs73173693
rs7035502
rs118126931
rs140729364
rs2275132
rs34085137
rs4634868
rs9314576
rs77611151
rs12938335
rs7971003
rs113165810
rs75688971
rs151257075
rs1717753
rs115500871
rs7280224
rs62143197
All
0.000
0.001
MR effect size for
'IST1 homolog' on 'eGFR'

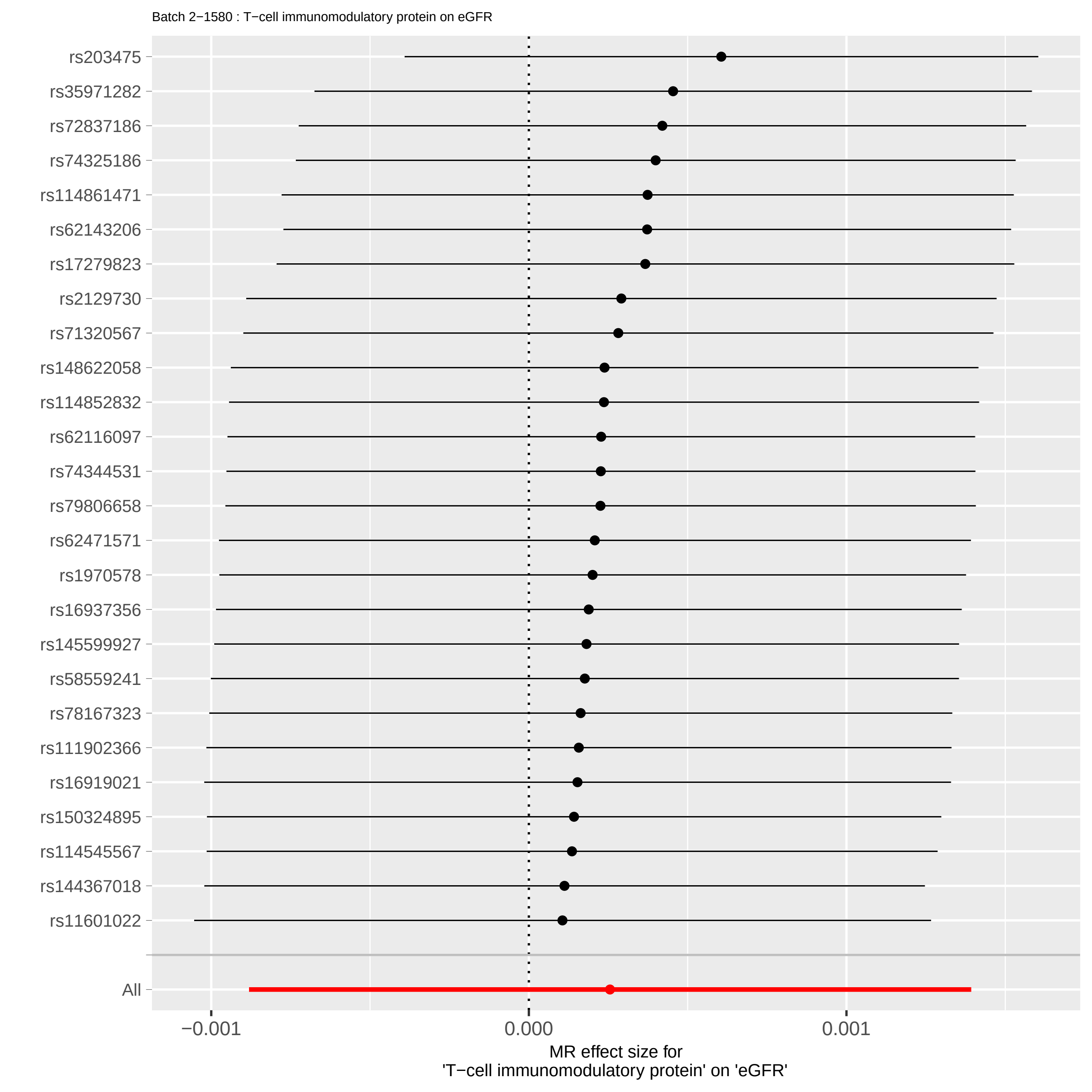

Batch 2−1580 : T−cell immunomodulatory protein on eGFR
rs203475
rs35971282
rs72837186
rs74325186
rs114861471
rs62143206
rs17279823
rs2129730
rs71320567
rs148622058
rs114852832
rs62116097
rs74344531
rs79806658
rs62471571
rs1970578
rs16937356
rs145599927
rs58559241
rs78167323
rs111902366
rs16919021
rs150324895
rs114545567
rs144367018
rs11601022
All
−0.001
0.000
0.001
MR effect size for
'T−cell immunomodulatory protein' on 'eGFR'

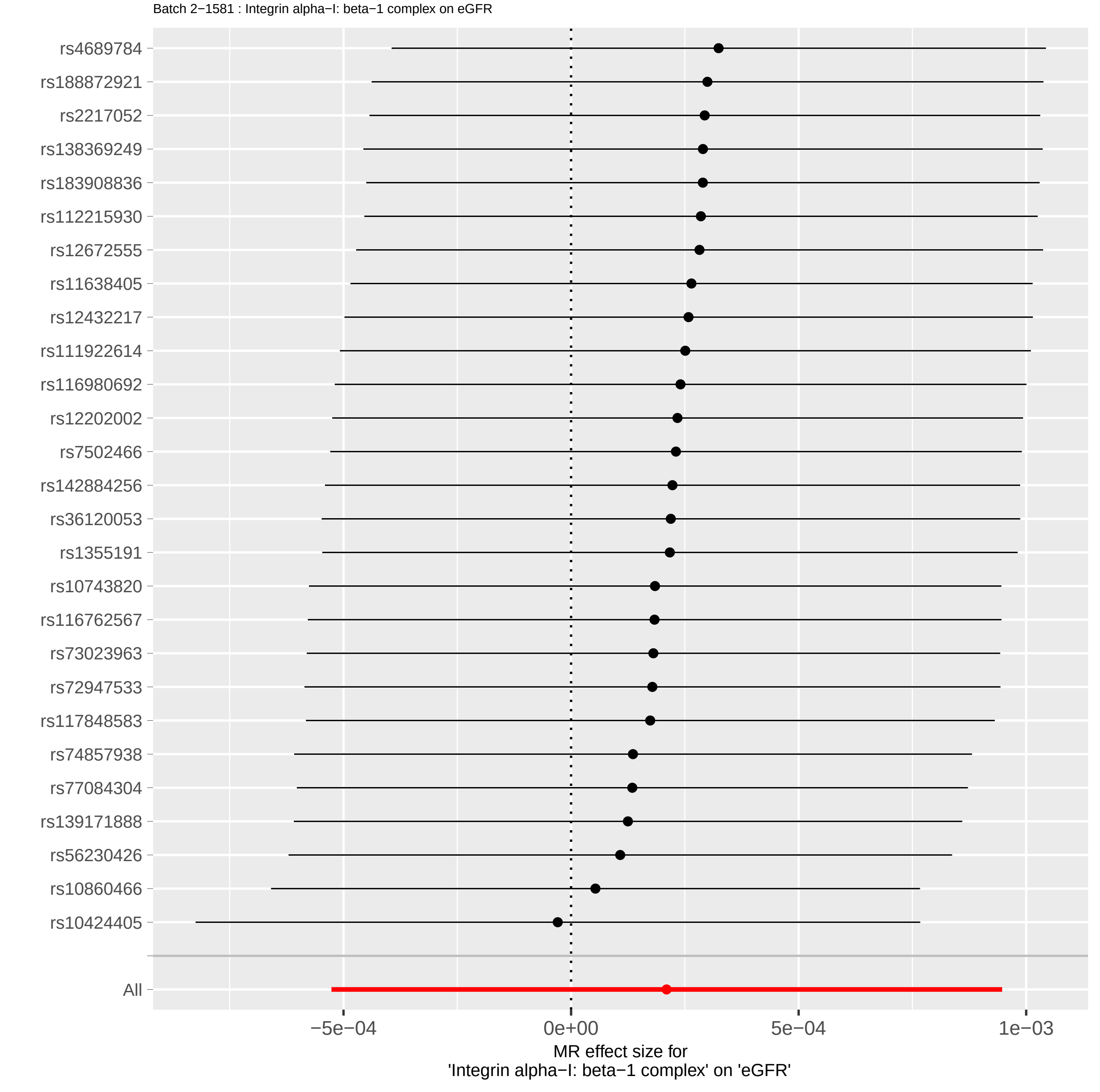

Batch 2−1581 : Integrin alpha−I: beta−1 complex on eGFR
rs4689784
rs188872921
rs2217052
rs138369249
rs183908836
rs112215930
rs12672555
rs11638405
rs12432217
rs111922614
rs116980692
rs12202002
rs7502466
rs142884256
rs36120053
rs1355191
rs10743820
rs116762567
rs73023963
rs72947533
rs117848583
rs74857938
rs77084304
rs139171888
rs56230426
rs10860466
rs10424405
All
−5e−04
0e+00
5e−04
1e−03
MR effect size for
'Integrin alpha−I: beta−1 complex' on 'eGFR'

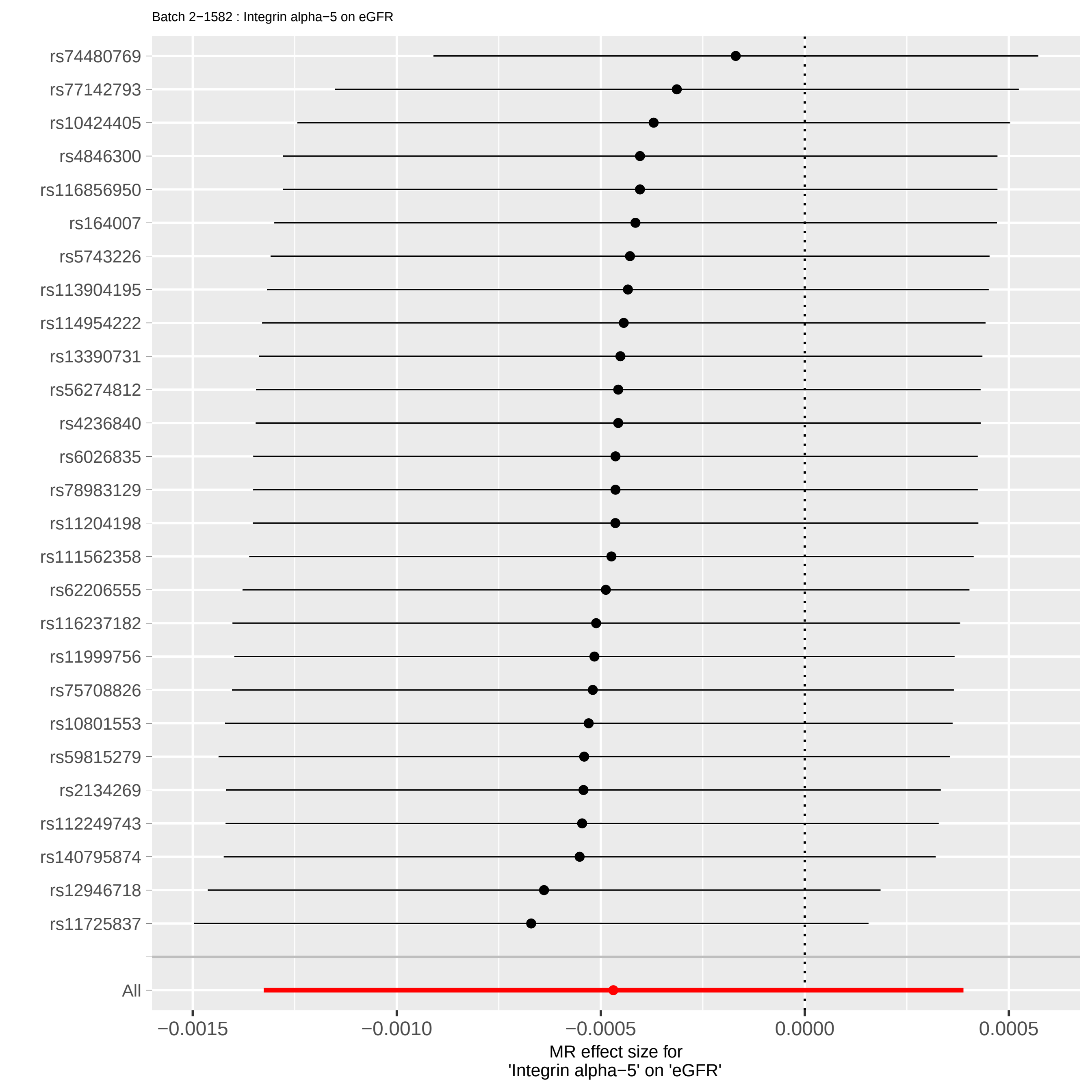

Batch 2−1582 : Integrin alpha−5 on eGFR
rs74480769
rs77142793
rs10424405
rs4846300
rs116856950
rs164007
rs5743226
rs113904195
rs114954222
rs13390731
rs56274812
rs4236840
rs6026835
rs78983129
rs11204198
rs111562358
rs62206555
rs116237182
rs11999756
rs75708826
rs10801553
rs59815279
rs2134269
rs112249743
rs140795874
rs12946718
rs11725837
All
−0.0015
−0.0010
−0.0005
0.0000
0.0005
MR effect size for
'Integrin alpha−5' on 'eGFR'

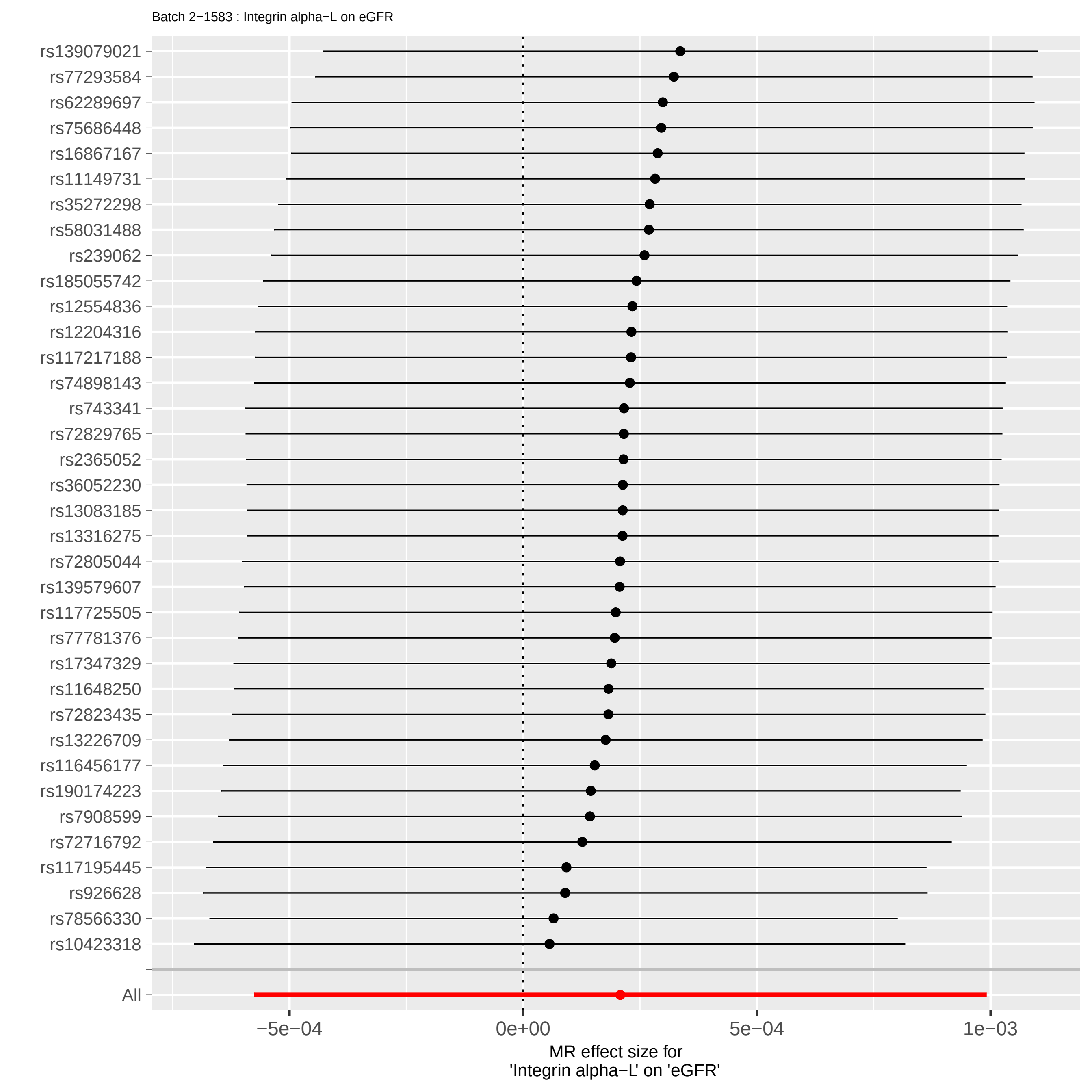

Batch 2−1583 : Integrin alpha−L on eGFR
rs139079021
rs77293584
rs62289697
rs75686448
rs16867167
rs11149731
rs35272298
rs58031488
rs239062
rs185055742
rs12554836
rs12204316
rs117217188
rs74898143
rs743341
rs72829765
rs2365052
rs36052230
rs13083185
rs13316275
rs72805044
rs139579607
rs117725505
rs77781376
rs17347329
rs11648250
rs72823435
rs13226709
rs116456177
rs190174223
rs7908599
rs72716792
rs117195445
rs926628
rs78566330
rs10423318
All
−5e−04
0e+00
5e−04
1e−03
MR effect size for
'Integrin alpha−L' on 'eGFR'

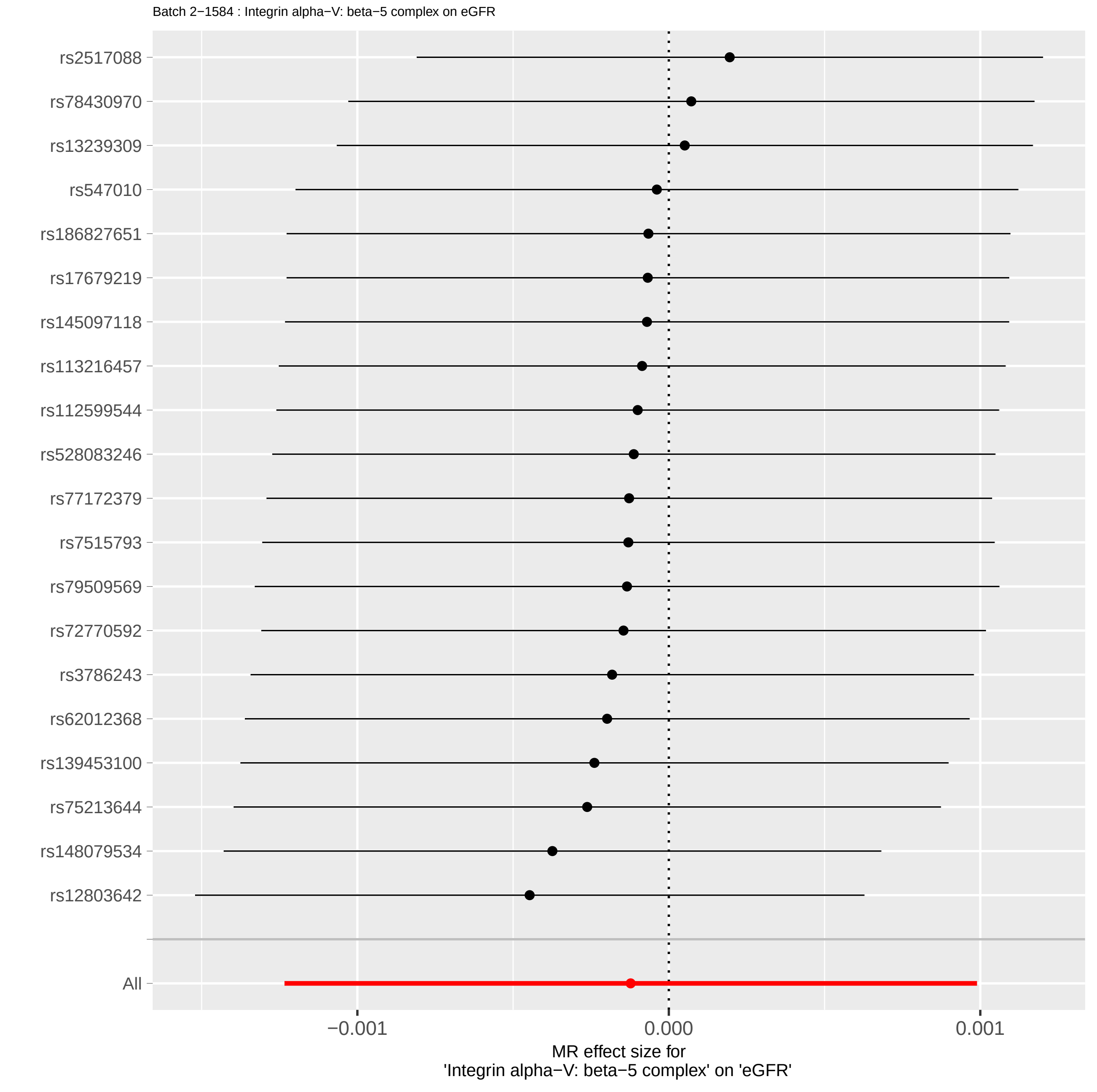

Batch 2−1584 : Integrin alpha−V: beta−5 complex on eGFR
rs2517088
rs78430970
rs13239309
rs547010
rs186827651
rs17679219
rs145097118
rs113216457
rs112599544
rs528083246
rs77172379
rs7515793
rs79509569
rs72770592
rs3786243
rs62012368
rs139453100
rs75213644
rs148079534
rs12803642
All
−0.001
0.000
0.001
MR effect size for
'Integrin alpha−V: beta−5 complex' on 'eGFR'

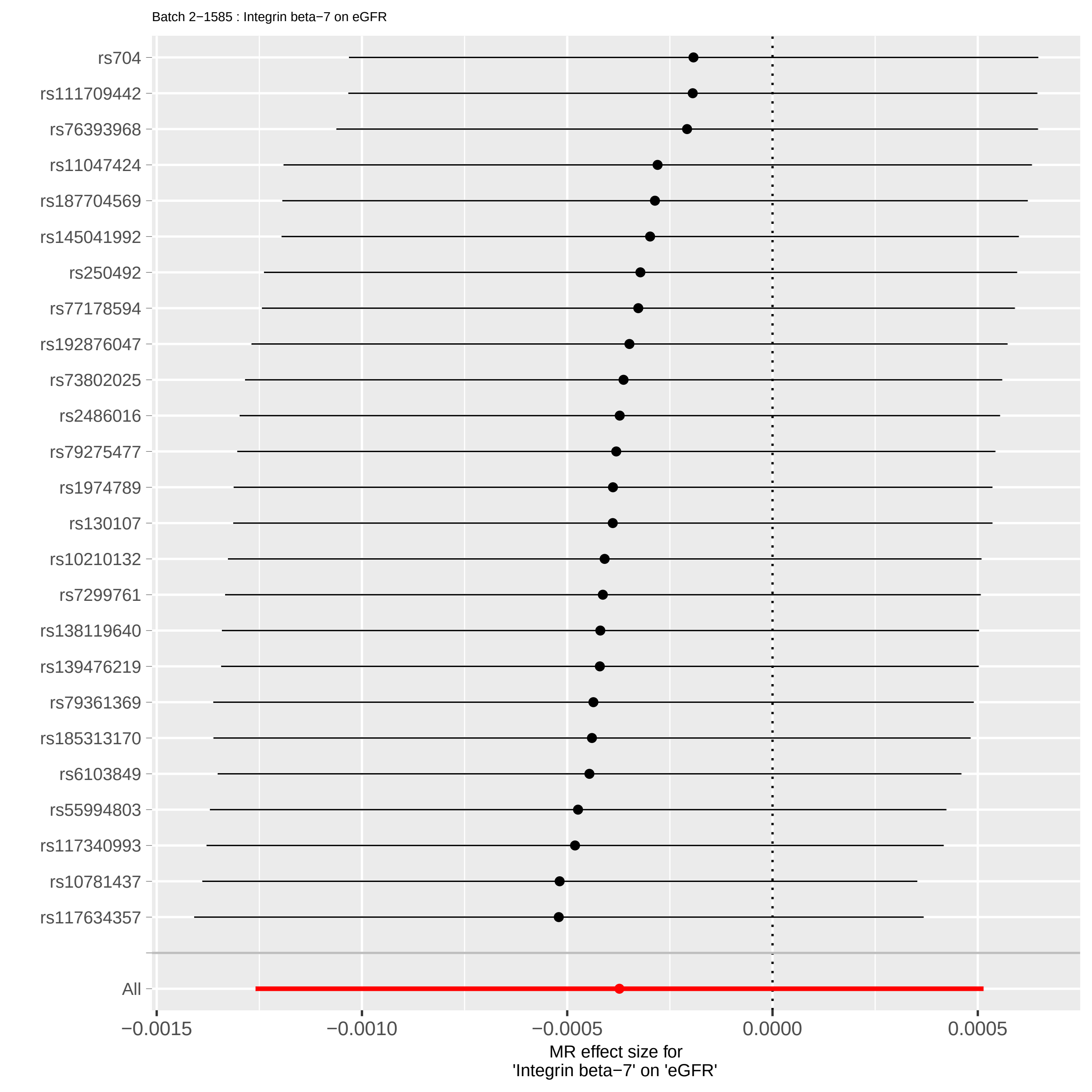

Batch 2−1585 : Integrin beta−7 on eGFR
rs704
rs111709442
rs76393968
rs11047424
rs187704569
rs145041992
rs250492
rs77178594
rs192876047
rs73802025
rs2486016
rs79275477
rs1974789
rs130107
rs10210132
rs7299761
rs138119640
rs139476219
rs79361369
rs185313170
rs6103849
rs55994803
rs117340993
rs10781437
rs117634357
All
−0.0015
−0.0010
−0.0005
0.0000
0.0005
MR effect size for
'Integrin beta−7' on 'eGFR'

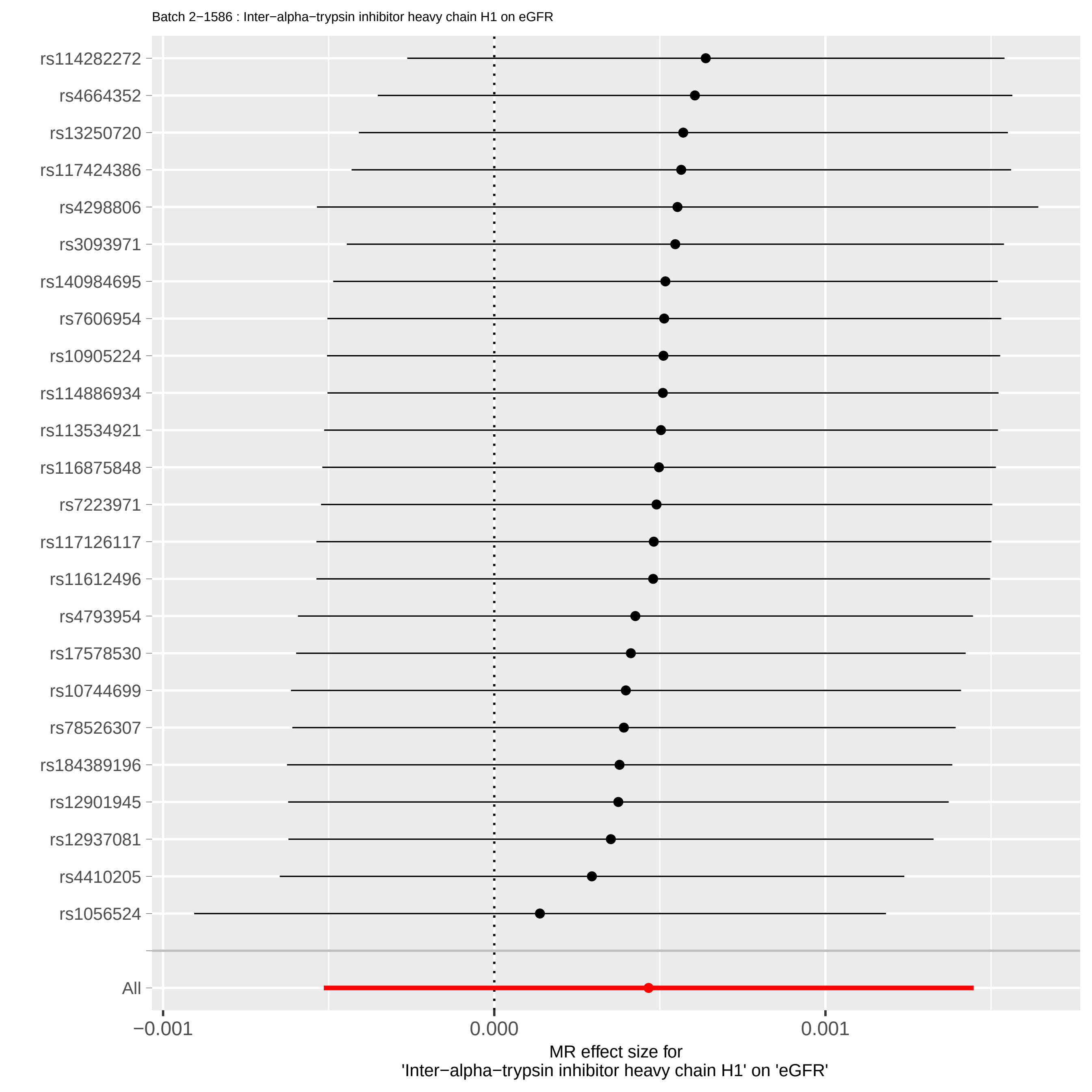

Batch 2−1586 : Inter−alpha−trypsin inhibitor heavy chain H1 on eGFR
rs114282272
rs4664352
rs13250720
rs117424386
rs4298806
rs3093971
rs140984695
rs7606954
rs10905224
rs114886934
rs113534921
rs116875848
rs7223971
rs117126117
rs11612496
rs4793954
rs17578530
rs10744699
rs78526307
rs184389196
rs12901945
rs12937081
rs4410205
rs1056524
All
−0.001
0.000
0.001
MR effect size for
'Inter−alpha−trypsin inhibitor heavy chain H1' on 'eGFR'

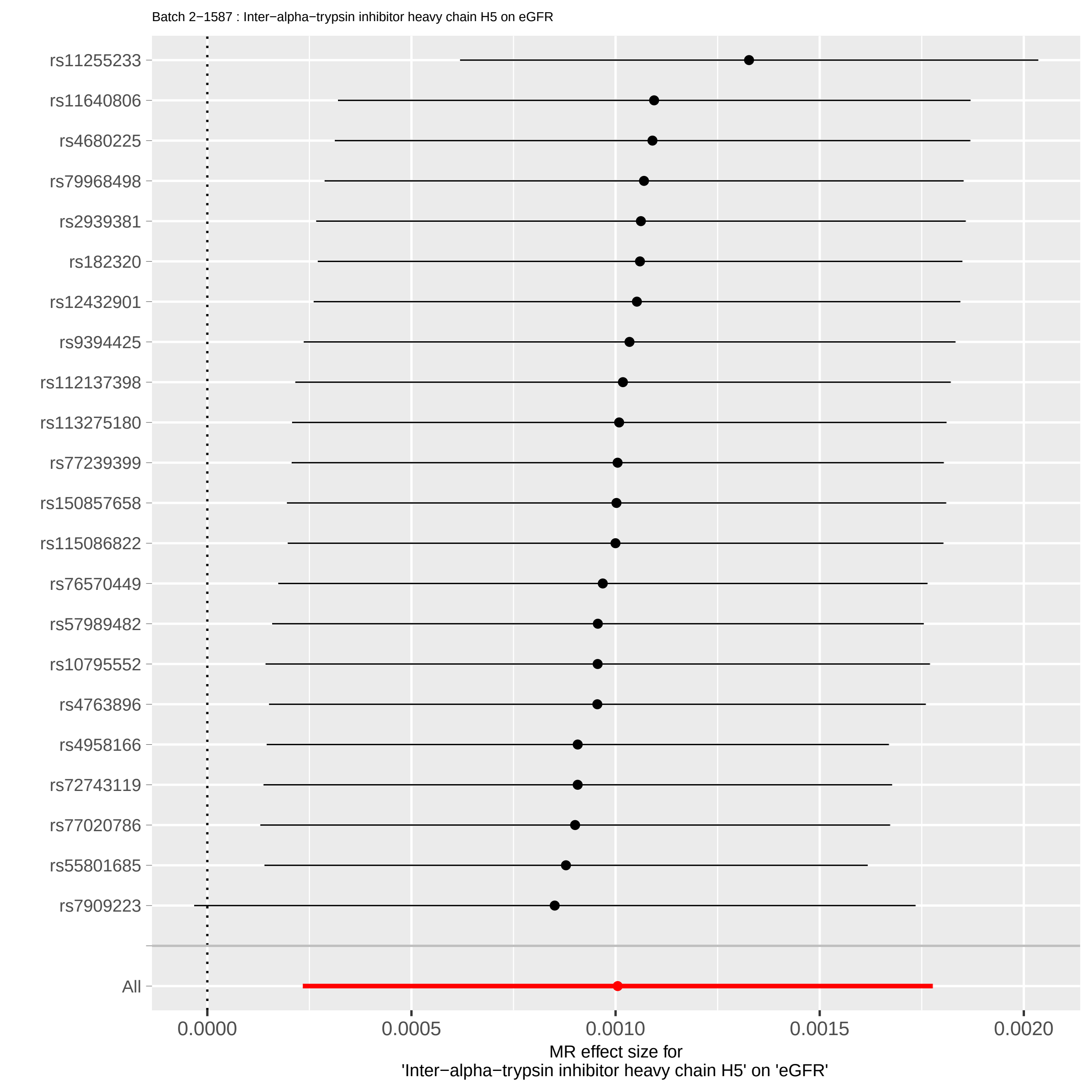

Batch 2−1587 : Inter−alpha−trypsin inhibitor heavy chain H5 on eGFR
rs11255233
rs11640806
rs4680225
rs79968498
rs2939381
rs182320
rs12432901
rs9394425
rs112137398
rs113275180
rs77239399
rs150857658
rs115086822
rs76570449
rs57989482
rs10795552
rs4763896
rs4958166
rs72743119
rs77020786
rs55801685
rs7909223
All
0.0000
0.0005
0.0010
0.0015
0.0020
MR effect size for
'Inter−alpha−trypsin inhibitor heavy chain H5' on 'eGFR'

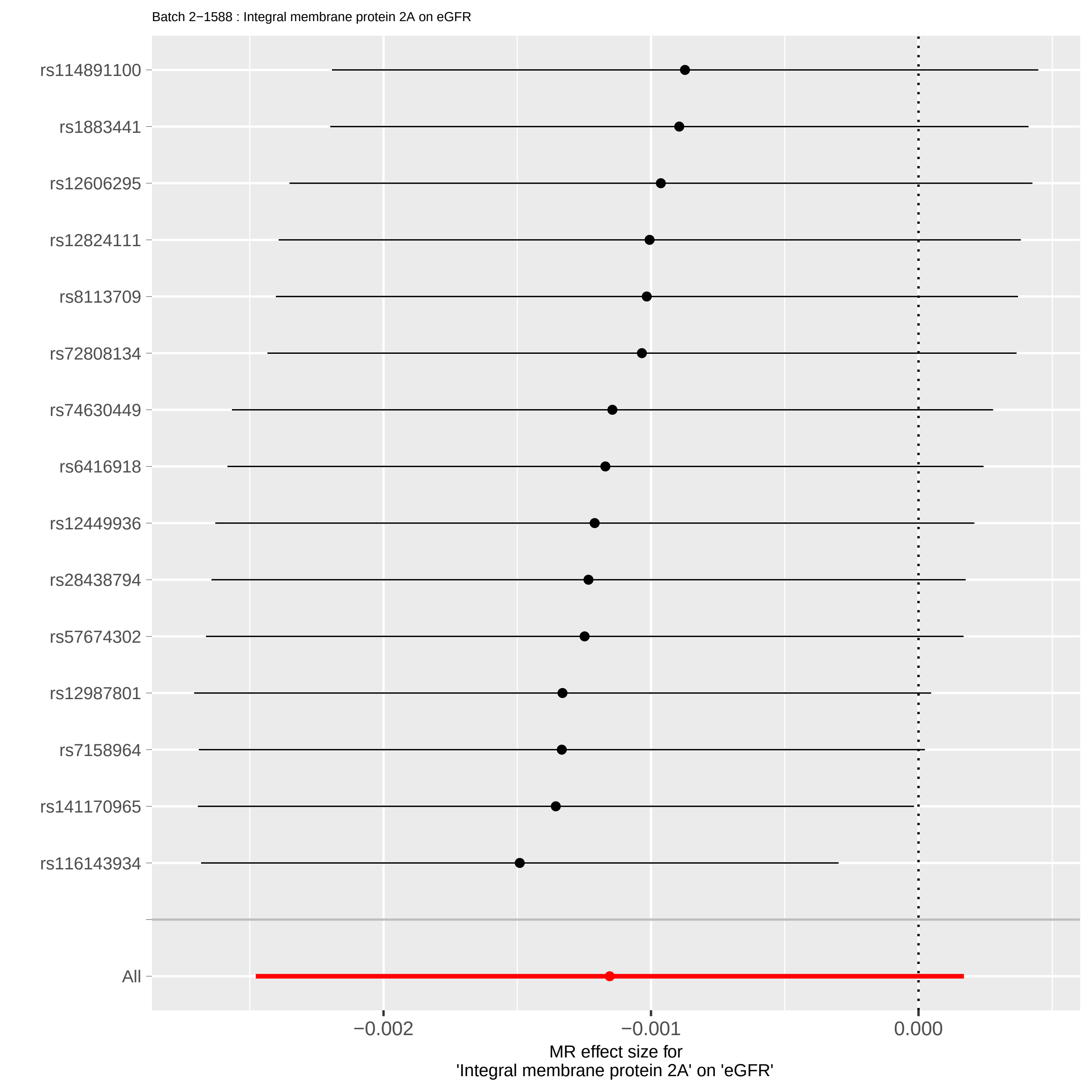

Batch 2−1588 : Integral membrane protein 2A on eGFR
rs114891100
rs1883441
rs12606295
rs12824111
rs8113709
rs72808134
rs74630449
rs6416918
rs12449936
rs28438794
rs57674302
rs12987801
rs7158964
rs141170965
rs116143934
All
−0.002
−0.001
0.000
MR effect size for
'Integral membrane protein 2A' on 'eGFR'

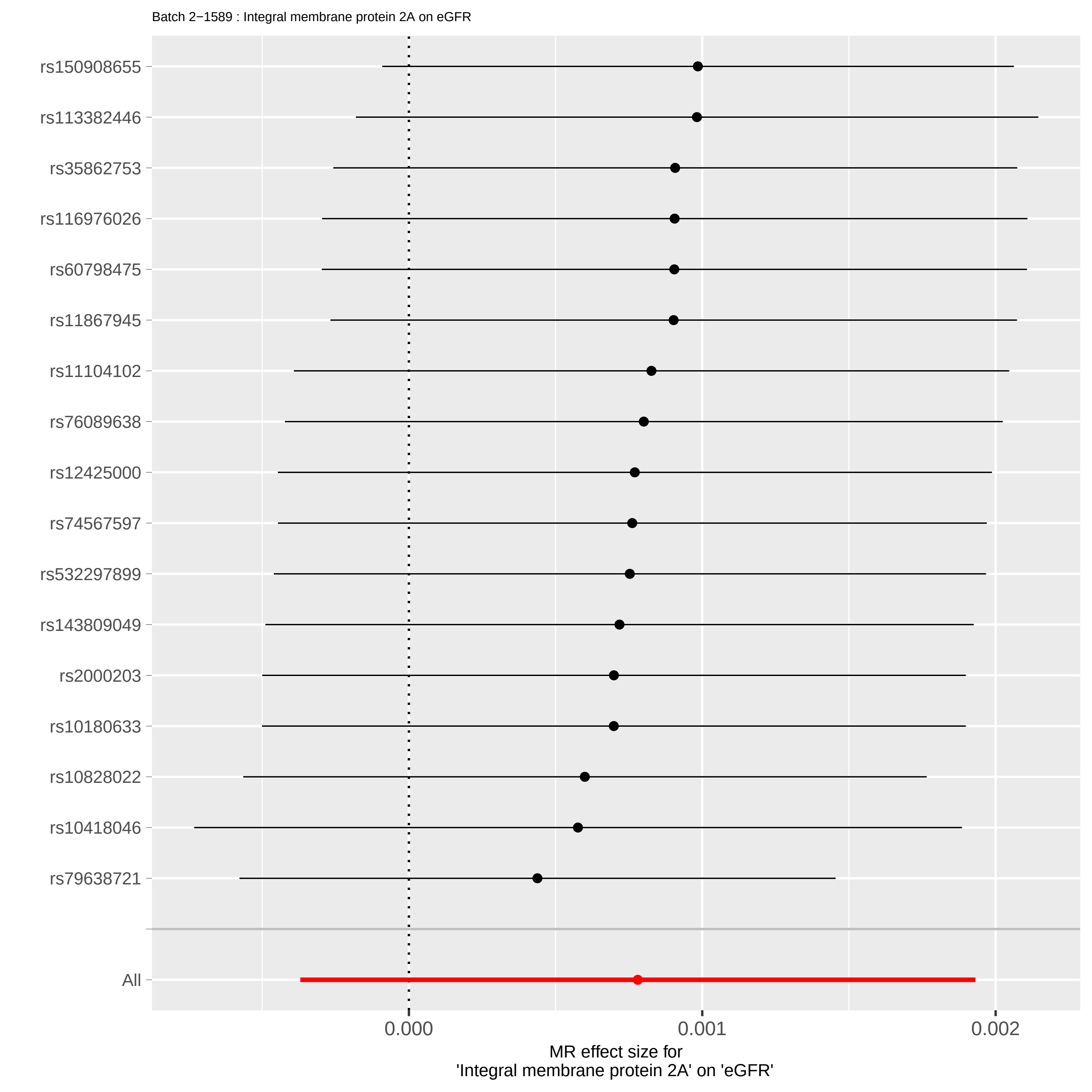

Batch 2−1589 : Integral membrane protein 2A on eGFR
rs150908655
rs113382446
rs35862753
rs116976026
rs60798475
rs11867945
rs11104102
rs76089638
rs12425000
rs74567597
rs532297899
rs143809049
rs2000203
rs10180633
rs10828022
rs10418046
rs79638721
All
0.000
0.001
0.002
MR effect size for
'Integral membrane protein 2A' on 'eGFR'

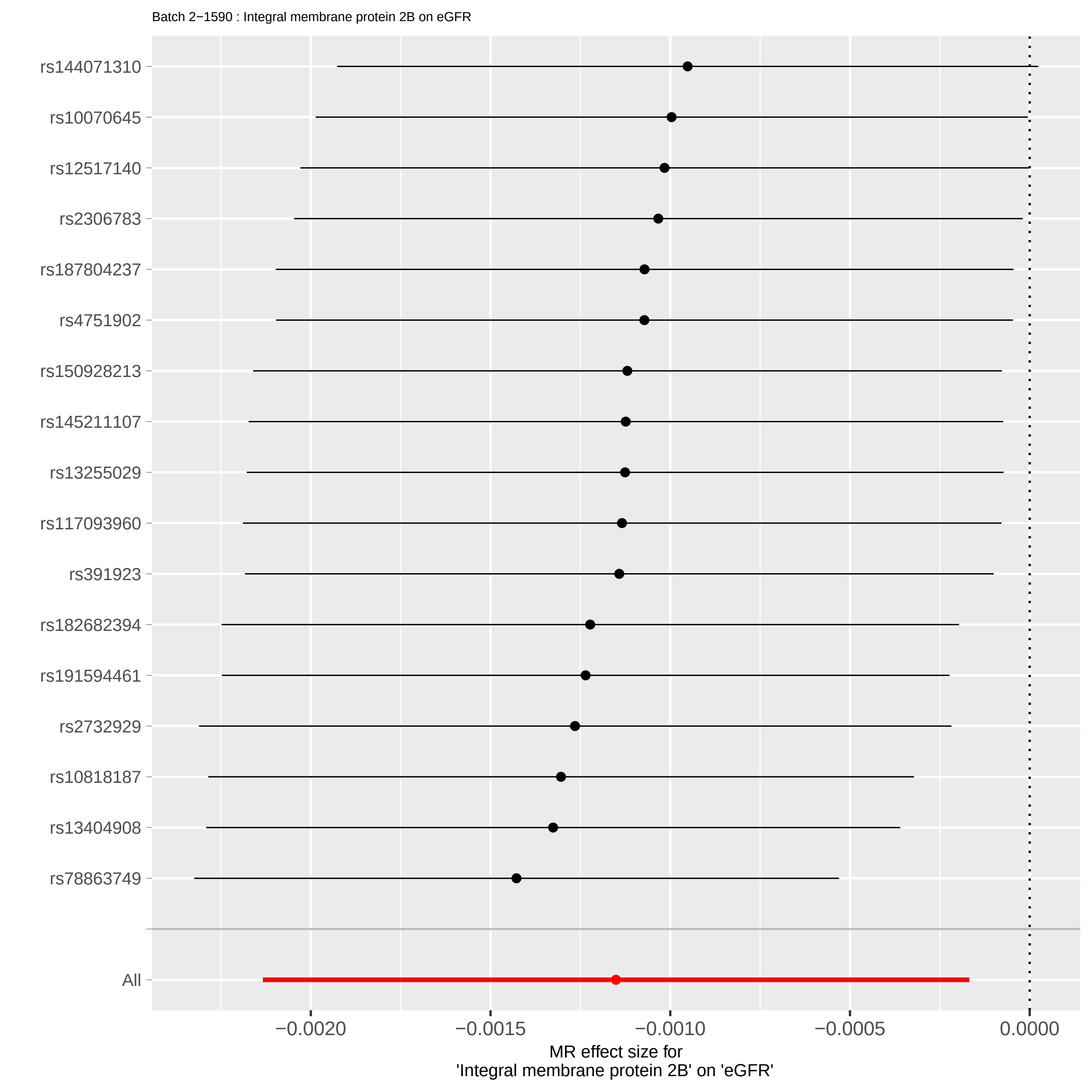

Batch 2−1590 : Integral membrane protein 2B on eGFR
rs144071310
rs10070645
rs12517140
rs2306783
rs187804237
rs4751902
rs150928213
rs145211107
rs13255029
rs117093960
rs391923
rs182682394
rs191594461
rs2732929
rs10818187
rs13404908
rs78863749
All
−0.0020
−0.0015
−0.0010
−0.0005
0.0000
MR effect size for
'Integral membrane protein 2B' on 'eGFR'

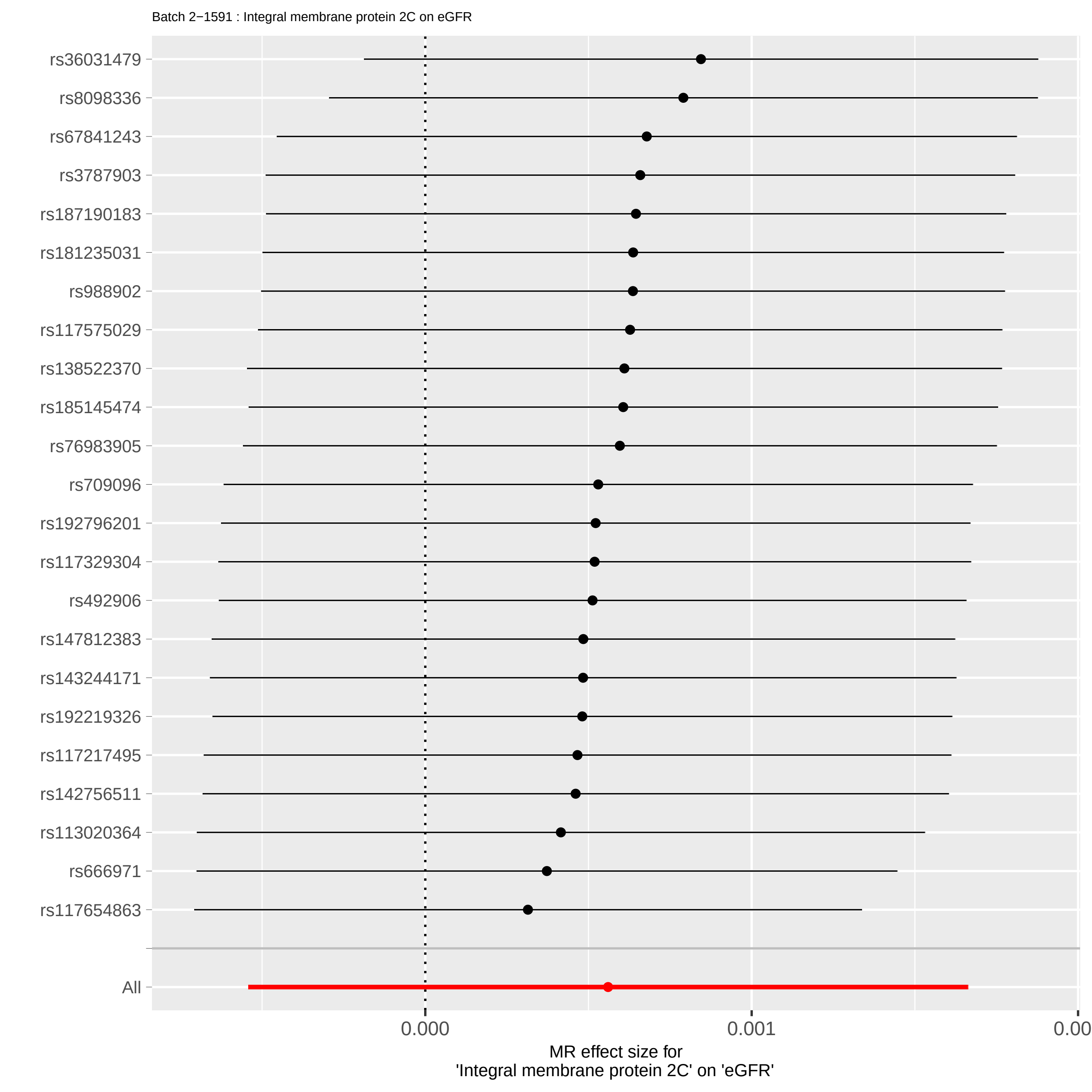

Batch 2−1591 : Integral membrane protein 2C on eGFR
rs36031479
rs8098336
rs67841243
rs3787903
rs187190183
rs181235031
rs988902
rs117575029
rs138522370
rs185145474
rs76983905
rs709096
rs192796201
rs117329304
rs492906
rs147812383
rs143244171
rs192219326
rs117217495
rs142756511
rs113020364
rs666971
rs117654863
All
0.000
0.001
0.00
MR effect size for
'Integral membrane protein 2C' on 'eGFR'

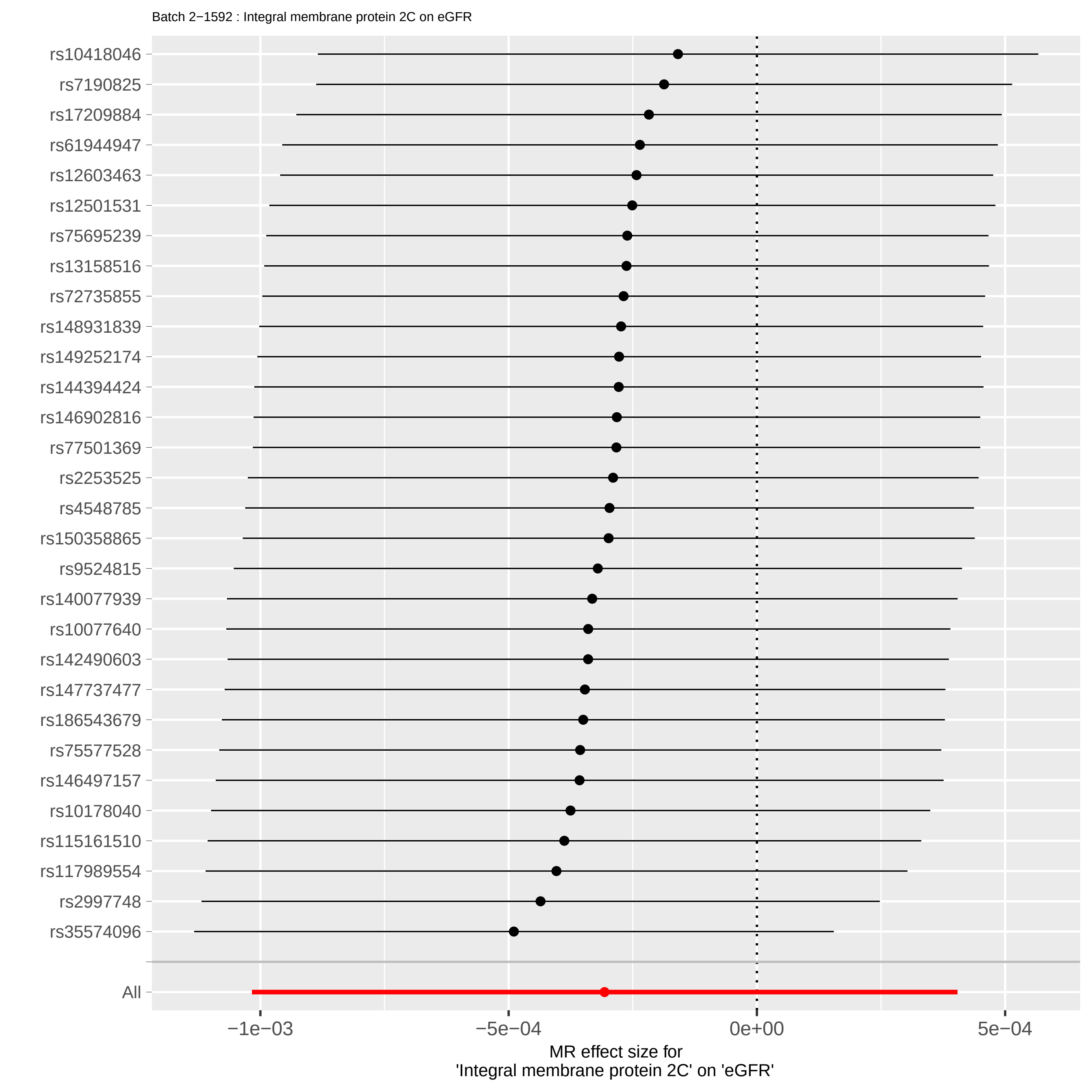

Batch 2−1592 : Integral membrane protein 2C on eGFR
rs10418046
rs7190825
rs17209884
rs61944947
rs12603463
rs12501531
rs75695239
rs13158516
rs72735855
rs148931839
rs149252174
rs144394424
rs146902816
rs77501369
rs2253525
rs4548785
rs150358865
rs9524815
rs140077939
rs10077640
rs142490603
rs147737477
rs186543679
rs75577528
rs146497157
rs10178040
rs115161510
rs117989554
rs2997748
rs35574096
All
−1e−03
−5e−04
0e+00
5e−04
MR effect size for
'Integral membrane protein 2C' on 'eGFR'

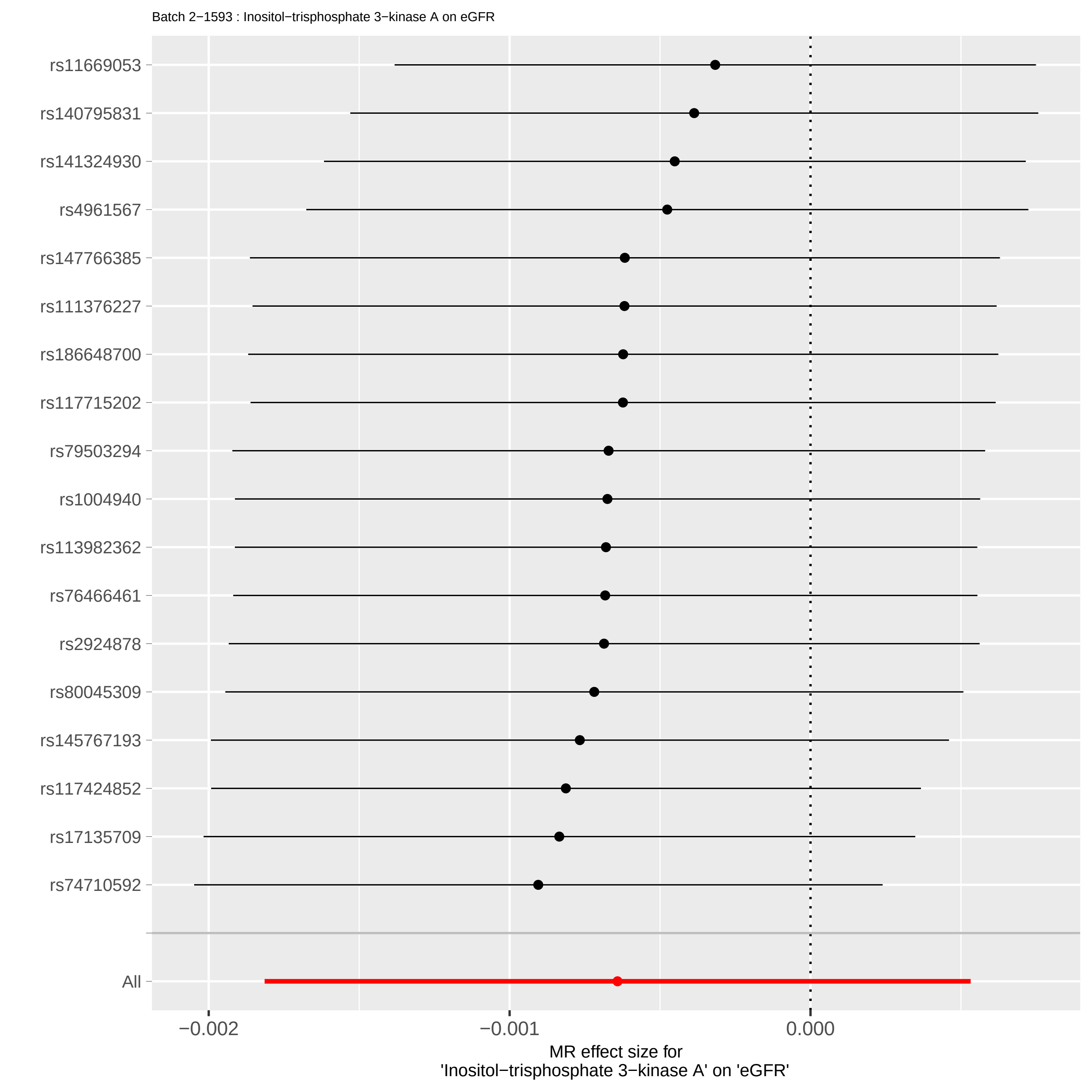

Batch 2−1593 : Inositol−trisphosphate 3−kinase A on eGFR
rs11669053
rs140795831
rs141324930
rs4961567
rs147766385
rs111376227
rs186648700
rs117715202
rs79503294
rs1004940
rs113982362
rs76466461
rs2924878
rs80045309
rs145767193
rs117424852
rs17135709
rs74710592
All
−0.002
−0.001
0.000
MR effect size for
'Inositol−trisphosphate 3−kinase A' on 'eGFR'

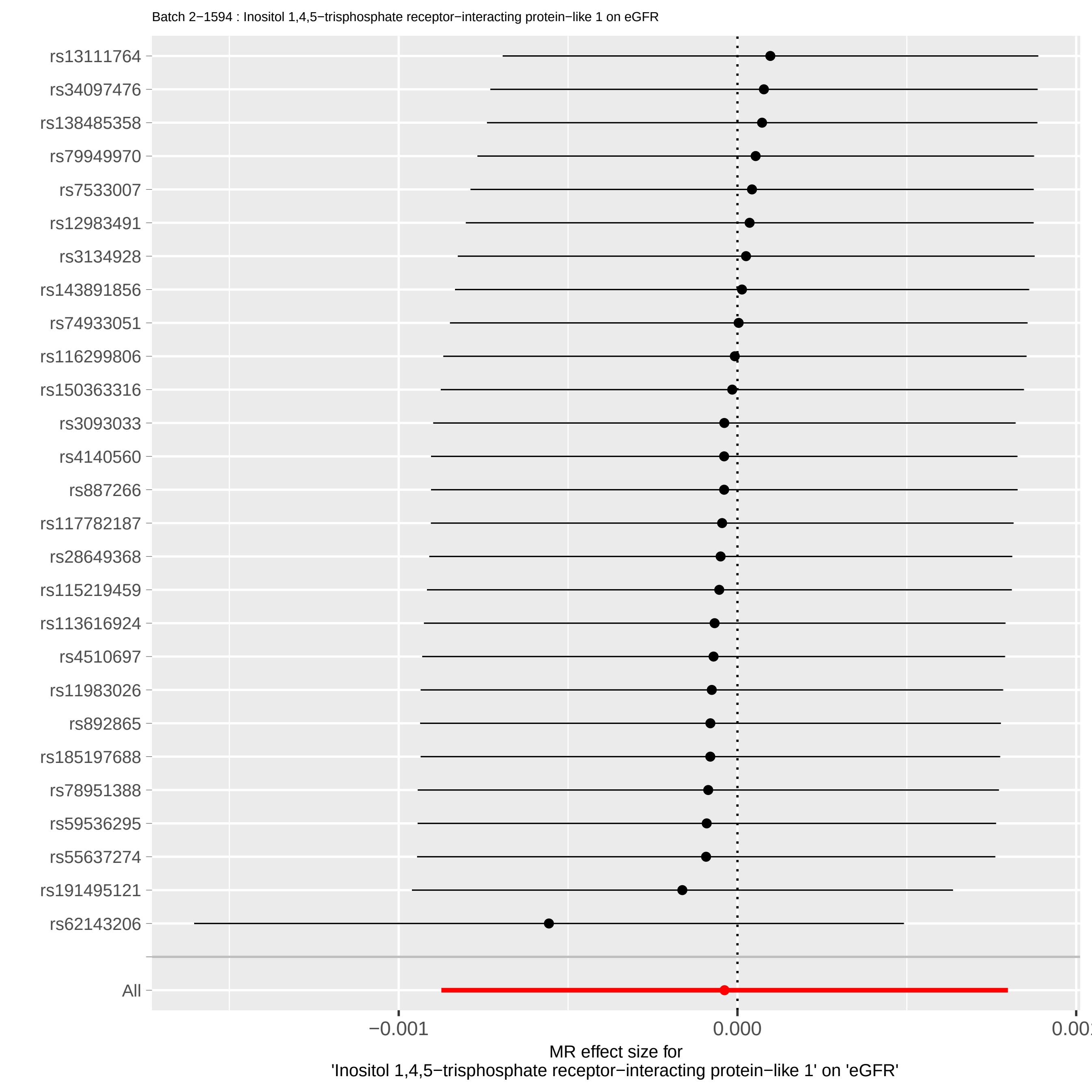
Batch 2−1594 : Inositol 1,4,5−trisphosphate receptor−interacting protein−like 1 on eGFR
rs13111764
rs34097476
rs138485358
rs79949970
rs7533007
rs12983491
rs3134928
rs143891856
rs74933051
rs116299806
rs150363316
rs3093033
rs4140560
rs887266
rs117782187
rs28649368
rs115219459
rs113616924
rs4510697
rs11983026
rs892865
rs185197688
rs78951388
rs59536295
rs55637274
rs191495121
rs62143206
All
−0.001
0.000
MR effect size for
'Inositol 1,4,5−trisphosphate receptor−interacting protein−like 1' on 'eGFR'

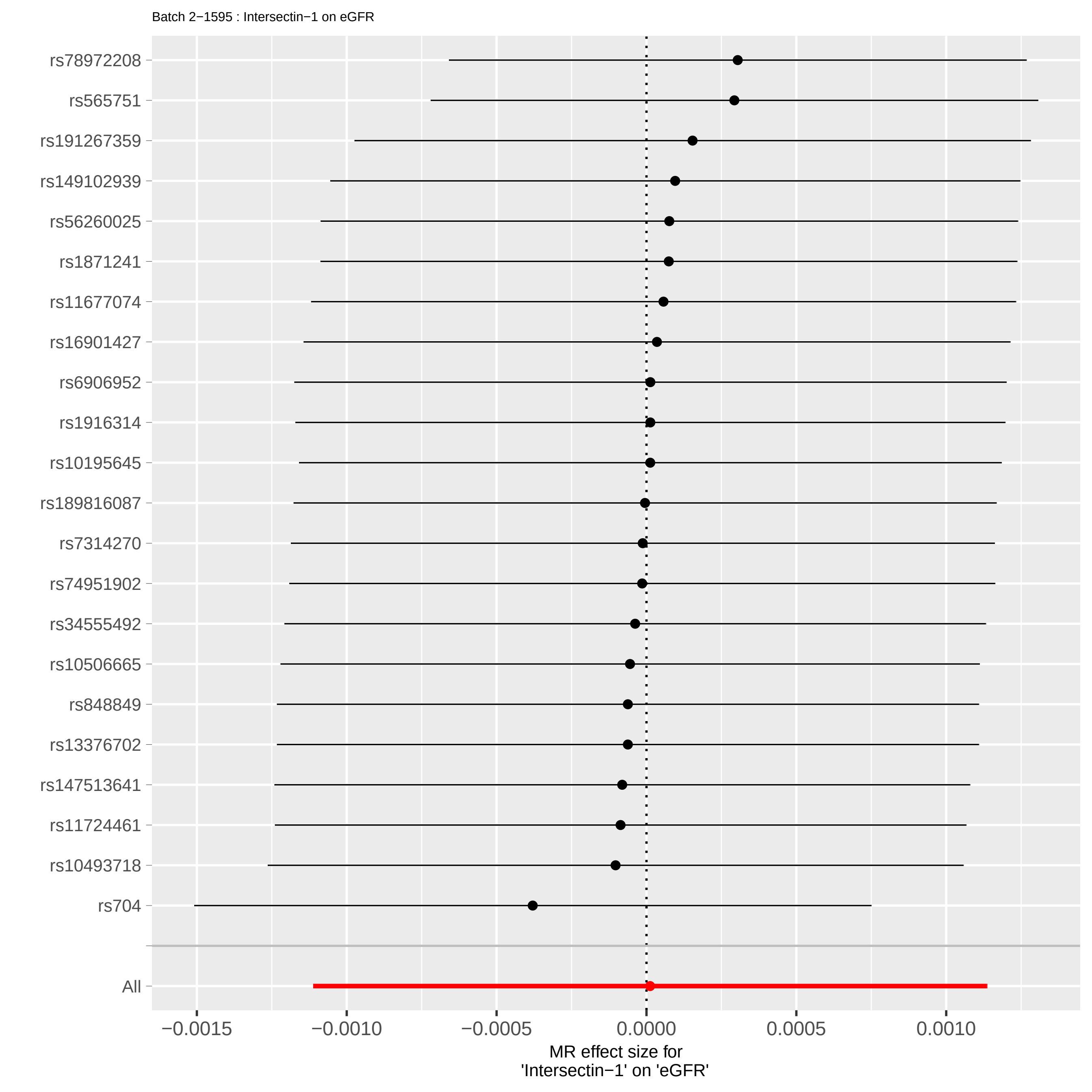

Batch 2−1595 : Intersectin−1 on eGFR
rs78972208
rs565751
rs191267359
rs149102939
rs56260025
rs1871241
rs11677074
rs16901427
rs6906952
rs1916314
rs10195645
rs189816087
rs7314270
rs74951902
rs34555492
rs10506665
rs848849
rs13376702
rs147513641
rs11724461
rs10493718
rs704
All
−0.0015
−0.0010
−0.0005
0.0000
0.0005
0.0010
MR effect size for
'Intersectin−1' on 'eGFR'

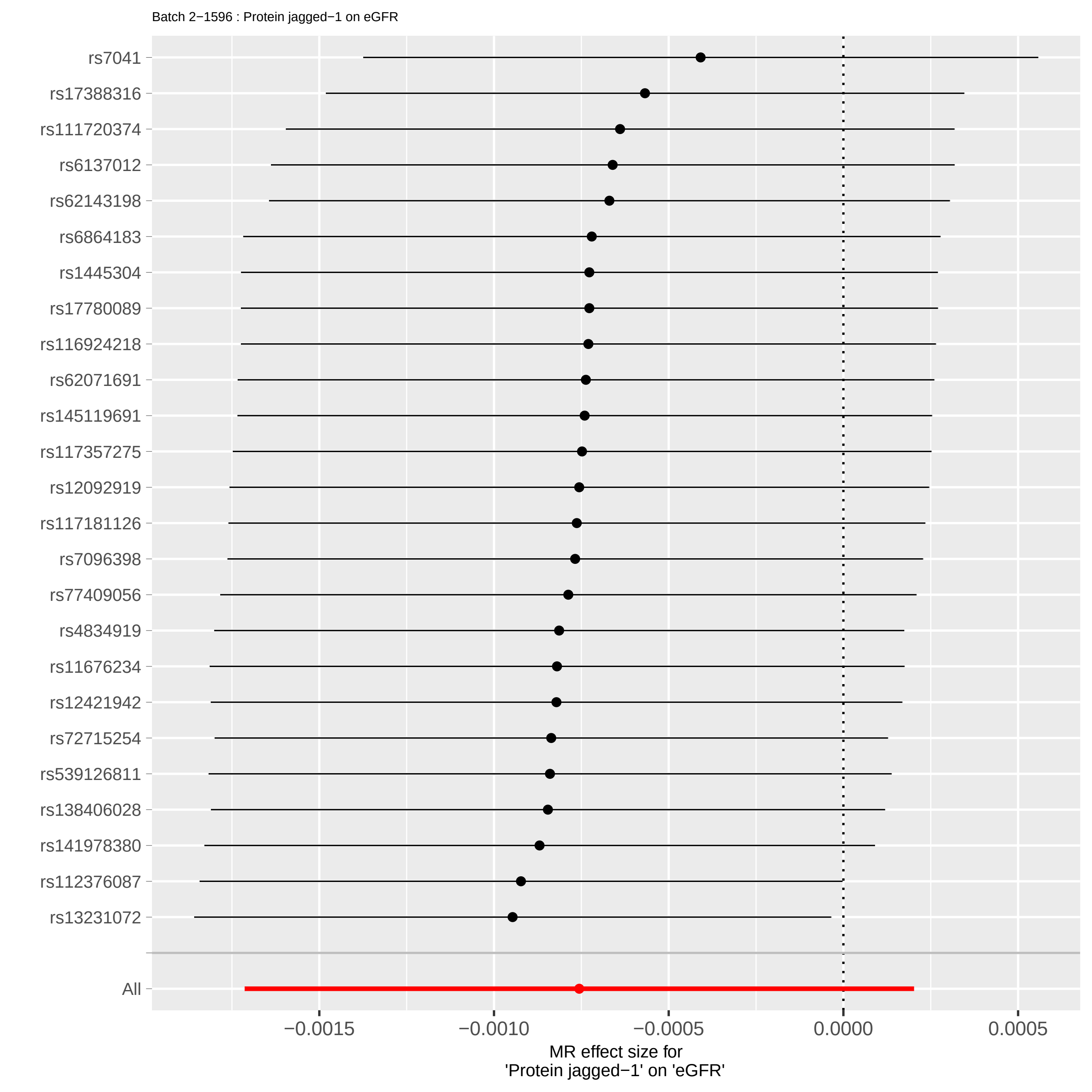

Batch 2−1596 : Protein jagged−1 on eGFR
rs7041
rs17388316
rs111720374
rs6137012
rs62143198
rs6864183
rs1445304
rs17780089
rs116924218
rs62071691
rs145119691
rs117357275
rs12092919
rs117181126
rs7096398
rs77409056
rs4834919
rs11676234
rs12421942
rs72715254
rs539126811
rs138406028
rs141978380
rs112376087
rs13231072
All
−0.0015
−0.0010
−0.0005
0.0000
0.0005
MR effect size for
'Protein jagged−1' on 'eGFR'

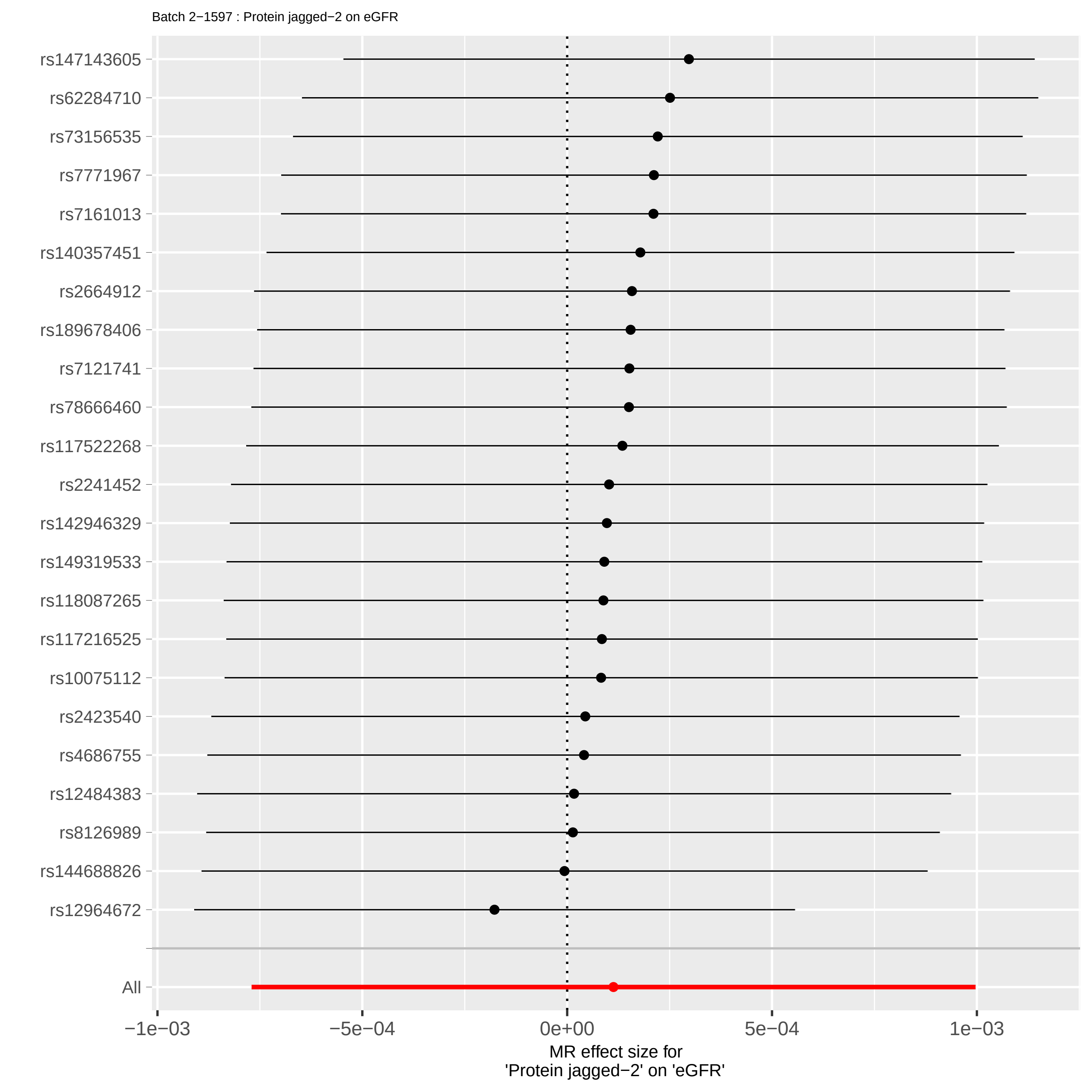

Batch 2−1597 : Protein jagged−2 on eGFR
rs147143605
rs62284710
rs73156535
rs7771967
rs7161013
rs140357451
rs2664912
rs189678406
rs7121741
rs78666460
rs117522268
rs2241452
rs142946329
rs149319533
rs118087265
rs117216525
rs10075112
rs2423540
rs4686755
rs12484383
rs8126989
rs144688826
rs12964672
All
−1e−03
−5e−04
0e+00
5e−04
1e−03
MR effect size for
'Protein jagged−2' on 'eGFR'

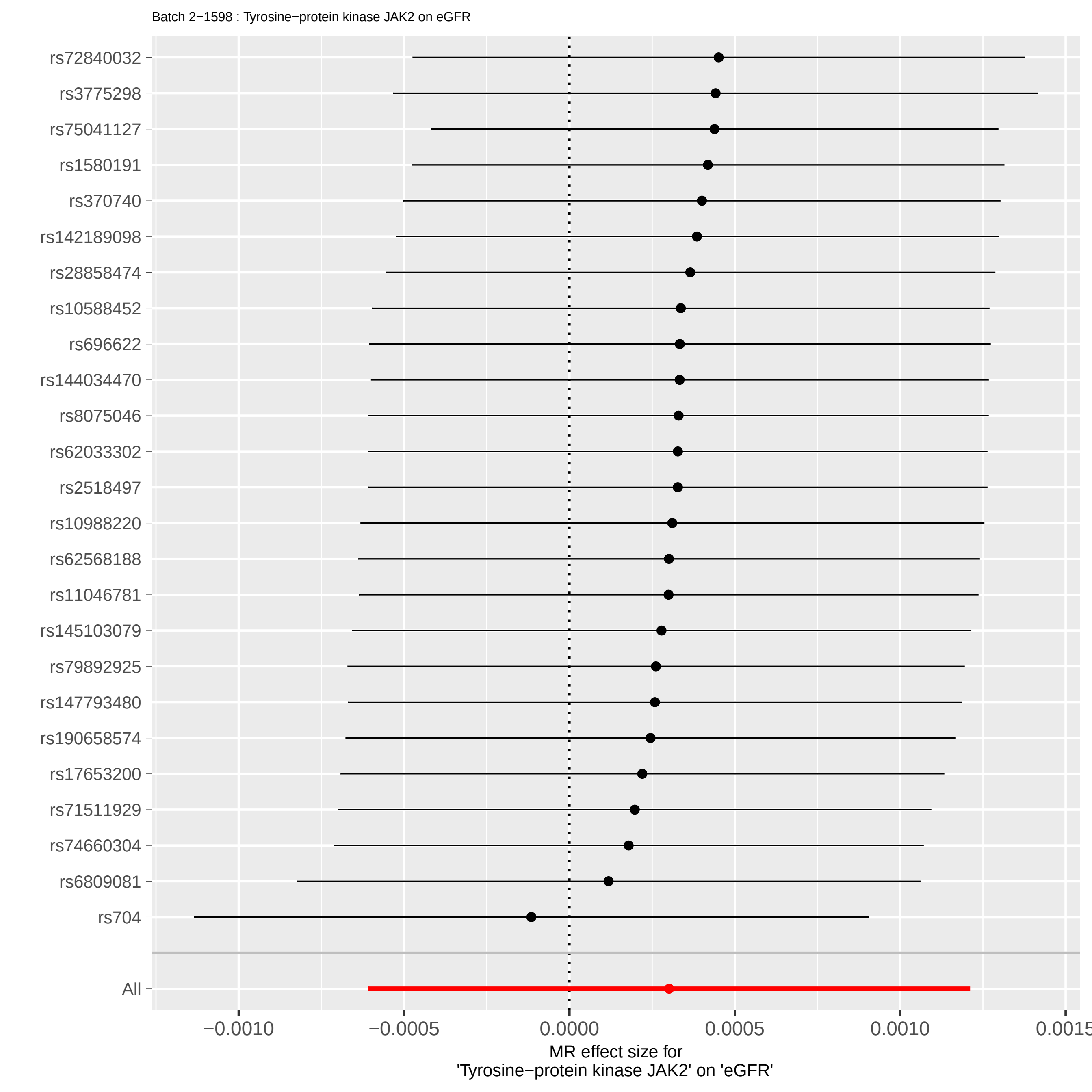

Batch 2−1598 : Tyrosine−protein kinase JAK2 on eGFR
rs72840032
rs3775298
rs75041127
rs1580191
rs370740
rs142189098
rs28858474
rs10588452
rs696622
rs144034470
rs8075046
rs62033302
rs2518497
rs10988220
rs62568188
rs11046781
rs145103079
rs79892925
rs147793480
rs190658574
rs17653200
rs71511929
rs74660304
rs6809081
rs704
All
−0.0010
−0.0005
0.0000
0.0005
0.0010
0.0015
MR effect size for
'Tyrosine−protein kinase JAK2' on 'eGFR'

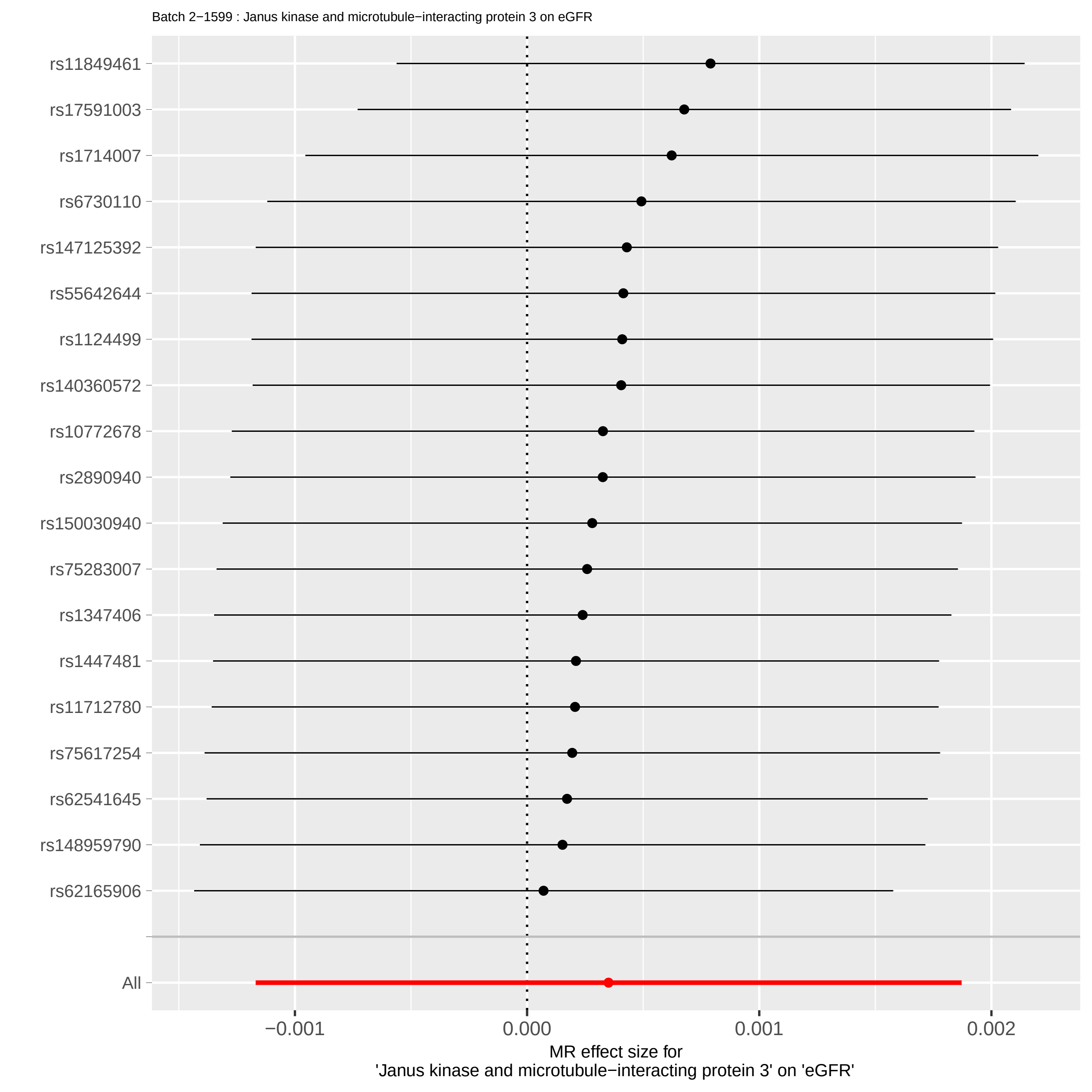
Batch 2−1599 : Janus kinase and microtubule−interacting protein 3 on eGFR
rs11849461
rs17591003
rs1714007
rs6730110
rs147125392
rs55642644
rs1124499
rs140360572
rs10772678
rs2890940
rs150030940
rs75283007
rs1347406
rs1447481
rs11712780
rs75617254
rs62541645
rs148959790
rs62165906
All
−0.001
0.000
0.001
0.002
MR effect size for
'Janus kinase and microtubule−interacting protein 3' on 'eGFR'

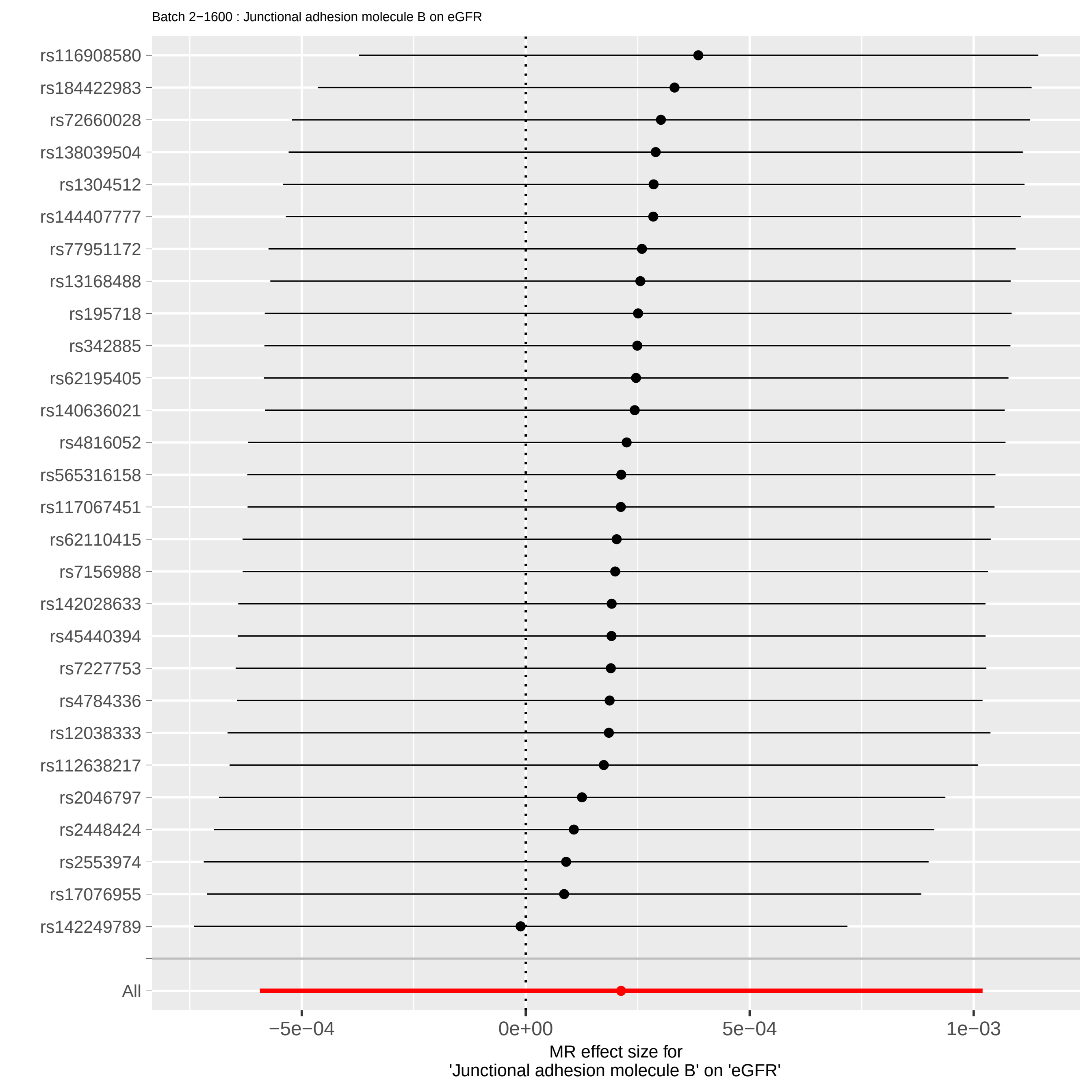
Batch 2−1600 : Junctional adhesion molecule B on eGFR
rs116908580
rs184422983
rs72660028
rs138039504
rs1304512
rs144407777
rs77951172
rs13168488
rs195718
rs342885
rs62195405
rs140636021
rs4816052
rs565316158
rs117067451
rs62110415
rs7156988
rs142028633
rs45440394
rs7227753
rs4784336
rs12038333
rs112638217
rs2046797
rs2448424
rs2553974
rs17076955
rs142249789
All
−5e−04
0e+00
5e−04
1e−03
MR effect size for
'Junctional adhesion molecule B' on 'eGFR'

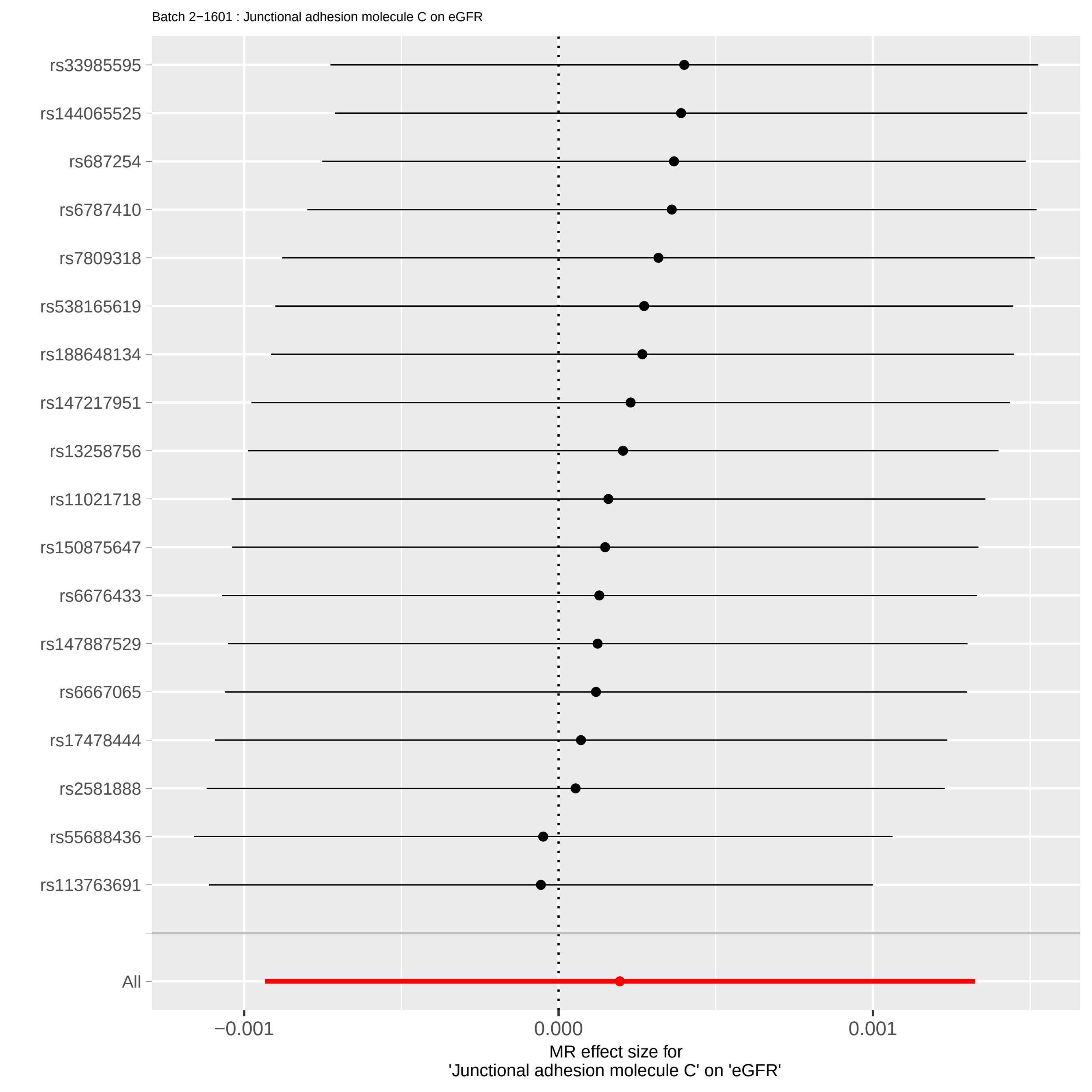

Batch 2−1601 : Junctional adhesion molecule C on eGFR
rs33985595
rs144065525
rs687254
rs6787410
rs7809318
rs538165619
rs188648134
rs147217951
rs13258756
rs11021718
rs150875647
rs6676433
rs147887529
rs6667065
rs17478444
rs2581888
rs55688436
rs113763691
All
−0.001
0.000
0.001
MR effect size for
'Junctional adhesion molecule C' on 'eGFR'

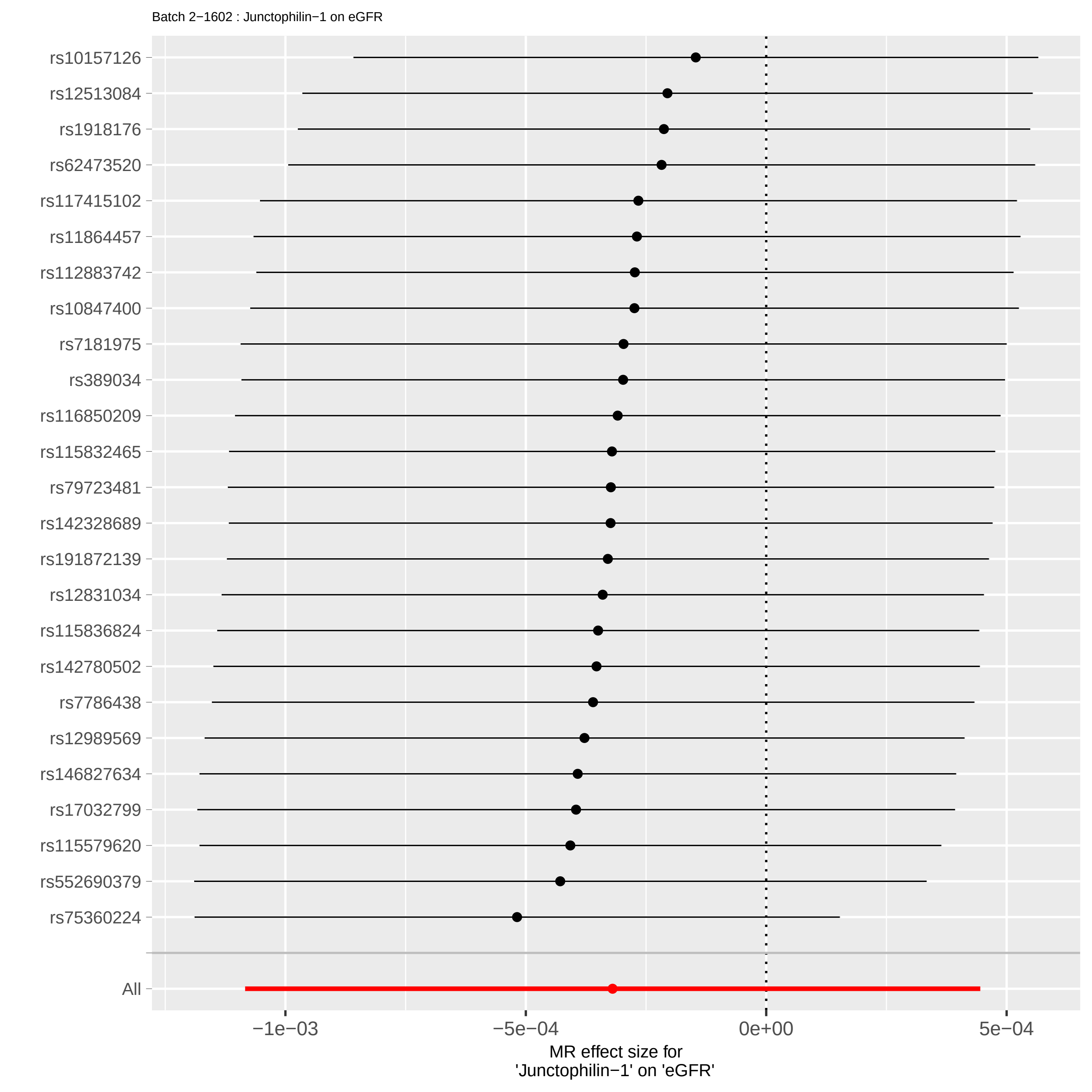

Batch 2−1602 : Junctophilin−1 on eGFR
rs10157126
rs12513084
rs1918176
rs62473520
rs117415102
rs11864457
rs112883742
rs10847400
rs7181975
rs389034
rs116850209
rs115832465
rs79723481
rs142328689
rs191872139
rs12831034
rs115836824
rs142780502
rs7786438
rs12989569
rs146827634
rs17032799
rs115579620
rs552690379
rs75360224
All
−1e−03
−5e−04
0e+00
5e−04
MR effect size for
'Junctophilin−1' on 'eGFR'

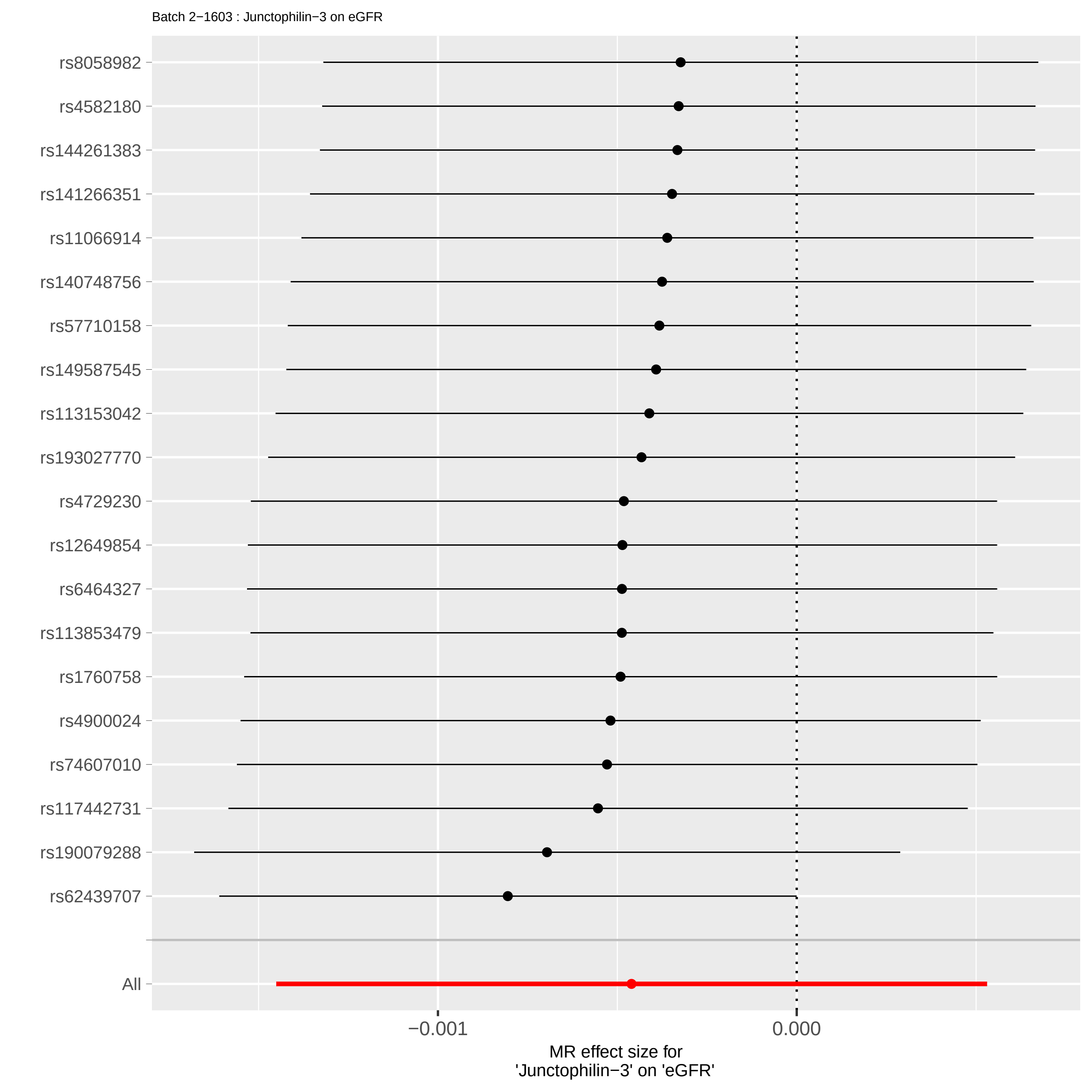

Batch 2−1603 : Junctophilin−3 on eGFR
rs8058982
rs4582180
rs144261383
rs141266351
rs11066914
rs140748756
rs57710158
rs149587545
rs113153042
rs193027770
rs4729230
rs12649854
rs6464327
rs113853479
rs1760758
rs4900024
rs74607010
rs117442731
rs190079288
rs62439707
All
−0.001
0.000
MR effect size for
'Junctophilin−3' on 'eGFR'

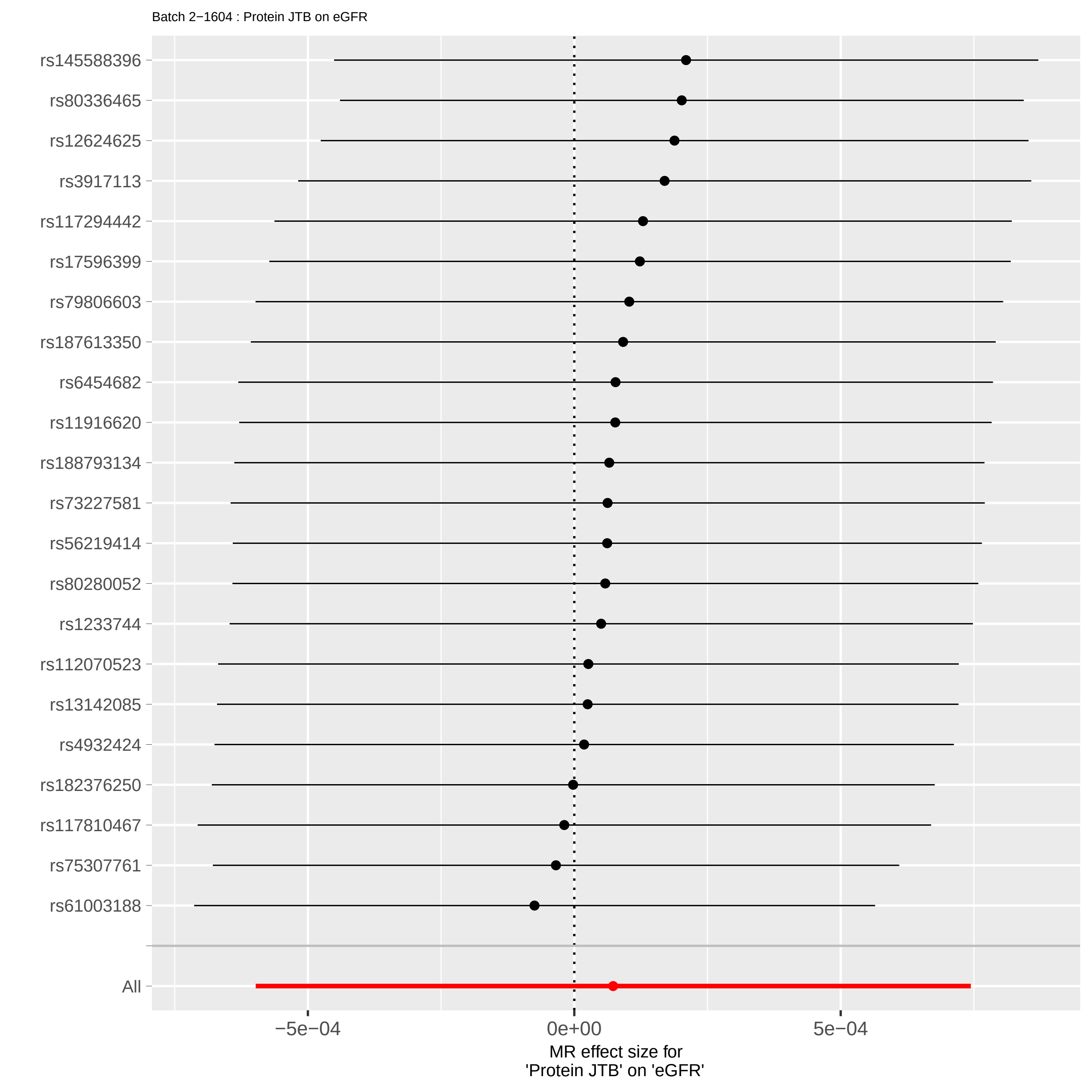
Batch 2−1604 : Protein JTB on eGFR
rs145588396
rs80336465
rs12624625
rs3917113
rs117294442
rs17596399
rs79806603
rs187613350
rs6454682
rs11916620
rs188793134
rs73227581
rs56219414
rs80280052
rs1233744
rs112070523
rs13142085
rs4932424
rs182376250
rs117810467
rs75307761
rs61003188
All
−5e−04
0e+00
5e−04
MR effect size for
'Protein JTB' on 'eGFR'

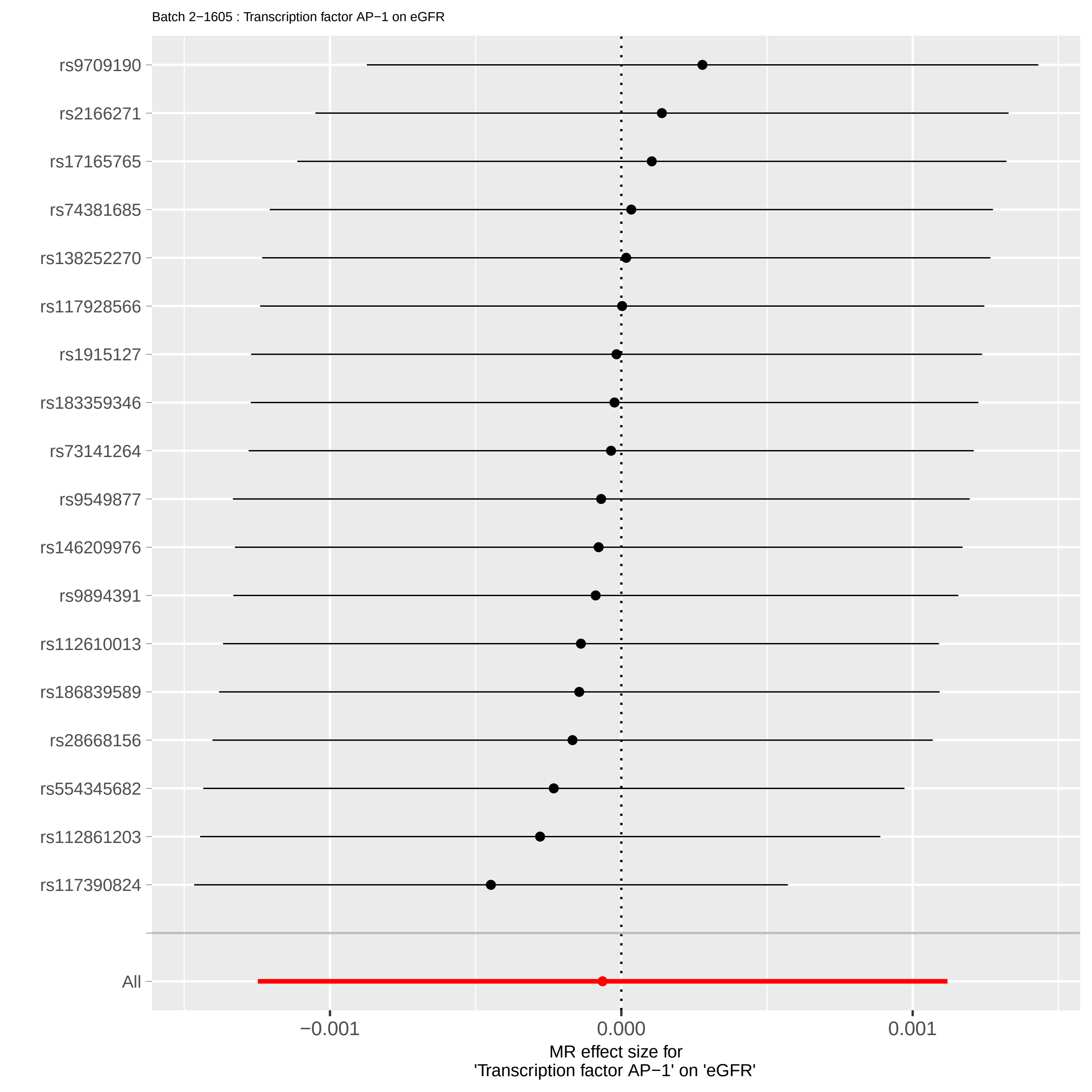

Batch 2−1605 : Transcription factor AP−1 on eGFR
rs9709190
rs2166271
rs17165765
rs74381685
rs138252270
rs117928566
rs1915127
rs183359346
rs73141264
rs9549877
rs146209976
rs9894391
rs112610013
rs186839589
rs28668156
rs554345682
rs112861203
rs117390824
All
−0.001
0.000
0.001
MR effect size for
'Transcription factor AP−1' on 'eGFR'

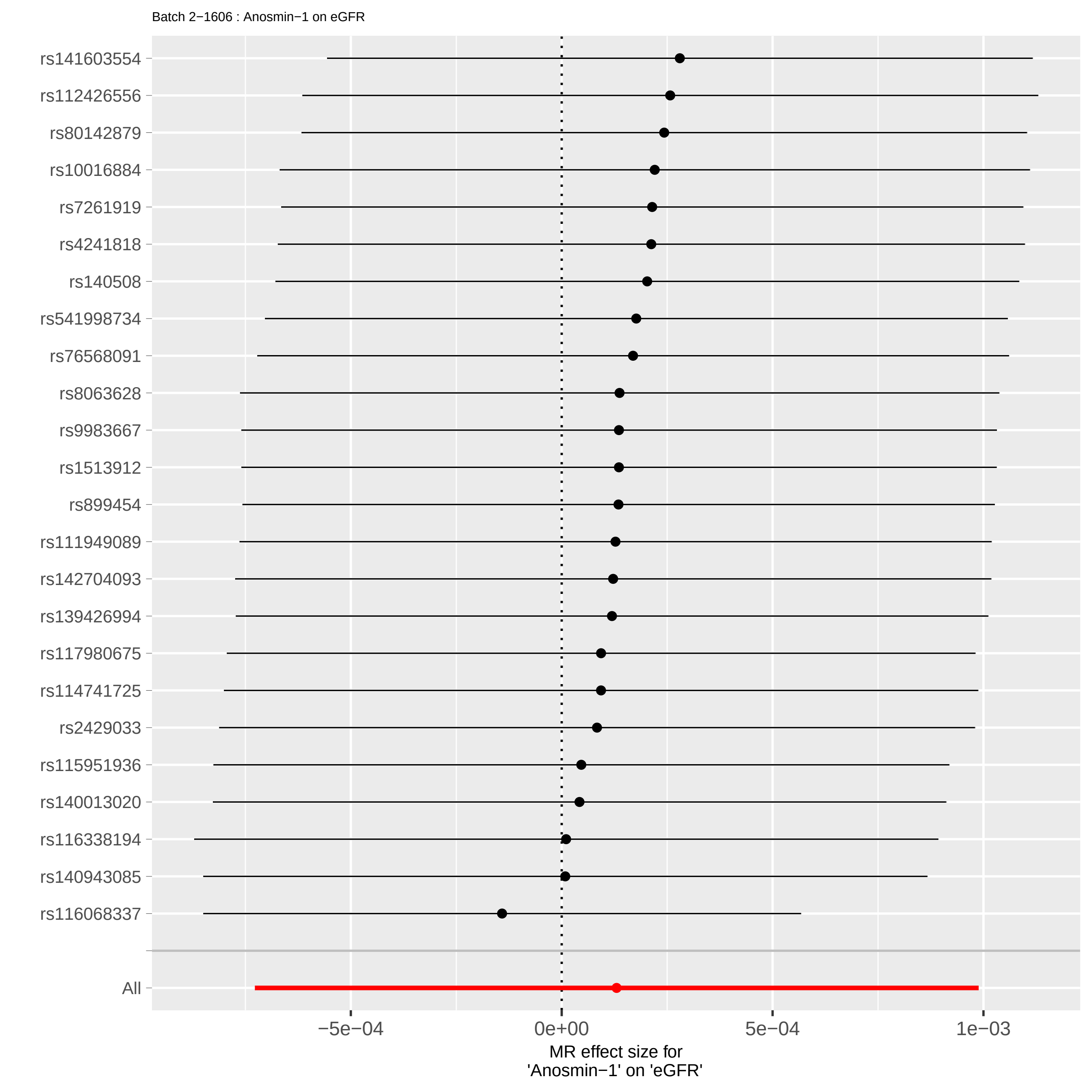

Batch 2−1606 : Anosmin−1 on eGFR
rs141603554
rs112426556
rs80142879
rs10016884
rs7261919
rs4241818
rs140508
rs541998734
rs76568091
rs8063628
rs9983667
rs1513912
rs899454
rs111949089
rs142704093
rs139426994
rs117980675
rs114741725
rs2429033
rs115951936
rs140013020
rs116338194
rs140943085
rs116068337
All
−5e−04
0e+00
5e−04
1e−03
MR effect size for
'Anosmin−1' on 'eGFR'

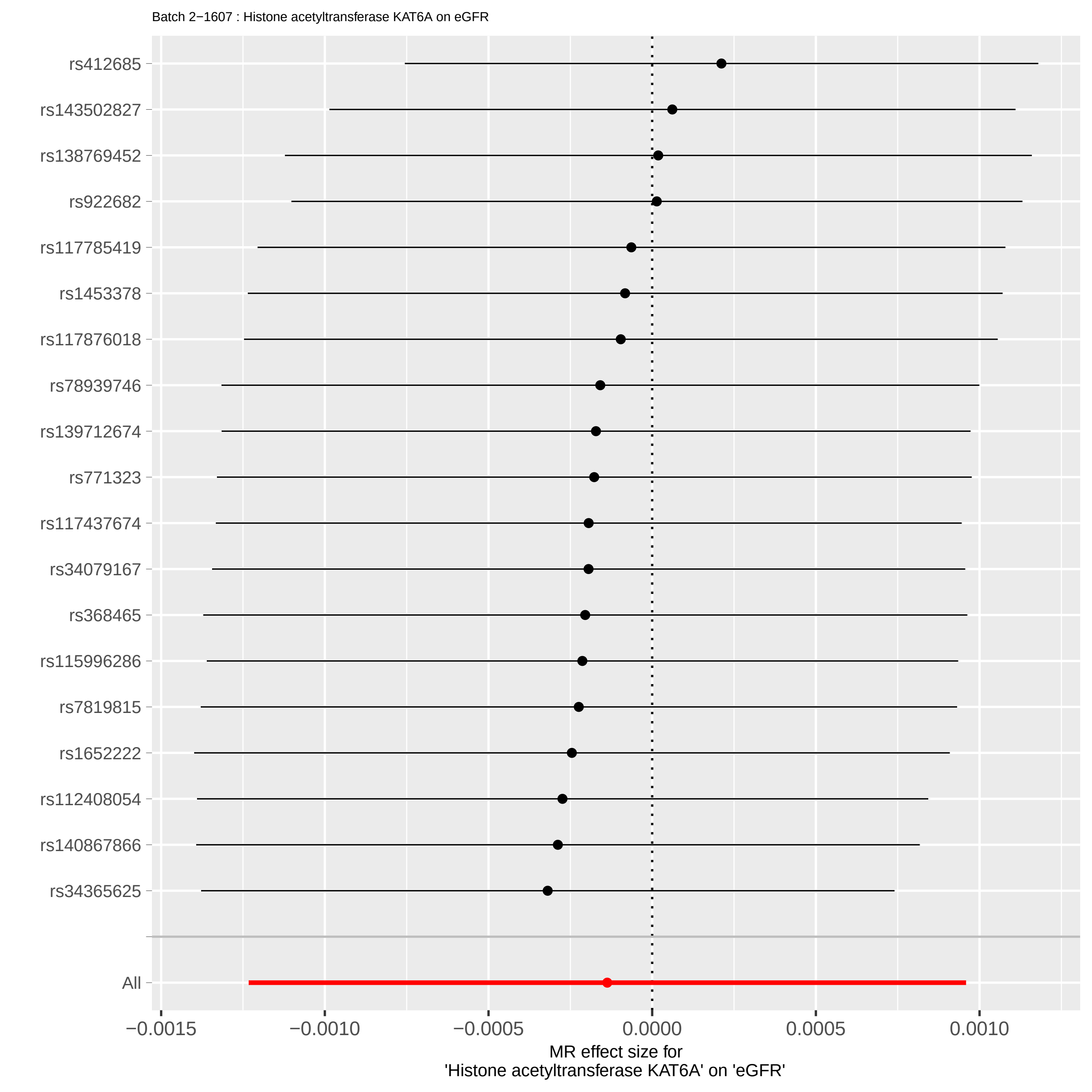

Batch 2−1607 : Histone acetyltransferase KAT6A on eGFR
rs412685
rs143502827
rs138769452
rs922682
rs117785419
rs1453378
rs117876018
rs78939746
rs139712674
rs771323
rs117437674
rs34079167
rs368465
rs115996286
rs7819815
rs1652222
rs112408054
rs140867866
rs34365625
All
−0.0015
−0.0010
−0.0005
0.0000
0.0005
0.0010
MR effect size for
'Histone acetyltransferase KAT6A' on 'eGFR'

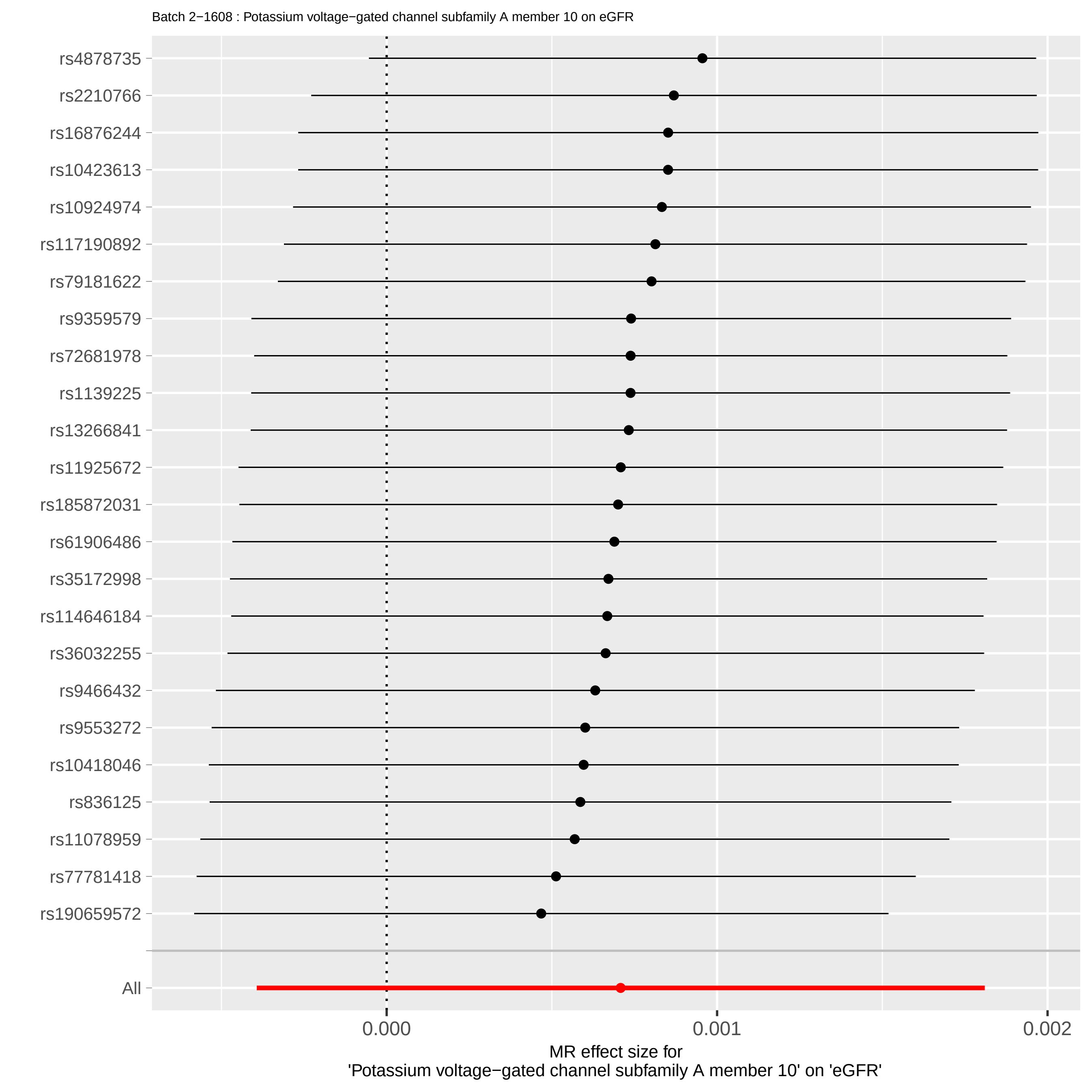

Batch 2−1608 : Potassium voltage−gated channel subfamily A member 10 on eGFR
rs4878735
rs2210766
rs16876244
rs10423613
rs10924974
rs117190892
rs79181622
rs9359579
rs72681978
rs1139225
rs13266841
rs11925672
rs185872031
rs61906486
rs35172998
rs114646184
rs36032255
rs9466432
rs9553272
rs10418046
rs836125
rs11078959
rs77781418
rs190659572
All
0.000
0.001
0.002
MR effect size for
'Potassium voltage−gated channel subfamily A member 10' on 'eGFR'

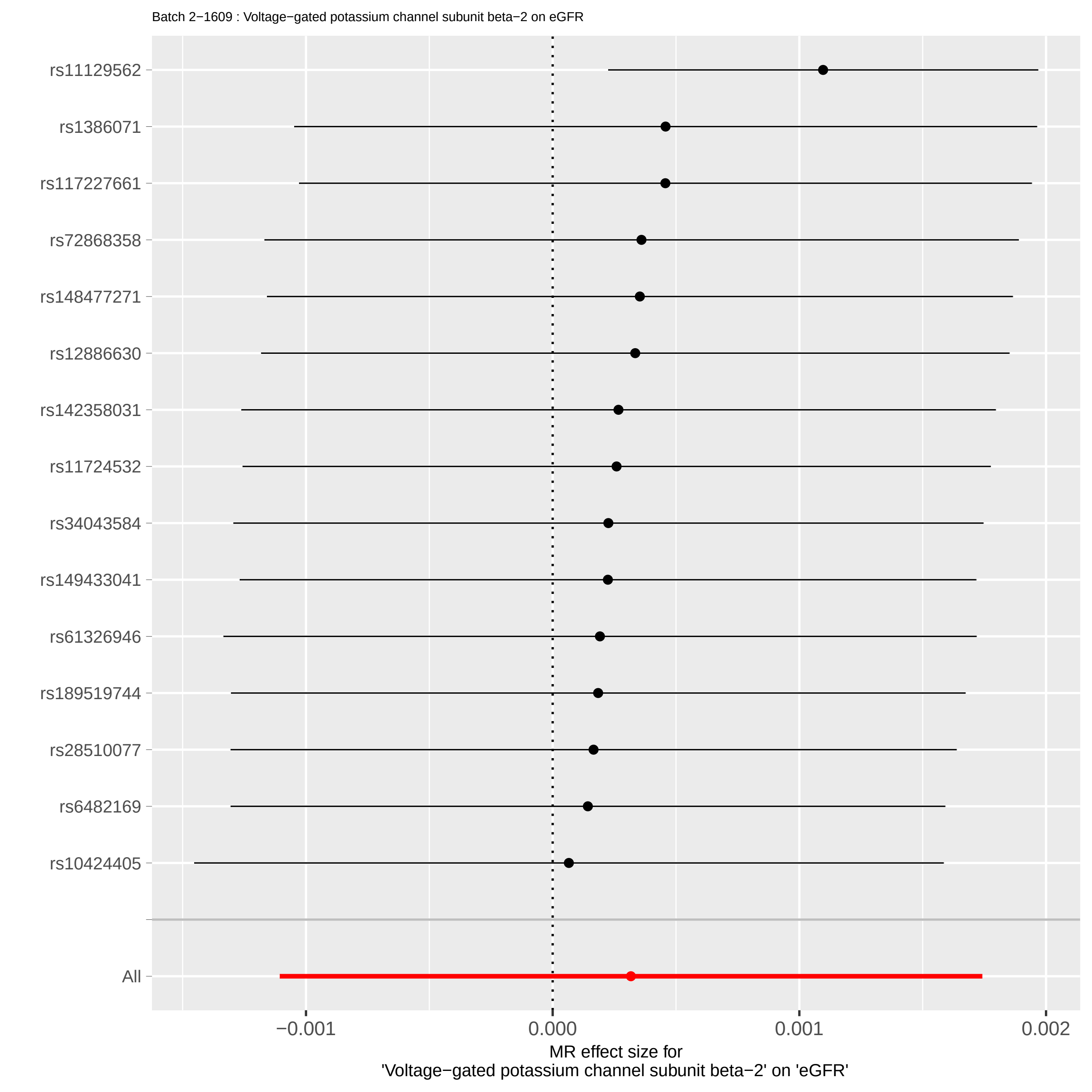

Batch 2−1609 : Voltage−gated potassium channel subunit beta−2 on eGFR
rs11129562
rs1386071
rs117227661
rs72868358
rs148477271
rs12886630
rs142358031
rs11724532
rs34043584
rs149433041
rs61326946
rs189519744
rs28510077
rs6482169
rs10424405
All
−0.001
0.000
0.001
0.002
MR effect size for
'Voltage−gated potassium channel subunit beta−2' on 'eGFR'

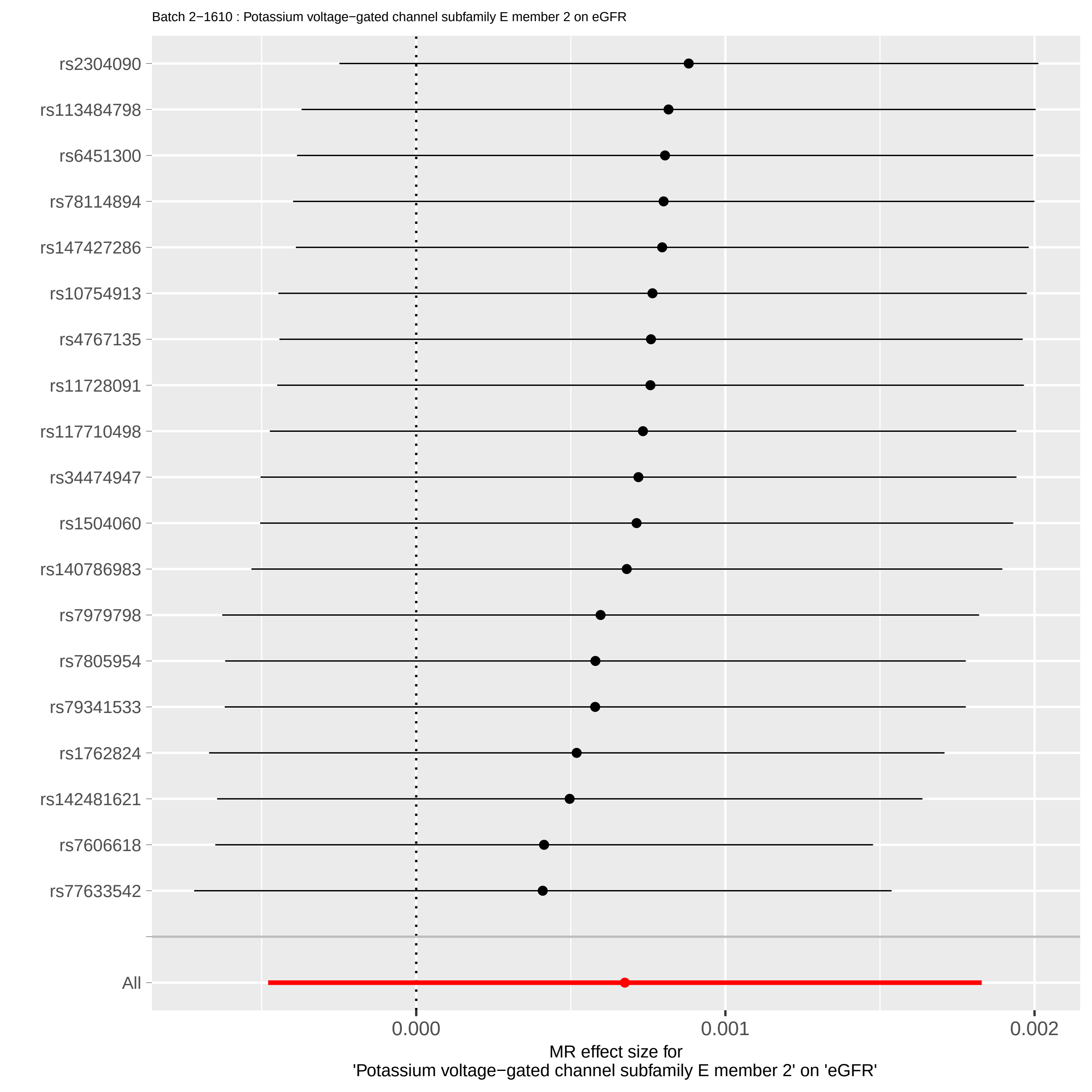

Batch 2−1610 : Potassium voltage−gated channel subfamily E member 2 on eGFR
rs2304090
rs113484798
rs6451300
rs78114894
rs147427286
rs10754913
rs4767135
rs11728091
rs117710498
rs34474947
rs1504060
rs140786983
rs7979798
rs7805954
rs79341533
rs1762824
rs142481621
rs7606618
rs77633542
All
0.000
0.001
0.002
MR effect size for
'Potassium voltage−gated channel subfamily E member 2' on 'eGFR'

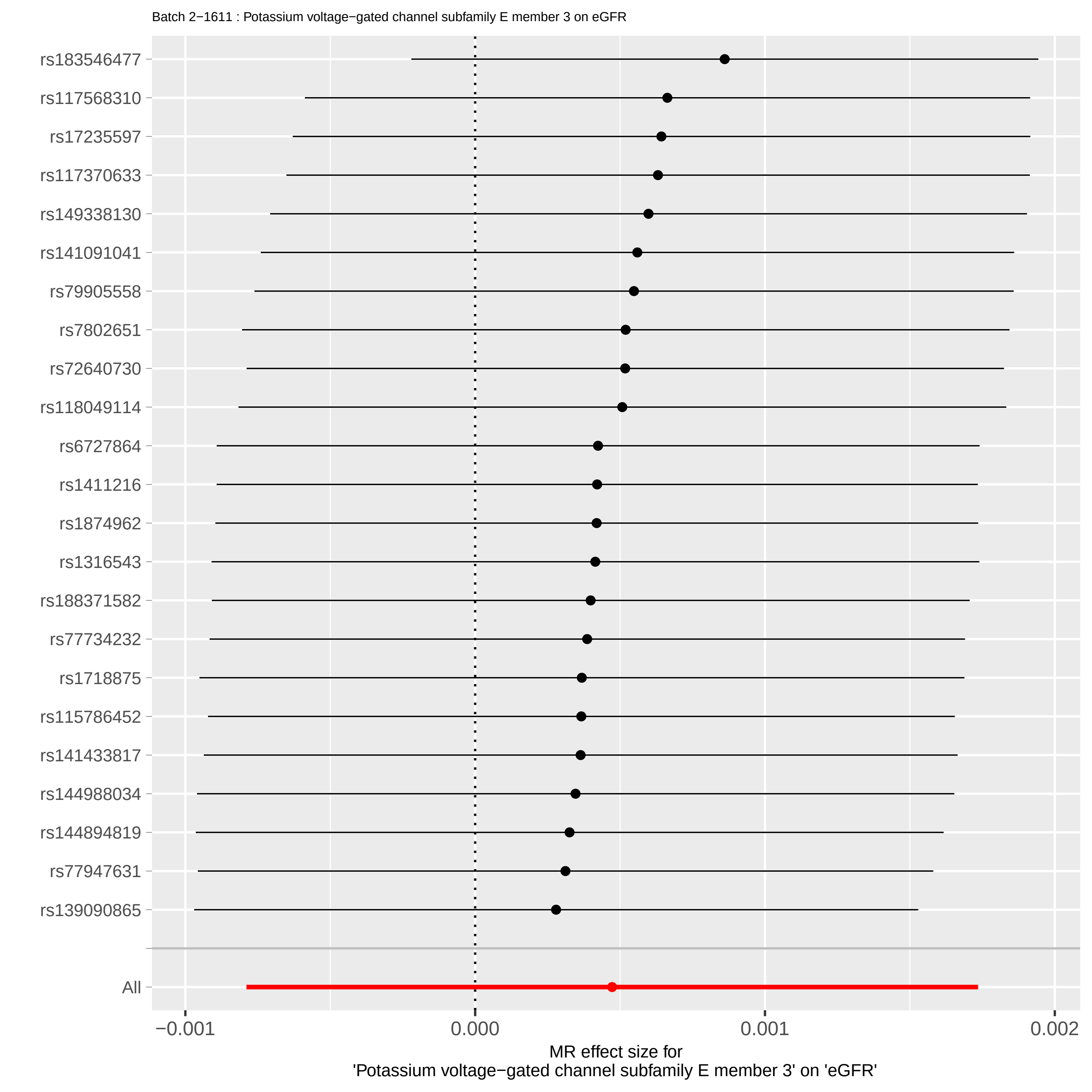
Batch 2−1611 : Potassium voltage−gated channel subfamily E member 3 on eGFR
rs183546477
rs117568310
rs17235597
rs117370633
rs149338130
rs141091041
rs79905558
rs7802651
rs72640730
rs118049114
rs6727864
rs1411216
rs1874962
rs1316543
rs188371582
rs77734232
rs1718875
rs115786452
rs141433817
rs144988034
rs144894819
rs77947631
rs139090865
All
−0.001
0.000
0.001
0.002
MR effect size for
'Potassium voltage−gated channel subfamily E member 3' on 'eGFR'

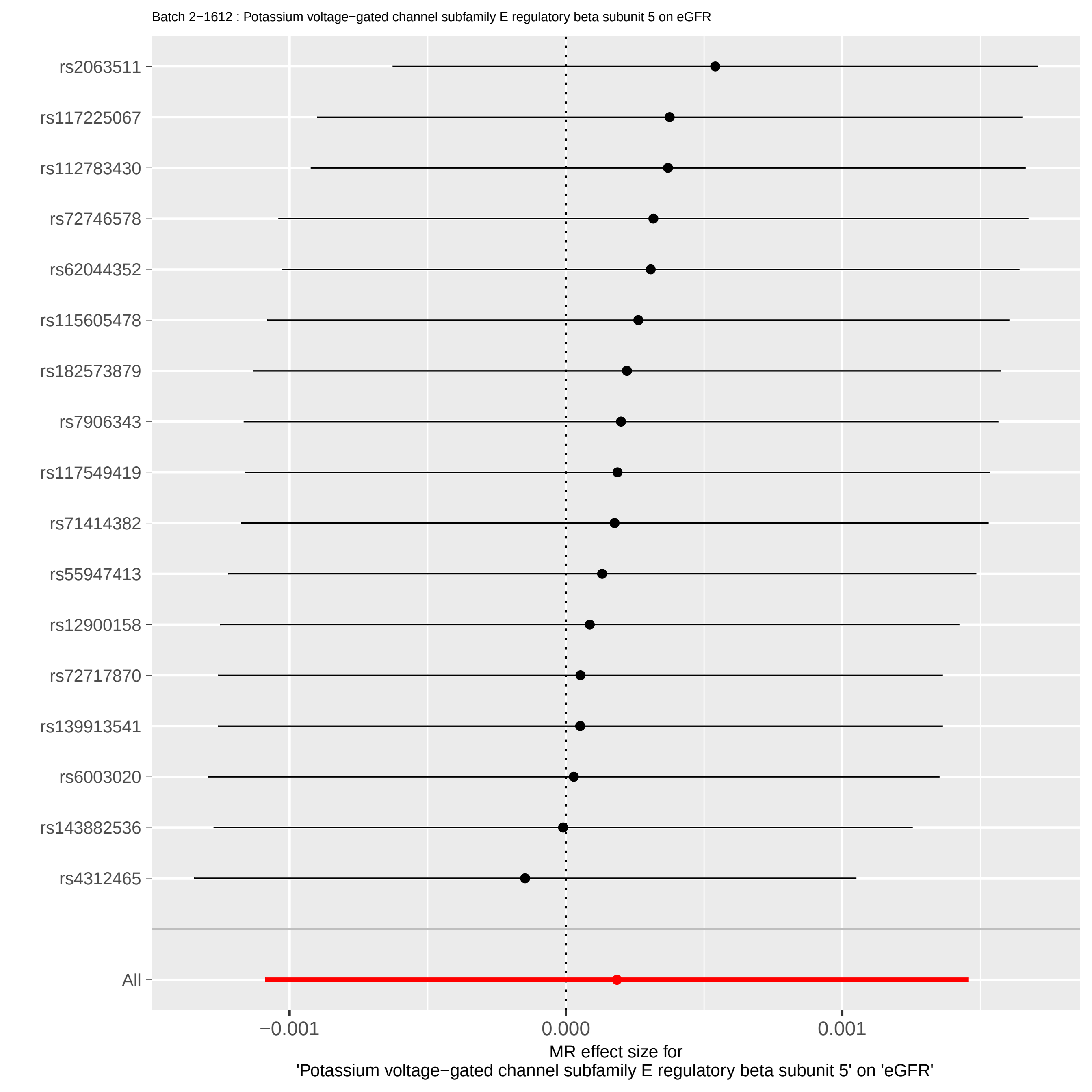

Batch 2−1612 : Potassium voltage−gated channel subfamily E regulatory beta subunit 5 on eGFR
rs2063511
rs117225067
rs112783430
rs72746578
rs62044352
rs115605478
rs182573879
rs7906343
rs117549419
rs71414382
rs55947413
rs12900158
rs72717870
rs139913541
rs6003020
rs143882536
rs4312465
All
−0.001
0.000
0.001
MR effect size for
'Potassium voltage−gated channel subfamily E regulatory beta subunit 5' on 'eGFR'

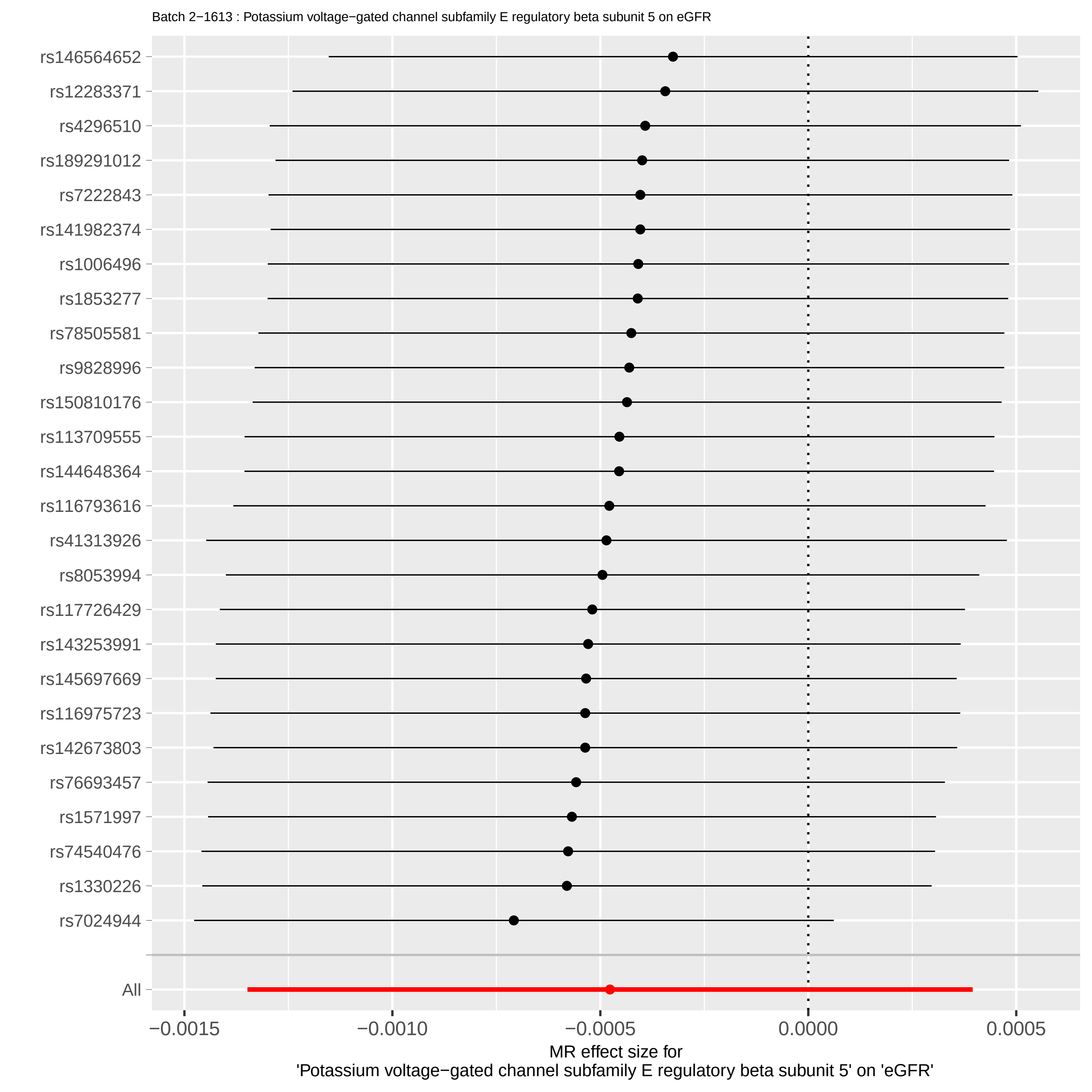
Batch 2−1613 : Potassium voltage−gated channel subfamily E regulatory beta subunit 5 on eGFR
rs146564652
rs12283371
rs4296510
rs189291012
rs7222843
rs141982374
rs1006496
rs1853277
rs78505581
rs9828996
rs150810176
rs113709555
rs144648364
rs116793616
rs41313926
rs8053994
rs117726429
rs143253991
rs145697669
rs116975723
rs142673803
rs76693457
rs1571997
rs74540476
rs1330226
rs7024944
All
−0.0015
−0.0010
−0.0005
0.0000
0.0005
MR effect size for
'Potassium voltage−gated channel subfamily E regulatory beta subunit 5' on 'eGFR'

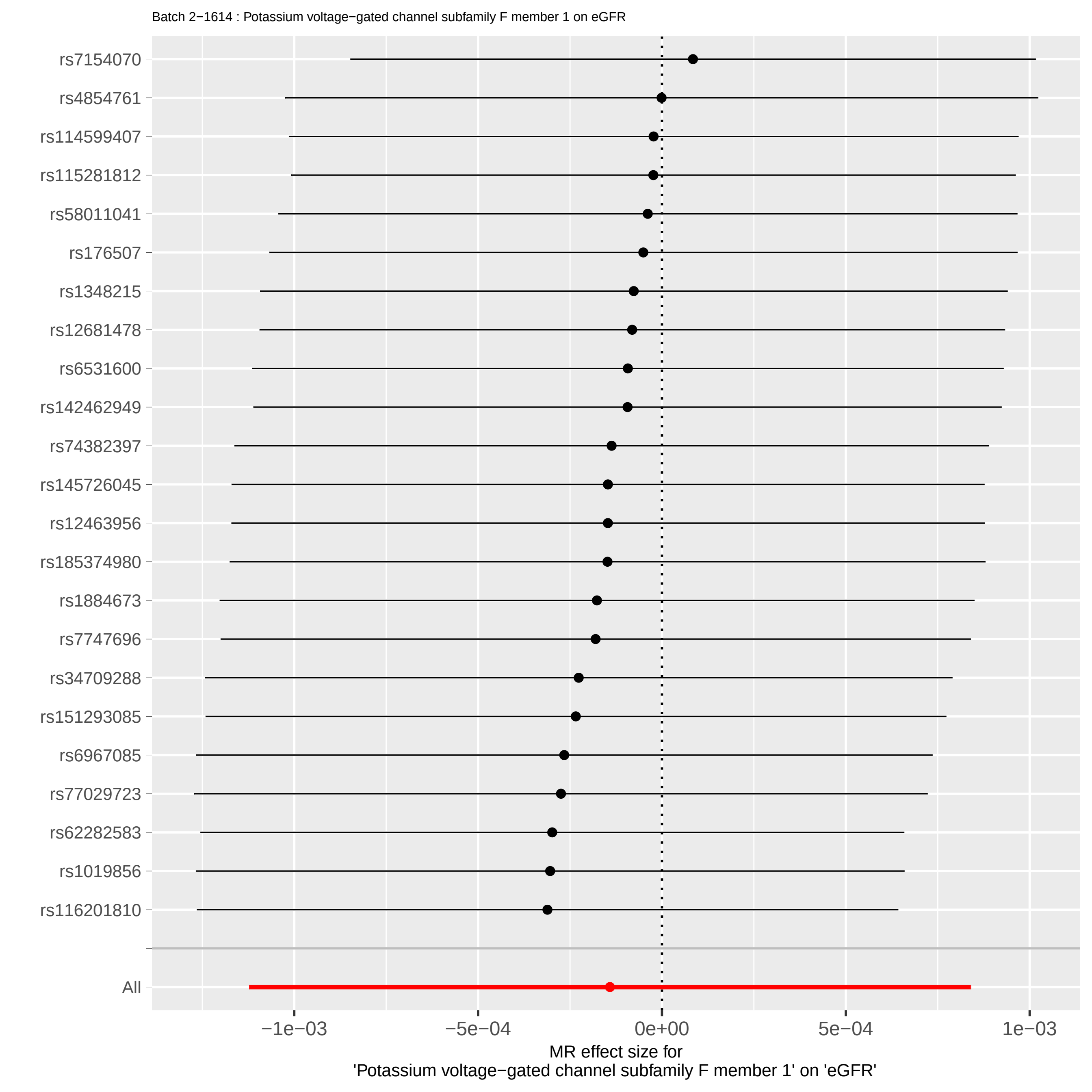

Batch 2−1614 : Potassium voltage−gated channel subfamily F member 1 on eGFR
rs7154070
rs4854761
rs114599407
rs115281812
rs58011041
rs176507
rs1348215
rs12681478
rs6531600
rs142462949
rs74382397
rs145726045
rs12463956
rs185374980
rs1884673
rs7747696
rs34709288
rs151293085
rs6967085
rs77029723
rs62282583
rs1019856
rs116201810
All
−1e−03
−5e−04
0e+00
5e−04
1e−03
MR effect size for
'Potassium voltage−gated channel subfamily F member 1' on 'eGFR'

Batch 2−1615 : Potassium voltage−gated channel subfamily G member 4 on eGFR

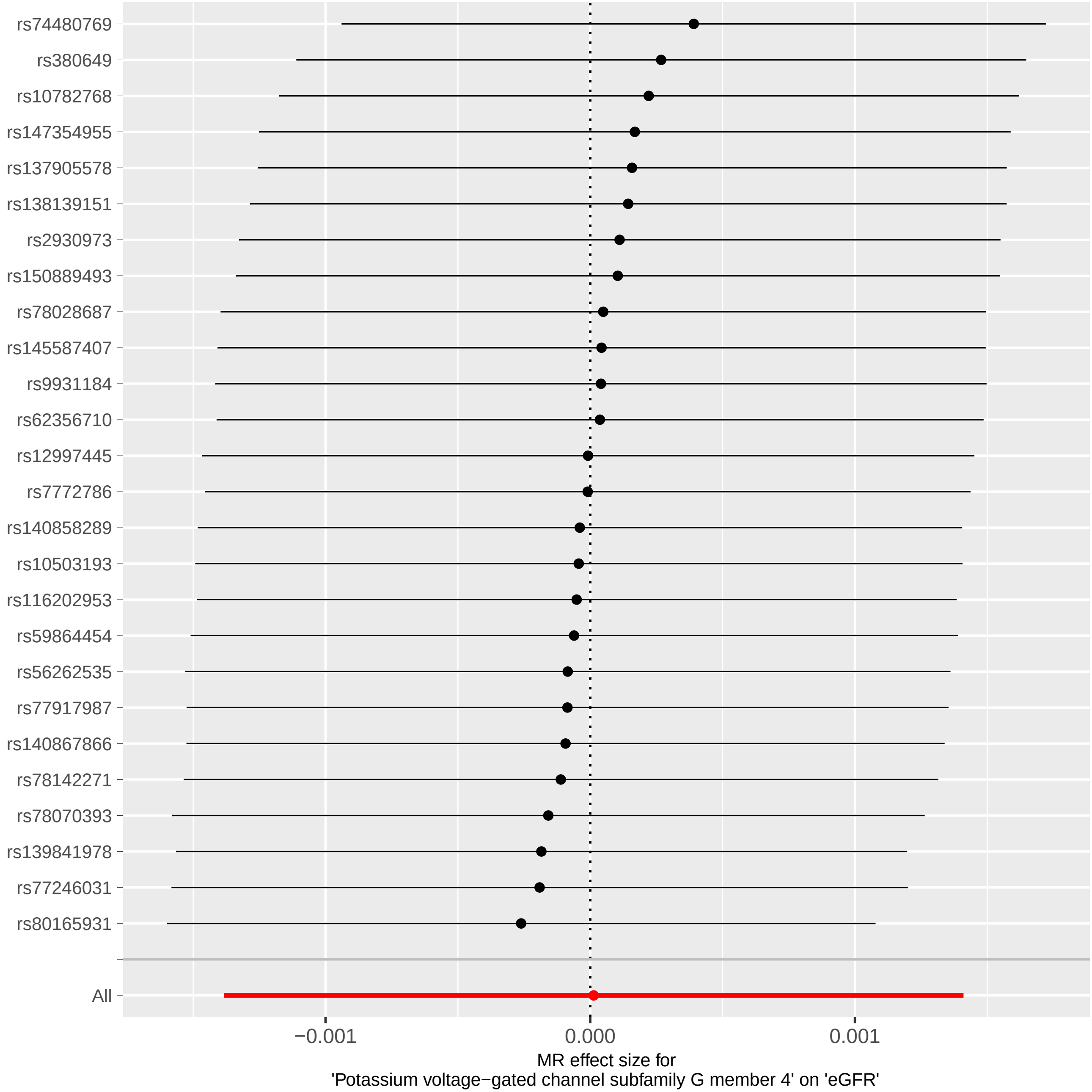

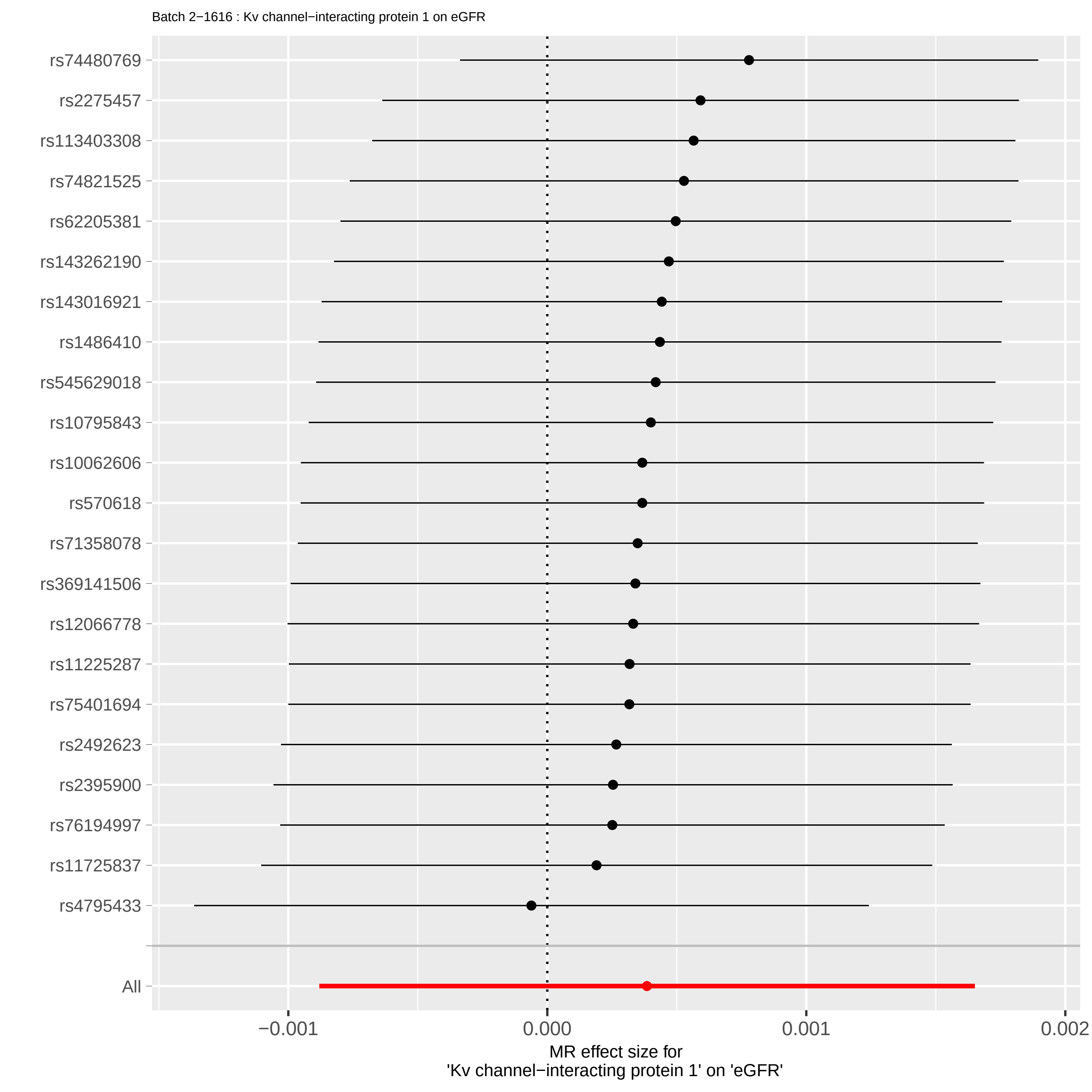
Batch 2−1616 : Kv channel−interacting protein 1 on eGFR
rs74480769
rs2275457
rs113403308
rs74821525
rs62205381
rs143262190
rs143016921
rs1486410
rs545629018
rs10795843
rs10062606
rs570618
rs71358078
rs369141506
rs12066778
rs11225287
rs75401694
rs2492623
rs2395900
rs76194997
rs11725837
rs4795433
All
−0.001
0.000
0.001
0.002
MR effect size for
'Kv channel−interacting protein 1' on 'eGFR'

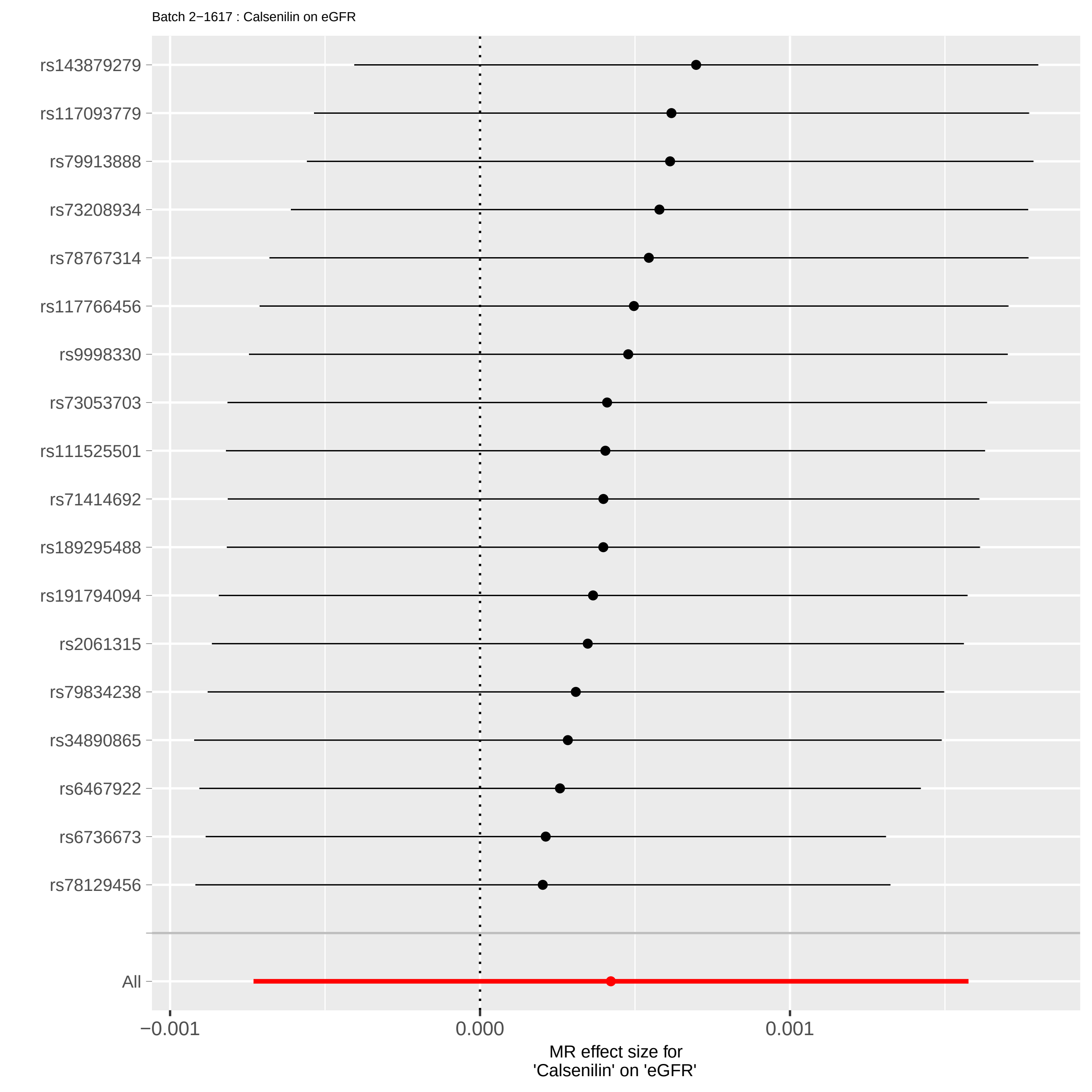
Batch 2−1617 : Calsenilin on eGFR
rs143879279
rs117093779
rs79913888
rs73208934
rs78767314
rs117766456
rs9998330
rs73053703
rs111525501
rs71414692
rs189295488
rs191794094
rs2061315
rs79834238
rs34890865
rs6467922
rs6736673
rs78129456
All
−0.001
0.000
0.001
MR effect size for
'Calsenilin' on 'eGFR'

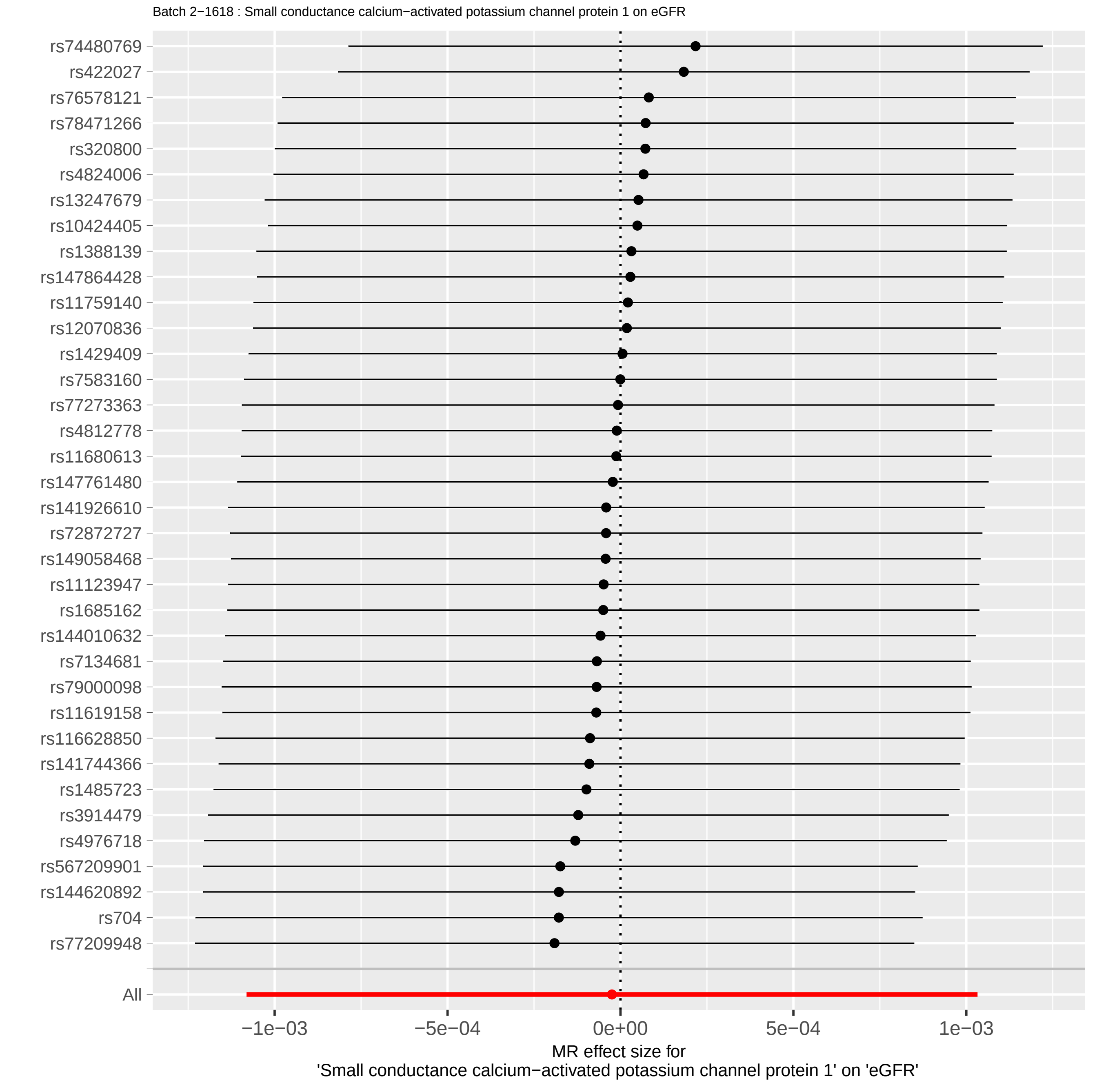

Batch 2−1618 : Small conductance calcium−activated potassium channel protein 1 on eGFR
rs74480769
rs422027
rs76578121
rs78471266
rs320800
rs4824006
rs13247679
rs10424405
rs1388139
rs147864428
rs11759140
rs12070836
rs1429409
rs7583160
rs77273363
rs4812778
rs11680613
rs147761480
rs141926610
rs72872727
rs149058468
rs11123947
rs1685162
rs144010632
rs7134681
rs79000098
rs11619158
rs116628850
rs141744366
rs1485723
rs3914479
rs4976718
rs567209901
rs144620892
rs704
rs77209948
All
−1e−03
−5e−04
0e+00
5e−04
1e−03
MR effect size for
'Small conductance calcium−activated potassium channel protein 1' on 'eGFR'

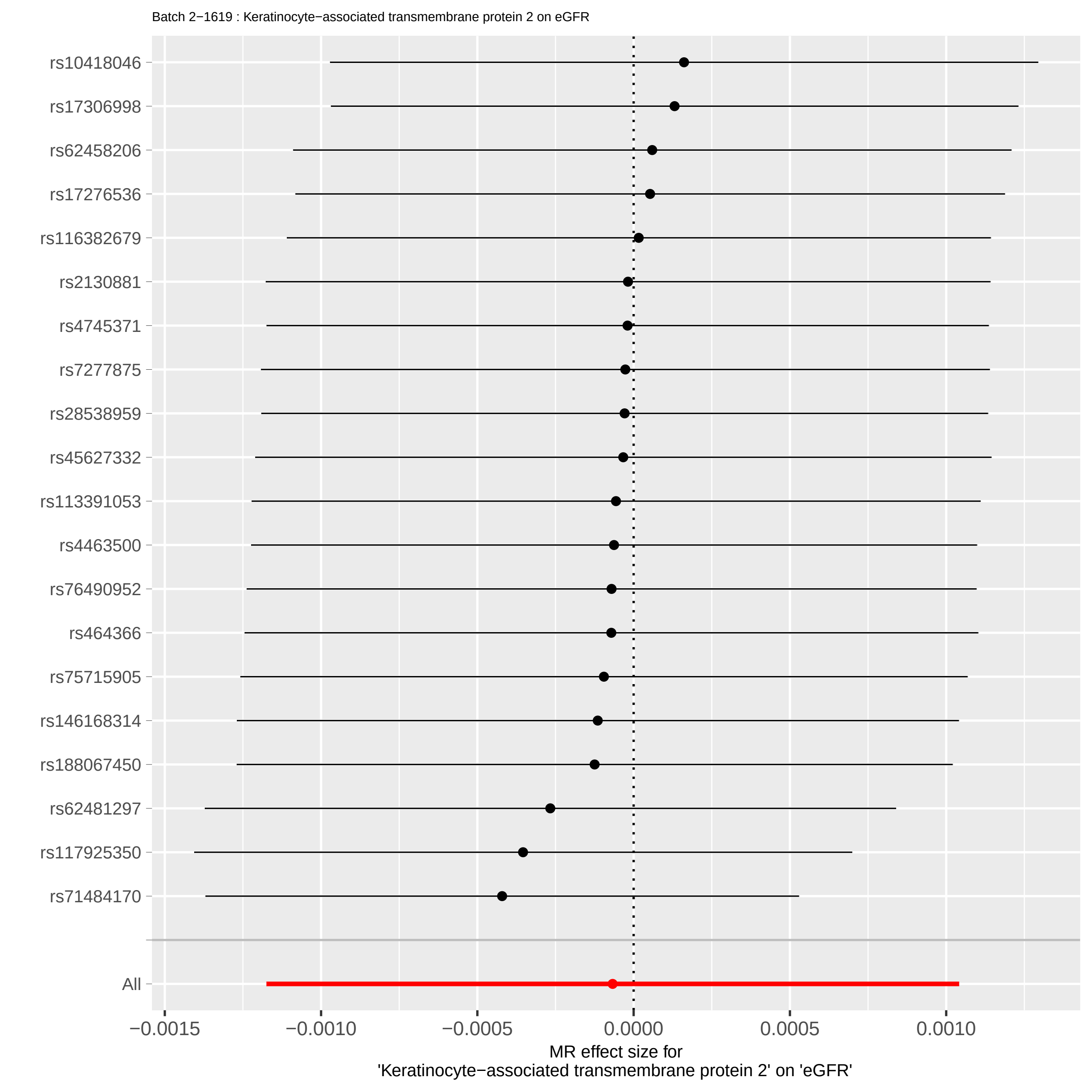
Batch 2−1619 : Keratinocyte−associated transmembrane protein 2 on eGFR
rs10418046
rs17306998
rs62458206
rs17276536
rs116382679
rs2130881
rs4745371
rs7277875
rs28538959
rs45627332
rs113391053
rs4463500
rs76490952
rs464366
rs75715905
rs146168314
rs188067450
rs62481297
rs117925350
rs71484170
All
−0.0015
−0.0010
−0.0005
0.0000
0.0005
0.0010
MR effect size for
'Keratinocyte−associated transmembrane protein 2' on 'eGFR'

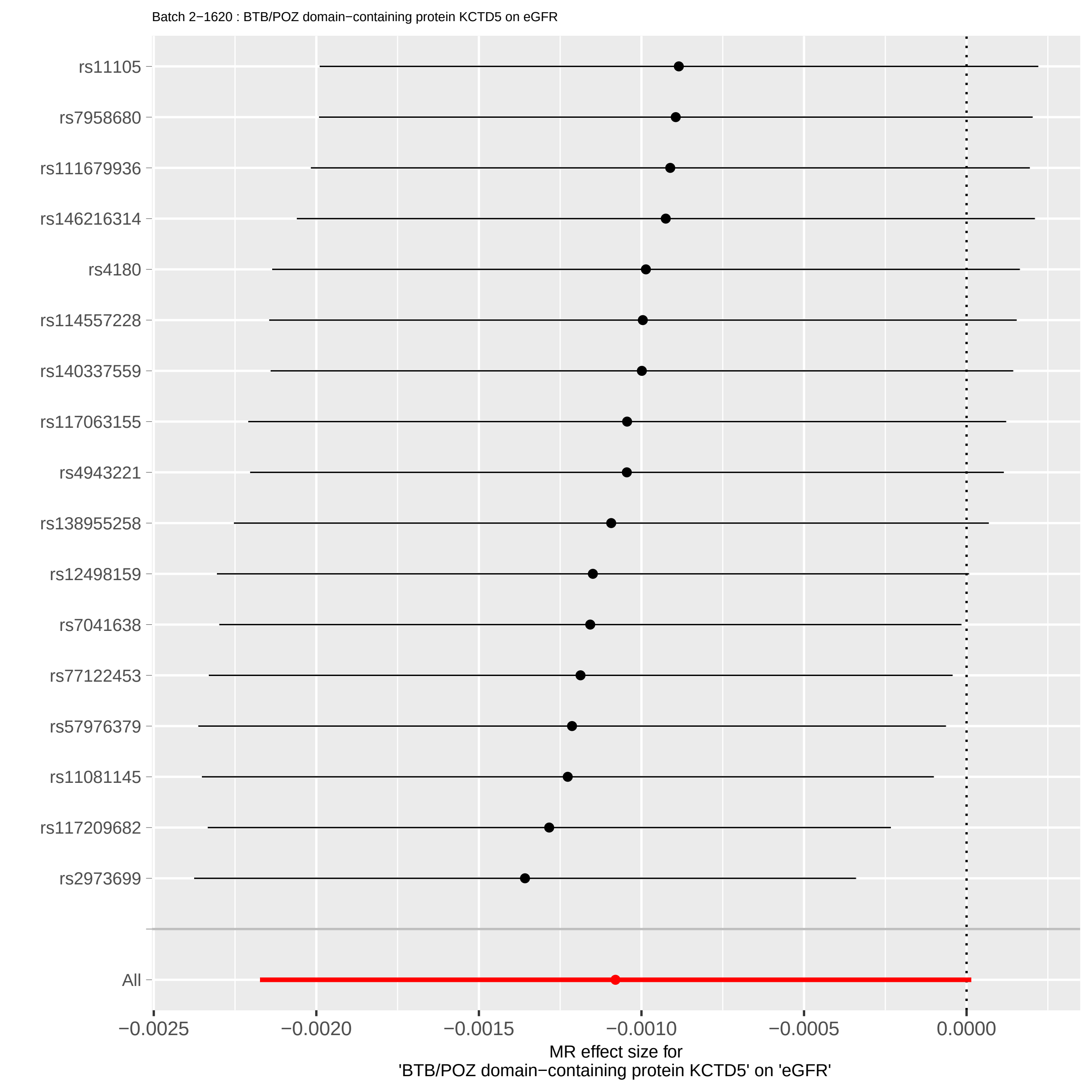

Batch 2−1620 : BTB/POZ domain−containing protein KCTD5 on eGFR
rs11105
rs7958680
rs111679936
rs146216314
rs4180
rs114557228
rs140337559
rs117063155
rs4943221
rs138955258
rs12498159
rs7041638
rs77122453
rs57976379
rs11081145
rs117209682
rs2973699
All
−0.0025
−0.0020
−0.0015
−0.0010
−0.0005
0.0000
MR effect size for
'BTB/POZ domain−containing protein KCTD5' on 'eGFR'

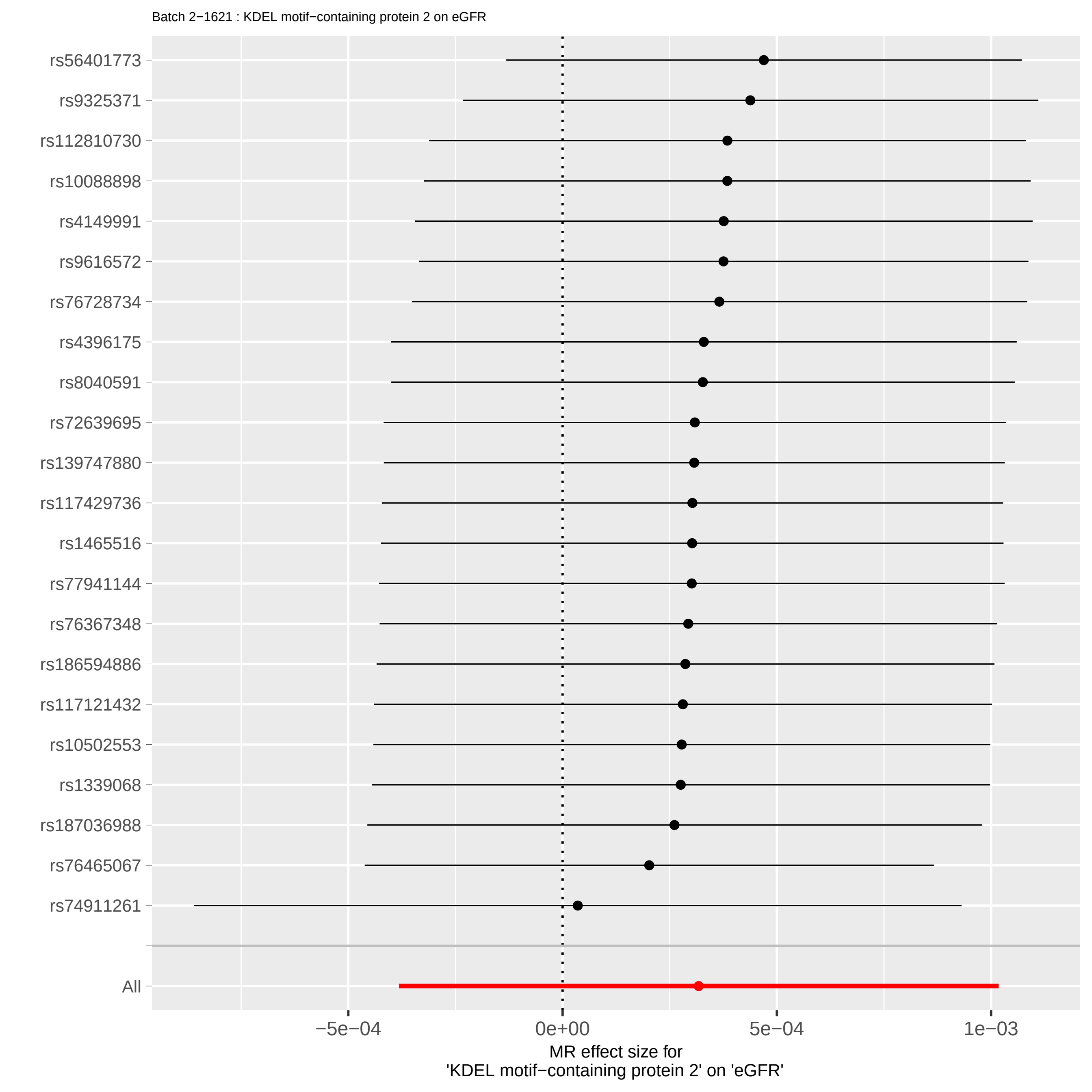

Batch 2−1621 : KDEL motif−containing protein 2 on eGFR
rs56401773
rs9325371
rs112810730
rs10088898
rs4149991
rs9616572
rs76728734
rs4396175
rs8040591
rs72639695
rs139747880
rs117429736
rs1465516
rs77941144
rs76367348
rs186594886
rs117121432
rs10502553
rs1339068
rs187036988
rs76465067
rs74911261
All
−5e−04
0e+00
5e−04
1e−03
MR effect size for
'KDEL motif−containing protein 2' on 'eGFR'

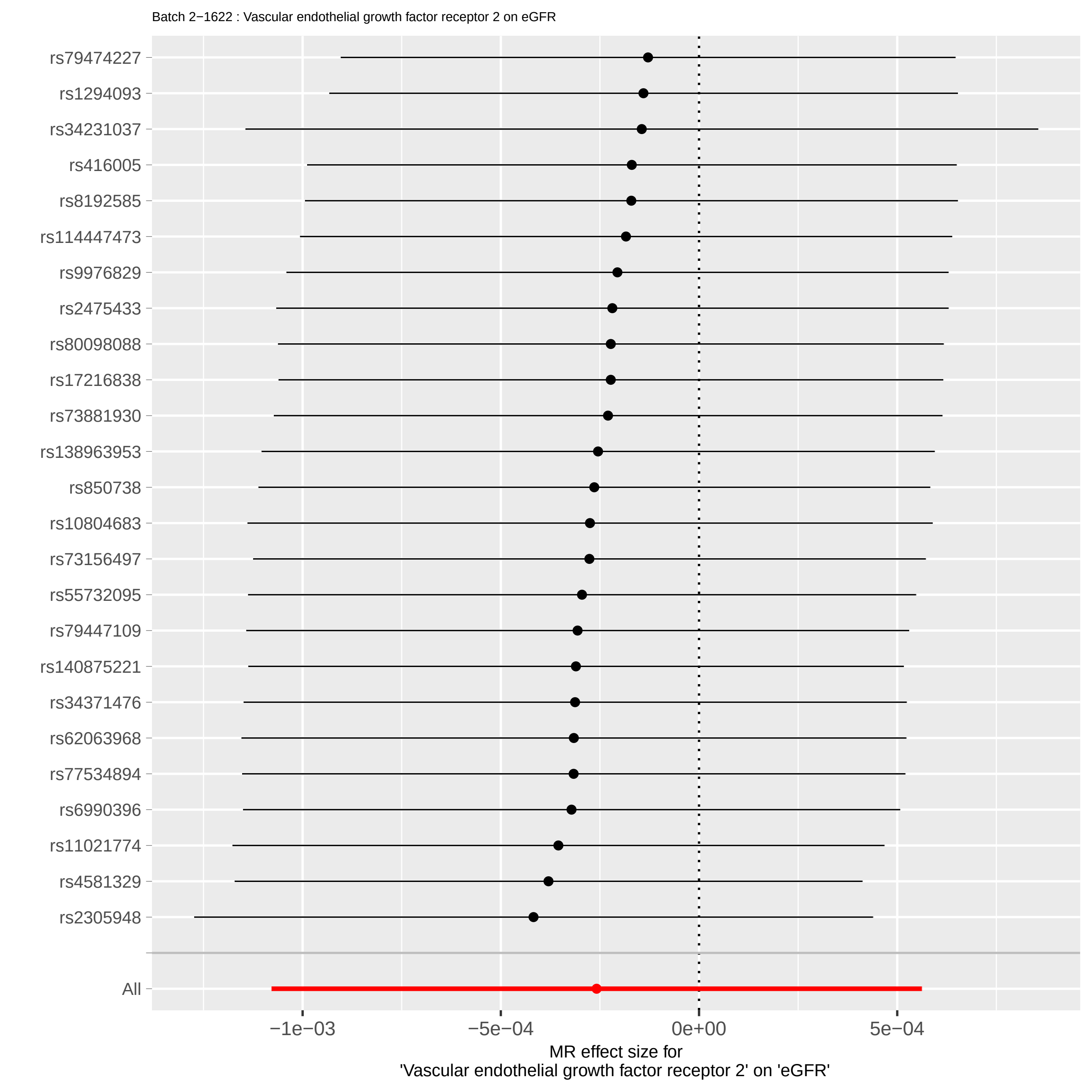
Batch 2−1622 : Vascular endothelial growth factor receptor 2 on eGFR
rs79474227
rs1294093
rs34231037
rs416005
rs8192585
rs114447473
rs9976829
rs2475433
rs80098088
rs17216838
rs73881930
rs138963953
rs850738
rs10804683
rs73156497
rs55732095
rs79447109
rs140875221
rs34371476
rs62063968
rs77534894
rs6990396
rs11021774
rs4581329
rs2305948
All
−1e−03
−5e−04
0e+00
5e−04
MR effect size for
'Vascular endothelial growth factor receptor 2' on 'eGFR'

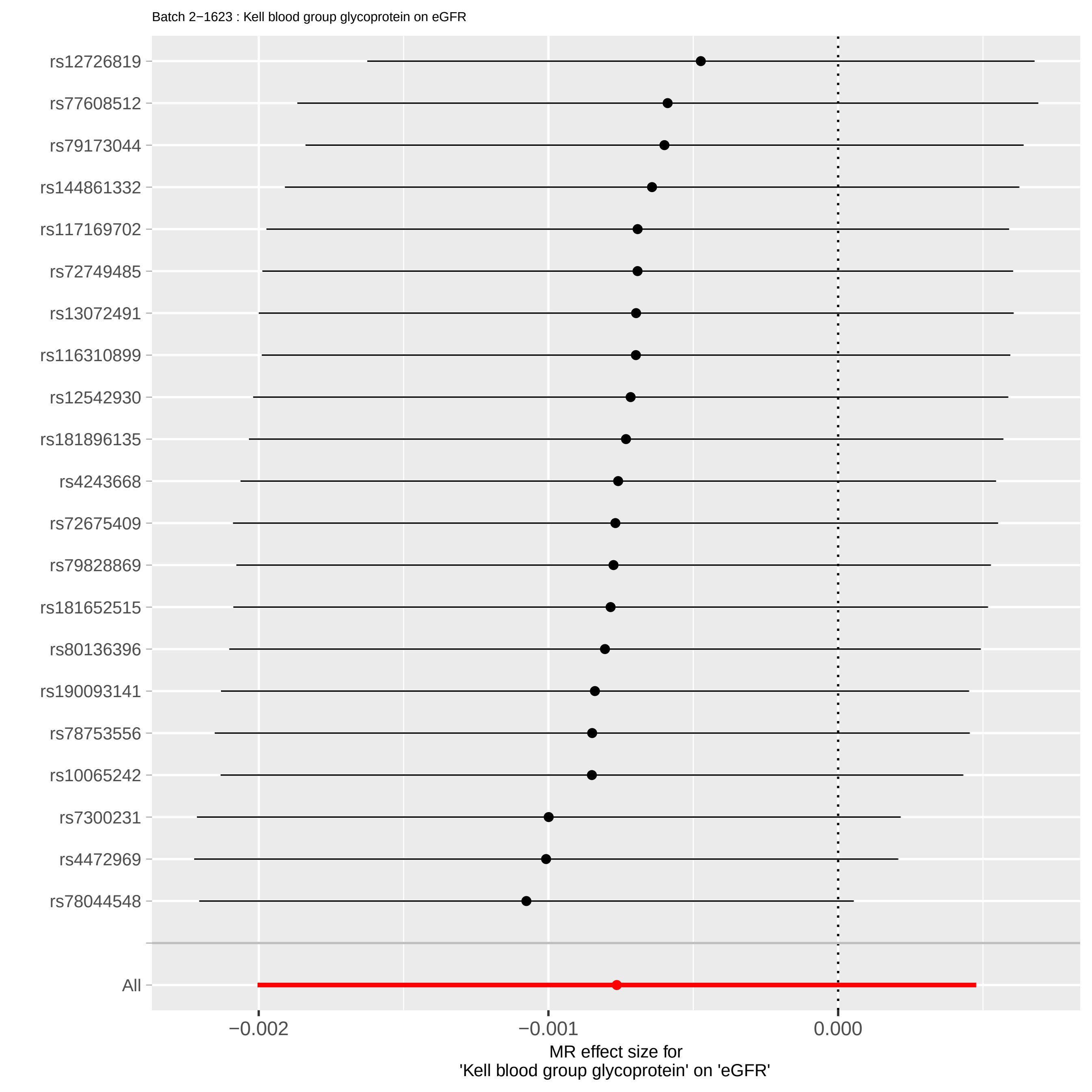
Batch 2−1623 : Kell blood group glycoprotein on eGFR
rs12726819
rs77608512
rs79173044
rs144861332
rs117169702
rs72749485
rs13072491
rs116310899
rs12542930
rs181896135
rs4243668
rs72675409
rs79828869
rs181652515
rs80136396
rs190093141
rs78753556
rs10065242
rs7300231
rs4472969
rs78044548
All
−0.002
−0.001
0.000
MR effect size for
'Kell blood group glycoprotein' on 'eGFR'

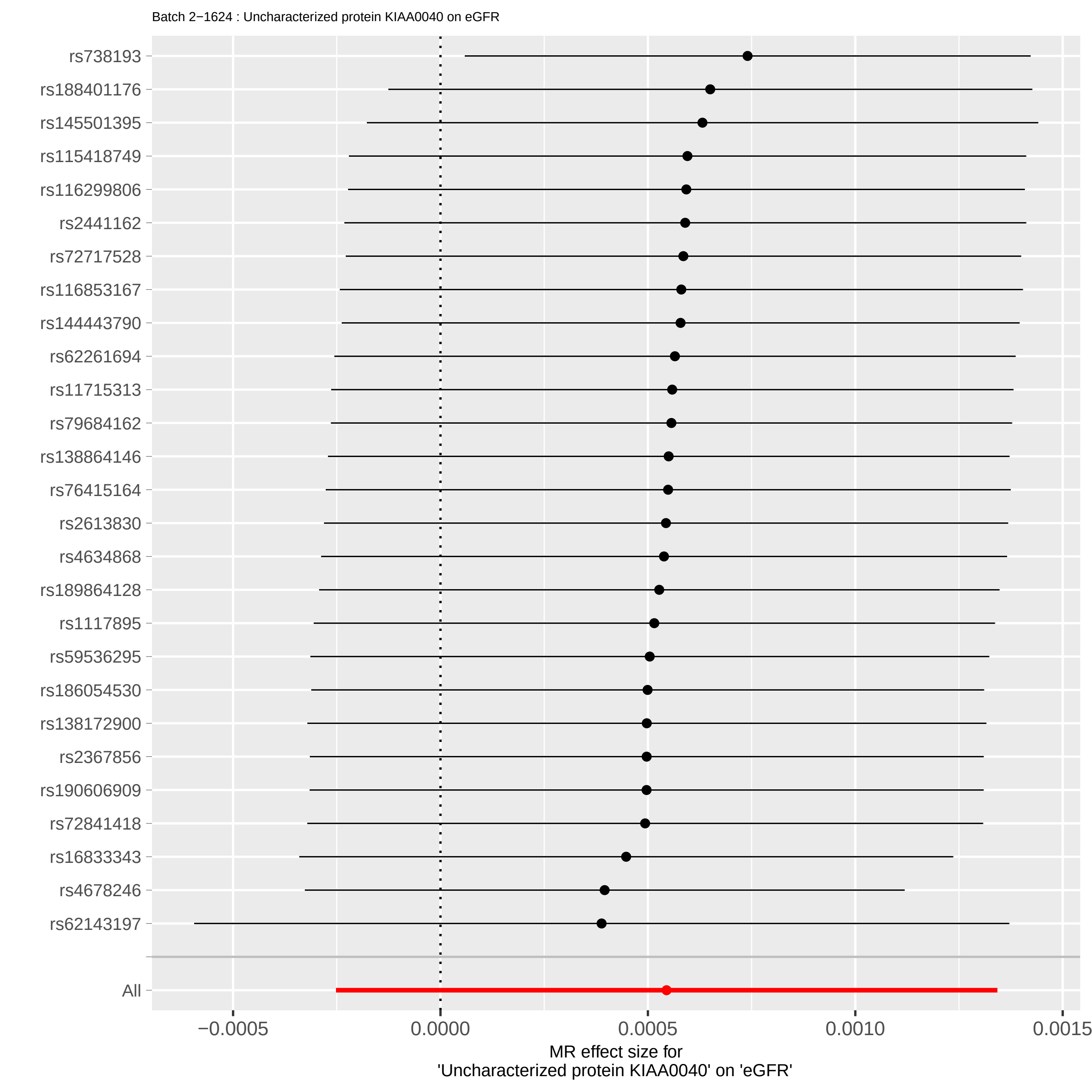

Batch 2−1624 : Uncharacterized protein KIAA0040 on eGFR
rs738193
rs188401176
rs145501395
rs115418749
rs116299806
rs2441162
rs72717528
rs116853167
rs144443790
rs62261694
rs11715313
rs79684162
rs138864146
rs76415164
rs2613830
rs4634868
rs189864128
rs1117895
rs59536295
rs186054530
rs138172900
rs2367856
rs190606909
rs72841418
rs16833343
rs4678246
rs62143197
All
−0.0005
0.0000
0.0005
0.0010
0.0015
MR effect size for
'Uncharacterized protein KIAA0040' on 'eGFR'

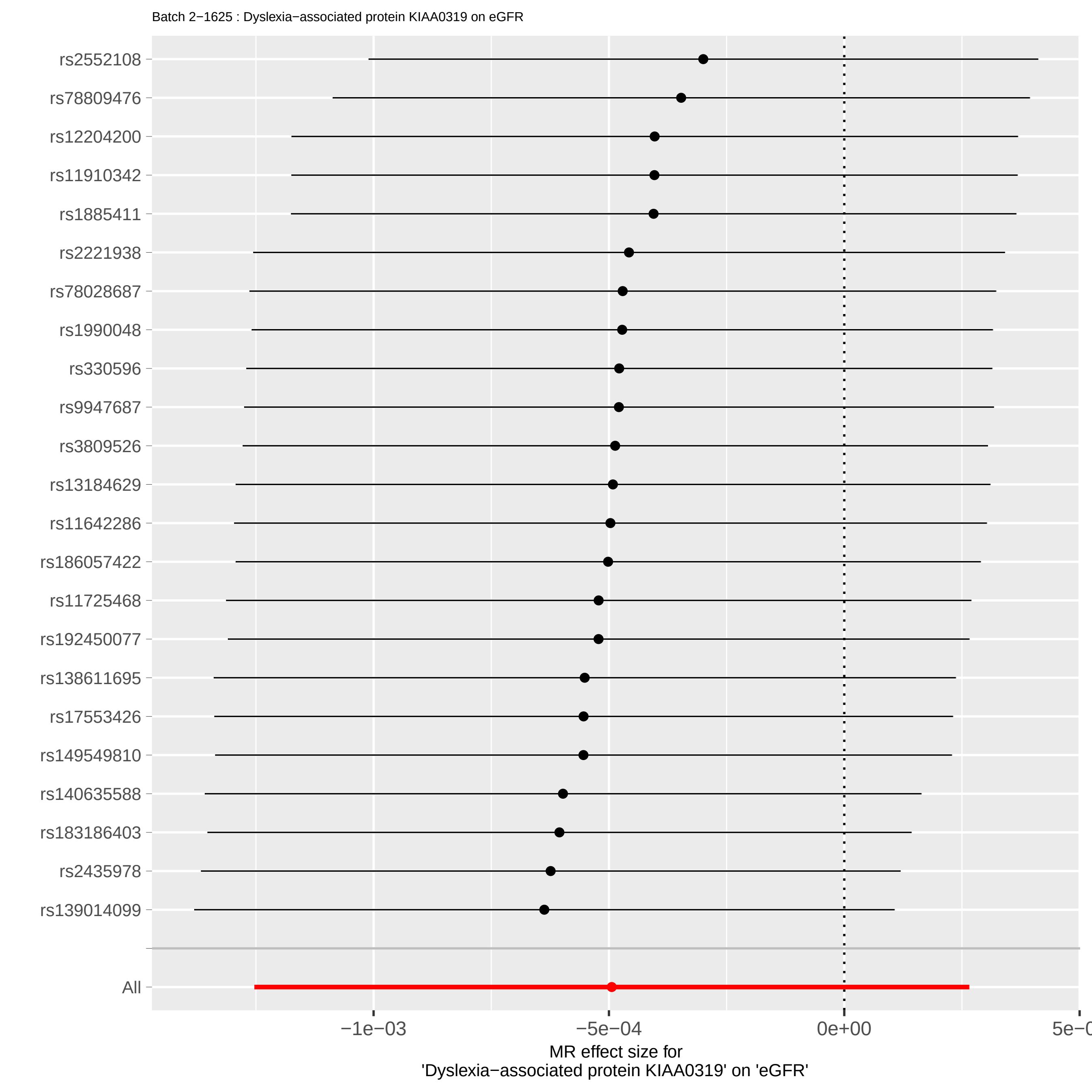

Batch 2−1625 : Dyslexia−associated protein KIAA0319 on eGFR
rs2552108
rs78809476
rs12204200
rs11910342
rs1885411
rs2221938
rs78028687
rs1990048
rs330596
rs9947687
rs3809526
rs13184629
rs11642286
rs186057422
rs11725468
rs192450077
rs138611695
rs17553426
rs149549810
rs140635588
rs183186403
rs2435978
rs139014099
All
−1e−03
−5e−04
0e+00
MR effect size for
'Dyslexia−associated protein KIAA0319' on 'eGFR'

Batch 2−1626 : Dyslexia−associated protein KIAA0319−like protein on eGFR

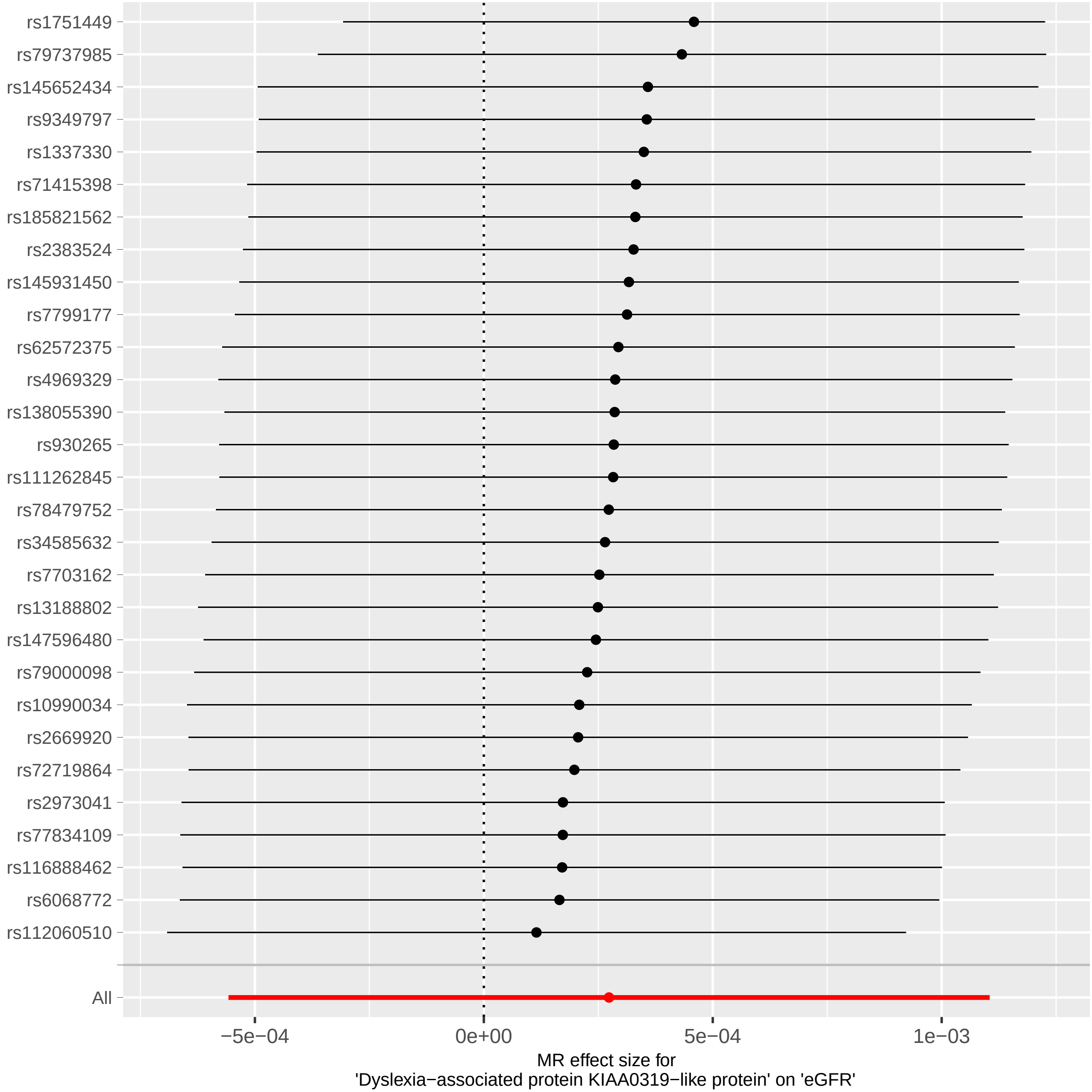

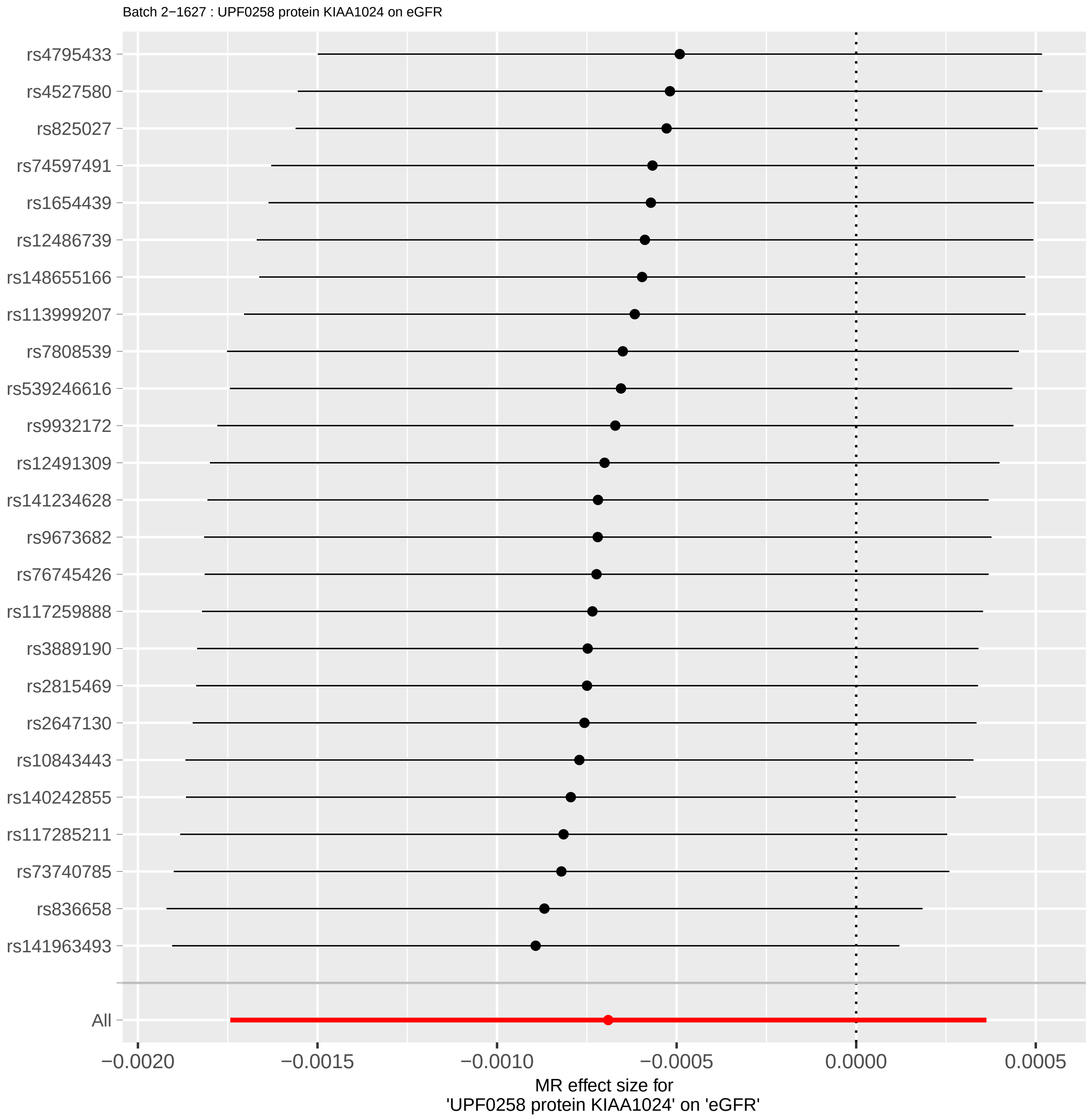

Batch 2−1627 : UPF0258 protein KIAA1024 on eGFR
rs4795433
rs4527580
rs825027
rs74597491
rs1654439
rs12486739
rs148655166
rs113999207
rs7808539
rs539246616
rs9932172
rs12491309
rs141234628
rs9673682
rs76745426
rs117259888
rs3889190
rs2815469
rs2647130
rs10843443
rs140242855
rs117285211
rs73740785
rs836658
rs141963493
All
−0.0020
−0.0015
−0.0010
−0.0005
0.0000
0.0005
MR effect size for
'UPF0258 protein KIAA1024' on 'eGFR'

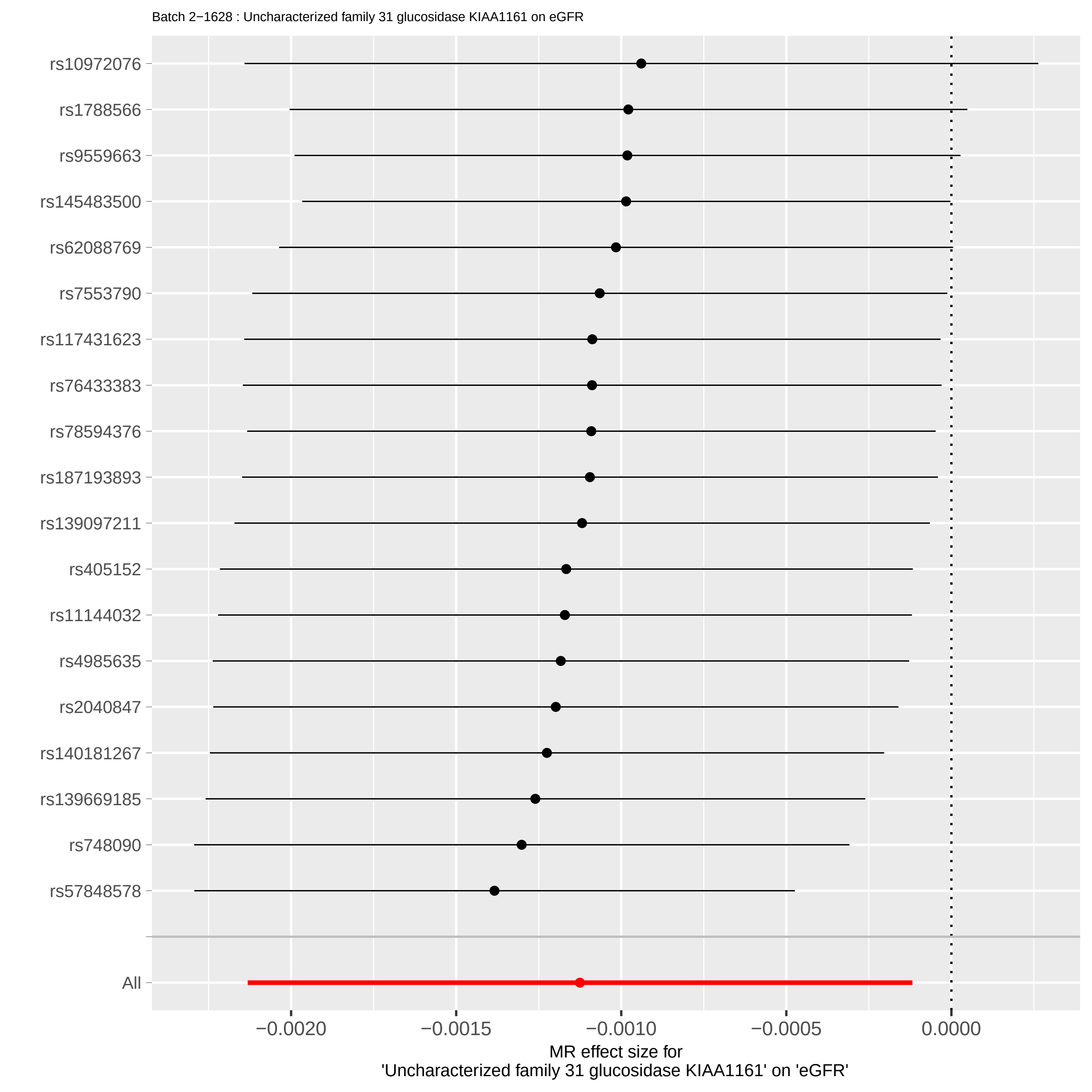

Batch 2−1628 : Uncharacterized family 31 glucosidase KIAA1161 on eGFR
rs10972076
rs1788566
rs9559663
rs145483500
rs62088769
rs7553790
rs117431623
rs76433383
rs78594376
rs187193893
rs139097211
rs405152
rs11144032
rs4985635
rs2040847
rs140181267
rs139669185
rs748090
rs57848578
All
−0.0020
−0.0015
−0.0010
−0.0005
0.0000
MR effect size for
'Uncharacterized family 31 glucosidase KIAA1161' on 'eGFR'

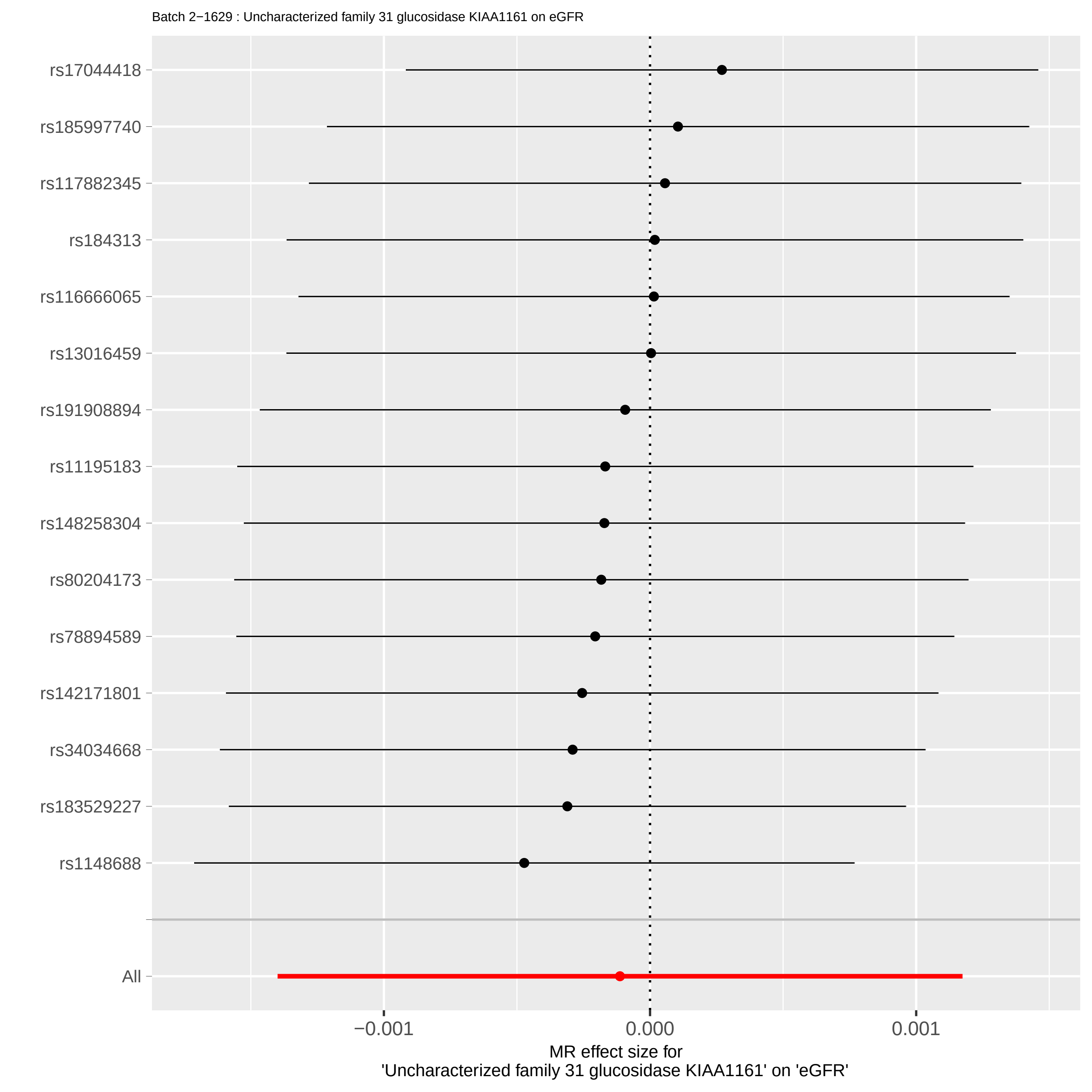

Batch 2−1629 : Uncharacterized family 31 glucosidase KIAA1161 on eGFR
rs17044418
rs185997740
rs117882345
rs184313
rs116666065
rs13016459
rs191908894
rs11195183
rs148258304
rs80204173
rs78894589
rs142171801
rs34034668
rs183529227
rs1148688
All
−0.001
0.000
0.001
MR effect size for
'Uncharacterized family 31 glucosidase KIAA1161' on 'eGFR'

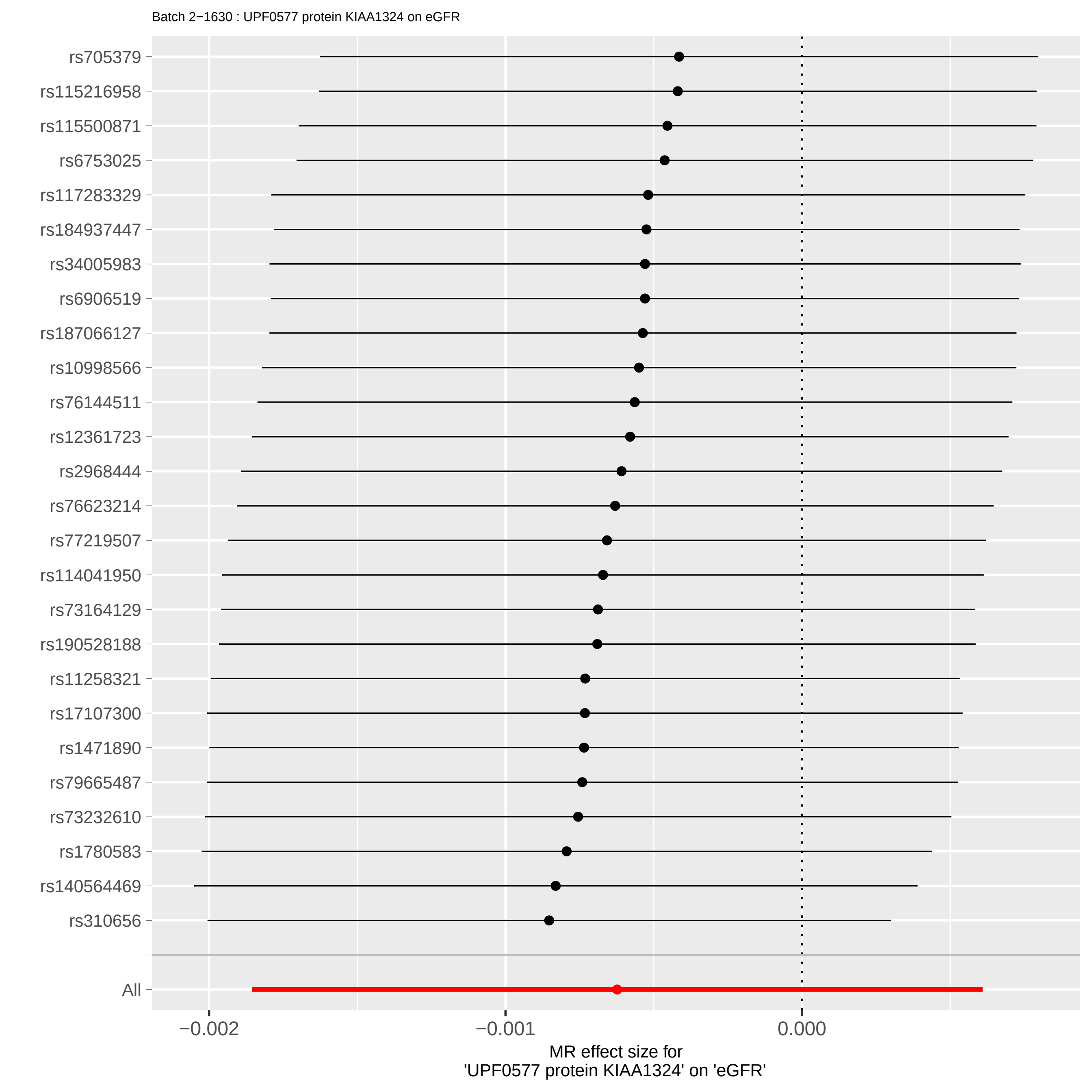

Batch 2−1630 : UPF0577 protein KIAA1324 on eGFR
rs705379
rs115216958
rs115500871
rs6753025
rs117283329
rs184937447
rs34005983
rs6906519
rs187066127
rs10998566
rs76144511
rs12361723
rs2968444
rs76623214
rs77219507
rs114041950
rs73164129
rs190528188
rs11258321
rs17107300
rs1471890
rs79665487
rs73232610
rs1780583
rs140564469
rs310656
All
−0.002
−0.001
0.000
MR effect size for
'UPF0577 protein KIAA1324' on 'eGFR'

Batch 2−1631 : UPF0577 protein KIAA1324 on eGFR

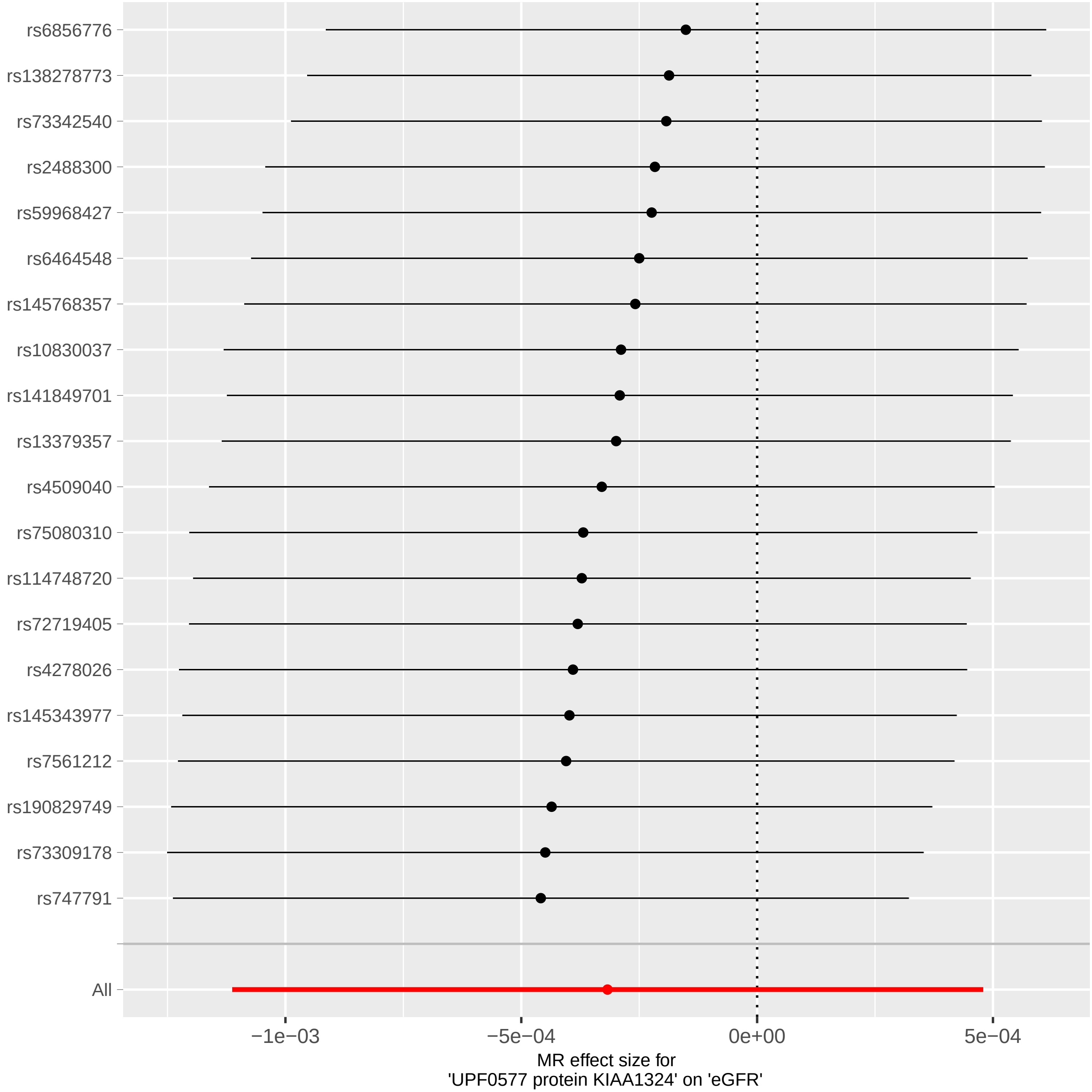

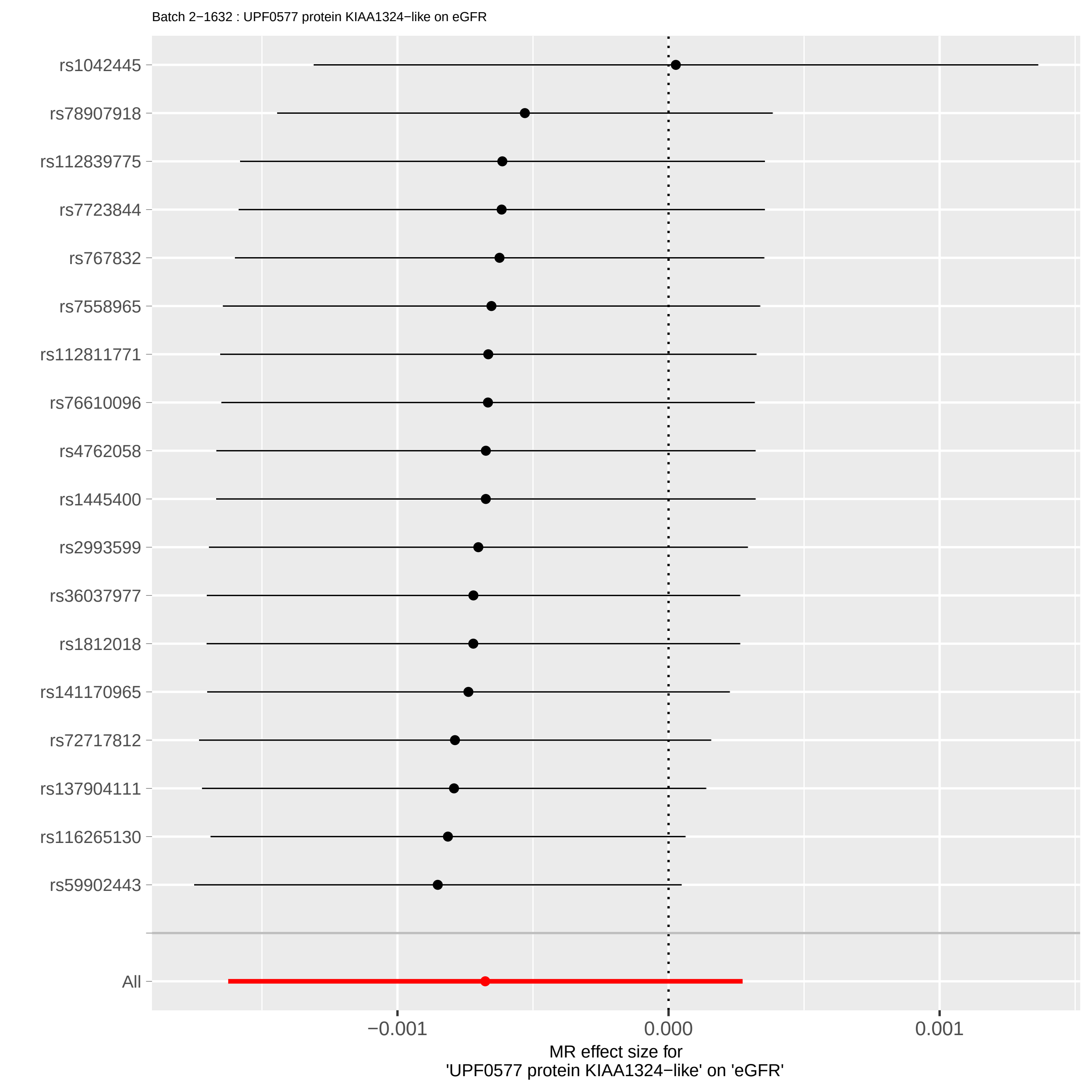

Batch 2−1632 : UPF0577 protein KIAA1324−like on eGFR
rs1042445
rs78907918
rs112839775
rs7723844
rs767832
rs7558965
rs112811771
rs76610096
rs4762058
rs1445400
rs2993599
rs36037977
rs1812018
rs141170965
rs72717812
rs137904111
rs116265130
rs59902443
All
−0.001
0.000
0.001
MR effect size for
'UPF0577 protein KIAA1324−like' on 'eGFR'

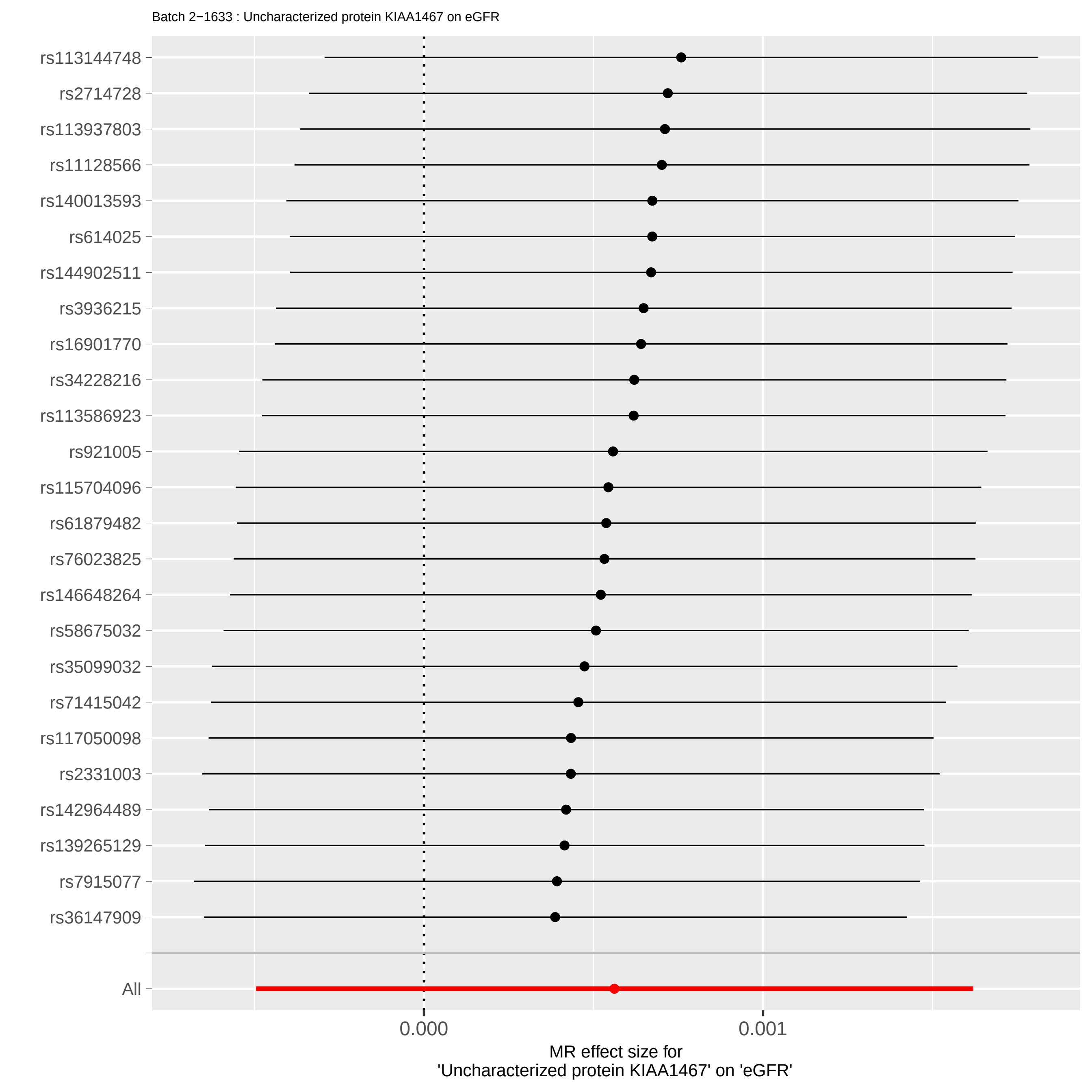

Batch 2−1633 : Uncharacterized protein KIAA1467 on eGFR
rs113144748
rs2714728
rs113937803
rs11128566
rs140013593
rs614025
rs144902511
rs3936215
rs16901770
rs34228216
rs113586923
rs921005
rs115704096
rs61879482
rs76023825
rs146648264
rs58675032
rs35099032
rs71415042
rs117050098
rs2331003
rs142964489
rs139265129
rs7915077
rs36147909
All
0.000
0.001
MR effect size for
'Uncharacterized protein KIAA1467' on 'eGFR'

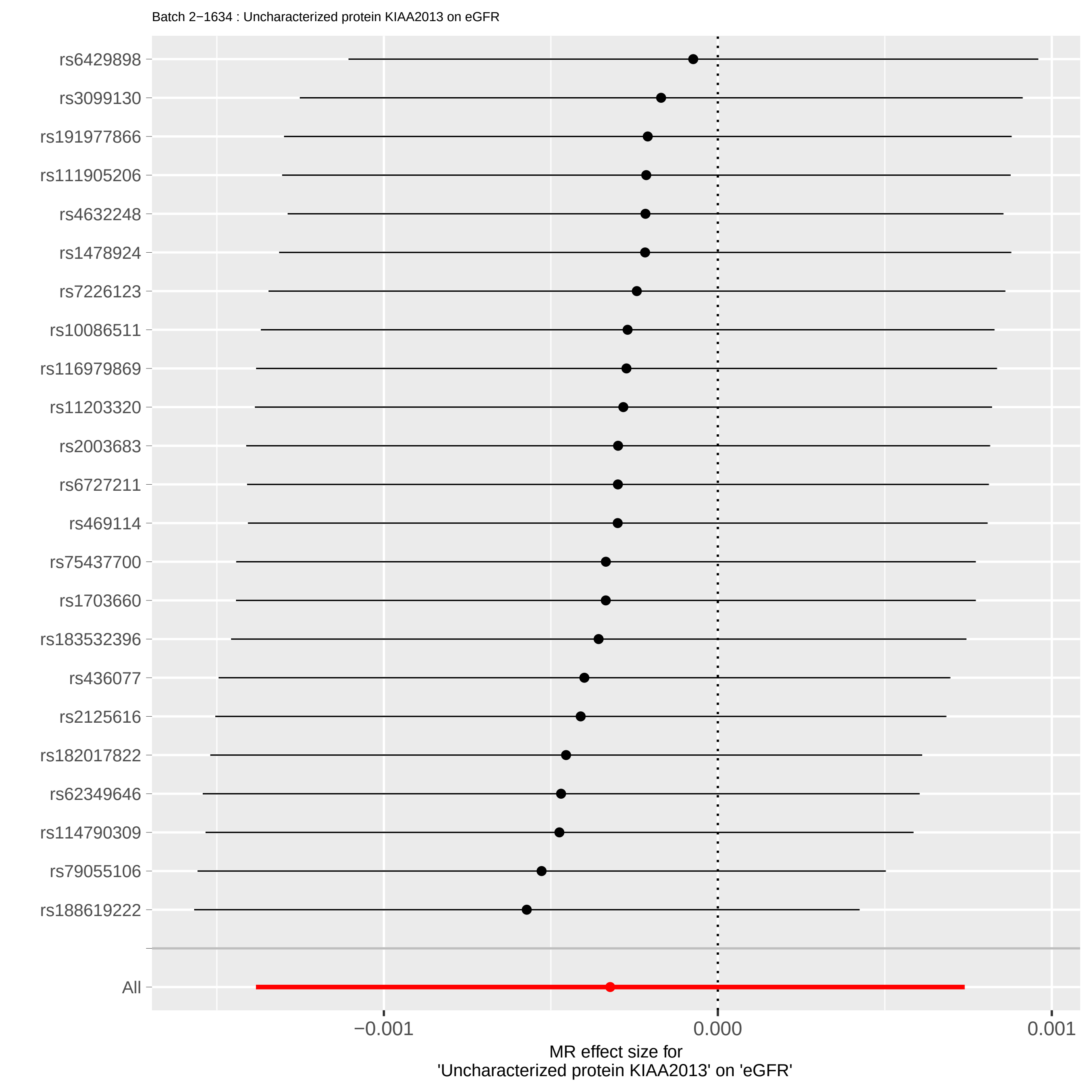

Batch 2−1634 : Uncharacterized protein KIAA2013 on eGFR
rs6429898
rs3099130
rs191977866
rs111905206
rs4632248
rs1478924
rs7226123
rs10086511
rs116979869
rs11203320
rs2003683
rs6727211
rs469114
rs75437700
rs1703660
rs183532396
rs436077
rs2125616
rs182017822
rs62349646
rs114790309
rs79055106
rs188619222
All
−0.001
0.000
0.001
MR effect size for
'Uncharacterized protein KIAA2013' on 'eGFR'

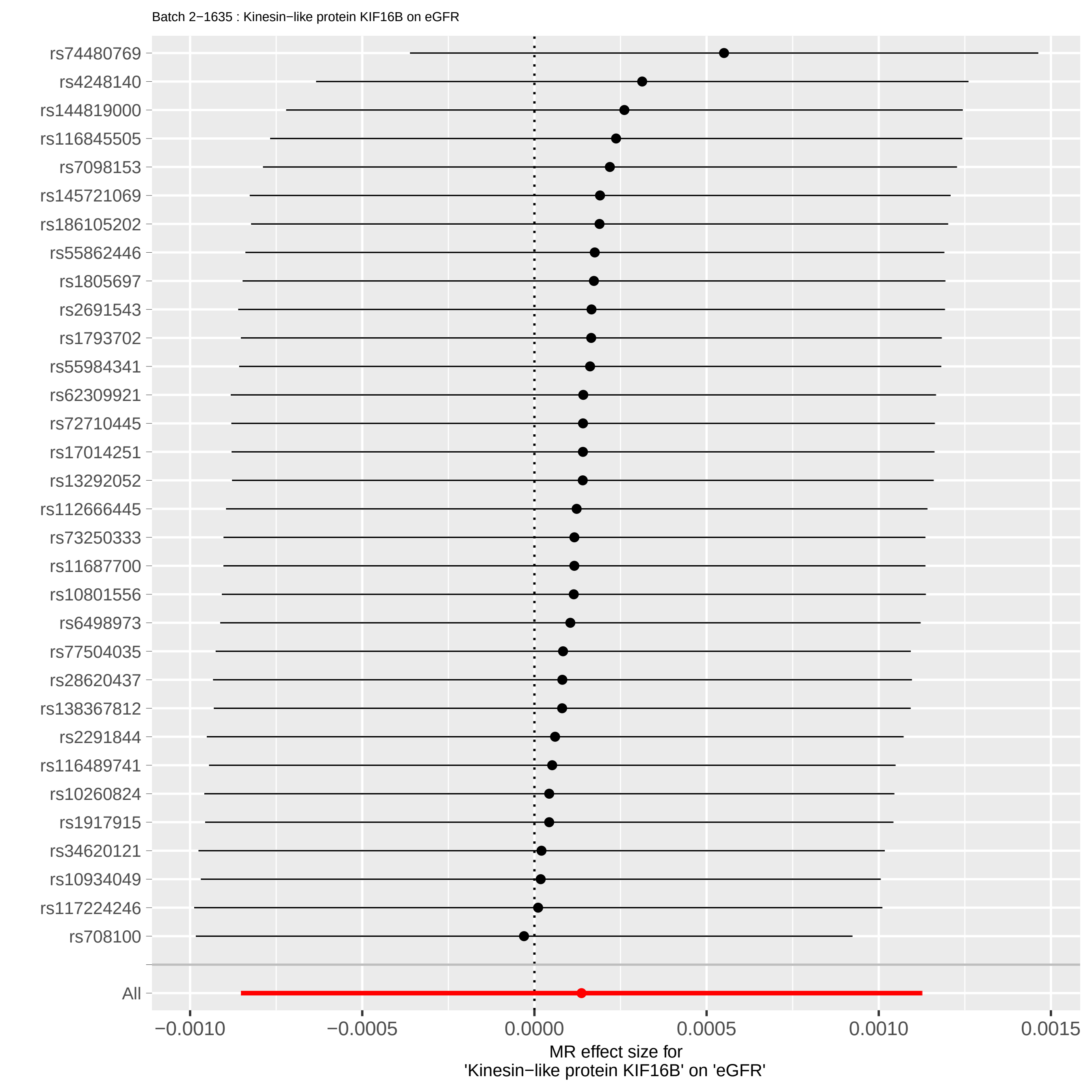

Batch 2−1635 : Kinesin−like protein KIF16B on eGFR
rs74480769
rs4248140
rs144819000
rs116845505
rs7098153
rs145721069
rs186105202
rs55862446
rs1805697
rs2691543
rs1793702
rs55984341
rs62309921
rs72710445
rs17014251
rs13292052
rs112666445
rs73250333
rs11687700
rs10801556
rs6498973
rs77504035
rs28620437
rs138367812
rs2291844
rs116489741
rs10260824
rs1917915
rs34620121
rs10934049
rs117224246
rs708100
All
−0.0010
−0.0005
0.0000
0.0005
0.0010
0.0015
MR effect size for
'Kinesin−like protein KIF16B' on 'eGFR'

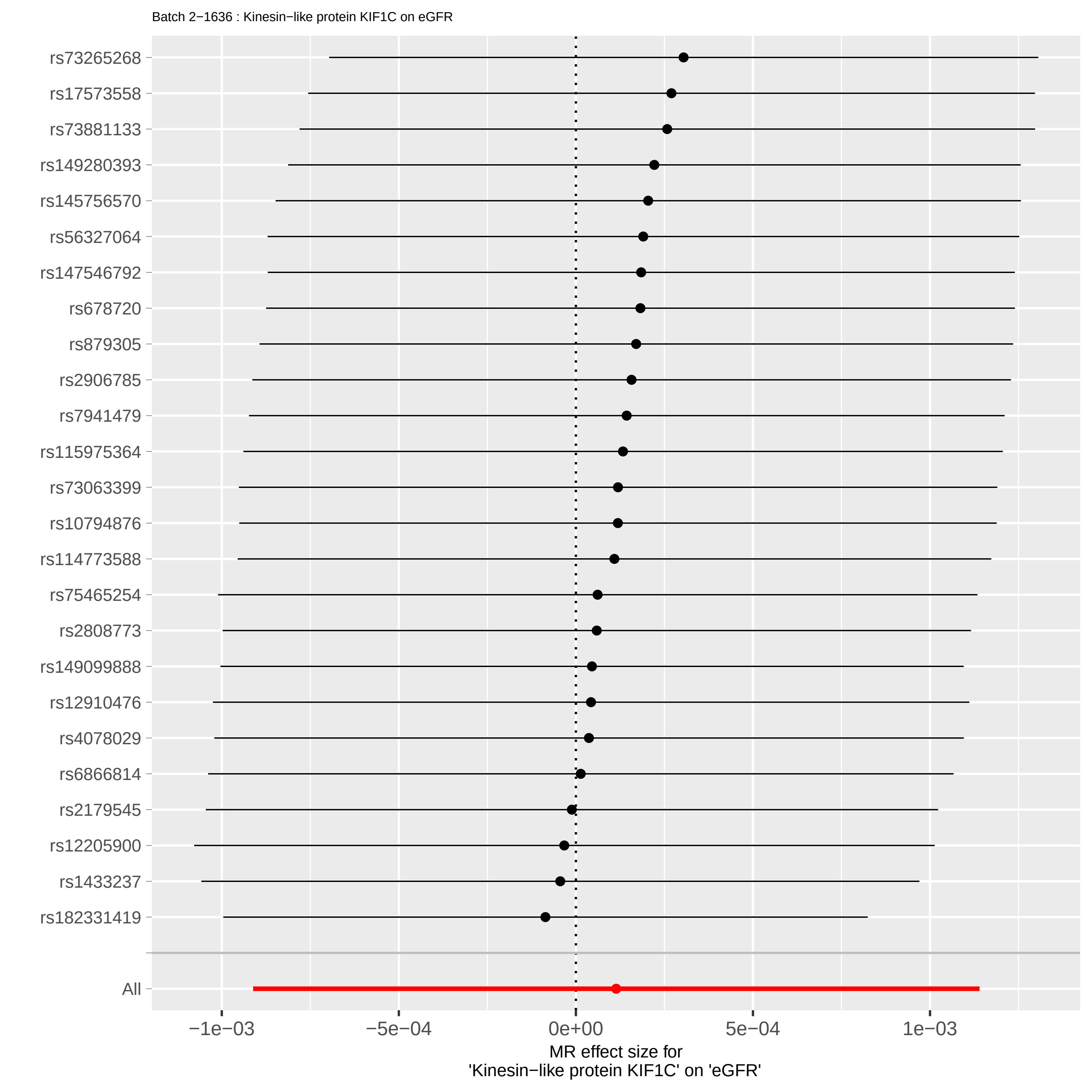
Batch 2−1636 : Kinesin−like protein KIF1C on eGFR
rs73265268
rs17573558
rs73881133
rs149280393
rs145756570
rs56327064
rs147546792
rs678720
rs879305
rs2906785
rs7941479
rs115975364
rs73063399
rs10794876
rs114773588
rs75465254
rs2808773
rs149099888
rs12910476
rs4078029
rs6866814
rs2179545
rs12205900
rs1433237
rs182331419
All
−1e−03
−5e−04
0e+00
5e−04
1e−03
MR effect size for
'Kinesin−like protein KIF1C' on 'eGFR'

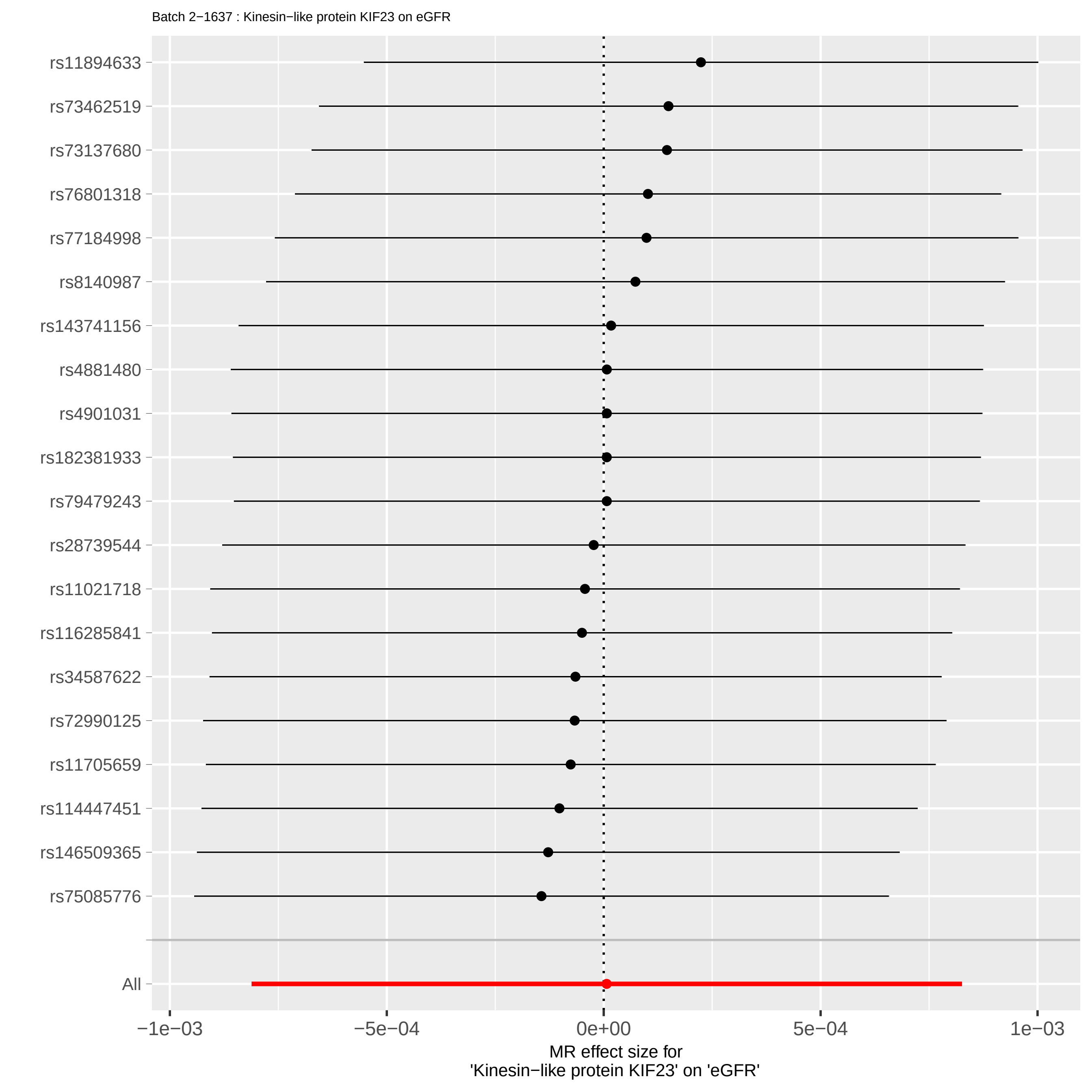

Batch 2−1637 : Kinesin−like protein KIF23 on eGFR
rs11894633
rs73462519
rs73137680
rs76801318
rs77184998
rs8140987
rs143741156
rs4881480
rs4901031
rs182381933
rs79479243
rs28739544
rs11021718
rs116285841
rs34587622
rs72990125
rs11705659
rs114447451
rs146509365
rs75085776
All
−1e−03
−5e−04
0e+00
5e−04
1e−03
MR effect size for
'Kinesin−like protein KIF23' on 'eGFR'

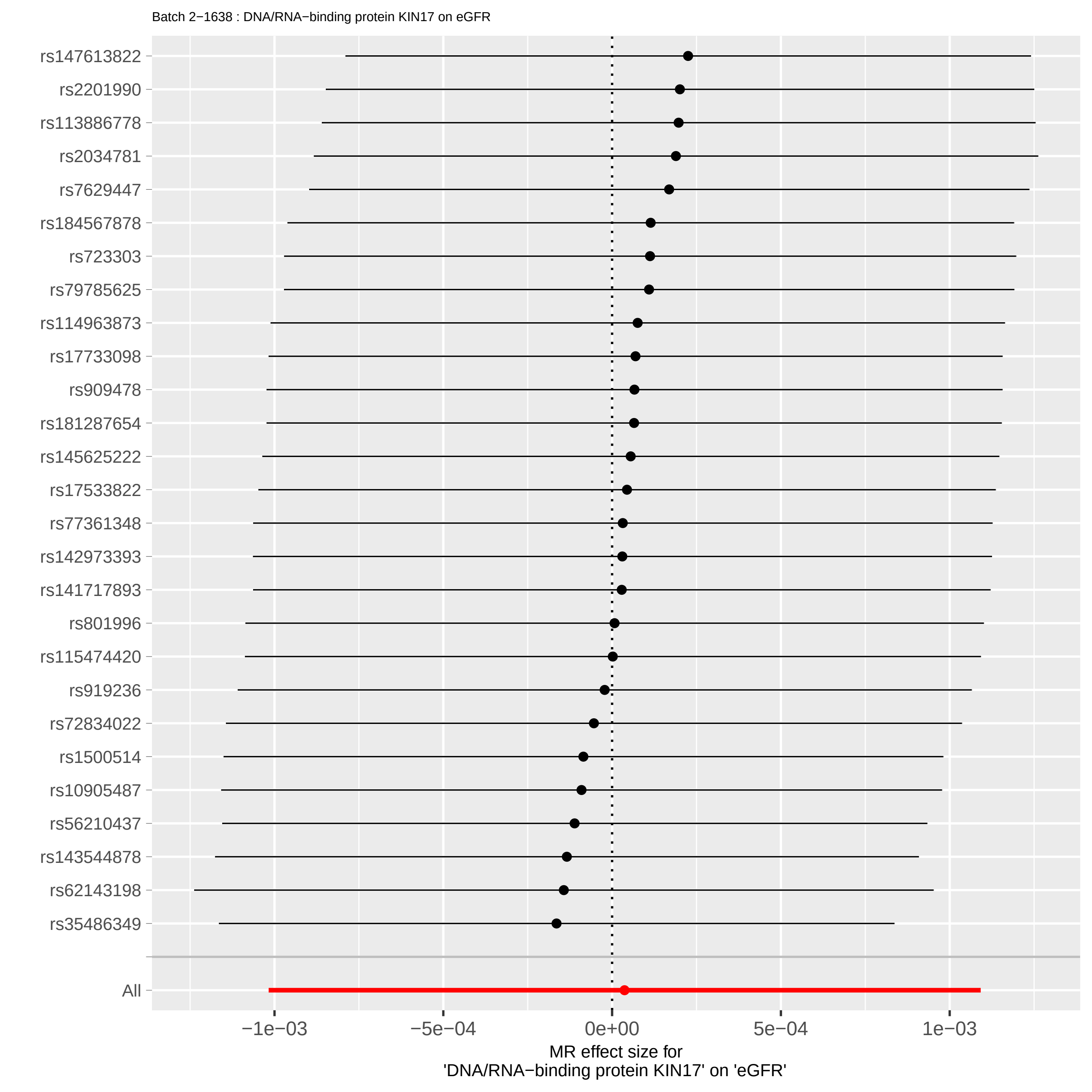
Batch 2−1638 : DNA/RNA−binding protein KIN17 on eGFR
rs147613822
rs2201990
rs113886778
rs2034781
rs7629447
rs184567878
rs723303
rs79785625
rs114963873
rs17733098
rs909478
rs181287654
rs145625222
rs17533822
rs77361348
rs142973393
rs141717893
rs801996
rs115474420
rs919236
rs72834022
rs1500514
rs10905487
rs56210437
rs143544878
rs62143198
rs35486349
All
−1e−03
−5e−04
0e+00
5e−04
1e−03
MR effect size for
'DNA/RNA−binding protein KIN17' on 'eGFR'

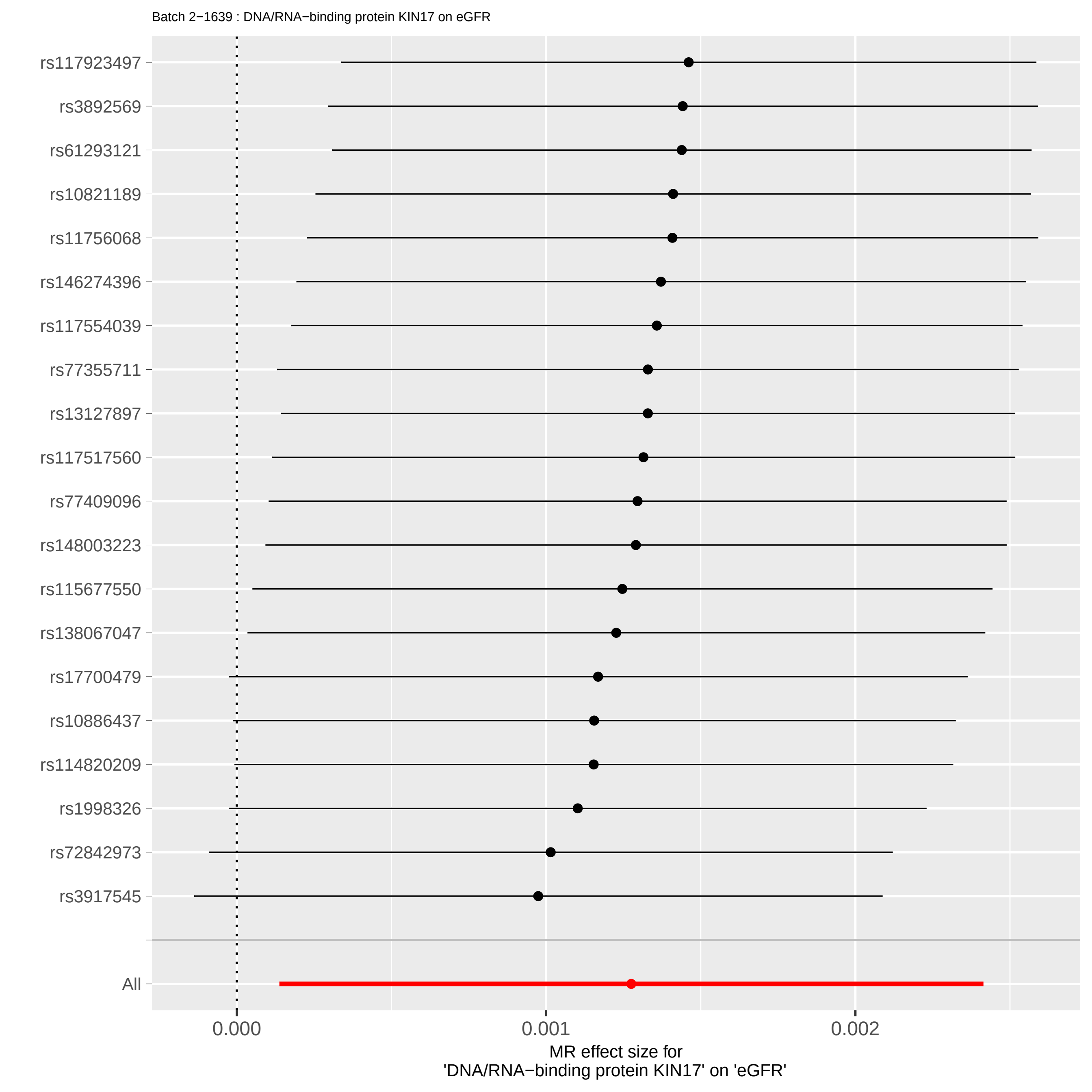

Batch 2−1639 : DNA/RNA−binding protein KIN17 on eGFR
rs117923497
rs3892569
rs61293121
rs10821189
rs11756068
rs146274396
rs117554039
rs77355711
rs13127897
rs117517560
rs77409096
rs148003223
rs115677550
rs138067047
rs17700479
rs10886437
rs114820209
rs1998326
rs72842973
rs3917545
All
0.000
0.001
0.002
MR effect size for
'DNA/RNA−binding protein KIN17' on 'eGFR'

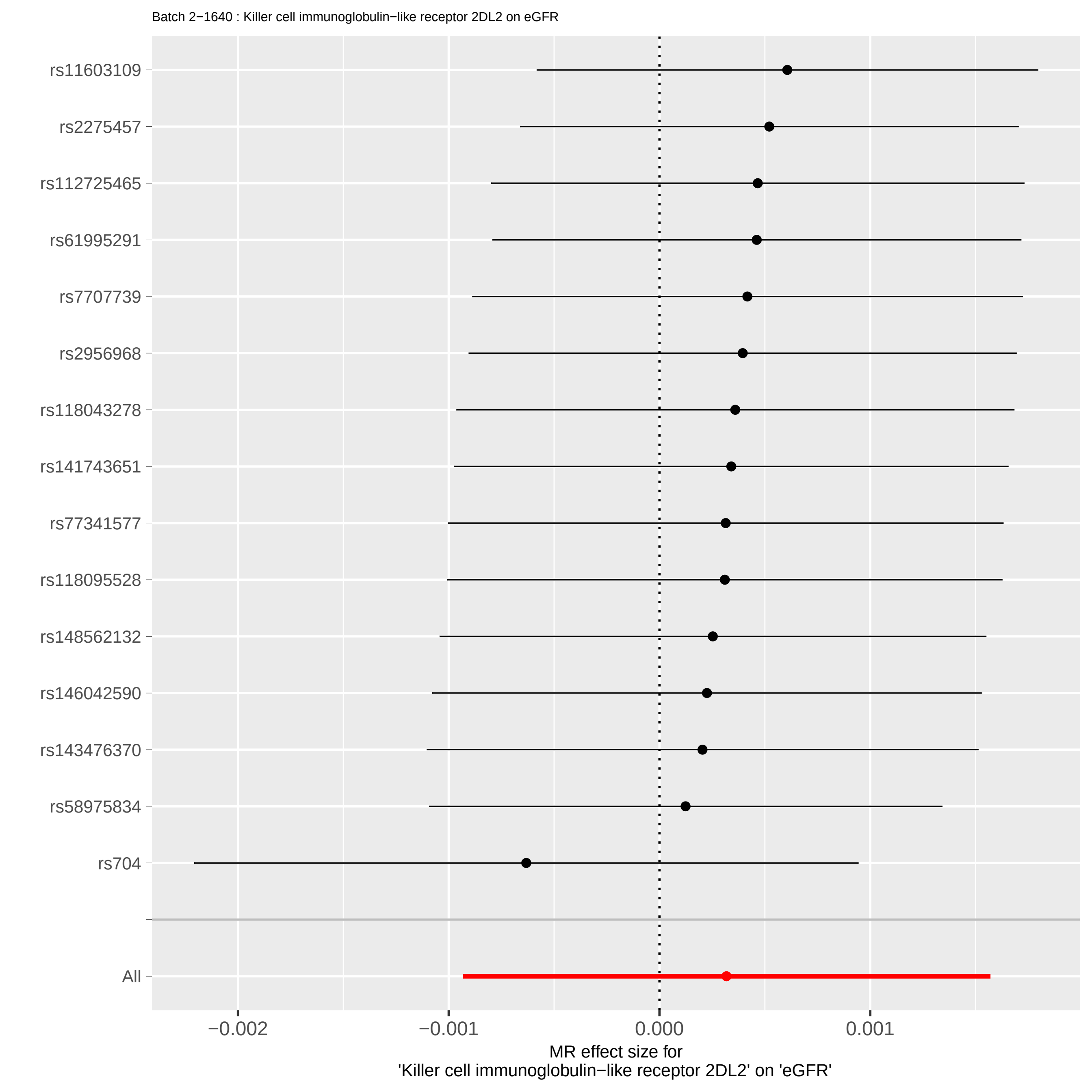

Batch 2−1640 : Killer cell immunoglobulin−like receptor 2DL2 on eGFR
rs11603109
rs2275457
rs112725465
rs61995291
rs7707739
rs2956968
rs118043278
rs141743651
rs77341577
rs118095528
rs148562132
rs146042590
rs143476370
rs58975834
rs704
All
−0.002
−0.001
0.000
0.001
MR effect size for
'Killer cell immunoglobulin−like receptor 2DL2' on 'eGFR'

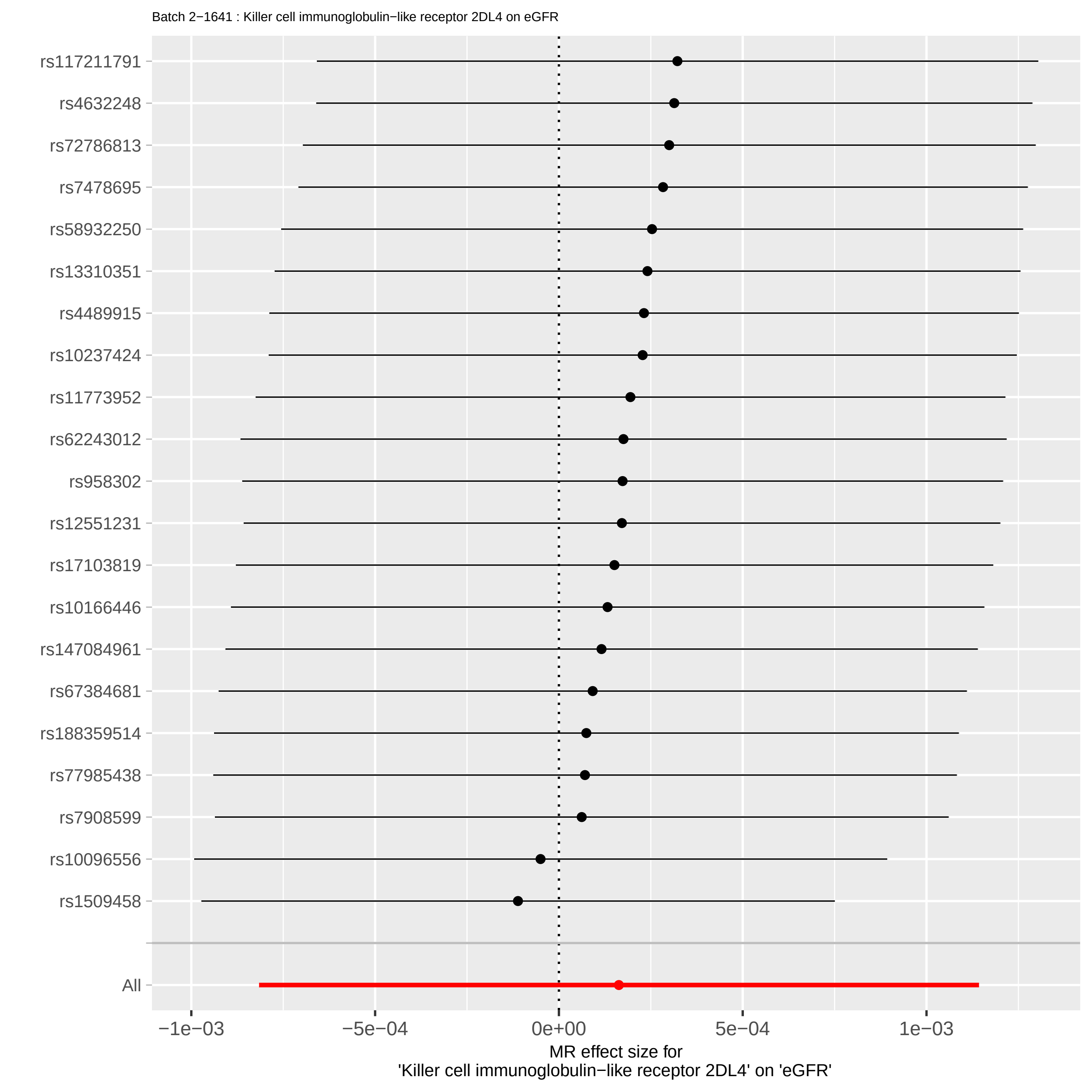
Batch 2−1641 : Killer cell immunoglobulin−like receptor 2DL4 on eGFR
rs117211791
rs4632248
rs72786813
rs7478695
rs58932250
rs13310351
rs4489915
rs10237424
rs11773952
rs62243012
rs958302
rs12551231
rs17103819
rs10166446
rs147084961
rs67384681
rs188359514
rs77985438
rs7908599
rs10096556
rs1509458
All
−1e−03
−5e−04
0e+00
5e−04
1e−03
MR effect size for
'Killer cell immunoglobulin−like receptor 2DL4' on 'eGFR'

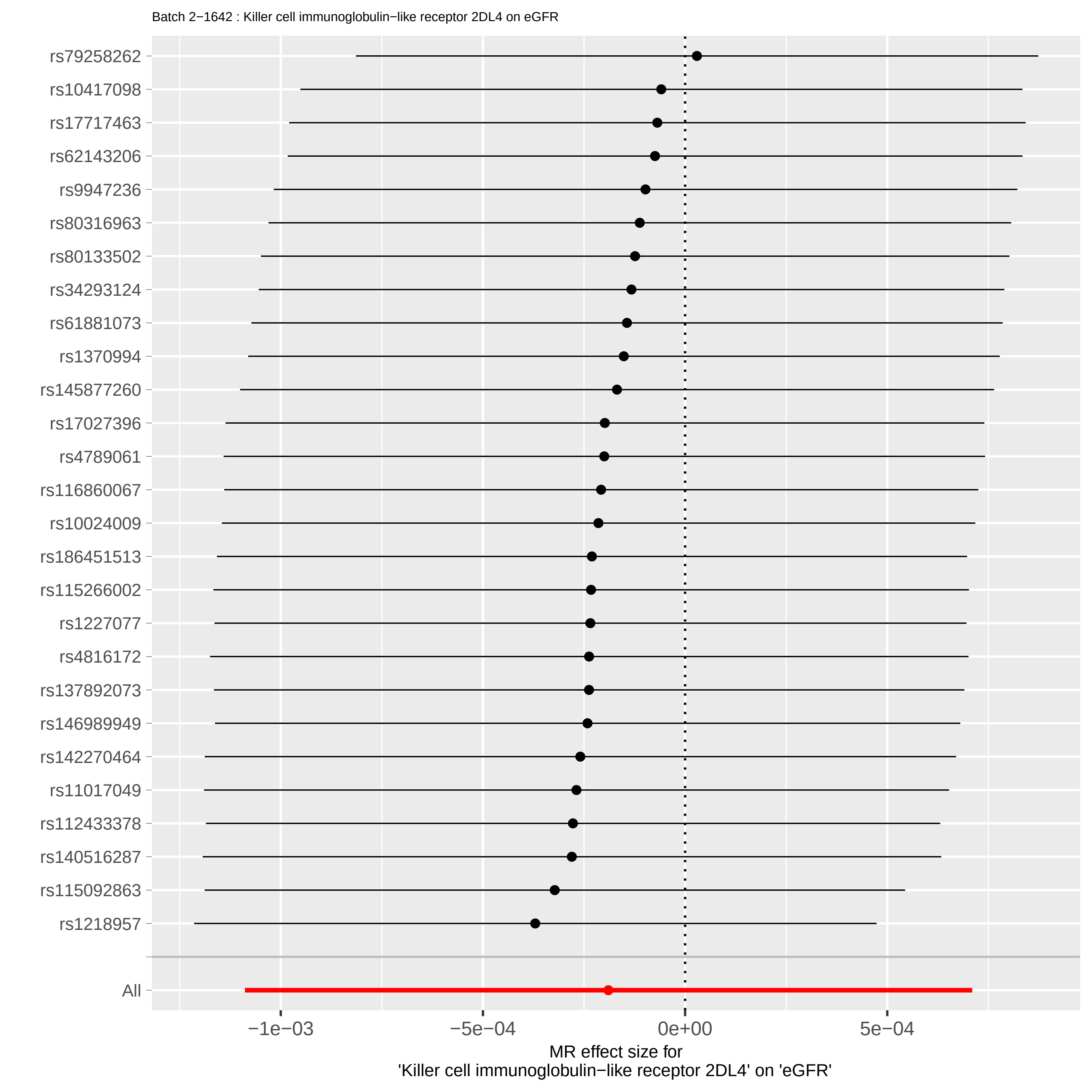

Batch 2−1642 : Killer cell immunoglobulin−like receptor 2DL4 on eGFR
rs79258262
rs10417098
rs17717463
rs62143206
rs9947236
rs80316963
rs80133502
rs34293124
rs61881073
rs1370994
rs145877260
rs17027396
rs4789061
rs116860067
rs10024009
rs186451513
rs115266002
rs1227077
rs4816172
rs137892073
rs146989949
rs142270464
rs11017049
rs112433378
rs140516287
rs115092863
rs1218957
All
−1e−03
−5e−04
0e+00
5e−04
MR effect size for
'Killer cell immunoglobulin−like receptor 2DL4' on 'eGFR'

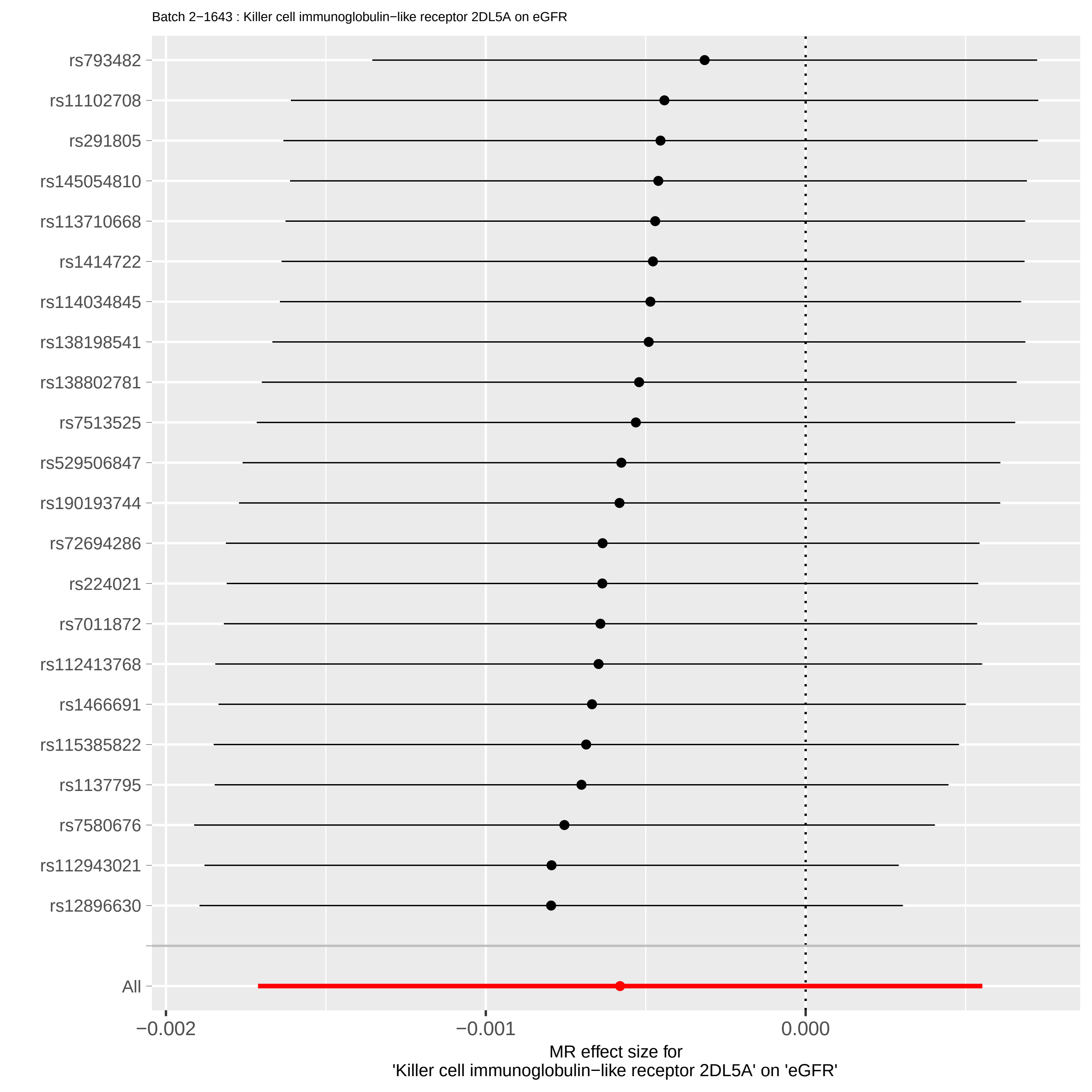
Batch 2−1643 : Killer cell immunoglobulin−like receptor 2DL5A on eGFR
rs793482
rs11102708
rs291805
rs145054810
rs113710668
rs1414722
rs114034845
rs138198541
rs138802781
rs7513525
rs529506847
rs190193744
rs72694286
rs224021
rs7011872
rs112413768
rs1466691
rs115385822
rs1137795
rs7580676
rs112943021
rs12896630
All
−0.002
−0.001
0.000
MR effect size for
'Killer cell immunoglobulin−like receptor 2DL5A' on 'eGFR'

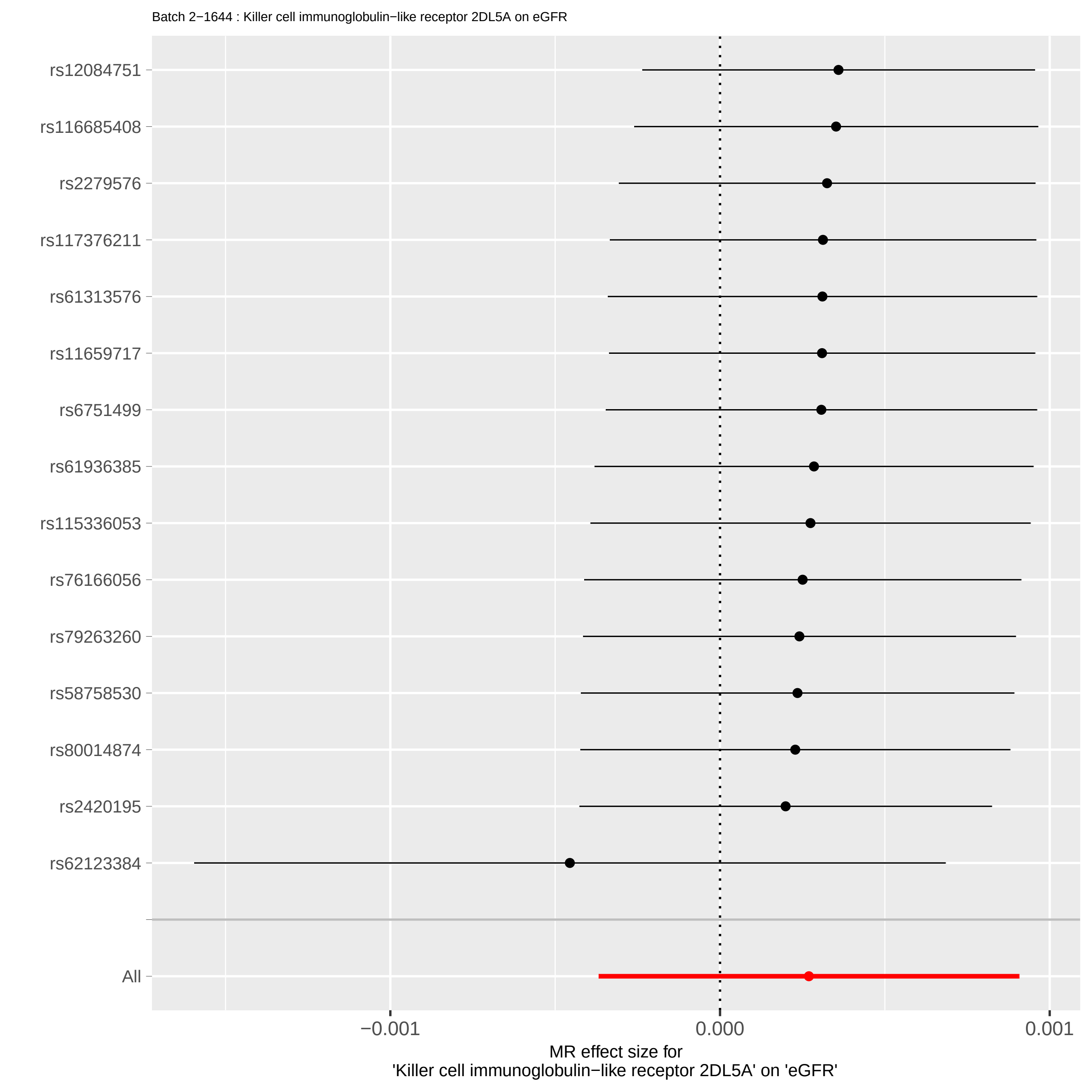

Batch 2−1644 : Killer cell immunoglobulin−like receptor 2DL5A on eGFR
rs12084751
rs116685408
rs2279576
rs117376211
rs61313576
rs11659717
rs6751499
rs61936385
rs115336053
rs76166056
rs79263260
rs58758530
rs80014874
rs2420195
rs62123384
All
−0.001
0.000
0.001
MR effect size for
'Killer cell immunoglobulin−like receptor 2DL5A' on 'eGFR'

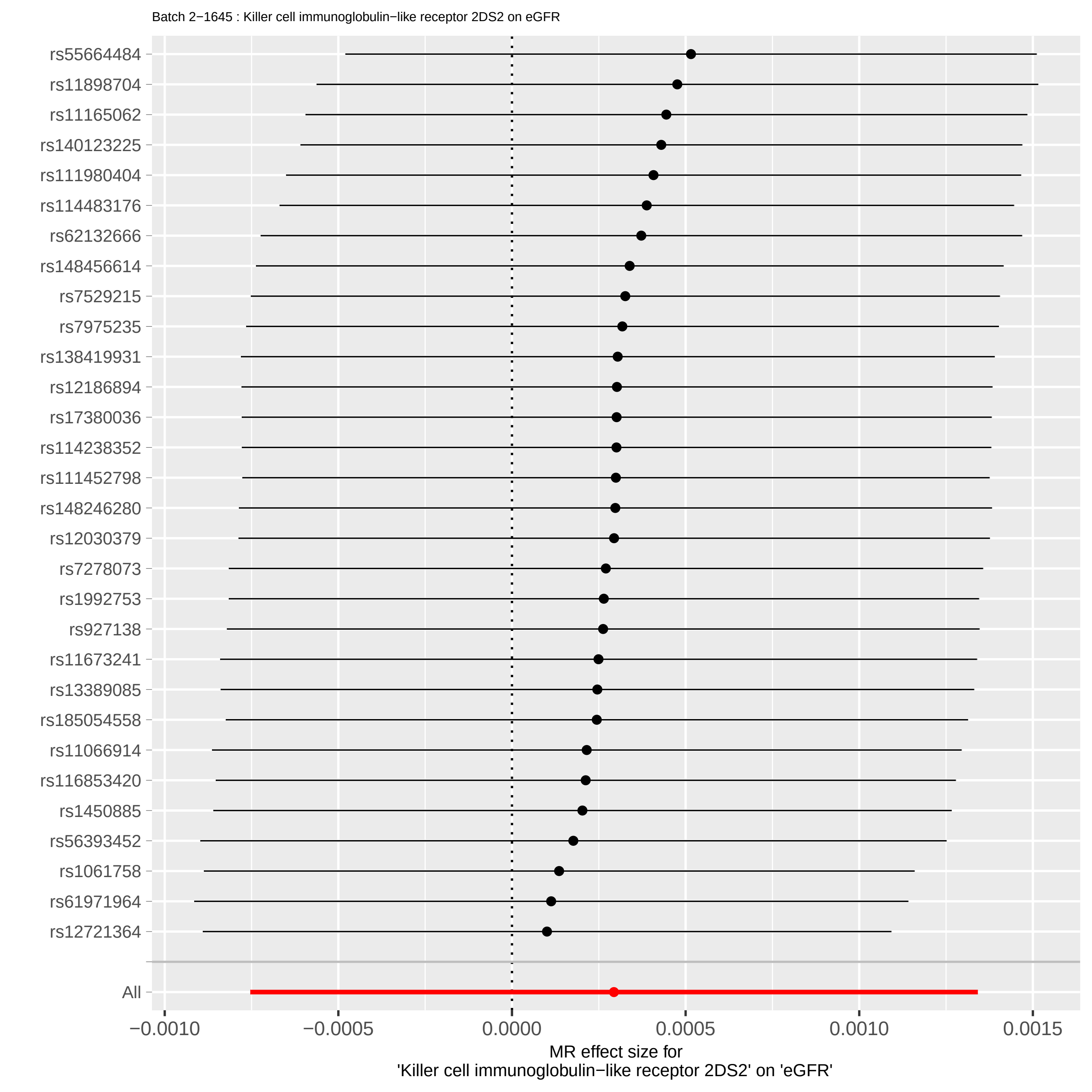

Batch 2−1645 : Killer cell immunoglobulin−like receptor 2DS2 on eGFR
rs55664484
rs11898704
rs11165062
rs140123225
rs111980404
rs114483176
rs62132666
rs148456614
rs7529215
rs7975235
rs138419931
rs12186894
rs17380036
rs114238352
rs111452798
rs148246280
rs12030379
rs7278073
rs1992753
rs927138
rs11673241
rs13389085
rs185054558
rs11066914
rs116853420
rs1450885
rs56393452
rs1061758
rs61971964
rs12721364
All
−0.0010
−0.0005
0.0000
0.0005
0.0010
0.0015
MR effect size for
'Killer cell immunoglobulin−like receptor 2DS2' on 'eGFR'

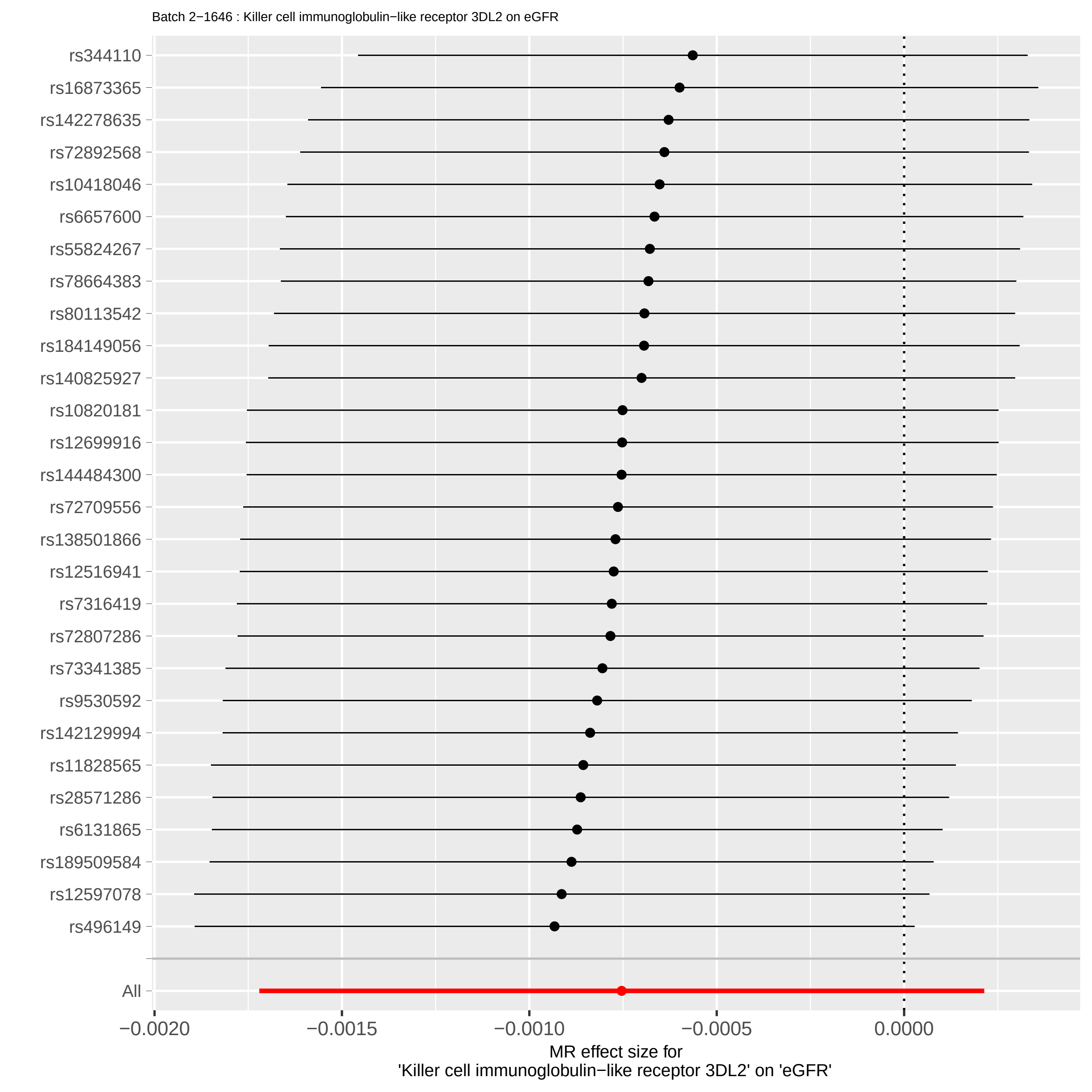

Batch 2−1646 : Killer cell immunoglobulin−like receptor 3DL2 on eGFR
rs344110
rs16873365
rs142278635
rs72892568
rs10418046
rs6657600
rs55824267
rs78664383
rs80113542
rs184149056
rs140825927
rs10820181
rs12699916
rs144484300
rs72709556
rs138501866
rs12516941
rs7316419
rs72807286
rs73341385
rs9530592
rs142129994
rs11828565
rs28571286
rs6131865
rs189509584
rs12597078
rs496149
All
−0.0020
−0.0015
−0.0010
−0.0005
0.0000
MR effect size for
'Killer cell immunoglobulin−like receptor 3DL2' on 'eGFR'

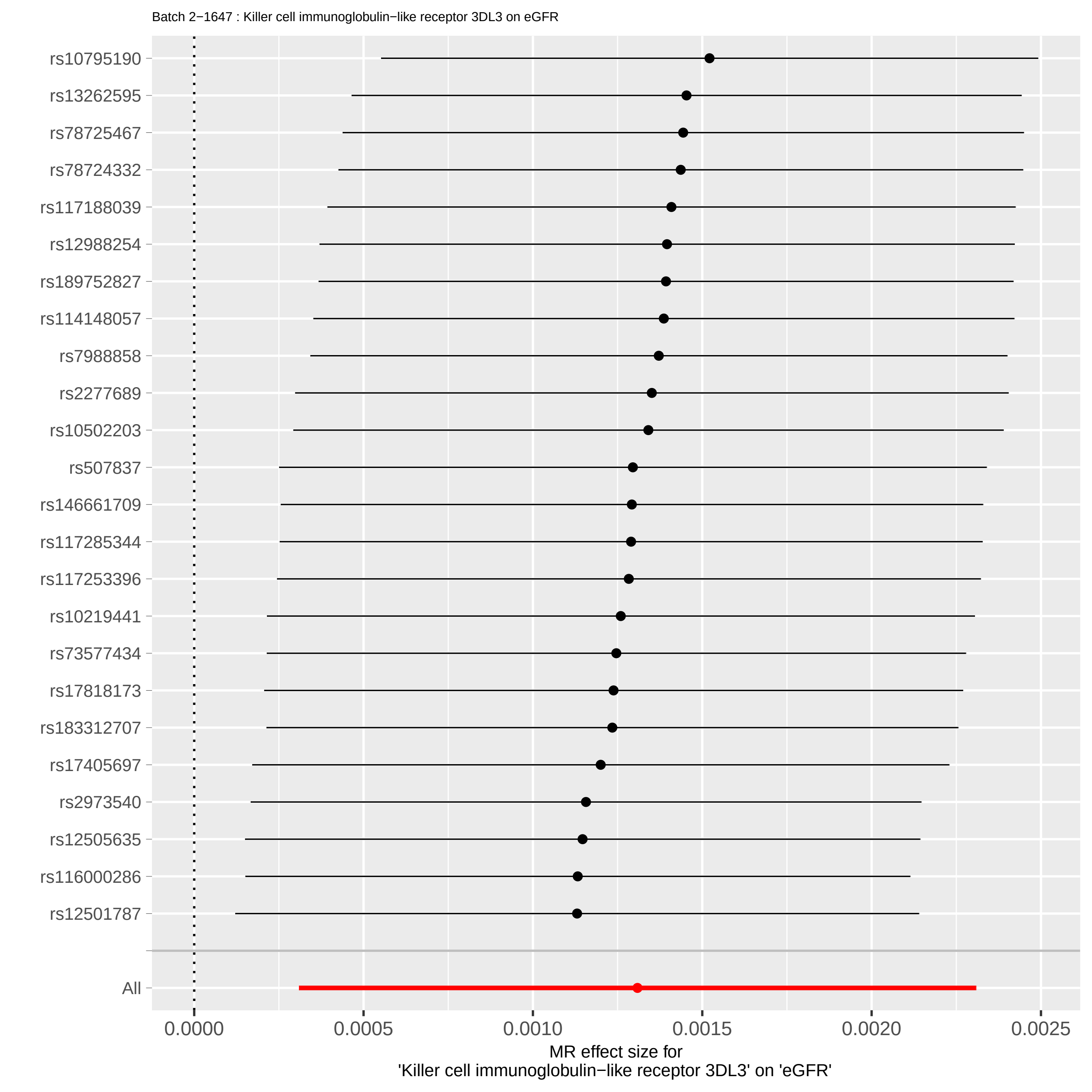

Batch 2−1647 : Killer cell immunoglobulin−like receptor 3DL3 on eGFR
rs10795190
rs13262595
rs78725467
rs78724332
rs117188039
rs12988254
rs189752827
rs114148057
rs7988858
rs2277689
rs10502203
rs507837
rs146661709
rs117285344
rs117253396
rs10219441
rs73577434
rs17818173
rs183312707
rs17405697
rs2973540
rs12505635
rs116000286
rs12501787
All
0.0000
0.0005
0.0010
0.0015
0.0020
0.0025
MR effect size for
'Killer cell immunoglobulin−like receptor 3DL3' on 'eGFR'

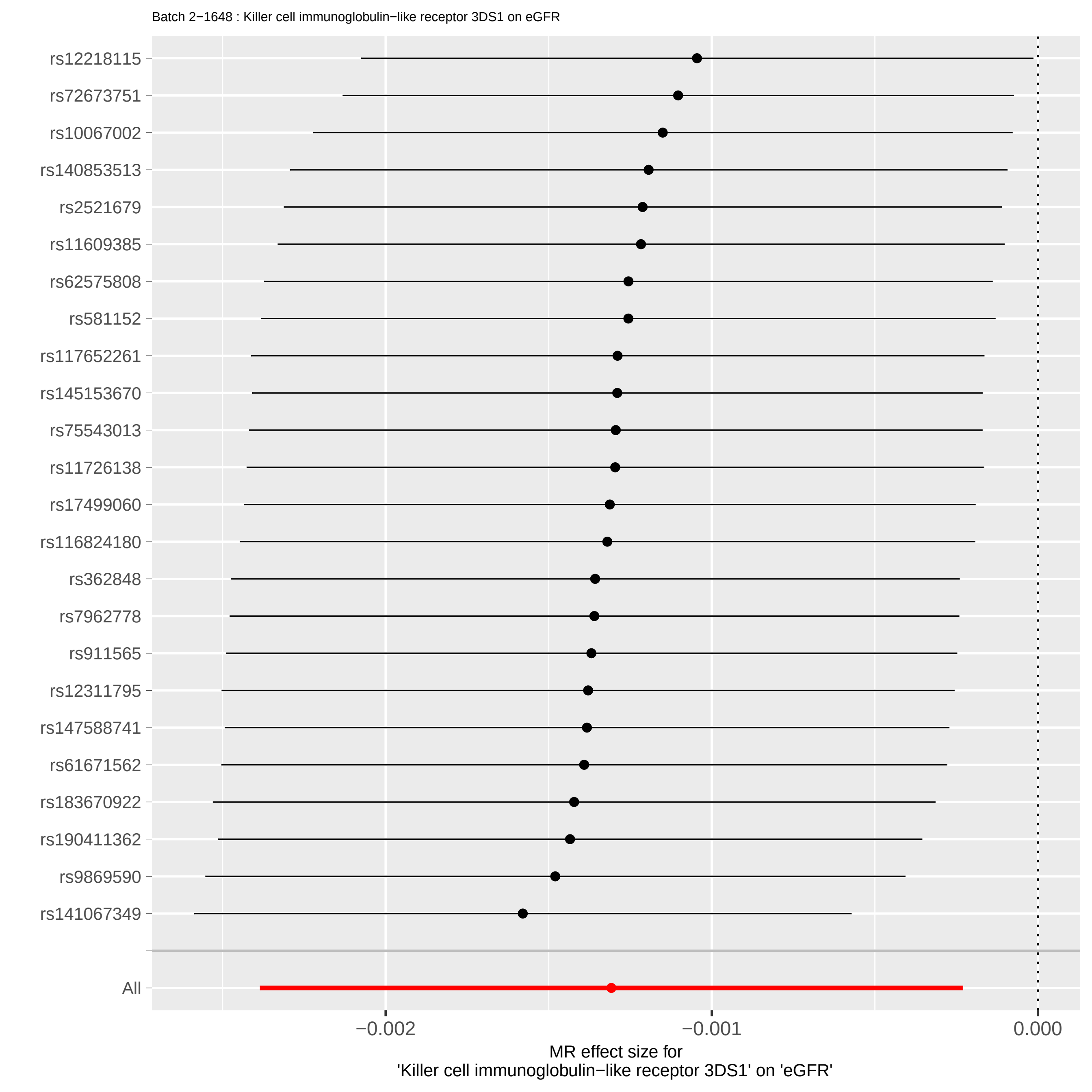
Batch 2−1648 : Killer cell immunoglobulin−like receptor 3DS1 on eGFR
rs12218115
rs72673751
rs10067002
rs140853513
rs2521679
rs11609385
rs62575808
rs581152
rs117652261
rs145153670
rs75543013
rs11726138
rs17499060
rs116824180
rs362848
rs7962778
rs911565
rs12311795
rs147588741
rs61671562
rs183670922
rs190411362
rs9869590
rs141067349
All
−0.002
−0.001
0.000
MR effect size for
'Killer cell immunoglobulin−like receptor 3DS1' on 'eGFR'

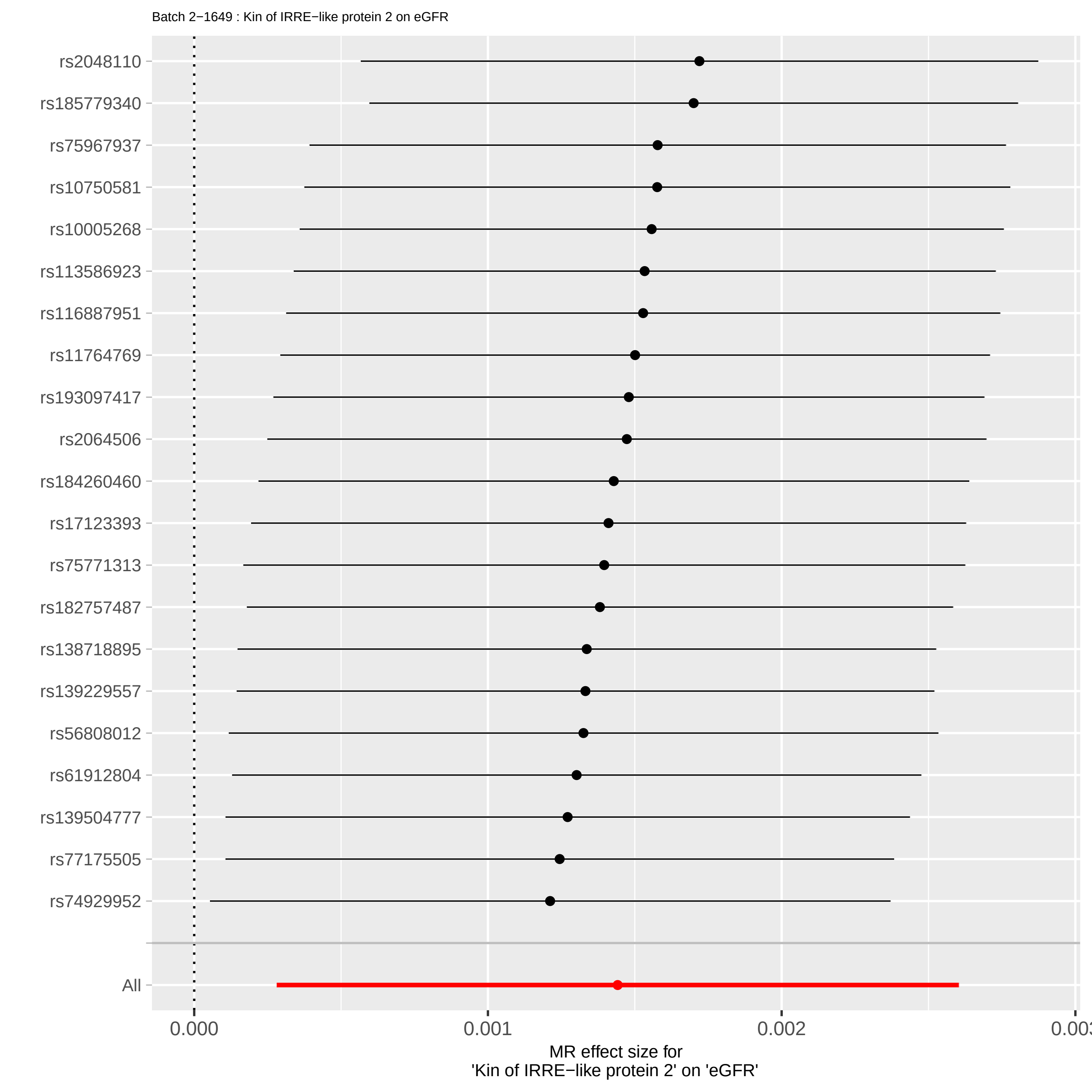

Batch 2−1649 : Kin of IRRE−like protein 2 on eGFR
rs2048110
rs185779340
rs75967937
rs10750581
rs10005268
rs113586923
rs116887951
rs11764769
rs193097417
rs2064506
rs184260460
rs17123393
rs75771313
rs182757487
rs138718895
rs139229557
rs56808012
rs61912804
rs139504777
rs77175505
rs74929952
All
0.000
0.001
0.002
MR effect size for
'Kin of IRRE−like protein 2' on 'eGFR'

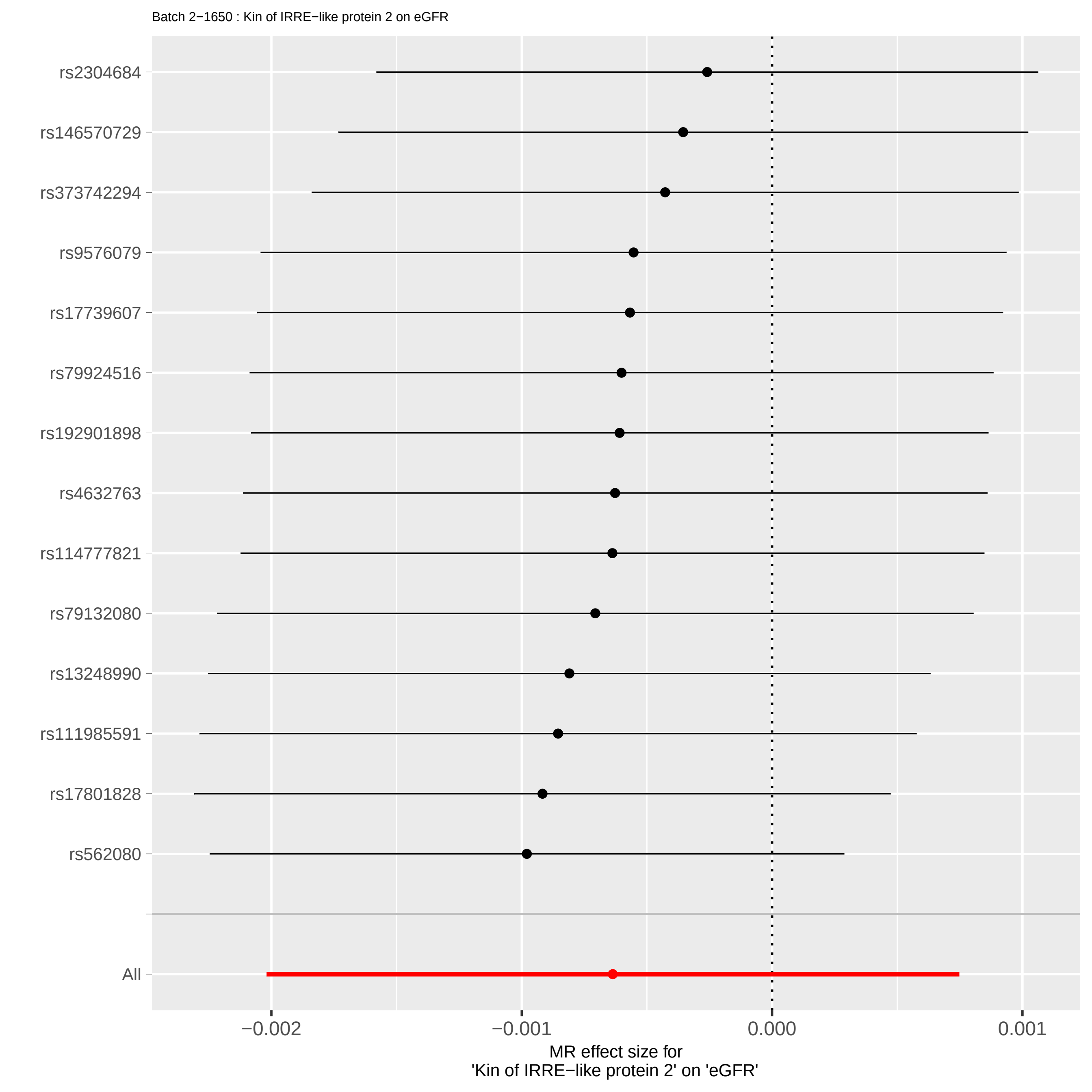
Batch 2−1650 : Kin of IRRE−like protein 2 on eGFR
rs2304684
rs146570729
rs373742294
rs9576079
rs17739607
rs79924516
rs192901898
rs4632763
rs114777821
rs79132080
rs13248990
rs111985591
rs17801828
rs562080
All
−0.002
−0.001
0.000
0.001
MR effect size for
'Kin of IRRE−like protein 2' on 'eGFR'

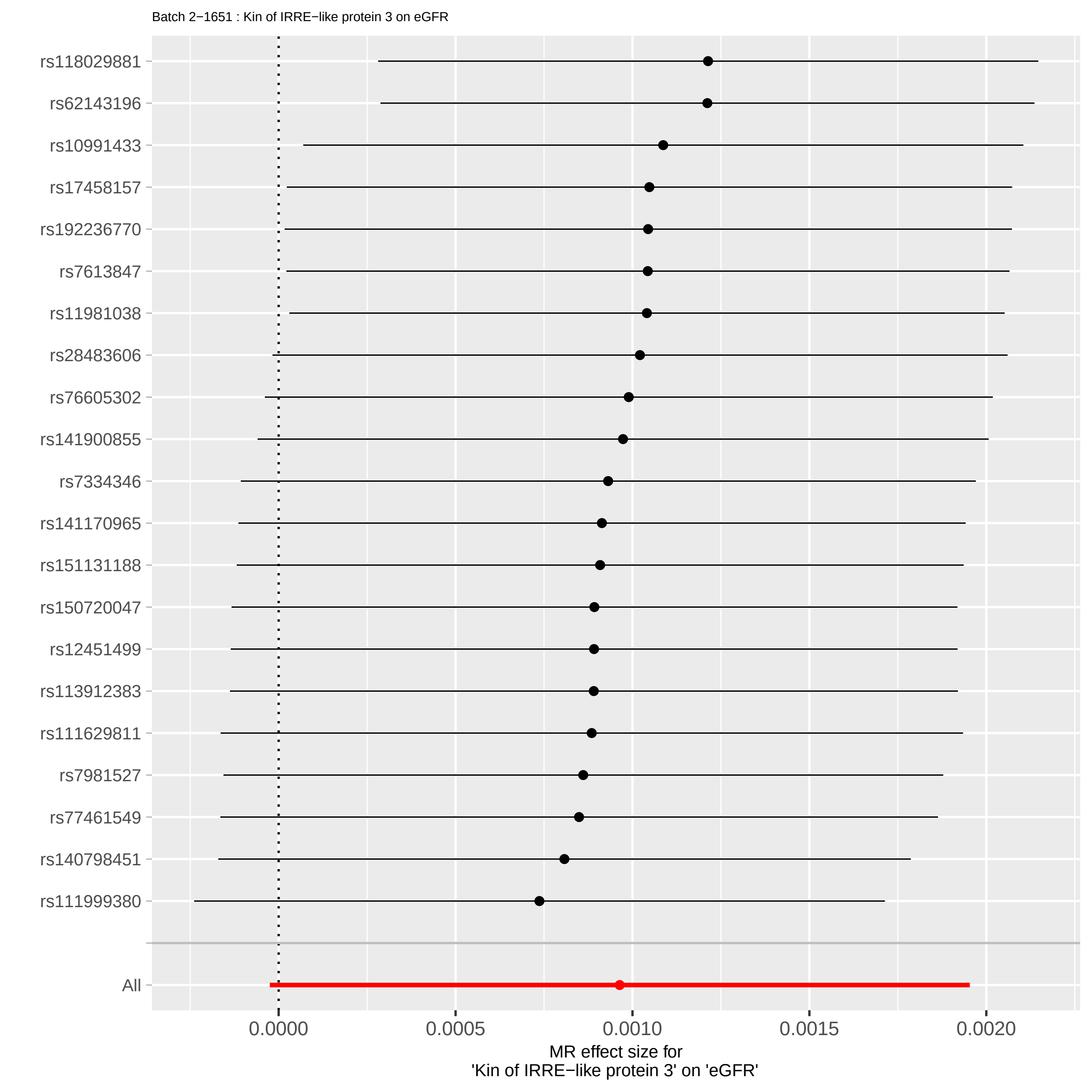
Batch 2−1651 : Kin of IRRE−like protein 3 on eGFR
rs118029881
rs62143196
rs10991433
rs17458157
rs192236770
rs7613847
rs11981038
rs28483606
rs76605302
rs141900855
rs7334346
rs141170965
rs151131188
rs150720047
rs12451499
rs113912383
rs111629811
rs7981527
rs77461549
rs140798451
rs111999380
All
0.0000
0.0005
0.0010
0.0015
0.0020
MR effect size for
'Kin of IRRE−like protein 3' on 'eGFR'

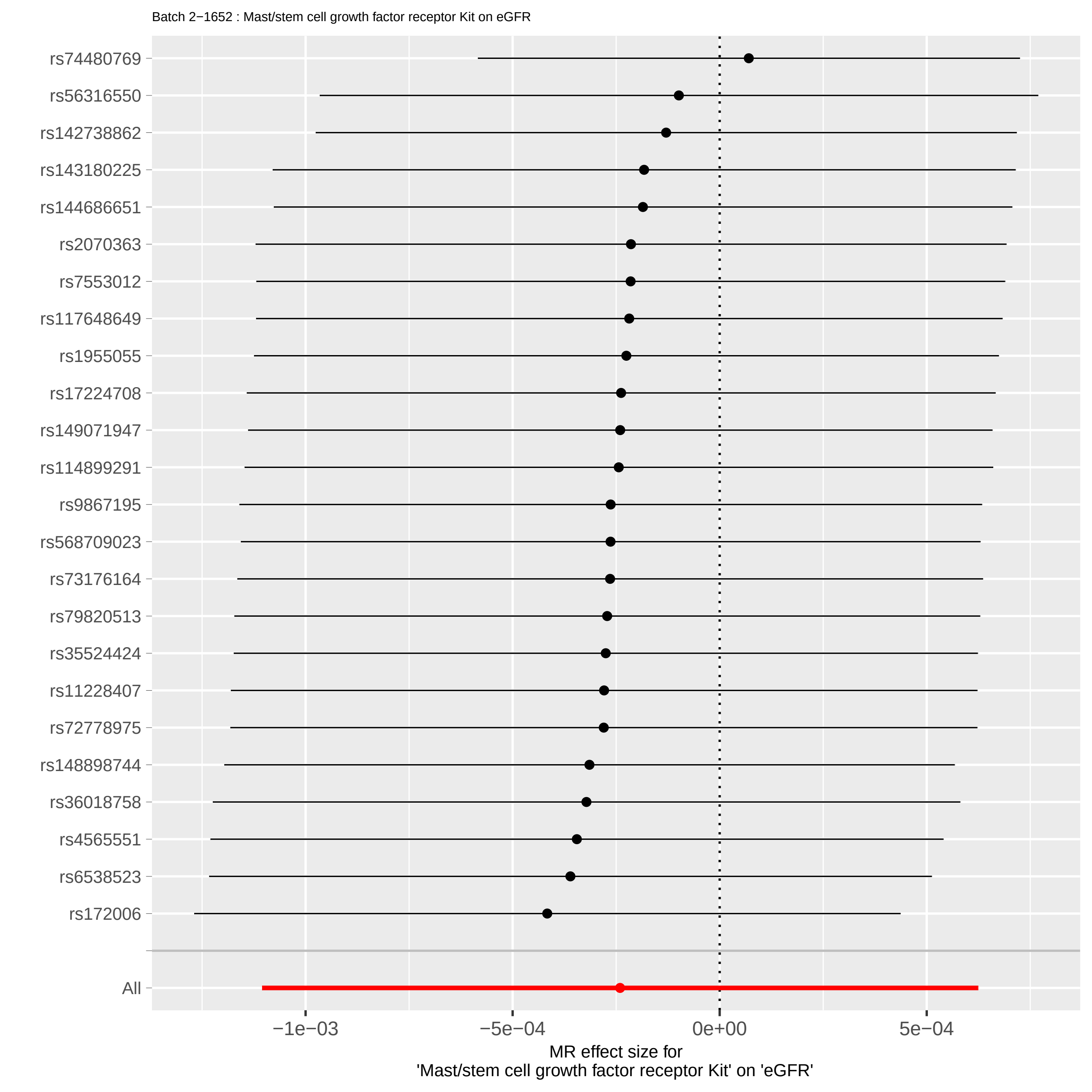

Batch 2−1652 : Mast/stem cell growth factor receptor Kit on eGFR
rs74480769
rs56316550
rs142738862
rs143180225
rs144686651
rs2070363
rs7553012
rs117648649
rs1955055
rs17224708
rs149071947
rs114899291
rs9867195
rs568709023
rs73176164
rs79820513
rs35524424
rs11228407
rs72778975
rs148898744
rs36018758
rs4565551
rs6538523
rs172006
All
−1e−03
−5e−04
0e+00
5e−04
MR effect size for
'Mast/stem cell growth factor receptor Kit' on 'eGFR'

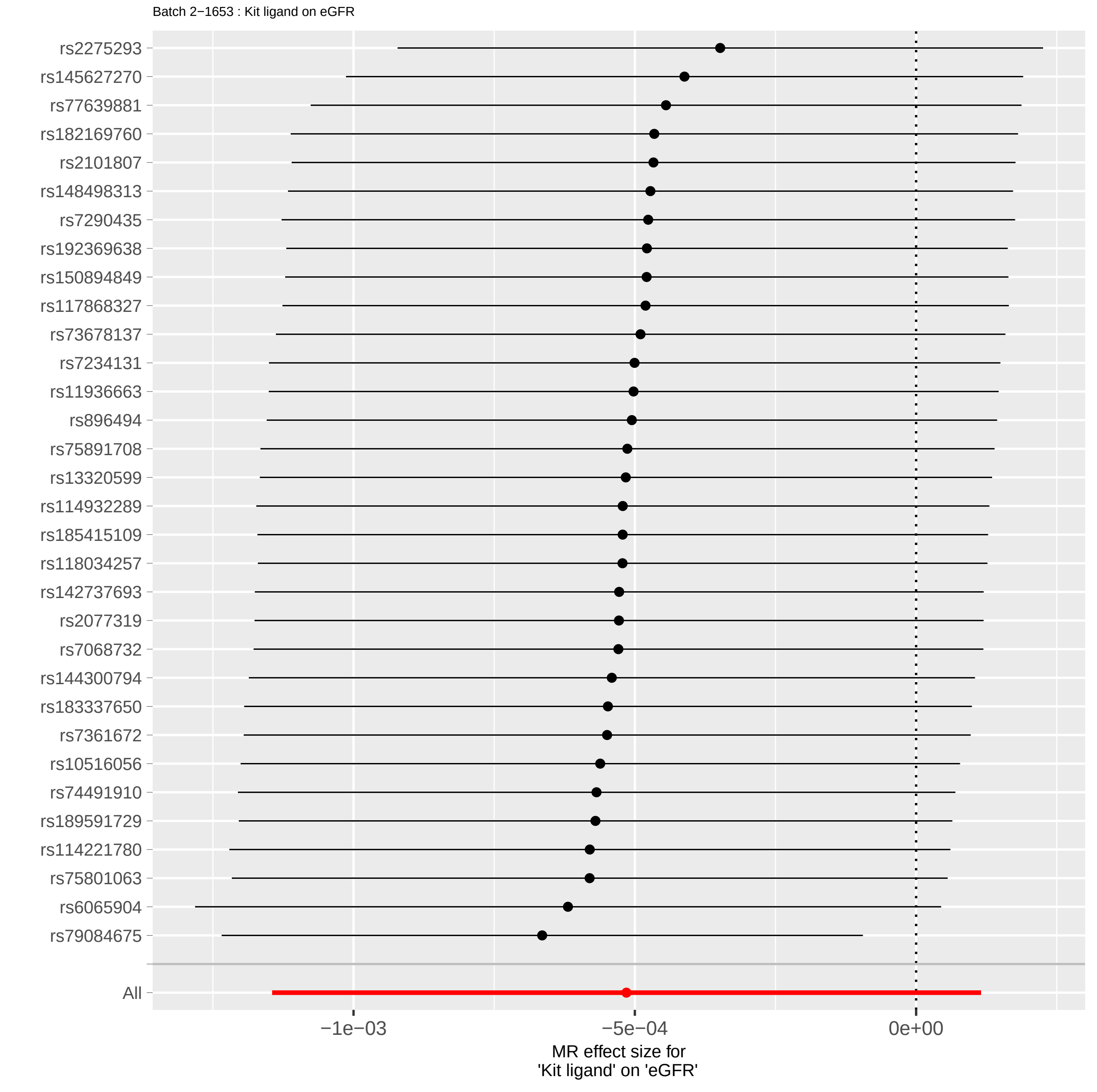

Batch 2−1653 : Kit ligand on eGFR
rs2275293
rs145627270
rs77639881
rs182169760
rs2101807
rs148498313
rs7290435
rs192369638
rs150894849
rs117868327
rs73678137
rs7234131
rs11936663
rs896494
rs75891708
rs13320599
rs114932289
rs185415109
rs118034257
rs142737693
rs2077319
rs7068732
rs144300794
rs183337650
rs7361672
rs10516056
rs74491910
rs189591729
rs114221780
rs75801063
rs6065904
rs79084675
All
−1e−03
−5e−04
0e+00
MR effect size for
'Kit ligand' on 'eGFR'

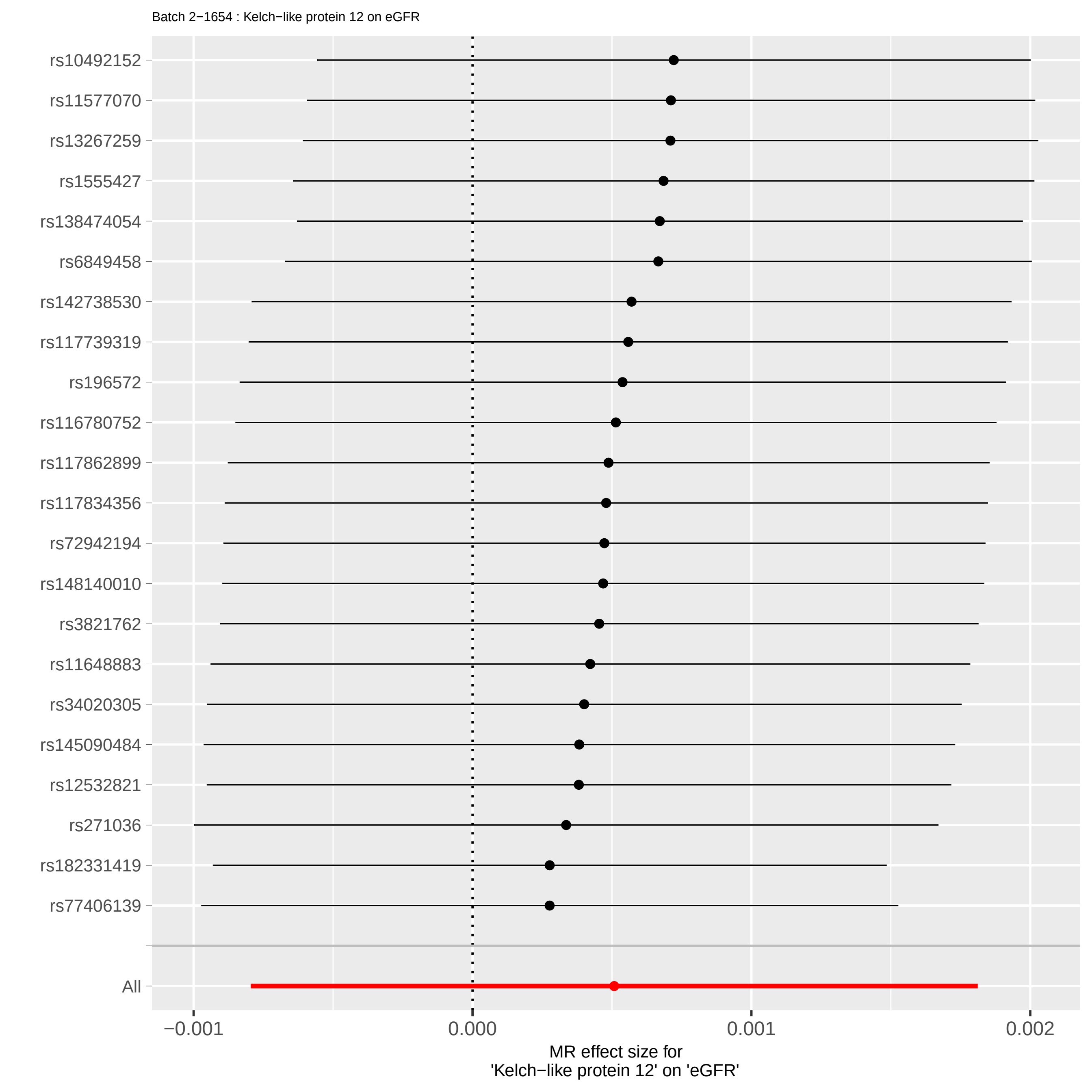
Batch 2−1654 : Kelch−like protein 12 on eGFR
rs10492152
rs11577070
rs13267259
rs1555427
rs138474054
rs6849458
rs142738530
rs117739319
rs196572
rs116780752
rs117862899
rs117834356
rs72942194
rs148140010
rs3821762
rs11648883
rs34020305
rs145090484
rs12532821
rs271036
rs182331419
rs77406139
All
−0.001
0.000
0.001
0.002
MR effect size for
'Kelch−like protein 12' on 'eGFR'

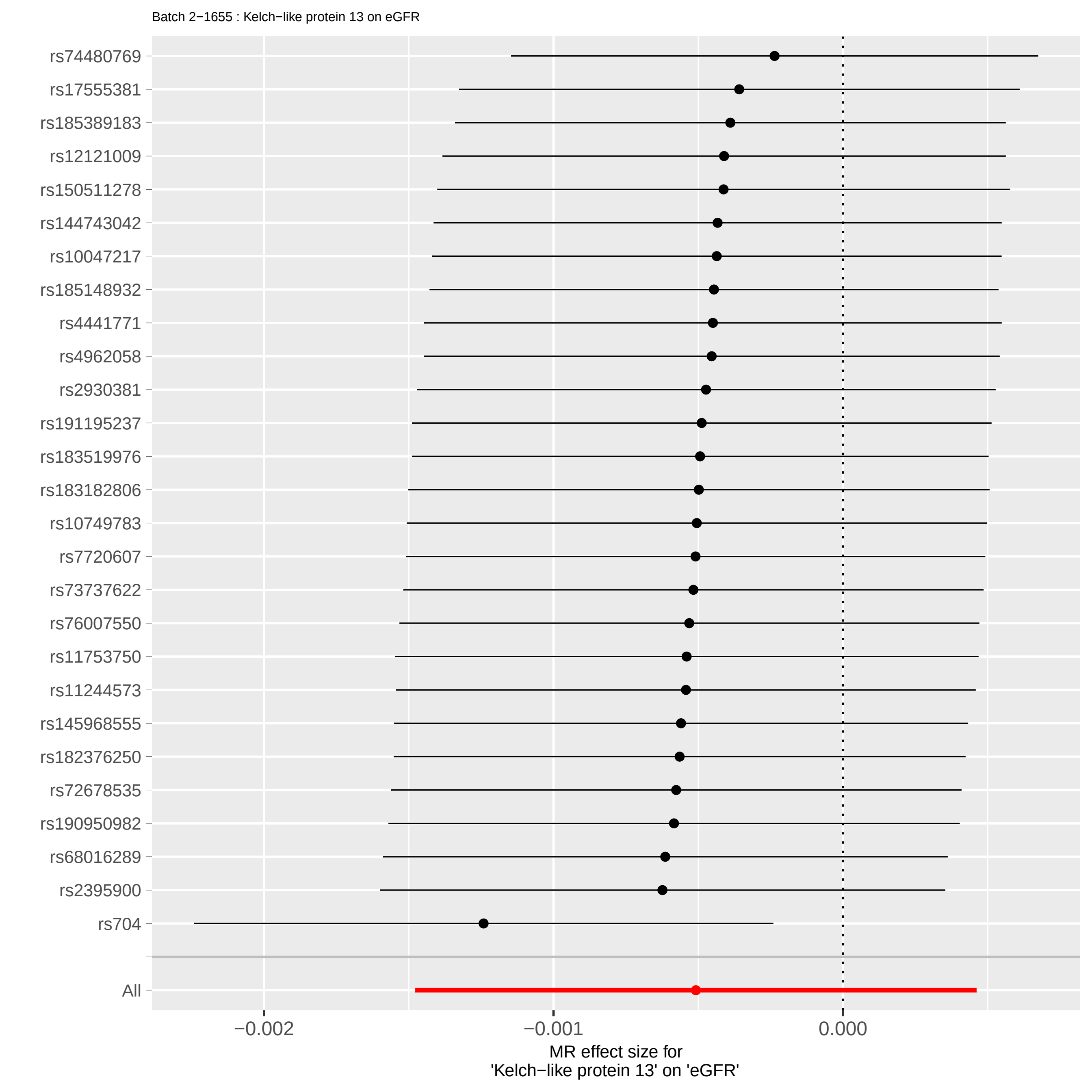

Batch 2−1655 : Kelch−like protein 13 on eGFR
rs74480769
rs17555381
rs185389183
rs12121009
rs150511278
rs144743042
rs10047217
rs185148932
rs4441771
rs4962058
rs2930381
rs191195237
rs183519976
rs183182806
rs10749783
rs7720607
rs73737622
rs76007550
rs11753750
rs11244573
rs145968555
rs182376250
rs72678535
rs190950982
rs68016289
rs2395900
rs704
All
−0.002
−0.001
0.000
MR effect size for
'Kelch−like protein 13' on 'eGFR'

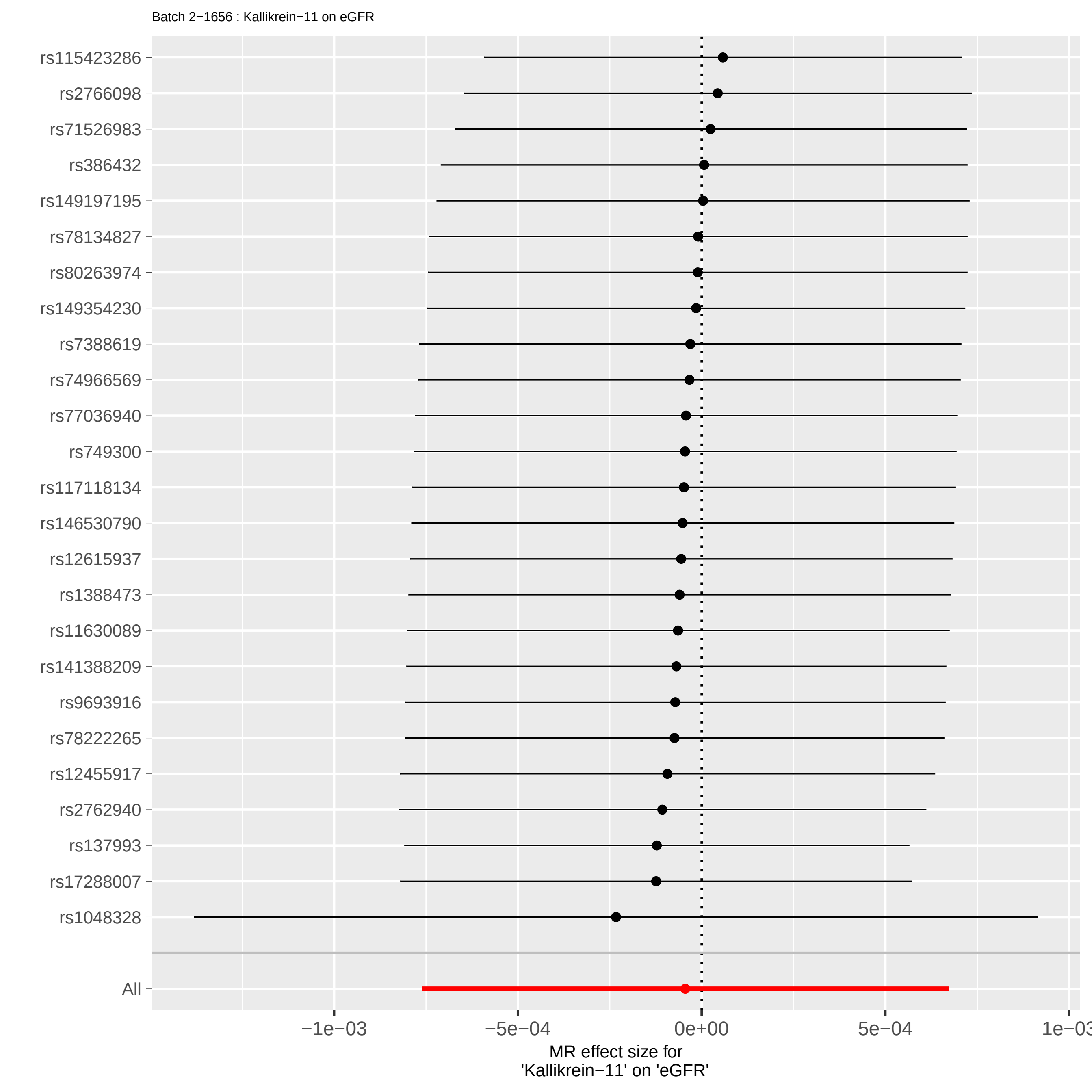

Batch 2−1656 : Kallikrein−11 on eGFR
rs115423286
rs2766098
rs71526983
rs386432
rs149197195
rs78134827
rs80263974
rs149354230
rs7388619
rs74966569
rs77036940
rs749300
rs117118134
rs146530790
rs12615937
rs1388473
rs11630089
rs141388209
rs9693916
rs78222265
rs12455917
rs2762940
rs137993
rs17288007
rs1048328
All
−1e−03
−5e−04
0e+00
5e−04
1e−03
MR effect size for
'Kallikrein−11' on 'eGFR'

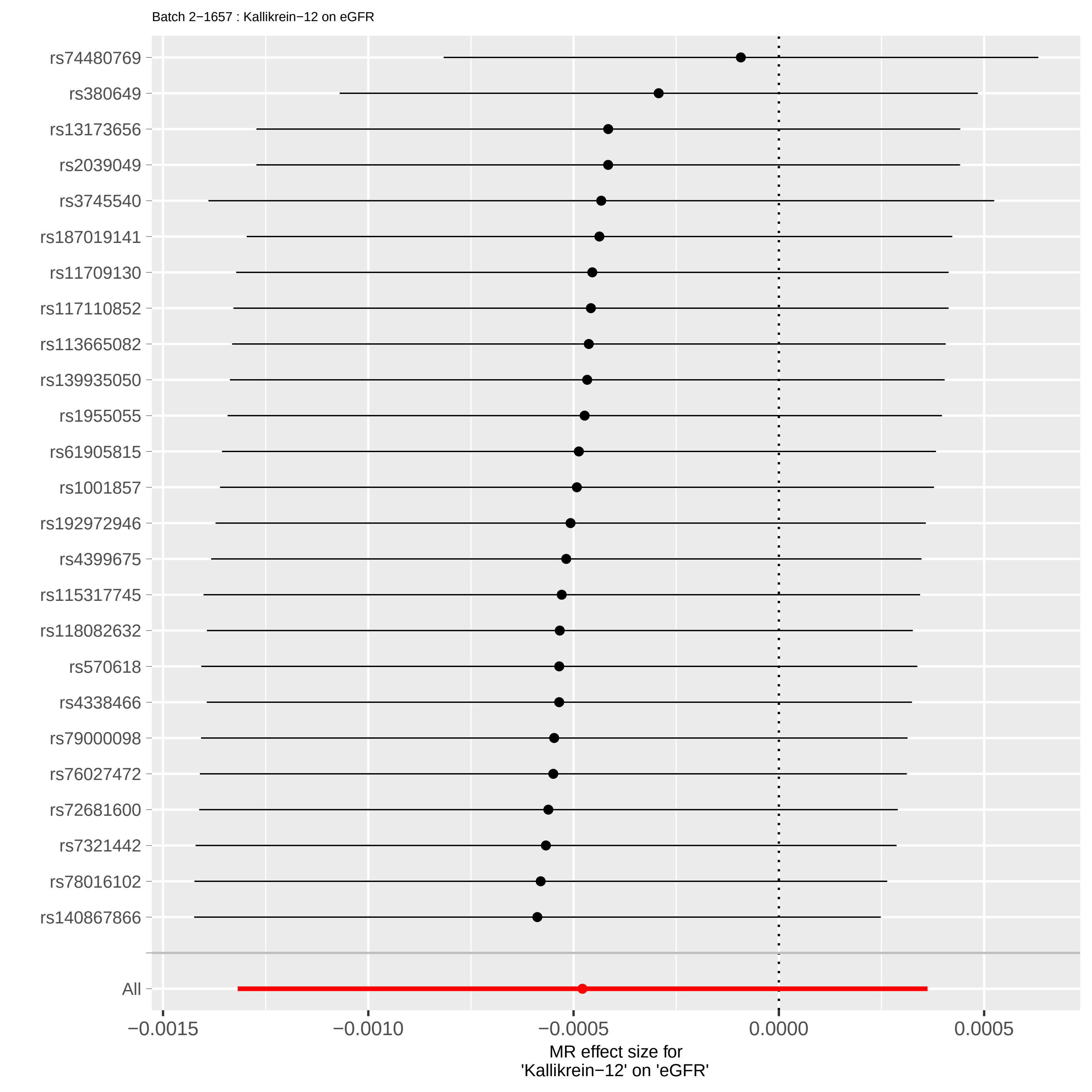

Batch 2−1657 : Kallikrein−12 on eGFR
rs74480769
rs380649
rs13173656
rs2039049
rs3745540
rs187019141
rs11709130
rs117110852
rs113665082
rs139935050
rs1955055
rs61905815
rs1001857
rs192972946
rs4399675
rs115317745
rs118082632
rs570618
rs4338466
rs79000098
rs76027472
rs72681600
rs7321442
rs78016102
rs140867866
All
−0.0015
−0.0010
−0.0005
0.0000
0.0005
MR effect size for
'Kallikrein−12' on 'eGFR'

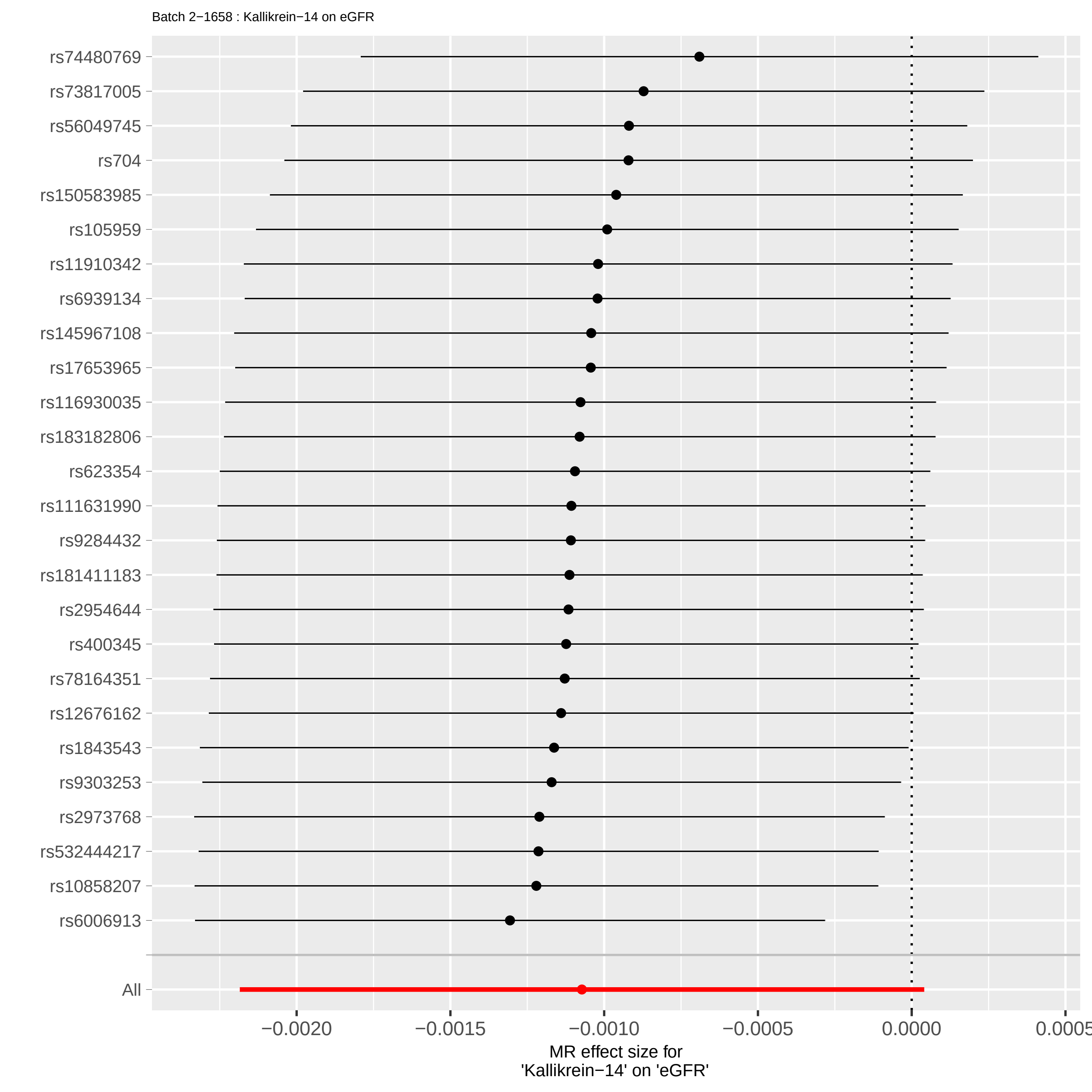

Batch 2−1658 : Kallikrein−14 on eGFR
rs74480769
rs73817005
rs56049745
rs704
rs150583985
rs105959
rs11910342
rs6939134
rs145967108
rs17653965
rs116930035
rs183182806
rs623354
rs111631990
rs9284432
rs181411183
rs2954644
rs400345
rs78164351
rs12676162
rs1843543
rs9303253
rs2973768
rs532444217
rs10858207
rs6006913
All
−0.0020
−0.0015
−0.0010
−0.0005
0.0000
MR effect size for
'Kallikrein−14' on 'eGFR'

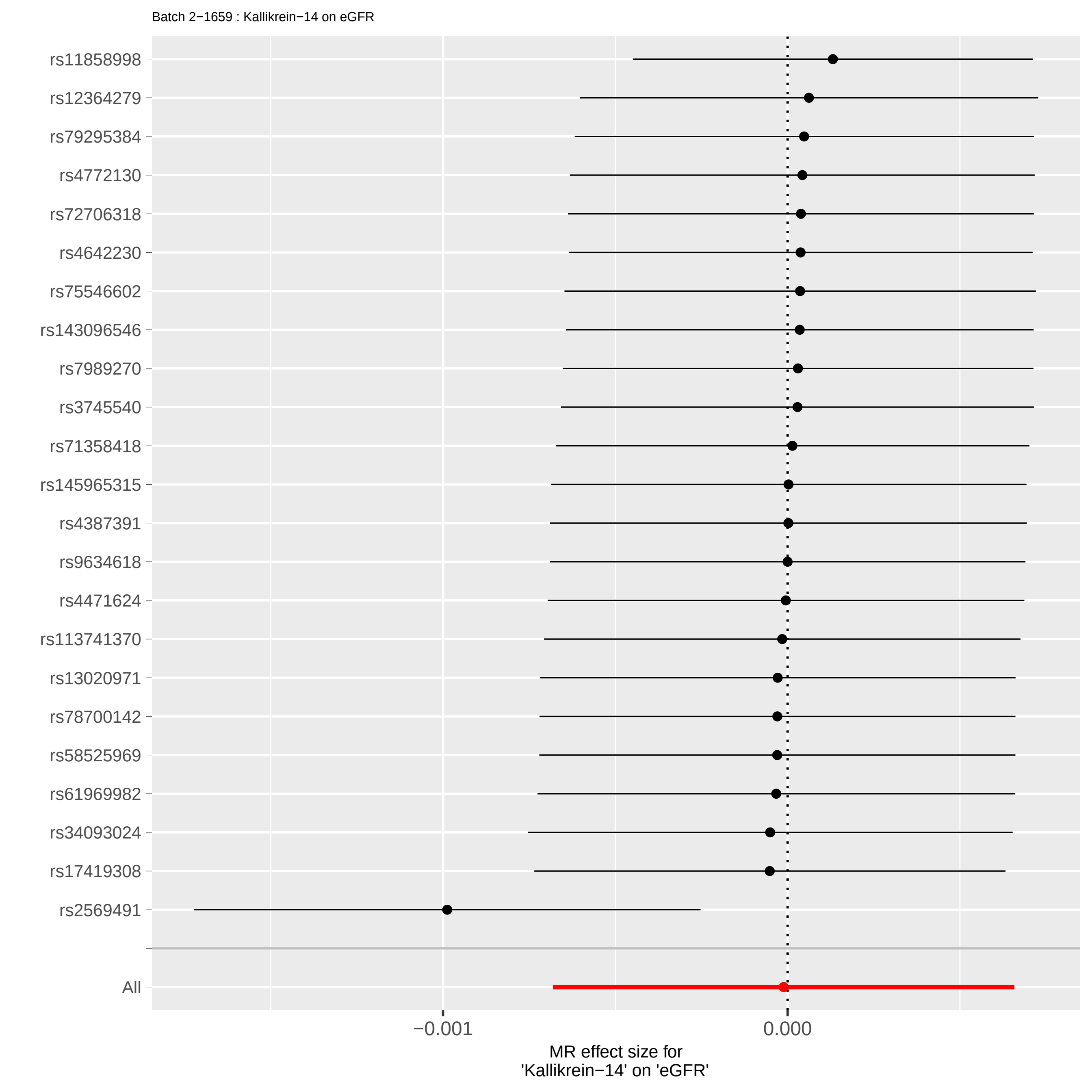

Batch 2−1659 : Kallikrein−14 on eGFR
rs11858998
rs12364279
rs79295384
rs4772130
rs72706318
rs4642230
rs75546602
rs143096546
rs7989270
rs3745540
rs71358418
rs145965315
rs4387391
rs9634618
rs4471624
rs113741370
rs13020971
rs78700142
rs58525969
rs61969982
rs34093024
rs17419308
rs2569491
All
−0.001
0.000
MR effect size for
'Kallikrein−14' on 'eGFR'

Batch 2−1660 : Kallikrein−15 on eGFR

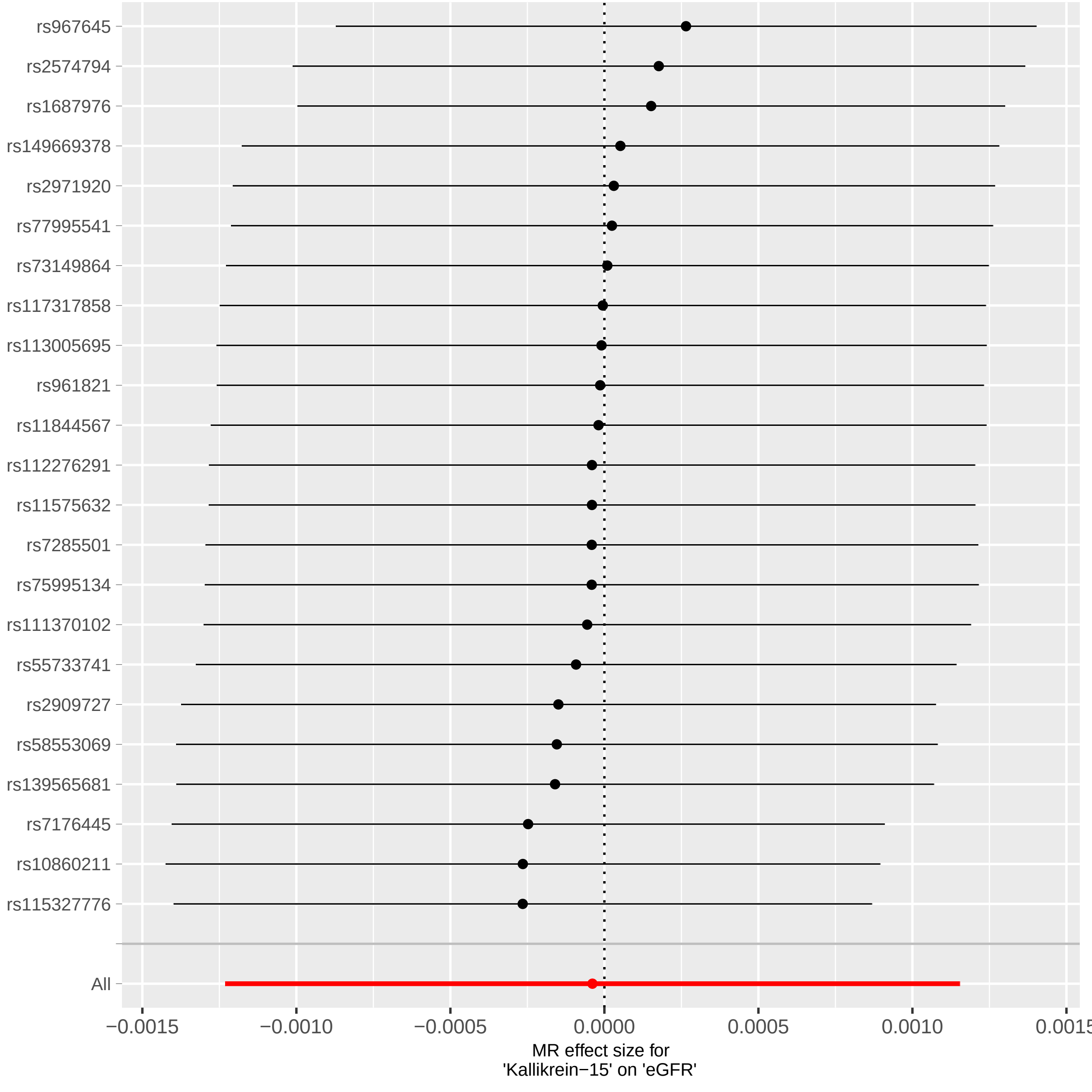

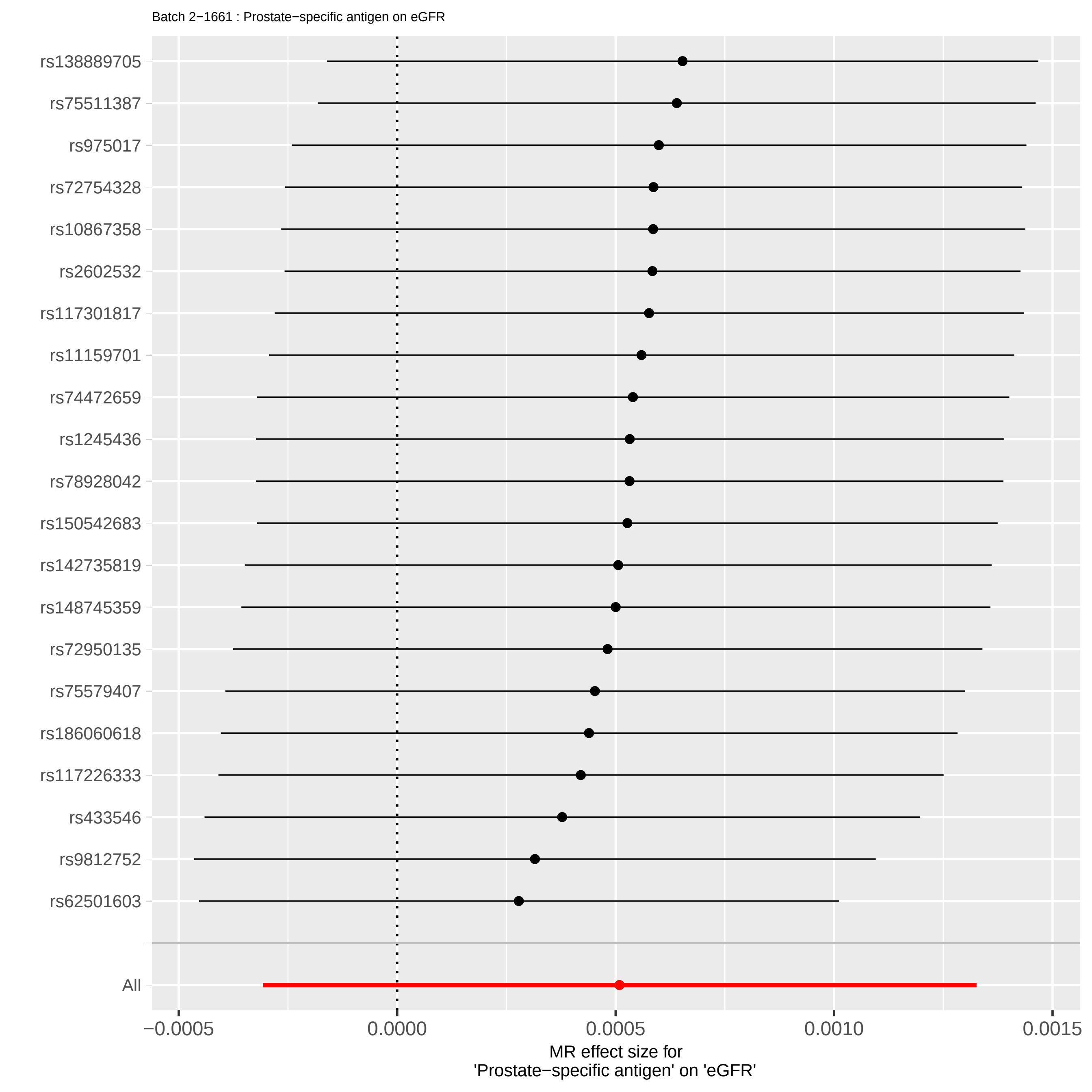
Batch 2−1661 : Prostate−specific antigen on eGFR
rs138889705
rs75511387
rs975017
rs72754328
rs10867358
rs2602532
rs117301817
rs11159701
rs74472659
rs1245436
rs78928042
rs150542683
rs142735819
rs148745359
rs72950135
rs75579407
rs186060618
rs117226333
rs433546
rs9812752
rs62501603
All
−0.0005
0.0000
0.0005
0.0010
0.0015
MR effect size for
'Prostate−specific antigen' on 'eGFR'

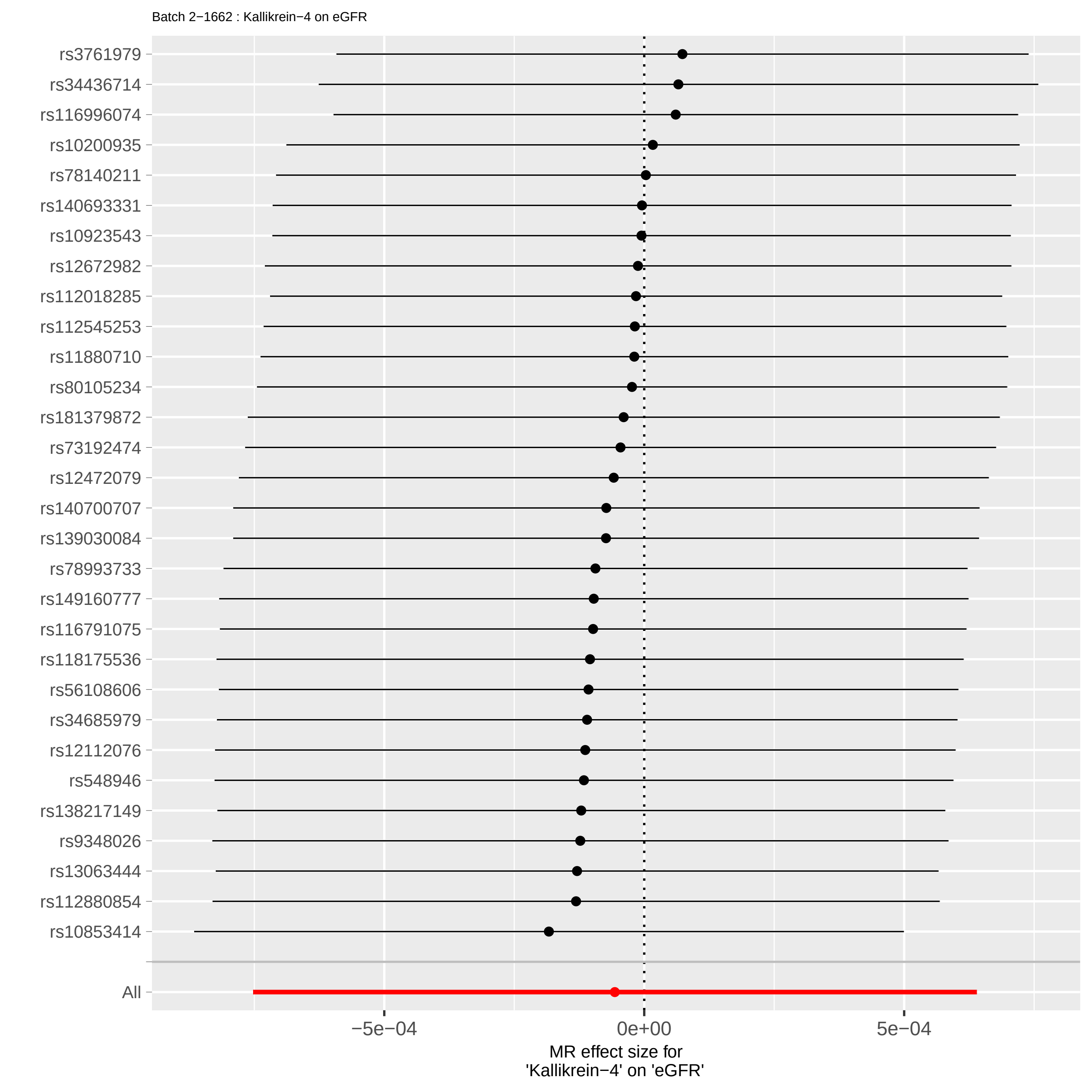

Batch 2−1662 : Kallikrein−4 on eGFR
rs3761979
rs34436714
rs116996074
rs10200935
rs78140211
rs140693331
rs10923543
rs12672982
rs112018285
rs112545253
rs11880710
rs80105234
rs181379872
rs73192474
rs12472079
rs140700707
rs139030084
rs78993733
rs149160777
rs116791075
rs118175536
rs56108606
rs34685979
rs12112076
rs548946
rs138217149
rs9348026
rs13063444
rs112880854
rs10853414
All
−5e−04
0e+00
5e−04
MR effect size for
'Kallikrein−4' on 'eGFR'

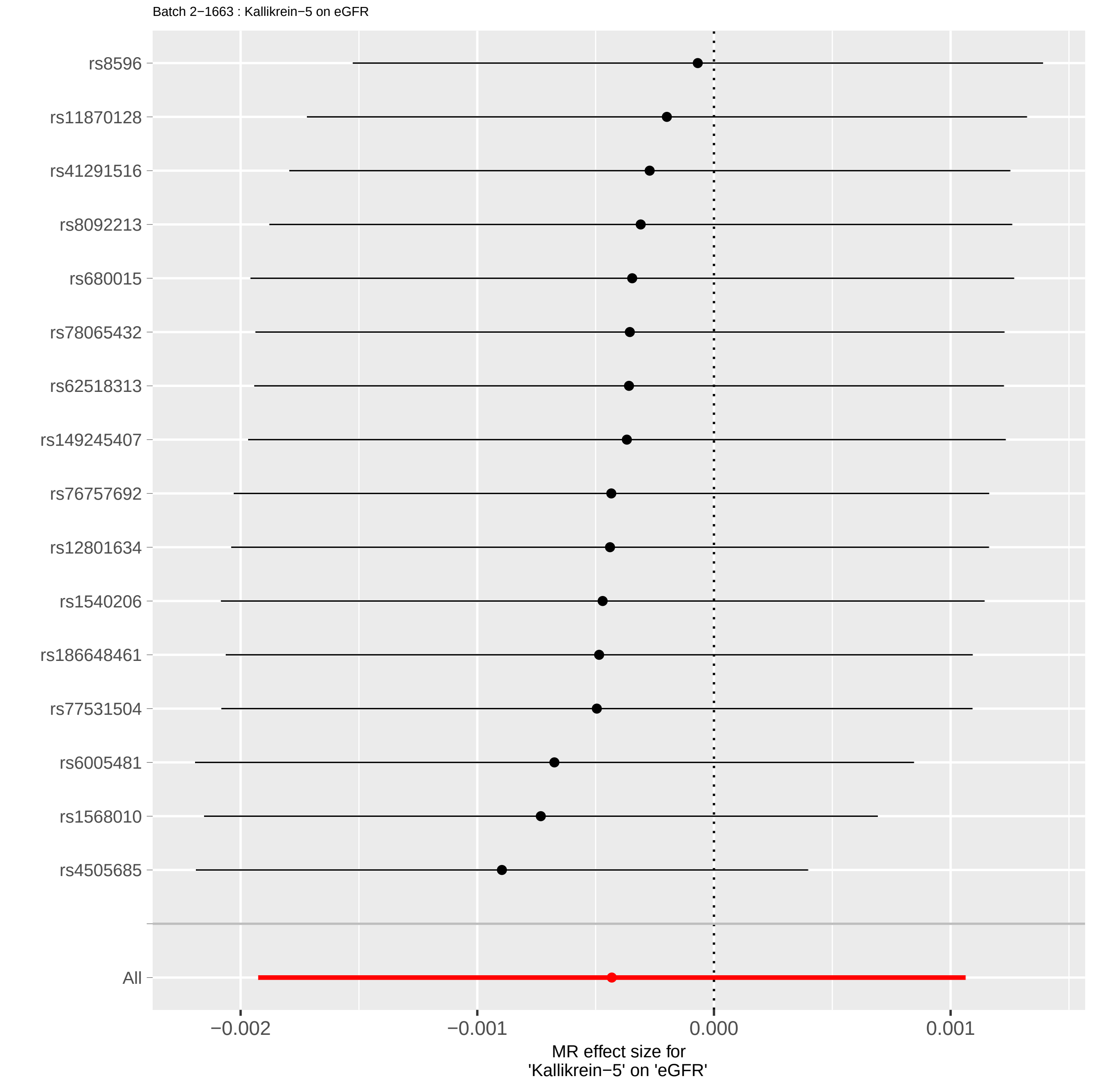

Batch 2−1663 : Kallikrein−5 on eGFR
rs8596
rs11870128
rs41291516
rs8092213
rs680015
rs78065432
rs62518313
rs149245407
rs76757692
rs12801634
rs1540206
rs186648461
rs77531504
rs6005481
rs1568010
rs4505685
All
−0.002
−0.001
0.000
0.001
MR effect size for
'Kallikrein−5' on 'eGFR'

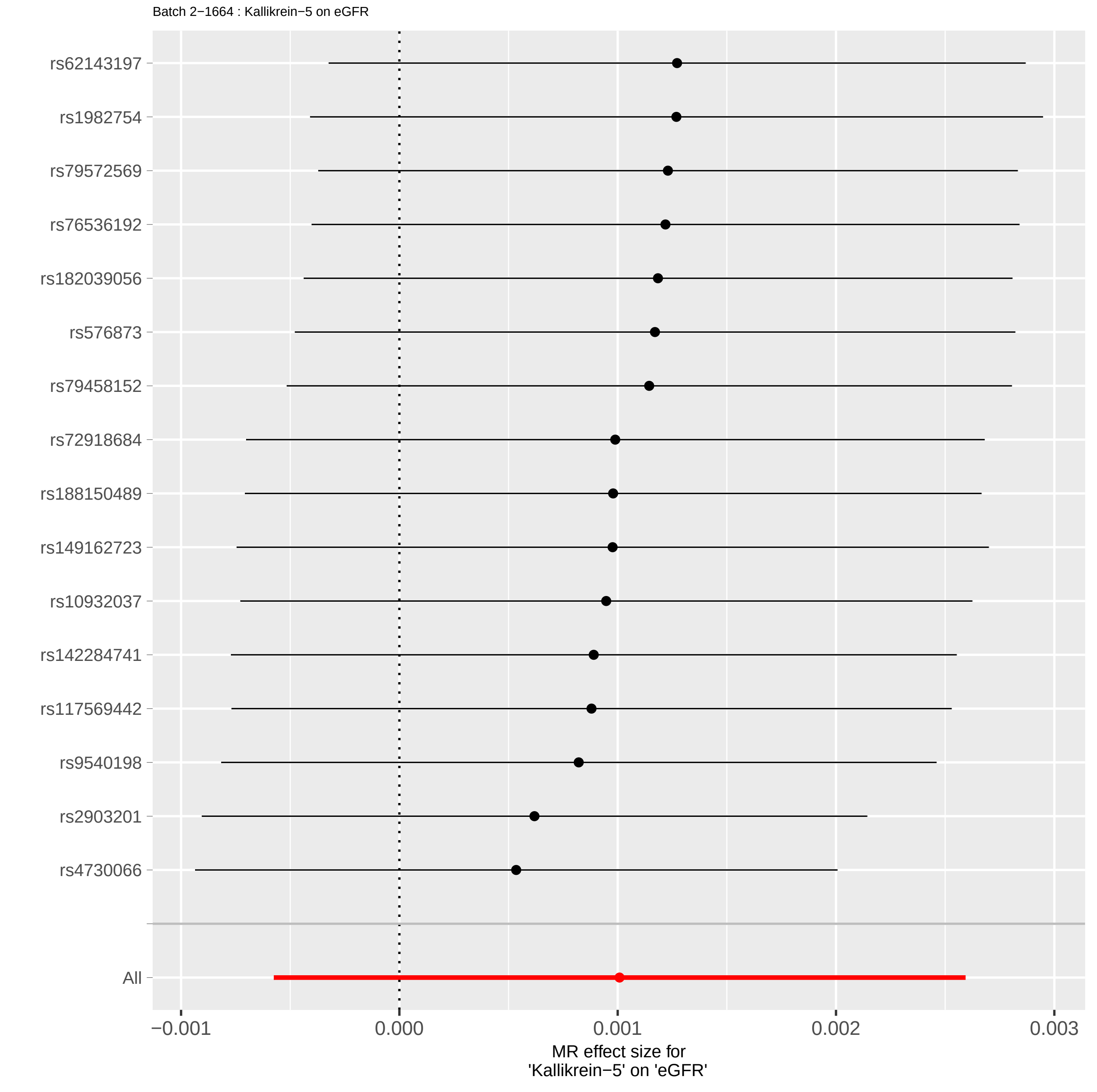
Batch 2−1664 : Kallikrein−5 on eGFR
rs62143197
rs1982754
rs79572569
rs76536192
rs182039056
rs576873
rs79458152
rs72918684
rs188150489
rs149162723
rs10932037
rs142284741
rs117569442
rs9540198
rs2903201
rs4730066
All
−0.001
0.000
0.001
0.002
0.003
MR effect size for
'Kallikrein−5' on 'eGFR'

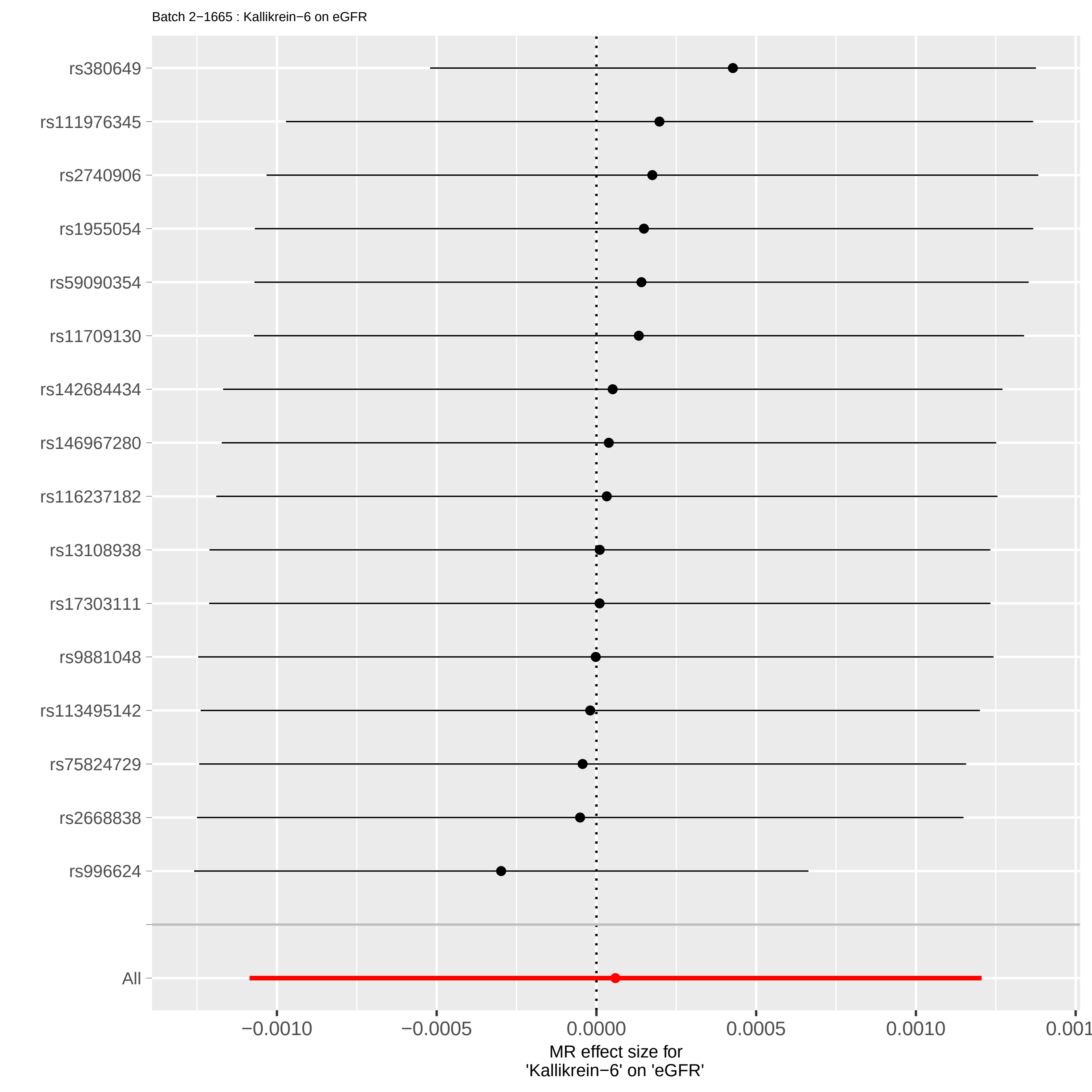

Batch 2−1665 : Kallikrein−6 on eGFR
rs380649
rs111976345
rs2740906
rs1955054
rs59090354
rs11709130
rs142684434
rs146967280
rs116237182
rs13108938
rs17303111
rs9881048
rs113495142
rs75824729
rs2668838
rs996624
All
−0.0010
−0.0005
0.0000
0.0005
0.0010
MR effect size for
'Kallikrein−6' on 'eGFR'

Batch 2−1666 : Kallikrein−7 on eGFR

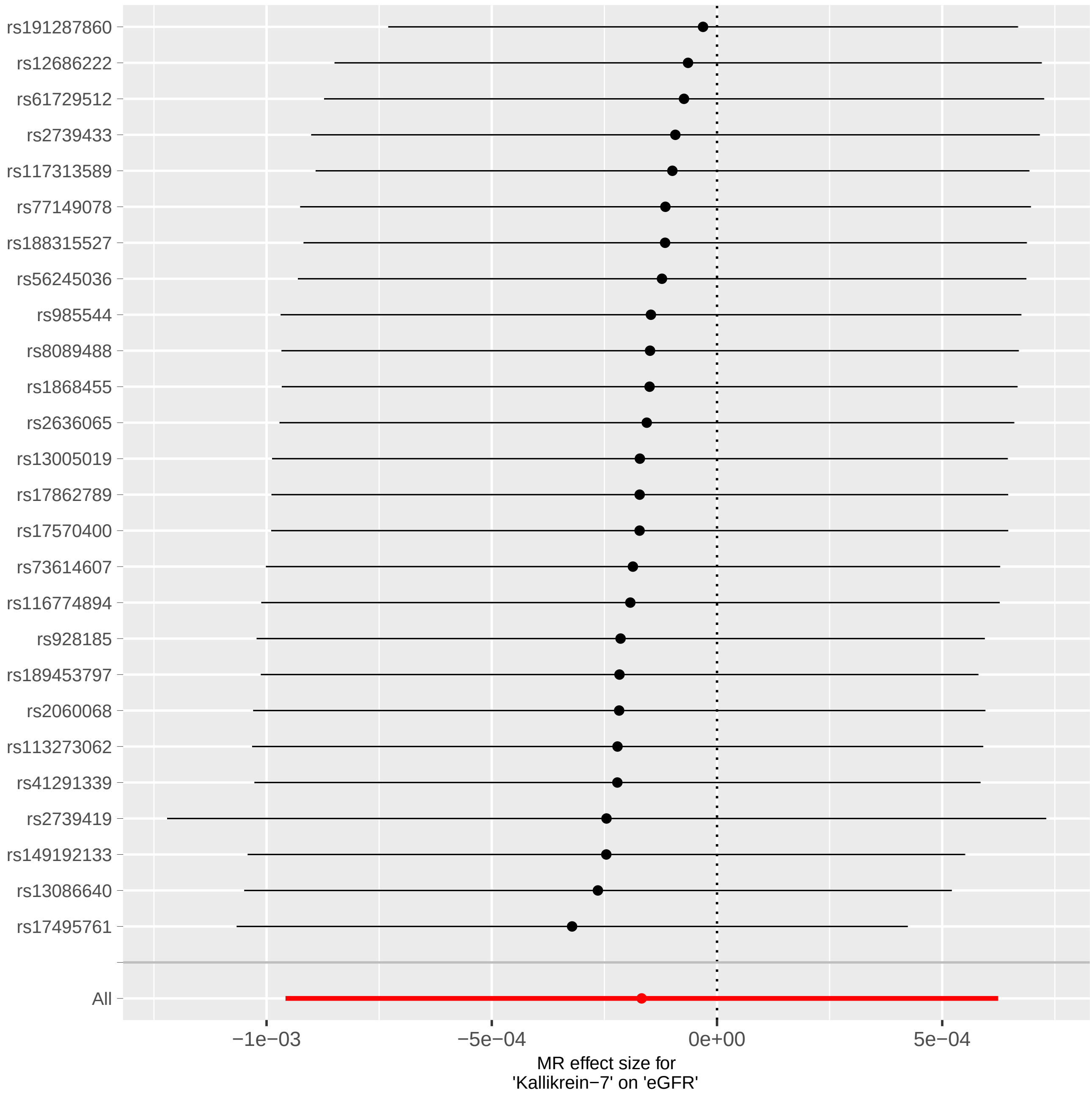

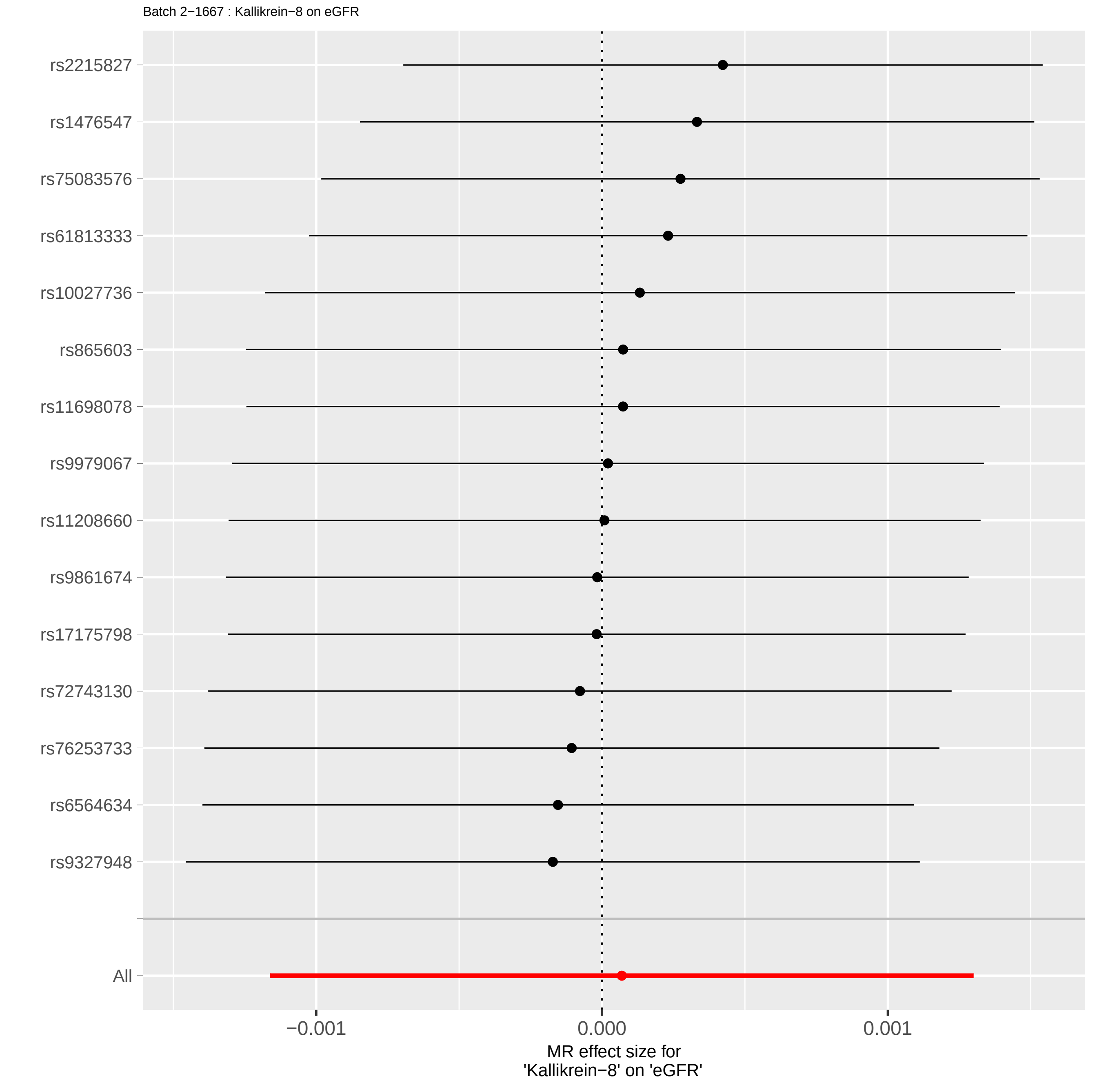
Batch 2−1667 : Kallikrein−8 on eGFR
rs2215827
rs1476547
rs75083576
rs61813333
rs10027736
rs865603
rs11698078
rs9979067
rs11208660
rs9861674
rs17175798
rs72743130
rs76253733
rs6564634
rs9327948
All
−0.001
0.000
0.001
MR effect size for
'Kallikrein−8' on 'eGFR'

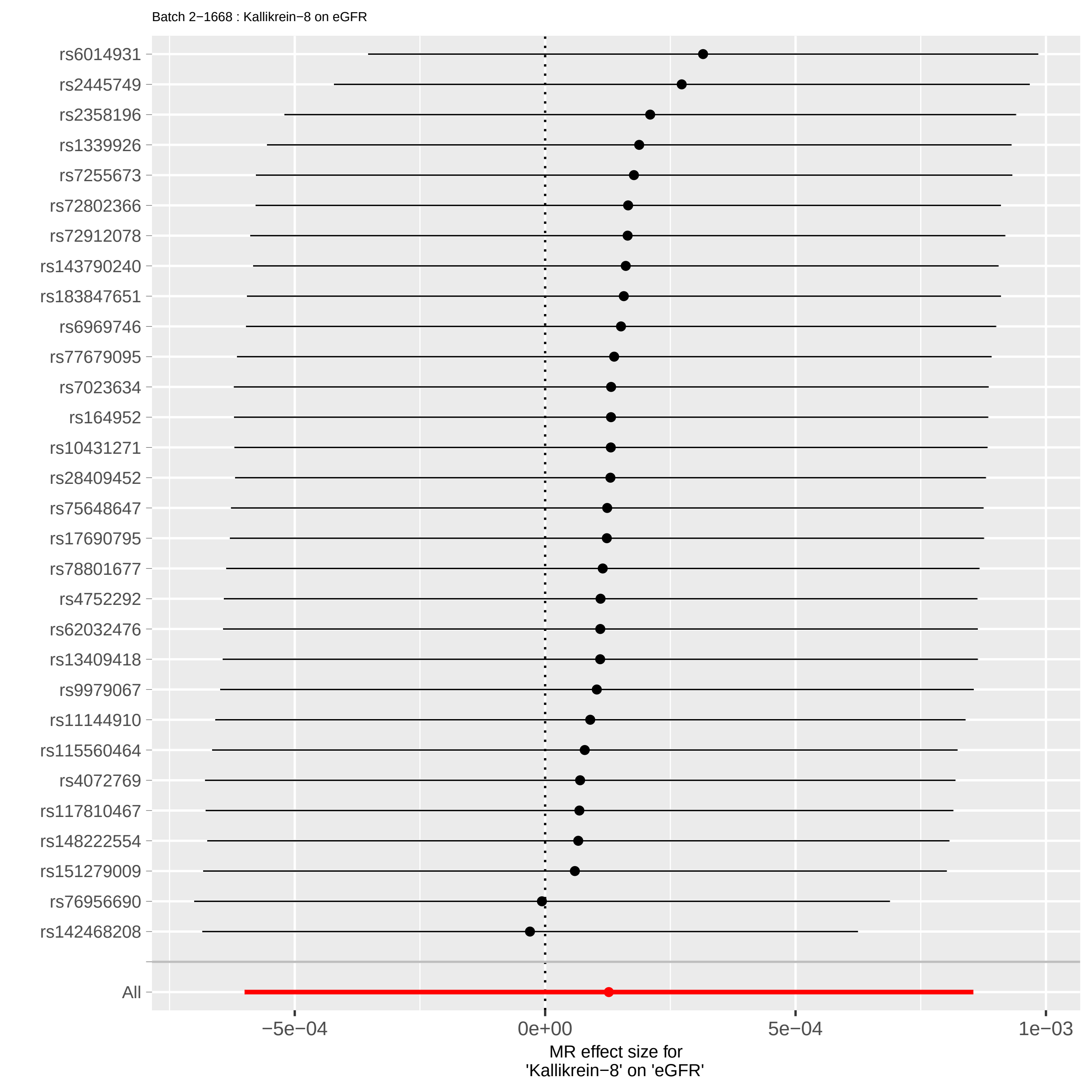

Batch 2−1668 : Kallikrein−8 on eGFR
rs6014931
rs2445749
rs2358196
rs1339926
rs7255673
rs72802366
rs72912078
rs143790240
rs183847651
rs6969746
rs77679095
rs7023634
rs164952
rs10431271
rs28409452
rs75648647
rs17690795
rs78801677
rs4752292
rs62032476
rs13409418
rs9979067
rs11144910
rs115560464
rs4072769
rs117810467
rs148222554
rs151279009
rs76956690
rs142468208
All
−5e−04
0e+00
5e−04
1e−03
MR effect size for
'Kallikrein−8' on 'eGFR'

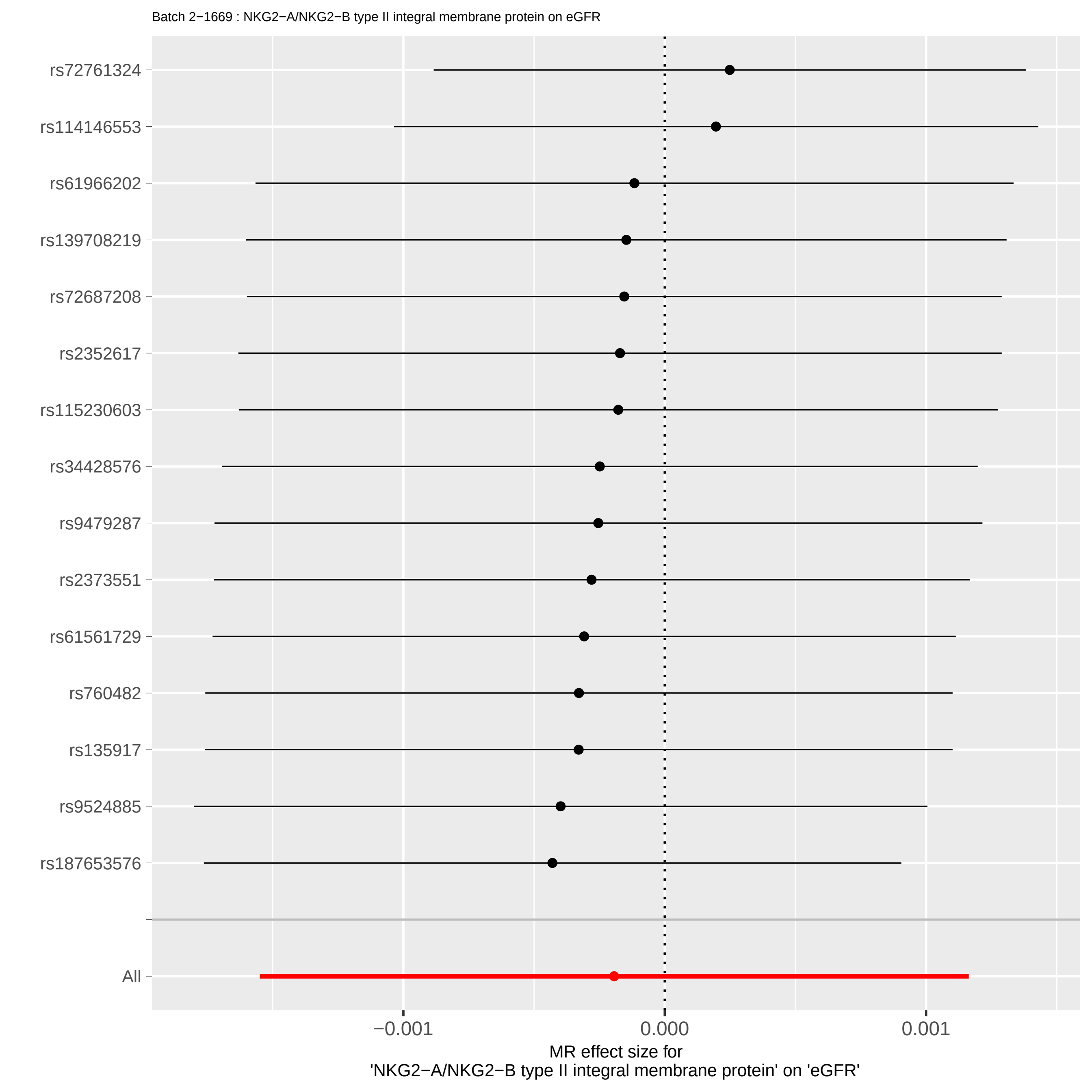

Batch 2−1669 : NKG2−A/NKG2−B type II integral membrane protein on eGFR
rs72761324
rs114146553
rs61966202
rs139708219
rs72687208
rs2352617
rs115230603
rs34428576
rs9479287
rs2373551
rs61561729
rs760482
rs135917
rs9524885
rs187653576
All
−0.001
0.000
0.001
MR effect size for
'NKG2−A/NKG2−B type II integral membrane protein' on 'eGFR'

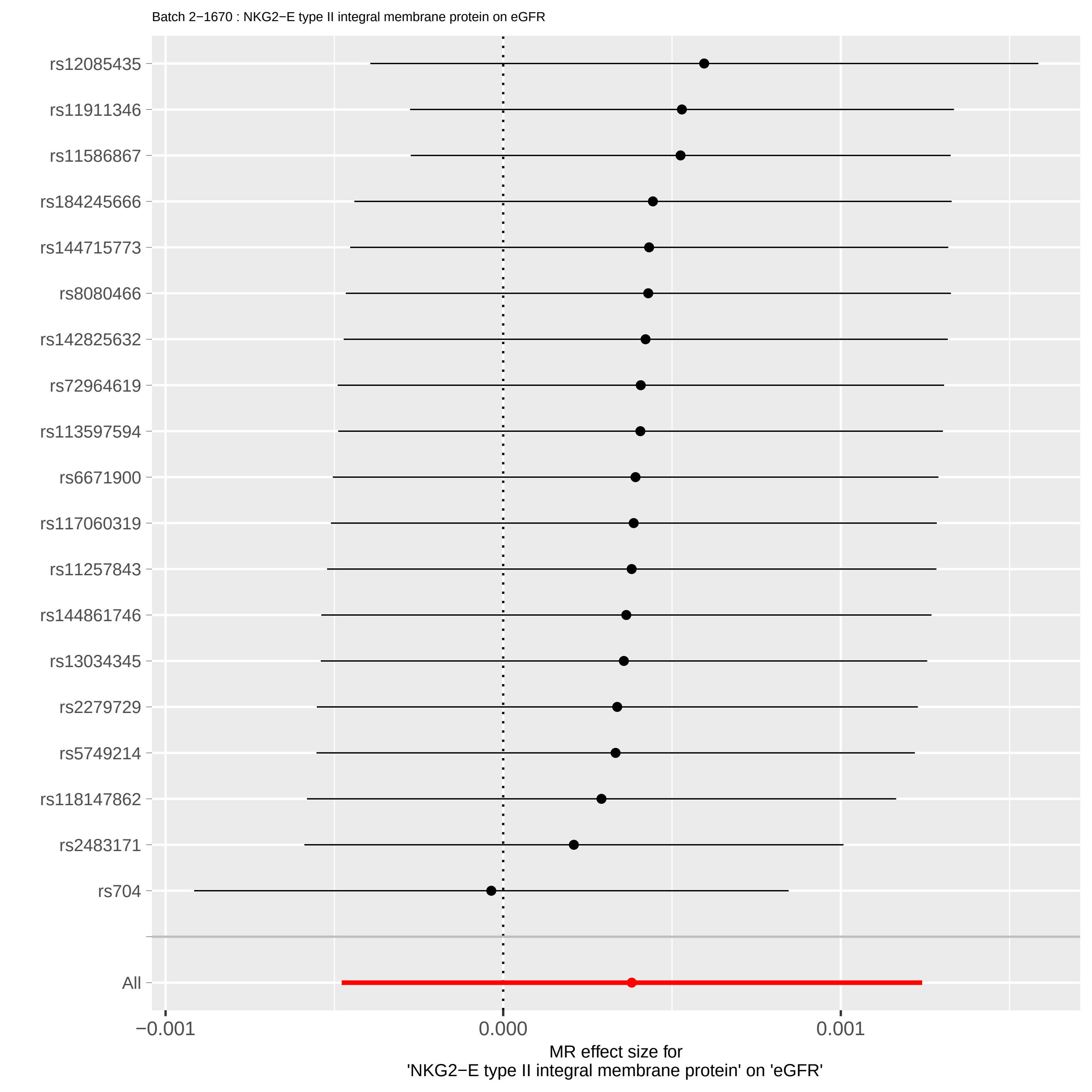

Batch 2−1670 : NKG2−E type II integral membrane protein on eGFR
rs12085435
rs11911346
rs11586867
rs184245666
rs144715773
rs8080466
rs142825632
rs72964619
rs113597594
rs6671900
rs117060319
rs11257843
rs144861746
rs13034345
rs2279729
rs5749214
rs118147862
rs2483171
rs704
All
−0.001
0.000
0.001
MR effect size for
'NKG2−E type II integral membrane protein' on 'eGFR'

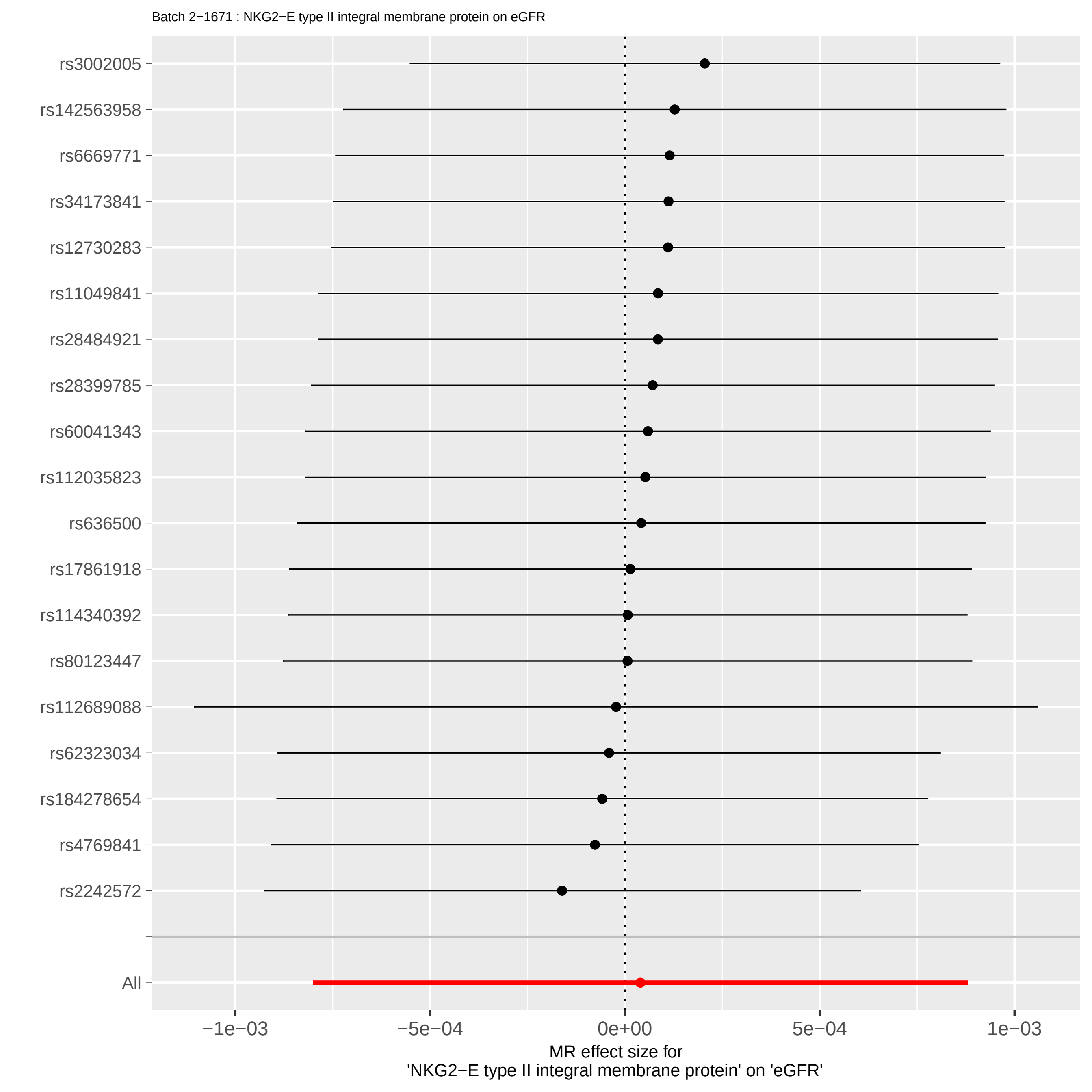
Batch 2−1671 : NKG2−E type II integral membrane protein on eGFR
rs3002005
rs142563958
rs6669771
rs34173841
rs12730283
rs11049841
rs28484921
rs28399785
rs60041343
rs112035823
rs636500
rs17861918
rs114340392
rs80123447
rs112689088
rs62323034
rs184278654
rs4769841
rs2242572
All
−1e−03
−5e−04
0e+00
5e−04
1e−03
MR effect size for
'NKG2−E type II integral membrane protein' on 'eGFR'

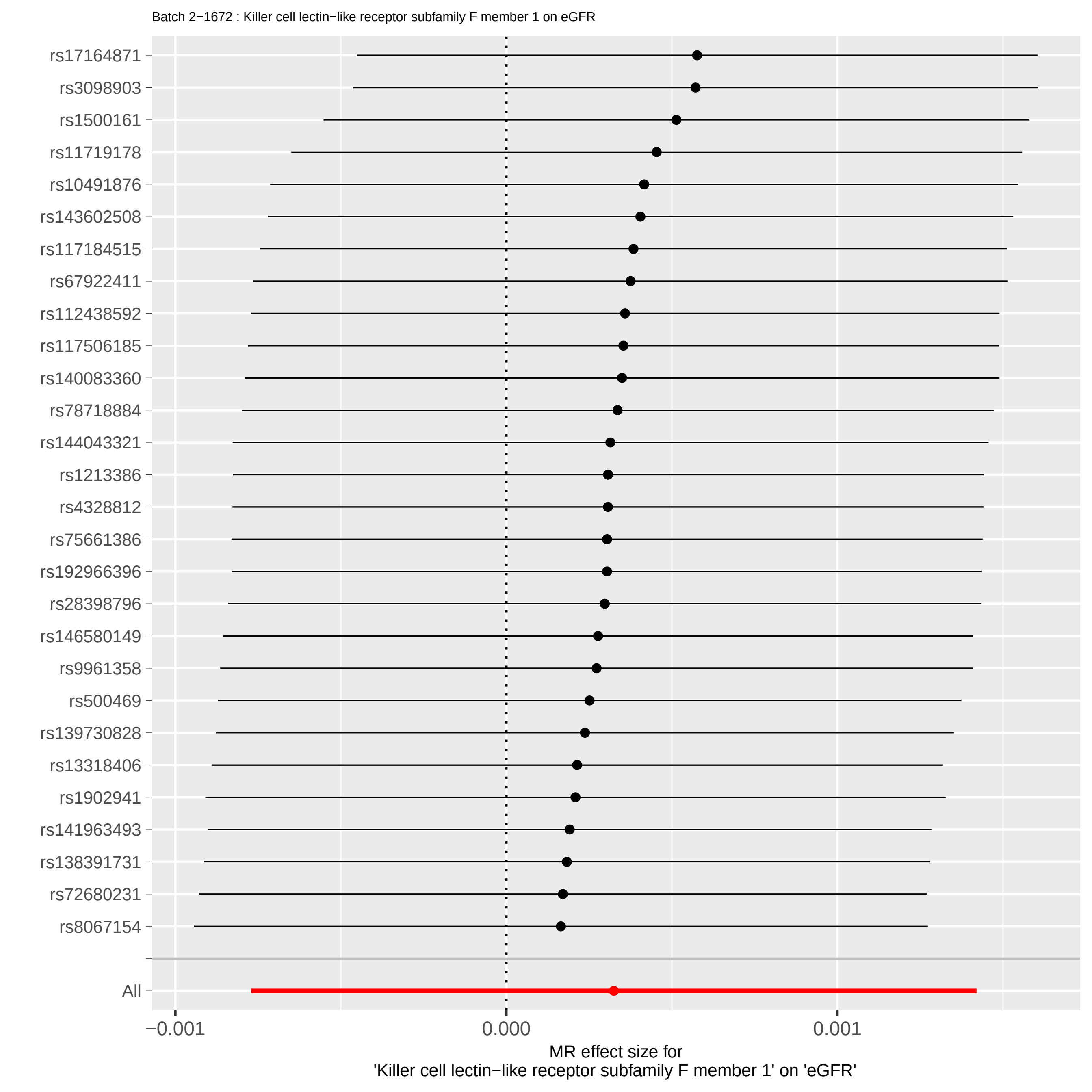

Batch 2−1672 : Killer cell lectin−like receptor subfamily F member 1 on eGFR
rs17164871
rs3098903
rs1500161
rs11719178
rs10491876
rs143602508
rs117184515
rs67922411
rs112438592
rs117506185
rs140083360
rs78718884
rs144043321
rs1213386
rs4328812
rs75661386
rs192966396
rs28398796
rs146580149
rs9961358
rs500469
rs139730828
rs13318406
rs1902941
rs141963493
rs138391731
rs72680231
rs8067154
All
−0.001
0.000
0.001
MR effect size for
'Killer cell lectin−like receptor subfamily F member 1' on 'eGFR'

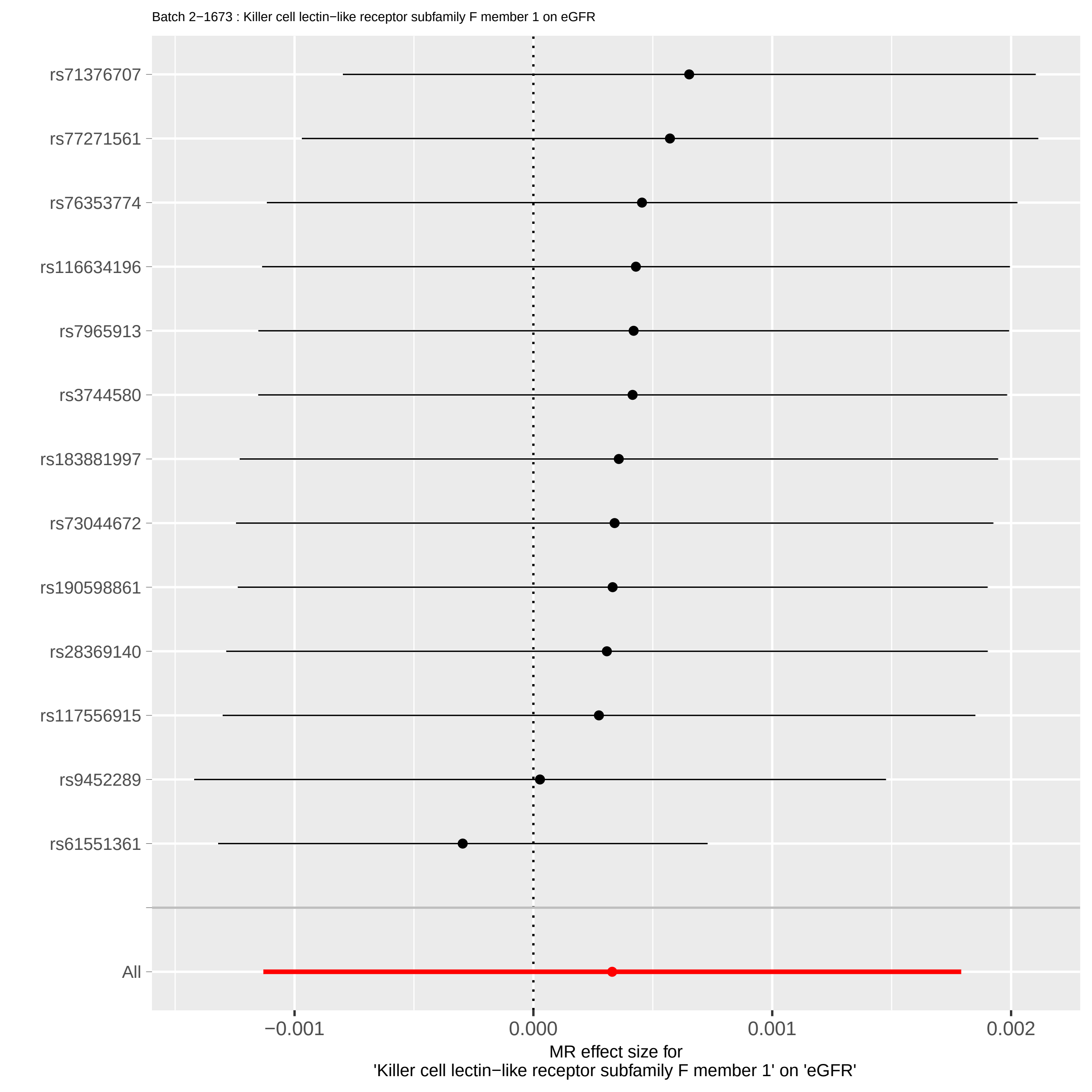

Batch 2−1673 : Killer cell lectin−like receptor subfamily F member 1 on eGFR
rs71376707
rs77271561
rs76353774
rs116634196
rs7965913
rs3744580
rs183881997
rs73044672
rs190598861
rs28369140
rs117556915
rs9452289
rs61551361
All
−0.001
0.000
0.001
0.002
MR effect size for
'Killer cell lectin−like receptor subfamily F member 1' on 'eGFR'

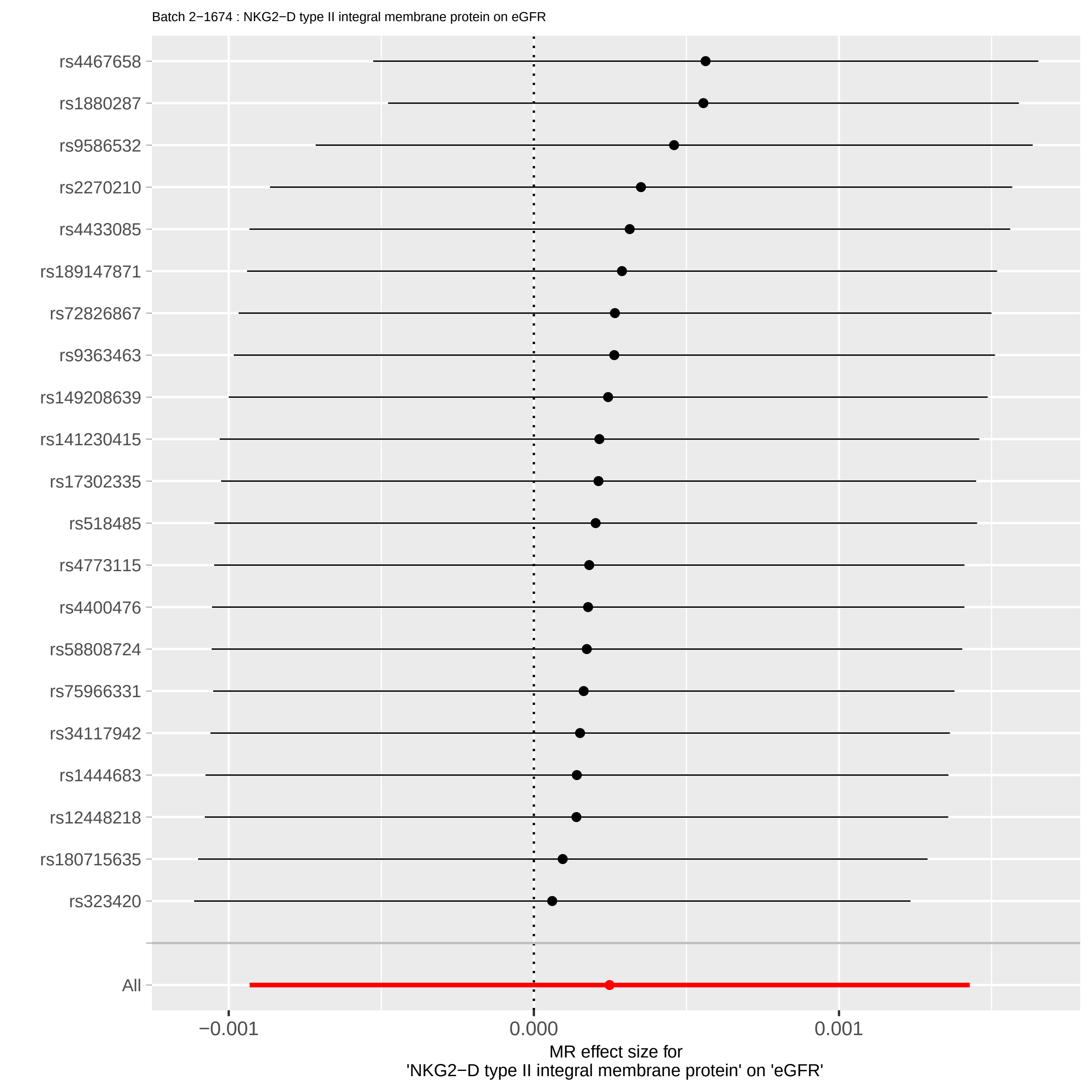

Batch 2−1674 : NKG2−D type II integral membrane protein on eGFR
rs4467658
rs1880287
rs9586532
rs2270210
rs4433085
rs189147871
rs72826867
rs9363463
rs149208639
rs141230415
rs17302335
rs518485
rs4773115
rs4400476
rs58808724
rs75966331
rs34117942
rs1444683
rs12448218
rs180715635
rs323420
All
−0.001
0.000
0.001
MR effect size for
'NKG2−D type II integral membrane protein' on 'eGFR'

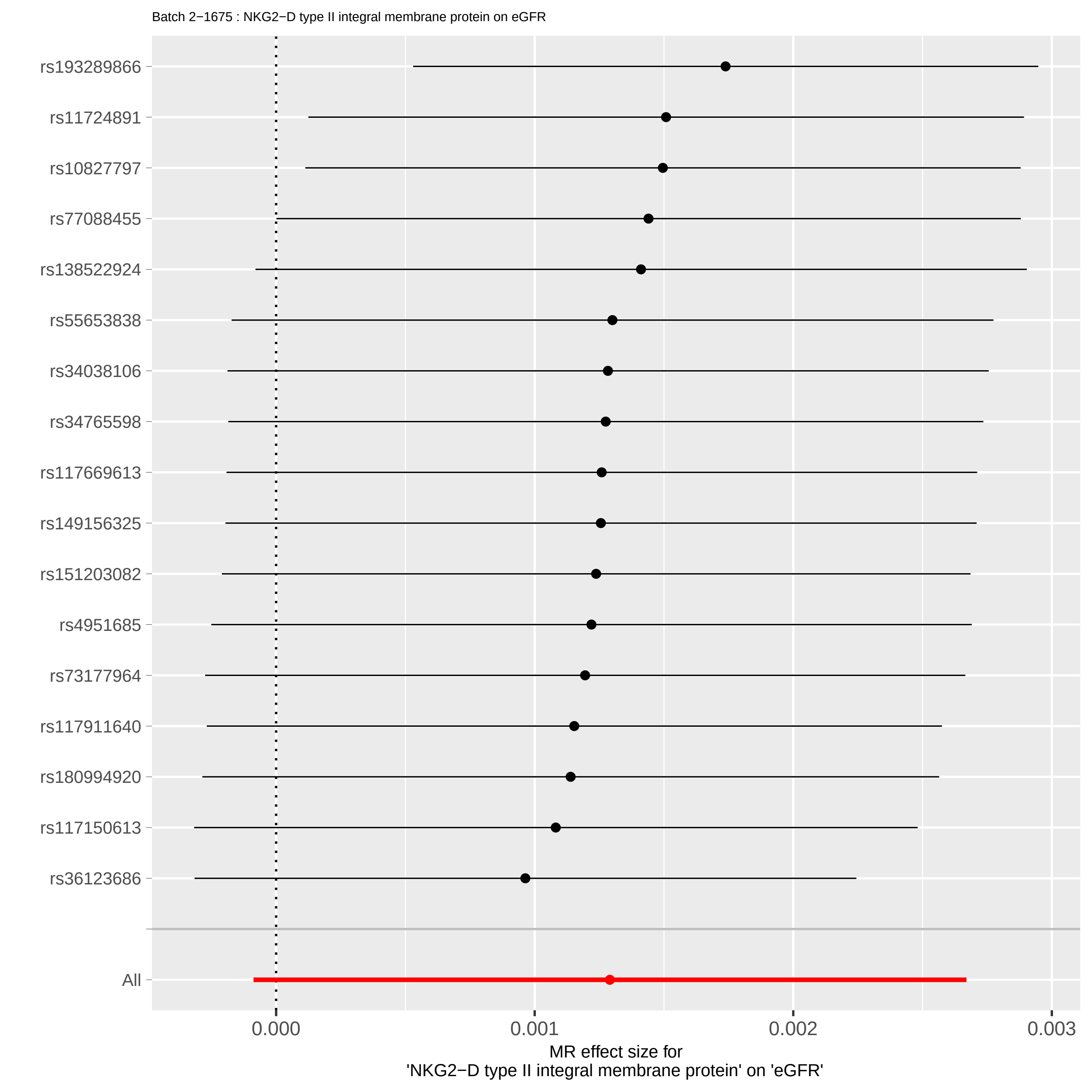
Batch 2−1675 : NKG2−D type II integral membrane protein on eGFR
rs193289866
rs11724891
rs10827797
rs77088455
rs138522924
rs55653838
rs34038106
rs34765598
rs117669613
rs149156325
rs151203082
rs4951685
rs73177964
rs117911640
rs180994920
rs117150613
rs36123686
All
0.000
0.001
0.002
0.003
MR effect size for
'NKG2−D type II integral membrane protein' on 'eGFR'

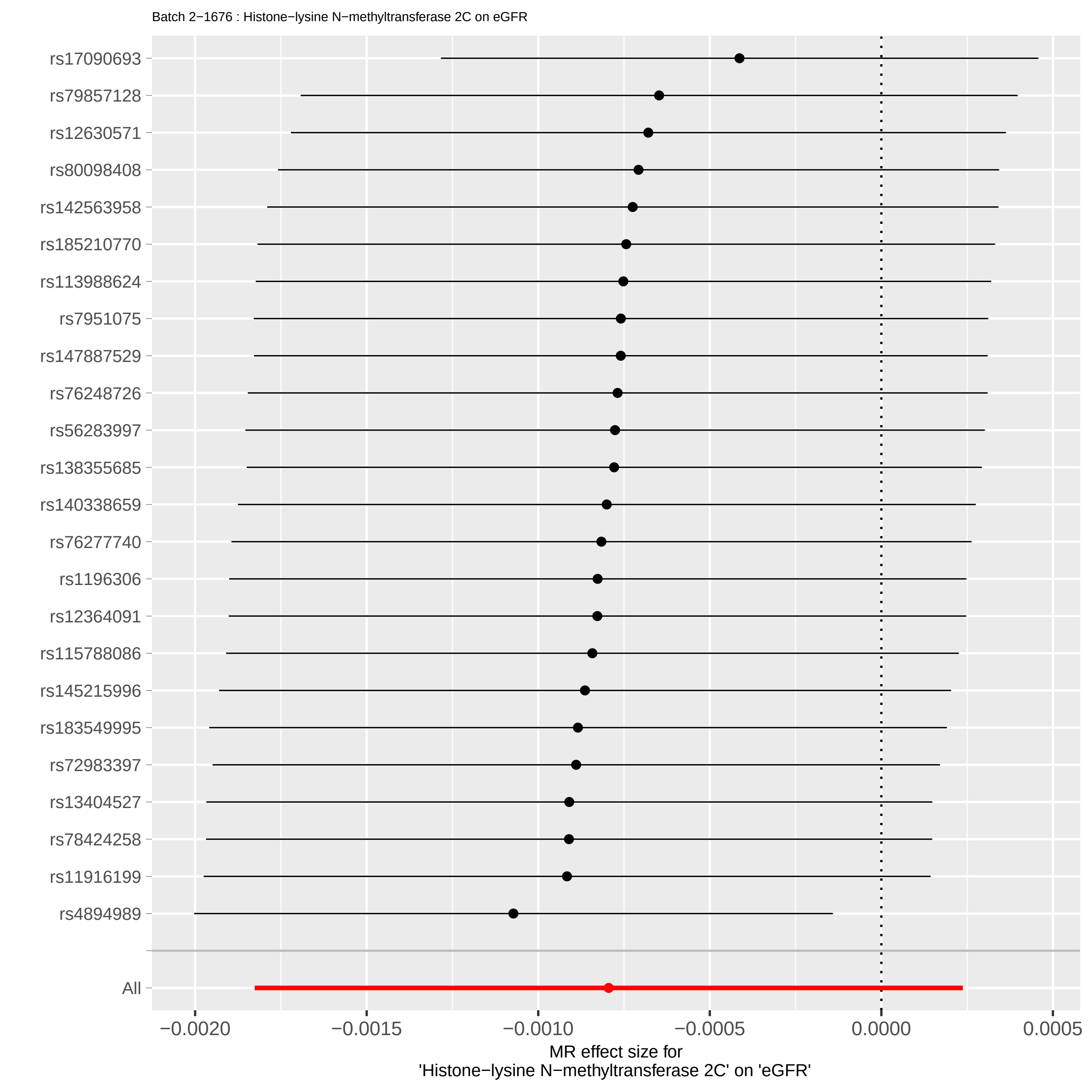

Batch 2−1676 : Histone−lysine N−methyltransferase 2C on eGFR
rs17090693
rs79857128
rs12630571
rs80098408
rs142563958
rs185210770
rs113988624
rs7951075
rs147887529
rs76248726
rs56283997
rs138355685
rs140338659
rs76277740
rs1196306
rs12364091
rs115788086
rs145215996
rs183549995
rs72983397
rs13404527
rs78424258
rs11916199
rs4894989
All
−0.0020
−0.0015
−0.0010
−0.0005
0.0000
0.0005
MR effect size for
'Histone−lysine N−methyltransferase 2C' on 'eGFR'

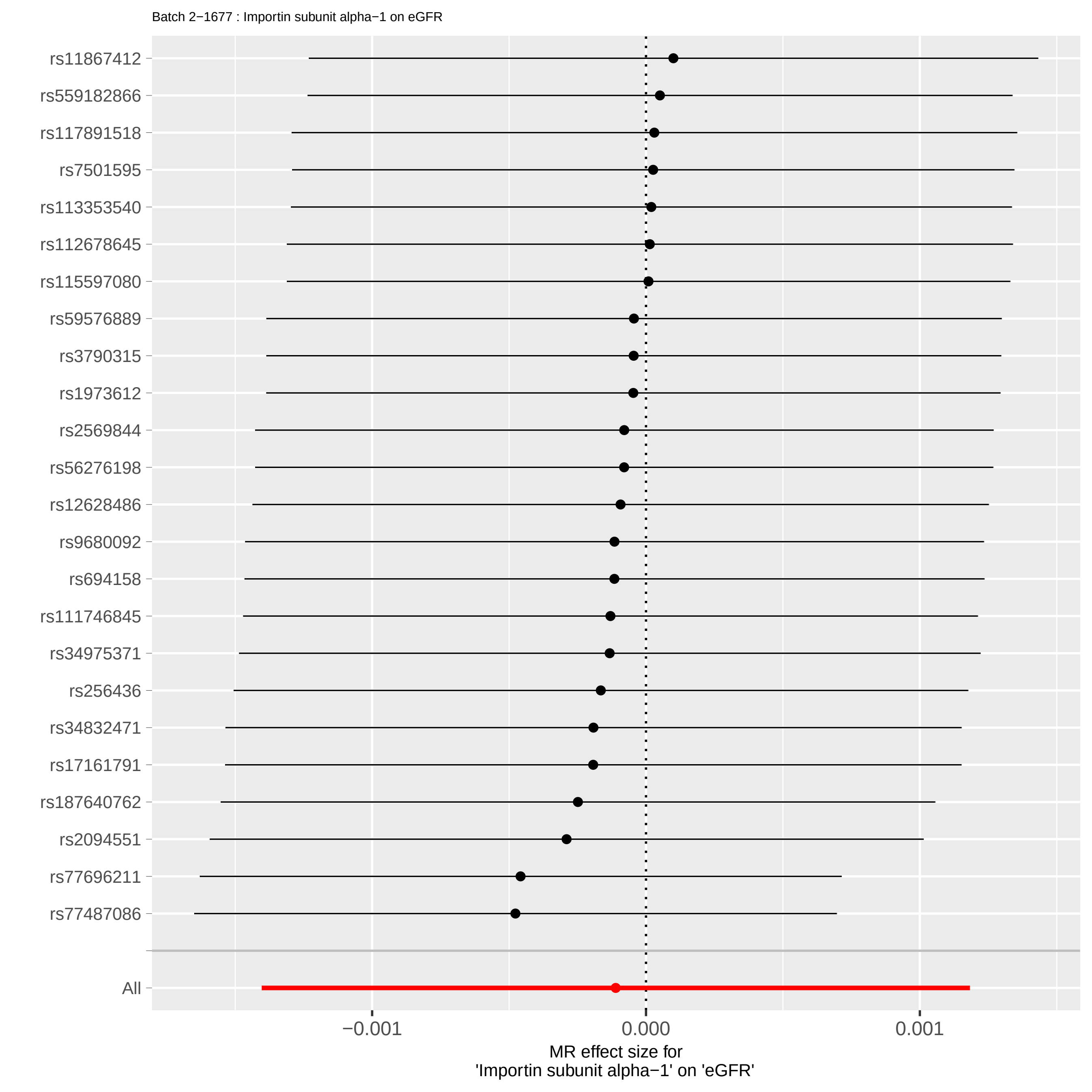

Batch 2−1677 : Importin subunit alpha−1 on eGFR
rs11867412
rs559182866
rs117891518
rs7501595
rs113353540
rs112678645
rs115597080
rs59576889
rs3790315
rs1973612
rs2569844
rs56276198
rs12628486
rs9680092
rs694158
rs111746845
rs34975371
rs256436
rs34832471
rs17161791
rs187640762
rs2094551
rs77696211
rs77487086
All
−0.001
0.000
0.001
MR effect size for
'Importin subunit alpha−1' on 'eGFR'

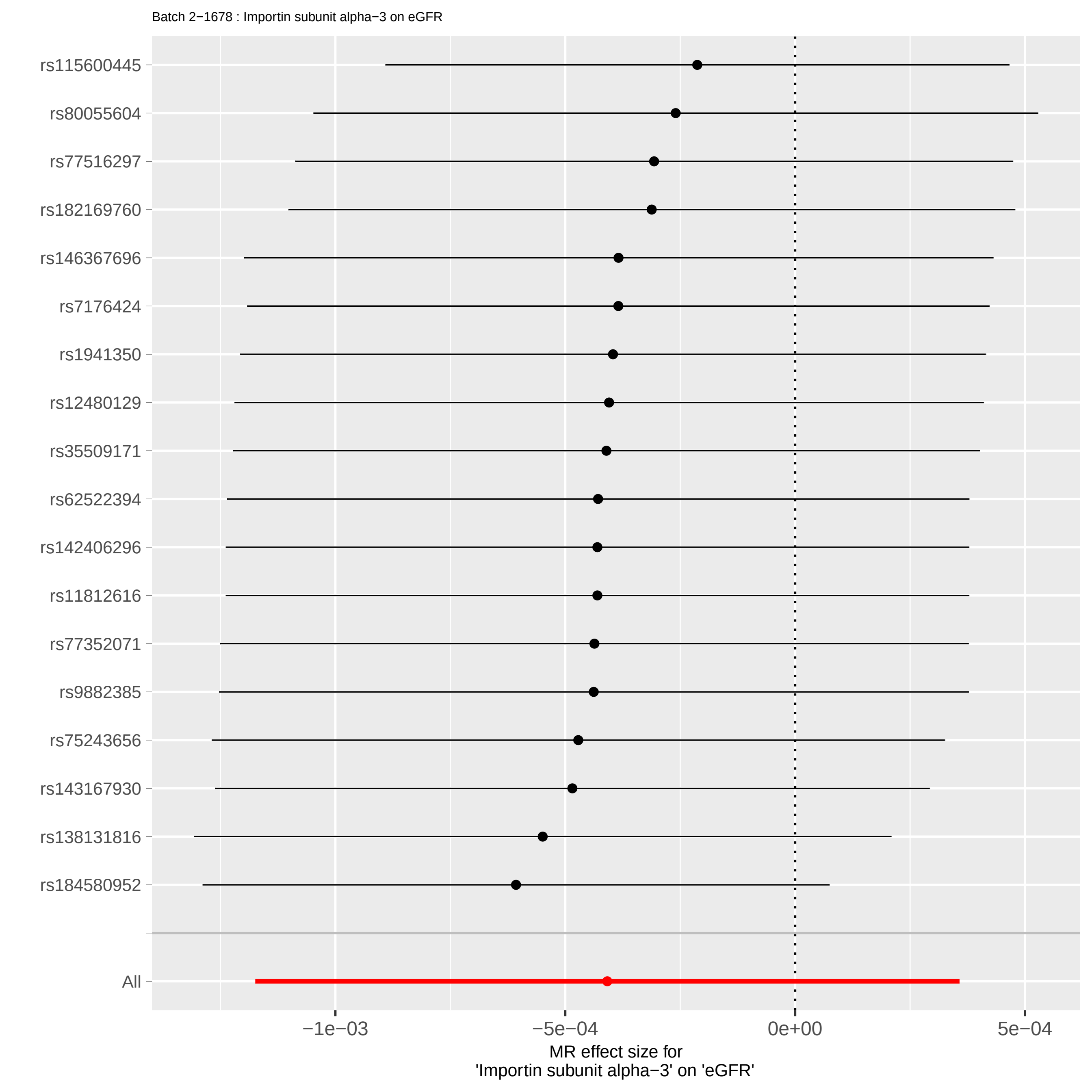

Batch 2−1678 : Importin subunit alpha−3 on eGFR
rs115600445
rs80055604
rs77516297
rs182169760
rs146367696
rs7176424
rs1941350
rs12480129
rs35509171
rs62522394
rs142406296
rs11812616
rs77352071
rs9882385
rs75243656
rs143167930
rs138131816
rs184580952
All
−1e−03
−5e−04
0e+00
5e−04
MR effect size for
'Importin subunit alpha−3' on 'eGFR'

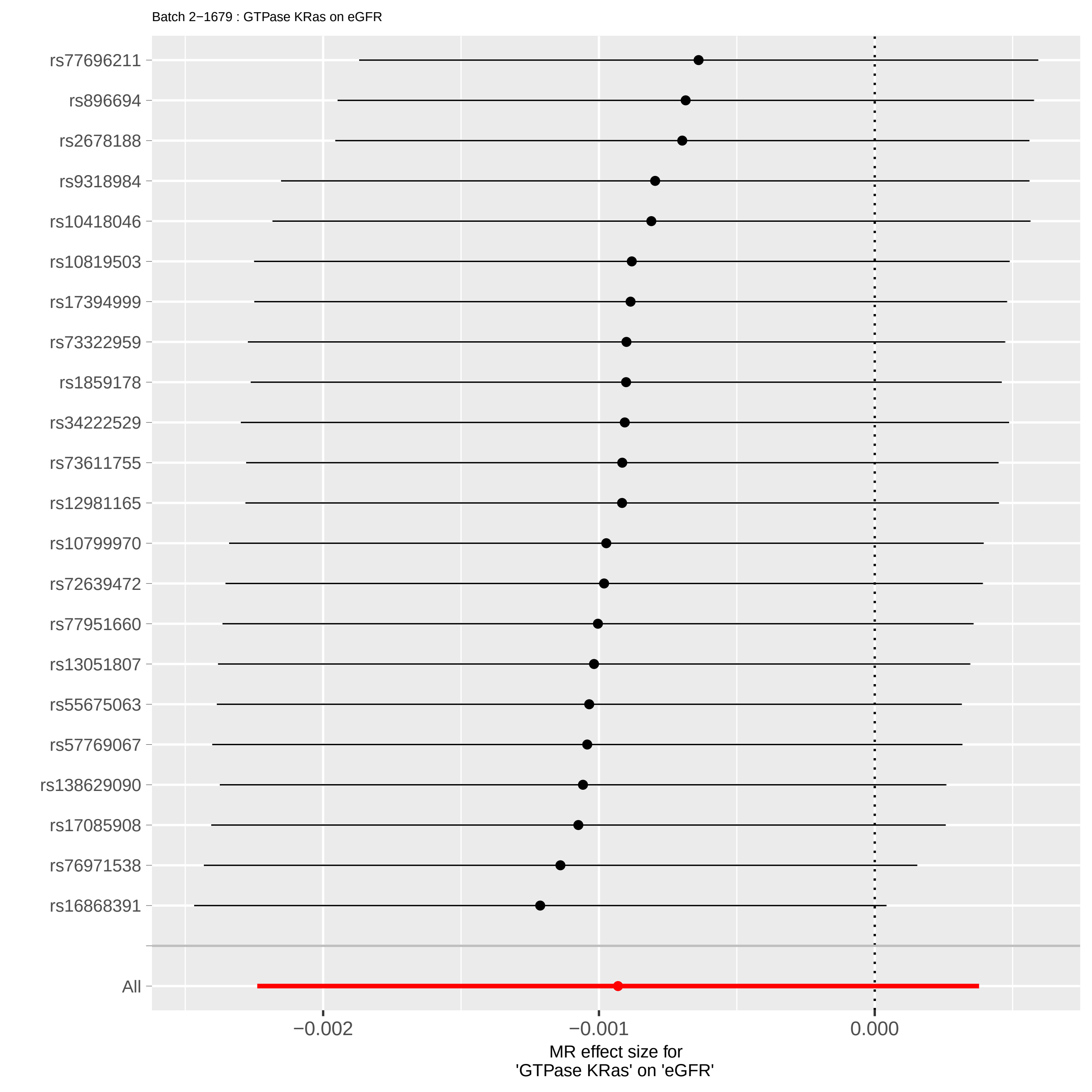

Batch 2−1679 : GTPase KRas on eGFR
rs77696211
rs896694
rs2678188
rs9318984
rs10418046
rs10819503
rs17394999
rs73322959
rs1859178
rs34222529
rs73611755
rs12981165
rs10799970
rs72639472
rs77951660
rs13051807
rs55675063
rs57769067
rs138629090
rs17085908
rs76971538
rs16868391
All
−0.002
−0.001
0.000
MR effect size for
'GTPase KRas' on 'eGFR'

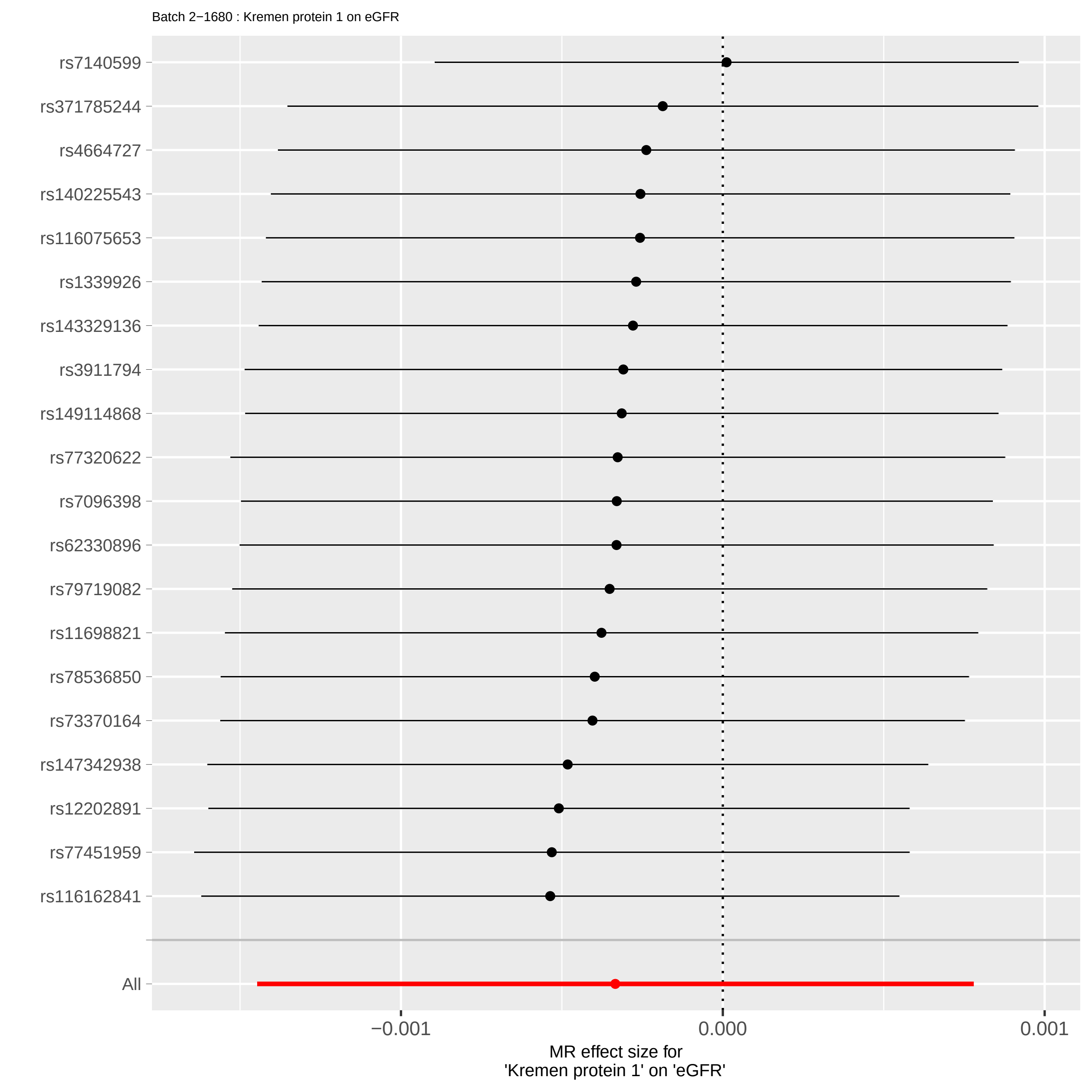
Batch 2−1680 : Kremen protein 1 on eGFR
rs7140599
rs371785244
rs4664727
rs140225543
rs116075653
rs1339926
rs143329136
rs3911794
rs149114868
rs77320622
rs7096398
rs62330896
rs79719082
rs11698821
rs78536850
rs73370164
rs147342938
rs12202891
rs77451959
rs116162841
All
−0.001
0.000
0.001
MR effect size for
'Kremen protein 1' on 'eGFR'

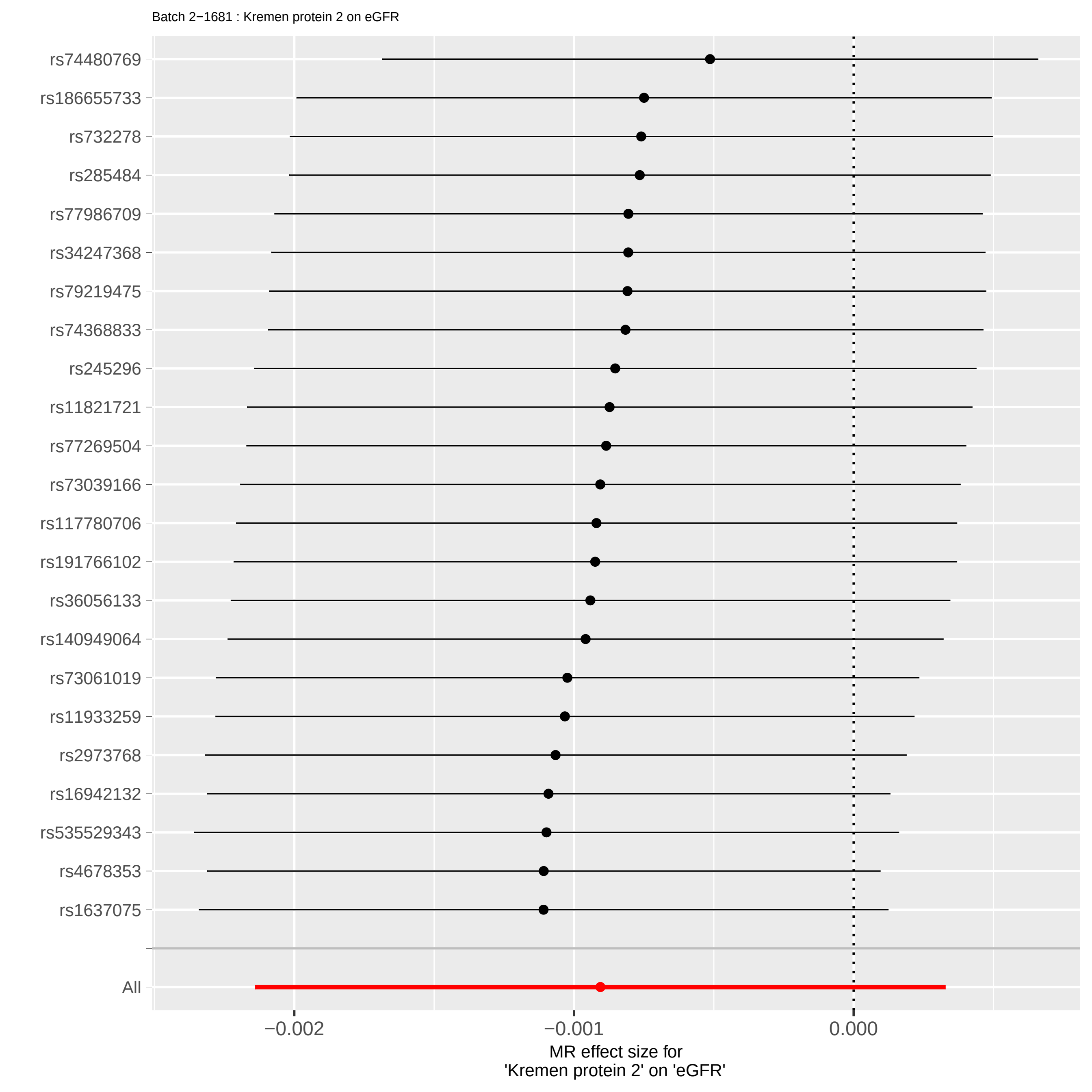

Batch 2−1681 : Kremen protein 2 on eGFR
rs74480769
rs186655733
rs732278
rs285484
rs77986709
rs34247368
rs79219475
rs74368833
rs245296
rs11821721
rs77269504
rs73039166
rs117780706
rs191766102
rs36056133
rs140949064
rs73061019
rs11933259
rs2973768
rs16942132
rs535529343
rs4678353
rs1637075
All
−0.002
−0.001
0.000
MR effect size for
'Kremen protein 2' on 'eGFR'

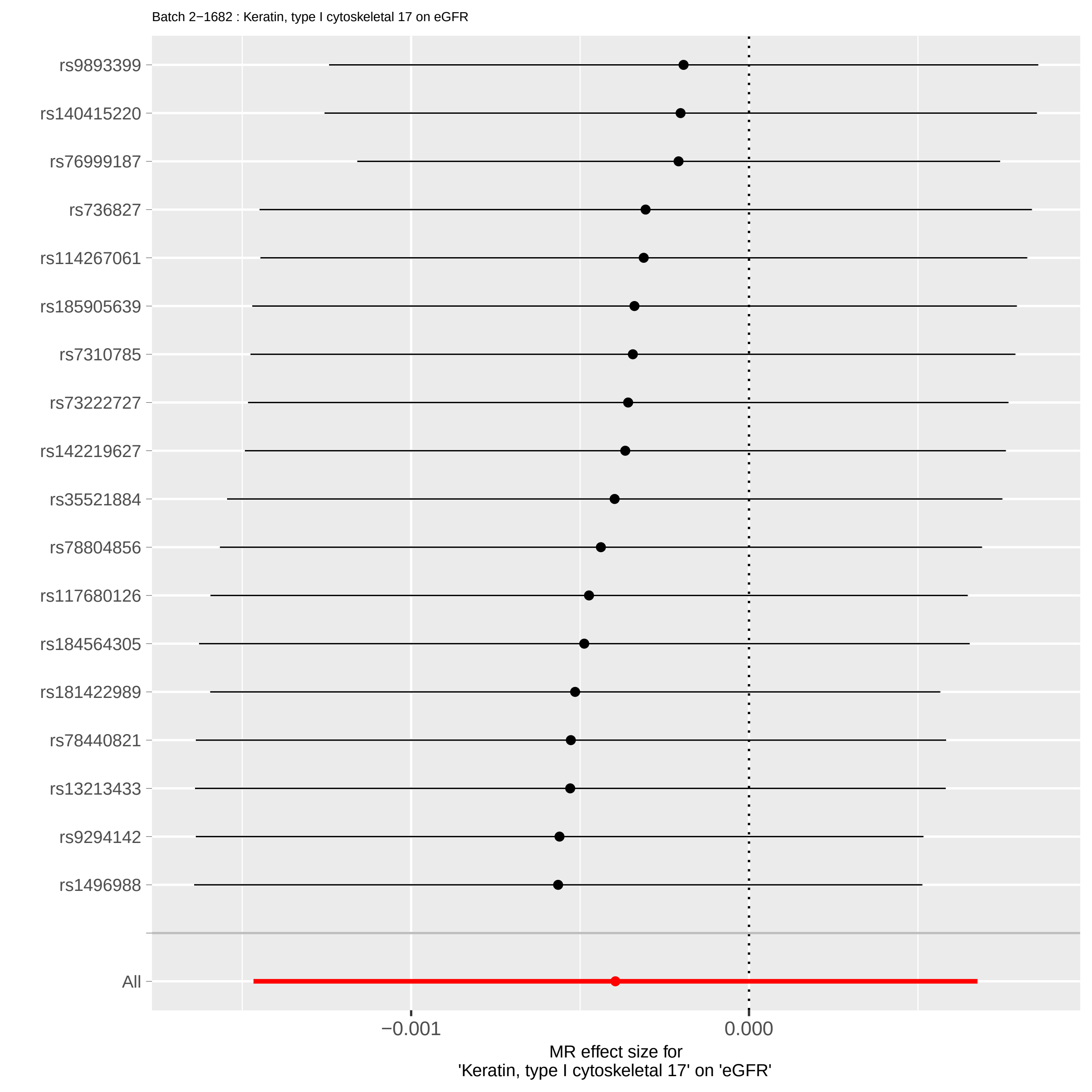

Batch 2−1682 : Keratin, type I cytoskeletal 17 on eGFR
rs9893399
rs140415220
rs76999187
rs736827
rs114267061
rs185905639
rs7310785
rs73222727
rs142219627
rs35521884
rs78804856
rs117680126
rs184564305
rs181422989
rs78440821
rs13213433
rs9294142
rs1496988
All
−0.001
0.000
MR effect size for
'Keratin, type I cytoskeletal 17' on 'eGFR'

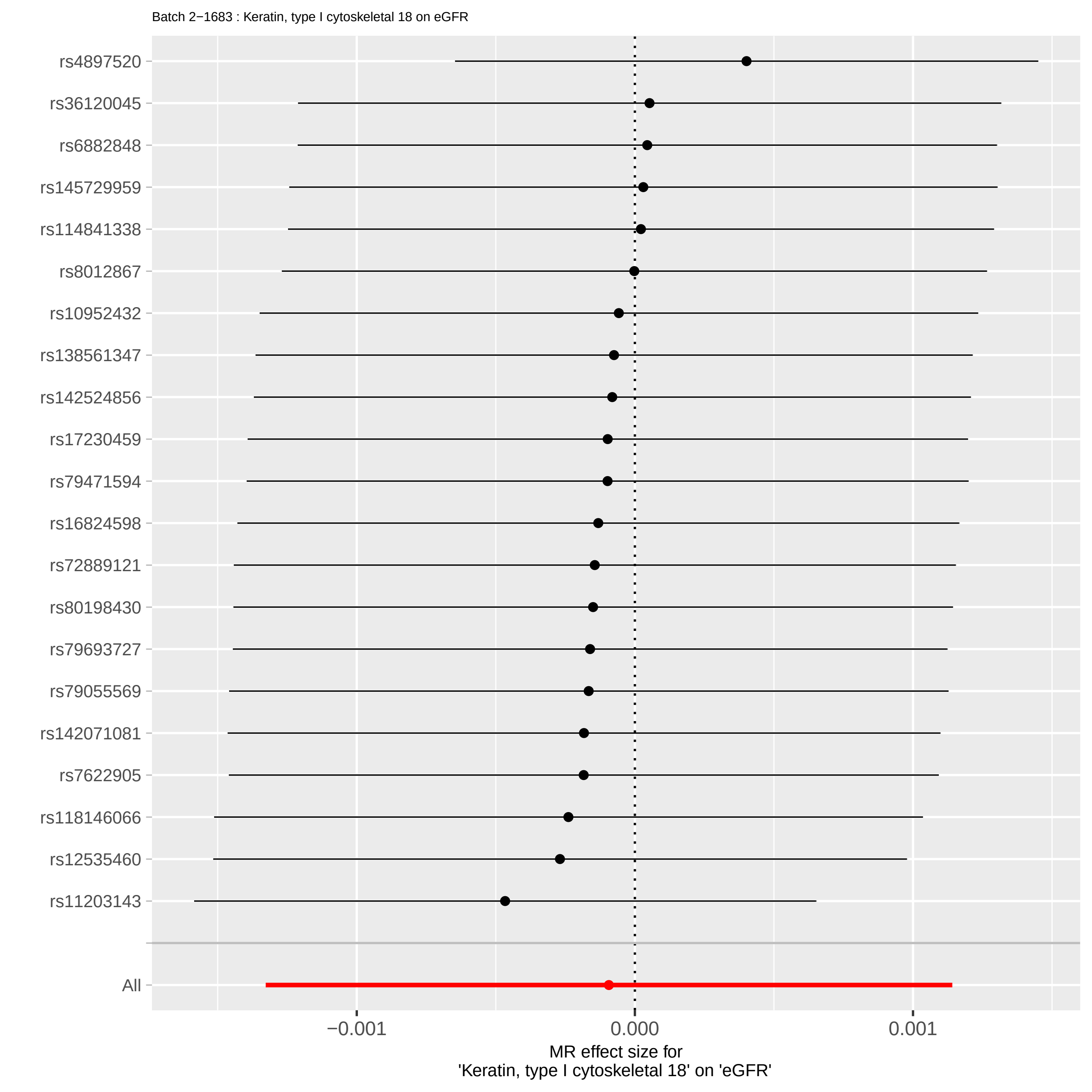
Batch 2−1683 : Keratin, type I cytoskeletal 18 on eGFR
rs4897520
rs36120045
rs6882848
rs145729959
rs114841338
rs8012867
rs10952432
rs138561347
rs142524856
rs17230459
rs79471594
rs16824598
rs72889121
rs80198430
rs79693727
rs79055569
rs142071081
rs7622905
rs118146066
rs12535460
rs11203143
All
−0.001
0.000
0.001
MR effect size for
'Keratin, type I cytoskeletal 18' on 'eGFR'

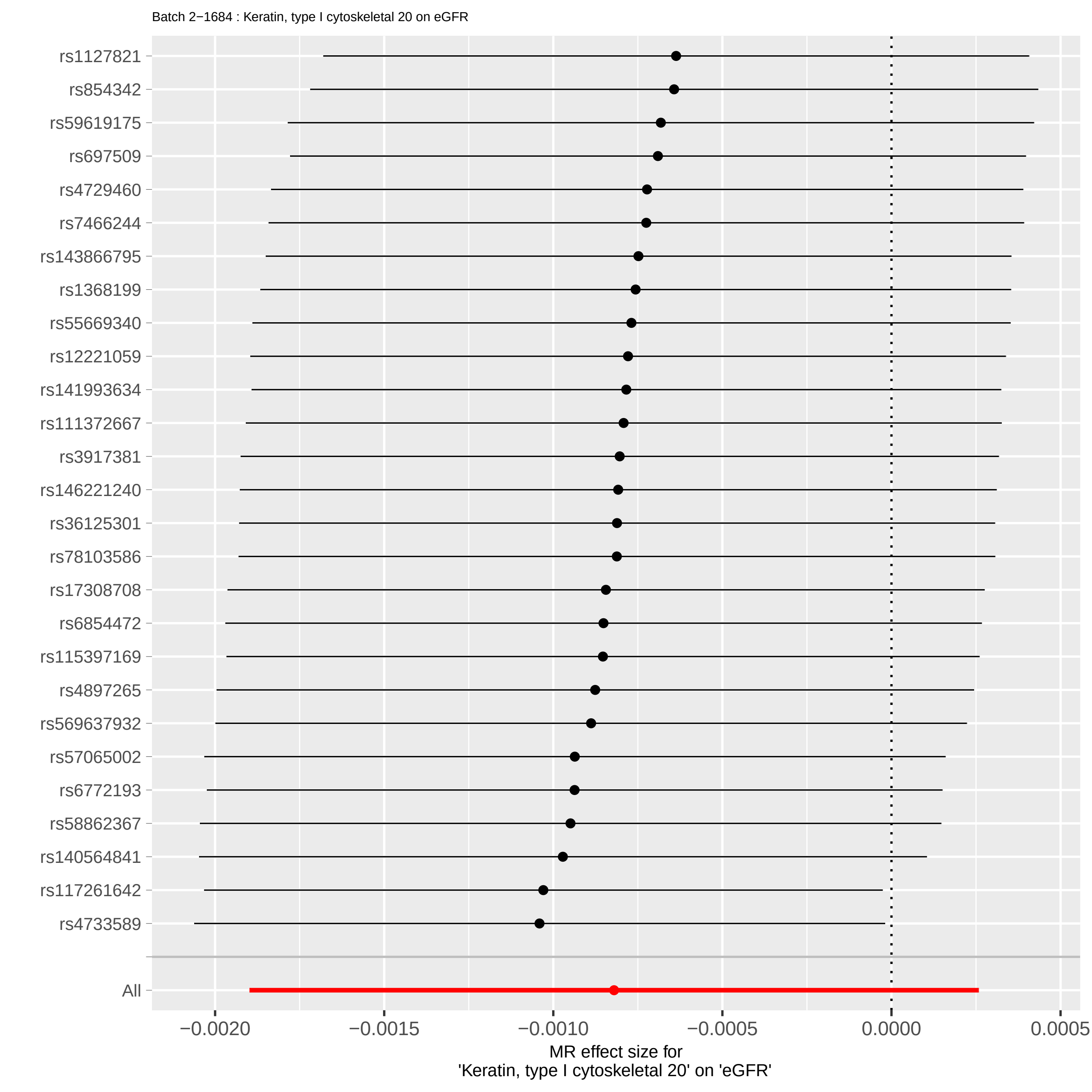
Batch 2−1684 : Keratin, type I cytoskeletal 20 on eGFR
rs1127821
rs854342
rs59619175
rs697509
rs4729460
rs7466244
rs143866795
rs1368199
rs55669340
rs12221059
rs141993634
rs111372667
rs3917381
rs146221240
rs36125301
rs78103586
rs17308708
rs6854472
rs115397169
rs4897265
rs569637932
rs57065002
rs6772193
rs58862367
rs140564841
rs117261642
rs4733589
All
−0.0020
−0.0015
−0.0010
−0.0005
0.0000
0.0005
MR effect size for
'Keratin, type I cytoskeletal 20' on 'eGFR'

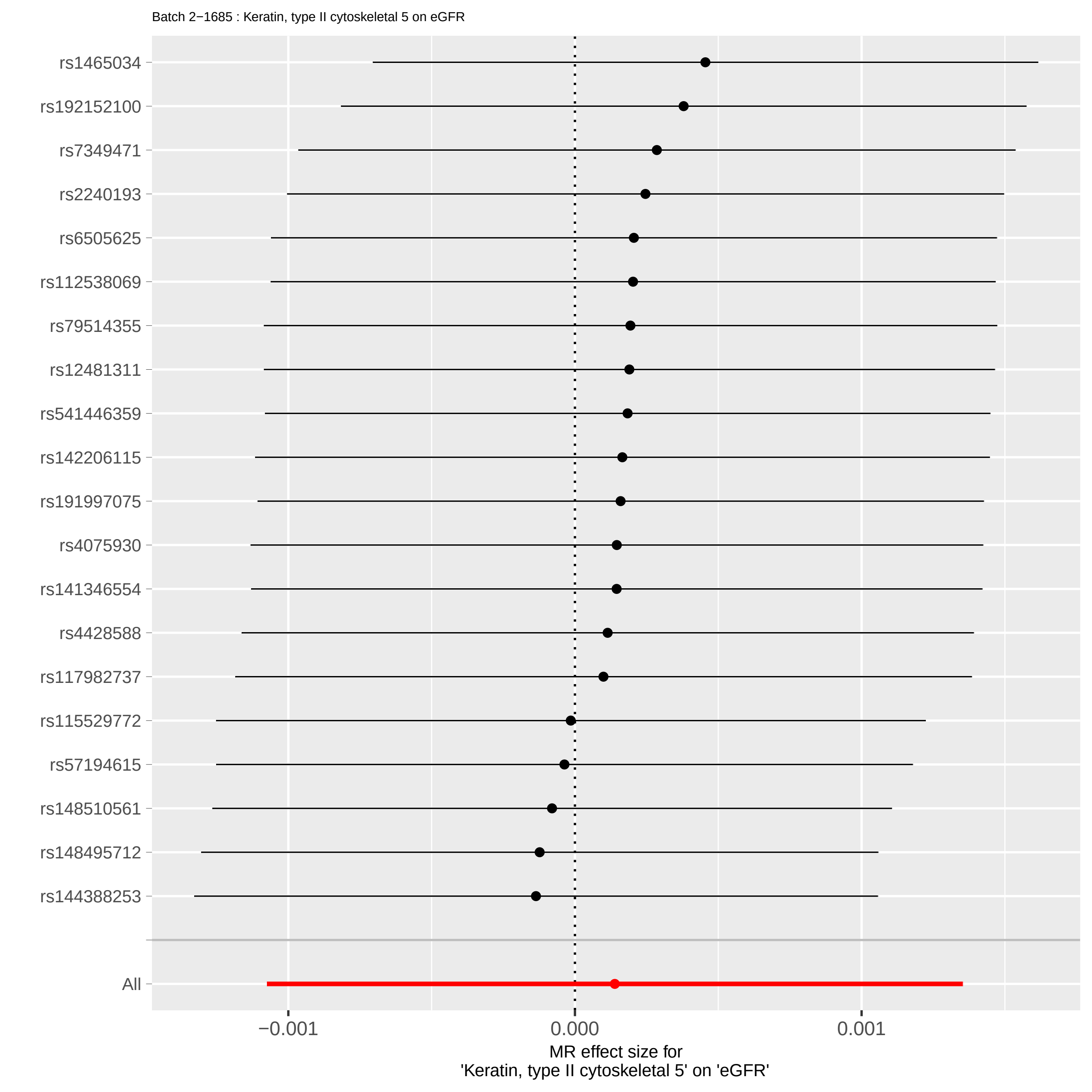

Batch 2−1685 : Keratin, type II cytoskeletal 5 on eGFR
rs1465034
rs192152100
rs7349471
rs2240193
rs6505625
rs112538069
rs79514355
rs12481311
rs541446359
rs142206115
rs191997075
rs4075930
rs141346554
rs4428588
rs117982737
rs115529772
rs57194615
rs148510561
rs148495712
rs144388253
All
−0.001
0.000
0.001
MR effect size for
'Keratin, type II cytoskeletal 5' on 'eGFR'

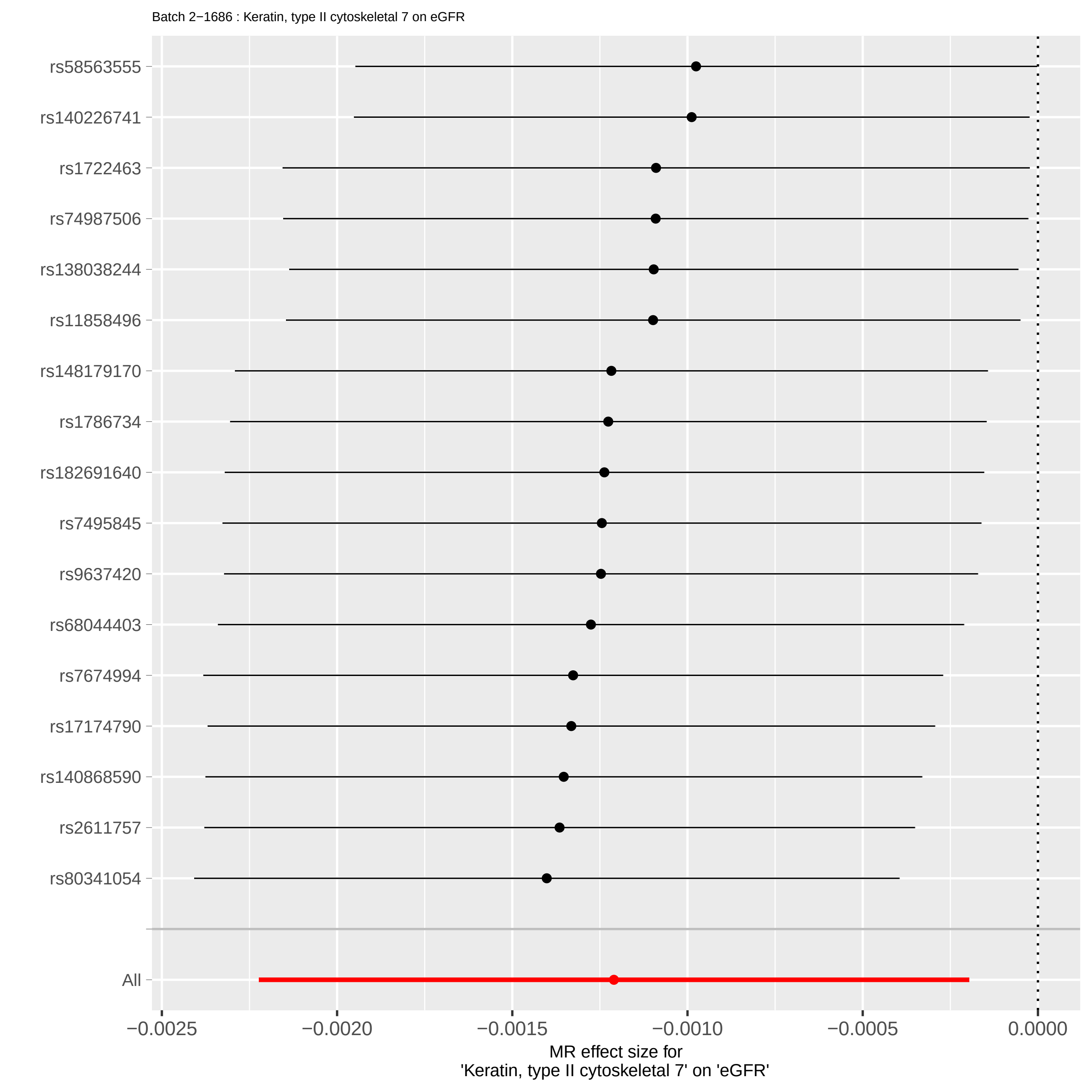

Batch 2−1686 : Keratin, type II cytoskeletal 7 on eGFR
rs58563555
rs140226741
rs1722463
rs74987506
rs138038244
rs11858496
rs148179170
rs1786734
rs182691640
rs7495845
rs9637420
rs68044403
rs7674994
rs17174790
rs140868590
rs2611757
rs80341054
All
−0.0025
−0.0020
−0.0015
−0.0010
−0.0005
0.0000
MR effect size for
'Keratin, type II cytoskeletal 7' on 'eGFR'

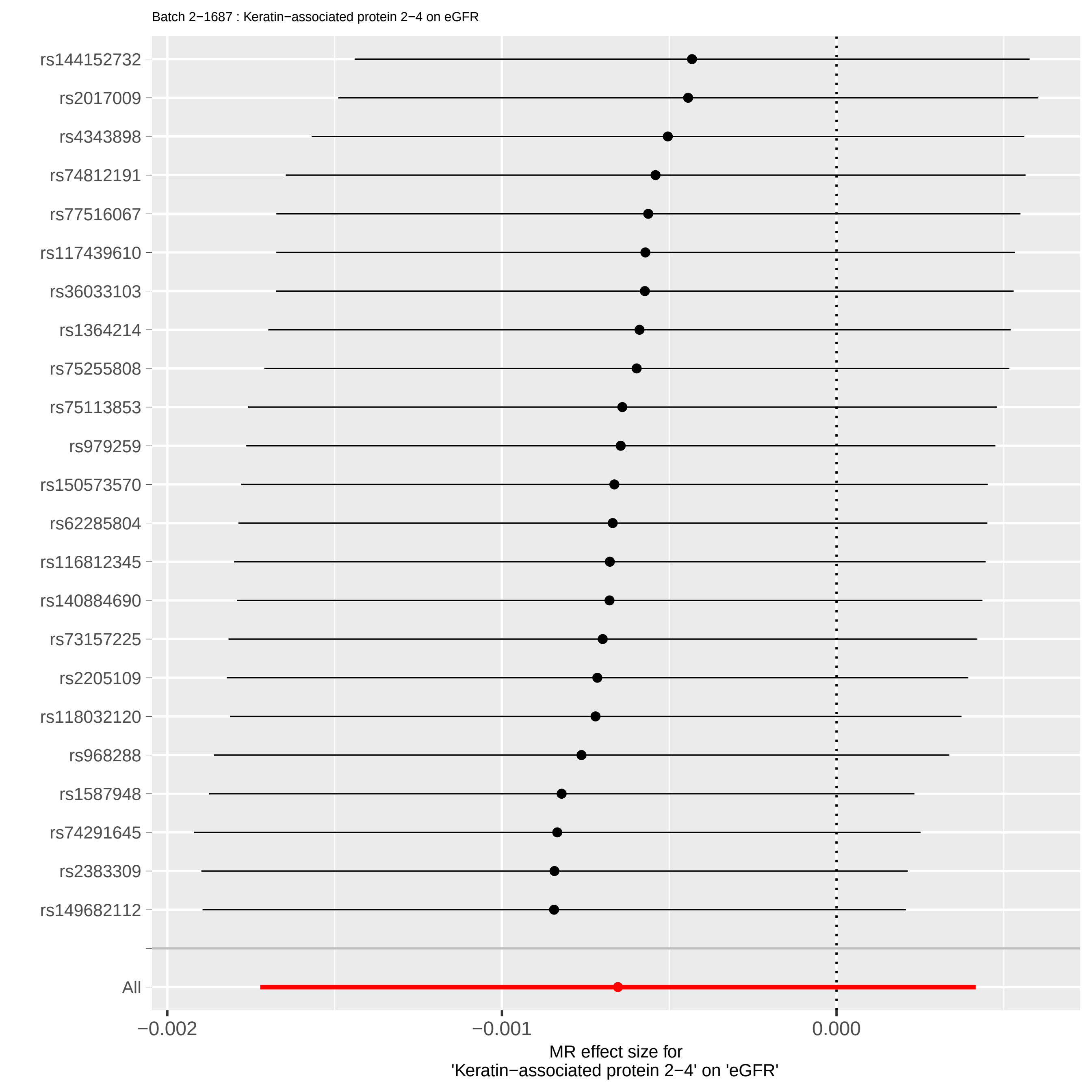
Batch 2−1687 : Keratin−associated protein 2−4 on eGFR
rs144152732
rs2017009
rs4343898
rs74812191
rs77516067
rs117439610
rs36033103
rs1364214
rs75255808
rs75113853
rs979259
rs150573570
rs62285804
rs116812345
rs140884690
rs73157225
rs2205109
rs118032120
rs968288
rs1587948
rs74291645
rs2383309
rs149682112
All
−0.002
−0.001
0.000
MR effect size for
'Keratin−associated protein 2−4' on 'eGFR'

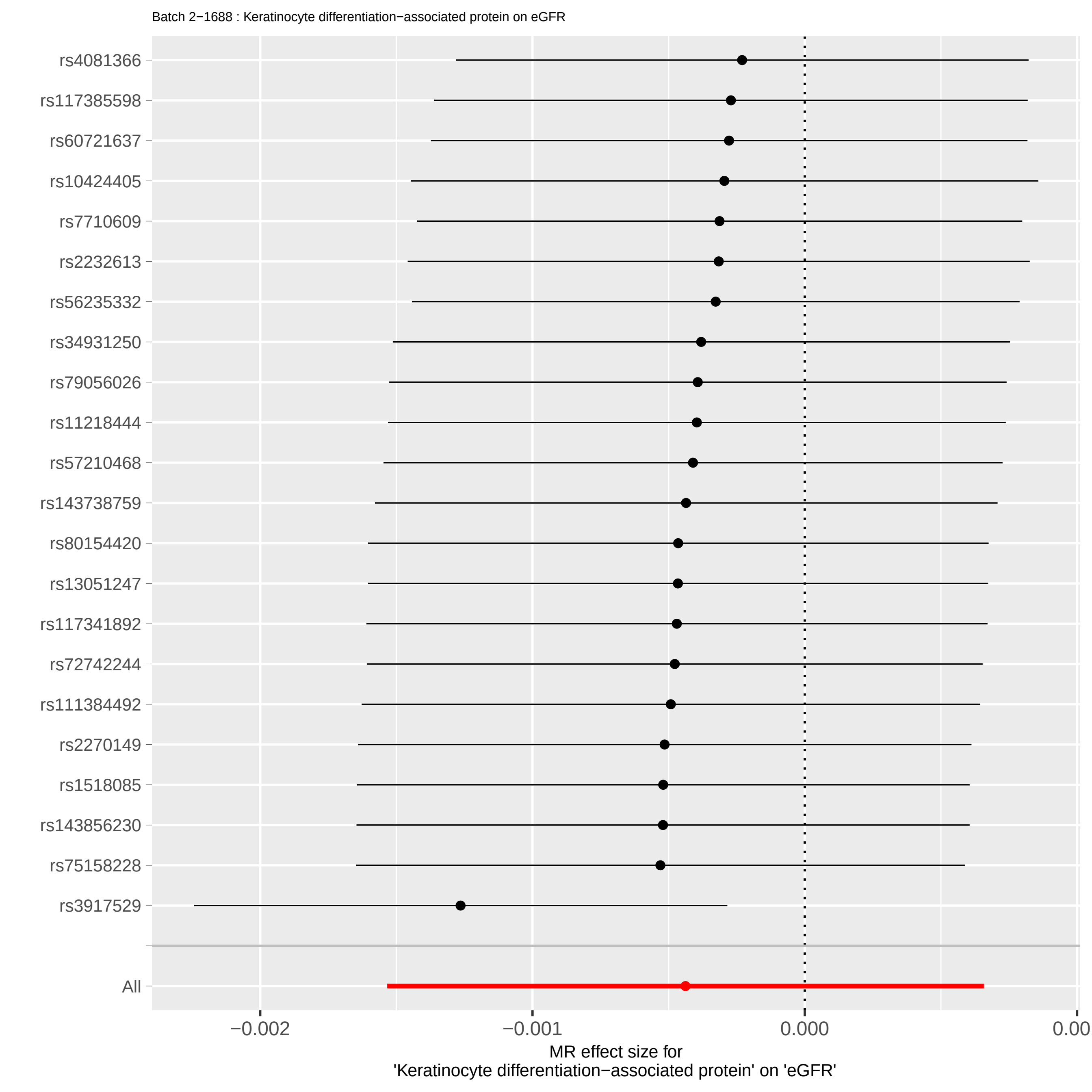

Batch 2−1688 : Keratinocyte differentiation−associated protein on eGFR
rs4081366
rs117385598
rs60721637
rs10424405
rs7710609
rs2232613
rs56235332
rs34931250
rs79056026
rs11218444
rs57210468
rs143738759
rs80154420
rs13051247
rs117341892
rs72742244
rs111384492
rs2270149
rs1518085
rs143856230
rs75158228
rs3917529
All
−0.002
−0.001
0.000
0.00
MR effect size for
'Keratinocyte differentiation−associated protein' on 'eGFR'

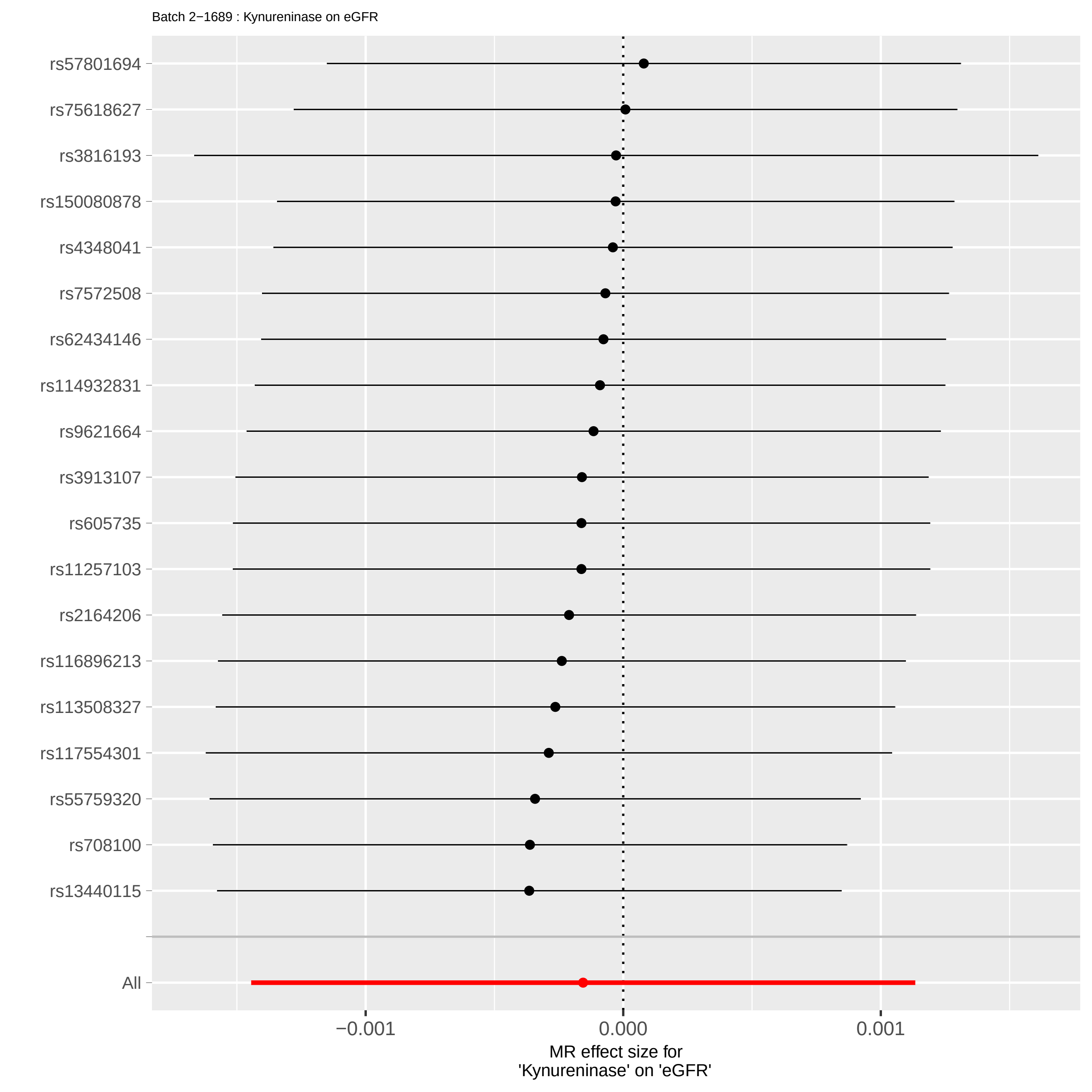

Batch 2−1689 : Kynureninase on eGFR
rs57801694
rs75618627
rs3816193
rs150080878
rs4348041
rs7572508
rs62434146
rs114932831
rs9621664
rs3913107
rs605735
rs11257103
rs2164206
rs116896213
rs113508327
rs117554301
rs55759320
rs708100
rs13440115
All
−0.001
0.000
0.001
MR effect size for
'Kynureninase' on 'eGFR'

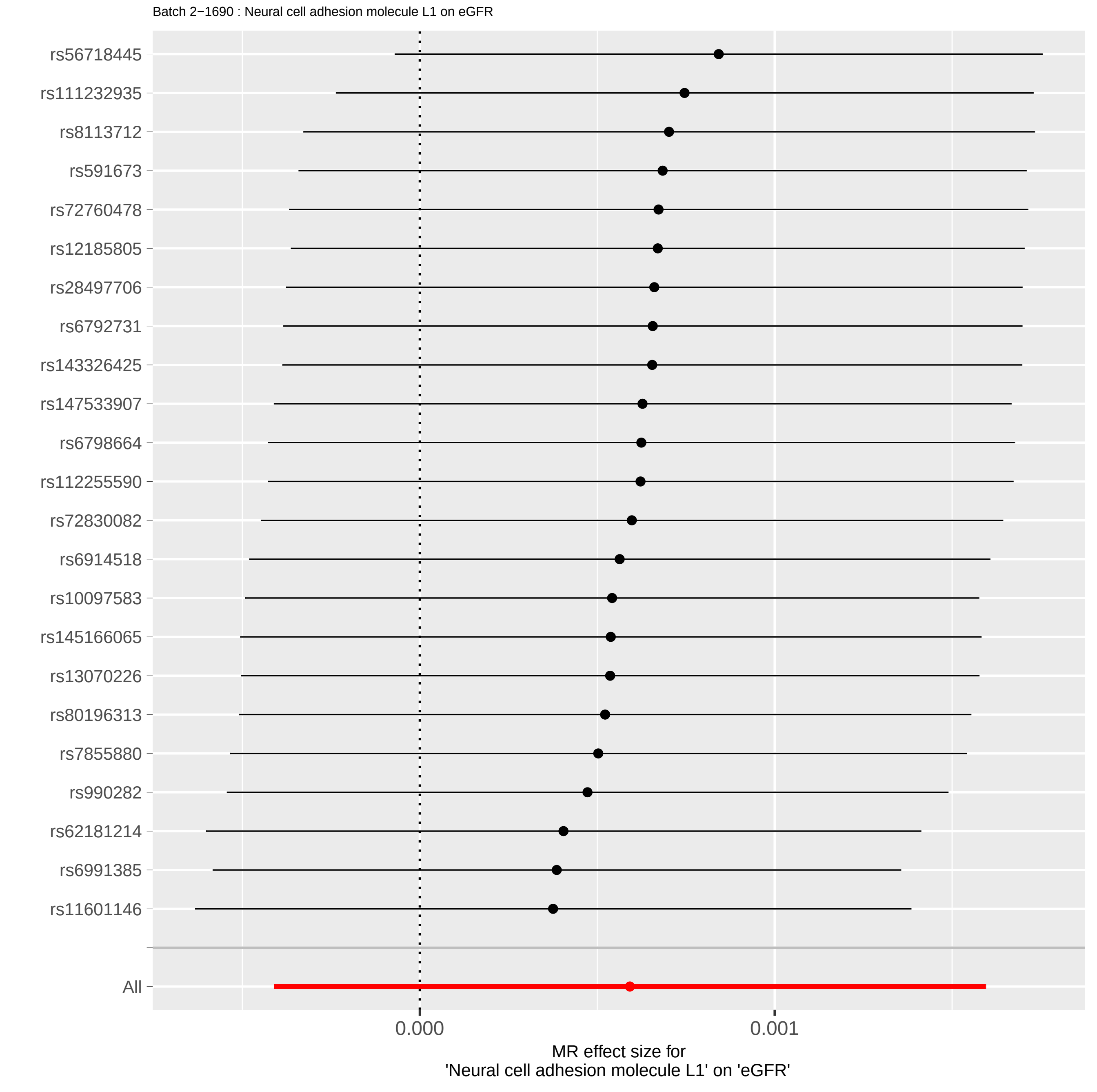

Batch 2−1690 : Neural cell adhesion molecule L1 on eGFR
rs56718445
rs111232935
rs8113712
rs591673
rs72760478
rs12185805
rs28497706
rs6792731
rs143326425
rs147533907
rs6798664
rs112255590
rs72830082
rs6914518
rs10097583
rs145166065
rs13070226
rs80196313
rs7855880
rs990282
rs62181214
rs6991385
rs11601146
All
0.000
0.001
MR effect size for
'Neural cell adhesion molecule L1' on 'eGFR'

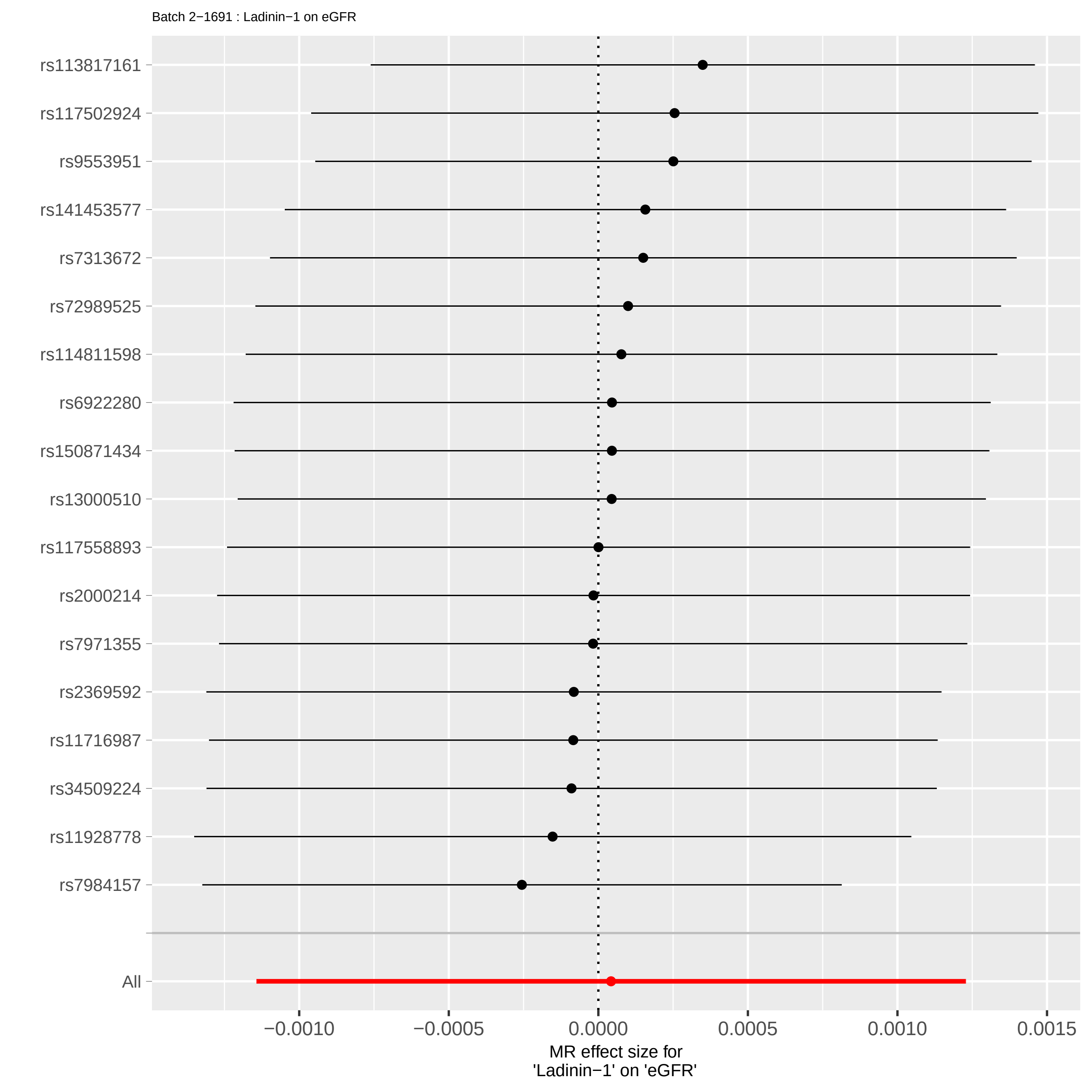

Batch 2−1691 : Ladinin−1 on eGFR
rs113817161
rs117502924
rs9553951
rs141453577
rs7313672
rs72989525
rs114811598
rs6922280
rs150871434
rs13000510
rs117558893
rs2000214
rs7971355
rs2369592
rs11716987
rs34509224
rs11928778
rs7984157
All
−0.0010
−0.0005
0.0000
0.0005
0.0010
0.0015
MR effect size for
'Ladinin−1' on 'eGFR'

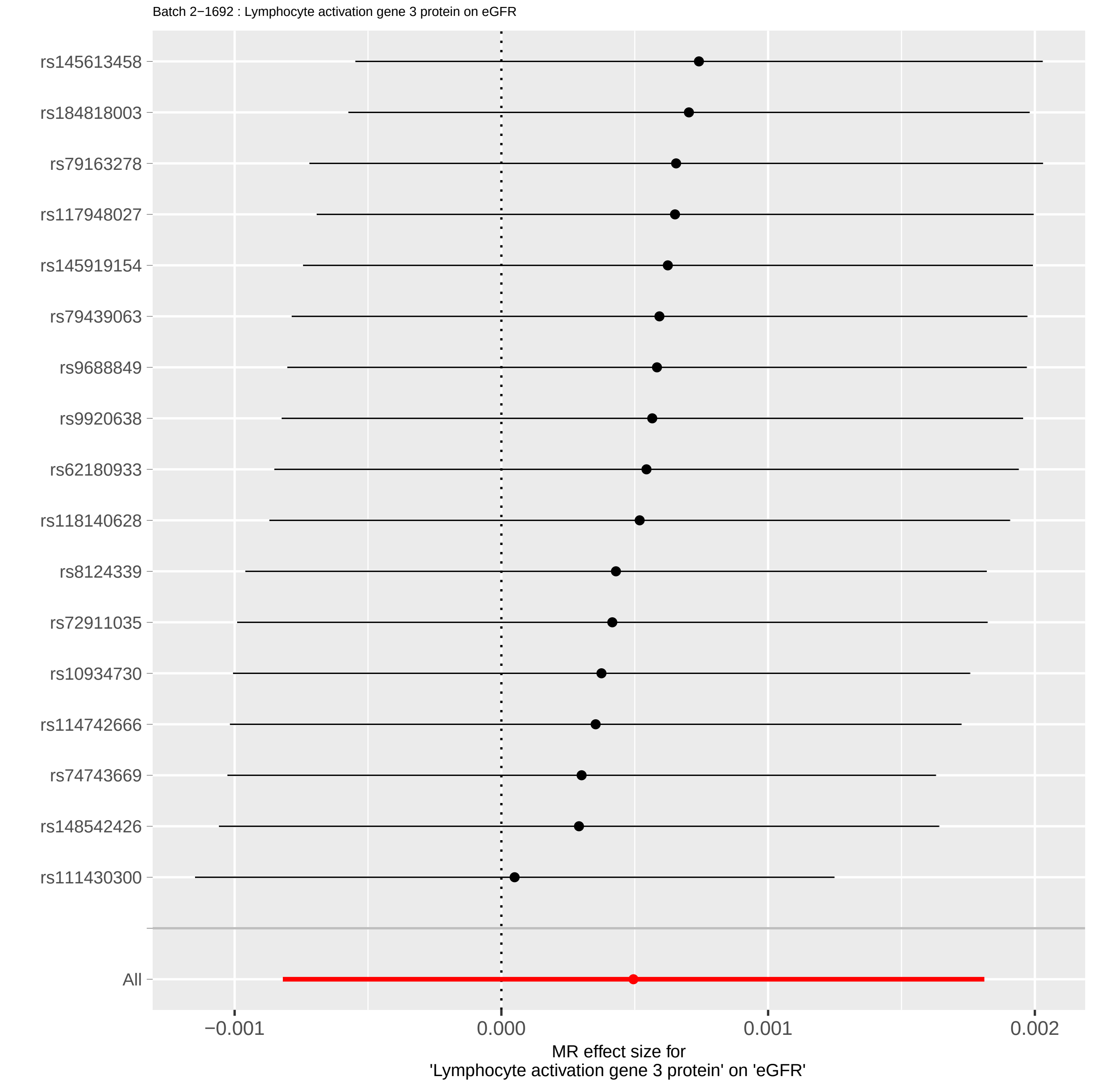

Batch 2−1692 : Lymphocyte activation gene 3 protein on eGFR
rs145613458
rs184818003
rs79163278
rs117948027
rs145919154
rs79439063
rs9688849
rs9920638
rs62180933
rs118140628
rs8124339
rs72911035
rs10934730
rs114742666
rs74743669
rs148542426
rs111430300
All
−0.001
0.000
0.001
0.002
MR effect size for
'Lymphocyte activation gene 3 protein' on 'eGFR'

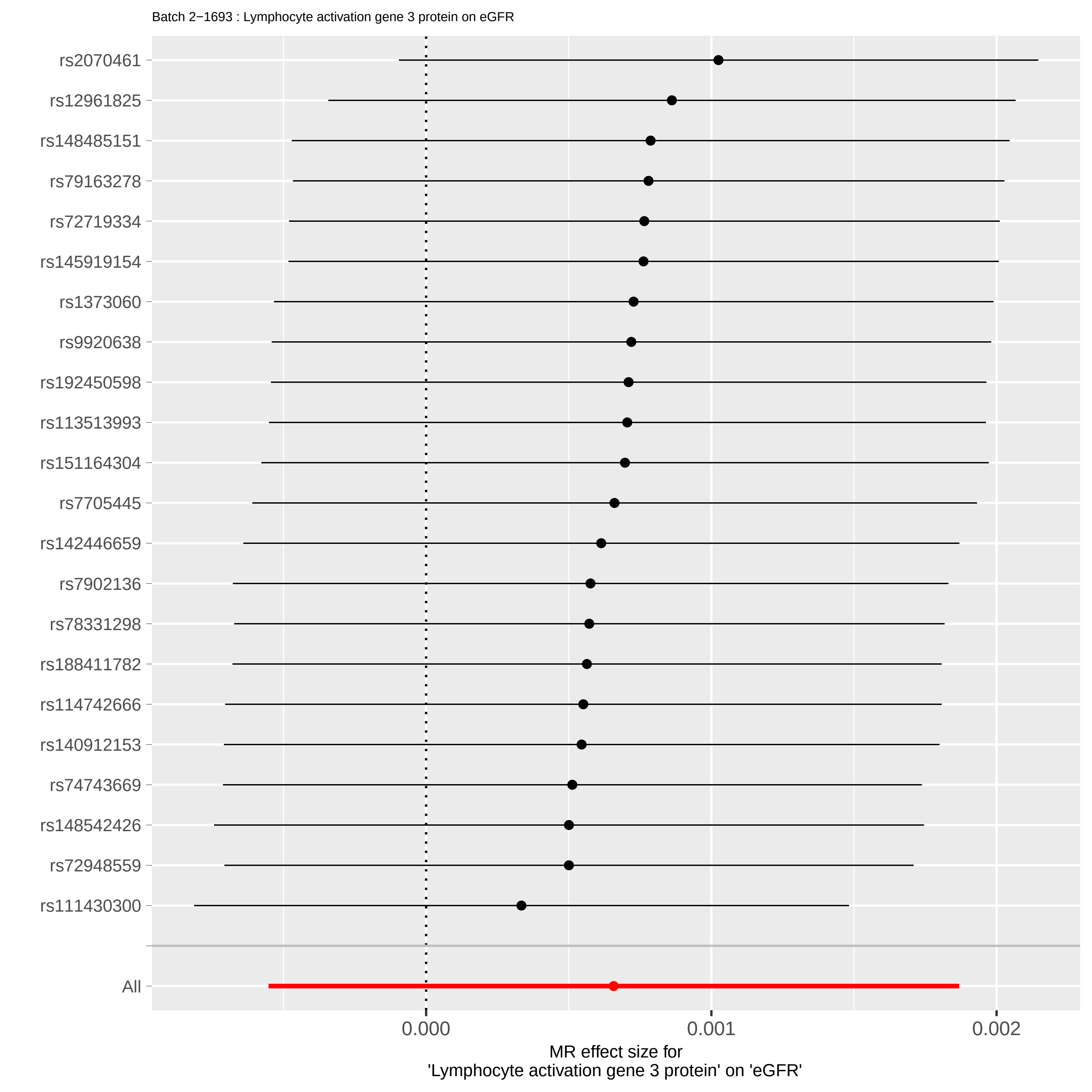
Batch 2−1693 : Lymphocyte activation gene 3 protein on eGFR
rs2070461
rs12961825
rs148485151
rs79163278
rs72719334
rs145919154
rs1373060
rs9920638
rs192450598
rs113513993
rs151164304
rs7705445
rs142446659
rs7902136
rs78331298
rs188411782
rs114742666
rs140912153
rs74743669
rs148542426
rs72948559
rs111430300
All
0.000
0.001
0.002
MR effect size for
'Lymphocyte activation gene 3 protein' on 'eGFR'

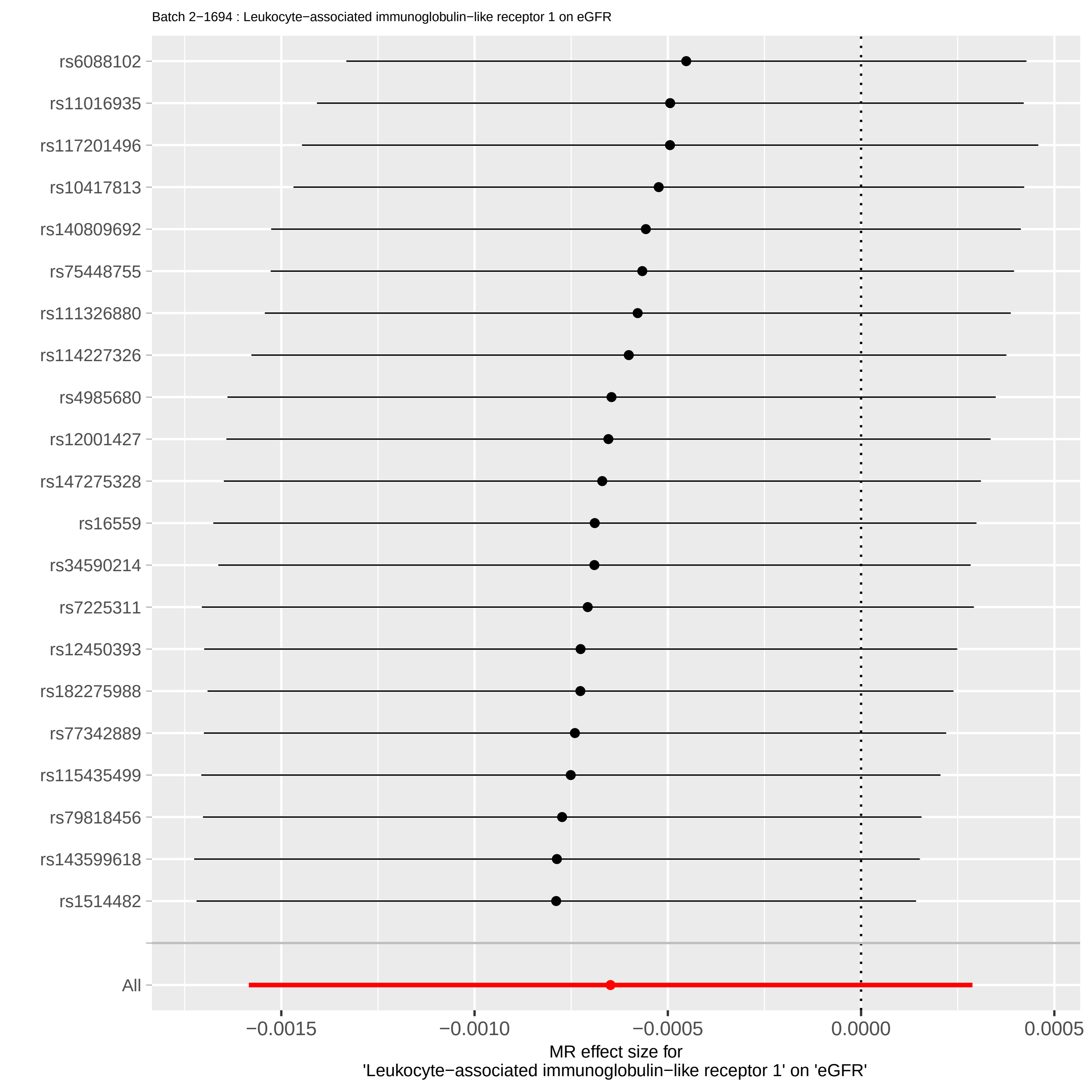

Batch 2−1694 : Leukocyte−associated immunoglobulin−like receptor 1 on eGFR
rs6088102
rs11016935
rs117201496
rs10417813
rs140809692
rs75448755
rs111326880
rs114227326
rs4985680
rs12001427
rs147275328
rs16559
rs34590214
rs7225311
rs12450393
rs182275988
rs77342889
rs115435499
rs79818456
rs143599618
rs1514482
All
−0.0015
−0.0010
−0.0005
0.0000
0.0005
MR effect size for
'Leukocyte−associated immunoglobulin−like receptor 1' on 'eGFR'

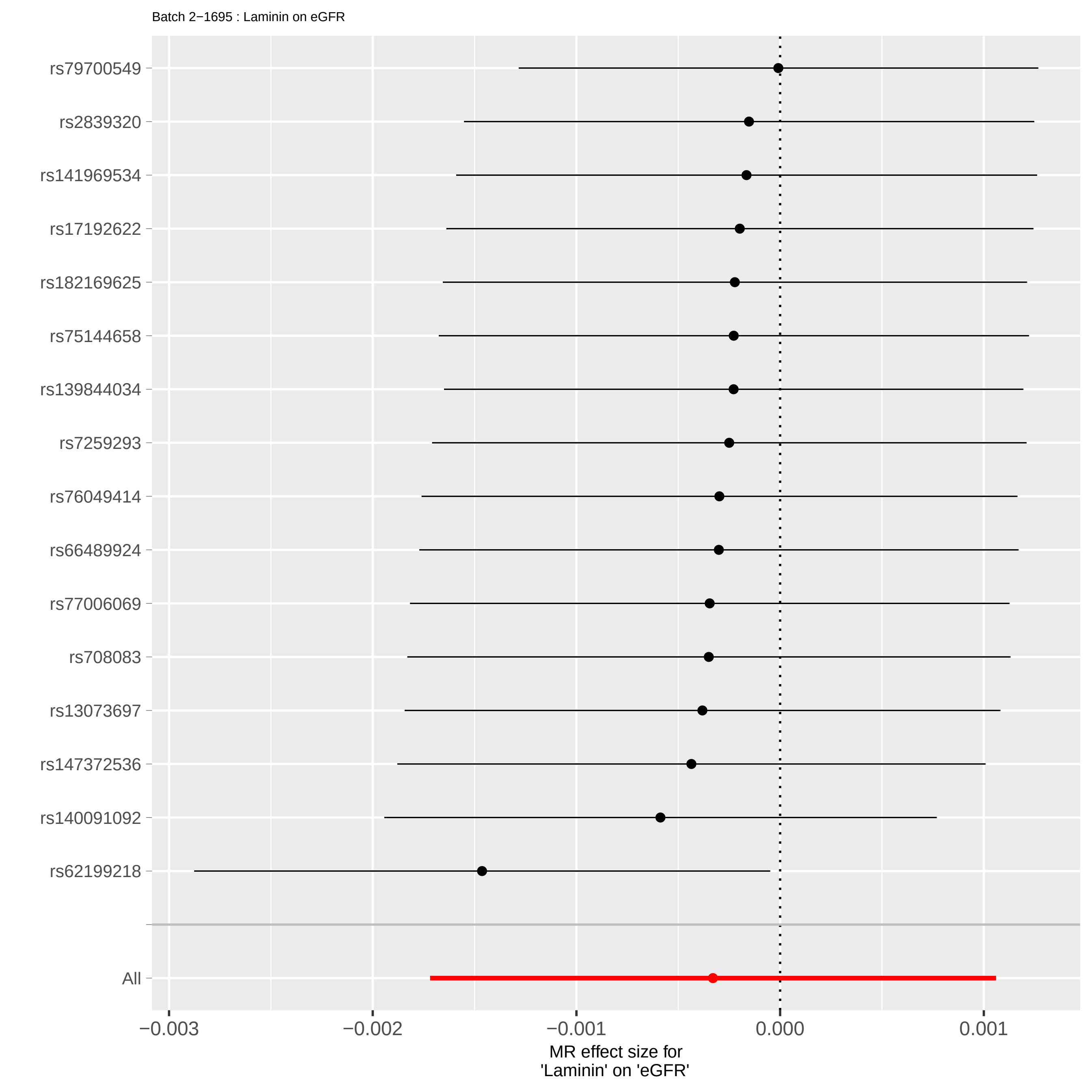
Batch 2−1695 : Laminin on eGFR
rs79700549
rs2839320
rs141969534
rs17192622
rs182169625
rs75144658
rs139844034
rs7259293
rs76049414
rs66489924
rs77006069
rs708083
rs13073697
rs147372536
rs140091092
rs62199218
All
−0.003
−0.002
−0.001
0.000
0.001
MR effect size for
'Laminin' on 'eGFR'

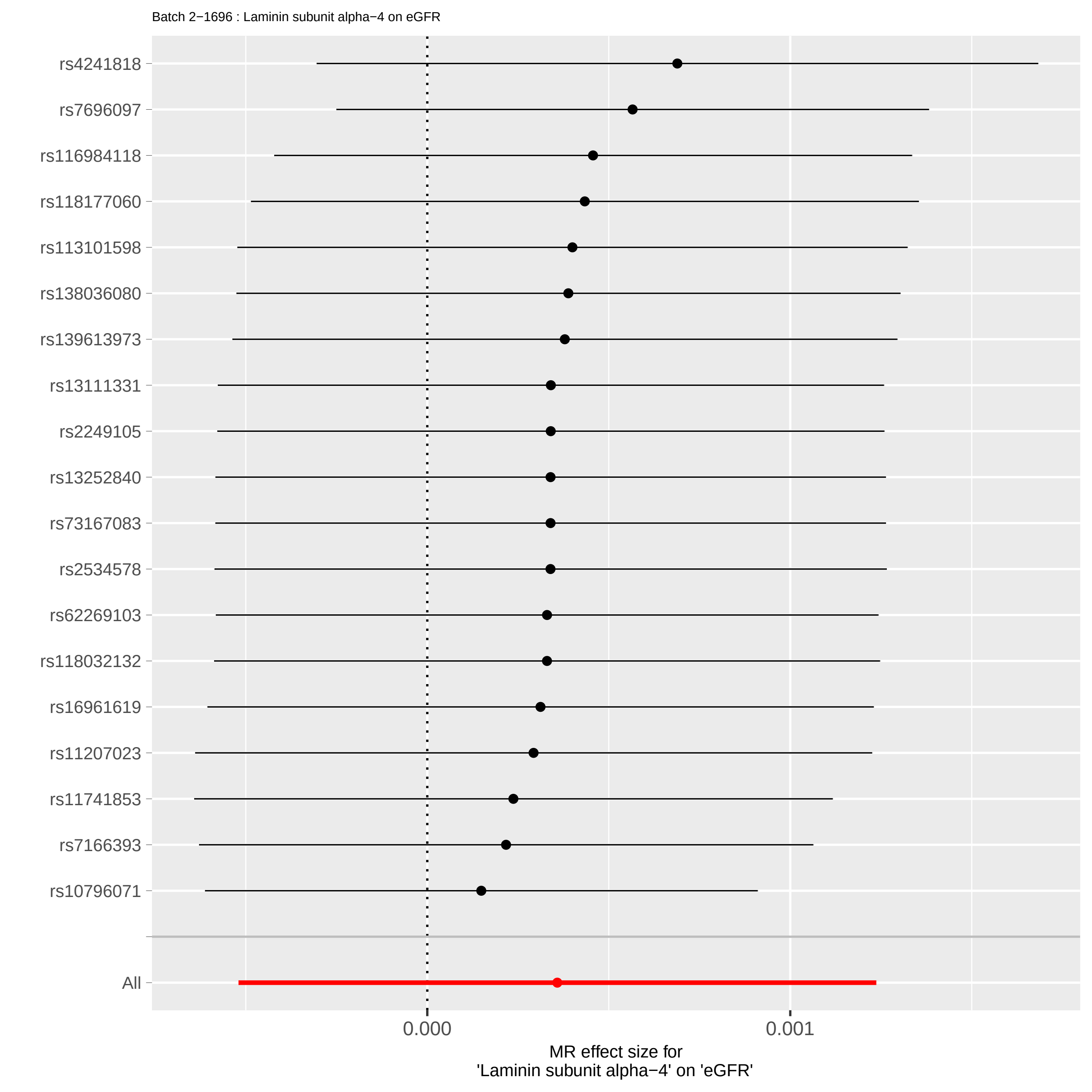

Batch 2−1696 : Laminin subunit alpha−4 on eGFR
rs4241818
rs7696097
rs116984118
rs118177060
rs113101598
rs138036080
rs139613973
rs13111331
rs2249105
rs13252840
rs73167083
rs2534578
rs62269103
rs118032132
rs16961619
rs11207023
rs11741853
rs7166393
rs10796071
All
0.000
0.001
MR effect size for
'Laminin subunit alpha−4' on 'eGFR'

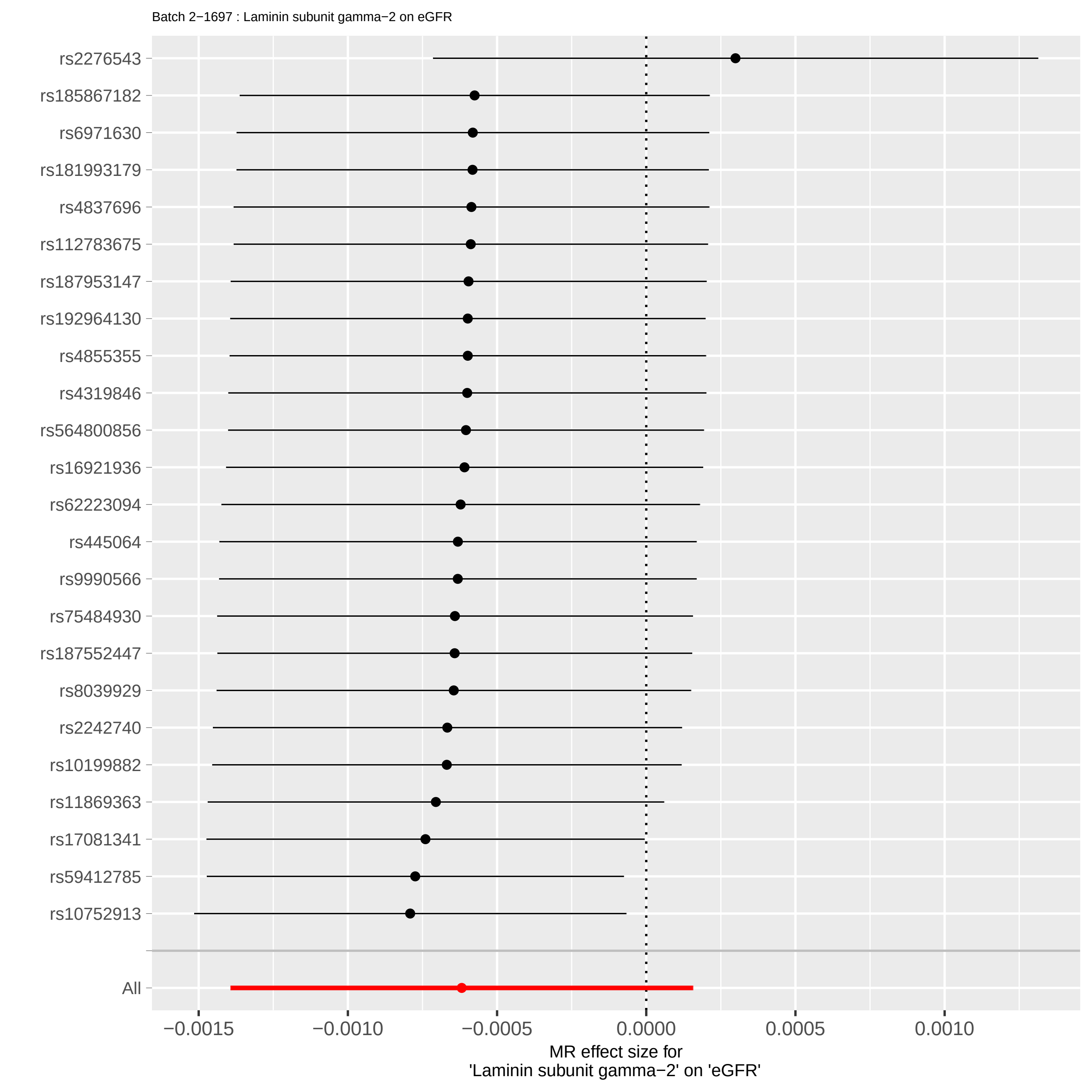

Batch 2−1697 : Laminin subunit gamma−2 on eGFR
rs2276543
rs185867182
rs6971630
rs181993179
rs4837696
rs112783675
rs187953147
rs192964130
rs4855355
rs4319846
rs564800856
rs16921936
rs62223094
rs445064
rs9990566
rs75484930
rs187552447
rs8039929
rs2242740
rs10199882
rs11869363
rs17081341
rs59412785
rs10752913
All
−0.0015
−0.0010
−0.0005
0.0000
0.0005
0.0010
MR effect size for
'Laminin subunit gamma−2' on 'eGFR'

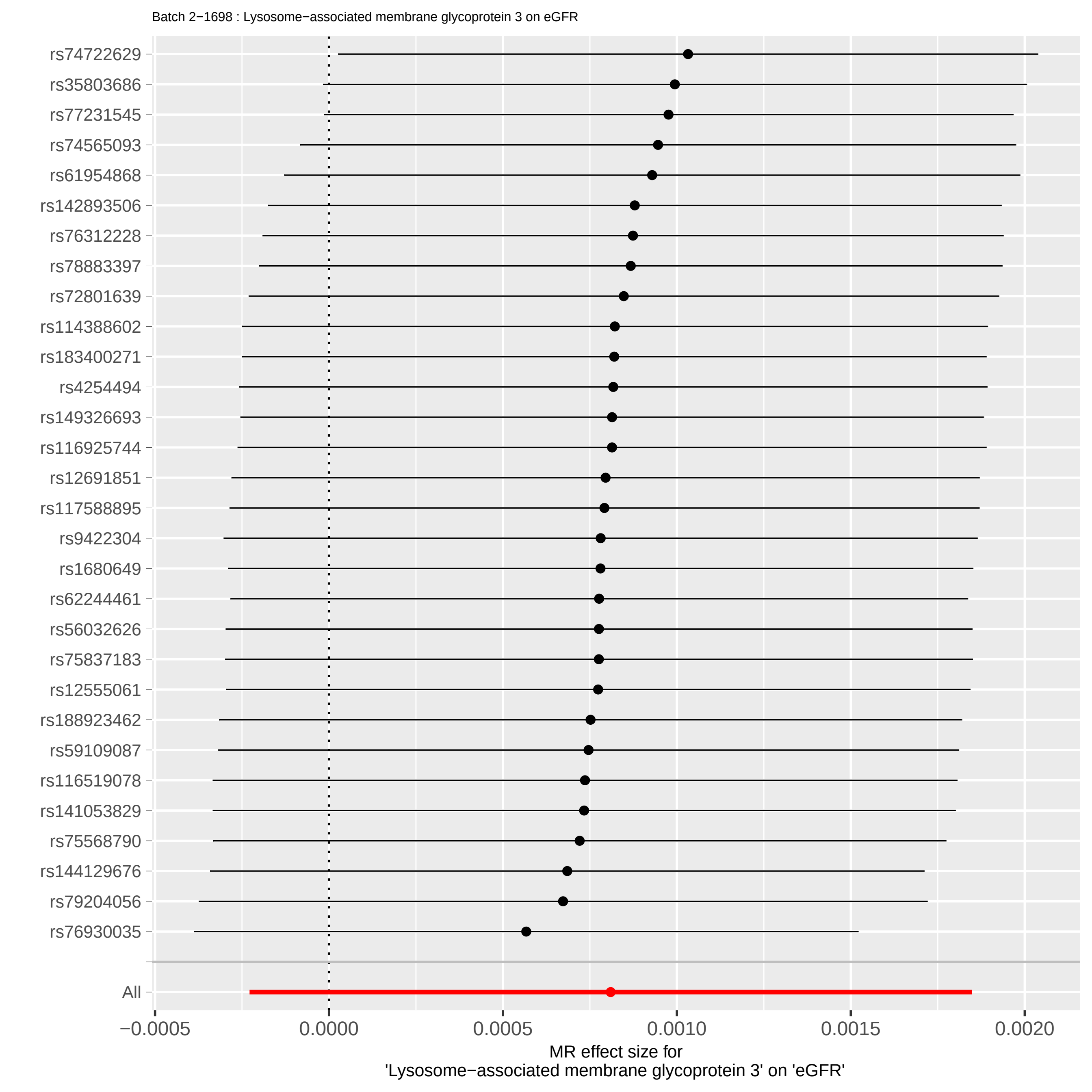
Batch 2−1698 : Lysosome−associated membrane glycoprotein 3 on eGFR
rs74722629
rs35803686
rs77231545
rs74565093
rs61954868
rs142893506
rs76312228
rs78883397
rs72801639
rs114388602
rs183400271
rs4254494
rs149326693
rs116925744
rs12691851
rs117588895
rs9422304
rs1680649
rs62244461
rs56032626
rs75837183
rs12555061
rs188923462
rs59109087
rs116519078
rs141053829
rs75568790
rs144129676
rs79204056
rs76930035
All
−0.0005
0.0000
0.0005
0.0010
0.0015
0.0020
MR effect size for
'Lysosome−associated membrane glycoprotein 3' on 'eGFR'

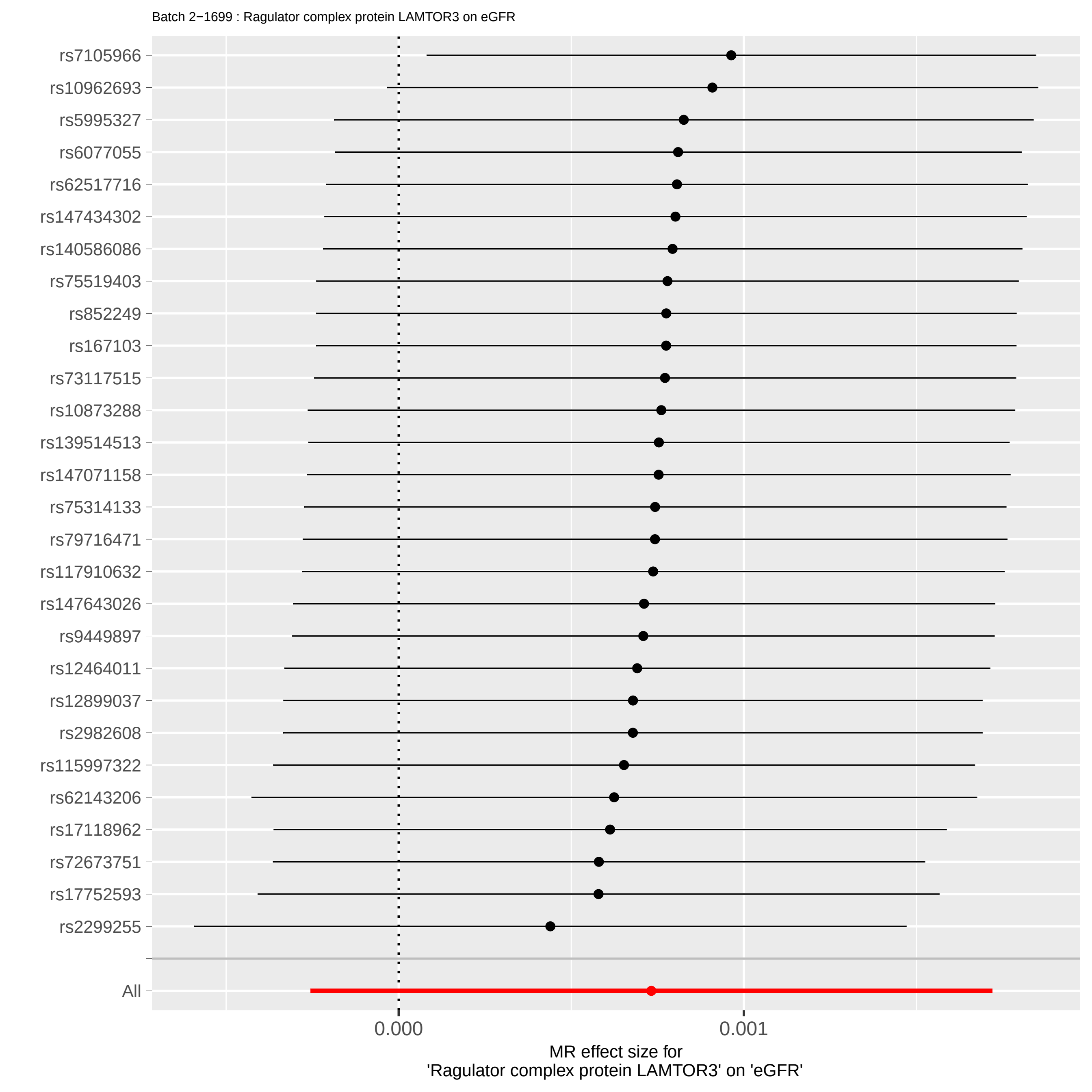

Batch 2−1699 : Ragulator complex protein LAMTOR3 on eGFR
rs7105966
rs10962693
rs5995327
rs6077055
rs62517716
rs147434302
rs140586086
rs75519403
rs852249
rs167103
rs73117515
rs10873288
rs139514513
rs147071158
rs75314133
rs79716471
rs117910632
rs147643026
rs9449897
rs12464011
rs12899037
rs2982608
rs115997322
rs62143206
rs17118962
rs72673751
rs17752593
rs2299255
All
0.000
0.001
MR effect size for
'Ragulator complex protein LAMTOR3' on 'eGFR'

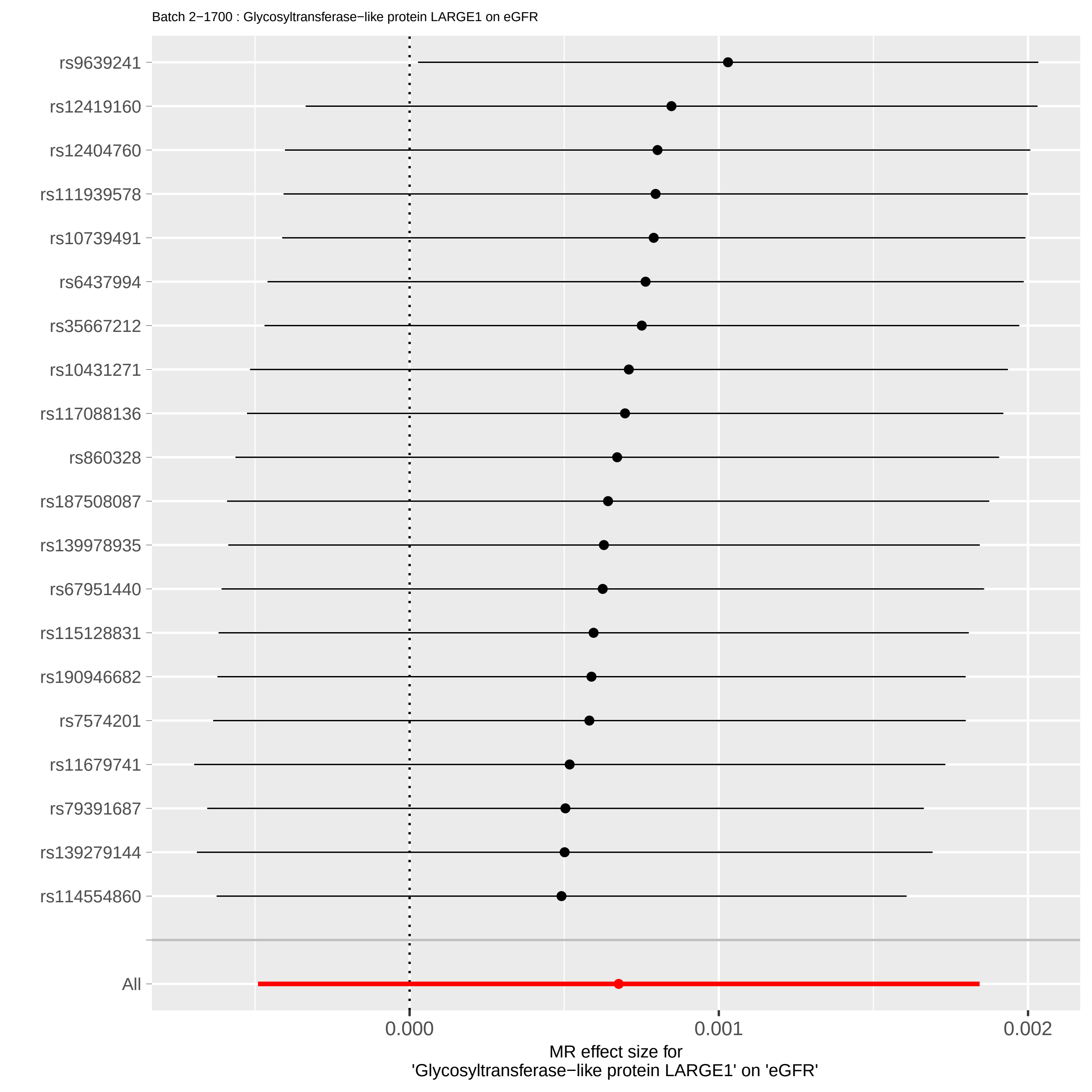
Batch 2−1700 : Glycosyltransferase−like protein LARGE1 on eGFR
rs9639241
rs12419160
rs12404760
rs111939578
rs10739491
rs6437994
rs35667212
rs10431271
rs117088136
rs860328
rs187508087
rs139978935
rs67951440
rs115128831
rs190946682
rs7574201
rs11679741
rs79391687
rs139279144
rs114554860
All
0.000
0.001
0.002
MR effect size for
'Glycosyltransferase−like protein LARGE1' on 'eGFR'

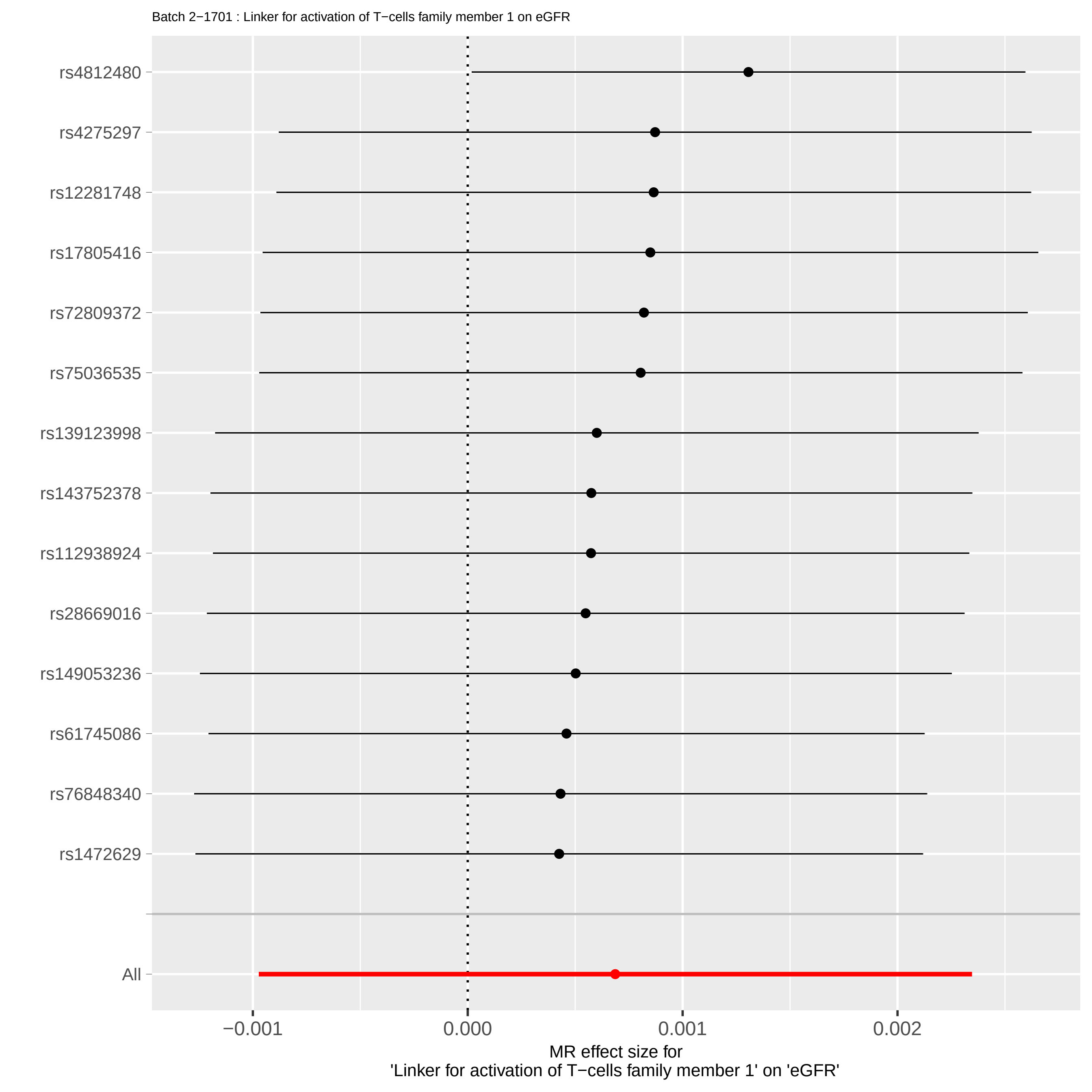
Batch 2−1701 : Linker for activation of T−cells family member 1 on eGFR
rs4812480
rs4275297
rs12281748
rs17805416
rs72809372
rs75036535
rs139123998
rs143752378
rs112938924
rs28669016
rs149053236
rs61745086
rs76848340
rs1472629
All
−0.001
0.000
0.001
0.002
MR effect size for
'Linker for activation of T−cells family member 1' on 'eGFR'

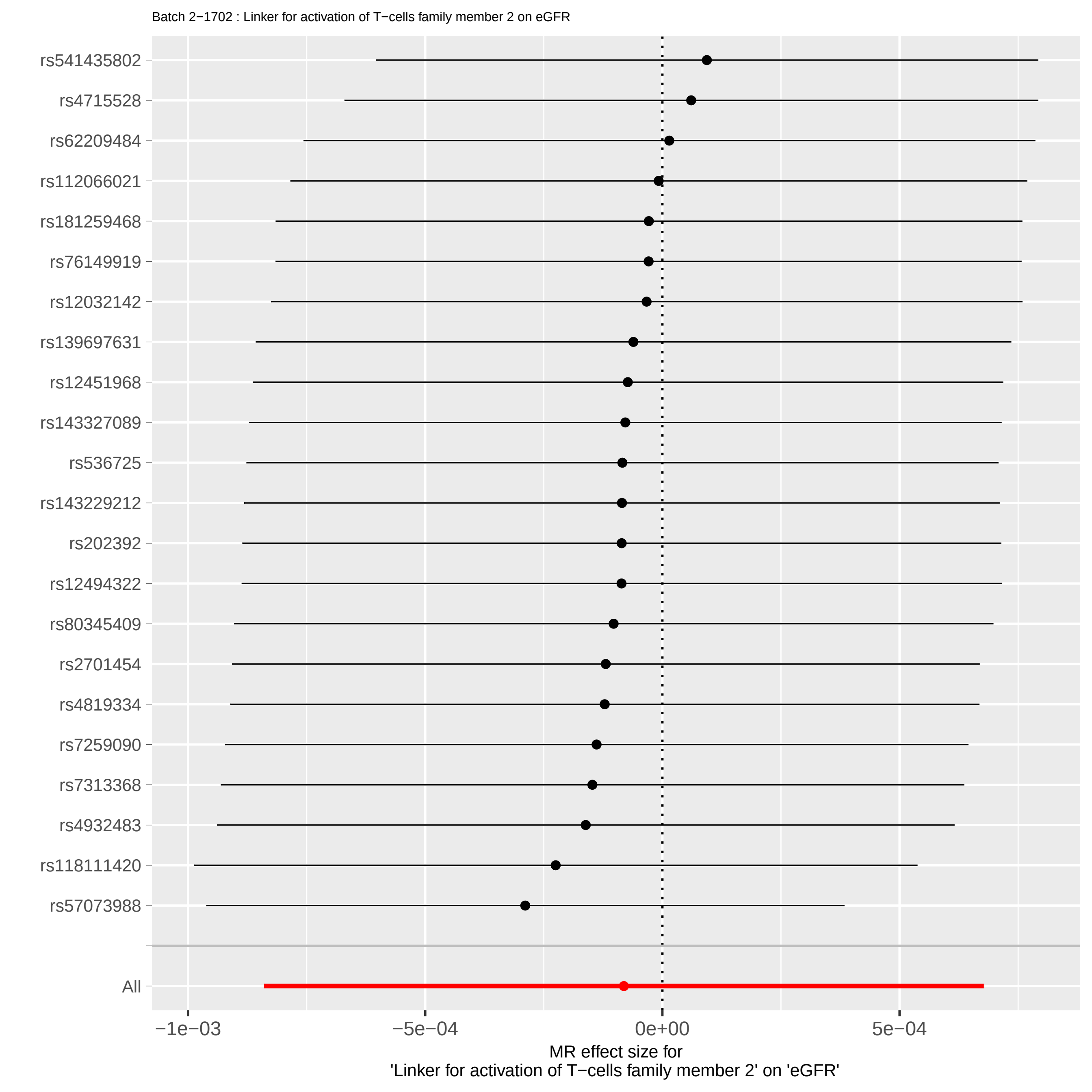
Batch 2−1702 : Linker for activation of T−cells family member 2 on eGFR
rs541435802
rs4715528
rs62209484
rs112066021
rs181259468
rs76149919
rs12032142
rs139697631
rs12451968
rs143327089
rs536725
rs143229212
rs202392
rs12494322
rs80345409
rs2701454
rs4819334
rs7259090
rs7313368
rs4932483
rs118111420
rs57073988
All
−1e−03
−5e−04
0e+00
5e−04
MR effect size for
'Linker for activation of T−cells family member 2' on 'eGFR'

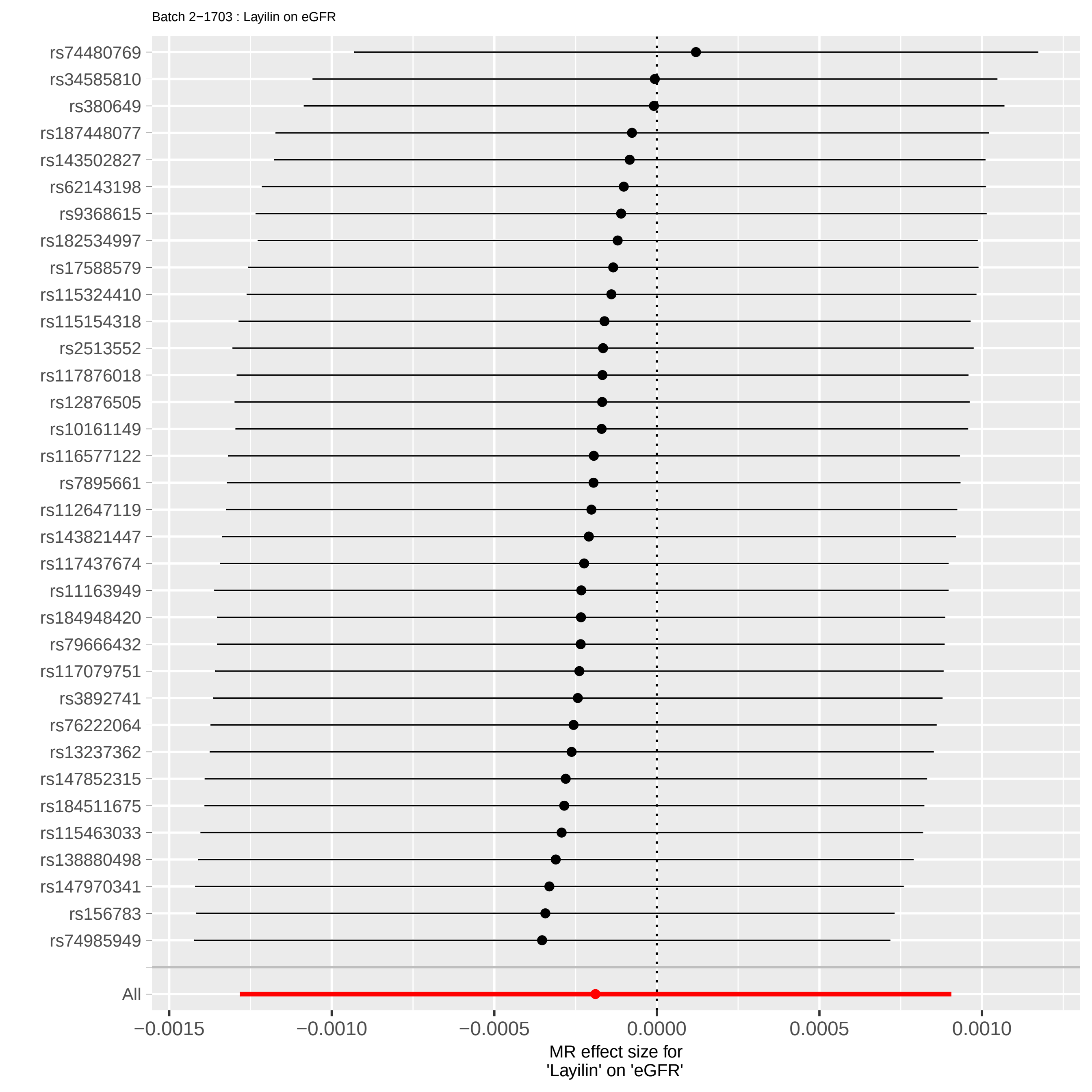

Batch 2−1703 : Layilin on eGFR
rs74480769
rs34585810
rs380649
rs187448077
rs143502827
rs62143198
rs9368615
rs182534997
rs17588579
rs115324410
rs115154318
rs2513552
rs117876018
rs12876505
rs10161149
rs116577122
rs7895661
rs112647119
rs143821447
rs117437674
rs11163949
rs184948420
rs79666432
rs117079751
rs3892741
rs76222064
rs13237362
rs147852315
rs184511675
rs115463033
rs138880498
rs147970341
rs156783
rs74985949
All
−0.0015
−0.0010
−0.0005
0.0000
0.0005
0.0010
MR effect size for
'Layilin' on 'eGFR'

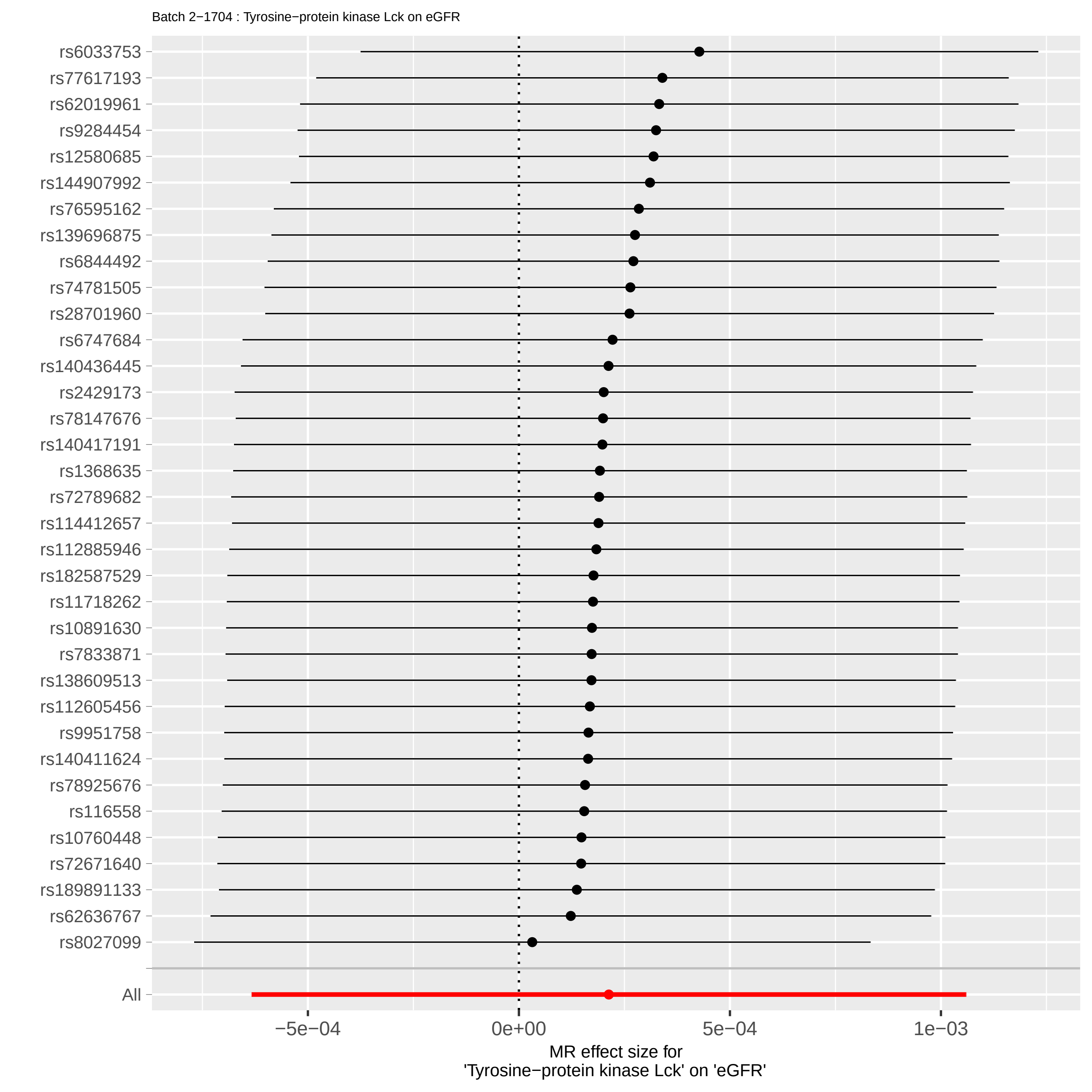

Batch 2−1704 : Tyrosine−protein kinase Lck on eGFR
rs6033753
rs77617193
rs62019961
rs9284454
rs12580685
rs144907992
rs76595162
rs139696875
rs6844492
rs74781505
rs28701960
rs6747684
rs140436445
rs2429173
rs78147676
rs140417191
rs1368635
rs72789682
rs114412657
rs112885946
rs182587529
rs11718262
rs10891630
rs7833871
rs138609513
rs112605456
rs9951758
rs140411624
rs78925676
rs116558
rs10760448
rs72671640
rs189891133
rs62636767
rs8027099
All
−5e−04
0e+00
5e−04
1e−03
MR effect size for
'Tyrosine−protein kinase Lck' on 'eGFR'

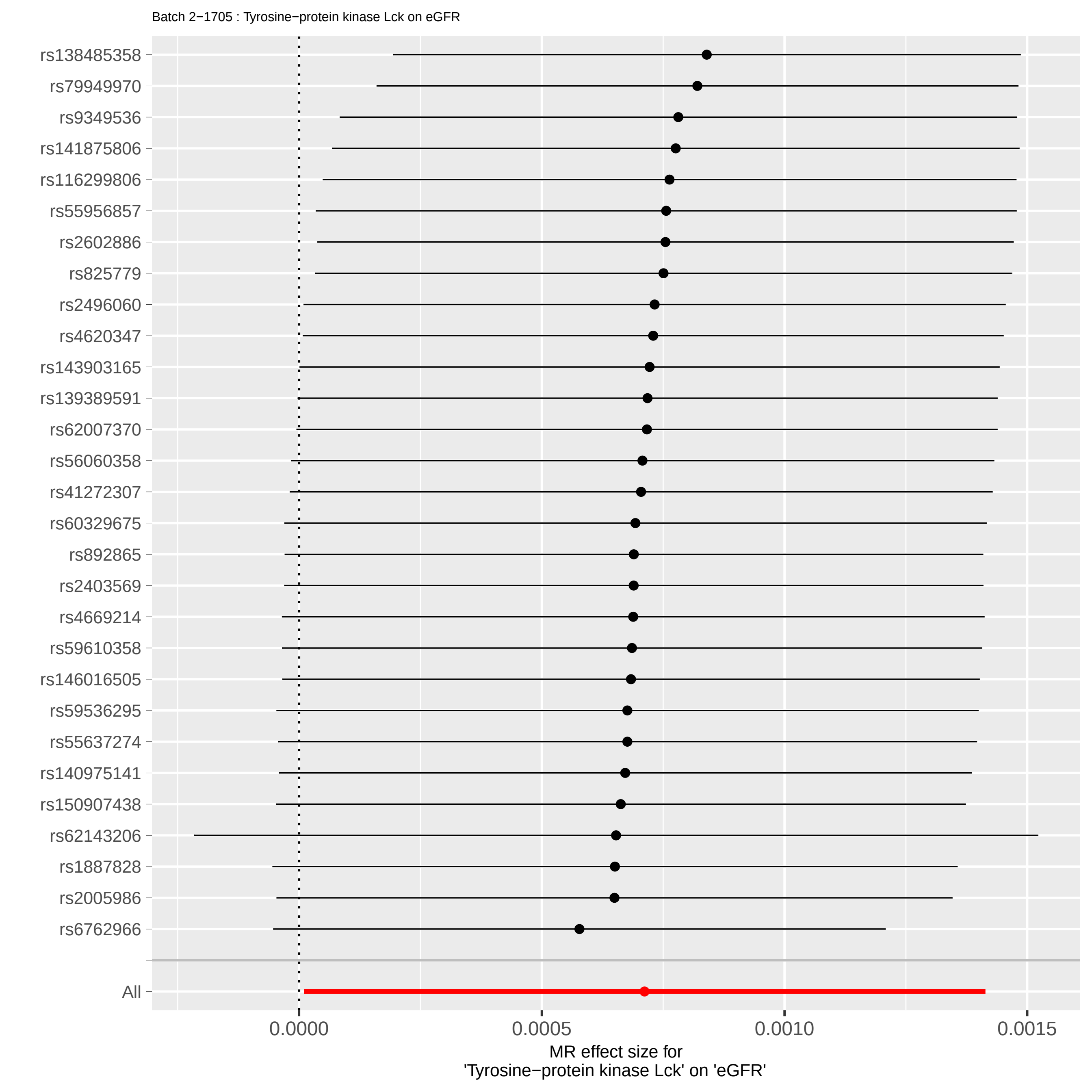

Batch 2−1705 : Tyrosine−protein kinase Lck on eGFR
rs138485358
rs79949970
rs9349536
rs141875806
rs116299806
rs55956857
rs2602886
rs825779
rs2496060
rs4620347
rs143903165
rs139389591
rs62007370
rs56060358
rs41272307
rs60329675
rs892865
rs2403569
rs4669214
rs59610358
rs146016505
rs59536295
rs55637274
rs140975141
rs150907438
rs62143206
rs1887828
rs2005986
rs6762966
All
0.0000
0.0005
0.0010
0.0015
MR effect size for
'Tyrosine−protein kinase Lck' on 'eGFR'

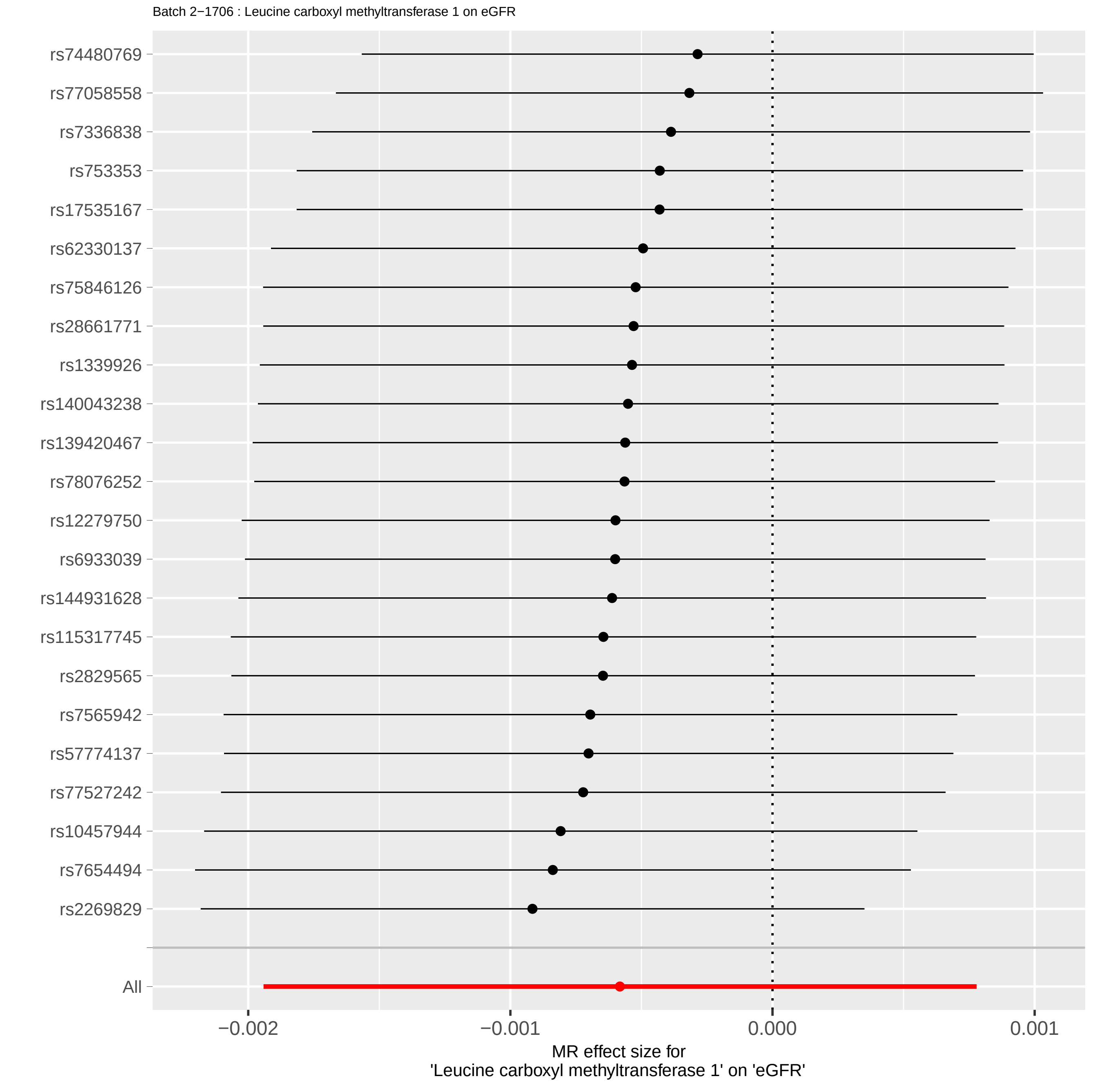

Batch 2−1706 : Leucine carboxyl methyltransferase 1 on eGFR
rs74480769
rs77058558
rs7336838
rs753353
rs17535167
rs62330137
rs75846126
rs28661771
rs1339926
rs140043238
rs139420467
rs78076252
rs12279750
rs6933039
rs144931628
rs115317745
rs2829565
rs7565942
rs57774137
rs77527242
rs10457944
rs7654494
rs2269829
All
−0.002
−0.001
0.000
0.001
MR effect size for
'Leucine carboxyl methyltransferase 1' on 'eGFR'

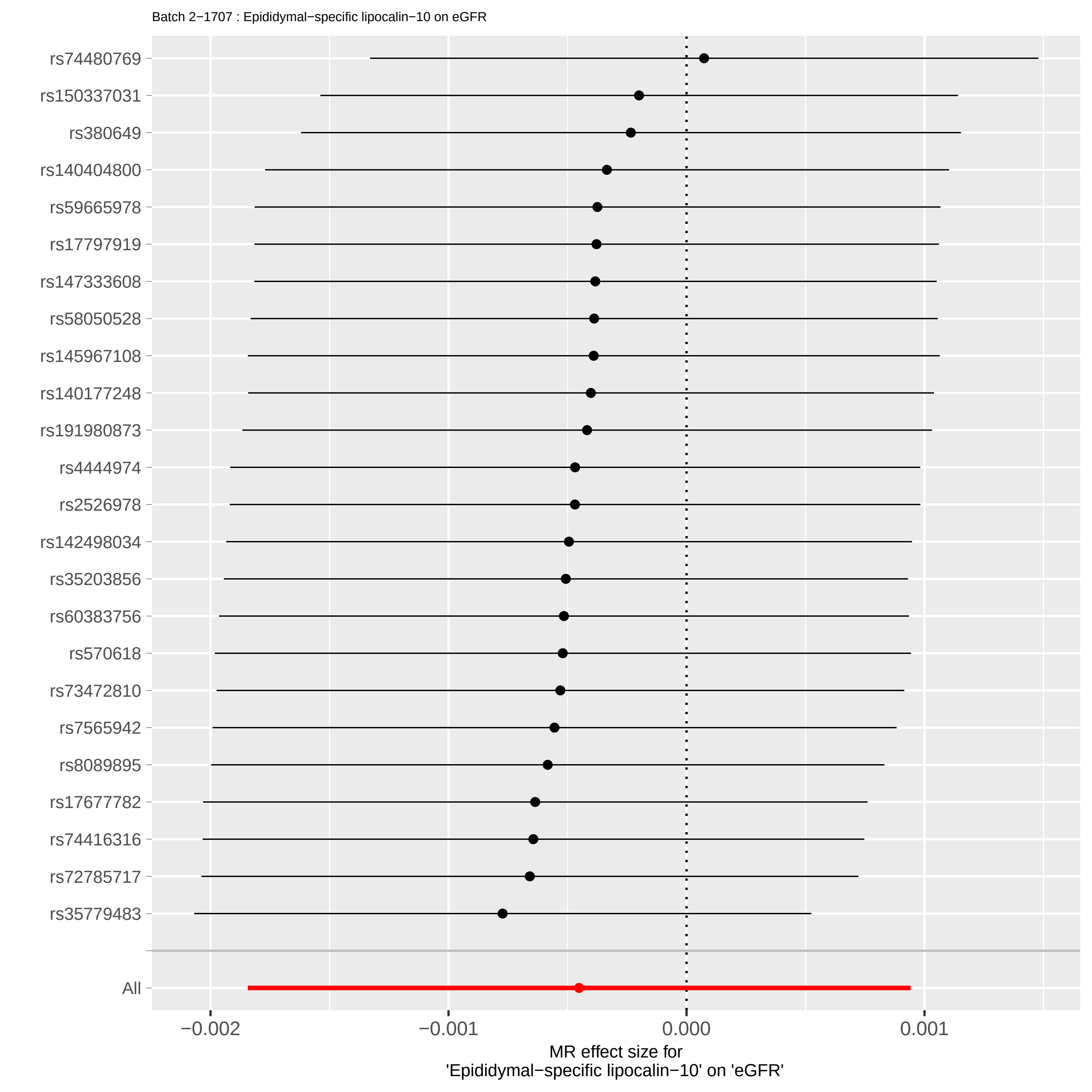

Batch 2−1707 : Epididymal−specific lipocalin−10 on eGFR
rs74480769
rs150337031
rs380649
rs140404800
rs59665978
rs17797919
rs147333608
rs58050528
rs145967108
rs140177248
rs191980873
rs4444974
rs2526978
rs142498034
rs35203856
rs60383756
rs570618
rs73472810
rs7565942
rs8089895
rs17677782
rs74416316
rs72785717
rs35779483
All
−0.002
−0.001
0.000
0.001
MR effect size for
'Epididymal−specific lipocalin−10' on 'eGFR'

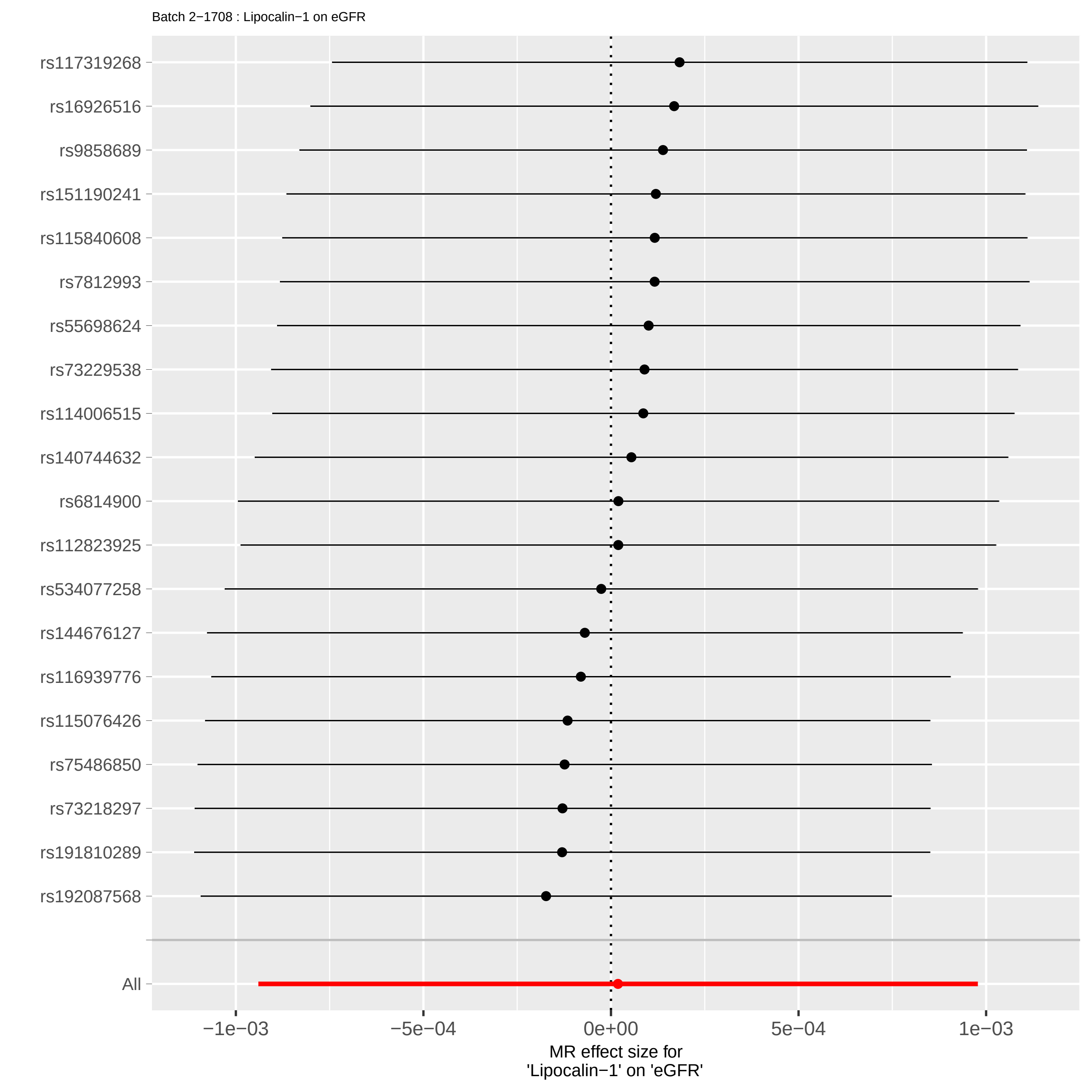

Batch 2−1708 : Lipocalin−1 on eGFR
rs117319268
rs16926516
rs9858689
rs151190241
rs115840608
rs7812993
rs55698624
rs73229538
rs114006515
rs140744632
rs6814900
rs112823925
rs534077258
rs144676127
rs116939776
rs115076426
rs75486850
rs73218297
rs191810289
rs192087568
All
−1e−03
−5e−04
0e+00
5e−04
1e−03
MR effect size for
'Lipocalin−1' on 'eGFR'

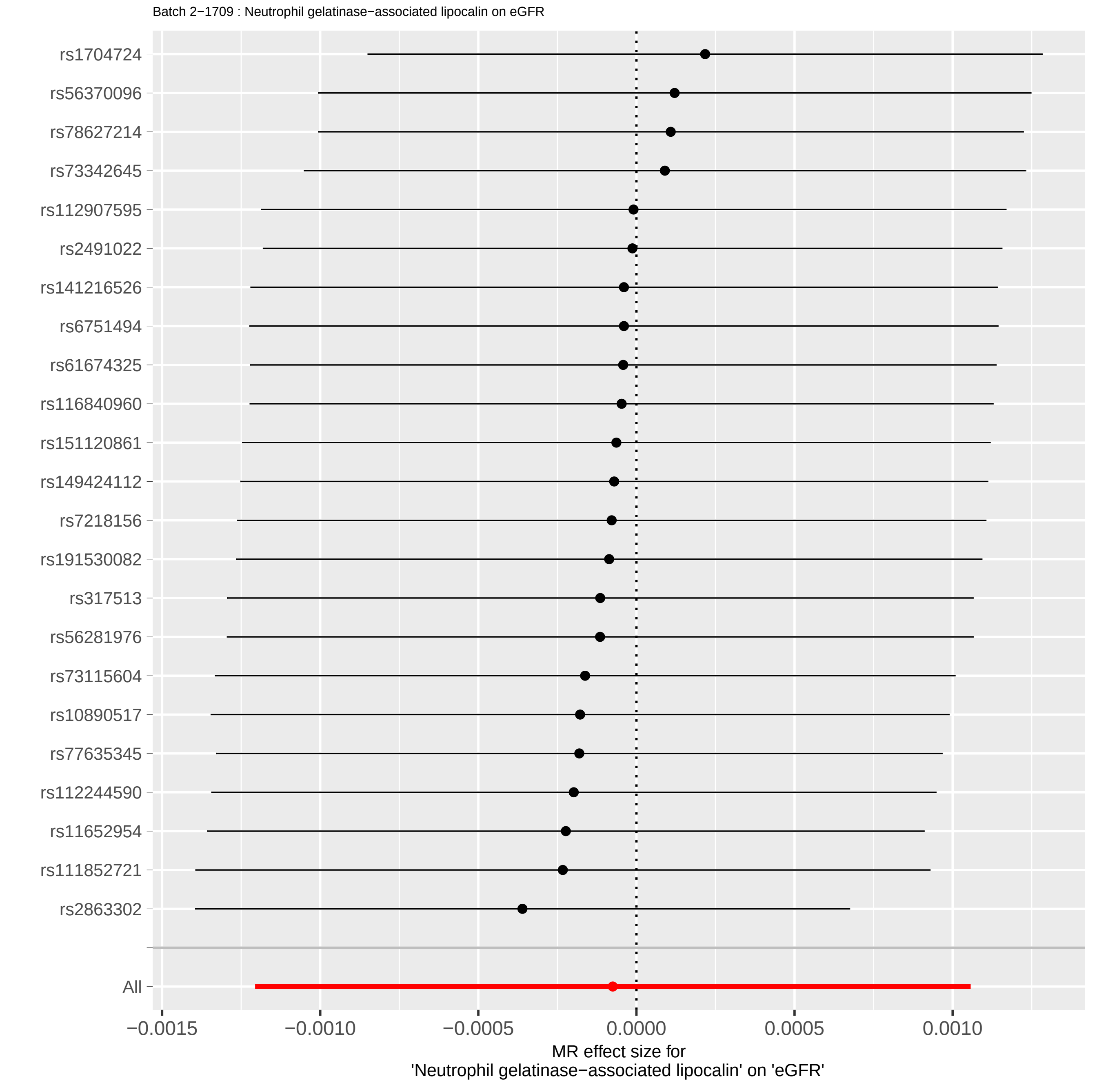

Batch 2−1709 : Neutrophil gelatinase−associated lipocalin on eGFR
rs1704724
rs56370096
rs78627214
rs73342645
rs112907595
rs2491022
rs141216526
rs6751494
rs61674325
rs116840960
rs151120861
rs149424112
rs7218156
rs191530082
rs317513
rs56281976
rs73115604
rs10890517
rs77635345
rs112244590
rs11652954
rs111852721
rs2863302
All
−0.0015
−0.0010
−0.0005
0.0000
0.0005
0.0010
MR effect size for
'Neutrophil gelatinase−associated lipocalin' on 'eGFR'

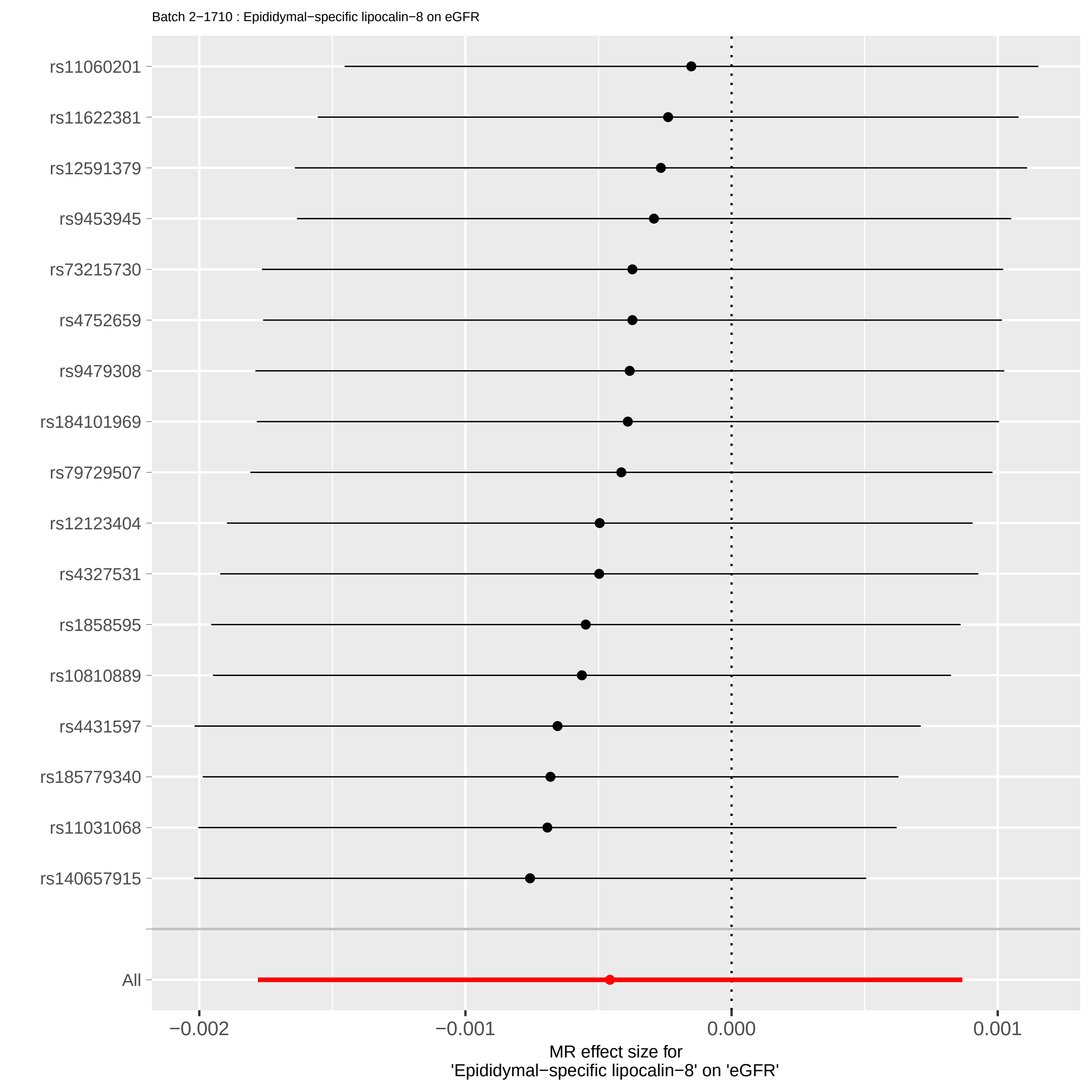

Batch 2−1710 : Epididymal−specific lipocalin−8 on eGFR
rs11060201
rs11622381
rs12591379
rs9453945
rs73215730
rs4752659
rs9479308
rs184101969
rs79729507
rs12123404
rs4327531
rs1858595
rs10810889
rs4431597
rs185779340
rs11031068
rs140657915
All
−0.002
−0.001
0.000
0.001
MR effect size for
'Epididymal−specific lipocalin−8' on 'eGFR'

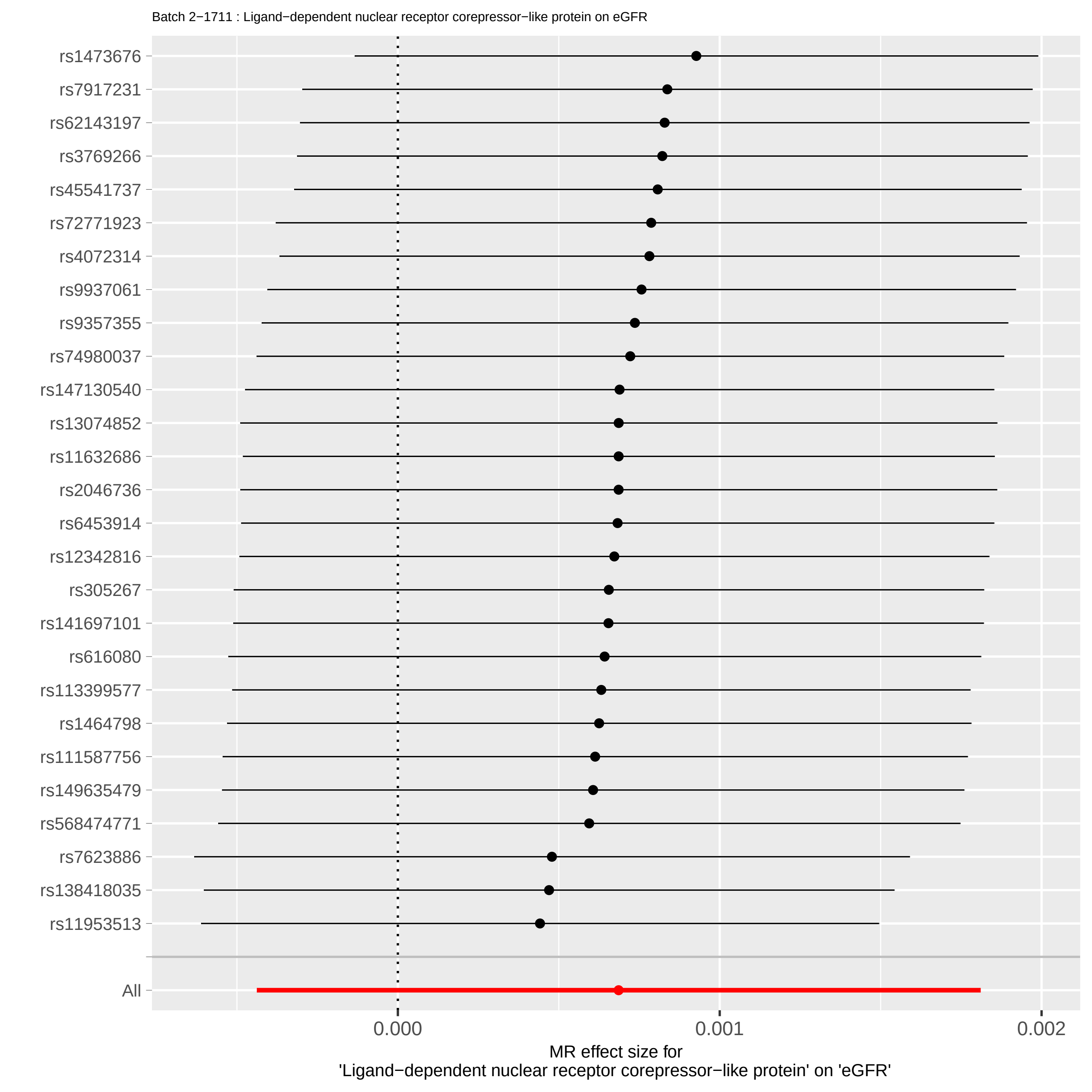

Batch 2−1711 : Ligand−dependent nuclear receptor corepressor−like protein on eGFR
rs1473676
rs7917231
rs62143197
rs3769266
rs45541737
rs72771923
rs4072314
rs9937061
rs9357355
rs74980037
rs147130540
rs13074852
rs11632686
rs2046736
rs6453914
rs12342816
rs305267
rs141697101
rs616080
rs113399577
rs1464798
rs111587756
rs149635479
rs568474771
rs7623886
rs138418035
rs11953513
All
0.000
0.001
0.002
MR effect size for
'Ligand−dependent nuclear receptor corepressor−like protein' on 'eGFR'

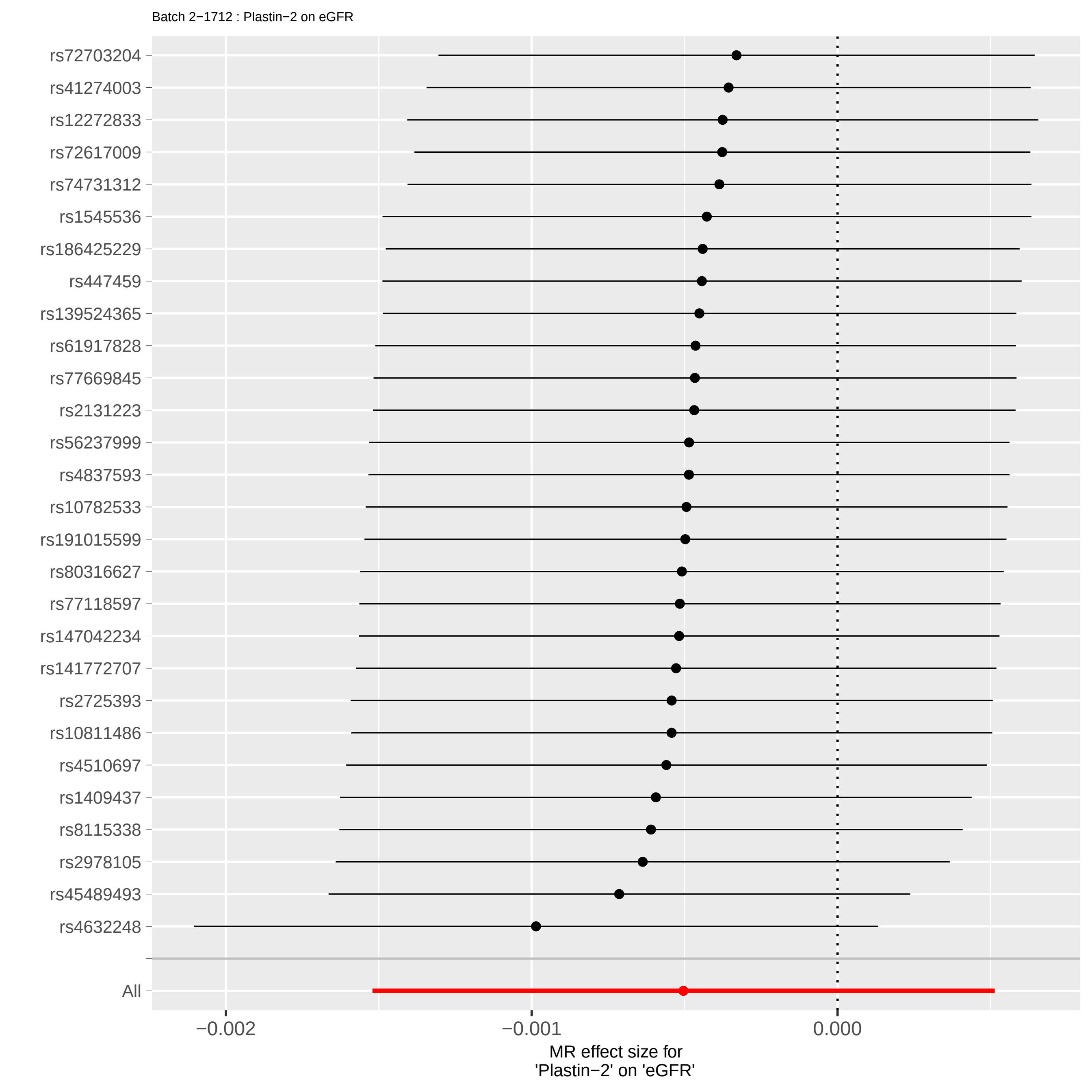
Batch 2−1712 : Plastin−2 on eGFR
rs72703204
rs41274003
rs12272833
rs72617009
rs74731312
rs1545536
rs186425229
rs447459
rs139524365
rs61917828
rs77669845
rs2131223
rs56237999
rs4837593
rs10782533
rs191015599
rs80316627
rs77118597
rs147042234
rs141772707
rs2725393
rs10811486
rs4510697
rs1409437
rs8115338
rs2978105
rs45489493
rs4632248
All
−0.002
−0.001
0.000
MR effect size for
'Plastin−2' on 'eGFR'

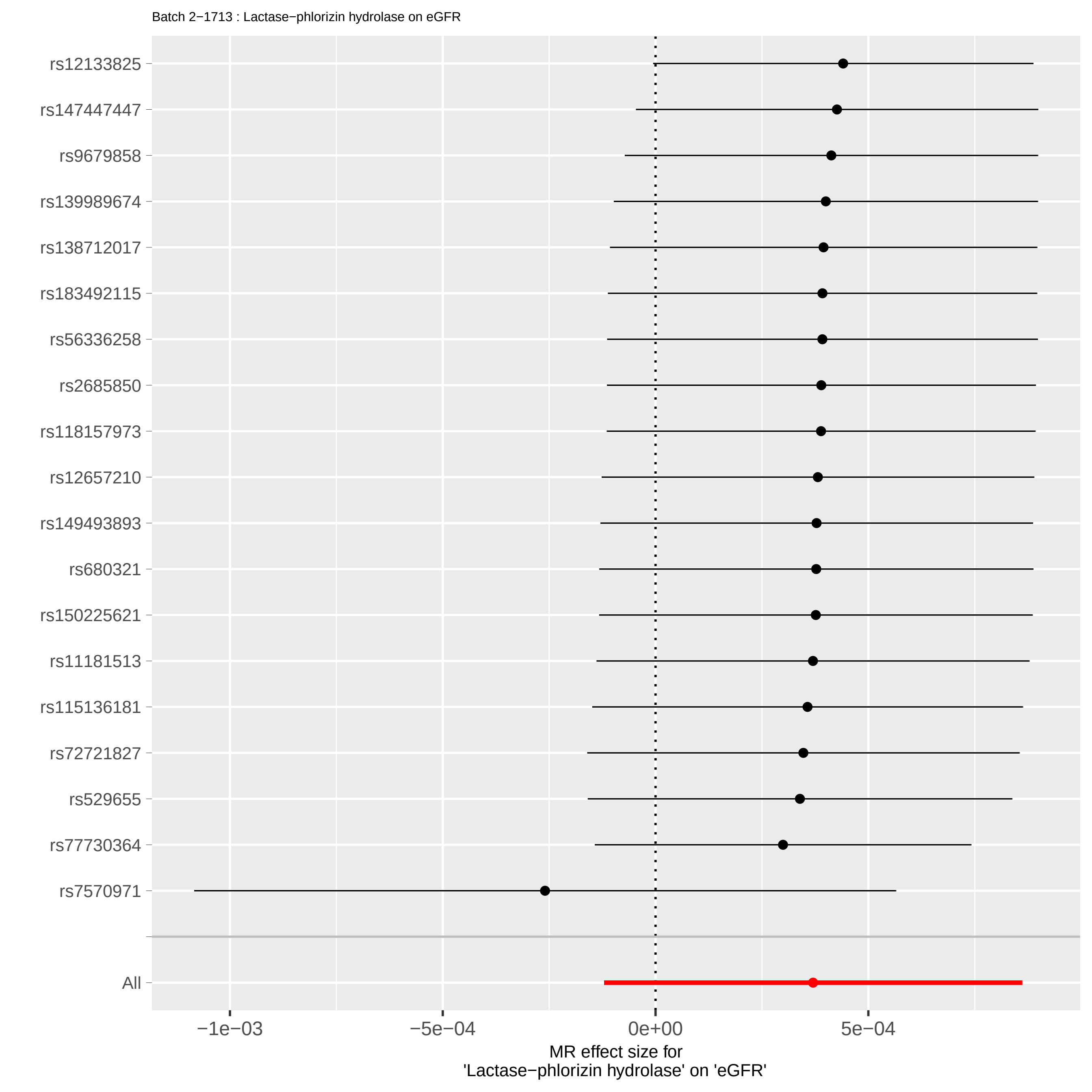

Batch 2−1713 : Lactase−phlorizin hydrolase on eGFR
rs12133825
rs147447447
rs9679858
rs139989674
rs138712017
rs183492115
rs56336258
rs2685850
rs118157973
rs12657210
rs149493893
rs680321
rs150225621
rs11181513
rs115136181
rs72721827
rs529655
rs77730364
rs7570971
All
−1e−03
−5e−04
0e+00
5e−04
MR effect size for
'Lactase−phlorizin hydrolase' on 'eGFR'

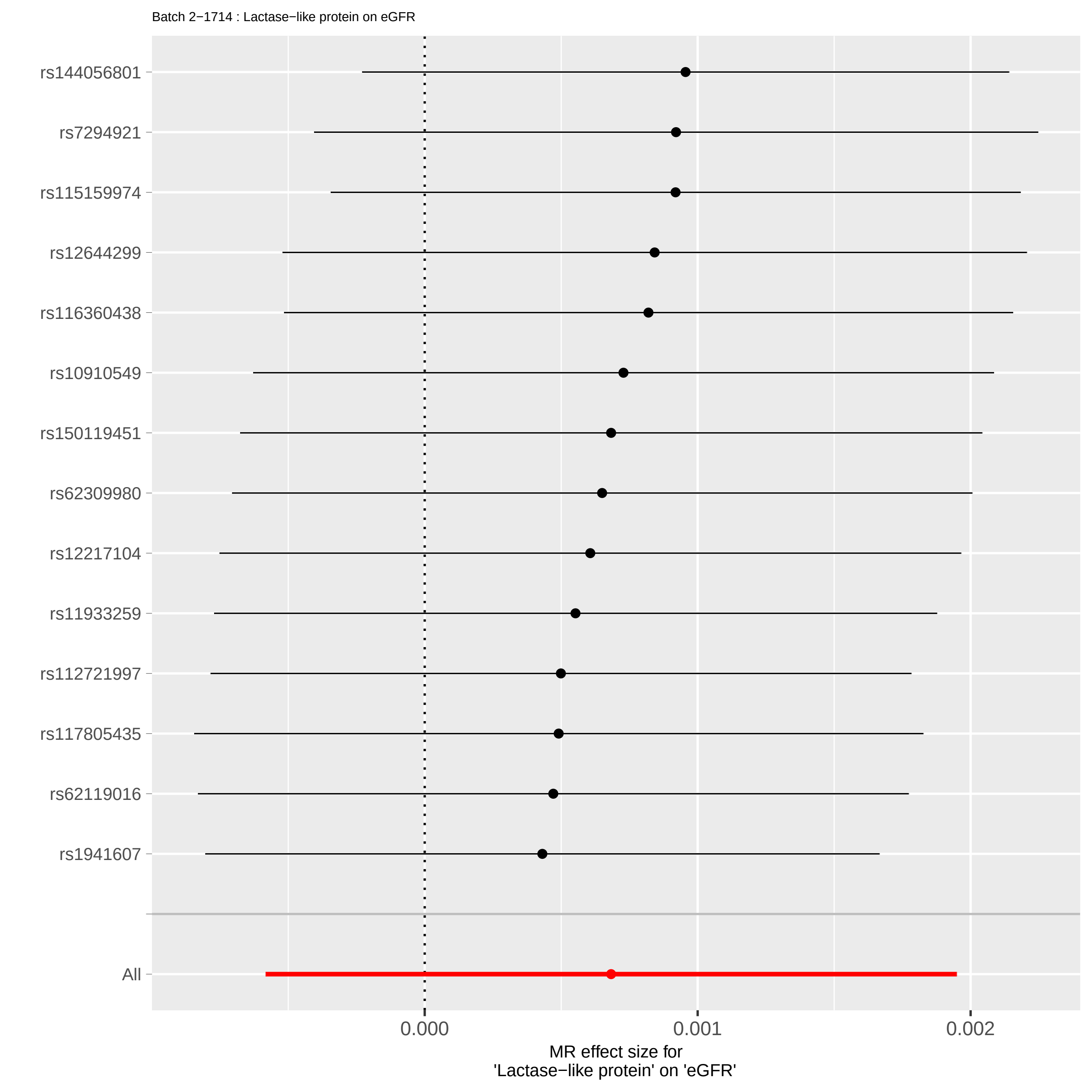

Batch 2−1714 : Lactase−like protein on eGFR
rs144056801
rs7294921
rs115159974
rs12644299
rs116360438
rs10910549
rs150119451
rs62309980
rs12217104
rs11933259
rs112721997
rs117805435
rs62119016
rs1941607
All
0.000
0.001
0.002
MR effect size for
'Lactase−like protein' on 'eGFR'

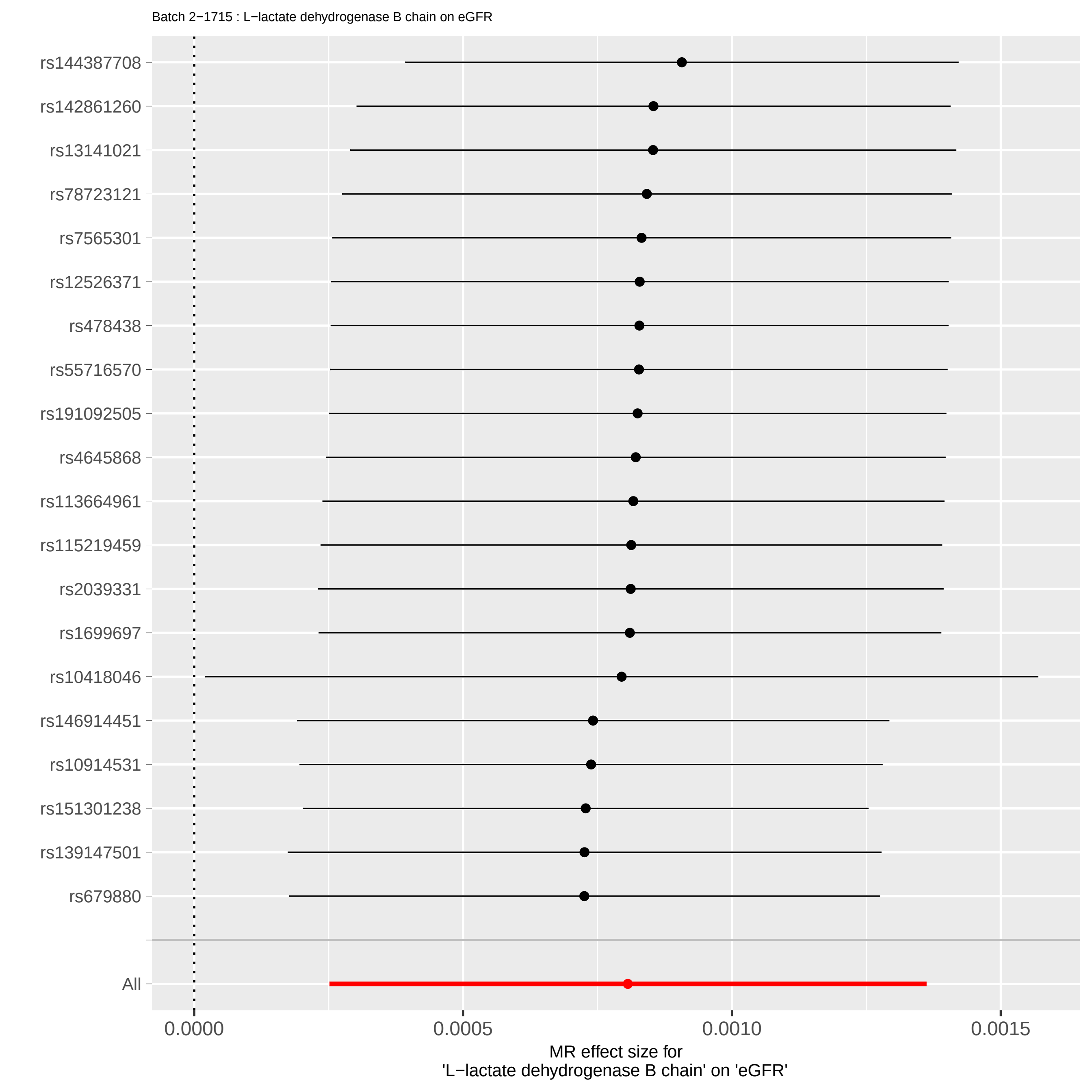
Batch 2−1715 : L−lactate dehydrogenase B chain on eGFR
rs144387708
rs142861260
rs13141021
rs78723121
rs7565301
rs12526371
rs478438
rs55716570
rs191092505
rs4645868
rs113664961
rs115219459
rs2039331
rs1699697
rs10418046
rs146914451
rs10914531
rs151301238
rs139147501
rs679880
All
0.0000
0.0005
0.0010
0.0015
MR effect size for
'L−lactate dehydrogenase B chain' on 'eGFR'

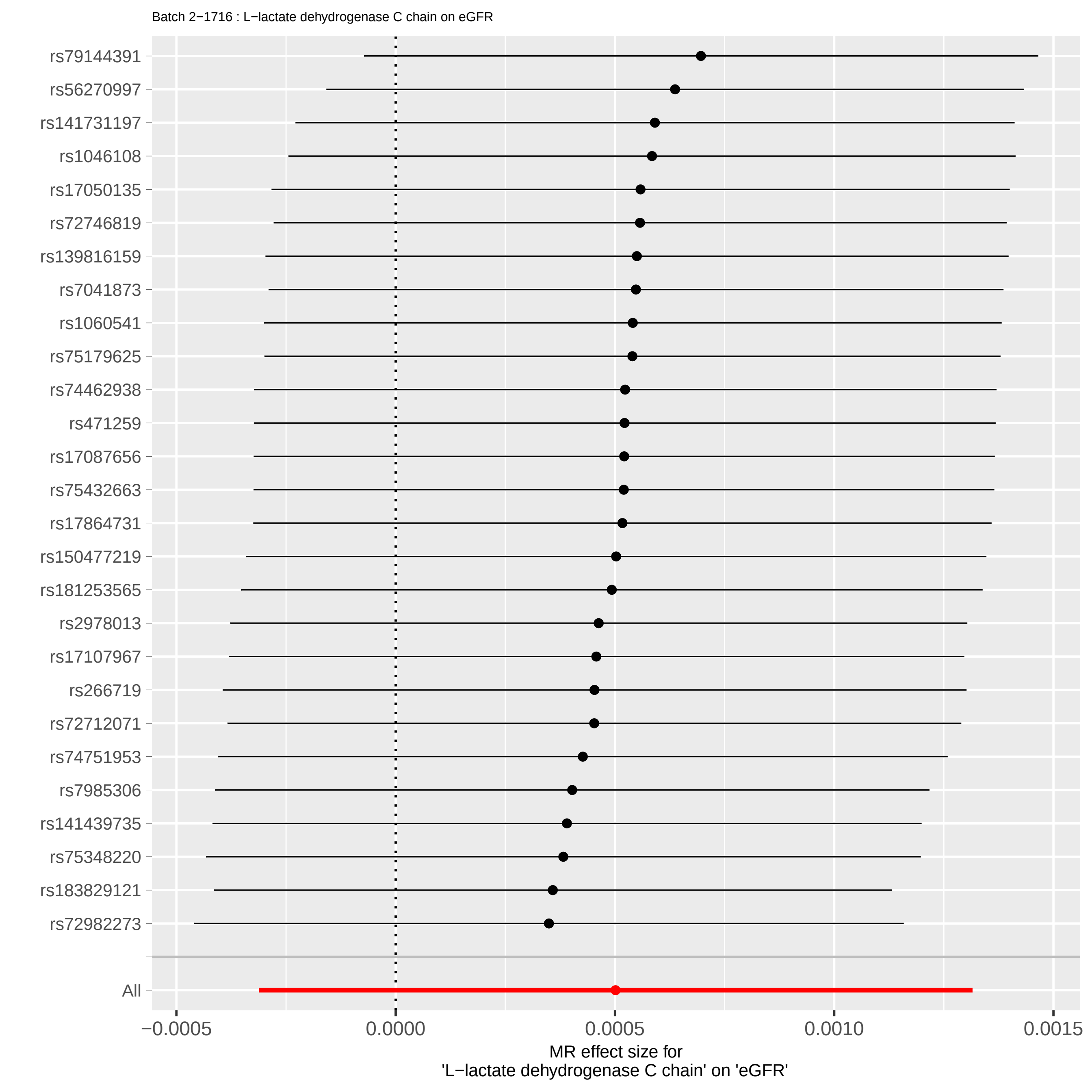
Batch 2−1716 : L−lactate dehydrogenase C chain on eGFR
rs79144391
rs56270997
rs141731197
rs1046108
rs17050135
rs72746819
rs139816159
rs7041873
rs1060541
rs75179625
rs74462938
rs471259
rs17087656
rs75432663
rs17864731
rs150477219
rs181253565
rs2978013
rs17107967
rs266719
rs72712071
rs74751953
rs7985306
rs141439735
rs75348220
rs183829121
rs72982273
All
−0.0005
0.0000
0.0005
0.0010
0.0015
MR effect size for
'L−lactate dehydrogenase C chain' on 'eGFR'

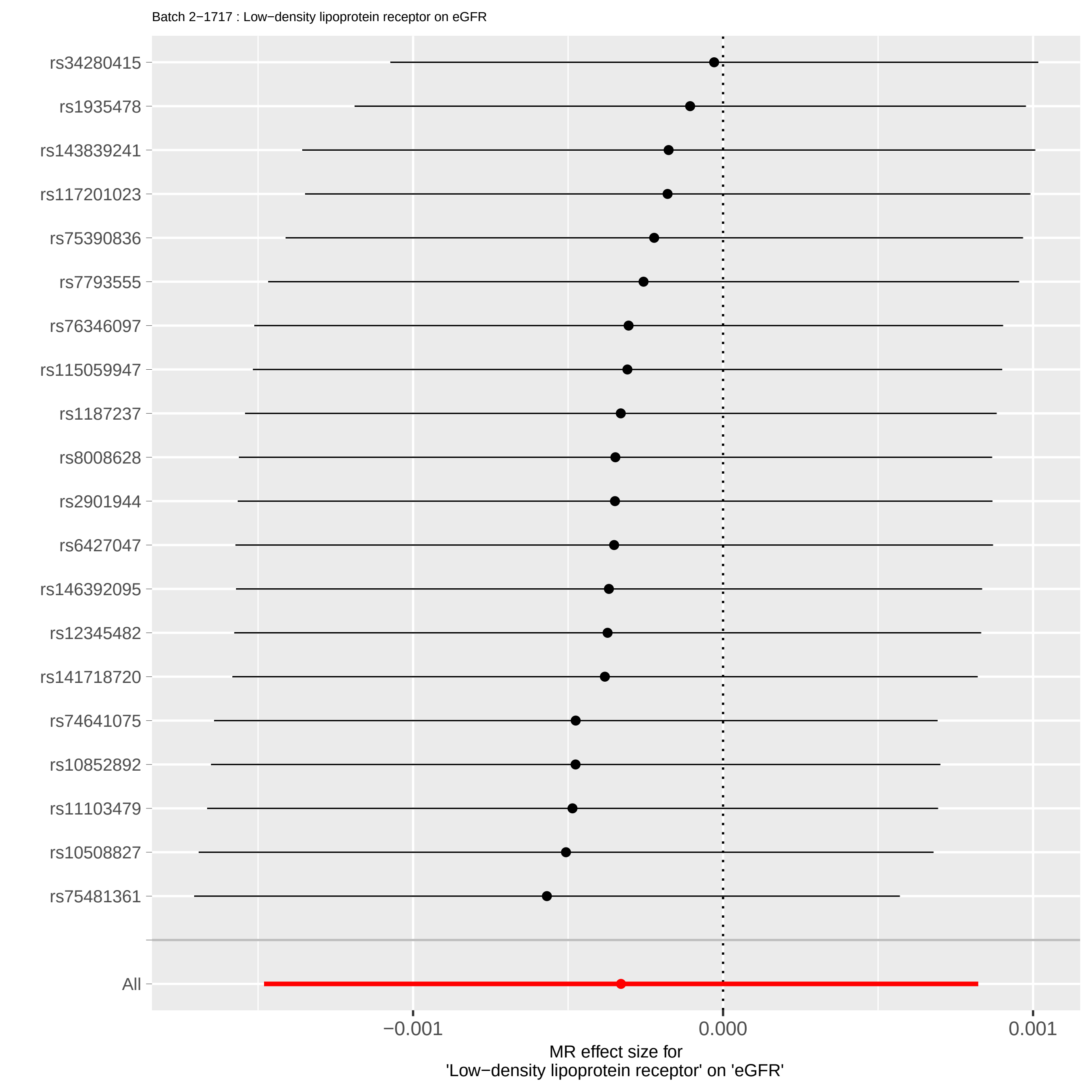
Batch 2−1717 : Low−density lipoprotein receptor on eGFR
rs34280415
rs1935478
rs143839241
rs117201023
rs75390836
rs7793555
rs76346097
rs115059947
rs1187237
rs8008628
rs2901944
rs6427047
rs146392095
rs12345482
rs141718720
rs74641075
rs10852892
rs11103479
rs10508827
rs75481361
All
−0.001
0.000
0.001
MR effect size for
'Low−density lipoprotein receptor' on 'eGFR'

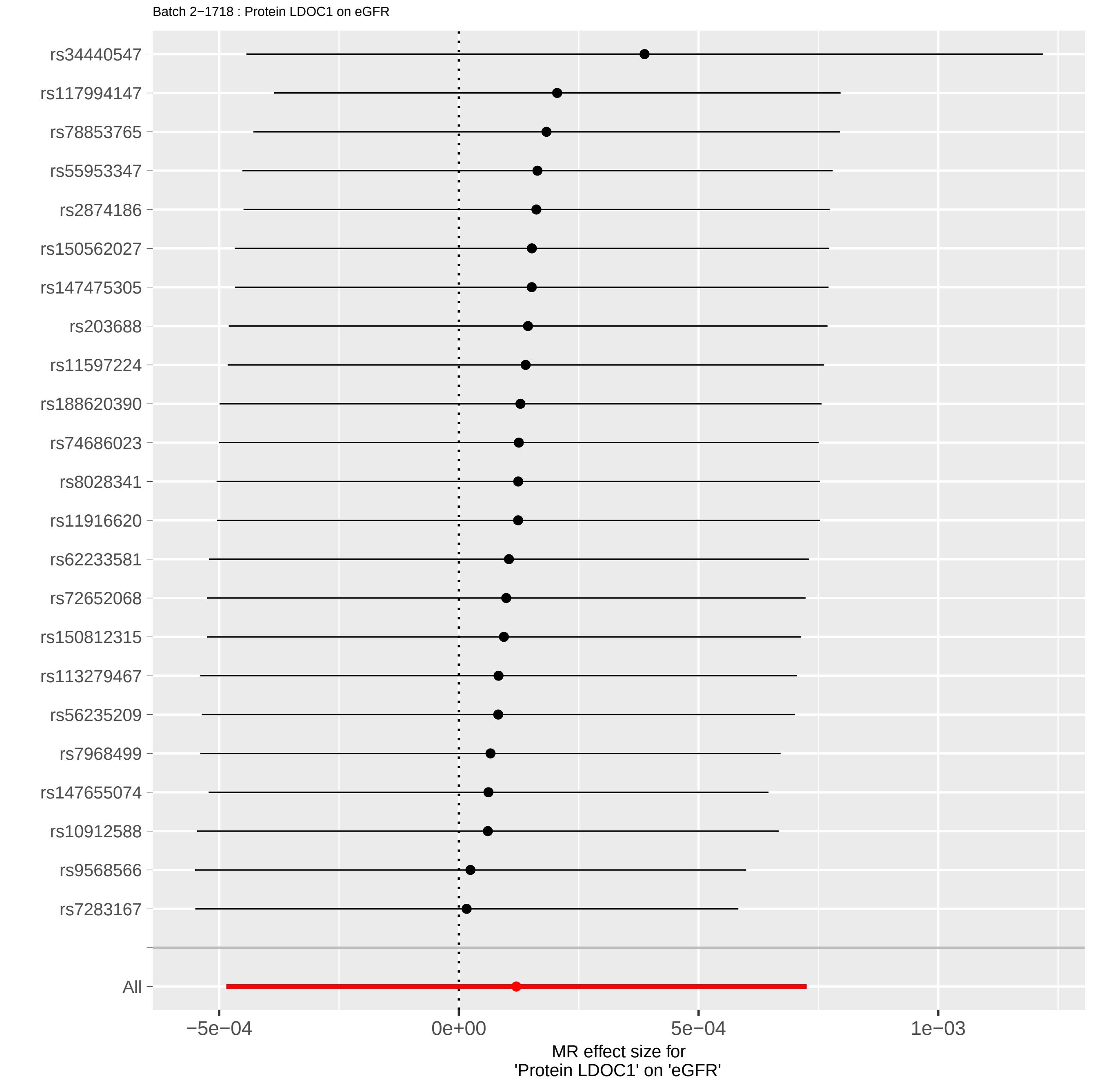

Batch 2−1718 : Protein LDOC1 on eGFR
rs34440547
rs117994147
rs78853765
rs55953347
rs2874186
rs150562027
rs147475305
rs203688
rs11597224
rs188620390
rs74686023
rs8028341
rs11916620
rs62233581
rs72652068
rs150812315
rs113279467
rs56235209
rs7968499
rs147655074
rs10912588
rs9568566
rs7283167
All
−5e−04
0e+00
5e−04
1e−03
MR effect size for
'Protein LDOC1' on 'eGFR'

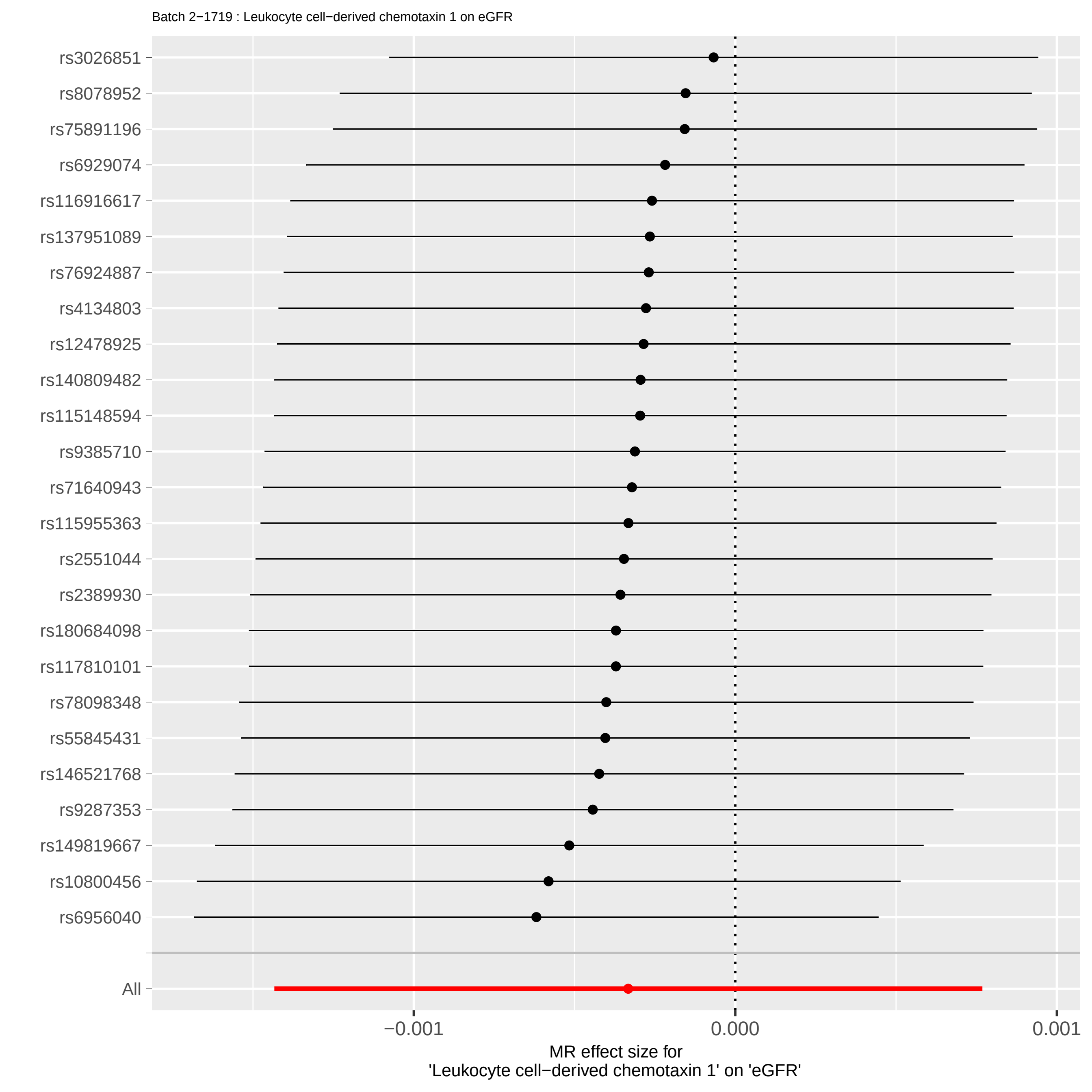

Batch 2−1719 : Leukocyte cell−derived chemotaxin 1 on eGFR
rs3026851
rs8078952
rs75891196
rs6929074
rs116916617
rs137951089
rs76924887
rs4134803
rs12478925
rs140809482
rs115148594
rs9385710
rs71640943
rs115955363
rs2551044
rs2389930
rs180684098
rs117810101
rs78098348
rs55845431
rs146521768
rs9287353
rs149819667
rs10800456
rs6956040
All
−0.001
0.000
0.001
MR effect size for
'Leukocyte cell−derived chemotaxin 1' on 'eGFR'

Batch 2−1720 : LEM domain−containing protein 1 on eGFR

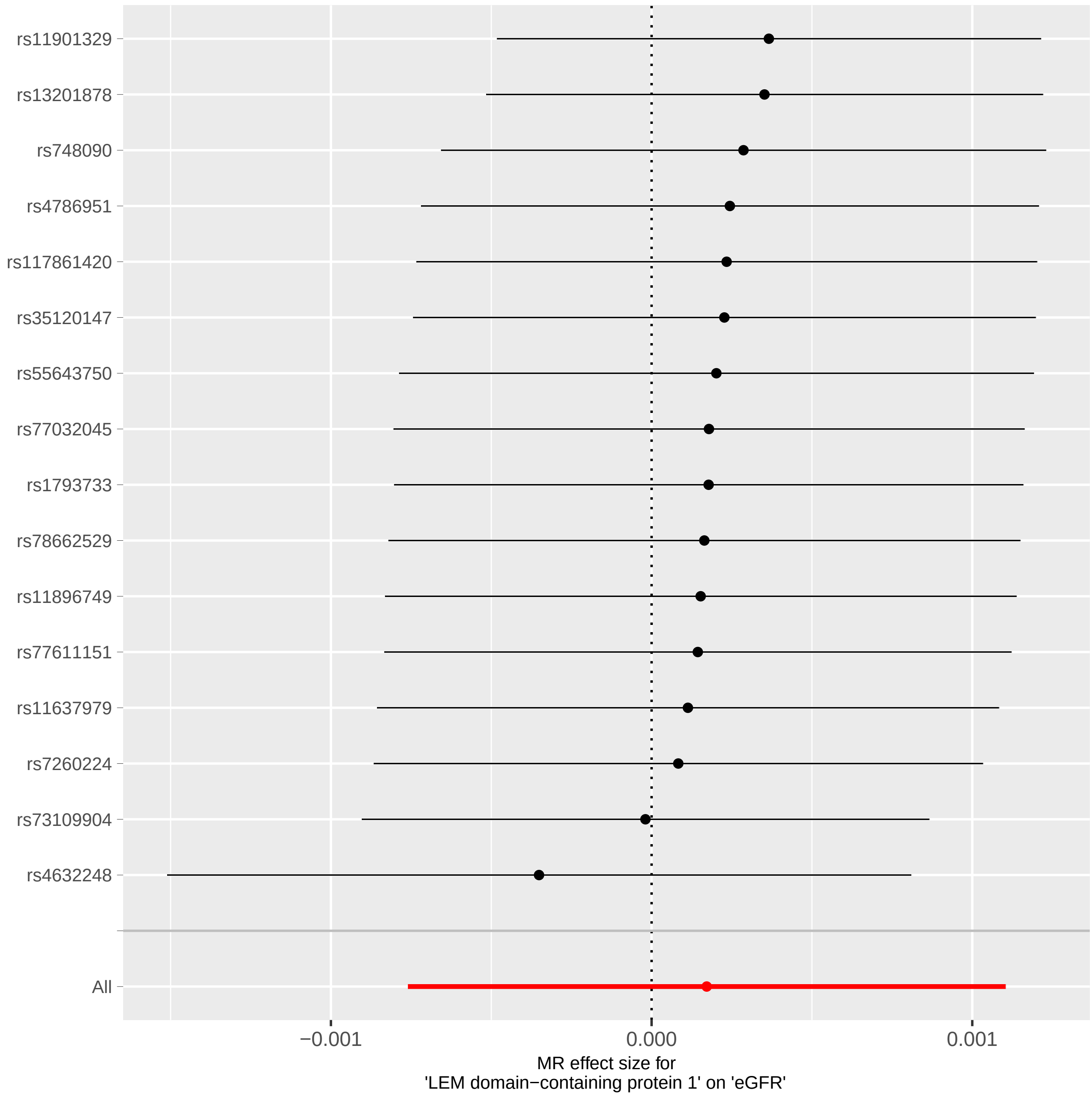

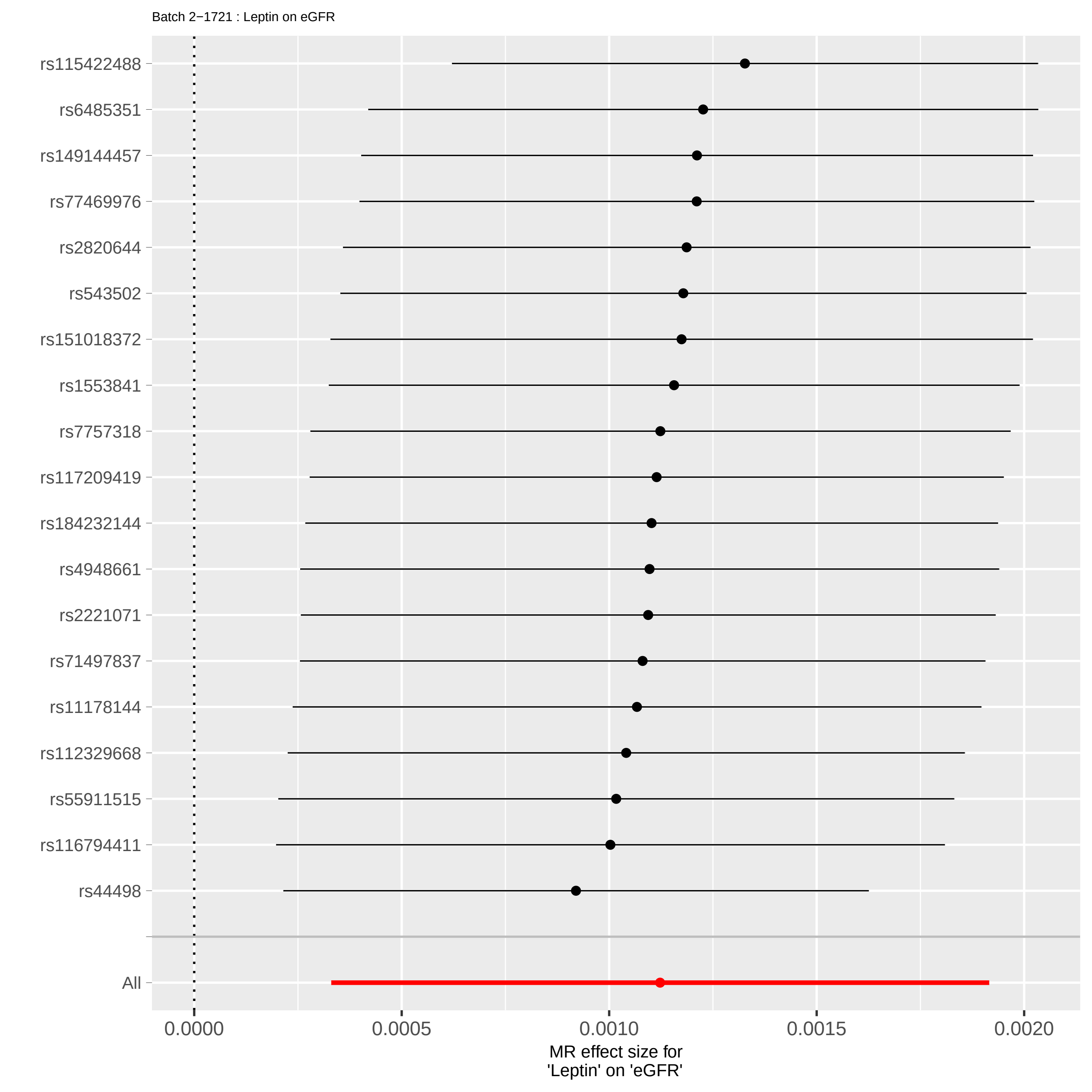

Batch 2−1721 : Leptin on eGFR
rs115422488
rs6485351
rs149144457
rs77469976
rs2820644
rs543502
rs151018372
rs1553841
rs7757318
rs117209419
rs184232144
rs4948661
rs2221071
rs71497837
rs11178144
rs112329668
rs55911515
rs116794411
rs44498
All
0.0000
0.0005
0.0010
0.0015
0.0020
MR effect size for
'Leptin' on 'eGFR'

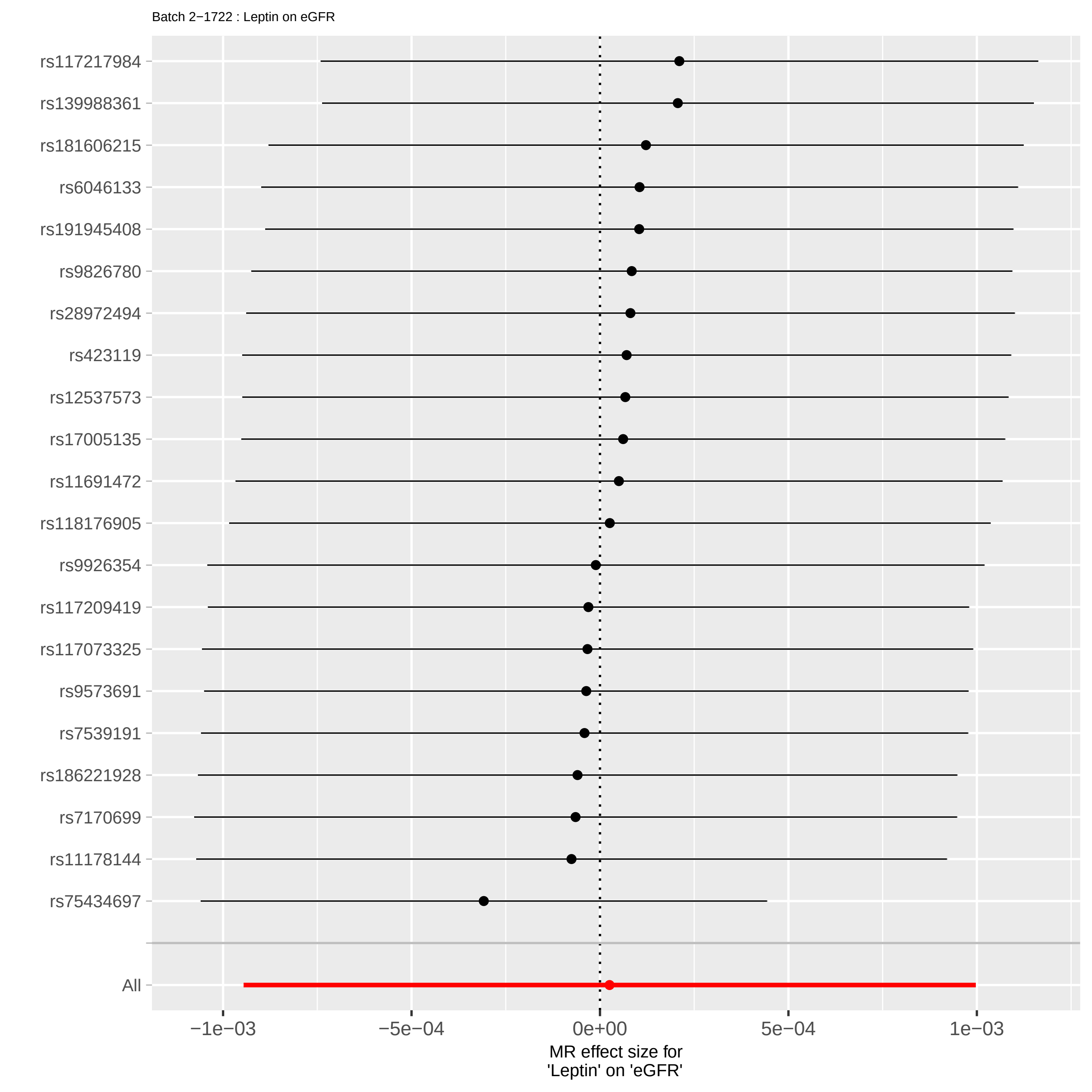

Batch 2−1722 : Leptin on eGFR
rs117217984
rs139988361
rs181606215
rs6046133
rs191945408
rs9826780
rs28972494
rs423119
rs12537573
rs17005135
rs11691472
rs118176905
rs9926354
rs117209419
rs117073325
rs9573691
rs7539191
rs186221928
rs7170699
rs11178144
rs75434697
All
−1e−03
−5e−04
0e+00
5e−04
1e−03
MR effect size for
'Leptin' on 'eGFR'

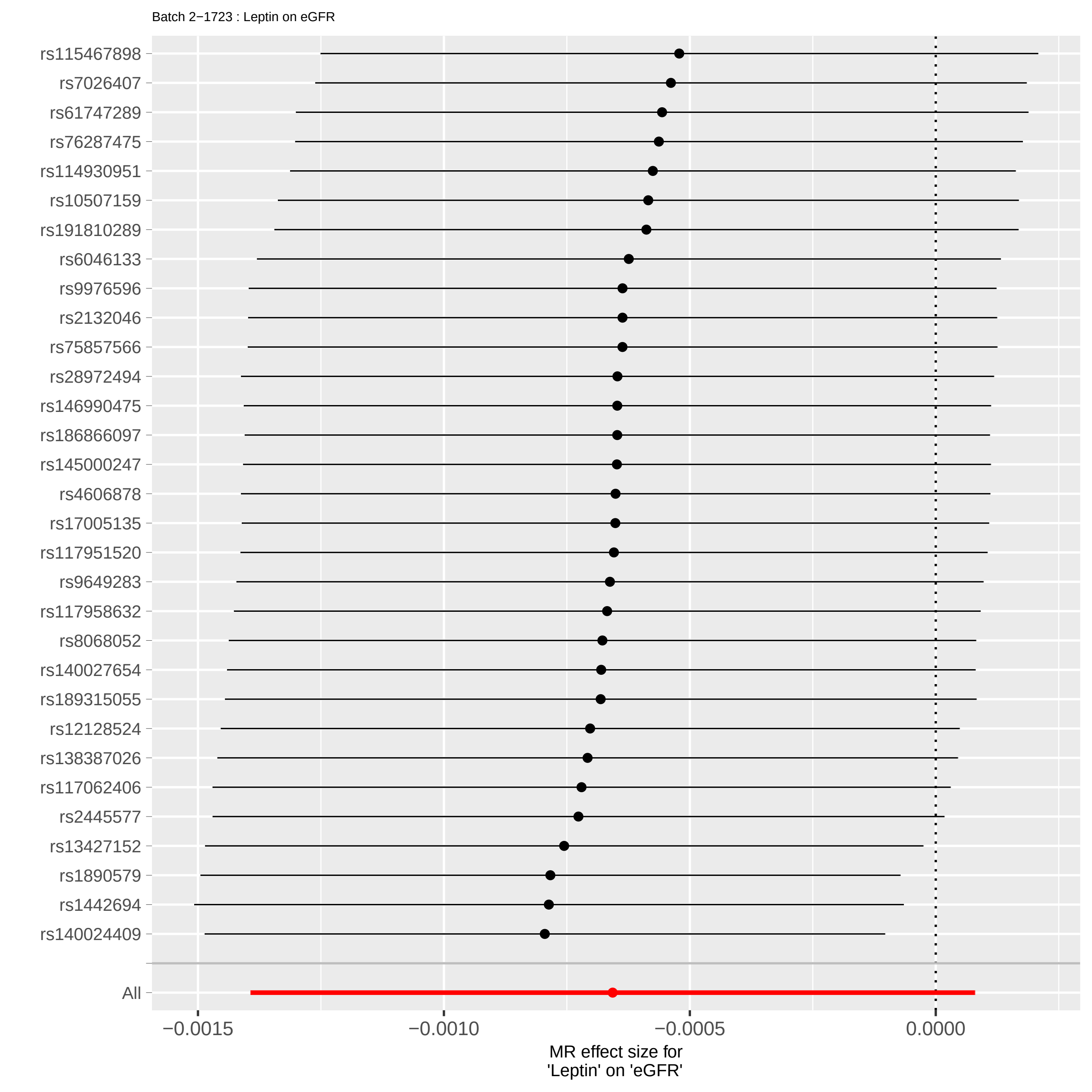
Batch 2−1723 : Leptin on eGFR
rs115467898
rs7026407
rs61747289
rs76287475
rs114930951
rs10507159
rs191810289
rs6046133
rs9976596
rs2132046
rs75857566
rs28972494
rs146990475
rs186866097
rs145000247
rs4606878
rs17005135
rs117951520
rs9649283
rs117958632
rs8068052
rs140027654
rs189315055
rs12128524
rs138387026
rs117062406
rs2445577
rs13427152
rs1890579
rs1442694
rs140024409
All
−0.0015
−0.0010
−0.0005
0.0000
MR effect size for
'Leptin' on 'eGFR'

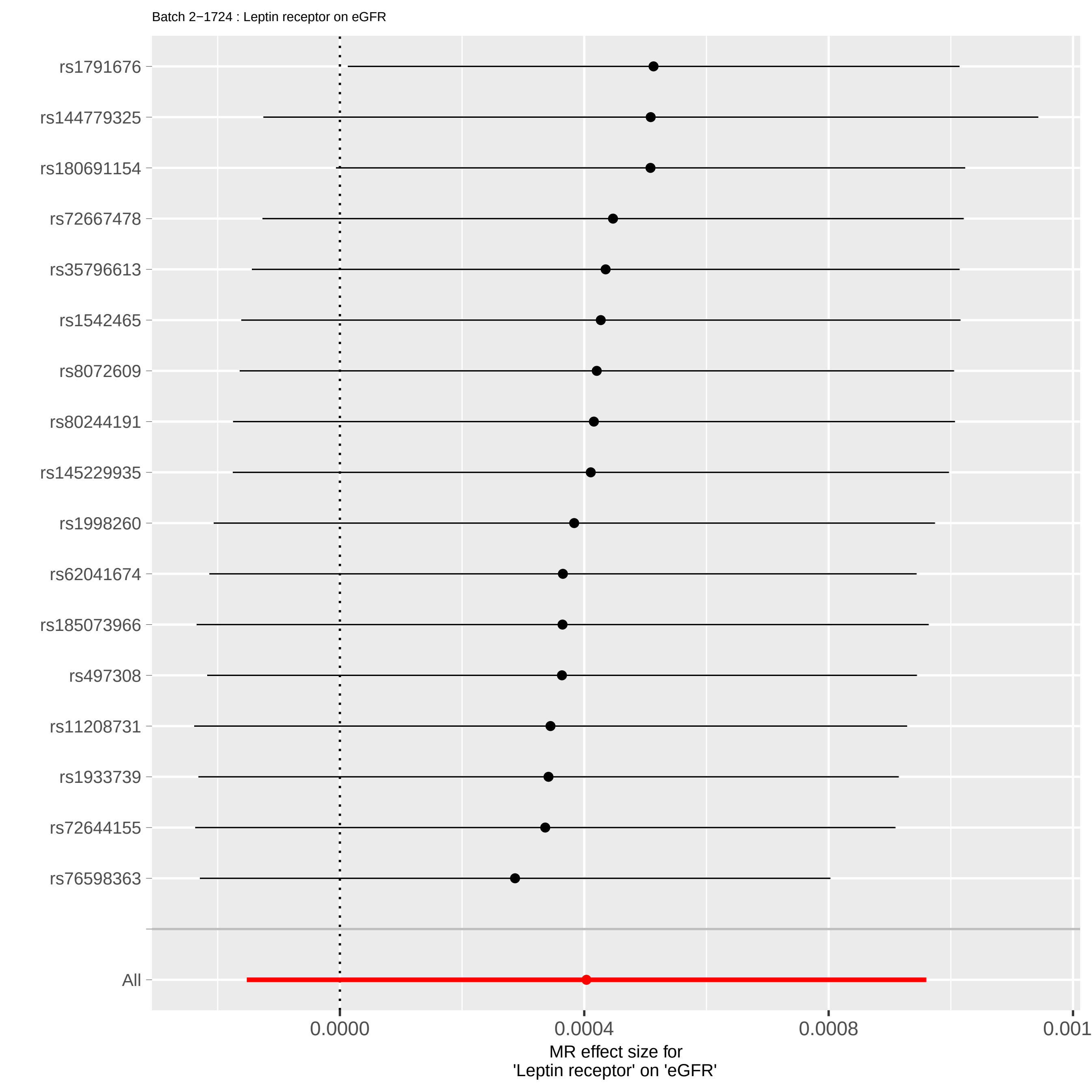

Batch 2−1724 : Leptin receptor on eGFR
rs1791676
rs144779325
rs180691154
rs72667478
rs35796613
rs1542465
rs8072609
rs80244191
rs145229935
rs1998260
rs62041674
rs185073966
rs497308
rs11208731
rs1933739
rs72644155
rs76598363
All
0.0000
0.0004
0.0008
0.001
MR effect size for
'Leptin receptor' on 'eGFR'

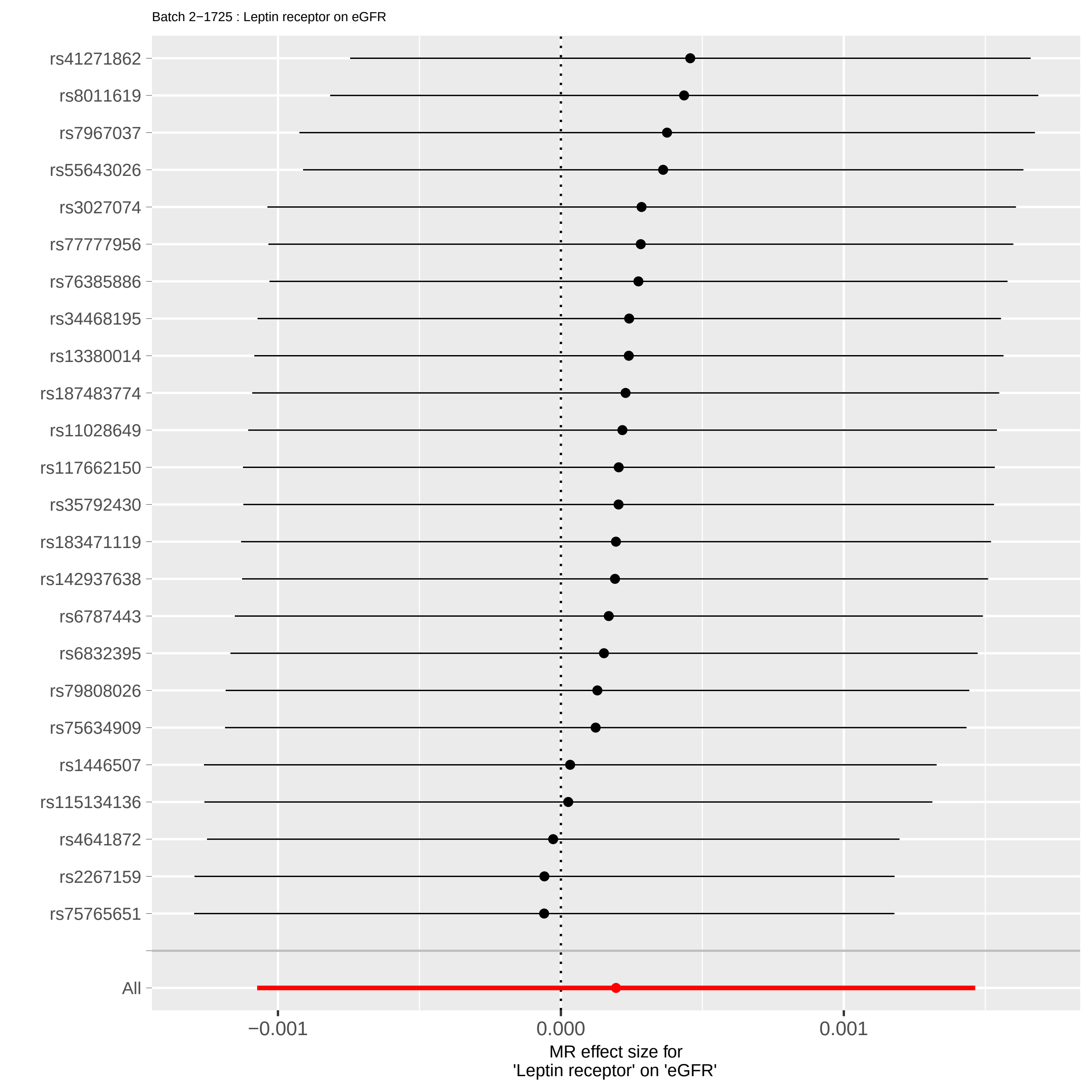
Batch 2−1725 : Leptin receptor on eGFR
rs41271862
rs8011619
rs7967037
rs55643026
rs3027074
rs77777956
rs76385886
rs34468195
rs13380014
rs187483774
rs11028649
rs117662150
rs35792430
rs183471119
rs142937638
rs6787443
rs6832395
rs79808026
rs75634909
rs1446507
rs115134136
rs4641872
rs2267159
rs75765651
All
−0.001
0.000
0.001
MR effect size for
'Leptin receptor' on 'eGFR'

Batch 2−1726 : Galectin−1 on eGFR

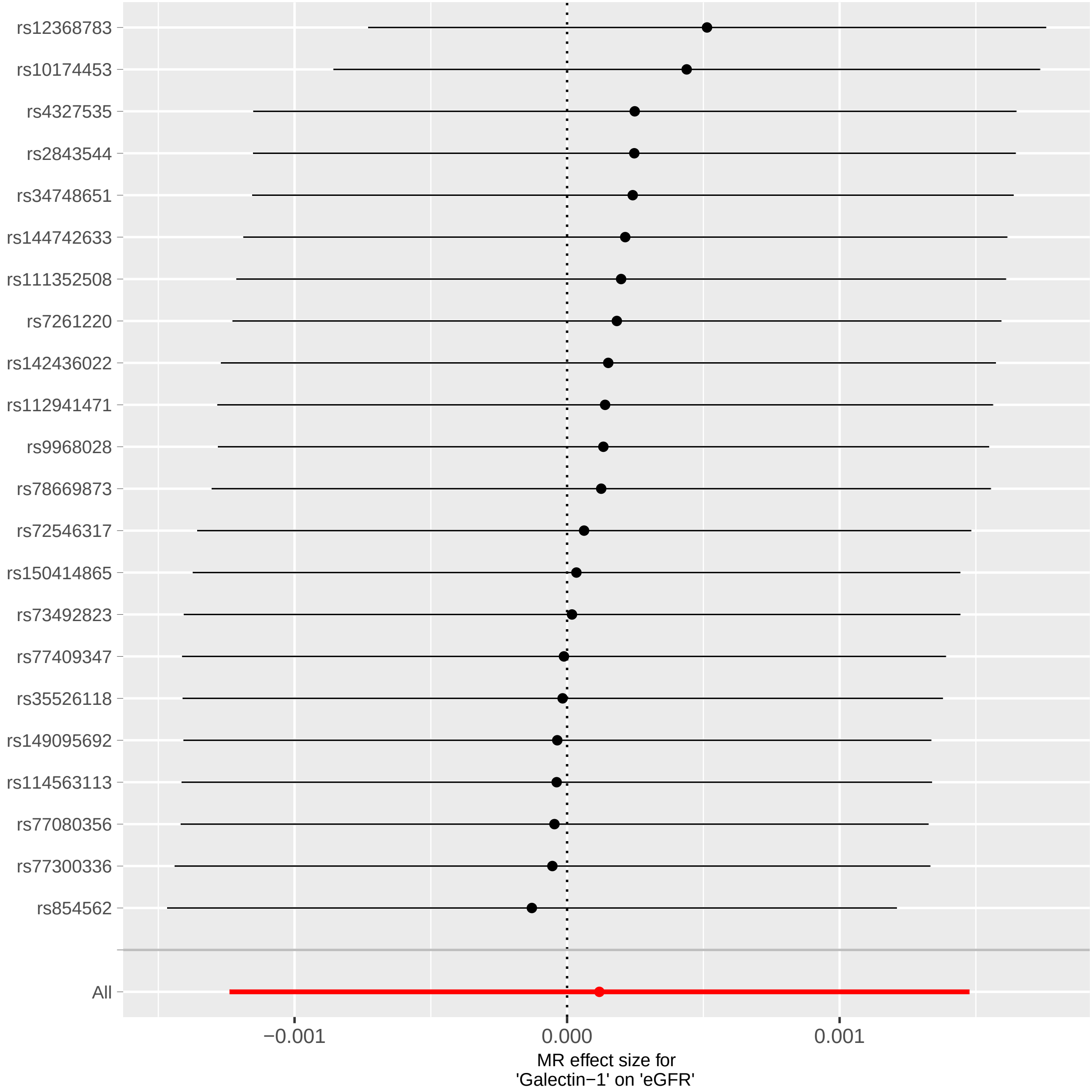

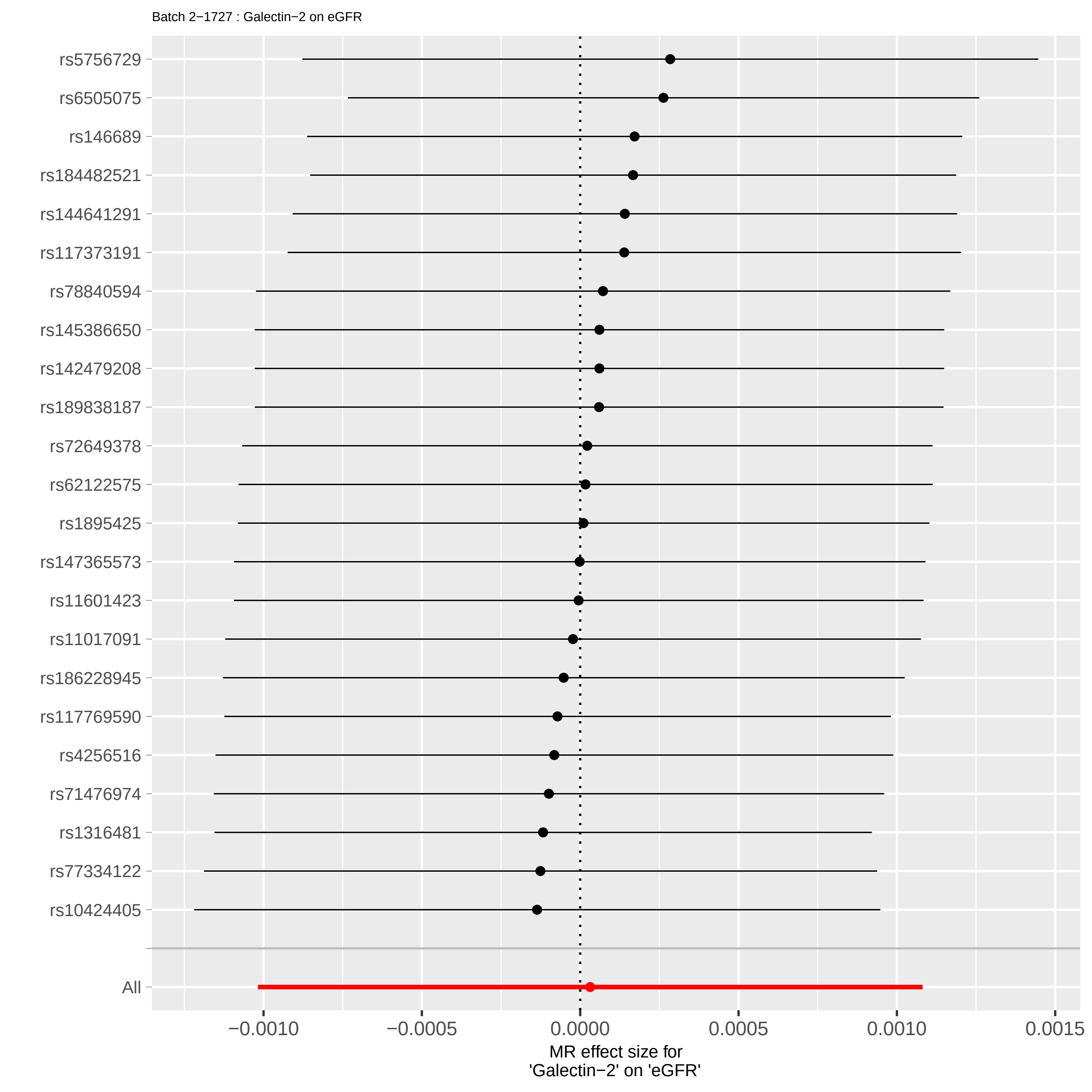

Batch 2−1727 : Galectin−2 on eGFR
rs5756729
rs6505075
rs146689
rs184482521
rs144641291
rs117373191
rs78840594
rs145386650
rs142479208
rs189838187
rs72649378
rs62122575
rs1895425
rs147365573
rs11601423
rs11017091
rs186228945
rs117769590
rs4256516
rs71476974
rs1316481
rs77334122
rs10424405
All
−0.0010
−0.0005
0.0000
0.0005
0.0010
0.0015
MR effect size for
'Galectin−2' on 'eGFR'

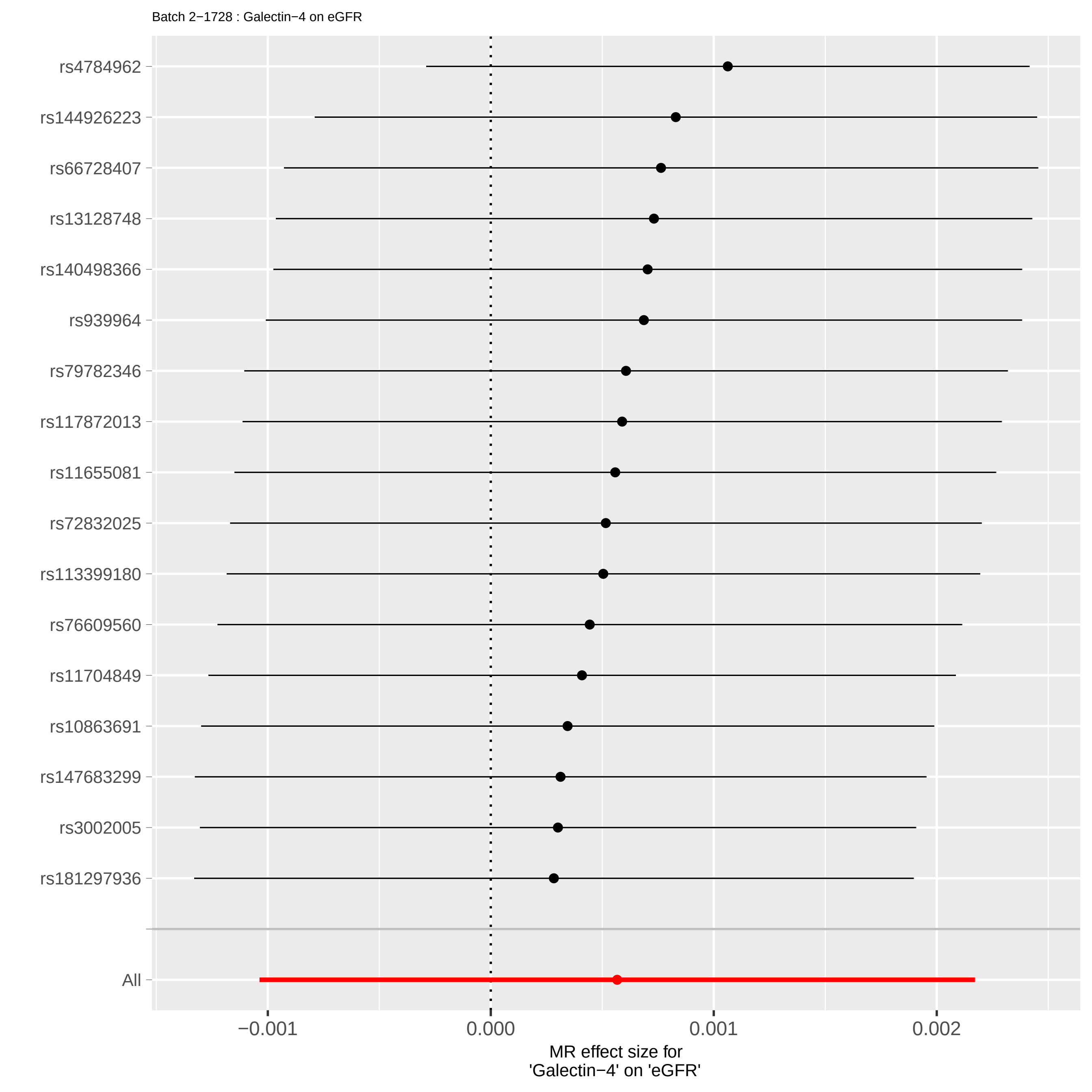

Batch 2−1728 : Galectin−4 on eGFR
rs4784962
rs144926223
rs66728407
rs13128748
rs140498366
rs939964
rs79782346
rs117872013
rs11655081
rs72832025
rs113399180
rs76609560
rs11704849
rs10863691
rs147683299
rs3002005
rs181297936
All
−0.001
0.000
0.001
0.002
MR effect size for
'Galectin−4' on 'eGFR'

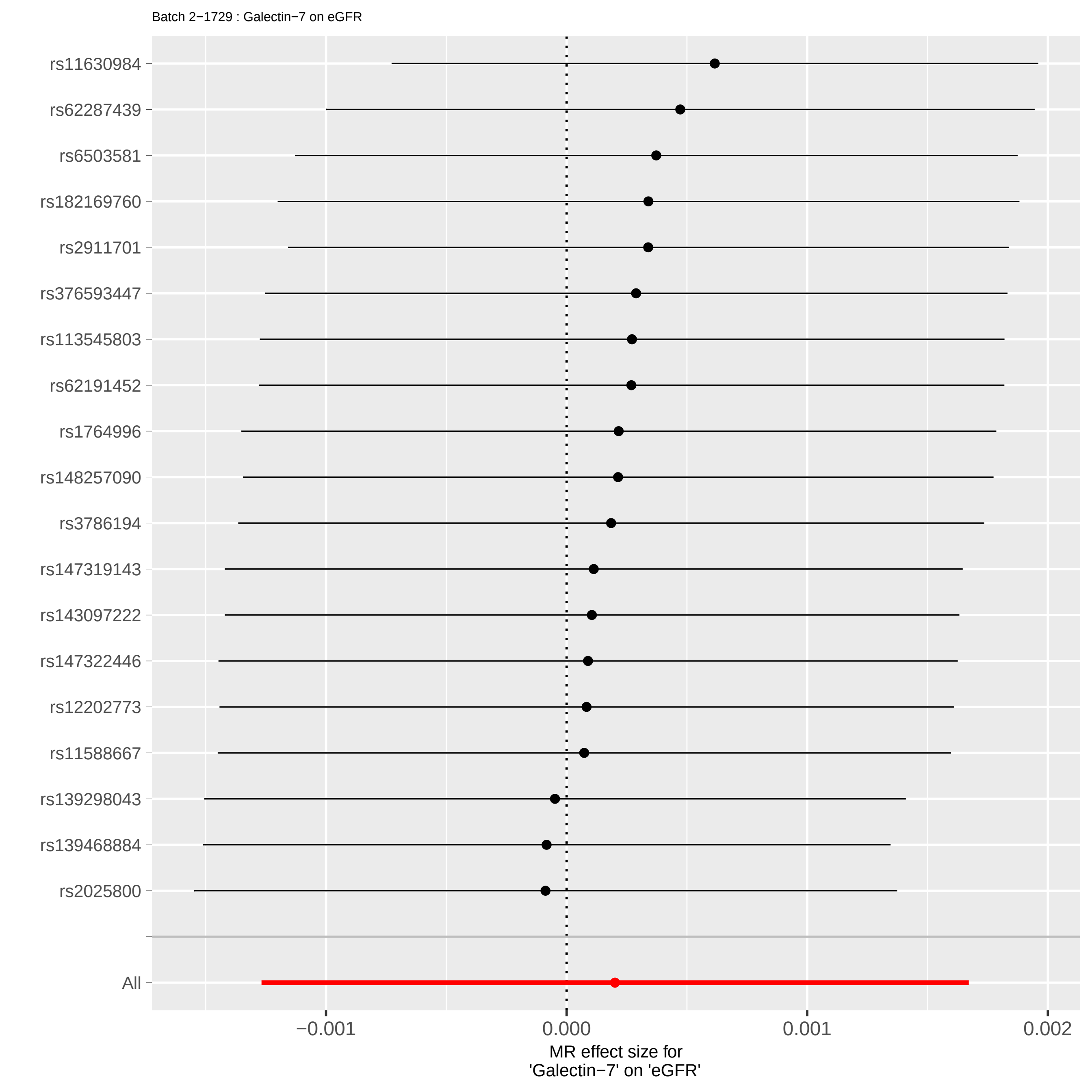

Batch 2−1729 : Galectin−7 on eGFR
rs11630984
rs62287439
rs6503581
rs182169760
rs2911701
rs376593447
rs113545803
rs62191452
rs1764996
rs148257090
rs3786194
rs147319143
rs143097222
rs147322446
rs12202773
rs11588667
rs139298043
rs139468884
rs2025800
All
−0.001
0.000
0.001
0.002
MR effect size for
'Galectin−7' on 'eGFR'

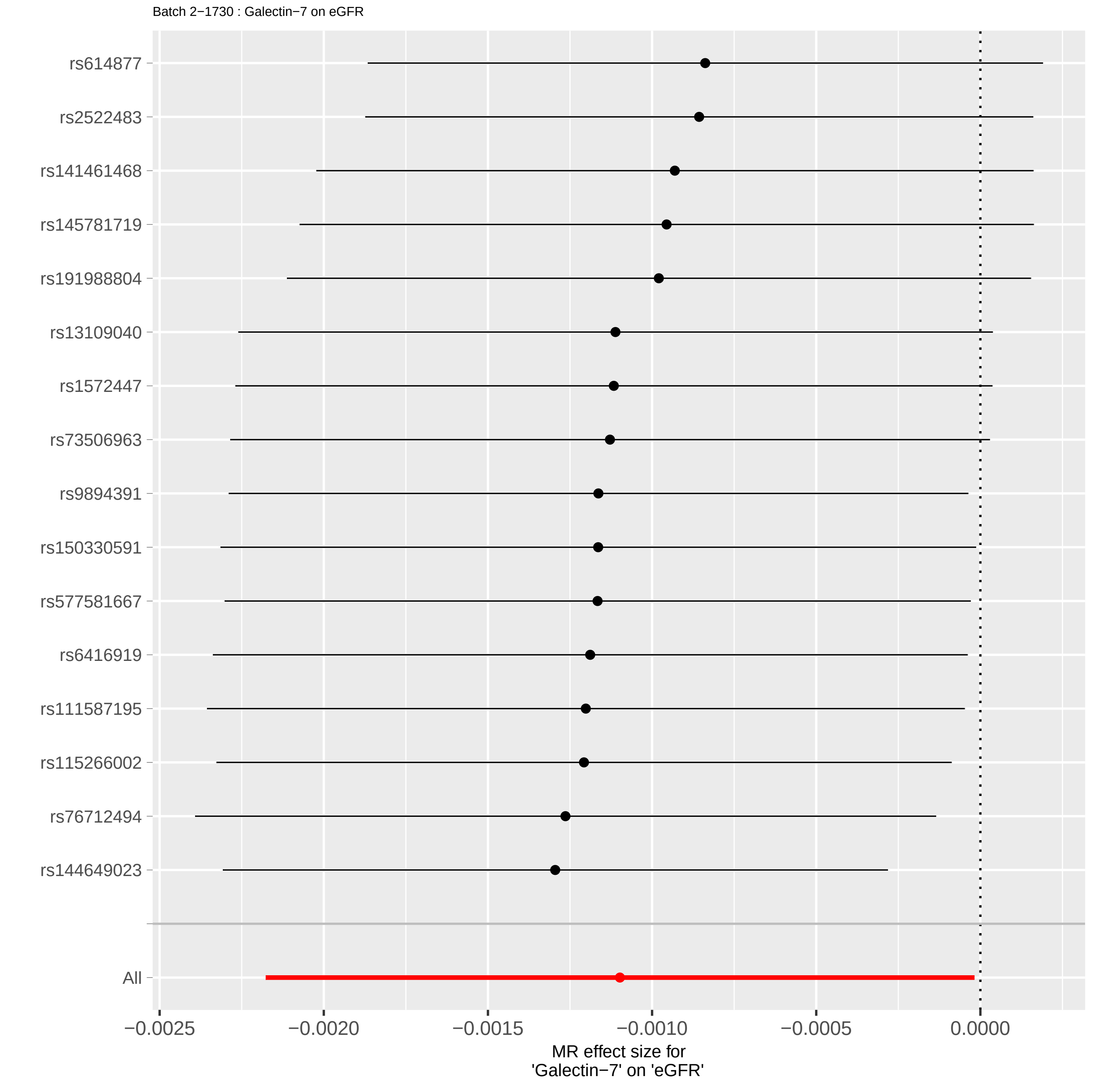
Batch 2−1730 : Galectin−7 on eGFR
rs614877
rs2522483
rs141461468
rs145781719
rs191988804
rs13109040
rs1572447
rs73506963
rs9894391
rs150330591
rs577581667
rs6416919
rs111587195
rs115266002
rs76712494
rs144649023
All
−0.0025
−0.0020
−0.0015
−0.0010
−0.0005
0.0000
MR effect size for
'Galectin−7' on 'eGFR'

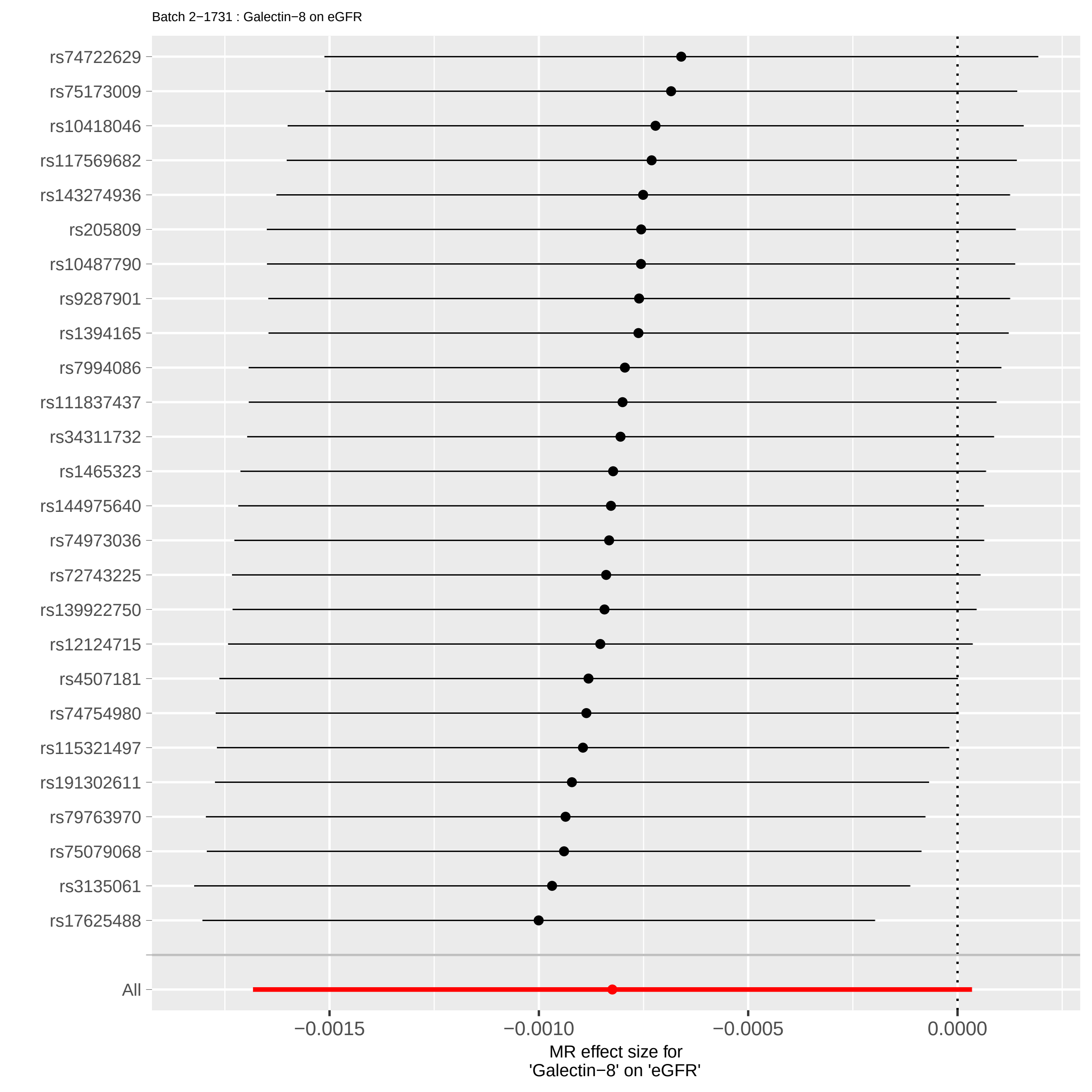

Batch 2−1731 : Galectin−8 on eGFR
rs74722629
rs75173009
rs10418046
rs117569682
rs143274936
rs205809
rs10487790
rs9287901
rs1394165
rs7994086
rs111837437
rs34311732
rs1465323
rs144975640
rs74973036
rs72743225
rs139922750
rs12124715
rs4507181
rs74754980
rs115321497
rs191302611
rs79763970
rs75079068
rs3135061
rs17625488
All
−0.0015
−0.0010
−0.0005
0.0000
MR effect size for
'Galectin−8' on 'eGFR'

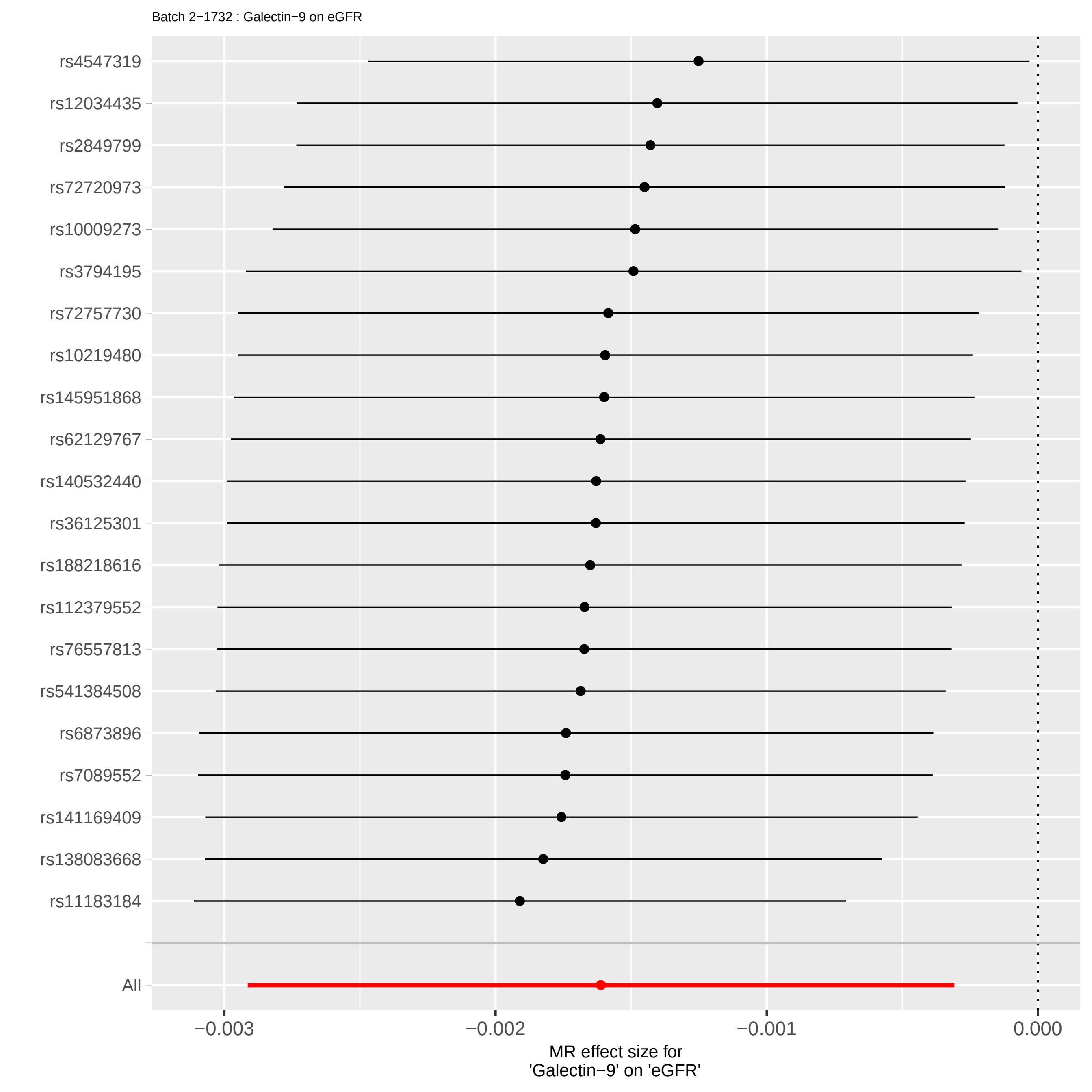

Batch 2−1732 : Galectin−9 on eGFR
rs4547319
rs12034435
rs2849799
rs72720973
rs10009273
rs3794195
rs72757730
rs10219480
rs145951868
rs62129767
rs140532440
rs36125301
rs188218616
rs112379552
rs76557813
rs541384508
rs6873896
rs7089552
rs141169409
rs138083668
rs11183184
All
−0.003
−0.002
−0.001
0.000
MR effect size for
'Galectin−9' on 'eGFR'

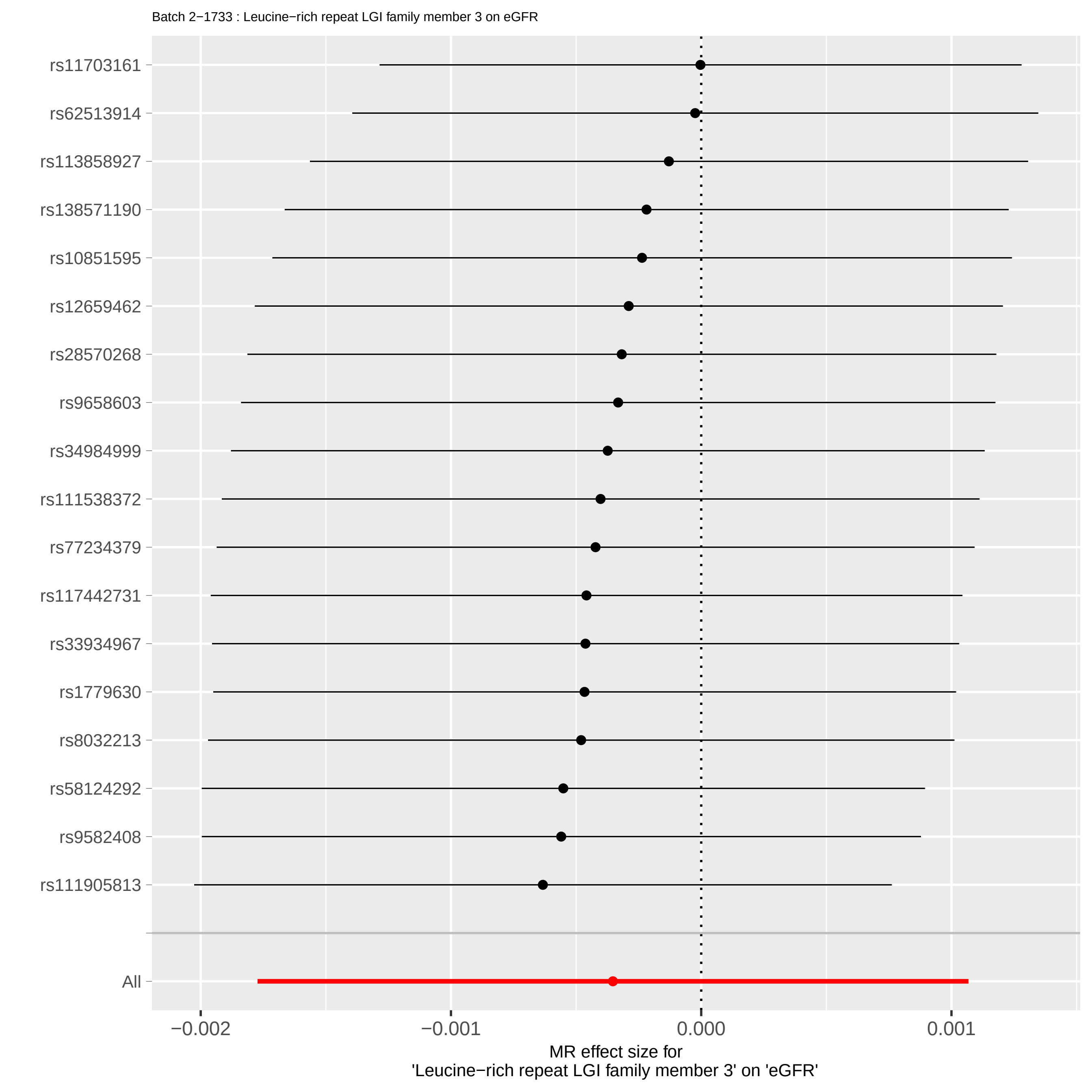

Batch 2−1733 : Leucine−rich repeat LGI family member 3 on eGFR
rs11703161
rs62513914
rs113858927
rs138571190
rs10851595
rs12659462
rs28570268
rs9658603
rs34984999
rs111538372
rs77234379
rs117442731
rs33934967
rs1779630
rs8032213
rs58124292
rs9582408
rs111905813
All
−0.002
−0.001
0.000
0.001
MR effect size for
'Leucine−rich repeat LGI family member 3' on 'eGFR'

Batch 2−1734 : Legumain on eGFR

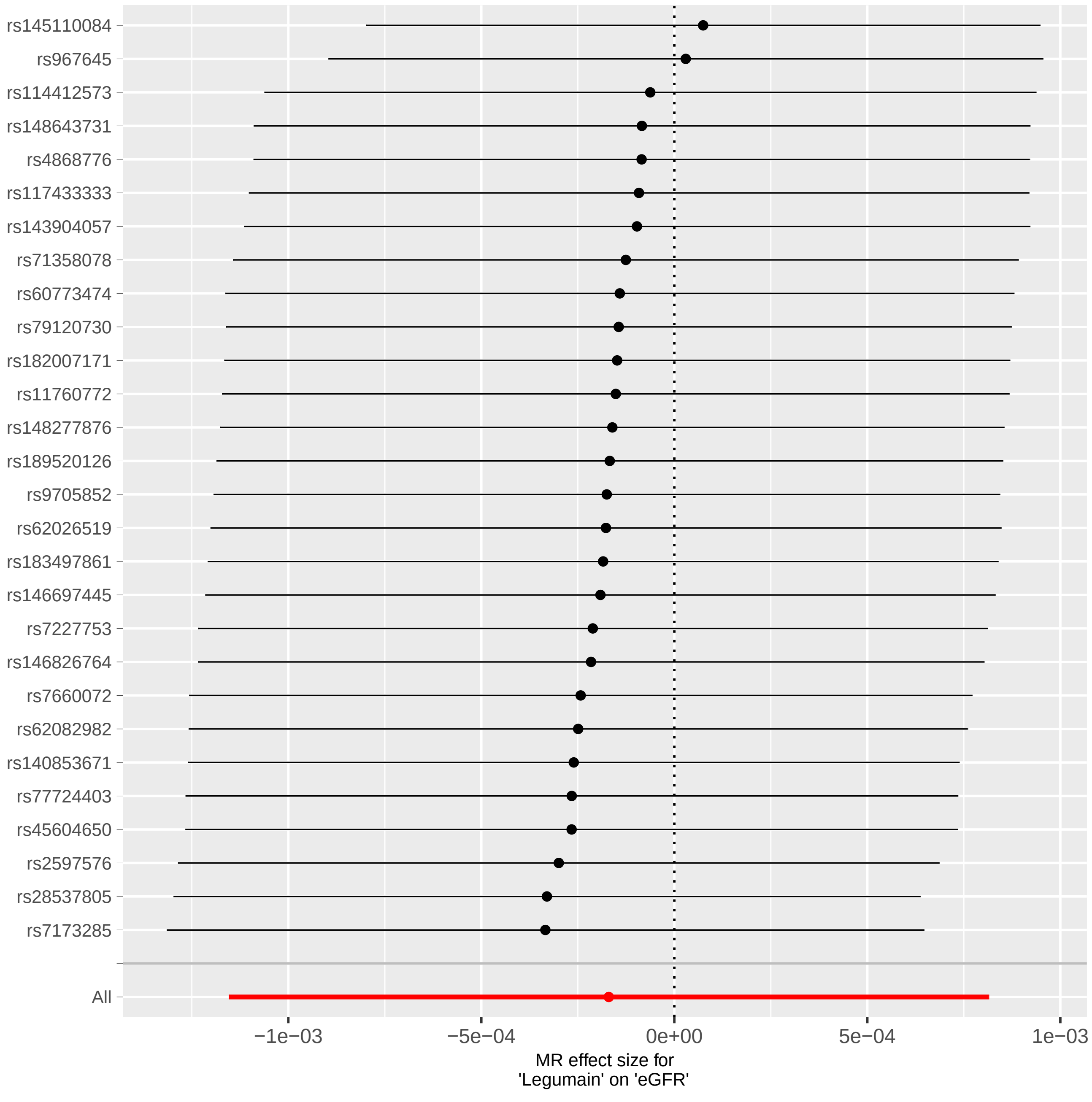

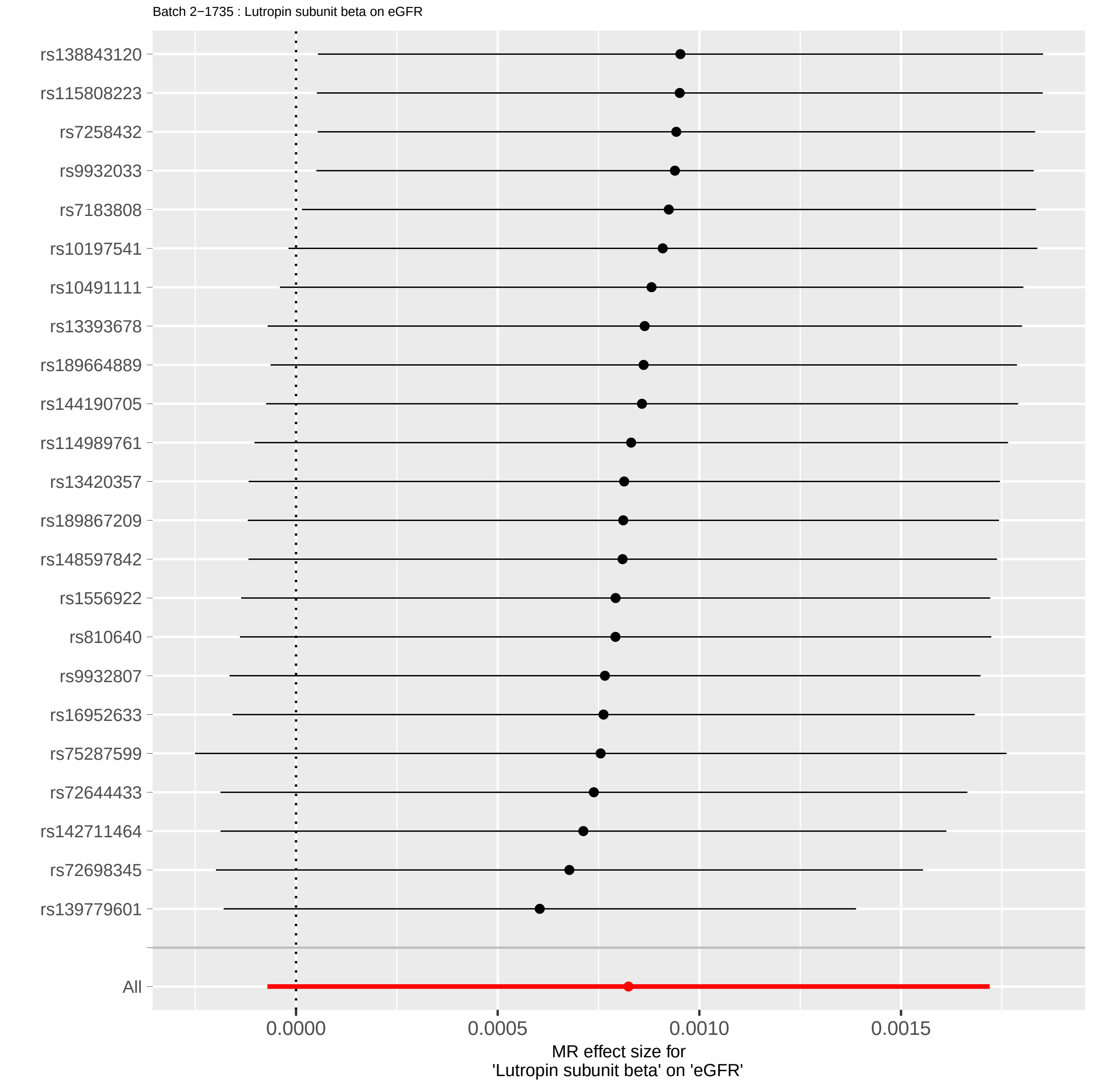

Batch 2−1735 : Lutropin subunit beta on eGFR
rs138843120
rs115808223
rs7258432
rs9932033
rs7183808
rs10197541
rs10491111
rs13393678
rs189664889
rs144190705
rs114989761
rs13420357
rs189867209
rs148597842
rs1556922
rs810640
rs9932807
rs16952633
rs75287599
rs72644433
rs142711464
rs72698345
rs139779601
All
0.0000
0.0005
0.0010
0.0015
MR effect size for
'Lutropin subunit beta' on 'eGFR'

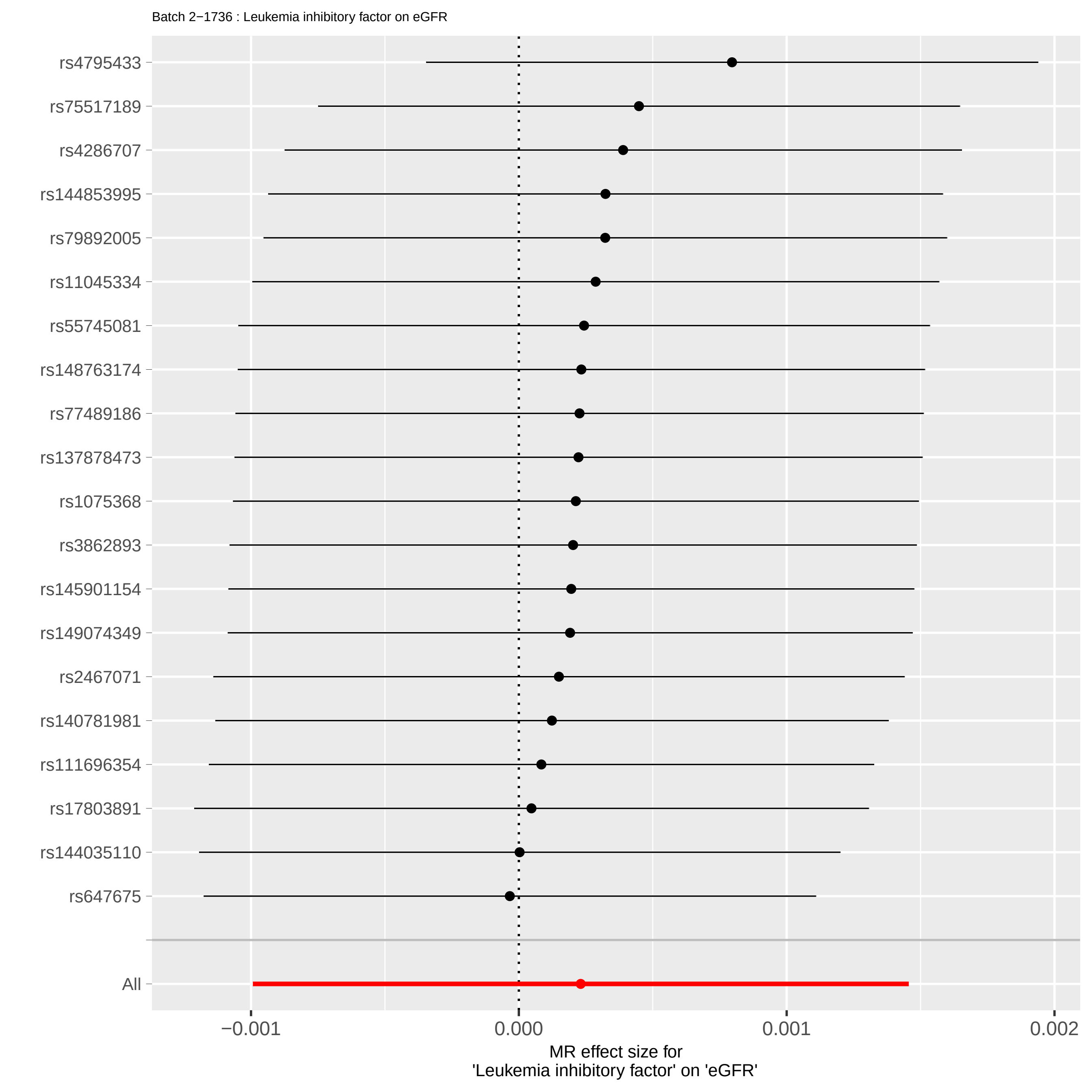
Batch 2−1736 : Leukemia inhibitory factor on eGFR
rs4795433
rs75517189
rs4286707
rs144853995
rs79892005
rs11045334
rs55745081
rs148763174
rs77489186
rs137878473
rs1075368
rs3862893
rs145901154
rs149074349
rs2467071
rs140781981
rs111696354
rs17803891
rs144035110
rs647675
All
−0.001
0.000
0.001
0.002
MR effect size for
'Leukemia inhibitory factor' on 'eGFR'

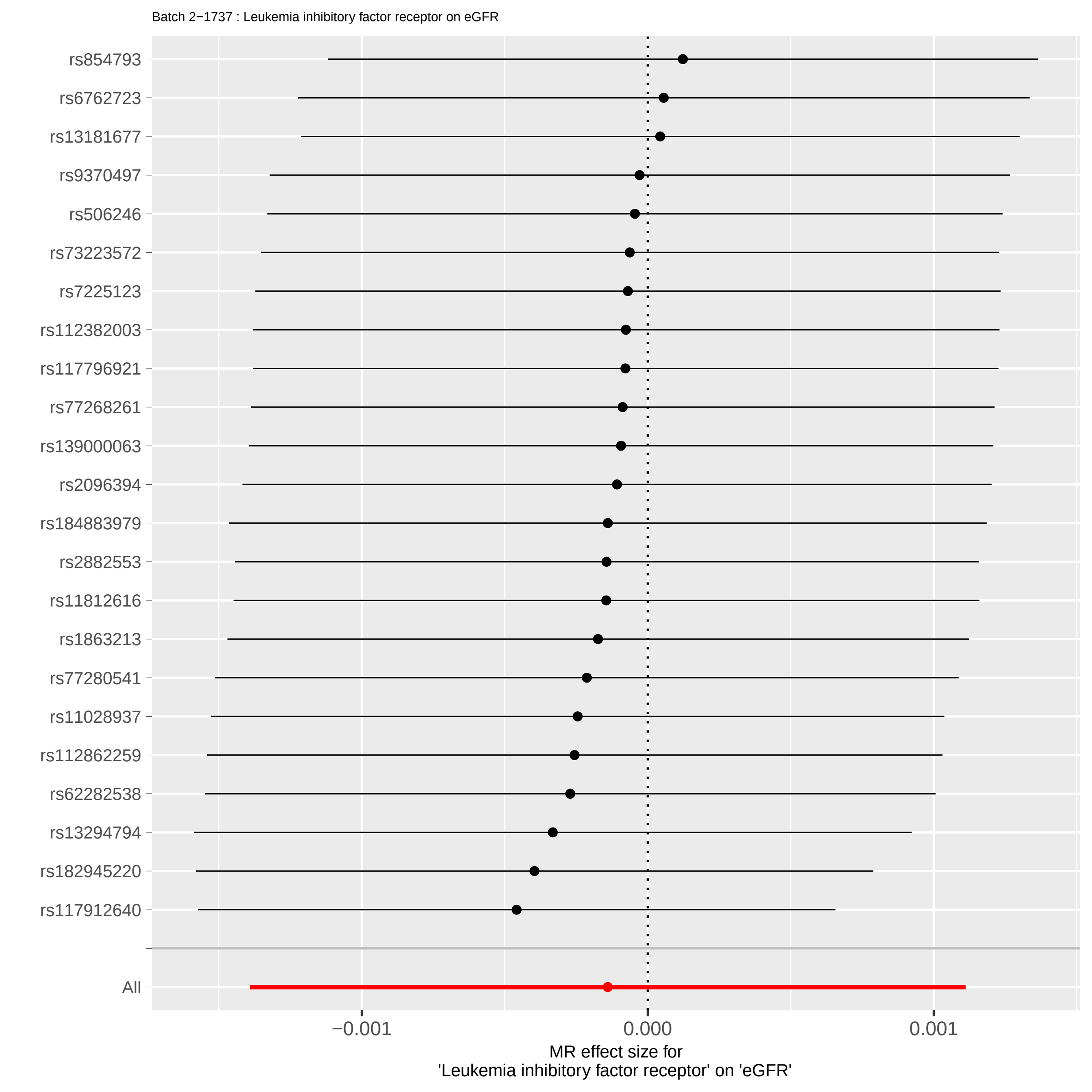

Batch 2−1737 : Leukemia inhibitory factor receptor on eGFR
rs854793
rs6762723
rs13181677
rs9370497
rs506246
rs73223572
rs7225123
rs112382003
rs117796921
rs77268261
rs139000063
rs2096394
rs184883979
rs2882553
rs11812616
rs1863213
rs77280541
rs11028937
rs112862259
rs62282538
rs13294794
rs182945220
rs117912640
All
−0.001
0.000
0.001
MR effect size for
'Leukemia inhibitory factor receptor' on 'eGFR'

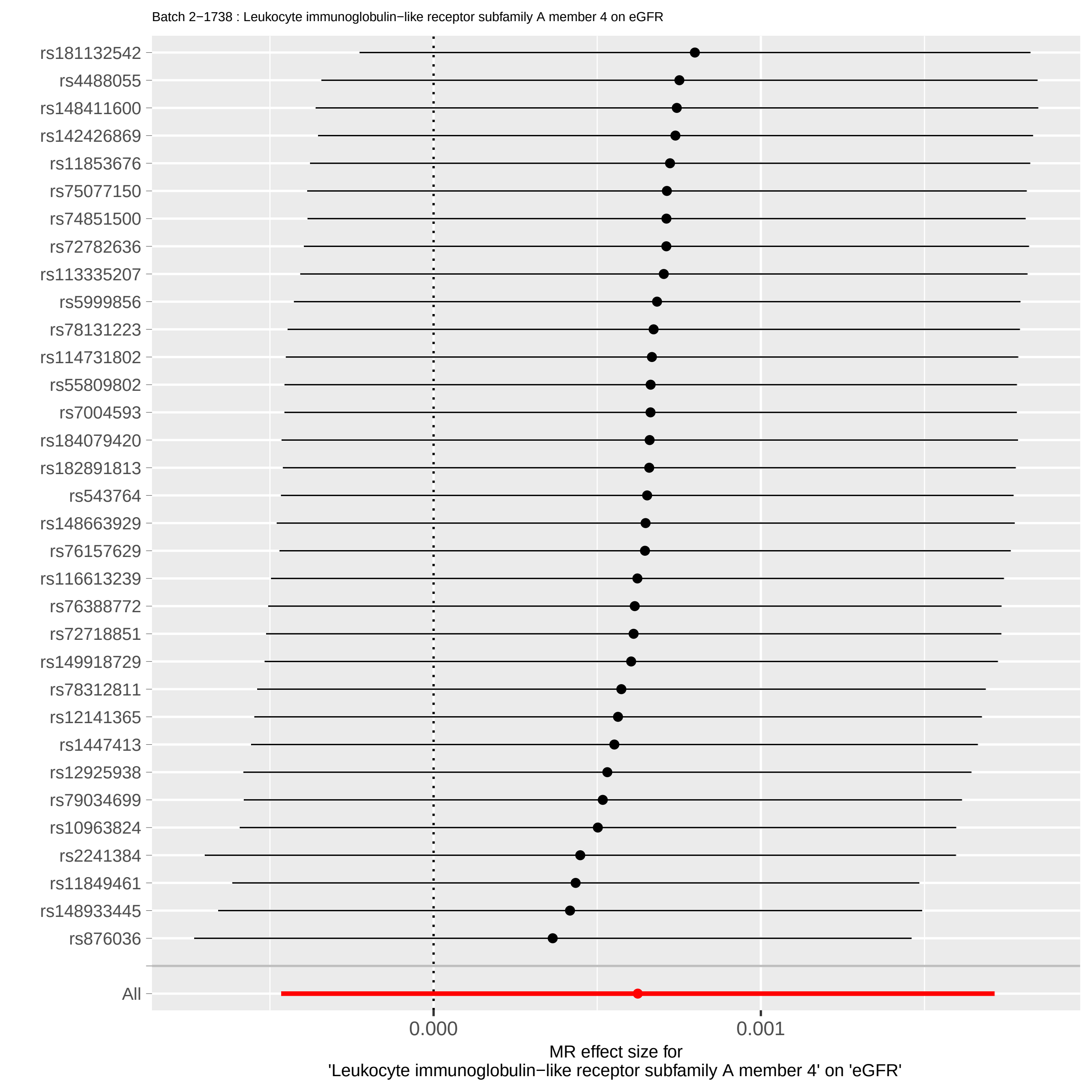
Batch 2−1738 : Leukocyte immunoglobulin−like receptor subfamily A member 4 on eGFR
rs181132542
rs4488055
rs148411600
rs142426869
rs11853676
rs75077150
rs74851500
rs72782636
rs113335207
rs5999856
rs78131223
rs114731802
rs55809802
rs7004593
rs184079420
rs182891813
rs543764
rs148663929
rs76157629
rs116613239
rs76388772
rs72718851
rs149918729
rs78312811
rs12141365
rs1447413
rs12925938
rs79034699
rs10963824
rs2241384
rs11849461
rs148933445
rs876036
All
0.000
0.001
MR effect size for
'Leukocyte immunoglobulin−like receptor subfamily A member 4' on 'eGFR'

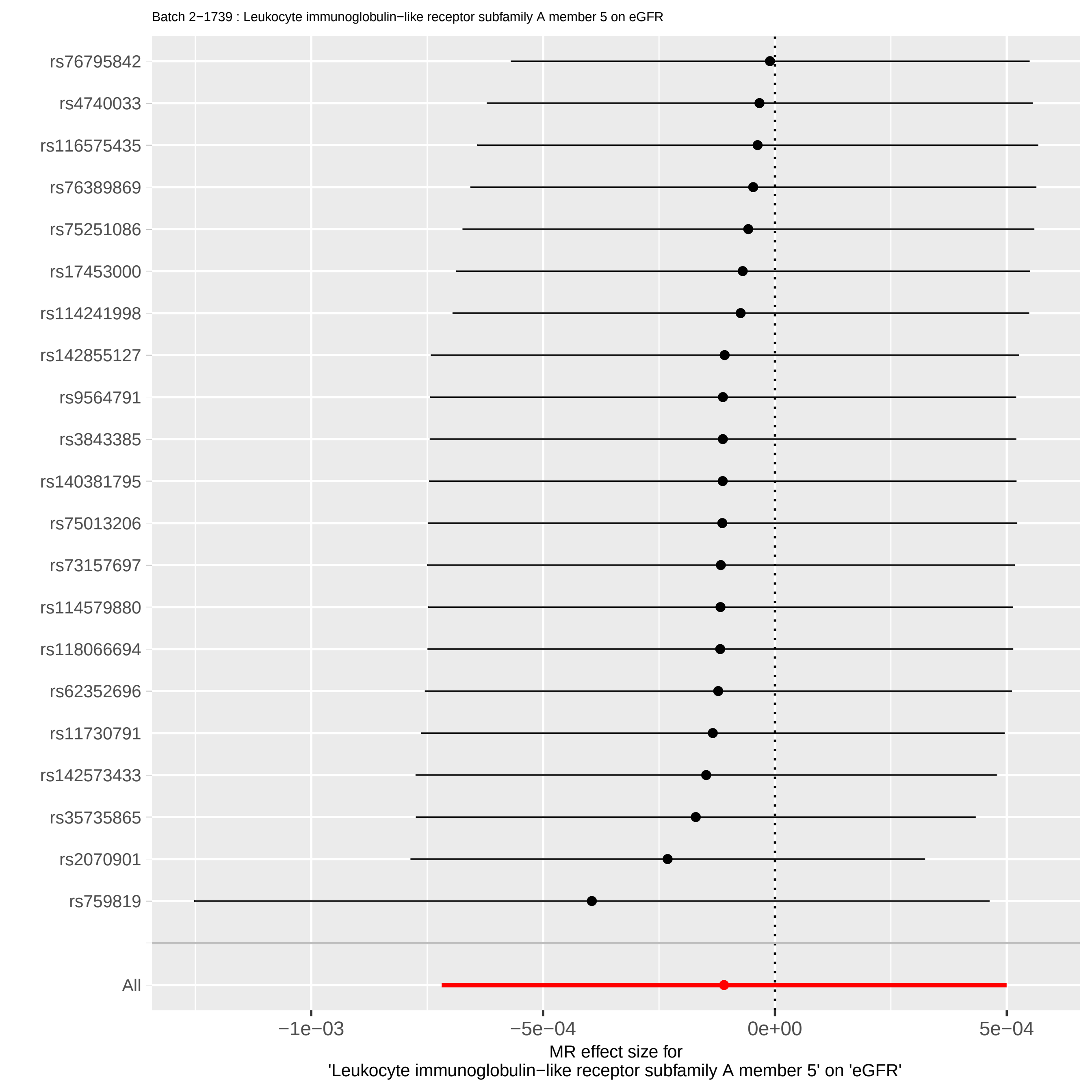
Batch 2−1739 : Leukocyte immunoglobulin−like receptor subfamily A member 5 on eGFR
rs76795842
rs4740033
rs116575435
rs76389869
rs75251086
rs17453000
rs114241998
rs142855127
rs9564791
rs3843385
rs140381795
rs75013206
rs73157697
rs114579880
rs118066694
rs62352696
rs11730791
rs142573433
rs35735865
rs2070901
rs759819
All
−1e−03
−5e−04
0e+00
5e−04
MR effect size for
'Leukocyte immunoglobulin−like receptor subfamily A member 5' on 'eGFR'

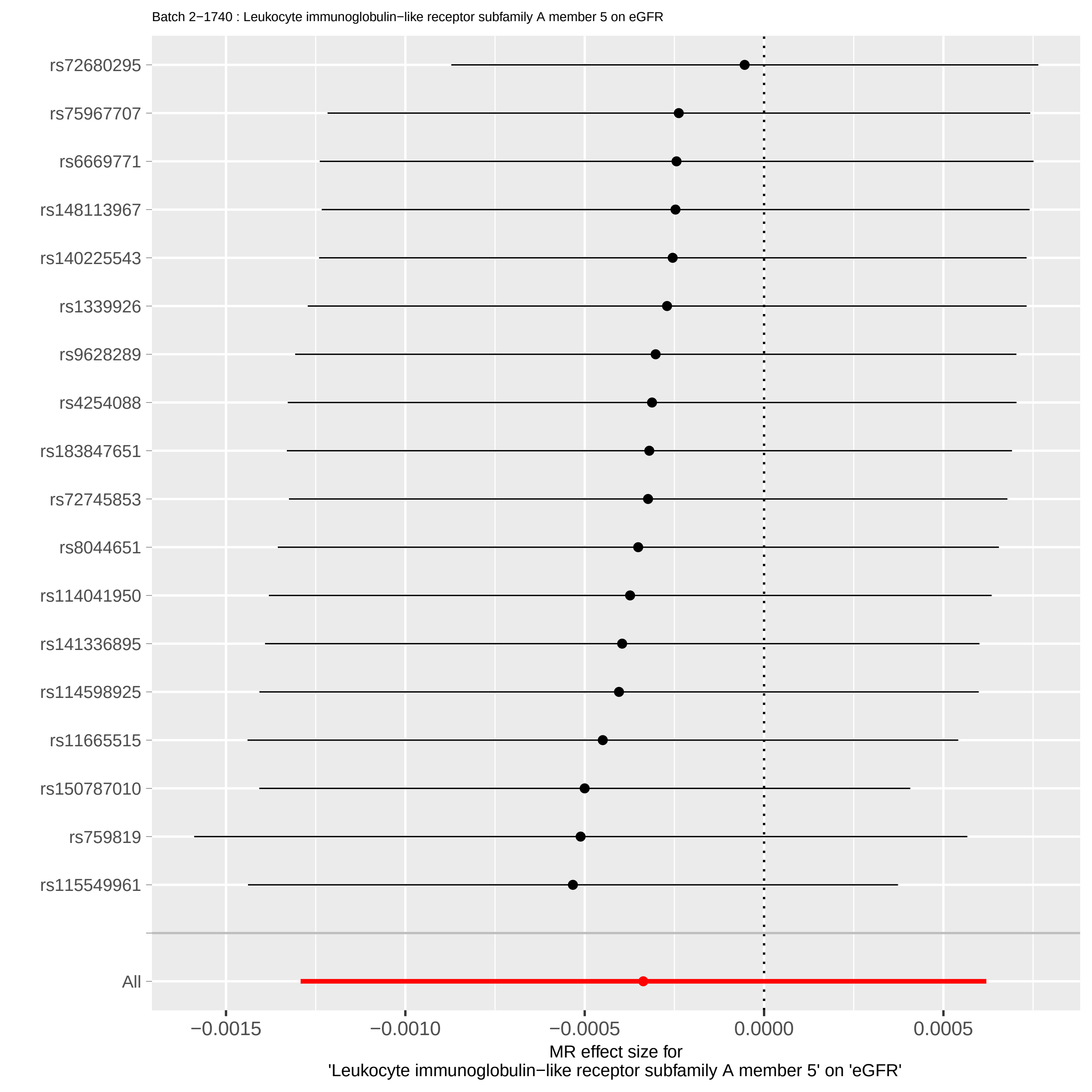

Batch 2−1740 : Leukocyte immunoglobulin−like receptor subfamily A member 5 on eGFR
rs72680295
rs75967707
rs6669771
rs148113967
rs140225543
rs1339926
rs9628289
rs4254088
rs183847651
rs72745853
rs8044651
rs114041950
rs141336895
rs114598925
rs11665515
rs150787010
rs759819
rs115549961
All
−0.0015
−0.0010
−0.0005
0.0000
0.0005
MR effect size for
'Leukocyte immunoglobulin−like receptor subfamily A member 5' on 'eGFR'

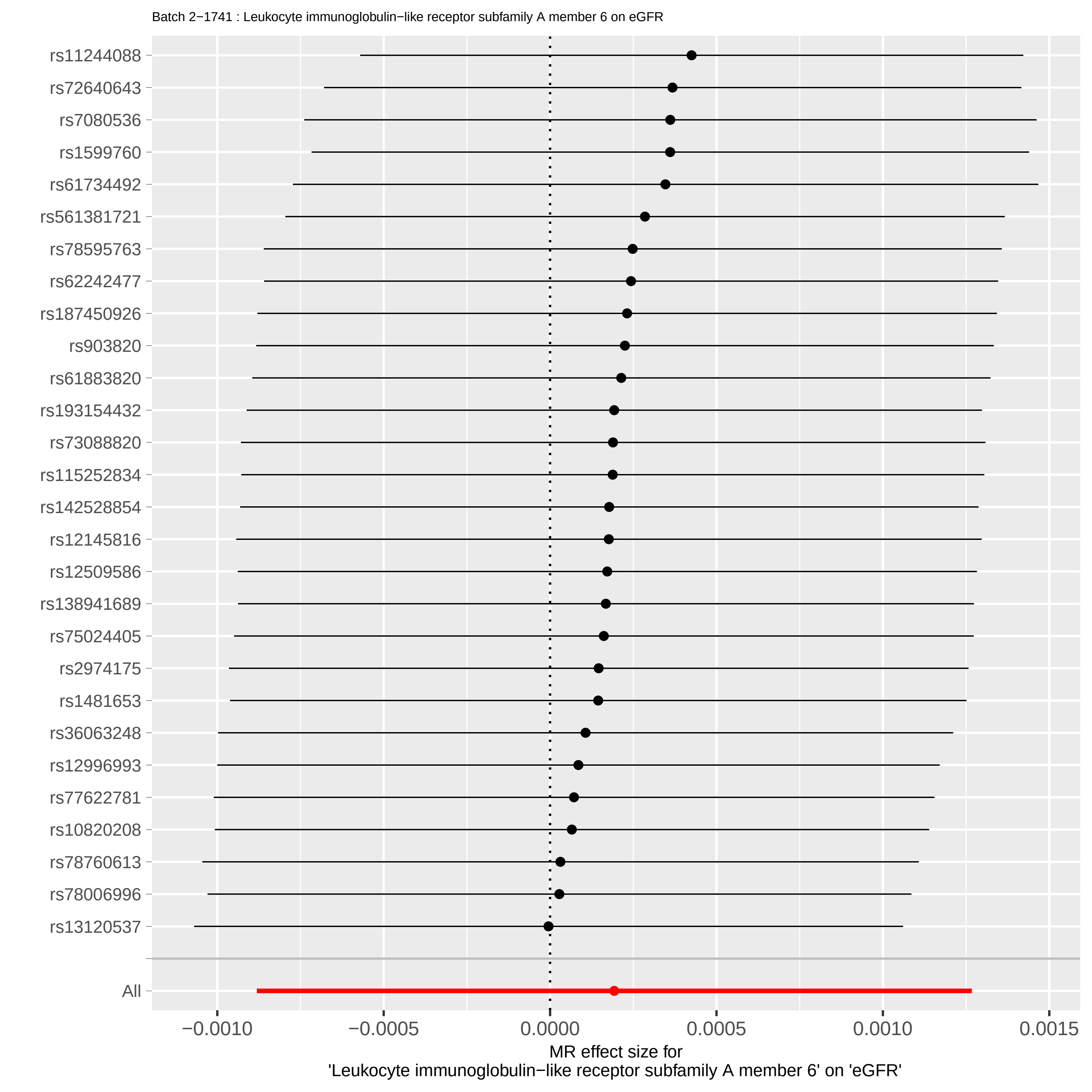

Batch 2−1741 : Leukocyte immunoglobulin−like receptor subfamily A member 6 on eGFR
rs11244088
rs72640643
rs7080536
rs1599760
rs61734492
rs561381721
rs78595763
rs62242477
rs187450926
rs903820
rs61883820
rs193154432
rs73088820
rs115252834
rs142528854
rs12145816
rs12509586
rs138941689
rs75024405
rs2974175
rs1481653
rs36063248
rs12996993
rs77622781
rs10820208
rs78760613
rs78006996
rs13120537
All
−0.0010
−0.0005
0.0000
0.0005
0.0010
0.0015
MR effect size for
'Leukocyte immunoglobulin−like receptor subfamily A member 6' on 'eGFR'

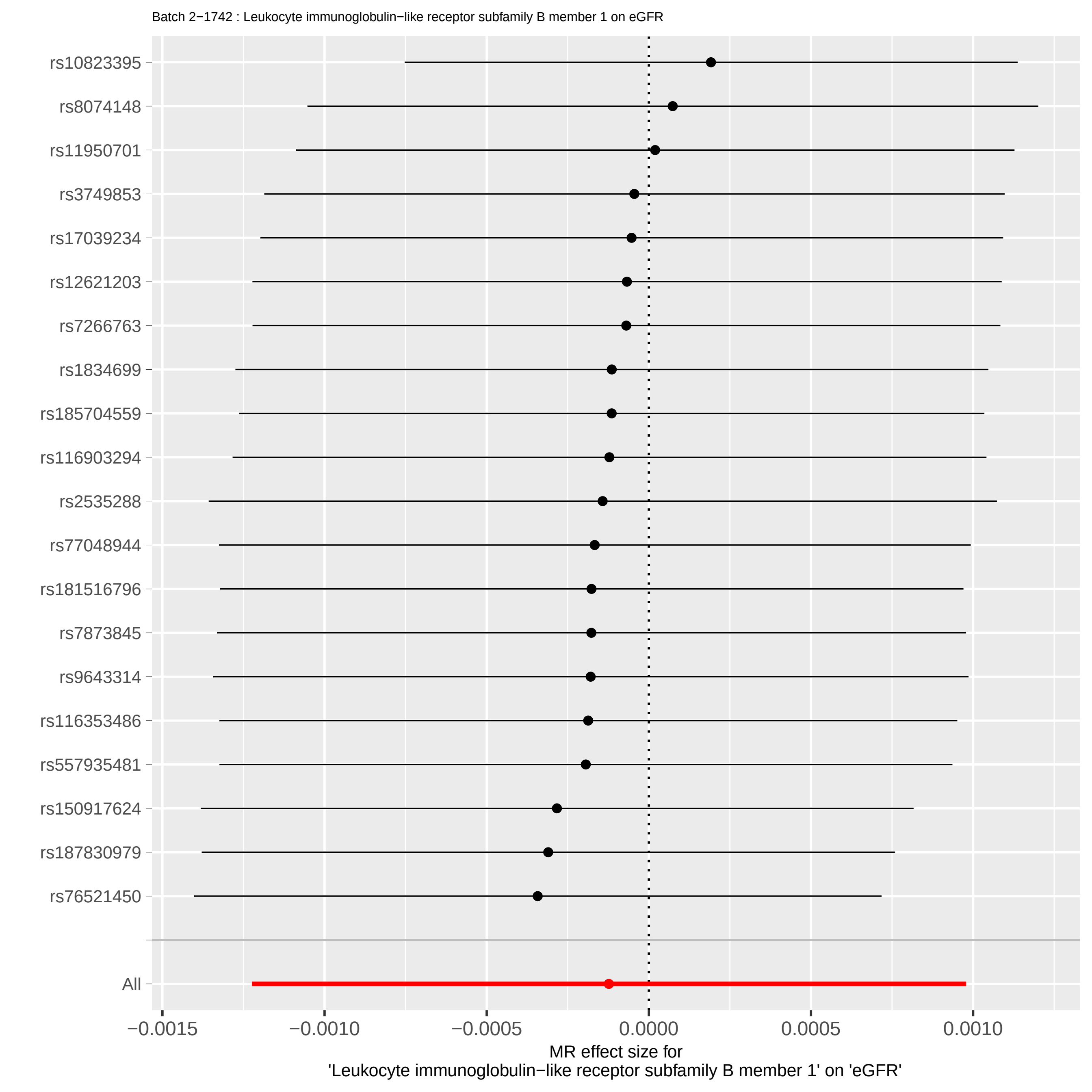

Batch 2−1742 : Leukocyte immunoglobulin−like receptor subfamily B member 1 on eGFR
rs10823395
rs8074148
rs11950701
rs3749853
rs17039234
rs12621203
rs7266763
rs1834699
rs185704559
rs116903294
rs2535288
rs77048944
rs181516796
rs7873845
rs9643314
rs116353486
rs557935481
rs150917624
rs187830979
rs76521450
All
−0.0015
−0.0010
−0.0005
0.0000
0.0005
0.0010
MR effect size for
'Leukocyte immunoglobulin−like receptor subfamily B member 1' on 'eGFR'

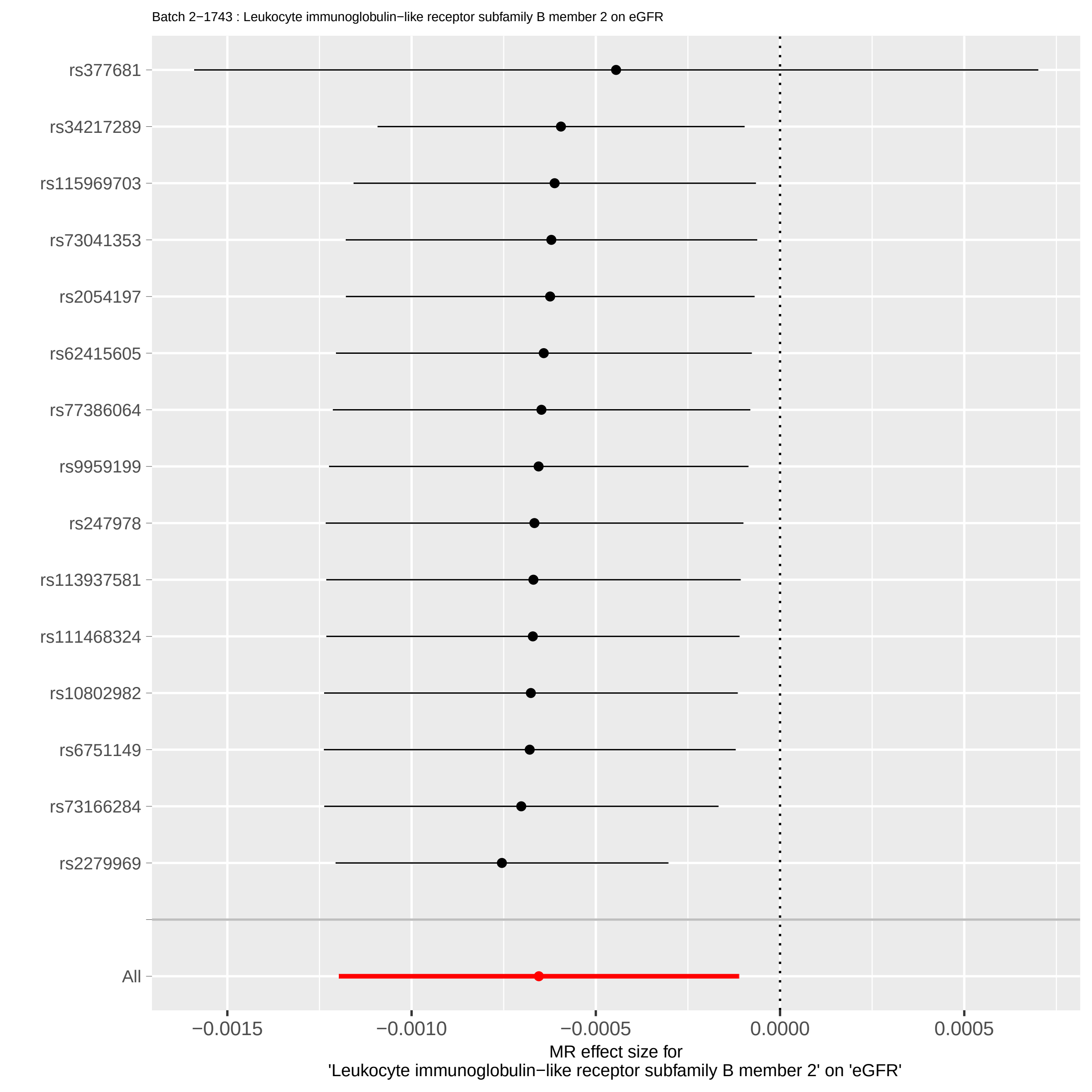

Batch 2−1743 : Leukocyte immunoglobulin−like receptor subfamily B member 2 on eGFR
rs377681
rs34217289
rs115969703
rs73041353
rs2054197
rs62415605
rs77386064
rs9959199
rs247978
rs113937581
rs111468324
rs10802982
rs6751149
rs73166284
rs2279969
All
−0.0015
−0.0010
−0.0005
0.0000
0.0005
MR effect size for
'Leukocyte immunoglobulin−like receptor subfamily B member 2' on 'eGFR'

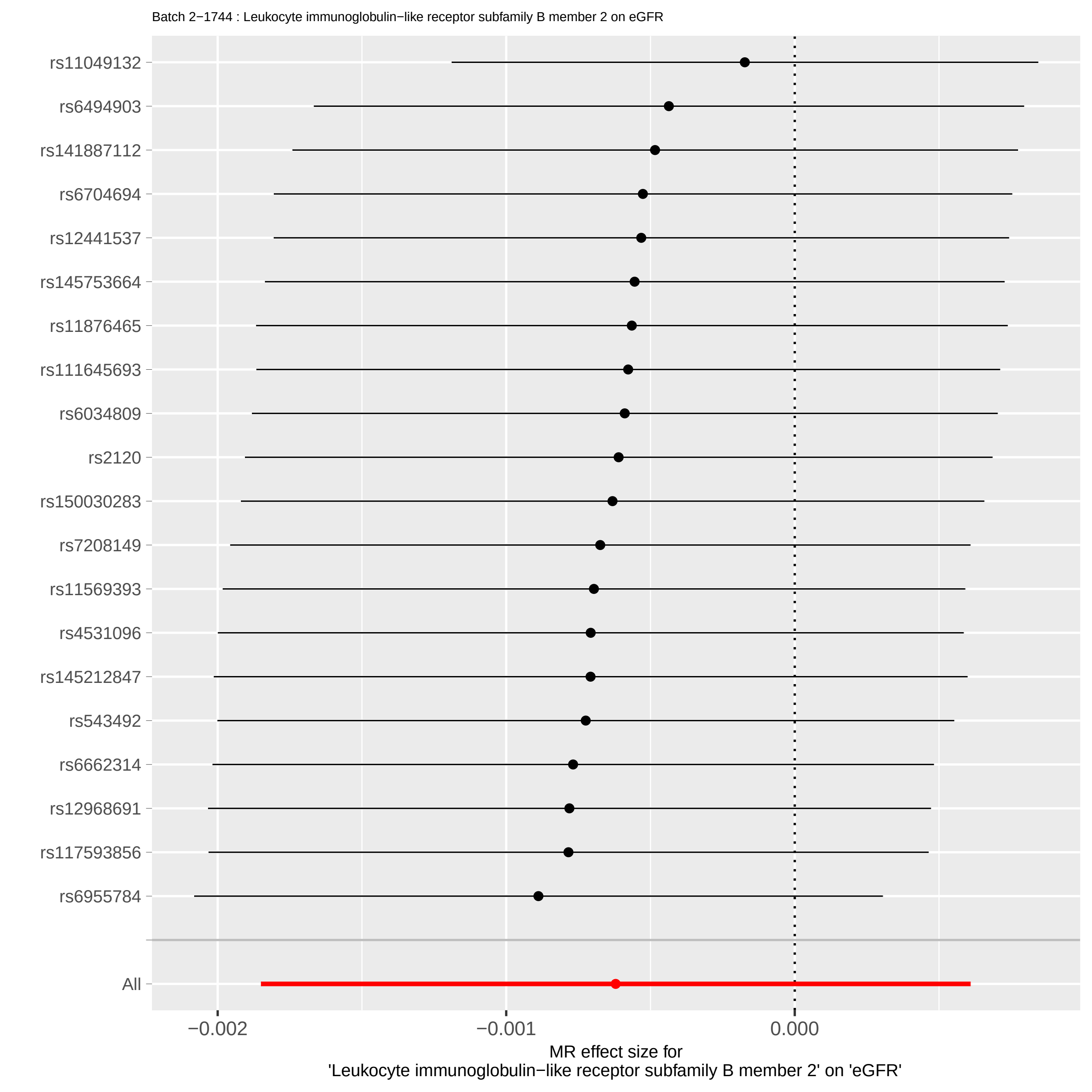

Batch 2−1744 : Leukocyte immunoglobulin−like receptor subfamily B member 2 on eGFR
rs11049132
rs6494903
rs141887112
rs6704694
rs12441537
rs145753664
rs11876465
rs111645693
rs6034809
rs2120
rs150030283
rs7208149
rs11569393
rs4531096
rs145212847
rs543492
rs6662314
rs12968691
rs117593856
rs6955784
All
−0.002
−0.001
0.000
MR effect size for
'Leukocyte immunoglobulin−like receptor subfamily B member 2' on 'eGFR'

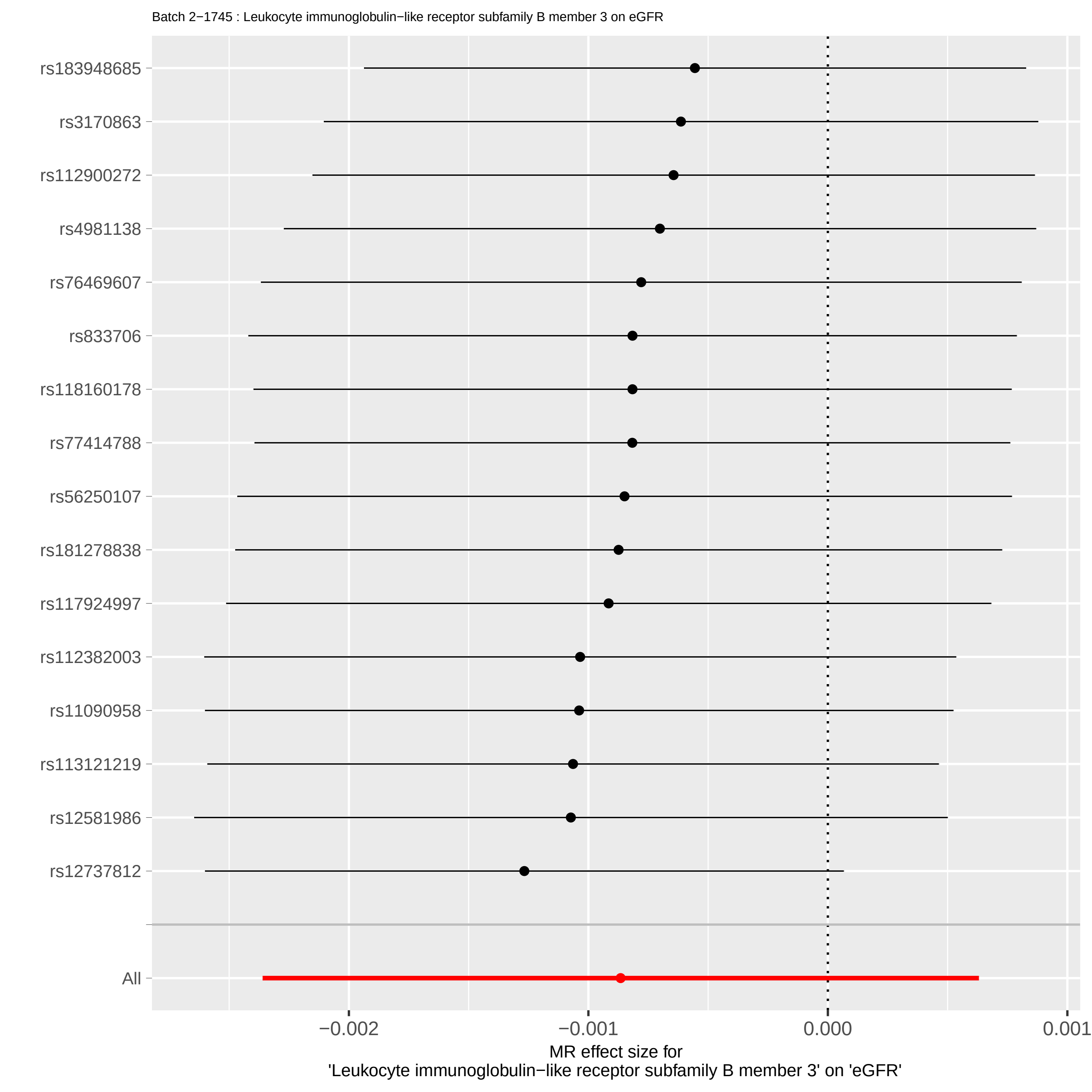

Batch 2−1745 : Leukocyte immunoglobulin−like receptor subfamily B member 3 on eGFR
rs183948685
rs3170863
rs112900272
rs4981138
rs76469607
rs833706
rs118160178
rs77414788
rs56250107
rs181278838
rs117924997
rs112382003
rs11090958
rs113121219
rs12581986
rs12737812
All
−0.002
−0.001
0.000
0.001
MR effect size for
'Leukocyte immunoglobulin−like receptor subfamily B member 3' on 'eGFR'

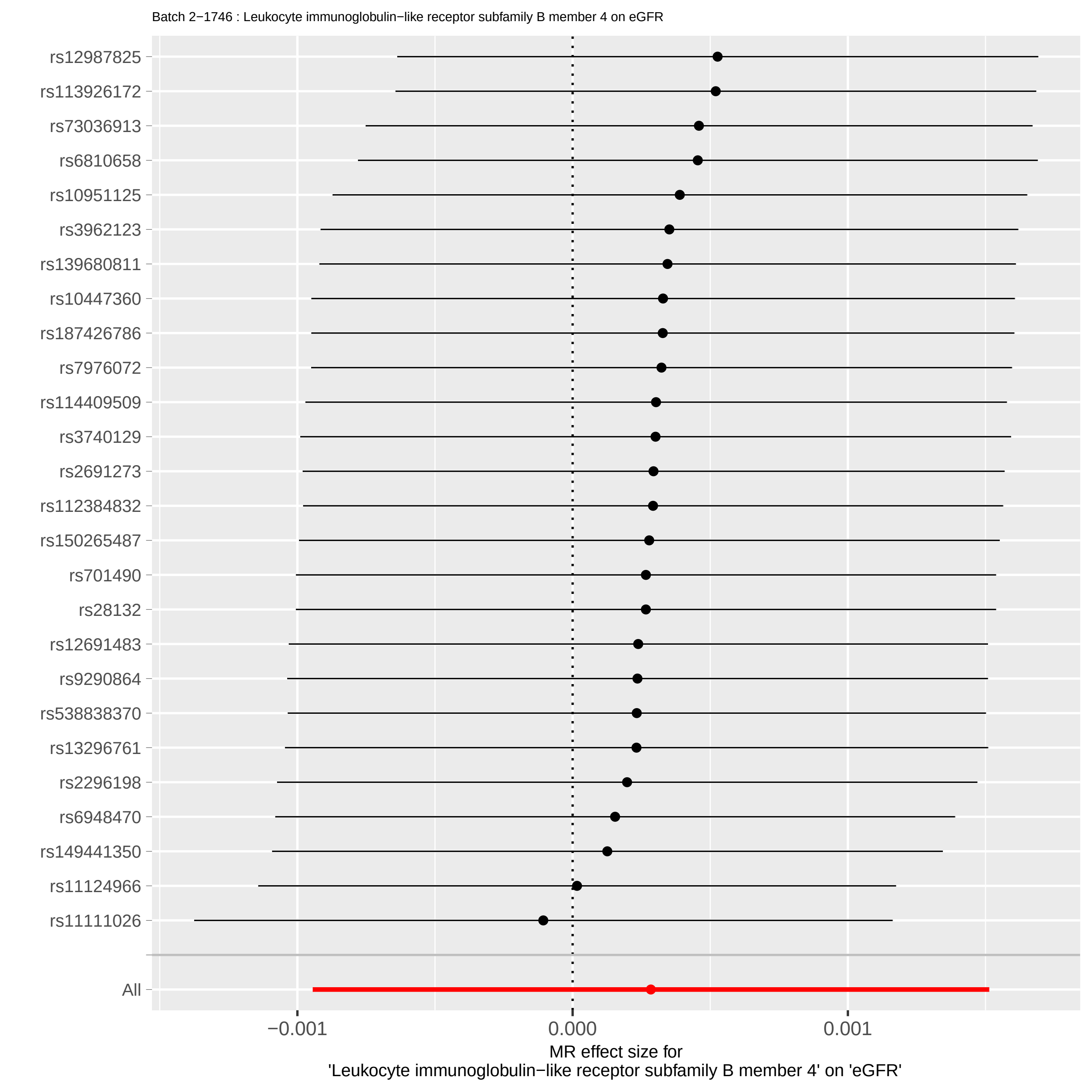

Batch 2−1746 : Leukocyte immunoglobulin−like receptor subfamily B member 4 on eGFR
rs12987825
rs113926172
rs73036913
rs6810658
rs10951125
rs3962123
rs139680811
rs10447360
rs187426786
rs7976072
rs114409509
rs3740129
rs2691273
rs112384832
rs150265487
rs701490
rs28132
rs12691483
rs9290864
rs538838370
rs13296761
rs2296198
rs6948470
rs149441350
rs11124966
rs11111026
All
−0.001
0.000
0.001
MR effect size for
'Leukocyte immunoglobulin−like receptor subfamily B member 4' on 'eGFR'

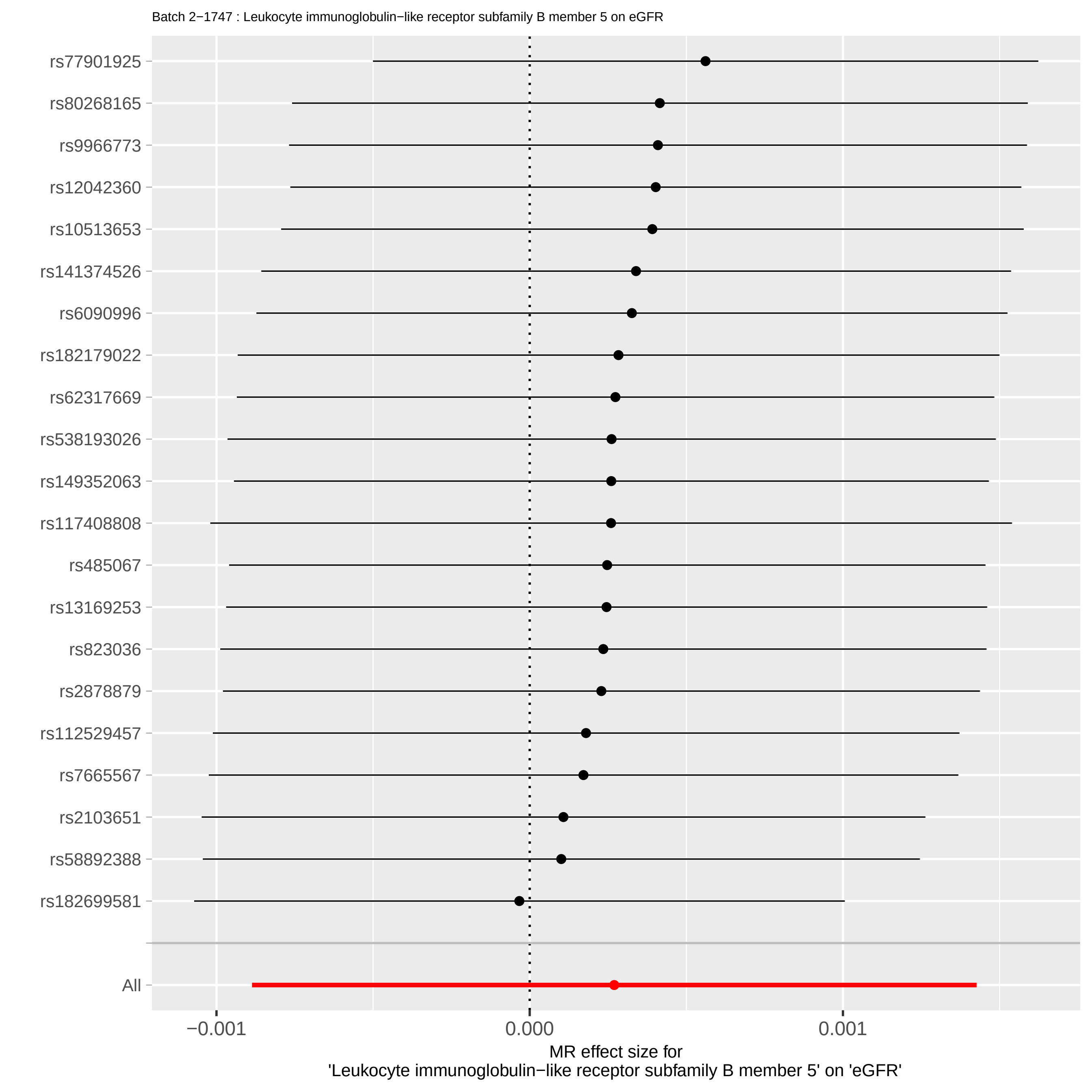
Batch 2−1747 : Leukocyte immunoglobulin−like receptor subfamily B member 5 on eGFR
rs77901925
rs80268165
rs9966773
rs12042360
rs10513653
rs141374526
rs6090996
rs182179022
rs62317669
rs538193026
rs149352063
rs117408808
rs485067
rs13169253
rs823036
rs2878879
rs112529457
rs7665567
rs2103651
rs58892388
rs182699581
All
−0.001
0.000
0.001
MR effect size for
'Leukocyte immunoglobulin−like receptor subfamily B member 5' on 'eGFR'

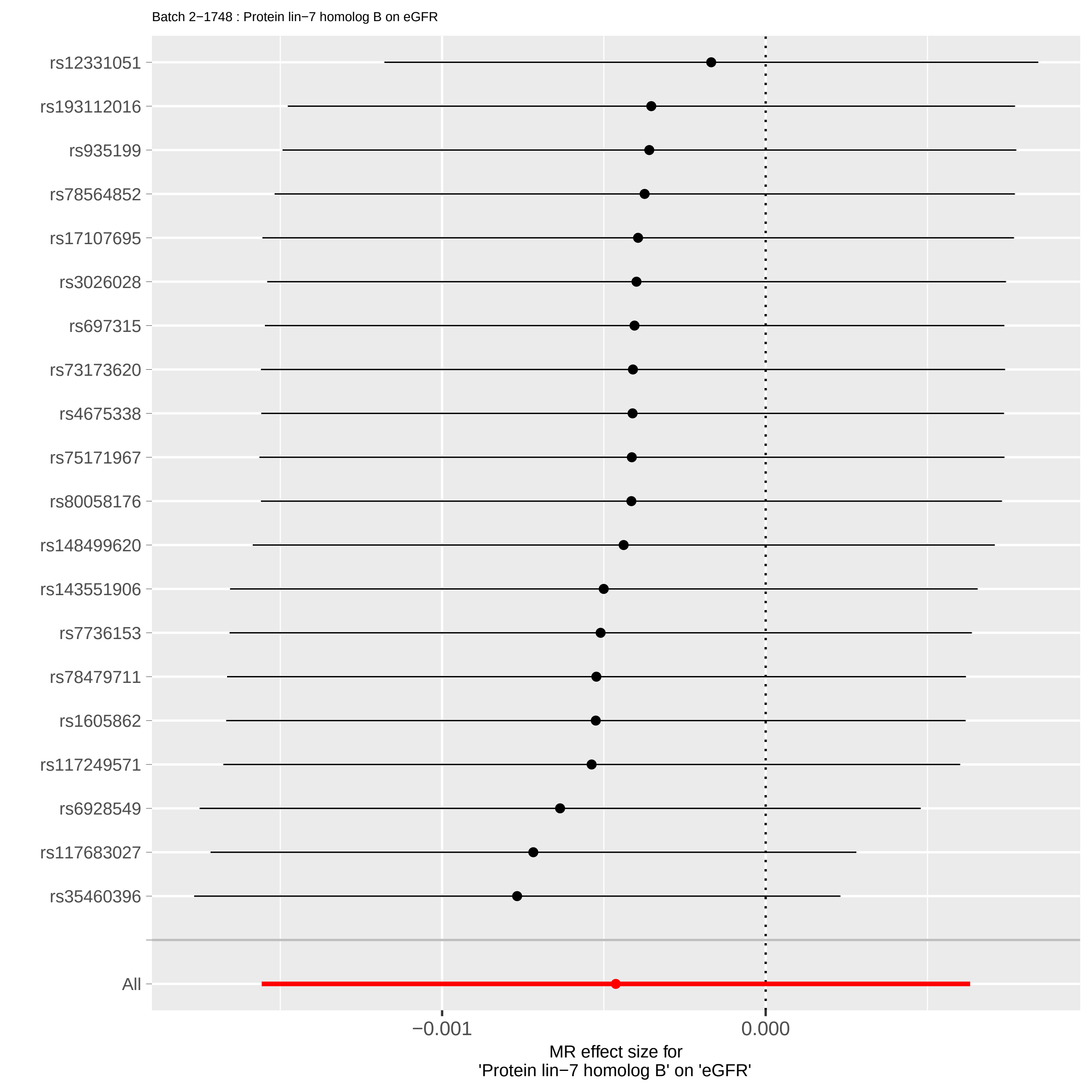
Batch 2−1748 : Protein lin−7 homolog B on eGFR
rs12331051
rs193112016
rs935199
rs78564852
rs17107695
rs3026028
rs697315
rs73173620
rs4675338
rs75171967
rs80058176
rs148499620
rs143551906
rs7736153
rs78479711
rs1605862
rs117249571
rs6928549
rs117683027
rs35460396
All
−0.001
0.000
MR effect size for
'Protein lin−7 homolog B' on 'eGFR'

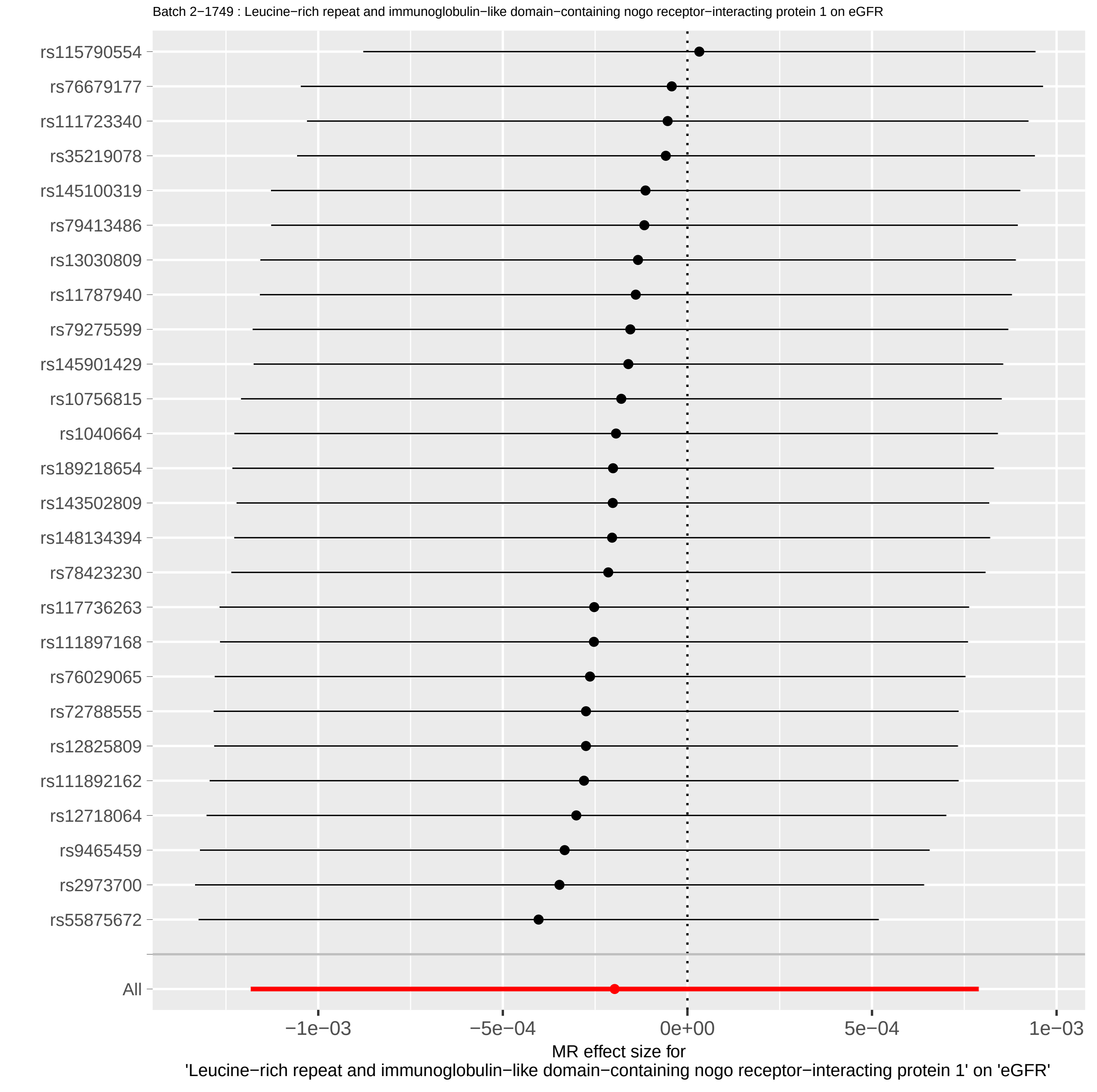
Batch 2−1749 : Leucine−rich repeat and immunoglobulin−like domain−containing nogo receptor−interacting protein 1 on eGFR
rs115790554
rs76679177
rs111723340
rs35219078
rs145100319
rs79413486
rs13030809
rs11787940
rs79275599
rs145901429
rs10756815
rs1040664
rs189218654
rs143502809
rs148134394
rs78423230
rs117736263
rs111897168
rs76029065
rs72788555
rs12825809
rs111892162
rs12718064
rs9465459
rs2973700
rs55875672
All
−1e−03
−5e−04
0e+00
5e−04
1e−03
MR effect size for
'Leucine−rich repeat and immunoglobulin−like domain−containing nogo receptor−interacting protein 1' on 'eGFR'

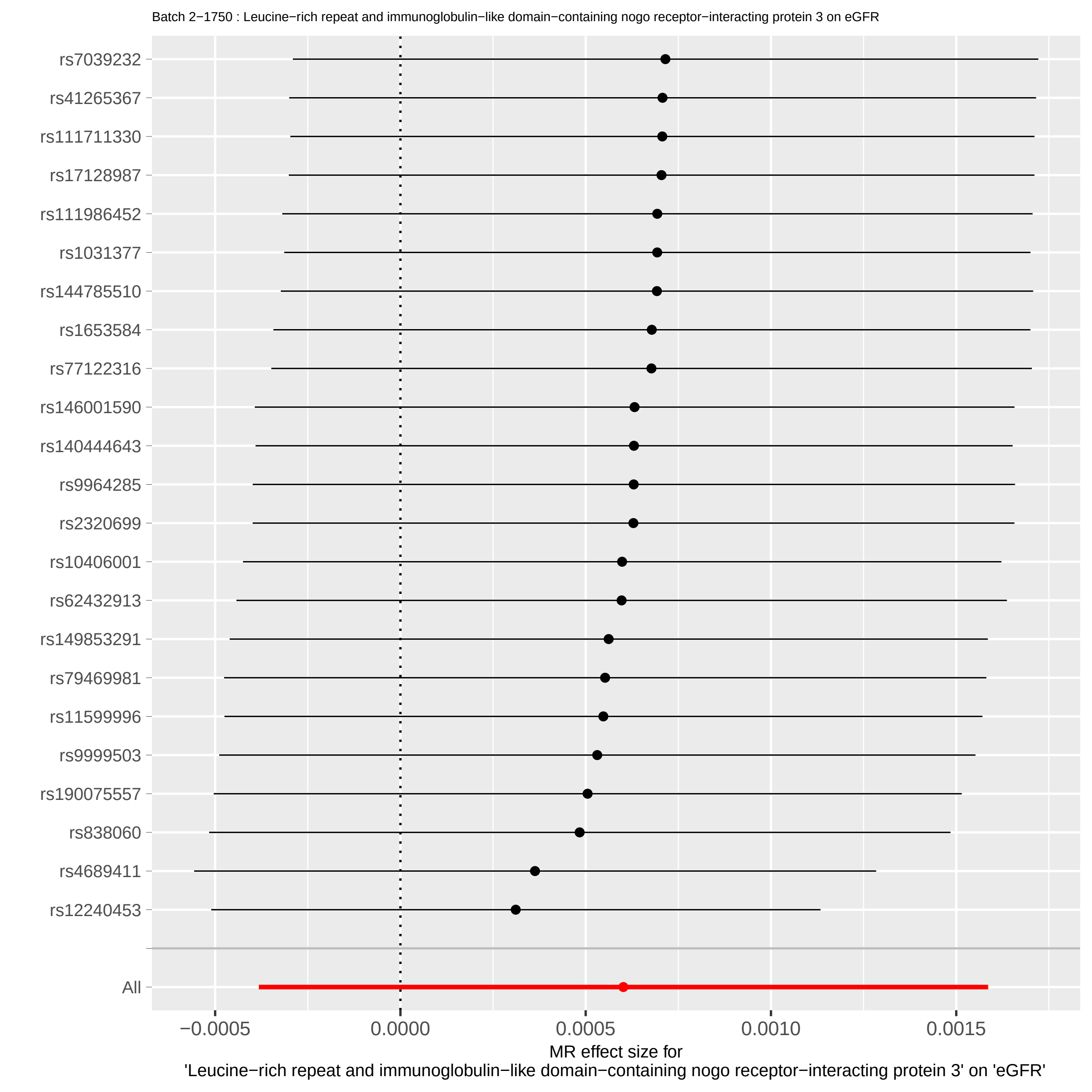

Batch 2−1750 : Leucine−rich repeat and immunoglobulin−like domain−containing nogo receptor−interacting protein 3 on eGFR
rs7039232
rs41265367
rs111711330
rs17128987
rs111986452
rs1031377
rs144785510
rs1653584
rs77122316
rs146001590
rs140444643
rs9964285
rs2320699
rs10406001
rs62432913
rs149853291
rs79469981
rs11599996
rs9999503
rs190075557
rs838060
rs4689411
rs12240453
All
−0.0005
0.0000
0.0005
0.0010
0.0015
MR effect size for
'Leucine−rich repeat and immunoglobulin−like domain−containing nogo receptor−interacting protein 3' on 'eGFR'

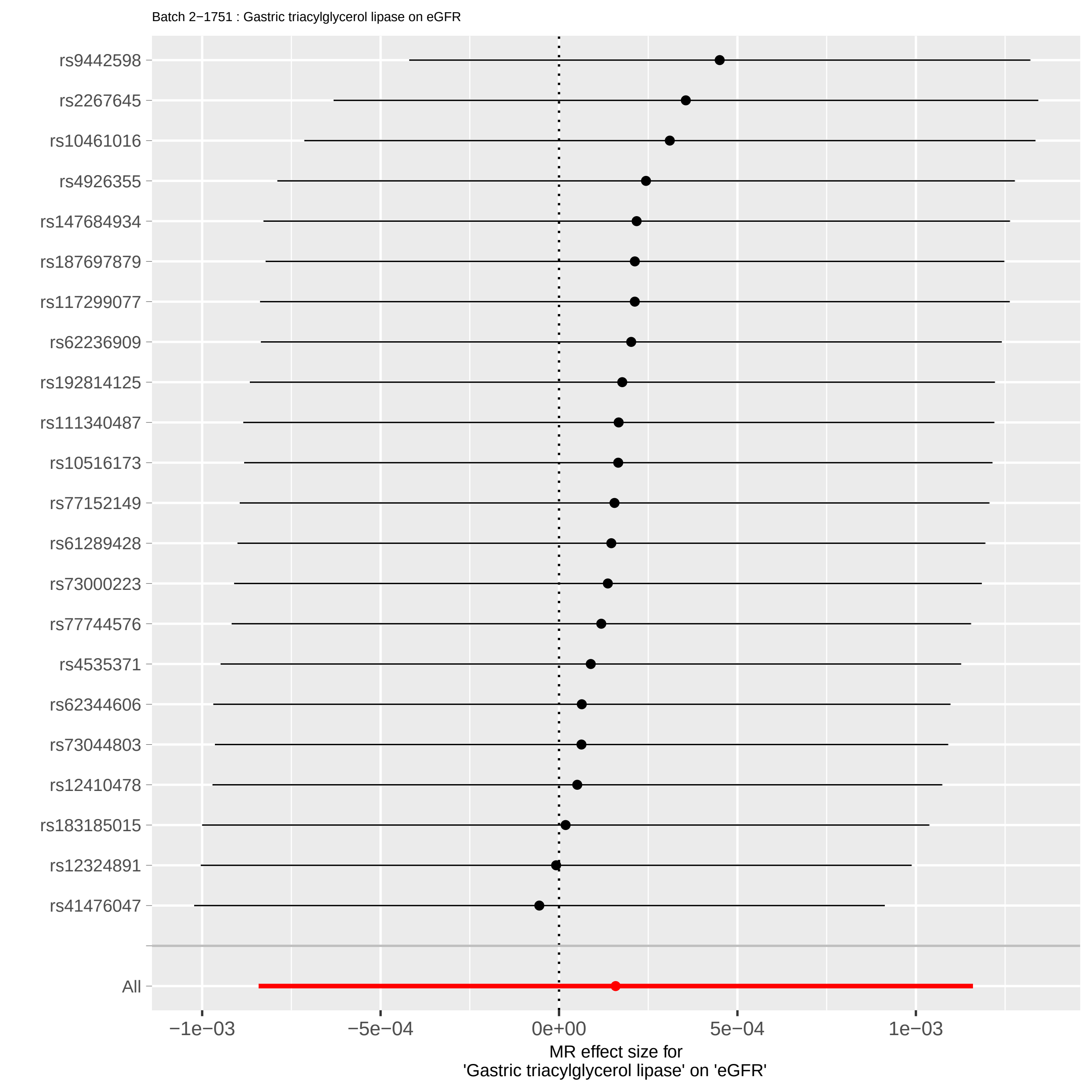
Batch 2−1751 : Gastric triacylglycerol lipase on eGFR
rs9442598
rs2267645
rs10461016
rs4926355
rs147684934
rs187697879
rs117299077
rs62236909
rs192814125
rs111340487
rs10516173
rs77152149
rs61289428
rs73000223
rs77744576
rs4535371
rs62344606
rs73044803
rs12410478
rs183185015
rs12324891
rs41476047
All
−1e−03
−5e−04
0e+00
5e−04
1e−03
MR effect size for
'Gastric triacylglycerol lipase' on 'eGFR'

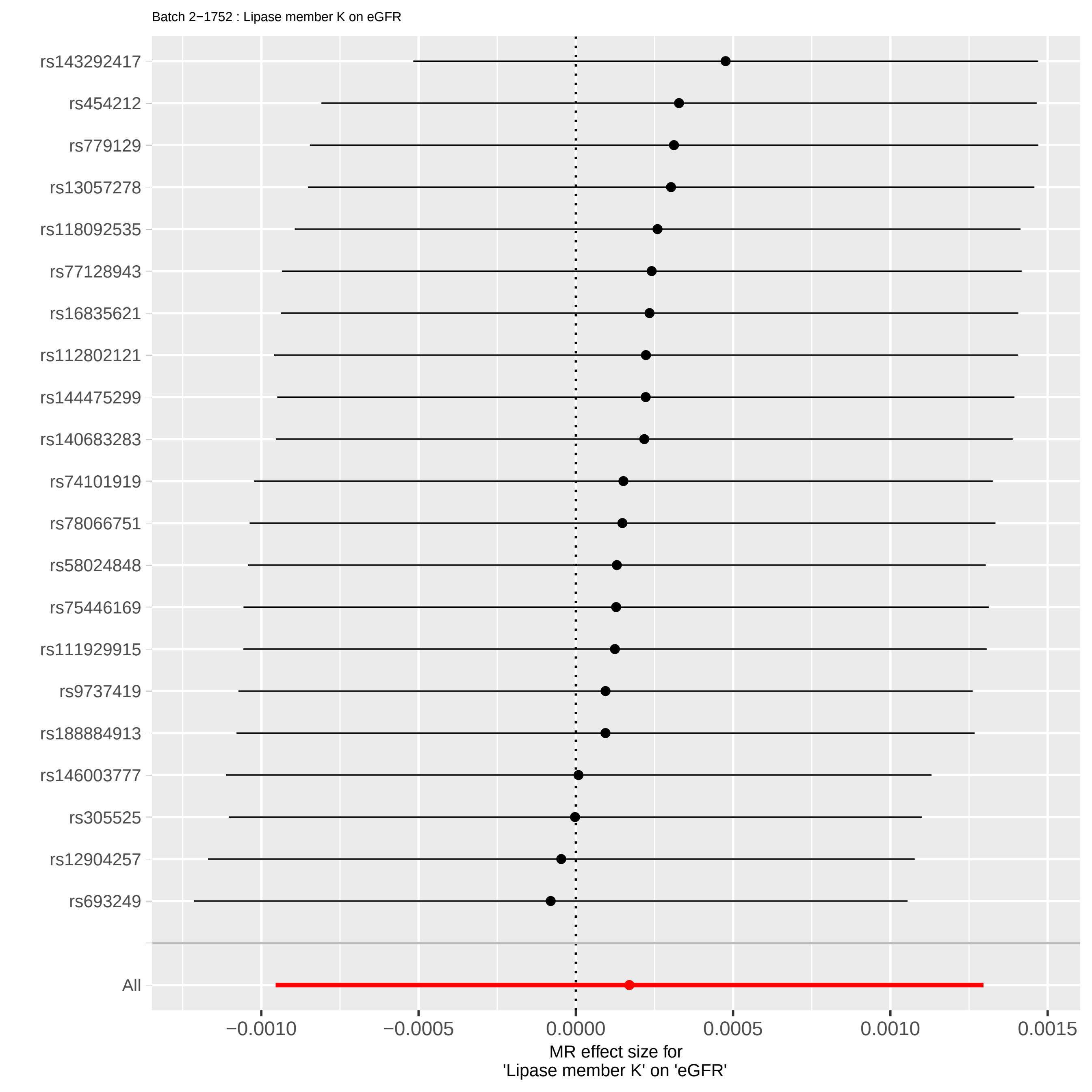

Batch 2−1752 : Lipase member K on eGFR
rs143292417
rs454212
rs779129
rs13057278
rs118092535
rs77128943
rs16835621
rs112802121
rs144475299
rs140683283
rs74101919
rs78066751
rs58024848
rs75446169
rs111929915
rs9737419
rs188884913
rs146003777
rs305525
rs12904257
rs693249
All
−0.0010
−0.0005
0.0000
0.0005
0.0010
0.0015
MR effect size for
'Lipase member K' on 'eGFR'

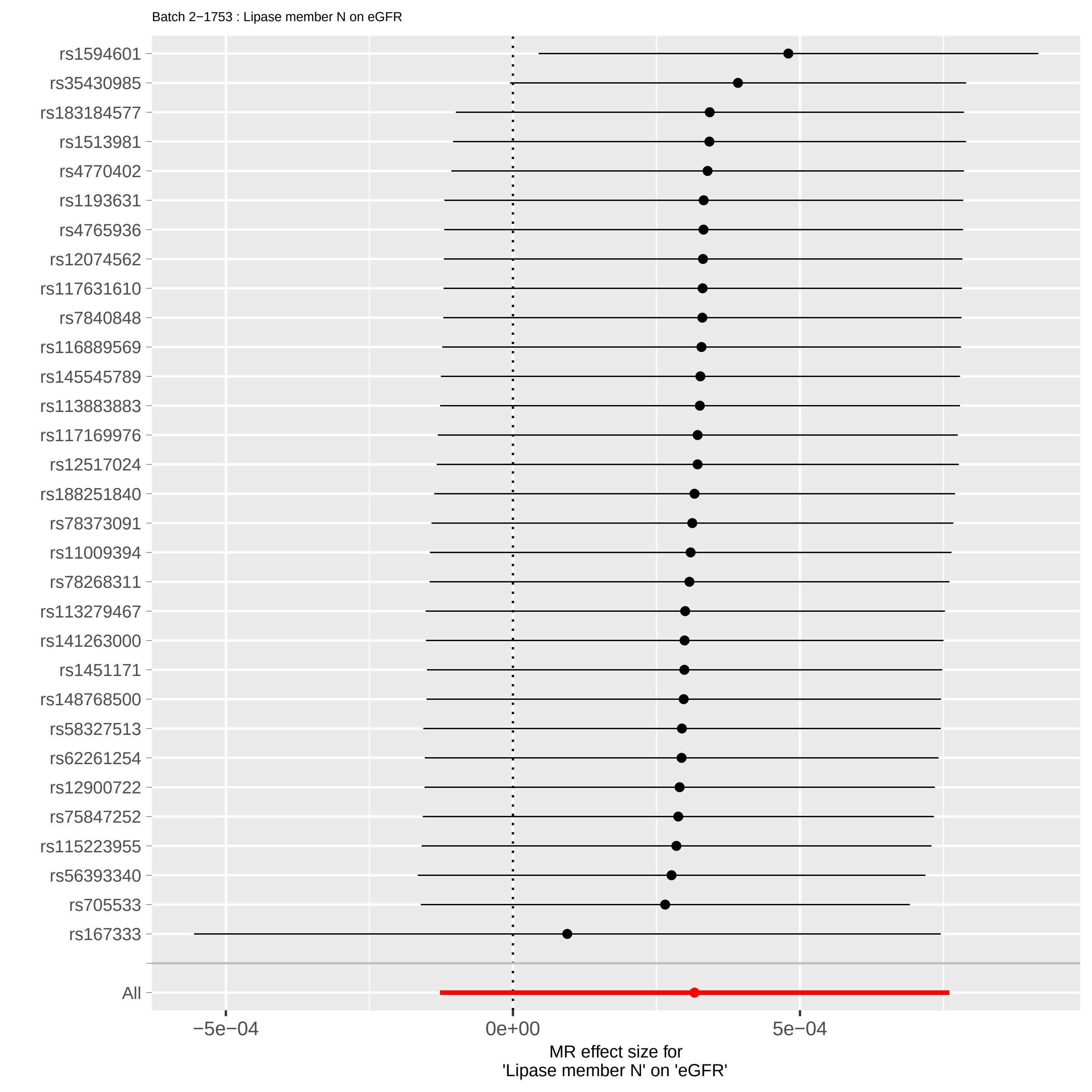

Batch 2−1753 : Lipase member N on eGFR
rs1594601
rs35430985
rs183184577
rs1513981
rs4770402
rs1193631
rs4765936
rs12074562
rs117631610
rs7840848
rs116889569
rs145545789
rs113883883
rs117169976
rs12517024
rs188251840
rs78373091
rs11009394
rs78268311
rs113279467
rs141263000
rs1451171
rs148768500
rs58327513
rs62261254
rs12900722
rs75847252
rs115223955
rs56393340
rs705533
rs167333
All
−5e−04
0e+00
5e−04
MR effect size for
'Lipase member N' on 'eGFR'

Batch 2−1754 : Vesicular integral−membrane protein VIP36 on eGFR

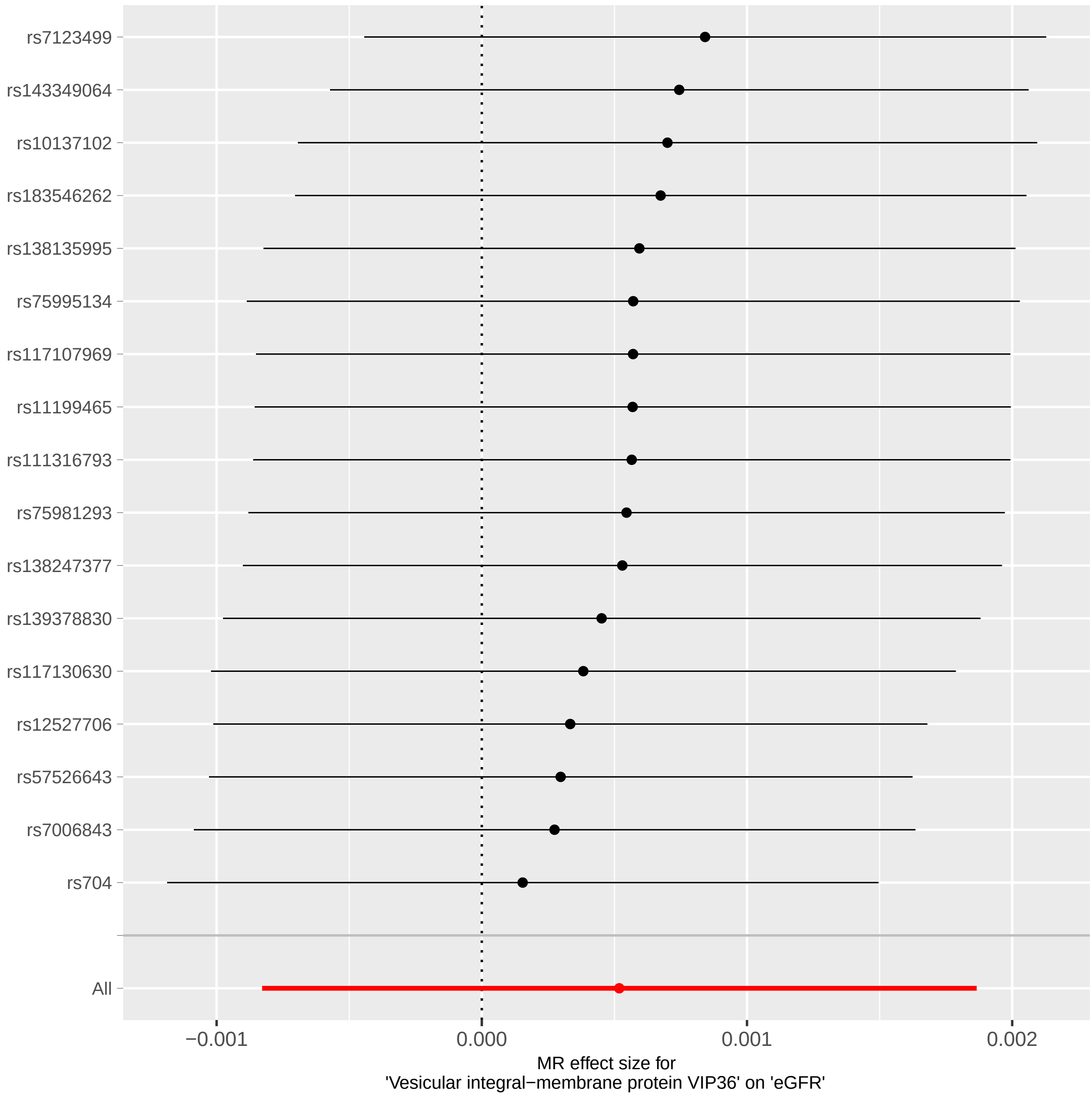

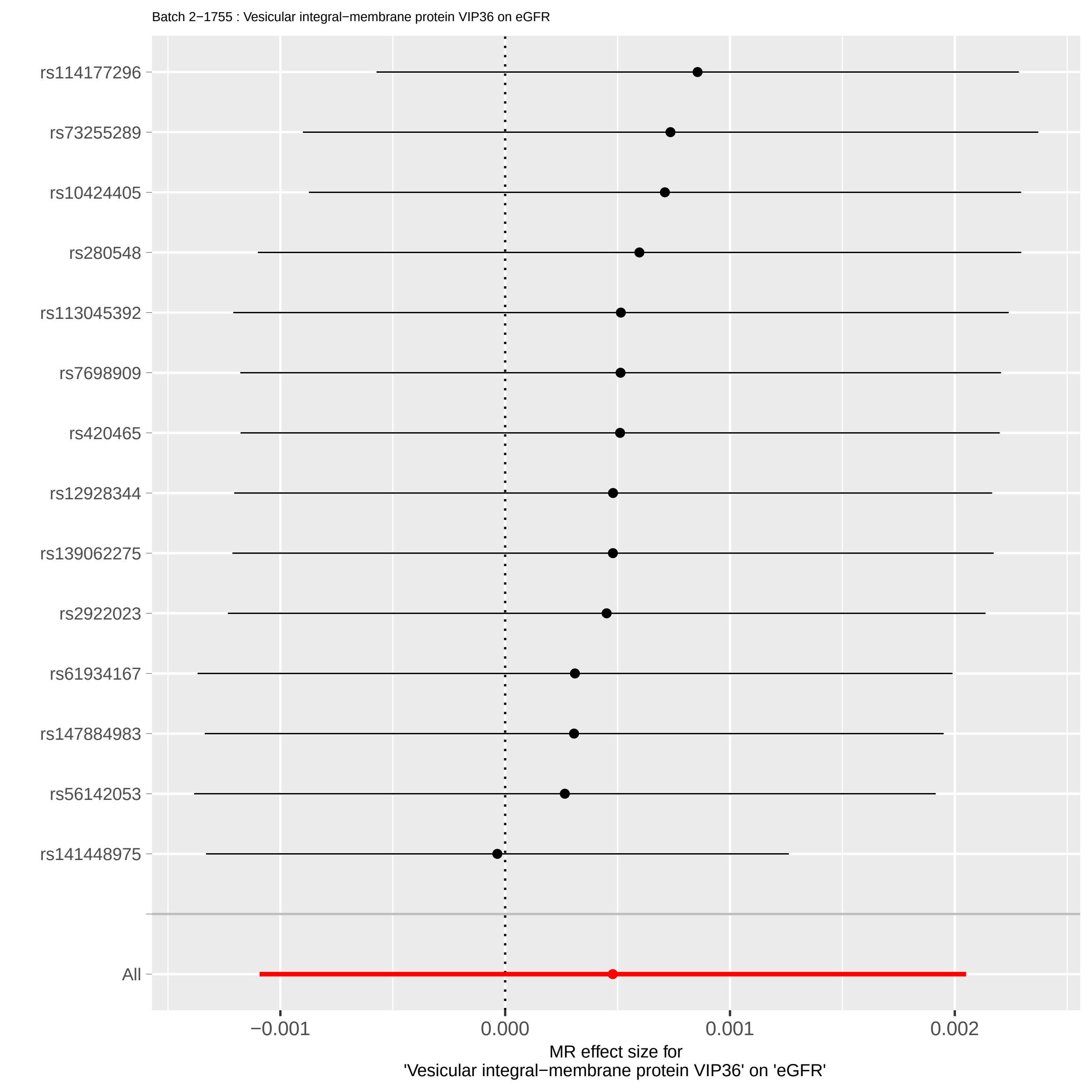

Batch 2−1755 : Vesicular integral−membrane protein VIP36 on eGFR
rs114177296
rs73255289
rs10424405
rs280548
rs113045392
rs7698909
rs420465
rs12928344
rs139062275
rs2922023
rs61934167
rs147884983
rs56142053
rs141448975
All
−0.001
0.000
0.001
0.002
MR effect size for
'Vesicular integral−membrane protein VIP36' on 'eGFR'

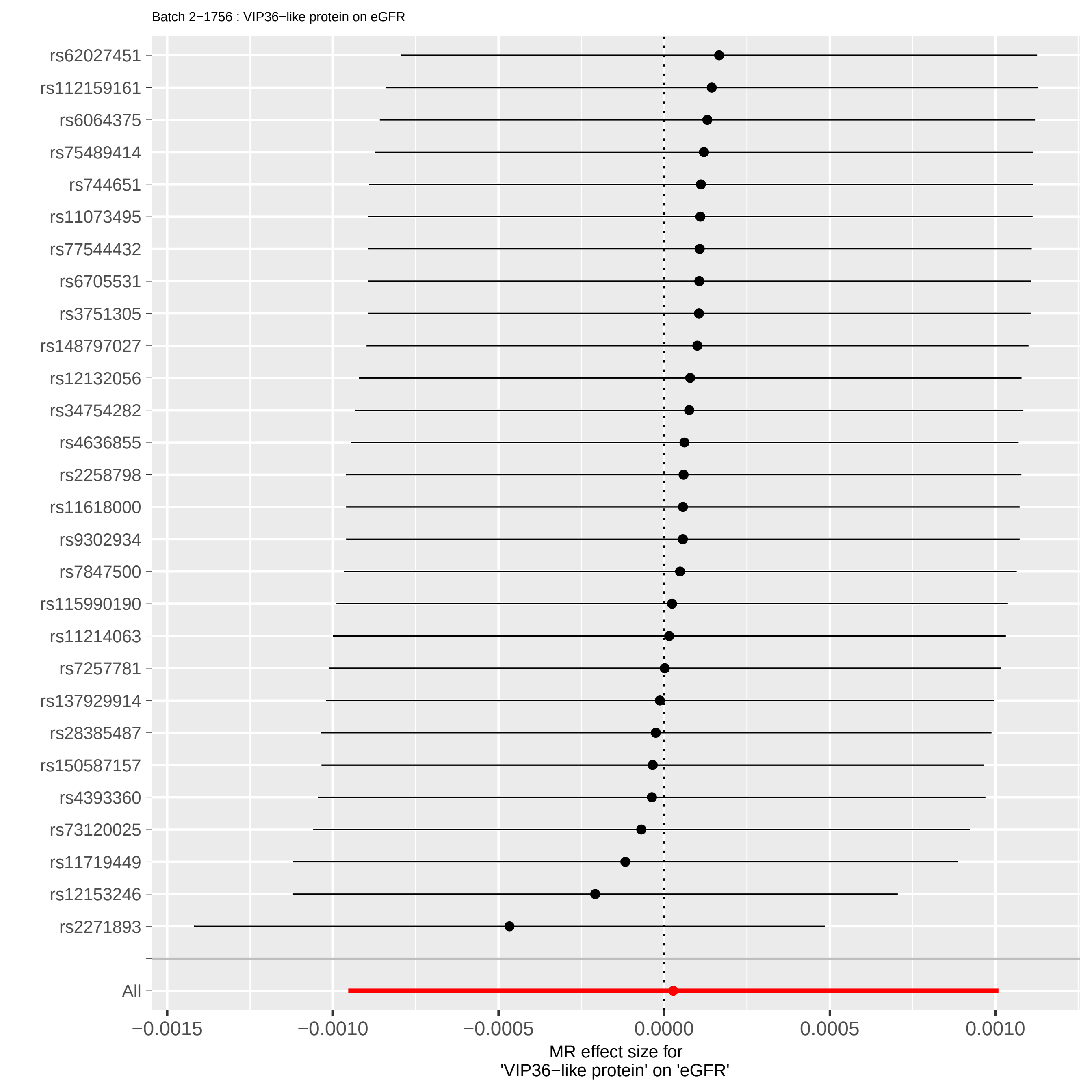

Batch 2−1756 : VIP36−like protein on eGFR
rs62027451
rs112159161
rs6064375
rs75489414
rs744651
rs11073495
rs77544432
rs6705531
rs3751305
rs148797027
rs12132056
rs34754282
rs4636855
rs2258798
rs11618000
rs9302934
rs7847500
rs115990190
rs11214063
rs7257781
rs137929914
rs28385487
rs150587157
rs4393360
rs73120025
rs11719449
rs12153246
rs2271893
All
−0.0015
−0.0010
−0.0005
0.0000
0.0005
0.0010
MR effect size for
'VIP36−like protein' on 'eGFR'

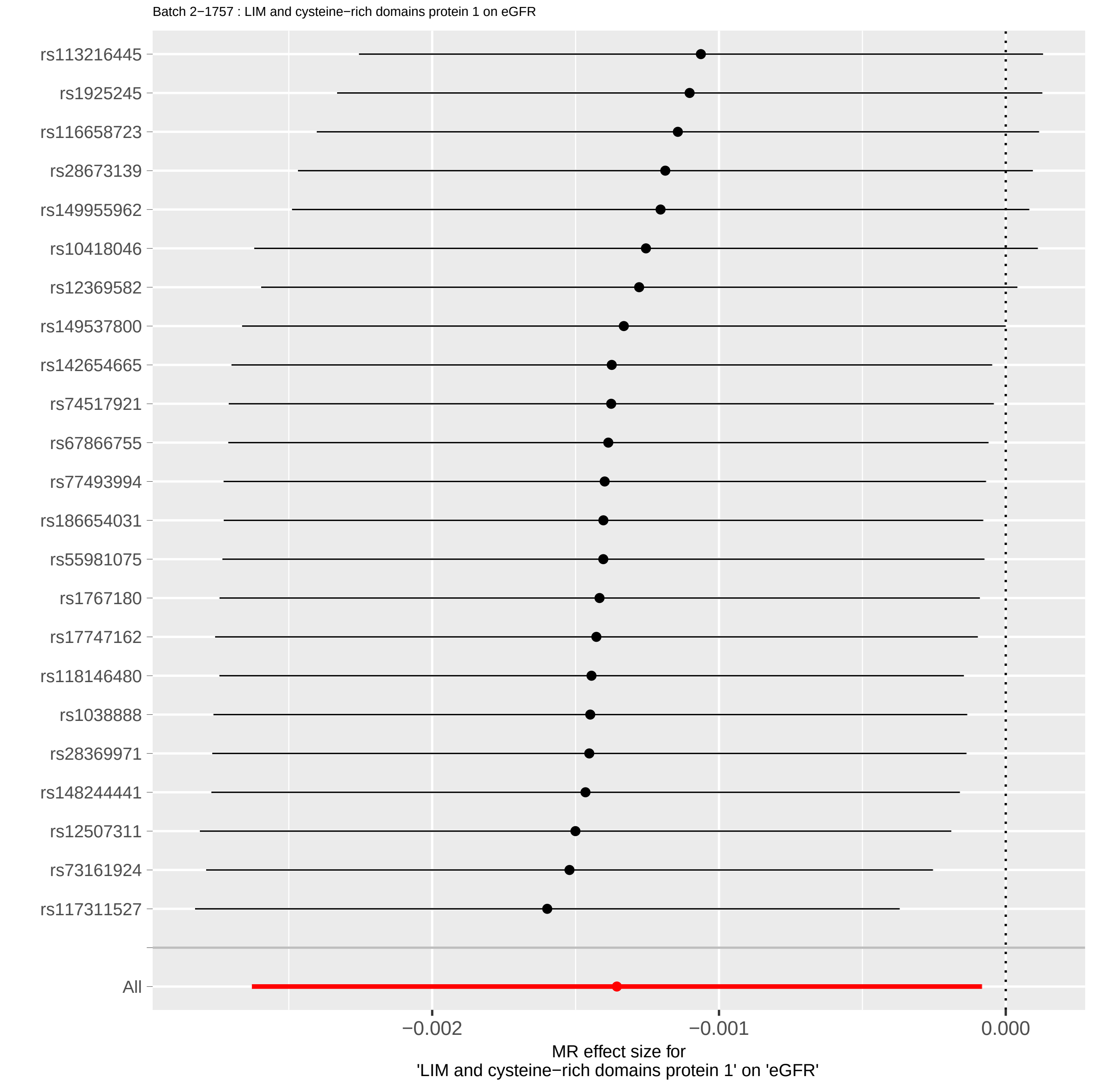

Batch 2−1757 : LIM and cysteine−rich domains protein 1 on eGFR
rs113216445
rs1925245
rs116658723
rs28673139
rs149955962
rs10418046
rs12369582
rs149537800
rs142654665
rs74517921
rs67866755
rs77493994
rs186654031
rs55981075
rs1767180
rs17747162
rs118146480
rs1038888
rs28369971
rs148244441
rs12507311
rs73161924
rs117311527
All
−0.002
−0.001
0.000
MR effect size for
'LIM and cysteine−rich domains protein 1' on 'eGFR'

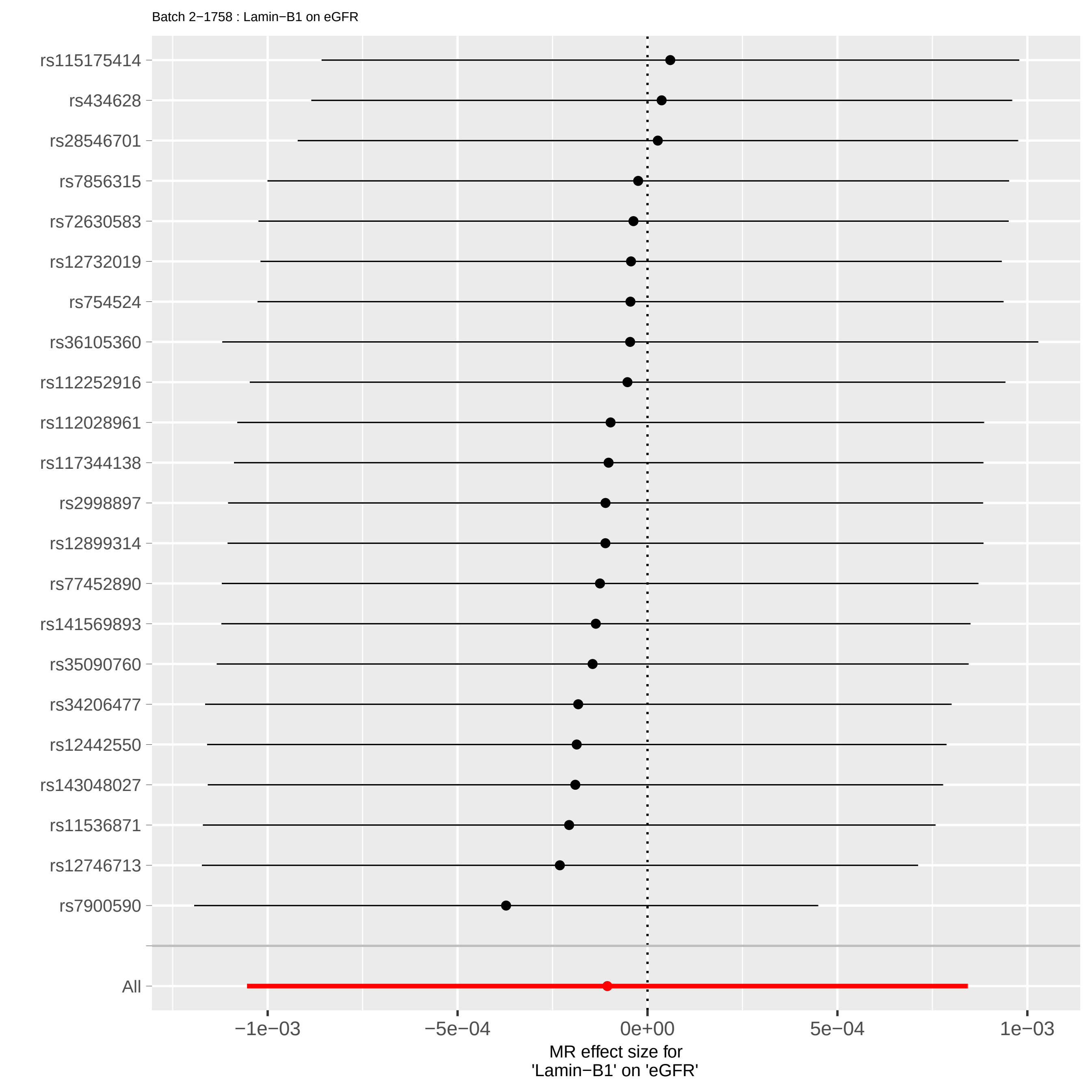

Batch 2−1758 : Lamin−B1 on eGFR
rs115175414
rs434628
rs28546701
rs7856315
rs72630583
rs12732019
rs754524
rs36105360
rs112252916
rs112028961
rs117344138
rs2998897
rs12899314
rs77452890
rs141569893
rs35090760
rs34206477
rs12442550
rs143048027
rs11536871
rs12746713
rs7900590
All
−1e−03
−5e−04
0e+00
5e−04
1e−03
MR effect size for
'Lamin−B1' on 'eGFR'

Batch 2−1759 : Leiomodin−1 on eGFR

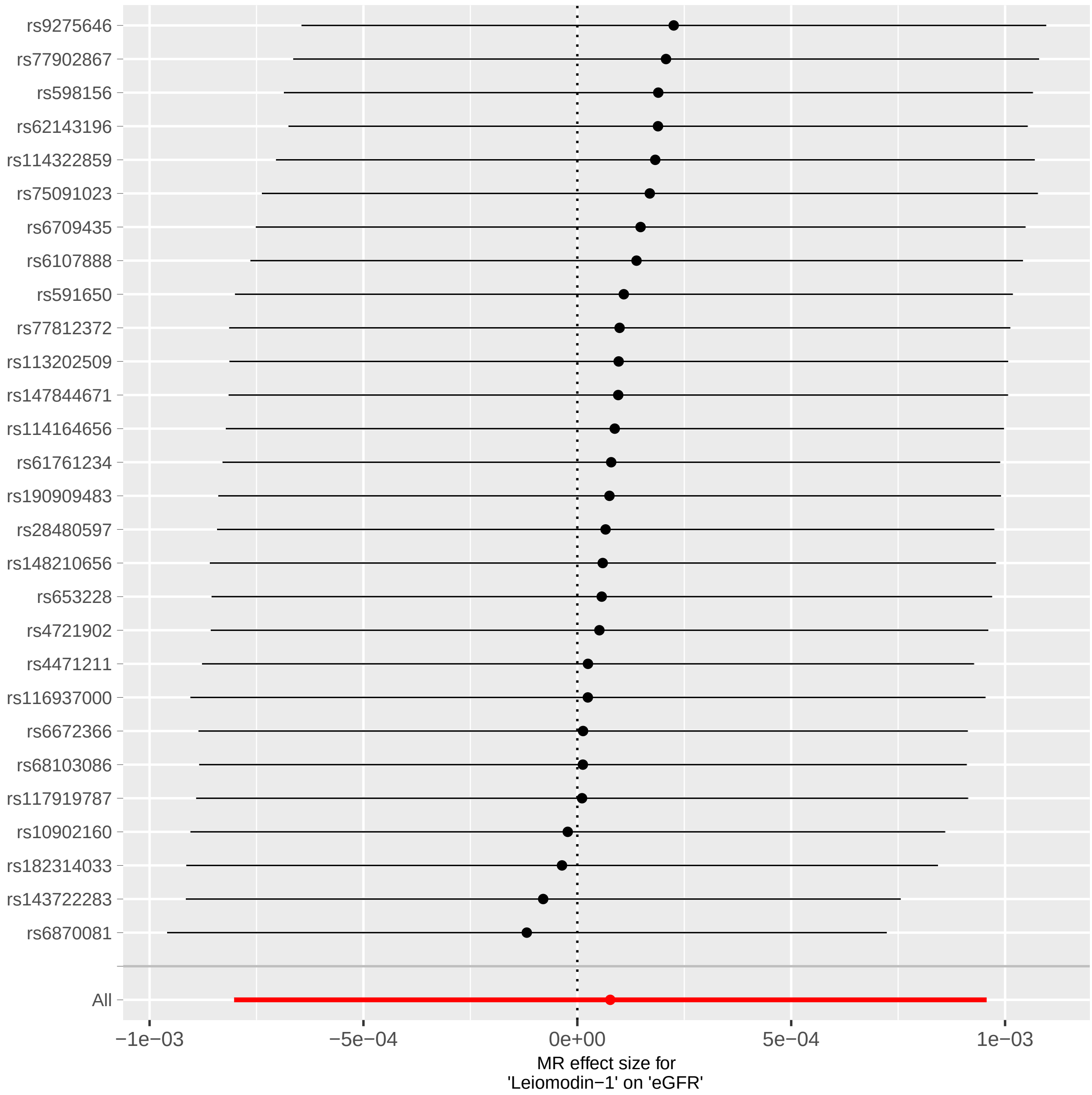

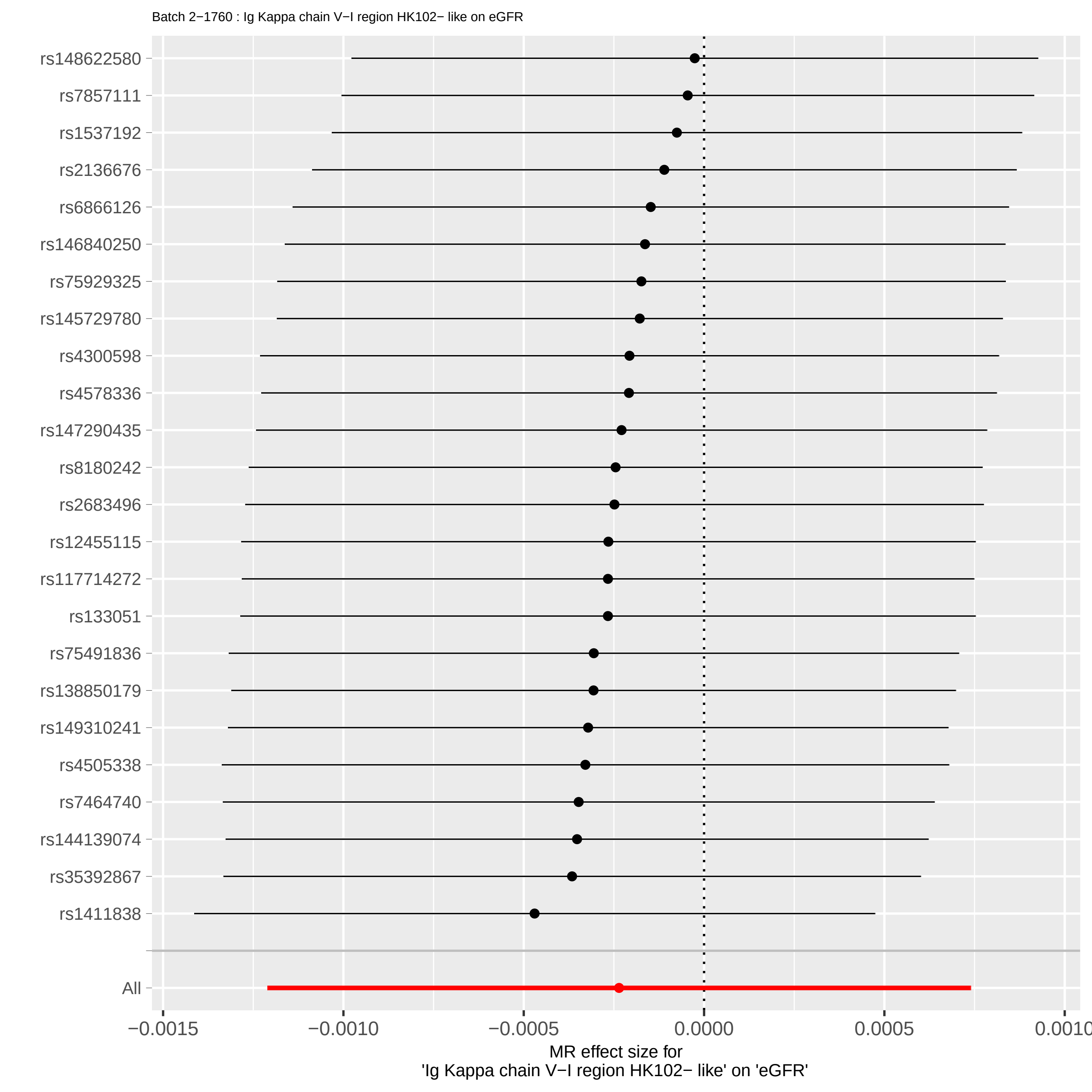

Batch 2−1760 : Ig Kappa chain V−I region HK102− like on eGFR
rs148622580
rs7857111
rs1537192
rs2136676
rs6866126
rs146840250
rs75929325
rs145729780
rs4300598
rs4578336
rs147290435
rs8180242
rs2683496
rs12455115
rs117714272
rs133051
rs75491836
rs138850179
rs149310241
rs4505338
rs7464740
rs144139074
rs35392867
rs1411838
All
−0.0015
−0.0010
−0.0005
0.0000
0.0005
0.0010
MR effect size for
'Ig Kappa chain V−I region HK102− like' on 'eGFR'

Batch 2−1761 : Lon protease homolog, mitochondrial on eGFR

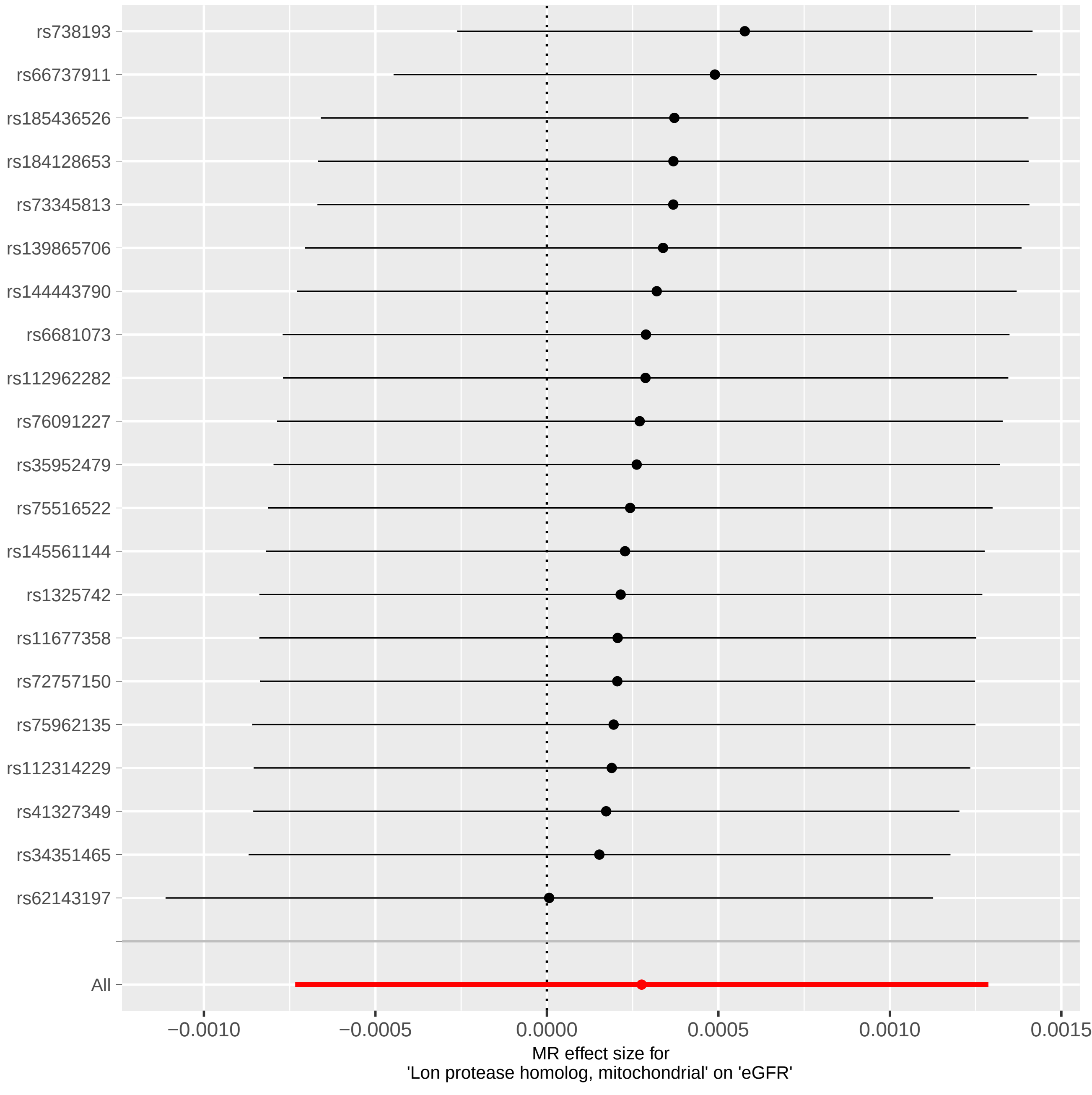

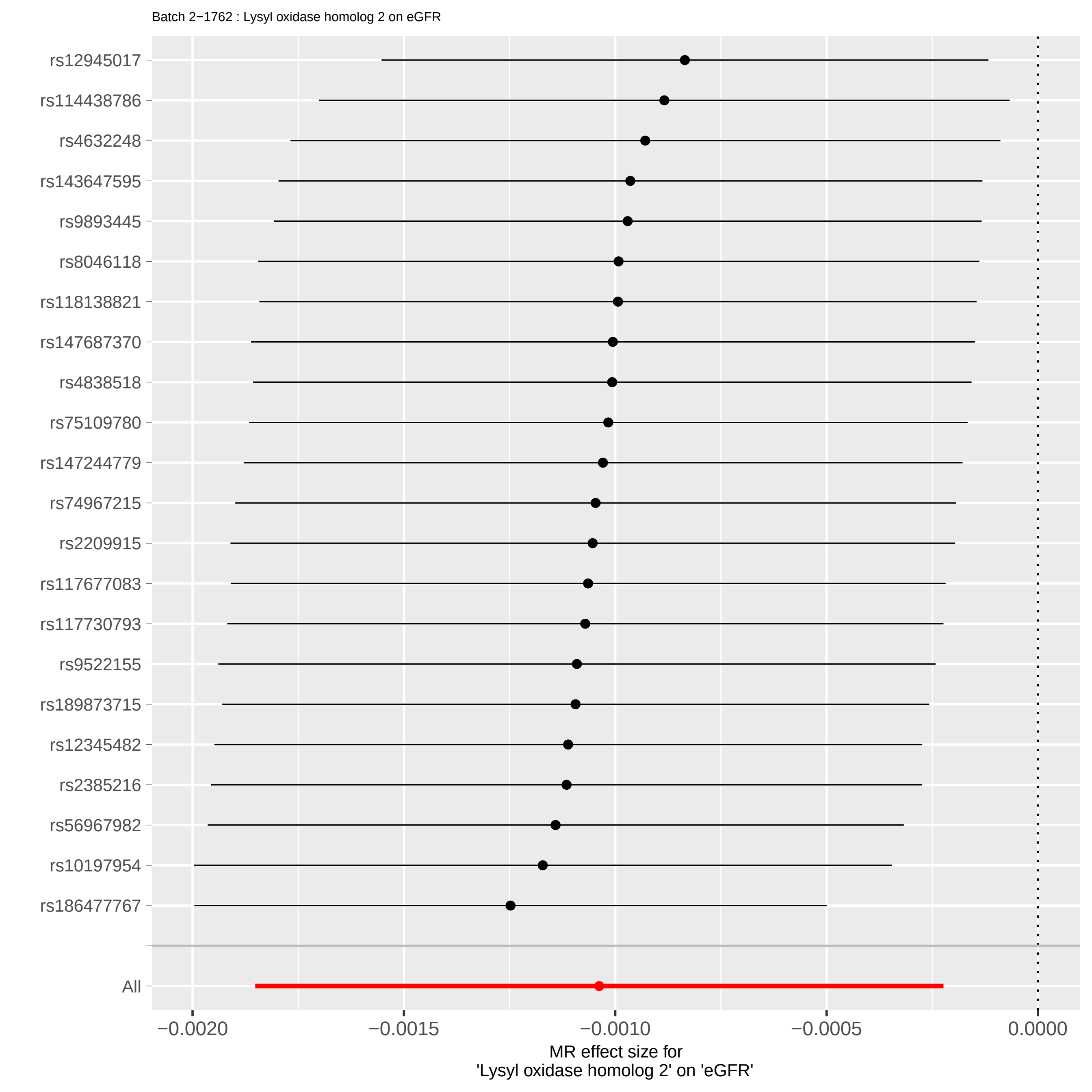

Batch 2−1762 : Lysyl oxidase homolog 2 on eGFR
rs12945017
rs114438786
rs4632248
rs143647595
rs9893445
rs8046118
rs118138821
rs147687370
rs4838518
rs75109780
rs147244779
rs74967215
rs2209915
rs117677083
rs117730793
rs9522155
rs189873715
rs12345482
rs2385216
rs56967982
rs10197954
rs186477767
All
−0.0020
−0.0015
−0.0010
−0.0005
0.0000
MR effect size for
'Lysyl oxidase homolog 2' on 'eGFR'

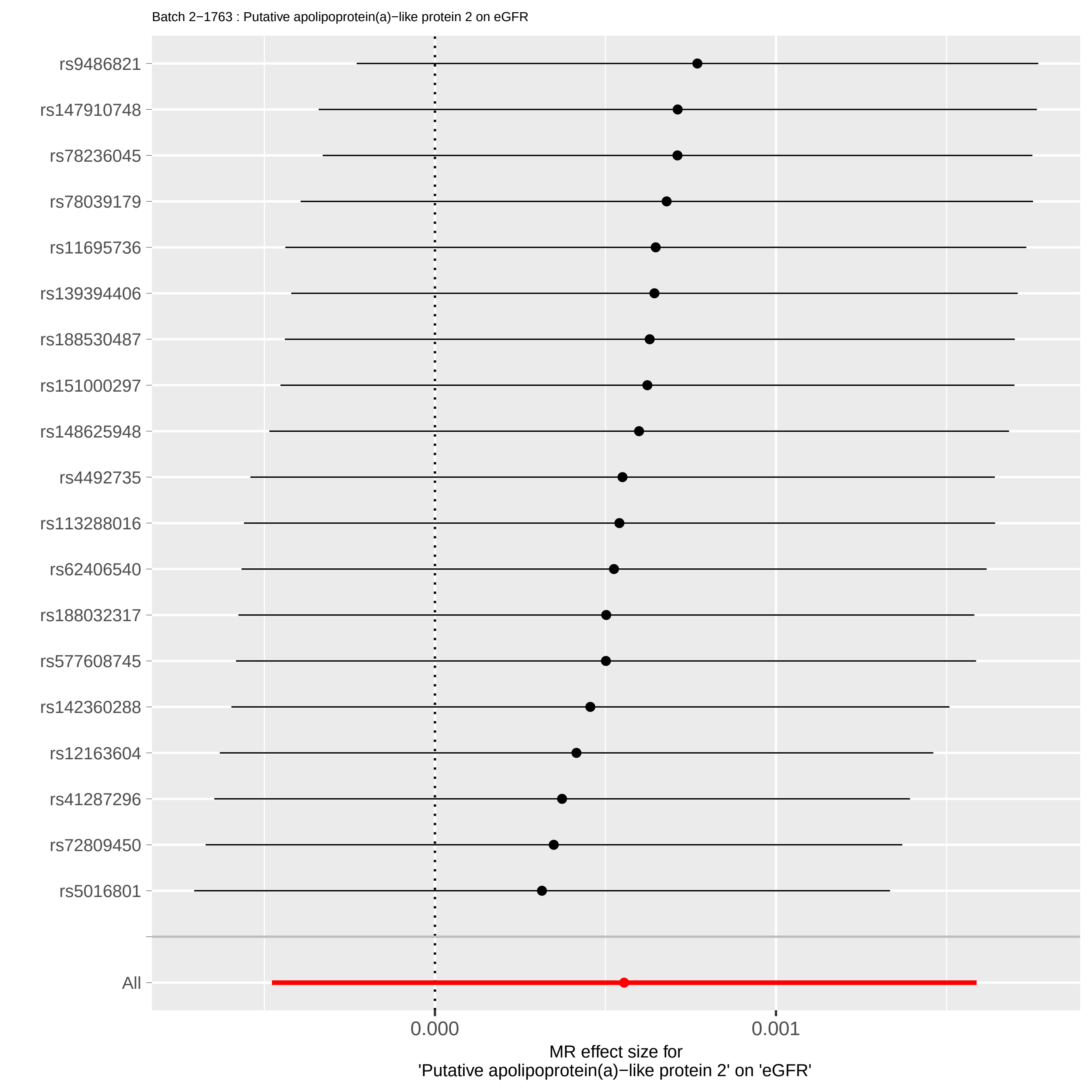
Batch 2−1763 : Putative apolipoprotein(a)−like protein 2 on eGFR
rs9486821
rs147910748
rs78236045
rs78039179
rs11695736
rs139394406
rs188530487
rs151000297
rs148625948
rs4492735
rs113288016
rs62406540
rs188032317
rs577608745
rs142360288
rs12163604
rs41287296
rs72809450
rs5016801
All
0.000
0.001
MR effect size for
'Putative apolipoprotein(a)−like protein 2' on 'eGFR'

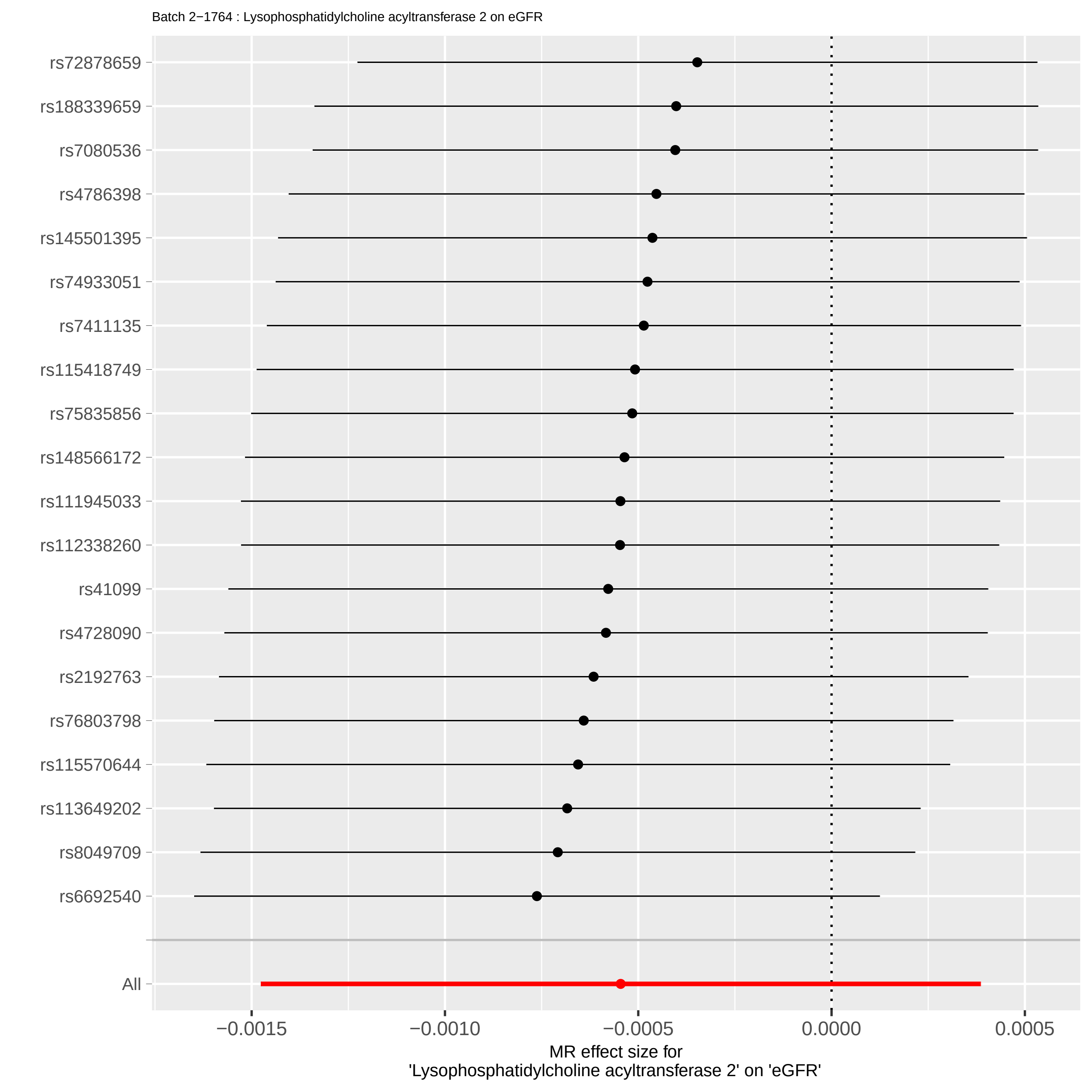

Batch 2−1764 : Lysophosphatidylcholine acyltransferase 2 on eGFR
rs72878659
rs188339659
rs7080536
rs4786398
rs145501395
rs74933051
rs7411135
rs115418749
rs75835856
rs148566172
rs111945033
rs112338260
rs41099
rs4728090
rs2192763
rs76803798
rs115570644
rs113649202
rs8049709
rs6692540
All
−0.0015
−0.0010
−0.0005
0.0000
0.0005
MR effect size for
'Lysophosphatidylcholine acyltransferase 2' on 'eGFR'

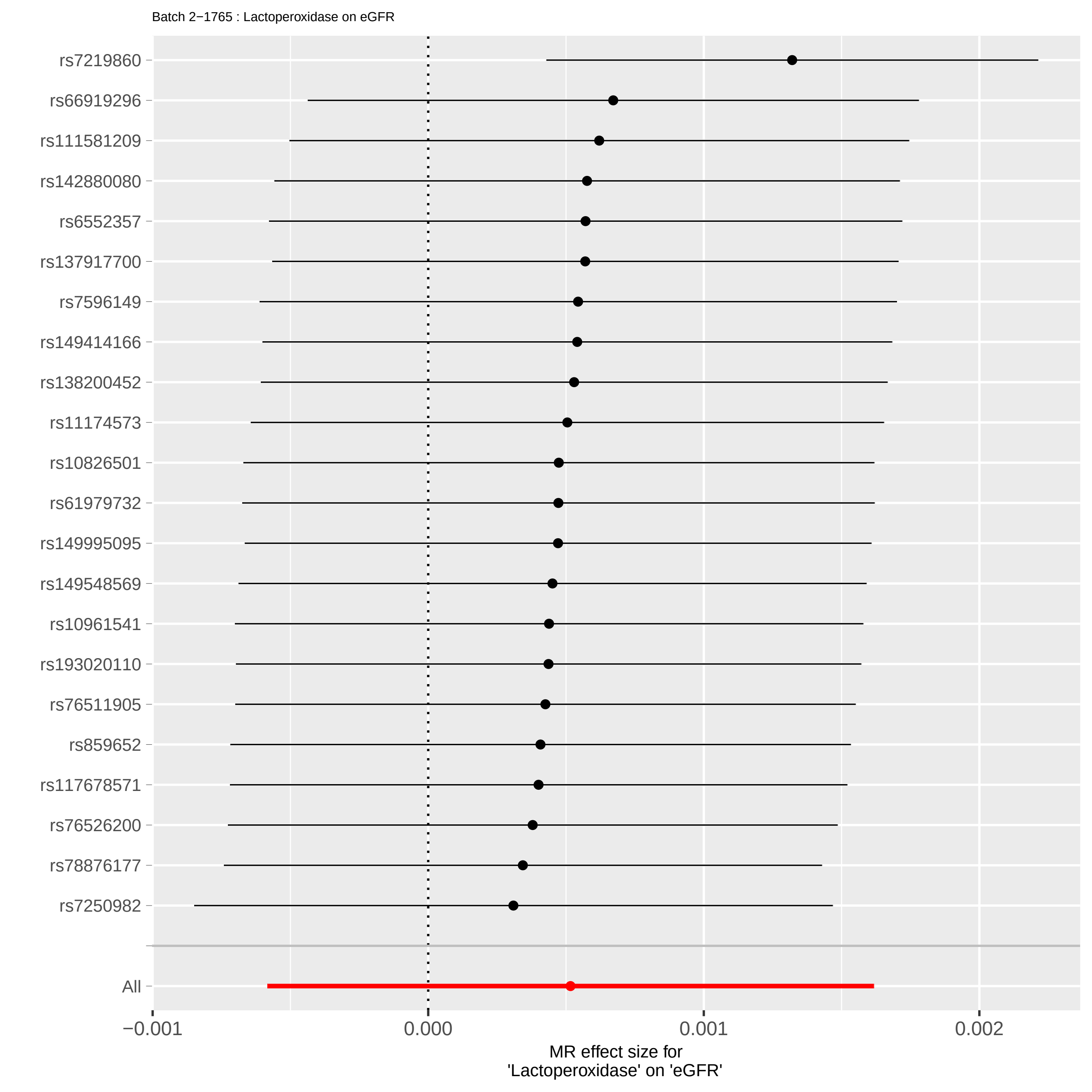

Batch 2−1765 : Lactoperoxidase on eGFR
rs7219860
rs66919296
rs111581209
rs142880080
rs6552357
rs137917700
rs7596149
rs149414166
rs138200452
rs11174573
rs10826501
rs61979732
rs149995095
rs149548569
rs10961541
rs193020110
rs76511905
rs859652
rs117678571
rs76526200
rs78876177
rs7250982
All
−0.001
0.000
0.001
0.002
MR effect size for
'Lactoperoxidase' on 'eGFR'

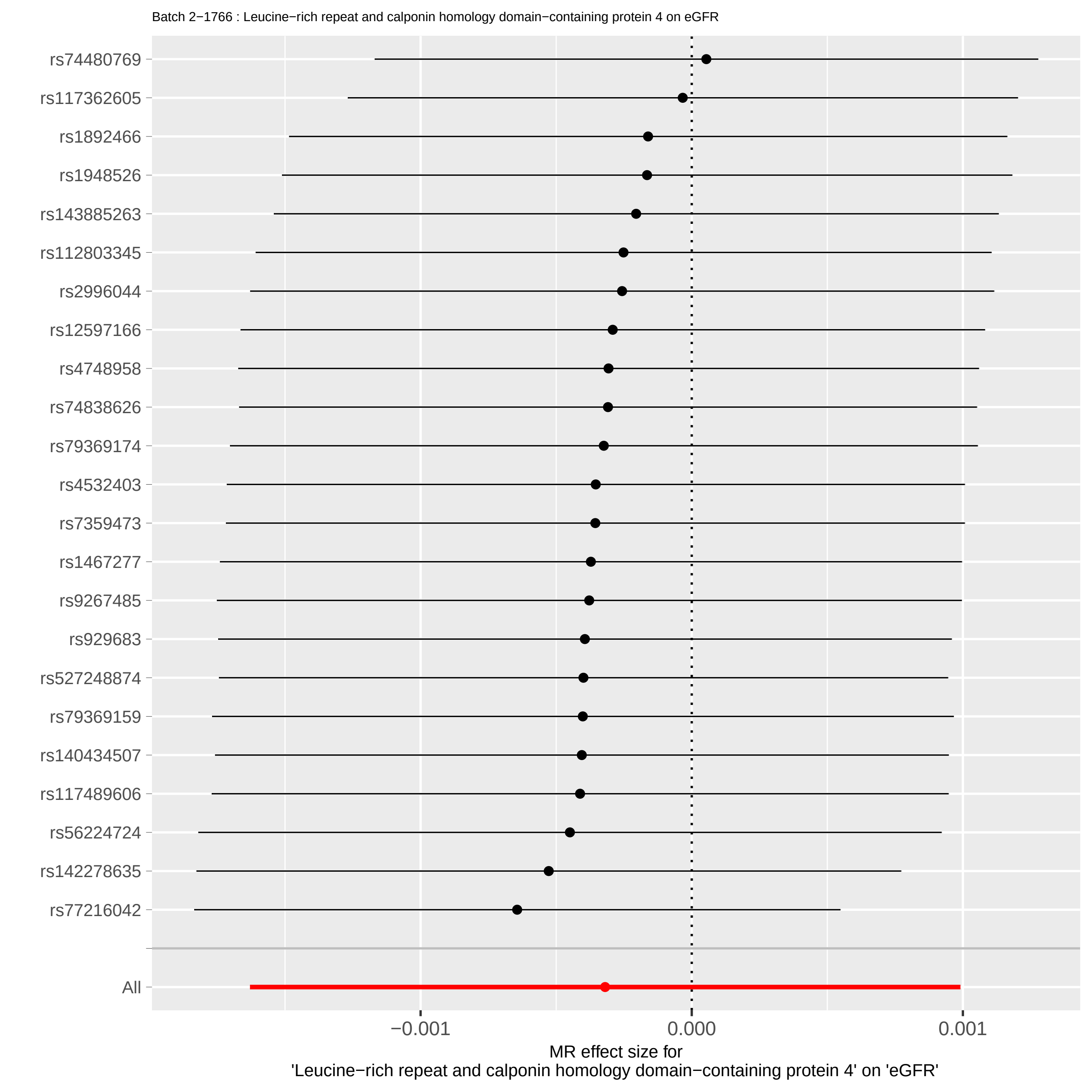

Batch 2−1766 : Leucine−rich repeat and calponin homology domain−containing protein 4 on eGFR
rs74480769
rs117362605
rs1892466
rs1948526
rs143885263
rs112803345
rs2996044
rs12597166
rs4748958
rs74838626
rs79369174
rs4532403
rs7359473
rs1467277
rs9267485
rs929683
rs527248874
rs79369159
rs140434507
rs117489606
rs56224724
rs142278635
rs77216042
All
−0.001
0.000
0.001
MR effect size for
'Leucine−rich repeat and calponin homology domain−containing protein 4' on 'eGFR'

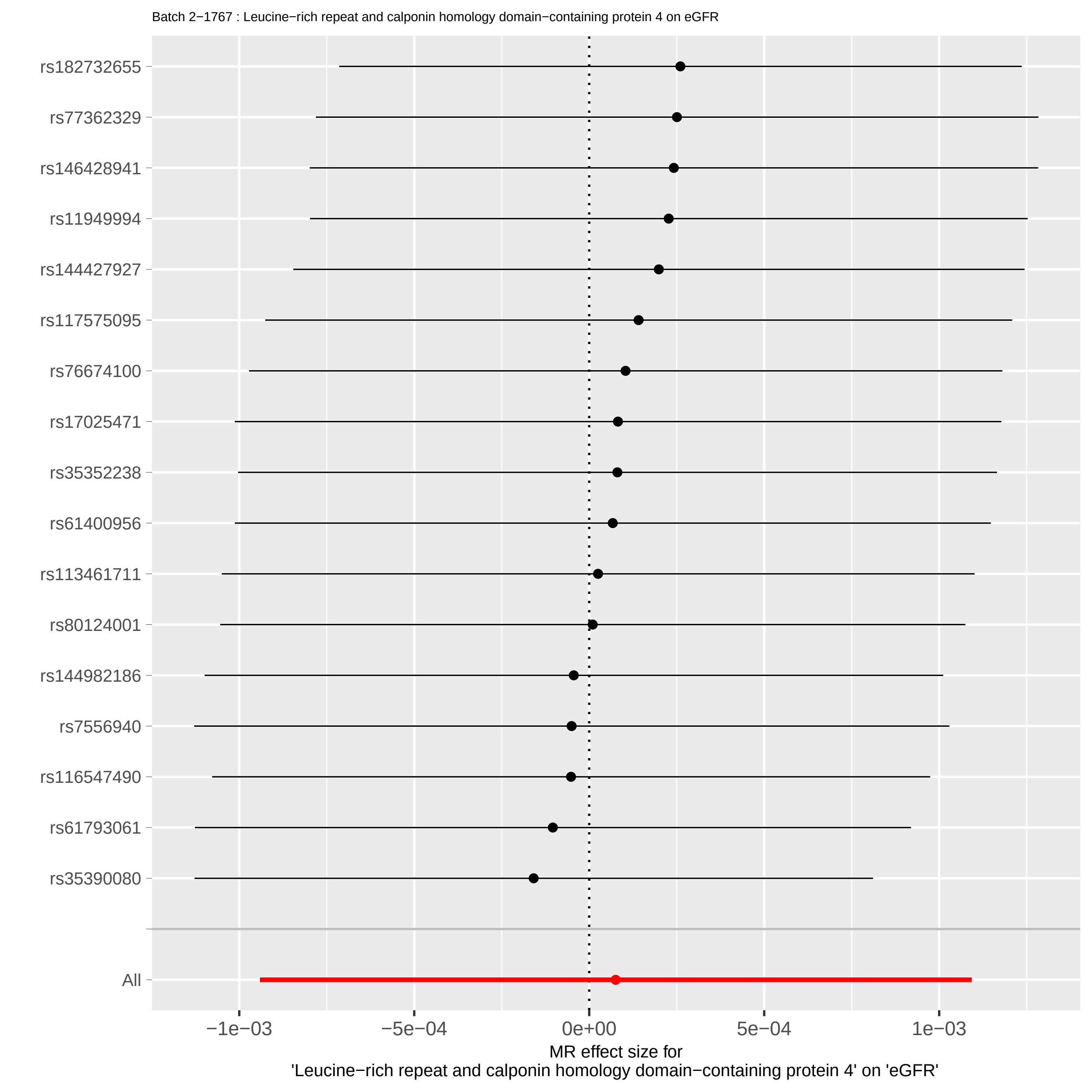

Batch 2−1767 : Leucine−rich repeat and calponin homology domain−containing protein 4 on eGFR
rs182732655
rs77362329
rs146428941
rs11949994
rs144427927
rs117575095
rs76674100
rs17025471
rs35352238
rs61400956
rs113461711
rs80124001
rs144982186
rs7556940
rs116547490
rs61793061
rs35390080
All
−1e−03
−5e−04
0e+00
5e−04
1e−03
MR effect size for
'Leucine−rich repeat and calponin homology domain−containing protein 4' on 'eGFR'

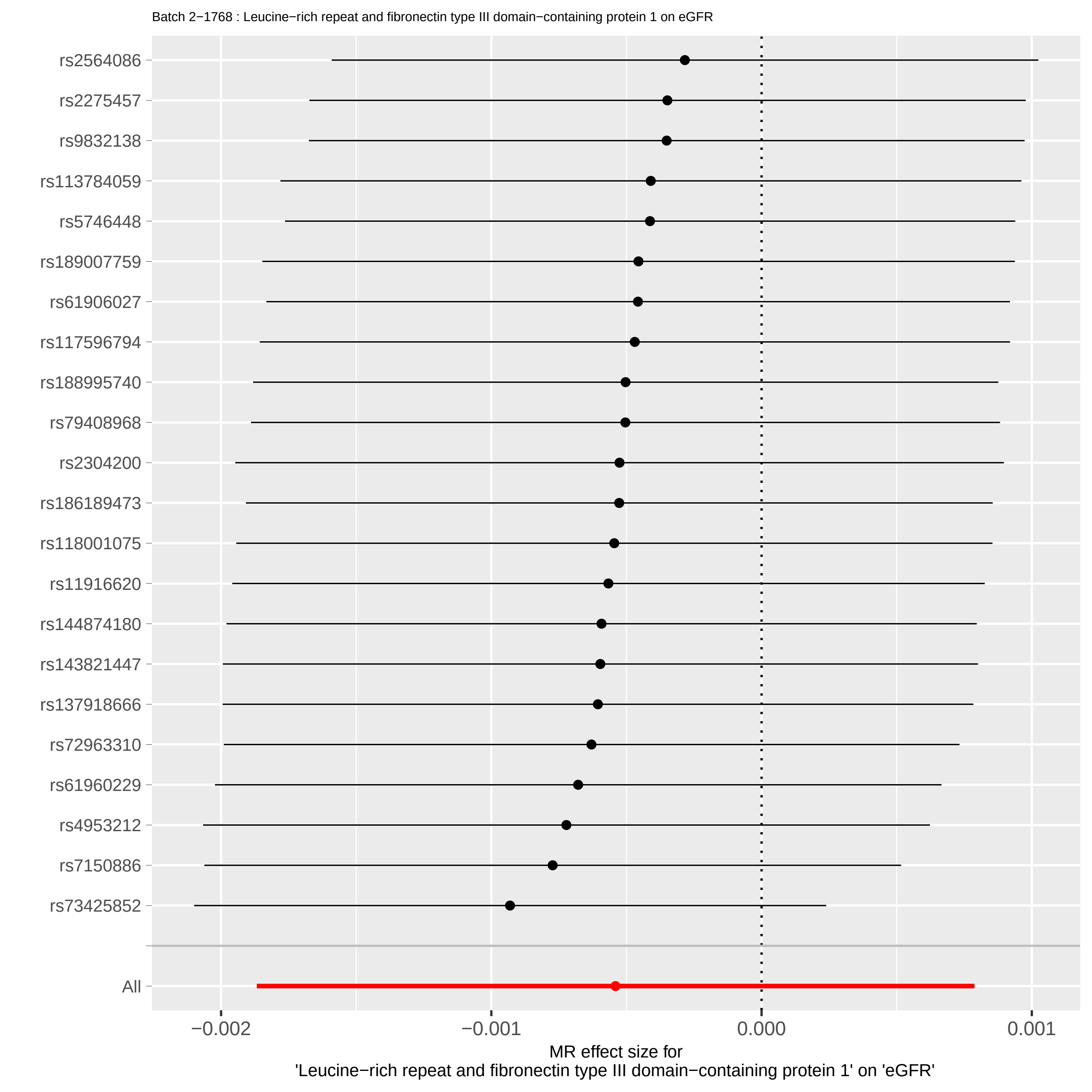

Batch 2−1768 : Leucine−rich repeat and fibronectin type III domain−containing protein 1 on eGFR
rs2564086
rs2275457
rs9832138
rs113784059
rs5746448
rs189007759
rs61906027
rs117596794
rs188995740
rs79408968
rs2304200
rs186189473
rs118001075
rs11916620
rs144874180
rs143821447
rs137918666
rs72963310
rs61960229
rs4953212
rs7150886
rs73425852
All
−0.002
−0.001
0.000
0.001
MR effect size for
'Leucine−rich repeat and fibronectin type III domain−containing protein 1' on 'eGFR'

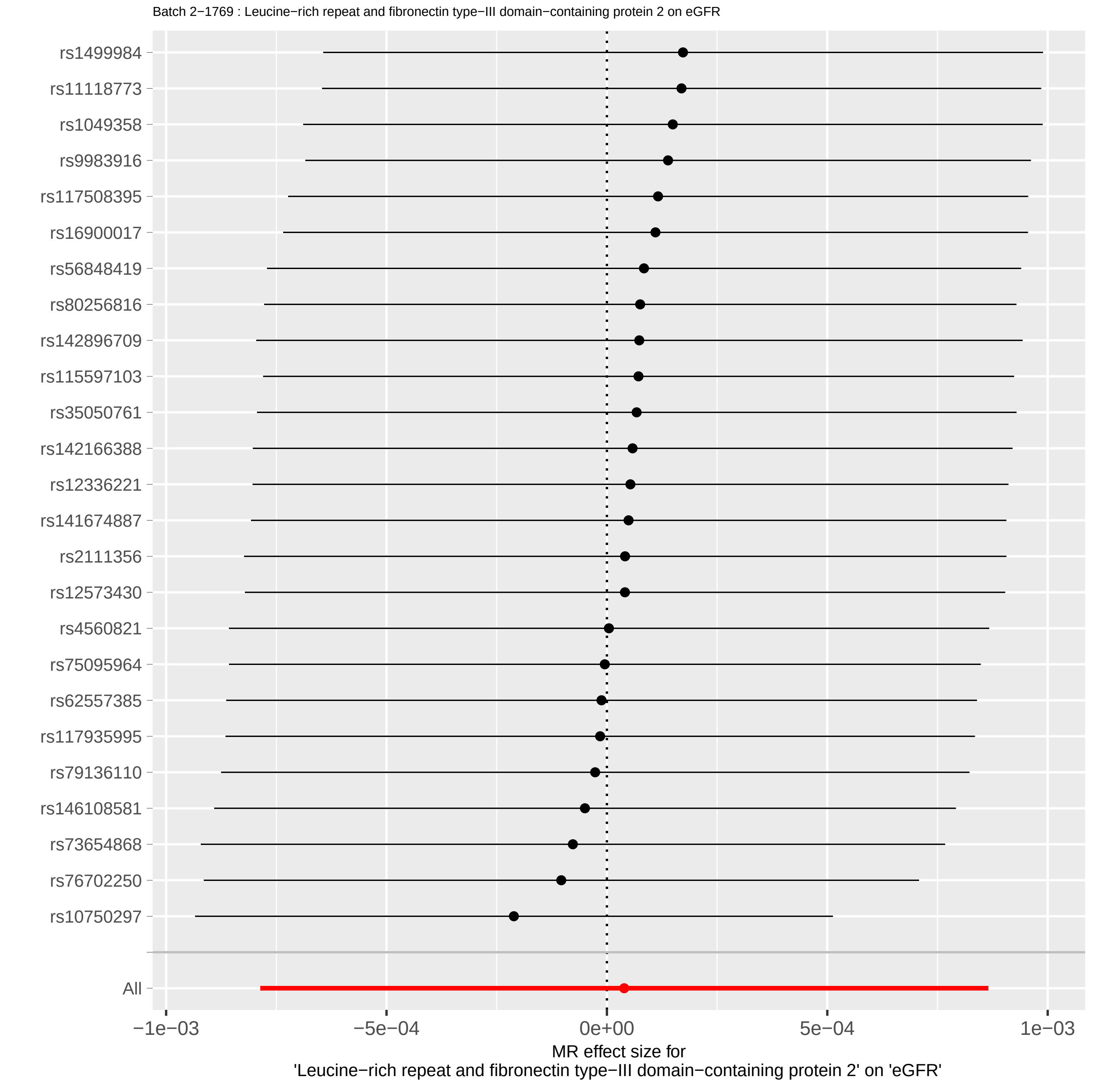

Batch 2−1769 : Leucine−rich repeat and fibronectin type−III domain−containing protein 2 on eGFR
rs1499984
rs11118773
rs1049358
rs9983916
rs117508395
rs16900017
rs56848419
rs80256816
rs142896709
rs115597103
rs35050761
rs142166388
rs12336221
rs141674887
rs2111356
rs12573430
rs4560821
rs75095964
rs62557385
rs117935995
rs79136110
rs146108581
rs73654868
rs76702250
rs10750297
All
−1e−03
−5e−04
0e+00
5e−04
1e−03
MR effect size for
'Leucine−rich repeat and fibronectin type−III domain−containing protein 2' on 'eGFR'

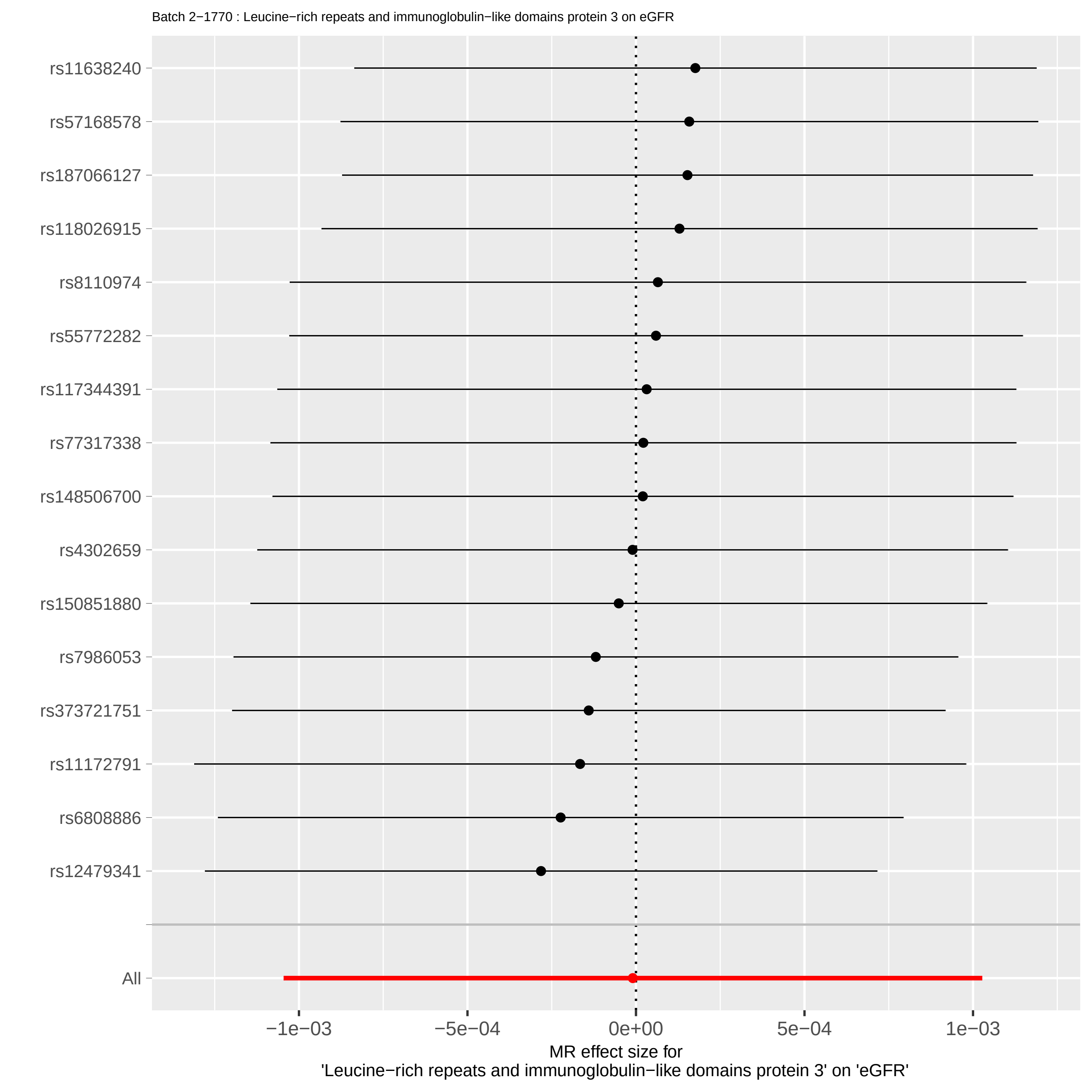

Batch 2−1770 : Leucine−rich repeats and immunoglobulin−like domains protein 3 on eGFR
rs11638240
rs57168578
rs187066127
rs118026915
rs8110974
rs55772282
rs117344391
rs77317338
rs148506700
rs4302659
rs150851880
rs7986053
rs373721751
rs11172791
rs6808886
rs12479341
All
−1e−03
−5e−04
0e+00
5e−04
1e−03
MR effect size for
'Leucine−rich repeats and immunoglobulin−like domains protein 3' on 'eGFR'

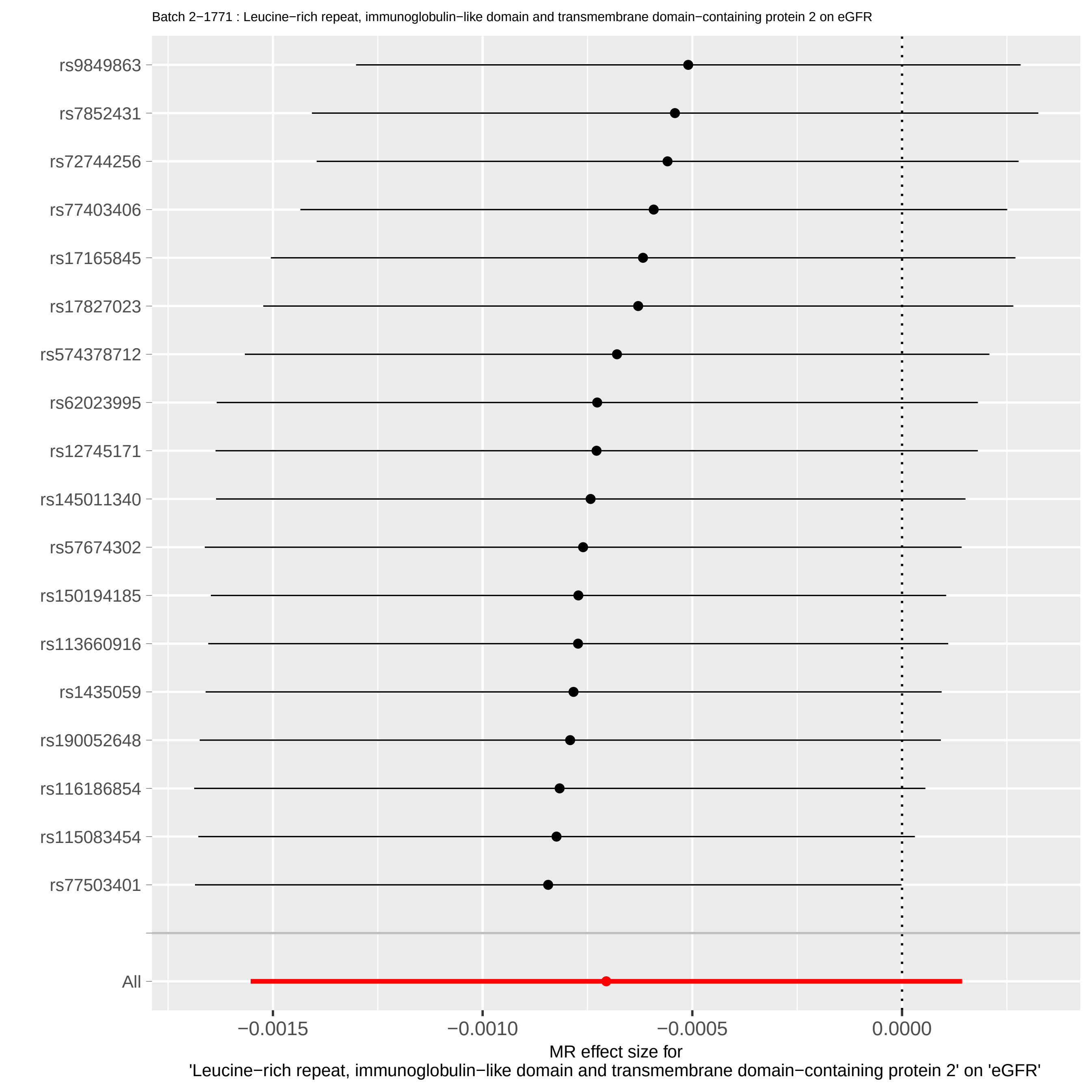
Batch 2−1771 : Leucine−rich repeat, immunoglobulin−like domain and transmembrane domain−containing protein 2 on eGFR
rs9849863
rs7852431
rs72744256
rs77403406
rs17165845
rs17827023
rs574378712
rs62023995
rs12745171
rs145011340
rs57674302
rs150194185
rs113660916
rs1435059
rs190052648
rs116186854
rs115083454
rs77503401
All
−0.0015
−0.0010
−0.0005
0.0000
MR effect size for
'Leucine−rich repeat, immunoglobulin−like domain and transmembrane domain−containing protein 2' on 'eGFR'

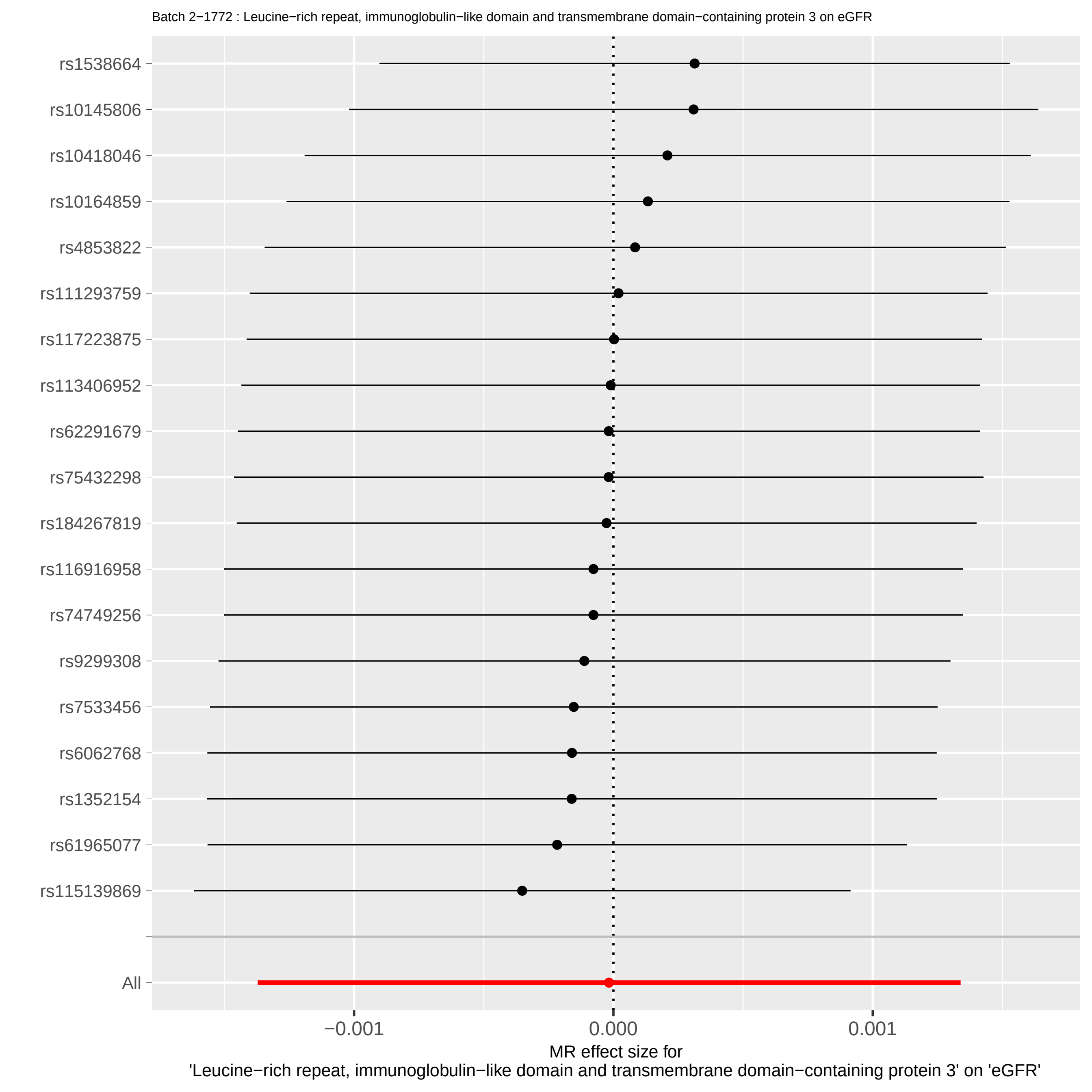
Batch 2−1772 : Leucine−rich repeat, immunoglobulin−like domain and transmembrane domain−containing protein 3 on eGFR
rs1538664
rs10145806
rs10418046
rs10164859
rs4853822
rs111293759
rs117223875
rs113406952
rs62291679
rs75432298
rs184267819
rs116916958
rs74749256
rs9299308
rs7533456
rs6062768
rs1352154
rs61965077
rs115139869
All
−0.001
0.000
0.001
MR effect size for
'Leucine−rich repeat, immunoglobulin−like domain and transmembrane domain−containing protein 3' on 'eGFR'

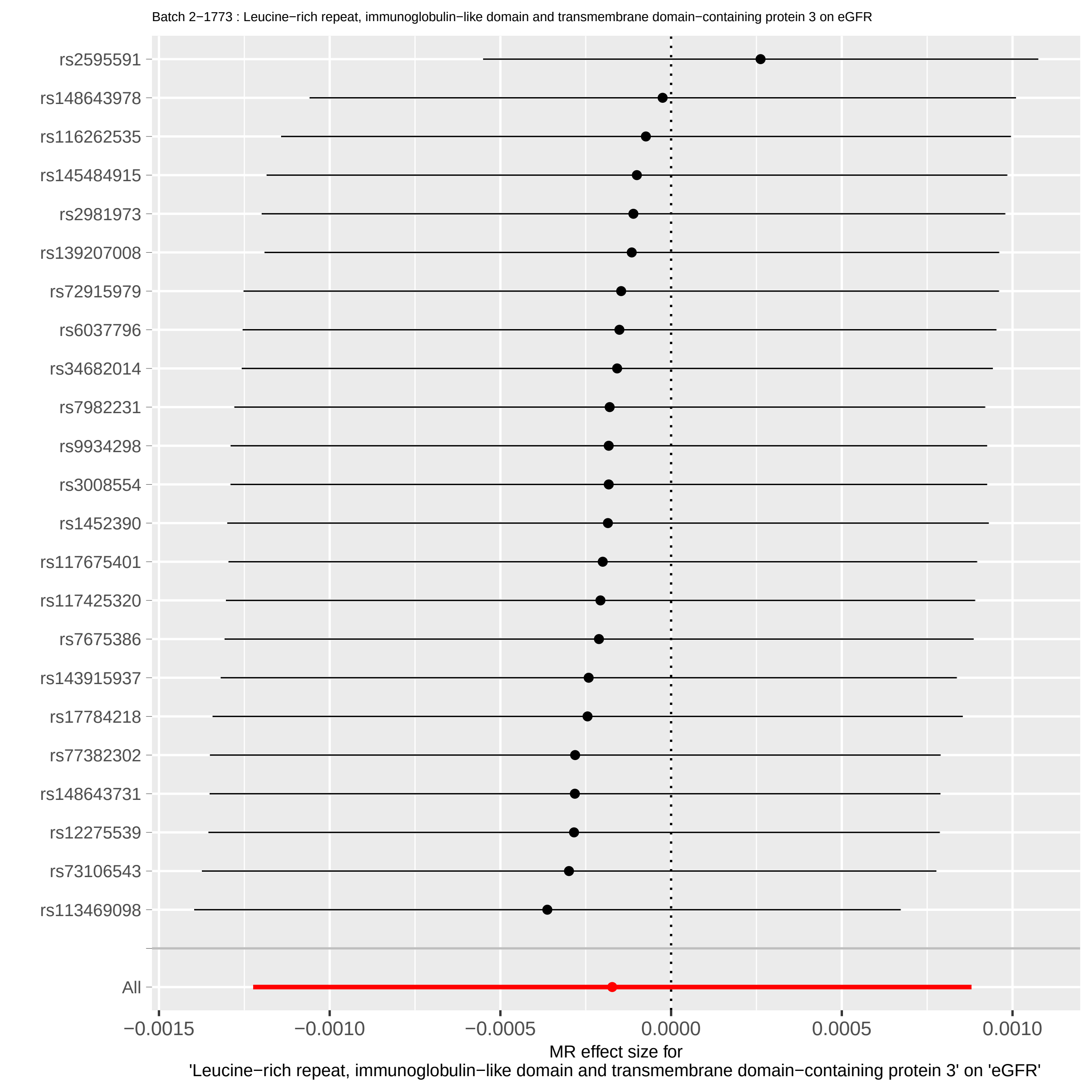

Batch 2−1773 : Leucine−rich repeat, immunoglobulin−like domain and transmembrane domain−containing protein 3 on eGFR
rs2595591
rs148643978
rs116262535
rs145484915
rs2981973
rs139207008
rs72915979
rs6037796
rs34682014
rs7982231
rs9934298
rs3008554
rs1452390
rs117675401
rs117425320
rs7675386
rs143915937
rs17784218
rs77382302
rs148643731
rs12275539
rs73106543
rs113469098
All
−0.0015
−0.0010
−0.0005
0.0000
0.0005
0.0010
MR effect size for
'Leucine−rich repeat, immunoglobulin−like domain and transmembrane domain−containing protein 3' on 'eGFR'

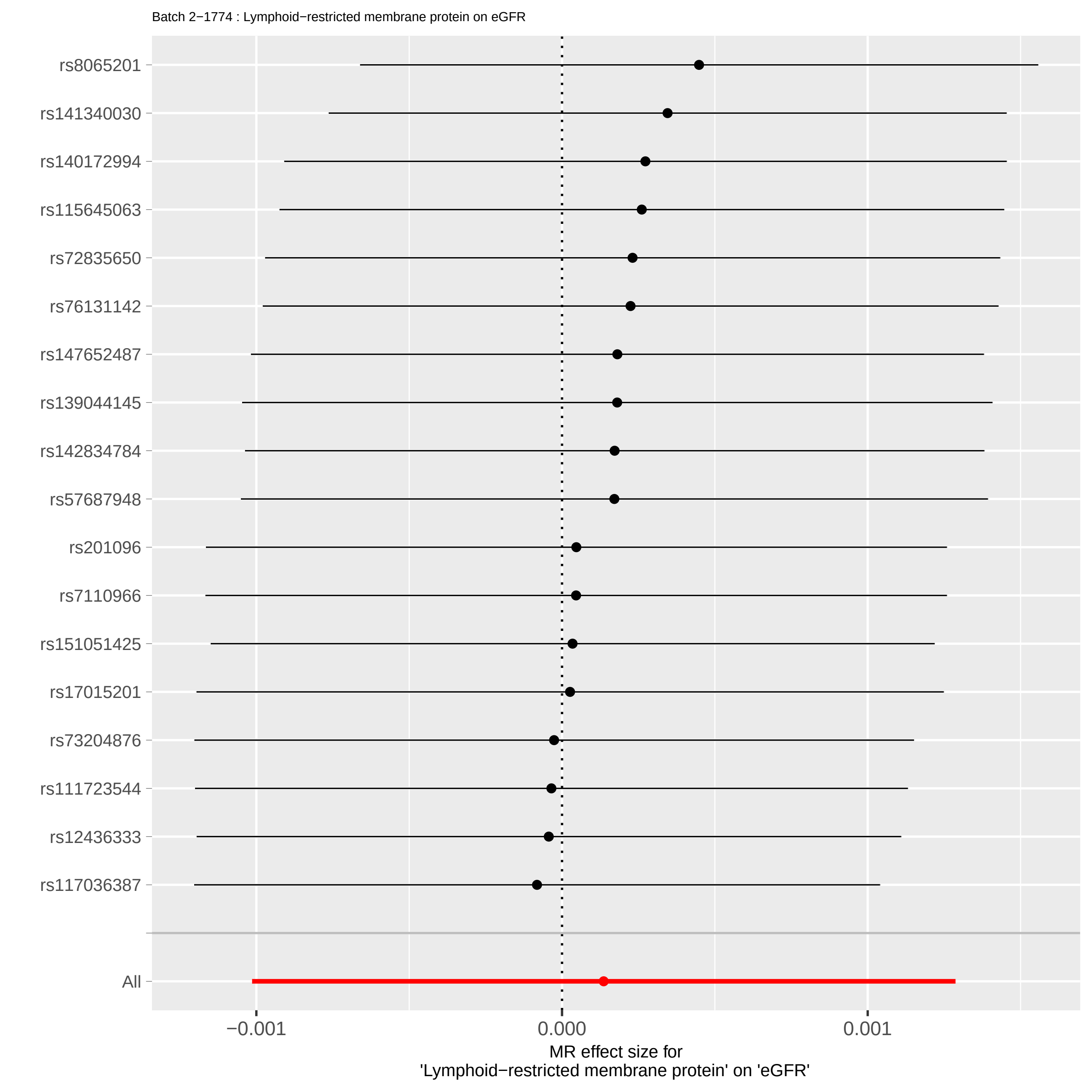
Batch 2−1774 : Lymphoid−restricted membrane protein on eGFR
rs8065201
rs141340030
rs140172994
rs115645063
rs72835650
rs76131142
rs147652487
rs139044145
rs142834784
rs57687948
rs201096
rs7110966
rs151051425
rs17015201
rs73204876
rs111723544
rs12436333
rs117036387
All
−0.001
0.000
0.001
MR effect size for
'Lymphoid−restricted membrane protein' on 'eGFR'

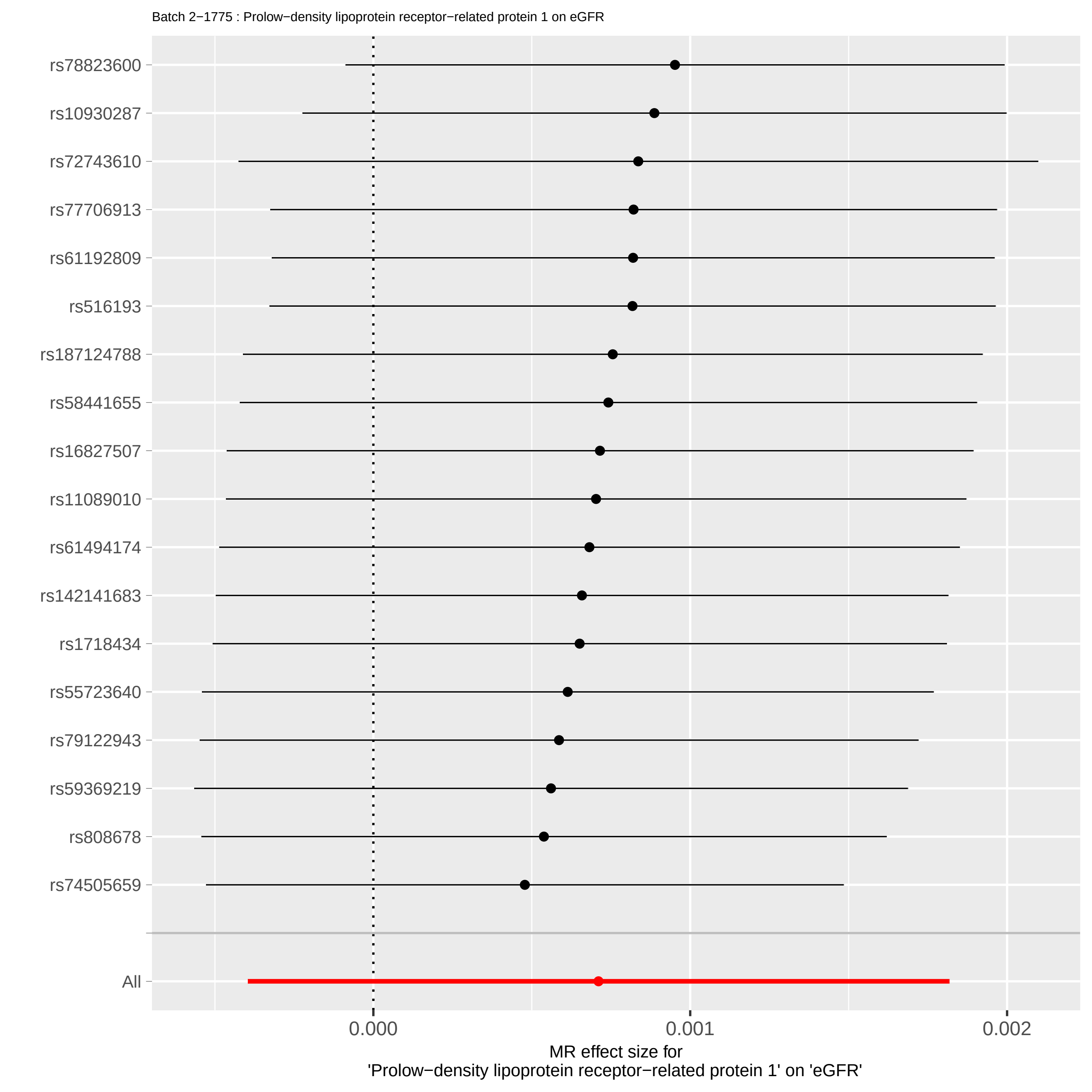

Batch 2−1775 : Prolow−density lipoprotein receptor−related protein 1 on eGFR
rs78823600
rs10930287
rs72743610
rs77706913
rs61192809
rs516193
rs187124788
rs58441655
rs16827507
rs11089010
rs61494174
rs142141683
rs1718434
rs55723640
rs79122943
rs59369219
rs808678
rs74505659
All
0.000
0.001
0.002
MR effect size for
'Prolow−density lipoprotein receptor−related protein 1' on 'eGFR'

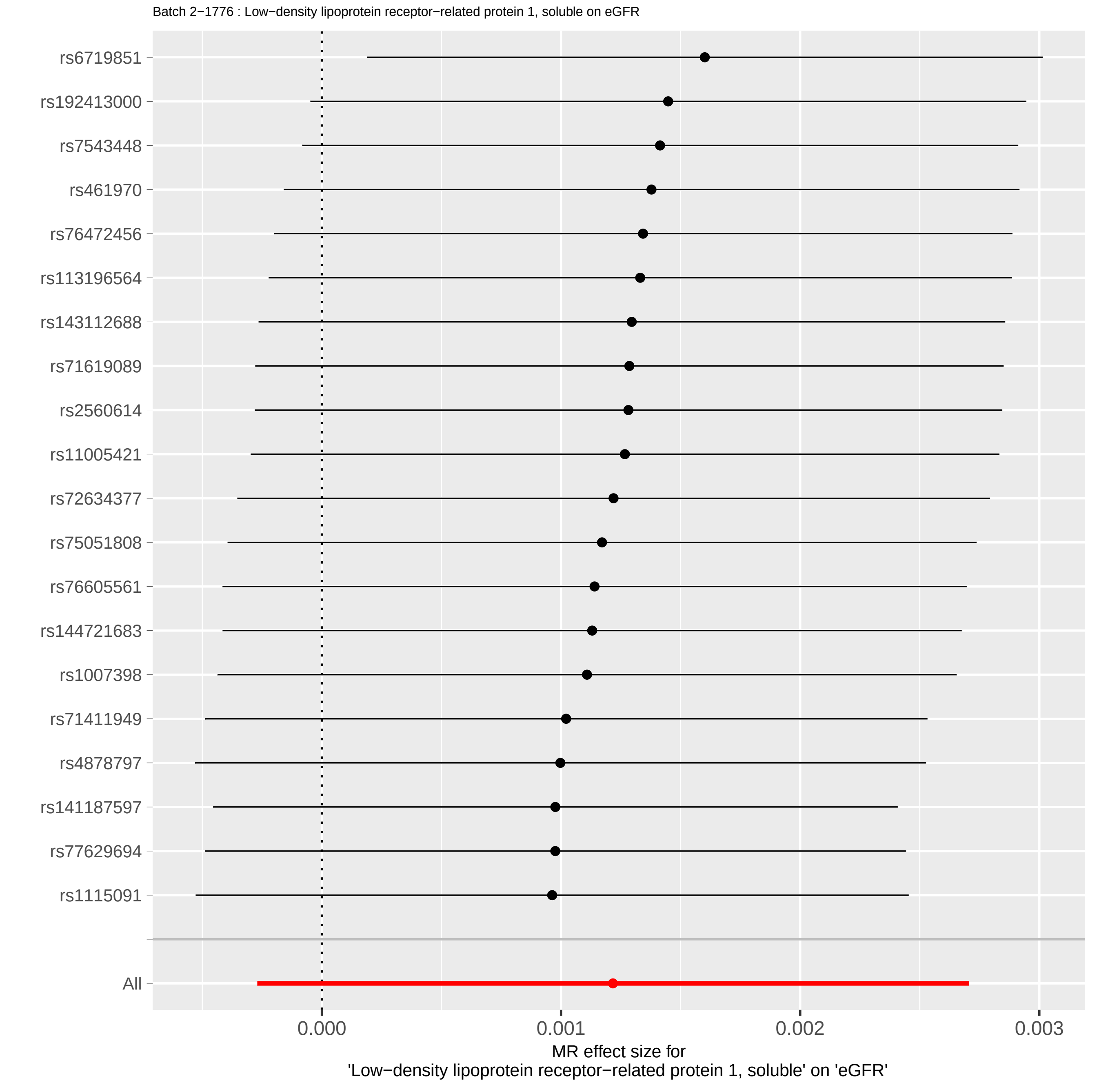
Batch 2−1776 : Low−density lipoprotein receptor−related protein 1, soluble on eGFR
rs6719851
rs192413000
rs7543448
rs461970
rs76472456
rs113196564
rs143112688
rs71619089
rs2560614
rs11005421
rs72634377
rs75051808
rs76605561
rs144721683
rs1007398
rs71411949
rs4878797
rs141187597
rs77629694
rs1115091
All
0.000
0.001
0.002
0.003
MR effect size for
'Low−density lipoprotein receptor−related protein 1, soluble' on 'eGFR'

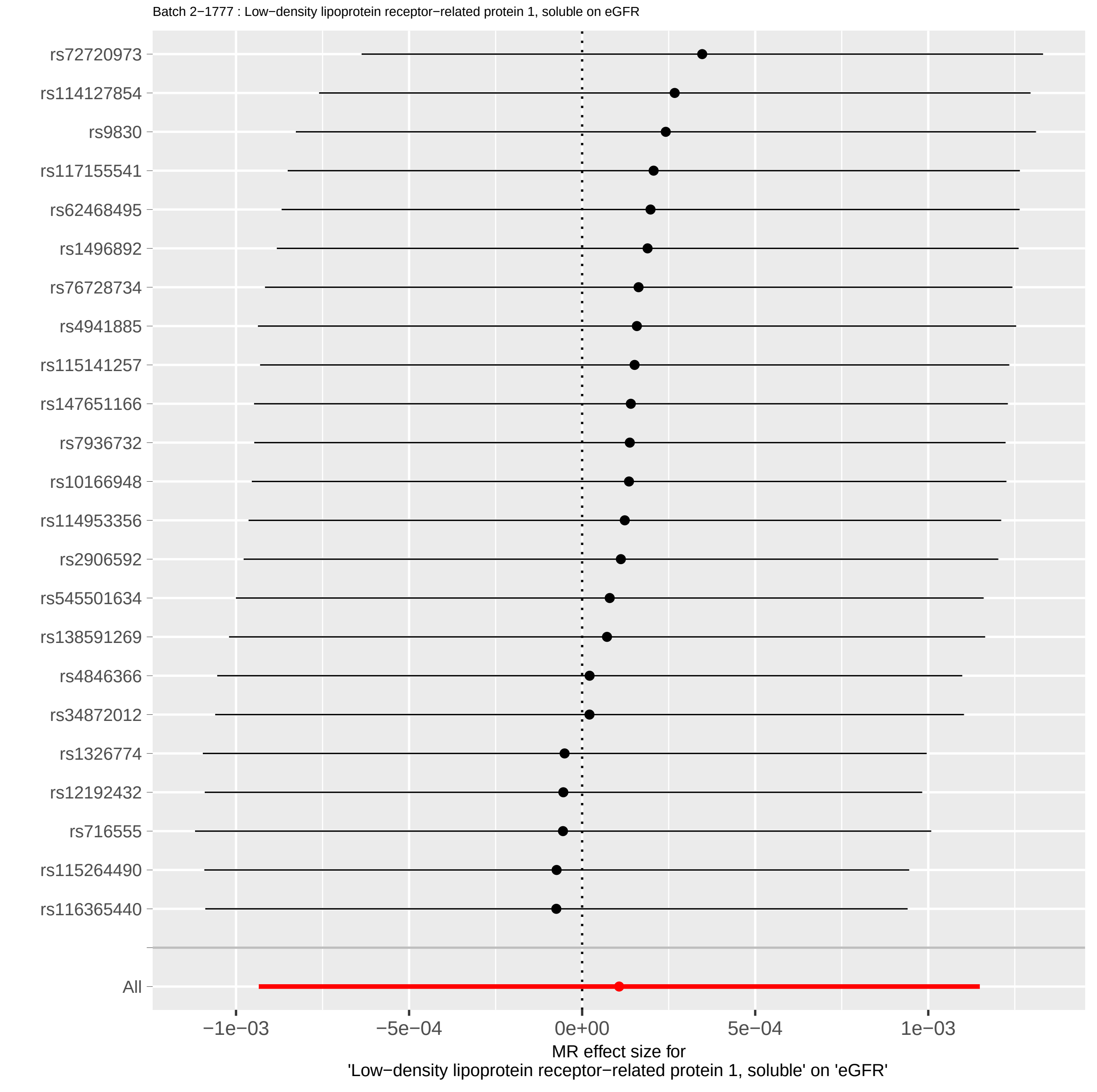

Batch 2−1777 : Low−density lipoprotein receptor−related protein 1, soluble on eGFR
rs72720973
rs114127854
rs9830
rs117155541
rs62468495
rs1496892
rs76728734
rs4941885
rs115141257
rs147651166
rs7936732
rs10166948
rs114953356
rs2906592
rs545501634
rs138591269
rs4846366
rs34872012
rs1326774
rs12192432
rs716555
rs115264490
rs116365440
All
−1e−03
−5e−04
0e+00
5e−04
1e−03
MR effect size for
'Low−density lipoprotein receptor−related protein 1, soluble' on 'eGFR'

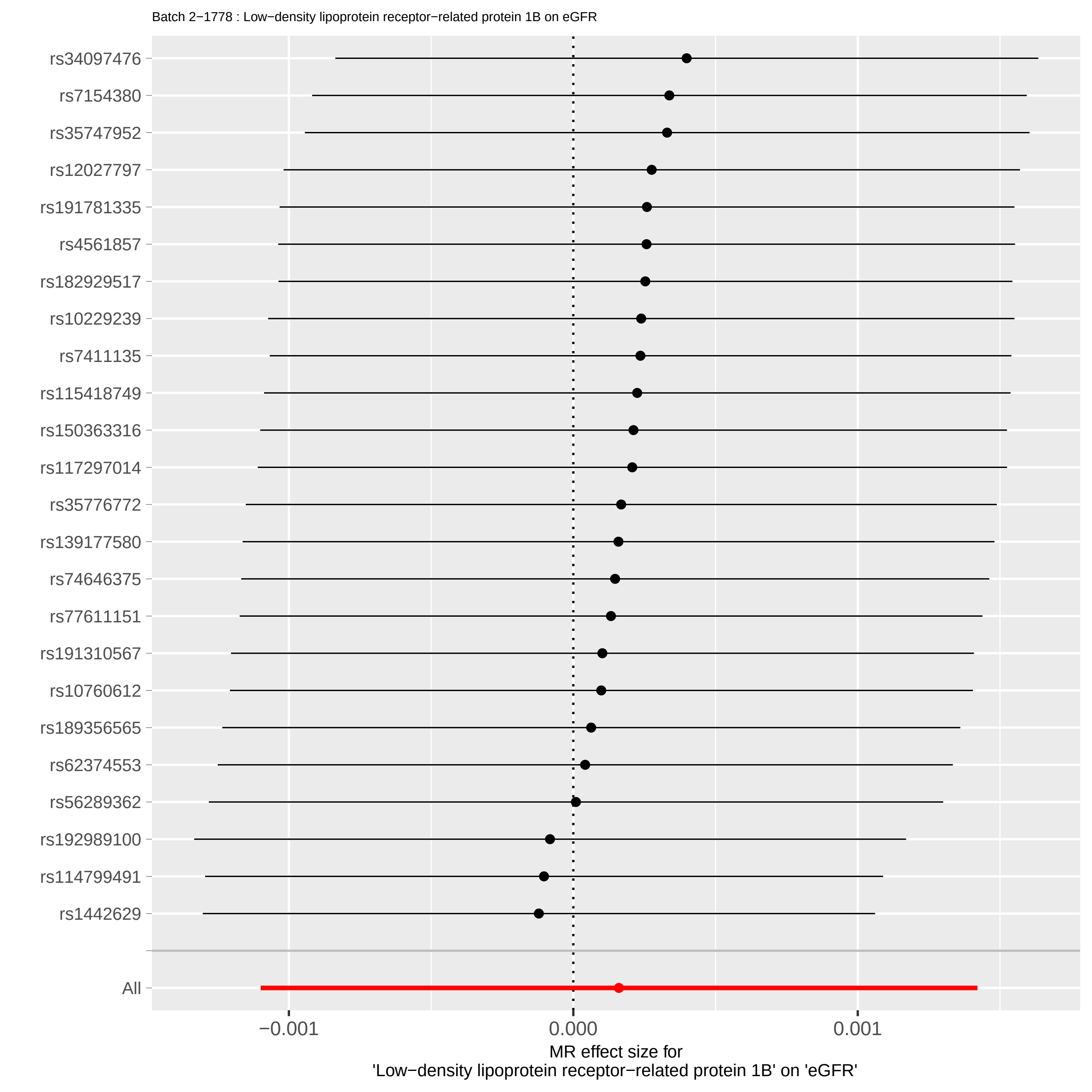

Batch 2−1778 : Low−density lipoprotein receptor−related protein 1B on eGFR
rs34097476
rs7154380
rs35747952
rs12027797
rs191781335
rs4561857
rs182929517
rs10229239
rs7411135
rs115418749
rs150363316
rs117297014
rs35776772
rs139177580
rs74646375
rs77611151
rs191310567
rs10760612
rs189356565
rs62374553
rs56289362
rs192989100
rs114799491
rs1442629
All
−0.001
0.000
0.001
MR effect size for
'Low−density lipoprotein receptor−related protein 1B' on 'eGFR'

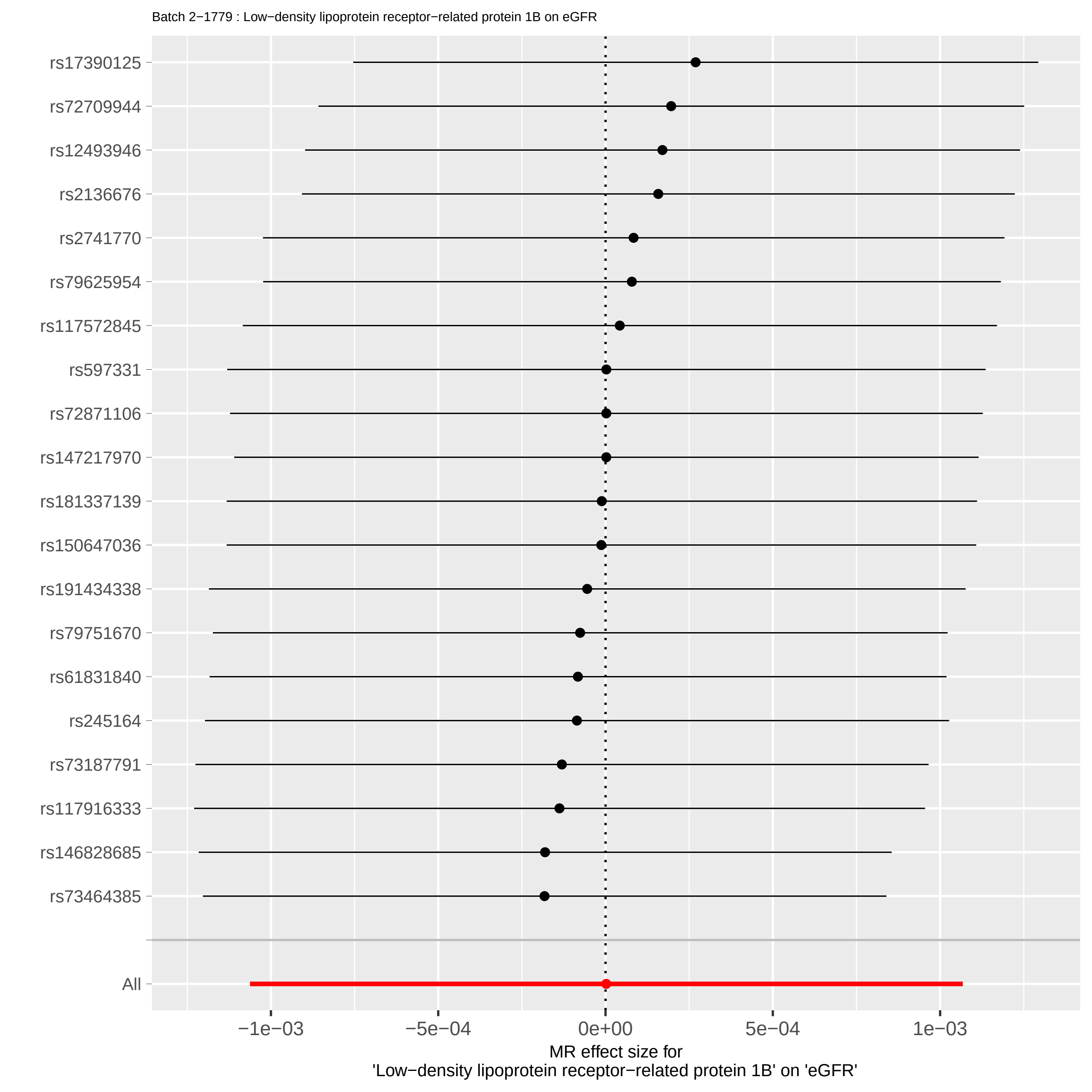
Batch 2−1779 : Low−density lipoprotein receptor−related protein 1B on eGFR
rs17390125
rs72709944
rs12493946
rs2136676
rs2741770
rs79625954
rs117572845
rs597331
rs72871106
rs147217970
rs181337139
rs150647036
rs191434338
rs79751670
rs61831840
rs245164
rs73187791
rs117916333
rs146828685
rs73464385
All
−1e−03
−5e−04
0e+00
5e−04
1e−03
MR effect size for
'Low−density lipoprotein receptor−related protein 1B' on 'eGFR'

Batch 2−1780 : Low−density lipoprotein receptor−related protein 8 on eGFR

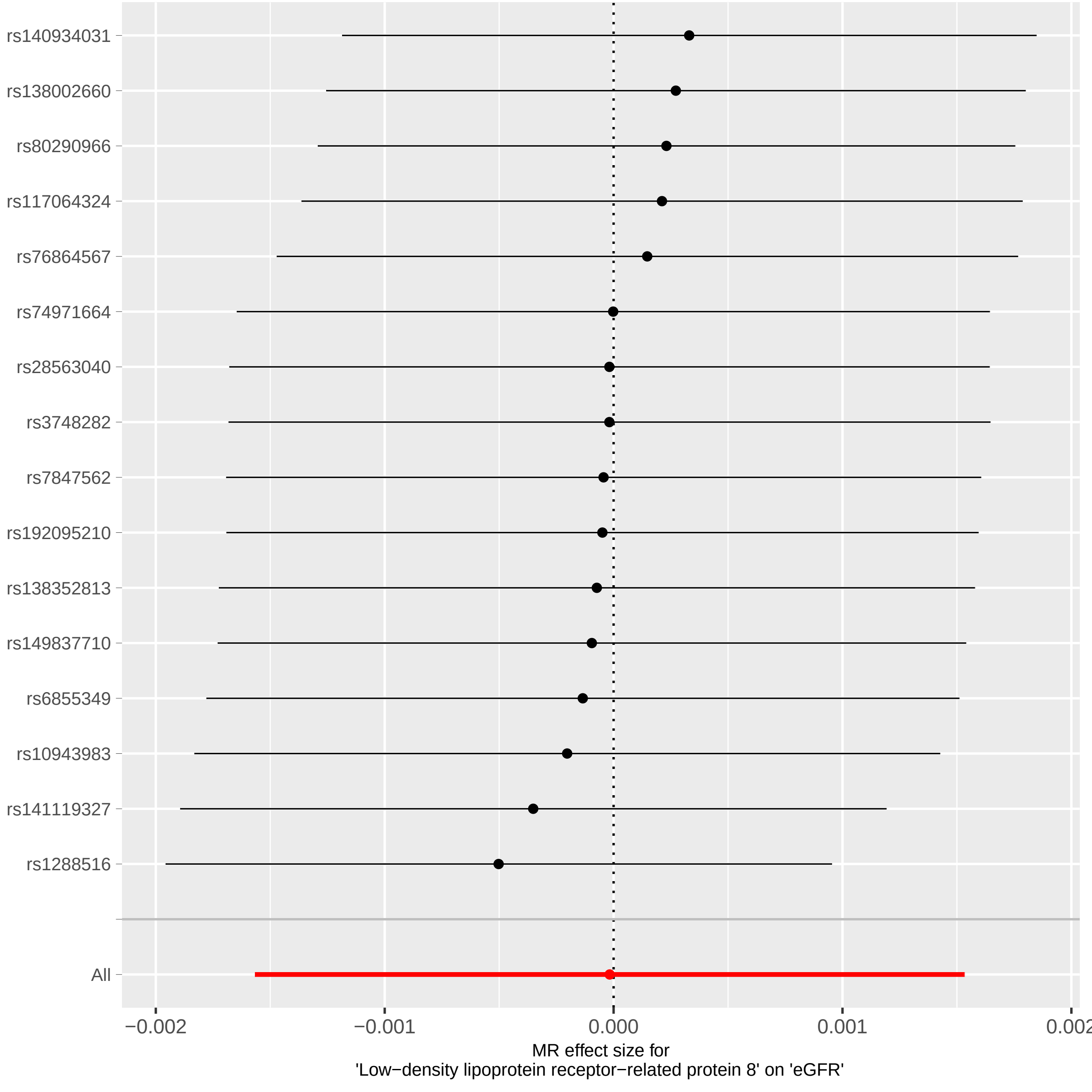

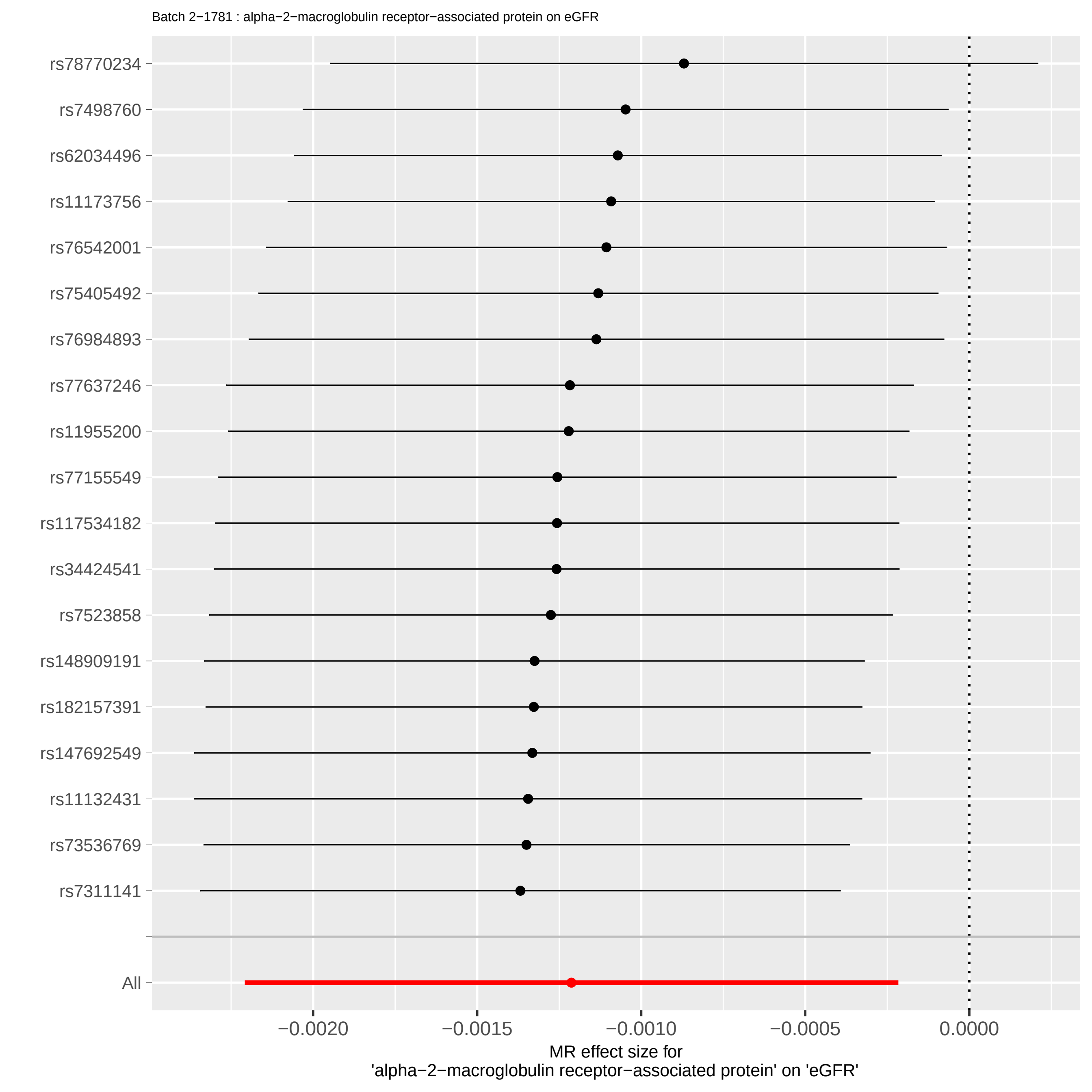
Batch 2−1781 : alpha−2−macroglobulin receptor−associated protein on eGFR
rs78770234
rs7498760
rs62034496
rs11173756
rs76542001
rs75405492
rs76984893
rs77637246
rs11955200
rs77155549
rs117534182
rs34424541
rs7523858
rs148909191
rs182157391
rs147692549
rs11132431
rs73536769
rs7311141
All
−0.0020
−0.0015
−0.0010
−0.0005
0.0000
MR effect size for
'alpha−2−macroglobulin receptor−associated protein' on 'eGFR'

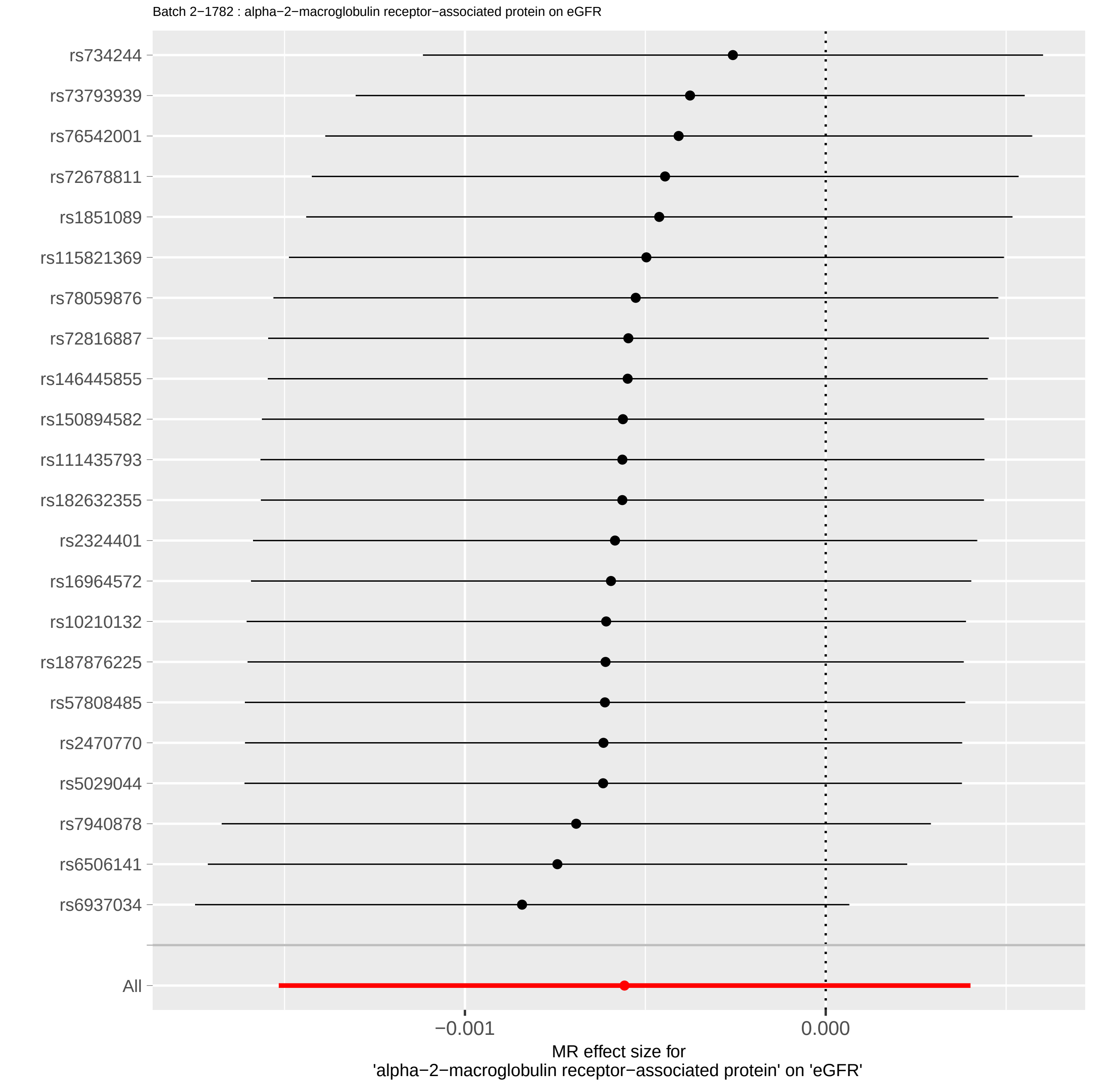
Batch 2−1782 : alpha−2−macroglobulin receptor−associated protein on eGFR
rs734244
rs73793939
rs76542001
rs72678811
rs1851089
rs115821369
rs78059876
rs72816887
rs146445855
rs150894582
rs111435793
rs182632355
rs2324401
rs16964572
rs10210132
rs187876225
rs57808485
rs2470770
rs5029044
rs7940878
rs6506141
rs6937034
All
−0.001
0.000
MR effect size for
'alpha−2−macroglobulin receptor−associated protein' on 'eGFR'

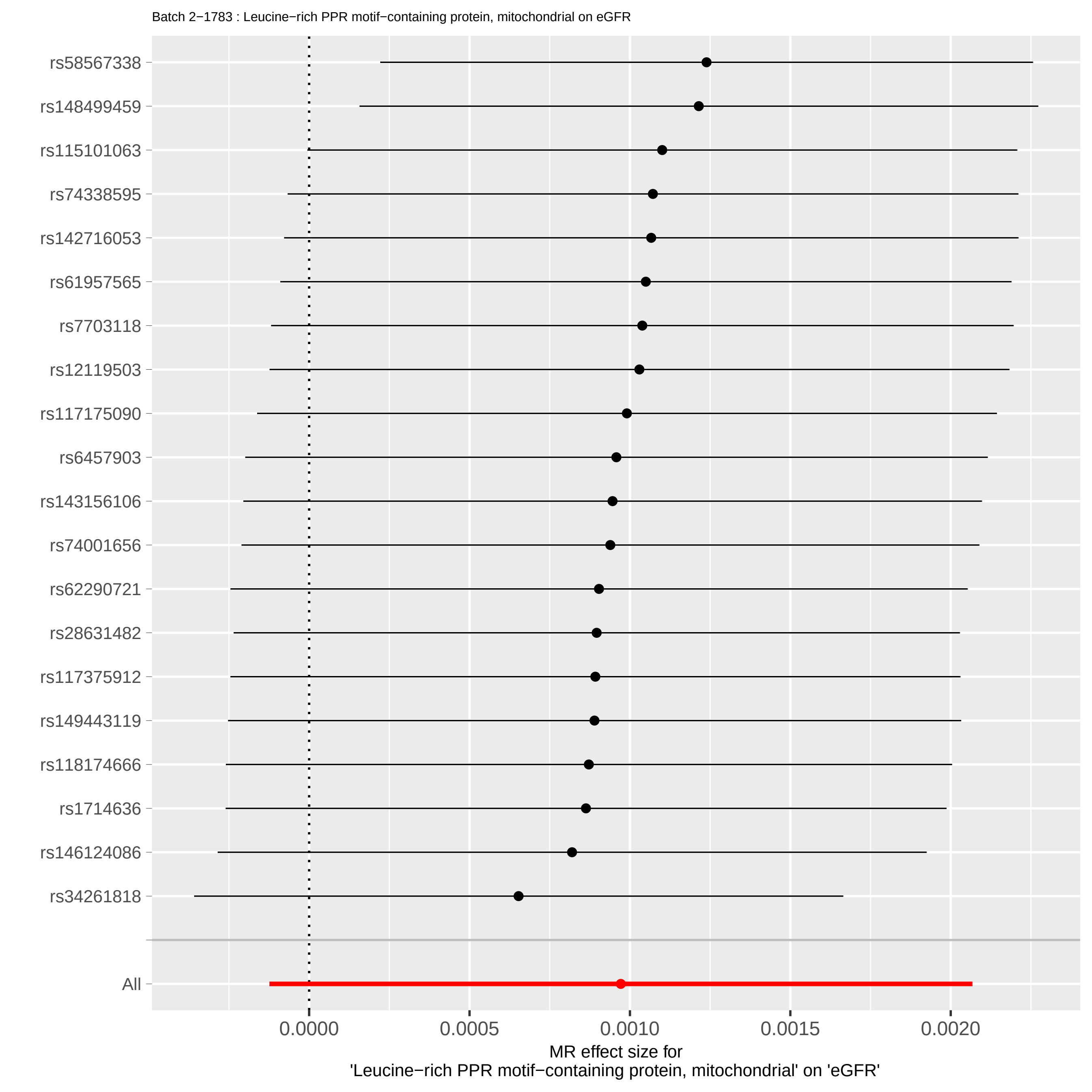
Batch 2−1783 : Leucine−rich PPR motif−containing protein, mitochondrial on eGFR
rs58567338
rs148499459
rs115101063
rs74338595
rs142716053
rs61957565
rs7703118
rs12119503
rs117175090
rs6457903
rs143156106
rs74001656
rs62290721
rs28631482
rs117375912
rs149443119
rs118174666
rs1714636
rs146124086
rs34261818
All
0.0000
0.0005
0.0010
0.0015
0.0020
MR effect size for
'Leucine−rich PPR motif−containing protein, mitochondrial' on 'eGFR'

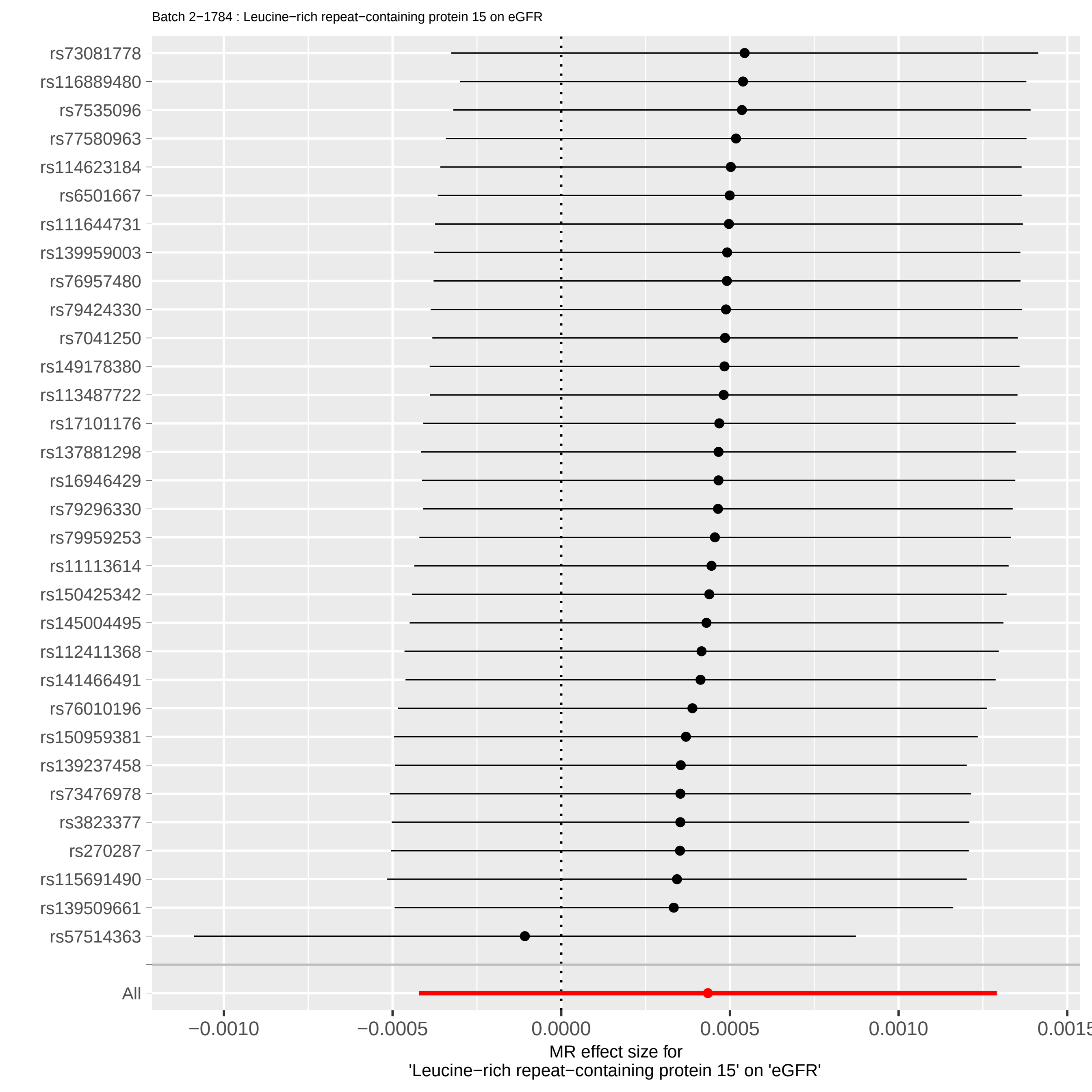
Batch 2−1784 : Leucine−rich repeat−containing protein 15 on eGFR
rs73081778
rs116889480
rs7535096
rs77580963
rs114623184
rs6501667
rs111644731
rs139959003
rs76957480
rs79424330
rs7041250
rs149178380
rs113487722
rs17101176
rs137881298
rs16946429
rs79296330
rs79959253
rs11113614
rs150425342
rs145004495
rs112411368
rs141466491
rs76010196
rs150959381
rs139237458
rs73476978
rs3823377
rs270287
rs115691490
rs139509661
rs57514363
All
−0.0010
−0.0005
0.0000
0.0005
0.0010
MR effect size for
'Leucine−rich repeat−containing protein 15' on 'eGFR'

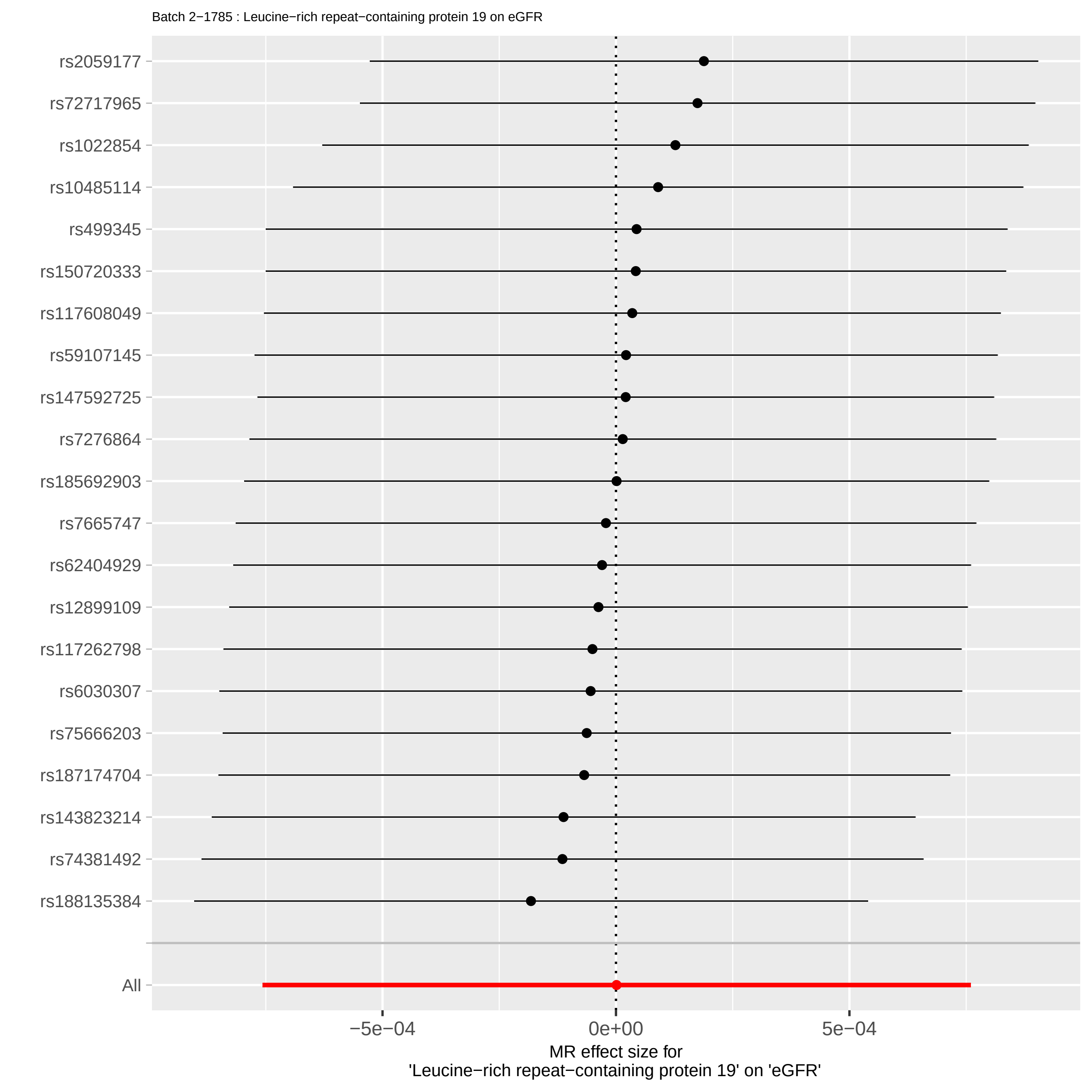

Batch 2−1785 : Leucine−rich repeat−containing protein 19 on eGFR
rs2059177
rs72717965
rs1022854
rs10485114
rs499345
rs150720333
rs117608049
rs59107145
rs147592725
rs7276864
rs185692903
rs7665747
rs62404929
rs12899109
rs117262798
rs6030307
rs75666203
rs187174704
rs143823214
rs74381492
rs188135384
All
−5e−04
0e+00
5e−04
MR effect size for
'Leucine−rich repeat−containing protein 19' on 'eGFR'

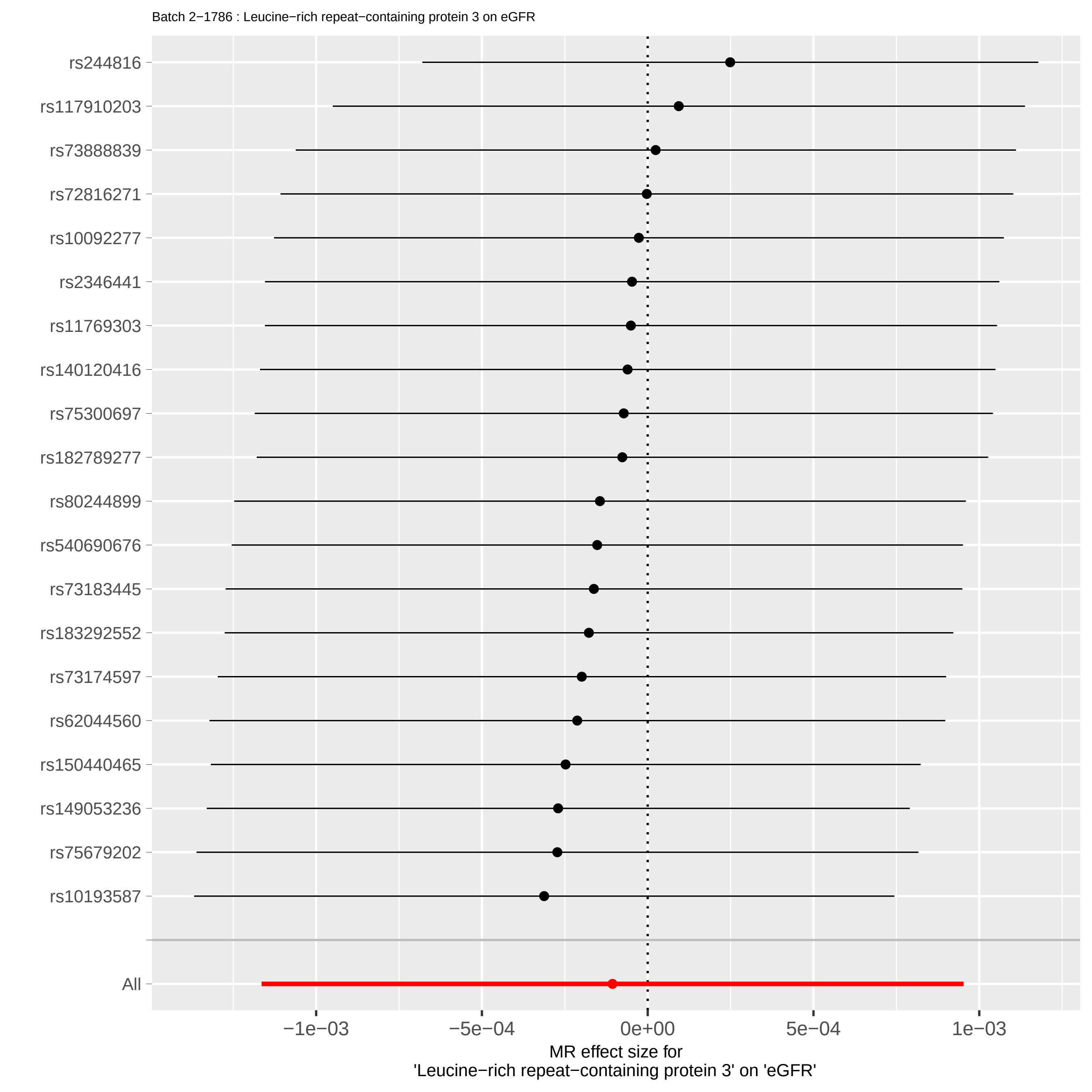
Batch 2−1786 : Leucine−rich repeat−containing protein 3 on eGFR
rs244816
rs117910203
rs73888839
rs72816271
rs10092277
rs2346441
rs11769303
rs140120416
rs75300697
rs182789277
rs80244899
rs540690676
rs73183445
rs183292552
rs73174597
rs62044560
rs150440465
rs149053236
rs75679202
rs10193587
All
−1e−03
−5e−04
0e+00
5e−04
1e−03
MR effect size for
'Leucine−rich repeat−containing protein 3' on 'eGFR'

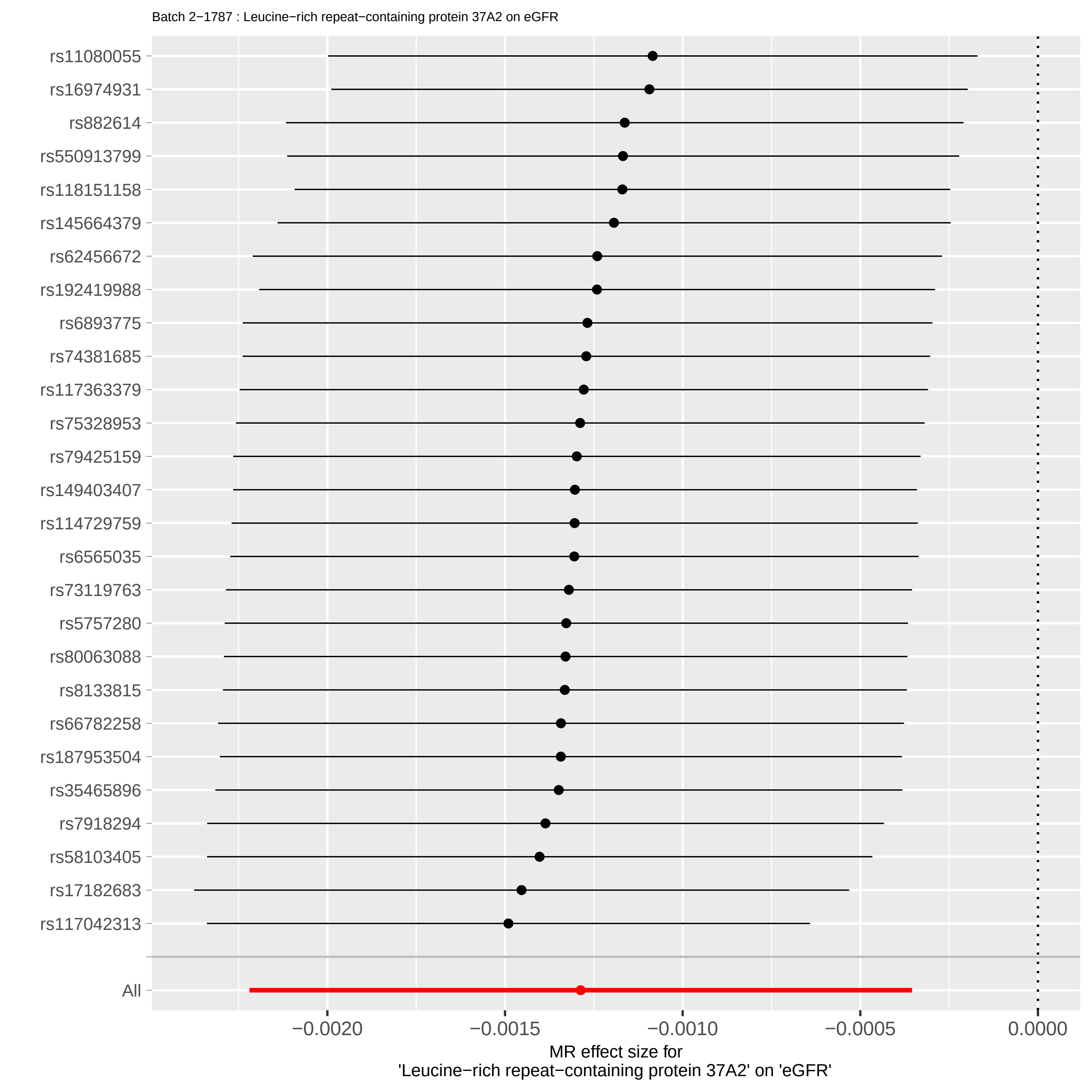
Batch 2−1787 : Leucine−rich repeat−containing protein 37A2 on eGFR
rs11080055
rs16974931
rs882614
rs550913799
rs118151158
rs145664379
rs62456672
rs192419988
rs6893775
rs74381685
rs117363379
rs75328953
rs79425159
rs149403407
rs114729759
rs6565035
rs73119763
rs5757280
rs80063088
rs8133815
rs66782258
rs187953504
rs35465896
rs7918294
rs58103405
rs17182683
rs117042313
All
−0.0020
−0.0015
−0.0010
−0.0005
0.0000
MR effect size for
'Leucine−rich repeat−containing protein 37A2' on 'eGFR'

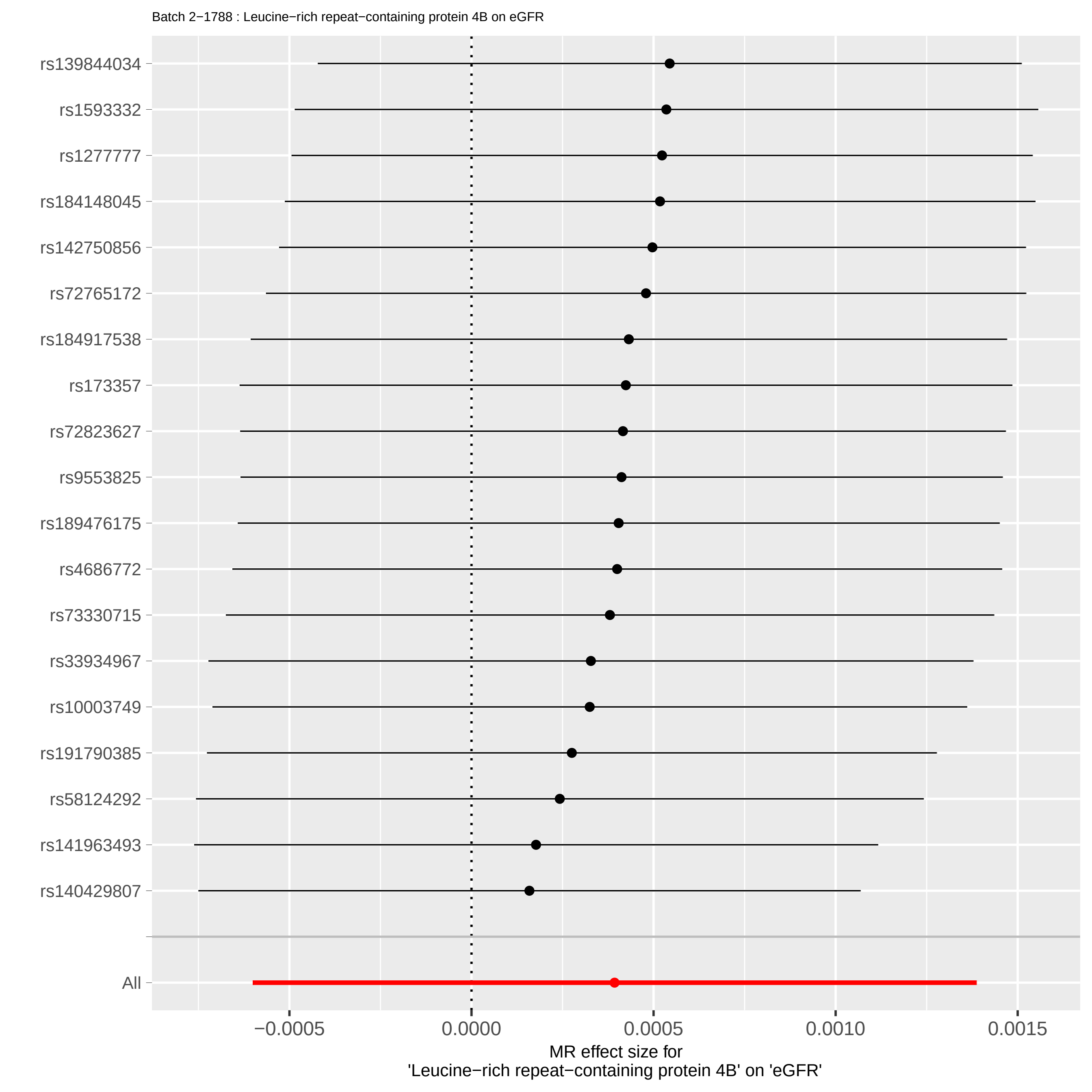

Batch 2−1788 : Leucine−rich repeat−containing protein 4B on eGFR
rs139844034
rs1593332
rs1277777
rs184148045
rs142750856
rs72765172
rs184917538
rs173357
rs72823627
rs9553825
rs189476175
rs4686772
rs73330715
rs33934967
rs10003749
rs191790385
rs58124292
rs141963493
rs140429807
All
−0.0005
0.0000
0.0005
0.0010
0.0015
MR effect size for
'Leucine−rich repeat−containing protein 4B' on 'eGFR'

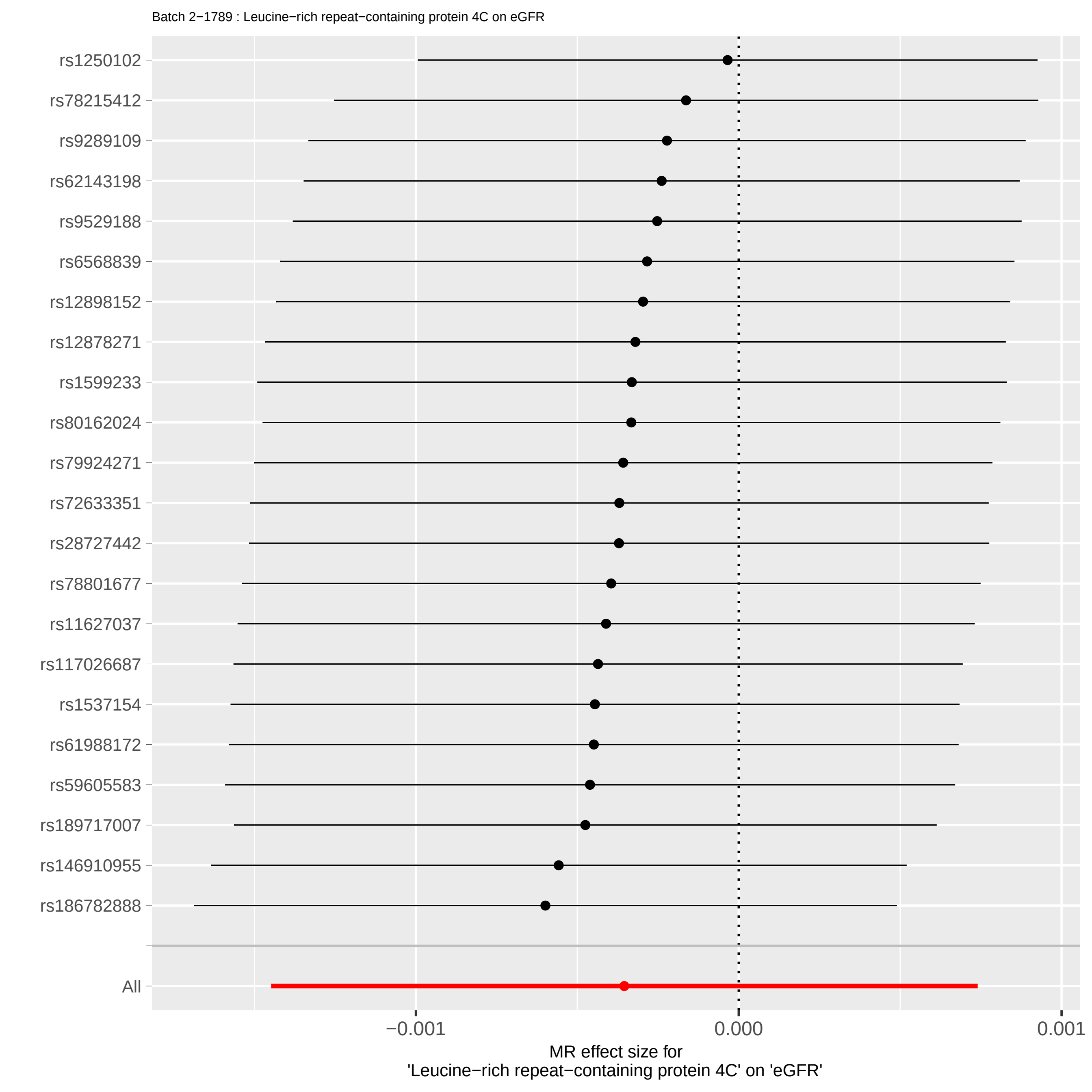

Batch 2−1789 : Leucine−rich repeat−containing protein 4C on eGFR
rs1250102
rs78215412
rs9289109
rs62143198
rs9529188
rs6568839
rs12898152
rs12878271
rs1599233
rs80162024
rs79924271
rs72633351
rs28727442
rs78801677
rs11627037
rs117026687
rs1537154
rs61988172
rs59605583
rs189717007
rs146910955
rs186782888
All
−0.001
0.000
0.001
MR effect size for
'Leucine−rich repeat−containing protein 4C' on 'eGFR'

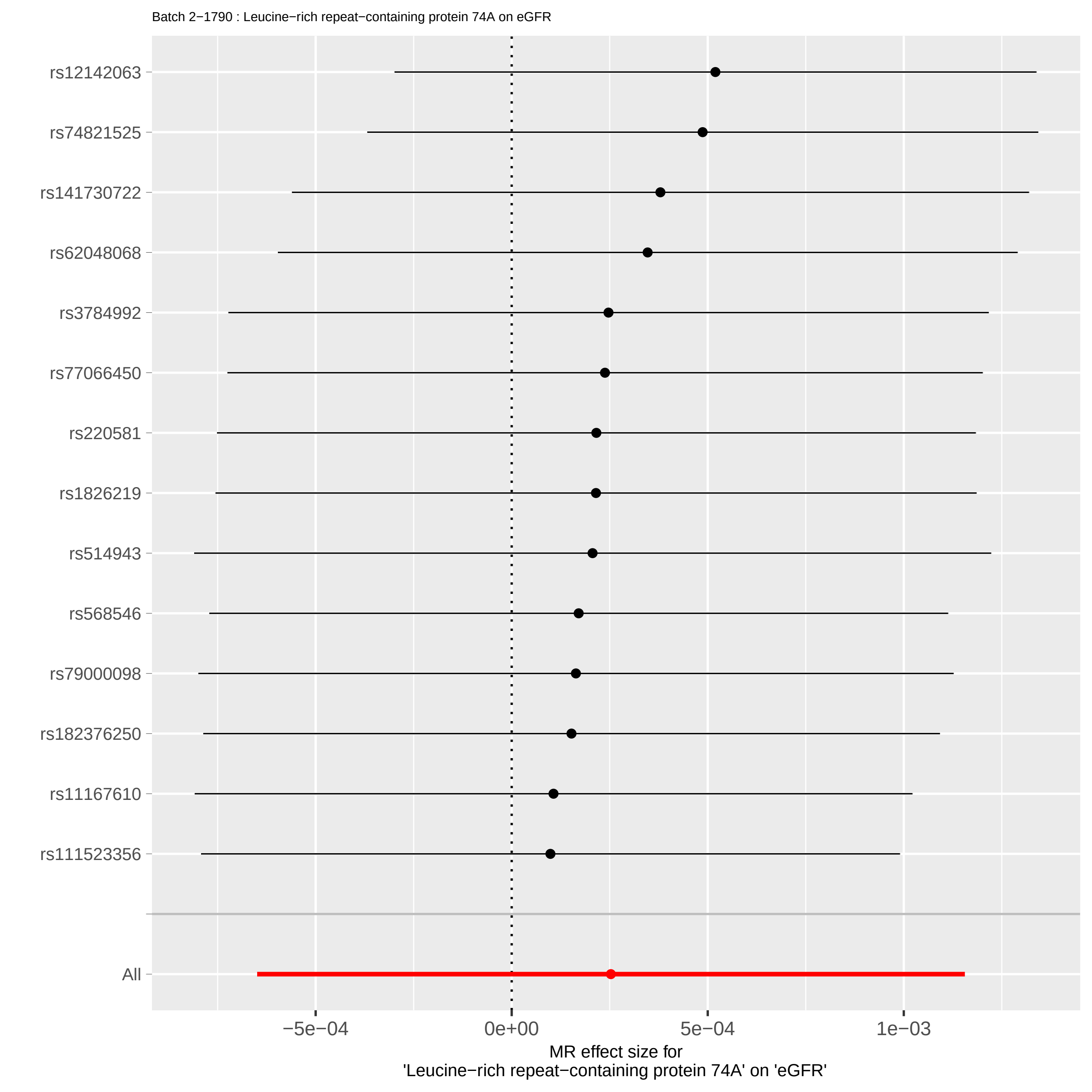

Batch 2−1790 : Leucine−rich repeat−containing protein 74A on eGFR
rs12142063
rs74821525
rs141730722
rs62048068
rs3784992
rs77066450
rs220581
rs1826219
rs514943
rs568546
rs79000098
rs182376250
rs11167610
rs111523356
All
−5e−04
0e+00
5e−04
1e−03
MR effect size for
'Leucine−rich repeat−containing protein 74A' on 'eGFR'

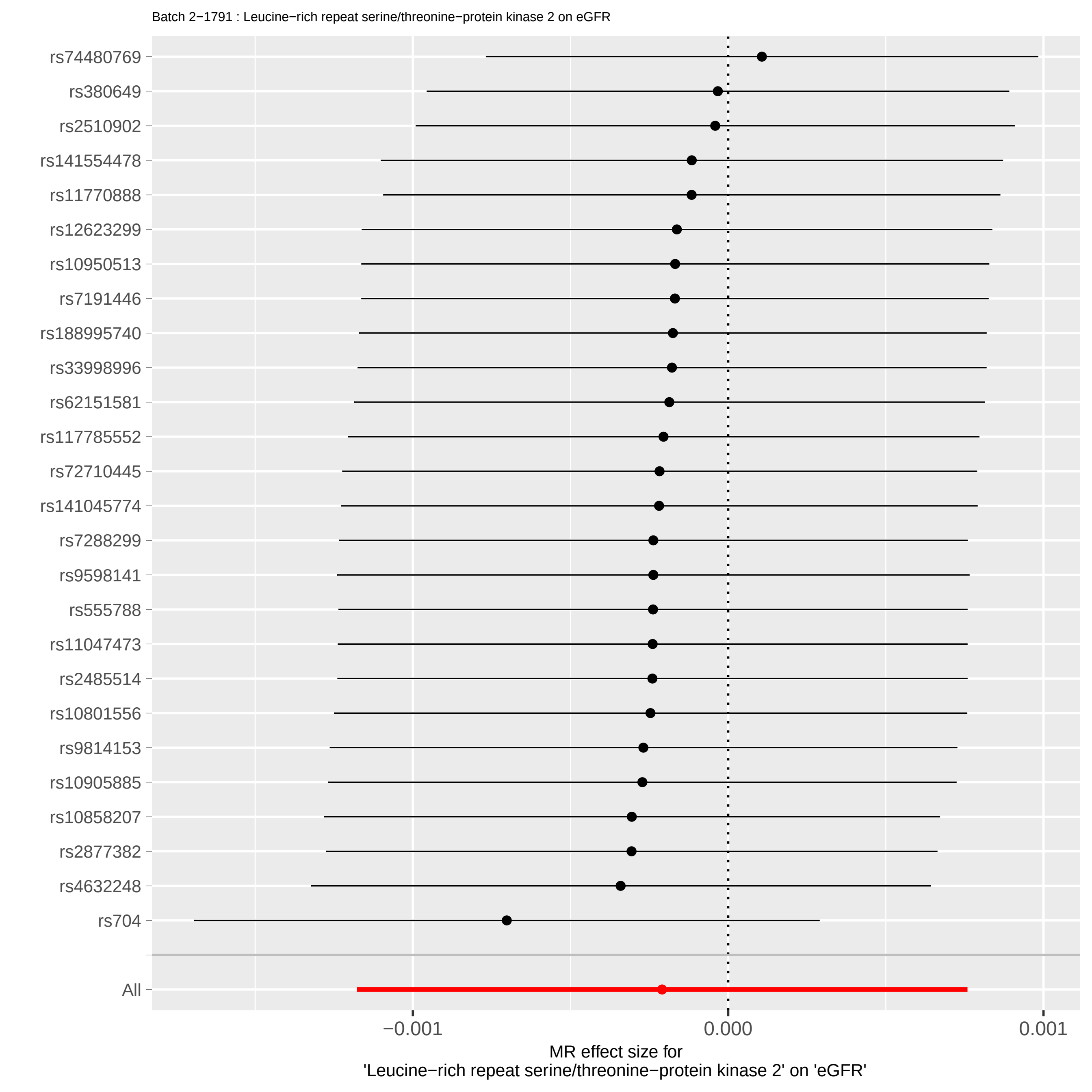

Batch 2−1791 : Leucine−rich repeat serine/threonine−protein kinase 2 on eGFR
rs74480769
rs380649
rs2510902
rs141554478
rs11770888
rs12623299
rs10950513
rs7191446
rs188995740
rs33998996
rs62151581
rs117785552
rs72710445
rs141045774
rs7288299
rs9598141
rs555788
rs11047473
rs2485514
rs10801556
rs9814153
rs10905885
rs10858207
rs2877382
rs4632248
rs704
All
−0.001
0.000
0.001
MR effect size for
'Leucine−rich repeat serine/threonine−protein kinase 2' on 'eGFR'

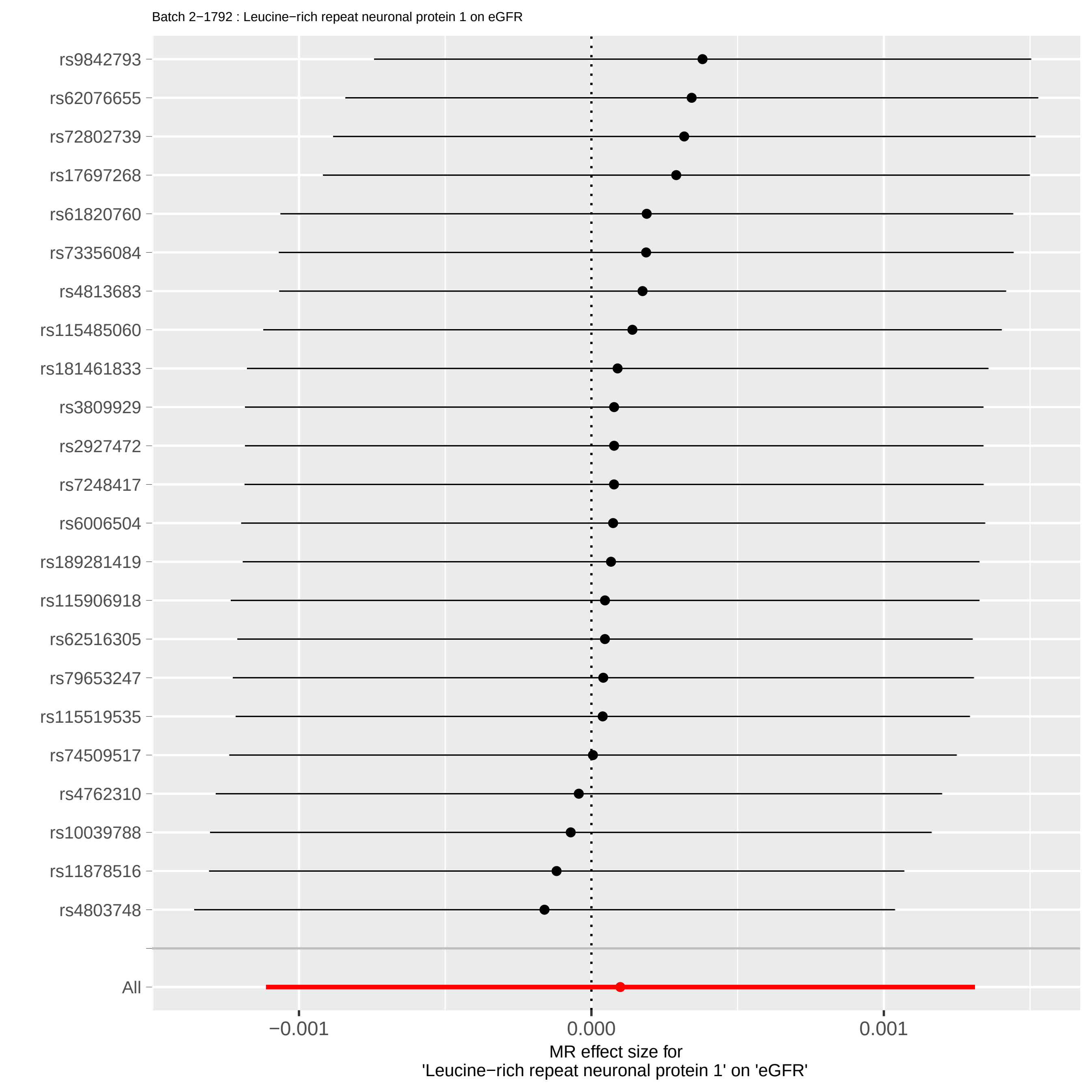

Batch 2−1792 : Leucine−rich repeat neuronal protein 1 on eGFR
rs9842793
rs62076655
rs72802739
rs17697268
rs61820760
rs73356084
rs4813683
rs115485060
rs181461833
rs3809929
rs2927472
rs7248417
rs6006504
rs189281419
rs115906918
rs62516305
rs79653247
rs115519535
rs74509517
rs4762310
rs10039788
rs11878516
rs4803748
All
−0.001
0.000
0.001
MR effect size for
'Leucine−rich repeat neuronal protein 1' on 'eGFR'

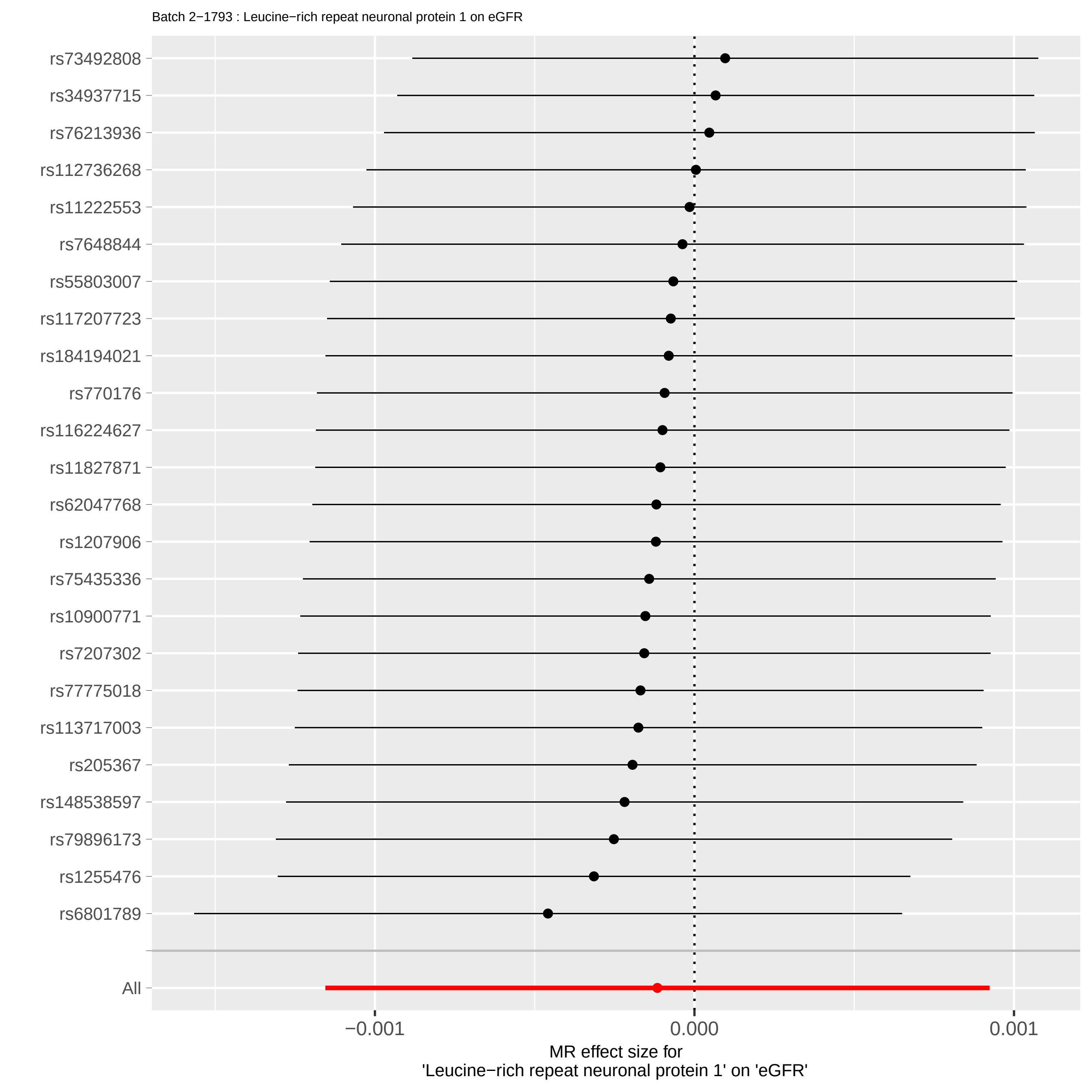

Batch 2−1793 : Leucine−rich repeat neuronal protein 1 on eGFR
rs73492808
rs34937715
rs76213936
rs112736268
rs11222553
rs7648844
rs55803007
rs117207723
rs184194021
rs770176
rs116224627
rs11827871
rs62047768
rs1207906
rs75435336
rs10900771
rs7207302
rs77775018
rs113717003
rs205367
rs148538597
rs79896173
rs1255476
rs6801789
All
−0.001
0.000
0.001
MR effect size for
'Leucine−rich repeat neuronal protein 1' on 'eGFR'

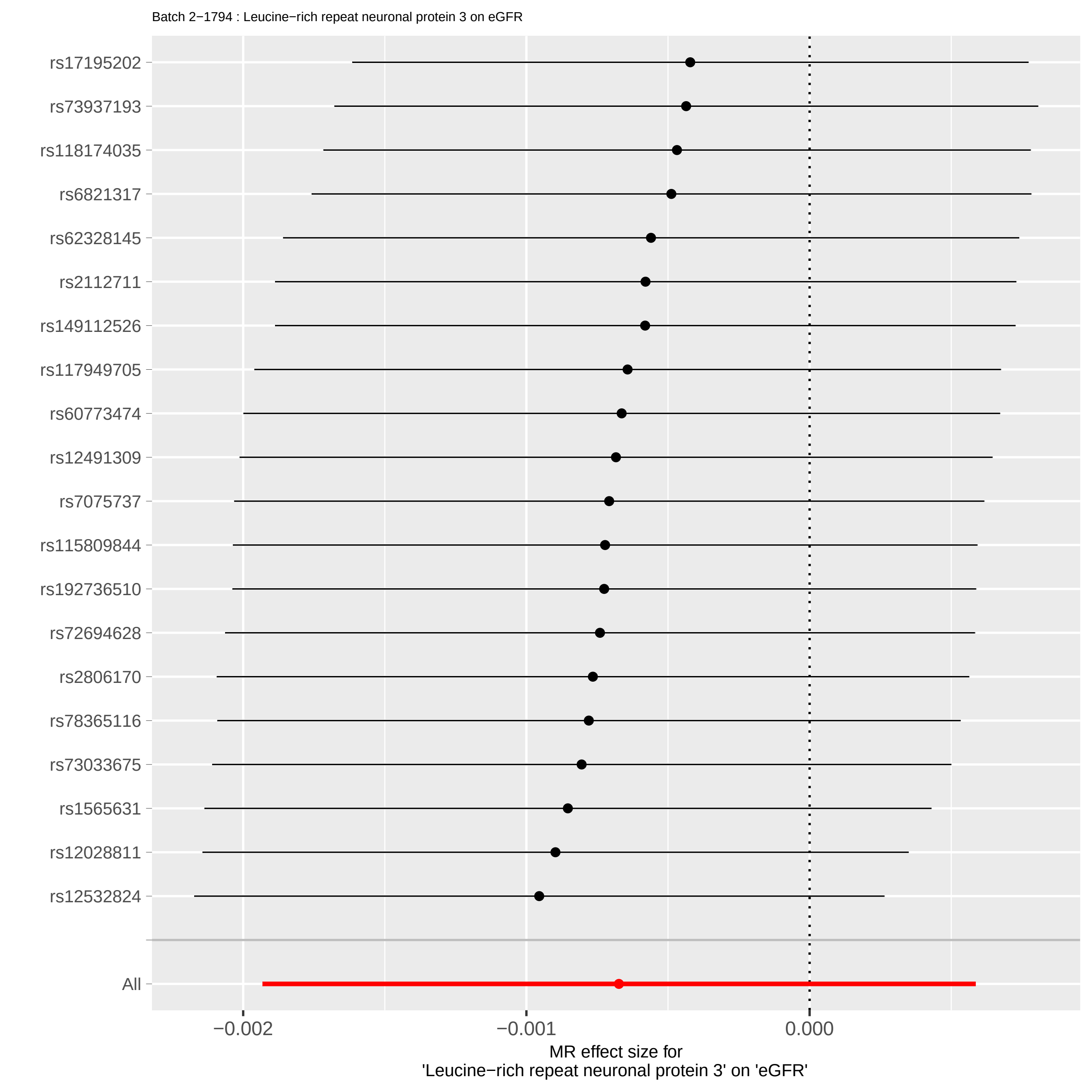

Batch 2−1794 : Leucine−rich repeat neuronal protein 3 on eGFR
rs17195202
rs73937193
rs118174035
rs6821317
rs62328145
rs2112711
rs149112526
rs117949705
rs60773474
rs12491309
rs7075737
rs115809844
rs192736510
rs72694628
rs2806170
rs78365116
rs73033675
rs1565631
rs12028811
rs12532824
All
−0.002
−0.001
0.000
MR effect size for
'Leucine−rich repeat neuronal protein 3' on 'eGFR'

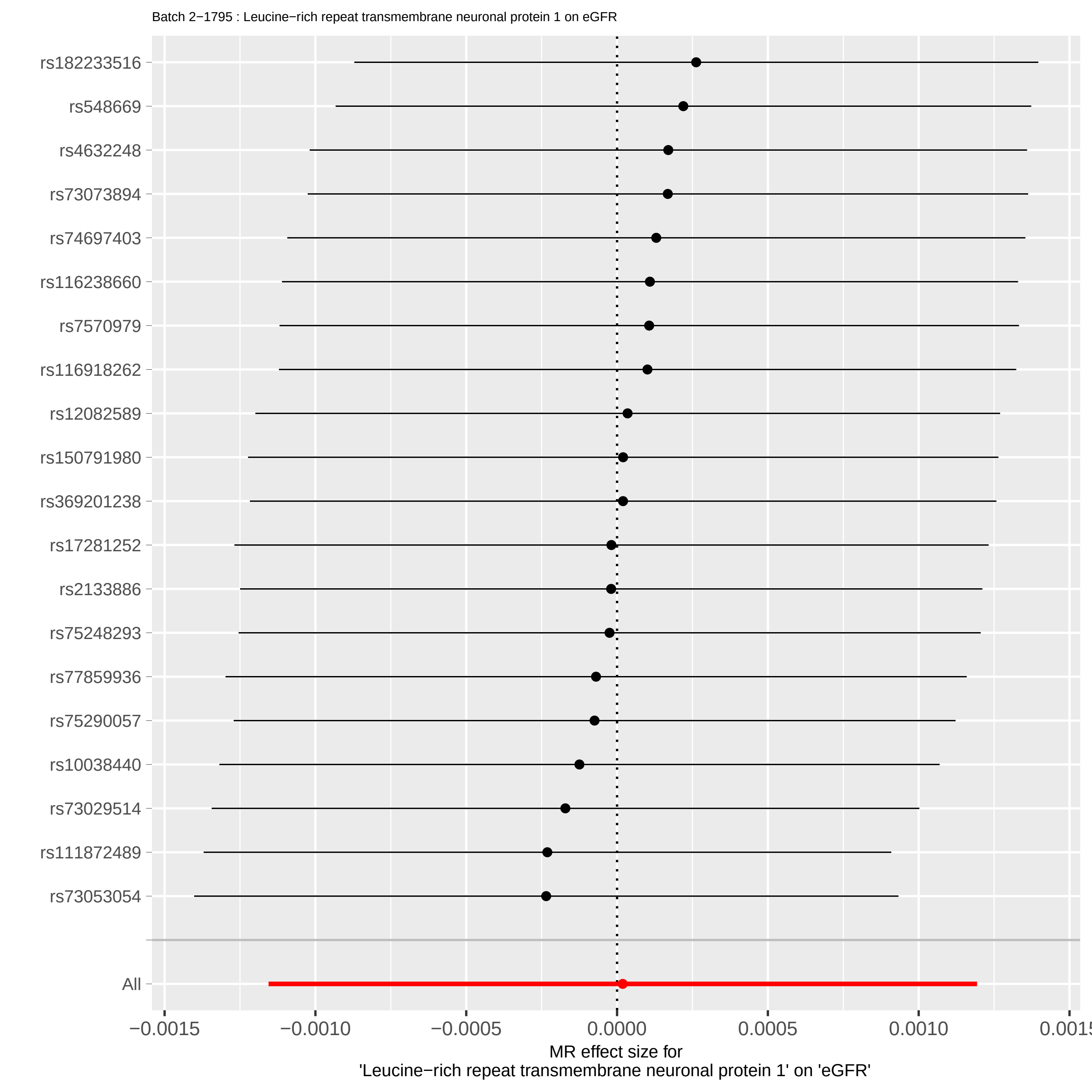

Batch 2−1795 : Leucine−rich repeat transmembrane neuronal protein 1 on eGFR
rs182233516
rs548669
rs4632248
rs73073894
rs74697403
rs116238660
rs7570979
rs116918262
rs12082589
rs150791980
rs369201238
rs17281252
rs2133886
rs75248293
rs77859936
rs75290057
rs10038440
rs73029514
rs111872489
rs73053054
All
−0.0015
−0.0010
−0.0005
0.0000
0.0005
0.0010
MR effect size for
'Leucine−rich repeat transmembrane neuronal protein 1' on 'eGFR'

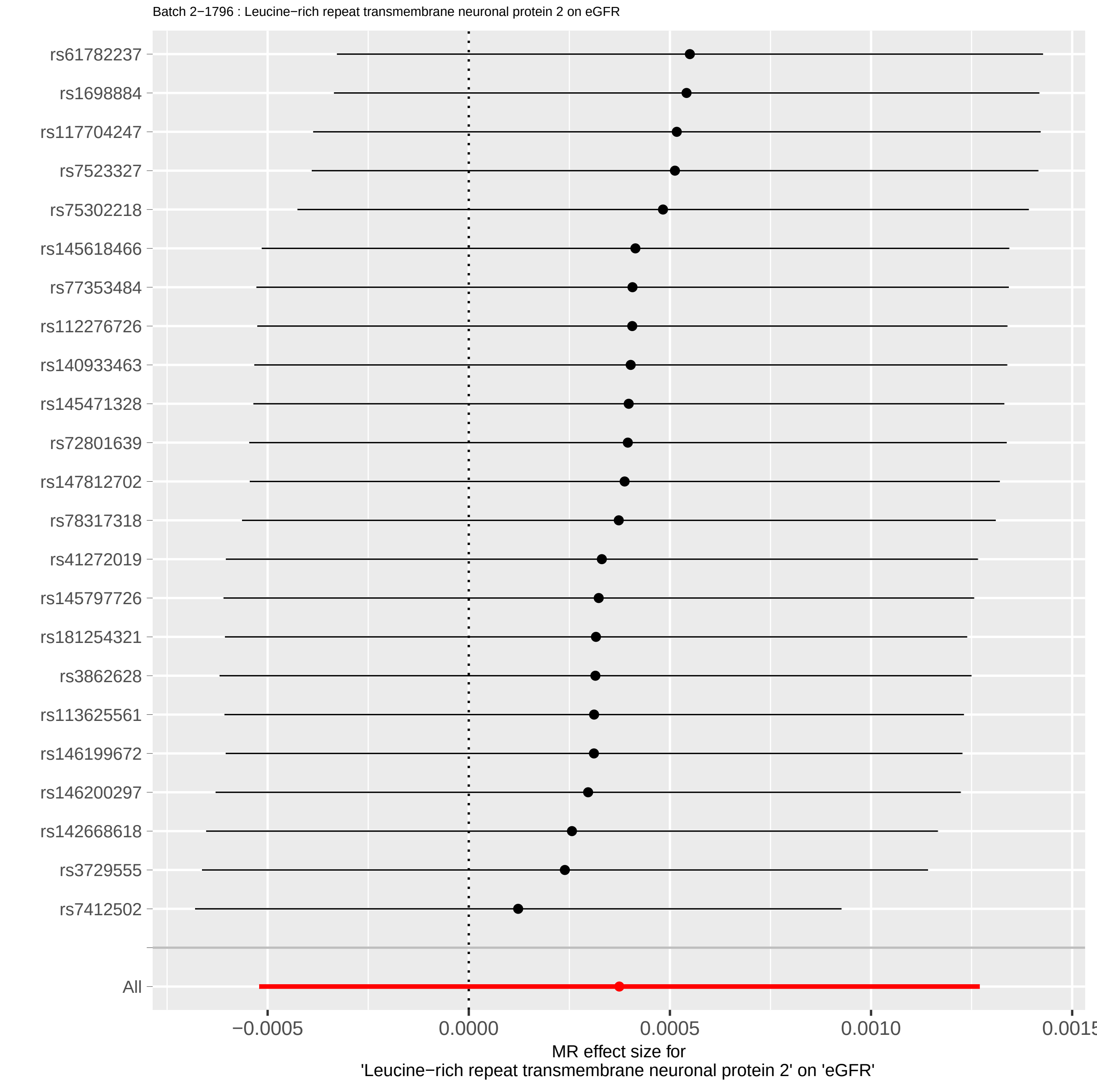

Batch 2−1796 : Leucine−rich repeat transmembrane neuronal protein 2 on eGFR
rs61782237
rs1698884
rs117704247
rs7523327
rs75302218
rs145618466
rs77353484
rs112276726
rs140933463
rs145471328
rs72801639
rs147812702
rs78317318
rs41272019
rs145797726
rs181254321
rs3862628
rs113625561
rs146199672
rs146200297
rs142668618
rs3729555
rs7412502
All
−0.0005
0.0000
0.0005
0.0010
0.0015
MR effect size for
'Leucine−rich repeat transmembrane neuronal protein 2' on 'eGFR'

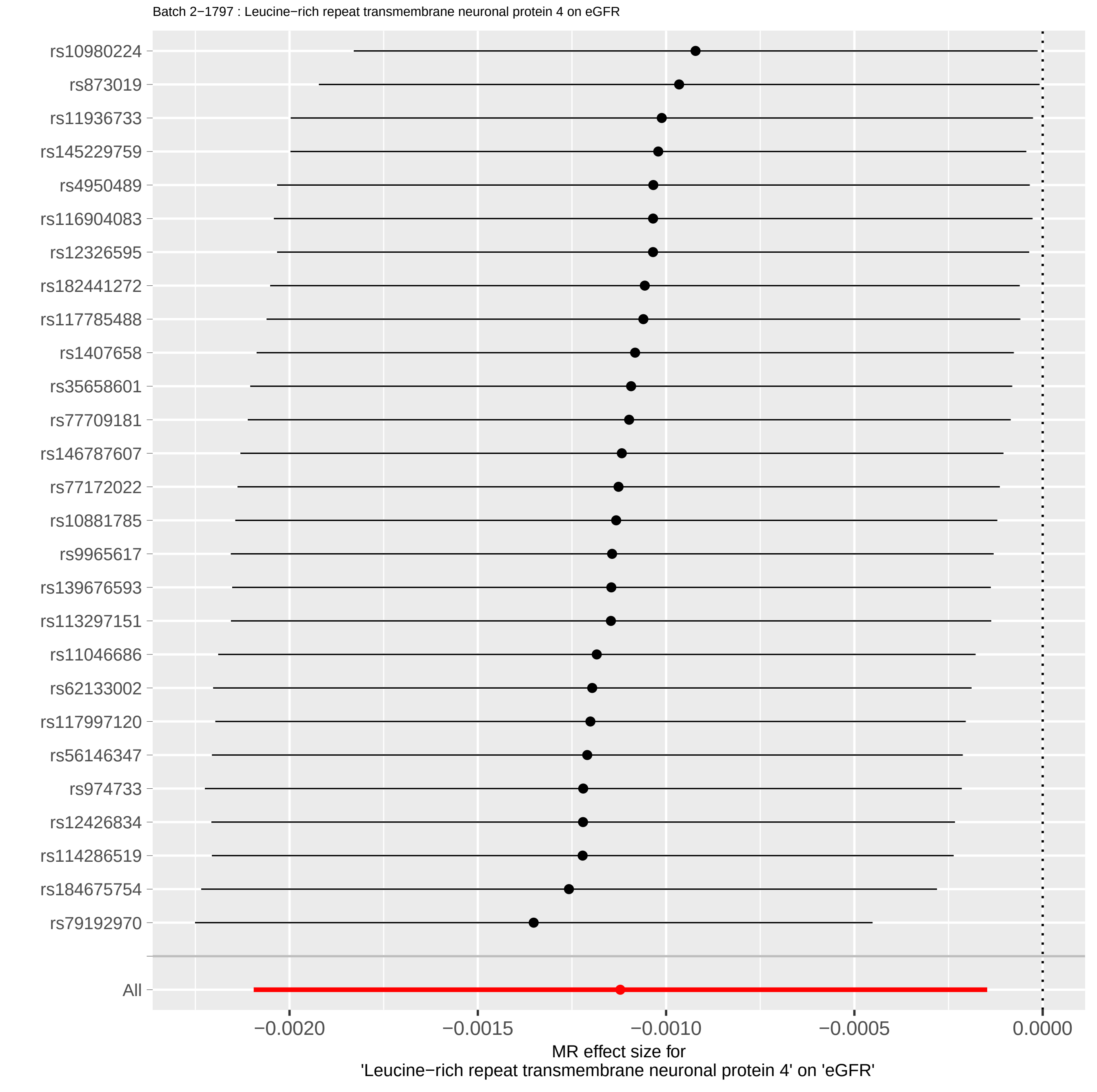

Batch 2−1797 : Leucine−rich repeat transmembrane neuronal protein 4 on eGFR
rs10980224
rs873019
rs11936733
rs145229759
rs4950489
rs116904083
rs12326595
rs182441272
rs117785488
rs1407658
rs35658601
rs77709181
rs146787607
rs77172022
rs10881785
rs9965617
rs139676593
rs113297151
rs11046686
rs62133002
rs117997120
rs56146347
rs974733
rs12426834
rs114286519
rs184675754
rs79192970
All
−0.0020
−0.0015
−0.0010
−0.0005
0.0000
MR effect size for
'Leucine−rich repeat transmembrane neuronal protein 4' on 'eGFR'

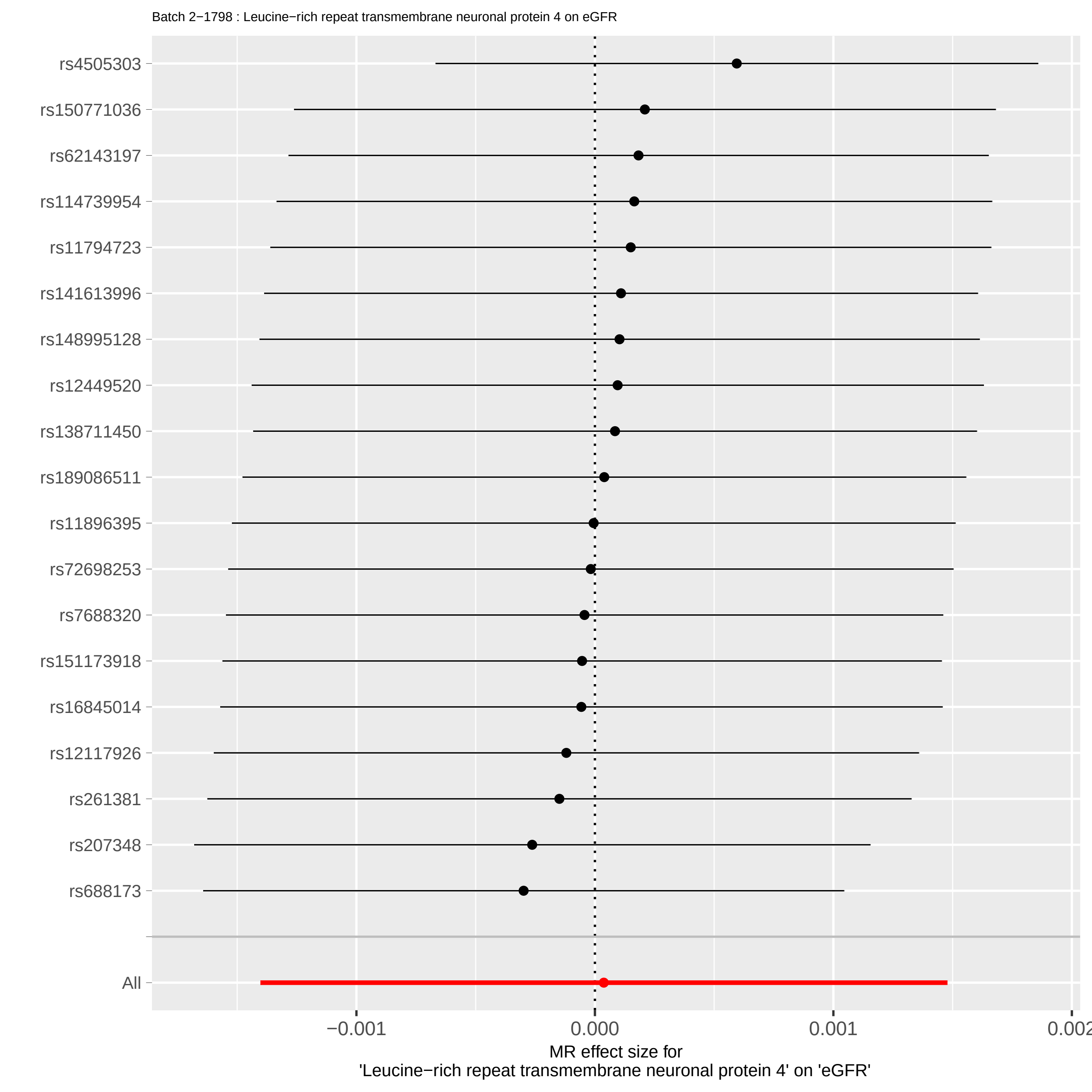

Batch 2−1798 : Leucine−rich repeat transmembrane neuronal protein 4 on eGFR
rs4505303
rs150771036
rs62143197
rs114739954
rs11794723
rs141613996
rs148995128
rs12449520
rs138711450
rs189086511
rs11896395
rs72698253
rs7688320
rs151173918
rs16845014
rs12117926
rs261381
rs207348
rs688173
All
−0.001
0.000
0.001
MR effect size for
'Leucine−rich repeat transmembrane neuronal protein 4' on 'eGFR'

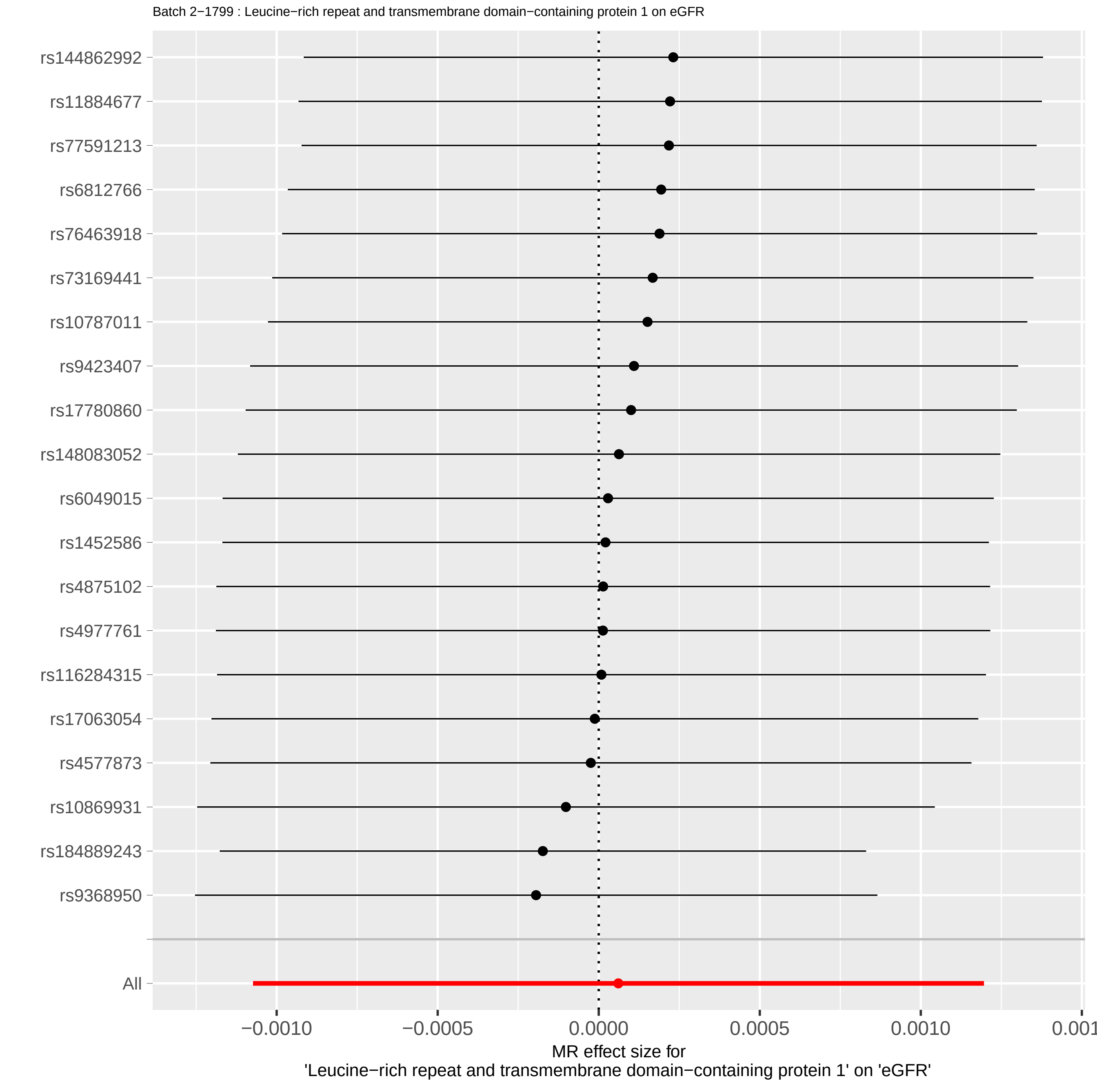

Batch 2−1799 : Leucine−rich repeat and transmembrane domain−containing protein 1 on eGFR
rs144862992
rs11884677
rs77591213
rs6812766
rs76463918
rs73169441
rs10787011
rs9423407
rs17780860
rs148083052
rs6049015
rs1452586
rs4875102
rs4977761
rs116284315
rs17063054
rs4577873
rs10869931
rs184889243
rs9368950
All
−0.0010
−0.0005
0.0000
0.0005
0.0010
MR effect size for
'Leucine−rich repeat and transmembrane domain−containing protein 1' on 'eGFR'

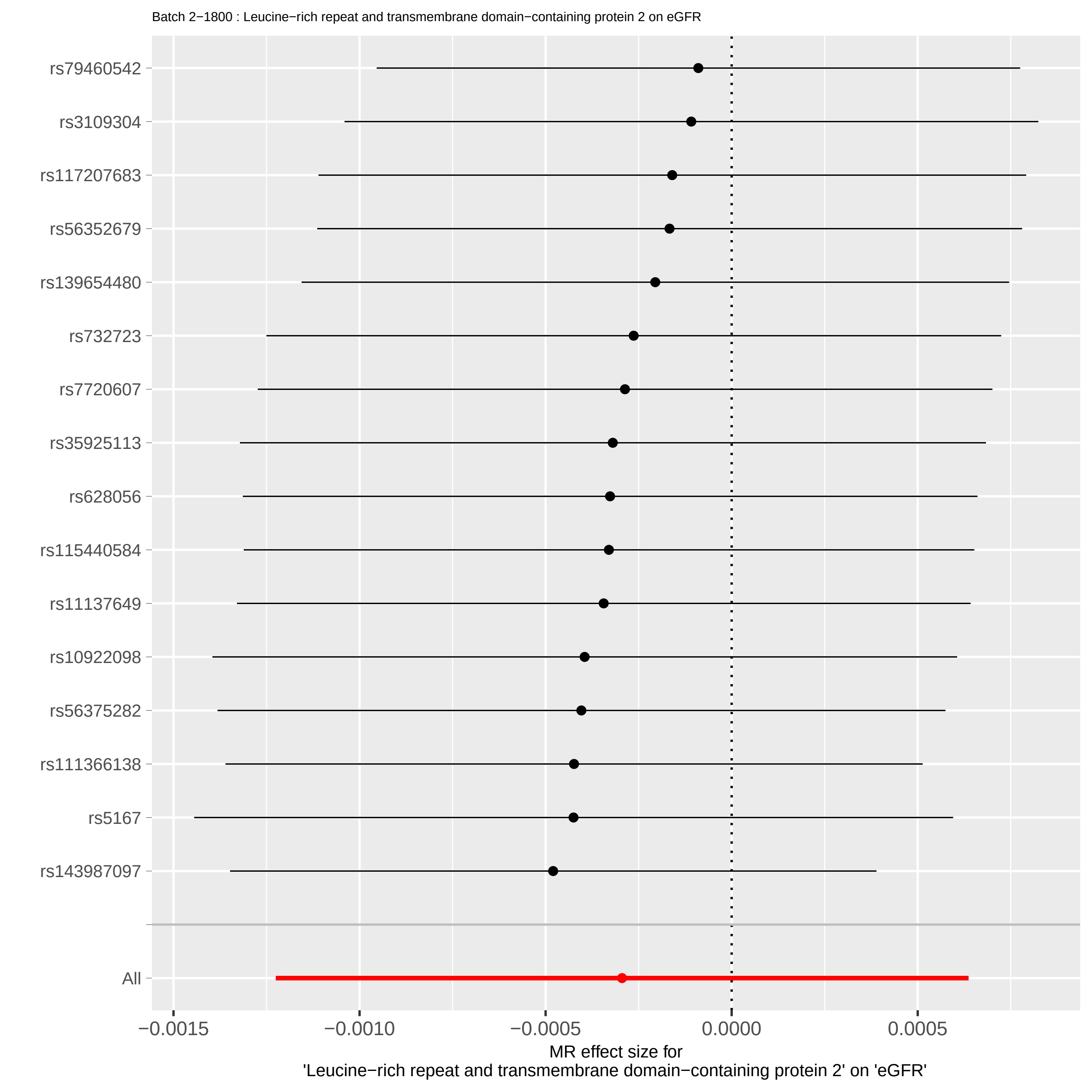
Batch 2−1800 : Leucine−rich repeat and transmembrane domain−containing protein 2 on eGFR
rs79460542
rs3109304
rs117207683
rs56352679
rs139654480
rs732723
rs7720607
rs35925113
rs628056
rs115440584
rs11137649
rs10922098
rs56375282
rs111366138
rs5167
rs143987097
All
−0.0015
−0.0010
−0.0005
0.0000
0.0005
MR effect size for
'Leucine−rich repeat and transmembrane domain−containing protein 2' on 'eGFR'

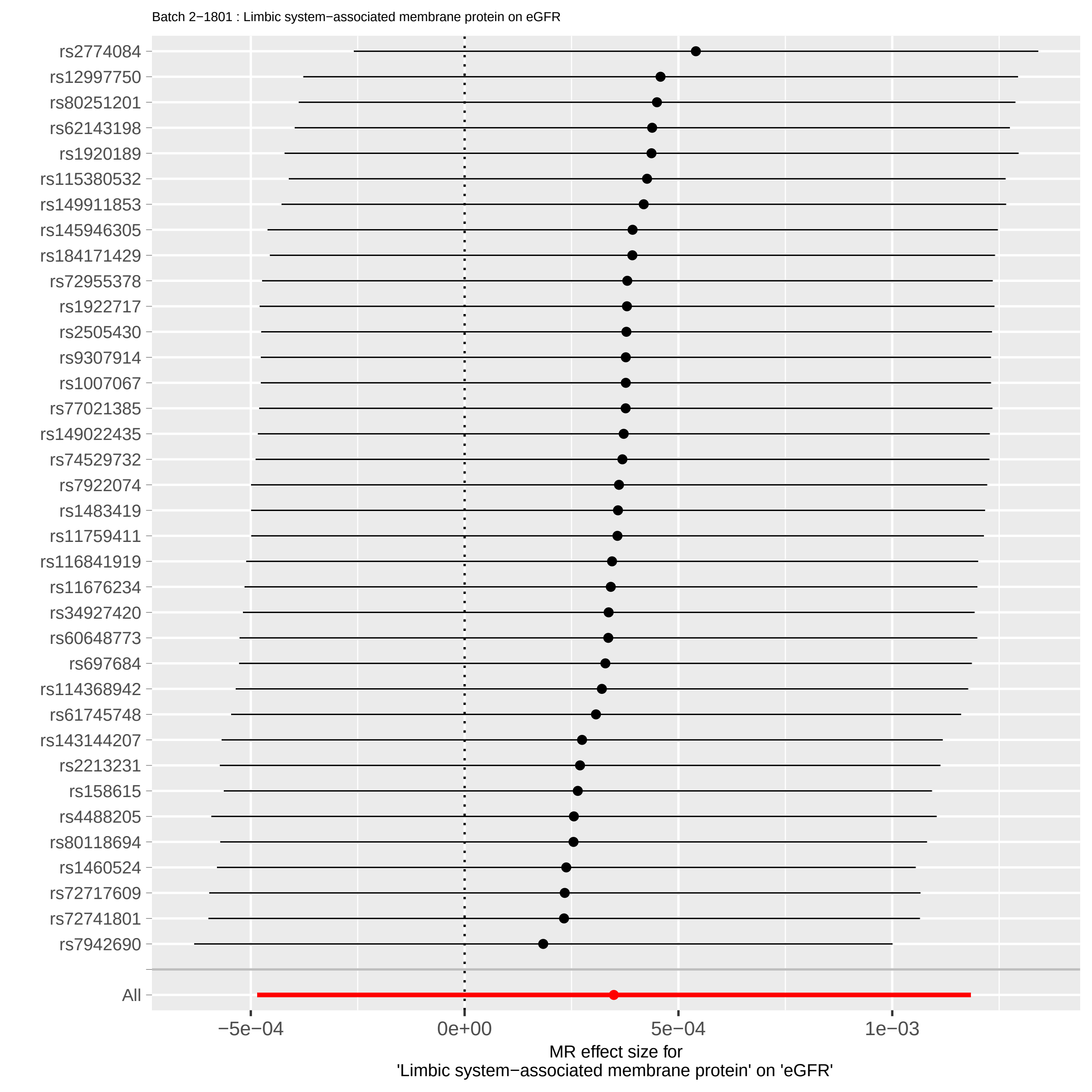

Batch 2−1801 : Limbic system−associated membrane protein on eGFR
rs2774084
rs12997750
rs80251201
rs62143198
rs1920189
rs115380532
rs149911853
rs145946305
rs184171429
rs72955378
rs1922717
rs2505430
rs9307914
rs1007067
rs77021385
rs149022435
rs74529732
rs7922074
rs1483419
rs11759411
rs116841919
rs11676234
rs34927420
rs60648773
rs697684
rs114368942
rs61745748
rs143144207
rs2213231
rs158615
rs4488205
rs80118694
rs1460524
rs72717609
rs72741801
rs7942690
All
−5e−04
0e+00
5e−04
1e−03
MR effect size for
'Limbic system−associated membrane protein' on 'eGFR'

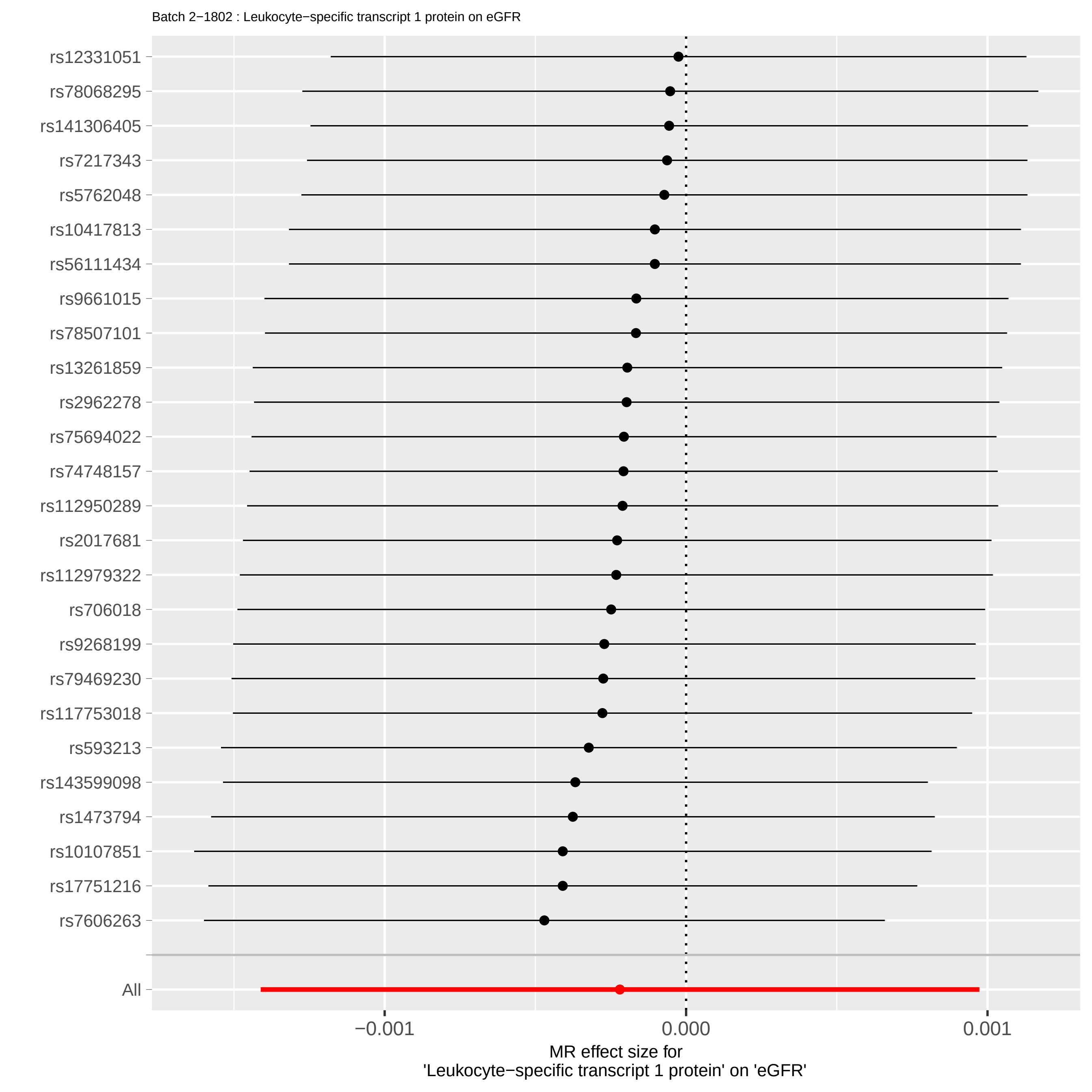

Batch 2−1802 : Leukocyte−specific transcript 1 protein on eGFR
rs12331051
rs78068295
rs141306405
rs7217343
rs5762048
rs10417813
rs56111434
rs9661015
rs78507101
rs13261859
rs2962278
rs75694022
rs74748157
rs112950289
rs2017681
rs112979322
rs706018
rs9268199
rs79469230
rs117753018
rs593213
rs143599098
rs1473794
rs10107851
rs17751216
rs7606263
All
−0.001
0.000
0.001
MR effect size for
'Leukocyte−specific transcript 1 protein' on 'eGFR'

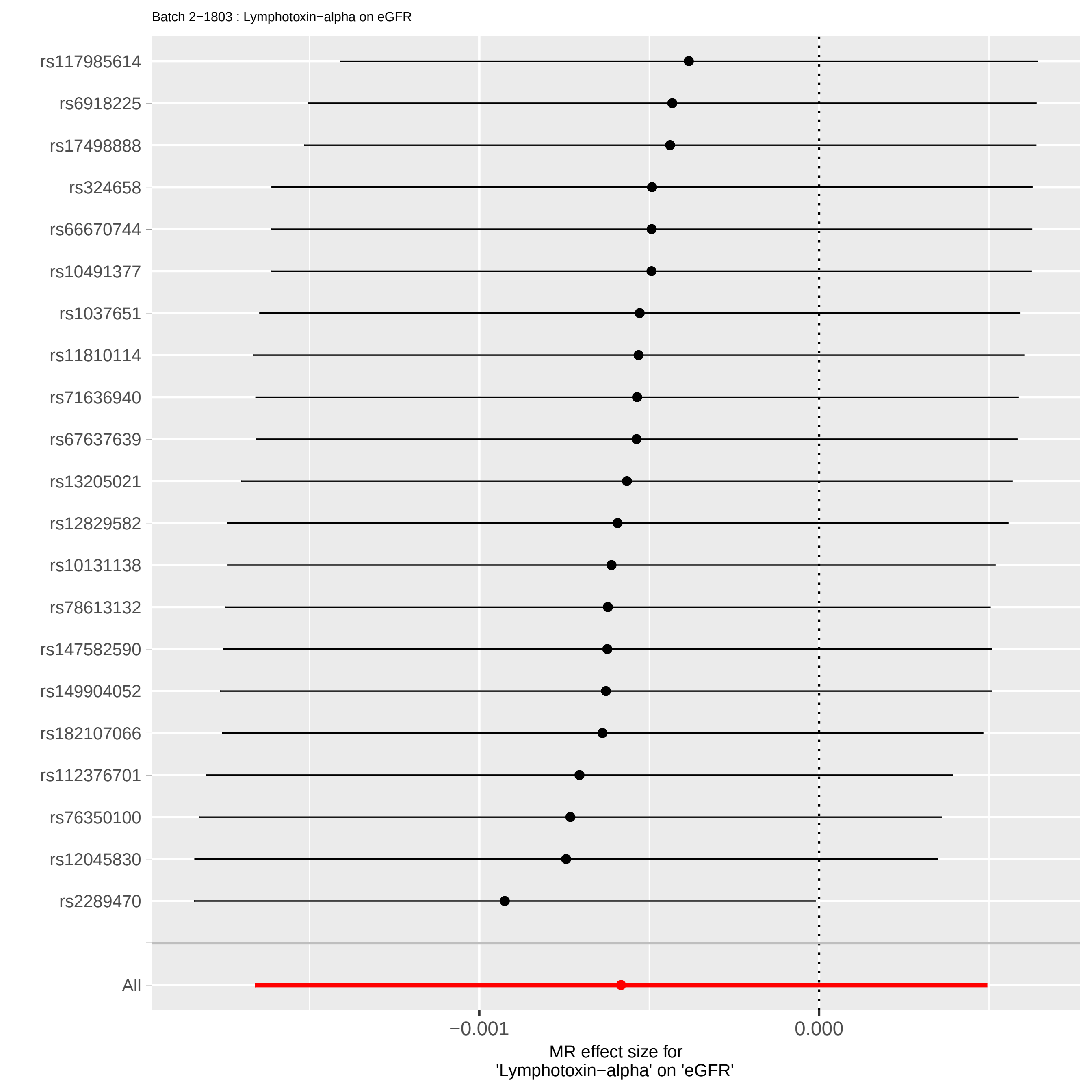
Batch 2−1803 : Lymphotoxin−alpha on eGFR
rs117985614
rs6918225
rs17498888
rs324658
rs66670744
rs10491377
rs1037651
rs11810114
rs71636940
rs67637639
rs13205021
rs12829582
rs10131138
rs78613132
rs147582590
rs149904052
rs182107066
rs112376701
rs76350100
rs12045830
rs2289470
All
−0.001
0.000
MR effect size for
'Lymphotoxin−alpha' on 'eGFR'

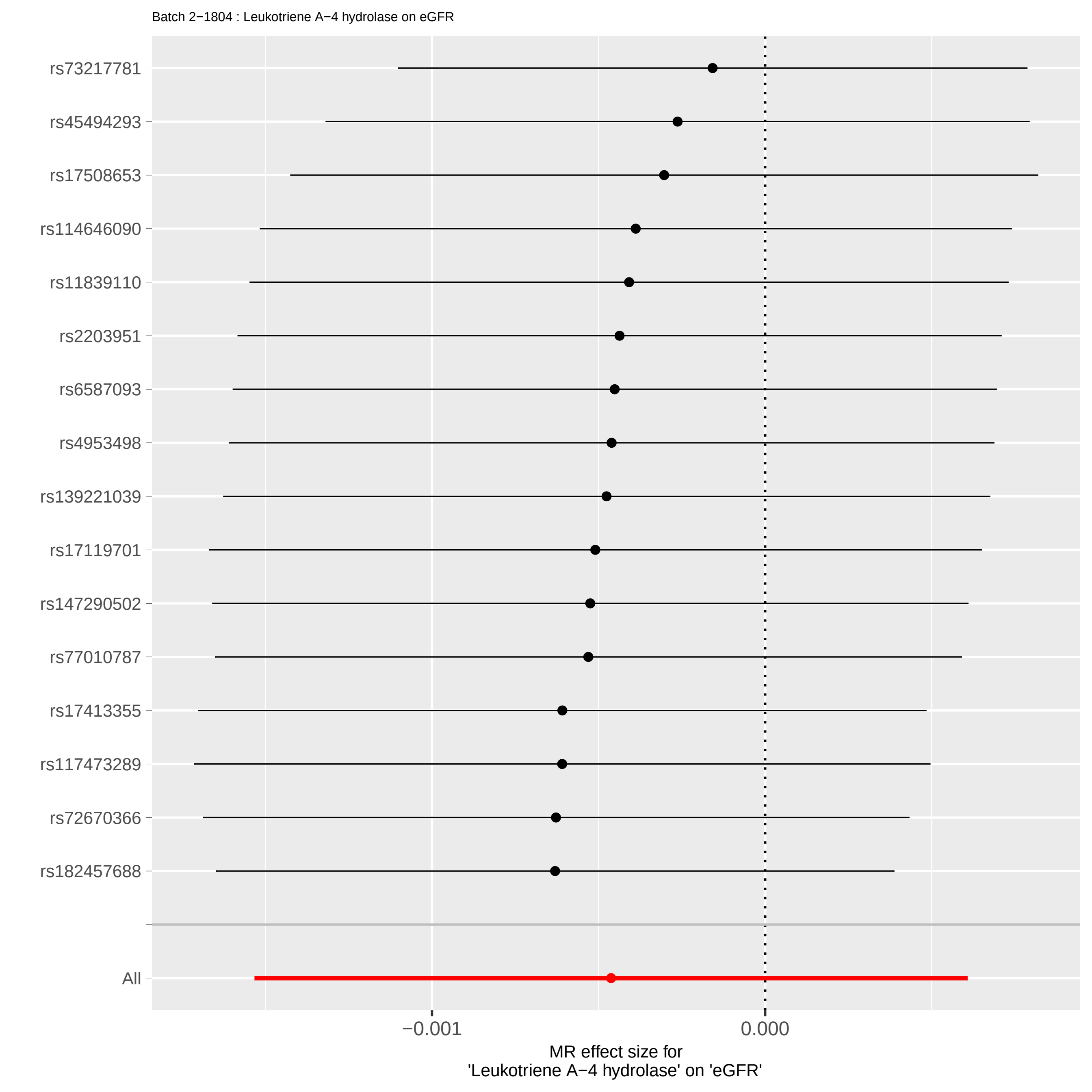
Batch 2−1804 : Leukotriene A−4 hydrolase on eGFR
rs73217781
rs45494293
rs17508653
rs114646090
rs11839110
rs2203951
rs6587093
rs4953498
rs139221039
rs17119701
rs147290502
rs77010787
rs17413355
rs117473289
rs72670366
rs182457688
All
−0.001
0.000
MR effect size for
'Leukotriene A−4 hydrolase' on 'eGFR'

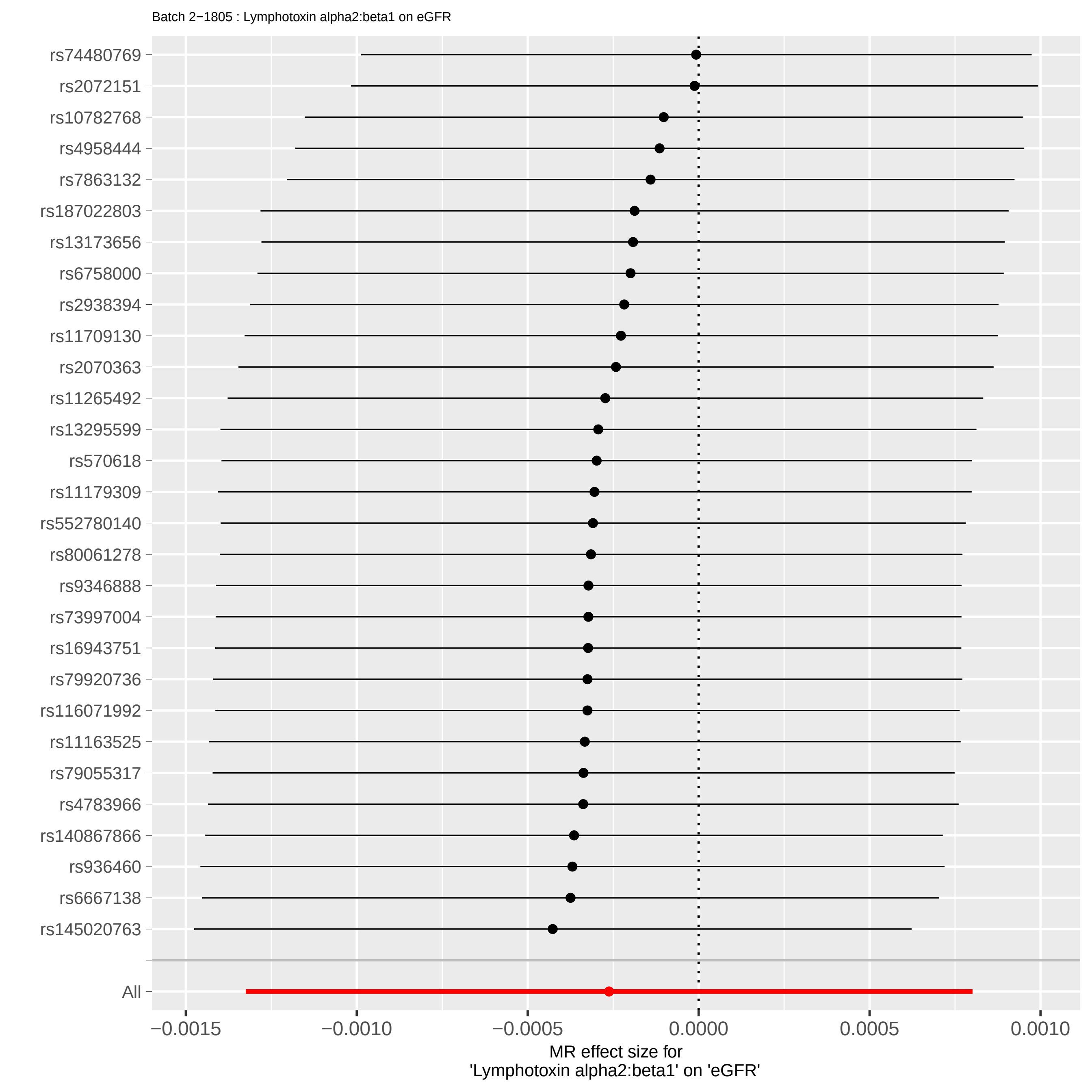

Batch 2−1805 : Lymphotoxin alpha2:beta1 on eGFR
rs74480769
rs2072151
rs10782768
rs4958444
rs7863132
rs187022803
rs13173656
rs6758000
rs2938394
rs11709130
rs2070363
rs11265492
rs13295599
rs570618
rs11179309
rs552780140
rs80061278
rs9346888
rs73997004
rs16943751
rs79920736
rs116071992
rs11163525
rs79055317
rs4783966
rs140867866
rs936460
rs6667138
rs145020763
All
−0.0015
−0.0010
−0.0005
0.0000
0.0005
0.0010
MR effect size for
'Lymphotoxin alpha2:beta1' on 'eGFR'

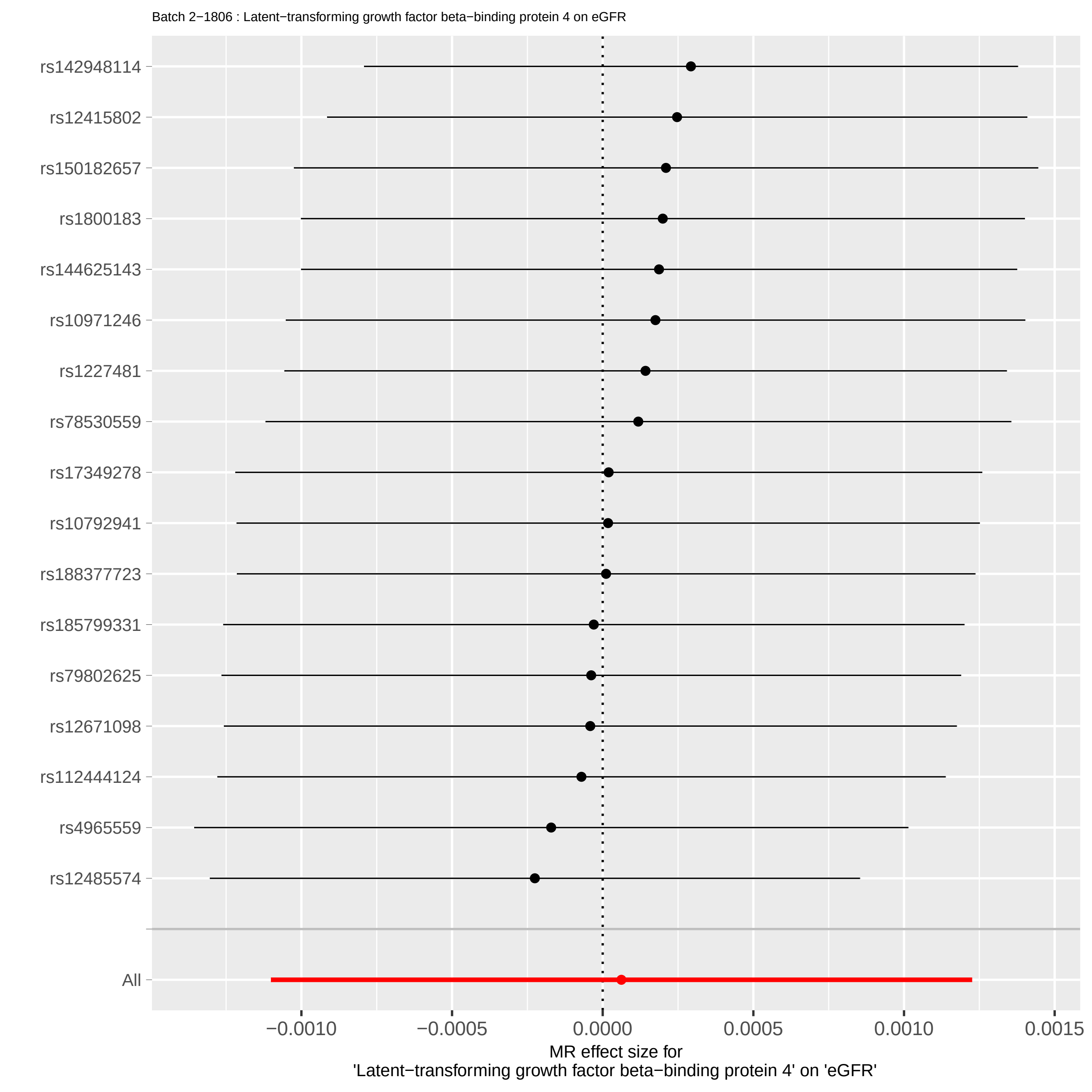

Batch 2−1806 : Latent−transforming growth factor beta−binding protein 4 on eGFR
rs142948114
rs12415802
rs150182657
rs1800183
rs144625143
rs10971246
rs1227481
rs78530559
rs17349278
rs10792941
rs188377723
rs185799331
rs79802625
rs12671098
rs112444124
rs4965559
rs12485574
All
−0.0010
−0.0005
0.0000
0.0005
0.0010
0.0015
MR effect size for
'Latent−transforming growth factor beta−binding protein 4' on 'eGFR'

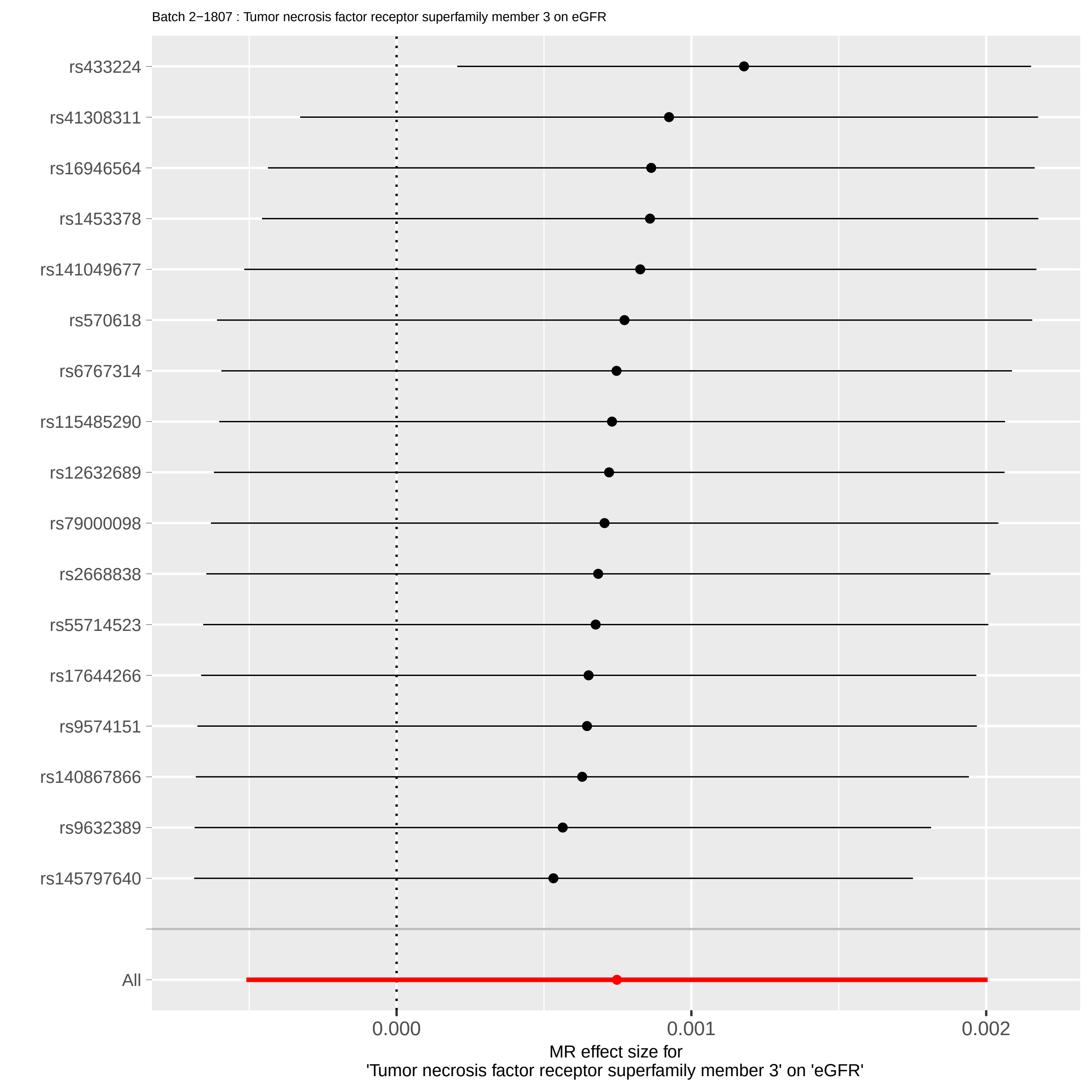
Batch 2−1807 : Tumor necrosis factor receptor superfamily member 3 on eGFR
rs433224
rs41308311
rs16946564
rs1453378
rs141049677
rs570618
rs6767314
rs115485290
rs12632689
rs79000098
rs2668838
rs55714523
rs17644266
rs9574151
rs140867866
rs9632389
rs145797640
All
0.000
0.001
0.002
MR effect size for
'Tumor necrosis factor receptor superfamily member 3' on 'eGFR'

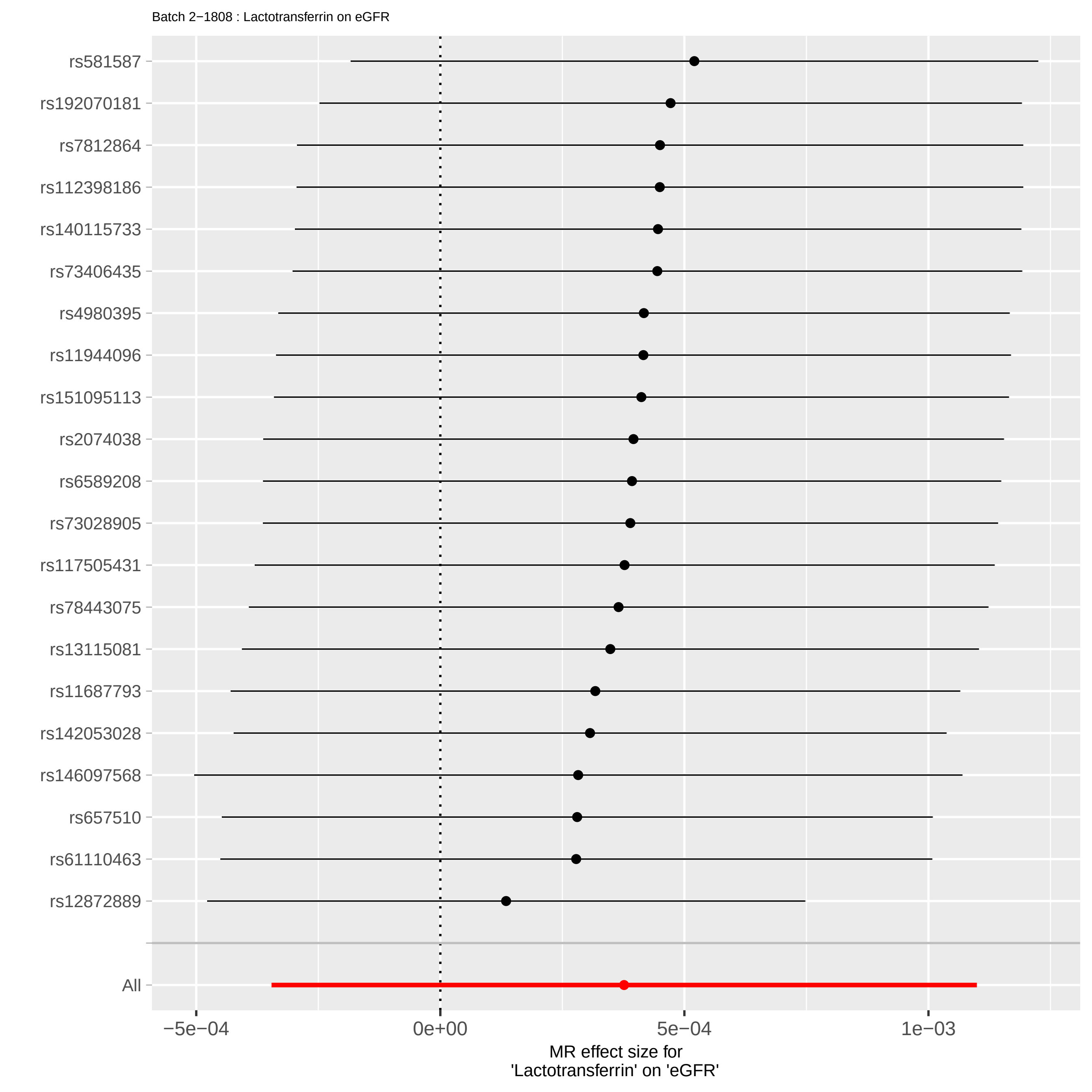

Batch 2−1808 : Lactotransferrin on eGFR
rs581587
rs192070181
rs7812864
rs112398186
rs140115733
rs73406435
rs4980395
rs11944096
rs151095113
rs2074038
rs6589208
rs73028905
rs117505431
rs78443075
rs13115081
rs11687793
rs142053028
rs146097568
rs657510
rs61110463
rs12872889
All
−5e−04
0e+00
5e−04
1e−03
MR effect size for
'Lactotransferrin' on 'eGFR'

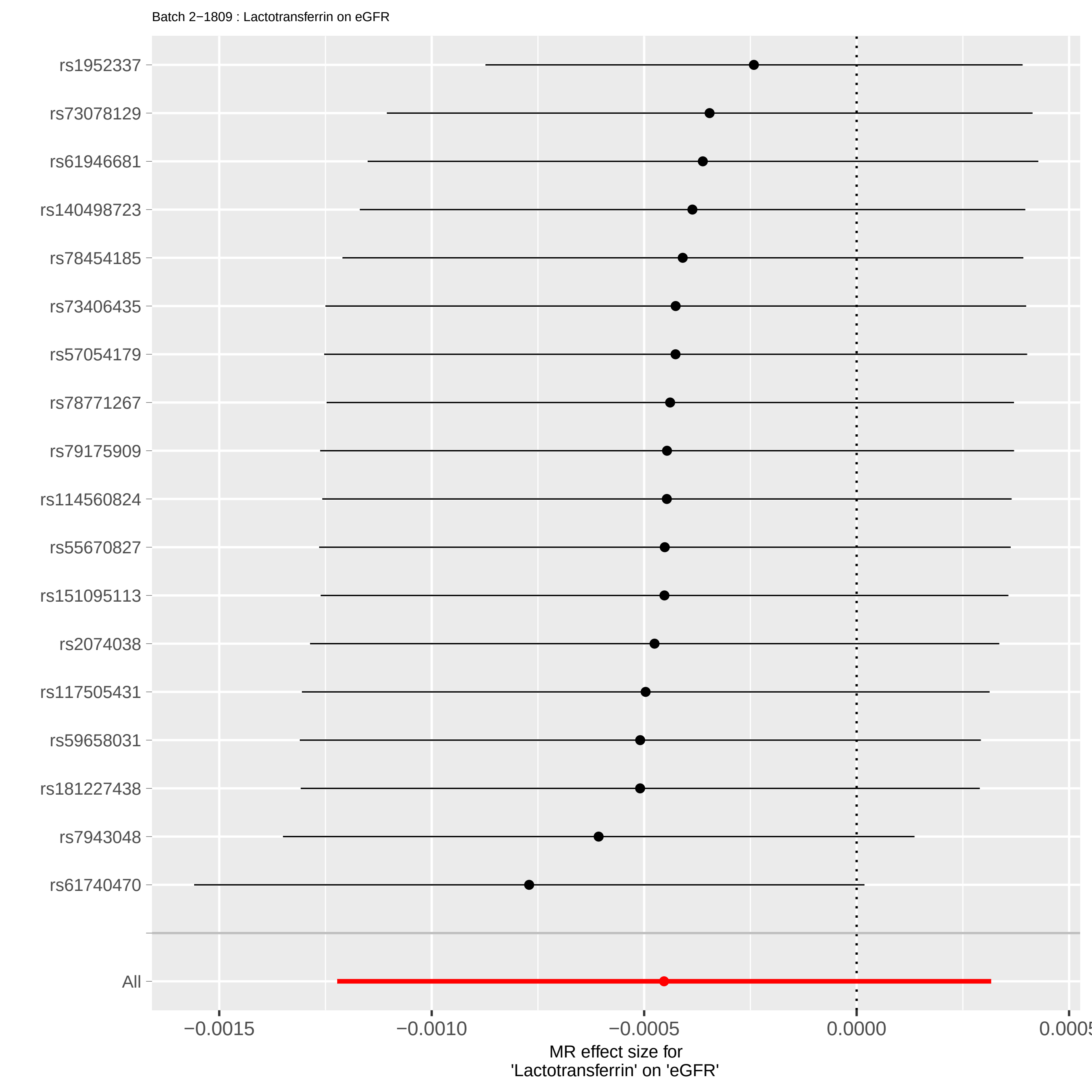

Batch 2−1809 : Lactotransferrin on eGFR
rs1952337
rs73078129
rs61946681
rs140498723
rs78454185
rs73406435
rs57054179
rs78771267
rs79175909
rs114560824
rs55670827
rs151095113
rs2074038
rs117505431
rs59658031
rs181227438
rs7943048
rs61740470
All
−0.0015
−0.0010
−0.0005
0.0000
MR effect size for
'Lactotransferrin' on 'eGFR'

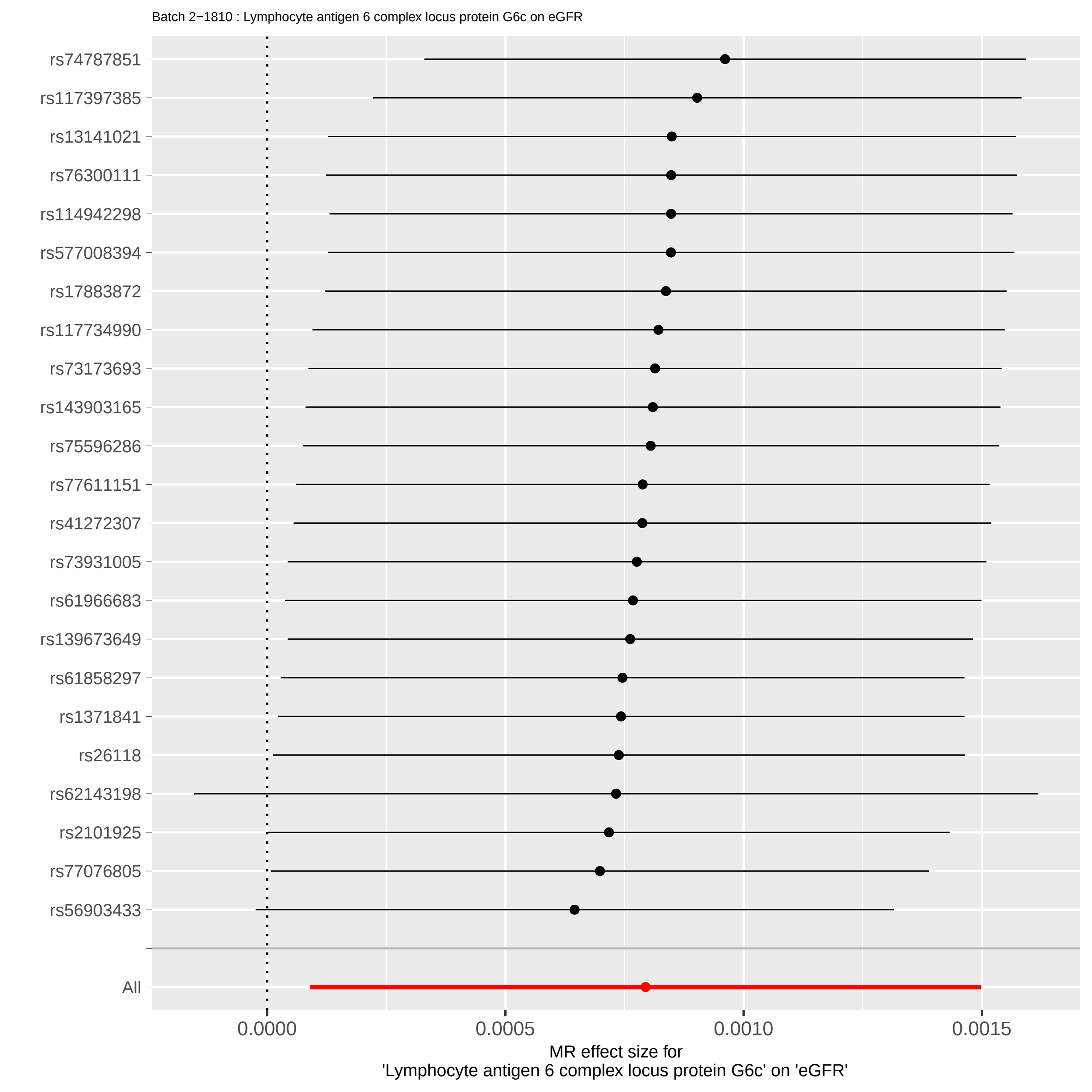

Batch 2−1810 : Lymphocyte antigen 6 complex locus protein G6c on eGFR
rs74787851
rs117397385
rs13141021
rs76300111
rs114942298
rs577008394
rs17883872
rs117734990
rs73173693
rs143903165
rs75596286
rs77611151
rs41272307
rs73931005
rs61966683
rs139673649
rs61858297
rs1371841
rs26118
rs62143198
rs2101925
rs77076805
rs56903433
All
0.0000
0.0005
0.0010
0.0015
MR effect size for
'Lymphocyte antigen 6 complex locus protein G6c' on 'eGFR'

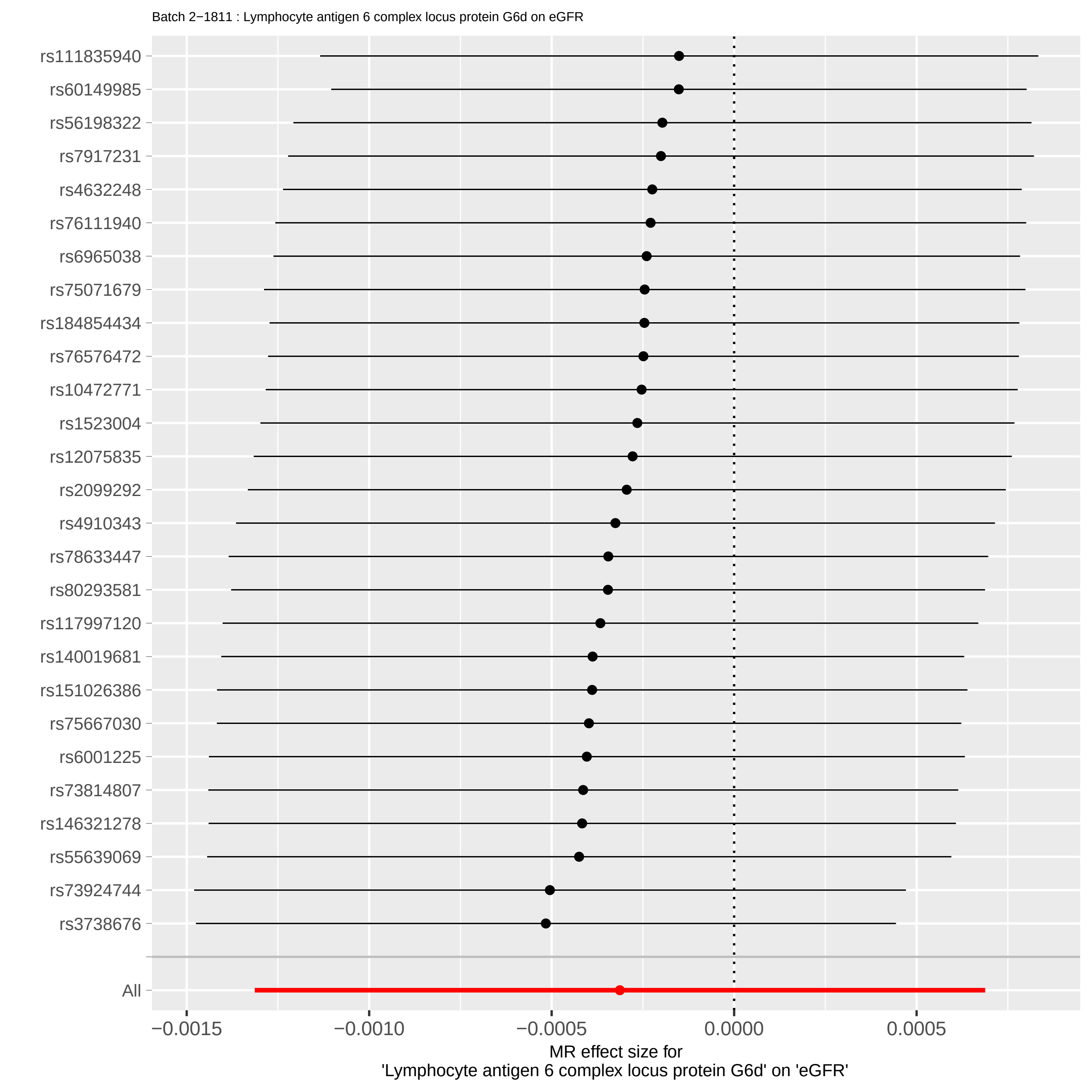

Batch 2−1811 : Lymphocyte antigen 6 complex locus protein G6d on eGFR
rs111835940
rs60149985
rs56198322
rs7917231
rs4632248
rs76111940
rs6965038
rs75071679
rs184854434
rs76576472
rs10472771
rs1523004
rs12075835
rs2099292
rs4910343
rs78633447
rs80293581
rs117997120
rs140019681
rs151026386
rs75667030
rs6001225
rs73814807
rs146321278
rs55639069
rs73924744
rs3738676
All
−0.0015
−0.0010
−0.0005
0.0000
0.0005
MR effect size for
'Lymphocyte antigen 6 complex locus protein G6d' on 'eGFR'

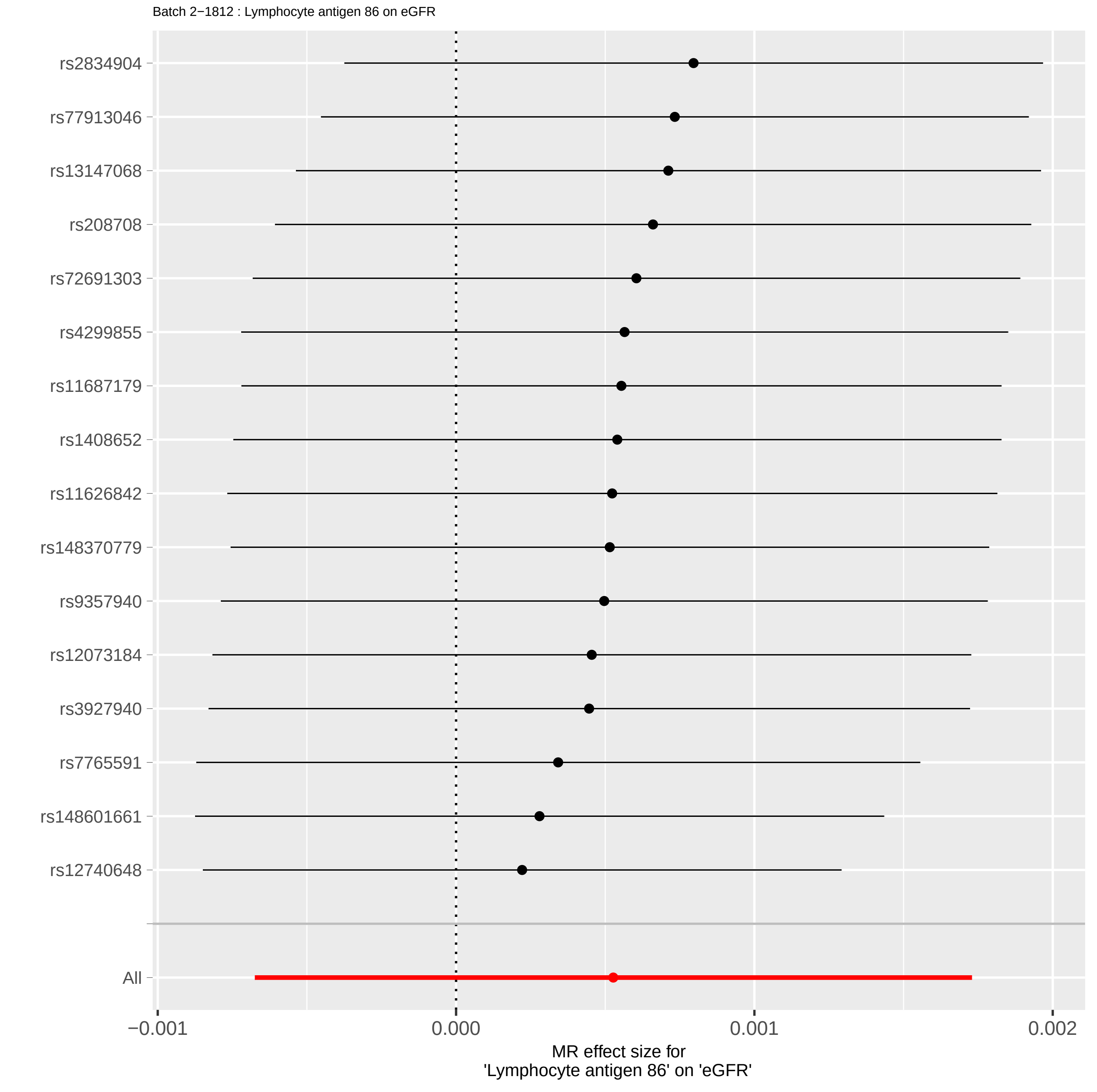
Batch 2−1812 : Lymphocyte antigen 86 on eGFR
rs2834904
rs77913046
rs13147068
rs208708
rs72691303
rs4299855
rs11687179
rs1408652
rs11626842
rs148370779
rs9357940
rs12073184
rs3927940
rs7765591
rs148601661
rs12740648
All
−0.001
0.000
0.001
0.002
MR effect size for
'Lymphocyte antigen 86' on 'eGFR'

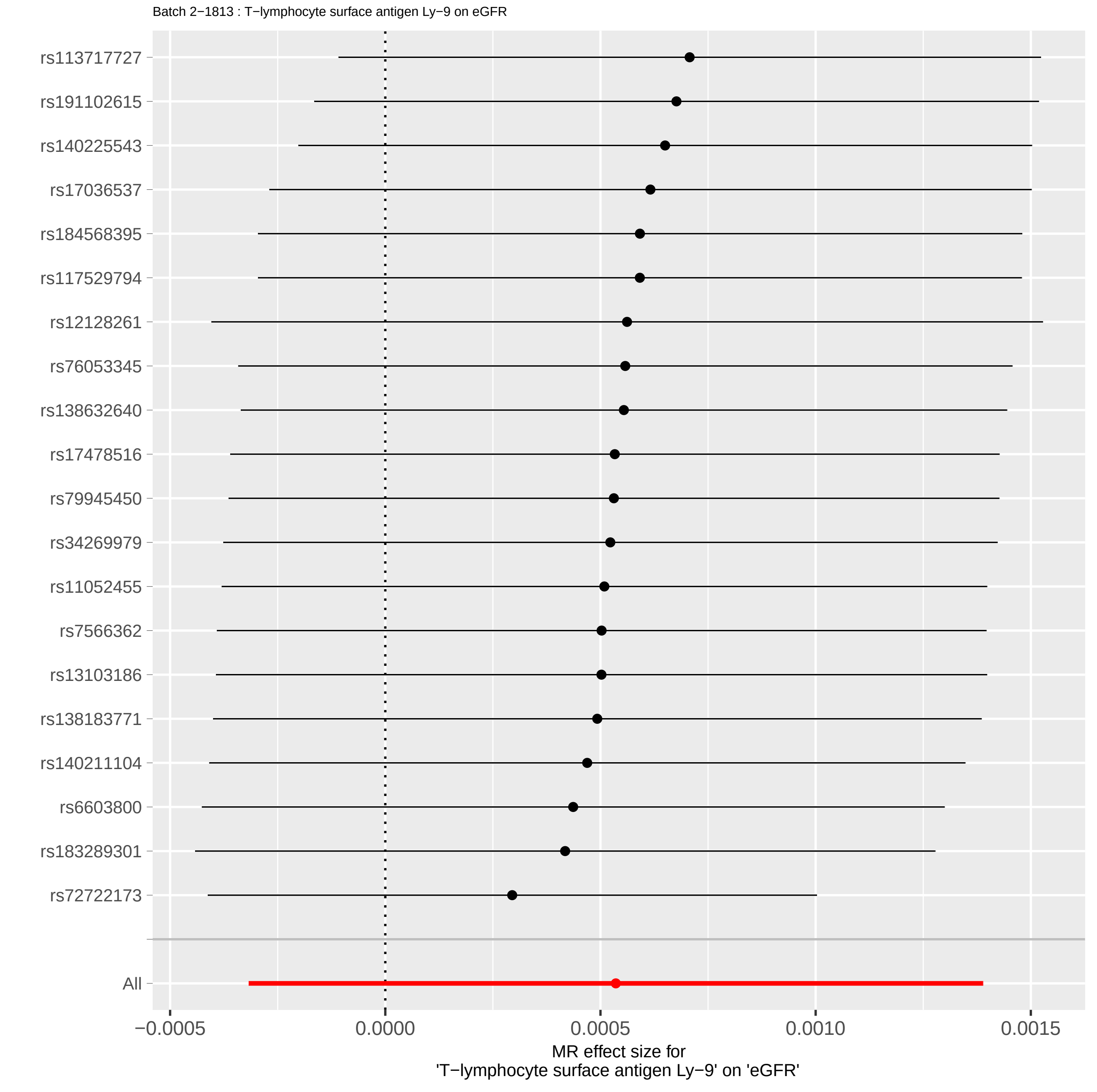
Batch 2−1813 : T−lymphocyte surface antigen Ly−9 on eGFR
rs113717727
rs191102615
rs140225543
rs17036537
rs184568395
rs117529794
rs12128261
rs76053345
rs138632640
rs17478516
rs79945450
rs34269979
rs11052455
rs7566362
rs13103186
rs138183771
rs140211104
rs6603800
rs183289301
rs72722173
All
−0.0005
0.0000
0.0005
0.0010
0.0015
MR effect size for
'T−lymphocyte surface antigen Ly−9' on 'eGFR'

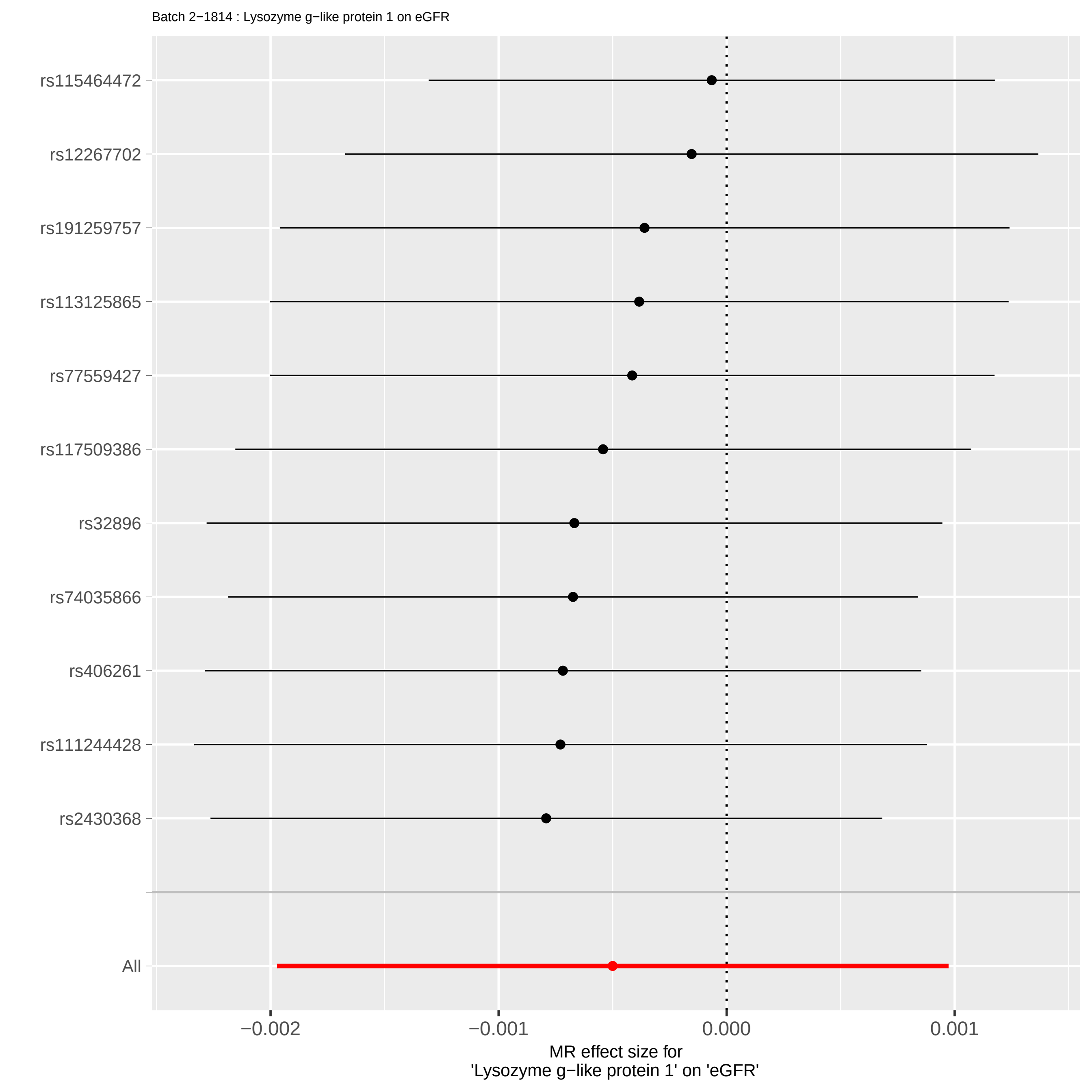

Batch 2−1814 : Lysozyme g−like protein 1 on eGFR
rs115464472
rs12267702
rs191259757
rs113125865
rs77559427
rs117509386
rs32896
rs74035866
rs406261
rs111244428
rs2430368
All
−0.002
−0.001
0.000
0.001
MR effect size for
'Lysozyme g−like protein 1' on 'eGFR'

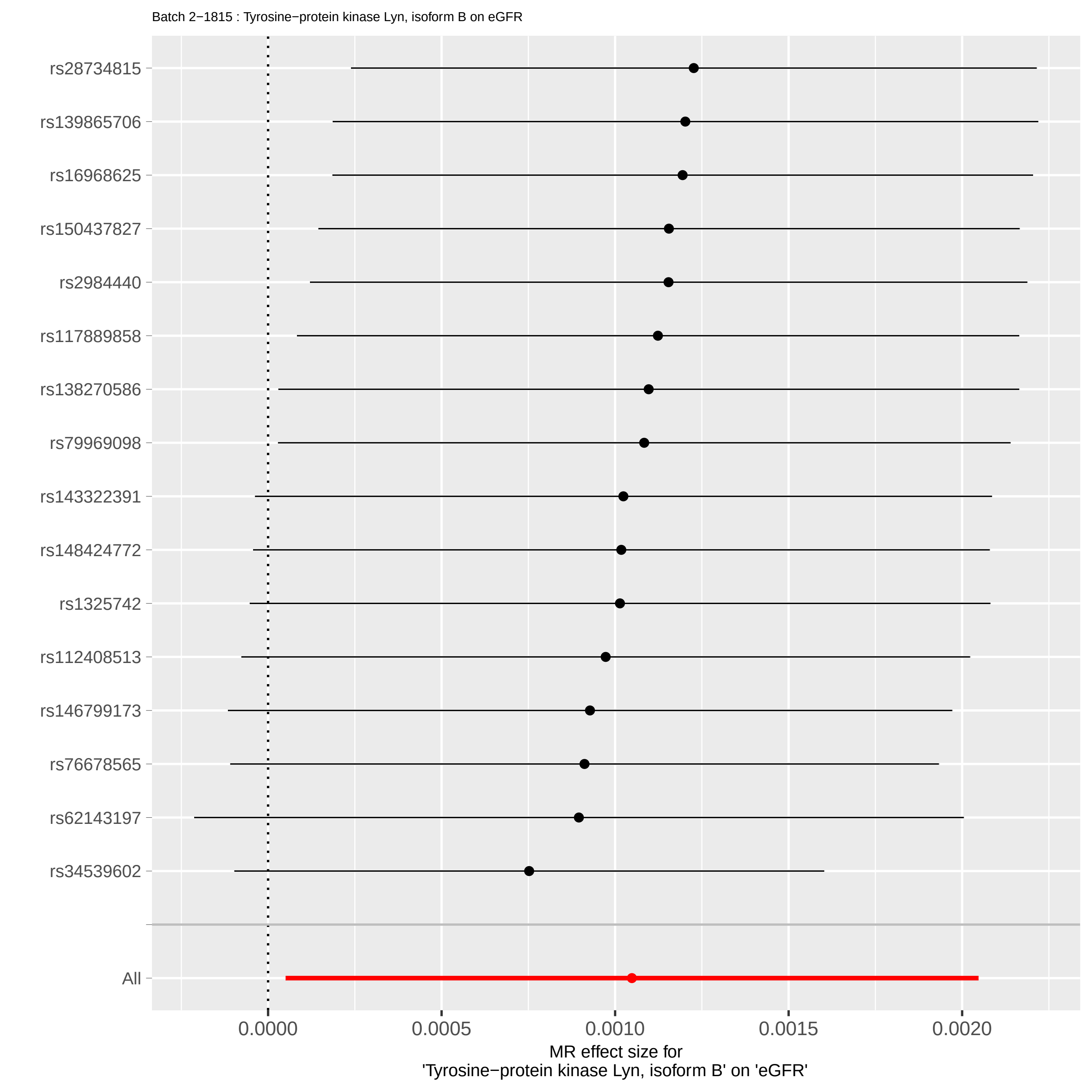

Batch 2−1815 : Tyrosine−protein kinase Lyn, isoform B on eGFR
rs28734815
rs139865706
rs16968625
rs150437827
rs2984440
rs117889858
rs138270586
rs79969098
rs143322391
rs148424772
rs1325742
rs112408513
rs146799173
rs76678565
rs62143197
rs34539602
All
0.0000
0.0005
0.0010
0.0015
0.0020
MR effect size for
'Tyrosine−protein kinase Lyn, isoform B' on 'eGFR'

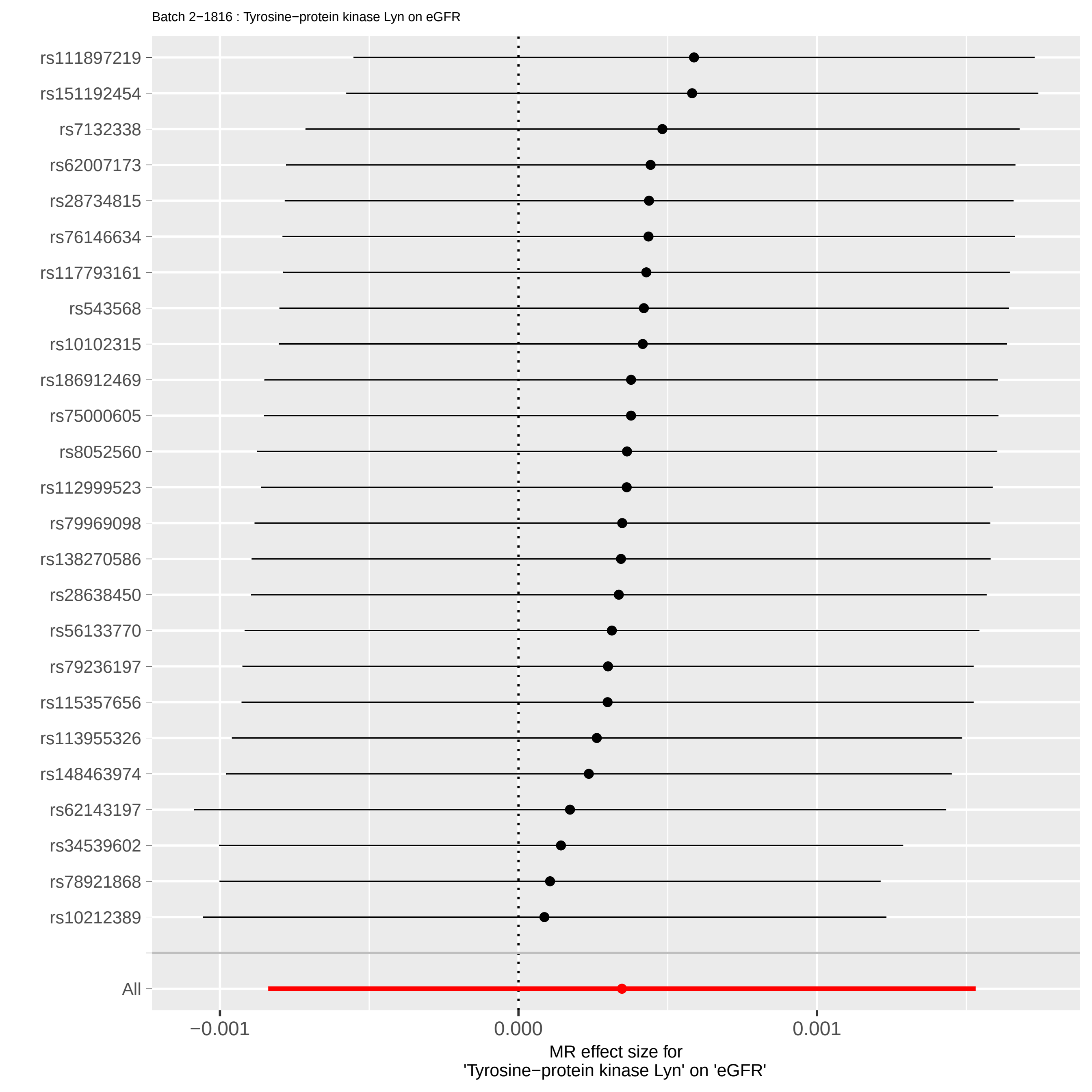

Batch 2−1816 : Tyrosine−protein kinase Lyn on eGFR
rs111897219
rs151192454
rs7132338
rs62007173
rs28734815
rs76146634
rs117793161
rs543568
rs10102315
rs186912469
rs75000605
rs8052560
rs112999523
rs79969098
rs138270586
rs28638450
rs56133770
rs79236197
rs115357656
rs113955326
rs148463974
rs62143197
rs34539602
rs78921868
rs10212389
All
−0.001
0.000
0.001
MR effect size for
'Tyrosine−protein kinase Lyn' on 'eGFR'

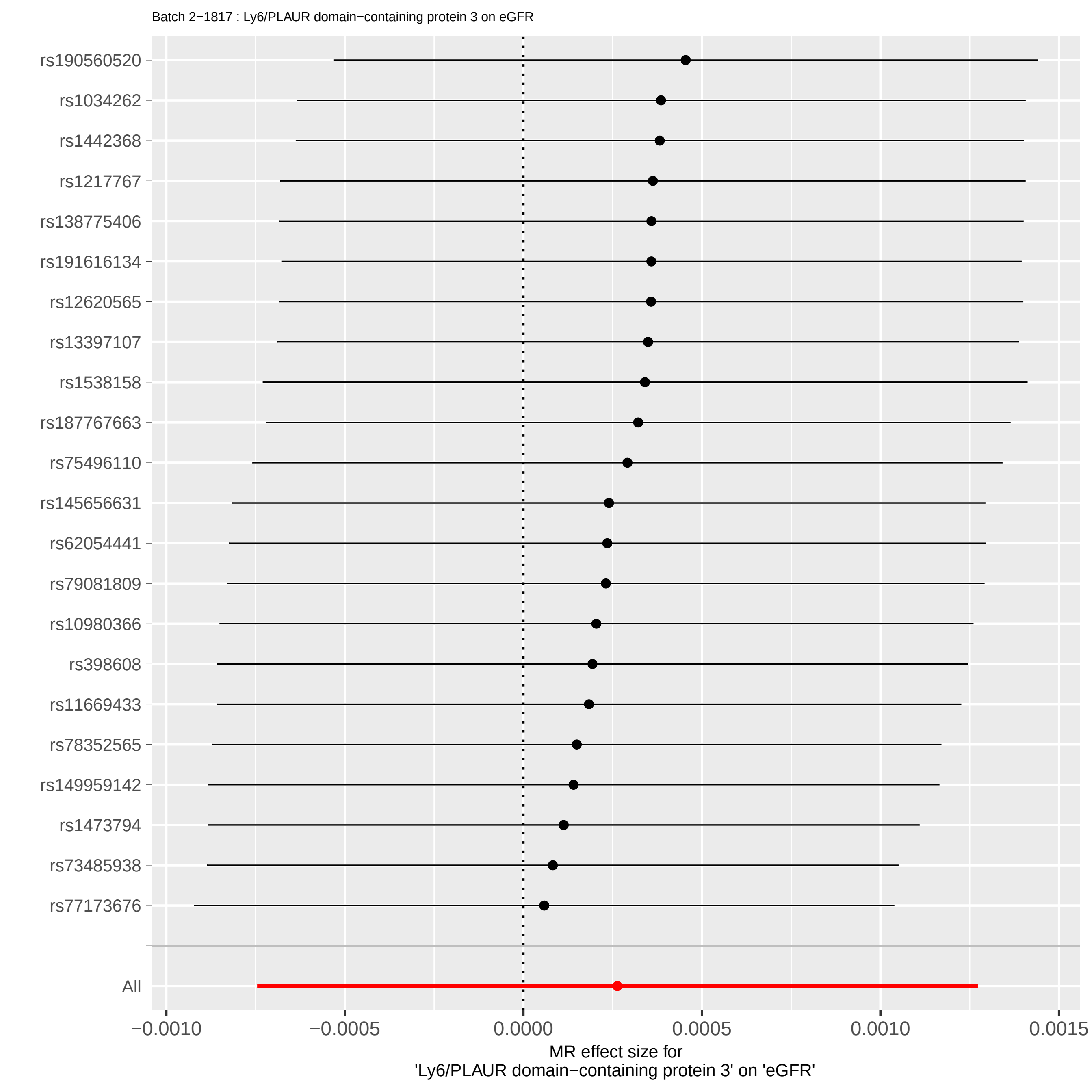

Batch 2−1817 : Ly6/PLAUR domain−containing protein 3 on eGFR
rs190560520
rs1034262
rs1442368
rs1217767
rs138775406
rs191616134
rs12620565
rs13397107
rs1538158
rs187767663
rs75496110
rs145656631
rs62054441
rs79081809
rs10980366
rs398608
rs11669433
rs78352565
rs149959142
rs1473794
rs73485938
rs77173676
All
−0.0010
−0.0005
0.0000
0.0005
0.0010
0.0015
MR effect size for
'Ly6/PLAUR domain−containing protein 3' on 'eGFR'

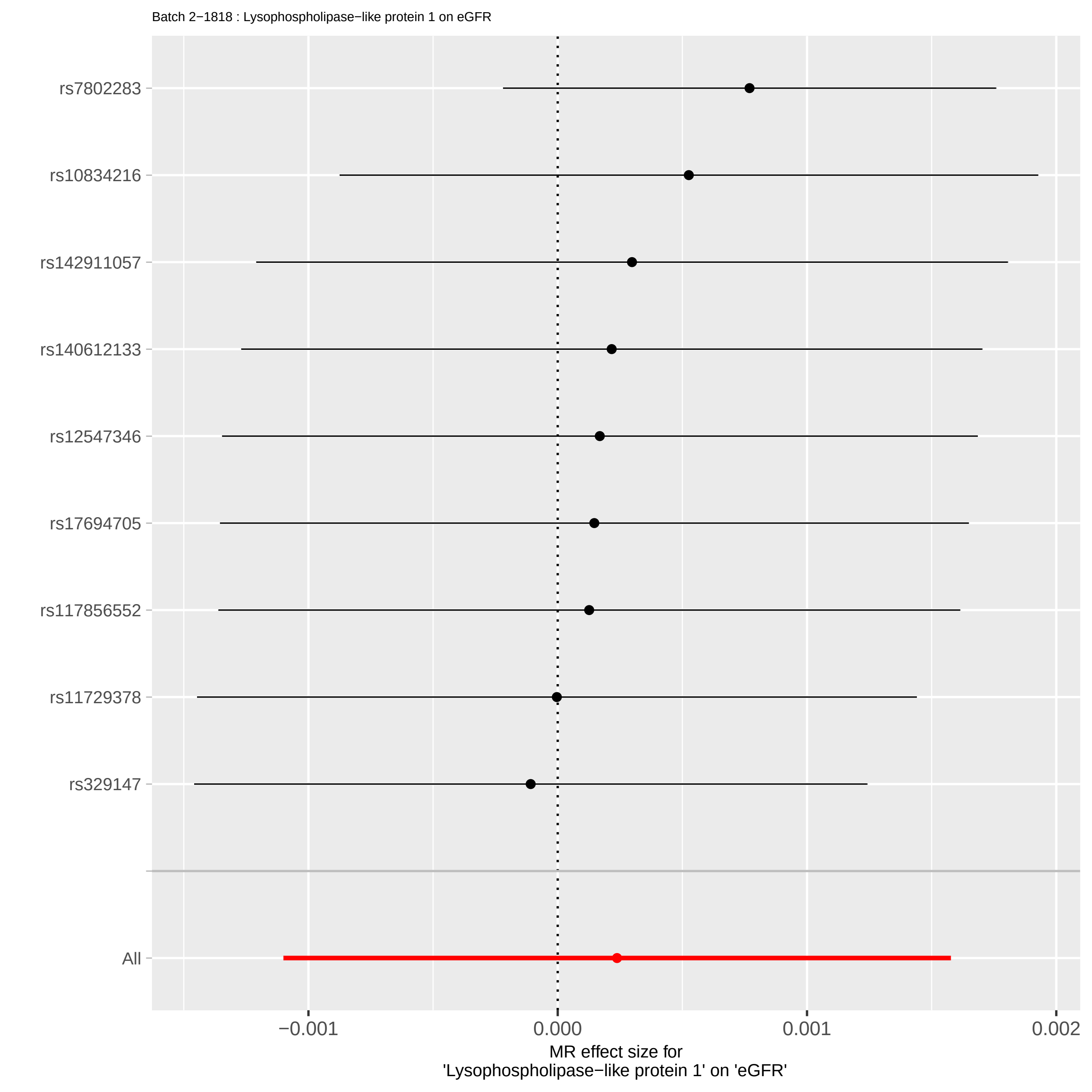

Batch 2−1818 : Lysophospholipase−like protein 1 on eGFR
rs7802283
rs10834216
rs142911057
rs140612133
rs12547346
rs17694705
rs117856552
rs11729378
rs329147
All
−0.001
0.000
0.001
0.002
MR effect size for
'Lysophospholipase−like protein 1' on 'eGFR'

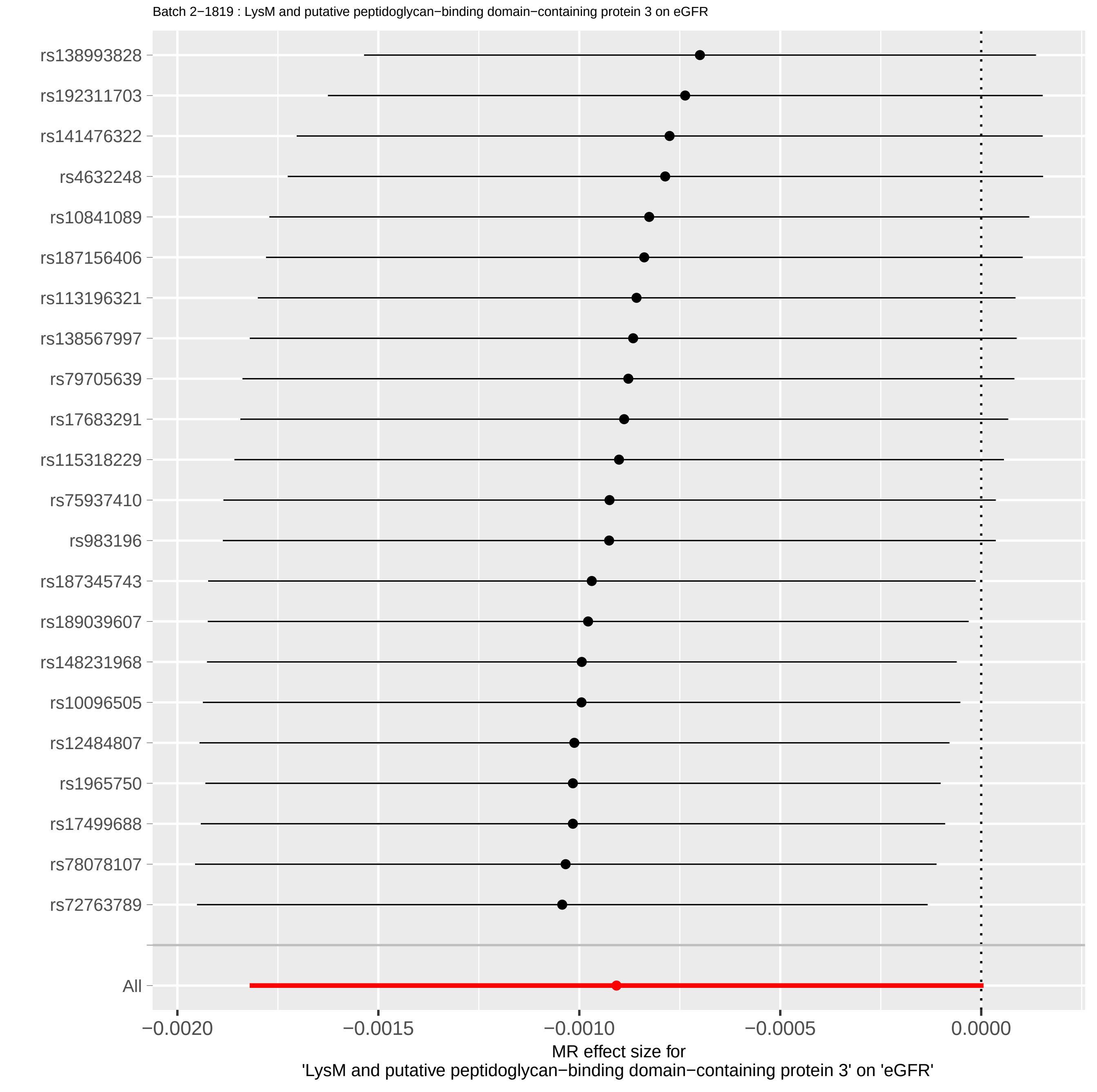
Batch 2−1819 : LysM and putative peptidoglycan−binding domain−containing protein 3 on eGFR
rs138993828
rs192311703
rs141476322
rs4632248
rs10841089
rs187156406
rs113196321
rs138567997
rs79705639
rs17683291
rs115318229
rs75937410
rs983196
rs187345743
rs189039607
rs148231968
rs10096505
rs12484807
rs1965750
rs17499688
rs78078107
rs72763789
All
−0.0020
−0.0015
−0.0010
−0.0005
0.0000
MR effect size for
'LysM and putative peptidoglycan−binding domain−containing protein 3' on 'eGFR'

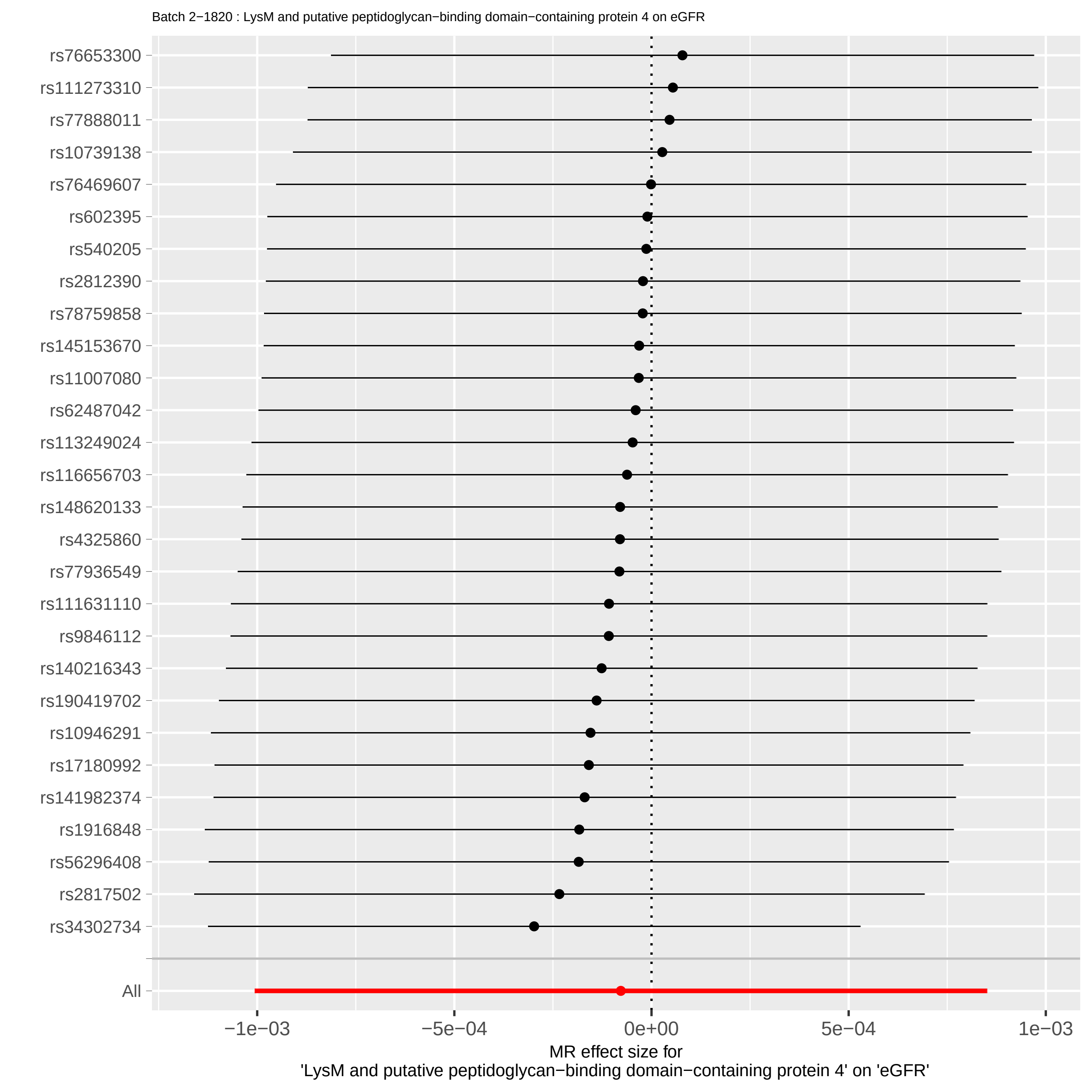
Batch 2−1820 : LysM and putative peptidoglycan−binding domain−containing protein 4 on eGFR
rs76653300
rs111273310
rs77888011
rs10739138
rs76469607
rs602395
rs540205
rs2812390
rs78759858
rs145153670
rs11007080
rs62487042
rs113249024
rs116656703
rs148620133
rs4325860
rs77936549
rs111631110
rs9846112
rs140216343
rs190419702
rs10946291
rs17180992
rs141982374
rs1916848
rs56296408
rs2817502
rs34302734
All
−1e−03
−5e−04
0e+00
5e−04
1e−03
MR effect size for
'LysM and putative peptidoglycan−binding domain−containing protein 4' on 'eGFR'

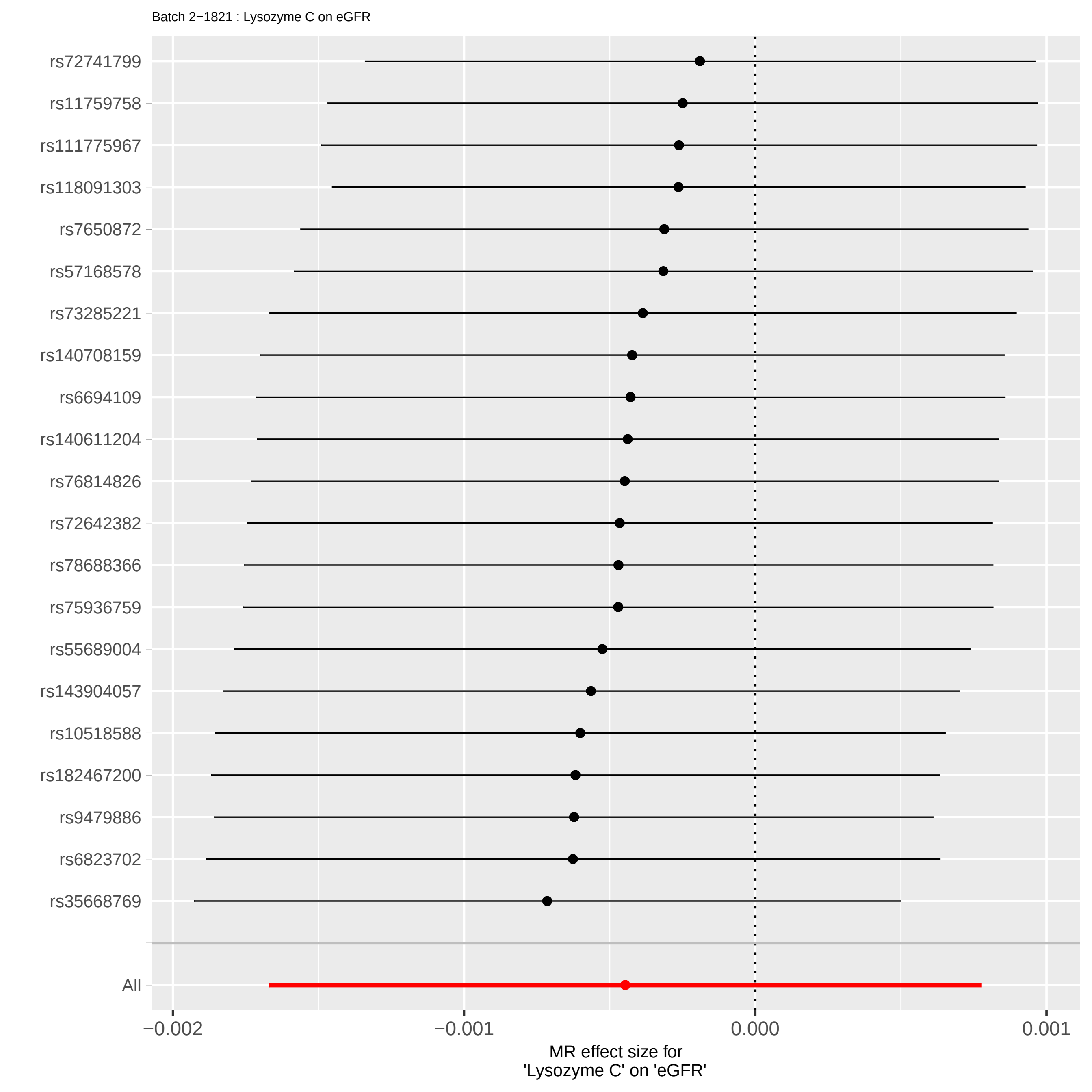

Batch 2−1821 : Lysozyme C on eGFR
rs72741799
rs11759758
rs111775967
rs118091303
rs7650872
rs57168578
rs73285221
rs140708159
rs6694109
rs140611204
rs76814826
rs72642382
rs78688366
rs75936759
rs55689004
rs143904057
rs10518588
rs182467200
rs9479886
rs6823702
rs35668769
All
−0.002
−0.001
0.000
0.001
MR effect size for
'Lysozyme C' on 'eGFR'

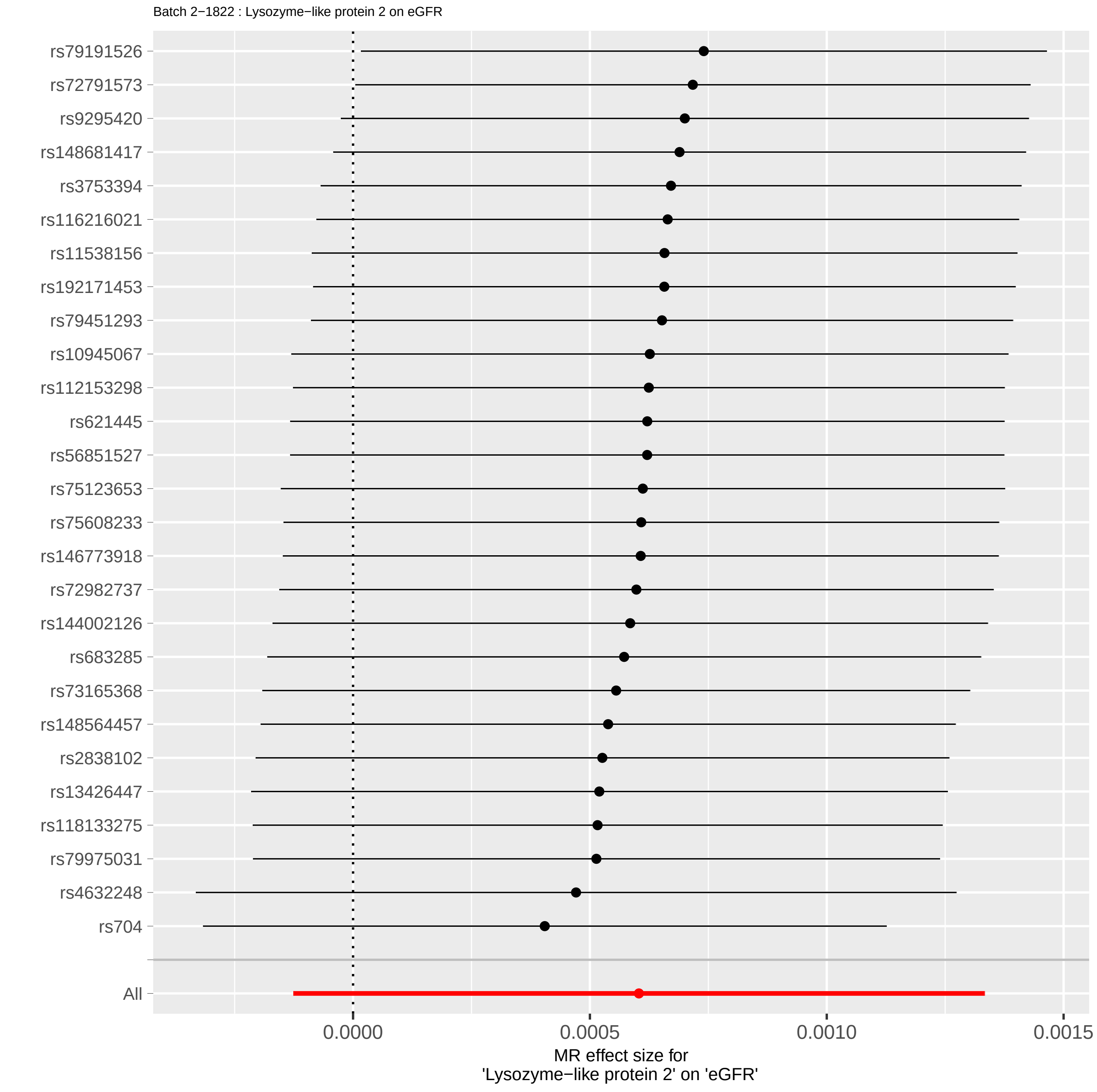

Batch 2−1822 : Lysozyme−like protein 2 on eGFR
rs79191526
rs72791573
rs9295420
rs148681417
rs3753394
rs116216021
rs11538156
rs192171453
rs79451293
rs10945067
rs112153298
rs621445
rs56851527
rs75123653
rs75608233
rs146773918
rs72982737
rs144002126
rs683285
rs73165368
rs148564457
rs2838102
rs13426447
rs118133275
rs79975031
rs4632248
rs704
All
0.0000
0.0005
0.0010
0.0015
MR effect size for
'Lysozyme−like protein 2' on 'eGFR'

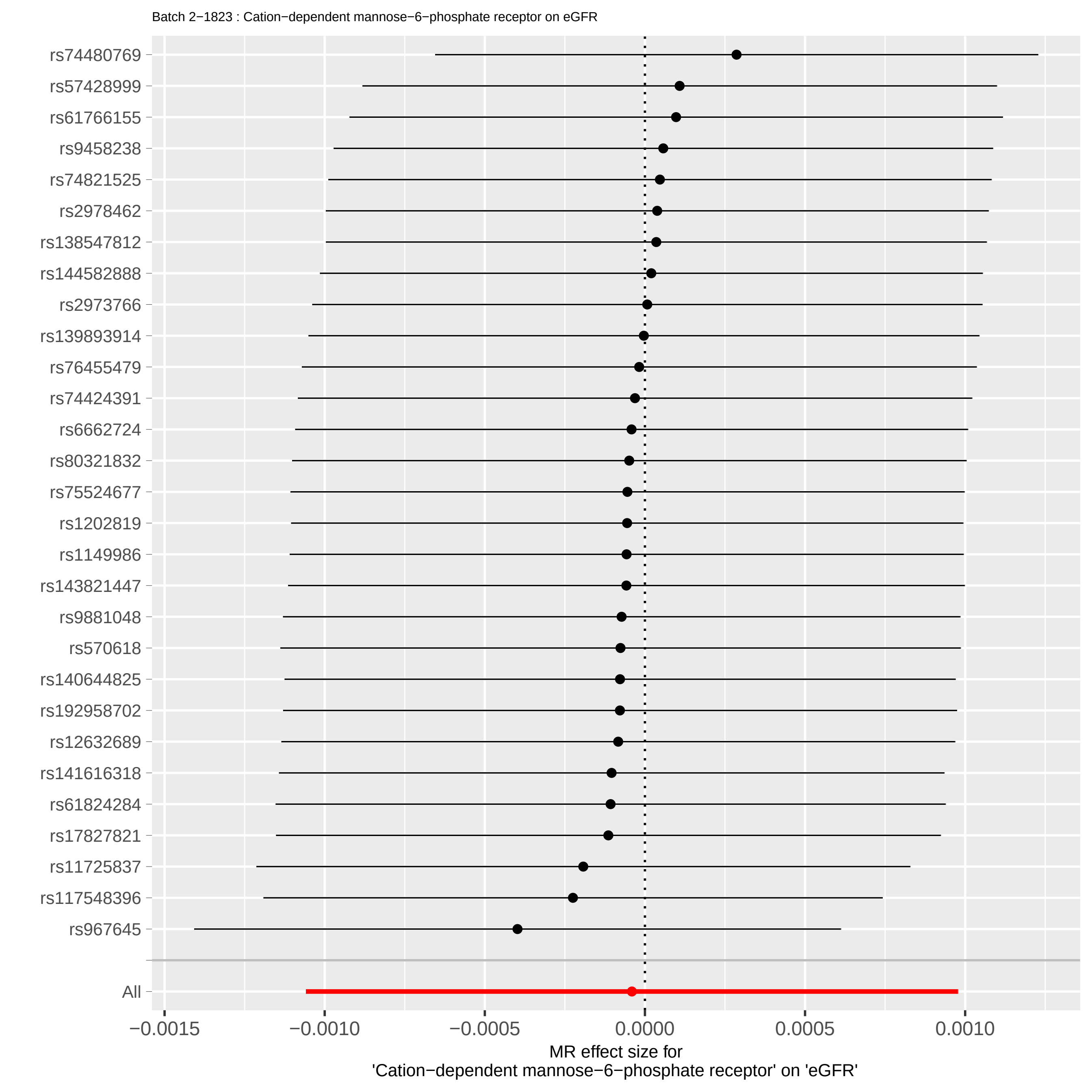

Batch 2−1823 : Cation−dependent mannose−6−phosphate receptor on eGFR
rs74480769
rs57428999
rs61766155
rs9458238
rs74821525
rs2978462
rs138547812
rs144582888
rs2973766
rs139893914
rs76455479
rs74424391
rs6662724
rs80321832
rs75524677
rs1202819
rs1149986
rs143821447
rs9881048
rs570618
rs140644825
rs192958702
rs12632689
rs141616318
rs61824284
rs17827821
rs11725837
rs117548396
rs967645
All
−0.0015
−0.0010
−0.0005
0.0000
0.0005
0.0010
MR effect size for
'Cation−dependent mannose−6−phosphate receptor' on 'eGFR'

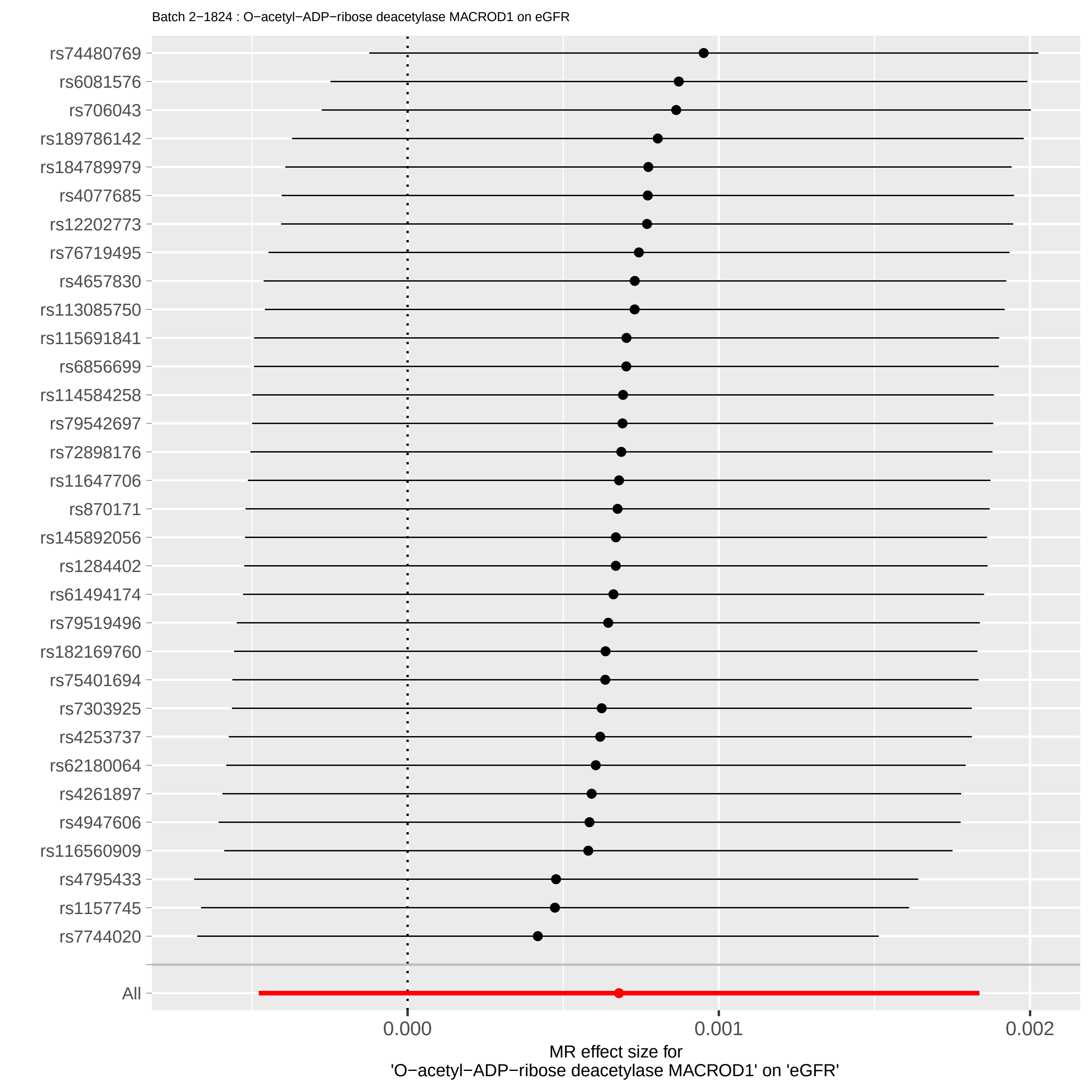

Batch 2−1824 : O−acetyl−ADP−ribose deacetylase MACROD1 on eGFR
rs74480769
rs6081576
rs706043
rs189786142
rs184789979
rs4077685
rs12202773
rs76719495
rs4657830
rs113085750
rs115691841
rs6856699
rs114584258
rs79542697
rs72898176
rs11647706
rs870171
rs145892056
rs1284402
rs61494174
rs79519496
rs182169760
rs75401694
rs7303925
rs4253737
rs62180064
rs4261897
rs4947606
rs116560909
rs4795433
rs1157745
rs7744020
All
0.000
0.001
0.002
MR effect size for
'O−acetyl−ADP−ribose deacetylase MACROD1' on 'eGFR'

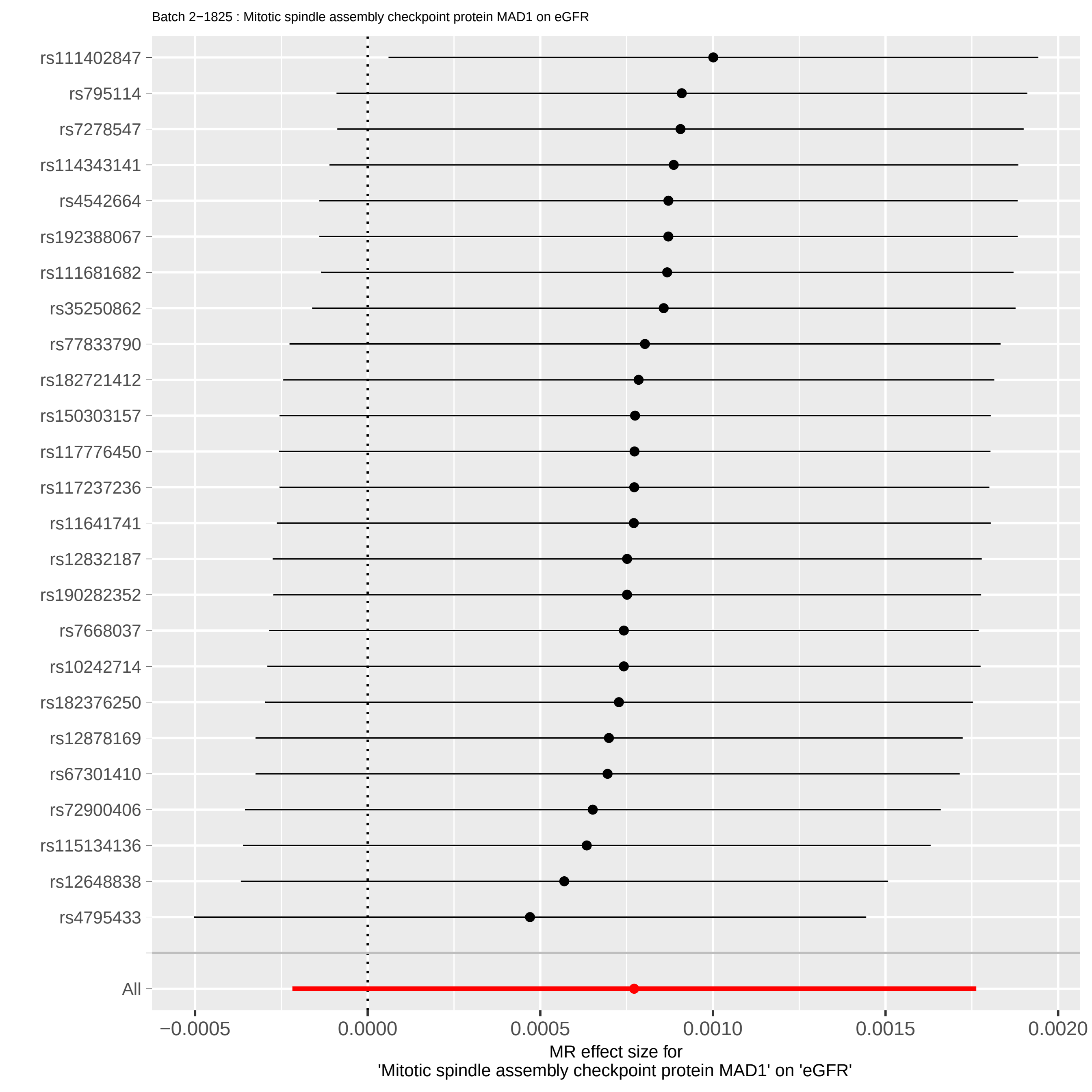

Batch 2−1825 : Mitotic spindle assembly checkpoint protein MAD1 on eGFR
rs111402847
rs795114
rs7278547
rs114343141
rs4542664
rs192388067
rs111681682
rs35250862
rs77833790
rs182721412
rs150303157
rs117776450
rs117237236
rs11641741
rs12832187
rs190282352
rs7668037
rs10242714
rs182376250
rs12878169
rs67301410
rs72900406
rs115134136
rs12648838
rs4795433
All
−0.0005
0.0000
0.0005
0.0010
0.0015
0.0020
MR effect size for
'Mitotic spindle assembly checkpoint protein MAD1' on 'eGFR'

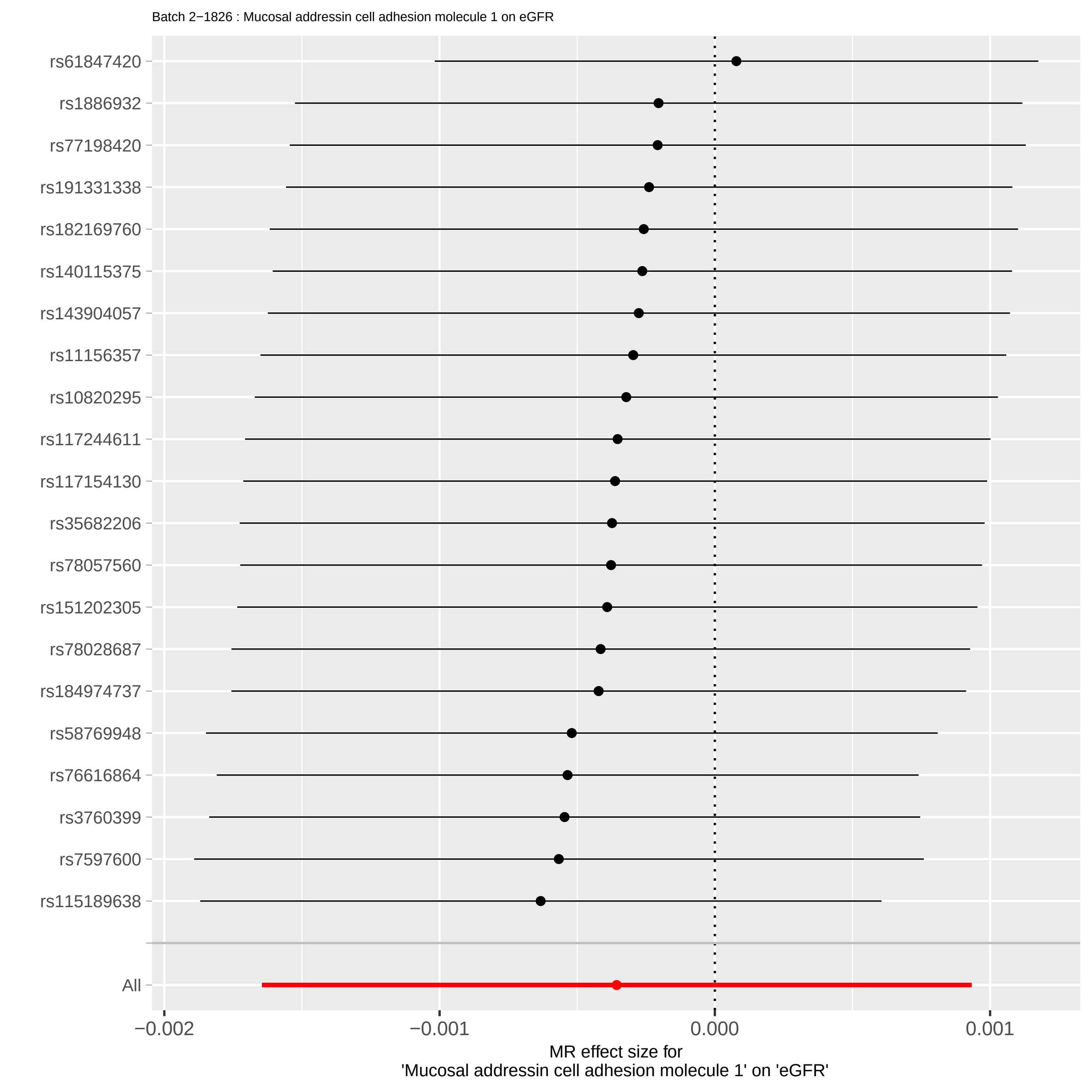

Batch 2−1826 : Mucosal addressin cell adhesion molecule 1 on eGFR
rs61847420
rs1886932
rs77198420
rs191331338
rs182169760
rs140115375
rs143904057
rs11156357
rs10820295
rs117244611
rs117154130
rs35682206
rs78057560
rs151202305
rs78028687
rs184974737
rs58769948
rs76616864
rs3760399
rs7597600
rs115189638
All
−0.002
−0.001
0.000
0.001
MR effect size for
'Mucosal addressin cell adhesion molecule 1' on 'eGFR'

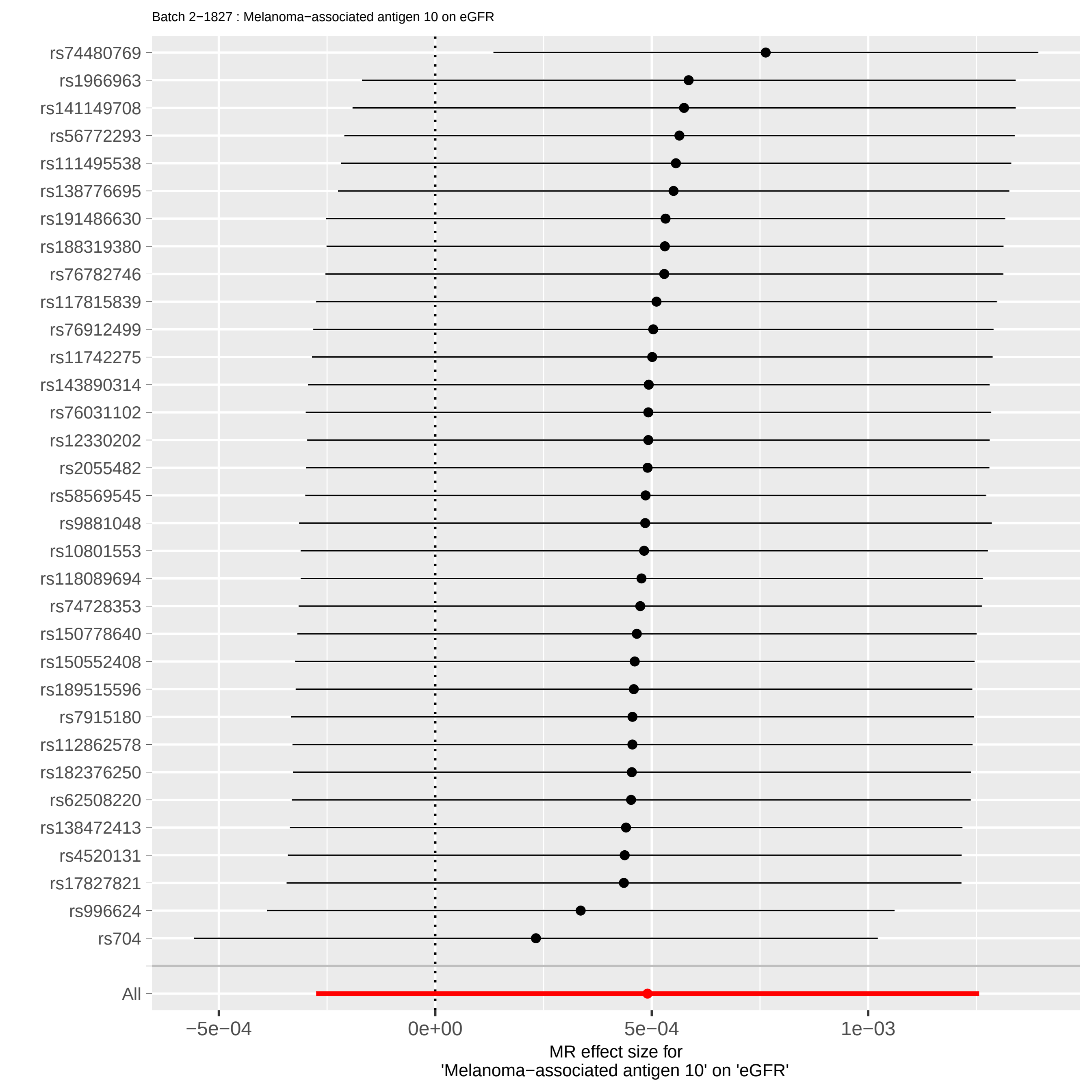

Batch 2−1827 : Melanoma−associated antigen 10 on eGFR
rs74480769
rs1966963
rs141149708
rs56772293
rs111495538
rs138776695
rs191486630
rs188319380
rs76782746
rs117815839
rs76912499
rs11742275
rs143890314
rs76031102
rs12330202
rs2055482
rs58569545
rs9881048
rs10801553
rs118089694
rs74728353
rs150778640
rs150552408
rs189515596
rs7915180
rs112862578
rs182376250
rs62508220
rs138472413
rs4520131
rs17827821
rs996624
rs704
All
−5e−04
0e+00
5e−04
1e−03
MR effect size for
'Melanoma−associated antigen 10' on 'eGFR'

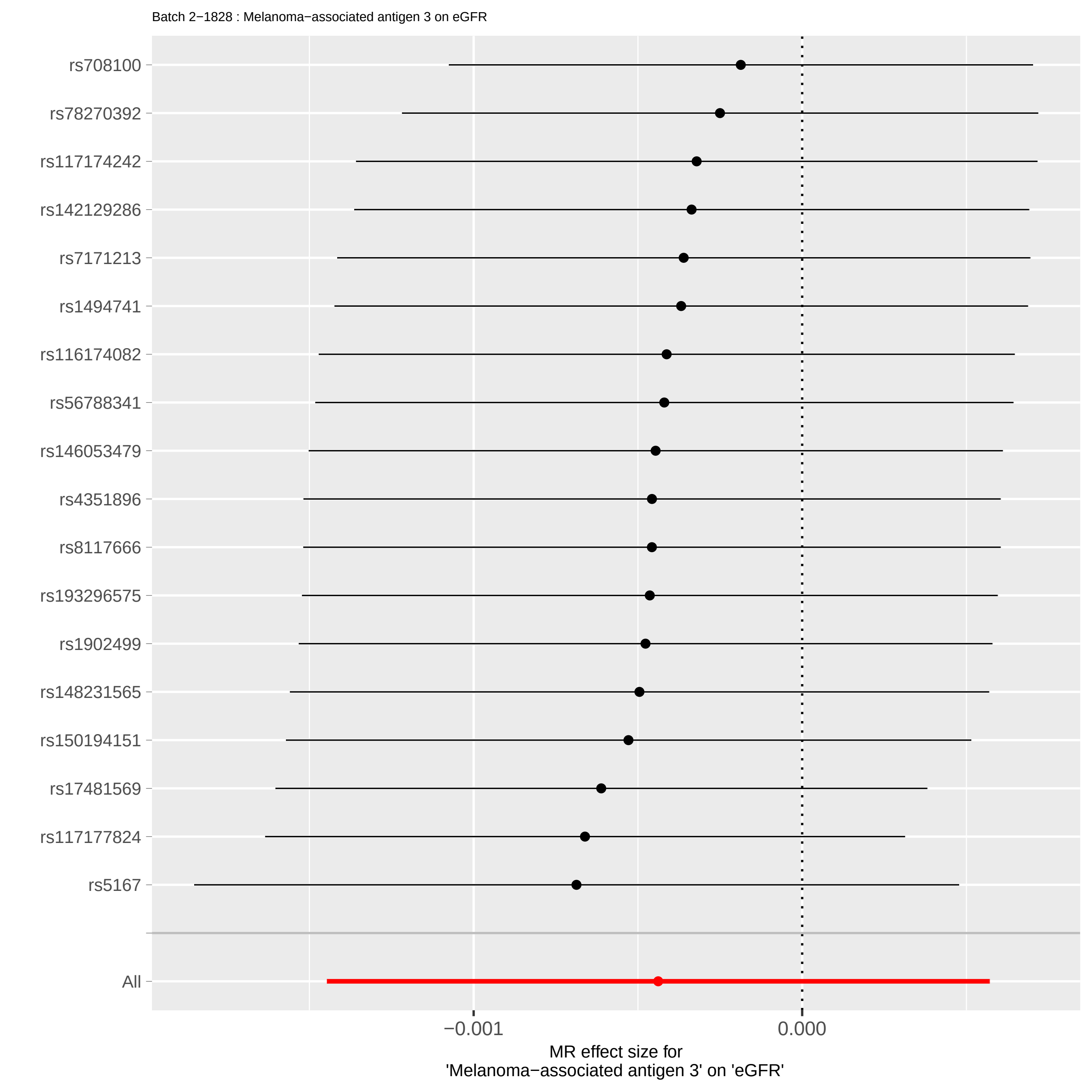

Batch 2−1828 : Melanoma−associated antigen 3 on eGFR
rs708100
rs78270392
rs117174242
rs142129286
rs7171213
rs1494741
rs116174082
rs56788341
rs146053479
rs4351896
rs8117666
rs193296575
rs1902499
rs148231565
rs150194151
rs17481569
rs117177824
rs5167
All
−0.001
0.000
MR effect size for
'Melanoma−associated antigen 3' on 'eGFR'

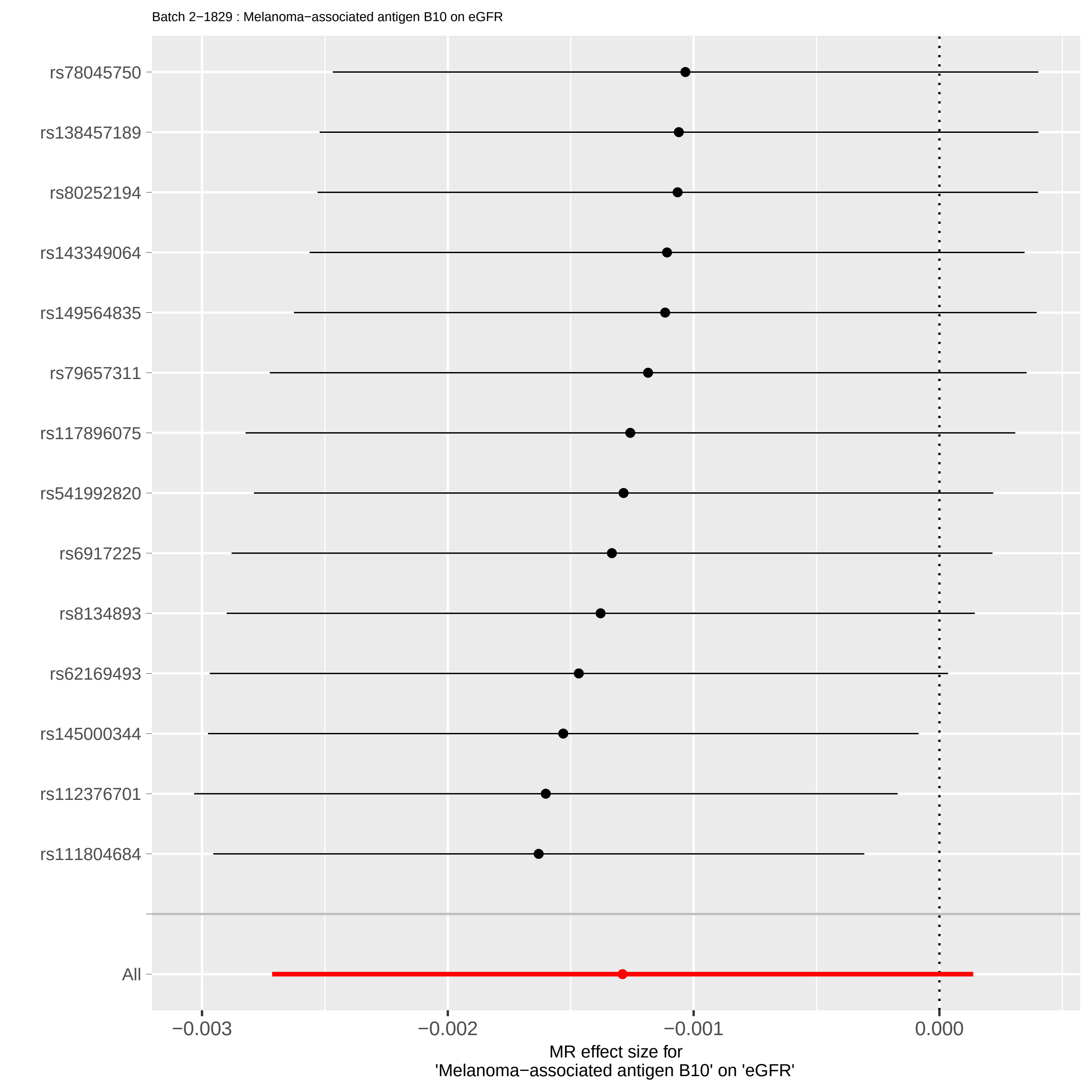

Batch 2−1829 : Melanoma−associated antigen B10 on eGFR
rs78045750
rs138457189
rs80252194
rs143349064
rs149564835
rs79657311
rs117896075
rs541992820
rs6917225
rs8134893
rs62169493
rs145000344
rs112376701
rs111804684
All
−0.003
−0.002
−0.001
0.000
MR effect size for
'Melanoma−associated antigen B10' on 'eGFR'

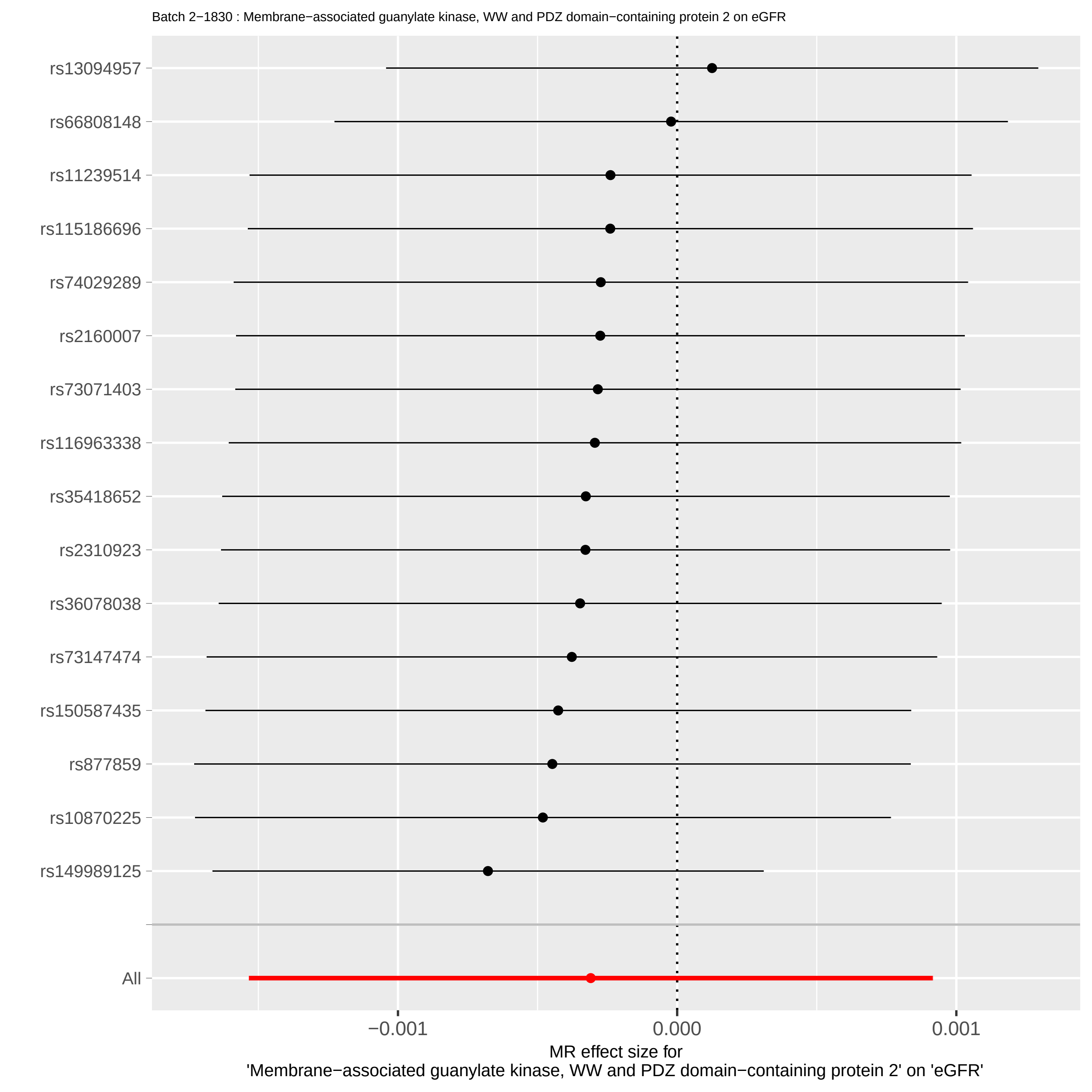

Batch 2−1830 : Membrane−associated guanylate kinase, WW and PDZ domain−containing protein 2 on eGFR
rs13094957
rs66808148
rs11239514
rs115186696
rs74029289
rs2160007
rs73071403
rs116963338
rs35418652
rs2310923
rs36078038
rs73147474
rs150587435
rs877859
rs10870225
rs149989125
All
−0.001
0.000
0.001
MR effect size for
'Membrane−associated guanylate kinase, WW and PDZ domain−containing protein 2' on 'eGFR'

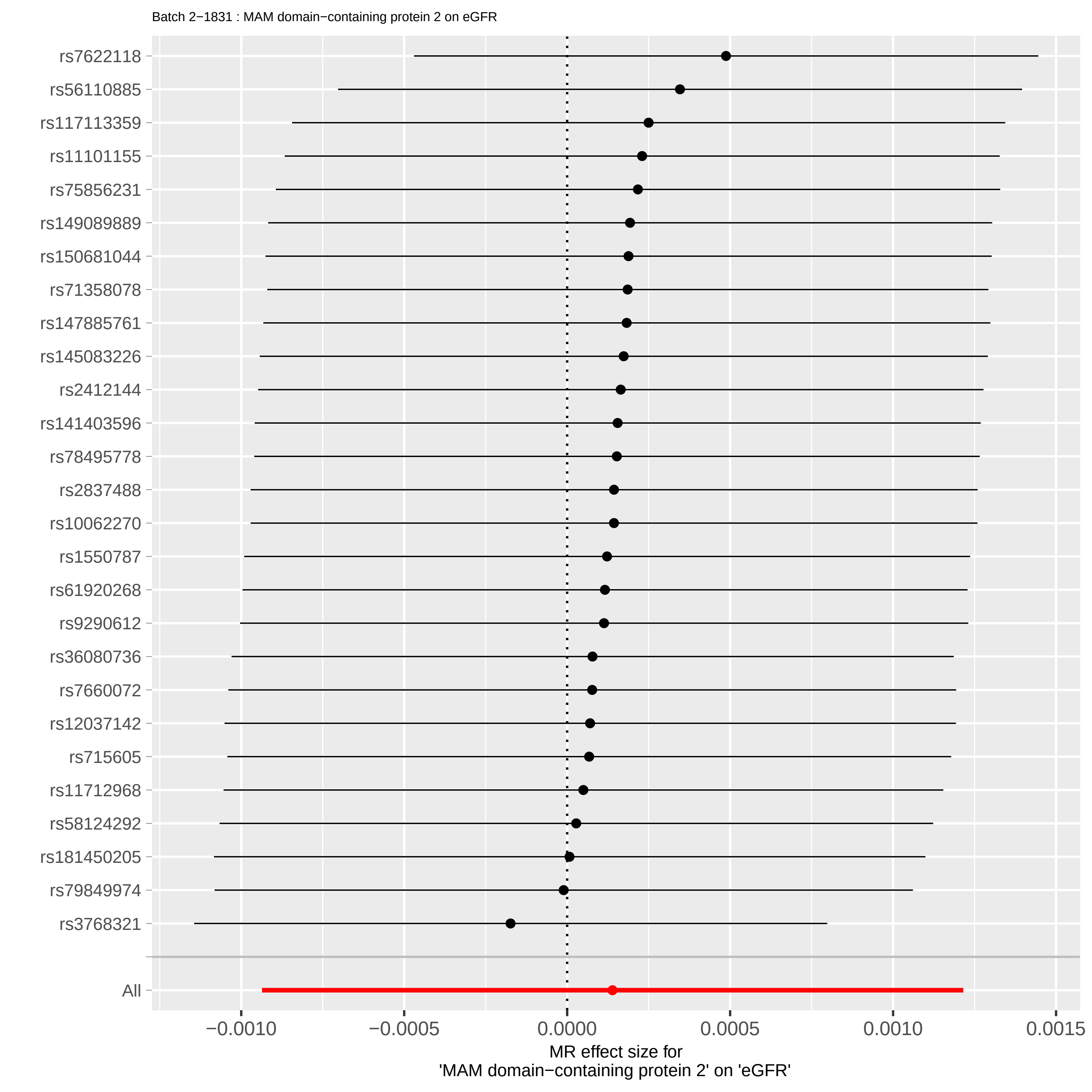

Batch 2−1831 : MAM domain−containing protein 2 on eGFR
rs7622118
rs56110885
rs117113359
rs11101155
rs75856231
rs149089889
rs150681044
rs71358078
rs147885761
rs145083226
rs2412144
rs141403596
rs78495778
rs2837488
rs10062270
rs1550787
rs61920268
rs9290612
rs36080736
rs7660072
rs12037142
rs715605
rs11712968
rs58124292
rs181450205
rs79849974
rs3768321
All
−0.0010
−0.0005
0.0000
0.0005
0.0010
0.0015
MR effect size for
'MAM domain−containing protein 2' on 'eGFR'

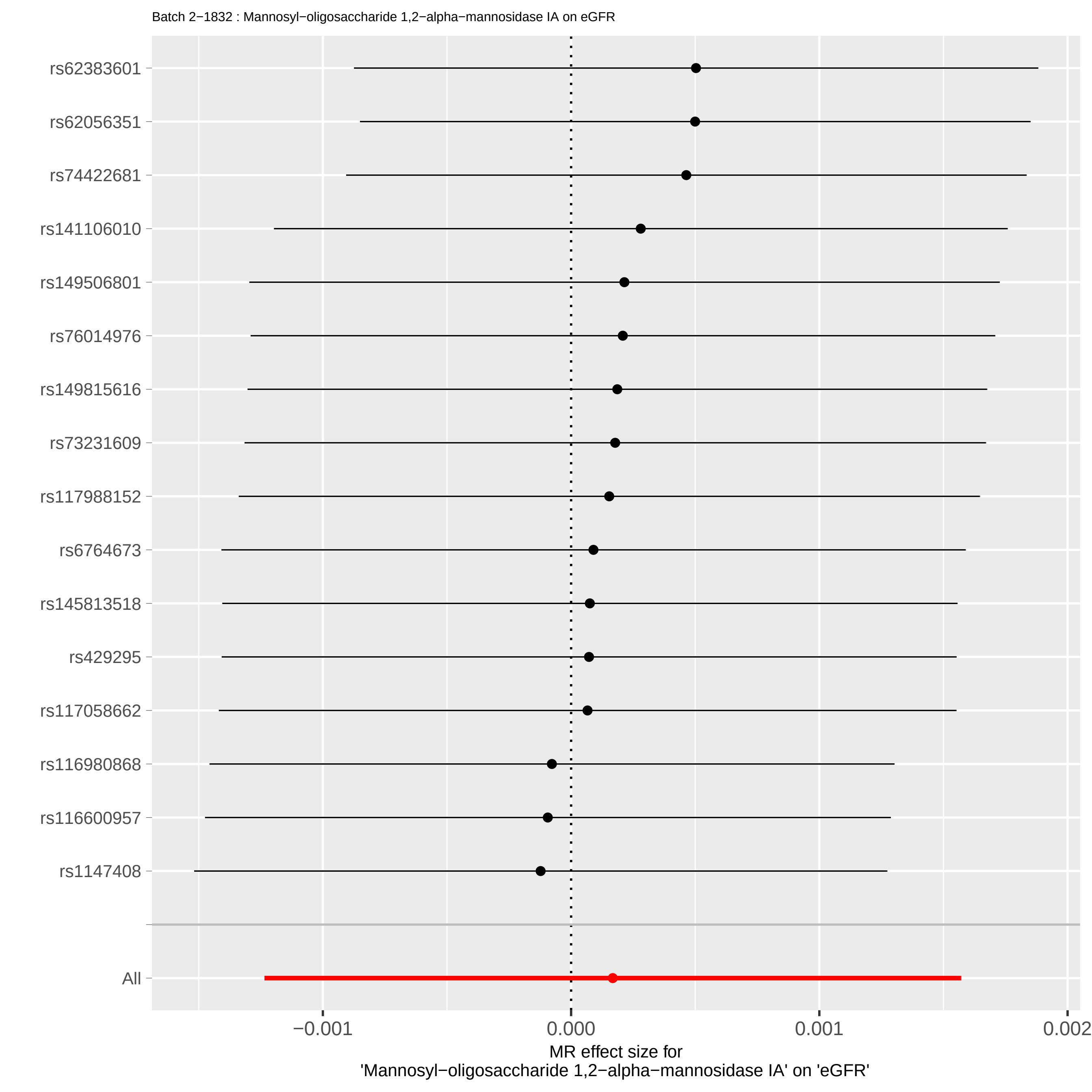

Batch 2−1832 : Mannosyl−oligosaccharide 1,2−alpha−mannosidase IA on eGFR
rs62383601
rs62056351
rs74422681
rs141106010
rs149506801
rs76014976
rs149815616
rs73231609
rs117988152
rs6764673
rs145813518
rs429295
rs117058662
rs116980868
rs116600957
rs1147408
All
−0.001
0.000
0.001
0.002
MR effect size for
'Mannosyl−oligosaccharide 1,2−alpha−mannosidase IA' on 'eGFR'

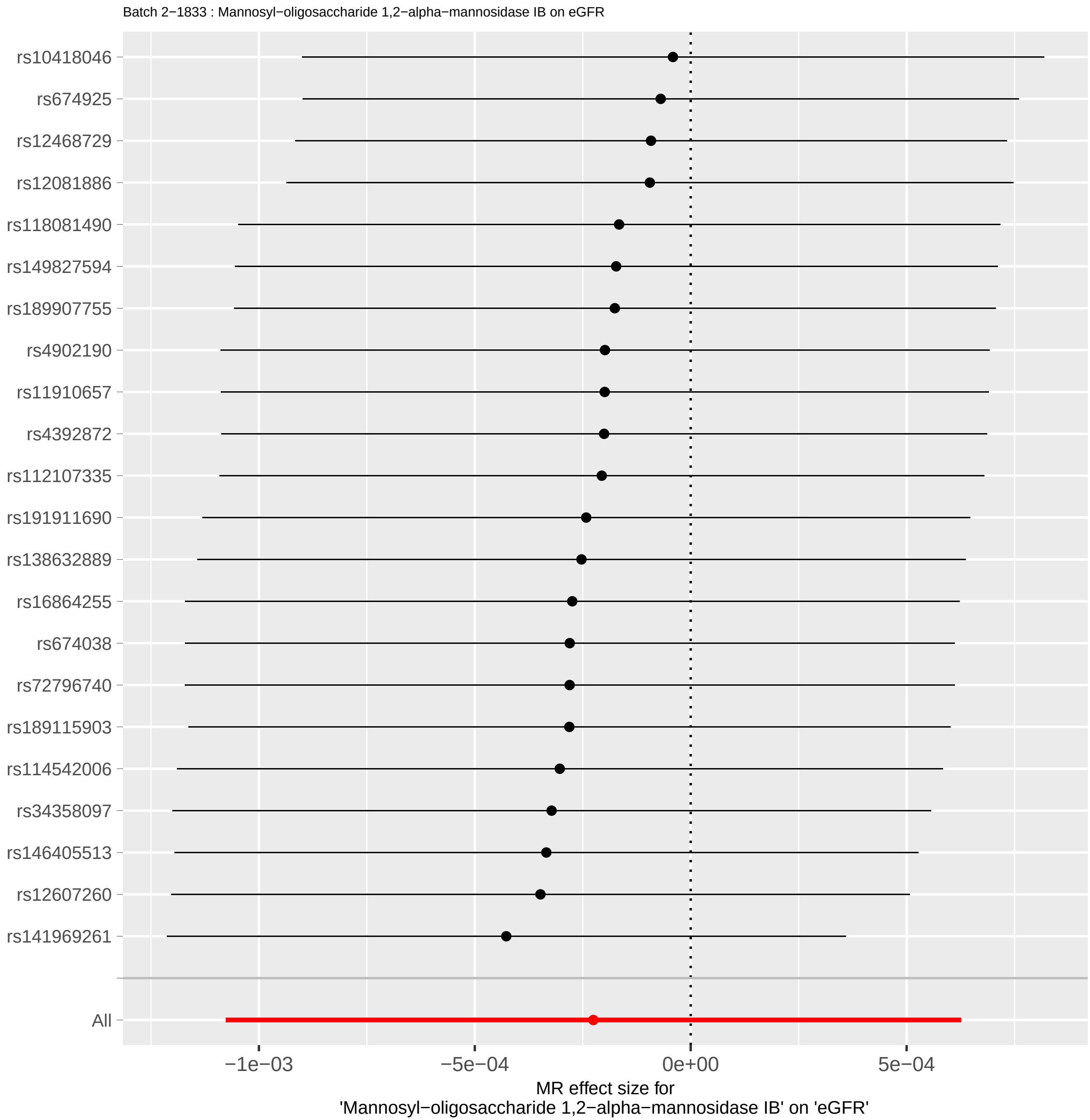

Batch 2−1833 : Mannosyl−oligosaccharide 1,2−alpha−mannosidase IB on eGFR
rs10418046
rs674925
rs12468729
rs12081886
rs118081490
rs149827594
rs189907755
rs4902190
rs11910657
rs4392872
rs112107335
rs191911690
rs138632889
rs16864255
rs674038
rs72796740
rs189115903
rs114542006
rs34358097
rs146405513
rs12607260
rs141969261
All
−1e−03
−5e−04
0e+00
5e−04
MR effect size for
'Mannosyl−oligosaccharide 1,2−alpha−mannosidase IB' on 'eGFR'

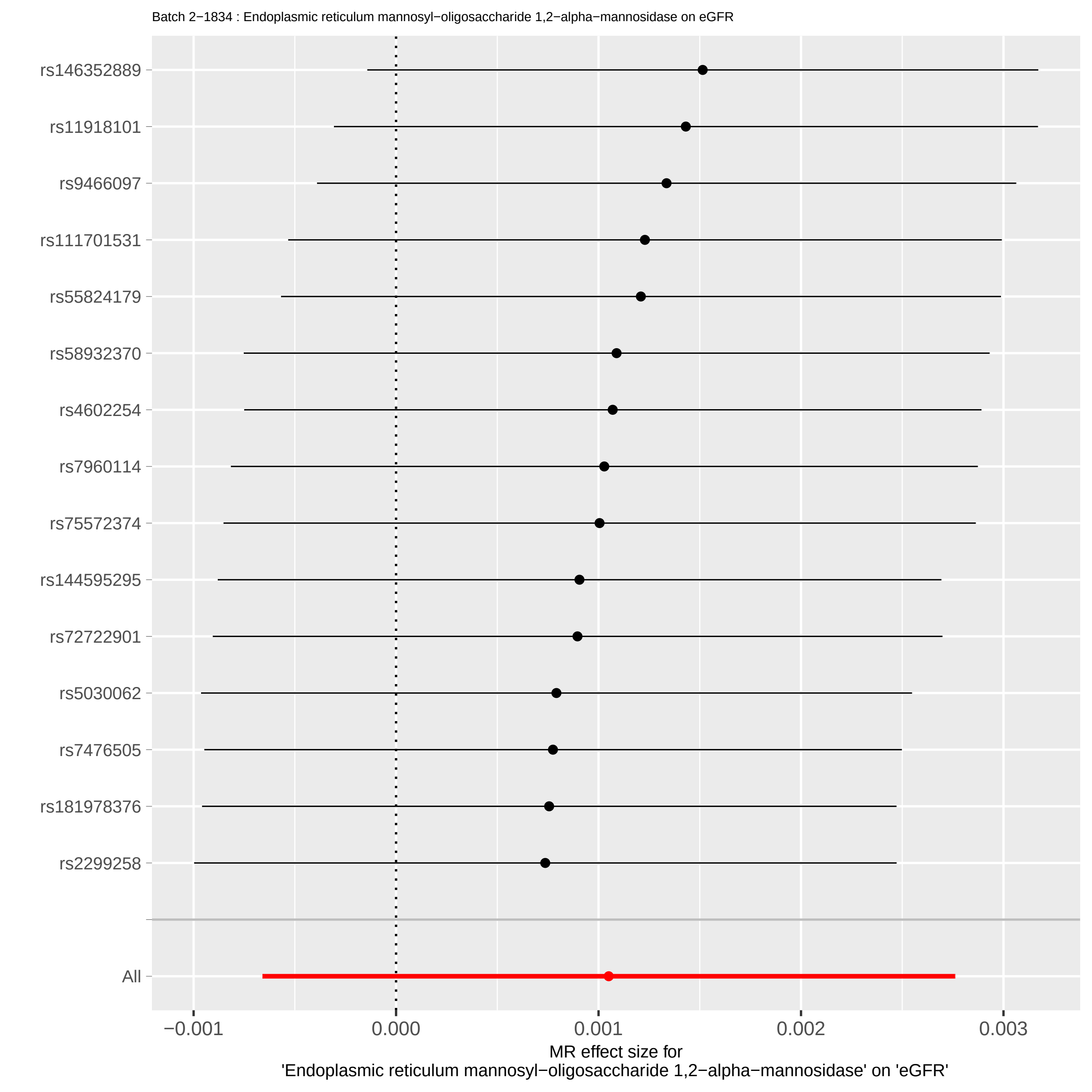

Batch 2−1834 : Endoplasmic reticulum mannosyl−oligosaccharide 1,2−alpha−mannosidase on eGFR
rs146352889
rs11918101
rs9466097
rs111701531
rs55824179
rs58932370
rs4602254
rs7960114
rs75572374
rs144595295
rs72722901
rs5030062
rs7476505
rs181978376
rs2299258
All
−0.001
0.000
0.001
0.002
0.003
MR effect size for
'Endoplasmic reticulum mannosyl−oligosaccharide 1,2−alpha−mannosidase' on 'eGFR'

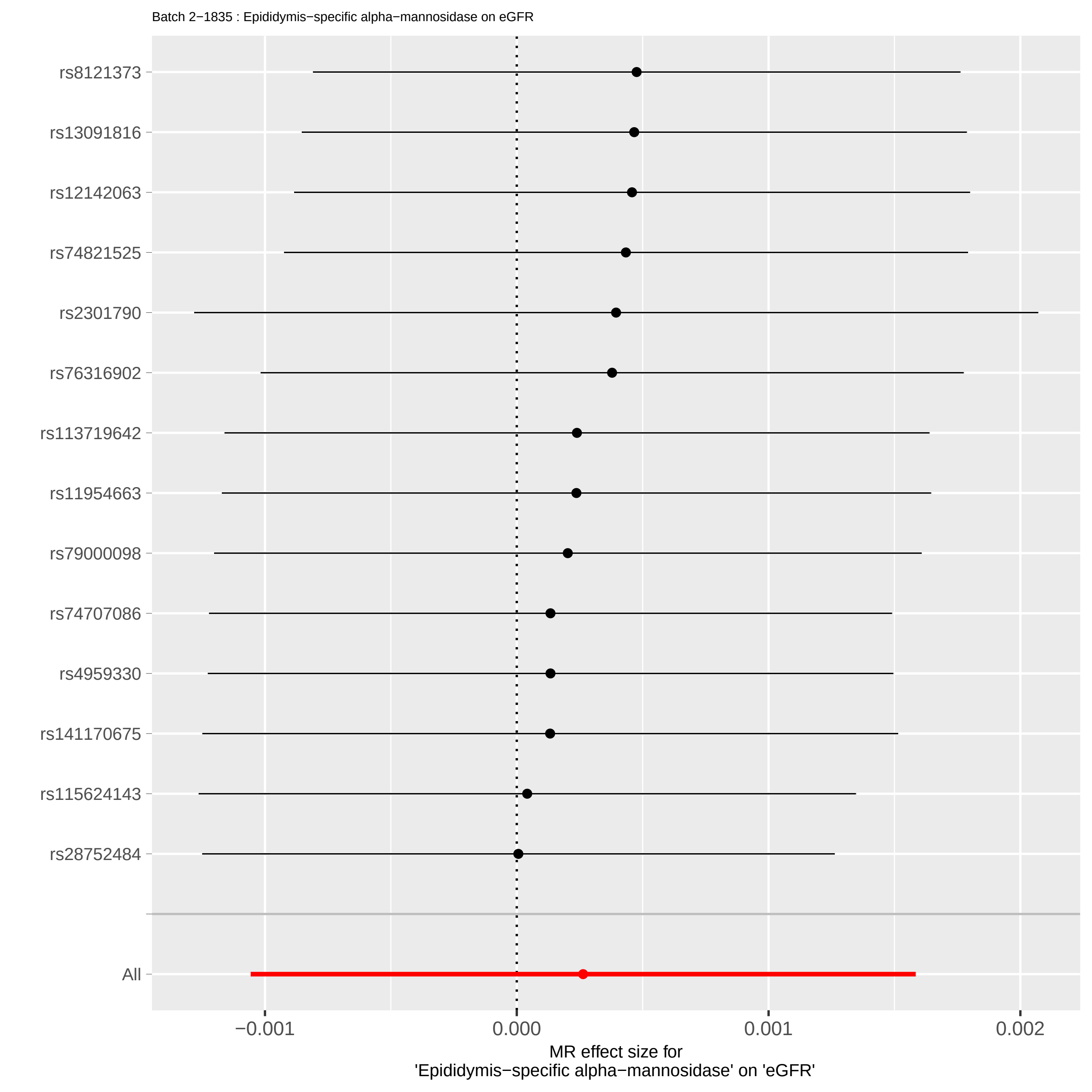
Batch 2−1835 : Epididymis−specific alpha−mannosidase on eGFR
rs8121373
rs13091816
rs12142063
rs74821525
rs2301790
rs76316902
rs113719642
rs11954663
rs79000098
rs74707086
rs4959330
rs141170675
rs115624143
rs28752484
All
−0.001
0.000
0.001
0.002
MR effect size for
'Epididymis−specific alpha−mannosidase' on 'eGFR'

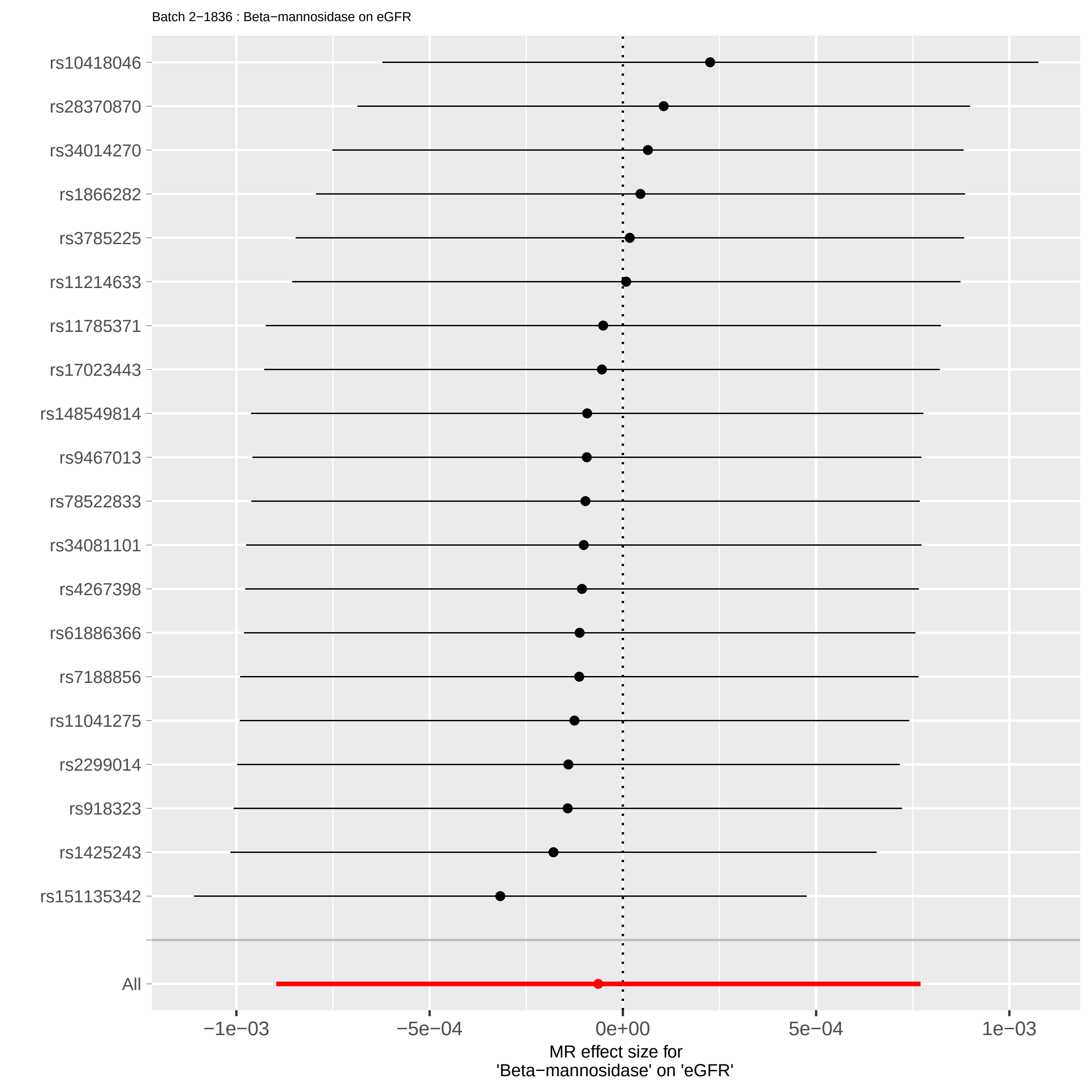

Batch 2−1836 : Beta−mannosidase on eGFR
rs10418046
rs28370870
rs34014270
rs1866282
rs3785225
rs11214633
rs11785371
rs17023443
rs148549814
rs9467013
rs78522833
rs34081101
rs4267398
rs61886366
rs7188856
rs11041275
rs2299014
rs918323
rs1425243
rs151135342
All
−1e−03
−5e−04
0e+00
5e−04
1e−03
MR effect size for
'Beta−mannosidase' on 'eGFR'

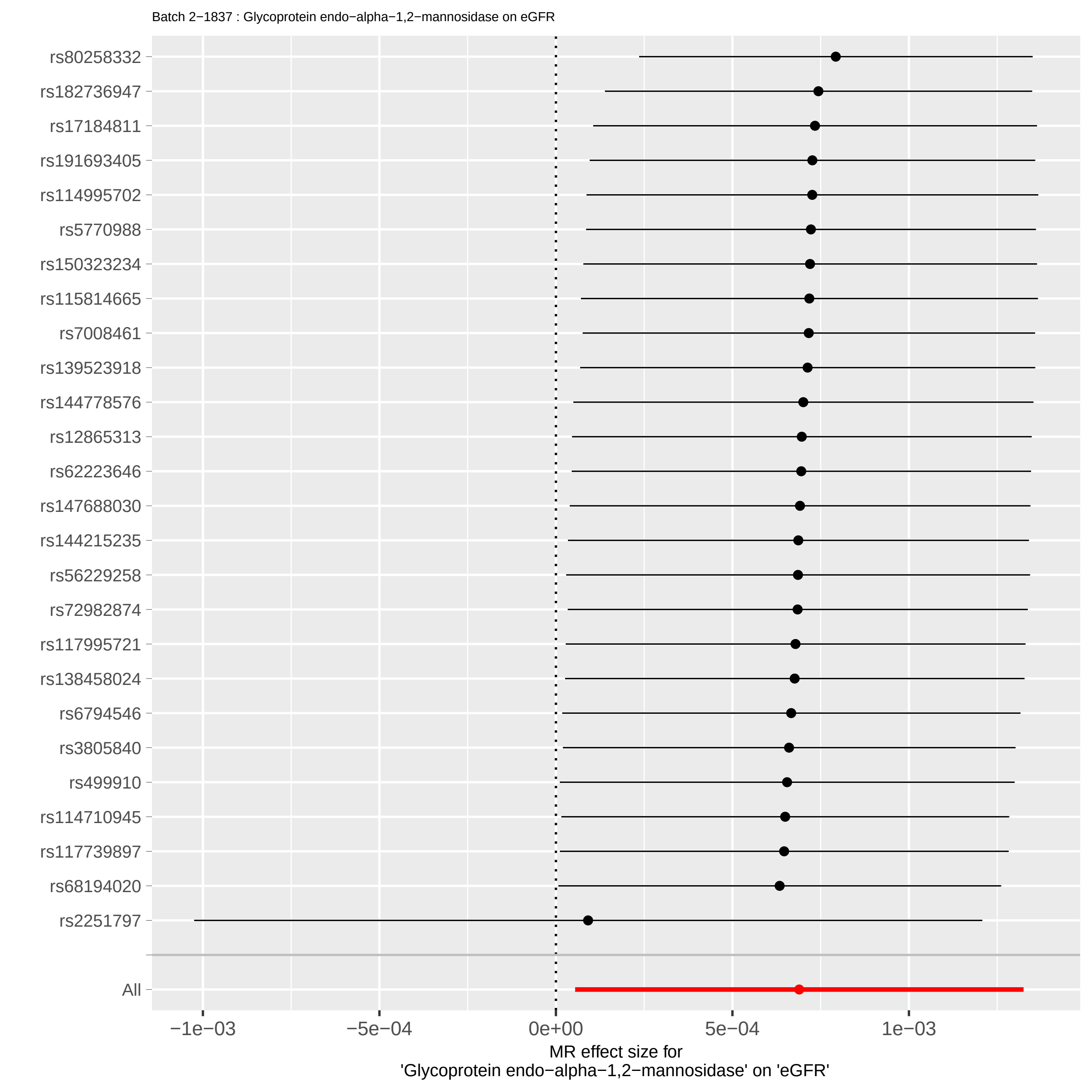

Batch 2−1837 : Glycoprotein endo−alpha−1,2−mannosidase on eGFR
rs80258332
rs182736947
rs17184811
rs191693405
rs114995702
rs5770988
rs150323234
rs115814665
rs7008461
rs139523918
rs144778576
rs12865313
rs62223646
rs147688030
rs144215235
rs56229258
rs72982874
rs117995721
rs138458024
rs6794546
rs3805840
rs499910
rs114710945
rs117739897
rs68194020
rs2251797
All
−1e−03
−5e−04
0e+00
5e−04
1e−03
MR effect size for
'Glycoprotein endo−alpha−1,2−mannosidase' on 'eGFR'

Batch 2−1838 : MANSC domain−containing protein 1 on eGFR

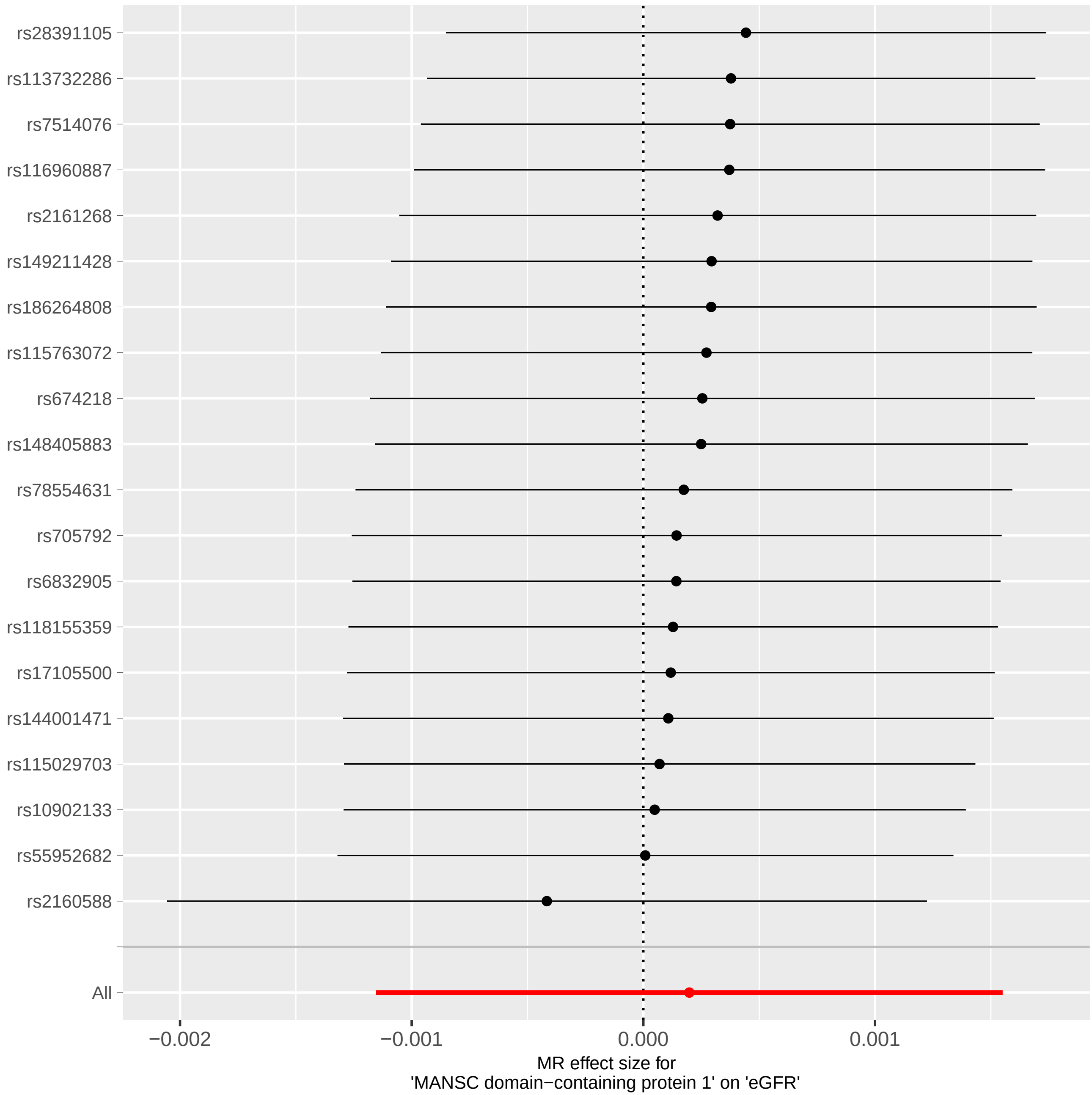

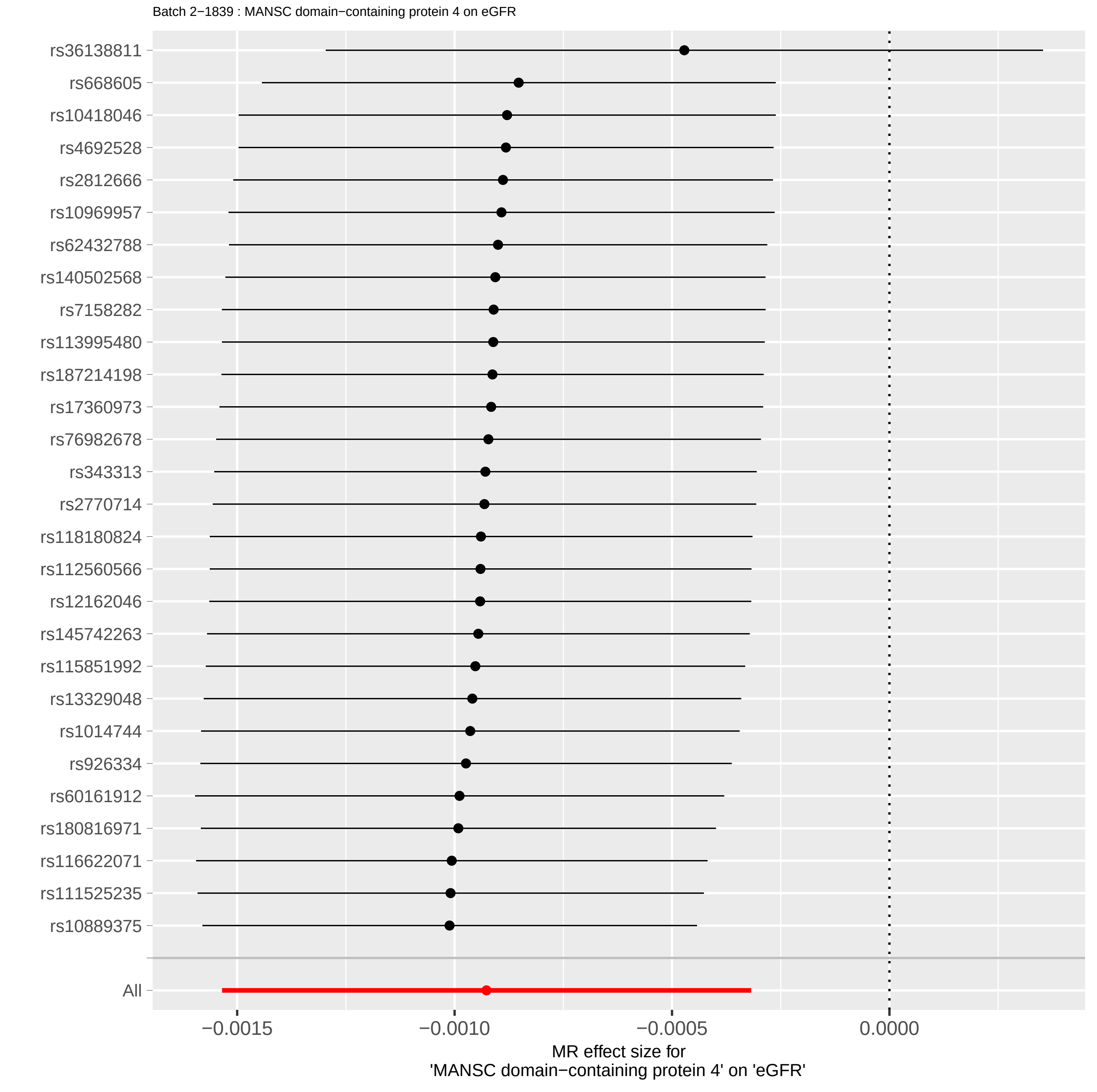

Batch 2−1839 : MANSC domain−containing protein 4 on eGFR
rs36138811
rs668605
rs10418046
rs4692528
rs2812666
rs10969957
rs62432788
rs140502568
rs7158282
rs113995480
rs187214198
rs17360973
rs76982678
rs343313
rs2770714
rs118180824
rs112560566
rs12162046
rs145742263
rs115851992
rs13329048
rs1014744
rs926334
rs60161912
rs180816971
rs116622071
rs111525235
rs10889375
All
−0.0015
−0.0010
−0.0005
0.0000
MR effect size for
'MANSC domain−containing protein 4' on 'eGFR'

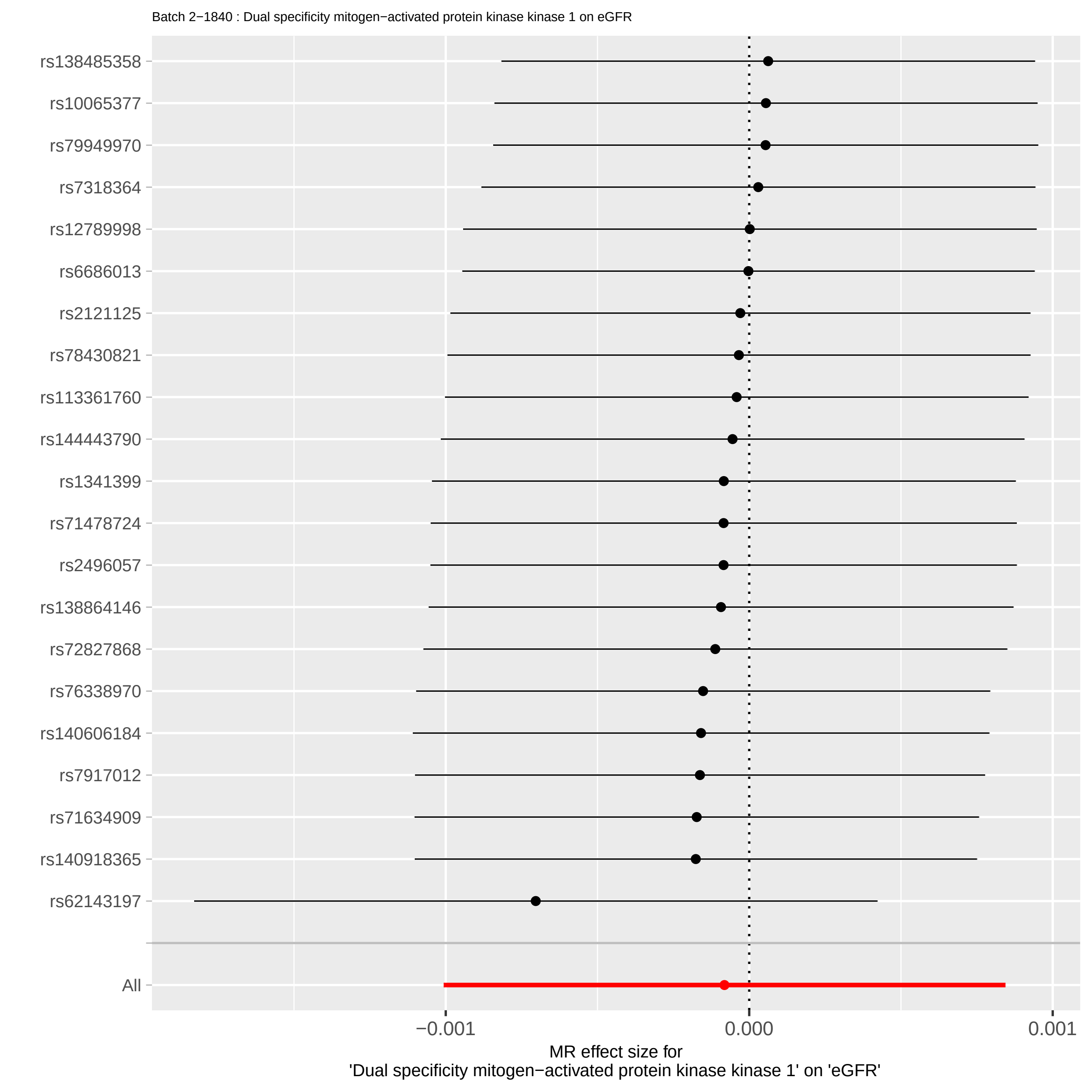

Batch 2−1840 : Dual specificity mitogen−activated protein kinase kinase 1 on eGFR
rs138485358
rs10065377
rs79949970
rs7318364
rs12789998
rs6686013
rs2121125
rs78430821
rs113361760
rs144443790
rs1341399
rs71478724
rs2496057
rs138864146
rs72827868
rs76338970
rs140606184
rs7917012
rs71634909
rs140918365
rs62143197
All
−0.001
0.000
0.001
MR effect size for
'Dual specificity mitogen−activated protein kinase kinase 1' on 'eGFR'

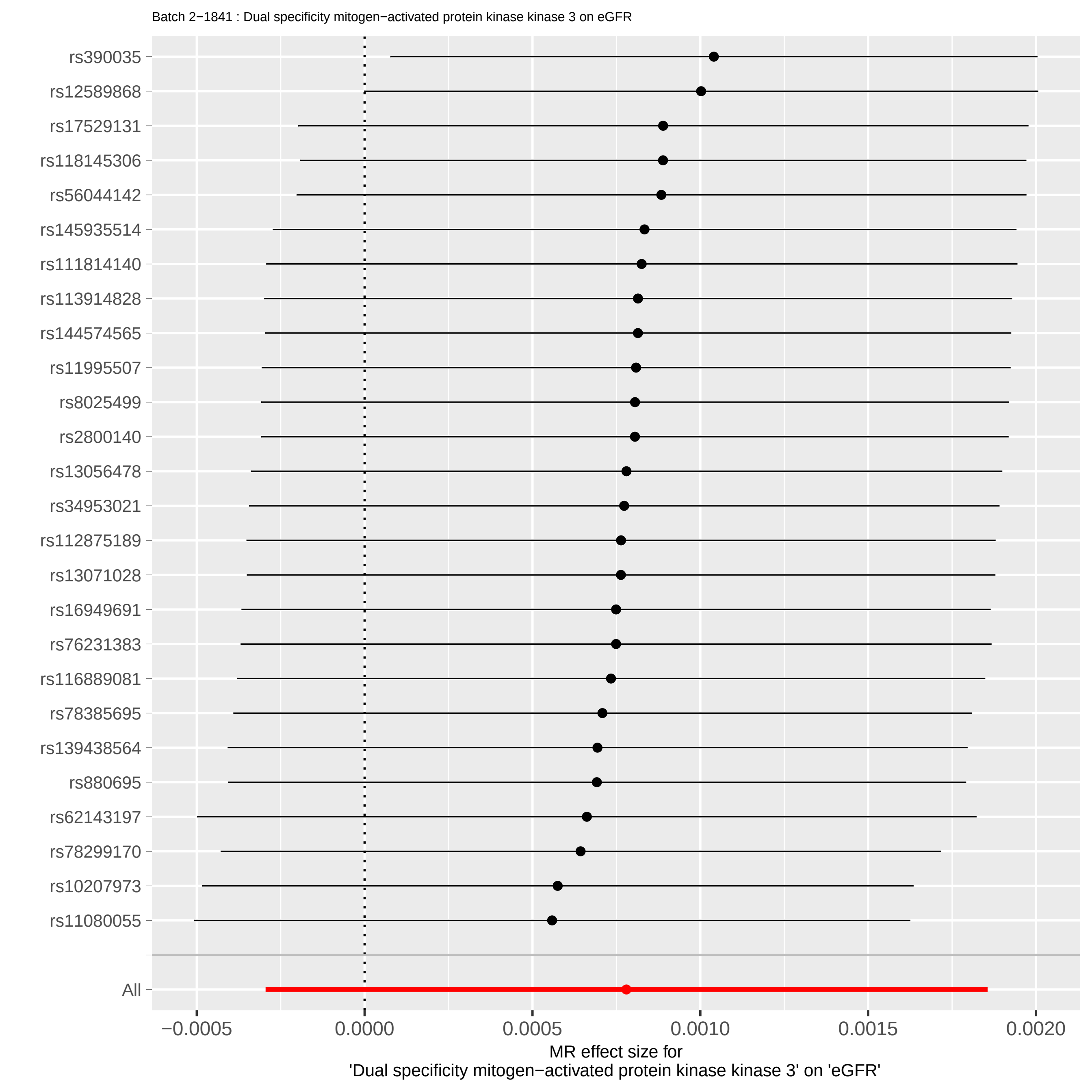

Batch 2−1841 : Dual specificity mitogen−activated protein kinase kinase 3 on eGFR
rs390035
rs12589868
rs17529131
rs118145306
rs56044142
rs145935514
rs111814140
rs113914828
rs144574565
rs11995507
rs8025499
rs2800140
rs13056478
rs34953021
rs112875189
rs13071028
rs16949691
rs76231383
rs116889081
rs78385695
rs139438564
rs880695
rs62143197
rs78299170
rs10207973
rs11080055
All
−0.0005
0.0000
0.0005
0.0010
0.0015
0.0020
MR effect size for
'Dual specificity mitogen−activated protein kinase kinase 3' on 'eGFR'

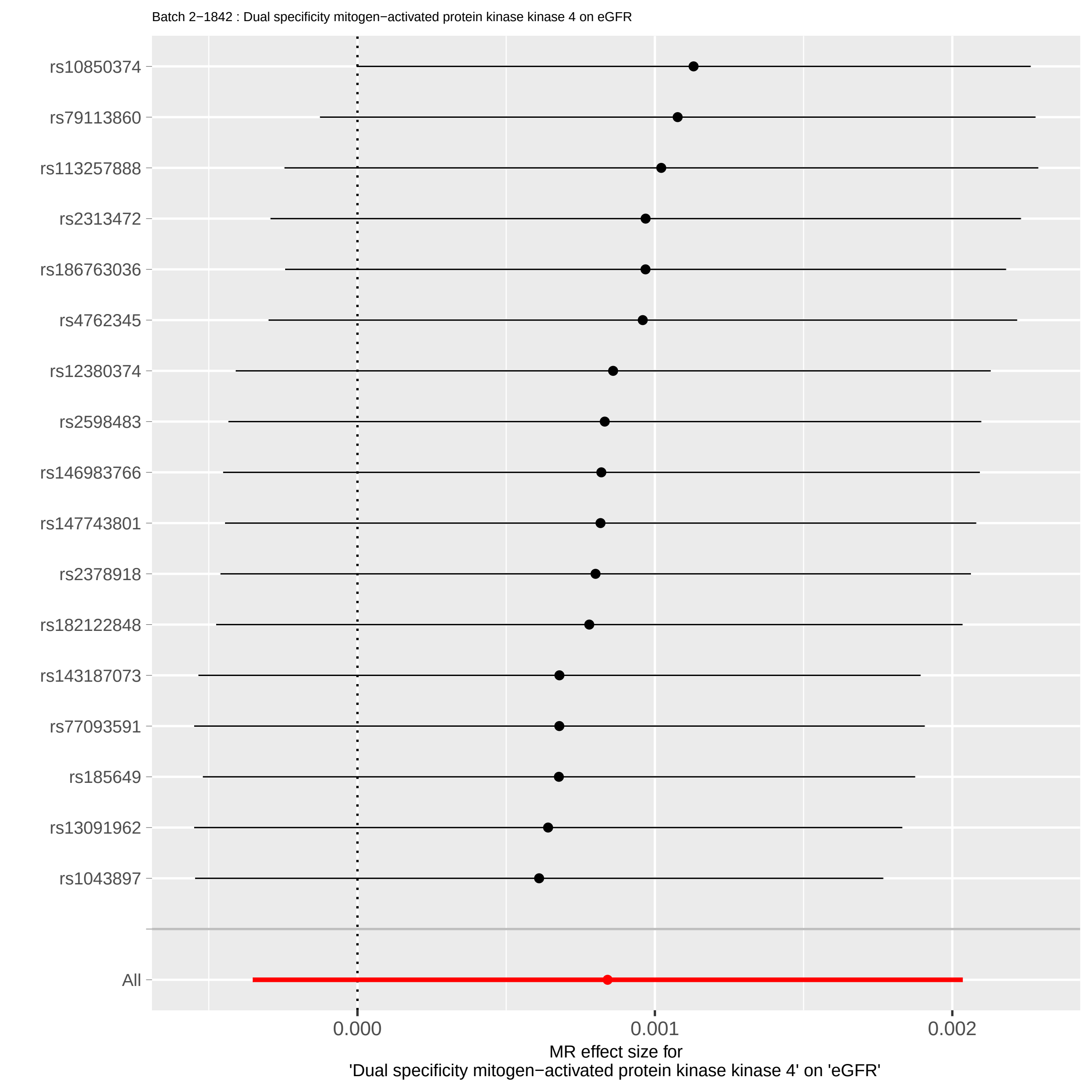

Batch 2−1842 : Dual specificity mitogen−activated protein kinase kinase 4 on eGFR
rs10850374
rs79113860
rs113257888
rs2313472
rs186763036
rs4762345
rs12380374
rs2598483
rs146983766
rs147743801
rs2378918
rs182122848
rs143187073
rs77093591
rs185649
rs13091962
rs1043897
All
0.000
0.001
0.002
MR effect size for
'Dual specificity mitogen−activated protein kinase kinase 4' on 'eGFR'

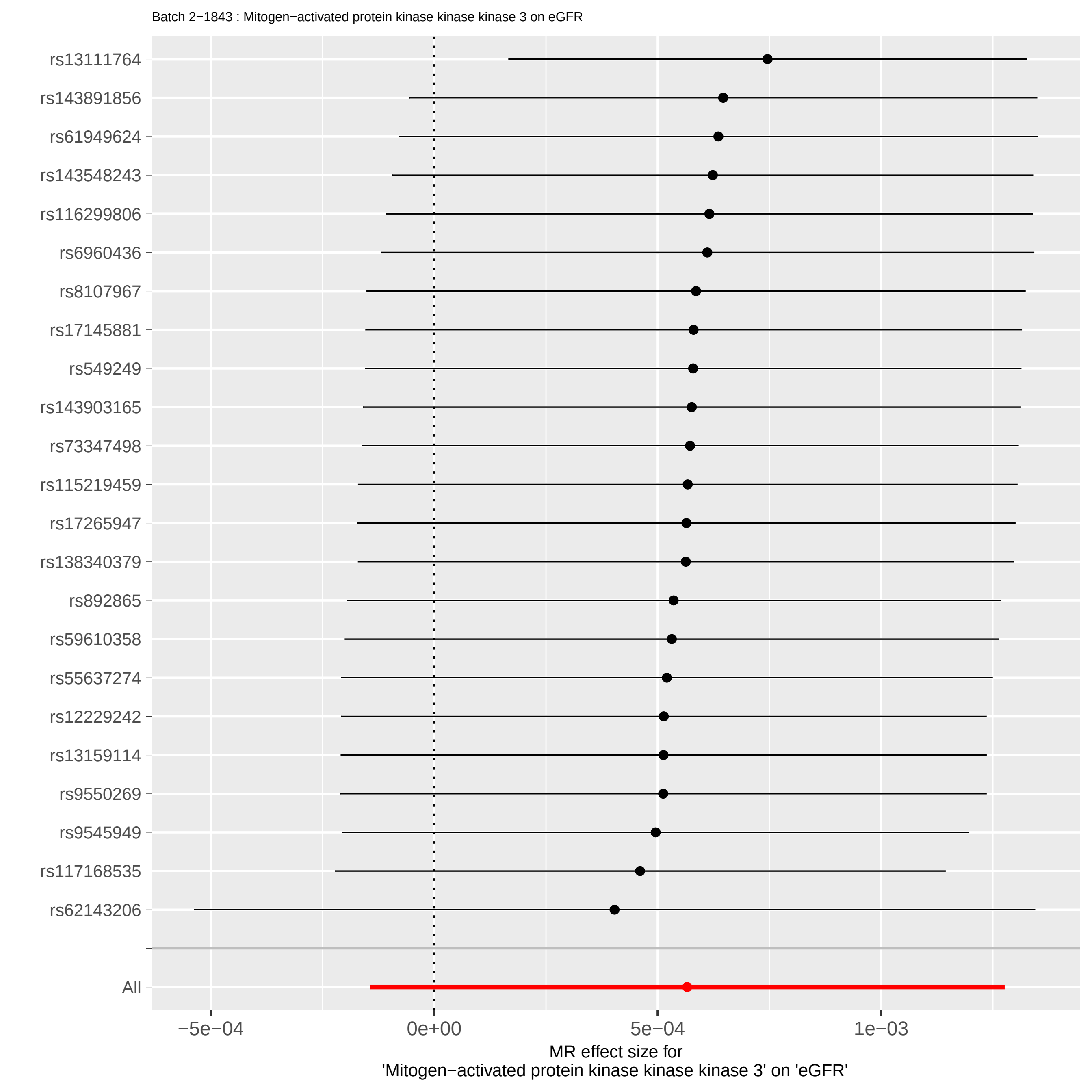

Batch 2−1843 : Mitogen−activated protein kinase kinase kinase 3 on eGFR
rs13111764
rs143891856
rs61949624
rs143548243
rs116299806
rs6960436
rs8107967
rs17145881
rs549249
rs143903165
rs73347498
rs115219459
rs17265947
rs138340379
rs892865
rs59610358
rs55637274
rs12229242
rs13159114
rs9550269
rs9545949
rs117168535
rs62143206
All
−5e−04
0e+00
5e−04
1e−03
MR effect size for
'Mitogen−activated protein kinase kinase kinase 3' on 'eGFR'

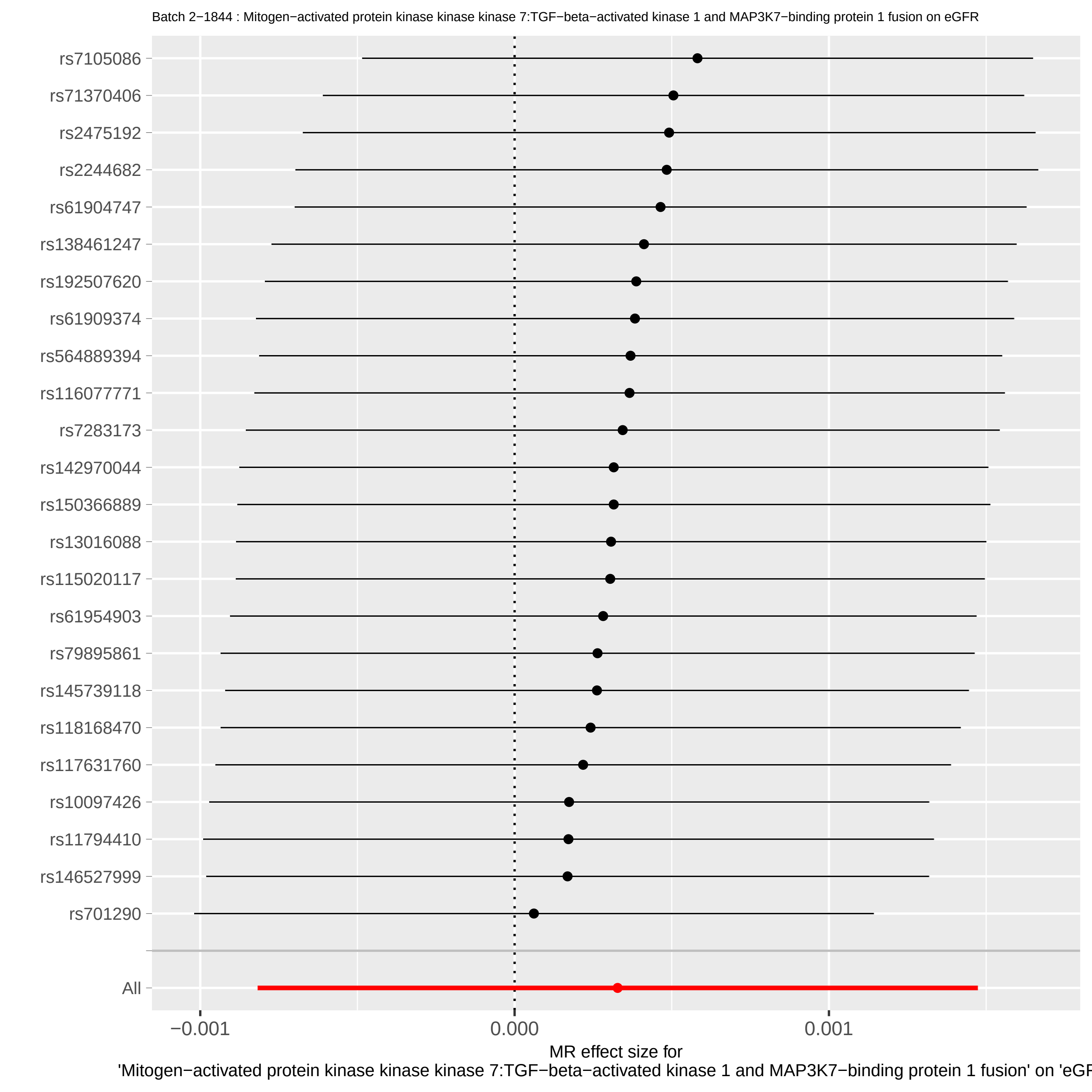

Batch 2−1844 : Mitogen−activated protein kinase kinase kinase 7:TGF−beta−activated kinase 1 and MAP3K7−binding protein 1 fusion on eGFR
rs7105086
rs71370406
rs2475192
rs2244682
rs61904747
rs138461247
rs192507620
rs61909374
rs564889394
rs116077771
rs7283173
rs142970044
rs150366889
rs13016088
rs115020117
rs61954903
rs79895861
rs145739118
rs118168470
rs117631760
rs10097426
rs11794410
rs146527999
rs701290
All
−0.001
0.000
0.001
MR effect size for
'Mitogen−activated protein kinase kinase kinase 7:TGF−beta−activated kinase 1 and MAP3K7−binding protein 1 fusion' on 'eGF

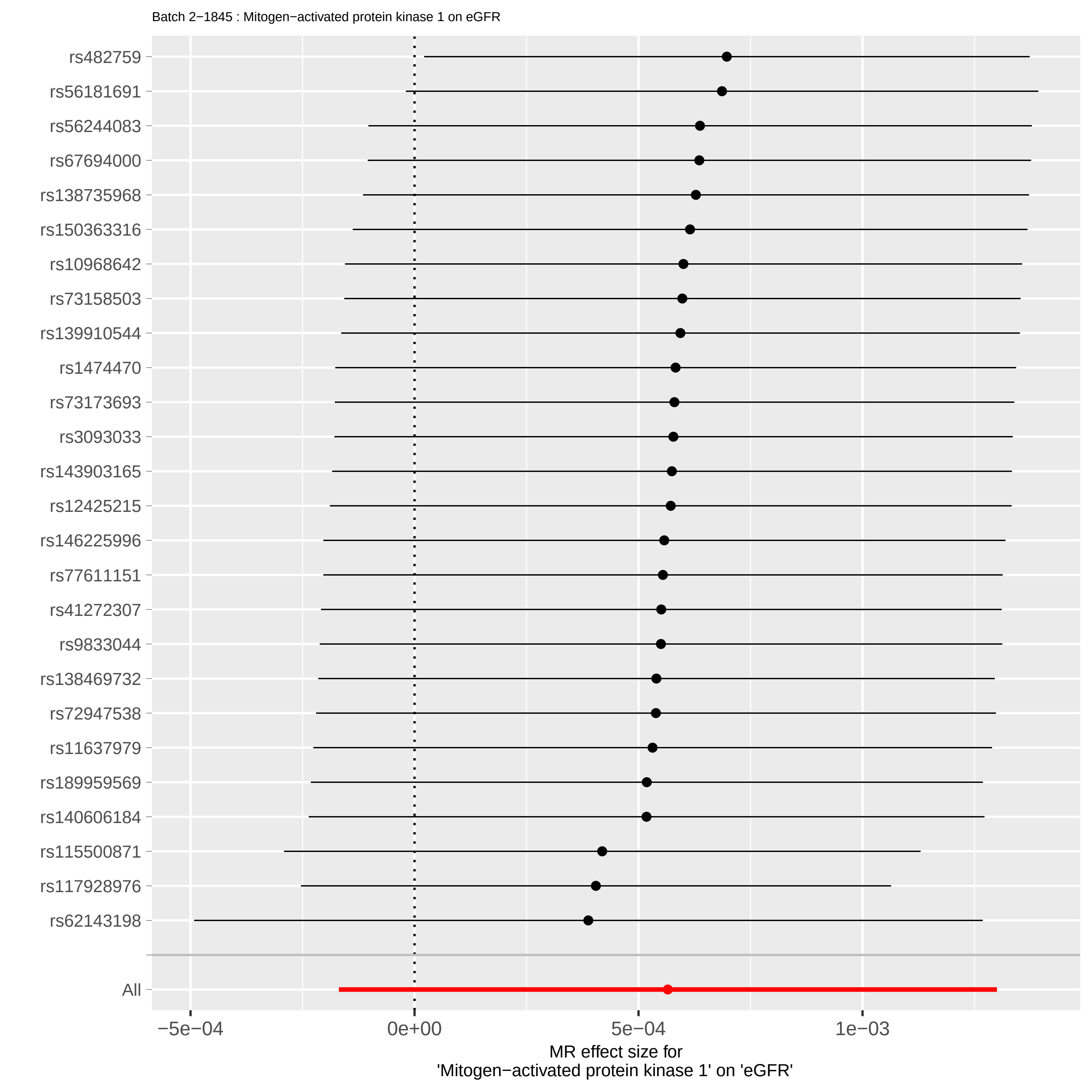

Batch 2−1845 : Mitogen−activated protein kinase 1 on eGFR
rs482759
rs56181691
rs56244083
rs67694000
rs138735968
rs150363316
rs10968642
rs73158503
rs139910544
rs1474470
rs73173693
rs3093033
rs143903165
rs12425215
rs146225996
rs77611151
rs41272307
rs9833044
rs138469732
rs72947538
rs11637979
rs189959569
rs140606184
rs115500871
rs117928976
rs62143198
All
−5e−04
0e+00
5e−04
1e−03
MR effect size for
'Mitogen−activated protein kinase 1' on 'eGFR'

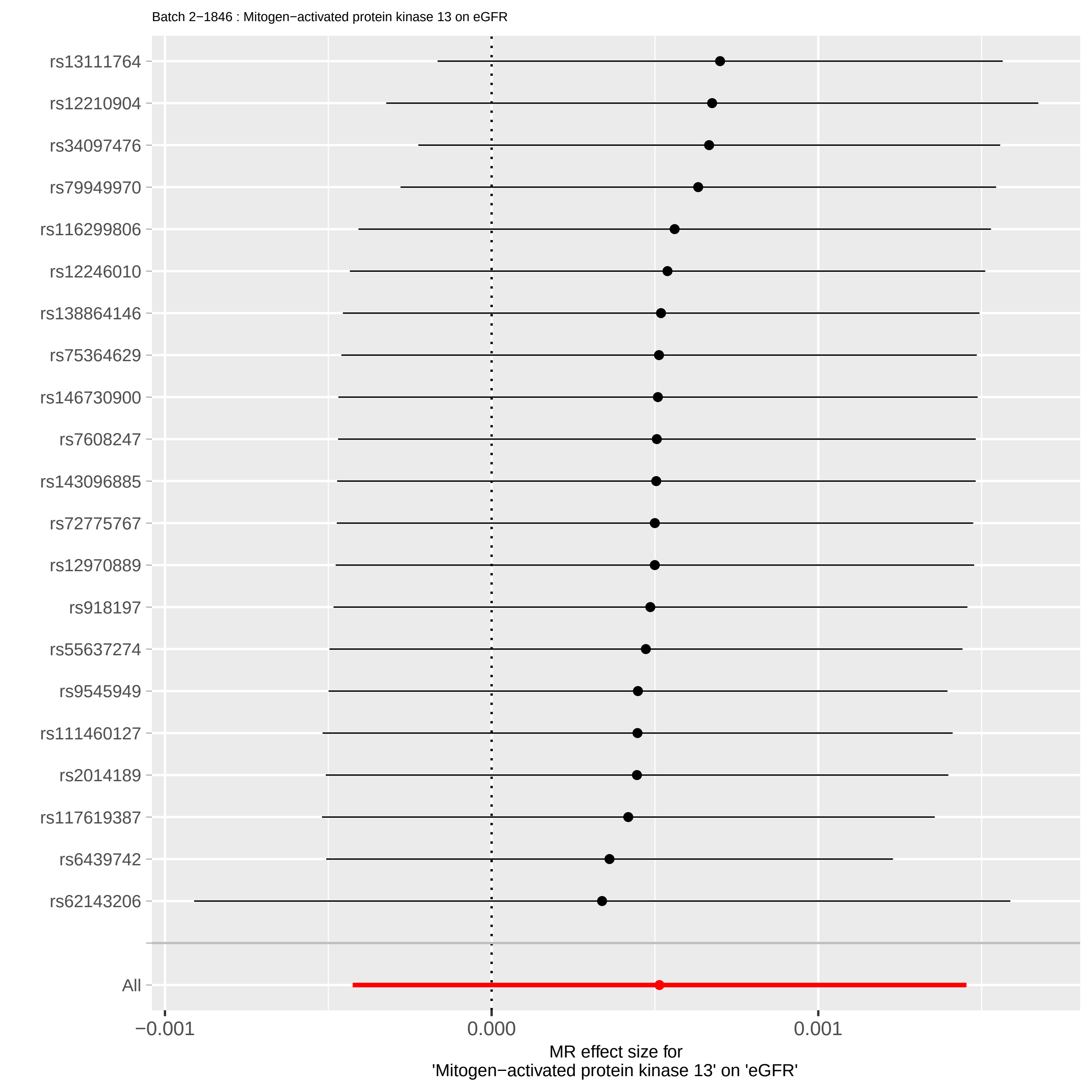
Batch 2−1846 : Mitogen−activated protein kinase 13 on eGFR
rs13111764
rs12210904
rs34097476
rs79949970
rs116299806
rs12246010
rs138864146
rs75364629
rs146730900
rs7608247
rs143096885
rs72775767
rs12970889
rs918197
rs55637274
rs9545949
rs111460127
rs2014189
rs117619387
rs6439742
rs62143206
All
−0.001
0.000
0.001
MR effect size for
'Mitogen−activated protein kinase 13' on 'eGFR'

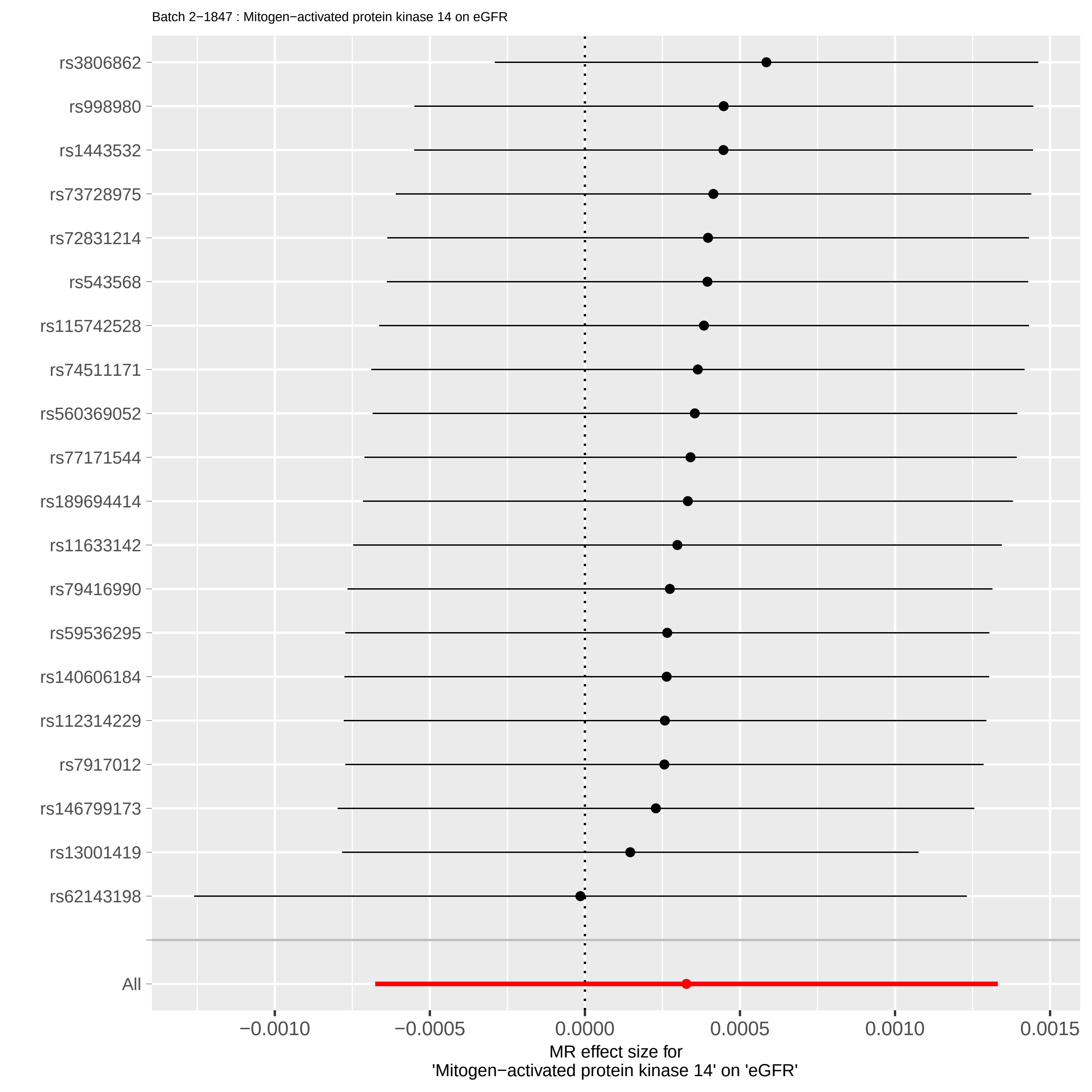

Batch 2−1847 : Mitogen−activated protein kinase 14 on eGFR
rs3806862
rs998980
rs1443532
rs73728975
rs72831214
rs543568
rs115742528
rs74511171
rs560369052
rs77171544
rs189694414
rs11633142
rs79416990
rs59536295
rs140606184
rs112314229
rs7917012
rs146799173
rs13001419
rs62143198
All
−0.0010
−0.0005
0.0000
0.0005
0.0010
0.0015
MR effect size for
'Mitogen−activated protein kinase 14' on 'eGFR'

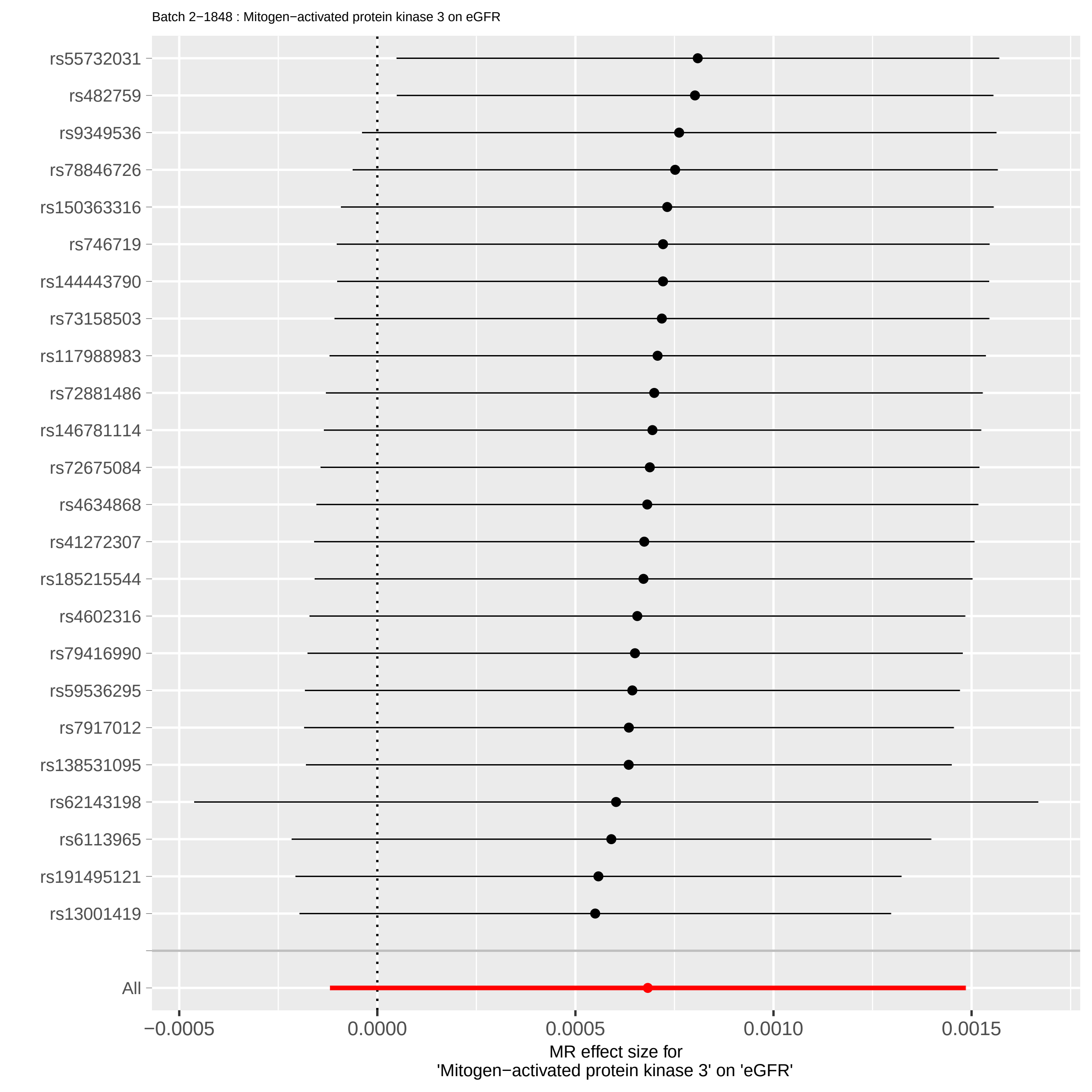

Batch 2−1848 : Mitogen−activated protein kinase 3 on eGFR
rs55732031
rs482759
rs9349536
rs78846726
rs150363316
rs746719
rs144443790
rs73158503
rs117988983
rs72881486
rs146781114
rs72675084
rs4634868
rs41272307
rs185215544
rs4602316
rs79416990
rs59536295
rs7917012
rs138531095
rs62143198
rs6113965
rs191495121
rs13001419
All
−0.0005
0.0000
0.0005
0.0010
0.0015
MR effect size for
'Mitogen−activated protein kinase 3' on 'eGFR'

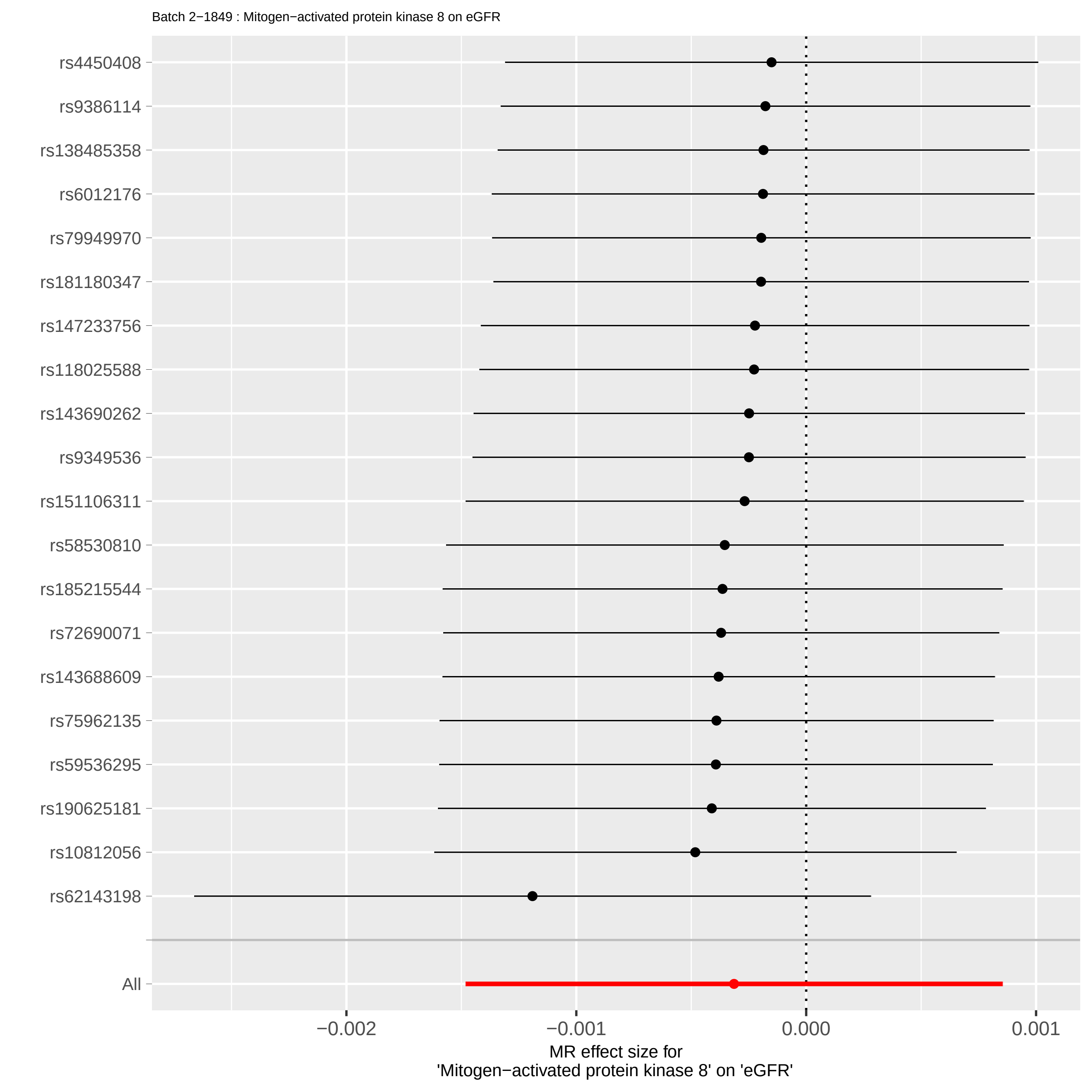

Batch 2−1849 : Mitogen−activated protein kinase 8 on eGFR
rs4450408
rs9386114
rs138485358
rs6012176
rs79949970
rs181180347
rs147233756
rs118025588
rs143690262
rs9349536
rs151106311
rs58530810
rs185215544
rs72690071
rs143688609
rs75962135
rs59536295
rs190625181
rs10812056
rs62143198
All
−0.002
−0.001
0.000
0.001
MR effect size for
'Mitogen−activated protein kinase 8' on 'eGFR'

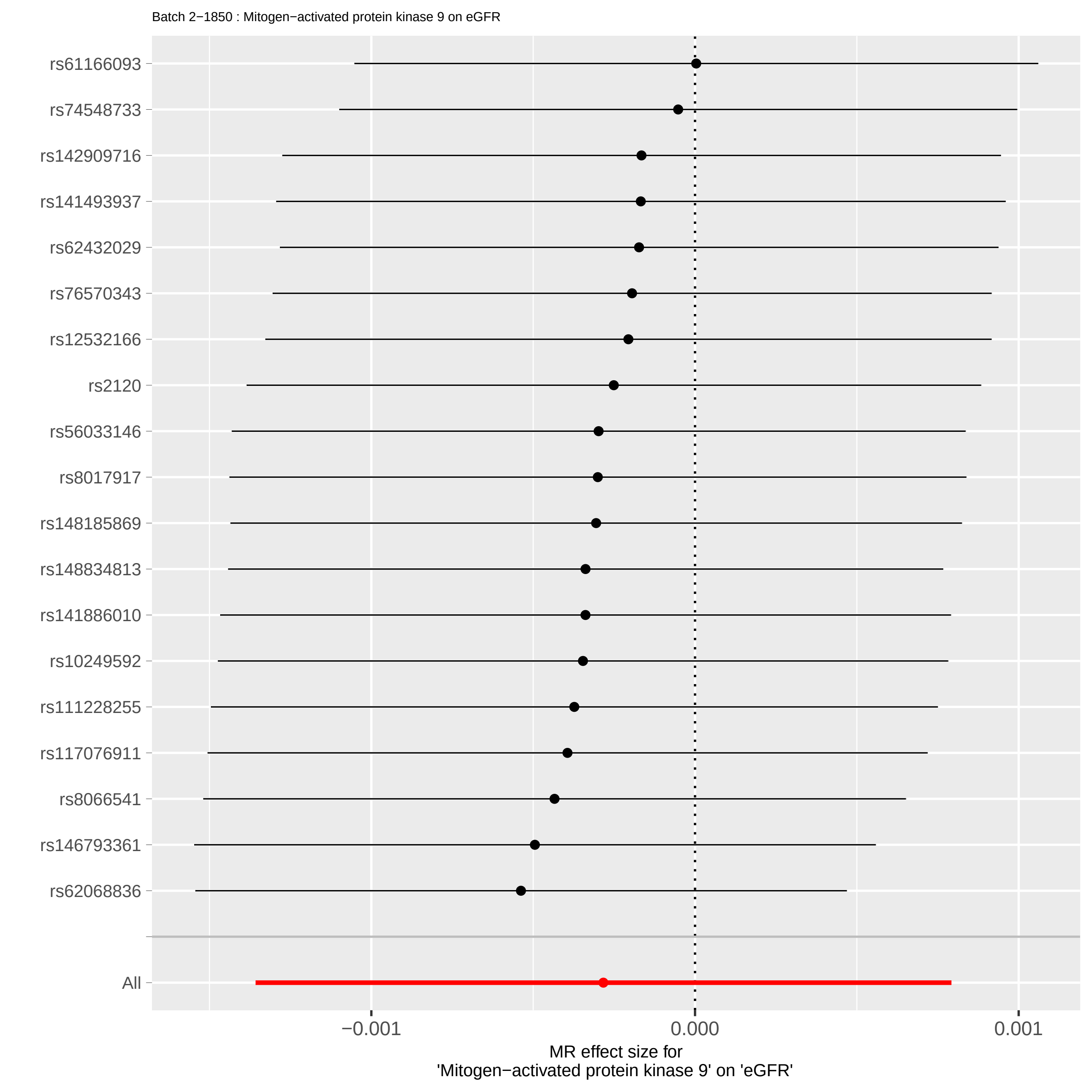

Batch 2−1850 : Mitogen−activated protein kinase 9 on eGFR
rs61166093
rs74548733
rs142909716
rs141493937
rs62432029
rs76570343
rs12532166
rs2120
rs56033146
rs8017917
rs148185869
rs148834813
rs141886010
rs10249592
rs111228255
rs117076911
rs8066541
rs146793361
rs62068836
All
−0.001
0.000
0.001
MR effect size for
'Mitogen−activated protein kinase 9' on 'eGFR'

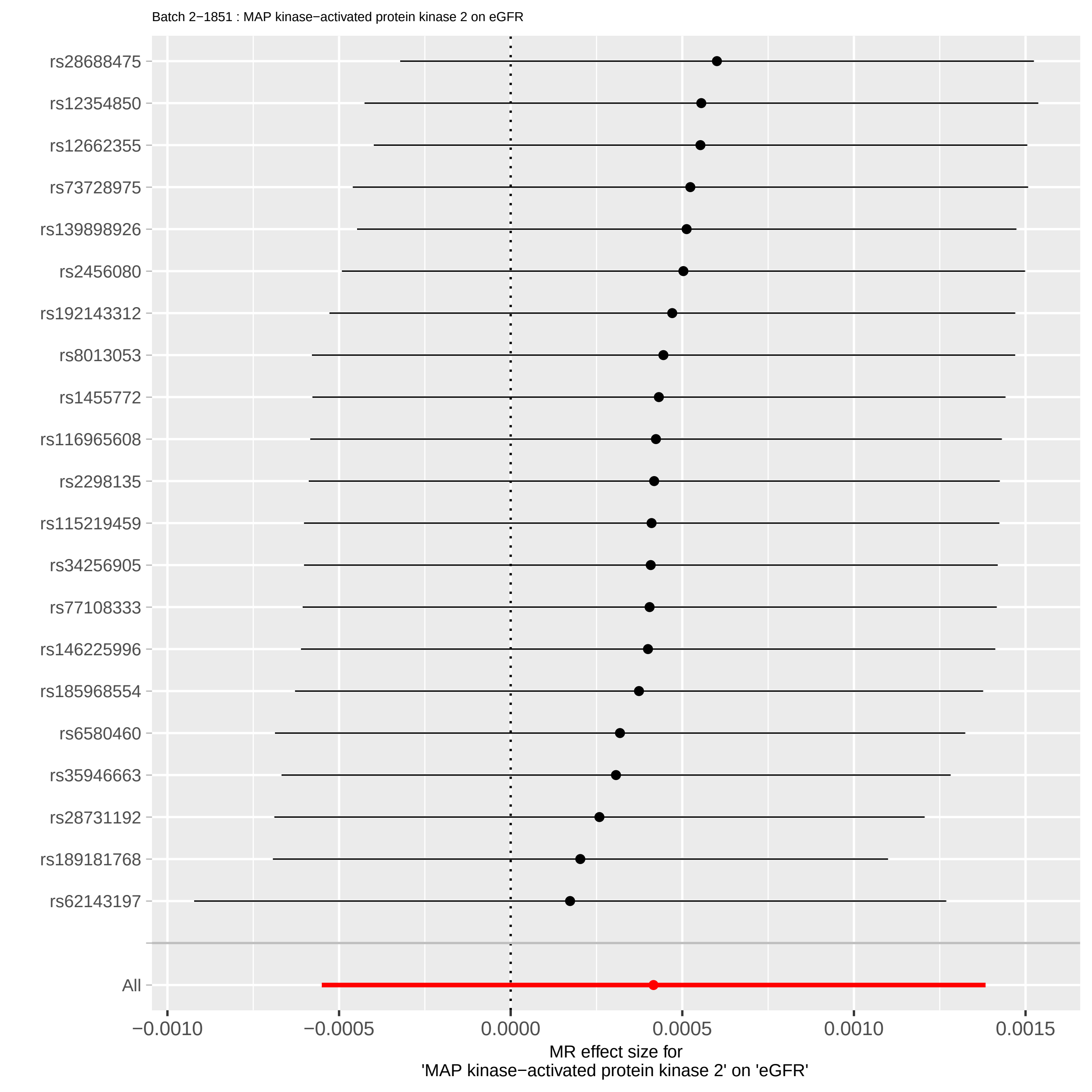

Batch 2−1851 : MAP kinase−activated protein kinase 2 on eGFR
rs28688475
rs12354850
rs12662355
rs73728975
rs139898926
rs2456080
rs192143312
rs8013053
rs1455772
rs116965608
rs2298135
rs115219459
rs34256905
rs77108333
rs146225996
rs185968554
rs6580460
rs35946663
rs28731192
rs189181768
rs62143197
All
−0.0010
−0.0005
0.0000
0.0005
0.0010
0.0015
MR effect size for
'MAP kinase−activated protein kinase 2' on 'eGFR'

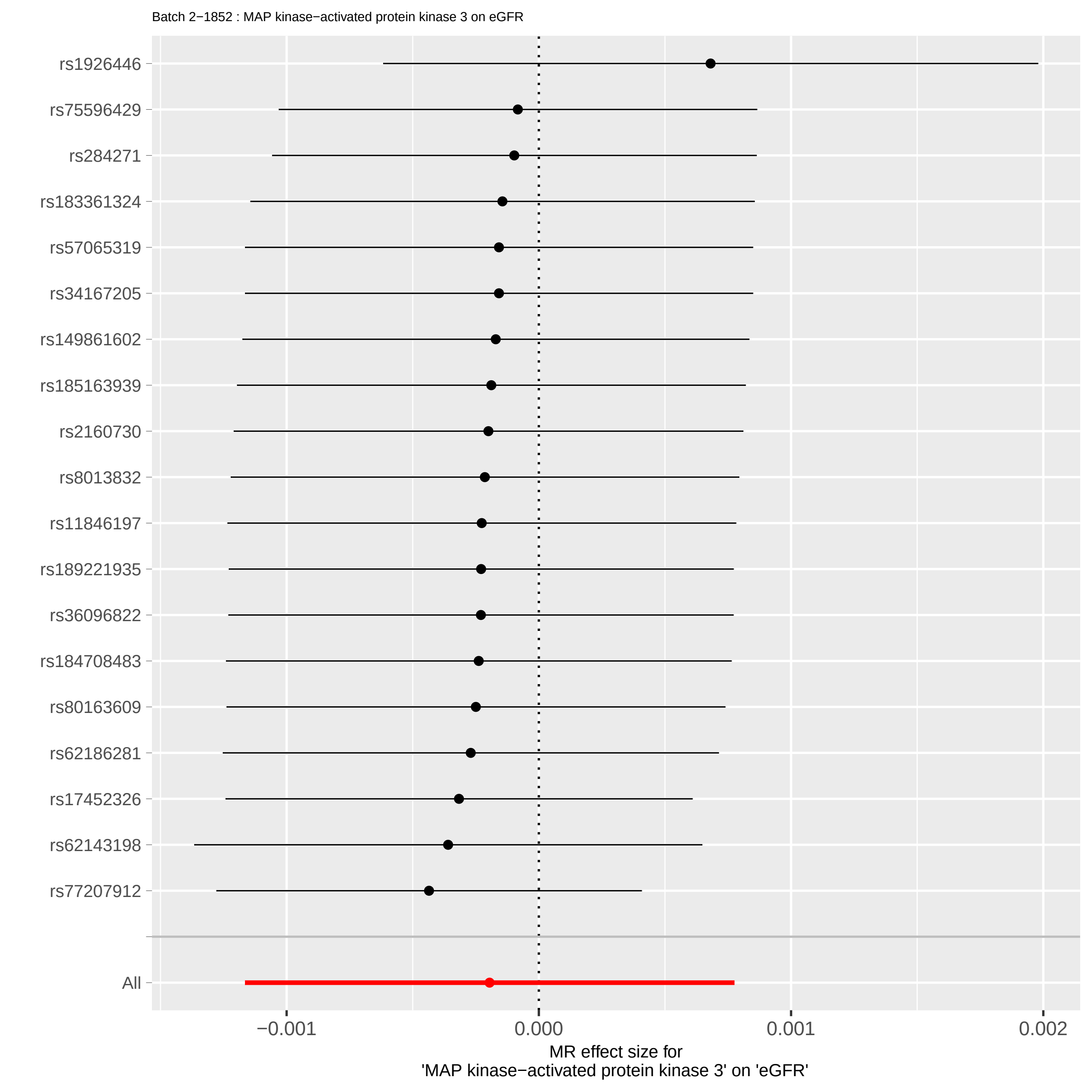
Batch 2−1852 : MAP kinase−activated protein kinase 3 on eGFR
rs1926446
rs75596429
rs284271
rs183361324
rs57065319
rs34167205
rs149861602
rs185163939
rs2160730
rs8013832
rs11846197
rs189221935
rs36096822
rs184708483
rs80163609
rs62186281
rs17452326
rs62143198
rs77207912
All
−0.001
0.000
0.001
0.002
MR effect size for
'MAP kinase−activated protein kinase 3' on 'eGFR'

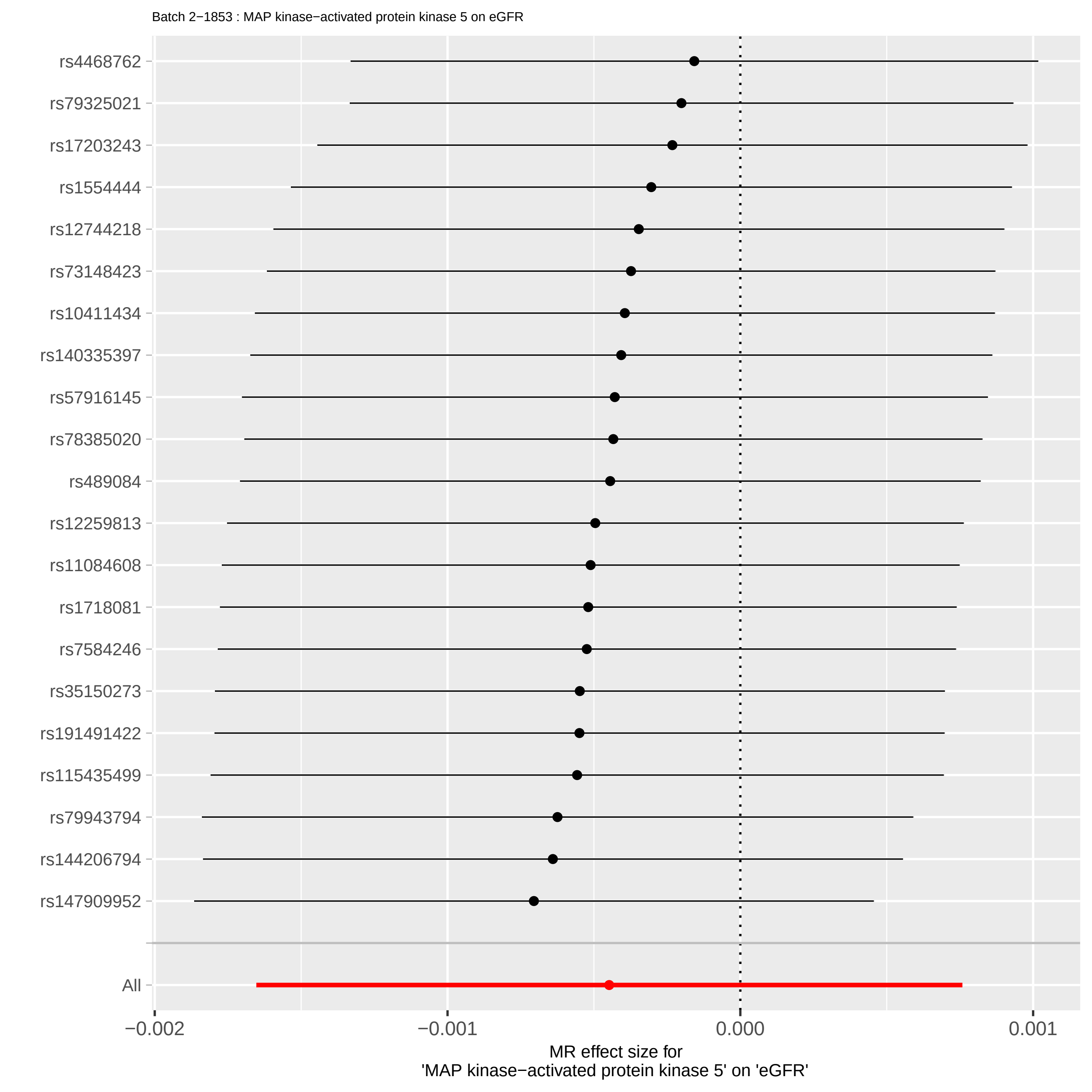

Batch 2−1853 : MAP kinase−activated protein kinase 5 on eGFR
rs4468762
rs79325021
rs17203243
rs1554444
rs12744218
rs73148423
rs10411434
rs140335397
rs57916145
rs78385020
rs489084
rs12259813
rs11084608
rs1718081
rs7584246
rs35150273
rs191491422
rs115435499
rs79943794
rs144206794
rs147909952
All
−0.002
−0.001
0.000
0.001
MR effect size for
'MAP kinase−activated protein kinase 5' on 'eGFR'

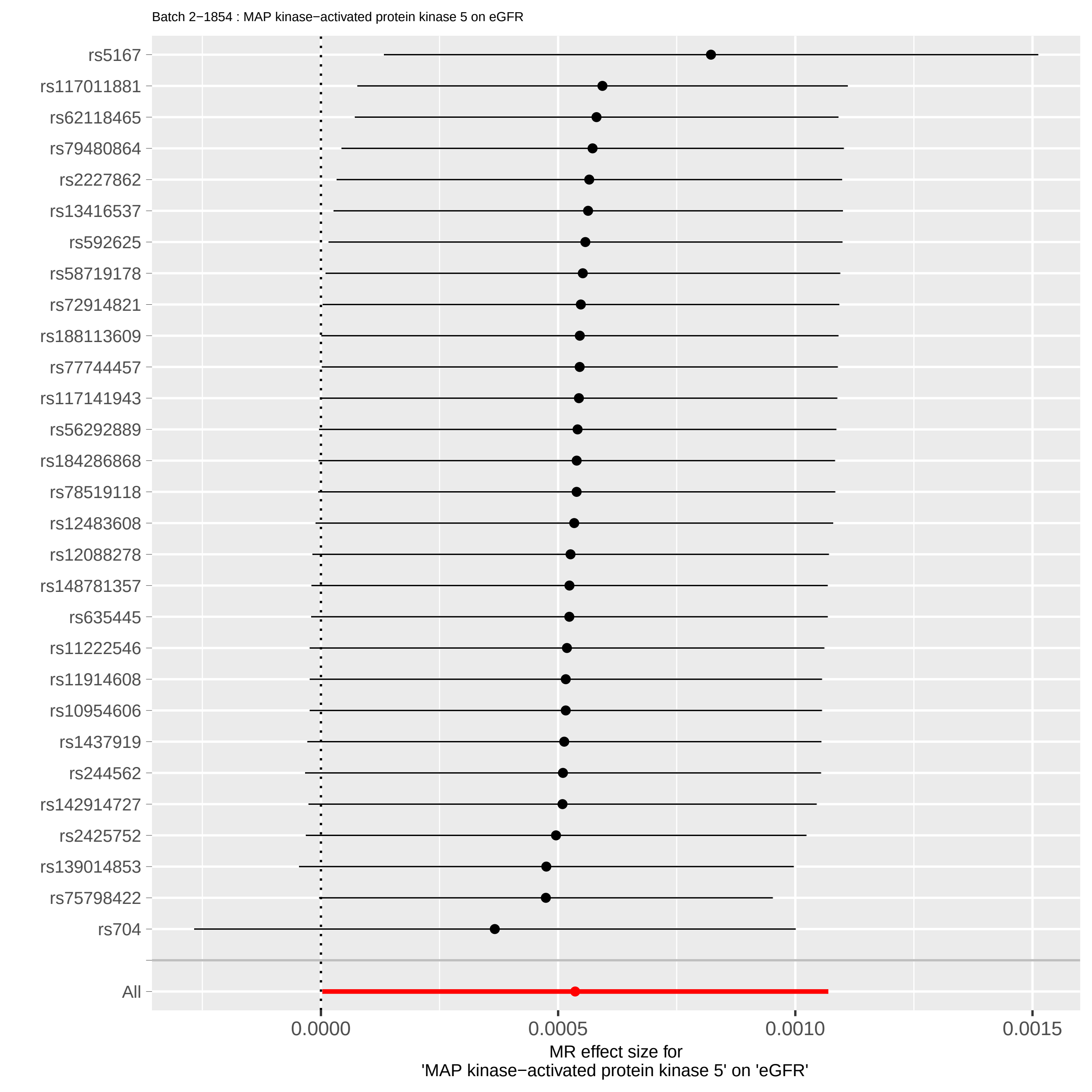

Batch 2−1854 : MAP kinase−activated protein kinase 5 on eGFR
rs5167
rs117011881
rs62118465
rs79480864
rs2227862
rs13416537
rs592625
rs58719178
rs72914821
rs188113609
rs77744457
rs117141943
rs56292889
rs184286868
rs78519118
rs12483608
rs12088278
rs148781357
rs635445
rs11222546
rs11914608
rs10954606
rs1437919
rs244562
rs142914727
rs2425752
rs139014853
rs75798422
rs704
All
0.0000
0.0005
0.0010
0.0015
MR effect size for
'MAP kinase−activated protein kinase 5' on 'eGFR'

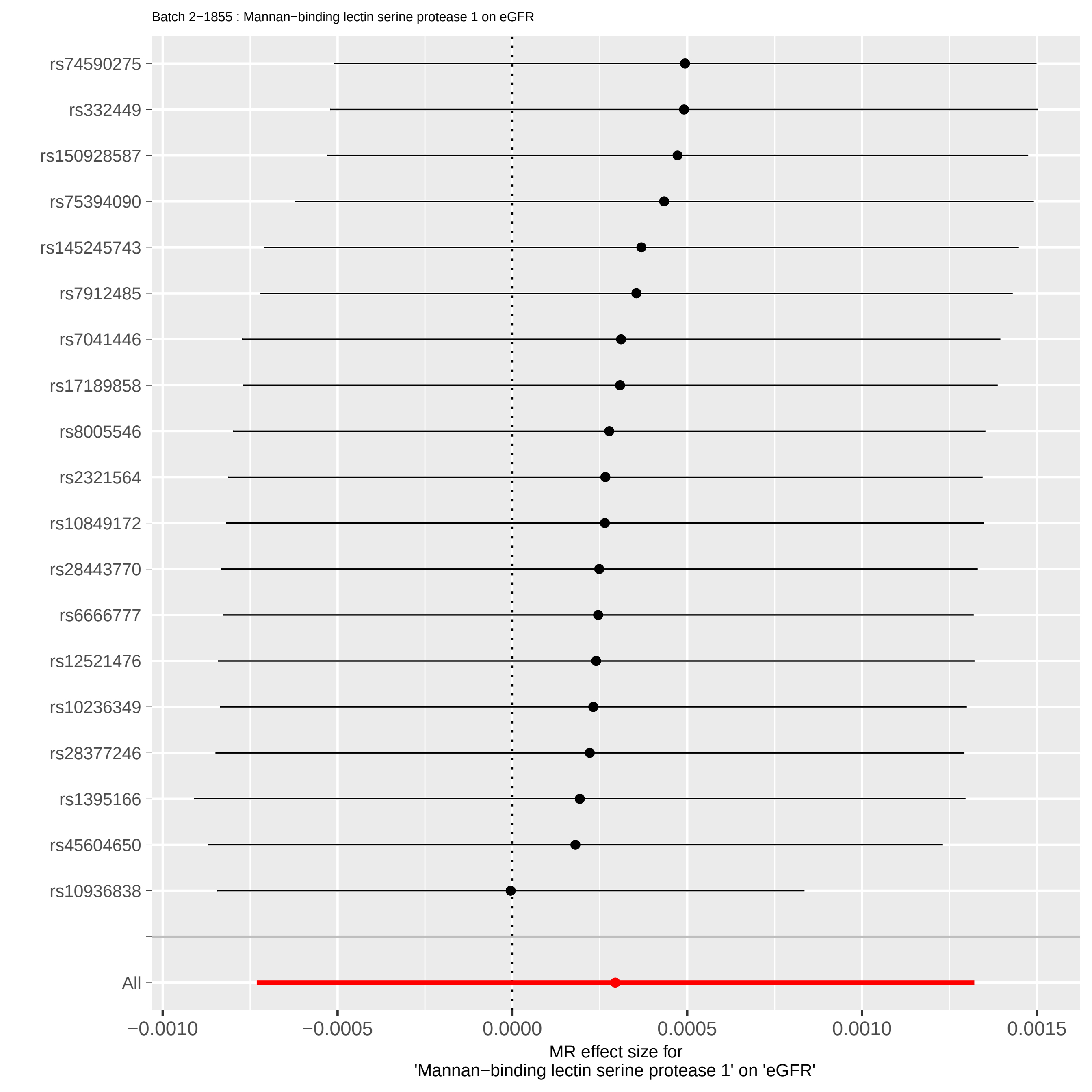

Batch 2−1855 : Mannan−binding lectin serine protease 1 on eGFR
rs74590275
rs332449
rs150928587
rs75394090
rs145245743
rs7912485
rs7041446
rs17189858
rs8005546
rs2321564
rs10849172
rs28443770
rs6666777
rs12521476
rs10236349
rs28377246
rs1395166
rs45604650
rs10936838
All
−0.0010
−0.0005
0.0000
0.0005
0.0010
0.0015
MR effect size for
'Mannan−binding lectin serine protease 1' on 'eGFR'

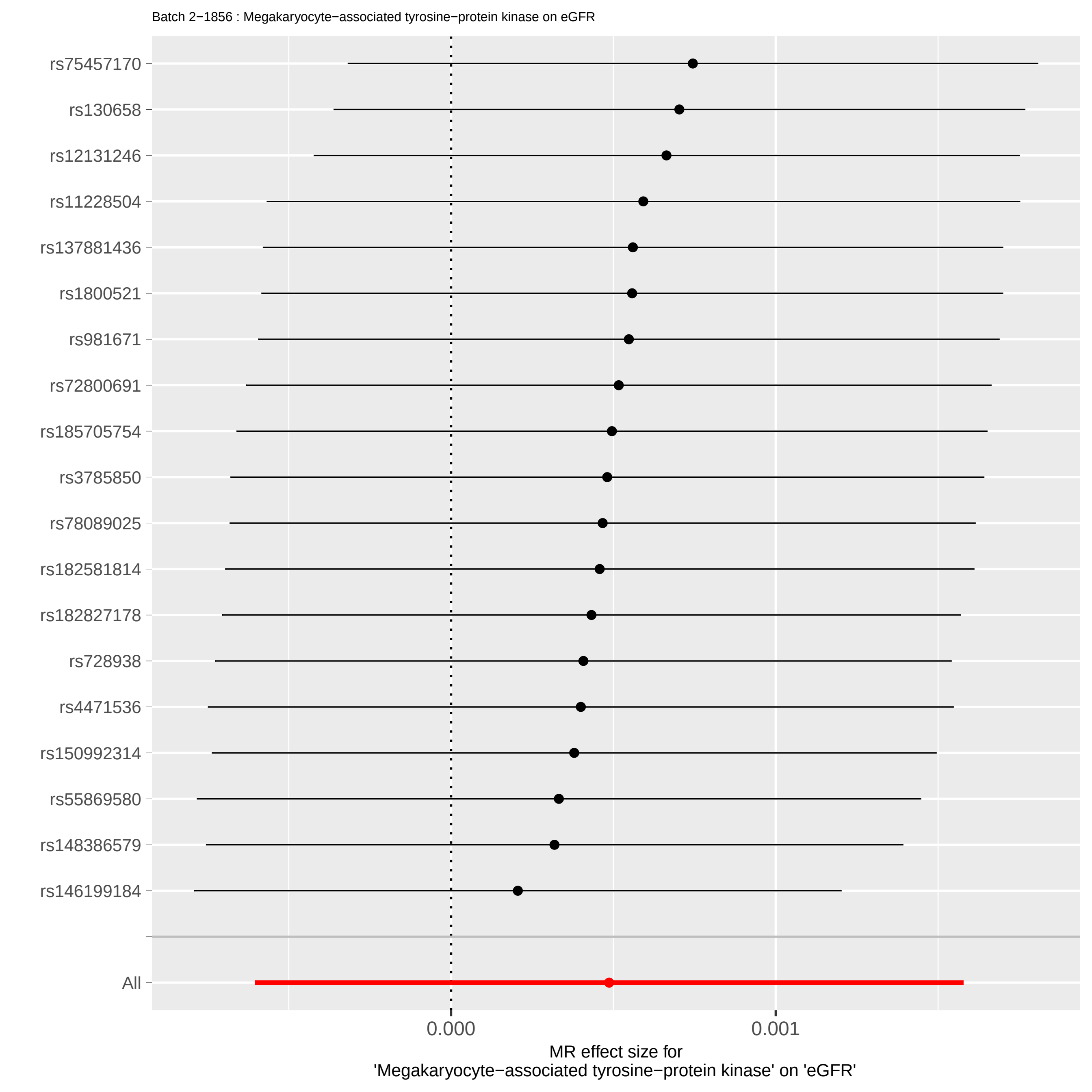

Batch 2−1856 : Megakaryocyte−associated tyrosine−protein kinase on eGFR
rs75457170
rs130658
rs12131246
rs11228504
rs137881436
rs1800521
rs981671
rs72800691
rs185705754
rs3785850
rs78089025
rs182581814
rs182827178
rs728938
rs4471536
rs150992314
rs55869580
rs148386579
rs146199184
All
0.000
0.001
MR effect size for
'Megakaryocyte−associated tyrosine−protein kinase' on 'eGFR'

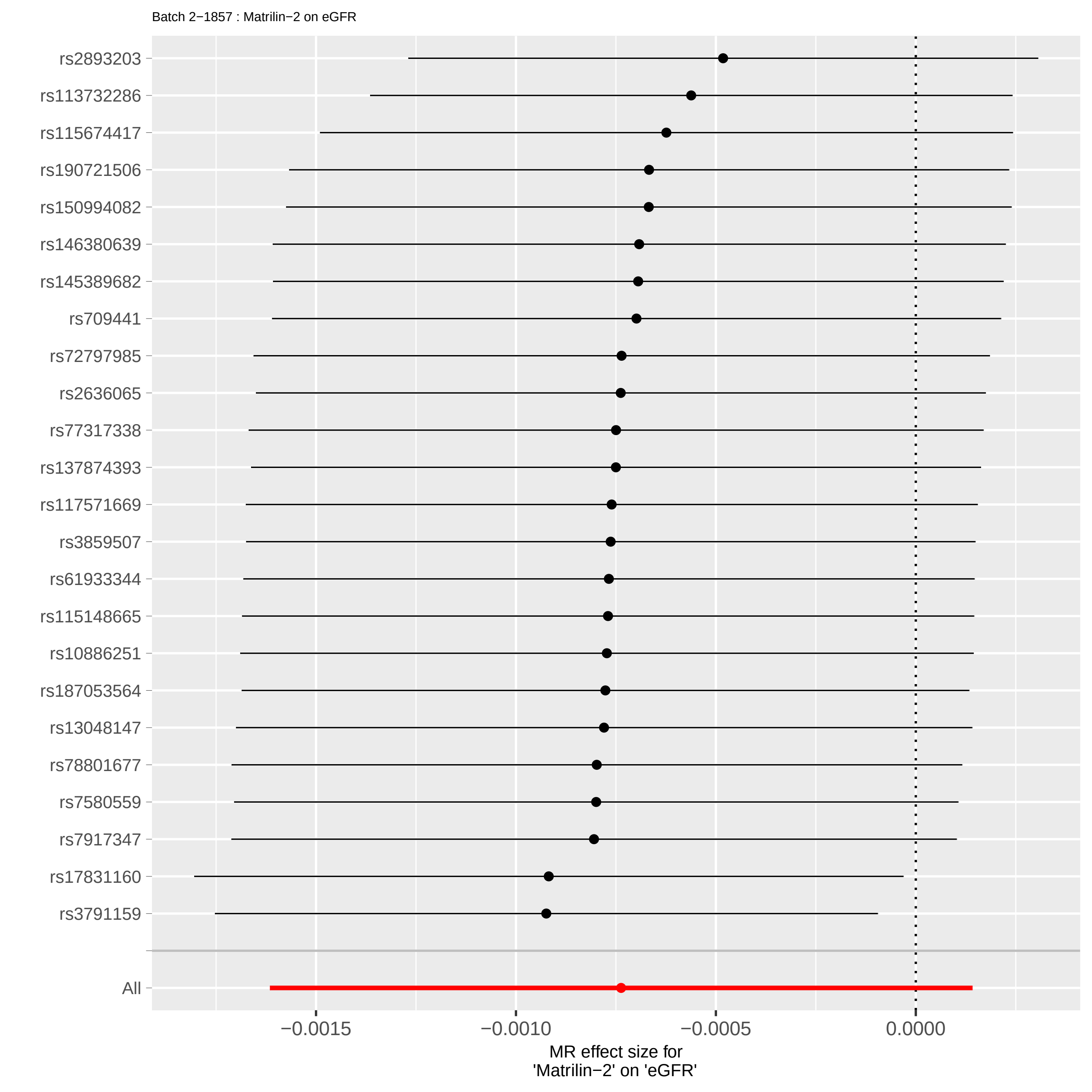

Batch 2−1857 : Matrilin−2 on eGFR
rs2893203
rs113732286
rs115674417
rs190721506
rs150994082
rs146380639
rs145389682
rs709441
rs72797985
rs2636065
rs77317338
rs137874393
rs117571669
rs3859507
rs61933344
rs115148665
rs10886251
rs187053564
rs13048147
rs78801677
rs7580559
rs7917347
rs17831160
rs3791159
All
−0.0015
−0.0010
−0.0005
0.0000
MR effect size for
'Matrilin−2' on 'eGFR'

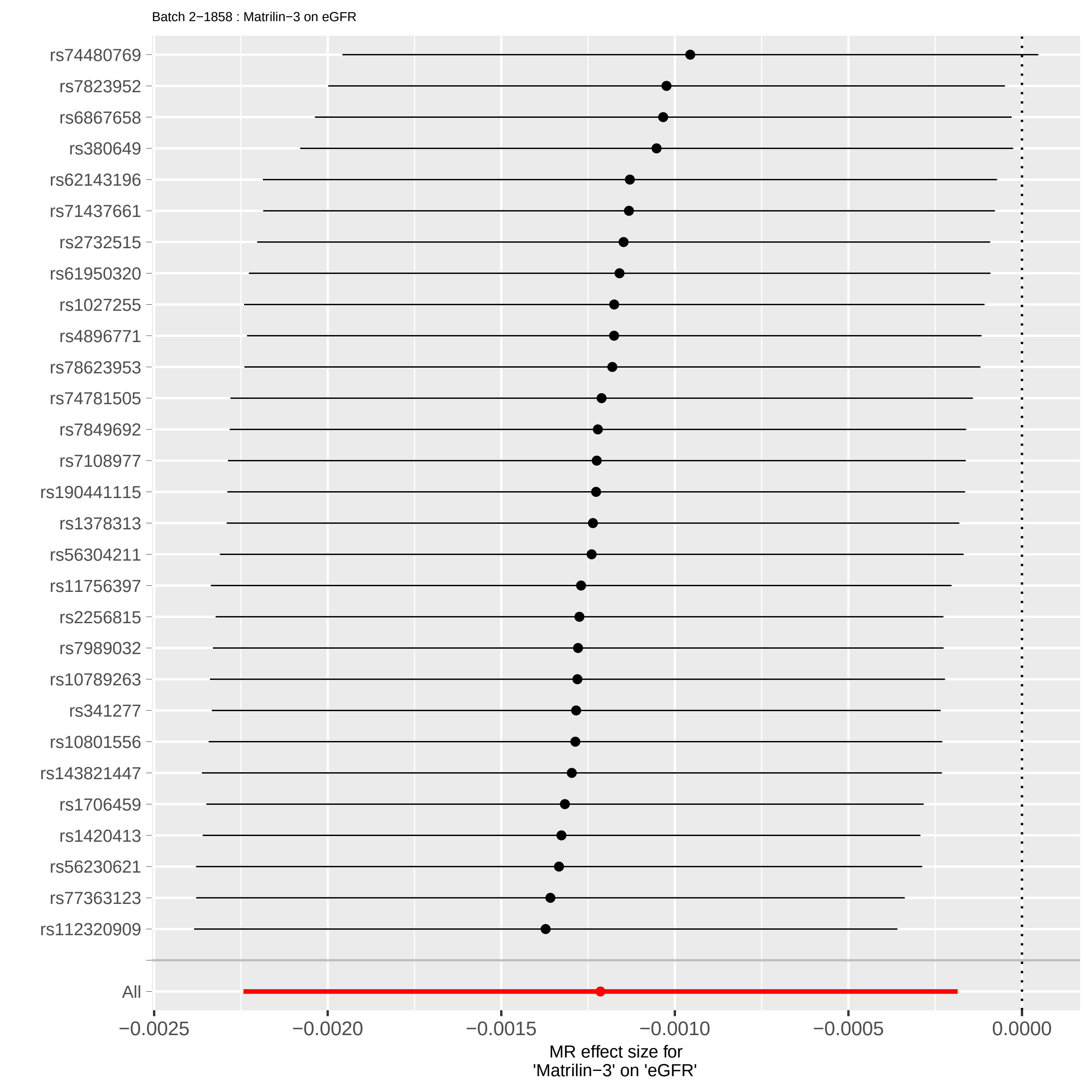

Batch 2−1858 : Matrilin−3 on eGFR
rs74480769
rs7823952
rs6867658
rs380649
rs62143196
rs71437661
rs2732515
rs61950320
rs1027255
rs4896771
rs78623953
rs74781505
rs7849692
rs7108977
rs190441115
rs1378313
rs56304211
rs11756397
rs2256815
rs7989032
rs10789263
rs341277
rs10801556
rs143821447
rs1706459
rs1420413
rs56230621
rs77363123
rs112320909
All
−0.0025
−0.0020
−0.0015
−0.0010
−0.0005
0.0000
MR effect size for
'Matrilin−3' on 'eGFR'

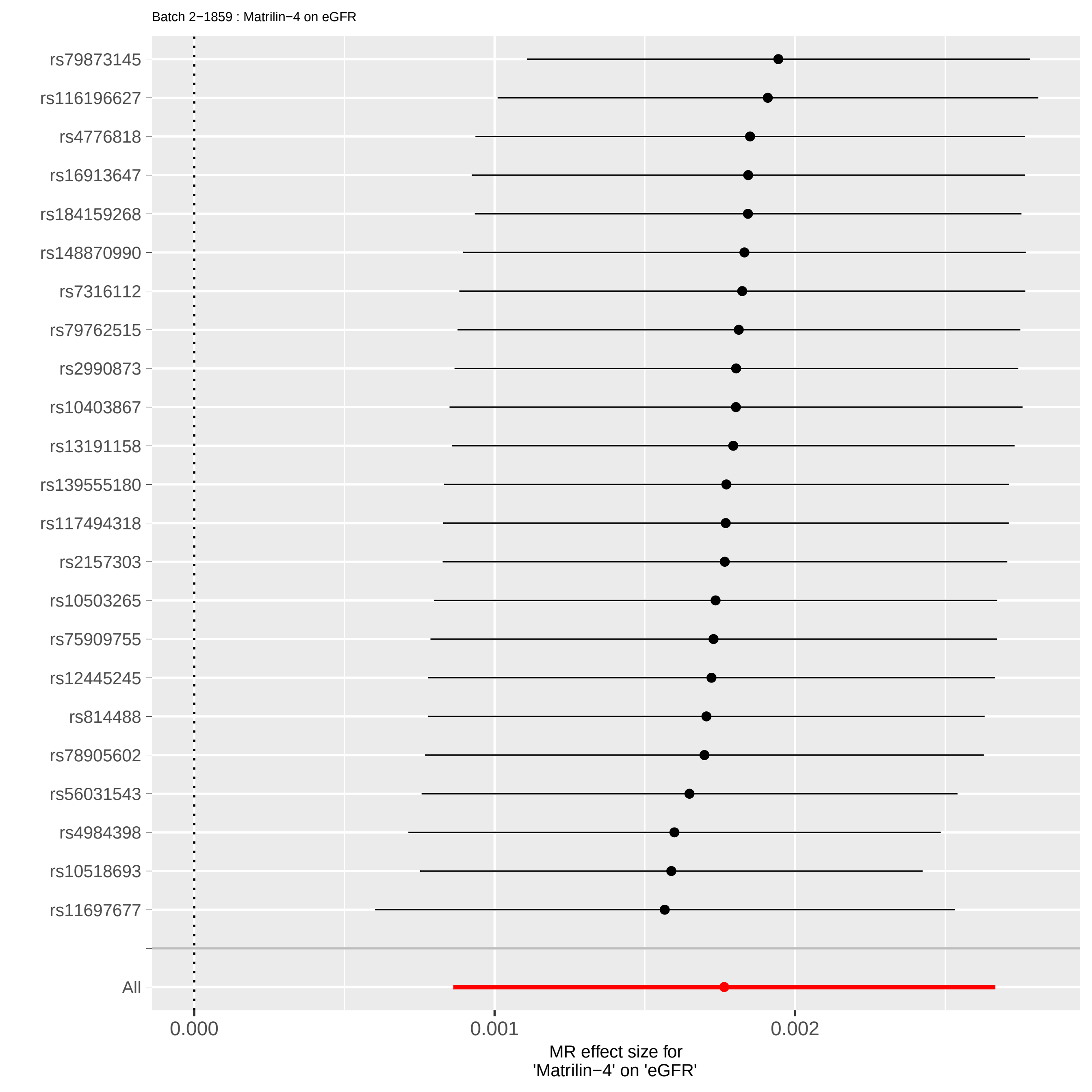
Batch 2−1859 : Matrilin−4 on eGFR
rs79873145
rs116196627
rs4776818
rs16913647
rs184159268
rs148870990
rs7316112
rs79762515
rs2990873
rs10403867
rs13191158
rs139555180
rs117494318
rs2157303
rs10503265
rs75909755
rs12445245
rs814488
rs78905602
rs56031543
rs4984398
rs10518693
rs11697677
All
0.000
0.001
0.002
MR effect size for
'Matrilin−4' on 'eGFR'

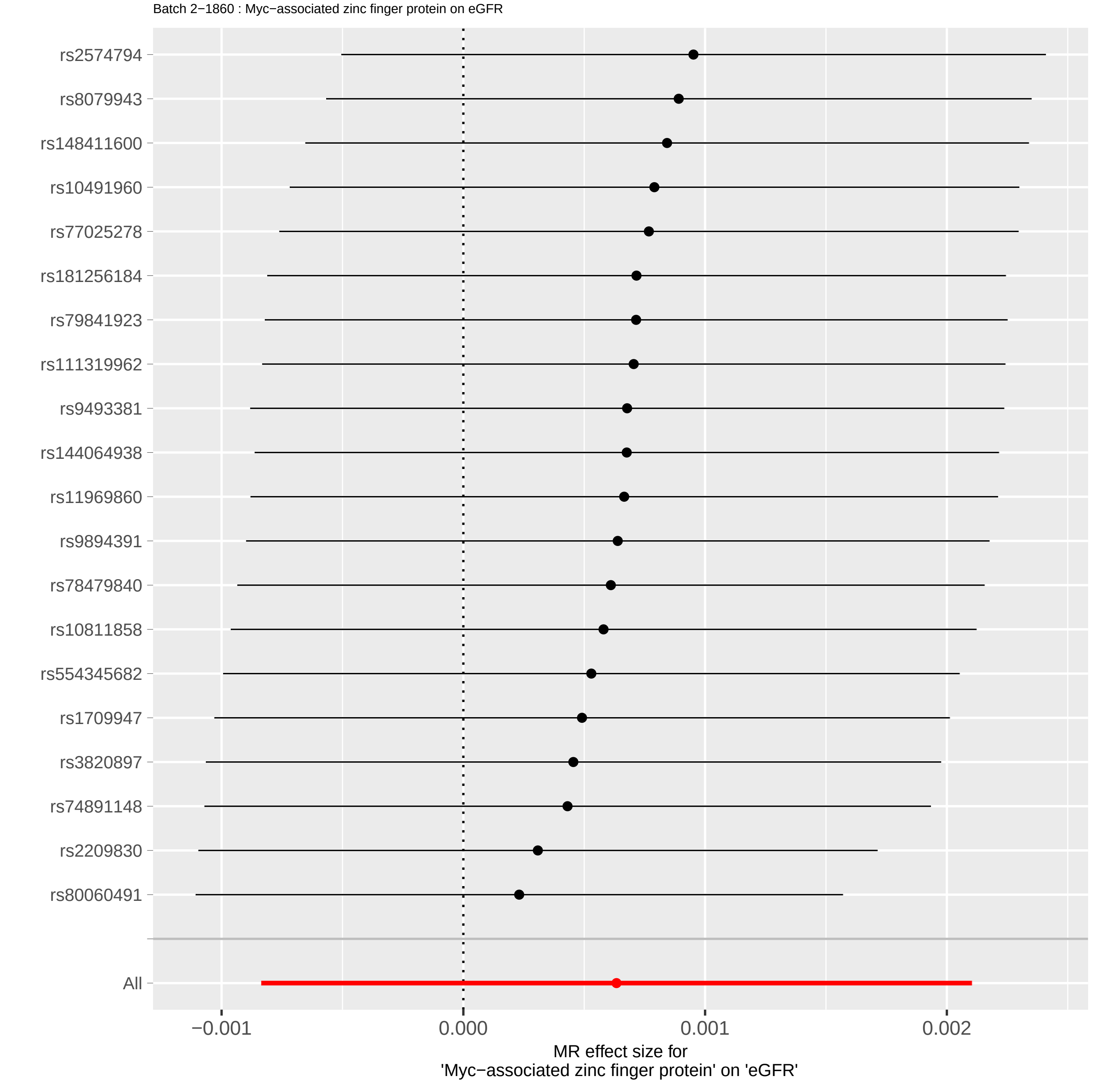
Batch 2−1860 : Myc−associated zinc finger protein on eGFR
rs2574794
rs8079943
rs148411600
rs10491960
rs77025278
rs181256184
rs79841923
rs111319962
rs9493381
rs144064938
rs11969860
rs9894391
rs78479840
rs10811858
rs554345682
rs1709947
rs3820897
rs74891148
rs2209830
rs80060491
All
−0.001
0.000
0.001
0.002
MR effect size for
'Myc−associated zinc finger protein' on 'eGFR'

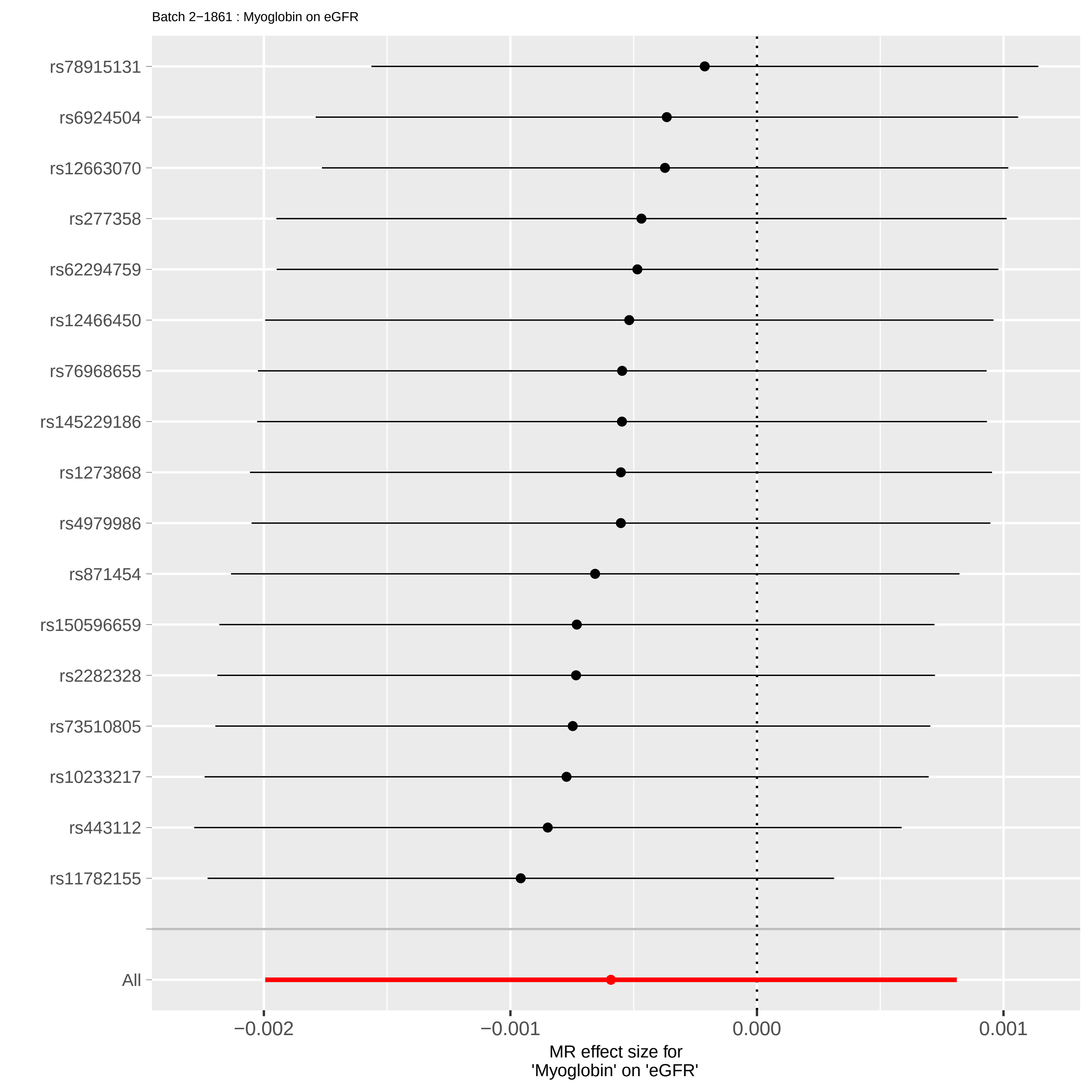

Batch 2−1861 : Myoglobin on eGFR
rs78915131
rs6924504
rs12663070
rs277358
rs62294759
rs12466450
rs76968655
rs145229186
rs1273868
rs4979986
rs871454
rs150596659
rs2282328
rs73510805
rs10233217
rs443112
rs11782155
All
−0.002
−0.001
0.000
0.001
MR effect size for
'Myoglobin' on 'eGFR'

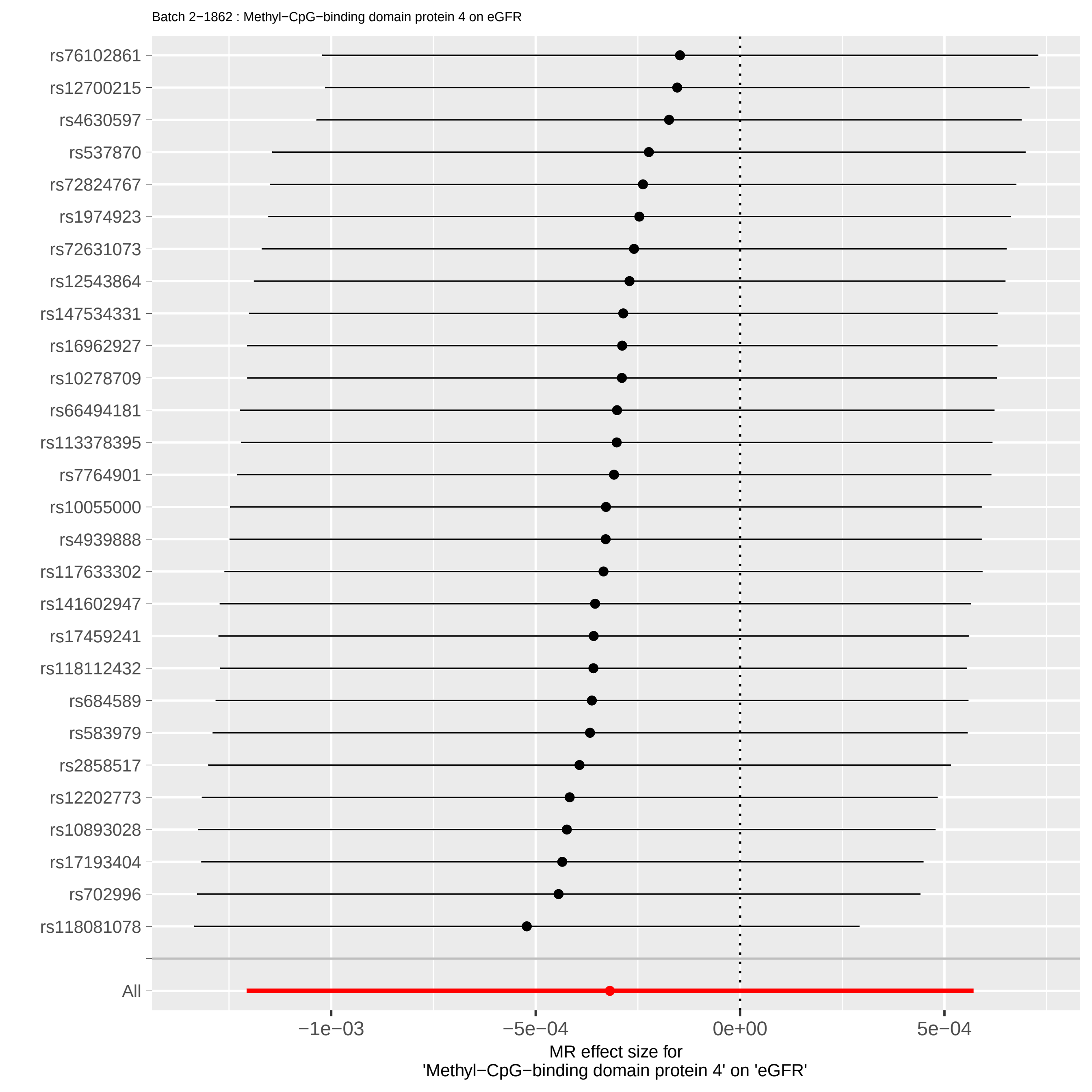

Batch 2−1862 : Methyl−CpG−binding domain protein 4 on eGFR
rs76102861
rs12700215
rs4630597
rs537870
rs72824767
rs1974923
rs72631073
rs12543864
rs147534331
rs16962927
rs10278709
rs66494181
rs113378395
rs7764901
rs10055000
rs4939888
rs117633302
rs141602947
rs17459241
rs118112432
rs684589
rs583979
rs2858517
rs12202773
rs10893028
rs17193404
rs702996
rs118081078
All
−1e−03
−5e−04
0e+00
5e−04
MR effect size for
'Methyl−CpG−binding domain protein 4' on 'eGFR'

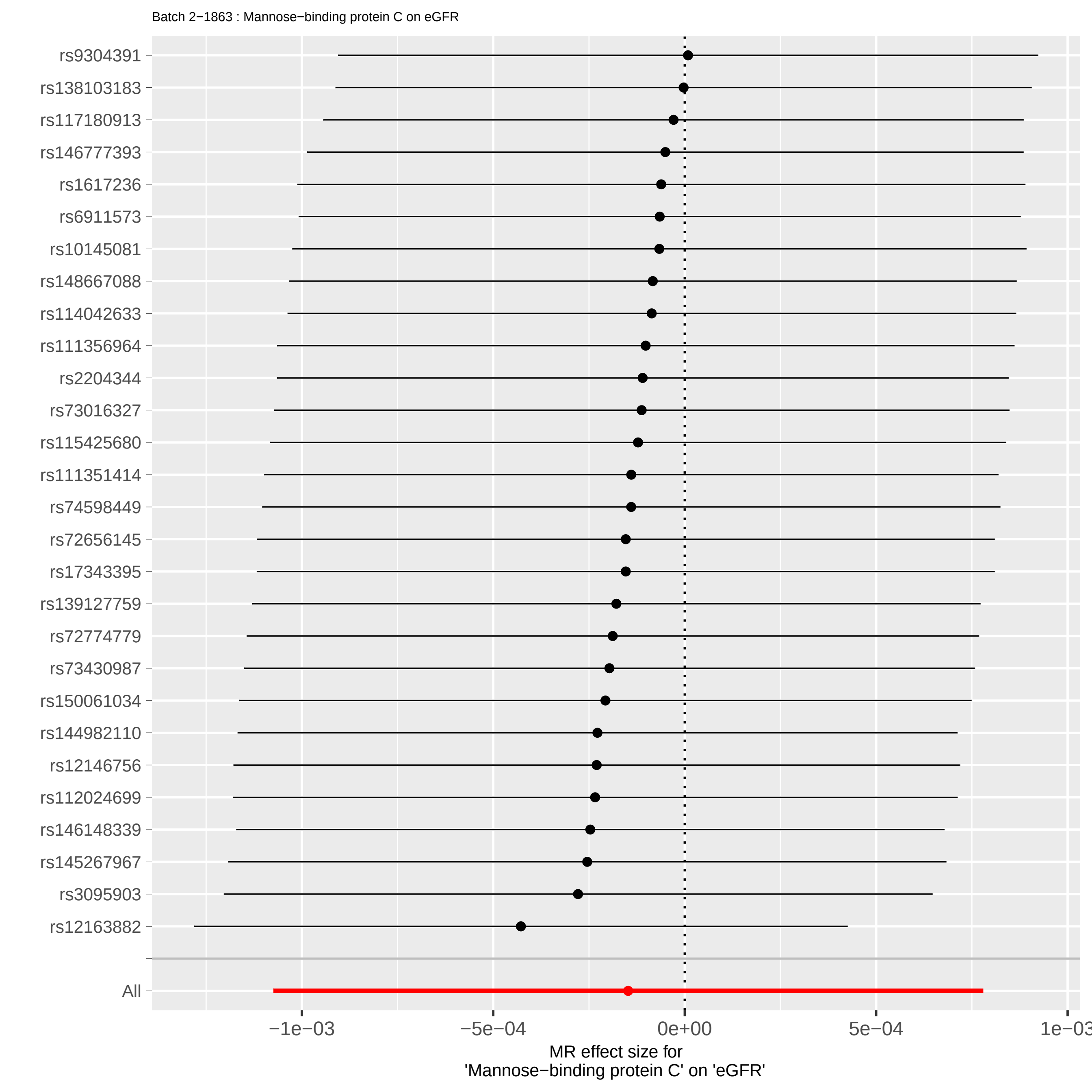
Batch 2−1863 : Mannose−binding protein C on eGFR
rs9304391
rs138103183
rs117180913
rs146777393
rs1617236
rs6911573
rs10145081
rs148667088
rs114042633
rs111356964
rs2204344
rs73016327
rs115425680
rs111351414
rs74598449
rs72656145
rs17343395
rs139127759
rs72774779
rs73430987
rs150061034
rs144982110
rs12146756
rs112024699
rs146148339
rs145267967
rs3095903
rs12163882
All
−1e−03
−5e−04
0e+00
5e−04
1e−03
MR effect size for
'Mannose−binding protein C' on 'eGFR'

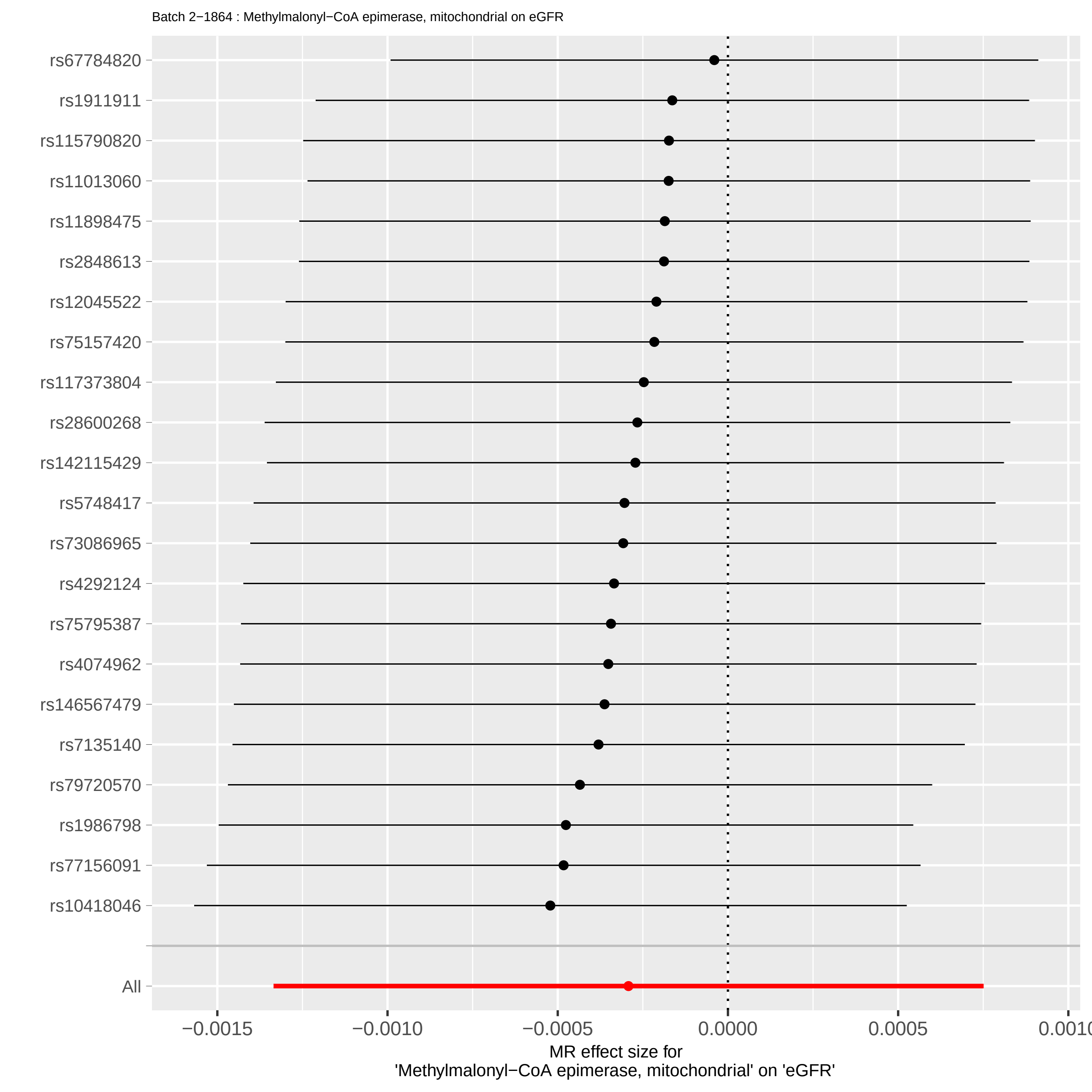

Batch 2−1864 : Methylmalonyl−CoA epimerase, mitochondrial on eGFR
rs67784820
rs1911911
rs115790820
rs11013060
rs11898475
rs2848613
rs12045522
rs75157420
rs117373804
rs28600268
rs142115429
rs5748417
rs73086965
rs4292124
rs75795387
rs4074962
rs146567479
rs7135140
rs79720570
rs1986798
rs77156091
rs10418046
All
−0.0015
−0.0010
−0.0005
0.0000
0.0005
MR effect size for
'Methylmalonyl−CoA epimerase, mitochondrial' on 'eGFR'

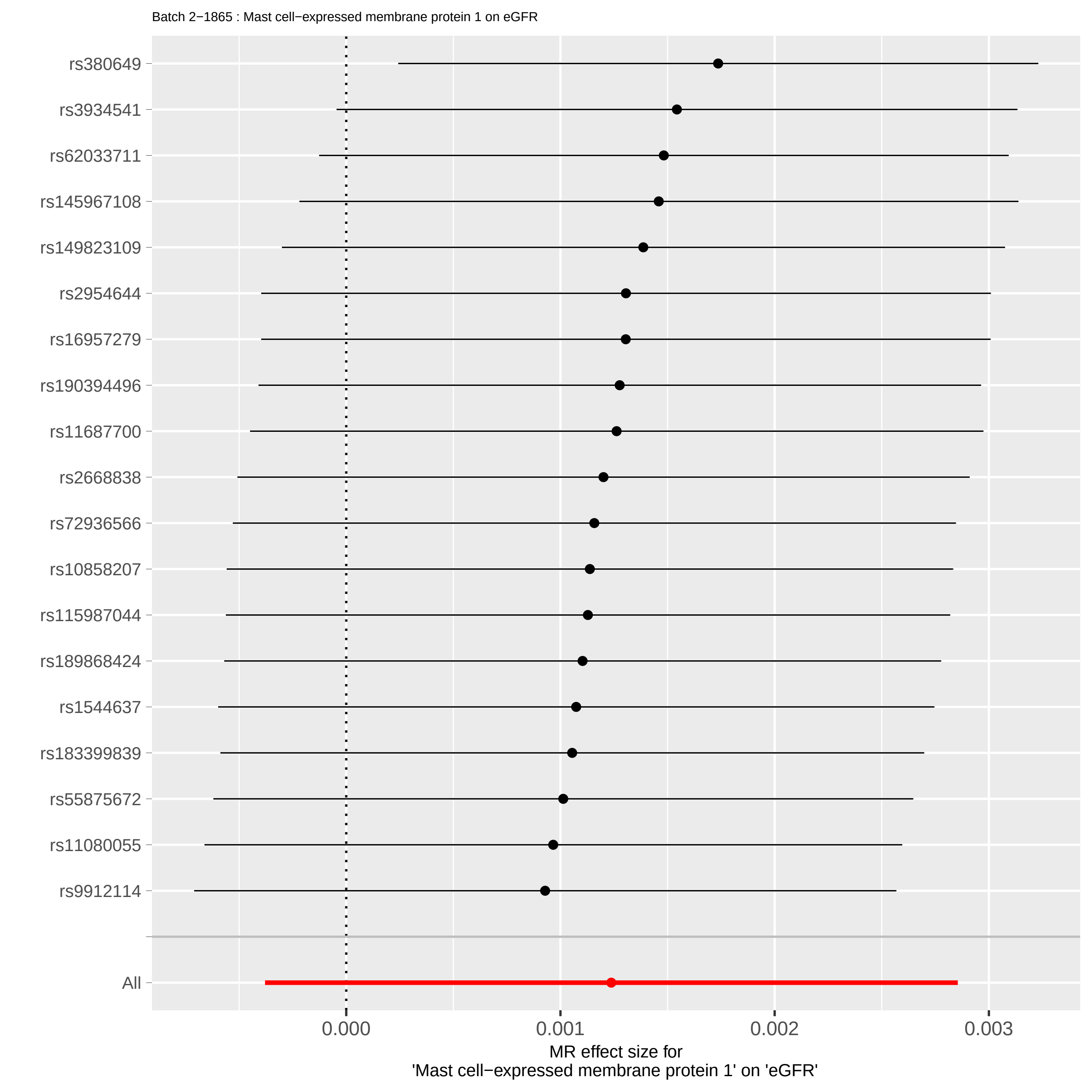

Batch 2−1865 : Mast cell−expressed membrane protein 1 on eGFR
rs380649
rs3934541
rs62033711
rs145967108
rs149823109
rs2954644
rs16957279
rs190394496
rs11687700
rs2668838
rs72936566
rs10858207
rs115987044
rs189868424
rs1544637
rs183399839
rs55875672
rs11080055
rs9912114
All
0.000
0.001
0.002
0.003
MR effect size for
'Mast cell−expressed membrane protein 1' on 'eGFR'

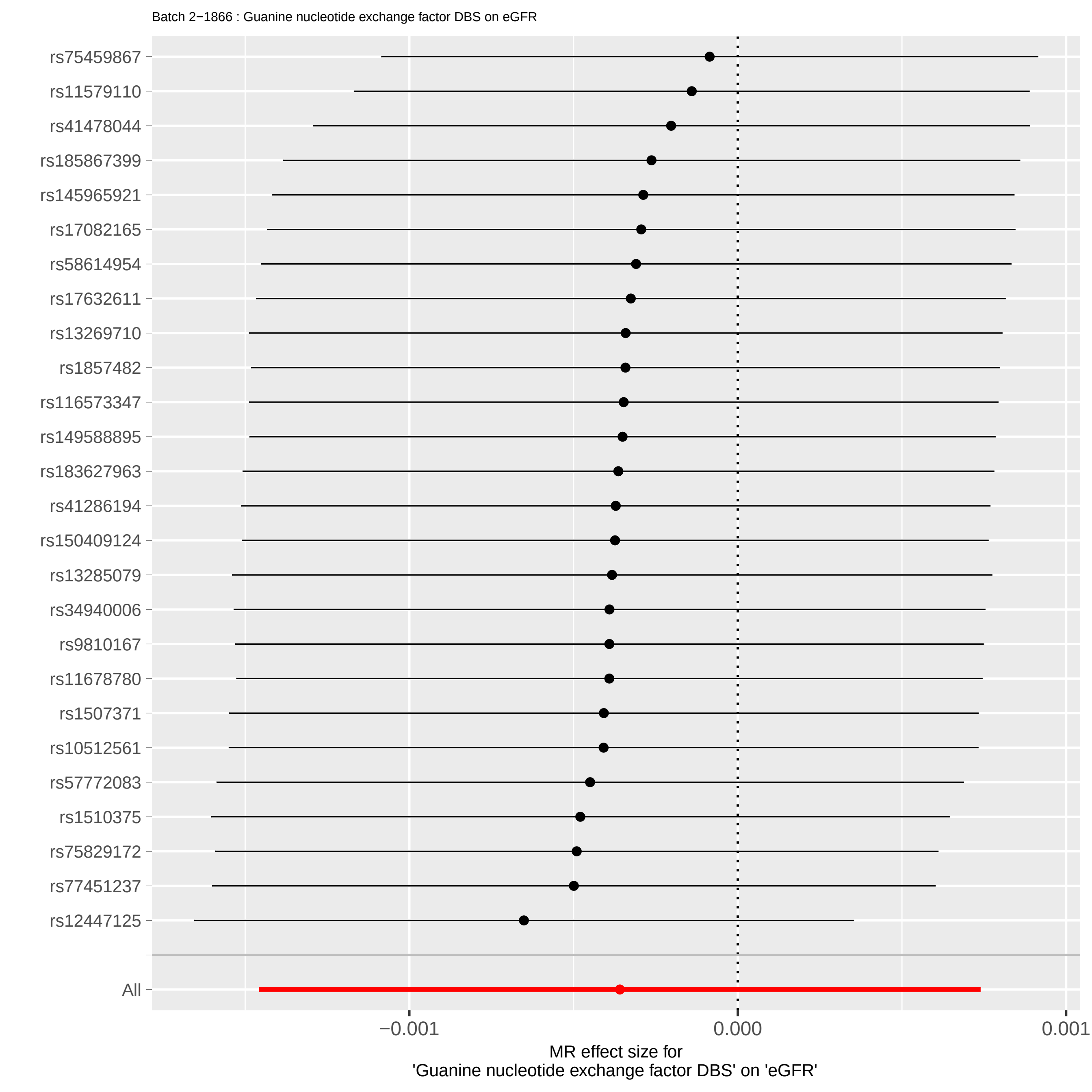

Batch 2−1866 : Guanine nucleotide exchange factor DBS on eGFR
rs75459867
rs11579110
rs41478044
rs185867399
rs145965921
rs17082165
rs58614954
rs17632611
rs13269710
rs1857482
rs116573347
rs149588895
rs183627963
rs41286194
rs150409124
rs13285079
rs34940006
rs9810167
rs11678780
rs1507371
rs10512561
rs57772083
rs1510375
rs75829172
rs77451237
rs12447125
All
−0.001
0.000
0.001
MR effect size for
'Guanine nucleotide exchange factor DBS' on 'eGFR'

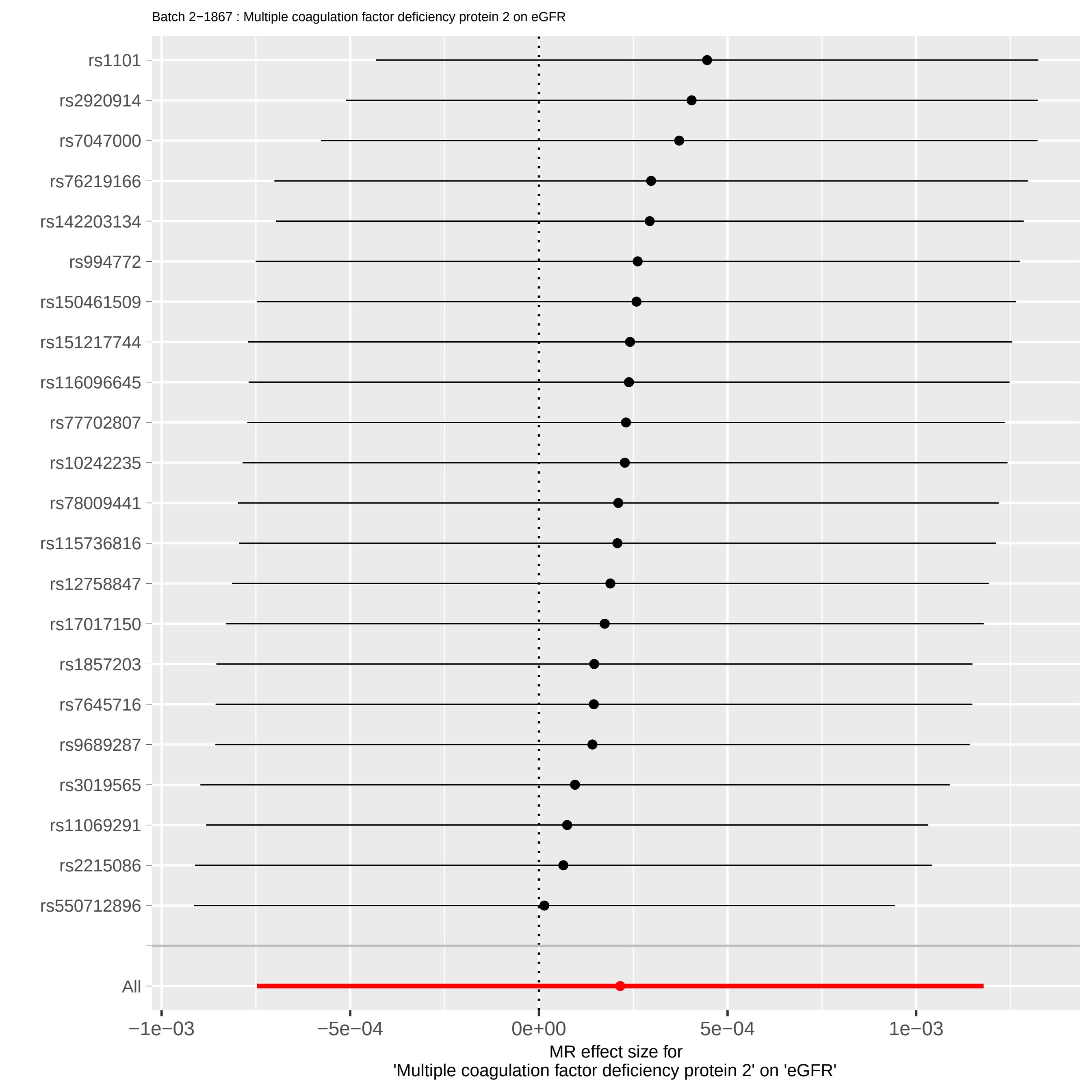

Batch 2−1867 : Multiple coagulation factor deficiency protein 2 on eGFR
rs1101
rs2920914
rs7047000
rs76219166
rs142203134
rs994772
rs150461509
rs151217744
rs116096645
rs77702807
rs10242235
rs78009441
rs115736816
rs12758847
rs17017150
rs1857203
rs7645716
rs9689287
rs3019565
rs11069291
rs2215086
rs550712896
All
−1e−03
−5e−04
0e+00
5e−04
1e−03
MR effect size for
'Multiple coagulation factor deficiency protein 2' on 'eGFR'

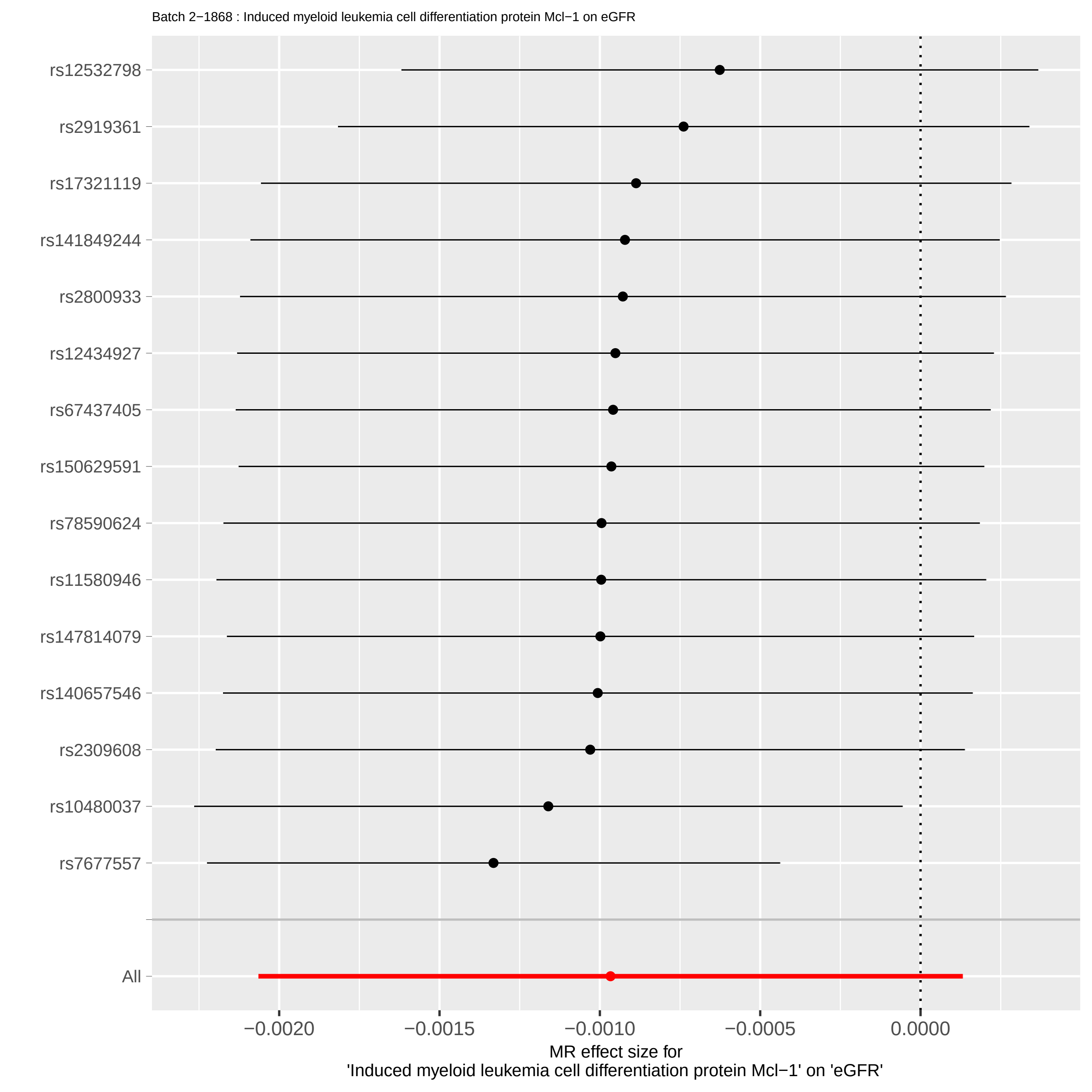

Batch 2−1868 : Induced myeloid leukemia cell differentiation protein Mcl−1 on eGFR
rs12532798
rs2919361
rs17321119
rs141849244
rs2800933
rs12434927
rs67437405
rs150629591
rs78590624
rs11580946
rs147814079
rs140657546
rs2309608
rs10480037
rs7677557
All
−0.0020
−0.0015
−0.0010
−0.0005
0.0000
MR effect size for
'Induced myeloid leukemia cell differentiation protein Mcl−1' on 'eGFR'

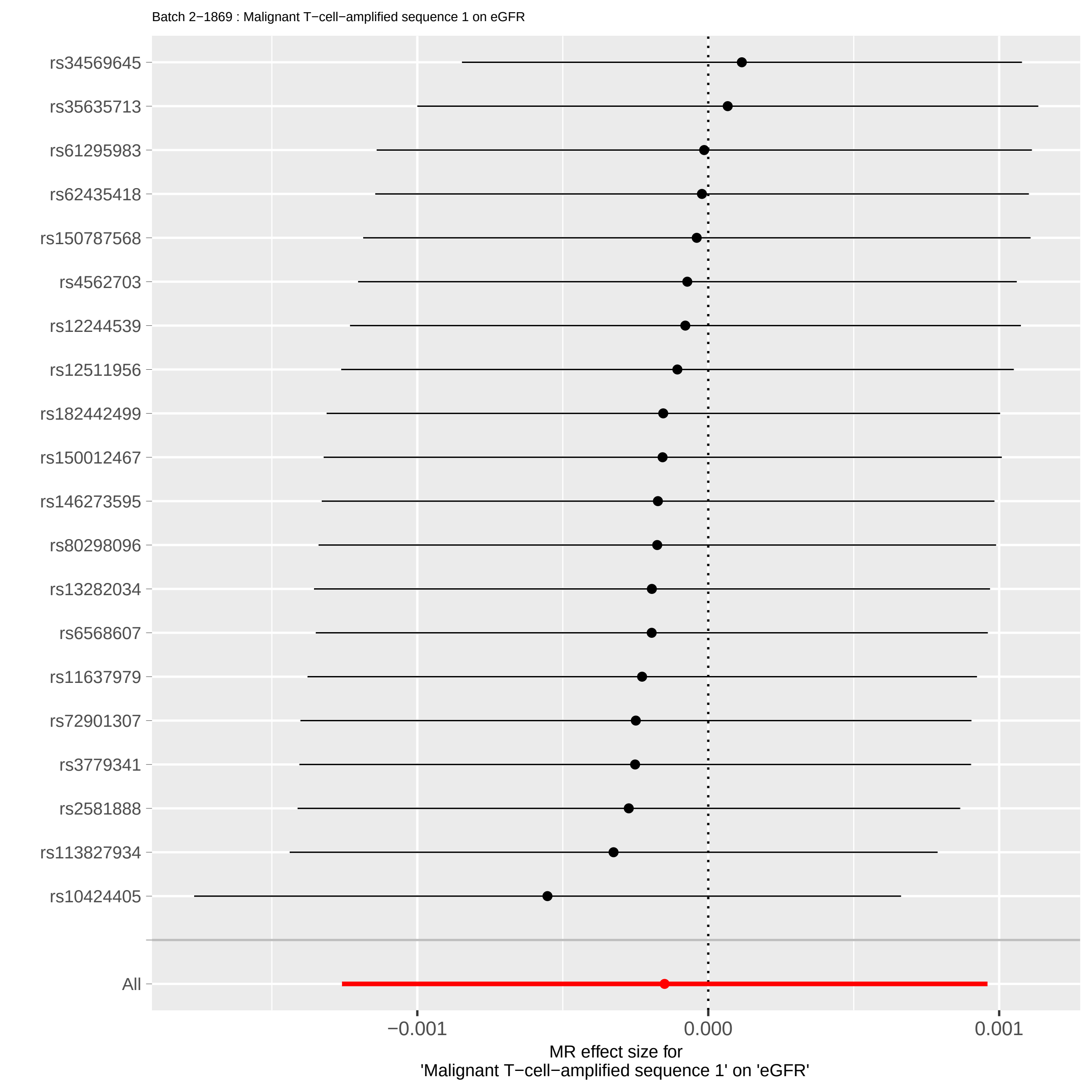
Batch 2−1869 : Malignant T−cell−amplified sequence 1 on eGFR
rs34569645
rs35635713
rs61295983
rs62435418
rs150787568
rs4562703
rs12244539
rs12511956
rs182442499
rs150012467
rs146273595
rs80298096
rs13282034
rs6568607
rs11637979
rs72901307
rs3779341
rs2581888
rs113827934
rs10424405
All
−0.001
0.000
0.001
MR effect size for
'Malignant T−cell−amplified sequence 1' on 'eGFR'

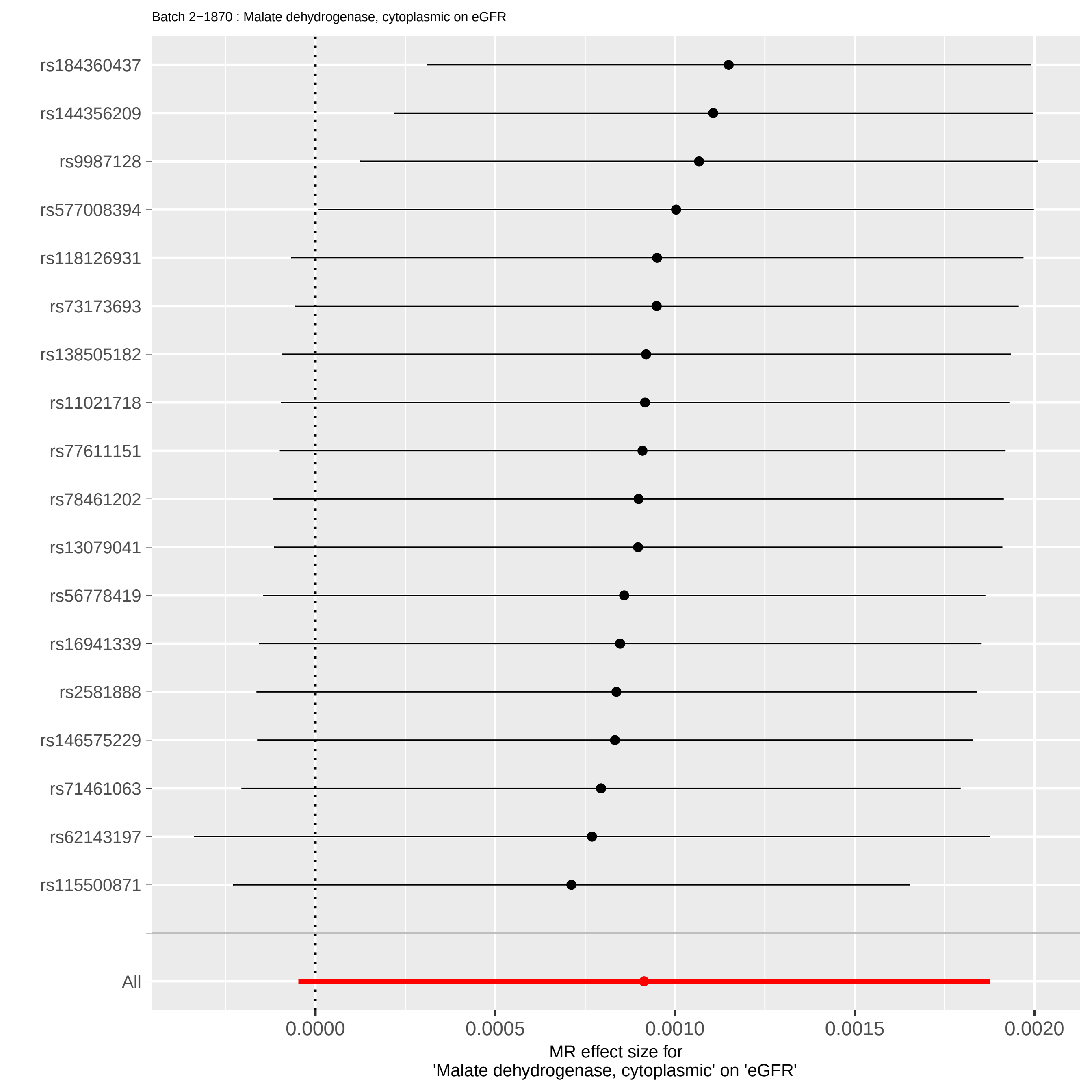

Batch 2−1870 : Malate dehydrogenase, cytoplasmic on eGFR
rs184360437
rs144356209
rs9987128
rs577008394
rs118126931
rs73173693
rs138505182
rs11021718
rs77611151
rs78461202
rs13079041
rs56778419
rs16941339
rs2581888
rs146575229
rs71461063
rs62143197
rs115500871
All
0.0000
0.0005
0.0010
0.0015
0.0020
MR effect size for
'Malate dehydrogenase, cytoplasmic' on 'eGFR'

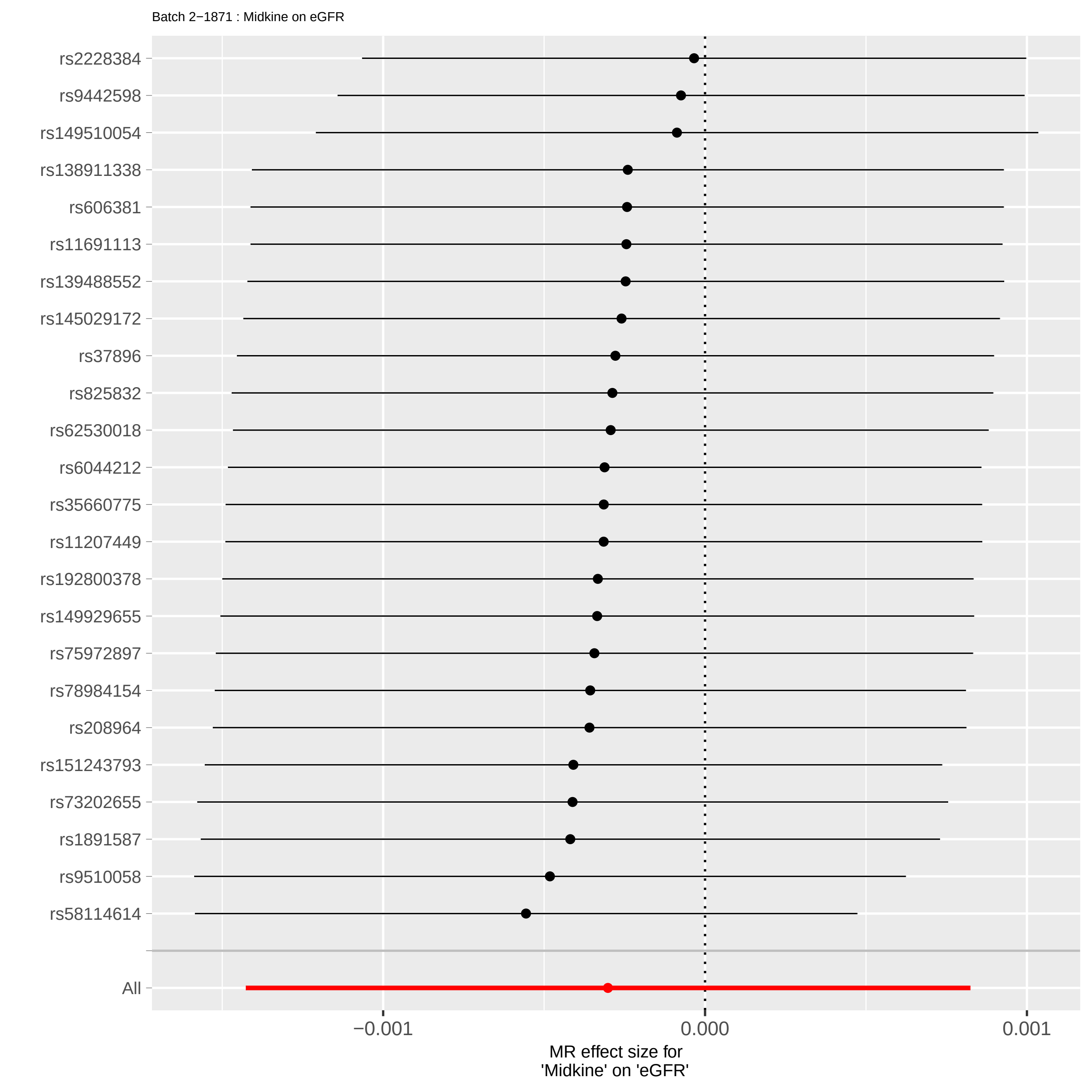
Batch 2−1871 : Midkine on eGFR
rs2228384
rs9442598
rs149510054
rs138911338
rs606381
rs11691113
rs139488552
rs145029172
rs37896
rs825832
rs62530018
rs6044212
rs35660775
rs11207449
rs192800378
rs149929655
rs75972897
rs78984154
rs208964
rs151243793
rs73202655
rs1891587
rs9510058
rs58114614
All
−0.001
0.000
0.001
MR effect size for
'Midkine' on 'eGFR'

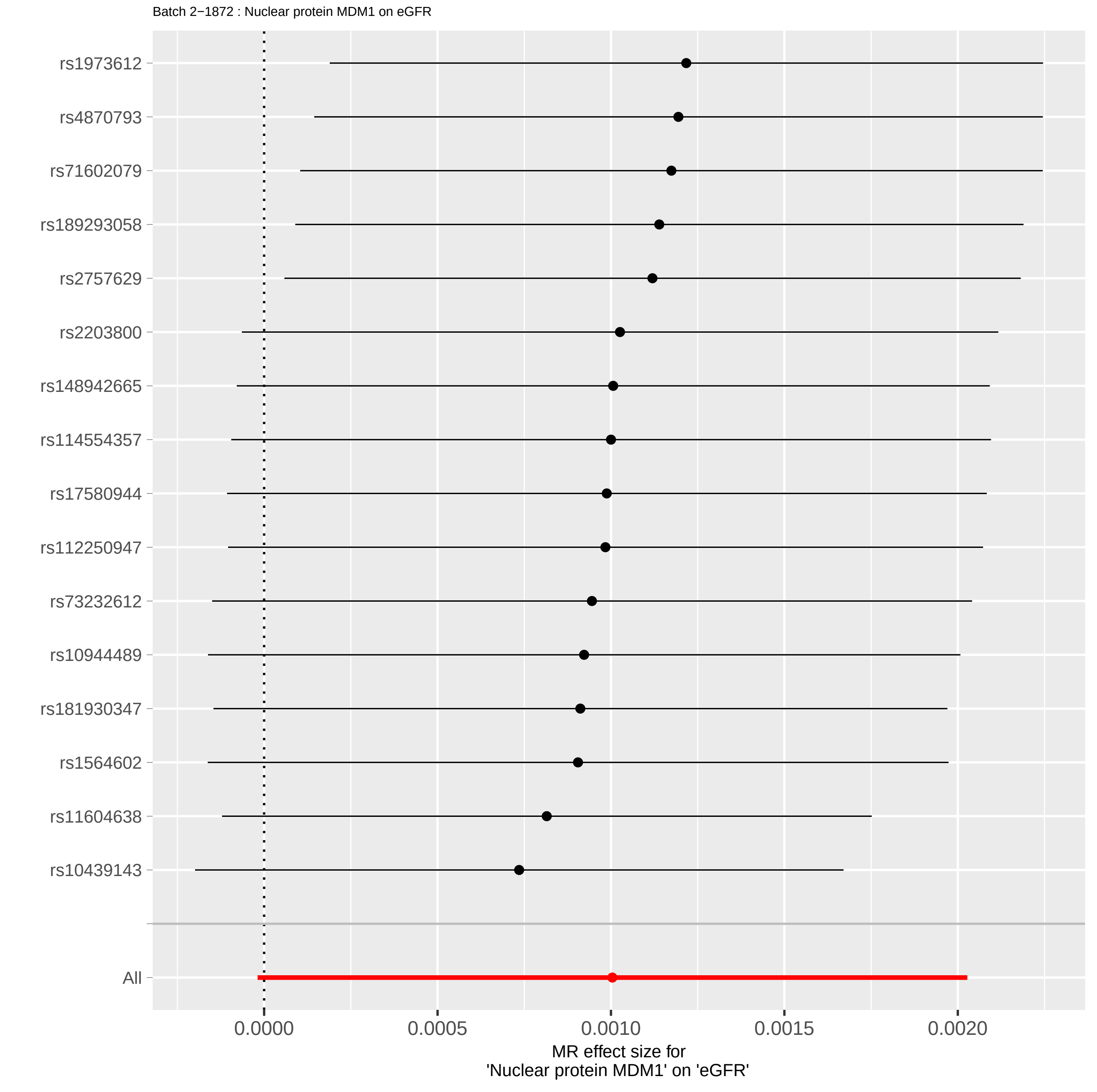

Batch 2−1872 : Nuclear protein MDM1 on eGFR
rs1973612
rs4870793
rs71602079
rs189293058
rs2757629
rs2203800
rs148942665
rs114554357
rs17580944
rs112250947
rs73232612
rs10944489
rs181930347
rs1564602
rs11604638
rs10439143
All
0.0000
0.0005
0.0010
0.0015
0.0020
MR effect size for
'Nuclear protein MDM1' on 'eGFR'

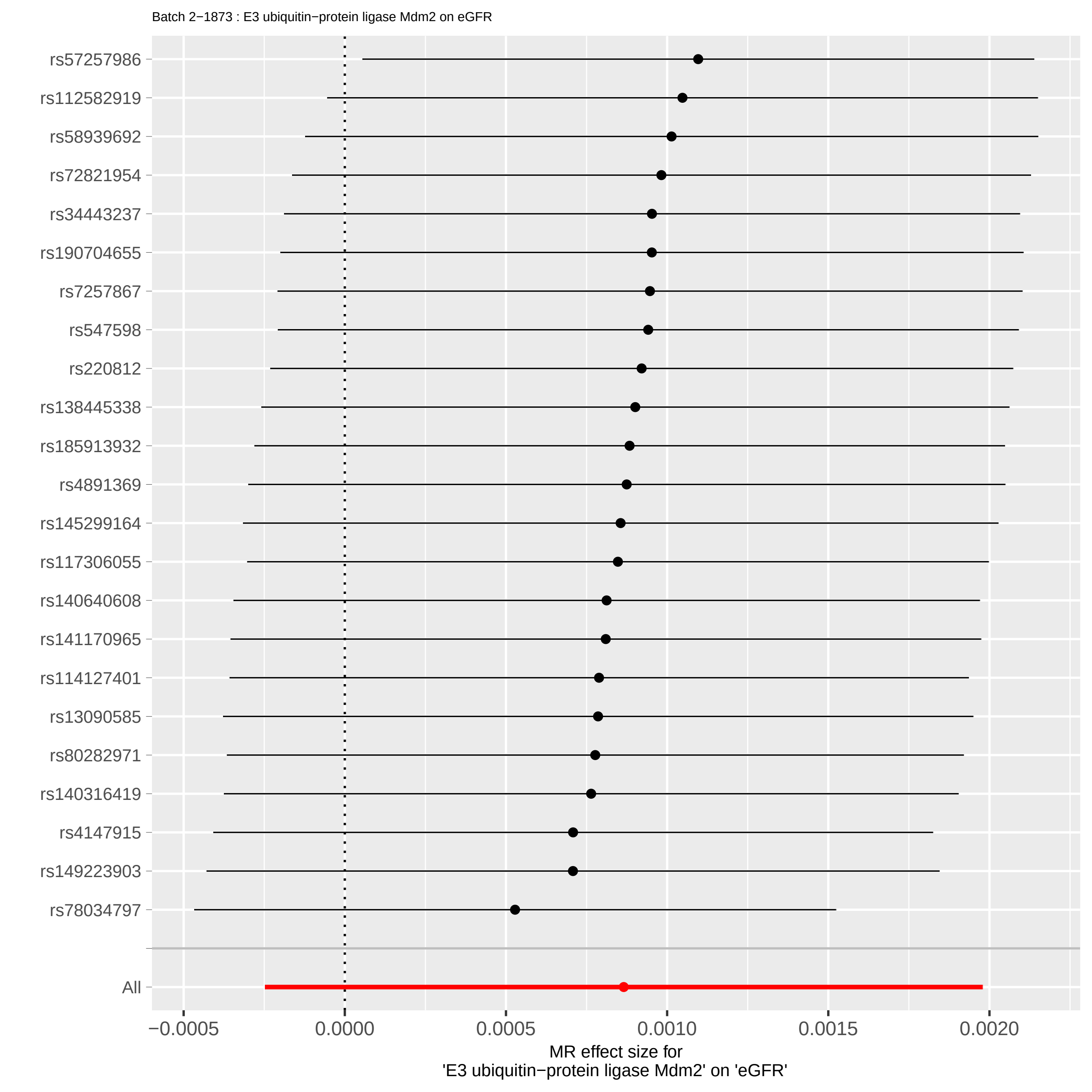

Batch 2−1873 : E3 ubiquitin−protein ligase Mdm2 on eGFR
rs57257986
rs112582919
rs58939692
rs72821954
rs34443237
rs190704655
rs7257867
rs547598
rs220812
rs138445338
rs185913932
rs4891369
rs145299164
rs117306055
rs140640608
rs141170965
rs114127401
rs13090585
rs80282971
rs140316419
rs4147915
rs149223903
rs78034797
All
−0.0005
0.0000
0.0005
0.0010
0.0015
0.0020
MR effect size for
'E3 ubiquitin−protein ligase Mdm2' on 'eGFR'

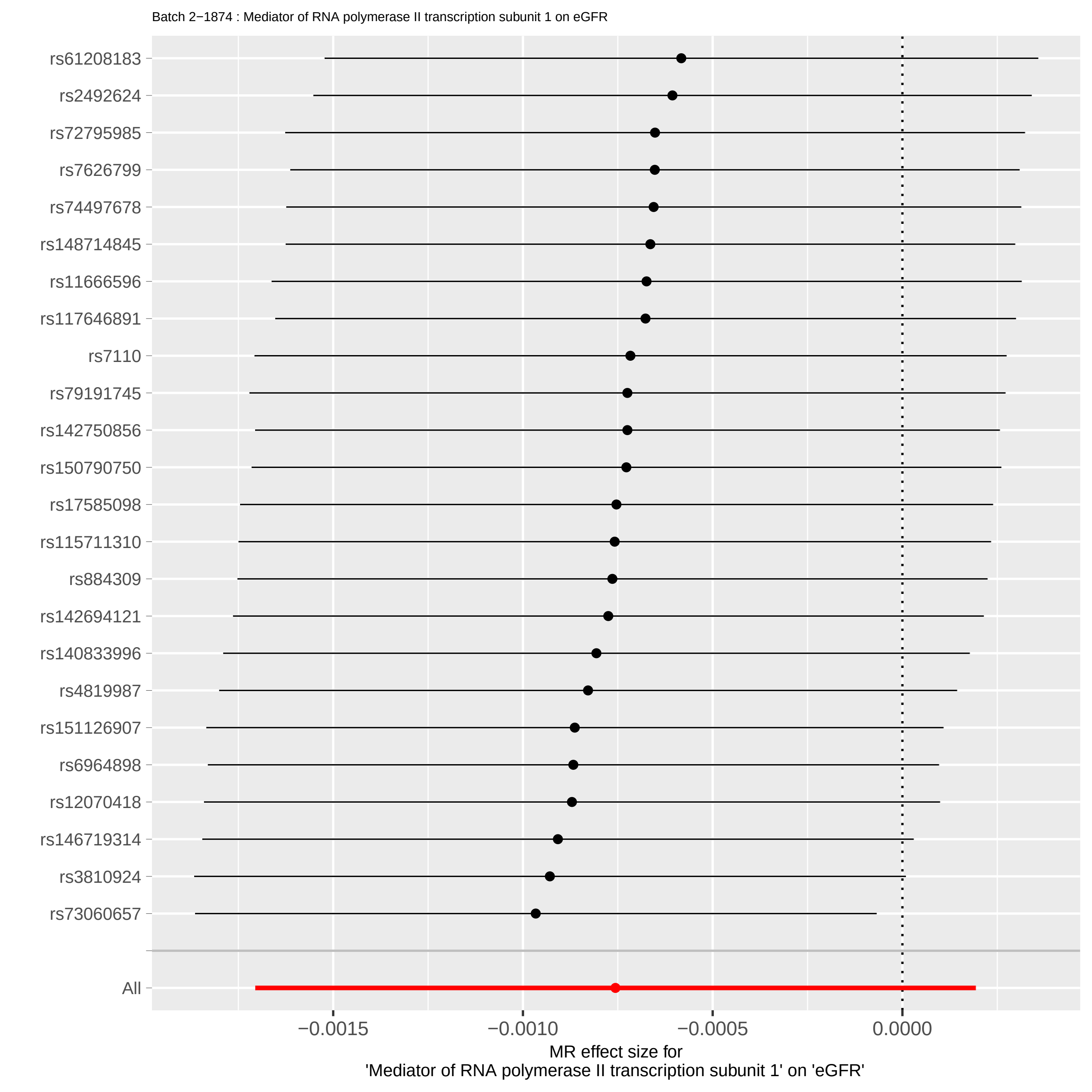
Batch 2−1874 : Mediator of RNA polymerase II transcription subunit 1 on eGFR
rs61208183
rs2492624
rs72795985
rs7626799
rs74497678
rs148714845
rs11666596
rs117646891
rs7110
rs79191745
rs142750856
rs150790750
rs17585098
rs115711310
rs884309
rs142694121
rs140833996
rs4819987
rs151126907
rs6964898
rs12070418
rs146719314
rs3810924
rs73060657
All
−0.0015
−0.0010
−0.0005
0.0000
MR effect size for
'Mediator of RNA polymerase II transcription subunit 1' on 'eGFR'

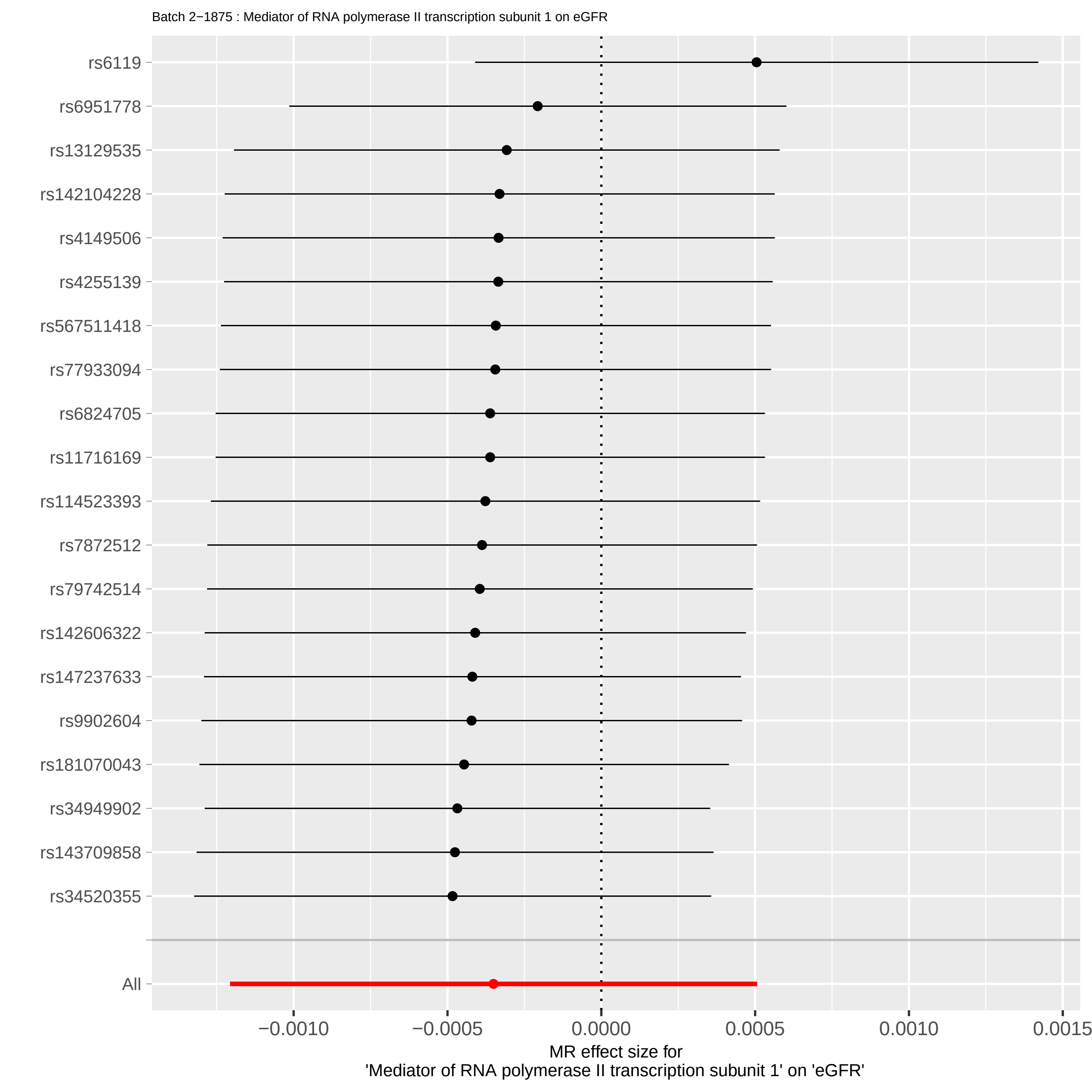
Batch 2−1875 : Mediator of RNA polymerase II transcription subunit 1 on eGFR
rs6119
rs6951778
rs13129535
rs142104228
rs4149506
rs4255139
rs567511418
rs77933094
rs6824705
rs11716169
rs114523393
rs7872512
rs79742514
rs142606322
rs147237633
rs9902604
rs181070043
rs34949902
rs143709858
rs34520355
All
−0.0010
−0.0005
0.0000
0.0005
0.0010
0.0015
MR effect size for
'Mediator of RNA polymerase II transcription subunit 1' on 'eGFR'

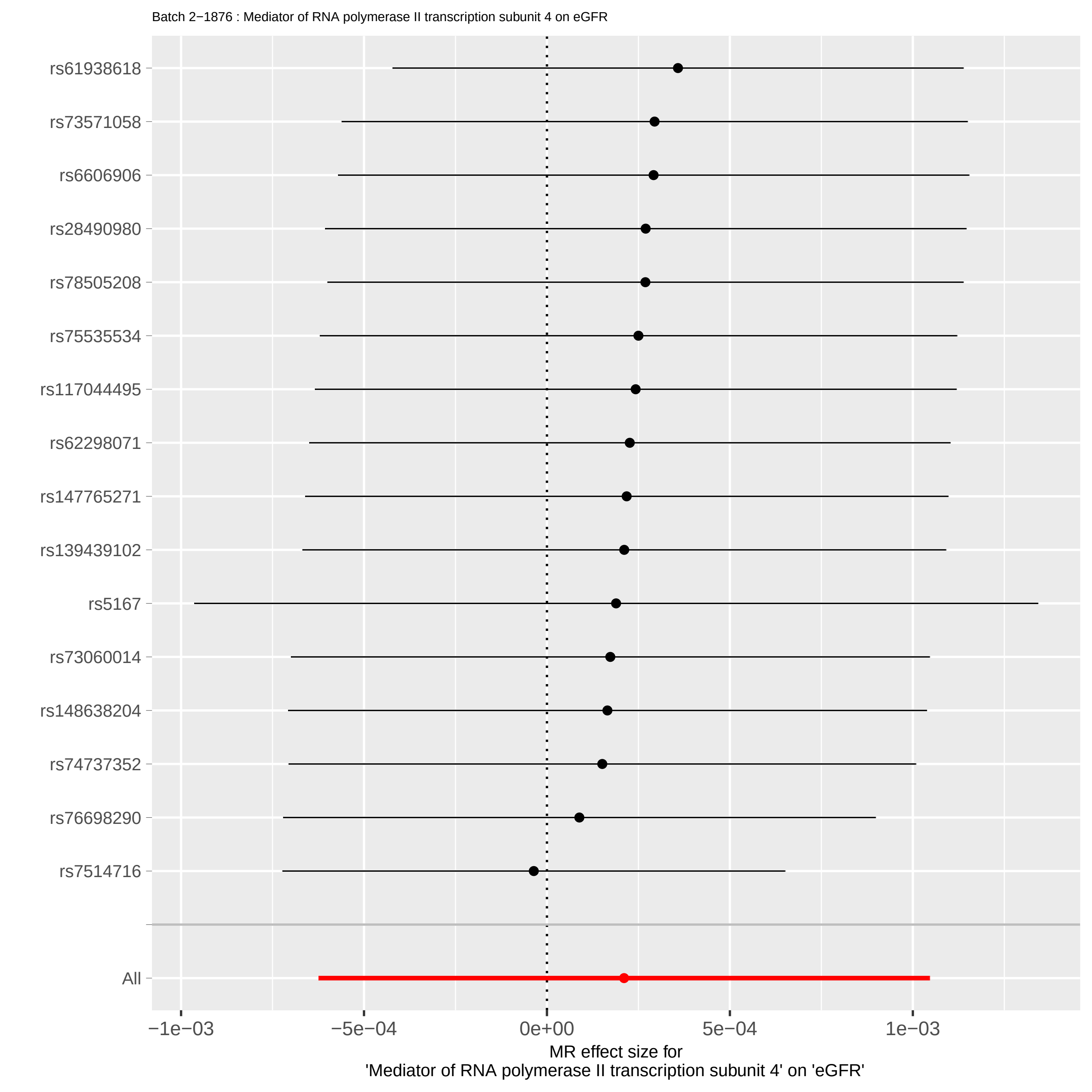

Batch 2−1876 : Mediator of RNA polymerase II transcription subunit 4 on eGFR
rs61938618
rs73571058
rs6606906
rs28490980
rs78505208
rs75535534
rs117044495
rs62298071
rs147765271
rs139439102
rs5167
rs73060014
rs148638204
rs74737352
rs76698290
rs7514716
All
−1e−03
−5e−04
0e+00
5e−04
1e−03
MR effect size for
'Mediator of RNA polymerase II transcription subunit 4' on 'eGFR'

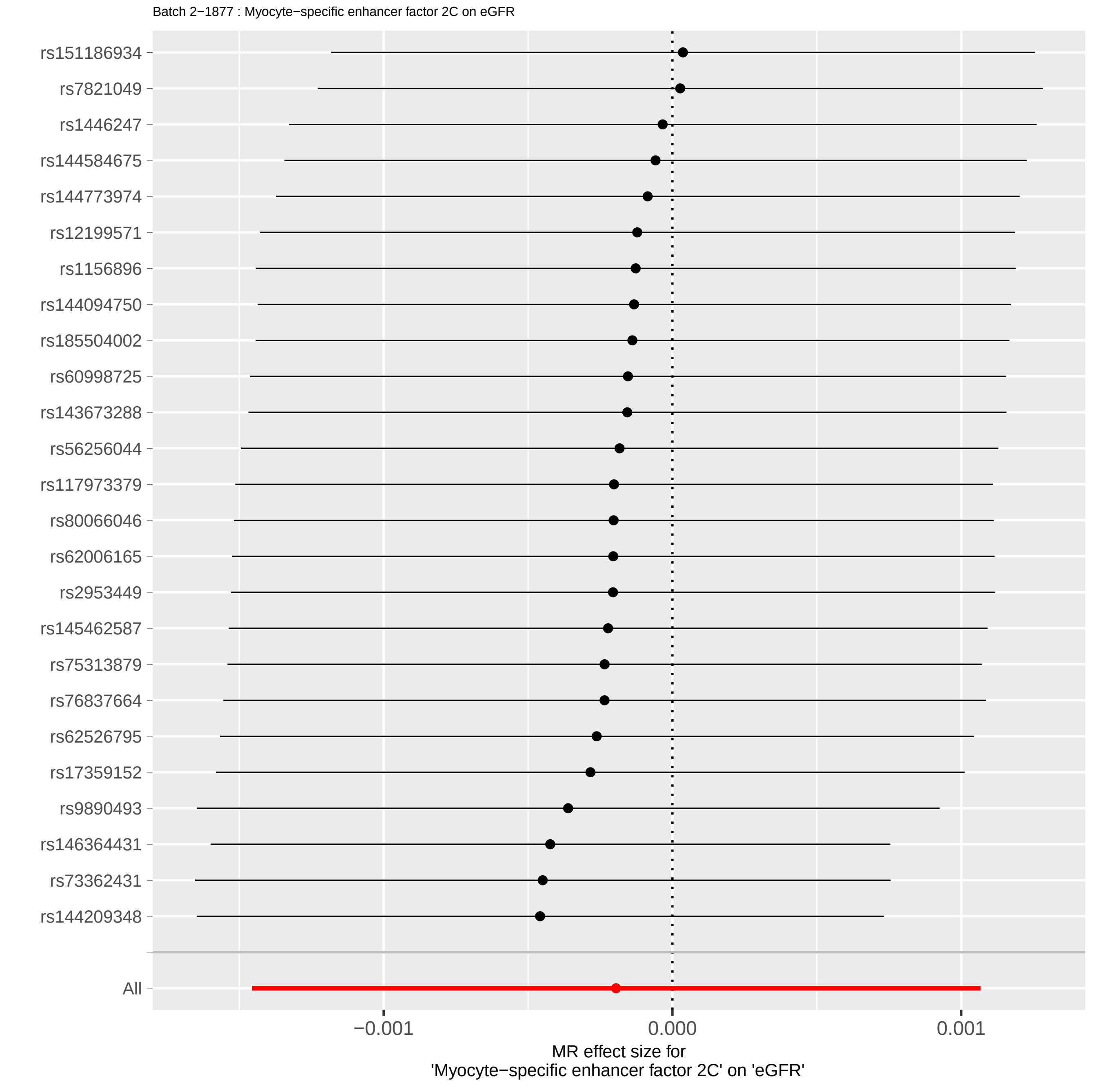

Batch 2−1877 : Myocyte−specific enhancer factor 2C on eGFR
rs151186934
rs7821049
rs1446247
rs144584675
rs144773974
rs12199571
rs1156896
rs144094750
rs185504002
rs60998725
rs143673288
rs56256044
rs117973379
rs80066046
rs62006165
rs2953449
rs145462587
rs75313879
rs76837664
rs62526795
rs17359152
rs9890493
rs146364431
rs73362431
rs144209348
All
−0.001
0.000
0.001
MR effect size for
'Myocyte−specific enhancer factor 2C' on 'eGFR'

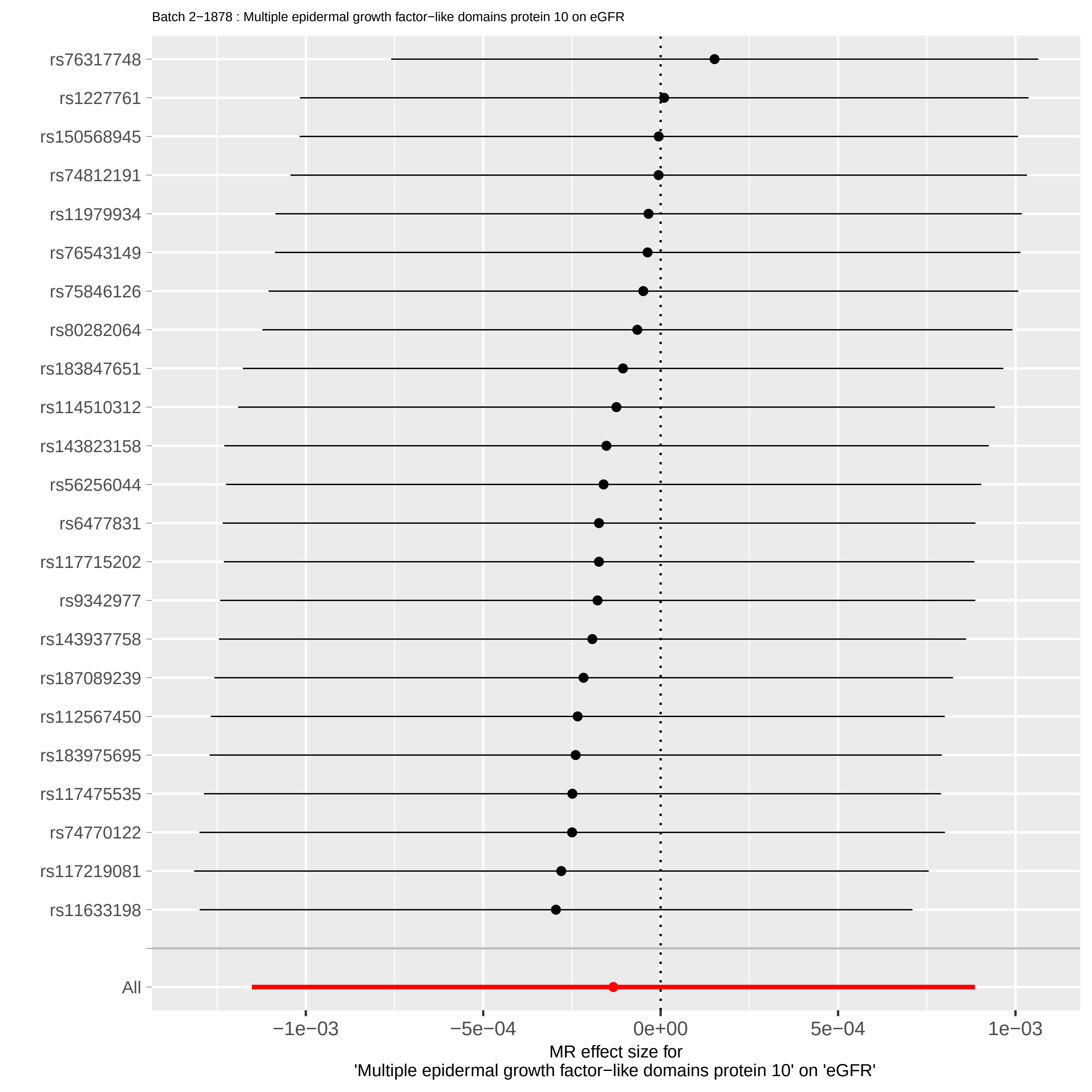
Batch 2−1878 : Multiple epidermal growth factor−like domains protein 10 on eGFR
rs76317748
rs1227761
rs150568945
rs74812191
rs11979934
rs76543149
rs75846126
rs80282064
rs183847651
rs114510312
rs143823158
rs56256044
rs6477831
rs117715202
rs9342977
rs143937758
rs187089239
rs112567450
rs183975695
rs117475535
rs74770122
rs117219081
rs11633198
All
−1e−03
−5e−04
0e+00
5e−04
1e−03
MR effect size for
'Multiple epidermal growth factor−like domains protein 10' on 'eGFR'

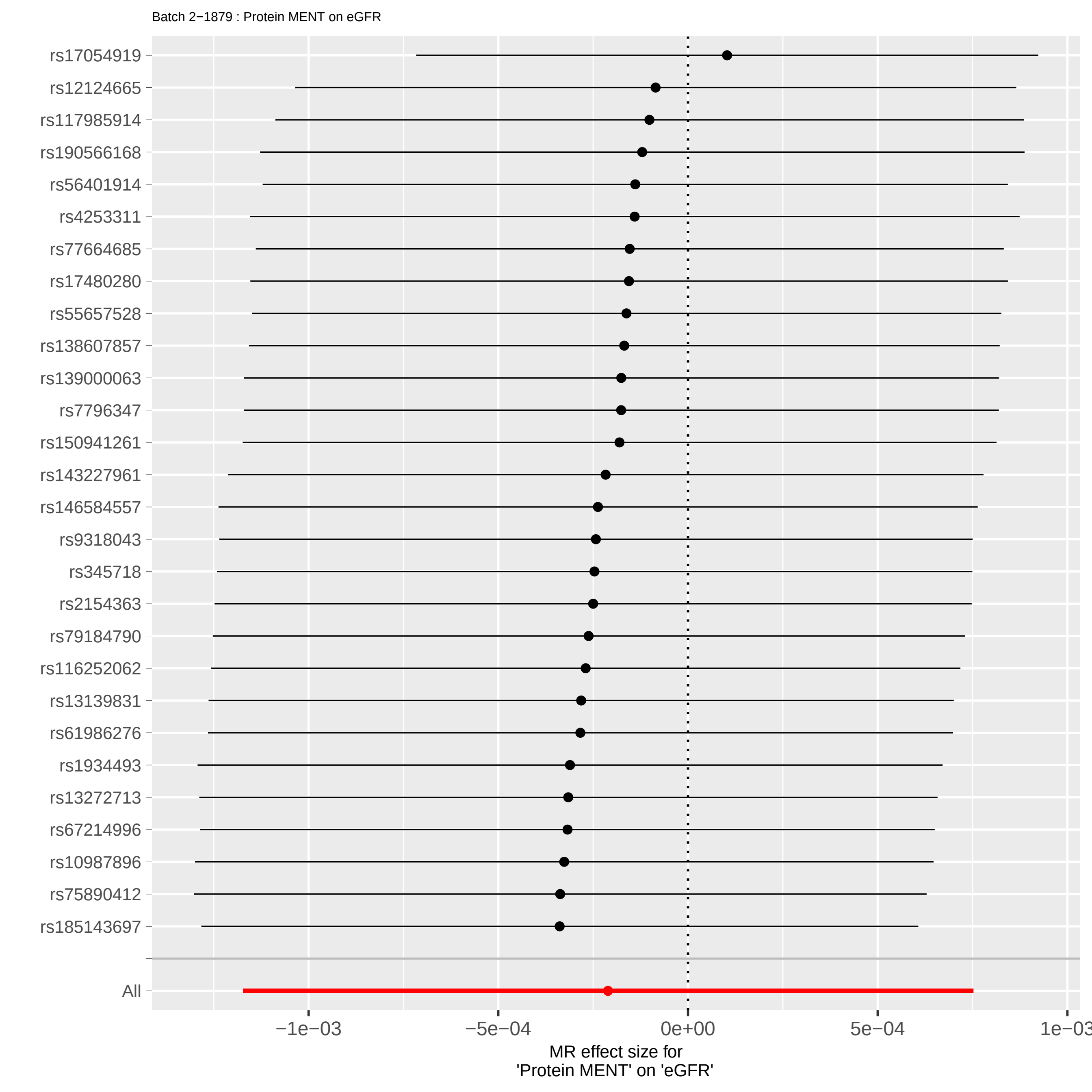
Batch 2−1879 : Protein MENT on eGFR
rs17054919
rs12124665
rs117985914
rs190566168
rs56401914
rs4253311
rs77664685
rs17480280
rs55657528
rs138607857
rs139000063
rs7796347
rs150941261
rs143227961
rs146584557
rs9318043
rs345718
rs2154363
rs79184790
rs116252062
rs13139831
rs61986276
rs1934493
rs13272713
rs67214996
rs10987896
rs75890412
rs185143697
All
−1e−03
−5e−04
0e+00
5e−04
1e−03
MR effect size for
'Protein MENT' on 'eGFR'

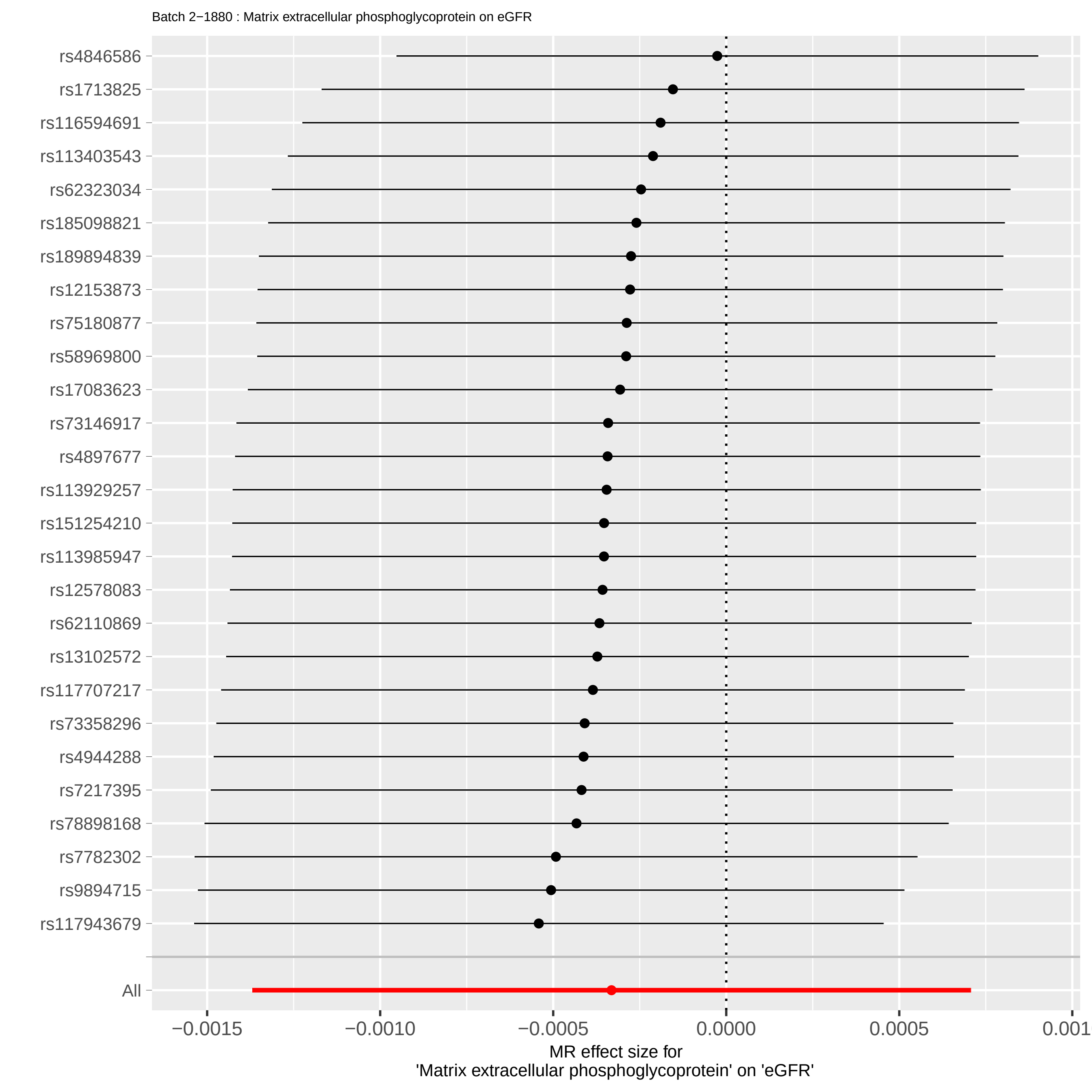

Batch 2−1880 : Matrix extracellular phosphoglycoprotein on eGFR
rs4846586
rs1713825
rs116594691
rs113403543
rs62323034
rs185098821
rs189894839
rs12153873
rs75180877
rs58969800
rs17083623
rs73146917
rs4897677
rs113929257
rs151254210
rs113985947
rs12578083
rs62110869
rs13102572
rs117707217
rs73358296
rs4944288
rs7217395
rs78898168
rs7782302
rs9894715
rs117943679
All
−0.0015
−0.0010
−0.0005
0.0000
0.0005
0.001
MR effect size for
'Matrix extracellular phosphoglycoprotein' on 'eGFR'

Batch 2−1881 : LDLR chaperone MESD on eGFR

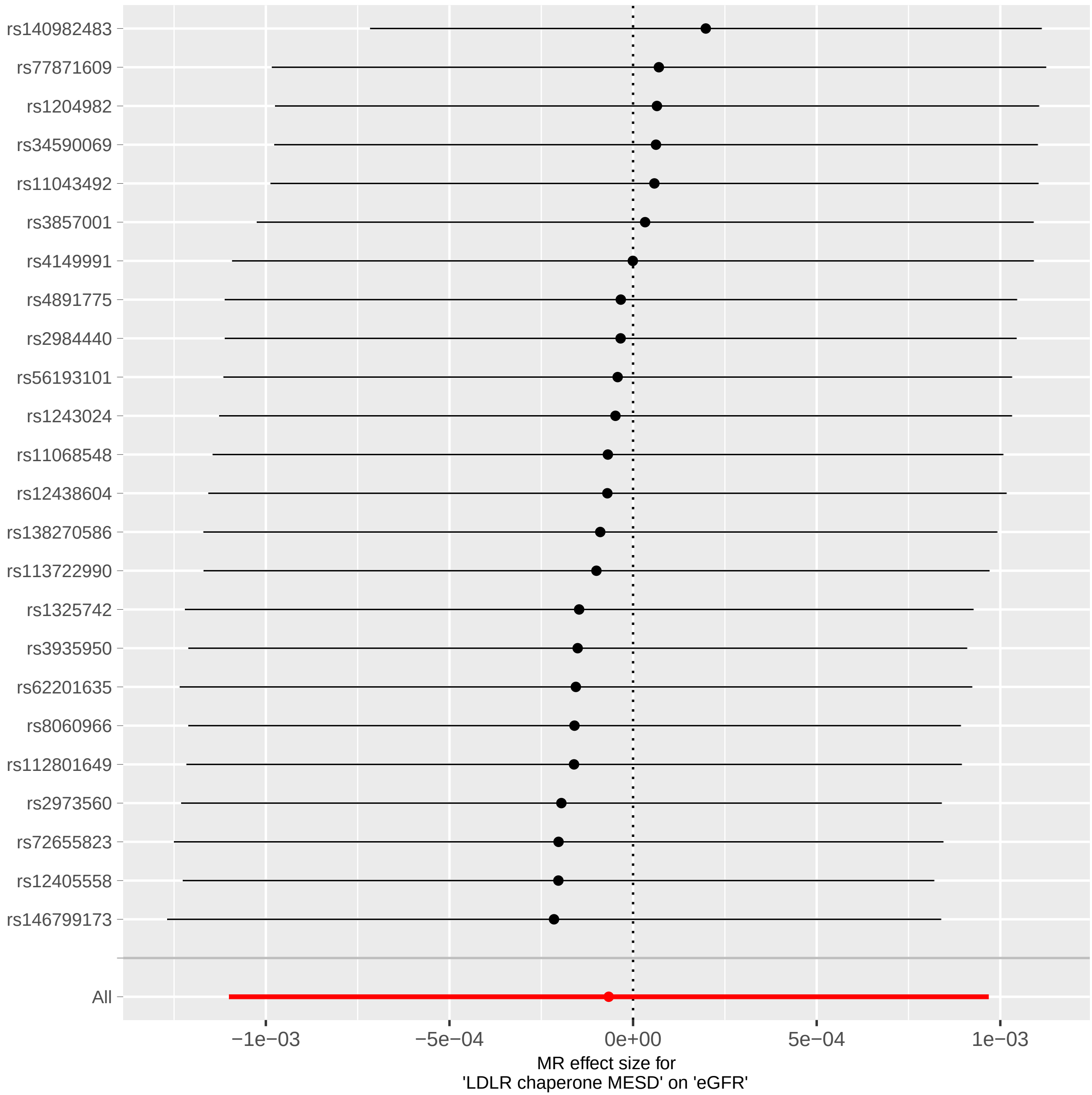

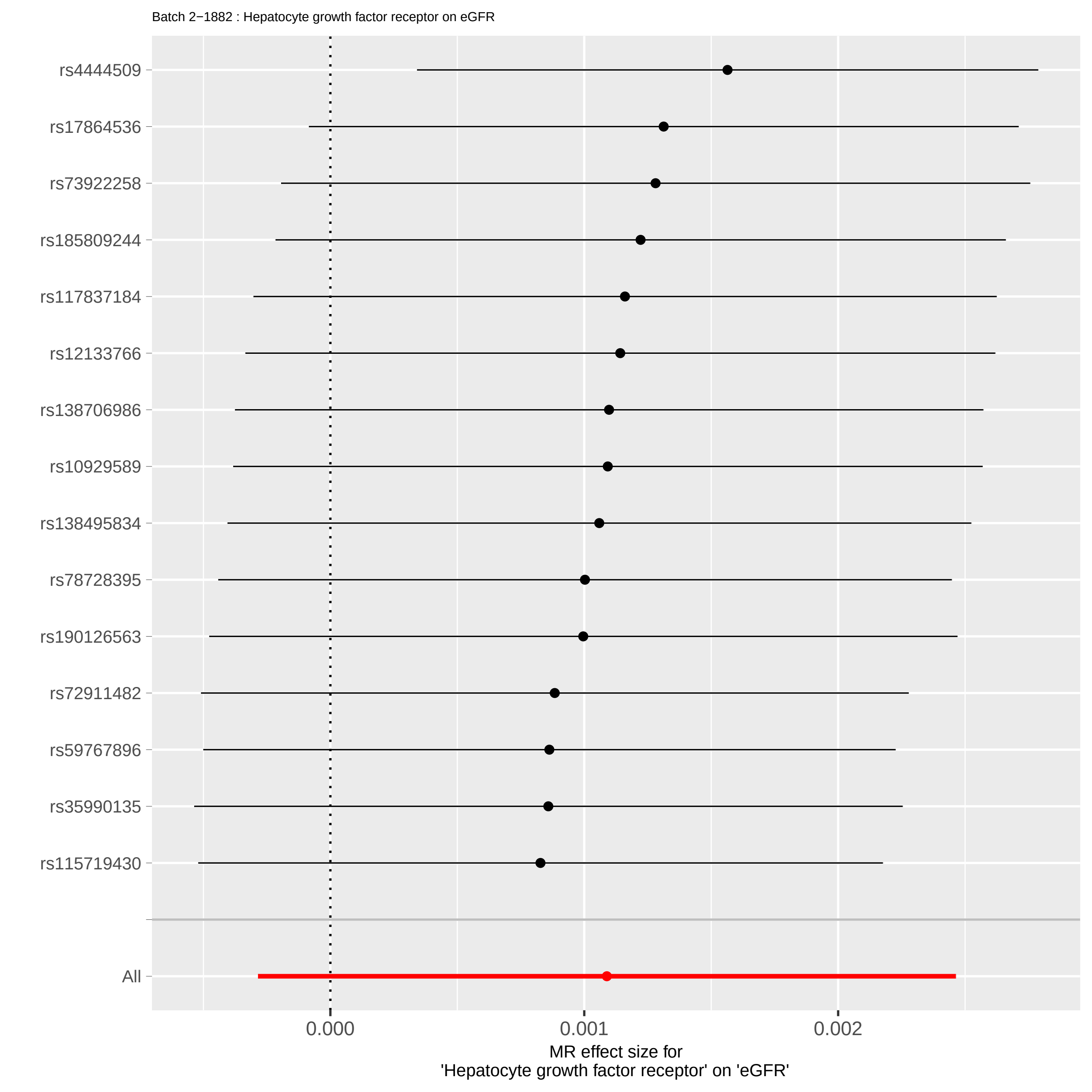

Batch 2−1882 : Hepatocyte growth factor receptor on eGFR
rs4444509
rs17864536
rs73922258
rs185809244
rs117837184
rs12133766
rs138706986
rs10929589
rs138495834
rs78728395
rs190126563
rs72911482
rs59767896
rs35990135
rs115719430
All
0.000
0.001
0.002
MR effect size for
'Hepatocyte growth factor receptor' on 'eGFR'

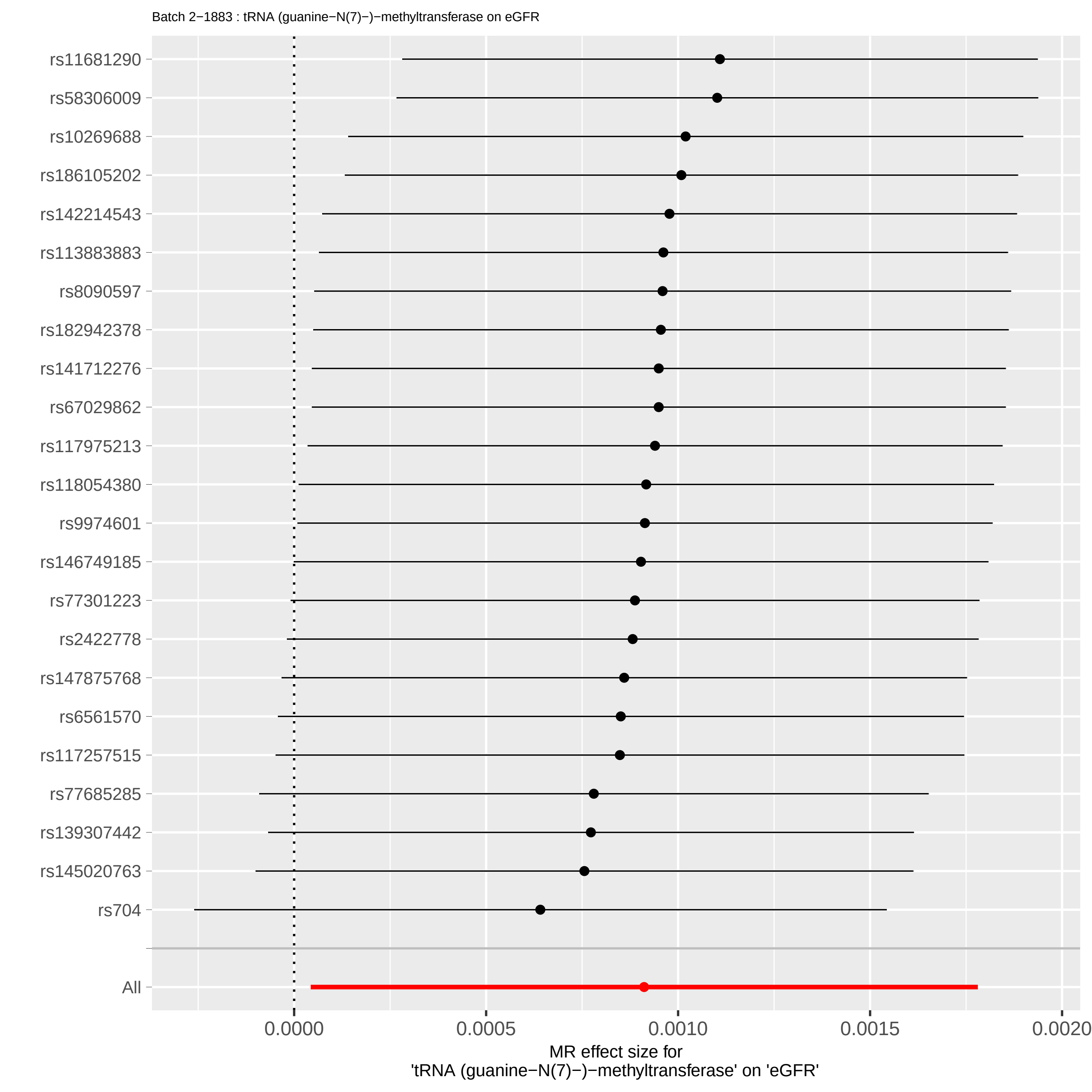
Batch 2−1883 : tRNA (guanine−N(7)−)−methyltransferase on eGFR
rs11681290
rs58306009
rs10269688
rs186105202
rs142214543
rs113883883
rs8090597
rs182942378
rs141712276
rs67029862
rs117975213
rs118054380
rs9974601
rs146749185
rs77301223
rs2422778
rs147875768
rs6561570
rs117257515
rs77685285
rs139307442
rs145020763
rs704
All
0.0000
0.0005
0.0010
0.0015
0.0020
MR effect size for
'tRNA (guanine−N(7)−)−methyltransferase' on 'eGFR'

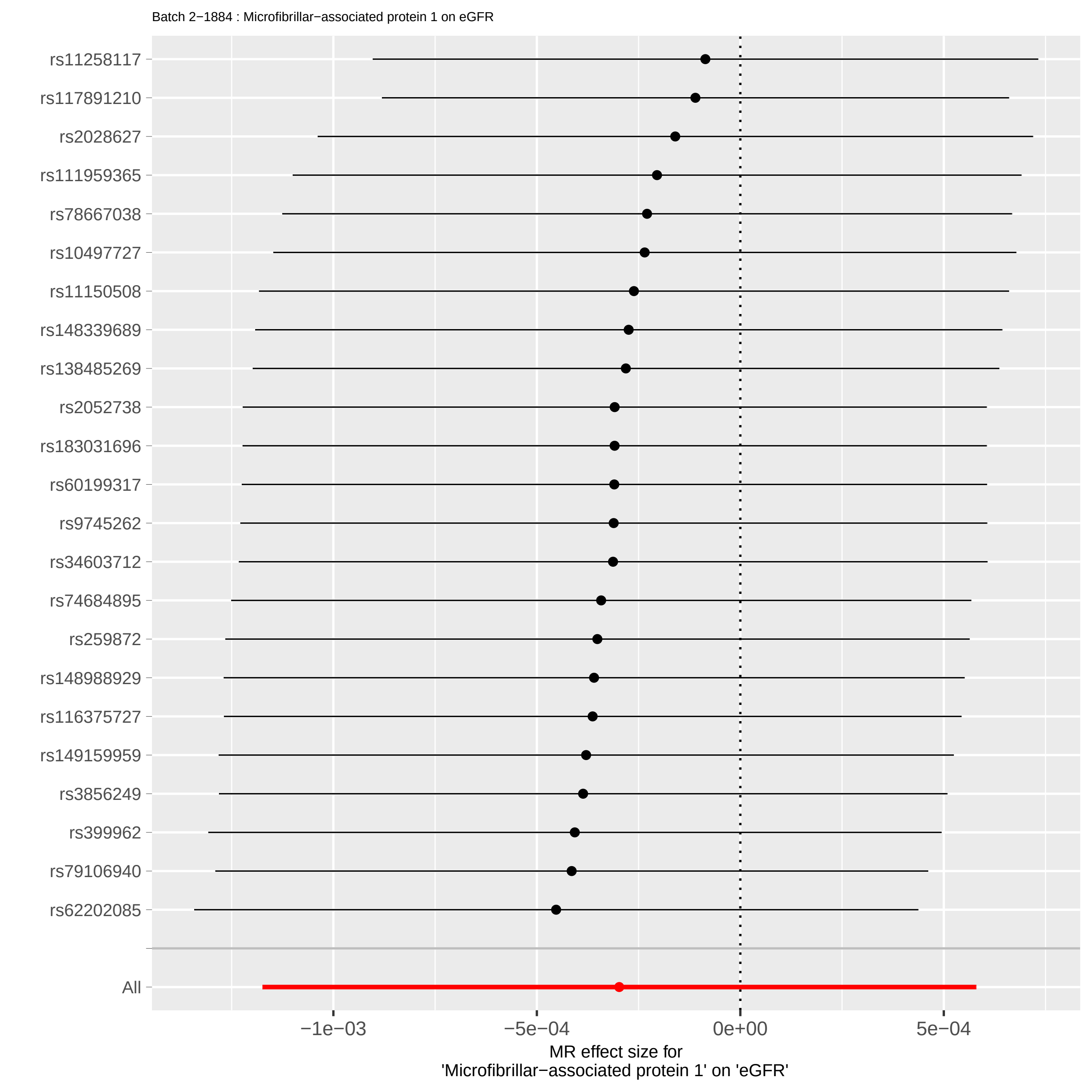
Batch 2−1884 : Microfibrillar−associated protein 1 on eGFR
rs11258117
rs117891210
rs2028627
rs111959365
rs78667038
rs10497727
rs11150508
rs148339689
rs138485269
rs2052738
rs183031696
rs60199317
rs9745262
rs34603712
rs74684895
rs259872
rs148988929
rs116375727
rs149159959
rs3856249
rs399962
rs79106940
rs62202085
All
−1e−03
−5e−04
0e+00
5e−04
MR effect size for
'Microfibrillar−associated protein 1' on 'eGFR'

Batch 2−1885 : Microfibrillar−associated protein 2 on eGFR

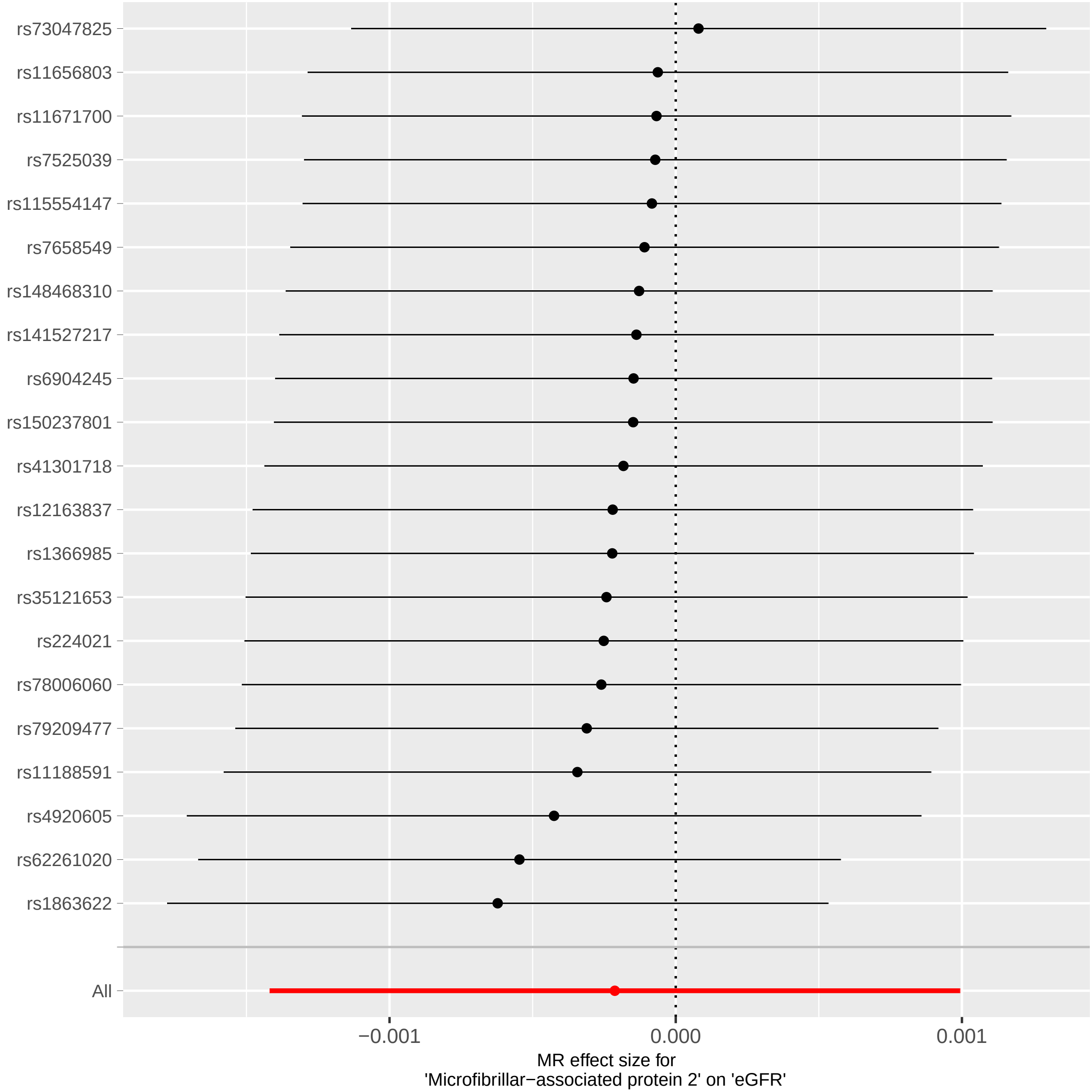

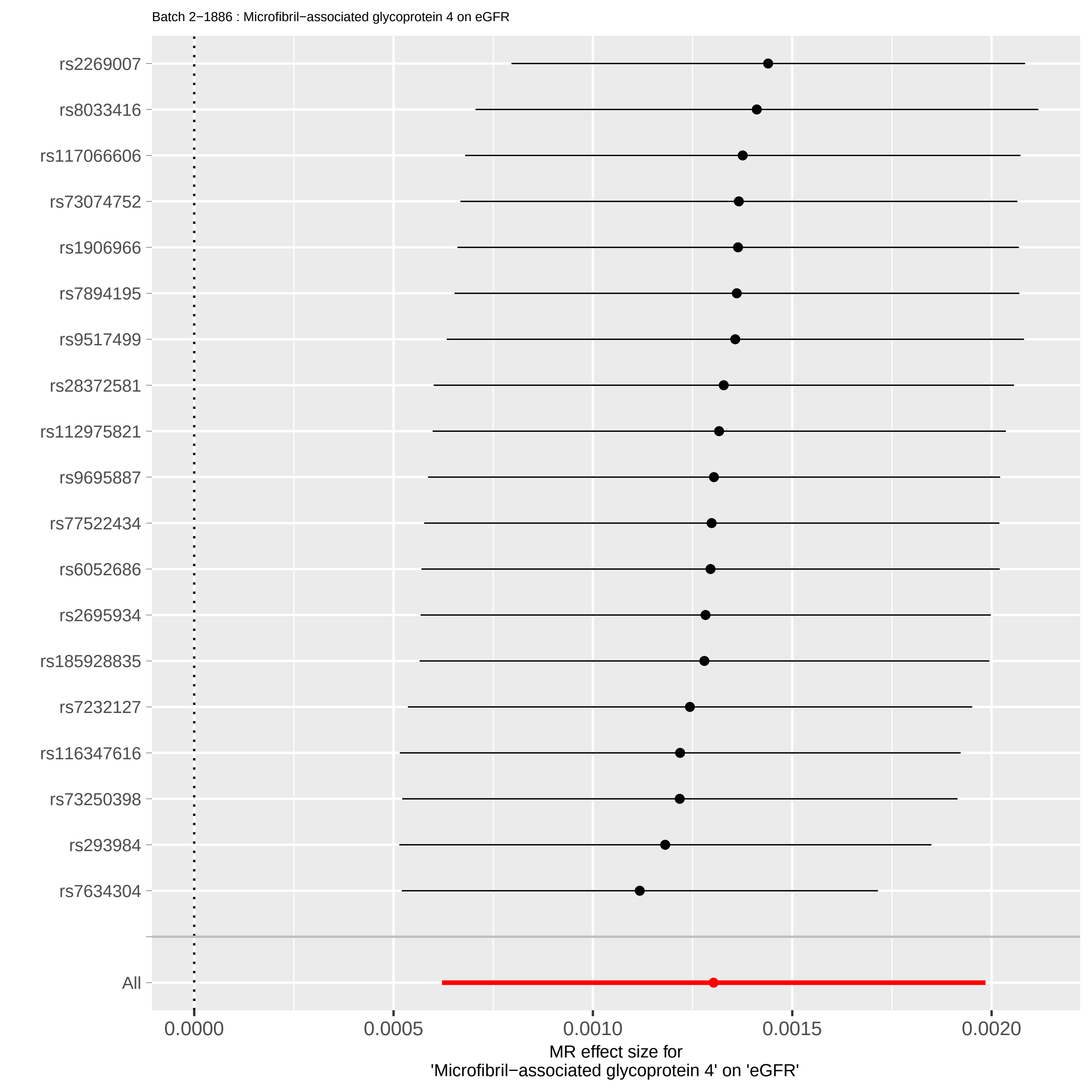

Batch 2−1886 : Microfibril−associated glycoprotein 4 on eGFR
rs2269007
rs8033416
rs117066606
rs73074752
rs1906966
rs7894195
rs9517499
rs28372581
rs112975821
rs9695887
rs77522434
rs6052686
rs2695934
rs185928835
rs7232127
rs116347616
rs73250398
rs293984
rs7634304
All
0.0000
0.0005
0.0010
0.0015
0.0020
MR effect size for
'Microfibril−associated glycoprotein 4' on 'eGFR'

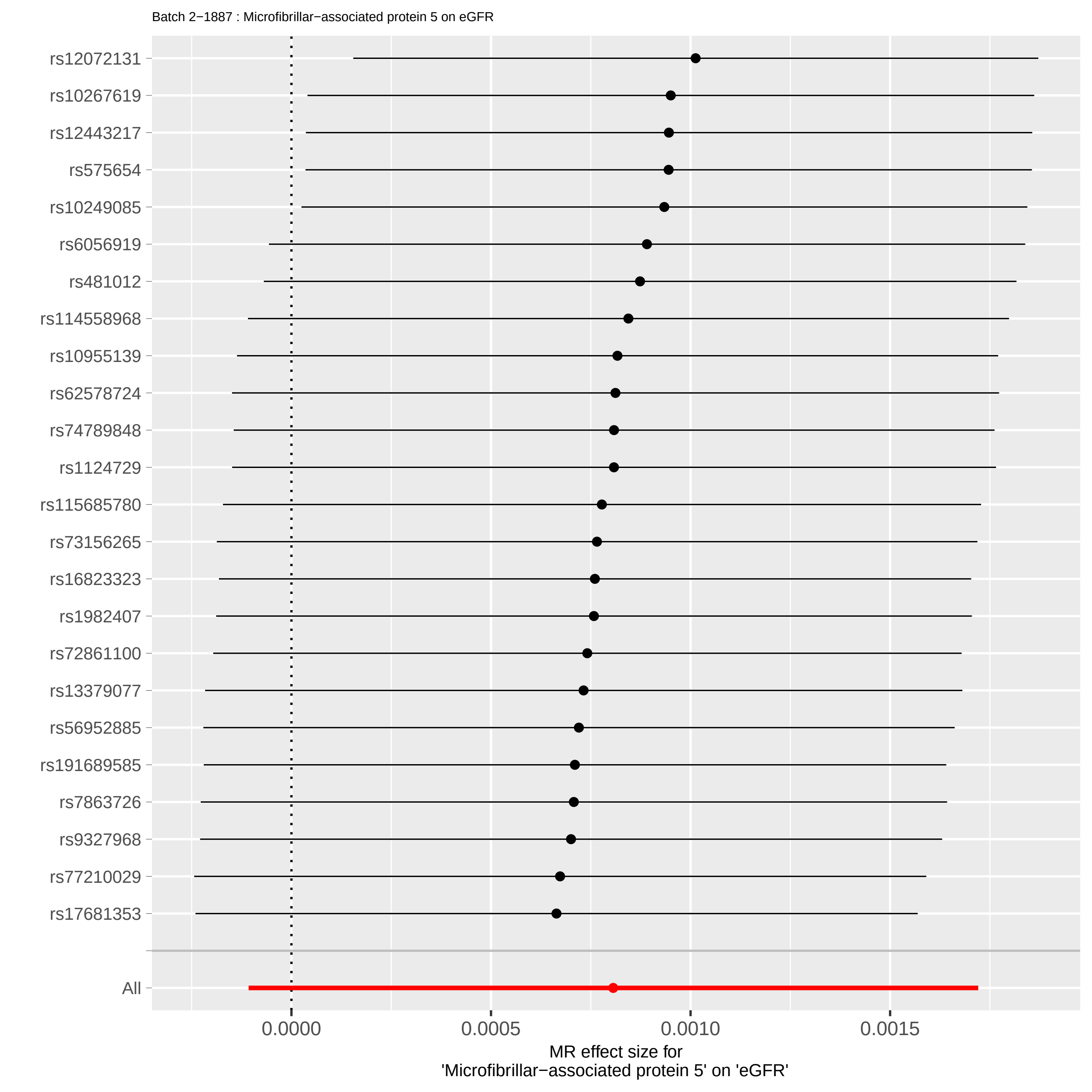

Batch 2−1887 : Microfibrillar−associated protein 5 on eGFR
rs12072131
rs10267619
rs12443217
rs575654
rs10249085
rs6056919
rs481012
rs114558968
rs10955139
rs62578724
rs74789848
rs1124729
rs115685780
rs73156265
rs16823323
rs1982407
rs72861100
rs13379077
rs56952885
rs191689585
rs7863726
rs9327968
rs77210029
rs17681353
All
0.0000
0.0005
0.0010
0.0015
MR effect size for
'Microfibrillar−associated protein 5' on 'eGFR'

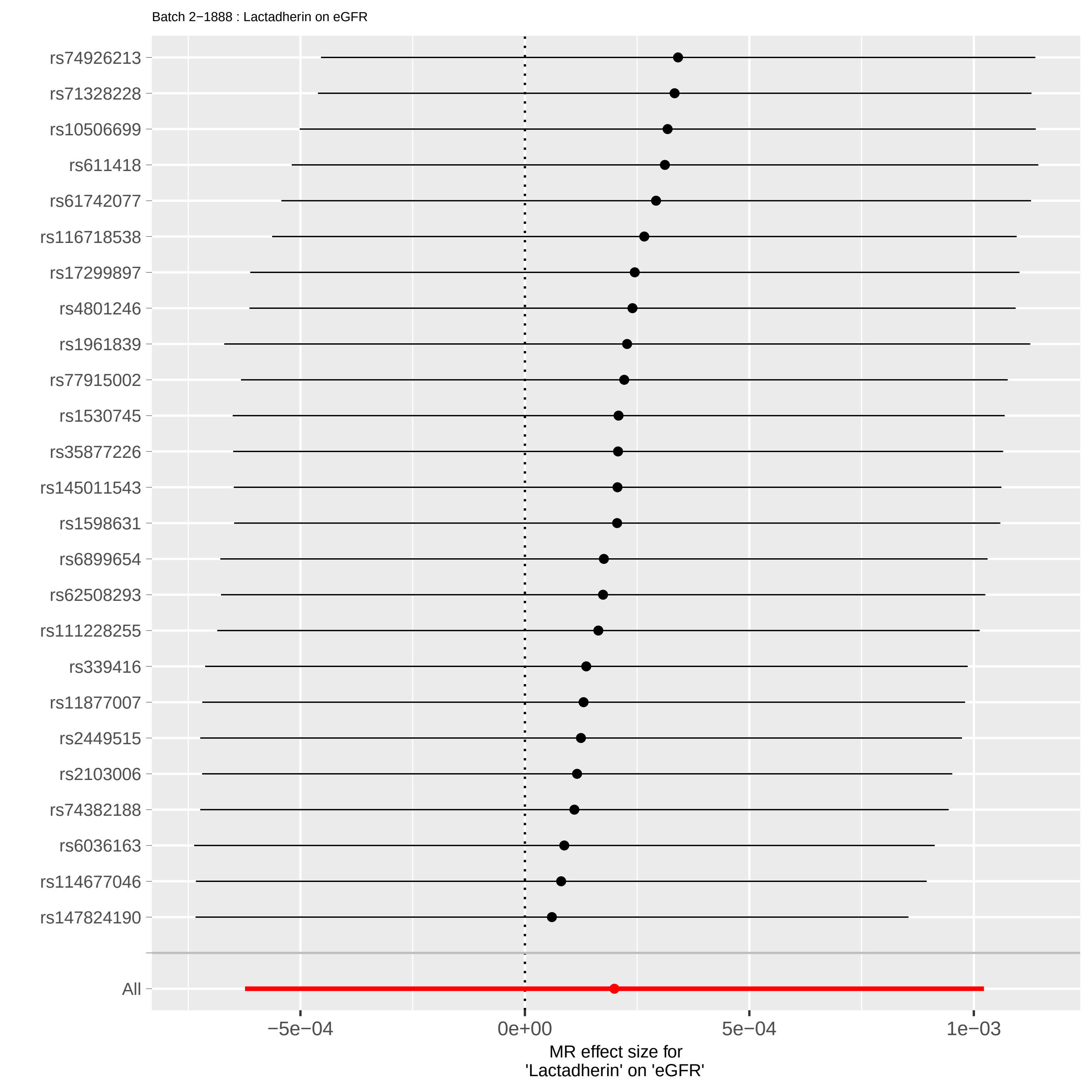
Batch 2−1888 : Lactadherin on eGFR
rs74926213
rs71328228
rs10506699
rs611418
rs61742077
rs116718538
rs17299897
rs4801246
rs1961839
rs77915002
rs1530745
rs35877226
rs145011543
rs1598631
rs6899654
rs62508293
rs111228255
rs339416
rs11877007
rs2449515
rs2103006
rs74382188
rs6036163
rs114677046
rs147824190
All
−5e−04
0e+00
5e−04
1e−03
MR effect size for
'Lactadherin' on 'eGFR'

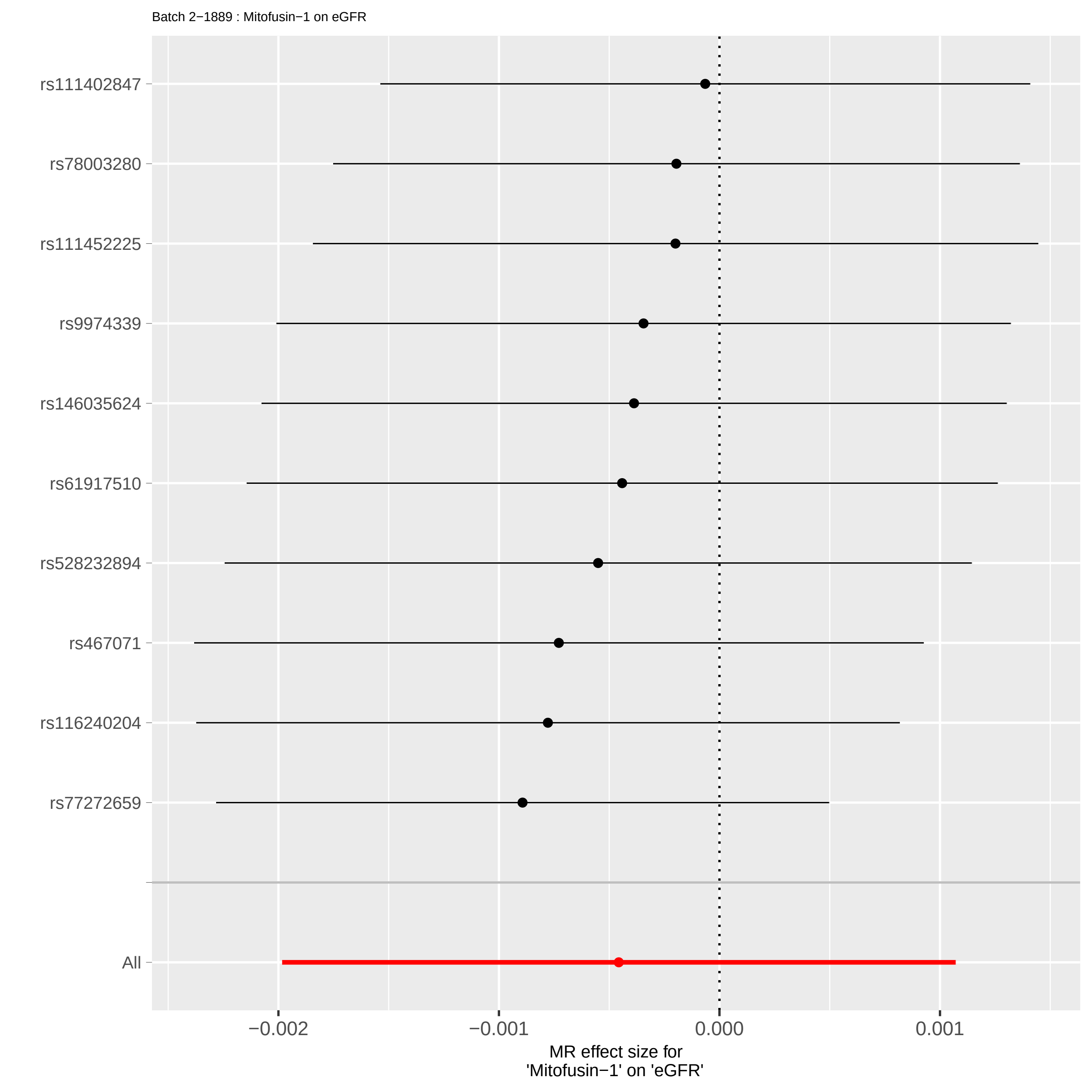

Batch 2−1889 : Mitofusin−1 on eGFR
rs111402847
rs78003280
rs111452225
rs9974339
rs146035624
rs61917510
rs528232894
rs467071
rs116240204
rs77272659
All
−0.002
−0.001
0.000
0.001
MR effect size for
'Mitofusin−1' on 'eGFR'

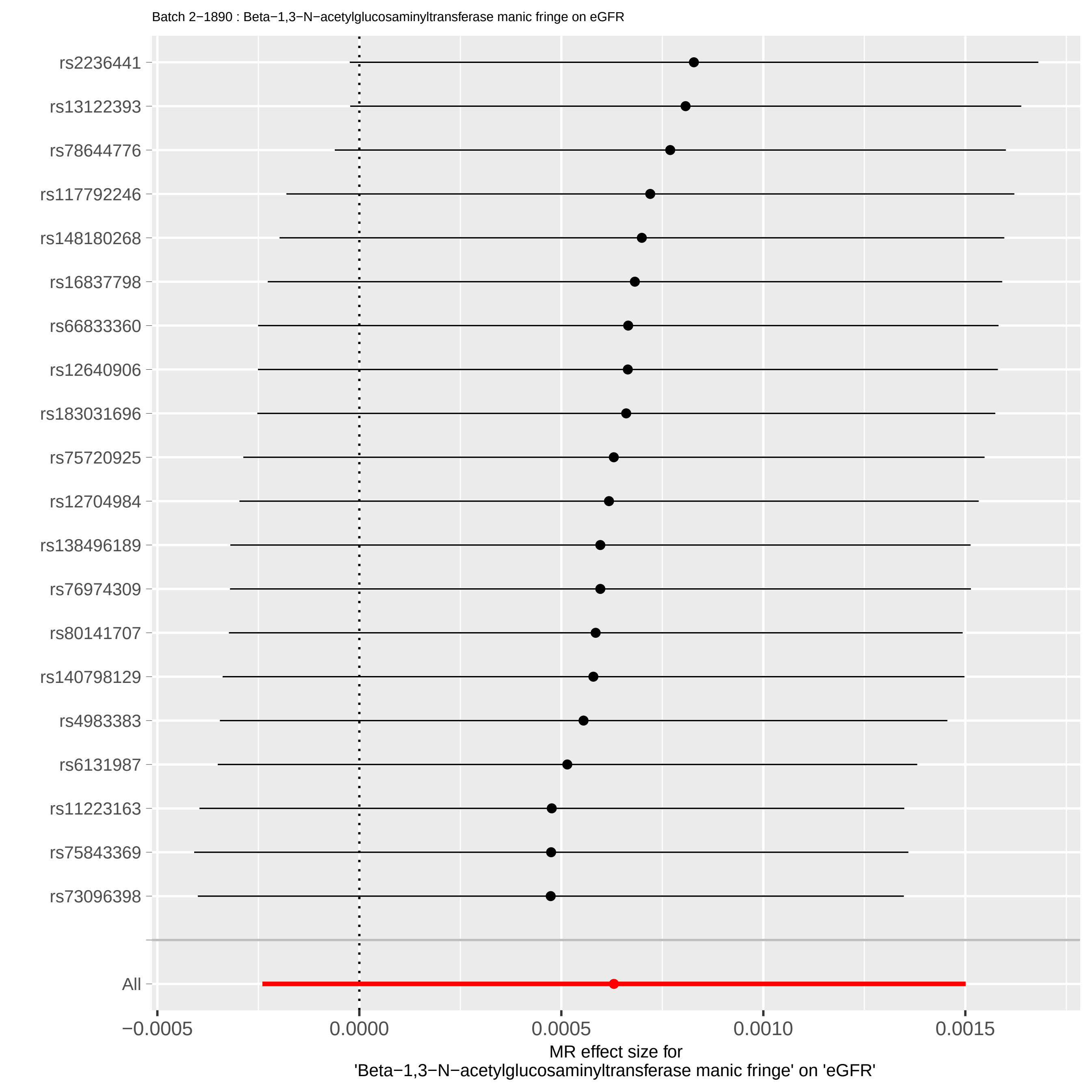

Batch 2−1890 : Beta−1,3−N−acetylglucosaminyltransferase manic fringe on eGFR
rs2236441
rs13122393
rs78644776
rs117792246
rs148180268
rs16837798
rs66833360
rs12640906
rs183031696
rs75720925
rs12704984
rs138496189
rs76974309
rs80141707
rs140798129
rs4983383
rs6131987
rs11223163
rs75843369
rs73096398
All
−0.0005
0.0000
0.0005
0.0010
0.0015
MR effect size for
'Beta−1,3−N−acetylglucosaminyltransferase manic fringe' on 'eGFR'

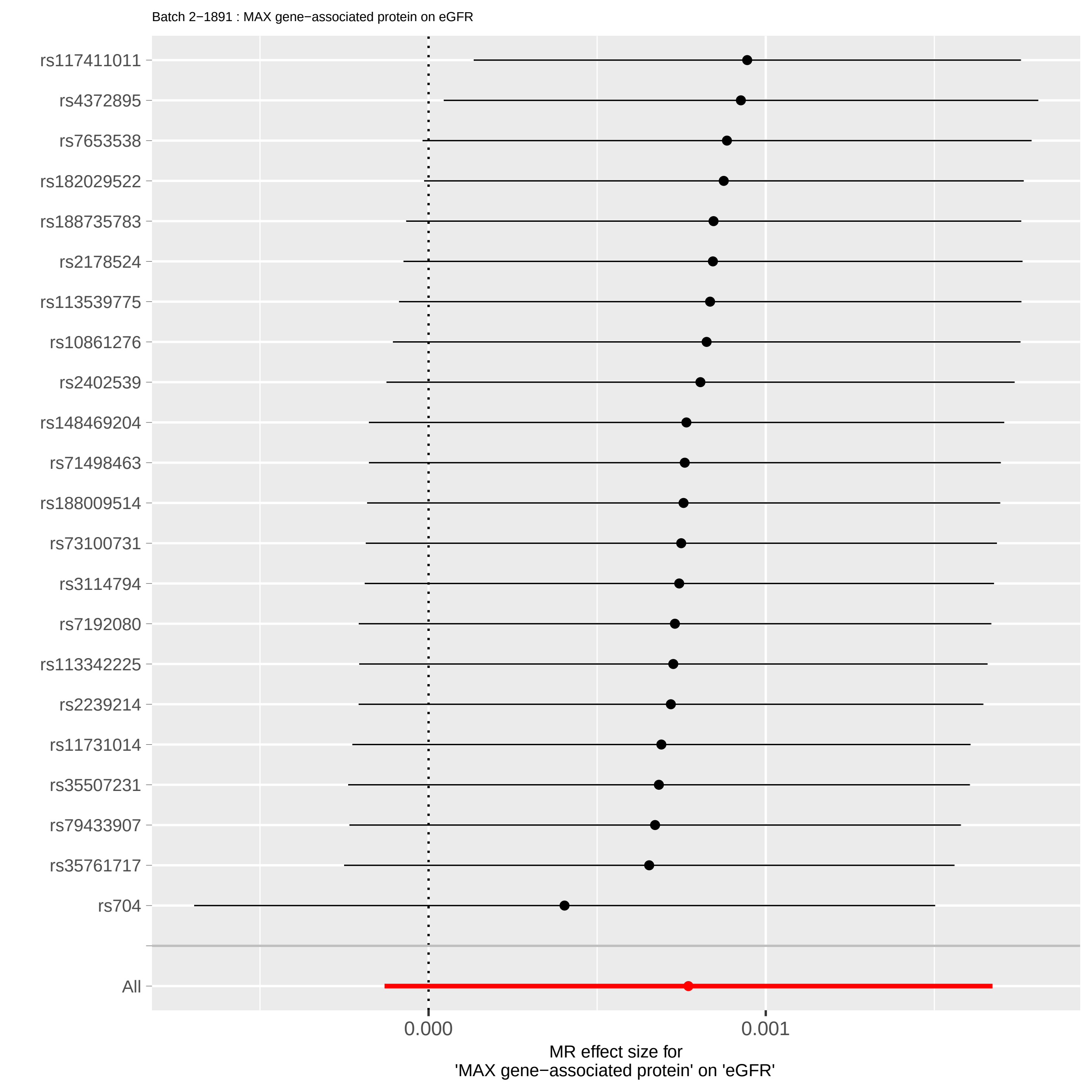

Batch 2−1891 : MAX gene−associated protein on eGFR
rs117411011
rs4372895
rs7653538
rs182029522
rs188735783
rs2178524
rs113539775
rs10861276
rs2402539
rs148469204
rs71498463
rs188009514
rs73100731
rs3114794
rs7192080
rs113342225
rs2239214
rs11731014
rs35507231
rs79433907
rs35761717
rs704
All
0.000
0.001
MR effect size for
'MAX gene−associated protein' on 'eGFR'

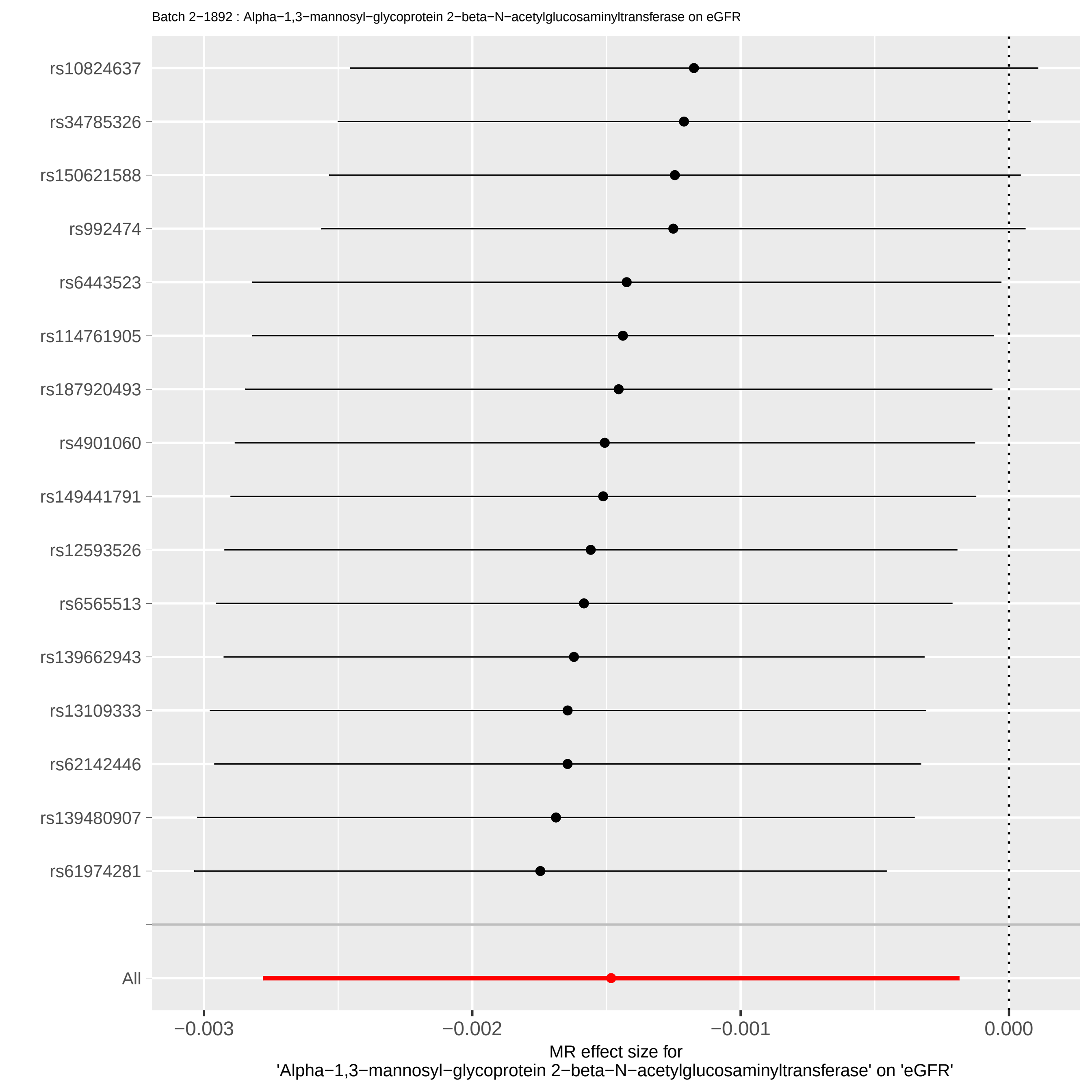

Batch 2−1892 : Alpha−1,3−mannosyl−glycoprotein 2−beta−N−acetylglucosaminyltransferase on eGFR
rs10824637
rs34785326
rs150621588
rs992474
rs6443523
rs114761905
rs187920493
rs4901060
rs149441791
rs12593526
rs6565513
rs139662943
rs13109333
rs62142446
rs139480907
rs61974281
All
−0.003
−0.002
−0.001
0.000
MR effect size for
'Alpha−1,3−mannosyl−glycoprotein 2−beta−N−acetylglucosaminyltransferase' on 'eGFR'

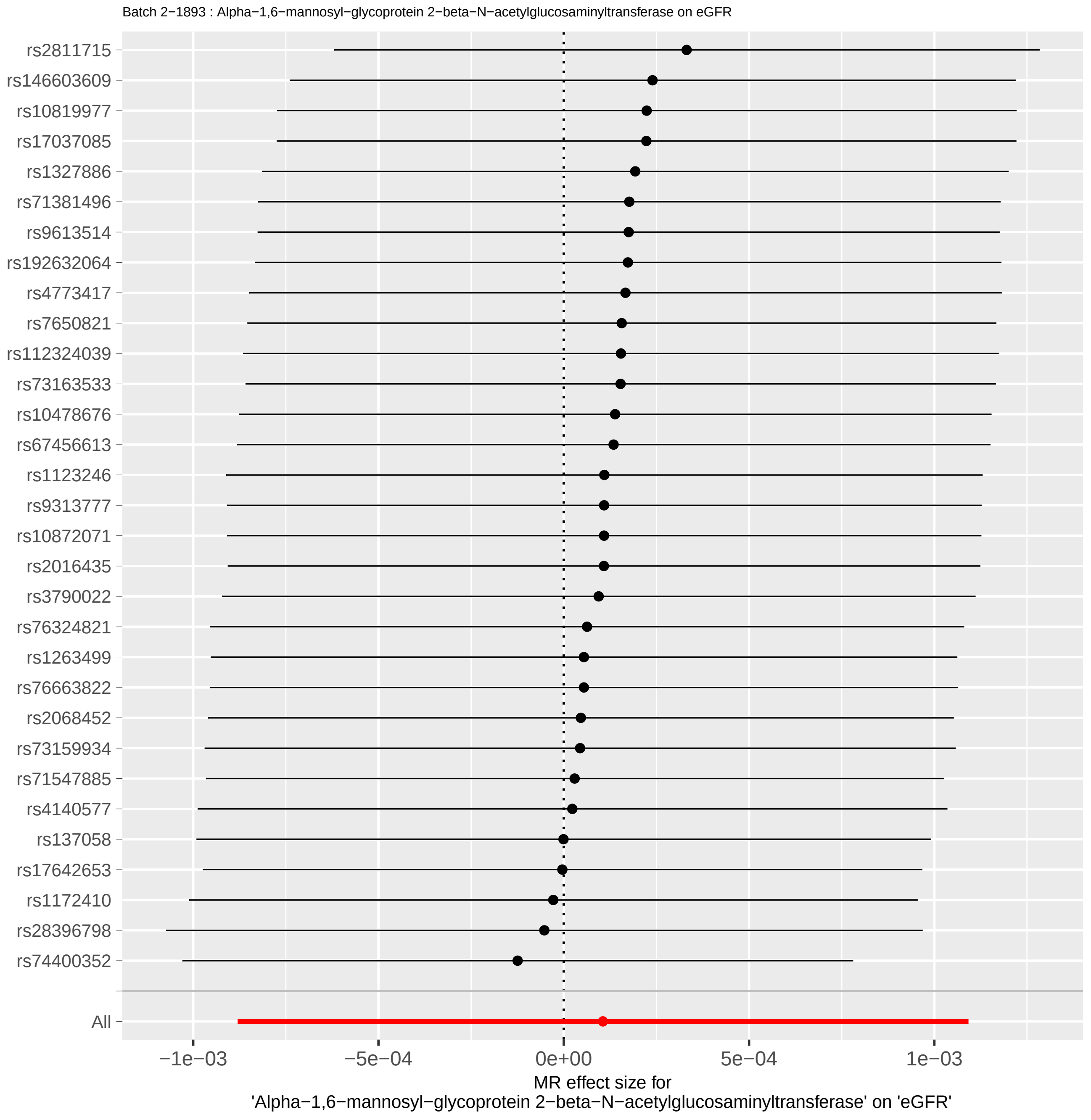
Batch 2−1893 : Alpha−1,6−mannosyl−glycoprotein 2−beta−N−acetylglucosaminyltransferase on eGFR
rs2811715
rs146603609
rs10819977
rs17037085
rs1327886
rs71381496
rs9613514
rs192632064
rs4773417
rs7650821
rs112324039
rs73163533
rs10478676
rs67456613
rs1123246
rs9313777
rs10872071
rs2016435
rs3790022
rs76324821
rs1263499
rs76663822
rs2068452
rs73159934
rs71547885
rs4140577
rs137058
rs17642653
rs1172410
rs28396798
rs74400352
All
−1e−03
−5e−04
0e+00
5e−04
1e−03
MR effect size for
'Alpha−1,6−mannosyl−glycoprotein 2−beta−N−acetylglucosaminyltransferase' on 'eGFR'

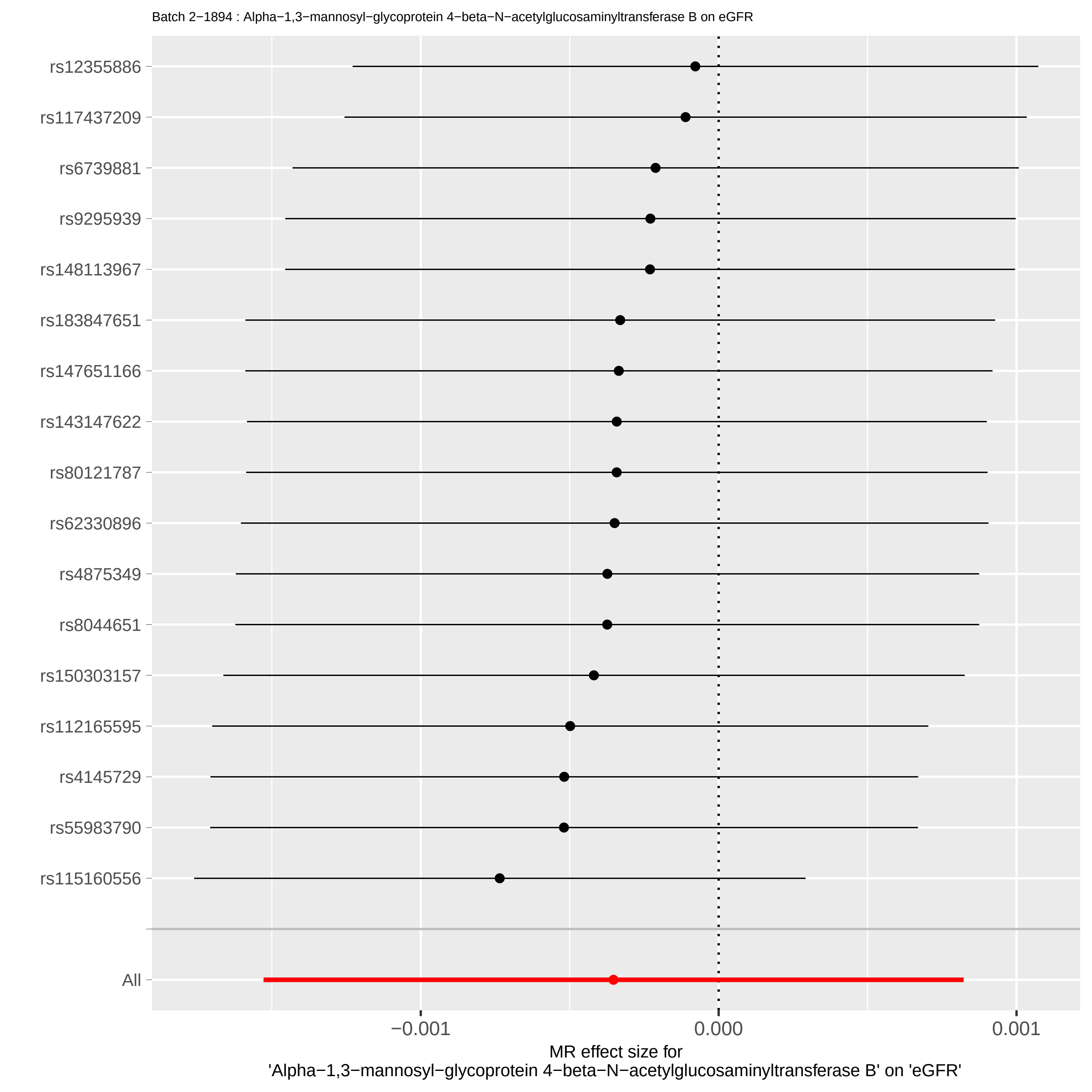
Batch 2−1894 : Alpha−1,3−mannosyl−glycoprotein 4−beta−N−acetylglucosaminyltransferase B on eGFR
rs12355886
rs117437209
rs6739881
rs9295939
rs148113967
rs183847651
rs147651166
rs143147622
rs80121787
rs62330896
rs4875349
rs8044651
rs150303157
rs112165595
rs4145729
rs55983790
rs115160556
All
−0.001
0.000
0.001
MR effect size for
'Alpha−1,3−mannosyl−glycoprotein 4−beta−N−acetylglucosaminyltransferase B' on 'eGFR'

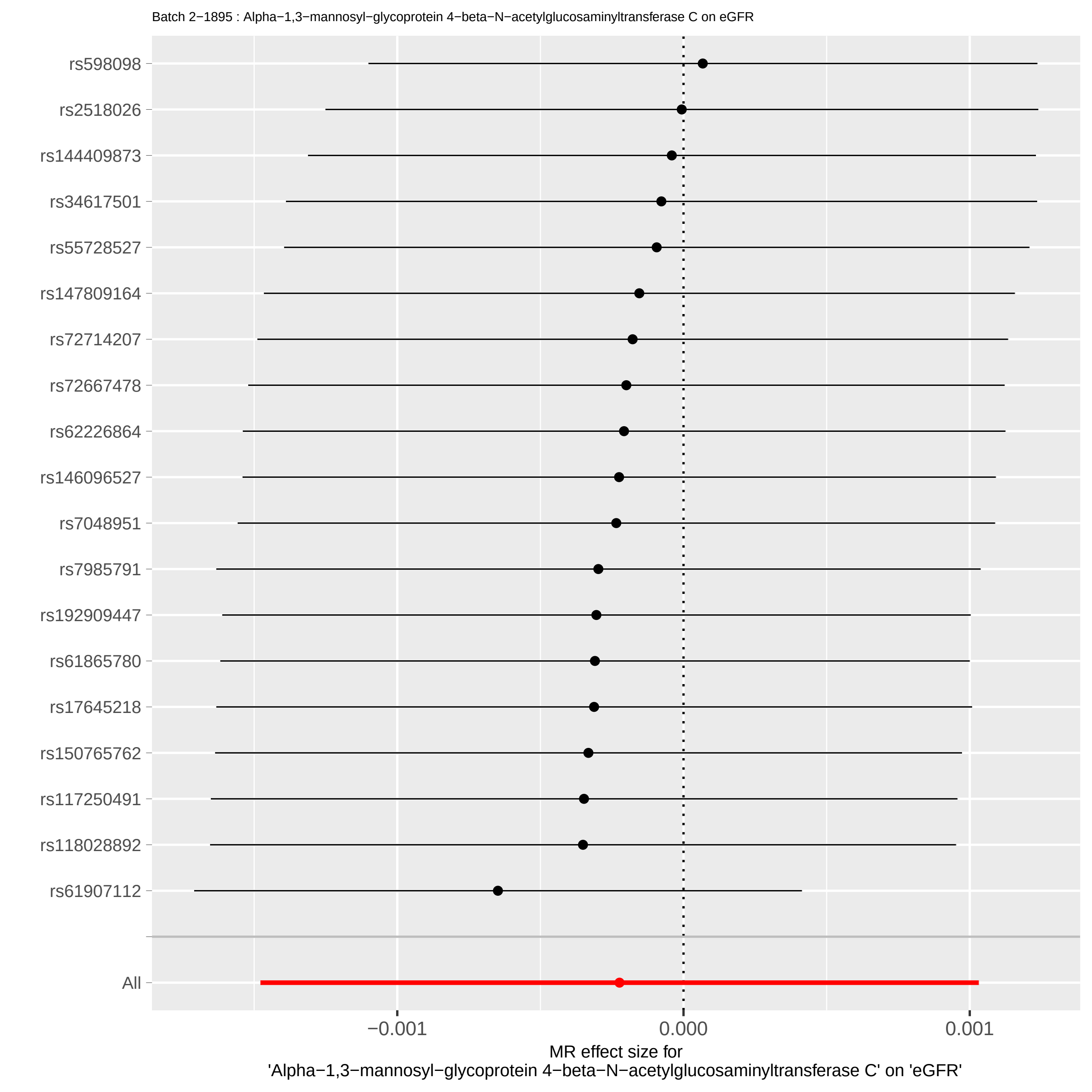

Batch 2−1895 : Alpha−1,3−mannosyl−glycoprotein 4−beta−N−acetylglucosaminyltransferase C on eGFR
rs598098
rs2518026
rs144409873
rs34617501
rs55728527
rs147809164
rs72714207
rs72667478
rs62226864
rs146096527
rs7048951
rs7985791
rs192909447
rs61865780
rs17645218
rs150765762
rs117250491
rs118028892
rs61907112
All
−0.001
0.000
0.001
MR effect size for
'Alpha−1,3−mannosyl−glycoprotein 4−beta−N−acetylglucosaminyltransferase C' on 'eGFR'

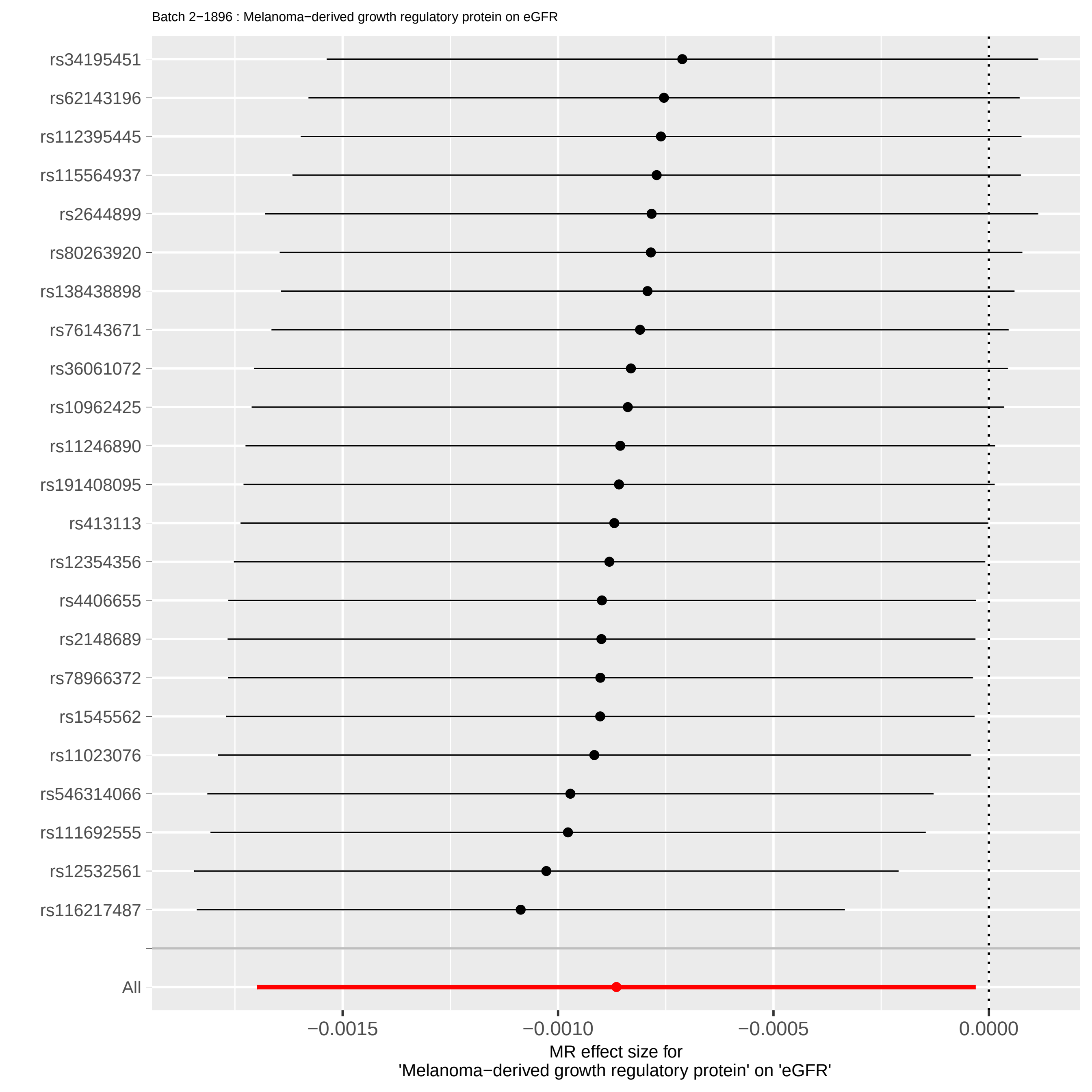

Batch 2−1896 : Melanoma−derived growth regulatory protein on eGFR
rs34195451
rs62143196
rs112395445
rs115564937
rs2644899
rs80263920
rs138438898
rs76143671
rs36061072
rs10962425
rs11246890
rs191408095
rs413113
rs12354356
rs4406655
rs2148689
rs78966372
rs1545562
rs11023076
rs546314066
rs111692555
rs12532561
rs116217487
All
−0.0015
−0.0010
−0.0005
0.0000
MR effect size for
'Melanoma−derived growth regulatory protein' on 'eGFR'

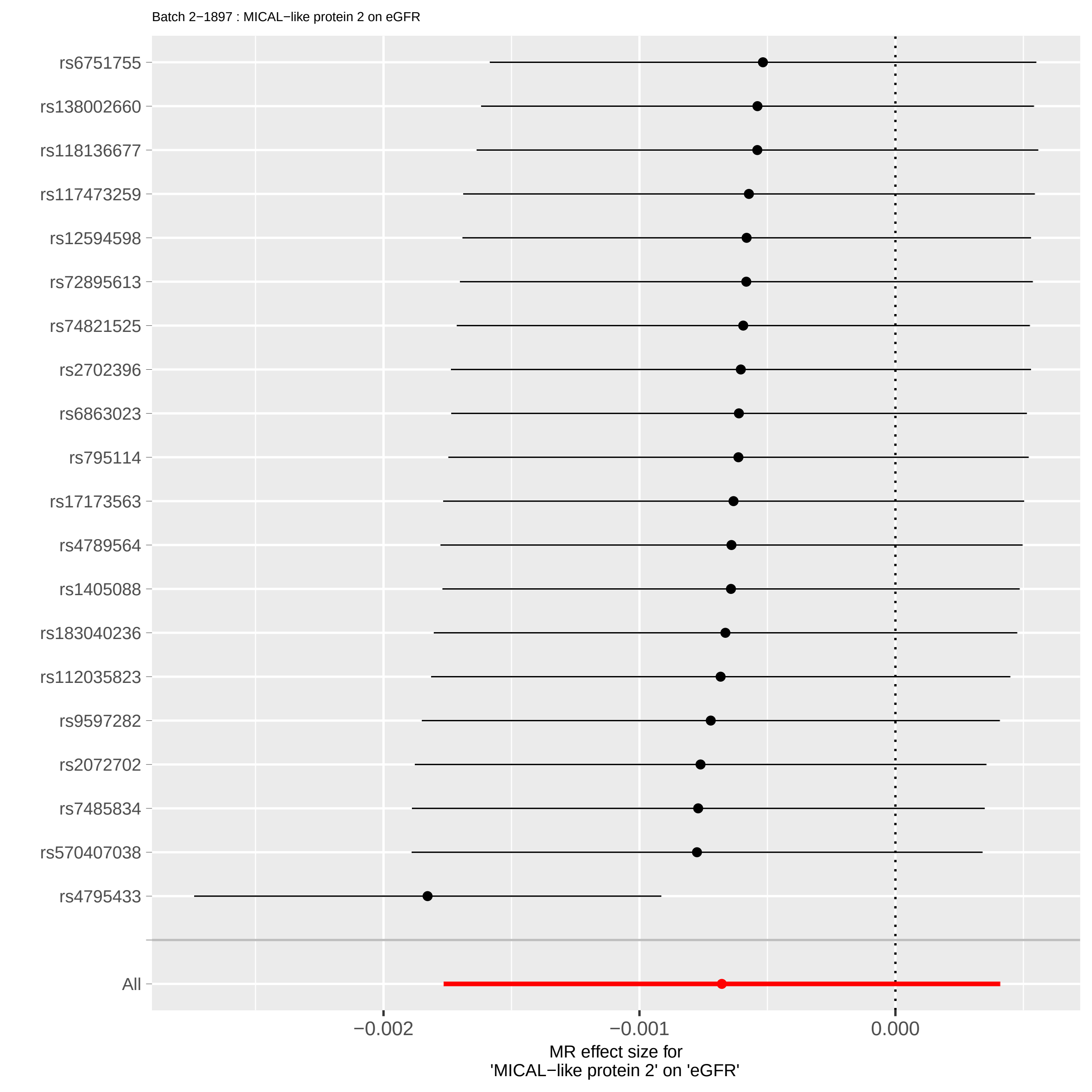

Batch 2−1897 : MICAL−like protein 2 on eGFR
rs6751755
rs138002660
rs118136677
rs117473259
rs12594598
rs72895613
rs74821525
rs2702396
rs6863023
rs795114
rs17173563
rs4789564
rs1405088
rs183040236
rs112035823
rs9597282
rs2072702
rs7485834
rs570407038
rs4795433
All
−0.002
−0.001
0.000
MR effect size for
'MICAL−like protein 2' on 'eGFR'

Batch 2−1898 : MHC class I polypeptide−related sequence B on eGFR

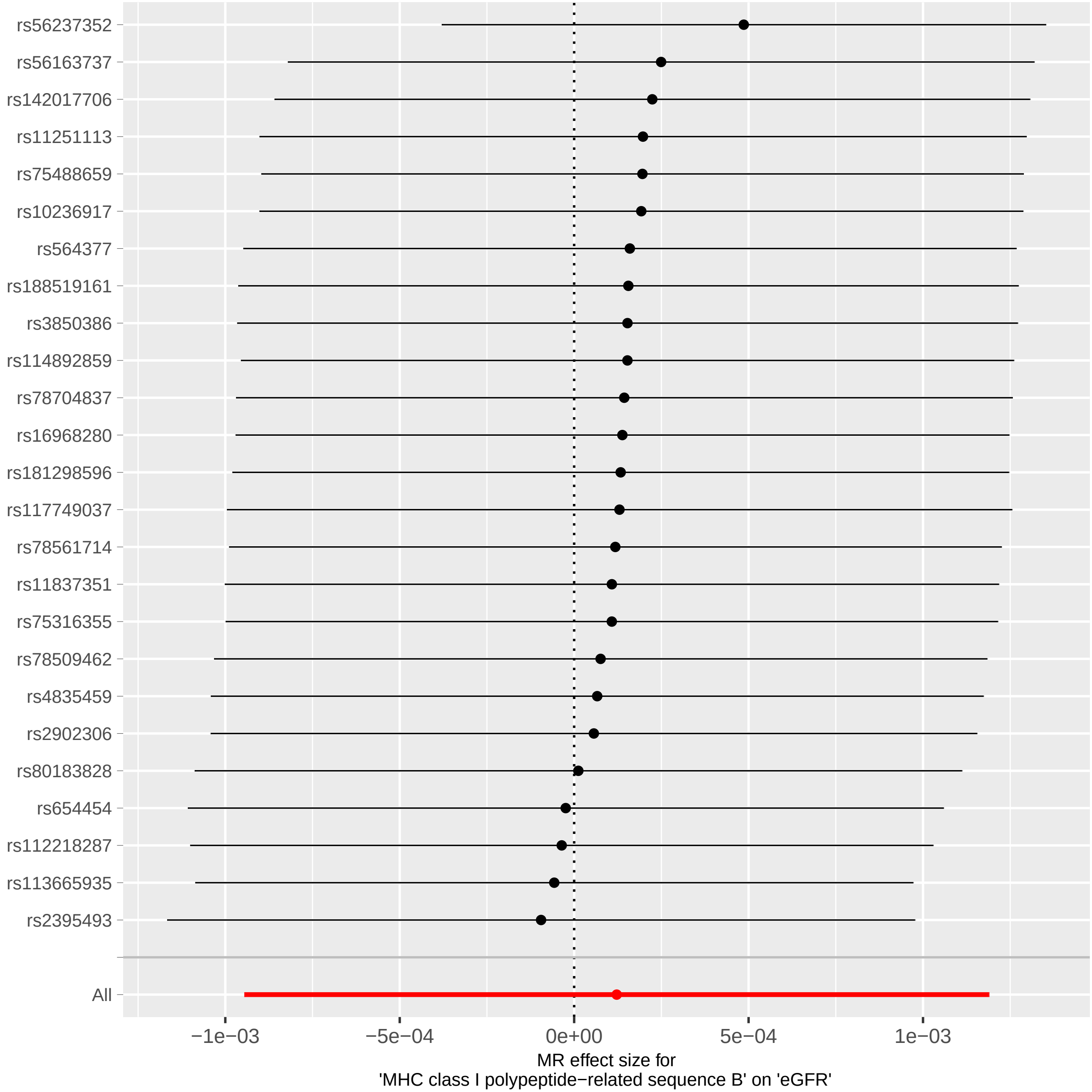

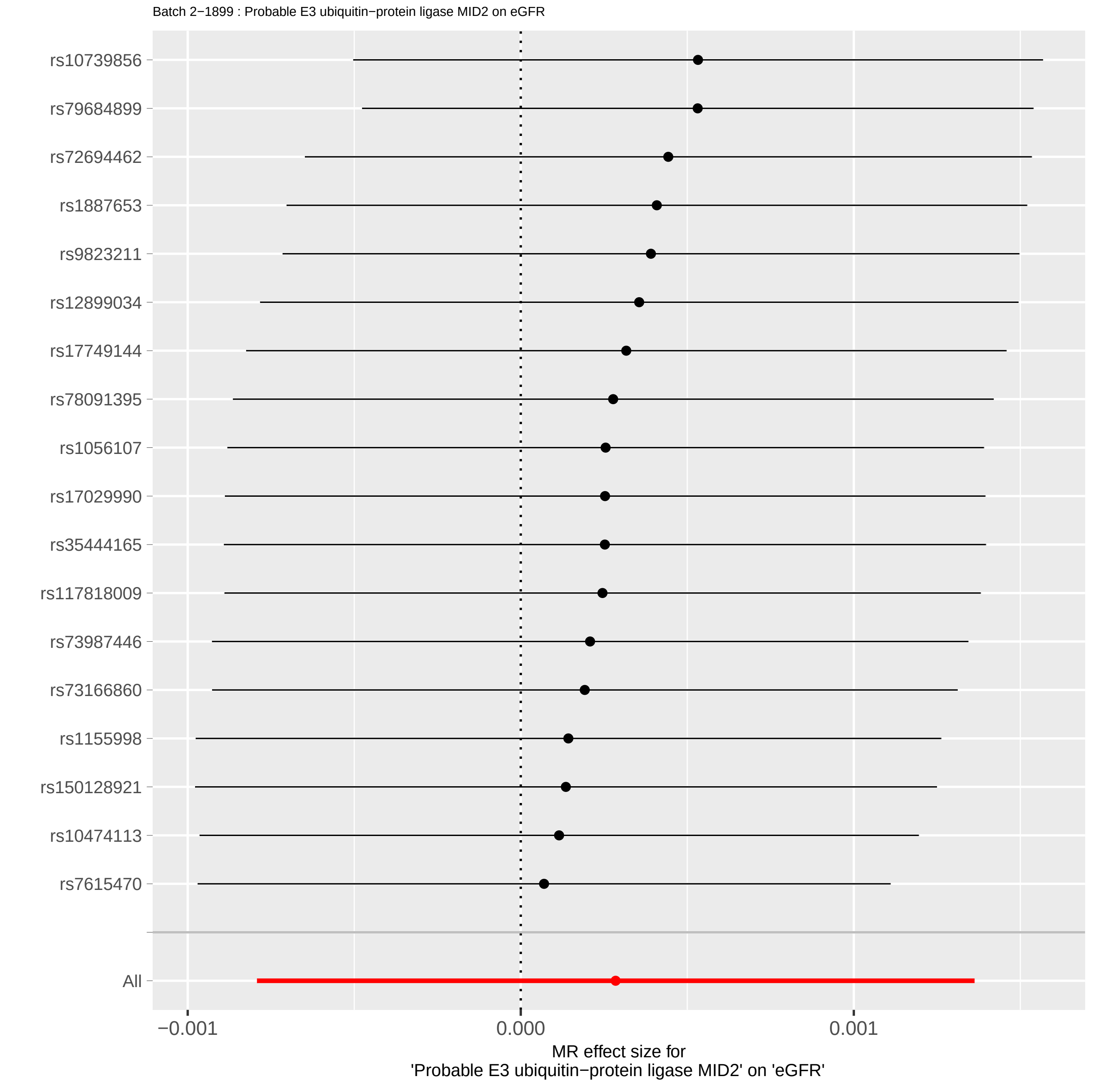

Batch 2−1899 : Probable E3 ubiquitin−protein ligase MID2 on eGFR
rs10739856
rs79684899
rs72694462
rs1887653
rs9823211
rs12899034
rs17749144
rs78091395
rs1056107
rs17029990
rs35444165
rs117818009
rs73987446
rs73166860
rs1155998
rs150128921
rs10474113
rs7615470
All
−0.001
0.000
0.001
MR effect size for
'Probable E3 ubiquitin−protein ligase MID2' on 'eGFR'

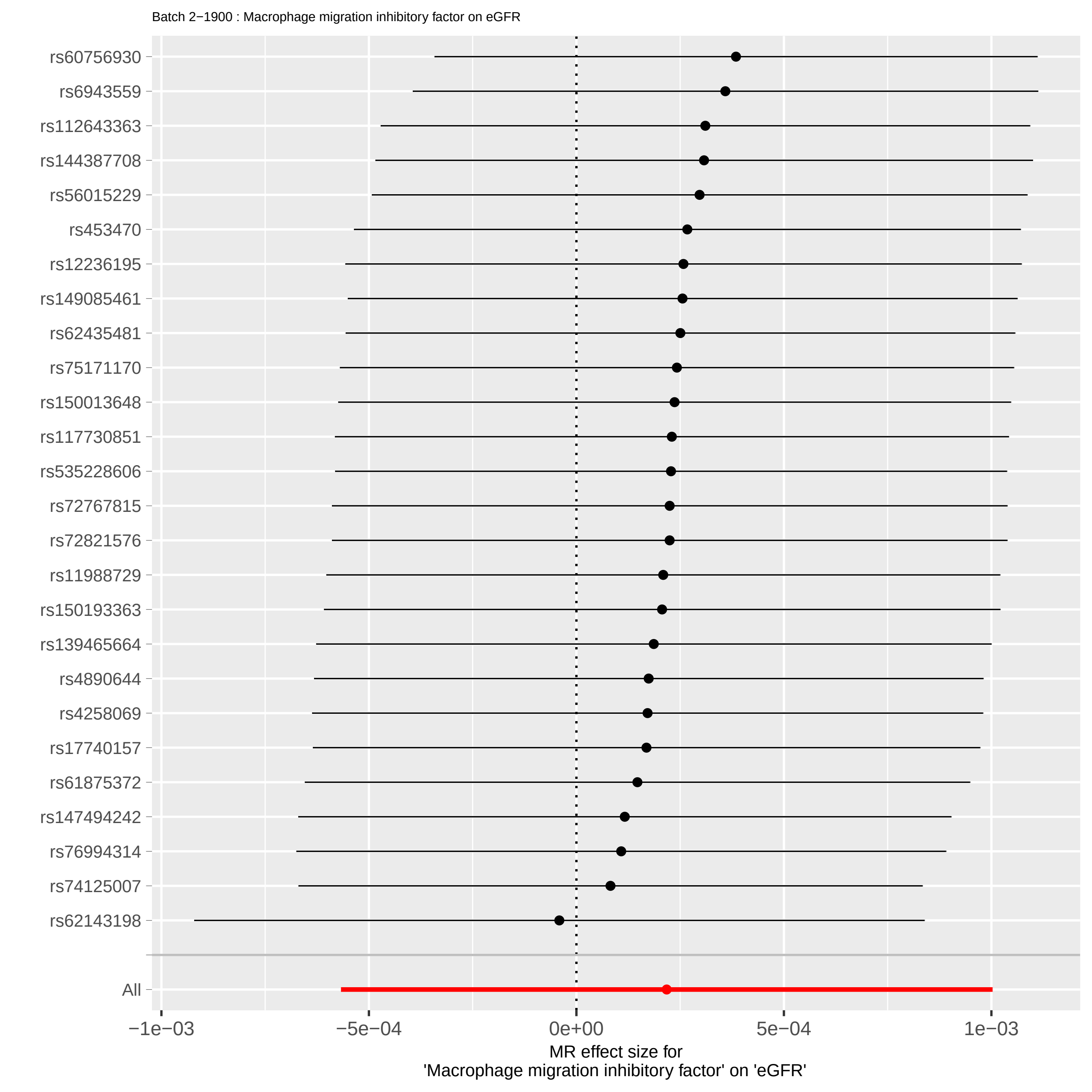

Batch 2−1900 : Macrophage migration inhibitory factor on eGFR
rs60756930
rs6943559
rs112643363
rs144387708
rs56015229
rs453470
rs12236195
rs149085461
rs62435481
rs75171170
rs150013648
rs117730851
rs535228606
rs72767815
rs72821576
rs11988729
rs150193363
rs139465664
rs4890644
rs4258069
rs17740157
rs61875372
rs147494242
rs76994314
rs74125007
rs62143198
All
−1e−03
−5e−04
0e+00
5e−04
1e−03
MR effect size for
'Macrophage migration inhibitory factor' on 'eGFR'

Batch 2−1901 : Allergin−1 on eGFR

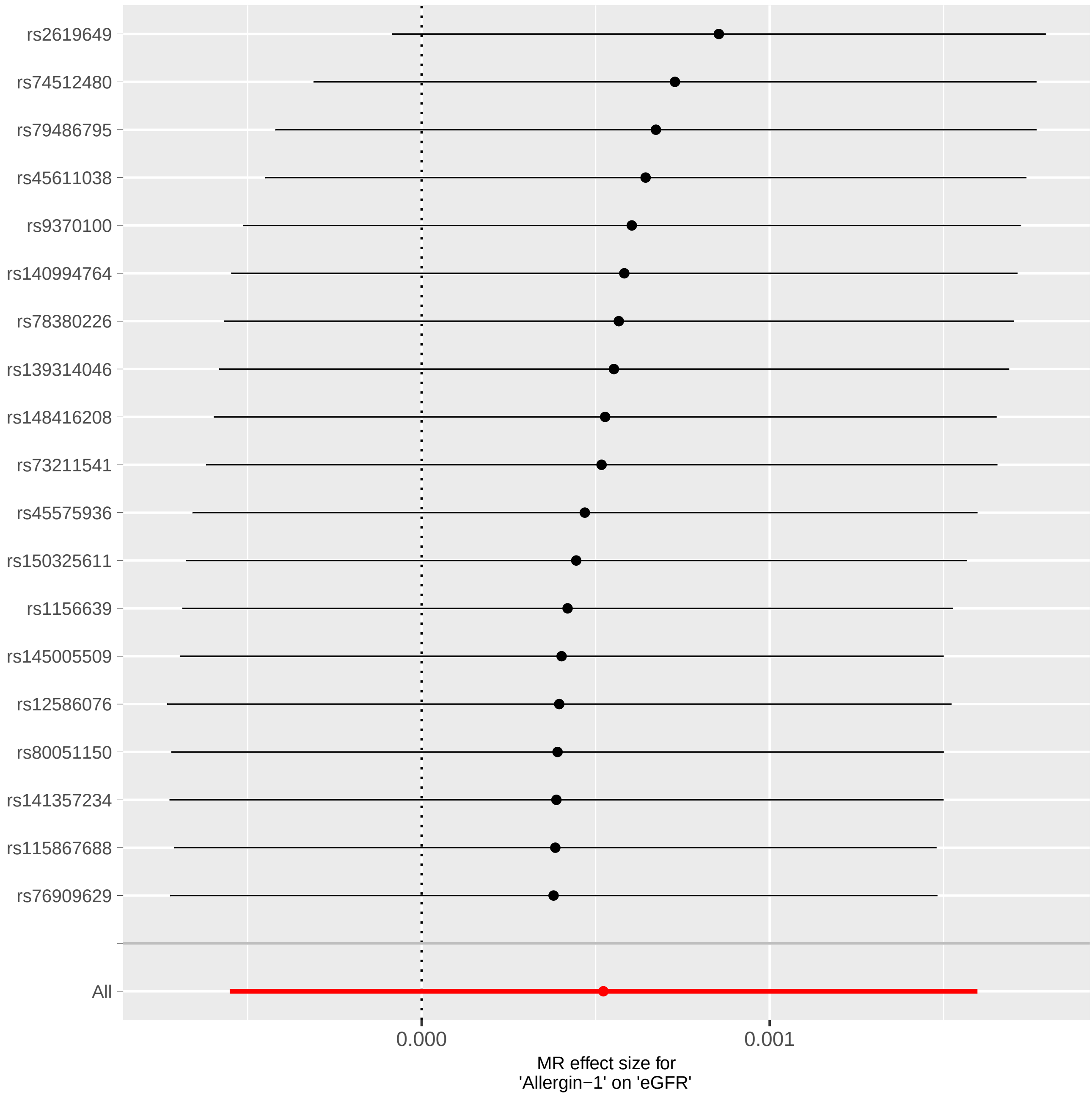

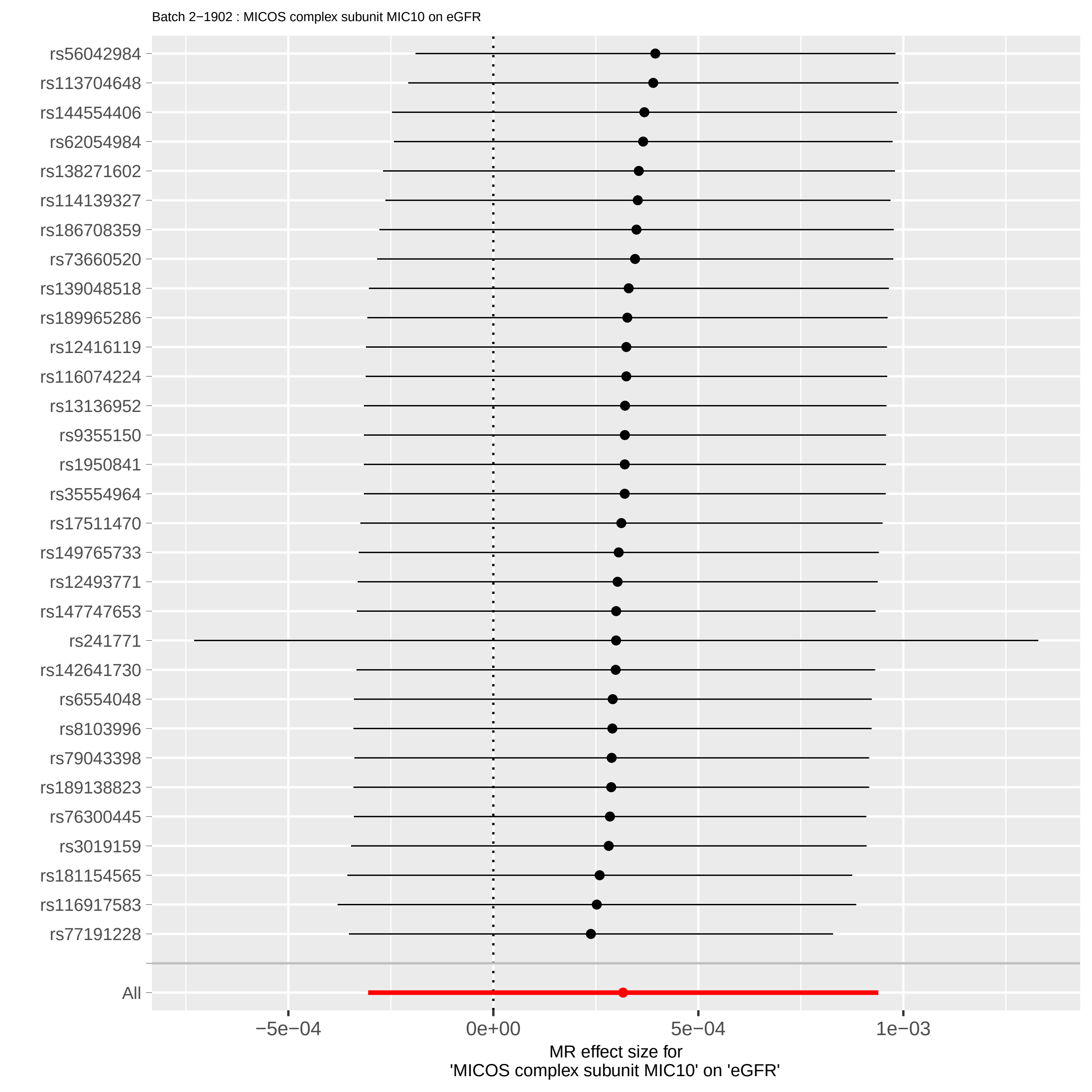

Batch 2−1902 : MICOS complex subunit MIC10 on eGFR
rs56042984
rs113704648
rs144554406
rs62054984
rs138271602
rs114139327
rs186708359
rs73660520
rs139048518
rs189965286
rs12416119
rs116074224
rs13136952
rs9355150
rs1950841
rs35554964
rs17511470
rs149765733
rs12493771
rs147747653
rs241771
rs142641730
rs6554048
rs8103996
rs79043398
rs189138823
rs76300445
rs3019159
rs181154565
rs116917583
rs77191228
All
−5e−04
0e+00
5e−04
1e−03
MR effect size for
'MICOS complex subunit MIC10' on 'eGFR'

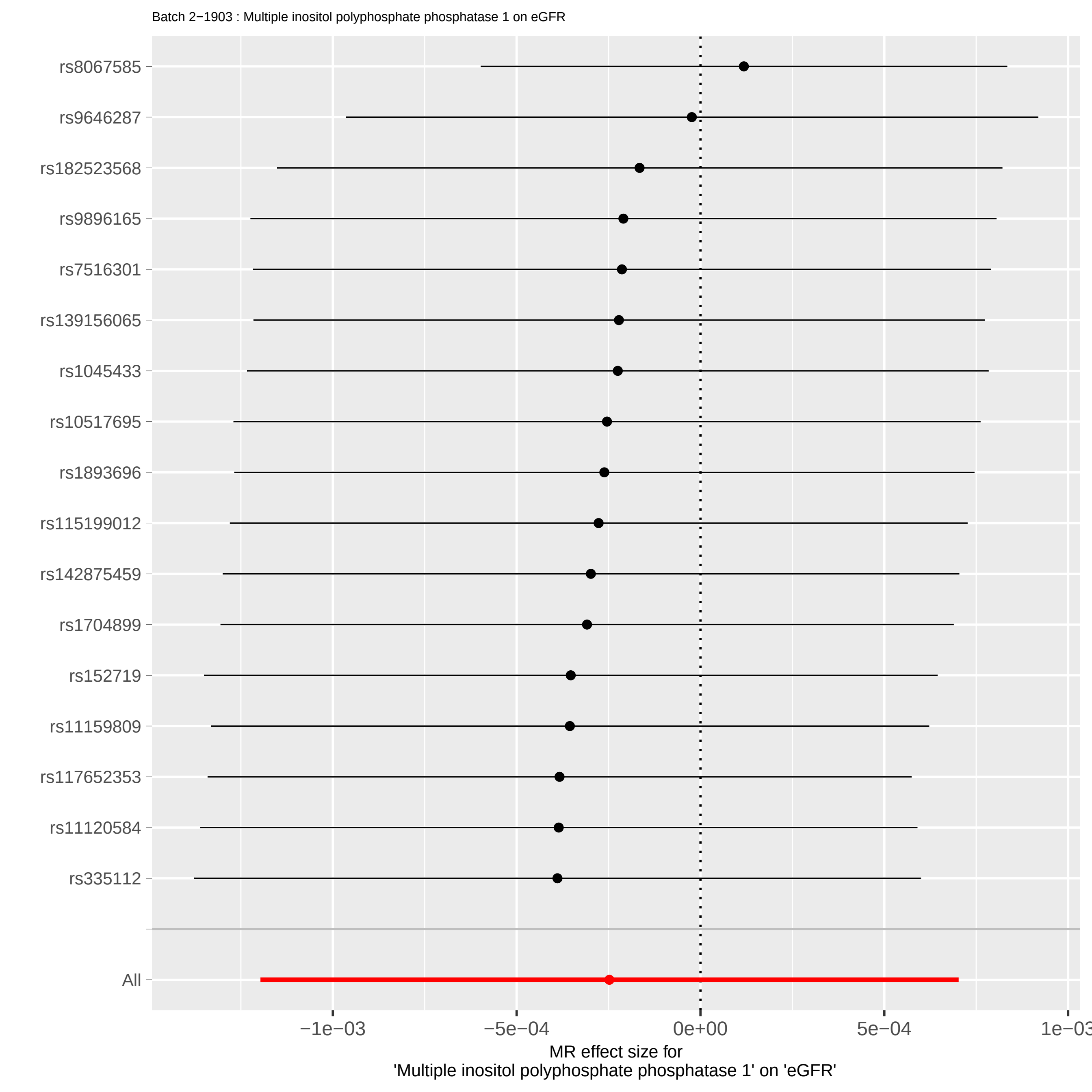
Batch 2−1903 : Multiple inositol polyphosphate phosphatase 1 on eGFR
rs8067585
rs9646287
rs182523568
rs9896165
rs7516301
rs139156065
rs1045433
rs10517695
rs1893696
rs115199012
rs142875459
rs1704899
rs152719
rs11159809
rs117652353
rs11120584
rs335112
All
−1e−03
−5e−04
0e+00
5e−04
1e−03
MR effect size for
'Multiple inositol polyphosphate phosphatase 1' on 'eGFR'

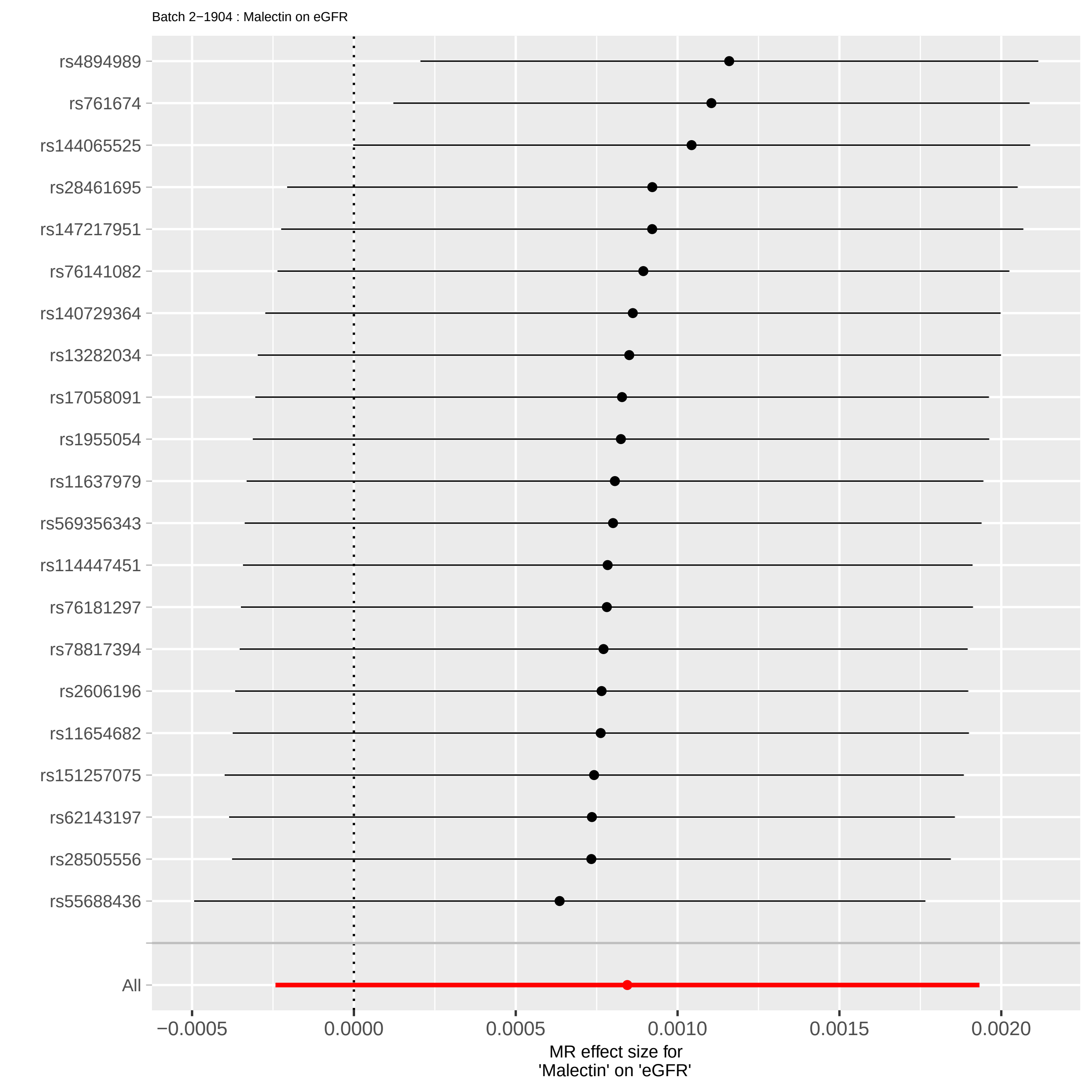

Batch 2−1904 : Malectin on eGFR
rs4894989
rs761674
rs144065525
rs28461695
rs147217951
rs76141082
rs140729364
rs13282034
rs17058091
rs1955054
rs11637979
rs569356343
rs114447451
rs76181297
rs78817394
rs2606196
rs11654682
rs151257075
rs62143197
rs28505556
rs55688436
All
−0.0005
0.0000
0.0005
0.0010
0.0015
0.0020
MR effect size for
'Malectin' on 'eGFR'

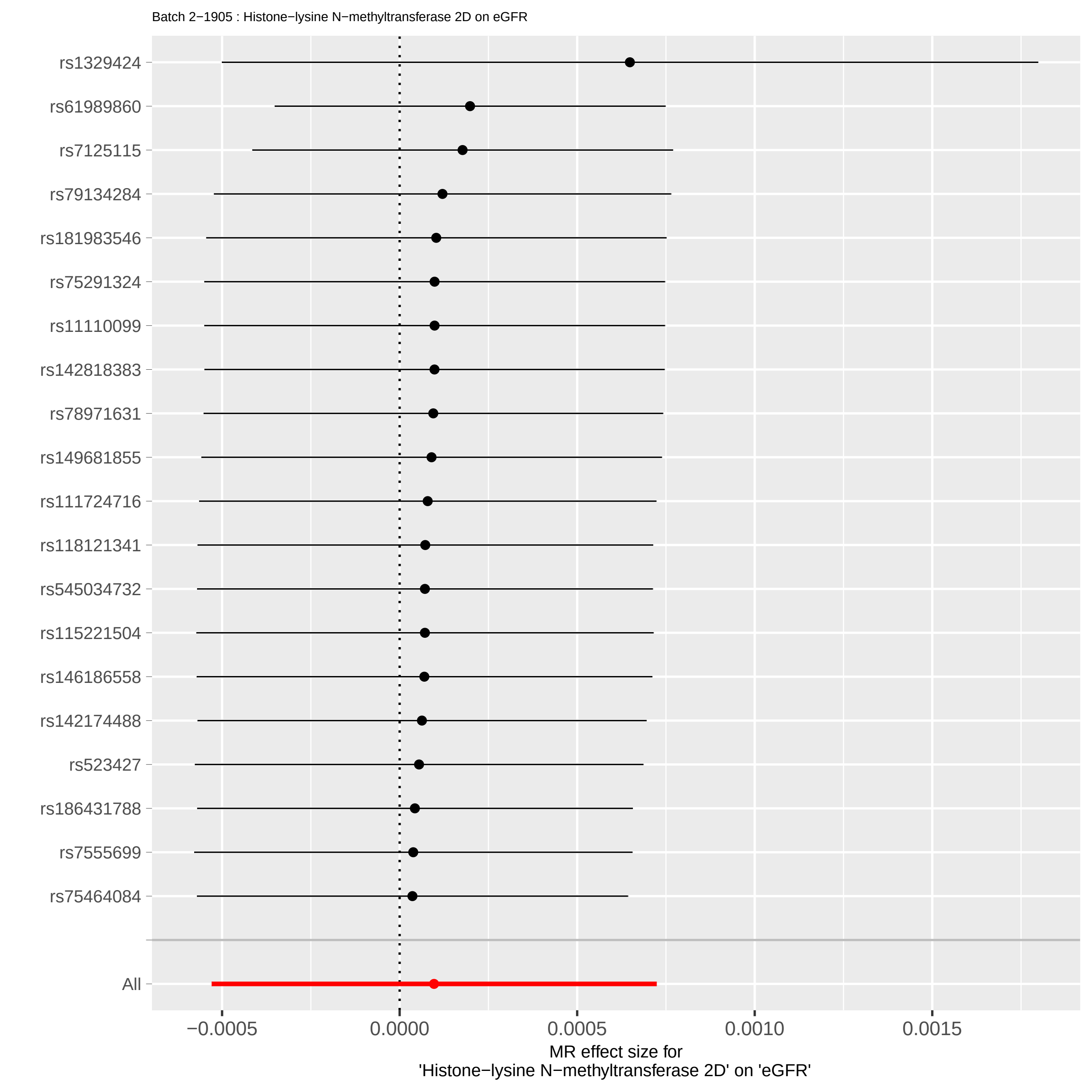
Batch 2−1905 : Histone−lysine N−methyltransferase 2D on eGFR
rs1329424
rs61989860
rs7125115
rs79134284
rs181983546
rs75291324
rs11110099
rs142818383
rs78971631
rs149681855
rs111724716
rs118121341
rs545034732
rs115221504
rs146186558
rs142174488
rs523427
rs186431788
rs7555699
rs75464084
All
−0.0005
0.0000
0.0005
0.0010
0.0015
MR effect size for
'Histone−lysine N−methyltransferase 2D' on 'eGFR'

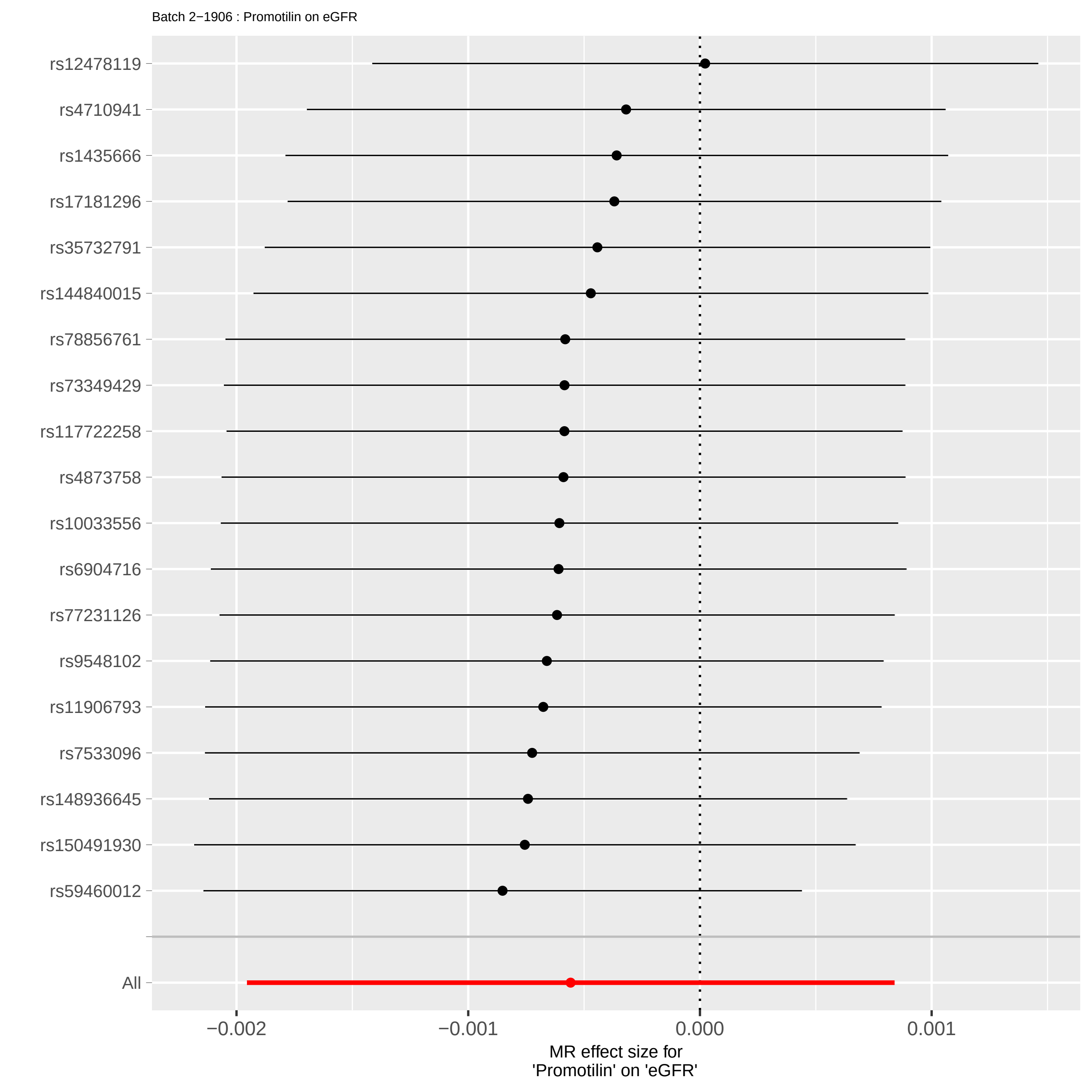
Batch 2−1906 : Promotilin on eGFR
rs12478119
rs4710941
rs1435666
rs17181296
rs35732791
rs144840015
rs78856761
rs73349429
rs117722258
rs4873758
rs10033556
rs6904716
rs77231126
rs9548102
rs11906793
rs7533096
rs148936645
rs150491930
rs59460012
All
−0.002
−0.001
0.000
0.001
MR effect size for
'Promotilin' on 'eGFR'

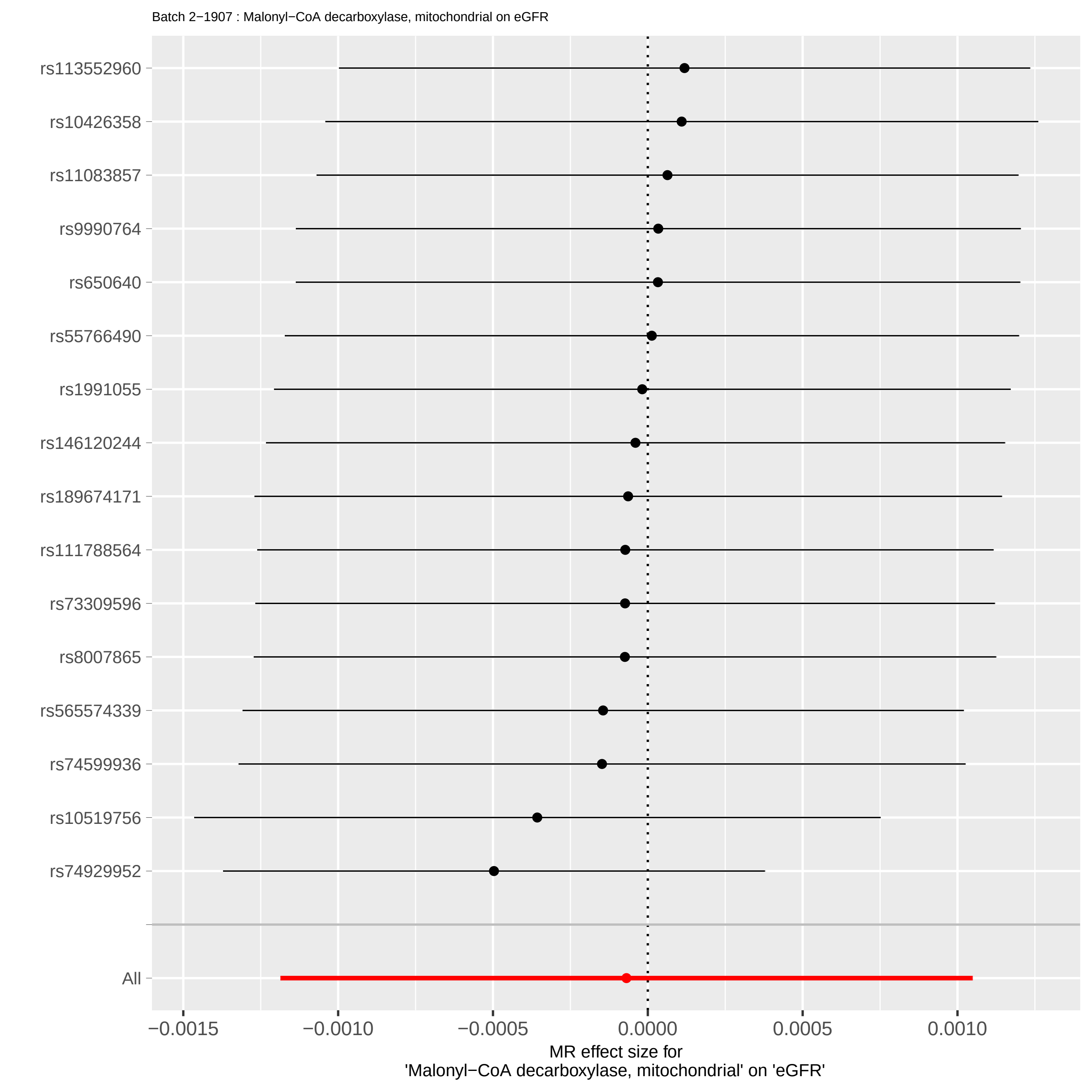

Batch 2−1907 : Malonyl−CoA decarboxylase, mitochondrial on eGFR
rs113552960
rs10426358
rs11083857
rs9990764
rs650640
rs55766490
rs1991055
rs146120244
rs189674171
rs111788564
rs73309596
rs8007865
rs565574339
rs74599936
rs10519756
rs74929952
All
−0.0015
−0.0010
−0.0005
0.0000
0.0005
0.0010
MR effect size for
'Malonyl−CoA decarboxylase, mitochondrial' on 'eGFR'

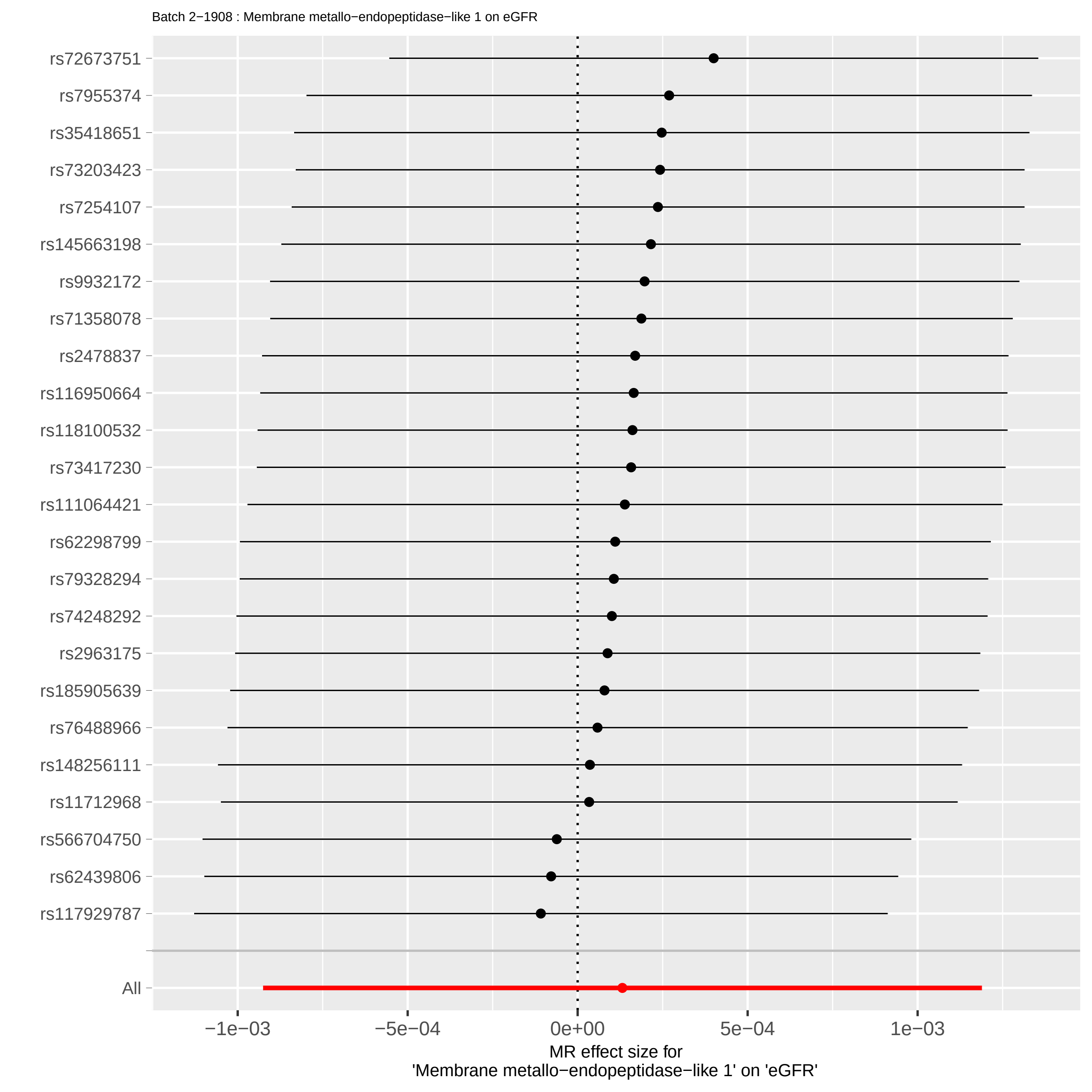

Batch 2−1908 : Membrane metallo−endopeptidase−like 1 on eGFR
rs72673751
rs7955374
rs35418651
rs73203423
rs7254107
rs145663198
rs9932172
rs71358078
rs2478837
rs116950664
rs118100532
rs73417230
rs111064421
rs62298799
rs79328294
rs74248292
rs2963175
rs185905639
rs76488966
rs148256111
rs11712968
rs566704750
rs62439806
rs117929787
All
−1e−03
−5e−04
0e+00
5e−04
1e−03
MR effect size for
'Membrane metallo−endopeptidase−like 1' on 'eGFR'

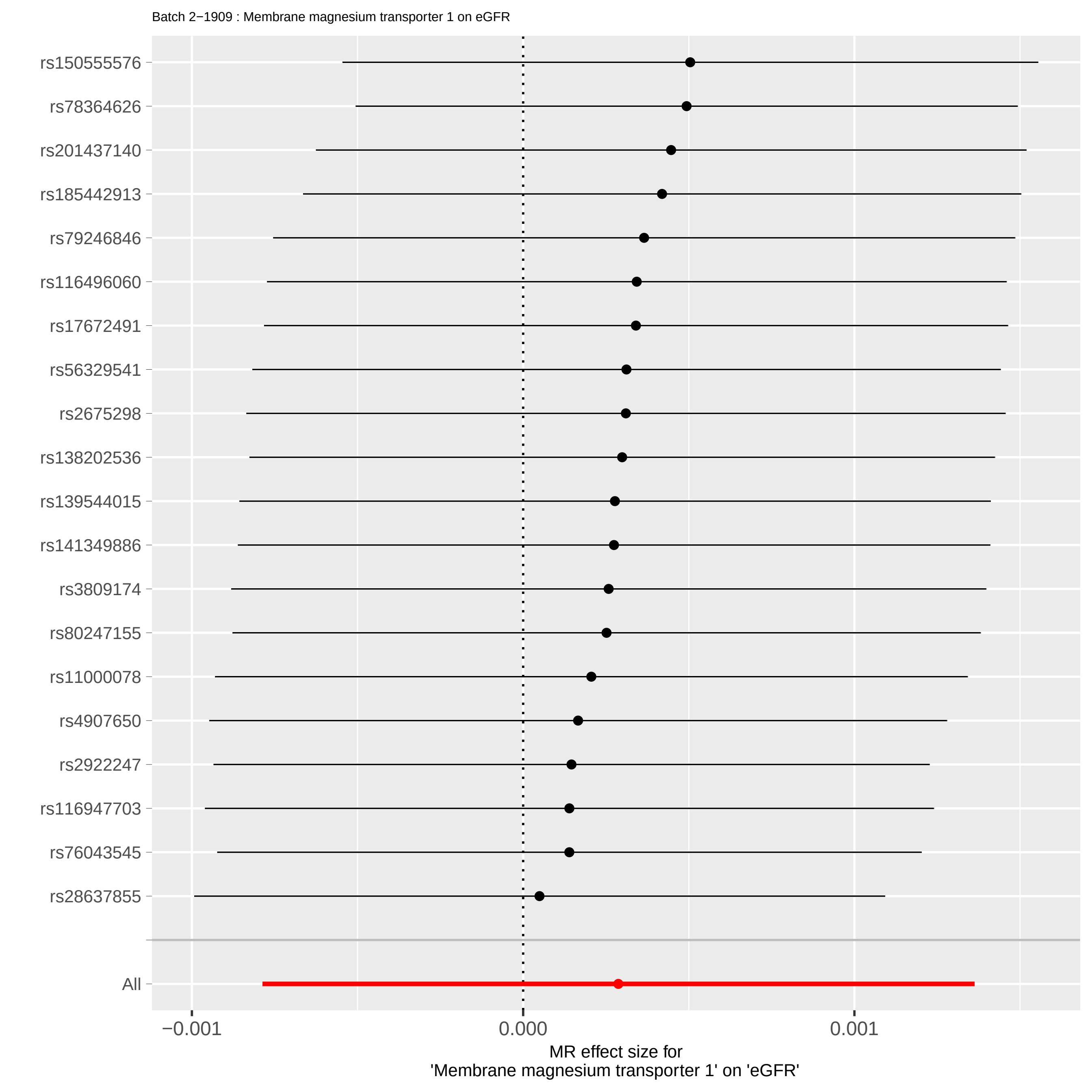

Batch 2−1909 : Membrane magnesium transporter 1 on eGFR
rs150555576
rs78364626
rs201437140
rs185442913
rs79246846
rs116496060
rs17672491
rs56329541
rs2675298
rs138202536
rs139544015
rs141349886
rs3809174
rs80247155
rs11000078
rs4907650
rs2922247
rs116947703
rs76043545
rs28637855
All
−0.001
0.000
0.001
MR effect size for
'Membrane magnesium transporter 1' on 'eGFR'

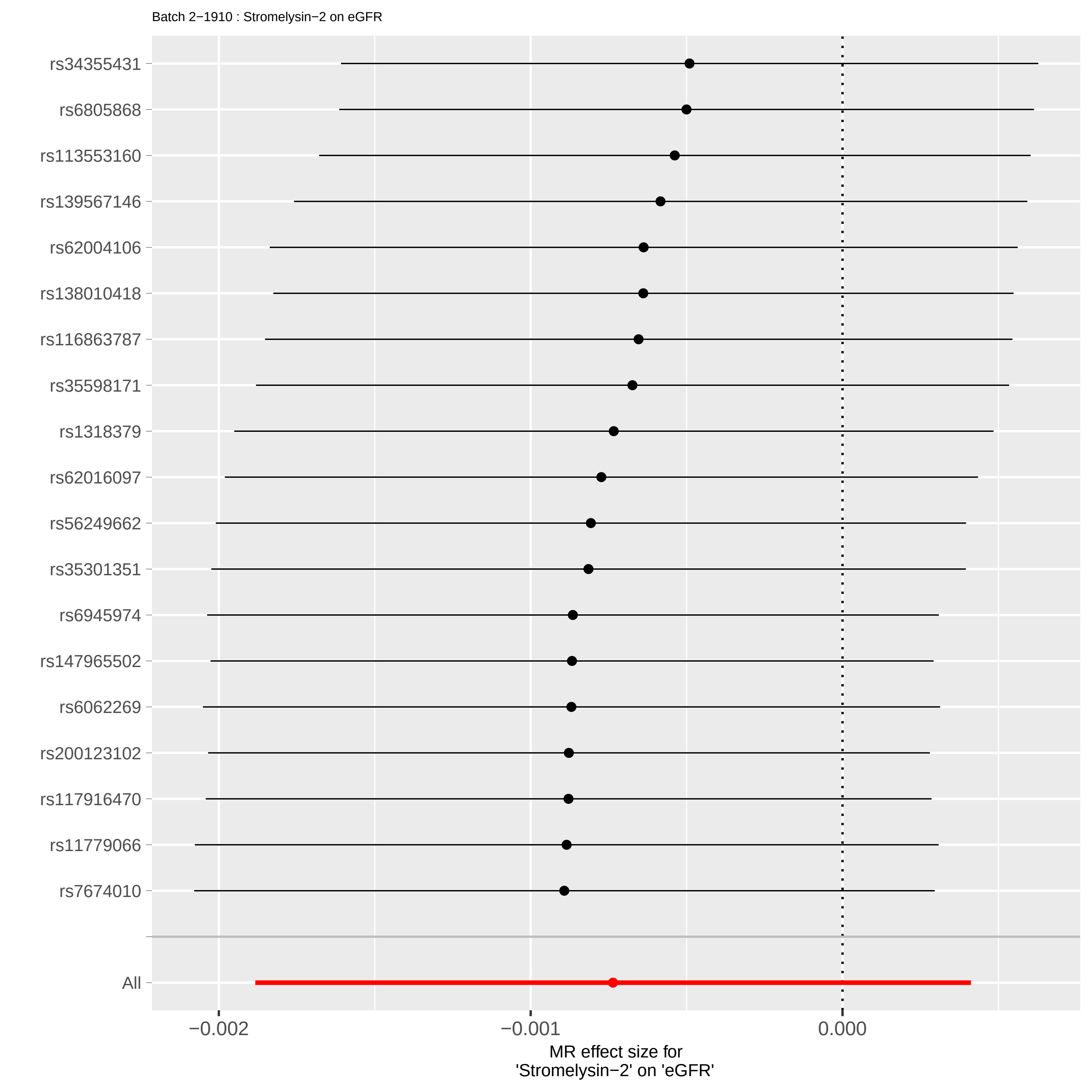

Batch 2−1910 : Stromelysin−2 on eGFR
rs34355431
rs6805868
rs113553160
rs139567146
rs62004106
rs138010418
rs116863787
rs35598171
rs1318379
rs62016097
rs56249662
rs35301351
rs6945974
rs147965502
rs6062269
rs200123102
rs117916470
rs11779066
rs7674010
All
−0.002
−0.001
0.000
MR effect size for
'Stromelysin−2' on 'eGFR'

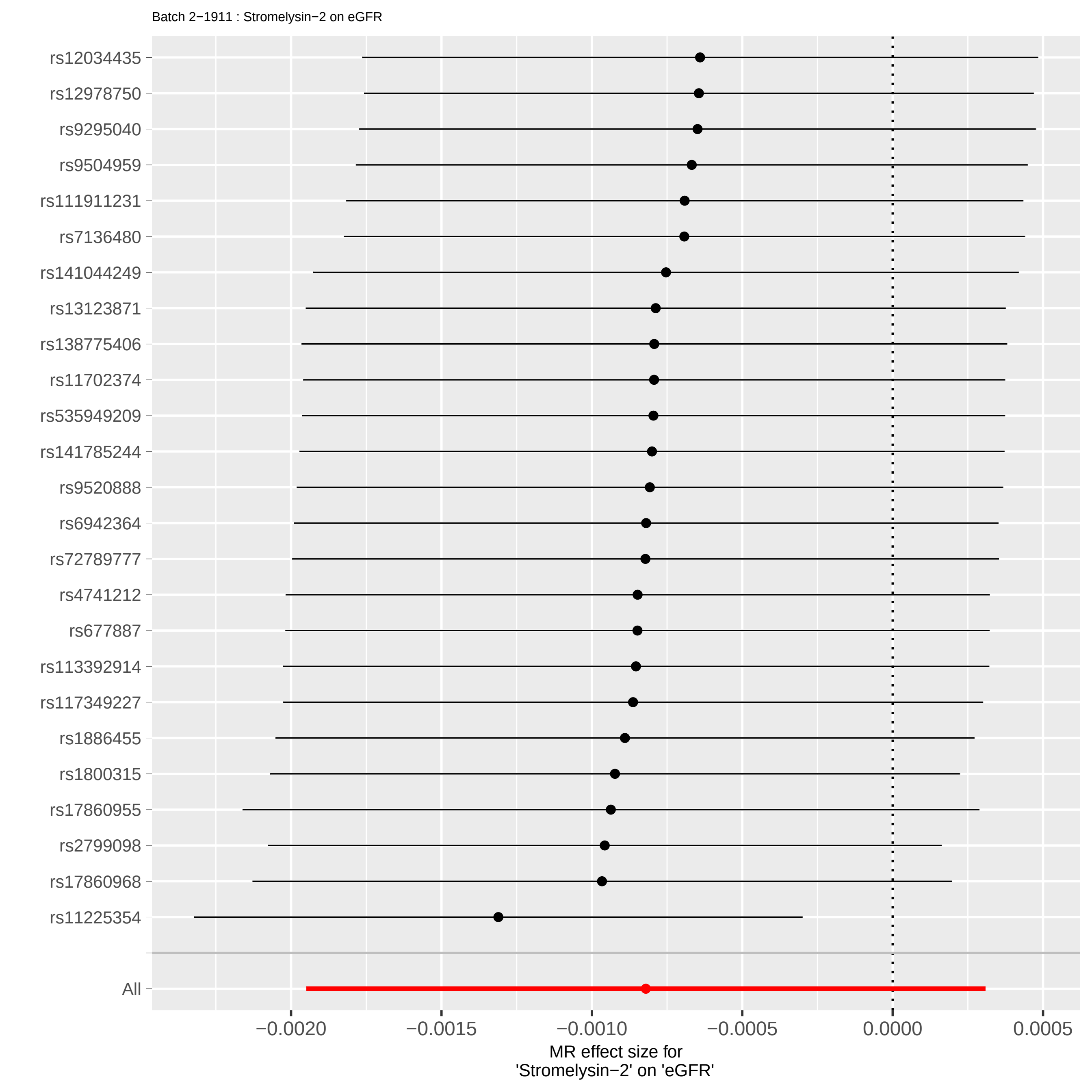

Batch 2−1911 : Stromelysin−2 on eGFR
rs12034435
rs12978750
rs9295040
rs9504959
rs111911231
rs7136480
rs141044249
rs13123871
rs138775406
rs11702374
rs535949209
rs141785244
rs9520888
rs6942364
rs72789777
rs4741212
rs677887
rs113392914
rs117349227
rs1886455
rs1800315
rs17860955
rs2799098
rs17860968
rs11225354
All
−0.0020
−0.0015
−0.0010
−0.0005
0.0000
0.0005
MR effect size for
'Stromelysin−2' on 'eGFR'

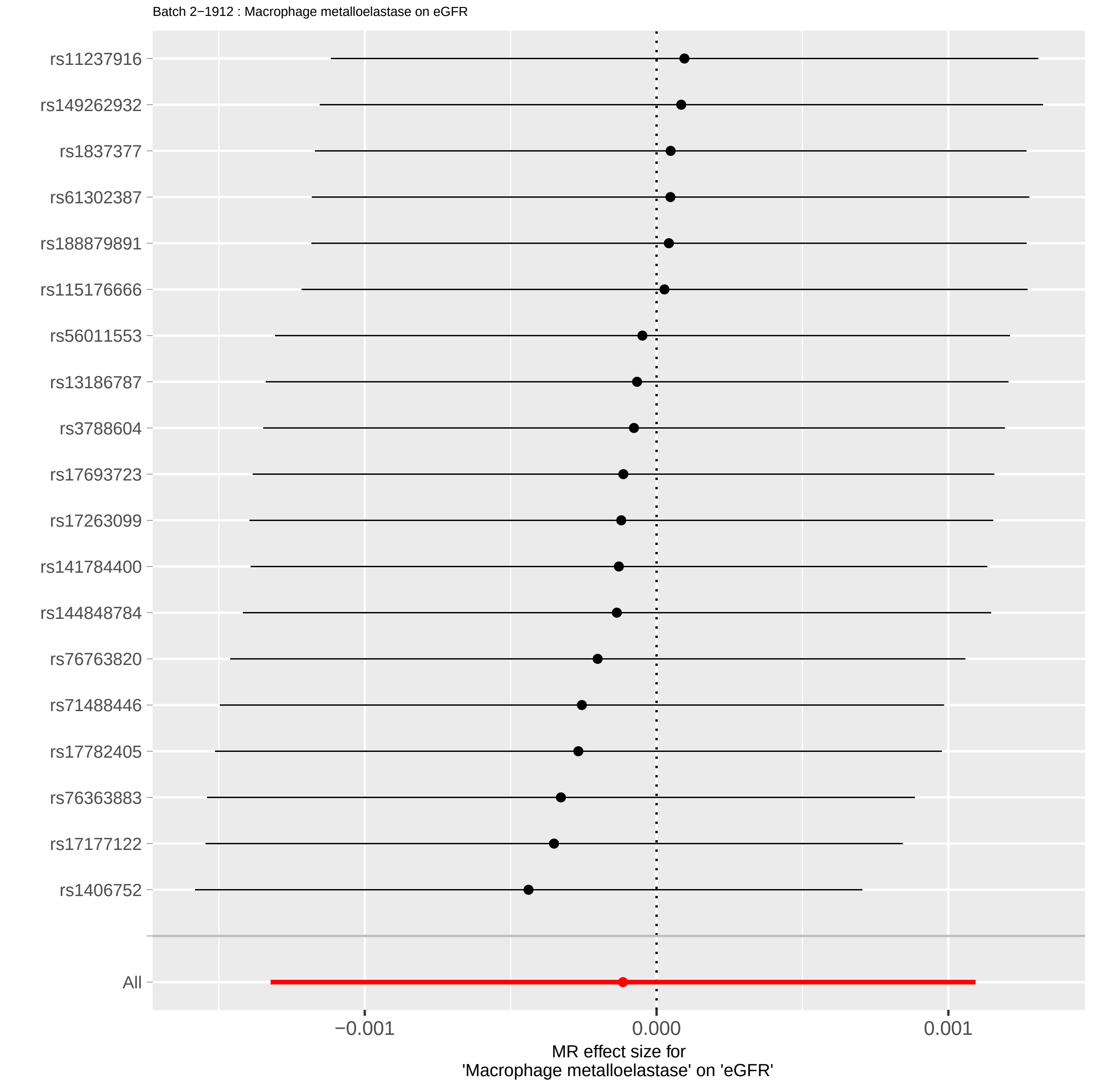

Batch 2−1912 : Macrophage metalloelastase on eGFR
rs11237916
rs149262932
rs1837377
rs61302387
rs188879891
rs115176666
rs56011553
rs13186787
rs3788604
rs17693723
rs17263099
rs141784400
rs144848784
rs76763820
rs71488446
rs17782405
rs76363883
rs17177122
rs1406752
All
−0.001
0.000
0.001
MR effect size for
'Macrophage metalloelastase' on 'eGFR'

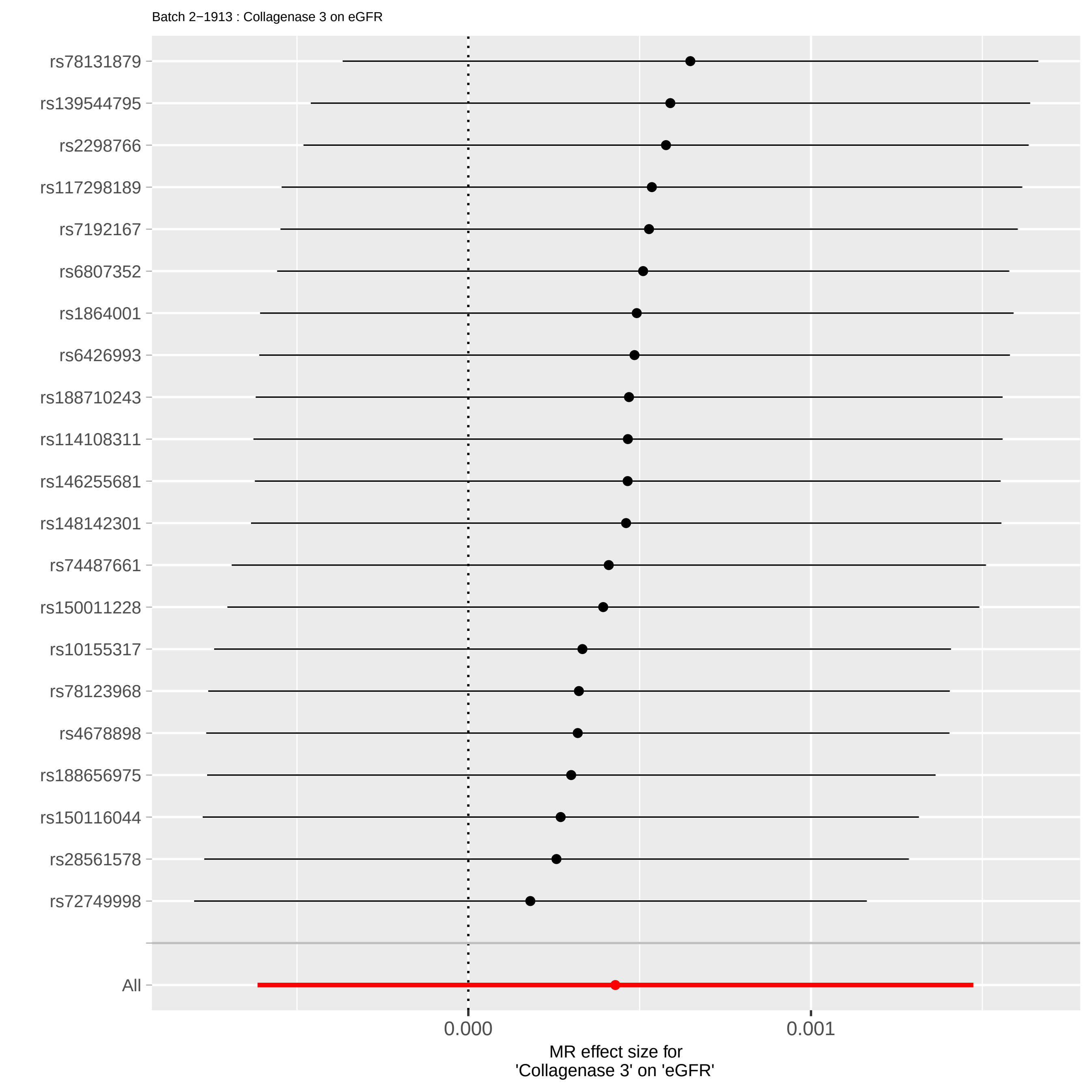

Batch 2−1913 : Collagenase 3 on eGFR
rs78131879
rs139544795
rs2298766
rs117298189
rs7192167
rs6807352
rs1864001
rs6426993
rs188710243
rs114108311
rs146255681
rs148142301
rs74487661
rs150011228
rs10155317
rs78123968
rs4678898
rs188656975
rs150116044
rs28561578
rs72749998
All
0.000
0.001
MR effect size for
'Collagenase 3' on 'eGFR'

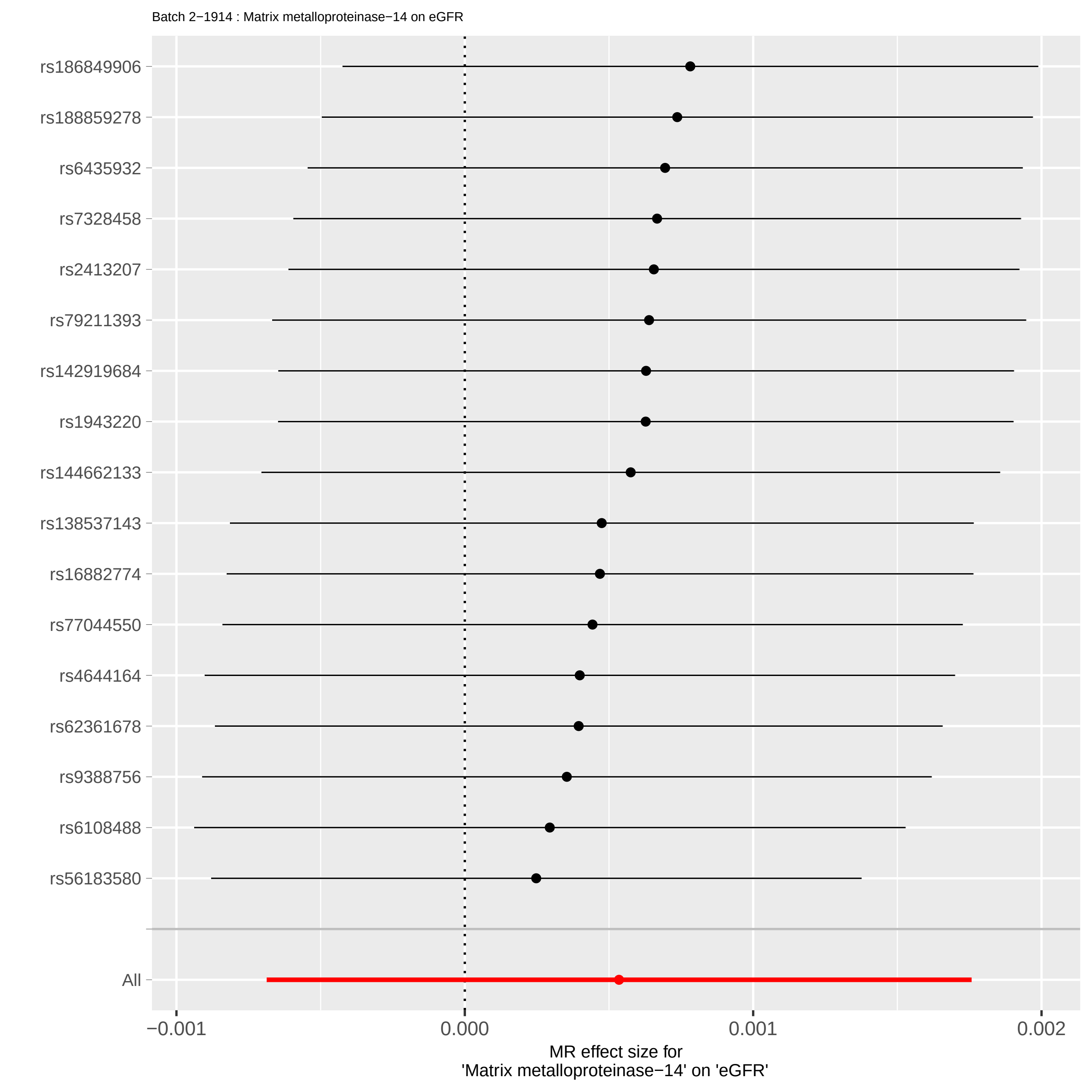
Batch 2−1914 : Matrix metalloproteinase−14 on eGFR
rs186849906
rs188859278
rs6435932
rs7328458
rs2413207
rs79211393
rs142919684
rs1943220
rs144662133
rs138537143
rs16882774
rs77044550
rs4644164
rs62361678
rs9388756
rs6108488
rs56183580
All
−0.001
0.000
0.001
0.002
MR effect size for
'Matrix metalloproteinase−14' on 'eGFR'

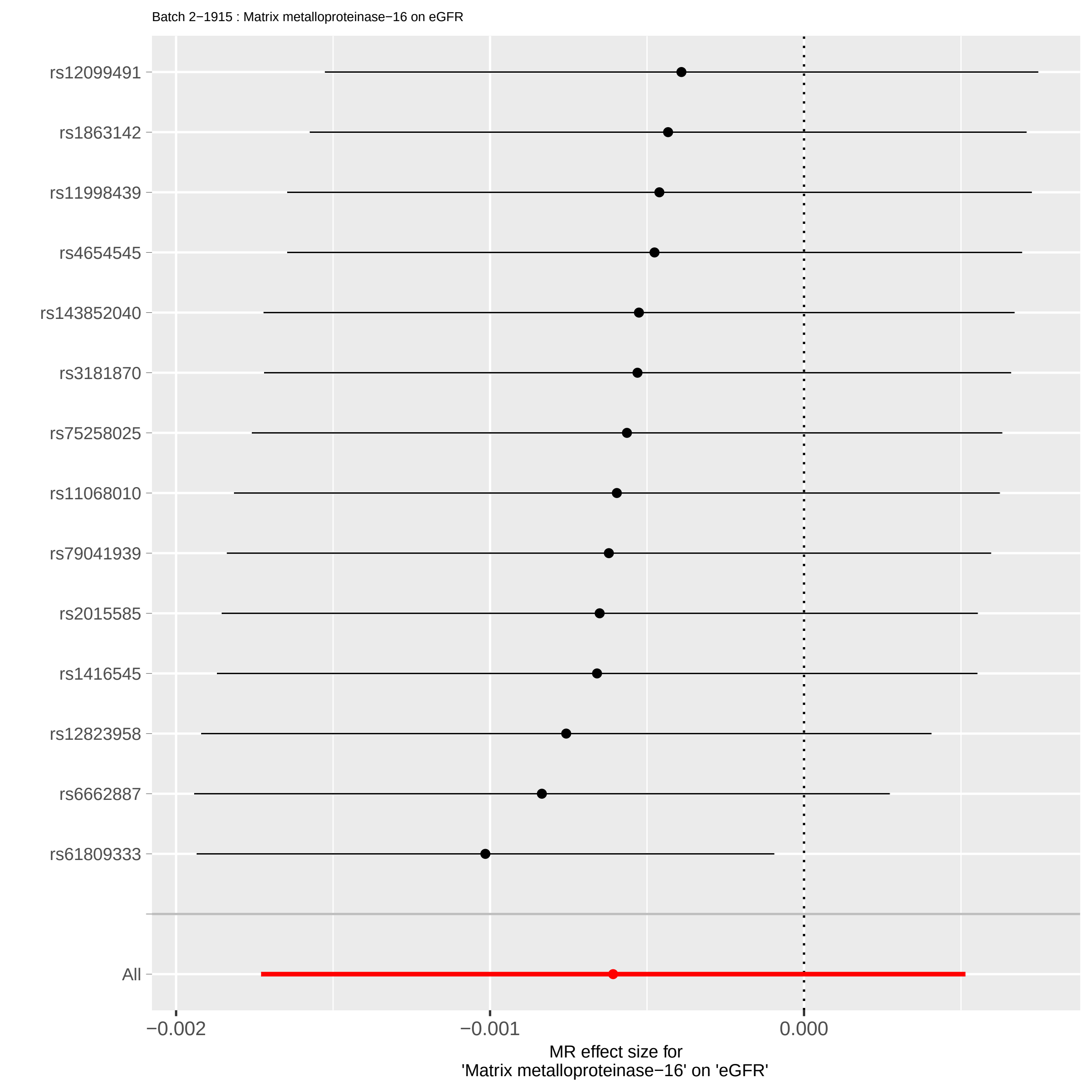

Batch 2−1915 : Matrix metalloproteinase−16 on eGFR
rs12099491
rs1863142
rs11998439
rs4654545
rs143852040
rs3181870
rs75258025
rs11068010
rs79041939
rs2015585
rs1416545
rs12823958
rs6662887
rs61809333
All
−0.002
−0.001
0.000
MR effect size for
'Matrix metalloproteinase−16' on 'eGFR'

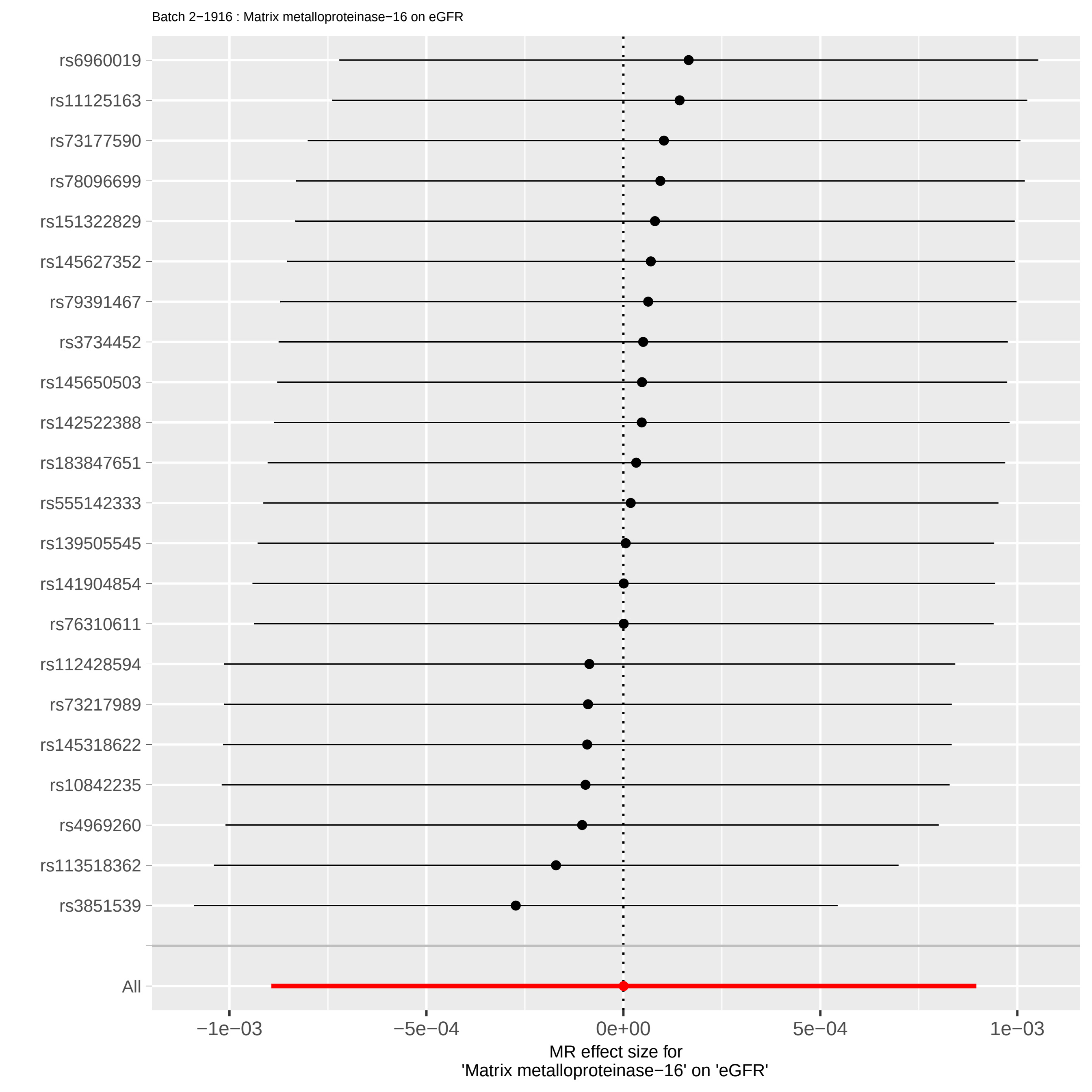

Batch 2−1916 : Matrix metalloproteinase−16 on eGFR
rs6960019
rs11125163
rs73177590
rs78096699
rs151322829
rs145627352
rs79391467
rs3734452
rs145650503
rs142522388
rs183847651
rs555142333
rs139505545
rs141904854
rs76310611
rs112428594
rs73217989
rs145318622
rs10842235
rs4969260
rs113518362
rs3851539
All
−1e−03
−5e−04
0e+00
5e−04
1e−03
MR effect size for
'Matrix metalloproteinase−16' on 'eGFR'

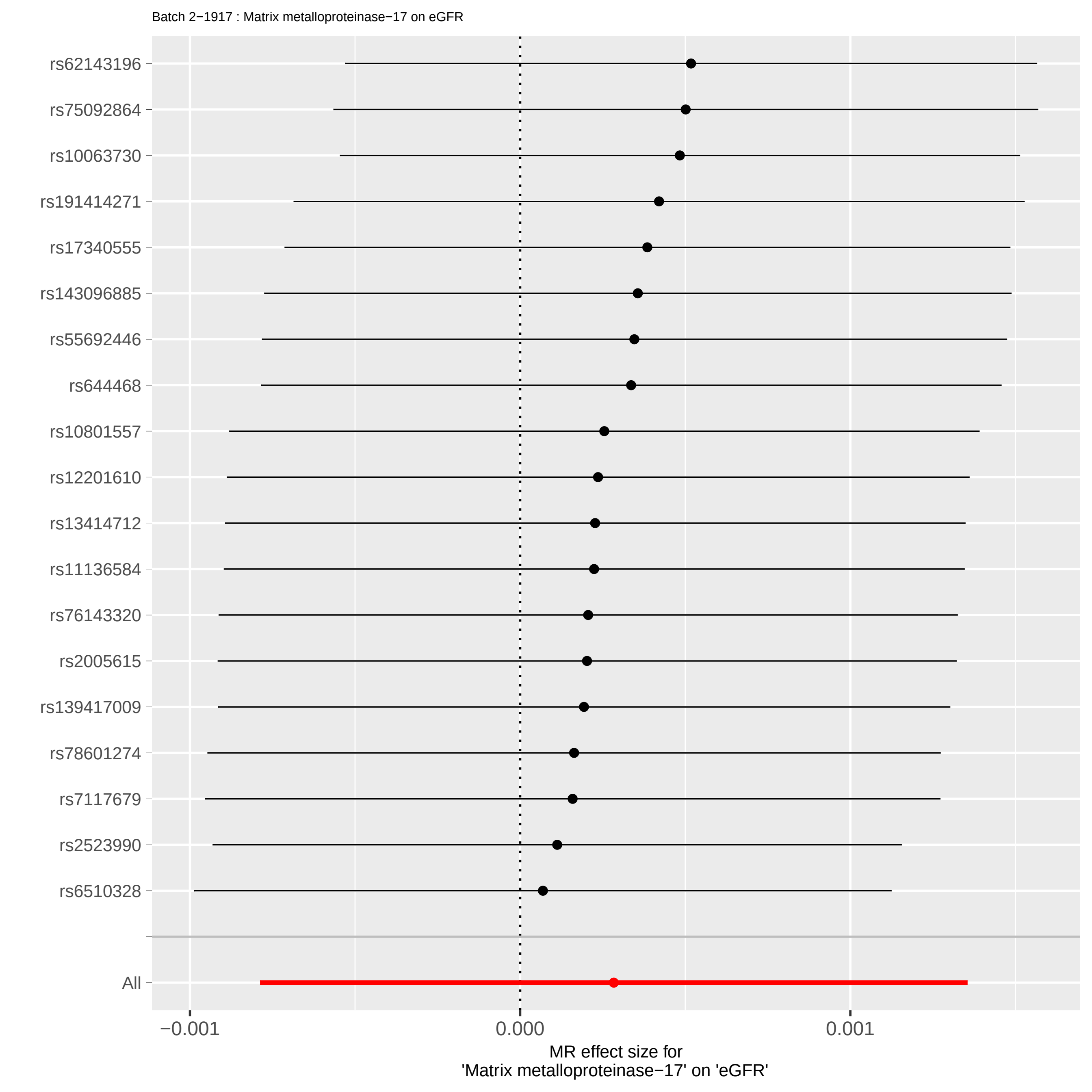
Batch 2−1917 : Matrix metalloproteinase−17 on eGFR
rs62143196
rs75092864
rs10063730
rs191414271
rs17340555
rs143096885
rs55692446
rs644468
rs10801557
rs12201610
rs13414712
rs11136584
rs76143320
rs2005615
rs139417009
rs78601274
rs7117679
rs2523990
rs6510328
All
−0.001
0.000
0.001
MR effect size for
'Matrix metalloproteinase−17' on 'eGFR'

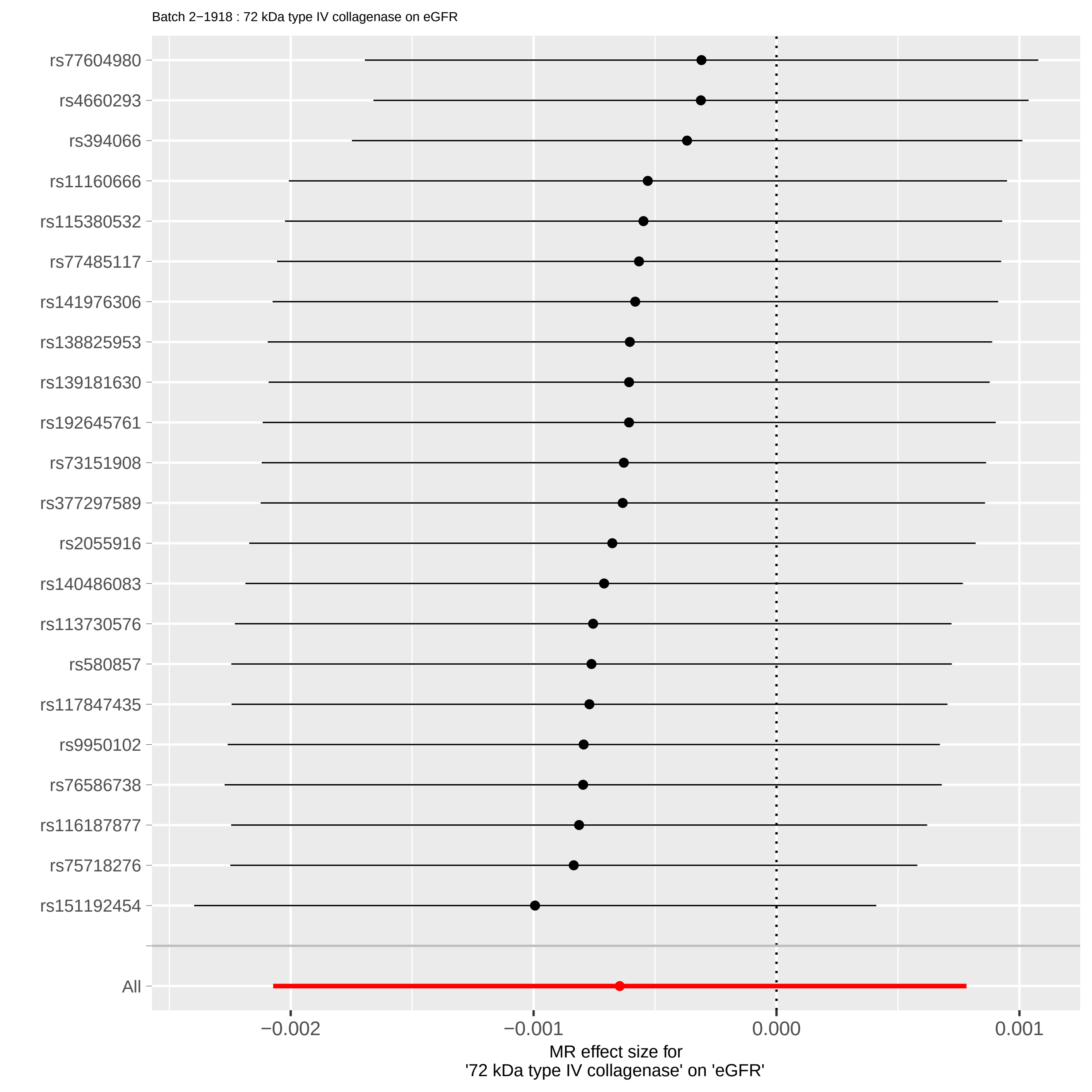

Batch 2−1918 : 72 kDa type IV collagenase on eGFR
rs77604980
rs4660293
rs394066
rs11160666
rs115380532
rs77485117
rs141976306
rs138825953
rs139181630
rs192645761
rs73151908
rs377297589
rs2055916
rs140486083
rs113730576
rs580857
rs117847435
rs9950102
rs76586738
rs116187877
rs75718276
rs151192454
All
−0.002
−0.001
0.000
0.001
MR effect size for
'72 kDa type IV collagenase' on 'eGFR'

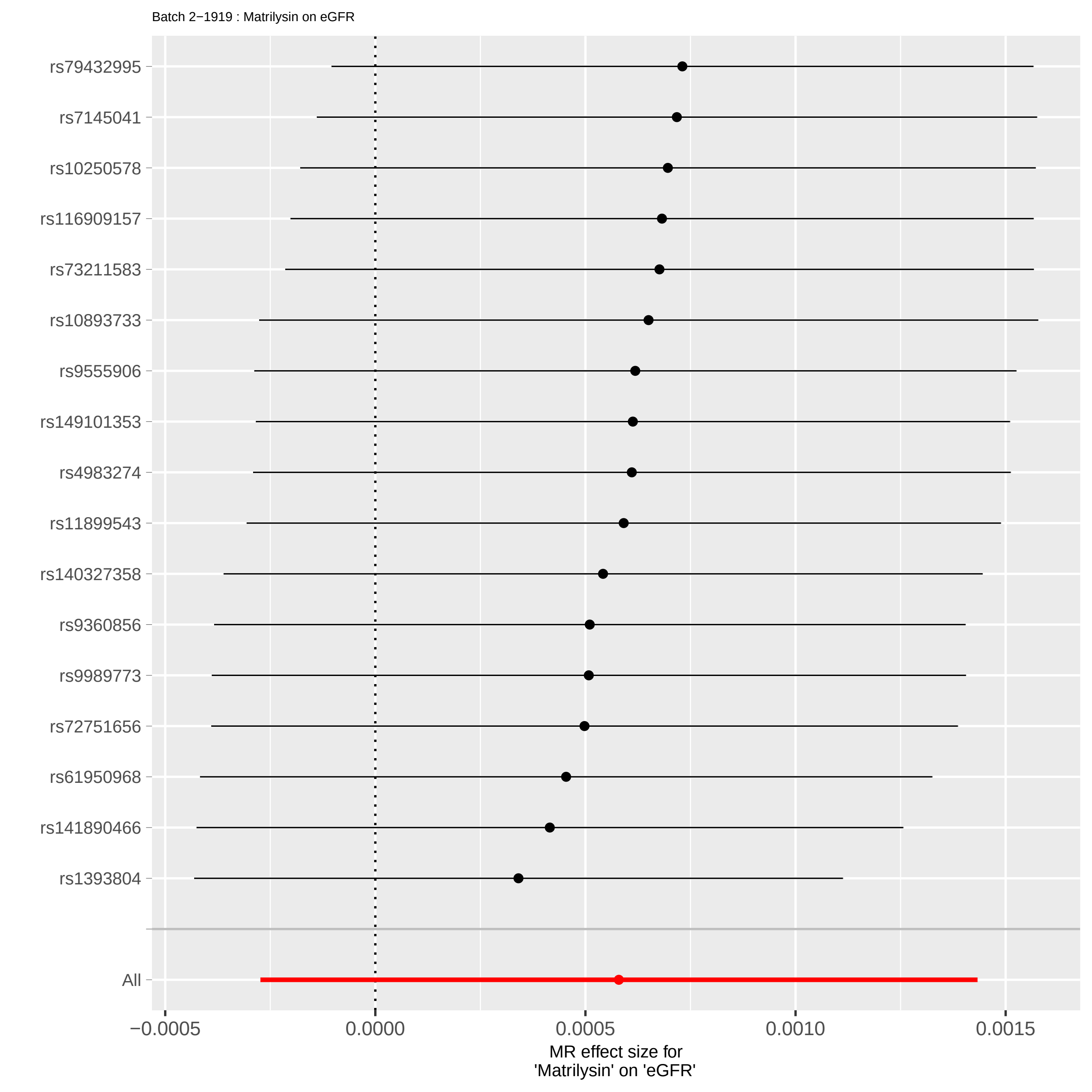

Batch 2−1919 : Matrilysin on eGFR
rs79432995
rs7145041
rs10250578
rs116909157
rs73211583
rs10893733
rs9555906
rs149101353
rs4983274
rs11899543
rs140327358
rs9360856
rs9989773
rs72751656
rs61950968
rs141890466
rs1393804
All
−0.0005
0.0000
0.0005
0.0010
0.0015
MR effect size for
'Matrilysin' on 'eGFR'

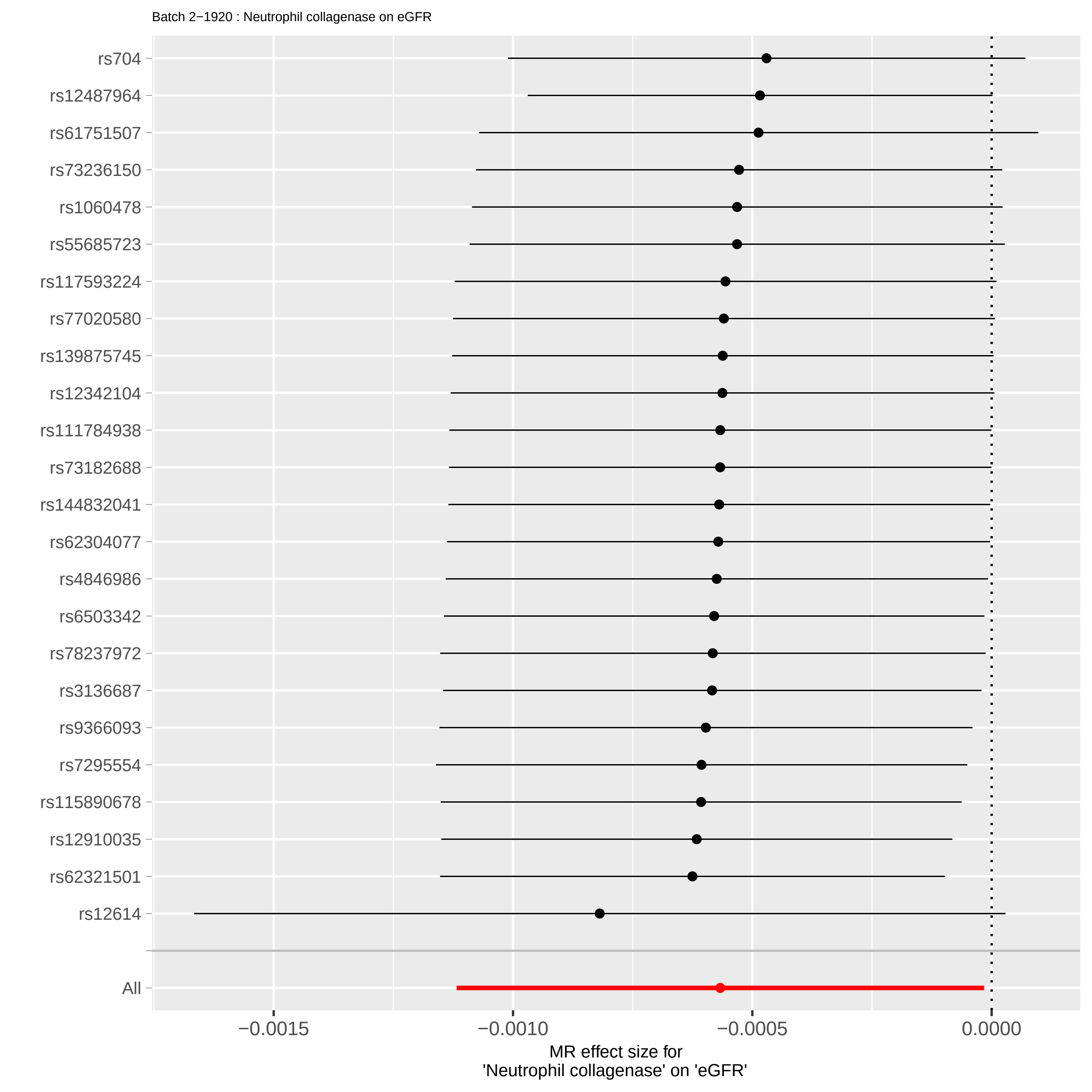

Batch 2−1920 : Neutrophil collagenase on eGFR
rs704
rs12487964
rs61751507
rs73236150
rs1060478
rs55685723
rs117593224
rs77020580
rs139875745
rs12342104
rs111784938
rs73182688
rs144832041
rs62304077
rs4846986
rs6503342
rs78237972
rs3136687
rs9366093
rs7295554
rs115890678
rs12910035
rs62321501
rs12614
All
−0.0015
−0.0010
−0.0005
0.0000
MR effect size for
'Neutrophil collagenase' on 'eGFR'

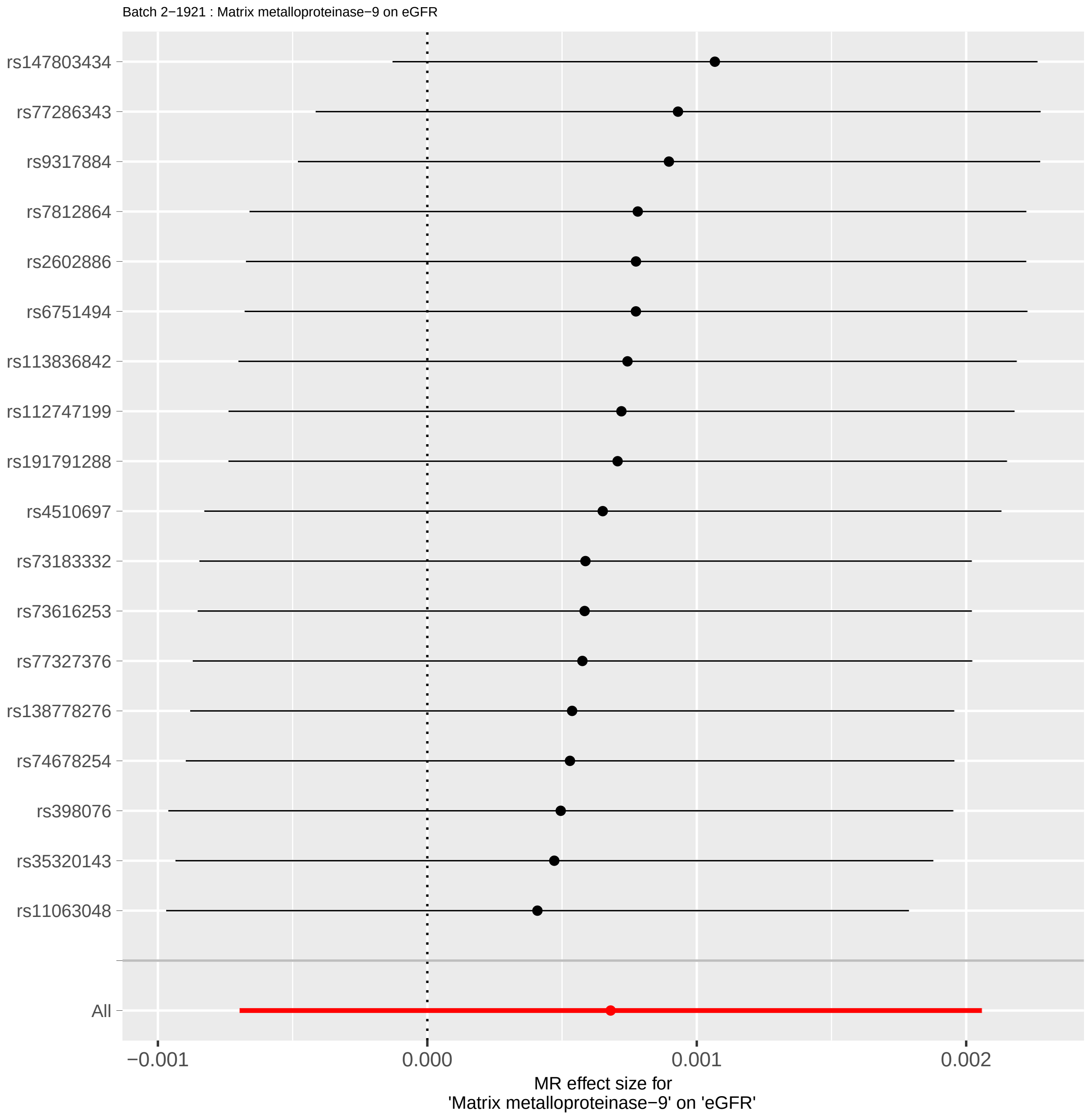

Batch 2−1921 : Matrix metalloproteinase−9 on eGFR
rs147803434
rs77286343
rs9317884
rs7812864
rs2602886
rs6751494
rs113836842
rs112747199
rs191791288
rs4510697
rs73183332
rs73616253
rs77327376
rs138778276
rs74678254
rs398076
rs35320143
rs11063048
All
−0.001
0.000
0.001
0.002
MR effect size for
'Matrix metalloproteinase−9' on 'eGFR'

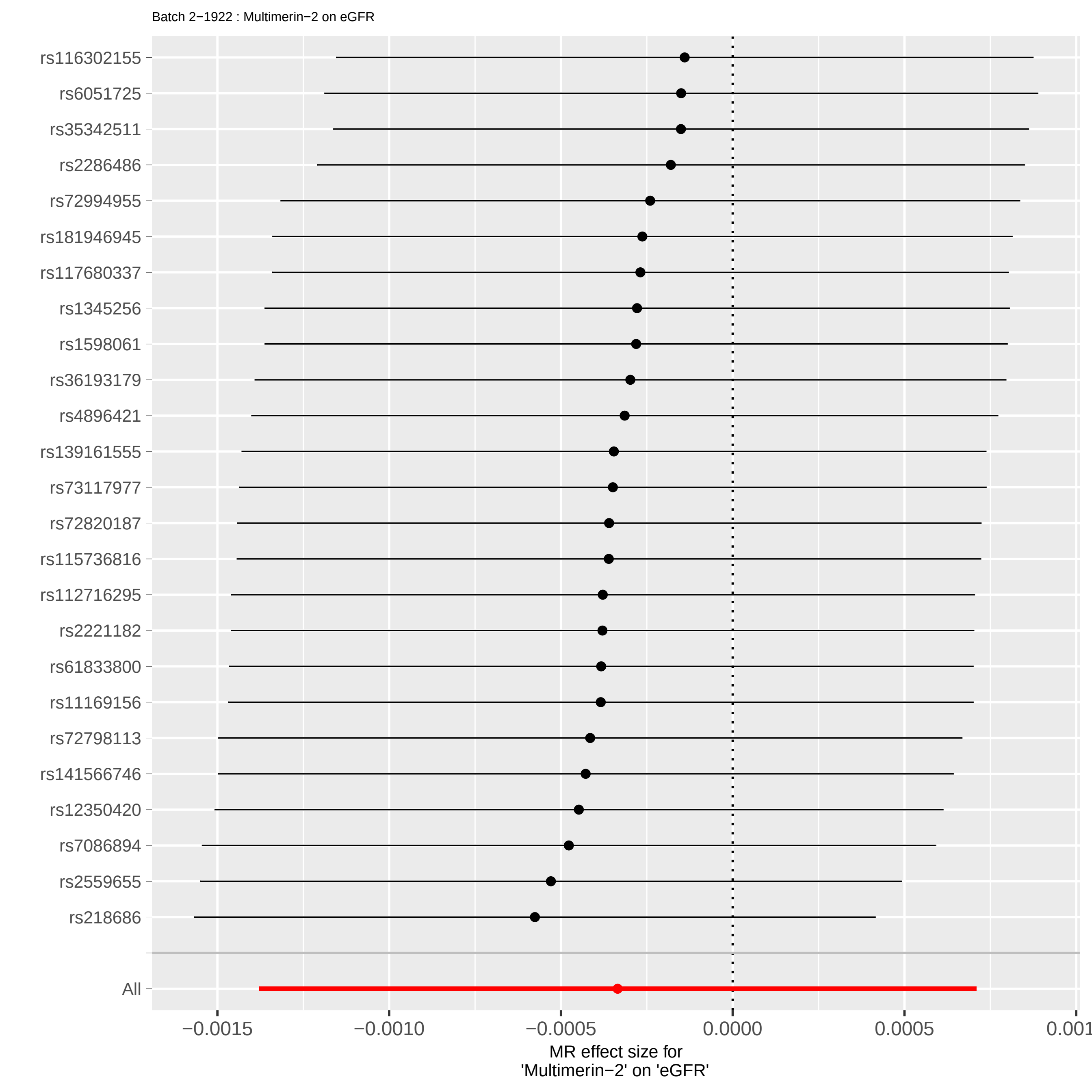

Batch 2−1922 : Multimerin−2 on eGFR
rs116302155
rs6051725
rs35342511
rs2286486
rs72994955
rs181946945
rs117680337
rs1345256
rs1598061
rs36193179
rs4896421
rs139161555
rs73117977
rs72820187
rs115736816
rs112716295
rs2221182
rs61833800
rs11169156
rs72798113
rs141566746
rs12350420
rs7086894
rs2559655
rs218686
All
−0.0015
−0.0010
−0.0005
0.0000
0.0005
0.001
MR effect size for
'Multimerin−2' on 'eGFR'

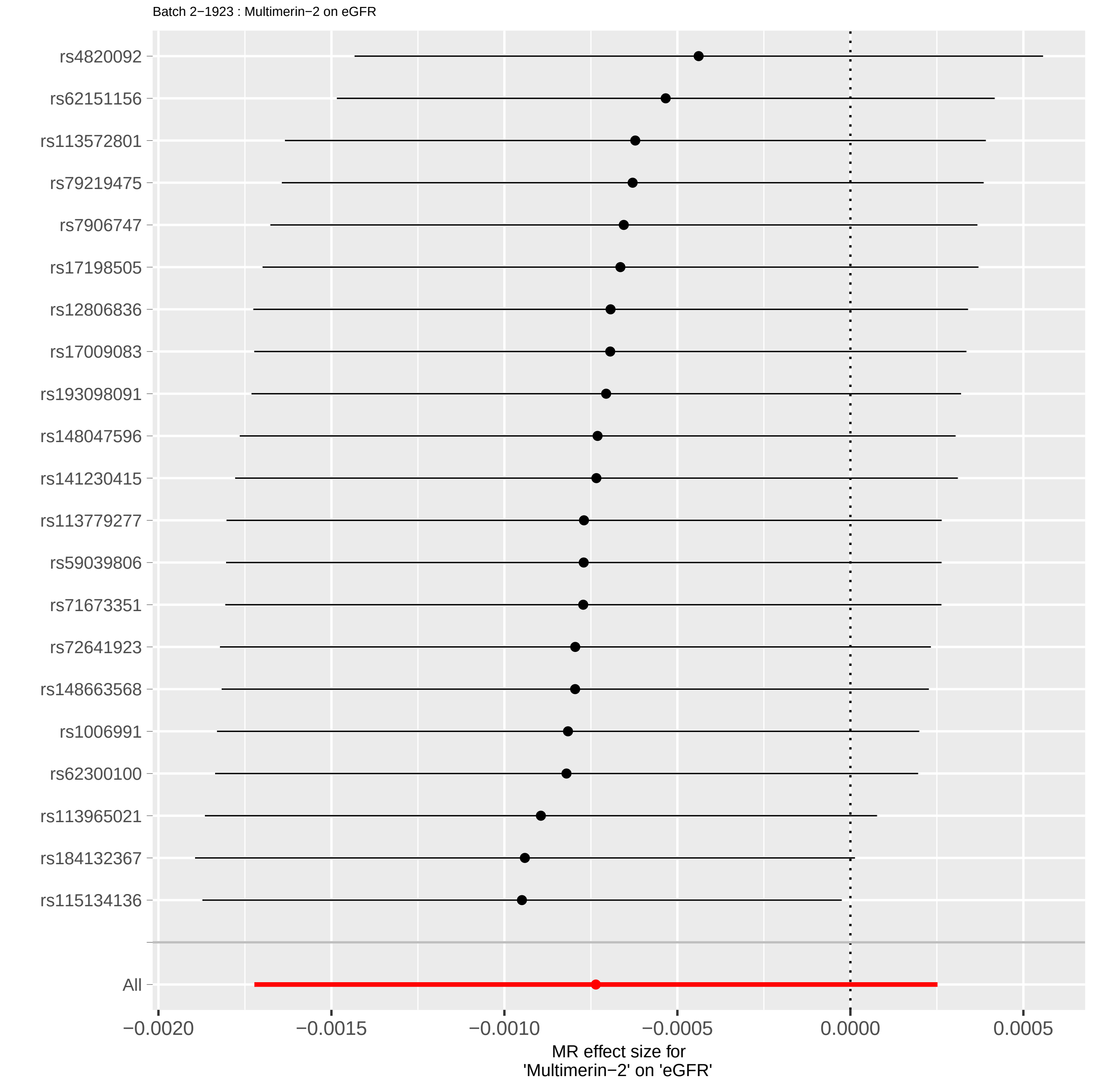

Batch 2−1923 : Multimerin−2 on eGFR
rs4820092
rs62151156
rs113572801
rs79219475
rs7906747
rs17198505
rs12806836
rs17009083
rs193098091
rs148047596
rs141230415
rs113779277
rs59039806
rs71673351
rs72641923
rs148663568
rs1006991
rs62300100
rs113965021
rs184132367
rs115134136
All
−0.0020
−0.0015
−0.0010
−0.0005
0.0000
0.0005
MR effect size for
'Multimerin−2' on 'eGFR'

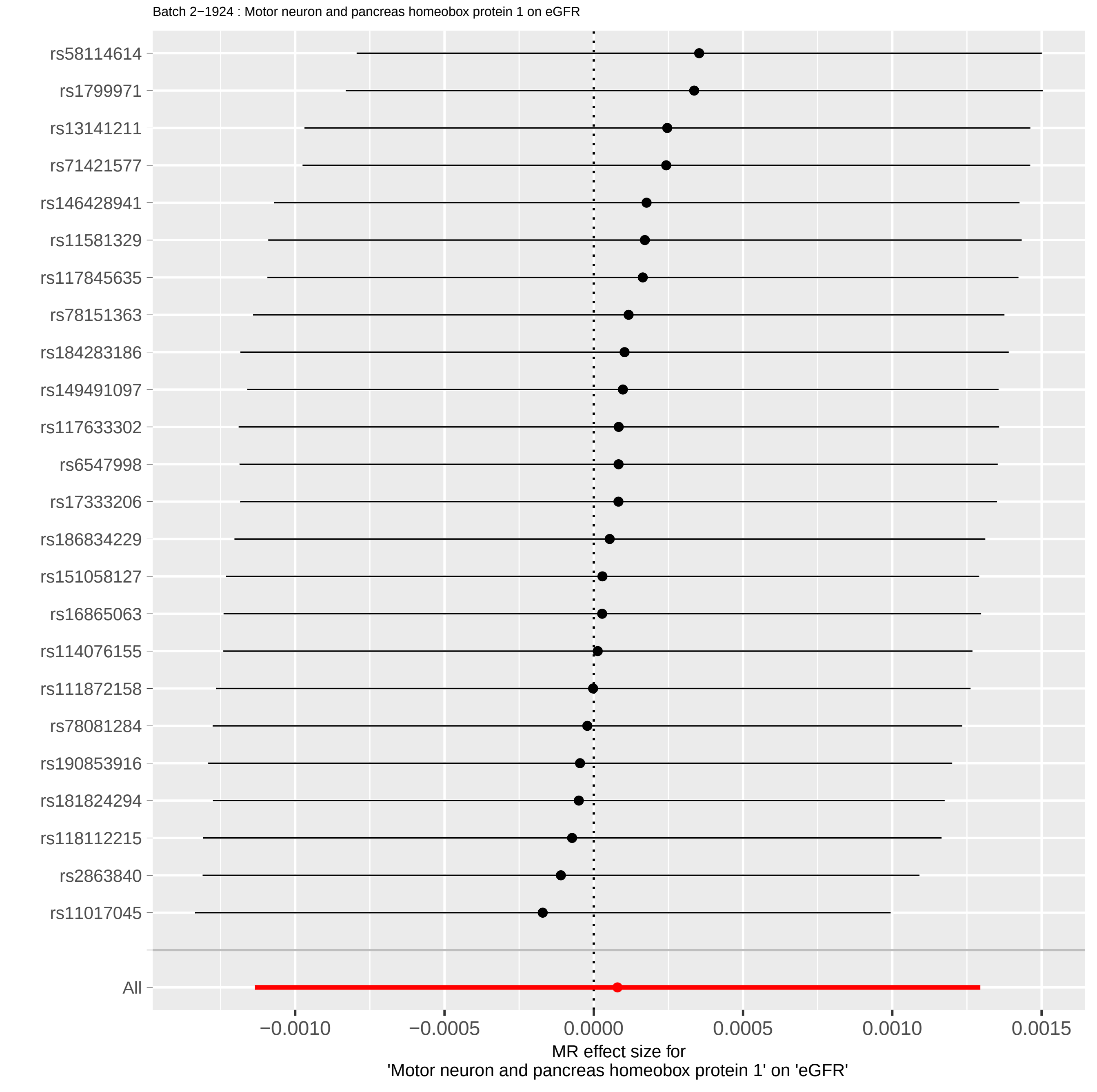
Batch 2−1924 : Motor neuron and pancreas homeobox protein 1 on eGFR
rs58114614
rs1799971
rs13141211
rs71421577
rs146428941
rs11581329
rs117845635
rs78151363
rs184283186
rs149491097
rs117633302
rs6547998
rs17333206
rs186834229
rs151058127
rs16865063
rs114076155
rs111872158
rs78081284
rs190853916
rs181824294
rs118112215
rs2863840
rs11017045
All
−0.0010
−0.0005
0.0000
0.0005
0.0010
0.0015
MR effect size for
'Motor neuron and pancreas homeobox protein 1' on 'eGFR'

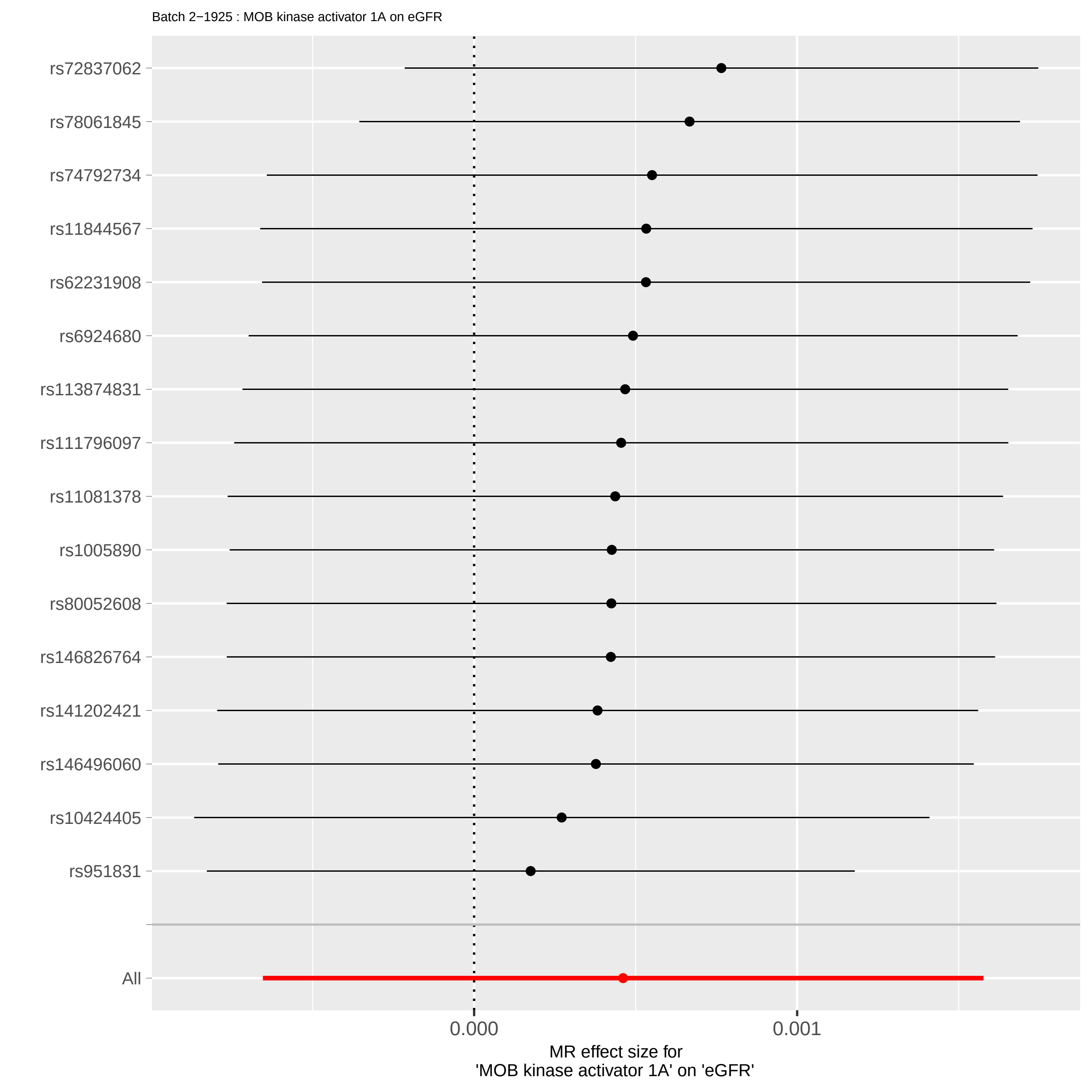

Batch 2−1925 : MOB kinase activator 1A on eGFR
rs72837062
rs78061845
rs74792734
rs11844567
rs62231908
rs6924680
rs113874831
rs111796097
rs11081378
rs1005890
rs80052608
rs146826764
rs141202421
rs146496060
rs10424405
rs951831
All
0.000
0.001
MR effect size for
'MOB kinase activator 1A' on 'eGFR'

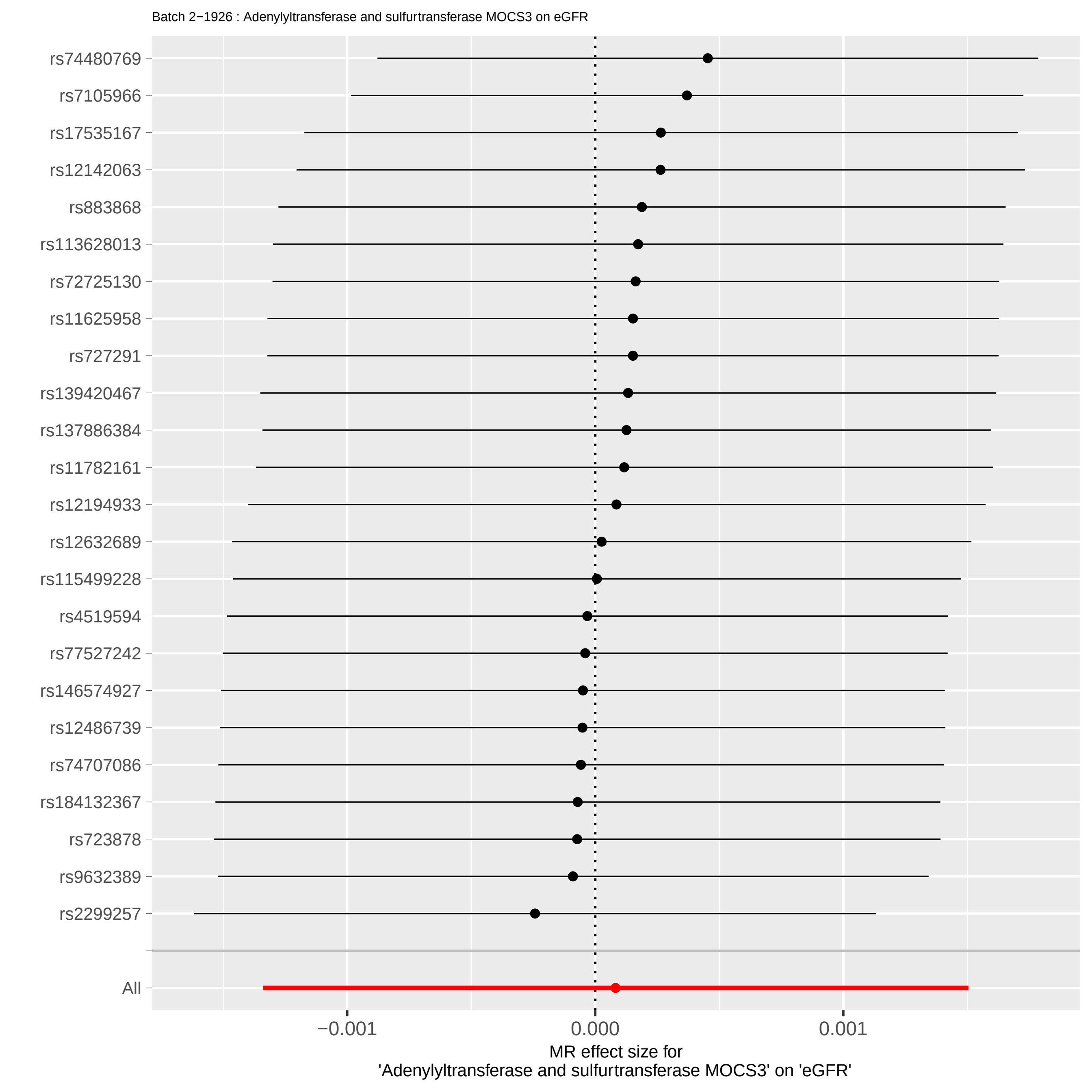
Batch 2−1926 : Adenylyltransferase and sulfurtransferase MOCS3 on eGFR
rs74480769
rs7105966
rs17535167
rs12142063
rs883868
rs113628013
rs72725130
rs11625958
rs727291
rs139420467
rs137886384
rs11782161
rs12194933
rs12632689
rs115499228
rs4519594
rs77527242
rs146574927
rs12486739
rs74707086
rs184132367
rs723878
rs9632389
rs2299257
All
−0.001
0.000
0.001
MR effect size for
'Adenylyltransferase and sulfurtransferase MOCS3' on 'eGFR'

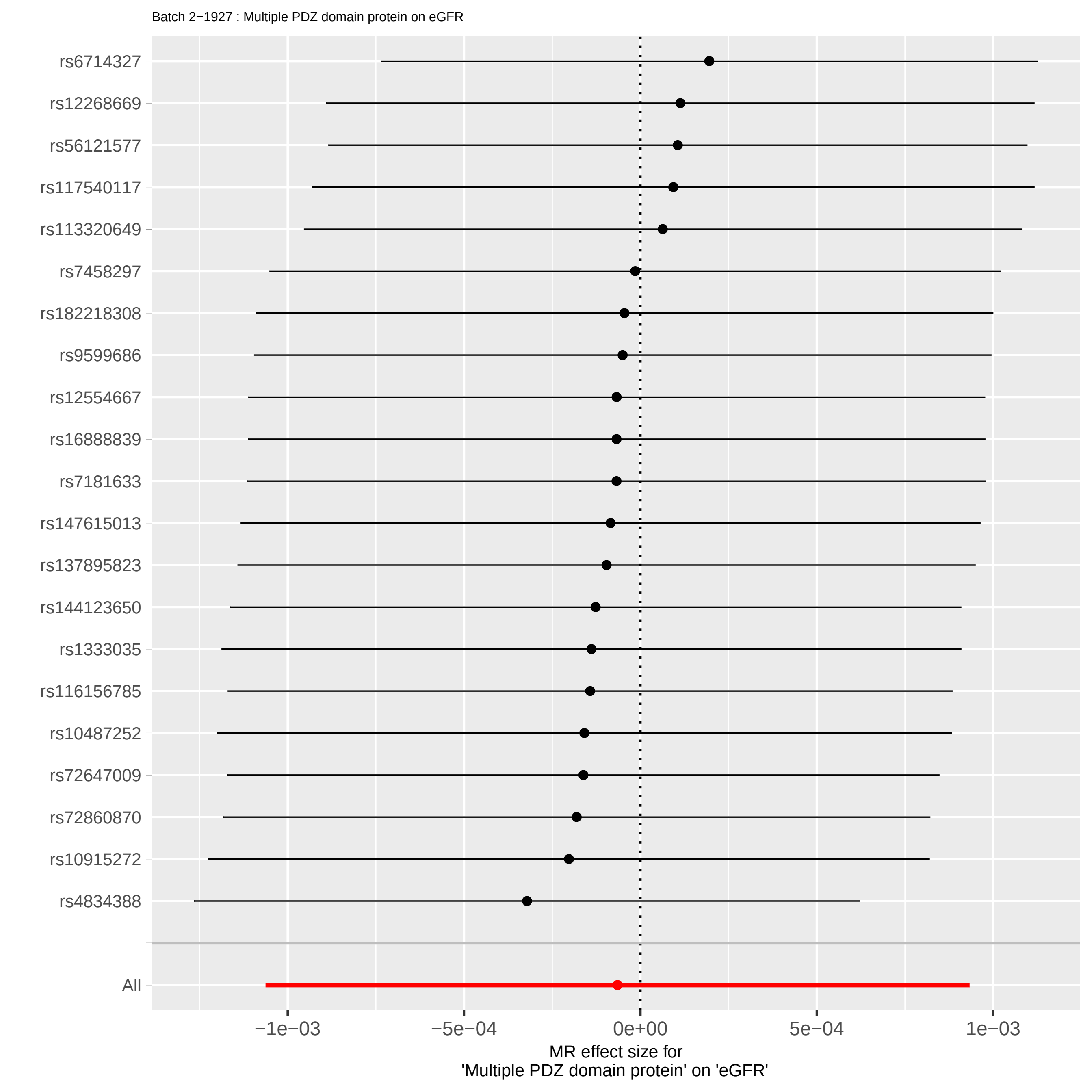

Batch 2−1927 : Multiple PDZ domain protein on eGFR
rs6714327
rs12268669
rs56121577
rs117540117
rs113320649
rs7458297
rs182218308
rs9599686
rs12554667
rs16888839
rs7181633
rs147615013
rs137895823
rs144123650
rs1333035
rs116156785
rs10487252
rs72647009
rs72860870
rs10915272
rs4834388
All
−1e−03
−5e−04
0e+00
5e−04
1e−03
MR effect size for
'Multiple PDZ domain protein' on 'eGFR'

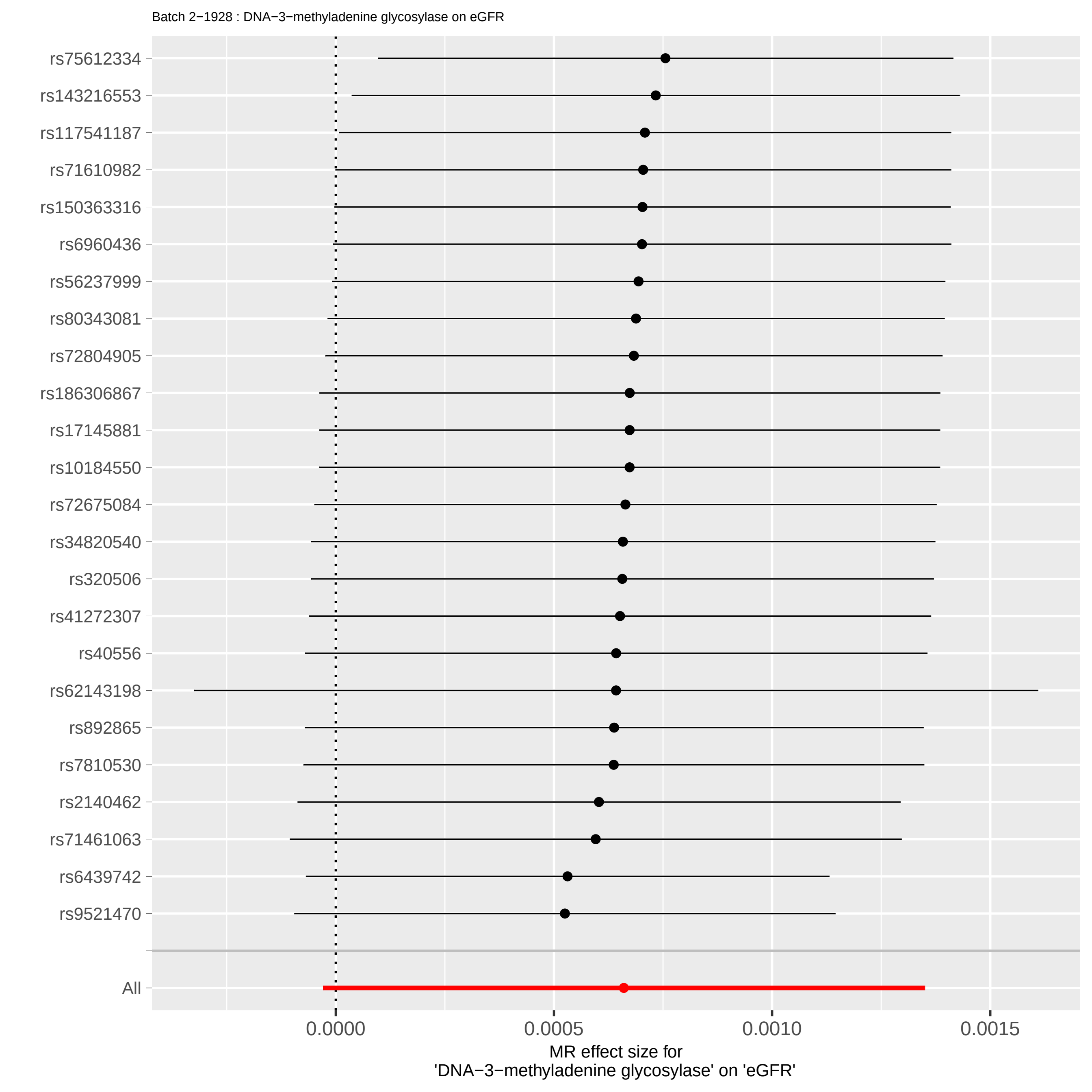
Batch 2−1928 : DNA−3−methyladenine glycosylase on eGFR
rs75612334
rs143216553
rs117541187
rs71610982
rs150363316
rs6960436
rs56237999
rs80343081
rs72804905
rs186306867
rs17145881
rs10184550
rs72675084
rs34820540
rs320506
rs41272307
rs40556
rs62143198
rs892865
rs7810530
rs2140462
rs71461063
rs6439742
rs9521470
All
0.0000
0.0005
0.0010
0.0015
MR effect size for
'DNA−3−methyladenine glycosylase' on 'eGFR'

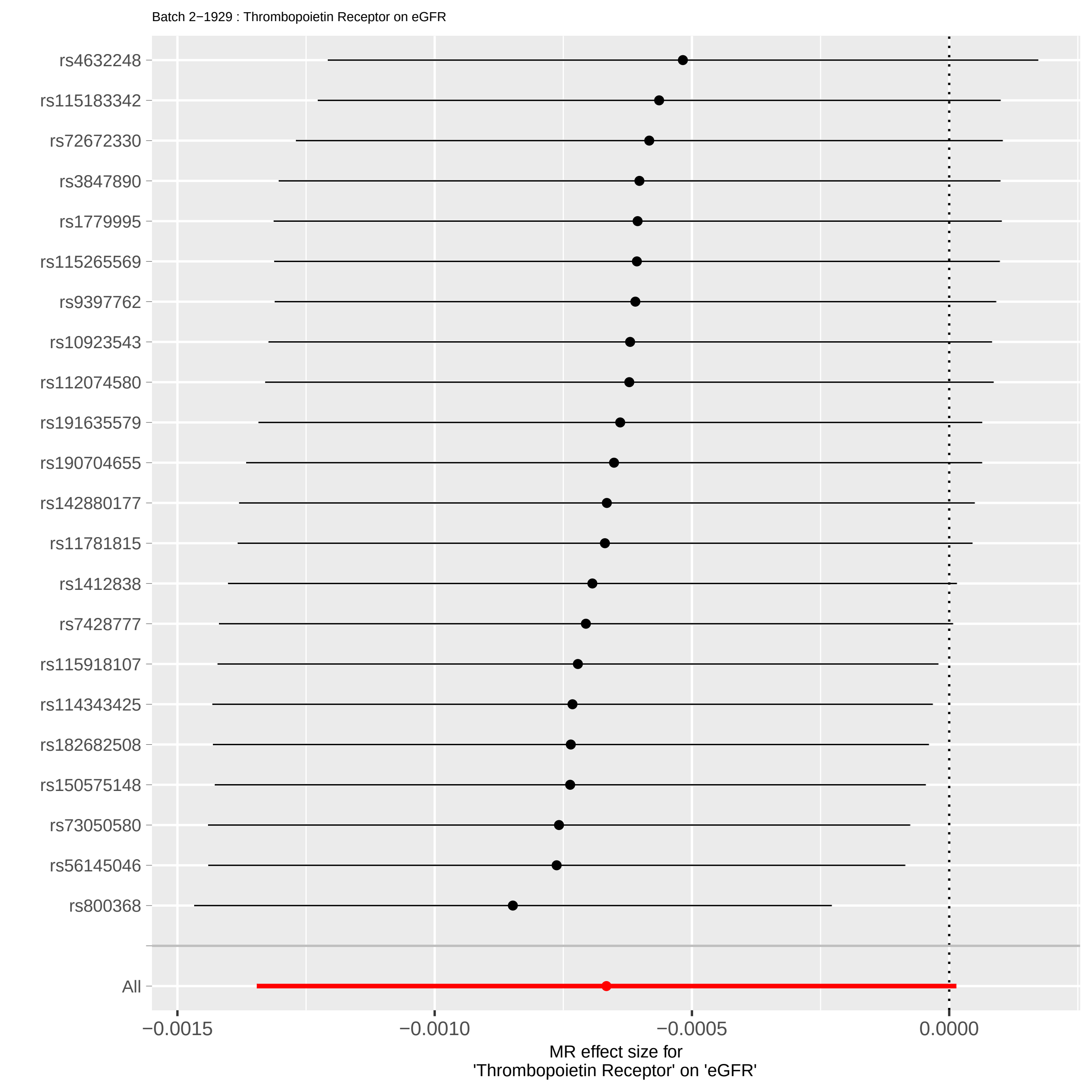

Batch 2−1929 : Thrombopoietin Receptor on eGFR
rs4632248
rs115183342
rs72672330
rs3847890
rs1779995
rs115265569
rs9397762
rs10923543
rs112074580
rs191635579
rs190704655
rs142880177
rs11781815
rs1412838
rs7428777
rs115918107
rs114343425
rs182682508
rs150575148
rs73050580
rs56145046
rs800368
All
−0.0015
−0.0010
−0.0005
0.0000
MR effect size for
'Thrombopoietin Receptor' on 'eGFR'

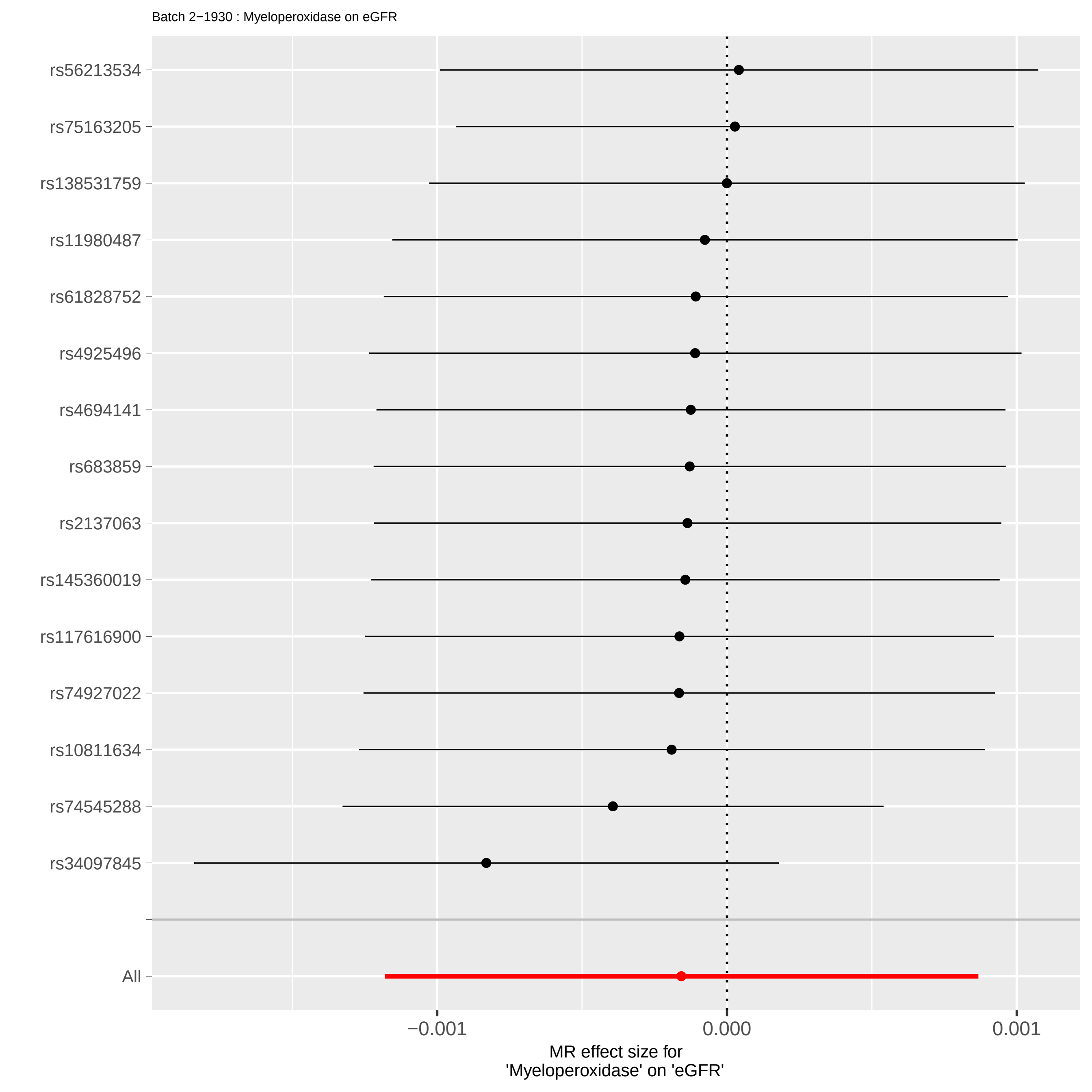
Batch 2−1930 : Myeloperoxidase on eGFR
rs56213534
rs75163205
rs138531759
rs11980487
rs61828752
rs4925496
rs4694141
rs683859
rs2137063
rs145360019
rs117616900
rs74927022
rs10811634
rs74545288
rs34097845
All
−0.001
0.000
0.001
MR effect size for
'Myeloperoxidase' on 'eGFR'

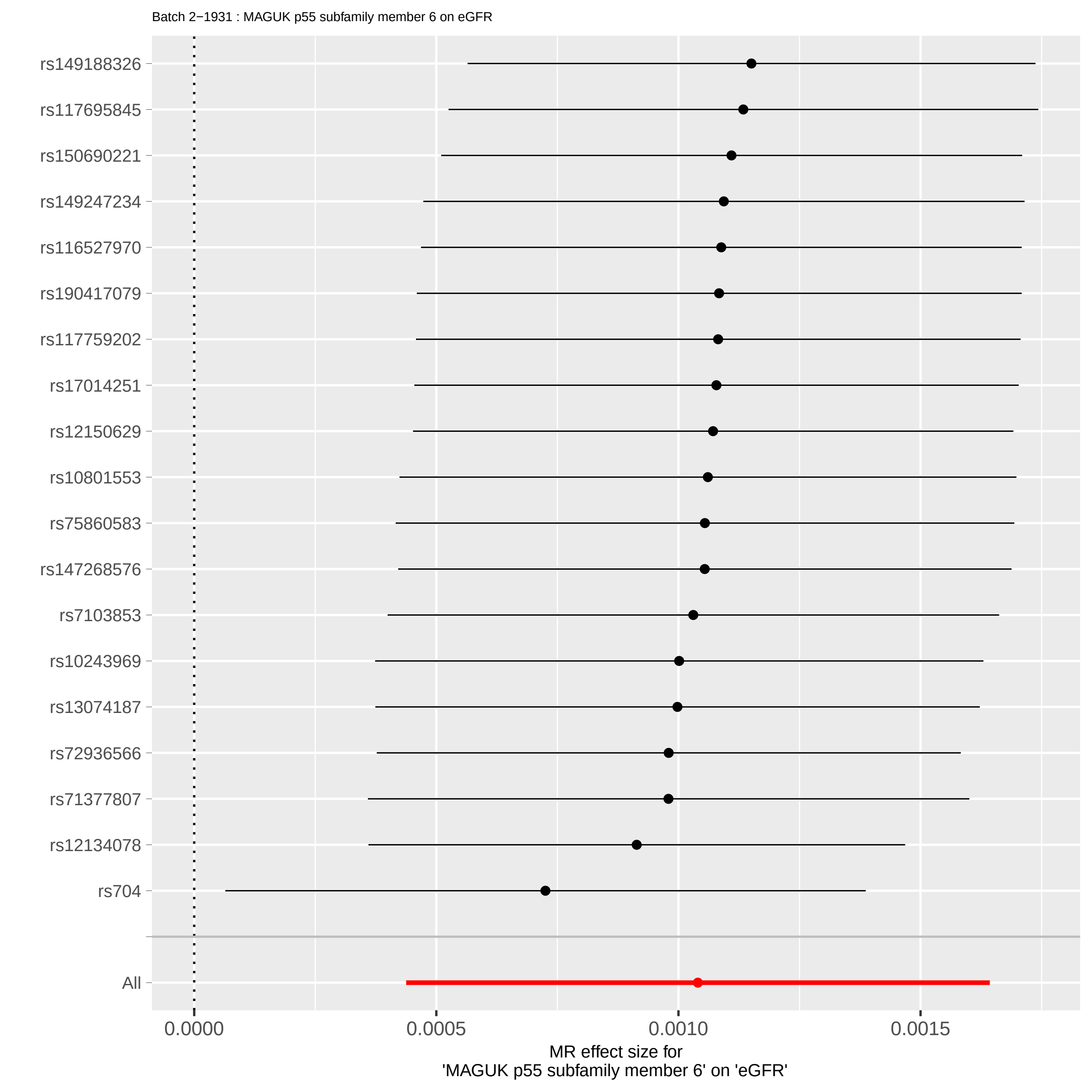

Batch 2−1931 : MAGUK p55 subfamily member 6 on eGFR
rs149188326
rs117695845
rs150690221
rs149247234
rs116527970
rs190417079
rs117759202
rs17014251
rs12150629
rs10801553
rs75860583
rs147268576
rs7103853
rs10243969
rs13074187
rs72936566
rs71377807
rs12134078
rs704
All
0.0000
0.0005
0.0010
0.0015
MR effect size for
'MAGUK p55 subfamily member 6' on 'eGFR'

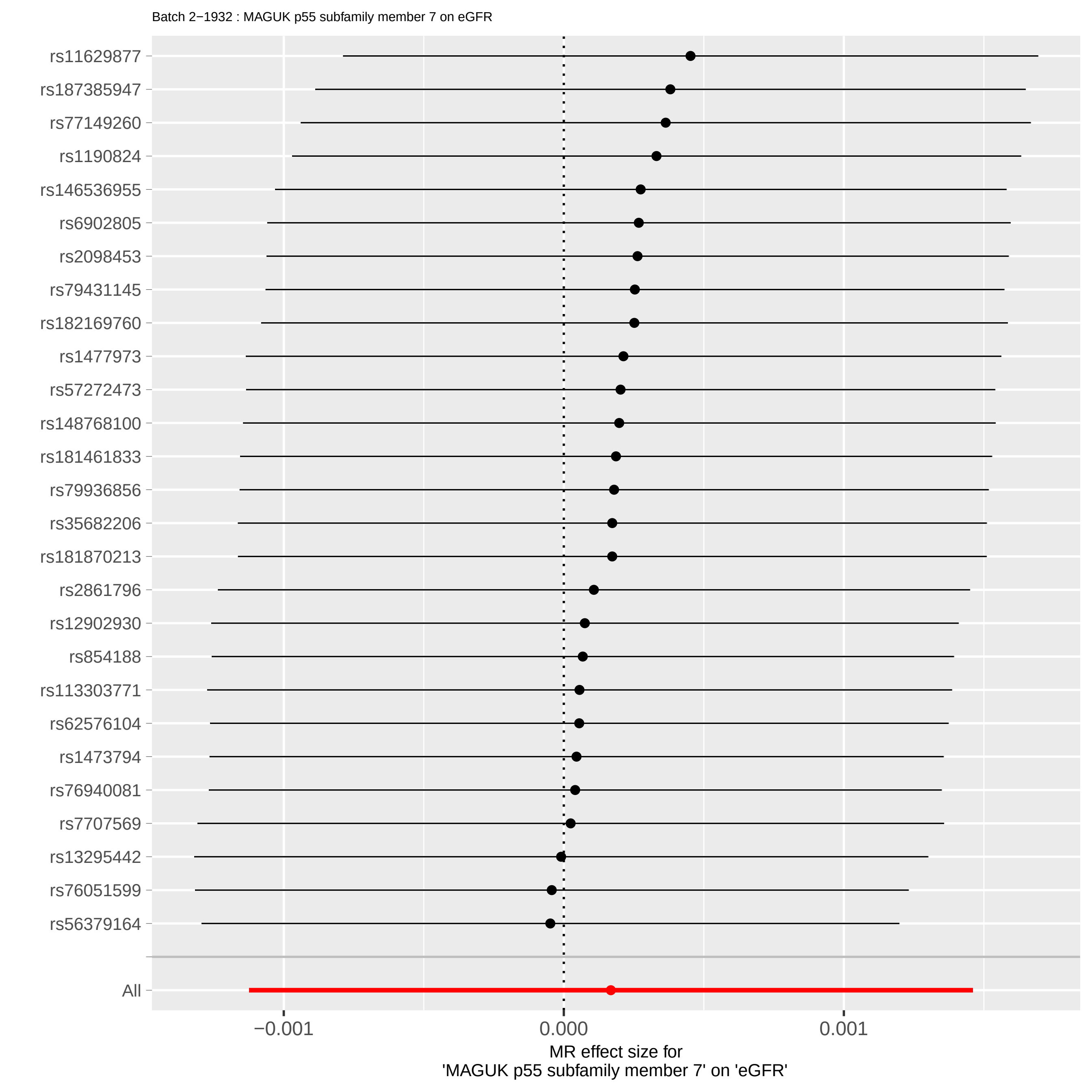

Batch 2−1932 : MAGUK p55 subfamily member 7 on eGFR
rs11629877
rs187385947
rs77149260
rs1190824
rs146536955
rs6902805
rs2098453
rs79431145
rs182169760
rs1477973
rs57272473
rs148768100
rs181461833
rs79936856
rs35682206
rs181870213
rs2861796
rs12902930
rs854188
rs113303771
rs62576104
rs1473794
rs76940081
rs7707569
rs13295442
rs76051599
rs56379164
All
−0.001
0.000
0.001
MR effect size for
'MAGUK p55 subfamily member 7' on 'eGFR'

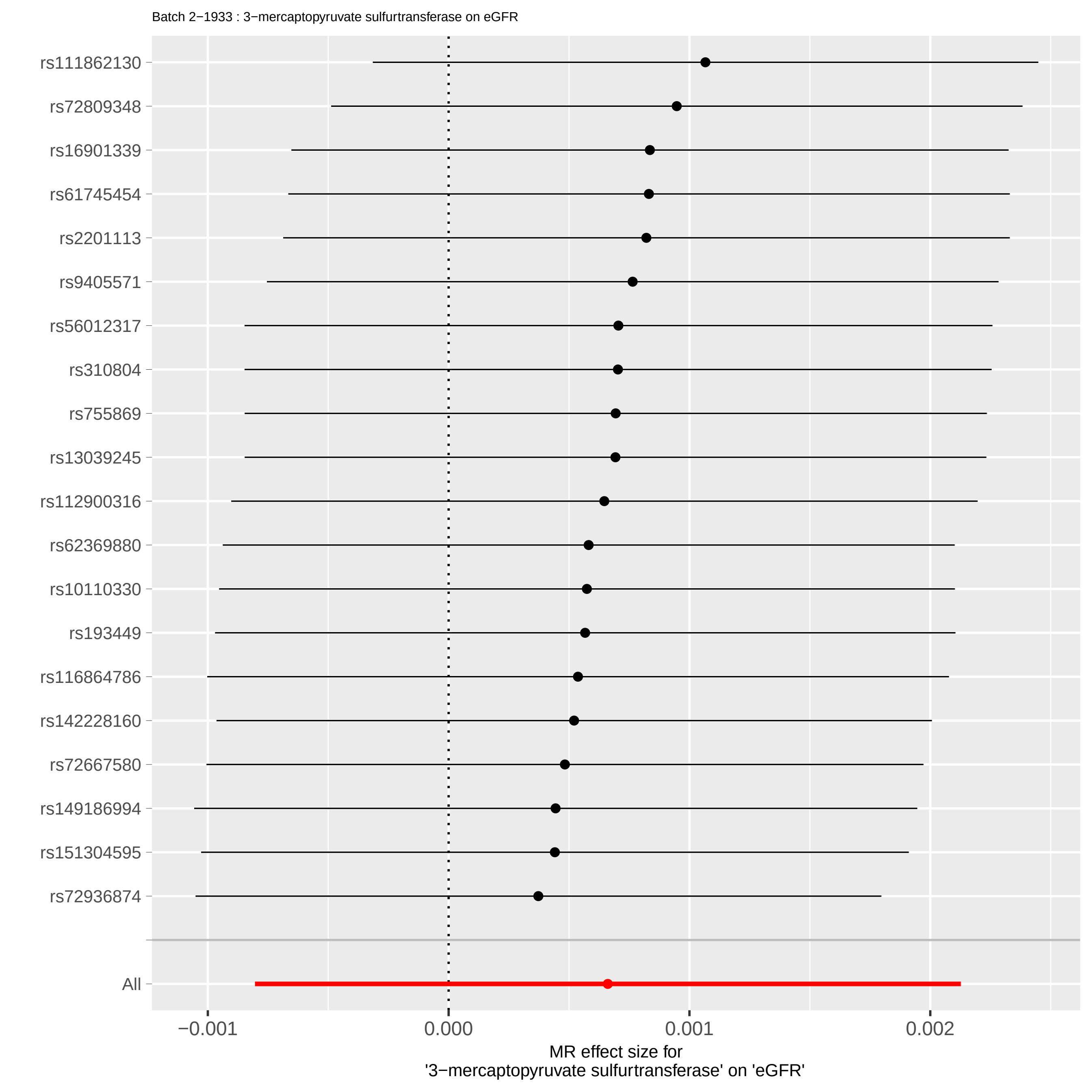
Batch 2−1933 : 3−mercaptopyruvate sulfurtransferase on eGFR
rs111862130
rs72809348
rs16901339
rs61745454
rs2201113
rs9405571
rs56012317
rs310804
rs755869
rs13039245
rs112900316
rs62369880
rs10110330
rs193449
rs116864786
rs142228160
rs72667580
rs149186994
rs151304595
rs72936874
All
−0.001
0.000
0.001
0.002
MR effect size for
'3−mercaptopyruvate sulfurtransferase' on 'eGFR'

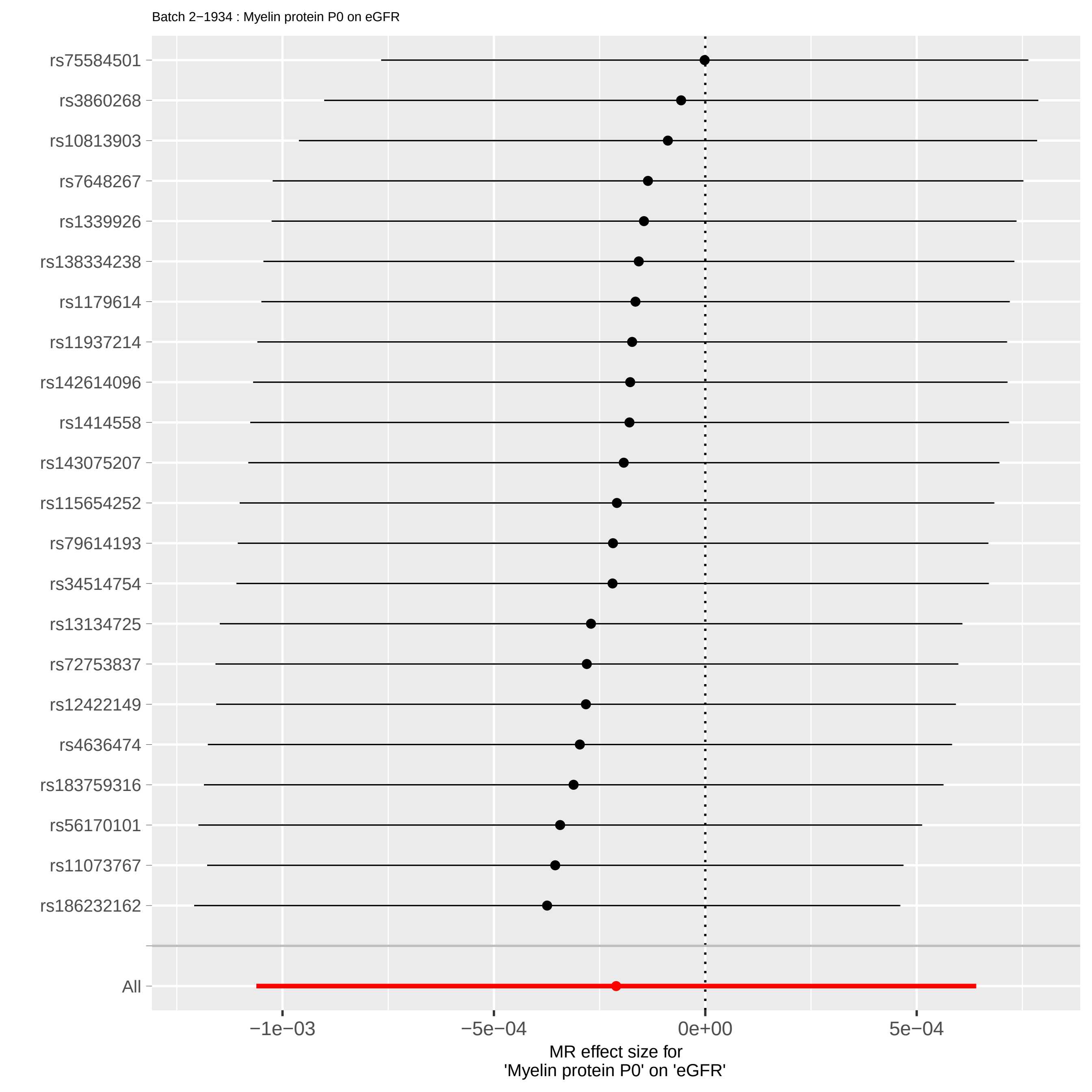

Batch 2−1934 : Myelin protein P0 on eGFR
rs75584501
rs3860268
rs10813903
rs7648267
rs1339926
rs138334238
rs1179614
rs11937214
rs142614096
rs1414558
rs143075207
rs115654252
rs79614193
rs34514754
rs13134725
rs72753837
rs12422149
rs4636474
rs183759316
rs56170101
rs11073767
rs186232162
All
−1e−03
−5e−04
0e+00
5e−04
MR effect size for
'Myelin protein P0' on 'eGFR'

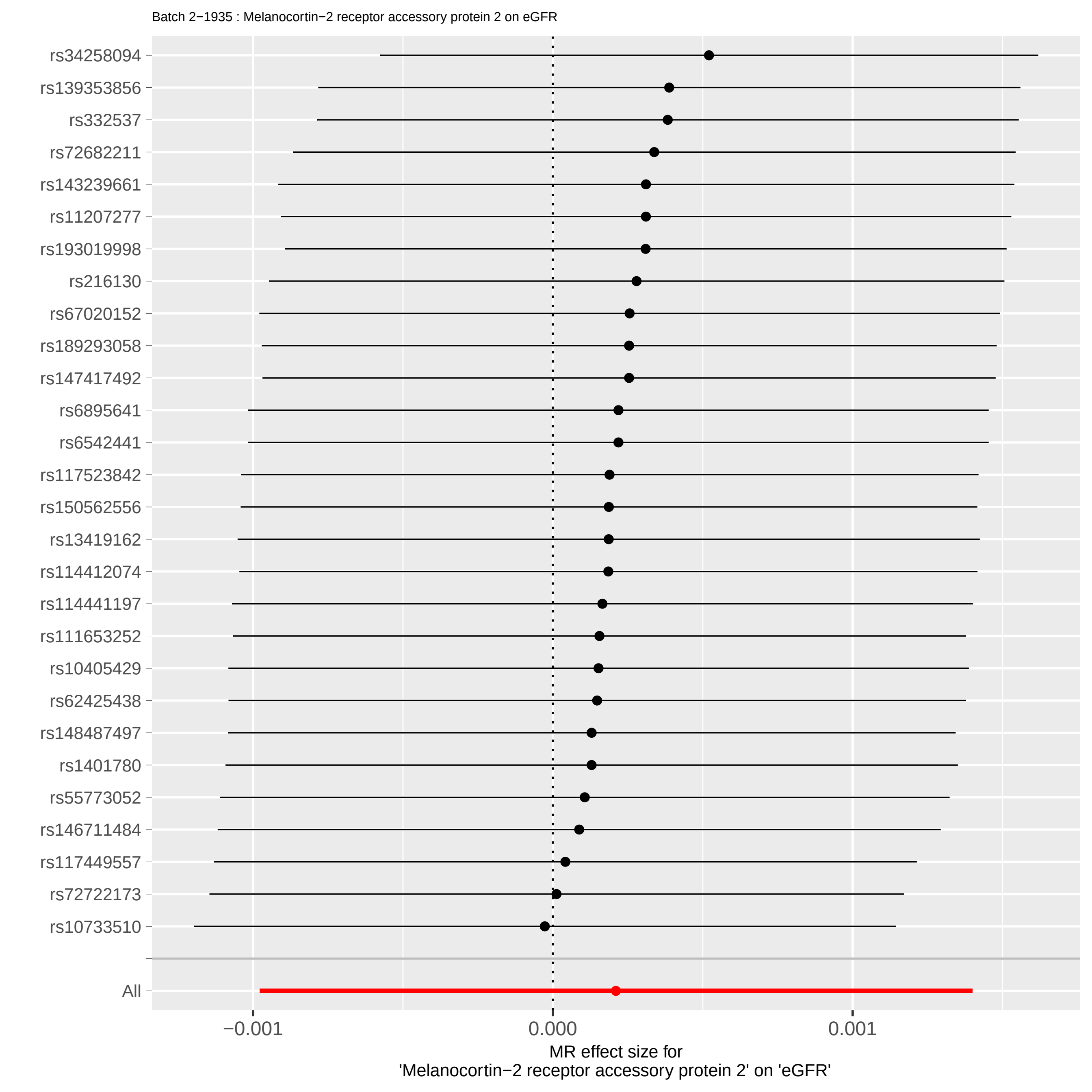

Batch 2−1935 : Melanocortin−2 receptor accessory protein 2 on eGFR
rs34258094
rs139353856
rs332537
rs72682211
rs143239661
rs11207277
rs193019998
rs216130
rs67020152
rs189293058
rs147417492
rs6895641
rs6542441
rs117523842
rs150562556
rs13419162
rs114412074
rs114441197
rs111653252
rs10405429
rs62425438
rs148487497
rs1401780
rs55773052
rs146711484
rs117449557
rs72722173
rs10733510
All
−0.001
0.000
0.001
MR effect size for
'Melanocortin−2 receptor accessory protein 2' on 'eGFR'

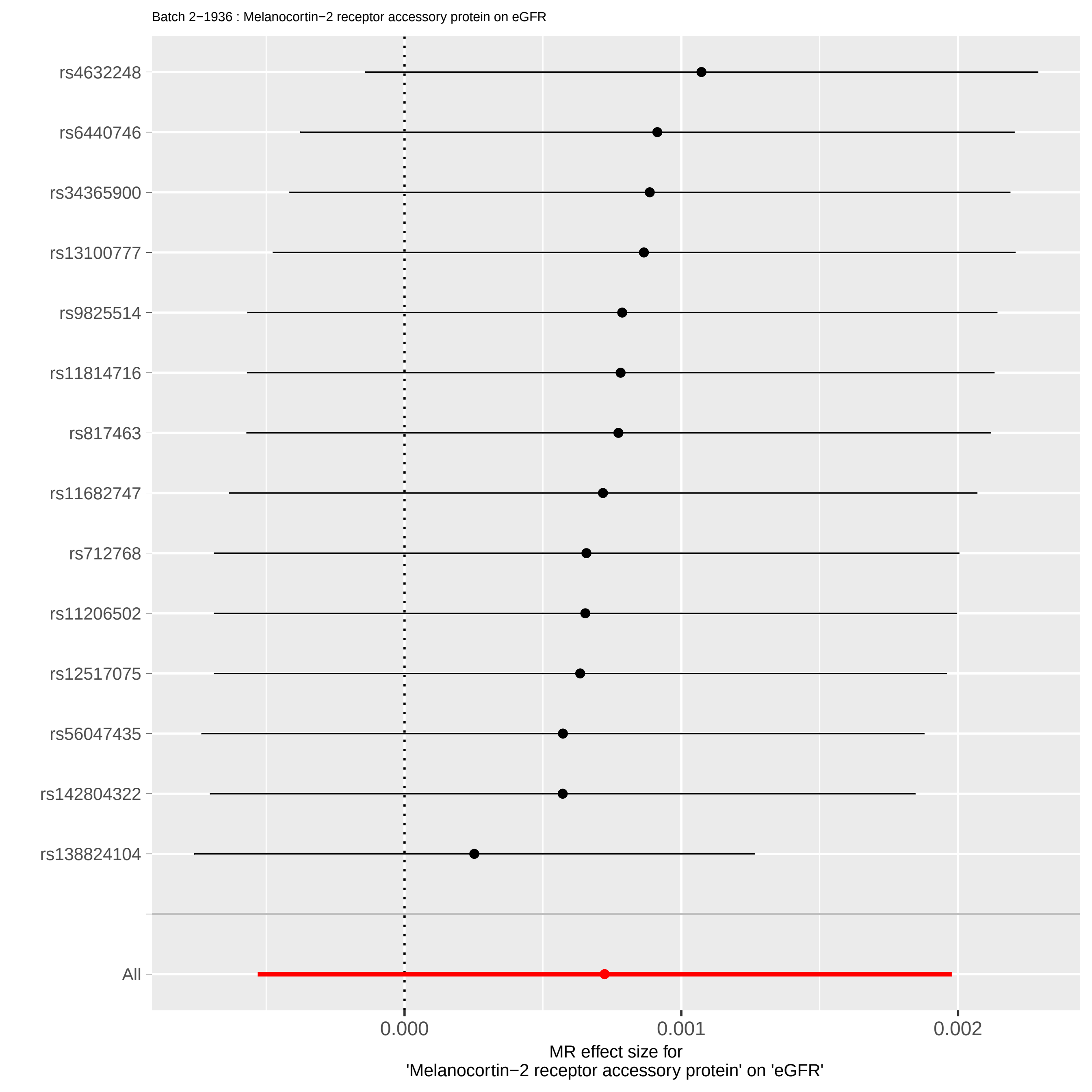

Batch 2−1936 : Melanocortin−2 receptor accessory protein on eGFR
rs4632248
rs6440746
rs34365900
rs13100777
rs9825514
rs11814716
rs817463
rs11682747
rs712768
rs11206502
rs12517075
rs56047435
rs142804322
rs138824104
All
0.000
0.001
0.002
MR effect size for
'Melanocortin−2 receptor accessory protein' on 'eGFR'

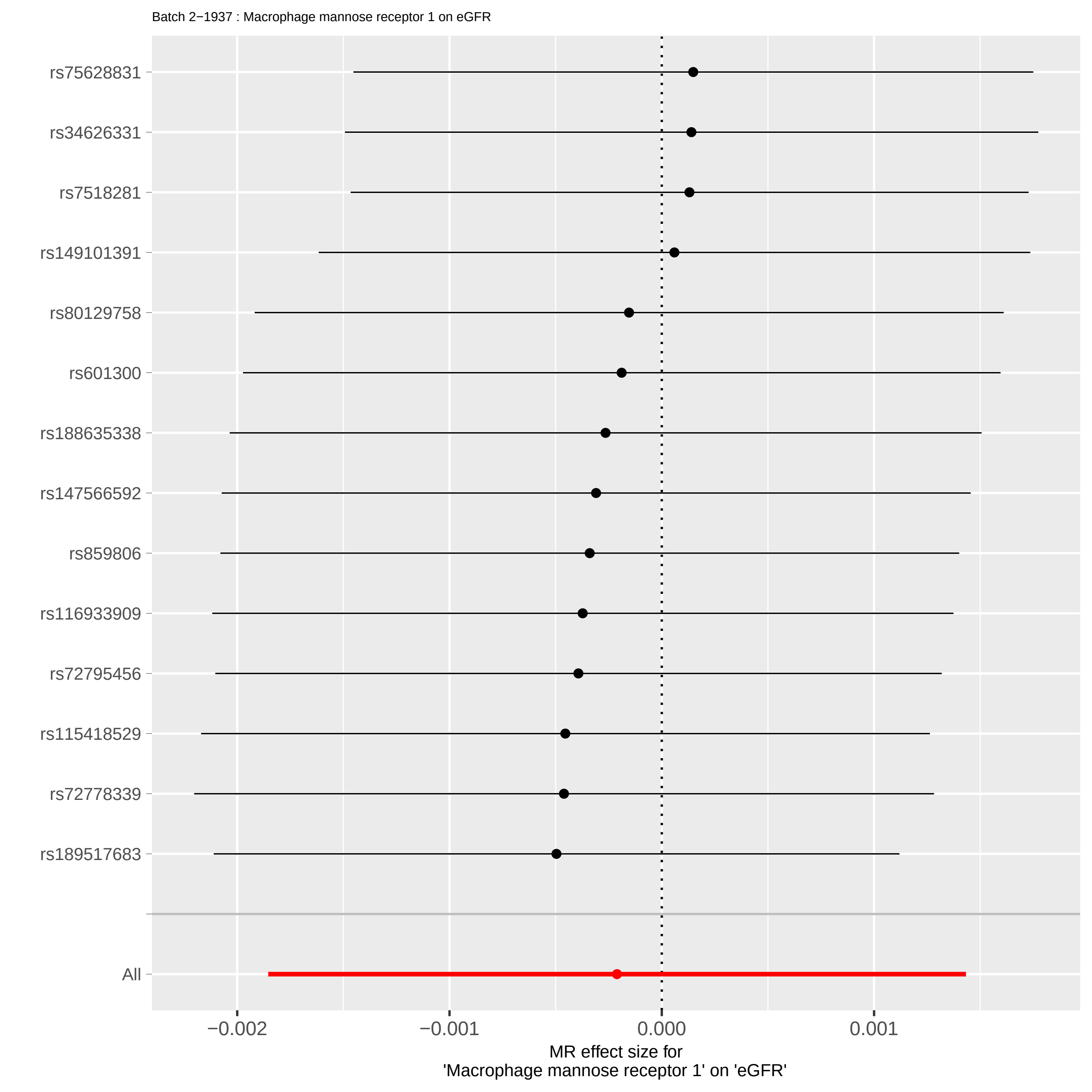

Batch 2−1937 : Macrophage mannose receptor 1 on eGFR
rs75628831
rs34626331
rs7518281
rs149101391
rs80129758
rs601300
rs188635338
rs147566592
rs859806
rs116933909
rs72795456
rs115418529
rs72778339
rs189517683
All
−0.002
−0.001
0.000
0.001
MR effect size for
'Macrophage mannose receptor 1' on 'eGFR'

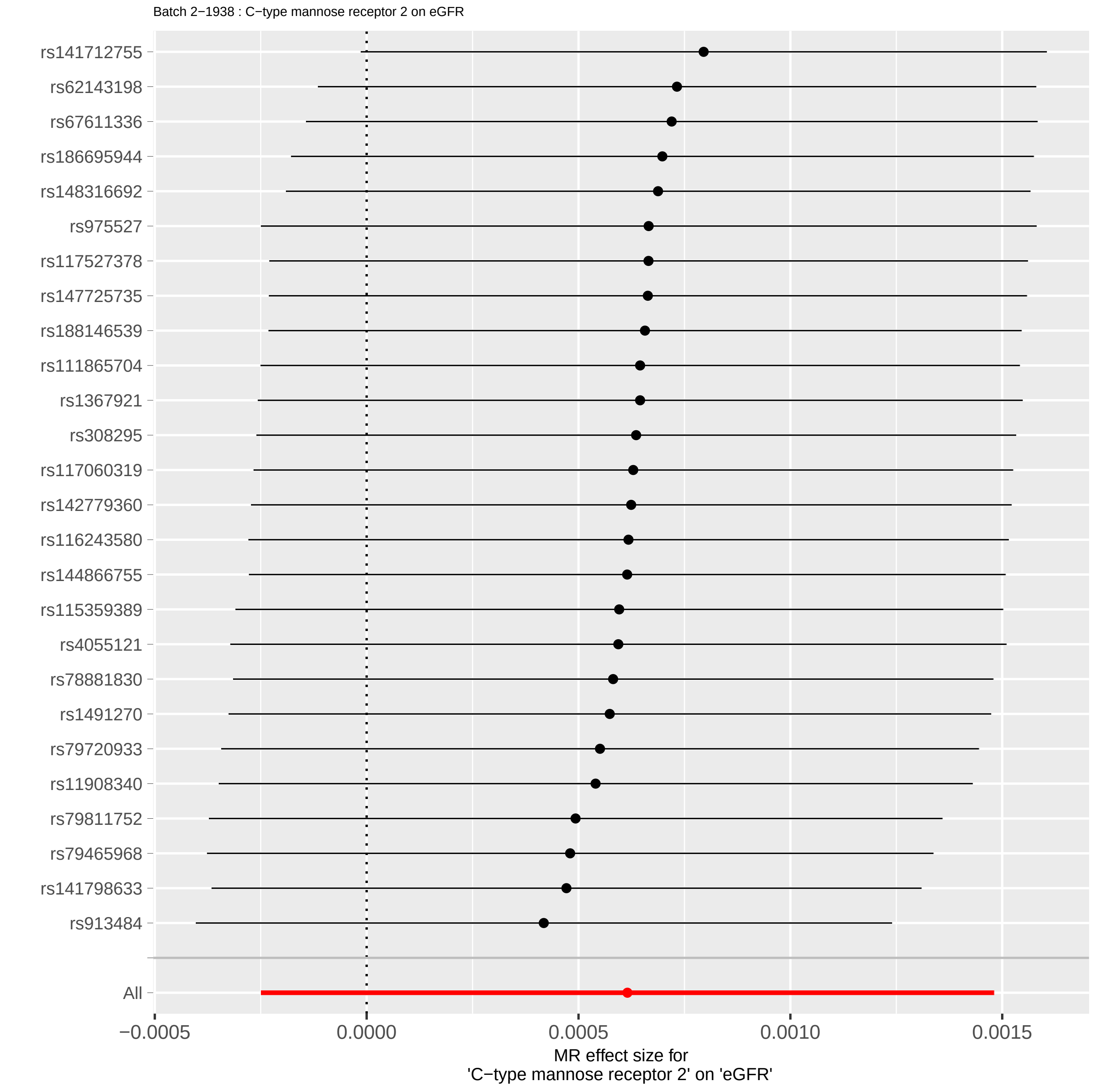

Batch 2−1938 : C−type mannose receptor 2 on eGFR
rs141712755
rs62143198
rs67611336
rs186695944
rs148316692
rs975527
rs117527378
rs147725735
rs188146539
rs111865704
rs1367921
rs308295
rs117060319
rs142779360
rs116243580
rs144866755
rs115359389
rs4055121
rs78881830
rs1491270
rs79720933
rs11908340
rs79811752
rs79465968
rs141798633
rs913484
All
−0.0005
0.0000
0.0005
0.0010
0.0015
MR effect size for
'C−type mannose receptor 2' on 'eGFR'

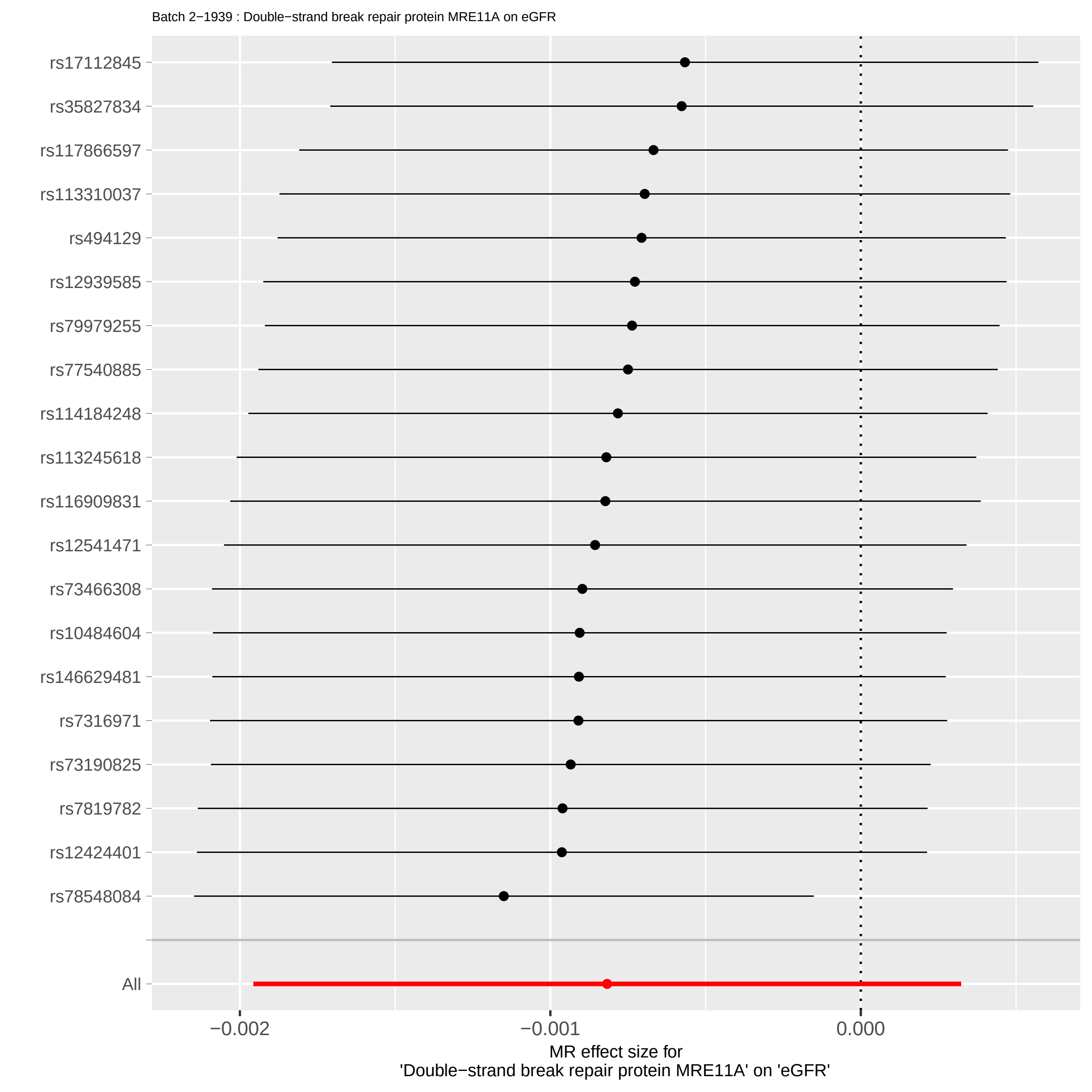

Batch 2−1939 : Double−strand break repair protein MRE11A on eGFR
rs17112845
rs35827834
rs117866597
rs113310037
rs494129
rs12939585
rs79979255
rs77540885
rs114184248
rs113245618
rs116909831
rs12541471
rs73466308
rs10484604
rs146629481
rs7316971
rs73190825
rs7819782
rs12424401
rs78548084
All
−0.002
−0.001
0.000
MR effect size for
'Double−strand break repair protein MRE11A' on 'eGFR'

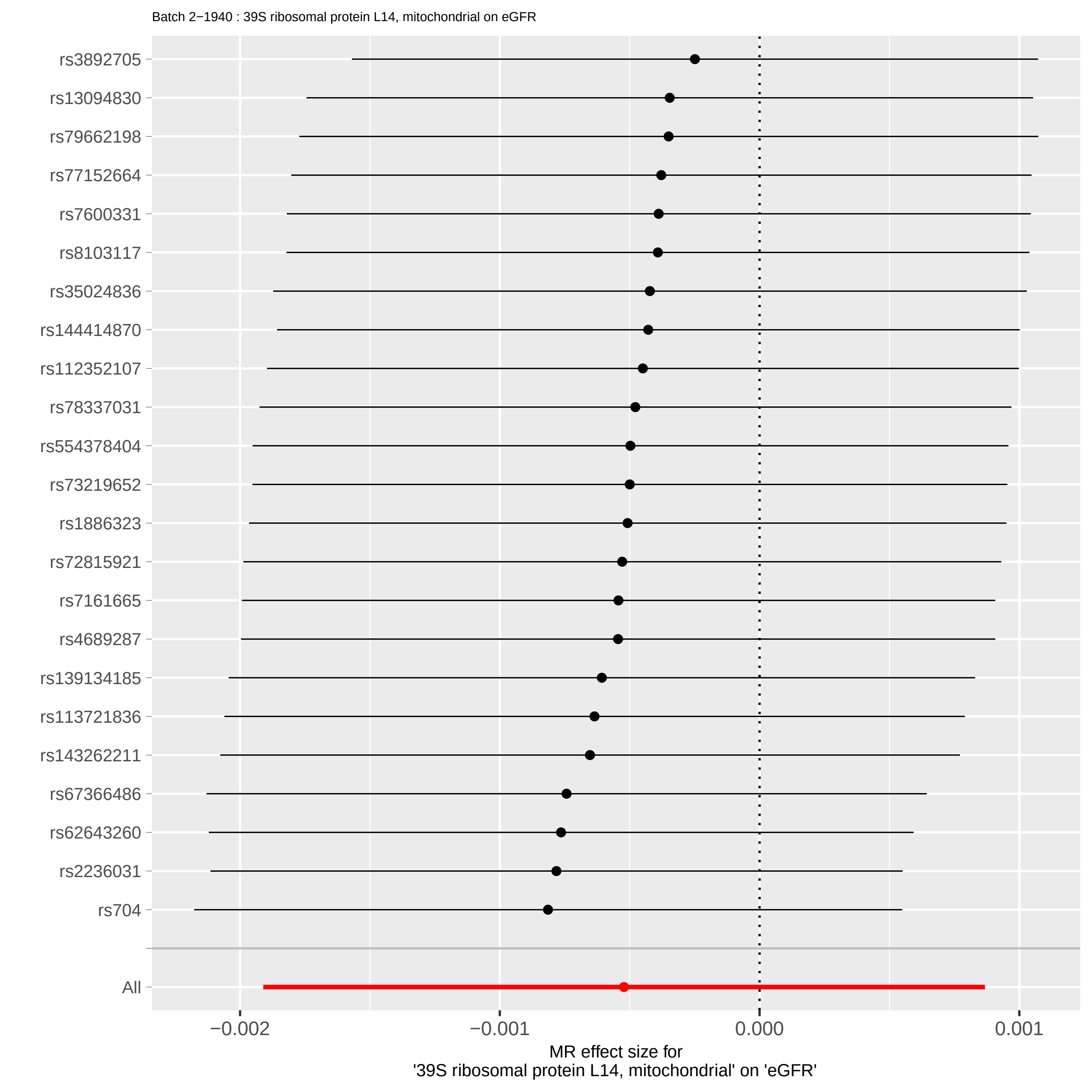
Batch 2−1940 : 39S ribosomal protein L14, mitochondrial on eGFR
rs3892705
rs13094830
rs79662198
rs77152664
rs7600331
rs8103117
rs35024836
rs144414870
rs112352107
rs78337031
rs554378404
rs73219652
rs1886323
rs72815921
rs7161665
rs4689287
rs139134185
rs113721836
rs143262211
rs67366486
rs62643260
rs2236031
rs704
All
−0.002
−0.001
0.000
0.001
MR effect size for
'39S ribosomal protein L14, mitochondrial' on 'eGFR'

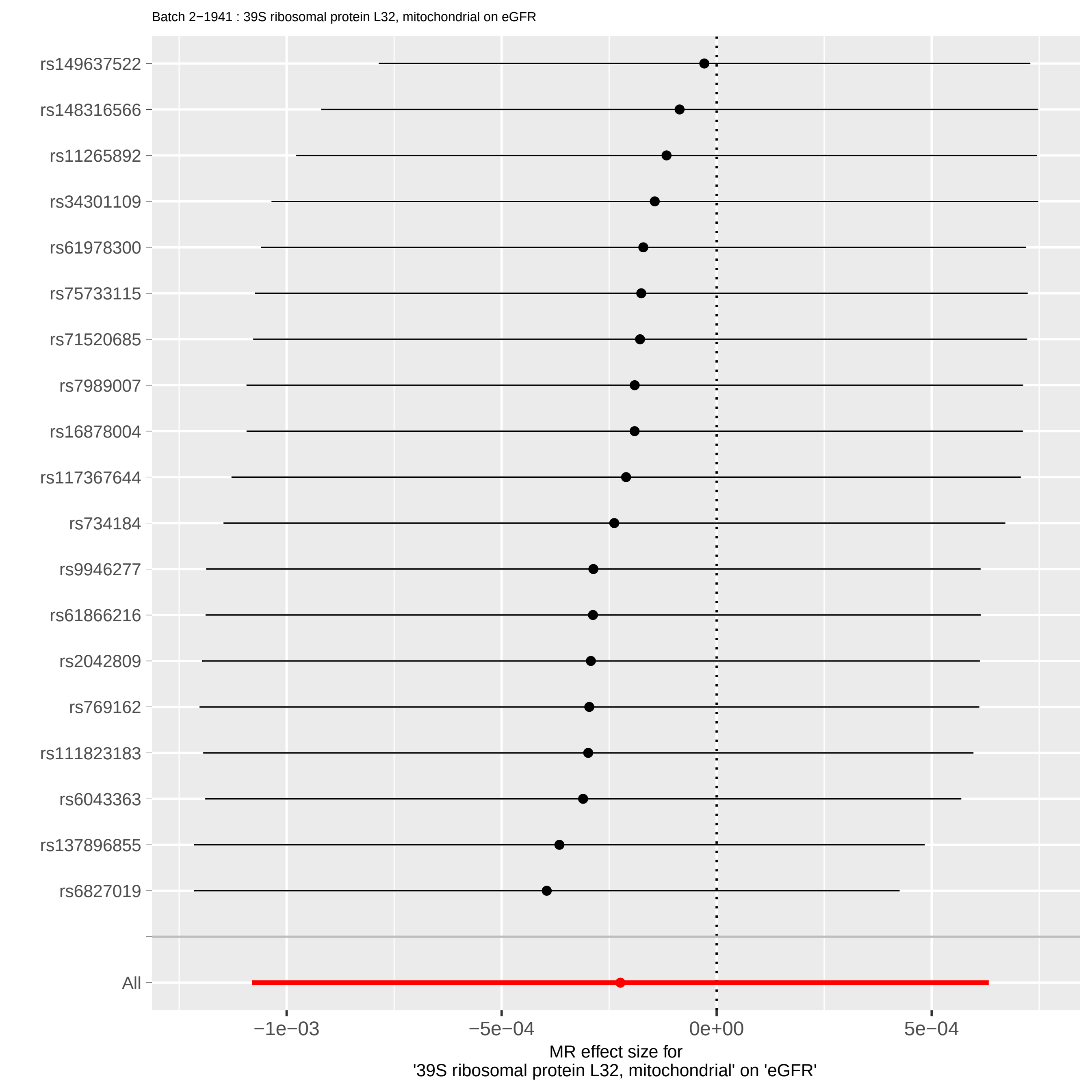
Batch 2−1941 : 39S ribosomal protein L32, mitochondrial on eGFR
rs149637522
rs148316566
rs11265892
rs34301109
rs61978300
rs75733115
rs71520685
rs7989007
rs16878004
rs117367644
rs734184
rs9946277
rs61866216
rs2042809
rs769162
rs111823183
rs6043363
rs137896855
rs6827019
All
−1e−03
−5e−04
0e+00
5e−04
MR effect size for
'39S ribosomal protein L32, mitochondrial' on 'eGFR'

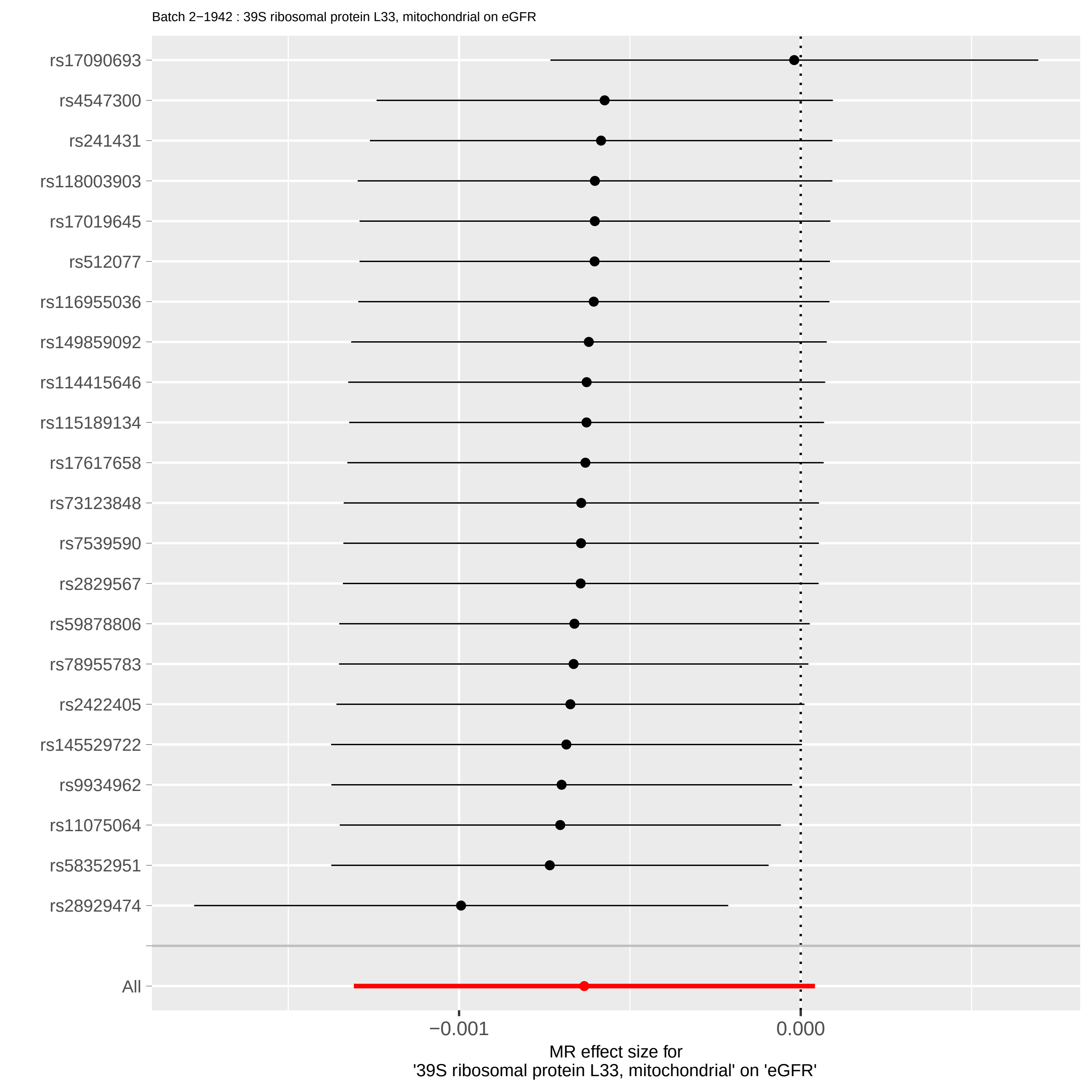

Batch 2−1942 : 39S ribosomal protein L33, mitochondrial on eGFR
rs17090693
rs4547300
rs241431
rs118003903
rs17019645
rs512077
rs116955036
rs149859092
rs114415646
rs115189134
rs17617658
rs73123848
rs7539590
rs2829567
rs59878806
rs78955783
rs2422405
rs145529722
rs9934962
rs11075064
rs58352951
rs28929474
All
−0.001
0.000
MR effect size for
'39S ribosomal protein L33, mitochondrial' on 'eGFR'

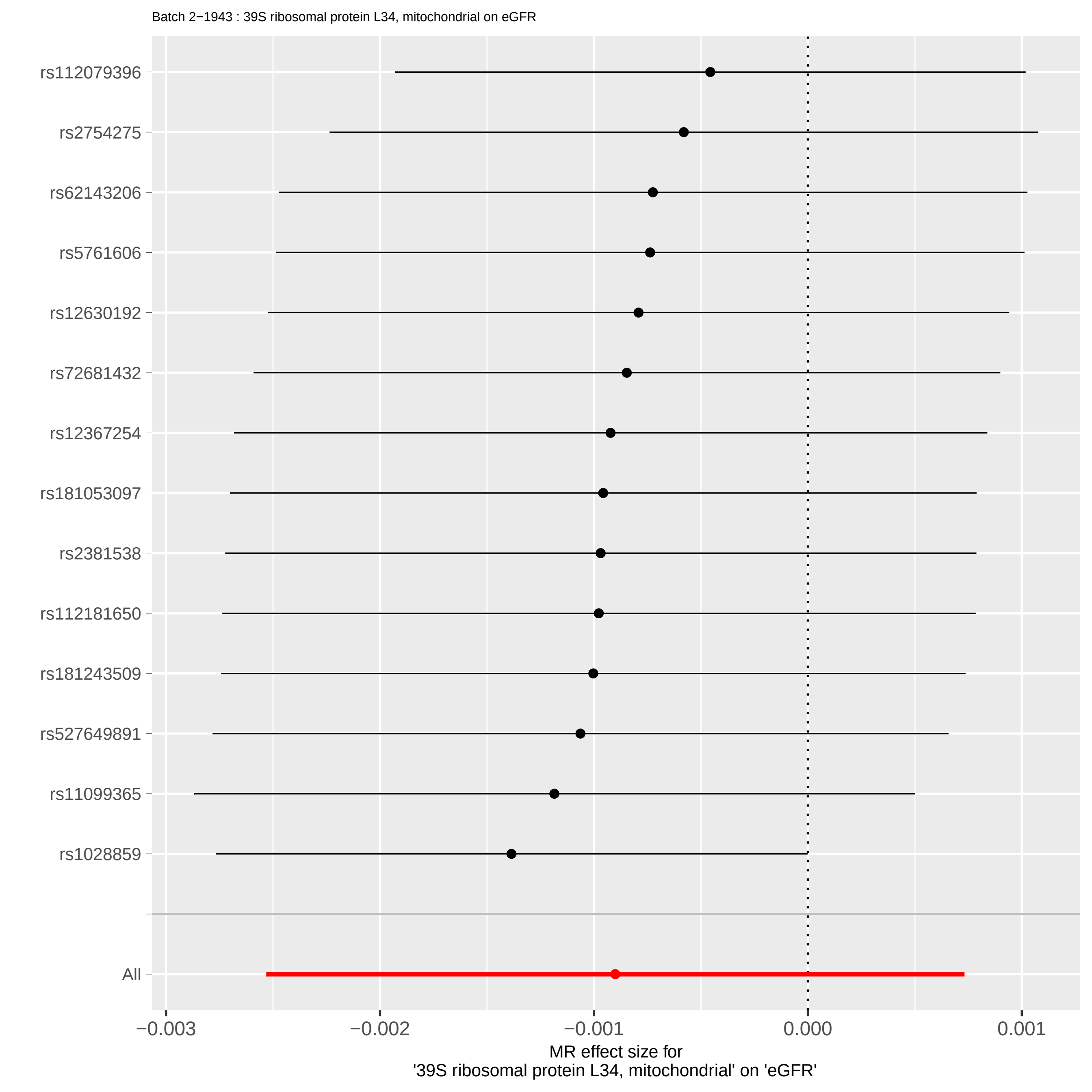
Batch 2−1943 : 39S ribosomal protein L34, mitochondrial on eGFR
rs112079396
rs2754275
rs62143206
rs5761606
rs12630192
rs72681432
rs12367254
rs181053097
rs2381538
rs112181650
rs181243509
rs527649891
rs11099365
rs1028859
All
−0.003
−0.002
−0.001
0.000
0.001
MR effect size for
'39S ribosomal protein L34, mitochondrial' on 'eGFR'

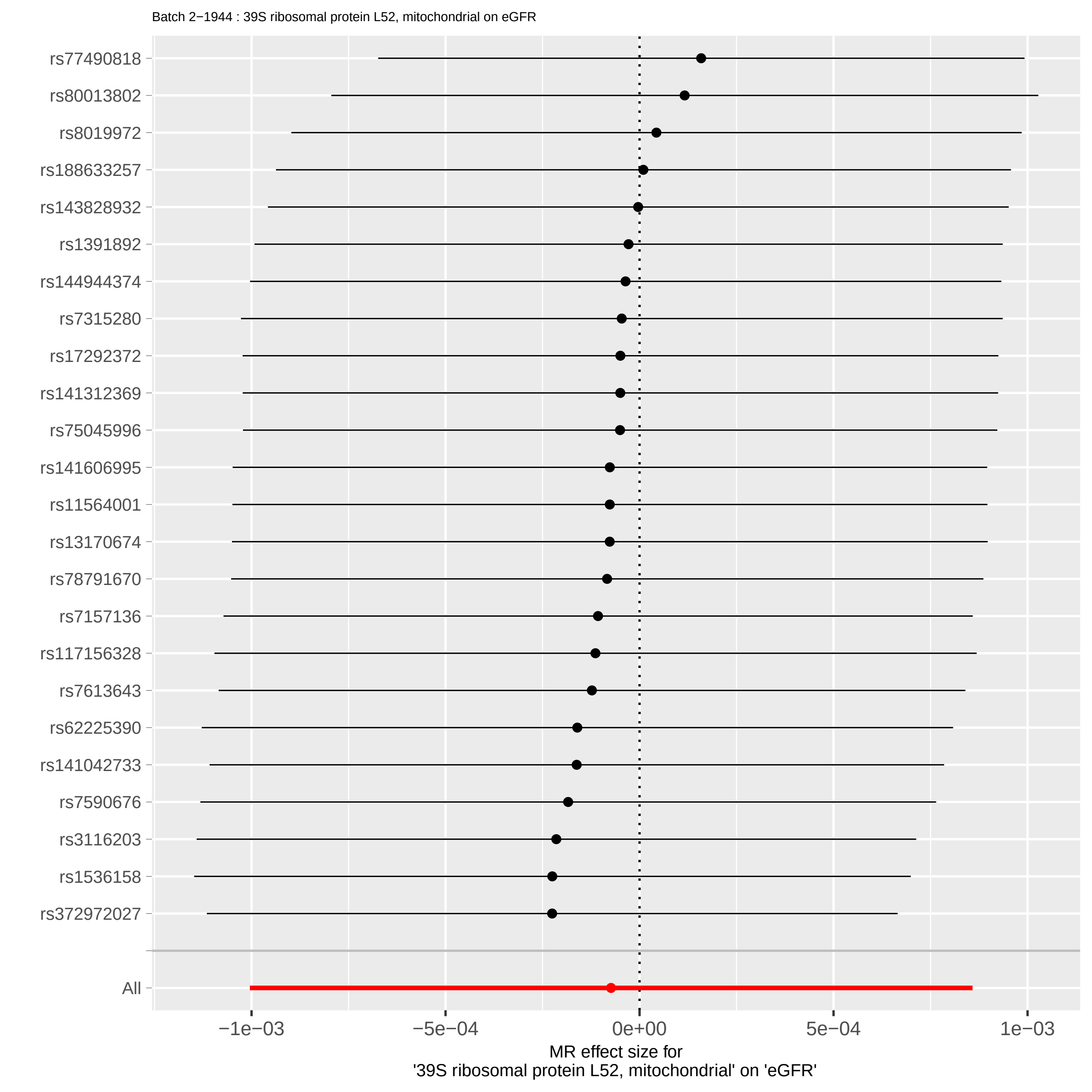
Batch 2−1944 : 39S ribosomal protein L52, mitochondrial on eGFR
rs77490818
rs80013802
rs8019972
rs188633257
rs143828932
rs1391892
rs144944374
rs7315280
rs17292372
rs141312369
rs75045996
rs141606995
rs11564001
rs13170674
rs78791670
rs7157136
rs117156328
rs7613643
rs62225390
rs141042733
rs7590676
rs3116203
rs1536158
rs372972027
All
−1e−03
−5e−04
0e+00
5e−04
1e−03
MR effect size for
'39S ribosomal protein L52, mitochondrial' on 'eGFR'

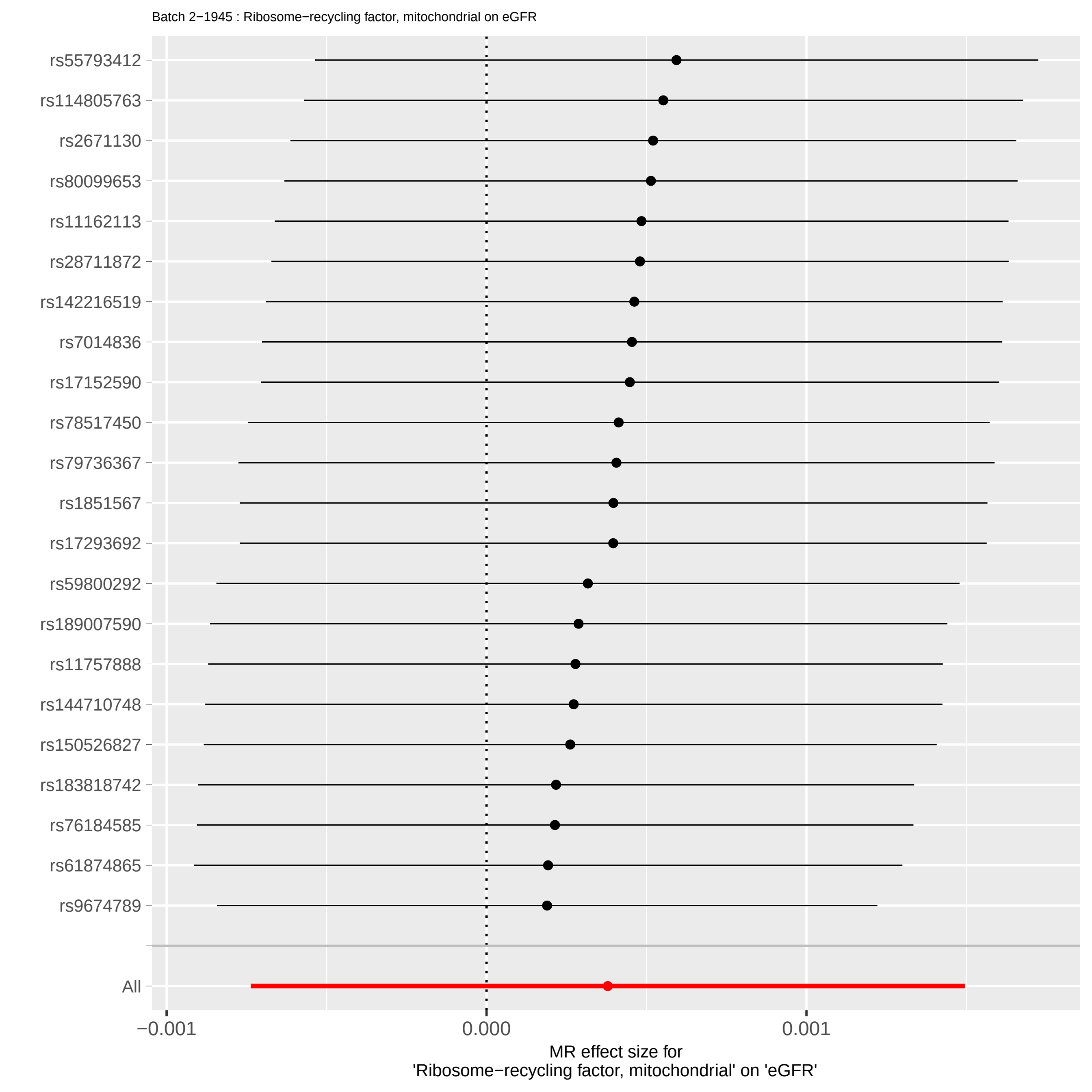

Batch 2−1945 : Ribosome−recycling factor, mitochondrial on eGFR
rs55793412
rs114805763
rs2671130
rs80099653
rs11162113
rs28711872
rs142216519
rs7014836
rs17152590
rs78517450
rs79736367
rs1851567
rs17293692
rs59800292
rs189007590
rs11757888
rs144710748
rs150526827
rs183818742
rs76184585
rs61874865
rs9674789
All
−0.001
0.000
0.001
MR effect size for
'Ribosome−recycling factor, mitochondrial' on 'eGFR'

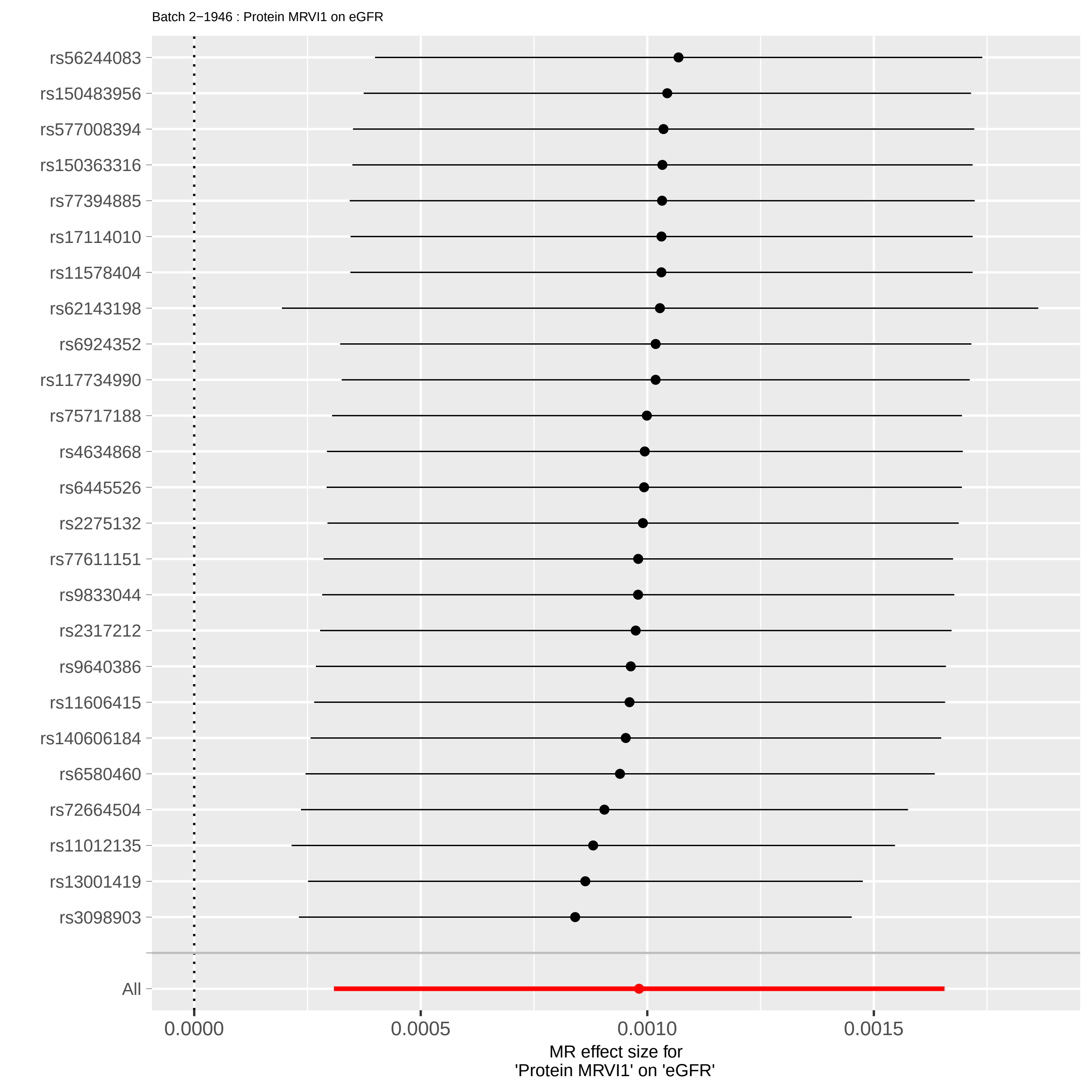
Batch 2−1946 : Protein MRVI1 on eGFR
rs56244083
rs150483956
rs577008394
rs150363316
rs77394885
rs17114010
rs11578404
rs62143198
rs6924352
rs117734990
rs75717188
rs4634868
rs6445526
rs2275132
rs77611151
rs9833044
rs2317212
rs9640386
rs11606415
rs140606184
rs6580460
rs72664504
rs11012135
rs13001419
rs3098903
All
0.0000
0.0005
0.0010
0.0015
MR effect size for
'Protein MRVI1' on 'eGFR'

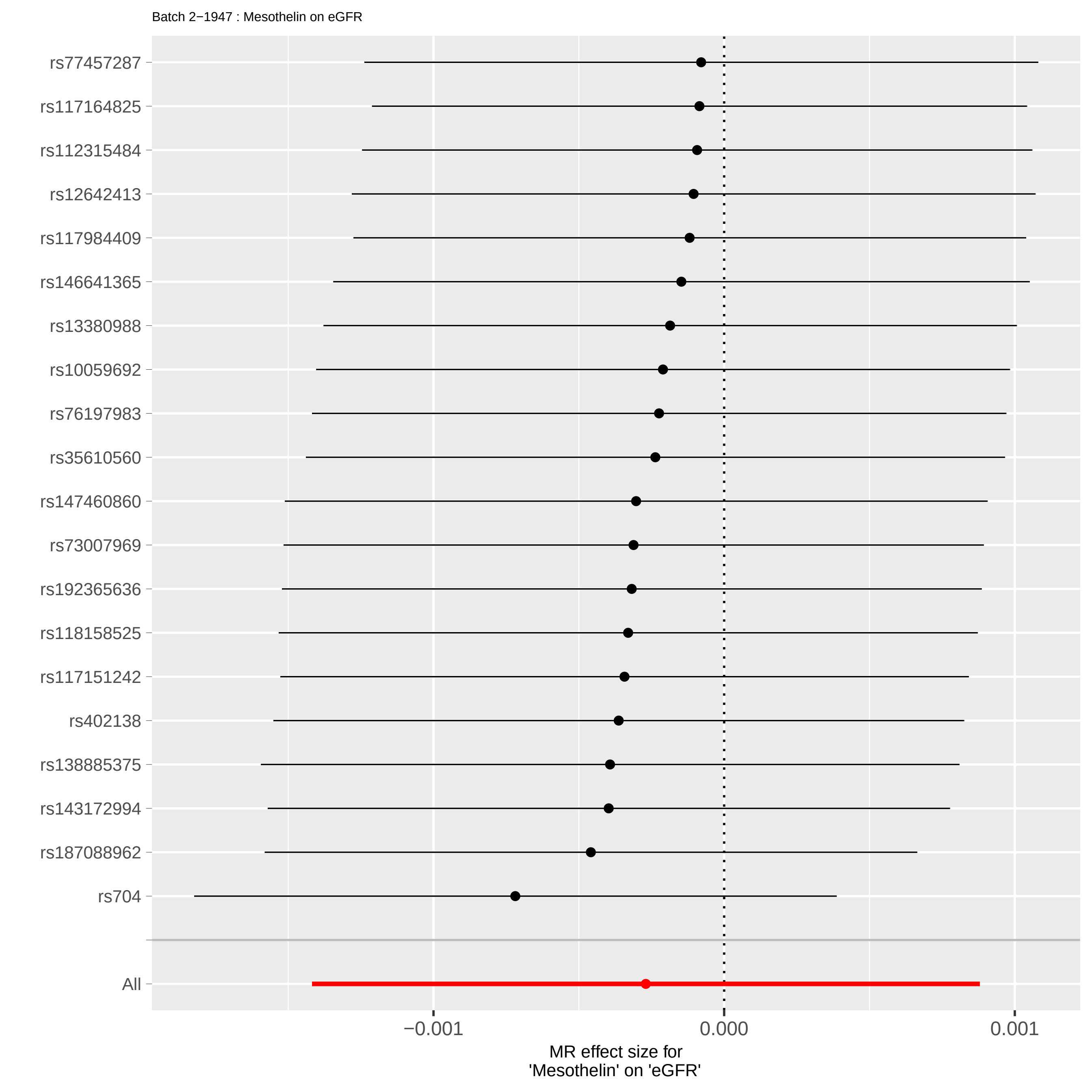
Batch 2−1947 : Mesothelin on eGFR
rs77457287
rs117164825
rs112315484
rs12642413
rs117984409
rs146641365
rs13380988
rs10059692
rs76197983
rs35610560
rs147460860
rs73007969
rs192365636
rs118158525
rs117151242
rs402138
rs138885375
rs143172994
rs187088962
rs704
All
−0.001
0.000
0.001
MR effect size for
'Mesothelin' on 'eGFR'

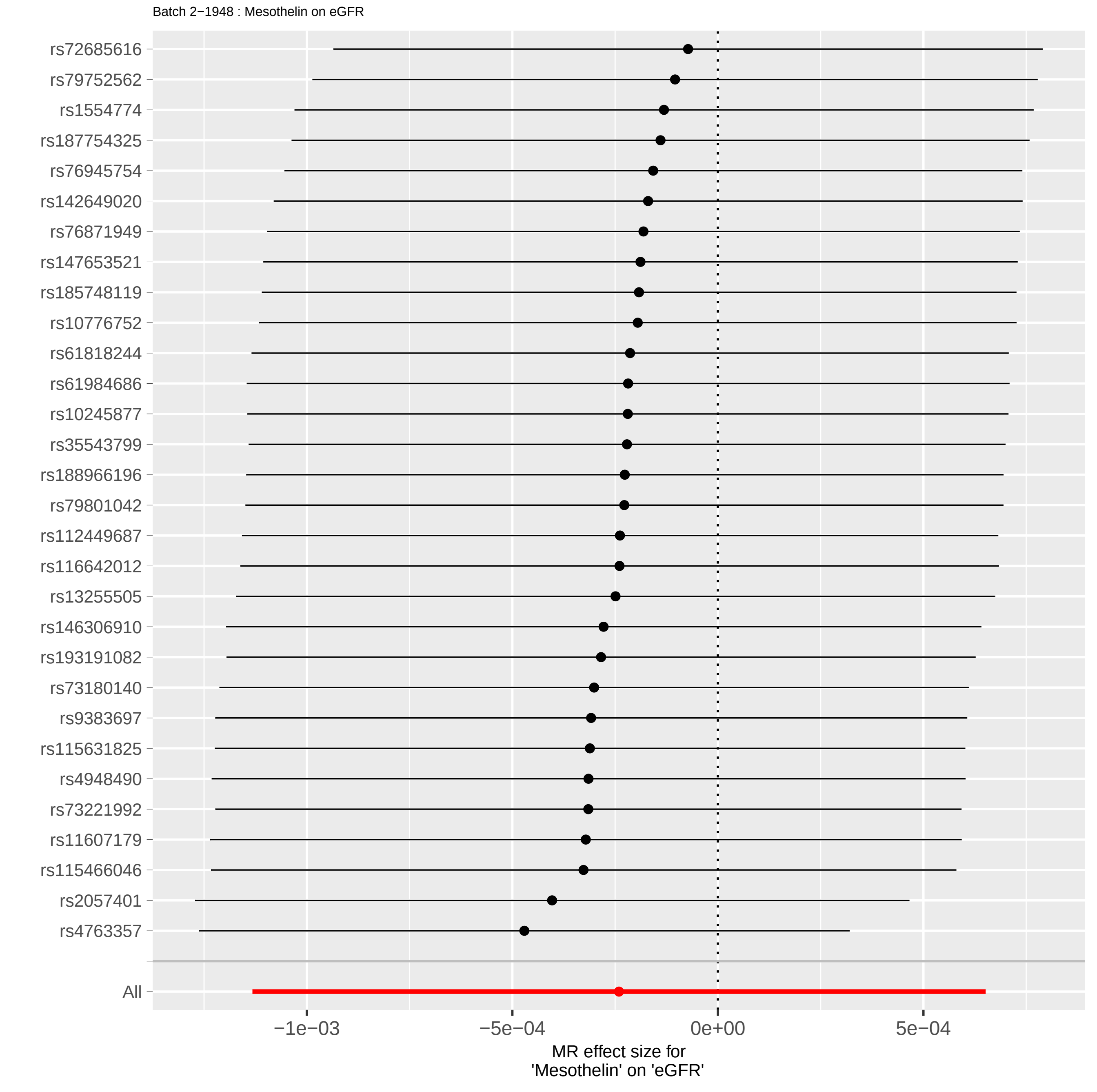

Batch 2−1948 : Mesothelin on eGFR
rs72685616
rs79752562
rs1554774
rs187754325
rs76945754
rs142649020
rs76871949
rs147653521
rs185748119
rs10776752
rs61818244
rs61984686
rs10245877
rs35543799
rs188966196
rs79801042
rs112449687
rs116642012
rs13255505
rs146306910
rs193191082
rs73180140
rs9383697
rs115631825
rs4948490
rs73221992
rs11607179
rs115466046
rs2057401
rs4763357
All
−1e−03
−5e−04
0e+00
5e−04
MR effect size for
'Mesothelin' on 'eGFR'

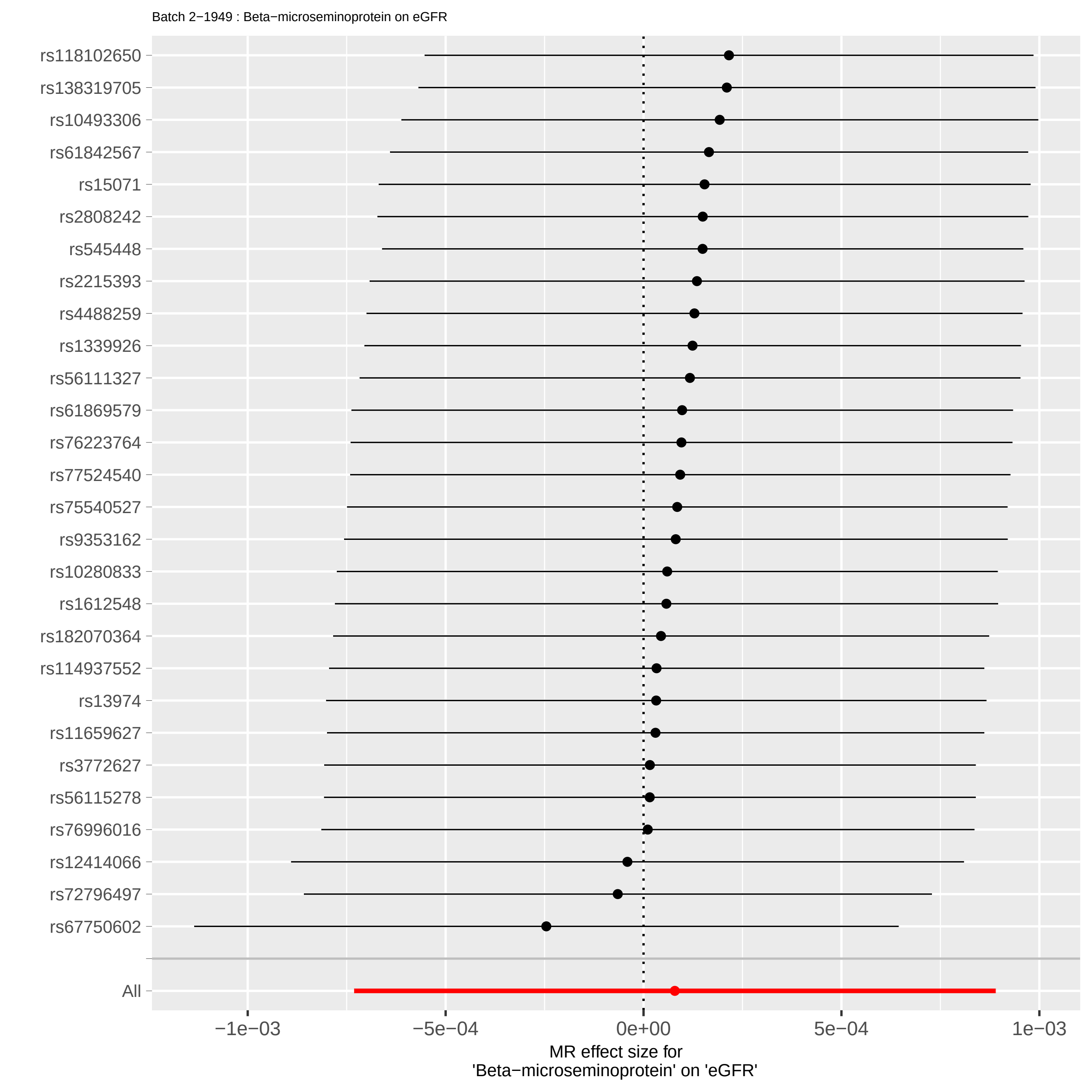

Batch 2−1949 : Beta−microseminoprotein on eGFR
rs118102650
rs138319705
rs10493306
rs61842567
rs15071
rs2808242
rs545448
rs2215393
rs4488259
rs1339926
rs56111327
rs61869579
rs76223764
rs77524540
rs75540527
rs9353162
rs10280833
rs1612548
rs182070364
rs114937552
rs13974
rs11659627
rs3772627
rs56115278
rs76996016
rs12414066
rs72796497
rs67750602
All
−1e−03
−5e−04
0e+00
5e−04
1e−03
MR effect size for
'Beta−microseminoprotein' on 'eGFR'

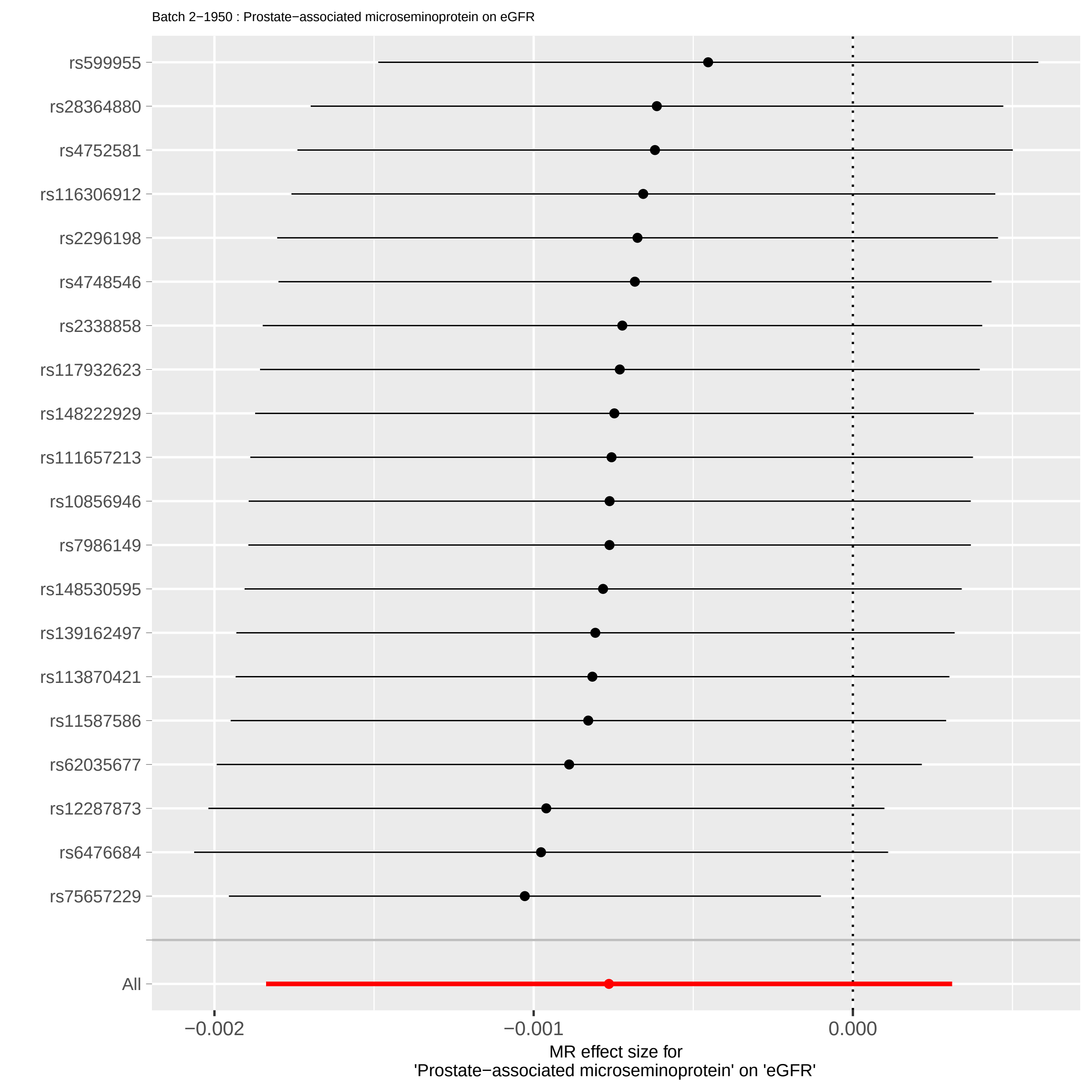

Batch 2−1950 : Prostate−associated microseminoprotein on eGFR
rs599955
rs28364880
rs4752581
rs116306912
rs2296198
rs4748546
rs2338858
rs117932623
rs148222929
rs111657213
rs10856946
rs7986149
rs148530595
rs139162497
rs113870421
rs11587586
rs62035677
rs12287873
rs6476684
rs75657229
All
−0.002
−0.001
0.000
MR effect size for
'Prostate−associated microseminoprotein' on 'eGFR'

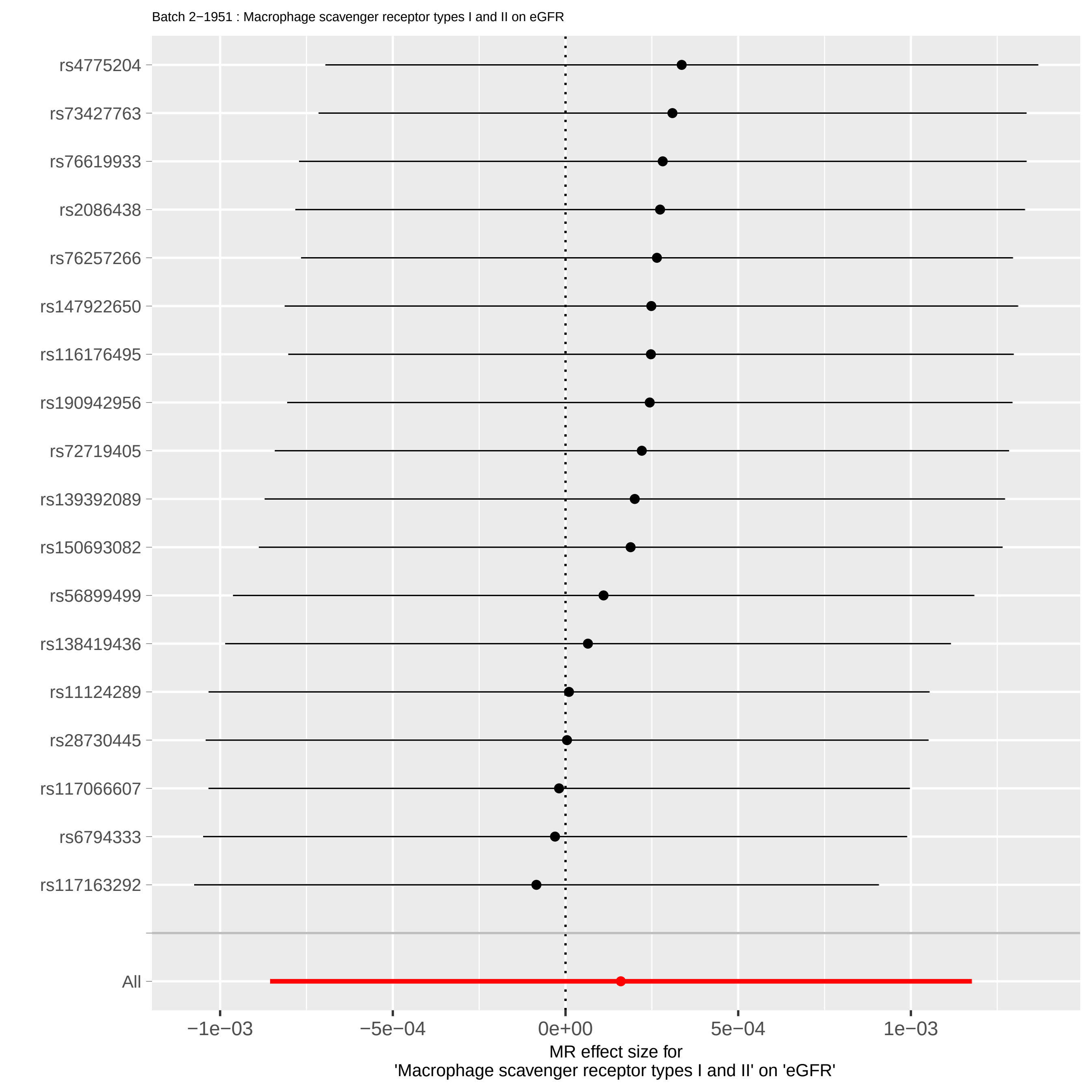

Batch 2−1951 : Macrophage scavenger receptor types I and II on eGFR
rs4775204
rs73427763
rs76619933
rs2086438
rs76257266
rs147922650
rs116176495
rs190942956
rs72719405
rs139392089
rs150693082
rs56899499
rs138419436
rs11124289
rs28730445
rs117066607
rs6794333
rs117163292
All
−1e−03
−5e−04
0e+00
5e−04
1e−03
MR effect size for
'Macrophage scavenger receptor types I and II' on 'eGFR'

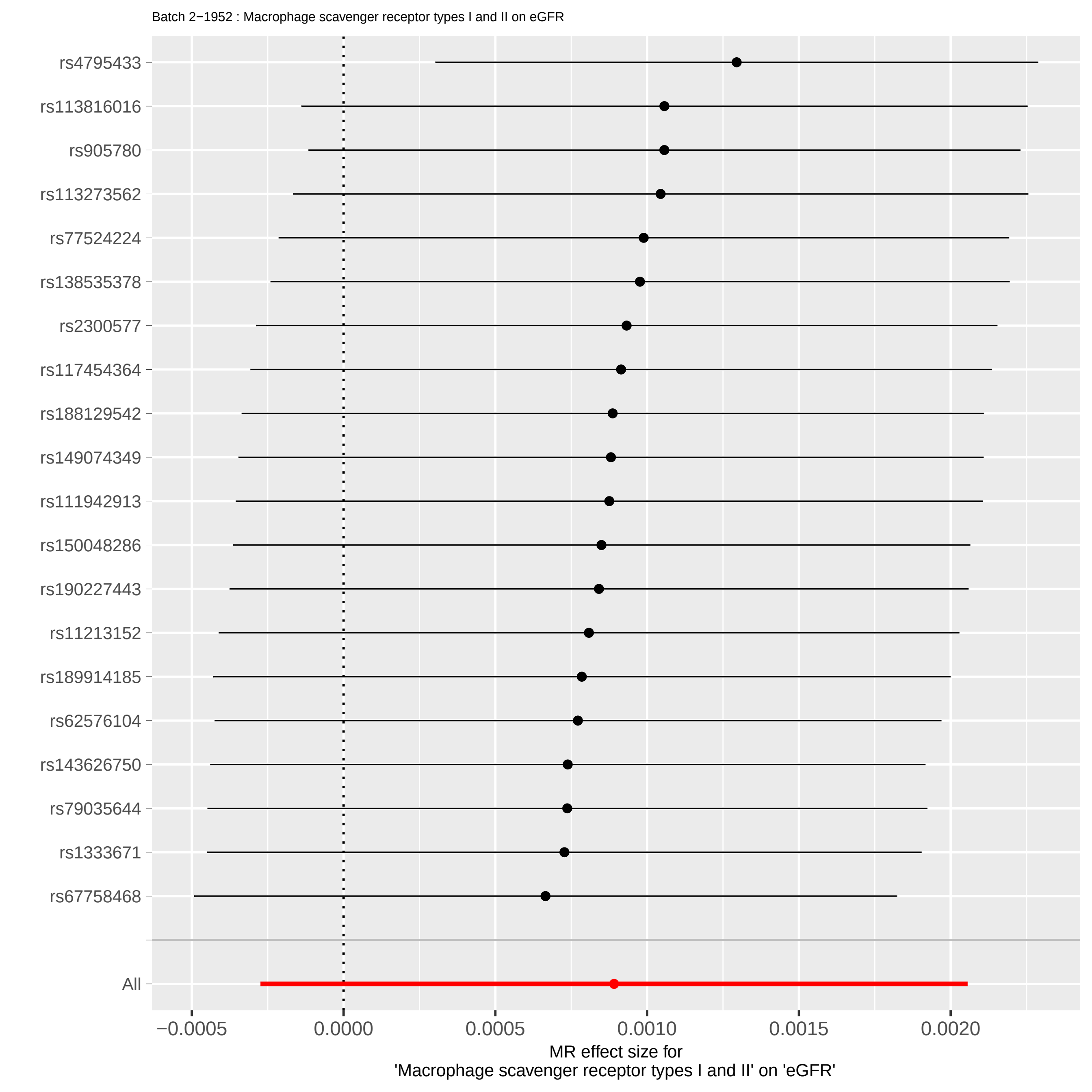

Batch 2−1952 : Macrophage scavenger receptor types I and II on eGFR
rs4795433
rs113816016
rs905780
rs113273562
rs77524224
rs138535378
rs2300577
rs117454364
rs188129542
rs149074349
rs111942913
rs150048286
rs190227443
rs11213152
rs189914185
rs62576104
rs143626750
rs79035644
rs1333671
rs67758468
All
−0.0005
0.0000
0.0005
0.0010
0.0015
0.0020
MR effect size for
'Macrophage scavenger receptor types I and II' on 'eGFR'

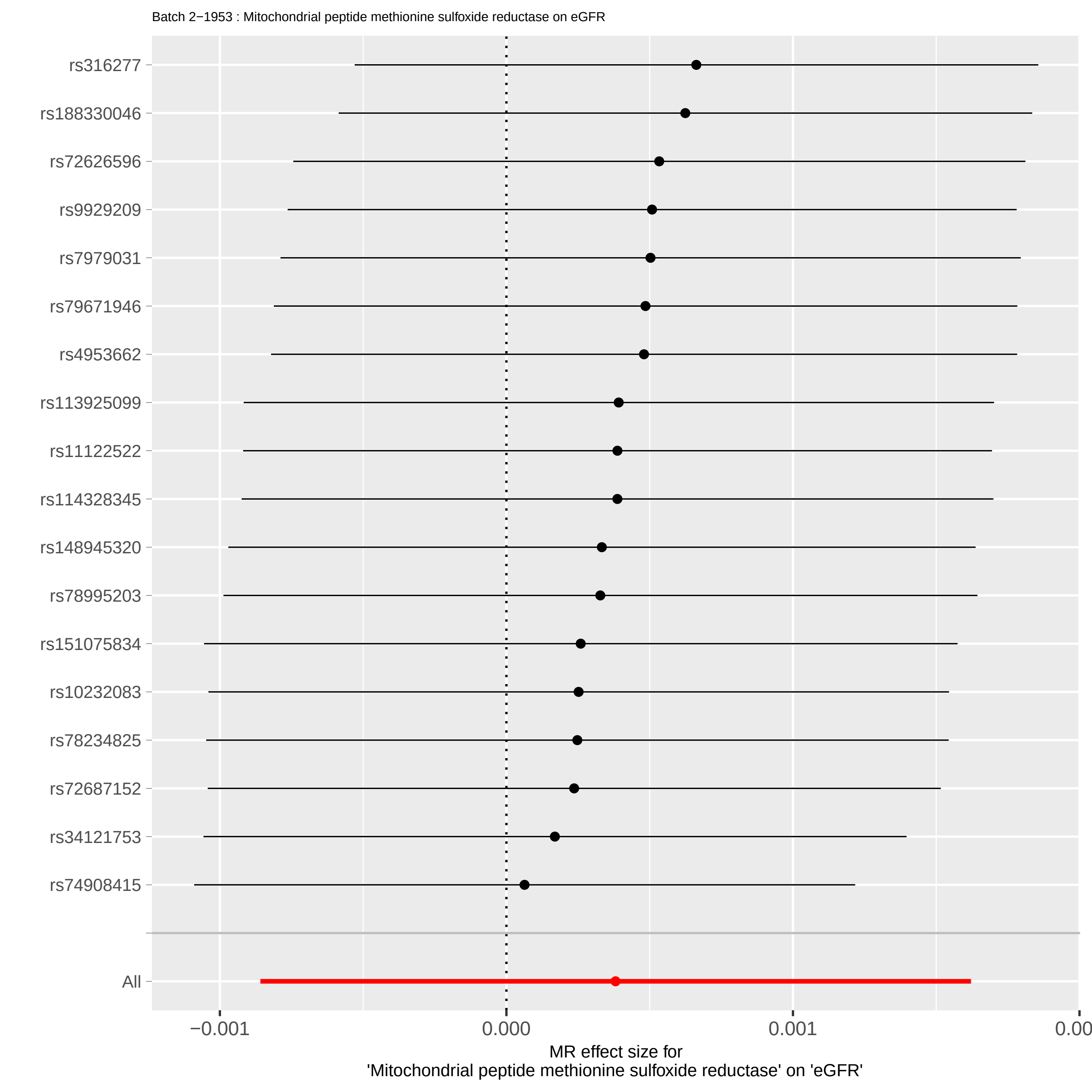
Batch 2−1953 : Mitochondrial peptide methionine sulfoxide reductase on eGFR
rs316277
rs188330046
rs72626596
rs9929209
rs7979031
rs79671946
rs4953662
rs113925099
rs11122522
rs114328345
rs148945320
rs78995203
rs151075834
rs10232083
rs78234825
rs72687152
rs34121753
rs74908415
All
−0.001
0.000
0.001
MR effect size for
'Mitochondrial peptide methionine sulfoxide reductase' on 'eGFR'

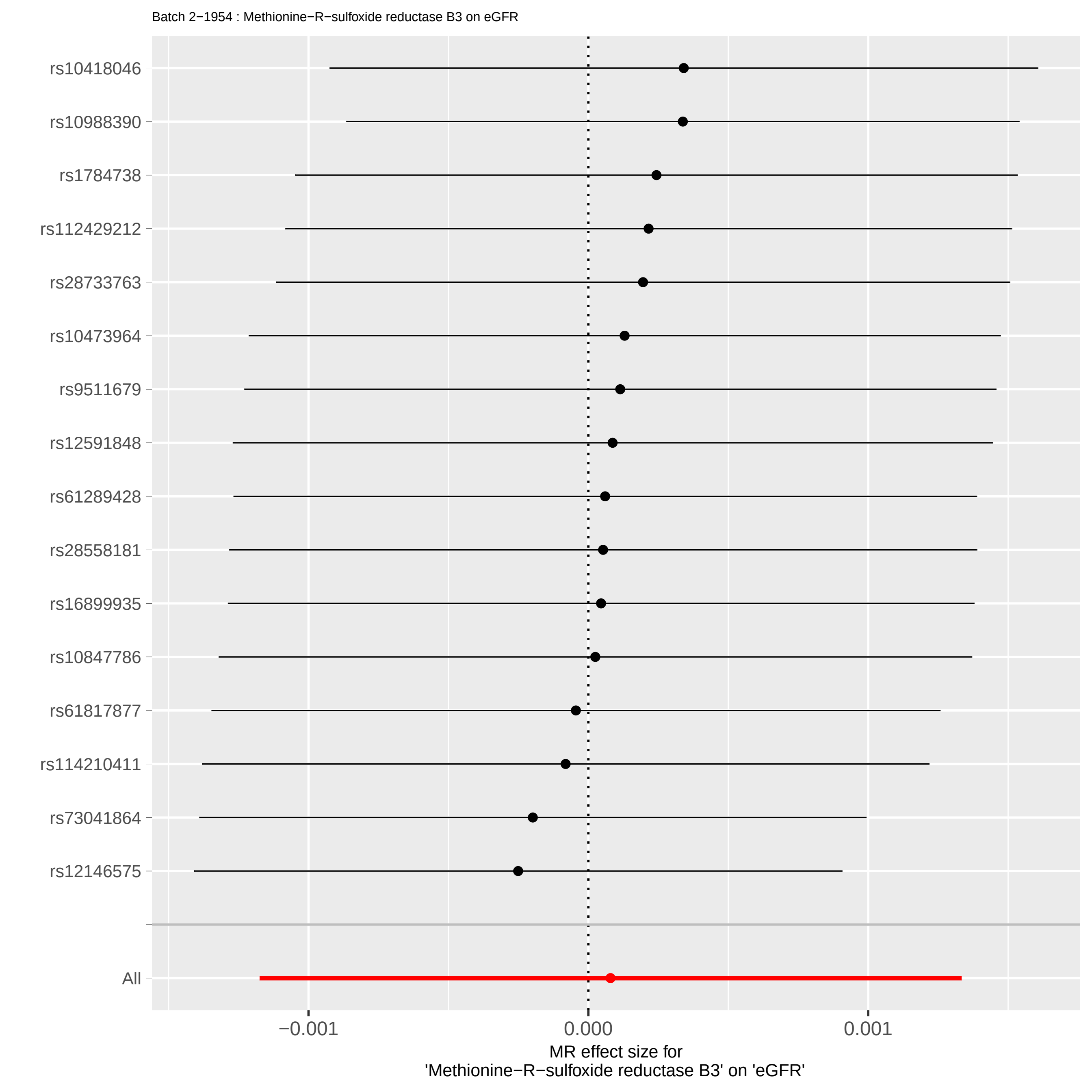

Batch 2−1954 : Methionine−R−sulfoxide reductase B3 on eGFR
rs10418046
rs10988390
rs1784738
rs112429212
rs28733763
rs10473964
rs9511679
rs12591848
rs61289428
rs28558181
rs16899935
rs10847786
rs61817877
rs114210411
rs73041864
rs12146575
All
−0.001
0.000
0.001
MR effect size for
'Methionine−R−sulfoxide reductase B3' on 'eGFR'

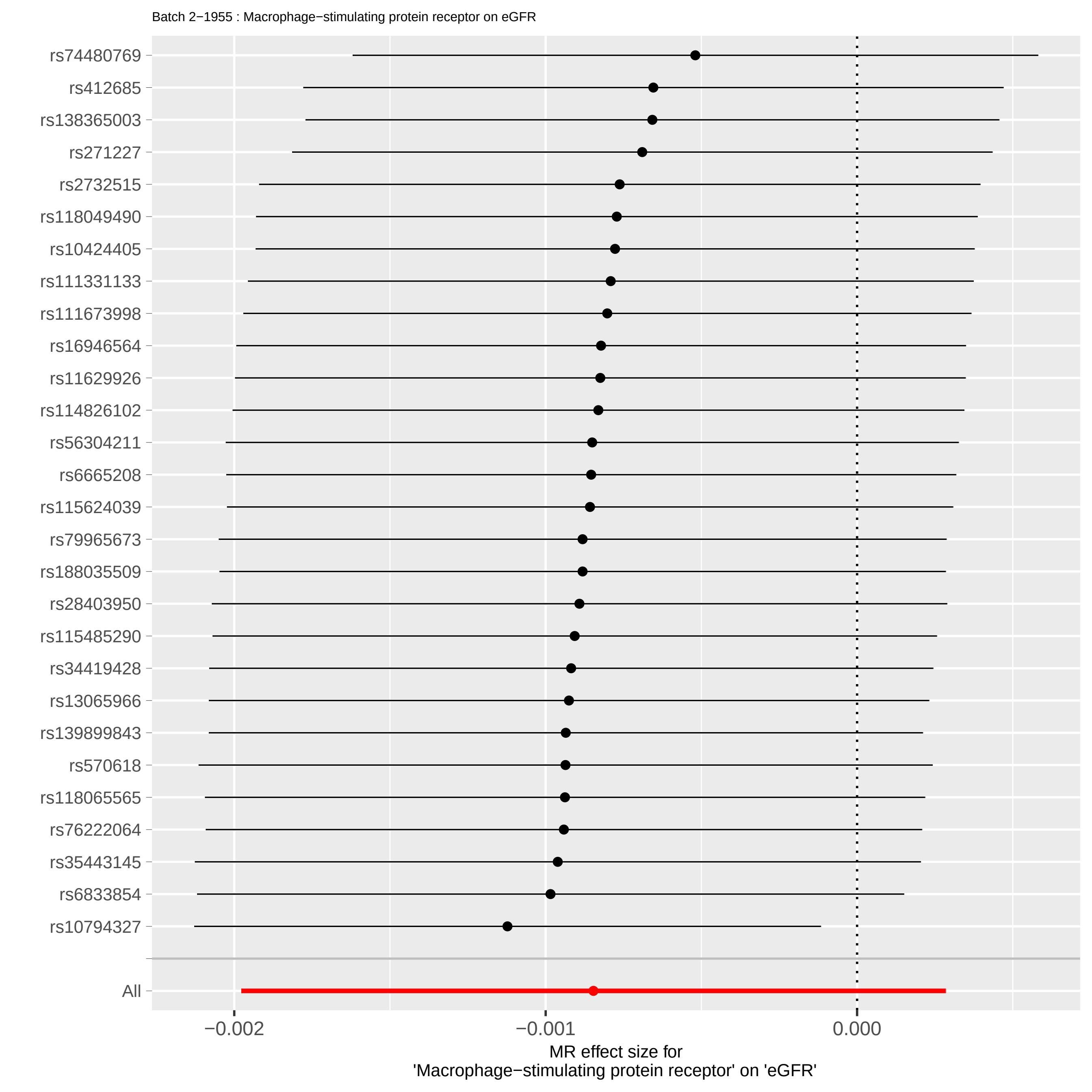

Batch 2−1955 : Macrophage−stimulating protein receptor on eGFR
rs74480769
rs412685
rs138365003
rs271227
rs2732515
rs118049490
rs10424405
rs111331133
rs111673998
rs16946564
rs11629926
rs114826102
rs56304211
rs6665208
rs115624039
rs79965673
rs188035509
rs28403950
rs115485290
rs34419428
rs13065966
rs139899843
rs570618
rs118065565
rs76222064
rs35443145
rs6833854
rs10794327
All
−0.002
−0.001
0.000
MR effect size for
'Macrophage−stimulating protein receptor' on 'eGFR'

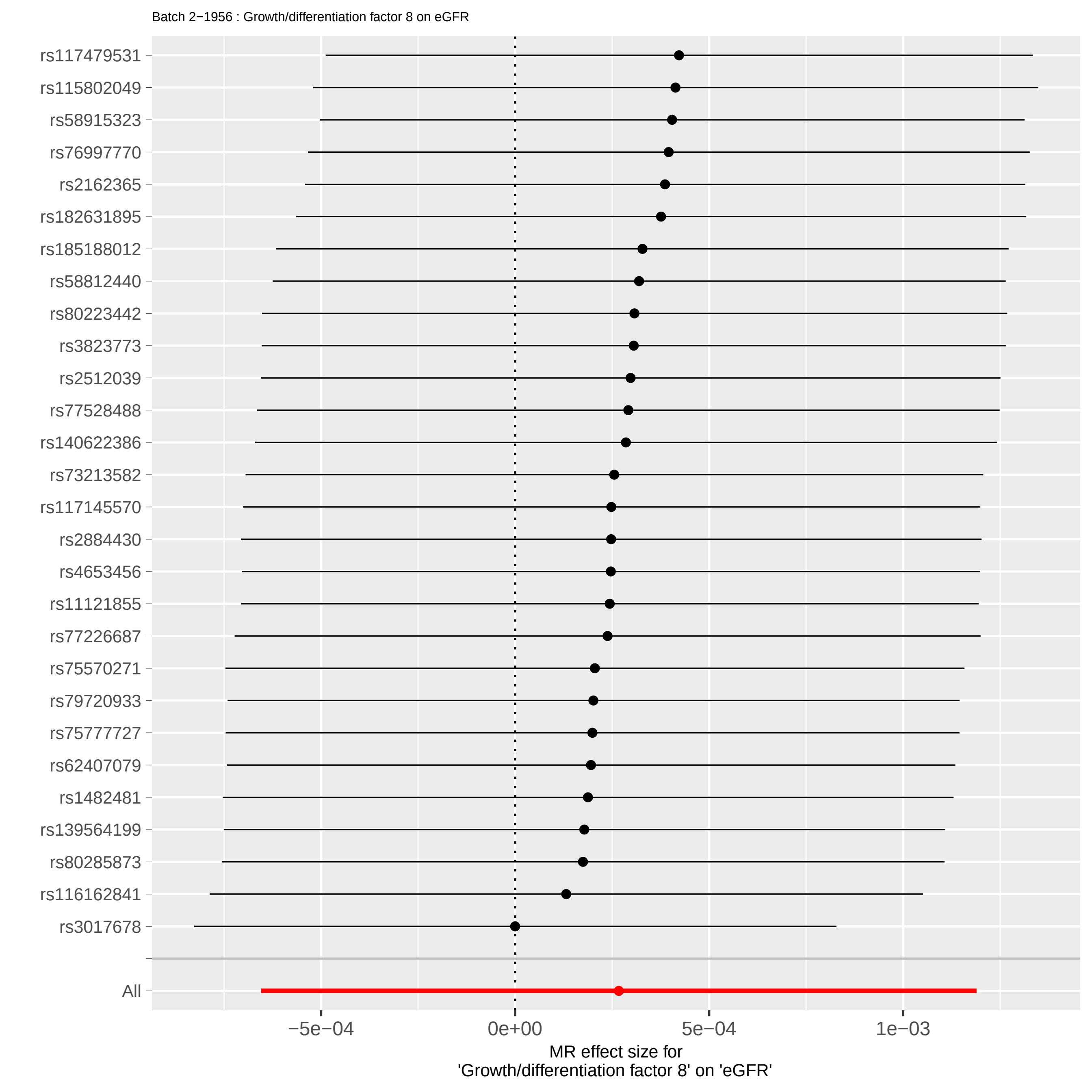

Batch 2−1956 : Growth/differentiation factor 8 on eGFR
rs117479531
rs115802049
rs58915323
rs76997770
rs2162365
rs182631895
rs185188012
rs58812440
rs80223442
rs3823773
rs2512039
rs77528488
rs140622386
rs73213582
rs117145570
rs2884430
rs4653456
rs11121855
rs77226687
rs75570271
rs79720933
rs75777727
rs62407079
rs1482481
rs139564199
rs80285873
rs116162841
rs3017678
All
−5e−04
0e+00
5e−04
1e−03
MR effect size for
'Growth/differentiation factor 8' on 'eGFR'

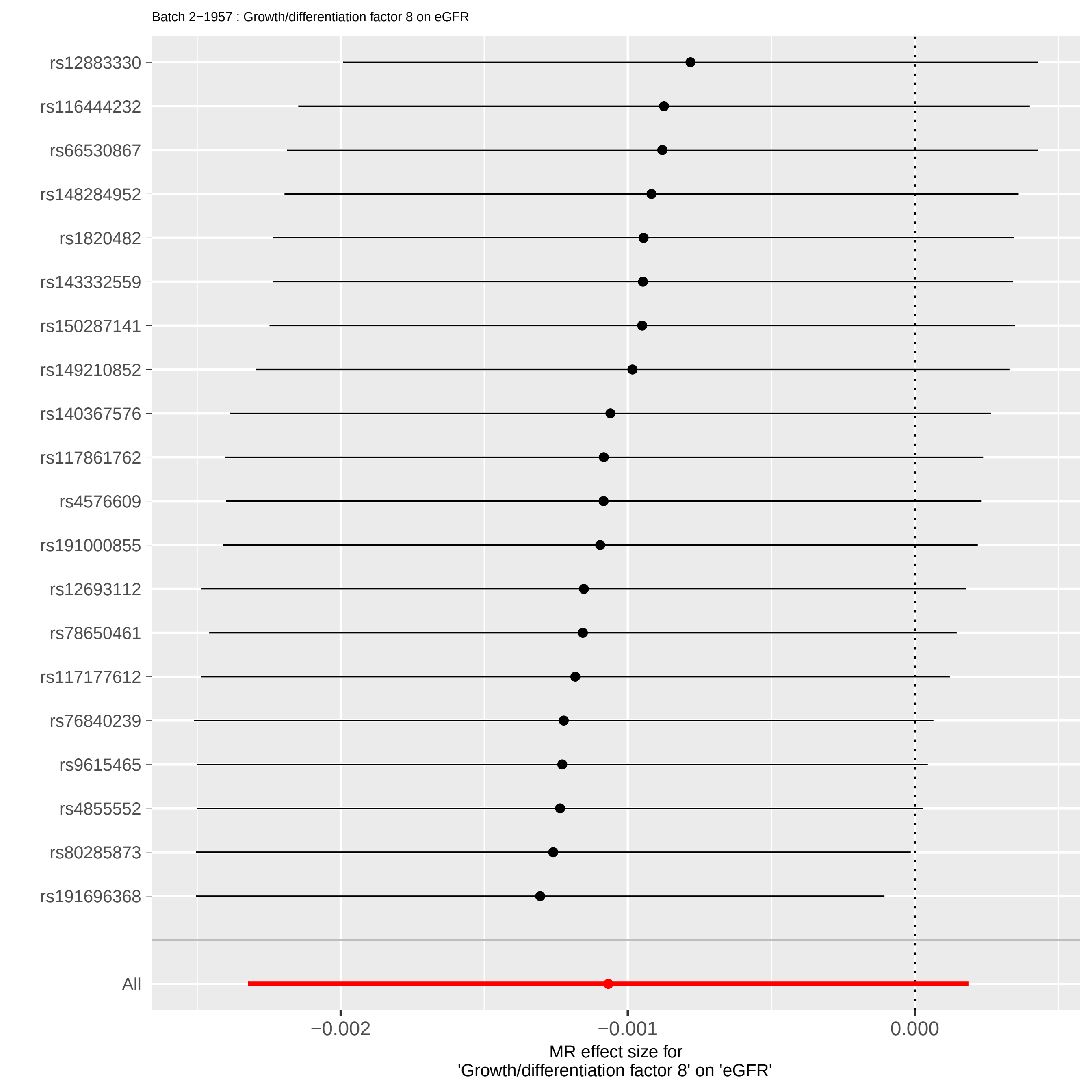

Batch 2−1957 : Growth/differentiation factor 8 on eGFR
rs12883330
rs116444232
rs66530867
rs148284952
rs1820482
rs143332559
rs150287141
rs149210852
rs140367576
rs117861762
rs4576609
rs191000855
rs12693112
rs78650461
rs117177612
rs76840239
rs9615465
rs4855552
rs80285873
rs191696368
All
−0.002
−0.001
0.000
MR effect size for
'Growth/differentiation factor 8' on 'eGFR'

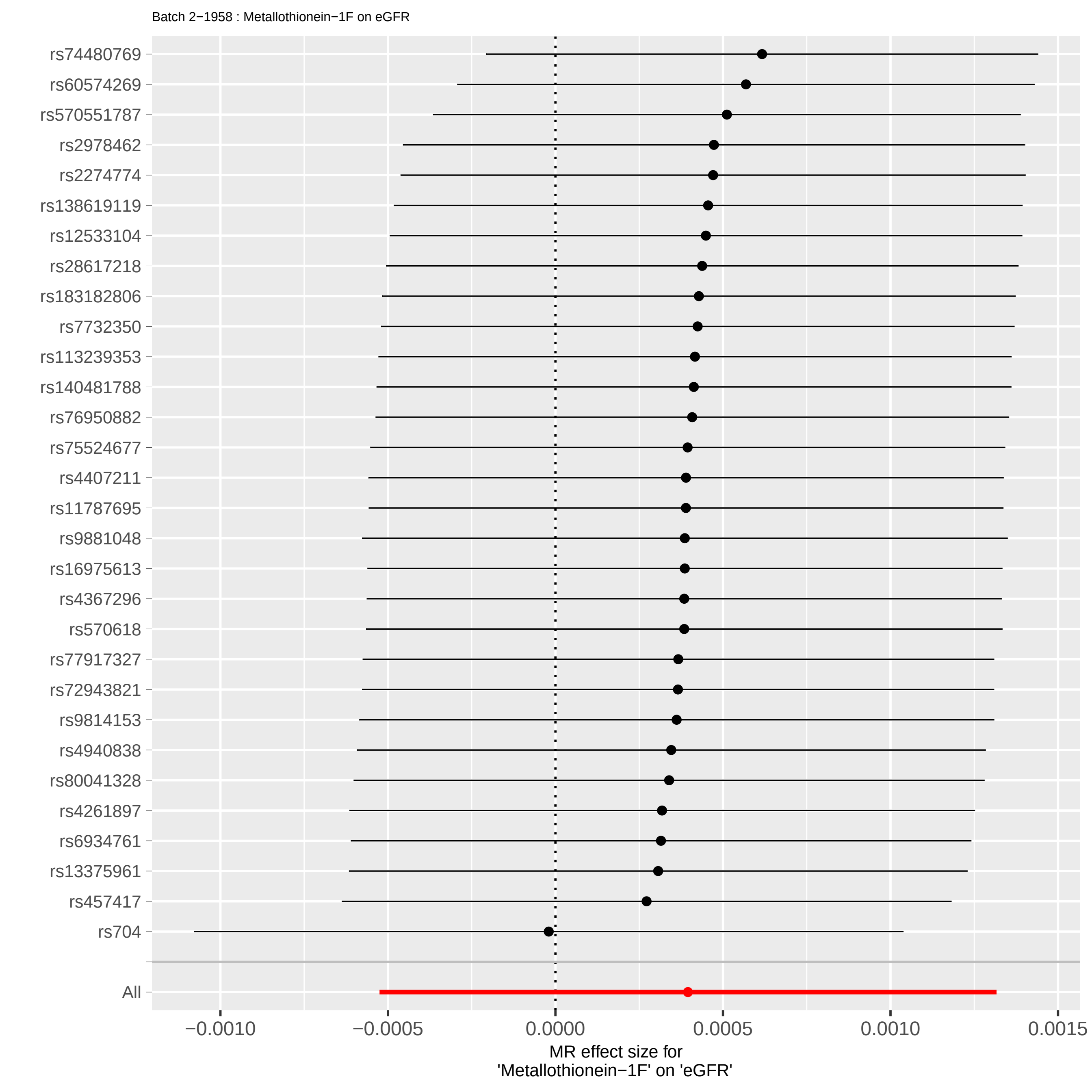

Batch 2−1958 : Metallothionein−1F on eGFR
rs74480769
rs60574269
rs570551787
rs2978462
rs2274774
rs138619119
rs12533104
rs28617218
rs183182806
rs7732350
rs113239353
rs140481788
rs76950882
rs75524677
rs4407211
rs11787695
rs9881048
rs16975613
rs4367296
rs570618
rs77917327
rs72943821
rs9814153
rs4940838
rs80041328
rs4261897
rs6934761
rs13375961
rs457417
rs704
All
−0.0010
−0.0005
0.0000
0.0005
0.0010
0.0015
MR effect size for
'Metallothionein−1F' on 'eGFR'

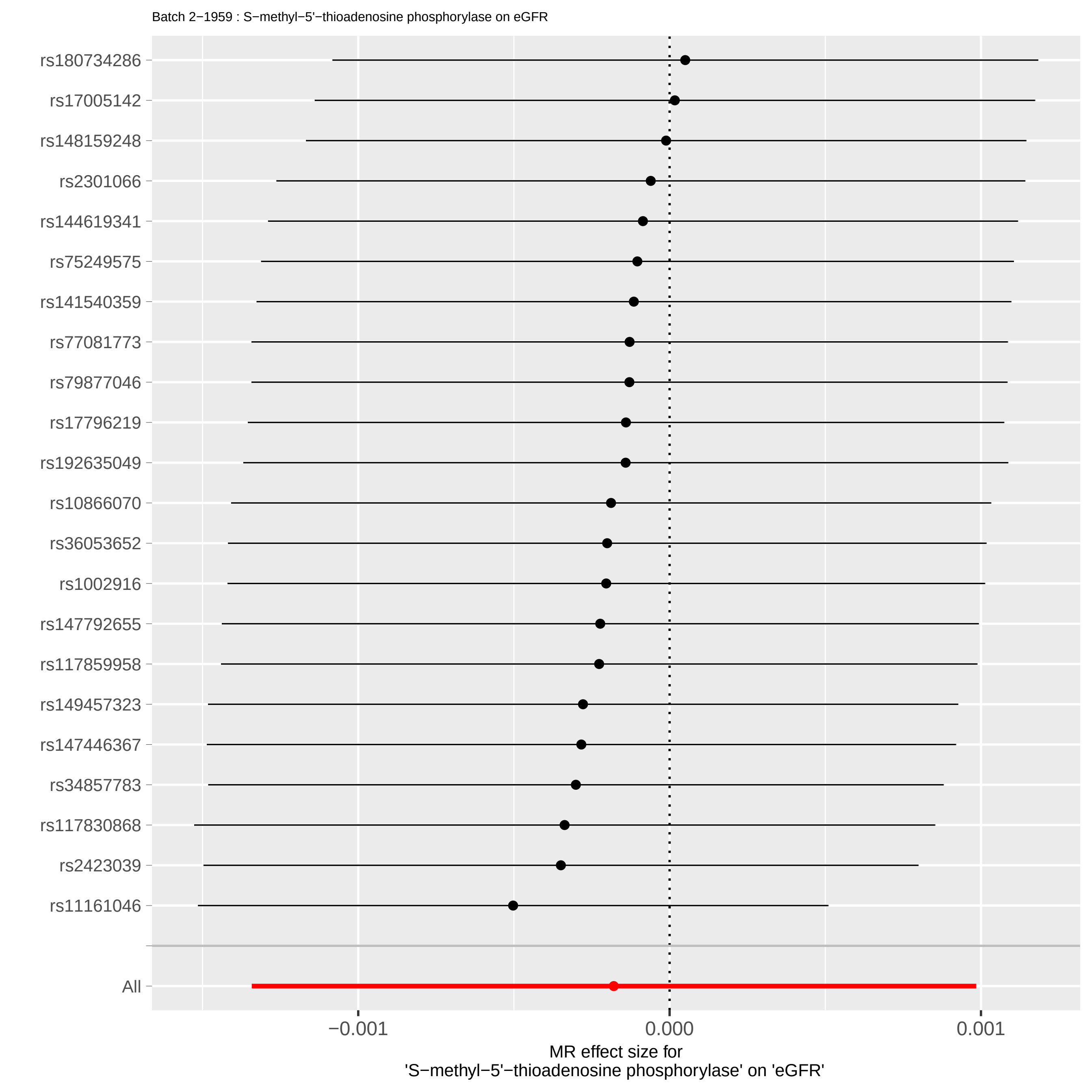

Batch 2−1959 : S−methyl−5'−thioadenosine phosphorylase on eGFR
rs180734286
rs17005142
rs148159248
rs2301066
rs144619341
rs75249575
rs141540359
rs77081773
rs79877046
rs17796219
rs192635049
rs10866070
rs36053652
rs1002916
rs147792655
rs117859958
rs149457323
rs147446367
rs34857783
rs117830868
rs2423039
rs11161046
All
−0.001
0.000
0.001
MR effect size for
'S−methyl−5'−thioadenosine phosphorylase' on 'eGFR'

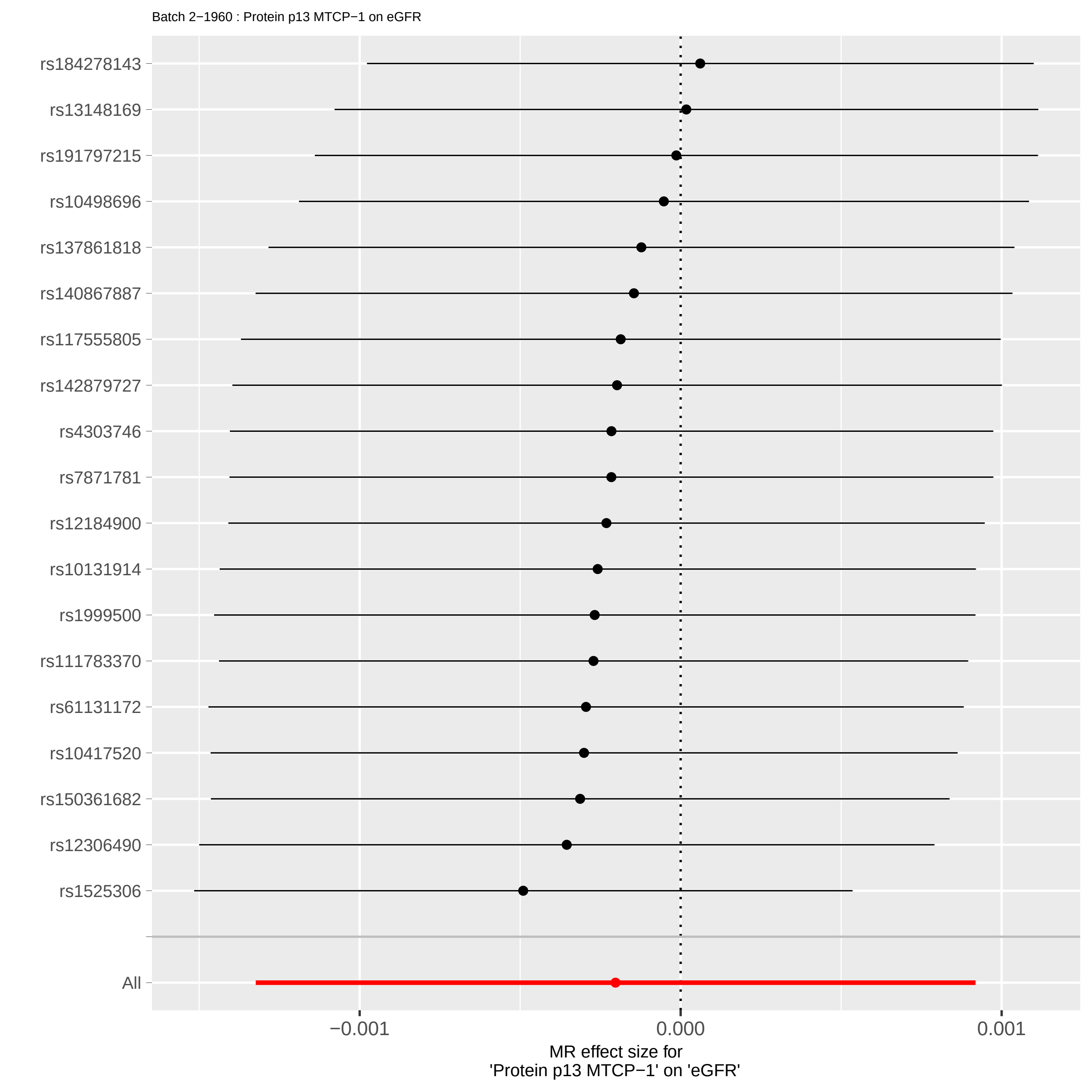

Batch 2−1960 : Protein p13 MTCP−1 on eGFR
rs184278143
rs13148169
rs191797215
rs10498696
rs137861818
rs140867887
rs117555805
rs142879727
rs4303746
rs7871781
rs12184900
rs10131914
rs1999500
rs111783370
rs61131172
rs10417520
rs150361682
rs12306490
rs1525306
All
−0.001
0.000
0.001
MR effect size for
'Protein p13 MTCP−1' on 'eGFR'

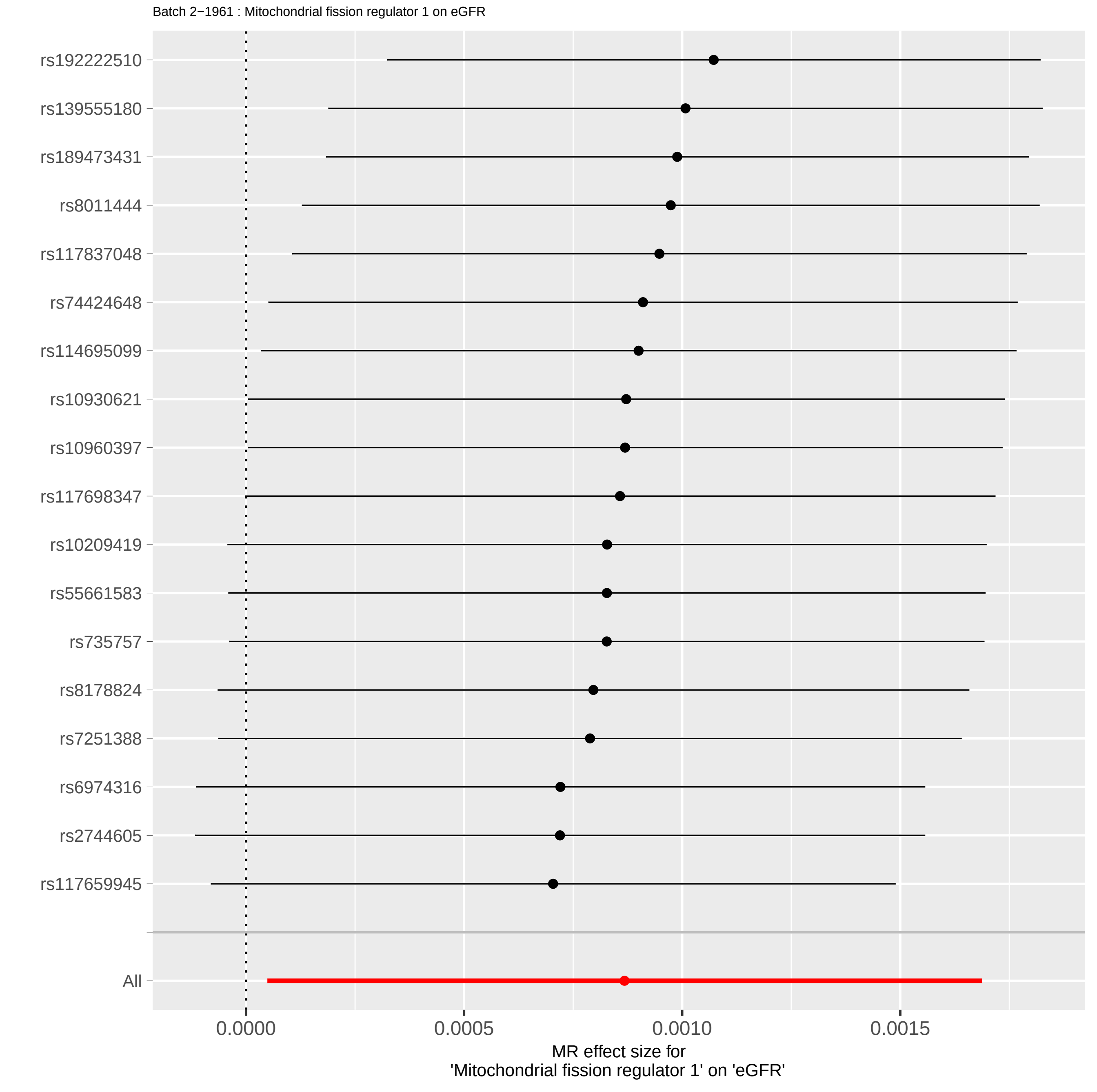

Batch 2−1961 : Mitochondrial fission regulator 1 on eGFR
rs192222510
rs139555180
rs189473431
rs8011444
rs117837048
rs74424648
rs114695099
rs10930621
rs10960397
rs117698347
rs10209419
rs55661583
rs735757
rs8178824
rs7251388
rs6974316
rs2744605
rs117659945
All
0.0000
0.0005
0.0010
0.0015
MR effect size for
'Mitochondrial fission regulator 1' on 'eGFR'

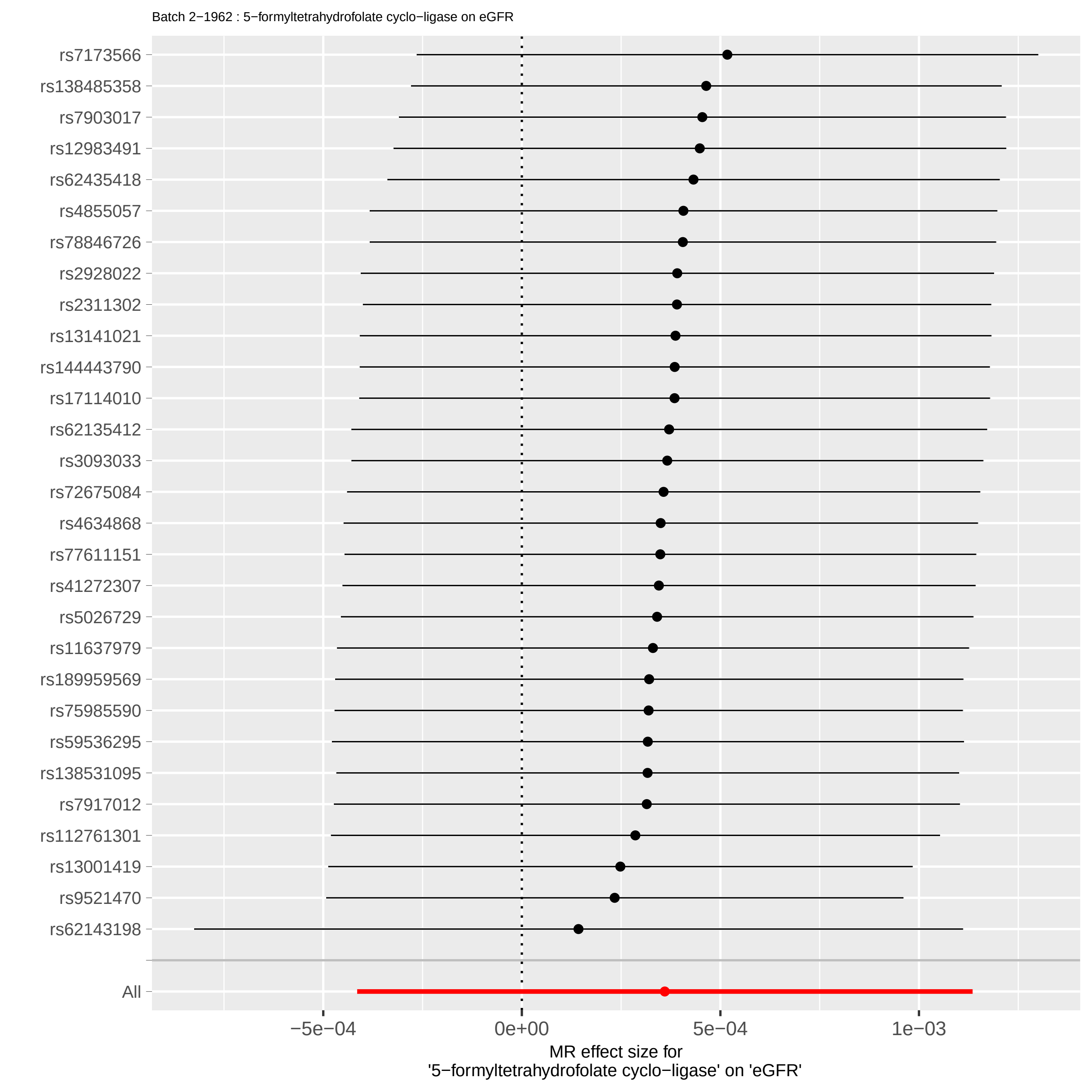
Batch 2−1962 : 5−formyltetrahydrofolate cyclo−ligase on eGFR
rs7173566
rs138485358
rs7903017
rs12983491
rs62435418
rs4855057
rs78846726
rs2928022
rs2311302
rs13141021
rs144443790
rs17114010
rs62135412
rs3093033
rs72675084
rs4634868
rs77611151
rs41272307
rs5026729
rs11637979
rs189959569
rs75985590
rs59536295
rs138531095
rs7917012
rs112761301
rs13001419
rs9521470
rs62143198
All
−5e−04
0e+00
5e−04
1e−03
MR effect size for
'5−formyltetrahydrofolate cyclo−ligase' on 'eGFR'

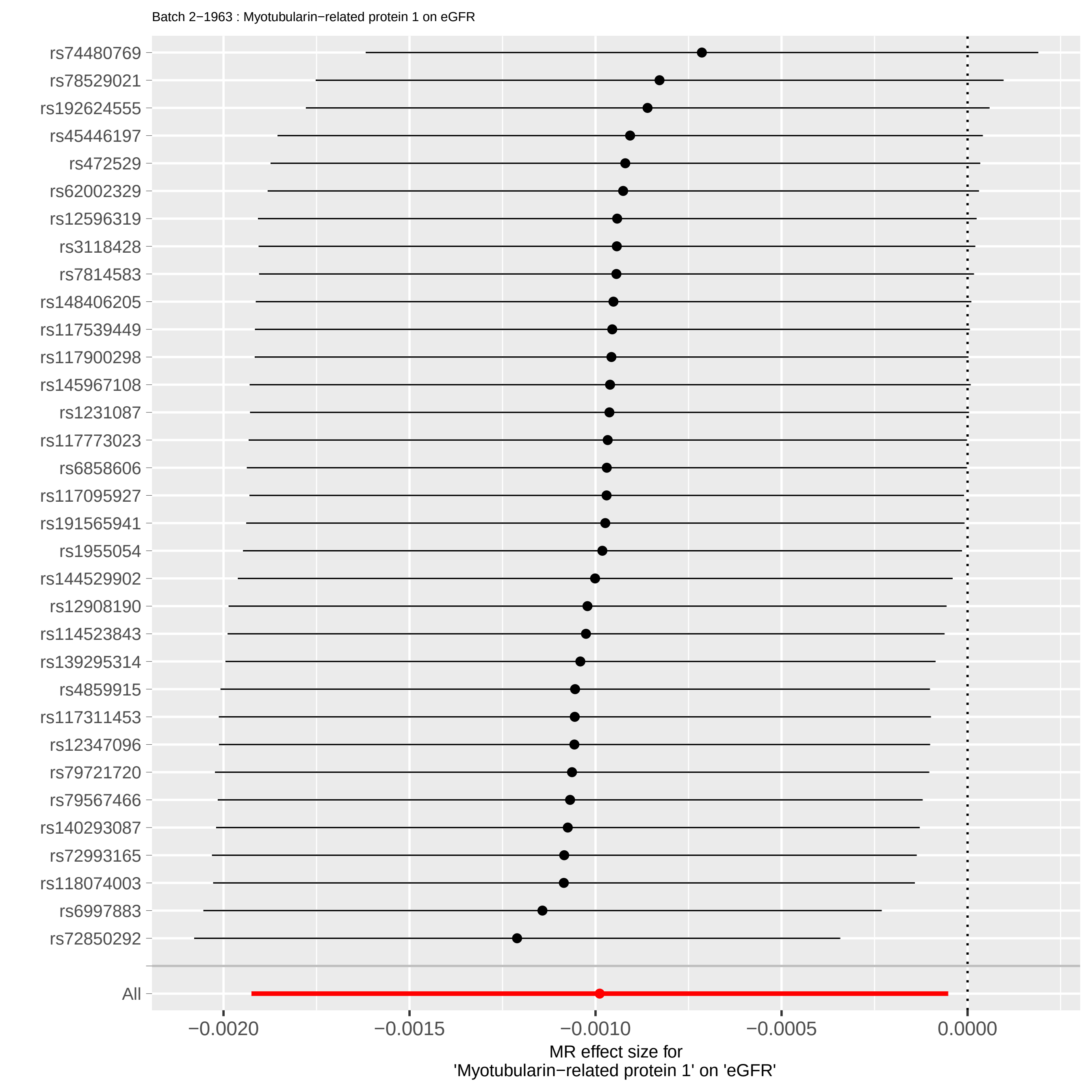

Batch 2−1963 : Myotubularin−related protein 1 on eGFR
rs74480769
rs78529021
rs192624555
rs45446197
rs472529
rs62002329
rs12596319
rs3118428
rs7814583
rs148406205
rs117539449
rs117900298
rs145967108
rs1231087
rs117773023
rs6858606
rs117095927
rs191565941
rs1955054
rs144529902
rs12908190
rs114523843
rs139295314
rs4859915
rs117311453
rs12347096
rs79721720
rs79567466
rs140293087
rs72993165
rs118074003
rs6997883
rs72850292
All
−0.0020
−0.0015
−0.0010
−0.0005
0.0000
MR effect size for
'Myotubularin−related protein 1' on 'eGFR'

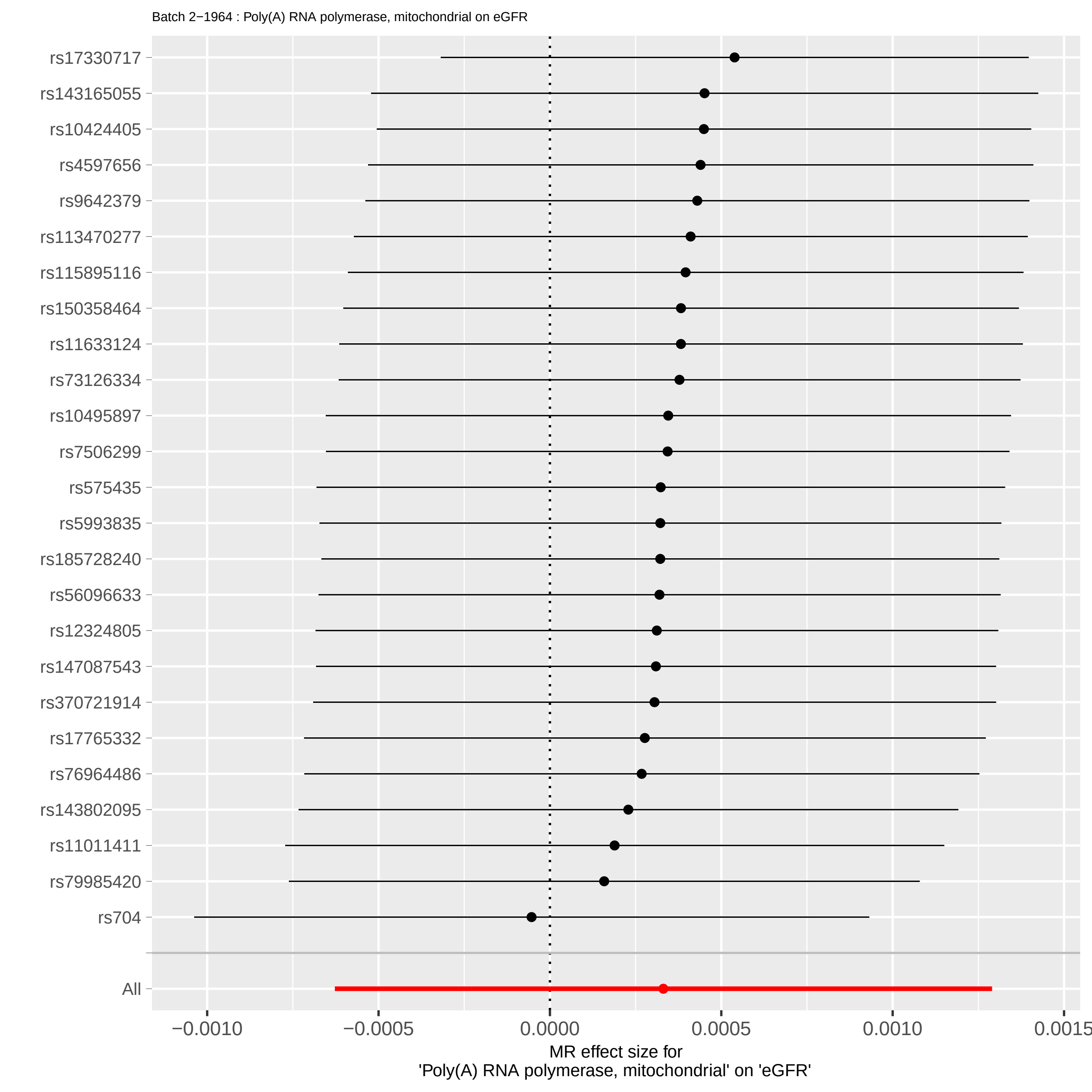

Batch 2−1964 : Poly(A) RNA polymerase, mitochondrial on eGFR
rs17330717
rs143165055
rs10424405
rs4597656
rs9642379
rs113470277
rs115895116
rs150358464
rs11633124
rs73126334
rs10495897
rs7506299
rs575435
rs5993835
rs185728240
rs56096633
rs12324805
rs147087543
rs370721914
rs17765332
rs76964486
rs143802095
rs11011411
rs79985420
rs704
All
−0.0010
−0.0005
0.0000
0.0005
0.0010
0.0015
MR effect size for
'Poly(A) RNA polymerase, mitochondrial' on 'eGFR'

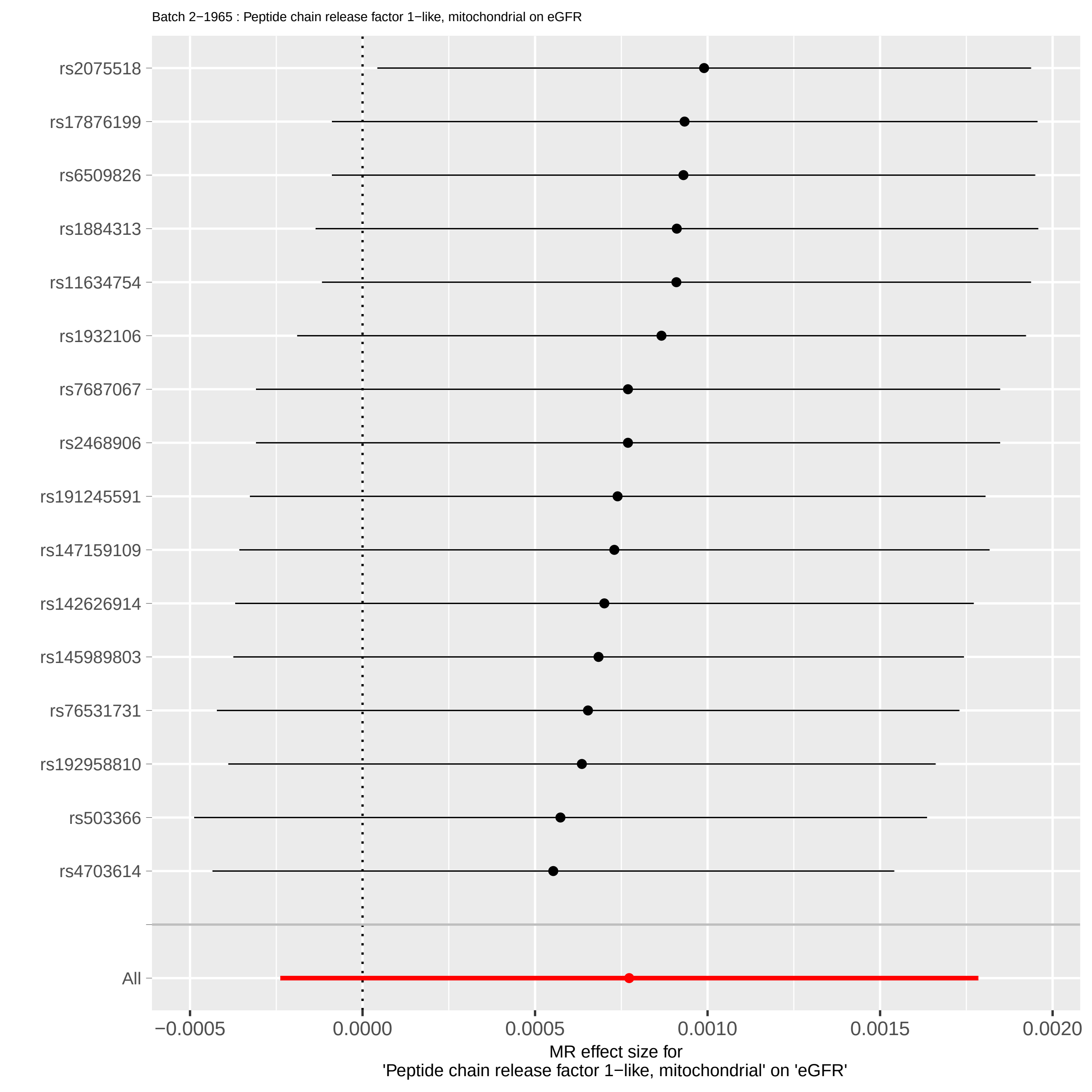

Batch 2−1965 : Peptide chain release factor 1−like, mitochondrial on eGFR
rs2075518
rs17876199
rs6509826
rs1884313
rs11634754
rs1932106
rs7687067
rs2468906
rs191245591
rs147159109
rs142626914
rs145989803
rs76531731
rs192958810
rs503366
rs4703614
All
−0.0005
0.0000
0.0005
0.0010
0.0015
0.0020
MR effect size for
'Peptide chain release factor 1−like, mitochondrial' on 'eGFR'

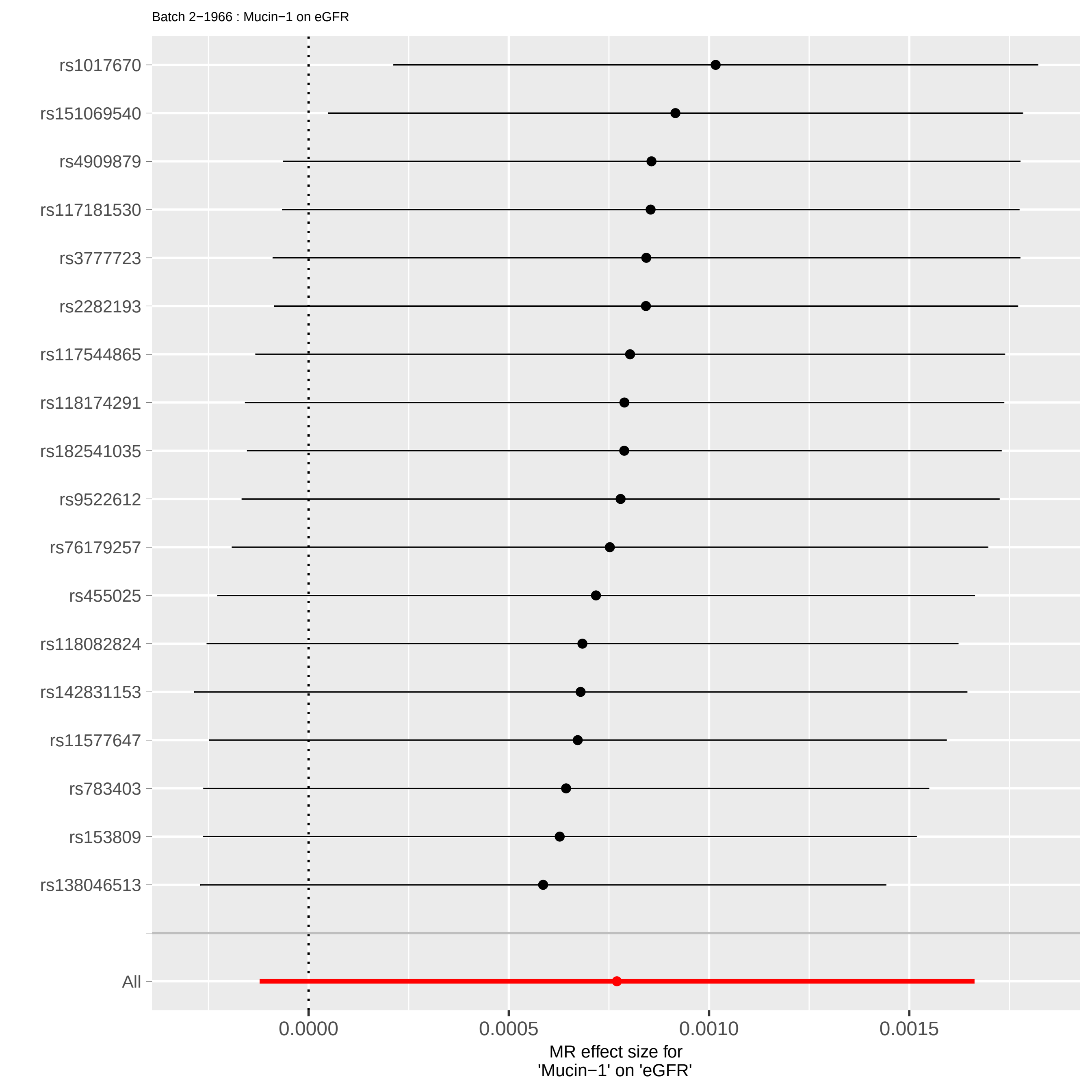
Batch 2−1966 : Mucin−1 on eGFR
rs1017670
rs151069540
rs4909879
rs117181530
rs3777723
rs2282193
rs117544865
rs118174291
rs182541035
rs9522612
rs76179257
rs455025
rs118082824
rs142831153
rs11577647
rs783403
rs153809
rs138046513
All
0.0000
0.0005
0.0010
0.0015
MR effect size for
'Mucin−1' on 'eGFR'

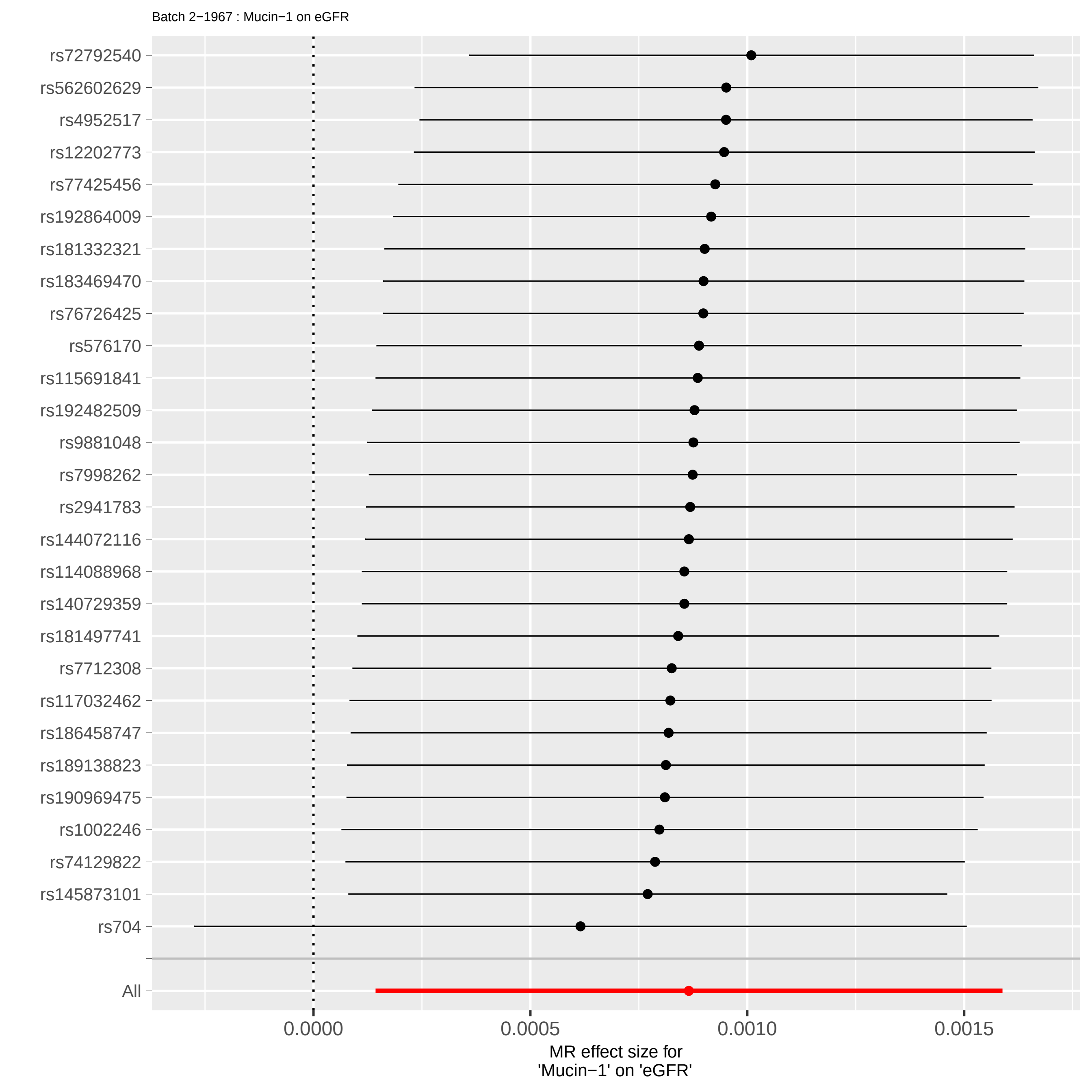

Batch 2−1967 : Mucin−1 on eGFR
rs72792540
rs562602629
rs4952517
rs12202773
rs77425456
rs192864009
rs181332321
rs183469470
rs76726425
rs576170
rs115691841
rs192482509
rs9881048
rs7998262
rs2941783
rs144072116
rs114088968
rs140729359
rs181497741
rs7712308
rs117032462
rs186458747
rs189138823
rs190969475
rs1002246
rs74129822
rs145873101
rs704
All
0.0000
0.0005
0.0010
0.0015
MR effect size for
'Mucin−1' on 'eGFR'

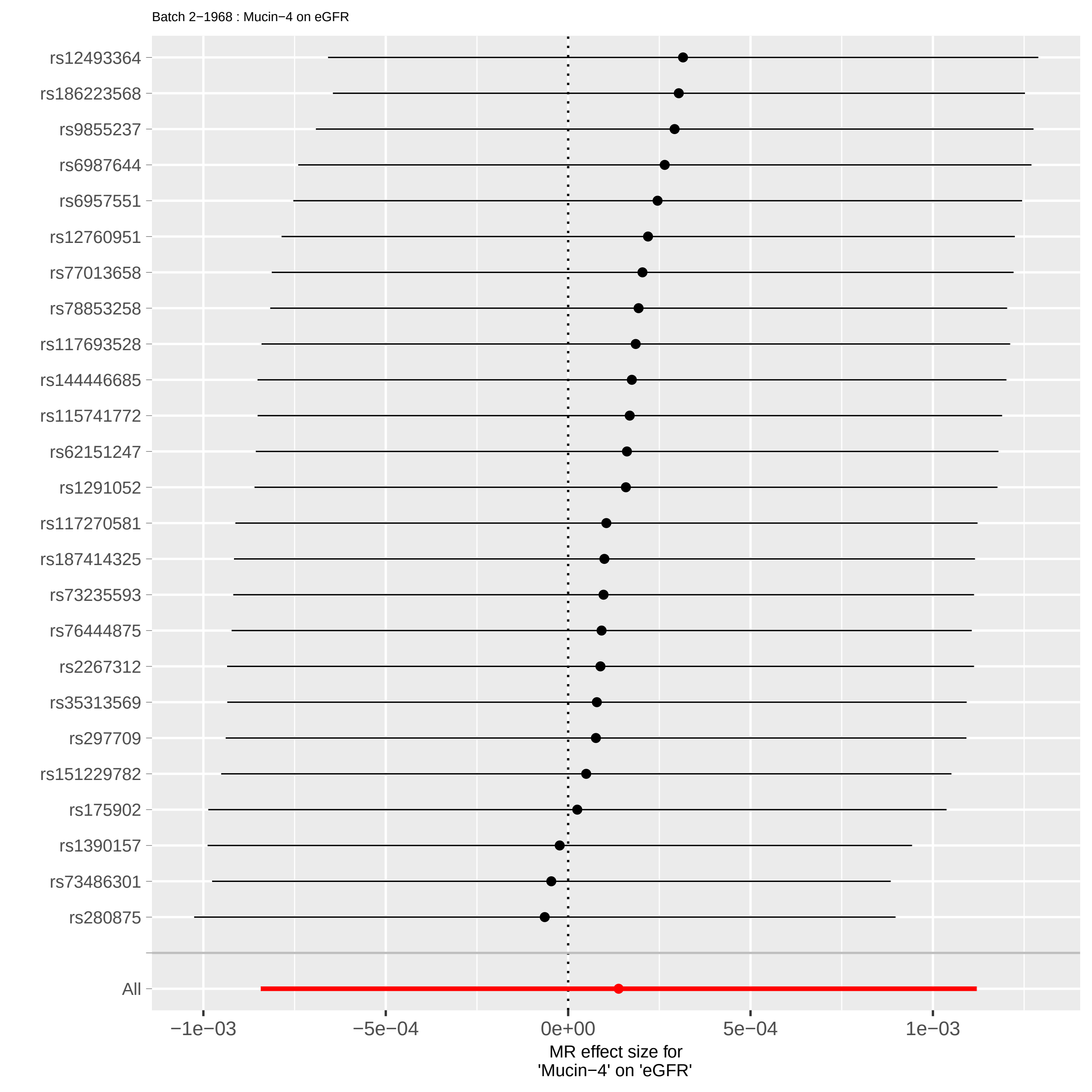

Batch 2−1968 : Mucin−4 on eGFR
rs12493364
rs186223568
rs9855237
rs6987644
rs6957551
rs12760951
rs77013658
rs78853258
rs117693528
rs144446685
rs115741772
rs62151247
rs1291052
rs117270581
rs187414325
rs73235593
rs76444875
rs2267312
rs35313569
rs297709
rs151229782
rs175902
rs1390157
rs73486301
rs280875
All
−1e−03
−5e−04
0e+00
5e−04
1e−03
MR effect size for
'Mucin−4' on 'eGFR'

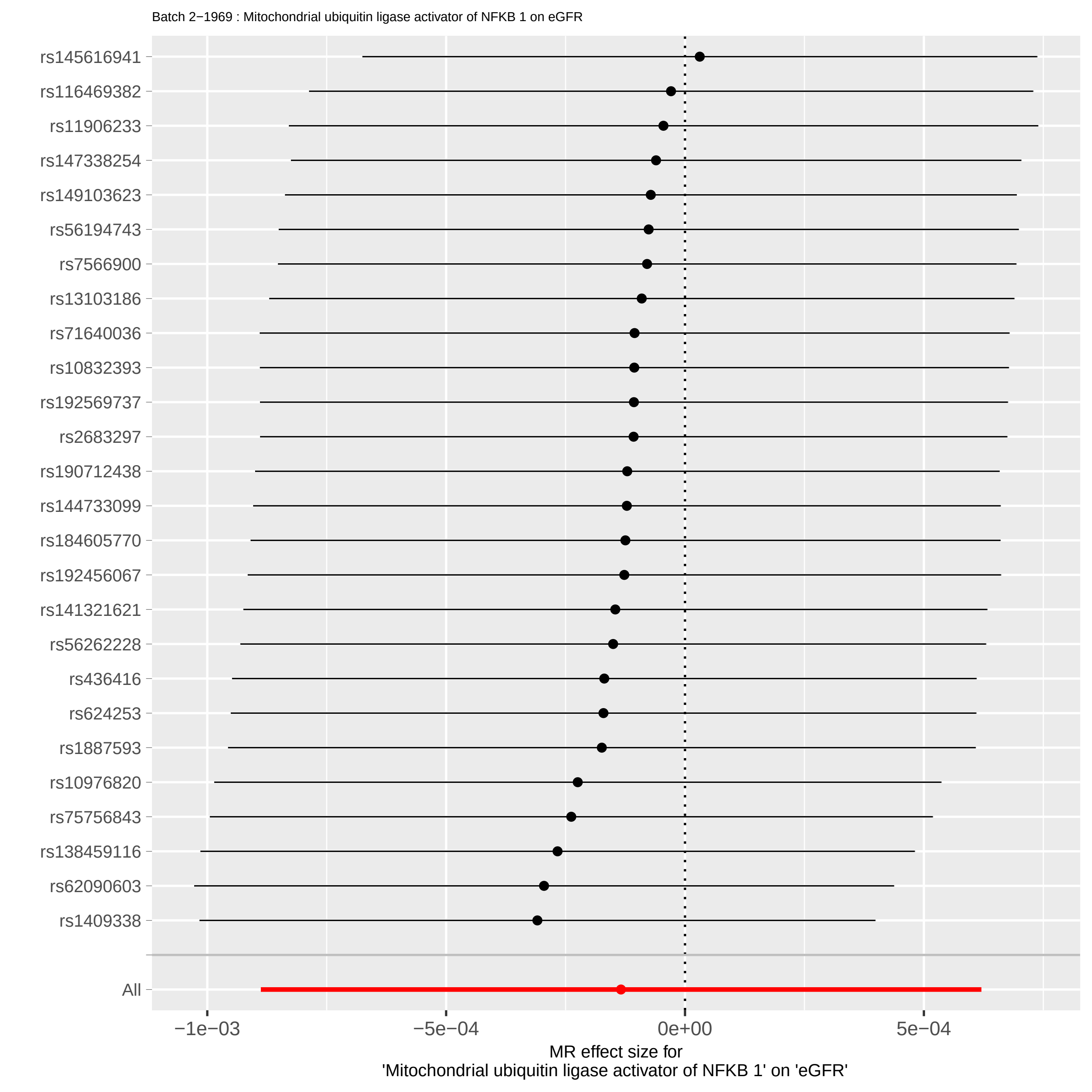

Batch 2−1969 : Mitochondrial ubiquitin ligase activator of NFKB 1 on eGFR
rs145616941
rs116469382
rs11906233
rs147338254
rs149103623
rs56194743
rs7566900
rs13103186
rs71640036
rs10832393
rs192569737
rs2683297
rs190712438
rs144733099
rs184605770
rs192456067
rs141321621
rs56262228
rs436416
rs624253
rs1887593
rs10976820
rs75756843
rs138459116
rs62090603
rs1409338
All
−1e−03
−5e−04
0e+00
5e−04
MR effect size for
'Mitochondrial ubiquitin ligase activator of NFKB 1' on 'eGFR'

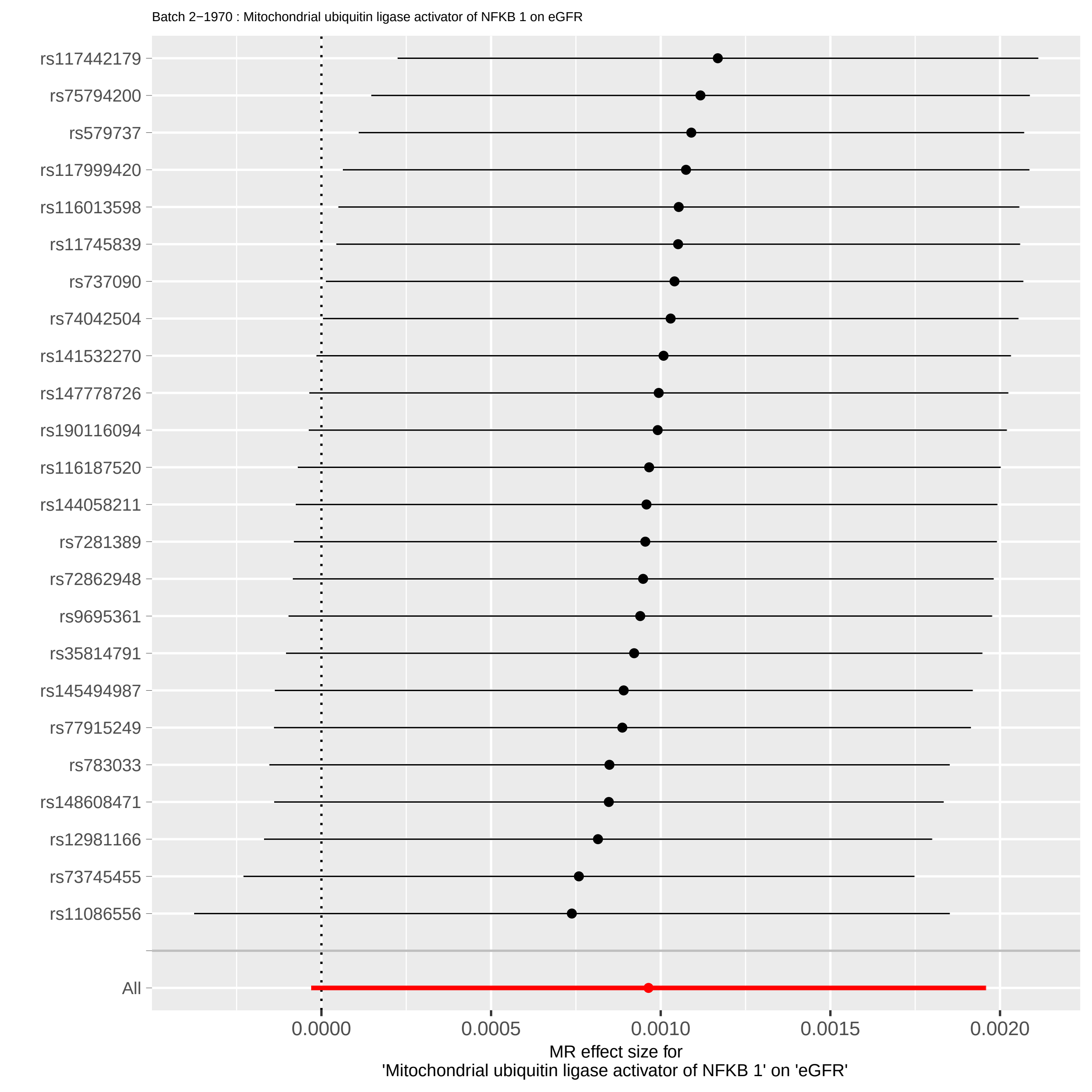

Batch 2−1970 : Mitochondrial ubiquitin ligase activator of NFKB 1 on eGFR
rs117442179
rs75794200
rs579737
rs117999420
rs116013598
rs11745839
rs737090
rs74042504
rs141532270
rs147778726
rs190116094
rs116187520
rs144058211
rs7281389
rs72862948
rs9695361
rs35814791
rs145494987
rs77915249
rs783033
rs148608471
rs12981166
rs73745455
rs11086556
All
0.0000
0.0005
0.0010
0.0015
0.0020
MR effect size for
'Mitochondrial ubiquitin ligase activator of NFKB 1' on 'eGFR'

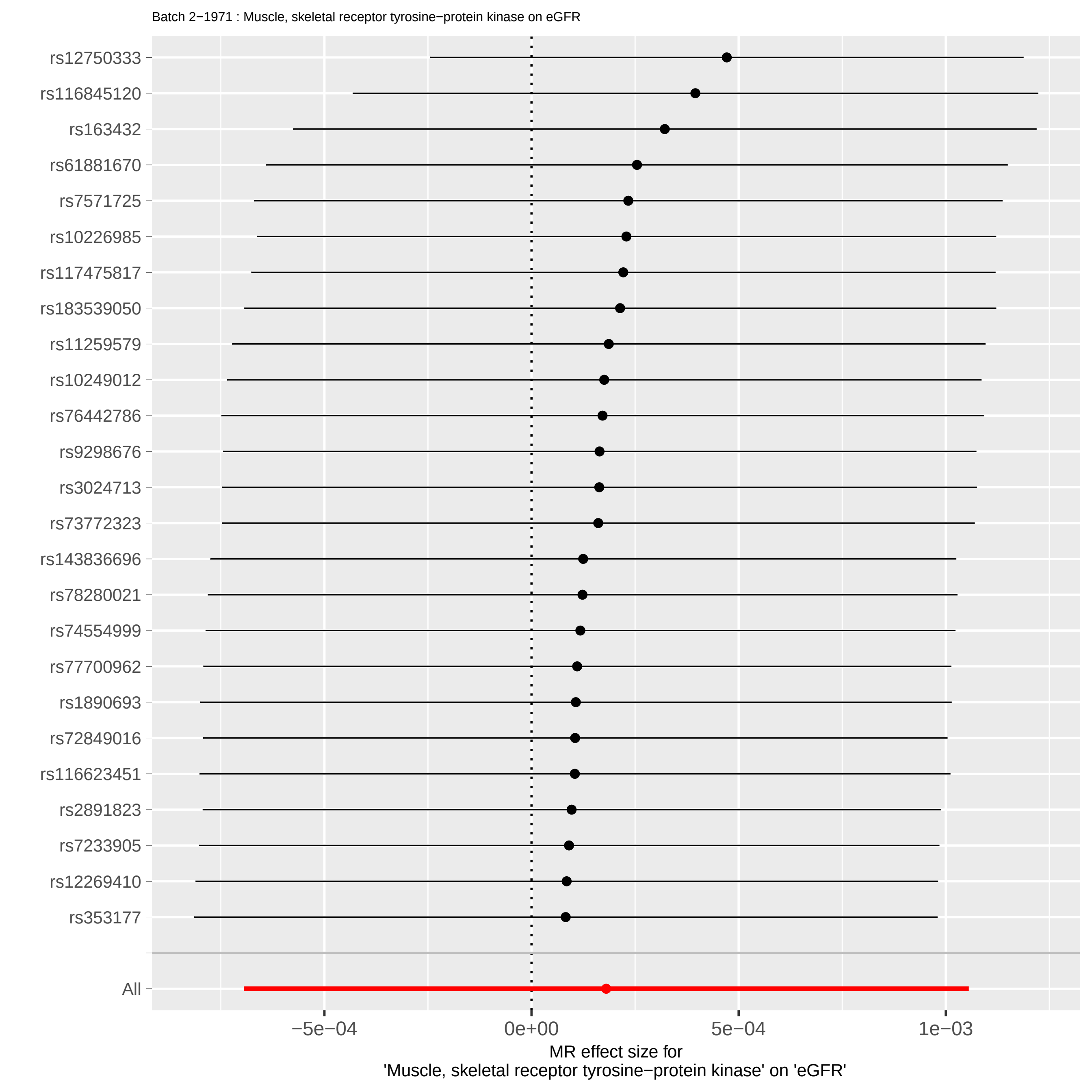

Batch 2−1971 : Muscle, skeletal receptor tyrosine−protein kinase on eGFR
rs12750333
rs116845120
rs163432
rs61881670
rs7571725
rs10226985
rs117475817
rs183539050
rs11259579
rs10249012
rs76442786
rs9298676
rs3024713
rs73772323
rs143836696
rs78280021
rs74554999
rs77700962
rs1890693
rs72849016
rs116623451
rs2891823
rs7233905
rs12269410
rs353177
All
−5e−04
0e+00
5e−04
1e−03
MR effect size for
'Muscle, skeletal receptor tyrosine−protein kinase' on 'eGFR'

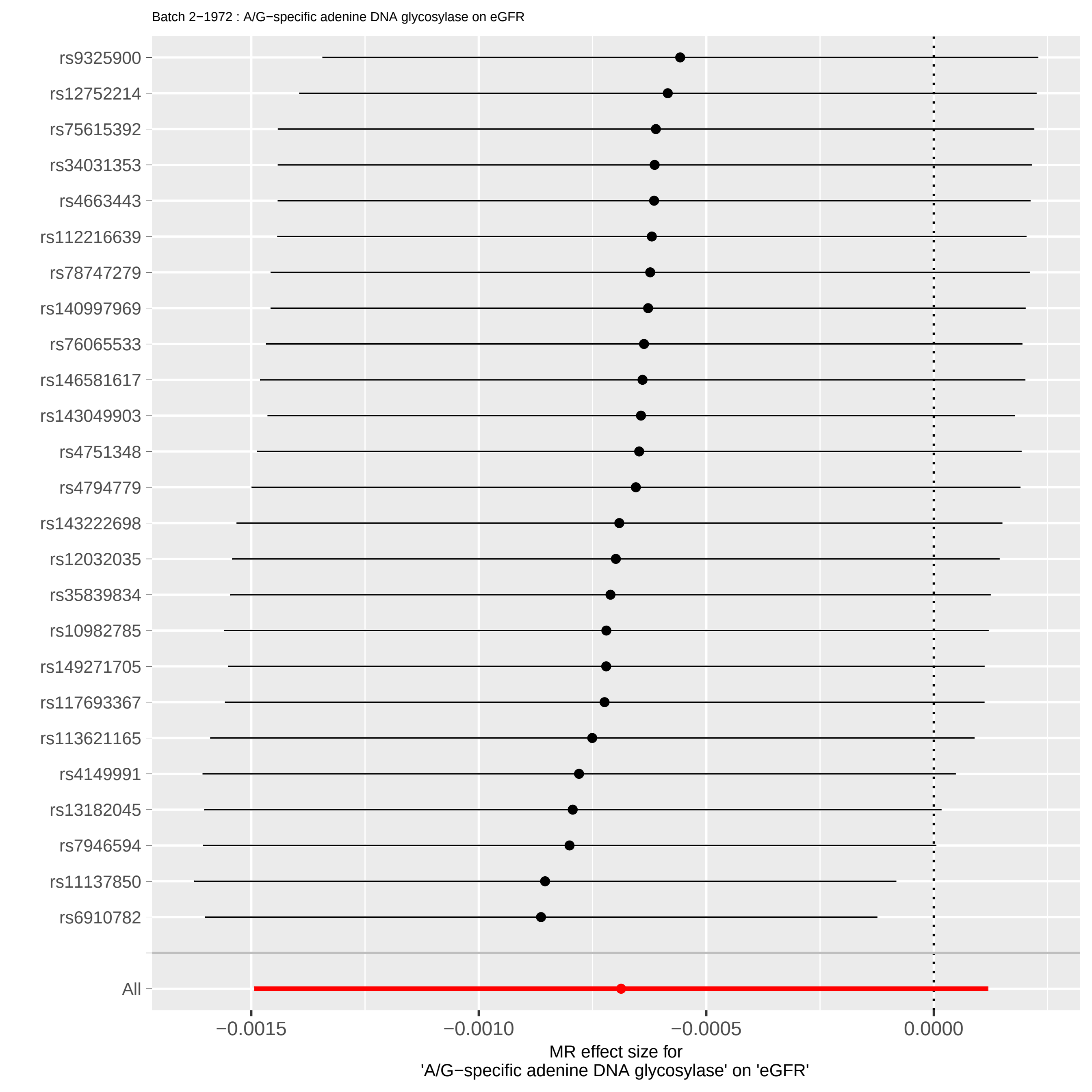

Batch 2−1972 : A/G−specific adenine DNA glycosylase on eGFR
rs9325900
rs12752214
rs75615392
rs34031353
rs4663443
rs112216639
rs78747279
rs140997969
rs76065533
rs146581617
rs143049903
rs4751348
rs4794779
rs143222698
rs12032035
rs35839834
rs10982785
rs149271705
rs117693367
rs113621165
rs4149991
rs13182045
rs7946594
rs11137850
rs6910782
All
−0.0015
−0.0010
−0.0005
0.0000
MR effect size for
'A/G−specific adenine DNA glycosylase' on 'eGFR'

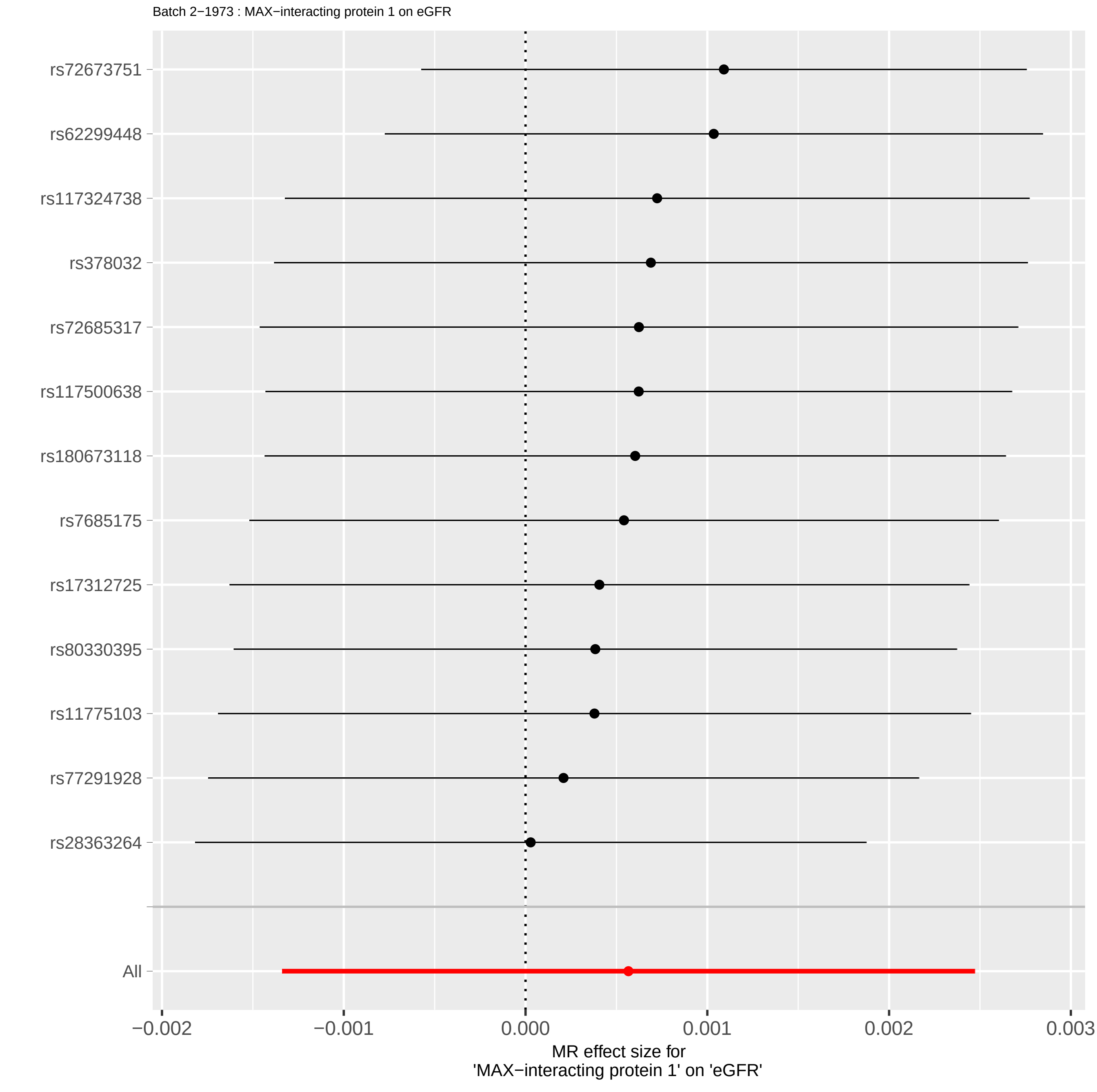

Batch 2−1973 : MAX−interacting protein 1 on eGFR
rs72673751
rs62299448
rs117324738
rs378032
rs72685317
rs117500638
rs180673118
rs7685175
rs17312725
rs80330395
rs11775103
rs77291928
rs28363264
All
−0.002
−0.001
0.000
0.001
0.002
0.003
MR effect size for
'MAX−interacting protein 1' on 'eGFR'

Batch 2−1974 : Matrix−remodeling−associated protein 7 on eGFR

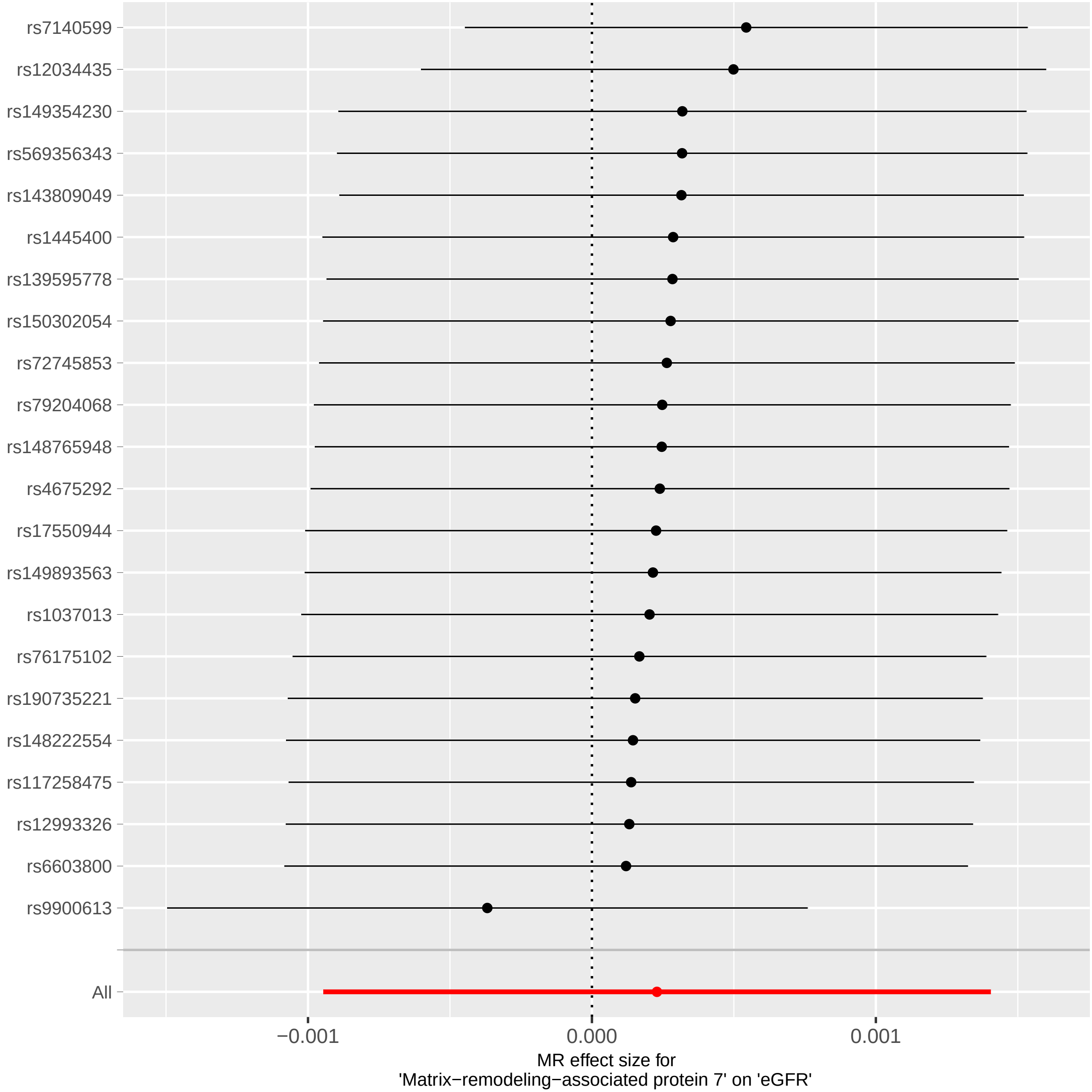

Batch 2−1975 : Matrix−remodeling−associated protein 8 on eGFR

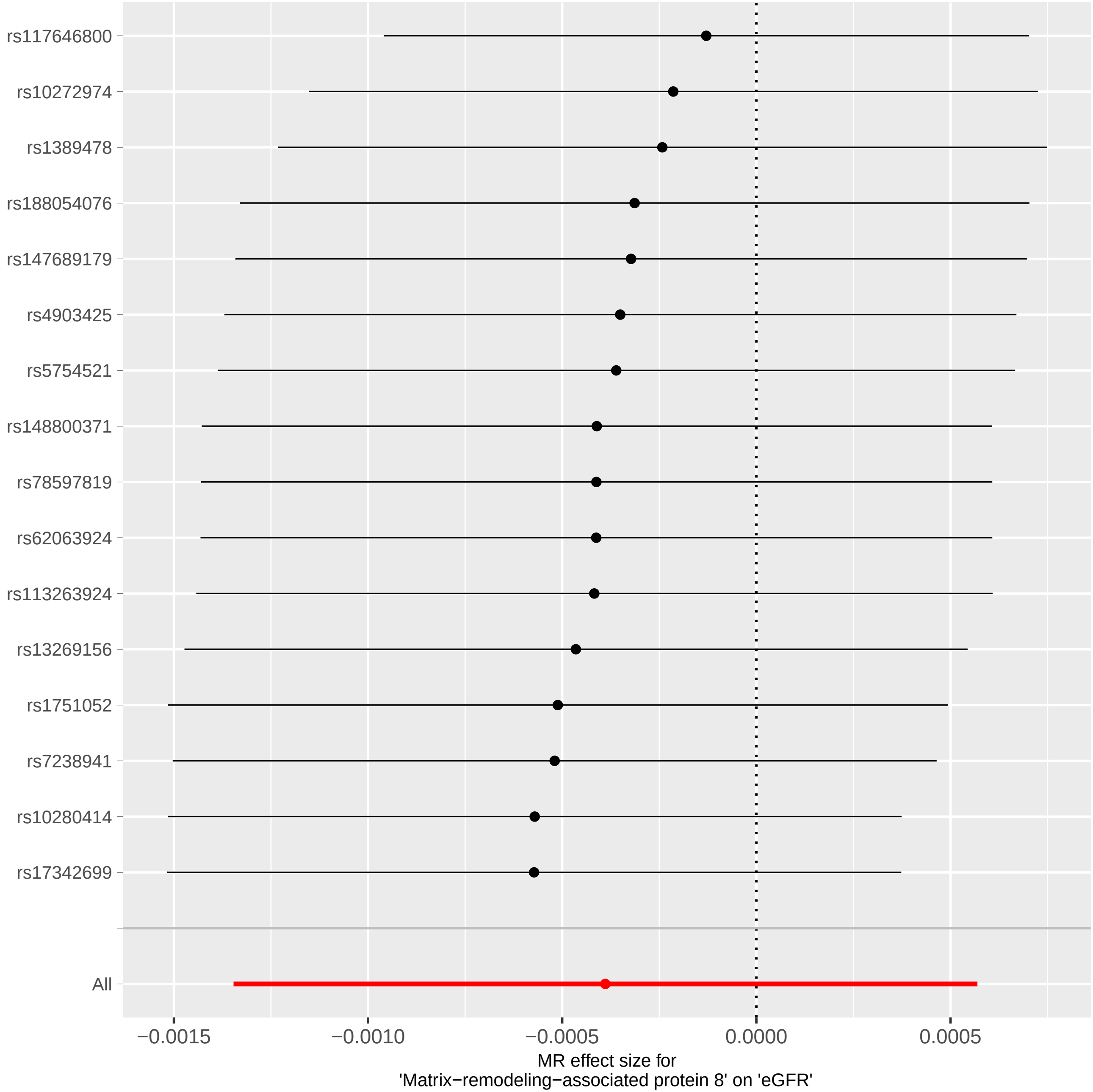

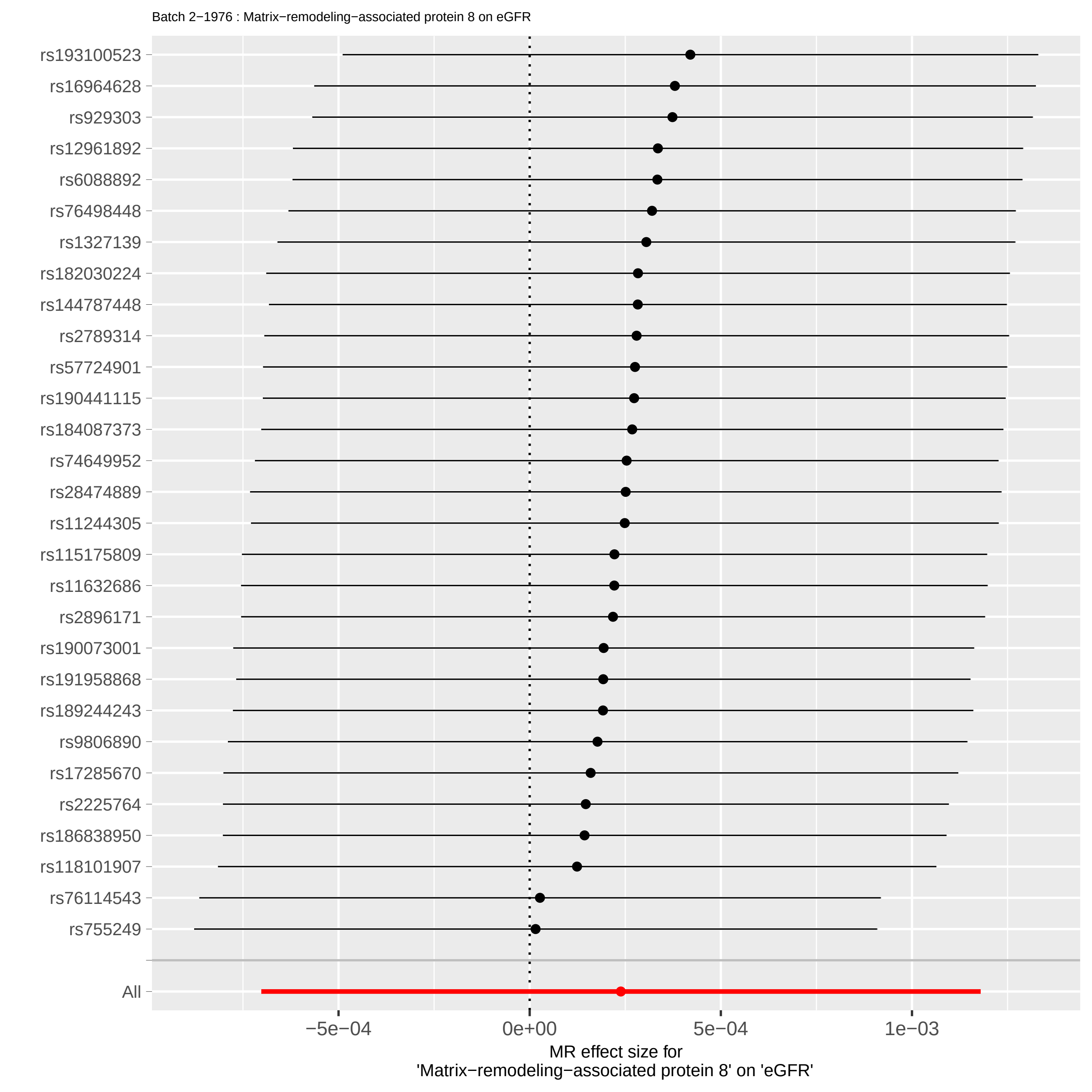

Batch 2−1976 : Matrix−remodeling−associated protein 8 on eGFR
rs193100523
rs16964628
rs929303
rs12961892
rs6088892
rs76498448
rs1327139
rs182030224
rs144787448
rs2789314
rs57724901
rs190441115
rs184087373
rs74649952
rs28474889
rs11244305
rs115175809
rs11632686
rs2896171
rs190073001
rs191958868
rs189244243
rs9806890
rs17285670
rs2225764
rs186838950
rs118101907
rs76114543
rs755249
All
−5e−04
0e+00
5e−04
1e−03
MR effect size for
'Matrix−remodeling−associated protein 8' on 'eGFR'

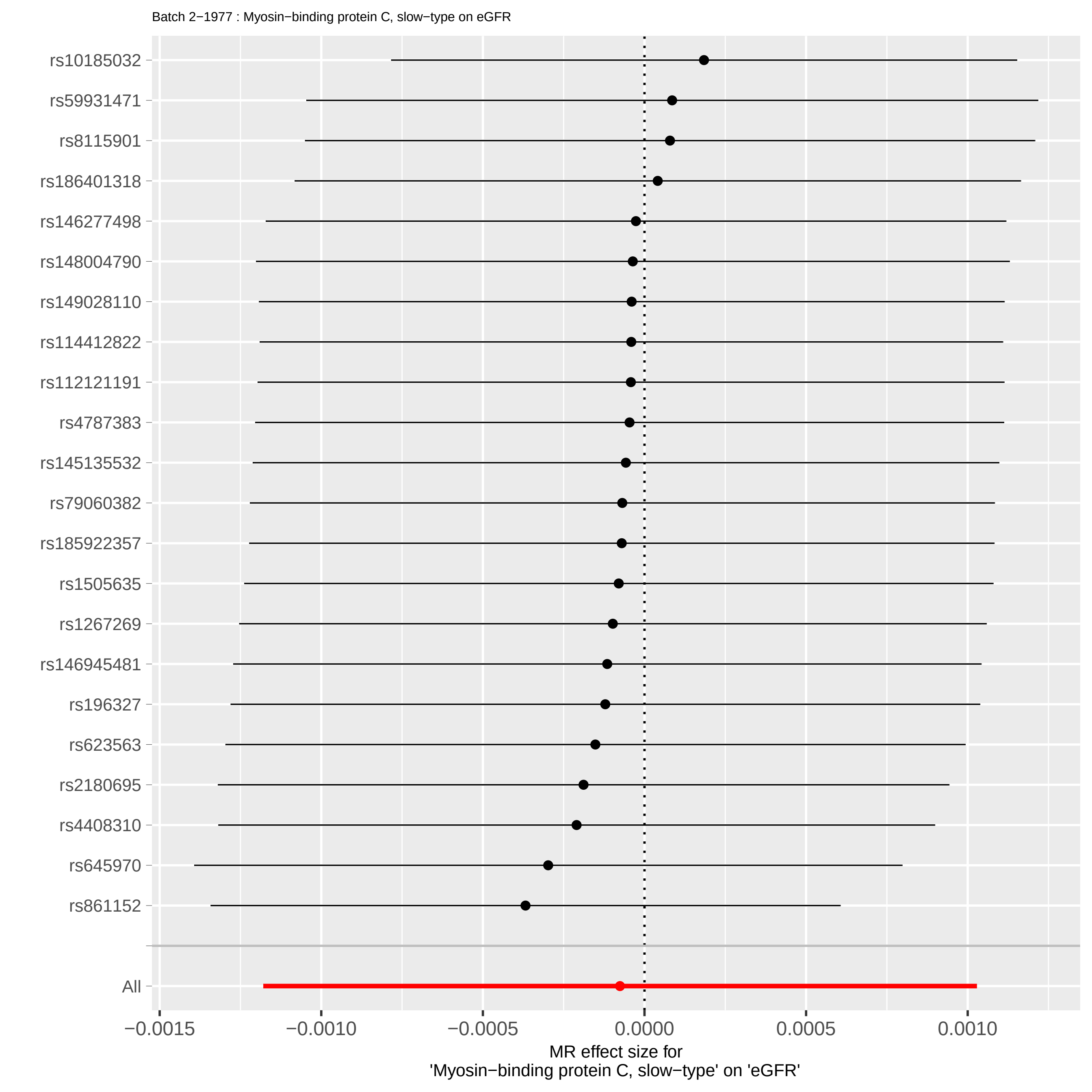
Batch 2−1977 : Myosin−binding protein C, slow−type on eGFR
rs10185032
rs59931471
rs8115901
rs186401318
rs146277498
rs148004790
rs149028110
rs114412822
rs112121191
rs4787383
rs145135532
rs79060382
rs185922357
rs1505635
rs1267269
rs146945481
rs196327
rs623563
rs2180695
rs4408310
rs645970
rs861152
All
−0.0015
−0.0010
−0.0005
0.0000
0.0005
0.0010
MR effect size for
'Myosin−binding protein C, slow−type' on 'eGFR'

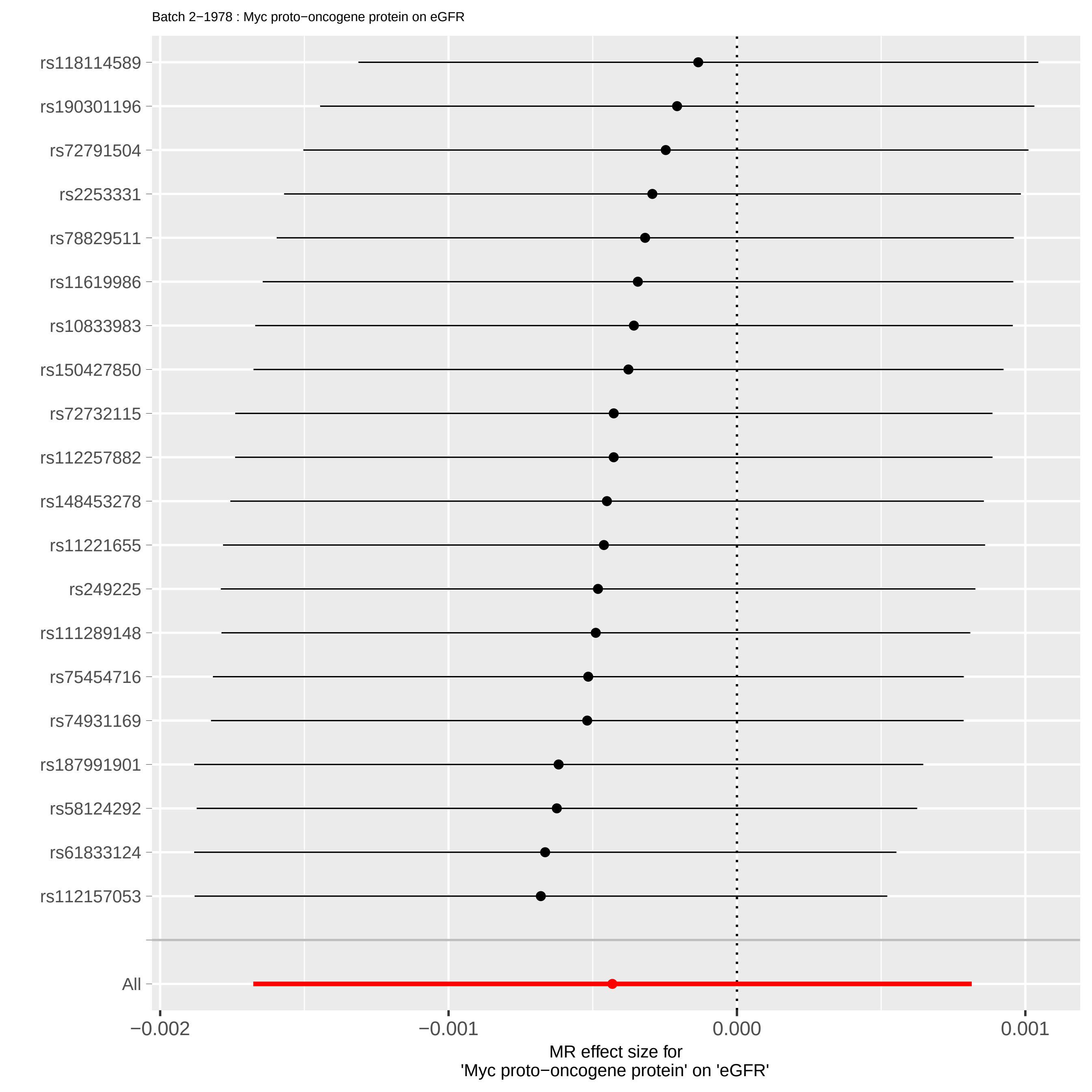

Batch 2−1978 : Myc proto−oncogene protein on eGFR
rs118114589
rs190301196
rs72791504
rs2253331
rs78829511
rs11619986
rs10833983
rs150427850
rs72732115
rs112257882
rs148453278
rs11221655
rs249225
rs111289148
rs75454716
rs74931169
rs187991901
rs58124292
rs61833124
rs112157053
All
−0.002
−0.001
0.000
0.001
MR effect size for
'Myc proto−oncogene protein' on 'eGFR'

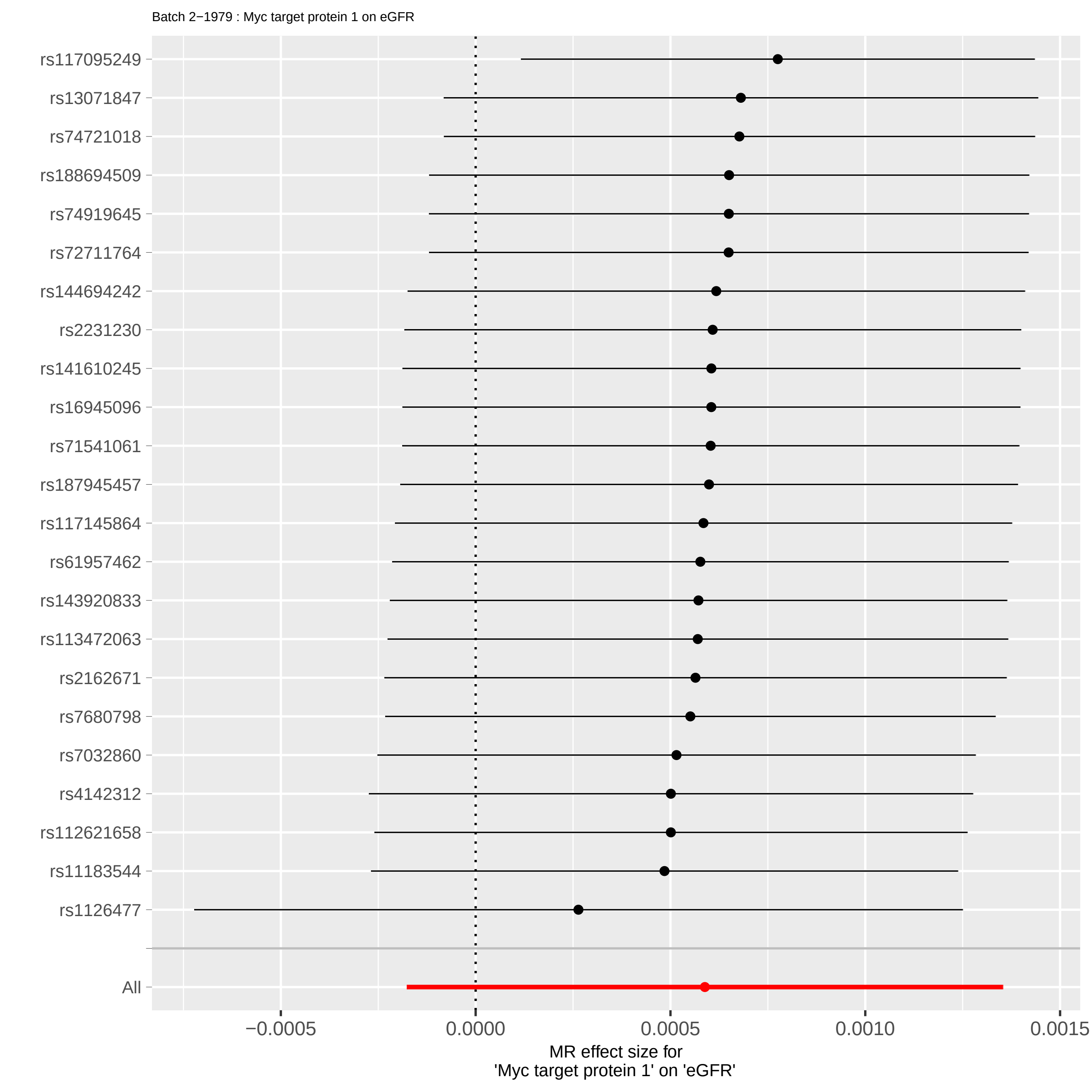

Batch 2−1979 : Myc target protein 1 on eGFR
rs117095249
rs13071847
rs74721018
rs188694509
rs74919645
rs72711764
rs144694242
rs2231230
rs141610245
rs16945096
rs71541061
rs187945457
rs117145864
rs61957462
rs143920833
rs113472063
rs2162671
rs7680798
rs7032860
rs4142312
rs112621658
rs11183544
rs1126477
All
−0.0005
0.0000
0.0005
0.0010
0.0015
MR effect size for
'Myc target protein 1' on 'eGFR'

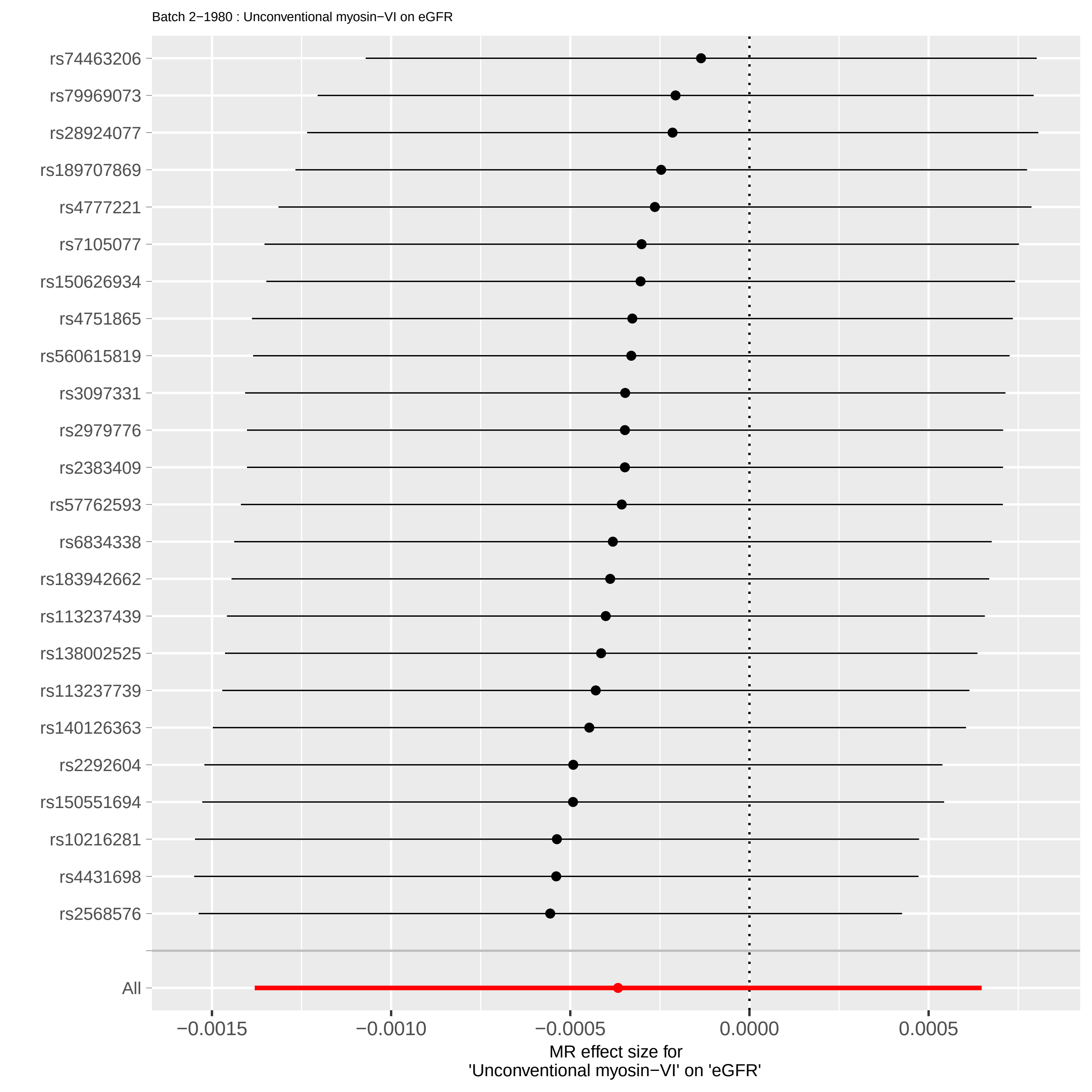

Batch 2−1980 : Unconventional myosin−VI on eGFR
rs74463206
rs79969073
rs28924077
rs189707869
rs4777221
rs7105077
rs150626934
rs4751865
rs560615819
rs3097331
rs2979776
rs2383409
rs57762593
rs6834338
rs183942662
rs113237439
rs138002525
rs113237739
rs140126363
rs2292604
rs150551694
rs10216281
rs4431698
rs2568576
All
−0.0015
−0.0010
−0.0005
0.0000
0.0005
MR effect size for
'Unconventional myosin−VI' on 'eGFR'

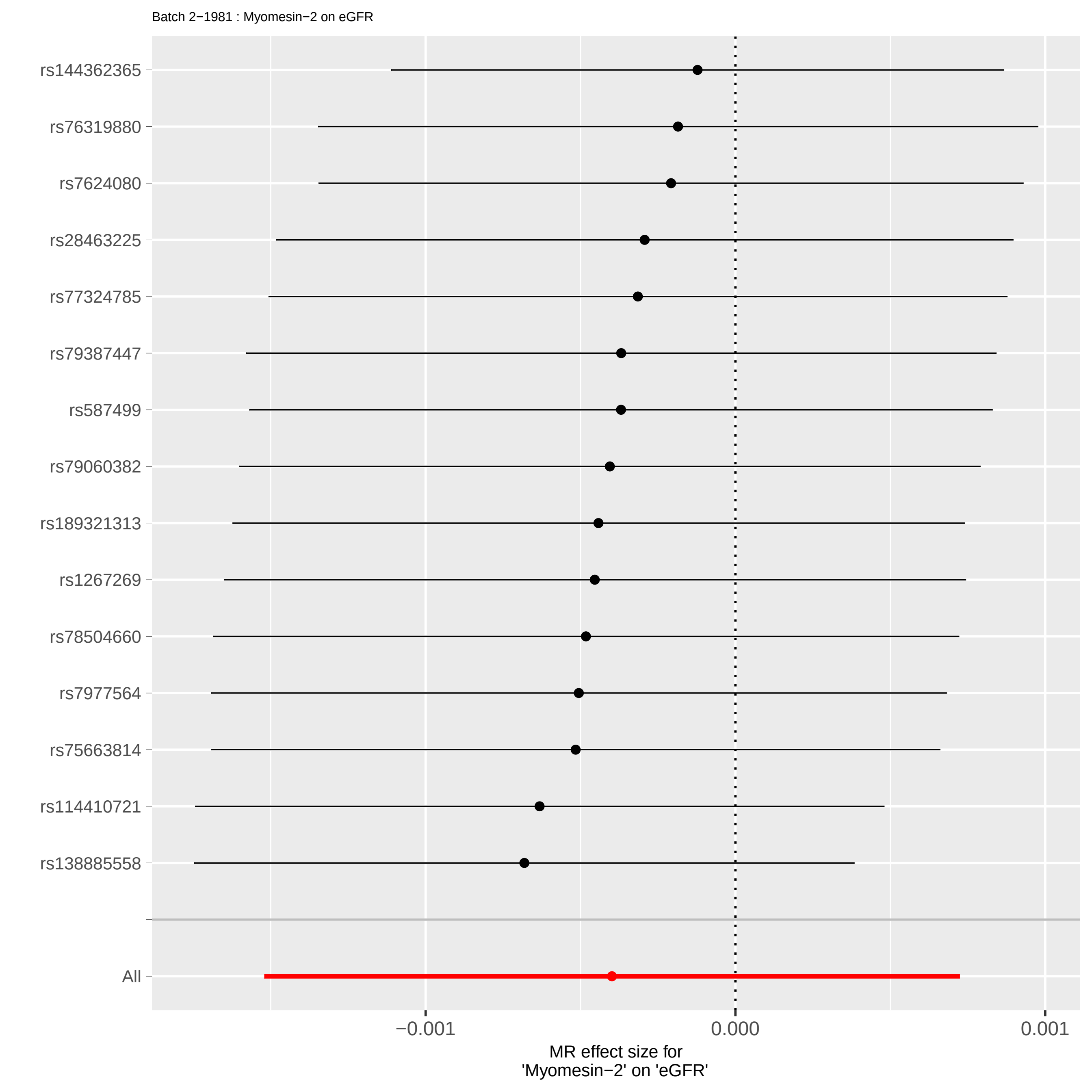
Batch 2−1981 : Myomesin−2 on eGFR
rs144362365
rs76319880
rs7624080
rs28463225
rs77324785
rs79387447
rs587499
rs79060382
rs189321313
rs1267269
rs78504660
rs7977564
rs75663814
rs114410721
rs138885558
All
−0.001
0.000
0.001
MR effect size for
'Myomesin−2' on 'eGFR'

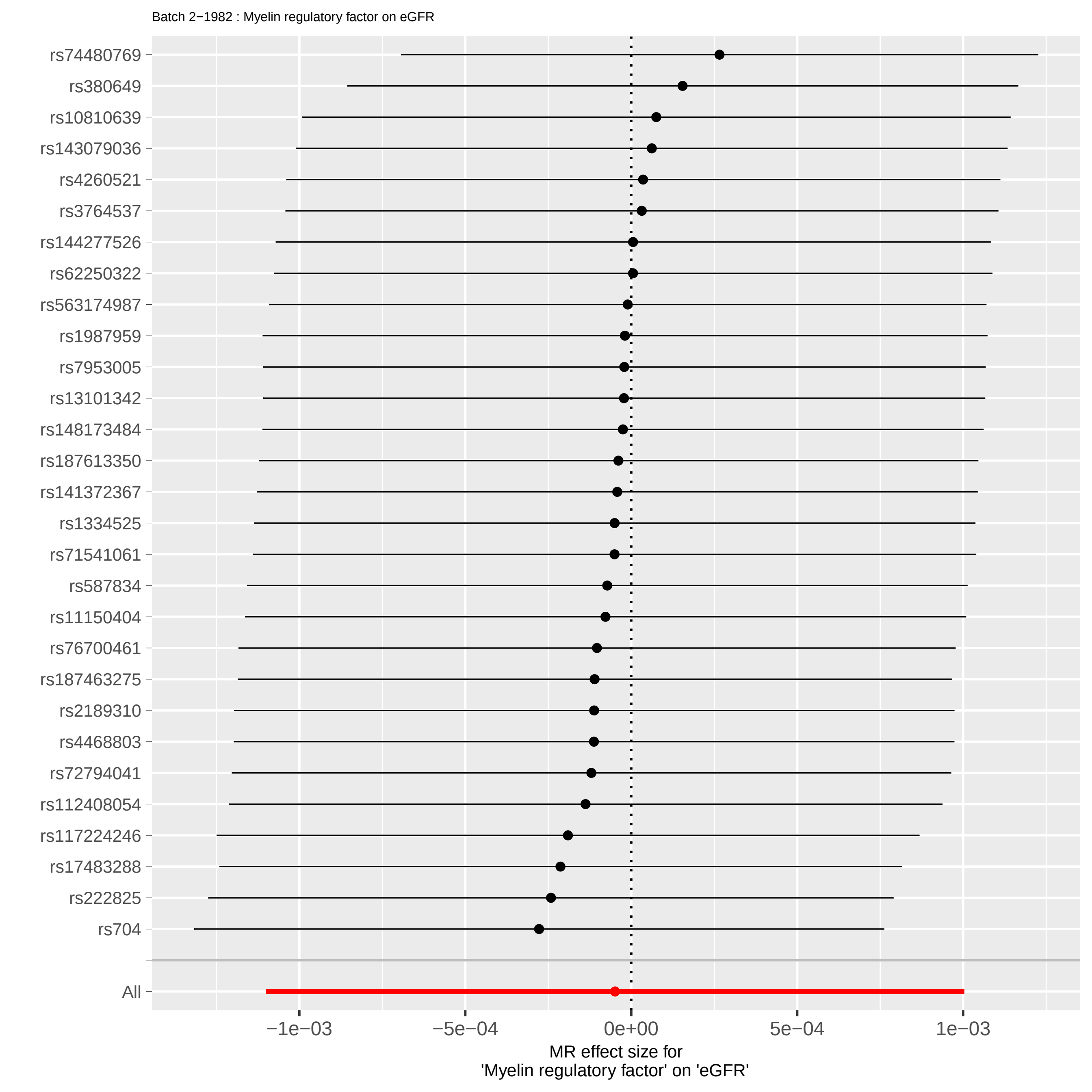
Batch 2−1982 : Myelin regulatory factor on eGFR
rs74480769
rs380649
rs10810639
rs143079036
rs4260521
rs3764537
rs144277526
rs62250322
rs563174987
rs1987959
rs7953005
rs13101342
rs148173484
rs187613350
rs141372367
rs1334525
rs71541061
rs587834
rs11150404
rs76700461
rs187463275
rs2189310
rs4468803
rs72794041
rs112408054
rs117224246
rs17483288
rs222825
rs704
All
−1e−03
−5e−04
0e+00
5e−04
1e−03
MR effect size for
'Myelin regulatory factor' on 'eGFR'

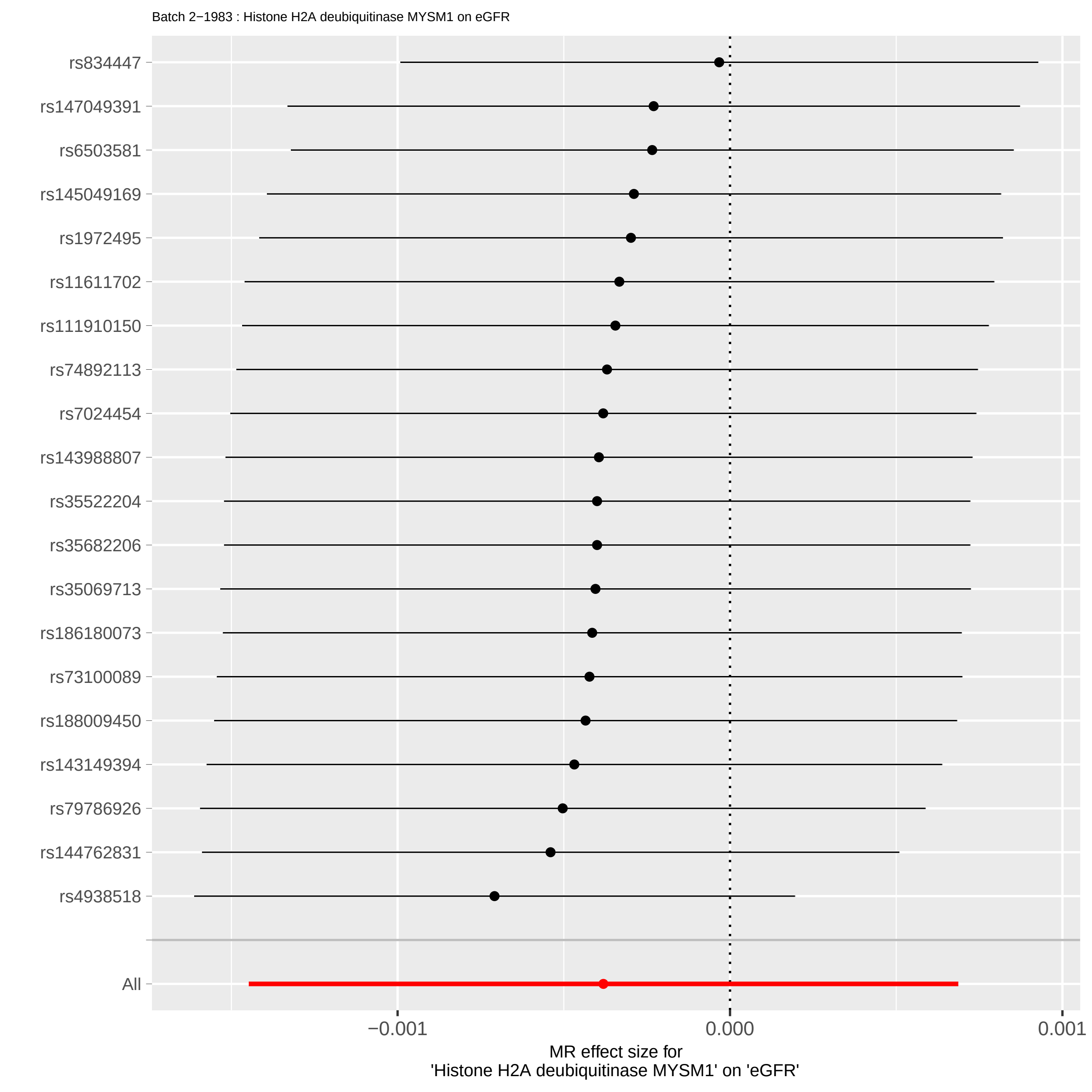

Batch 2−1983 : Histone H2A deubiquitinase MYSM1 on eGFR
rs834447
rs147049391
rs6503581
rs145049169
rs1972495
rs11611702
rs111910150
rs74892113
rs7024454
rs143988807
rs35522204
rs35682206
rs35069713
rs186180073
rs73100089
rs188009450
rs143149394
rs79786926
rs144762831
rs4938518
All
−0.001
0.000
0.001
MR effect size for
'Histone H2A deubiquitinase MYSM1' on 'eGFR'

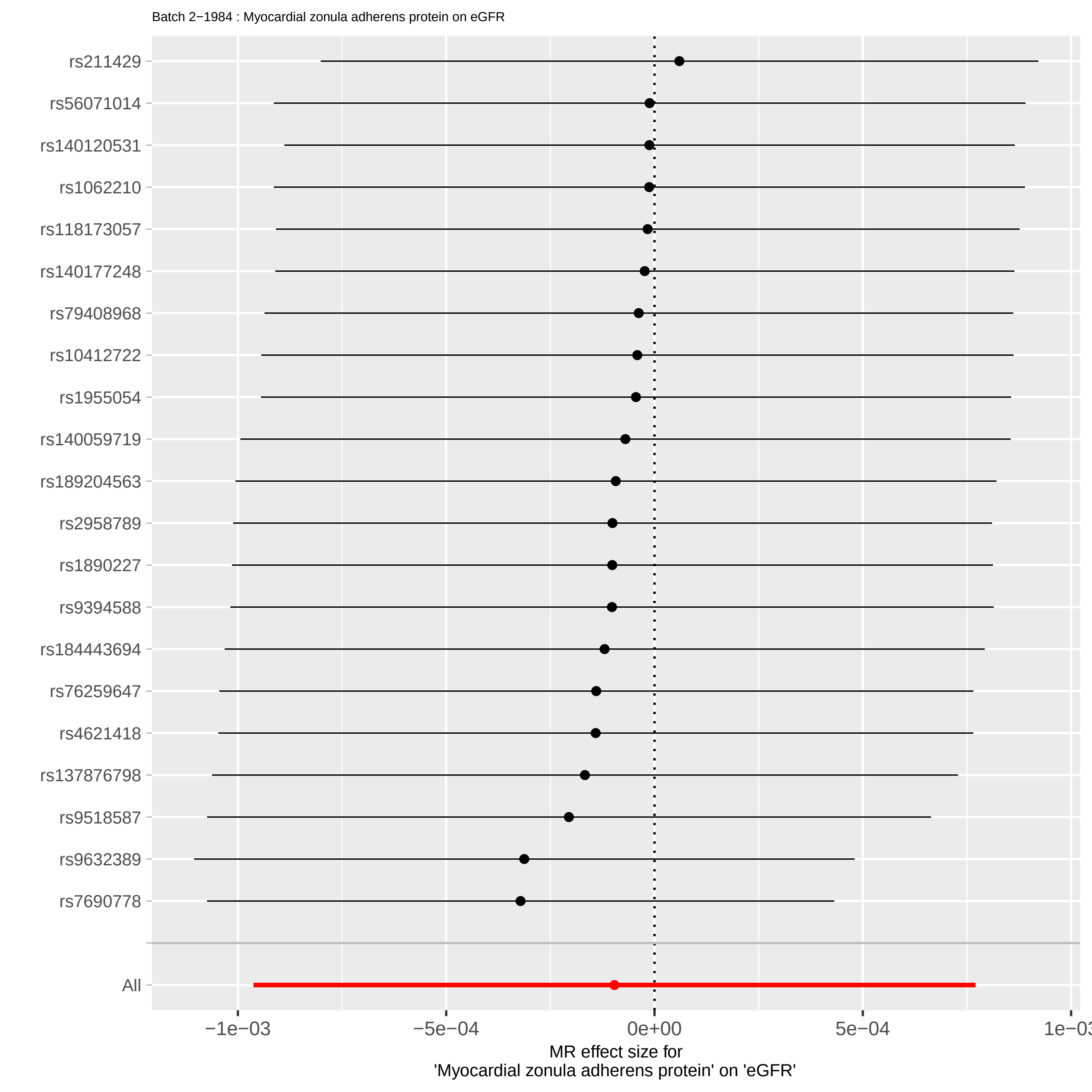
Batch 2−1984 : Myocardial zonula adherens protein on eGFR
rs211429
rs56071014
rs140120531
rs1062210
rs118173057
rs140177248
rs79408968
rs10412722
rs1955054
rs140059719
rs189204563
rs2958789
rs1890227
rs9394588
rs184443694
rs76259647
rs4621418
rs137876798
rs9518587
rs9632389
rs7690778
All
−1e−03
−5e−04
0e+00
5e−04
MR effect size for
'Myocardial zonula adherens protein' on 'eGFR'

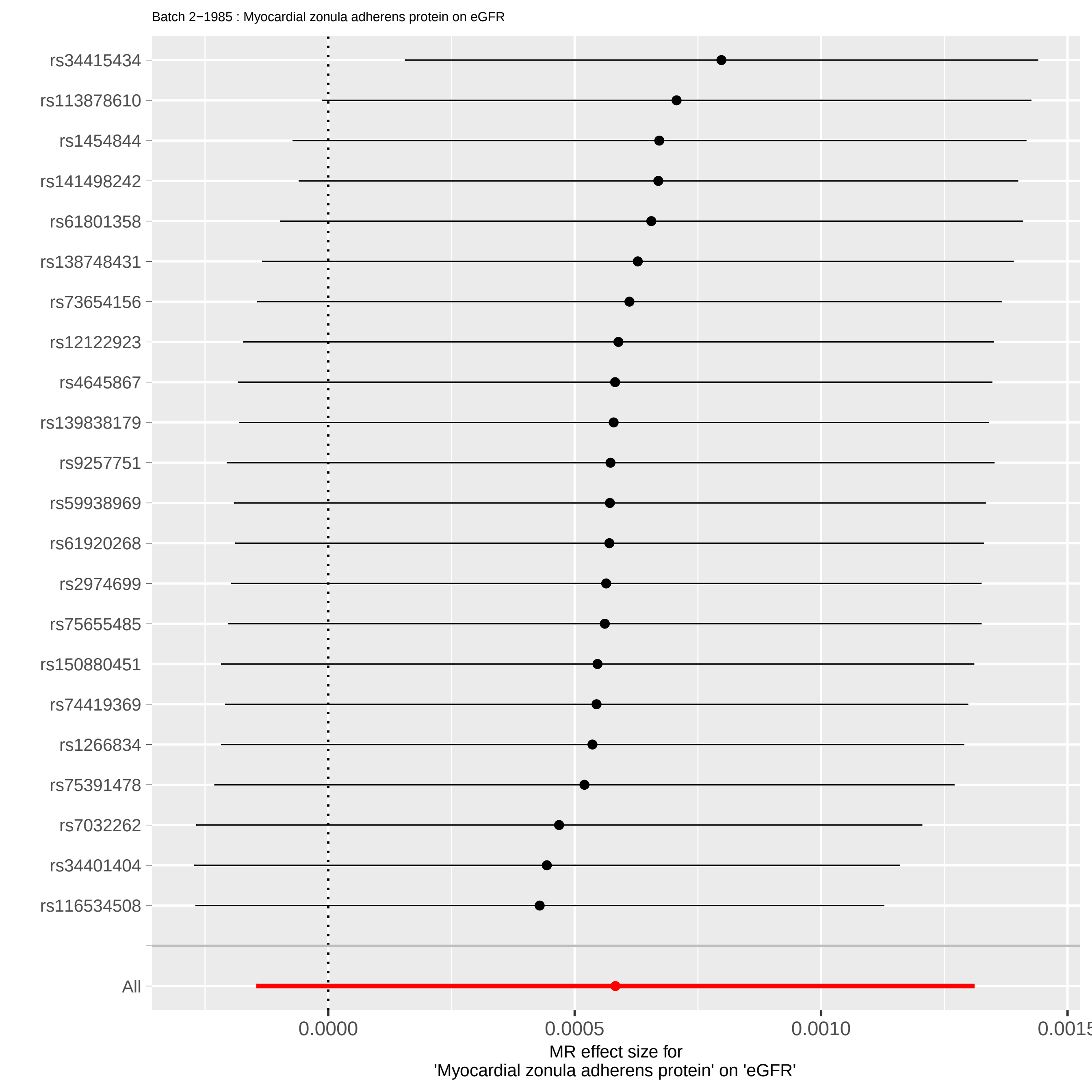
Batch 2−1985 : Myocardial zonula adherens protein on eGFR
rs34415434
rs113878610
rs1454844
rs141498242
rs61801358
rs138748431
rs73654156
rs12122923
rs4645867
rs139838179
rs9257751
rs59938969
rs61920268
rs2974699
rs75655485
rs150880451
rs74419369
rs1266834
rs75391478
rs7032262
rs34401404
rs116534508
All
0.0000
0.0005
0.0010
0.0015
MR effect size for
'Myocardial zonula adherens protein' on 'eGFR'

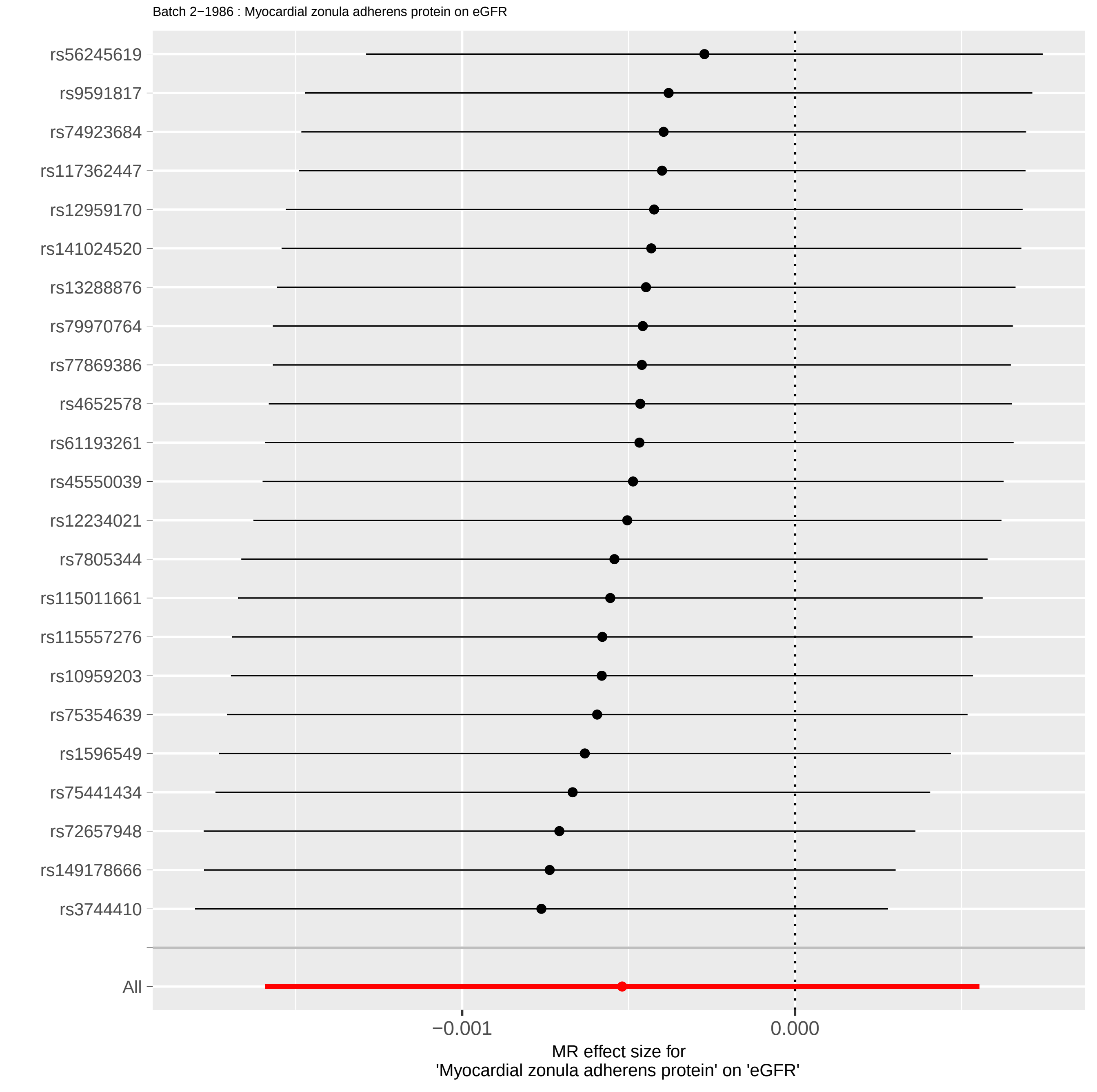
Batch 2−1986 : Myocardial zonula adherens protein on eGFR
rs56245619
rs9591817
rs74923684
rs117362447
rs12959170
rs141024520
rs13288876
rs79970764
rs77869386
rs4652578
rs61193261
rs45550039
rs12234021
rs7805344
rs115011661
rs115557276
rs10959203
rs75354639
rs1596549
rs75441434
rs72657948
rs149178666
rs3744410
All
−0.001
0.000
MR effect size for
'Myocardial zonula adherens protein' on 'eGFR'

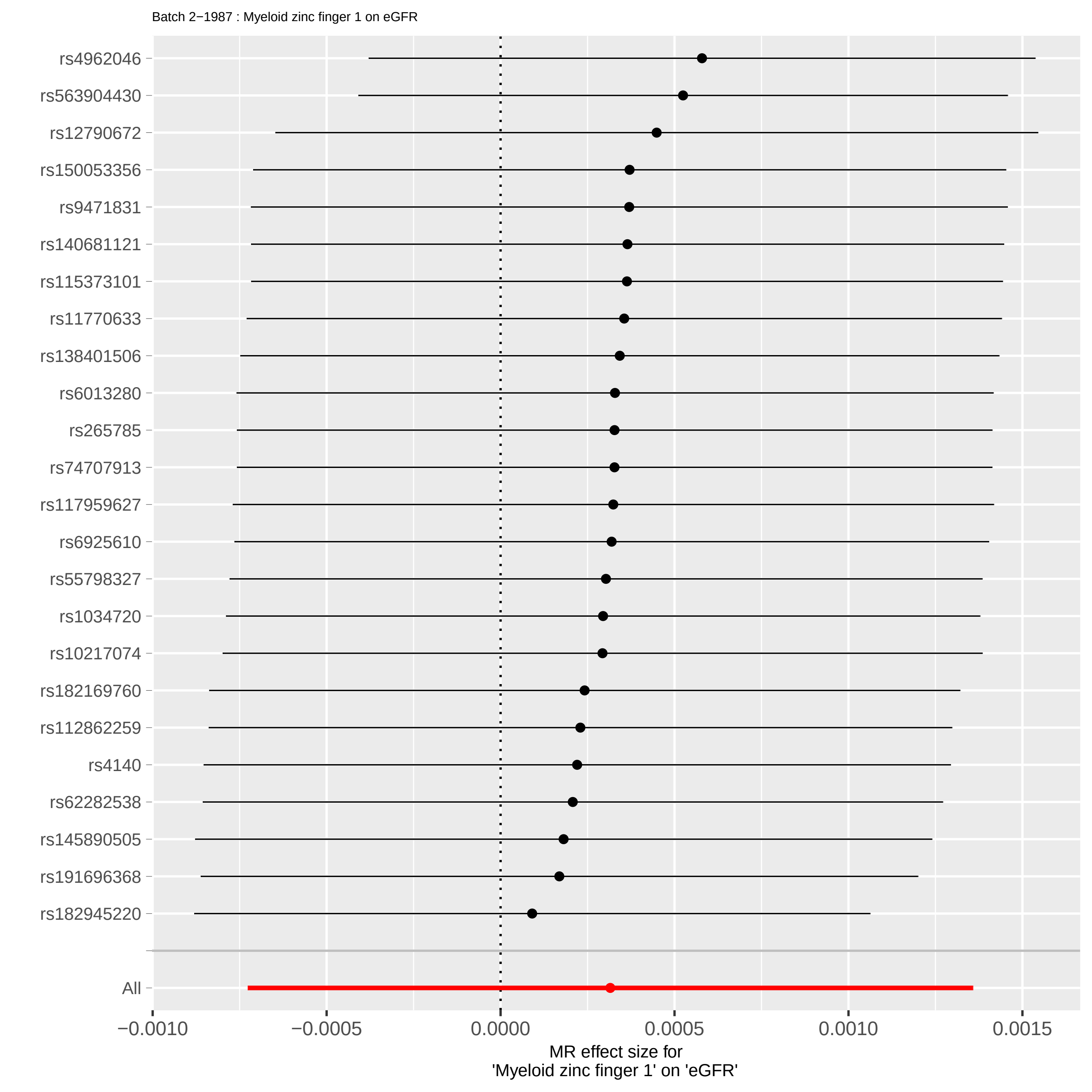

Batch 2−1987 : Myeloid zinc finger 1 on eGFR
rs4962046
rs563904430
rs12790672
rs150053356
rs9471831
rs140681121
rs115373101
rs11770633
rs138401506
rs6013280
rs265785
rs74707913
rs117959627
rs6925610
rs55798327
rs1034720
rs10217074
rs182169760
rs112862259
rs4140
rs62282538
rs145890505
rs191696368
rs182945220
All
−0.0010
−0.0005
0.0000
0.0005
0.0010
0.0015
MR effect size for
'Myeloid zinc finger 1' on 'eGFR'

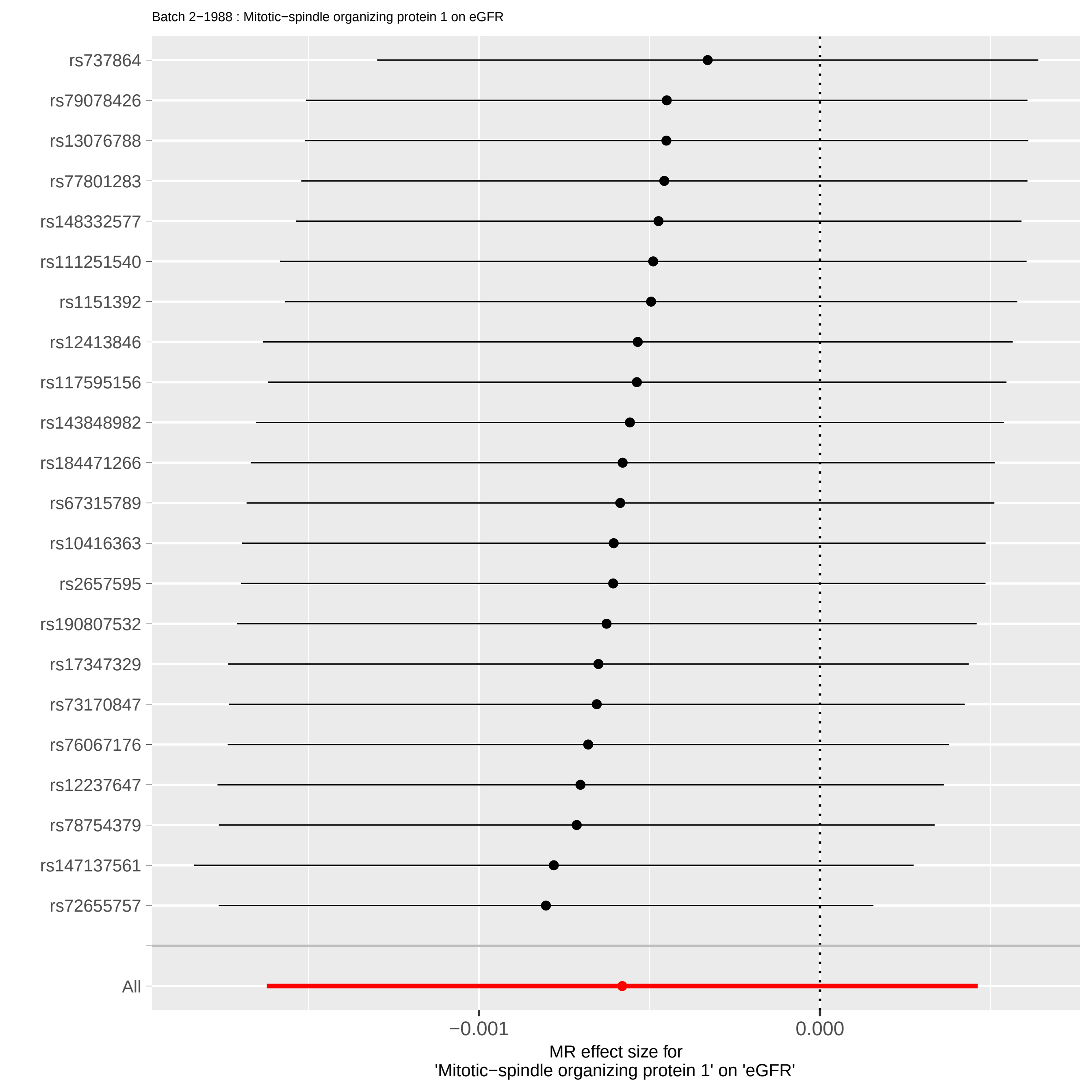

Batch 2−1988 : Mitotic−spindle organizing protein 1 on eGFR
rs737864
rs79078426
rs13076788
rs77801283
rs148332577
rs111251540
rs1151392
rs12413846
rs117595156
rs143848982
rs184471266
rs67315789
rs10416363
rs2657595
rs190807532
rs17347329
rs73170847
rs76067176
rs12237647
rs78754379
rs147137561
rs72655757
All
−0.001
0.000
MR effect size for
'Mitotic−spindle organizing protein 1' on 'eGFR'

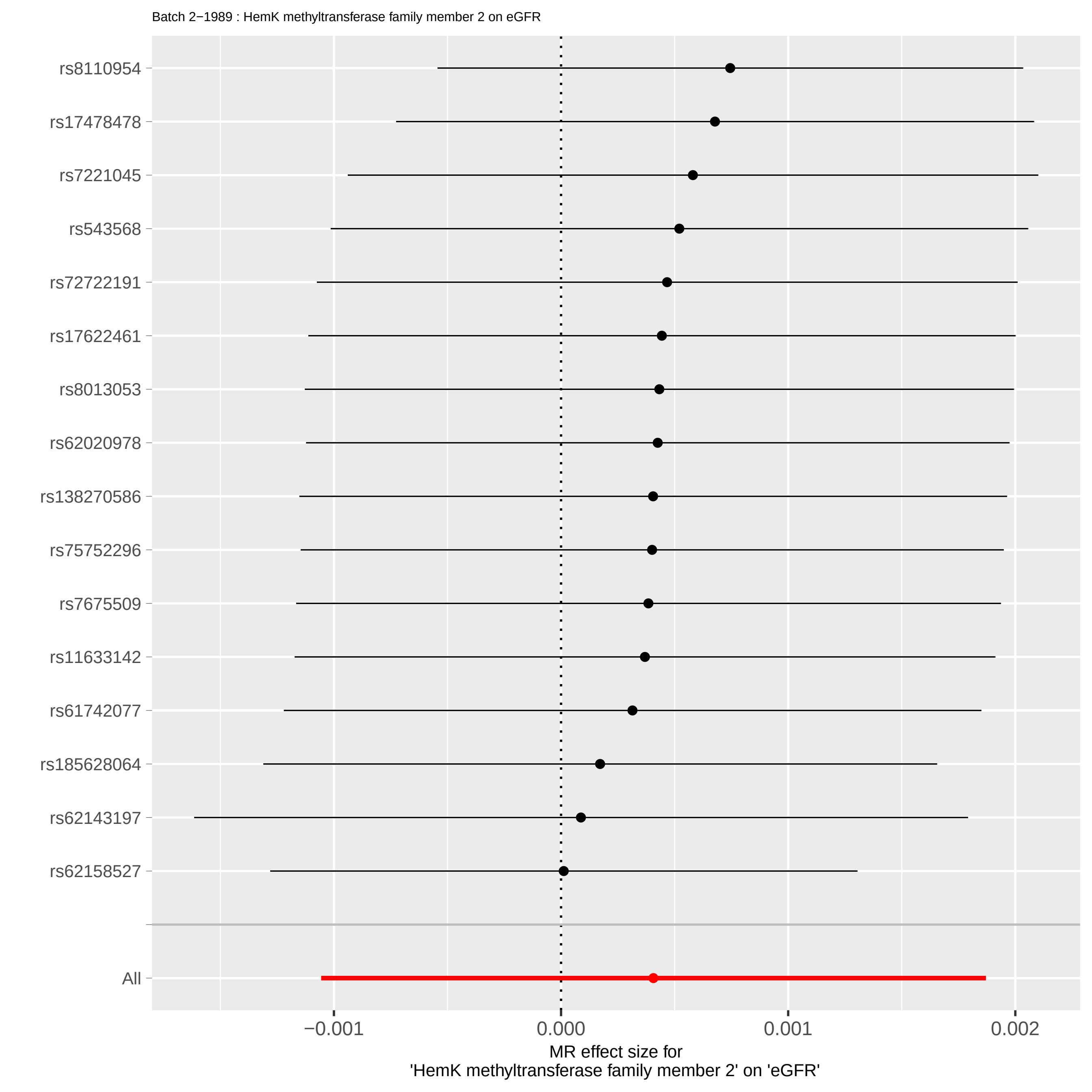

Batch 2−1989 : HemK methyltransferase family member 2 on eGFR
rs8110954
rs17478478
rs7221045
rs543568
rs72722191
rs17622461
rs8013053
rs62020978
rs138270586
rs75752296
rs7675509
rs11633142
rs61742077
rs185628064
rs62143197
rs62158527
All
−0.001
0.000
0.001
0.002
MR effect size for
'HemK methyltransferase family member 2' on 'eGFR'

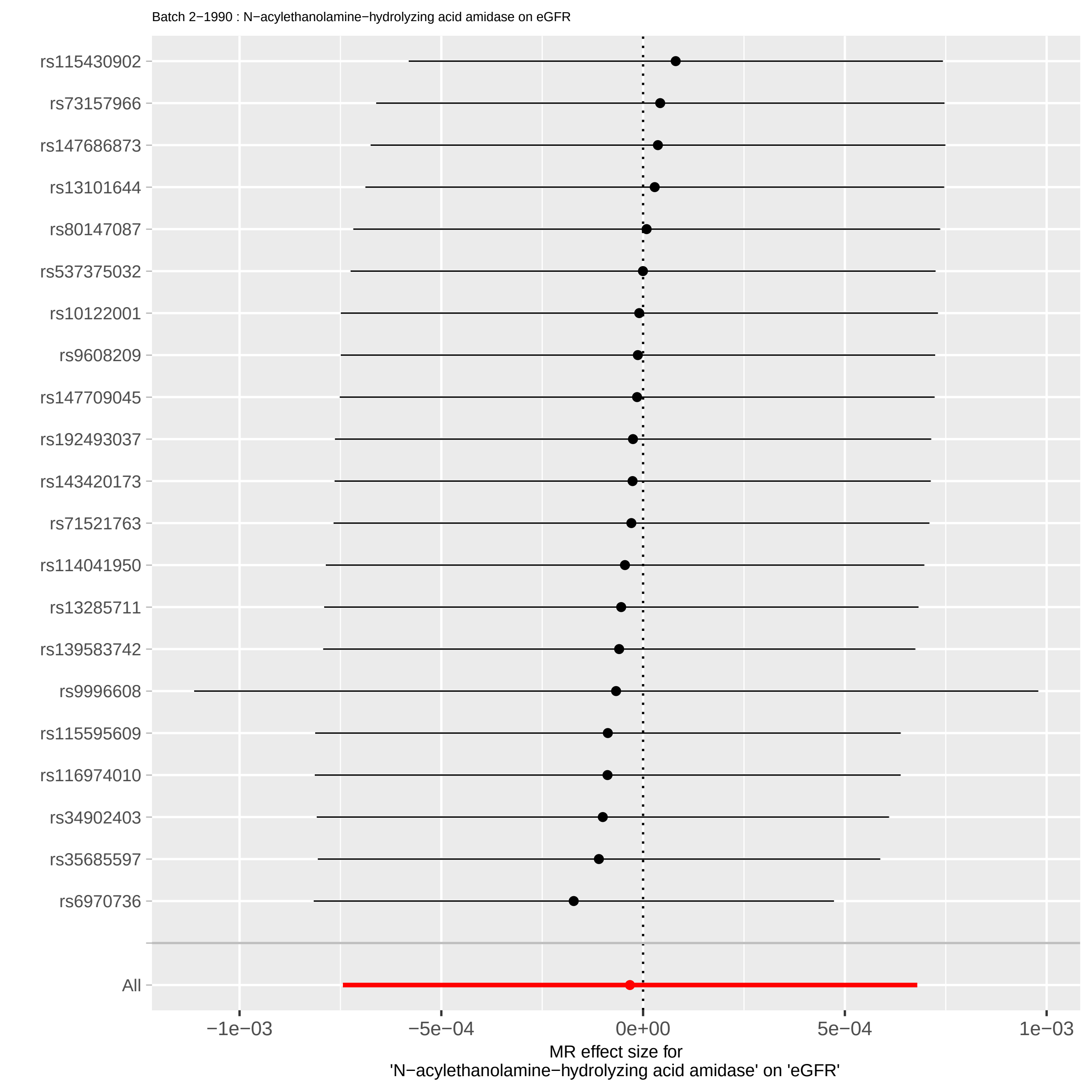

Batch 2−1990 : N−acylethanolamine−hydrolyzing acid amidase on eGFR
rs115430902
rs73157966
rs147686873
rs13101644
rs80147087
rs537375032
rs10122001
rs9608209
rs147709045
rs192493037
rs143420173
rs71521763
rs114041950
rs13285711
rs139583742
rs9996608
rs115595609
rs116974010
rs34902403
rs35685597
rs6970736
All
−1e−03
−5e−04
0e+00
5e−04
1e−03
MR effect size for
'N−acylethanolamine−hydrolyzing acid amidase' on 'eGFR'

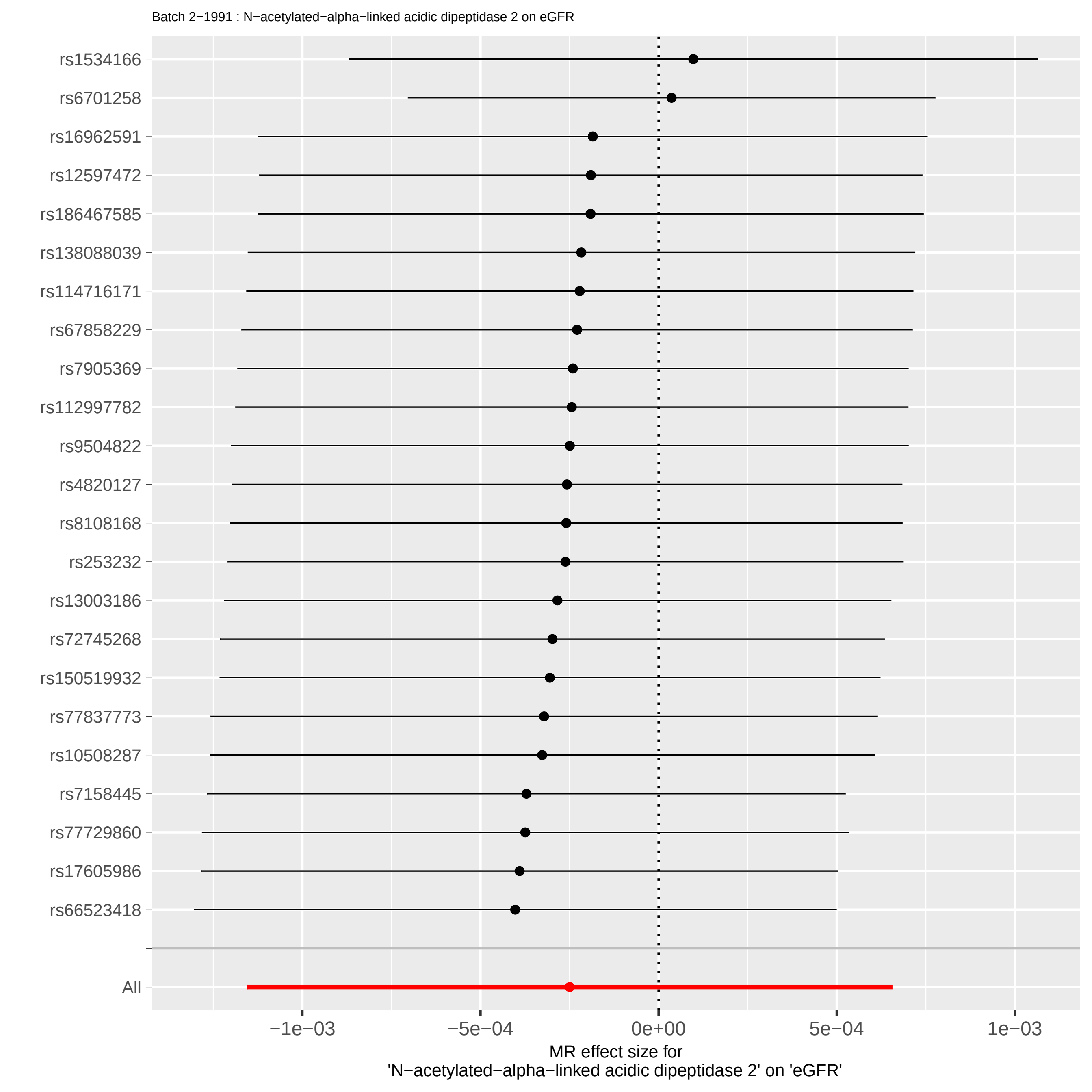
Batch 2−1991 : N−acetylated−alpha−linked acidic dipeptidase 2 on eGFR
rs1534166
rs6701258
rs16962591
rs12597472
rs186467585
rs138088039
rs114716171
rs67858229
rs7905369
rs112997782
rs9504822
rs4820127
rs8108168
rs253232
rs13003186
rs72745268
rs150519932
rs77837773
rs10508287
rs7158445
rs77729860
rs17605986
rs66523418
All
−1e−03
−5e−04
0e+00
5e−04
1e−03
MR effect size for
'N−acetylated−alpha−linked acidic dipeptidase 2' on 'eGFR'

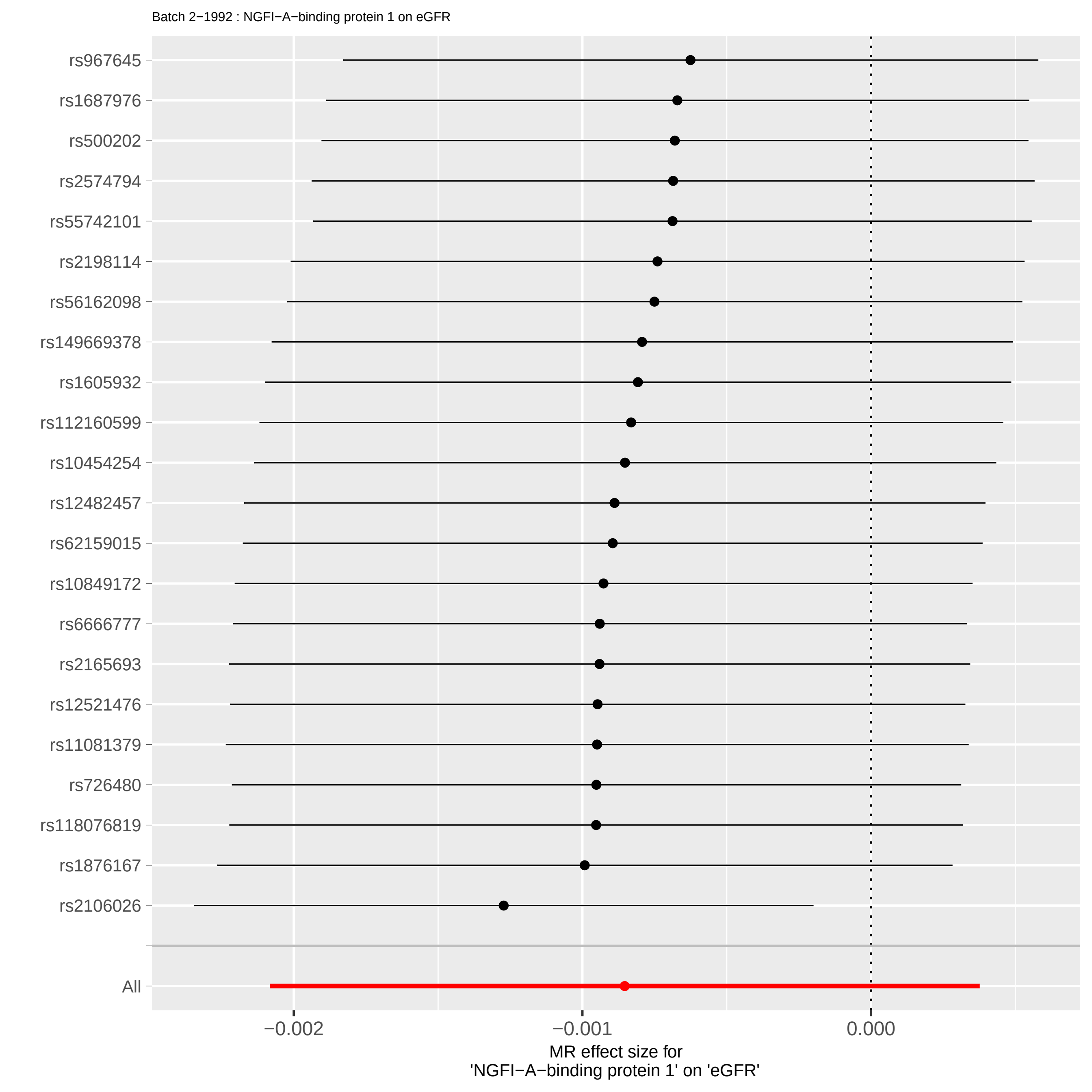
Batch 2−1992 : NGFI−A−binding protein 1 on eGFR
rs967645
rs1687976
rs500202
rs2574794
rs55742101
rs2198114
rs56162098
rs149669378
rs1605932
rs112160599
rs10454254
rs12482457
rs62159015
rs10849172
rs6666777
rs2165693
rs12521476
rs11081379
rs726480
rs118076819
rs1876167
rs2106026
All
−0.002
−0.001
0.000
MR effect size for
'NGFI−A−binding protein 1' on 'eGFR'

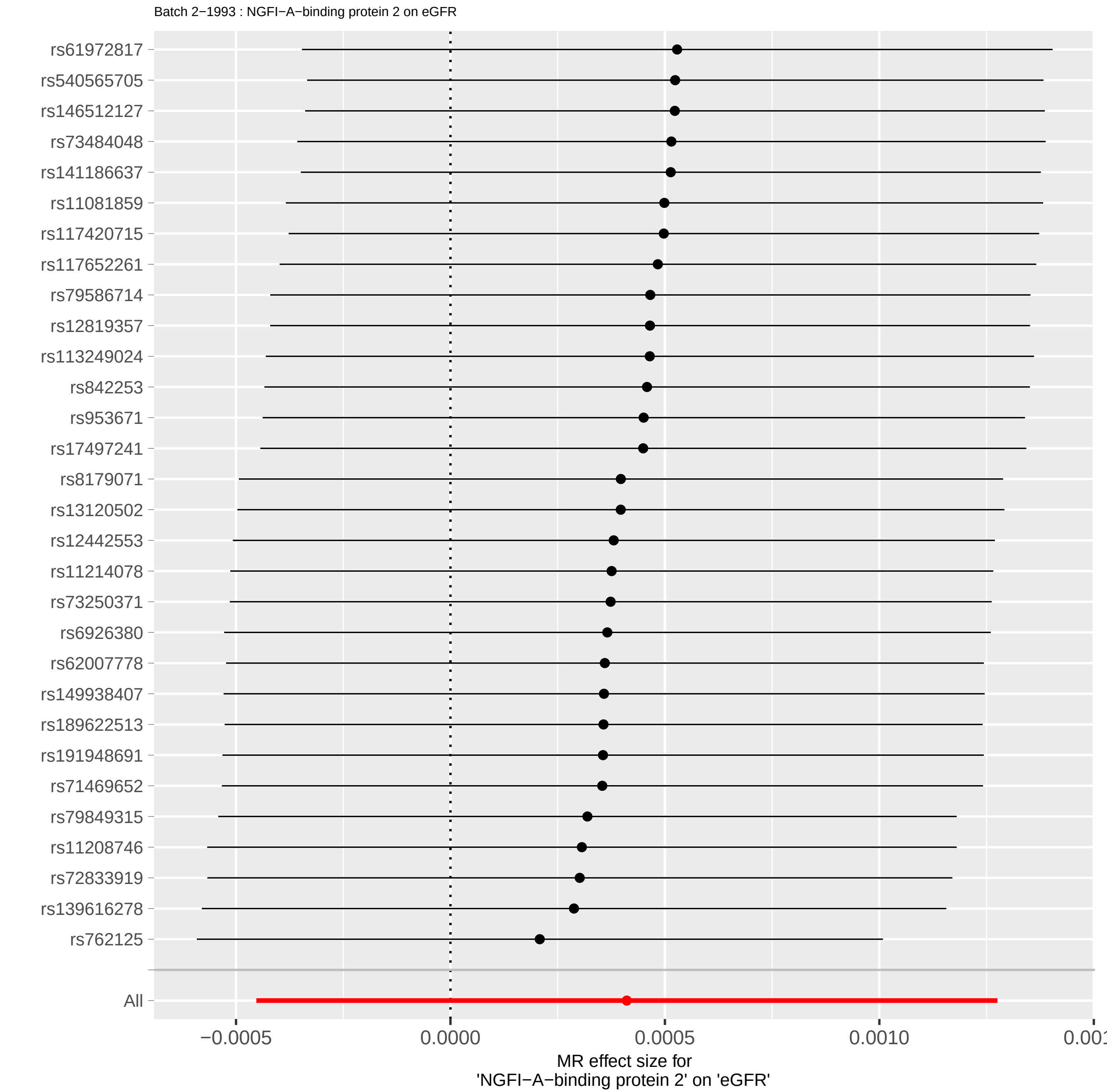

Batch 2−1993 : NGFI−A−binding protein 2 on eGFR
rs61972817
rs540565705
rs146512127
rs73484048
rs141186637
rs11081859
rs117420715
rs117652261
rs79586714
rs12819357
rs113249024
rs842253
rs953671
rs17497241
rs8179071
rs13120502
rs12442553
rs11214078
rs73250371
rs6926380
rs62007778
rs149938407
rs189622513
rs191948691
rs71469652
rs79849315
rs11208746
rs72833919
rs139616278
rs762125
All
−0.0005
0.0000
0.0005
0.0010
MR effect size for
'NGFI−A−binding protein 2' on 'eGFR'

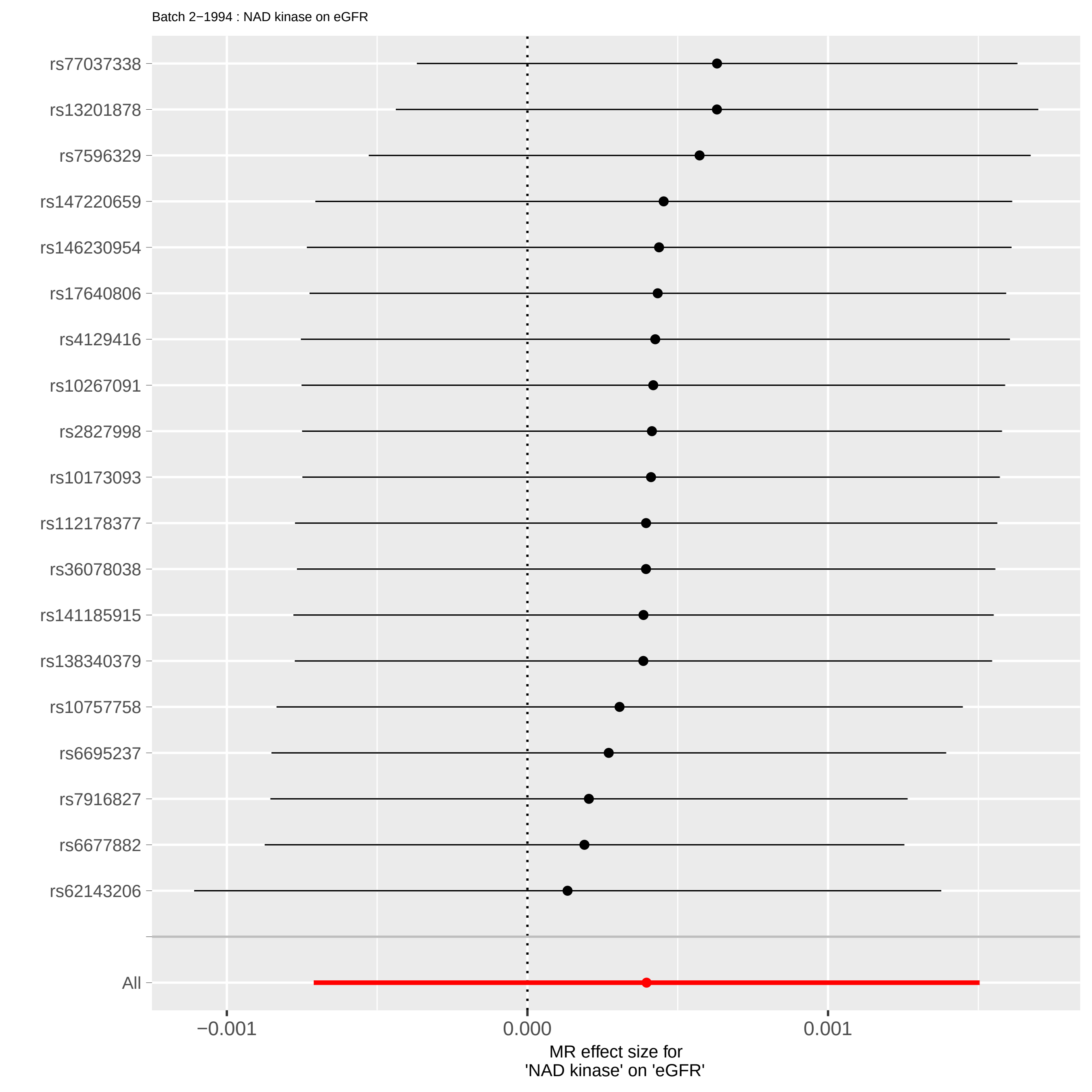

Batch 2−1994 : NAD kinase on eGFR
rs77037338
rs13201878
rs7596329
rs147220659
rs146230954
rs17640806
rs4129416
rs10267091
rs2827998
rs10173093
rs112178377
rs36078038
rs141185915
rs138340379
rs10757758
rs6695237
rs7916827
rs6677882
rs62143206
All
−0.001
0.000
0.001
MR effect size for
'NAD kinase' on 'eGFR'

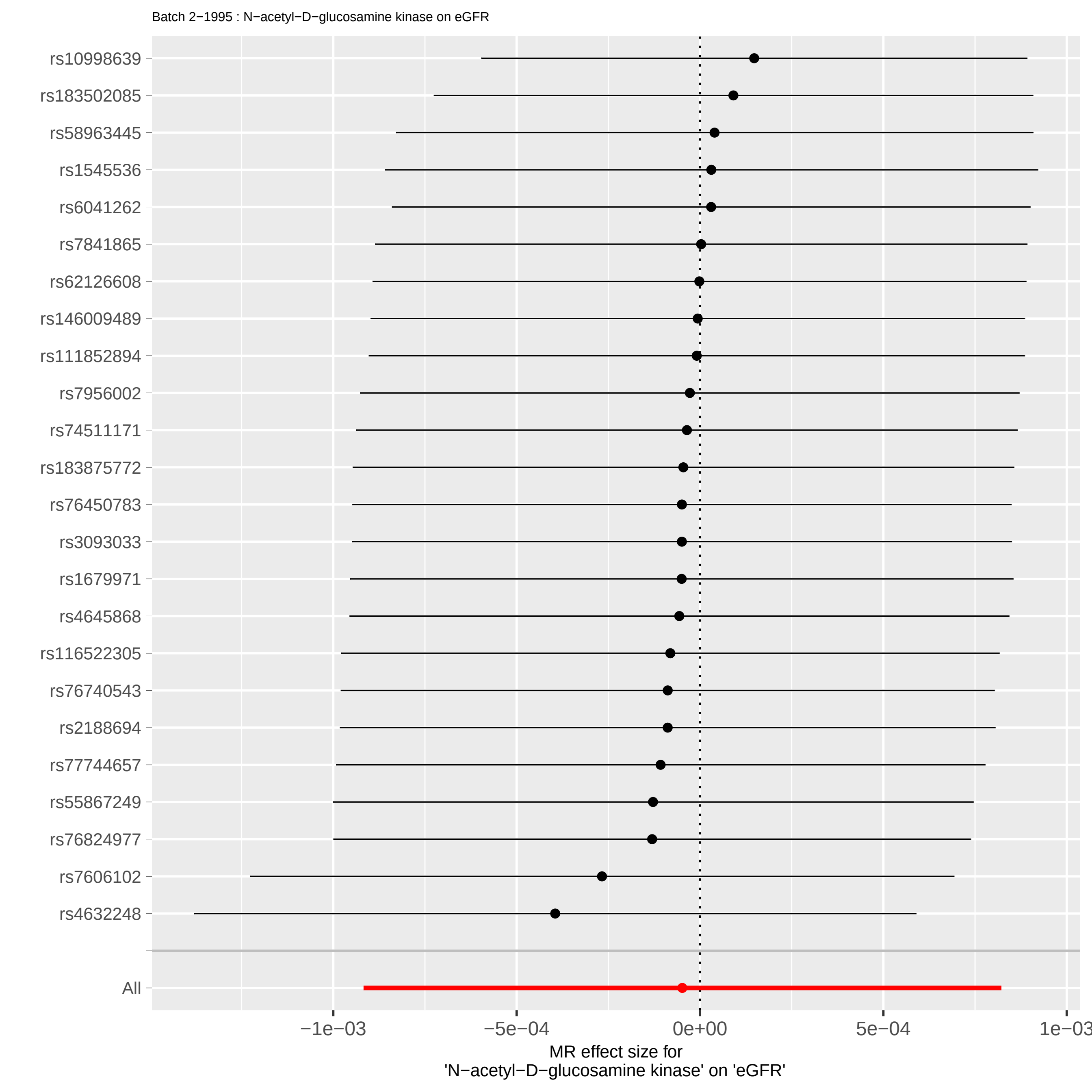
Batch 2−1995 : N−acetyl−D−glucosamine kinase on eGFR
rs10998639
rs183502085
rs58963445
rs1545536
rs6041262
rs7841865
rs62126608
rs146009489
rs111852894
rs7956002
rs74511171
rs183875772
rs76450783
rs3093033
rs1679971
rs4645868
rs116522305
rs76740543
rs2188694
rs77744657
rs55867249
rs76824977
rs7606102
rs4632248
All
−1e−03
−5e−04
0e+00
5e−04
1e−03
MR effect size for
'N−acetyl−D−glucosamine kinase' on 'eGFR'

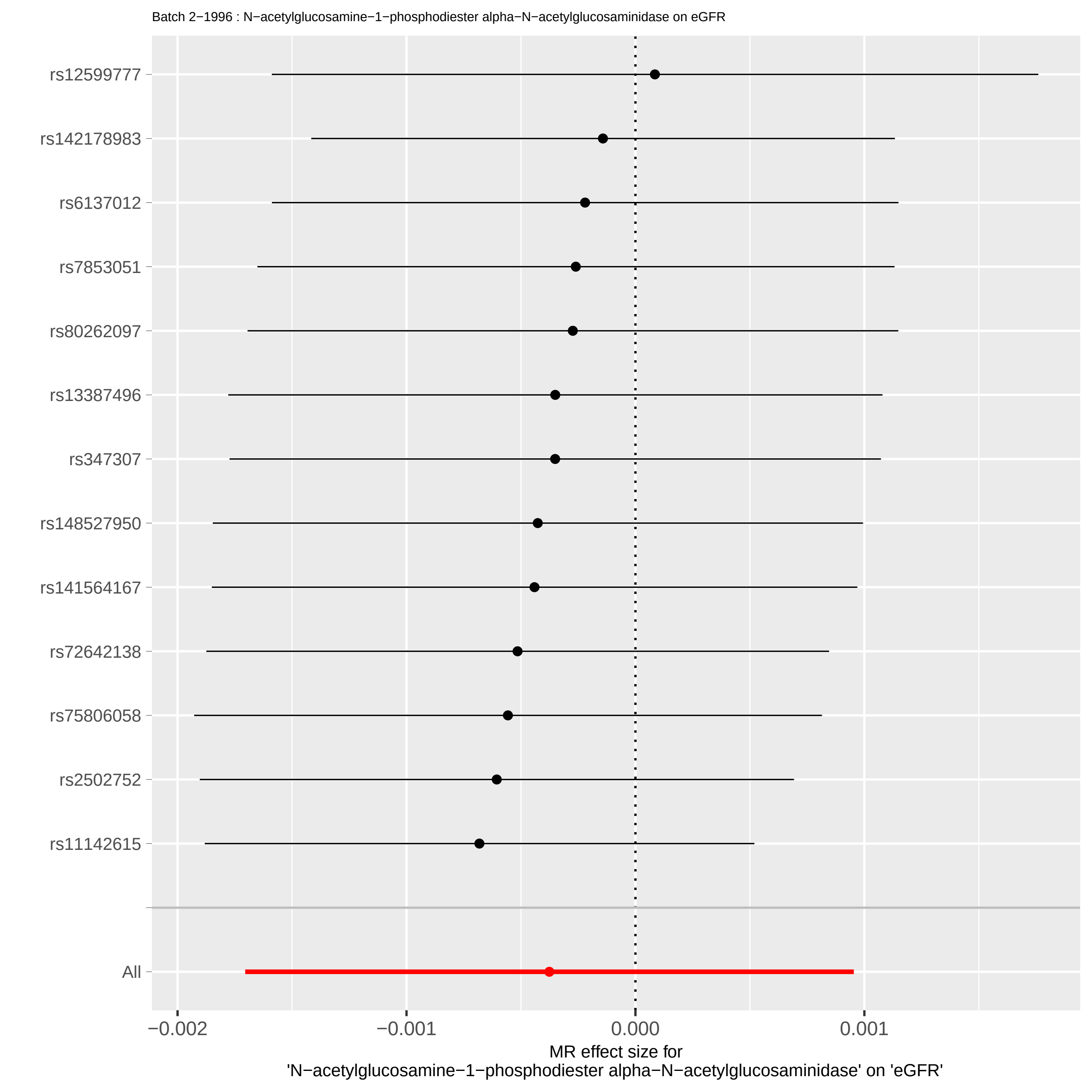

Batch 2−1996 : N−acetylglucosamine−1−phosphodiester alpha−N−acetylglucosaminidase on eGFR
rs12599777
rs142178983
rs6137012
rs7853051
rs80262097
rs13387496
rs347307
rs148527950
rs141564167
rs72642138
rs75806058
rs2502752
rs11142615
All
−0.002
−0.001
0.000
0.001
MR effect size for
'N−acetylglucosamine−1−phosphodiester alpha−N−acetylglucosaminidase' on 'eGFR'

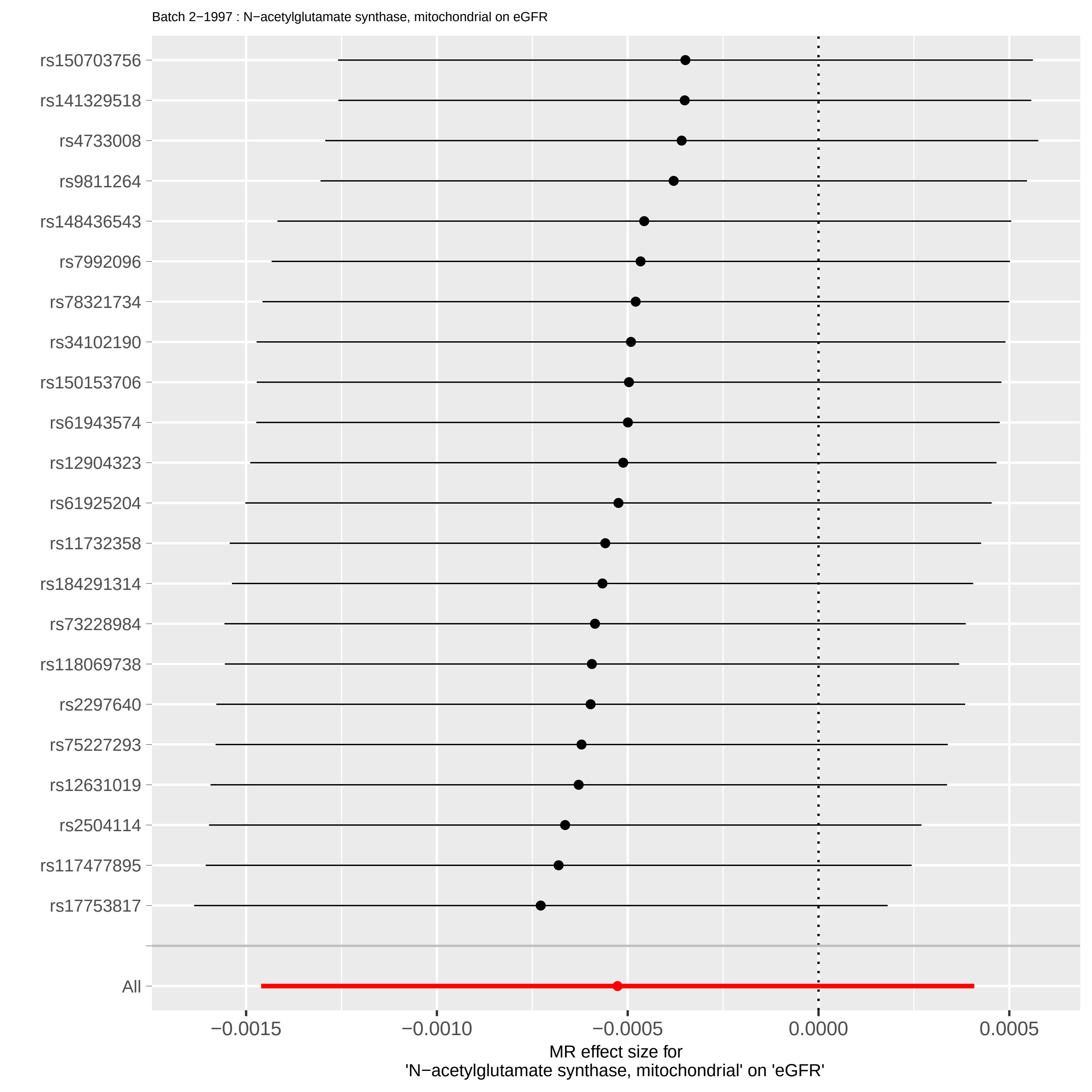
Batch 2−1997 : N−acetylglutamate synthase, mitochondrial on eGFR
rs150703756
rs141329518
rs4733008
rs9811264
rs148436543
rs7992096
rs78321734
rs34102190
rs150153706
rs61943574
rs12904323
rs61925204
rs11732358
rs184291314
rs73228984
rs118069738
rs2297640
rs75227293
rs12631019
rs2504114
rs117477895
rs17753817
All
−0.0015
−0.0010
−0.0005
0.0000
0.0005
MR effect size for
'N−acetylglutamate synthase, mitochondrial' on 'eGFR'

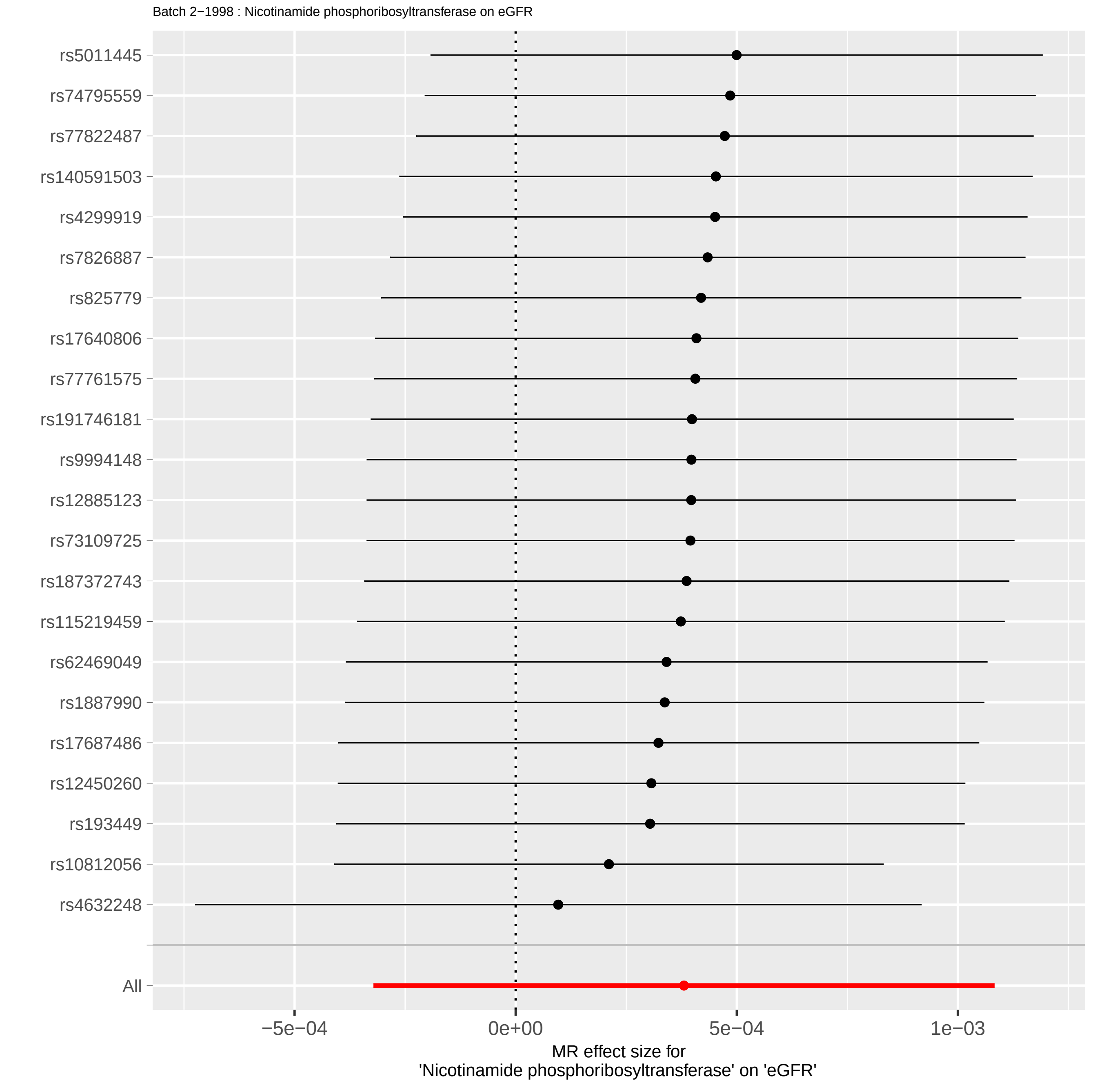

Batch 2−1998 : Nicotinamide phosphoribosyltransferase on eGFR
rs5011445
rs74795559
rs77822487
rs140591503
rs4299919
rs7826887
rs825779
rs17640806
rs77761575
rs191746181
rs9994148
rs12885123
rs73109725
rs187372743
rs115219459
rs62469049
rs1887990
rs17687486
rs12450260
rs193449
rs10812056
rs4632248
All
−5e−04
0e+00
5e−04
1e−03
MR effect size for
'Nicotinamide phosphoribosyltransferase' on 'eGFR'

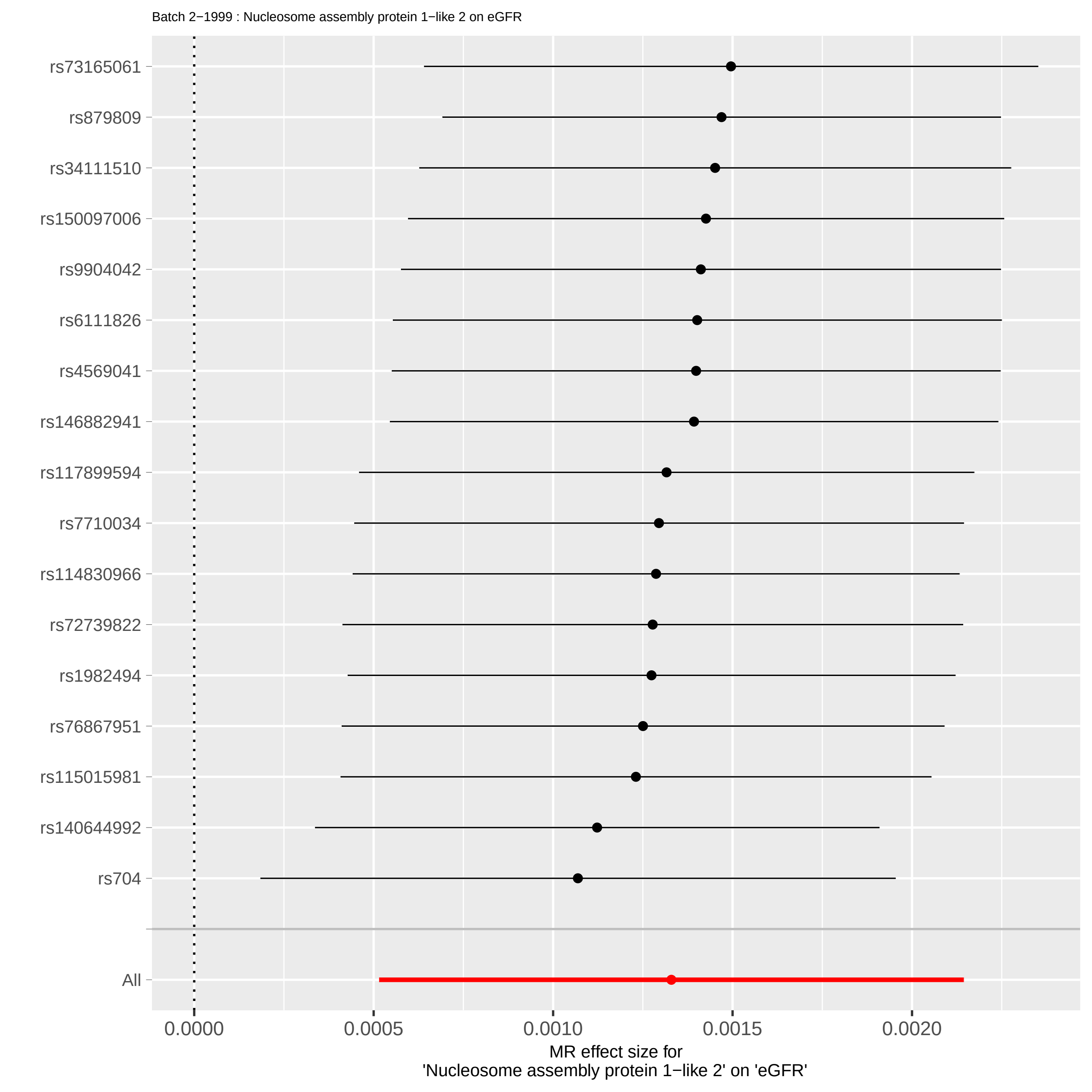

Batch 2−1999 : Nucleosome assembly protein 1−like 2 on eGFR
rs73165061
rs879809
rs34111510
rs150097006
rs9904042
rs6111826
rs4569041
rs146882941
rs117899594
rs7710034
rs114830966
rs72739822
rs1982494
rs76867951
rs115015981
rs140644992
rs704
All
0.0000
0.0005
0.0010
0.0015
0.0020
MR effect size for
'Nucleosome assembly protein 1−like 2' on 'eGFR'

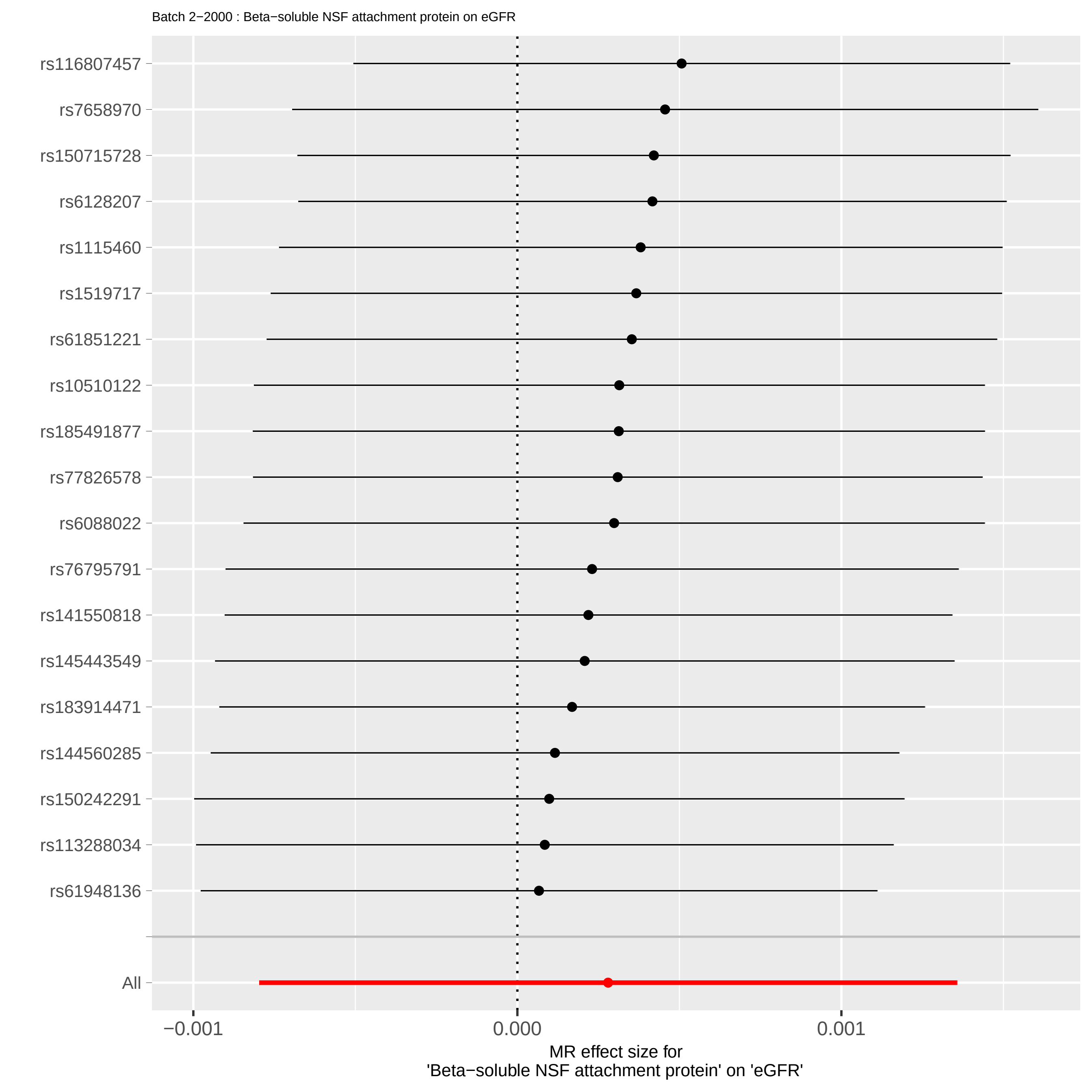

Batch 2−2000 : Beta−soluble NSF attachment protein on eGFR
rs116807457
rs7658970
rs150715728
rs6128207
rs1115460
rs1519717
rs61851221
rs10510122
rs185491877
rs77826578
rs6088022
rs76795791
rs141550818
rs145443549
rs183914471
rs144560285
rs150242291
rs113288034
rs61948136
All
−0.001
0.000
0.001
MR effect size for
'Beta−soluble NSF attachment protein' on 'eGFR'

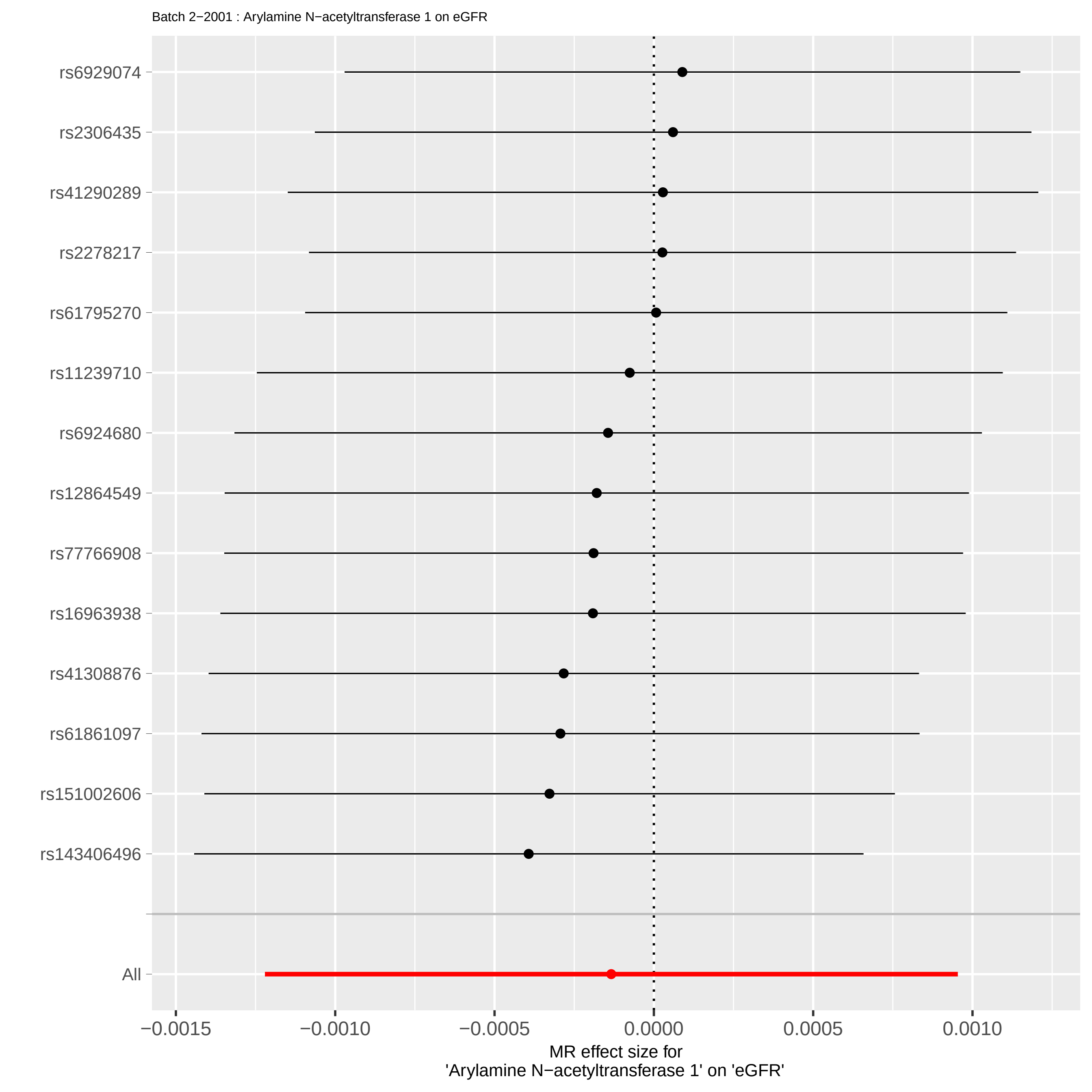

Batch 2−2001 : Arylamine N−acetyltransferase 1 on eGFR
rs6929074
rs2306435
rs41290289
rs2278217
rs61795270
rs11239710
rs6924680
rs12864549
rs77766908
rs16963938
rs41308876
rs61861097
rs151002606
rs143406496
All
−0.0015
−0.0010
−0.0005
0.0000
0.0005
0.0010
MR effect size for
'Arylamine N−acetyltransferase 1' on 'eGFR'

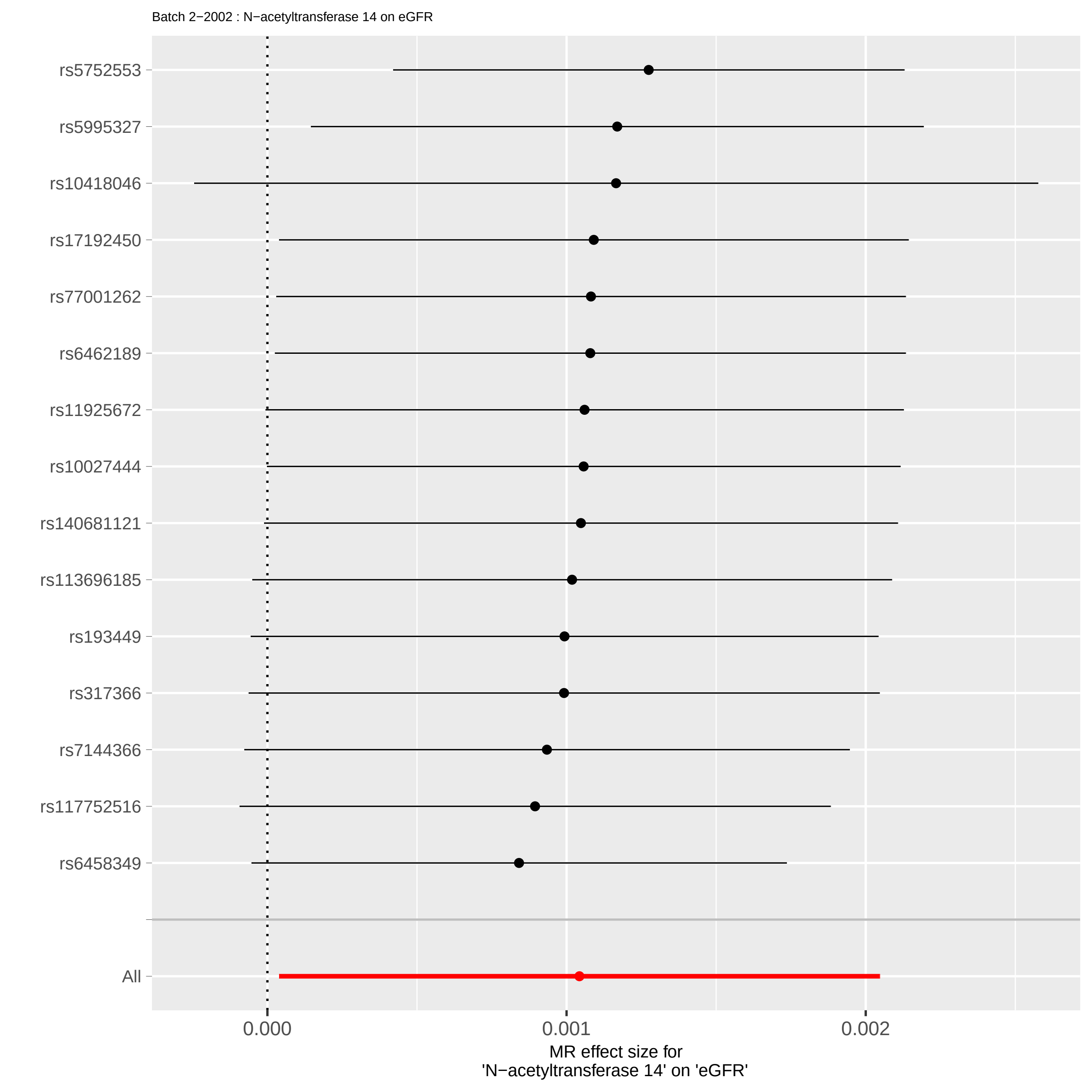

Batch 2−2002 : N−acetyltransferase 14 on eGFR
rs5752553
rs5995327
rs10418046
rs17192450
rs77001262
rs6462189
rs11925672
rs10027444
rs140681121
rs113696185
rs193449
rs317366
rs7144366
rs117752516
rs6458349
All
0.000
0.001
0.002
MR effect size for
'N−acetyltransferase 14' on 'eGFR'

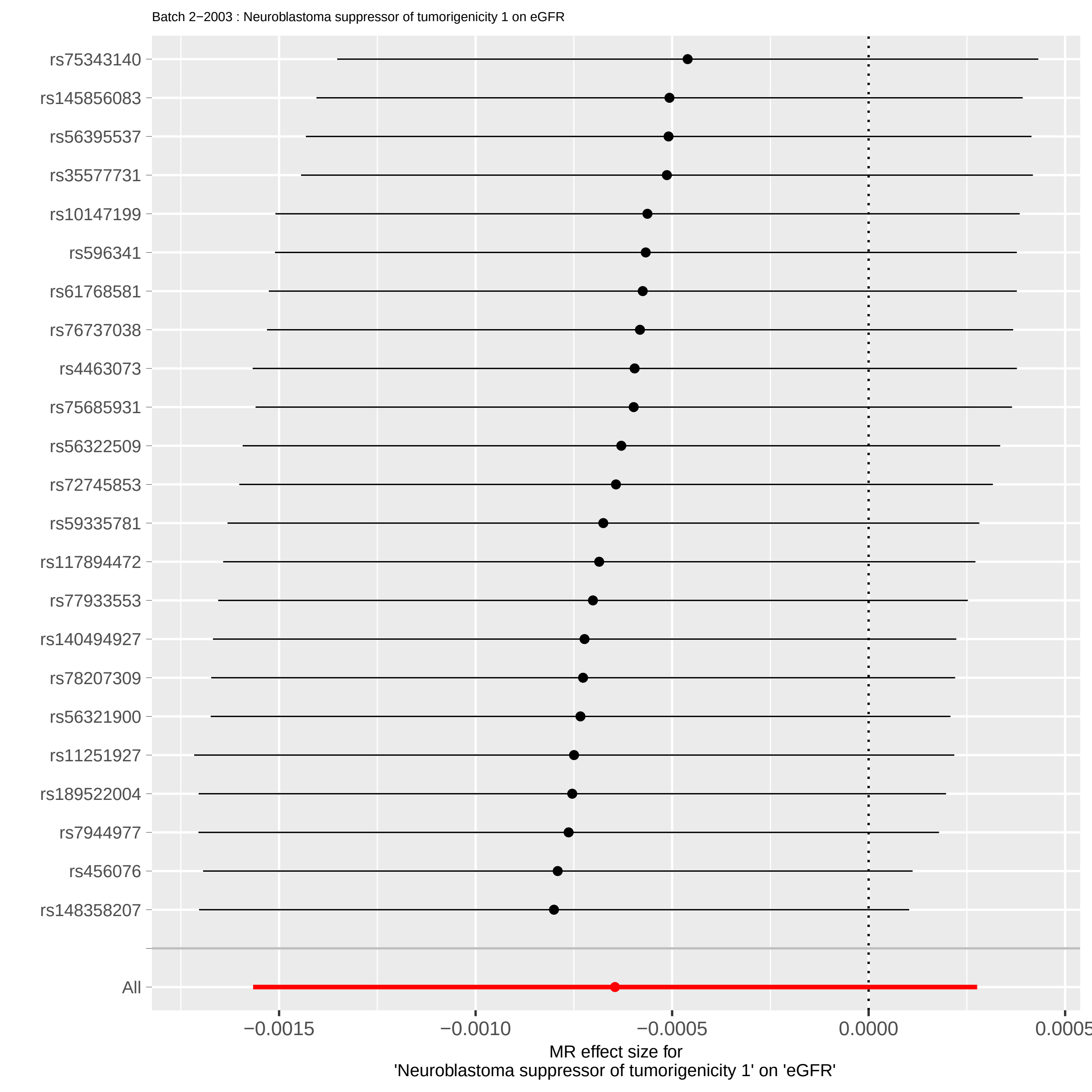

Batch 2−2003 : Neuroblastoma suppressor of tumorigenicity 1 on eGFR
rs75343140
rs145856083
rs56395537
rs35577731
rs10147199
rs596341
rs61768581
rs76737038
rs4463073
rs75685931
rs56322509
rs72745853
rs59335781
rs117894472
rs77933553
rs140494927
rs78207309
rs56321900
rs11251927
rs189522004
rs7944977
rs456076
rs148358207
All
−0.0015
−0.0010
−0.0005
0.0000
0.0005
MR effect size for
'Neuroblastoma suppressor of tumorigenicity 1' on 'eGFR'

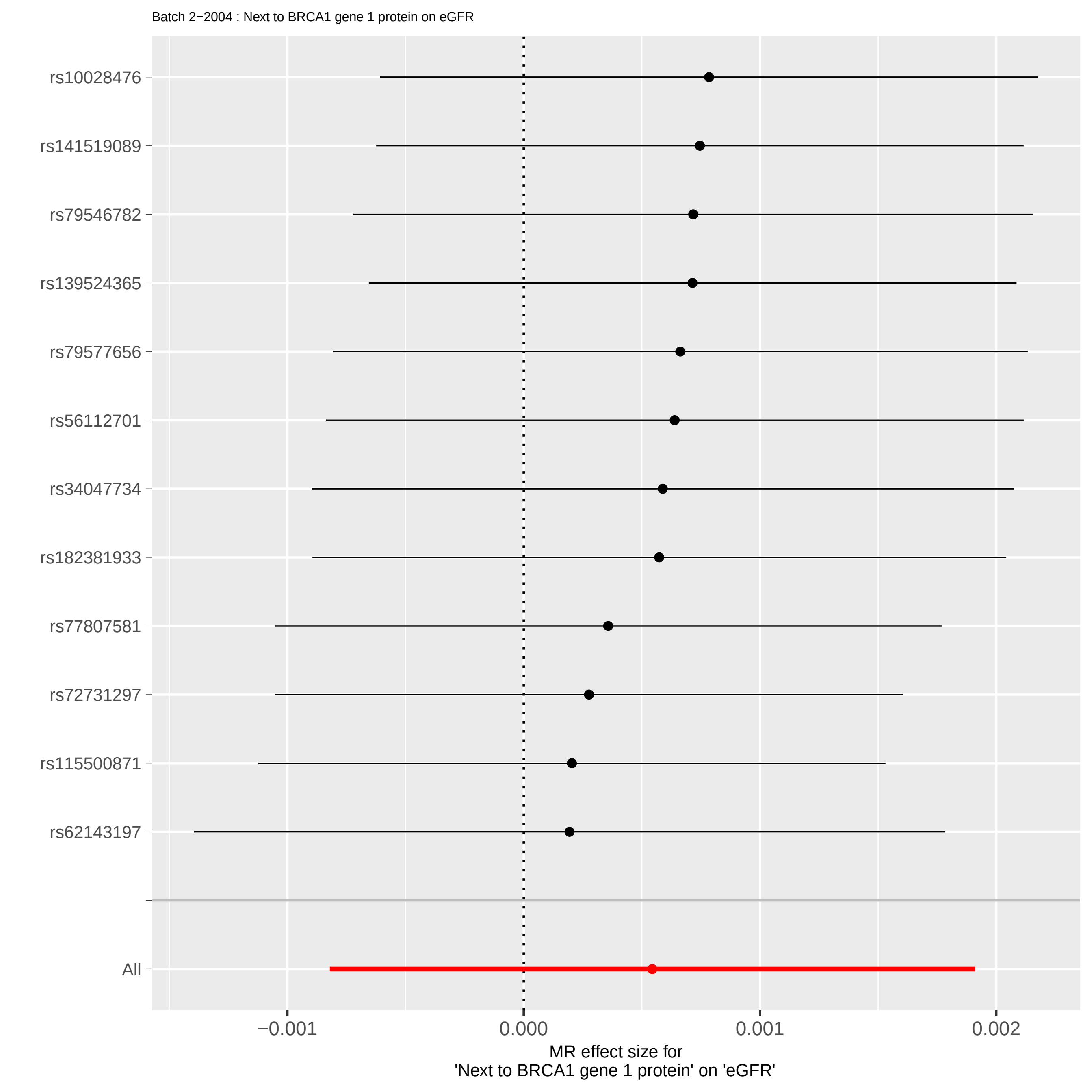
Batch 2−2004 : Next to BRCA1 gene 1 protein on eGFR
rs10028476
rs141519089
rs79546782
rs139524365
rs79577656
rs56112701
rs34047734
rs182381933
rs77807581
rs72731297
rs115500871
rs62143197
All
−0.001
0.000
0.001
0.002
MR effect size for
'Next to BRCA1 gene 1 protein' on 'eGFR'

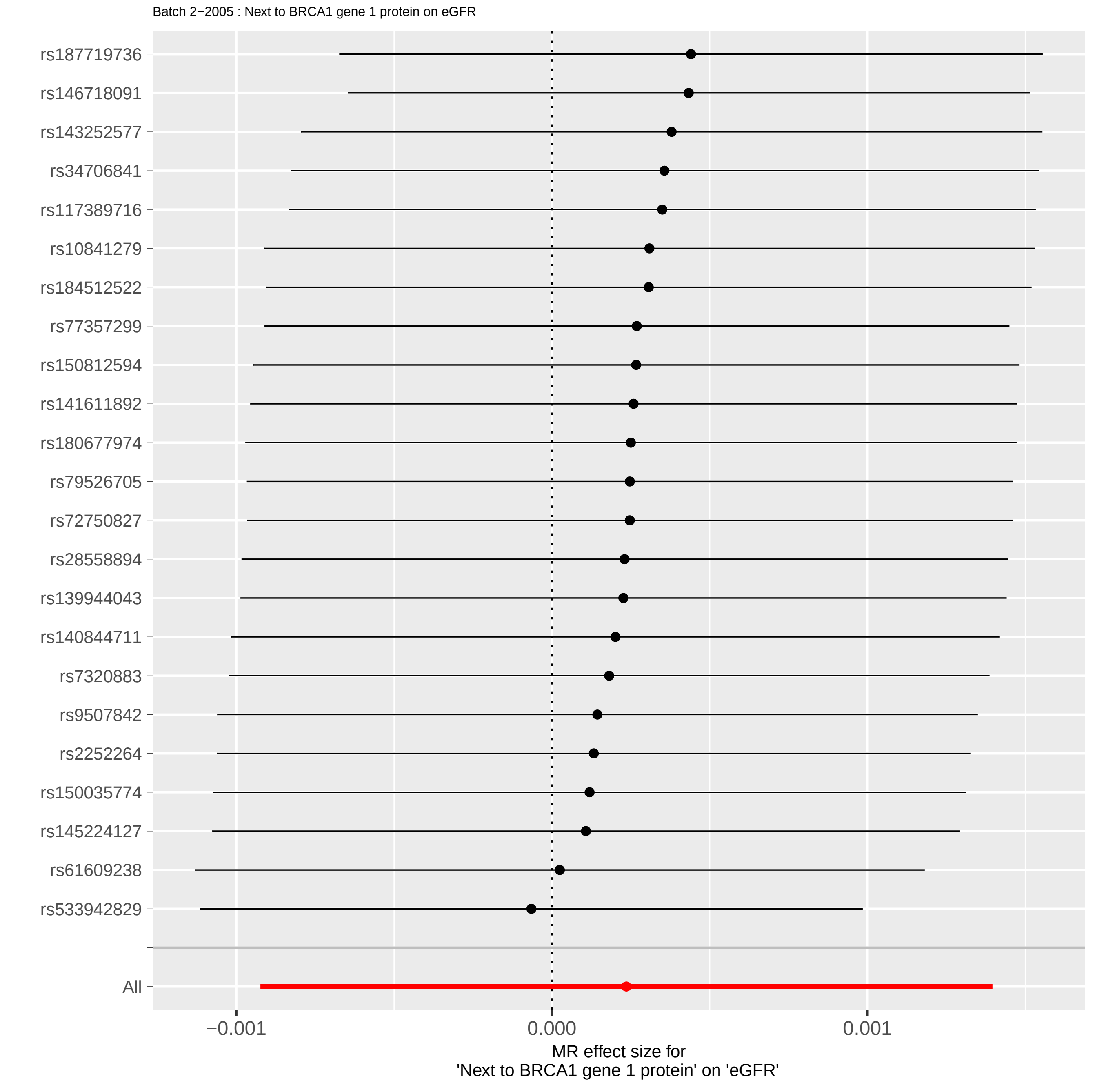
Batch 2−2005 : Next to BRCA1 gene 1 protein on eGFR
rs187719736
rs146718091
rs143252577
rs34706841
rs117389716
rs10841279
rs184512522
rs77357299
rs150812594
rs141611892
rs180677974
rs79526705
rs72750827
rs28558894
rs139944043
rs140844711
rs7320883
rs9507842
rs2252264
rs150035774
rs145224127
rs61609238
rs533942829
All
−0.001
0.000
0.001
MR effect size for
'Next to BRCA1 gene 1 protein' on 'eGFR'

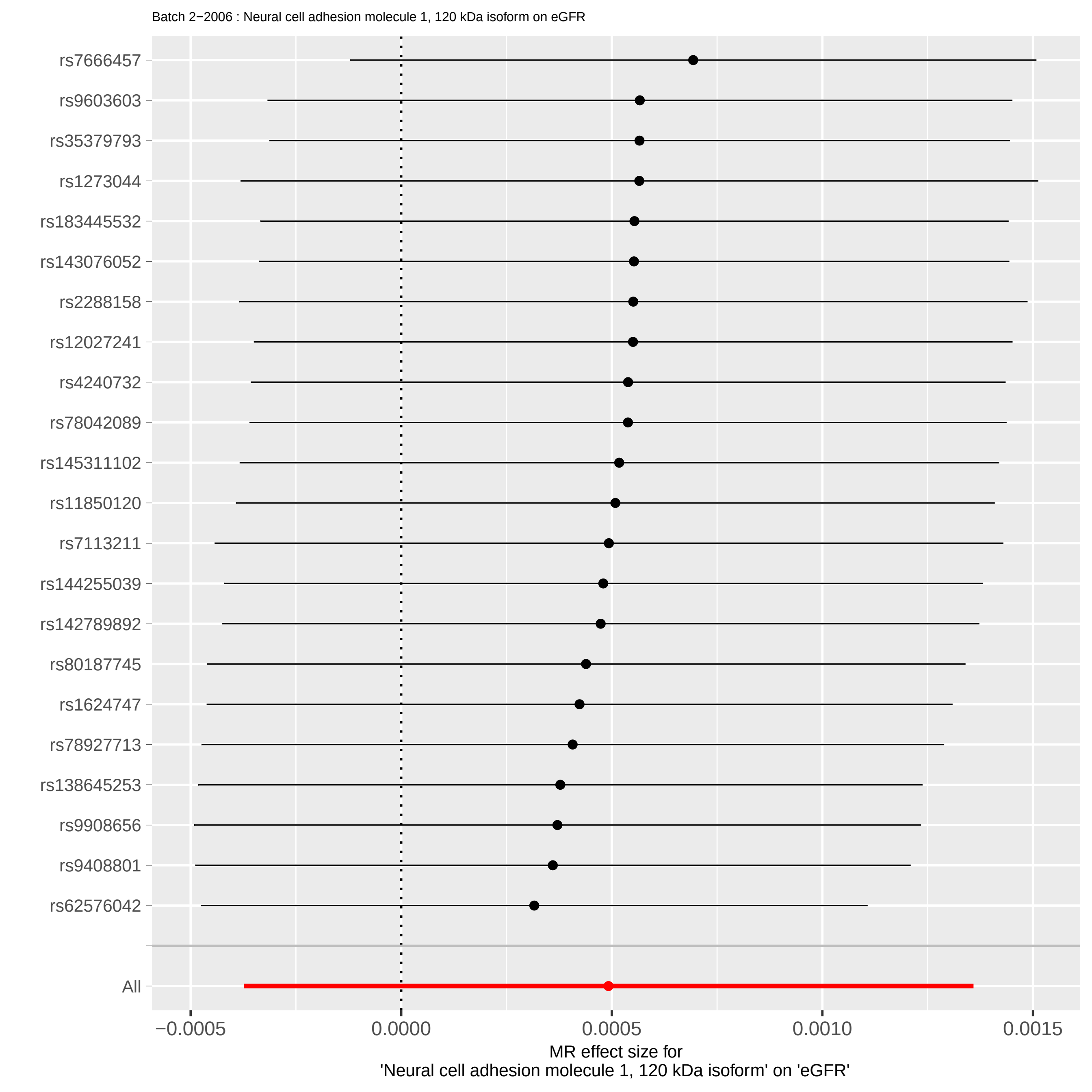

Batch 2−2006 : Neural cell adhesion molecule 1, 120 kDa isoform on eGFR
rs7666457
rs9603603
rs35379793
rs1273044
rs183445532
rs143076052
rs2288158
rs12027241
rs4240732
rs78042089
rs145311102
rs11850120
rs7113211
rs144255039
rs142789892
rs80187745
rs1624747
rs78927713
rs138645253
rs9908656
rs9408801
rs62576042
All
−0.0005
0.0000
0.0005
0.0010
0.0015
MR effect size for
'Neural cell adhesion molecule 1, 120 kDa isoform' on 'eGFR'

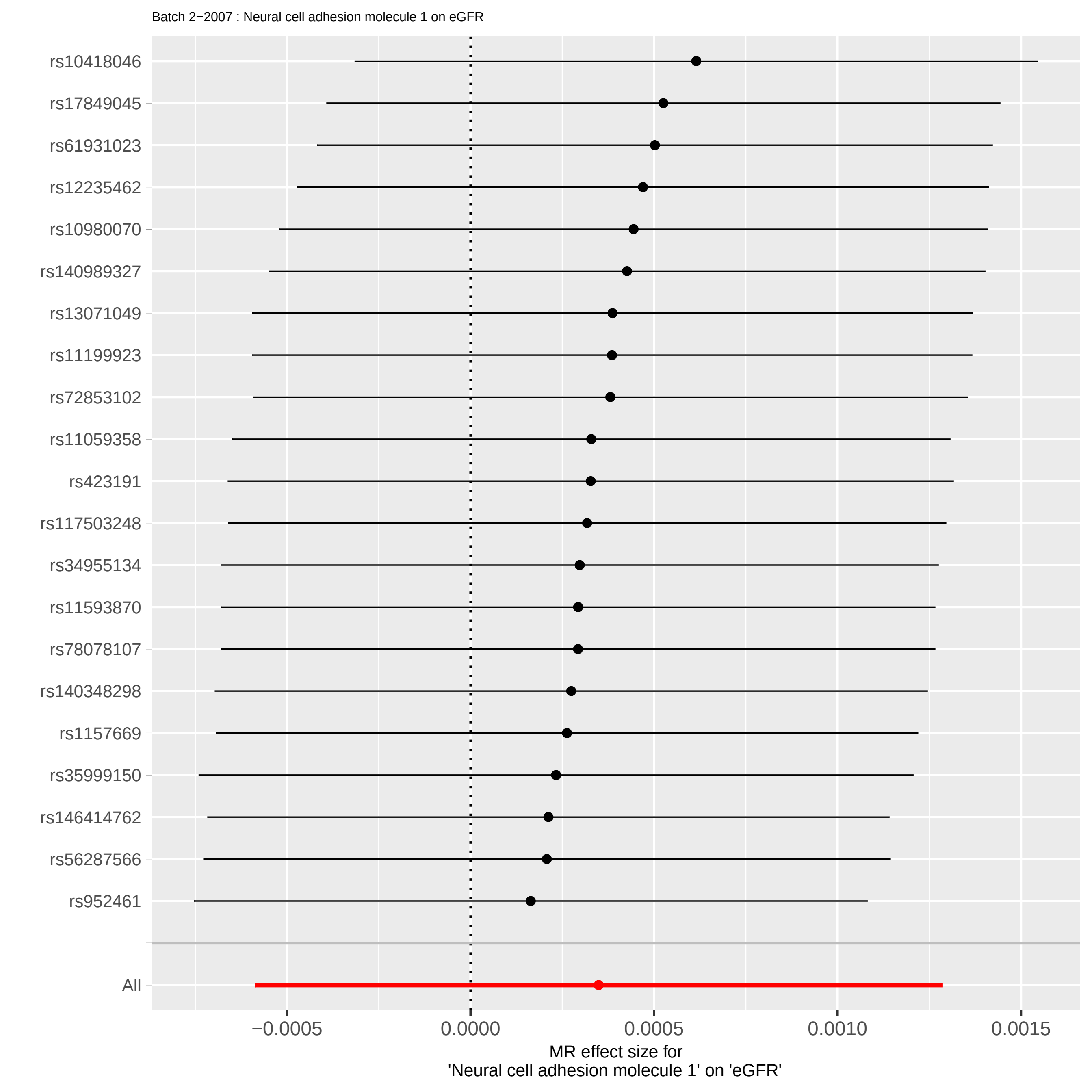

Batch 2−2007 : Neural cell adhesion molecule 1 on eGFR
rs10418046
rs17849045
rs61931023
rs12235462
rs10980070
rs140989327
rs13071049
rs11199923
rs72853102
rs11059358
rs423191
rs117503248
rs34955134
rs11593870
rs78078107
rs140348298
rs1157669
rs35999150
rs146414762
rs56287566
rs952461
All
−0.0005
0.0000
0.0005
0.0010
0.0015
MR effect size for
'Neural cell adhesion molecule 1' on 'eGFR'

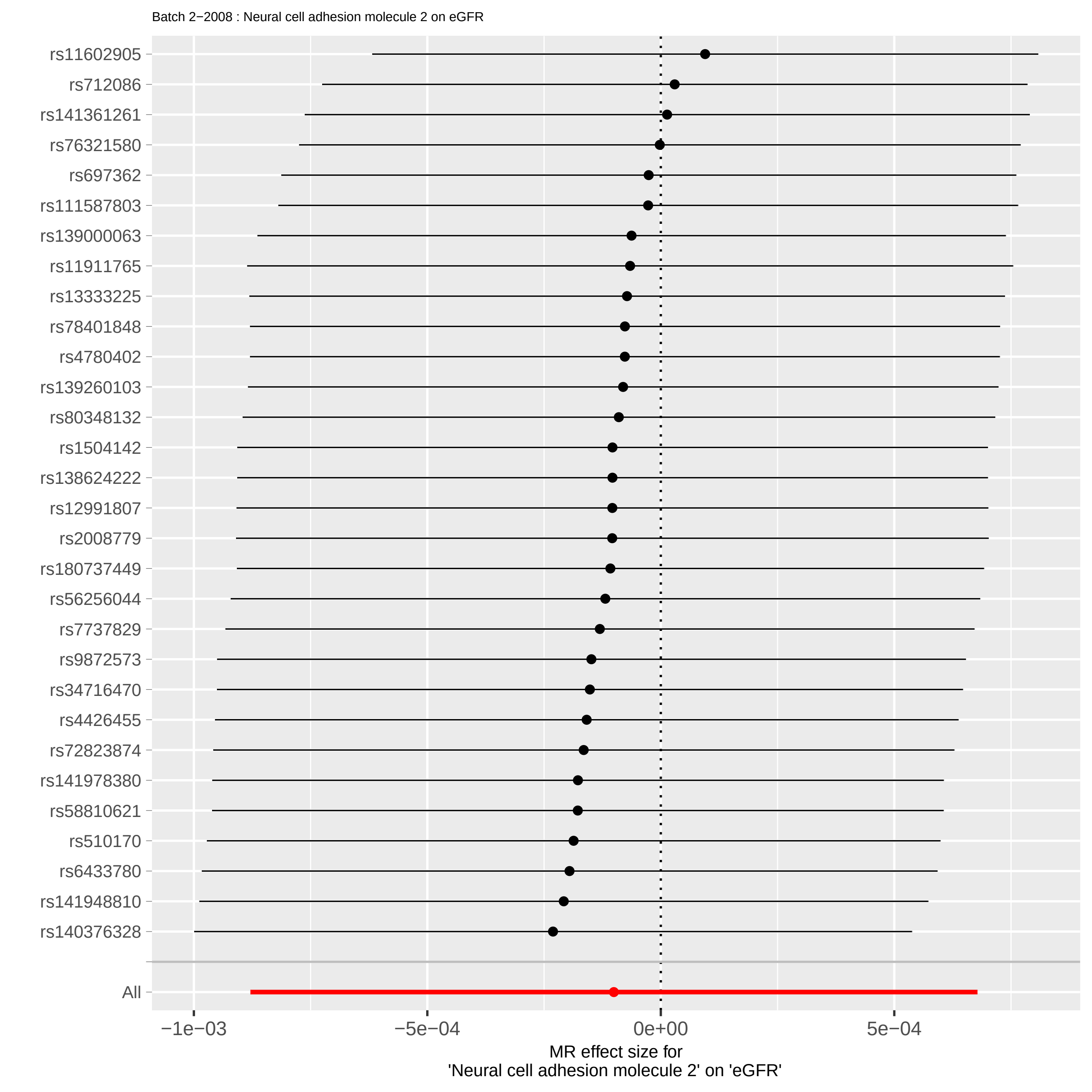

Batch 2−2008 : Neural cell adhesion molecule 2 on eGFR
rs11602905
rs712086
rs141361261
rs76321580
rs697362
rs111587803
rs139000063
rs11911765
rs13333225
rs78401848
rs4780402
rs139260103
rs80348132
rs1504142
rs138624222
rs12991807
rs2008779
rs180737449
rs56256044
rs7737829
rs9872573
rs34716470
rs4426455
rs72823874
rs141978380
rs58810621
rs510170
rs6433780
rs141948810
rs140376328
All
−1e−03
−5e−04
0e+00
5e−04
MR effect size for
'Neural cell adhesion molecule 2' on 'eGFR'

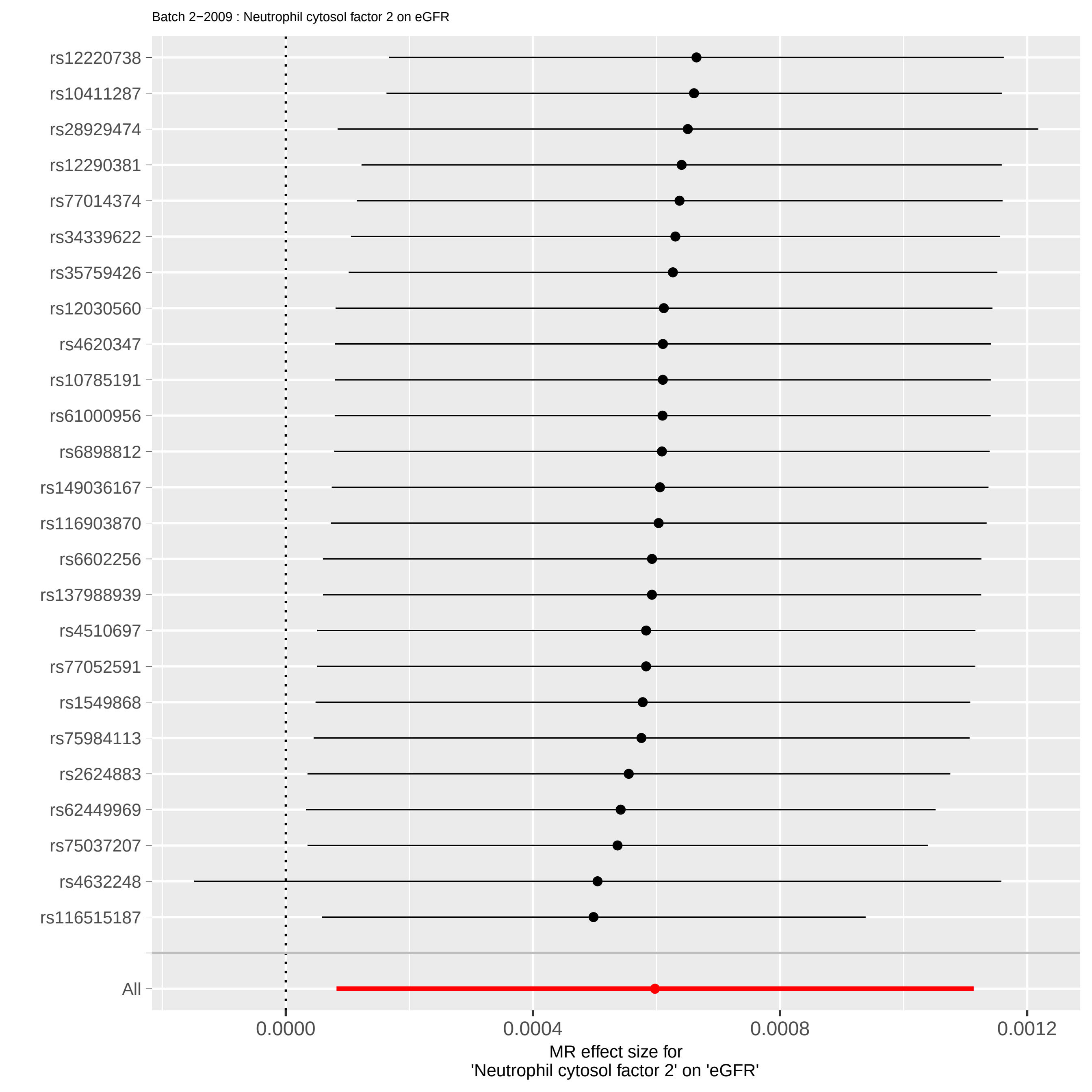

Batch 2−2009 : Neutrophil cytosol factor 2 on eGFR
rs12220738
rs10411287
rs28929474
rs12290381
rs77014374
rs34339622
rs35759426
rs12030560
rs4620347
rs10785191
rs61000956
rs6898812
rs149036167
rs116903870
rs6602256
rs137988939
rs4510697
rs77052591
rs1549868
rs75984113
rs2624883
rs62449969
rs75037207
rs4632248
rs116515187
All
0.0000
0.0004
0.0008
0.0012
MR effect size for
'Neutrophil cytosol factor 2' on 'eGFR'

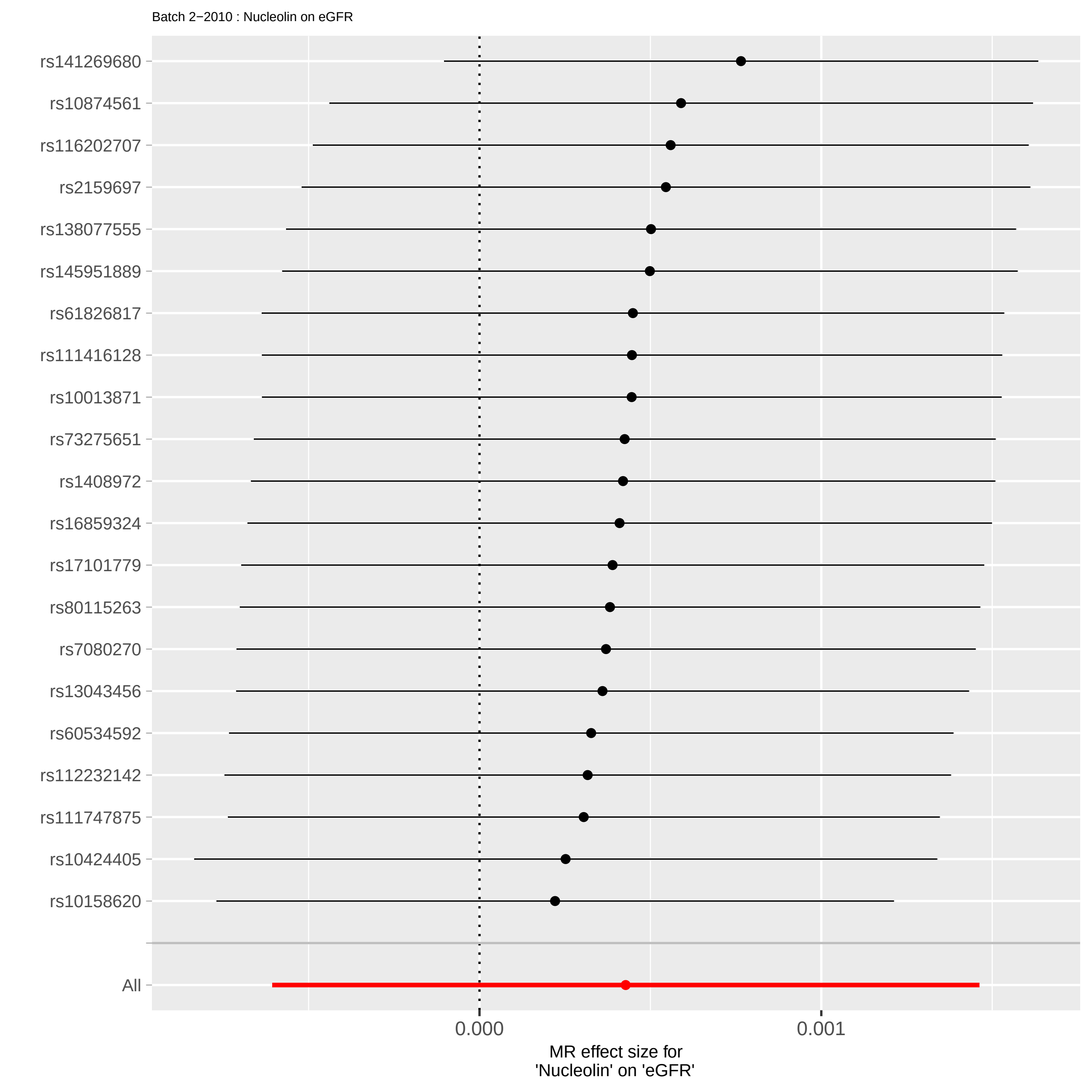
Batch 2−2010 : Nucleolin on eGFR
rs141269680
rs10874561
rs116202707
rs2159697
rs138077555
rs145951889
rs61826817
rs111416128
rs10013871
rs73275651
rs1408972
rs16859324
rs17101779
rs80115263
rs7080270
rs13043456
rs60534592
rs112232142
rs111747875
rs10424405
rs10158620
All
0.000
0.001
MR effect size for
'Nucleolin' on 'eGFR'

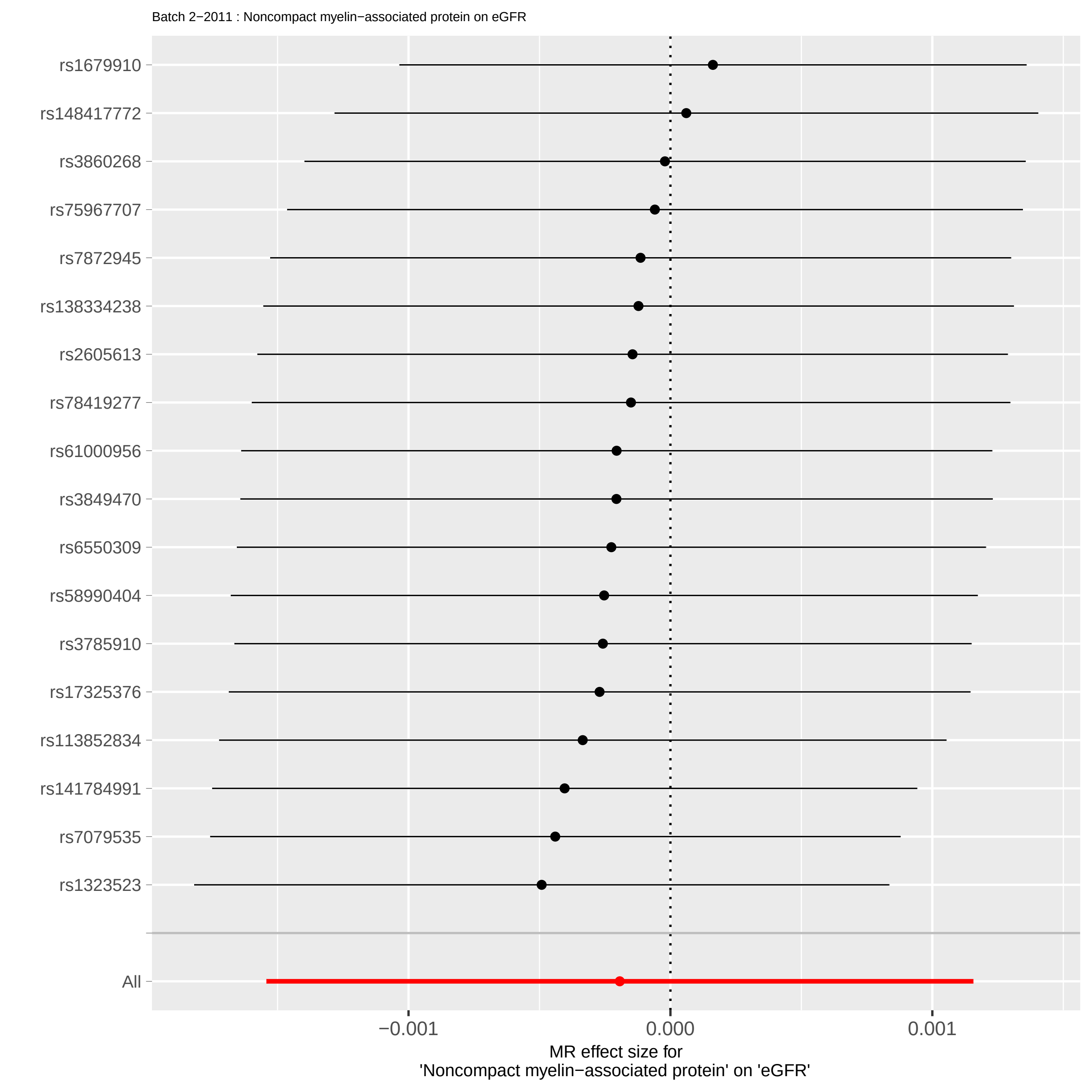

Batch 2−2011 : Noncompact myelin−associated protein on eGFR
rs1679910
rs148417772
rs3860268
rs75967707
rs7872945
rs138334238
rs2605613
rs78419277
rs61000956
rs3849470
rs6550309
rs58990404
rs3785910
rs17325376
rs113852834
rs141784991
rs7079535
rs1323523
All
−0.001
0.000
0.001
MR effect size for
'Noncompact myelin−associated protein' on 'eGFR'

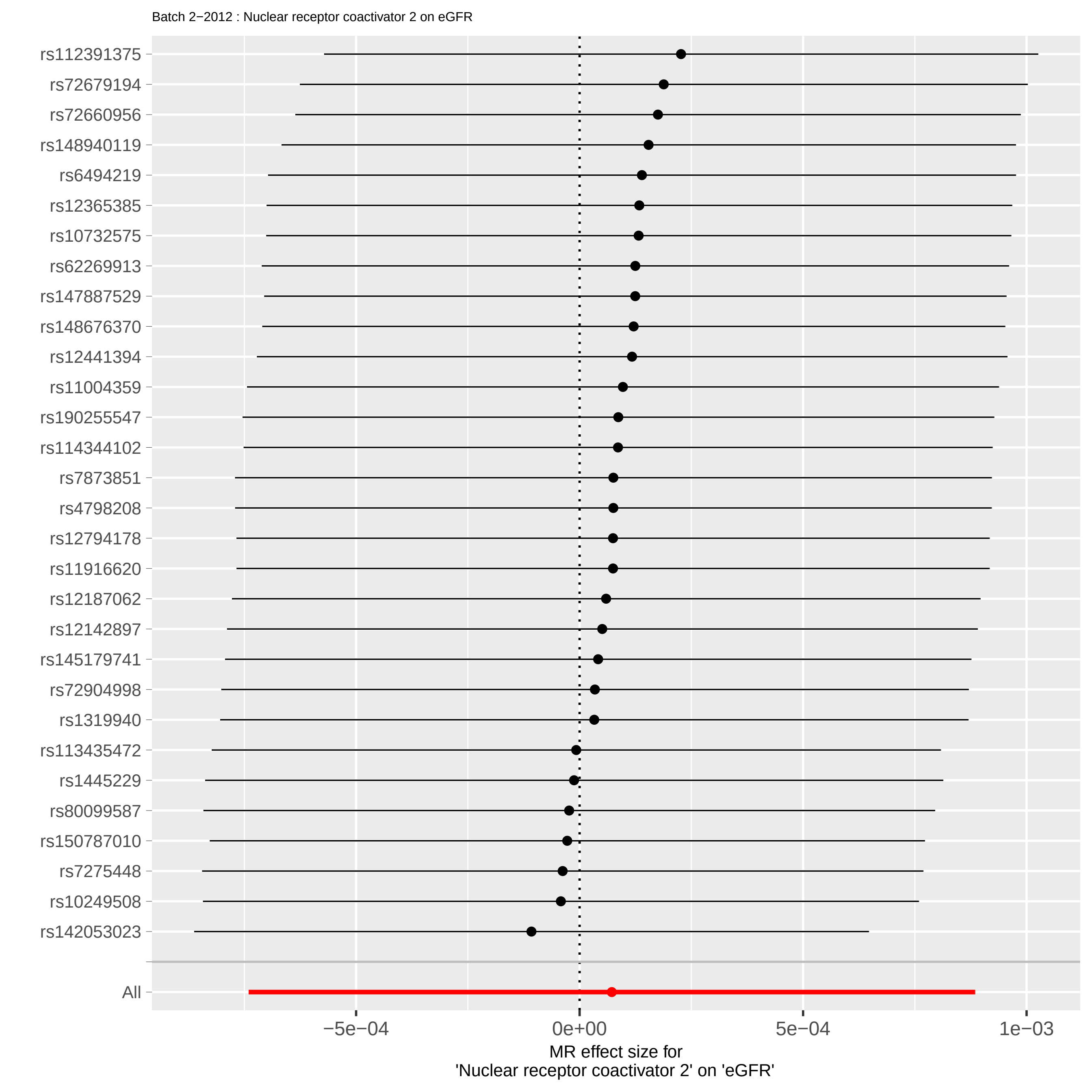
Batch 2−2012 : Nuclear receptor coactivator 2 on eGFR
rs112391375
rs72679194
rs72660956
rs148940119
rs6494219
rs12365385
rs10732575
rs62269913
rs147887529
rs148676370
rs12441394
rs11004359
rs190255547
rs114344102
rs7873851
rs4798208
rs12794178
rs11916620
rs12187062
rs12142897
rs145179741
rs72904998
rs1319940
rs113435472
rs1445229
rs80099587
rs150787010
rs7275448
rs10249508
rs142053023
All
−5e−04
0e+00
5e−04
1e−03
MR effect size for
'Nuclear receptor coactivator 2' on 'eGFR'

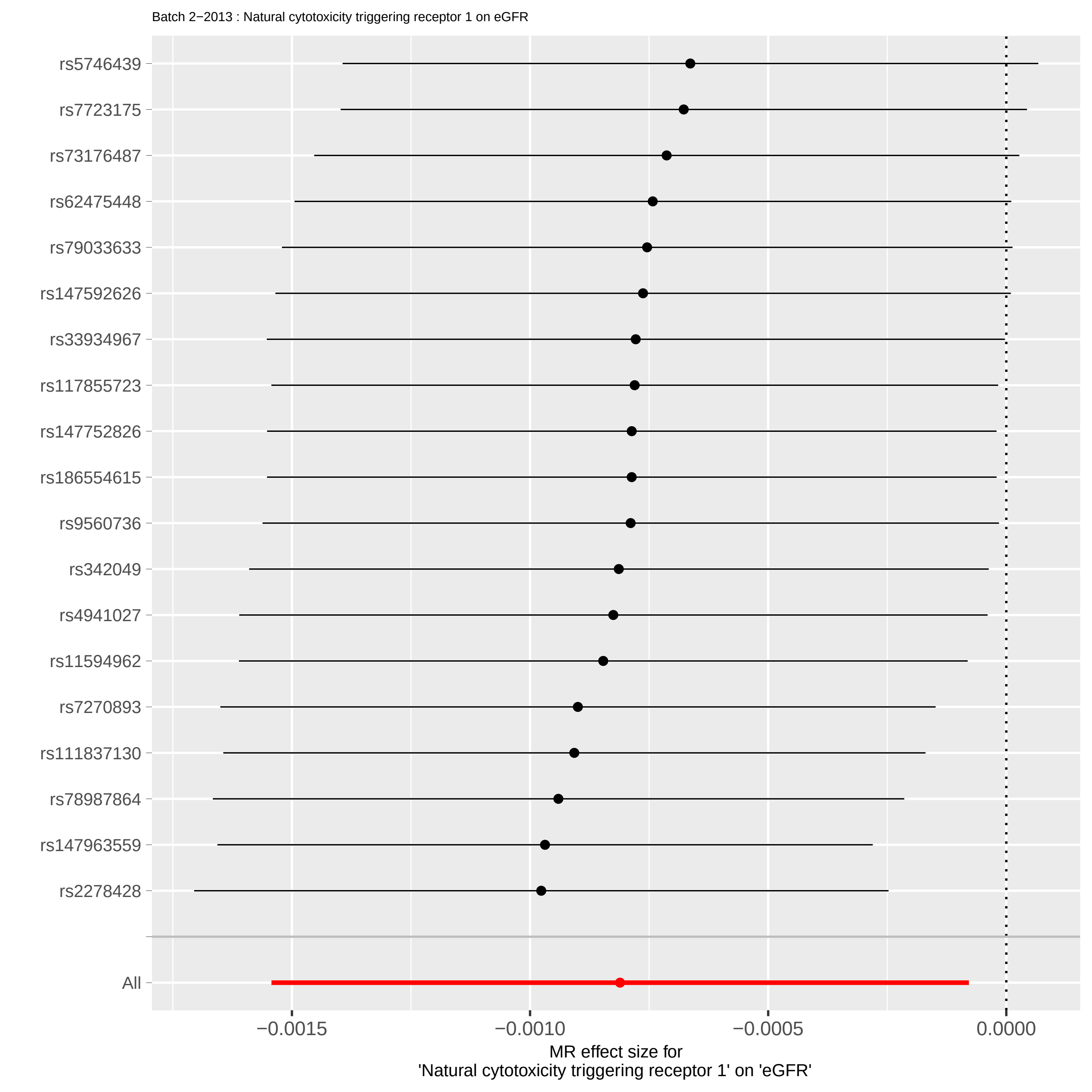
Batch 2−2013 : Natural cytotoxicity triggering receptor 1 on eGFR
rs5746439
rs7723175
rs73176487
rs62475448
rs79033633
rs147592626
rs33934967
rs117855723
rs147752826
rs186554615
rs9560736
rs342049
rs4941027
rs11594962
rs7270893
rs111837130
rs78987864
rs147963559
rs2278428
All
−0.0015
−0.0010
−0.0005
0.0000
MR effect size for
'Natural cytotoxicity triggering receptor 1' on 'eGFR'

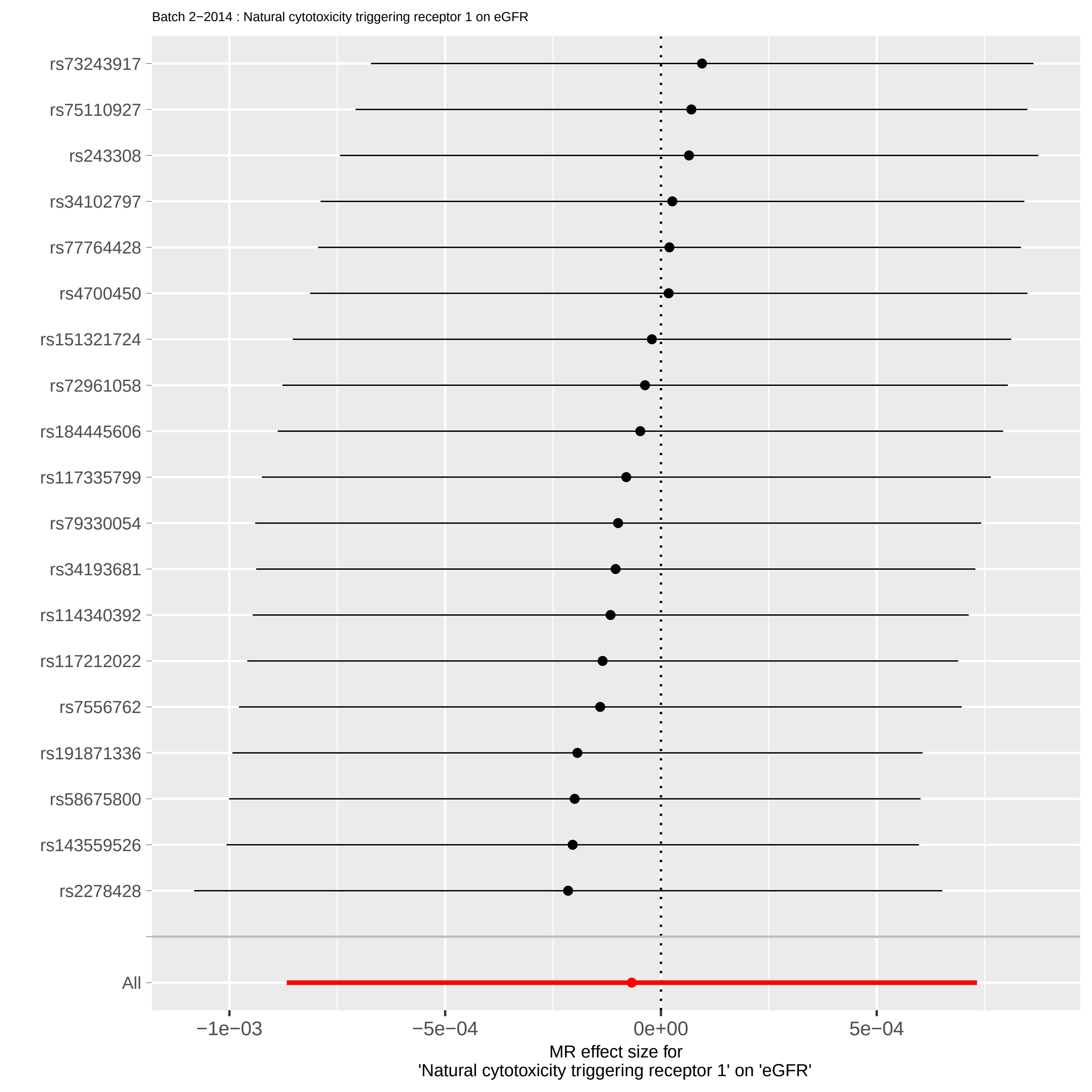

Batch 2−2014 : Natural cytotoxicity triggering receptor 1 on eGFR
rs73243917
rs75110927
rs243308
rs34102797
rs77764428
rs4700450
rs151321724
rs72961058
rs184445606
rs117335799
rs79330054
rs34193681
rs114340392
rs117212022
rs7556762
rs191871336
rs58675800
rs143559526
rs2278428
All
−1e−03
−5e−04
0e+00
5e−04
MR effect size for
'Natural cytotoxicity triggering receptor 1' on 'eGFR'

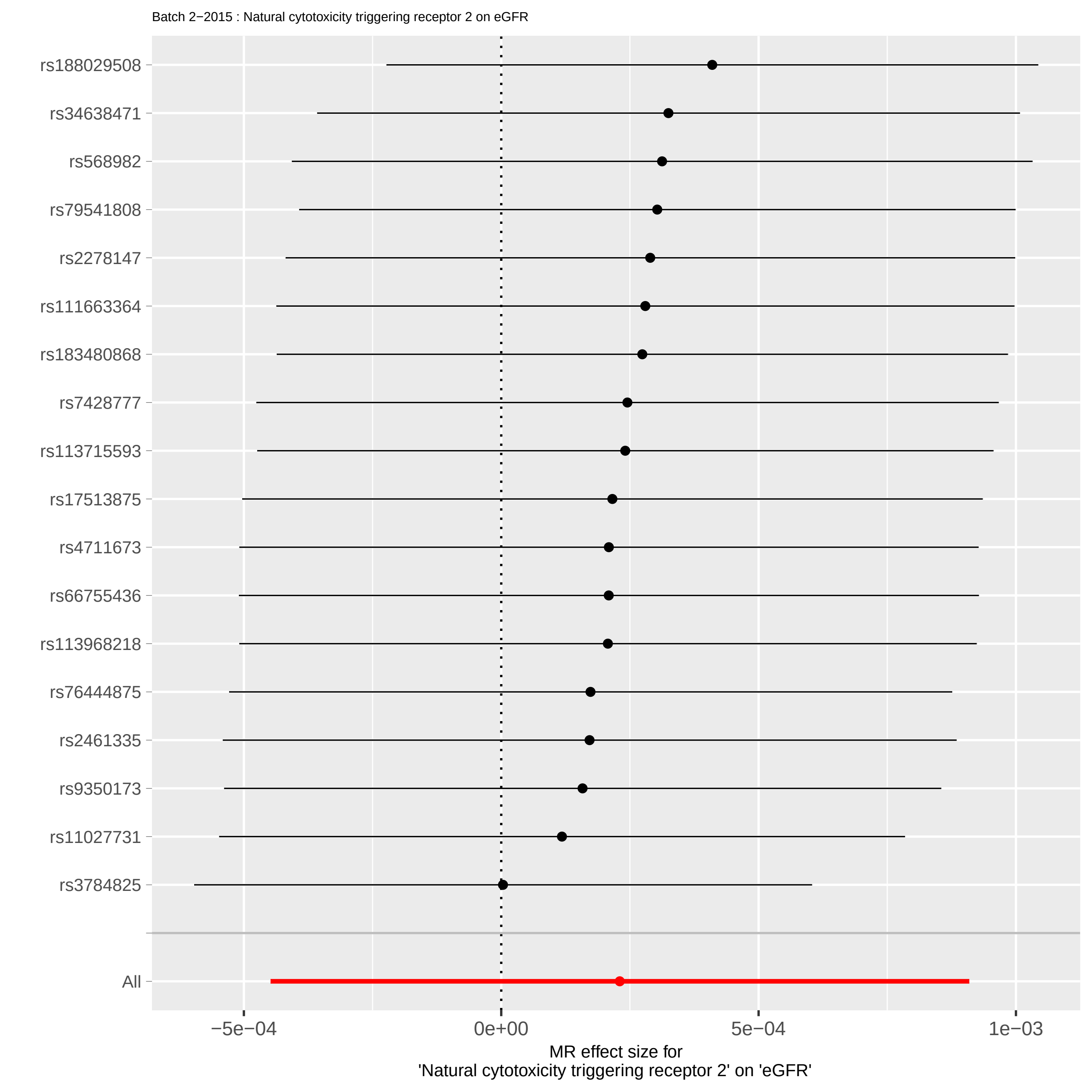

Batch 2−2015 : Natural cytotoxicity triggering receptor 2 on eGFR
rs188029508
rs34638471
rs568982
rs79541808
rs2278147
rs111663364
rs183480868
rs7428777
rs113715593
rs17513875
rs4711673
rs66755436
rs113968218
rs76444875
rs2461335
rs9350173
rs11027731
rs3784825
All
−5e−04
0e+00
5e−04
1e−03
MR effect size for
'Natural cytotoxicity triggering receptor 2' on 'eGFR'

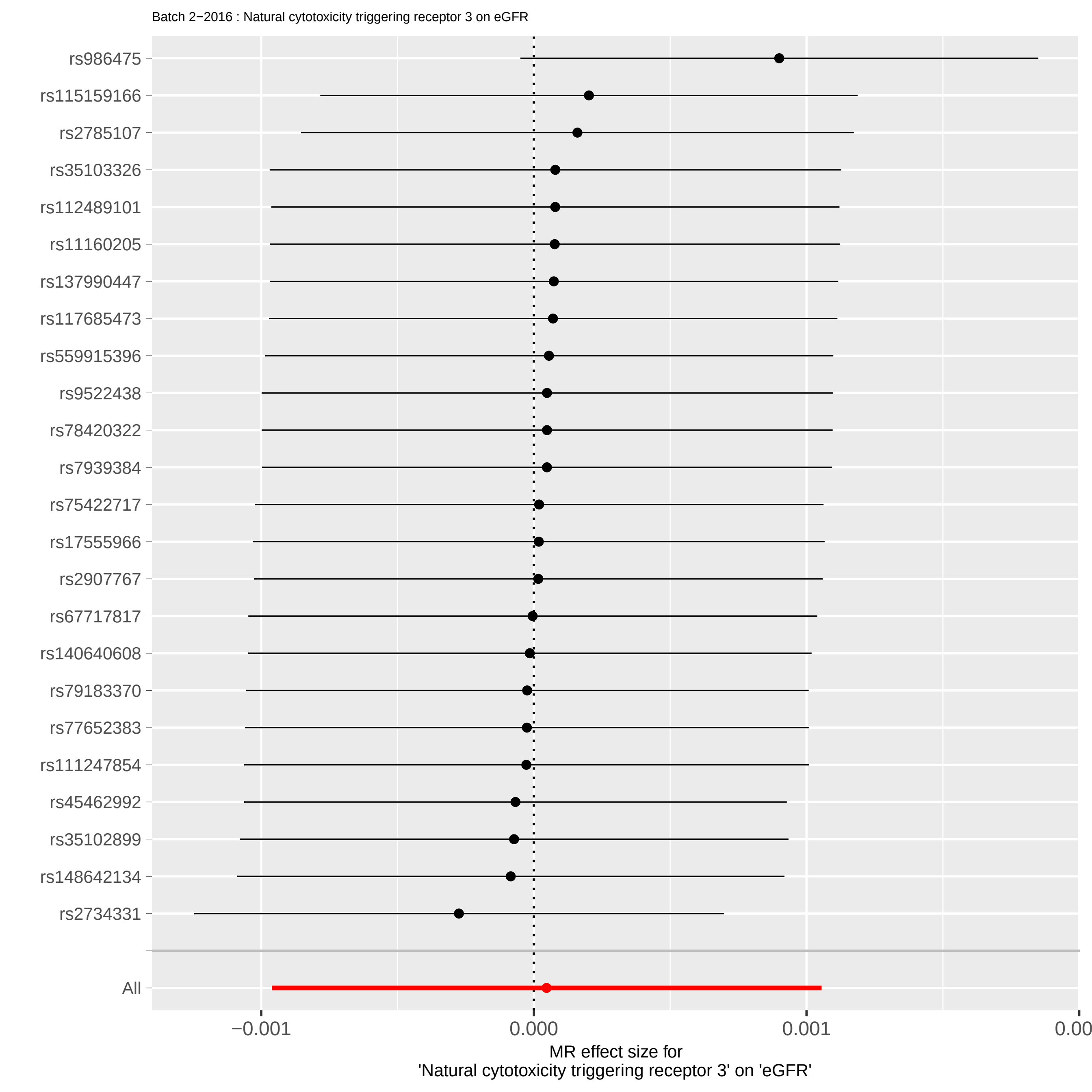

Batch 2−2016 : Natural cytotoxicity triggering receptor 3 on eGFR
rs986475
rs115159166
rs2785107
rs35103326
rs112489101
rs11160205
rs137990447
rs117685473
rs559915396
rs9522438
rs78420322
rs7939384
rs75422717
rs17555966
rs2907767
rs67717817
rs140640608
rs79183370
rs77652383
rs111247854
rs45462992
rs35102899
rs148642134
rs2734331
All
−0.001
0.000
0.001
0.00
MR effect size for
'Natural cytotoxicity triggering receptor 3' on 'eGFR'

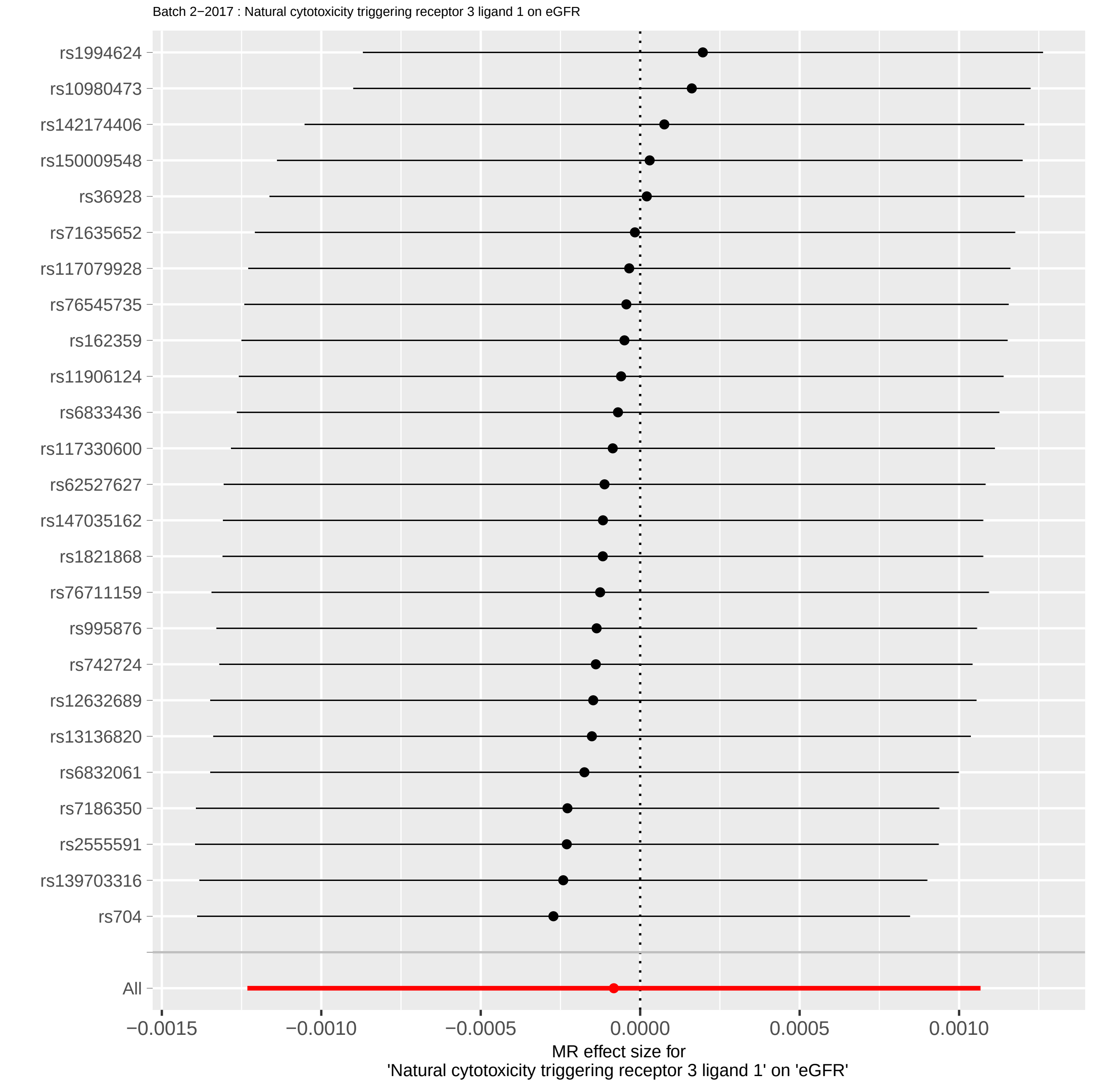
Batch 2−2017 : Natural cytotoxicity triggering receptor 3 ligand 1 on eGFR
rs1994624
rs10980473
rs142174406
rs150009548
rs36928
rs71635652
rs117079928
rs76545735
rs162359
rs11906124
rs6833436
rs117330600
rs62527627
rs147035162
rs1821868
rs76711159
rs995876
rs742724
rs12632689
rs13136820
rs6832061
rs7186350
rs2555591
rs139703316
rs704
All
−0.0015
−0.0010
−0.0005
0.0000
0.0005
0.0010
MR effect size for
'Natural cytotoxicity triggering receptor 3 ligand 1' on 'eGFR'

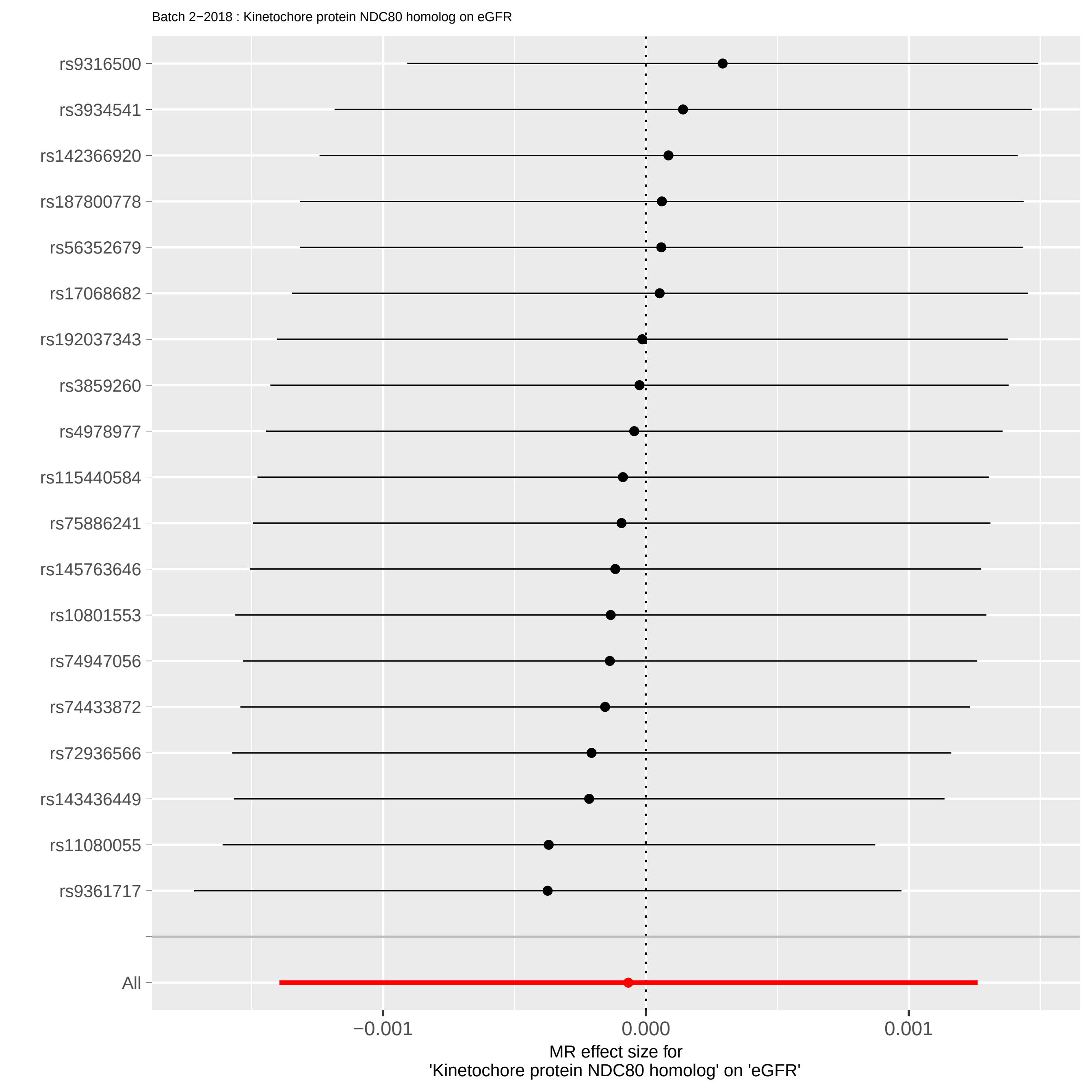

Batch 2−2018 : Kinetochore protein NDC80 homolog on eGFR
rs9316500
rs3934541
rs142366920
rs187800778
rs56352679
rs17068682
rs192037343
rs3859260
rs4978977
rs115440584
rs75886241
rs145763646
rs10801553
rs74947056
rs74433872
rs72936566
rs143436449
rs11080055
rs9361717
All
−0.001
0.000
0.001
MR effect size for
'Kinetochore protein NDC80 homolog' on 'eGFR'

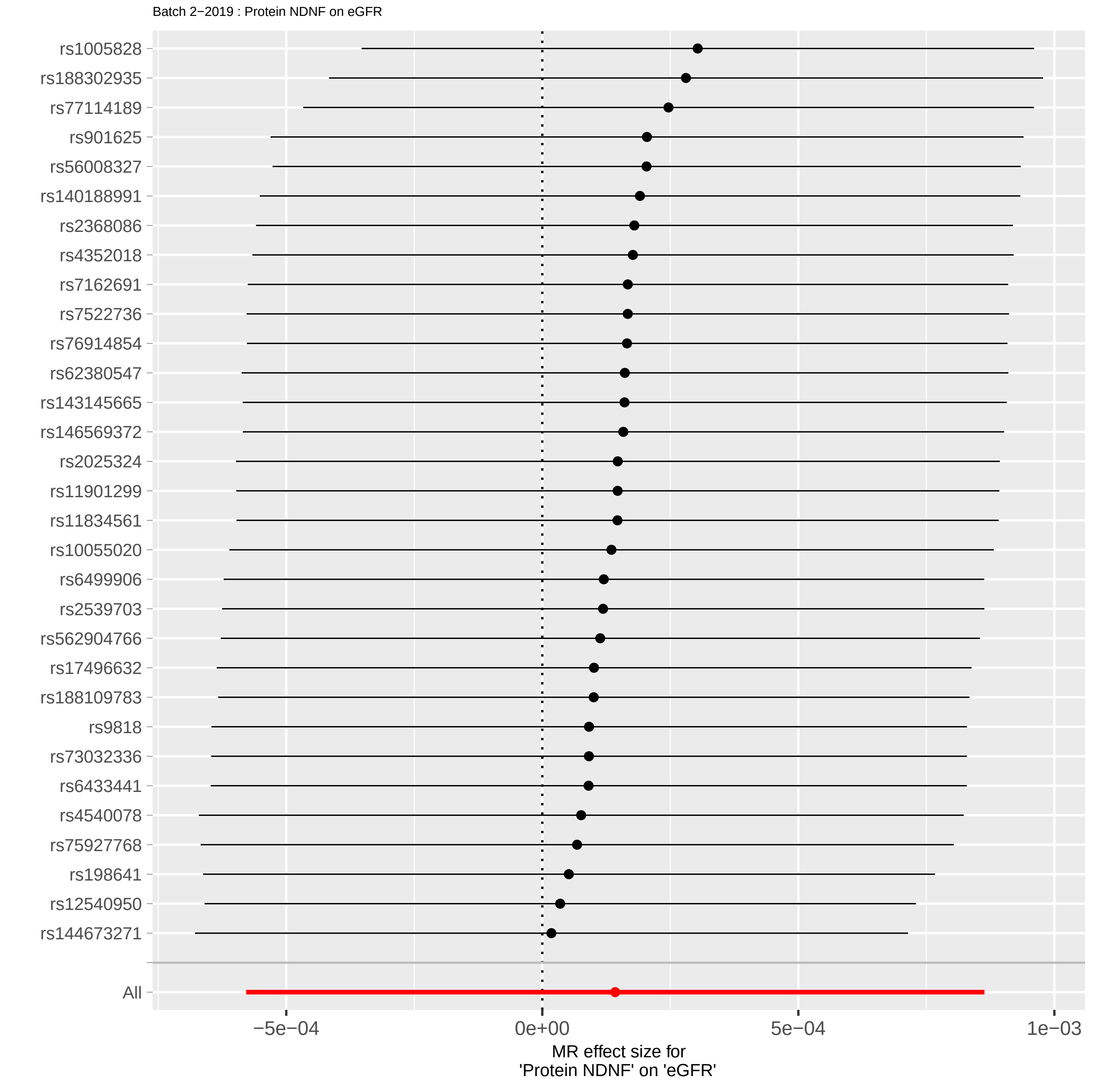
Batch 2−2019 : Protein NDNF on eGFR
rs1005828
rs188302935
rs77114189
rs901625
rs56008327
rs140188991
rs2368086
rs4352018
rs7162691
rs7522736
rs76914854
rs62380547
rs143145665
rs146569372
rs2025324
rs11901299
rs11834561
rs10055020
rs6499906
rs2539703
rs562904766
rs17496632
rs188109783
rs9818
rs73032336
rs6433441
rs4540078
rs75927768
rs198641
rs12540950
rs144673271
All
−5e−04
0e+00
5e−04
1e−03
MR effect size for
'Protein NDNF' on 'eGFR'

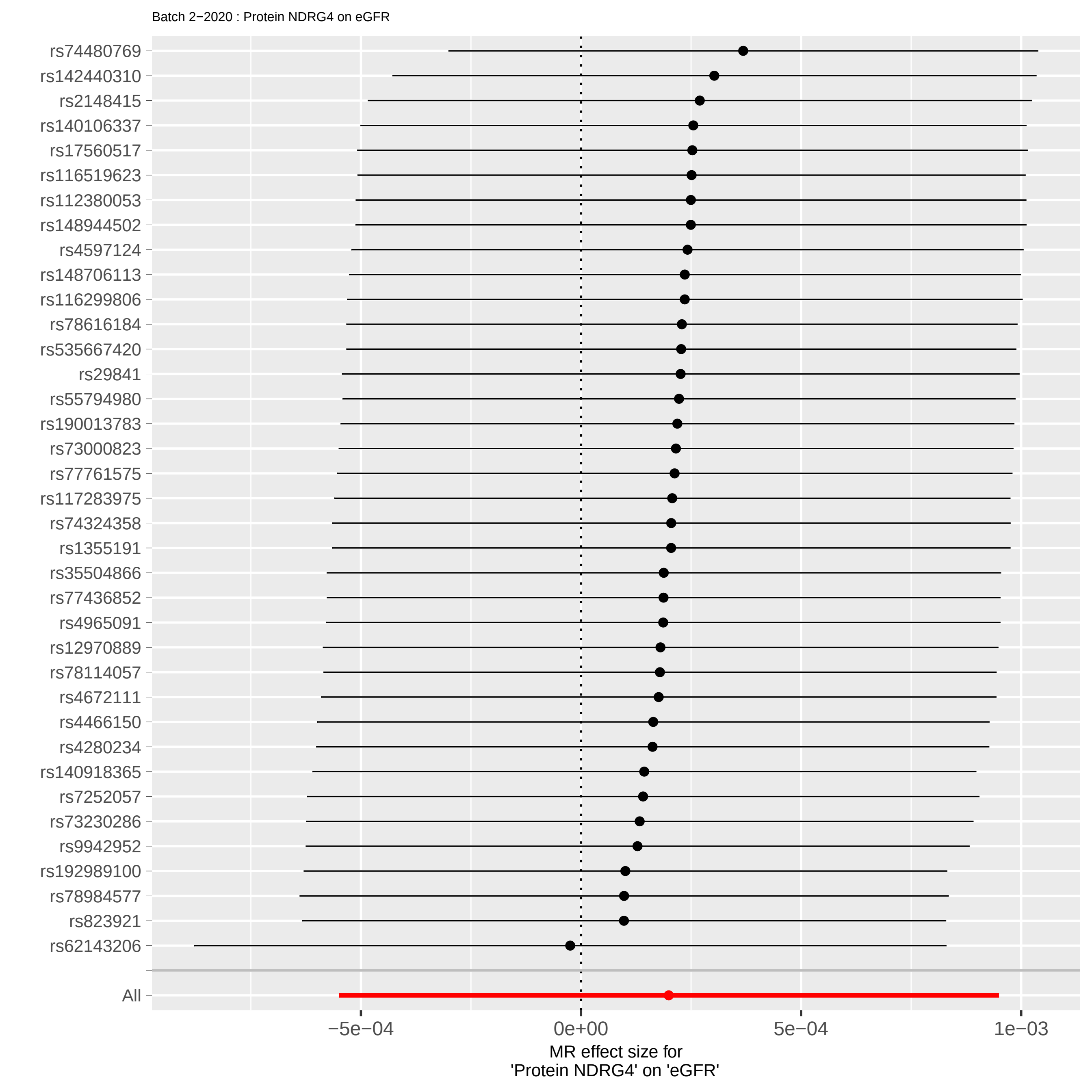

Batch 2−2020 : Protein NDRG4 on eGFR
rs74480769
rs142440310
rs2148415
rs140106337
rs17560517
rs116519623
rs112380053
rs148944502
rs4597124
rs148706113
rs116299806
rs78616184
rs535667420
rs29841
rs55794980
rs190013783
rs73000823
rs77761575
rs117283975
rs74324358
rs1355191
rs35504866
rs77436852
rs4965091
rs12970889
rs78114057
rs4672111
rs4466150
rs4280234
rs140918365
rs7252057
rs73230286
rs9942952
rs192989100
rs78984577
rs823921
rs62143206
All
−5e−04
0e+00
5e−04
1e−03
MR effect size for
'Protein NDRG4' on 'eGFR'

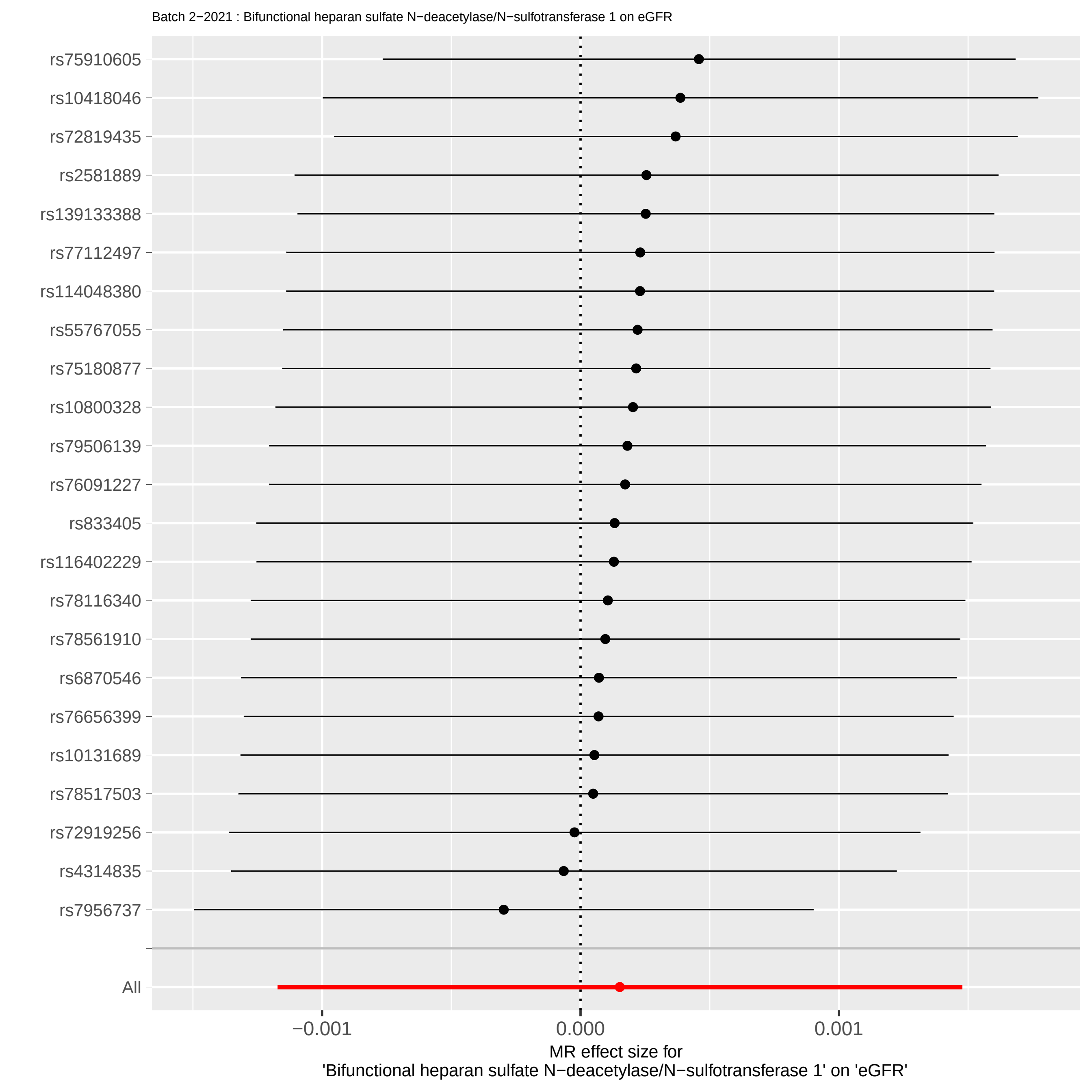

Batch 2−2021 : Bifunctional heparan sulfate N−deacetylase/N−sulfotransferase 1 on eGFR
rs75910605
rs10418046
rs72819435
rs2581889
rs139133388
rs77112497
rs114048380
rs55767055
rs75180877
rs10800328
rs79506139
rs76091227
rs833405
rs116402229
rs78116340
rs78561910
rs6870546
rs76656399
rs10131689
rs78517503
rs72919256
rs4314835
rs7956737
All
−0.001
0.000
0.001
MR effect size for
'Bifunctional heparan sulfate N−deacetylase/N−sulfotransferase 1' on 'eGFR'

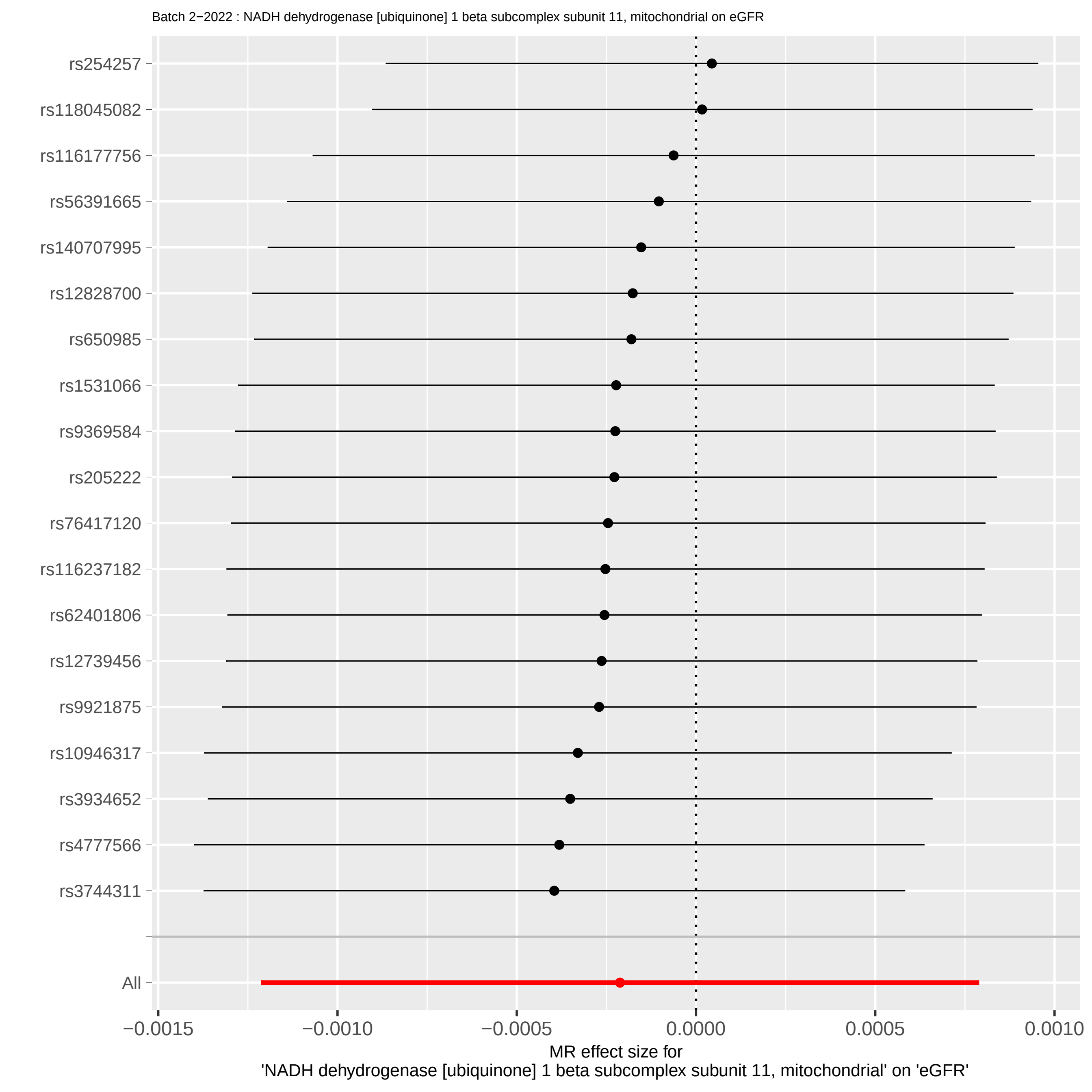

Batch 2−2022 : NADH dehydrogenase [ubiquinone] 1 beta subcomplex subunit 11, mitochondrial on eGFR
rs254257
rs118045082
rs116177756
rs56391665
rs140707995
rs12828700
rs650985
rs1531066
rs9369584
rs205222
rs76417120
rs116237182
rs62401806
rs12739456
rs9921875
rs10946317
rs3934652
rs4777566
rs3744311
All
−0.0015
−0.0010
−0.0005
0.0000
0.0005
0.0010
MR effect size for
'NADH dehydrogenase [ubiquinone] 1 beta subcomplex subunit 11, mitochondrial' on 'eGFR'

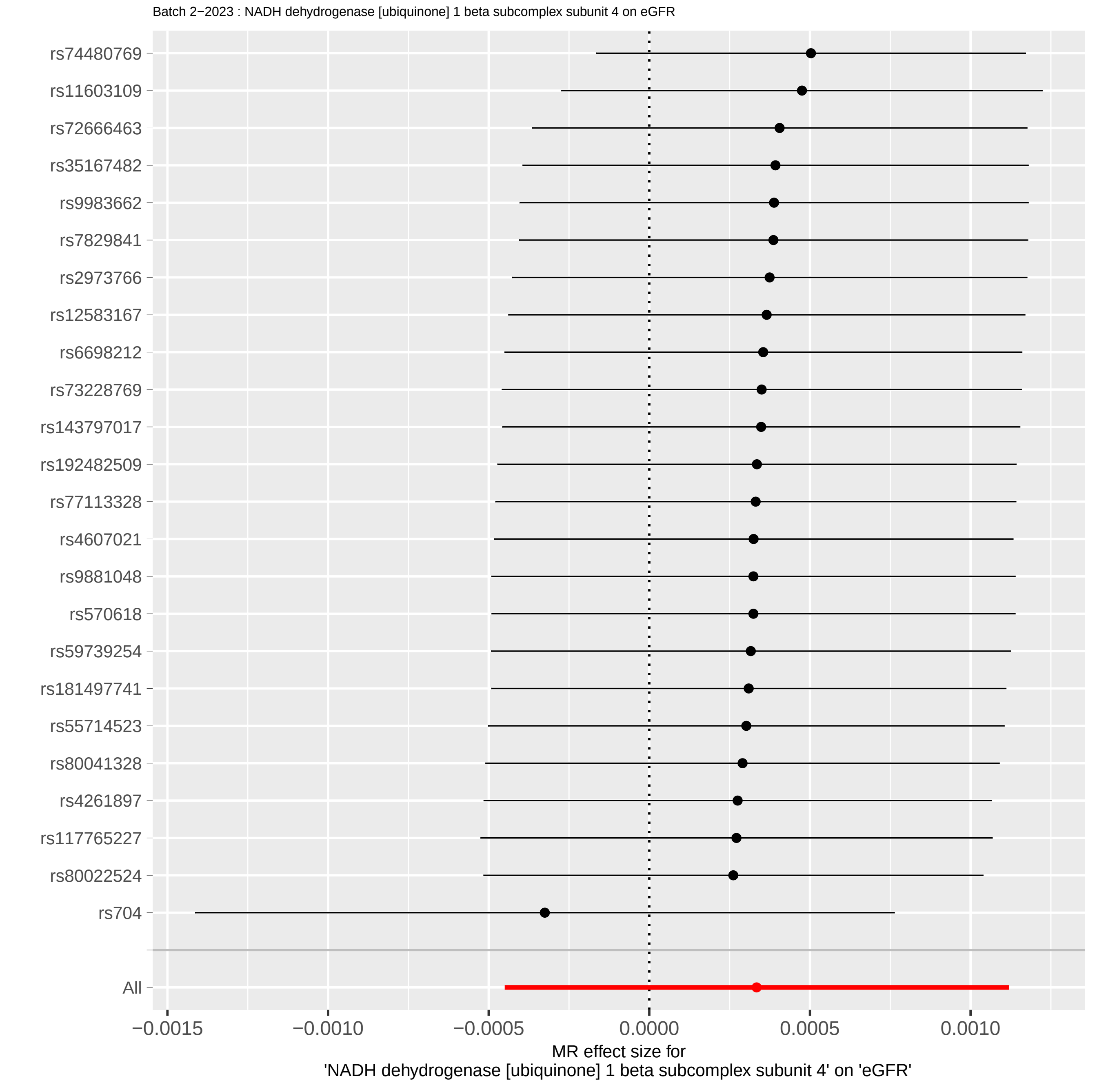

Batch 2−2023 : NADH dehydrogenase [ubiquinone] 1 beta subcomplex subunit 4 on eGFR
rs74480769
rs11603109
rs72666463
rs35167482
rs9983662
rs7829841
rs2973766
rs12583167
rs6698212
rs73228769
rs143797017
rs192482509
rs77113328
rs4607021
rs9881048
rs570618
rs59739254
rs181497741
rs55714523
rs80041328
rs4261897
rs117765227
rs80022524
rs704
All
−0.0015
−0.0010
−0.0005
0.0000
0.0005
0.0010
MR effect size for
'NADH dehydrogenase [ubiquinone] 1 beta subcomplex subunit 4' on 'eGFR'

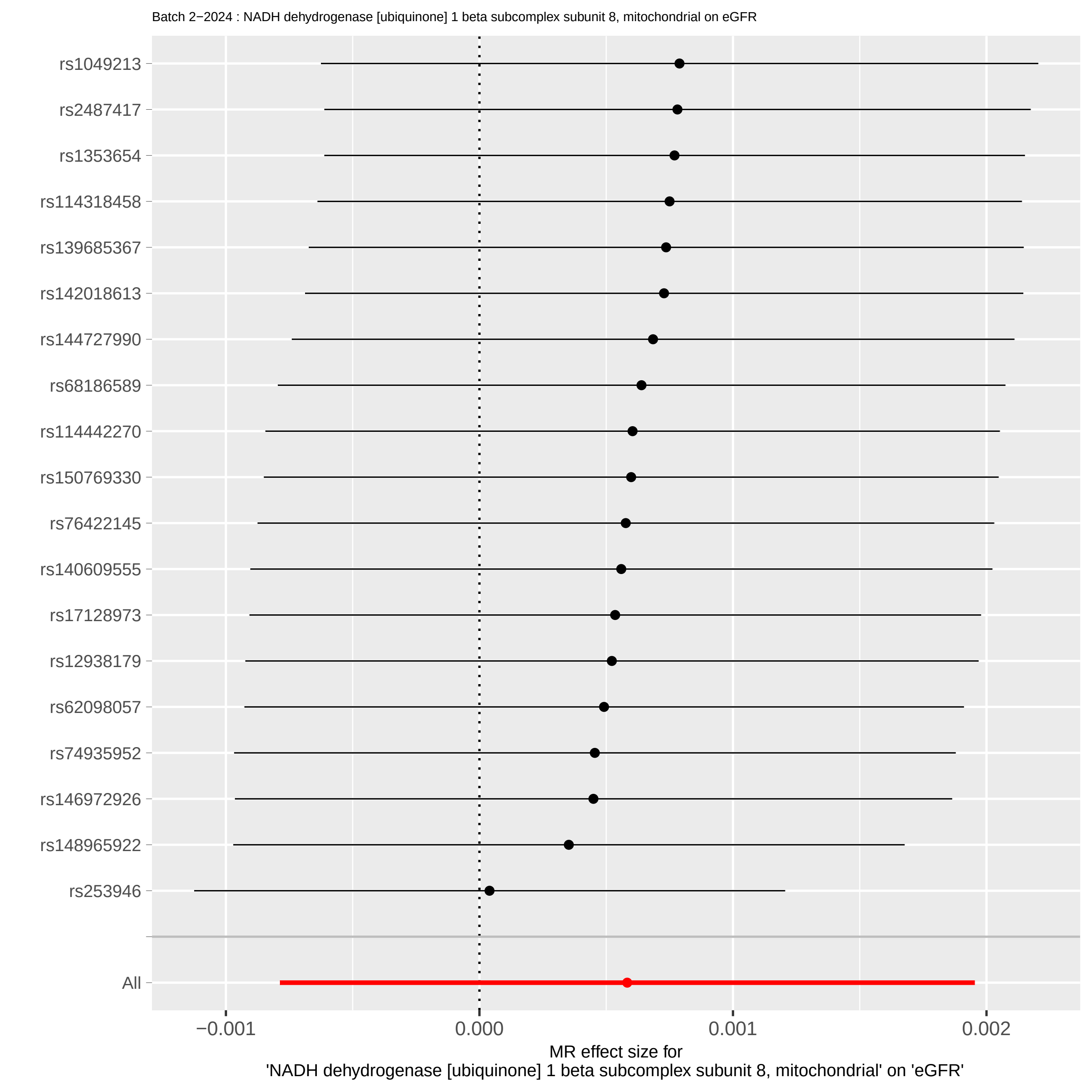

Batch 2−2024 : NADH dehydrogenase [ubiquinone] 1 beta subcomplex subunit 8, mitochondrial on eGFR
rs1049213
rs2487417
rs1353654
rs114318458
rs139685367
rs142018613
rs144727990
rs68186589
rs114442270
rs150769330
rs76422145
rs140609555
rs17128973
rs12938179
rs62098057
rs74935952
rs146972926
rs148965922
rs253946
All
−0.001
0.000
0.001
0.002
MR effect size for
'NADH dehydrogenase [ubiquinone] 1 beta subcomplex subunit 8, mitochondrial' on 'eGFR'

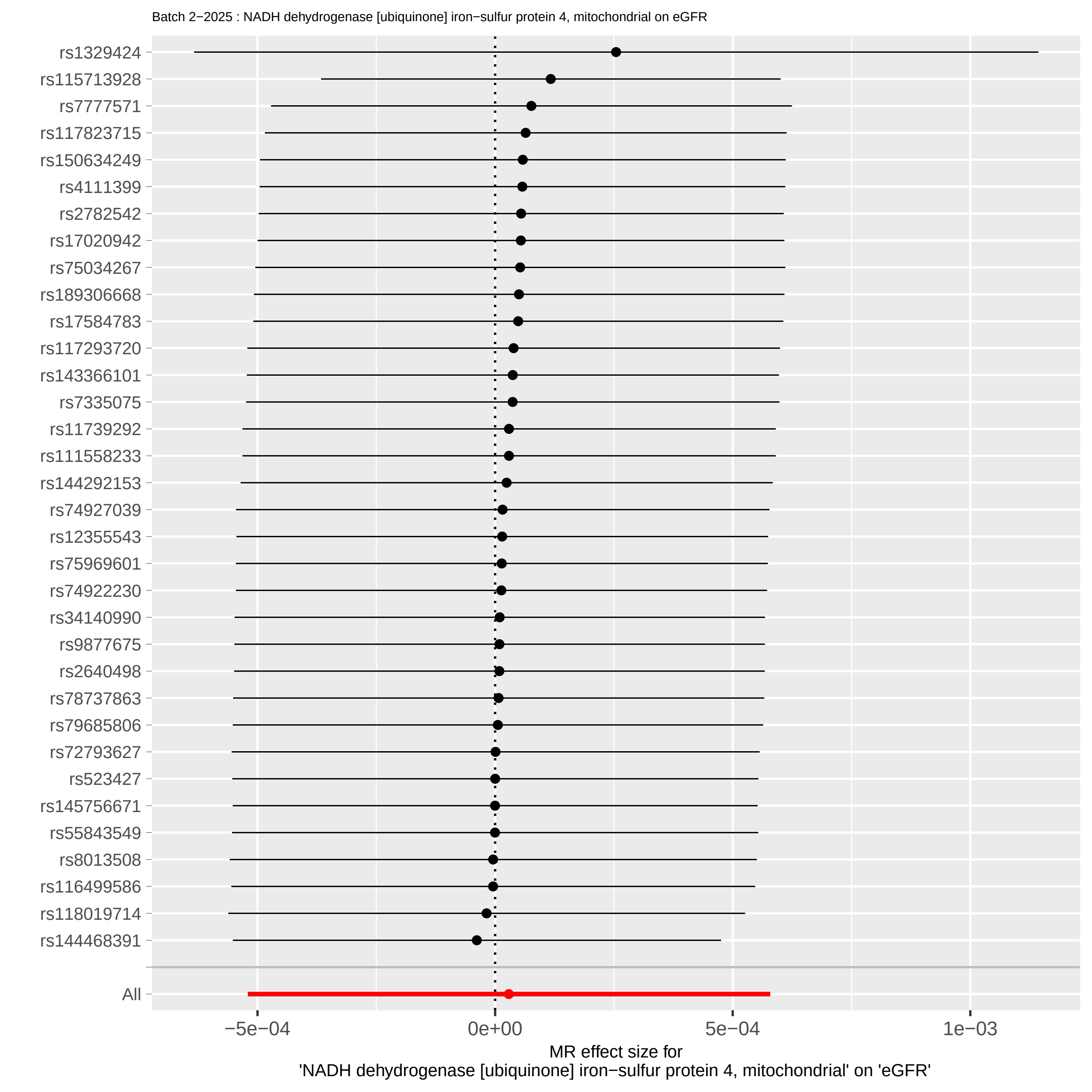

Batch 2−2025 : NADH dehydrogenase [ubiquinone] iron−sulfur protein 4, mitochondrial on eGFR
rs1329424
rs115713928
rs7777571
rs117823715
rs150634249
rs4111399
rs2782542
rs17020942
rs75034267
rs189306668
rs17584783
rs117293720
rs143366101
rs7335075
rs11739292
rs111558233
rs144292153
rs74927039
rs12355543
rs75969601
rs74922230
rs34140990
rs9877675
rs2640498
rs78737863
rs79685806
rs72793627
rs523427
rs145756671
rs55843549
rs8013508
rs116499586
rs118019714
rs144468391
All
−5e−04
0e+00
5e−04
1e−03
MR effect size for
'NADH dehydrogenase [ubiquinone] iron−sulfur protein 4, mitochondrial' on 'eGFR'

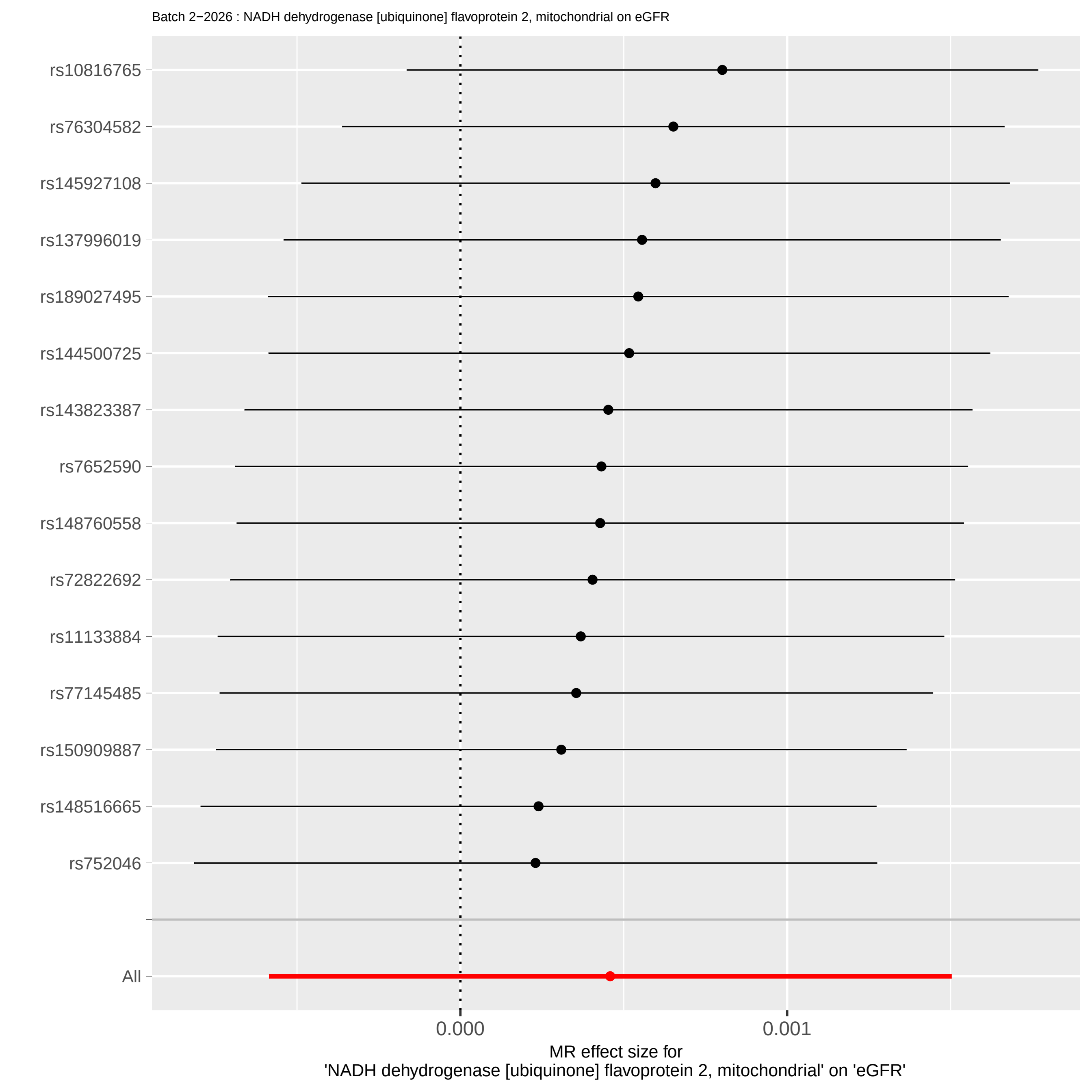
Batch 2−2026 : NADH dehydrogenase [ubiquinone] flavoprotein 2, mitochondrial on eGFR
rs10816765
rs76304582
rs145927108
rs137996019
rs189027495
rs144500725
rs143823387
rs7652590
rs148760558
rs72822692
rs11133884
rs77145485
rs150909887
rs148516665
rs752046
All
0.000
0.001
MR effect size for
'NADH dehydrogenase [ubiquinone] flavoprotein 2, mitochondrial' on 'eGFR'

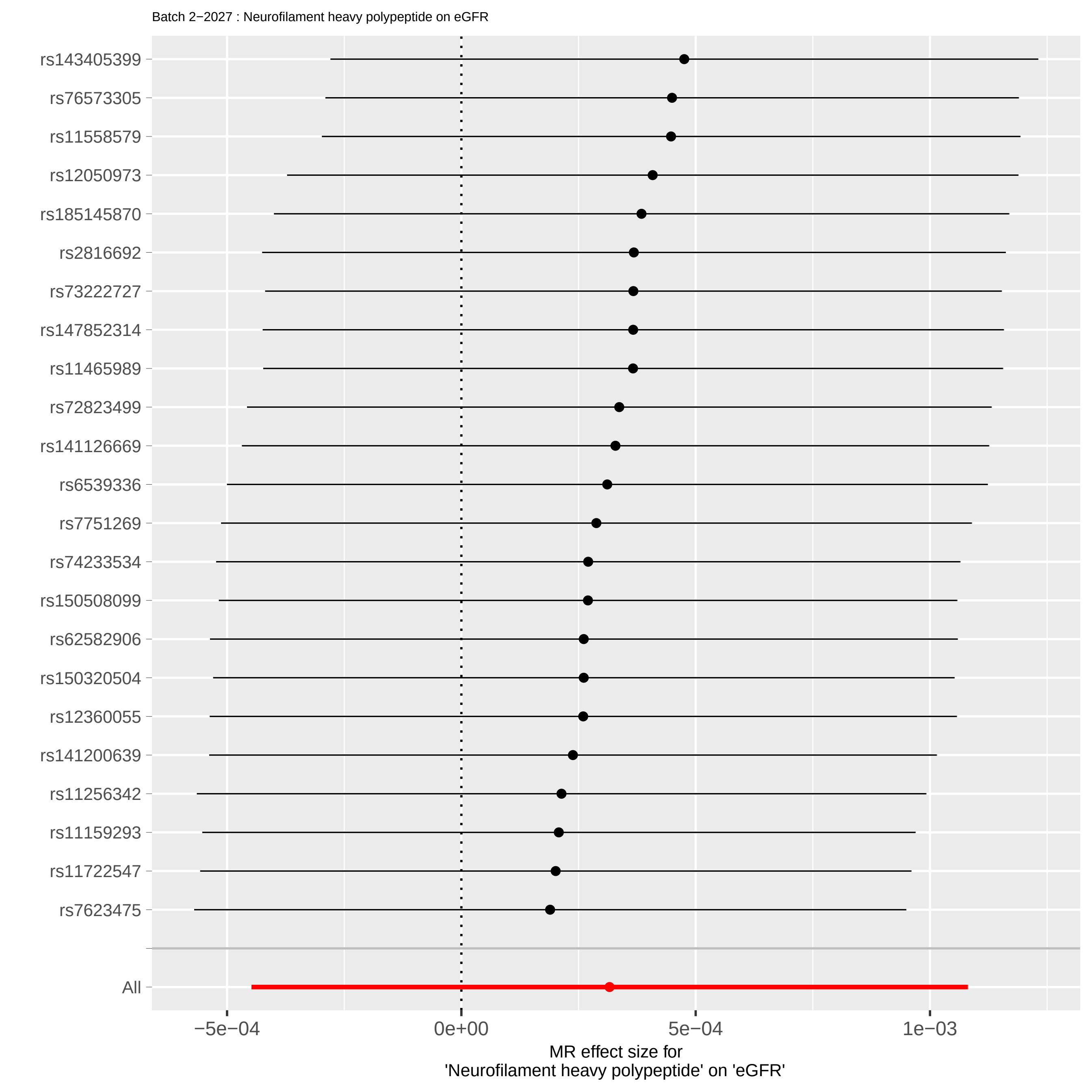
Batch 2−2027 : Neurofilament heavy polypeptide on eGFR
rs143405399
rs76573305
rs11558579
rs12050973
rs185145870
rs2816692
rs73222727
rs147852314
rs11465989
rs72823499
rs141126669
rs6539336
rs7751269
rs74233534
rs150508099
rs62582906
rs150320504
rs12360055
rs141200639
rs11256342
rs11159293
rs11722547
rs7623475
All
−5e−04
0e+00
5e−04
1e−03
MR effect size for
'Neurofilament heavy polypeptide' on 'eGFR'

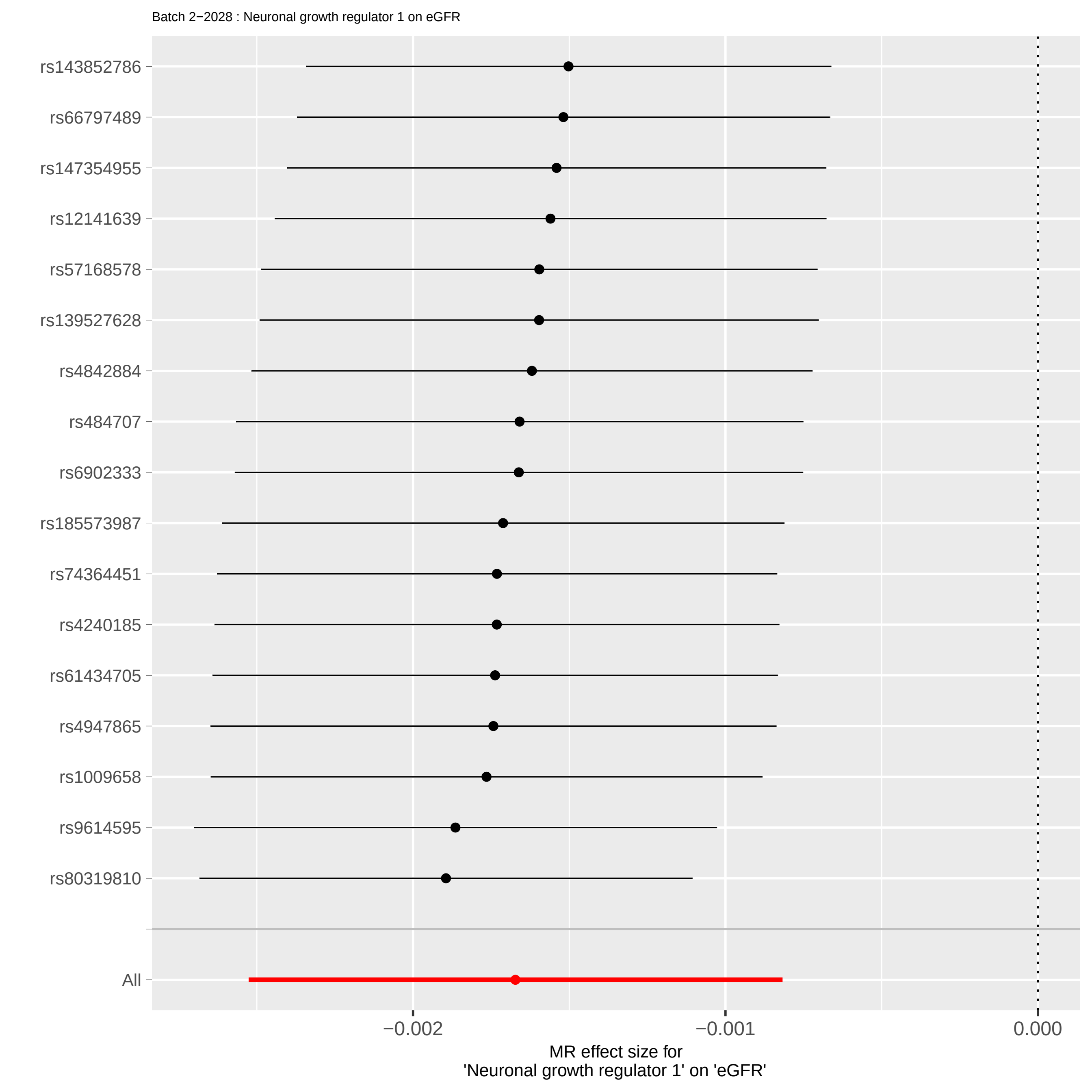

Batch 2−2028 : Neuronal growth regulator 1 on eGFR
rs143852786
rs66797489
rs147354955
rs12141639
rs57168578
rs139527628
rs4842884
rs484707
rs6902333
rs185573987
rs74364451
rs4240185
rs61434705
rs4947865
rs1009658
rs9614595
rs80319810
All
−0.002
−0.001
0.000
MR effect size for
'Neuronal growth regulator 1' on 'eGFR'

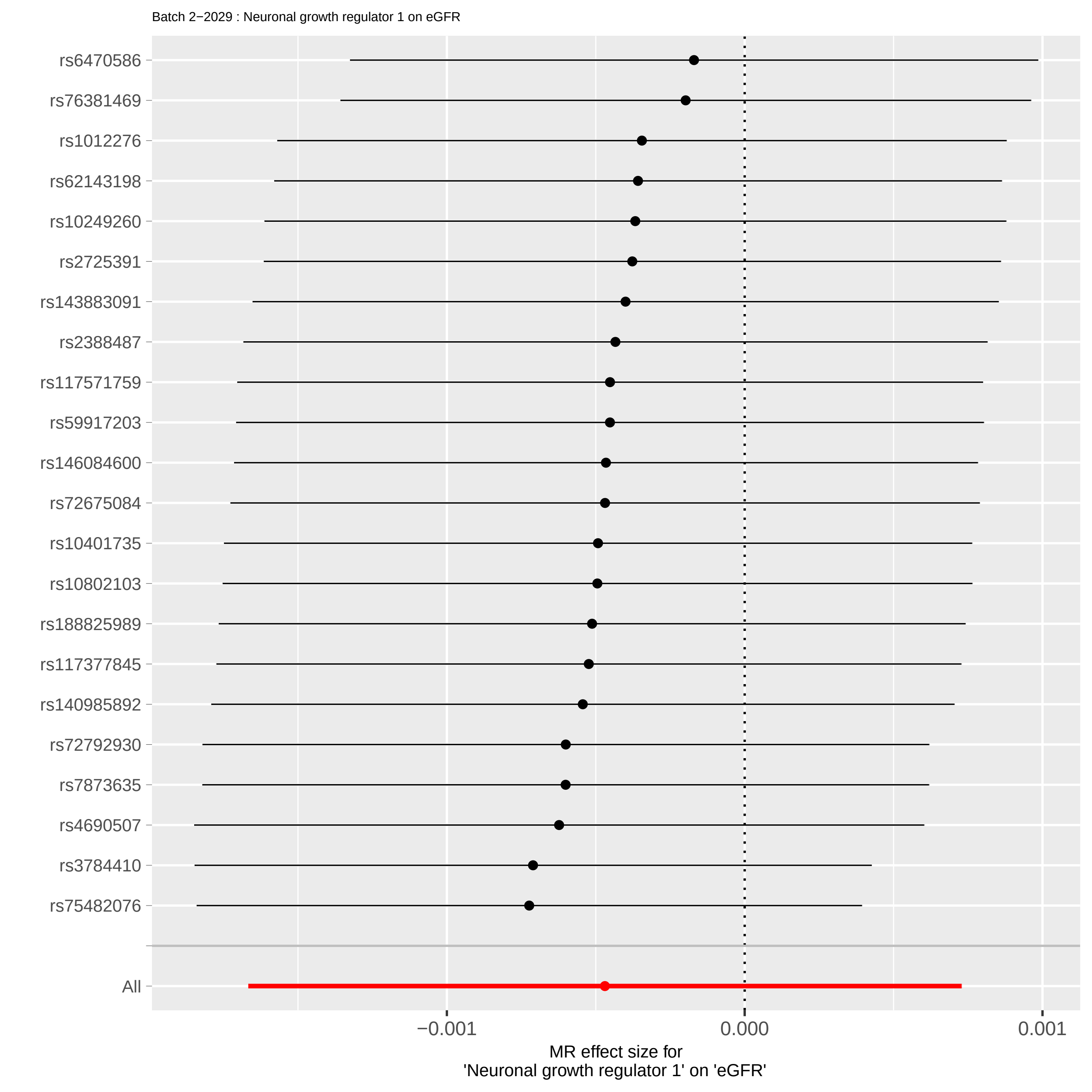

Batch 2−2029 : Neuronal growth regulator 1 on eGFR
rs6470586
rs76381469
rs1012276
rs62143198
rs10249260
rs2725391
rs143883091
rs2388487
rs117571759
rs59917203
rs146084600
rs72675084
rs10401735
rs10802103
rs188825989
rs117377845
rs140985892
rs72792930
rs7873635
rs4690507
rs3784410
rs75482076
All
−0.001
0.000
0.001
MR effect size for
'Neuronal growth regulator 1' on 'eGFR'

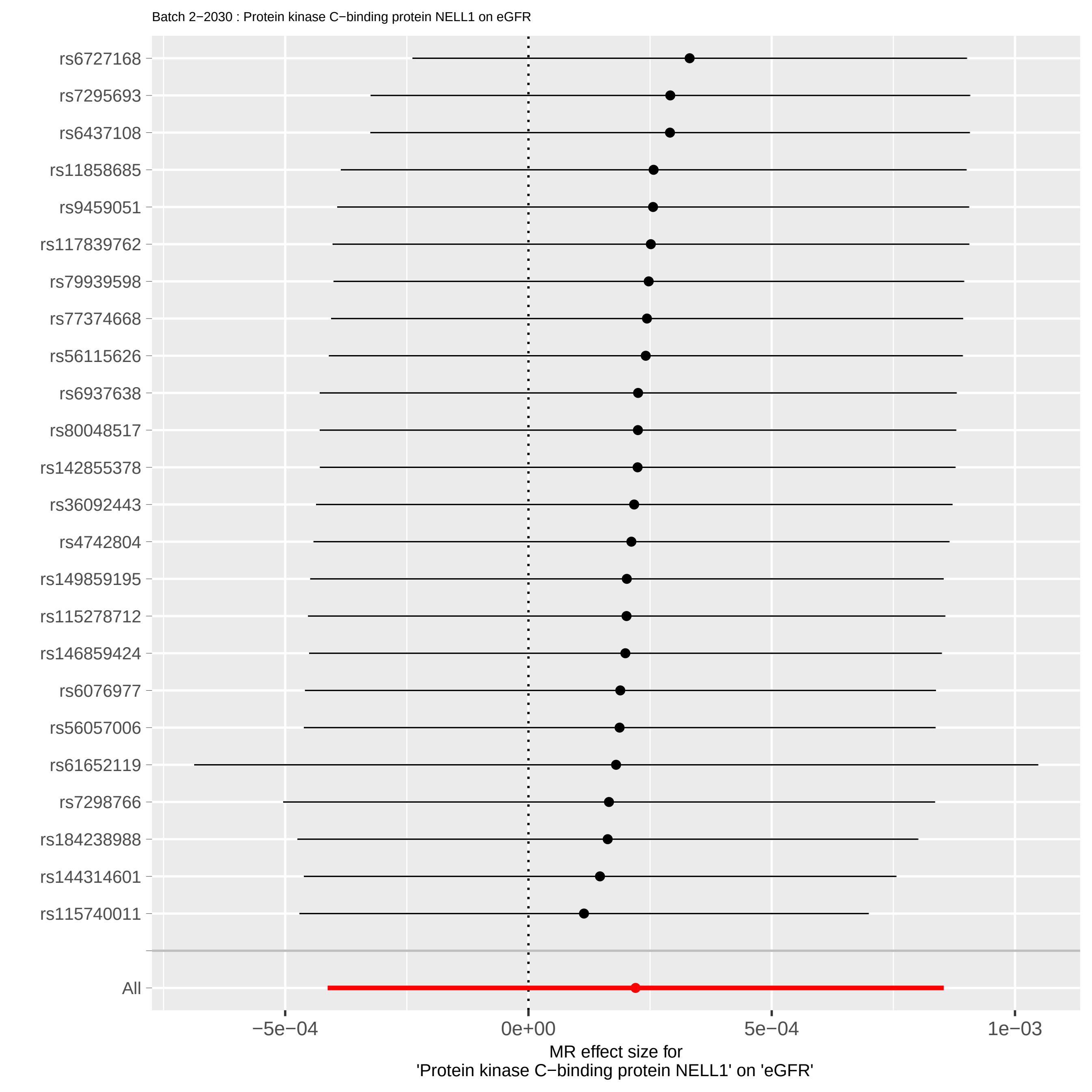

Batch 2−2030 : Protein kinase C−binding protein NELL1 on eGFR
rs6727168
rs7295693
rs6437108
rs11858685
rs9459051
rs117839762
rs79939598
rs77374668
rs56115626
rs6937638
rs80048517
rs142855378
rs36092443
rs4742804
rs149859195
rs115278712
rs146859424
rs6076977
rs56057006
rs61652119
rs7298766
rs184238988
rs144314601
rs115740011
All
−5e−04
0e+00
5e−04
1e−03
MR effect size for
'Protein kinase C−binding protein NELL1' on 'eGFR'

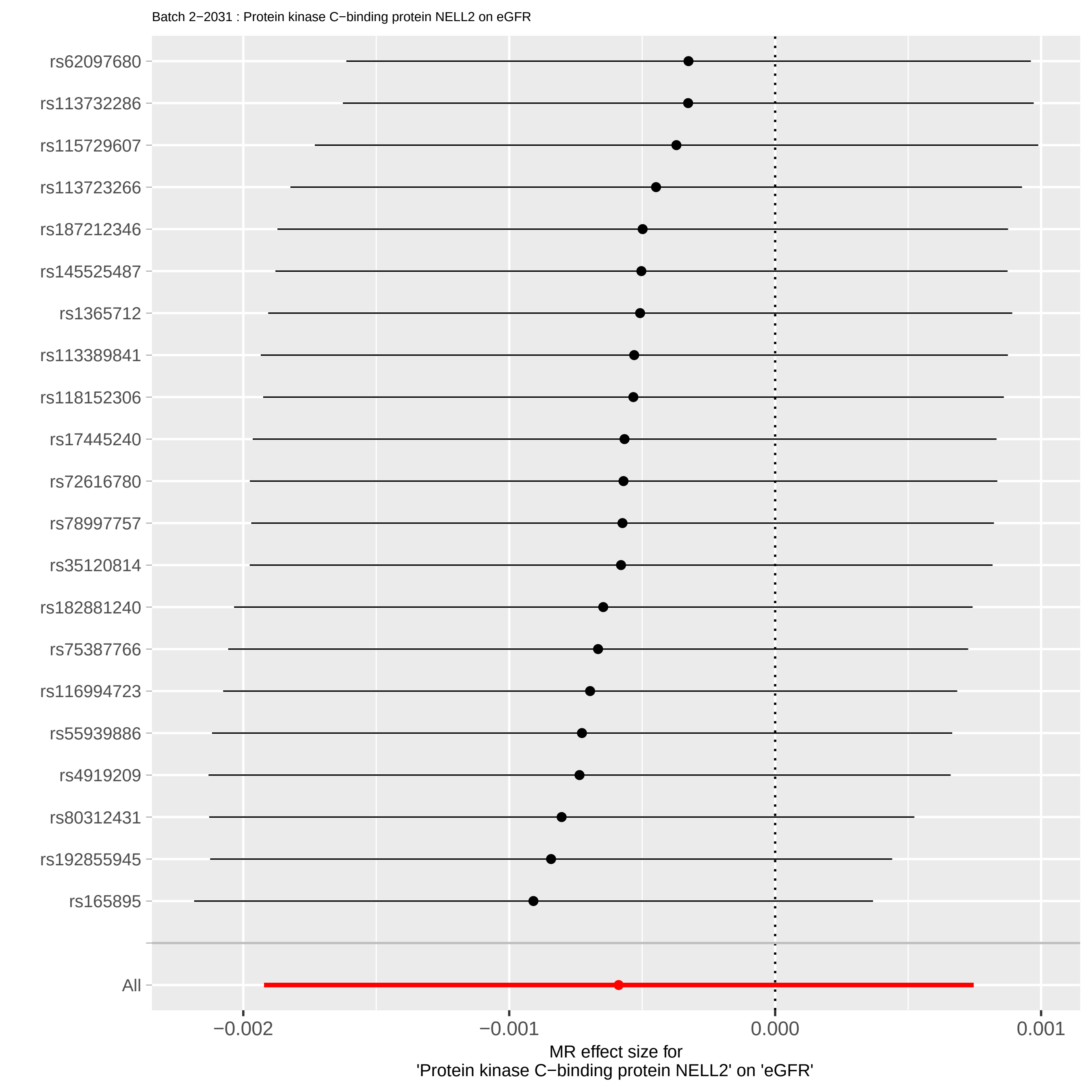

Batch 2−2031 : Protein kinase C−binding protein NELL2 on eGFR
rs62097680
rs113732286
rs115729607
rs113723266
rs187212346
rs145525487
rs1365712
rs113389841
rs118152306
rs17445240
rs72616780
rs78997757
rs35120814
rs182881240
rs75387766
rs116994723
rs55939886
rs4919209
rs80312431
rs192855945
rs165895
All
−0.002
−0.001
0.000
0.001
MR effect size for
'Protein kinase C−binding protein NELL2' on 'eGFR'

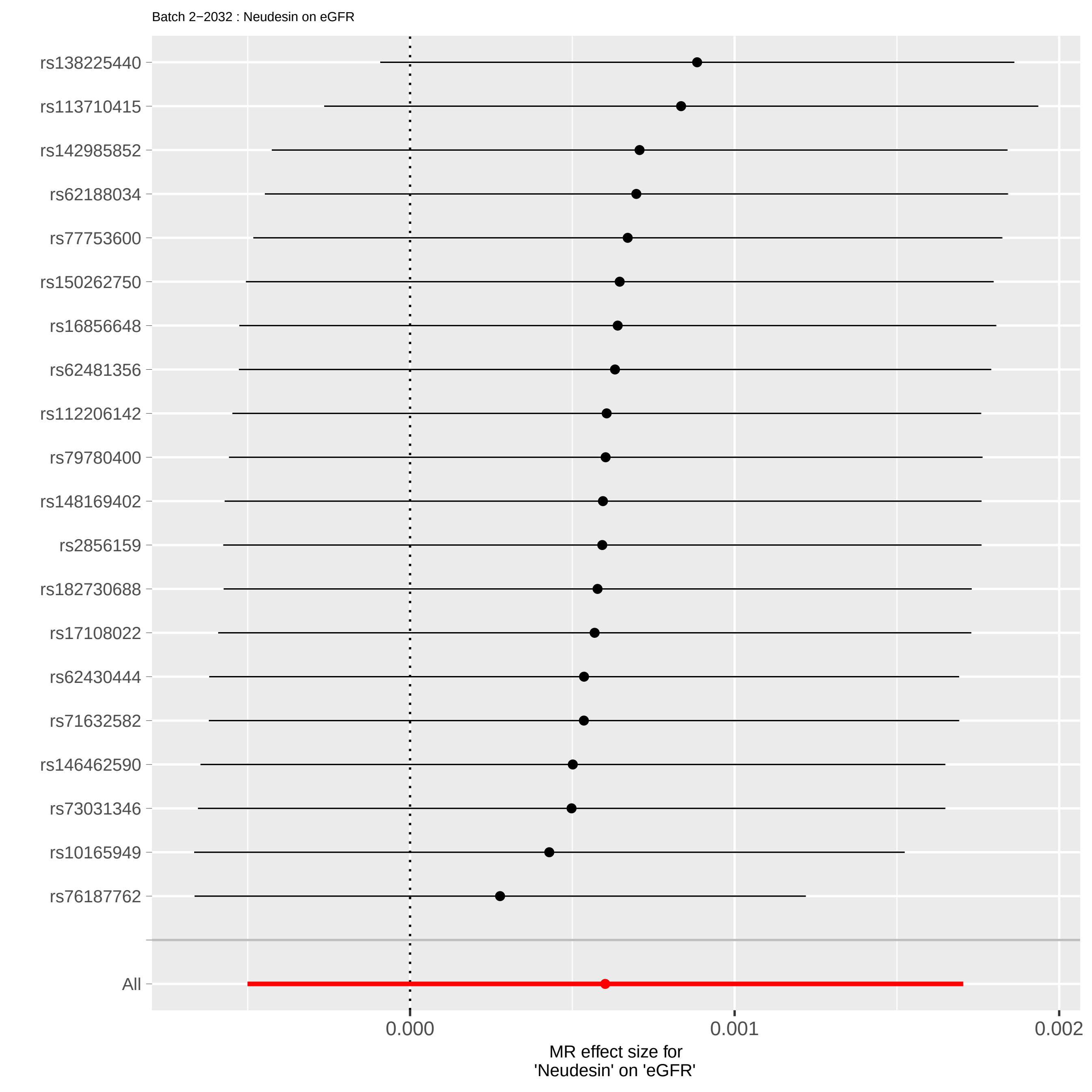
Batch 2−2032 : Neudesin on eGFR
rs138225440
rs113710415
rs142985852
rs62188034
rs77753600
rs150262750
rs16856648
rs62481356
rs112206142
rs79780400
rs148169402
rs2856159
rs182730688
rs17108022
rs62430444
rs71632582
rs146462590
rs73031346
rs10165949
rs76187762
All
0.000
0.001
0.002
MR effect size for
'Neudesin' on 'eGFR'

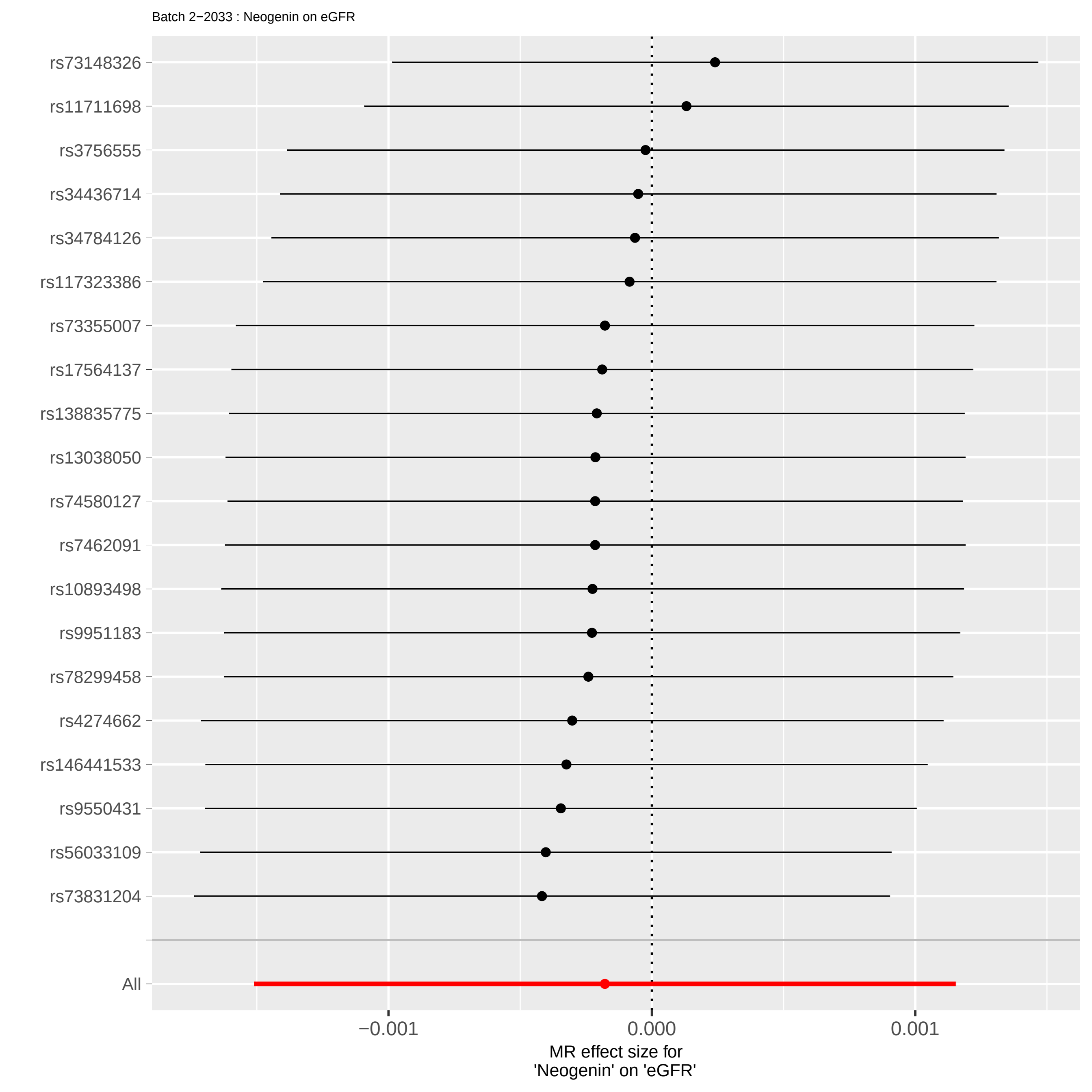

Batch 2−2033 : Neogenin on eGFR
rs73148326
rs11711698
rs3756555
rs34436714
rs34784126
rs117323386
rs73355007
rs17564137
rs138835775
rs13038050
rs74580127
rs7462091
rs10893498
rs9951183
rs78299458
rs4274662
rs146441533
rs9550431
rs56033109
rs73831204
All
−0.001
0.000
0.001
MR effect size for
'Neogenin' on 'eGFR'

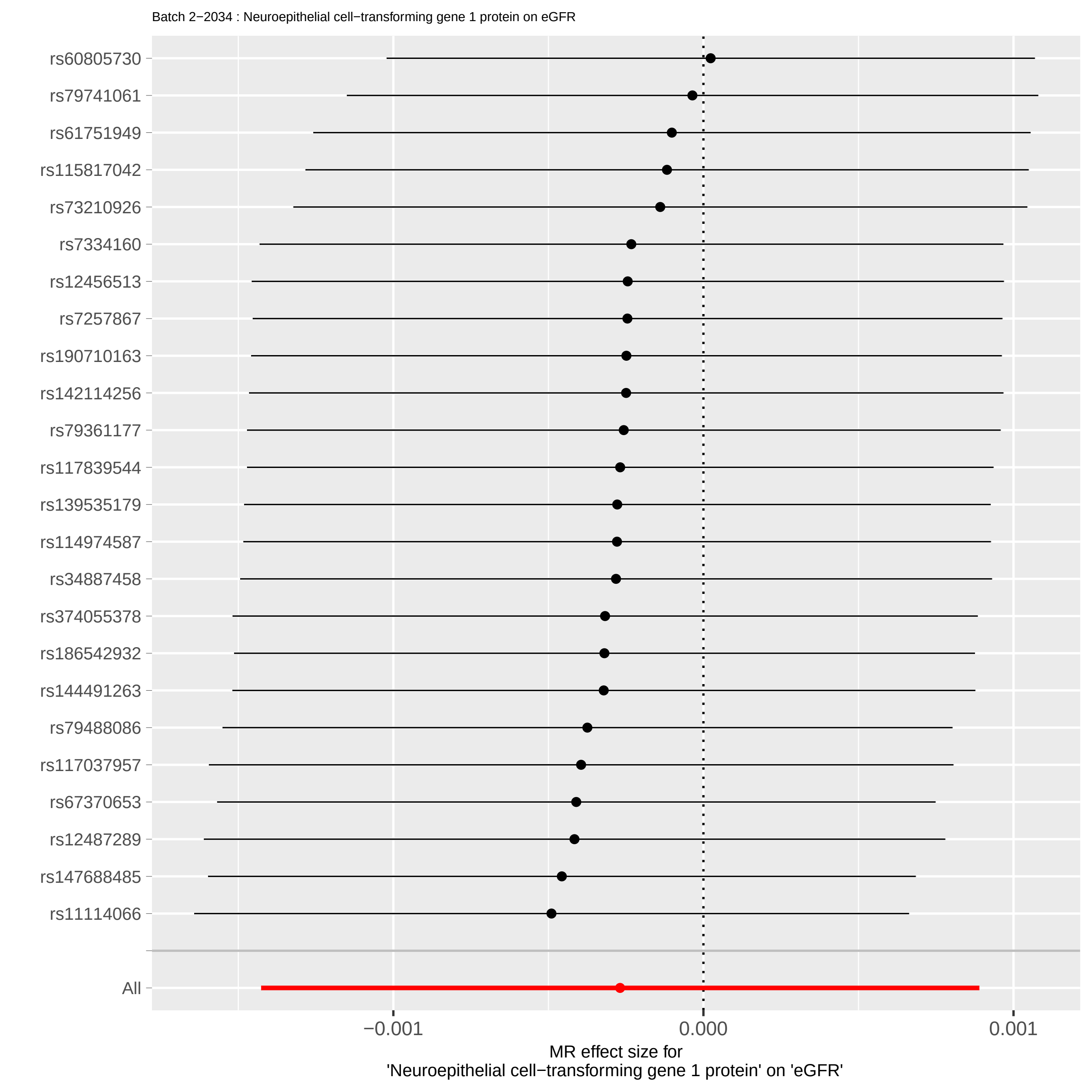

Batch 2−2034 : Neuroepithelial cell−transforming gene 1 protein on eGFR
rs60805730
rs79741061
rs61751949
rs115817042
rs73210926
rs7334160
rs12456513
rs7257867
rs190710163
rs142114256
rs79361177
rs117839544
rs139535179
rs114974587
rs34887458
rs374055378
rs186542932
rs144491263
rs79488086
rs117037957
rs67370653
rs12487289
rs147688485
rs11114066
All
−0.001
0.000
0.001
MR effect size for
'Neuroepithelial cell−transforming gene 1 protein' on 'eGFR'

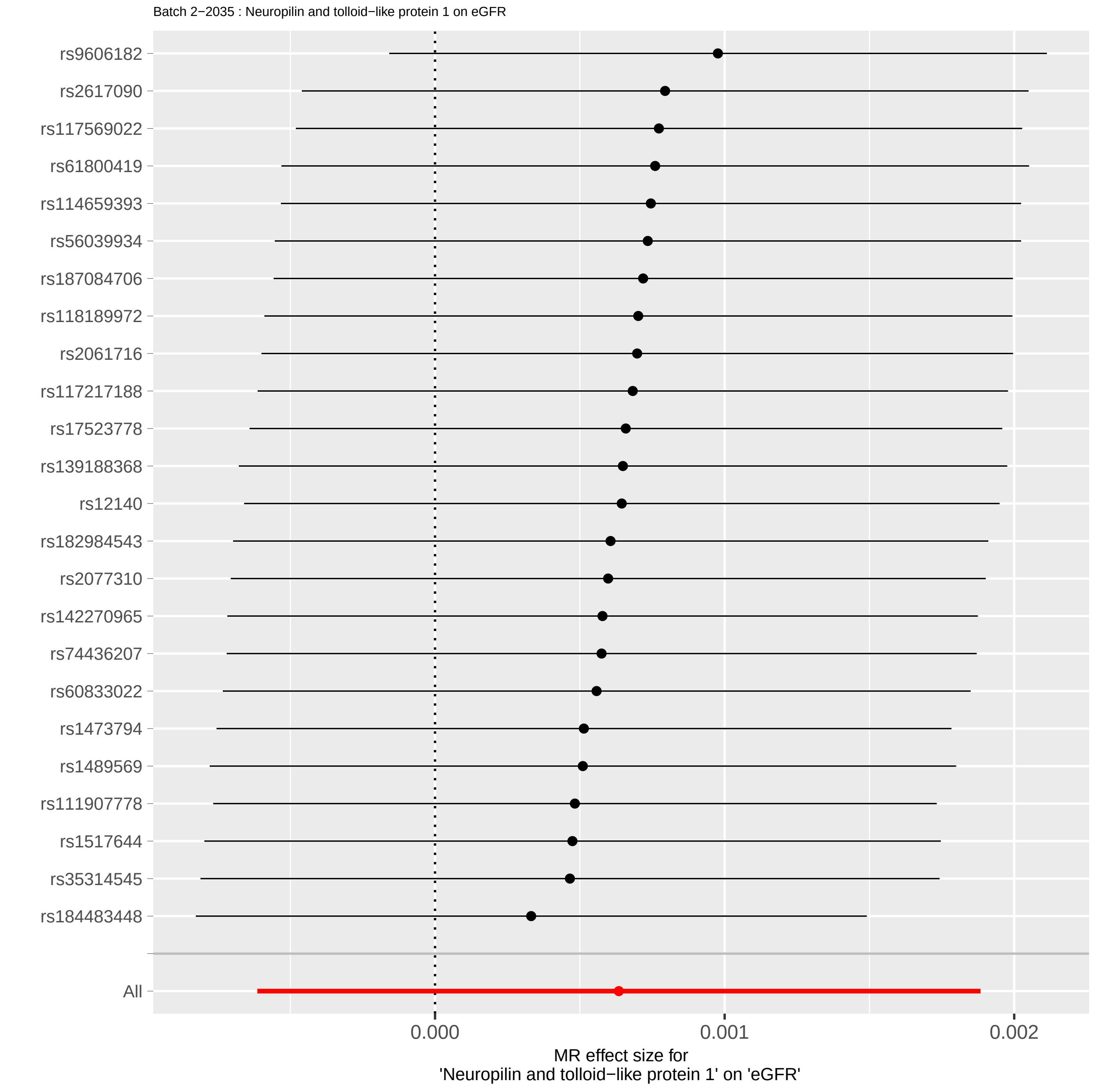
Batch 2−2035 : Neuropilin and tolloid−like protein 1 on eGFR
rs9606182
rs2617090
rs117569022
rs61800419
rs114659393
rs56039934
rs187084706
rs118189972
rs2061716
rs117217188
rs17523778
rs139188368
rs12140
rs182984543
rs2077310
rs142270965
rs74436207
rs60833022
rs1473794
rs1489569
rs111907778
rs1517644
rs35314545
rs184483448
All
0.000
0.001
0.002
MR effect size for
'Neuropilin and tolloid−like protein 1' on 'eGFR'

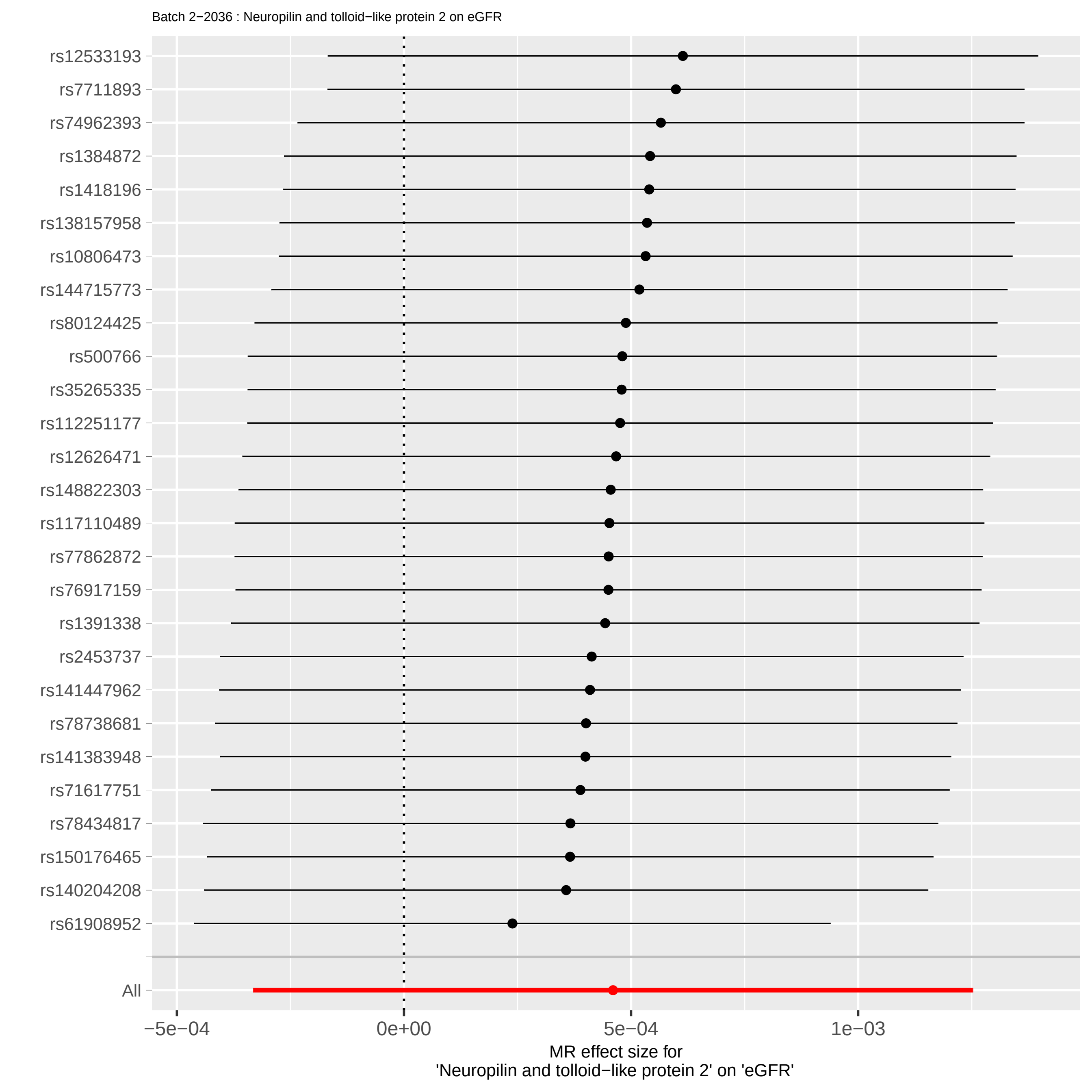

Batch 2−2036 : Neuropilin and tolloid−like protein 2 on eGFR
rs12533193
rs7711893
rs74962393
rs1384872
rs1418196
rs138157958
rs10806473
rs144715773
rs80124425
rs500766
rs35265335
rs112251177
rs12626471
rs148822303
rs117110489
rs77862872
rs76917159
rs1391338
rs2453737
rs141447962
rs78738681
rs141383948
rs71617751
rs78434817
rs150176465
rs140204208
rs61908952
All
−5e−04
0e+00
5e−04
1e−03
MR effect size for
'Neuropilin and tolloid−like protein 2' on 'eGFR'

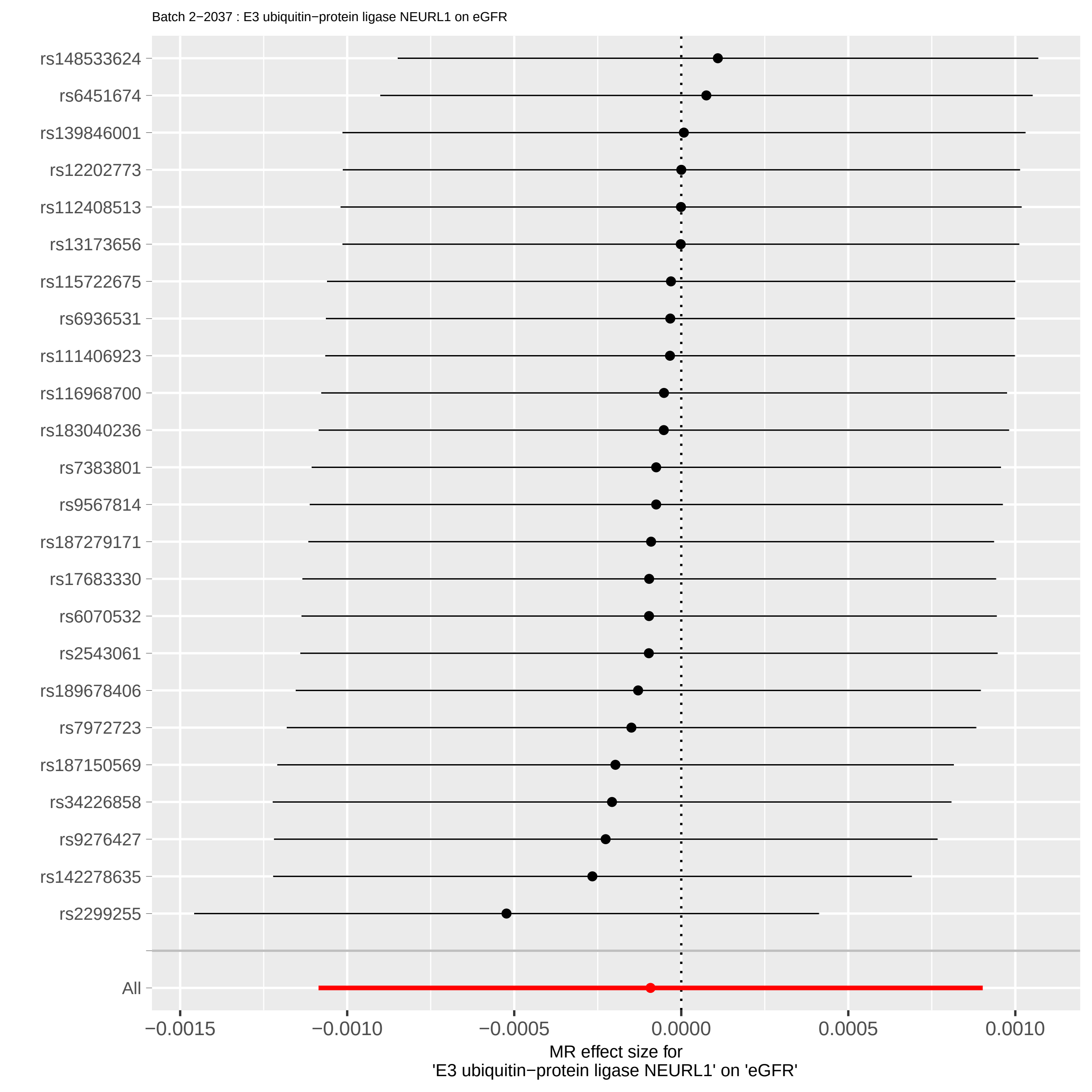

Batch 2−2037 : E3 ubiquitin−protein ligase NEURL1 on eGFR
rs148533624
rs6451674
rs139846001
rs12202773
rs112408513
rs13173656
rs115722675
rs6936531
rs111406923
rs116968700
rs183040236
rs7383801
rs9567814
rs187279171
rs17683330
rs6070532
rs2543061
rs189678406
rs7972723
rs187150569
rs34226858
rs9276427
rs142278635
rs2299255
All
−0.0015
−0.0010
−0.0005
0.0000
0.0005
0.0010
MR effect size for
'E3 ubiquitin−protein ligase NEURL1' on 'eGFR'

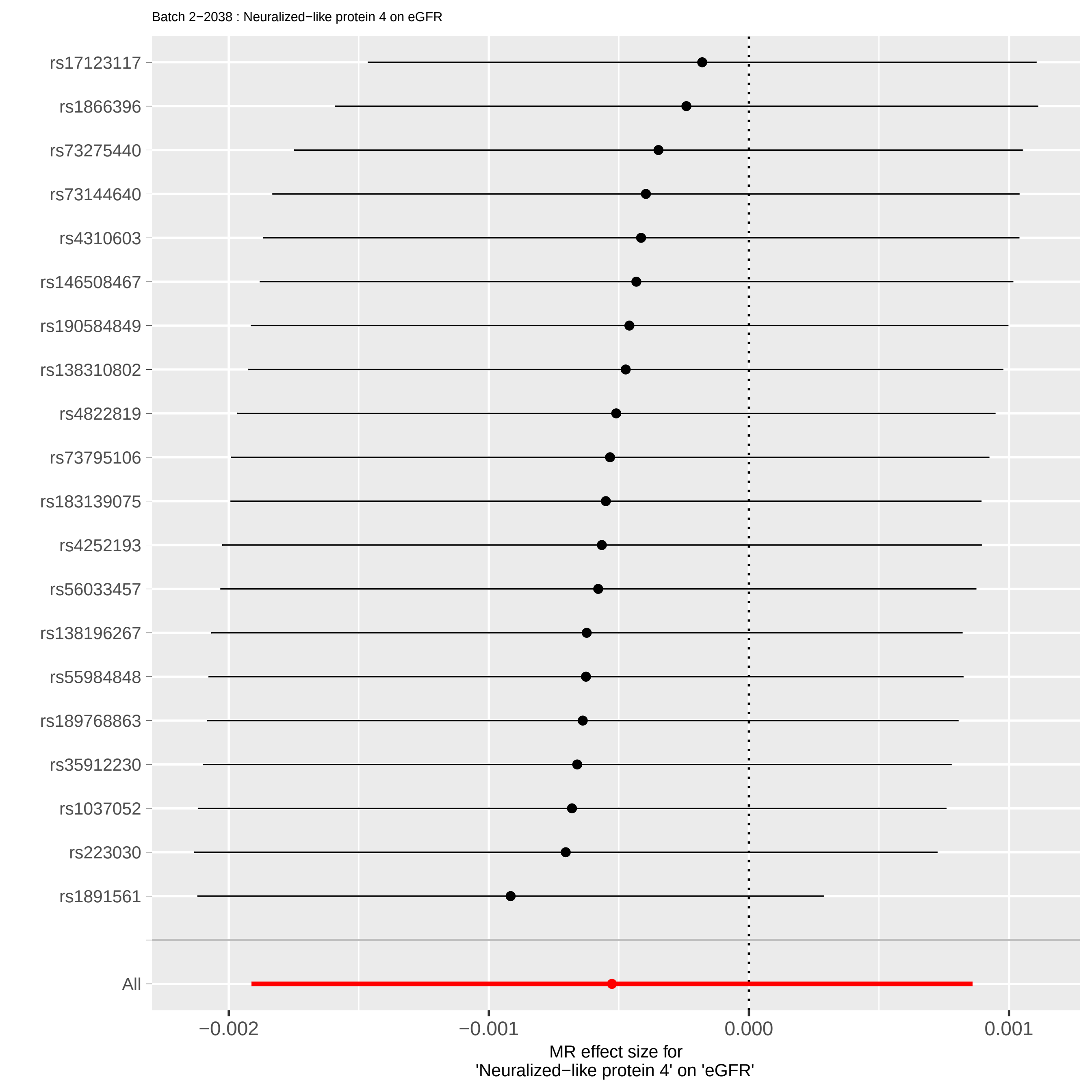

Batch 2−2038 : Neuralized−like protein 4 on eGFR
rs17123117
rs1866396
rs73275440
rs73144640
rs4310603
rs146508467
rs190584849
rs138310802
rs4822819
rs73795106
rs183139075
rs4252193
rs56033457
rs138196267
rs55984848
rs189768863
rs35912230
rs1037052
rs223030
rs1891561
All
−0.002
−0.001
0.000
0.001
MR effect size for
'Neuralized−like protein 4' on 'eGFR'

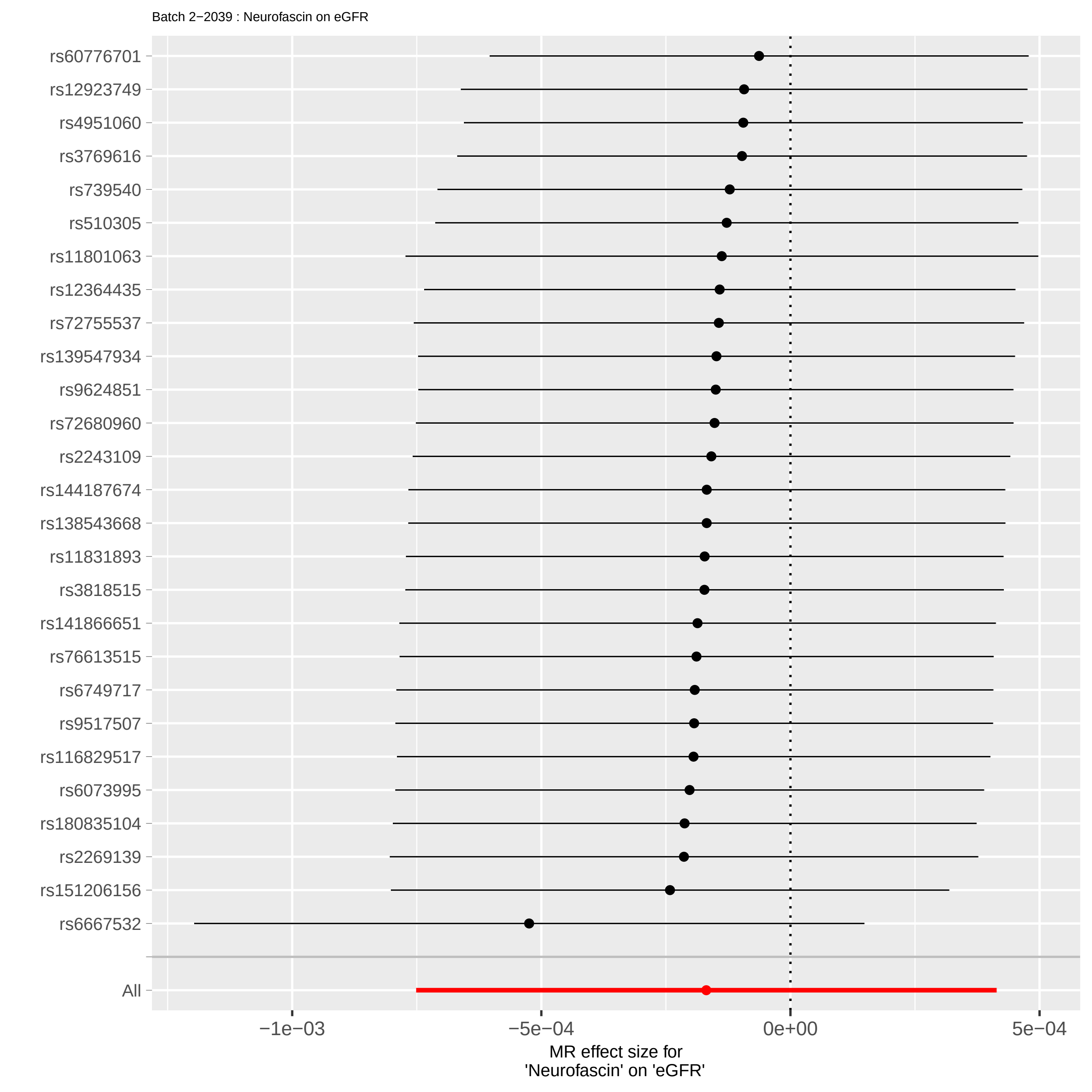
Batch 2−2039 : Neurofascin on eGFR
rs60776701
rs12923749
rs4951060
rs3769616
rs739540
rs510305
rs11801063
rs12364435
rs72755537
rs139547934
rs9624851
rs72680960
rs2243109
rs144187674
rs138543668
rs11831893
rs3818515
rs141866651
rs76613515
rs6749717
rs9517507
rs116829517
rs6073995
rs180835104
rs2269139
rs151206156
rs6667532
All
−1e−03
−5e−04
0e+00
5e−04
MR effect size for
'Neurofascin' on 'eGFR'

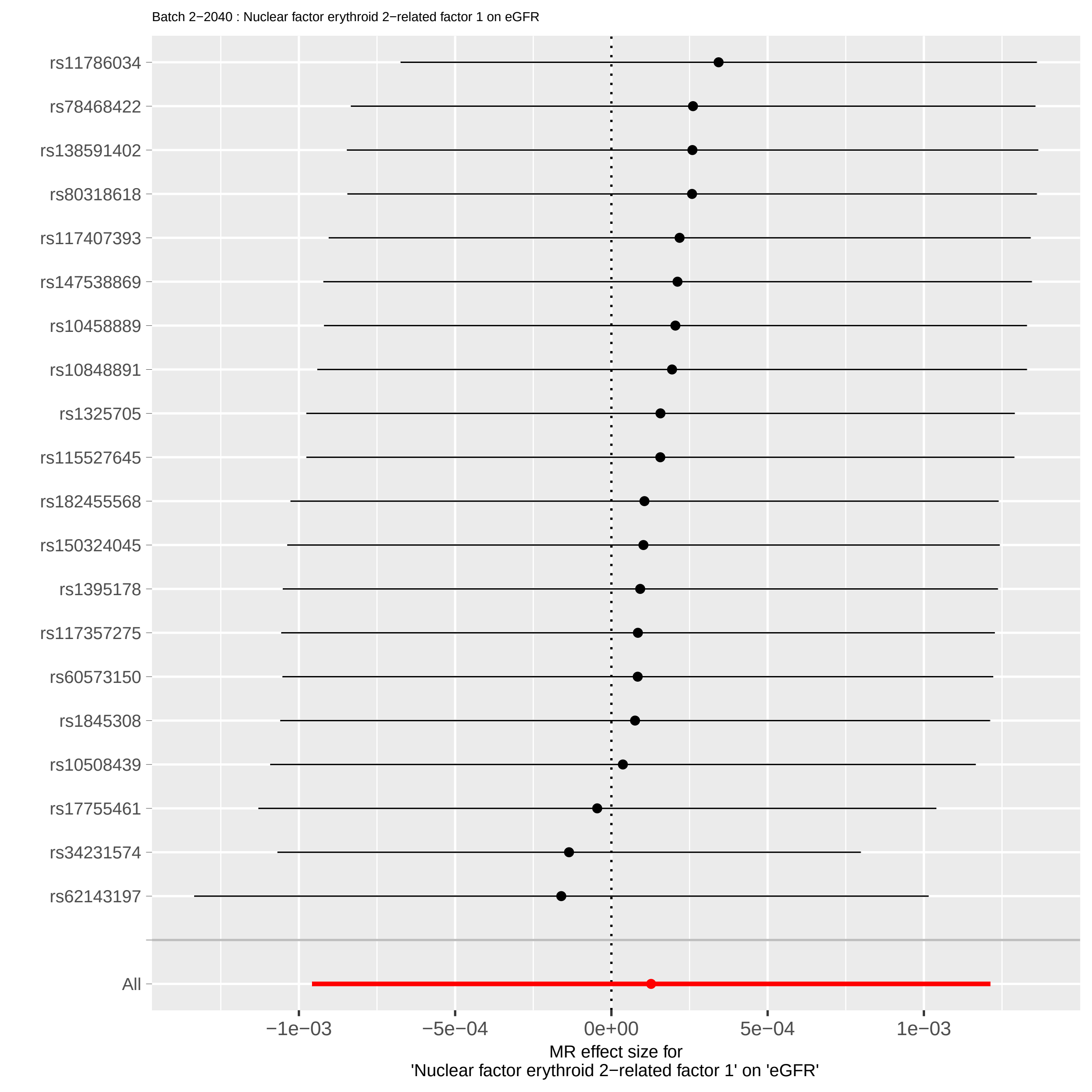

Batch 2−2040 : Nuclear factor erythroid 2−related factor 1 on eGFR
rs11786034
rs78468422
rs138591402
rs80318618
rs117407393
rs147538869
rs10458889
rs10848891
rs1325705
rs115527645
rs182455568
rs150324045
rs1395178
rs117357275
rs60573150
rs1845308
rs10508439
rs17755461
rs34231574
rs62143197
All
−1e−03
−5e−04
0e+00
5e−04
1e−03
MR effect size for
'Nuclear factor erythroid 2−related factor 1' on 'eGFR'

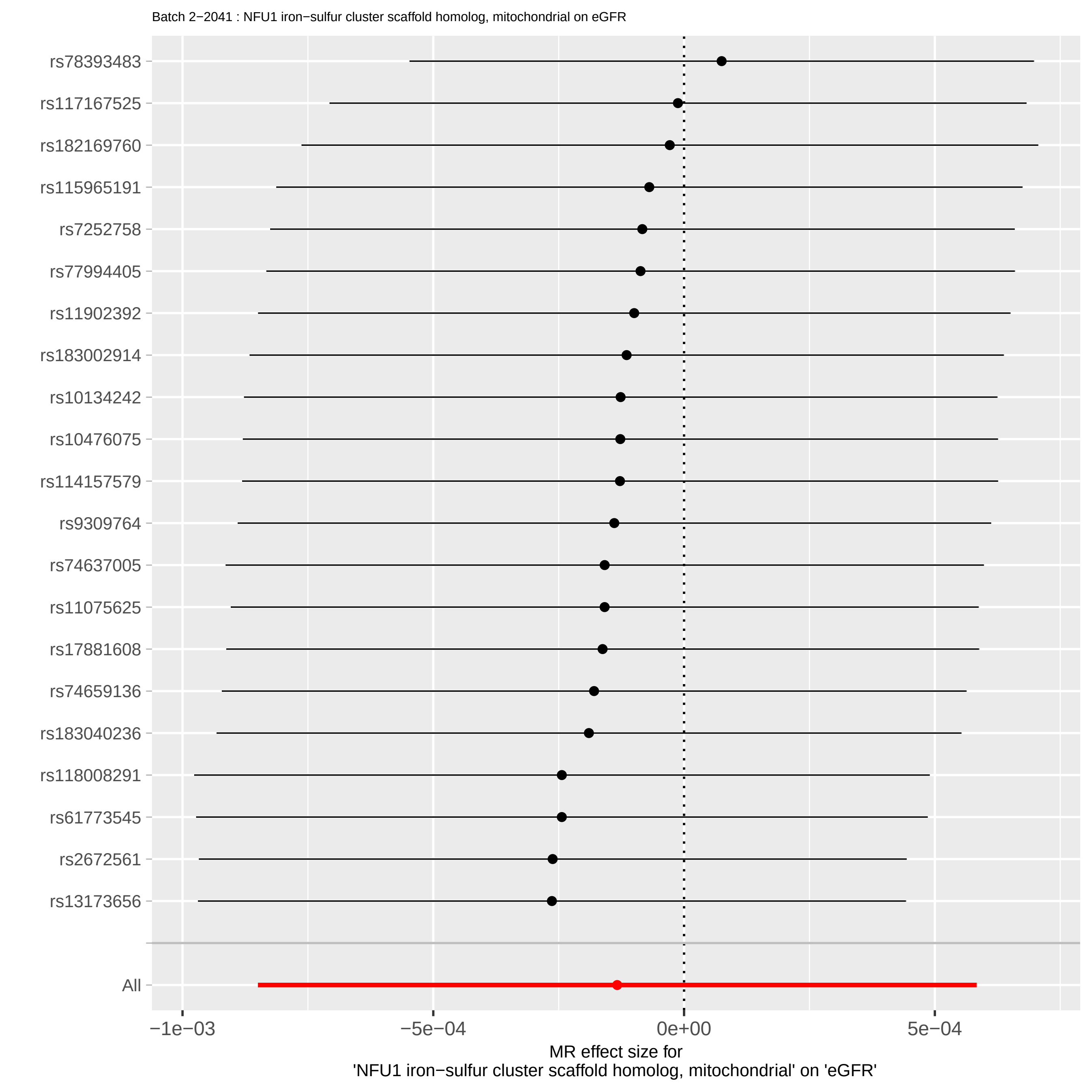

Batch 2−2041 : NFU1 iron−sulfur cluster scaffold homolog, mitochondrial on eGFR
rs78393483
rs117167525
rs182169760
rs115965191
rs7252758
rs77994405
rs11902392
rs183002914
rs10134242
rs10476075
rs114157579
rs9309764
rs74637005
rs11075625
rs17881608
rs74659136
rs183040236
rs118008291
rs61773545
rs2672561
rs13173656
All
−1e−03
−5e−04
0e+00
5e−04
MR effect size for
'NFU1 iron−sulfur cluster scaffold homolog, mitochondrial' on 'eGFR'

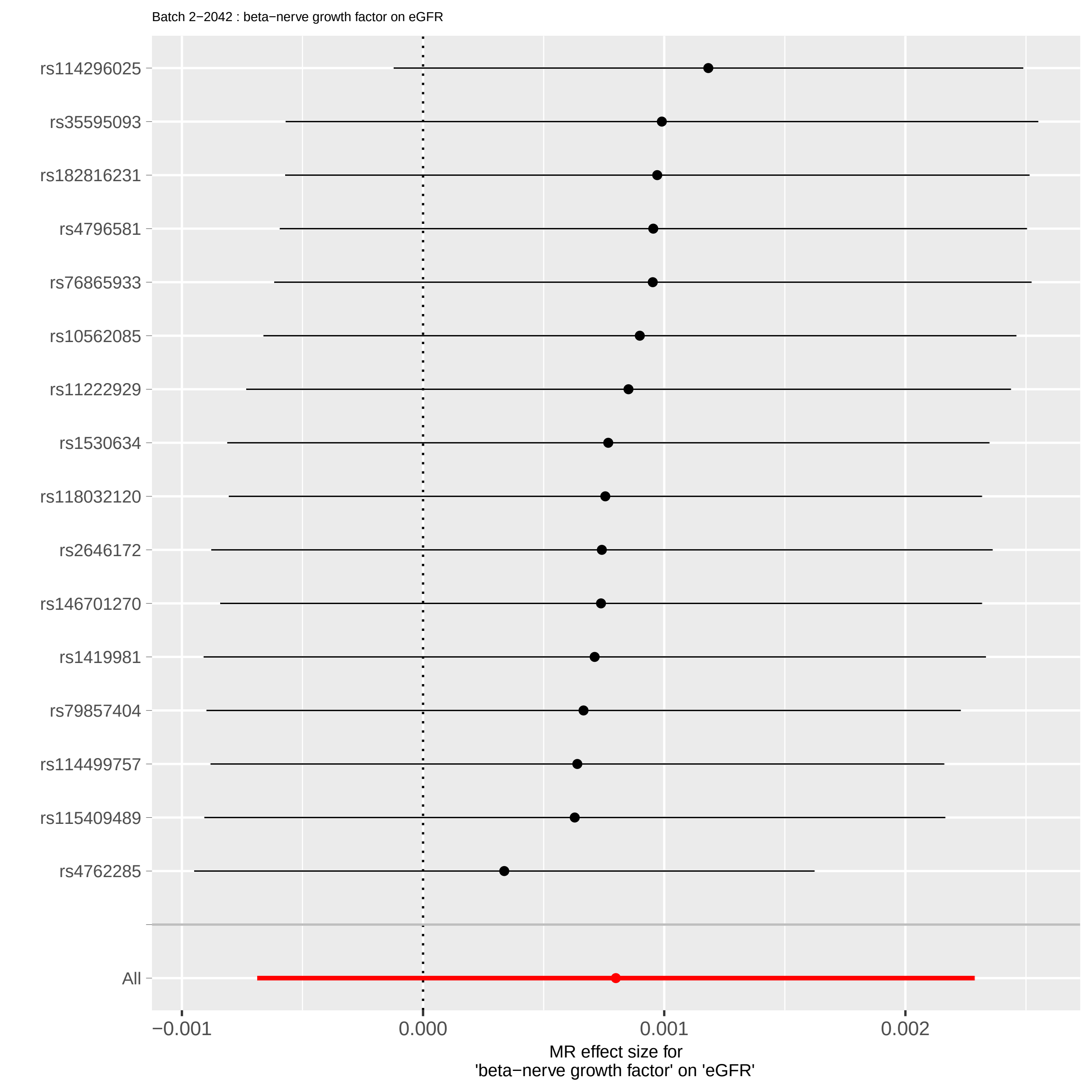

Batch 2−2042 : beta−nerve growth factor on eGFR
rs114296025
rs35595093
rs182816231
rs4796581
rs76865933
rs10562085
rs11222929
rs1530634
rs118032120
rs2646172
rs146701270
rs1419981
rs79857404
rs114499757
rs115409489
rs4762285
All
−0.001
0.000
0.001
0.002
MR effect size for
'beta−nerve growth factor' on 'eGFR'

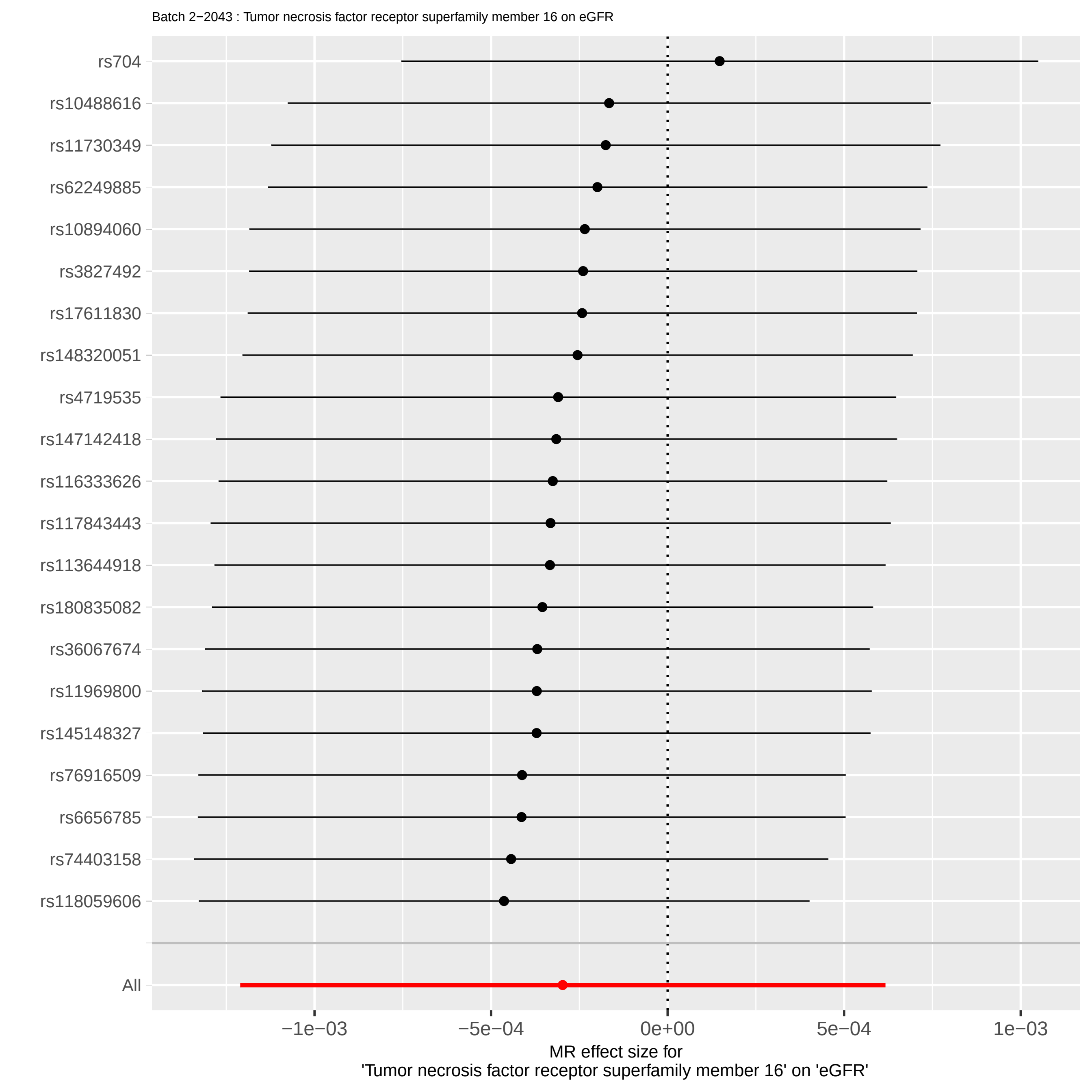

Batch 2−2043 : Tumor necrosis factor receptor superfamily member 16 on eGFR
rs704
rs10488616
rs11730349
rs62249885
rs10894060
rs3827492
rs17611830
rs148320051
rs4719535
rs147142418
rs116333626
rs117843443
rs113644918
rs180835082
rs36067674
rs11969800
rs145148327
rs76916509
rs6656785
rs74403158
rs118059606
All
−1e−03
−5e−04
0e+00
5e−04
1e−03
MR effect size for
'Tumor necrosis factor receptor superfamily member 16' on 'eGFR'

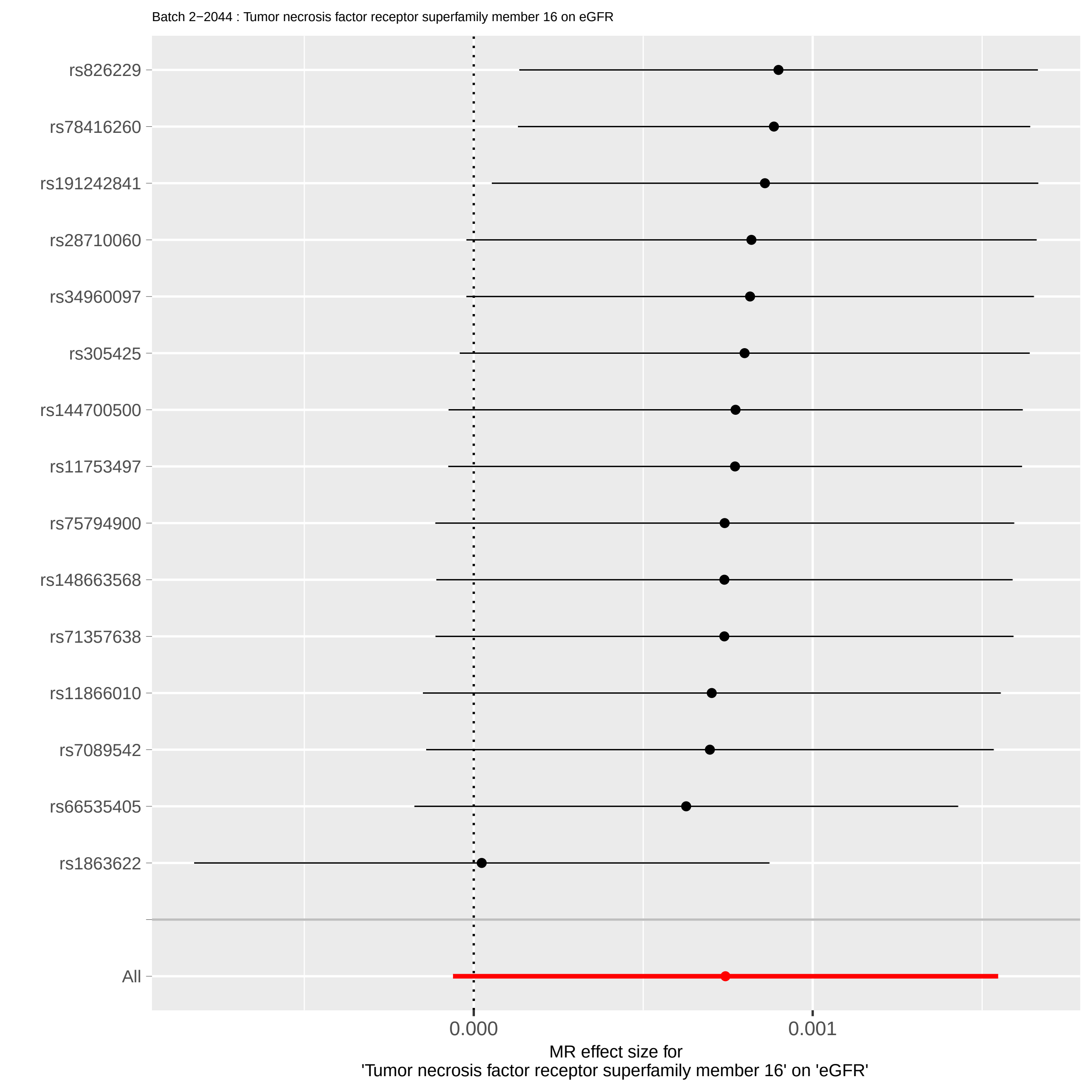
Batch 2−2044 : Tumor necrosis factor receptor superfamily member 16 on eGFR
rs826229
rs78416260
rs191242841
rs28710060
rs34960097
rs305425
rs144700500
rs11753497
rs75794900
rs148663568
rs71357638
rs11866010
rs7089542
rs66535405
rs1863622
All
0.000
0.001
MR effect size for
'Tumor necrosis factor receptor superfamily member 16' on 'eGFR'

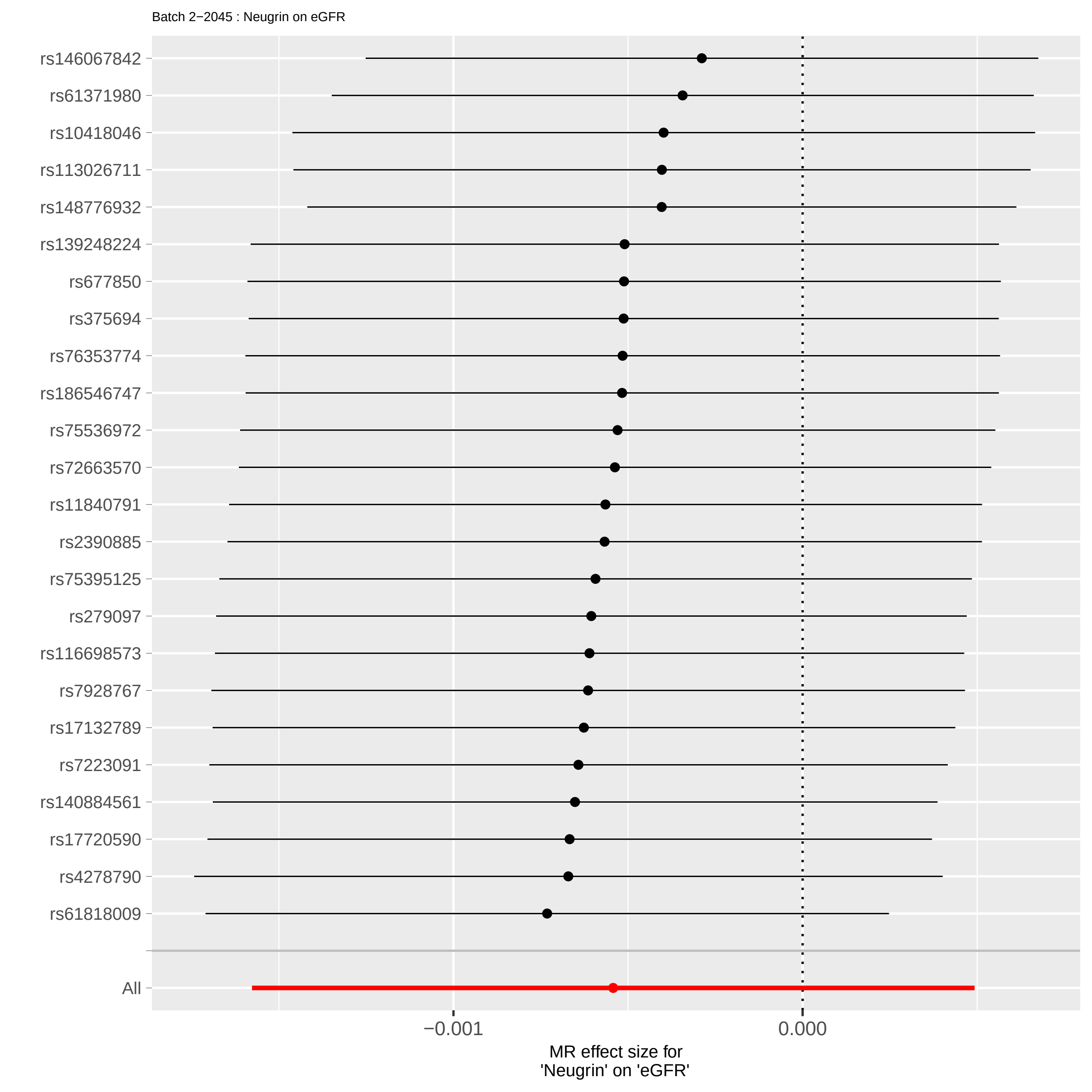

Batch 2−2045 : Neugrin on eGFR
rs146067842
rs61371980
rs10418046
rs113026711
rs148776932
rs139248224
rs677850
rs375694
rs76353774
rs186546747
rs75536972
rs72663570
rs11840791
rs2390885
rs75395125
rs279097
rs116698573
rs7928767
rs17132789
rs7223091
rs140884561
rs17720590
rs4278790
rs61818009
All
−0.001
0.000
MR effect size for
'Neugrin' on 'eGFR'

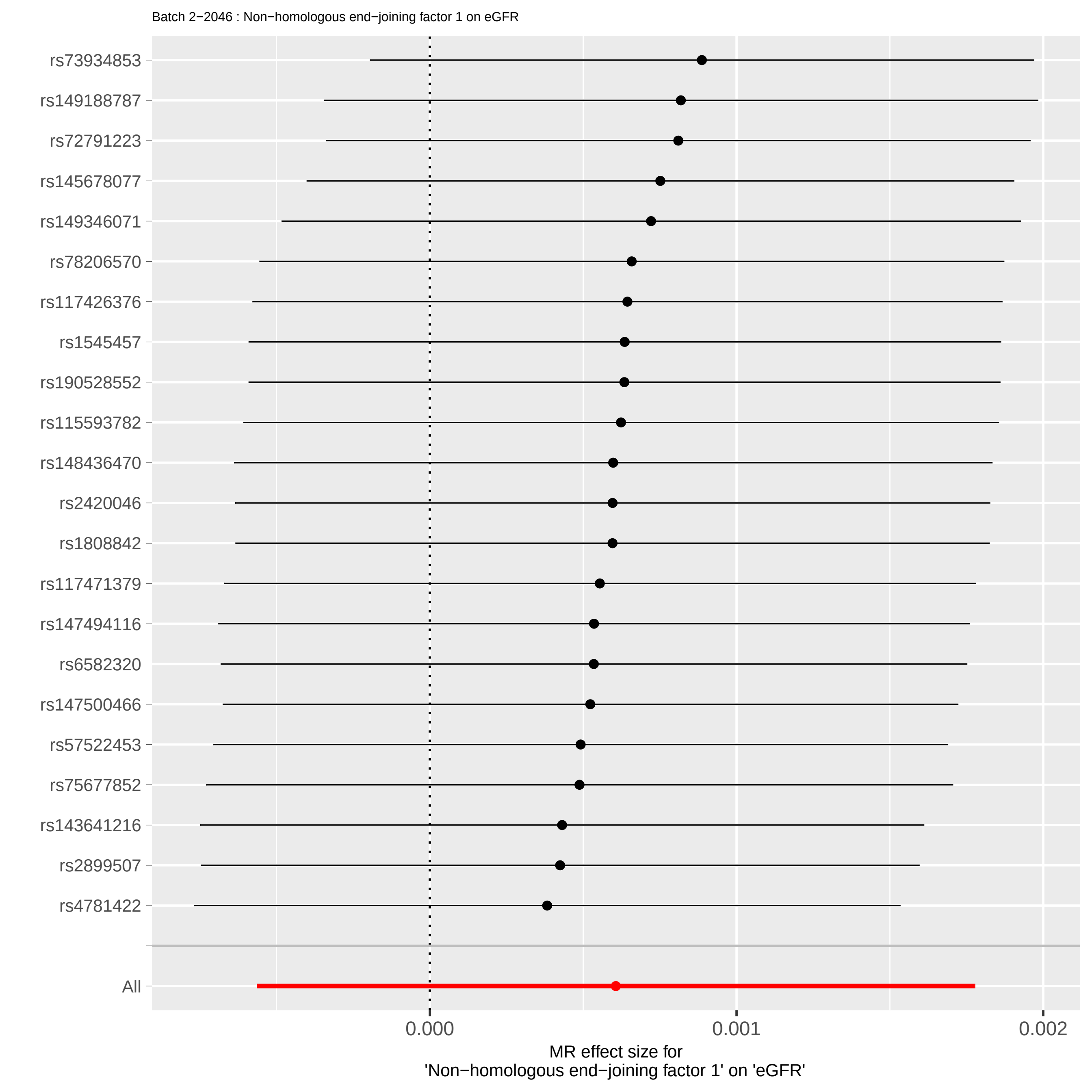

Batch 2−2046 : Non−homologous end−joining factor 1 on eGFR
rs73934853
rs149188787
rs72791223
rs145678077
rs149346071
rs78206570
rs117426376
rs1545457
rs190528552
rs115593782
rs148436470
rs2420046
rs1808842
rs117471379
rs147494116
rs6582320
rs147500466
rs57522453
rs75677852
rs143641216
rs2899507
rs4781422
All
0.000
0.001
0.002
MR effect size for
'Non−homologous end−joining factor 1' on 'eGFR'

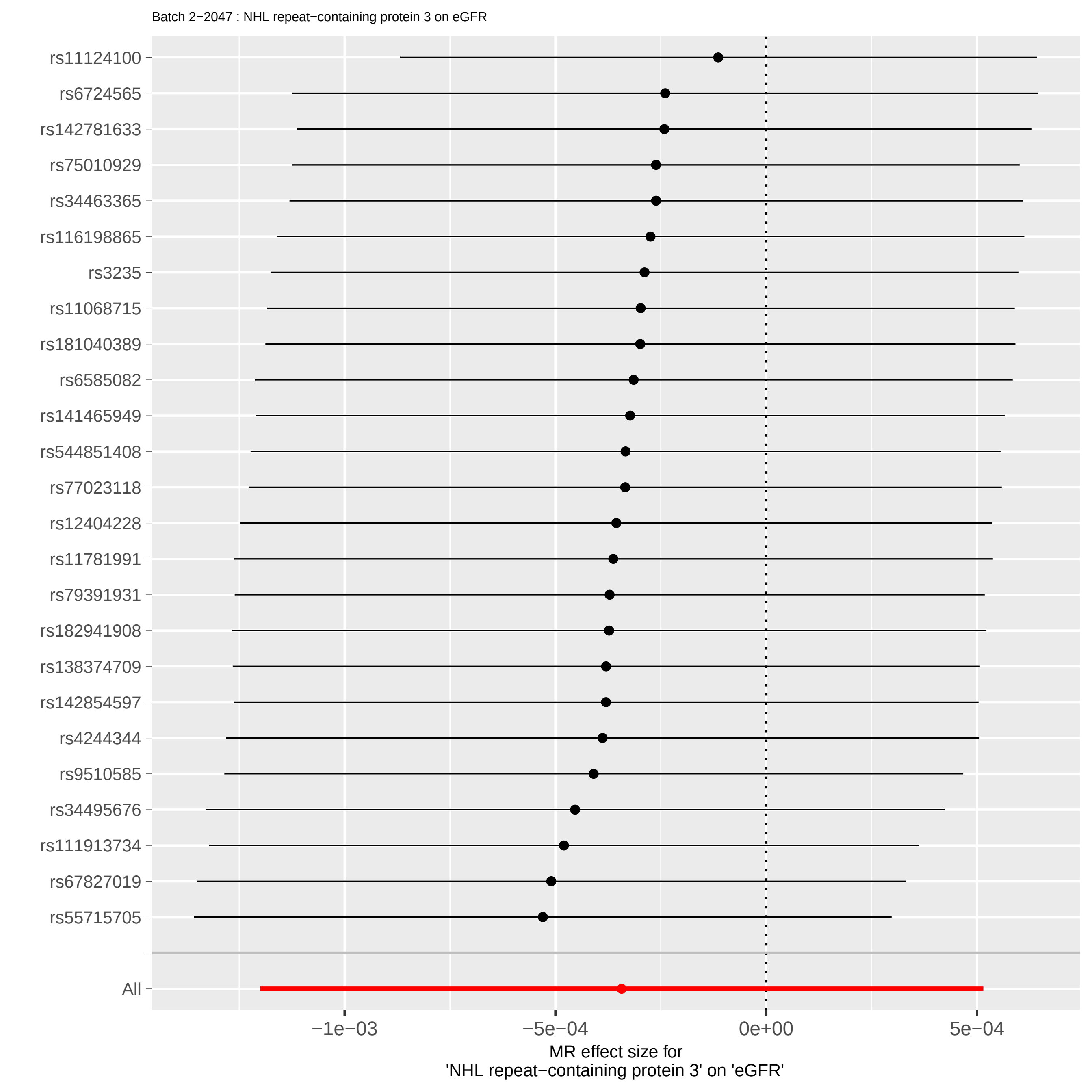

Batch 2−2047 : NHL repeat−containing protein 3 on eGFR
rs11124100
rs6724565
rs142781633
rs75010929
rs34463365
rs116198865
rs3235
rs11068715
rs181040389
rs6585082
rs141465949
rs544851408
rs77023118
rs12404228
rs11781991
rs79391931
rs182941908
rs138374709
rs142854597
rs4244344
rs9510585
rs34495676
rs111913734
rs67827019
rs55715705
All
−1e−03
−5e−04
0e+00
5e−04
MR effect size for
'NHL repeat−containing protein 3' on 'eGFR'

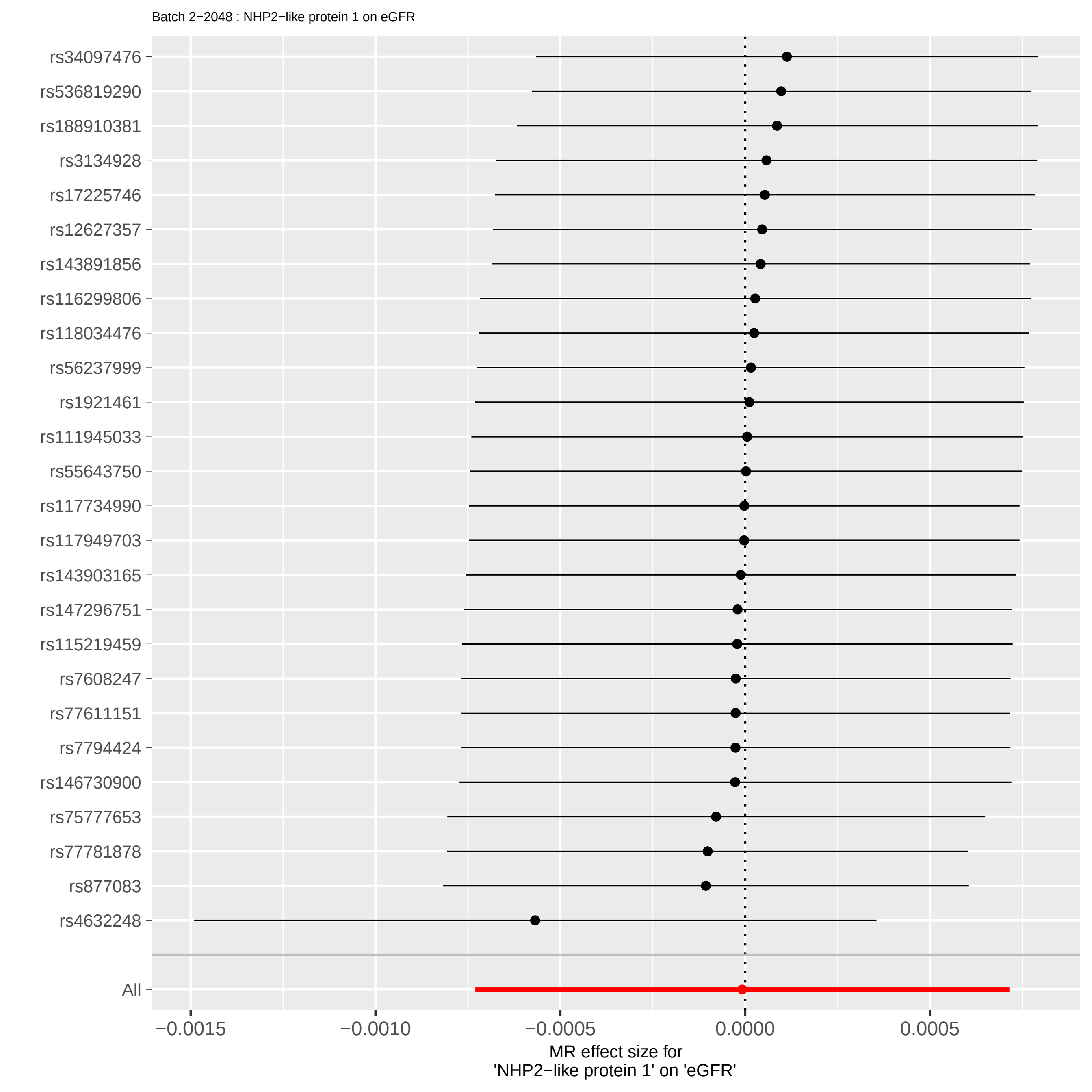

Batch 2−2048 : NHP2−like protein 1 on eGFR
rs34097476
rs536819290
rs188910381
rs3134928
rs17225746
rs12627357
rs143891856
rs116299806
rs118034476
rs56237999
rs1921461
rs111945033
rs55643750
rs117734990
rs117949703
rs143903165
rs147296751
rs115219459
rs7608247
rs77611151
rs7794424
rs146730900
rs75777653
rs77781878
rs877083
rs4632248
All
−0.0015
−0.0010
−0.0005
0.0000
0.0005
MR effect size for
'NHP2−like protein 1' on 'eGFR'

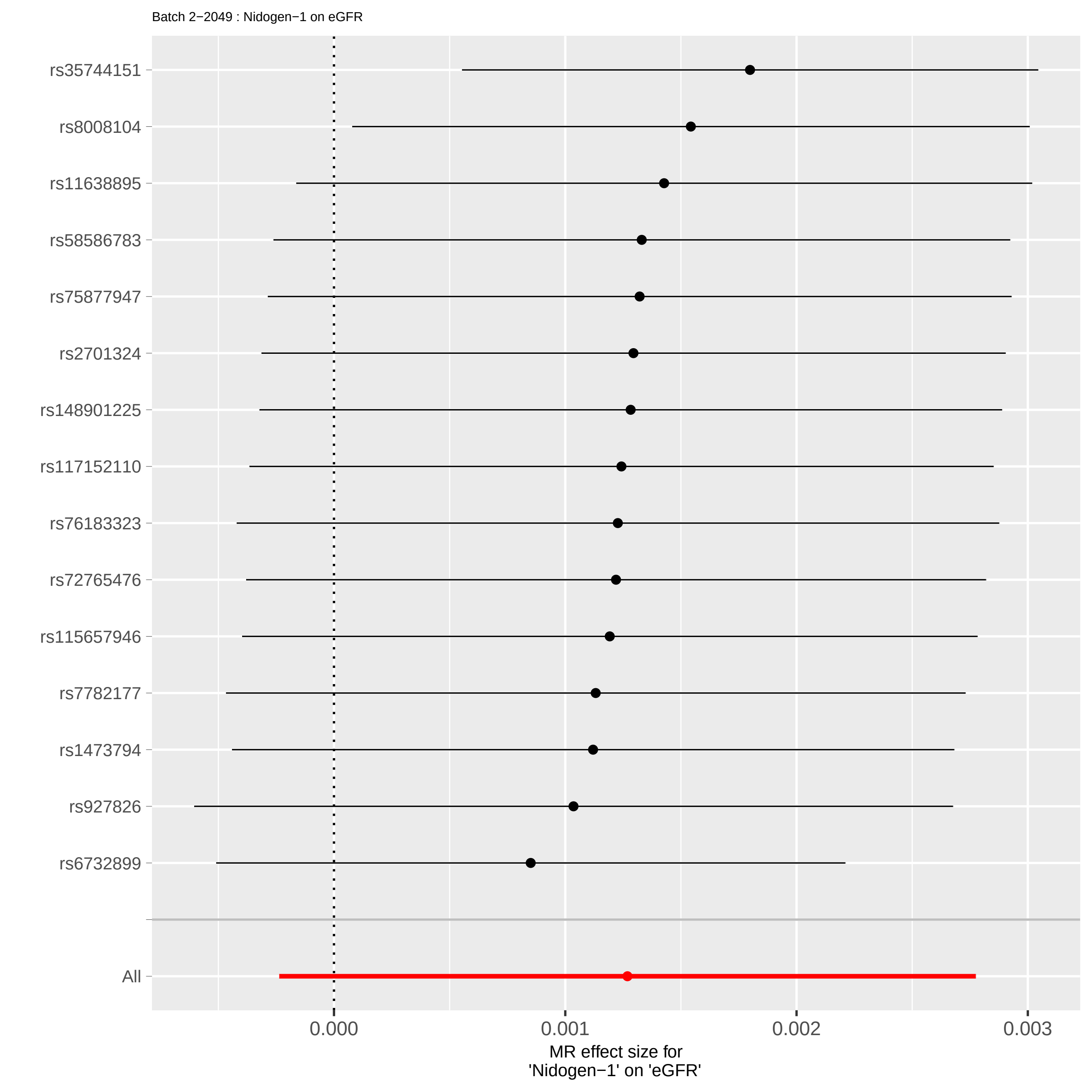

Batch 2−2049 : Nidogen−1 on eGFR
rs35744151
rs8008104
rs11638895
rs58586783
rs75877947
rs2701324
rs148901225
rs117152110
rs76183323
rs72765476
rs115657946
rs7782177
rs1473794
rs927826
rs6732899
All
0.000
0.001
0.002
0.003
MR effect size for
'Nidogen−1' on 'eGFR'

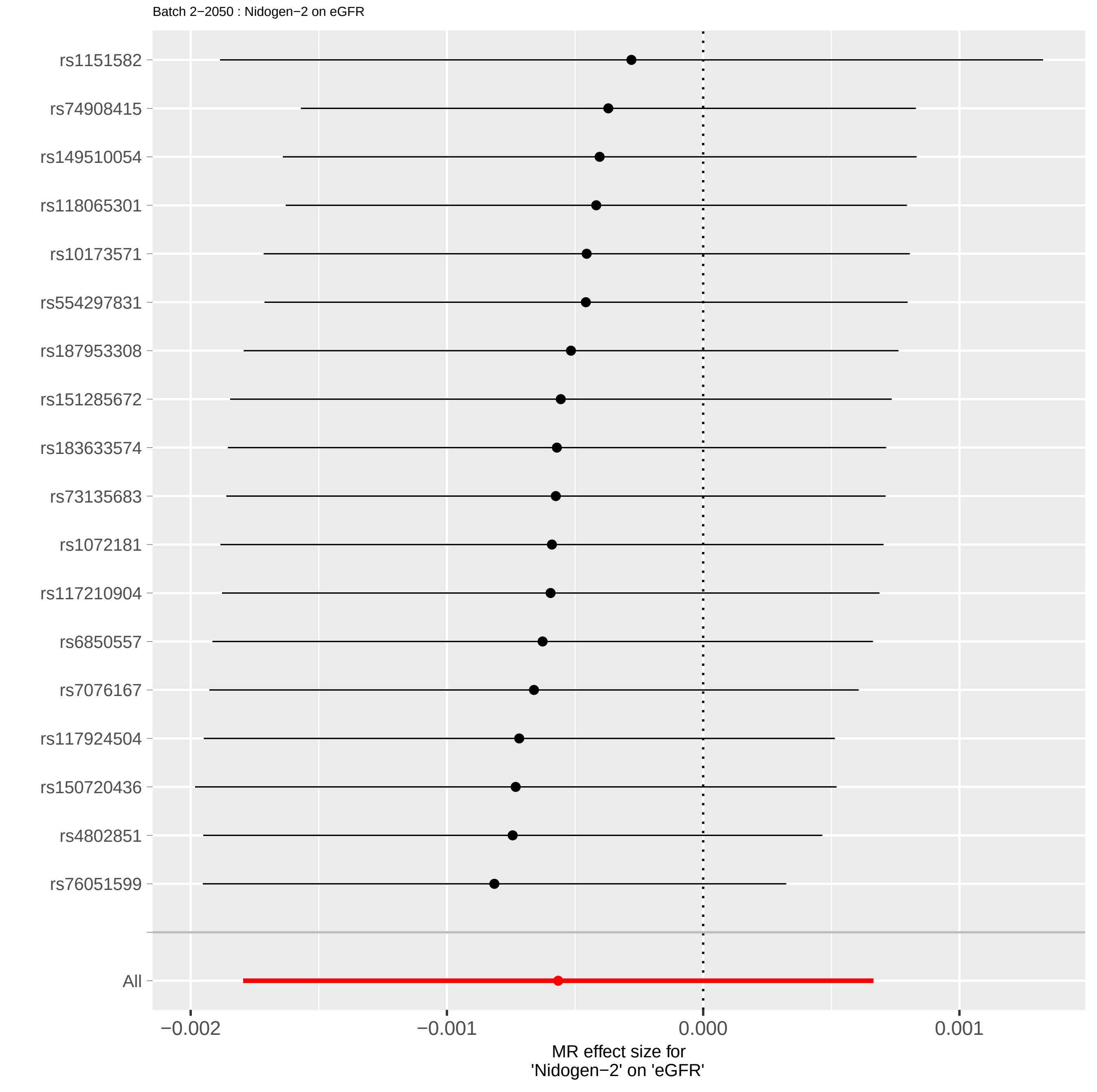

Batch 2−2050 : Nidogen−2 on eGFR
rs1151582
rs74908415
rs149510054
rs118065301
rs10173571
rs554297831
rs187953308
rs151285672
rs183633574
rs73135683
rs1072181
rs117210904
rs6850557
rs7076167
rs117924504
rs150720436
rs4802851
rs76051599
All
−0.002
−0.001
0.000
0.001
MR effect size for
'Nidogen−2' on 'eGFR'

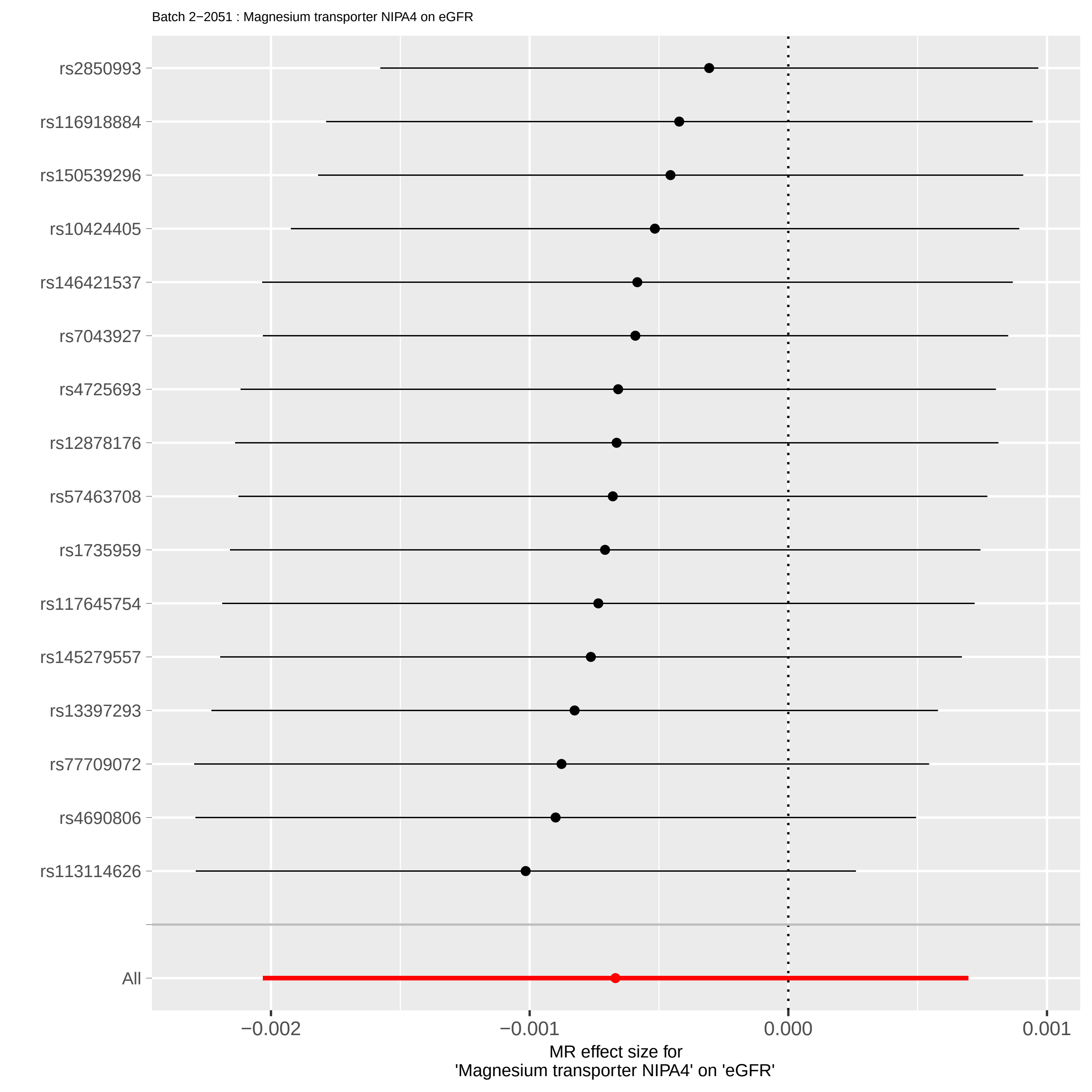

Batch 2−2051 : Magnesium transporter NIPA4 on eGFR
rs2850993
rs116918884
rs150539296
rs10424405
rs146421537
rs7043927
rs4725693
rs12878176
rs57463708
rs1735959
rs117645754
rs145279557
rs13397293
rs77709072
rs4690806
rs113114626
All
−0.002
−0.001
0.000
0.001
MR effect size for
'Magnesium transporter NIPA4' on 'eGFR'

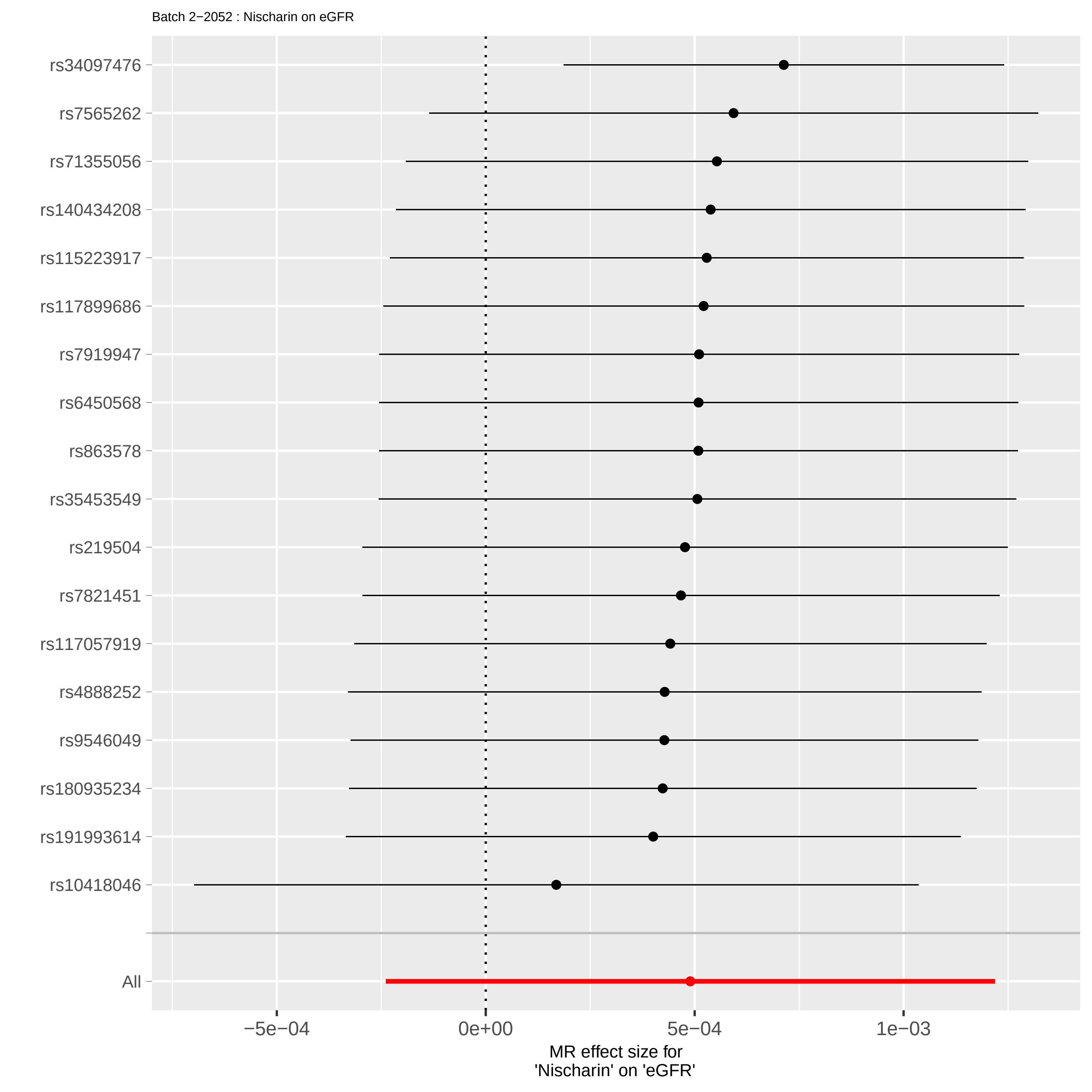

Batch 2−2052 : Nischarin on eGFR
rs34097476
rs7565262
rs71355056
rs140434208
rs115223917
rs117899686
rs7919947
rs6450568
rs863578
rs35453549
rs219504
rs7821451
rs117057919
rs4888252
rs9546049
rs180935234
rs191993614
rs10418046
All
−5e−04
0e+00
5e−04
1e−03
MR effect size for
'Nischarin' on 'eGFR'

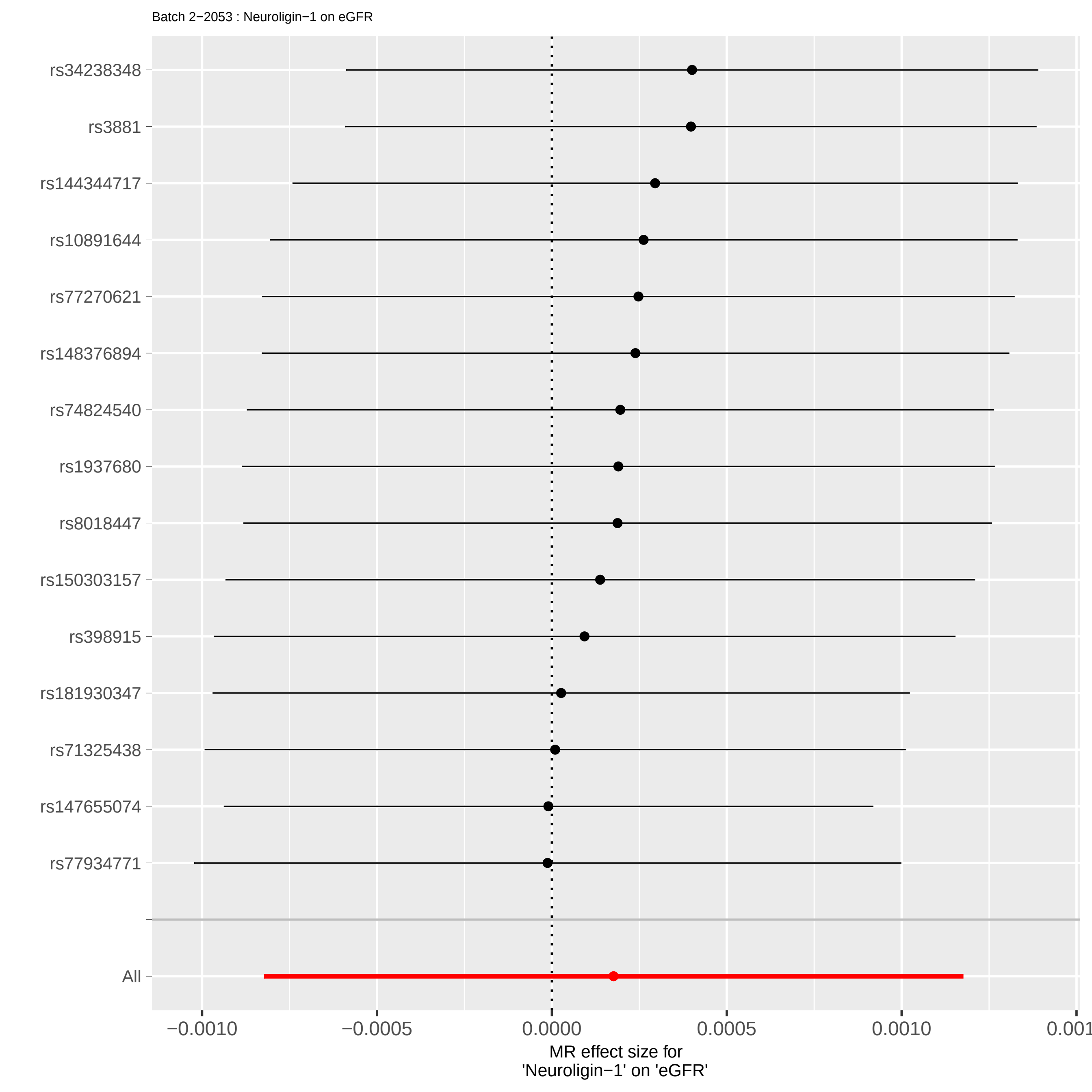
Batch 2−2053 : Neuroligin−1 on eGFR
rs34238348
rs3881
rs144344717
rs10891644
rs77270621
rs148376894
rs74824540
rs1937680
rs8018447
rs150303157
rs398915
rs181930347
rs71325438
rs147655074
rs77934771
All
−0.0010
−0.0005
0.0000
0.0005
0.0010
MR effect size for
'Neuroligin−1' on 'eGFR'

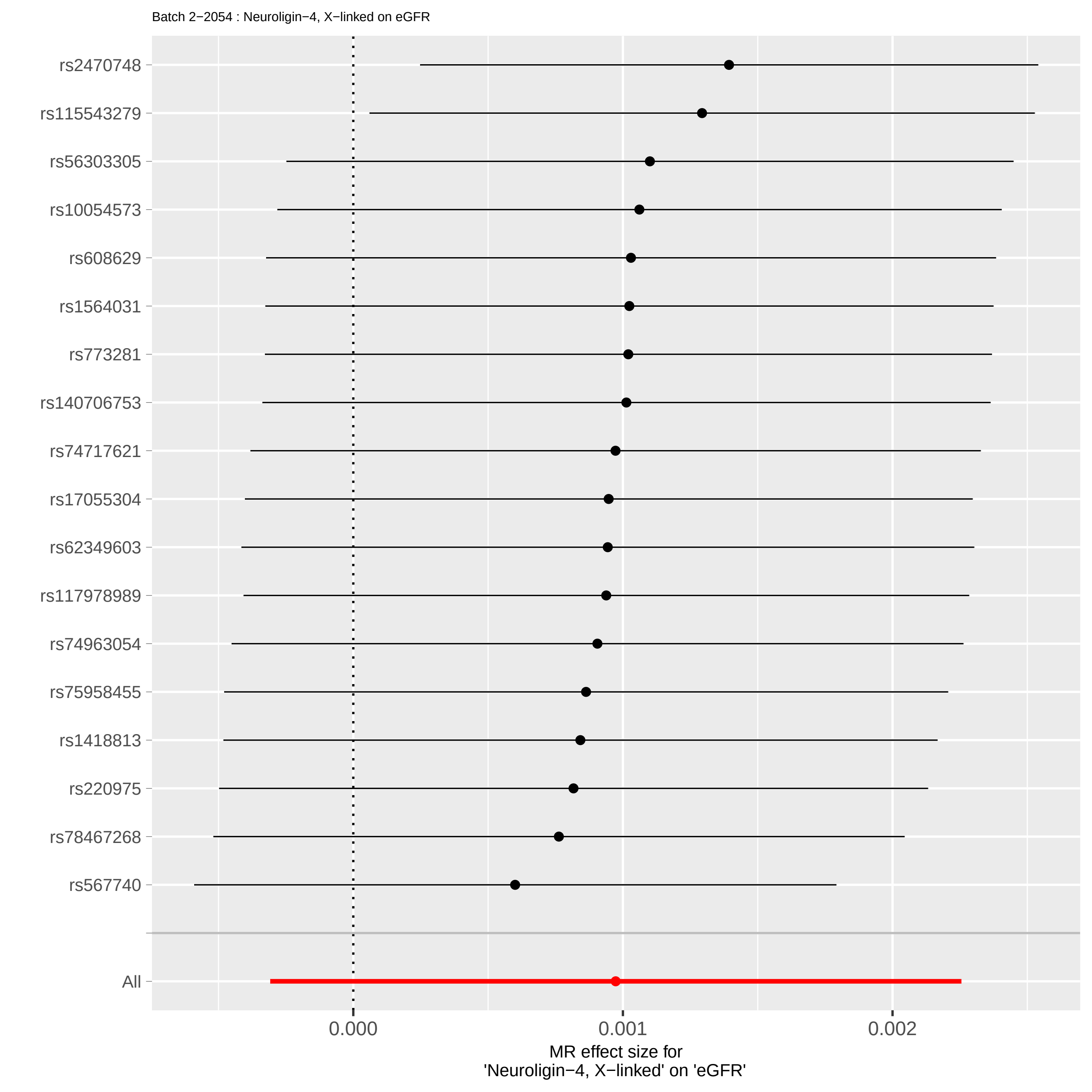

Batch 2−2054 : Neuroligin−4, X−linked on eGFR
rs2470748
rs115543279
rs56303305
rs10054573
rs608629
rs1564031
rs773281
rs140706753
rs74717621
rs17055304
rs62349603
rs117978989
rs74963054
rs75958455
rs1418813
rs220975
rs78467268
rs567740
All
0.000
0.001
0.002
MR effect size for
'Neuroligin−4, X−linked' on 'eGFR'

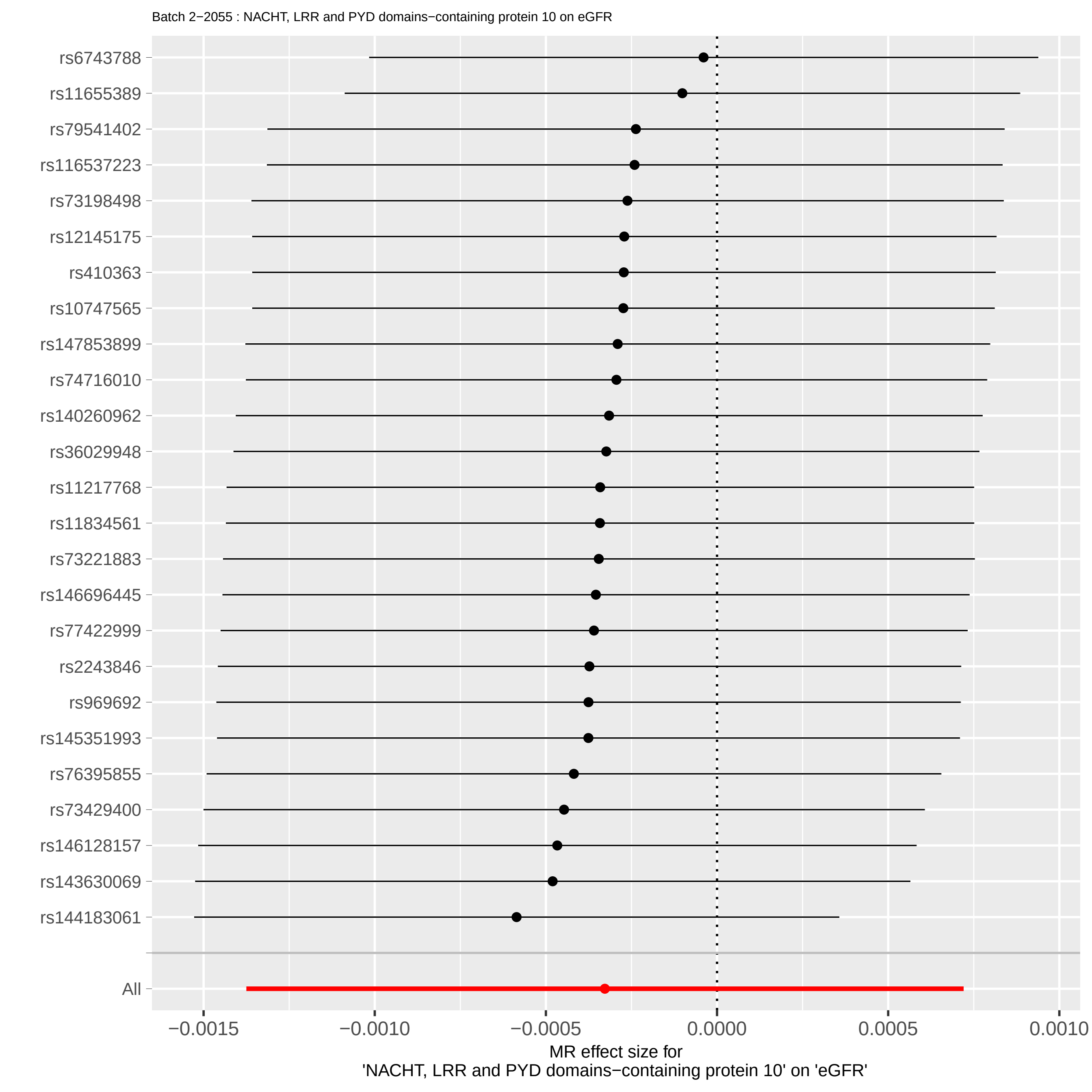

Batch 2−2055 : NACHT, LRR and PYD domains−containing protein 10 on eGFR
rs6743788
rs11655389
rs79541402
rs116537223
rs73198498
rs12145175
rs410363
rs10747565
rs147853899
rs74716010
rs140260962
rs36029948
rs11217768
rs11834561
rs73221883
rs146696445
rs77422999
rs2243846
rs969692
rs145351993
rs76395855
rs73429400
rs146128157
rs143630069
rs144183061
All
−0.0015
−0.0010
−0.0005
0.0000
0.0005
0.0010
MR effect size for
'NACHT, LRR and PYD domains−containing protein 10' on 'eGFR'

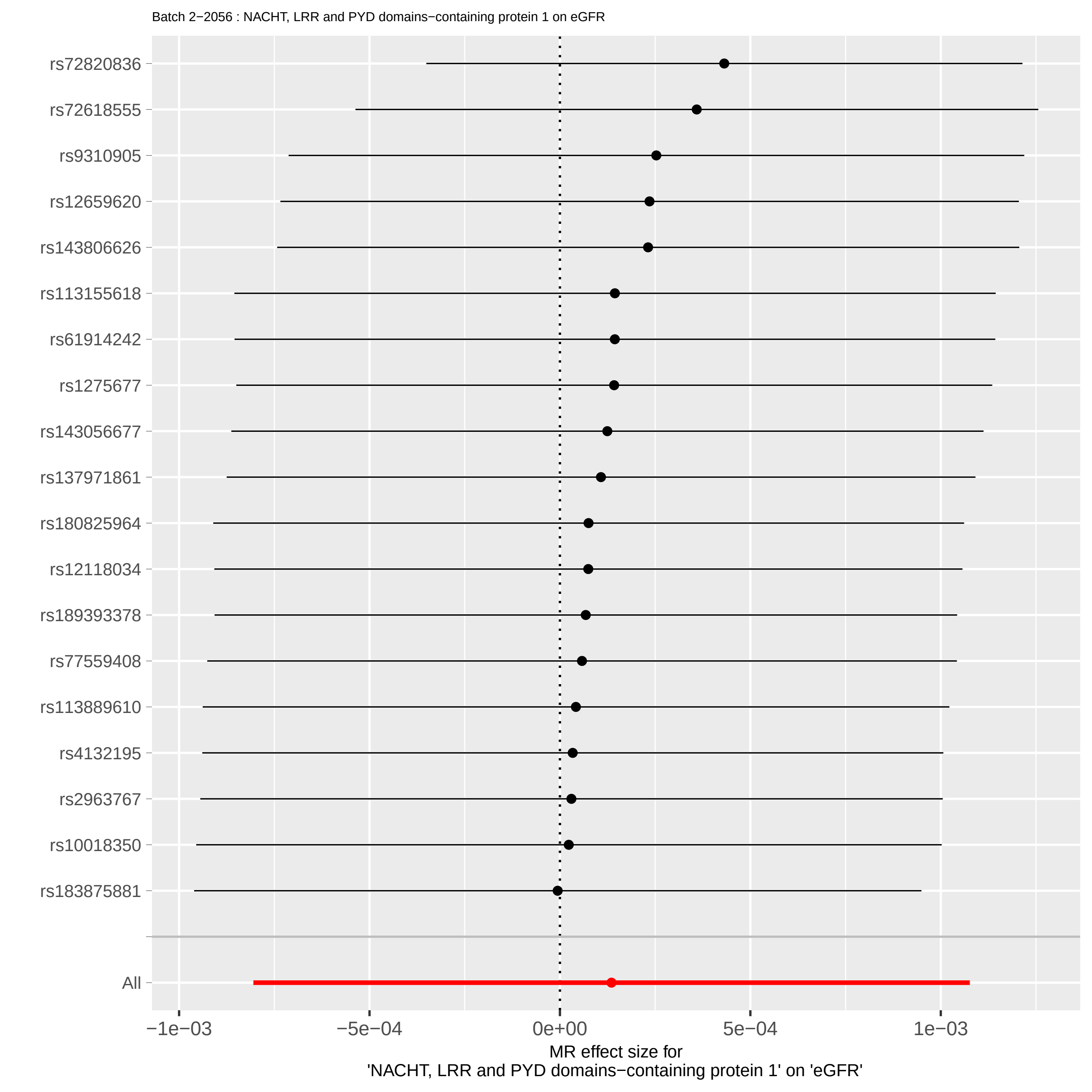

Batch 2−2056 : NACHT, LRR and PYD domains−containing protein 1 on eGFR
rs72820836
rs72618555
rs9310905
rs12659620
rs143806626
rs113155618
rs61914242
rs1275677
rs143056677
rs137971861
rs180825964
rs12118034
rs189393378
rs77559408
rs113889610
rs4132195
rs2963767
rs10018350
rs183875881
All
−1e−03
−5e−04
0e+00
5e−04
1e−03
MR effect size for
'NACHT, LRR and PYD domains−containing protein 1' on 'eGFR'

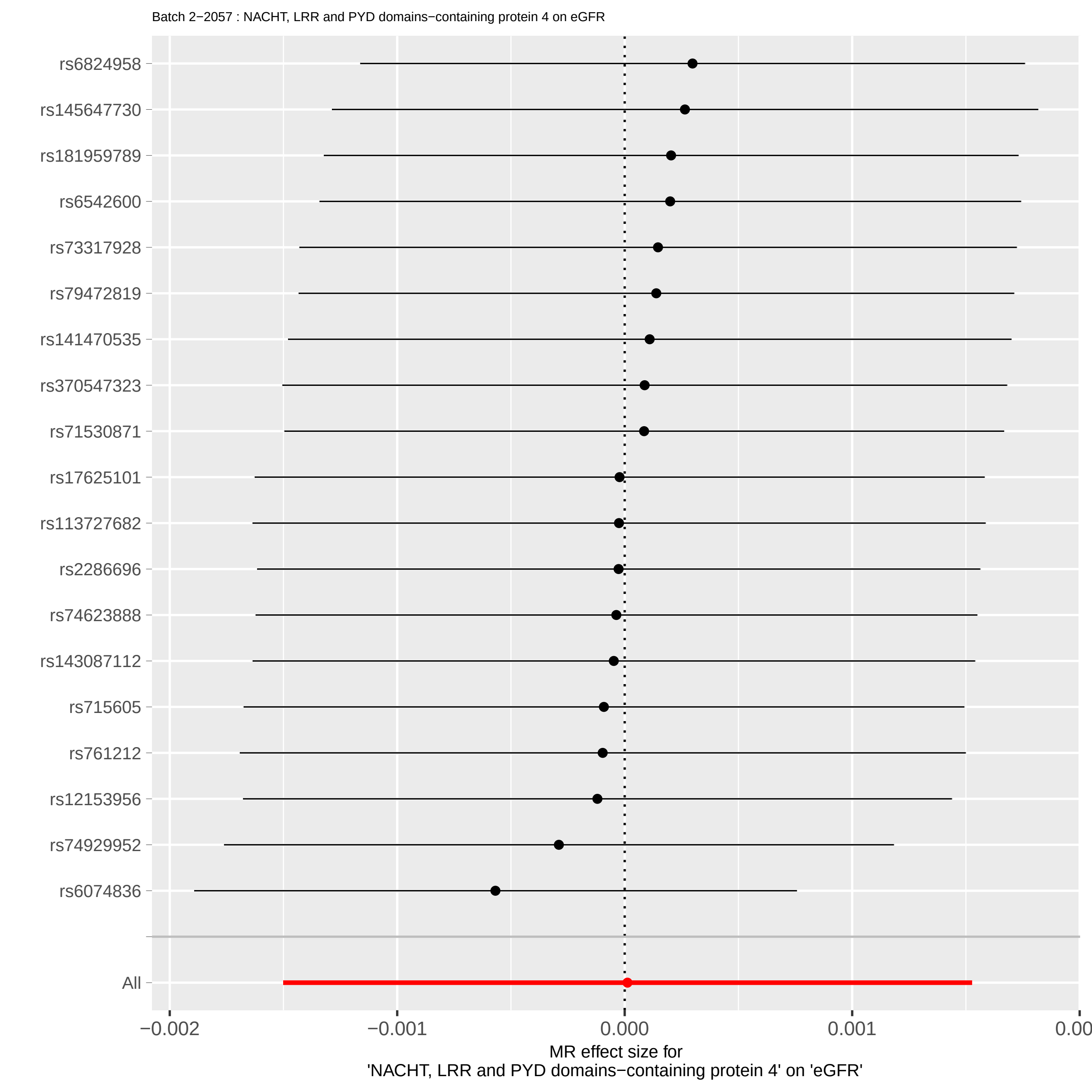

Batch 2−2057 : NACHT, LRR and PYD domains−containing protein 4 on eGFR
rs6824958
rs145647730
rs181959789
rs6542600
rs73317928
rs79472819
rs141470535
rs370547323
rs71530871
rs17625101
rs113727682
rs2286696
rs74623888
rs143087112
rs715605
rs761212
rs12153956
rs74929952
rs6074836
All
−0.002
−0.001
0.000
0.001
0.00
MR effect size for
'NACHT, LRR and PYD domains−containing protein 4' on 'eGFR'

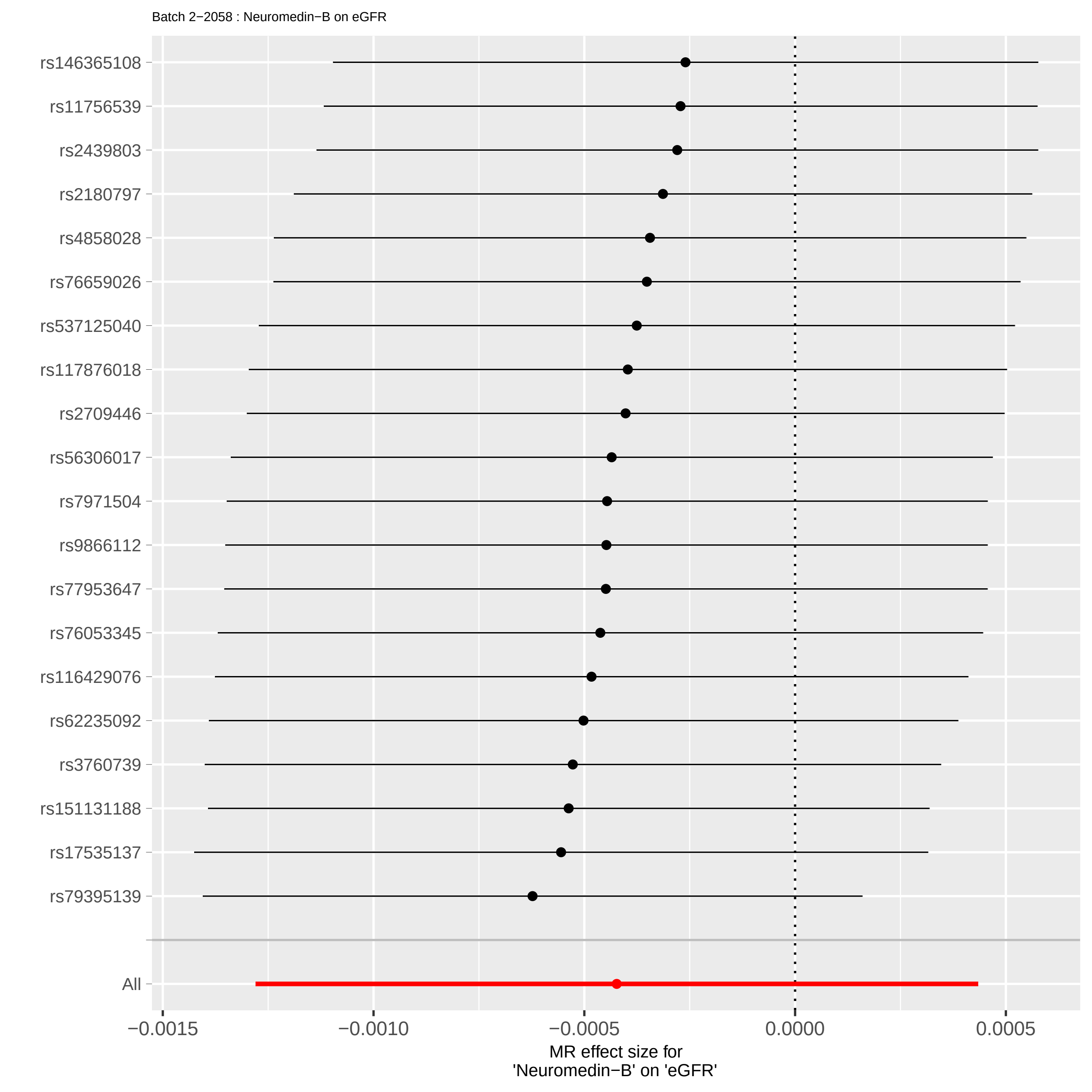
Batch 2−2058 : Neuromedin−B on eGFR
rs146365108
rs11756539
rs2439803
rs2180797
rs4858028
rs76659026
rs537125040
rs117876018
rs2709446
rs56306017
rs7971504
rs9866112
rs77953647
rs76053345
rs116429076
rs62235092
rs3760739
rs151131188
rs17535137
rs79395139
All
−0.0015
−0.0010
−0.0005
0.0000
0.0005
MR effect size for
'Neuromedin−B' on 'eGFR'

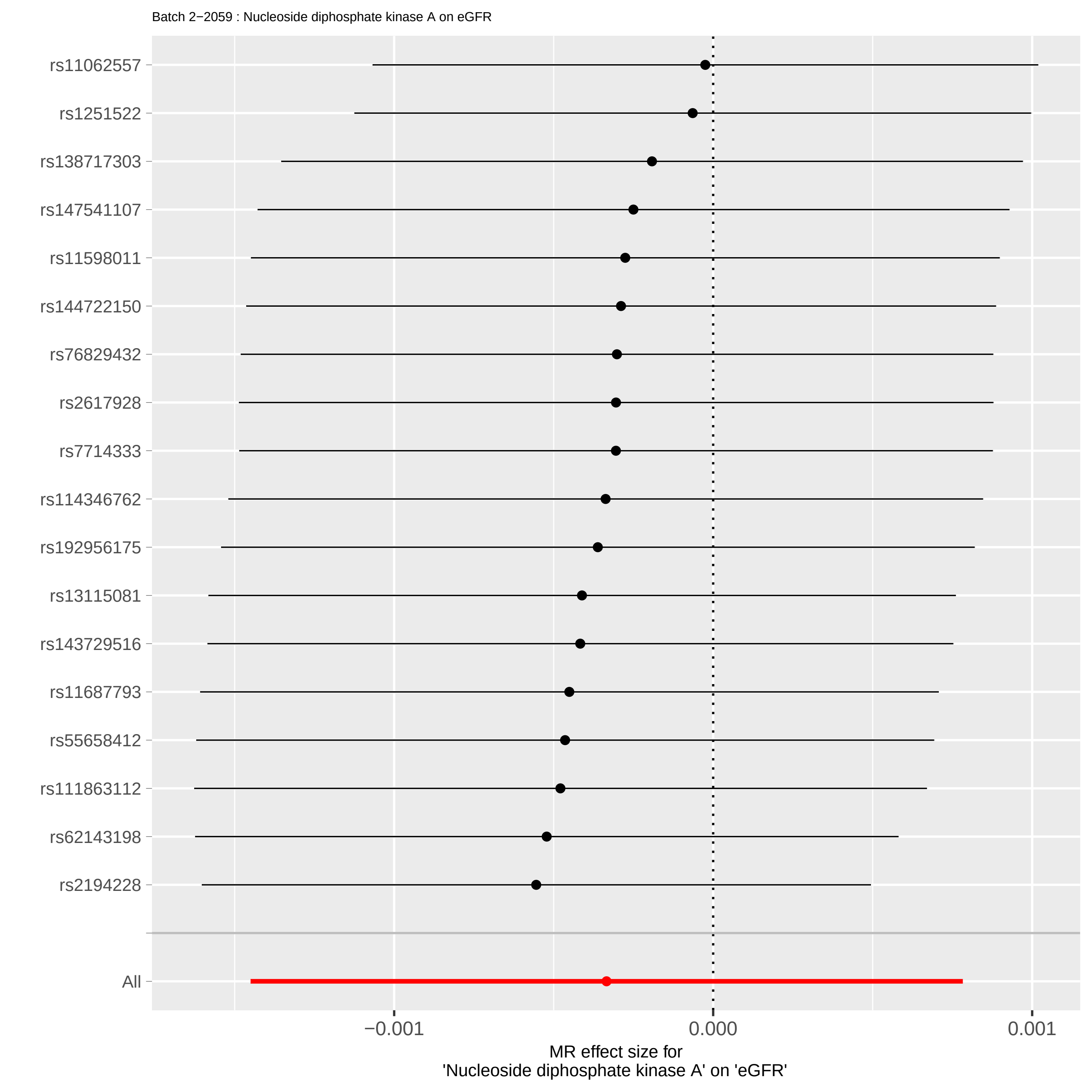

Batch 2−2059 : Nucleoside diphosphate kinase A on eGFR
rs11062557
rs1251522
rs138717303
rs147541107
rs11598011
rs144722150
rs76829432
rs2617928
rs7714333
rs114346762
rs192956175
rs13115081
rs143729516
rs11687793
rs55658412
rs111863112
rs62143198
rs2194228
All
−0.001
0.000
0.001
MR effect size for
'Nucleoside diphosphate kinase A' on 'eGFR'

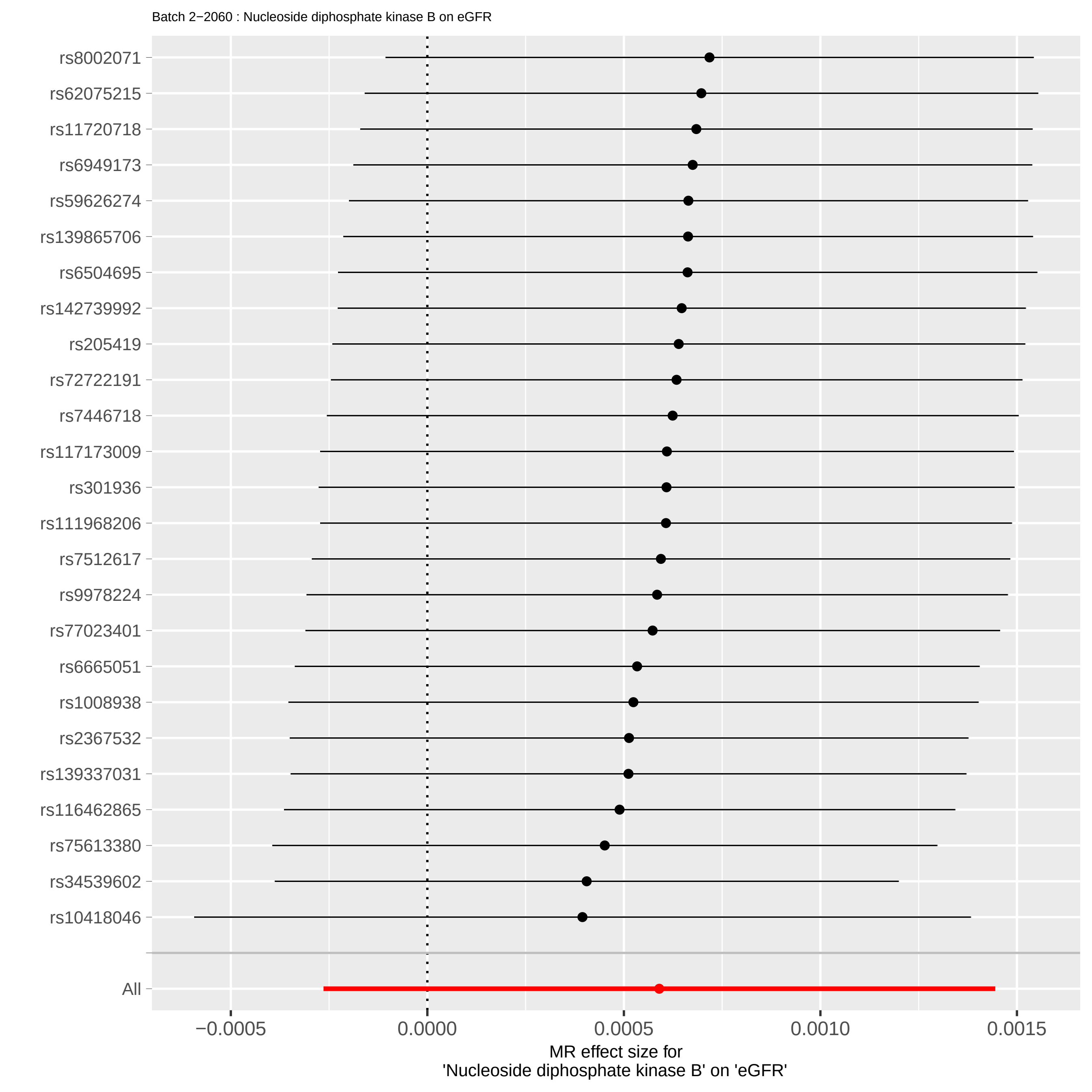

Batch 2−2060 : Nucleoside diphosphate kinase B on eGFR
rs8002071
rs62075215
rs11720718
rs6949173
rs59626274
rs139865706
rs6504695
rs142739992
rs205419
rs72722191
rs7446718
rs117173009
rs301936
rs111968206
rs7512617
rs9978224
rs77023401
rs6665051
rs1008938
rs2367532
rs139337031
rs116462865
rs75613380
rs34539602
rs10418046
All
−0.0005
0.0000
0.0005
0.0010
0.0015
MR effect size for
'Nucleoside diphosphate kinase B' on 'eGFR'

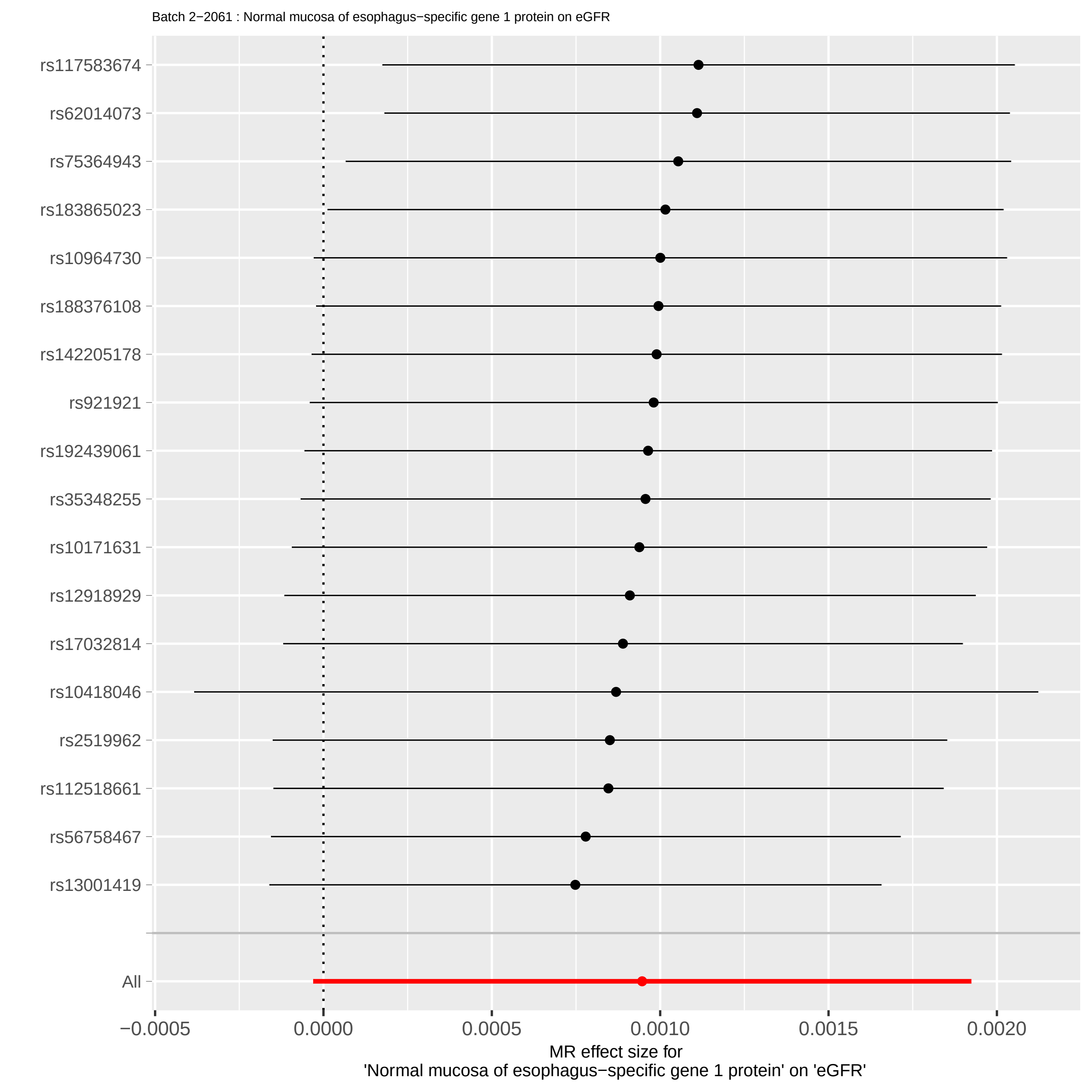

Batch 2−2061 : Normal mucosa of esophagus−specific gene 1 protein on eGFR
rs117583674
rs62014073
rs75364943
rs183865023
rs10964730
rs188376108
rs142205178
rs921921
rs192439061
rs35348255
rs10171631
rs12918929
rs17032814
rs10418046
rs2519962
rs112518661
rs56758467
rs13001419
All
−0.0005
0.0000
0.0005
0.0010
0.0015
0.0020
MR effect size for
'Normal mucosa of esophagus−specific gene 1 protein' on 'eGFR'

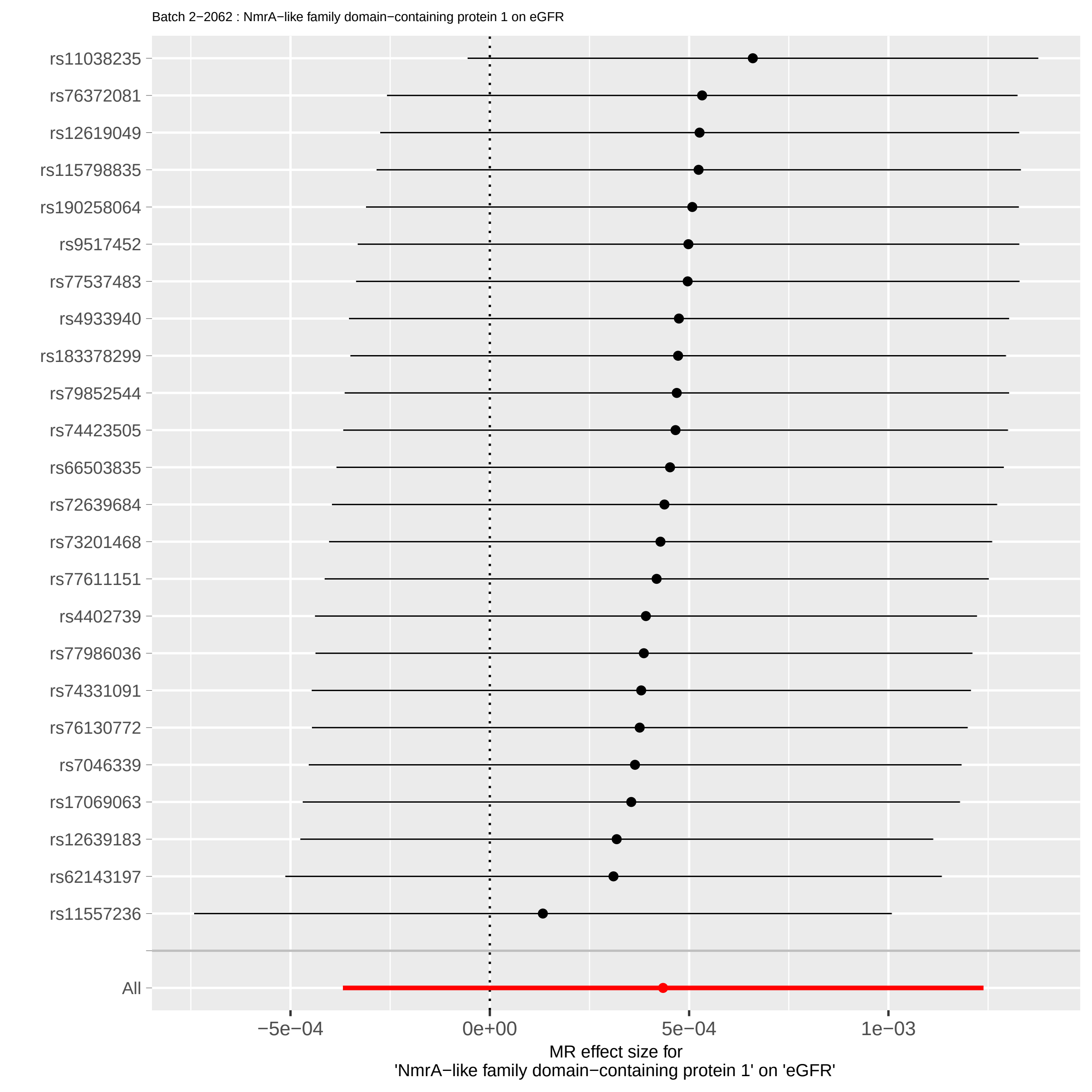

Batch 2−2062 : NmrA−like family domain−containing protein 1 on eGFR
rs11038235
rs76372081
rs12619049
rs115798835
rs190258064
rs9517452
rs77537483
rs4933940
rs183378299
rs79852544
rs74423505
rs66503835
rs72639684
rs73201468
rs77611151
rs4402739
rs77986036
rs74331091
rs76130772
rs7046339
rs17069063
rs12639183
rs62143197
rs11557236
All
−5e−04
0e+00
5e−04
1e−03
MR effect size for
'NmrA−like family domain−containing protein 1' on 'eGFR'

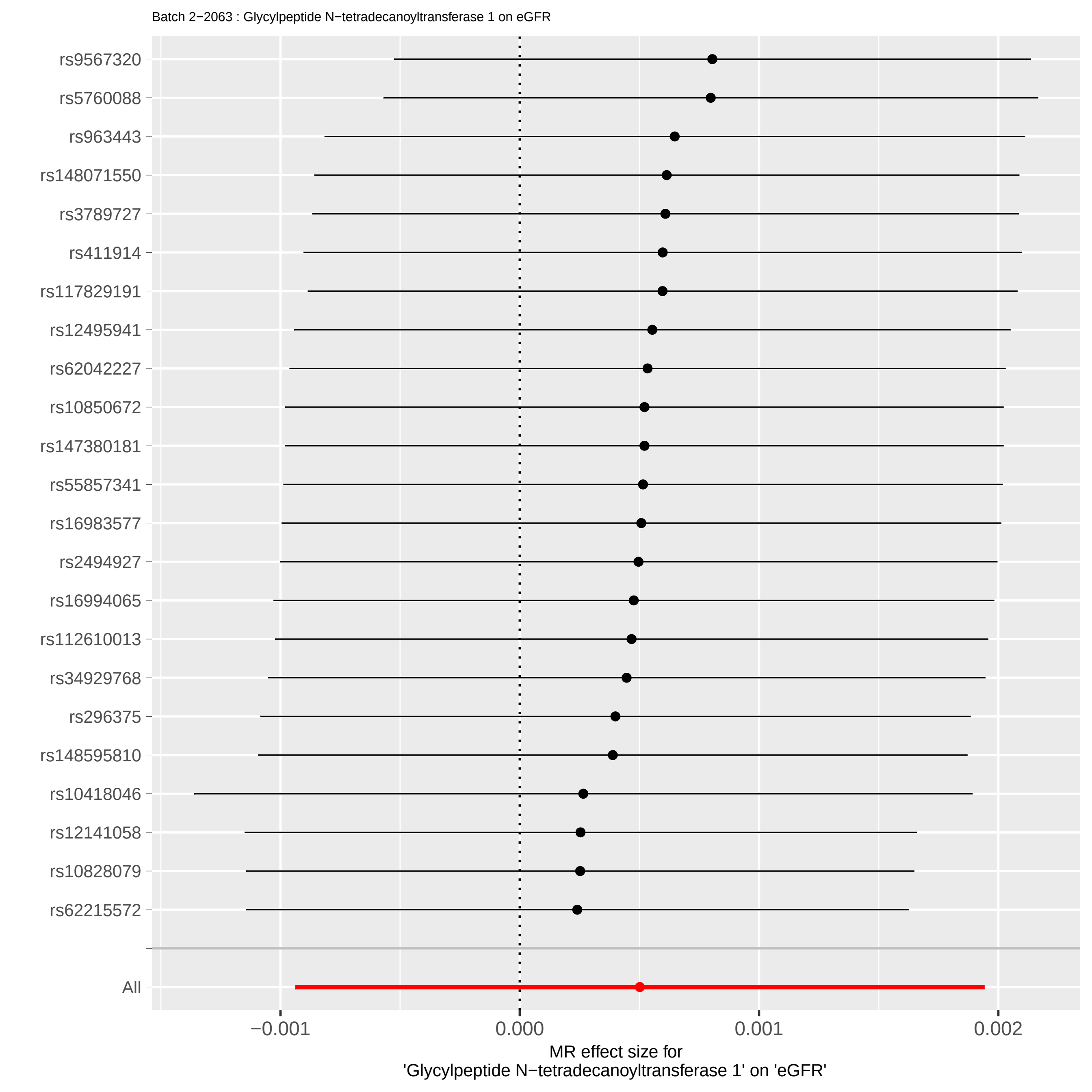
Batch 2−2063 : Glycylpeptide N−tetradecanoyltransferase 1 on eGFR
rs9567320
rs5760088
rs963443
rs148071550
rs3789727
rs411914
rs117829191
rs12495941
rs62042227
rs10850672
rs147380181
rs55857341
rs16983577
rs2494927
rs16994065
rs112610013
rs34929768
rs296375
rs148595810
rs10418046
rs12141058
rs10828079
rs62215572
All
−0.001
0.000
0.001
0.002
MR effect size for
'Glycylpeptide N−tetradecanoyltransferase 1' on 'eGFR'

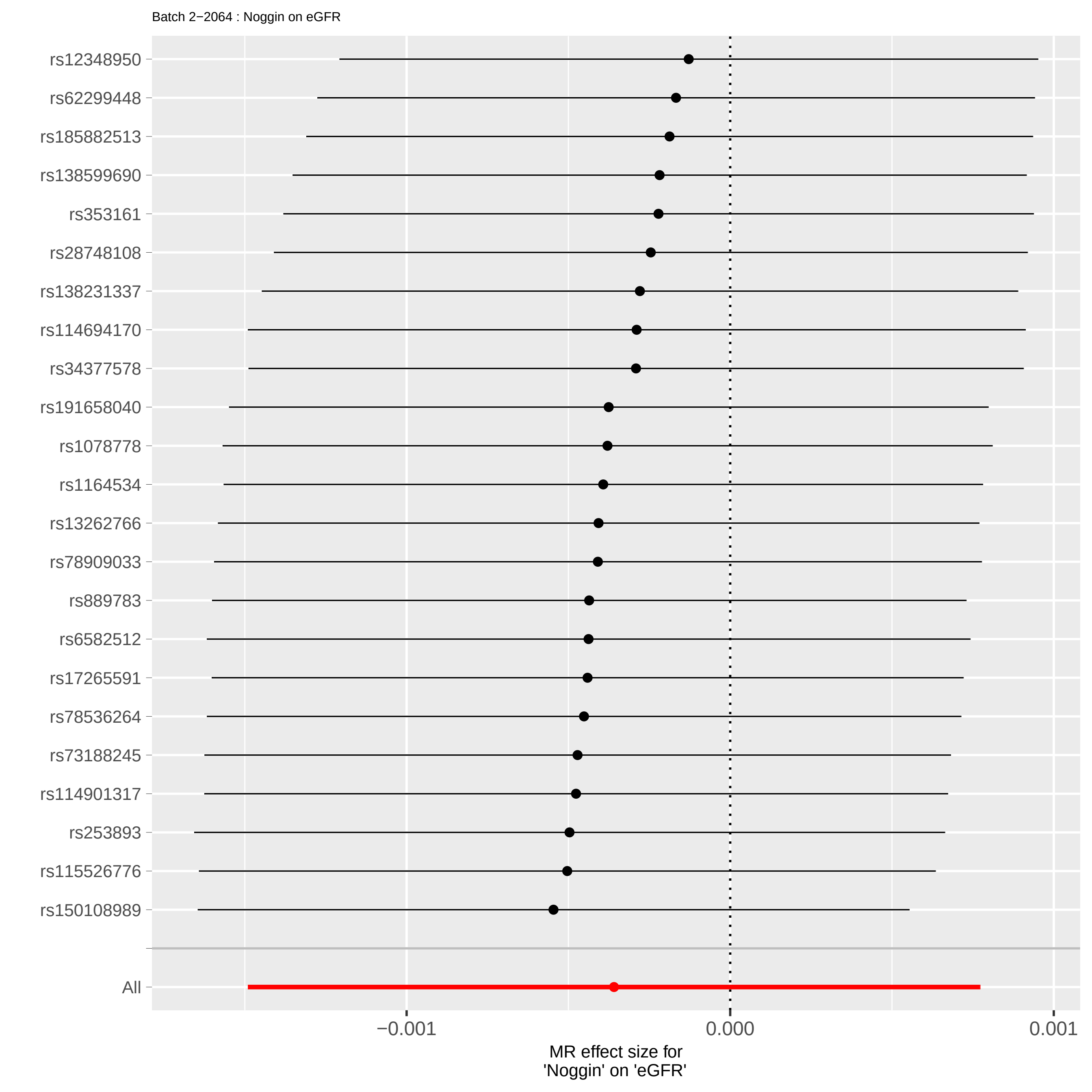

Batch 2−2064 : Noggin on eGFR
rs12348950
rs62299448
rs185882513
rs138599690
rs353161
rs28748108
rs138231337
rs114694170
rs34377578
rs191658040
rs1078778
rs1164534
rs13262766
rs78909033
rs889783
rs6582512
rs17265591
rs78536264
rs73188245
rs114901317
rs253893
rs115526776
rs150108989
All
−0.001
0.000
0.001
MR effect size for
'Noggin' on 'eGFR'

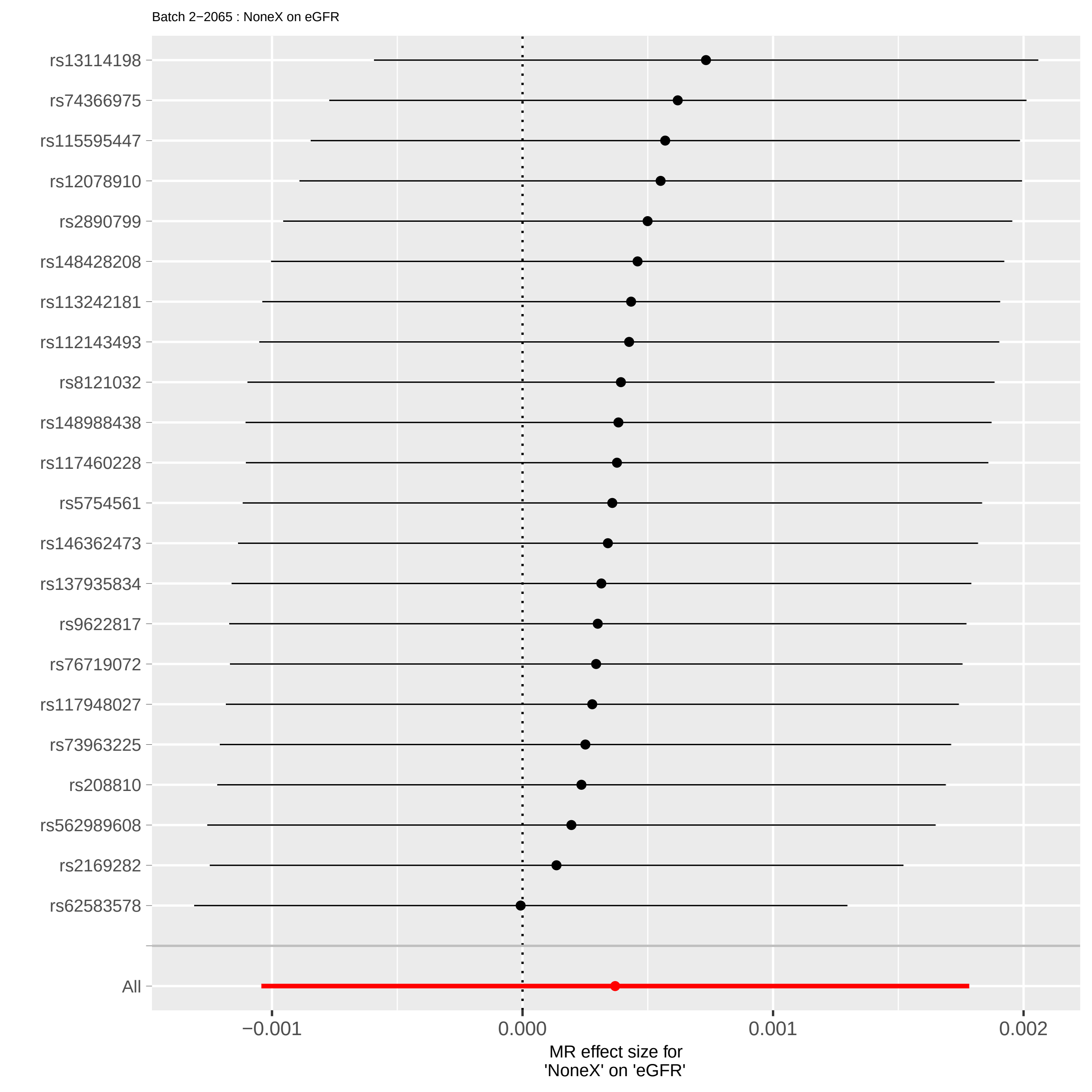
Batch 2−2065 : NoneX on eGFR
rs13114198
rs74366975
rs115595447
rs12078910
rs2890799
rs148428208
rs113242181
rs112143493
rs8121032
rs148988438
rs117460228
rs5754561
rs146362473
rs137935834
rs9622817
rs76719072
rs117948027
rs73963225
rs208810
rs562989608
rs2169282
rs62583578
All
−0.001
0.000
0.001
0.002
MR effect size for
'NoneX' on 'eGFR'

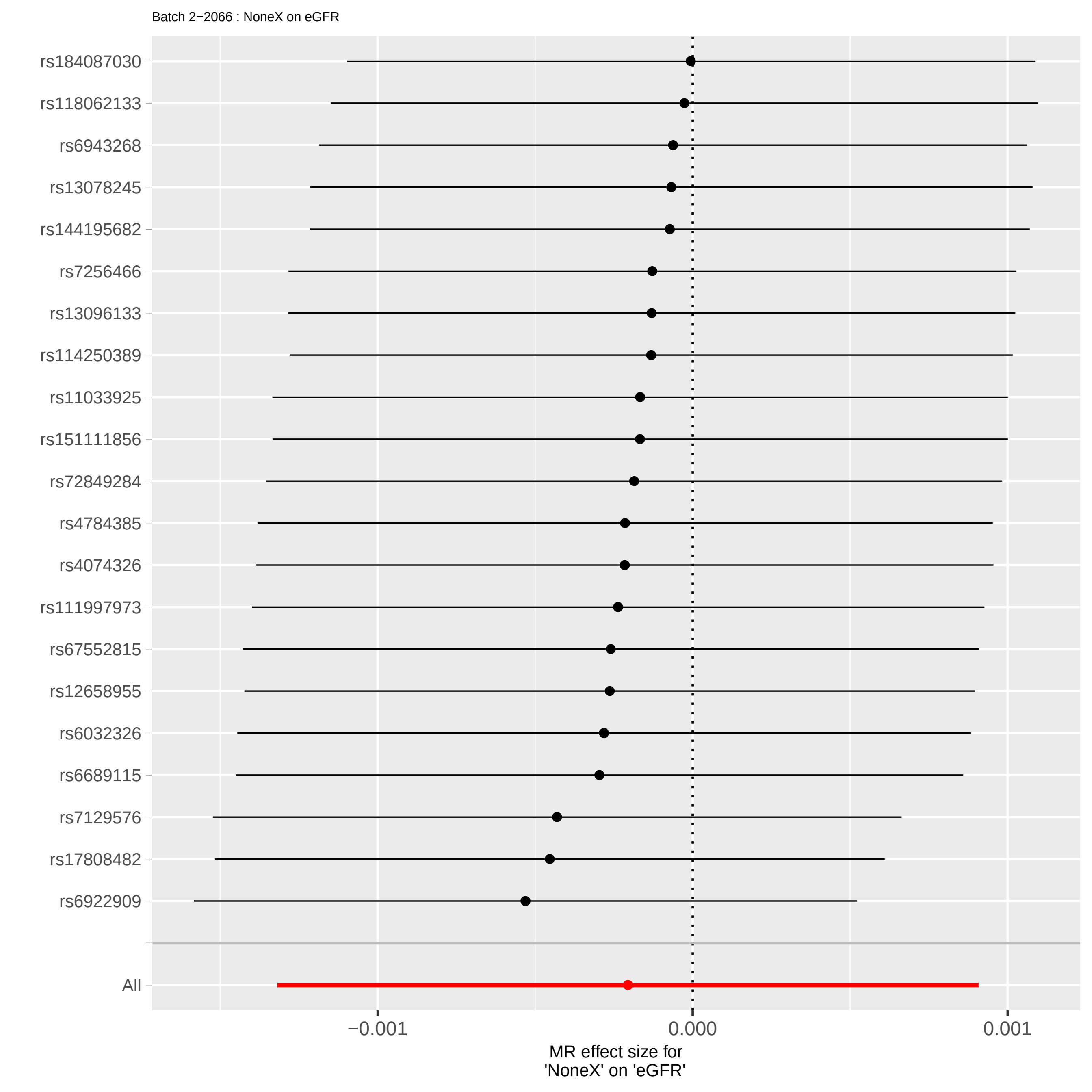

Batch 2−2066 : NoneX on eGFR
rs184087030
rs118062133
rs6943268
rs13078245
rs144195682
rs7256466
rs13096133
rs114250389
rs11033925
rs151111856
rs72849284
rs4784385
rs4074326
rs111997973
rs67552815
rs12658955
rs6032326
rs6689115
rs7129576
rs17808482
rs6922909
All
−0.001
0.000
0.001
MR effect size for
'NoneX' on 'eGFR'

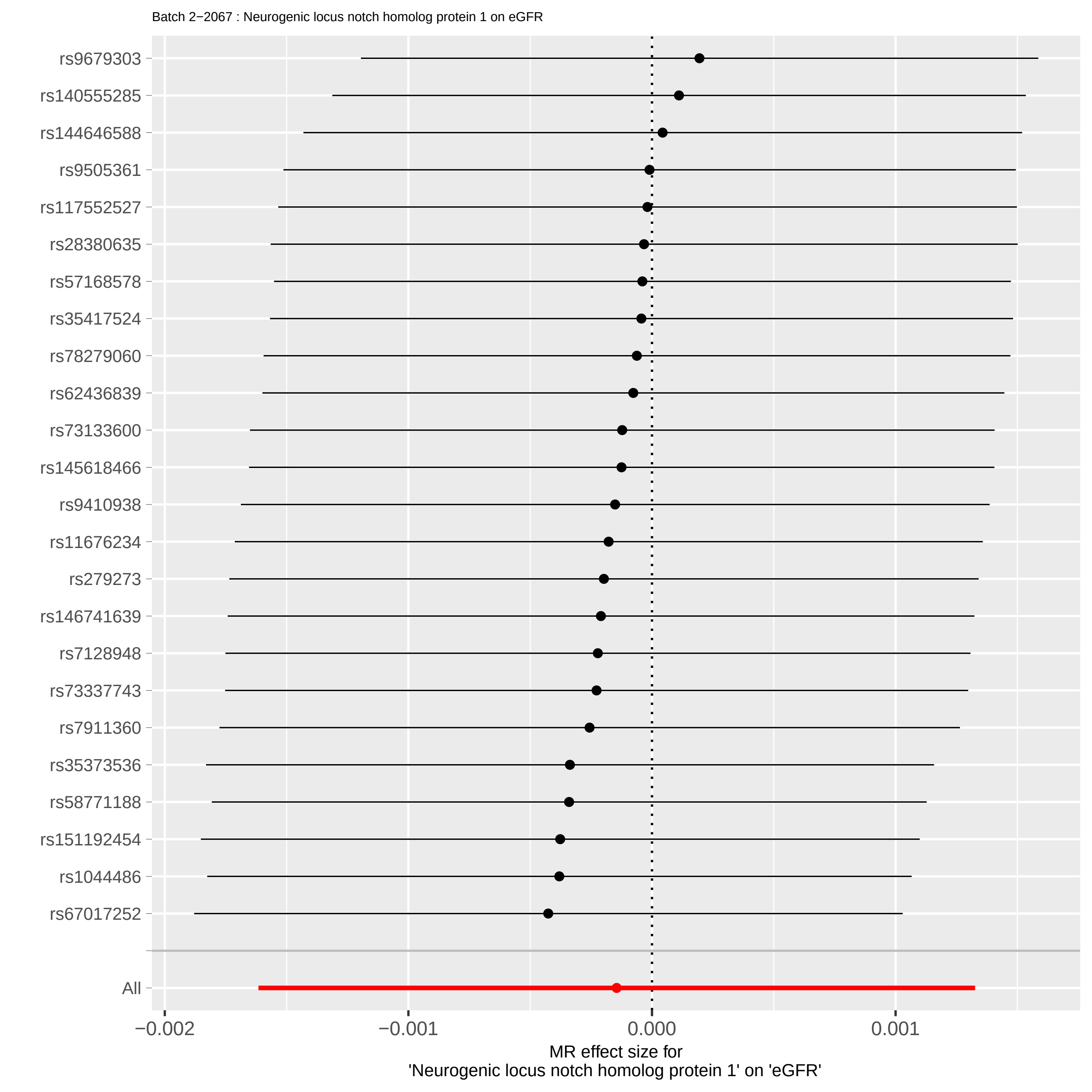

Batch 2−2067 : Neurogenic locus notch homolog protein 1 on eGFR
rs9679303
rs140555285
rs144646588
rs9505361
rs117552527
rs28380635
rs57168578
rs35417524
rs78279060
rs62436839
rs73133600
rs145618466
rs9410938
rs11676234
rs279273
rs146741639
rs7128948
rs73337743
rs7911360
rs35373536
rs58771188
rs151192454
rs1044486
rs67017252
All
−0.002
−0.001
0.000
0.001
MR effect size for
'Neurogenic locus notch homolog protein 1' on 'eGFR'

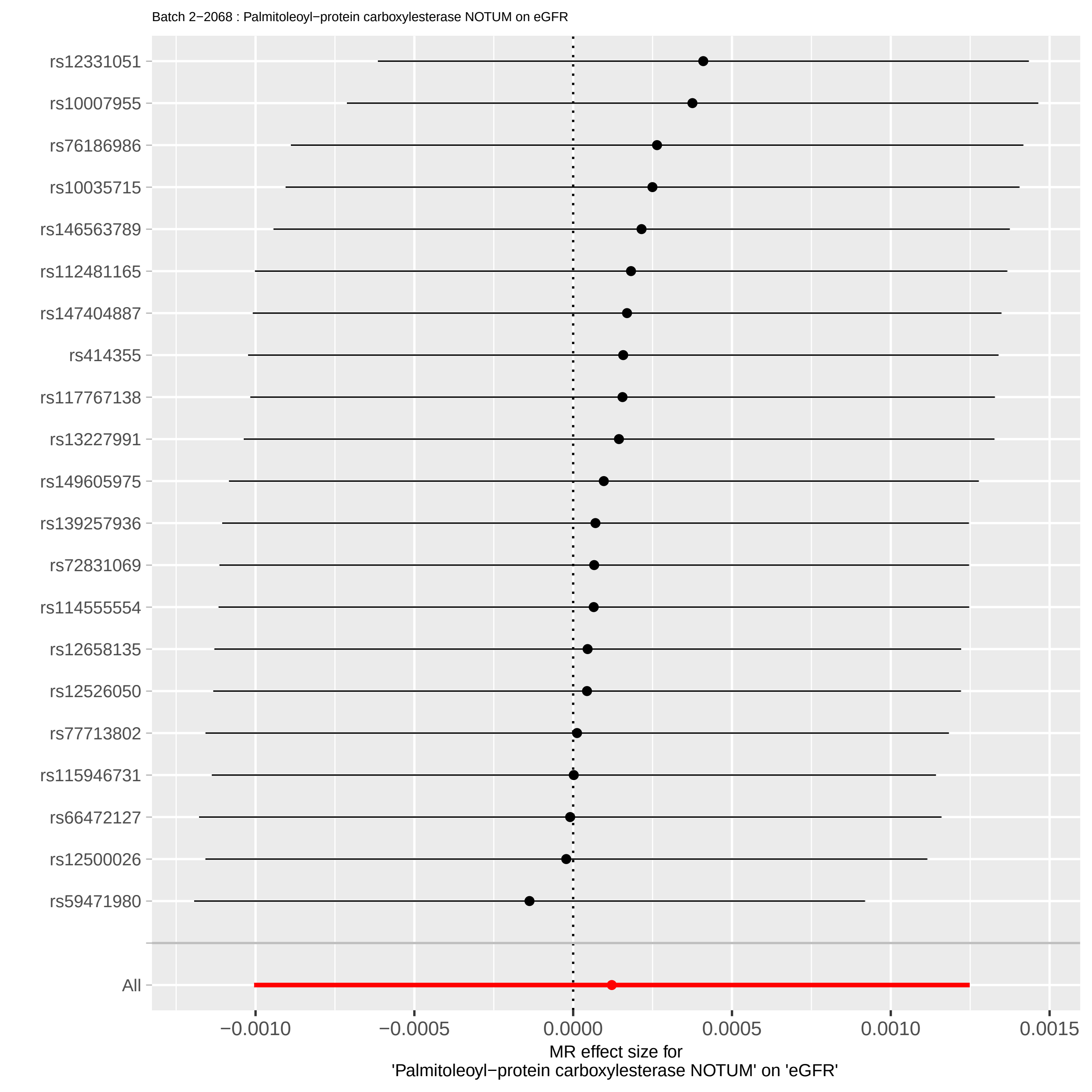

Batch 2−2068 : Palmitoleoyl−protein carboxylesterase NOTUM on eGFR
rs12331051
rs10007955
rs76186986
rs10035715
rs146563789
rs112481165
rs147404887
rs414355
rs117767138
rs13227991
rs149605975
rs139257936
rs72831069
rs114555554
rs12658135
rs12526050
rs77713802
rs115946731
rs66472127
rs12500026
rs59471980
All
−0.0010
−0.0005
0.0000
0.0005
0.0010
0.0015
MR effect size for
'Palmitoleoyl−protein carboxylesterase NOTUM' on 'eGFR'

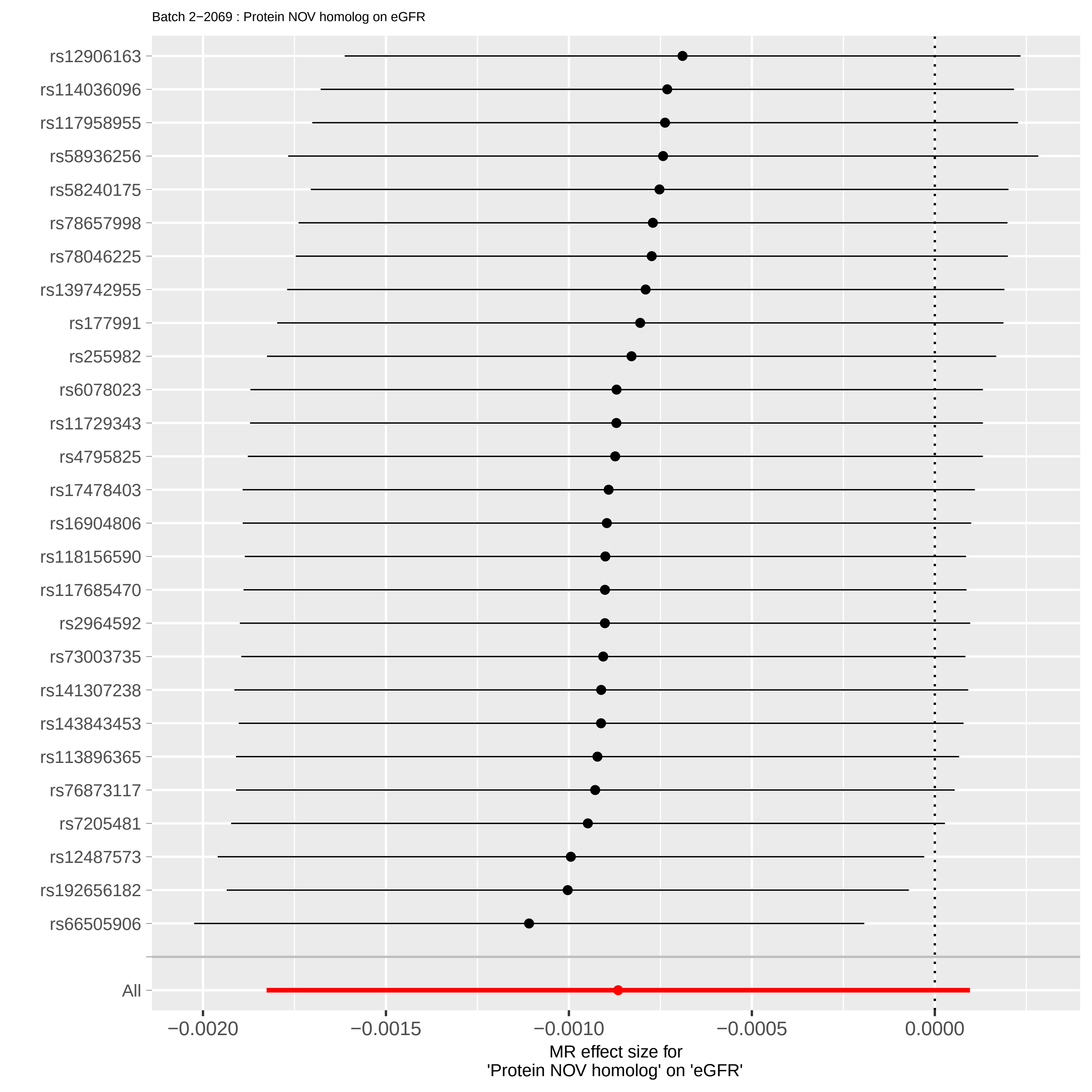

Batch 2−2069 : Protein NOV homolog on eGFR
rs12906163
rs114036096
rs117958955
rs58936256
rs58240175
rs78657998
rs78046225
rs139742955
rs177991
rs255982
rs6078023
rs11729343
rs4795825
rs17478403
rs16904806
rs118156590
rs117685470
rs2964592
rs73003735
rs141307238
rs143843453
rs113896365
rs76873117
rs7205481
rs12487573
rs192656182
rs66505906
All
−0.0020
−0.0015
−0.0010
−0.0005
0.0000
MR effect size for
'Protein NOV homolog' on 'eGFR'

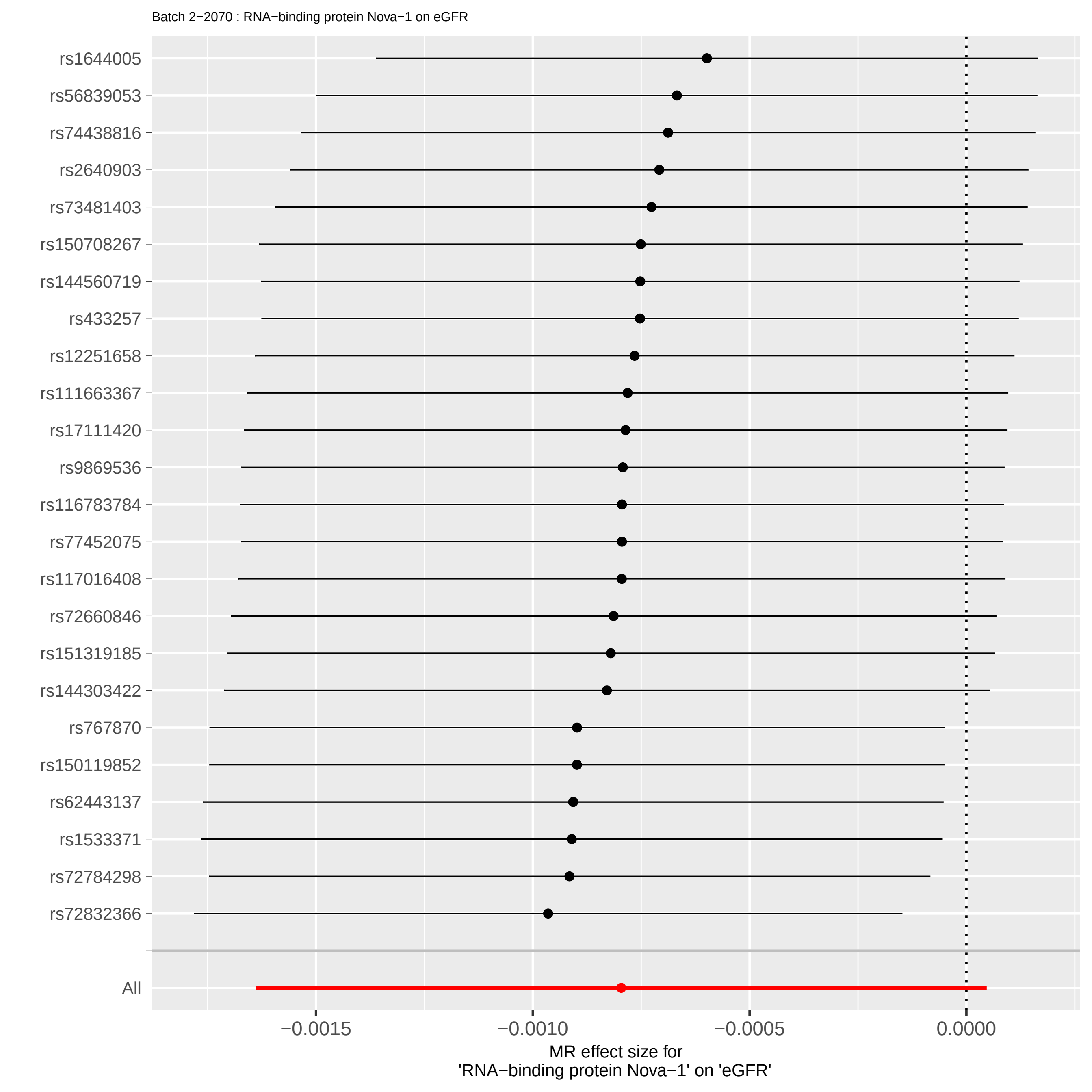

Batch 2−2070 : RNA−binding protein Nova−1 on eGFR
rs1644005
rs56839053
rs74438816
rs2640903
rs73481403
rs150708267
rs144560719
rs433257
rs12251658
rs111663367
rs17111420
rs9869536
rs116783784
rs77452075
rs117016408
rs72660846
rs151319185
rs144303422
rs767870
rs150119852
rs62443137
rs1533371
rs72784298
rs72832366
All
−0.0015
−0.0010
−0.0005
0.0000
MR effect size for
'RNA−binding protein Nova−1' on 'eGFR'

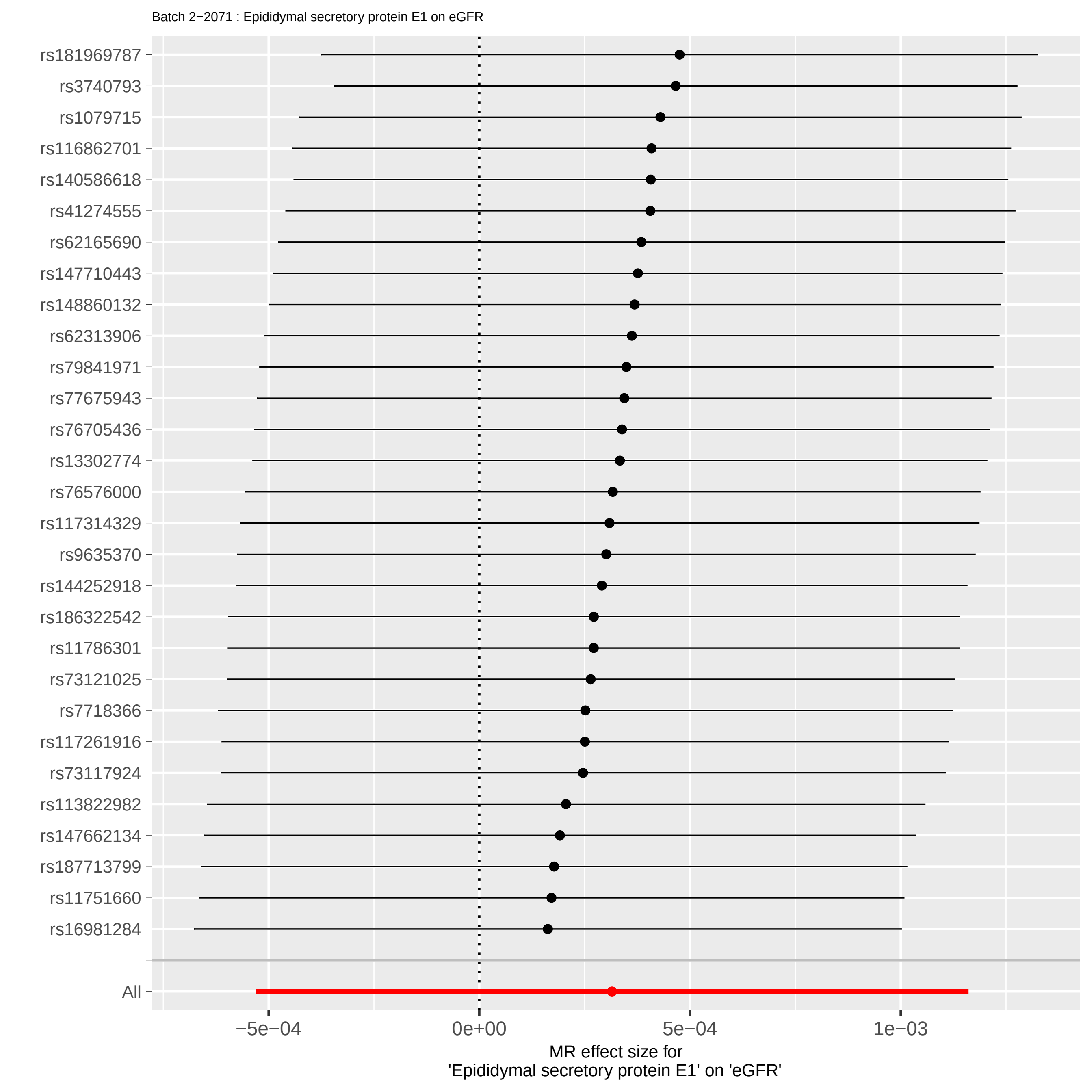

Batch 2−2071 : Epididymal secretory protein E1 on eGFR
rs181969787
rs3740793
rs1079715
rs116862701
rs140586618
rs41274555
rs62165690
rs147710443
rs148860132
rs62313906
rs79841971
rs77675943
rs76705436
rs13302774
rs76576000
rs117314329
rs9635370
rs144252918
rs186322542
rs11786301
rs73121025
rs7718366
rs117261916
rs73117924
rs113822982
rs147662134
rs187713799
rs11751660
rs16981284
All
−5e−04
0e+00
5e−04
1e−03
MR effect size for
'Epididymal secretory protein E1' on 'eGFR'

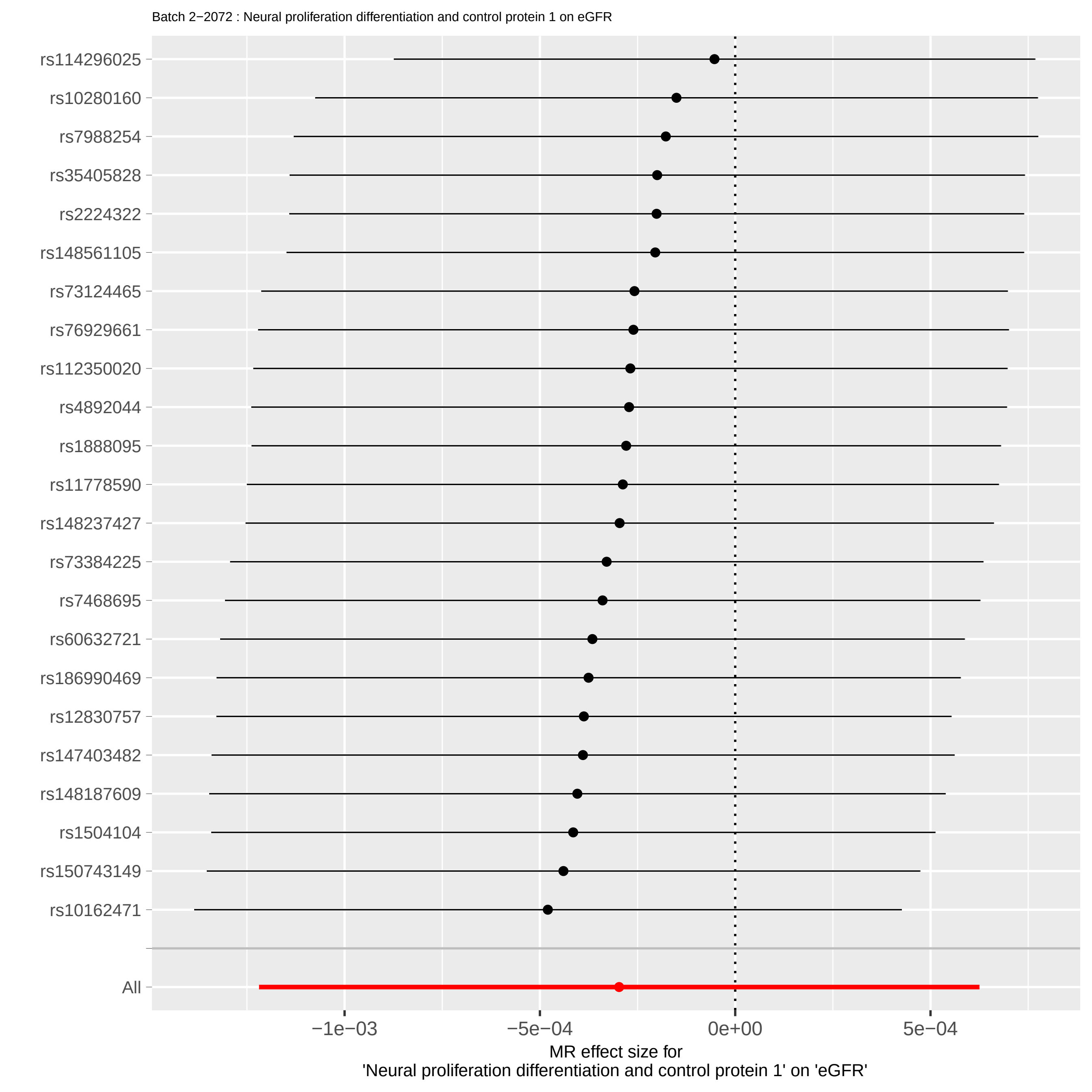
Batch 2−2072 : Neural proliferation differentiation and control protein 1 on eGFR
rs114296025
rs10280160
rs7988254
rs35405828
rs2224322
rs148561105
rs73124465
rs76929661
rs112350020
rs4892044
rs1888095
rs11778590
rs148237427
rs73384225
rs7468695
rs60632721
rs186990469
rs12830757
rs147403482
rs148187609
rs1504104
rs150743149
rs10162471
All
−1e−03
−5e−04
0e+00
5e−04
MR effect size for
'Neural proliferation differentiation and control protein 1' on 'eGFR'

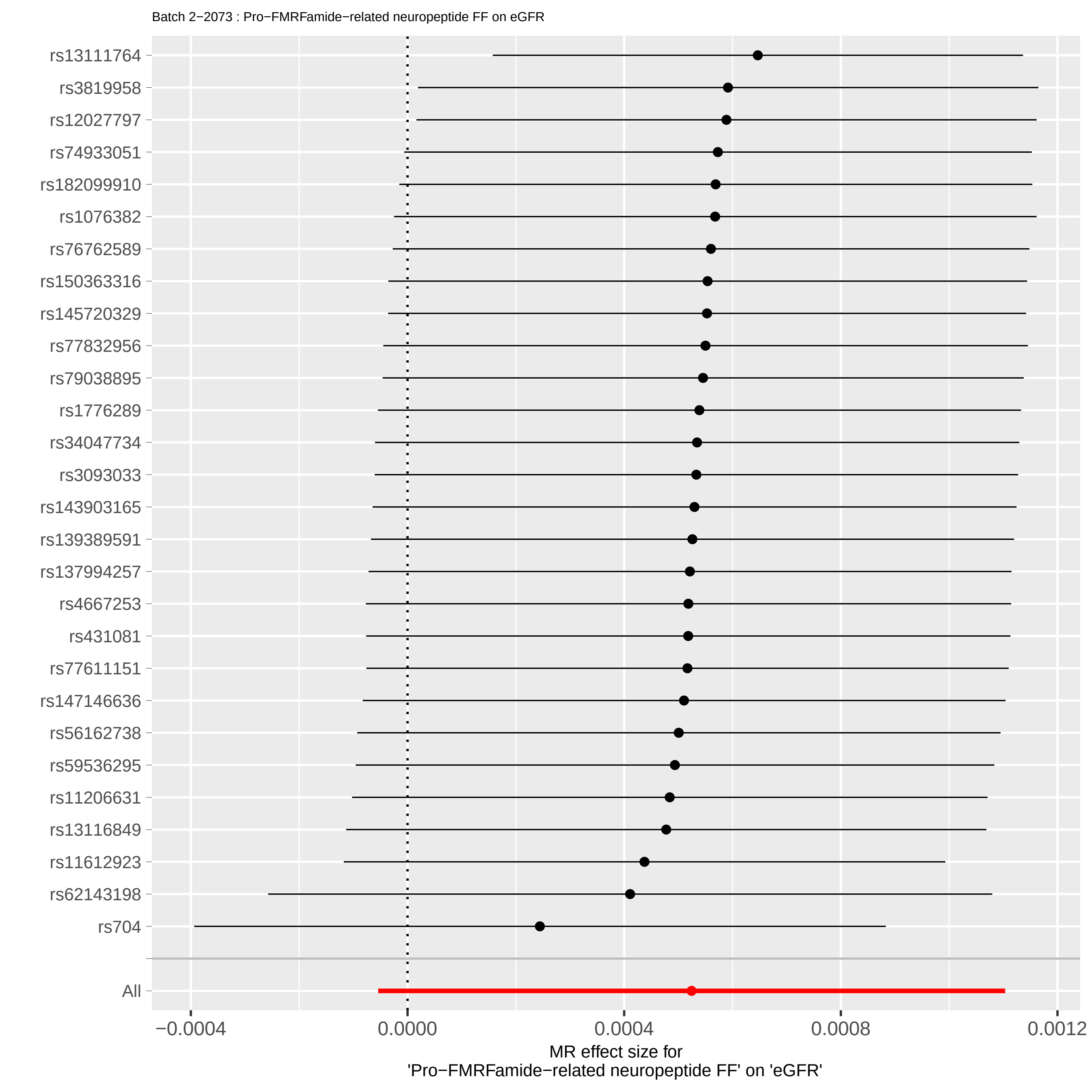

Batch 2−2073 : Pro−FMRFamide−related neuropeptide FF on eGFR
rs13111764
rs3819958
rs12027797
rs74933051
rs182099910
rs1076382
rs76762589
rs150363316
rs145720329
rs77832956
rs79038895
rs1776289
rs34047734
rs3093033
rs143903165
rs139389591
rs137994257
rs4667253
rs431081
rs77611151
rs147146636
rs56162738
rs59536295
rs11206631
rs13116849
rs11612923
rs62143198
rs704
All
−0.0004
0.0000
0.0004
0.0008
0.0012
MR effect size for
'Pro−FMRFamide−related neuropeptide FF' on 'eGFR'

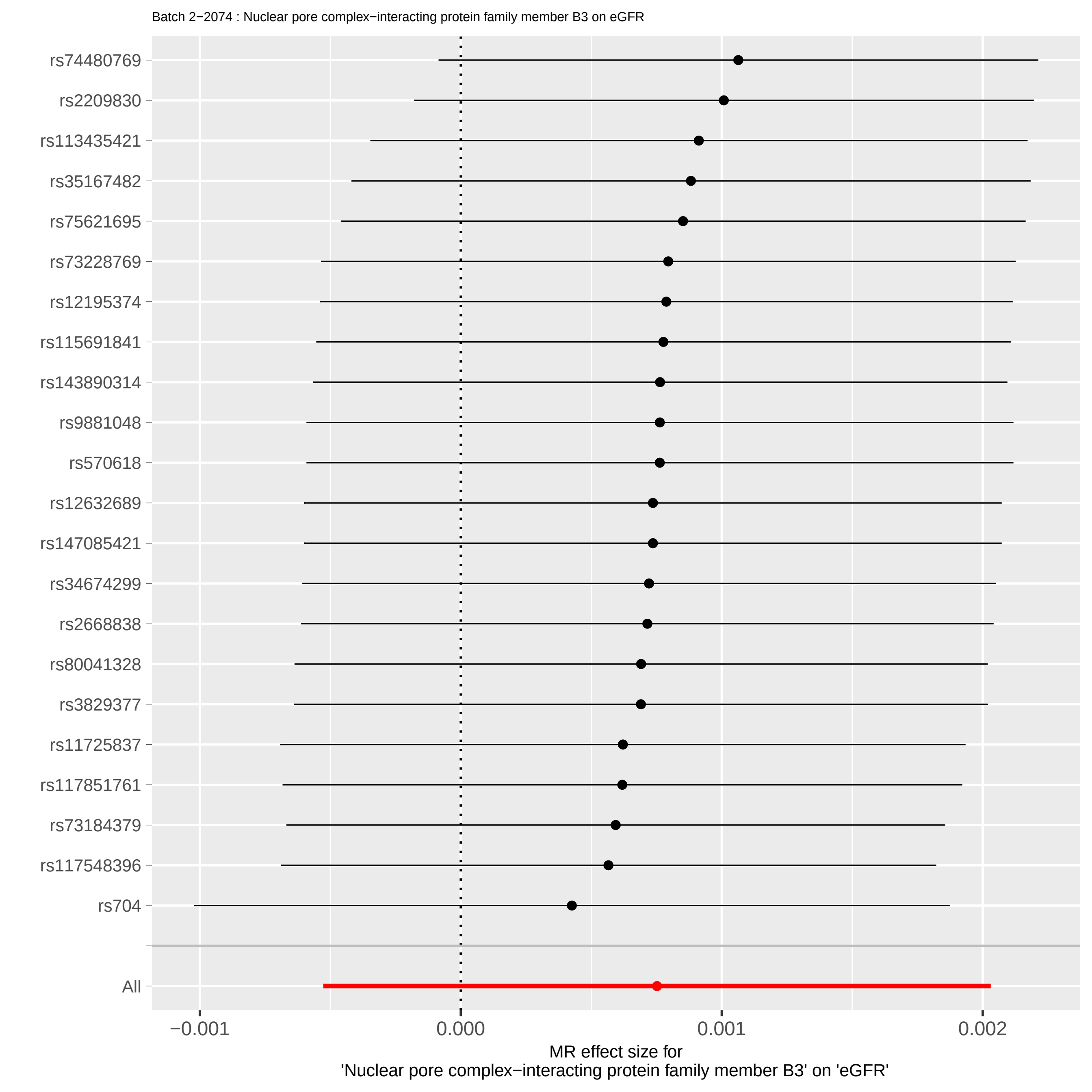

Batch 2−2074 : Nuclear pore complex−interacting protein family member B3 on eGFR
rs74480769
rs2209830
rs113435421
rs35167482
rs75621695
rs73228769
rs12195374
rs115691841
rs143890314
rs9881048
rs570618
rs12632689
rs147085421
rs34674299
rs2668838
rs80041328
rs3829377
rs11725837
rs117851761
rs73184379
rs117548396
rs704
All
−0.001
0.000
0.001
0.002
MR effect size for
'Nuclear pore complex−interacting protein family member B3' on 'eGFR'

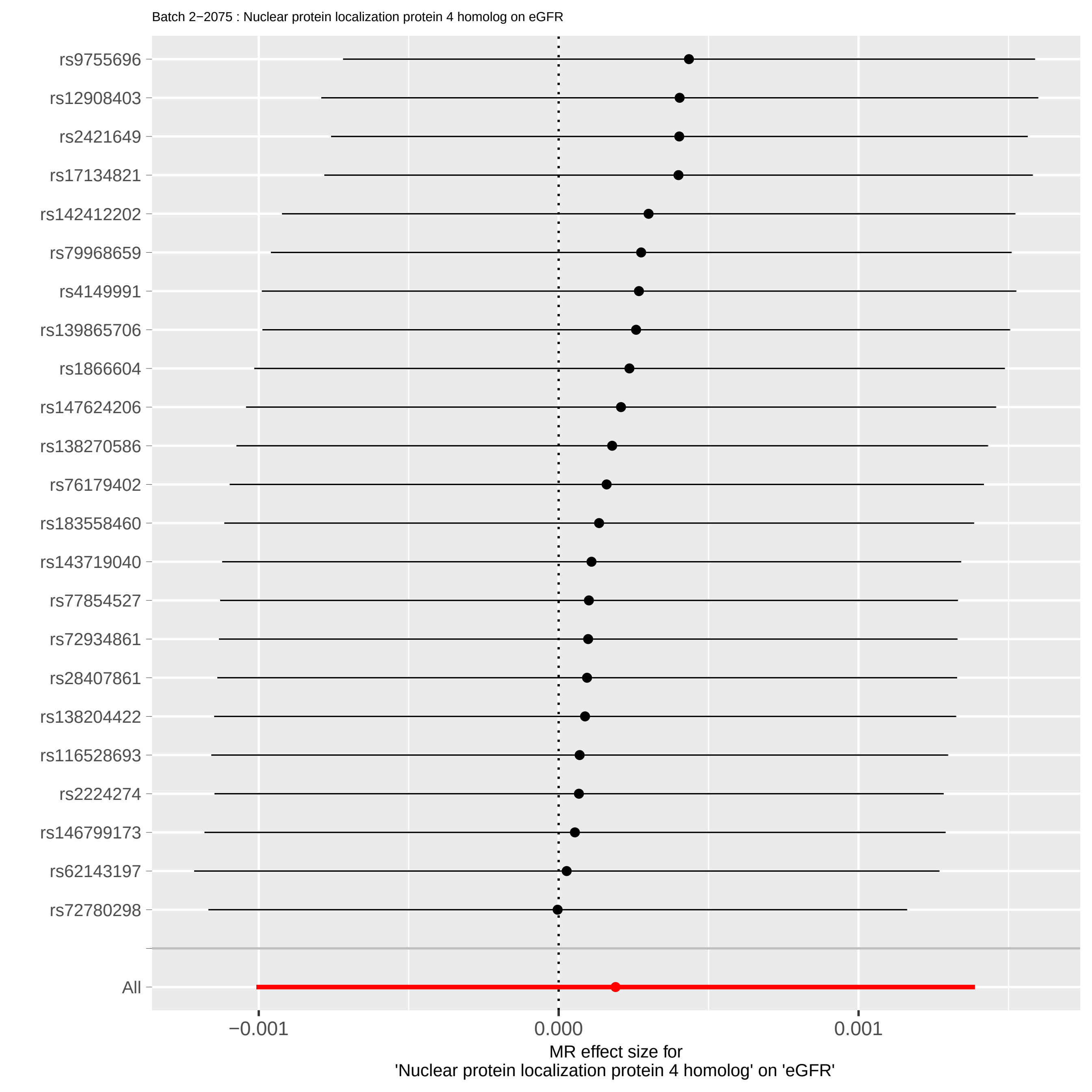

Batch 2−2075 : Nuclear protein localization protein 4 homolog on eGFR
rs9755696
rs12908403
rs2421649
rs17134821
rs142412202
rs79968659
rs4149991
rs139865706
rs1866604
rs147624206
rs138270586
rs76179402
rs183558460
rs143719040
rs77854527
rs72934861
rs28407861
rs138204422
rs116528693
rs2224274
rs146799173
rs62143197
rs72780298
All
−0.001
0.000
0.001
MR effect size for
'Nuclear protein localization protein 4 homolog' on 'eGFR'

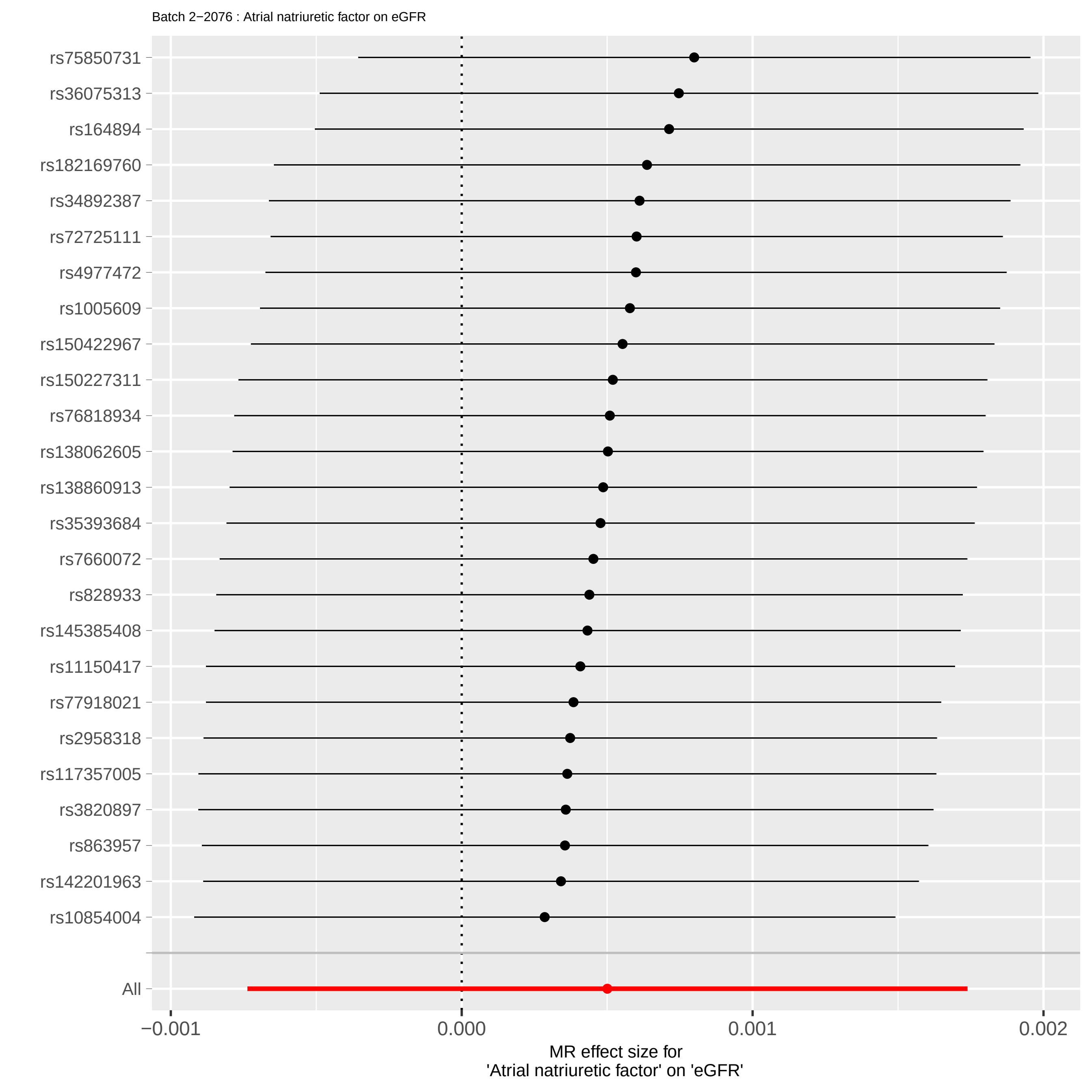
Batch 2−2076 : Atrial natriuretic factor on eGFR
rs75850731
rs36075313
rs164894
rs182169760
rs34892387
rs72725111
rs4977472
rs1005609
rs150422967
rs150227311
rs76818934
rs138062605
rs138860913
rs35393684
rs7660072
rs828933
rs145385408
rs11150417
rs77918021
rs2958318
rs117357005
rs3820897
rs863957
rs142201963
rs10854004
All
−0.001
0.000
0.001
0.002
MR effect size for
'Atrial natriuretic factor' on 'eGFR'

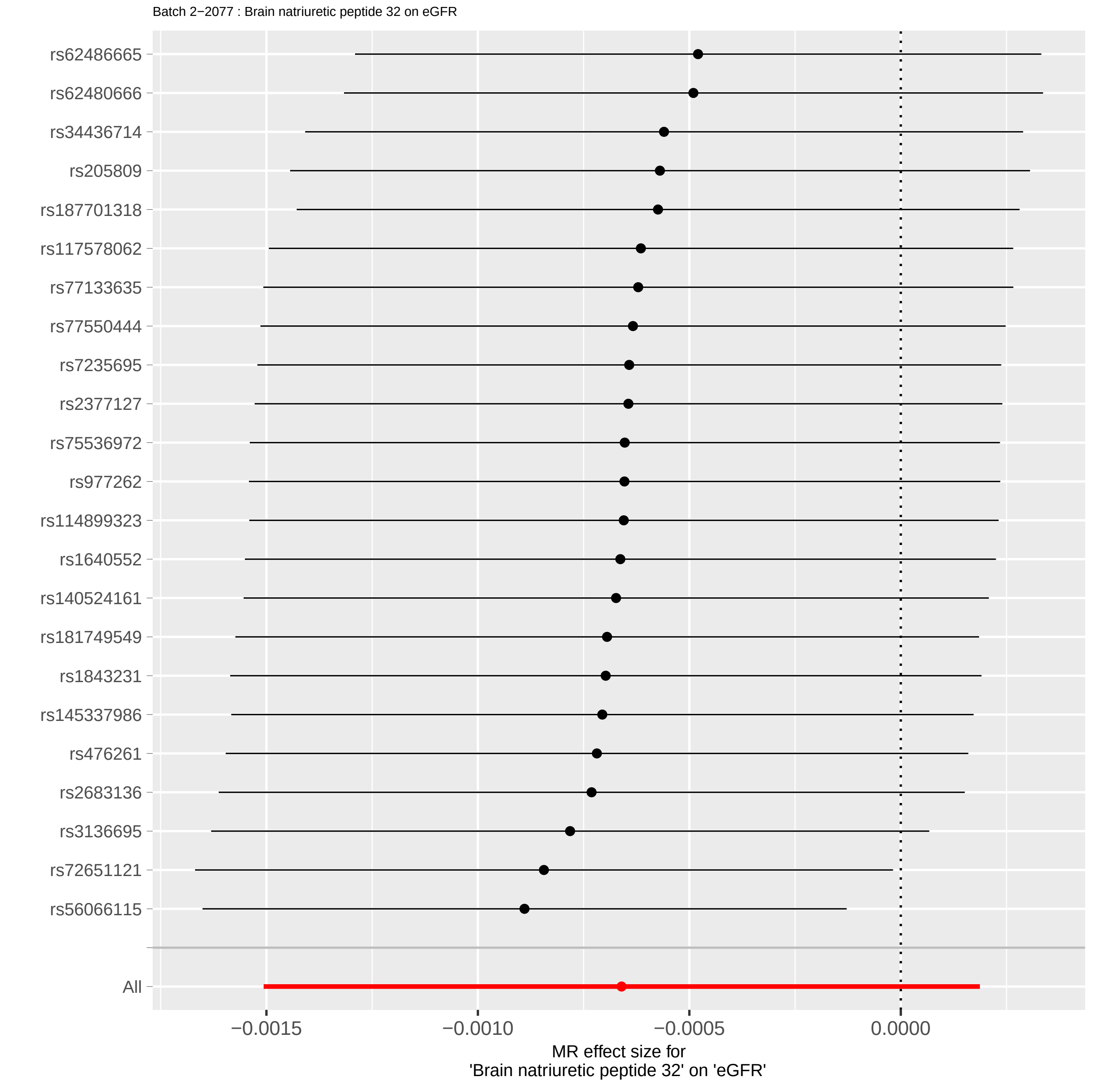

Batch 2−2077 : Brain natriuretic peptide 32 on eGFR
rs62486665
rs62480666
rs34436714
rs205809
rs187701318
rs117578062
rs77133635
rs77550444
rs7235695
rs2377127
rs75536972
rs977262
rs114899323
rs1640552
rs140524161
rs181749549
rs1843231
rs145337986
rs476261
rs2683136
rs3136695
rs72651121
rs56066115
All
−0.0015
−0.0010
−0.0005
0.0000
MR effect size for
'Brain natriuretic peptide 32' on 'eGFR'

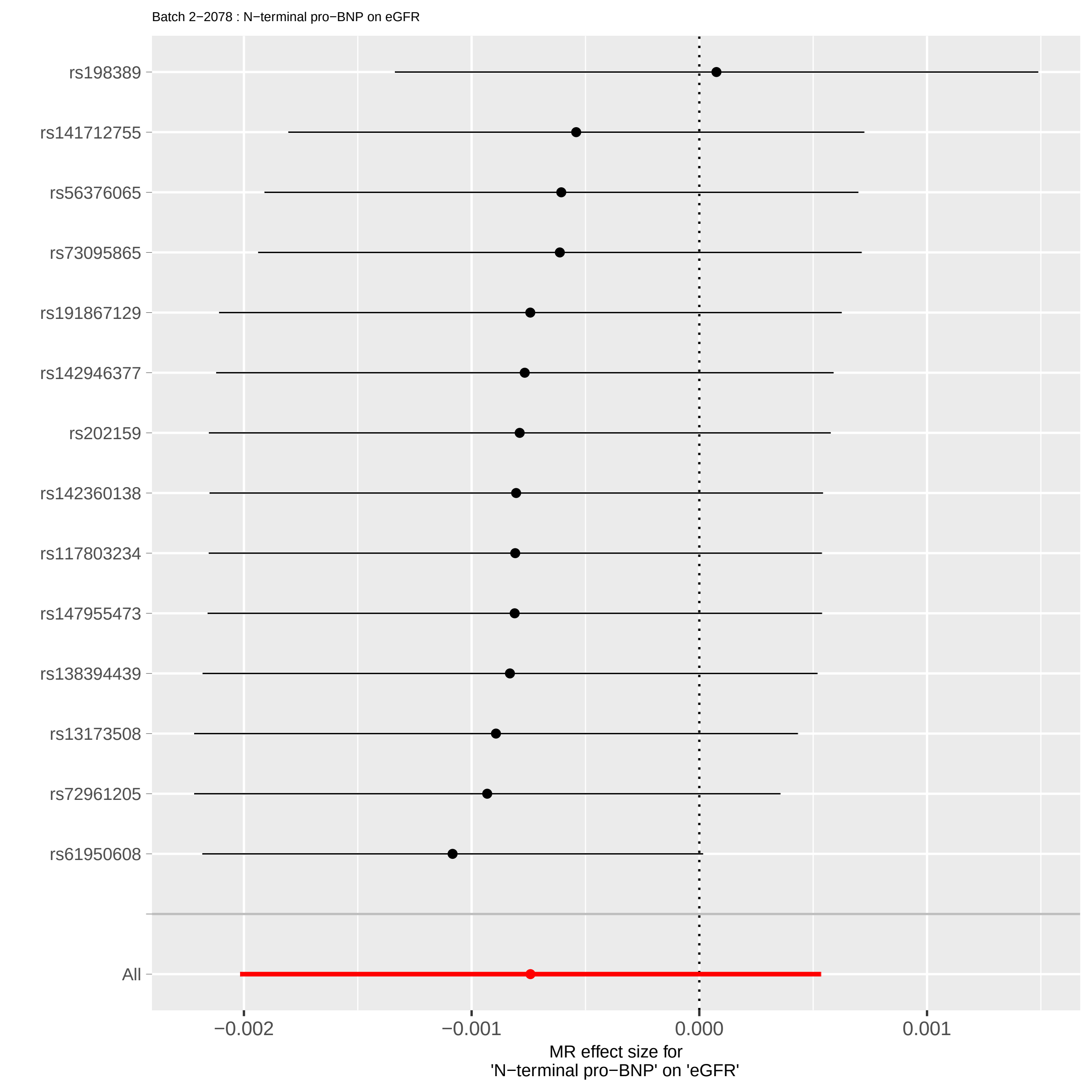

Batch 2−2078 : N−terminal pro−BNP on eGFR
rs198389
rs141712755
rs56376065
rs73095865
rs191867129
rs142946377
rs202159
rs142360138
rs117803234
rs147955473
rs138394439
rs13173508
rs72961205
rs61950608
All
−0.002
−0.001
0.000
0.001
MR effect size for
'N−terminal pro−BNP' on 'eGFR'

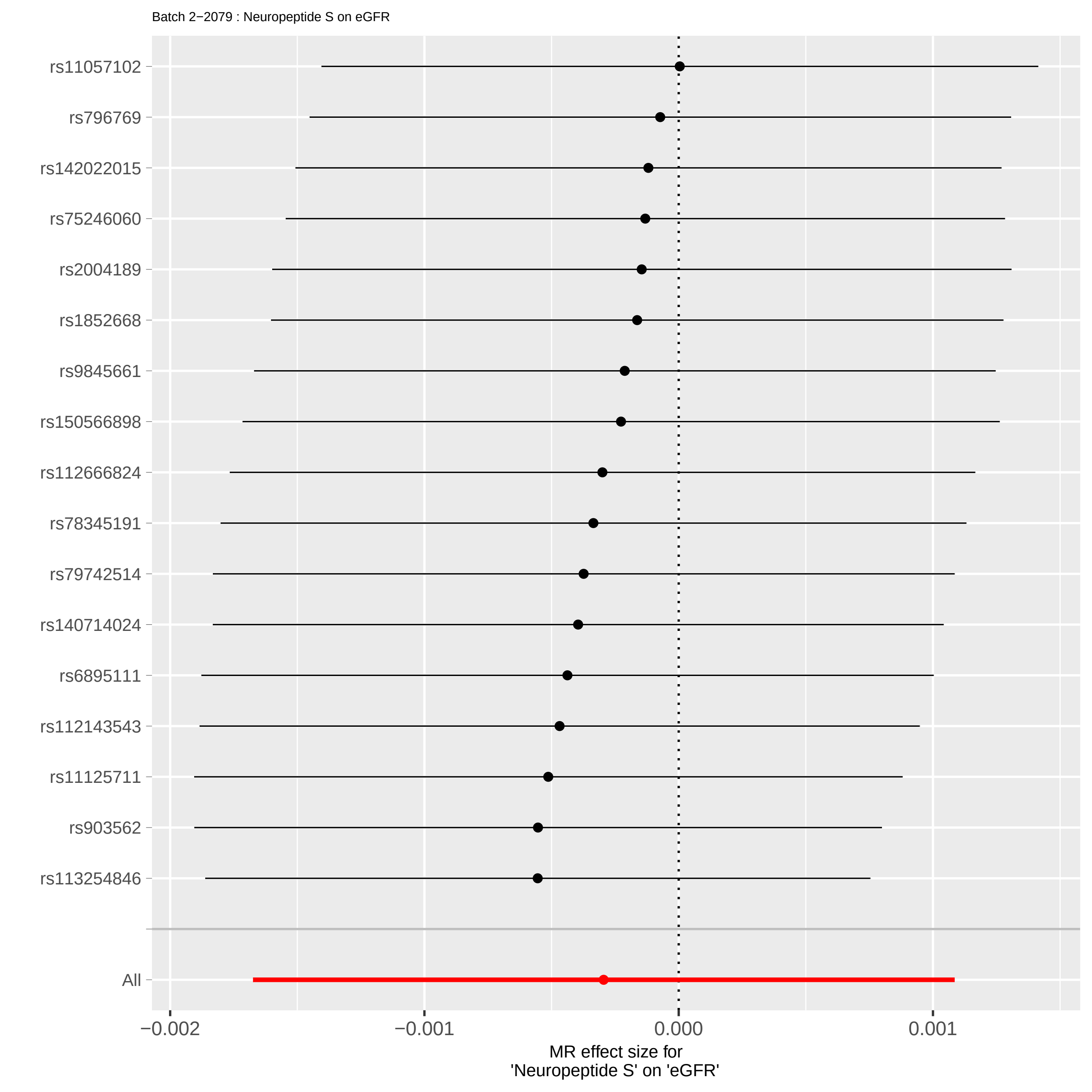

Batch 2−2079 : Neuropeptide S on eGFR
rs11057102
rs796769
rs142022015
rs75246060
rs2004189
rs1852668
rs9845661
rs150566898
rs112666824
rs78345191
rs79742514
rs140714024
rs6895111
rs112143543
rs11125711
rs903562
rs113254846
All
−0.002
−0.001
0.000
0.001
MR effect size for
'Neuropeptide S' on 'eGFR'

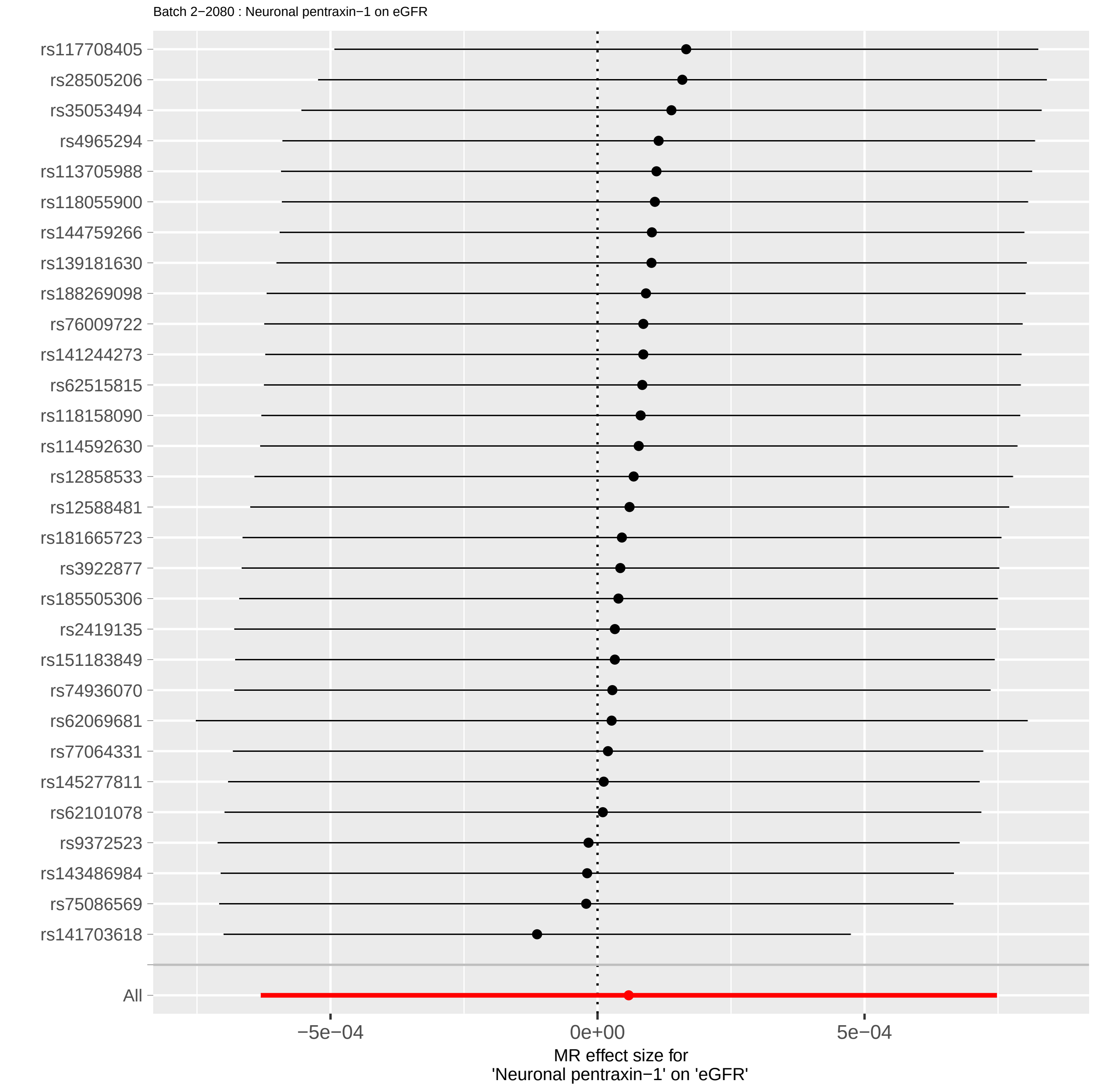

Batch 2−2080 : Neuronal pentraxin−1 on eGFR
rs117708405
rs28505206
rs35053494
rs4965294
rs113705988
rs118055900
rs144759266
rs139181630
rs188269098
rs76009722
rs141244273
rs62515815
rs118158090
rs114592630
rs12858533
rs12588481
rs181665723
rs3922877
rs185505306
rs2419135
rs151183849
rs74936070
rs62069681
rs77064331
rs145277811
rs62101078
rs9372523
rs143486984
rs75086569
rs141703618
All
−5e−04
0e+00
5e−04
MR effect size for
'Neuronal pentraxin−1' on 'eGFR'

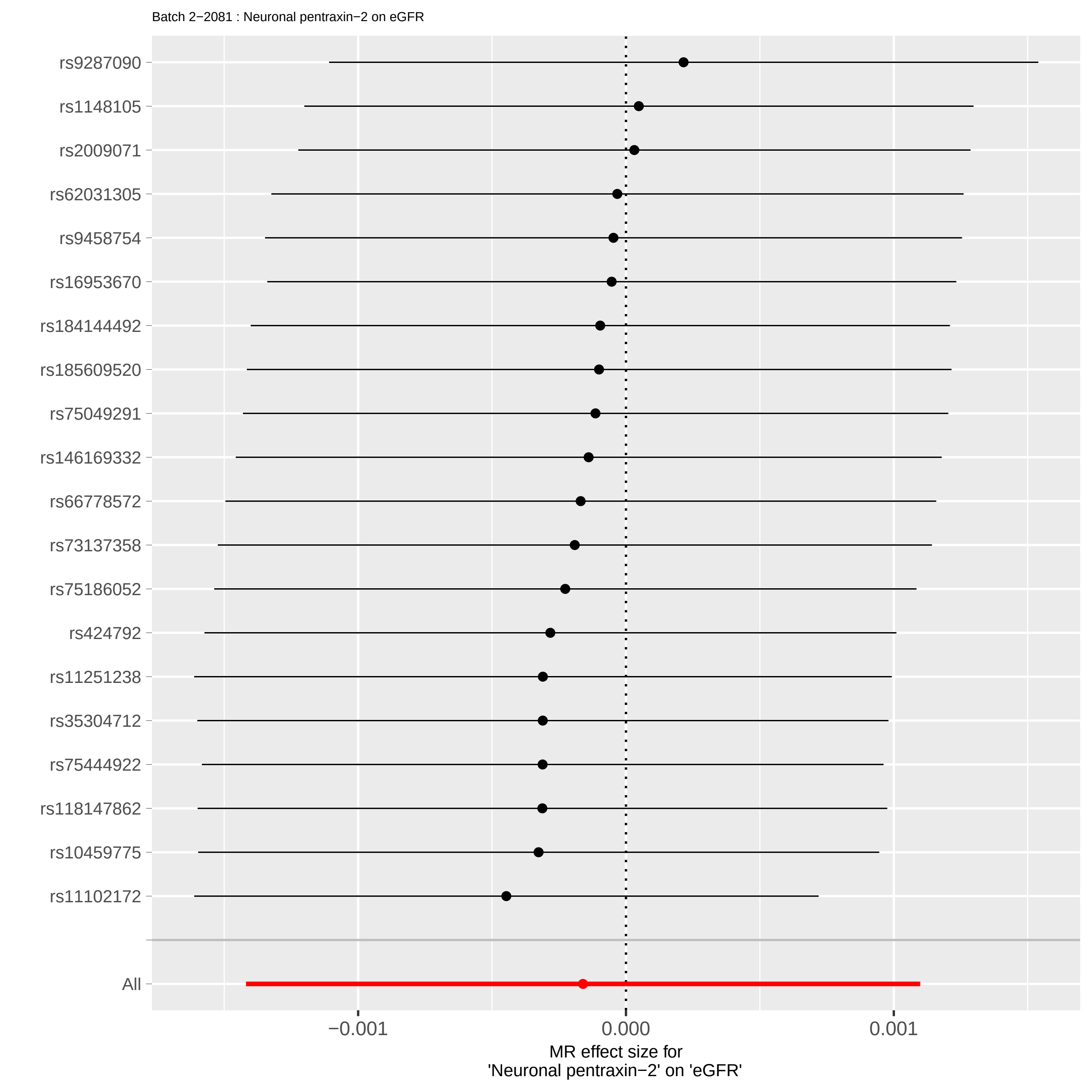

Batch 2−2081 : Neuronal pentraxin−2 on eGFR
rs9287090
rs1148105
rs2009071
rs62031305
rs9458754
rs16953670
rs184144492
rs185609520
rs75049291
rs146169332
rs66778572
rs73137358
rs75186052
rs424792
rs11251238
rs35304712
rs75444922
rs118147862
rs10459775
rs11102172
All
−0.001
0.000
0.001
MR effect size for
'Neuronal pentraxin−2' on 'eGFR'

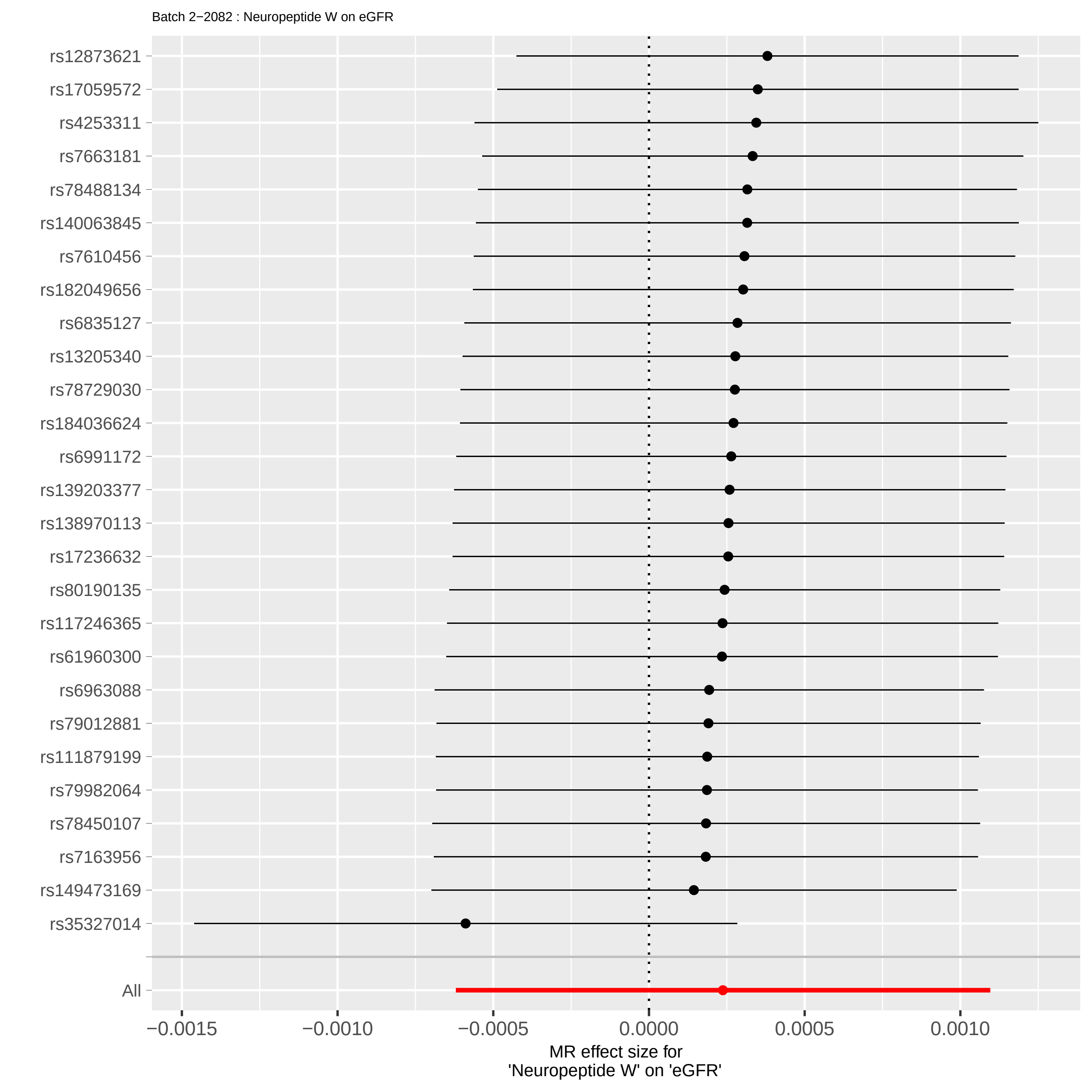

Batch 2−2082 : Neuropeptide W on eGFR
rs12873621
rs17059572
rs4253311
rs7663181
rs78488134
rs140063845
rs7610456
rs182049656
rs6835127
rs13205340
rs78729030
rs184036624
rs6991172
rs139203377
rs138970113
rs17236632
rs80190135
rs117246365
rs61960300
rs6963088
rs79012881
rs111879199
rs79982064
rs78450107
rs7163956
rs149473169
rs35327014
All
−0.0015
−0.0010
−0.0005
0.0000
0.0005
0.0010
MR effect size for
'Neuropeptide W' on 'eGFR'

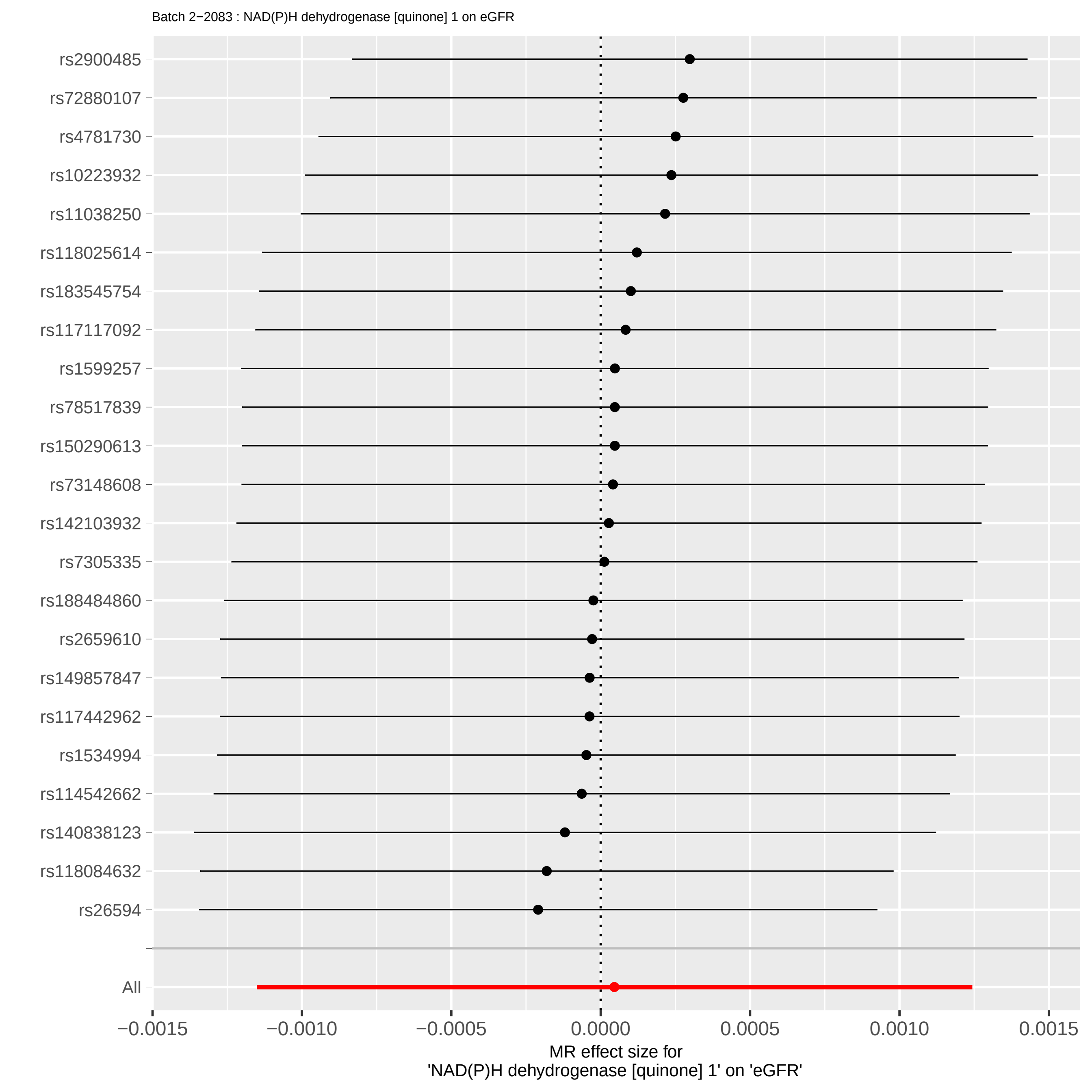

Batch 2−2083 : NAD(P)H dehydrogenase [quinone] 1 on eGFR
rs2900485
rs72880107
rs4781730
rs10223932
rs11038250
rs118025614
rs183545754
rs117117092
rs1599257
rs78517839
rs150290613
rs73148608
rs142103932
rs7305335
rs188484860
rs2659610
rs149857847
rs117442962
rs1534994
rs114542662
rs140838123
rs118084632
rs26594
All
−0.0015
−0.0010
−0.0005
0.0000
0.0005
0.0010
0.0015
MR effect size for
'NAD(P)H dehydrogenase [quinone] 1' on 'eGFR'

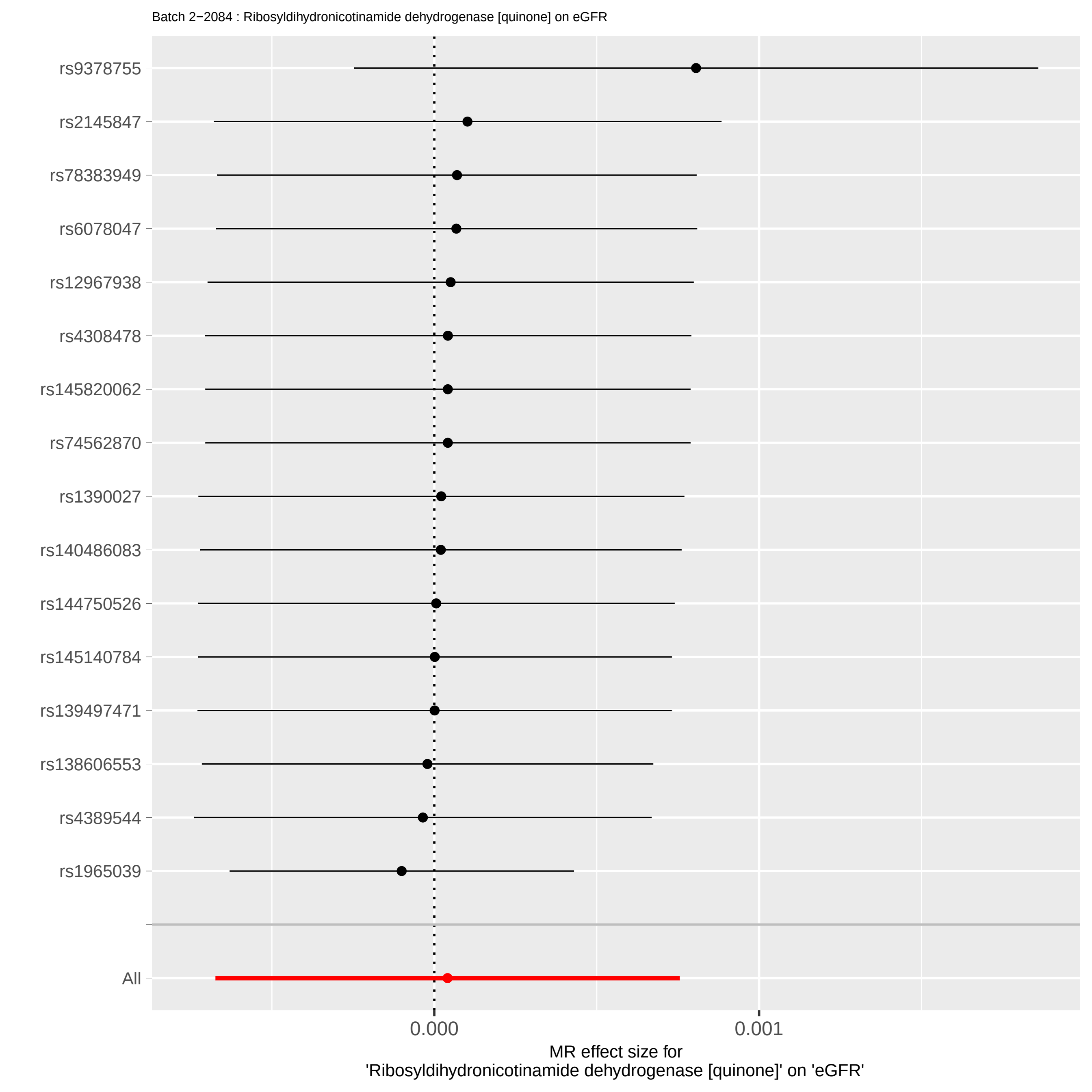

Batch 2−2084 : Ribosyldihydronicotinamide dehydrogenase [quinone] on eGFR
rs9378755
rs2145847
rs78383949
rs6078047
rs12967938
rs4308478
rs145820062
rs74562870
rs1390027
rs140486083
rs144750526
rs145140784
rs139497471
rs138606553
rs4389544
rs1965039
All
0.000
0.001
MR effect size for
'Ribosyldihydronicotinamide dehydrogenase [quinone]' on 'eGFR'

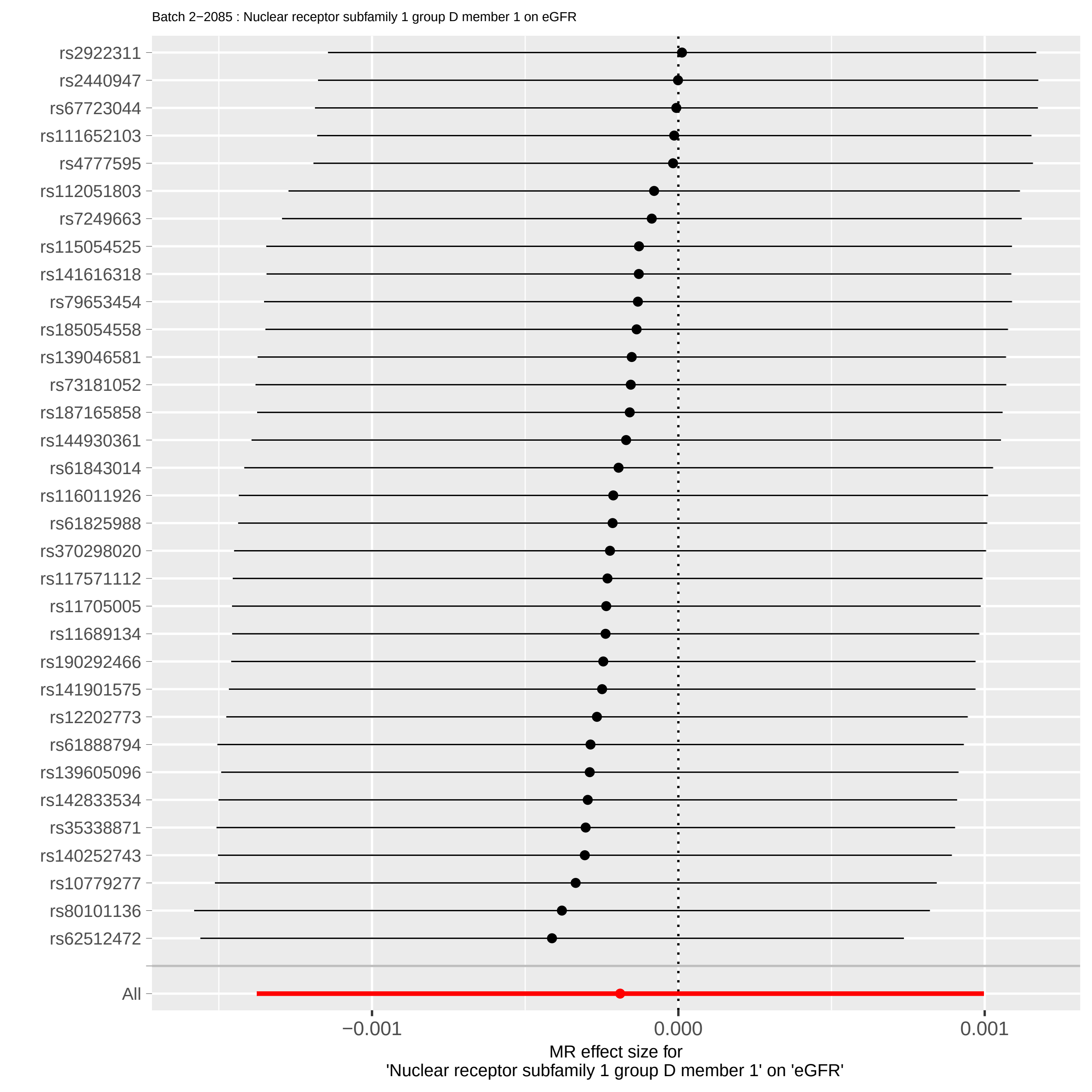
Batch 2−2085 : Nuclear receptor subfamily 1 group D member 1 on eGFR
rs2922311
rs2440947
rs67723044
rs111652103
rs4777595
rs112051803
rs7249663
rs115054525
rs141616318
rs79653454
rs185054558
rs139046581
rs73181052
rs187165858
rs144930361
rs61843014
rs116011926
rs61825988
rs370298020
rs117571112
rs11705005
rs11689134
rs190292466
rs141901575
rs12202773
rs61888794
rs139605096
rs142833534
rs35338871
rs140252743
rs10779277
rs80101136
rs62512472
All
−0.001
0.000
0.001
MR effect size for
'Nuclear receptor subfamily 1 group D member 1' on 'eGFR'

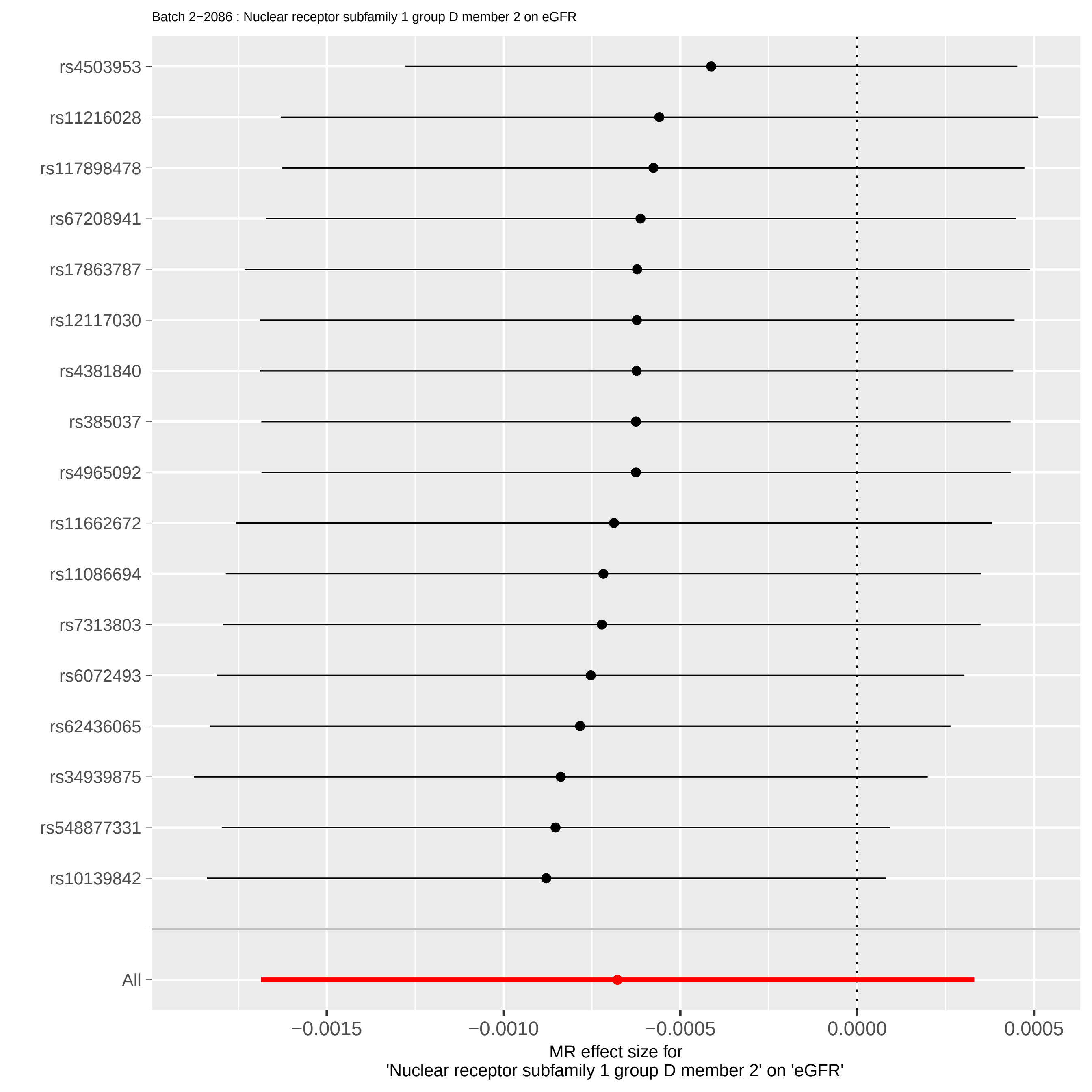

Batch 2−2086 : Nuclear receptor subfamily 1 group D member 2 on eGFR
rs4503953
rs11216028
rs117898478
rs67208941
rs17863787
rs12117030
rs4381840
rs385037
rs4965092
rs11662672
rs11086694
rs7313803
rs6072493
rs62436065
rs34939875
rs548877331
rs10139842
All
−0.0015
−0.0010
−0.0005
0.0000
0.0005
MR effect size for
'Nuclear receptor subfamily 1 group D member 2' on 'eGFR'

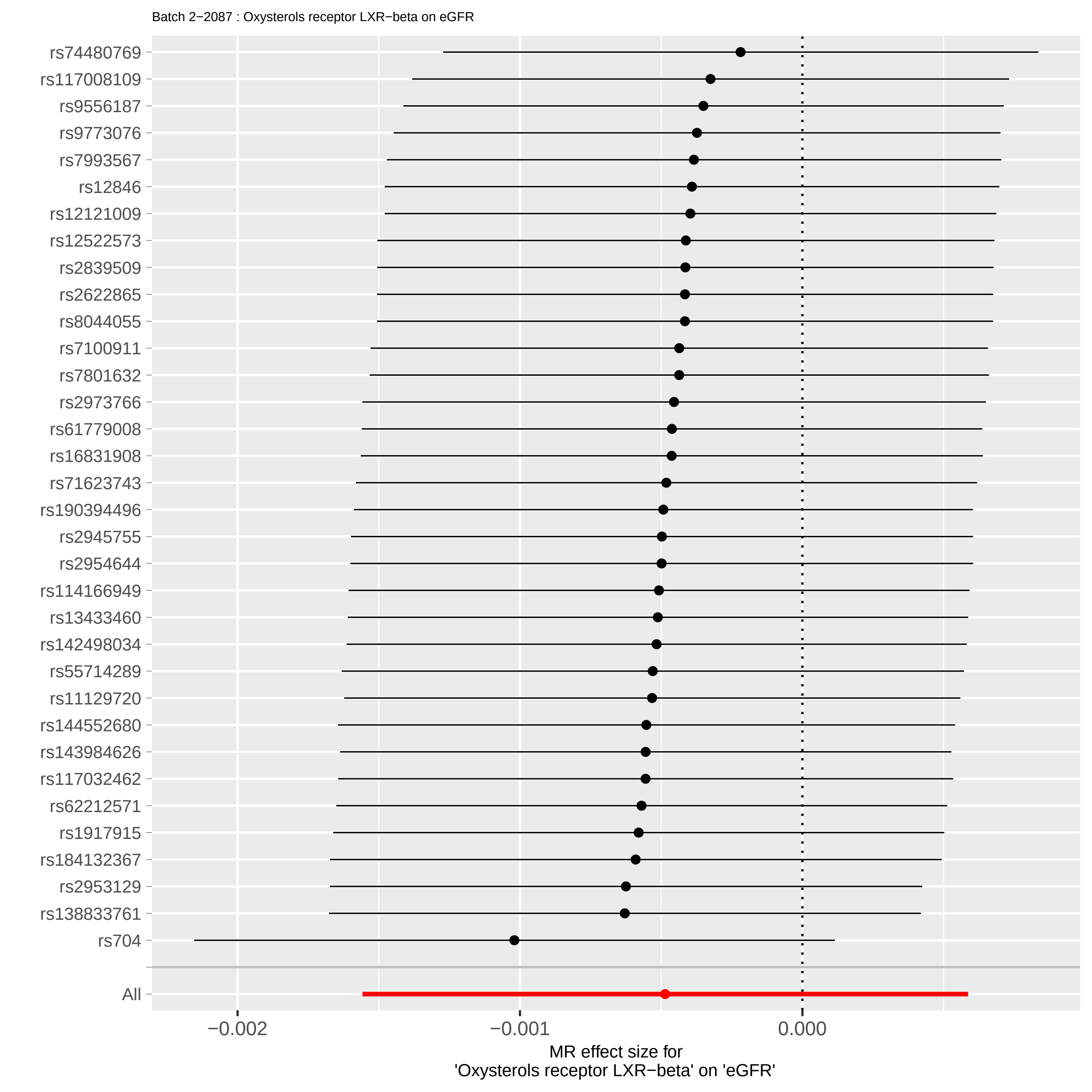
Batch 2−2087 : Oxysterols receptor LXR−beta on eGFR
rs74480769
rs117008109
rs9556187
rs9773076
rs7993567
rs12846
rs12121009
rs12522573
rs2839509
rs2622865
rs8044055
rs7100911
rs7801632
rs2973766
rs61779008
rs16831908
rs71623743
rs190394496
rs2945755
rs2954644
rs114166949
rs13433460
rs142498034
rs55714289
rs11129720
rs144552680
rs143984626
rs117032462
rs62212571
rs1917915
rs184132367
rs2953129
rs138833761
rs704
All
−0.002
−0.001
0.000
MR effect size for
'Oxysterols receptor LXR−beta' on 'eGFR'

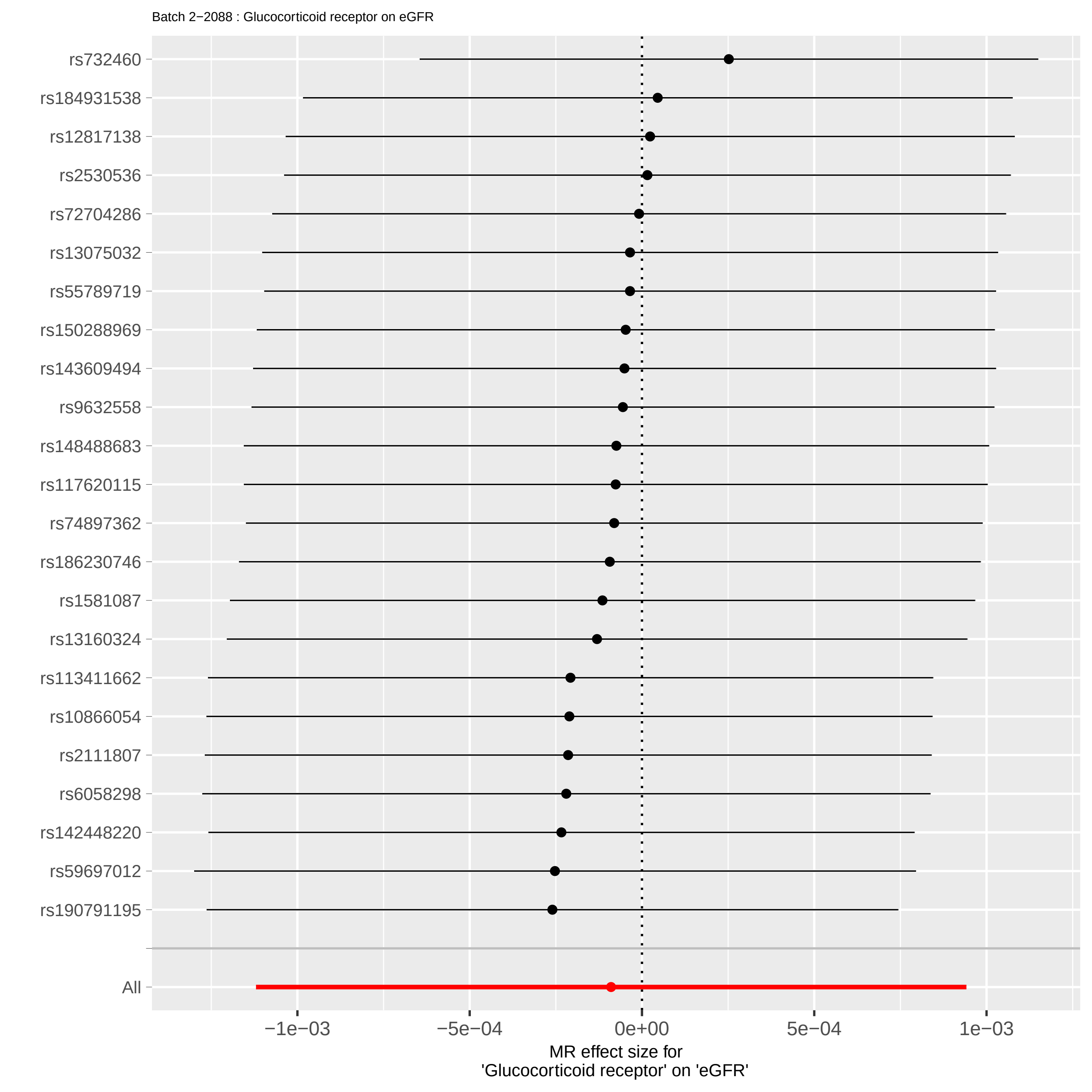

Batch 2−2088 : Glucocorticoid receptor on eGFR
rs732460
rs184931538
rs12817138
rs2530536
rs72704286
rs13075032
rs55789719
rs150288969
rs143609494
rs9632558
rs148488683
rs117620115
rs74897362
rs186230746
rs1581087
rs13160324
rs113411662
rs10866054
rs2111807
rs6058298
rs142448220
rs59697012
rs190791195
All
−1e−03
−5e−04
0e+00
5e−04
1e−03
MR effect size for
'Glucocorticoid receptor' on 'eGFR'

Batch 2−2089 : Mineralocorticoid receptor on eGFR

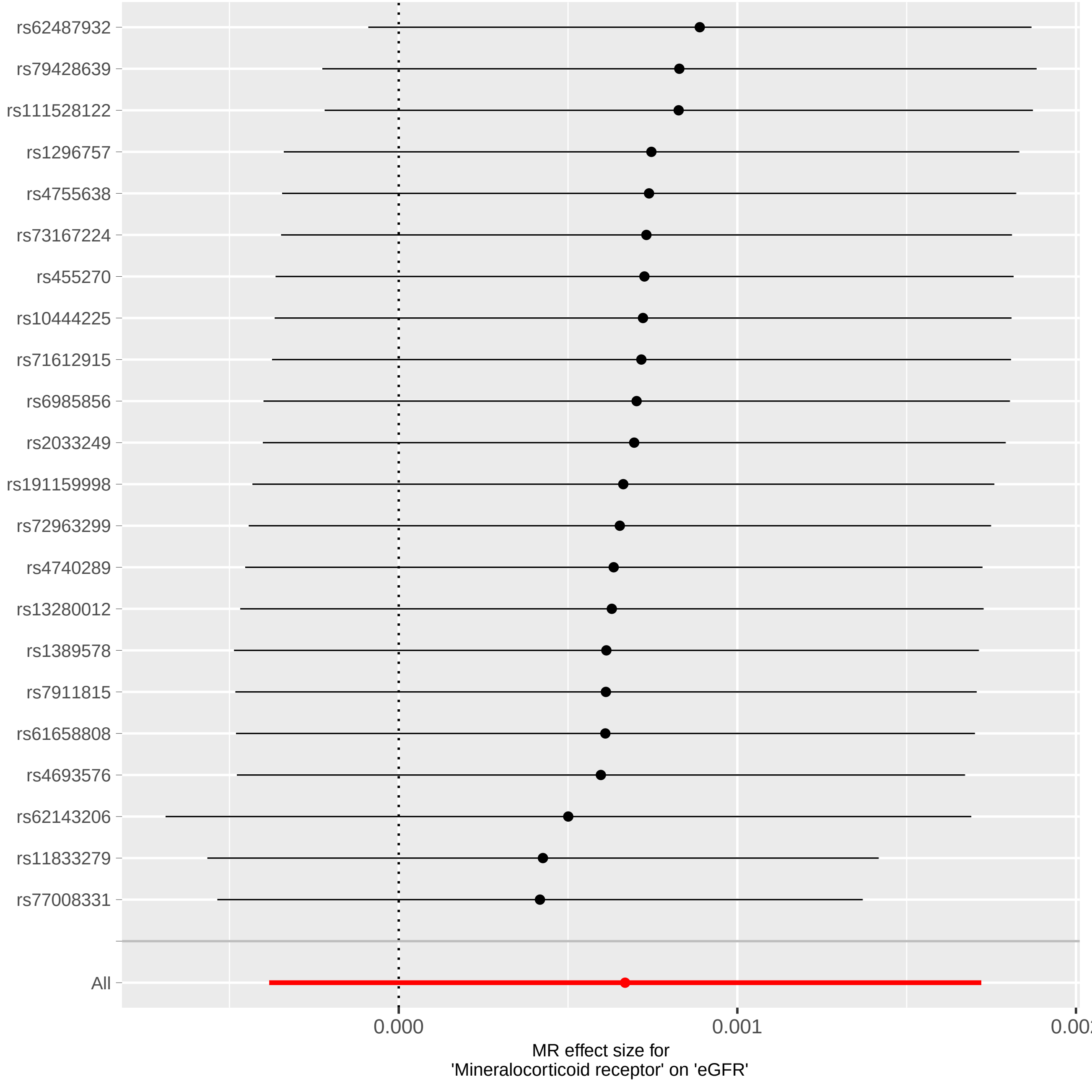

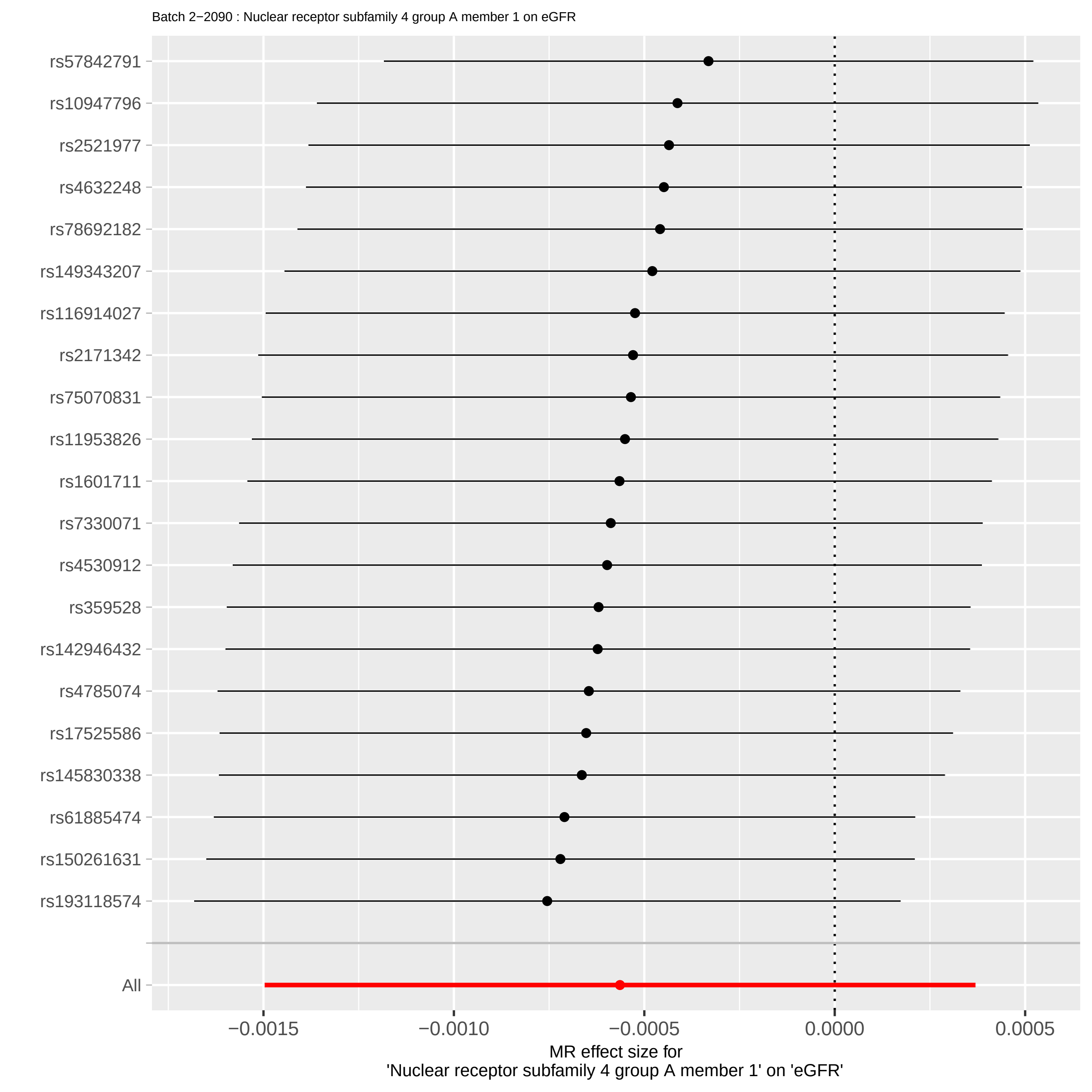

Batch 2−2090 : Nuclear receptor subfamily 4 group A member 1 on eGFR
rs57842791
rs10947796
rs2521977
rs4632248
rs78692182
rs149343207
rs116914027
rs2171342
rs75070831
rs11953826
rs1601711
rs7330071
rs4530912
rs359528
rs142946432
rs4785074
rs17525586
rs145830338
rs61885474
rs150261631
rs193118574
All
−0.0015
−0.0010
−0.0005
0.0000
0.0005
MR effect size for
'Nuclear receptor subfamily 4 group A member 1' on 'eGFR'

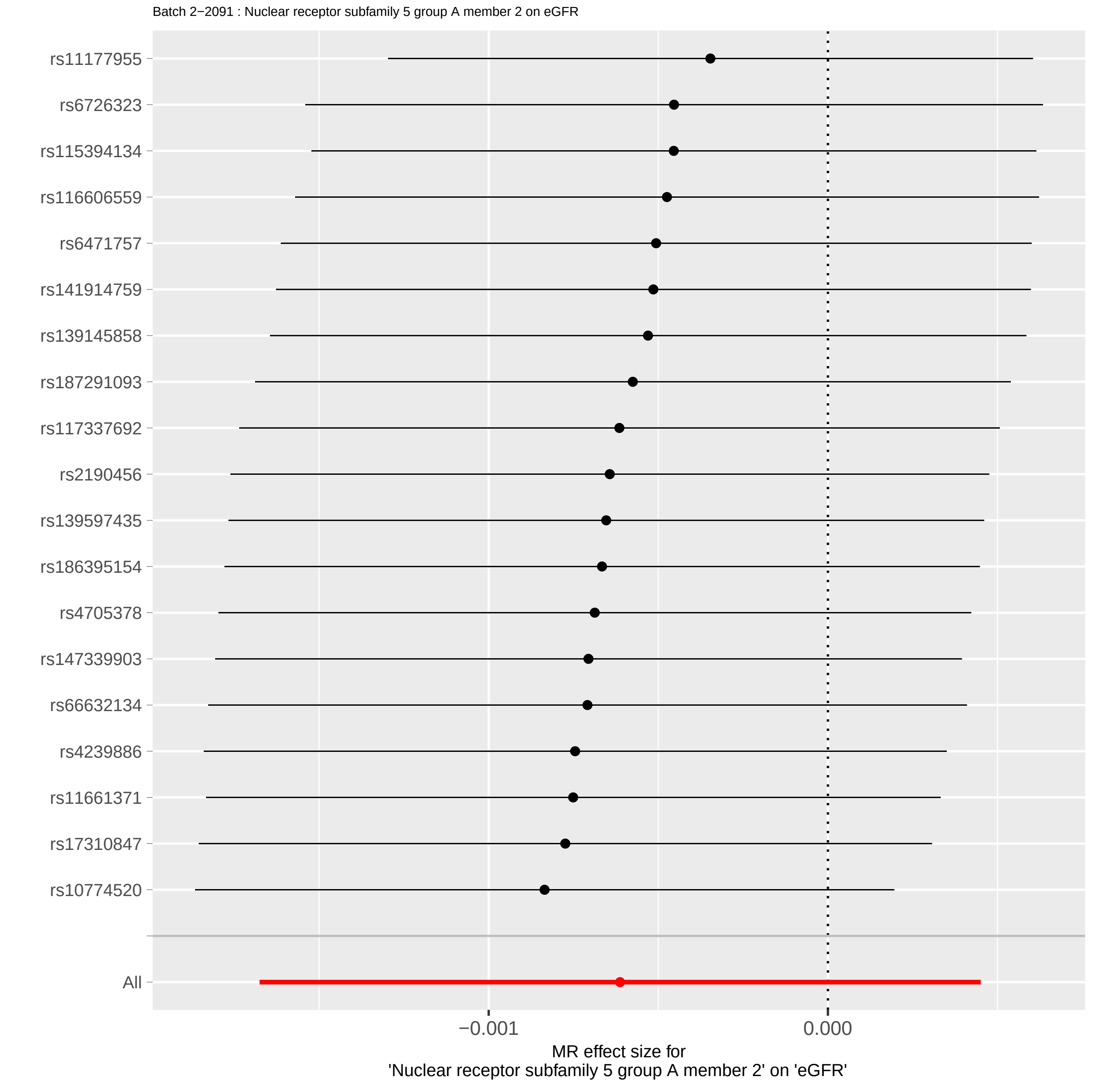
Batch 2−2091 : Nuclear receptor subfamily 5 group A member 2 on eGFR
rs11177955
rs6726323
rs115394134
rs116606559
rs6471757
rs141914759
rs139145858
rs187291093
rs117337692
rs2190456
rs139597435
rs186395154
rs4705378
rs147339903
rs66632134
rs4239886
rs11661371
rs17310847
rs10774520
All
−0.001
0.000
MR effect size for
'Nuclear receptor subfamily 5 group A member 2' on 'eGFR'

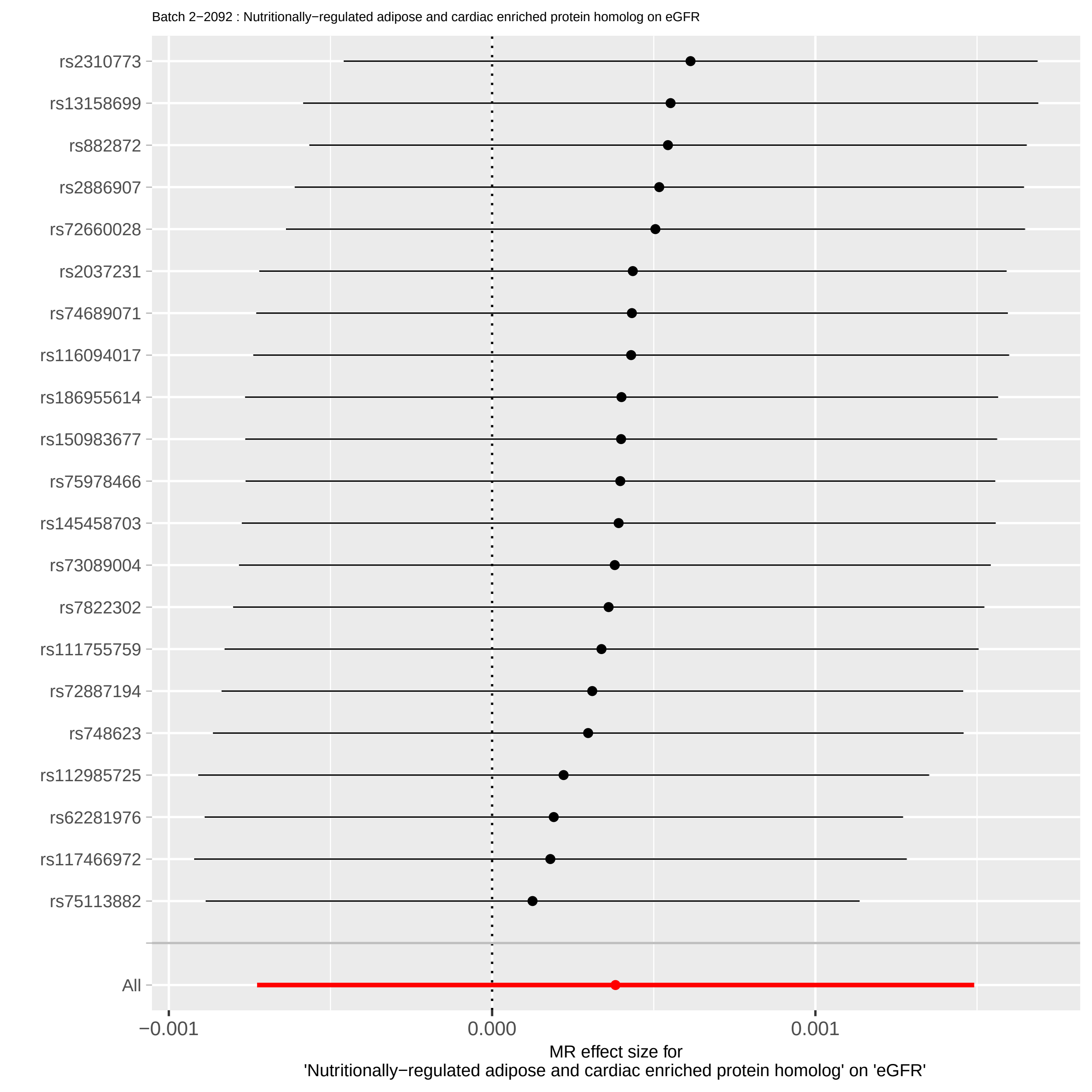
Batch 2−2092 : Nutritionally−regulated adipose and cardiac enriched protein homolog on eGFR
rs2310773
rs13158699
rs882872
rs2886907
rs72660028
rs2037231
rs74689071
rs116094017
rs186955614
rs150983677
rs75978466
rs145458703
rs73089004
rs7822302
rs111755759
rs72887194
rs748623
rs112985725
rs62281976
rs117466972
rs75113882
All
−0.001
0.000
0.001
MR effect size for
'Nutritionally−regulated adipose and cardiac enriched protein homolog' on 'eGFR'

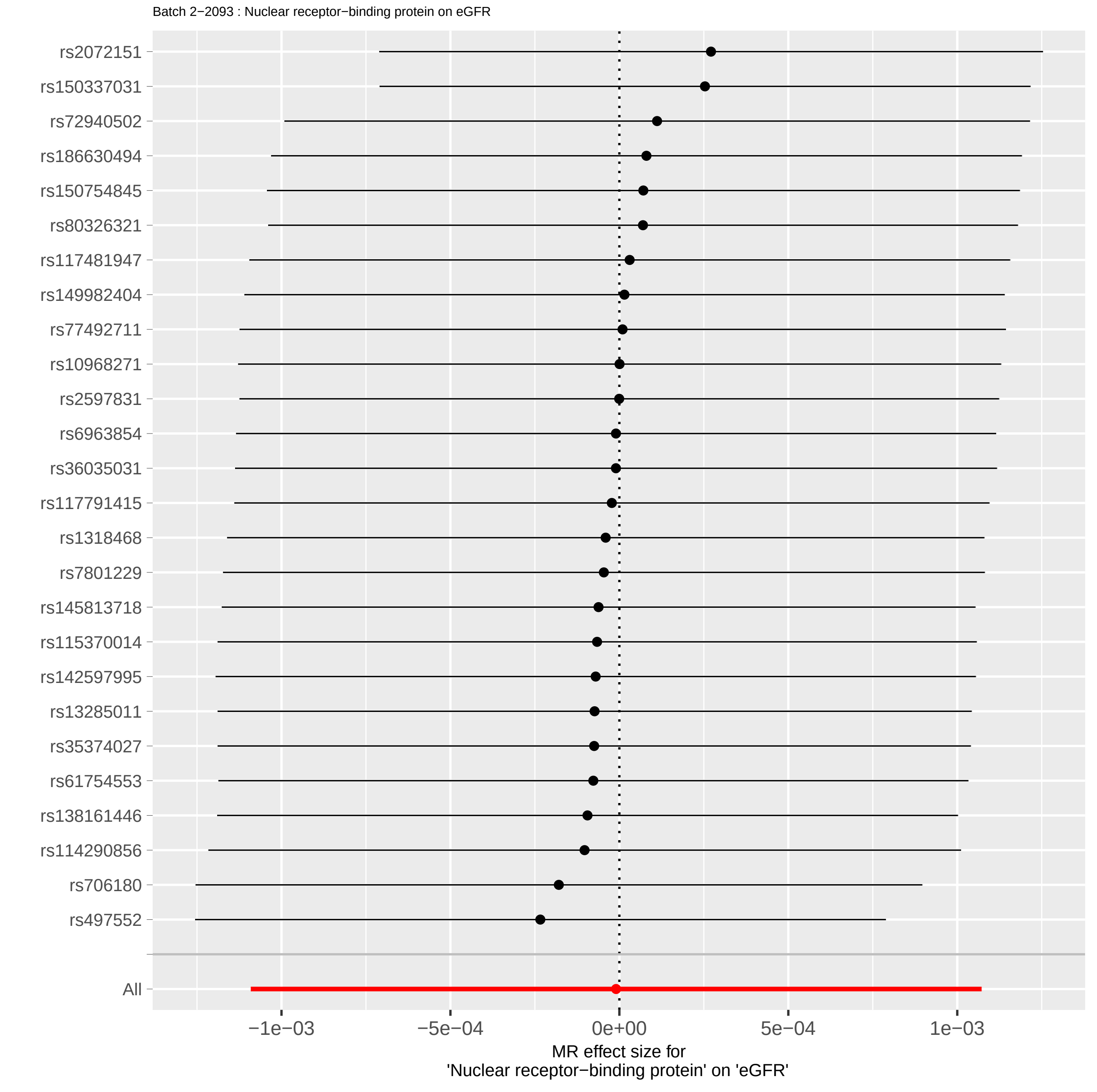
Batch 2−2093 : Nuclear receptor−binding protein on eGFR
rs2072151
rs150337031
rs72940502
rs186630494
rs150754845
rs80326321
rs117481947
rs149982404
rs77492711
rs10968271
rs2597831
rs6963854
rs36035031
rs117791415
rs1318468
rs7801229
rs145813718
rs115370014
rs142597995
rs13285011
rs35374027
rs61754553
rs138161446
rs114290856
rs706180
rs497552
All
−1e−03
−5e−04
0e+00
5e−04
1e−03
MR effect size for
'Nuclear receptor−binding protein' on 'eGFR'

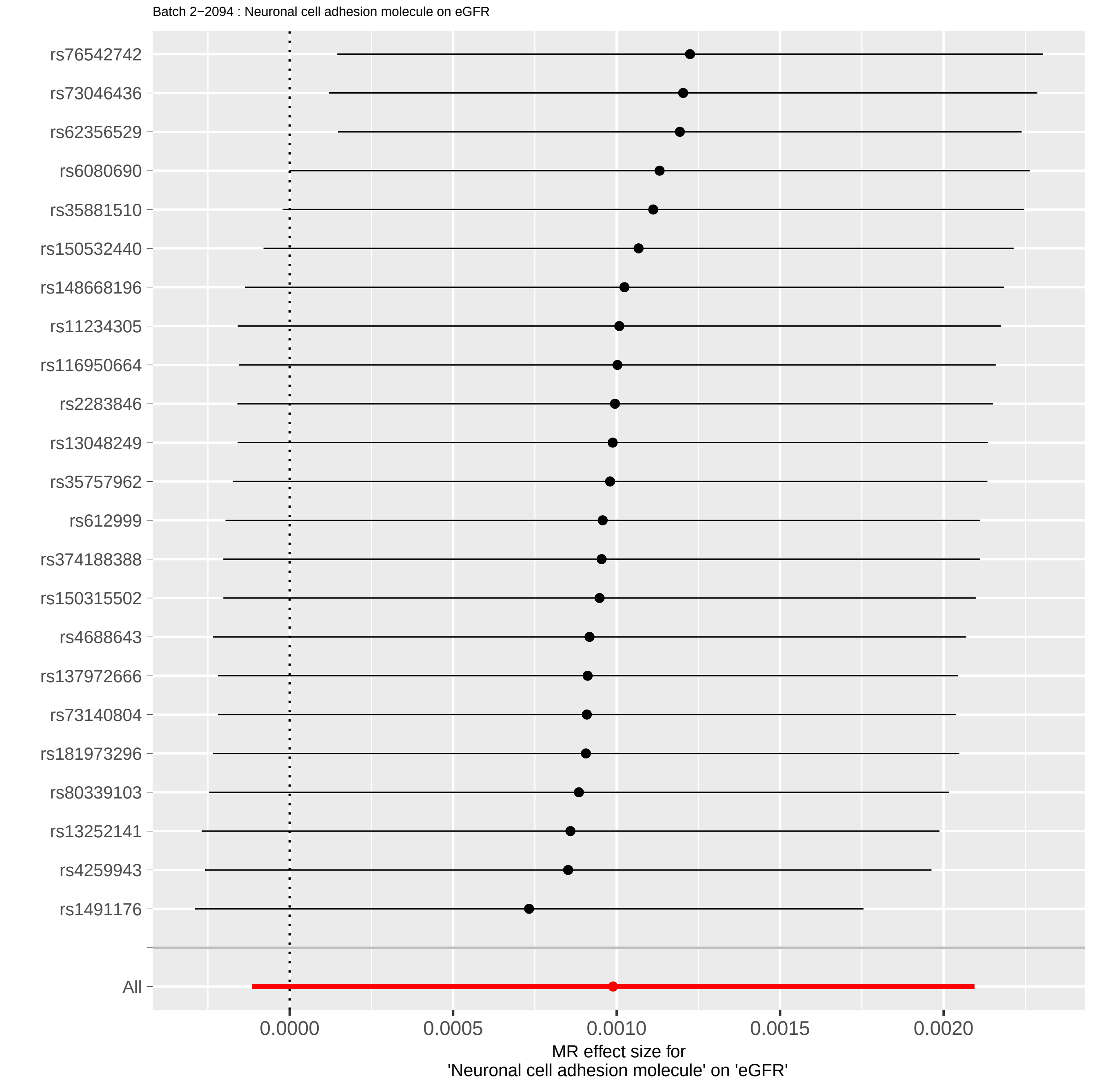

Batch 2−2094 : Neuronal cell adhesion molecule on eGFR
rs76542742
rs73046436
rs62356529
rs6080690
rs35881510
rs150532440
rs148668196
rs11234305
rs116950664
rs2283846
rs13048249
rs35757962
rs612999
rs374188388
rs150315502
rs4688643
rs137972666
rs73140804
rs181973296
rs80339103
rs13252141
rs4259943
rs1491176
All
0.0000
0.0005
0.0010
0.0015
0.0020
MR effect size for
'Neuronal cell adhesion molecule' on 'eGFR'

Batch 2−2095 : Neuregulin−1 on eGFR

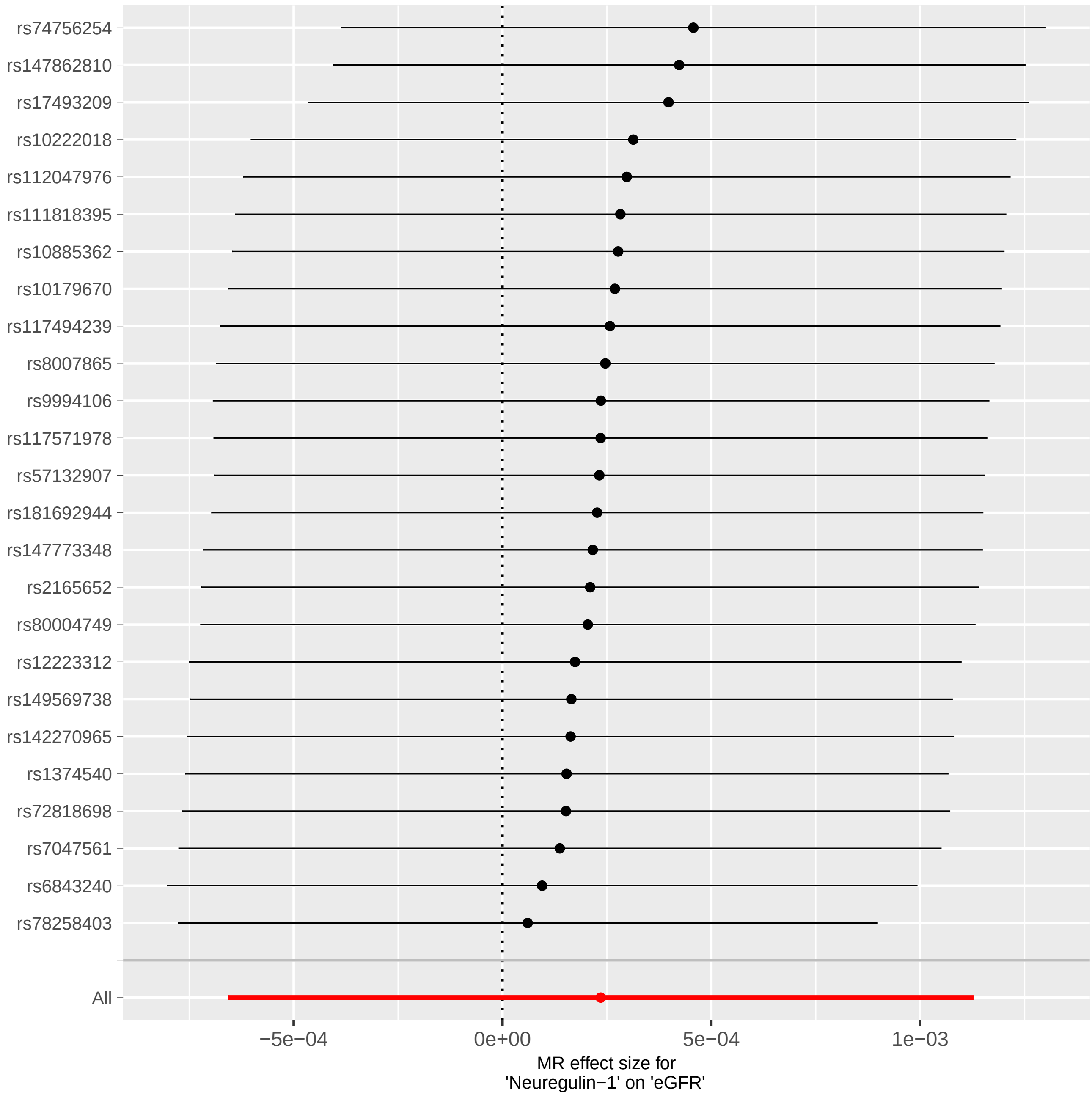

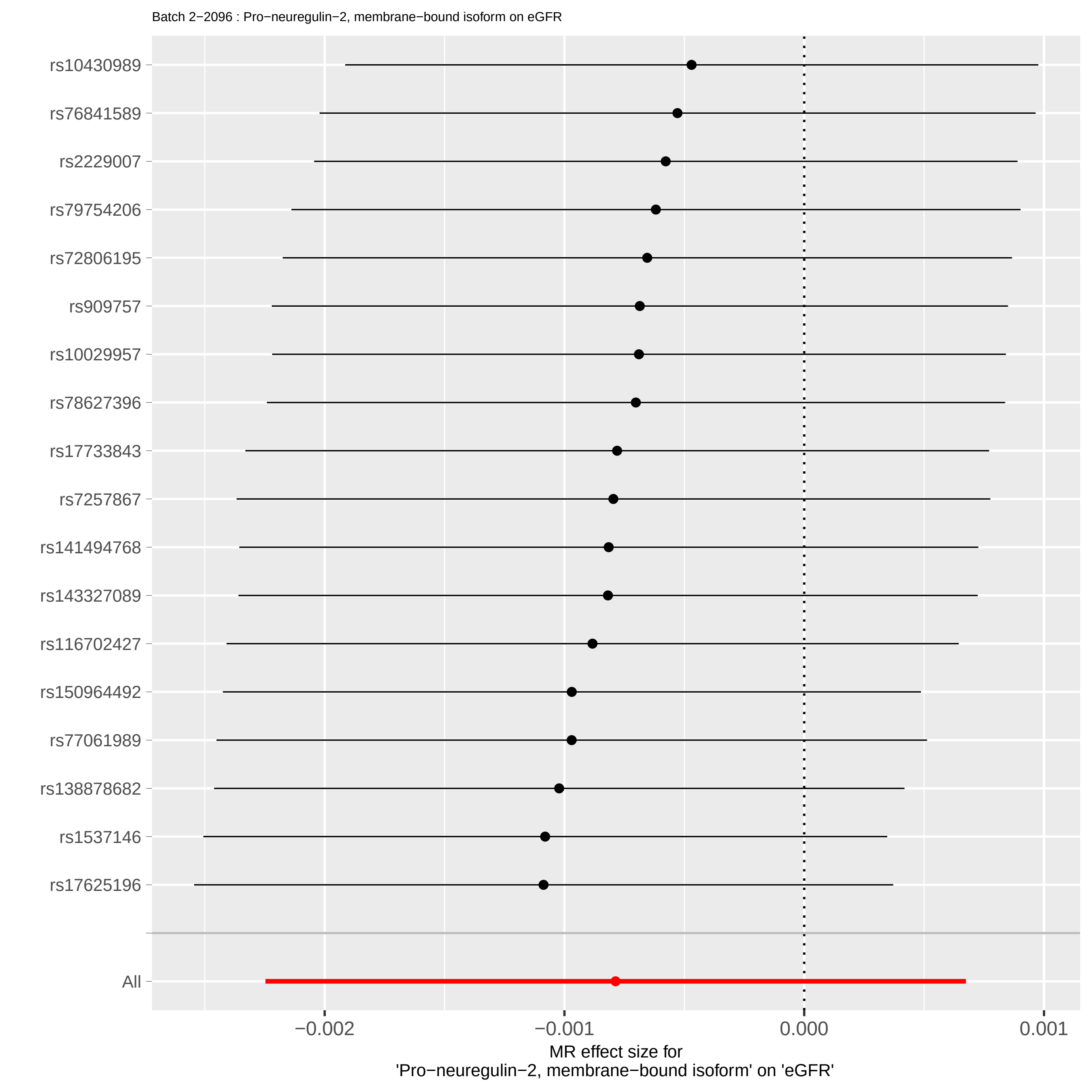

Batch 2−2096 : Pro−neuregulin−2, membrane−bound isoform on eGFR
rs10430989
rs76841589
rs2229007
rs79754206
rs72806195
rs909757
rs10029957
rs78627396
rs17733843
rs7257867
rs141494768
rs143327089
rs116702427
rs150964492
rs77061989
rs138878682
rs1537146
rs17625196
All
−0.002
−0.001
0.000
0.001
MR effect size for
'Pro−neuregulin−2, membrane−bound isoform' on 'eGFR'

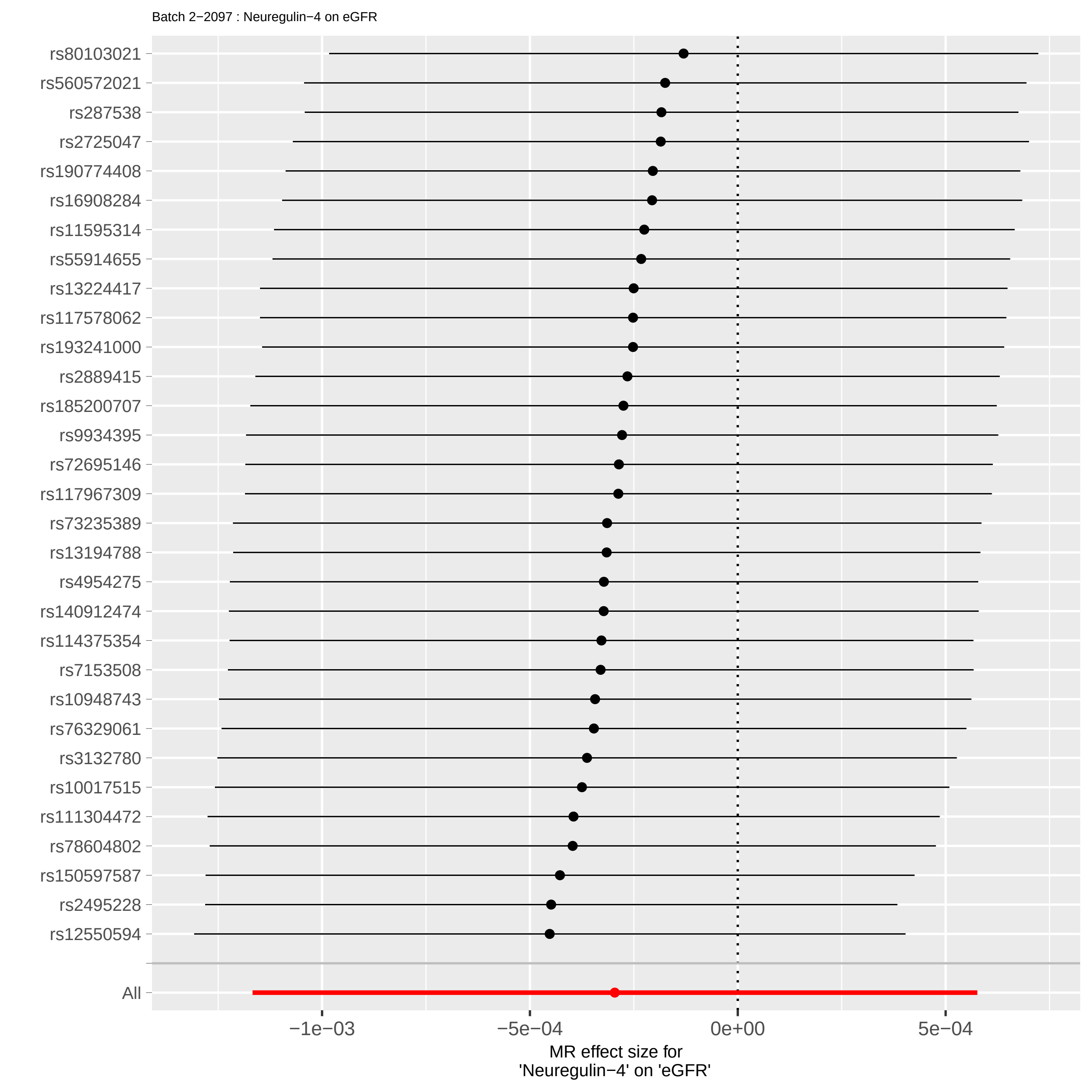

Batch 2−2097 : Neuregulin−4 on eGFR
rs80103021
rs560572021
rs287538
rs2725047
rs190774408
rs16908284
rs11595314
rs55914655
rs13224417
rs117578062
rs193241000
rs2889415
rs185200707
rs9934395
rs72695146
rs117967309
rs73235389
rs13194788
rs4954275
rs140912474
rs114375354
rs7153508
rs10948743
rs76329061
rs3132780
rs10017515
rs111304472
rs78604802
rs150597587
rs2495228
rs12550594
All
−1e−03
−5e−04
0e+00
5e−04
MR effect size for
'Neuregulin−4' on 'eGFR'

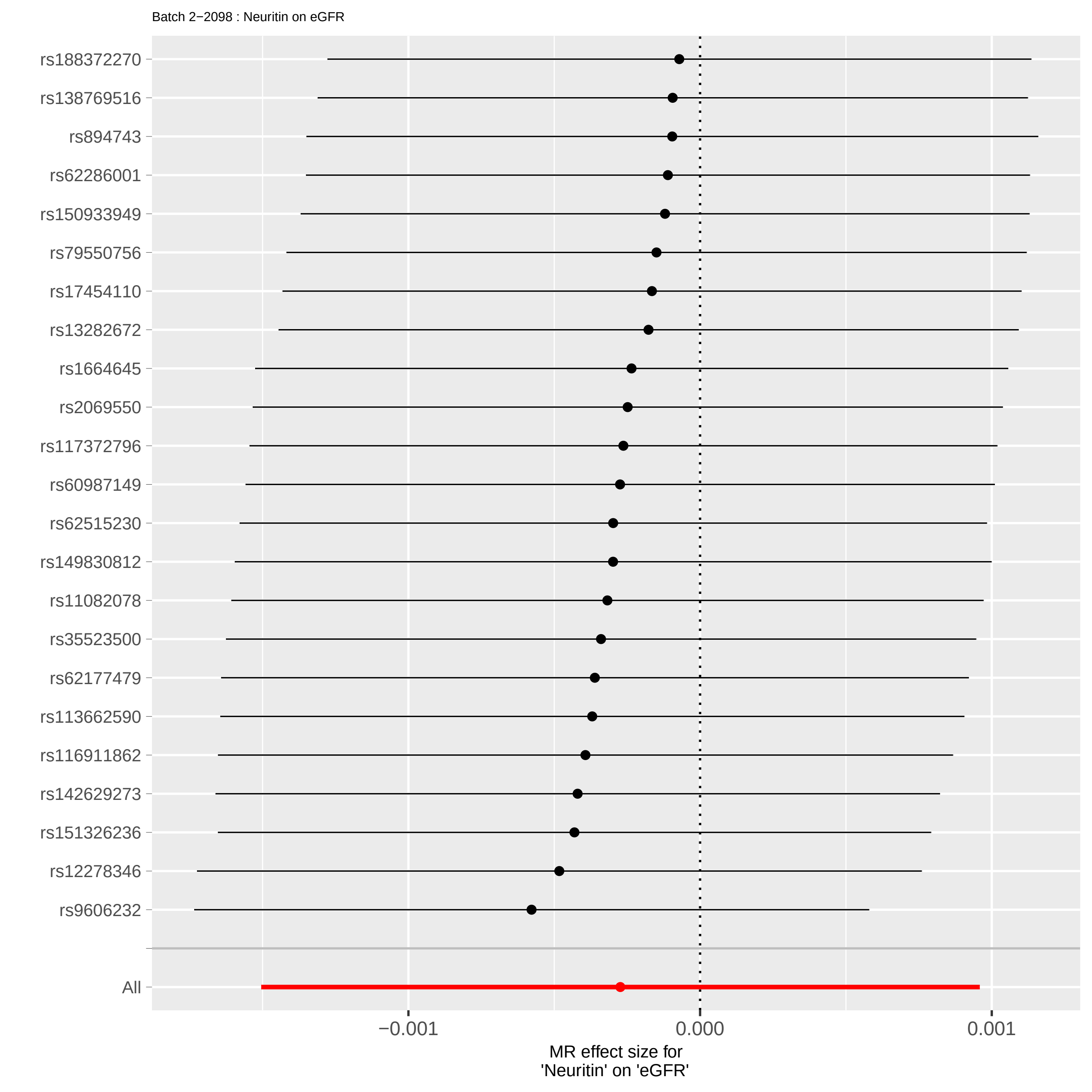

Batch 2−2098 : Neuritin on eGFR
rs188372270
rs138769516
rs894743
rs62286001
rs150933949
rs79550756
rs17454110
rs13282672
rs1664645
rs2069550
rs117372796
rs60987149
rs62515230
rs149830812
rs11082078
rs35523500
rs62177479
rs113662590
rs116911862
rs142629273
rs151326236
rs12278346
rs9606232
All
−0.001
0.000
0.001
MR effect size for
'Neuritin' on 'eGFR'

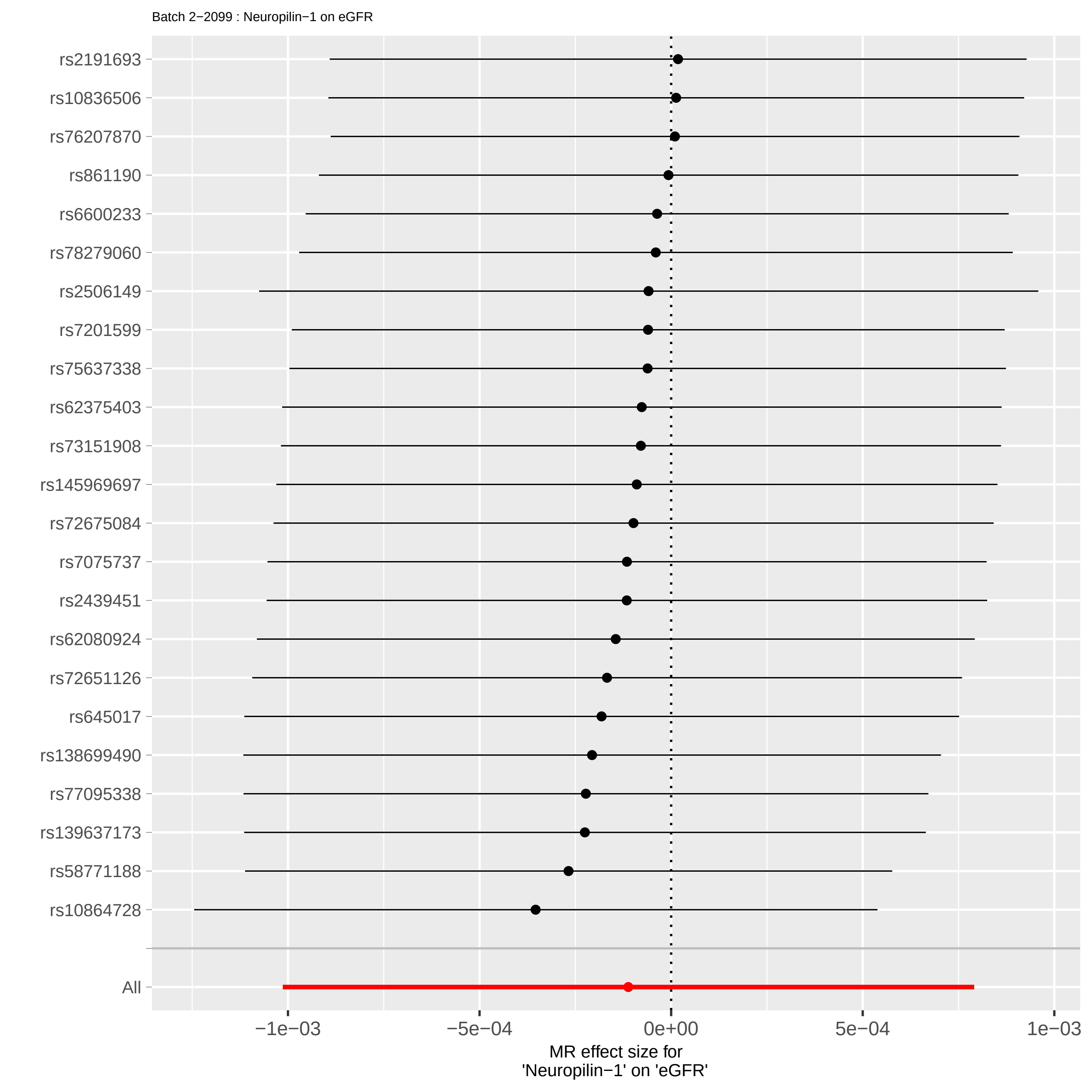
Batch 2−2099 : Neuropilin−1 on eGFR
rs2191693
rs10836506
rs76207870
rs861190
rs6600233
rs78279060
rs2506149
rs7201599
rs75637338
rs62375403
rs73151908
rs145969697
rs72675084
rs7075737
rs2439451
rs62080924
rs72651126
rs645017
rs138699490
rs77095338
rs139637173
rs58771188
rs10864728
All
−1e−03
−5e−04
0e+00
5e−04
1e−03
MR effect size for
'Neuropilin−1' on 'eGFR'

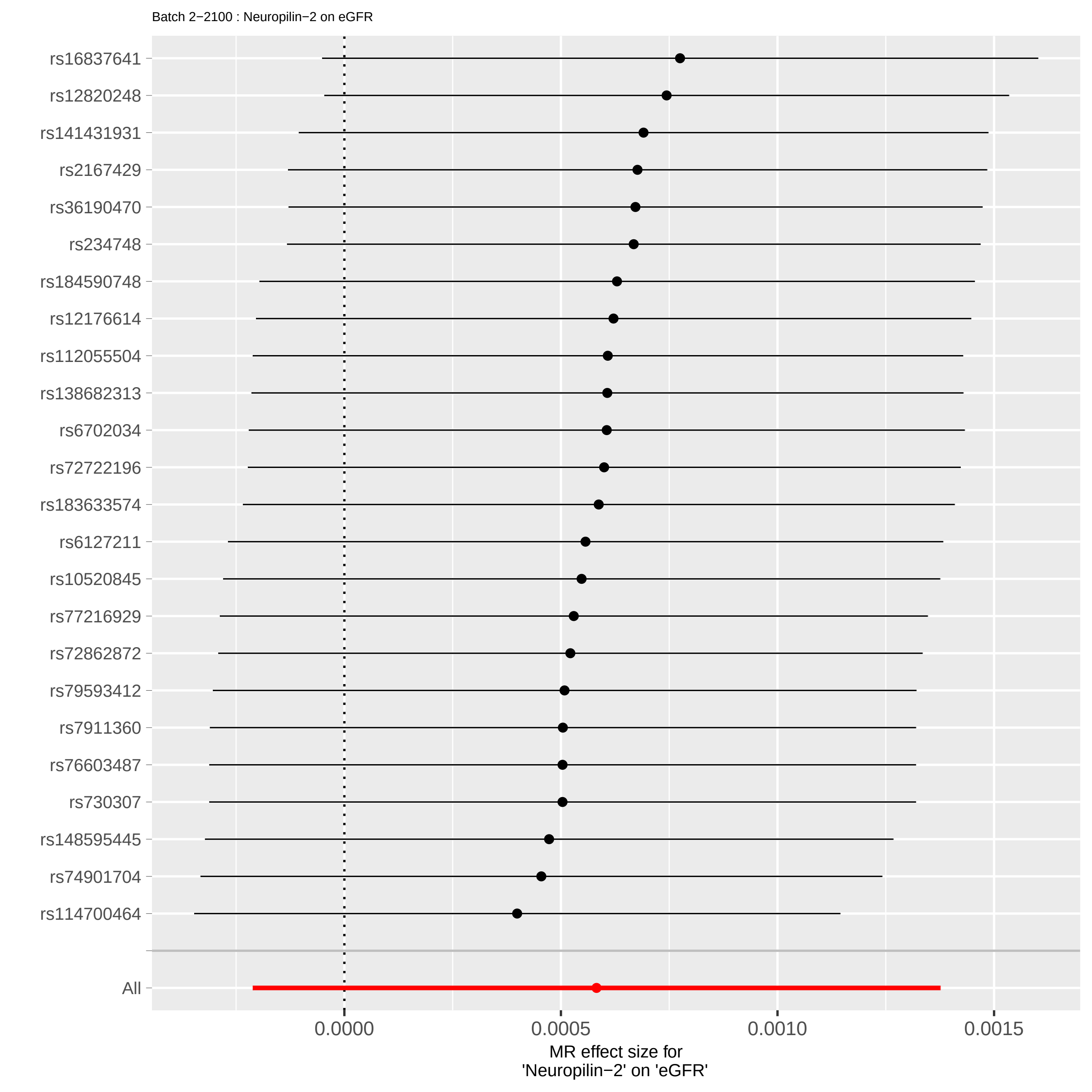

Batch 2−2100 : Neuropilin−2 on eGFR
rs16837641
rs12820248
rs141431931
rs2167429
rs36190470
rs234748
rs184590748
rs12176614
rs112055504
rs138682313
rs6702034
rs72722196
rs183633574
rs6127211
rs10520845
rs77216929
rs72862872
rs79593412
rs7911360
rs76603487
rs730307
rs148595445
rs74901704
rs114700464
All
0.0000
0.0005
0.0010
0.0015
MR effect size for
'Neuropilin−2' on 'eGFR'

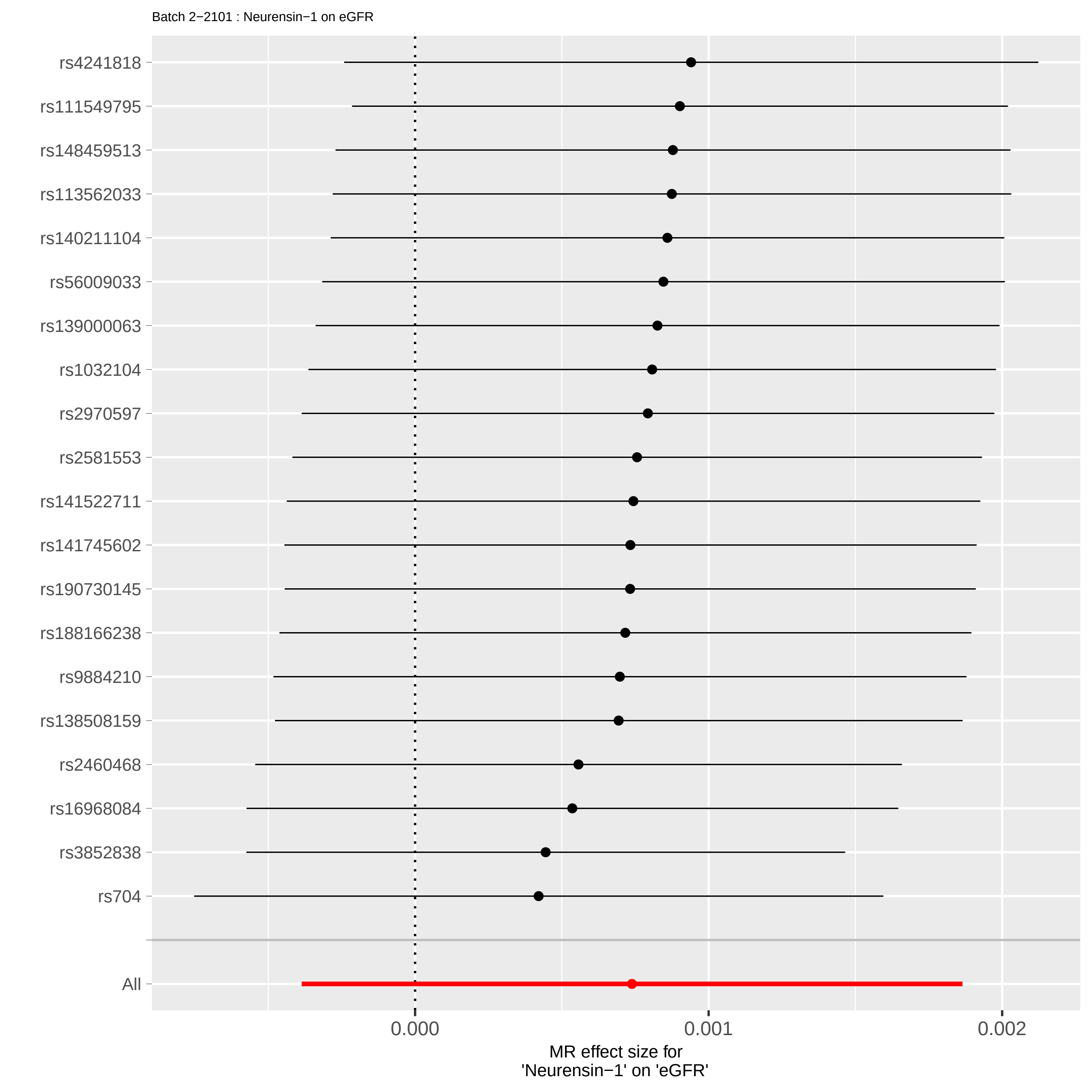

Batch 2−2101 : Neurensin−1 on eGFR
rs4241818
rs111549795
rs148459513
rs113562033
rs140211104
rs56009033
rs139000063
rs1032104
rs2970597
rs2581553
rs141522711
rs141745602
rs190730145
rs188166238
rs9884210
rs138508159
rs2460468
rs16968084
rs3852838
rs704
All
0.000
0.001
0.002
MR effect size for
'Neurensin−1' on 'eGFR'

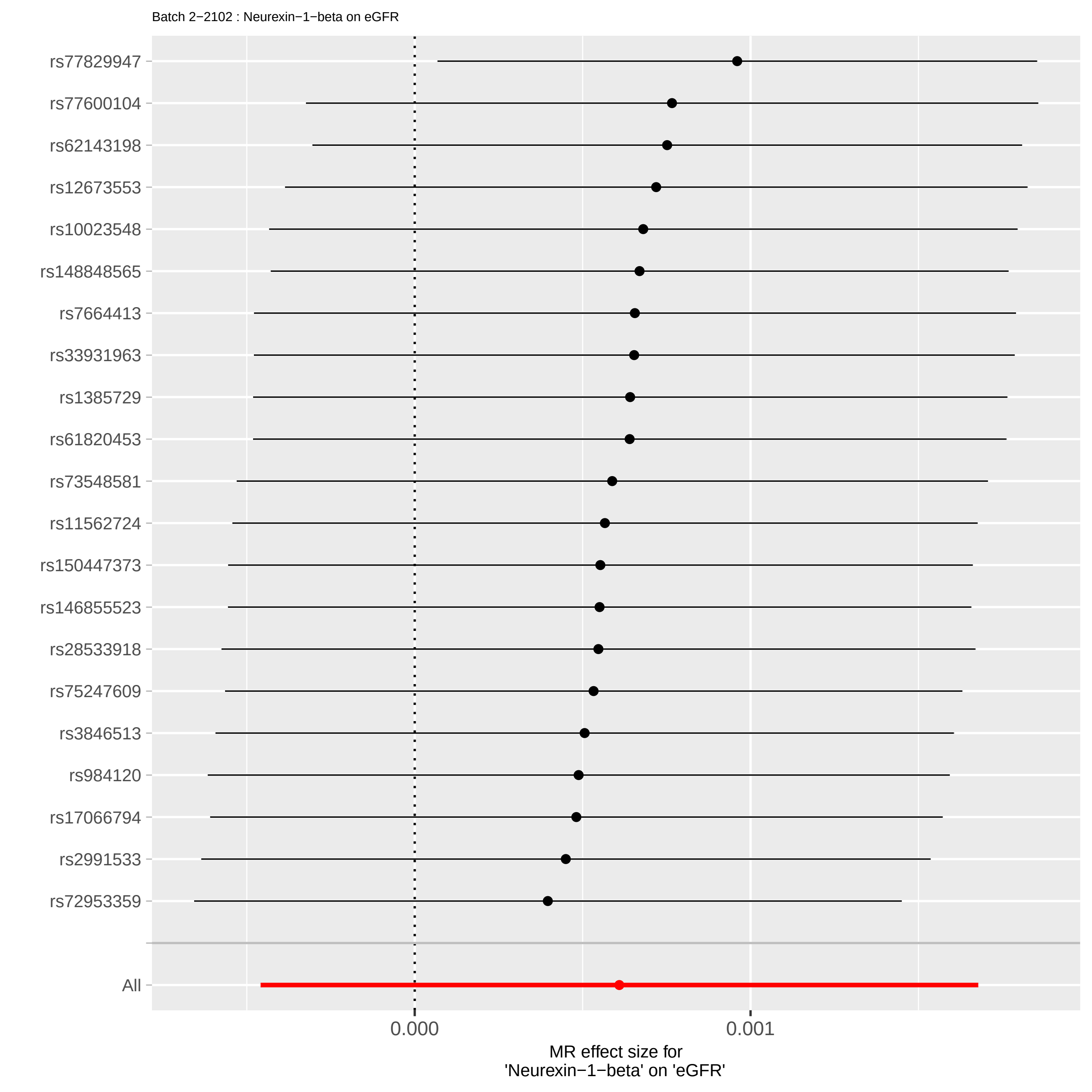
Batch 2−2102 : Neurexin−1−beta on eGFR
rs77829947
rs77600104
rs62143198
rs12673553
rs10023548
rs148848565
rs7664413
rs33931963
rs1385729
rs61820453
rs73548581
rs11562724
rs150447373
rs146855523
rs28533918
rs75247609
rs3846513
rs984120
rs17066794
rs2991533
rs72953359
All
0.000
0.001
MR effect size for
'Neurexin−1−beta' on 'eGFR'

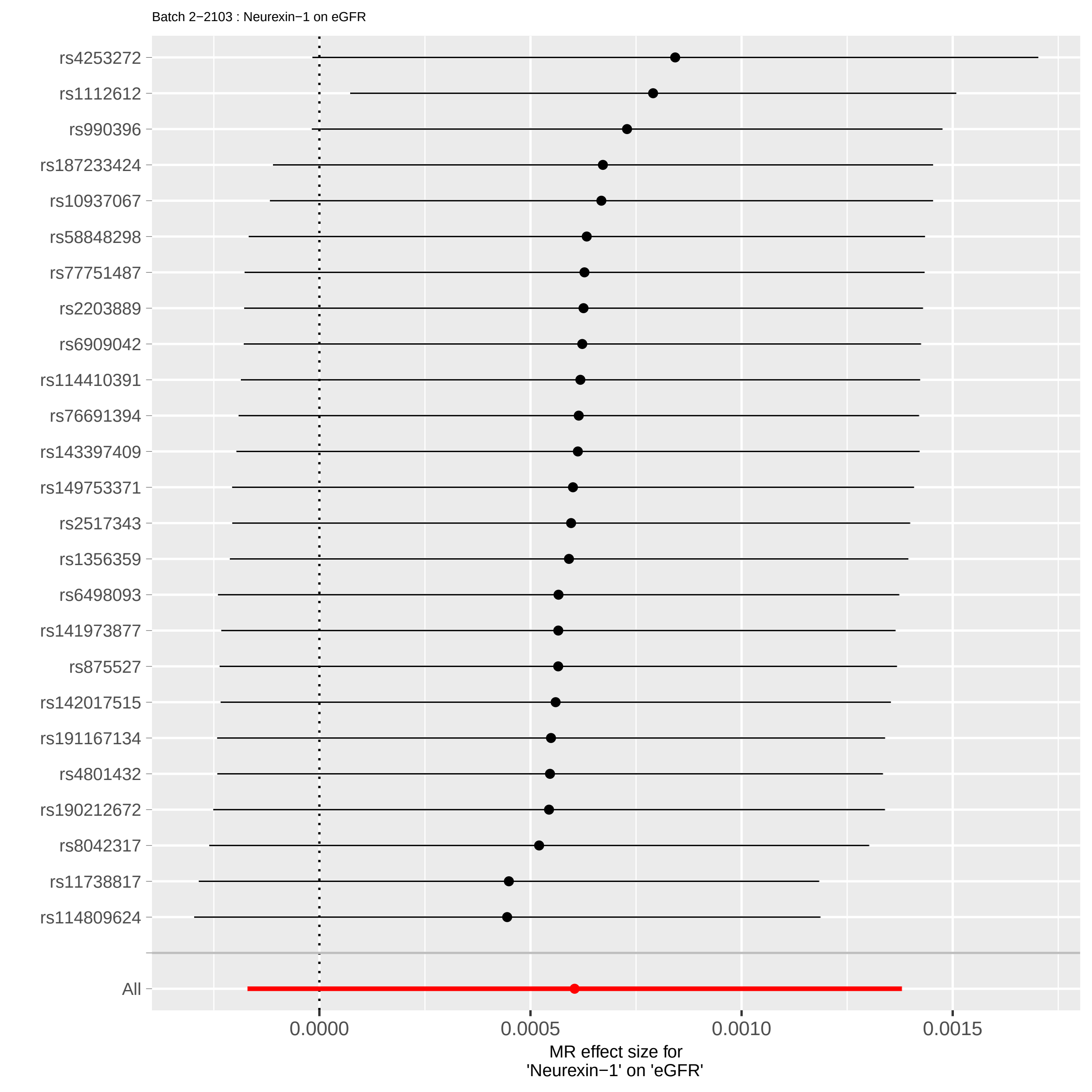
Batch 2−2103 : Neurexin−1 on eGFR
rs4253272
rs1112612
rs990396
rs187233424
rs10937067
rs58848298
rs77751487
rs2203889
rs6909042
rs114410391
rs76691394
rs143397409
rs149753371
rs2517343
rs1356359
rs6498093
rs141973877
rs875527
rs142017515
rs191167134
rs4801432
rs190212672
rs8042317
rs11738817
rs114809624
All
0.0000
0.0005
0.0010
0.0015
MR effect size for
'Neurexin−1' on 'eGFR'

Batch 2−2104 : Neurexin−2−beta on eGFR

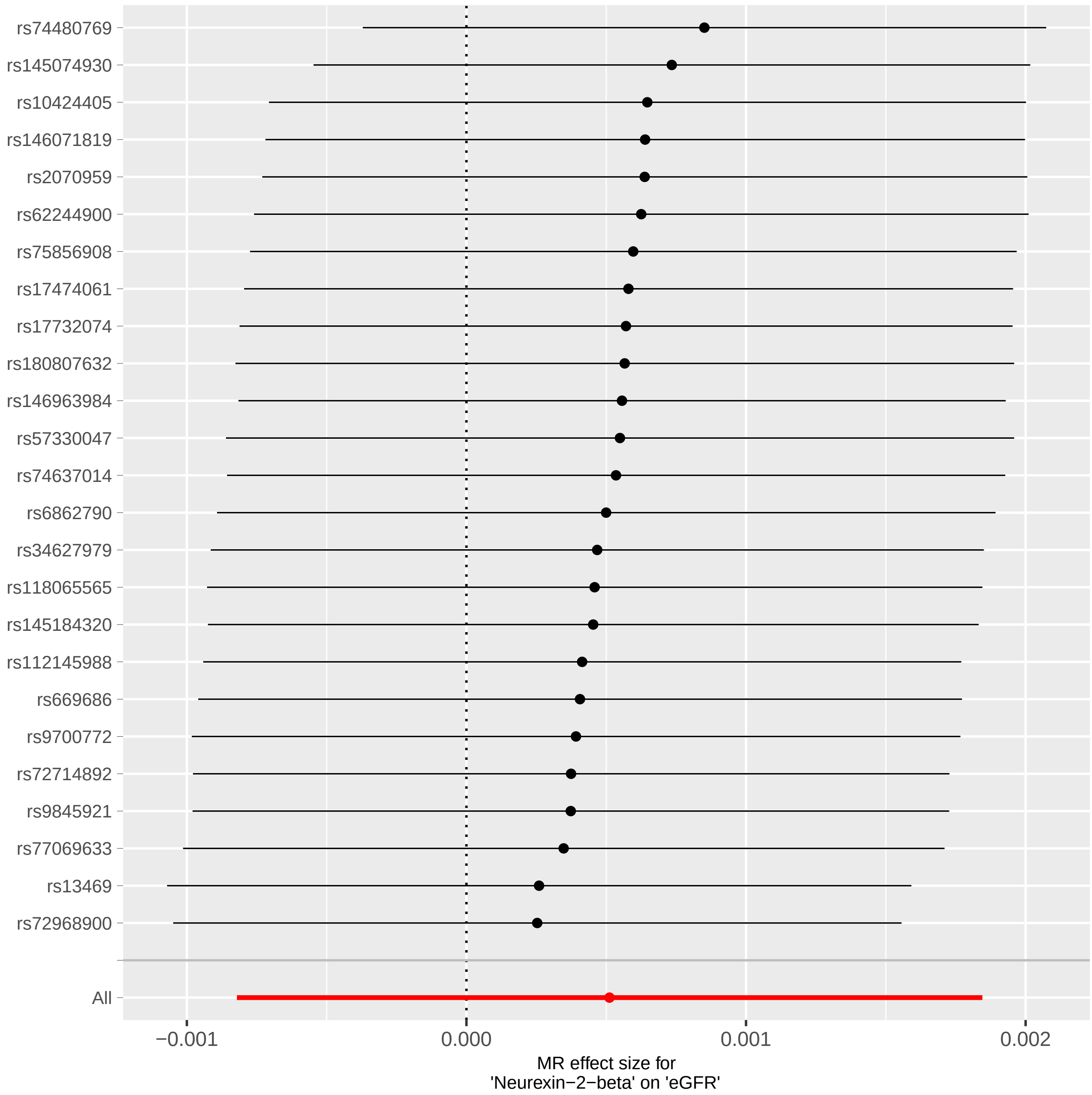

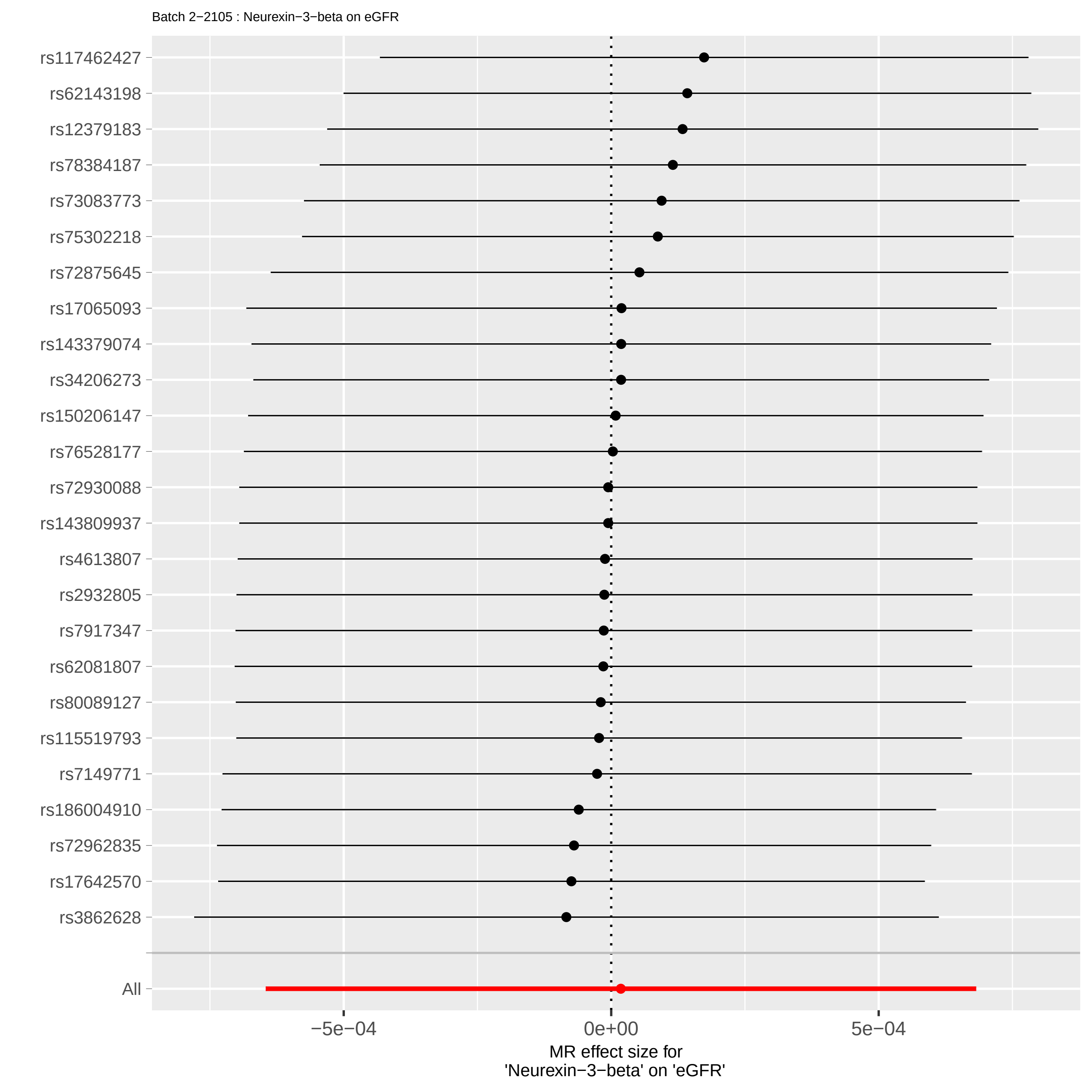
Batch 2−2105 : Neurexin−3−beta on eGFR
rs117462427
rs62143198
rs12379183
rs78384187
rs73083773
rs75302218
rs72875645
rs17065093
rs143379074
rs34206273
rs150206147
rs76528177
rs72930088
rs143809937
rs4613807
rs2932805
rs7917347
rs62081807
rs80089127
rs115519793
rs7149771
rs186004910
rs72962835
rs17642570
rs3862628
All
−5e−04
0e+00
5e−04
MR effect size for
'Neurexin−3−beta' on 'eGFR'

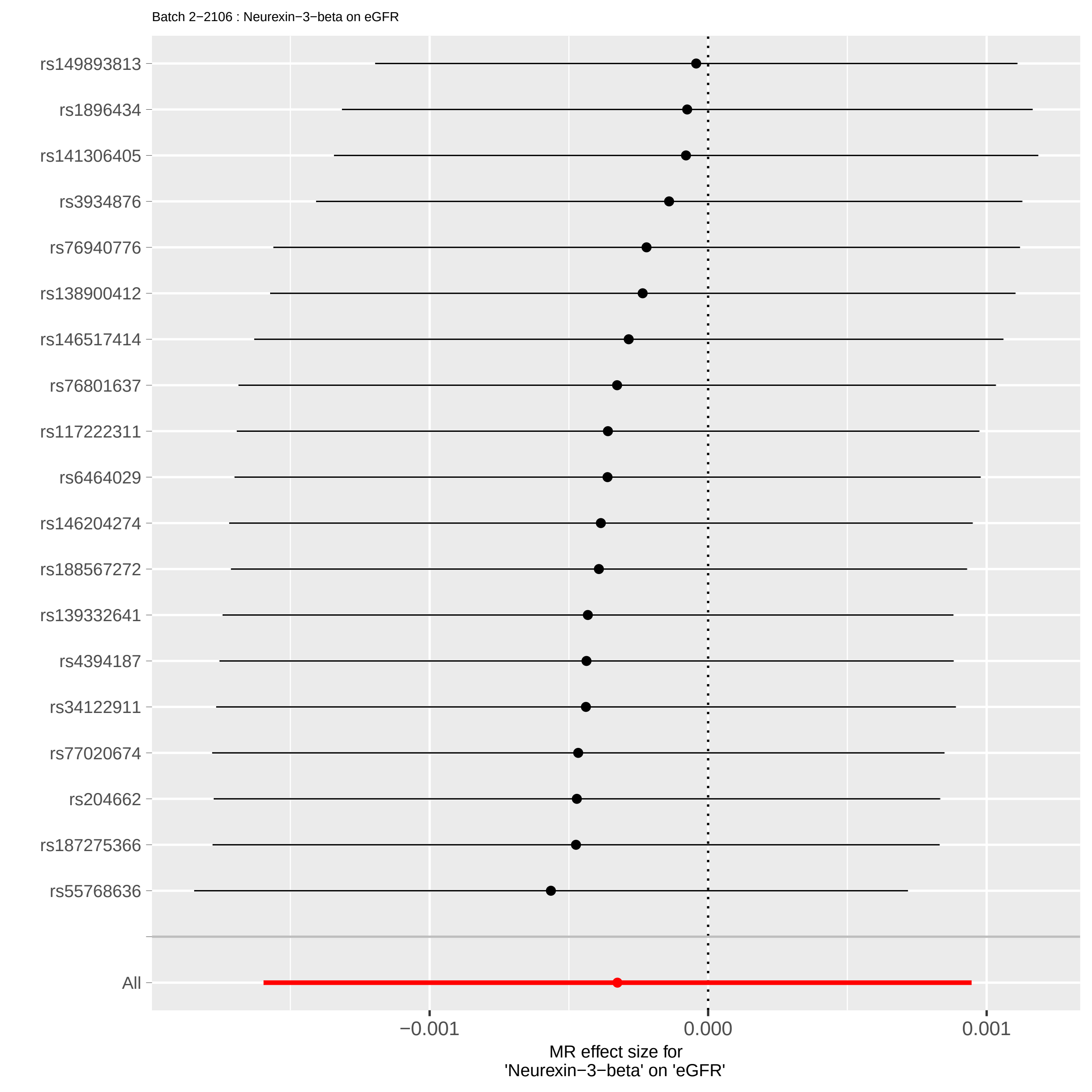
Batch 2−2106 : Neurexin−3−beta on eGFR
rs149893813
rs1896434
rs141306405
rs3934876
rs76940776
rs138900412
rs146517414
rs76801637
rs117222311
rs6464029
rs146204274
rs188567272
rs139332641
rs4394187
rs34122911
rs77020674
rs204662
rs187275366
rs55768636
All
−0.001
0.000
0.001
MR effect size for
'Neurexin−3−beta' on 'eGFR'

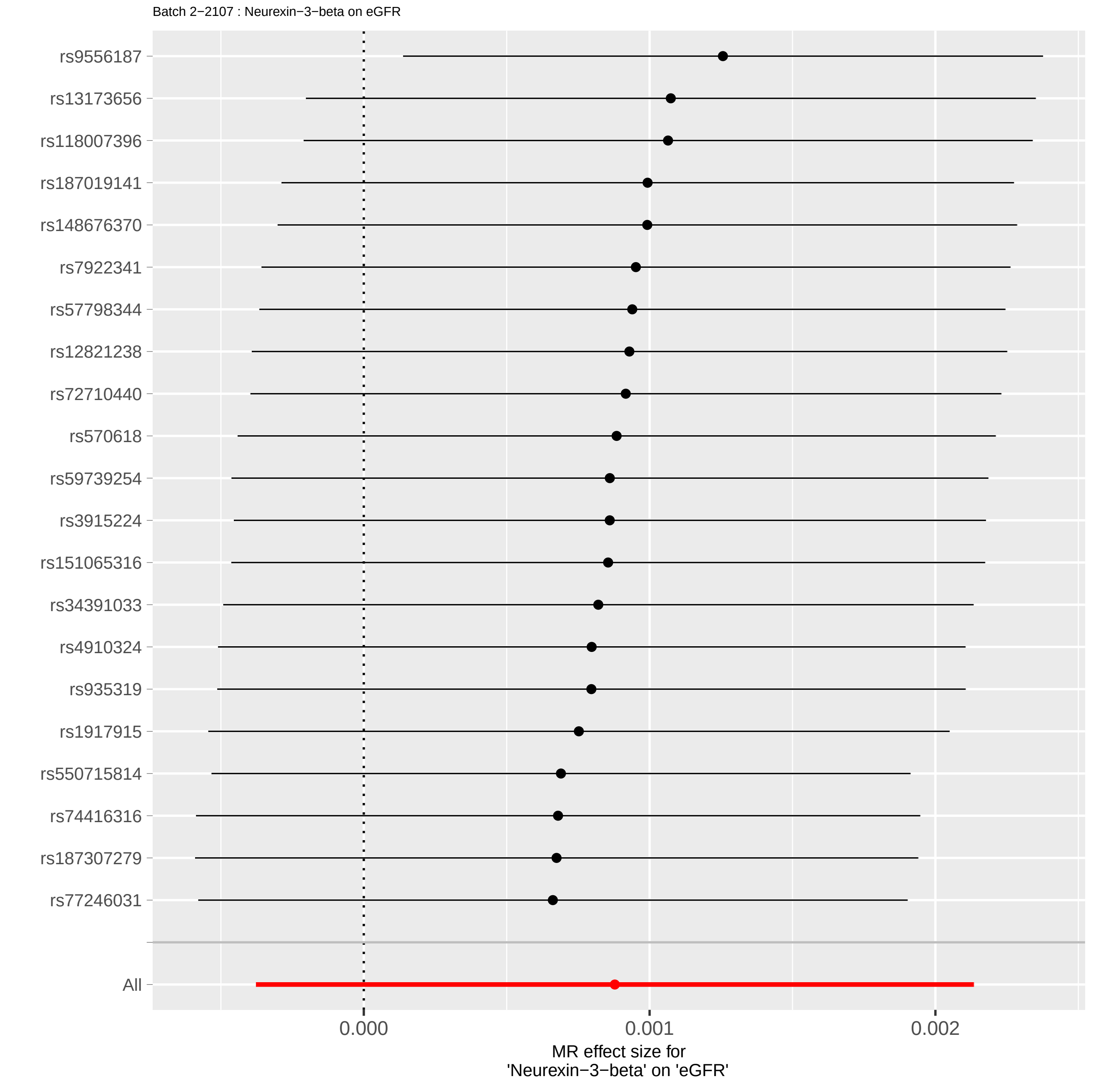

Batch 2−2107 : Neurexin−3−beta on eGFR
rs9556187
rs13173656
rs118007396
rs187019141
rs148676370
rs7922341
rs57798344
rs12821238
rs72710440
rs570618
rs59739254
rs3915224
rs151065316
rs34391033
rs4910324
rs935319
rs1917915
rs550715814
rs74416316
rs187307279
rs77246031
All
0.000
0.001
0.002
MR effect size for
'Neurexin−3−beta' on 'eGFR'

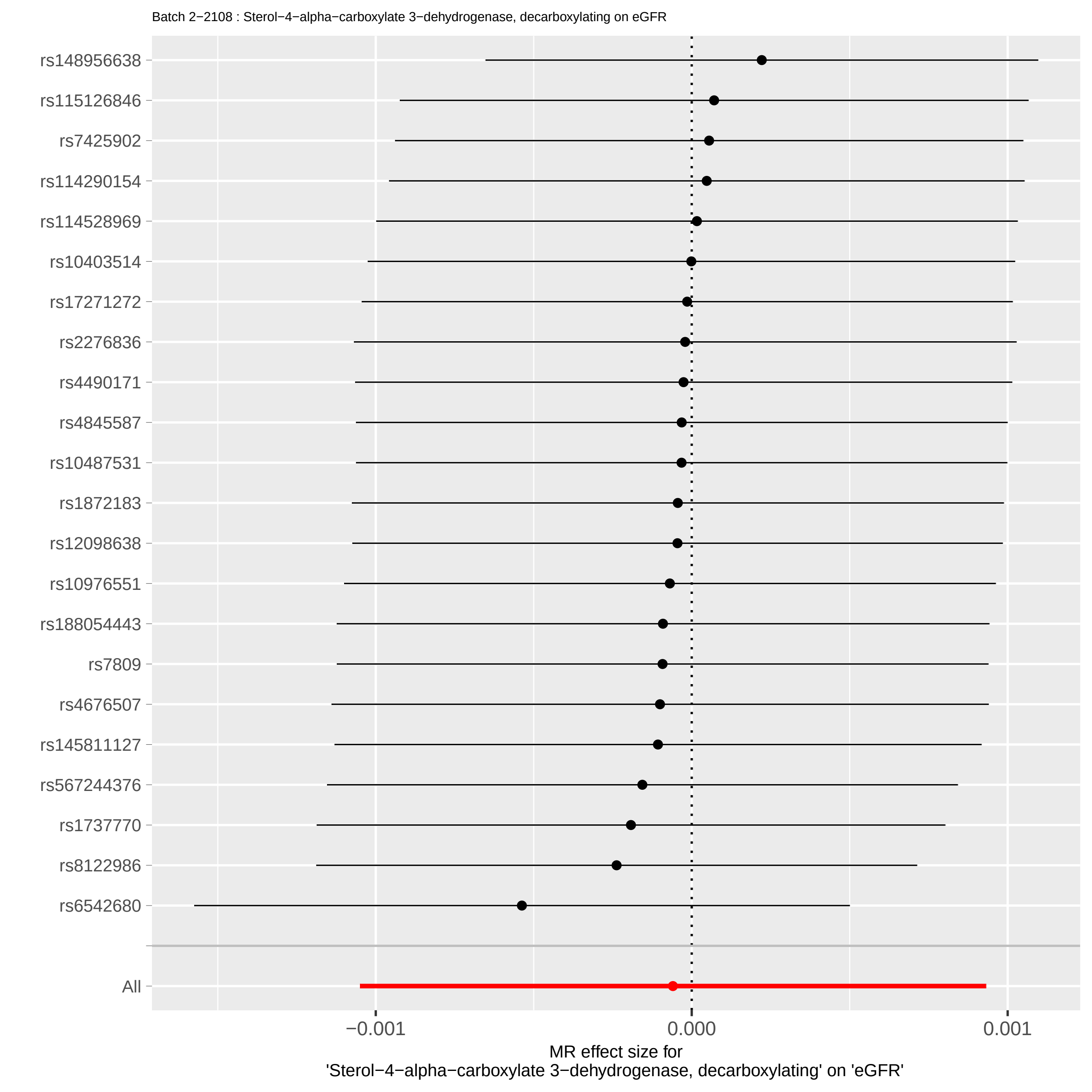

Batch 2−2108 : Sterol−4−alpha−carboxylate 3−dehydrogenase, decarboxylating on eGFR
rs148956638
rs115126846
rs7425902
rs114290154
rs114528969
rs10403514
rs17271272
rs2276836
rs4490171
rs4845587
rs10487531
rs1872183
rs12098638
rs10976551
rs188054443
rs7809
rs4676507
rs145811127
rs567244376
rs1737770
rs8122986
rs6542680
All
−0.001
0.000
0.001
MR effect size for
'Sterol−4−alpha−carboxylate 3−dehydrogenase, decarboxylating' on 'eGFR'

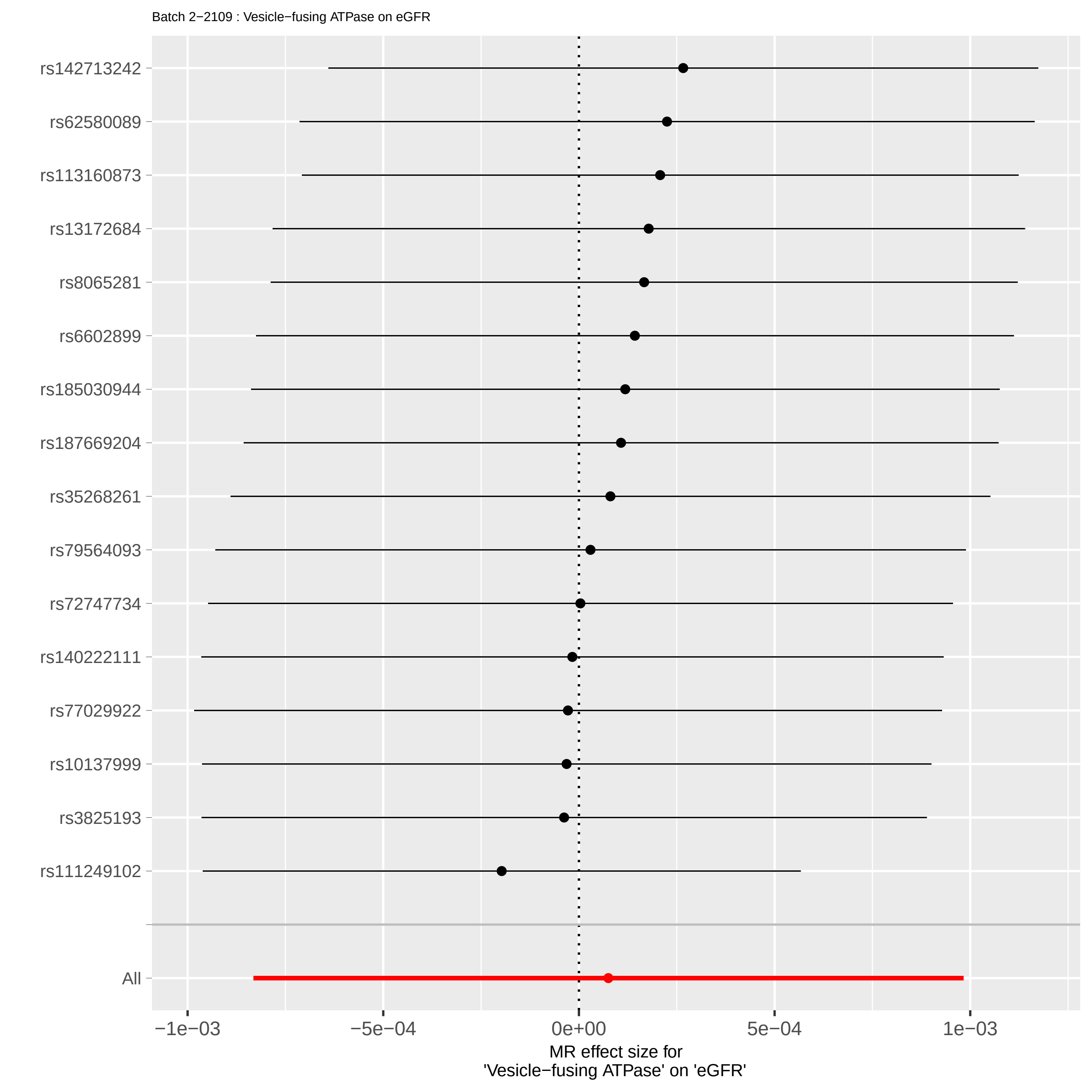

Batch 2−2109 : Vesicle−fusing ATPase on eGFR
rs142713242
rs62580089
rs113160873
rs13172684
rs8065281
rs6602899
rs185030944
rs187669204
rs35268261
rs79564093
rs72747734
rs140222111
rs77029922
rs10137999
rs3825193
rs111249102
All
−1e−03
−5e−04
0e+00
5e−04
1e−03
MR effect size for
'Vesicle−fusing ATPase' on 'eGFR'

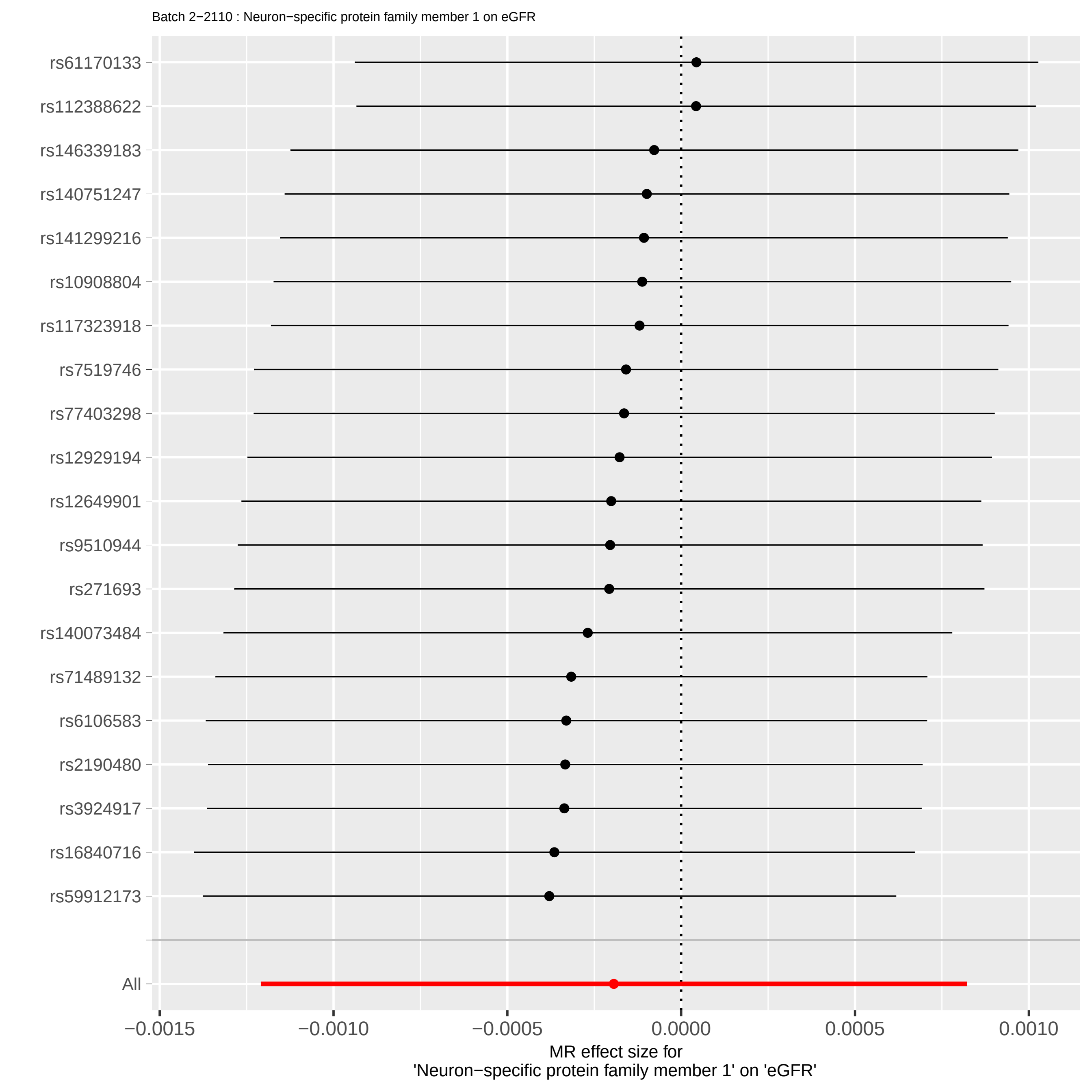

Batch 2−2110 : Neuron−specific protein family member 1 on eGFR
rs61170133
rs112388622
rs146339183
rs140751247
rs141299216
rs10908804
rs117323918
rs7519746
rs77403298
rs12929194
rs12649901
rs9510944
rs271693
rs140073484
rs71489132
rs6106583
rs2190480
rs3924917
rs16840716
rs59912173
All
−0.0015
−0.0010
−0.0005
0.0000
0.0005
0.0010
MR effect size for
'Neuron−specific protein family member 1' on 'eGFR'

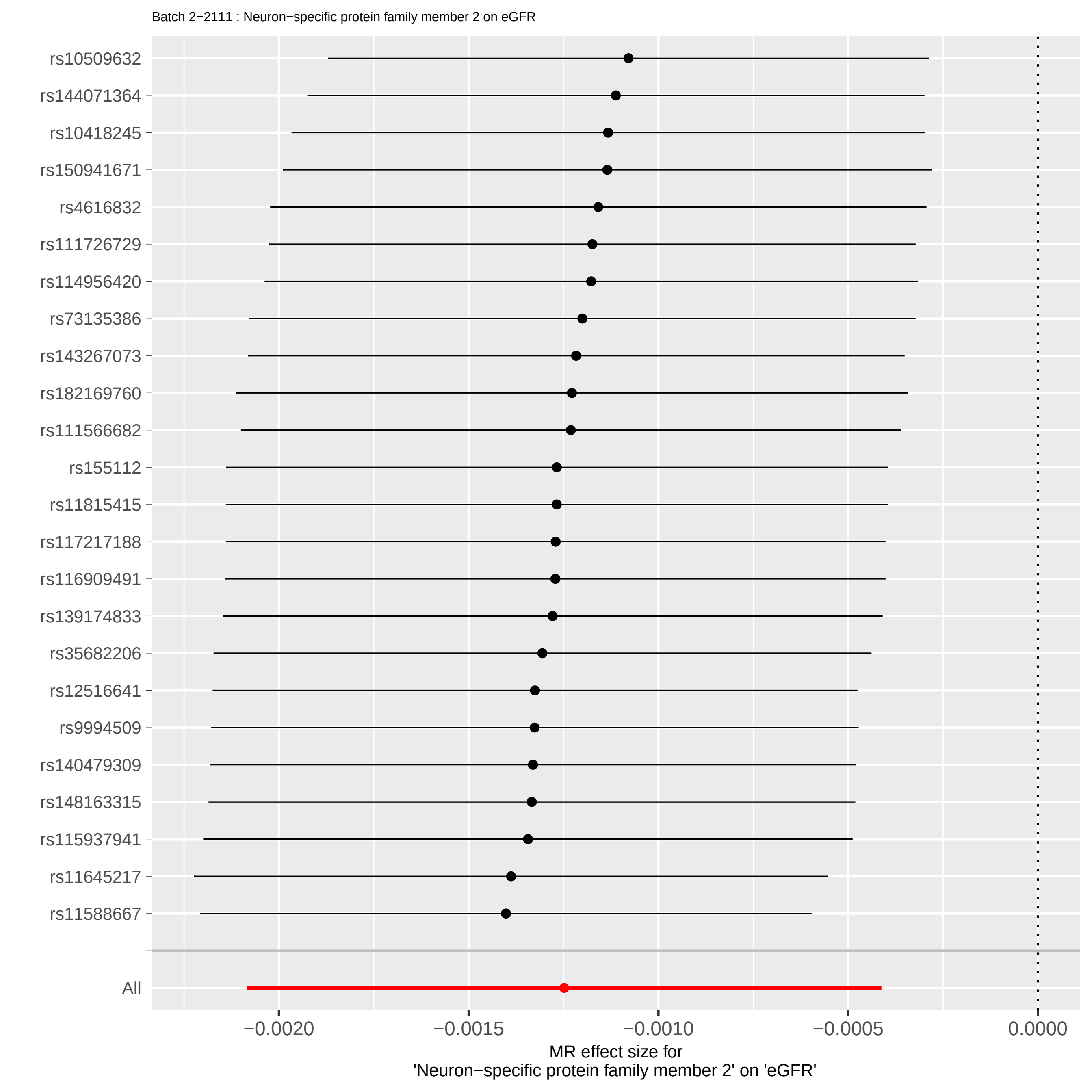
Batch 2−2111 : Neuron−specific protein family member 2 on eGFR
rs10509632
rs144071364
rs10418245
rs150941671
rs4616832
rs111726729
rs114956420
rs73135386
rs143267073
rs182169760
rs111566682
rs155112
rs11815415
rs117217188
rs116909491
rs139174833
rs35682206
rs12516641
rs9994509
rs140479309
rs148163315
rs115937941
rs11645217
rs11588667
All
−0.0020
−0.0015
−0.0010
−0.0005
0.0000
MR effect size for
'Neuron−specific protein family member 2' on 'eGFR'

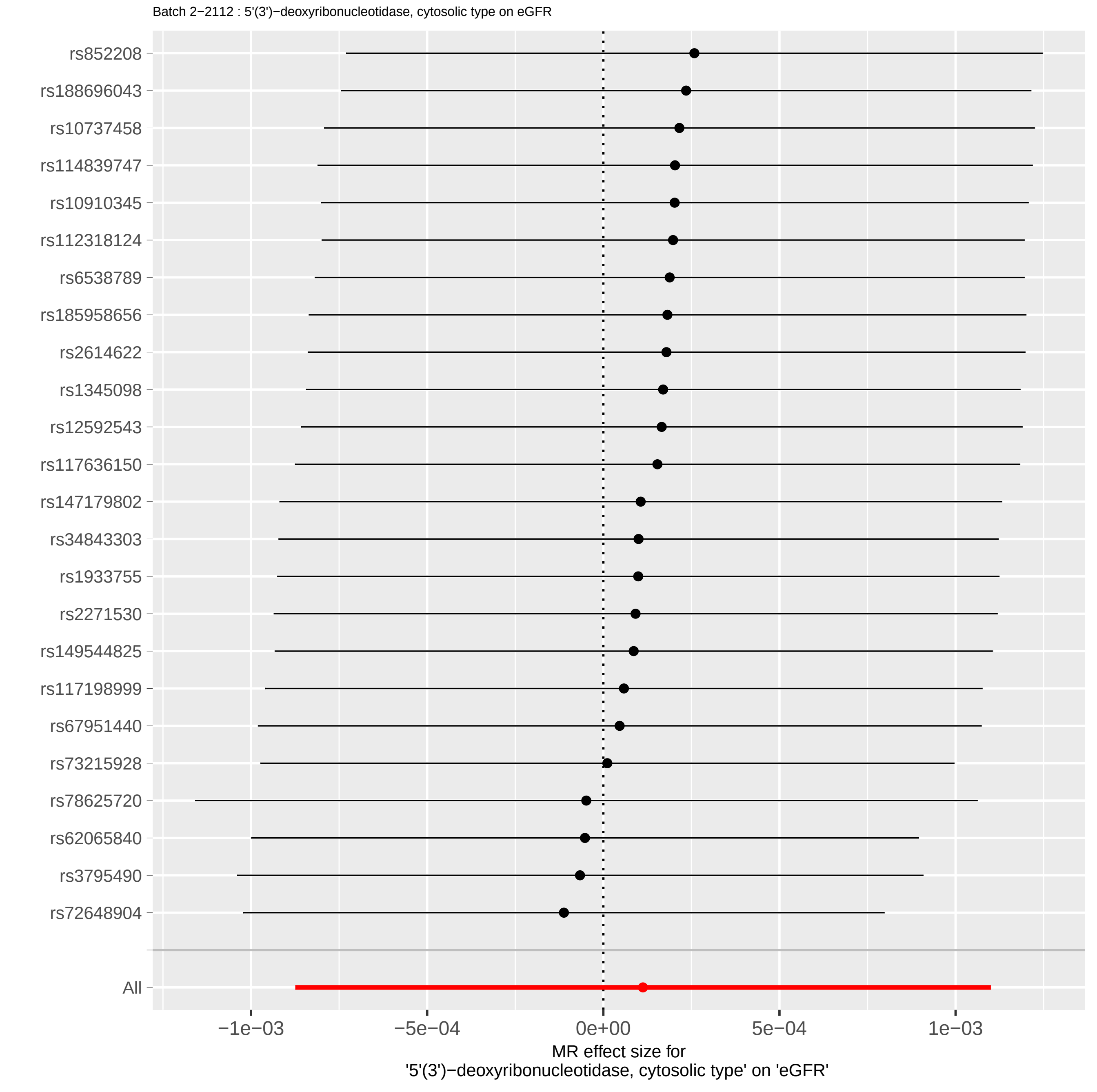

Batch 2−2112 : 5'(3')−deoxyribonucleotidase, cytosolic type on eGFR
rs852208
rs188696043
rs10737458
rs114839747
rs10910345
rs112318124
rs6538789
rs185958656
rs2614622
rs1345098
rs12592543
rs117636150
rs147179802
rs34843303
rs1933755
rs2271530
rs149544825
rs117198999
rs67951440
rs73215928
rs78625720
rs62065840
rs3795490
rs72648904
All
−1e−03
−5e−04
0e+00
5e−04
1e−03
MR effect size for
'5'(3')−deoxyribonucleotidase, cytosolic type' on 'eGFR'

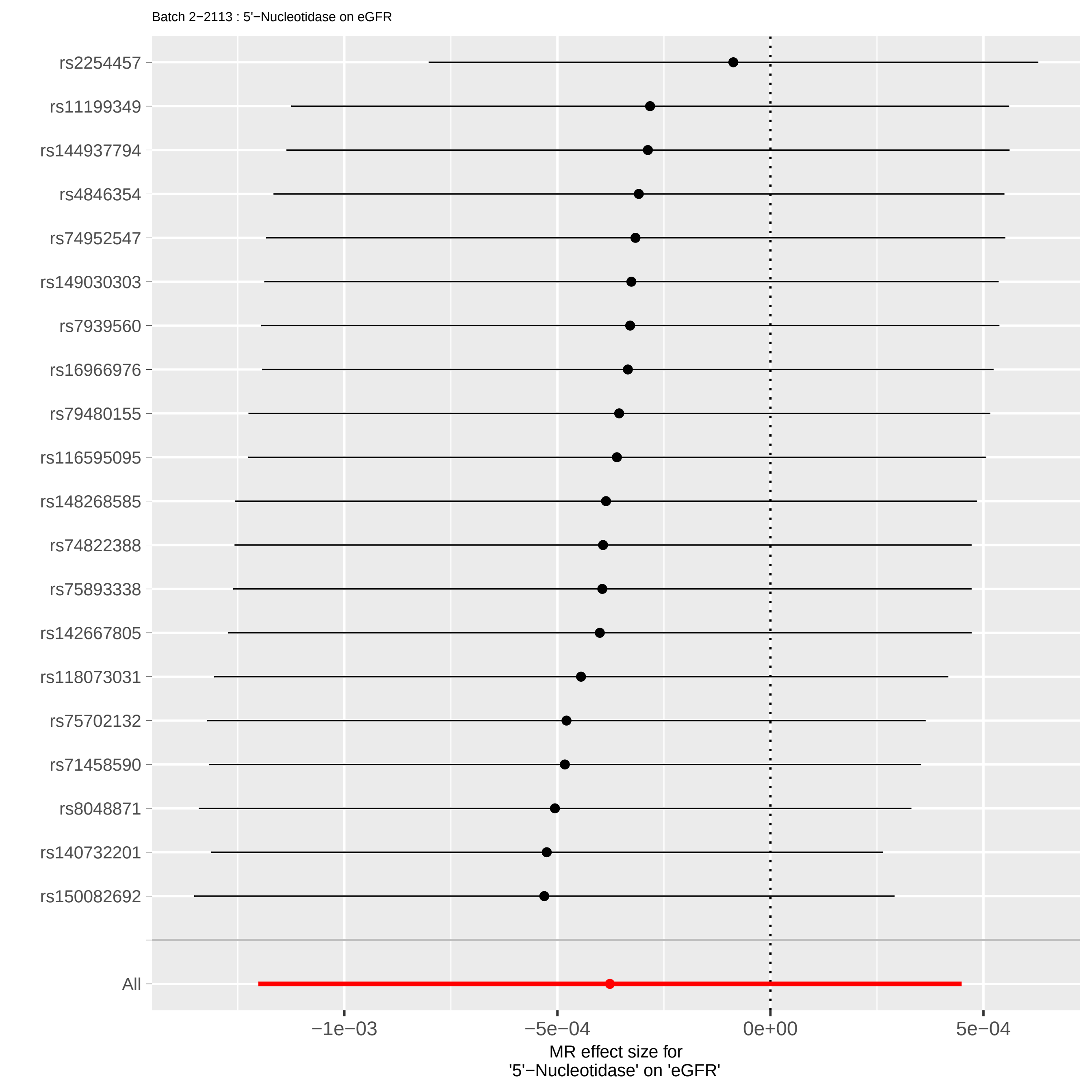

Batch 2−2113 : 5'−Nucleotidase on eGFR
rs2254457
rs11199349
rs144937794
rs4846354
rs74952547
rs149030303
rs7939560
rs16966976
rs79480155
rs116595095
rs148268585
rs74822388
rs75893338
rs142667805
rs118073031
rs75702132
rs71458590
rs8048871
rs140732201
rs150082692
All
−1e−03
−5e−04
0e+00
5e−04
MR effect size for
'5'−Nucleotidase' on 'eGFR'

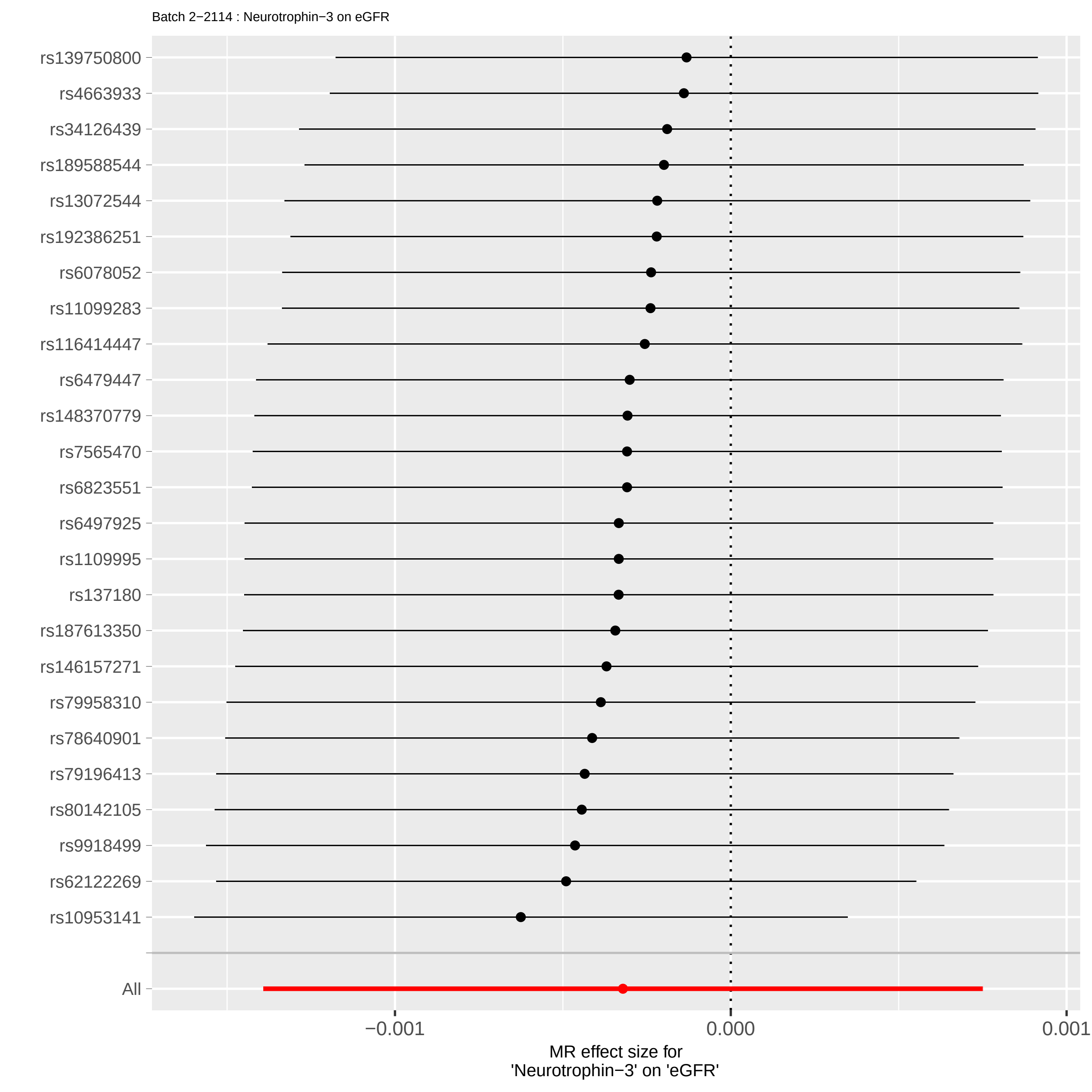

Batch 2−2114 : Neurotrophin−3 on eGFR
rs139750800
rs4663933
rs34126439
rs189588544
rs13072544
rs192386251
rs6078052
rs11099283
rs116414447
rs6479447
rs148370779
rs7565470
rs6823551
rs6497925
rs1109995
rs137180
rs187613350
rs146157271
rs79958310
rs78640901
rs79196413
rs80142105
rs9918499
rs62122269
rs10953141
All
−0.001
0.000
0.001
MR effect size for
'Neurotrophin−3' on 'eGFR'

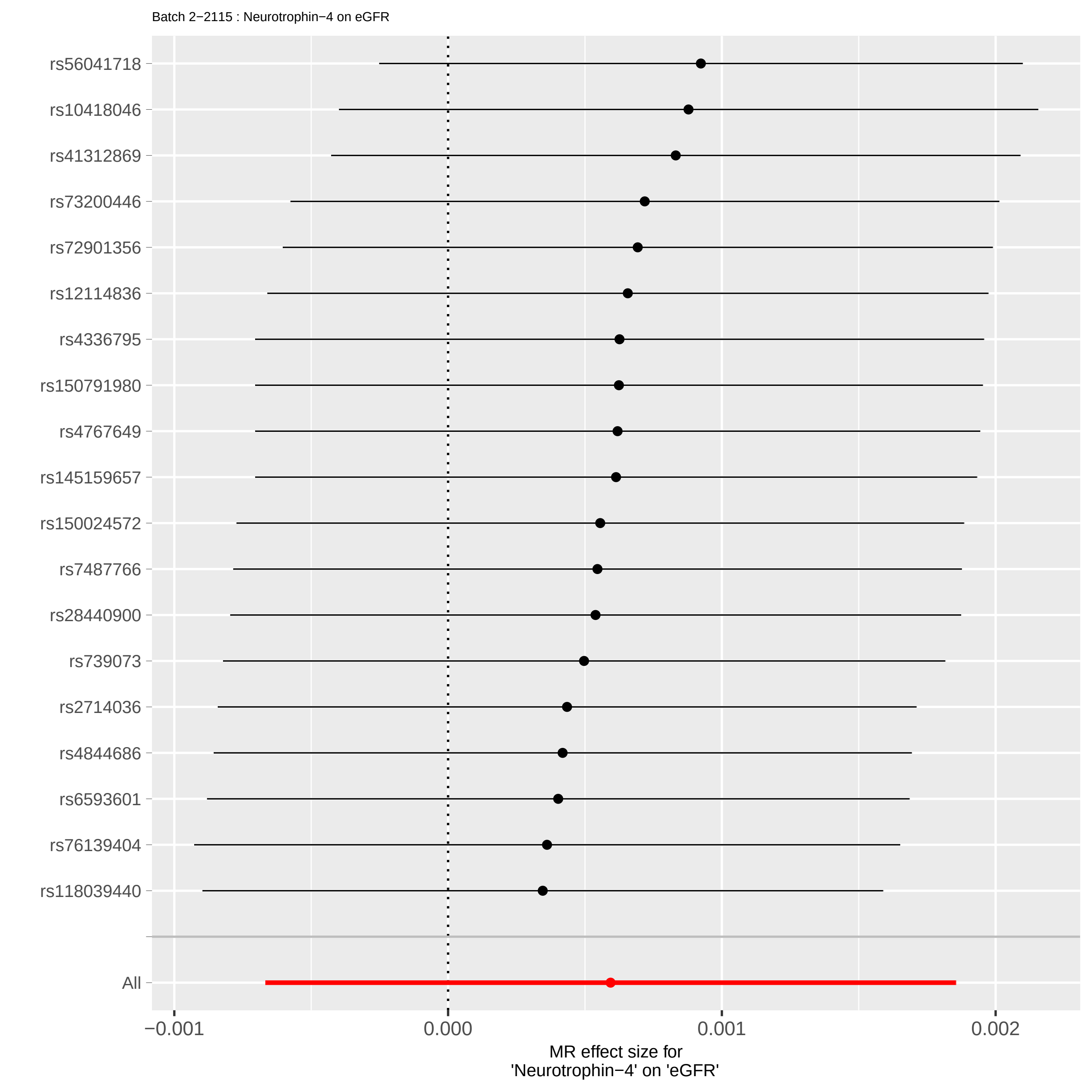

Batch 2−2115 : Neurotrophin−4 on eGFR
rs56041718
rs10418046
rs41312869
rs73200446
rs72901356
rs12114836
rs4336795
rs150791980
rs4767649
rs145159657
rs150024572
rs7487766
rs28440900
rs739073
rs2714036
rs4844686
rs6593601
rs76139404
rs118039440
All
−0.001
0.000
0.001
0.002
MR effect size for
'Neurotrophin−4' on 'eGFR'

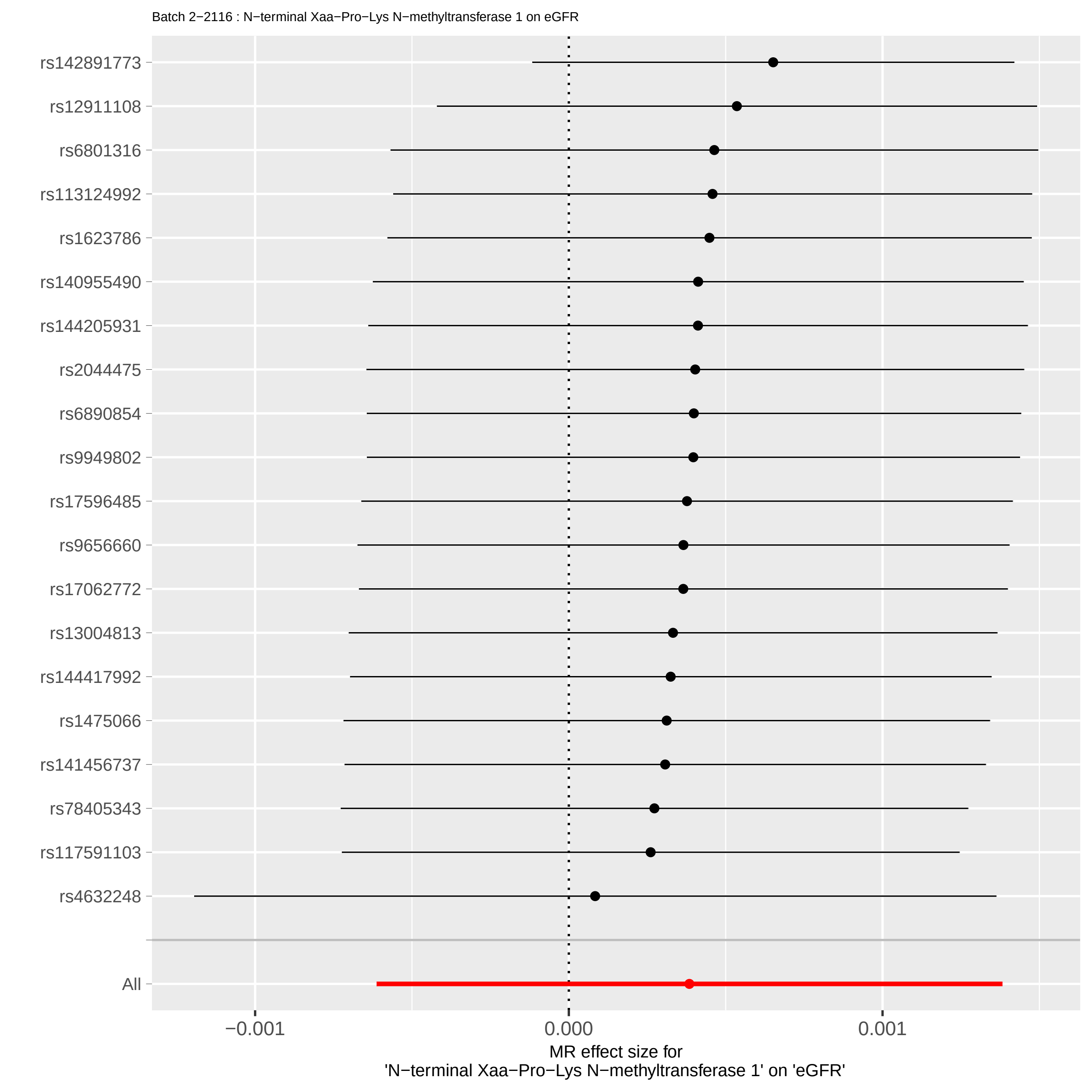
Batch 2−2116 : N−terminal Xaa−Pro−Lys N−methyltransferase 1 on eGFR
rs142891773
rs12911108
rs6801316
rs113124992
rs1623786
rs140955490
rs144205931
rs2044475
rs6890854
rs9949802
rs17596485
rs9656660
rs17062772
rs13004813
rs144417992
rs1475066
rs141456737
rs78405343
rs117591103
rs4632248
All
−0.001
0.000
0.001
MR effect size for
'N−terminal Xaa−Pro−Lys N−methyltransferase 1' on 'eGFR'

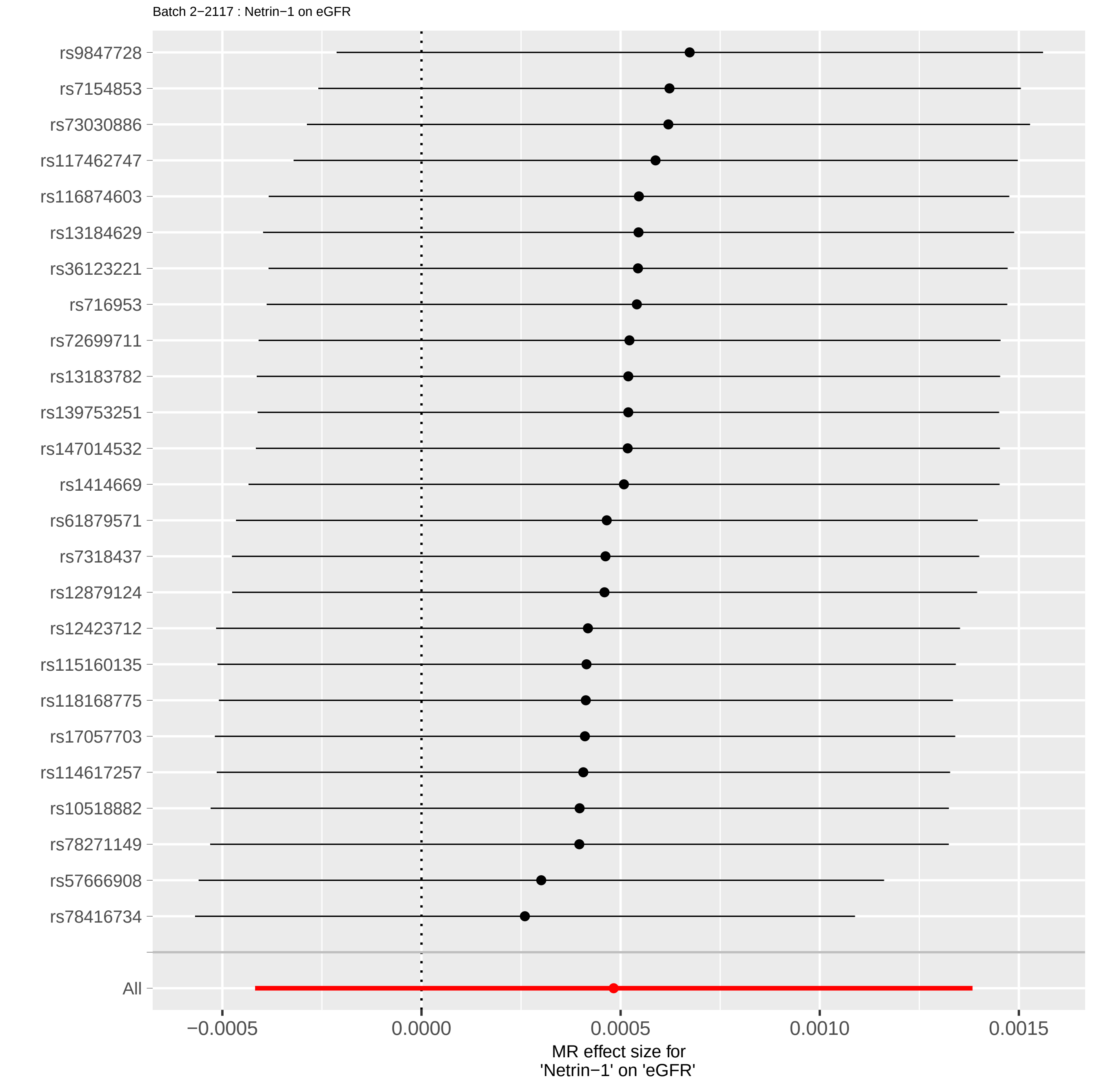
Batch 2−2117 : Netrin−1 on eGFR
rs9847728
rs7154853
rs73030886
rs117462747
rs116874603
rs13184629
rs36123221
rs716953
rs72699711
rs13183782
rs139753251
rs147014532
rs1414669
rs61879571
rs7318437
rs12879124
rs12423712
rs115160135
rs118168775
rs17057703
rs114617257
rs10518882
rs78271149
rs57666908
rs78416734
All
−0.0005
0.0000
0.0005
0.0010
0.0015
MR effect size for
'Netrin−1' on 'eGFR'

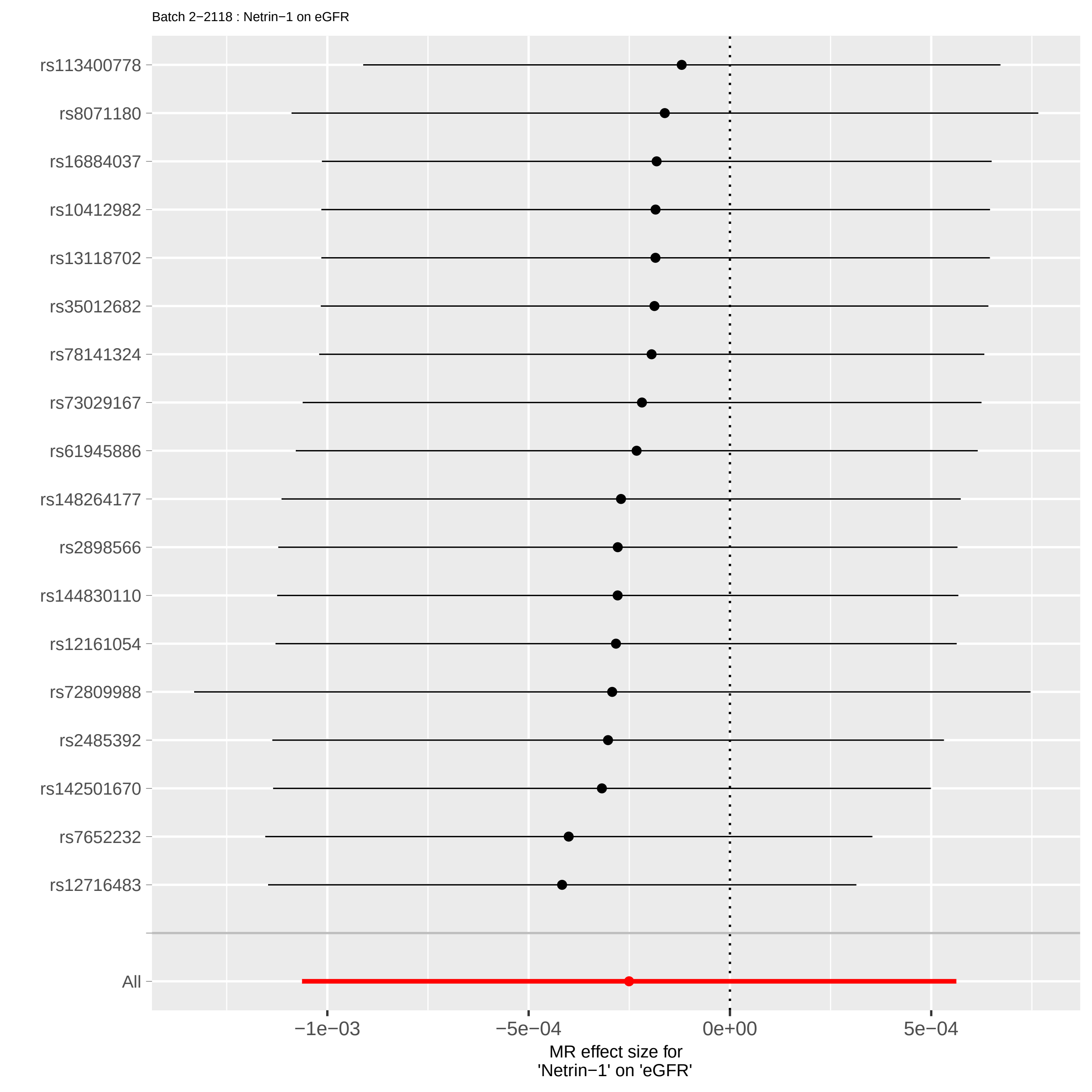

Batch 2−2118 : Netrin−1 on eGFR
rs113400778
rs8071180
rs16884037
rs10412982
rs13118702
rs35012682
rs78141324
rs73029167
rs61945886
rs148264177
rs2898566
rs144830110
rs12161054
rs72809988
rs2485392
rs142501670
rs7652232
rs12716483
All
−1e−03
−5e−04
0e+00
5e−04
MR effect size for
'Netrin−1' on 'eGFR'

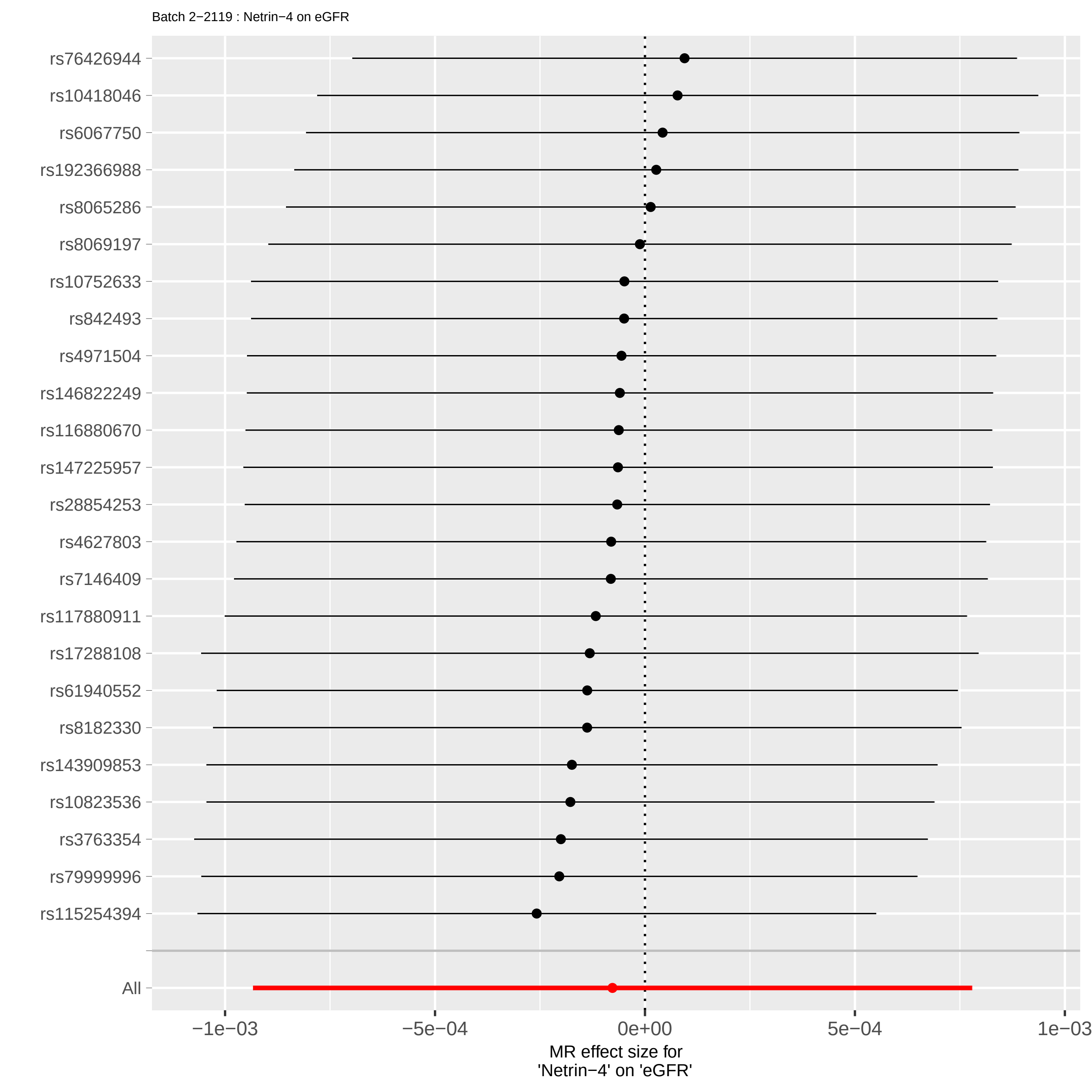

Batch 2−2119 : Netrin−4 on eGFR
rs76426944
rs10418046
rs6067750
rs192366988
rs8065286
rs8069197
rs10752633
rs842493
rs4971504
rs146822249
rs116880670
rs147225957
rs28854253
rs4627803
rs7146409
rs117880911
rs17288108
rs61940552
rs8182330
rs143909853
rs10823536
rs3763354
rs79999996
rs115254394
All
−1e−03
−5e−04
0e+00
5e−04
1e−03
MR effect size for
'Netrin−4' on 'eGFR'

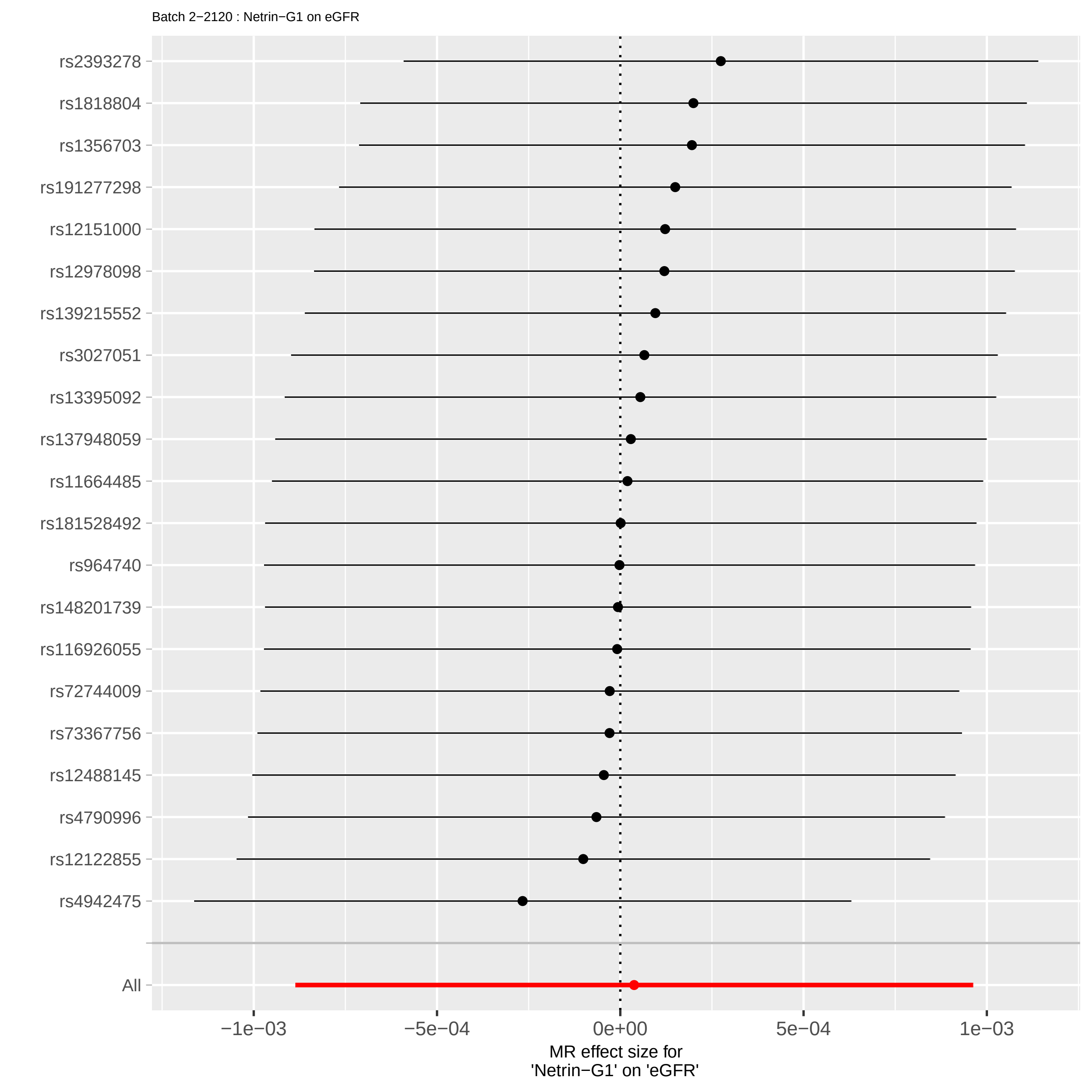

Batch 2−2120 : Netrin−G1 on eGFR
rs2393278
rs1818804
rs1356703
rs191277298
rs12151000
rs12978098
rs139215552
rs3027051
rs13395092
rs137948059
rs11664485
rs181528492
rs964740
rs148201739
rs116926055
rs72744009
rs73367756
rs12488145
rs4790996
rs12122855
rs4942475
All
−1e−03
−5e−04
0e+00
5e−04
1e−03
MR effect size for
'Netrin−G1' on 'eGFR'

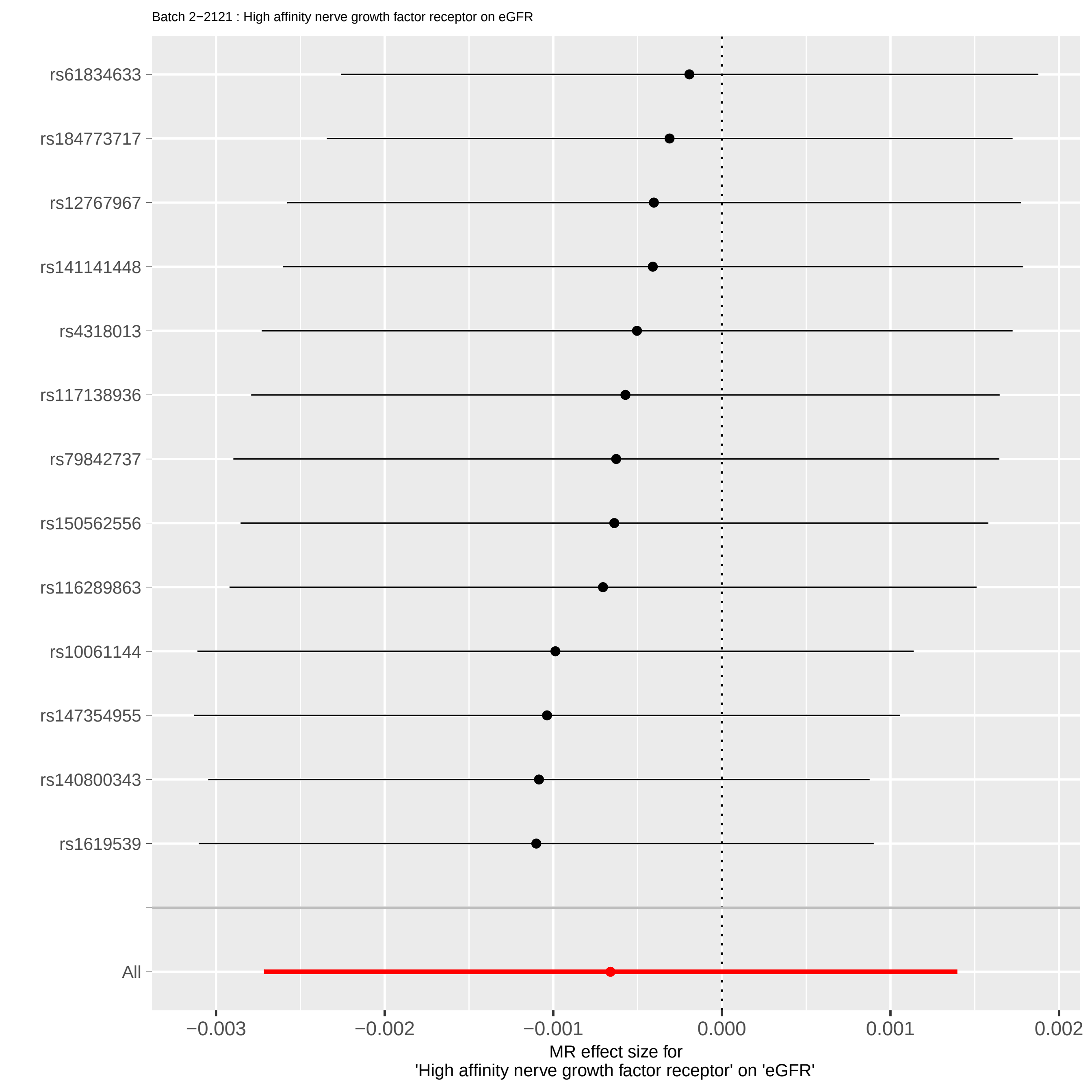
Batch 2−2121 : High affinity nerve growth factor receptor on eGFR
rs61834633
rs184773717
rs12767967
rs141141448
rs4318013
rs117138936
rs79842737
rs150562556
rs116289863
rs10061144
rs147354955
rs140800343
rs1619539
All
−0.003
−0.002
−0.001
0.000
0.001
0.002
MR effect size for
'High affinity nerve growth factor receptor' on 'eGFR'

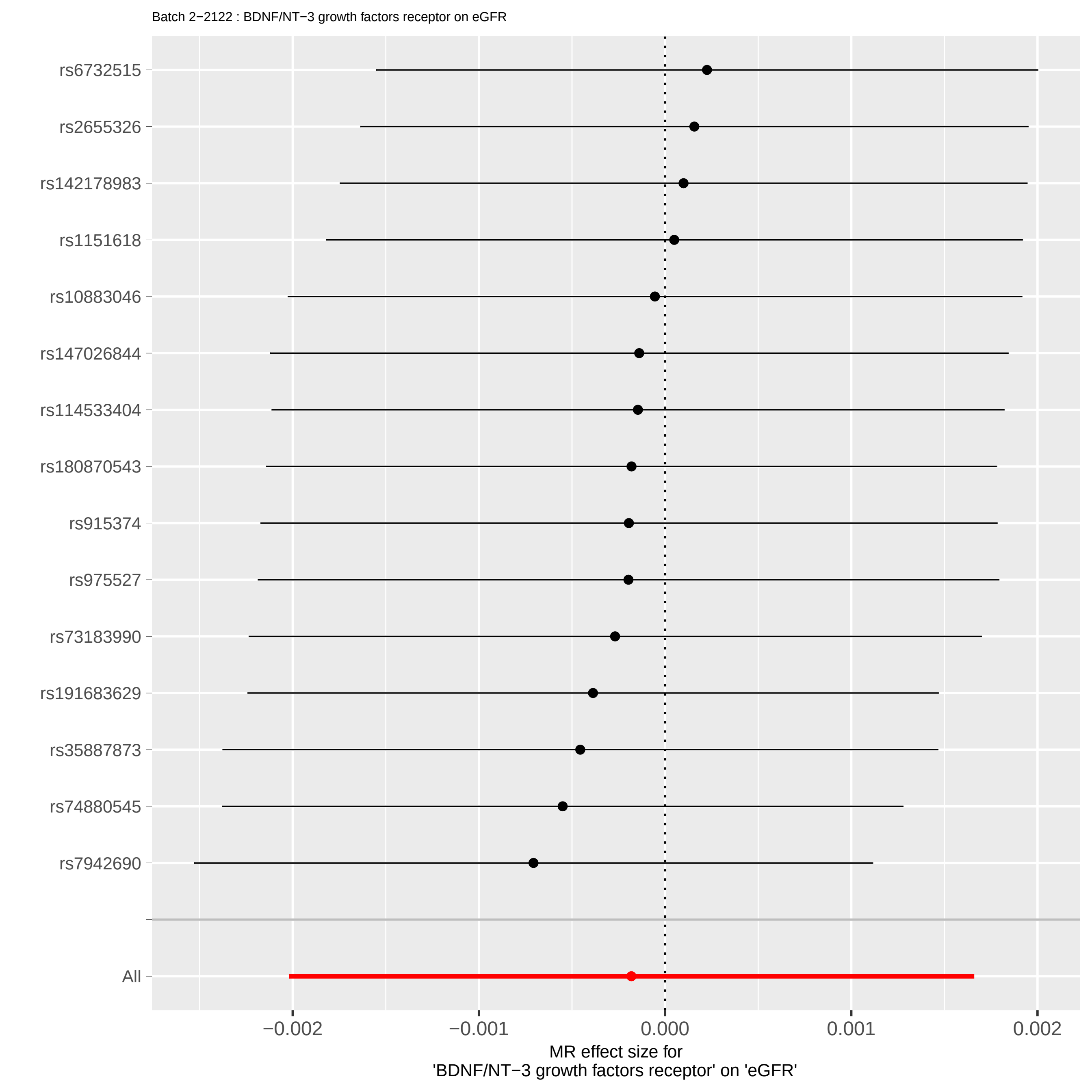

Batch 2−2122 : BDNF/NT−3 growth factors receptor on eGFR
rs6732515
rs2655326
rs142178983
rs1151618
rs10883046
rs147026844
rs114533404
rs180870543
rs915374
rs975527
rs73183990
rs191683629
rs35887873
rs74880545
rs7942690
All
−0.002
−0.001
0.000
0.001
0.002
MR effect size for
'BDNF/NT−3 growth factors receptor' on 'eGFR'

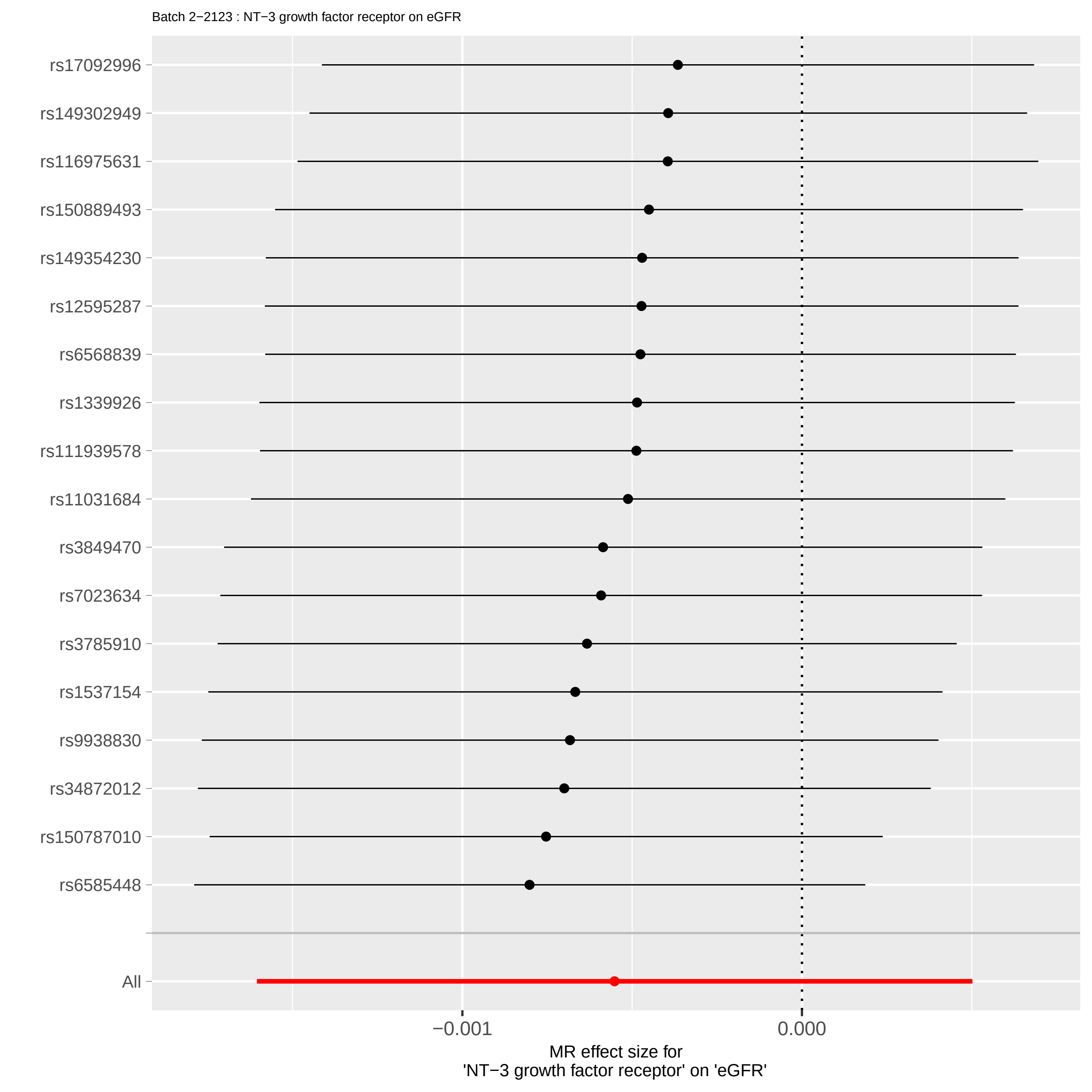

Batch 2−2123 : NT−3 growth factor receptor on eGFR
rs17092996
rs149302949
rs116975631
rs150889493
rs149354230
rs12595287
rs6568839
rs1339926
rs111939578
rs11031684
rs3849470
rs7023634
rs3785910
rs1537154
rs9938830
rs34872012
rs150787010
rs6585448
All
−0.001
0.000
MR effect size for
'NT−3 growth factor receptor' on 'eGFR'

Batch 2−2124 : Neurotensin/neuromedin N on eGFR

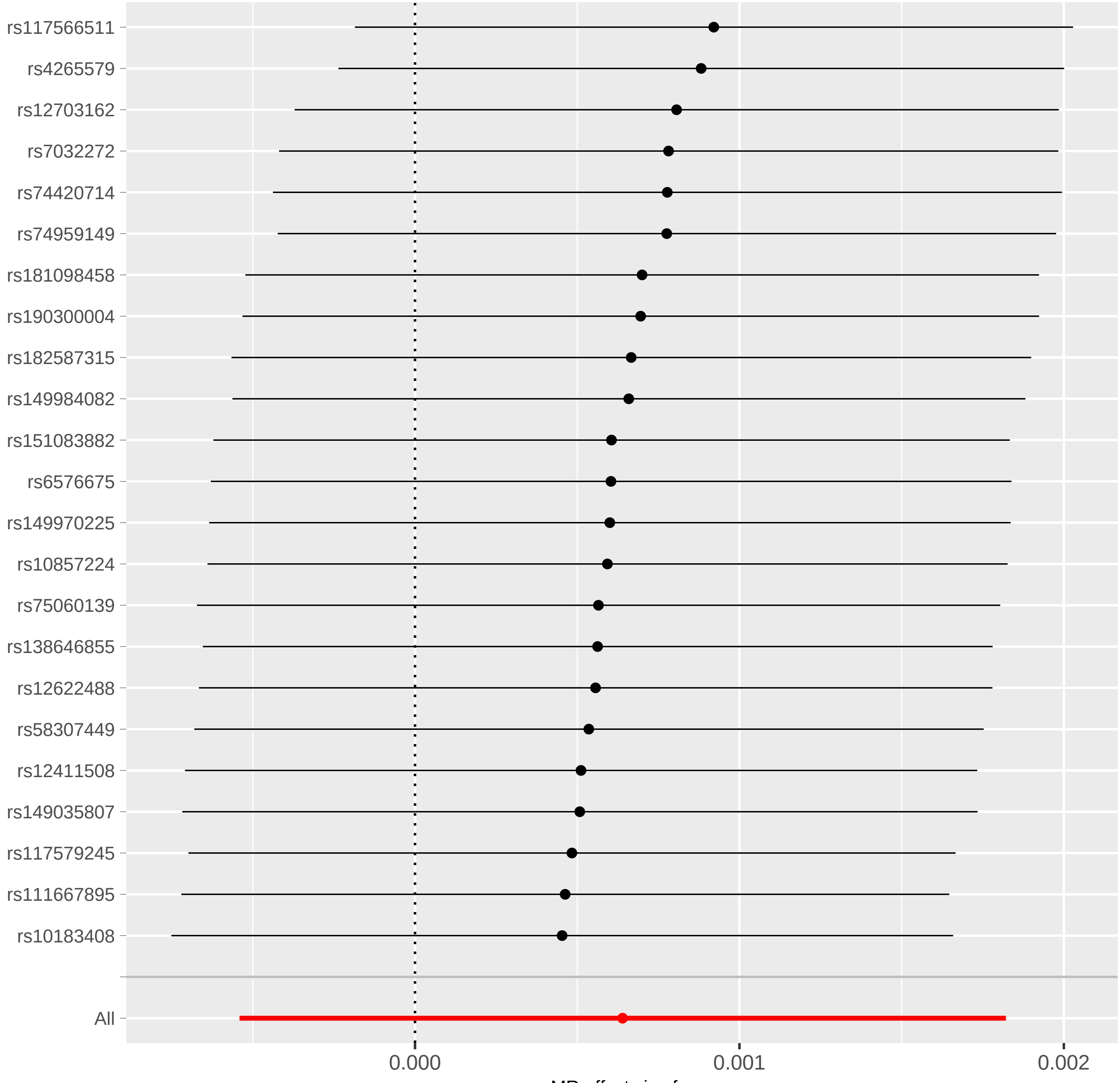

MR effect size for
'Neurotensin/neuromedin N' on 'eGFR'

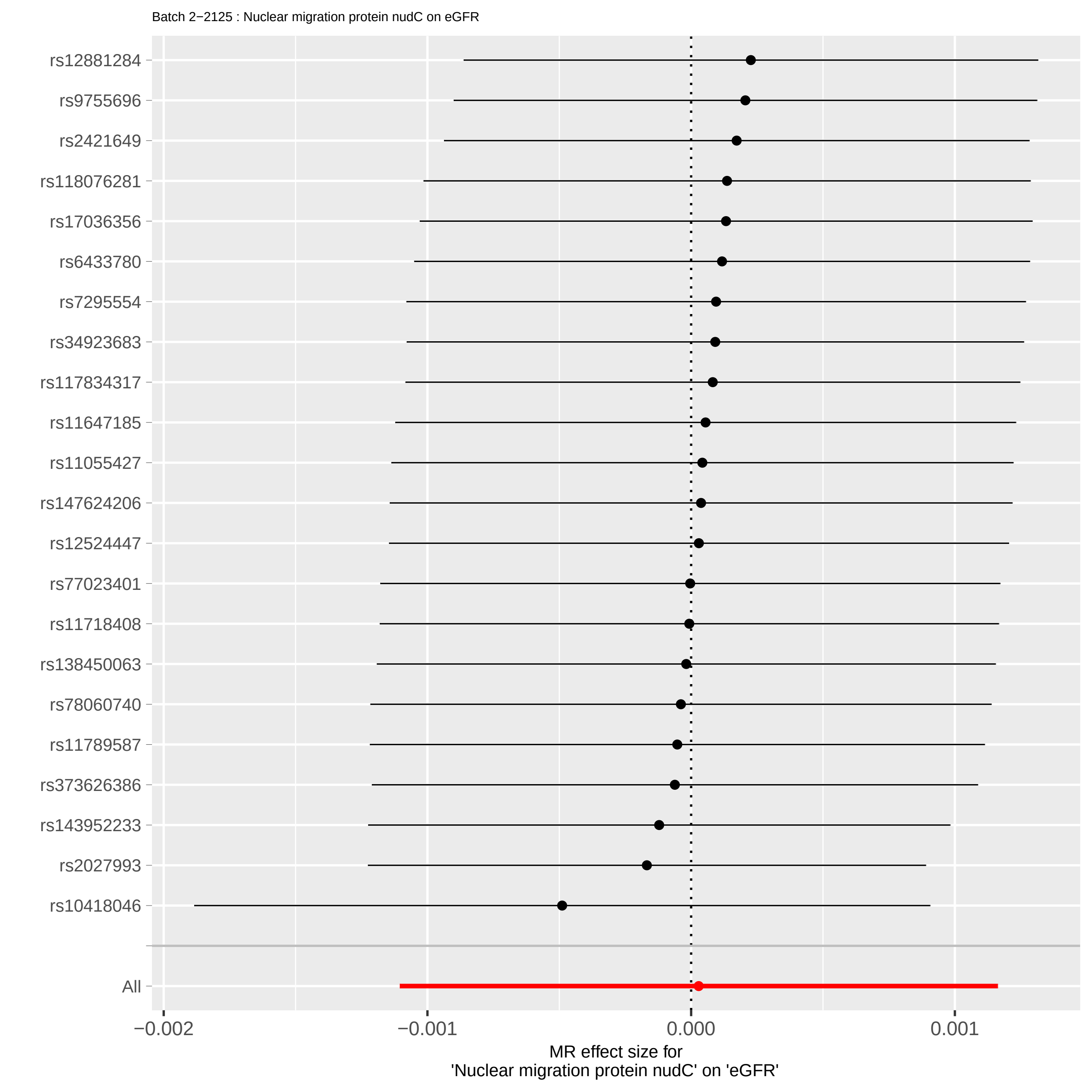

Batch 2−2125 : Nuclear migration protein nudC on eGFR
rs12881284
rs9755696
rs2421649
rs118076281
rs17036356
rs6433780
rs7295554
rs34923683
rs117834317
rs11647185
rs11055427
rs147624206
rs12524447
rs77023401
rs11718408
rs138450063
rs78060740
rs11789587
rs373626386
rs143952233
rs2027993
rs10418046
All
−0.002
−0.001
0.000
0.001
MR effect size for
'Nuclear migration protein nudC' on 'eGFR'

Batch 2−2126 : Peroxisomal NADH pyrophosphatase NUDT12 on eGFR

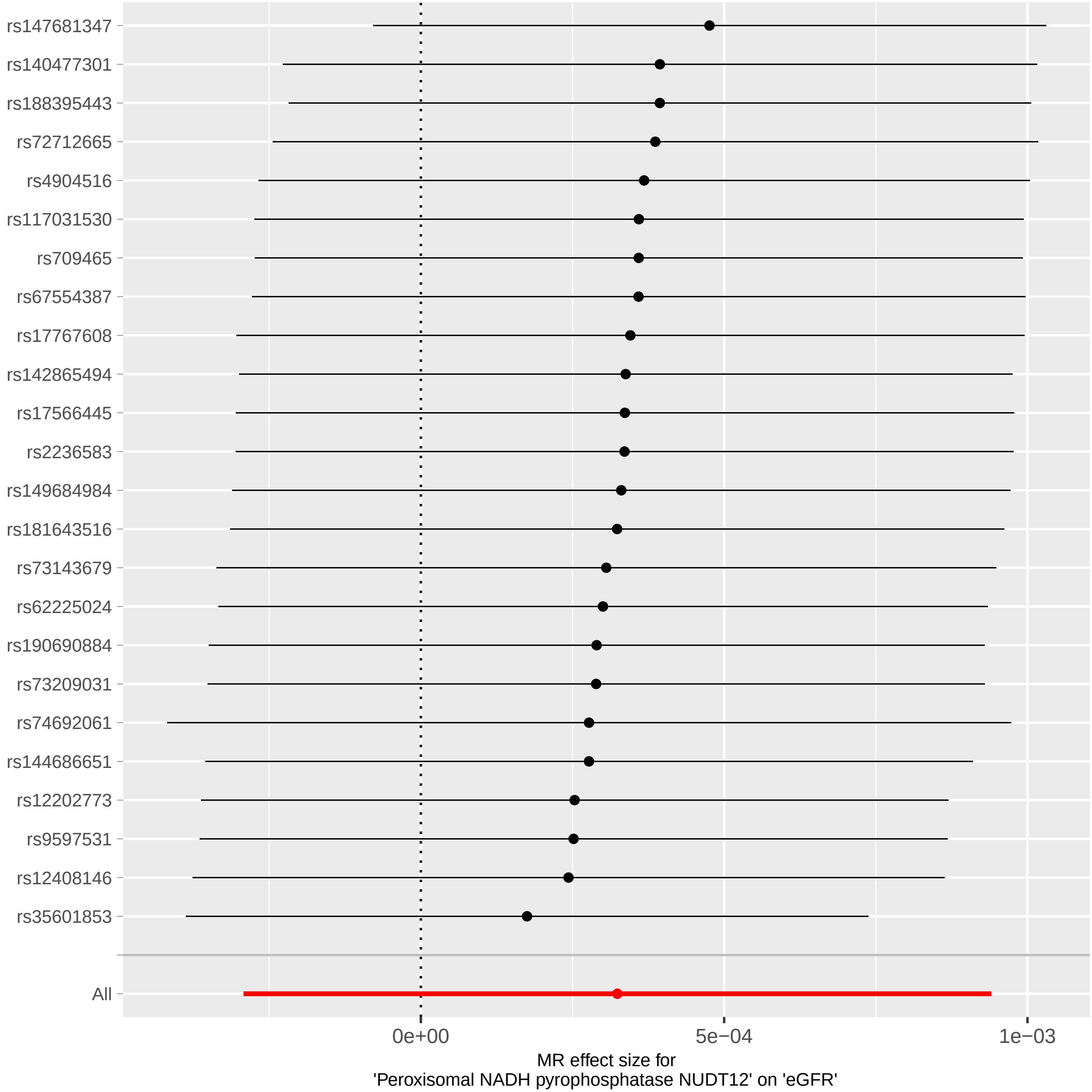

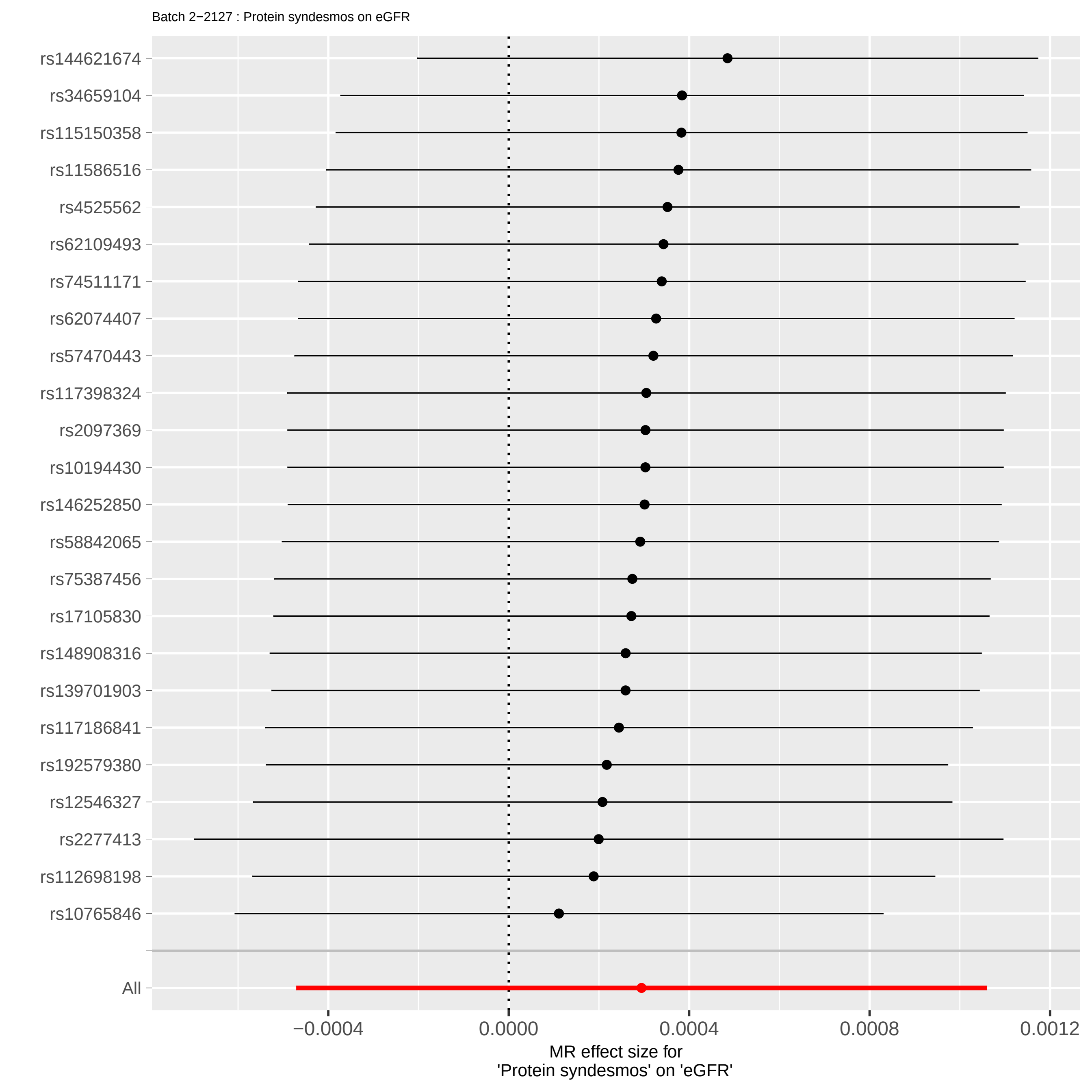

Batch 2−2127 : Protein syndesmos on eGFR
rs144621674
rs34659104
rs115150358
rs11586516
rs4525562
rs62109493
rs74511171
rs62074407
rs57470443
rs117398324
rs2097369
rs10194430
rs146252850
rs58842065
rs75387456
rs17105830
rs148908316
rs139701903
rs117186841
rs192579380
rs12546327
rs2277413
rs112698198
rs10765846
All
−0.0004
0.0000
0.0004
0.0008
0.0012
MR effect size for
'Protein syndesmos' on 'eGFR'

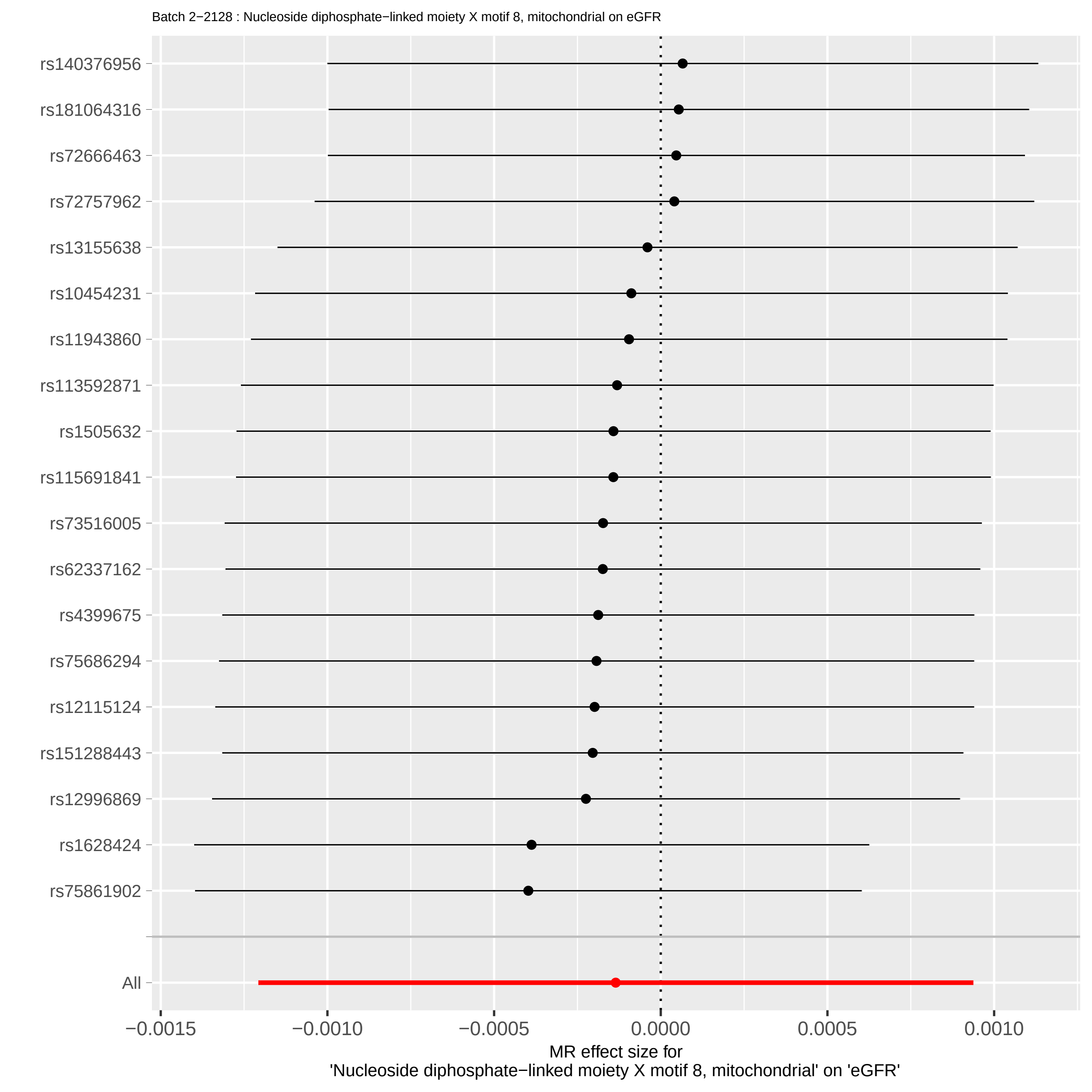
Batch 2−2128 : Nucleoside diphosphate−linked moiety X motif 8, mitochondrial on eGFR
rs140376956
rs181064316
rs72666463
rs72757962
rs13155638
rs10454231
rs11943860
rs113592871
rs1505632
rs115691841
rs73516005
rs62337162
rs4399675
rs75686294
rs12115124
rs151288443
rs12996869
rs1628424
rs75861902
All
−0.0015
−0.0010
−0.0005
0.0000
0.0005
0.0010
MR effect size for
'Nucleoside diphosphate−linked moiety X motif 8, mitochondrial' on 'eGFR'

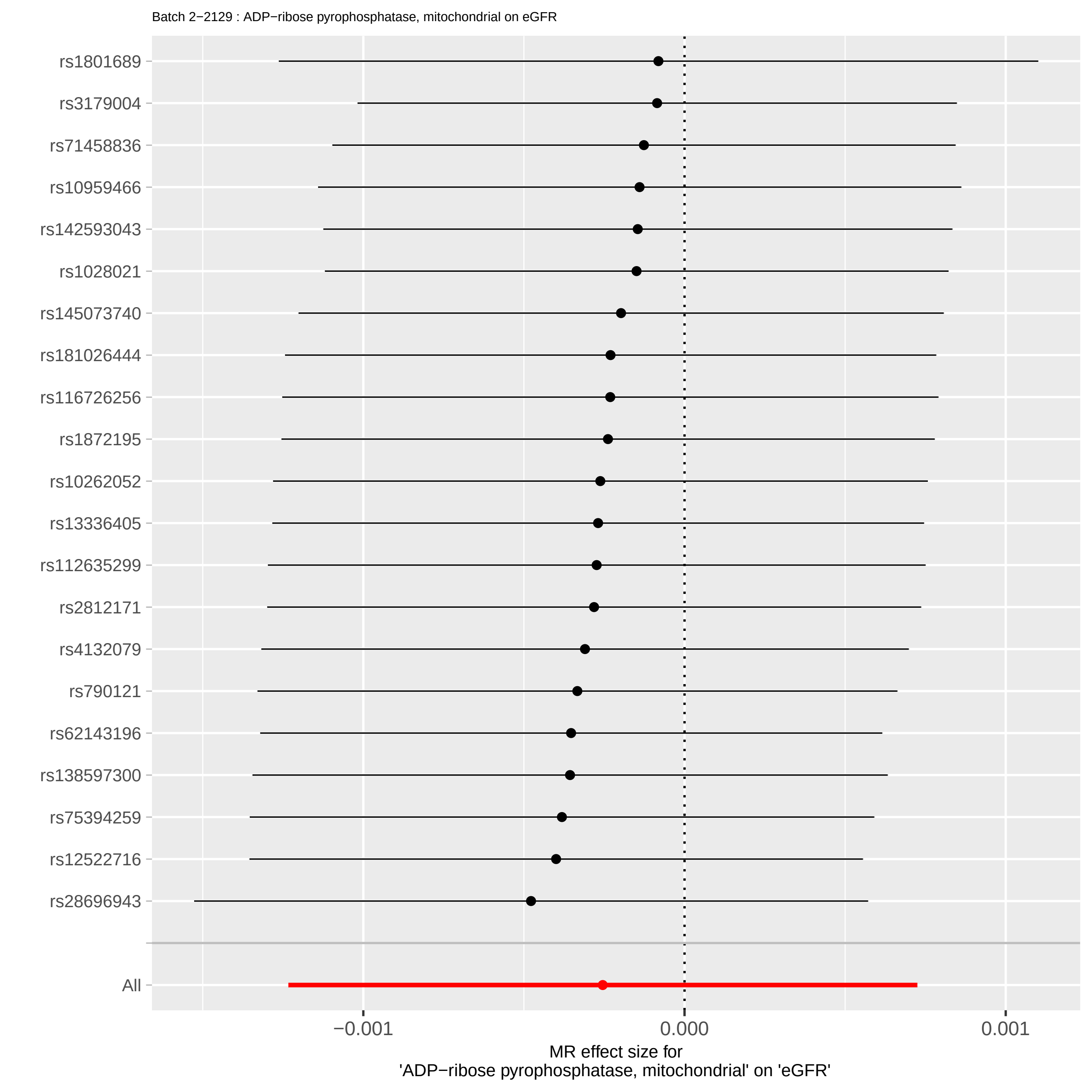

Batch 2−2129 : ADP−ribose pyrophosphatase, mitochondrial on eGFR
rs1801689
rs3179004
rs71458836
rs10959466
rs142593043
rs1028021
rs145073740
rs181026444
rs116726256
rs1872195
rs10262052
rs13336405
rs112635299
rs2812171
rs4132079
rs790121
rs62143196
rs138597300
rs75394259
rs12522716
rs28696943
All
−0.001
0.000
0.001
MR effect size for
'ADP−ribose pyrophosphatase, mitochondrial' on 'eGFR'

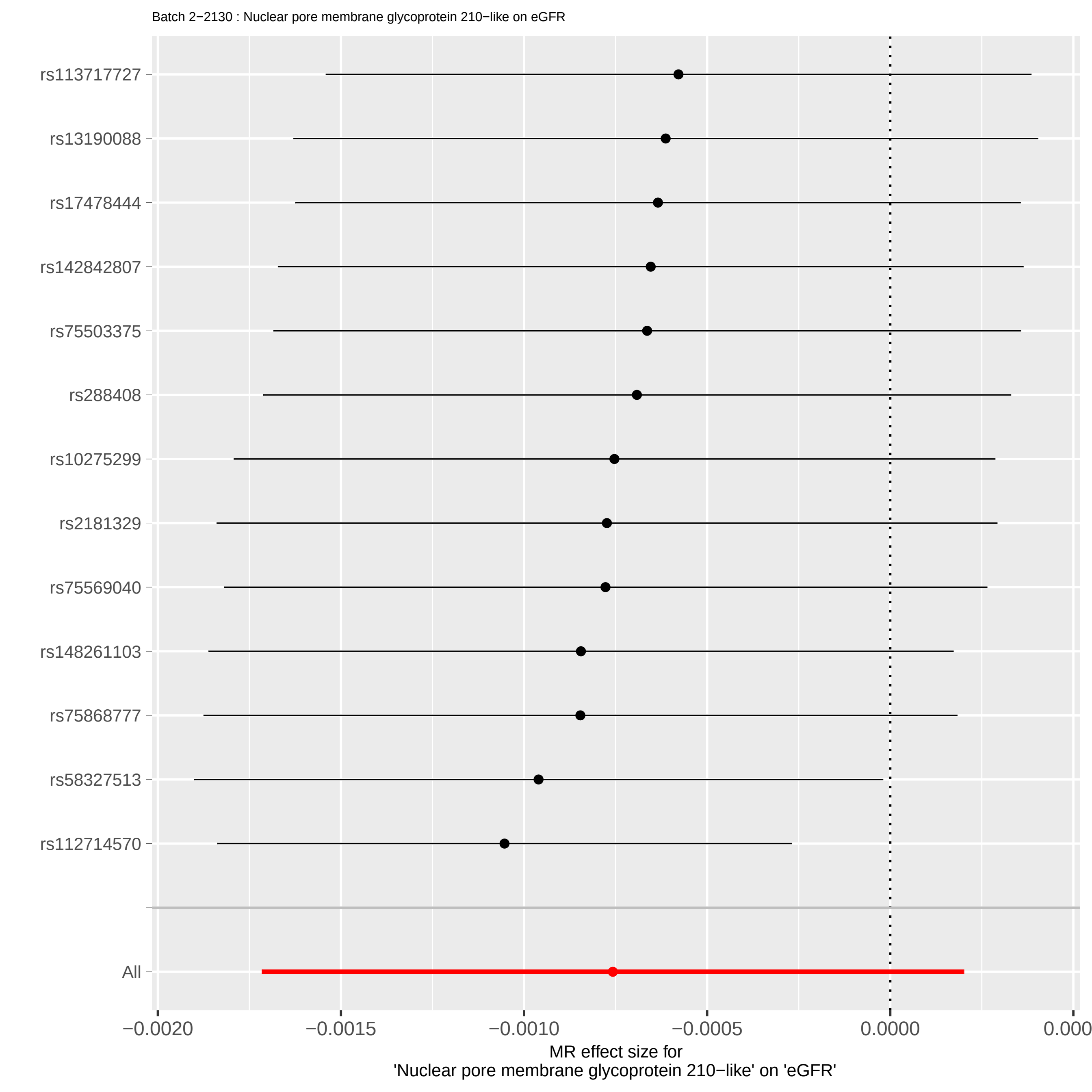

Batch 2−2130 : Nuclear pore membrane glycoprotein 210−like on eGFR
rs113717727
rs13190088
rs17478444
rs142842807
rs75503375
rs288408
rs10275299
rs2181329
rs75569040
rs148261103
rs75868777
rs58327513
rs112714570
All
−0.0020
−0.0015
−0.0010
−0.0005
0.0000
0.000
MR effect size for
'Nuclear pore membrane glycoprotein 210−like' on 'eGFR'

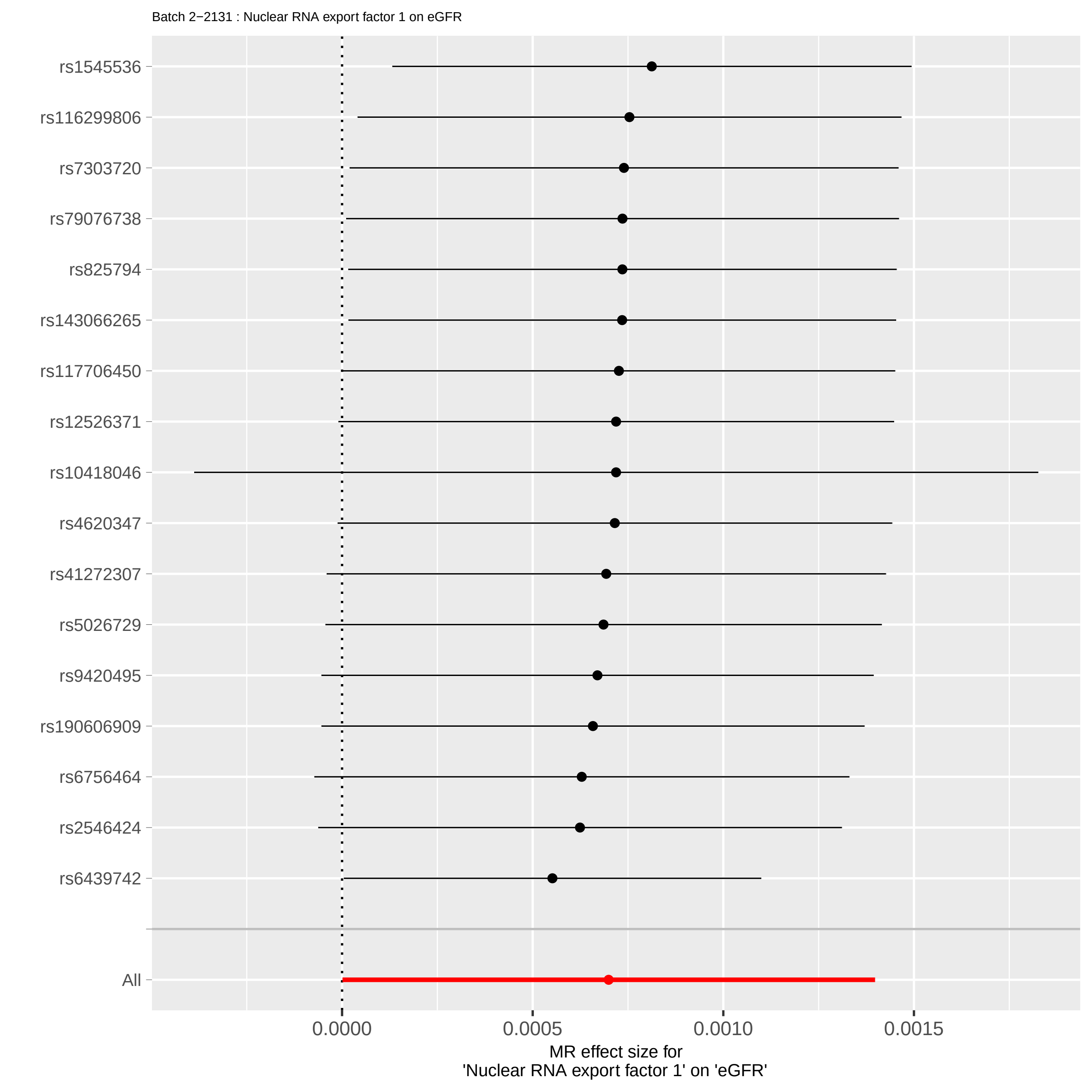
Batch 2−2131 : Nuclear RNA export factor 1 on eGFR
rs1545536
rs116299806
rs7303720
rs79076738
rs825794
rs143066265
rs117706450
rs12526371
rs10418046
rs4620347
rs41272307
rs5026729
rs9420495
rs190606909
rs6756464
rs2546424
rs6439742
All
0.0000
0.0005
0.0010
0.0015
MR effect size for
'Nuclear RNA export factor 1' on 'eGFR'

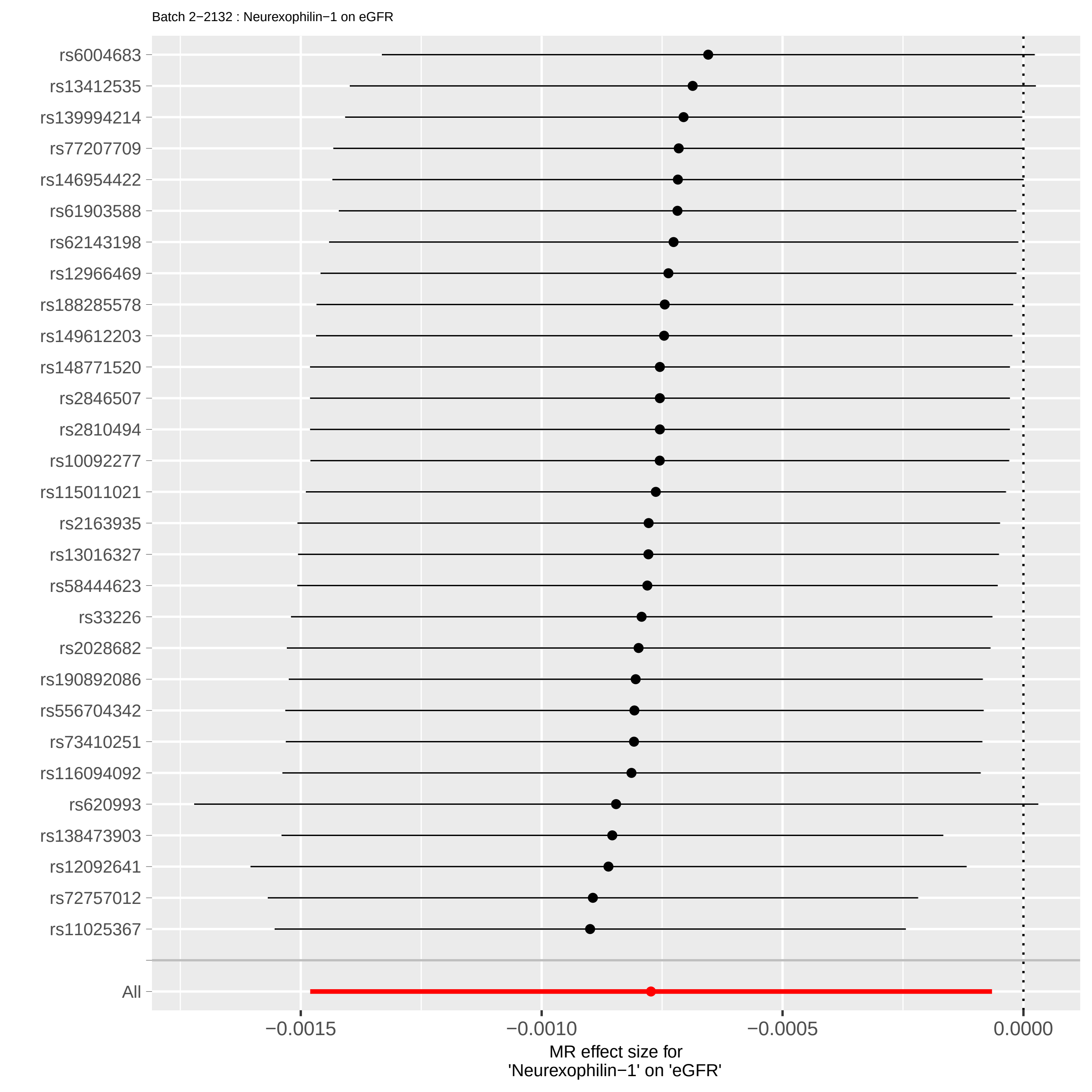

Batch 2−2132 : Neurexophilin−1 on eGFR
rs6004683
rs13412535
rs139994214
rs77207709
rs146954422
rs61903588
rs62143198
rs12966469
rs188285578
rs149612203
rs148771520
rs2846507
rs2810494
rs10092277
rs115011021
rs2163935
rs13016327
rs58444623
rs33226
rs2028682
rs190892086
rs556704342
rs73410251
rs116094092
rs620993
rs138473903
rs12092641
rs72757012
rs11025367
All
−0.0015
−0.0010
−0.0005
0.0000
MR effect size for
'Neurexophilin−1' on 'eGFR'

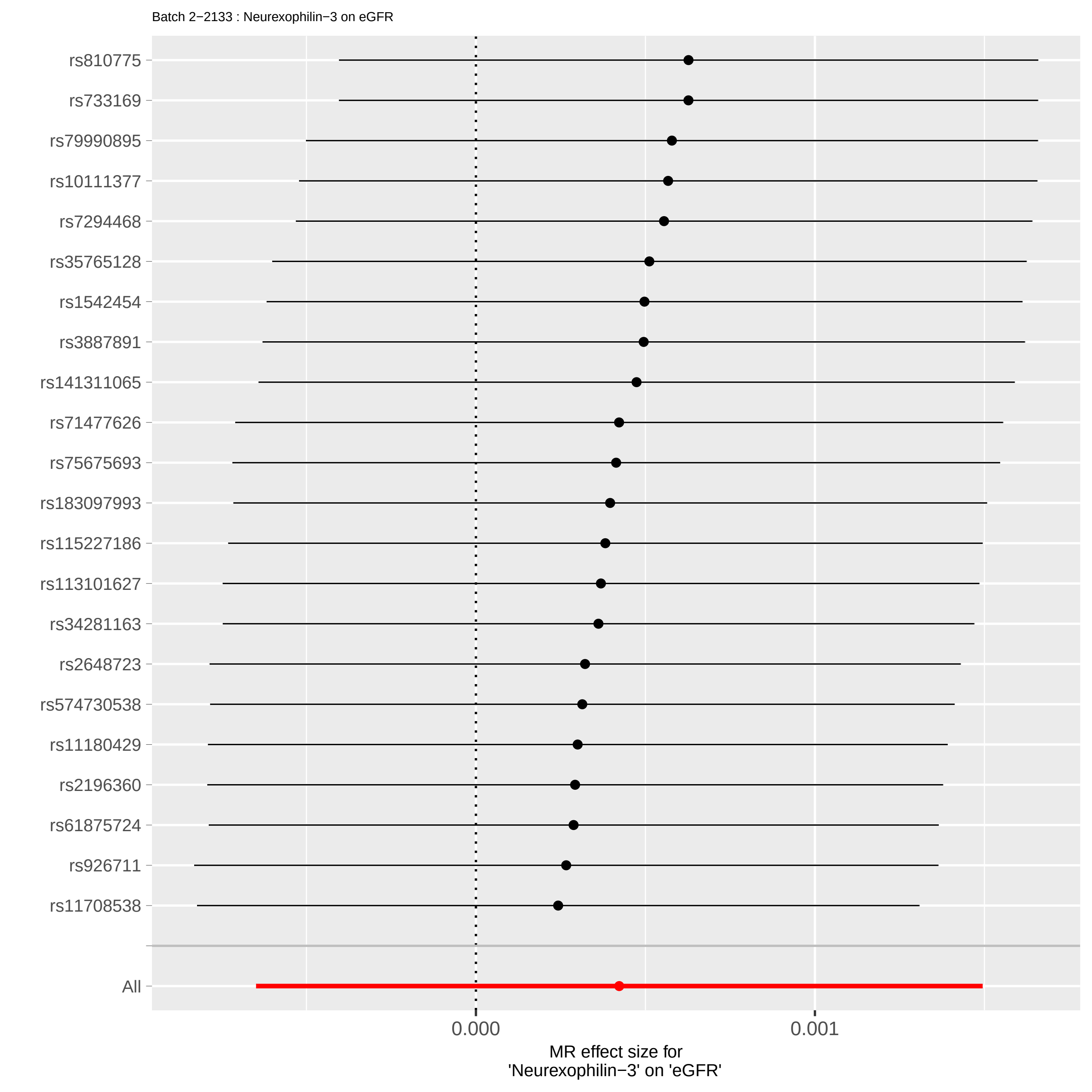

Batch 2−2133 : Neurexophilin−3 on eGFR
rs810775
rs733169
rs79990895
rs10111377
rs7294468
rs35765128
rs1542454
rs3887891
rs141311065
rs71477626
rs75675693
rs183097993
rs115227186
rs113101627
rs34281163
rs2648723
rs574730538
rs11180429
rs2196360
rs61875724
rs926711
rs11708538
All
0.000
0.001
MR effect size for
'Neurexophilin−3' on 'eGFR'

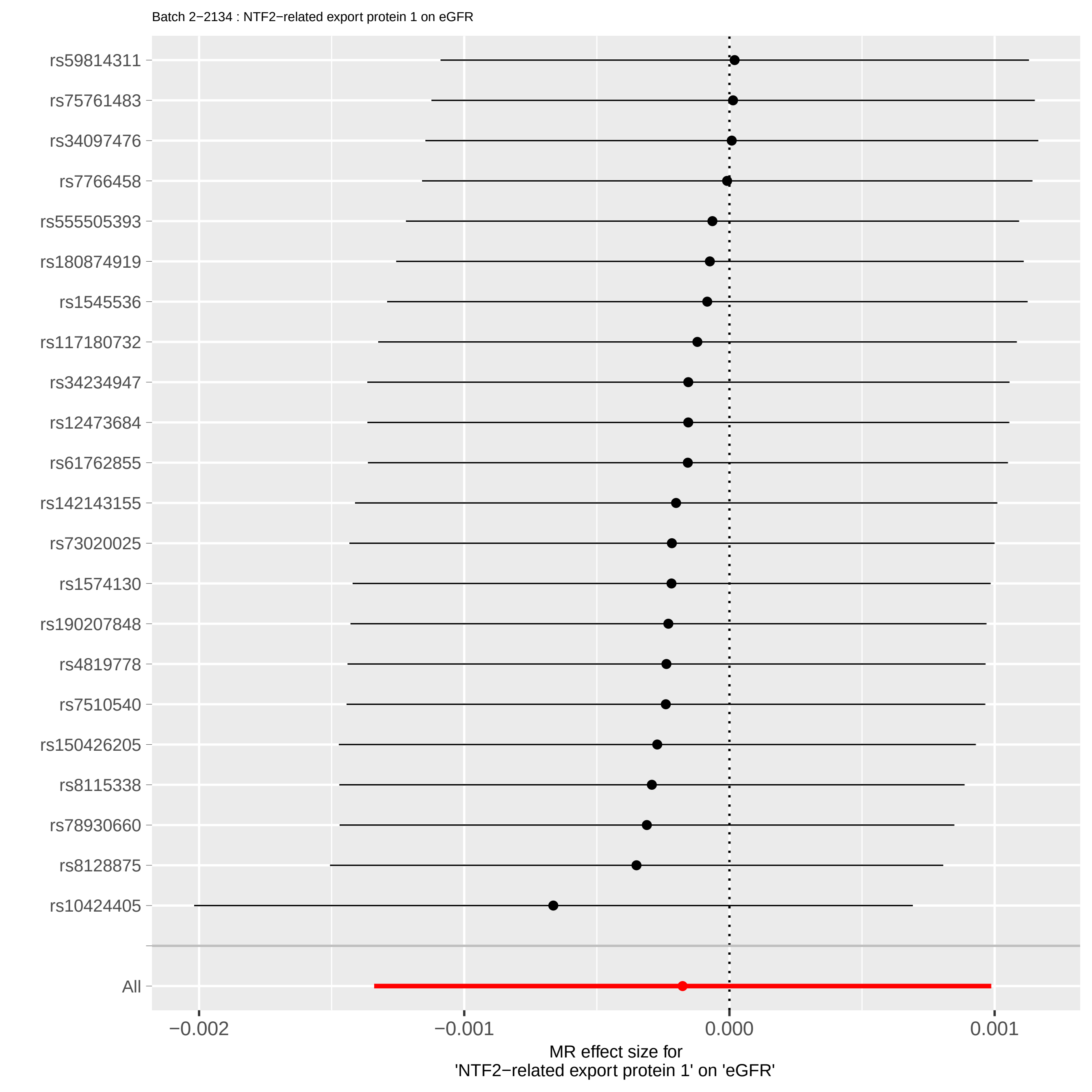

Batch 2−2134 : NTF2−related export protein 1 on eGFR
rs59814311
rs75761483
rs34097476
rs7766458
rs555505393
rs180874919
rs1545536
rs117180732
rs34234947
rs12473684
rs61762855
rs142143155
rs73020025
rs1574130
rs190207848
rs4819778
rs7510540
rs150426205
rs8115338
rs78930660
rs8128875
rs10424405
All
−0.002
−0.001
0.000
0.001
MR effect size for
'NTF2−related export protein 1' on 'eGFR'

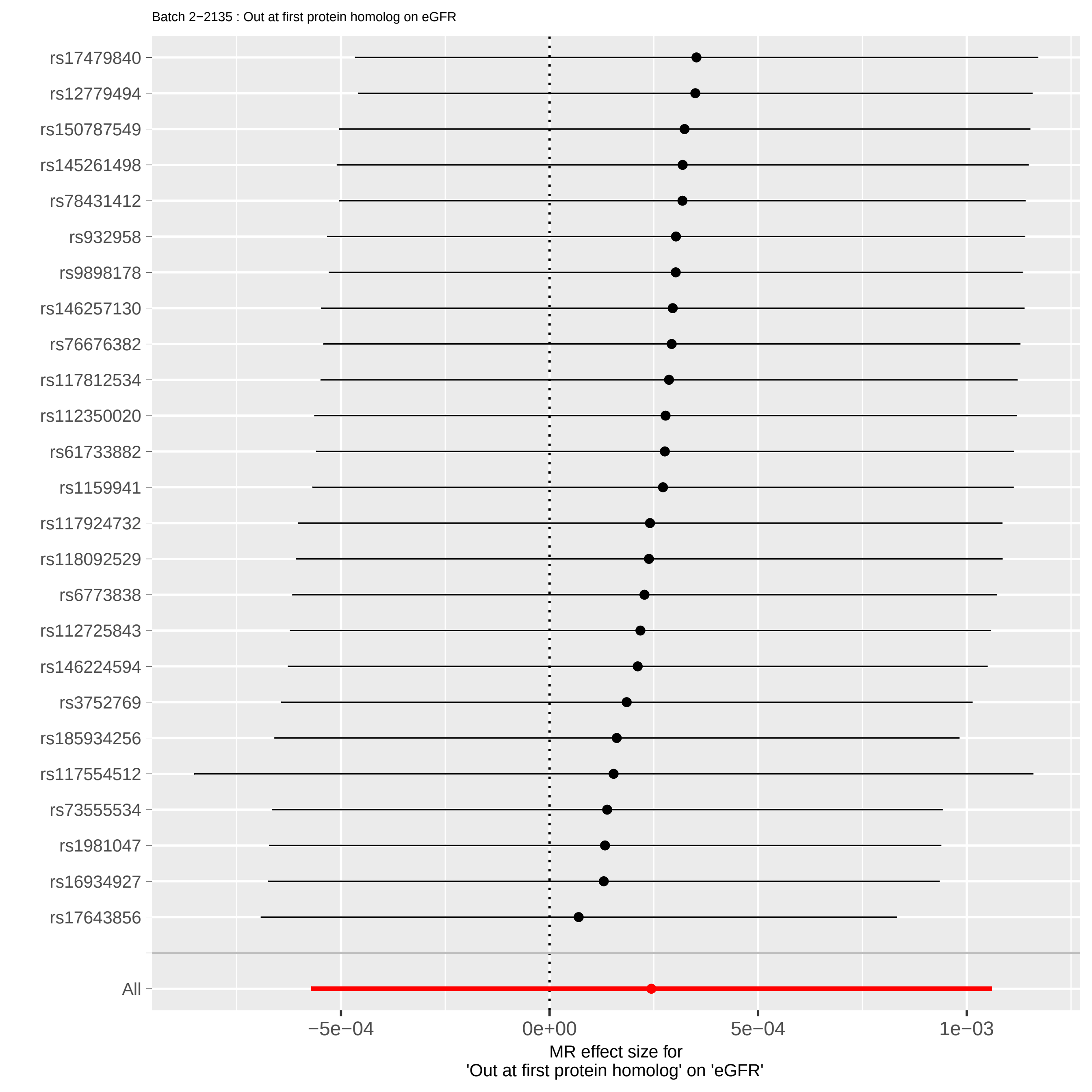

Batch 2−2135 : Out at first protein homolog on eGFR
rs17479840
rs12779494
rs150787549
rs145261498
rs78431412
rs932958
rs9898178
rs146257130
rs76676382
rs117812534
rs112350020
rs61733882
rs1159941
rs117924732
rs118092529
rs6773838
rs112725843
rs146224594
rs3752769
rs185934256
rs117554512
rs73555534
rs1981047
rs16934927
rs17643856
All
−5e−04
0e+00
5e−04
1e−03
MR effect size for
'Out at first protein homolog' on 'eGFR'

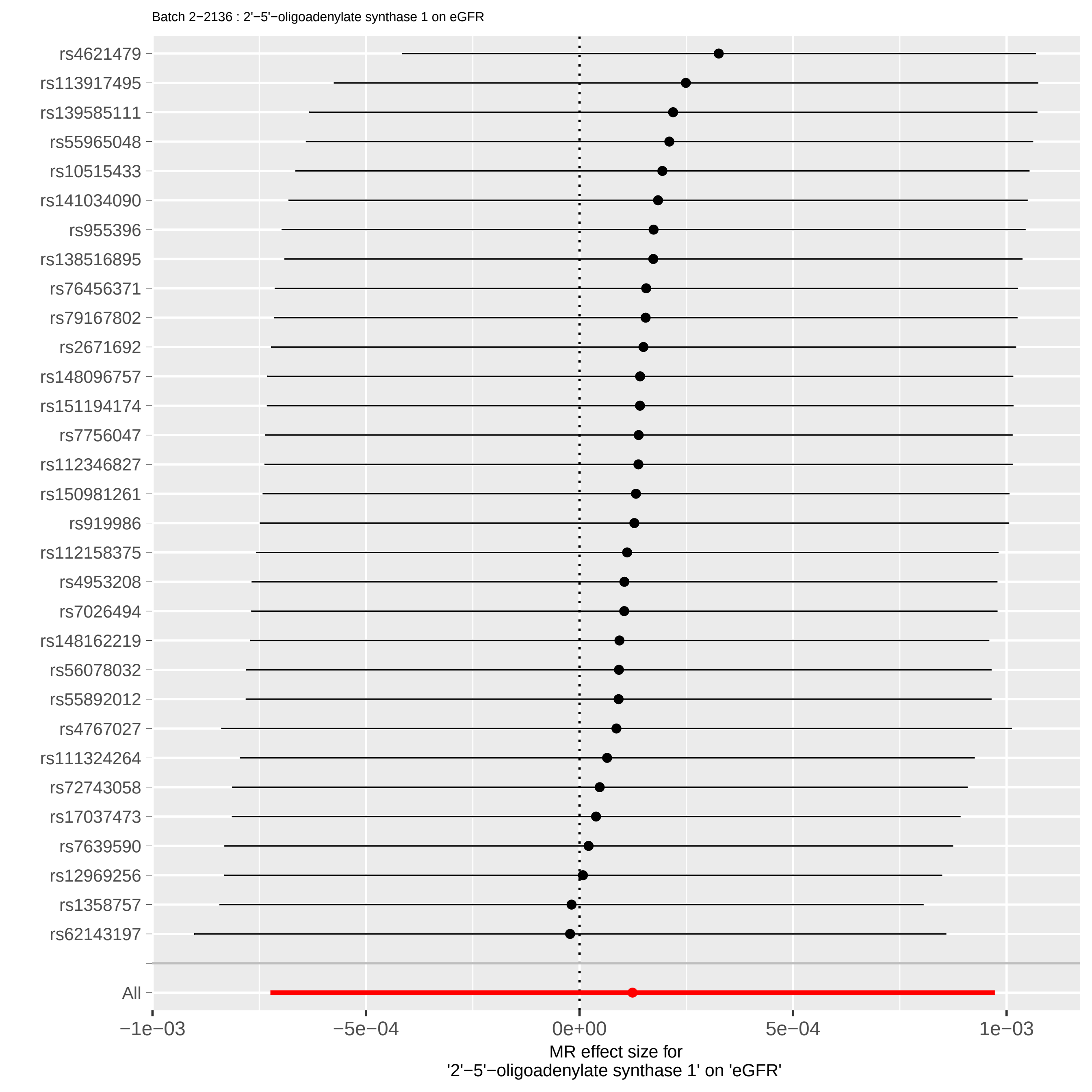

Batch 2−2136 : 2'−5'−oligoadenylate synthase 1 on eGFR
rs4621479
rs113917495
rs139585111
rs55965048
rs10515433
rs141034090
rs955396
rs138516895
rs76456371
rs79167802
rs2671692
rs148096757
rs151194174
rs7756047
rs112346827
rs150981261
rs919986
rs112158375
rs4953208
rs7026494
rs148162219
rs56078032
rs55892012
rs4767027
rs111324264
rs72743058
rs17037473
rs7639590
rs12969256
rs1358757
rs62143197
All
−1e−03
−5e−04
0e+00
5e−04
1e−03
MR effect size for
'2'−5'−oligoadenylate synthase 1' on 'eGFR'

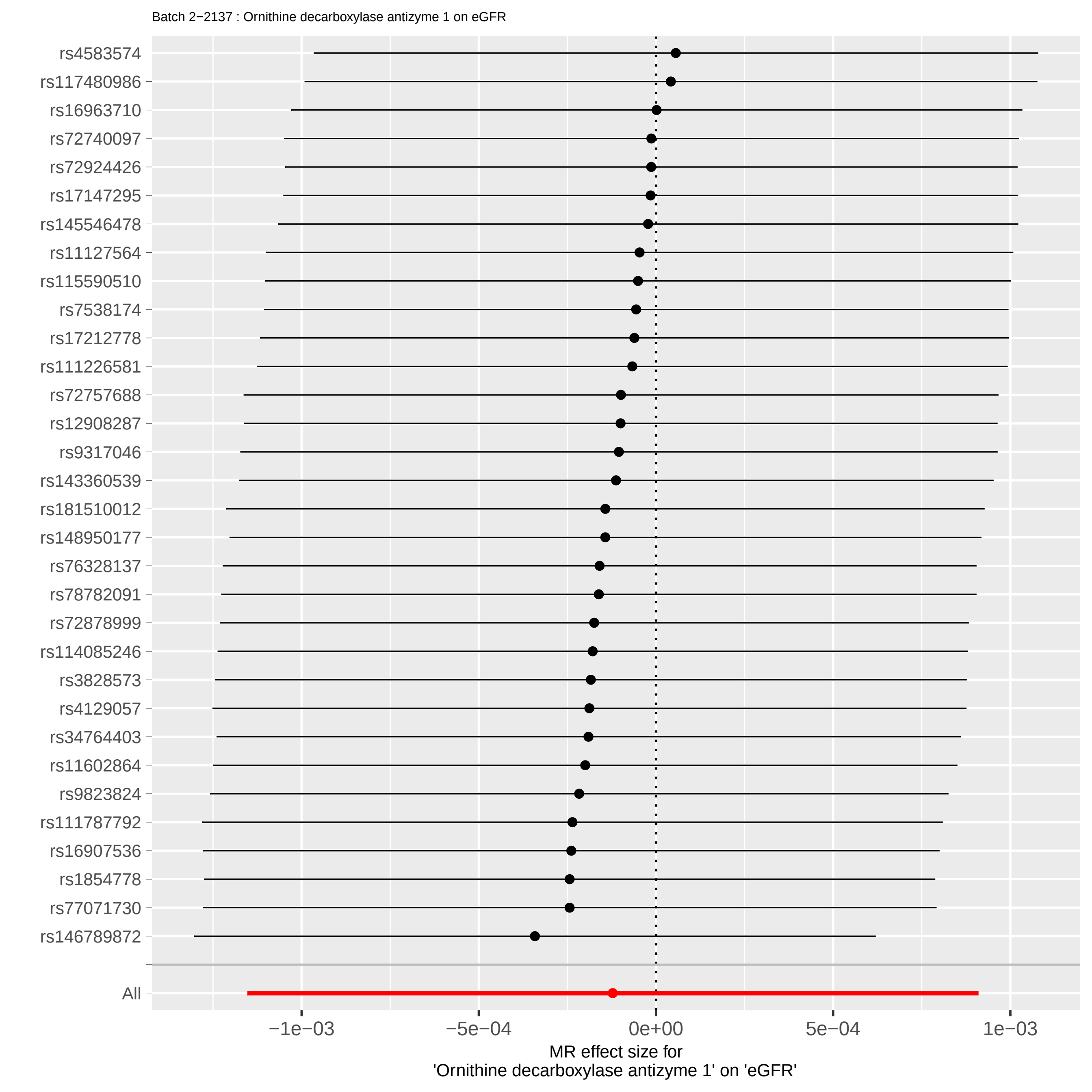

Batch 2−2137 : Ornithine decarboxylase antizyme 1 on eGFR
rs4583574
rs117480986
rs16963710
rs72740097
rs72924426
rs17147295
rs145546478
rs11127564
rs115590510
rs7538174
rs17212778
rs111226581
rs72757688
rs12908287
rs9317046
rs143360539
rs181510012
rs148950177
rs76328137
rs78782091
rs72878999
rs114085246
rs3828573
rs4129057
rs34764403
rs11602864
rs9823824
rs111787792
rs16907536
rs1854778
rs77071730
rs146789872
All
−1e−03
−5e−04
0e+00
5e−04
1e−03
MR effect size for
'Ornithine decarboxylase antizyme 1' on 'eGFR'

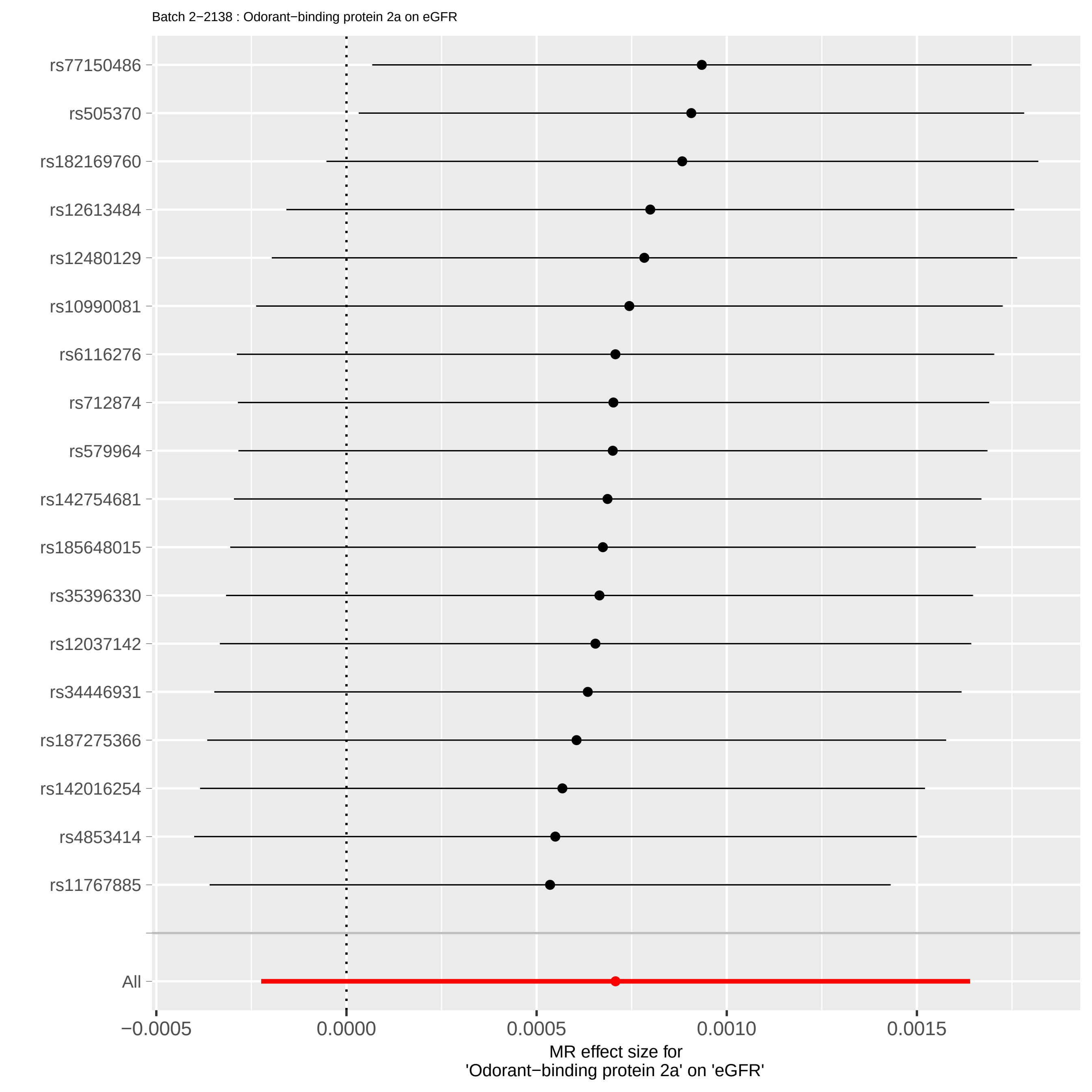

Batch 2−2138 : Odorant−binding protein 2a on eGFR
rs77150486
rs505370
rs182169760
rs12613484
rs12480129
rs10990081
rs6116276
rs712874
rs579964
rs142754681
rs185648015
rs35396330
rs12037142
rs34446931
rs187275366
rs142016254
rs4853414
rs11767885
All
−0.0005
0.0000
0.0005
0.0010
0.0015
MR effect size for
'Odorant−binding protein 2a' on 'eGFR'

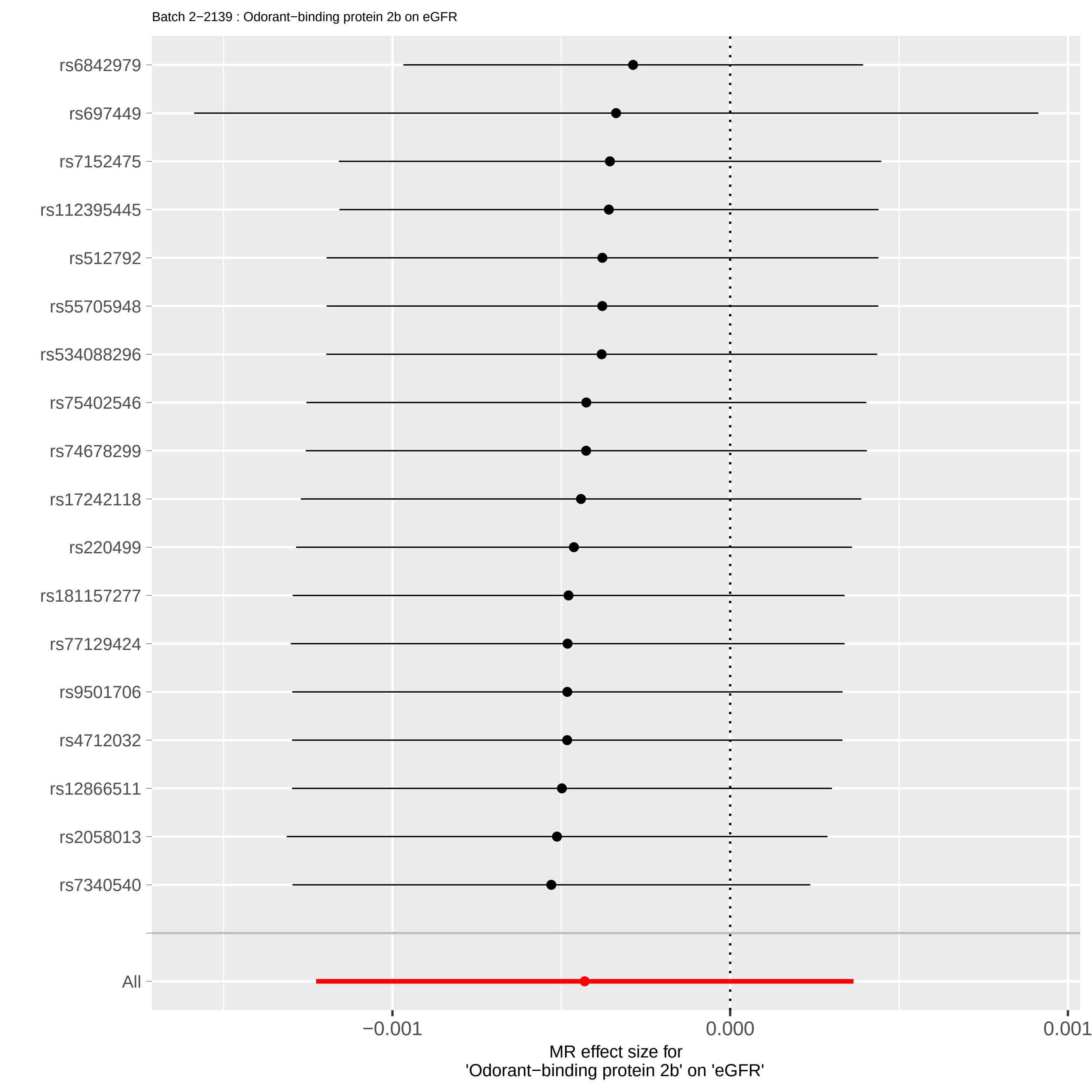
Batch 2−2139 : Odorant−binding protein 2b on eGFR
rs6842979
rs697449
rs7152475
rs112395445
rs512792
rs55705948
rs534088296
rs75402546
rs74678299
rs17242118
rs220499
rs181157277
rs77129424
rs9501706
rs4712032
rs12866511
rs2058013
rs7340540
All
−0.001
0.000
0.001
MR effect size for
'Odorant−binding protein 2b' on 'eGFR'

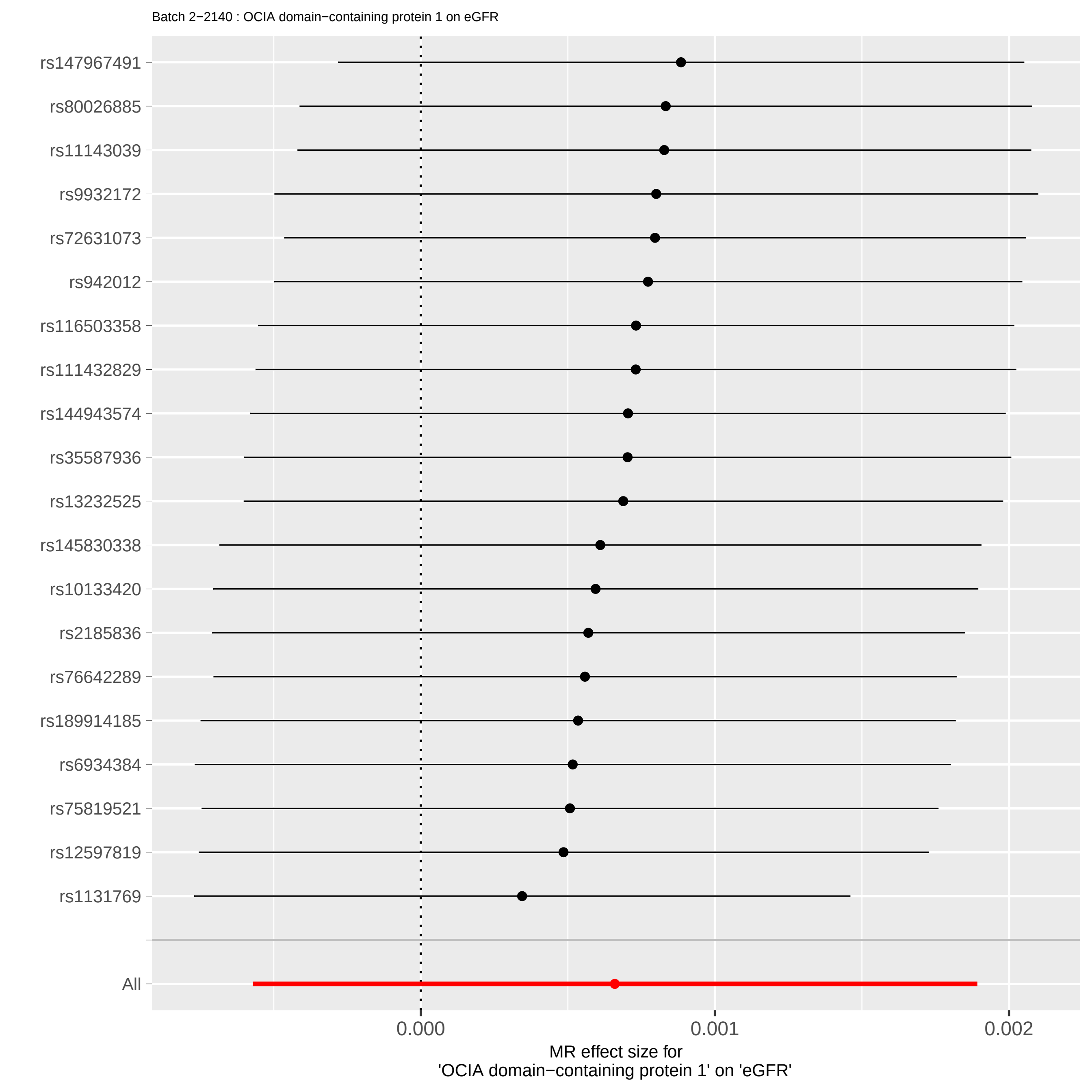

Batch 2−2140 : OCIA domain−containing protein 1 on eGFR
rs147967491
rs80026885
rs11143039
rs9932172
rs72631073
rs942012
rs116503358
rs111432829
rs144943574
rs35587936
rs13232525
rs145830338
rs10133420
rs2185836
rs76642289
rs189914185
rs6934384
rs75819521
rs12597819
rs1131769
All
0.000
0.001
0.002
MR effect size for
'OCIA domain−containing protein 1' on 'eGFR'

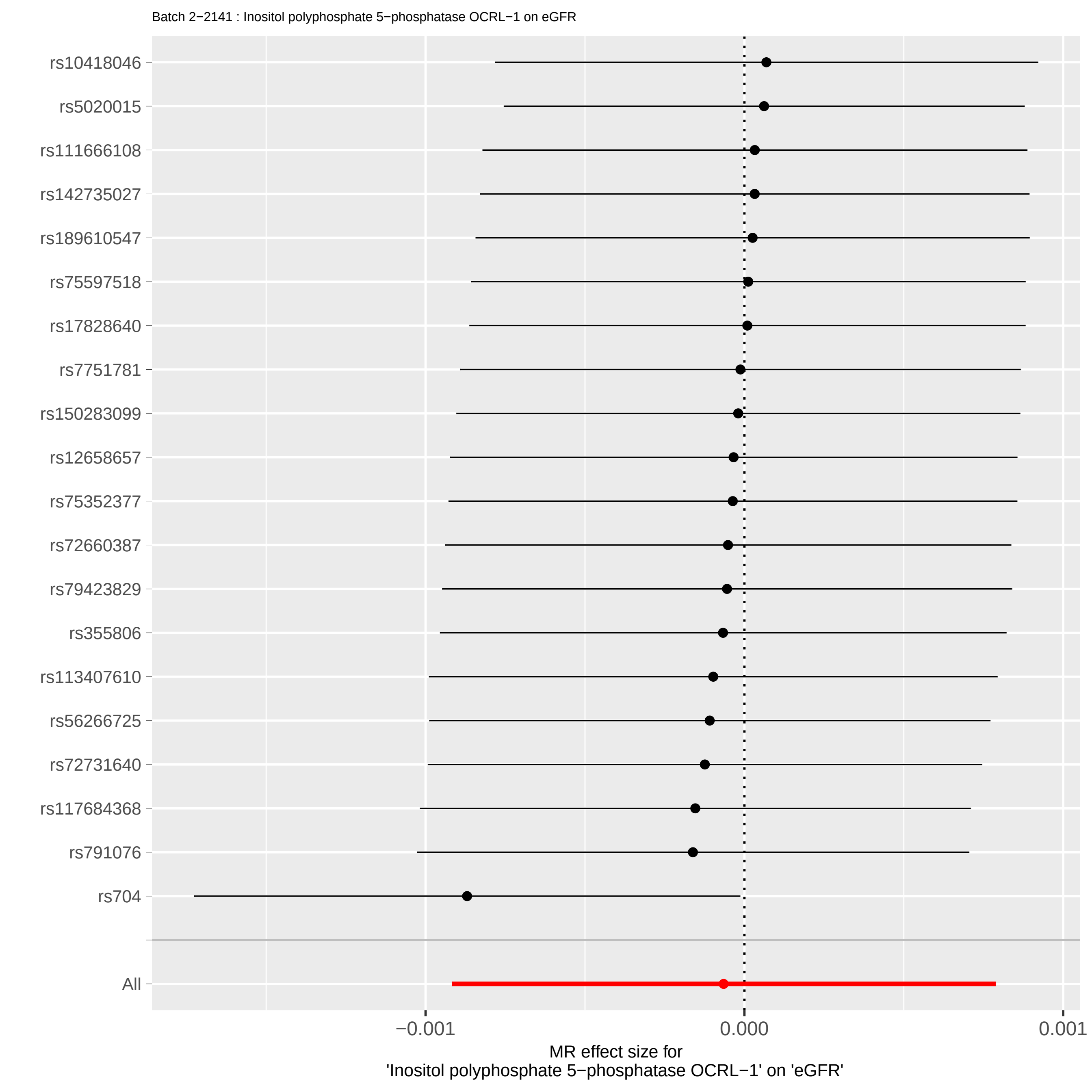
Batch 2−2141 : Inositol polyphosphate 5−phosphatase OCRL−1 on eGFR
rs10418046
rs5020015
rs111666108
rs142735027
rs189610547
rs75597518
rs17828640
rs7751781
rs150283099
rs12658657
rs75352377
rs72660387
rs79423829
rs355806
rs113407610
rs56266725
rs72731640
rs117684368
rs791076
rs704
All
−0.001
0.000
0.001
MR effect size for
'Inositol polyphosphate 5−phosphatase OCRL−1' on 'eGFR'

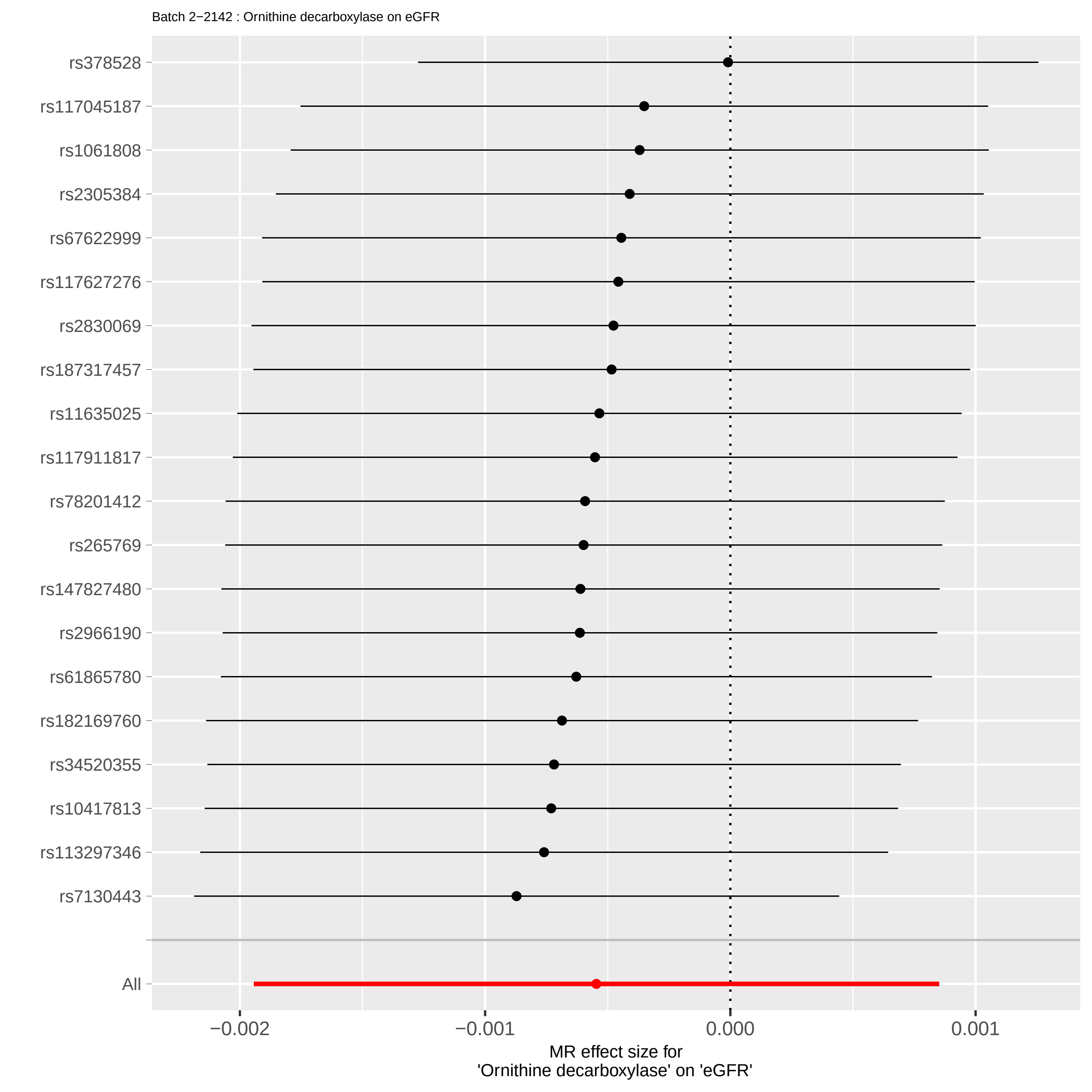

Batch 2−2142 : Ornithine decarboxylase on eGFR
rs378528
rs117045187
rs1061808
rs2305384
rs67622999
rs117627276
rs2830069
rs187317457
rs11635025
rs117911817
rs78201412
rs265769
rs147827480
rs2966190
rs61865780
rs182169760
rs34520355
rs10417813
rs113297346
rs7130443
All
−0.002
−0.001
0.000
0.001
MR effect size for
'Ornithine decarboxylase' on 'eGFR'

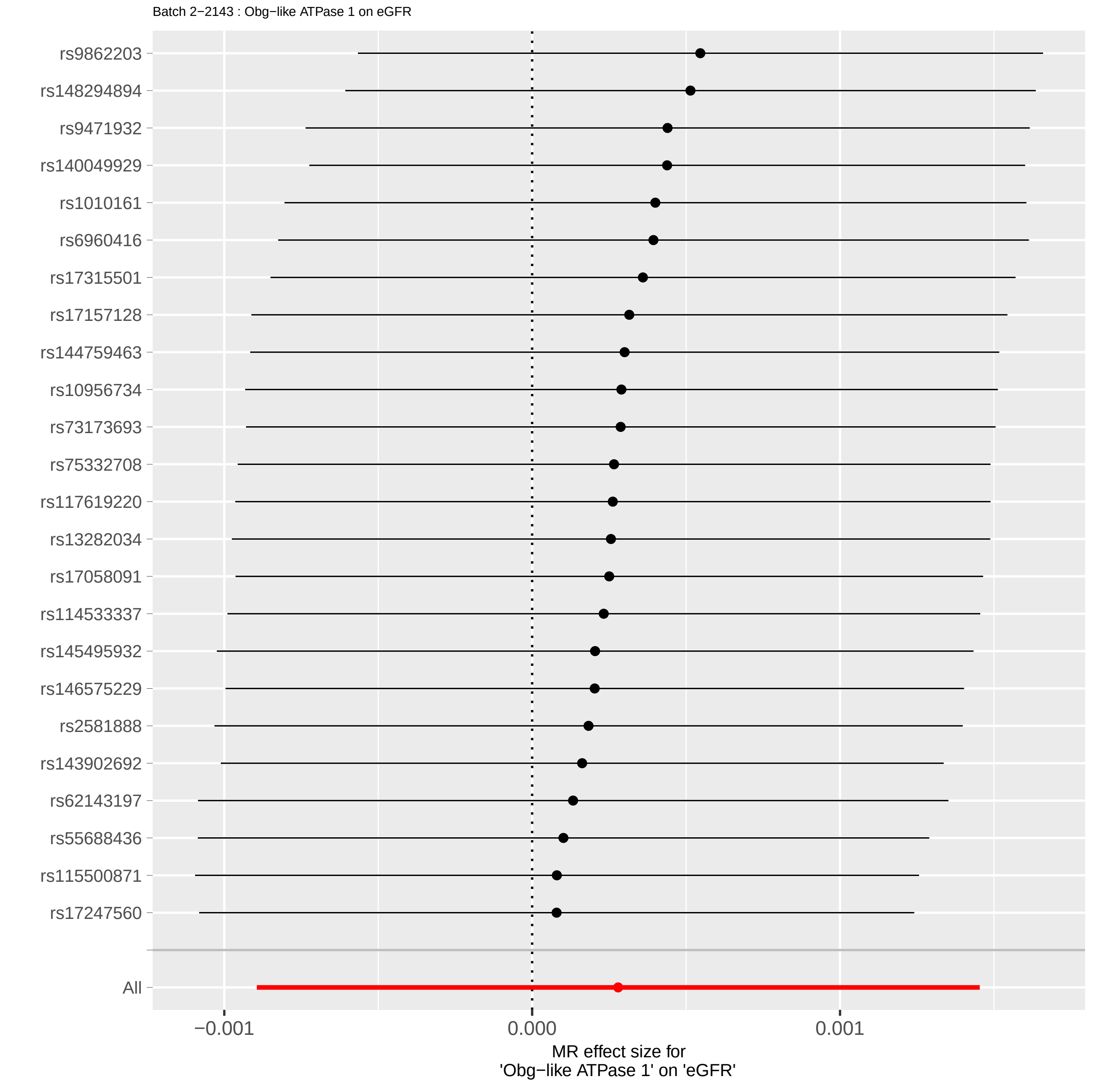
Batch 2−2143 : Obg−like ATPase 1 on eGFR
rs9862203
rs148294894
rs9471932
rs140049929
rs1010161
rs6960416
rs17315501
rs17157128
rs144759463
rs10956734
rs73173693
rs75332708
rs117619220
rs13282034
rs17058091
rs114533337
rs145495932
rs146575229
rs2581888
rs143902692
rs62143197
rs55688436
rs115500871
rs17247560
All
−0.001
0.000
0.001
MR effect size for
'Obg−like ATPase 1' on 'eGFR'

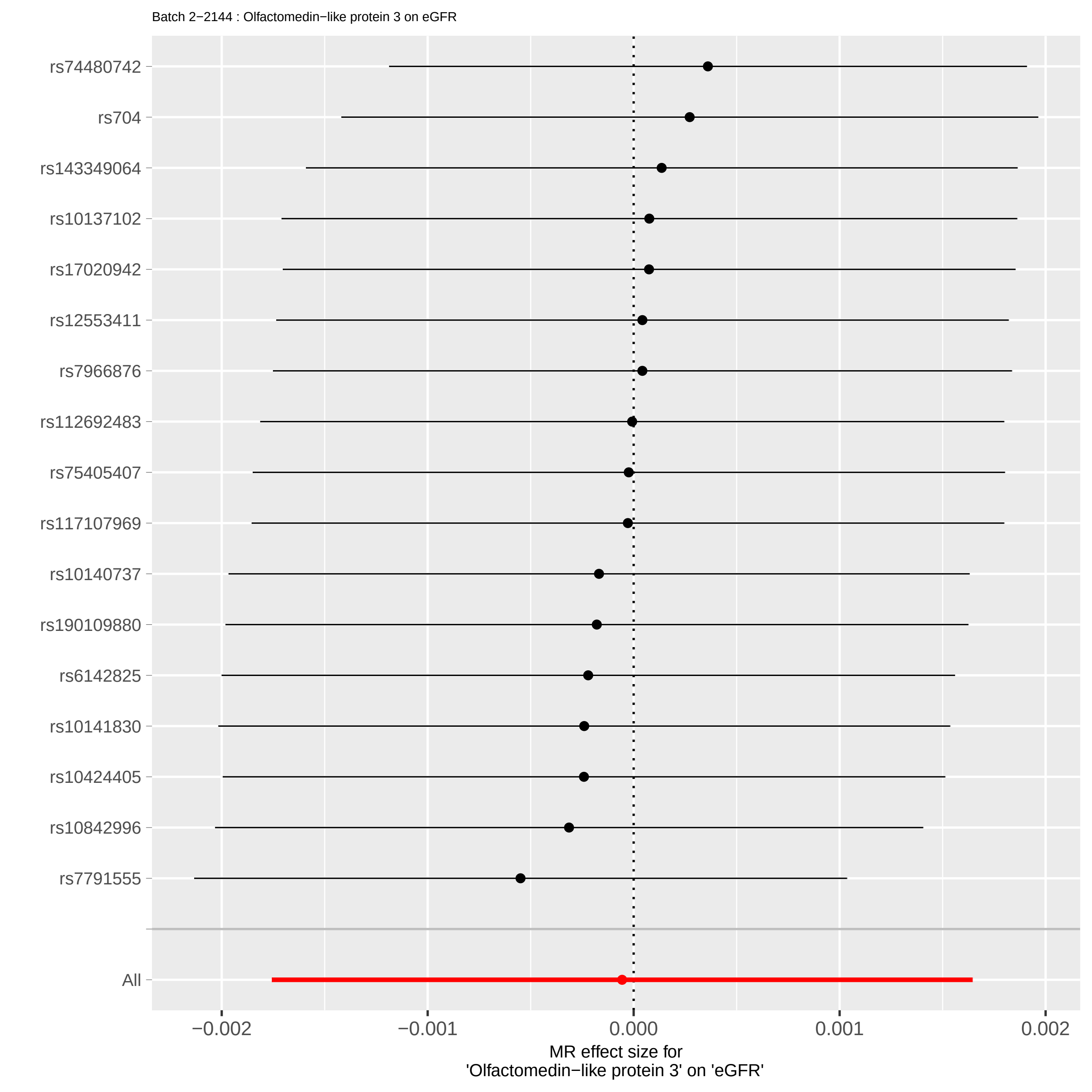

Batch 2−2144 : Olfactomedin−like protein 3 on eGFR
rs74480742
rs704
rs143349064
rs10137102
rs17020942
rs12553411
rs7966876
rs112692483
rs75405407
rs117107969
rs10140737
rs190109880
rs6142825
rs10141830
rs10424405
rs10842996
rs7791555
All
−0.002
−0.001
0.000
0.001
0.002
MR effect size for
'Olfactomedin−like protein 3' on 'eGFR'

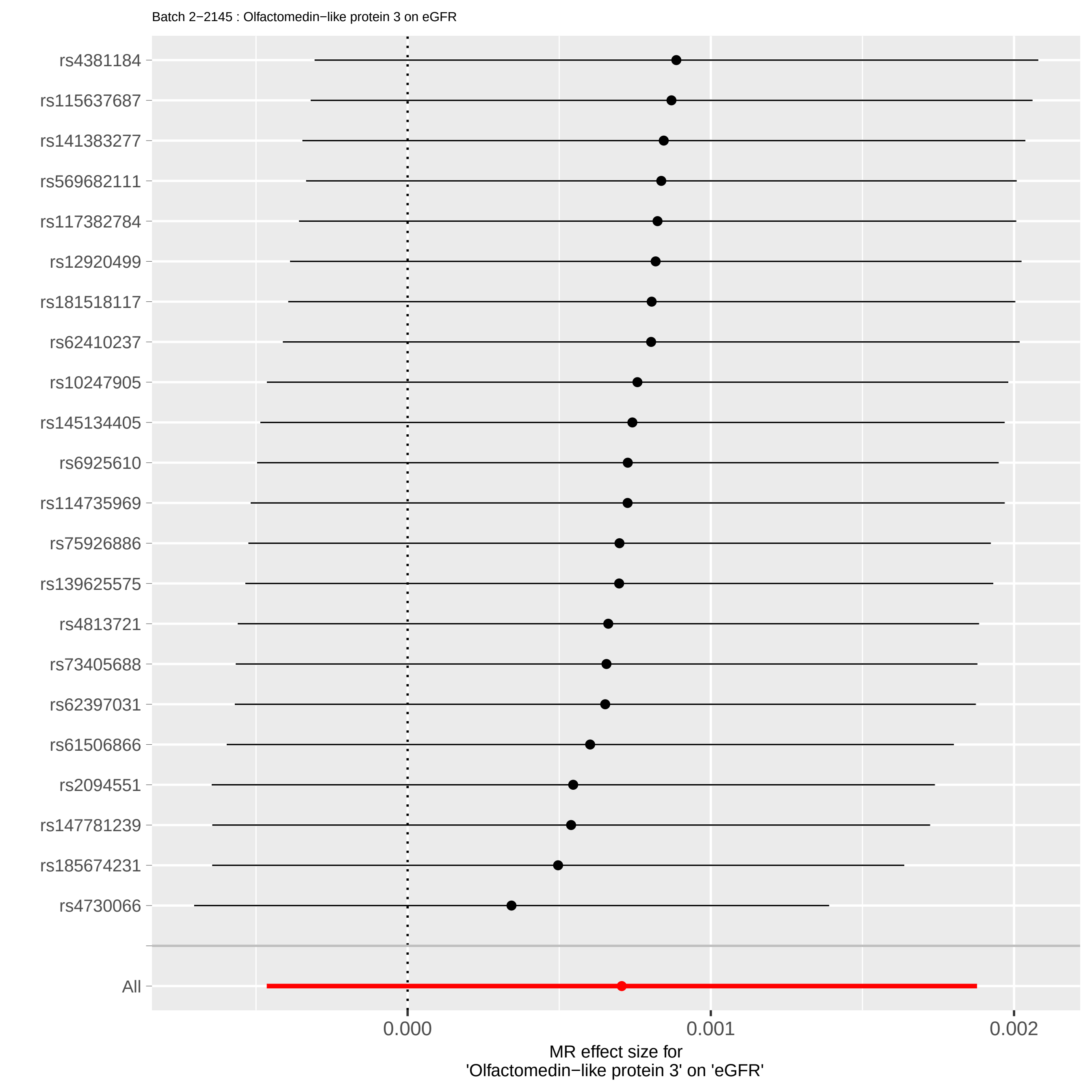

Batch 2−2145 : Olfactomedin−like protein 3 on eGFR
rs4381184
rs115637687
rs141383277
rs569682111
rs117382784
rs12920499
rs181518117
rs62410237
rs10247905
rs145134405
rs6925610
rs114735969
rs75926886
rs139625575
rs4813721
rs73405688
rs62397031
rs61506866
rs2094551
rs147781239
rs185674231
rs4730066
All
0.000
0.001
0.002
MR effect size for
'Olfactomedin−like protein 3' on 'eGFR'

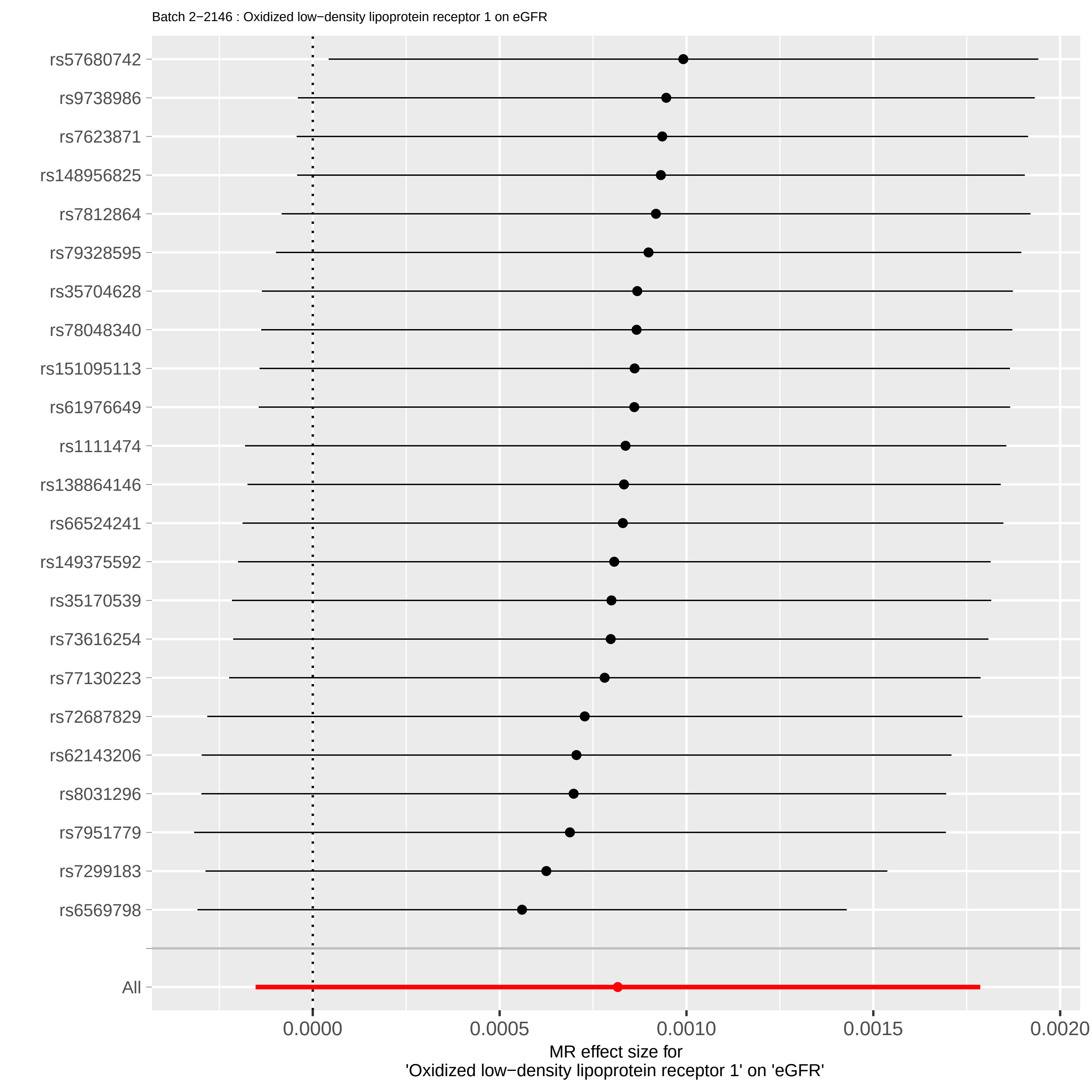

Batch 2−2146 : Oxidized low−density lipoprotein receptor 1 on eGFR
rs57680742
rs9738986
rs7623871
rs148956825
rs7812864
rs79328595
rs35704628
rs78048340
rs151095113
rs61976649
rs1111474
rs138864146
rs66524241
rs149375592
rs35170539
rs73616254
rs77130223
rs72687829
rs62143206
rs8031296
rs7951779
rs7299183
rs6569798
All
0.0000
0.0005
0.0010
0.0015
0.0020
MR effect size for
'Oxidized low−density lipoprotein receptor 1' on 'eGFR'

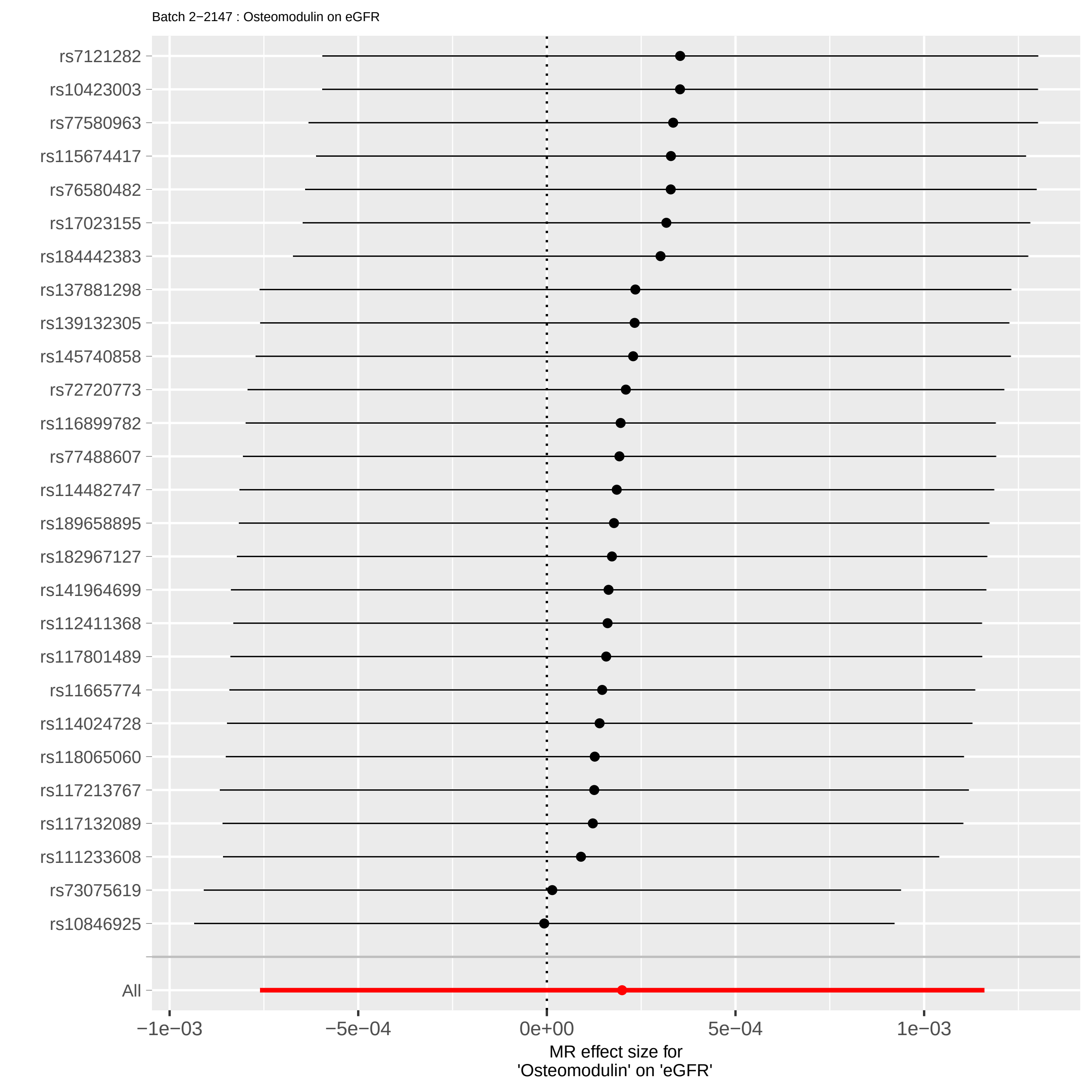

Batch 2−2147 : Osteomodulin on eGFR
rs7121282
rs10423003
rs77580963
rs115674417
rs76580482
rs17023155
rs184442383
rs137881298
rs139132305
rs145740858
rs72720773
rs116899782
rs77488607
rs114482747
rs189658895
rs182967127
rs141964699
rs112411368
rs117801489
rs11665774
rs114024728
rs118065060
rs117213767
rs117132089
rs111233608
rs73075619
rs10846925
All
−1e−03
−5e−04
0e+00
5e−04
1e−03
MR effect size for
'Osteomodulin' on 'eGFR'

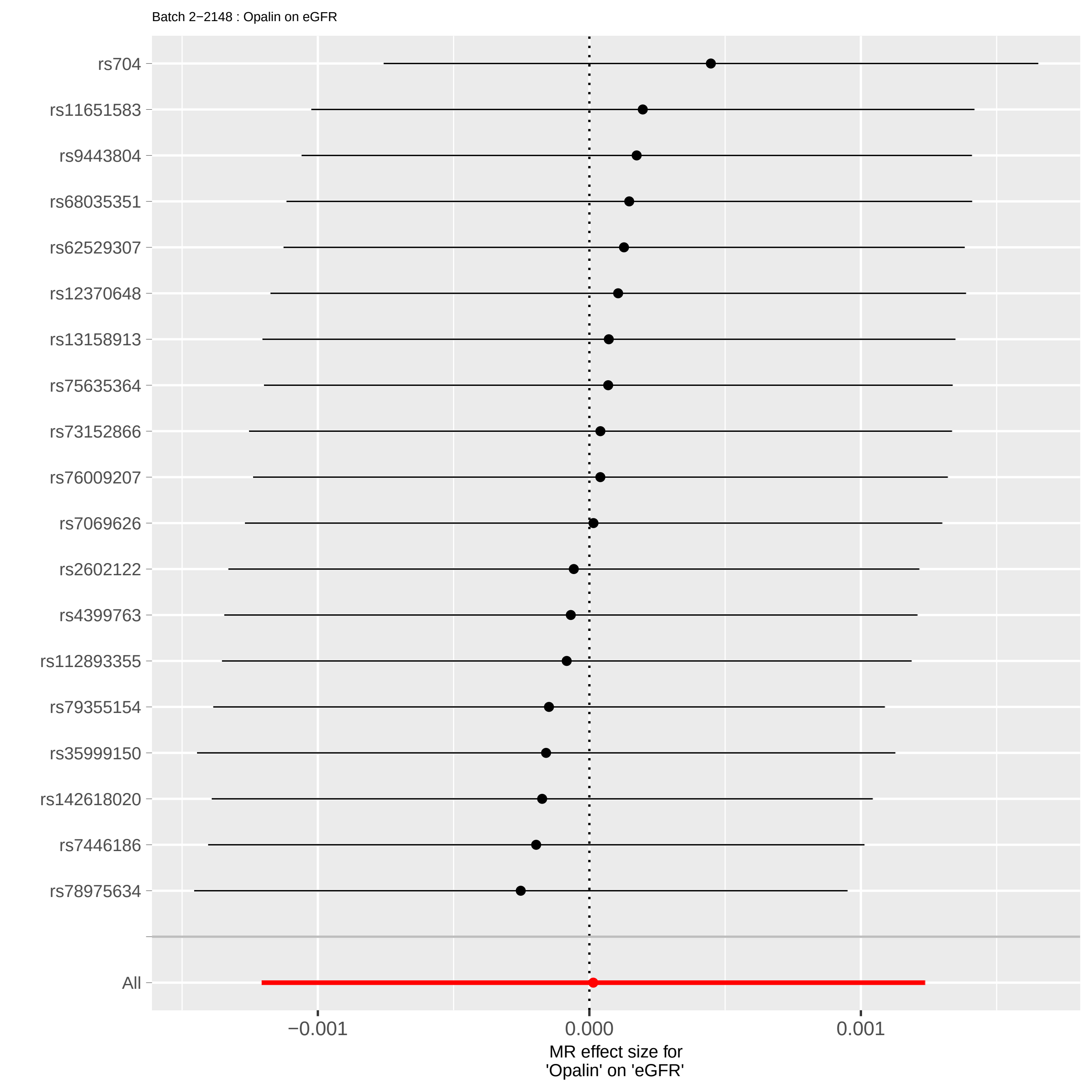
Batch 2−2148 : Opalin on eGFR
rs704
rs11651583
rs9443804
rs68035351
rs62529307
rs12370648
rs13158913
rs75635364
rs73152866
rs76009207
rs7069626
rs2602122
rs4399763
rs112893355
rs79355154
rs35999150
rs142618020
rs7446186
rs78975634
All
−0.001
0.000
0.001
MR effect size for
'Opalin' on 'eGFR'

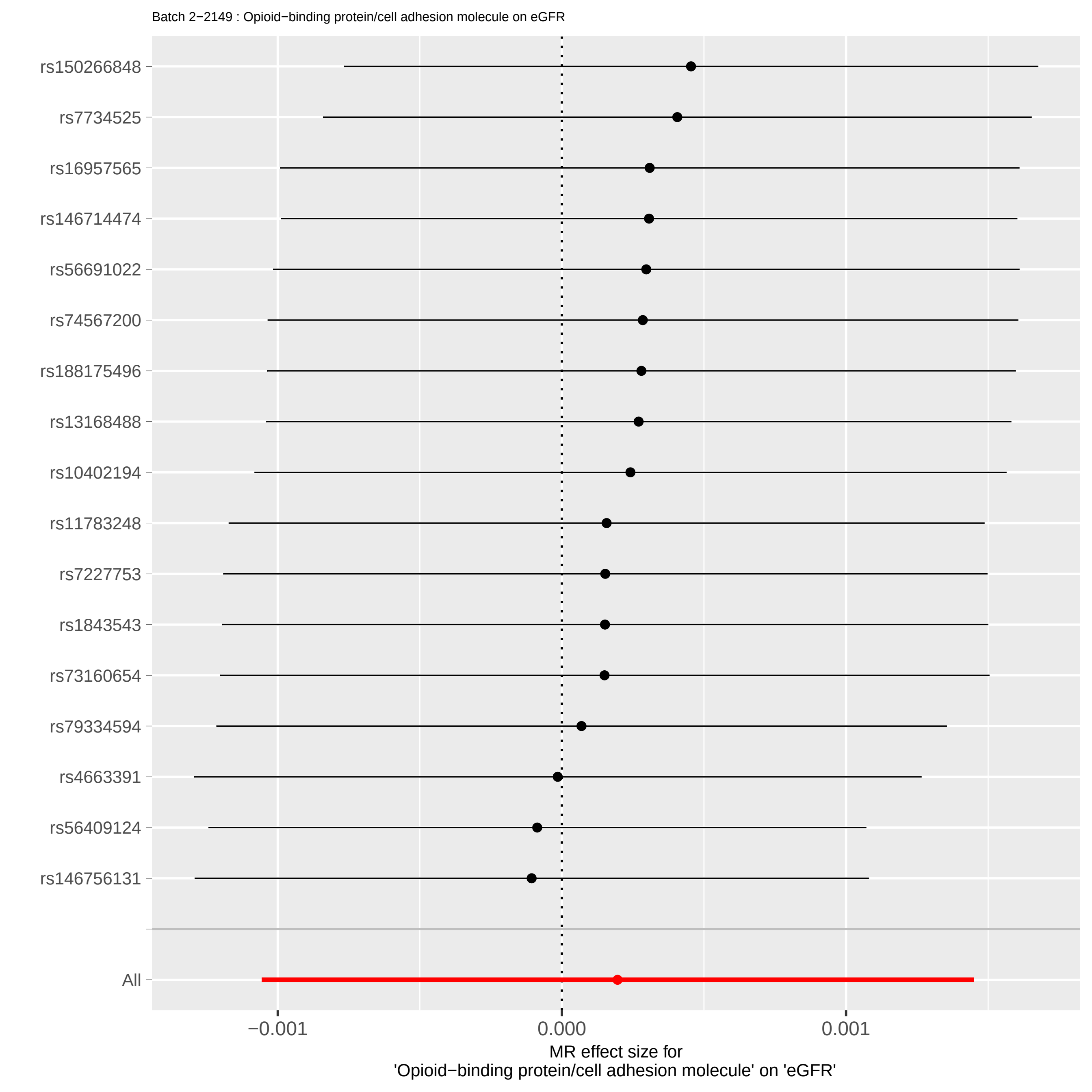
Batch 2−2149 : Opioid−binding protein/cell adhesion molecule on eGFR
rs150266848
rs7734525
rs16957565
rs146714474
rs56691022
rs74567200
rs188175496
rs13168488
rs10402194
rs11783248
rs7227753
rs1843543
rs73160654
rs79334594
rs4663391
rs56409124
rs146756131
All
−0.001
0.000
0.001
MR effect size for
'Opioid−binding protein/cell adhesion molecule' on 'eGFR'

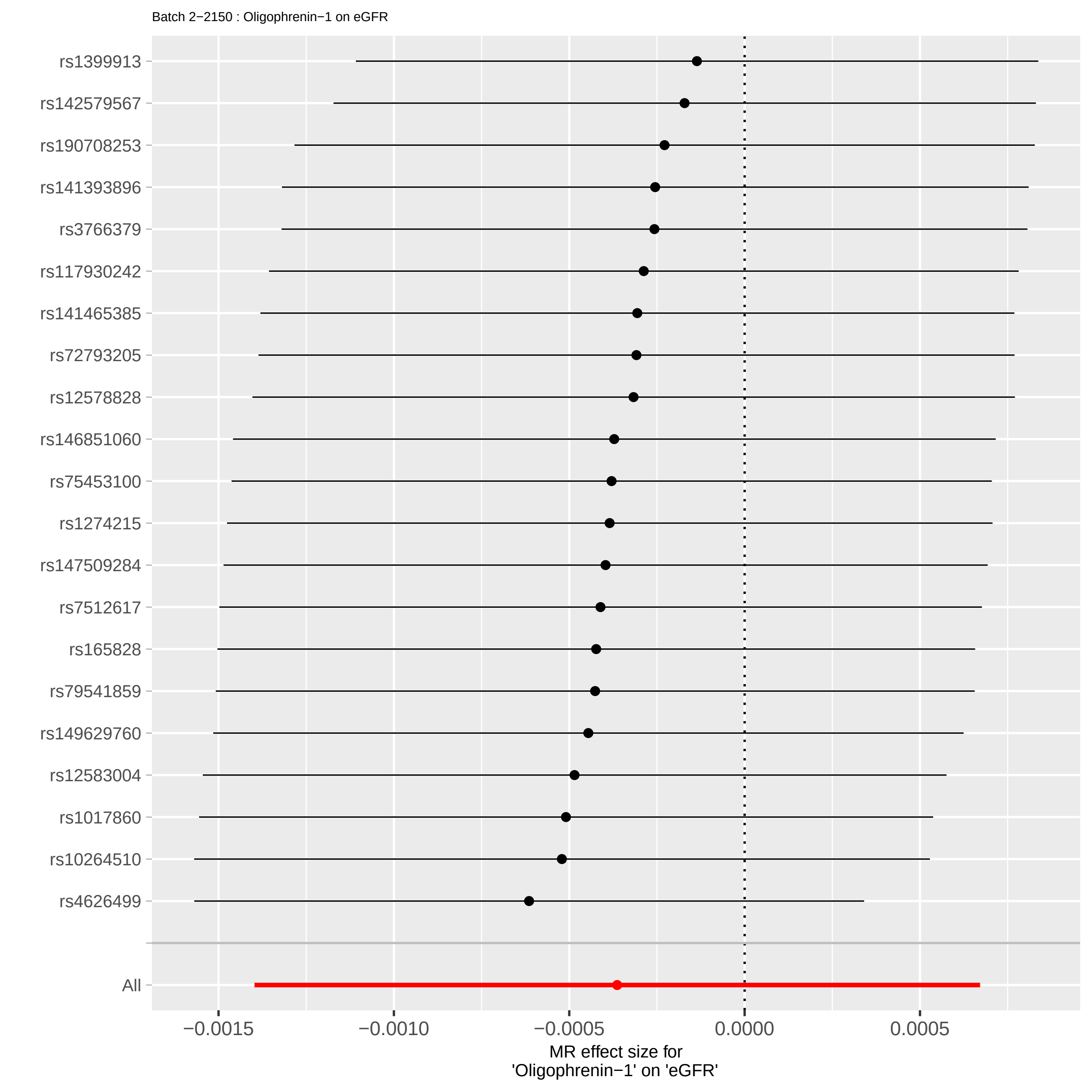

Batch 2−2150 : Oligophrenin−1 on eGFR
rs1399913
rs142579567
rs190708253
rs141393896
rs3766379
rs117930242
rs141465385
rs72793205
rs12578828
rs146851060
rs75453100
rs1274215
rs147509284
rs7512617
rs165828
rs79541859
rs149629760
rs12583004
rs1017860
rs10264510
rs4626499
All
−0.0015
−0.0010
−0.0005
0.0000
0.0005
MR effect size for
'Oligophrenin−1' on 'eGFR'

Batch 2−2151 : Origin recognition complex subunit 6 on eGFR

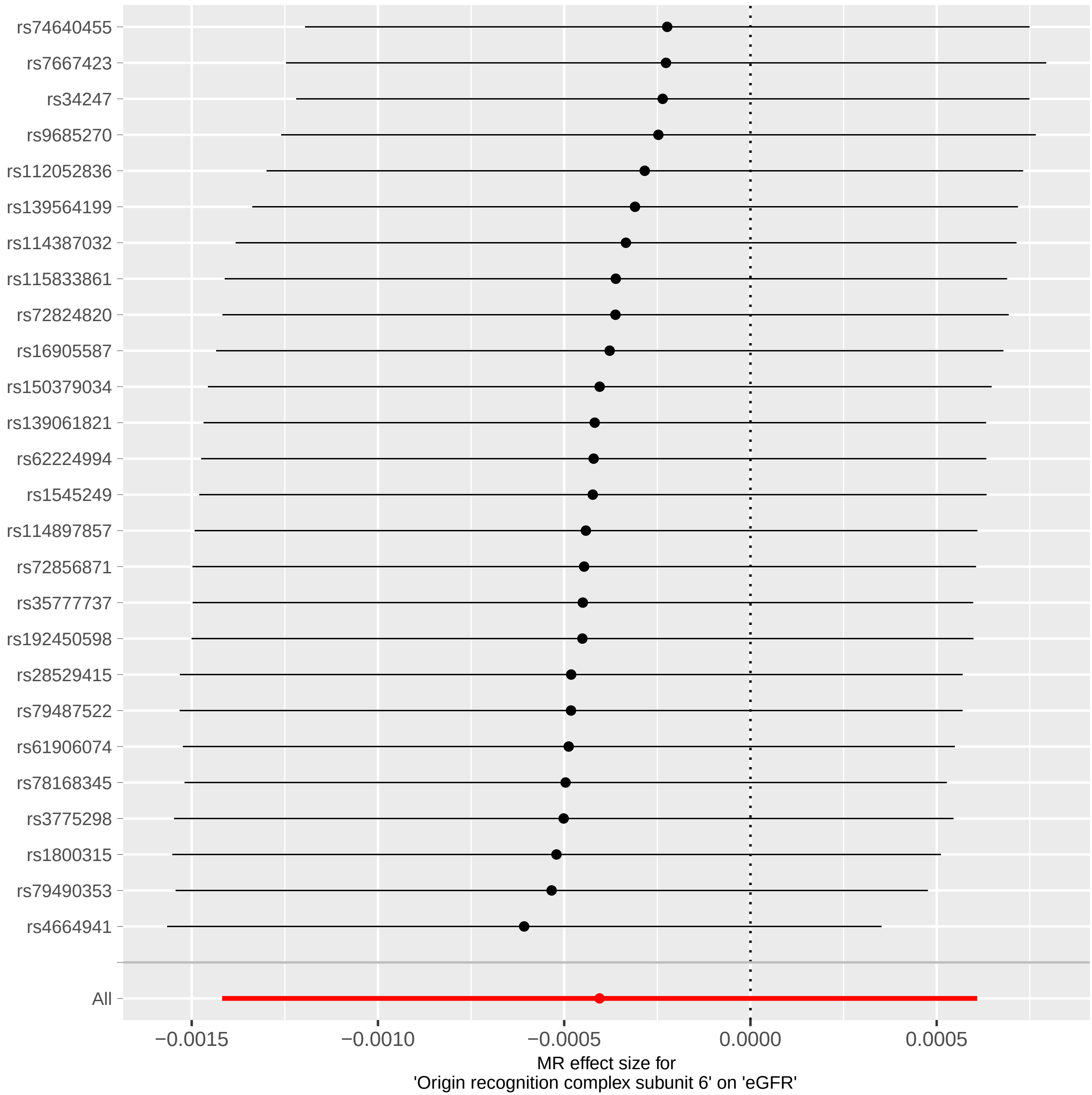

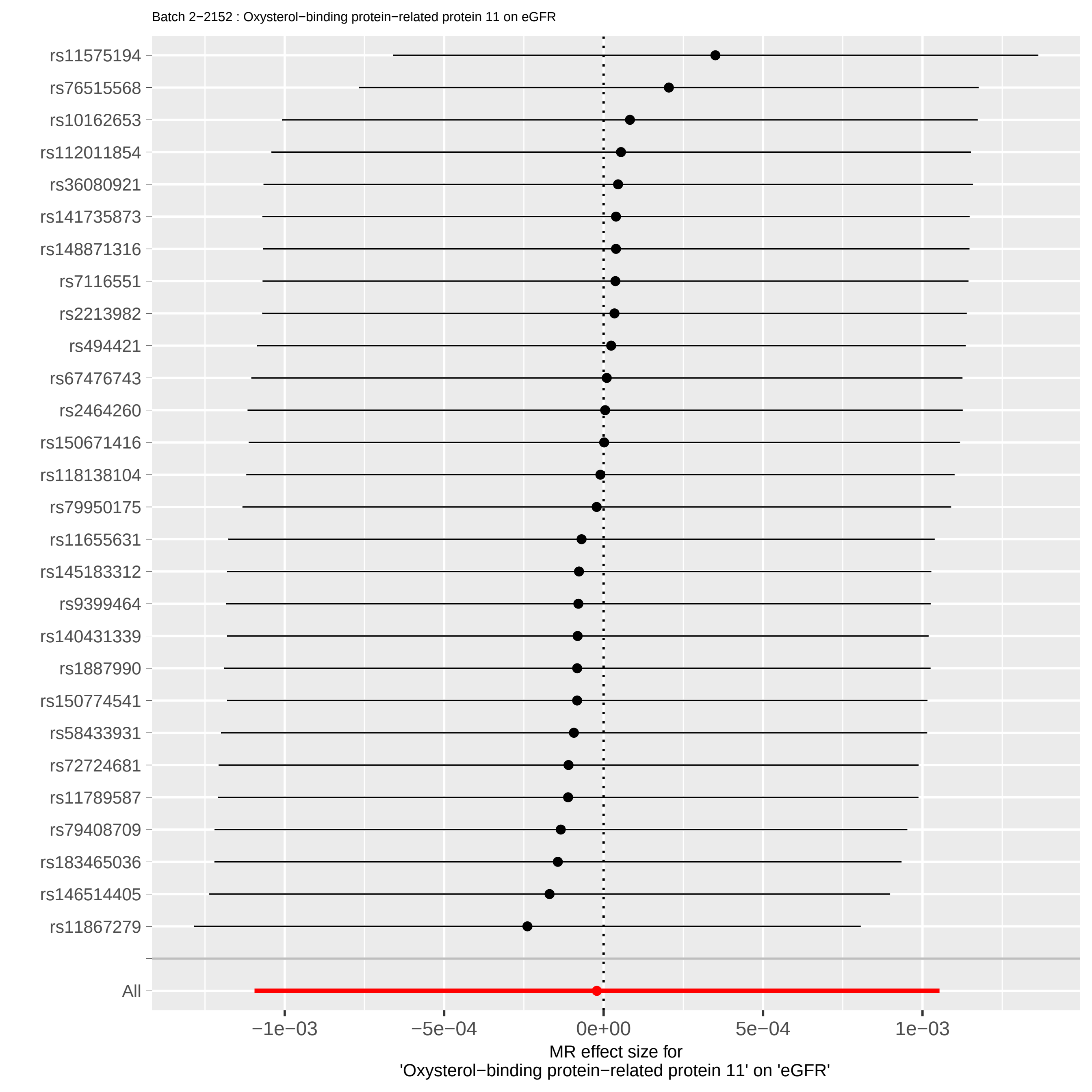

Batch 2−2152 : Oxysterol−binding protein−related protein 11 on eGFR
rs11575194
rs76515568
rs10162653
rs112011854
rs36080921
rs141735873
rs148871316
rs7116551
rs2213982
rs494421
rs67476743
rs2464260
rs150671416
rs118138104
rs79950175
rs11655631
rs145183312
rs9399464
rs140431339
rs1887990
rs150774541
rs58433931
rs72724681
rs11789587
rs79408709
rs183465036
rs146514405
rs11867279
All
−1e−03
−5e−04
0e+00
5e−04
1e−03
MR effect size for
'Oxysterol−binding protein−related protein 11' on 'eGFR'

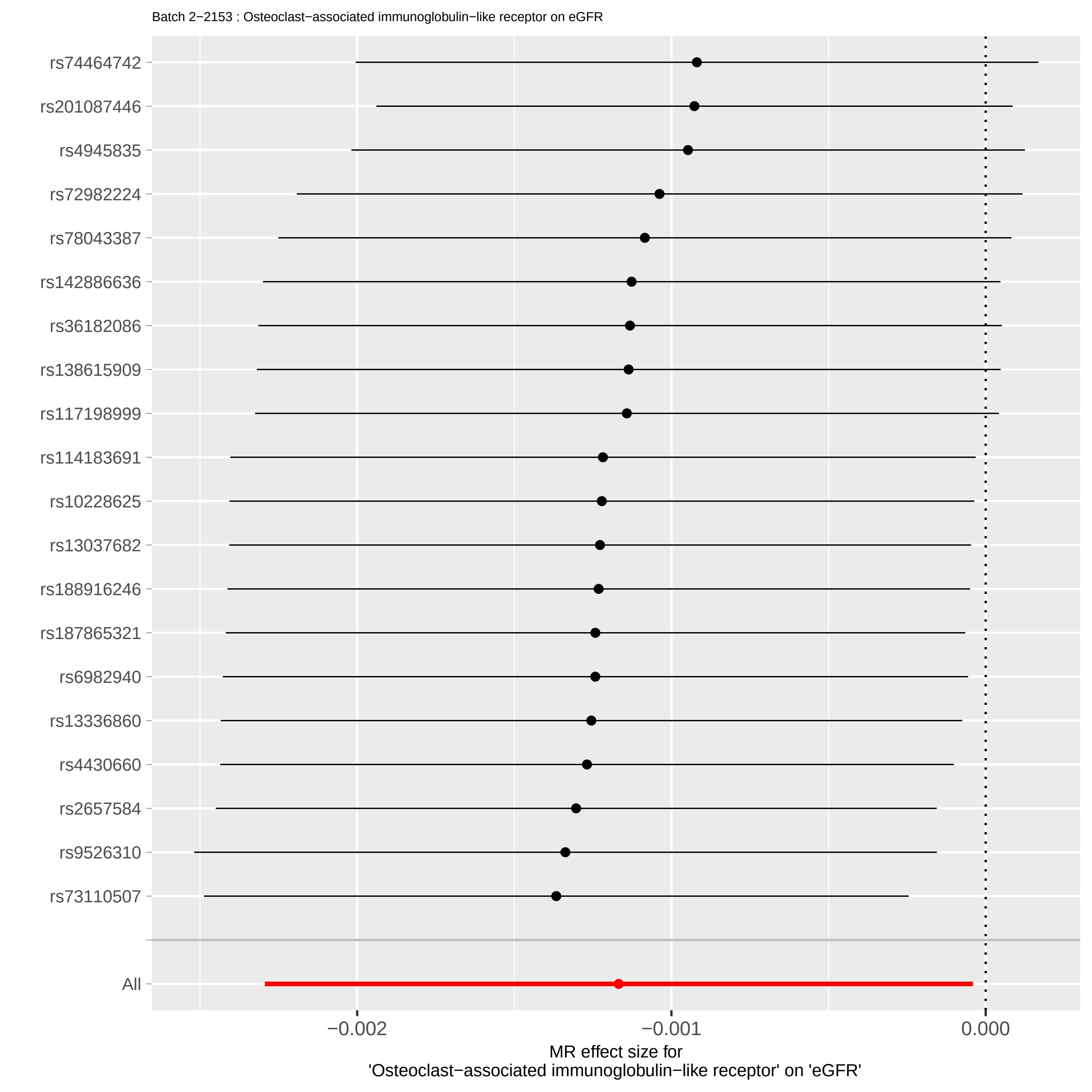
Batch 2−2153 : Osteoclast−associated immunoglobulin−like receptor on eGFR
rs74464742
rs201087446
rs4945835
rs72982224
rs78043387
rs142886636
rs36182086
rs138615909
rs117198999
rs114183691
rs10228625
rs13037682
rs188916246
rs187865321
rs6982940
rs13336860
rs4430660
rs2657584
rs9526310
rs73110507
All
−0.002
−0.001
0.000
MR effect size for
'Osteoclast−associated immunoglobulin−like receptor' on 'eGFR'

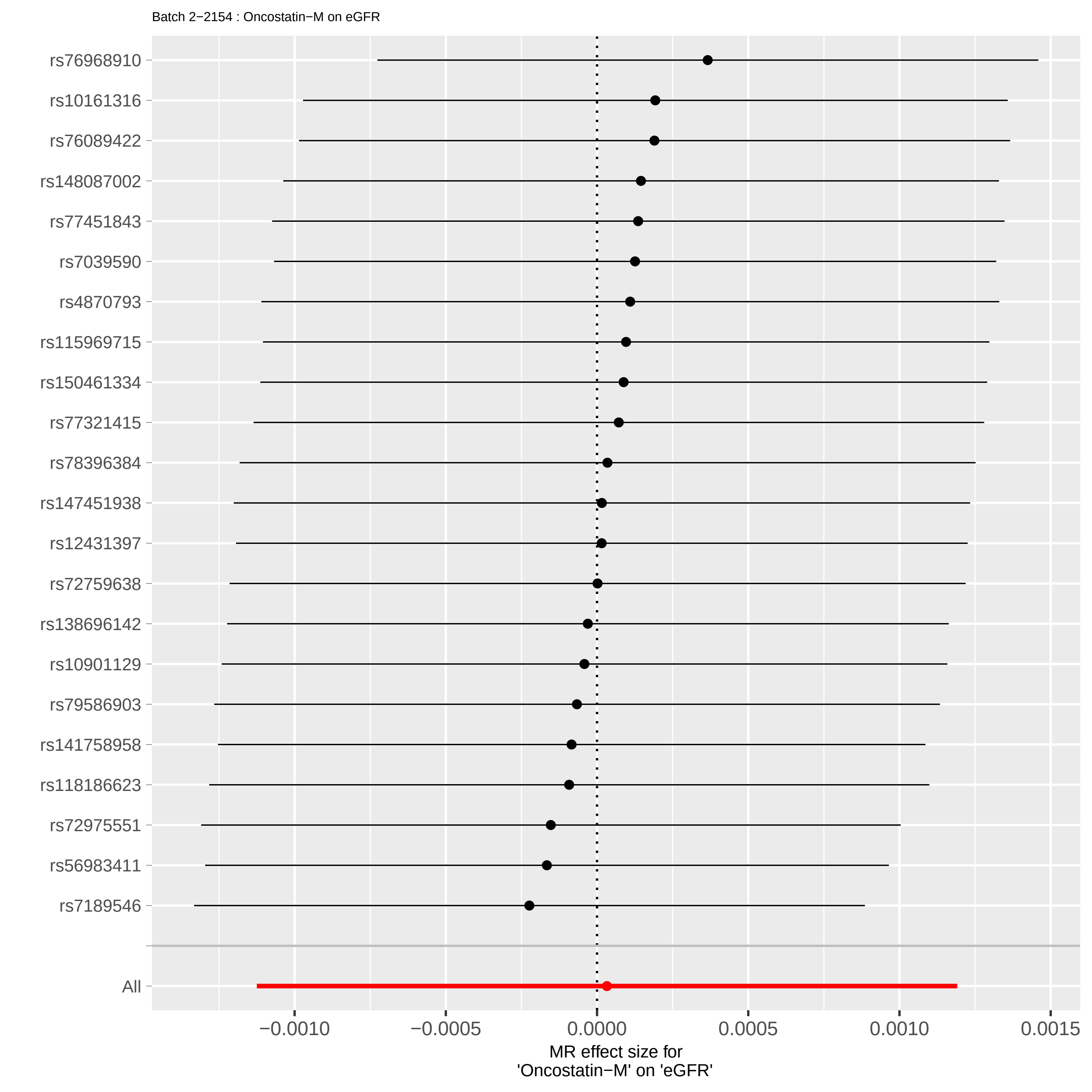

Batch 2−2154 : Oncostatin−M on eGFR
rs76968910
rs10161316
rs76089422
rs148087002
rs77451843
rs7039590
rs4870793
rs115969715
rs150461334
rs77321415
rs78396384
rs147451938
rs12431397
rs72759638
rs138696142
rs10901129
rs79586903
rs141758958
rs118186623
rs72975551
rs56983411
rs7189546
All
−0.0010
−0.0005
0.0000
0.0005
0.0010
0.0015
MR effect size for
'Oncostatin−M' on 'eGFR'

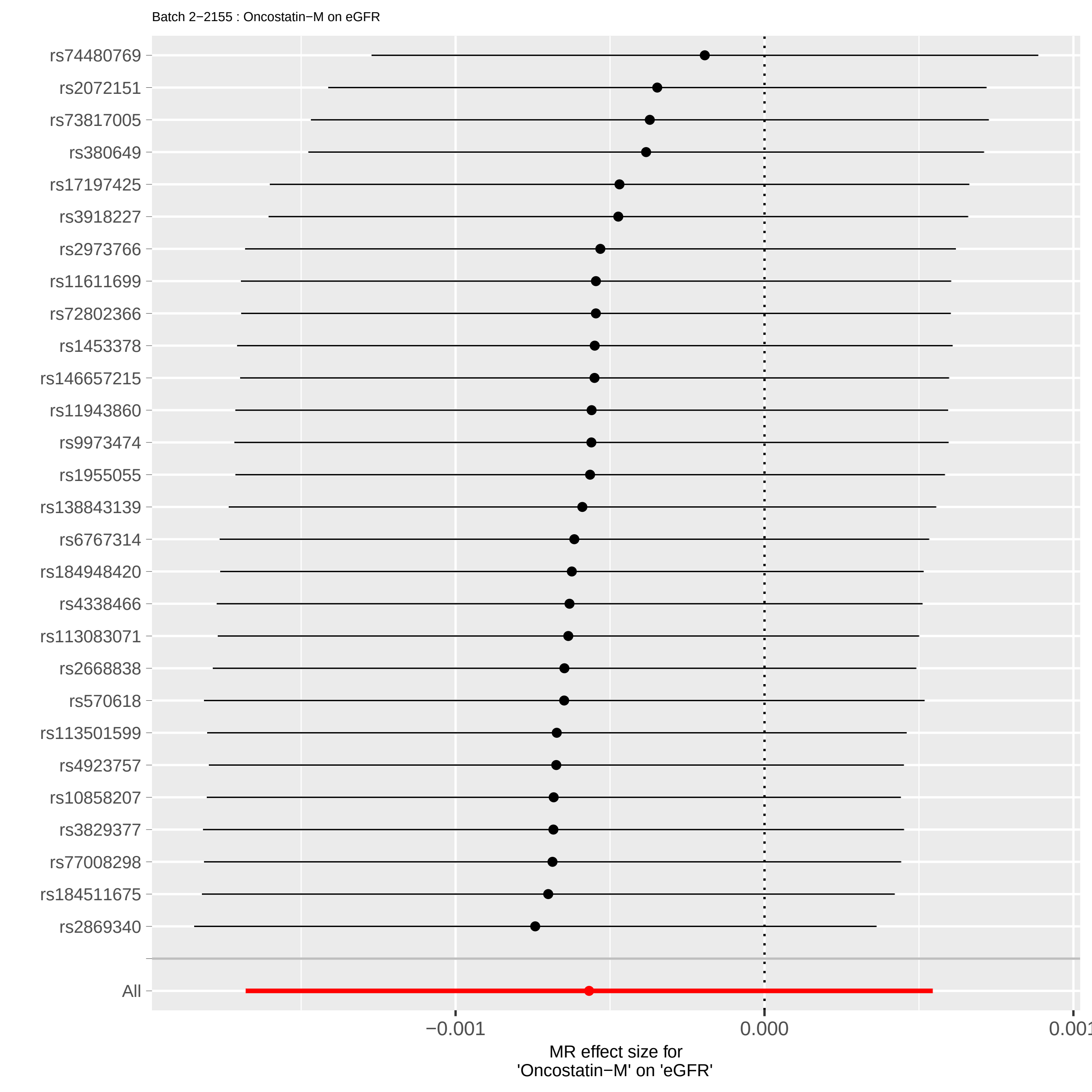

Batch 2−2155 : Oncostatin−M on eGFR
rs74480769
rs2072151
rs73817005
rs380649
rs17197425
rs3918227
rs2973766
rs11611699
rs72802366
rs1453378
rs146657215
rs11943860
rs9973474
rs1955055
rs138843139
rs6767314
rs184948420
rs4338466
rs113083071
rs2668838
rs570618
rs113501599
rs4923757
rs10858207
rs3829377
rs77008298
rs184511675
rs2869340
All
−0.001
0.000
0.001
MR effect size for
'Oncostatin−M' on 'eGFR'

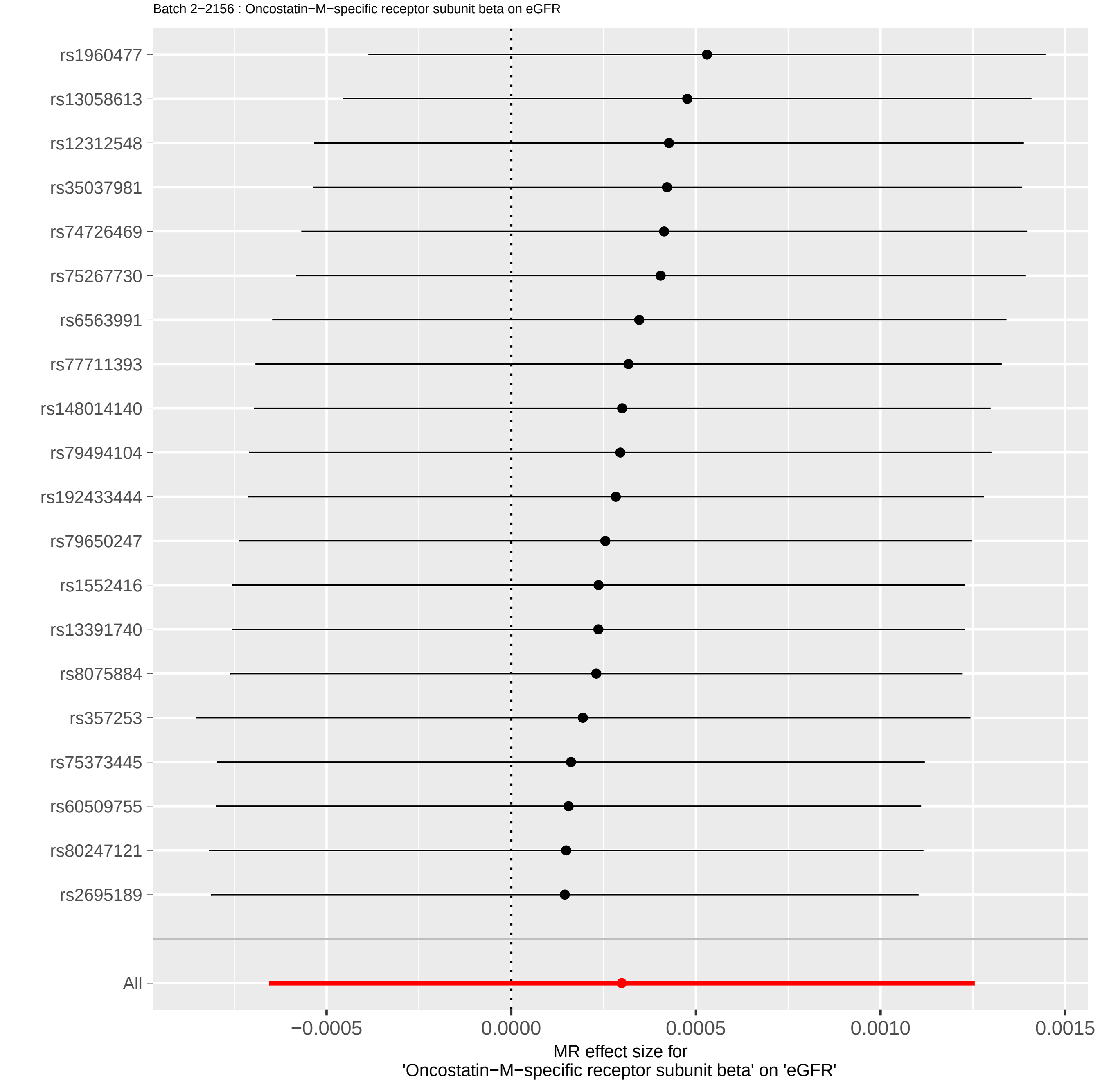

Batch 2−2156 : Oncostatin−M−specific receptor subunit beta on eGFR
rs1960477
rs13058613
rs12312548
rs35037981
rs74726469
rs75267730
rs6563991
rs77711393
rs148014140
rs79494104
rs192433444
rs79650247
rs1552416
rs13391740
rs8075884
rs357253
rs75373445
rs60509755
rs80247121
rs2695189
All
−0.0005
0.0000
0.0005
0.0010
0.0015
MR effect size for
'Oncostatin−M−specific receptor subunit beta' on 'eGFR'

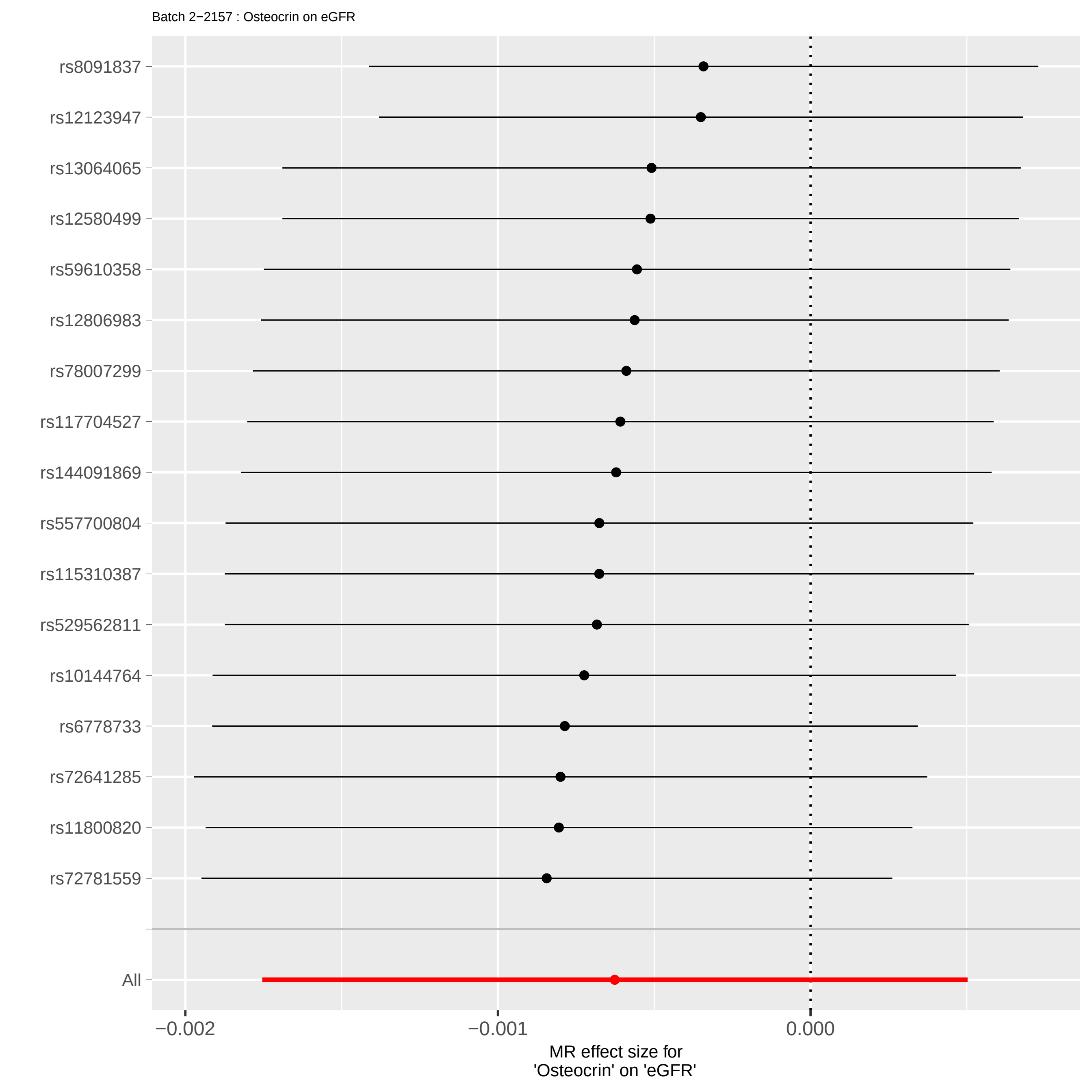
Batch 2−2157 : Osteocrin on eGFR
rs8091837
rs12123947
rs13064065
rs12580499
rs59610358
rs12806983
rs78007299
rs117704527
rs144091869
rs557700804
rs115310387
rs529562811
rs10144764
rs6778733
rs72641285
rs11800820
rs72781559
All
−0.002
−0.001
0.000
MR effect size for
'Osteocrin' on 'eGFR'

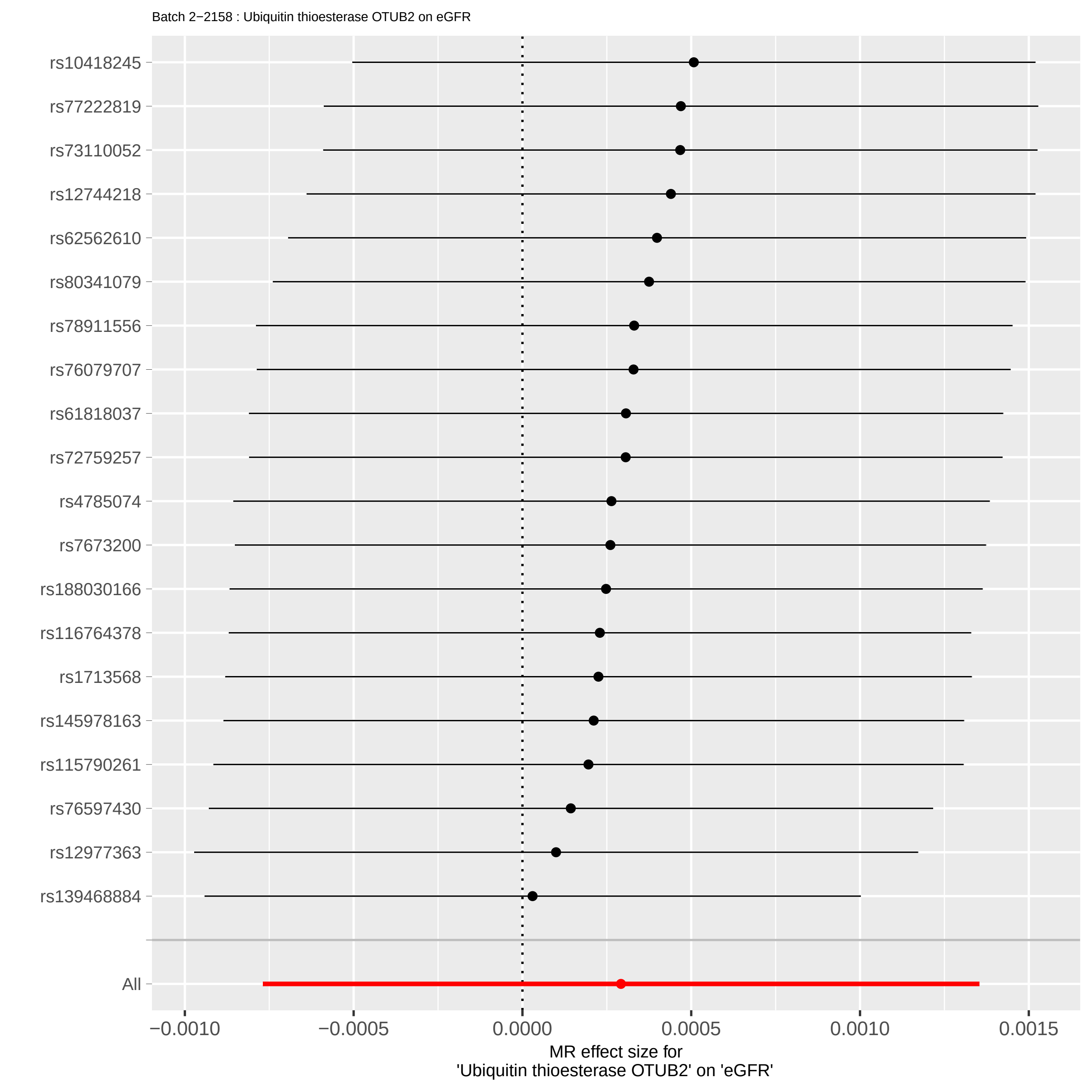

Batch 2−2158 : Ubiquitin thioesterase OTUB2 on eGFR
rs10418245
rs77222819
rs73110052
rs12744218
rs62562610
rs80341079
rs78911556
rs76079707
rs61818037
rs72759257
rs4785074
rs7673200
rs188030166
rs116764378
rs1713568
rs145978163
rs115790261
rs76597430
rs12977363
rs139468884
All
−0.0010
−0.0005
0.0000
0.0005
0.0010
0.0015
MR effect size for
'Ubiquitin thioesterase OTUB2' on 'eGFR'

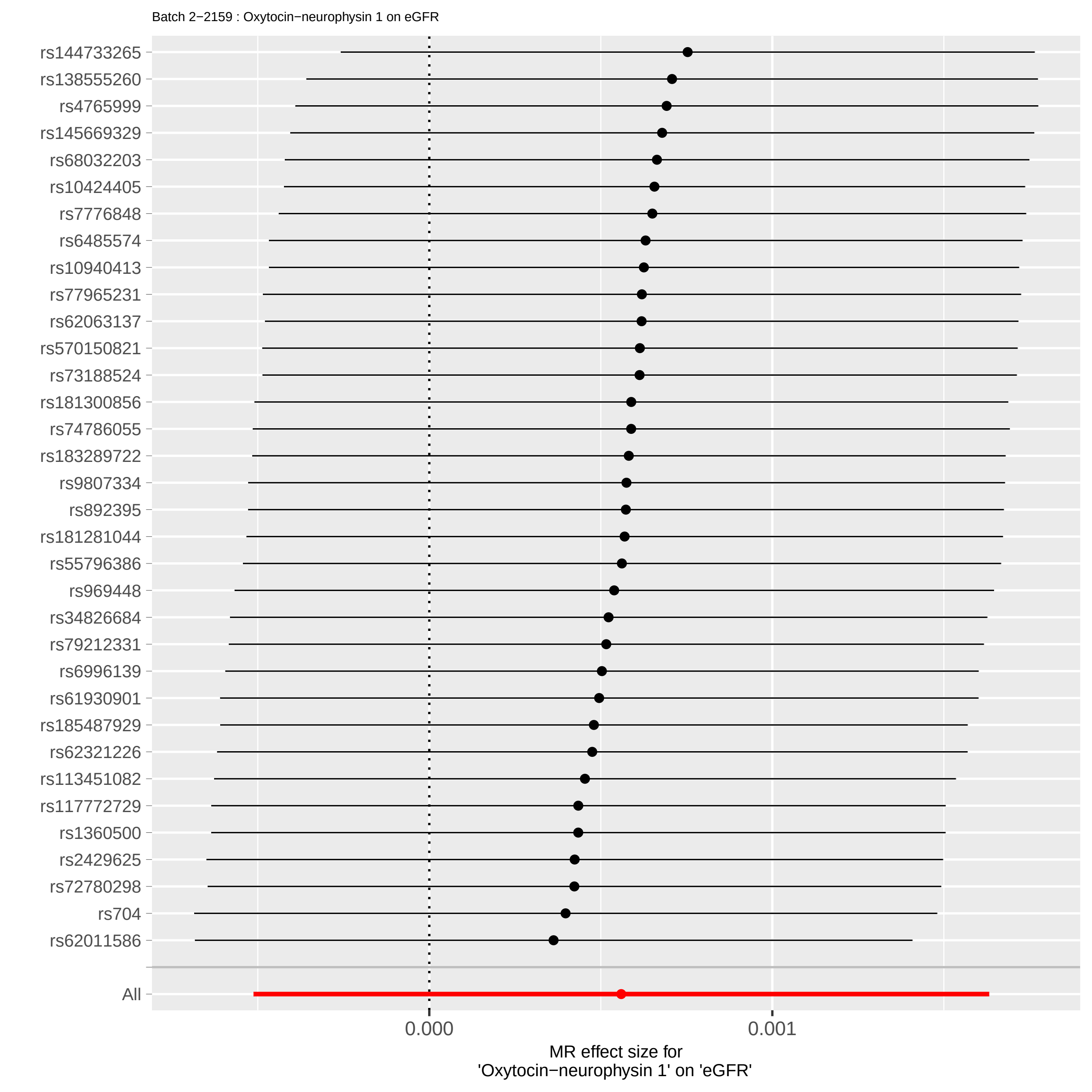

Batch 2−2159 : Oxytocin−neurophysin 1 on eGFR
rs144733265
rs138555260
rs4765999
rs145669329
rs68032203
rs10424405
rs7776848
rs6485574
rs10940413
rs77965231
rs62063137
rs570150821
rs73188524
rs181300856
rs74786055
rs183289722
rs9807334
rs892395
rs181281044
rs55796386
rs969448
rs34826684
rs79212331
rs6996139
rs61930901
rs185487929
rs62321226
rs113451082
rs117772729
rs1360500
rs2429625
rs72780298
rs704
rs62011586
All
0.000
0.001
MR effect size for
'Oxytocin−neurophysin 1' on 'eGFR'

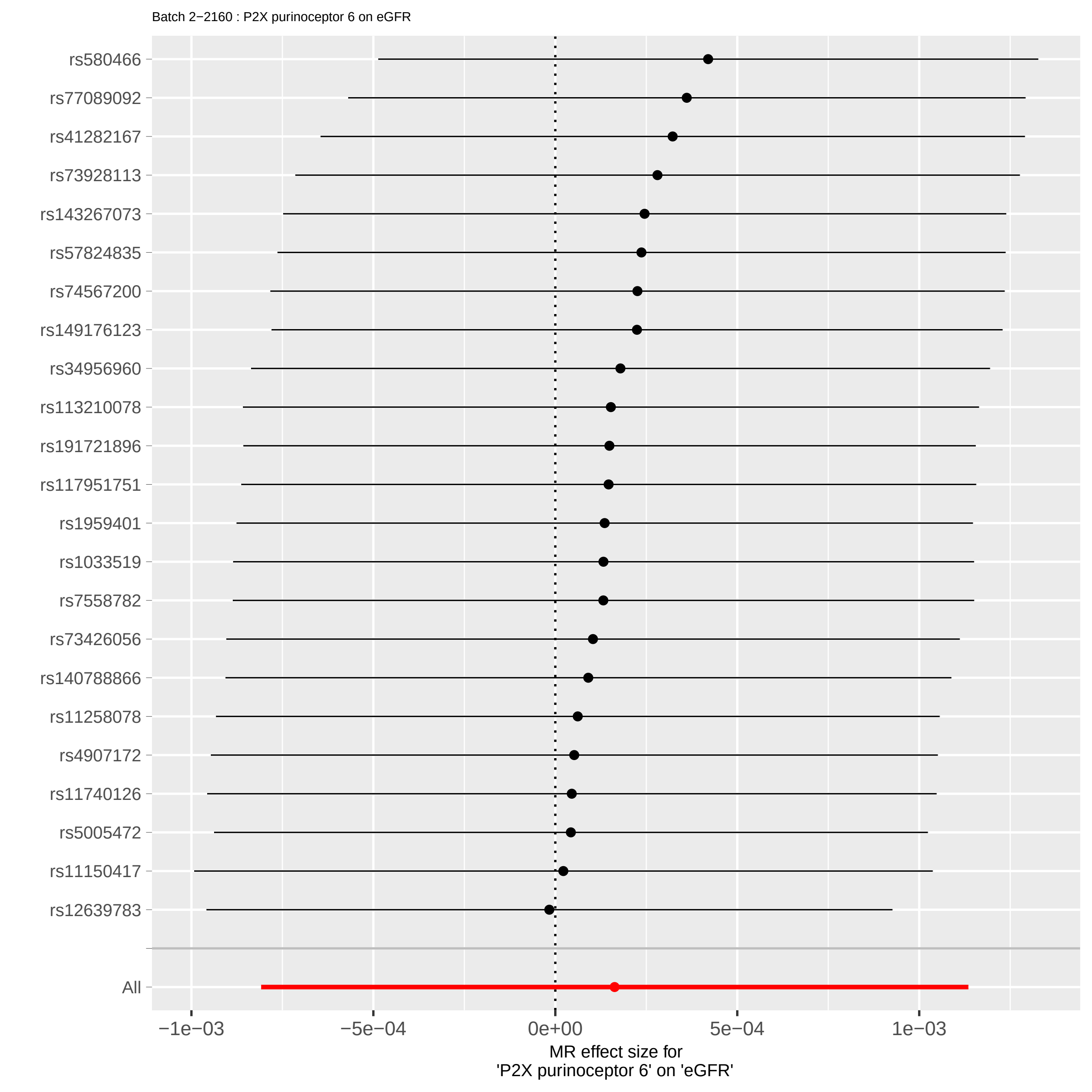

Batch 2−2160 : P2X purinoceptor 6 on eGFR
rs580466
rs77089092
rs41282167
rs73928113
rs143267073
rs57824835
rs74567200
rs149176123
rs34956960
rs113210078
rs191721896
rs117951751
rs1959401
rs1033519
rs7558782
rs73426056
rs140788866
rs11258078
rs4907172
rs11740126
rs5005472
rs11150417
rs12639783
All
−1e−03
−5e−04
0e+00
5e−04
1e−03
MR effect size for
'P2X purinoceptor 6' on 'eGFR'

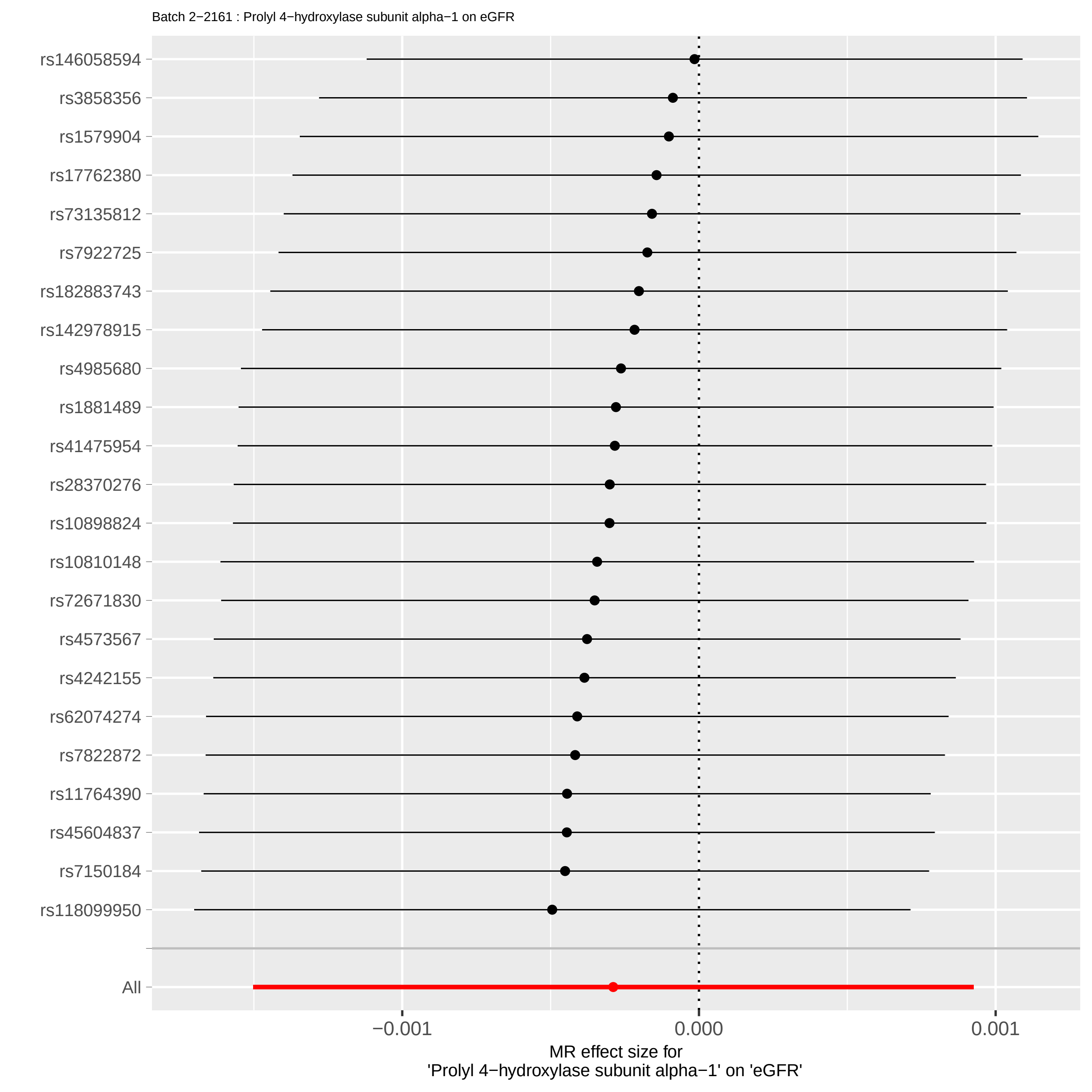
Batch 2−2161 : Prolyl 4−hydroxylase subunit alpha−1 on eGFR
rs146058594
rs3858356
rs1579904
rs17762380
rs73135812
rs7922725
rs182883743
rs142978915
rs4985680
rs1881489
rs41475954
rs28370276
rs10898824
rs10810148
rs72671830
rs4573567
rs4242155
rs62074274
rs7822872
rs11764390
rs45604837
rs7150184
rs118099950
All
−0.001
0.000
0.001
MR effect size for
'Prolyl 4−hydroxylase subunit alpha−1' on 'eGFR'

Batch 2−2162 : Prolyl 4−hydroxylase subunit alpha−2 on eGFR

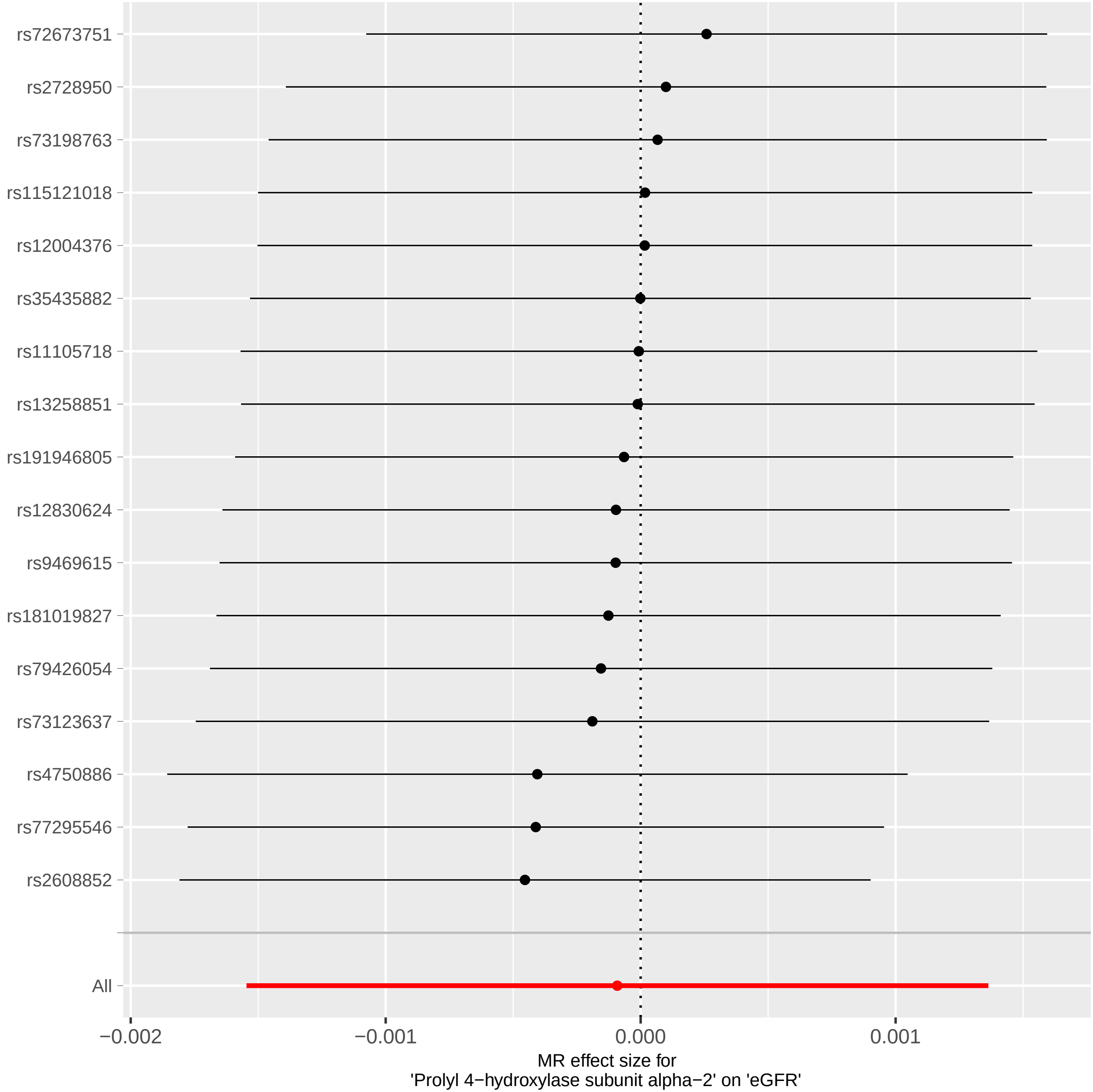

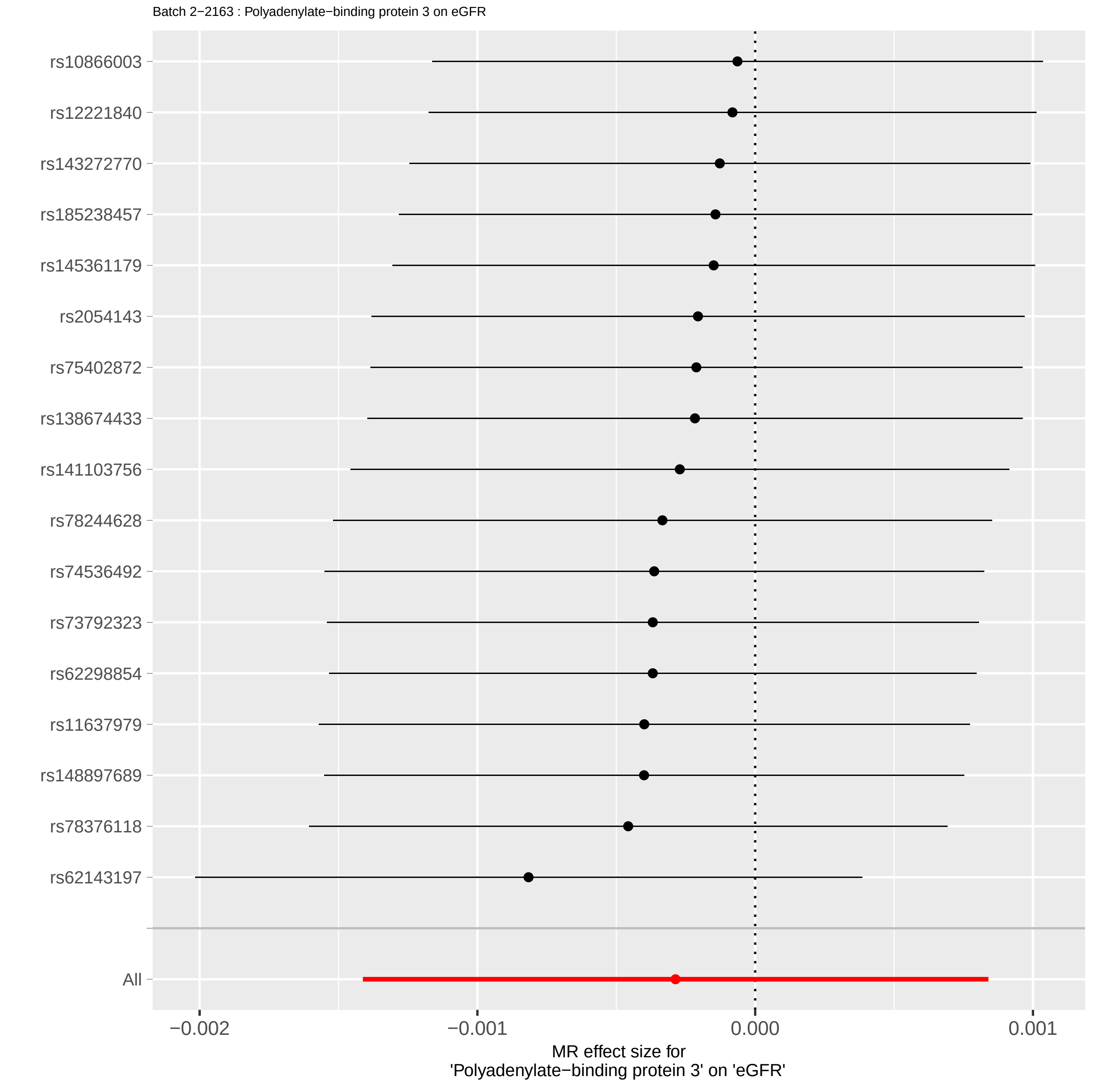
Batch 2−2163 : Polyadenylate−binding protein 3 on eGFR
rs10866003
rs12221840
rs143272770
rs185238457
rs145361179
rs2054143
rs75402872
rs138674433
rs141103756
rs78244628
rs74536492
rs73792323
rs62298854
rs11637979
rs148897689
rs78376118
rs62143197
All
−0.002
−0.001
0.000
0.001
MR effect size for
'Polyadenylate−binding protein 3' on 'eGFR'

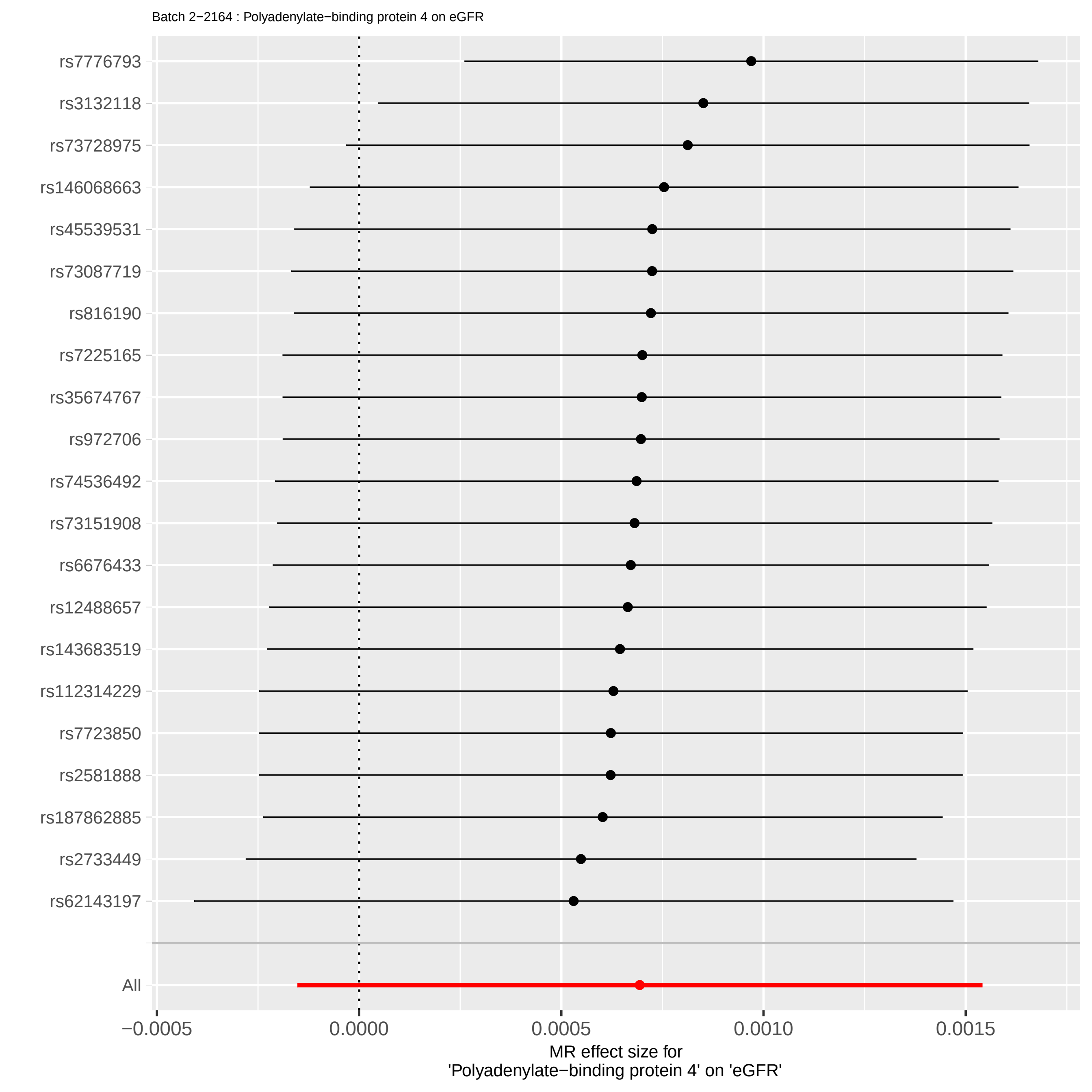

Batch 2−2164 : Polyadenylate−binding protein 4 on eGFR
rs7776793
rs3132118
rs73728975
rs146068663
rs45539531
rs73087719
rs816190
rs7225165
rs35674767
rs972706
rs74536492
rs73151908
rs6676433
rs12488657
rs143683519
rs112314229
rs7723850
rs2581888
rs187862885
rs2733449
rs62143197
All
−0.0005
0.0000
0.0005
0.0010
0.0015
MR effect size for
'Polyadenylate−binding protein 4' on 'eGFR'

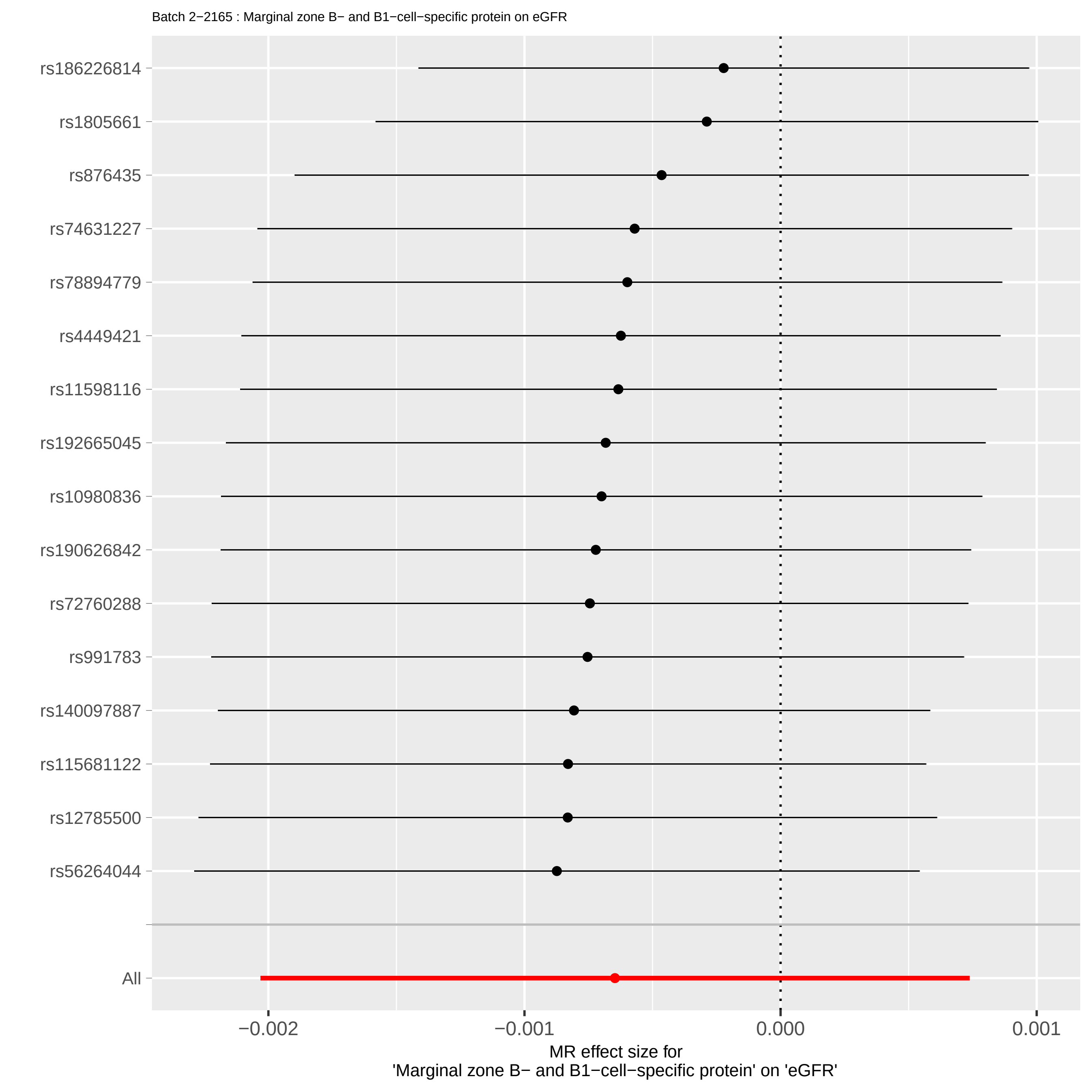
Batch 2−2165 : Marginal zone B− and B1−cell−specific protein on eGFR
rs186226814
rs1805661
rs876435
rs74631227
rs78894779
rs4449421
rs11598116
rs192665045
rs10980836
rs190626842
rs72760288
rs991783
rs140097887
rs115681122
rs12785500
rs56264044
All
−0.002
−0.001
0.000
0.001
MR effect size for
'Marginal zone B− and B1−cell−specific protein' on 'eGFR'

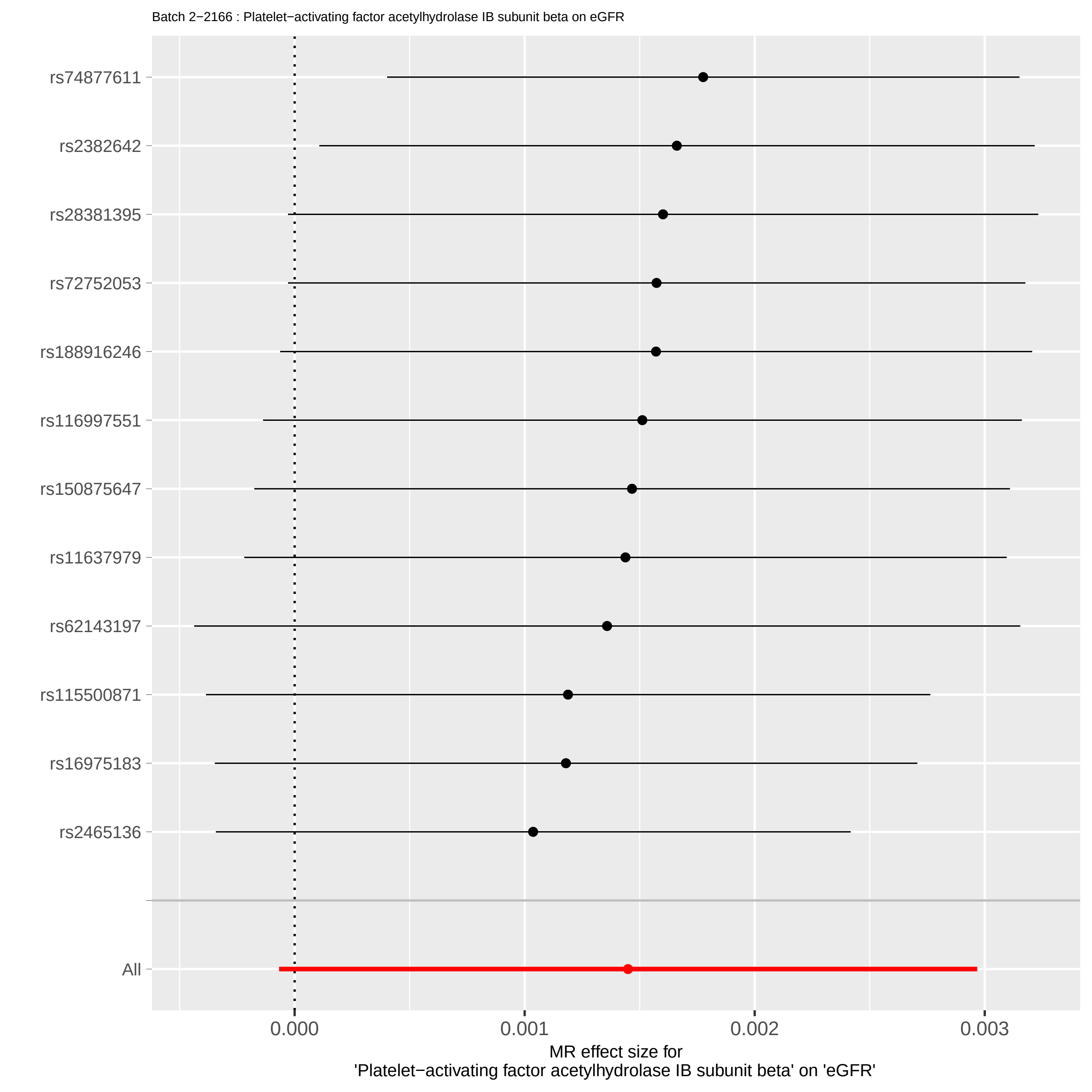

Batch 2−2166 : Platelet−activating factor acetylhydrolase IB subunit beta on eGFR
rs74877611
rs2382642
rs28381395
rs72752053
rs188916246
rs116997551
rs150875647
rs11637979
rs62143197
rs115500871
rs16975183
rs2465136
All
0.000
0.001
0.002
0.003
MR effect size for
'Platelet−activating factor acetylhydrolase IB subunit beta' on 'eGFR'

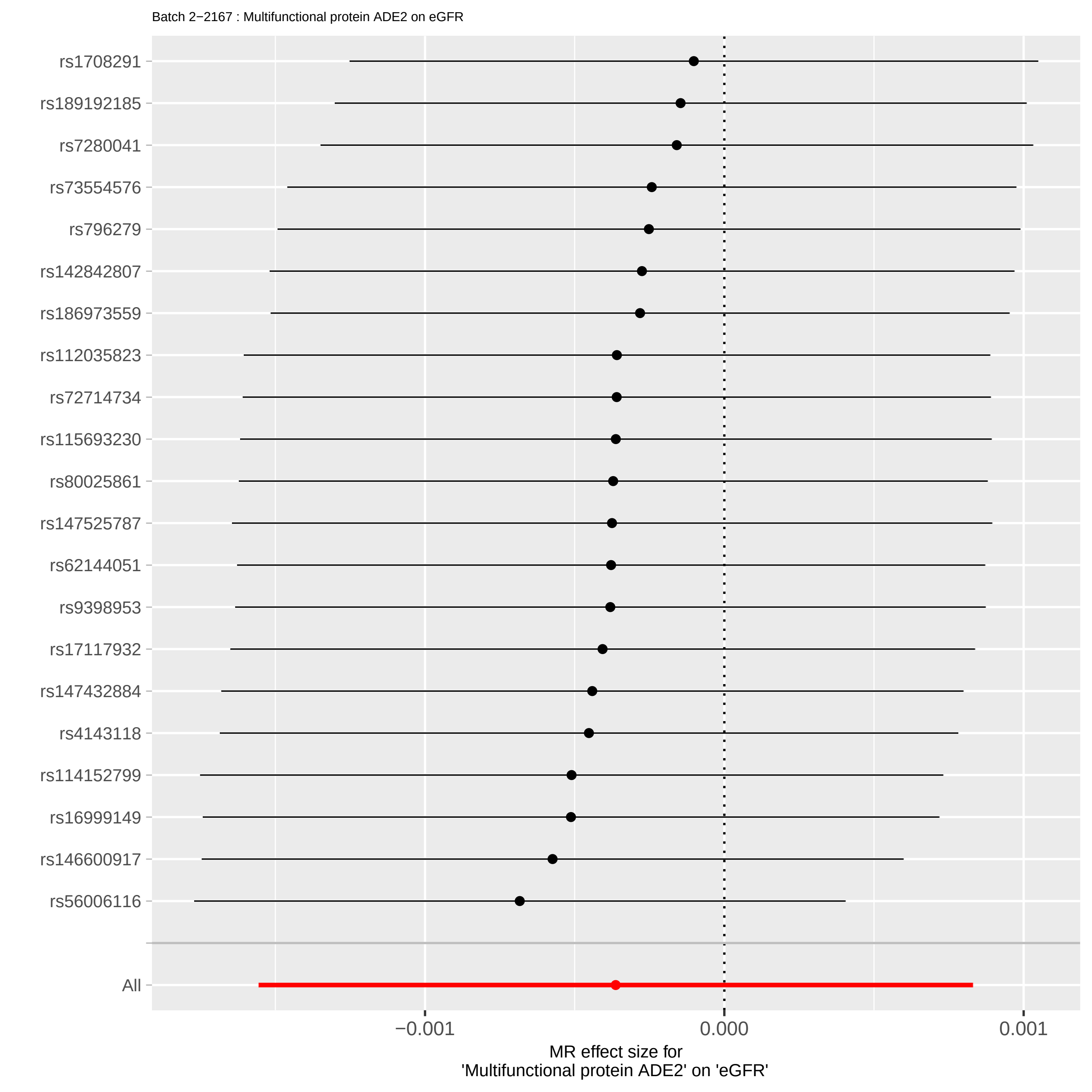

Batch 2−2167 : Multifunctional protein ADE2 on eGFR
rs1708291
rs189192185
rs7280041
rs73554576
rs796279
rs142842807
rs186973559
rs112035823
rs72714734
rs115693230
rs80025861
rs147525787
rs62144051
rs9398953
rs17117932
rs147432884
rs4143118
rs114152799
rs16999149
rs146600917
rs56006116
All
−0.001
0.000
0.001
MR effect size for
'Multifunctional protein ADE2' on 'eGFR'

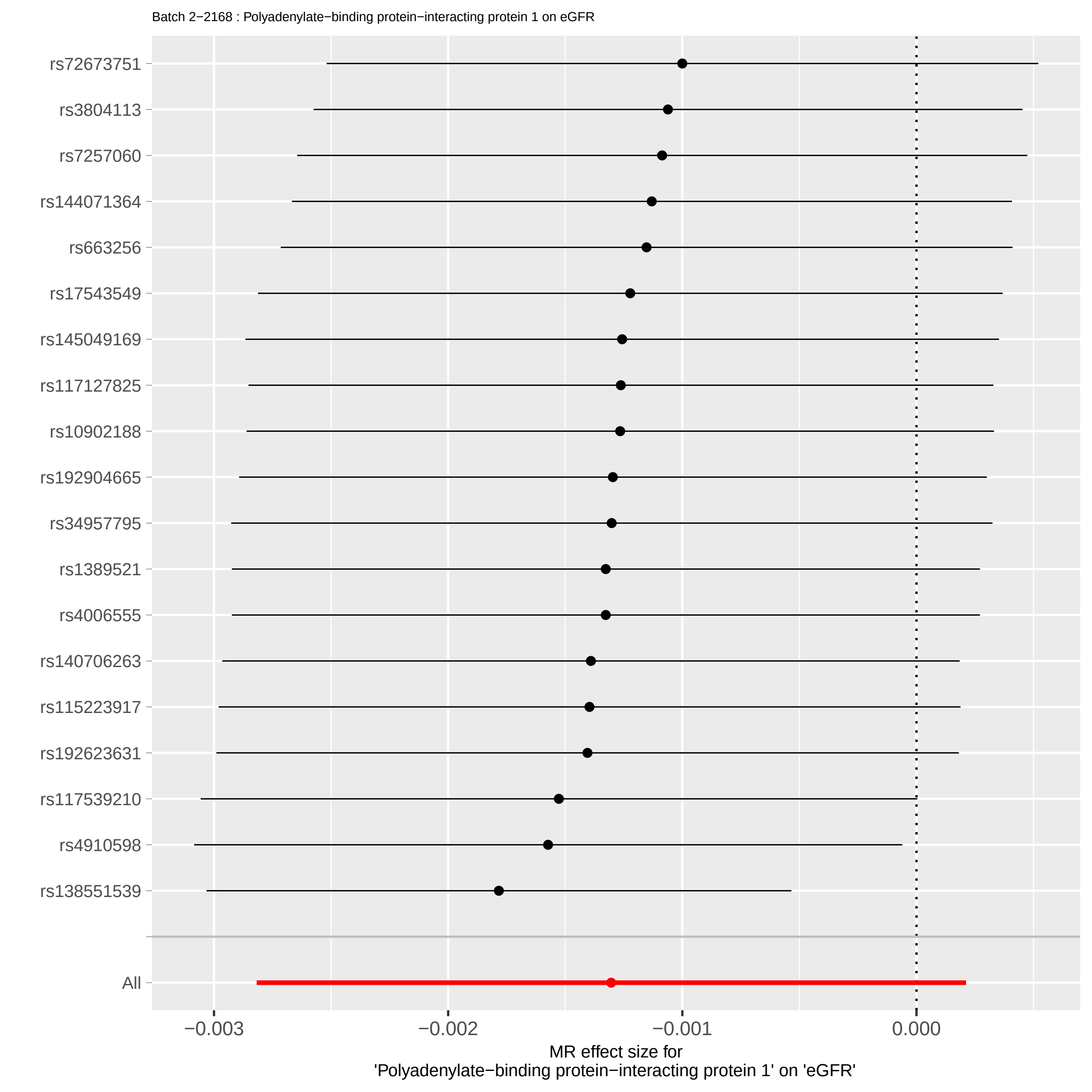

Batch 2−2168 : Polyadenylate−binding protein−interacting protein 1 on eGFR
rs72673751
rs3804113
rs7257060
rs144071364
rs663256
rs17543549
rs145049169
rs117127825
rs10902188
rs192904665
rs34957795
rs1389521
rs4006555
rs140706263
rs115223917
rs192623631
rs117539210
rs4910598
rs138551539
All
−0.003
−0.002
−0.001
0.000
MR effect size for
'Polyadenylate−binding protein−interacting protein 1' on 'eGFR'

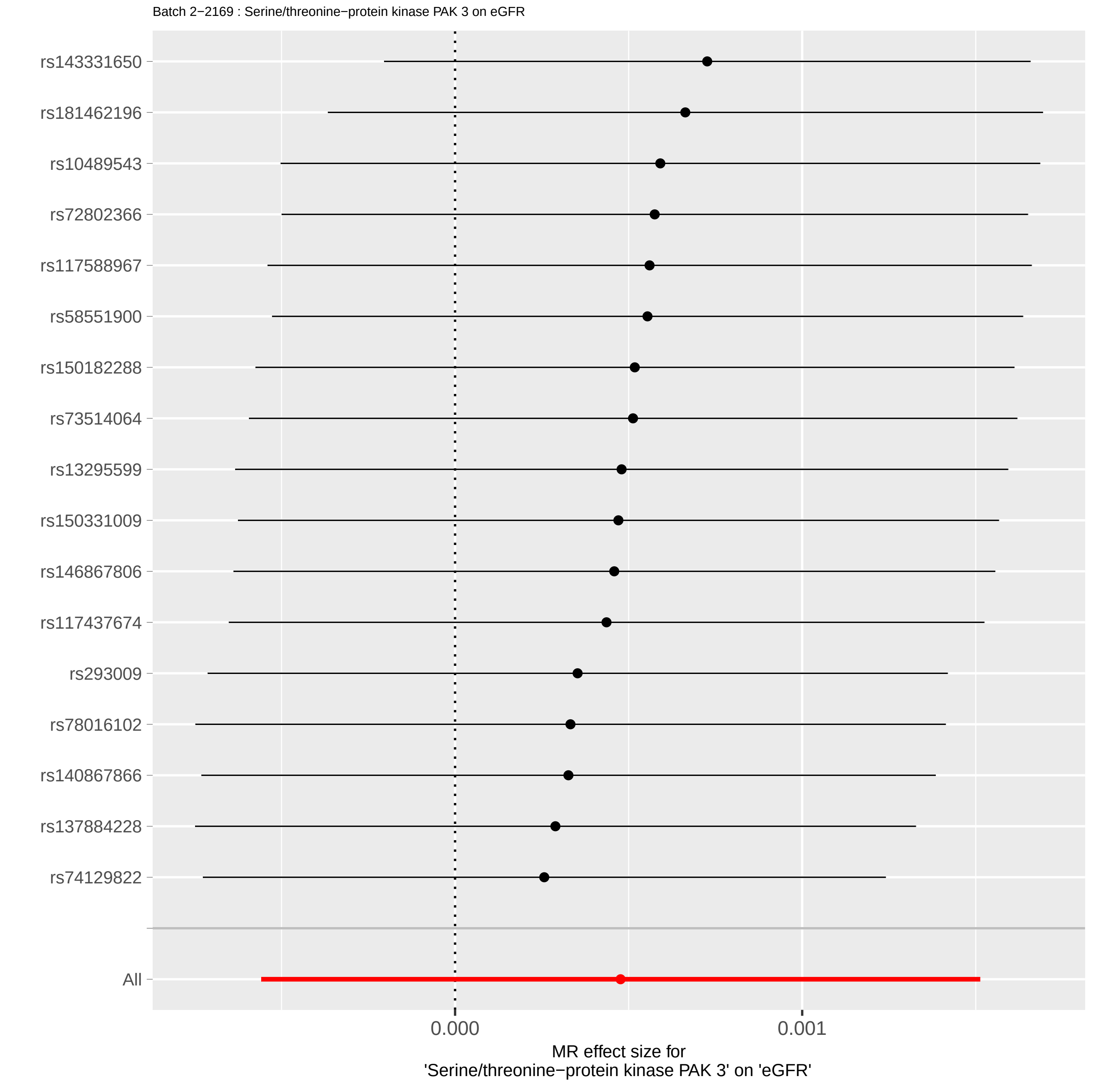

Batch 2−2169 : Serine/threonine−protein kinase PAK 3 on eGFR
rs143331650
rs181462196
rs10489543
rs72802366
rs117588967
rs58551900
rs150182288
rs73514064
rs13295599
rs150331009
rs146867806
rs117437674
rs293009
rs78016102
rs140867866
rs137884228
rs74129822
All
0.000
0.001
MR effect size for
'Serine/threonine−protein kinase PAK 3' on 'eGFR'

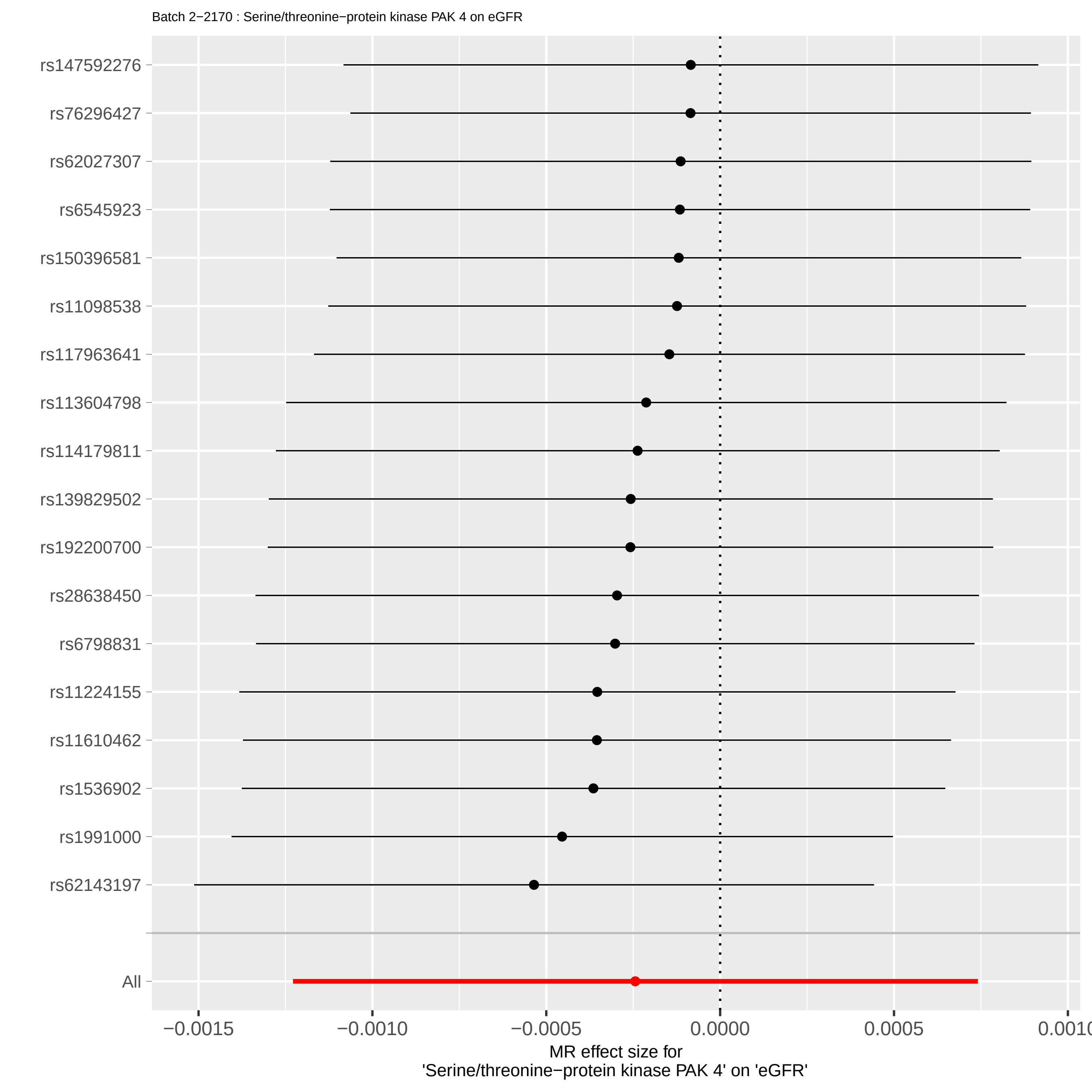

Batch 2−2170 : Serine/threonine−protein kinase PAK 4 on eGFR
rs147592276
rs76296427
rs62027307
rs6545923
rs150396581
rs11098538
rs117963641
rs113604798
rs114179811
rs139829502
rs192200700
rs28638450
rs6798831
rs11224155
rs11610462
rs1536902
rs1991000
rs62143197
All
−0.0015
−0.0010
−0.0005
0.0000
0.0005
MR effect size for
'Serine/threonine−protein kinase PAK 4' on 'eGFR'

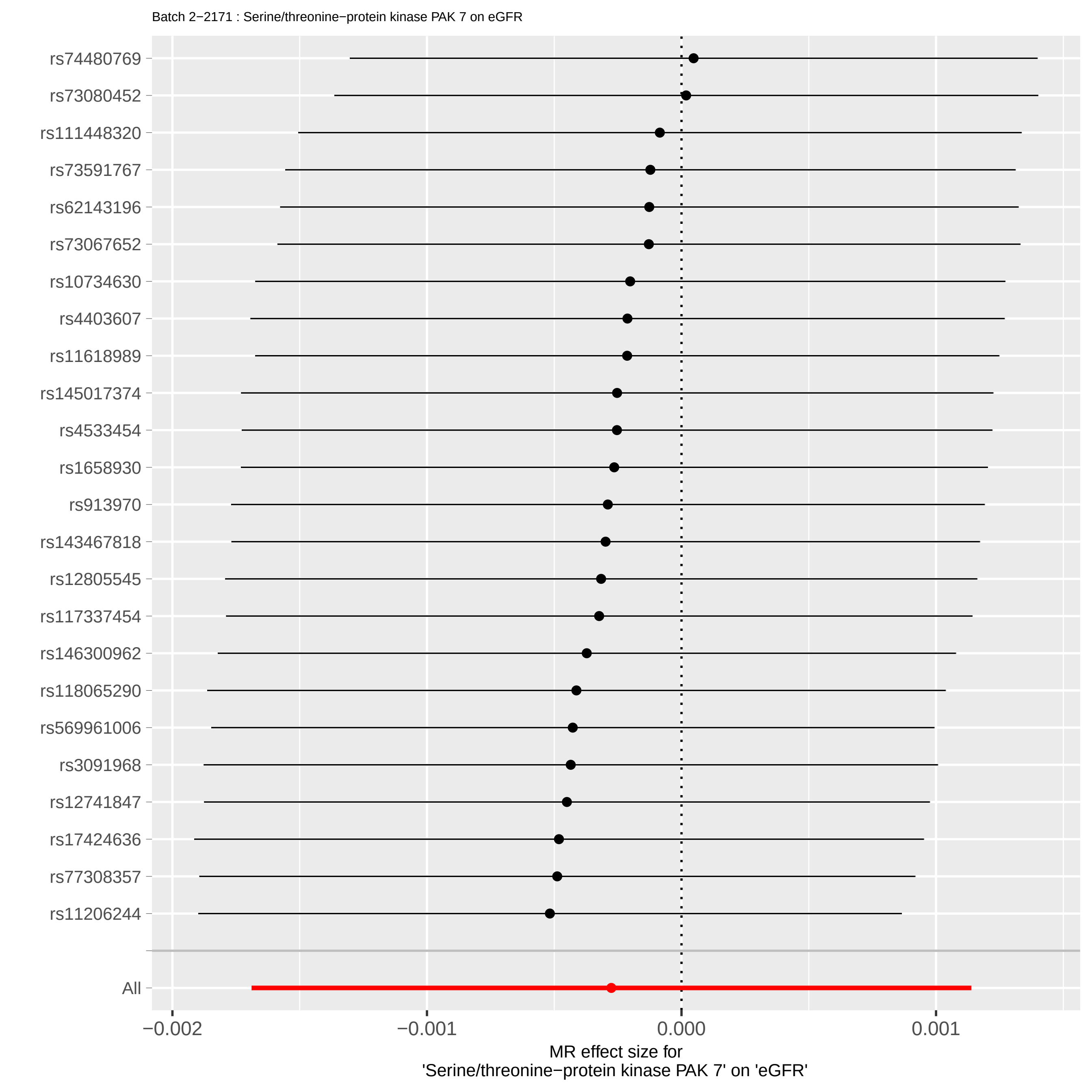

Batch 2−2171 : Serine/threonine−protein kinase PAK 7 on eGFR
rs74480769
rs73080452
rs111448320
rs73591767
rs62143196
rs73067652
rs10734630
rs4403607
rs11618989
rs145017374
rs4533454
rs1658930
rs913970
rs143467818
rs12805545
rs117337454
rs146300962
rs118065290
rs569961006
rs3091968
rs12741847
rs17424636
rs77308357
rs11206244
All
−0.002
−0.001
0.000
0.001
MR effect size for
'Serine/threonine−protein kinase PAK 7' on 'eGFR'

Batch 2−2172 : Peptidyl−glycine alpha−amidating monooxygenase on eGFR

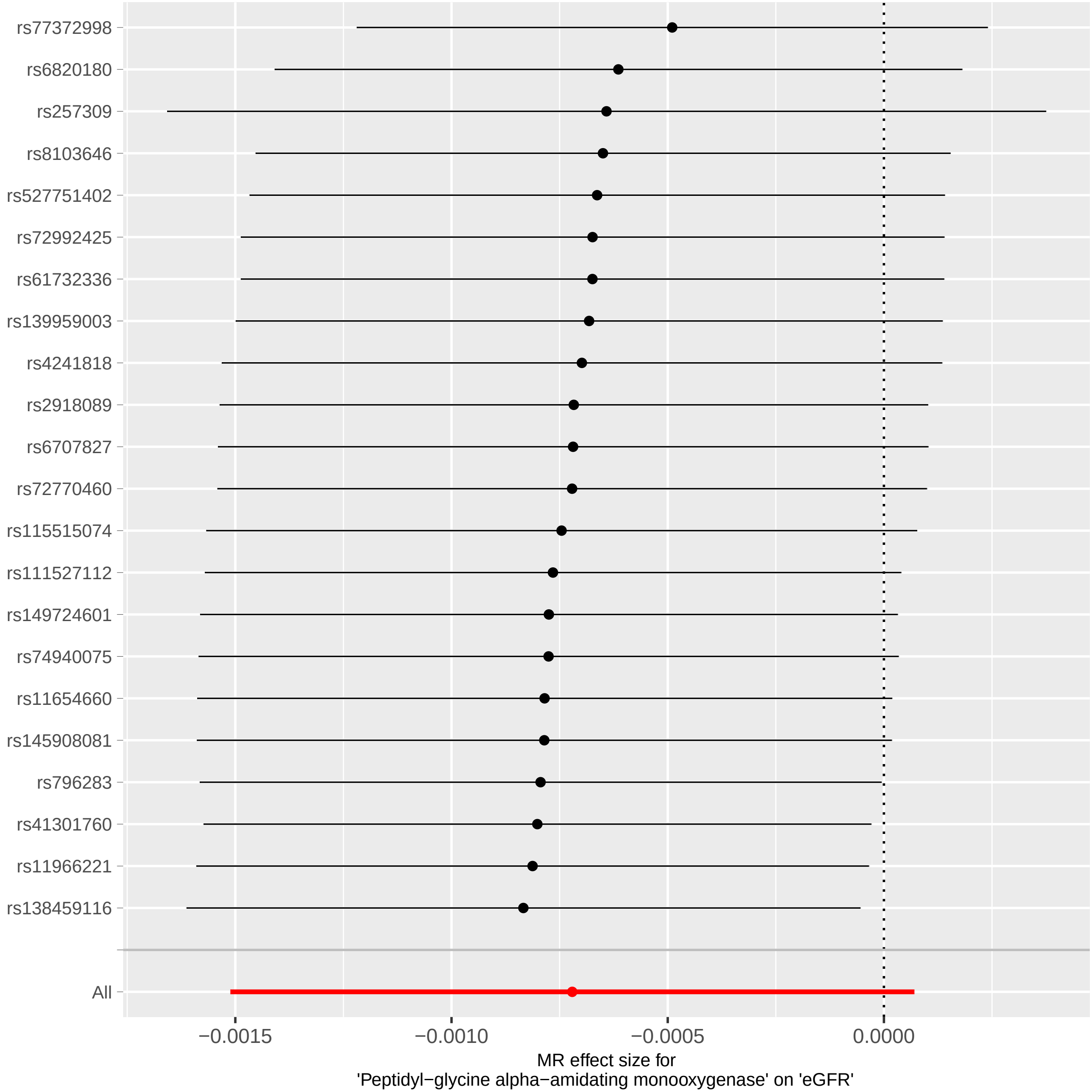

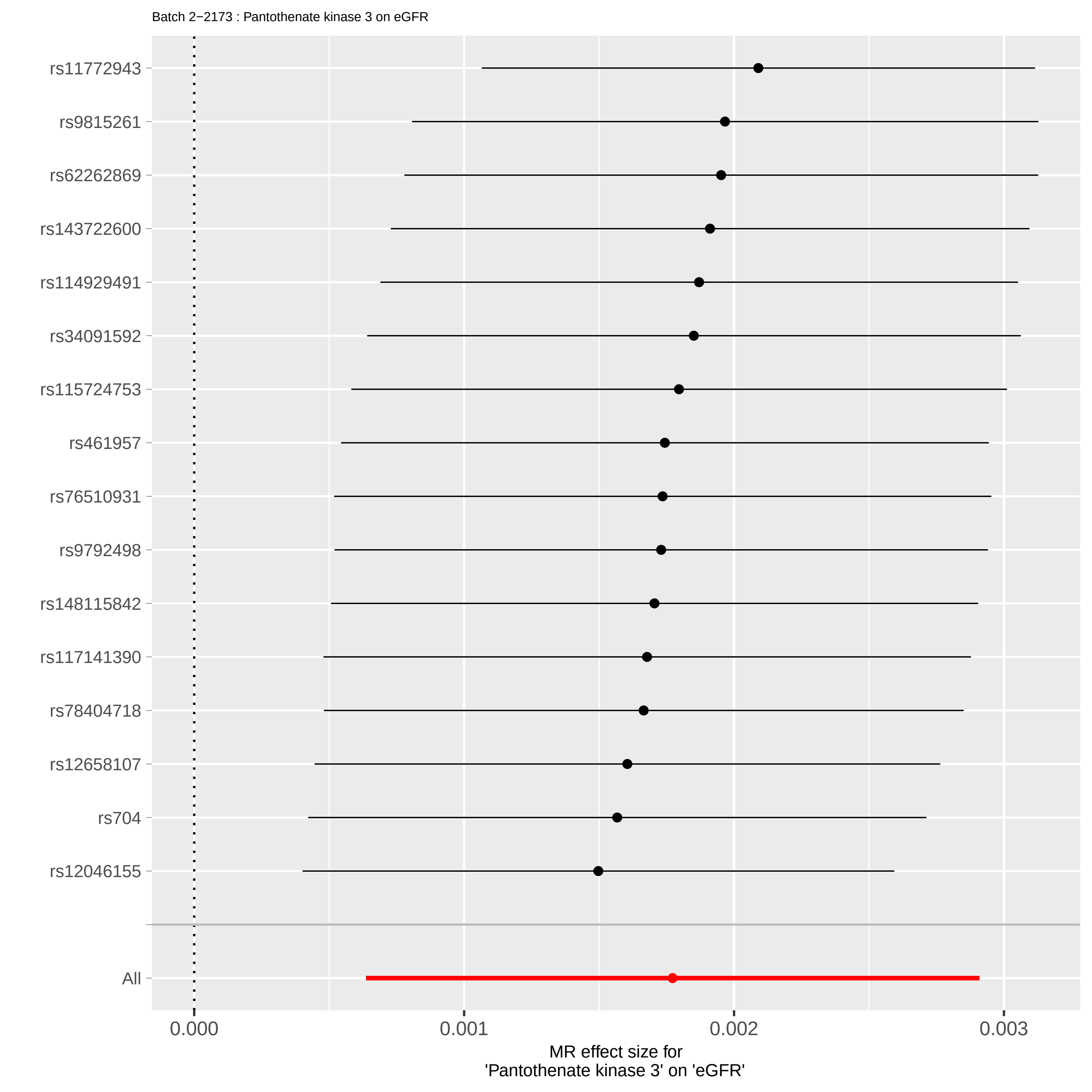
Batch 2−2173 : Pantothenate kinase 3 on eGFR
rs11772943
rs9815261
rs62262869
rs143722600
rs114929491
rs34091592
rs115724753
rs461957
rs76510931
rs9792498
rs148115842
rs117141390
rs78404718
rs12658107
rs704
rs12046155
All
0.000
0.001
0.002
0.003
MR effect size for
'Pantothenate kinase 3' on 'eGFR'

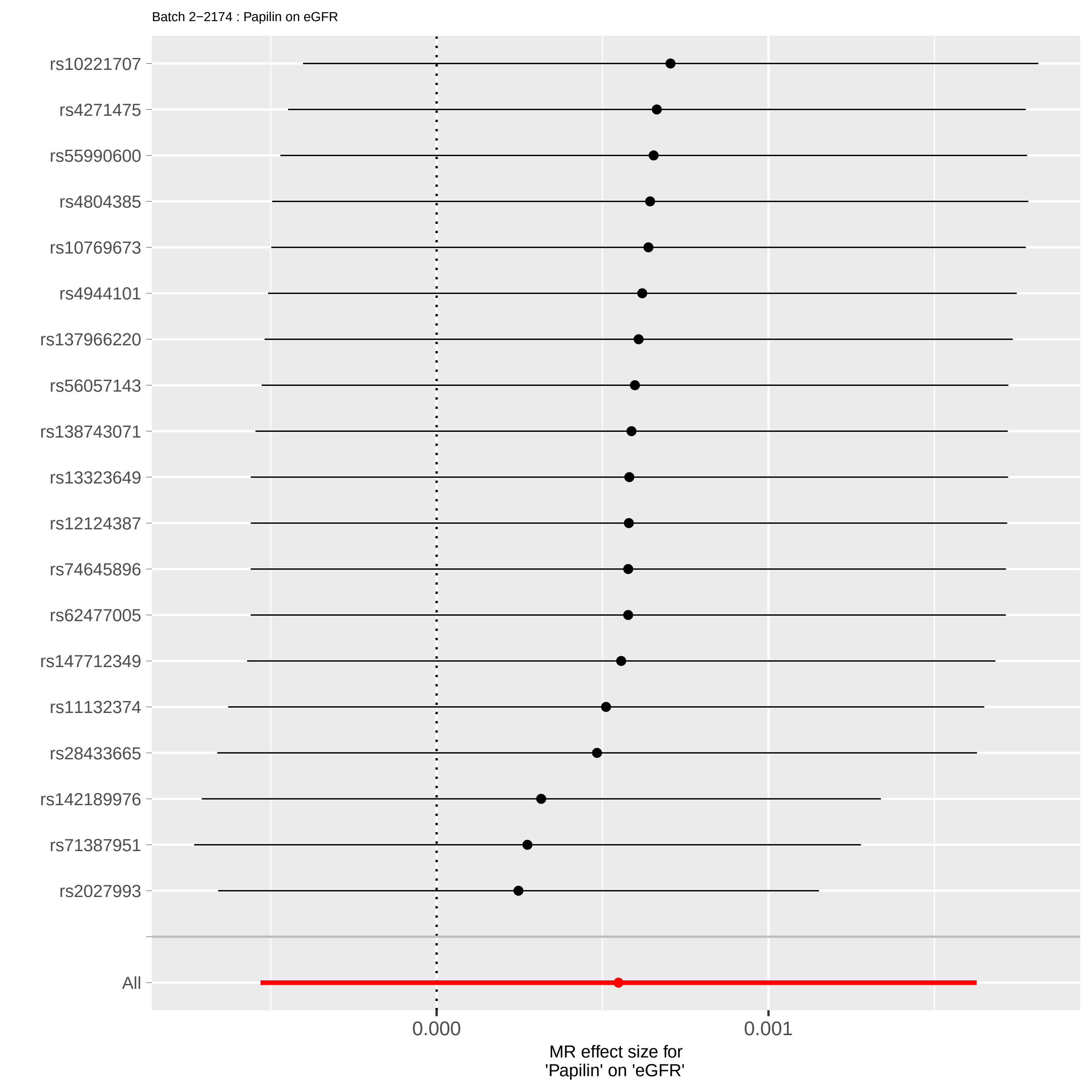

Batch 2−2174 : Papilin on eGFR
rs10221707
rs4271475
rs55990600
rs4804385
rs10769673
rs4944101
rs137966220
rs56057143
rs138743071
rs13323649
rs12124387
rs74645896
rs62477005
rs147712349
rs11132374
rs28433665
rs142189976
rs71387951
rs2027993
All
0.000
0.001
MR effect size for
'Papilin' on 'eGFR'

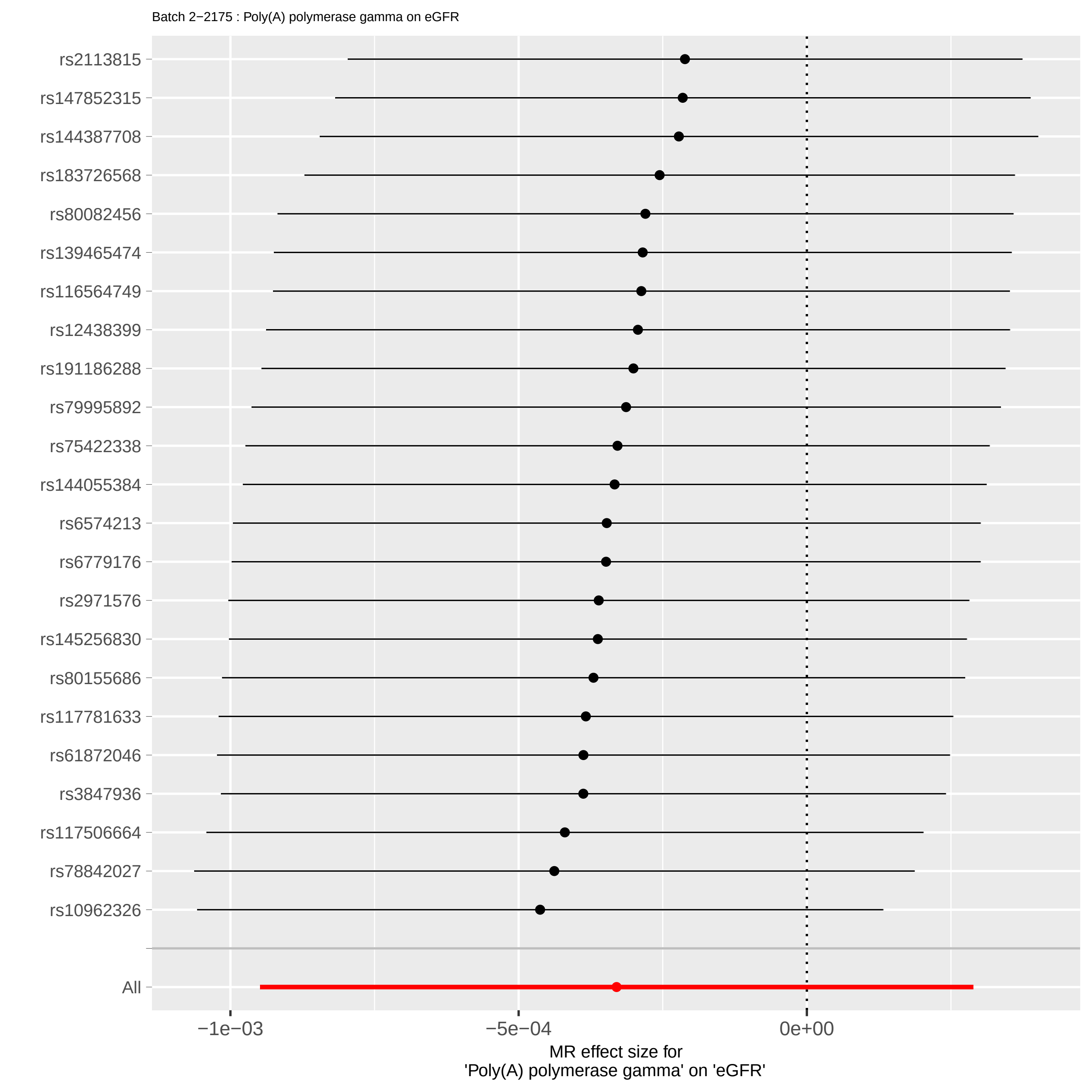

Batch 2−2175 : Poly(A) polymerase gamma on eGFR
rs2113815
rs147852315
rs144387708
rs183726568
rs80082456
rs139465474
rs116564749
rs12438399
rs191186288
rs79995892
rs75422338
rs144055384
rs6574213
rs6779176
rs2971576
rs145256830
rs80155686
rs117781633
rs61872046
rs3847936
rs117506664
rs78842027
rs10962326
All
−1e−03
−5e−04
0e+00
MR effect size for
'Poly(A) polymerase gamma' on 'eGFR'

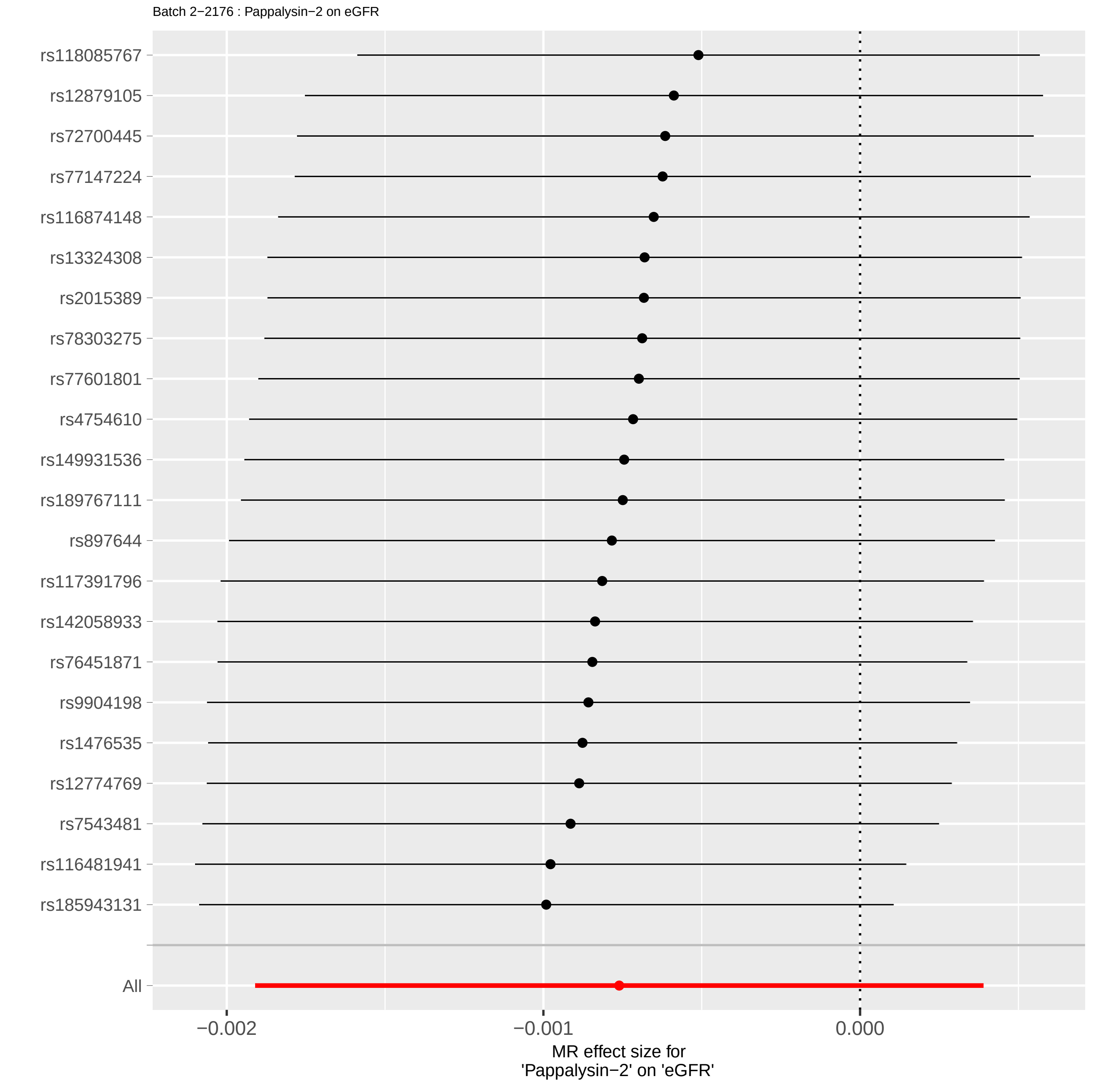

Batch 2−2176 : Pappalysin−2 on eGFR
rs118085767
rs12879105
rs72700445
rs77147224
rs116874148
rs13324308
rs2015389
rs78303275
rs77601801
rs4754610
rs149931536
rs189767111
rs897644
rs117391796
rs142058933
rs76451871
rs9904198
rs1476535
rs12774769
rs7543481
rs116481941
rs185943131
All
−0.002
−0.001
0.000
MR effect size for
'Pappalysin−2' on 'eGFR'

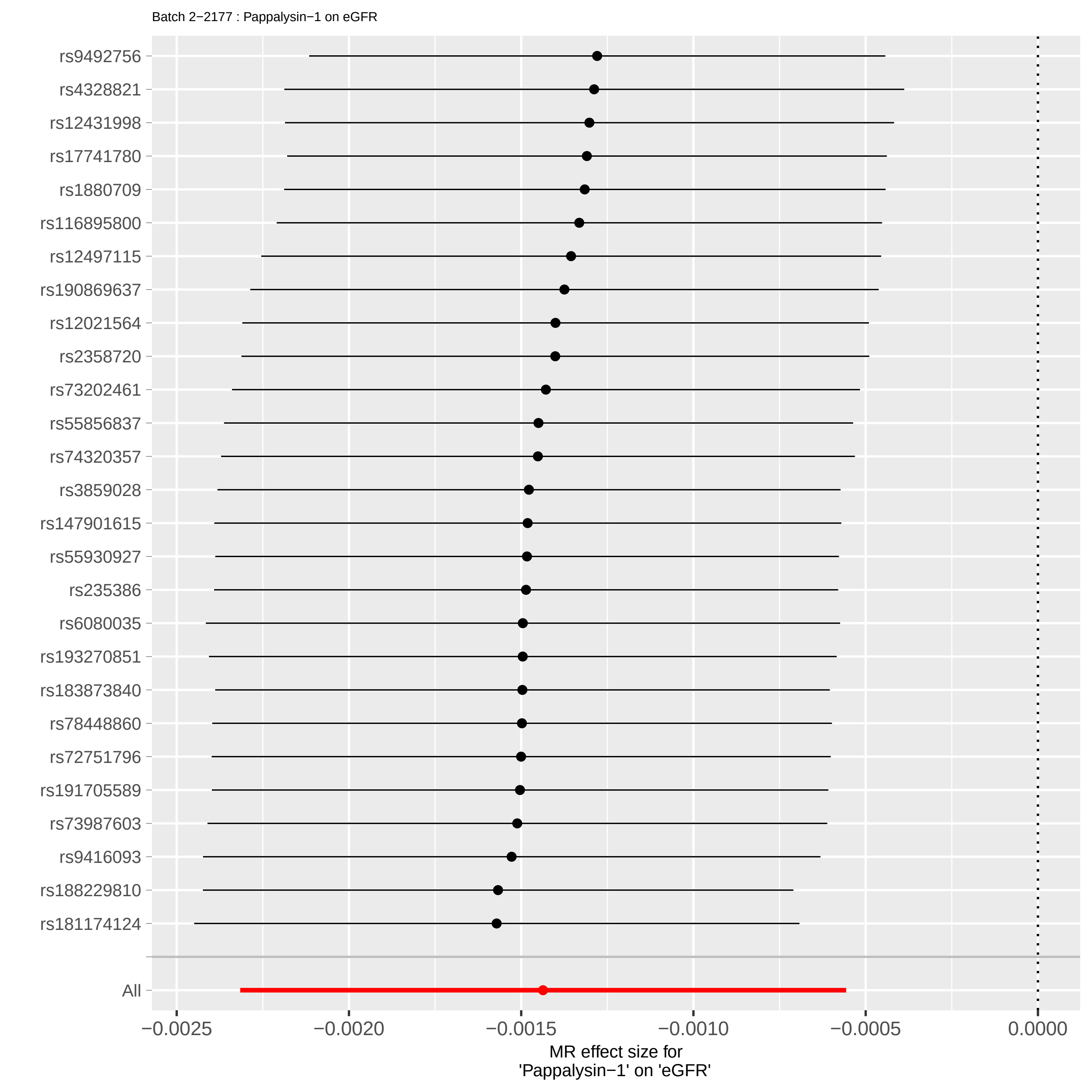

Batch 2−2177 : Pappalysin−1 on eGFR
rs9492756
rs4328821
rs12431998
rs17741780
rs1880709
rs116895800
rs12497115
rs190869637
rs12021564
rs2358720
rs73202461
rs55856837
rs74320357
rs3859028
rs147901615
rs55930927
rs235386
rs6080035
rs193270851
rs183873840
rs78448860
rs72751796
rs191705589
rs73987603
rs9416093
rs188229810
rs181174124
All
−0.0025
−0.0020
−0.0015
−0.0010
−0.0005
0.0000
MR effect size for
'Pappalysin−1' on 'eGFR'

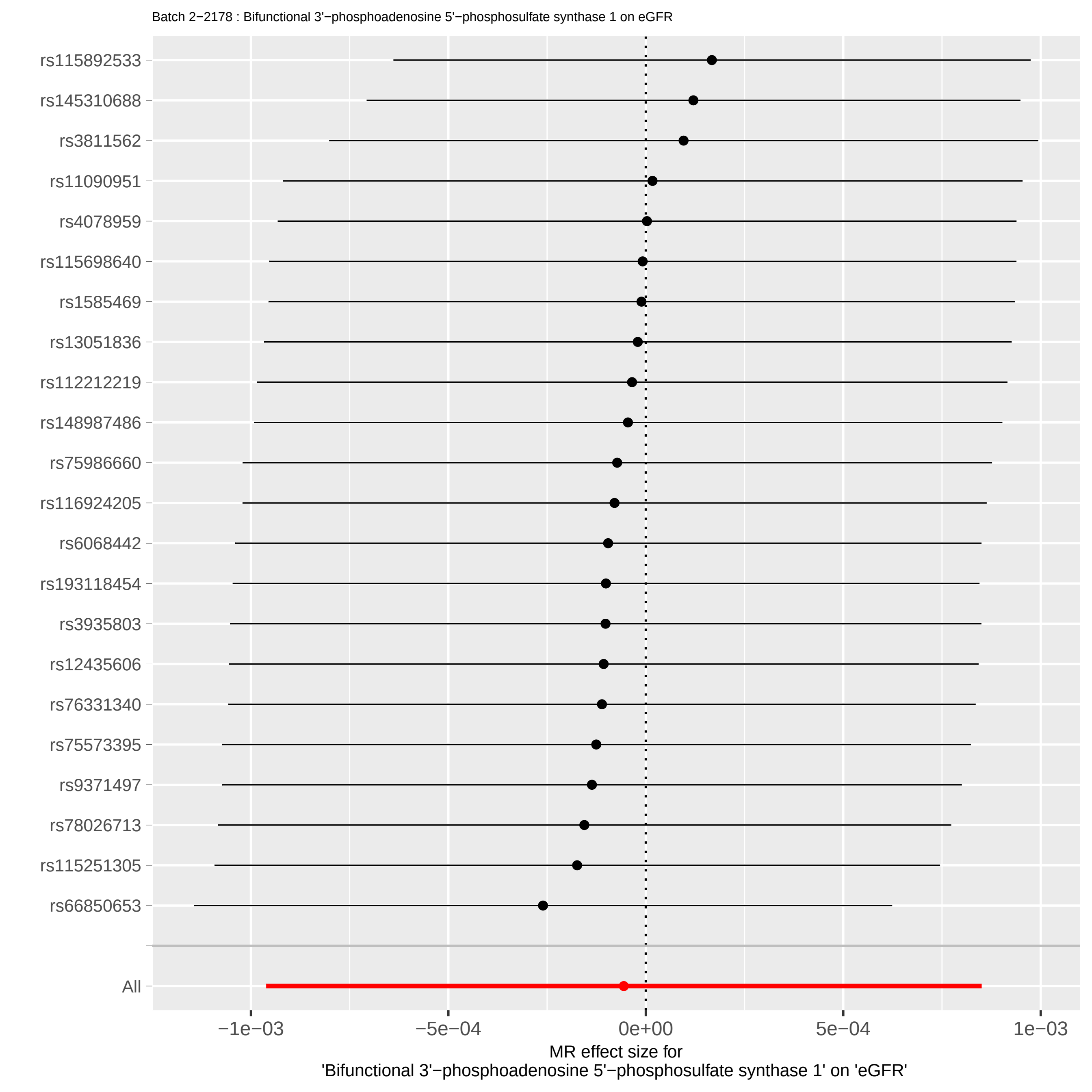

Batch 2−2178 : Bifunctional 3'−phosphoadenosine 5'−phosphosulfate synthase 1 on eGFR
rs115892533
rs145310688
rs3811562
rs11090951
rs4078959
rs115698640
rs1585469
rs13051836
rs112212219
rs148987486
rs75986660
rs116924205
rs6068442
rs193118454
rs3935803
rs12435606
rs76331340
rs75573395
rs9371497
rs78026713
rs115251305
rs66850653
All
−1e−03
−5e−04
0e+00
5e−04
1e−03
MR effect size for
'Bifunctional 3'−phosphoadenosine 5'−phosphosulfate synthase 1' on 'eGFR'

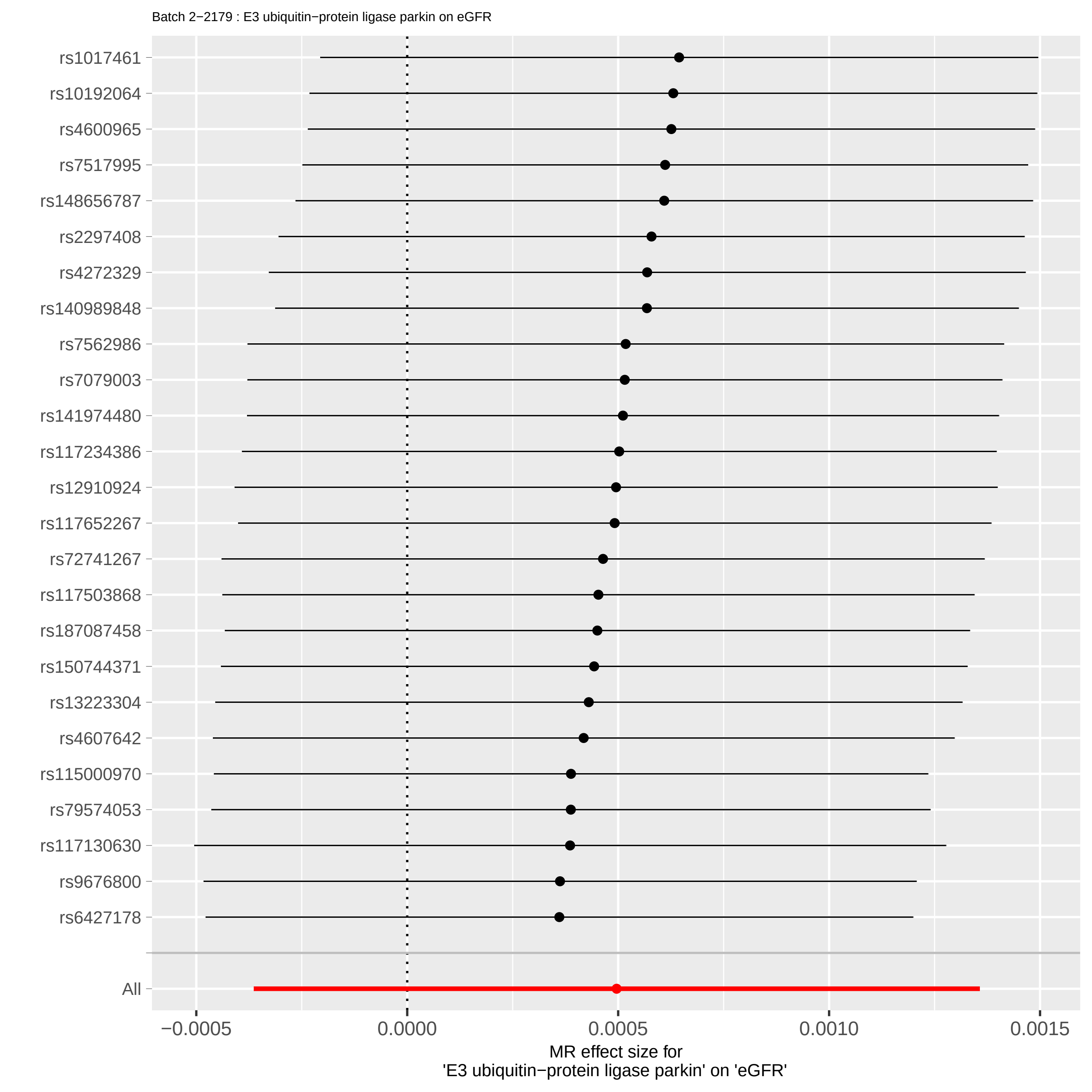

Batch 2−2179 : E3 ubiquitin−protein ligase parkin on eGFR
rs1017461
rs10192064
rs4600965
rs7517995
rs148656787
rs2297408
rs4272329
rs140989848
rs7562986
rs7079003
rs141974480
rs117234386
rs12910924
rs117652267
rs72741267
rs117503868
rs187087458
rs150744371
rs13223304
rs4607642
rs115000970
rs79574053
rs117130630
rs9676800
rs6427178
All
−0.0005
0.0000
0.0005
0.0010
0.0015
MR effect size for
'E3 ubiquitin−protein ligase parkin' on 'eGFR'

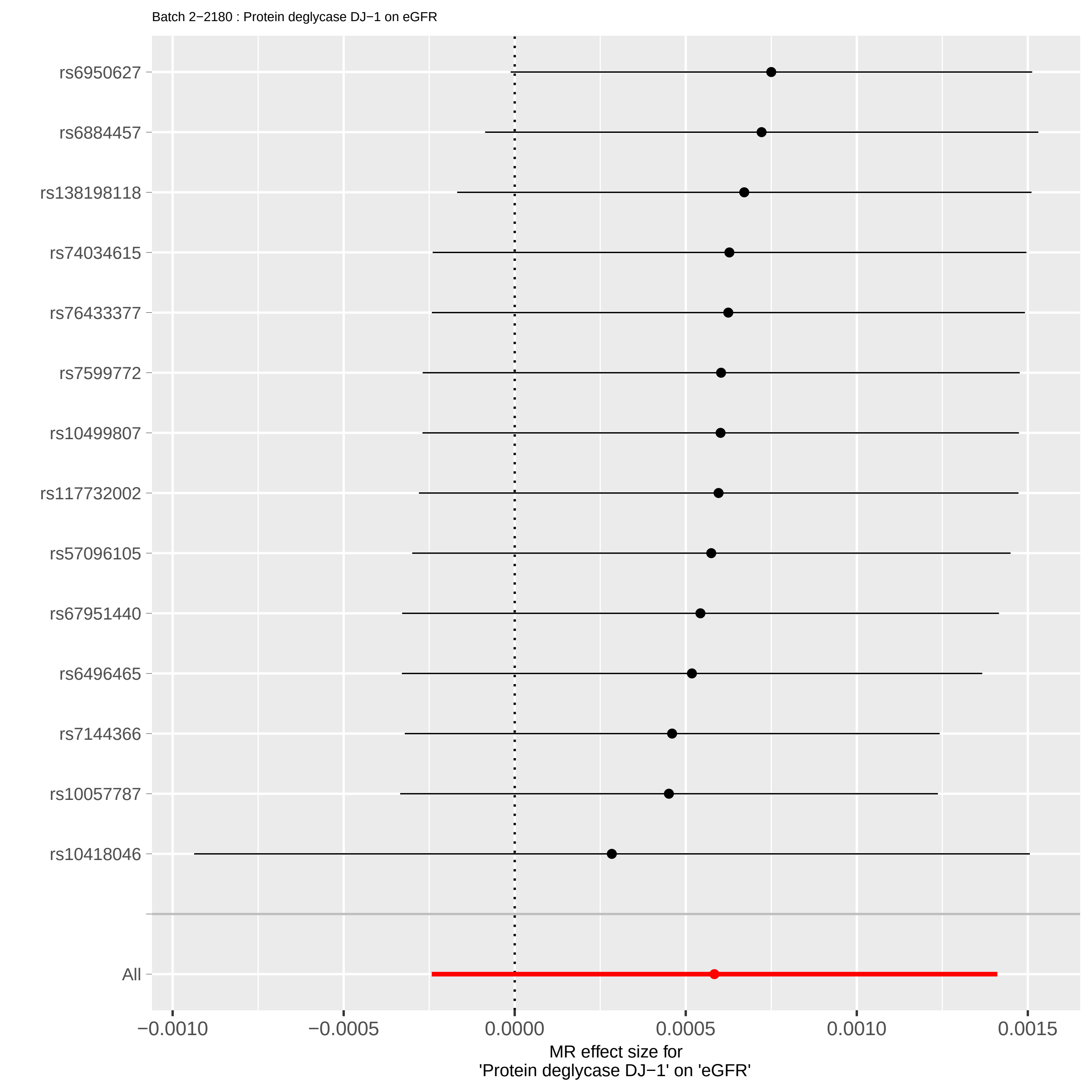

Batch 2−2180 : Protein deglycase DJ−1 on eGFR
rs6950627
rs6884457
rs138198118
rs74034615
rs76433377
rs7599772
rs10499807
rs117732002
rs57096105
rs67951440
rs6496465
rs7144366
rs10057787
rs10418046
All
−0.0010
−0.0005
0.0000
0.0005
0.0010
0.0015
MR effect size for
'Protein deglycase DJ−1' on 'eGFR'

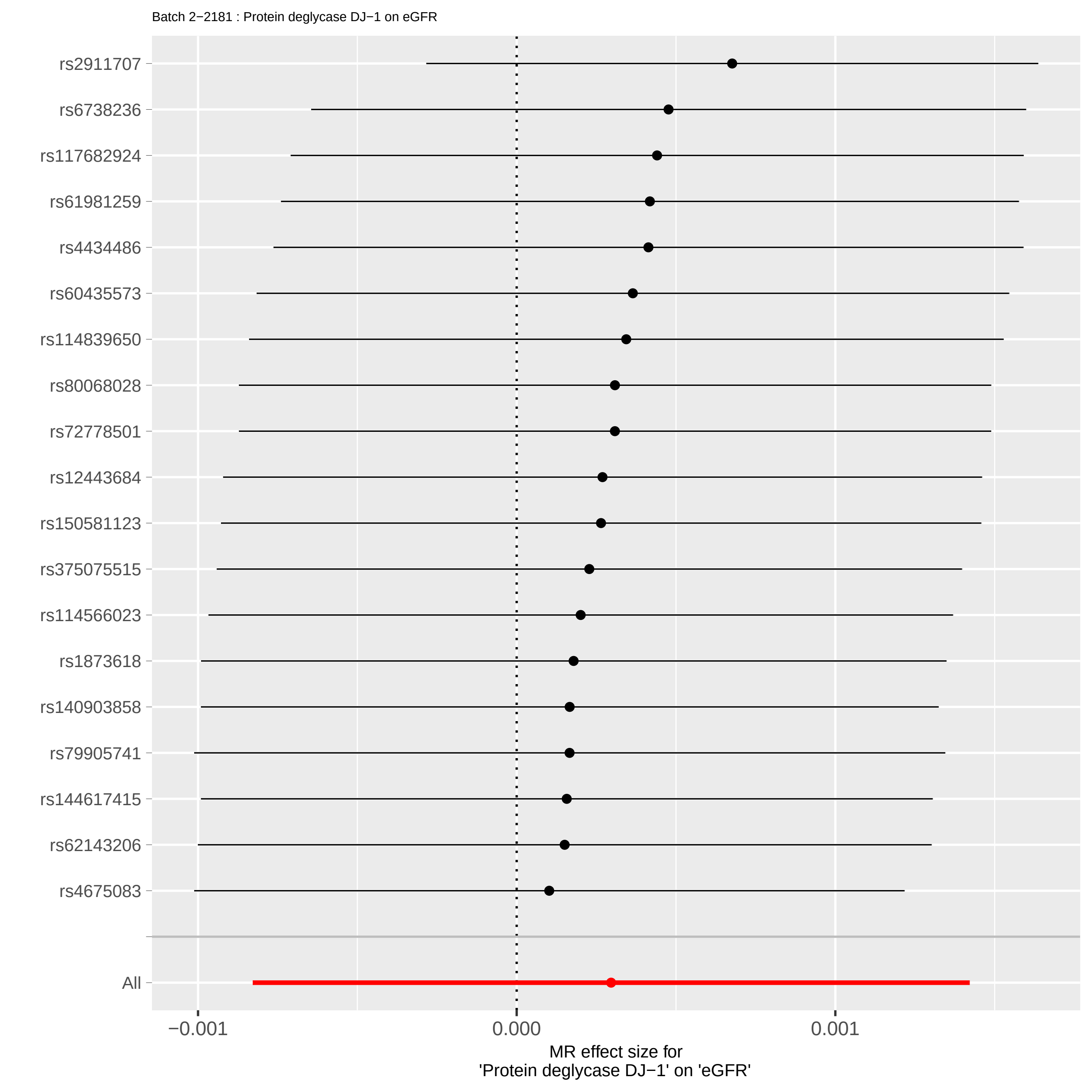
Batch 2−2181 : Protein deglycase DJ−1 on eGFR
rs2911707
rs6738236
rs117682924
rs61981259
rs4434486
rs60435573
rs114839650
rs80068028
rs72778501
rs12443684
rs150581123
rs375075515
rs114566023
rs1873618
rs140903858
rs79905741
rs144617415
rs62143206
rs4675083
All
−0.001
0.000
0.001
MR effect size for
'Protein deglycase DJ−1' on 'eGFR'

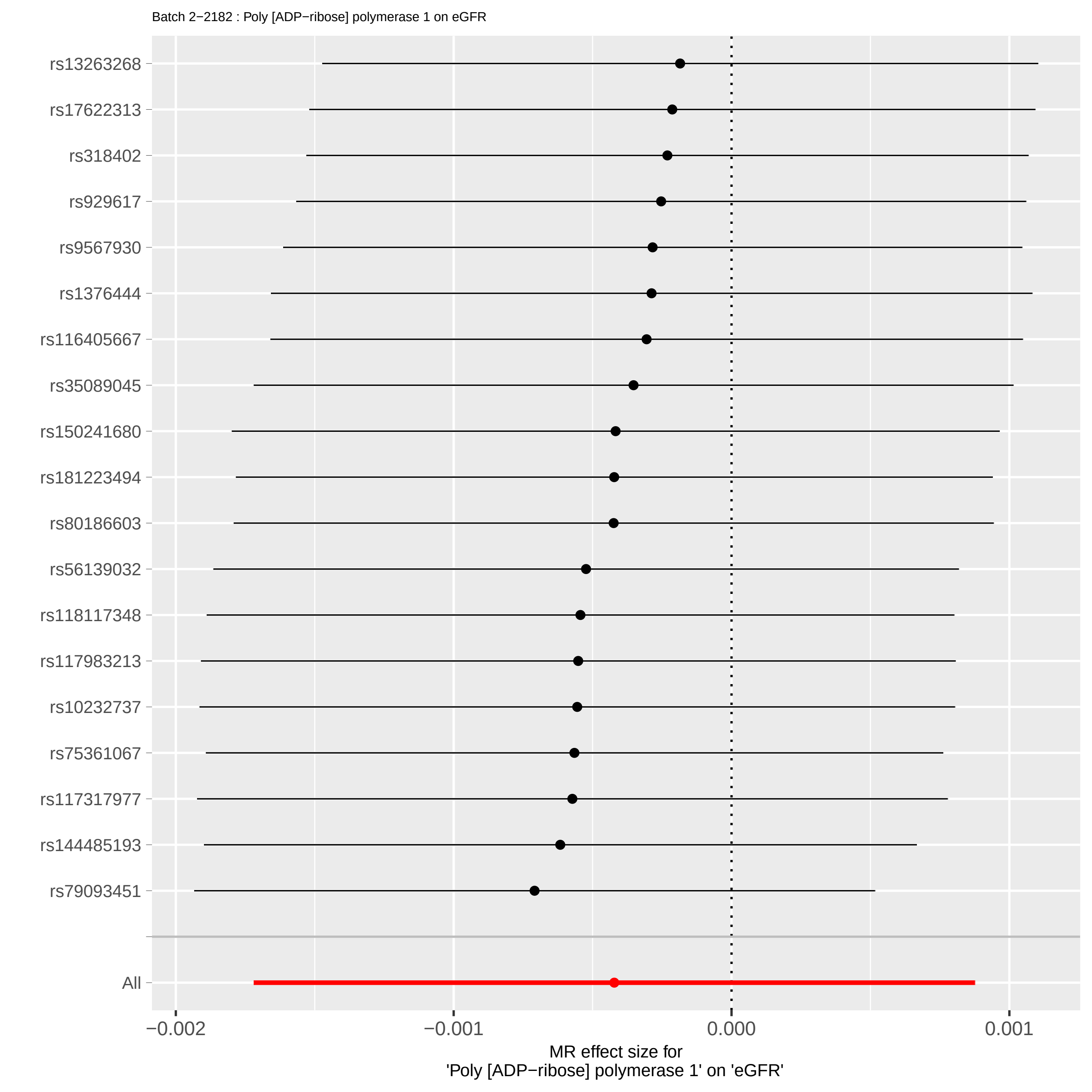

Batch 2−2182 : Poly [ADP−ribose] polymerase 1 on eGFR
rs13263268
rs17622313
rs318402
rs929617
rs9567930
rs1376444
rs116405667
rs35089045
rs150241680
rs181223494
rs80186603
rs56139032
rs118117348
rs117983213
rs10232737
rs75361067
rs117317977
rs144485193
rs79093451
All
−0.002
−0.001
0.000
0.001
MR effect size for
'Poly [ADP−ribose] polymerase 1' on 'eGFR'

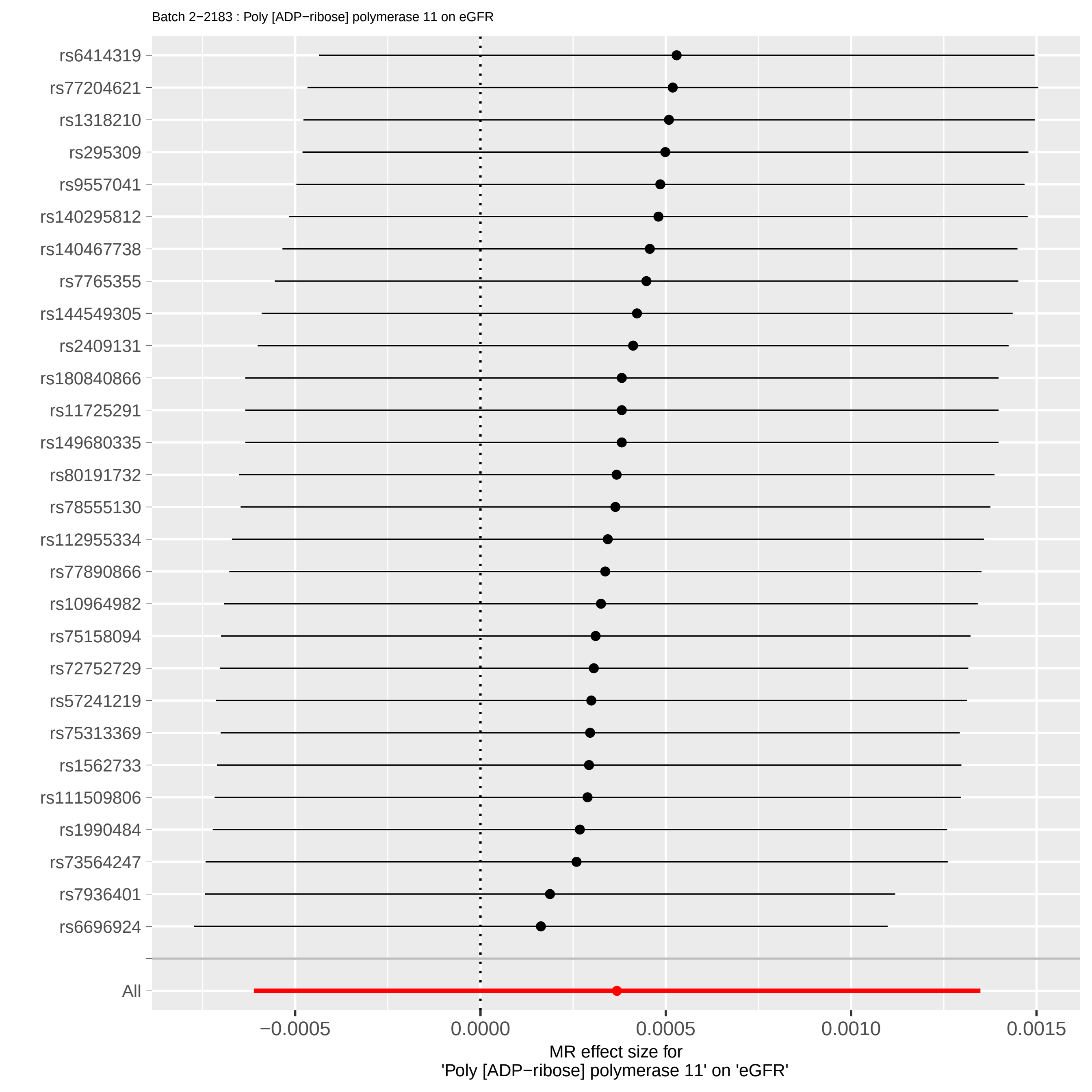
Batch 2−2183 : Poly [ADP−ribose] polymerase 11 on eGFR
rs6414319
rs77204621
rs1318210
rs295309
rs9557041
rs140295812
rs140467738
rs7765355
rs144549305
rs2409131
rs180840866
rs11725291
rs149680335
rs80191732
rs78555130
rs112955334
rs77890866
rs10964982
rs75158094
rs72752729
rs57241219
rs75313369
rs1562733
rs111509806
rs1990484
rs73564247
rs7936401
rs6696924
All
−0.0005
0.0000
0.0005
0.0010
0.0015
MR effect size for
'Poly [ADP−ribose] polymerase 11' on 'eGFR'

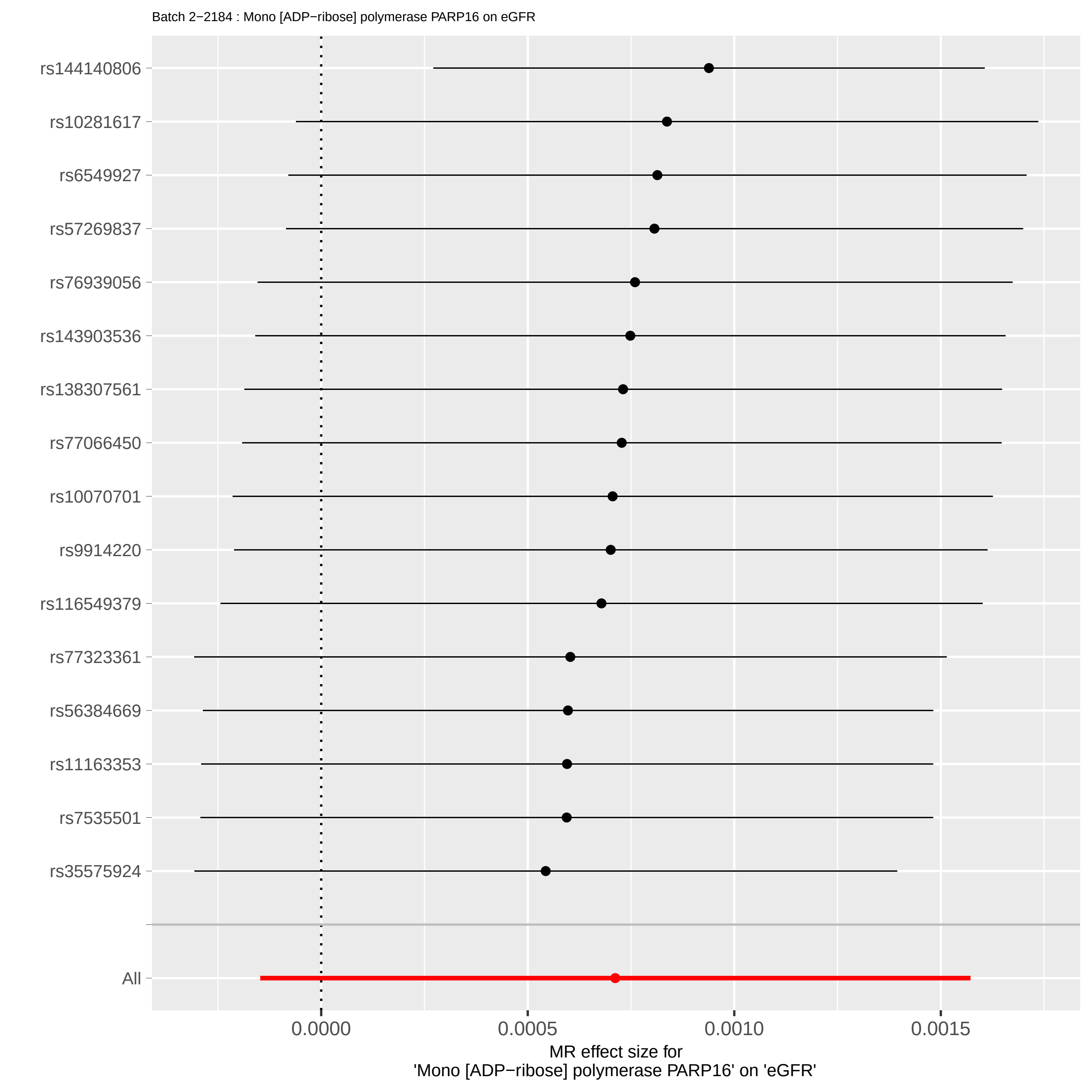

Batch 2−2184 : Mono [ADP−ribose] polymerase PARP16 on eGFR
rs144140806
rs10281617
rs6549927
rs57269837
rs76939056
rs143903536
rs138307561
rs77066450
rs10070701
rs9914220
rs116549379
rs77323361
rs56384669
rs11163353
rs7535501
rs35575924
All
0.0000
0.0005
0.0010
0.0015
MR effect size for
'Mono [ADP−ribose] polymerase PARP16' on 'eGFR'

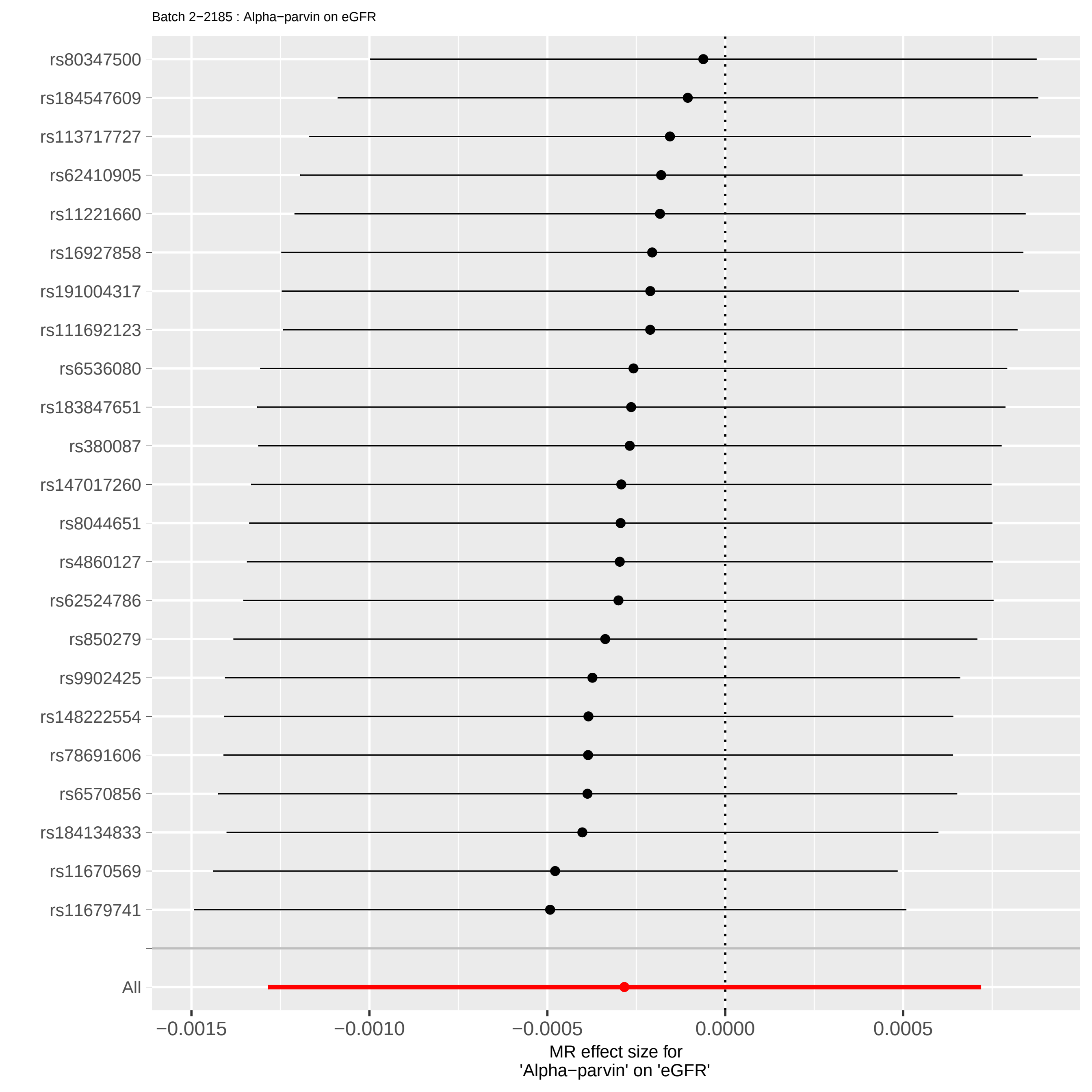

Batch 2−2185 : Alpha−parvin on eGFR
rs80347500
rs184547609
rs113717727
rs62410905
rs11221660
rs16927858
rs191004317
rs111692123
rs6536080
rs183847651
rs380087
rs147017260
rs8044651
rs4860127
rs62524786
rs850279
rs9902425
rs148222554
rs78691606
rs6570856
rs184134833
rs11670569
rs11679741
All
−0.0015
−0.0010
−0.0005
0.0000
0.0005
MR effect size for
'Alpha−parvin' on 'eGFR'

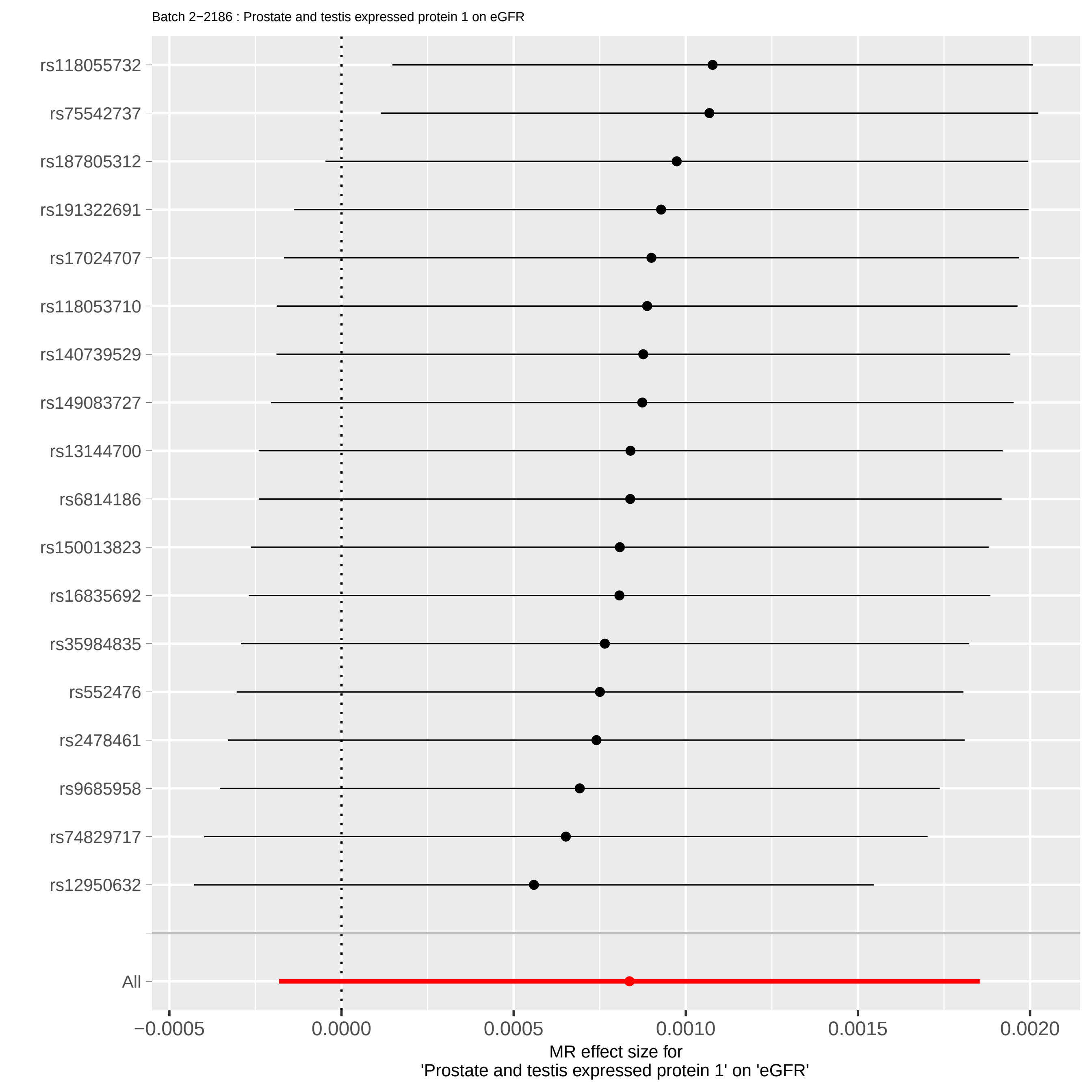

Batch 2−2186 : Prostate and testis expressed protein 1 on eGFR
rs118055732
rs75542737
rs187805312
rs191322691
rs17024707
rs118053710
rs140739529
rs149083727
rs13144700
rs6814186
rs150013823
rs16835692
rs35984835
rs552476
rs2478461
rs9685958
rs74829717
rs12950632
All
−0.0005
0.0000
0.0005
0.0010
0.0015
0.0020
MR effect size for
'Prostate and testis expressed protein 1' on 'eGFR'

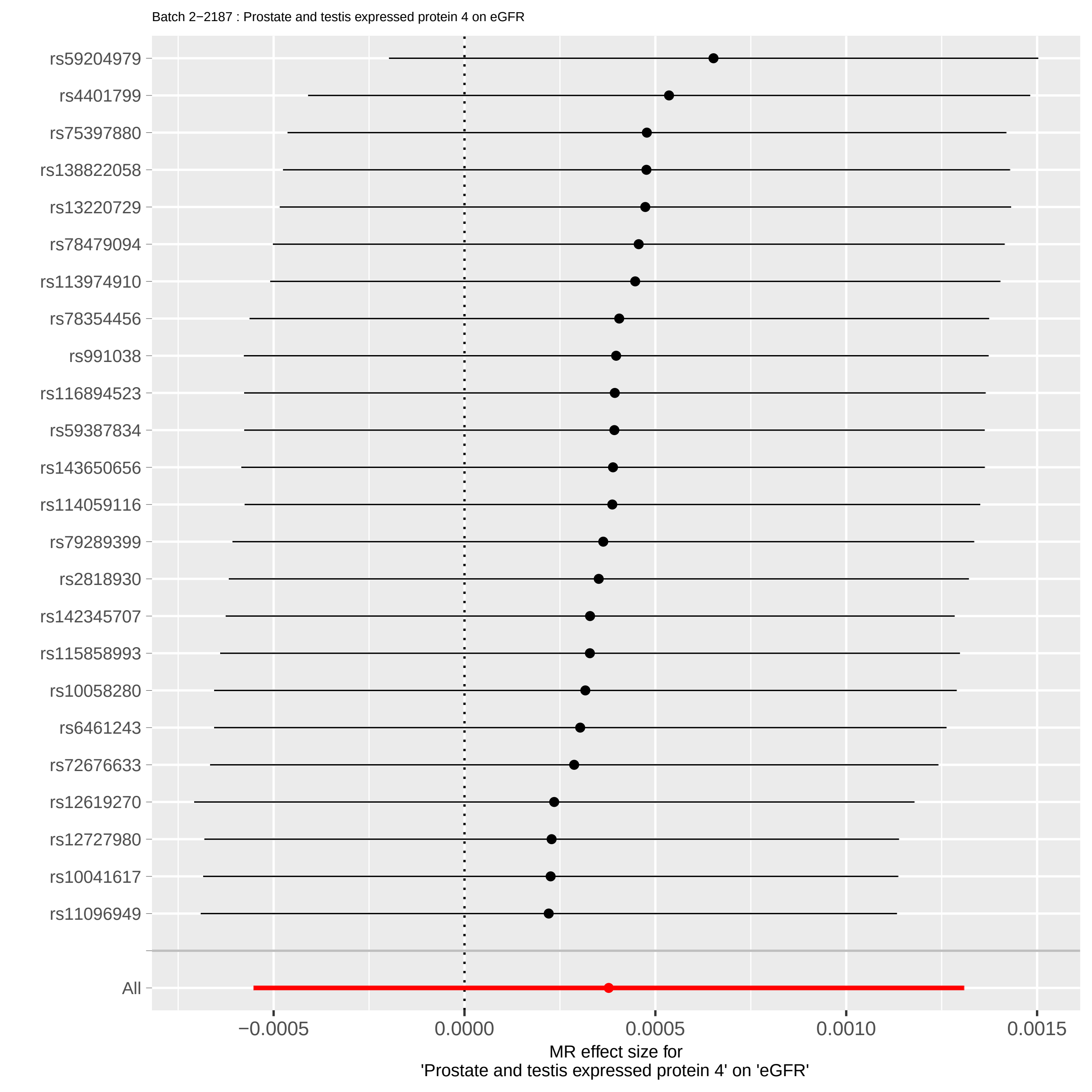
Batch 2−2187 : Prostate and testis expressed protein 4 on eGFR
rs59204979
rs4401799
rs75397880
rs138822058
rs13220729
rs78479094
rs113974910
rs78354456
rs991038
rs116894523
rs59387834
rs143650656
rs114059116
rs79289399
rs2818930
rs142345707
rs115858993
rs10058280
rs6461243
rs72676633
rs12619270
rs12727980
rs10041617
rs11096949
All
−0.0005
0.0000
0.0005
0.0010
0.0015
MR effect size for
'Prostate and testis expressed protein 4' on 'eGFR'

Batch 2−2188 : Paired box protein Pax−4 on eGFR

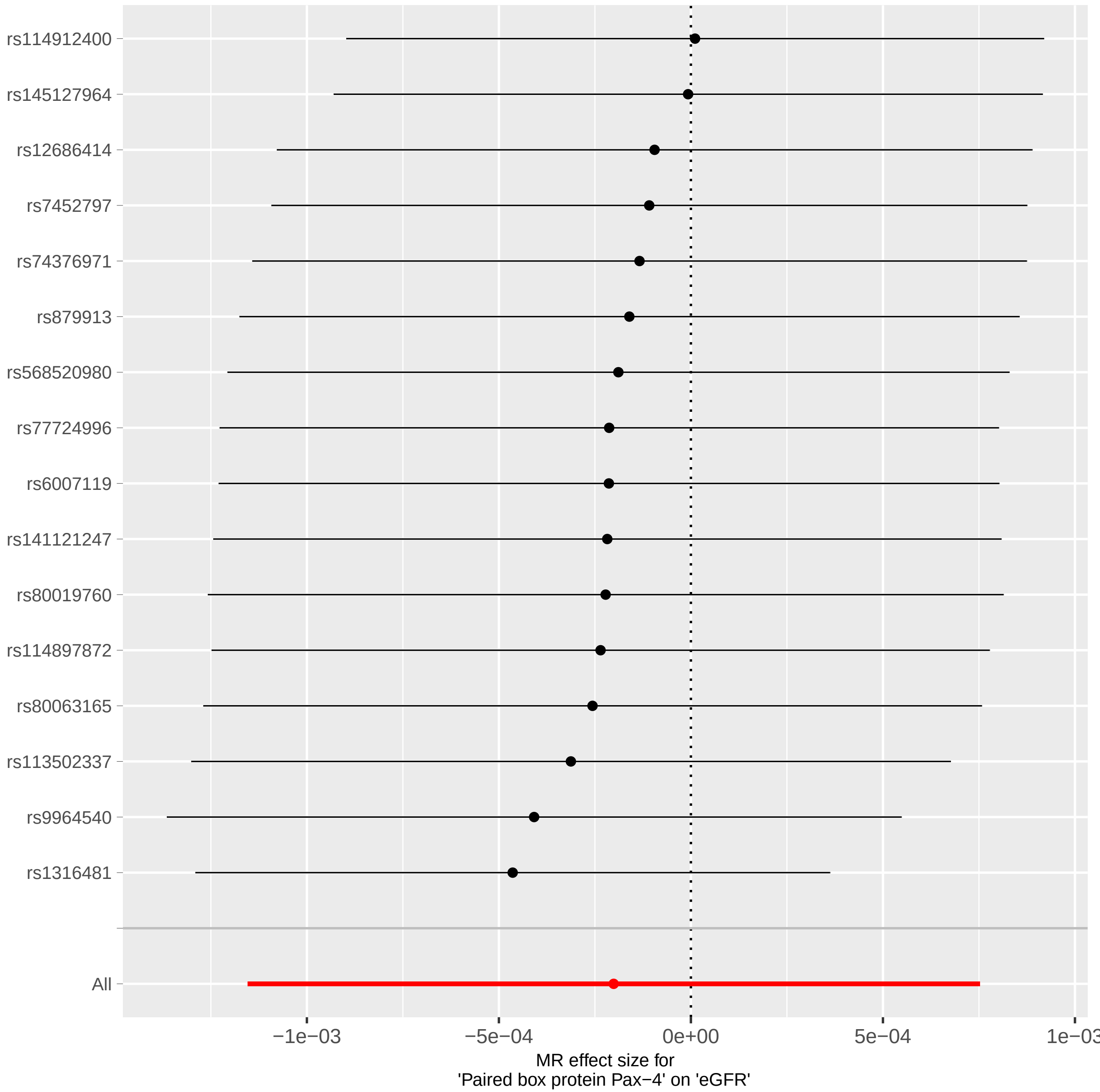

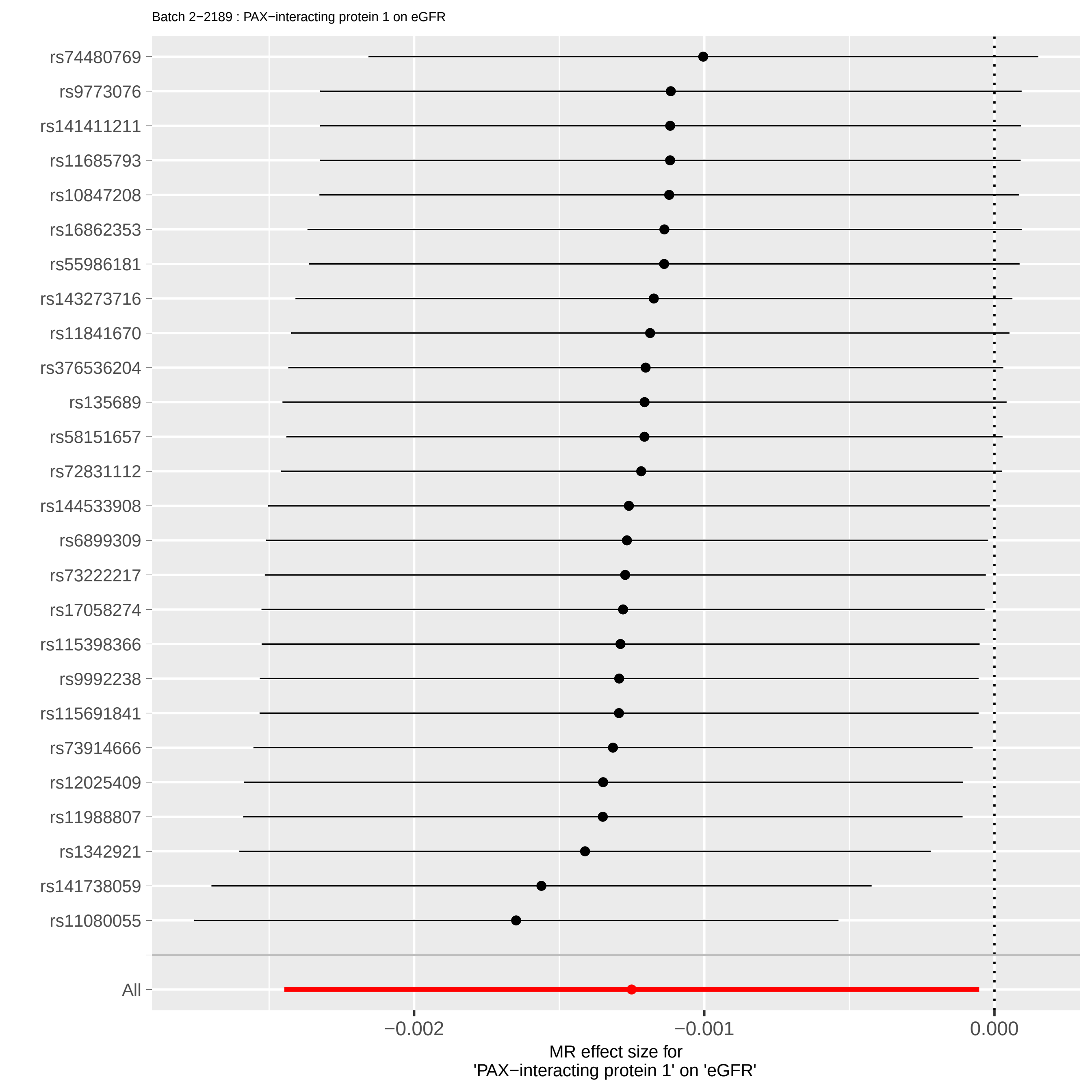
Batch 2−2189 : PAX−interacting protein 1 on eGFR
rs74480769
rs9773076
rs141411211
rs11685793
rs10847208
rs16862353
rs55986181
rs143273716
rs11841670
rs376536204
rs135689
rs58151657
rs72831112
rs144533908
rs6899309
rs73222217
rs17058274
rs115398366
rs9992238
rs115691841
rs73914666
rs12025409
rs11988807
rs1342921
rs141738059
rs11080055
All
−0.002
−0.001
0.000
MR effect size for
'PAX−interacting protein 1' on 'eGFR'

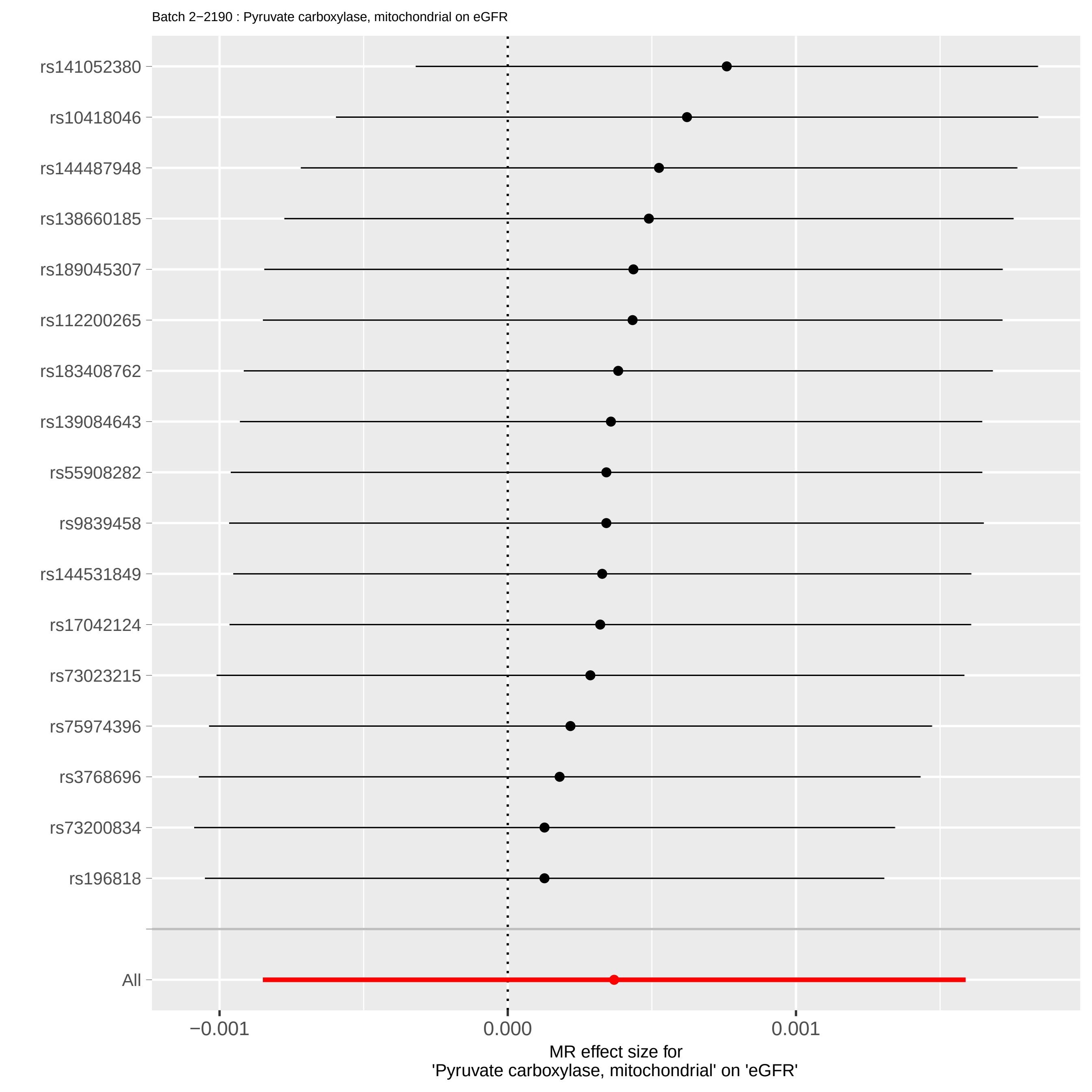

Batch 2−2190 : Pyruvate carboxylase, mitochondrial on eGFR
rs141052380
rs10418046
rs144487948
rs138660185
rs189045307
rs112200265
rs183408762
rs139084643
rs55908282
rs9839458
rs144531849
rs17042124
rs73023215
rs75974396
rs3768696
rs73200834
rs196818
All
−0.001
0.000
0.001
MR effect size for
'Pyruvate carboxylase, mitochondrial' on 'eGFR'

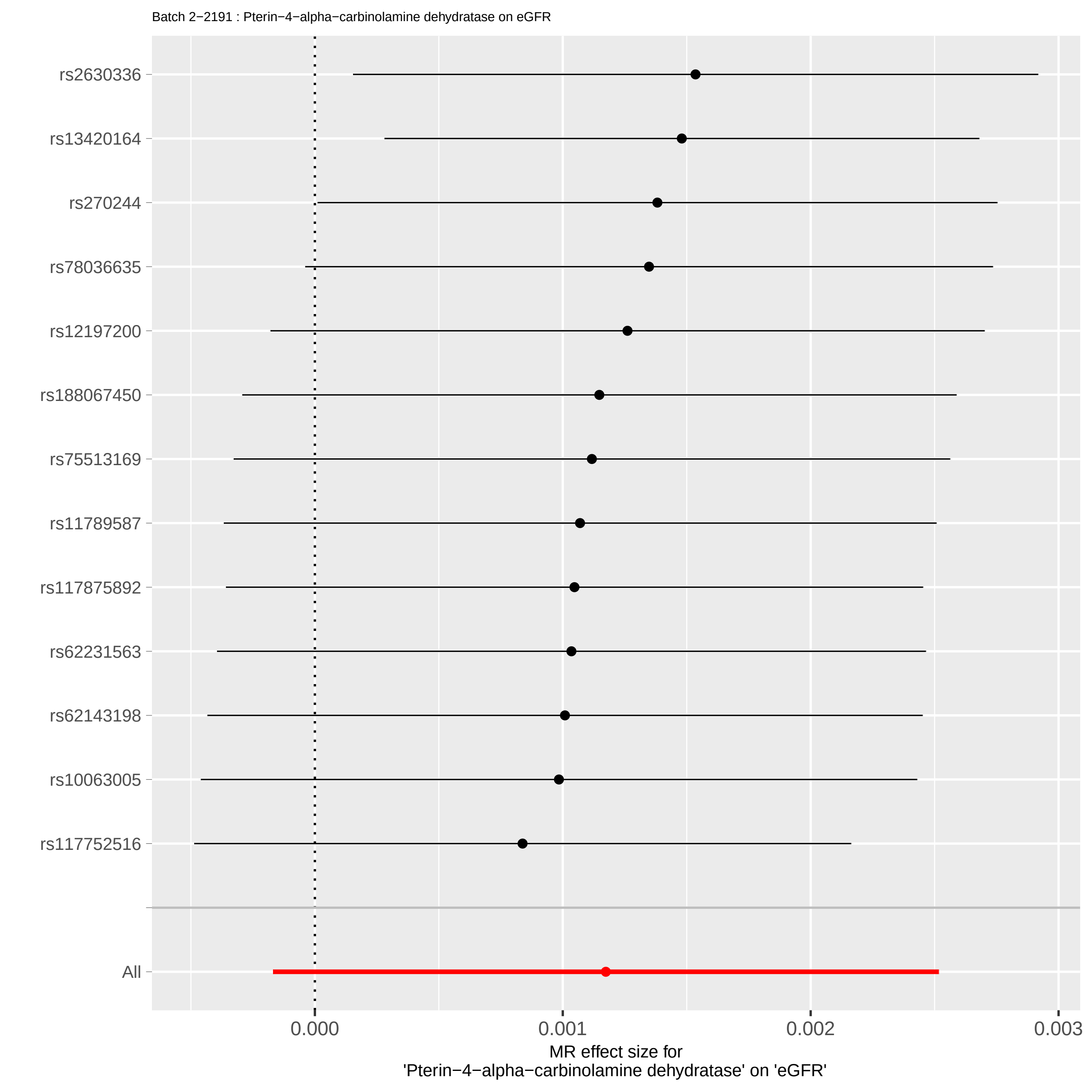
Batch 2−2191 : Pterin−4−alpha−carbinolamine dehydratase on eGFR
rs2630336
rs13420164
rs270244
rs78036635
rs12197200
rs188067450
rs75513169
rs11789587
rs117875892
rs62231563
rs62143198
rs10063005
rs117752516
All
0.000
0.001
0.002
0.003
MR effect size for
'Pterin−4−alpha−carbinolamine dehydratase' on 'eGFR'

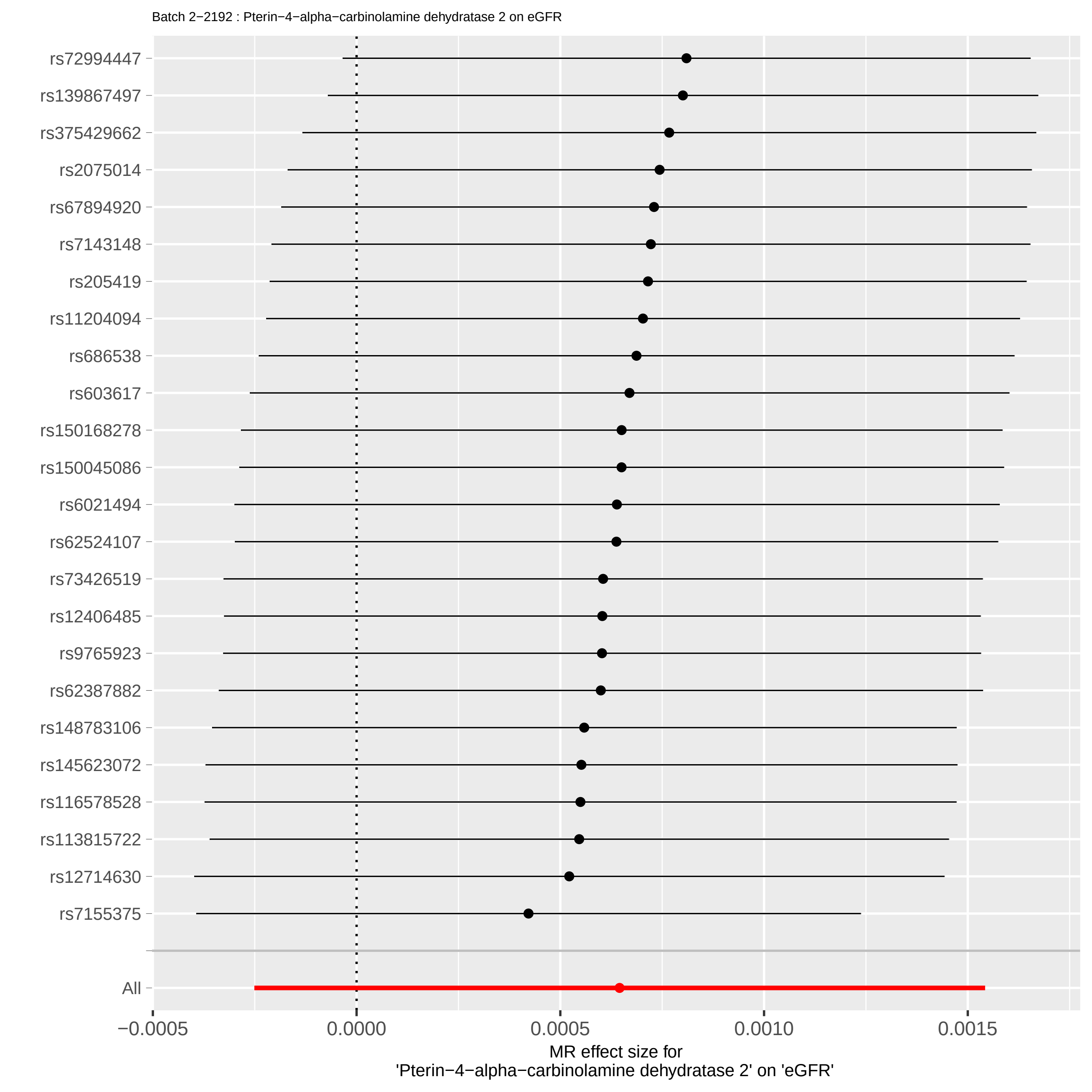

Batch 2−2192 : Pterin−4−alpha−carbinolamine dehydratase 2 on eGFR
rs72994447
rs139867497
rs375429662
rs2075014
rs67894920
rs7143148
rs205419
rs11204094
rs686538
rs603617
rs150168278
rs150045086
rs6021494
rs62524107
rs73426519
rs12406485
rs9765923
rs62387882
rs148783106
rs145623072
rs116578528
rs113815722
rs12714630
rs7155375
All
−0.0005
0.0000
0.0005
0.0010
0.0015
MR effect size for
'Pterin−4−alpha−carbinolamine dehydratase 2' on 'eGFR'

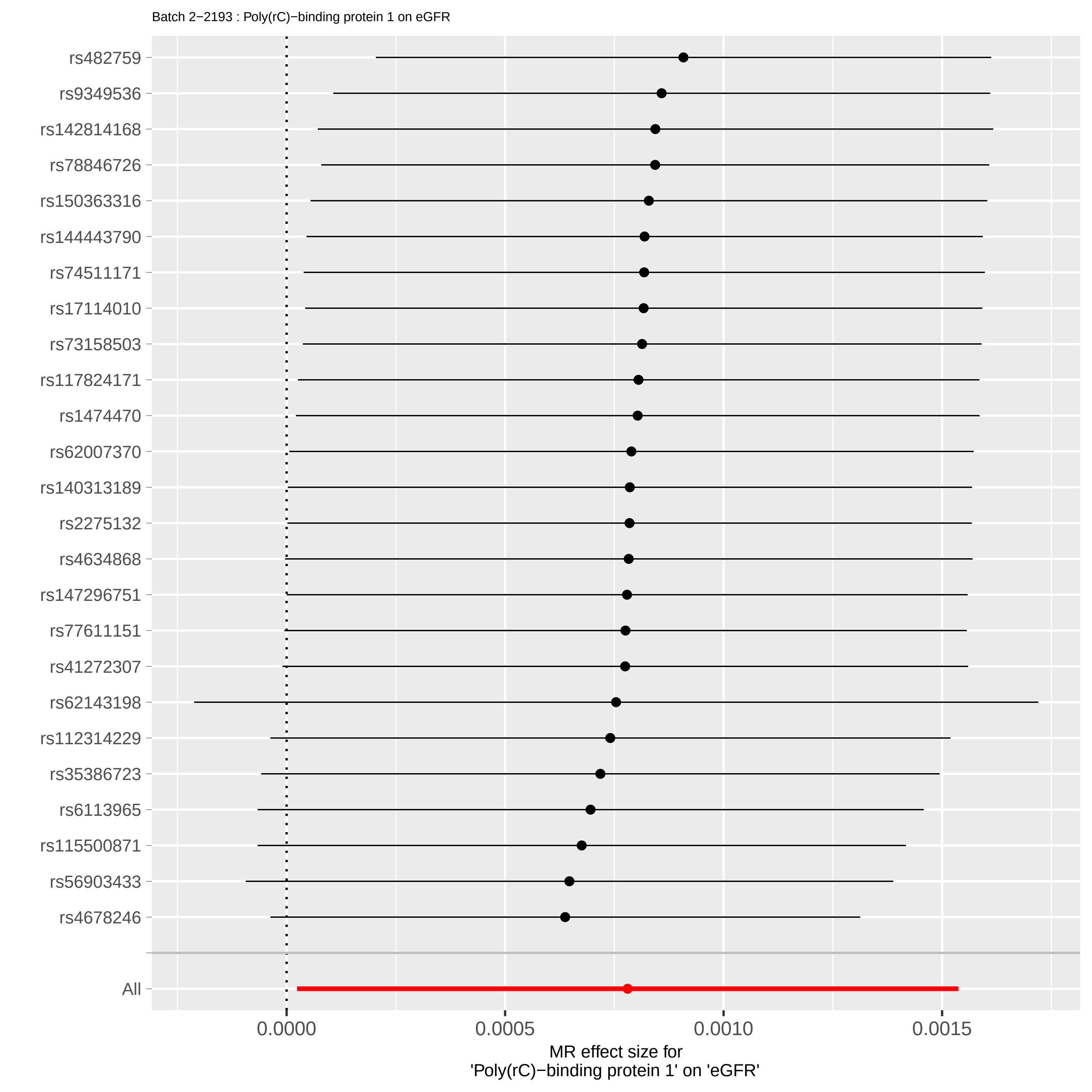

Batch 2−2193 : Poly(rC)−binding protein 1 on eGFR
rs482759
rs9349536
rs142814168
rs78846726
rs150363316
rs144443790
rs74511171
rs17114010
rs73158503
rs117824171
rs1474470
rs62007370
rs140313189
rs2275132
rs4634868
rs147296751
rs77611151
rs41272307
rs62143198
rs112314229
rs35386723
rs6113965
rs115500871
rs56903433
rs4678246
All
0.0000
0.0005
0.0010
0.0015
MR effect size for
'Poly(rC)−binding protein 1' on 'eGFR'

Batch 2−2194 : Protocadherin−10 on eGFR

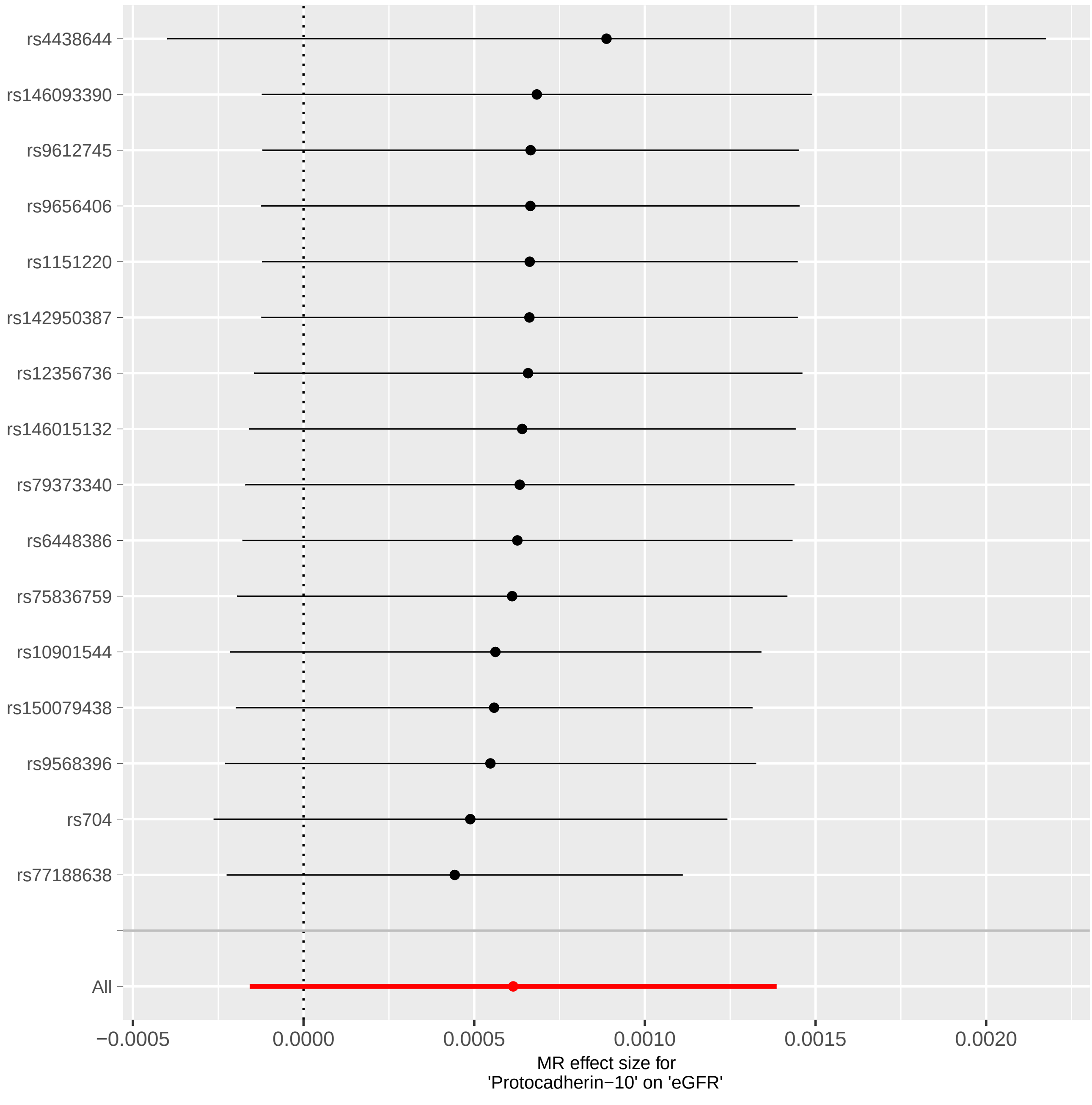

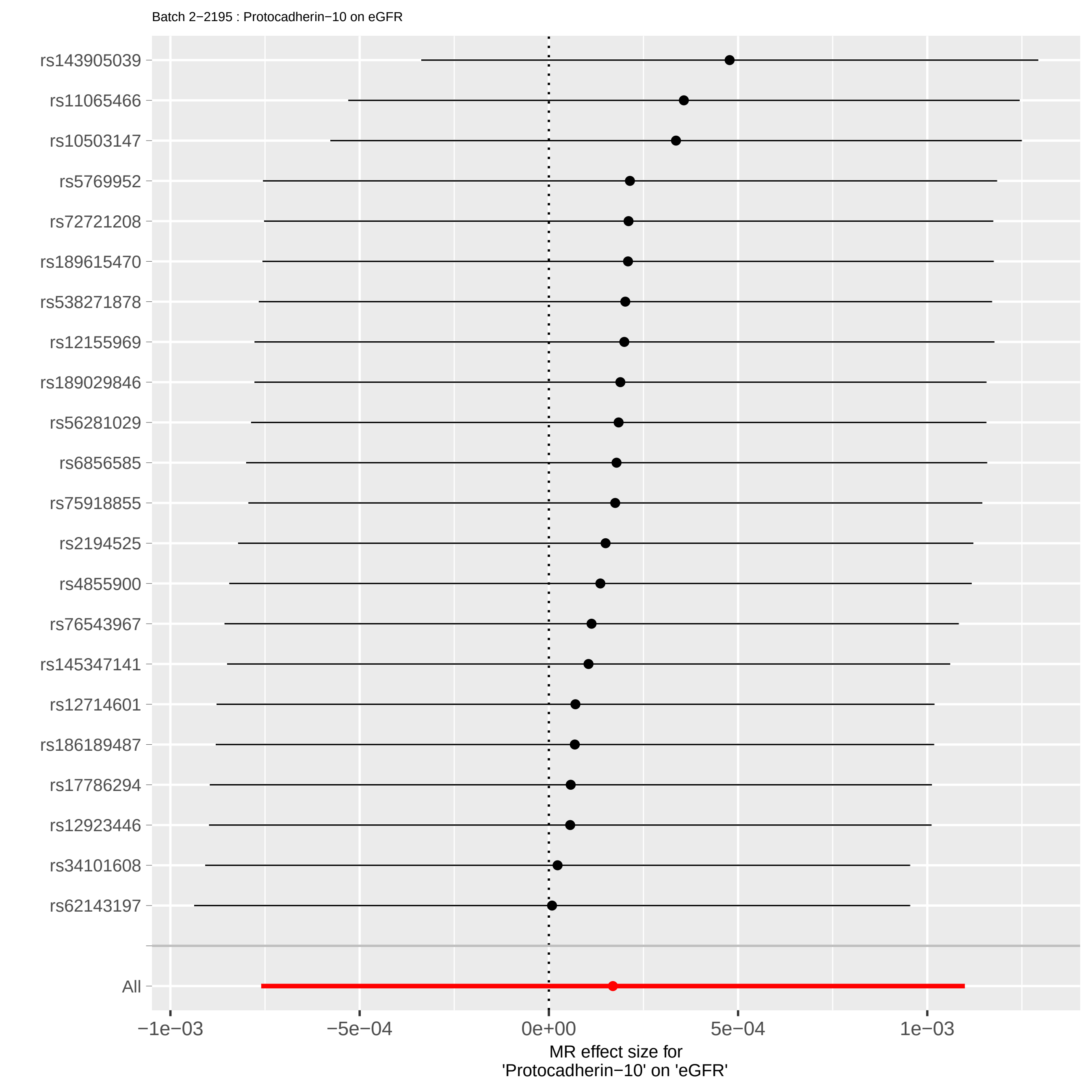

Batch 2−2195 : Protocadherin−10 on eGFR
rs143905039
rs11065466
rs10503147
rs5769952
rs72721208
rs189615470
rs538271878
rs12155969
rs189029846
rs56281029
rs6856585
rs75918855
rs2194525
rs4855900
rs76543967
rs145347141
rs12714601
rs186189487
rs17786294
rs12923446
rs34101608
rs62143197
All
−1e−03
−5e−04
0e+00
5e−04
1e−03
MR effect size for
'Protocadherin−10' on 'eGFR'

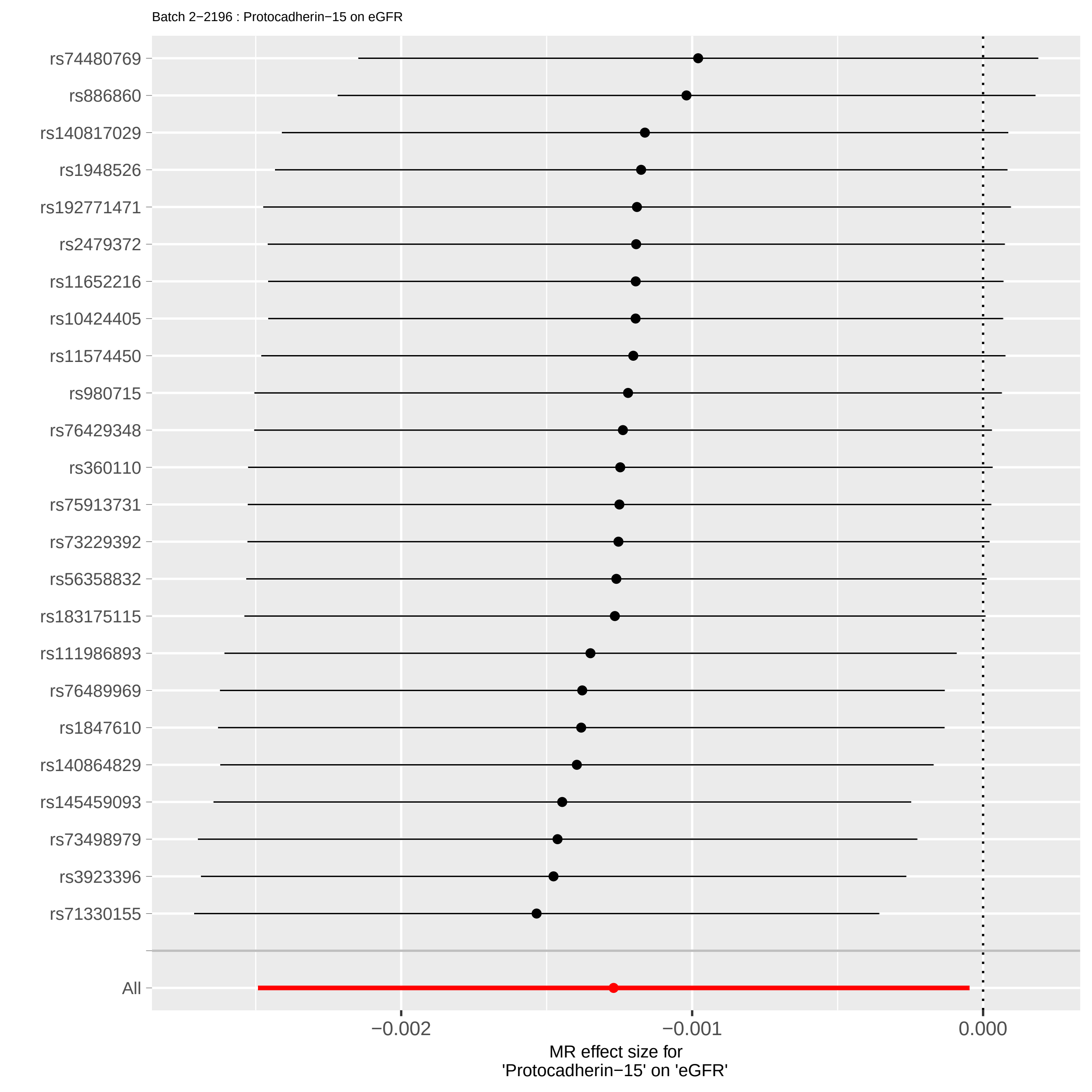
Batch 2−2196 : Protocadherin−15 on eGFR
rs74480769
rs886860
rs140817029
rs1948526
rs192771471
rs2479372
rs11652216
rs10424405
rs11574450
rs980715
rs76429348
rs360110
rs75913731
rs73229392
rs56358832
rs183175115
rs111986893
rs76489969
rs1847610
rs140864829
rs145459093
rs73498979
rs3923396
rs71330155
All
−0.002
−0.001
0.000
MR effect size for
'Protocadherin−15' on 'eGFR'

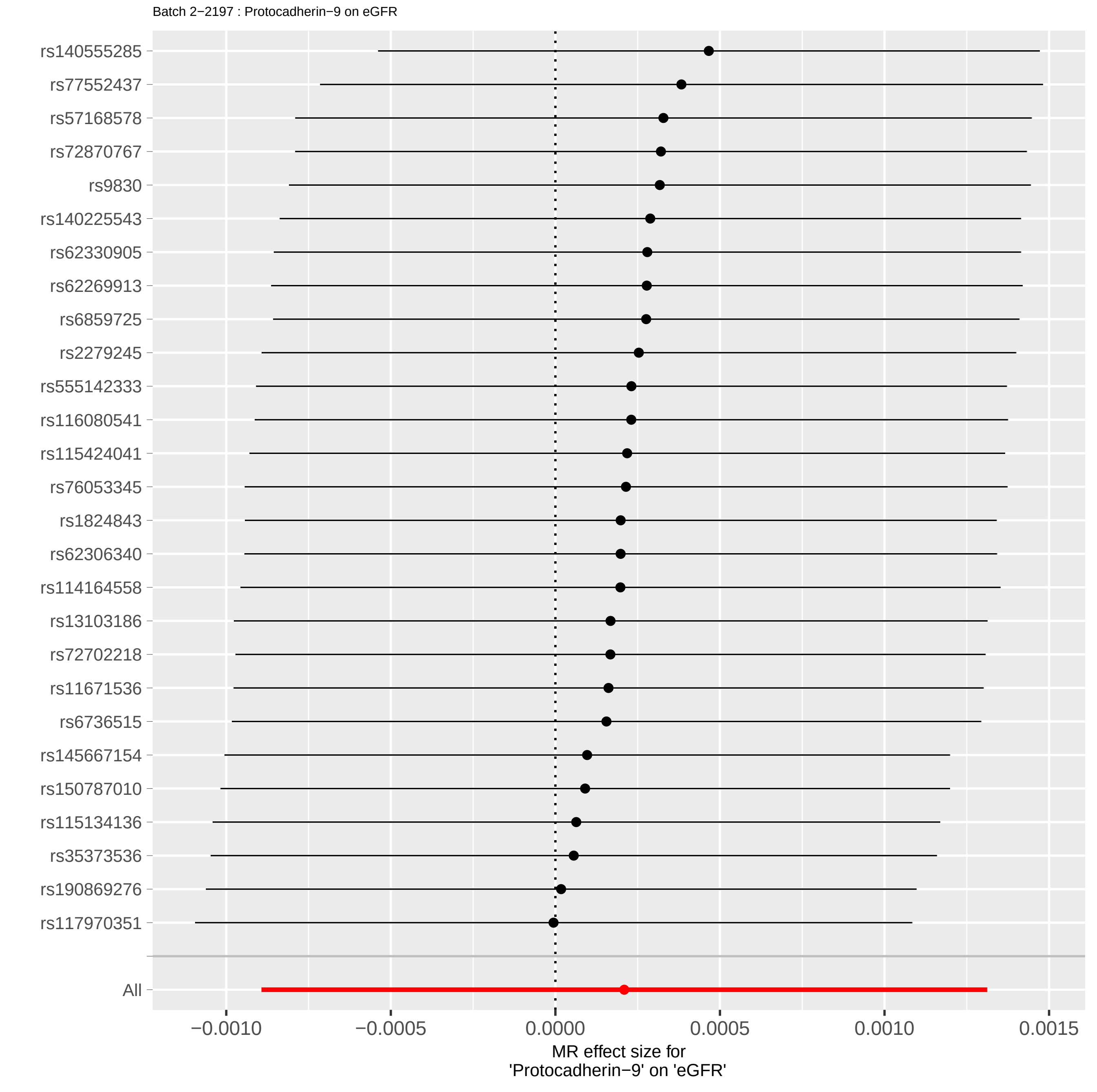
Batch 2−2197 : Protocadherin−9 on eGFR
rs140555285
rs77552437
rs57168578
rs72870767
rs9830
rs140225543
rs62330905
rs62269913
rs6859725
rs2279245
rs555142333
rs116080541
rs115424041
rs76053345
rs1824843
rs62306340
rs114164558
rs13103186
rs72702218
rs11671536
rs6736515
rs145667154
rs150787010
rs115134136
rs35373536
rs190869276
rs117970351
All
−0.0010
−0.0005
0.0000
0.0005
0.0010
0.0015
MR effect size for
'Protocadherin−9' on 'eGFR'

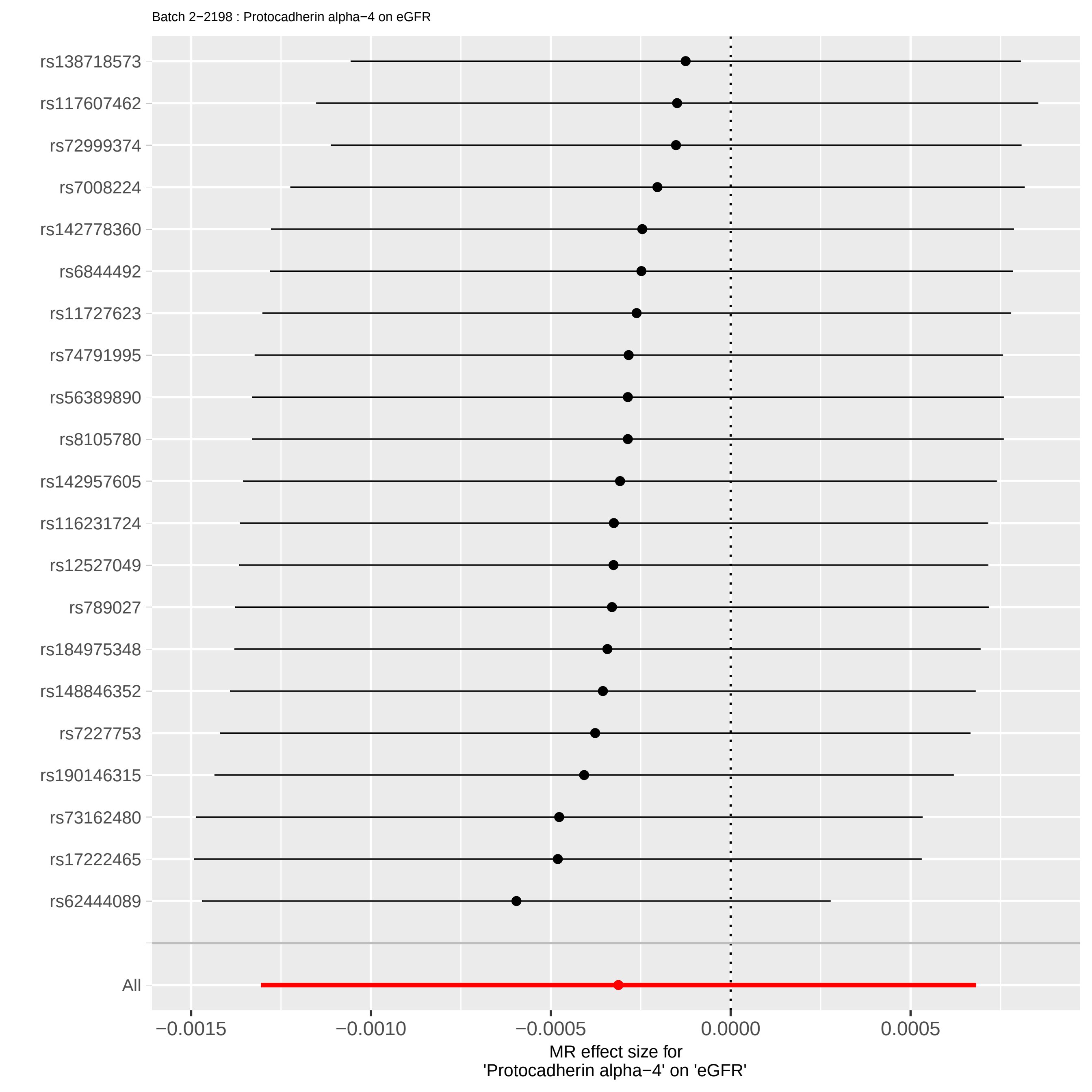
Batch 2−2198 : Protocadherin alpha−4 on eGFR
rs138718573
rs117607462
rs72999374
rs7008224
rs142778360
rs6844492
rs11727623
rs74791995
rs56389890
rs8105780
rs142957605
rs116231724
rs12527049
rs789027
rs184975348
rs148846352
rs7227753
rs190146315
rs73162480
rs17222465
rs62444089
All
−0.0015
−0.0010
−0.0005
0.0000
0.0005
MR effect size for
'Protocadherin alpha−4' on 'eGFR'

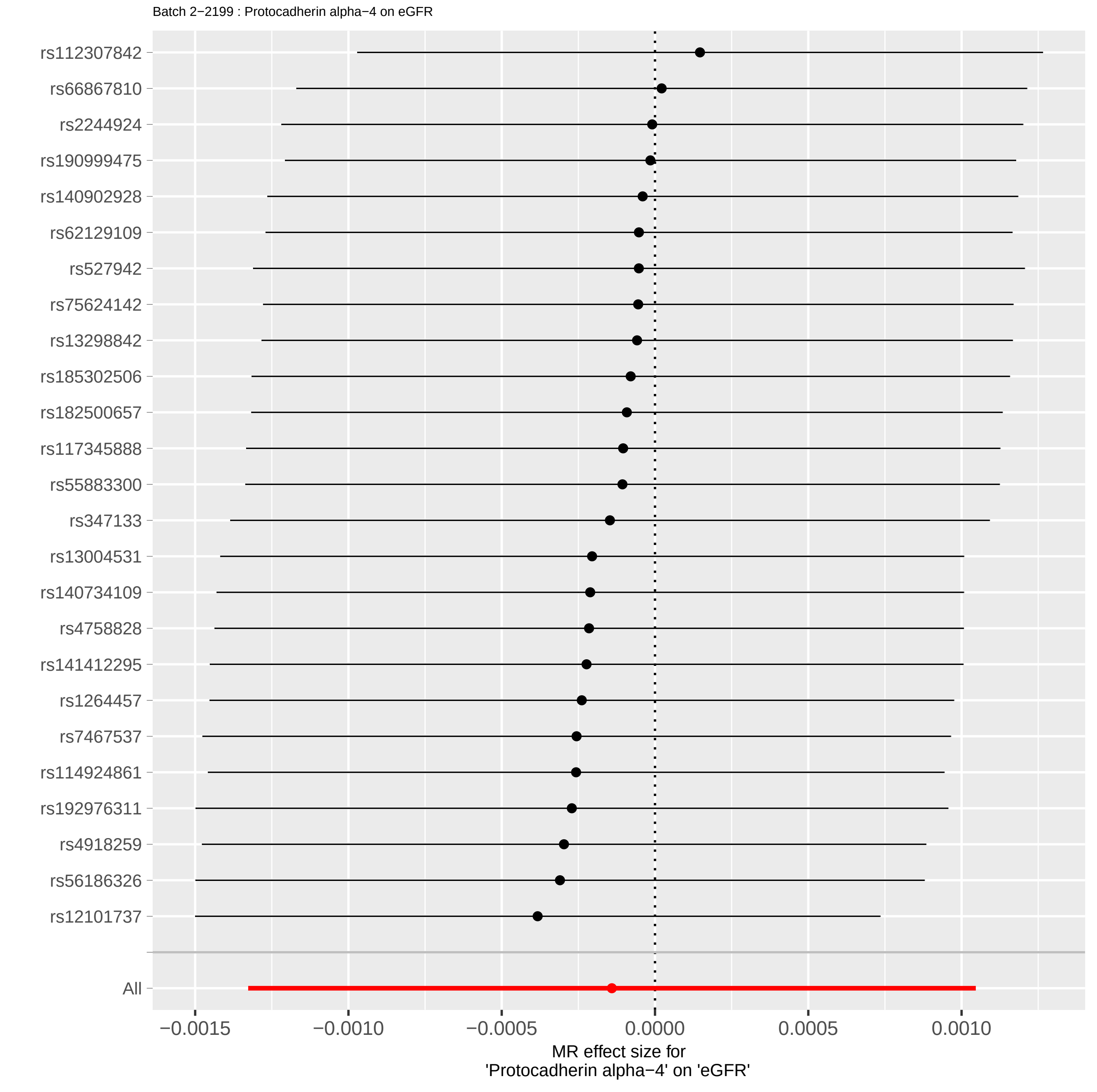

Batch 2−2199 : Protocadherin alpha−4 on eGFR
rs112307842
rs66867810
rs2244924
rs190999475
rs140902928
rs62129109
rs527942
rs75624142
rs13298842
rs185302506
rs182500657
rs117345888
rs55883300
rs347133
rs13004531
rs140734109
rs4758828
rs141412295
rs1264457
rs7467537
rs114924861
rs192976311
rs4918259
rs56186326
rs12101737
All
−0.0015
−0.0010
−0.0005
0.0000
0.0005
0.0010
MR effect size for
'Protocadherin alpha−4' on 'eGFR'

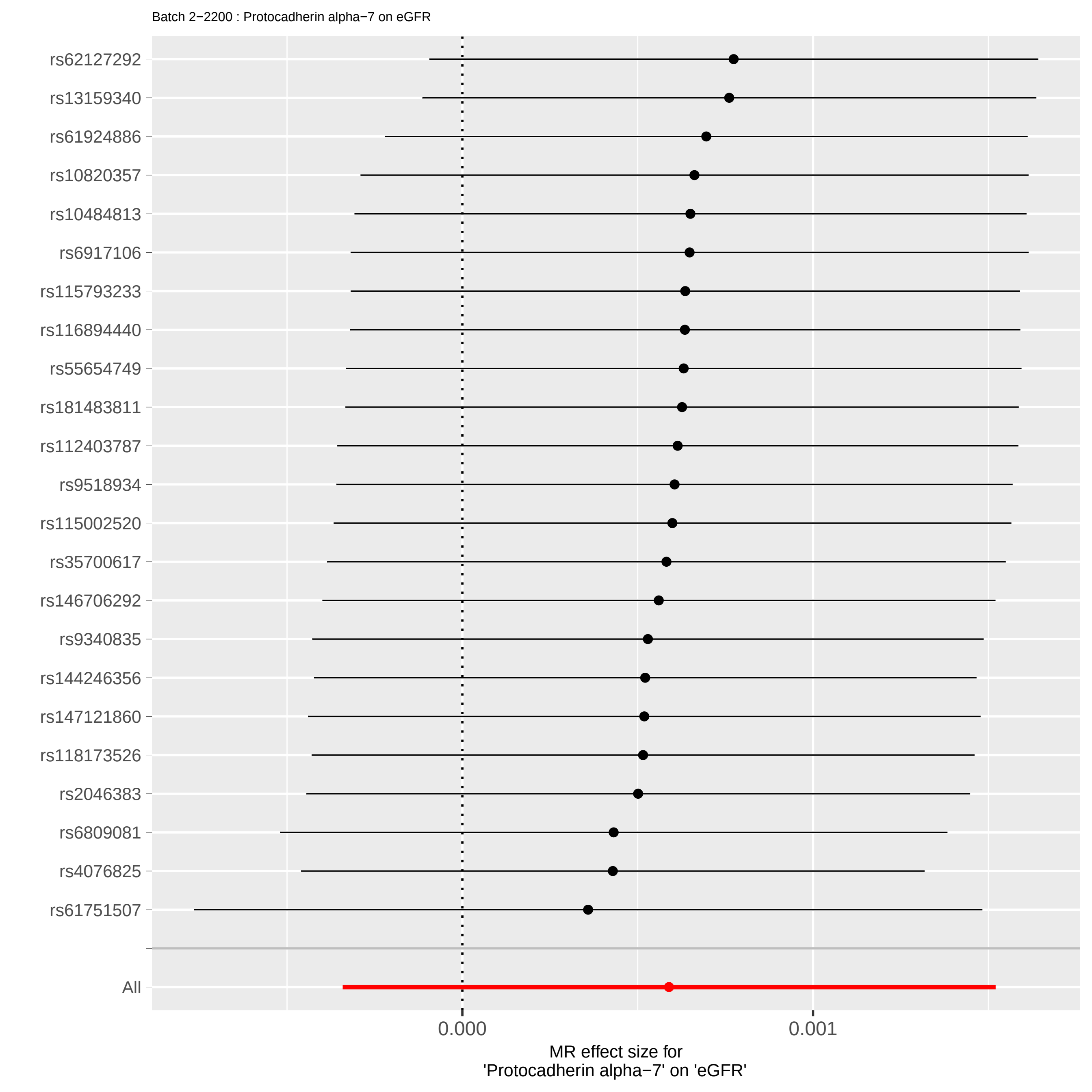

Batch 2−2200 : Protocadherin alpha−7 on eGFR
rs62127292
rs13159340
rs61924886
rs10820357
rs10484813
rs6917106
rs115793233
rs116894440
rs55654749
rs181483811
rs112403787
rs9518934
rs115002520
rs35700617
rs146706292
rs9340835
rs144246356
rs147121860
rs118173526
rs2046383
rs6809081
rs4076825
rs61751507
All
0.000
0.001
MR effect size for
'Protocadherin alpha−7' on 'eGFR'

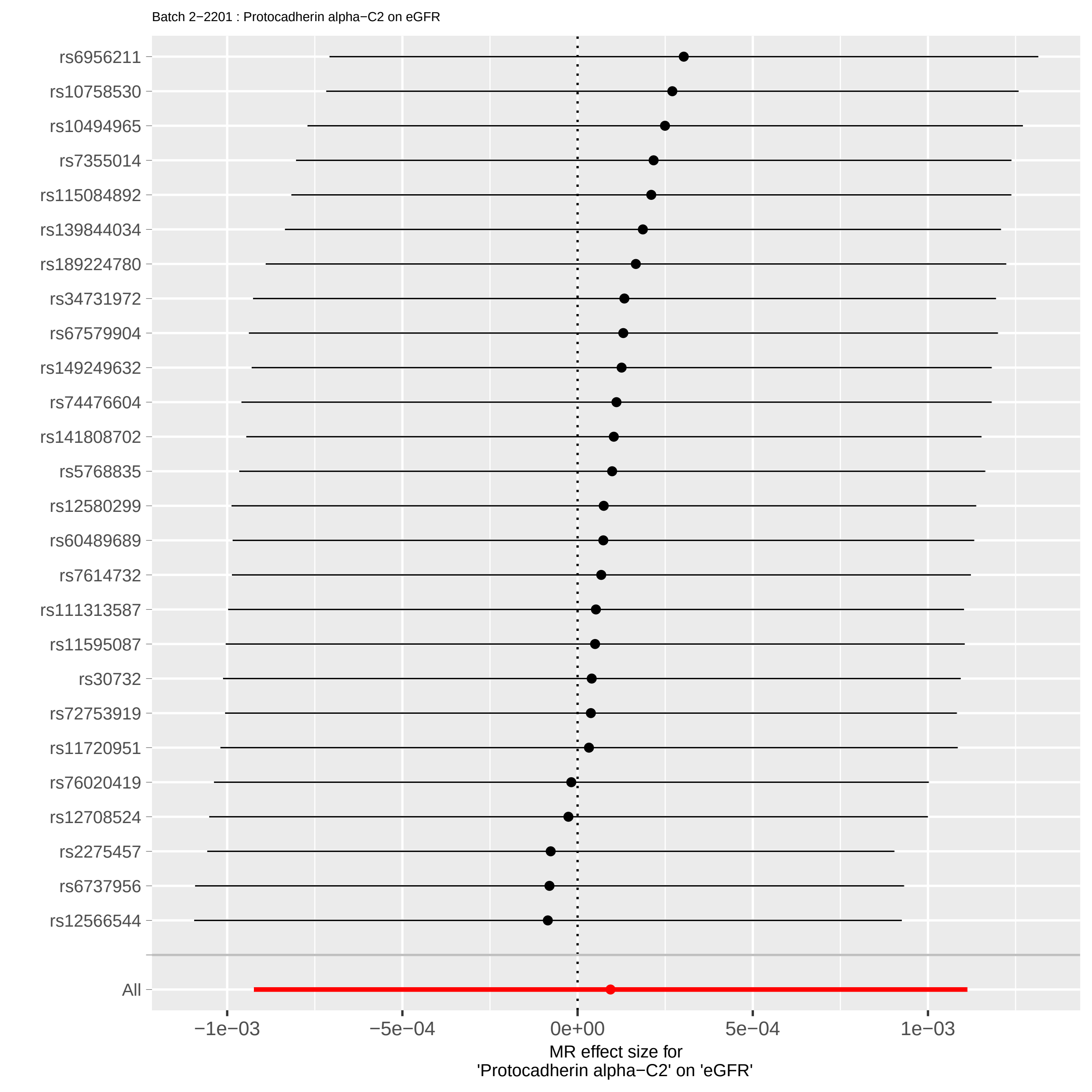
Batch 2−2201 : Protocadherin alpha−C2 on eGFR
rs6956211
rs10758530
rs10494965
rs7355014
rs115084892
rs139844034
rs189224780
rs34731972
rs67579904
rs149249632
rs74476604
rs141808702
rs5768835
rs12580299
rs60489689
rs7614732
rs111313587
rs11595087
rs30732
rs72753919
rs11720951
rs76020419
rs12708524
rs2275457
rs6737956
rs12566544
All
−1e−03
−5e−04
0e+00
5e−04
1e−03
MR effect size for
'Protocadherin alpha−C2' on 'eGFR'

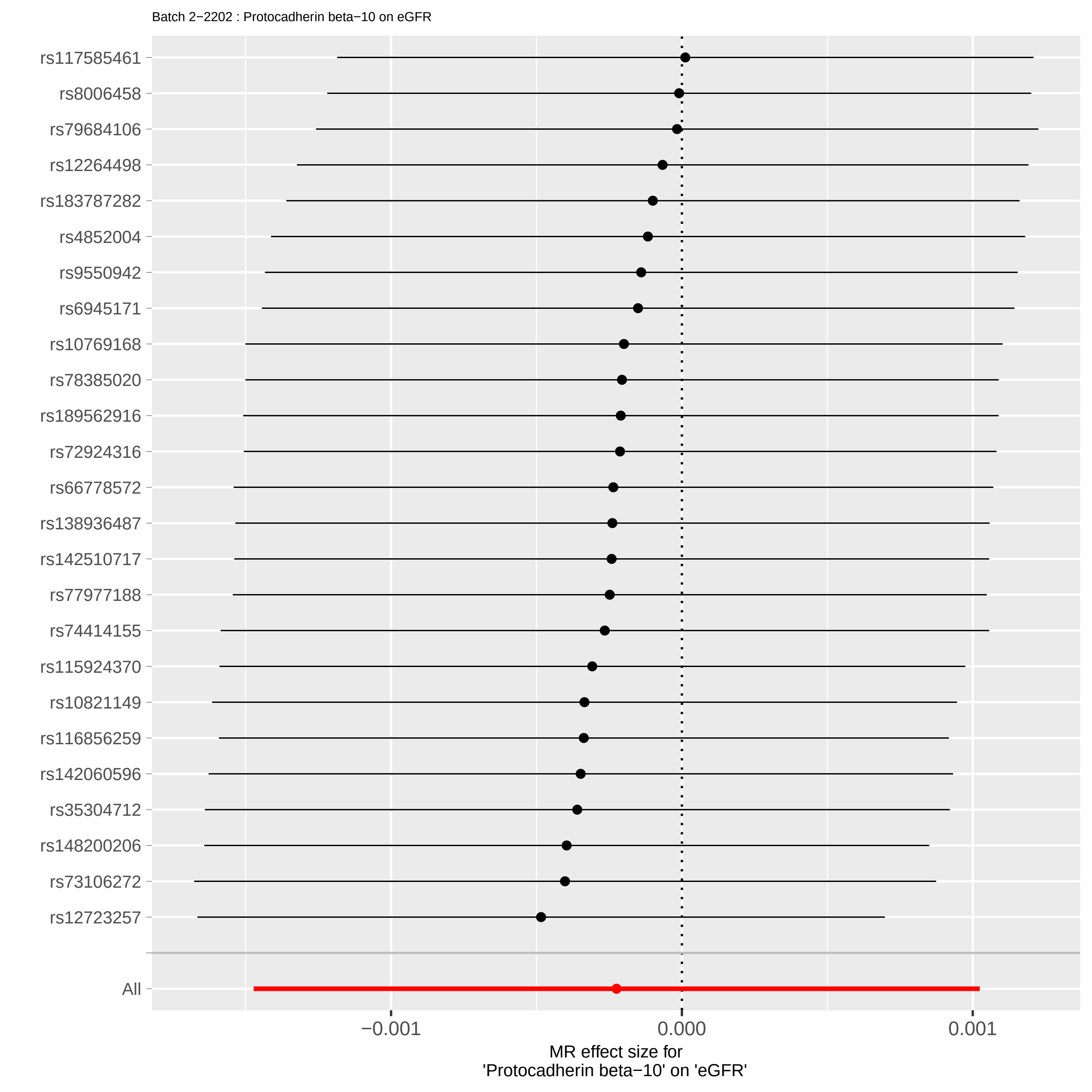
Batch 2−2202 : Protocadherin beta−10 on eGFR
rs117585461
rs8006458
rs79684106
rs12264498
rs183787282
rs4852004
rs9550942
rs6945171
rs10769168
rs78385020
rs189562916
rs72924316
rs66778572
rs138936487
rs142510717
rs77977188
rs74414155
rs115924370
rs10821149
rs116856259
rs142060596
rs35304712
rs148200206
rs73106272
rs12723257
All
−0.001
0.000
0.001
MR effect size for
'Protocadherin beta−10' on 'eGFR'

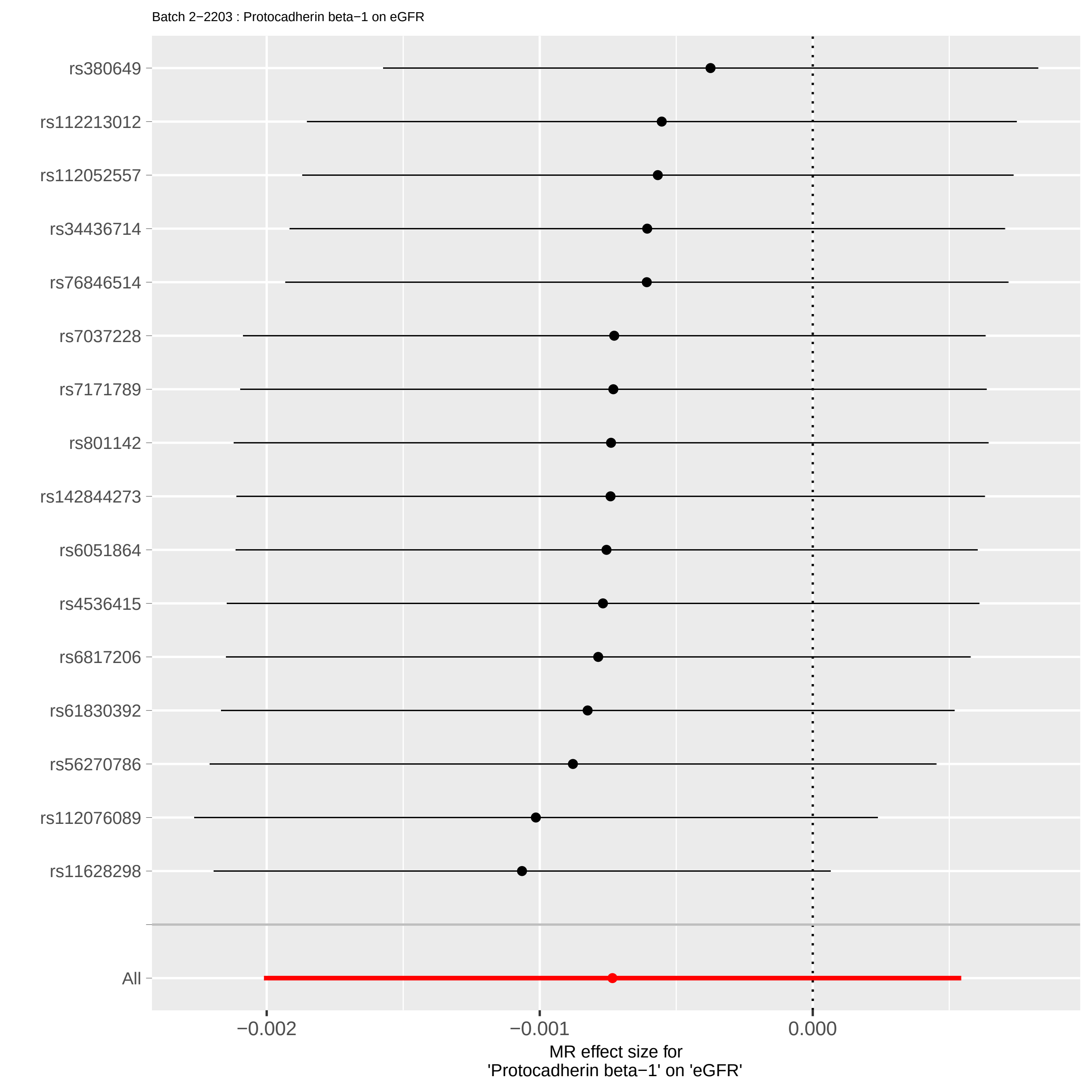

Batch 2−2203 : Protocadherin beta−1 on eGFR
rs380649
rs112213012
rs112052557
rs34436714
rs76846514
rs7037228
rs7171789
rs801142
rs142844273
rs6051864
rs4536415
rs6817206
rs61830392
rs56270786
rs112076089
rs11628298
All
−0.002
−0.001
0.000
MR effect size for
'Protocadherin beta−1' on 'eGFR'

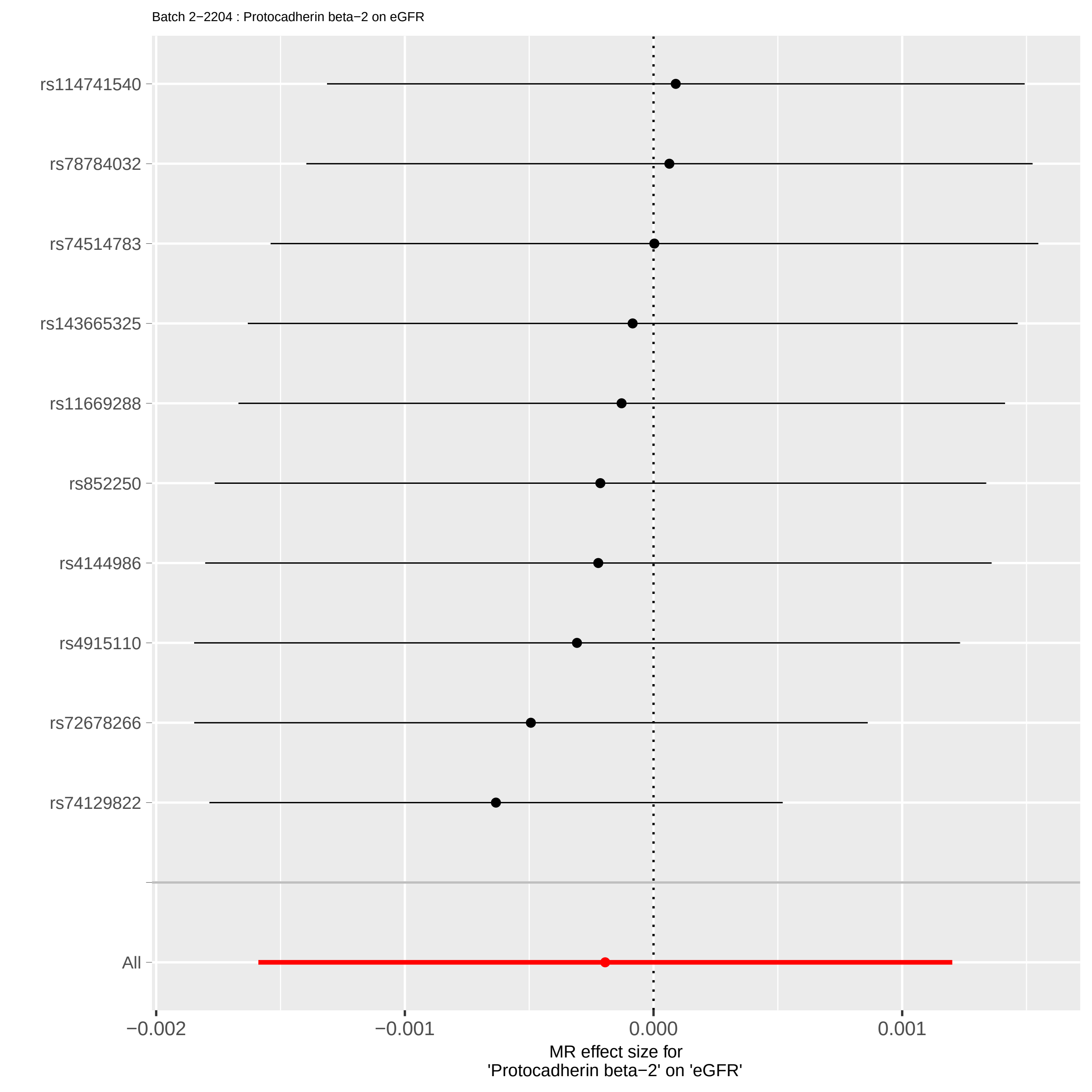

Batch 2−2204 : Protocadherin beta−2 on eGFR
rs114741540
rs78784032
rs74514783
rs143665325
rs11669288
rs852250
rs4144986
rs4915110
rs72678266
rs74129822
All
−0.002
−0.001
0.000
0.001
MR effect size for
'Protocadherin beta−2' on 'eGFR'

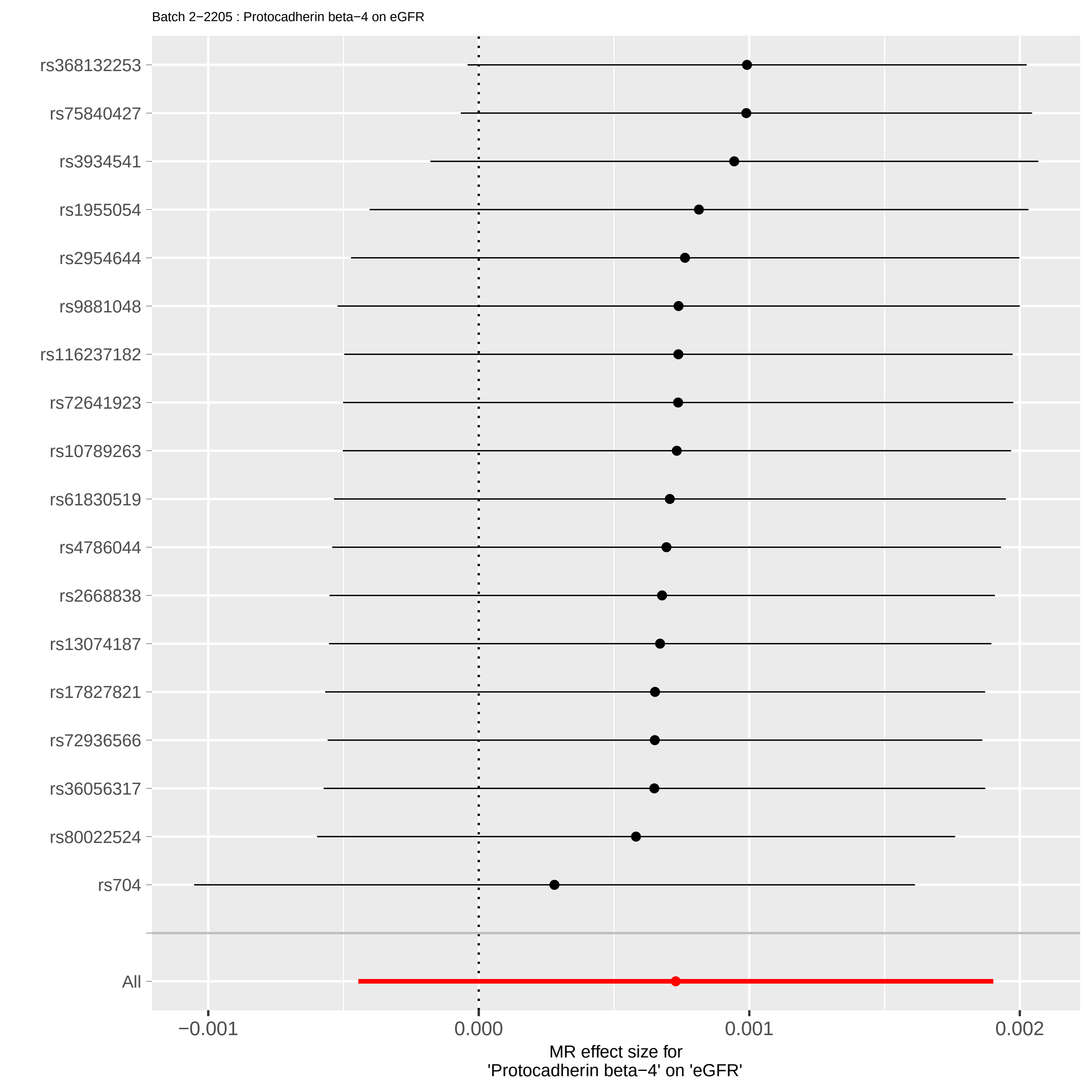
Batch 2−2205 : Protocadherin beta−4 on eGFR
rs368132253
rs75840427
rs3934541
rs1955054
rs2954644
rs9881048
rs116237182
rs72641923
rs10789263
rs61830519
rs4786044
rs2668838
rs13074187
rs17827821
rs72936566
rs36056317
rs80022524
rs704
All
−0.001
0.000
0.001
0.002
MR effect size for
'Protocadherin beta−4' on 'eGFR'

Batch 2−2206 : Protocadherin gamma−A8 on eGFR

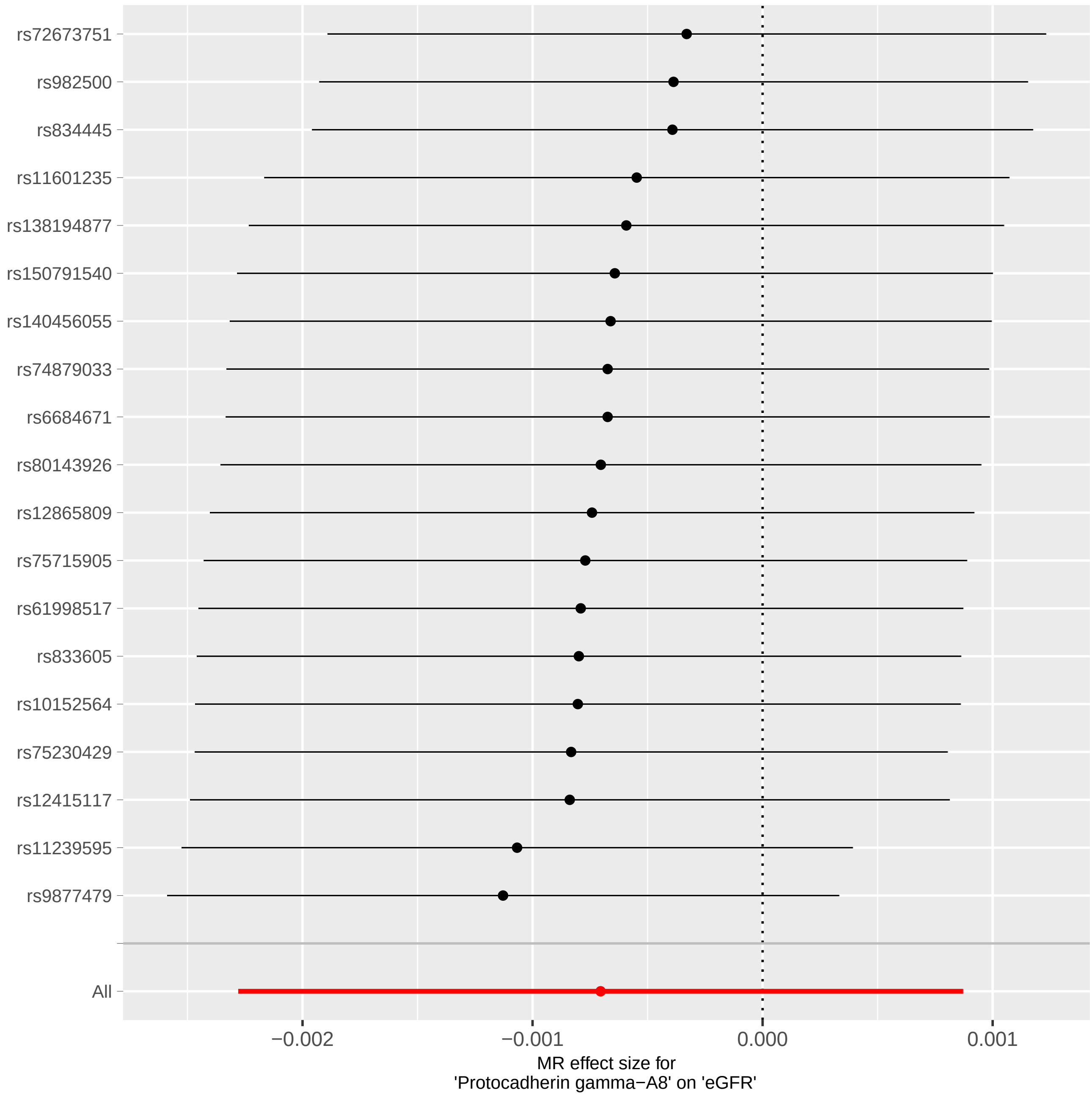

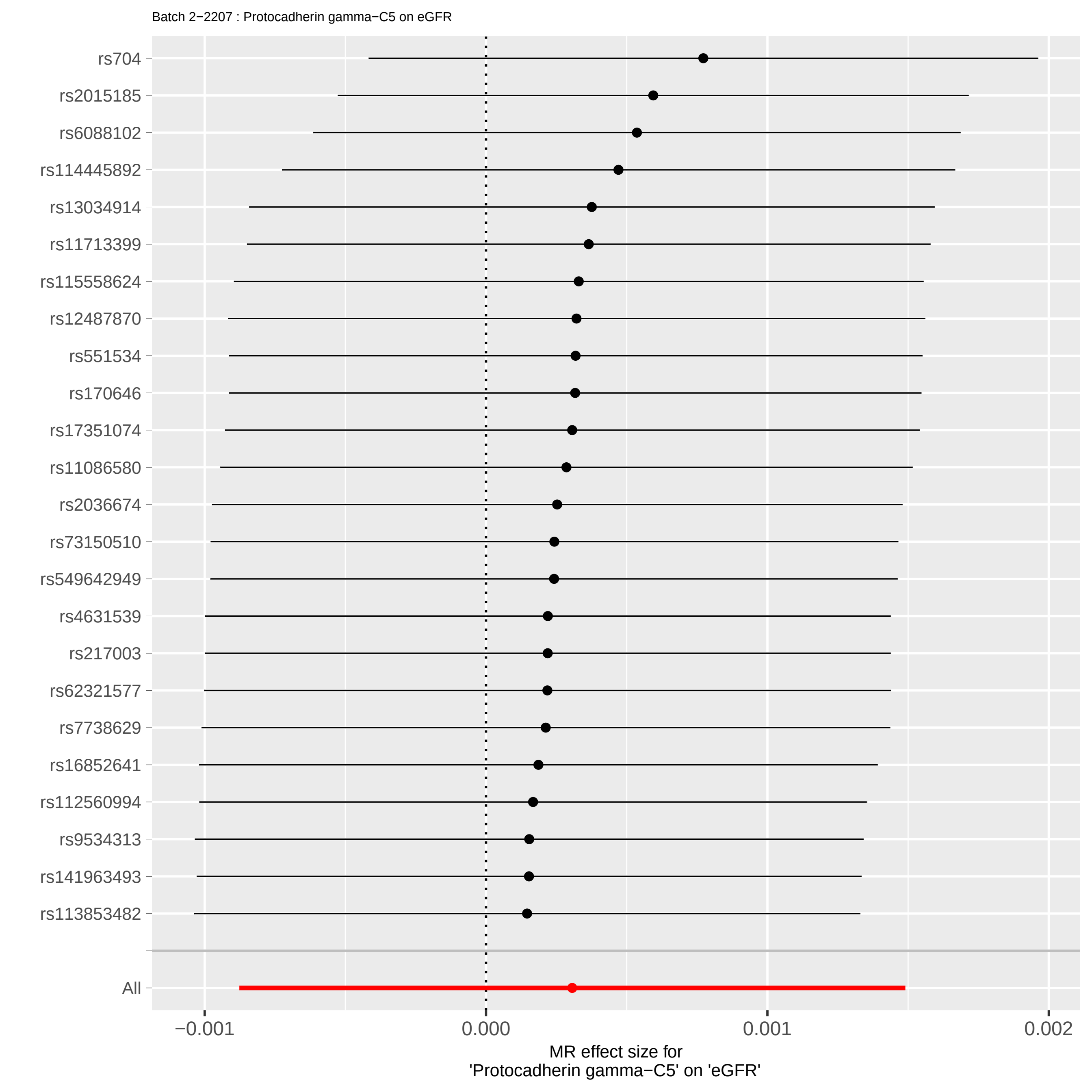
Batch 2−2207 : Protocadherin gamma−C5 on eGFR
rs704
rs2015185
rs6088102
rs114445892
rs13034914
rs11713399
rs115558624
rs12487870
rs551534
rs170646
rs17351074
rs11086580
rs2036674
rs73150510
rs549642949
rs4631539
rs217003
rs62321577
rs7738629
rs16852641
rs112560994
rs9534313
rs141963493
rs113853482
All
−0.001
0.000
0.001
0.002
MR effect size for
'Protocadherin gamma−C5' on 'eGFR'

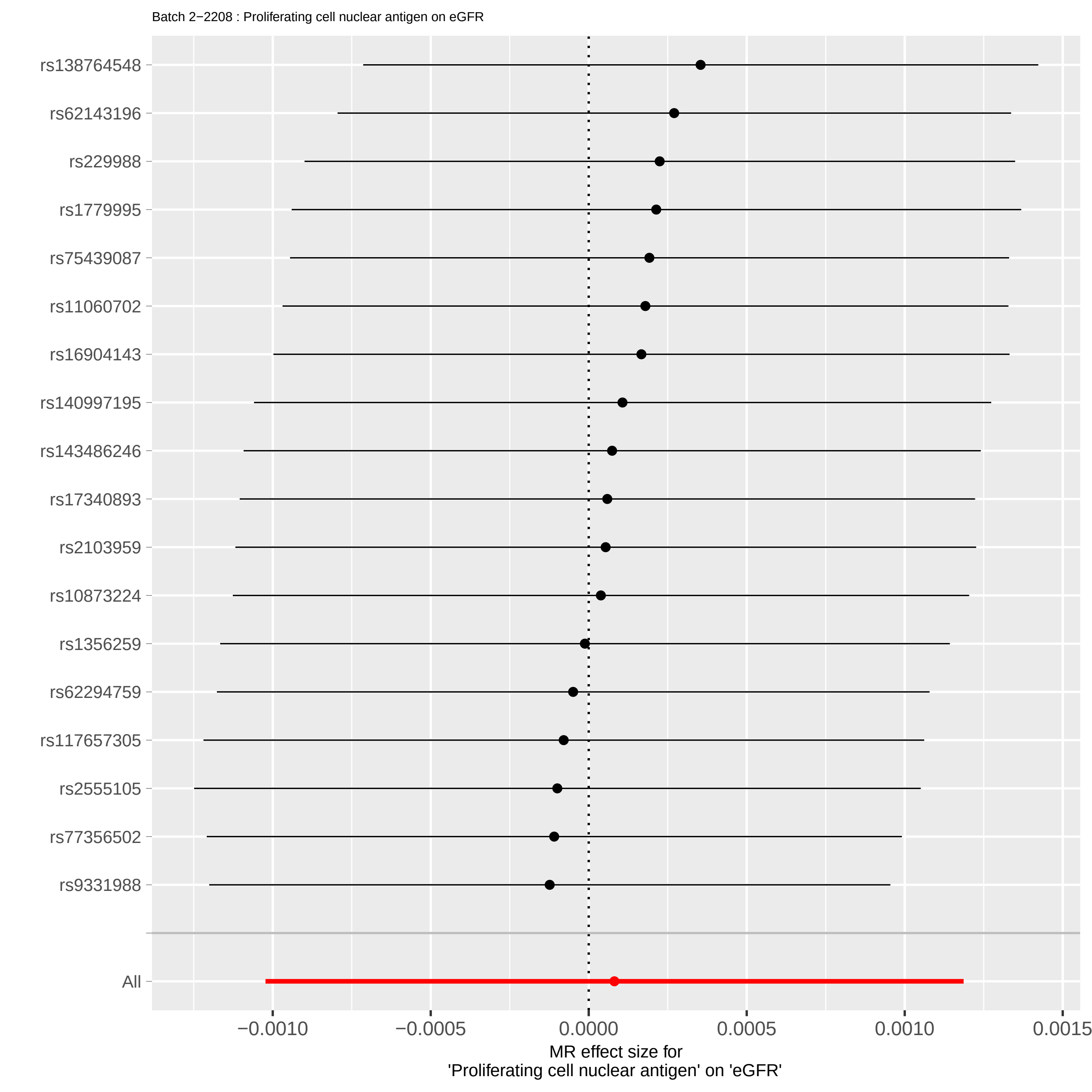

Batch 2−2208 : Proliferating cell nuclear antigen on eGFR
rs138764548
rs62143196
rs229988
rs1779995
rs75439087
rs11060702
rs16904143
rs140997195
rs143486246
rs17340893
rs2103959
rs10873224
rs1356259
rs62294759
rs117657305
rs2555105
rs77356502
rs9331988
All
−0.0010
−0.0005
0.0000
0.0005
0.0010
0.0015
MR effect size for
'Proliferating cell nuclear antigen' on 'eGFR'

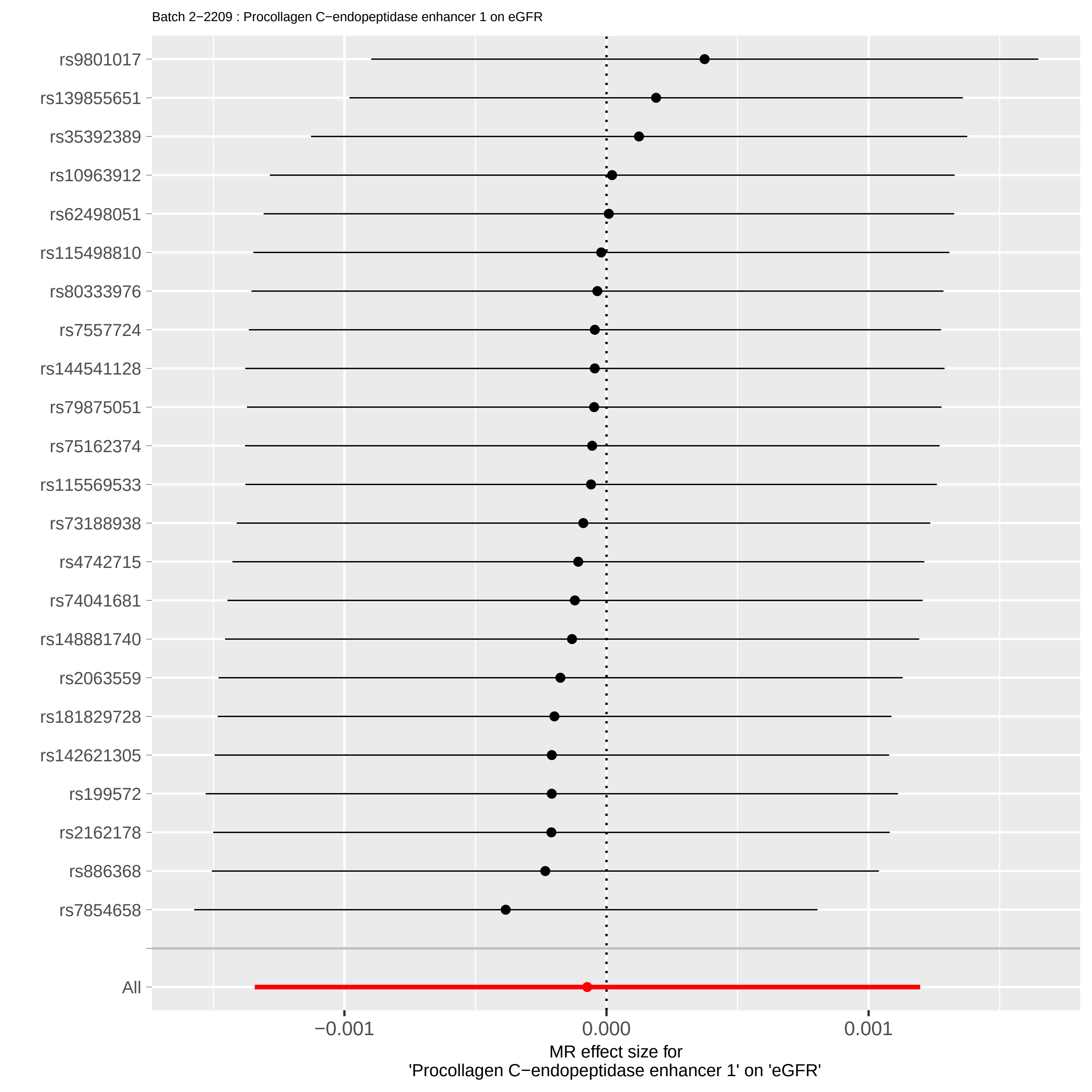
Batch 2−2209 : Procollagen C−endopeptidase enhancer 1 on eGFR
rs9801017
rs139855651
rs35392389
rs10963912
rs62498051
rs115498810
rs80333976
rs7557724
rs144541128
rs79875051
rs75162374
rs115569533
rs73188938
rs4742715
rs74041681
rs148881740
rs2063559
rs181829728
rs142621305
rs199572
rs2162178
rs886368
rs7854658
All
−0.001
0.000
0.001
MR effect size for
'Procollagen C−endopeptidase enhancer 1' on 'eGFR'

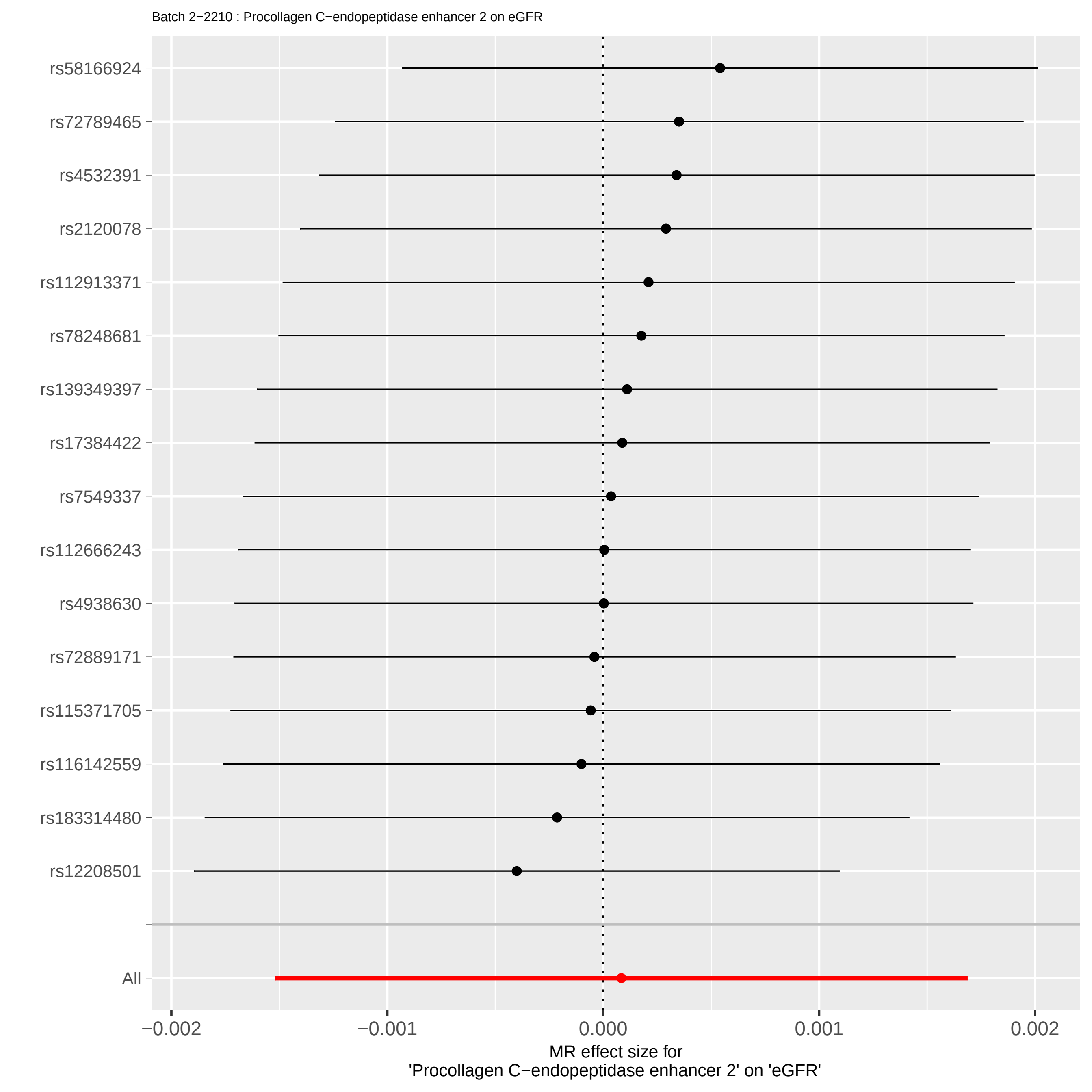
Batch 2−2210 : Procollagen C−endopeptidase enhancer 2 on eGFR
rs58166924
rs72789465
rs4532391
rs2120078
rs112913371
rs78248681
rs139349397
rs17384422
rs7549337
rs112666243
rs4938630
rs72889171
rs115371705
rs116142559
rs183314480
rs12208501
All
−0.002
−0.001
0.000
0.001
0.002
MR effect size for
'Procollagen C−endopeptidase enhancer 2' on 'eGFR'

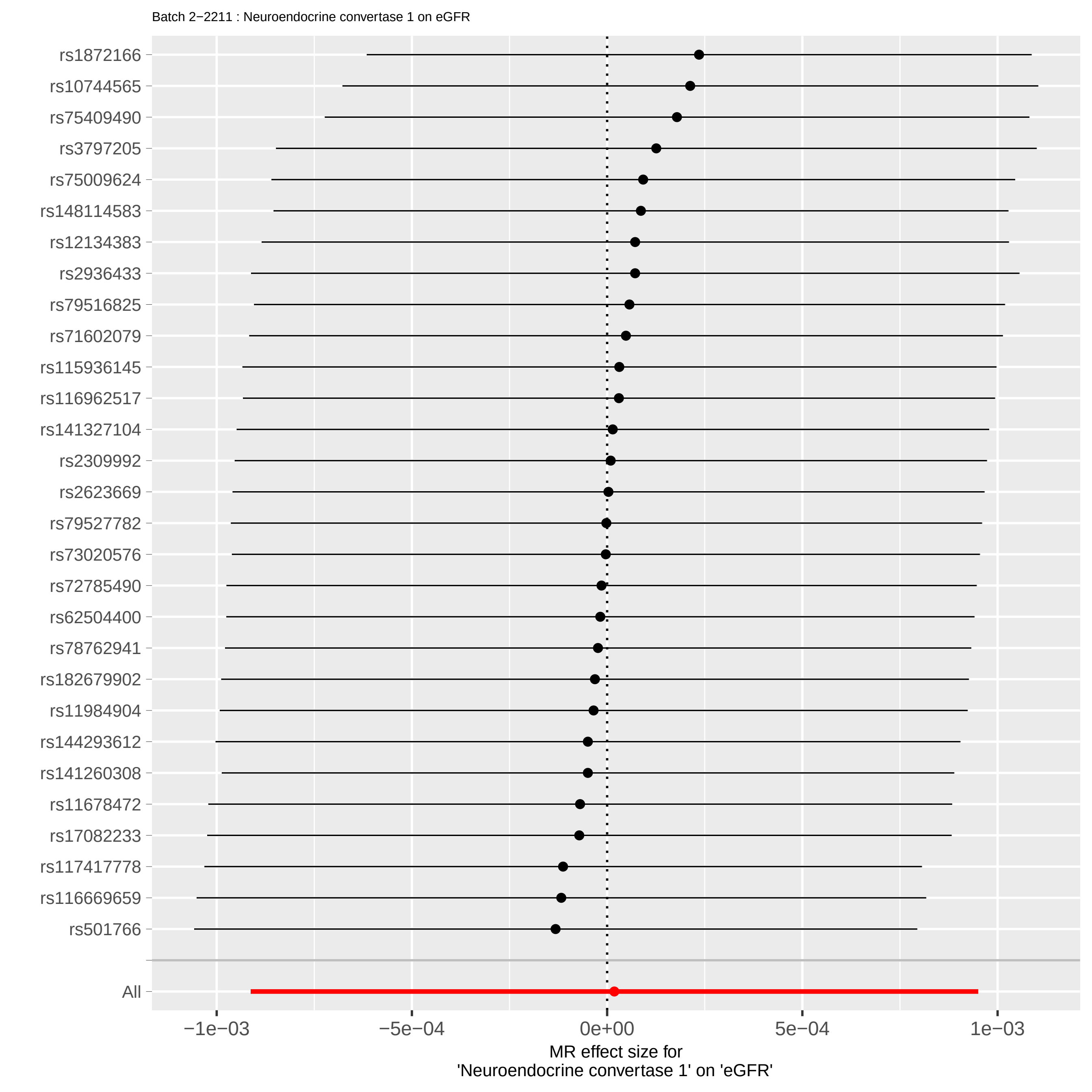

Batch 2−2211 : Neuroendocrine convertase 1 on eGFR
rs1872166
rs10744565
rs75409490
rs3797205
rs75009624
rs148114583
rs12134383
rs2936433
rs79516825
rs71602079
rs115936145
rs116962517
rs141327104
rs2309992
rs2623669
rs79527782
rs73020576
rs72785490
rs62504400
rs78762941
rs182679902
rs11984904
rs144293612
rs141260308
rs11678472
rs17082233
rs117417778
rs116669659
rs501766
All
−1e−03
−5e−04
0e+00
5e−04
1e−03
MR effect size for
'Neuroendocrine convertase 1' on 'eGFR'

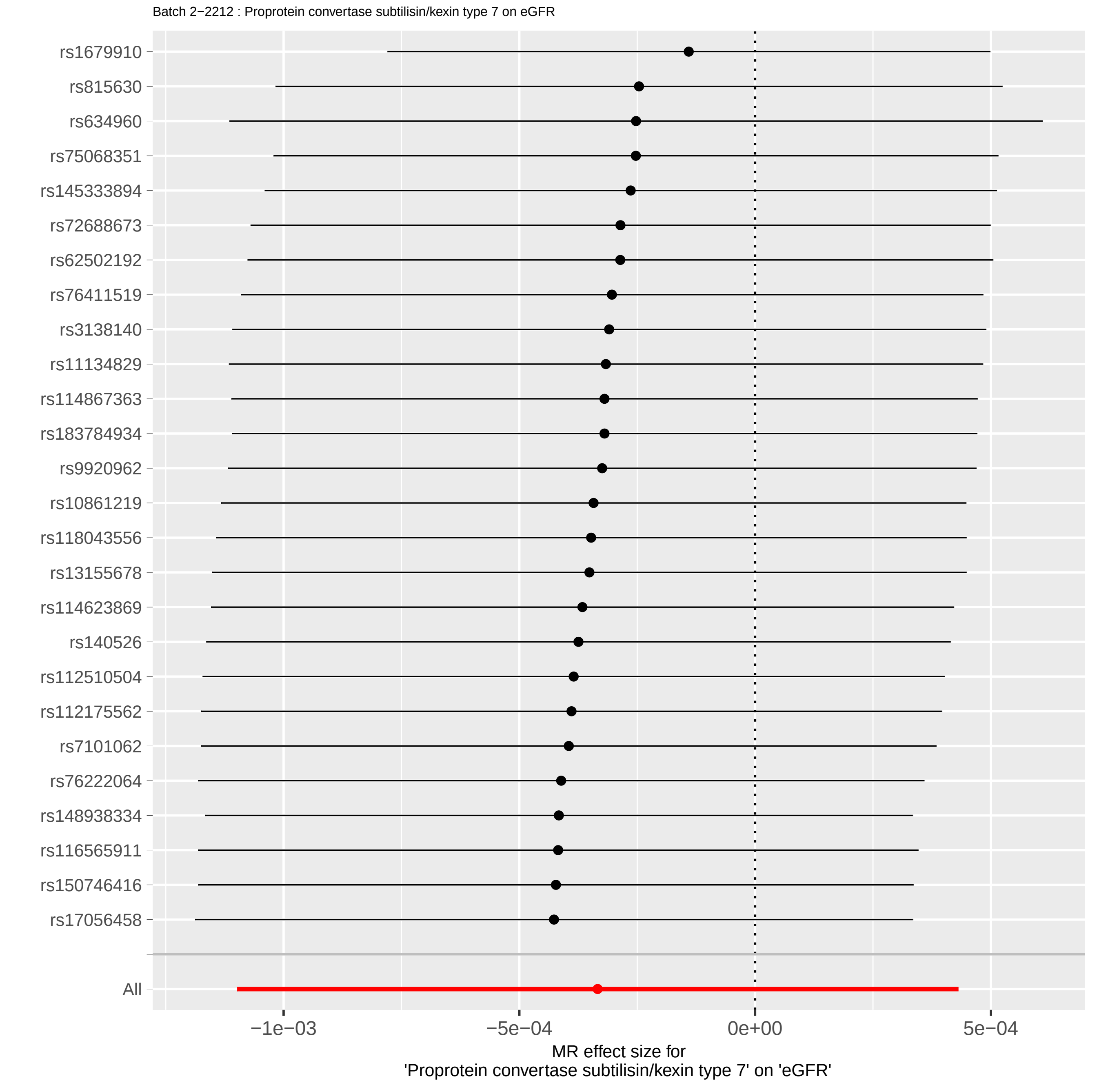

Batch 2−2212 : Proprotein convertase subtilisin/kexin type 7 on eGFR
rs1679910
rs815630
rs634960
rs75068351
rs145333894
rs72688673
rs62502192
rs76411519
rs3138140
rs11134829
rs114867363
rs183784934
rs9920962
rs10861219
rs118043556
rs13155678
rs114623869
rs140526
rs112510504
rs112175562
rs7101062
rs76222064
rs148938334
rs116565911
rs150746416
rs17056458
All
−1e−03
−5e−04
0e+00
5e−04
MR effect size for
'Proprotein convertase subtilisin/kexin type 7' on 'eGFR'

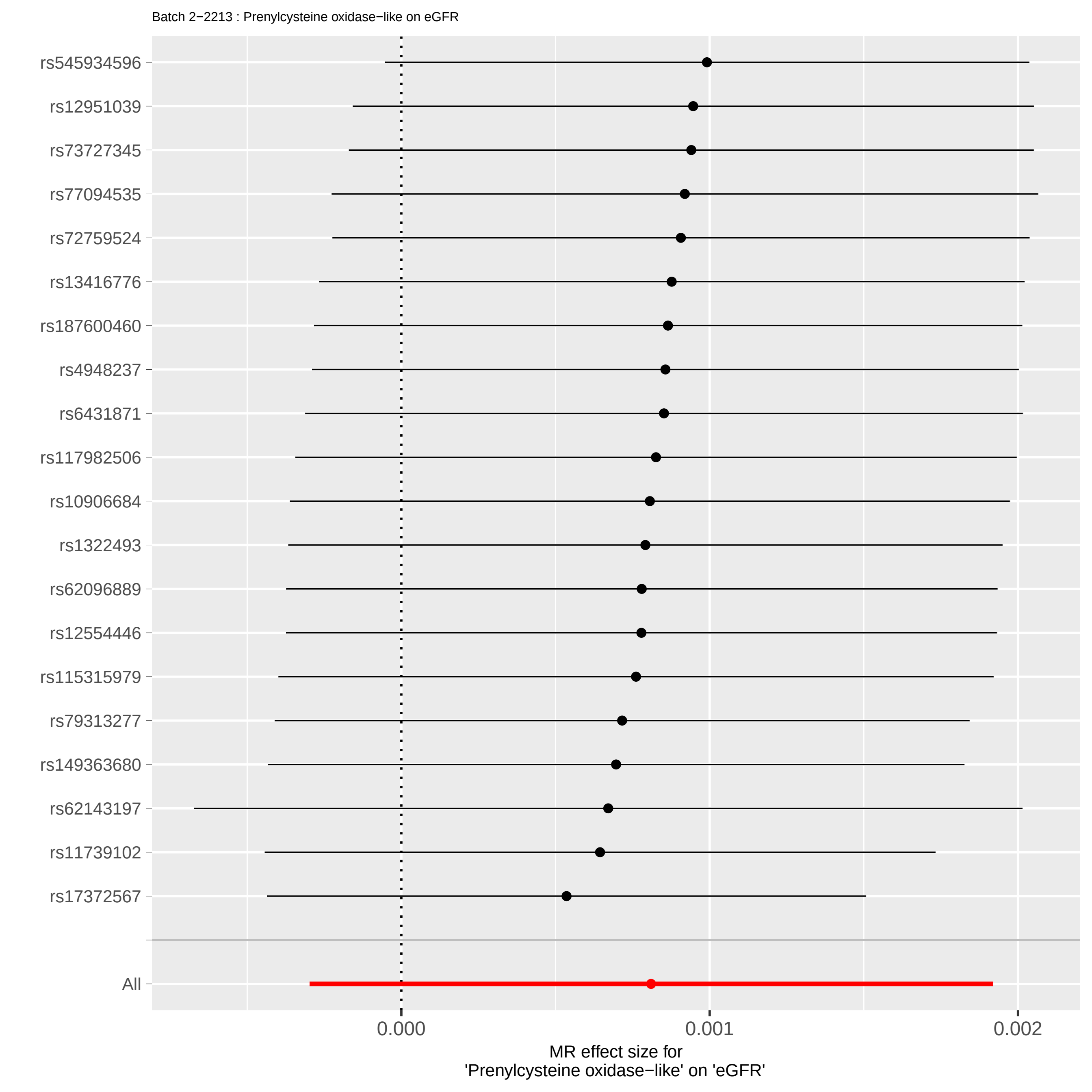

Batch 2−2213 : Prenylcysteine oxidase−like on eGFR
rs545934596
rs12951039
rs73727345
rs77094535
rs72759524
rs13416776
rs187600460
rs4948237
rs6431871
rs117982506
rs10906684
rs1322493
rs62096889
rs12554446
rs115315979
rs79313277
rs149363680
rs62143197
rs11739102
rs17372567
All
0.000
0.001
0.002
MR effect size for
'Prenylcysteine oxidase−like' on 'eGFR'

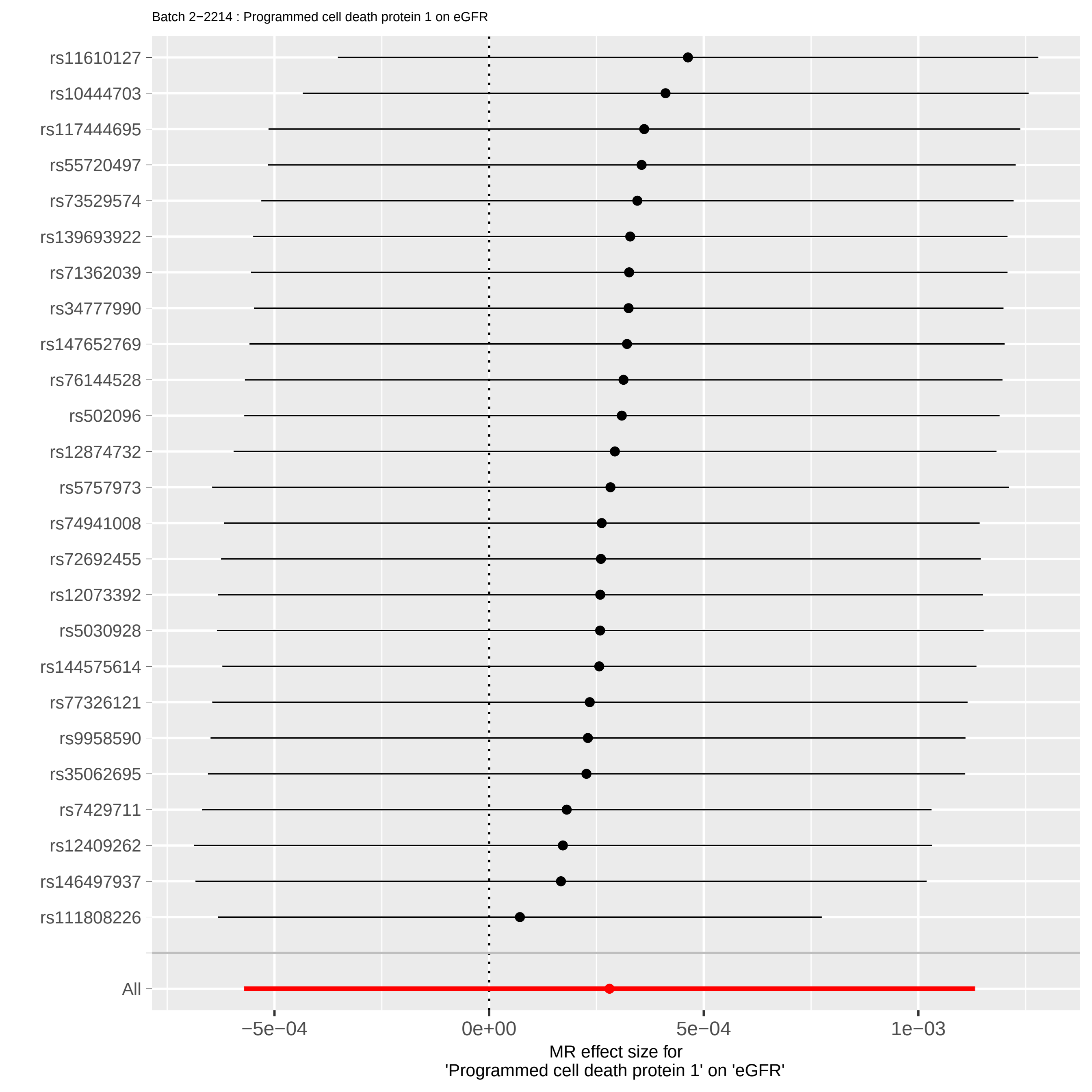

Batch 2−2214 : Programmed cell death protein 1 on eGFR
rs11610127
rs10444703
rs117444695
rs55720497
rs73529574
rs139693922
rs71362039
rs34777990
rs147652769
rs76144528
rs502096
rs12874732
rs5757973
rs74941008
rs72692455
rs12073392
rs5030928
rs144575614
rs77326121
rs9958590
rs35062695
rs7429711
rs12409262
rs146497937
rs111808226
All
−5e−04
0e+00
5e−04
1e−03
MR effect size for
'Programmed cell death protein 1' on 'eGFR'

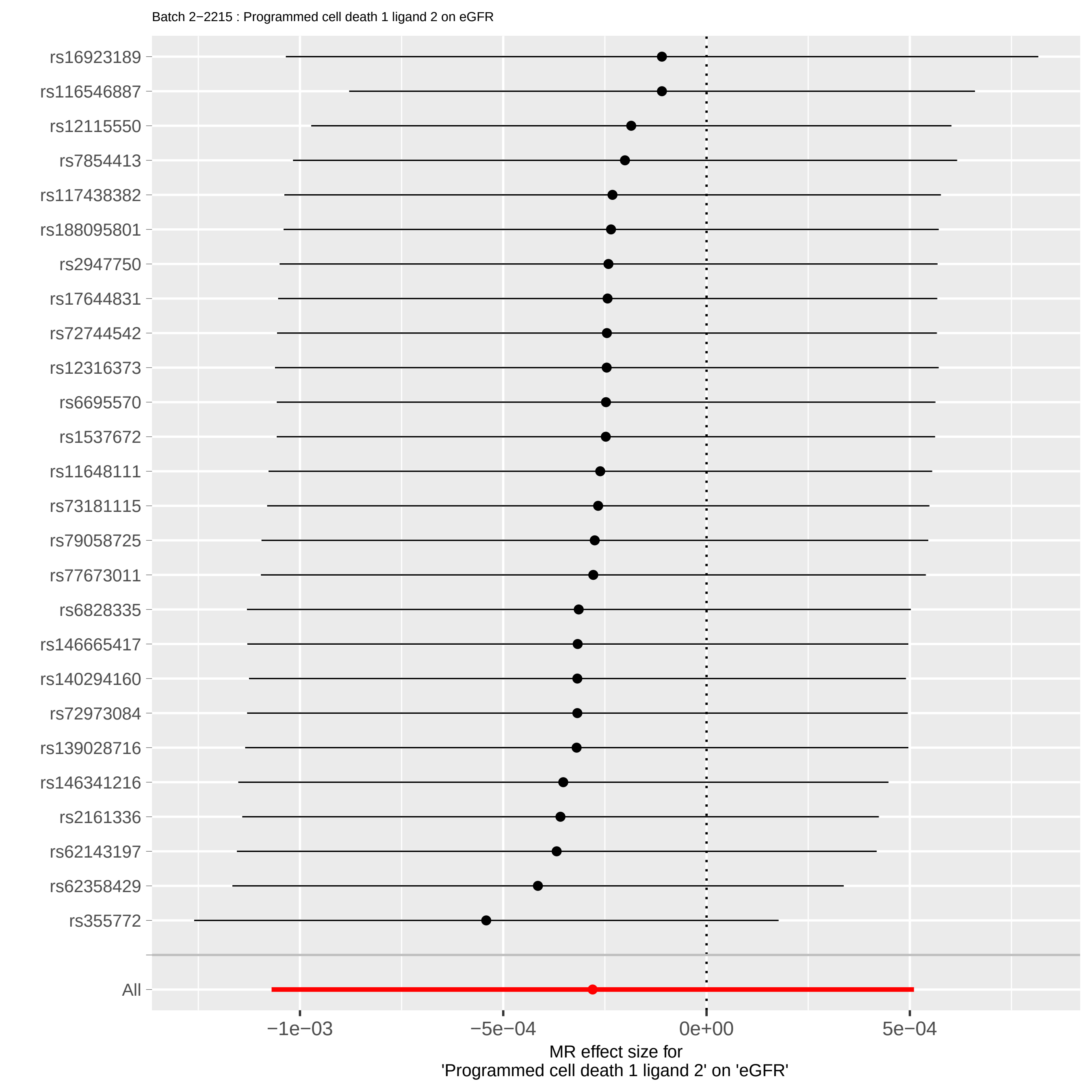

Batch 2−2215 : Programmed cell death 1 ligand 2 on eGFR
rs16923189
rs116546887
rs12115550
rs7854413
rs117438382
rs188095801
rs2947750
rs17644831
rs72744542
rs12316373
rs6695570
rs1537672
rs11648111
rs73181115
rs79058725
rs77673011
rs6828335
rs146665417
rs140294160
rs72973084
rs139028716
rs146341216
rs2161336
rs62143197
rs62358429
rs355772
All
−1e−03
−5e−04
0e+00
5e−04
MR effect size for
'Programmed cell death 1 ligand 2' on 'eGFR'

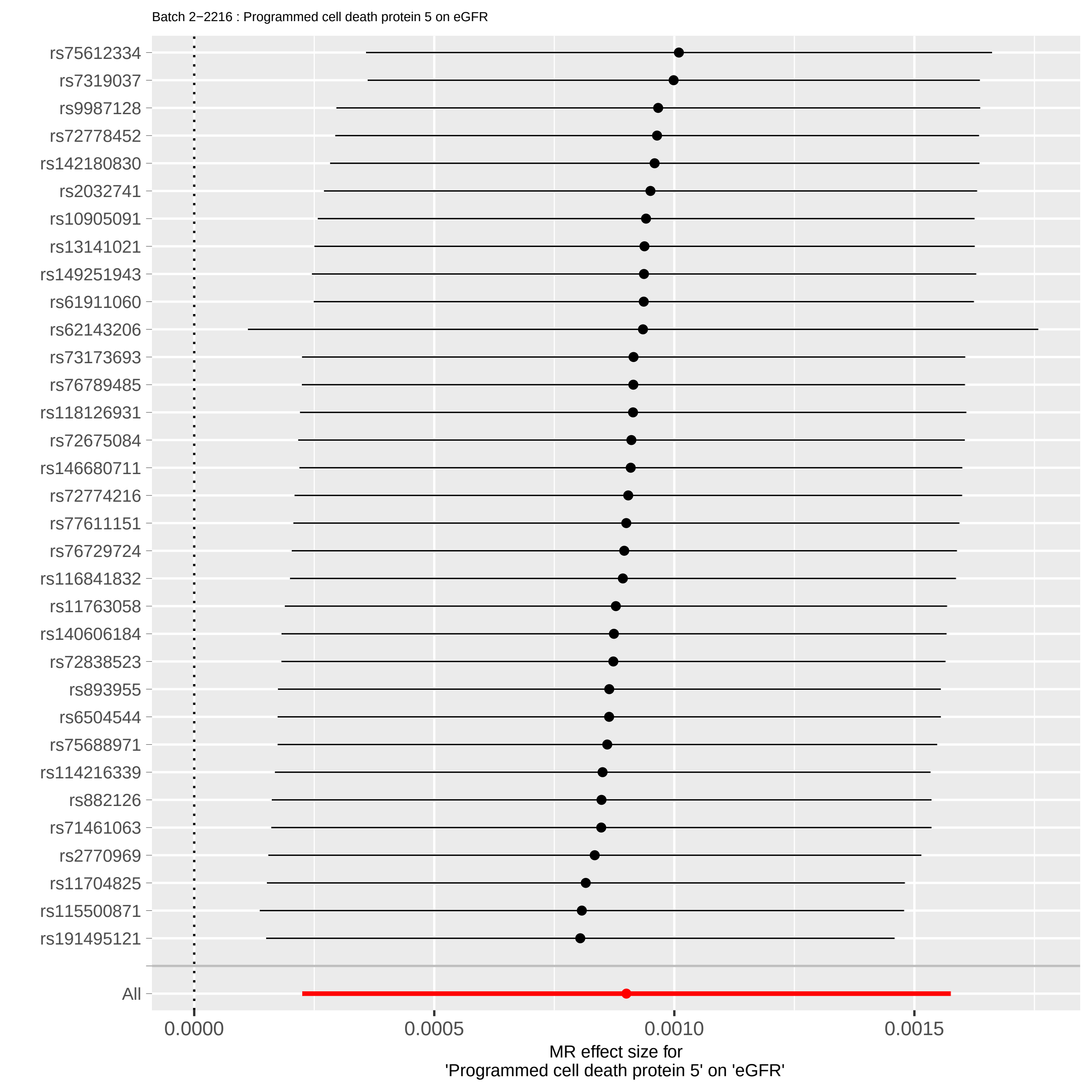

Batch 2−2216 : Programmed cell death protein 5 on eGFR
rs75612334
rs7319037
rs9987128
rs72778452
rs142180830
rs2032741
rs10905091
rs13141021
rs149251943
rs61911060
rs62143206
rs73173693
rs76789485
rs118126931
rs72675084
rs146680711
rs72774216
rs77611151
rs76729724
rs116841832
rs11763058
rs140606184
rs72838523
rs893955
rs6504544
rs75688971
rs114216339
rs882126
rs71461063
rs2770969
rs11704825
rs115500871
rs191495121
All
0.0000
0.0005
0.0010
0.0015
MR effect size for
'Programmed cell death protein 5' on 'eGFR'

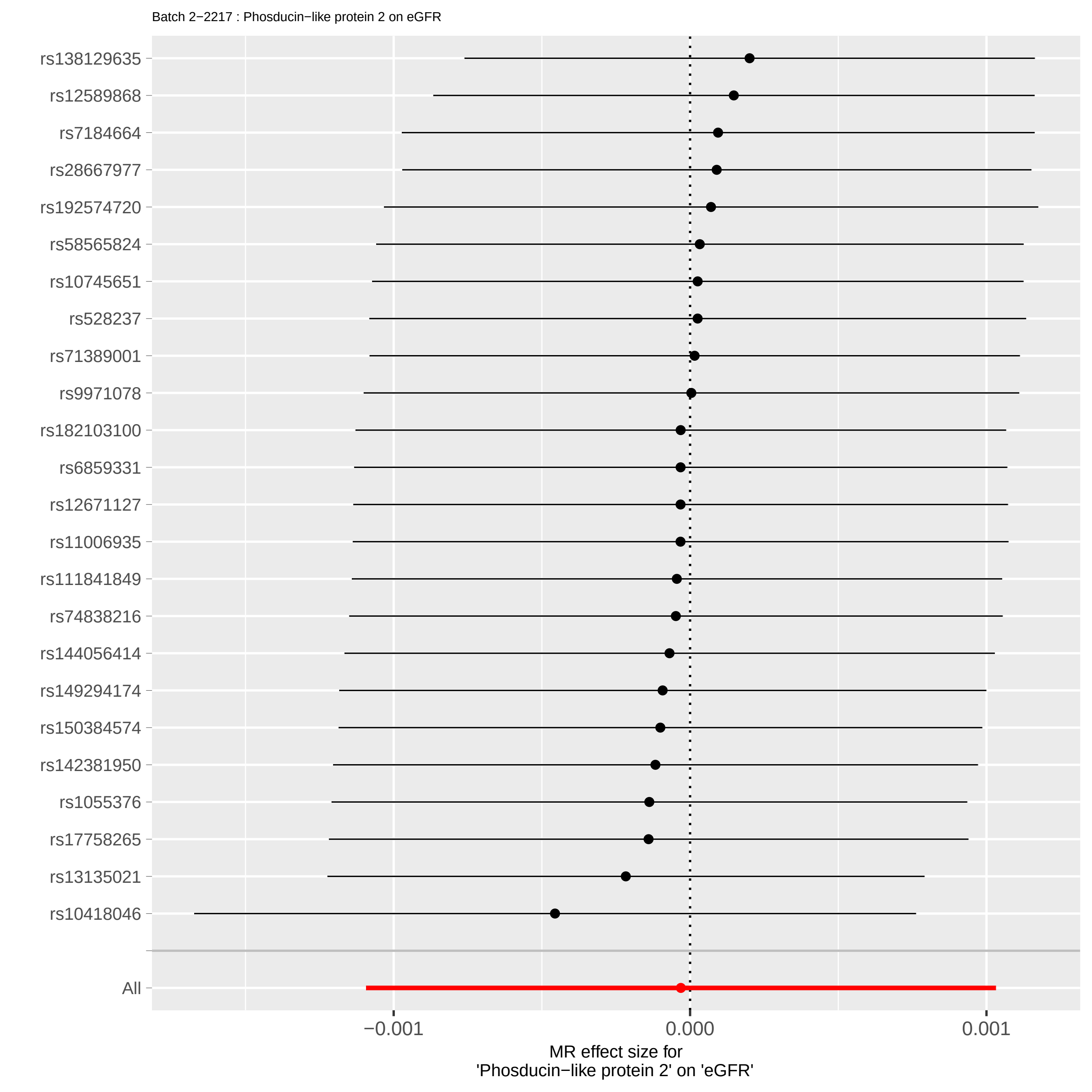

Batch 2−2217 : Phosducin−like protein 2 on eGFR
rs138129635
rs12589868
rs7184664
rs28667977
rs192574720
rs58565824
rs10745651
rs528237
rs71389001
rs9971078
rs182103100
rs6859331
rs12671127
rs11006935
rs111841849
rs74838216
rs144056414
rs149294174
rs150384574
rs142381950
rs1055376
rs17758265
rs13135021
rs10418046
All
−0.001
0.000
0.001
MR effect size for
'Phosducin−like protein 2' on 'eGFR'

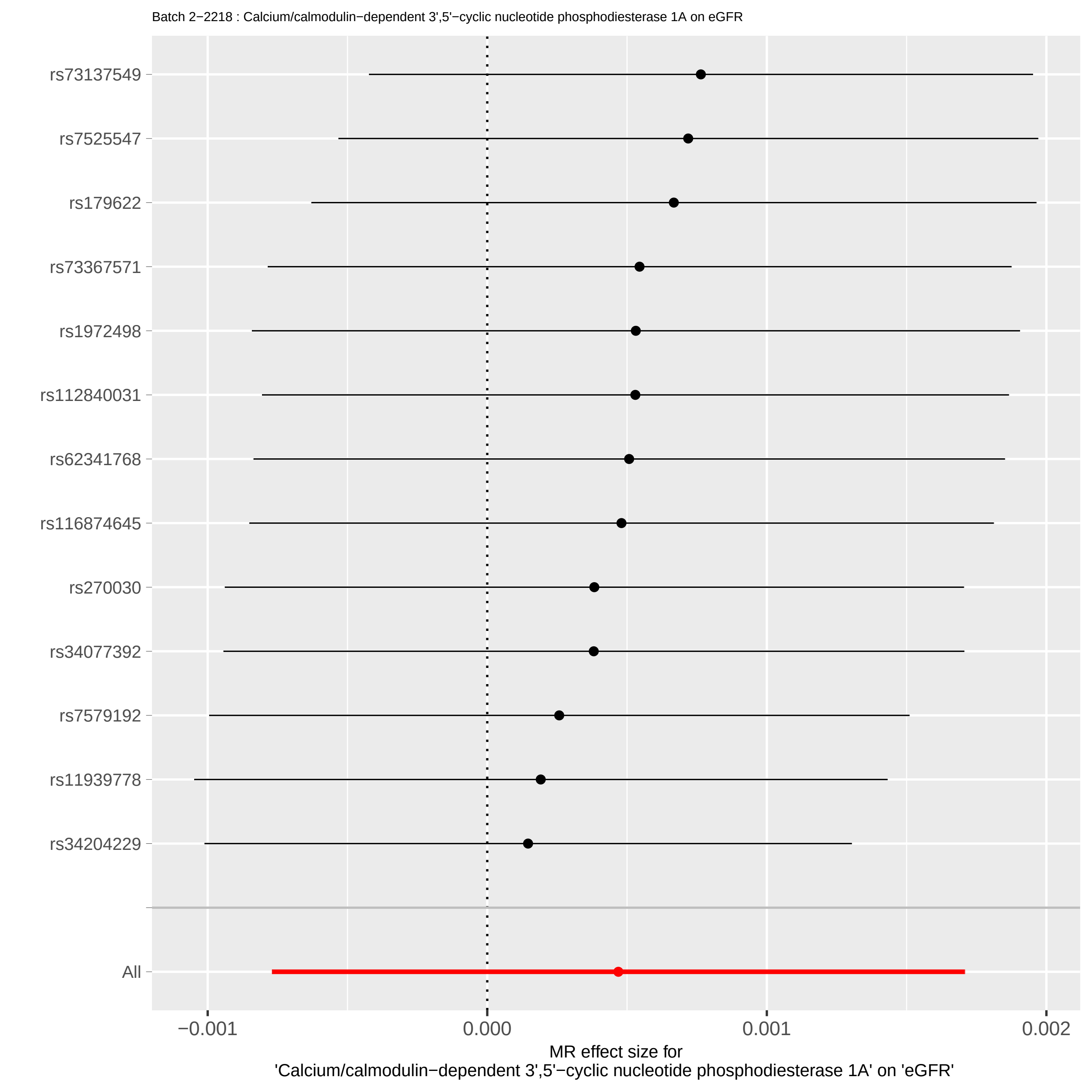
Batch 2−2218 : Calcium/calmodulin−dependent 3',5'−cyclic nucleotide phosphodiesterase 1A on eGFR
rs73137549
rs7525547
rs179622
rs73367571
rs1972498
rs112840031
rs62341768
rs116874645
rs270030
rs34077392
rs7579192
rs11939778
rs34204229
All
−0.001
0.000
0.001
0.002
MR effect size for
'Calcium/calmodulin−dependent 3',5'−cyclic nucleotide phosphodiesterase 1A' on 'eGFR'

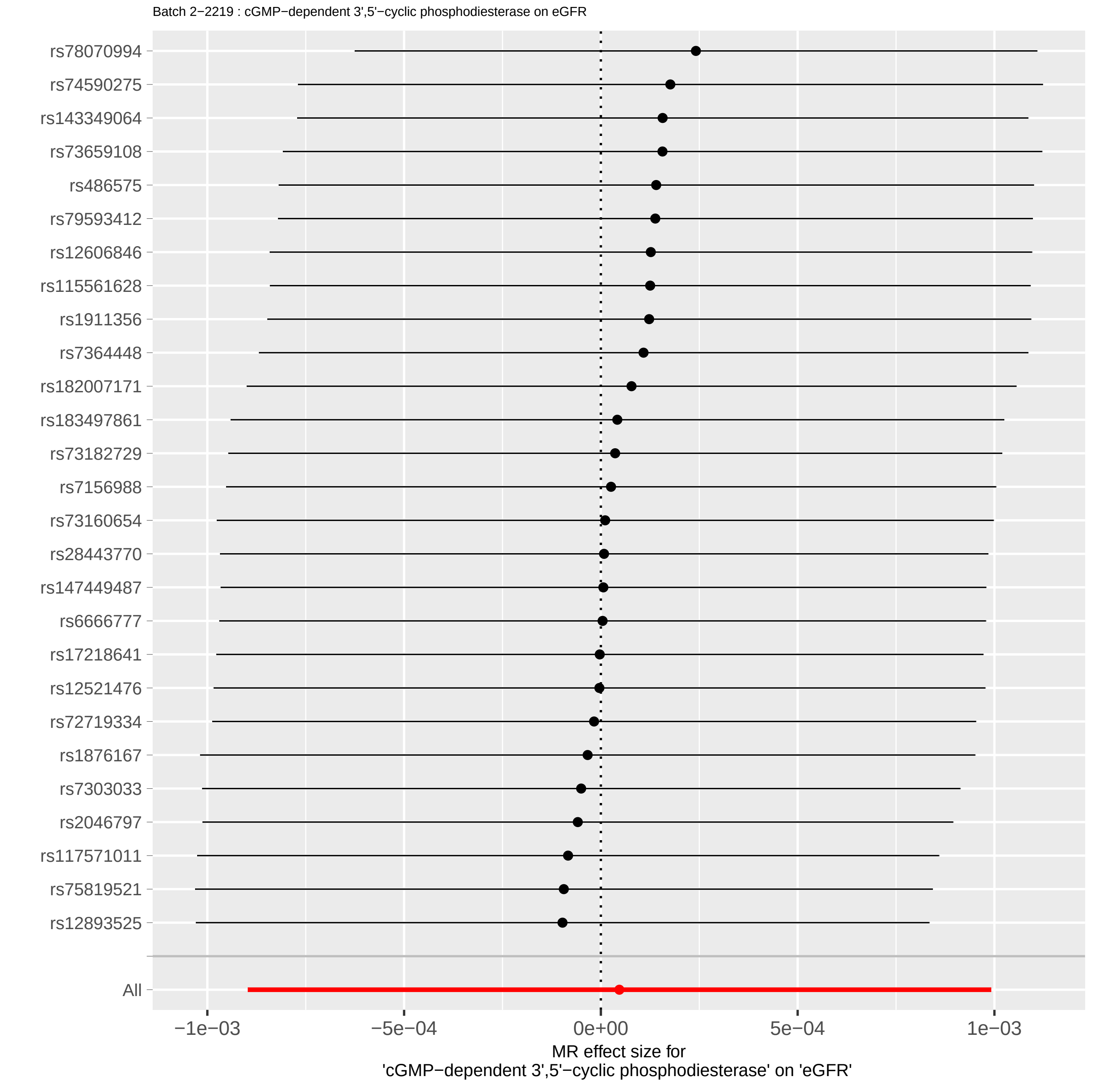

Batch 2−2219 : cGMP−dependent 3',5'−cyclic phosphodiesterase on eGFR
rs78070994
rs74590275
rs143349064
rs73659108
rs486575
rs79593412
rs12606846
rs115561628
rs1911356
rs7364448
rs182007171
rs183497861
rs73182729
rs7156988
rs73160654
rs28443770
rs147449487
rs6666777
rs17218641
rs12521476
rs72719334
rs1876167
rs7303033
rs2046797
rs117571011
rs75819521
rs12893525
All
−1e−03
−5e−04
0e+00
5e−04
1e−03
MR effect size for
'cGMP−dependent 3',5'−cyclic phosphodiesterase' on 'eGFR'

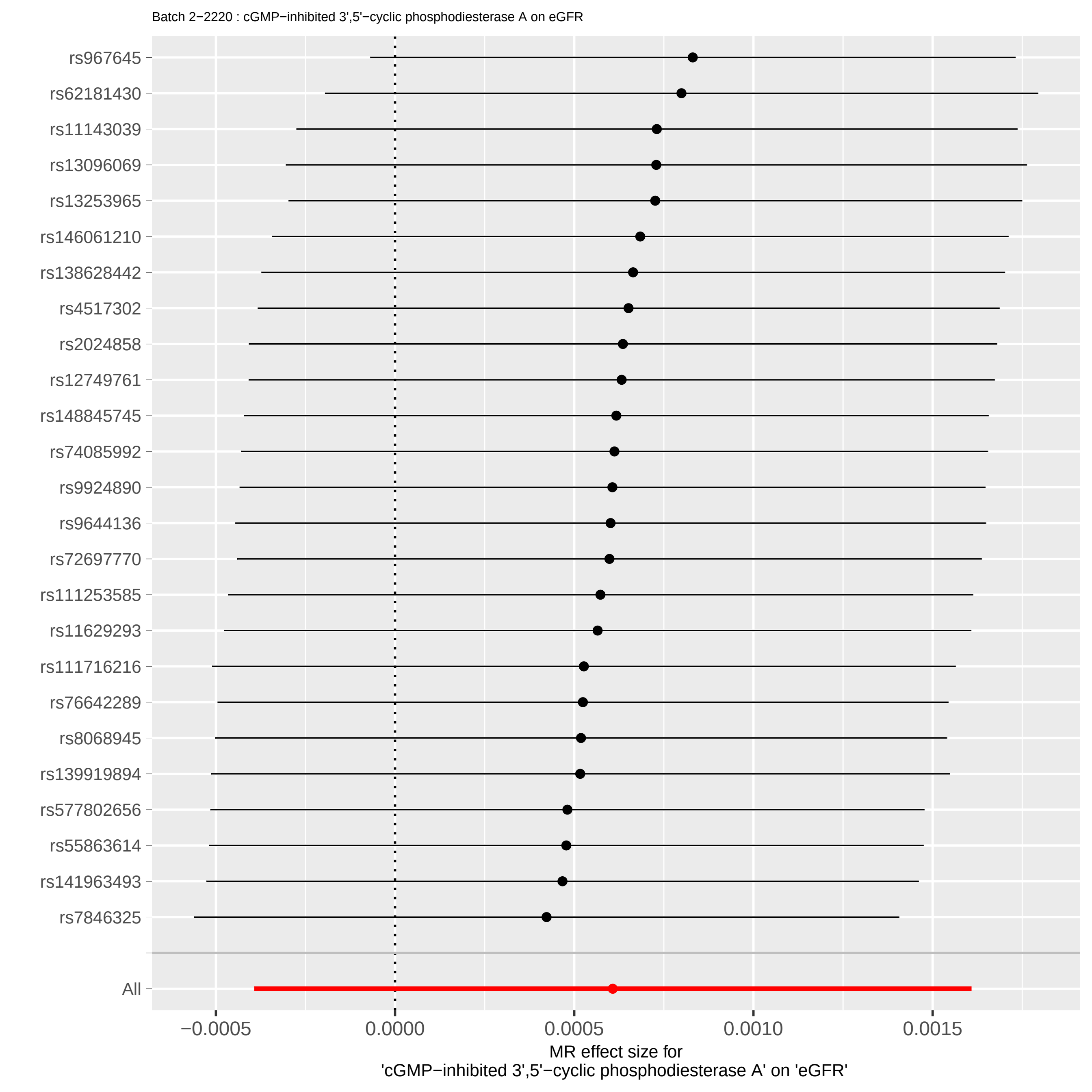

Batch 2−2220 : cGMP−inhibited 3',5'−cyclic phosphodiesterase A on eGFR
rs967645
rs62181430
rs11143039
rs13096069
rs13253965
rs146061210
rs138628442
rs4517302
rs2024858
rs12749761
rs148845745
rs74085992
rs9924890
rs9644136
rs72697770
rs111253585
rs11629293
rs111716216
rs76642289
rs8068945
rs139919894
rs577802656
rs55863614
rs141963493
rs7846325
All
−0.0005
0.0000
0.0005
0.0010
0.0015
MR effect size for
'cGMP−inhibited 3',5'−cyclic phosphodiesterase A' on 'eGFR'

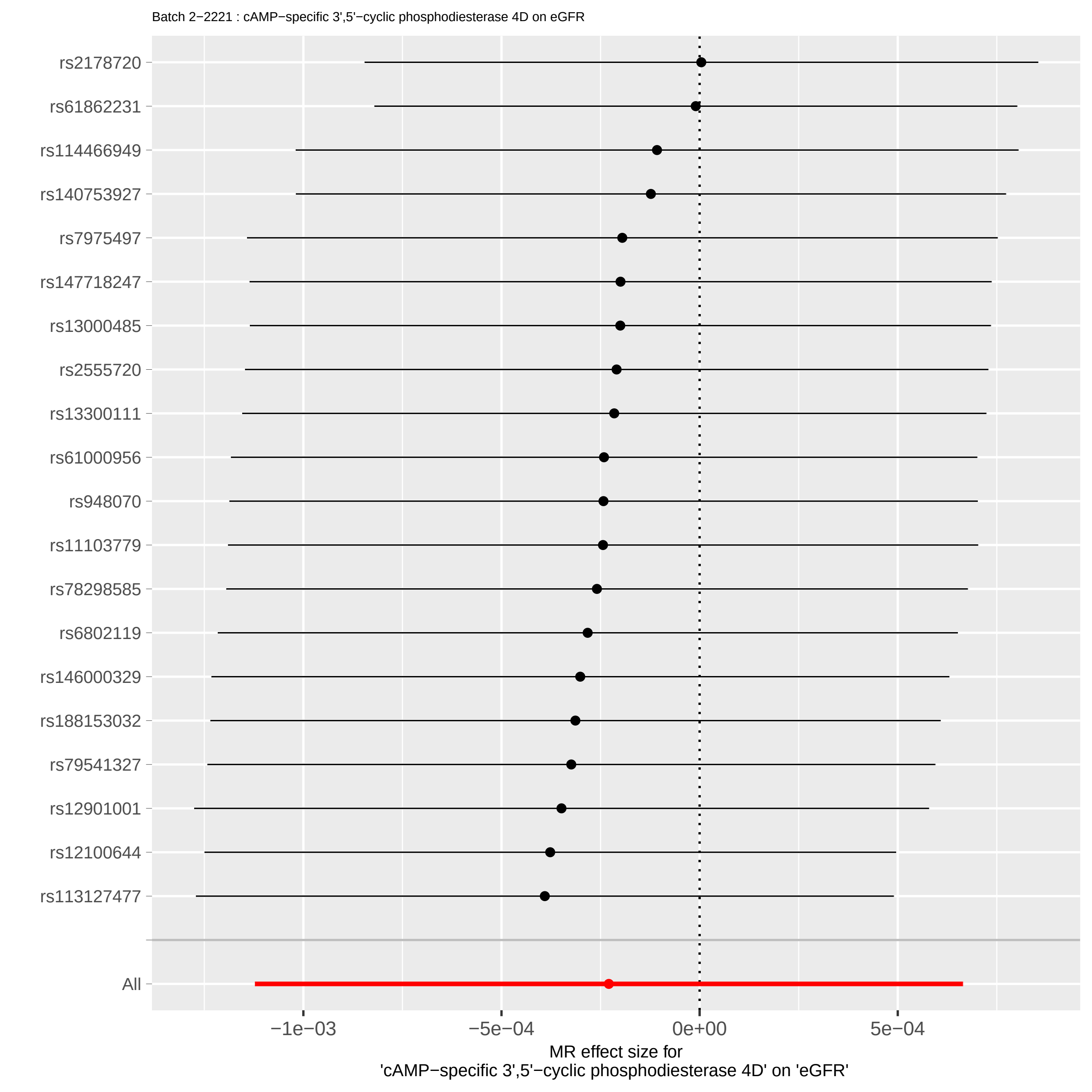
Batch 2−2221 : cAMP−specific 3',5'−cyclic phosphodiesterase 4D on eGFR
rs2178720
rs61862231
rs114466949
rs140753927
rs7975497
rs147718247
rs13000485
rs2555720
rs13300111
rs61000956
rs948070
rs11103779
rs78298585
rs6802119
rs146000329
rs188153032
rs79541327
rs12901001
rs12100644
rs113127477
All
−1e−03
−5e−04
0e+00
5e−04
MR effect size for
'cAMP−specific 3',5'−cyclic phosphodiesterase 4D' on 'eGFR'

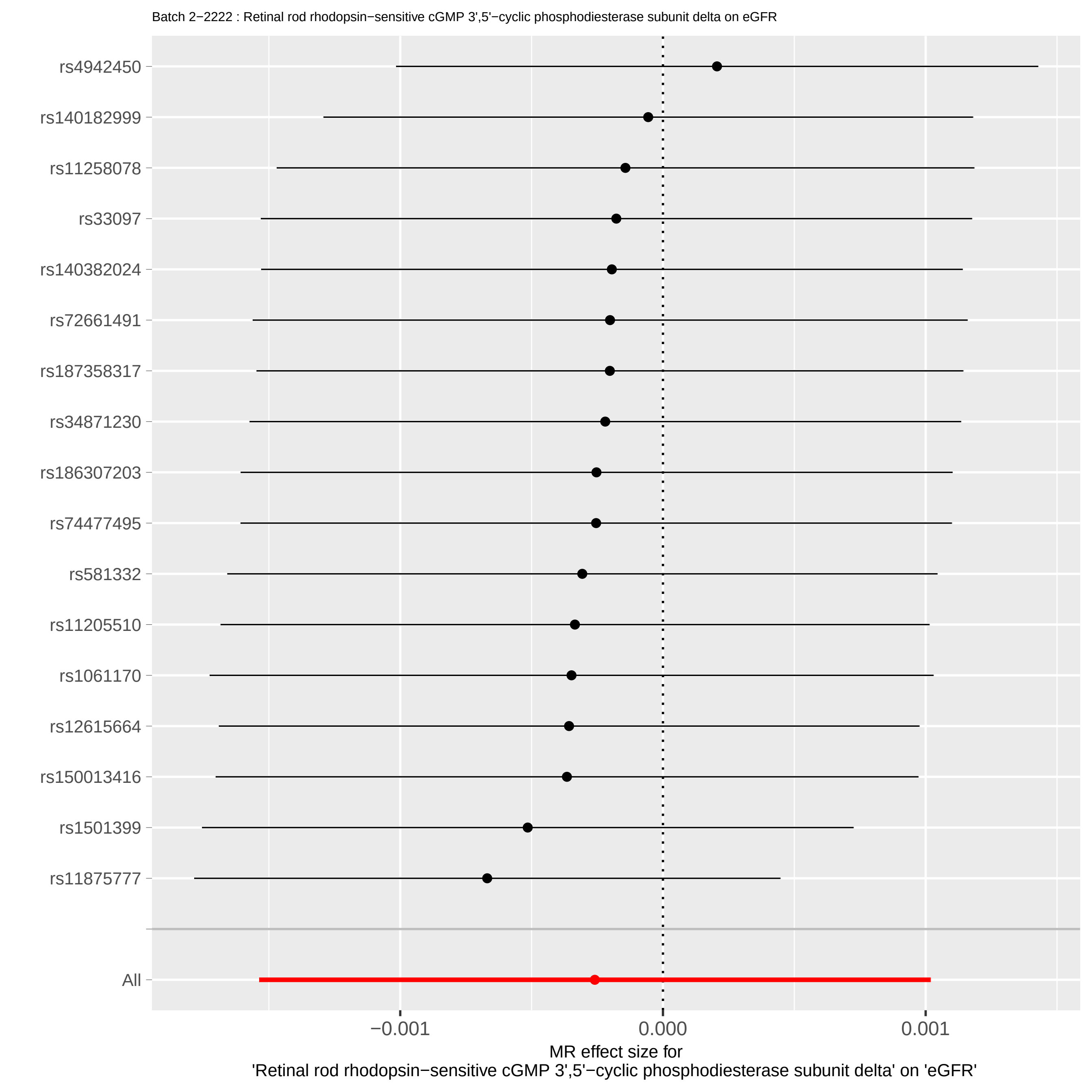

Batch 2−2222 : Retinal rod rhodopsin−sensitive cGMP 3',5'−cyclic phosphodiesterase subunit delta on eGFR
rs4942450
rs140182999
rs11258078
rs33097
rs140382024
rs72661491
rs187358317
rs34871230
rs186307203
rs74477495
rs581332
rs11205510
rs1061170
rs12615664
rs150013416
rs1501399
rs11875777
All
−0.001
0.000
0.001
MR effect size for
'Retinal rod rhodopsin−sensitive cGMP 3',5'−cyclic phosphodiesterase subunit delta' on 'eGFR'

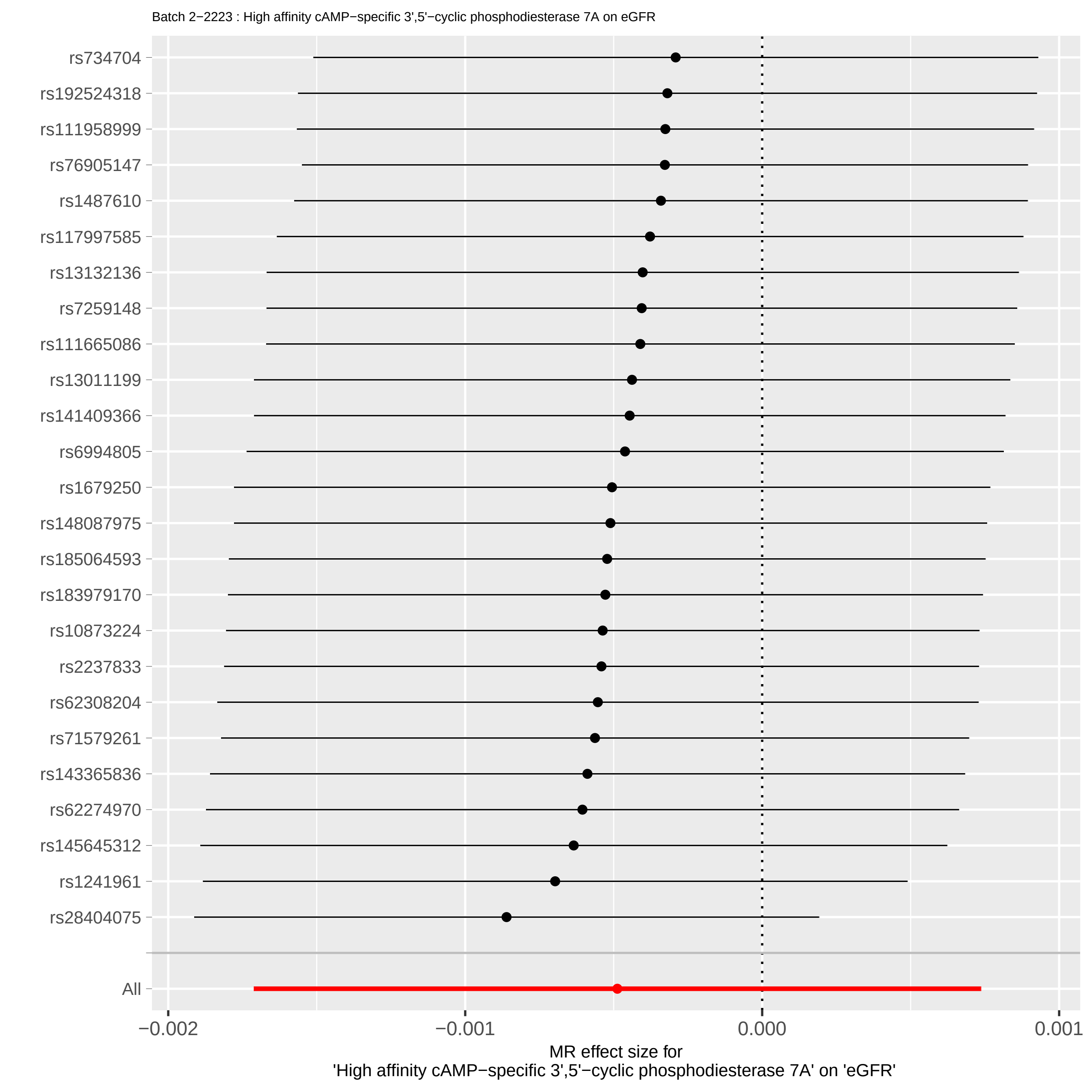

Batch 2−2223 : High affinity cAMP−specific 3',5'−cyclic phosphodiesterase 7A on eGFR
rs734704
rs192524318
rs111958999
rs76905147
rs1487610
rs117997585
rs13132136
rs7259148
rs111665086
rs13011199
rs141409366
rs6994805
rs1679250
rs148087975
rs185064593
rs183979170
rs10873224
rs2237833
rs62308204
rs71579261
rs143365836
rs62274970
rs145645312
rs1241961
rs28404075
All
−0.002
−0.001
0.000
0.001
MR effect size for
'High affinity cAMP−specific 3',5'−cyclic phosphodiesterase 7A' on 'eGFR'

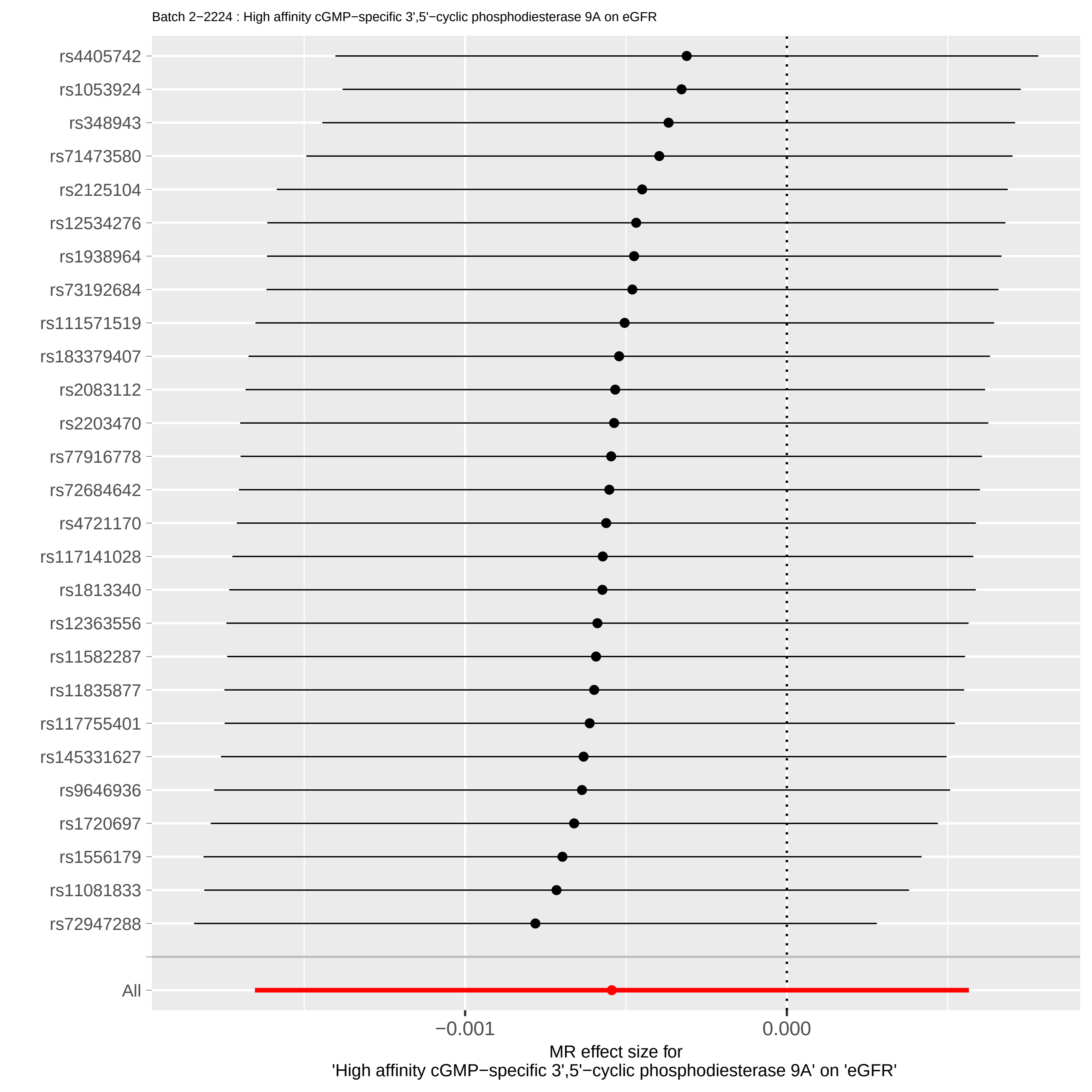

Batch 2−2224 : High affinity cGMP−specific 3',5'−cyclic phosphodiesterase 9A on eGFR
rs4405742
rs1053924
rs348943
rs71473580
rs2125104
rs12534276
rs1938964
rs73192684
rs111571519
rs183379407
rs2083112
rs2203470
rs77916778
rs72684642
rs4721170
rs117141028
rs1813340
rs12363556
rs11582287
rs11835877
rs117755401
rs145331627
rs9646936
rs1720697
rs1556179
rs11081833
rs72947288
All
−0.001
0.000
MR effect size for
'High affinity cGMP−specific 3',5'−cyclic phosphodiesterase 9A' on 'eGFR'

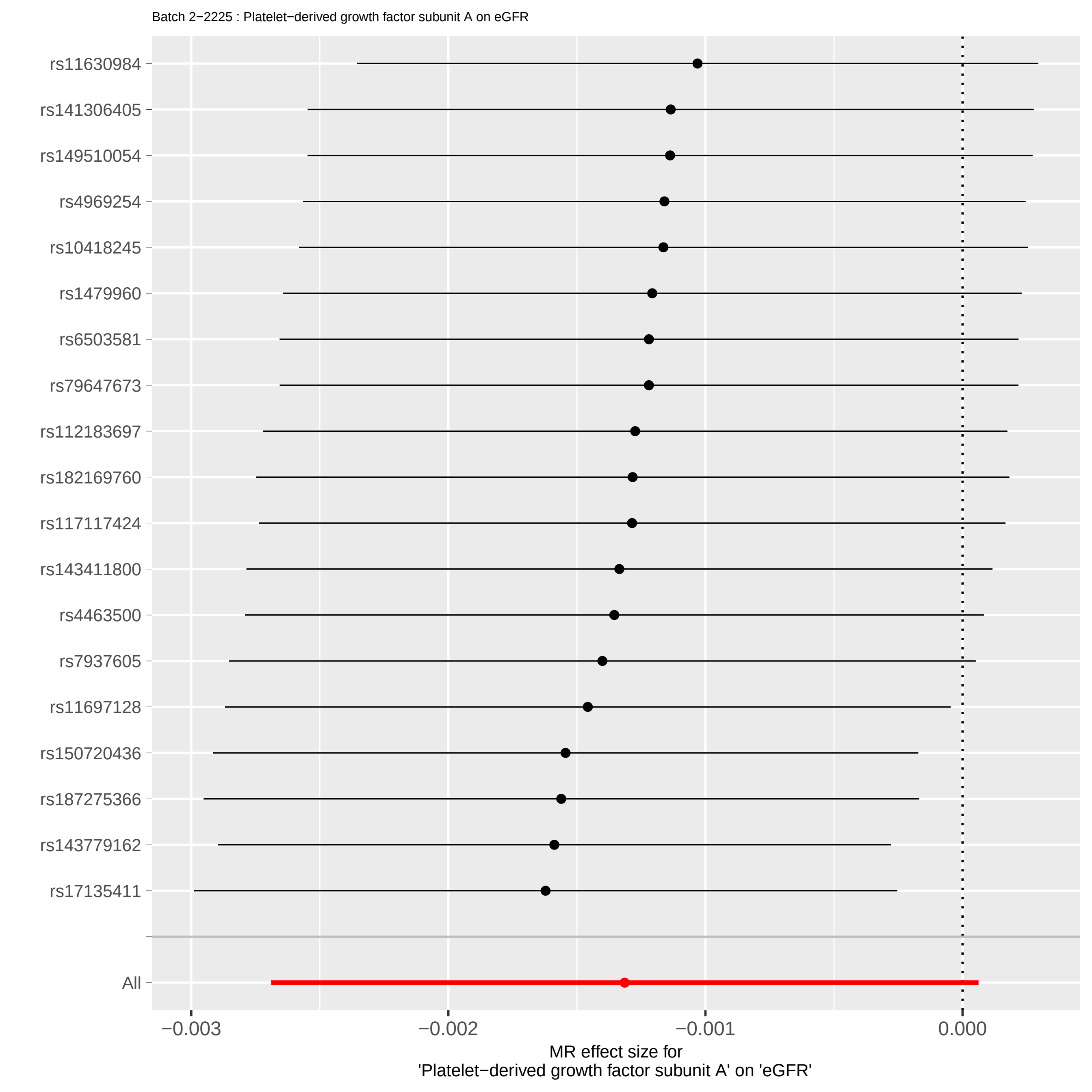
Batch 2−2225 : Platelet−derived growth factor subunit A on eGFR
rs11630984
rs141306405
rs149510054
rs4969254
rs10418245
rs1479960
rs6503581
rs79647673
rs112183697
rs182169760
rs117117424
rs143411800
rs4463500
rs7937605
rs11697128
rs150720436
rs187275366
rs143779162
rs17135411
All
−0.003
−0.002
−0.001
0.000
MR effect size for
'Platelet−derived growth factor subunit A' on 'eGFR'

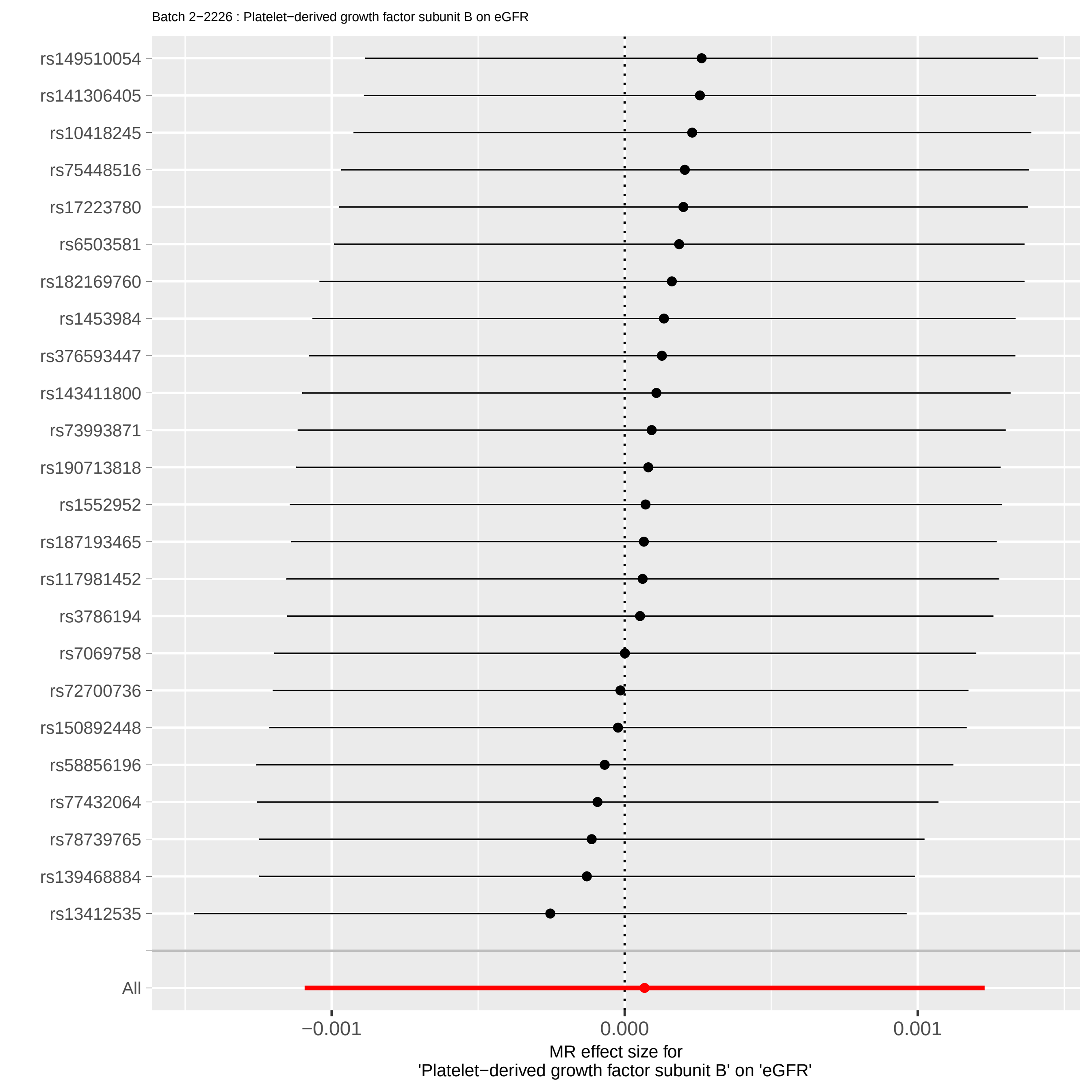

Batch 2−2226 : Platelet−derived growth factor subunit B on eGFR
rs149510054
rs141306405
rs10418245
rs75448516
rs17223780
rs6503581
rs182169760
rs1453984
rs376593447
rs143411800
rs73993871
rs190713818
rs1552952
rs187193465
rs117981452
rs3786194
rs7069758
rs72700736
rs150892448
rs58856196
rs77432064
rs78739765
rs139468884
rs13412535
All
−0.001
0.000
0.001
MR effect size for
'Platelet−derived growth factor subunit B' on 'eGFR'

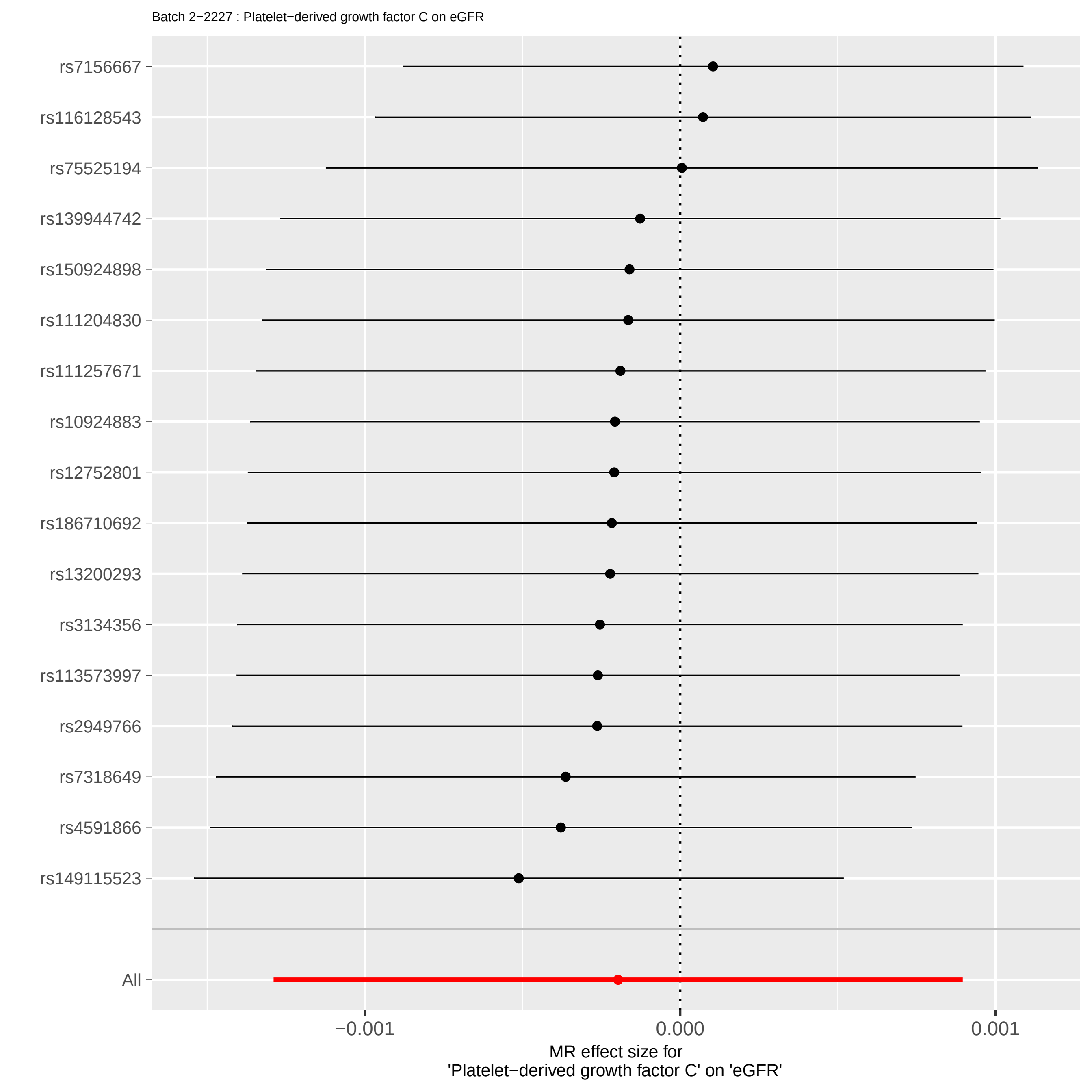
Batch 2−2227 : Platelet−derived growth factor C on eGFR
rs7156667
rs116128543
rs75525194
rs139944742
rs150924898
rs111204830
rs111257671
rs10924883
rs12752801
rs186710692
rs13200293
rs3134356
rs113573997
rs2949766
rs7318649
rs4591866
rs149115523
All
−0.001
0.000
0.001
MR effect size for
'Platelet−derived growth factor C' on 'eGFR'

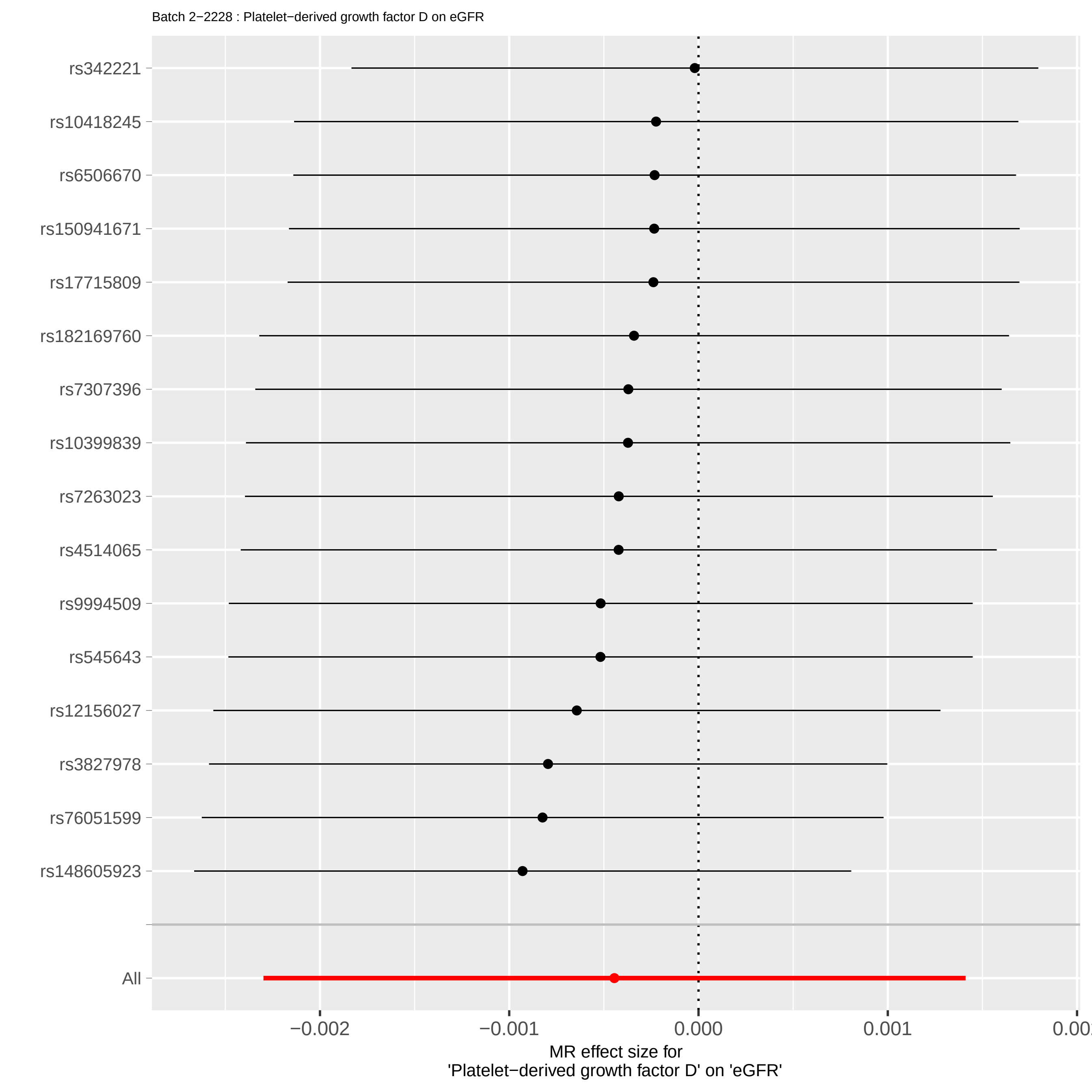

Batch 2−2228 : Platelet−derived growth factor D on eGFR
rs342221
rs10418245
rs6506670
rs150941671
rs17715809
rs182169760
rs7307396
rs10399839
rs7263023
rs4514065
rs9994509
rs545643
rs12156027
rs3827978
rs76051599
rs148605923
All
−0.002
−0.001
0.000
0.001
MR effect size for
'Platelet−derived growth factor D' on 'eGFR'

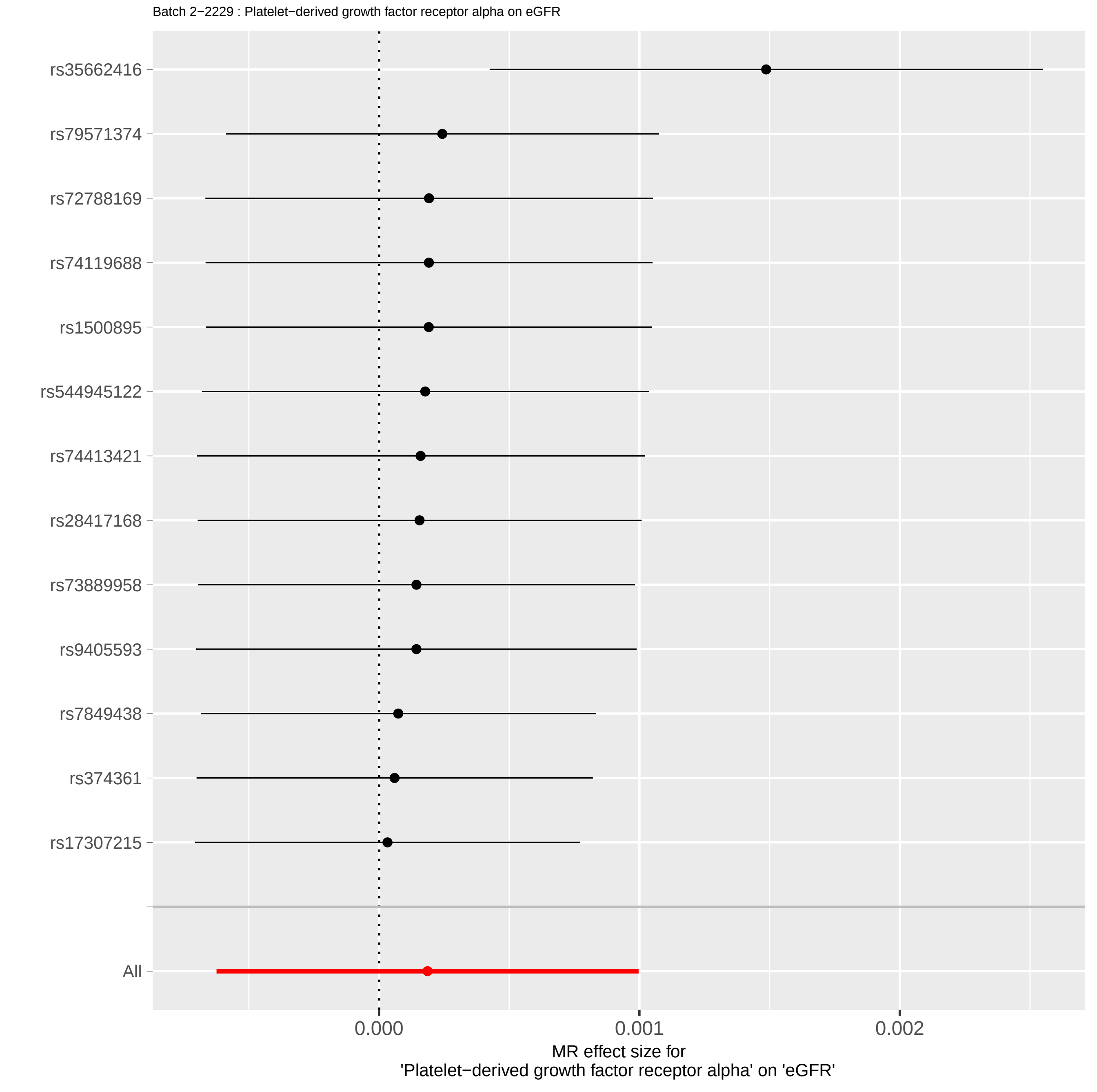
Batch 2−2229 : Platelet−derived growth factor receptor alpha on eGFR
rs35662416
rs79571374
rs72788169
rs74119688
rs1500895
rs544945122
rs74413421
rs28417168
rs73889958
rs9405593
rs7849438
rs374361
rs17307215
All
0.000
0.001
0.002
MR effect size for
'Platelet−derived growth factor receptor alpha' on 'eGFR'

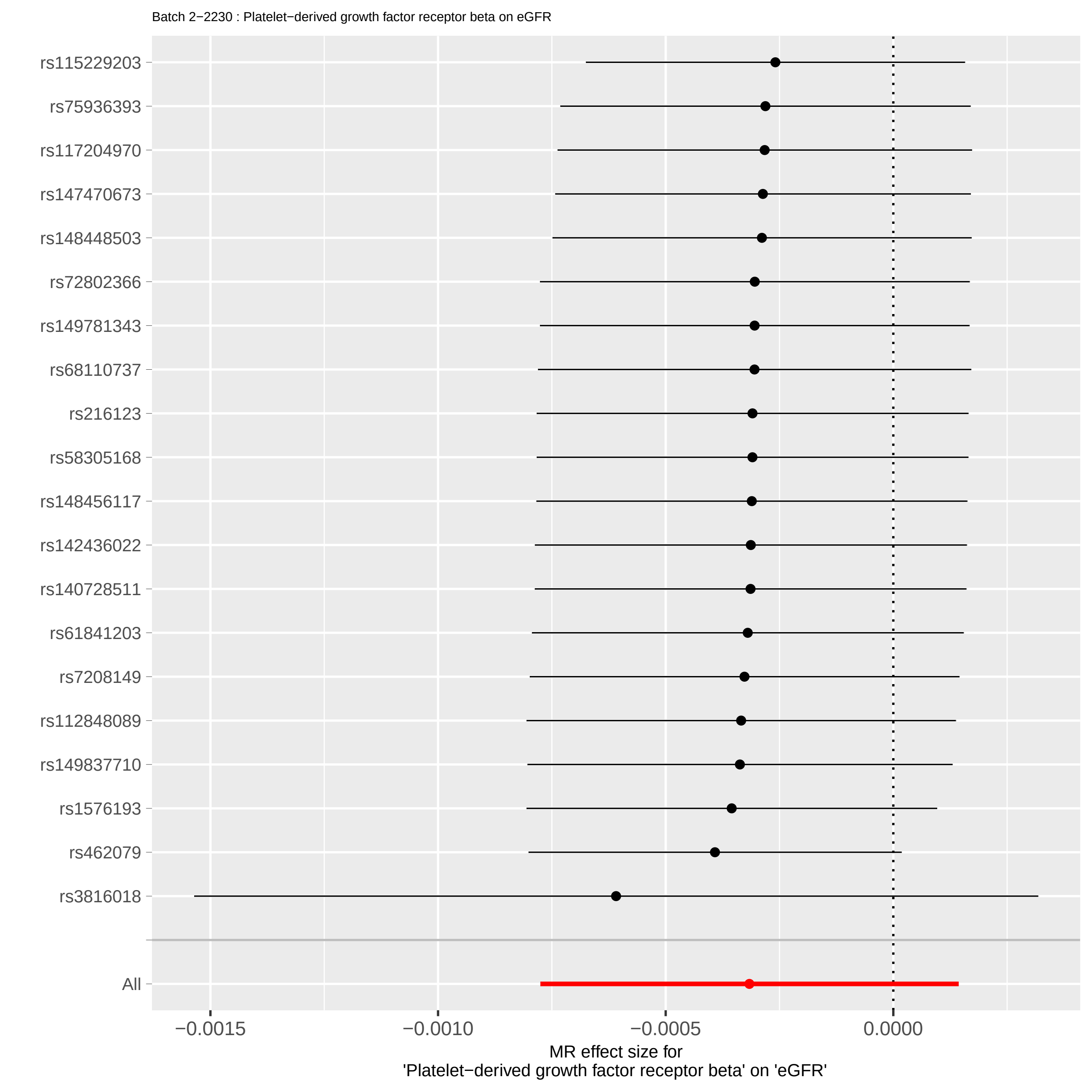

Batch 2−2230 : Platelet−derived growth factor receptor beta on eGFR
rs115229203
rs75936393
rs117204970
rs147470673
rs148448503
rs72802366
rs149781343
rs68110737
rs216123
rs58305168
rs148456117
rs142436022
rs140728511
rs61841203
rs7208149
rs112848089
rs149837710
rs1576193
rs462079
rs3816018
All
−0.0015
−0.0010
−0.0005
0.0000
MR effect size for
'Platelet−derived growth factor receptor beta' on 'eGFR'

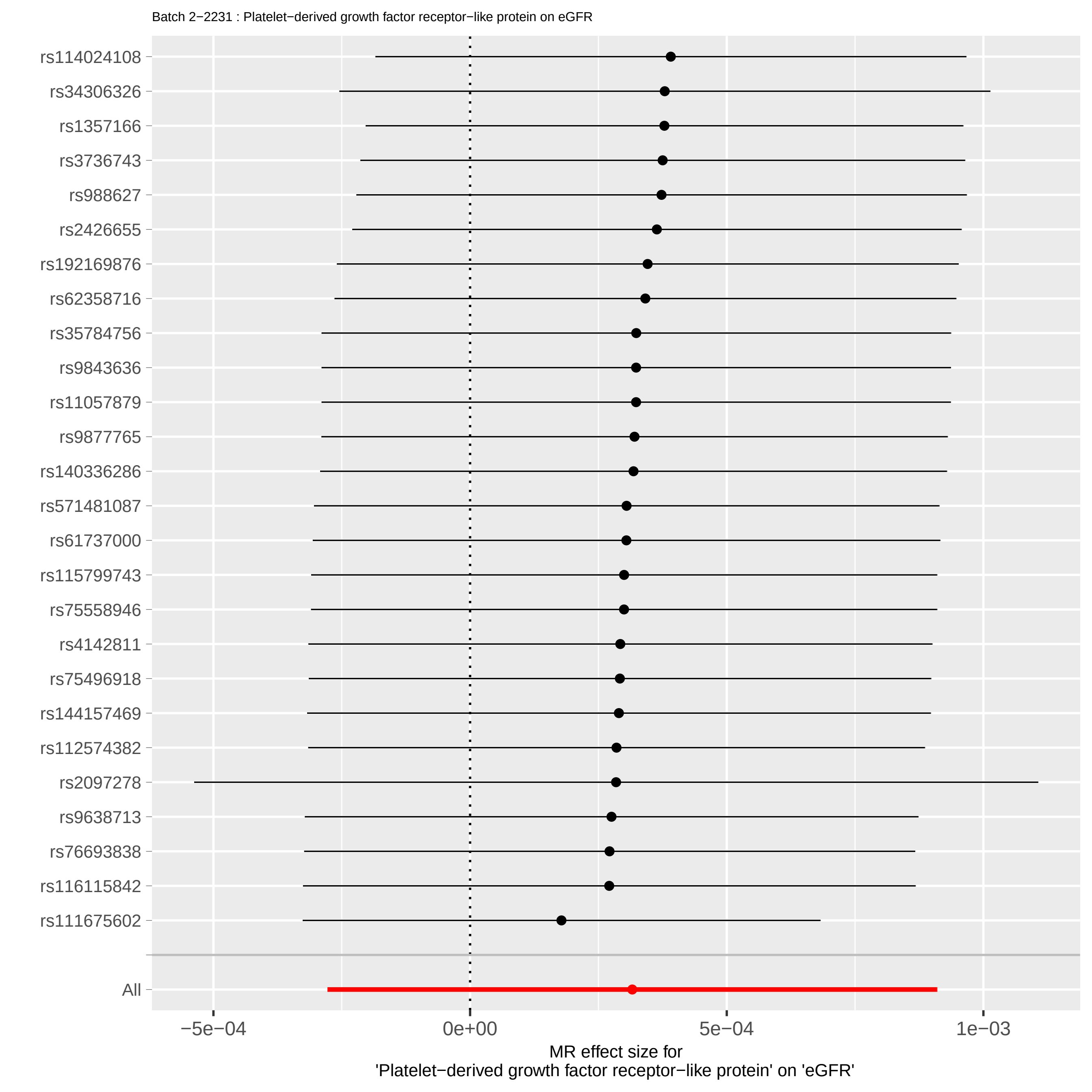

Batch 2−2231 : Platelet−derived growth factor receptor−like protein on eGFR
rs114024108
rs34306326
rs1357166
rs3736743
rs988627
rs2426655
rs192169876
rs62358716
rs35784756
rs9843636
rs11057879
rs9877765
rs140336286
rs571481087
rs61737000
rs115799743
rs75558946
rs4142811
rs75496918
rs144157469
rs112574382
rs2097278
rs9638713
rs76693838
rs116115842
rs111675602
All
−5e−04
0e+00
5e−04
1e−03
MR effect size for
'Platelet−derived growth factor receptor−like protein' on 'eGFR'

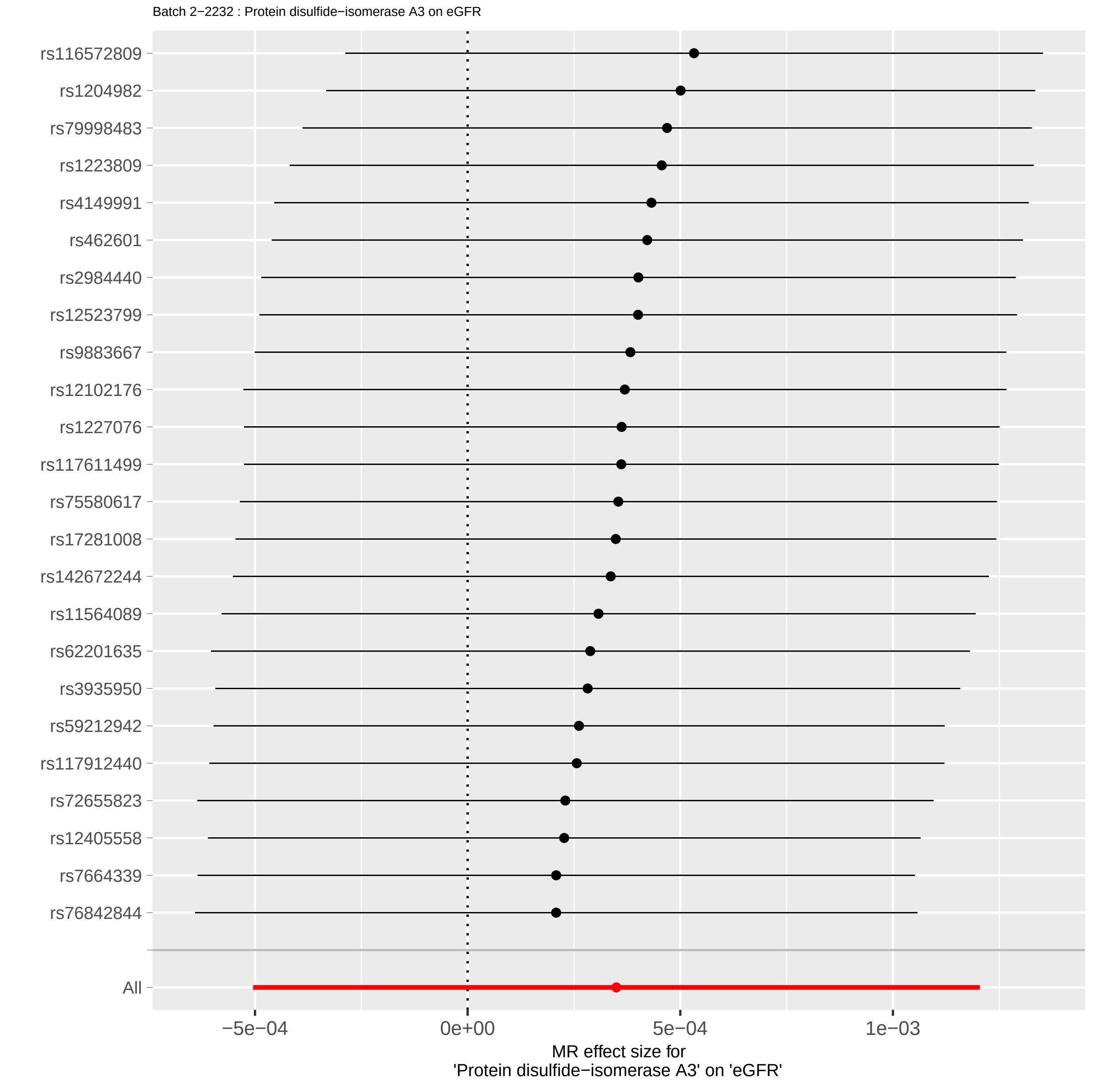

Batch 2−2232 : Protein disulfide−isomerase A3 on eGFR
rs116572809
rs1204982
rs79998483
rs1223809
rs4149991
rs462601
rs2984440
rs12523799
rs9883667
rs12102176
rs1227076
rs117611499
rs75580617
rs17281008
rs142672244
rs11564089
rs62201635
rs3935950
rs59212942
rs117912440
rs72655823
rs12405558
rs7664339
rs76842844
All
−5e−04
0e+00
5e−04
1e−03
MR effect size for
'Protein disulfide−isomerase A3' on 'eGFR'

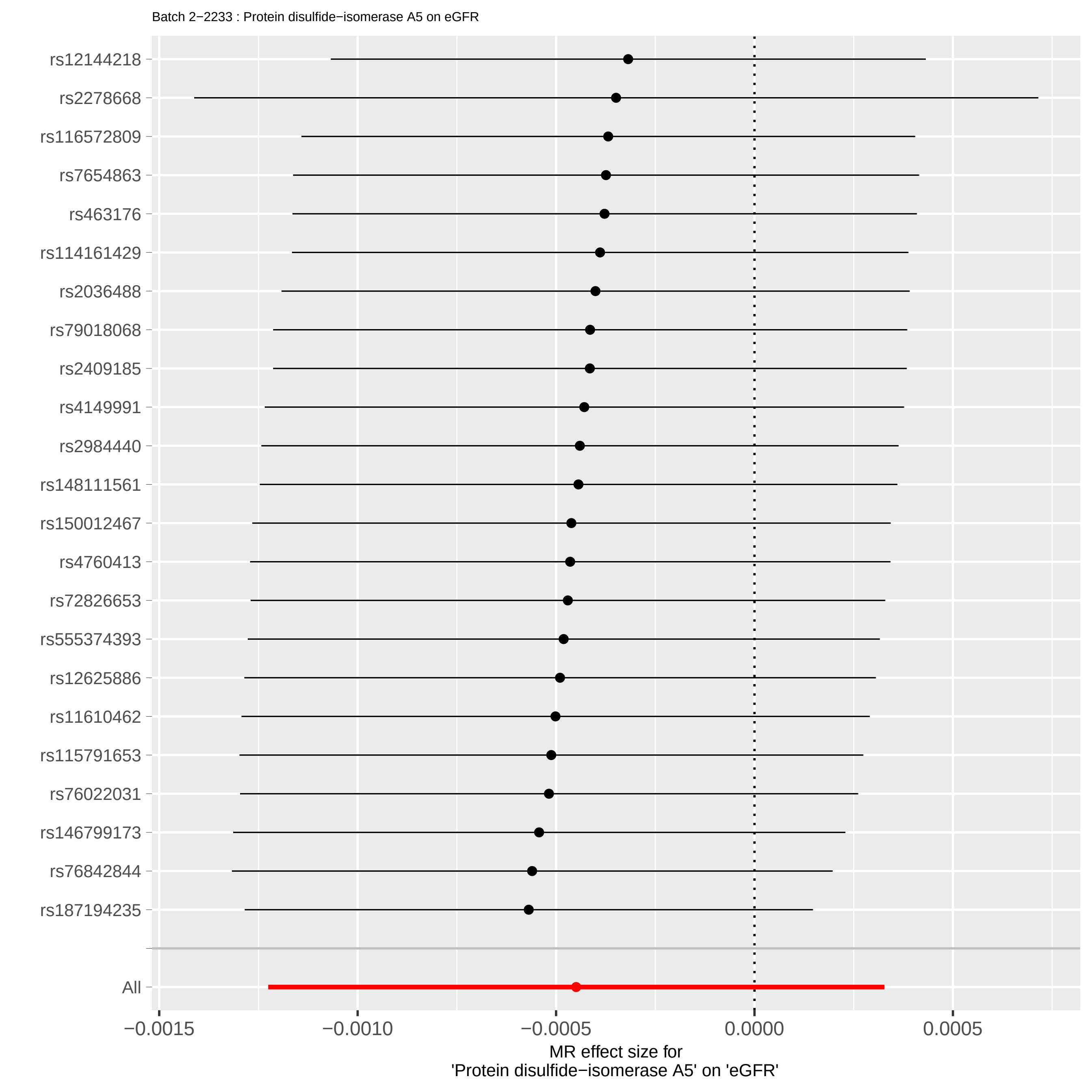

Batch 2−2233 : Protein disulfide−isomerase A5 on eGFR
rs12144218
rs2278668
rs116572809
rs7654863
rs463176
rs114161429
rs2036488
rs79018068
rs2409185
rs4149991
rs2984440
rs148111561
rs150012467
rs4760413
rs72826653
rs555374393
rs12625886
rs11610462
rs115791653
rs76022031
rs146799173
rs76842844
rs187194235
All
−0.0015
−0.0010
−0.0005
0.0000
0.0005
MR effect size for
'Protein disulfide−isomerase A5' on 'eGFR'

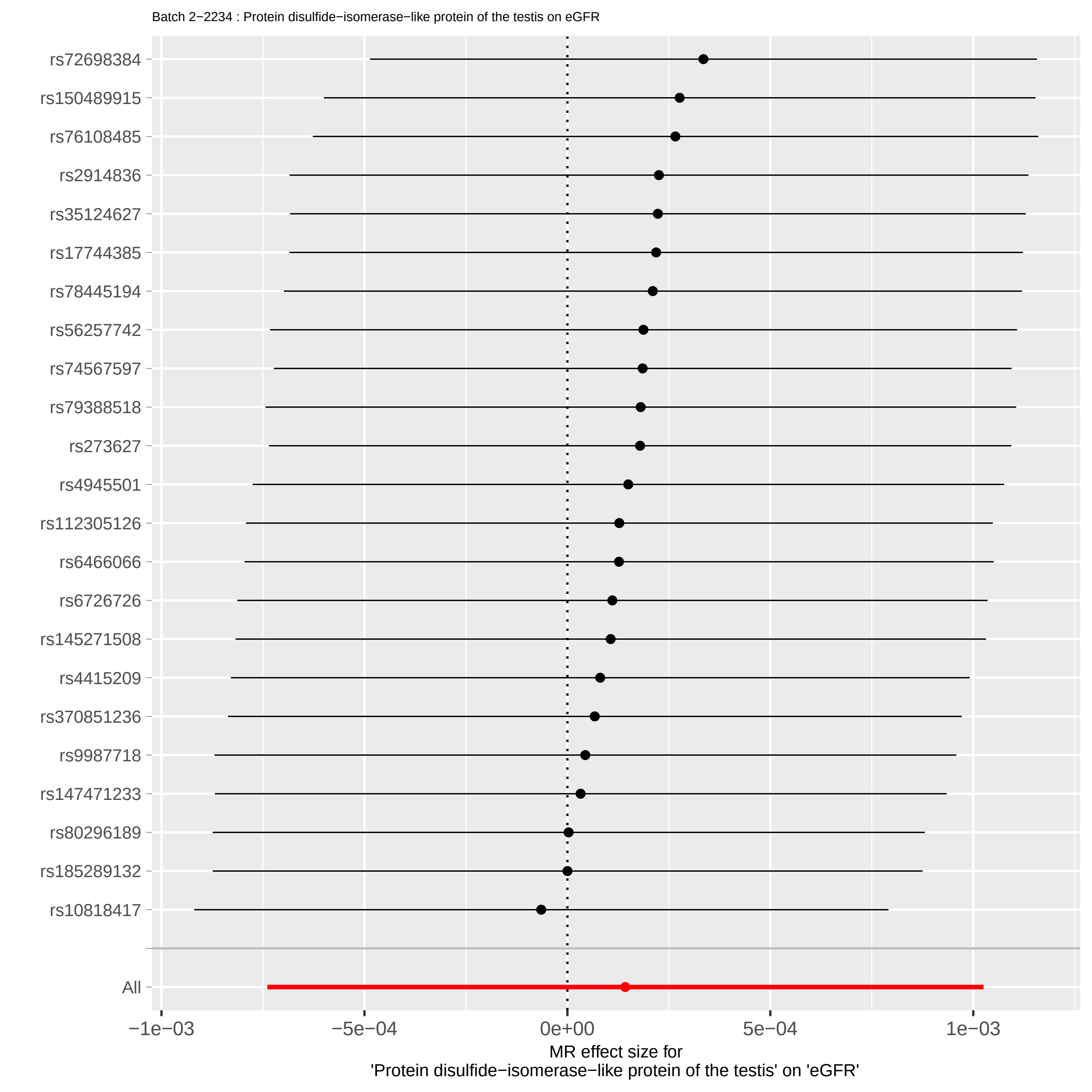

Batch 2−2234 : Protein disulfide−isomerase−like protein of the testis on eGFR
rs72698384
rs150489915
rs76108485
rs2914836
rs35124627
rs17744385
rs78445194
rs56257742
rs74567597
rs79388518
rs273627
rs4945501
rs112305126
rs6466066
rs6726726
rs145271508
rs4415209
rs370851236
rs9987718
rs147471233
rs80296189
rs185289132
rs10818417
All
−1e−03
−5e−04
0e+00
5e−04
1e−03
MR effect size for
'Protein disulfide−isomerase−like protein of the testis' on 'eGFR'

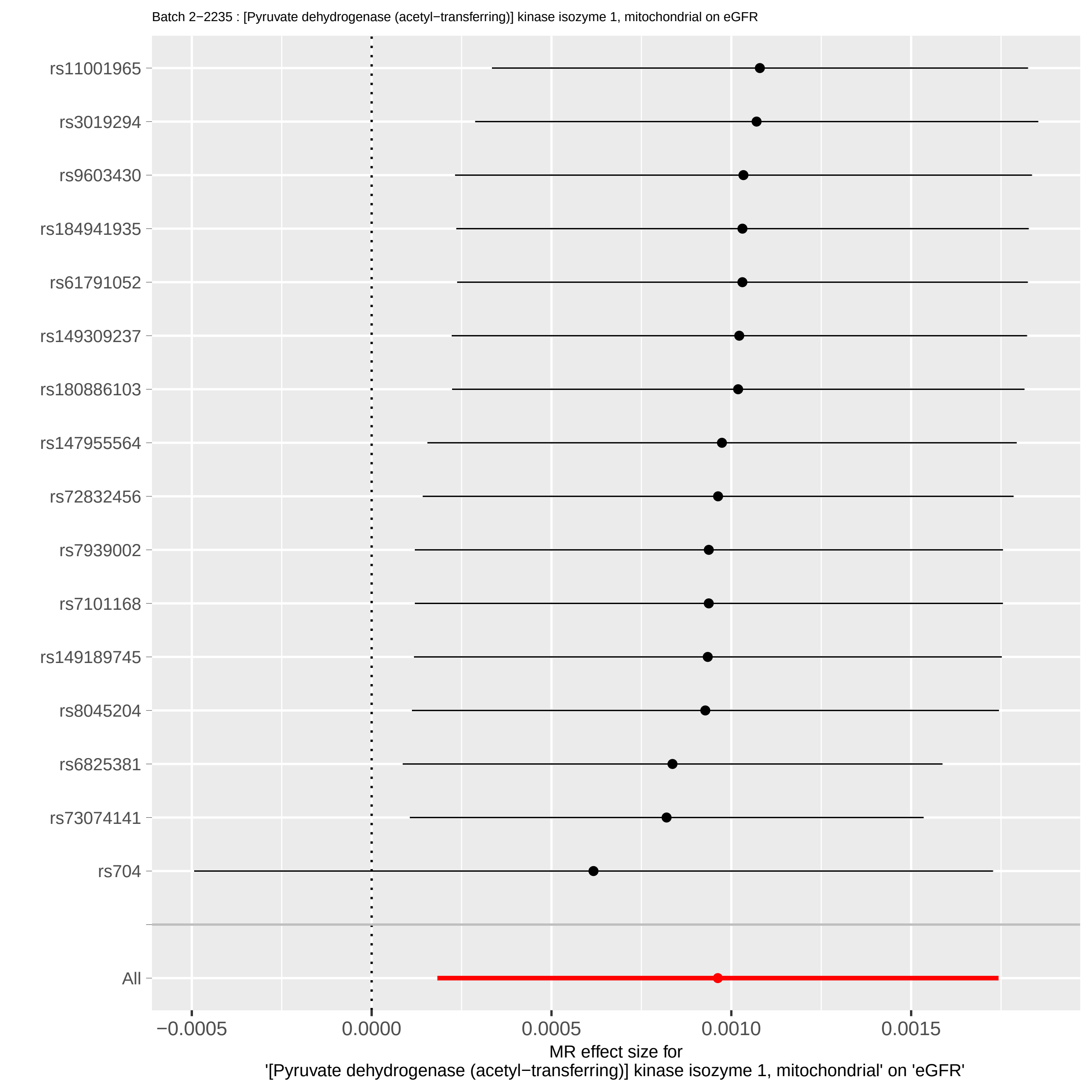
Batch 2−2235 : [Pyruvate dehydrogenase (acetyl−transferring)] kinase isozyme 1, mitochondrial on eGFR
rs11001965
rs3019294
rs9603430
rs184941935
rs61791052
rs149309237
rs180886103
rs147955564
rs72832456
rs7939002
rs7101168
rs149189745
rs8045204
rs6825381
rs73074141
rs704
All
−0.0005
0.0000
0.0005
0.0010
0.0015
MR effect size for
'[Pyruvate dehydrogenase (acetyl−transferring)] kinase isozyme 1, mitochondrial' on 'eGFR'

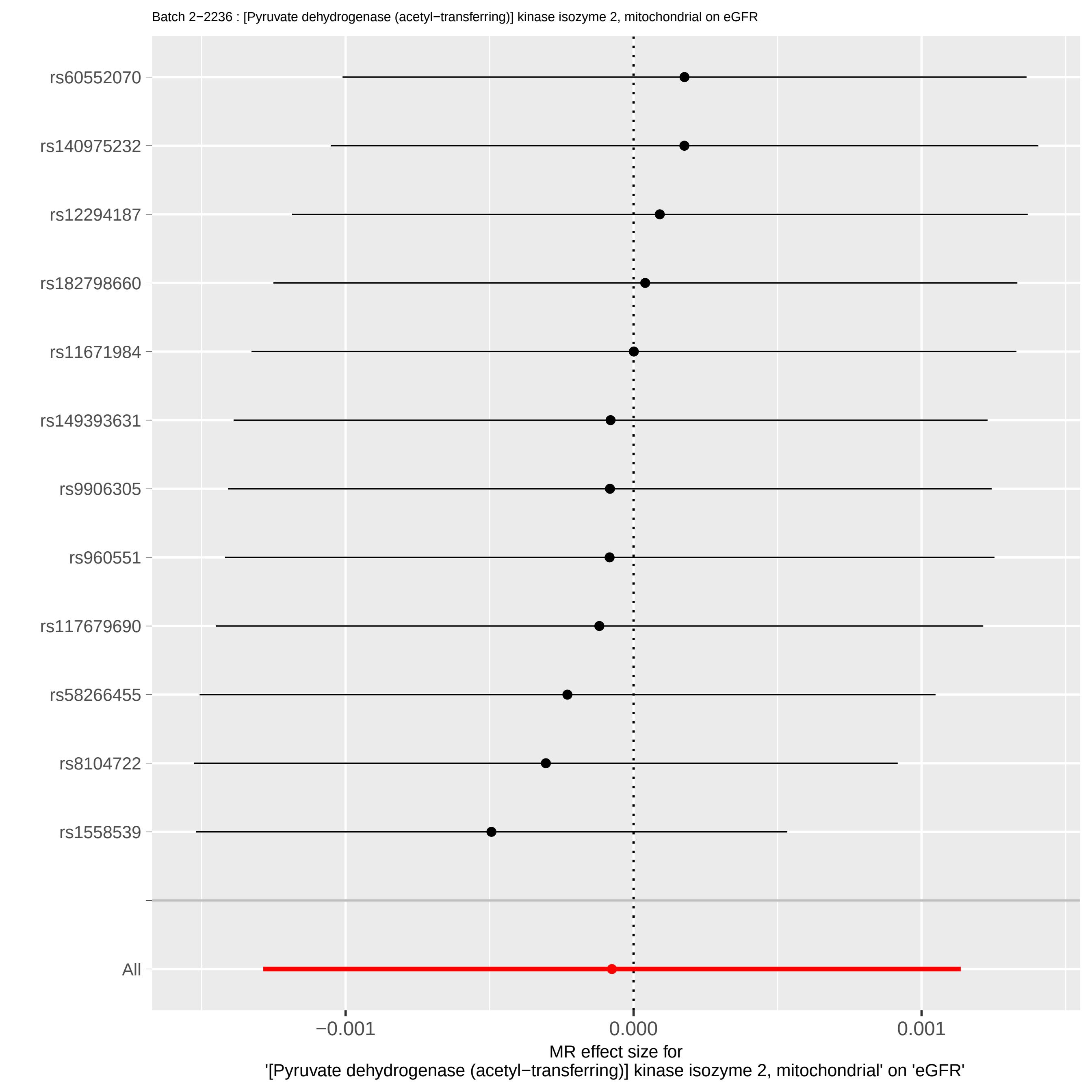

Batch 2−2236 : [Pyruvate dehydrogenase (acetyl−transferring)] kinase isozyme 2, mitochondrial on eGFR
rs60552070
rs140975232
rs12294187
rs182798660
rs11671984
rs149393631
rs9906305
rs960551
rs117679690
rs58266455
rs8104722
rs1558539
All
−0.001
0.000
0.001
MR effect size for
'[Pyruvate dehydrogenase (acetyl−transferring)] kinase isozyme 2, mitochondrial' on 'eGFR'

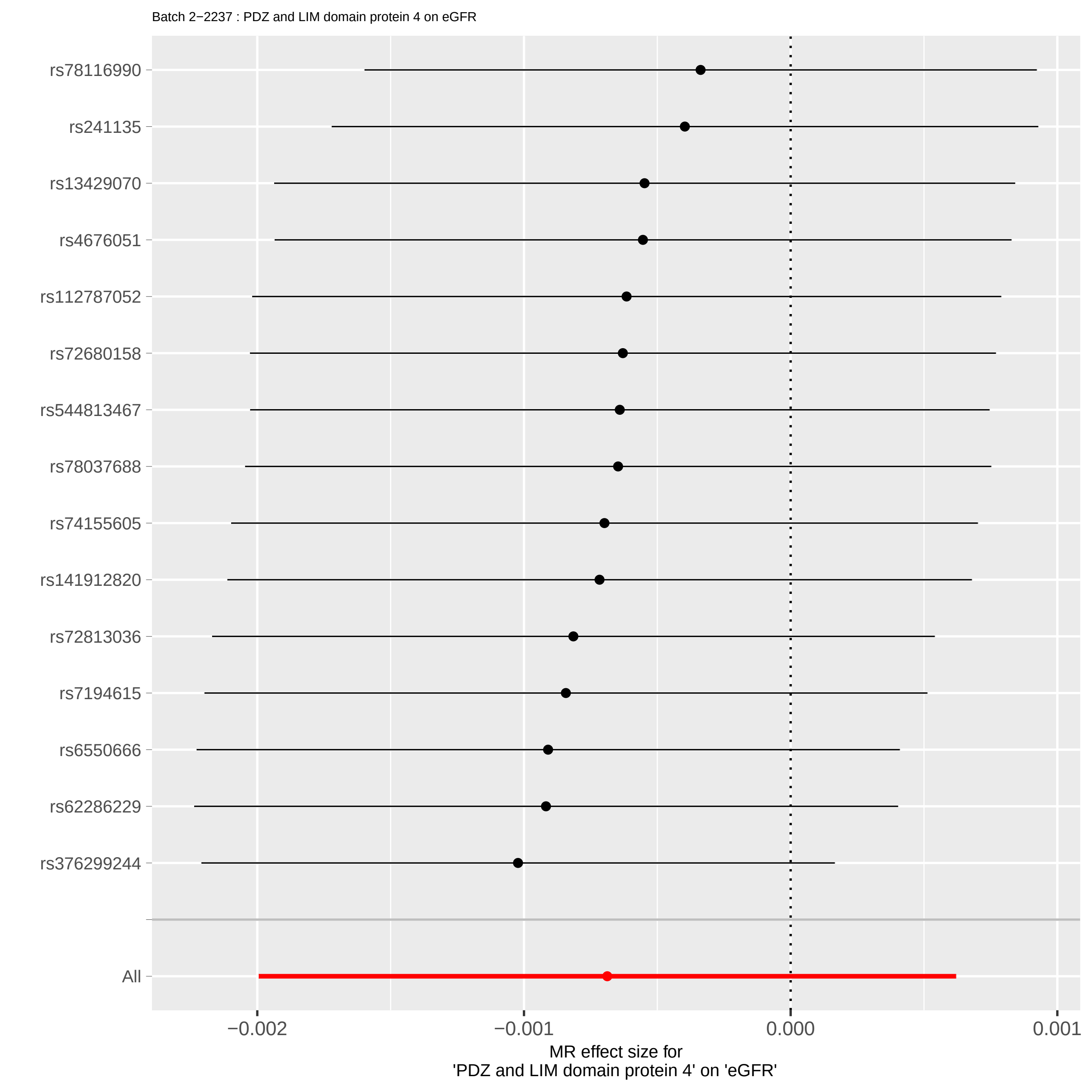

Batch 2−2237 : PDZ and LIM domain protein 4 on eGFR
rs78116990
rs241135
rs13429070
rs4676051
rs112787052
rs72680158
rs544813467
rs78037688
rs74155605
rs141912820
rs72813036
rs7194615
rs6550666
rs62286229
rs376299244
All
−0.002
−0.001
0.000
0.001
MR effect size for
'PDZ and LIM domain protein 4' on 'eGFR'

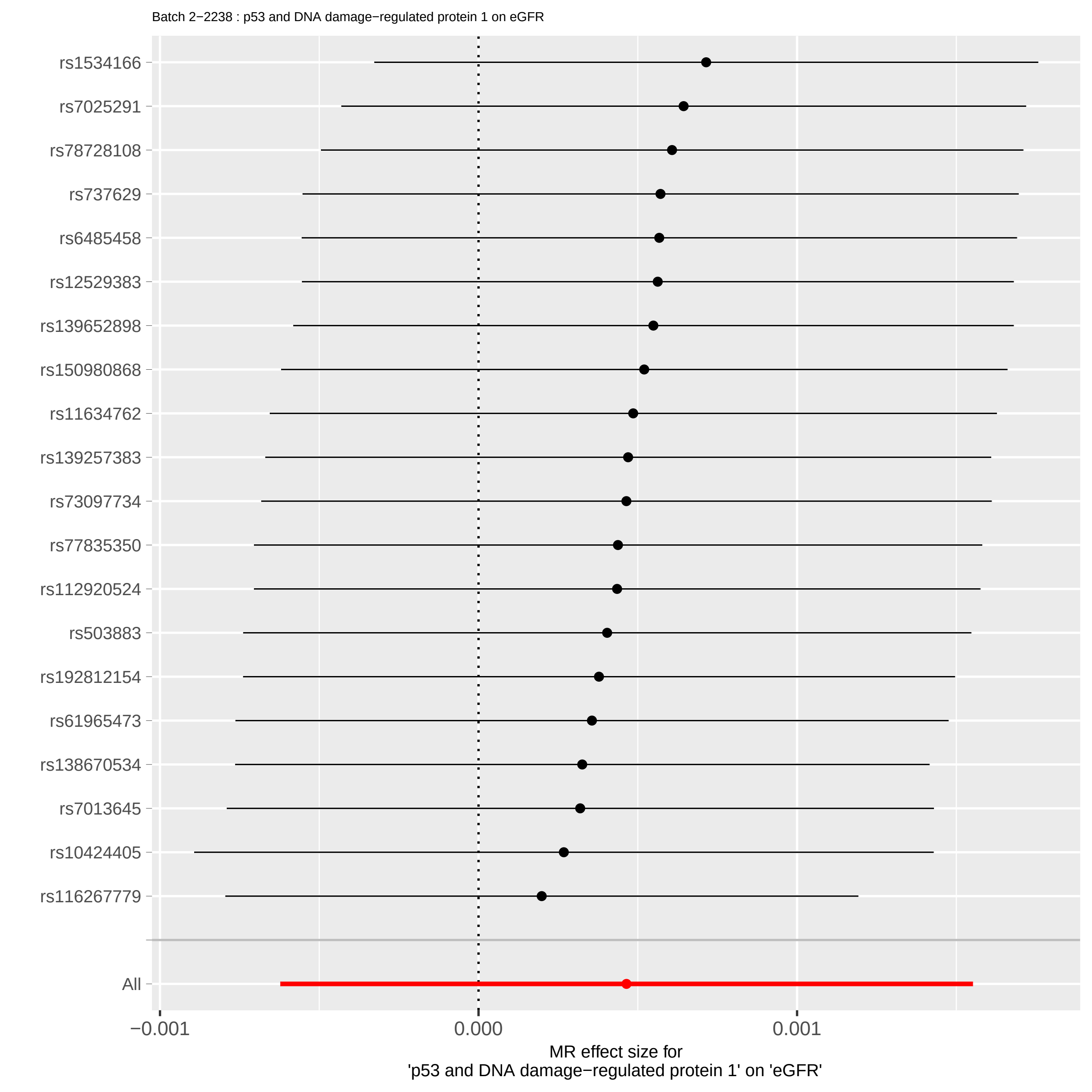
Batch 2−2238 : p53 and DNA damage−regulated protein 1 on eGFR
rs1534166
rs7025291
rs78728108
rs737629
rs6485458
rs12529383
rs139652898
rs150980868
rs11634762
rs139257383
rs73097734
rs77835350
rs112920524
rs503883
rs192812154
rs61965473
rs138670534
rs7013645
rs10424405
rs116267779
All
−0.001
0.000
0.001
MR effect size for
'p53 and DNA damage−regulated protein 1' on 'eGFR'

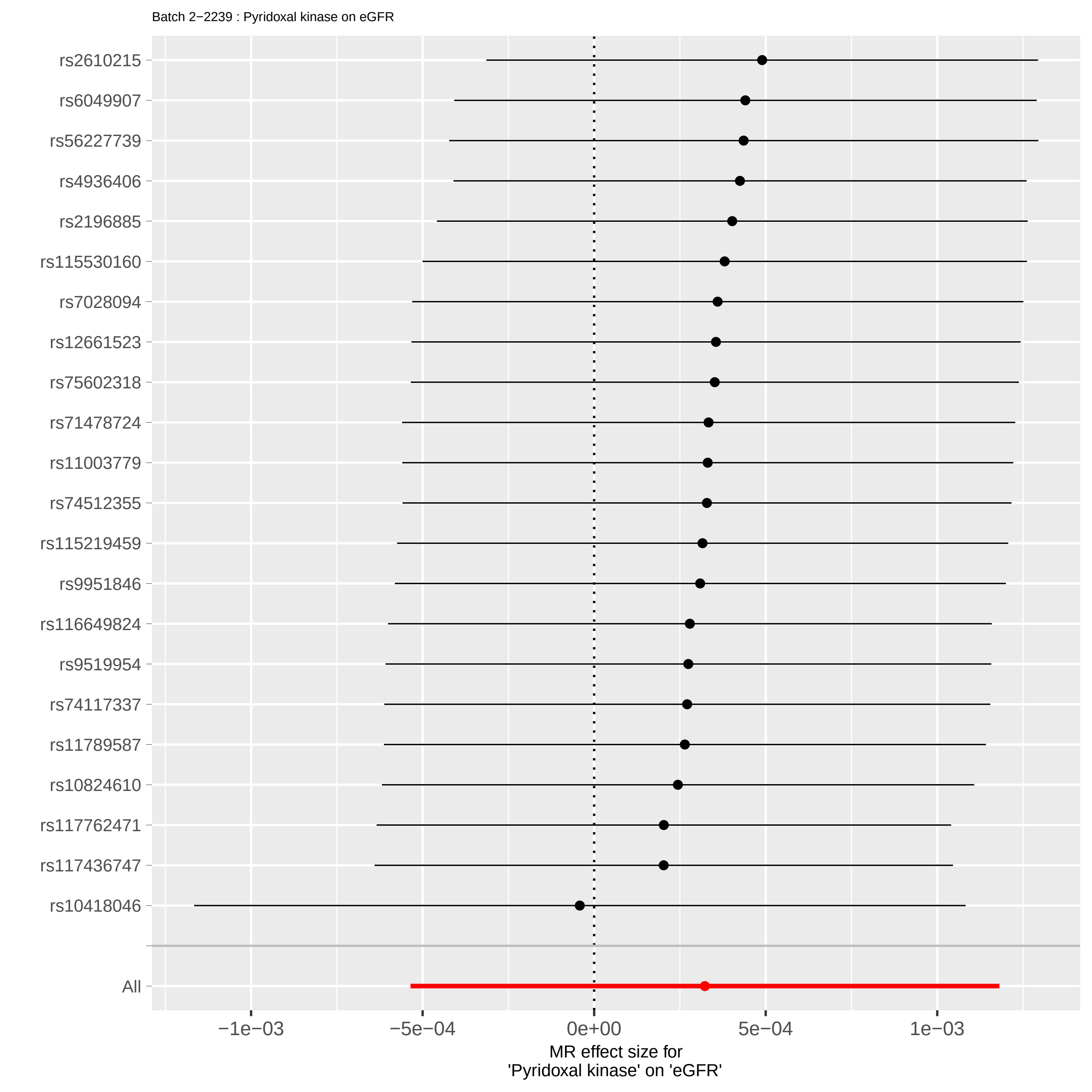

Batch 2−2239 : Pyridoxal kinase on eGFR
rs2610215
rs6049907
rs56227739
rs4936406
rs2196885
rs115530160
rs7028094
rs12661523
rs75602318
rs71478724
rs11003779
rs74512355
rs115219459
rs9951846
rs116649824
rs9519954
rs74117337
rs11789587
rs10824610
rs117762471
rs117436747
rs10418046
All
−1e−03
−5e−04
0e+00
5e−04
1e−03
MR effect size for
'Pyridoxal kinase' on 'eGFR'

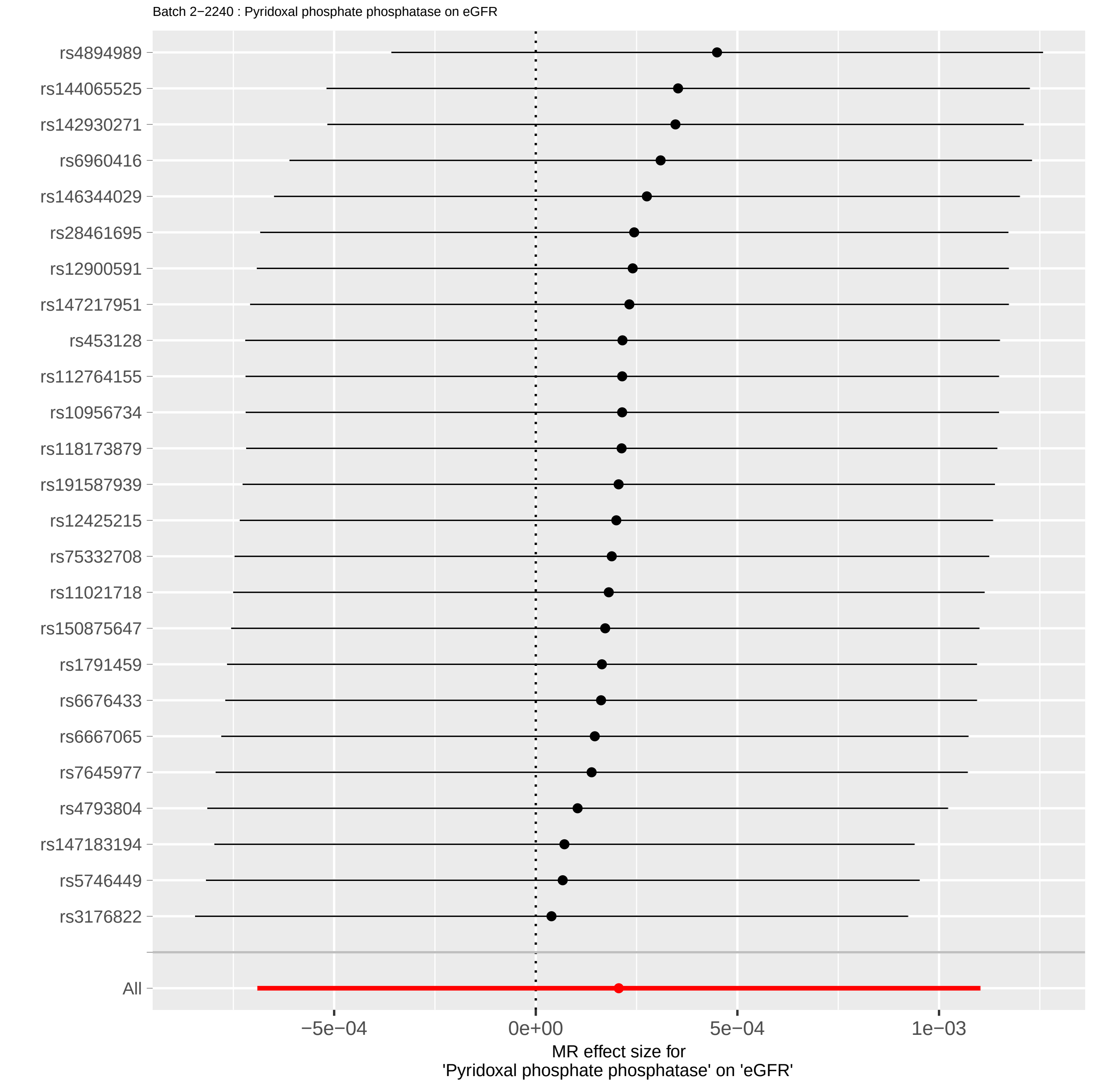

Batch 2−2240 : Pyridoxal phosphate phosphatase on eGFR
rs4894989
rs144065525
rs142930271
rs6960416
rs146344029
rs28461695
rs12900591
rs147217951
rs453128
rs112764155
rs10956734
rs118173879
rs191587939
rs12425215
rs75332708
rs11021718
rs150875647
rs1791459
rs6676433
rs6667065
rs7645977
rs4793804
rs147183194
rs5746449
rs3176822
All
−5e−04
0e+00
5e−04
1e−03
MR effect size for
'Pyridoxal phosphate phosphatase' on 'eGFR'

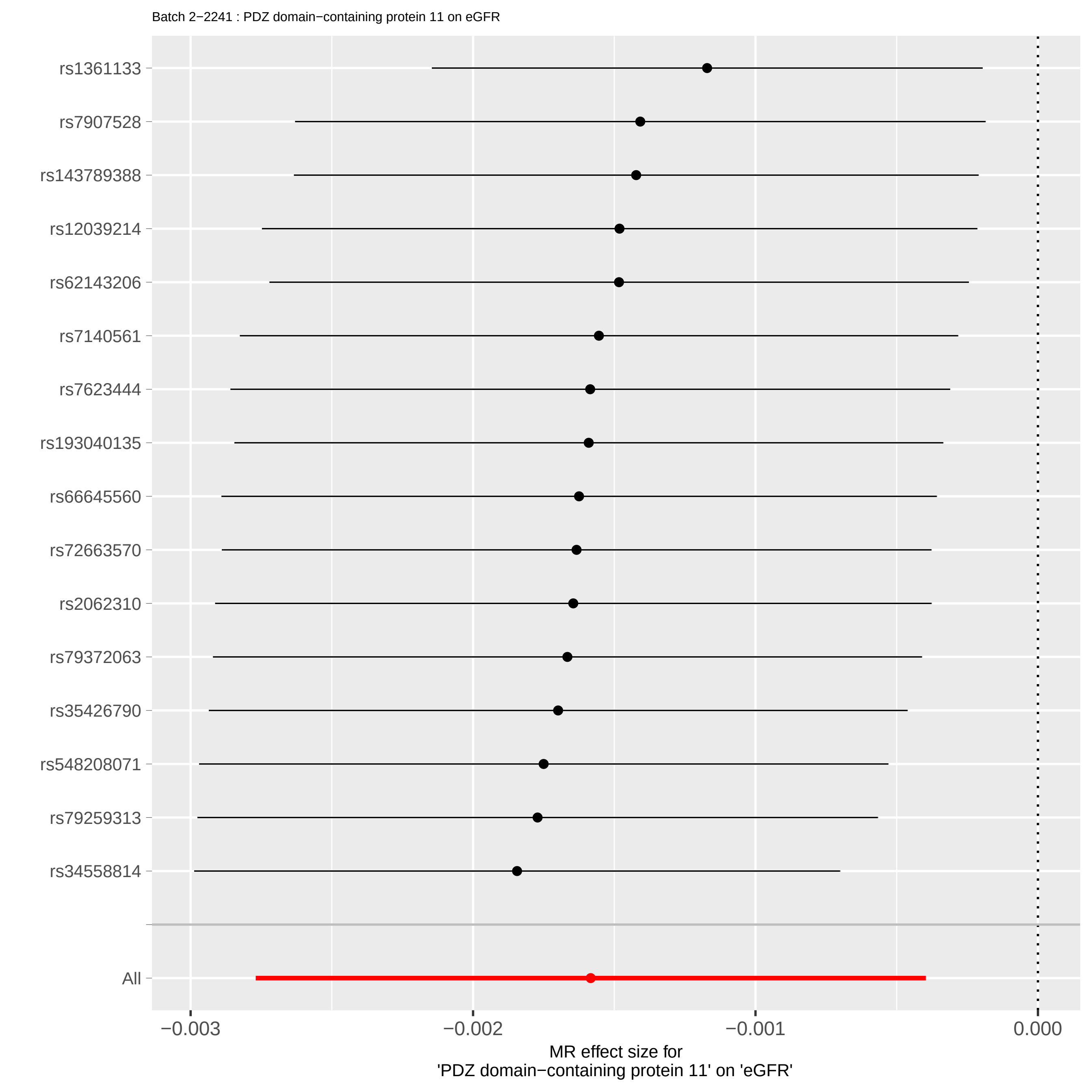

Batch 2−2241 : PDZ domain−containing protein 11 on eGFR
rs1361133
rs7907528
rs143789388
rs12039214
rs62143206
rs7140561
rs7623444
rs193040135
rs66645560
rs72663570
rs2062310
rs79372063
rs35426790
rs548208071
rs79259313
rs34558814
All
−0.003
−0.002
−0.001
0.000
MR effect size for
'PDZ domain−containing protein 11' on 'eGFR'

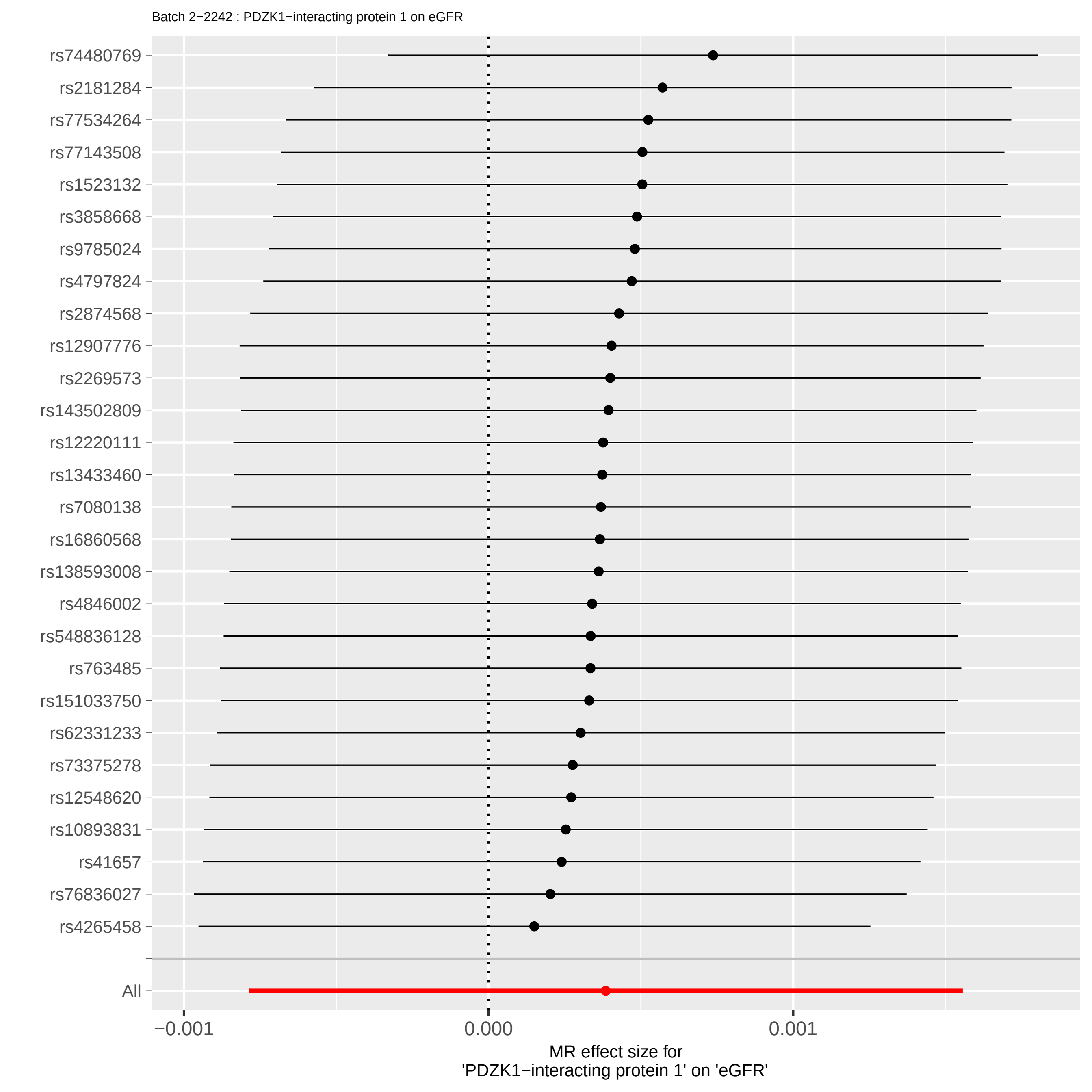

Batch 2−2242 : PDZK1−interacting protein 1 on eGFR
rs74480769
rs2181284
rs77534264
rs77143508
rs1523132
rs3858668
rs9785024
rs4797824
rs2874568
rs12907776
rs2269573
rs143502809
rs12220111
rs13433460
rs7080138
rs16860568
rs138593008
rs4846002
rs548836128
rs763485
rs151033750
rs62331233
rs73375278
rs12548620
rs10893831
rs41657
rs76836027
rs4265458
All
−0.001
0.000
0.001
MR effect size for
'PDZK1−interacting protein 1' on 'eGFR'

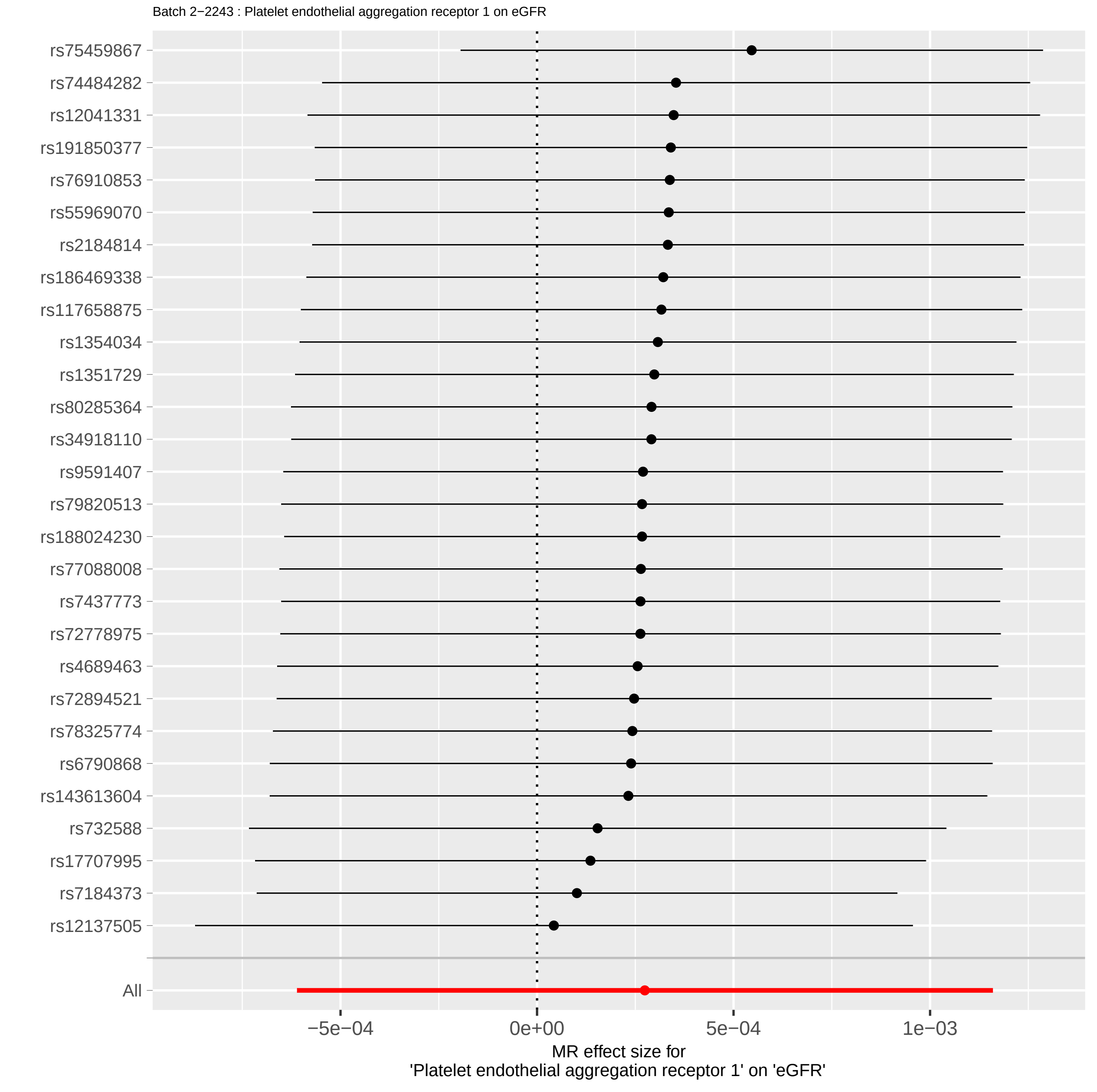
Batch 2−2243 : Platelet endothelial aggregation receptor 1 on eGFR
rs75459867
rs74484282
rs12041331
rs191850377
rs76910853
rs55969070
rs2184814
rs186469338
rs117658875
rs1354034
rs1351729
rs80285364
rs34918110
rs9591407
rs79820513
rs188024230
rs77088008
rs7437773
rs72778975
rs4689463
rs72894521
rs78325774
rs6790868
rs143613604
rs732588
rs17707995
rs7184373
rs12137505
All
−5e−04
0e+00
5e−04
1e−03
MR effect size for
'Platelet endothelial aggregation receptor 1' on 'eGFR'

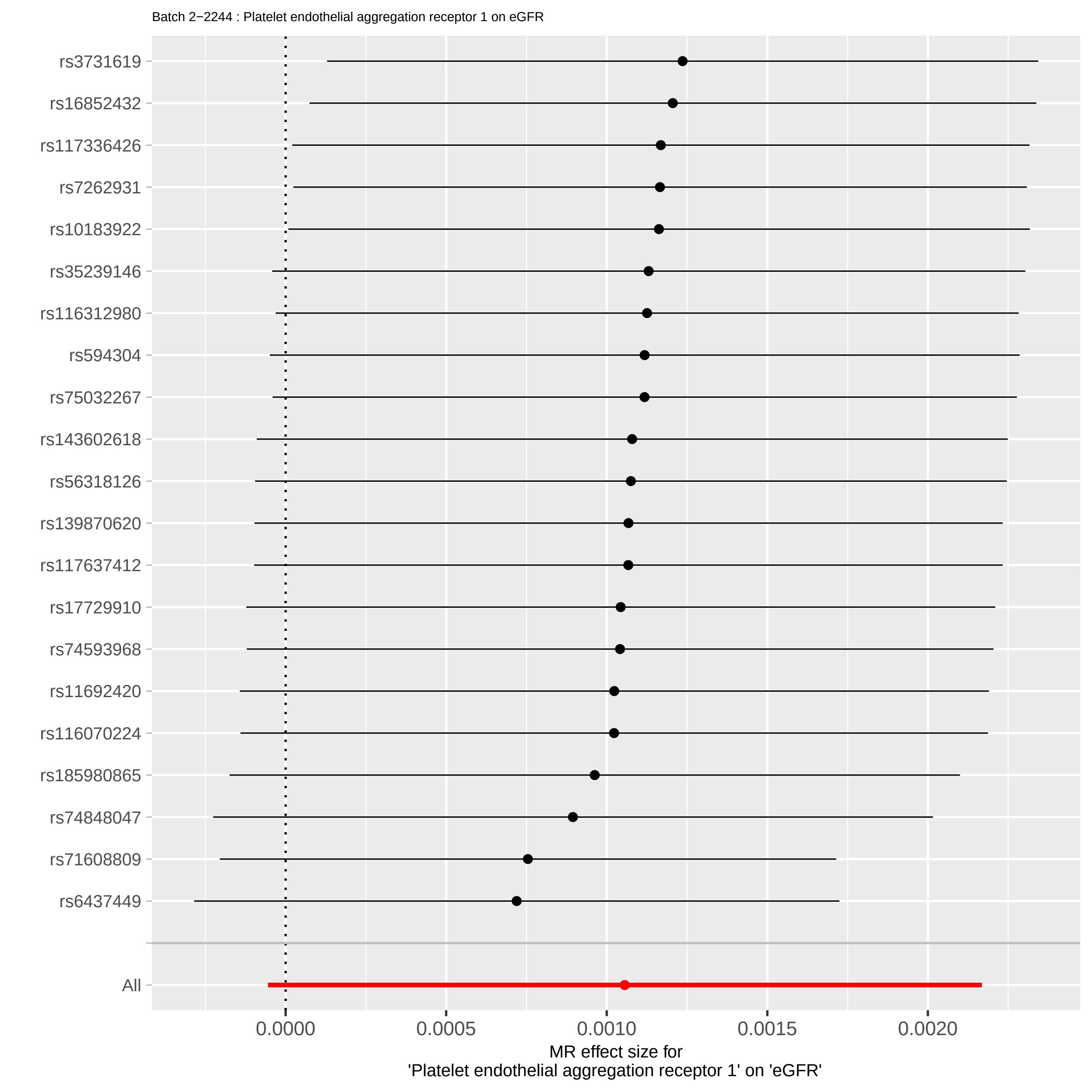
Batch 2−2244 : Platelet endothelial aggregation receptor 1 on eGFR
rs3731619
rs16852432
rs117336426
rs7262931
rs10183922
rs35239146
rs116312980
rs594304
rs75032267
rs143602618
rs56318126
rs139870620
rs117637412
rs17729910
rs74593968
rs11692420
rs116070224
rs185980865
rs74848047
rs71608809
rs6437449
All
0.0000
0.0005
0.0010
0.0015
0.0020
MR effect size for
'Platelet endothelial aggregation receptor 1' on 'eGFR'

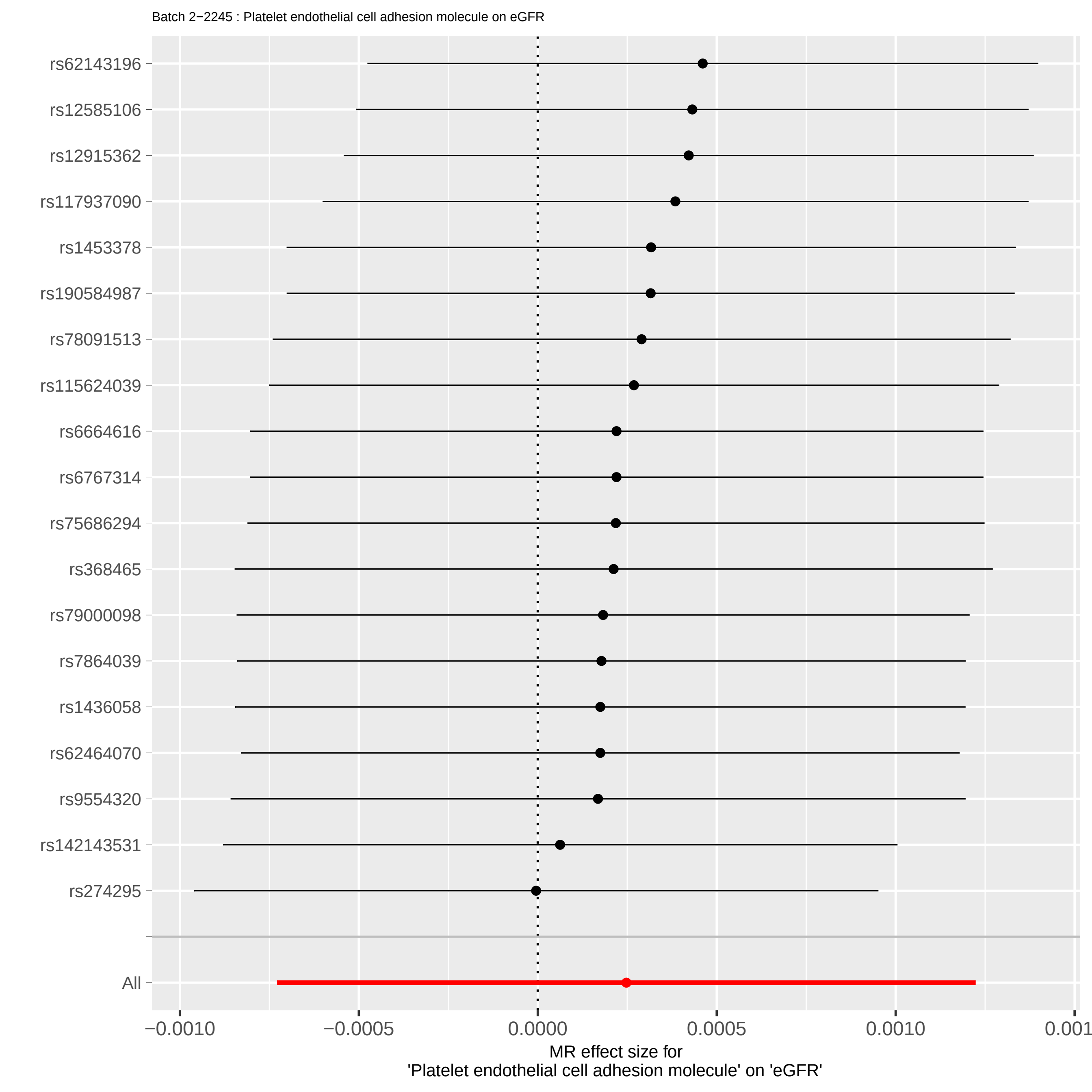

Batch 2−2245 : Platelet endothelial cell adhesion molecule on eGFR
rs62143196
rs12585106
rs12915362
rs117937090
rs1453378
rs190584987
rs78091513
rs115624039
rs6664616
rs6767314
rs75686294
rs368465
rs79000098
rs7864039
rs1436058
rs62464070
rs9554320
rs142143531
rs274295
All
−0.0010
−0.0005
0.0000
0.0005
0.0010
MR effect size for
'Platelet endothelial cell adhesion molecule' on 'eGFR'

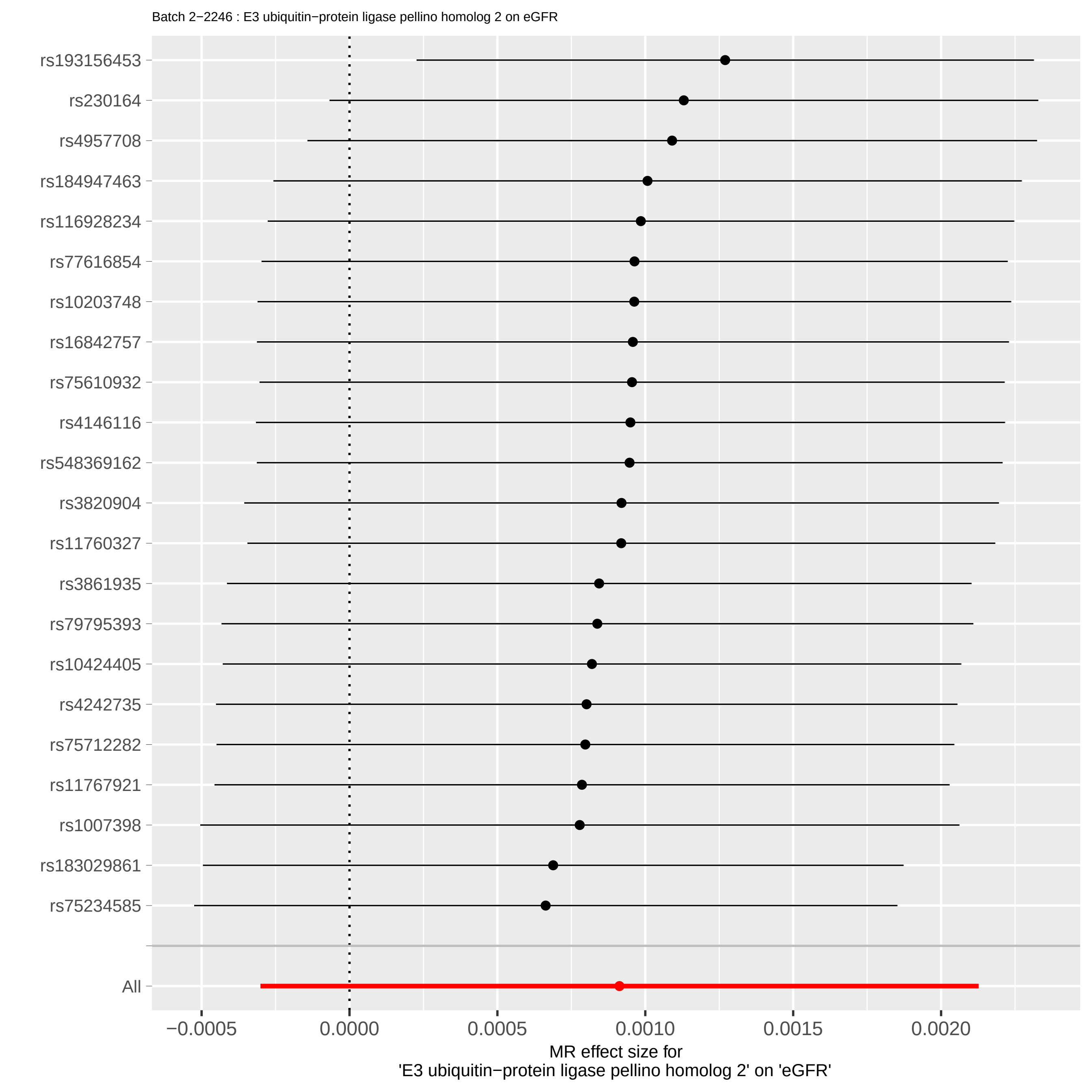

Batch 2−2246 : E3 ubiquitin−protein ligase pellino homolog 2 on eGFR
rs193156453
rs230164
rs4957708
rs184947463
rs116928234
rs77616854
rs10203748
rs16842757
rs75610932
rs4146116
rs548369162
rs3820904
rs11760327
rs3861935
rs79795393
rs10424405
rs4242735
rs75712282
rs11767921
rs1007398
rs183029861
rs75234585
All
−0.0005
0.0000
0.0005
0.0010
0.0015
0.0020
MR effect size for
'E3 ubiquitin−protein ligase pellino homolog 2' on 'eGFR'

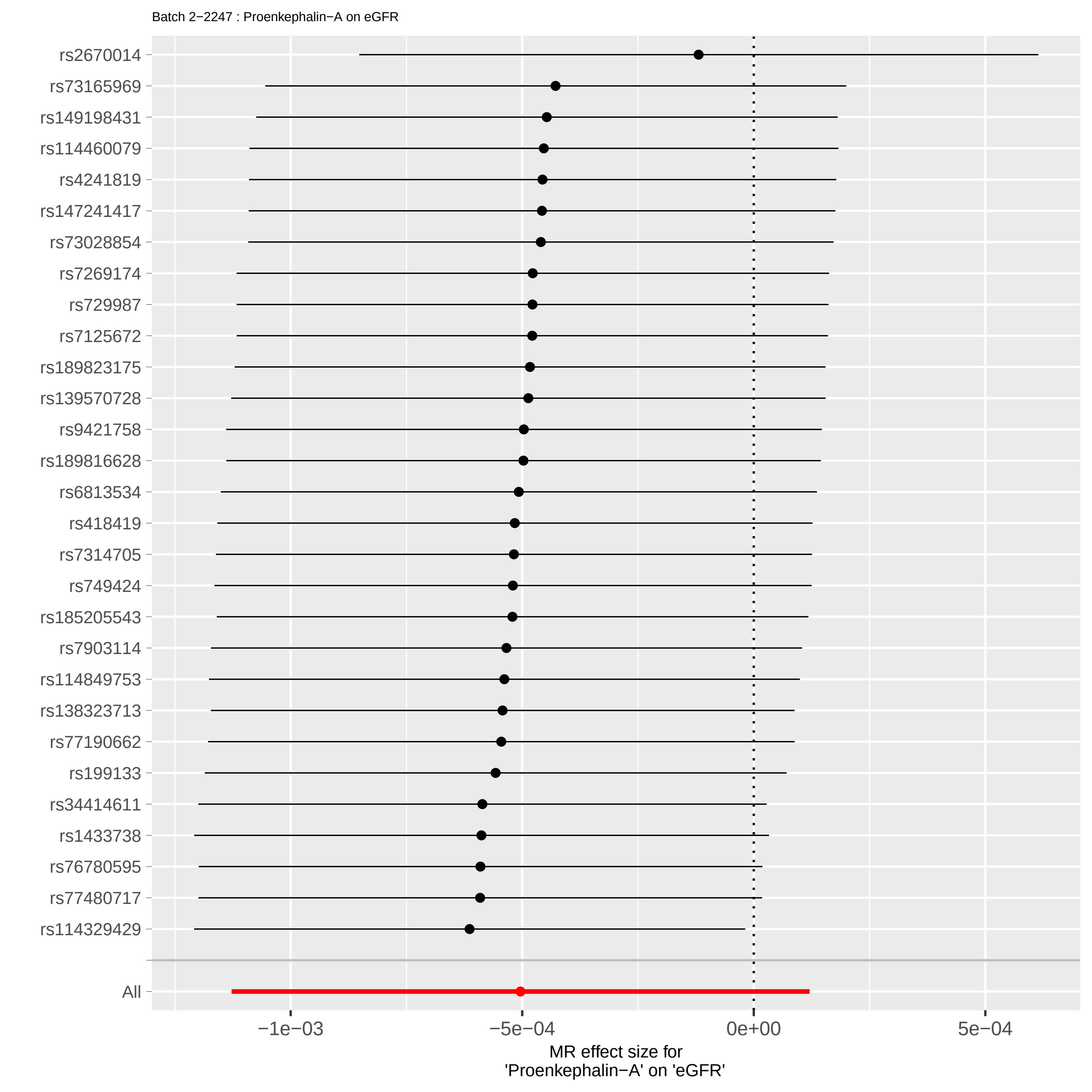

Batch 2−2247 : Proenkephalin−A on eGFR
rs2670014
rs73165969
rs149198431
rs114460079
rs4241819
rs147241417
rs73028854
rs7269174
rs729987
rs7125672
rs189823175
rs139570728
rs9421758
rs189816628
rs6813534
rs418419
rs7314705
rs749424
rs185205543
rs7903114
rs114849753
rs138323713
rs77190662
rs199133
rs34414611
rs1433738
rs76780595
rs77480717
rs114329429
All
−1e−03
−5e−04
0e+00
5e−04
MR effect size for
'Proenkephalin−A' on 'eGFR'

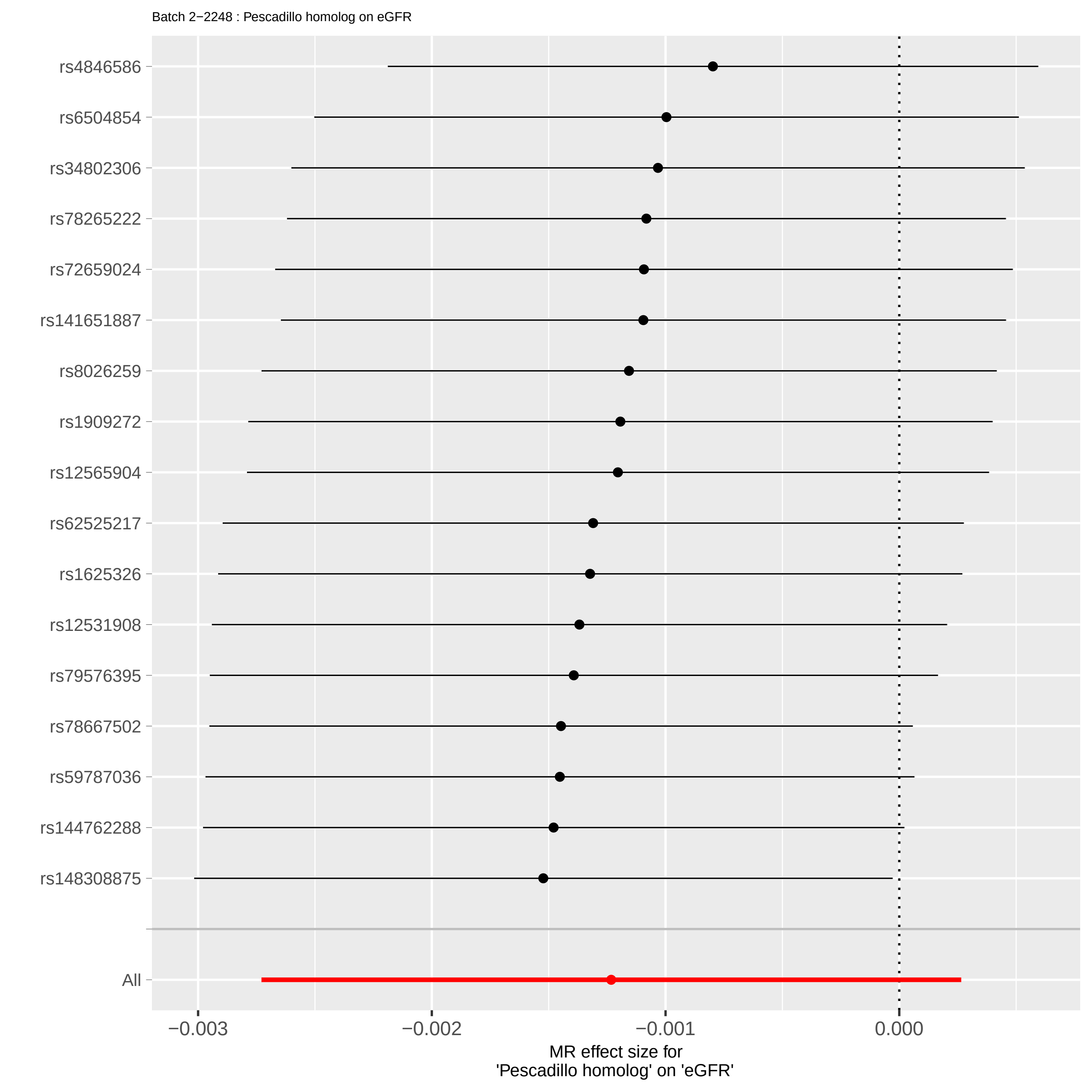

Batch 2−2248 : Pescadillo homolog on eGFR
rs4846586
rs6504854
rs34802306
rs78265222
rs72659024
rs141651887
rs8026259
rs1909272
rs12565904
rs62525217
rs1625326
rs12531908
rs79576395
rs78667502
rs59787036
rs144762288
rs148308875
All
−0.003
−0.002
−0.001
0.000
MR effect size for
'Pescadillo homolog' on 'eGFR'

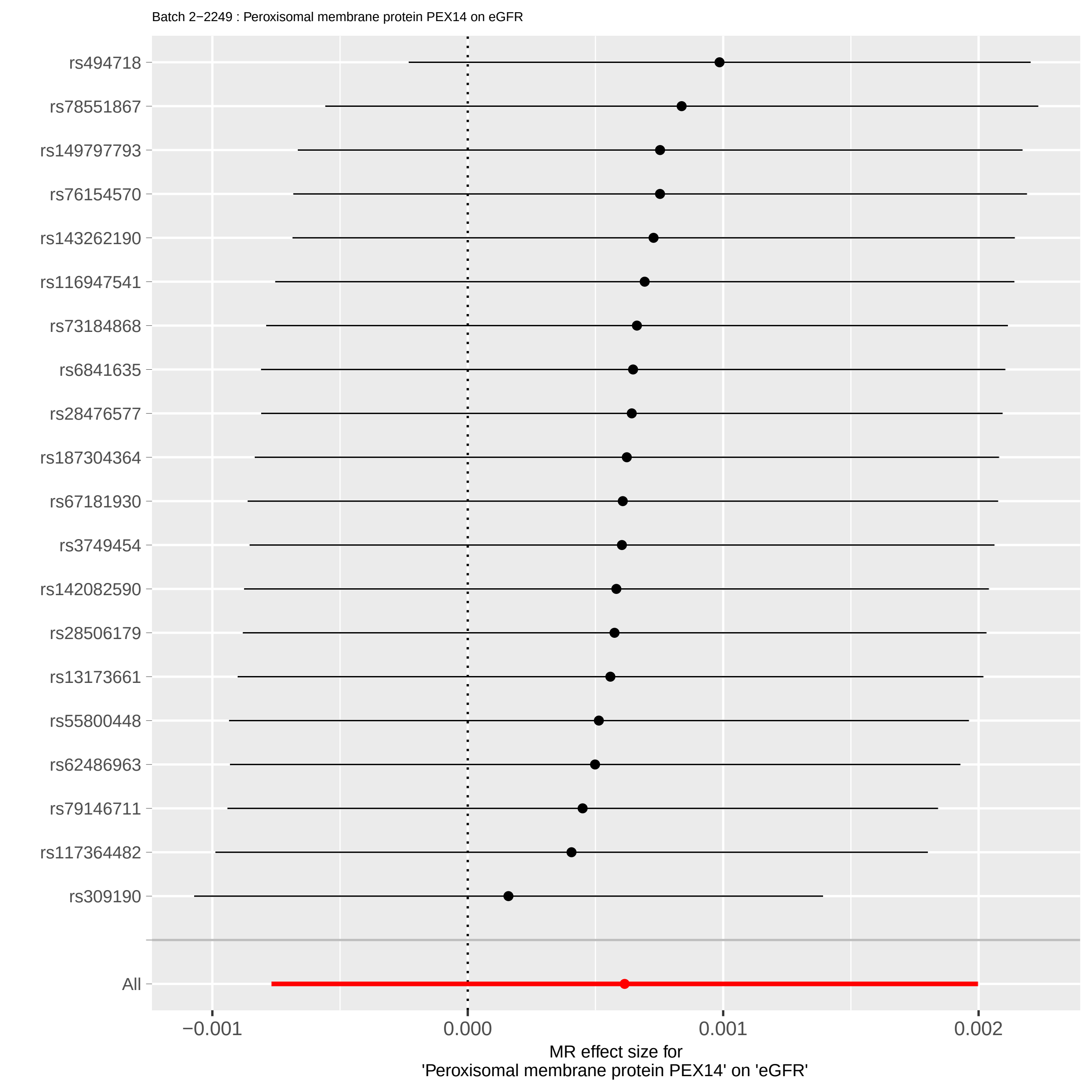
Batch 2−2249 : Peroxisomal membrane protein PEX14 on eGFR
rs494718
rs78551867
rs149797793
rs76154570
rs143262190
rs116947541
rs73184868
rs6841635
rs28476577
rs187304364
rs67181930
rs3749454
rs142082590
rs28506179
rs13173661
rs55800448
rs62486963
rs79146711
rs117364482
rs309190
All
−0.001
0.000
0.001
0.002
MR effect size for
'Peroxisomal membrane protein PEX14' on 'eGFR'

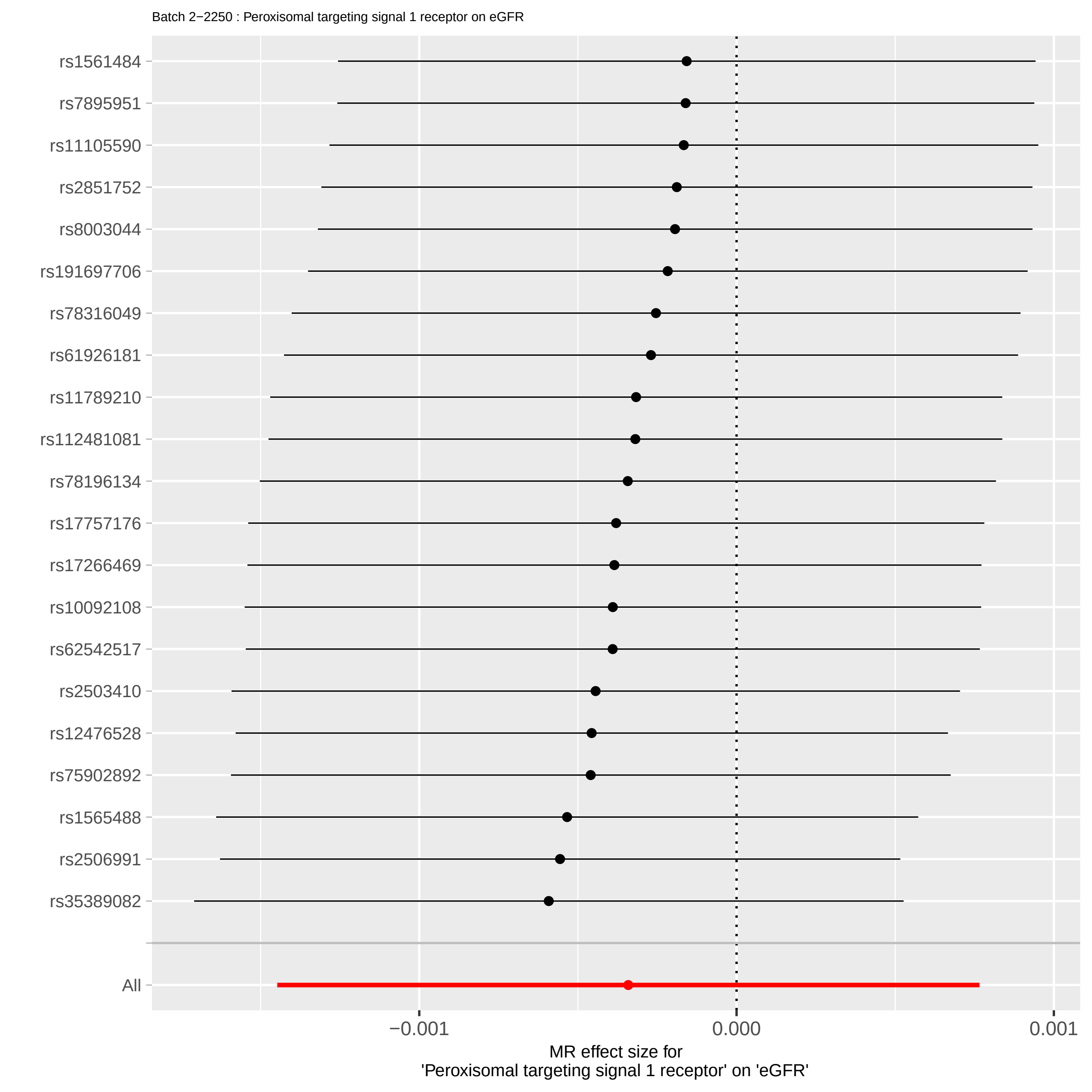

Batch 2−2250 : Peroxisomal targeting signal 1 receptor on eGFR
rs1561484
rs7895951
rs11105590
rs2851752
rs8003044
rs191697706
rs78316049
rs61926181
rs11789210
rs112481081
rs78196134
rs17757176
rs17266469
rs10092108
rs62542517
rs2503410
rs12476528
rs75902892
rs1565488
rs2506991
rs35389082
All
−0.001
0.000
0.001
MR effect size for
'Peroxisomal targeting signal 1 receptor' on 'eGFR'

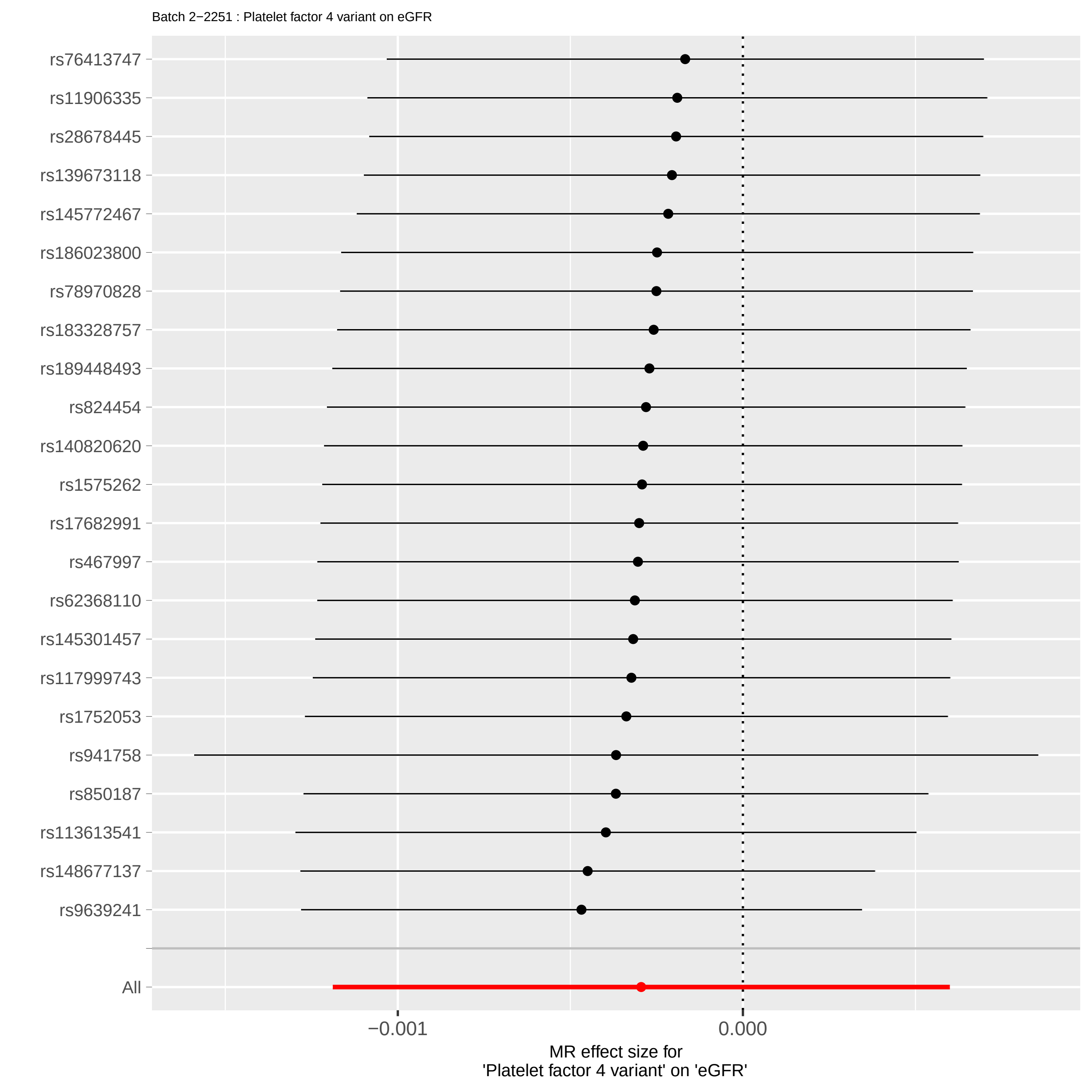

Batch 2−2251 : Platelet factor 4 variant on eGFR
rs76413747
rs11906335
rs28678445
rs139673118
rs145772467
rs186023800
rs78970828
rs183328757
rs189448493
rs824454
rs140820620
rs1575262
rs17682991
rs467997
rs62368110
rs145301457
rs117999743
rs1752053
rs941758
rs850187
rs113613541
rs148677137
rs9639241
All
−0.001
0.000
MR effect size for
'Platelet factor 4 variant' on 'eGFR'

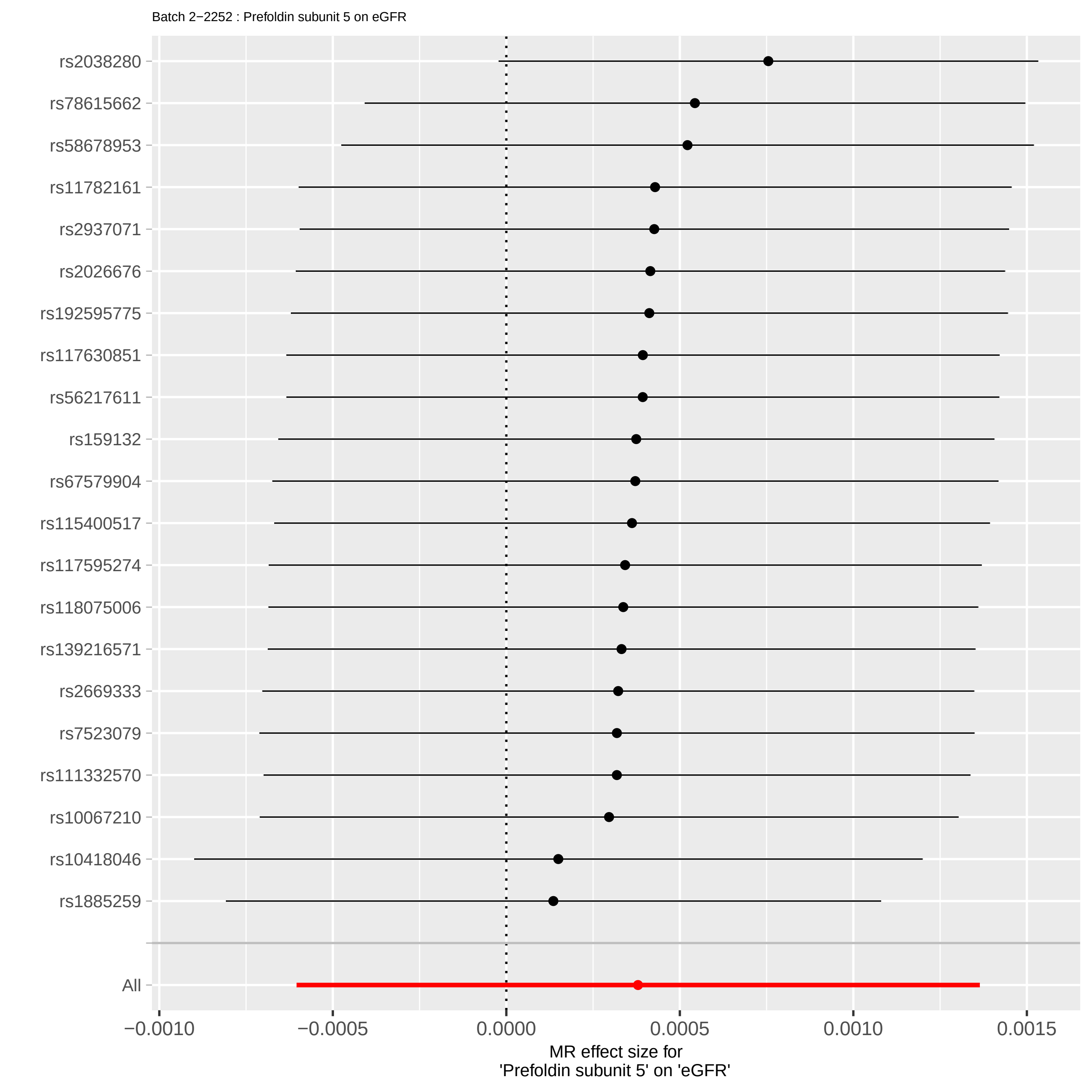

Batch 2−2252 : Prefoldin subunit 5 on eGFR
rs2038280
rs78615662
rs58678953
rs11782161
rs2937071
rs2026676
rs192595775
rs117630851
rs56217611
rs159132
rs67579904
rs115400517
rs117595274
rs118075006
rs139216571
rs2669333
rs7523079
rs111332570
rs10067210
rs10418046
rs1885259
All
−0.0010
−0.0005
0.0000
0.0005
0.0010
0.0015
MR effect size for
'Prefoldin subunit 5' on 'eGFR'

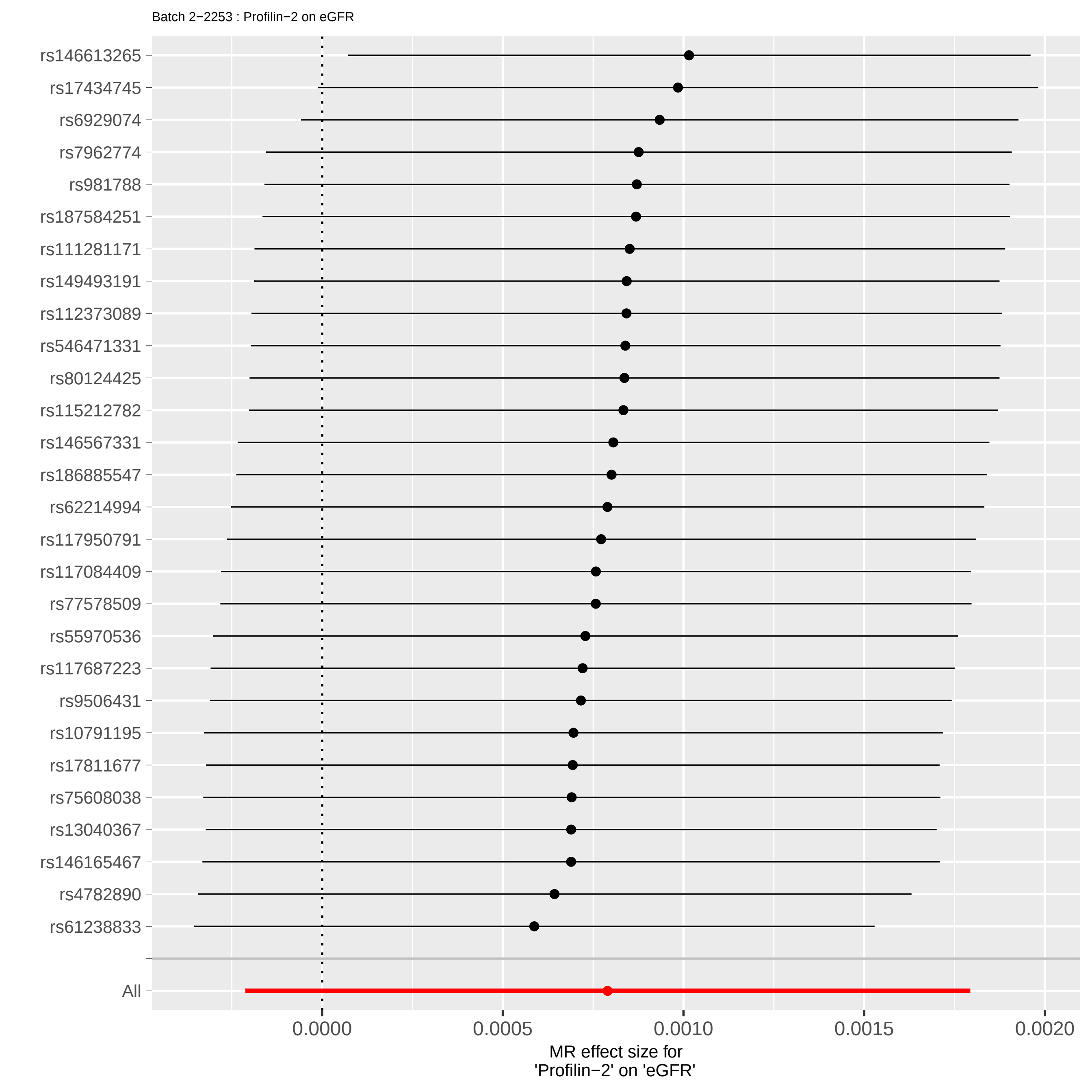

Batch 2−2253 : Profilin−2 on eGFR
rs146613265
rs17434745
rs6929074
rs7962774
rs981788
rs187584251
rs111281171
rs149493191
rs112373089
rs546471331
rs80124425
rs115212782
rs146567331
rs186885547
rs62214994
rs117950791
rs117084409
rs77578509
rs55970536
rs117687223
rs9506431
rs10791195
rs17811677
rs75608038
rs13040367
rs146165467
rs4782890
rs61238833
All
0.0000
0.0005
0.0010
0.0015
0.0020
MR effect size for
'Profilin−2' on 'eGFR'

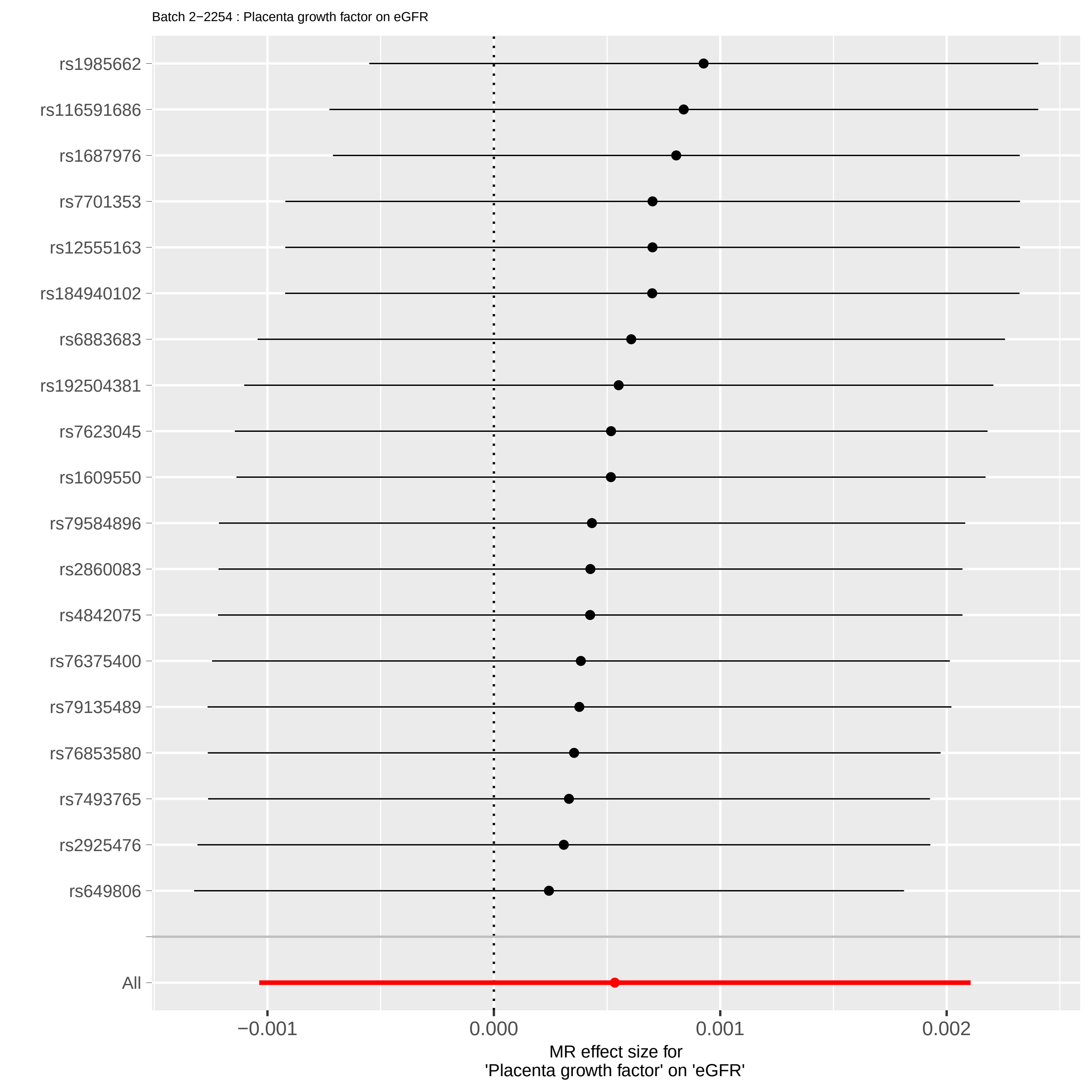
Batch 2−2254 : Placenta growth factor on eGFR
rs1985662
rs116591686
rs1687976
rs7701353
rs12555163
rs184940102
rs6883683
rs192504381
rs7623045
rs1609550
rs79584896
rs2860083
rs4842075
rs76375400
rs79135489
rs76853580
rs7493765
rs2925476
rs649806
All
−0.001
0.000
0.001
0.002
MR effect size for
'Placenta growth factor' on 'eGFR'

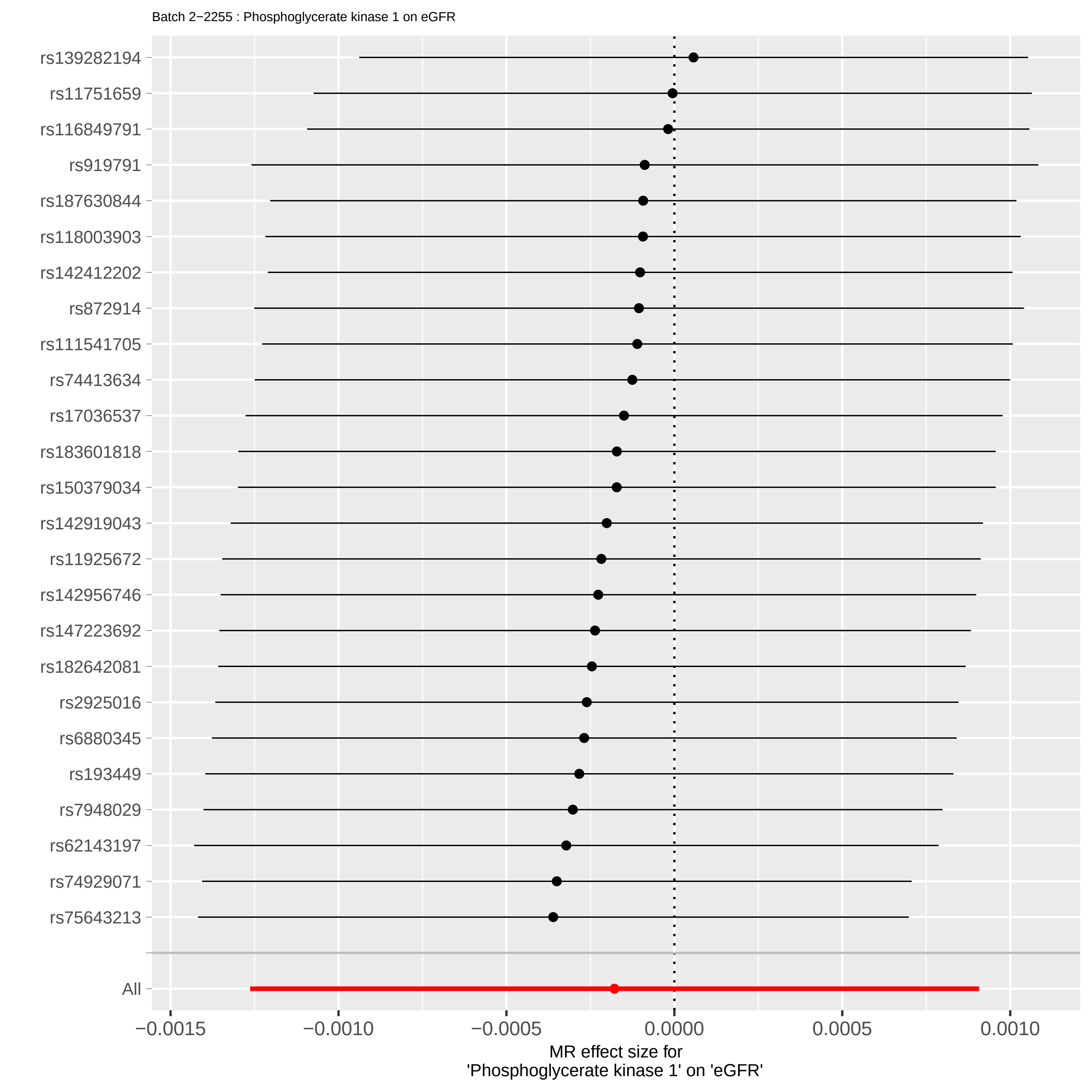
Batch 2−2255 : Phosphoglycerate kinase 1 on eGFR
rs139282194
rs11751659
rs116849791
rs919791
rs187630844
rs118003903
rs142412202
rs872914
rs111541705
rs74413634
rs17036537
rs183601818
rs150379034
rs142919043
rs11925672
rs142956746
rs147223692
rs182642081
rs2925016
rs6880345
rs193449
rs7948029
rs62143197
rs74929071
rs75643213
All
−0.0015
−0.0010
−0.0005
0.0000
0.0005
0.0010
MR effect size for
'Phosphoglycerate kinase 1' on 'eGFR'

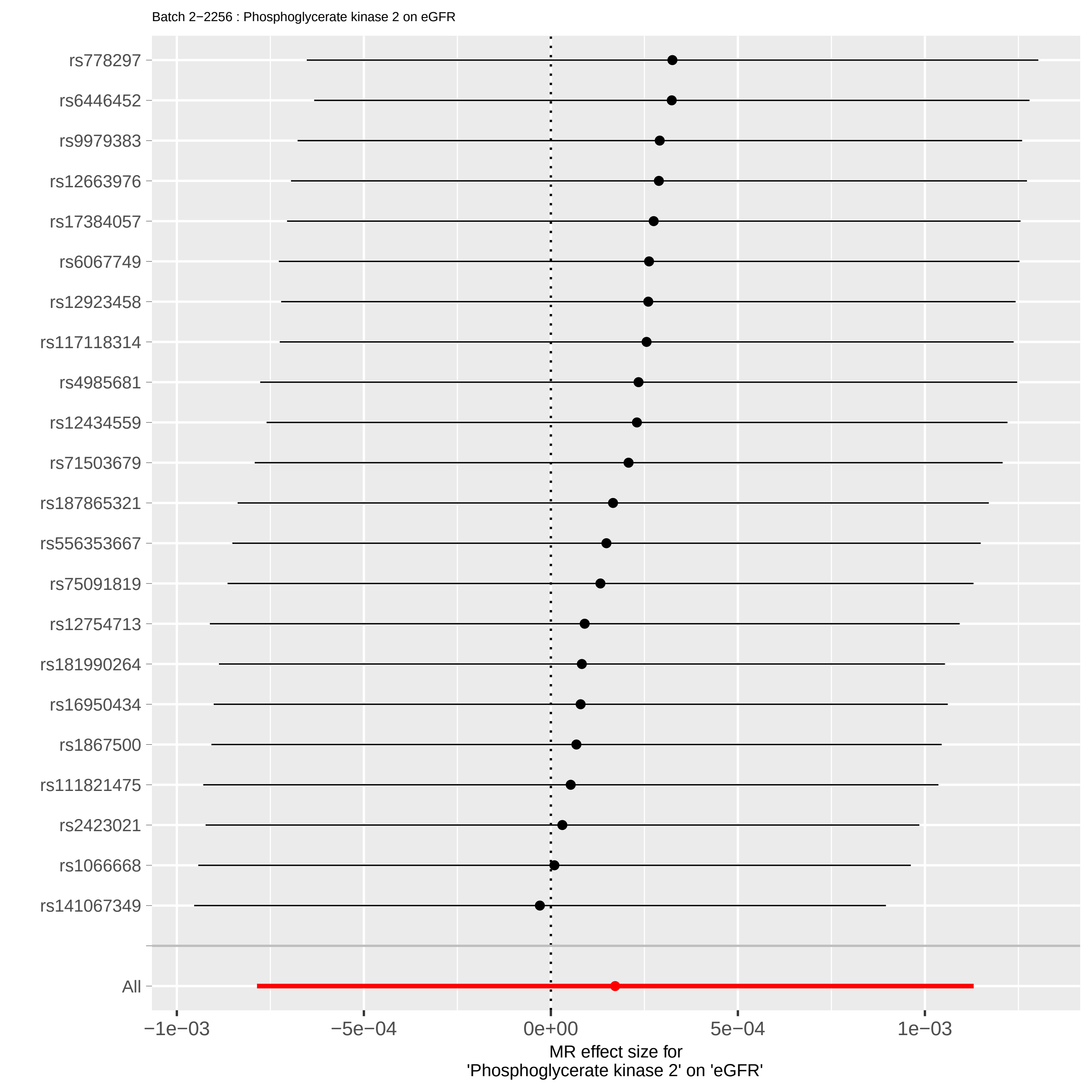

Batch 2−2256 : Phosphoglycerate kinase 2 on eGFR
rs778297
rs6446452
rs9979383
rs12663976
rs17384057
rs6067749
rs12923458
rs117118314
rs4985681
rs12434559
rs71503679
rs187865321
rs556353667
rs75091819
rs12754713
rs181990264
rs16950434
rs1867500
rs111821475
rs2423021
rs1066668
rs141067349
All
−1e−03
−5e−04
0e+00
5e−04
1e−03
MR effect size for
'Phosphoglycerate kinase 2' on 'eGFR'

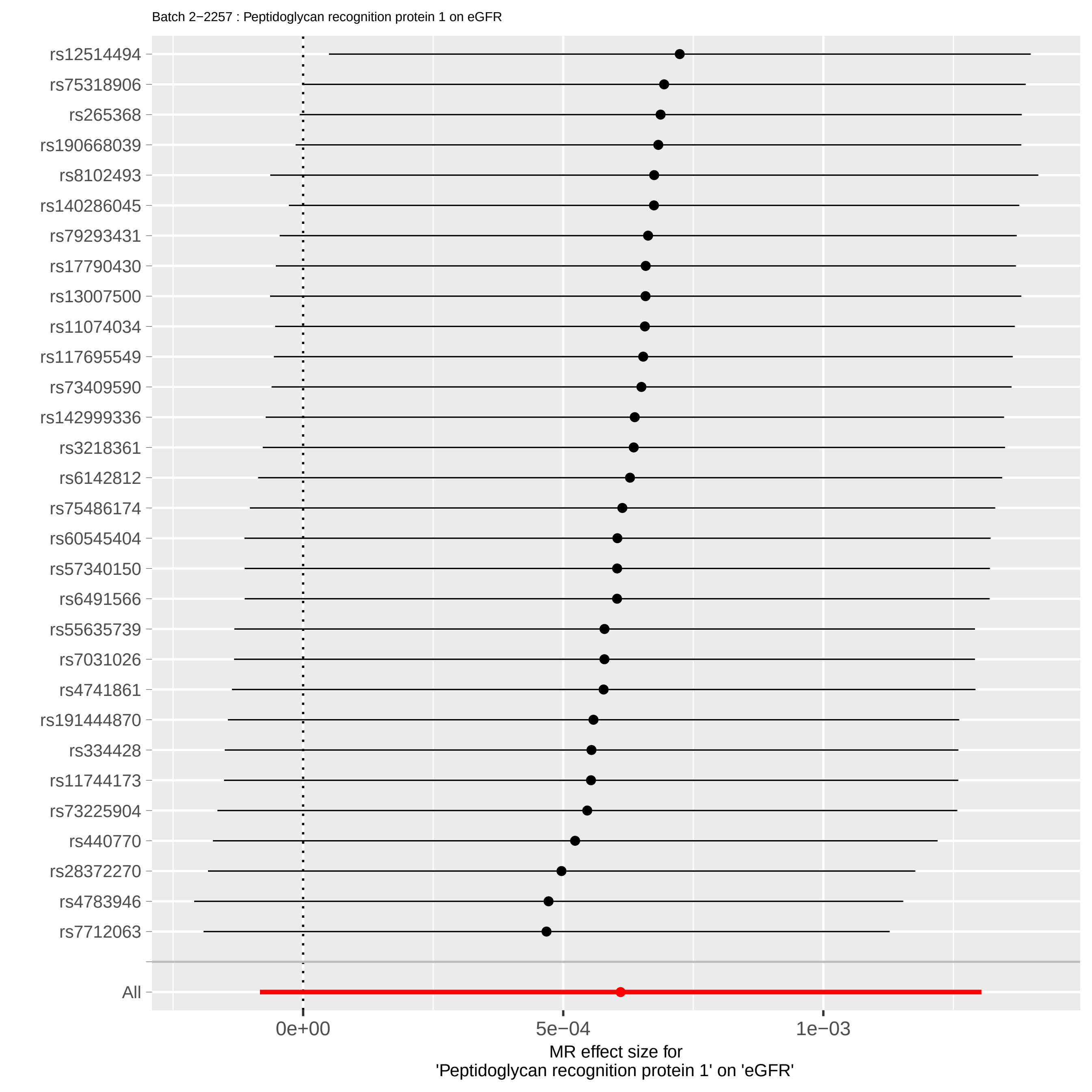

Batch 2−2257 : Peptidoglycan recognition protein 1 on eGFR
rs12514494
rs75318906
rs265368
rs190668039
rs8102493
rs140286045
rs79293431
rs17790430
rs13007500
rs11074034
rs117695549
rs73409590
rs142999336
rs3218361
rs6142812
rs75486174
rs60545404
rs57340150
rs6491566
rs55635739
rs7031026
rs4741861
rs191444870
rs334428
rs11744173
rs73225904
rs440770
rs28372270
rs4783946
rs7712063
All
0e+00
5e−04
1e−03
MR effect size for
'Peptidoglycan recognition protein 1' on 'eGFR'

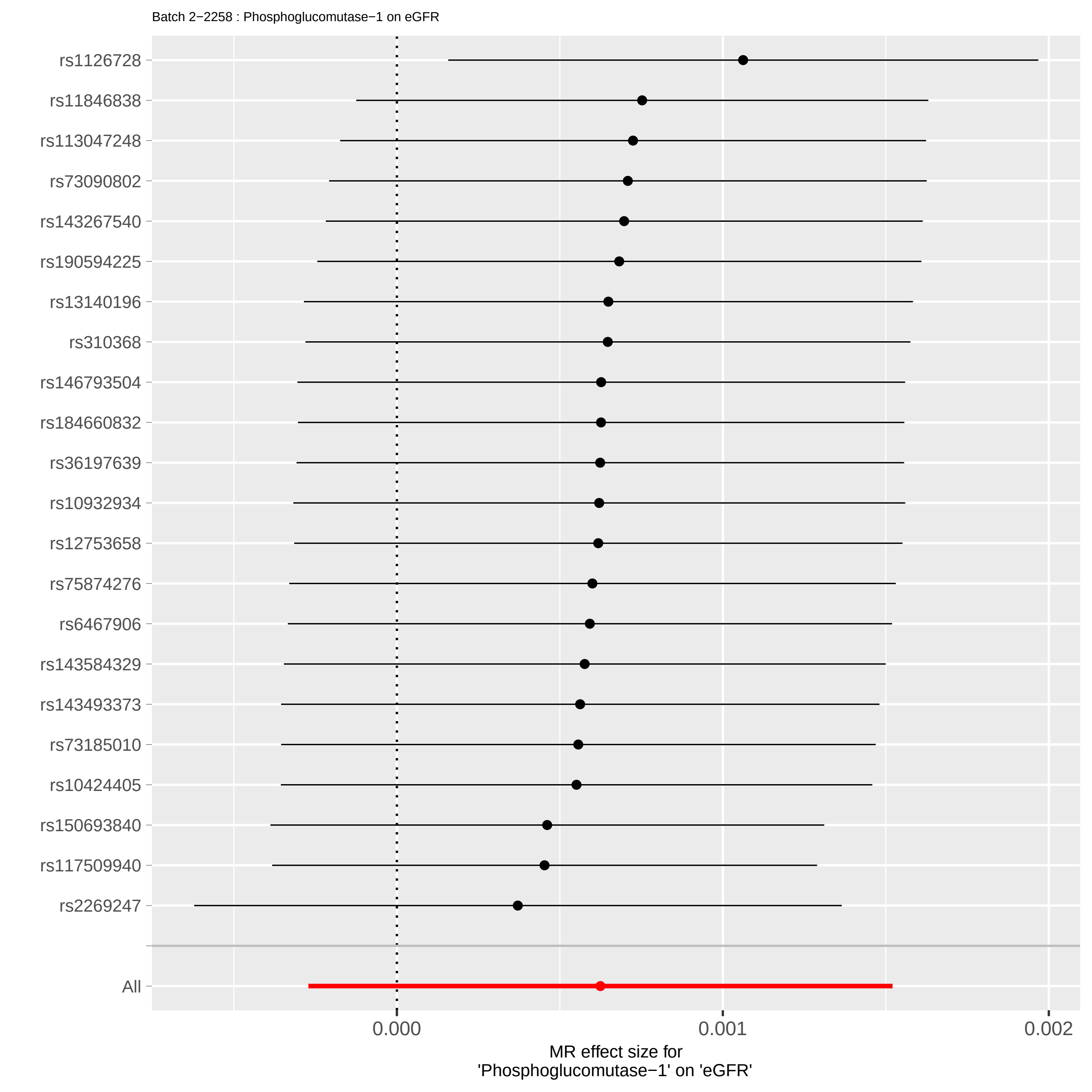

Batch 2−2258 : Phosphoglucomutase−1 on eGFR
rs1126728
rs11846838
rs113047248
rs73090802
rs143267540
rs190594225
rs13140196
rs310368
rs146793504
rs184660832
rs36197639
rs10932934
rs12753658
rs75874276
rs6467906
rs143584329
rs143493373
rs73185010
rs10424405
rs150693840
rs117509940
rs2269247
All
0.000
0.001
0.002
MR effect size for
'Phosphoglucomutase−1' on 'eGFR'

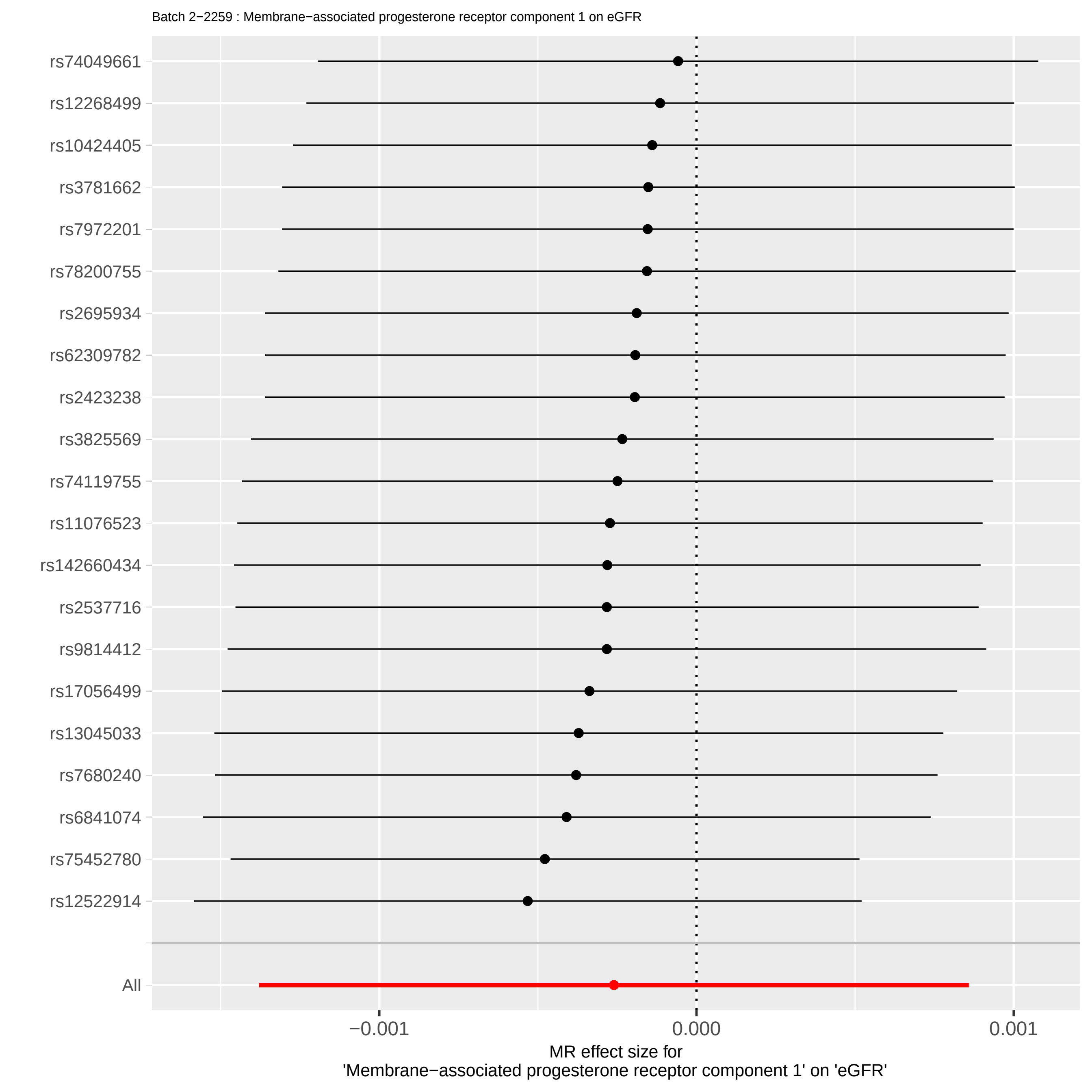

Batch 2−2259 : Membrane−associated progesterone receptor component 1 on eGFR
rs74049661
rs12268499
rs10424405
rs3781662
rs7972201
rs78200755
rs2695934
rs62309782
rs2423238
rs3825569
rs74119755
rs11076523
rs142660434
rs2537716
rs9814412
rs17056499
rs13045033
rs7680240
rs6841074
rs75452780
rs12522914
All
−0.001
0.000
0.001
MR effect size for
'Membrane−associated progesterone receptor component 1' on 'eGFR'

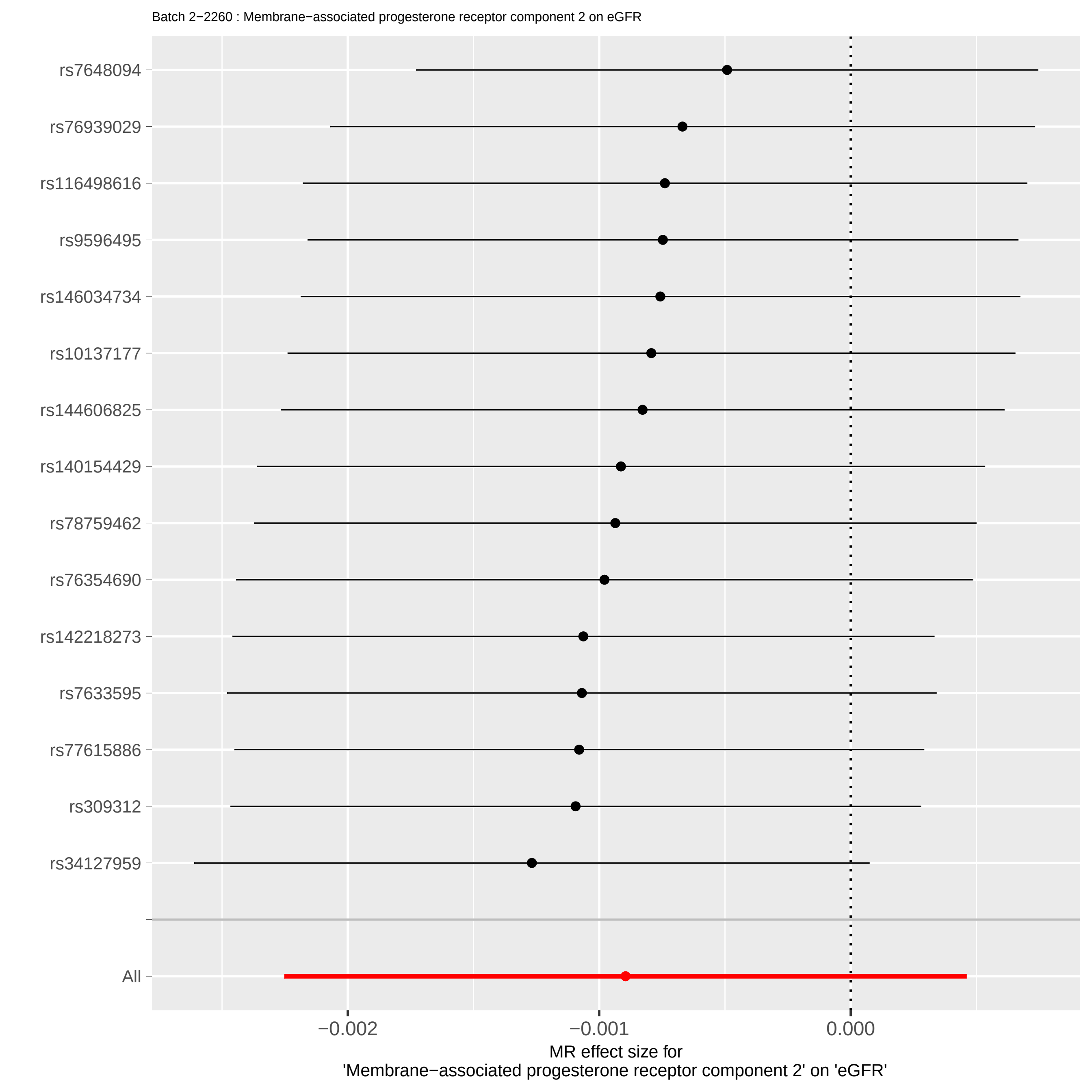

Batch 2−2260 : Membrane−associated progesterone receptor component 2 on eGFR
rs7648094
rs76939029
rs116498616
rs9596495
rs146034734
rs10137177
rs144606825
rs140154429
rs78759462
rs76354690
rs142218273
rs7633595
rs77615886
rs309312
rs34127959
All
−0.002
−0.001
0.000
MR effect size for
'Membrane−associated progesterone receptor component 2' on 'eGFR'

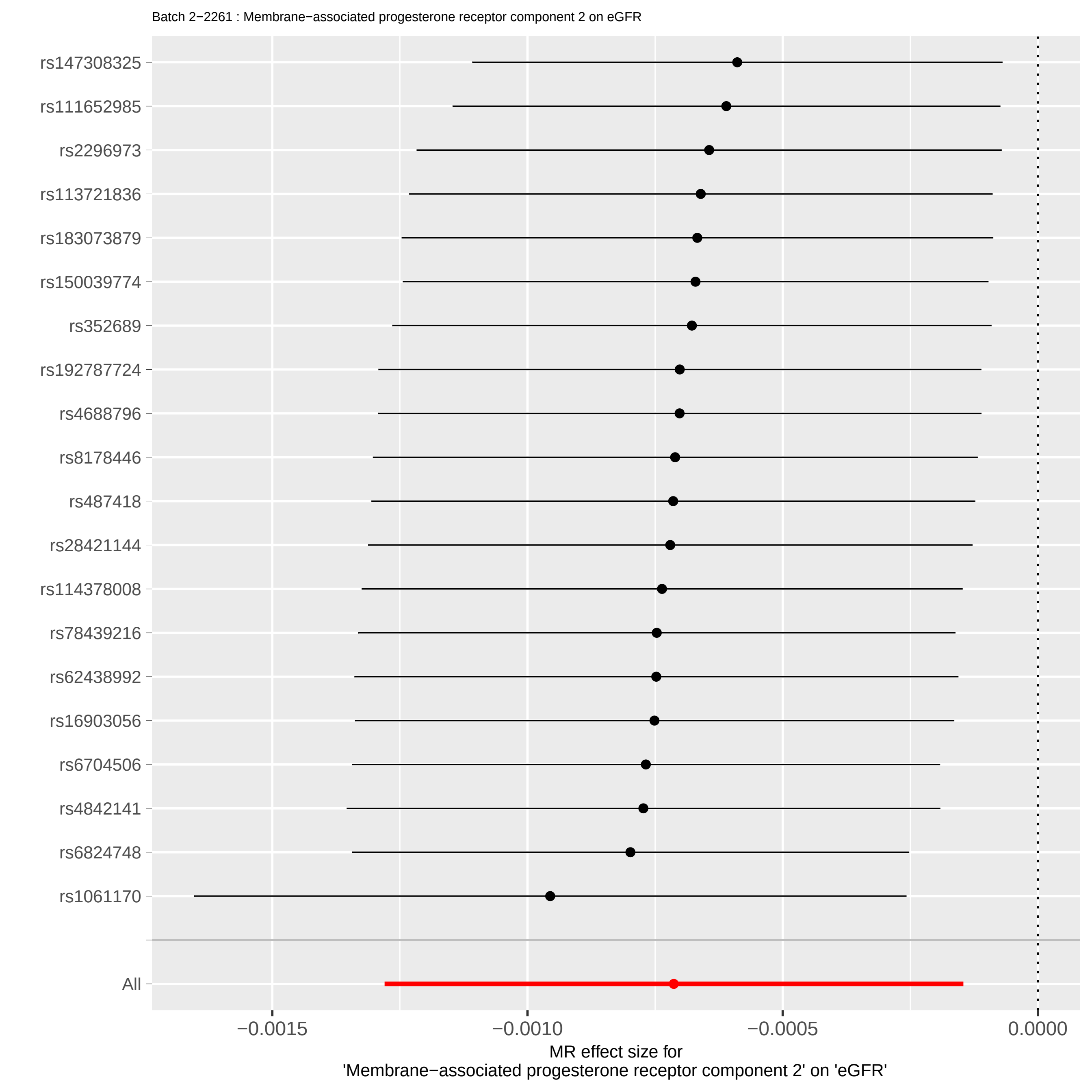

Batch 2−2261 : Membrane−associated progesterone receptor component 2 on eGFR
rs147308325
rs111652985
rs2296973
rs113721836
rs183073879
rs150039774
rs352689
rs192787724
rs4688796
rs8178446
rs487418
rs28421144
rs114378008
rs78439216
rs62438992
rs16903056
rs6704506
rs4842141
rs6824748
rs1061170
All
−0.0015
−0.0010
−0.0005
0.0000
MR effect size for
'Membrane−associated progesterone receptor component 2' on 'eGFR'

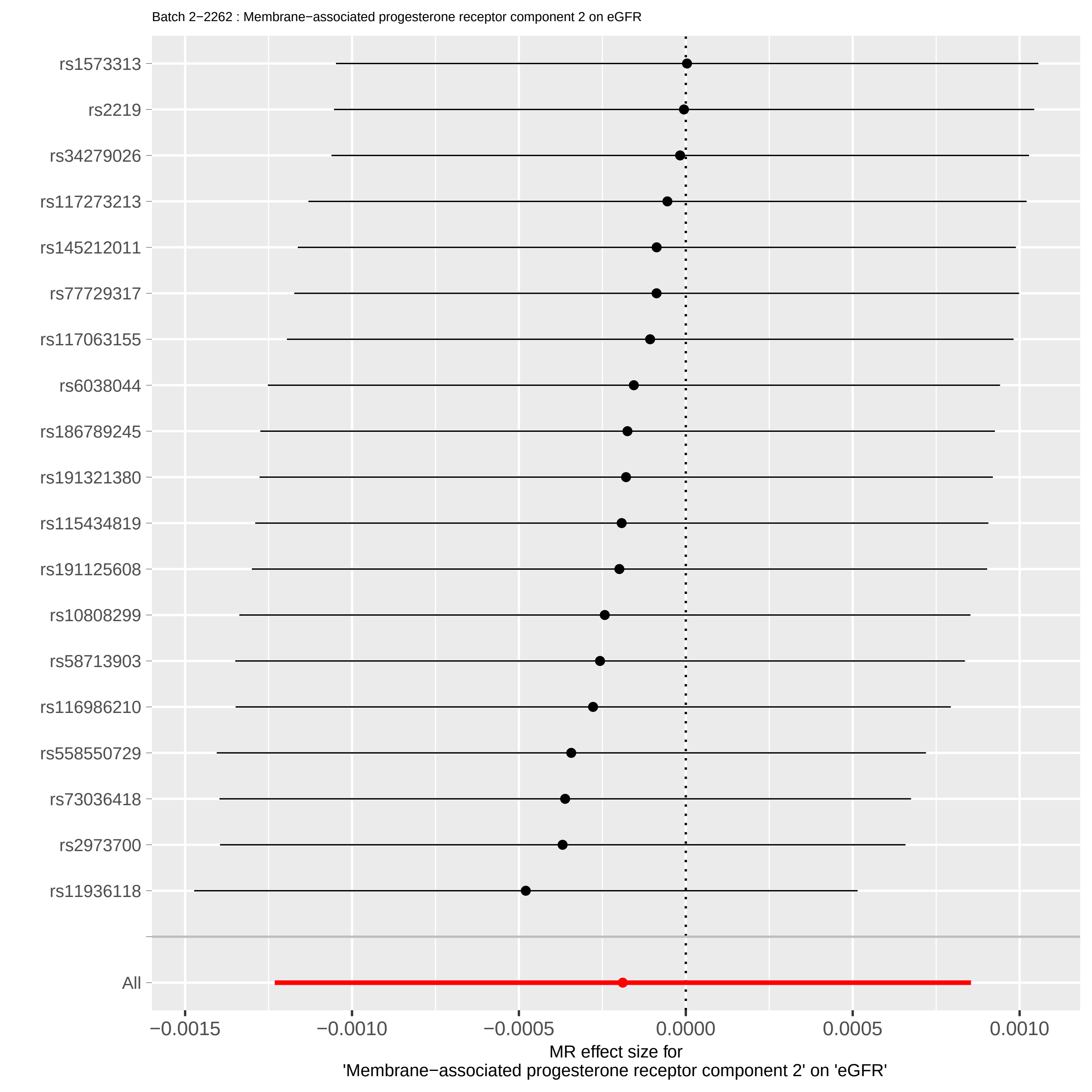

Batch 2−2262 : Membrane−associated progesterone receptor component 2 on eGFR
rs1573313
rs2219
rs34279026
rs117273213
rs145212011
rs77729317
rs117063155
rs6038044
rs186789245
rs191321380
rs115434819
rs191125608
rs10808299
rs58713903
rs116986210
rs558550729
rs73036418
rs2973700
rs11936118
All
−0.0015
−0.0010
−0.0005
0.0000
0.0005
0.0010
MR effect size for
'Membrane−associated progesterone receptor component 2' on 'eGFR'

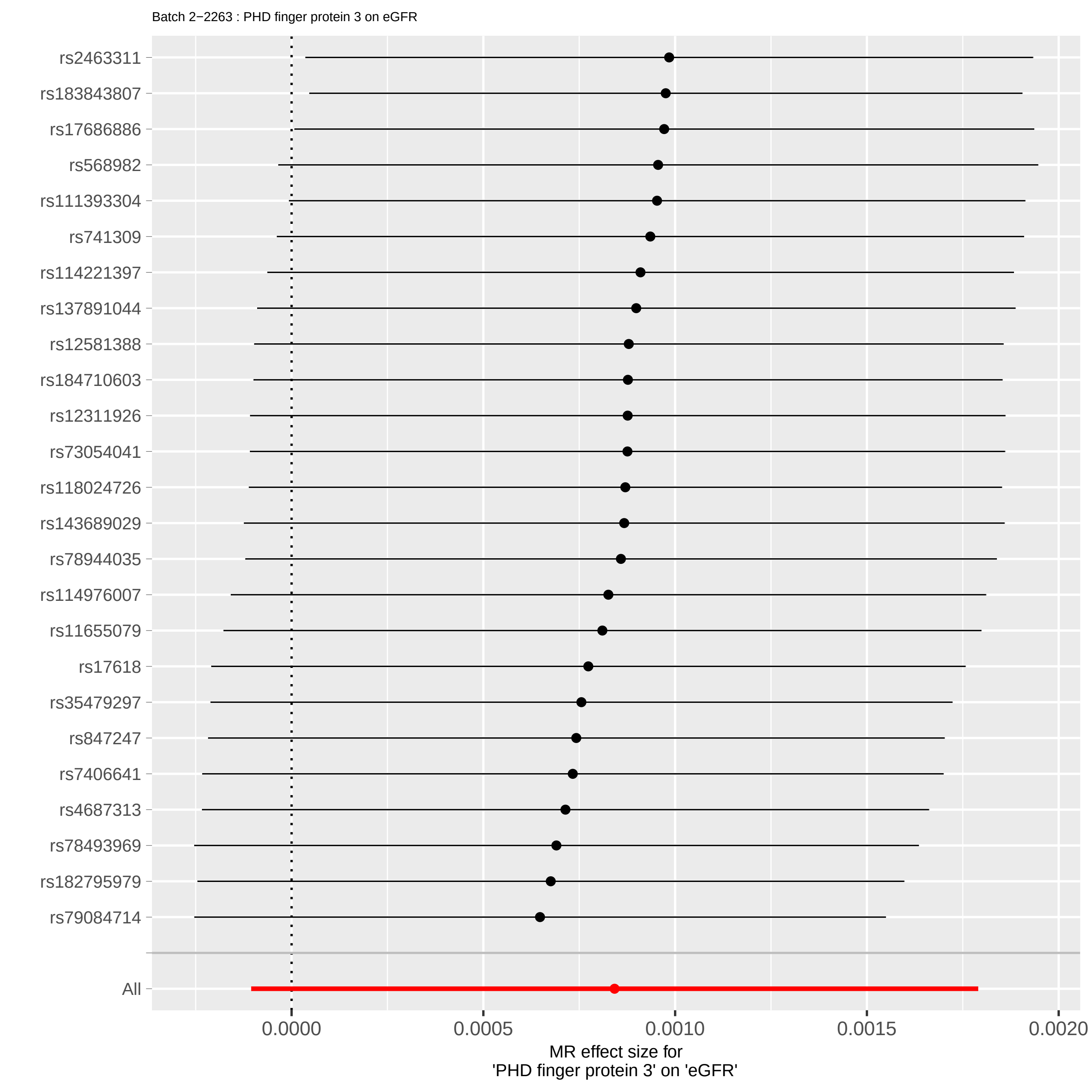

Batch 2−2263 : PHD finger protein 3 on eGFR
rs2463311
rs183843807
rs17686886
rs568982
rs111393304
rs741309
rs114221397
rs137891044
rs12581388
rs184710603
rs12311926
rs73054041
rs118024726
rs143689029
rs78944035
rs114976007
rs11655079
rs17618
rs35479297
rs847247
rs7406641
rs4687313
rs78493969
rs182795979
rs79084714
All
0.0000
0.0005
0.0010
0.0015
0.0020
MR effect size for
'PHD finger protein 3' on 'eGFR'

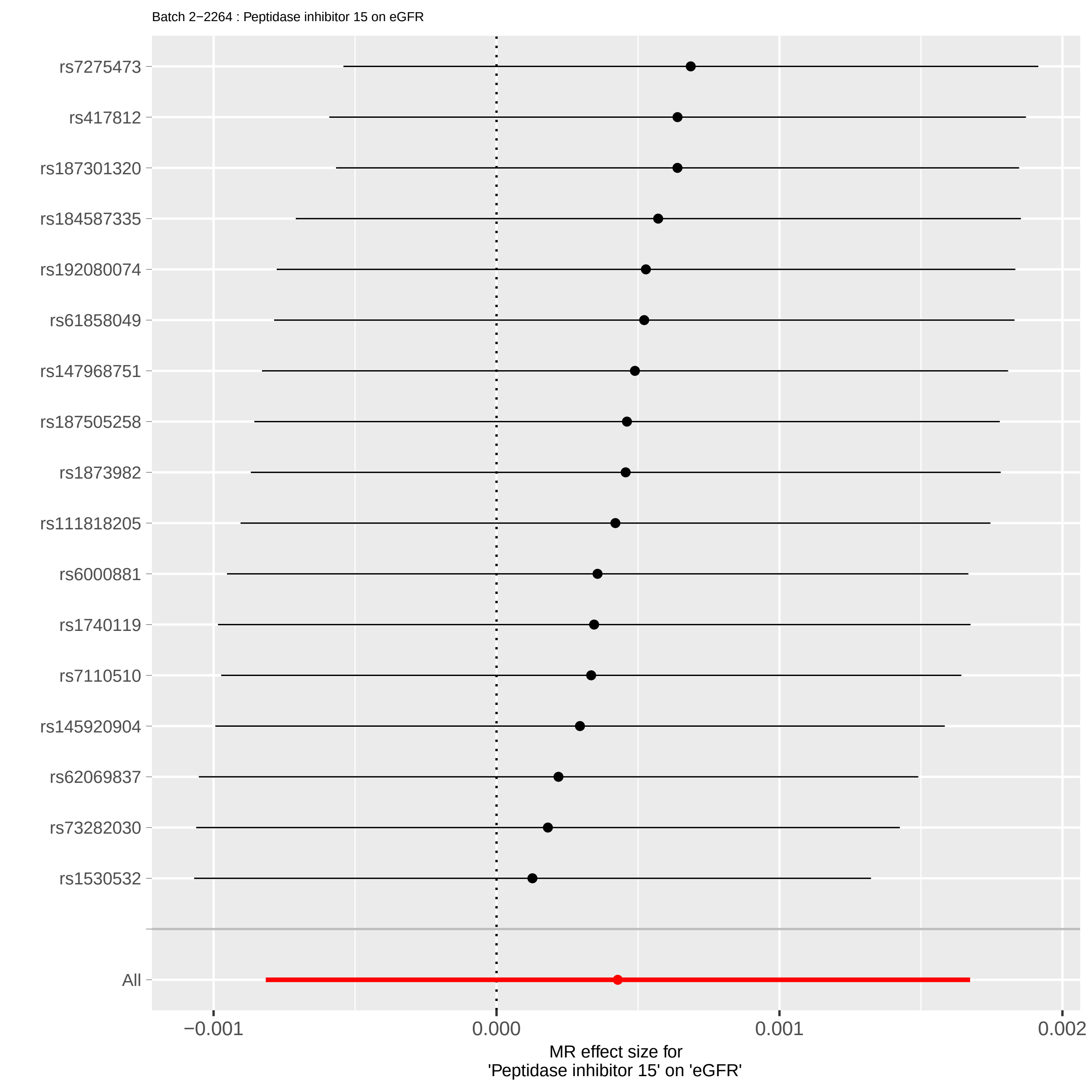
Batch 2−2264 : Peptidase inhibitor 15 on eGFR
rs7275473
rs417812
rs187301320
rs184587335
rs192080074
rs61858049
rs147968751
rs187505258
rs1873982
rs111818205
rs6000881
rs1740119
rs7110510
rs145920904
rs62069837
rs73282030
rs1530532
All
−0.001
0.000
0.001
0.002
MR effect size for
'Peptidase inhibitor 15' on 'eGFR'

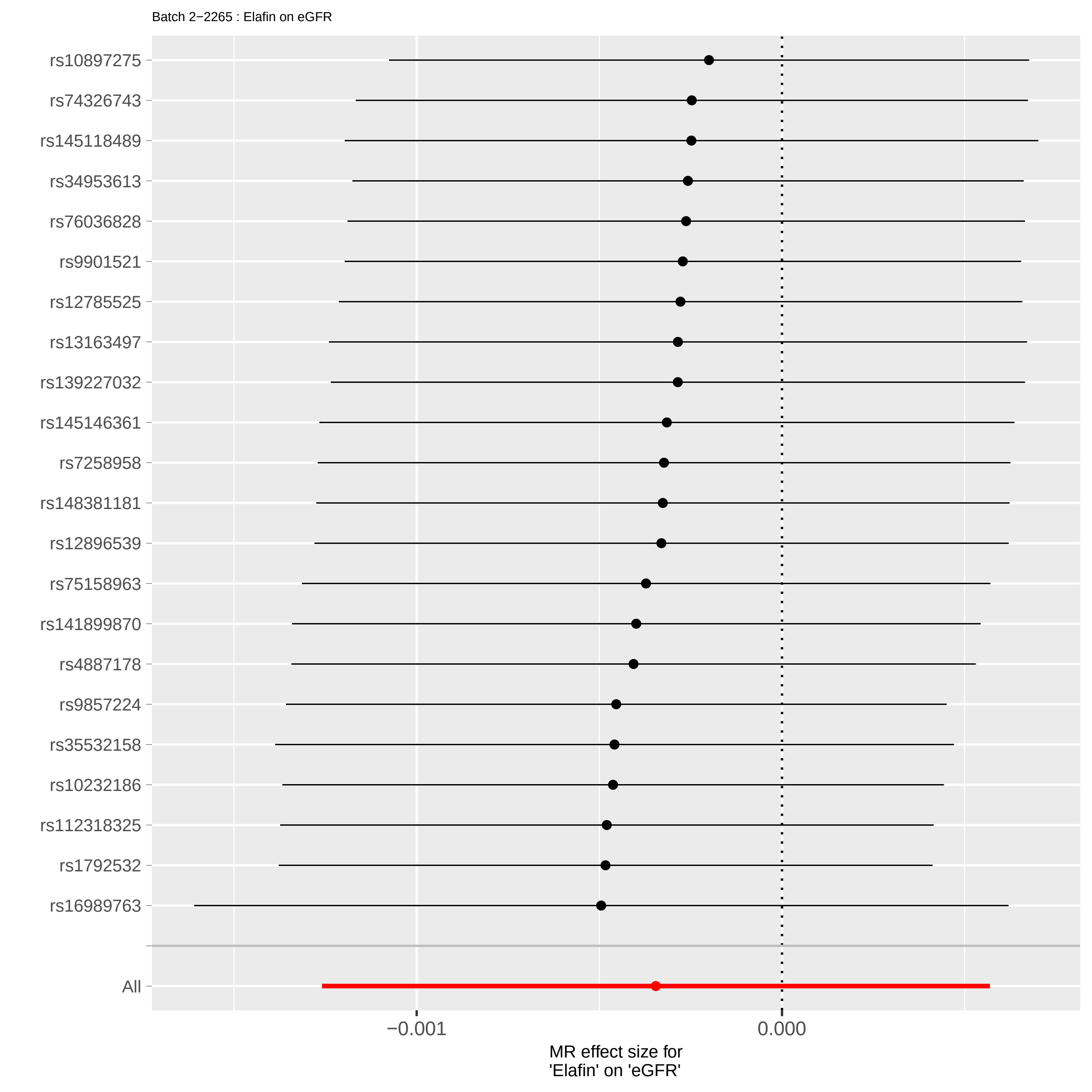
Batch 2−2265 : Elafin on eGFR
rs10897275
rs74326743
rs145118489
rs34953613
rs76036828
rs9901521
rs12785525
rs13163497
rs139227032
rs145146361
rs7258958
rs148381181
rs12896539
rs75158963
rs141899870
rs4887178
rs9857224
rs35532158
rs10232186
rs112318325
rs1792532
rs16989763
All
−0.001
0.000
MR effect size for
'Elafin' on 'eGFR'

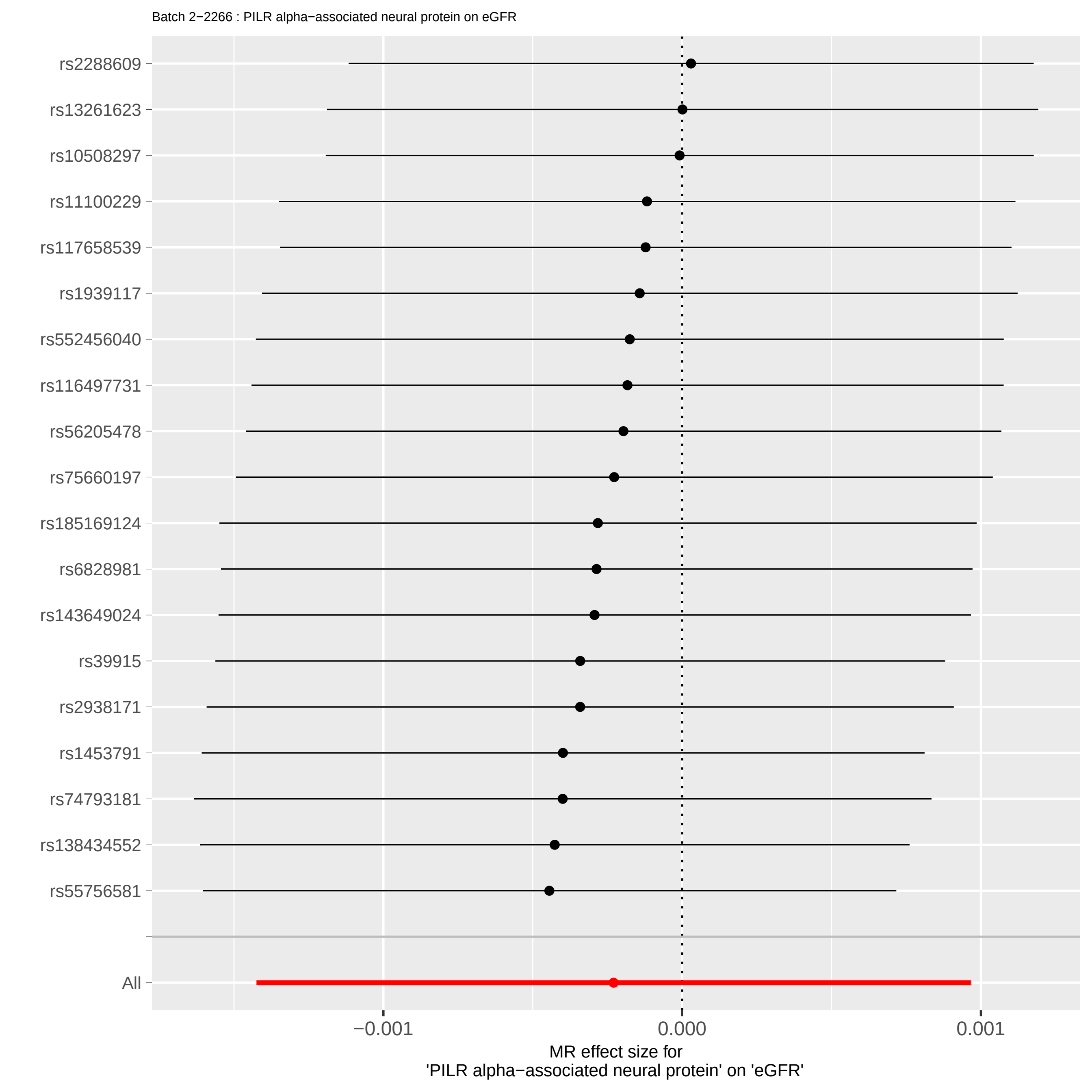
Batch 2−2266 : PILR alpha−associated neural protein on eGFR
rs2288609
rs13261623
rs10508297
rs11100229
rs117658539
rs1939117
rs552456040
rs116497731
rs56205478
rs75660197
rs185169124
rs6828981
rs143649024
rs39915
rs2938171
rs1453791
rs74793181
rs138434552
rs55756581
All
−0.001
0.000
0.001
MR effect size for
'PILR alpha−associated neural protein' on 'eGFR'

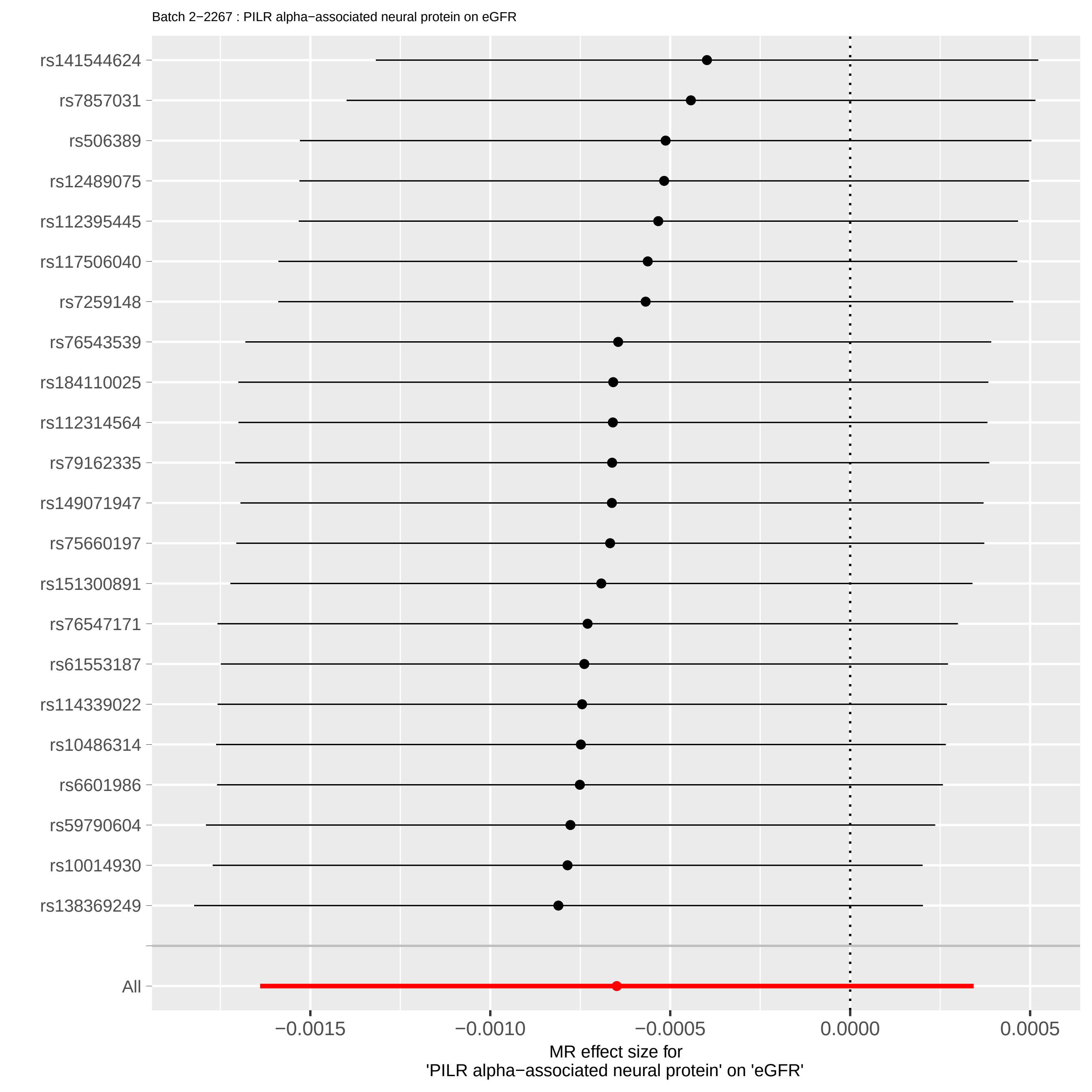

Batch 2−2267 : PILR alpha−associated neural protein on eGFR
rs141544624
rs7857031
rs506389
rs12489075
rs112395445
rs117506040
rs7259148
rs76543539
rs184110025
rs112314564
rs79162335
rs149071947
rs75660197
rs151300891
rs76547171
rs61553187
rs114339022
rs10486314
rs6601986
rs59790604
rs10014930
rs138369249
All
−0.0015
−0.0010
−0.0005
0.0000
0.0005
MR effect size for
'PILR alpha−associated neural protein' on 'eGFR'

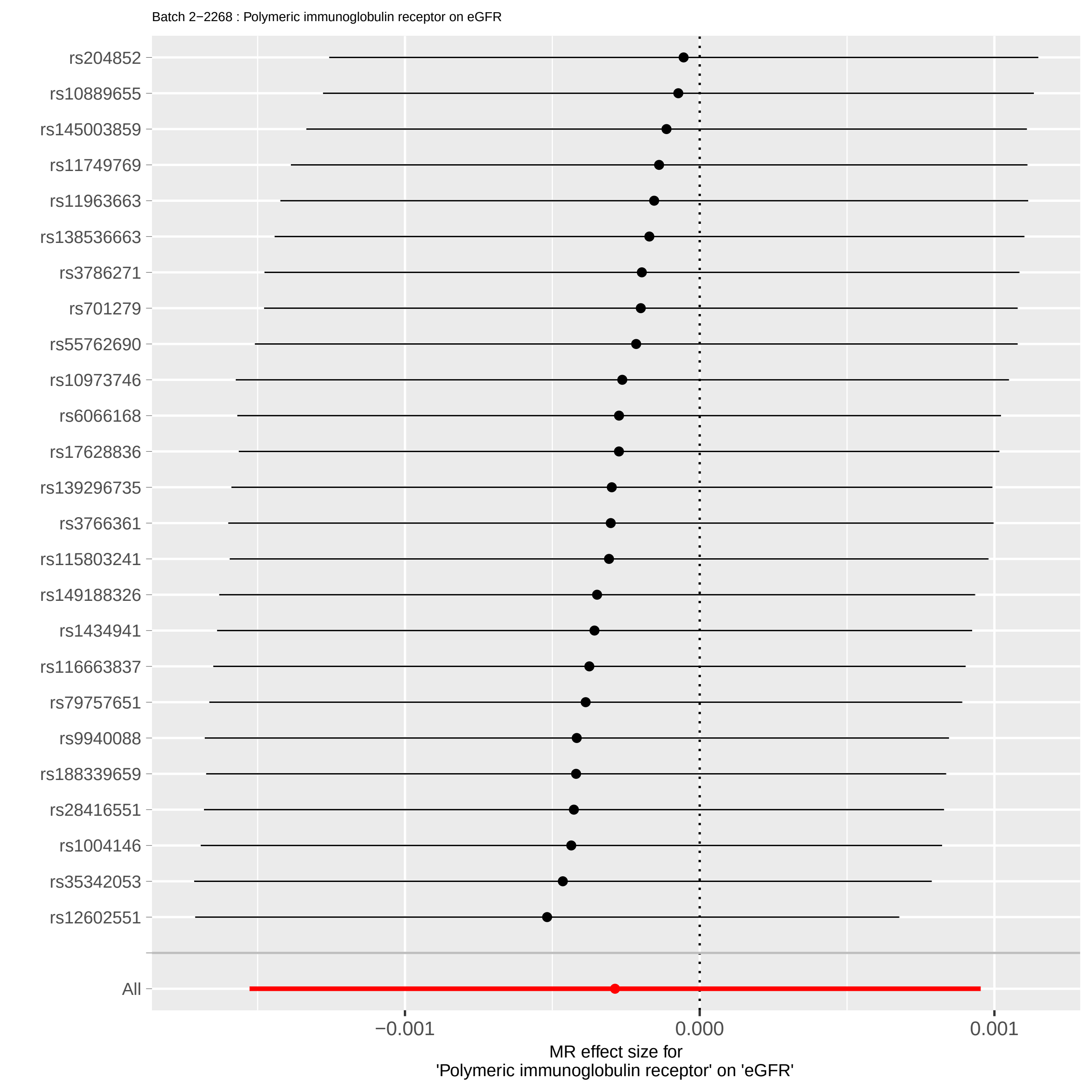
Batch 2−2268 : Polymeric immunoglobulin receptor on eGFR
rs204852
rs10889655
rs145003859
rs11749769
rs11963663
rs138536663
rs3786271
rs701279
rs55762690
rs10973746
rs6066168
rs17628836
rs139296735
rs3766361
rs115803241
rs149188326
rs1434941
rs116663837
rs79757651
rs9940088
rs188339659
rs28416551
rs1004146
rs35342053
rs12602551
All
−0.001
0.000
0.001
MR effect size for
'Polymeric immunoglobulin receptor' on 'eGFR'

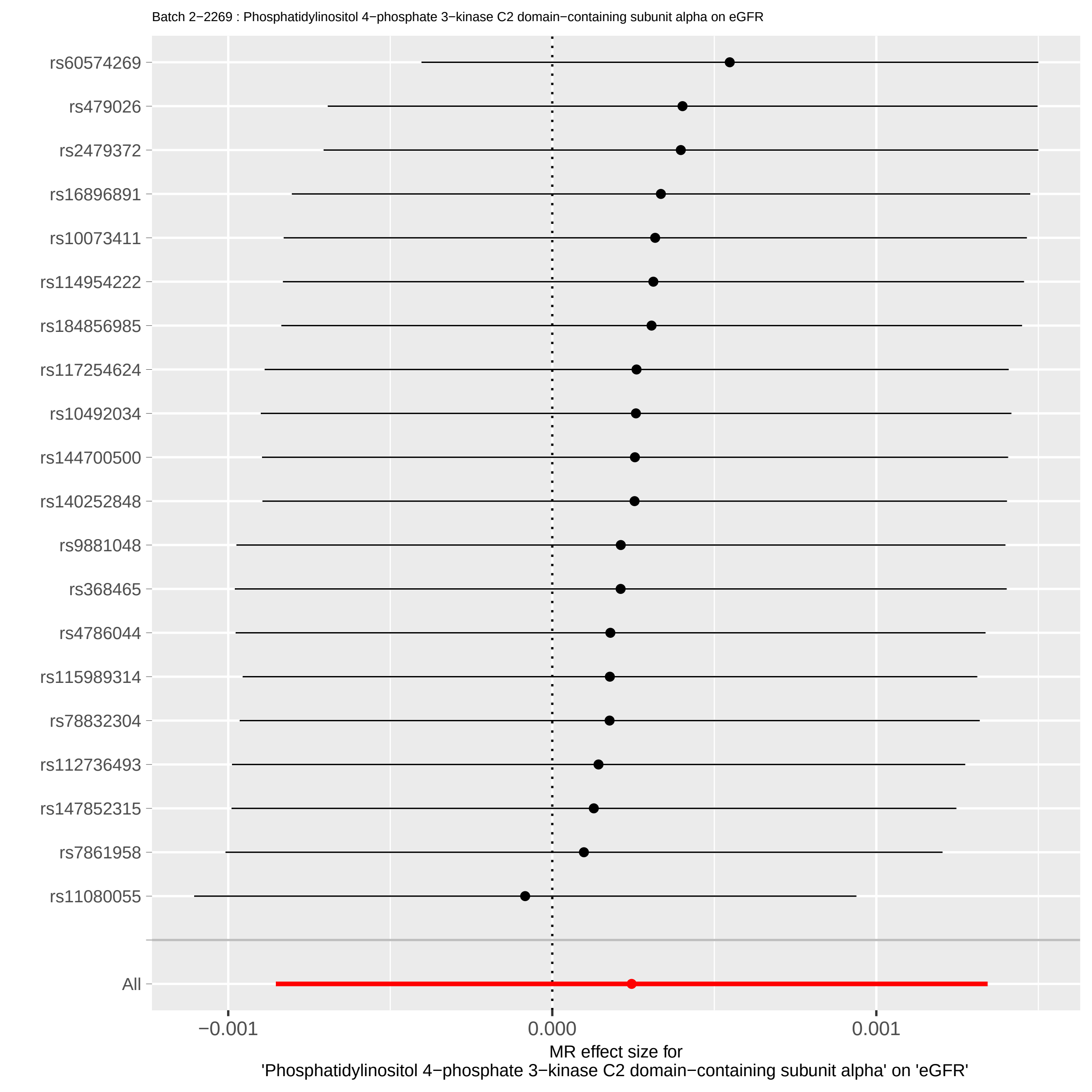

Batch 2−2269 : Phosphatidylinositol 4−phosphate 3−kinase C2 domain−containing subunit alpha on eGFR
rs60574269
rs479026
rs2479372
rs16896891
rs10073411
rs114954222
rs184856985
rs117254624
rs10492034
rs144700500
rs140252848
rs9881048
rs368465
rs4786044
rs115989314
rs78832304
rs112736493
rs147852315
rs7861958
rs11080055
All
−0.001
0.000
0.001
MR effect size for
'Phosphatidylinositol 4−phosphate 3−kinase C2 domain−containing subunit alpha' on 'eGFR'

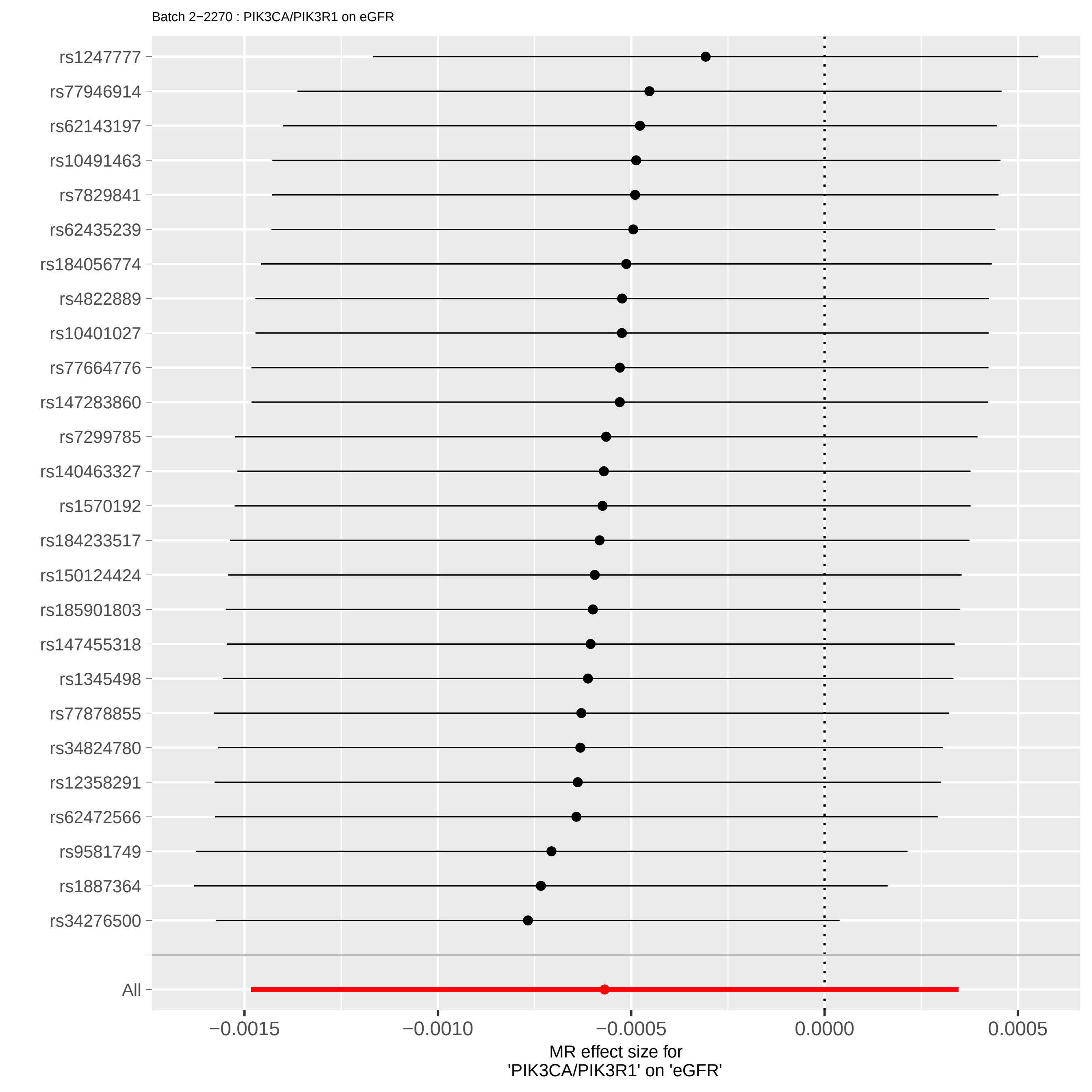

Batch 2−2270 : PIK3CA/PIK3R1 on eGFR
rs1247777
rs77946914
rs62143197
rs10491463
rs7829841
rs62435239
rs184056774
rs4822889
rs10401027
rs77664776
rs147283860
rs7299785
rs140463327
rs1570192
rs184233517
rs150124424
rs185901803
rs147455318
rs1345498
rs77878855
rs34824780
rs12358291
rs62472566
rs9581749
rs1887364
rs34276500
All
−0.0015
−0.0010
−0.0005
0.0000
0.0005
MR effect size for
'PIK3CA/PIK3R1' on 'eGFR'

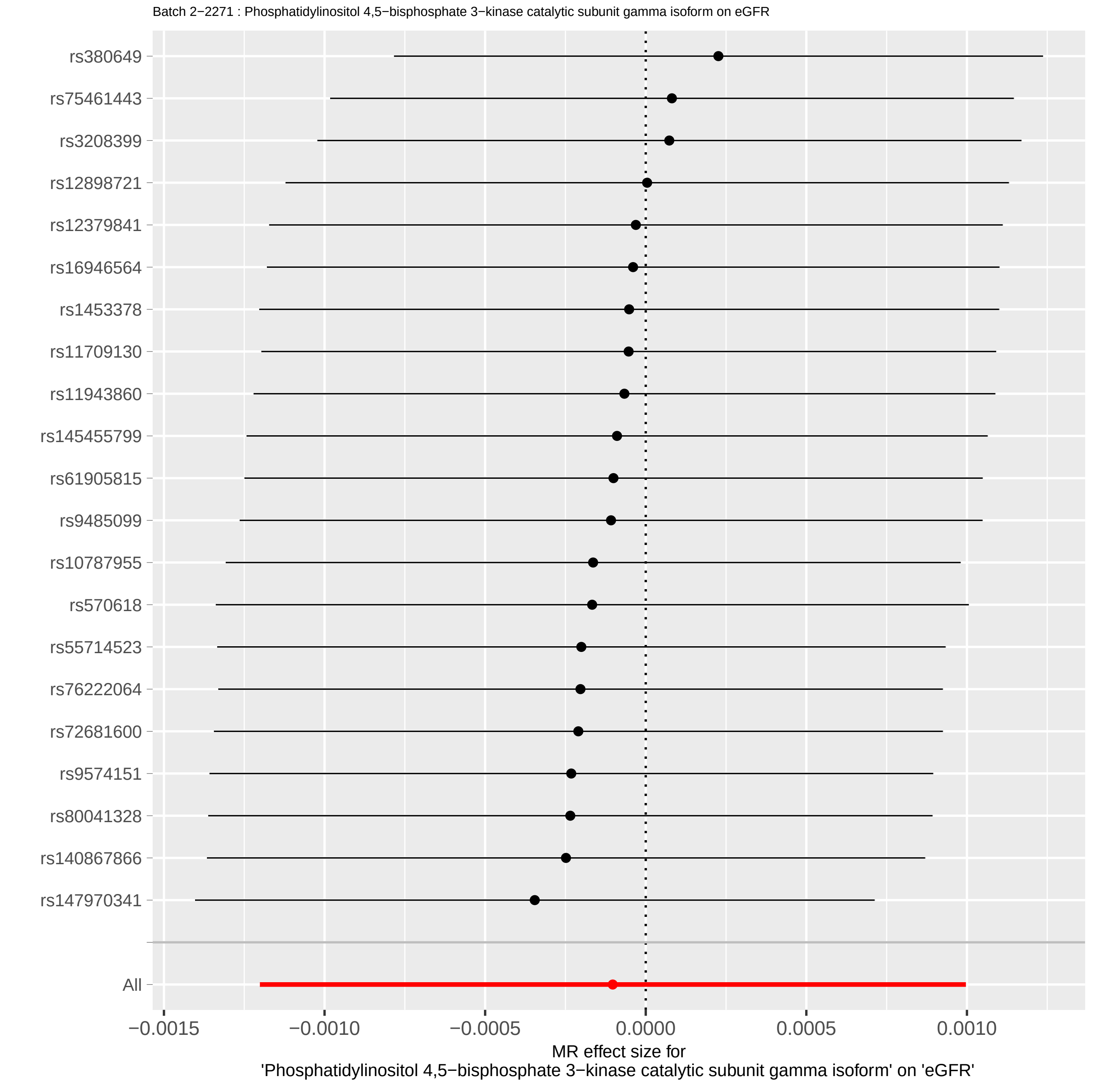

Batch 2−2271 : Phosphatidylinositol 4,5−bisphosphate 3−kinase catalytic subunit gamma isoform on eGFR
rs380649
rs75461443
rs3208399
rs12898721
rs12379841
rs16946564
rs1453378
rs11709130
rs11943860
rs145455799
rs61905815
rs9485099
rs10787955
rs570618
rs55714523
rs76222064
rs72681600
rs9574151
rs80041328
rs140867866
rs147970341
All
−0.0015
−0.0010
−0.0005
0.0000
0.0005
0.0010
MR effect size for
'Phosphatidylinositol 4,5−bisphosphate 3−kinase catalytic subunit gamma isoform' on 'eGFR'

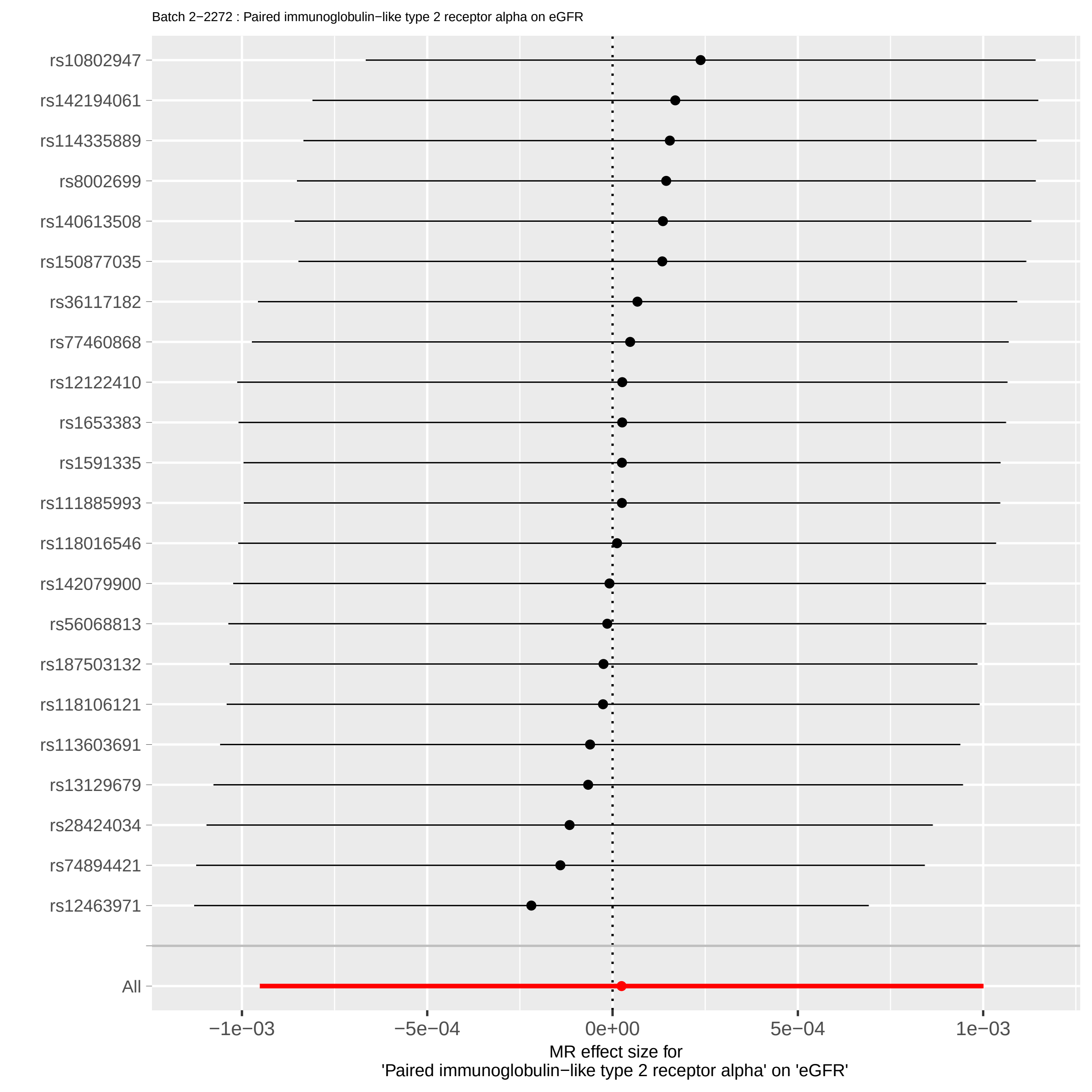

Batch 2−2272 : Paired immunoglobulin−like type 2 receptor alpha on eGFR
rs10802947
rs142194061
rs114335889
rs8002699
rs140613508
rs150877035
rs36117182
rs77460868
rs12122410
rs1653383
rs1591335
rs111885993
rs118016546
rs142079900
rs56068813
rs187503132
rs118106121
rs113603691
rs13129679
rs28424034
rs74894421
rs12463971
All
−1e−03
−5e−04
0e+00
5e−04
1e−03
MR effect size for
'Paired immunoglobulin−like type 2 receptor alpha' on 'eGFR'

Batch 2−2273 : Paired immunoglobulin−like type 2 receptor alpha on eGFR

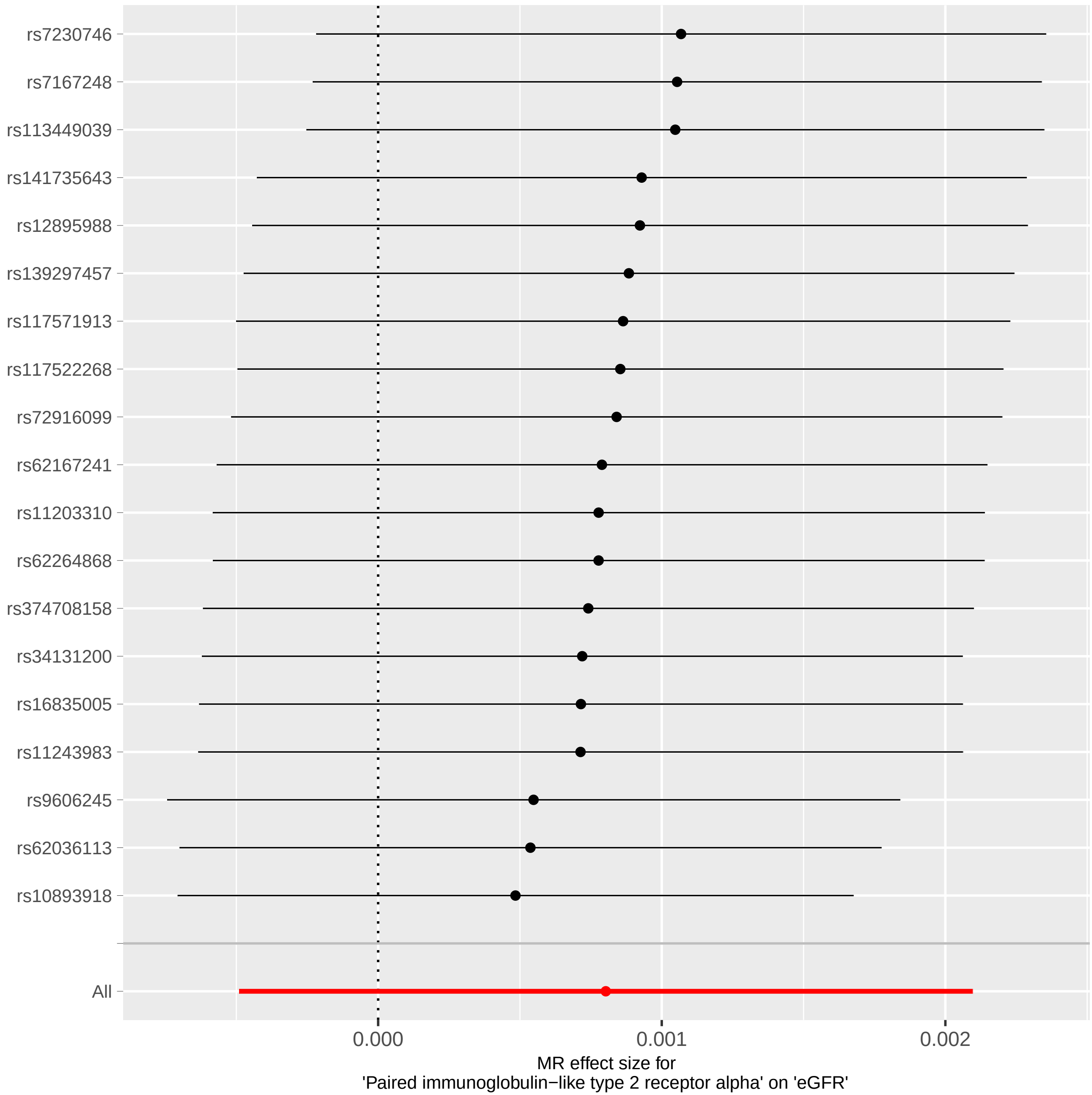

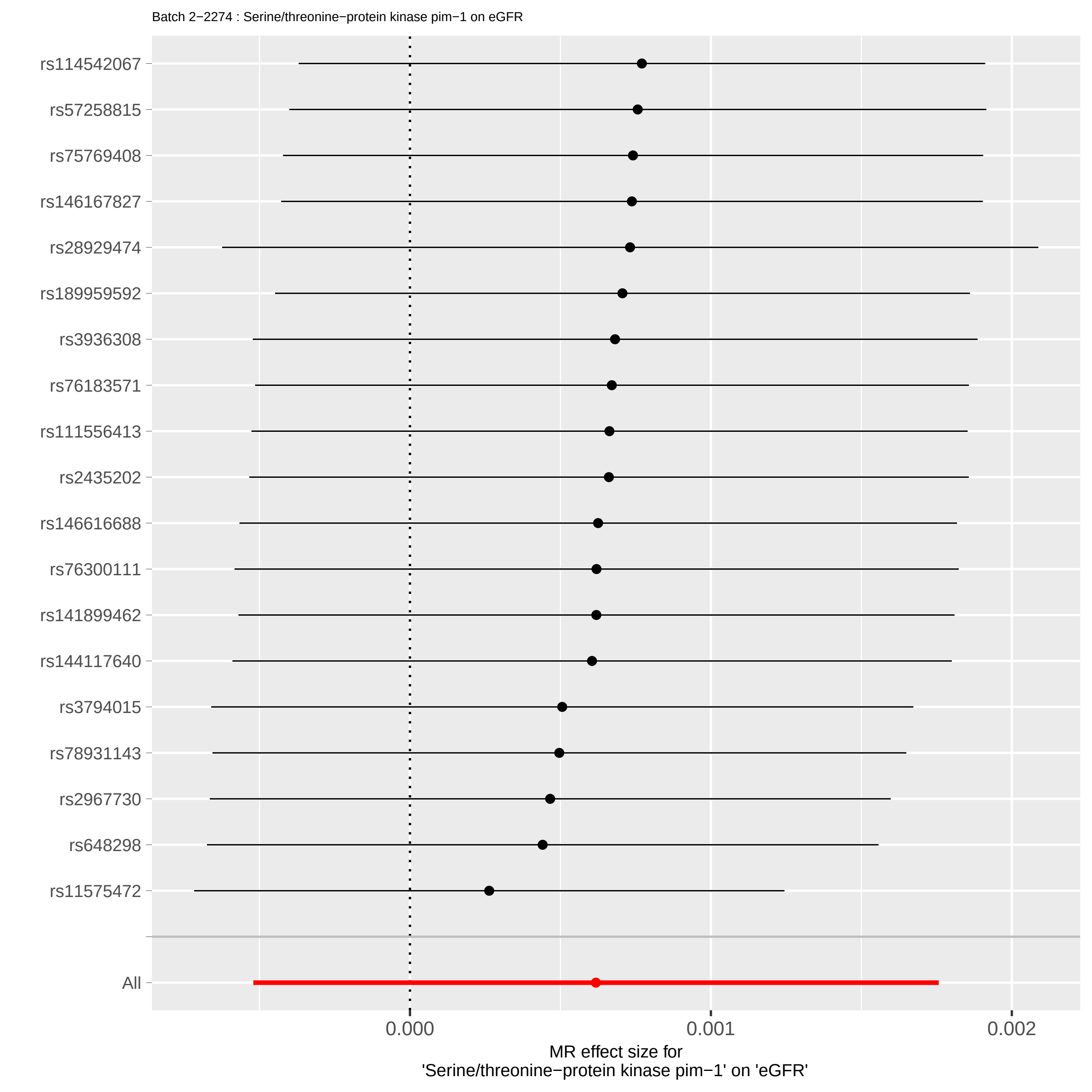

Batch 2−2274 : Serine/threonine−protein kinase pim−1 on eGFR
rs114542067
rs57258815
rs75769408
rs146167827
rs28929474
rs189959592
rs3936308
rs76183571
rs111556413
rs2435202
rs146616688
rs76300111
rs141899462
rs144117640
rs3794015
rs78931143
rs2967730
rs648298
rs11575472
All
0.000
0.001
0.002
MR effect size for
'Serine/threonine−protein kinase pim−1' on 'eGFR'

Batch 2−2275 : Peptidyl−prolyl cis−trans isomerase NIMA−interacting 4 on eGFR

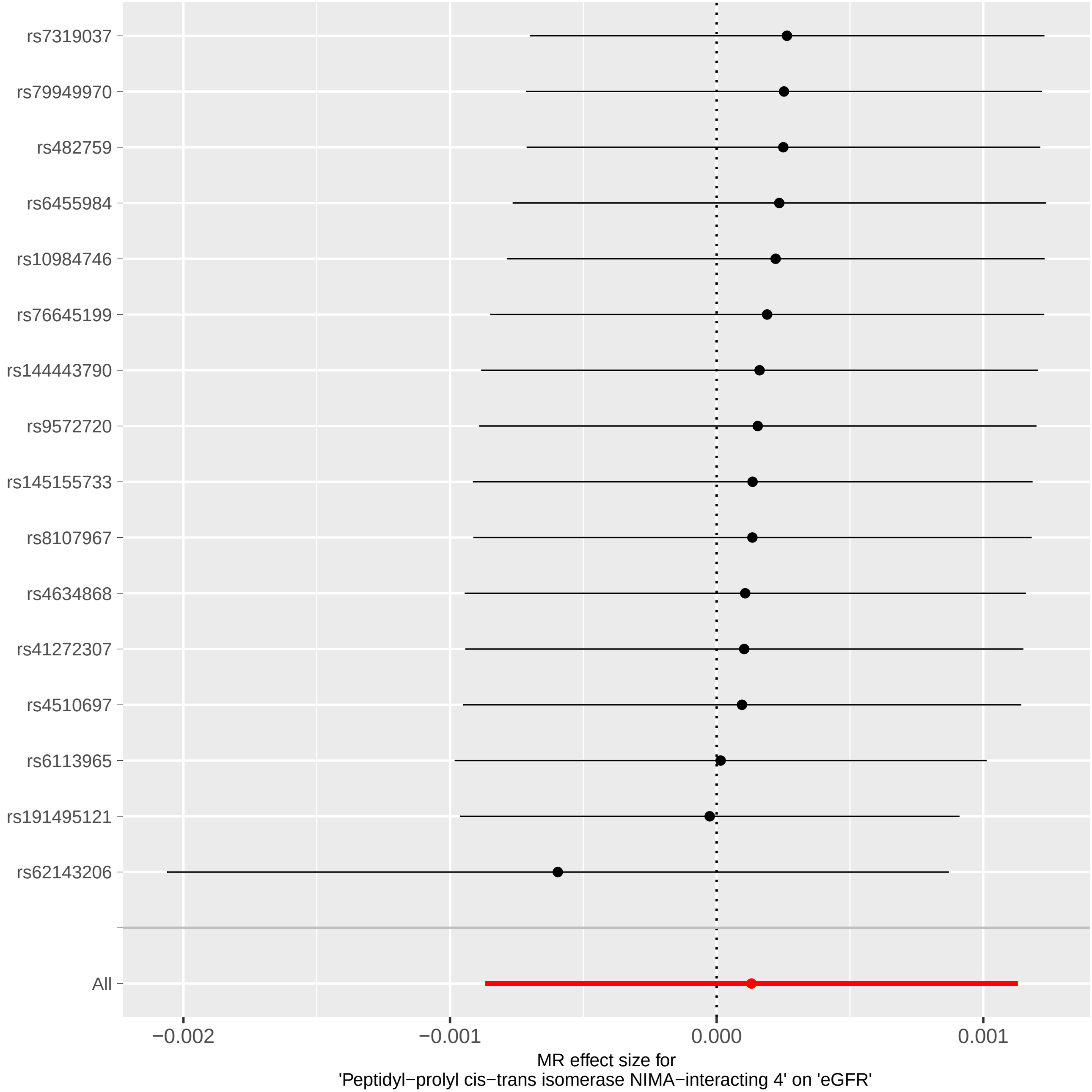

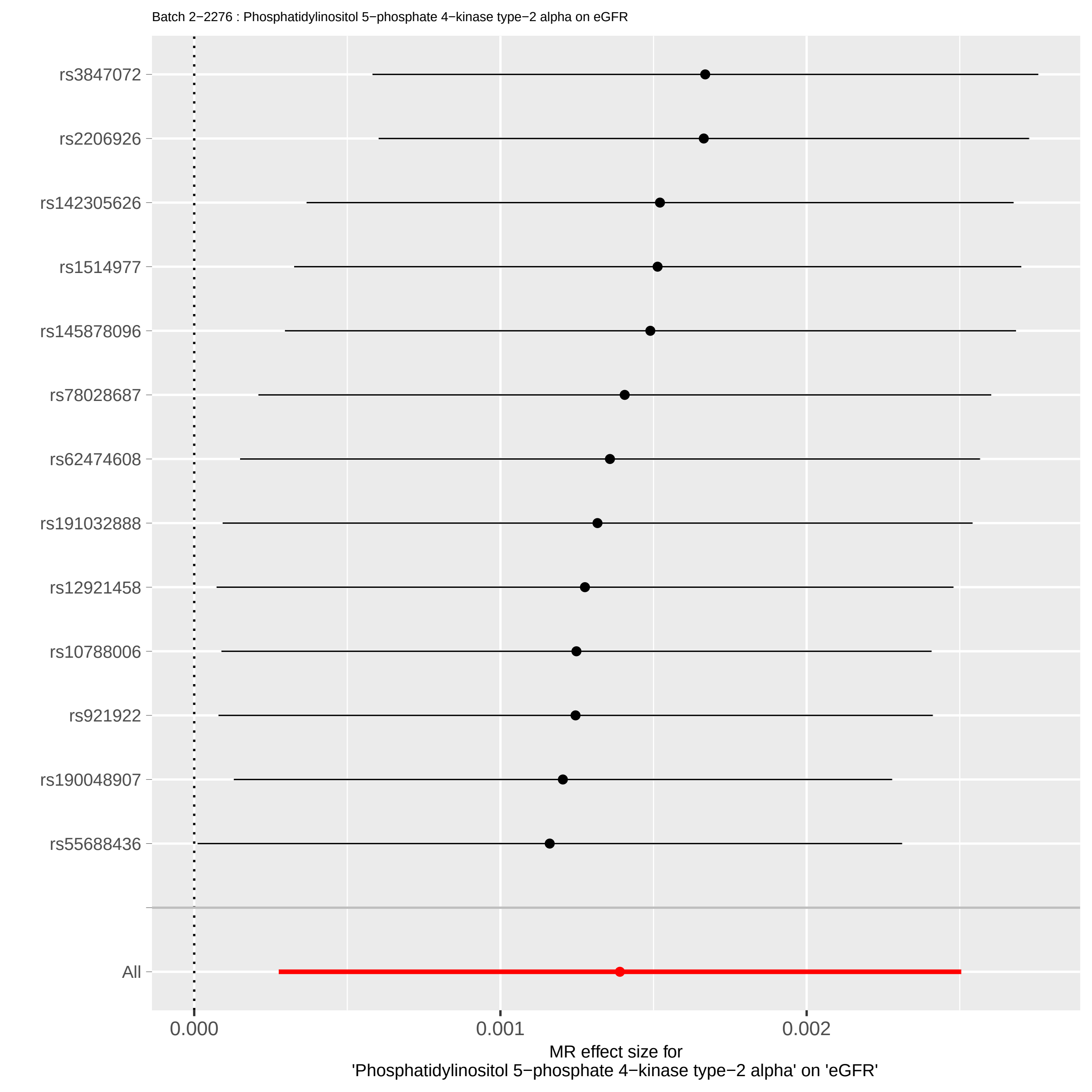

Batch 2−2276 : Phosphatidylinositol 5−phosphate 4−kinase type−2 alpha on eGFR
rs3847072
rs2206926
rs142305626
rs1514977
rs145878096
rs78028687
rs62474608
rs191032888
rs12921458
rs10788006
rs921922
rs190048907
rs55688436
All
0.000
0.001
0.002
MR effect size for
'Phosphatidylinositol 5−phosphate 4−kinase type−2 alpha' on 'eGFR'

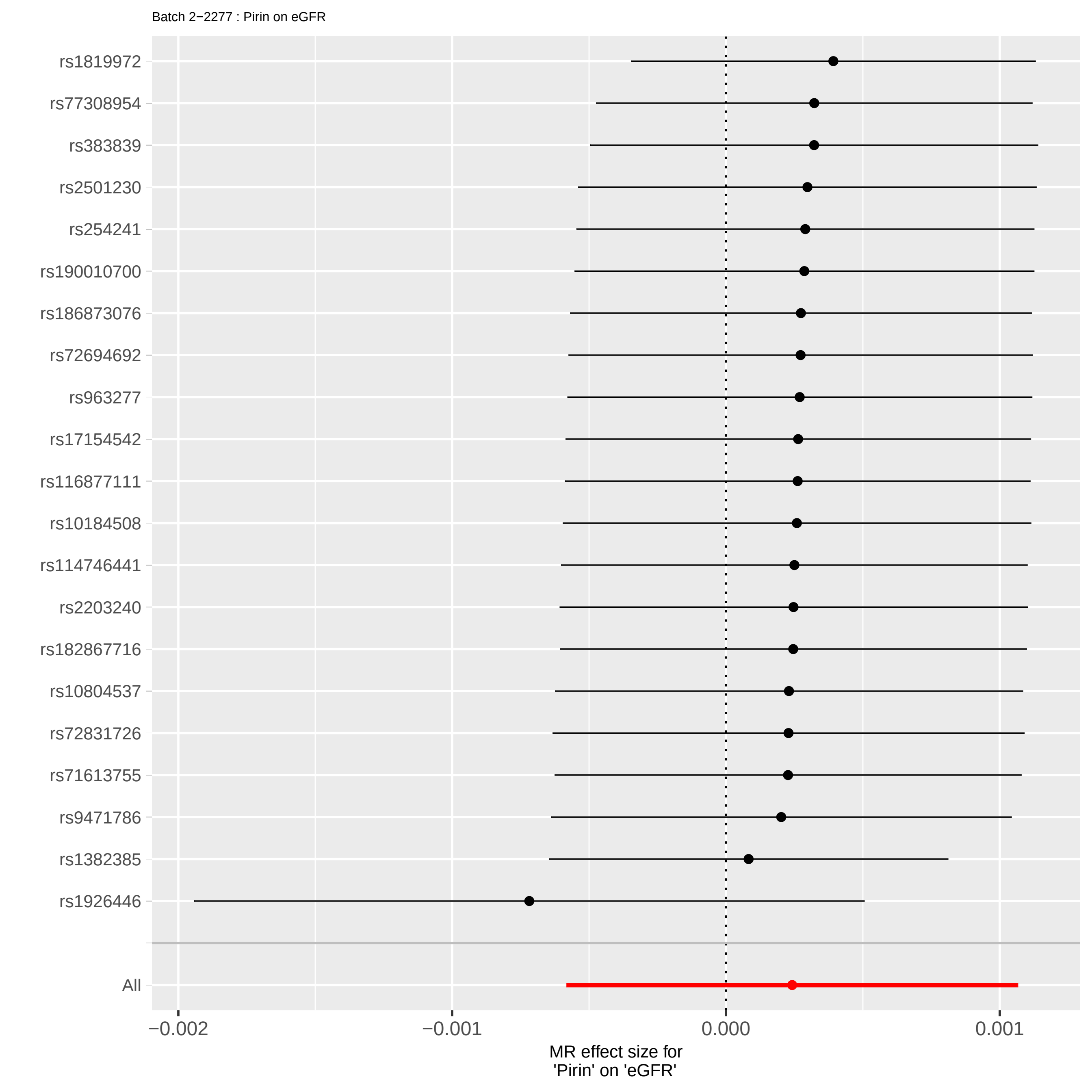

Batch 2−2277 : Pirin on eGFR
rs1819972
rs77308954
rs383839
rs2501230
rs254241
rs190010700
rs186873076
rs72694692
rs963277
rs17154542
rs116877111
rs10184508
rs114746441
rs2203240
rs182867716
rs10804537
rs72831726
rs71613755
rs9471786
rs1382385
rs1926446
All
−0.002
−0.001
0.000
0.001
MR effect size for
'Pirin' on 'eGFR'

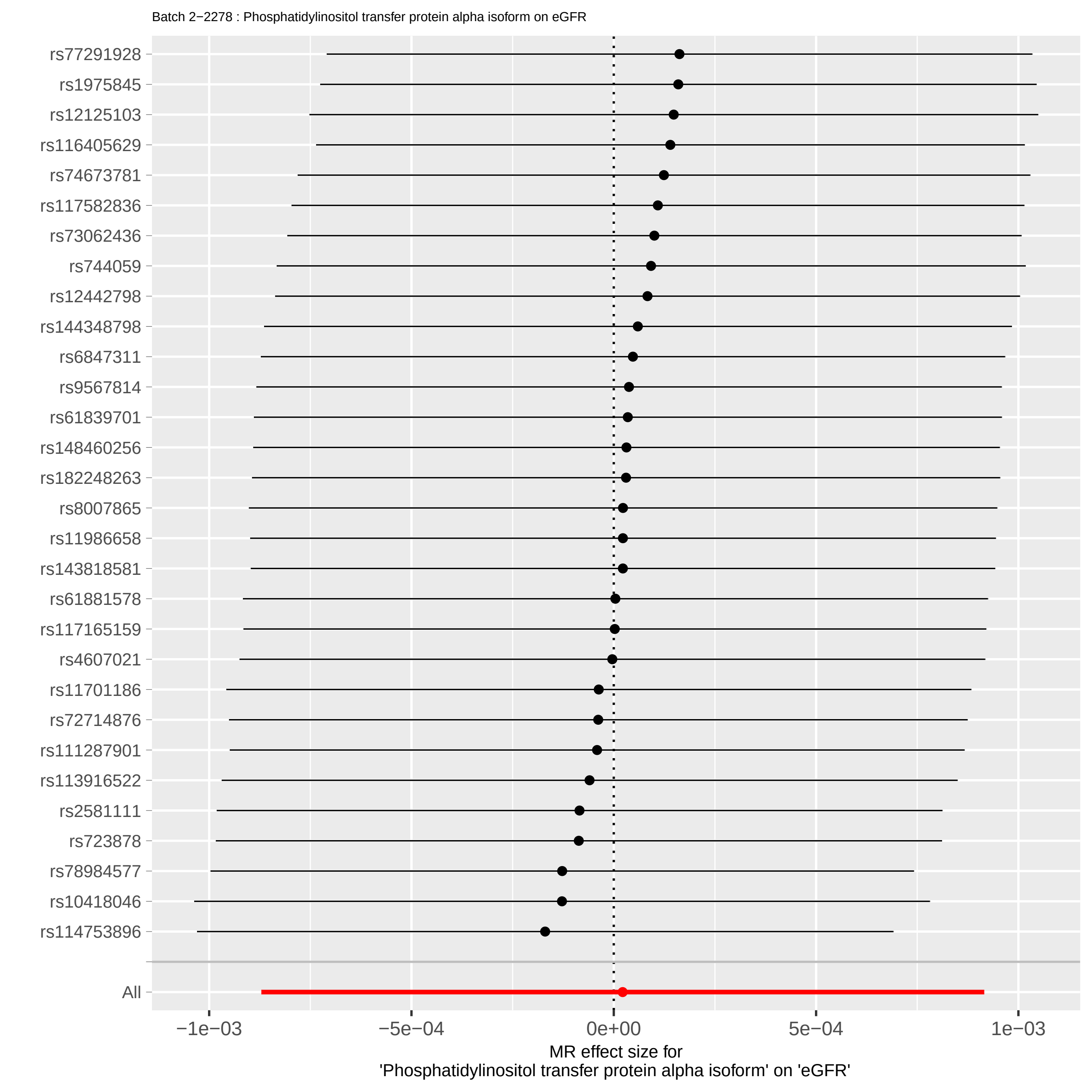
Batch 2−2278 : Phosphatidylinositol transfer protein alpha isoform on eGFR
rs77291928
rs1975845
rs12125103
rs116405629
rs74673781
rs117582836
rs73062436
rs744059
rs12442798
rs144348798
rs6847311
rs9567814
rs61839701
rs148460256
rs182248263
rs8007865
rs11986658
rs143818581
rs61881578
rs117165159
rs4607021
rs11701186
rs72714876
rs111287901
rs113916522
rs2581111
rs723878
rs78984577
rs10418046
rs114753896
All
−1e−03
−5e−04
0e+00
5e−04
1e−03
MR effect size for
'Phosphatidylinositol transfer protein alpha isoform' on 'eGFR'

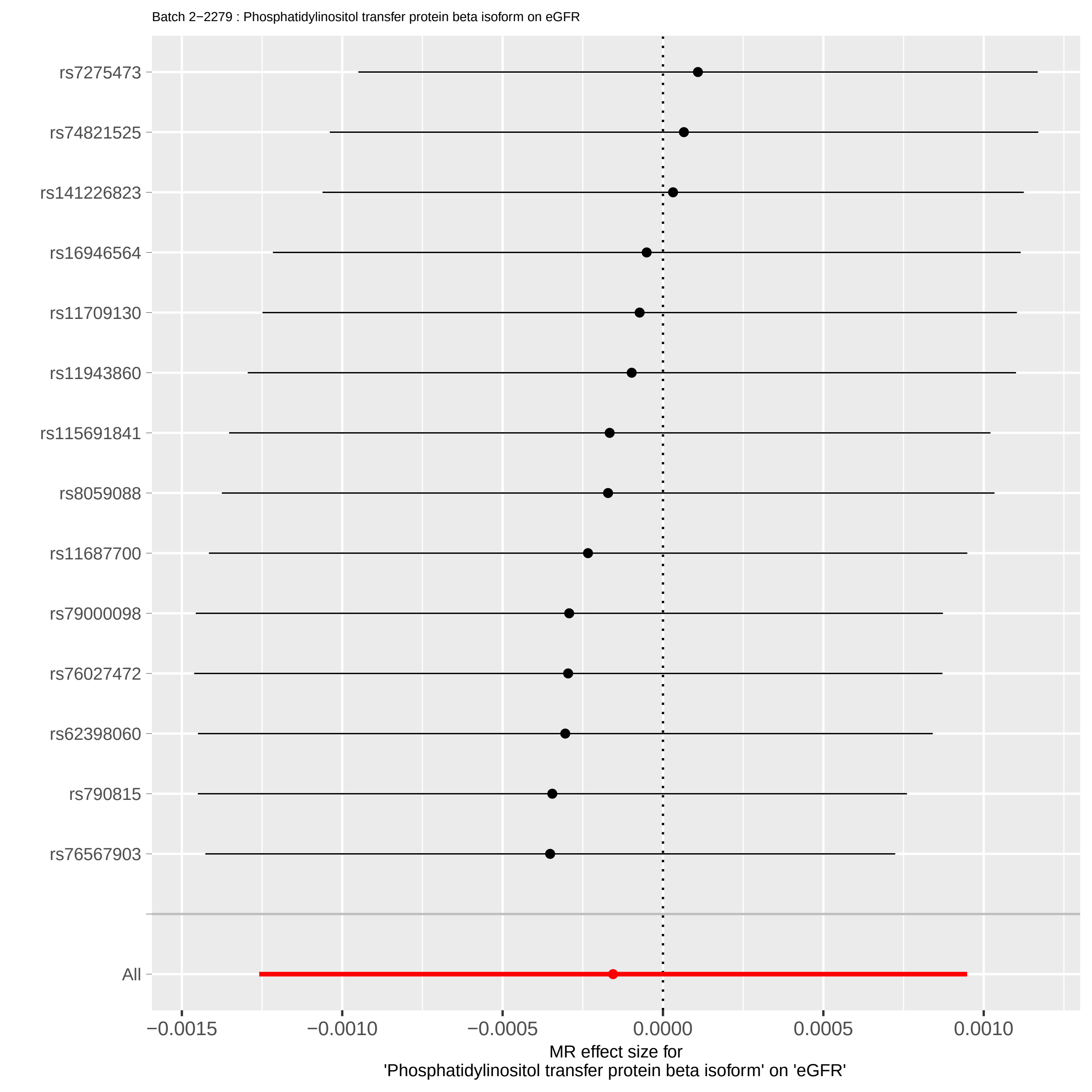

Batch 2−2279 : Phosphatidylinositol transfer protein beta isoform on eGFR
rs7275473
rs74821525
rs141226823
rs16946564
rs11709130
rs11943860
rs115691841
rs8059088
rs11687700
rs79000098
rs76027472
rs62398060
rs790815
rs76567903
All
−0.0015
−0.0010
−0.0005
0.0000
0.0005
0.0010
MR effect size for
'Phosphatidylinositol transfer protein beta isoform' on 'eGFR'

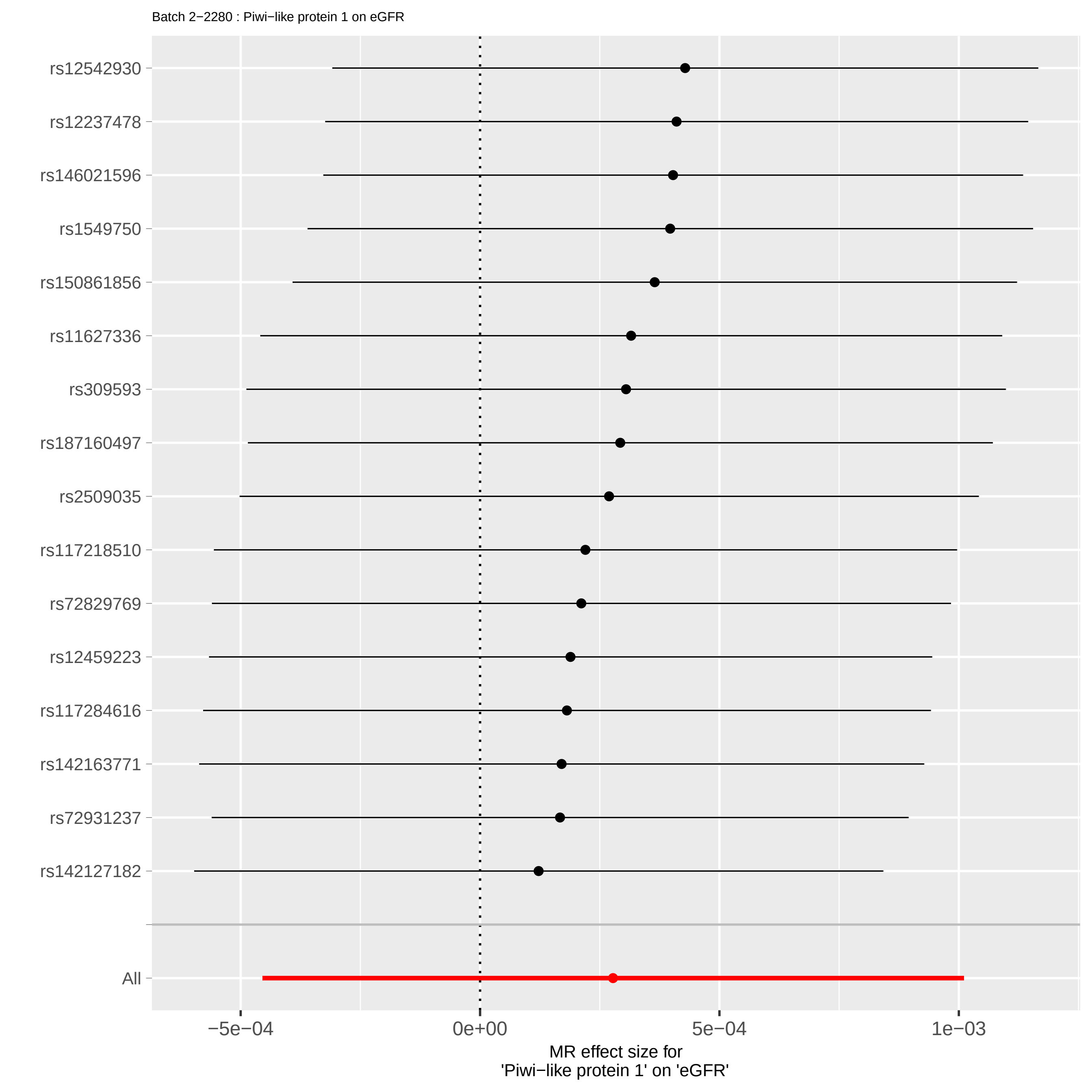

Batch 2−2280 : Piwi−like protein 1 on eGFR
rs12542930
rs12237478
rs146021596
rs1549750
rs150861856
rs11627336
rs309593
rs187160497
rs2509035
rs117218510
rs72829769
rs12459223
rs117284616
rs142163771
rs72931237
rs142127182
All
−5e−04
0e+00
5e−04
1e−03
MR effect size for
'Piwi−like protein 1' on 'eGFR'

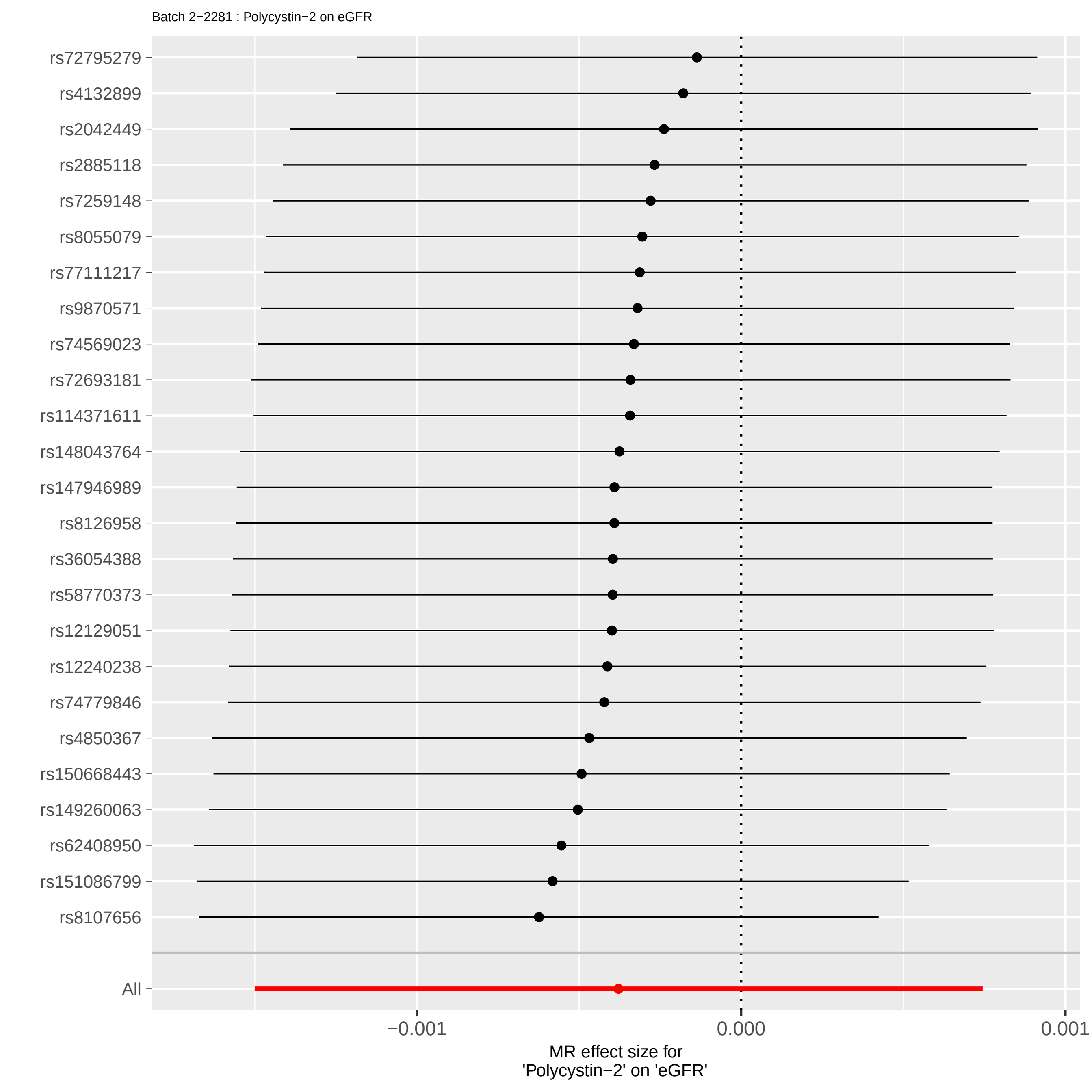

Batch 2−2281 : Polycystin−2 on eGFR
rs72795279
rs4132899
rs2042449
rs2885118
rs7259148
rs8055079
rs77111217
rs9870571
rs74569023
rs72693181
rs114371611
rs148043764
rs147946989
rs8126958
rs36054388
rs58770373
rs12129051
rs12240238
rs74779846
rs4850367
rs150668443
rs149260063
rs62408950
rs151086799
rs8107656
All
−0.001
0.000
0.001
MR effect size for
'Polycystin−2' on 'eGFR'

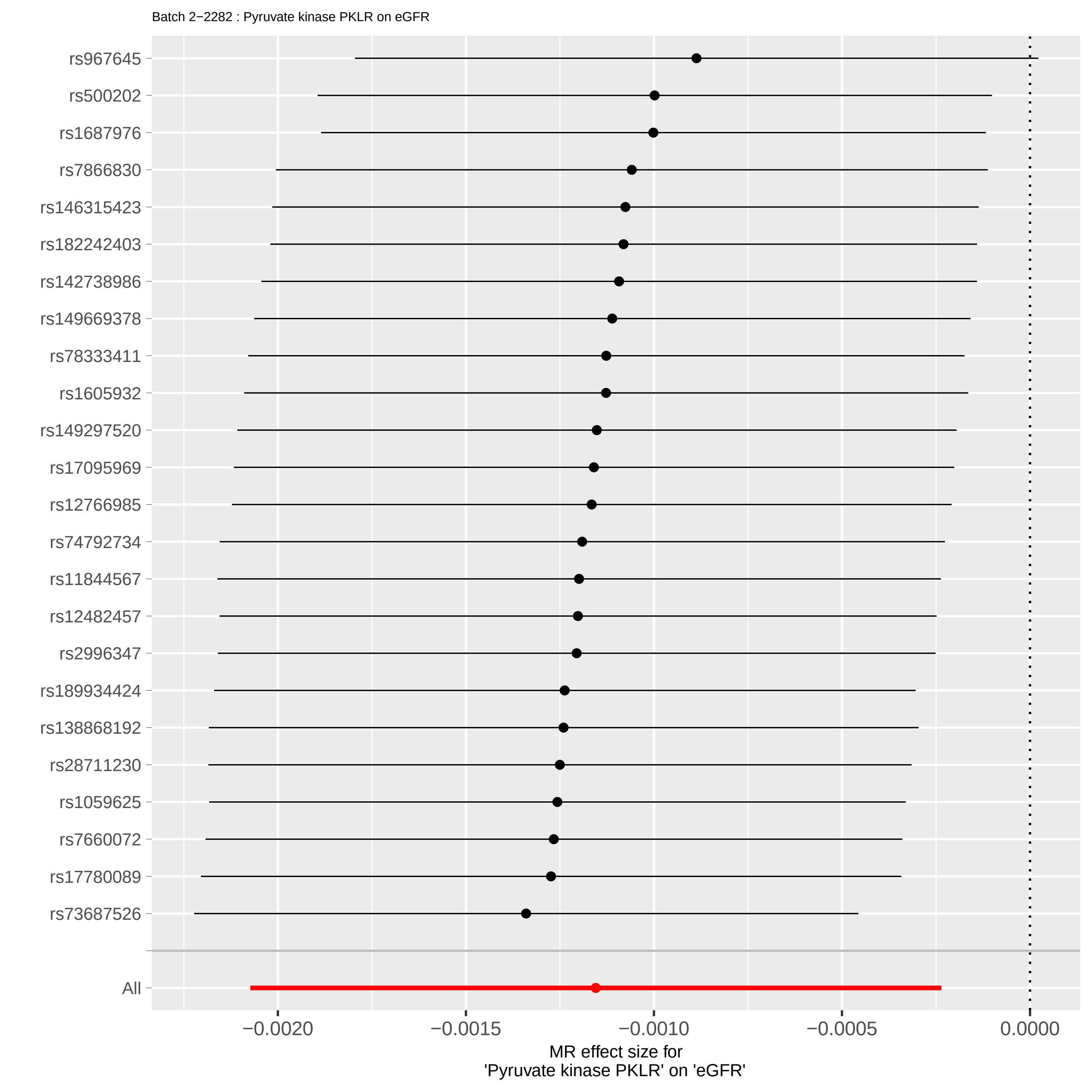

Batch 2−2282 : Pyruvate kinase PKLR on eGFR
rs967645
rs500202
rs1687976
rs7866830
rs146315423
rs182242403
rs142738986
rs149669378
rs78333411
rs1605932
rs149297520
rs17095969
rs12766985
rs74792734
rs11844567
rs12482457
rs2996347
rs189934424
rs138868192
rs28711230
rs1059625
rs7660072
rs17780089
rs73687526
All
−0.0020
−0.0015
−0.0010
−0.0005
0.0000
MR effect size for
'Pyruvate kinase PKLR' on 'eGFR'

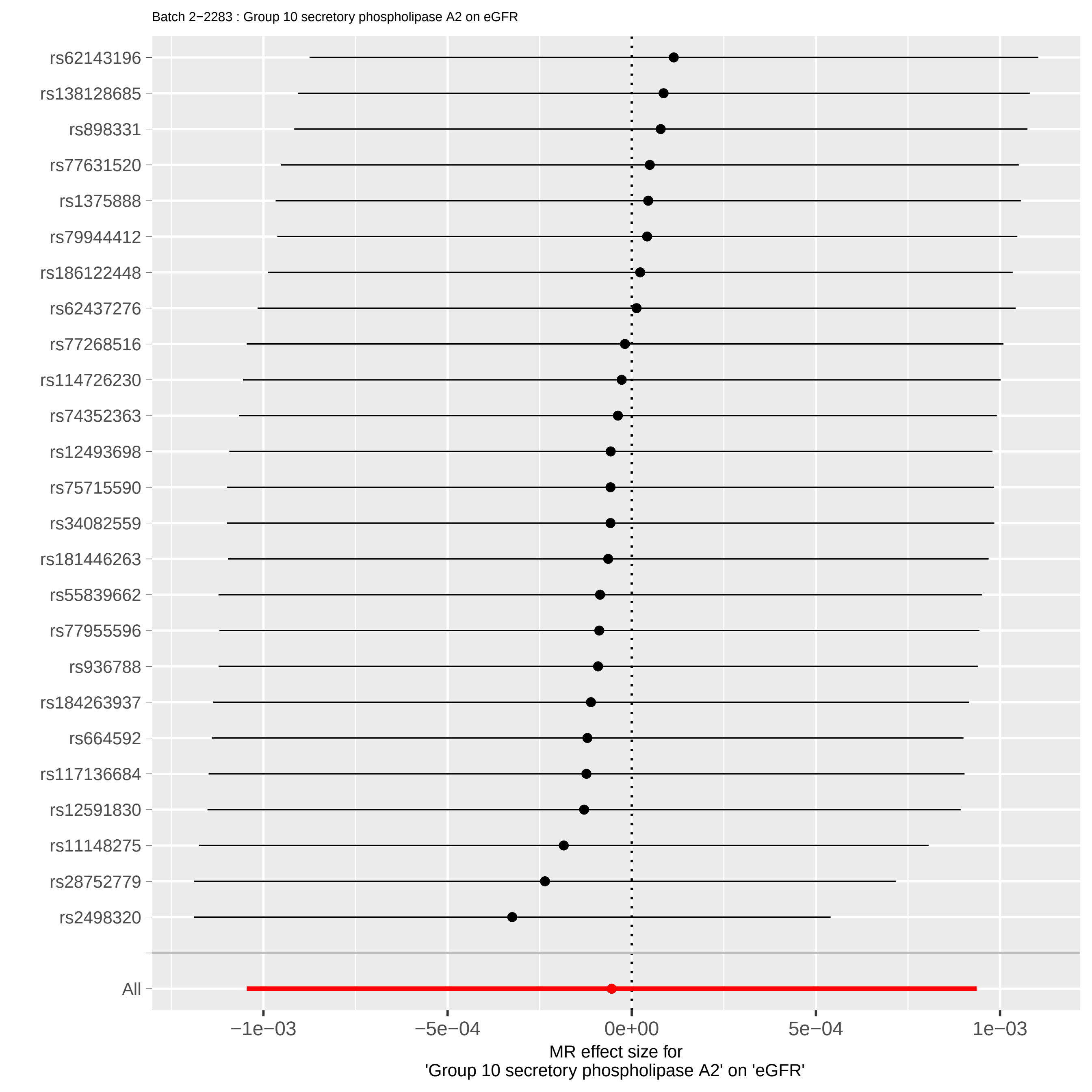

Batch 2−2283 : Group 10 secretory phospholipase A2 on eGFR
rs62143196
rs138128685
rs898331
rs77631520
rs1375888
rs79944412
rs186122448
rs62437276
rs77268516
rs114726230
rs74352363
rs12493698
rs75715590
rs34082559
rs181446263
rs55839662
rs77955596
rs936788
rs184263937
rs664592
rs117136684
rs12591830
rs11148275
rs28752779
rs2498320
All
−1e−03
−5e−04
0e+00
5e−04
1e−03
MR effect size for
'Group 10 secretory phospholipase A2' on 'eGFR'

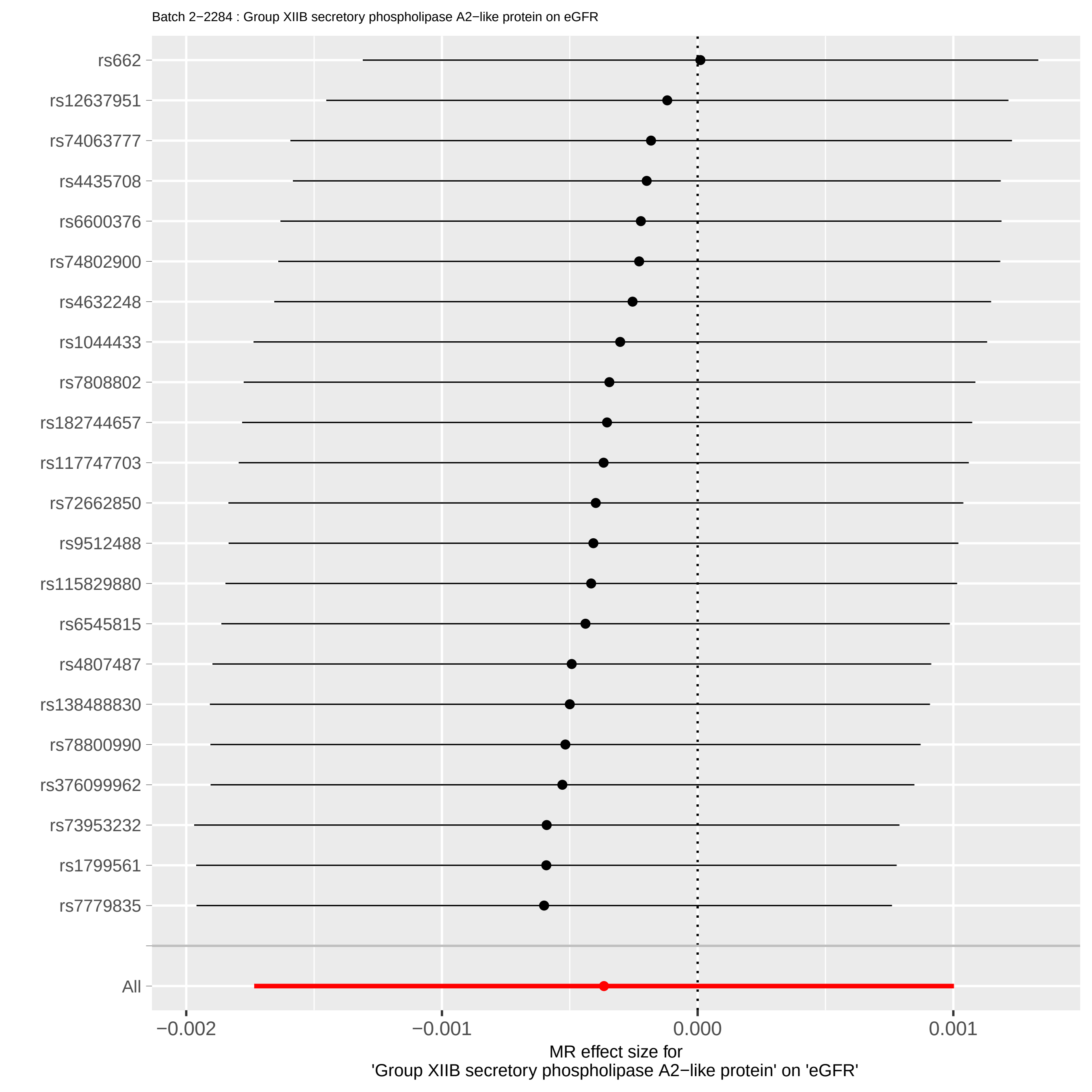

Batch 2−2284 : Group XIIB secretory phospholipase A2−like protein on eGFR
rs662
rs12637951
rs74063777
rs4435708
rs6600376
rs74802900
rs4632248
rs1044433
rs7808802
rs182744657
rs117747703
rs72662850
rs9512488
rs115829880
rs6545815
rs4807487
rs138488830
rs78800990
rs376099962
rs73953232
rs1799561
rs7779835
All
−0.002
−0.001
0.000
0.001
MR effect size for
'Group XIIB secretory phospholipase A2−like protein' on 'eGFR'

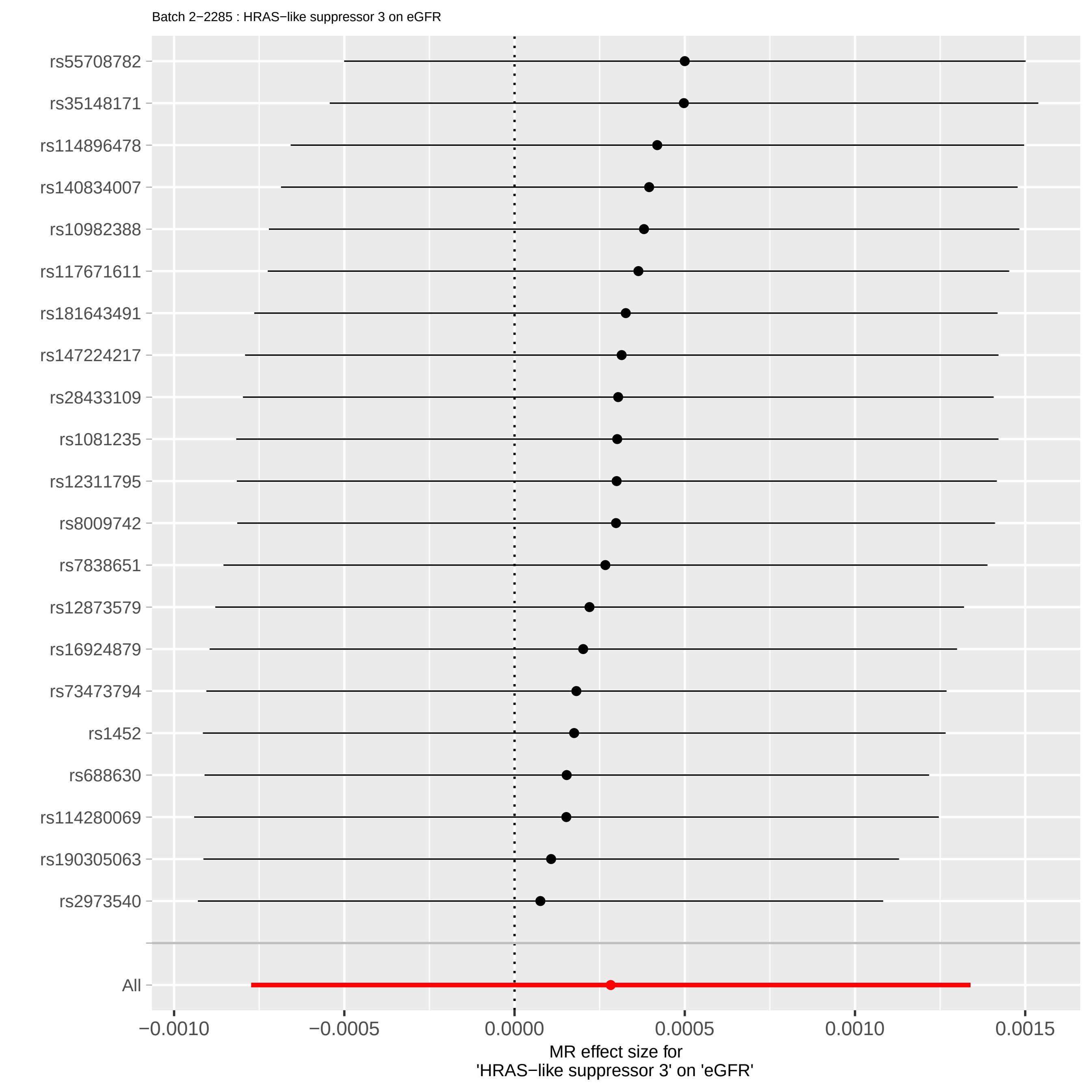

Batch 2−2285 : HRAS−like suppressor 3 on eGFR
rs55708782
rs35148171
rs114896478
rs140834007
rs10982388
rs117671611
rs181643491
rs147224217
rs28433109
rs1081235
rs12311795
rs8009742
rs7838651
rs12873579
rs16924879
rs73473794
rs1452
rs688630
rs114280069
rs190305063
rs2973540
All
−0.0010
−0.0005
0.0000
0.0005
0.0010
0.0015
MR effect size for
'HRAS−like suppressor 3' on 'eGFR'

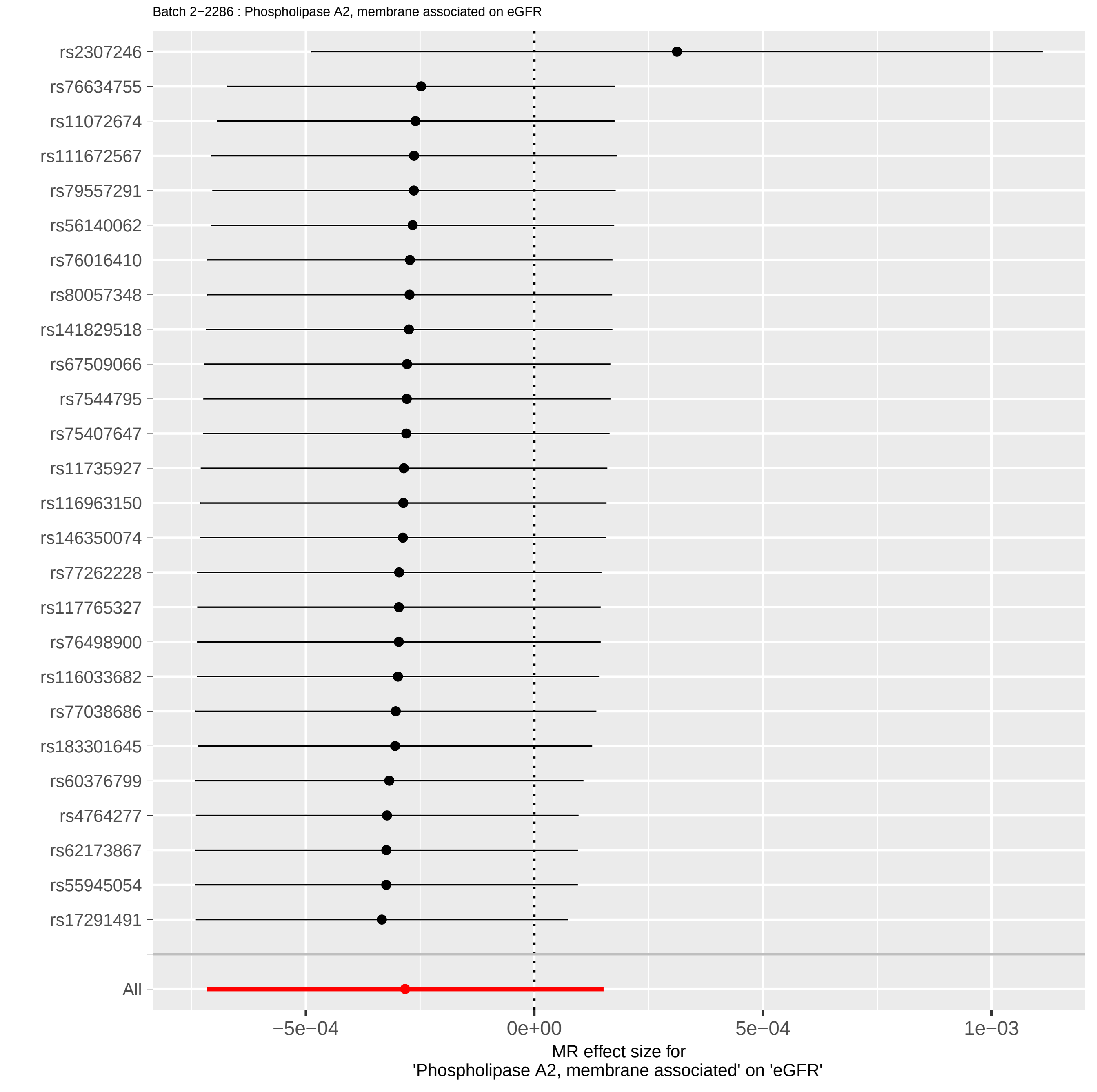

Batch 2−2286 : Phospholipase A2, membrane associated on eGFR
rs2307246
rs76634755
rs11072674
rs111672567
rs79557291
rs56140062
rs76016410
rs80057348
rs141829518
rs67509066
rs7544795
rs75407647
rs11735927
rs116963150
rs146350074
rs77262228
rs117765327
rs76498900
rs116033682
rs77038686
rs183301645
rs60376799
rs4764277
rs62173867
rs55945054
rs17291491
All
−5e−04
0e+00
5e−04
1e−03
MR effect size for
'Phospholipase A2, membrane associated' on 'eGFR'

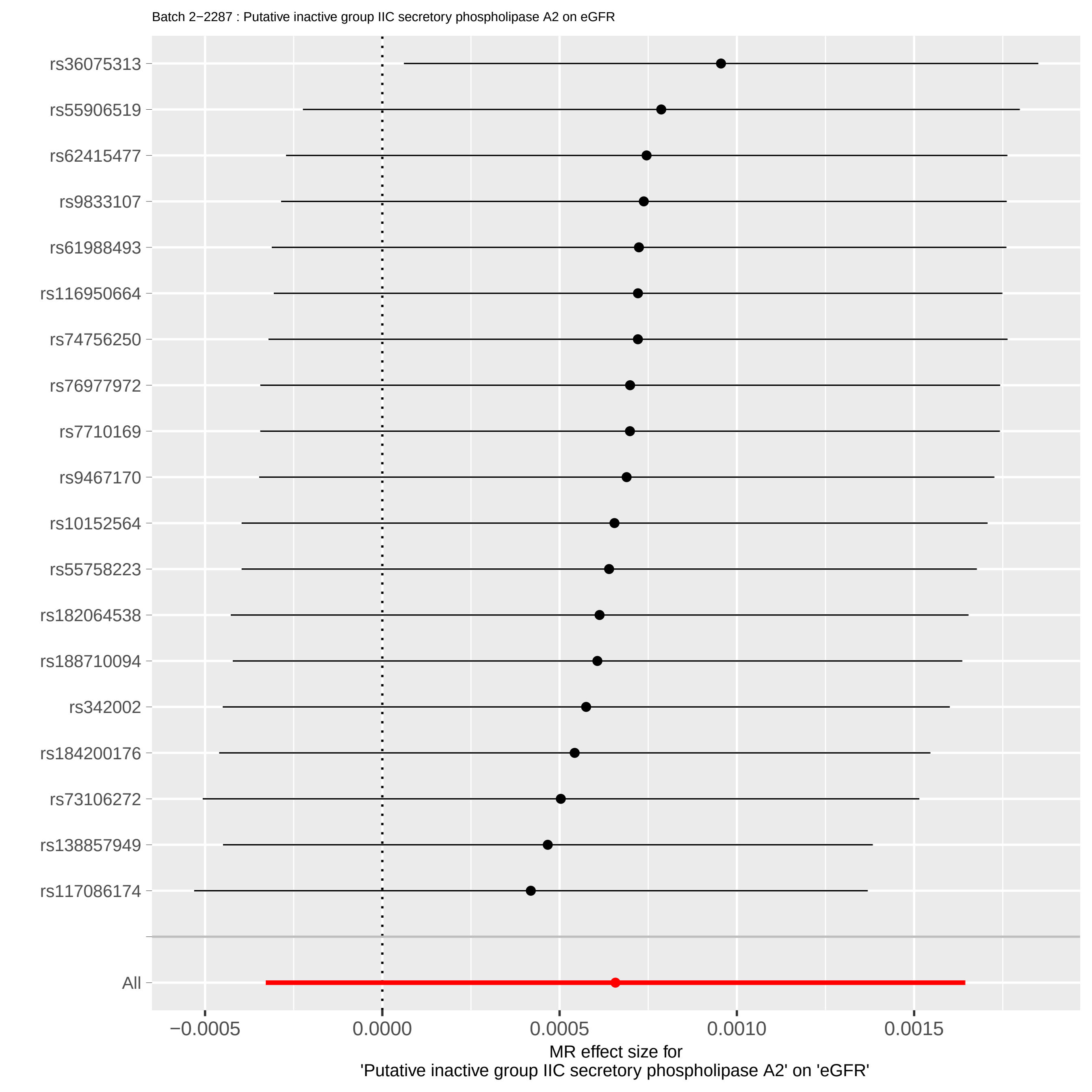

Batch 2−2287 : Putative inactive group IIC secretory phospholipase A2 on eGFR
rs36075313
rs55906519
rs62415477
rs9833107
rs61988493
rs116950664
rs74756250
rs76977972
rs7710169
rs9467170
rs10152564
rs55758223
rs182064538
rs188710094
rs342002
rs184200176
rs73106272
rs138857949
rs117086174
All
−0.0005
0.0000
0.0005
0.0010
0.0015
MR effect size for
'Putative inactive group IIC secretory phospholipase A2' on 'eGFR'

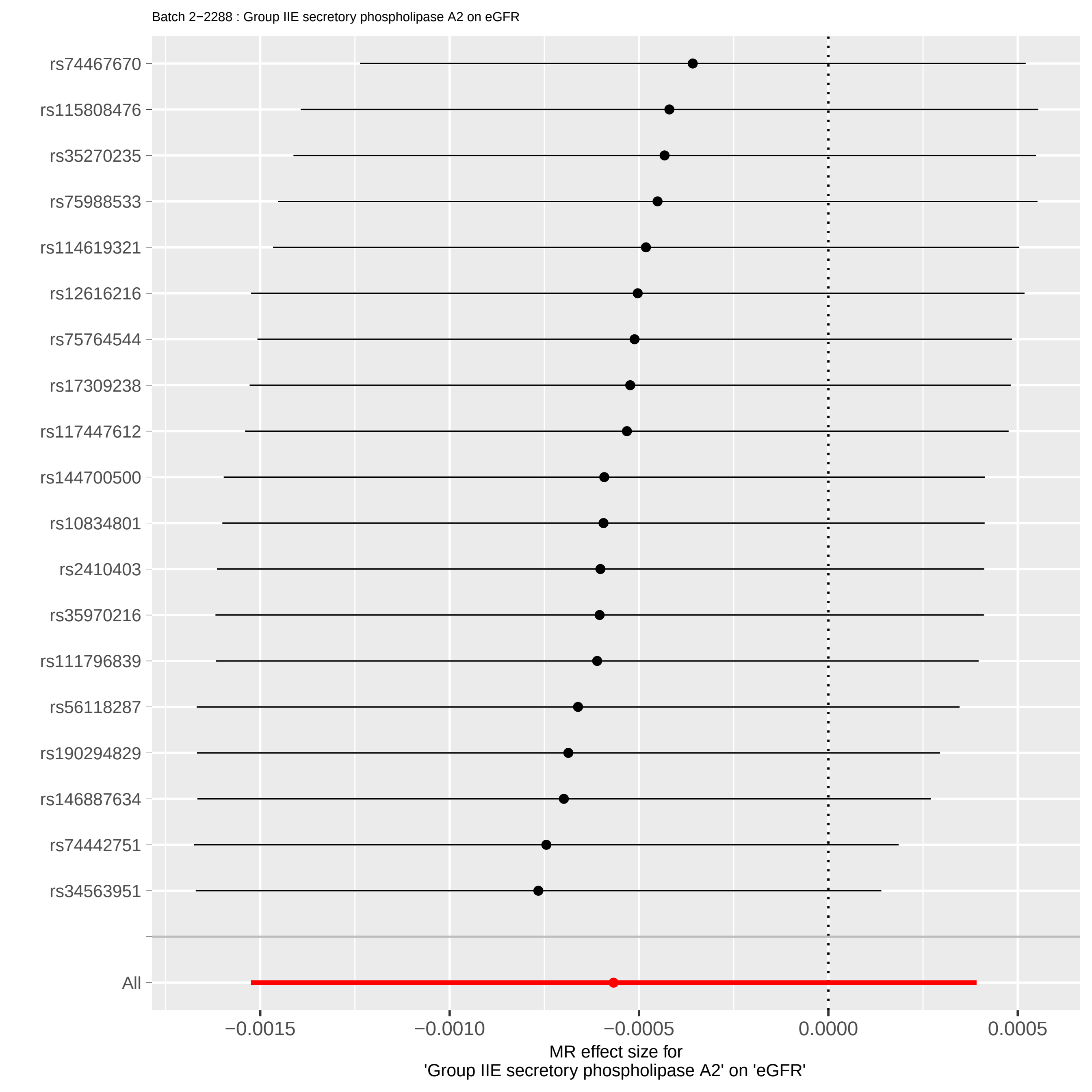

Batch 2−2288 : Group IIE secretory phospholipase A2 on eGFR
rs74467670
rs115808476
rs35270235
rs75988533
rs114619321
rs12616216
rs75764544
rs17309238
rs117447612
rs144700500
rs10834801
rs2410403
rs35970216
rs111796839
rs56118287
rs190294829
rs146887634
rs74442751
rs34563951
All
−0.0015
−0.0010
−0.0005
0.0000
0.0005
MR effect size for
'Group IIE secretory phospholipase A2' on 'eGFR'

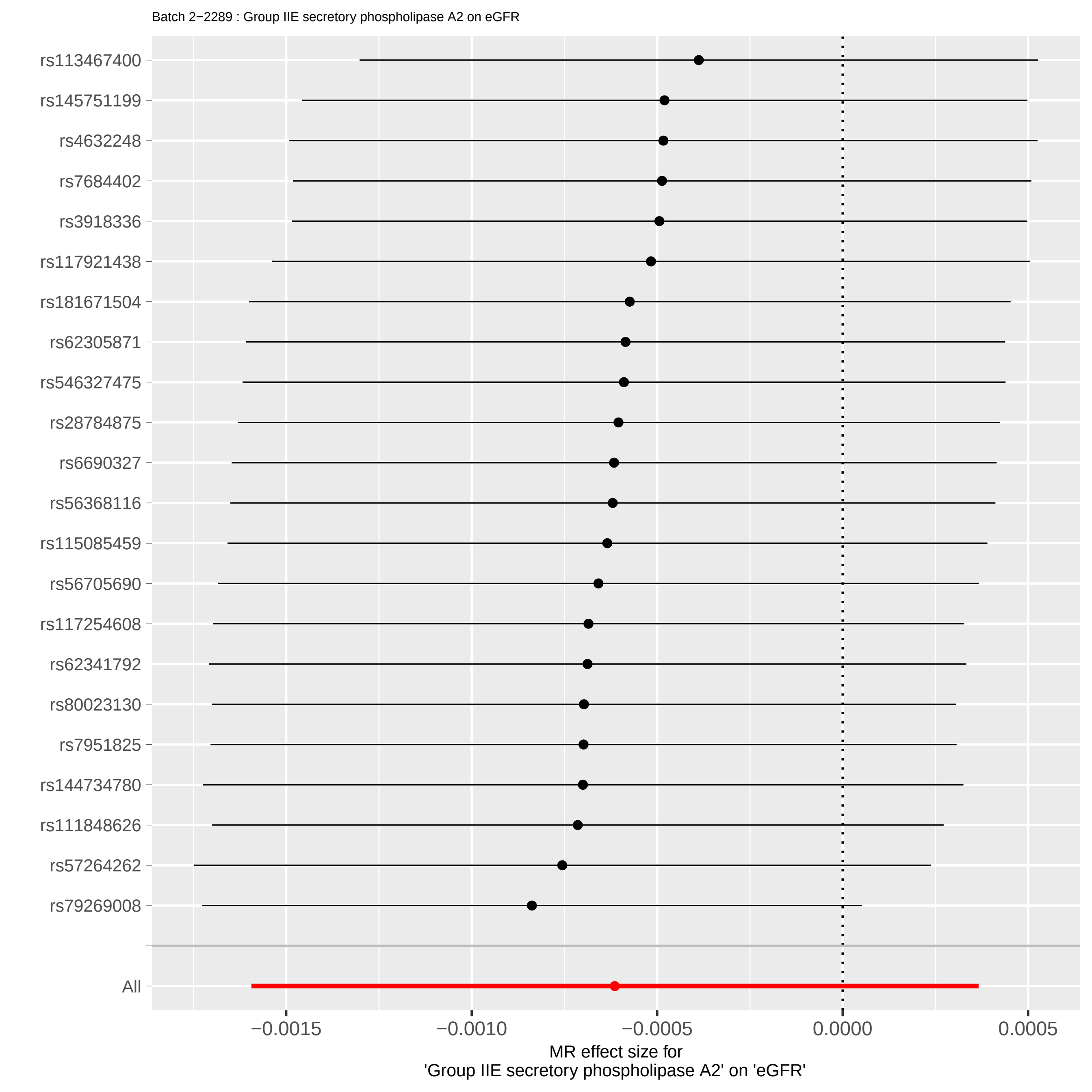

Batch 2−2289 : Group IIE secretory phospholipase A2 on eGFR
rs113467400
rs145751199
rs4632248
rs7684402
rs3918336
rs117921438
rs181671504
rs62305871
rs546327475
rs28784875
rs6690327
rs56368116
rs115085459
rs56705690
rs117254608
rs62341792
rs80023130
rs7951825
rs144734780
rs111848626
rs57264262
rs79269008
All
−0.0015
−0.0010
−0.0005
0.0000
0.0005
MR effect size for
'Group IIE secretory phospholipase A2' on 'eGFR'

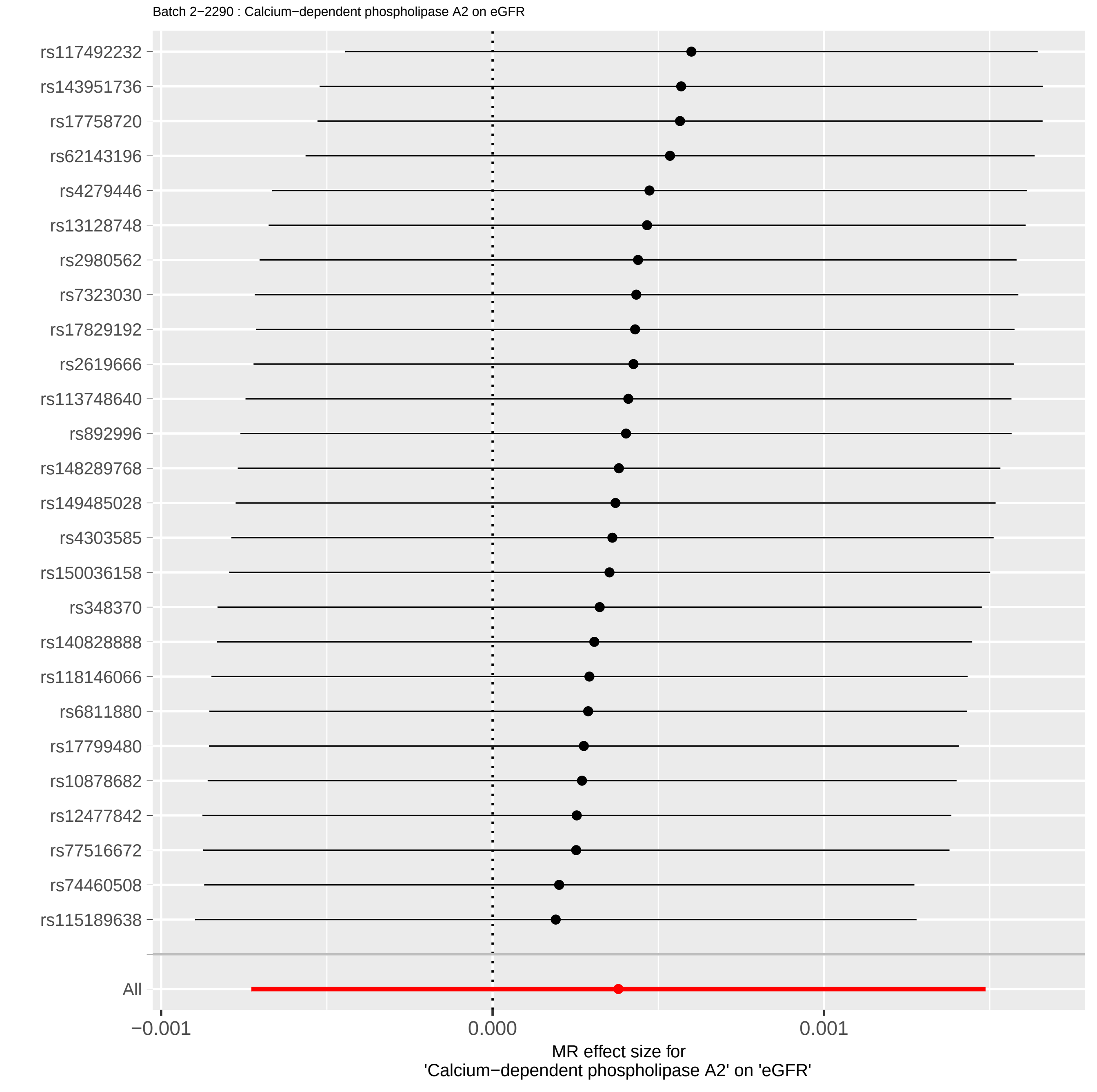

Batch 2−2290 : Calcium−dependent phospholipase A2 on eGFR
rs117492232
rs143951736
rs17758720
rs62143196
rs4279446
rs13128748
rs2980562
rs7323030
rs17829192
rs2619666
rs113748640
rs892996
rs148289768
rs149485028
rs4303585
rs150036158
rs348370
rs140828888
rs118146066
rs6811880
rs17799480
rs10878682
rs12477842
rs77516672
rs74460508
rs115189638
All
−0.001
0.000
0.001
MR effect size for
'Calcium−dependent phospholipase A2' on 'eGFR'

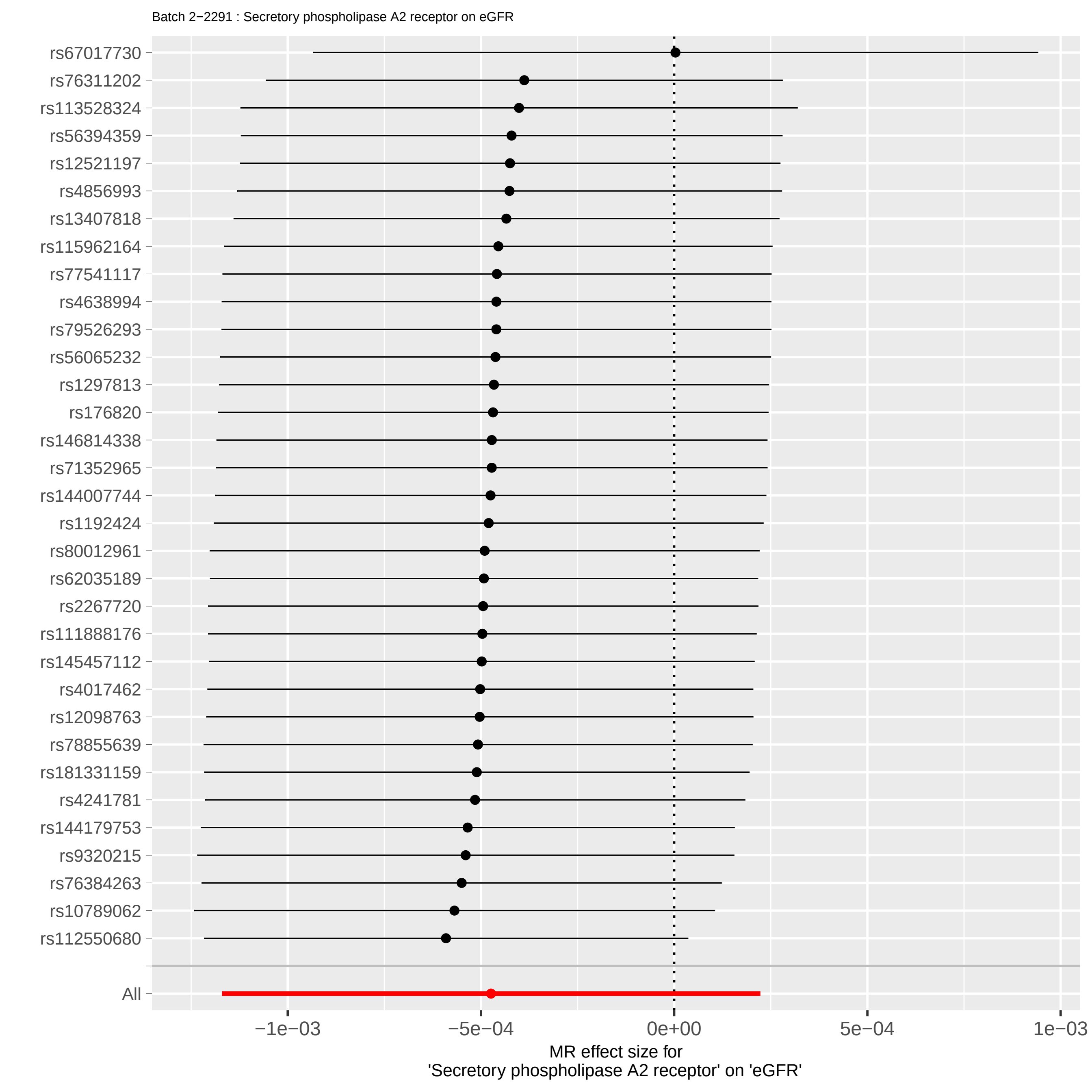
Batch 2−2291 : Secretory phospholipase A2 receptor on eGFR
rs67017730
rs76311202
rs113528324
rs56394359
rs12521197
rs4856993
rs13407818
rs115962164
rs77541117
rs4638994
rs79526293
rs56065232
rs1297813
rs176820
rs146814338
rs71352965
rs144007744
rs1192424
rs80012961
rs62035189
rs2267720
rs111888176
rs145457112
rs4017462
rs12098763
rs78855639
rs181331159
rs4241781
rs144179753
rs9320215
rs76384263
rs10789062
rs112550680
All
−1e−03
−5e−04
0e+00
5e−04
1e−03
MR effect size for
'Secretory phospholipase A2 receptor' on 'eGFR'

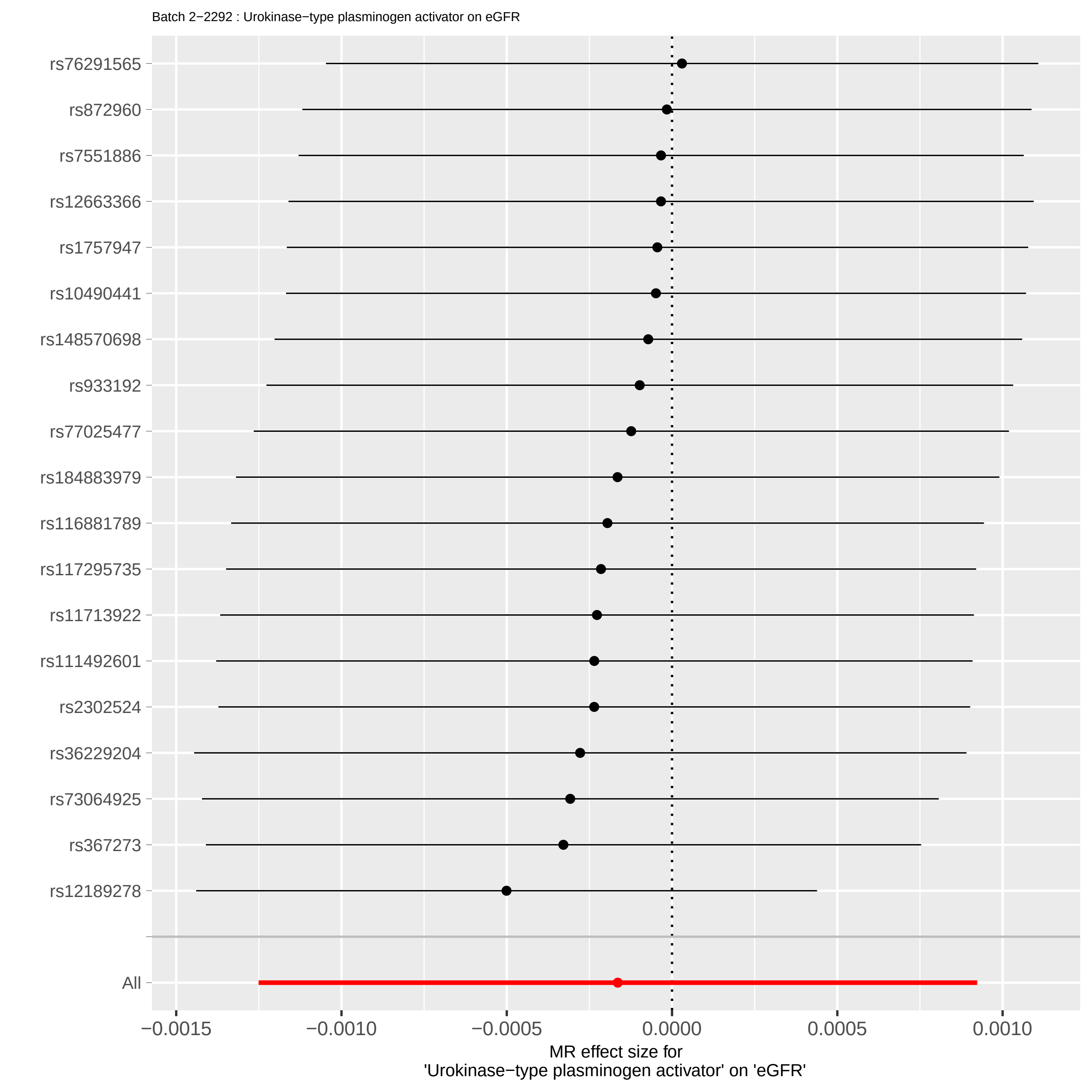

Batch 2−2292 : Urokinase−type plasminogen activator on eGFR
rs76291565
rs872960
rs7551886
rs12663366
rs1757947
rs10490441
rs148570698
rs933192
rs77025477
rs184883979
rs116881789
rs117295735
rs11713922
rs111492601
rs2302524
rs36229204
rs73064925
rs367273
rs12189278
All
−0.0015
−0.0010
−0.0005
0.0000
0.0005
0.0010
MR effect size for
'Urokinase−type plasminogen activator' on 'eGFR'

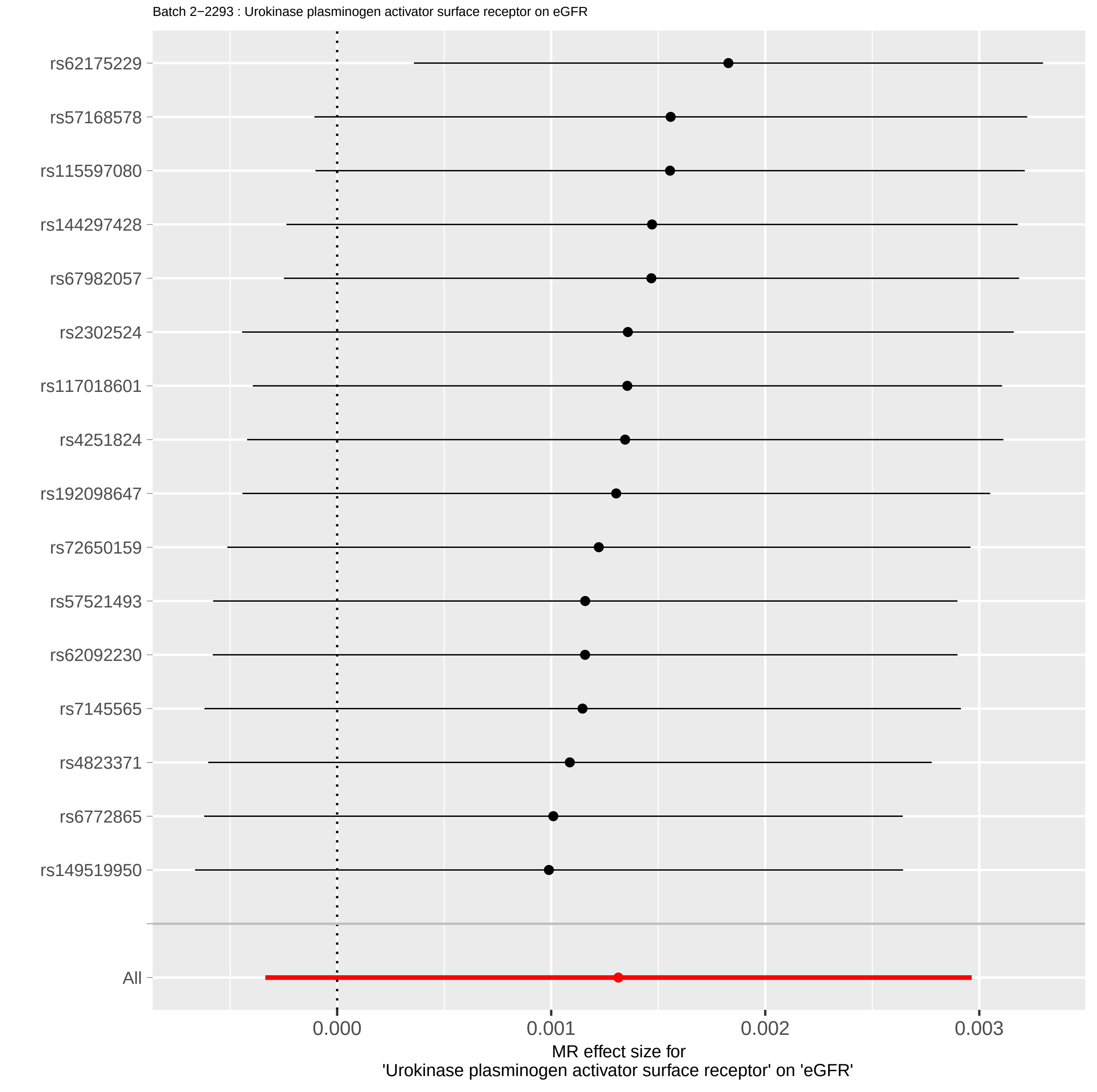

Batch 2−2293 : Urokinase plasminogen activator surface receptor on eGFR
rs62175229
rs57168578
rs115597080
rs144297428
rs67982057
rs2302524
rs117018601
rs4251824
rs192098647
rs72650159
rs57521493
rs62092230
rs7145565
rs4823371
rs6772865
rs149519950
All
0.000
0.001
0.002
0.003
MR effect size for
'Urokinase plasminogen activator surface receptor' on 'eGFR'

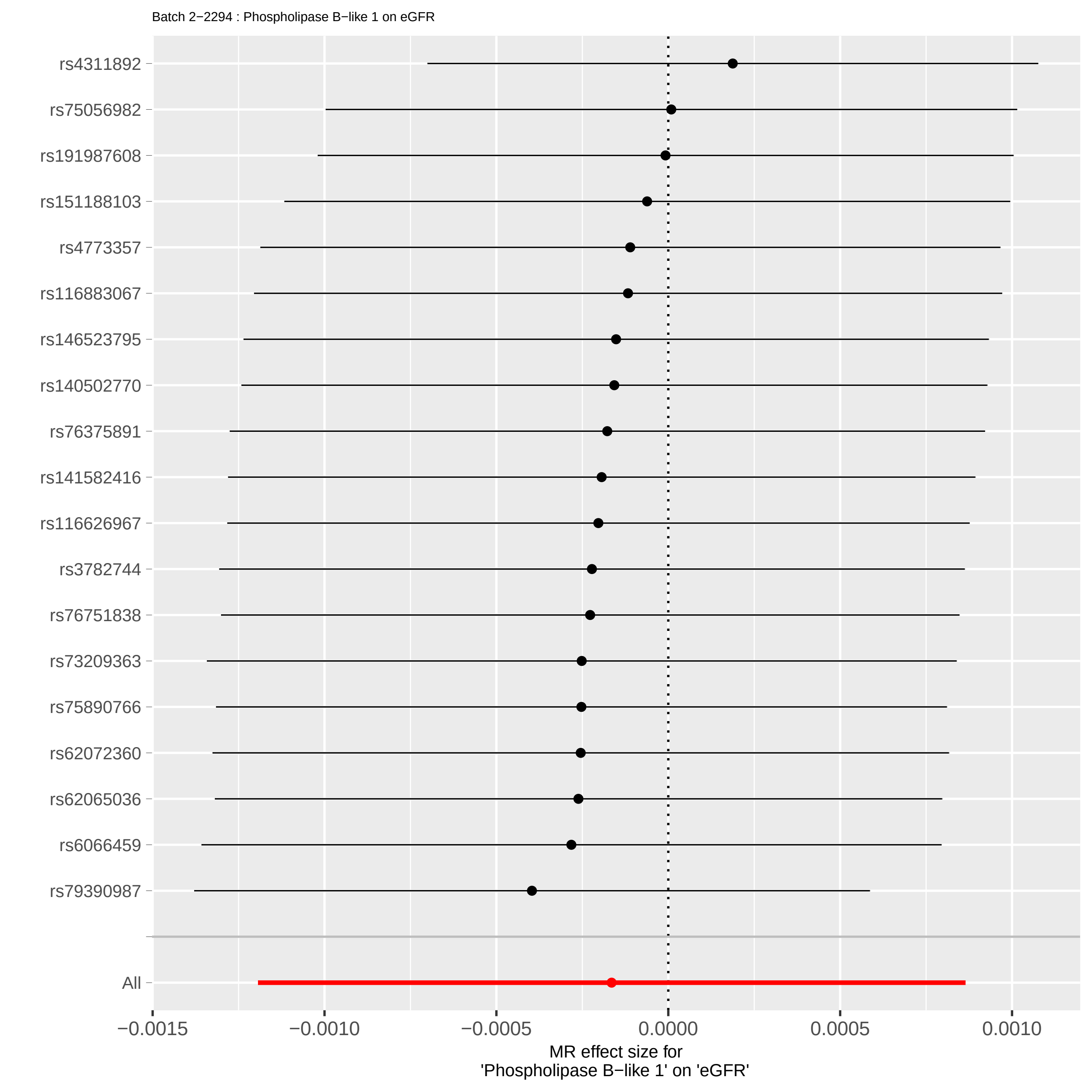

Batch 2−2294 : Phospholipase B−like 1 on eGFR
rs4311892
rs75056982
rs191987608
rs151188103
rs4773357
rs116883067
rs146523795
rs140502770
rs76375891
rs141582416
rs116626967
rs3782744
rs76751838
rs73209363
rs75890766
rs62072360
rs62065036
rs6066459
rs79390987
All
−0.0015
−0.0010
−0.0005
0.0000
0.0005
0.0010
MR effect size for
'Phospholipase B−like 1' on 'eGFR'

Batch 2−2295 : Putative phospholipase B−like 2 on eGFR

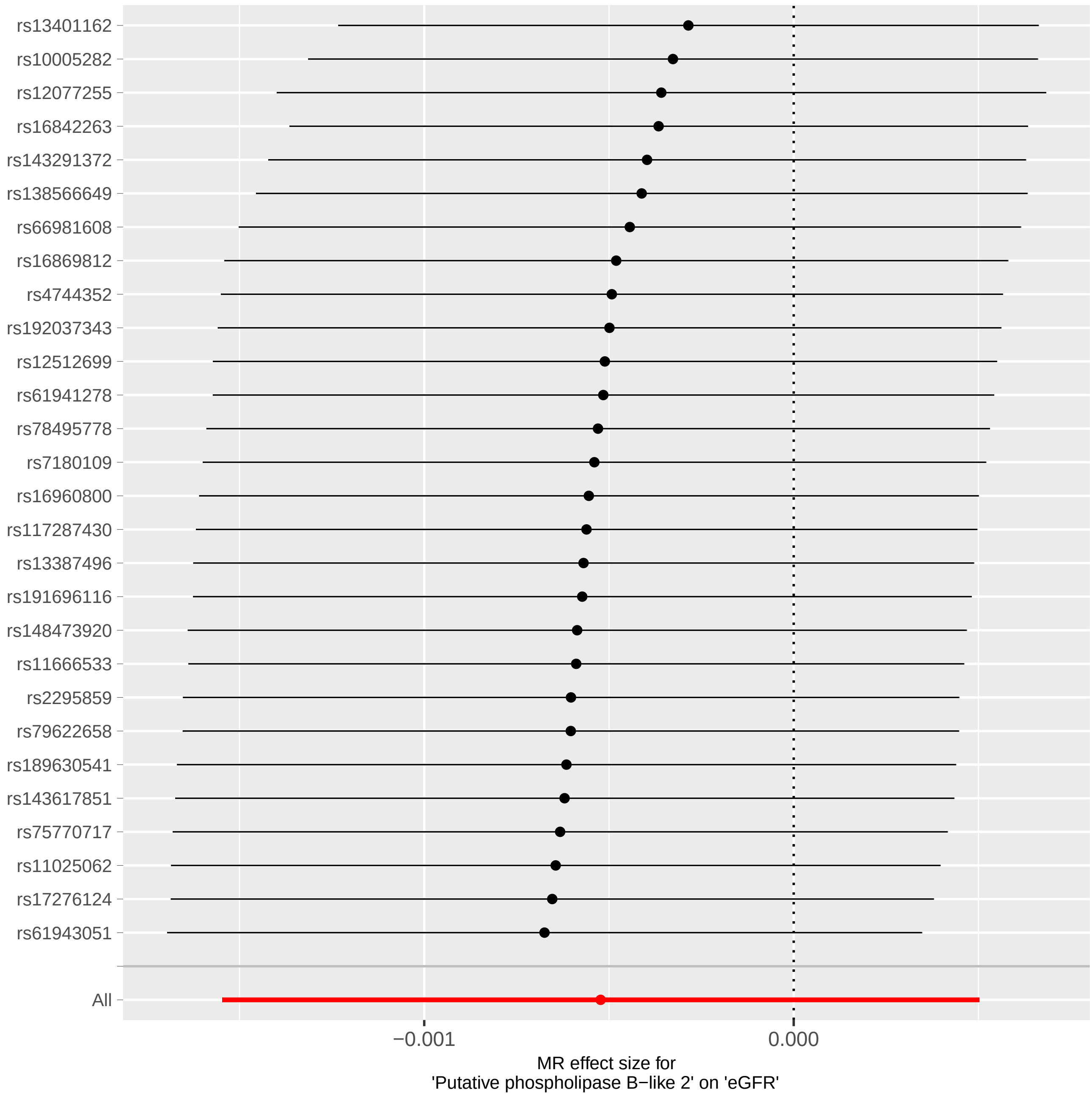

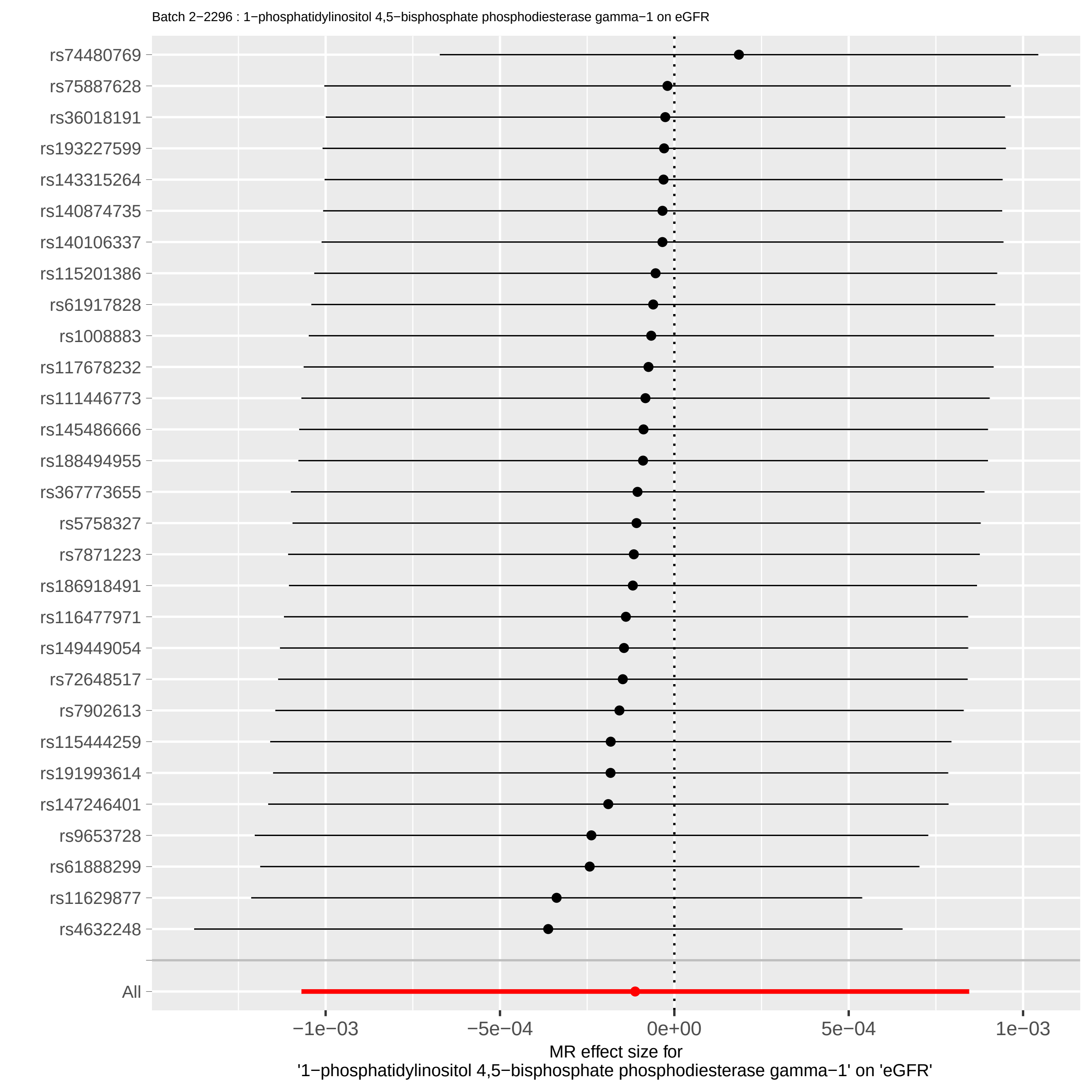

Batch 2−2296 : 1−phosphatidylinositol 4,5−bisphosphate phosphodiesterase gamma−1 on eGFR
rs74480769
rs75887628
rs36018191
rs193227599
rs143315264
rs140874735
rs140106337
rs115201386
rs61917828
rs1008883
rs117678232
rs111446773
rs145486666
rs188494955
rs367773655
rs5758327
rs7871223
rs186918491
rs116477971
rs149449054
rs72648517
rs7902613
rs115444259
rs191993614
rs147246401
rs9653728
rs61888299
rs11629877
rs4632248
All
−1e−03
−5e−04
0e+00
5e−04
1e−03
MR effect size for
'1−phosphatidylinositol 4,5−bisphosphate phosphodiesterase gamma−1' on 'eGFR'

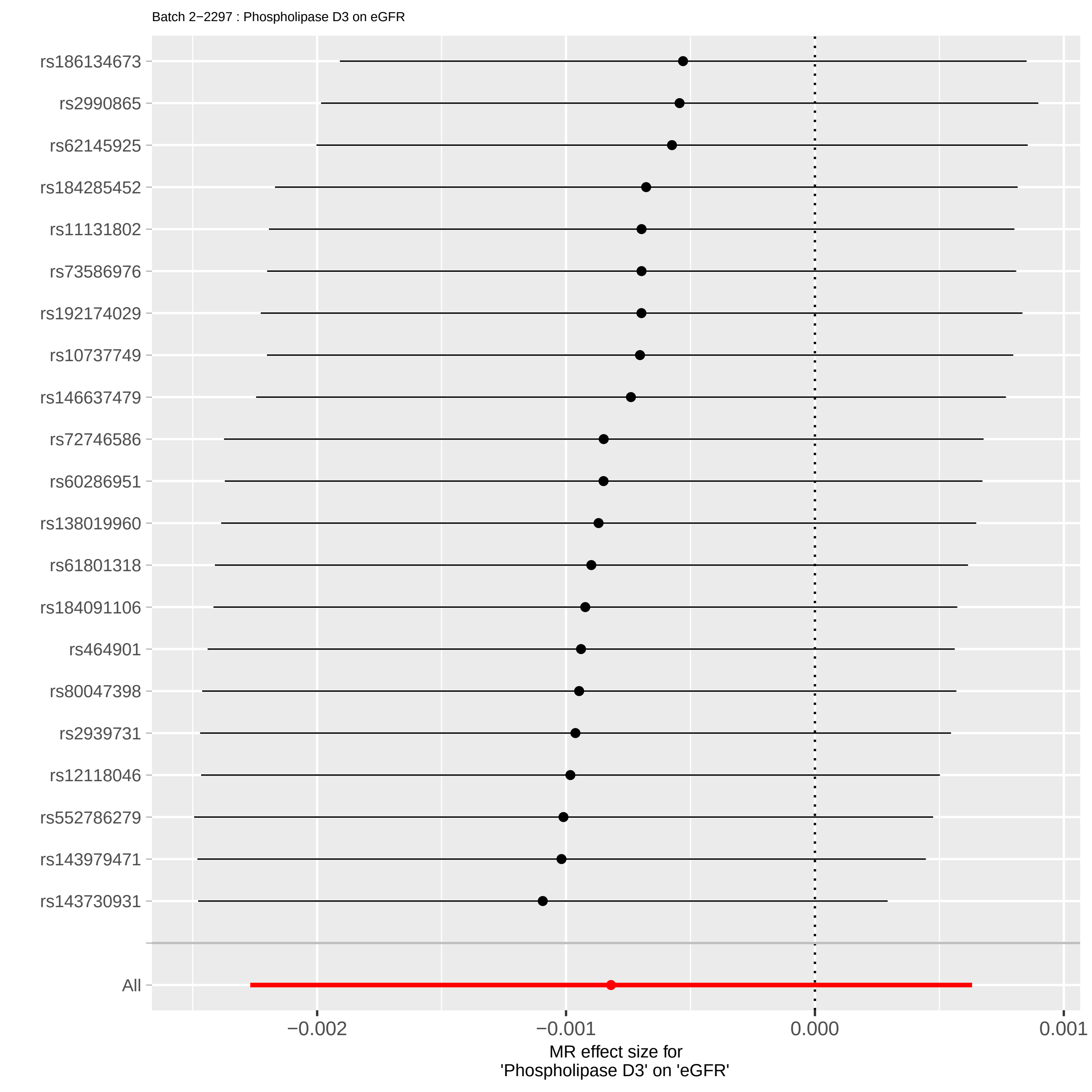

Batch 2−2297 : Phospholipase D3 on eGFR
rs186134673
rs2990865
rs62145925
rs184285452
rs11131802
rs73586976
rs192174029
rs10737749
rs146637479
rs72746586
rs60286951
rs138019960
rs61801318
rs184091106
rs464901
rs80047398
rs2939731
rs12118046
rs552786279
rs143979471
rs143730931
All
−0.002
−0.001
0.000
0.001
MR effect size for
'Phospholipase D3' on 'eGFR'

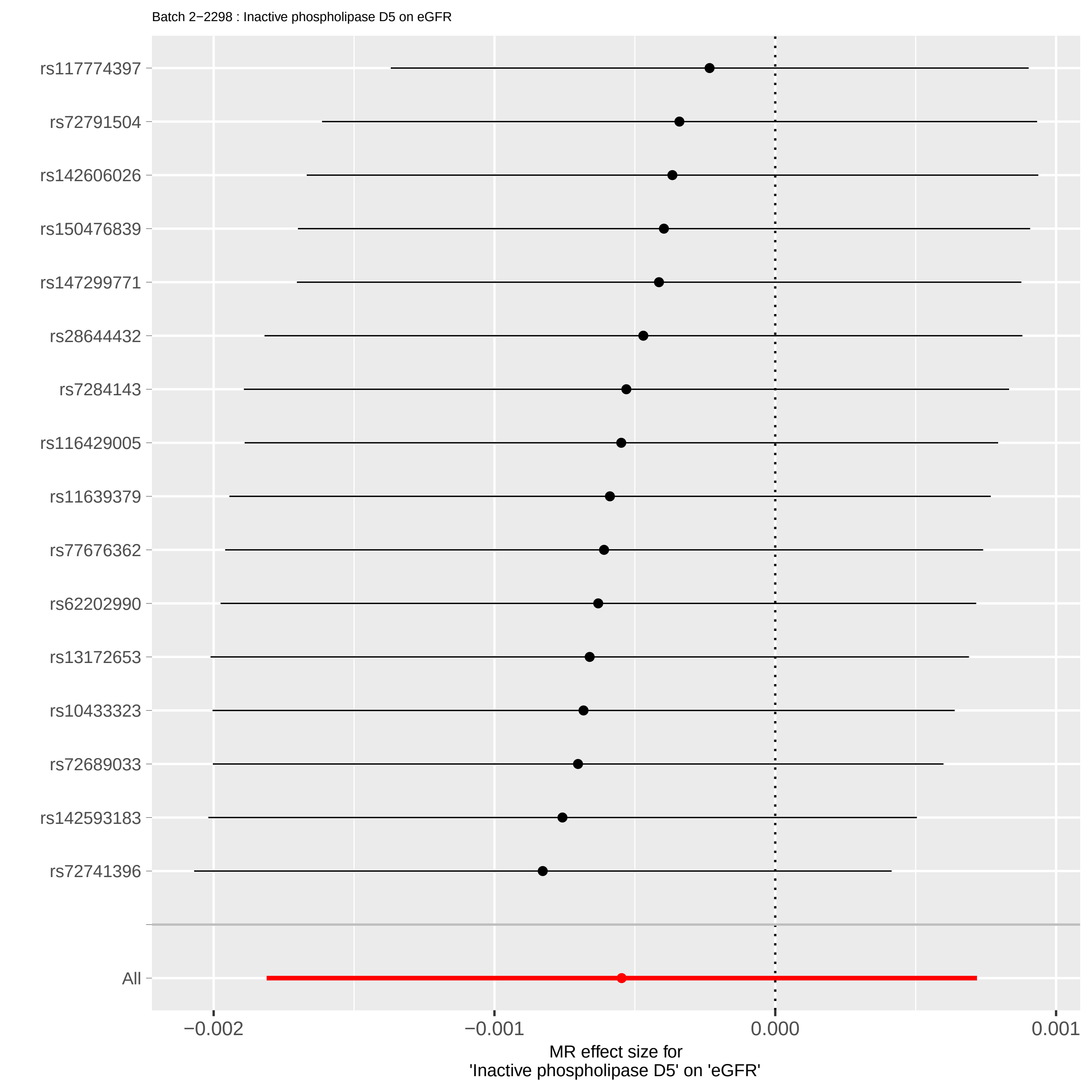

Batch 2−2298 : Inactive phospholipase D5 on eGFR
rs117774397
rs72791504
rs142606026
rs150476839
rs147299771
rs28644432
rs7284143
rs116429005
rs11639379
rs77676362
rs62202990
rs13172653
rs10433323
rs72689033
rs142593183
rs72741396
All
−0.002
−0.001
0.000
0.001
MR effect size for
'Inactive phospholipase D5' on 'eGFR'

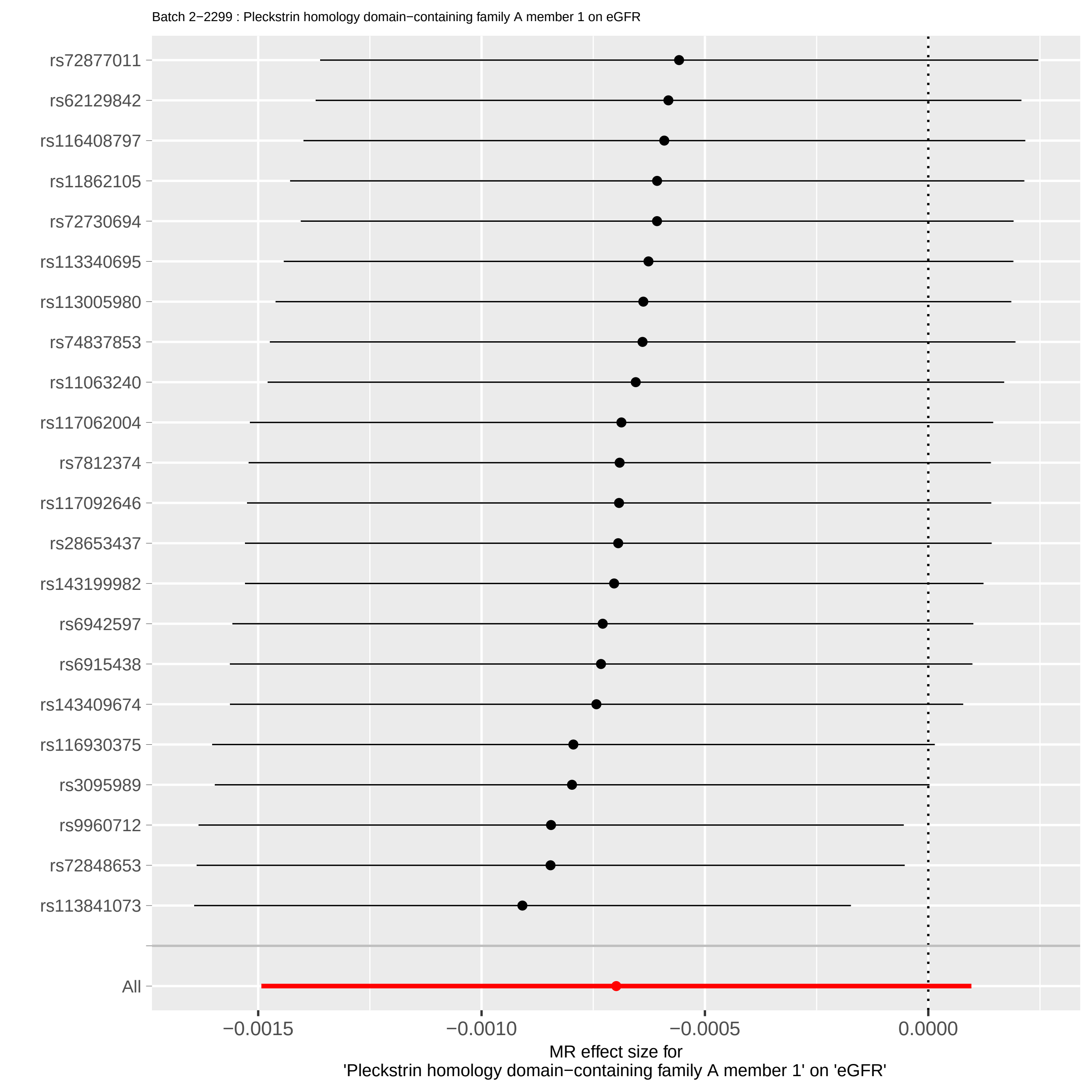

Batch 2−2299 : Pleckstrin homology domain−containing family A member 1 on eGFR
rs72877011
rs62129842
rs116408797
rs11862105
rs72730694
rs113340695
rs113005980
rs74837853
rs11063240
rs117062004
rs7812374
rs117092646
rs28653437
rs143199982
rs6942597
rs6915438
rs143409674
rs116930375
rs3095989
rs9960712
rs72848653
rs113841073
All
−0.0015
−0.0010
−0.0005
0.0000
MR effect size for
'Pleckstrin homology domain−containing family A member 1' on 'eGFR'

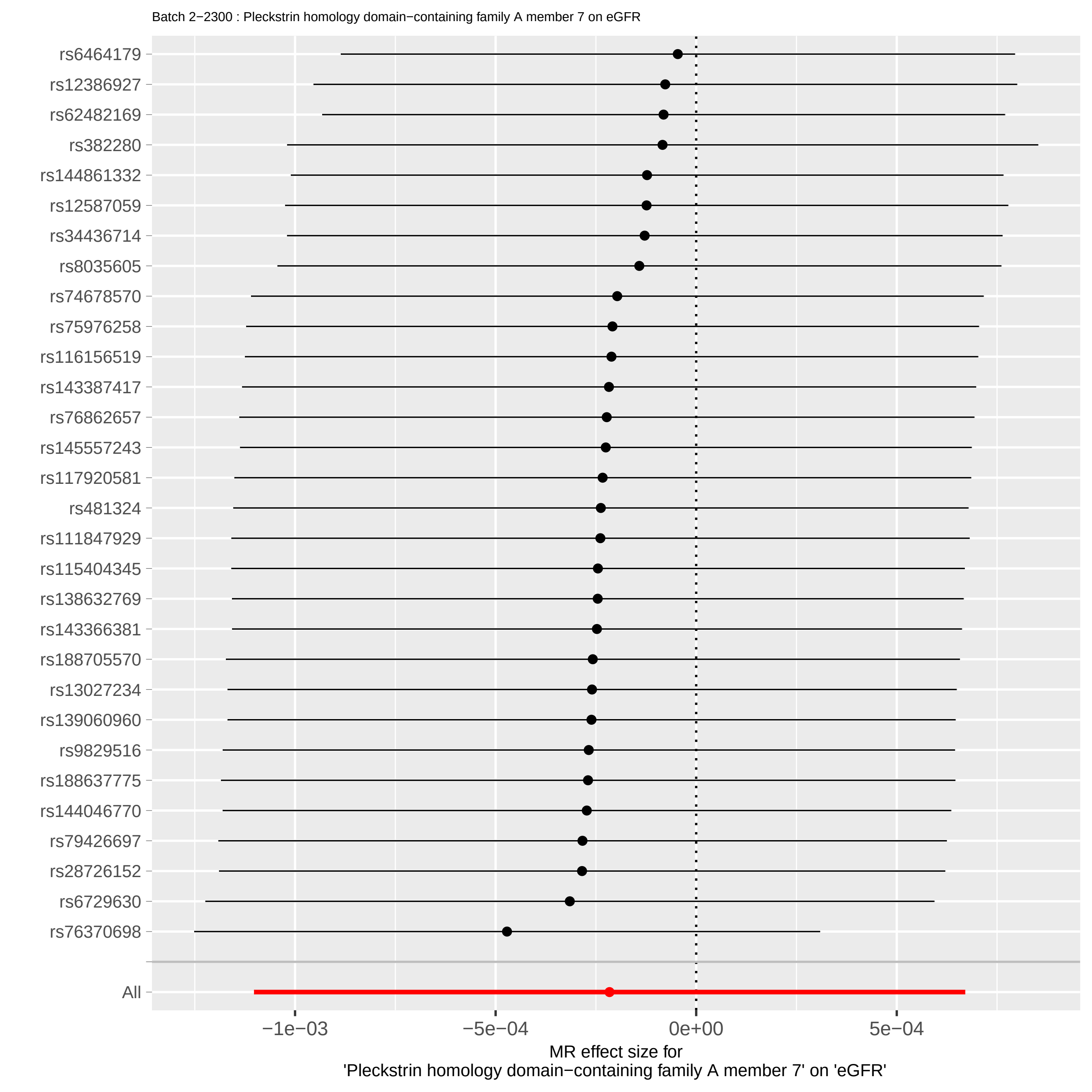

Batch 2−2300 : Pleckstrin homology domain−containing family A member 7 on eGFR
rs6464179
rs12386927
rs62482169
rs382280
rs144861332
rs12587059
rs34436714
rs8035605
rs74678570
rs75976258
rs116156519
rs143387417
rs76862657
rs145557243
rs117920581
rs481324
rs111847929
rs115404345
rs138632769
rs143366381
rs188705570
rs13027234
rs139060960
rs9829516
rs188637775
rs144046770
rs79426697
rs28726152
rs6729630
rs76370698
All
−1e−03
−5e−04
0e+00
5e−04
MR effect size for
'Pleckstrin homology domain−containing family A member 7' on 'eGFR'

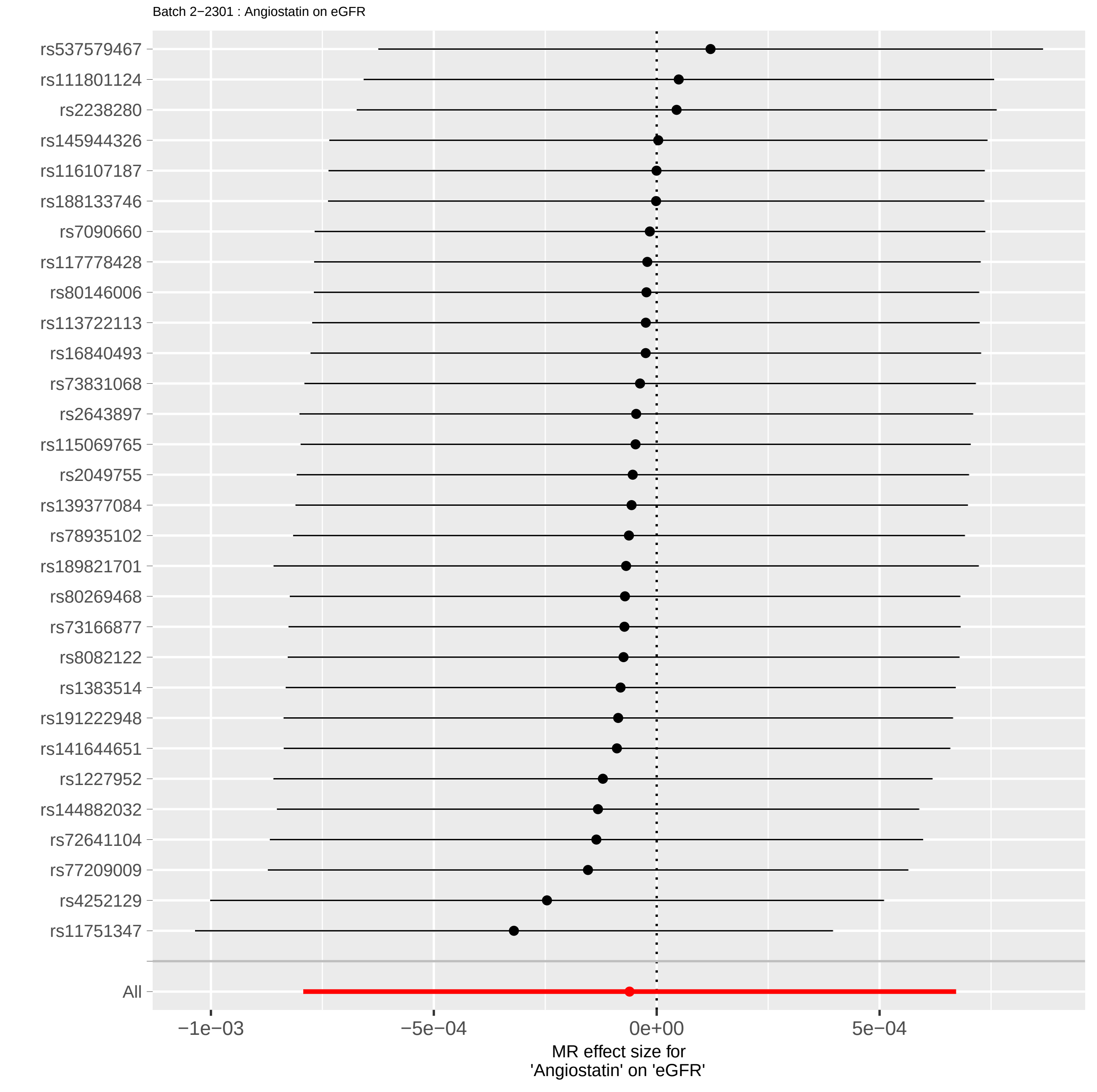

Batch 2−2301 : Angiostatin on eGFR
rs537579467
rs111801124
rs2238280
rs145944326
rs116107187
rs188133746
rs7090660
rs117778428
rs80146006
rs113722113
rs16840493
rs73831068
rs2643897
rs115069765
rs2049755
rs139377084
rs78935102
rs189821701
rs80269468
rs73166877
rs8082122
rs1383514
rs191222948
rs141644651
rs1227952
rs144882032
rs72641104
rs77209009
rs4252129
rs11751347
All
−1e−03
−5e−04
0e+00
5e−04
MR effect size for
'Angiostatin' on 'eGFR'

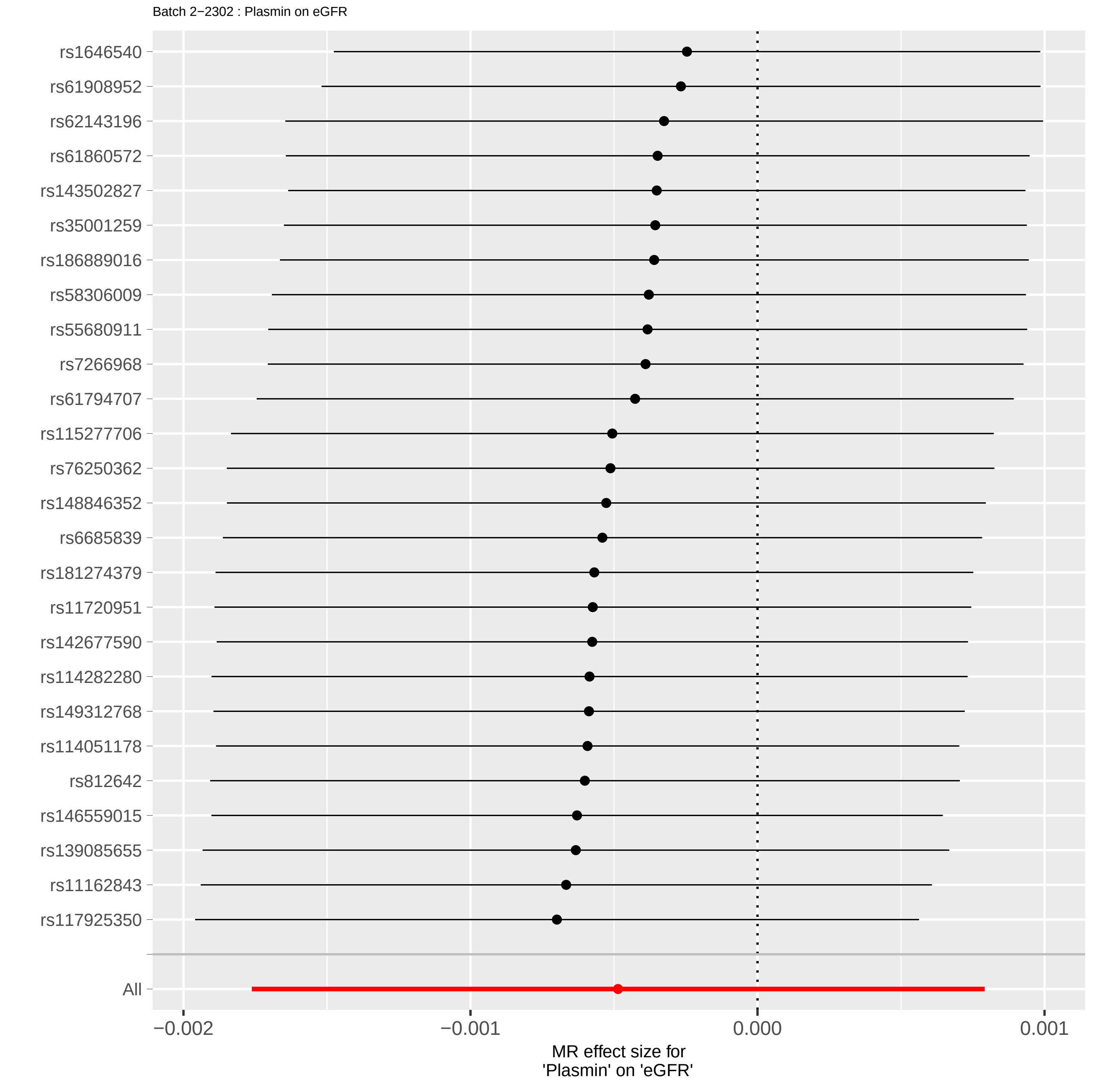

Batch 2−2302 : Plasmin on eGFR
rs1646540
rs61908952
rs62143196
rs61860572
rs143502827
rs35001259
rs186889016
rs58306009
rs55680911
rs7266968
rs61794707
rs115277706
rs76250362
rs148846352
rs6685839
rs181274379
rs11720951
rs142677590
rs114282280
rs149312768
rs114051178
rs812642
rs146559015
rs139085655
rs11162843
rs117925350
All
−0.002
−0.001
0.000
0.001
MR effect size for
'Plasmin' on 'eGFR'

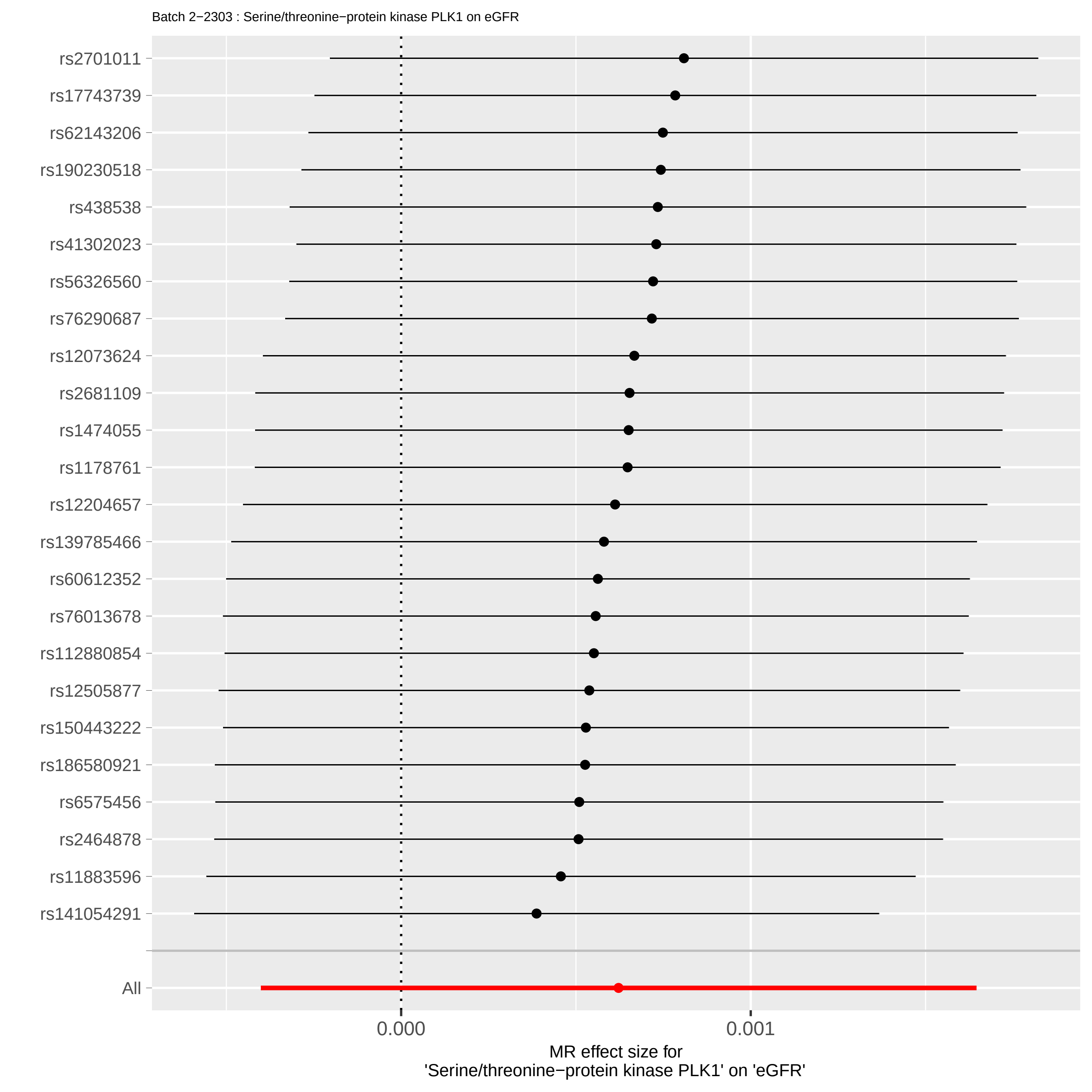

Batch 2−2303 : Serine/threonine−protein kinase PLK1 on eGFR
rs2701011
rs17743739
rs62143206
rs190230518
rs438538
rs41302023
rs56326560
rs76290687
rs12073624
rs2681109
rs1474055
rs1178761
rs12204657
rs139785466
rs60612352
rs76013678
rs112880854
rs12505877
rs150443222
rs186580921
rs6575456
rs2464878
rs11883596
rs141054291
All
0.000
0.001
MR effect size for
'Serine/threonine−protein kinase PLK1' on 'eGFR'

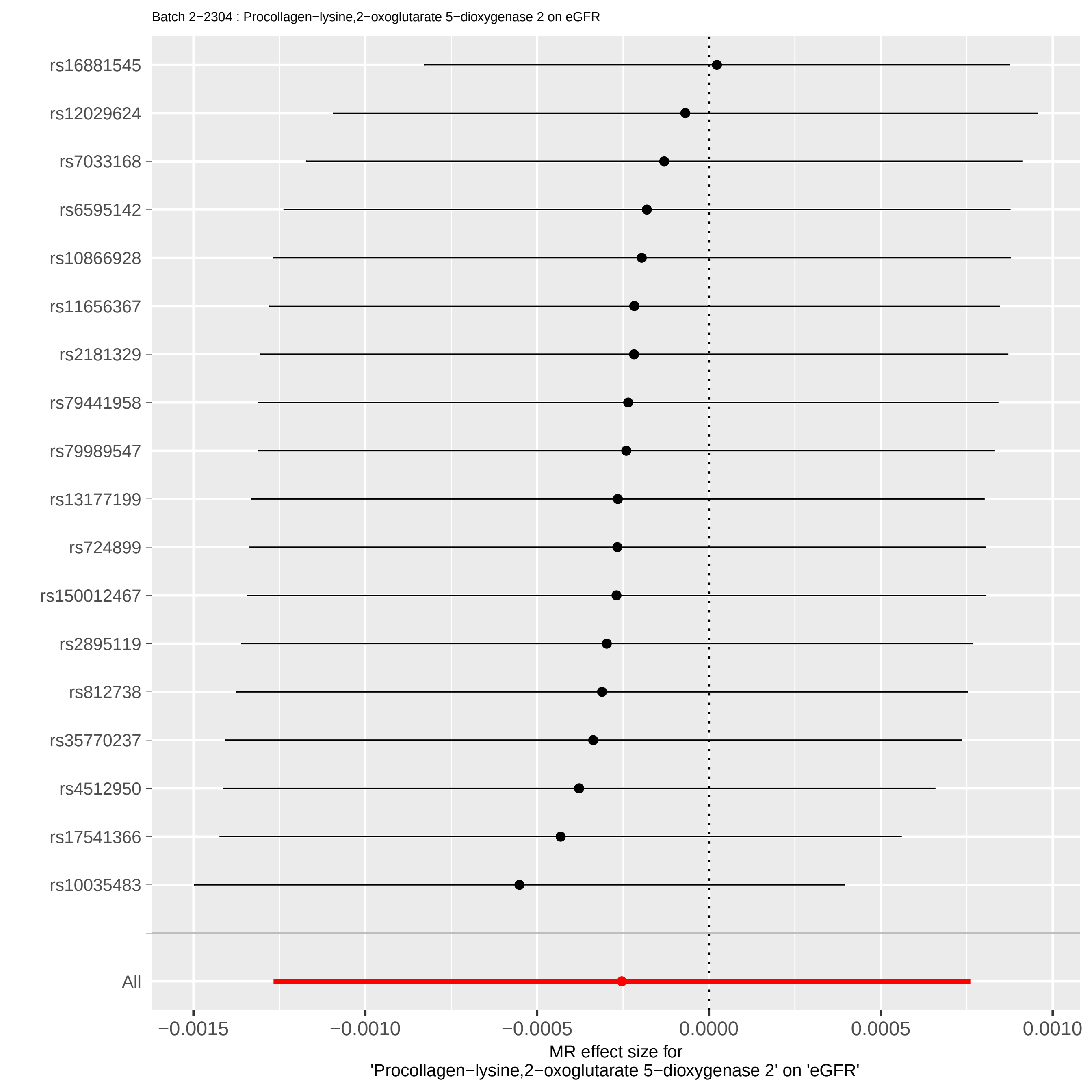
Batch 2−2304 : Procollagen−lysine,2−oxoglutarate 5−dioxygenase 2 on eGFR
rs16881545
rs12029624
rs7033168
rs6595142
rs10866928
rs11656367
rs2181329
rs79441958
rs79989547
rs13177199
rs724899
rs150012467
rs2895119
rs812738
rs35770237
rs4512950
rs17541366
rs10035483
All
−0.0015
−0.0010
−0.0005
0.0000
0.0005
0.0010
MR effect size for
'Procollagen−lysine,2−oxoglutarate 5−dioxygenase 2' on 'eGFR'

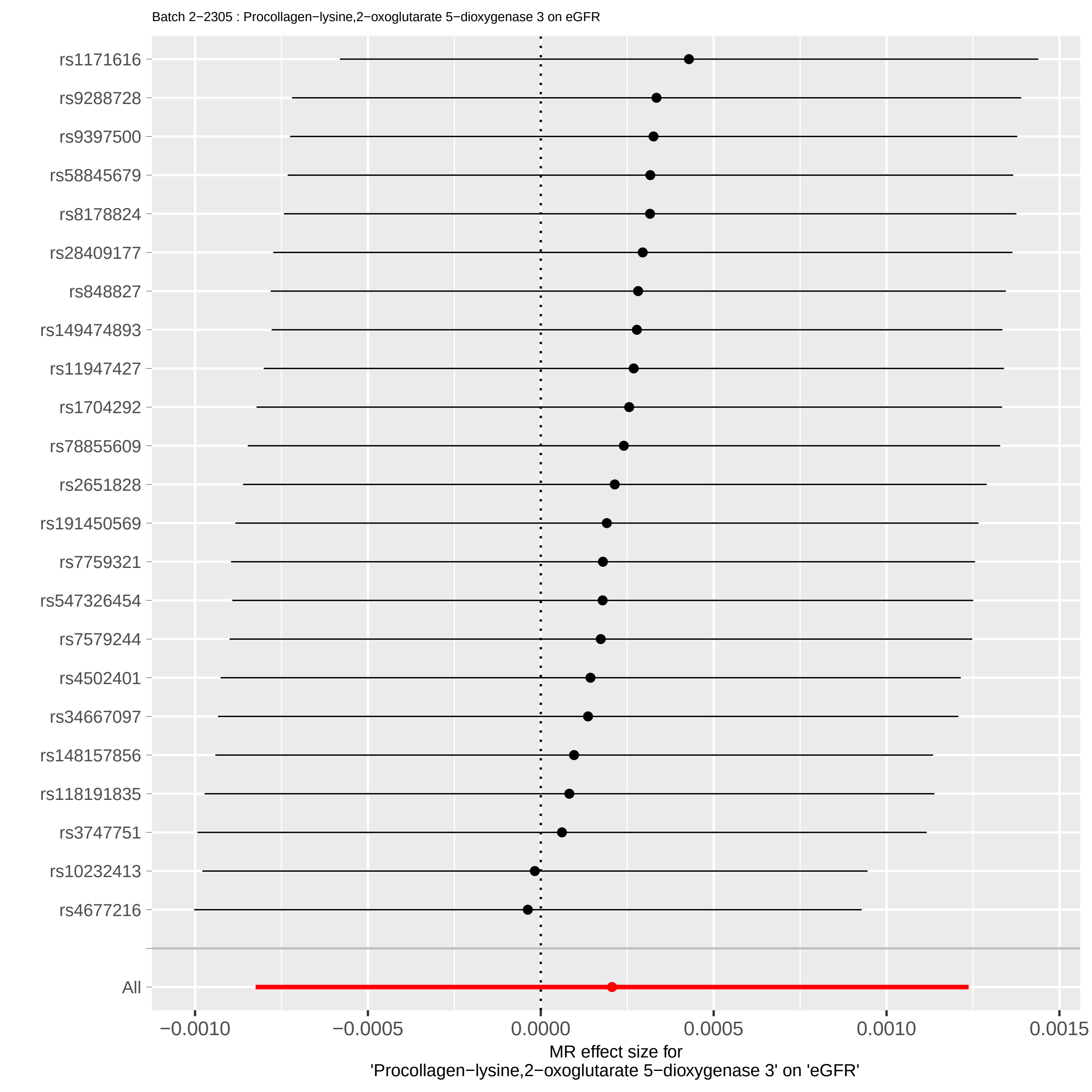

Batch 2−2305 : Procollagen−lysine,2−oxoglutarate 5−dioxygenase 3 on eGFR
rs1171616
rs9288728
rs9397500
rs58845679
rs8178824
rs28409177
rs848827
rs149474893
rs11947427
rs1704292
rs78855609
rs2651828
rs191450569
rs7759321
rs547326454
rs7579244
rs4502401
rs34667097
rs148157856
rs118191835
rs3747751
rs10232413
rs4677216
All
−0.0010
−0.0005
0.0000
0.0005
0.0010
0.0015
MR effect size for
'Procollagen−lysine,2−oxoglutarate 5−dioxygenase 3' on 'eGFR'

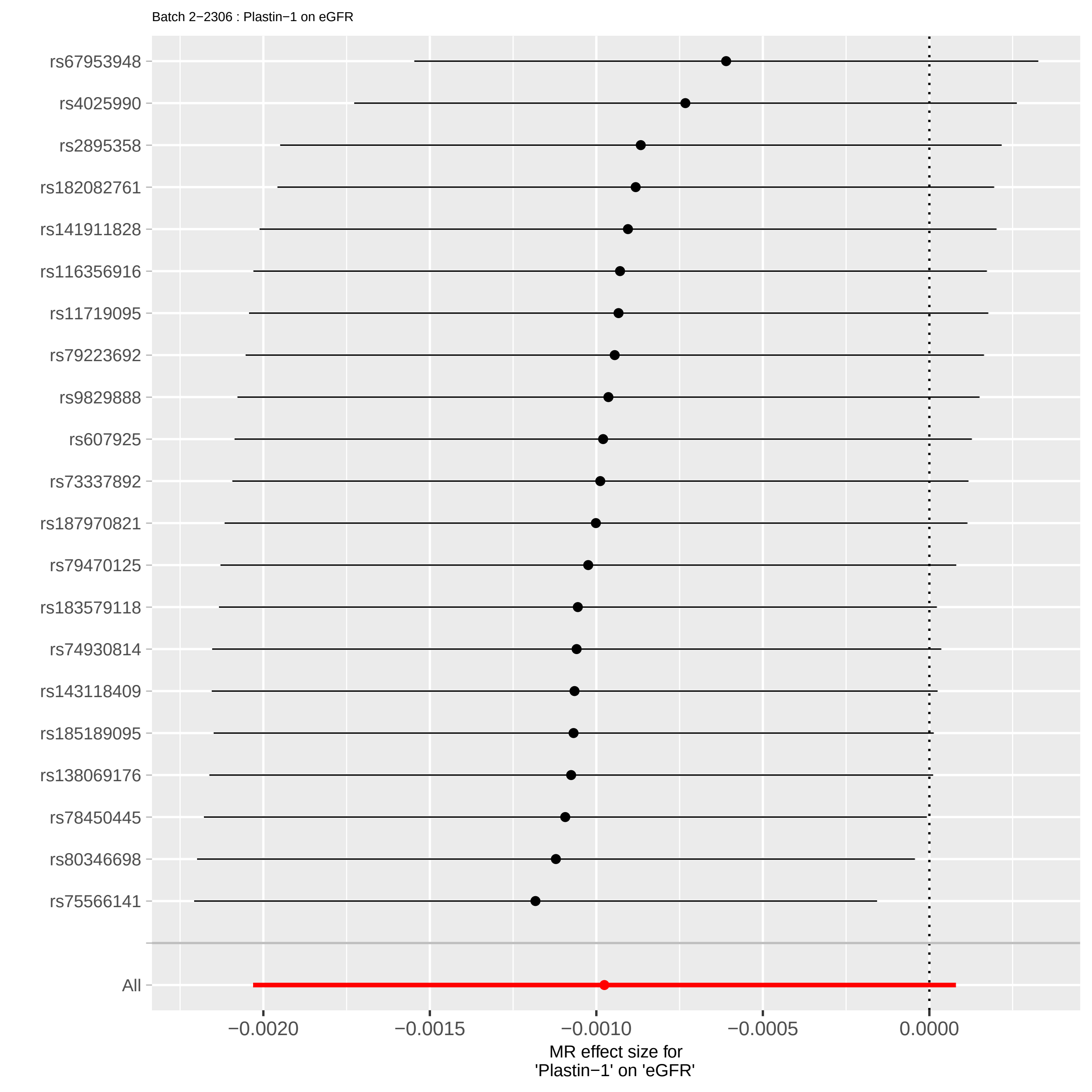

Batch 2−2306 : Plastin−1 on eGFR
rs67953948
rs4025990
rs2895358
rs182082761
rs141911828
rs116356916
rs11719095
rs79223692
rs9829888
rs607925
rs73337892
rs187970821
rs79470125
rs183579118
rs74930814
rs143118409
rs185189095
rs138069176
rs78450445
rs80346698
rs75566141
All
−0.0020
−0.0015
−0.0010
−0.0005
0.0000
MR effect size for
'Plastin−1' on 'eGFR'

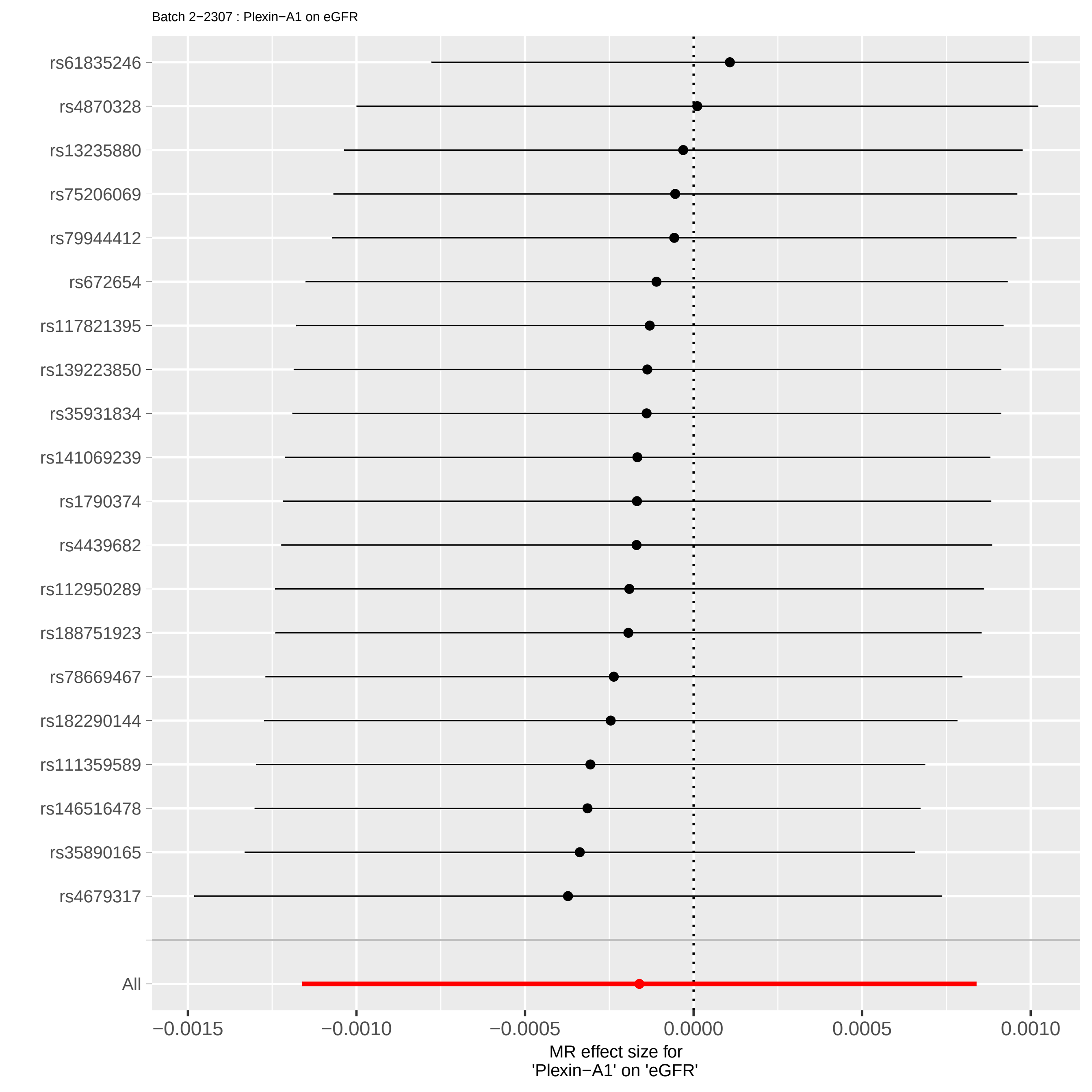

Batch 2−2307 : Plexin−A1 on eGFR
rs61835246
rs4870328
rs13235880
rs75206069
rs79944412
rs672654
rs117821395
rs139223850
rs35931834
rs141069239
rs1790374
rs4439682
rs112950289
rs188751923
rs78669467
rs182290144
rs111359589
rs146516478
rs35890165
rs4679317
All
−0.0015
−0.0010
−0.0005
0.0000
0.0005
0.0010
MR effect size for
'Plexin−A1' on 'eGFR'

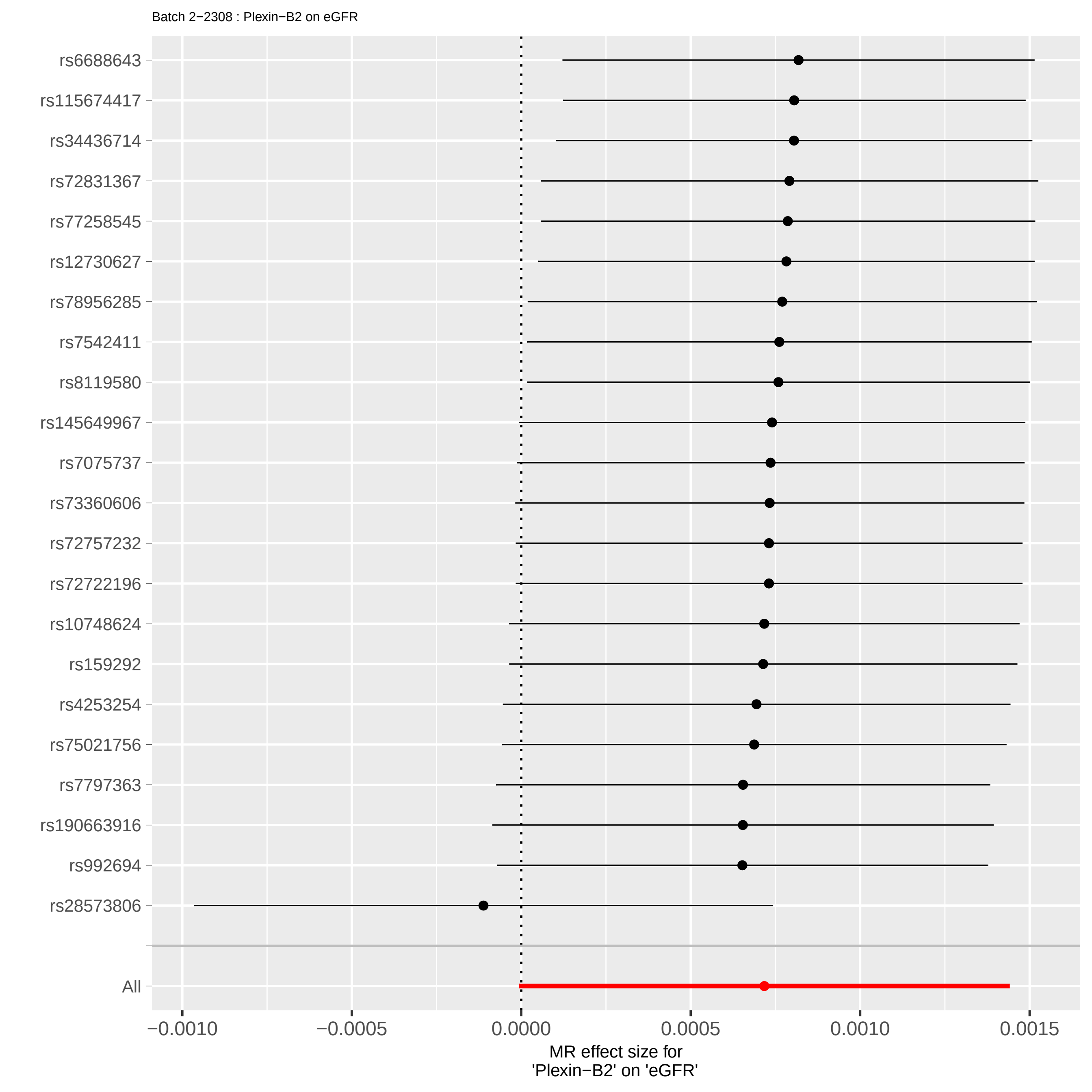

Batch 2−2308 : Plexin−B2 on eGFR
rs6688643
rs115674417
rs34436714
rs72831367
rs77258545
rs12730627
rs78956285
rs7542411
rs8119580
rs145649967
rs7075737
rs73360606
rs72757232
rs72722196
rs10748624
rs159292
rs4253254
rs75021756
rs7797363
rs190663916
rs992694
rs28573806
All
−0.0010
−0.0005
0.0000
0.0005
0.0010
0.0015
MR effect size for
'Plexin−B2' on 'eGFR'

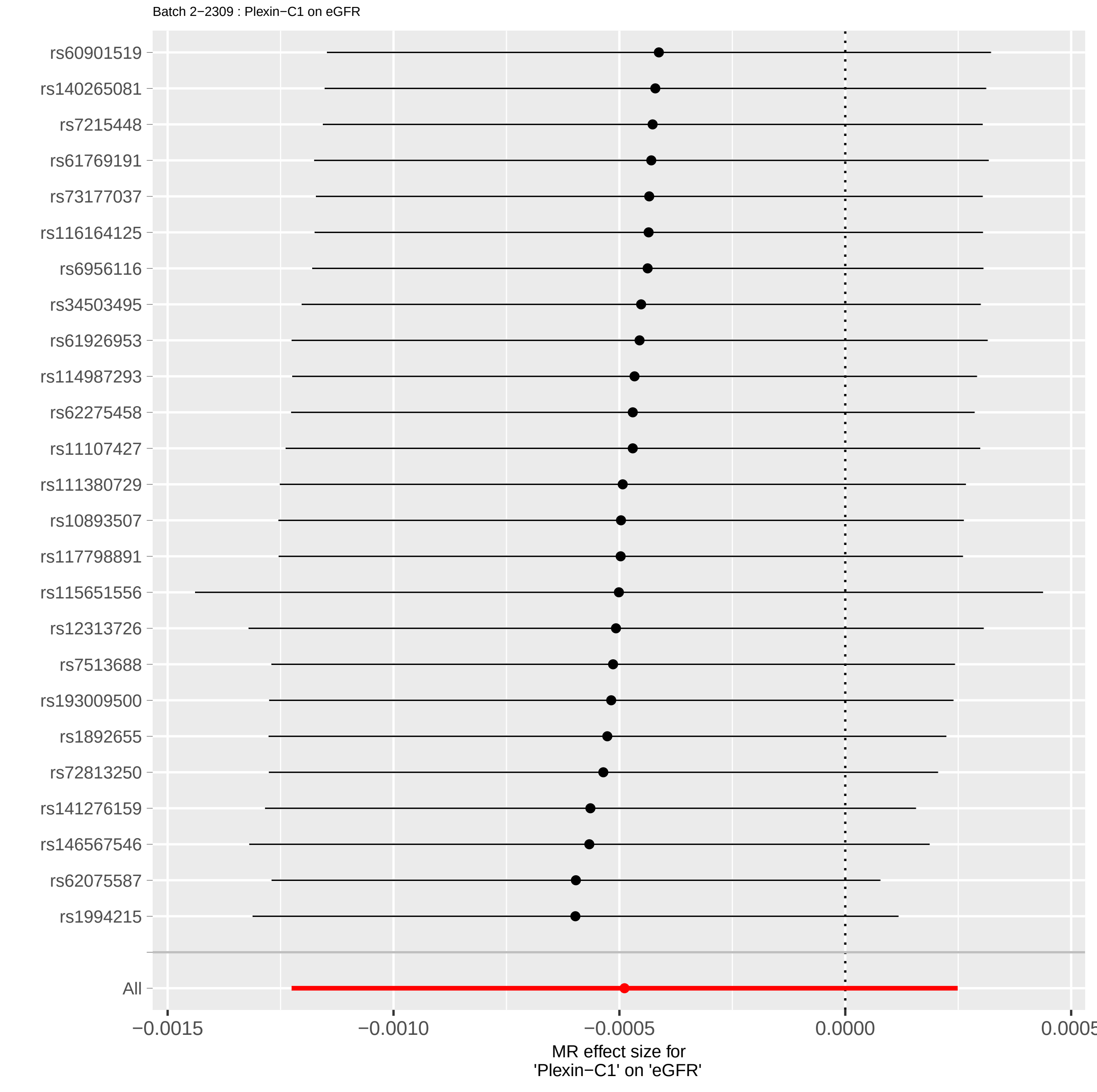

Batch 2−2309 : Plexin−C1 on eGFR
rs60901519
rs140265081
rs7215448
rs61769191
rs73177037
rs116164125
rs6956116
rs34503495
rs61926953
rs114987293
rs62275458
rs11107427
rs111380729
rs10893507
rs117798891
rs115651556
rs12313726
rs7513688
rs193009500
rs1892655
rs72813250
rs141276159
rs146567546
rs62075587
rs1994215
All
−0.0015
−0.0010
−0.0005
0.0000
0.0005
MR effect size for
'Plexin−C1' on 'eGFR'

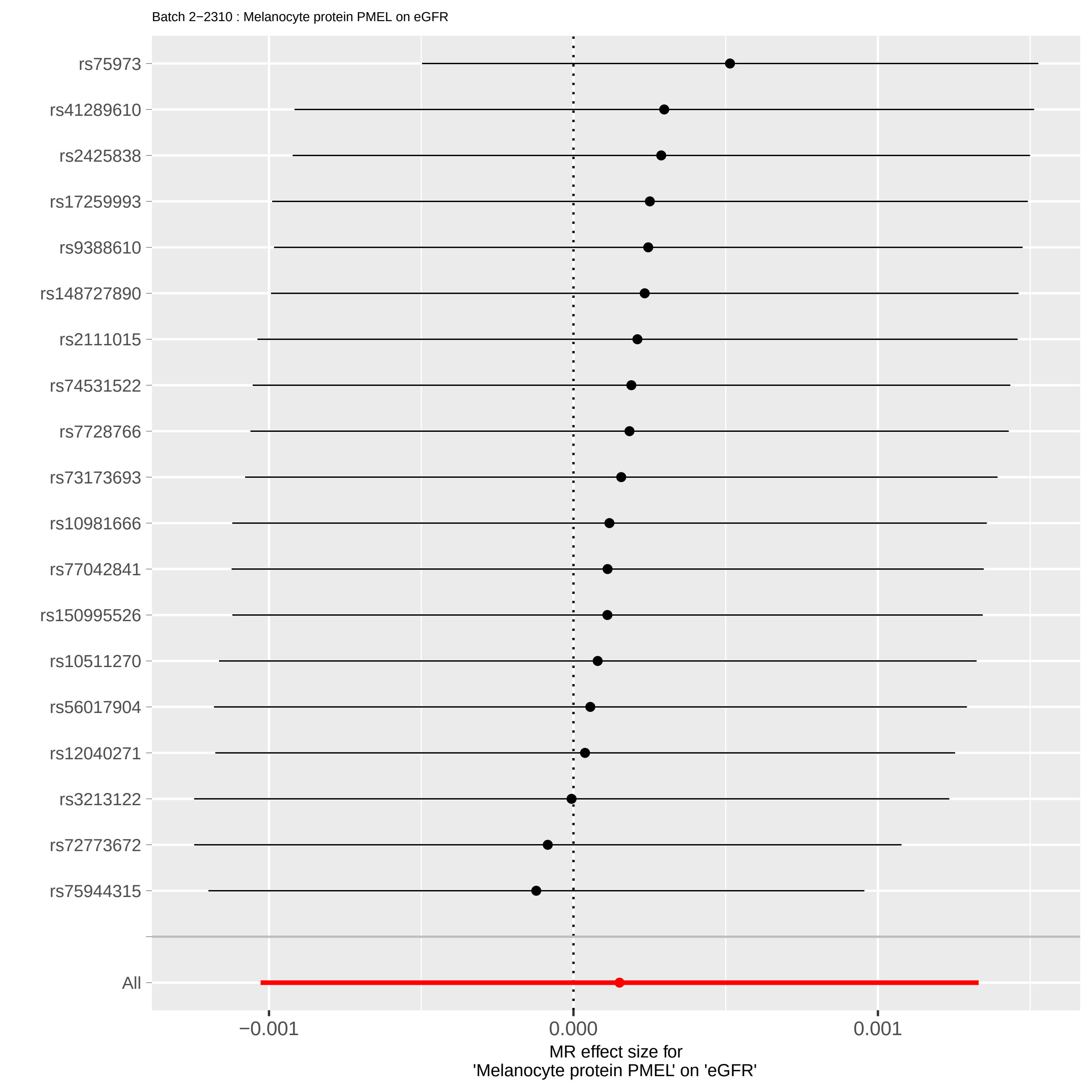

Batch 2−2310 : Melanocyte protein PMEL on eGFR
rs75973
rs41289610
rs2425838
rs17259993
rs9388610
rs148727890
rs2111015
rs74531522
rs7728766
rs73173693
rs10981666
rs77042841
rs150995526
rs10511270
rs56017904
rs12040271
rs3213122
rs72773672
rs75944315
All
−0.001
0.000
0.001
MR effect size for
'Melanocyte protein PMEL' on 'eGFR'

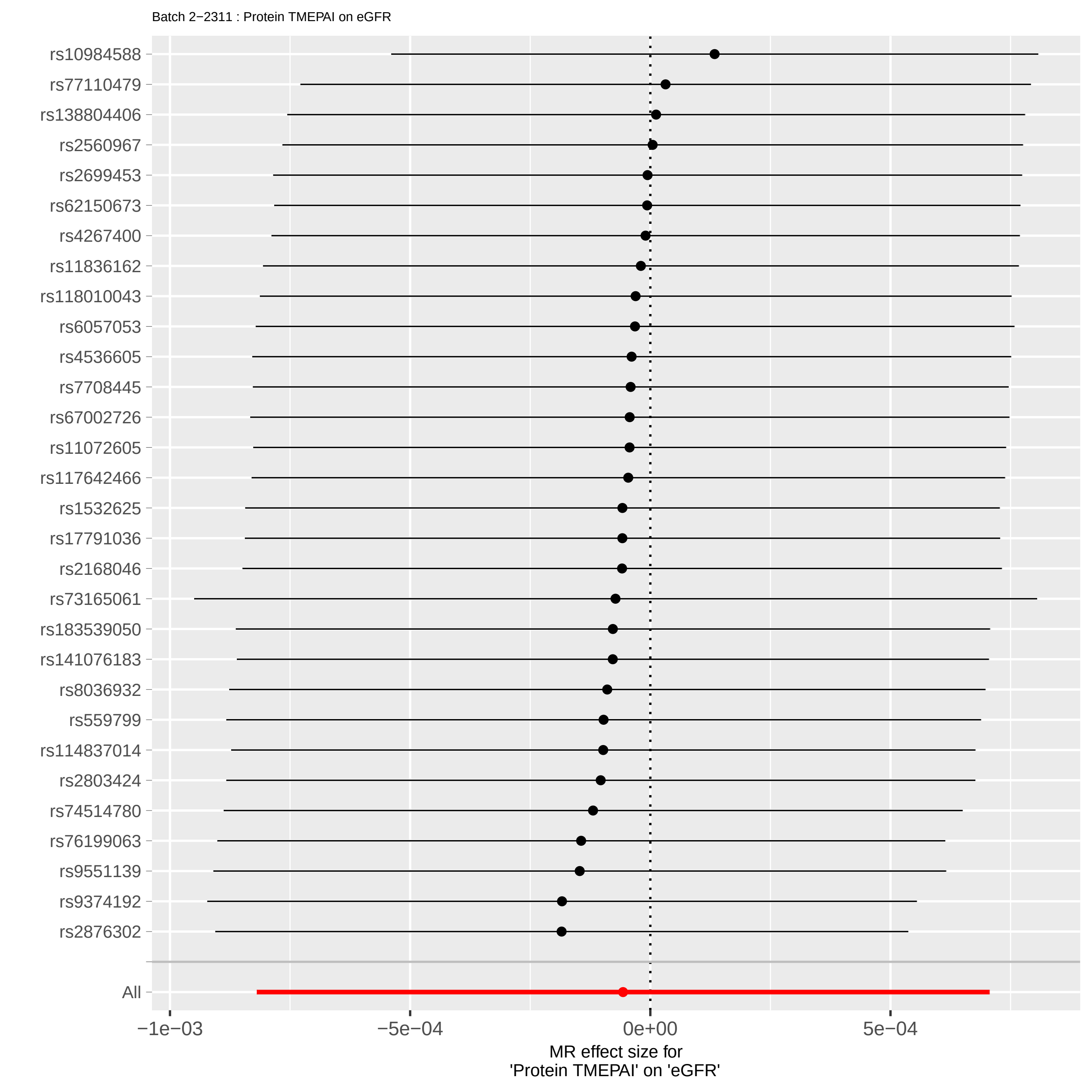

Batch 2−2311 : Protein TMEPAI on eGFR
rs10984588
rs77110479
rs138804406
rs2560967
rs2699453
rs62150673
rs4267400
rs11836162
rs118010043
rs6057053
rs4536605
rs7708445
rs67002726
rs11072605
rs117642466
rs1532625
rs17791036
rs2168046
rs73165061
rs183539050
rs141076183
rs8036932
rs559799
rs114837014
rs2803424
rs74514780
rs76199063
rs9551139
rs9374192
rs2876302
All
−1e−03
−5e−04
0e+00
5e−04
MR effect size for
'Protein TMEPAI' on 'eGFR'

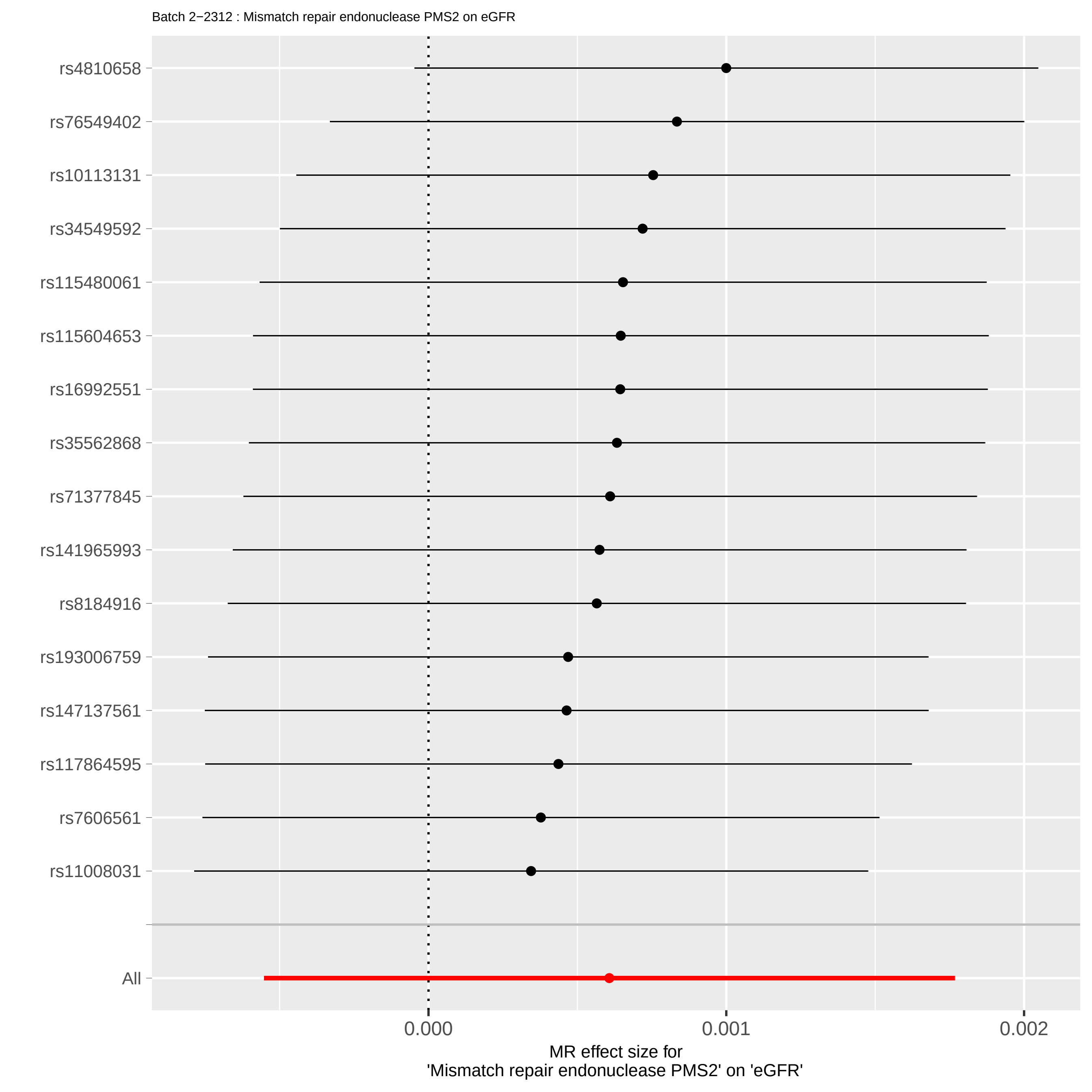
Batch 2−2312 : Mismatch repair endonuclease PMS2 on eGFR
rs4810658
rs76549402
rs10113131
rs34549592
rs115480061
rs115604653
rs16992551
rs35562868
rs71377845
rs141965993
rs8184916
rs193006759
rs147137561
rs117864595
rs7606561
rs11008031
All
0.000
0.001
0.002
MR effect size for
'Mismatch repair endonuclease PMS2' on 'eGFR'

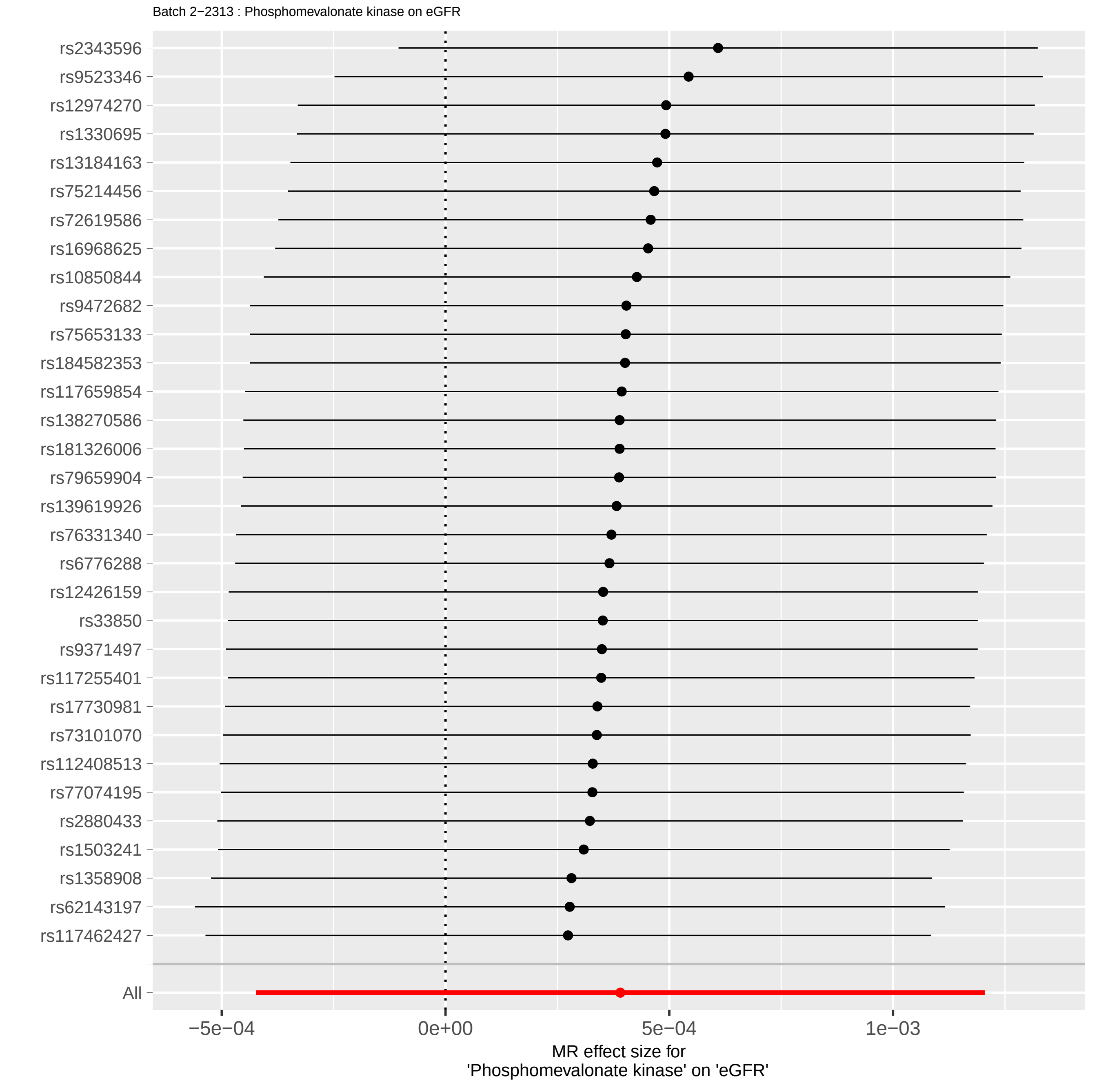

Batch 2−2313 : Phosphomevalonate kinase on eGFR
rs2343596
rs9523346
rs12974270
rs1330695
rs13184163
rs75214456
rs72619586
rs16968625
rs10850844
rs9472682
rs75653133
rs184582353
rs117659854
rs138270586
rs181326006
rs79659904
rs139619926
rs76331340
rs6776288
rs12426159
rs33850
rs9371497
rs117255401
rs17730981
rs73101070
rs112408513
rs77074195
rs2880433
rs1503241
rs1358908
rs62143197
rs117462427
All
−5e−04
0e+00
5e−04
1e−03
MR effect size for
'Phosphomevalonate kinase' on 'eGFR'

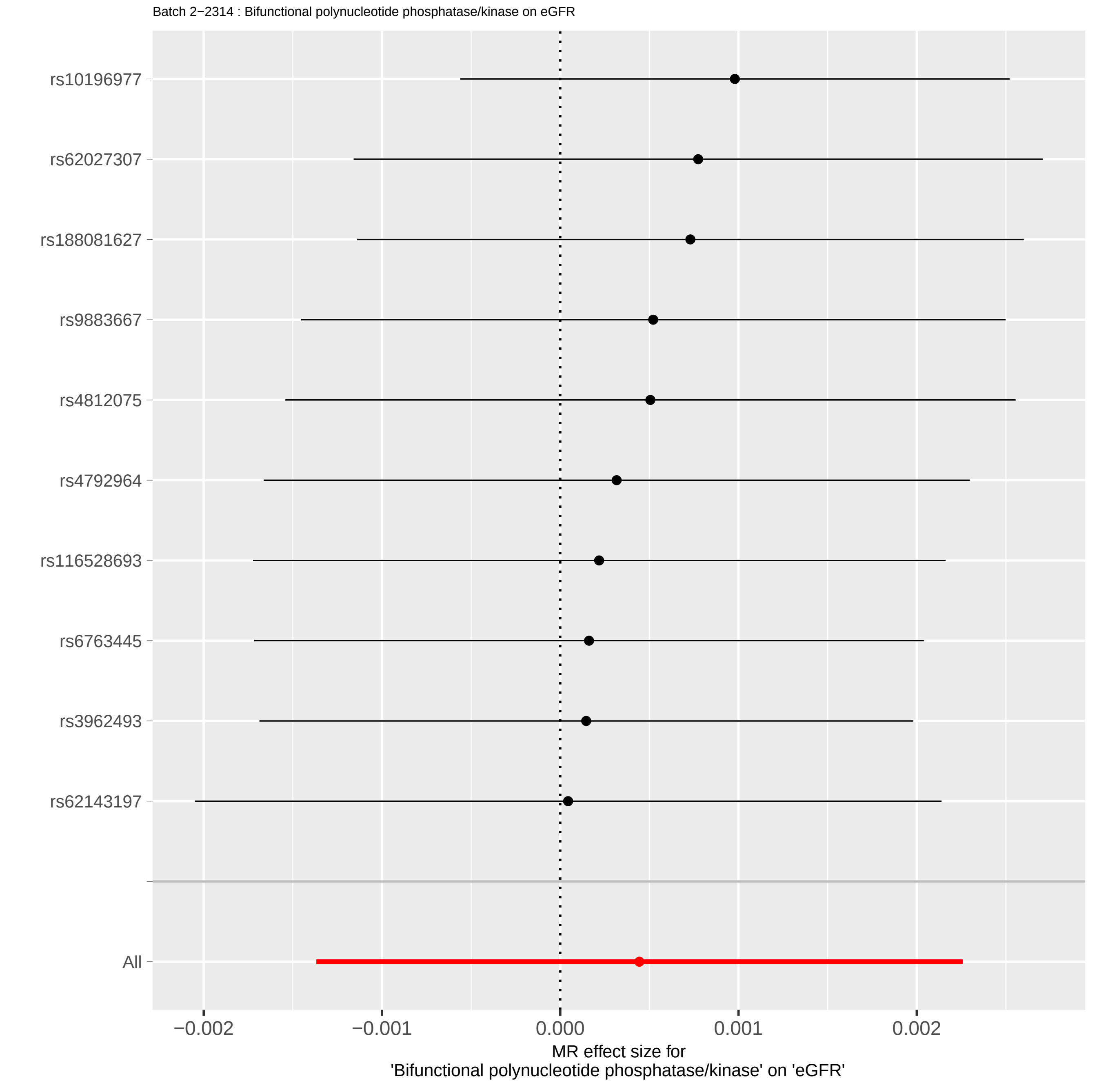
Batch 2−2314 : Bifunctional polynucleotide phosphatase/kinase on eGFR
rs10196977
rs62027307
rs188081627
rs9883667
rs4812075
rs4792964
rs116528693
rs6763445
rs3962493
rs62143197
All
−0.002
−0.001
0.000
0.001
0.002
MR effect size for
'Bifunctional polynucleotide phosphatase/kinase' on 'eGFR'

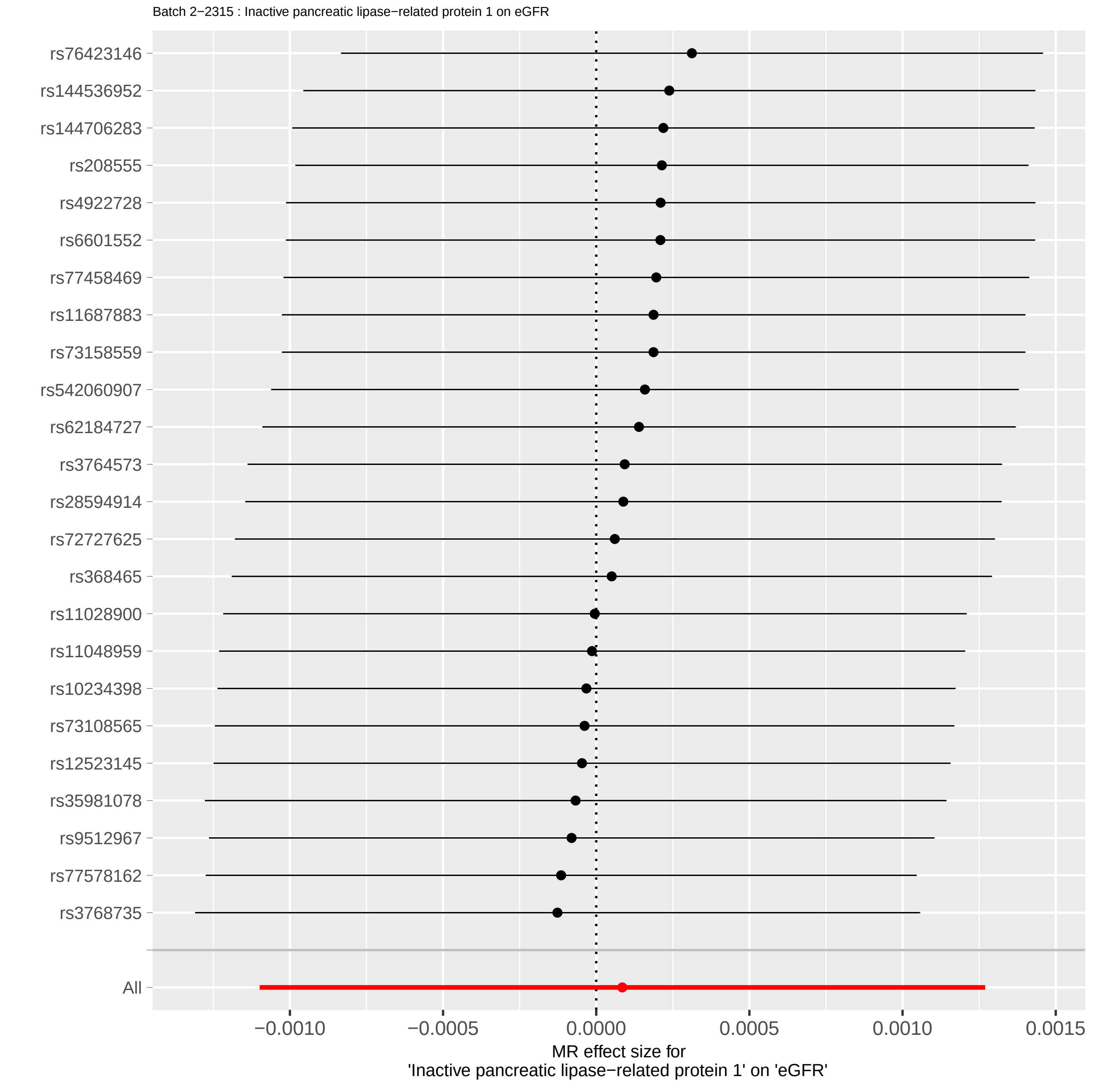

Batch 2−2315 : Inactive pancreatic lipase−related protein 1 on eGFR
rs76423146
rs144536952
rs144706283
rs208555
rs4922728
rs6601552
rs77458469
rs11687883
rs73158559
rs542060907
rs62184727
rs3764573
rs28594914
rs72727625
rs368465
rs11028900
rs11048959
rs10234398
rs73108565
rs12523145
rs35981078
rs9512967
rs77578162
rs3768735
All
−0.0010
−0.0005
0.0000
0.0005
0.0010
0.0015
MR effect size for
'Inactive pancreatic lipase−related protein 1' on 'eGFR'

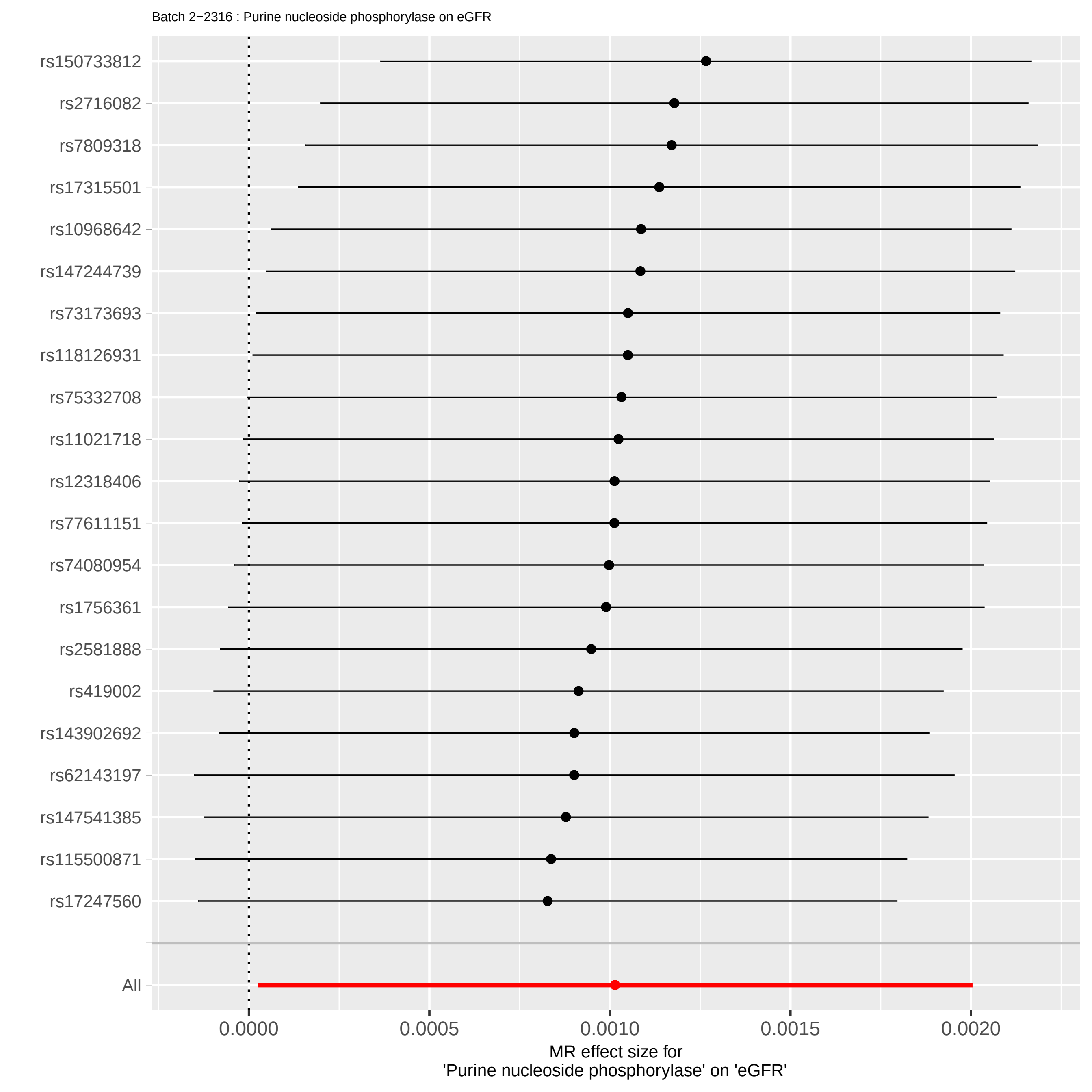

Batch 2−2316 : Purine nucleoside phosphorylase on eGFR
rs150733812
rs2716082
rs7809318
rs17315501
rs10968642
rs147244739
rs73173693
rs118126931
rs75332708
rs11021718
rs12318406
rs77611151
rs74080954
rs1756361
rs2581888
rs419002
rs143902692
rs62143197
rs147541385
rs115500871
rs17247560
All
0.0000
0.0005
0.0010
0.0015
0.0020
MR effect size for
'Purine nucleoside phosphorylase' on 'eGFR'

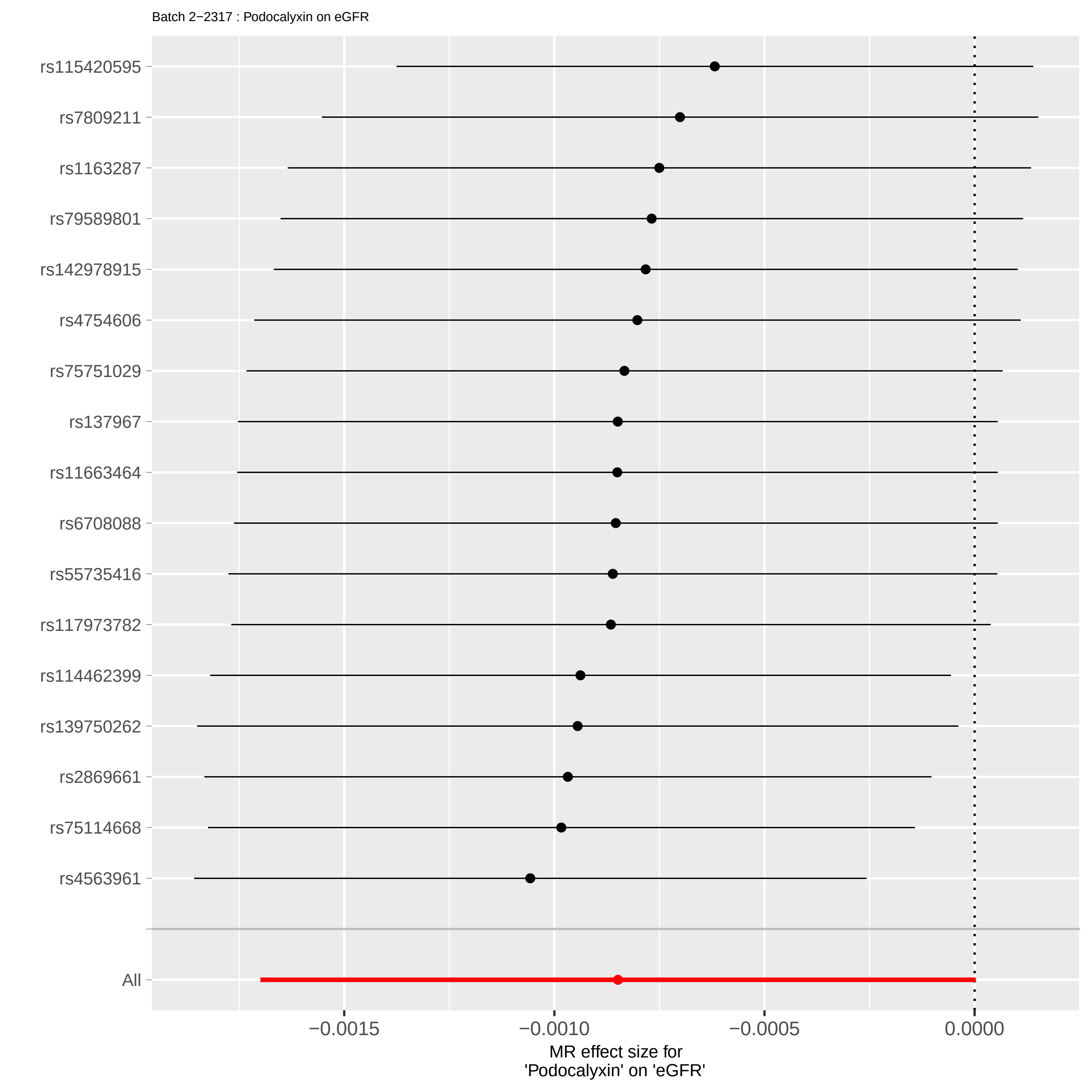

Batch 2−2317 : Podocalyxin on eGFR
rs115420595
rs7809211
rs1163287
rs79589801
rs142978915
rs4754606
rs75751029
rs137967
rs11663464
rs6708088
rs55735416
rs117973782
rs114462399
rs139750262
rs2869661
rs75114668
rs4563961
All
−0.0015
−0.0010
−0.0005
0.0000
MR effect size for
'Podocalyxin' on 'eGFR'

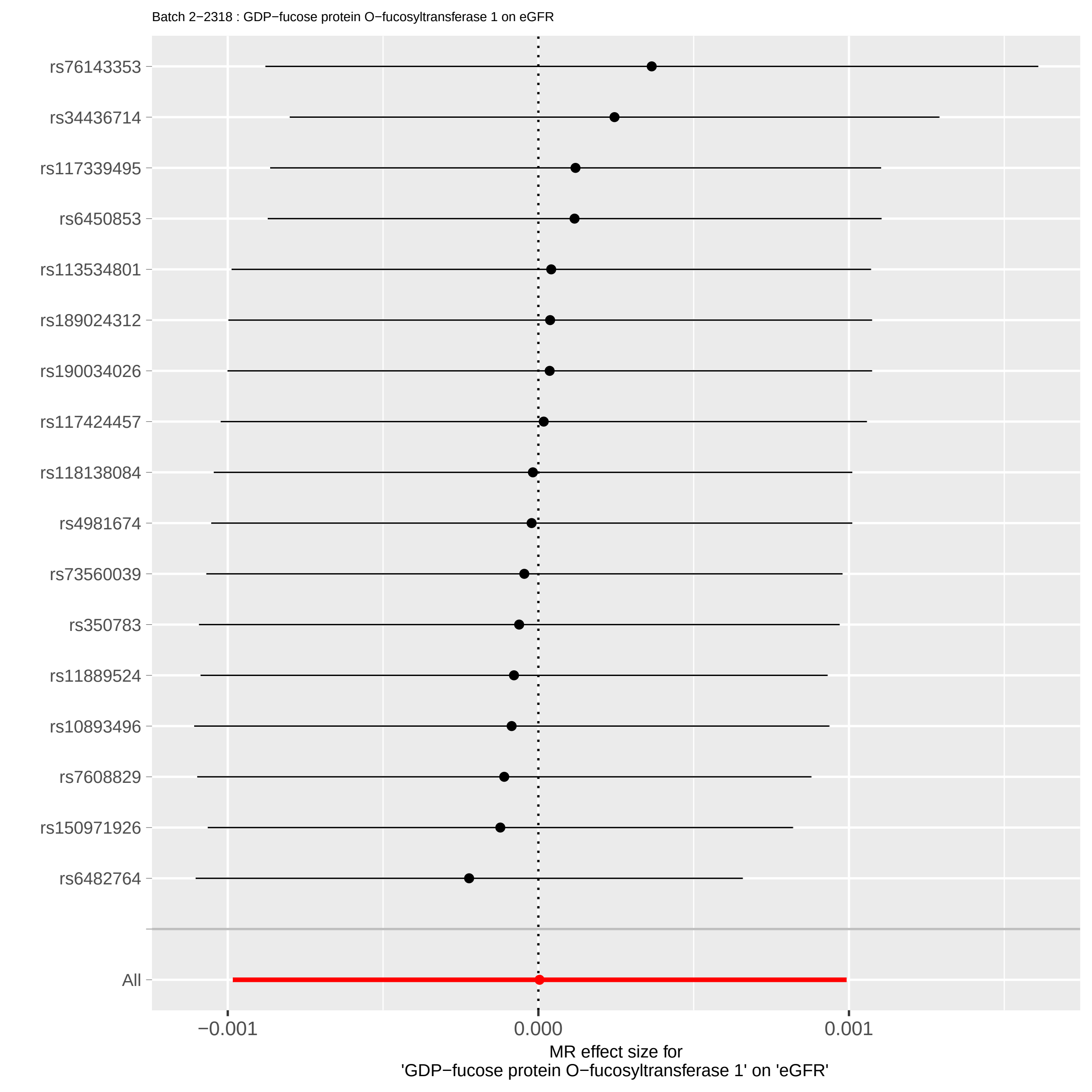

Batch 2−2318 : GDP−fucose protein O−fucosyltransferase 1 on eGFR
rs76143353
rs34436714
rs117339495
rs6450853
rs113534801
rs189024312
rs190034026
rs117424457
rs118138084
rs4981674
rs73560039
rs350783
rs11889524
rs10893496
rs7608829
rs150971926
rs6482764
All
−0.001
0.000
0.001
MR effect size for
'GDP−fucose protein O−fucosyltransferase 1' on 'eGFR'

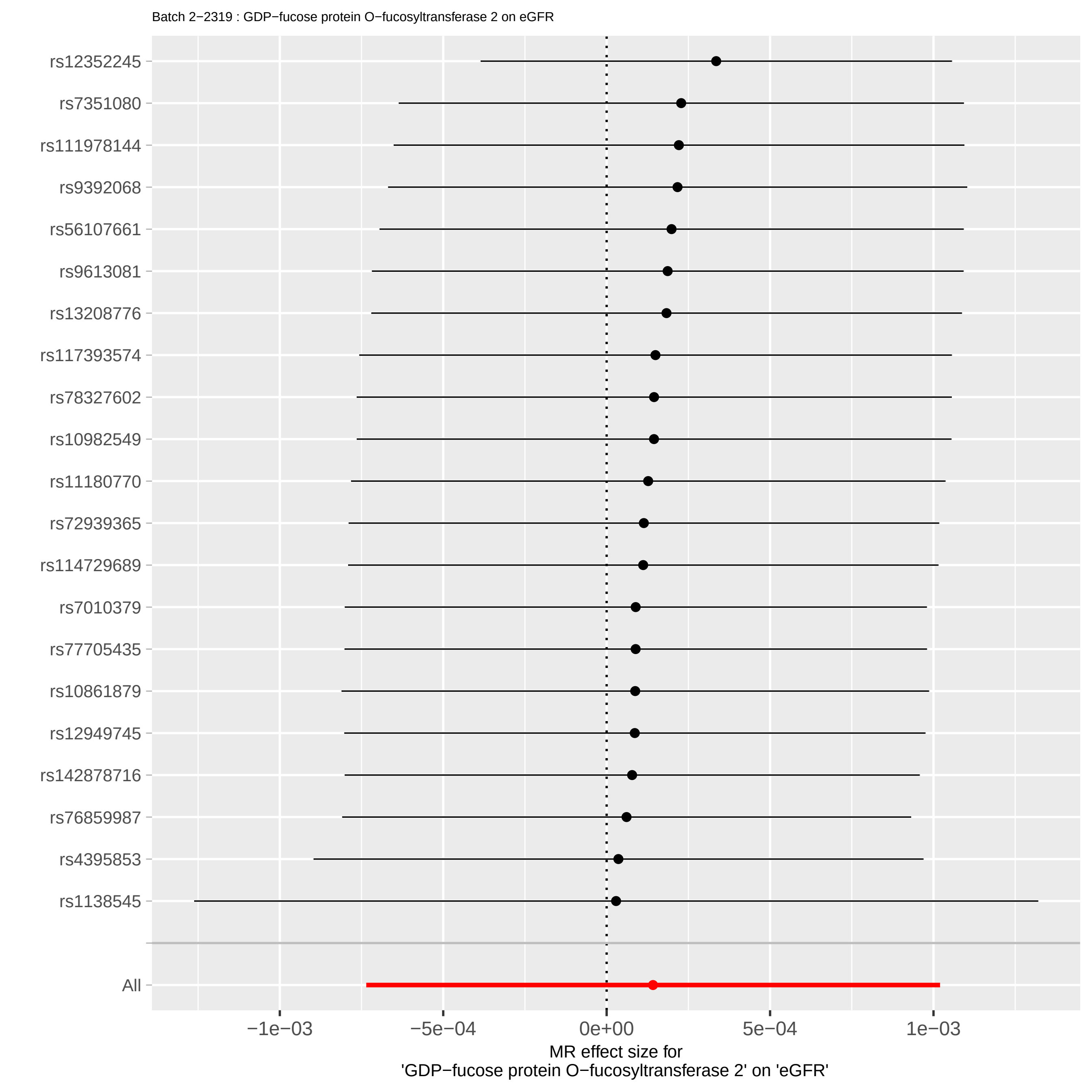
Batch 2−2319 : GDP−fucose protein O−fucosyltransferase 2 on eGFR
rs12352245
rs7351080
rs111978144
rs9392068
rs56107661
rs9613081
rs13208776
rs117393574
rs78327602
rs10982549
rs11180770
rs72939365
rs114729689
rs7010379
rs77705435
rs10861879
rs12949745
rs142878716
rs76859987
rs4395853
rs1138545
All
−1e−03
−5e−04
0e+00
5e−04
1e−03
MR effect size for
'GDP−fucose protein O−fucosyltransferase 2' on 'eGFR'

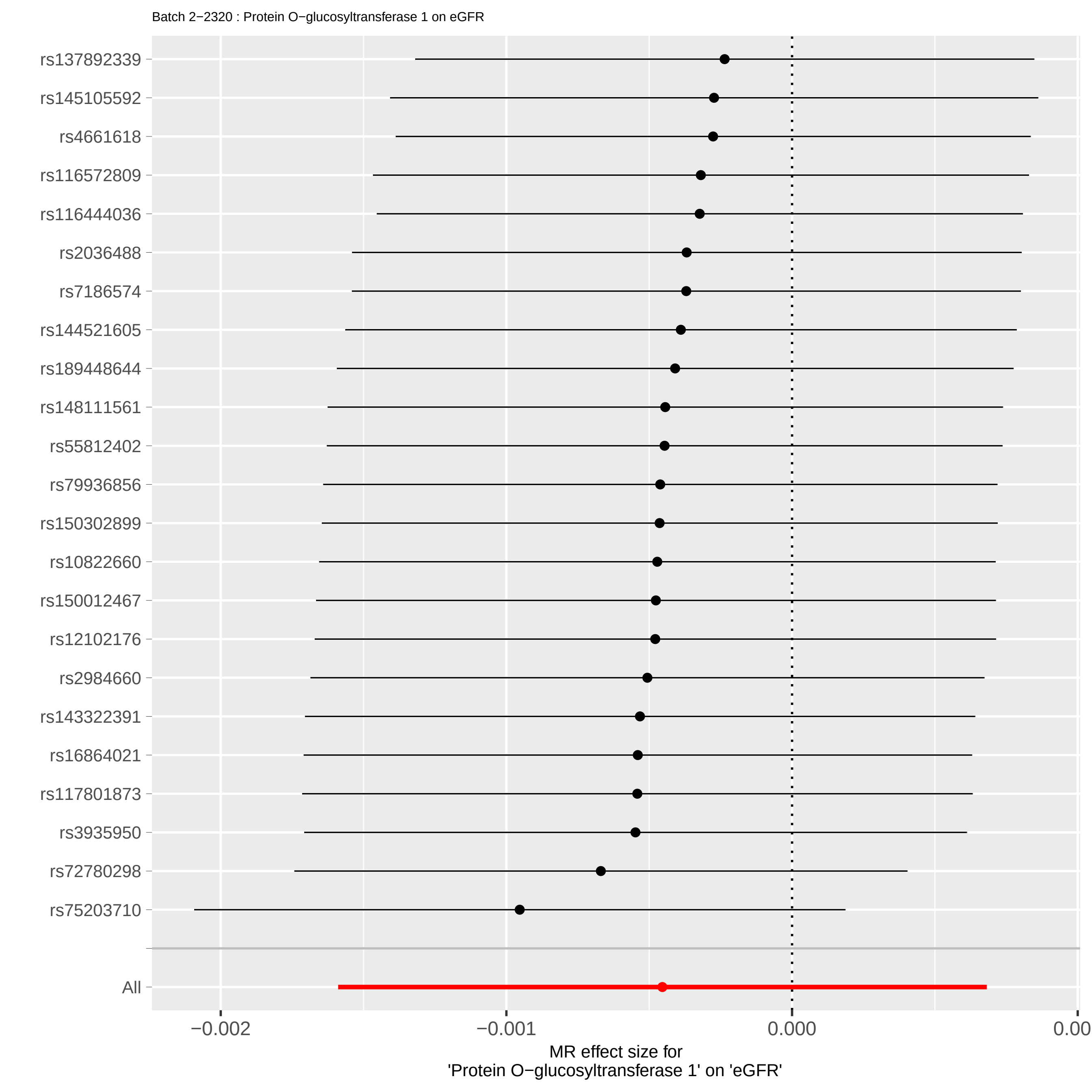
Batch 2−2320 : Protein O−glucosyltransferase 1 on eGFR
rs137892339
rs145105592
rs4661618
rs116572809
rs116444036
rs2036488
rs7186574
rs144521605
rs189448644
rs148111561
rs55812402
rs79936856
rs150302899
rs10822660
rs150012467
rs12102176
rs2984660
rs143322391
rs16864021
rs117801873
rs3935950
rs72780298
rs75203710
All
−0.002
−0.001
0.000
0.00
MR effect size for
'Protein O−glucosyltransferase 1' on 'eGFR'

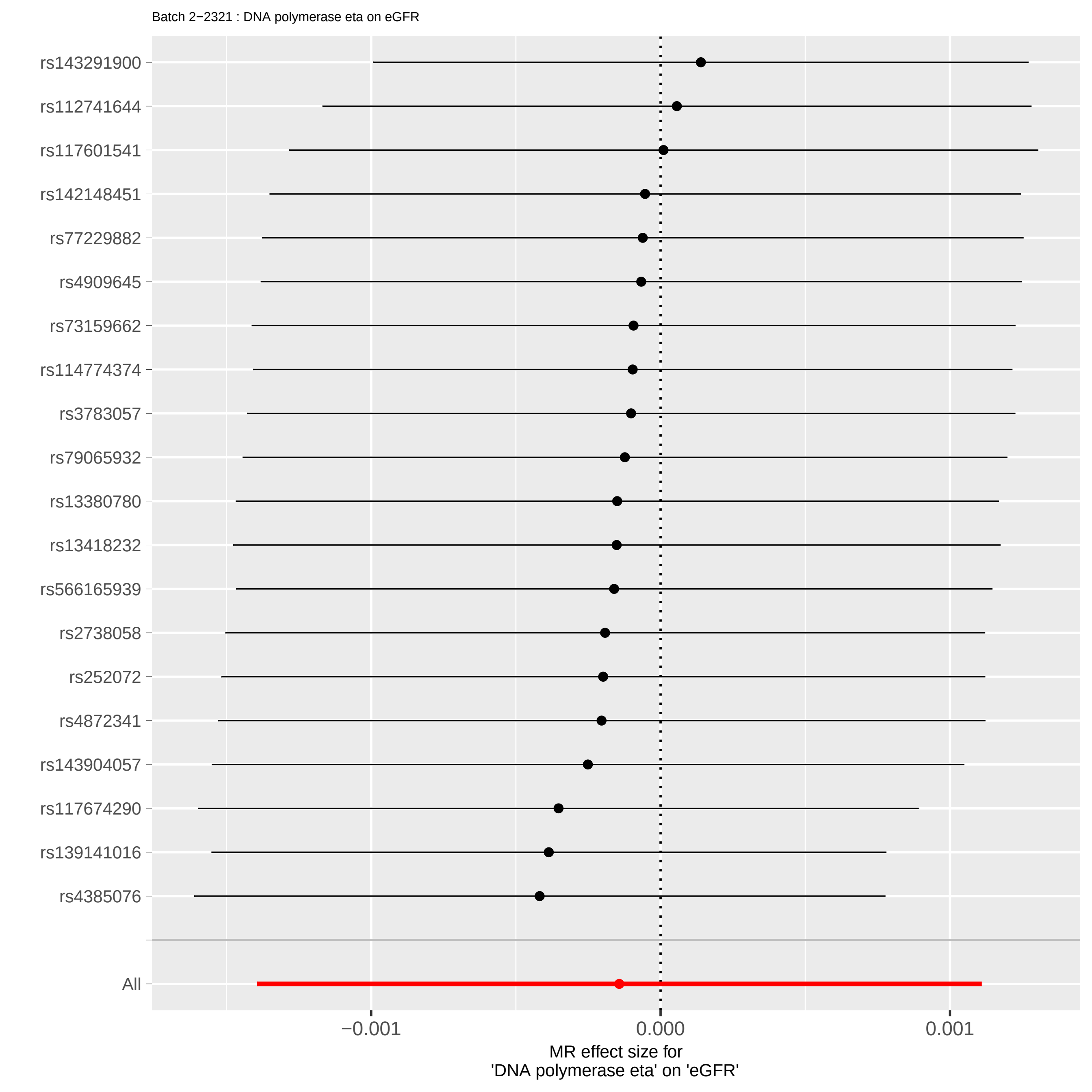
Batch 2−2321 : DNA polymerase eta on eGFR
rs143291900
rs112741644
rs117601541
rs142148451
rs77229882
rs4909645
rs73159662
rs114774374
rs3783057
rs79065932
rs13380780
rs13418232
rs566165939
rs2738058
rs252072
rs4872341
rs143904057
rs117674290
rs139141016
rs4385076
All
−0.001
0.000
0.001
MR effect size for
'DNA polymerase eta' on 'eGFR'

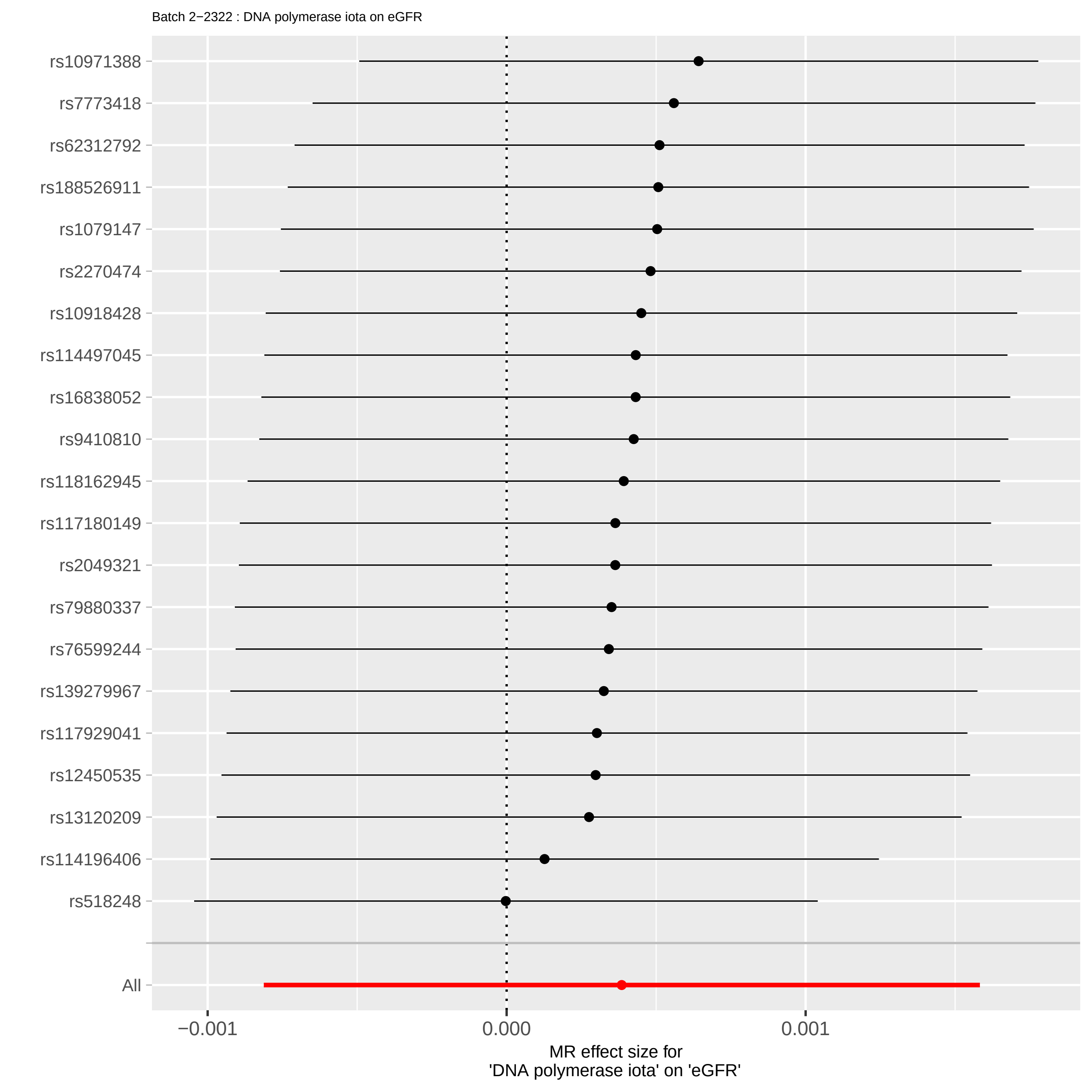

Batch 2−2322 : DNA polymerase iota on eGFR
rs10971388
rs7773418
rs62312792
rs188526911
rs1079147
rs2270474
rs10918428
rs114497045
rs16838052
rs9410810
rs118162945
rs117180149
rs2049321
rs79880337
rs76599244
rs139279967
rs117929041
rs12450535
rs13120209
rs114196406
rs518248
All
−0.001
0.000
0.001
MR effect size for
'DNA polymerase iota' on 'eGFR'

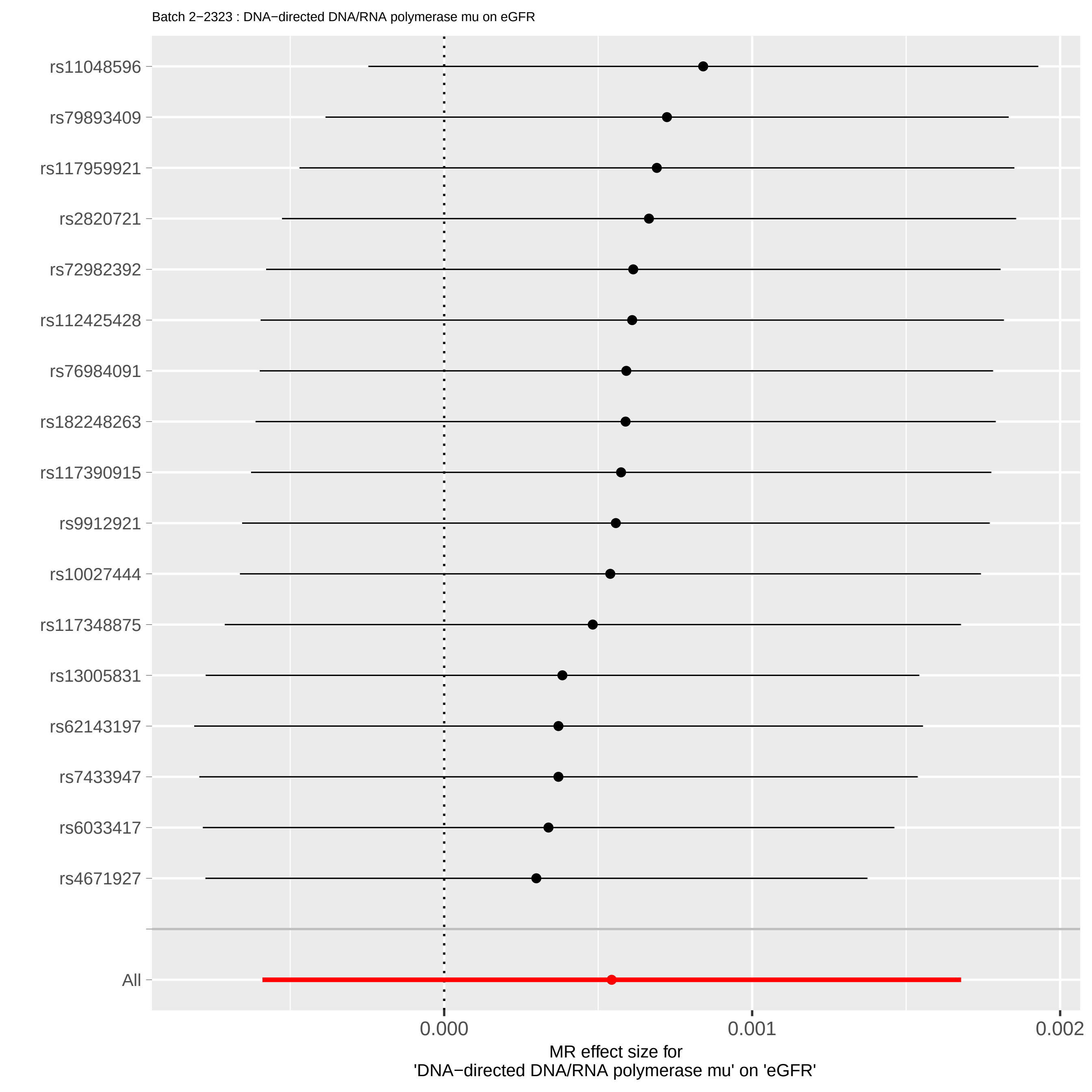
Batch 2−2323 : DNA−directed DNA/RNA polymerase mu on eGFR
rs11048596
rs79893409
rs117959921
rs2820721
rs72982392
rs112425428
rs76984091
rs182248263
rs117390915
rs9912921
rs10027444
rs117348875
rs13005831
rs62143197
rs7433947
rs6033417
rs4671927
All
0.000
0.001
0.002
MR effect size for
'DNA−directed DNA/RNA polymerase mu' on 'eGFR'

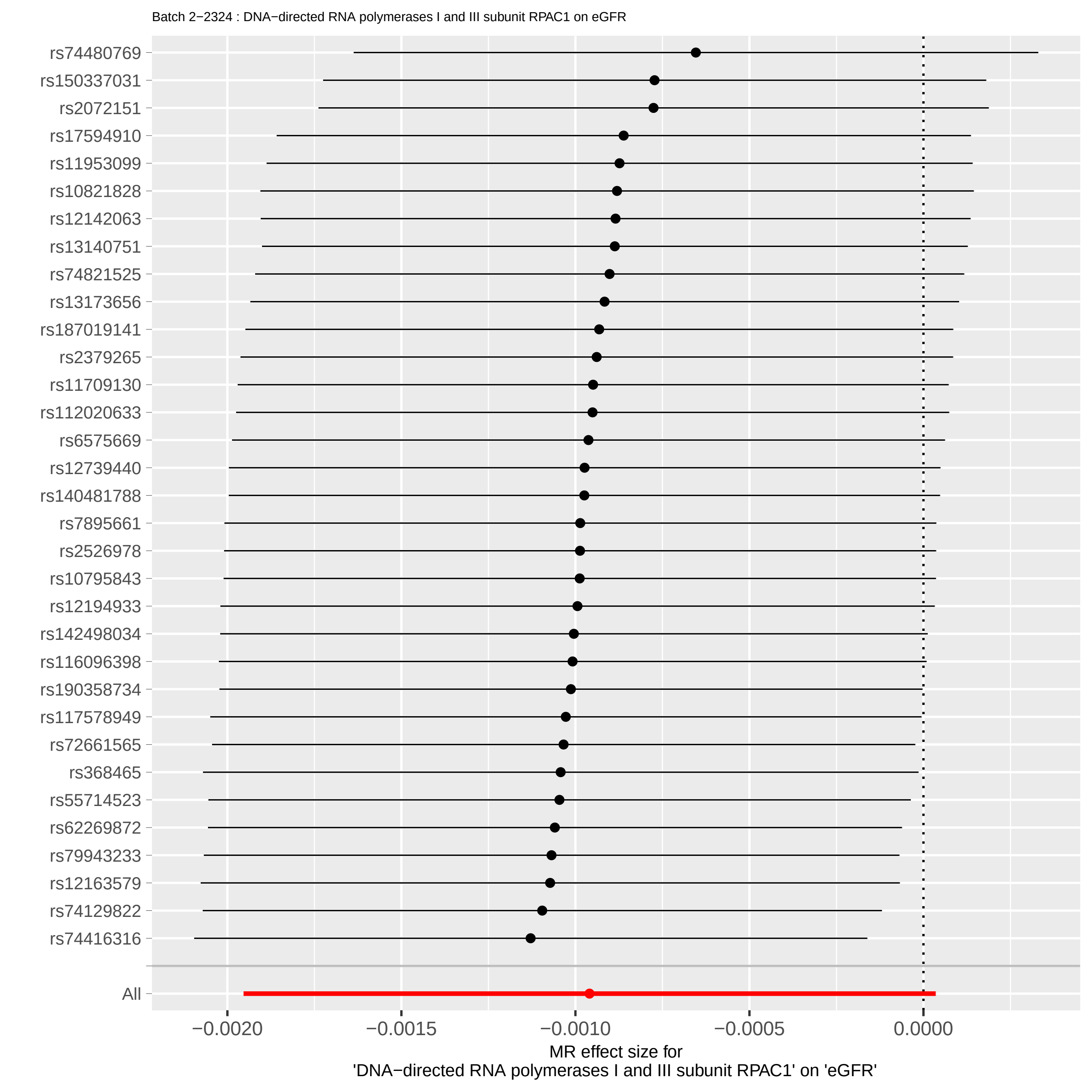

Batch 2−2324 : DNA−directed RNA polymerases I and III subunit RPAC1 on eGFR
rs74480769
rs150337031
rs2072151
rs17594910
rs11953099
rs10821828
rs12142063
rs13140751
rs74821525
rs13173656
rs187019141
rs2379265
rs11709130
rs112020633
rs6575669
rs12739440
rs140481788
rs7895661
rs2526978
rs10795843
rs12194933
rs142498034
rs116096398
rs190358734
rs117578949
rs72661565
rs368465
rs55714523
rs62269872
rs79943233
rs12163579
rs74129822
rs74416316
All
−0.0020
−0.0015
−0.0010
−0.0005
0.0000
MR effect size for
'DNA−directed RNA polymerases I and III subunit RPAC1' on 'eGFR'

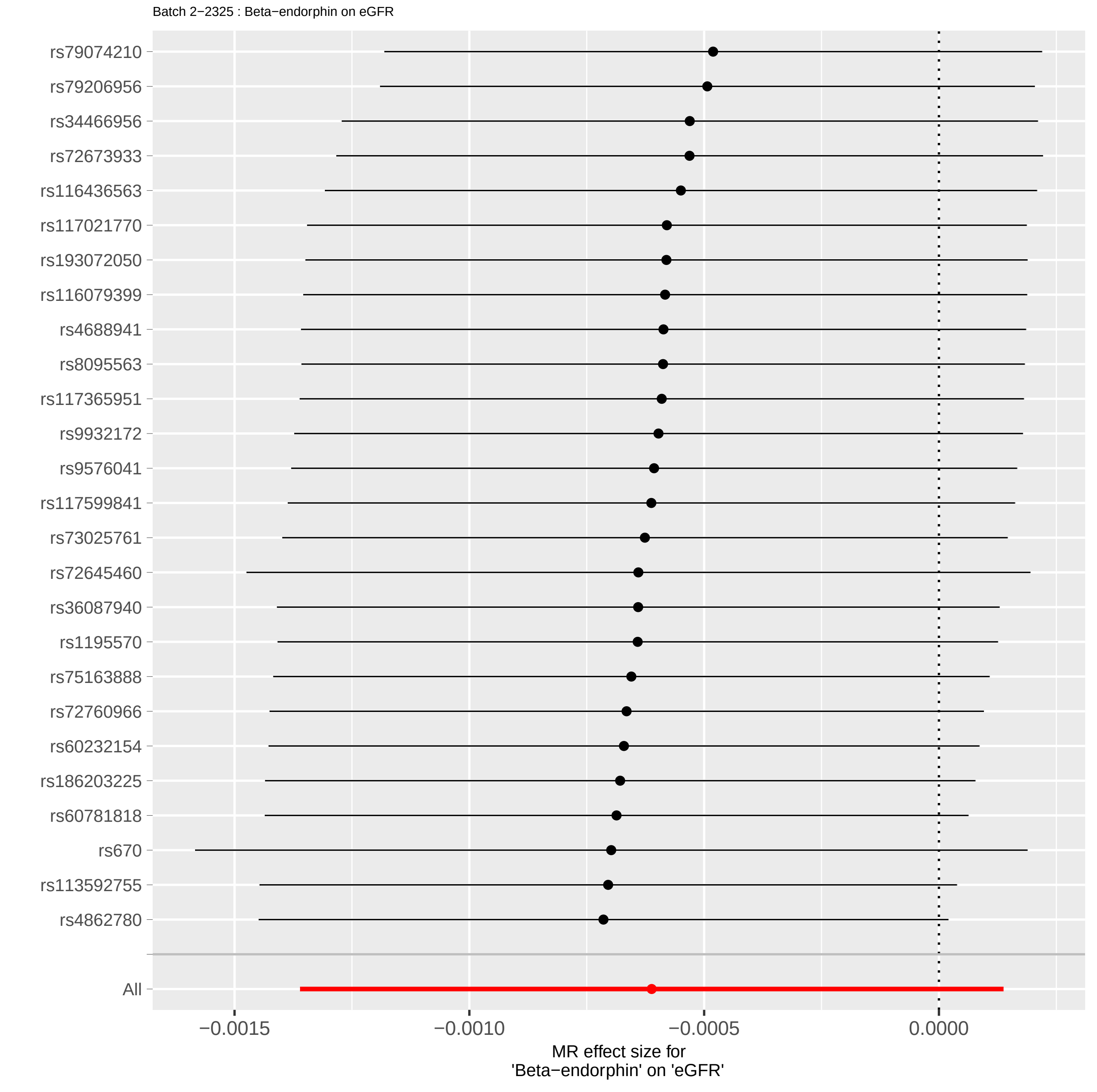

Batch 2−2325 : Beta−endorphin on eGFR
rs79074210
rs79206956
rs34466956
rs72673933
rs116436563
rs117021770
rs193072050
rs116079399
rs4688941
rs8095563
rs117365951
rs9932172
rs9576041
rs117599841
rs73025761
rs72645460
rs36087940
rs1195570
rs75163888
rs72760966
rs60232154
rs186203225
rs60781818
rs670
rs113592755
rs4862780
All
−0.0015
−0.0010
−0.0005
0.0000
MR effect size for
'Beta−endorphin' on 'eGFR'

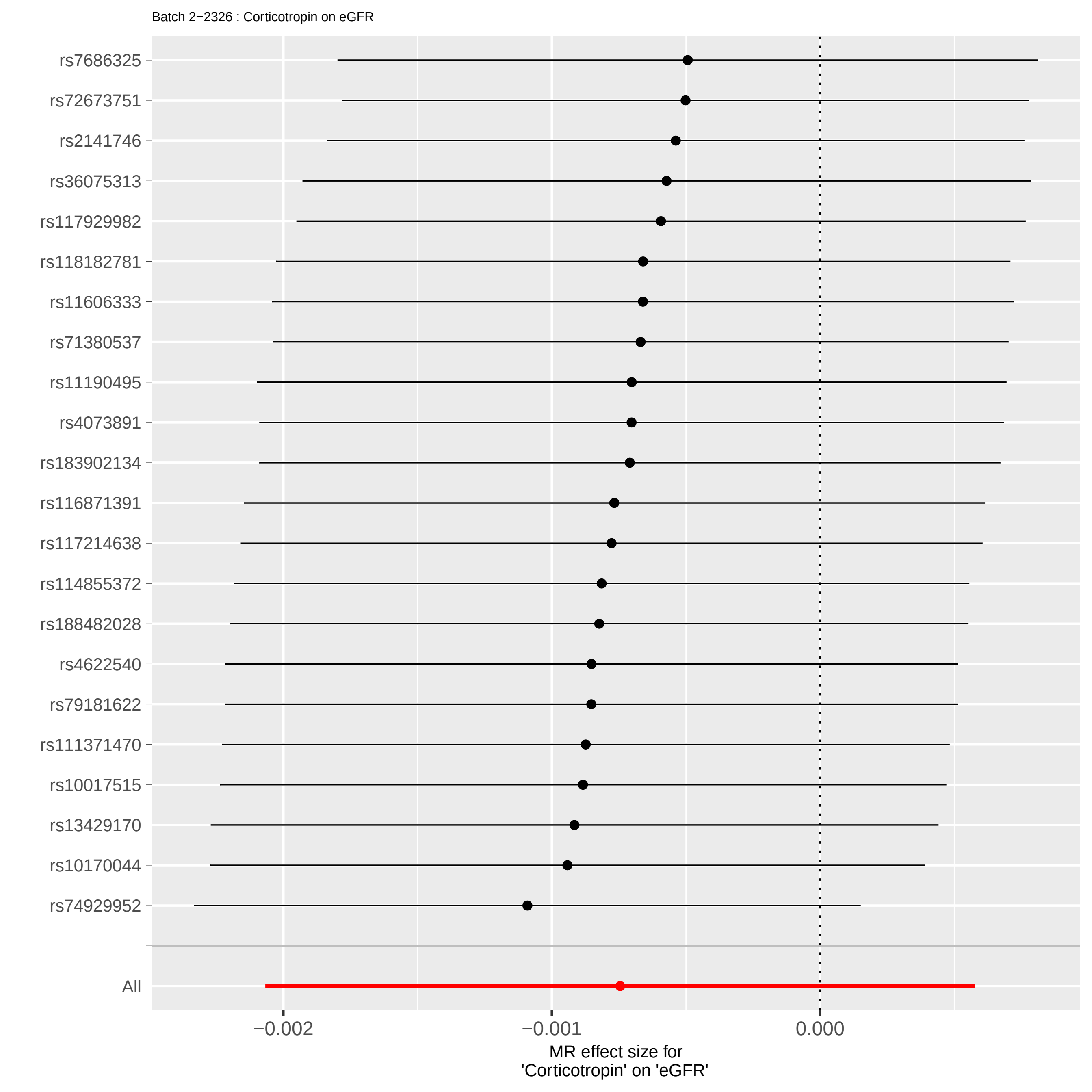

Batch 2−2326 : Corticotropin on eGFR
rs7686325
rs72673751
rs2141746
rs36075313
rs117929982
rs118182781
rs11606333
rs71380537
rs11190495
rs4073891
rs183902134
rs116871391
rs117214638
rs114855372
rs188482028
rs4622540
rs79181622
rs111371470
rs10017515
rs13429170
rs10170044
rs74929952
All
−0.002
−0.001
0.000
MR effect size for
'Corticotropin' on 'eGFR'

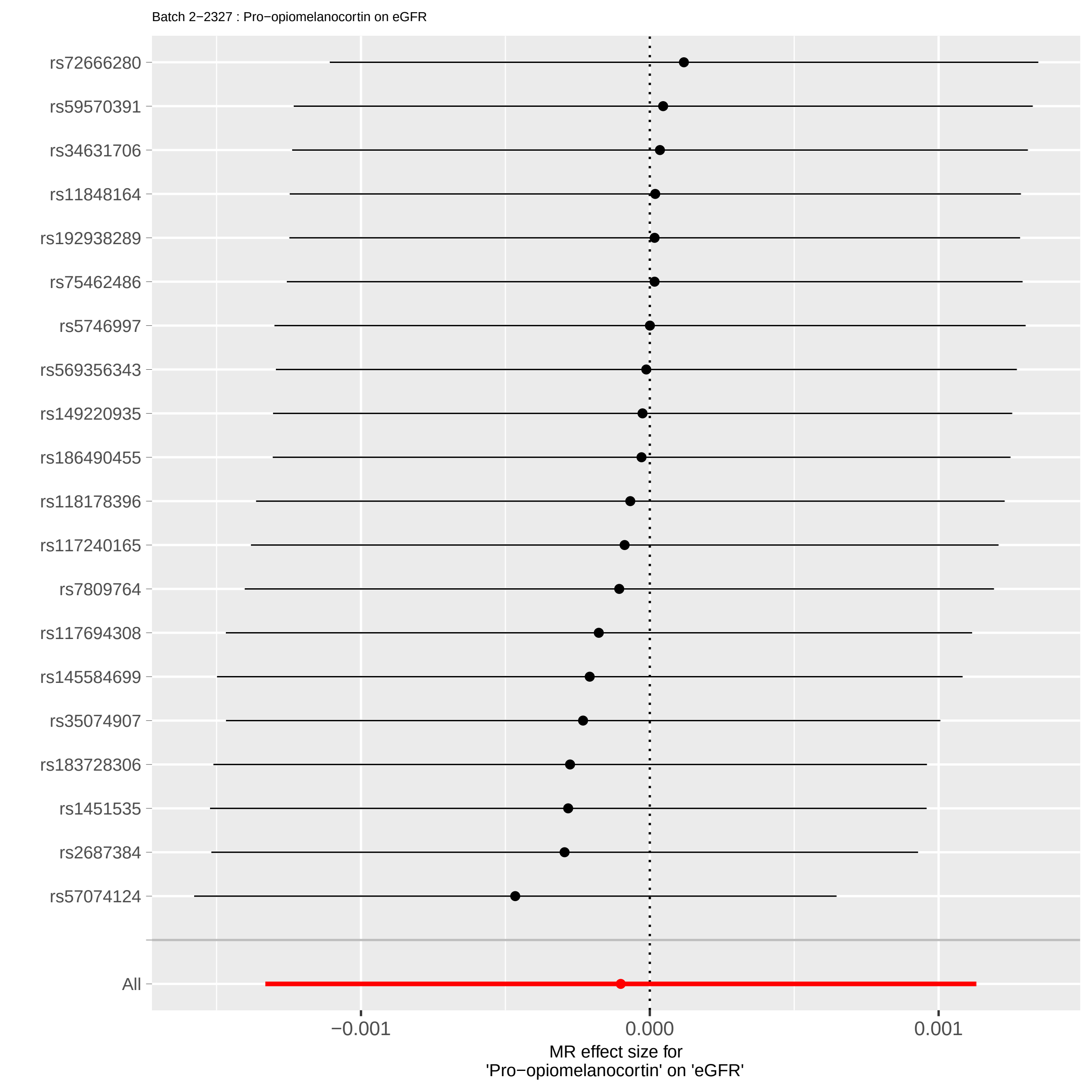

Batch 2−2327 : Pro−opiomelanocortin on eGFR
rs72666280
rs59570391
rs34631706
rs11848164
rs192938289
rs75462486
rs5746997
rs569356343
rs149220935
rs186490455
rs118178396
rs117240165
rs7809764
rs117694308
rs145584699
rs35074907
rs183728306
rs1451535
rs2687384
rs57074124
All
−0.001
0.000
0.001
MR effect size for
'Pro−opiomelanocortin' on 'eGFR'

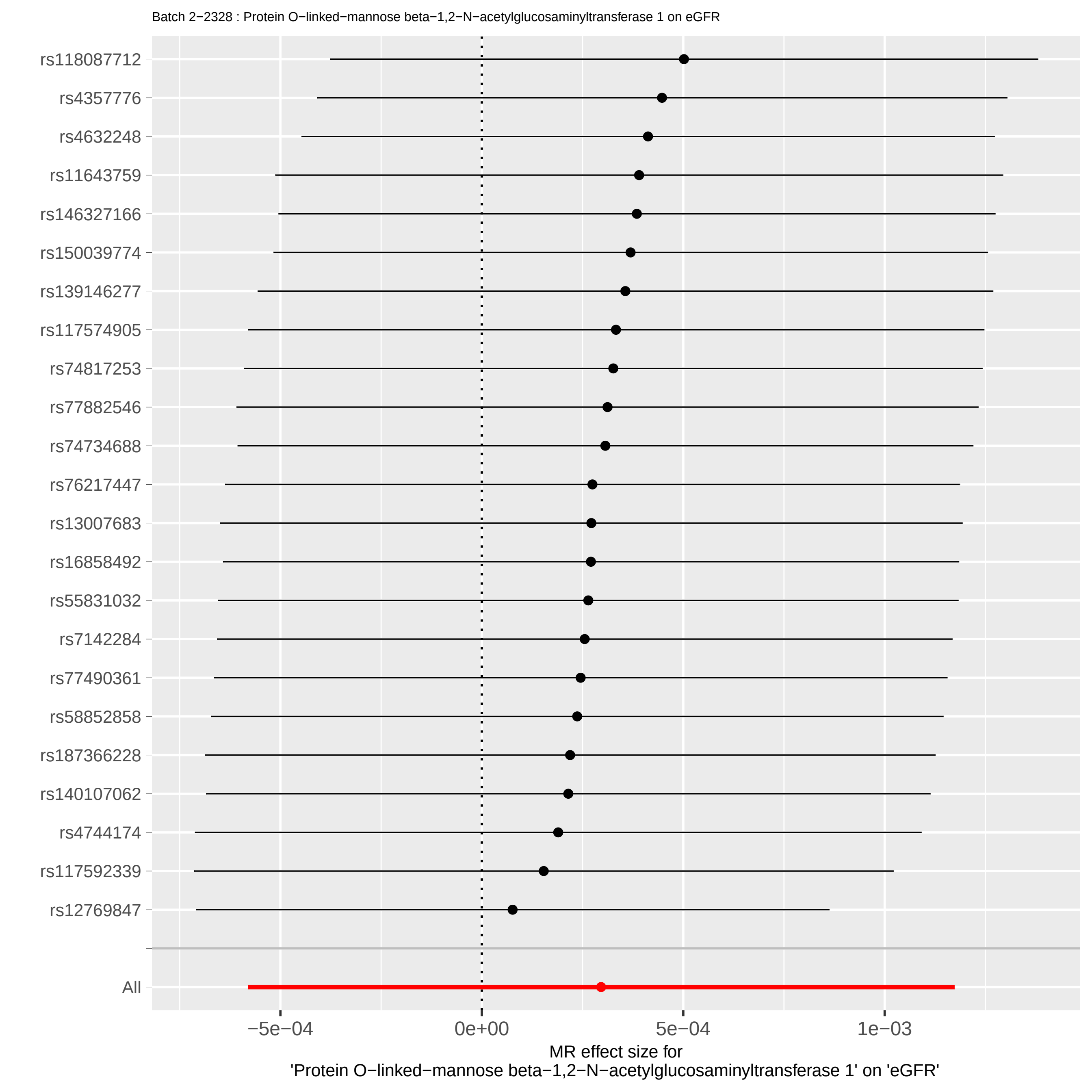

Batch 2−2328 : Protein O−linked−mannose beta−1,2−N−acetylglucosaminyltransferase 1 on eGFR
rs118087712
rs4357776
rs4632248
rs11643759
rs146327166
rs150039774
rs139146277
rs117574905
rs74817253
rs77882546
rs74734688
rs76217447
rs13007683
rs16858492
rs55831032
rs7142284
rs77490361
rs58852858
rs187366228
rs140107062
rs4744174
rs117592339
rs12769847
All
−5e−04
0e+00
5e−04
1e−03
MR effect size for
'Protein O−linked−mannose beta−1,2−N−acetylglucosaminyltransferase 1' on 'eGFR'

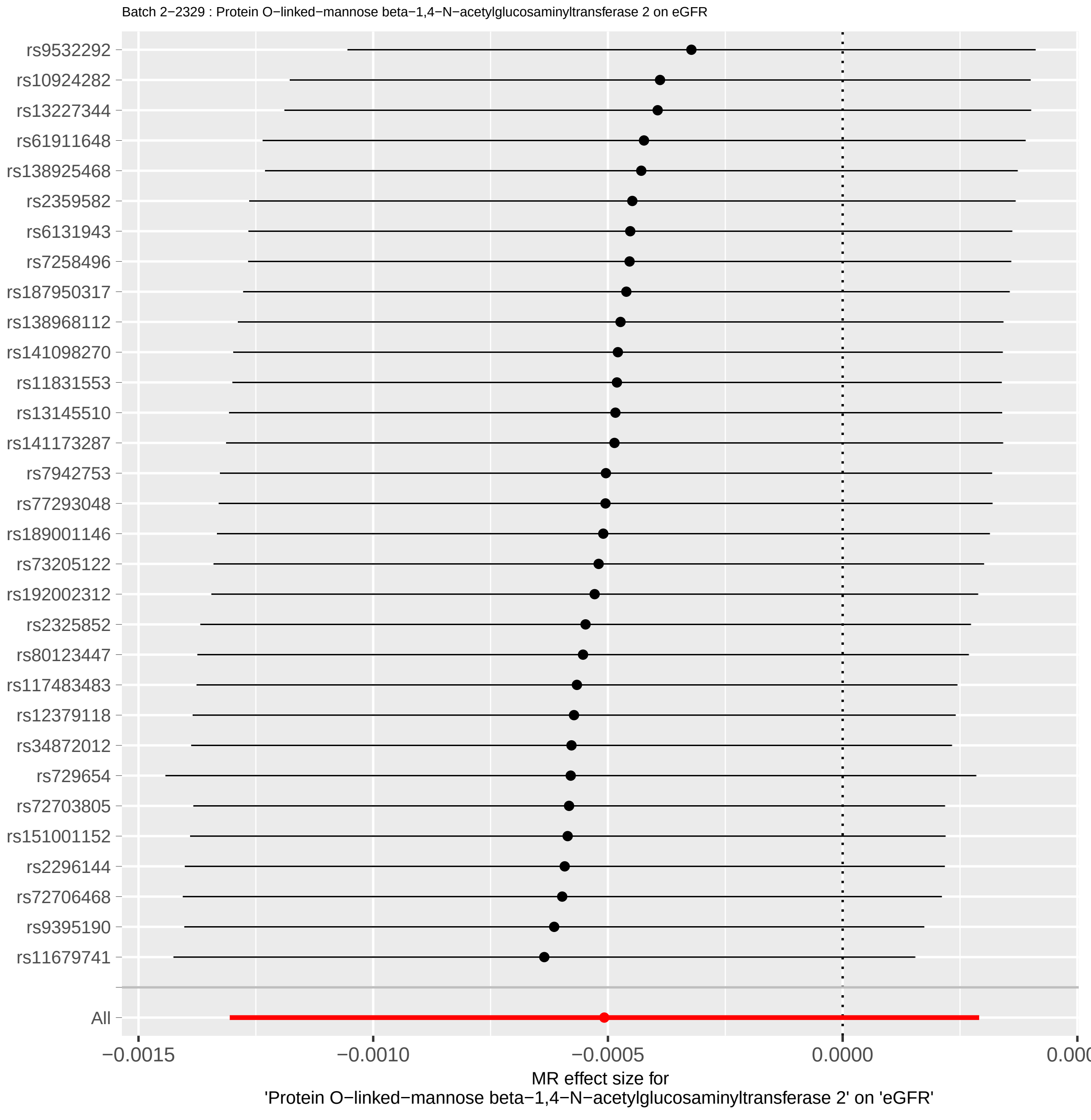
Batch 2−2329 : Protein O−linked−mannose beta−1,4−N−acetylglucosaminyltransferase 2 on eGFR
rs9532292
rs10924282
rs13227344
rs61911648
rs138925468
rs2359582
rs6131943
rs7258496
rs187950317
rs138968112
rs141098270
rs11831553
rs13145510
rs141173287
rs7942753
rs77293048
rs189001146
rs73205122
rs192002312
rs2325852
rs80123447
rs117483483
rs12379118
rs34872012
rs729654
rs72703805
rs151001152
rs2296144
rs72706468
rs9395190
rs11679741
All
−0.0015
−0.0010
−0.0005
0.0000
MR effect size for
'Protein O−linked−mannose beta−1,4−N−acetylglucosaminyltransferase 2' on 'eGFR'

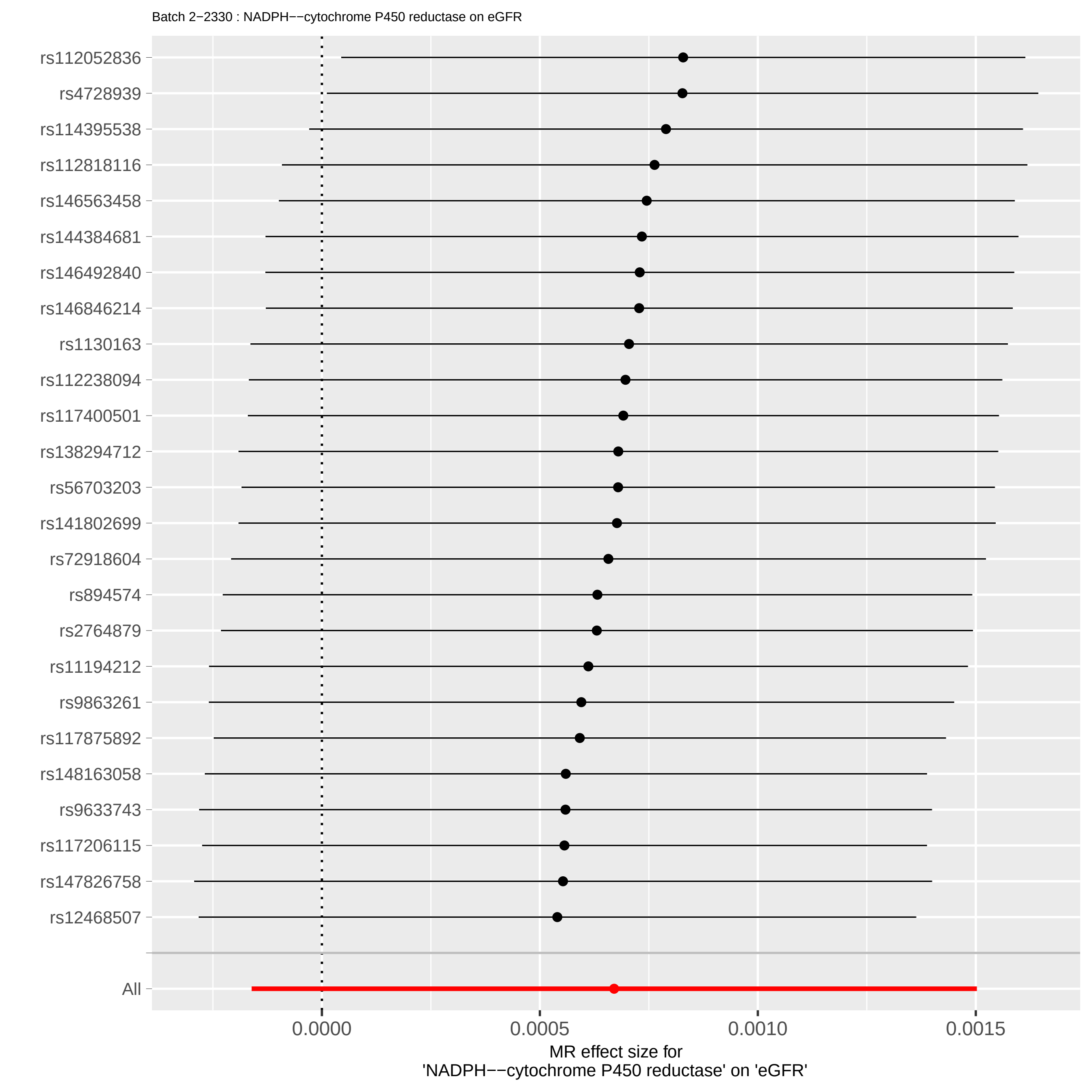

Batch 2−2330 : NADPH−−cytochrome P450 reductase on eGFR
rs112052836
rs4728939
rs114395538
rs112818116
rs146563458
rs144384681
rs146492840
rs146846214
rs1130163
rs112238094
rs117400501
rs138294712
rs56703203
rs141802699
rs72918604
rs894574
rs2764879
rs11194212
rs9863261
rs117875892
rs148163058
rs9633743
rs117206115
rs147826758
rs12468507
All
0.0000
0.0005
0.0010
0.0015
MR effect size for
'NADPH−−cytochrome P450 reductase' on 'eGFR'

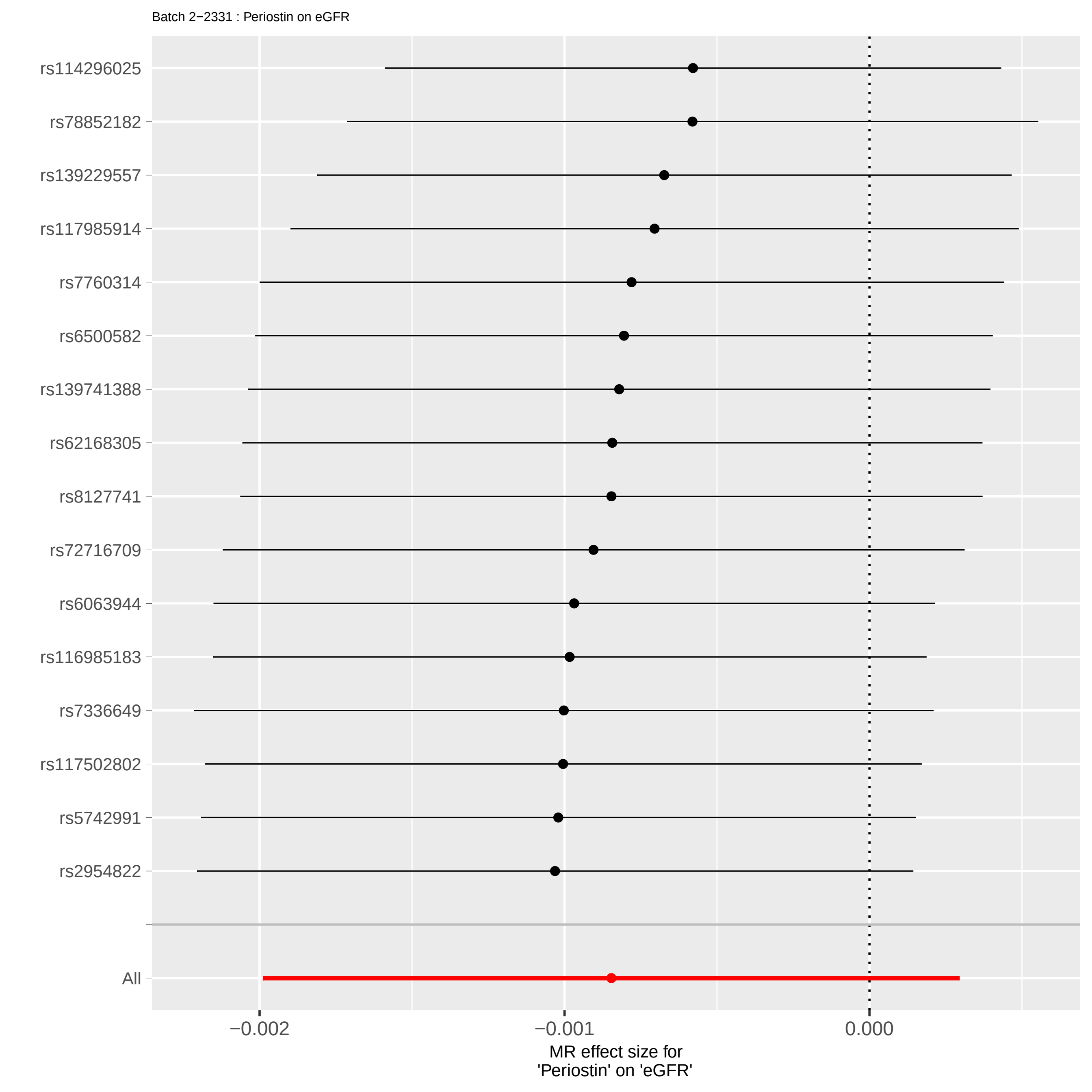

Batch 2−2331 : Periostin on eGFR
rs114296025
rs78852182
rs139229557
rs117985914
rs7760314
rs6500582
rs139741388
rs62168305
rs8127741
rs72716709
rs6063944
rs116985183
rs7336649
rs117502802
rs5742991
rs2954822
All
−0.002
−0.001
0.000
MR effect size for
'Periostin' on 'eGFR'

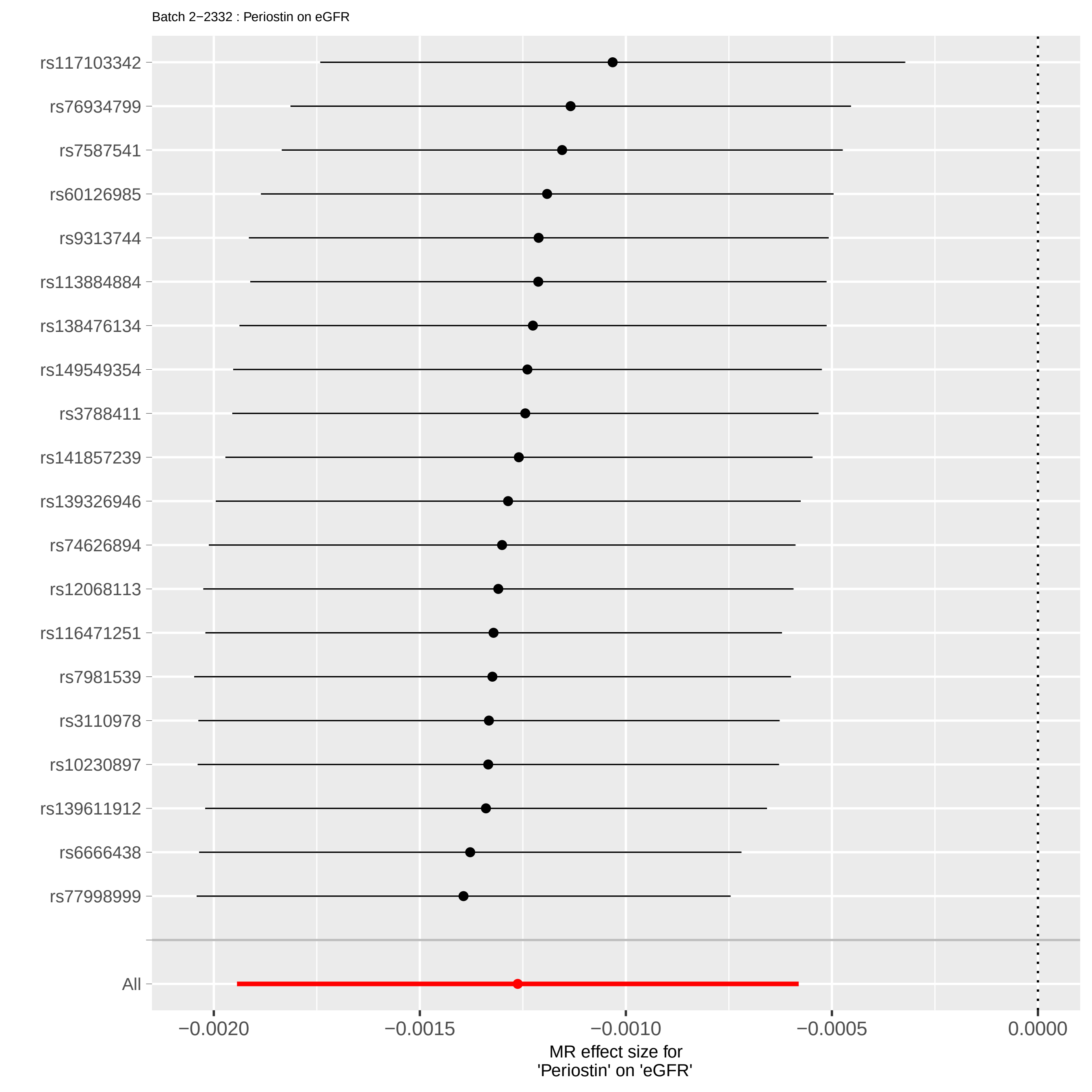

Batch 2−2332 : Periostin on eGFR
rs117103342
rs76934799
rs7587541
rs60126985
rs9313744
rs113884884
rs138476134
rs149549354
rs3788411
rs141857239
rs139326946
rs74626894
rs12068113
rs116471251
rs7981539
rs3110978
rs10230897
rs139611912
rs6666438
rs77998999
All
−0.0020
−0.0015
−0.0010
−0.0005
0.0000
MR effect size for
'Periostin' on 'eGFR'

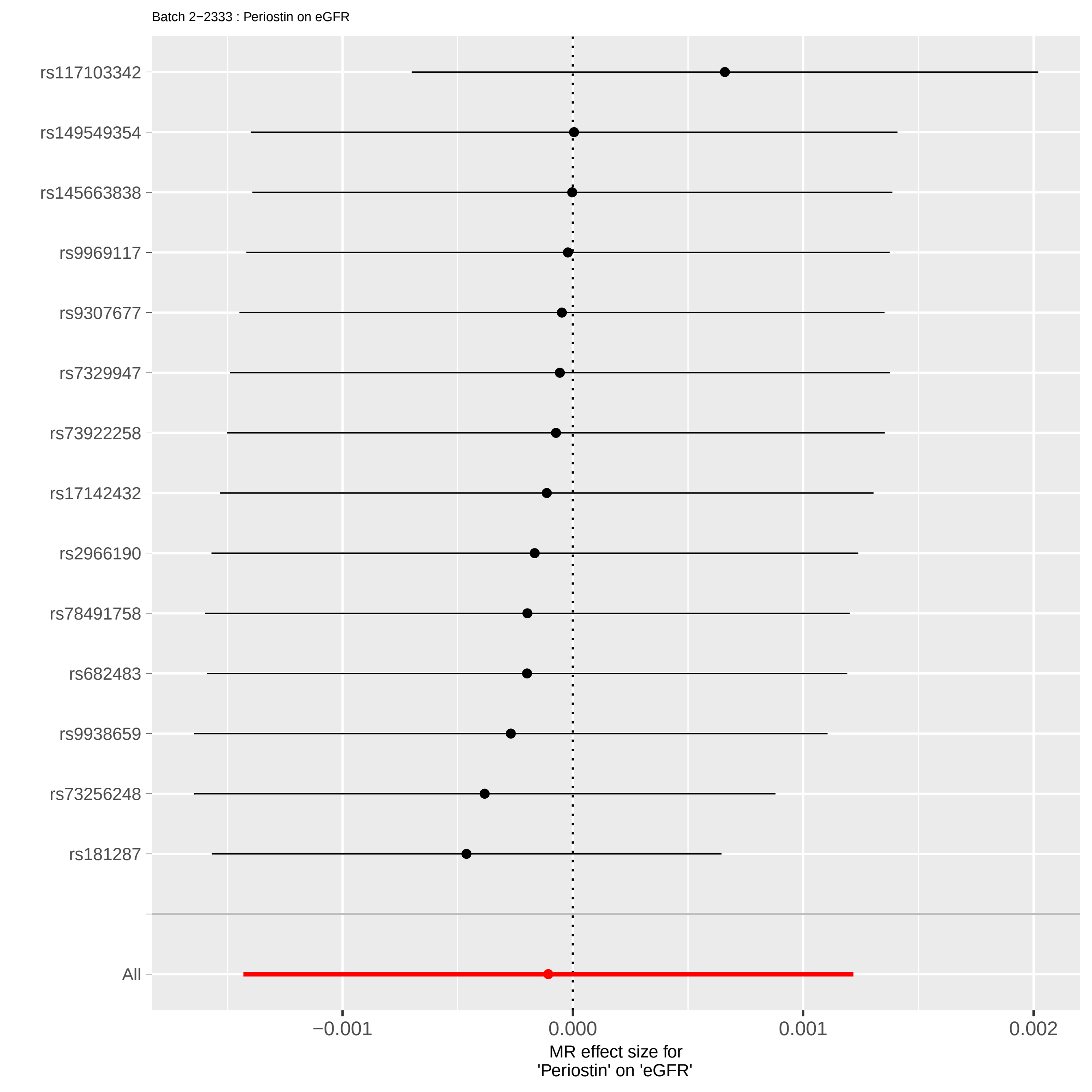
Batch 2−2333 : Periostin on eGFR
rs117103342
rs149549354
rs145663838
rs9969117
rs9307677
rs7329947
rs73922258
rs17142432
rs2966190
rs78491758
rs682483
rs9938659
rs73256248
rs181287
All
−0.001
0.000
0.001
0.002
MR effect size for
'Periostin' on 'eGFR'

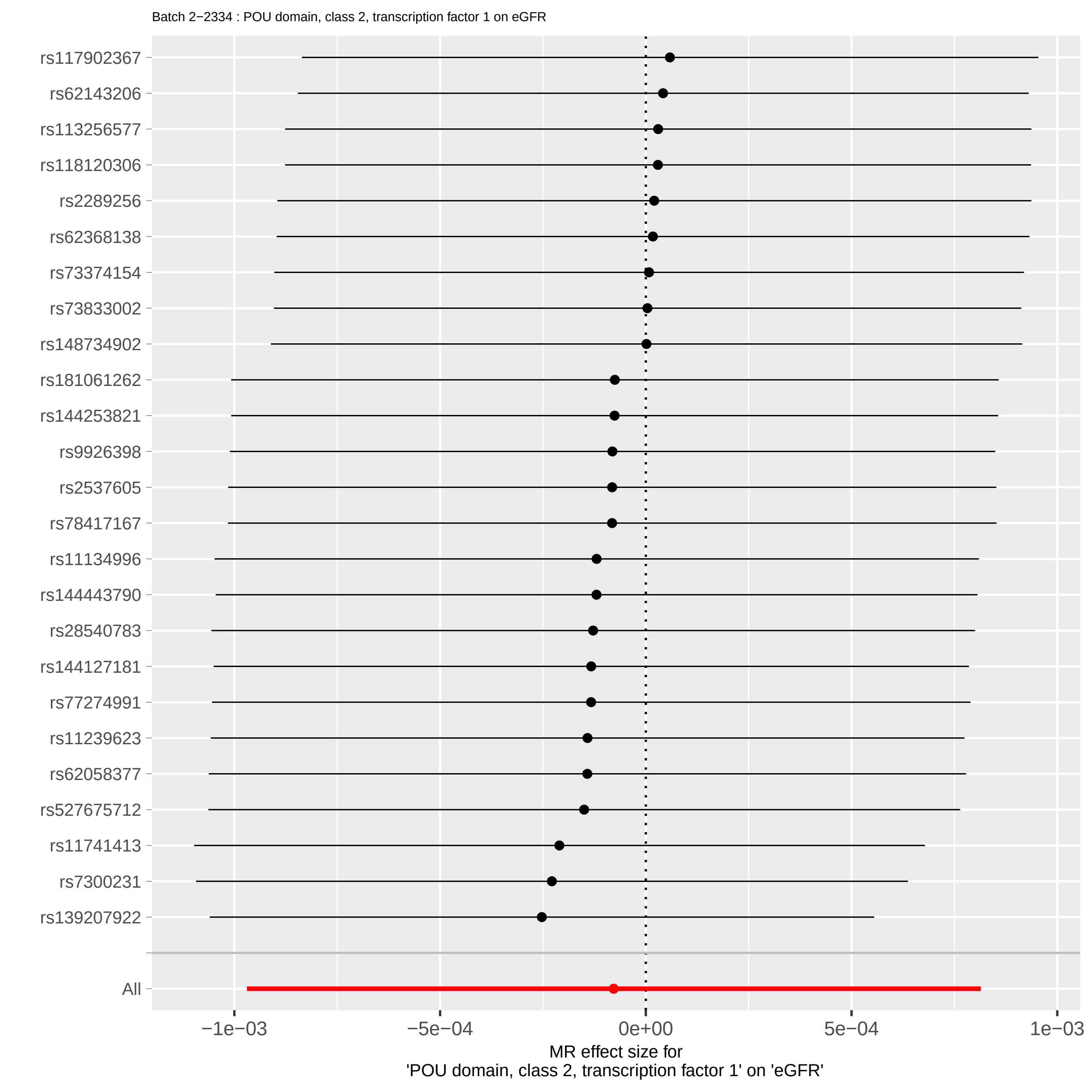

Batch 2−2334 : POU domain, class 2, transcription factor 1 on eGFR
rs117902367
rs62143206
rs113256577
rs118120306
rs2289256
rs62368138
rs73374154
rs73833002
rs148734902
rs181061262
rs144253821
rs9926398
rs2537605
rs78417167
rs11134996
rs144443790
rs28540783
rs144127181
rs77274991
rs11239623
rs62058377
rs527675712
rs11741413
rs7300231
rs139207922
All
−1e−03
−5e−04
0e+00
5e−04
1e−03
MR effect size for
'POU domain, class 2, transcription factor 1' on 'eGFR'

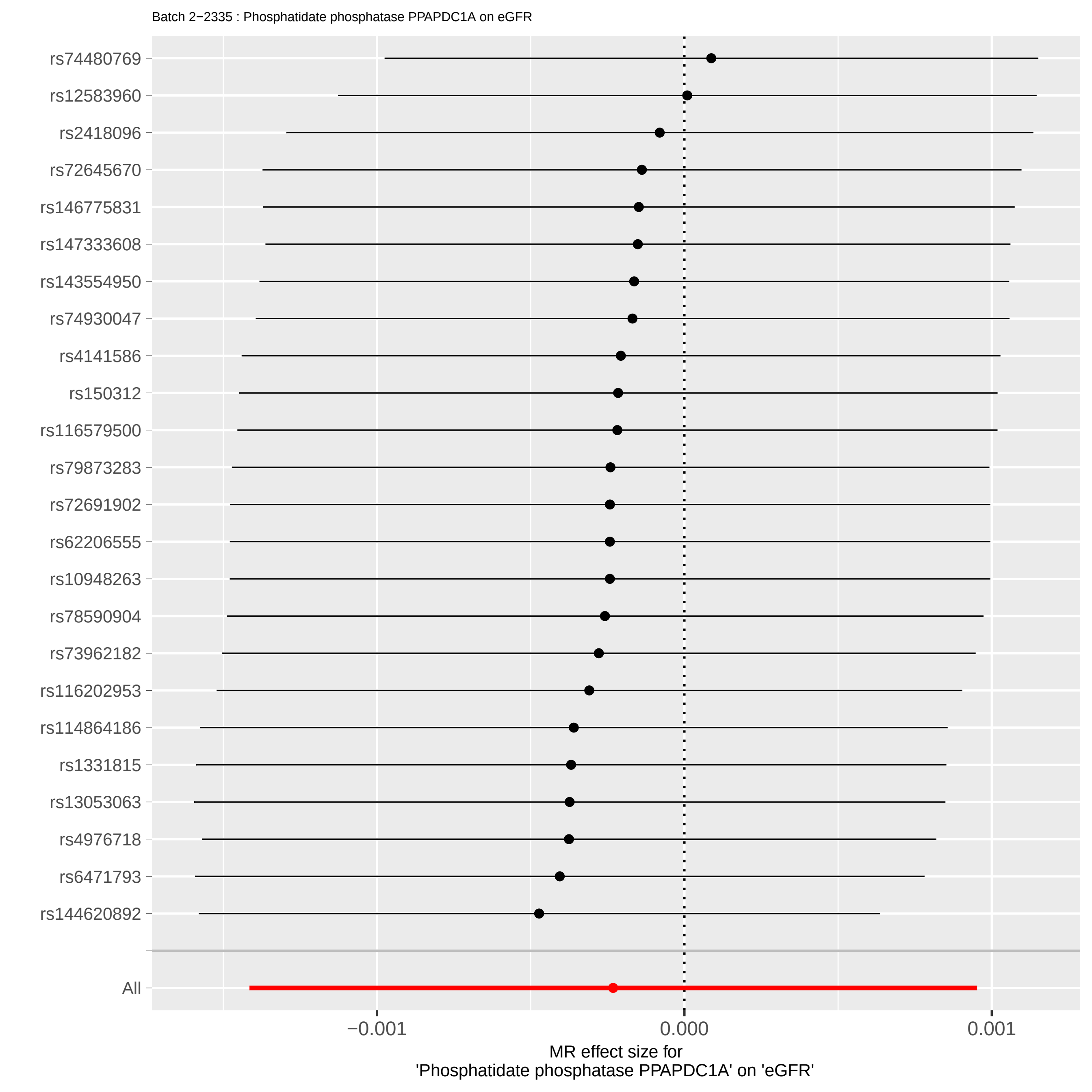

Batch 2−2335 : Phosphatidate phosphatase PPAPDC1A on eGFR
rs74480769
rs12583960
rs2418096
rs72645670
rs146775831
rs147333608
rs143554950
rs74930047
rs4141586
rs150312
rs116579500
rs79873283
rs72691902
rs62206555
rs10948263
rs78590904
rs73962182
rs116202953
rs114864186
rs1331815
rs13053063
rs4976718
rs6471793
rs144620892
All
−0.001
0.000
0.001
MR effect size for
'Phosphatidate phosphatase PPAPDC1A' on 'eGFR'

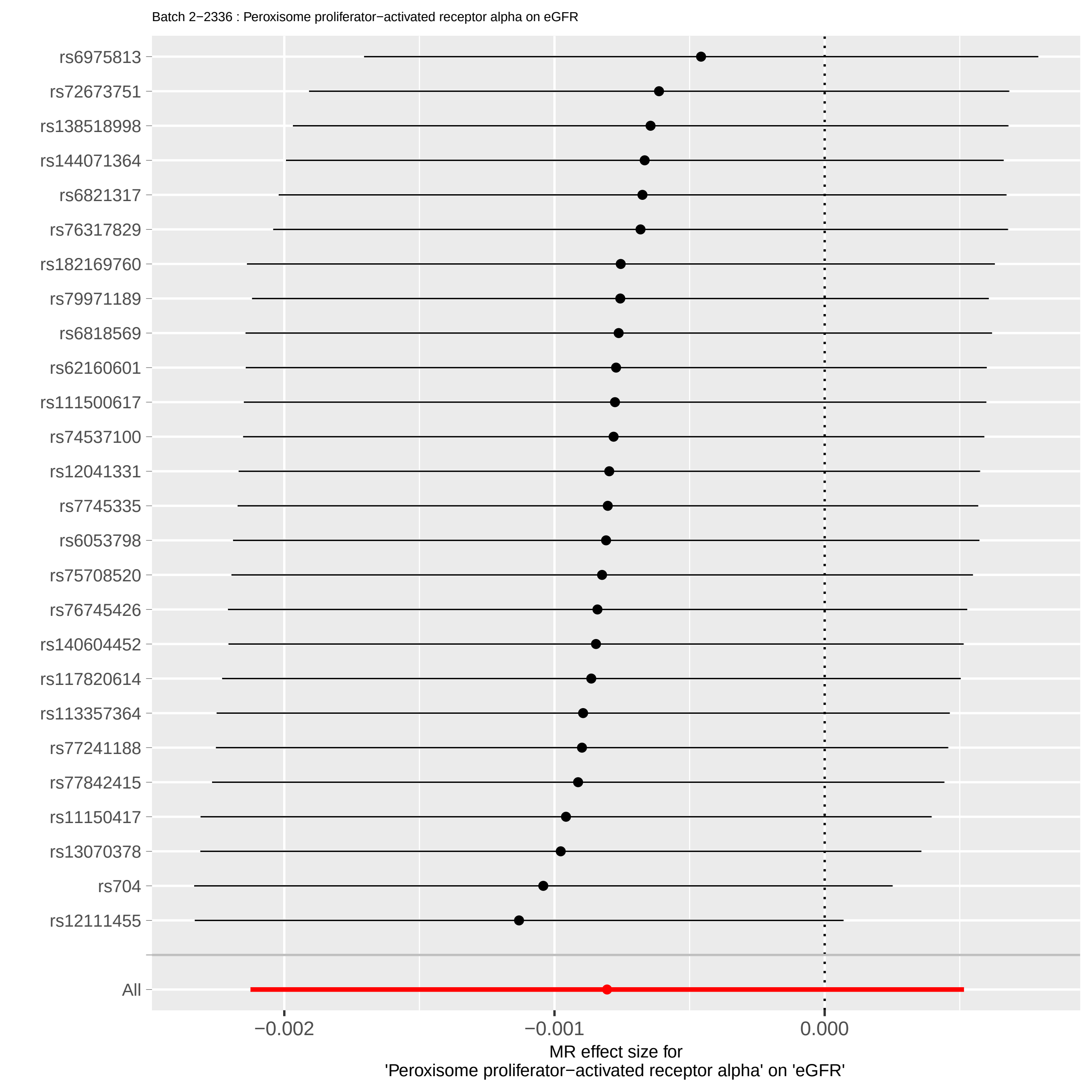
Batch 2−2336 : Peroxisome proliferator−activated receptor alpha on eGFR
rs6975813
rs72673751
rs138518998
rs144071364
rs6821317
rs76317829
rs182169760
rs79971189
rs6818569
rs62160601
rs111500617
rs74537100
rs12041331
rs7745335
rs6053798
rs75708520
rs76745426
rs140604452
rs117820614
rs113357364
rs77241188
rs77842415
rs11150417
rs13070378
rs704
rs12111455
All
−0.002
−0.001
0.000
MR effect size for
'Peroxisome proliferator−activated receptor alpha' on 'eGFR'

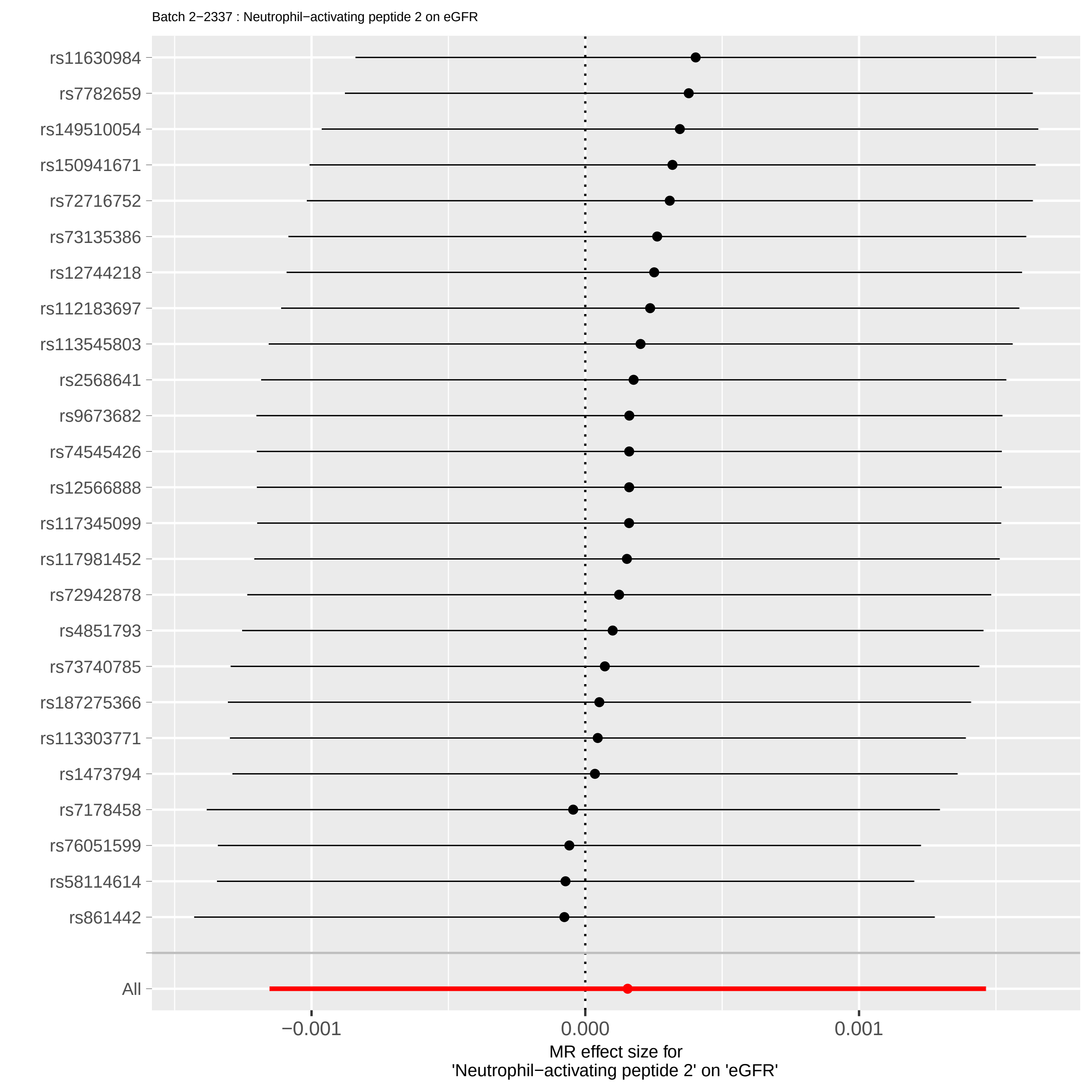

Batch 2−2337 : Neutrophil−activating peptide 2 on eGFR
rs11630984
rs7782659
rs149510054
rs150941671
rs72716752
rs73135386
rs12744218
rs112183697
rs113545803
rs2568641
rs9673682
rs74545426
rs12566888
rs117345099
rs117981452
rs72942878
rs4851793
rs73740785
rs187275366
rs113303771
rs1473794
rs7178458
rs76051599
rs58114614
rs861442
All
−0.001
0.000
0.001
MR effect size for
'Neutrophil−activating peptide 2' on 'eGFR'

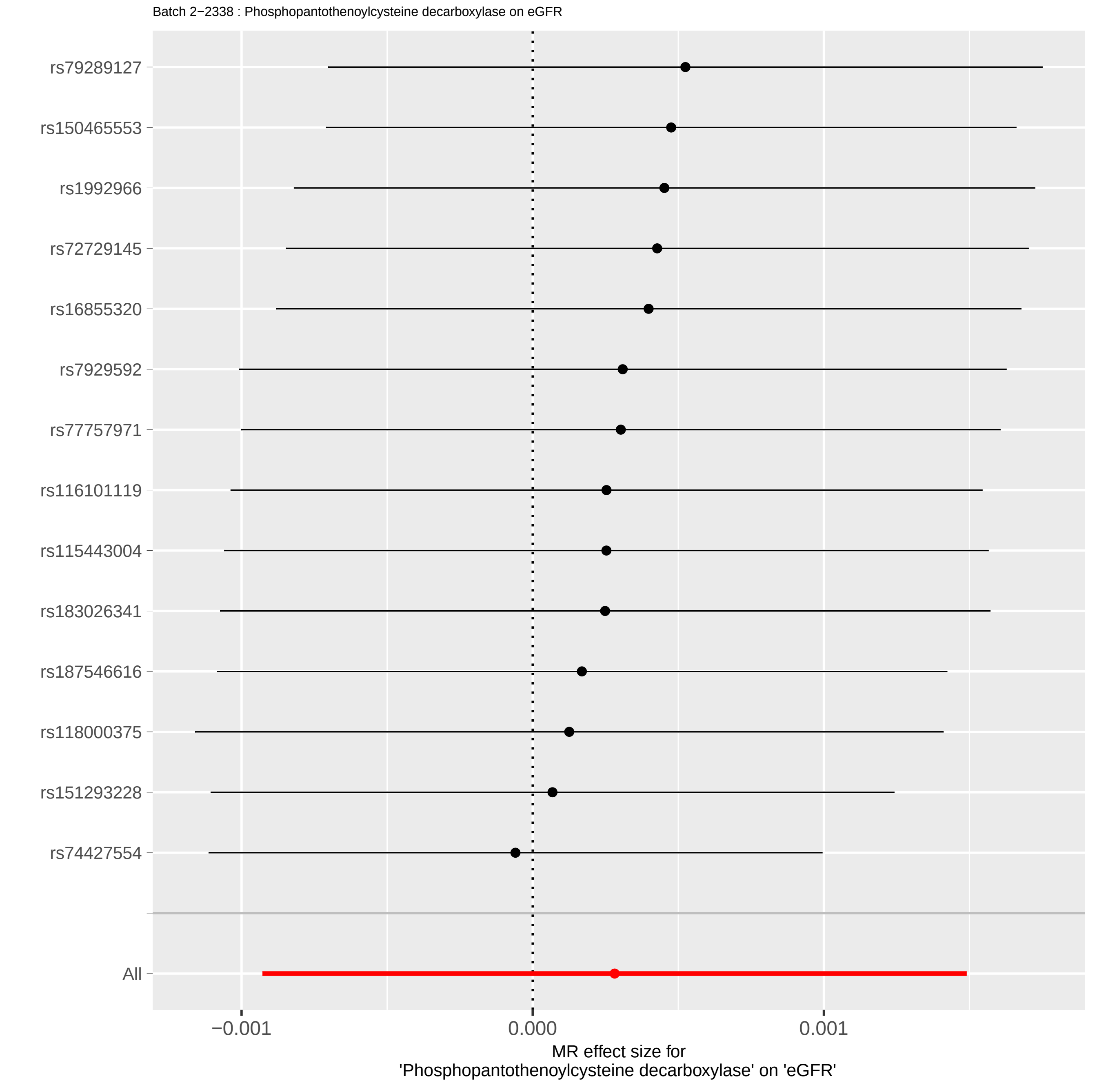

Batch 2−2338 : Phosphopantothenoylcysteine decarboxylase on eGFR
rs79289127
rs150465553
rs1992966
rs72729145
rs16855320
rs7929592
rs77757971
rs116101119
rs115443004
rs183026341
rs187546616
rs118000375
rs151293228
rs74427554
All
−0.001
0.000
0.001
MR effect size for
'Phosphopantothenoylcysteine decarboxylase' on 'eGFR'

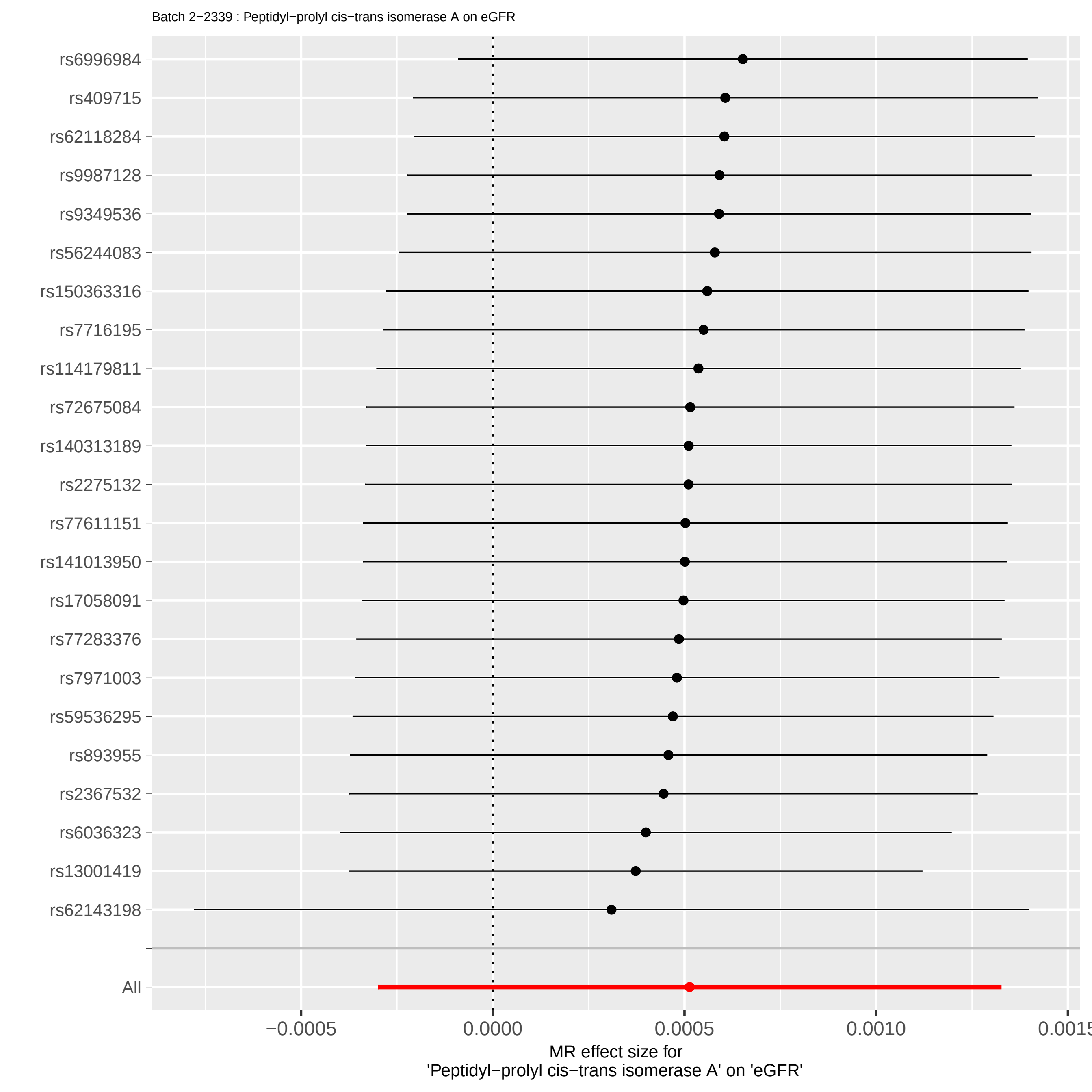

Batch 2−2339 : Peptidyl−prolyl cis−trans isomerase A on eGFR
rs6996984
rs409715
rs62118284
rs9987128
rs9349536
rs56244083
rs150363316
rs7716195
rs114179811
rs72675084
rs140313189
rs2275132
rs77611151
rs141013950
rs17058091
rs77283376
rs7971003
rs59536295
rs893955
rs2367532
rs6036323
rs13001419
rs62143198
All
−0.0005
0.0000
0.0005
0.0010
0.0015
MR effect size for
'Peptidyl−prolyl cis−trans isomerase A' on 'eGFR'

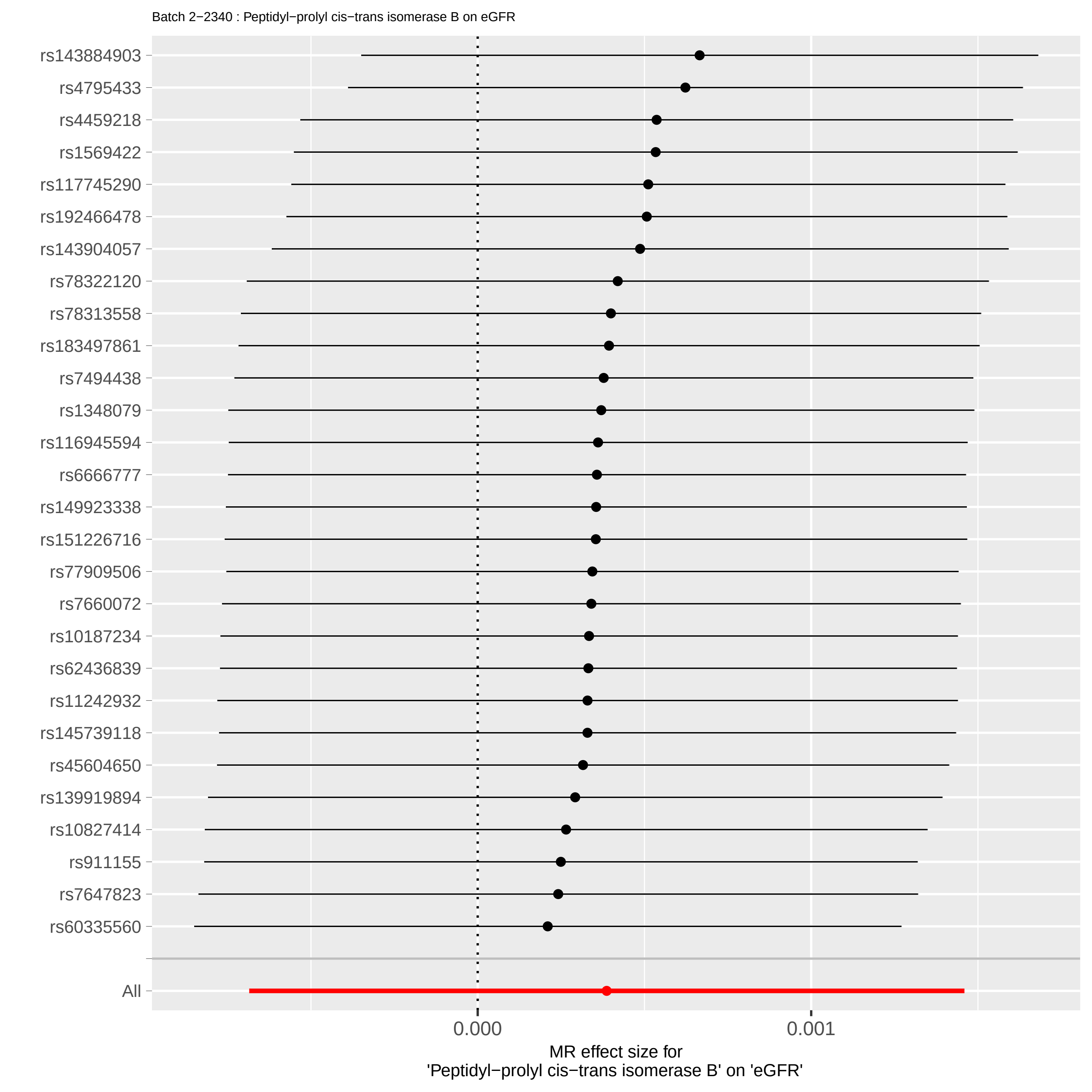

Batch 2−2340 : Peptidyl−prolyl cis−trans isomerase B on eGFR
rs143884903
rs4795433
rs4459218
rs1569422
rs117745290
rs192466478
rs143904057
rs78322120
rs78313558
rs183497861
rs7494438
rs1348079
rs116945594
rs6666777
rs149923338
rs151226716
rs77909506
rs7660072
rs10187234
rs62436839
rs11242932
rs145739118
rs45604650
rs139919894
rs10827414
rs911155
rs7647823
rs60335560
All
0.000
0.001
MR effect size for
'Peptidyl−prolyl cis−trans isomerase B' on 'eGFR'

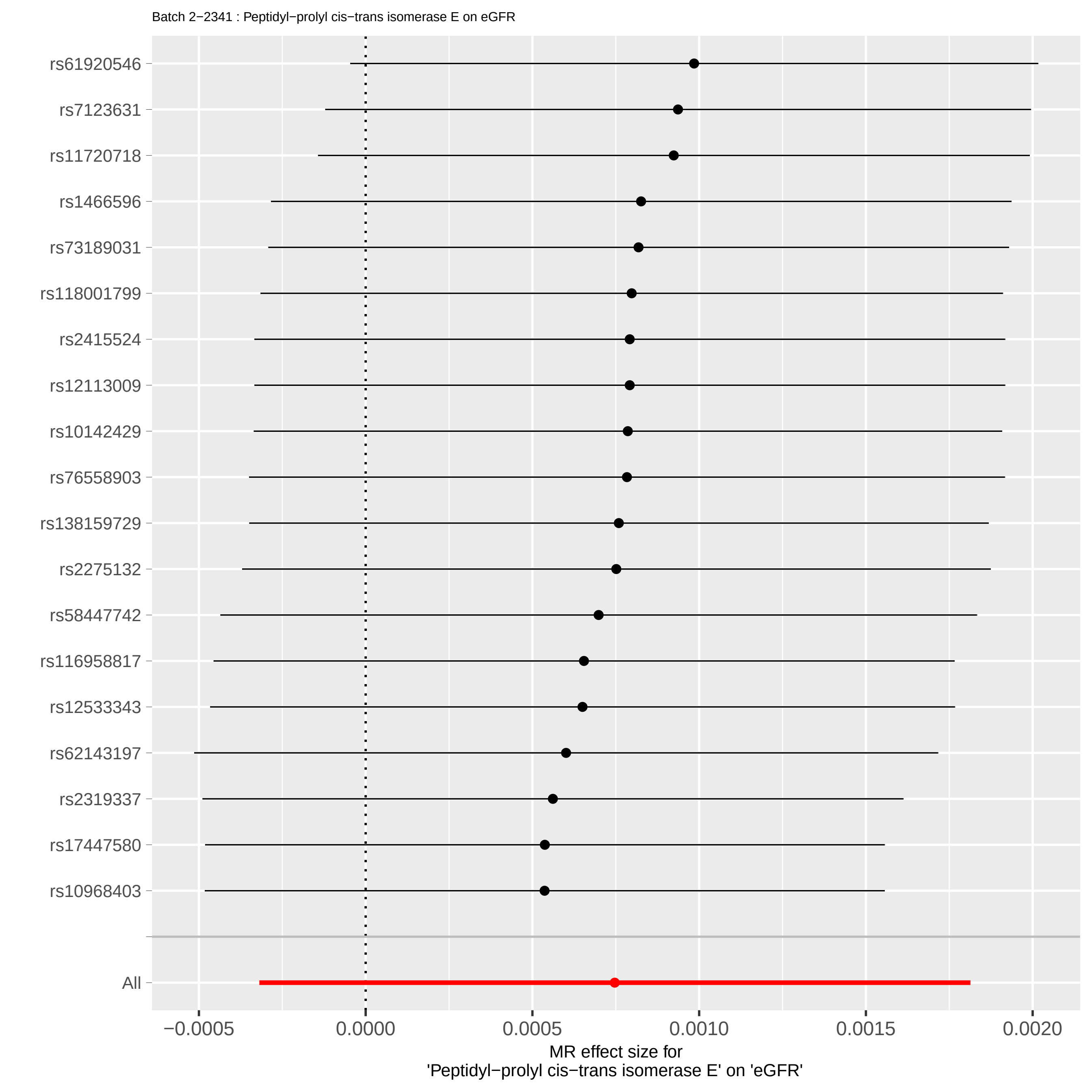
Batch 2−2341 : Peptidyl−prolyl cis−trans isomerase E on eGFR
rs61920546
rs7123631
rs11720718
rs1466596
rs73189031
rs118001799
rs2415524
rs12113009
rs10142429
rs76558903
rs138159729
rs2275132
rs58447742
rs116958817
rs12533343
rs62143197
rs2319337
rs17447580
rs10968403
All
−0.0005
0.0000
0.0005
0.0010
0.0015
0.0020
MR effect size for
'Peptidyl−prolyl cis−trans isomerase E' on 'eGFR'

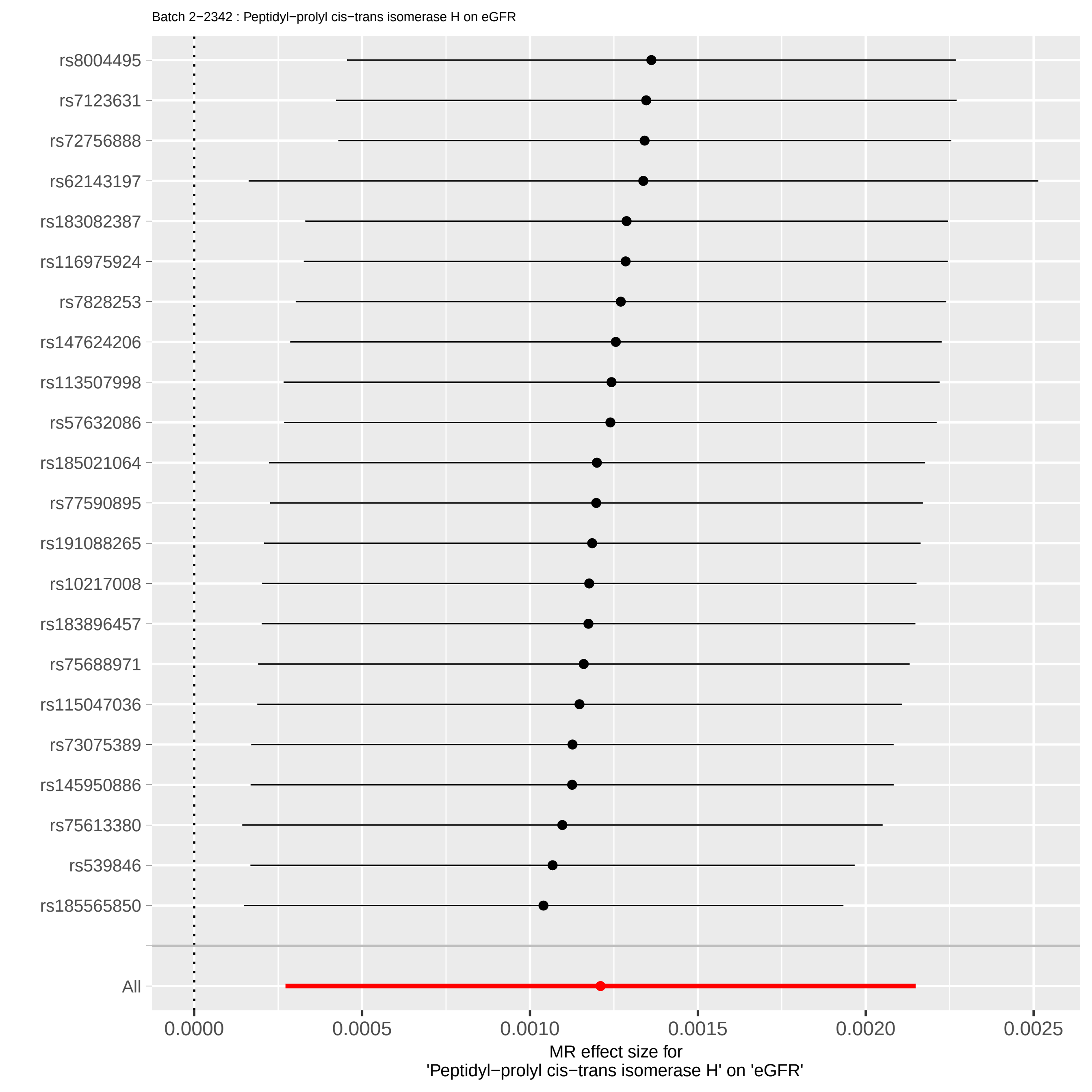
Batch 2−2342 : Peptidyl−prolyl cis−trans isomerase H on eGFR
rs8004495
rs7123631
rs72756888
rs62143197
rs183082387
rs116975924
rs7828253
rs147624206
rs113507998
rs57632086
rs185021064
rs77590895
rs191088265
rs10217008
rs183896457
rs75688971
rs115047036
rs73075389
rs145950886
rs75613380
rs539846
rs185565850
All
0.0000
0.0005
0.0010
0.0015
0.0020
0.0025
MR effect size for
'Peptidyl−prolyl cis−trans isomerase H' on 'eGFR'

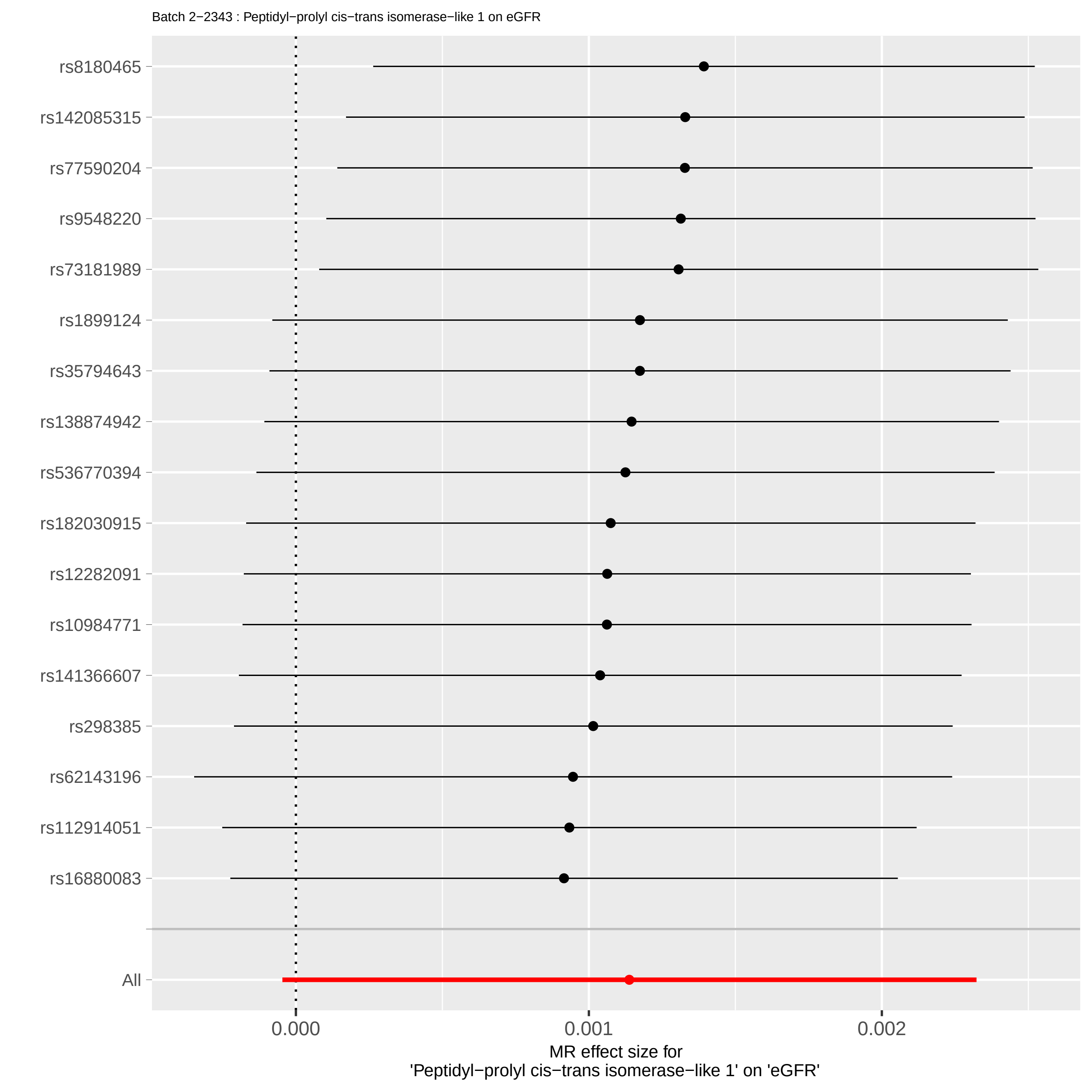

Batch 2−2343 : Peptidyl−prolyl cis−trans isomerase−like 1 on eGFR
rs8180465
rs142085315
rs77590204
rs9548220
rs73181989
rs1899124
rs35794643
rs138874942
rs536770394
rs182030915
rs12282091
rs10984771
rs141366607
rs298385
rs62143196
rs112914051
rs16880083
All
0.000
0.001
0.002
MR effect size for
'Peptidyl−prolyl cis−trans isomerase−like 1' on 'eGFR'

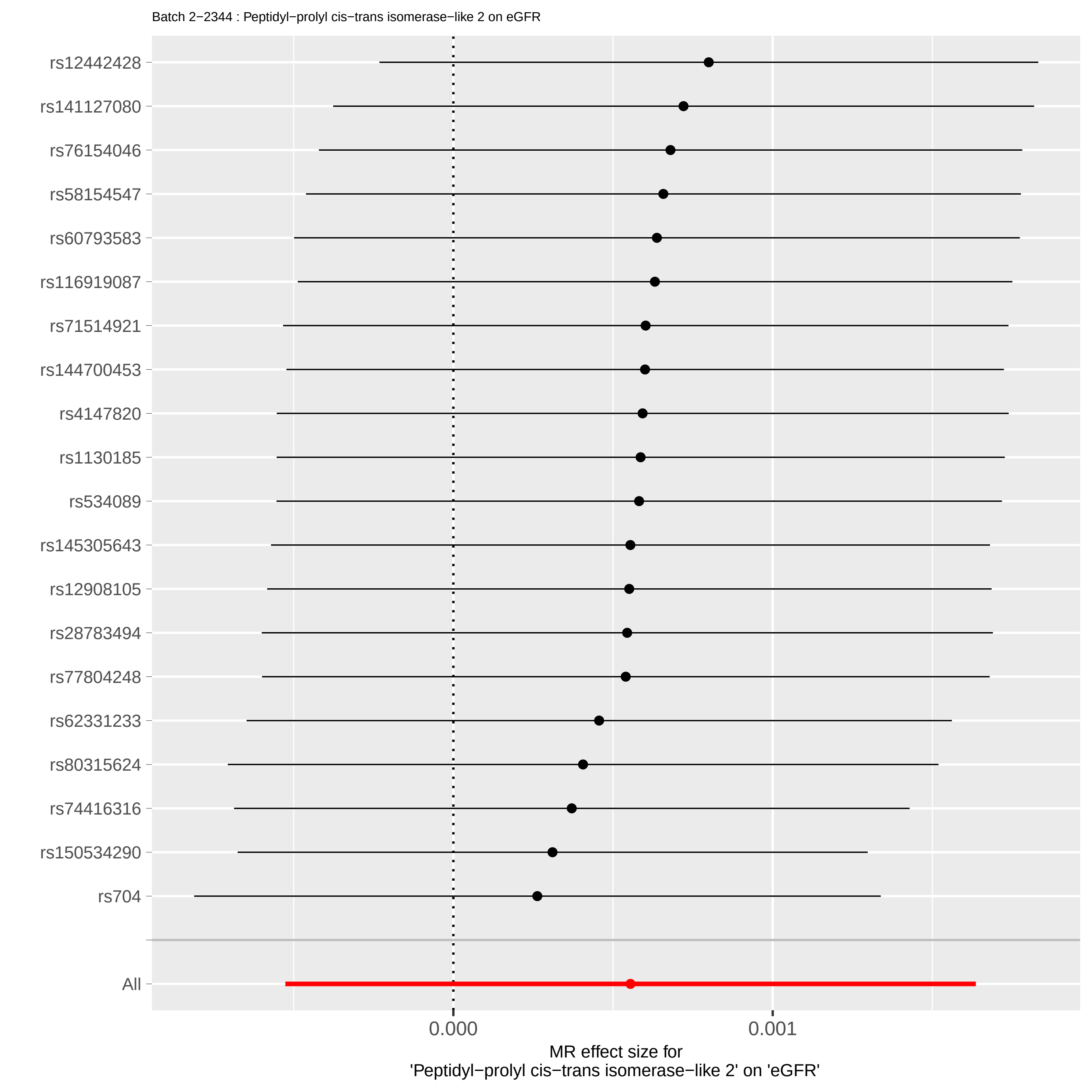

Batch 2−2344 : Peptidyl−prolyl cis−trans isomerase−like 2 on eGFR
rs12442428
rs141127080
rs76154046
rs58154547
rs60793583
rs116919087
rs71514921
rs144700453
rs4147820
rs1130185
rs534089
rs145305643
rs12908105
rs28783494
rs77804248
rs62331233
rs80315624
rs74416316
rs150534290
rs704
All
0.000
0.001
MR effect size for
'Peptidyl−prolyl cis−trans isomerase−like 2' on 'eGFR'

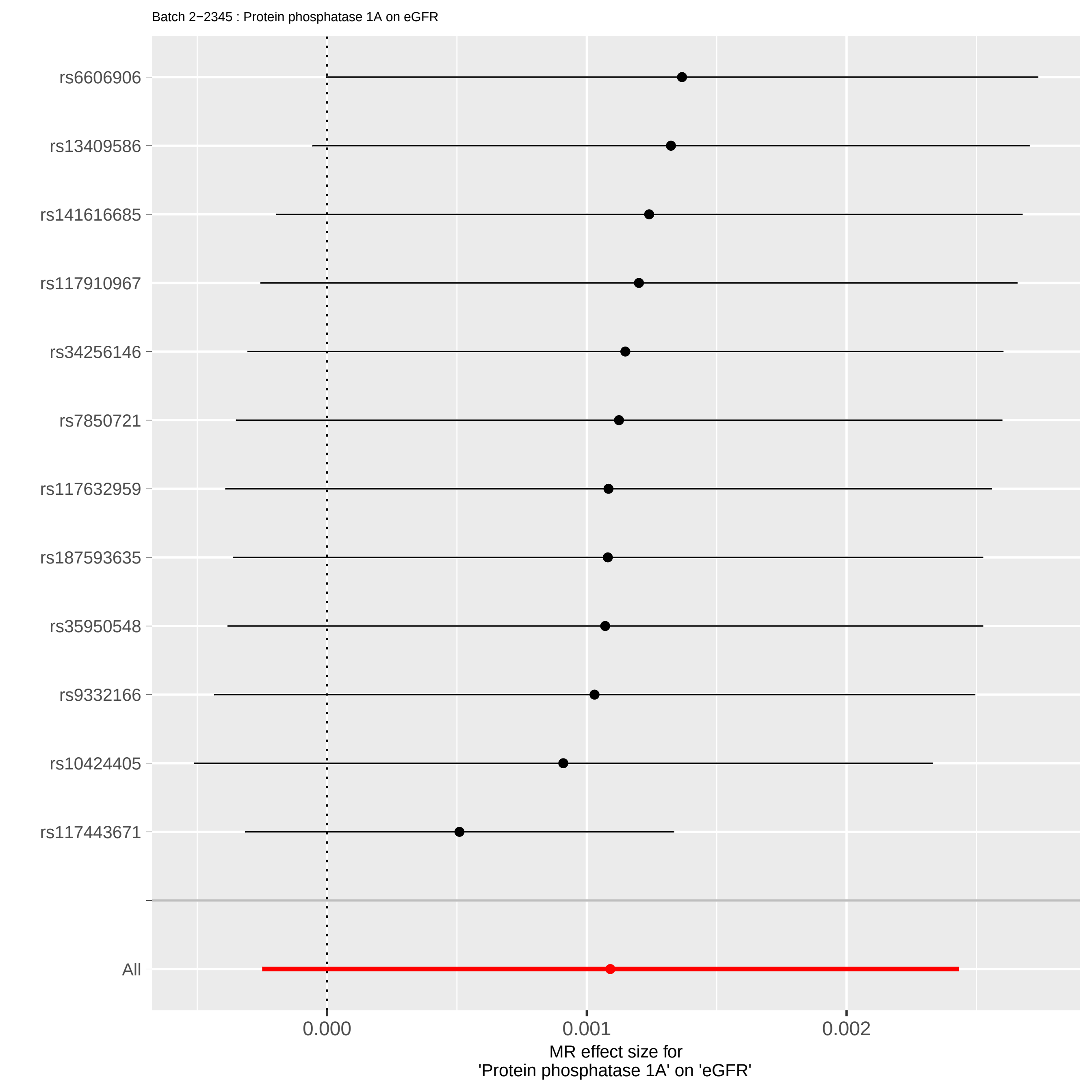
Batch 2−2345 : Protein phosphatase 1A on eGFR
rs6606906
rs13409586
rs141616685
rs117910967
rs34256146
rs7850721
rs117632959
rs187593635
rs35950548
rs9332166
rs10424405
rs117443671
All
0.000
0.001
0.002
MR effect size for
'Protein phosphatase 1A' on 'eGFR'

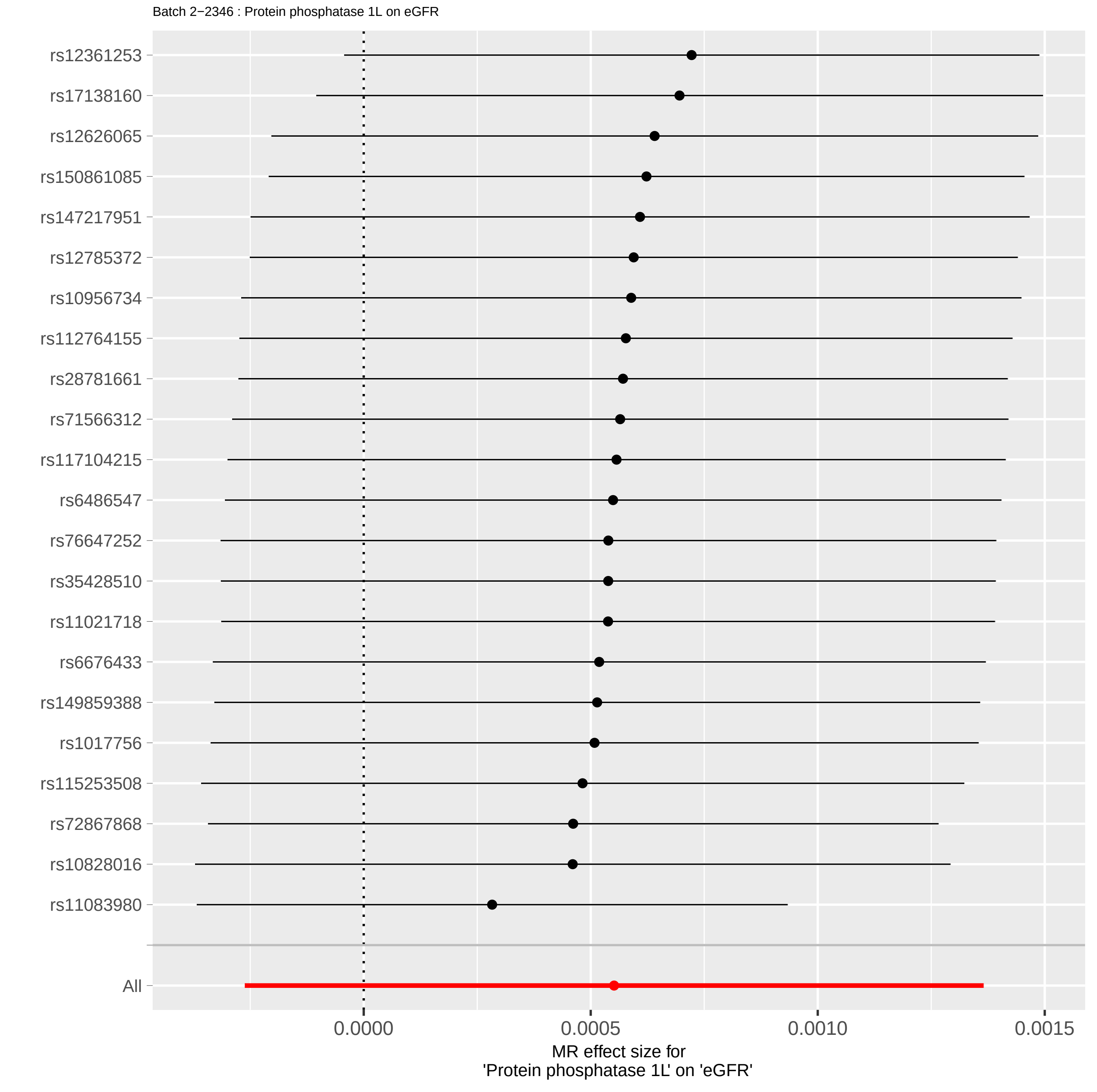
Batch 2−2346 : Protein phosphatase 1L on eGFR
rs12361253
rs17138160
rs12626065
rs150861085
rs147217951
rs12785372
rs10956734
rs112764155
rs28781661
rs71566312
rs117104215
rs6486547
rs76647252
rs35428510
rs11021718
rs6676433
rs149859388
rs1017756
rs115253508
rs72867868
rs10828016
rs11083980
All
0.0000
0.0005
0.0010
0.0015
MR effect size for
'Protein phosphatase 1L' on 'eGFR'

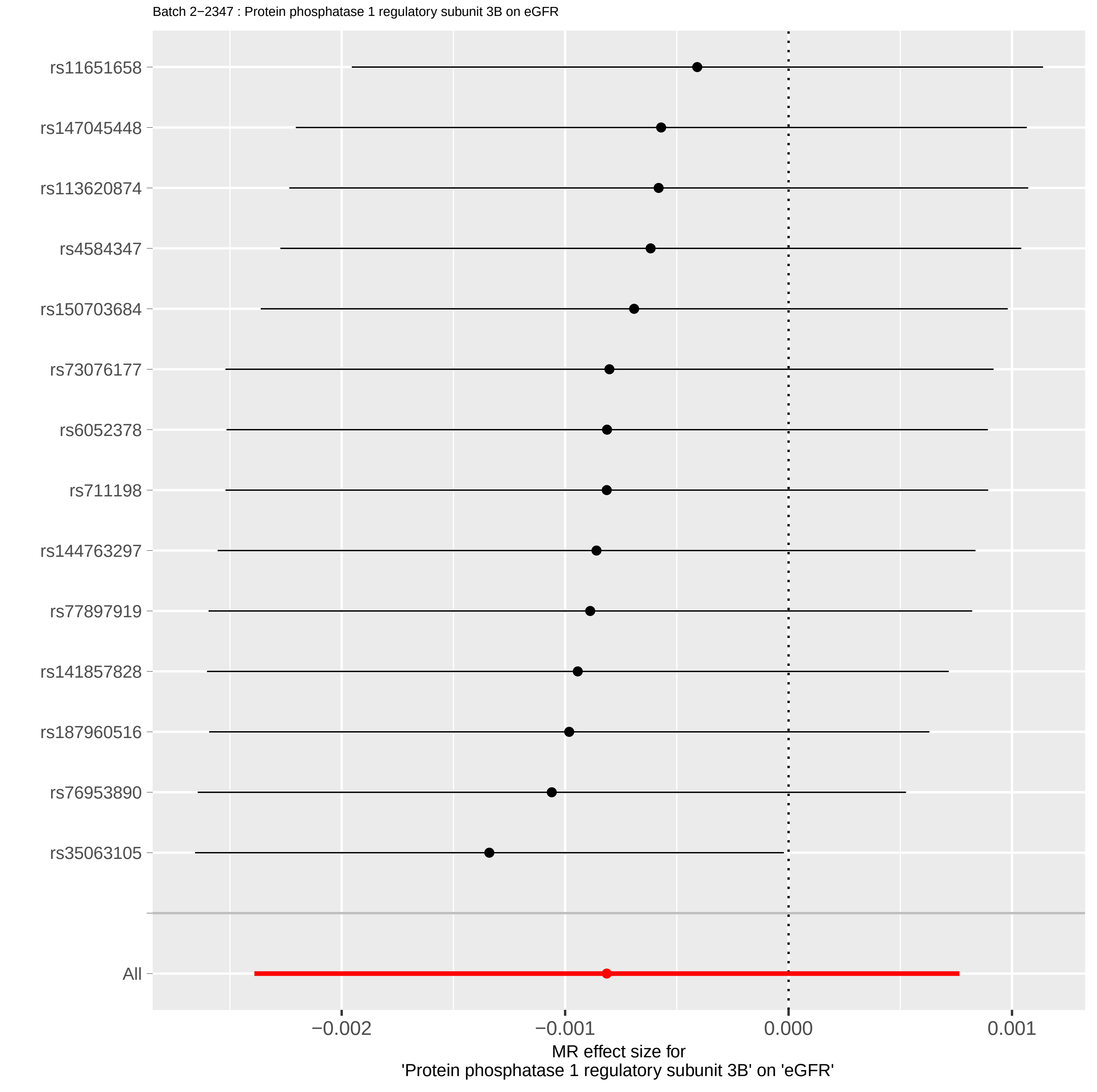
Batch 2−2347 : Protein phosphatase 1 regulatory subunit 3B on eGFR
rs11651658
rs147045448
rs113620874
rs4584347
rs150703684
rs73076177
rs6052378
rs711198
rs144763297
rs77897919
rs141857828
rs187960516
rs76953890
rs35063105
All
−0.002
−0.001
0.000
0.001
MR effect size for
'Protein phosphatase 1 regulatory subunit 3B' on 'eGFR'

Batch 2−2348 : Serine/threonine−protein phosphatase 2A 65 kDa regulatory subunit A alpha isoform on eGFR

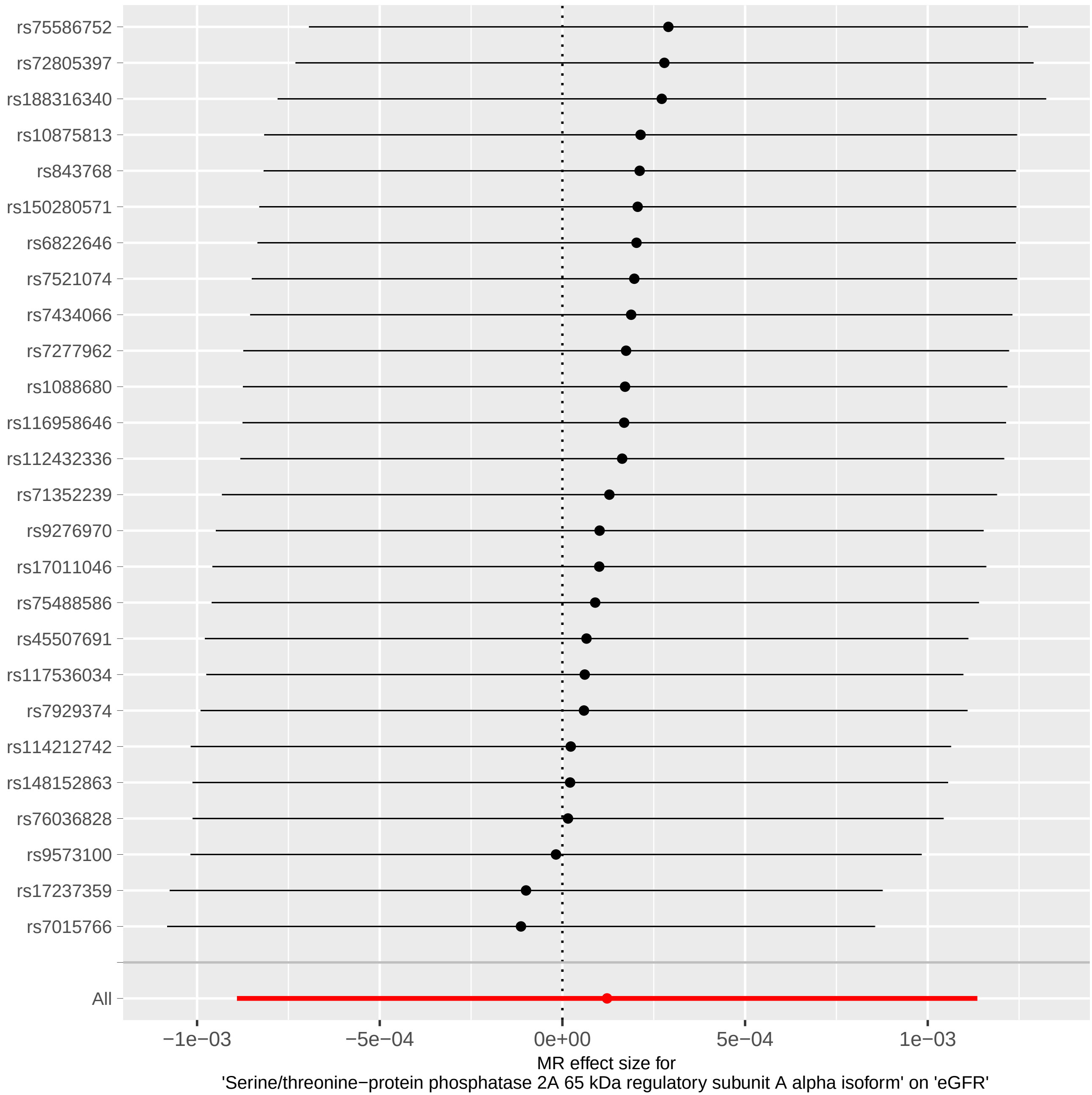

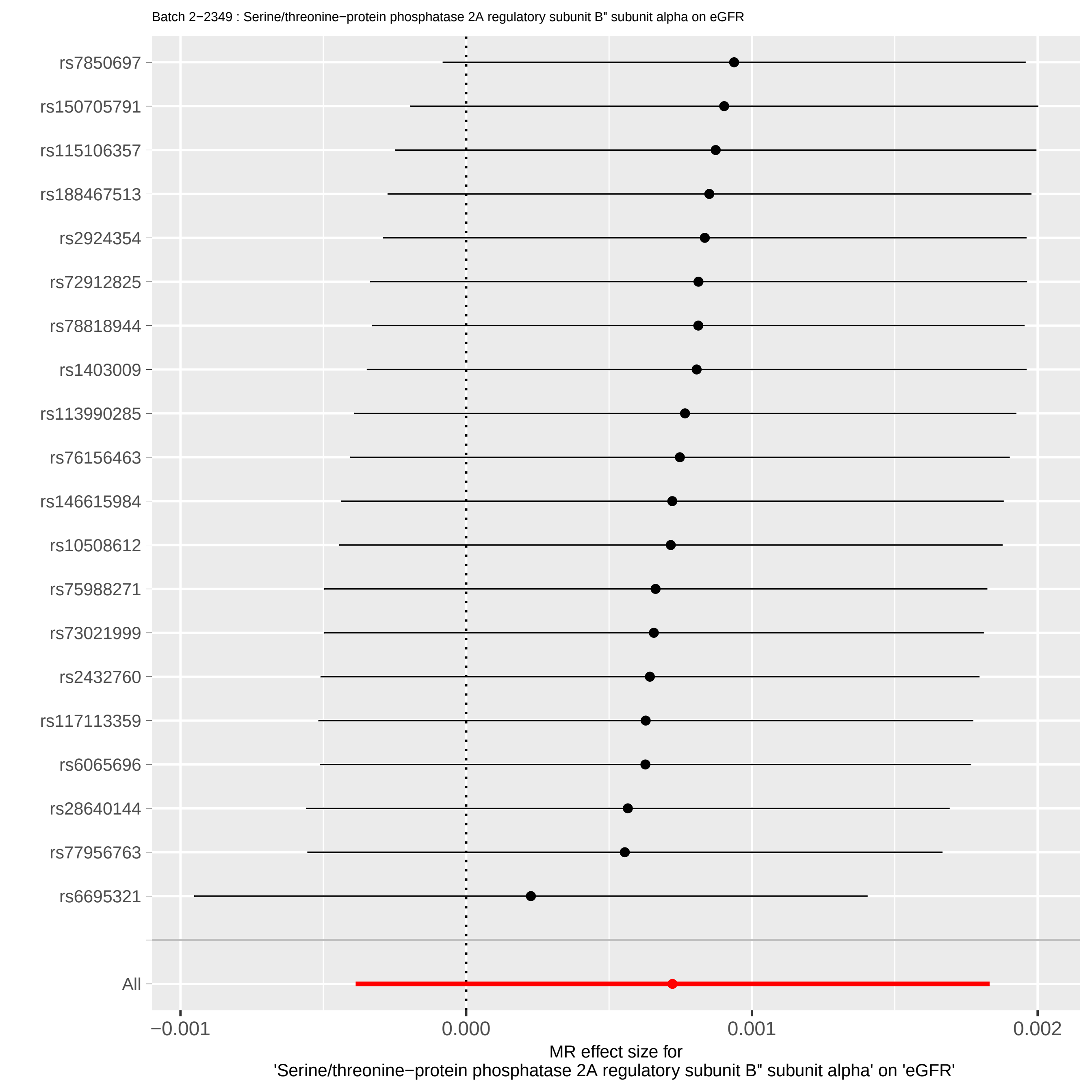

Batch 2−2349 : Serine/threonine−protein phosphatase 2A regulatory subunit B" subunit alpha on eGFR
rs7850697
rs150705791
rs115106357
rs188467513
rs2924354
rs72912825
rs78818944
rs1403009
rs113990285
rs76156463
rs146615984
rs10508612
rs75988271
rs73021999
rs2432760
rs117113359
rs6065696
rs28640144
rs77956763
rs6695321
All
−0.001
0.000
0.001
0.002
MR effect size for
'Serine/threonine−protein phosphatase 2A regulatory subunit B" subunit alpha' on 'eGFR'

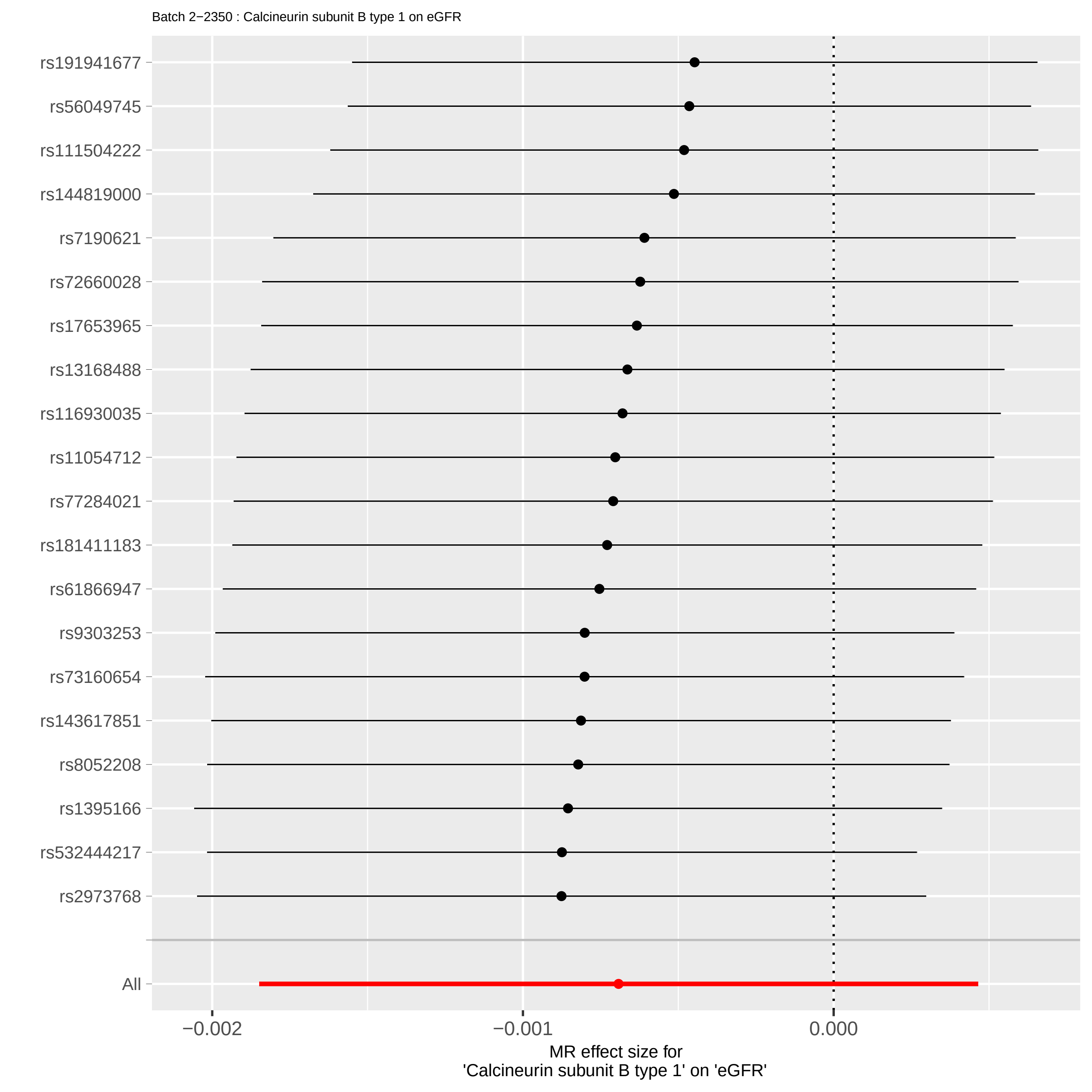

Batch 2−2350 : Calcineurin subunit B type 1 on eGFR
rs191941677
rs56049745
rs111504222
rs144819000
rs7190621
rs72660028
rs17653965
rs13168488
rs116930035
rs11054712
rs77284021
rs181411183
rs61866947
rs9303253
rs73160654
rs143617851
rs8052208
rs1395166
rs532444217
rs2973768
All
−0.002
−0.001
0.000
MR effect size for
'Calcineurin subunit B type 1' on 'eGFR'

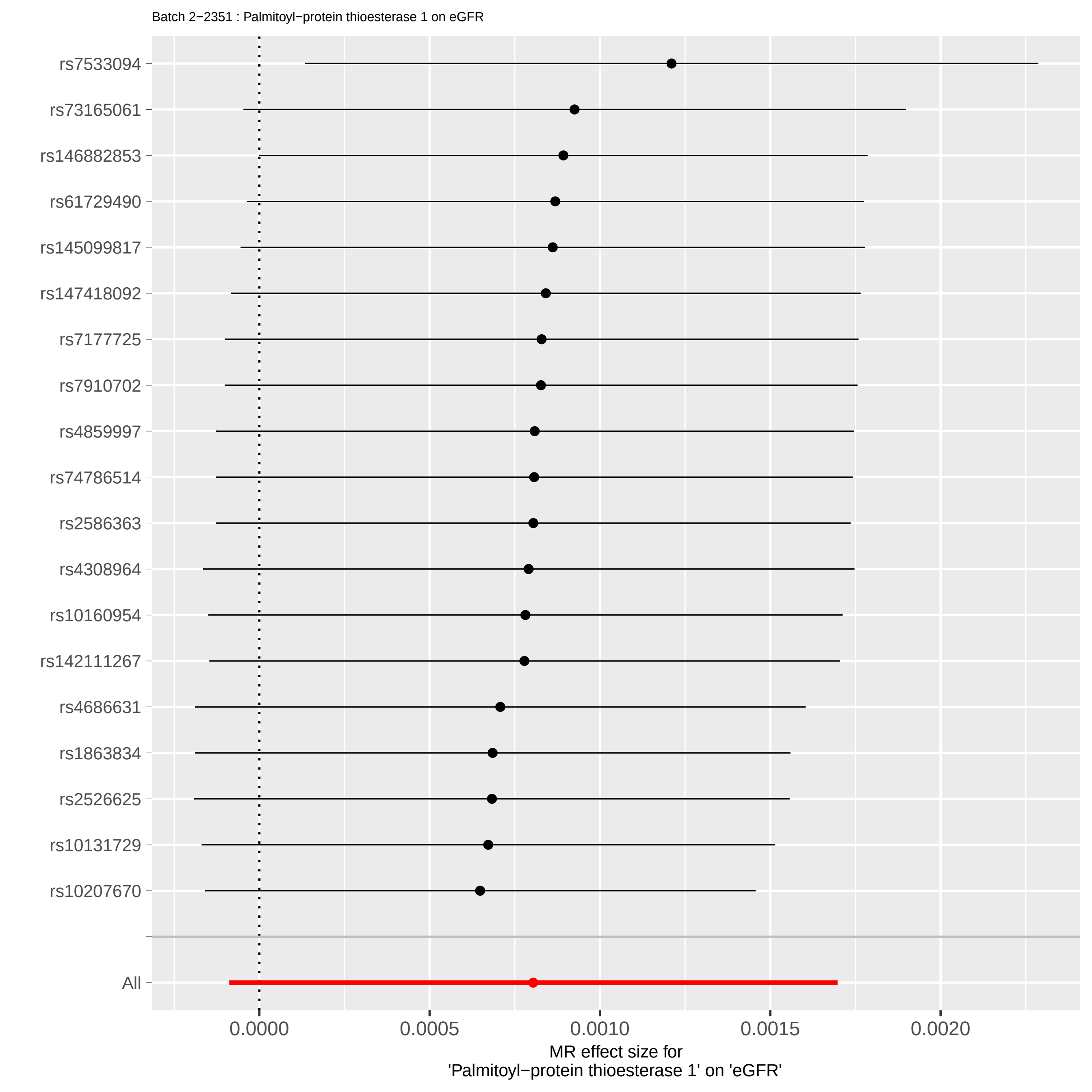
Batch 2−2351 : Palmitoyl−protein thioesterase 1 on eGFR
rs7533094
rs73165061
rs146882853
rs61729490
rs145099817
rs147418092
rs7177725
rs7910702
rs4859997
rs74786514
rs2586363
rs4308964
rs10160954
rs142111267
rs4686631
rs1863834
rs2526625
rs10131729
rs10207670
All
0.0000
0.0005
0.0010
0.0015
0.0020
MR effect size for
'Palmitoyl−protein thioesterase 1' on 'eGFR'

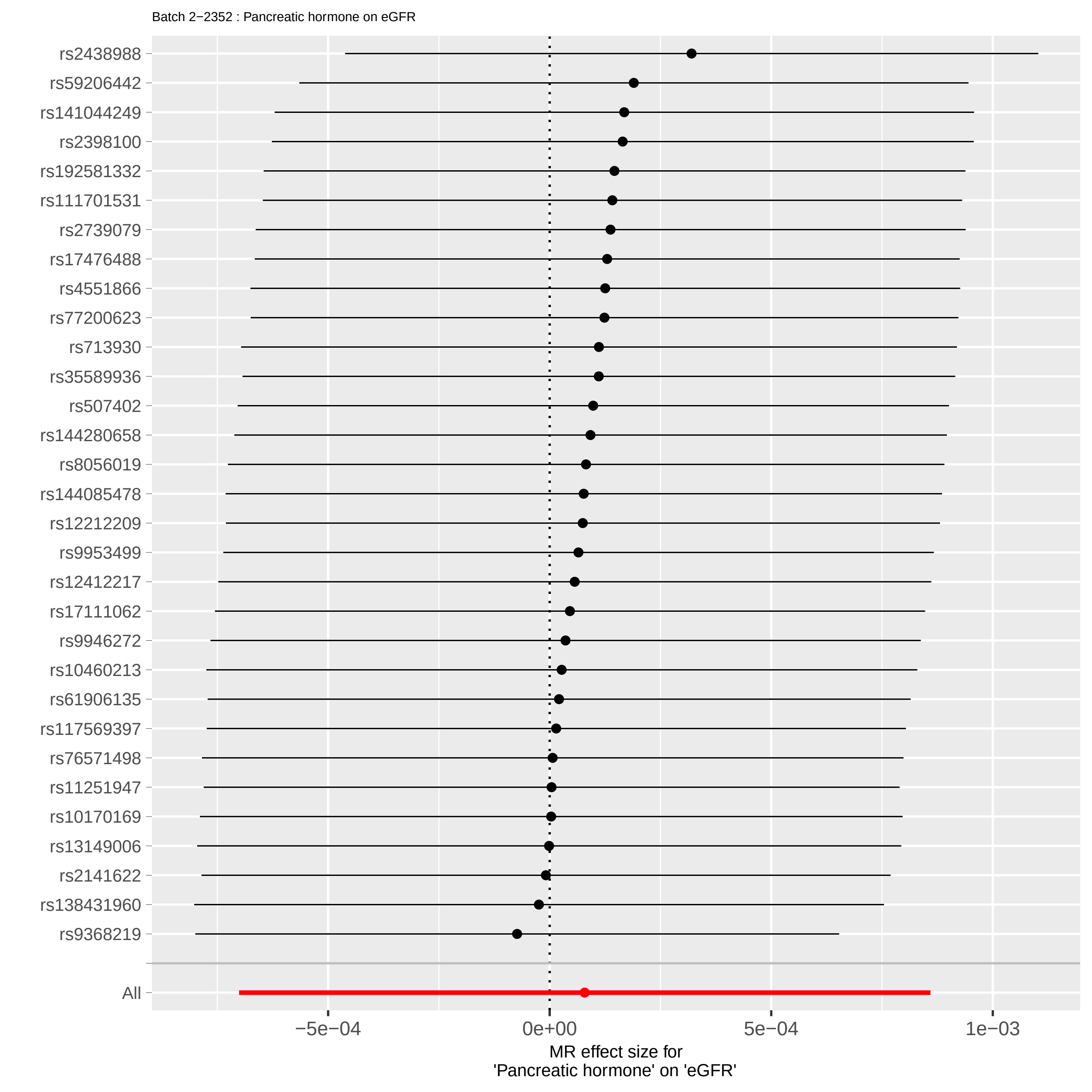

Batch 2−2352 : Pancreatic hormone on eGFR
rs2438988
rs59206442
rs141044249
rs2398100
rs192581332
rs111701531
rs2739079
rs17476488
rs4551866
rs77200623
rs713930
rs35589936
rs507402
rs144280658
rs8056019
rs144085478
rs12212209
rs9953499
rs12412217
rs17111062
rs9946272
rs10460213
rs61906135
rs117569397
rs76571498
rs11251947
rs10170169
rs13149006
rs2141622
rs138431960
rs9368219
All
−5e−04
0e+00
5e−04
1e−03
MR effect size for
'Pancreatic hormone' on 'eGFR'

Batch 2−2353 : Putative uncharacterized protein PQLC2L on eGFR

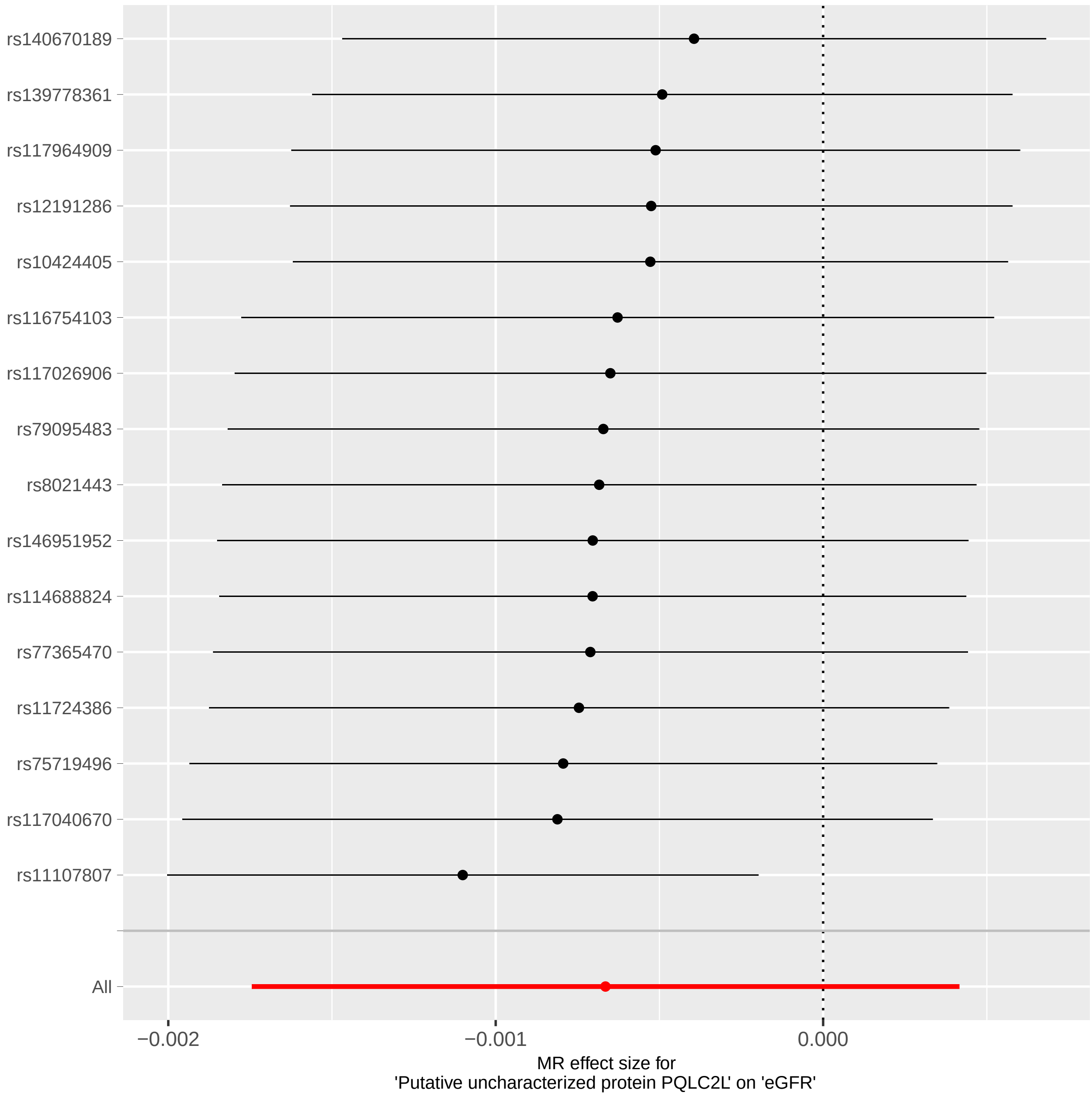

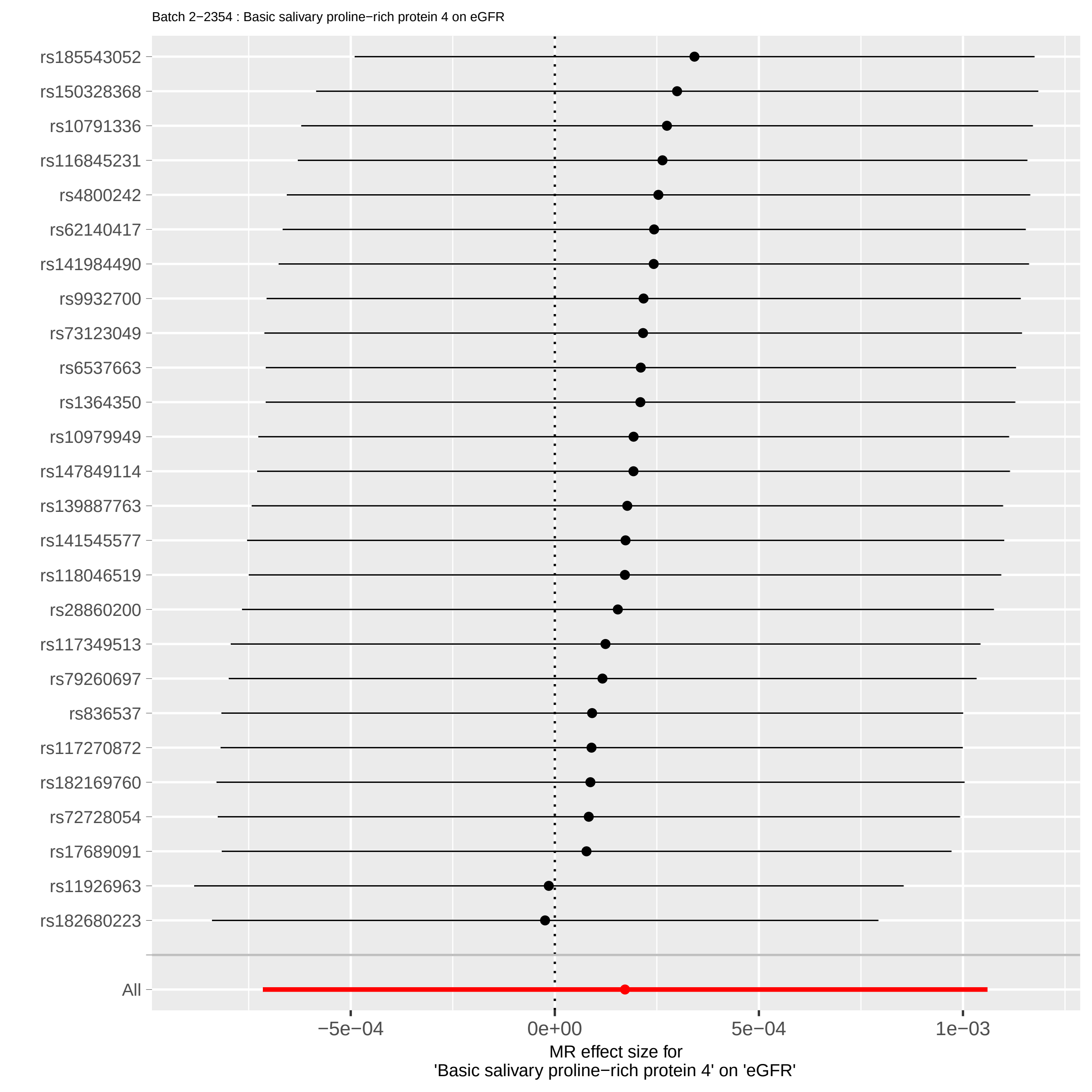

Batch 2−2354 : Basic salivary proline−rich protein 4 on eGFR
rs185543052
rs150328368
rs10791336
rs116845231
rs4800242
rs62140417
rs141984490
rs9932700
rs73123049
rs6537663
rs1364350
rs10979949
rs147849114
rs139887763
rs141545577
rs118046519
rs28860200
rs117349513
rs79260697
rs836537
rs117270872
rs182169760
rs72728054
rs17689091
rs11926963
rs182680223
All
−5e−04
0e+00
5e−04
1e−03
MR effect size for
'Basic salivary proline−rich protein 4' on 'eGFR'

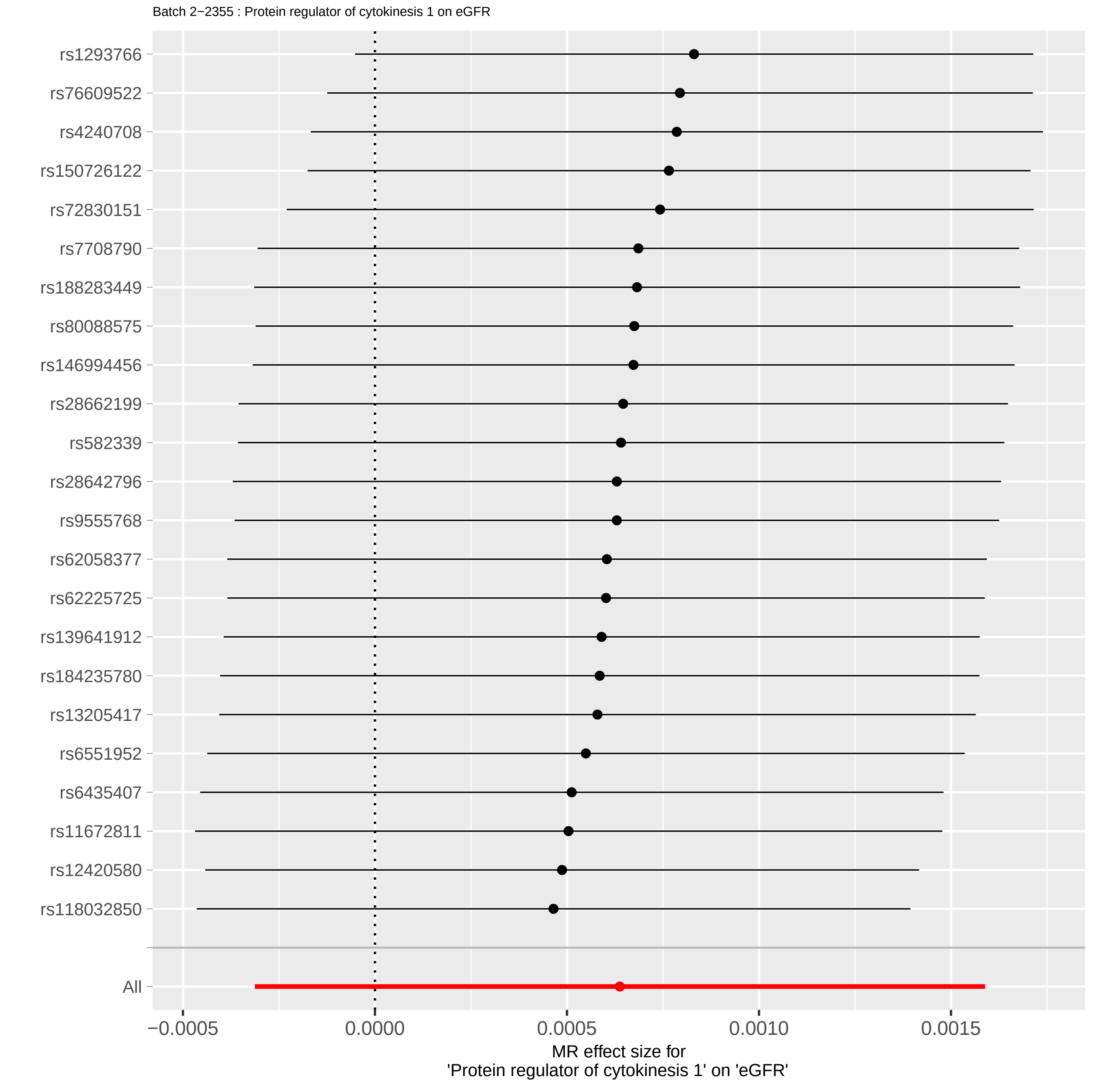

Batch 2−2355 : Protein regulator of cytokinesis 1 on eGFR
rs1293766
rs76609522
rs4240708
rs150726122
rs72830151
rs7708790
rs188283449
rs80088575
rs146994456
rs28662199
rs582339
rs28642796
rs9555768
rs62058377
rs62225725
rs139641912
rs184235780
rs13205417
rs6551952
rs6435407
rs11672811
rs12420580
rs118032850
All
−0.0005
0.0000
0.0005
0.0010
0.0015
MR effect size for
'Protein regulator of cytokinesis 1' on 'eGFR'

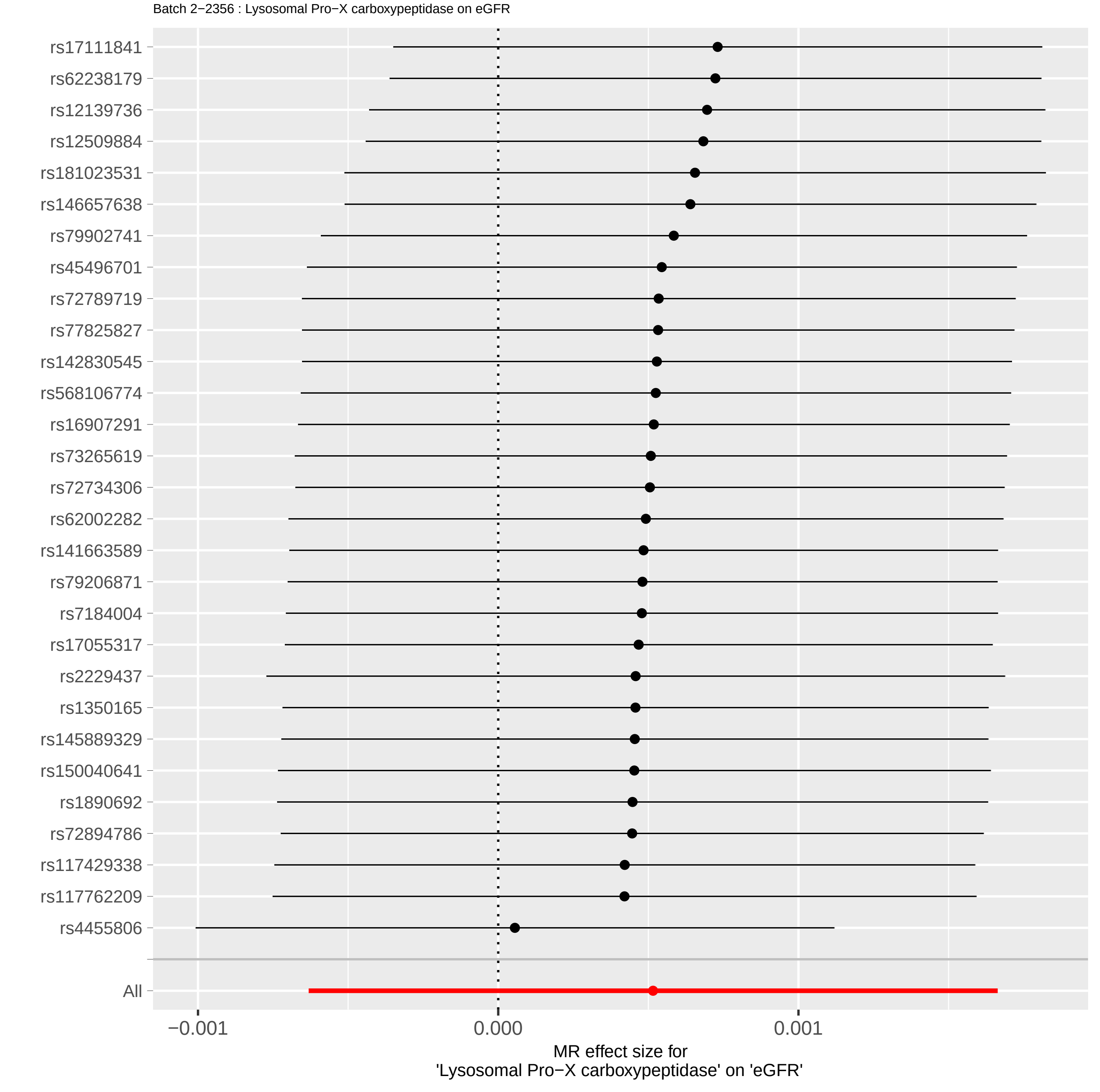

Batch 2−2356 : Lysosomal Pro−X carboxypeptidase on eGFR
rs17111841
rs62238179
rs12139736
rs12509884
rs181023531
rs146657638
rs79902741
rs45496701
rs72789719
rs77825827
rs142830545
rs568106774
rs16907291
rs73265619
rs72734306
rs62002282
rs141663589
rs79206871
rs7184004
rs17055317
rs2229437
rs1350165
rs145889329
rs150040641
rs1890692
rs72894786
rs117429338
rs117762209
rs4455806
All
−0.001
0.000
0.001
MR effect size for
'Lysosomal Pro−X carboxypeptidase' on 'eGFR'

Batch 2−2357 : PR domain zinc finger protein 1 on eGFR

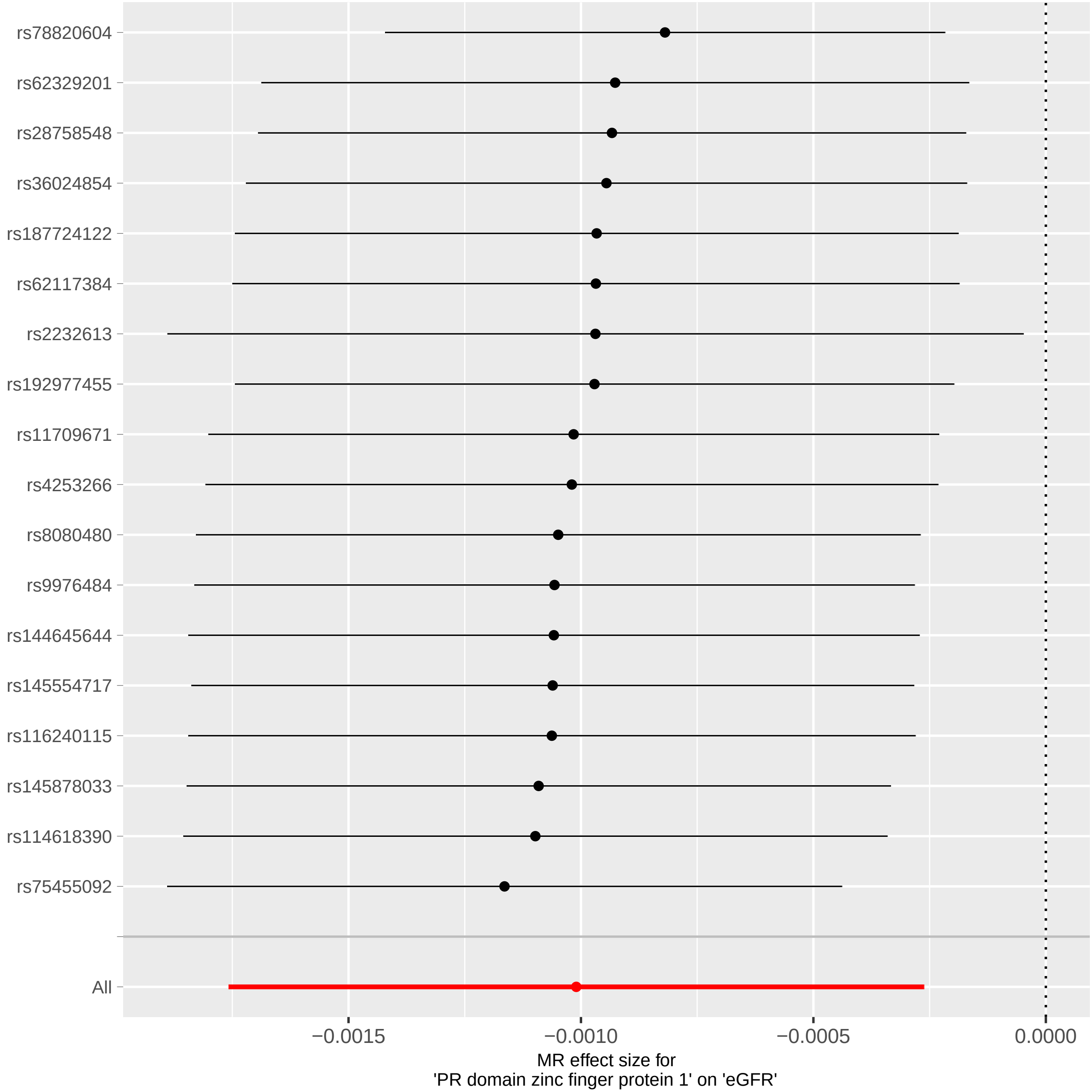

Batch 2−2358 : PR domain zinc finger protein 4 on eGFR

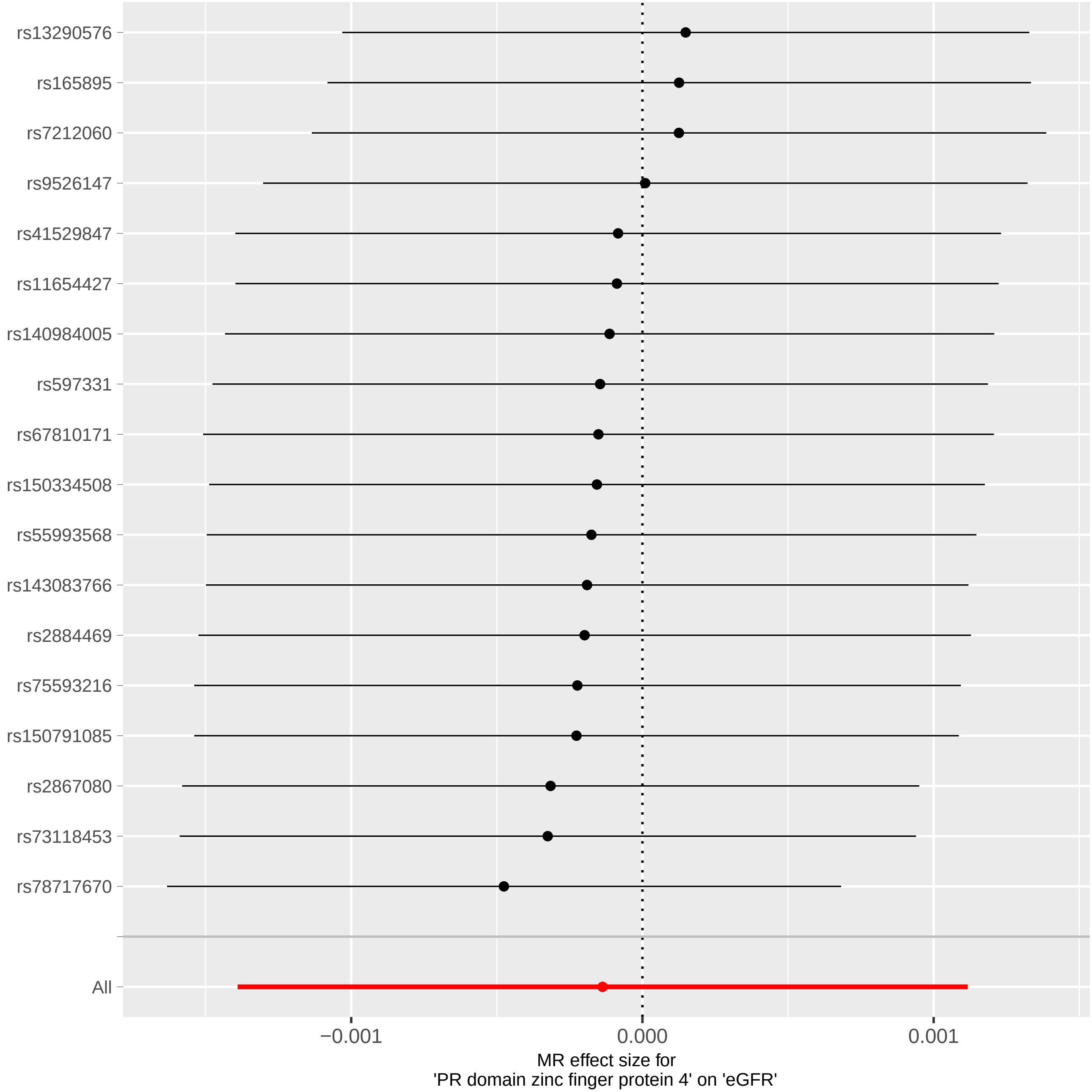

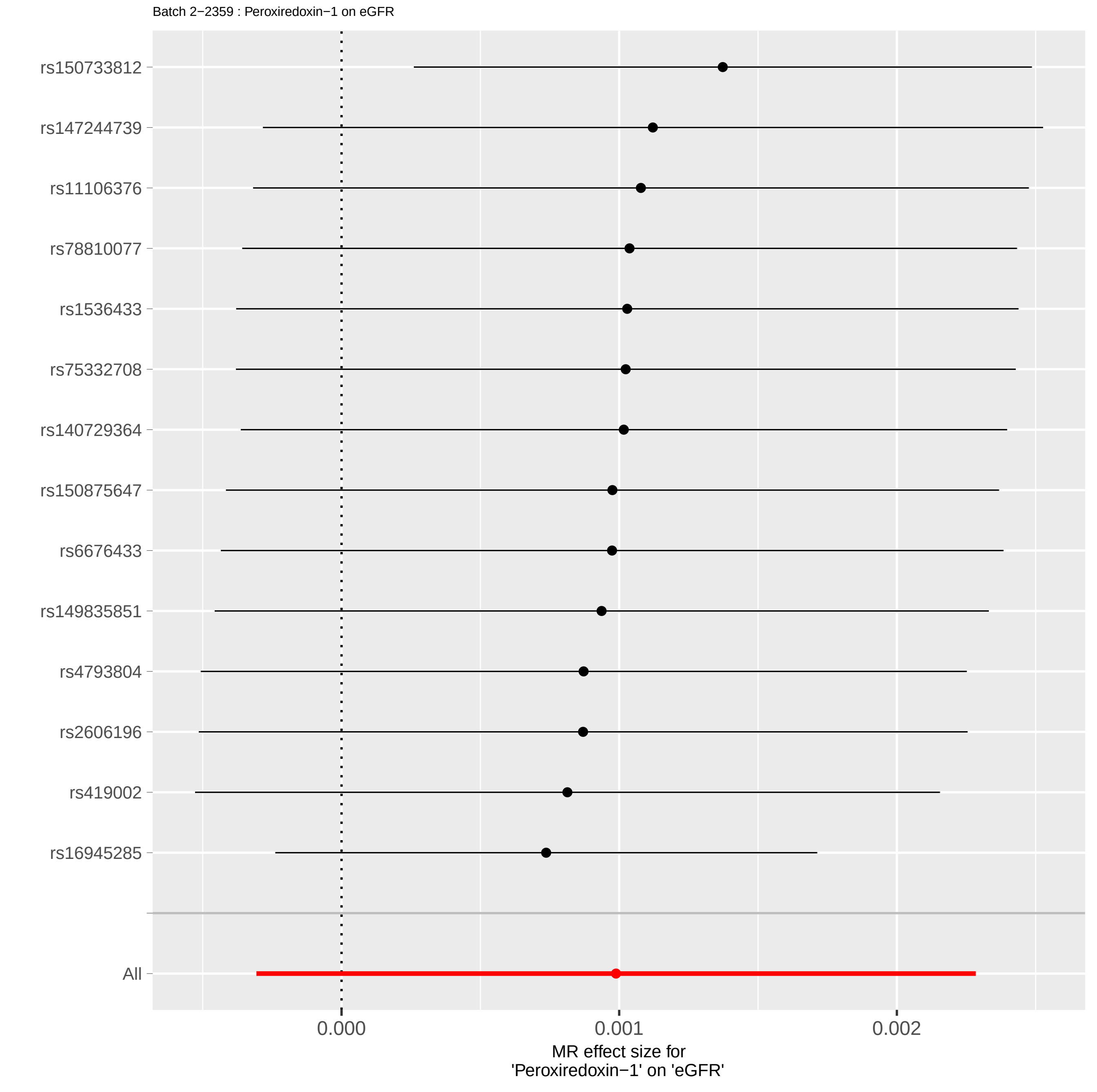
Batch 2−2359 : Peroxiredoxin−1 on eGFR
rs150733812
rs147244739
rs11106376
rs78810077
rs1536433
rs75332708
rs140729364
rs150875647
rs6676433
rs149835851
rs4793804
rs2606196
rs419002
rs16945285
All
0.000
0.001
0.002
MR effect size for
'Peroxiredoxin−1' on 'eGFR'

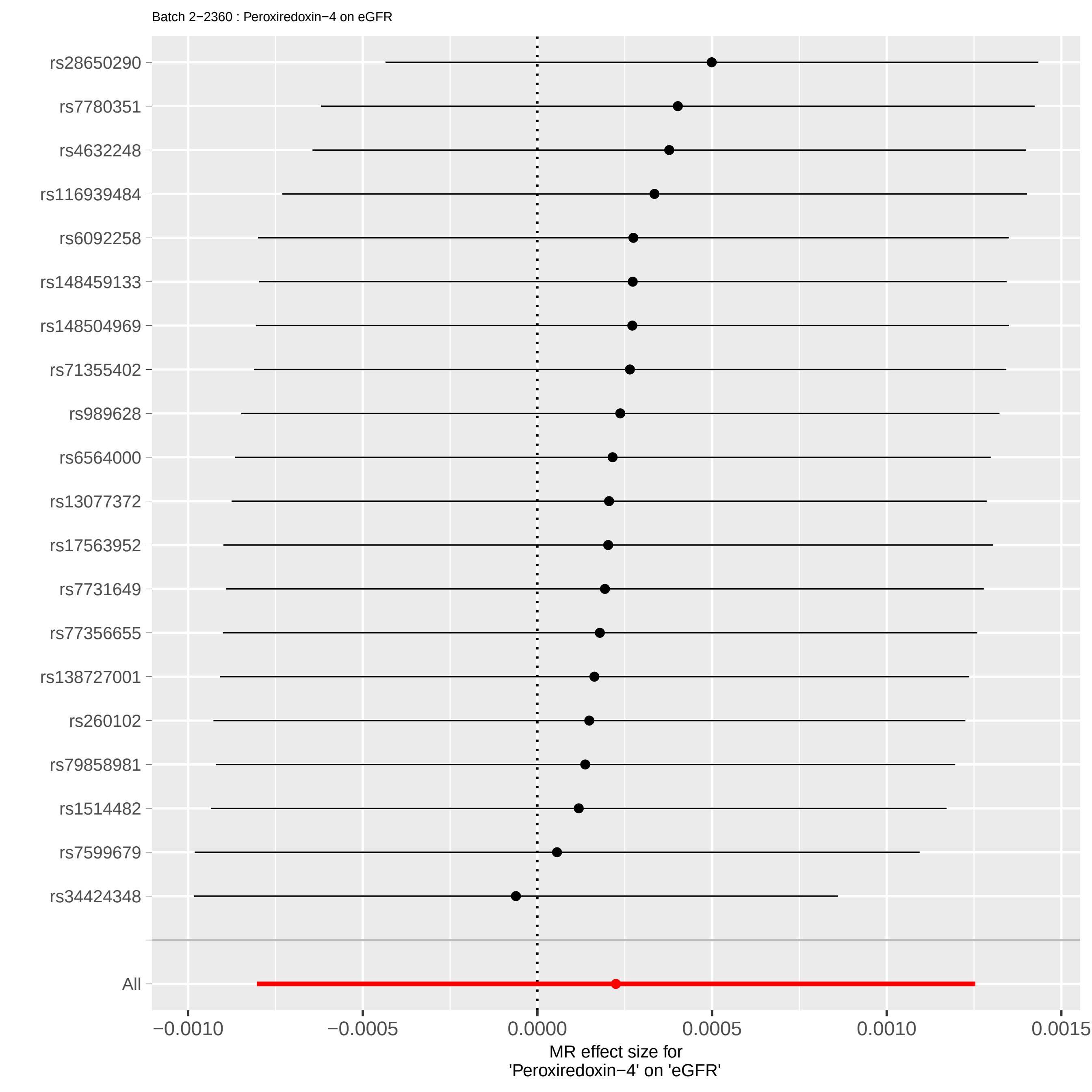

Batch 2−2360 : Peroxiredoxin−4 on eGFR
rs28650290
rs7780351
rs4632248
rs116939484
rs6092258
rs148459133
rs148504969
rs71355402
rs989628
rs6564000
rs13077372
rs17563952
rs7731649
rs77356655
rs138727001
rs260102
rs79858981
rs1514482
rs7599679
rs34424348
All
−0.0010
−0.0005
0.0000
0.0005
0.0010
0.0015
MR effect size for
'Peroxiredoxin−4' on 'eGFR'

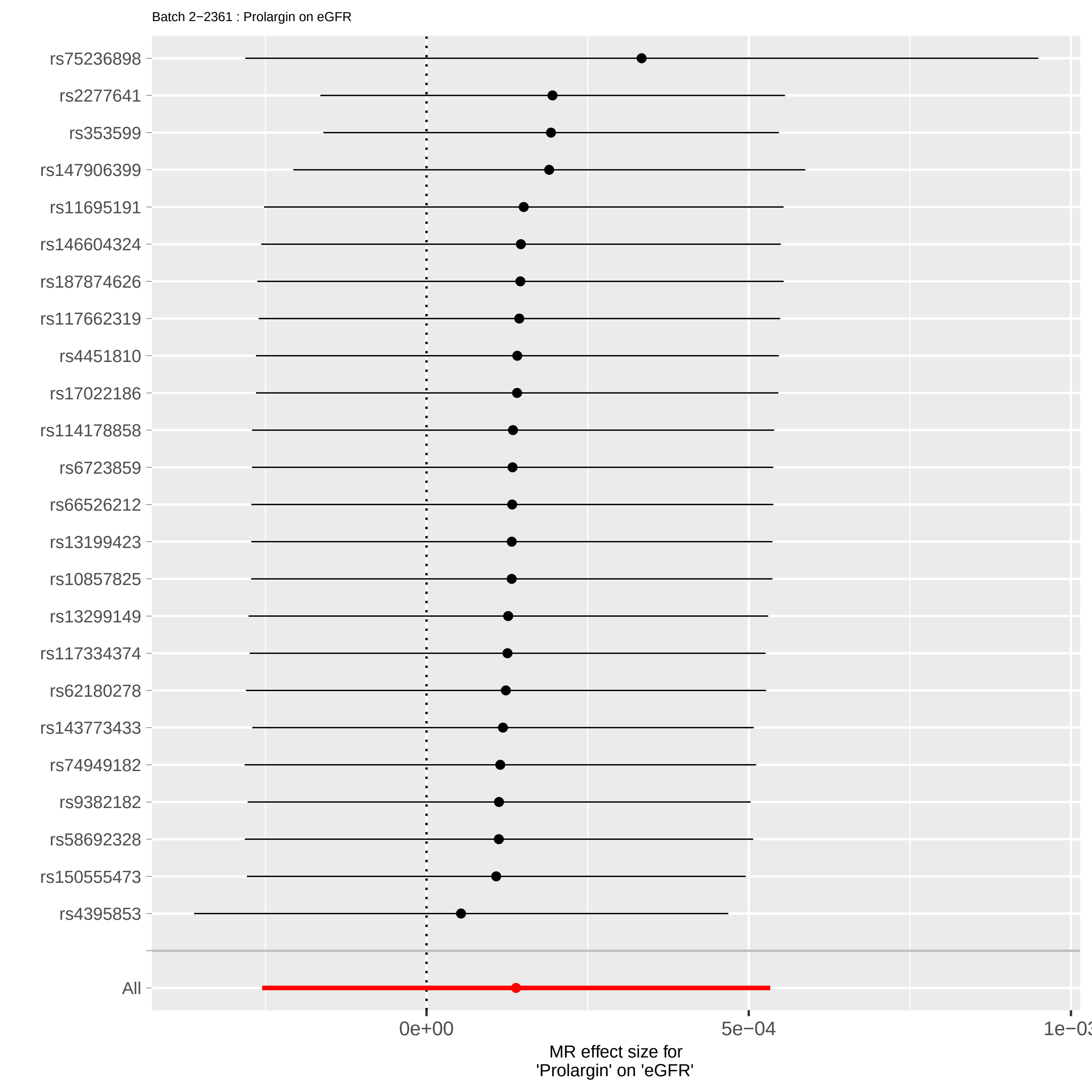
Batch 2−2361 : Prolargin on eGFR
rs75236898
rs2277641
rs353599
rs147906399
rs11695191
rs146604324
rs187874626
rs117662319
rs4451810
rs17022186
rs114178858
rs6723859
rs66526212
rs13199423
rs10857825
rs13299149
rs117334374
rs62180278
rs143773433
rs74949182
rs9382182
rs58692328
rs150555473
rs4395853
All
0e+00
5e−04
1e−03
MR effect size for
'Prolargin' on 'eGFR'

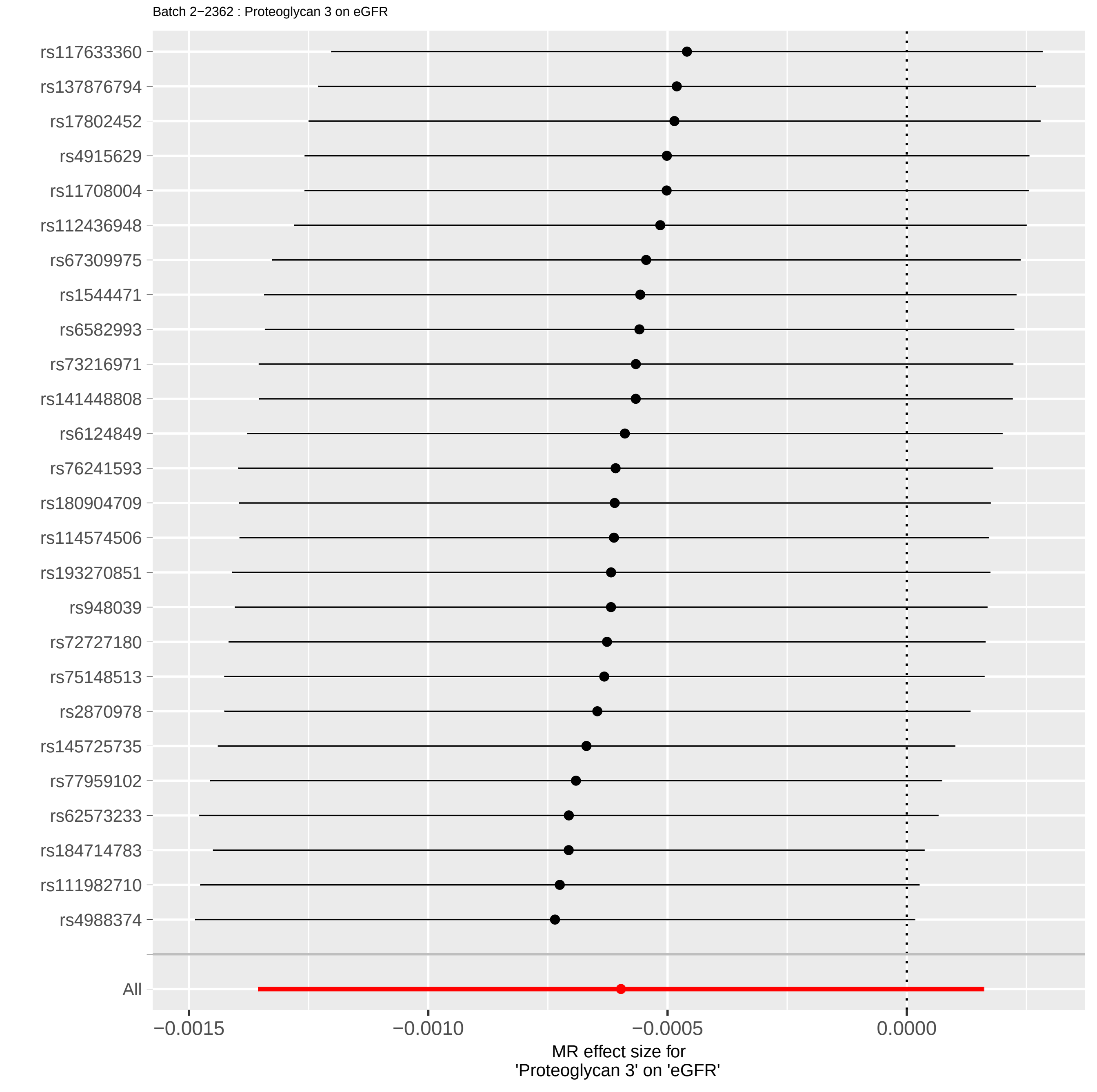

Batch 2−2362 : Proteoglycan 3 on eGFR
rs117633360
rs137876794
rs17802452
rs4915629
rs11708004
rs112436948
rs67309975
rs1544471
rs6582993
rs73216971
rs141448808
rs6124849
rs76241593
rs180904709
rs114574506
rs193270851
rs948039
rs72727180
rs75148513
rs2870978
rs145725735
rs77959102
rs62573233
rs184714783
rs111982710
rs4988374
All
−0.0015
−0.0010
−0.0005
0.0000
MR effect size for
'Proteoglycan 3' on 'eGFR'

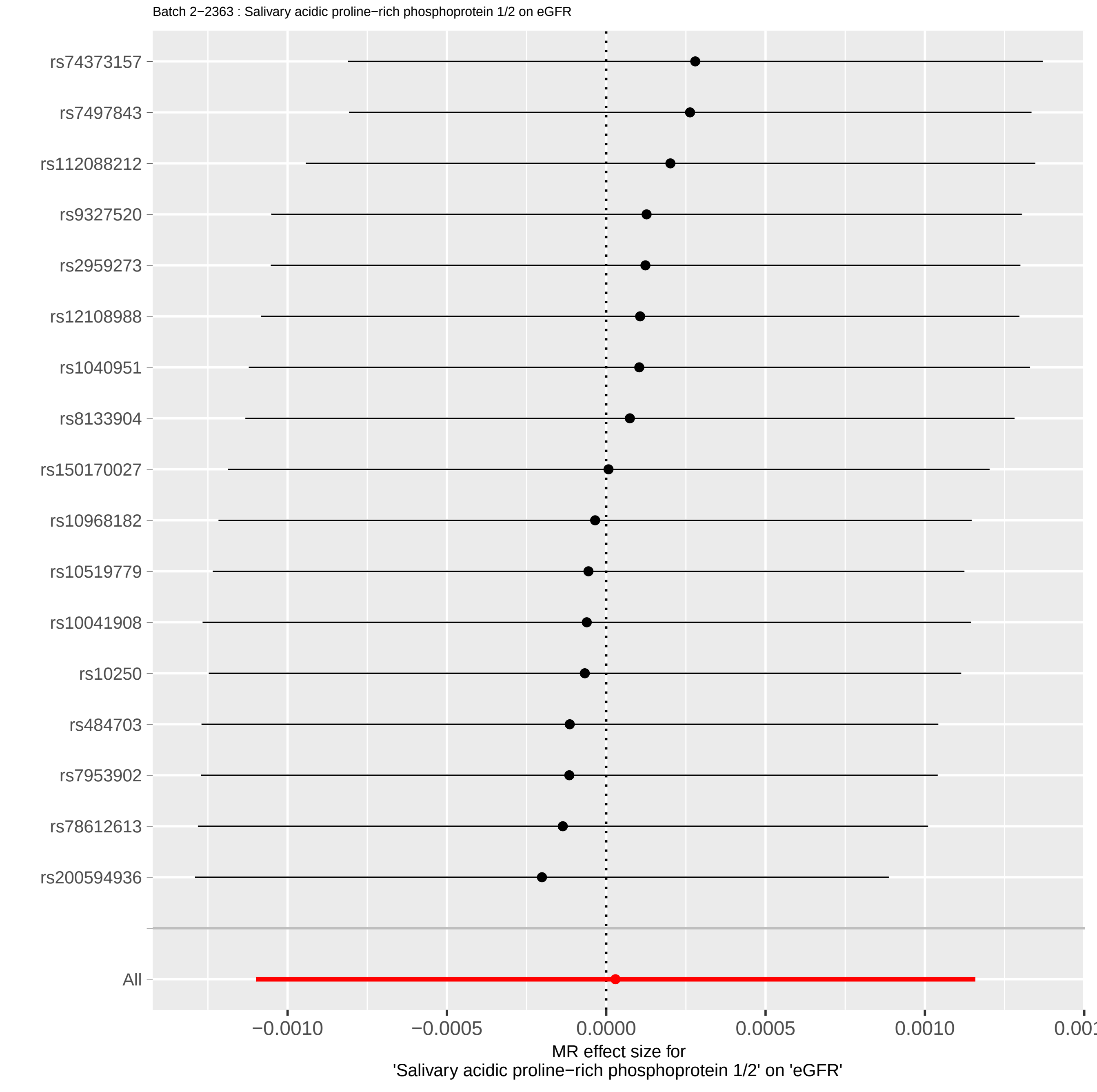

Batch 2−2363 : Salivary acidic proline−rich phosphoprotein 1/2 on eGFR
rs74373157
rs7497843
rs112088212
rs9327520
rs2959273
rs12108988
rs1040951
rs8133904
rs150170027
rs10968182
rs10519779
rs10041908
rs10250
rs484703
rs7953902
rs78612613
rs200594936
All
−0.0010
−0.0005
0.0000
0.0005
0.0010
MR effect size for
'Salivary acidic proline−rich phosphoprotein 1/2' on 'eGFR'

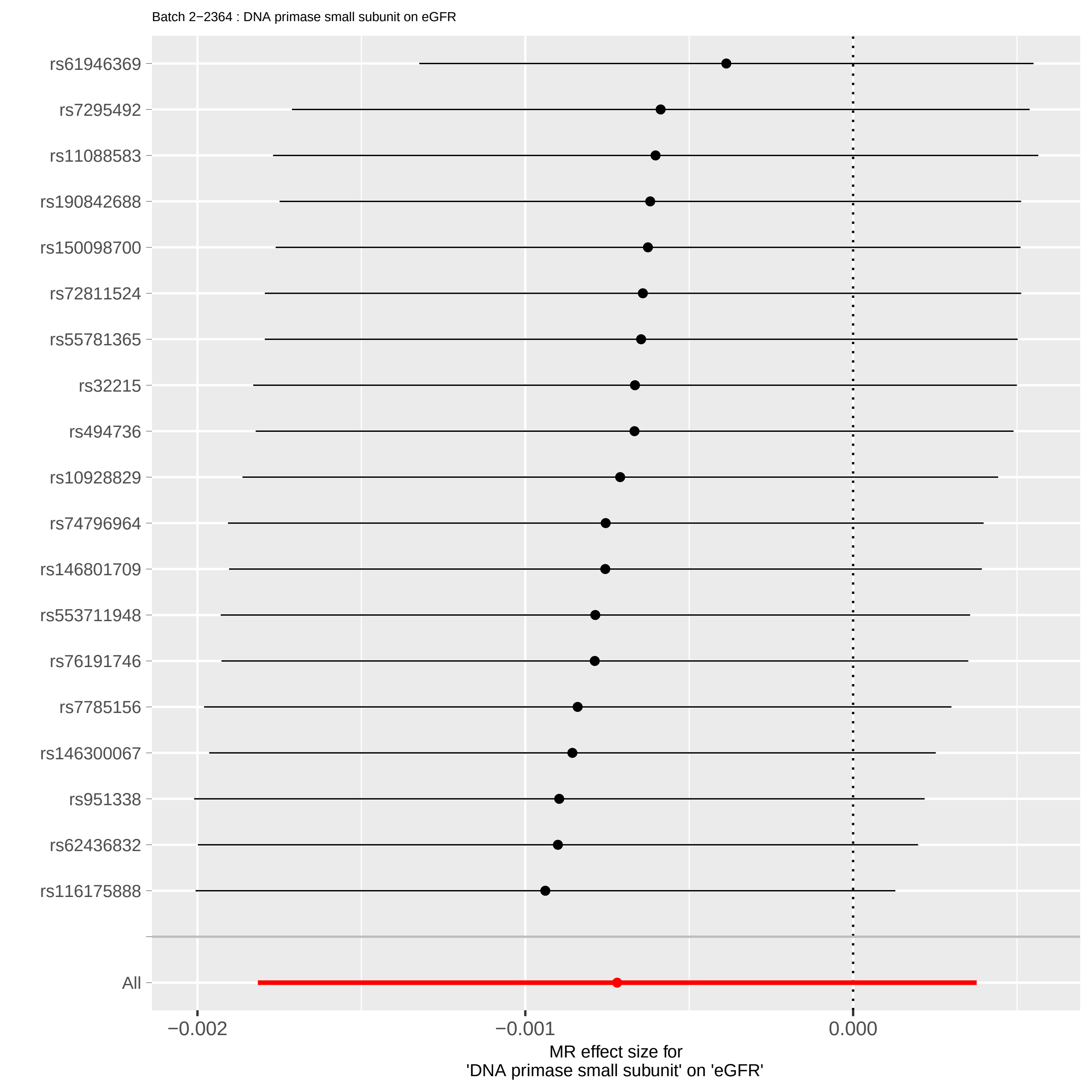

Batch 2−2364 : DNA primase small subunit on eGFR
rs61946369
rs7295492
rs11088583
rs190842688
rs150098700
rs72811524
rs55781365
rs32215
rs494736
rs10928829
rs74796964
rs146801709
rs553711948
rs76191746
rs7785156
rs146300067
rs951338
rs62436832
rs116175888
All
−0.002
−0.001
0.000
MR effect size for
'DNA primase small subunit' on 'eGFR'

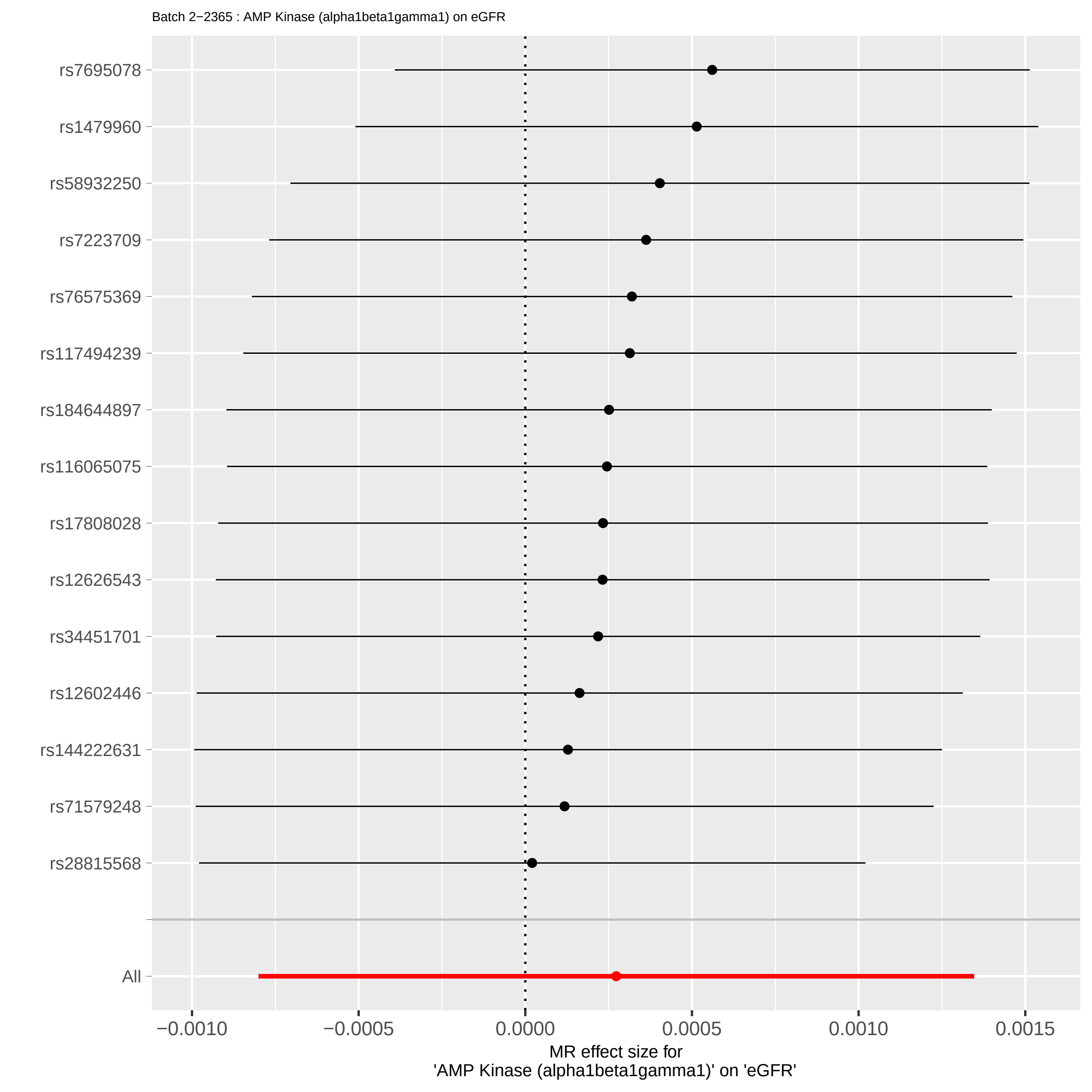

Batch 2−2365 : AMP Kinase (alpha1beta1gamma1) on eGFR
rs7695078
rs1479960
rs58932250
rs7223709
rs76575369
rs117494239
rs184644897
rs116065075
rs17808028
rs12626543
rs34451701
rs12602446
rs144222631
rs71579248
rs28815568
All
−0.0010
−0.0005
0.0000
0.0005
0.0010
0.0015
MR effect size for
'AMP Kinase (alpha1beta1gamma1)' on 'eGFR'

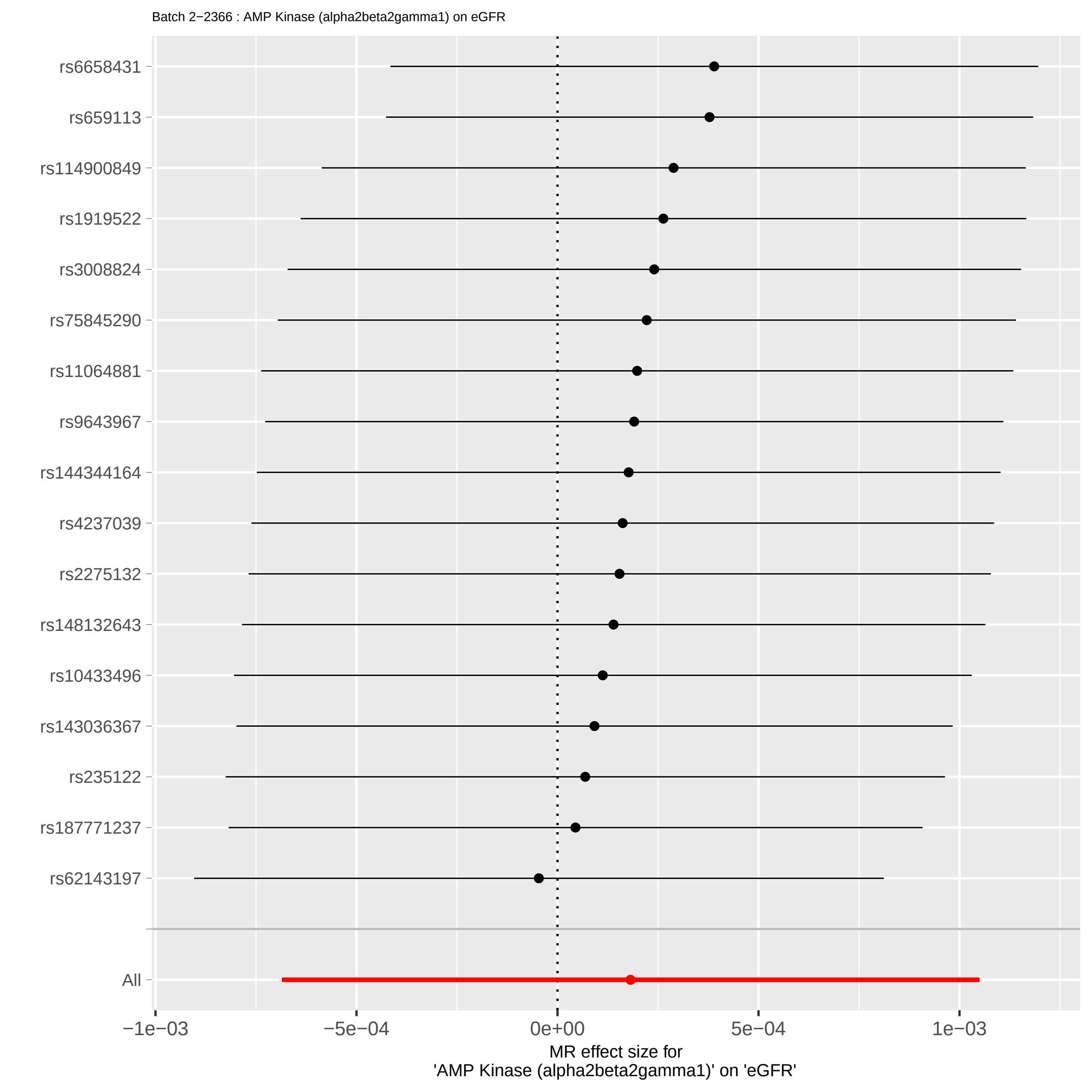

Batch 2−2366 : AMP Kinase (alpha2beta2gamma1) on eGFR
rs6658431
rs659113
rs114900849
rs1919522
rs3008824
rs75845290
rs11064881
rs9643967
rs144344164
rs4237039
rs2275132
rs148132643
rs10433496
rs143036367
rs235122
rs187771237
rs62143197
All
−1e−03
−5e−04
0e+00
5e−04
1e−03
MR effect size for
'AMP Kinase (alpha2beta2gamma1)' on 'eGFR'

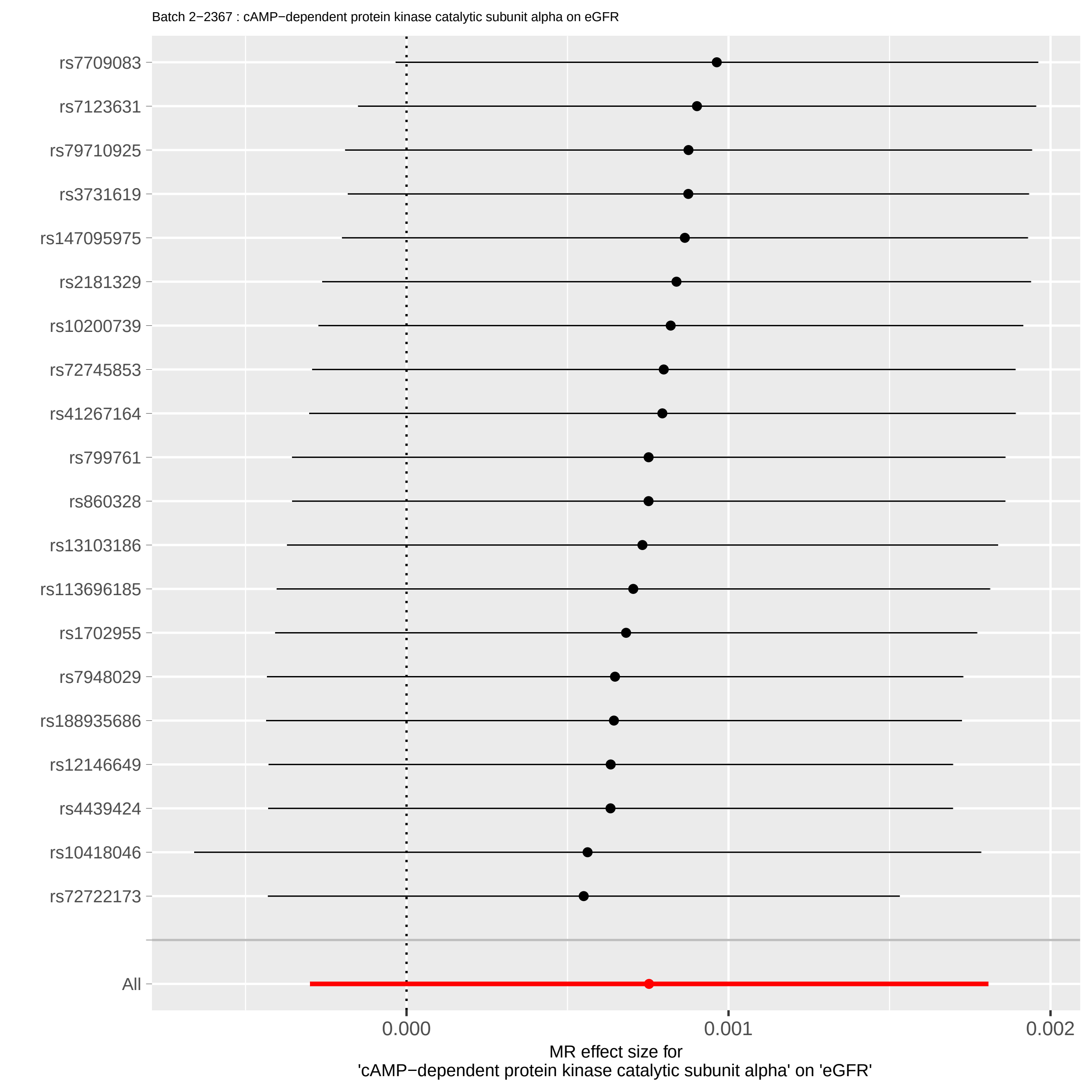

Batch 2−2367 : cAMP−dependent protein kinase catalytic subunit alpha on eGFR
rs7709083
rs7123631
rs79710925
rs3731619
rs147095975
rs2181329
rs10200739
rs72745853
rs41267164
rs799761
rs860328
rs13103186
rs113696185
rs1702955
rs7948029
rs188935686
rs12146649
rs4439424
rs10418046
rs72722173
All
0.000
0.001
0.002
MR effect size for
'cAMP−dependent protein kinase catalytic subunit alpha' on 'eGFR'

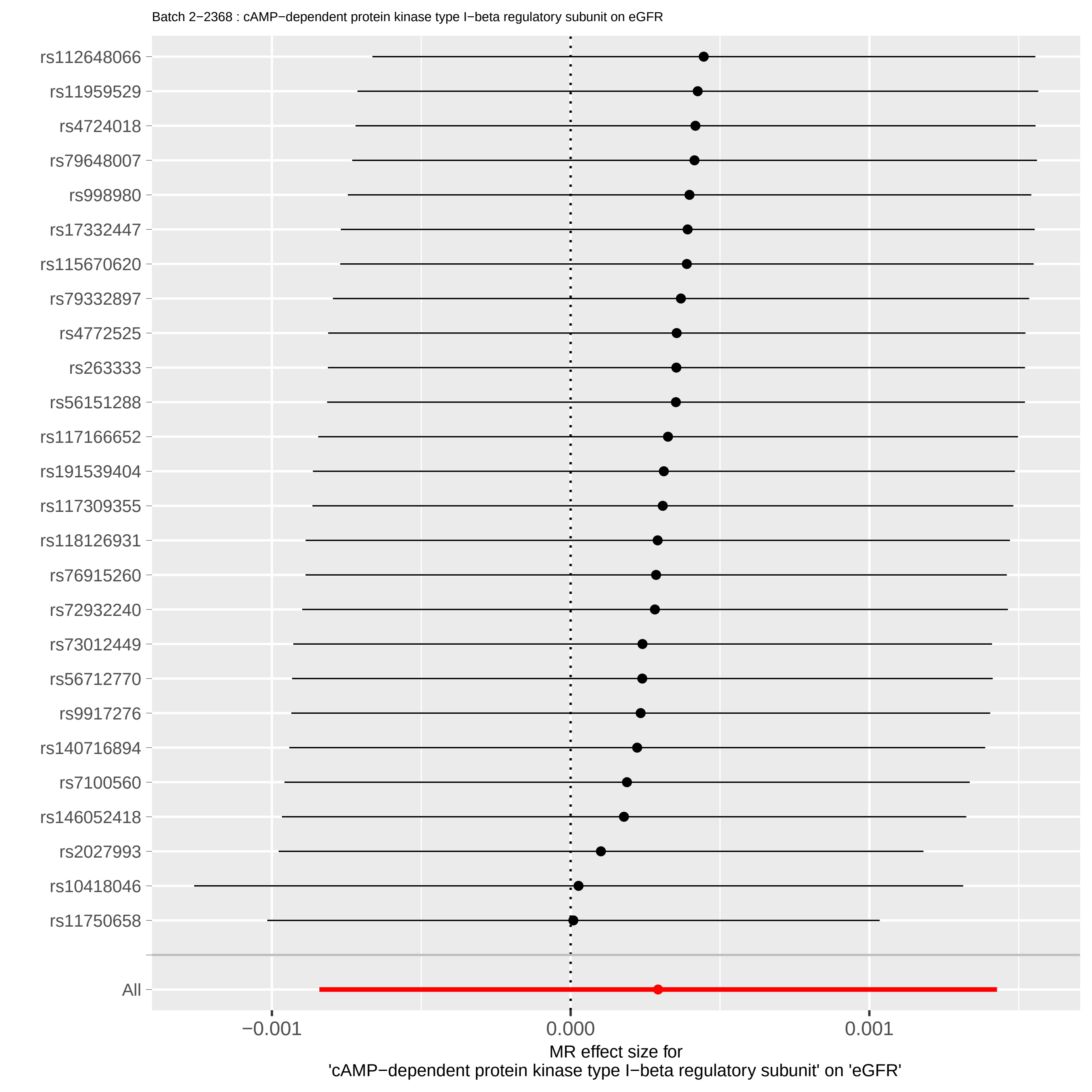

Batch 2−2368 : cAMP−dependent protein kinase type I−beta regulatory subunit on eGFR
rs112648066
rs11959529
rs4724018
rs79648007
rs998980
rs17332447
rs115670620
rs79332897
rs4772525
rs263333
rs56151288
rs117166652
rs191539404
rs117309355
rs118126931
rs76915260
rs72932240
rs73012449
rs56712770
rs9917276
rs140716894
rs7100560
rs146052418
rs2027993
rs10418046
rs11750658
All
−0.001
0.000
0.001
MR effect size for
'cAMP−dependent protein kinase type I−beta regulatory subunit' on 'eGFR'

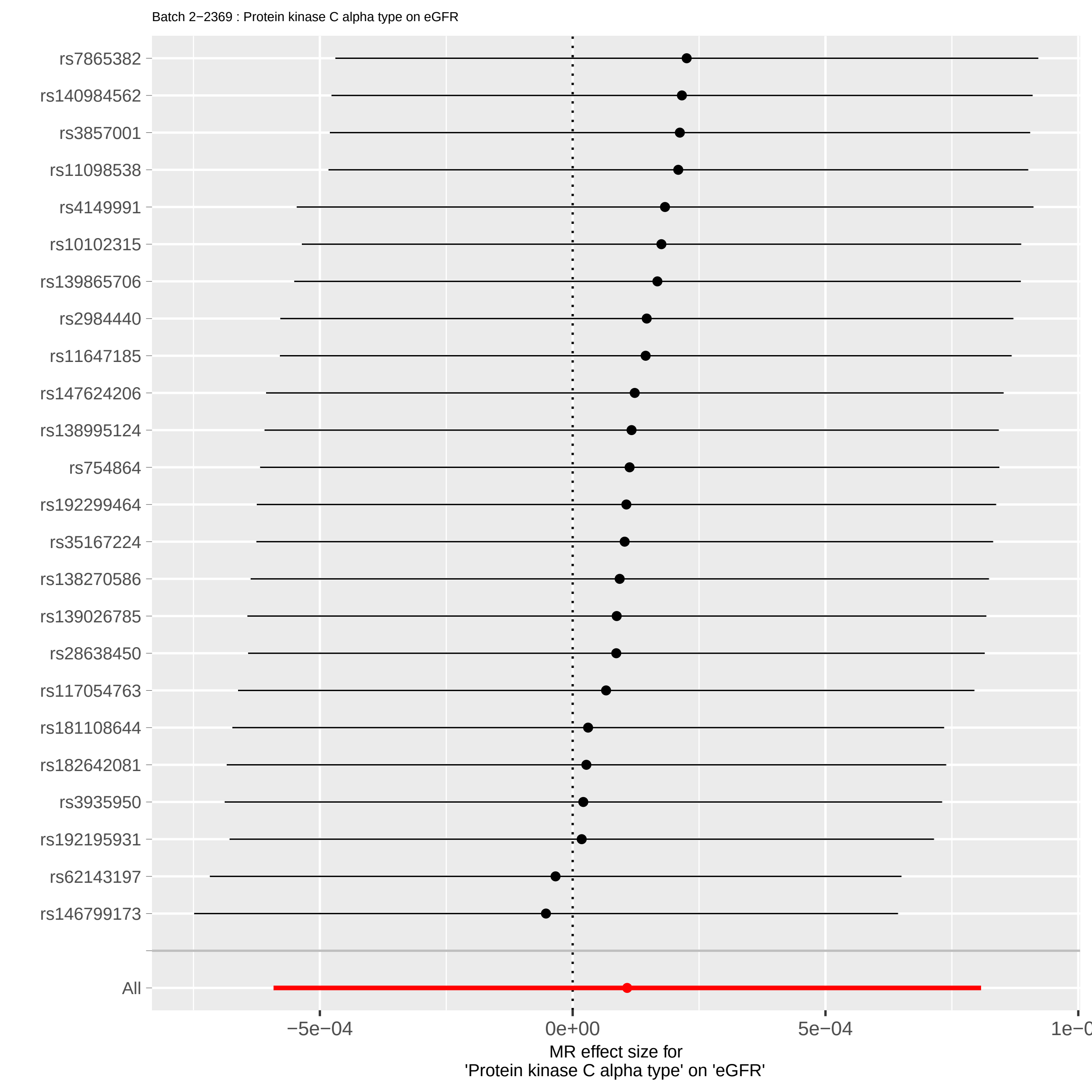

Batch 2−2369 : Protein kinase C alpha type on eGFR
rs7865382
rs140984562
rs3857001
rs11098538
rs4149991
rs10102315
rs139865706
rs2984440
rs11647185
rs147624206
rs138995124
rs754864
rs192299464
rs35167224
rs138270586
rs139026785
rs28638450
rs117054763
rs181108644
rs182642081
rs3935950
rs192195931
rs62143197
rs146799173
All
−5e−04
0e+00
5e−04
1e−0
MR effect size for
'Protein kinase C alpha type' on 'eGFR'

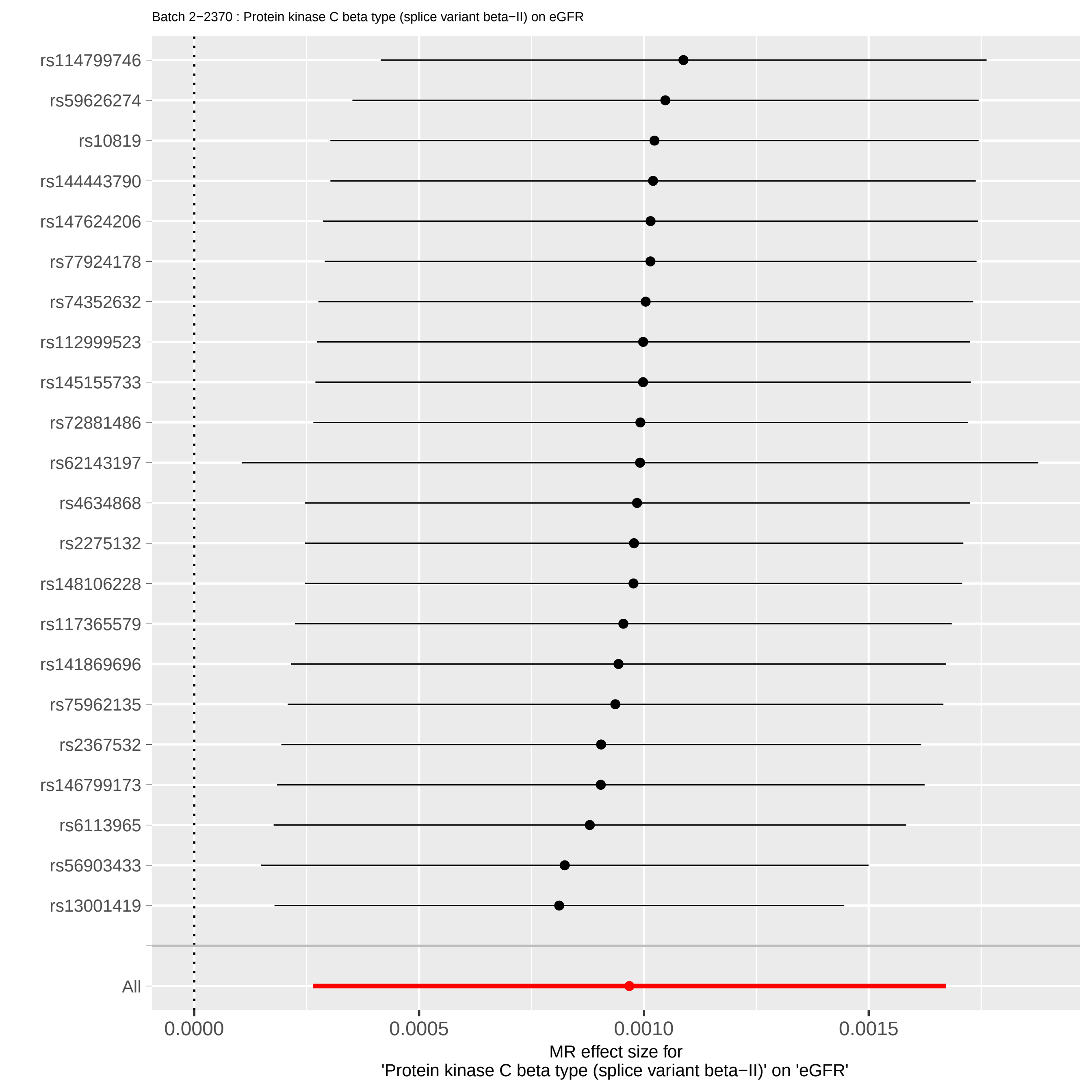

Batch 2−2370 : Protein kinase C beta type (splice variant beta−II) on eGFR
rs114799746
rs59626274
rs10819
rs144443790
rs147624206
rs77924178
rs74352632
rs112999523
rs145155733
rs72881486
rs62143197
rs4634868
rs2275132
rs148106228
rs117365579
rs141869696
rs75962135
rs2367532
rs146799173
rs6113965
rs56903433
rs13001419
All
0.0000
0.0005
0.0010
0.0015
MR effect size for
'Protein kinase C beta type (splice variant beta−II)' on 'eGFR'

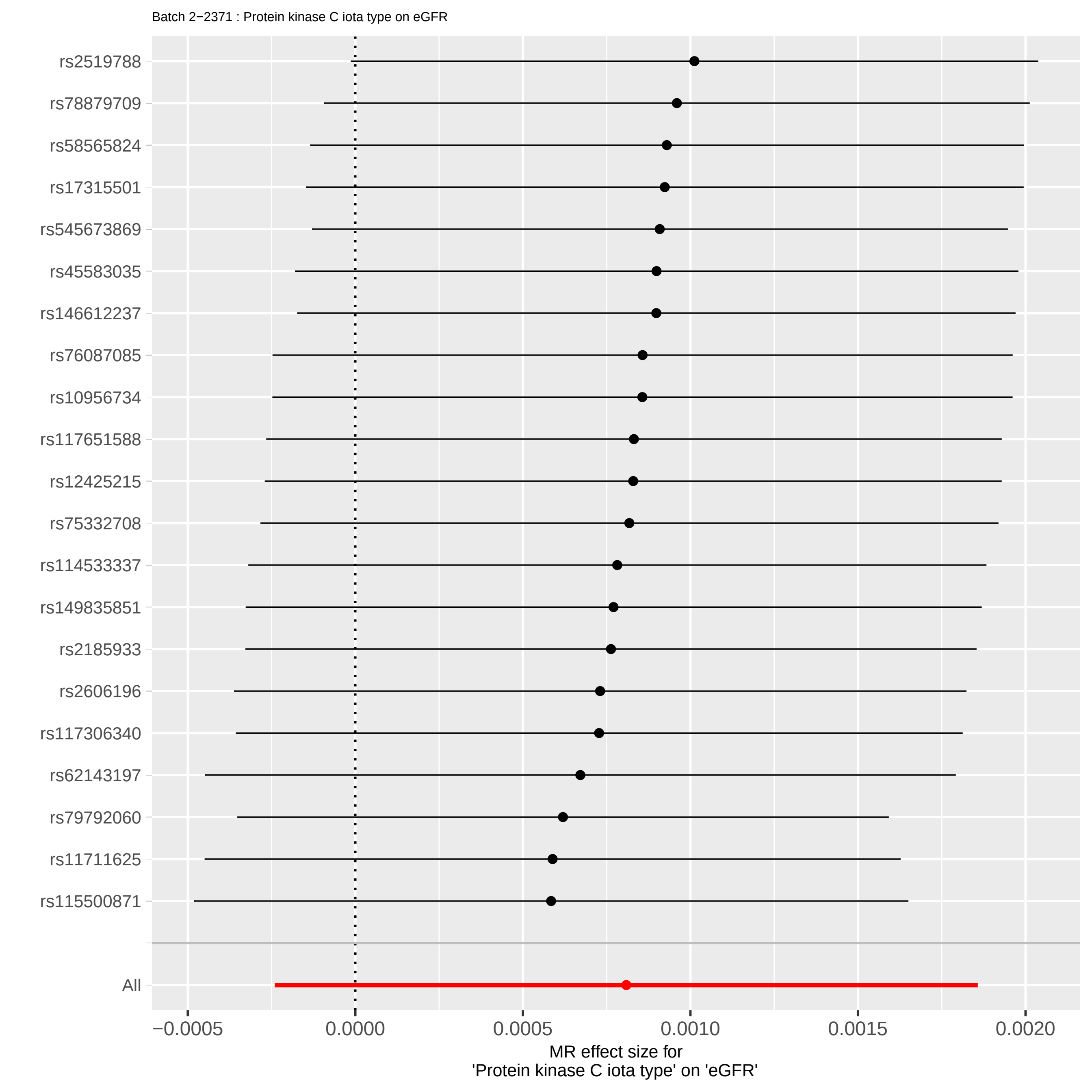
Batch 2−2371 : Protein kinase C iota type on eGFR
rs2519788
rs78879709
rs58565824
rs17315501
rs545673869
rs45583035
rs146612237
rs76087085
rs10956734
rs117651588
rs12425215
rs75332708
rs114533337
rs149835851
rs2185933
rs2606196
rs117306340
rs62143197
rs79792060
rs11711625
rs115500871
All
−0.0005
0.0000
0.0005
0.0010
0.0015
0.0020
MR effect size for
'Protein kinase C iota type' on 'eGFR'

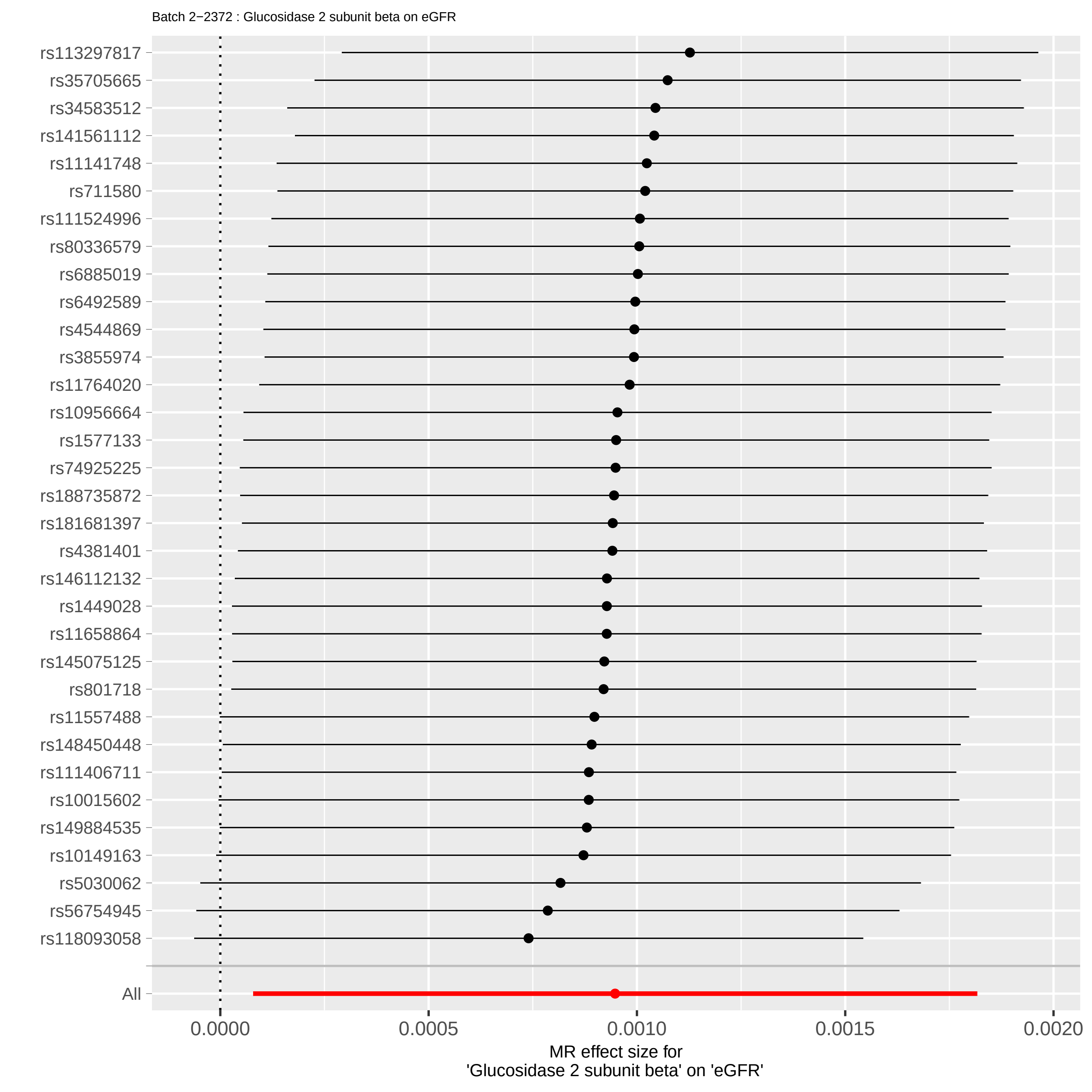
Batch 2−2372 : Glucosidase 2 subunit beta on eGFR
rs113297817
rs35705665
rs34583512
rs141561112
rs11141748
rs711580
rs111524996
rs80336579
rs6885019
rs6492589
rs4544869
rs3855974
rs11764020
rs10956664
rs1577133
rs74925225
rs188735872
rs181681397
rs4381401
rs146112132
rs1449028
rs11658864
rs145075125
rs801718
rs11557488
rs148450448
rs111406711
rs10015602
rs149884535
rs10149163
rs5030062
rs56754945
rs118093058
All
0.0000
0.0005
0.0010
0.0015
0.0020
MR effect size for
'Glucosidase 2 subunit beta' on 'eGFR'

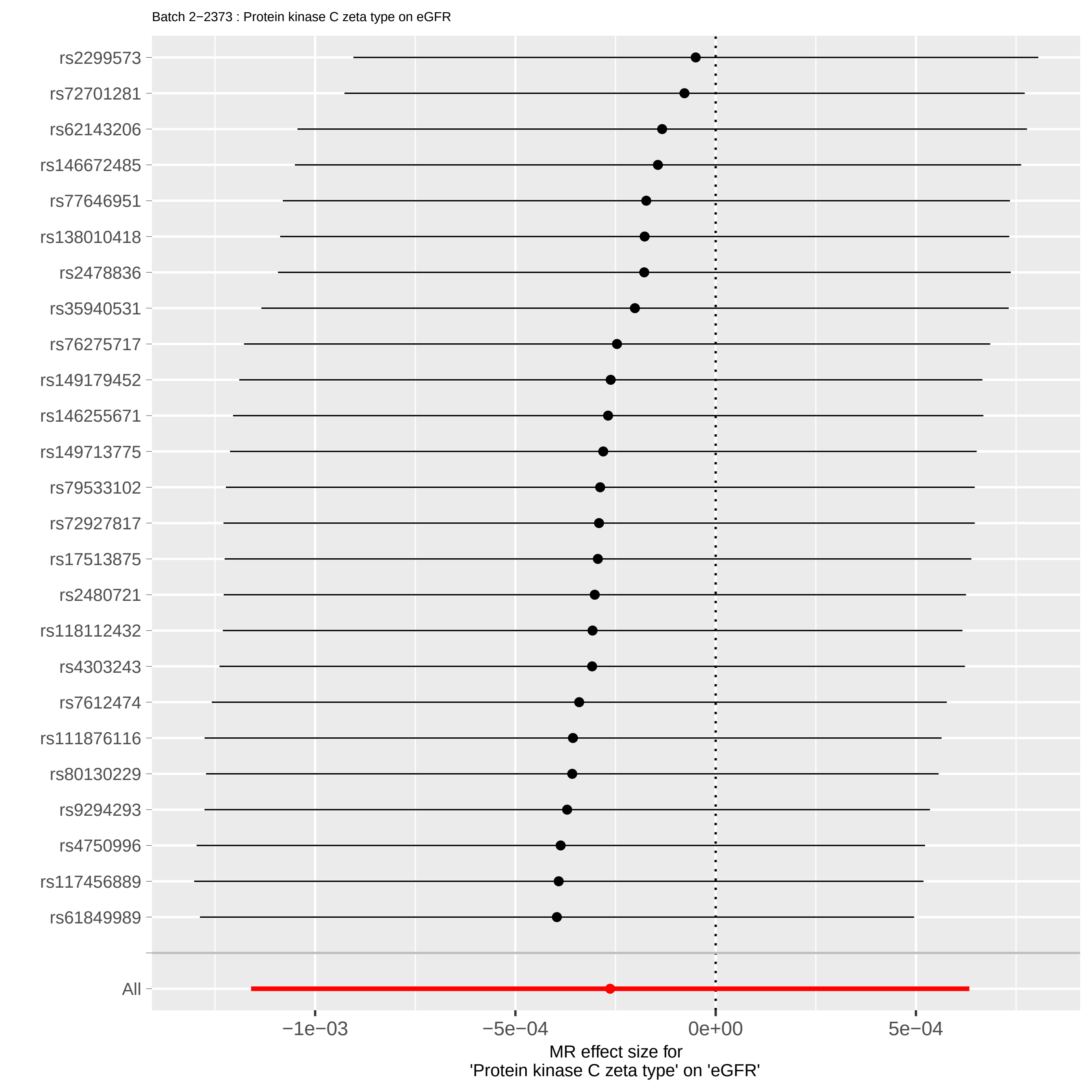

Batch 2−2373 : Protein kinase C zeta type on eGFR
rs2299573
rs72701281
rs62143206
rs146672485
rs77646951
rs138010418
rs2478836
rs35940531
rs76275717
rs149179452
rs146255671
rs149713775
rs79533102
rs72927817
rs17513875
rs2480721
rs118112432
rs4303243
rs7612474
rs111876116
rs80130229
rs9294293
rs4750996
rs117456889
rs61849989
All
−1e−03
−5e−04
0e+00
5e−04
MR effect size for
'Protein kinase C zeta type' on 'eGFR'

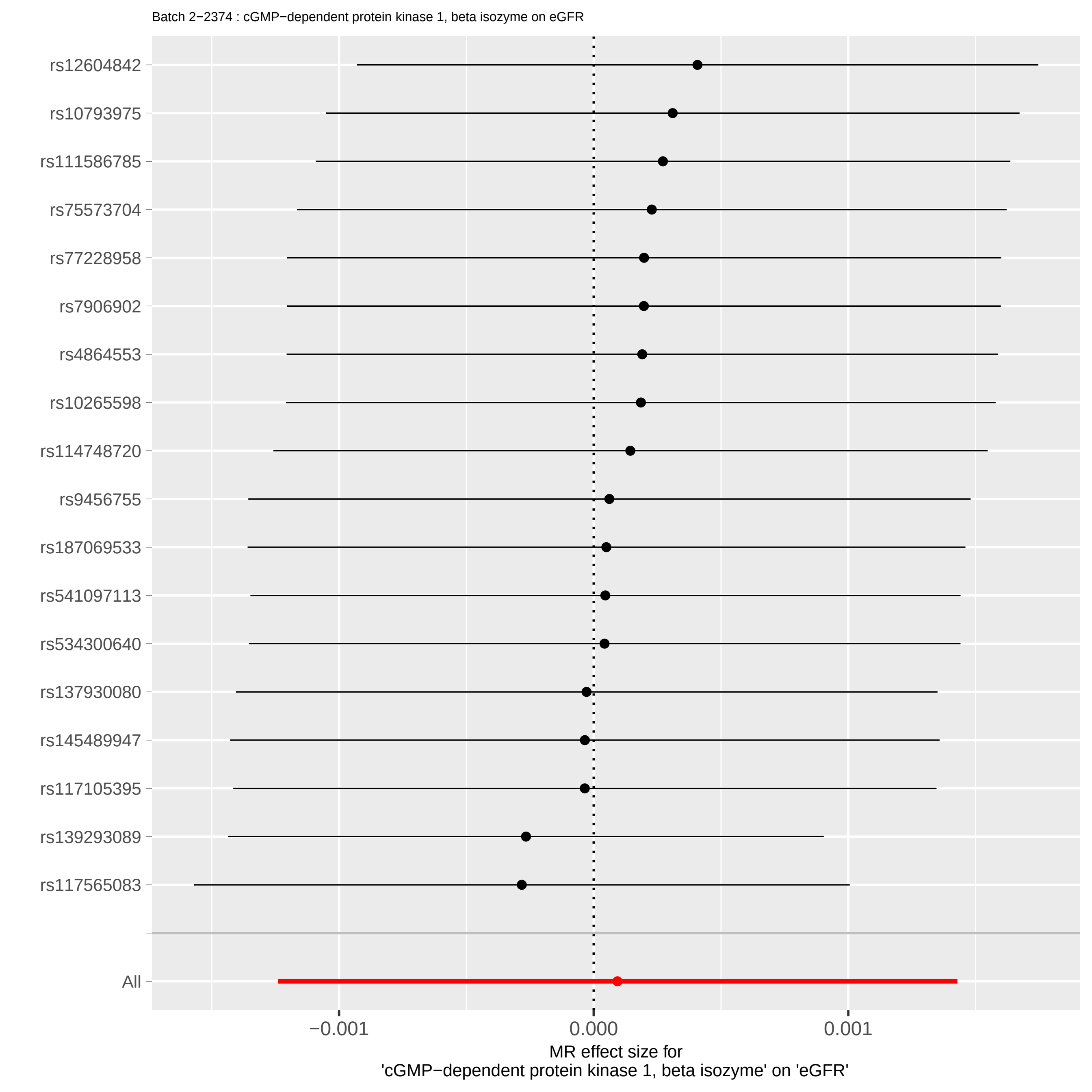

Batch 2−2374 : cGMP−dependent protein kinase 1, beta isozyme on eGFR
rs12604842
rs10793975
rs111586785
rs75573704
rs77228958
rs7906902
rs4864553
rs10265598
rs114748720
rs9456755
rs187069533
rs541097113
rs534300640
rs137930080
rs145489947
rs117105395
rs139293089
rs117565083
All
−0.001
0.000
0.001
MR effect size for
'cGMP−dependent protein kinase 1, beta isozyme' on 'eGFR'

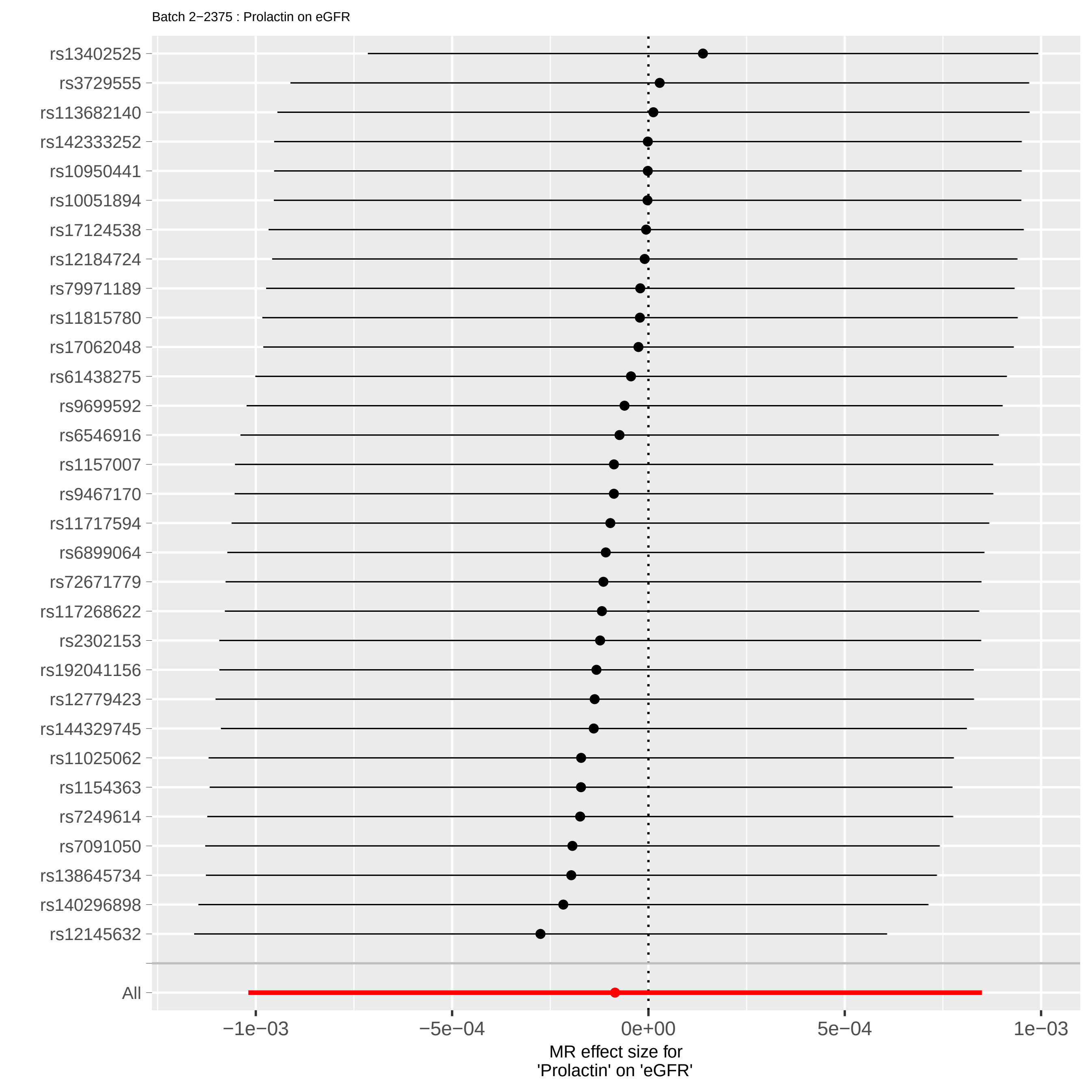
Batch 2−2375 : Prolactin on eGFR
rs13402525
rs3729555
rs113682140
rs142333252
rs10950441
rs10051894
rs17124538
rs12184724
rs79971189
rs11815780
rs17062048
rs61438275
rs9699592
rs6546916
rs1157007
rs9467170
rs11717594
rs6899064
rs72671779
rs117268622
rs2302153
rs192041156
rs12779423
rs144329745
rs11025062
rs1154363
rs7249614
rs7091050
rs138645734
rs140296898
rs12145632
All
−1e−03
−5e−04
0e+00
5e−04
1e−03
MR effect size for
'Prolactin' on 'eGFR'

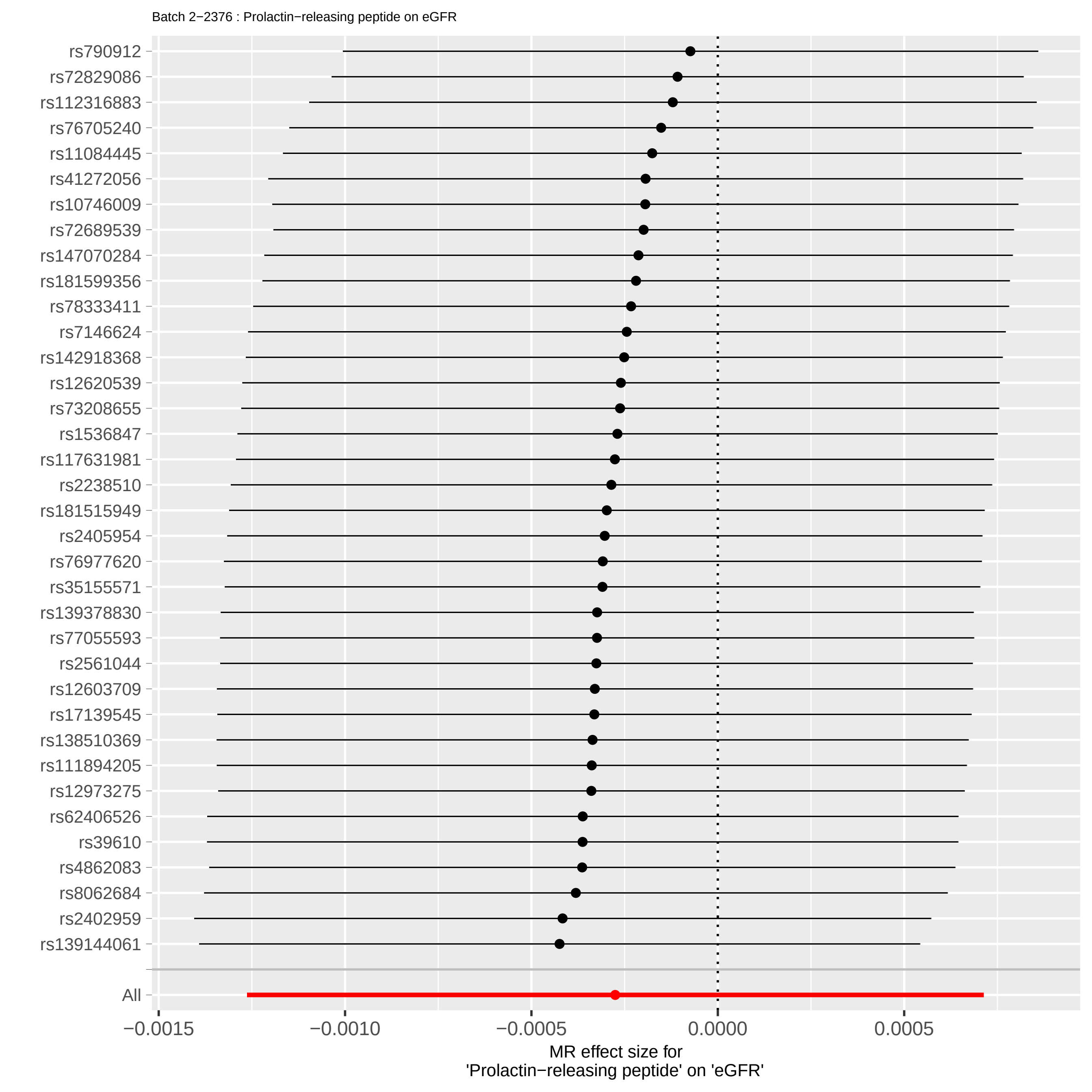

Batch 2−2376 : Prolactin−releasing peptide on eGFR
rs790912
rs72829086
rs112316883
rs76705240
rs11084445
rs41272056
rs10746009
rs72689539
rs147070284
rs181599356
rs78333411
rs7146624
rs142918368
rs12620539
rs73208655
rs1536847
rs117631981
rs2238510
rs181515949
rs2405954
rs76977620
rs35155571
rs139378830
rs77055593
rs2561044
rs12603709
rs17139545
rs138510369
rs111894205
rs12973275
rs62406526
rs39610
rs4862083
rs8062684
rs2402959
rs139144061
All
−0.0015
−0.0010
−0.0005
0.0000
0.0005
MR effect size for
'Prolactin−releasing peptide' on 'eGFR'

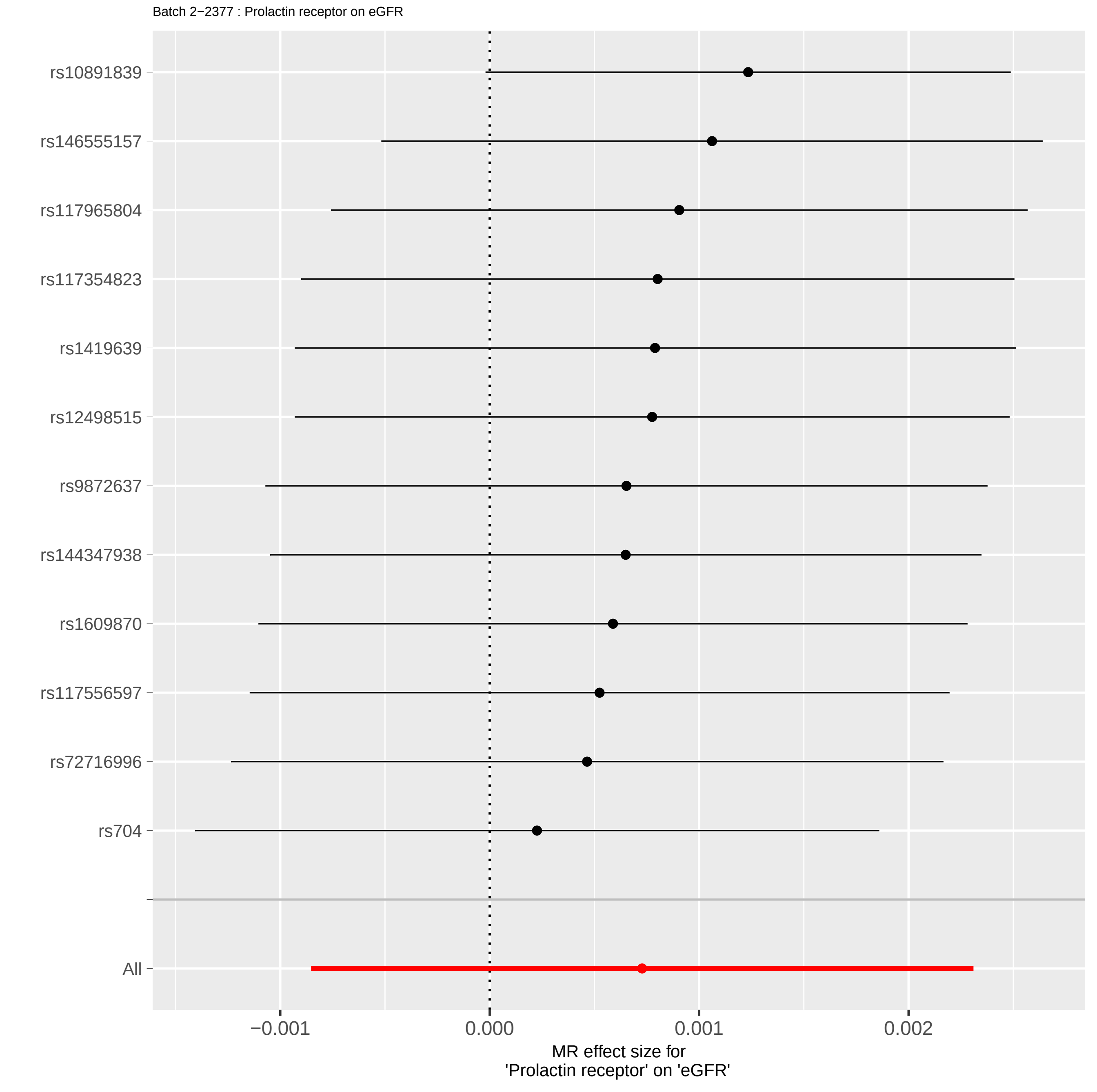

Batch 2−2377 : Prolactin receptor on eGFR
rs10891839
rs146555157
rs117965804
rs117354823
rs1419639
rs12498515
rs9872637
rs144347938
rs1609870
rs117556597
rs72716996
rs704
All
−0.001
0.000
0.001
0.002
MR effect size for
'Prolactin receptor' on 'eGFR'

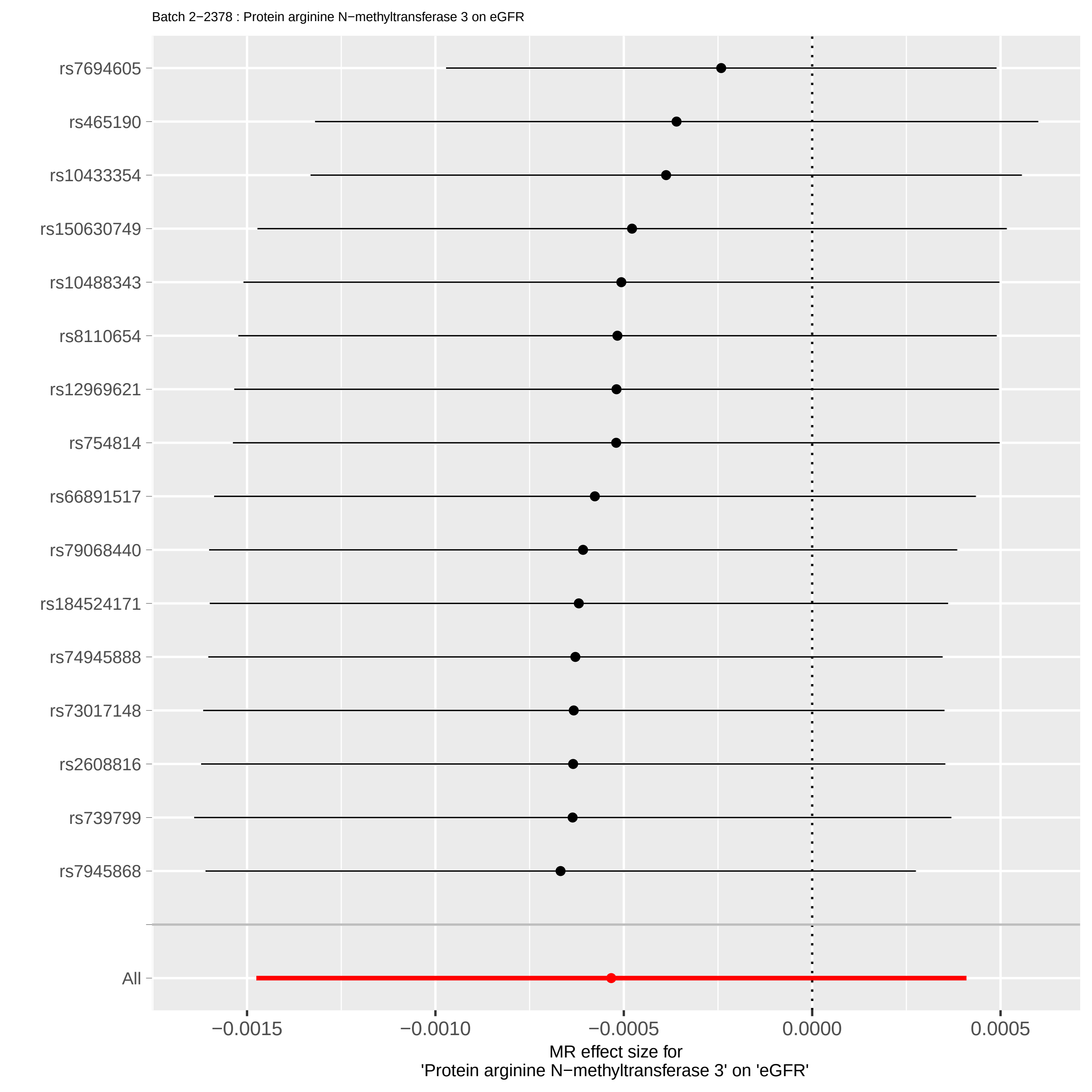

Batch 2−2378 : Protein arginine N−methyltransferase 3 on eGFR
rs7694605
rs465190
rs10433354
rs150630749
rs10488343
rs8110654
rs12969621
rs754814
rs66891517
rs79068440
rs184524171
rs74945888
rs73017148
rs2608816
rs739799
rs7945868
All
−0.0015
−0.0010
−0.0005
0.0000
0.0005
MR effect size for
'Protein arginine N−methyltransferase 3' on 'eGFR'

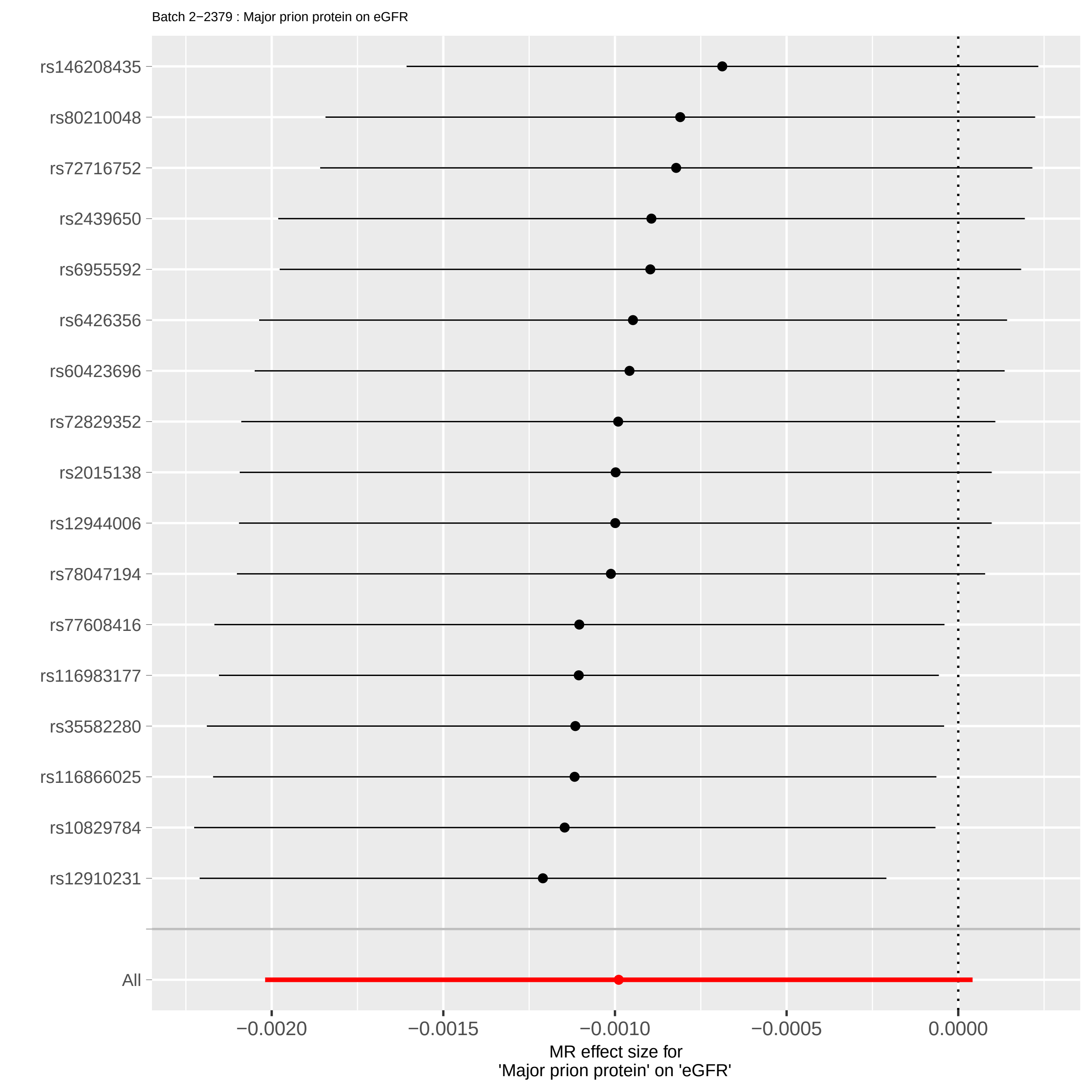

Batch 2−2379 : Major prion protein on eGFR
rs146208435
rs80210048
rs72716752
rs2439650
rs6955592
rs6426356
rs60423696
rs72829352
rs2015138
rs12944006
rs78047194
rs77608416
rs116983177
rs35582280
rs116866025
rs10829784
rs12910231
All
−0.0020
−0.0015
−0.0010
−0.0005
0.0000
MR effect size for
'Major prion protein' on 'eGFR'

Batch 2−2380 : Activated Protein C on eGFR

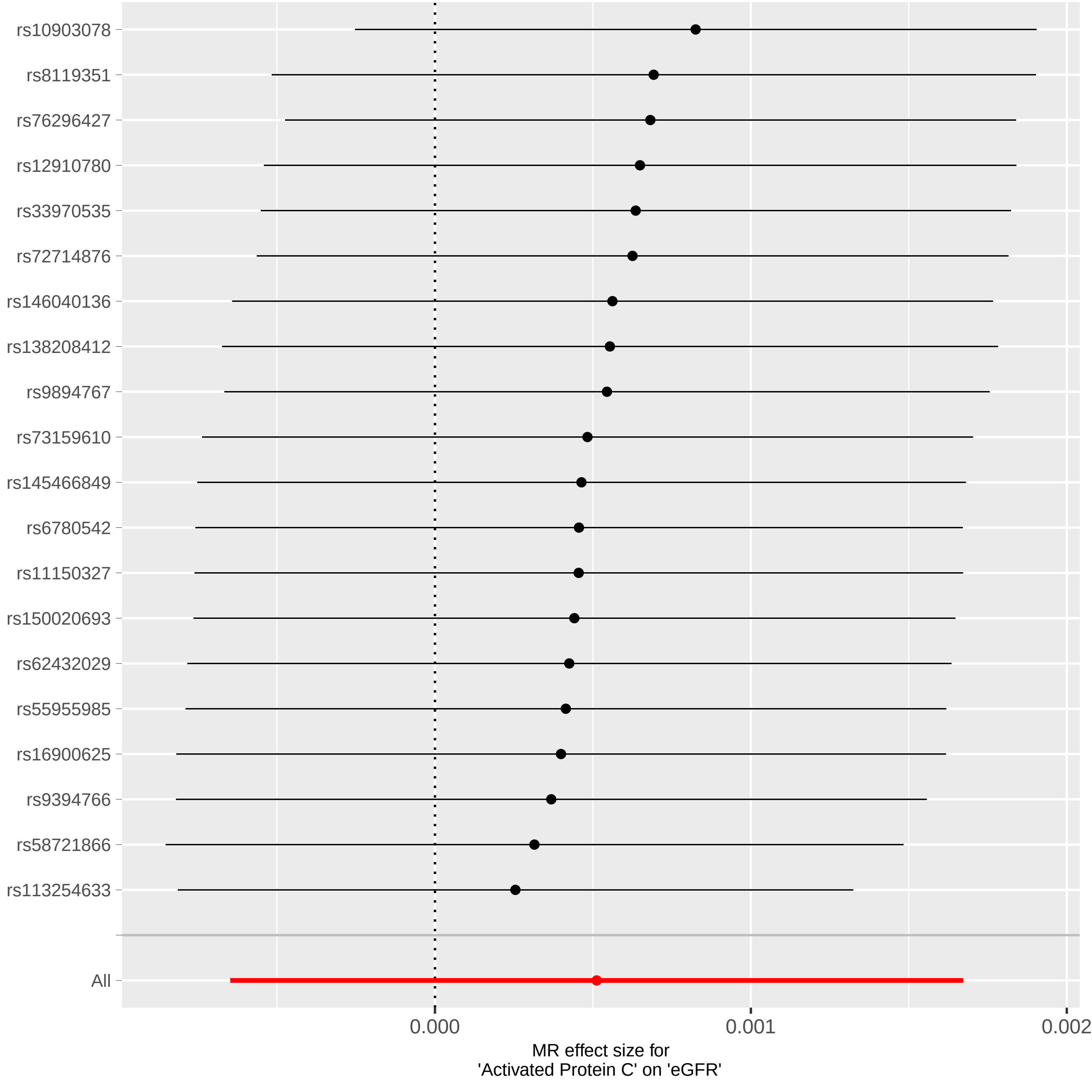

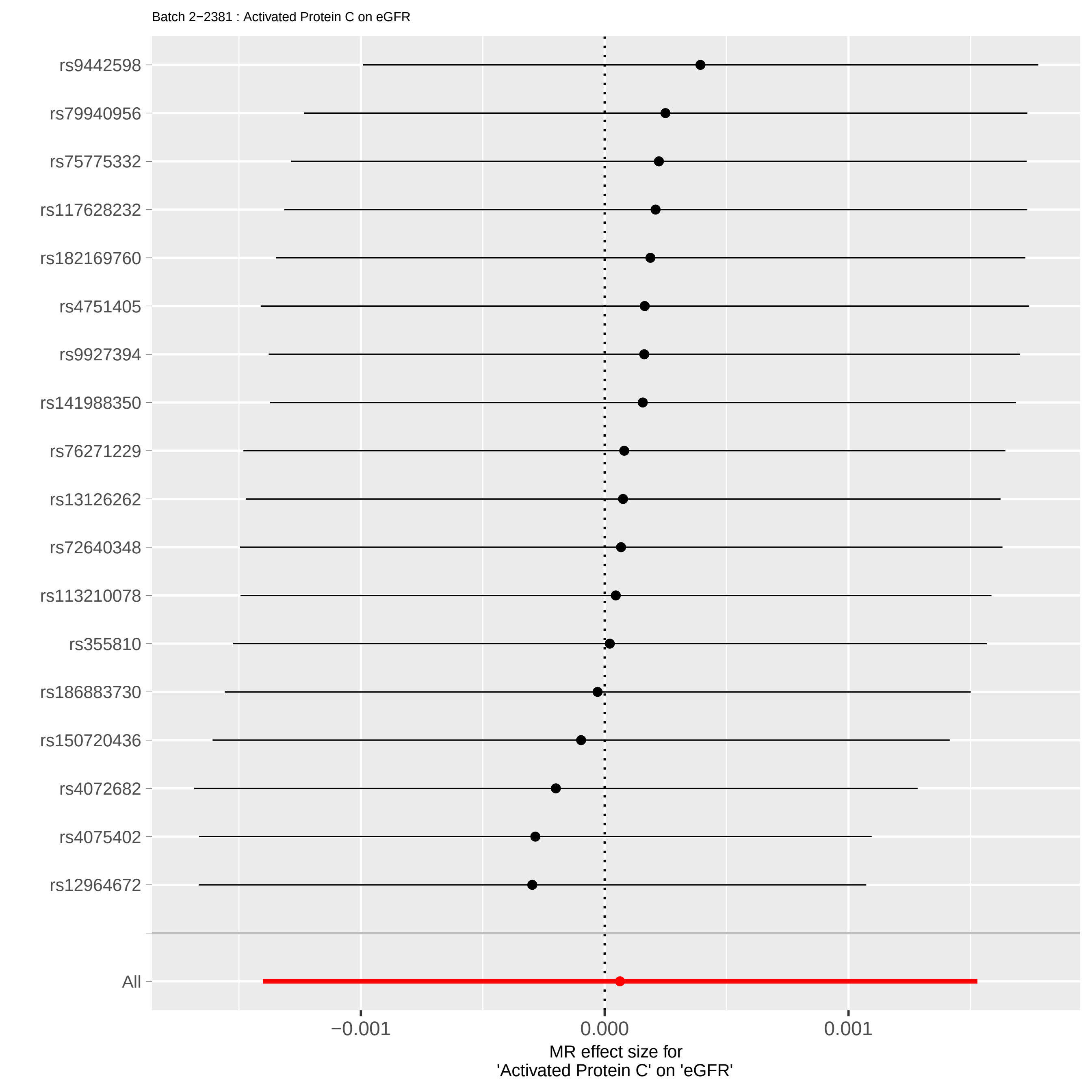

Batch 2−2381 : Activated Protein C on eGFR
rs9442598
rs79940956
rs75775332
rs117628232
rs182169760
rs4751405
rs9927394
rs141988350
rs76271229
rs13126262
rs72640348
rs113210078
rs355810
rs186883730
rs150720436
rs4072682
rs4075402
rs12964672
All
−0.001
0.000
0.001
MR effect size for
'Activated Protein C' on 'eGFR'

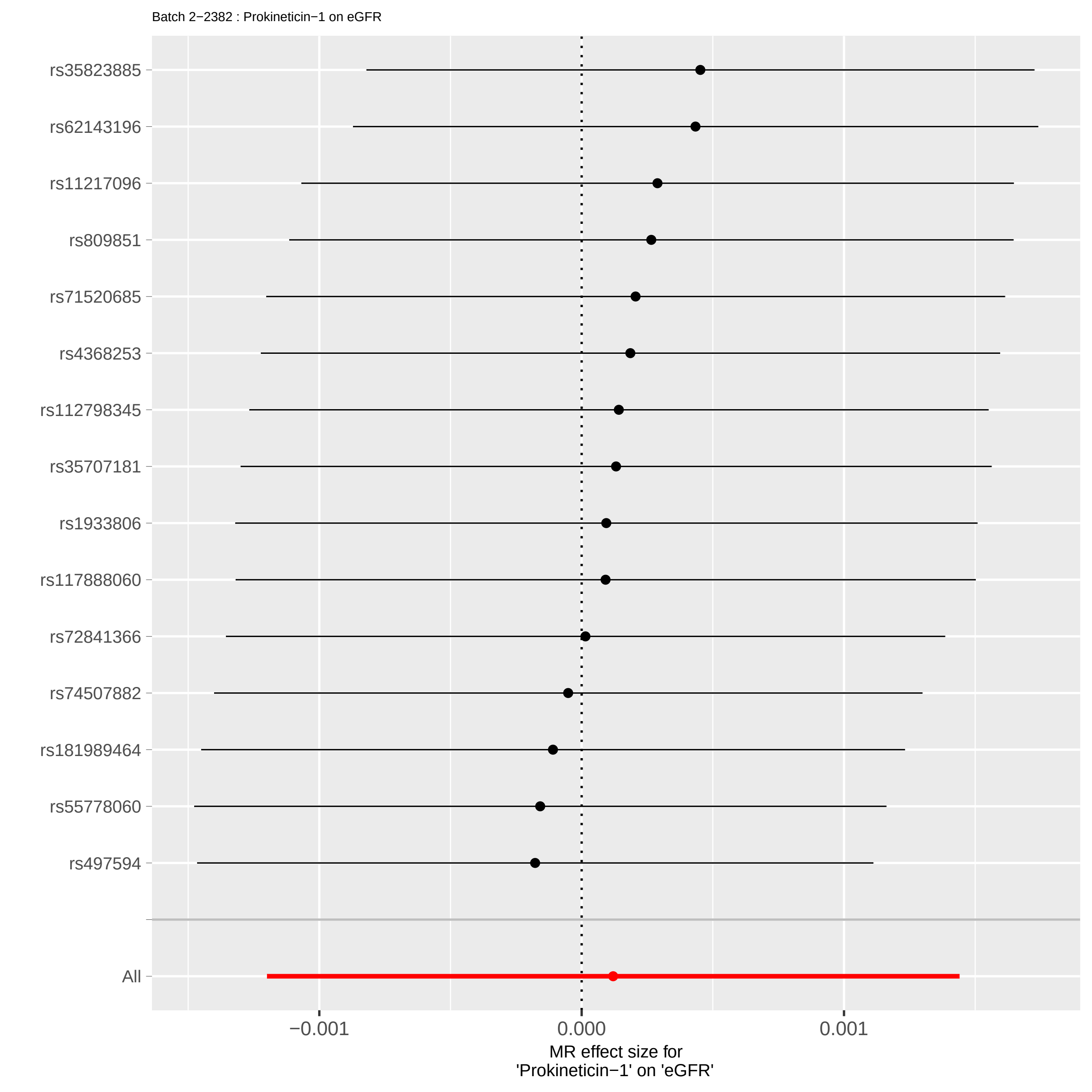

Batch 2−2382 : Prokineticin−1 on eGFR
rs35823885
rs62143196
rs11217096
rs809851
rs71520685
rs4368253
rs112798345
rs35707181
rs1933806
rs117888060
rs72841366
rs74507882
rs181989464
rs55778060
rs497594
All
−0.001
0.000
0.001
MR effect size for
'Prokineticin−1' on 'eGFR'

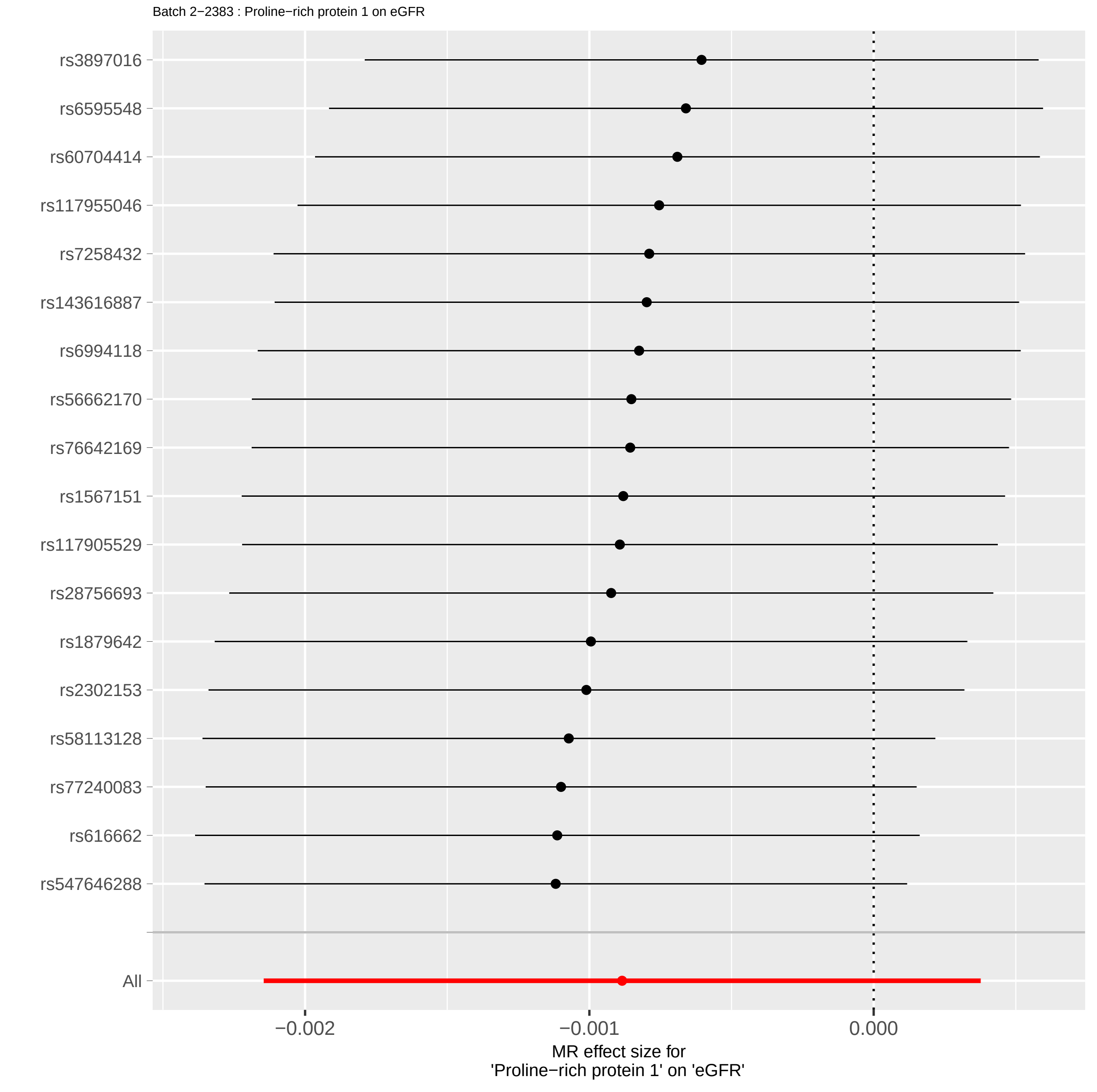

Batch 2−2383 : Proline−rich protein 1 on eGFR
rs3897016
rs6595548
rs60704414
rs117955046
rs7258432
rs143616887
rs6994118
rs56662170
rs76642169
rs1567151
rs117905529
rs28756693
rs1879642
rs2302153
rs58113128
rs77240083
rs616662
rs547646288
All
−0.002
−0.001
0.000
MR effect size for
'Proline−rich protein 1' on 'eGFR'

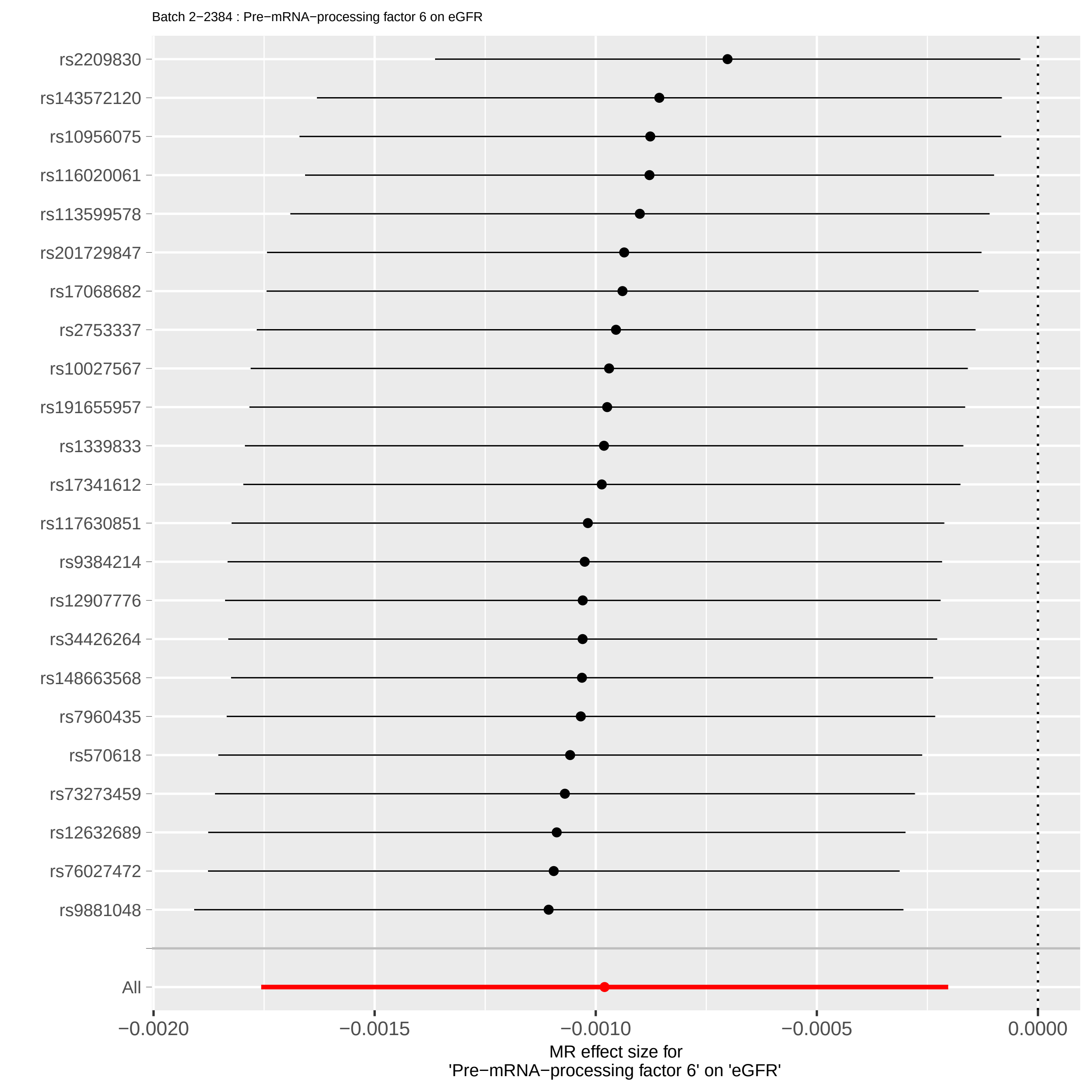

Batch 2−2384 : Pre−mRNA−processing factor 6 on eGFR
rs2209830
rs143572120
rs10956075
rs116020061
rs113599578
rs201729847
rs17068682
rs2753337
rs10027567
rs191655957
rs1339833
rs17341612
rs117630851
rs9384214
rs12907776
rs34426264
rs148663568
rs7960435
rs570618
rs73273459
rs12632689
rs76027472
rs9881048
All
−0.0020
−0.0015
−0.0010
−0.0005
0.0000
MR effect size for
'Pre−mRNA−processing factor 6' on 'eGFR'

Batch 2−2385 : Phosphoribosyl pyrophosphate synthase−associated protein 1 on eGFR

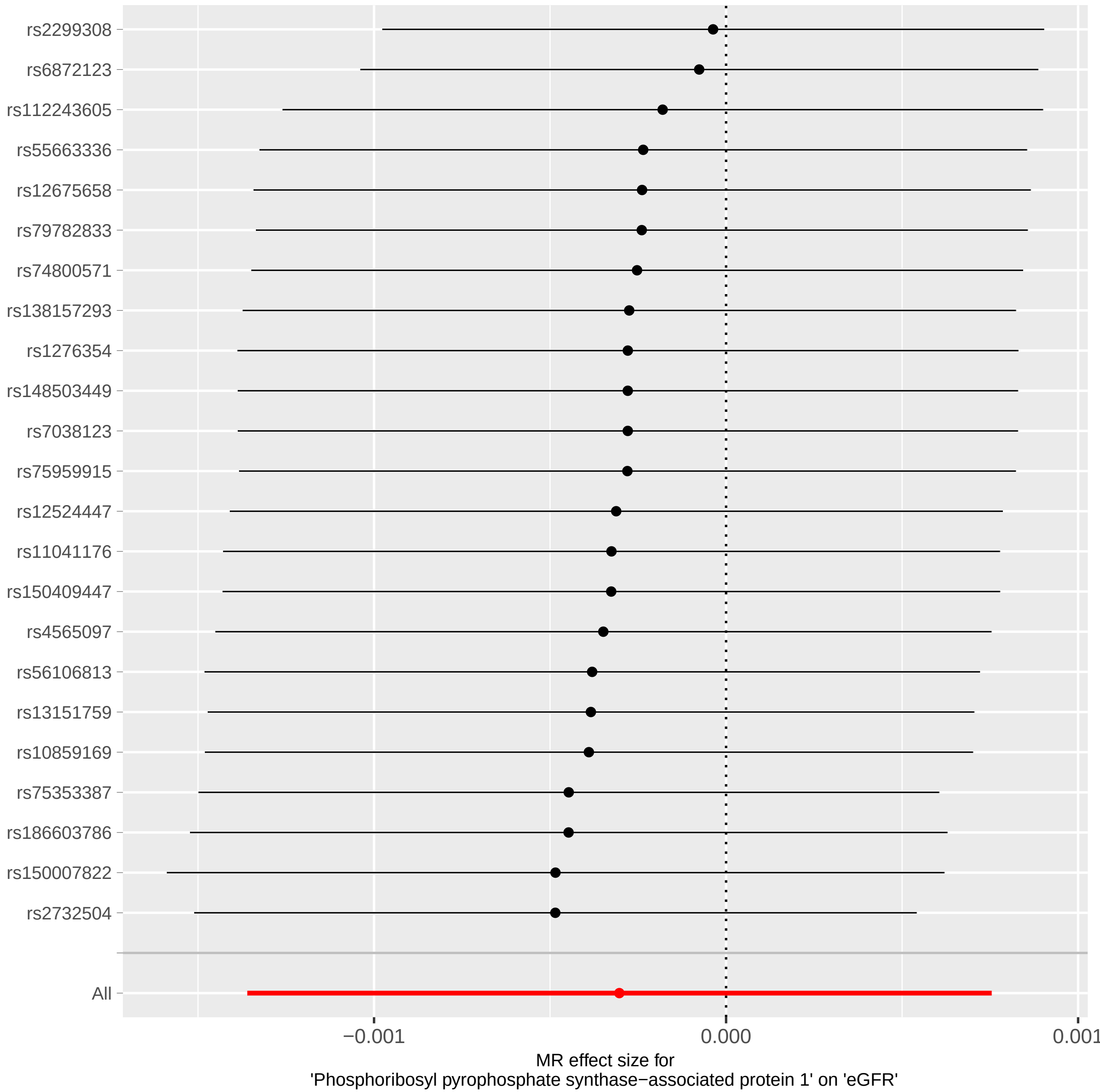

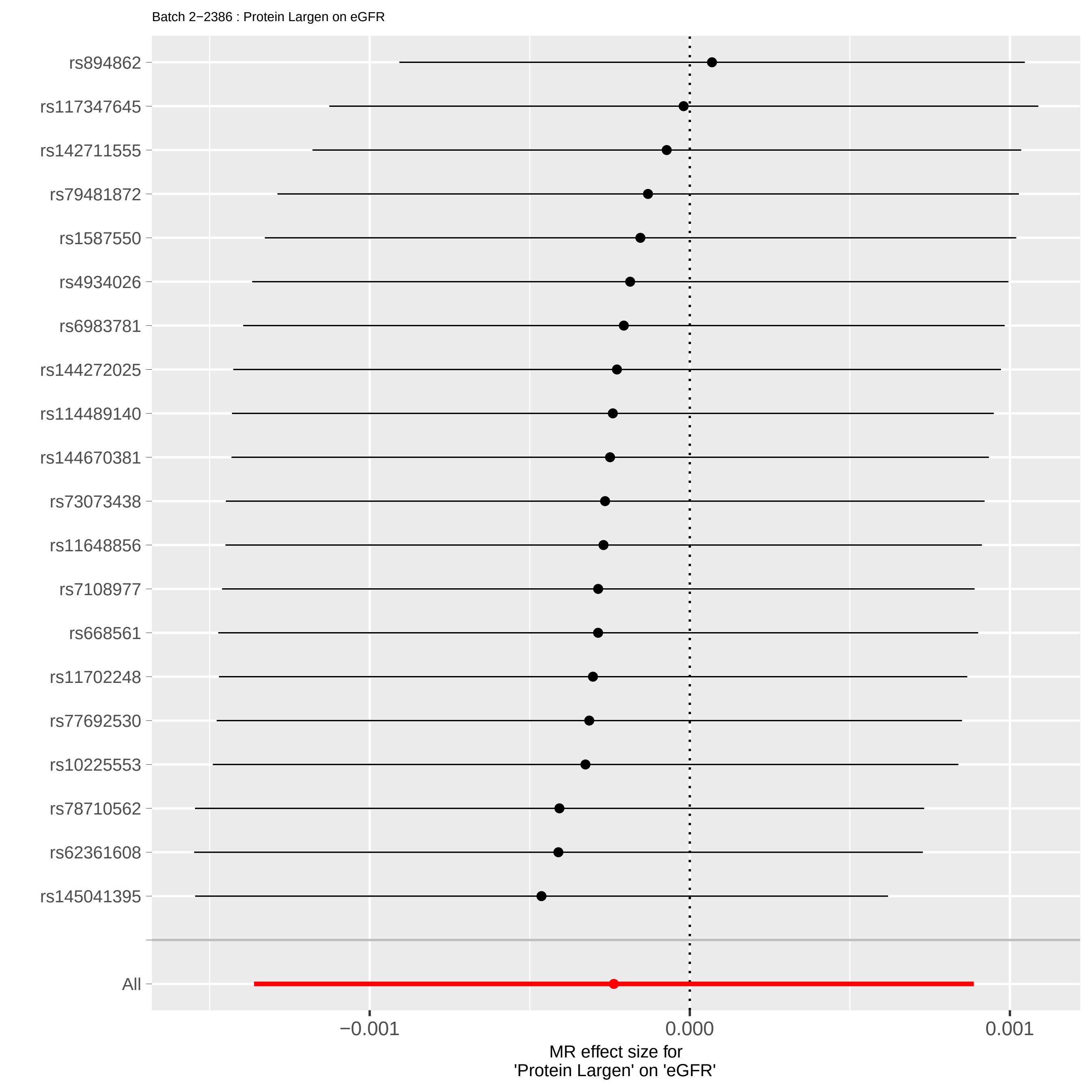

Batch 2−2386 : Protein Largen on eGFR
rs894862
rs117347645
rs142711555
rs79481872
rs1587550
rs4934026
rs6983781
rs144272025
rs114489140
rs144670381
rs73073438
rs11648856
rs7108977
rs668561
rs11702248
rs77692530
rs10225553
rs78710562
rs62361608
rs145041395
All
−0.001
0.000
0.001
MR effect size for
'Protein Largen' on 'eGFR'

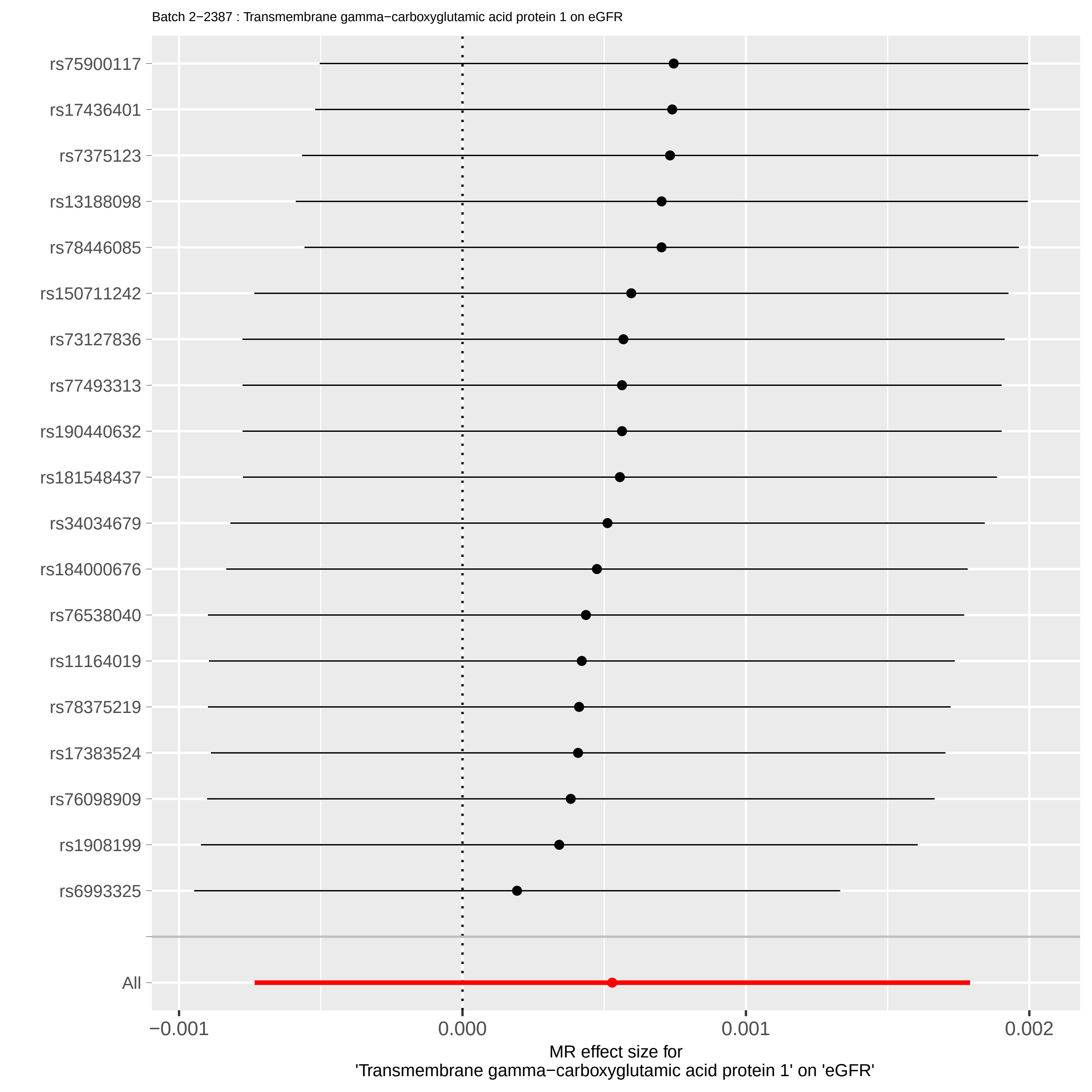

Batch 2−2387 : Transmembrane gamma−carboxyglutamic acid protein 1 on eGFR
rs75900117
rs17436401
rs7375123
rs13188098
rs78446085
rs150711242
rs73127836
rs77493313
rs190440632
rs181548437
rs34034679
rs184000676
rs76538040
rs11164019
rs78375219
rs17383524
rs76098909
rs1908199
rs6993325
All
−0.001
0.000
0.001
0.002
MR effect size for
'Transmembrane gamma−carboxyglutamic acid protein 1' on 'eGFR'

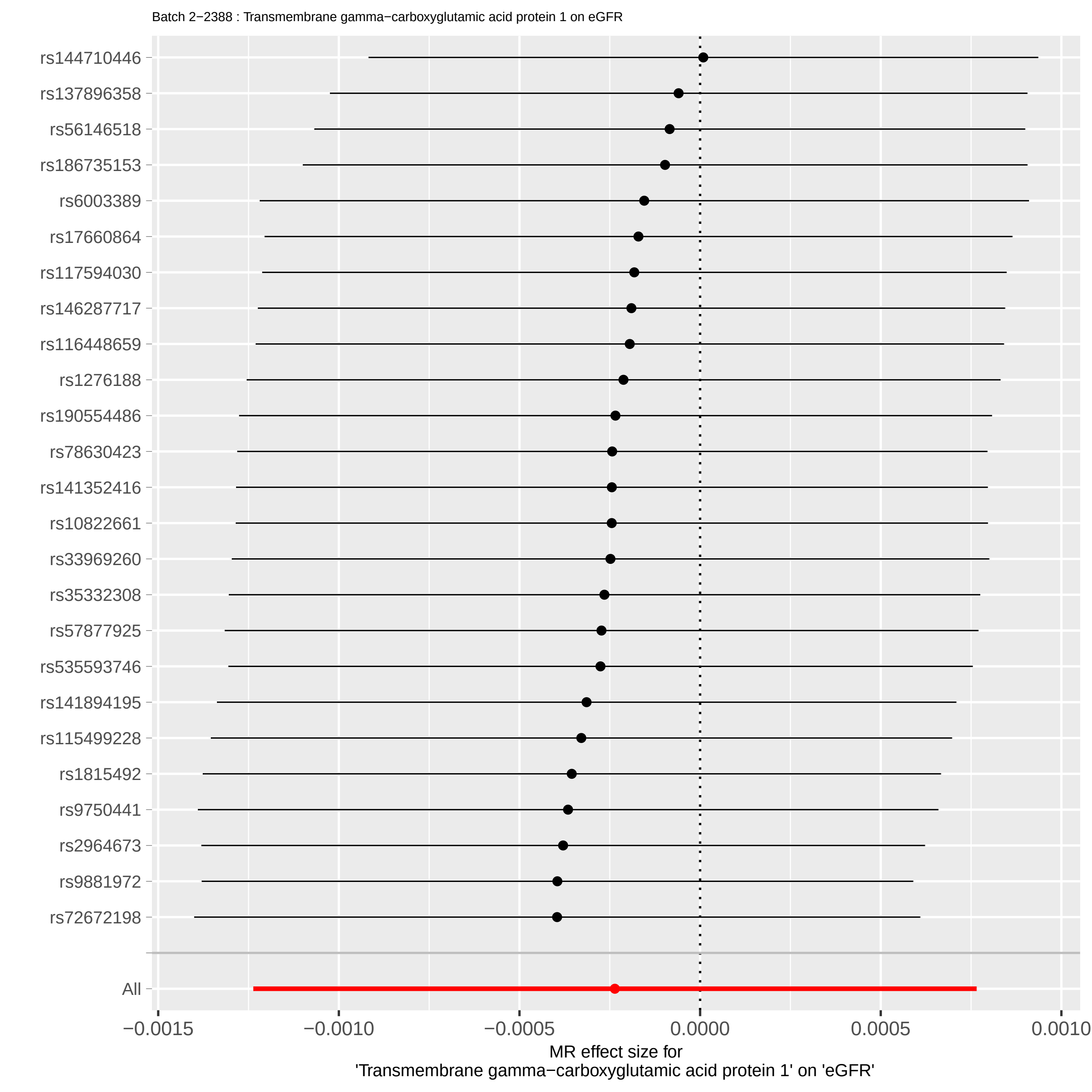

Batch 2−2388 : Transmembrane gamma−carboxyglutamic acid protein 1 on eGFR
rs144710446
rs137896358
rs56146518
rs186735153
rs6003389
rs17660864
rs117594030
rs146287717
rs116448659
rs1276188
rs190554486
rs78630423
rs141352416
rs10822661
rs33969260
rs35332308
rs57877925
rs535593746
rs141894195
rs115499228
rs1815492
rs9750441
rs2964673
rs9881972
rs72672198
All
−0.0015
−0.0010
−0.0005
0.0000
0.0005
0.0010
MR effect size for
'Transmembrane gamma−carboxyglutamic acid protein 1' on 'eGFR'

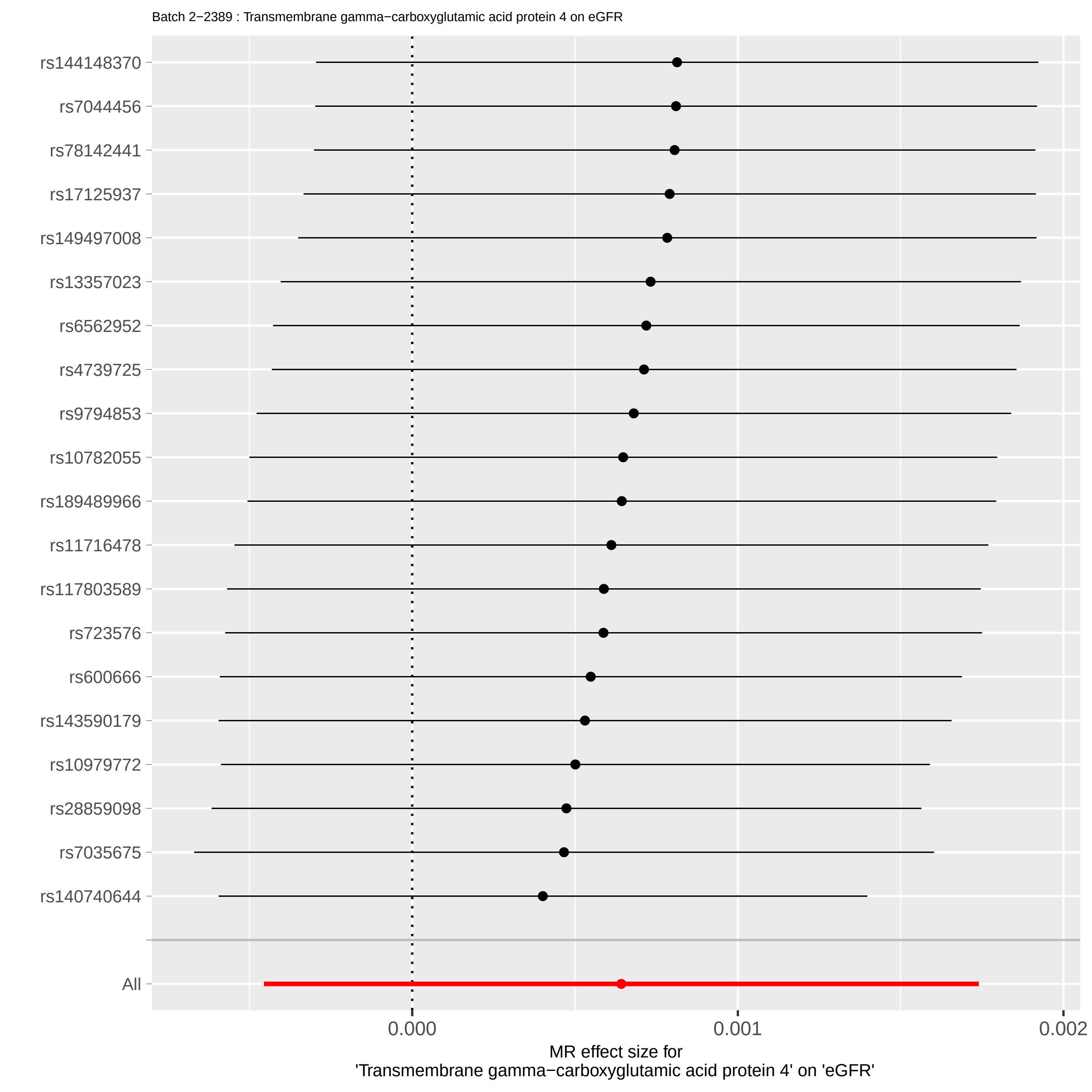

Batch 2−2389 : Transmembrane gamma−carboxyglutamic acid protein 4 on eGFR
rs144148370
rs7044456
rs78142441
rs17125937
rs149497008
rs13357023
rs6562952
rs4739725
rs9794853
rs10782055
rs189489966
rs11716478
rs117803589
rs723576
rs600666
rs143590179
rs10979772
rs28859098
rs7035675
rs140740644
All
0.000
0.001
0.002
MR effect size for
'Transmembrane gamma−carboxyglutamic acid protein 4' on 'eGFR'

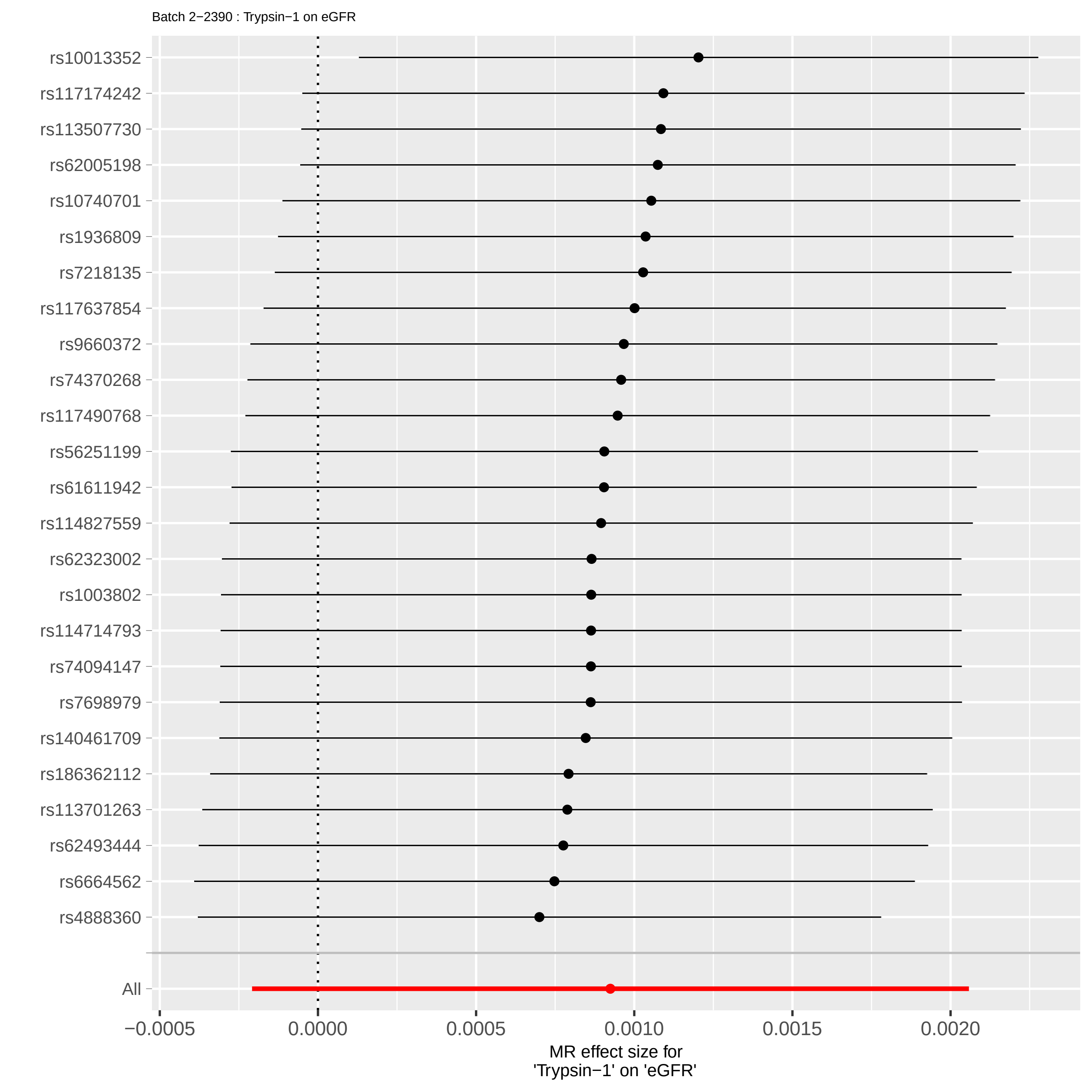

Batch 2−2390 : Trypsin−1 on eGFR
rs10013352
rs117174242
rs113507730
rs62005198
rs10740701
rs1936809
rs7218135
rs117637854
rs9660372
rs74370268
rs117490768
rs56251199
rs61611942
rs114827559
rs62323002
rs1003802
rs114714793
rs74094147
rs7698979
rs140461709
rs186362112
rs113701263
rs62493444
rs6664562
rs4888360
All
−0.0005
0.0000
0.0005
0.0010
0.0015
0.0020
MR effect size for
'Trypsin−1' on 'eGFR'

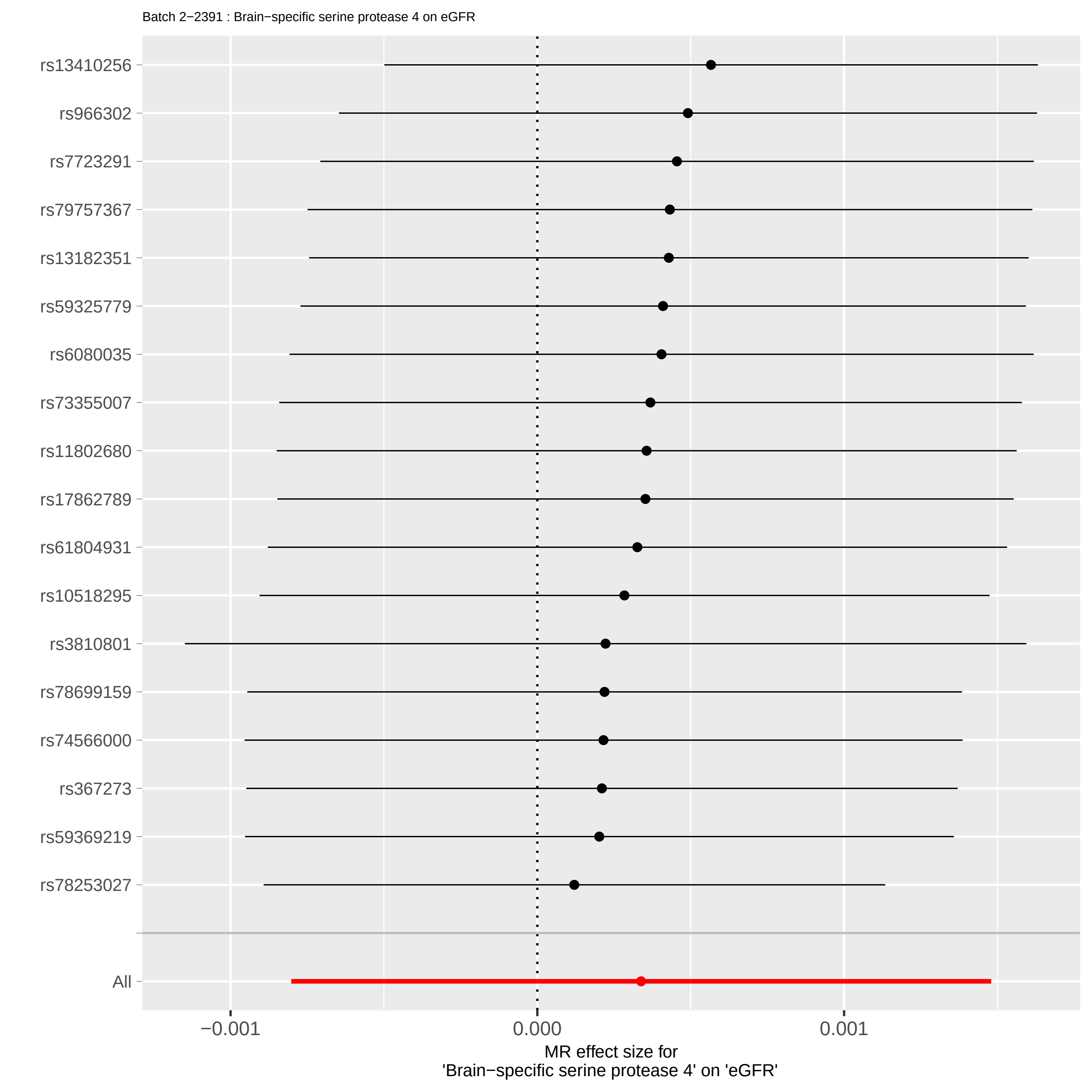
Batch 2−2391 : Brain−specific serine protease 4 on eGFR
rs13410256
rs966302
rs7723291
rs79757367
rs13182351
rs59325779
rs6080035
rs73355007
rs11802680
rs17862789
rs61804931
rs10518295
rs3810801
rs78699159
rs74566000
rs367273
rs59369219
rs78253027
All
−0.001
0.000
0.001
MR effect size for
'Brain−specific serine protease 4' on 'eGFR'

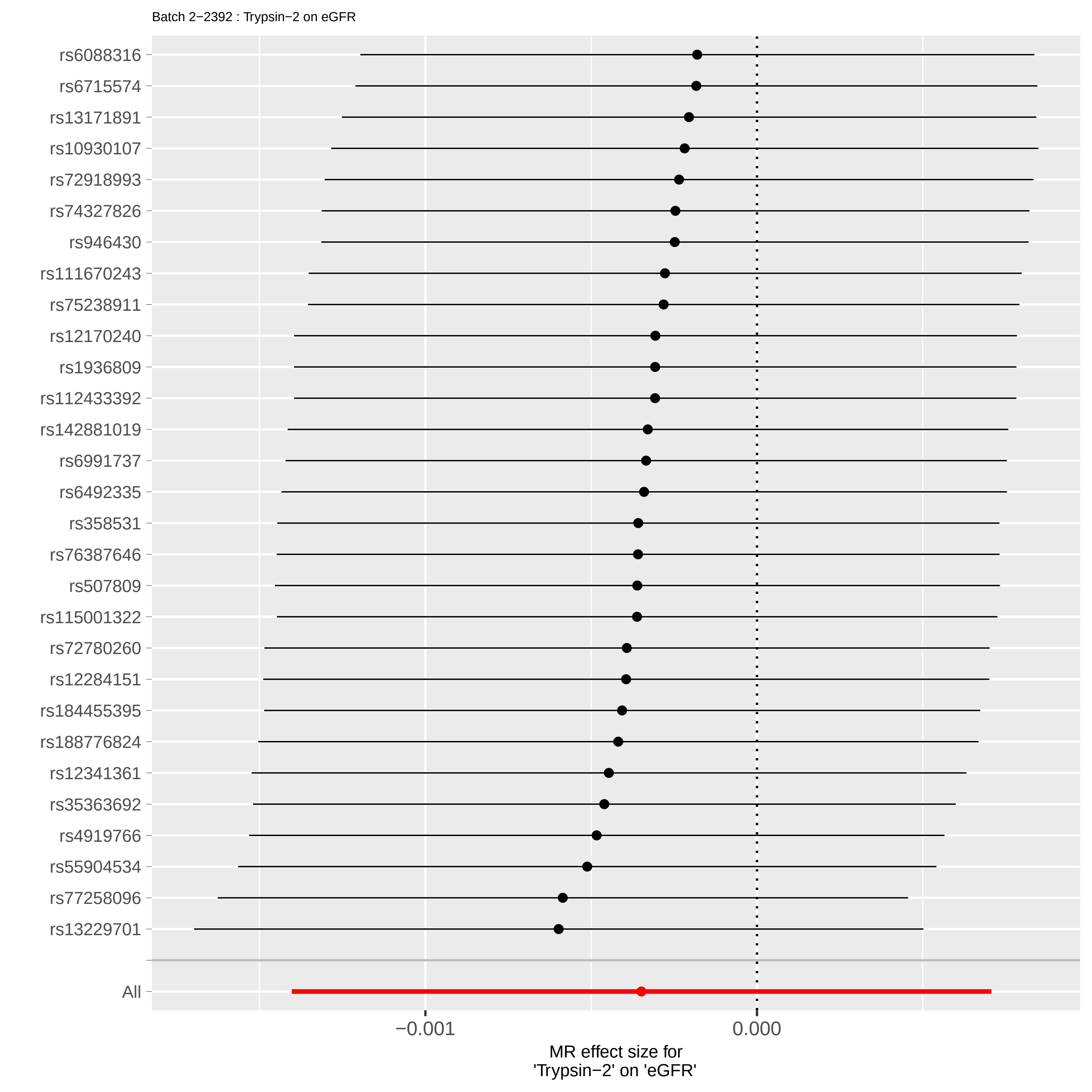
Batch 2−2392 : Trypsin−2 on eGFR
rs6088316
rs6715574
rs13171891
rs10930107
rs72918993
rs74327826
rs946430
rs111670243
rs75238911
rs12170240
rs1936809
rs112433392
rs142881019
rs6991737
rs6492335
rs358531
rs76387646
rs507809
rs115001322
rs72780260
rs12284151
rs184455395
rs188776824
rs12341361
rs35363692
rs4919766
rs55904534
rs77258096
rs13229701
All
−0.001
0.000
MR effect size for
'Trypsin−2' on 'eGFR'

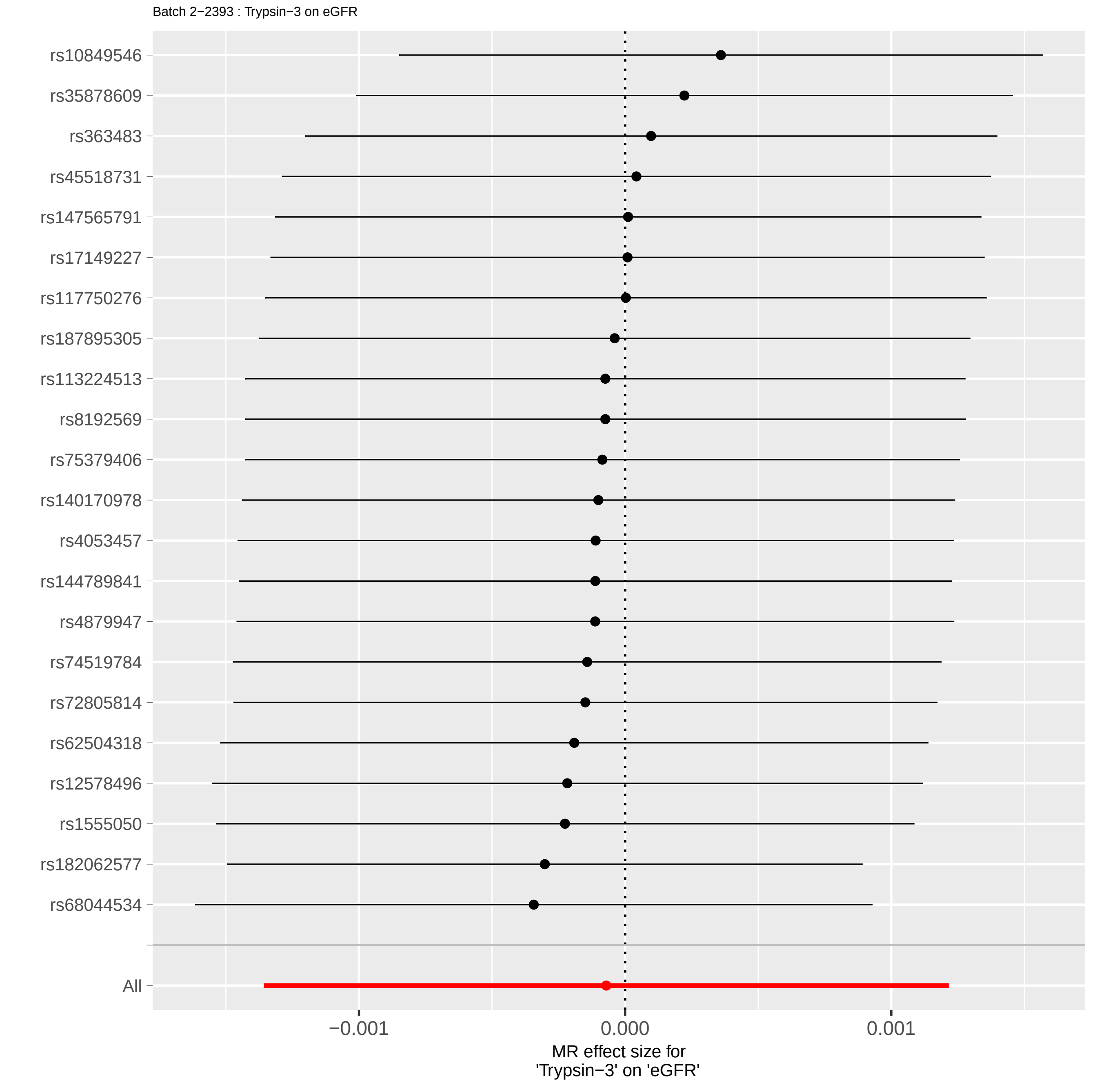

Batch 2−2393 : Trypsin−3 on eGFR
rs10849546
rs35878609
rs363483
rs45518731
rs147565791
rs17149227
rs117750276
rs187895305
rs113224513
rs8192569
rs75379406
rs140170978
rs4053457
rs144789841
rs4879947
rs74519784
rs72805814
rs62504318
rs12578496
rs1555050
rs182062577
rs68044534
All
−0.001
0.000
0.001
MR effect size for
'Trypsin−3' on 'eGFR'

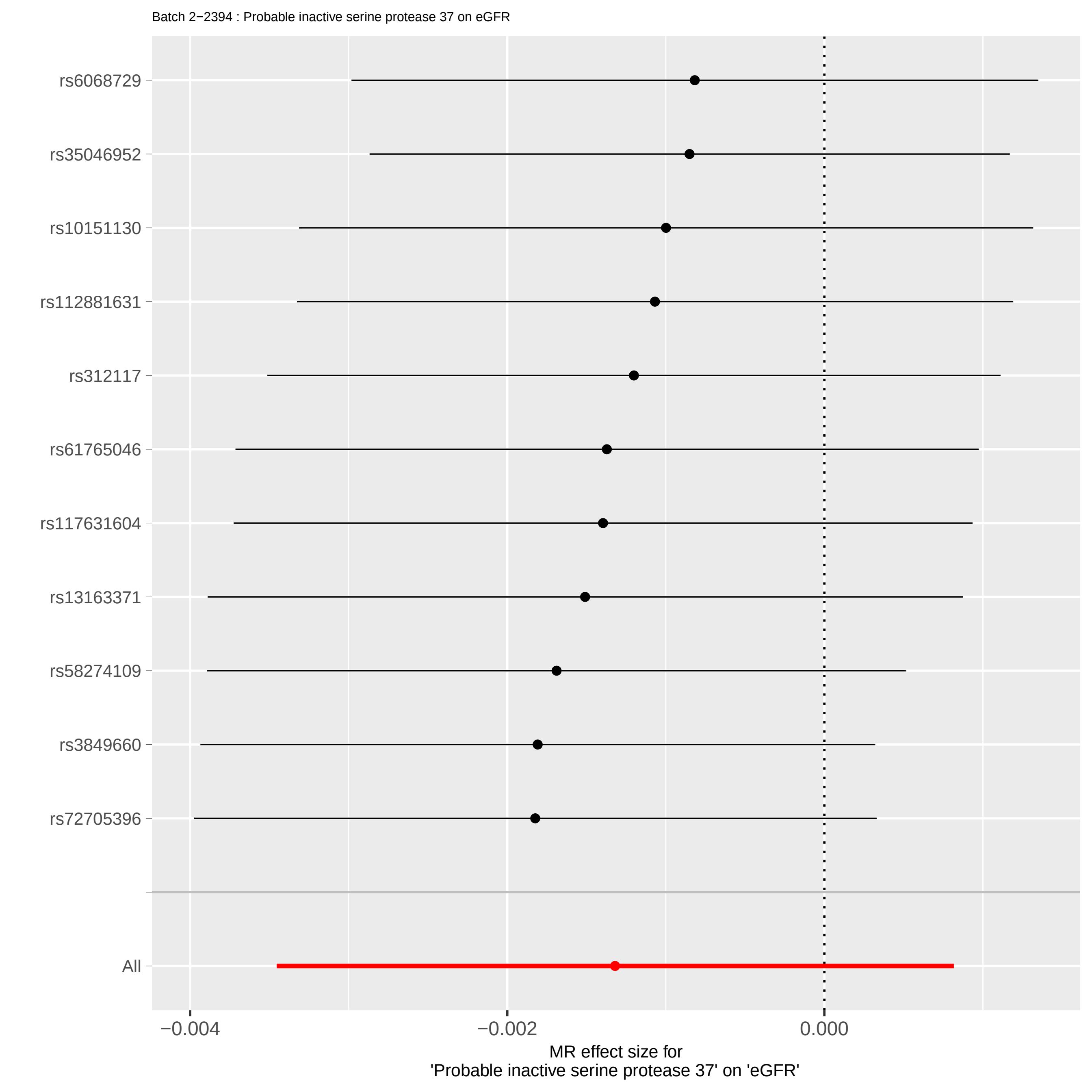
Batch 2−2394 : Probable inactive serine protease 37 on eGFR
rs6068729
rs35046952
rs10151130
rs112881631
rs312117
rs61765046
rs117631604
rs13163371
rs58274109
rs3849660
rs72705396
All
−0.004
−0.002
0.000
MR effect size for
'Probable inactive serine protease 37' on 'eGFR'

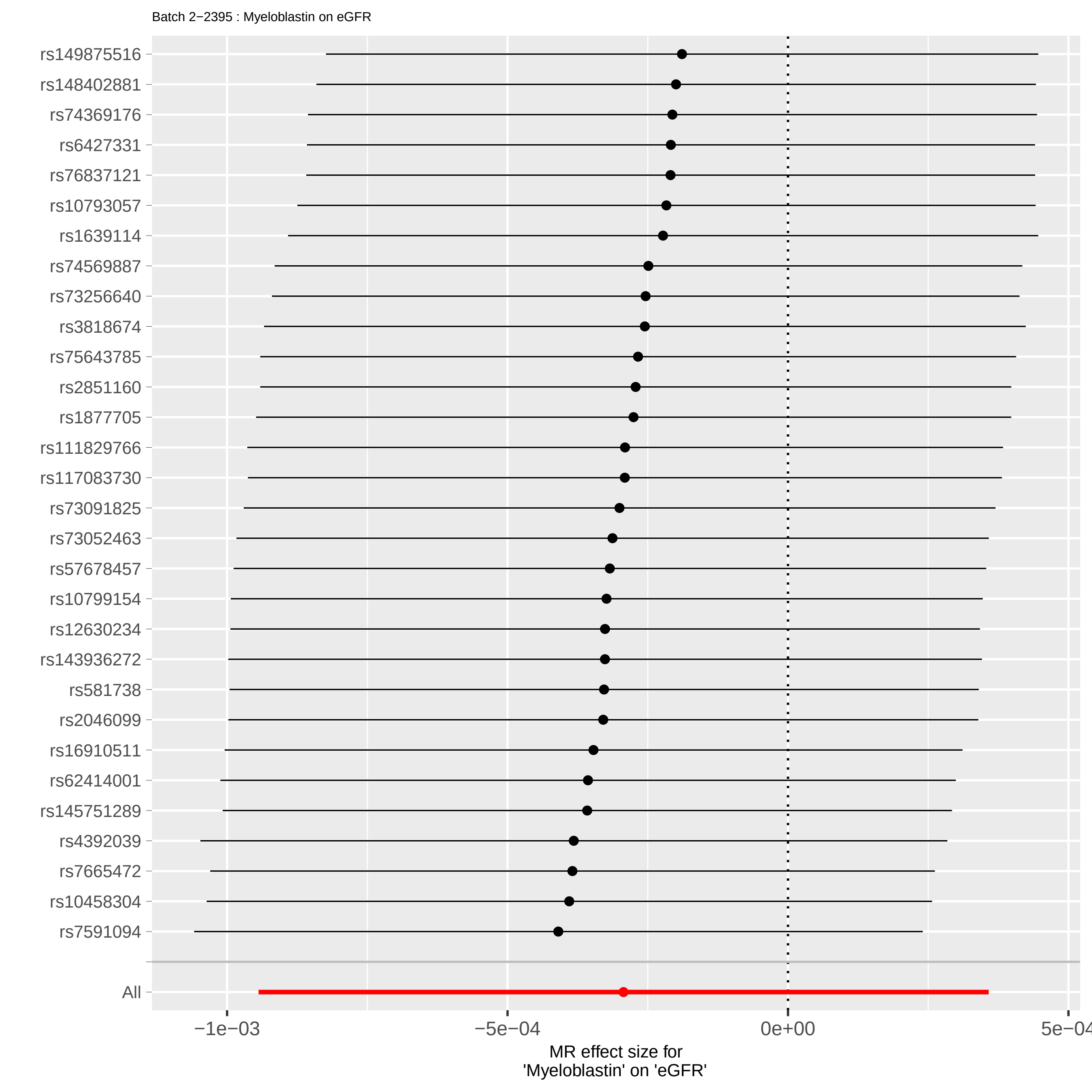
Batch 2−2395 : Myeloblastin on eGFR
rs149875516
rs148402881
rs74369176
rs6427331
rs76837121
rs10793057
rs1639114
rs74569887
rs73256640
rs3818674
rs75643785
rs2851160
rs1877705
rs111829766
rs117083730
rs73091825
rs73052463
rs57678457
rs10799154
rs12630234
rs143936272
rs581738
rs2046099
rs16910511
rs62414001
rs145751289
rs4392039
rs7665472
rs10458304
rs7591094
All
−1e−03
−5e−04
0e+00
5e−04
MR effect size for
'Myeloblastin' on 'eGFR'

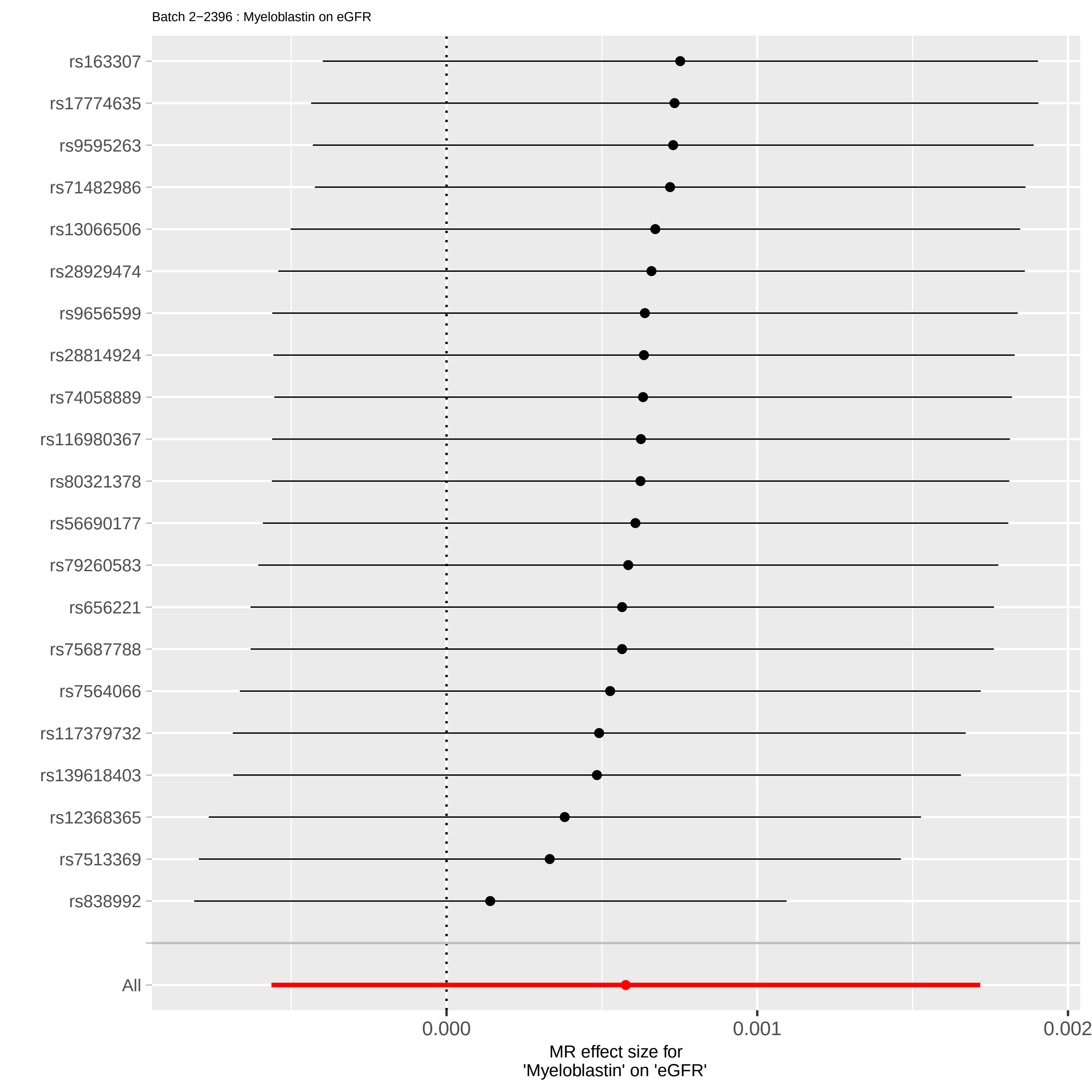

Batch 2−2396 : Myeloblastin on eGFR
rs163307
rs17774635
rs9595263
rs71482986
rs13066506
rs28929474
rs9656599
rs28814924
rs74058889
rs116980367
rs80321378
rs56690177
rs79260583
rs656221
rs75687788
rs7564066
rs117379732
rs139618403
rs12368365
rs7513369
rs838992
All
0.000
0.001
0.002
MR effect size for
'Myeloblastin' on 'eGFR'

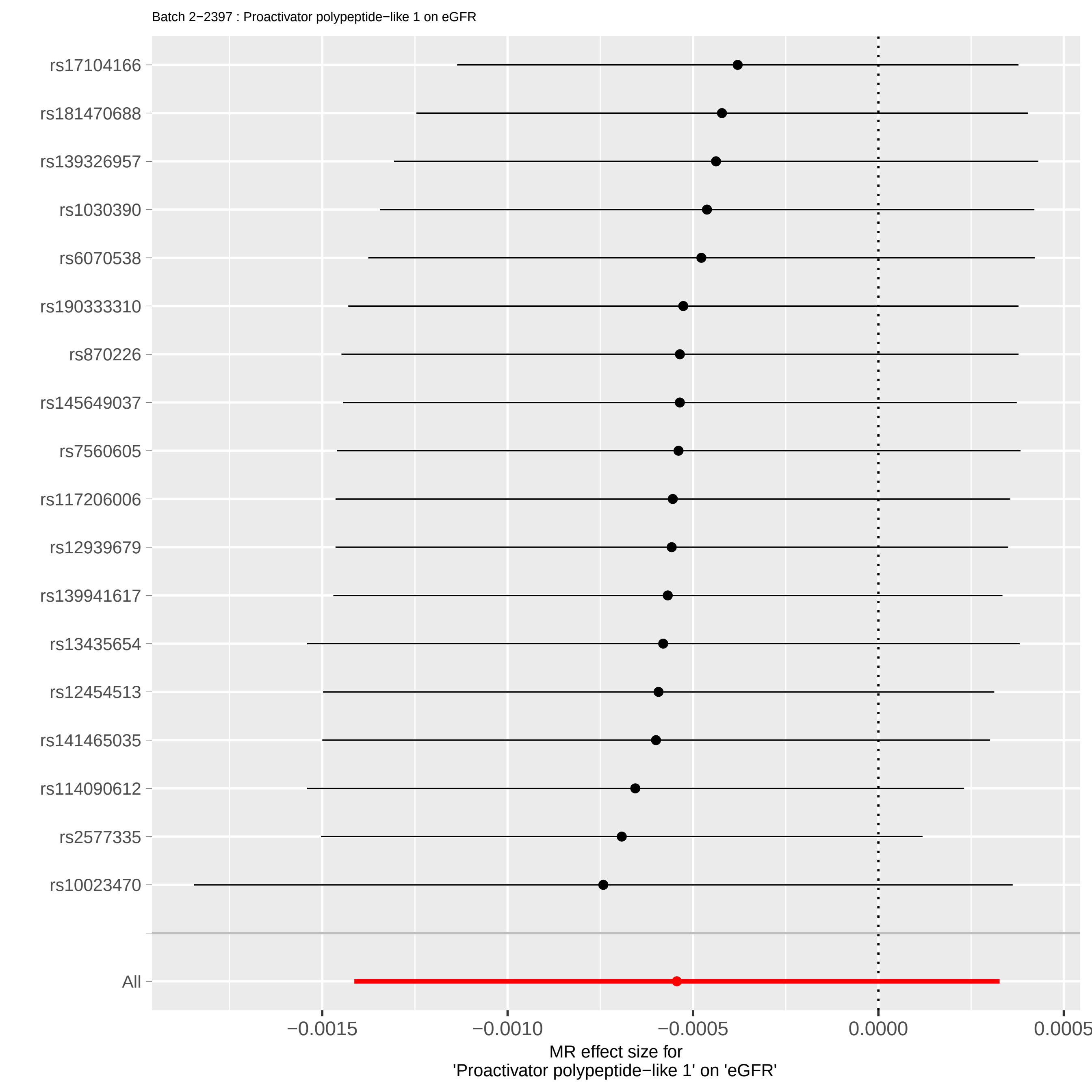

Batch 2−2397 : Proactivator polypeptide−like 1 on eGFR
rs17104166
rs181470688
rs139326957
rs1030390
rs6070538
rs190333310
rs870226
rs145649037
rs7560605
rs117206006
rs12939679
rs139941617
rs13435654
rs12454513
rs141465035
rs114090612
rs2577335
rs10023470
All
−0.0015
−0.0010
−0.0005
0.0000
0.0005
MR effect size for
'Proactivator polypeptide−like 1' on 'eGFR'

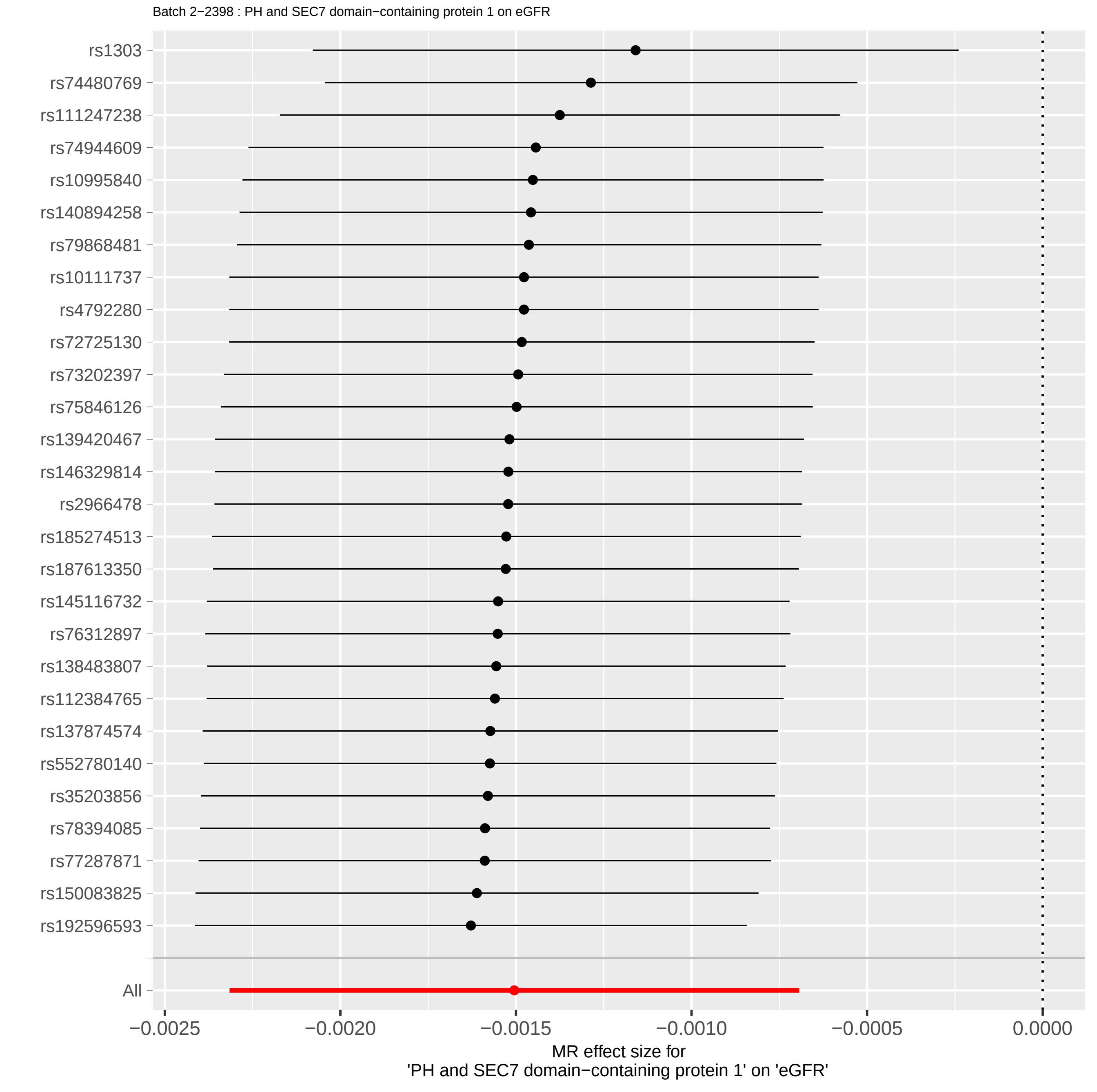
Batch 2−2398 : PH and SEC7 domain−containing protein 1 on eGFR
rs1303
rs74480769
rs111247238
rs74944609
rs10995840
rs140894258
rs79868481
rs10111737
rs4792280
rs72725130
rs73202397
rs75846126
rs139420467
rs146329814
rs2966478
rs185274513
rs187613350
rs145116732
rs76312897
rs138483807
rs112384765
rs137874574
rs552780140
rs35203856
rs78394085
rs77287871
rs150083825
rs192596593
All
−0.0025
−0.0020
−0.0015
−0.0010
−0.0005
0.0000
MR effect size for
'PH and SEC7 domain−containing protein 1' on 'eGFR'

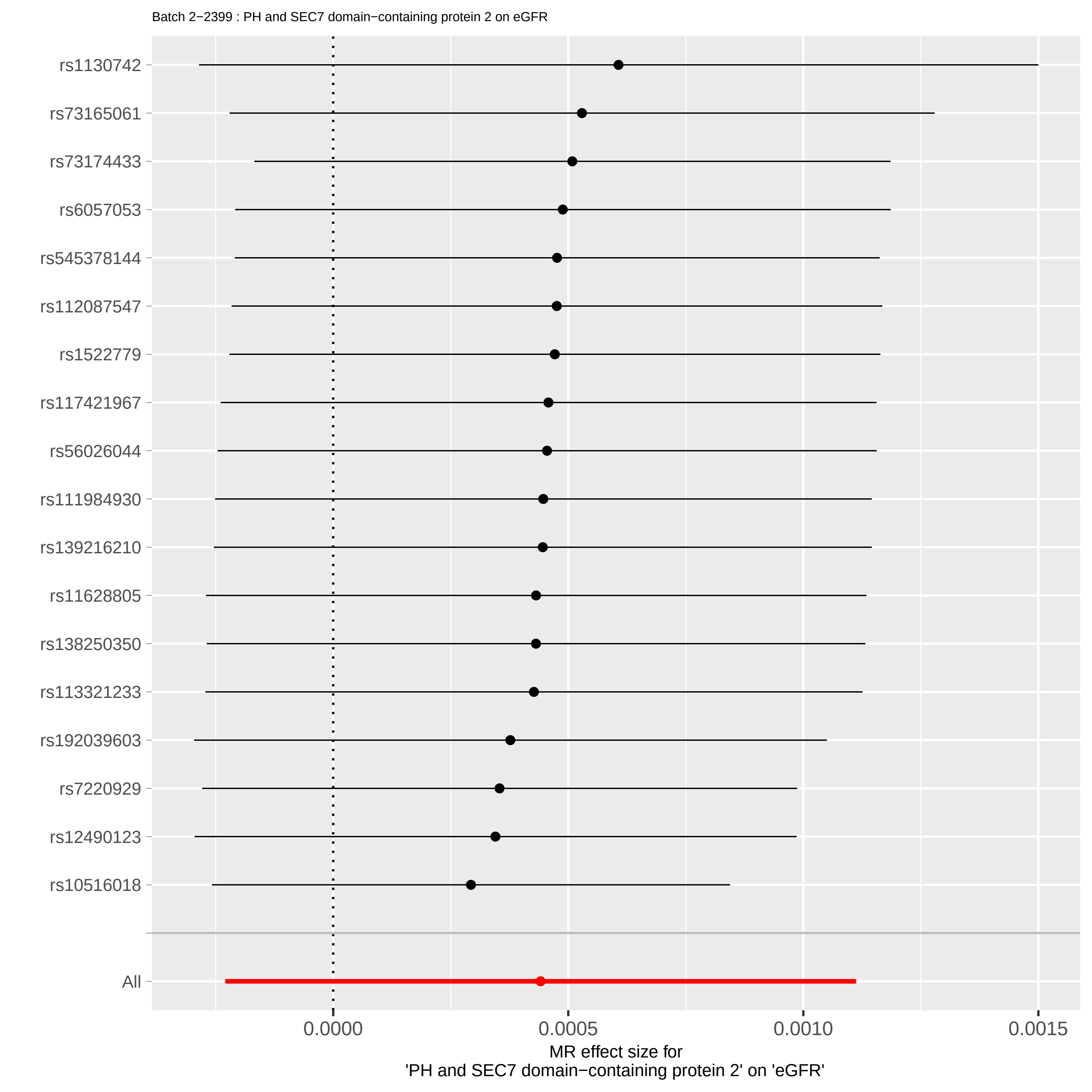

Batch 2−2399 : PH and SEC7 domain−containing protein 2 on eGFR
rs1130742
rs73165061
rs73174433
rs6057053
rs545378144
rs112087547
rs1522779
rs117421967
rs56026044
rs111984930
rs139216210
rs11628805
rs138250350
rs113321233
rs192039603
rs7220929
rs12490123
rs10516018
All
0.0000
0.0005
0.0010
0.0015
MR effect size for
'PH and SEC7 domain−containing protein 2' on 'eGFR'

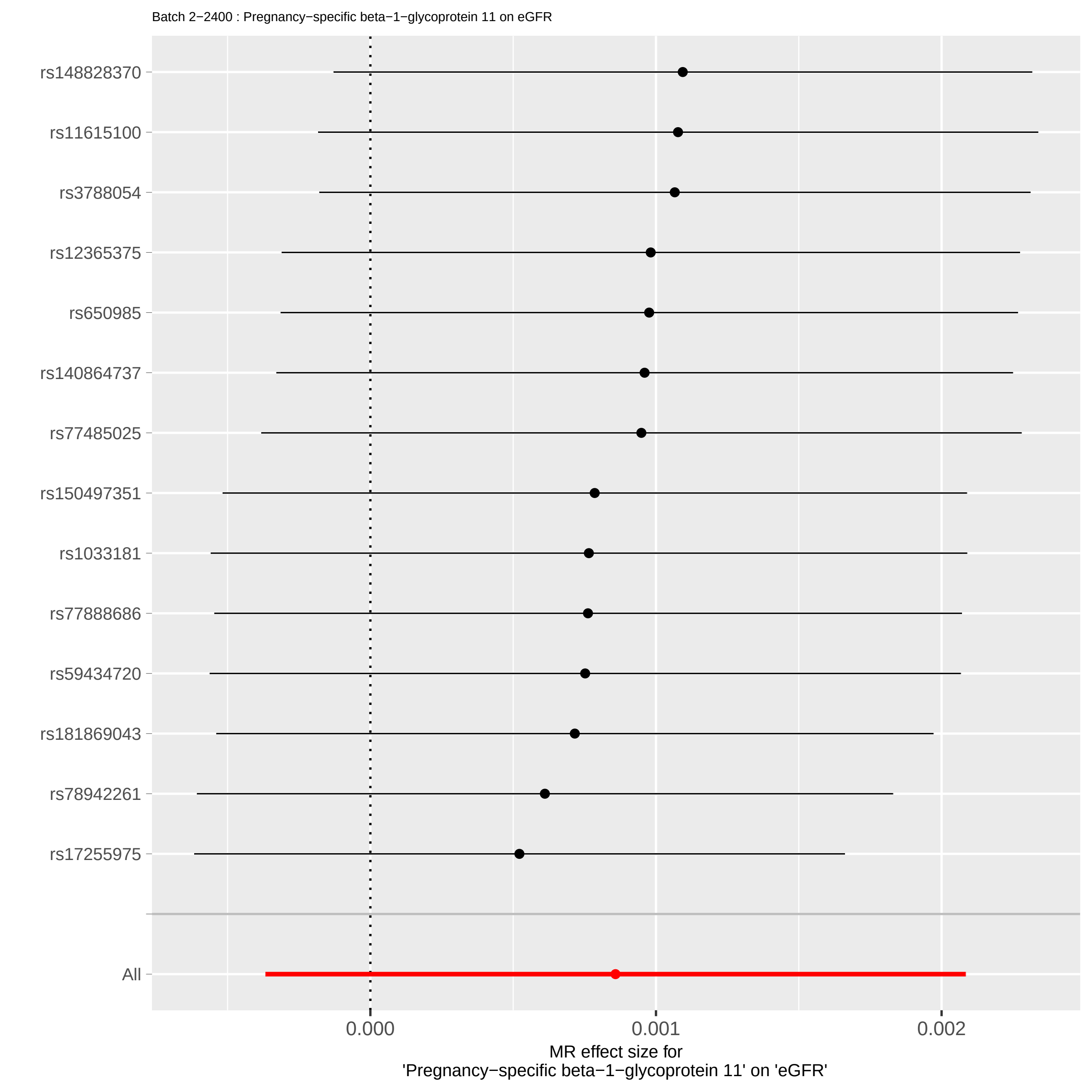

Batch 2−2400 : Pregnancy−specific beta−1−glycoprotein 11 on eGFR
rs148828370
rs11615100
rs3788054
rs12365375
rs650985
rs140864737
rs77485025
rs150497351
rs1033181
rs77888686
rs59434720
rs181869043
rs78942261
rs17255975
All
0.000
0.001
0.002
MR effect size for
'Pregnancy−specific beta−1−glycoprotein 11' on 'eGFR'

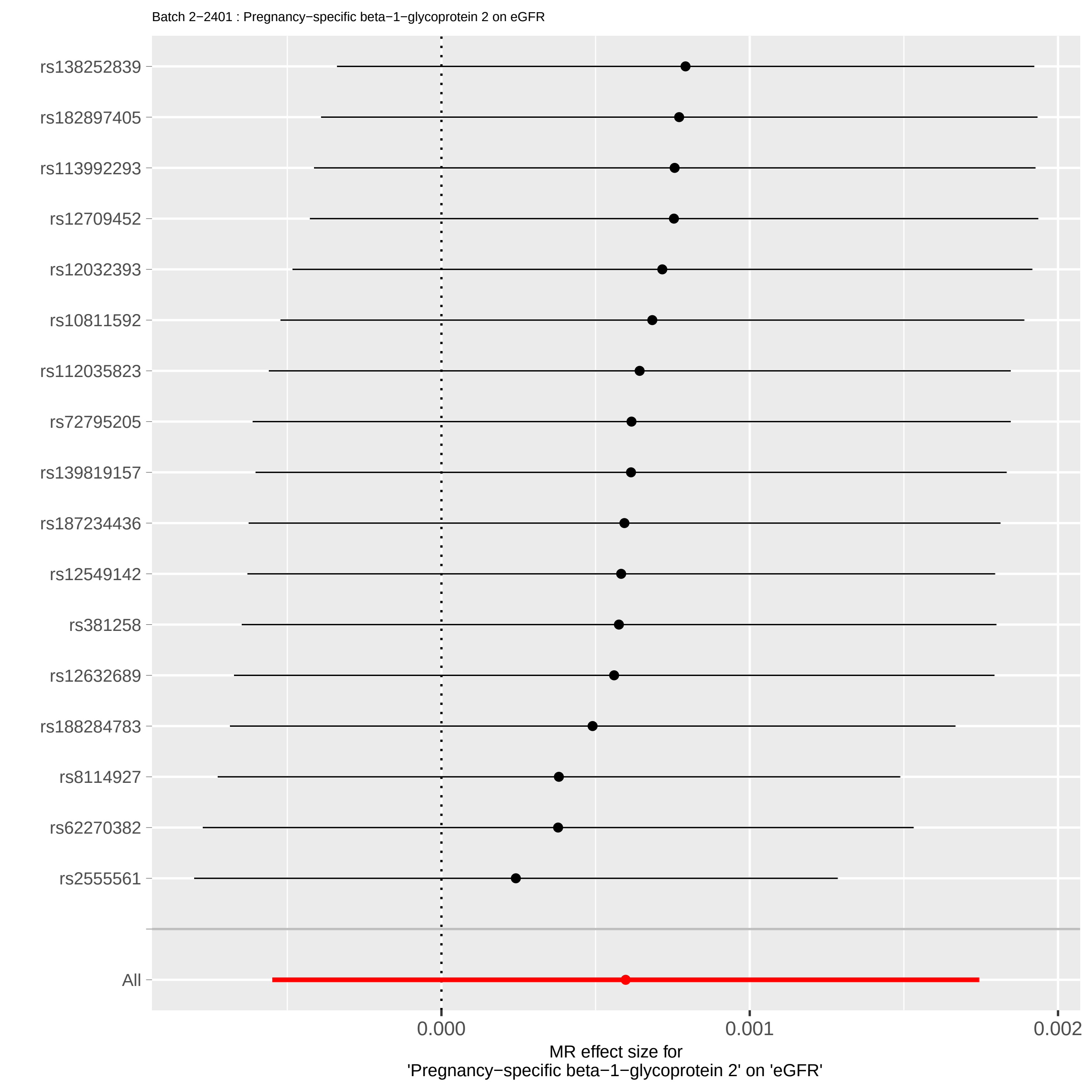

Batch 2−2401 : Pregnancy−specific beta−1−glycoprotein 2 on eGFR
rs138252839
rs182897405
rs113992293
rs12709452
rs12032393
rs10811592
rs112035823
rs72795205
rs139819157
rs187234436
rs12549142
rs381258
rs12632689
rs188284783
rs8114927
rs62270382
rs2555561
All
0.000
0.001
0.002
MR effect size for
'Pregnancy−specific beta−1−glycoprotein 2' on 'eGFR'

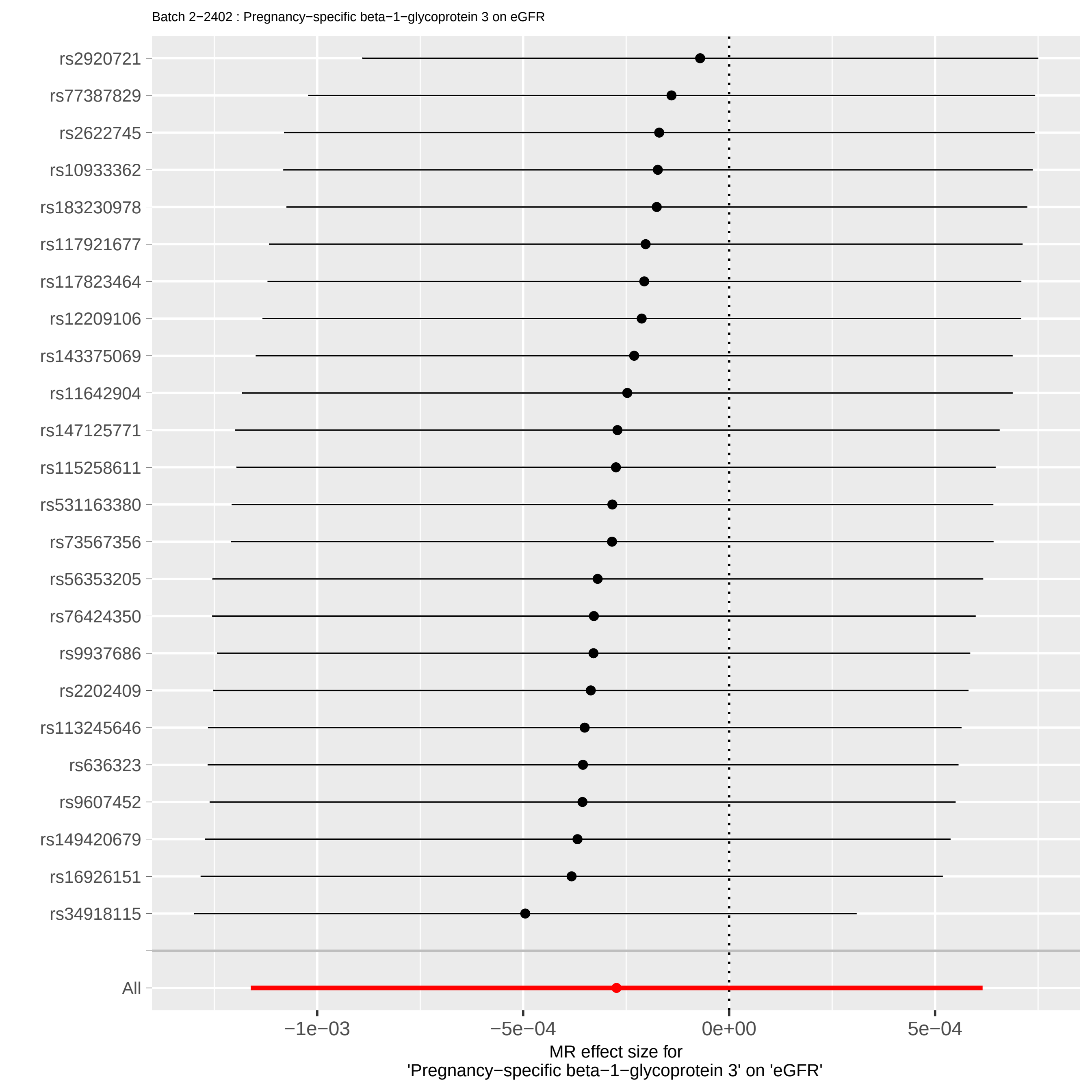

Batch 2−2402 : Pregnancy−specific beta−1−glycoprotein 3 on eGFR
rs2920721
rs77387829
rs2622745
rs10933362
rs183230978
rs117921677
rs117823464
rs12209106
rs143375069
rs11642904
rs147125771
rs115258611
rs531163380
rs73567356
rs56353205
rs76424350
rs9937686
rs2202409
rs113245646
rs636323
rs9607452
rs149420679
rs16926151
rs34918115
All
−1e−03
−5e−04
0e+00
5e−04
MR effect size for
'Pregnancy−specific beta−1−glycoprotein 3' on 'eGFR'

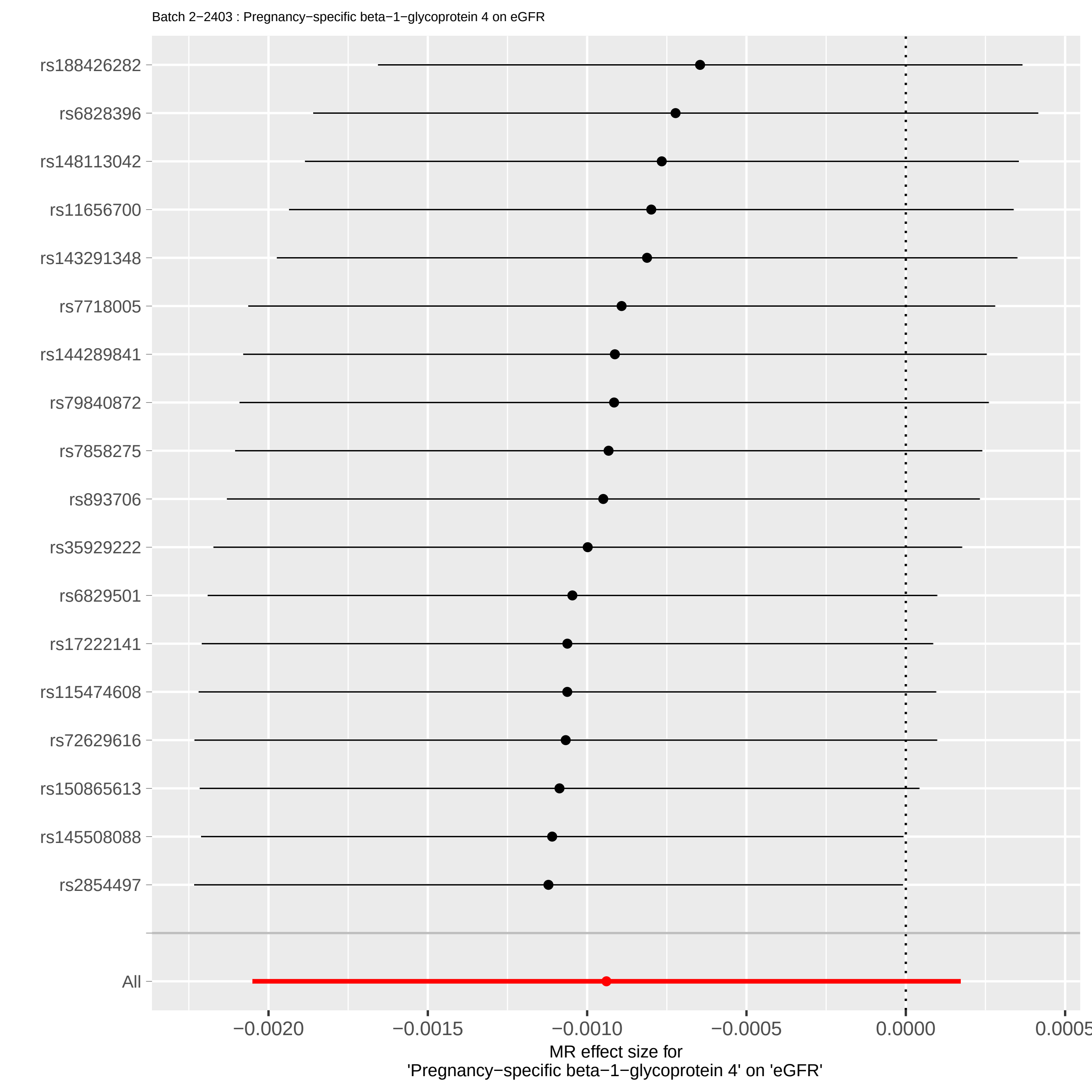

Batch 2−2403 : Pregnancy−specific beta−1−glycoprotein 4 on eGFR
rs188426282
rs6828396
rs148113042
rs11656700
rs143291348
rs7718005
rs144289841
rs79840872
rs7858275
rs893706
rs35929222
rs6829501
rs17222141
rs115474608
rs72629616
rs150865613
rs145508088
rs2854497
All
−0.0020
−0.0015
−0.0010
−0.0005
0.0000
0.0005
MR effect size for
'Pregnancy−specific beta−1−glycoprotein 4' on 'eGFR'

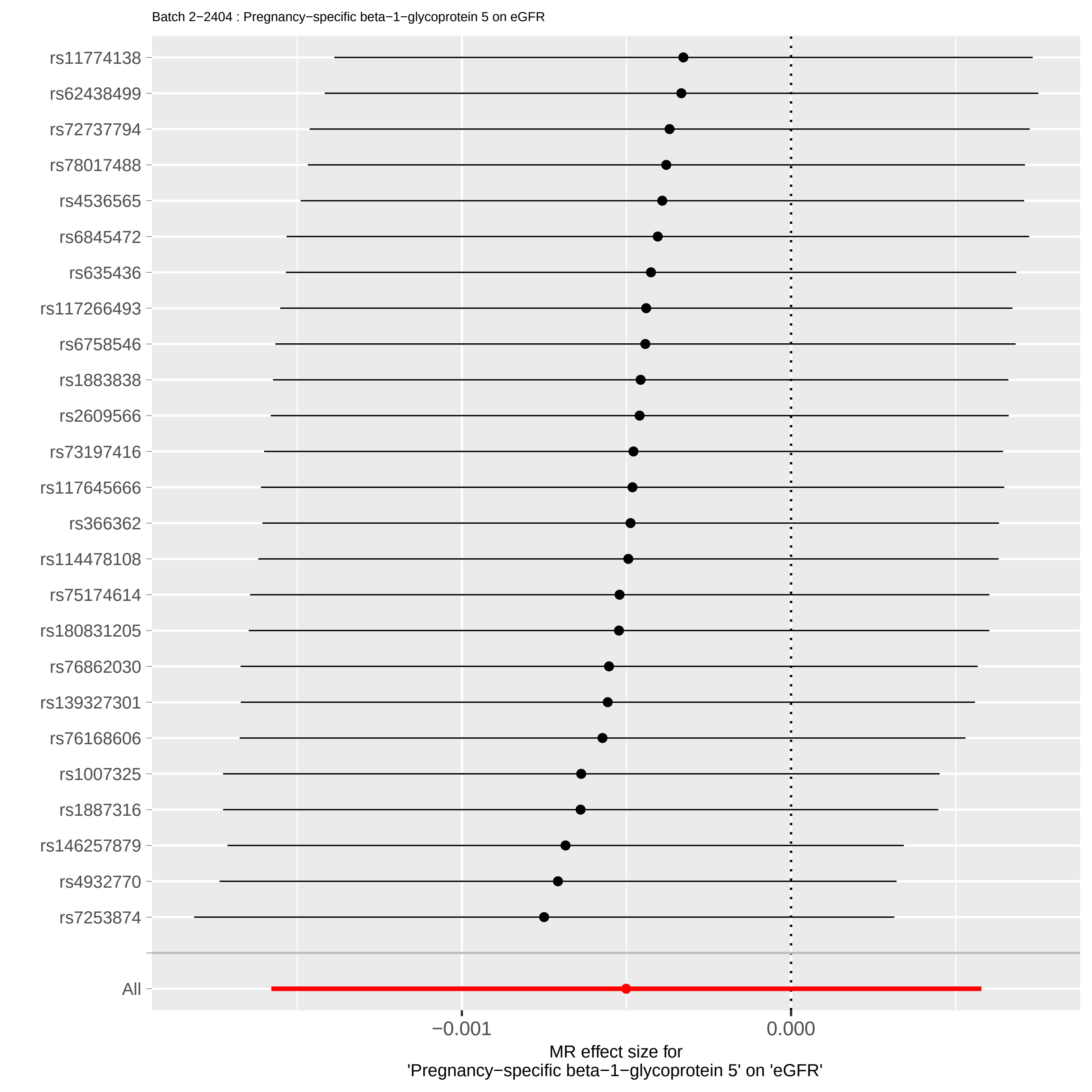

Batch 2−2404 : Pregnancy−specific beta−1−glycoprotein 5 on eGFR
rs11774138
rs62438499
rs72737794
rs78017488
rs4536565
rs6845472
rs635436
rs117266493
rs6758546
rs1883838
rs2609566
rs73197416
rs117645666
rs366362
rs114478108
rs75174614
rs180831205
rs76862030
rs139327301
rs76168606
rs1007325
rs1887316
rs146257879
rs4932770
rs7253874
All
−0.001
0.000
MR effect size for
'Pregnancy−specific beta−1−glycoprotein 5' on 'eGFR'

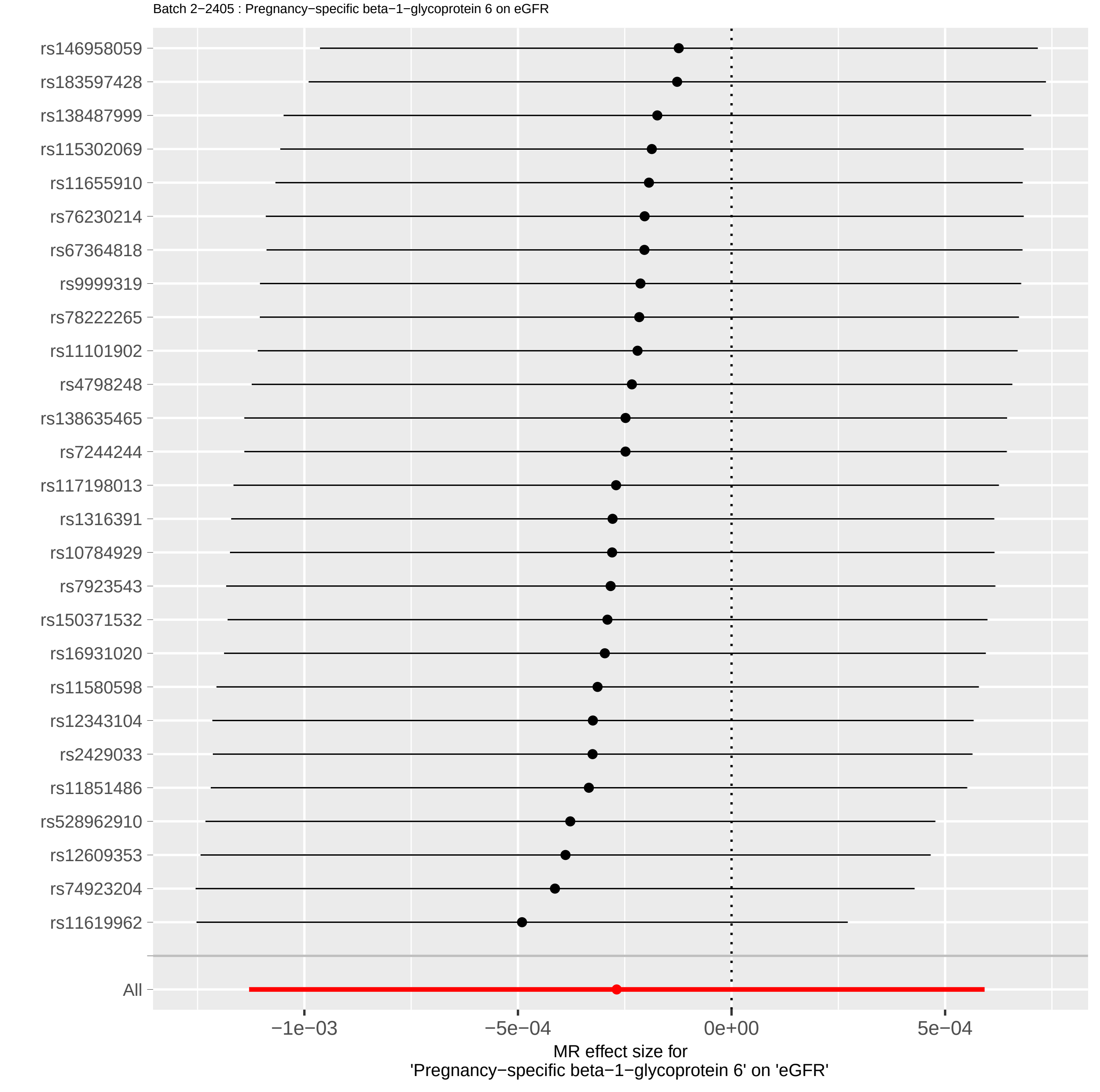

Batch 2−2405 : Pregnancy−specific beta−1−glycoprotein 6 on eGFR
rs146958059
rs183597428
rs138487999
rs115302069
rs11655910
rs76230214
rs67364818
rs9999319
rs78222265
rs11101902
rs4798248
rs138635465
rs7244244
rs117198013
rs1316391
rs10784929
rs7923543
rs150371532
rs16931020
rs11580598
rs12343104
rs2429033
rs11851486
rs528962910
rs12609353
rs74923204
rs11619962
All
−1e−03
−5e−04
0e+00
5e−04
MR effect size for
'Pregnancy−specific beta−1−glycoprotein 6' on 'eGFR'

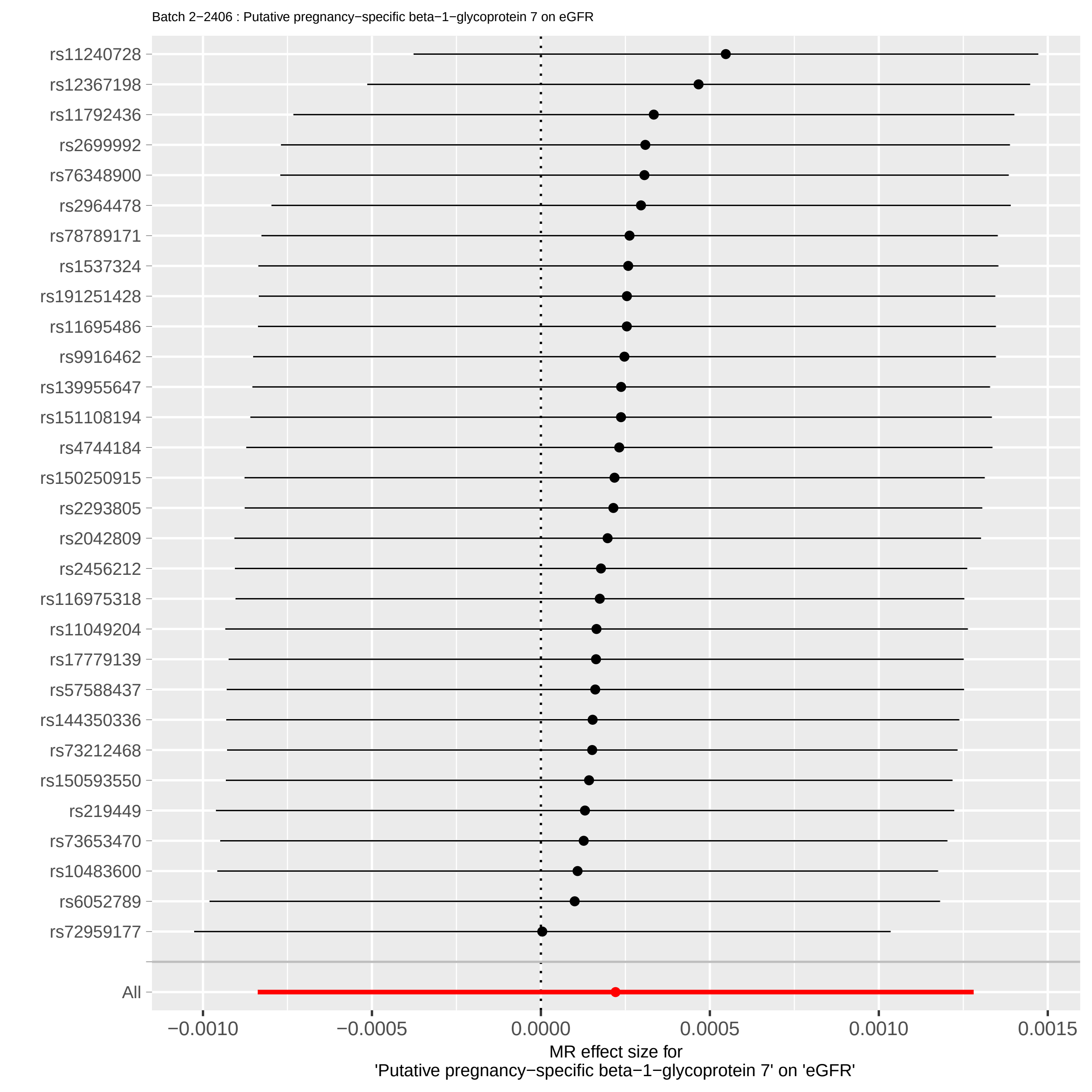

Batch 2−2406 : Putative pregnancy−specific beta−1−glycoprotein 7 on eGFR
rs11240728
rs12367198
rs11792436
rs2699992
rs76348900
rs2964478
rs78789171
rs1537324
rs191251428
rs11695486
rs9916462
rs139955647
rs151108194
rs4744184
rs150250915
rs2293805
rs2042809
rs2456212
rs116975318
rs11049204
rs17779139
rs57588437
rs144350336
rs73212468
rs150593550
rs219449
rs73653470
rs10483600
rs6052789
rs72959177
All
−0.0010
−0.0005
0.0000
0.0005
0.0010
0.0015
MR effect size for
'Putative pregnancy−specific beta−1−glycoprotein 7' on 'eGFR'

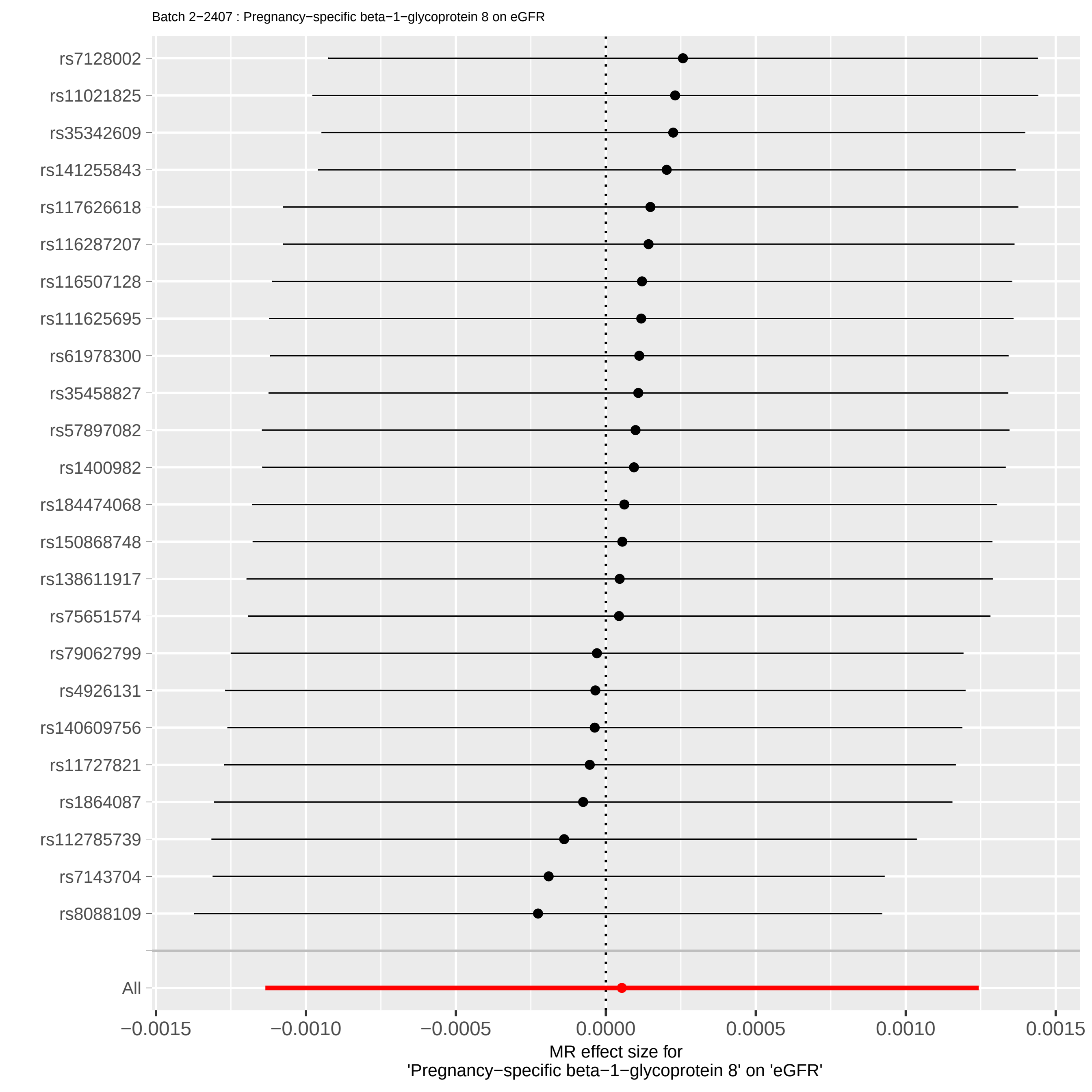

Batch 2−2407 : Pregnancy−specific beta−1−glycoprotein 8 on eGFR
rs7128002
rs11021825
rs35342609
rs141255843
rs117626618
rs116287207
rs116507128
rs111625695
rs61978300
rs35458827
rs57897082
rs1400982
rs184474068
rs150868748
rs138611917
rs75651574
rs79062799
rs4926131
rs140609756
rs11727821
rs1864087
rs112785739
rs7143704
rs8088109
All
−0.0015
−0.0010
−0.0005
0.0000
0.0005
0.0010
0.0015
MR effect size for
'Pregnancy−specific beta−1−glycoprotein 8' on 'eGFR'

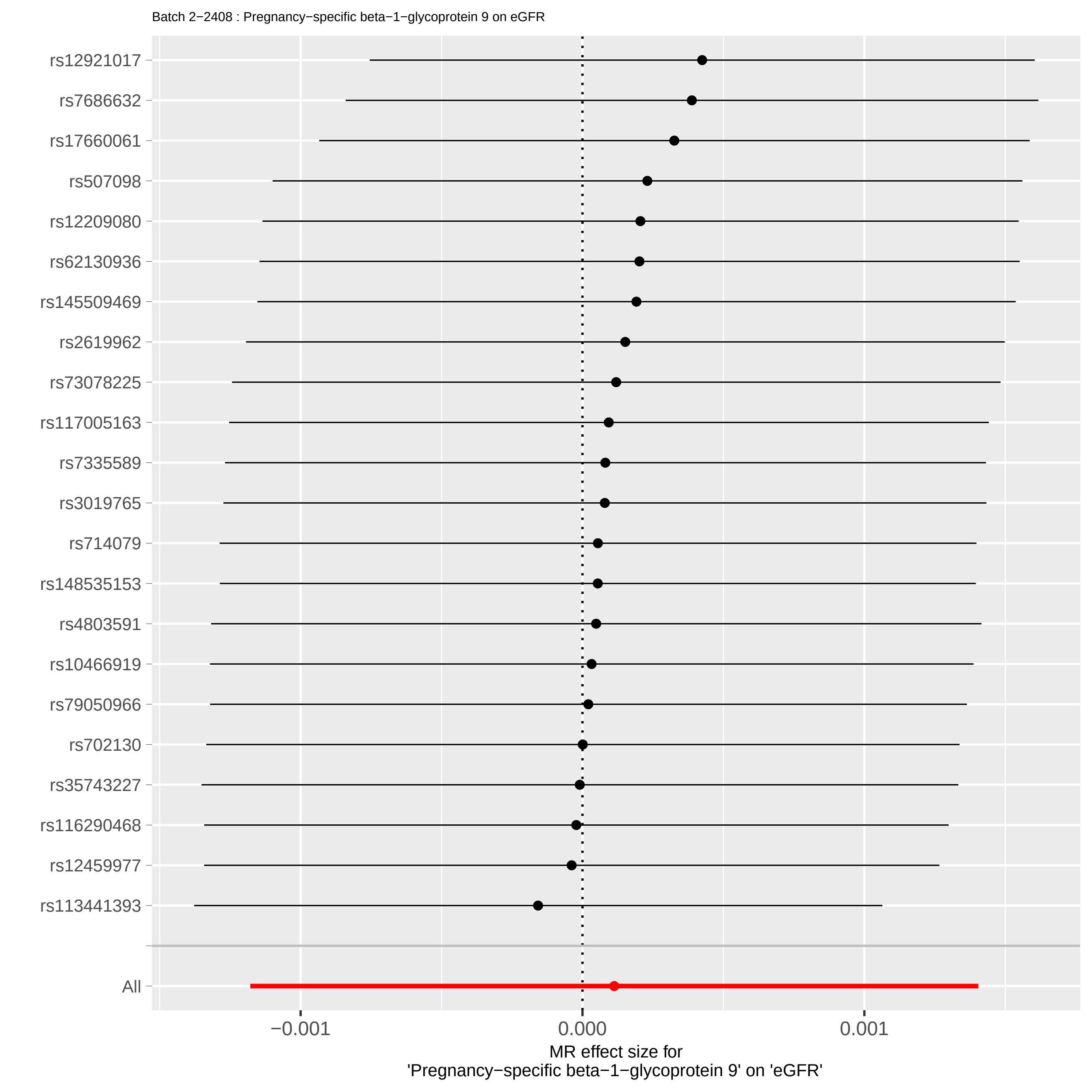

Batch 2−2408 : Pregnancy−specific beta−1−glycoprotein 9 on eGFR
rs12921017
rs7686632
rs17660061
rs507098
rs12209080
rs62130936
rs145509469
rs2619962
rs73078225
rs117005163
rs7335589
rs3019765
rs714079
rs148535153
rs4803591
rs10466919
rs79050966
rs702130
rs35743227
rs116290468
rs12459977
rs113441393
All
−0.001
0.000
0.001
MR effect size for
'Pregnancy−specific beta−1−glycoprotein 9' on 'eGFR'

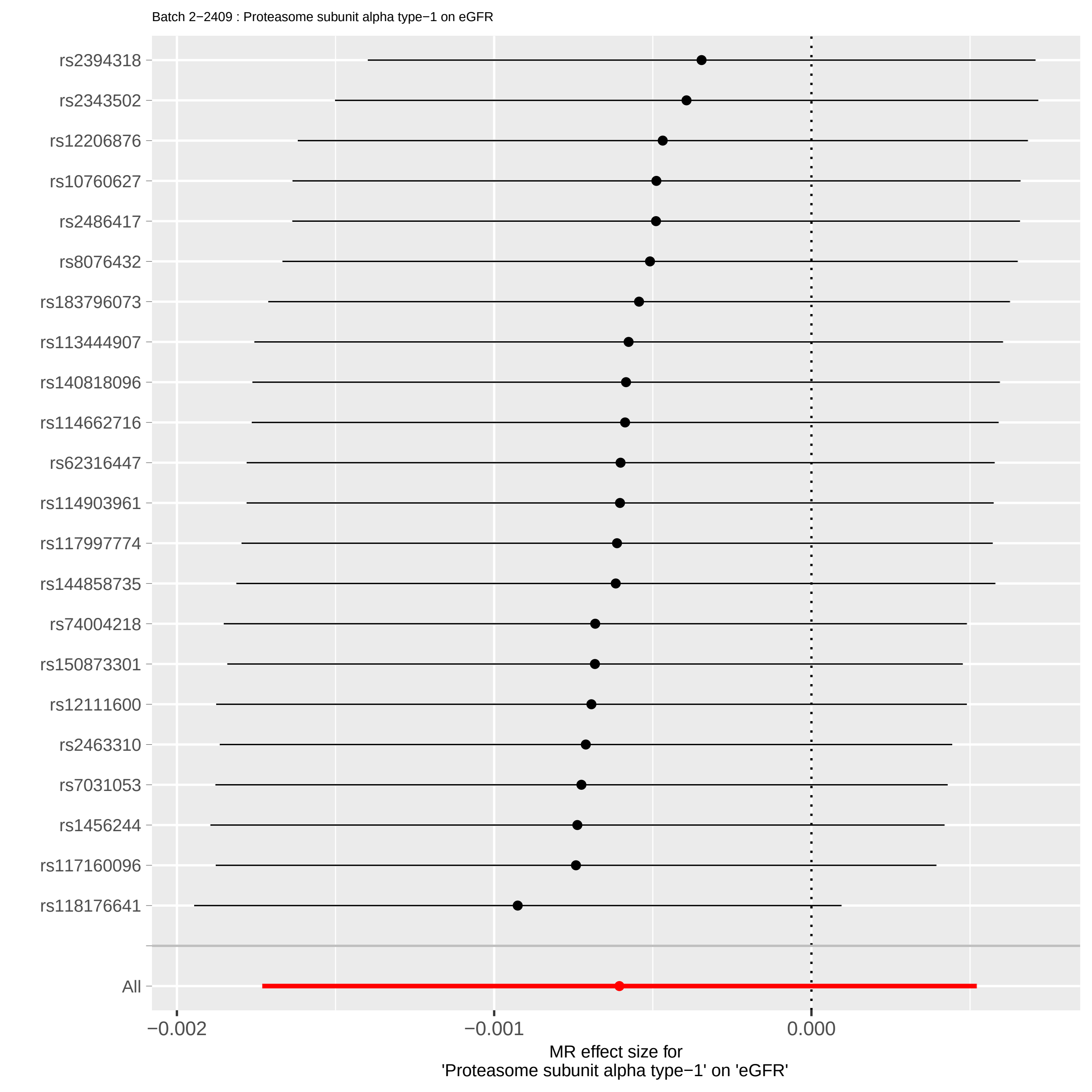

Batch 2−2409 : Proteasome subunit alpha type−1 on eGFR
rs2394318
rs2343502
rs12206876
rs10760627
rs2486417
rs8076432
rs183796073
rs113444907
rs140818096
rs114662716
rs62316447
rs114903961
rs117997774
rs144858735
rs74004218
rs150873301
rs12111600
rs2463310
rs7031053
rs1456244
rs117160096
rs118176641
All
−0.002
−0.001
0.000
MR effect size for
'Proteasome subunit alpha type−1' on 'eGFR'

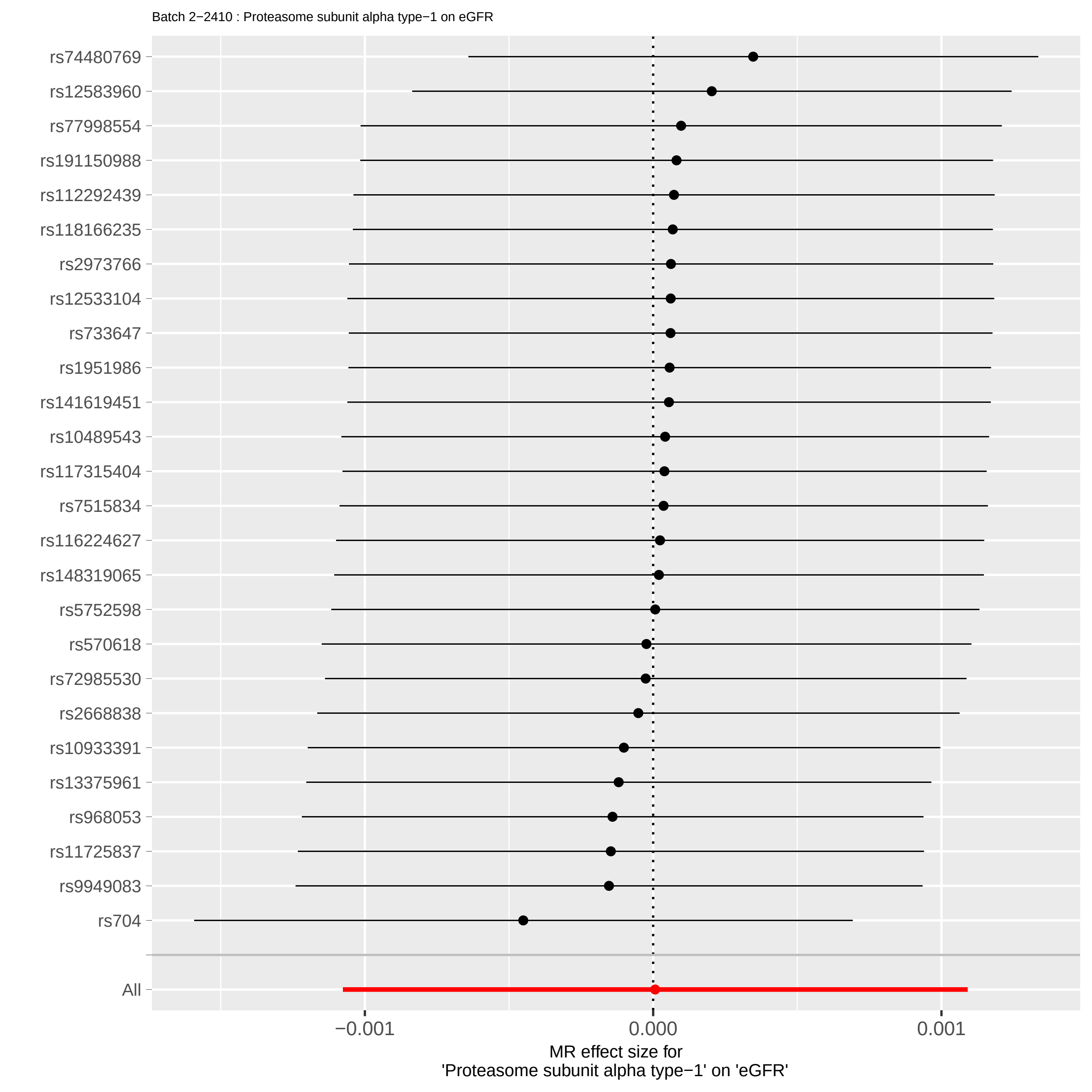

Batch 2−2410 : Proteasome subunit alpha type−1 on eGFR
rs74480769
rs12583960
rs77998554
rs191150988
rs112292439
rs118166235
rs2973766
rs12533104
rs733647
rs1951986
rs141619451
rs10489543
rs117315404
rs7515834
rs116224627
rs148319065
rs5752598
rs570618
rs72985530
rs2668838
rs10933391
rs13375961
rs968053
rs11725837
rs9949083
rs704
All
−0.001
0.000
0.001
MR effect size for
'Proteasome subunit alpha type−1' on 'eGFR'

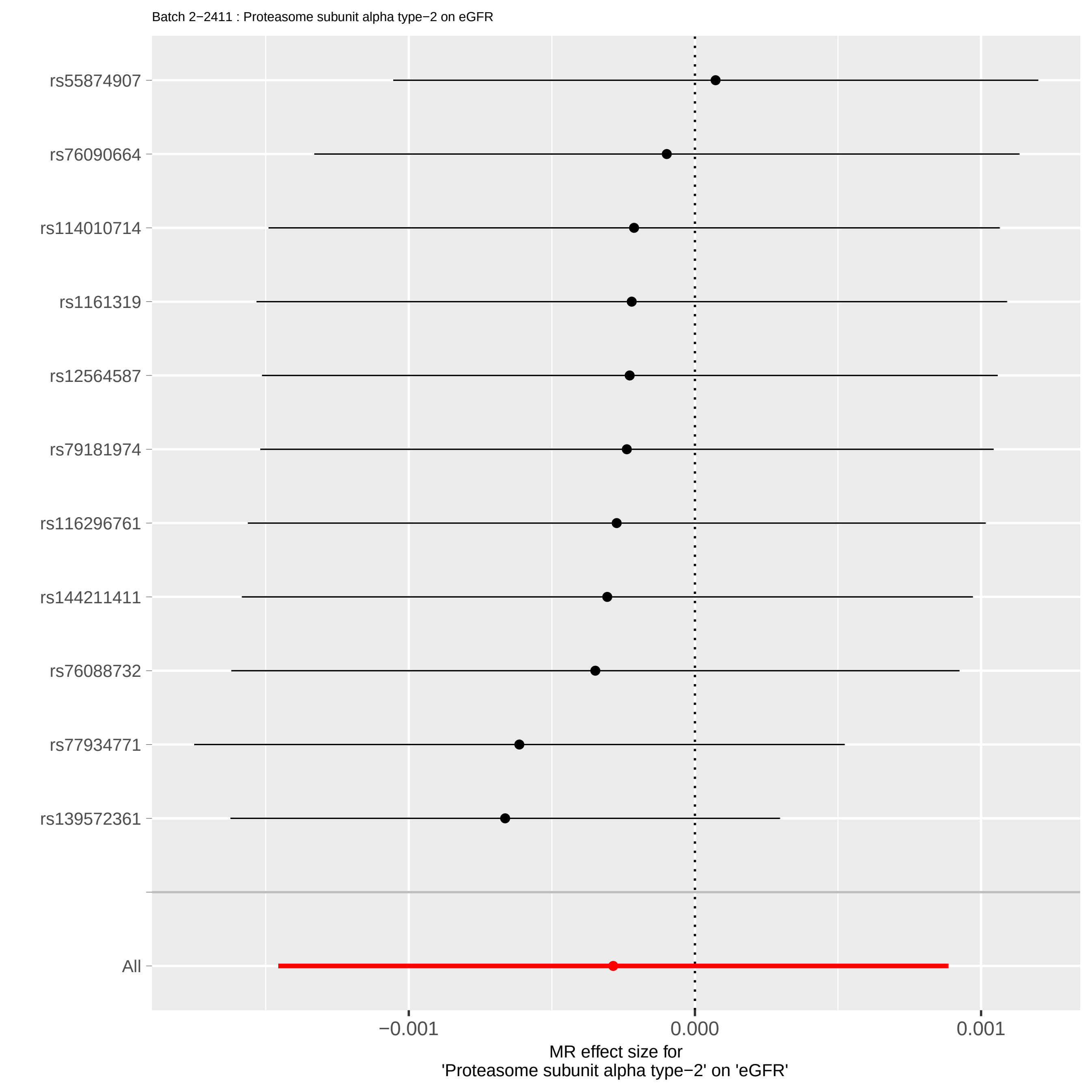

Batch 2−2411 : Proteasome subunit alpha type−2 on eGFR
rs55874907
rs76090664
rs114010714
rs1161319
rs12564587
rs79181974
rs116296761
rs144211411
rs76088732
rs77934771
rs139572361
All
−0.001
0.000
0.001
MR effect size for
'Proteasome subunit alpha type−2' on 'eGFR'

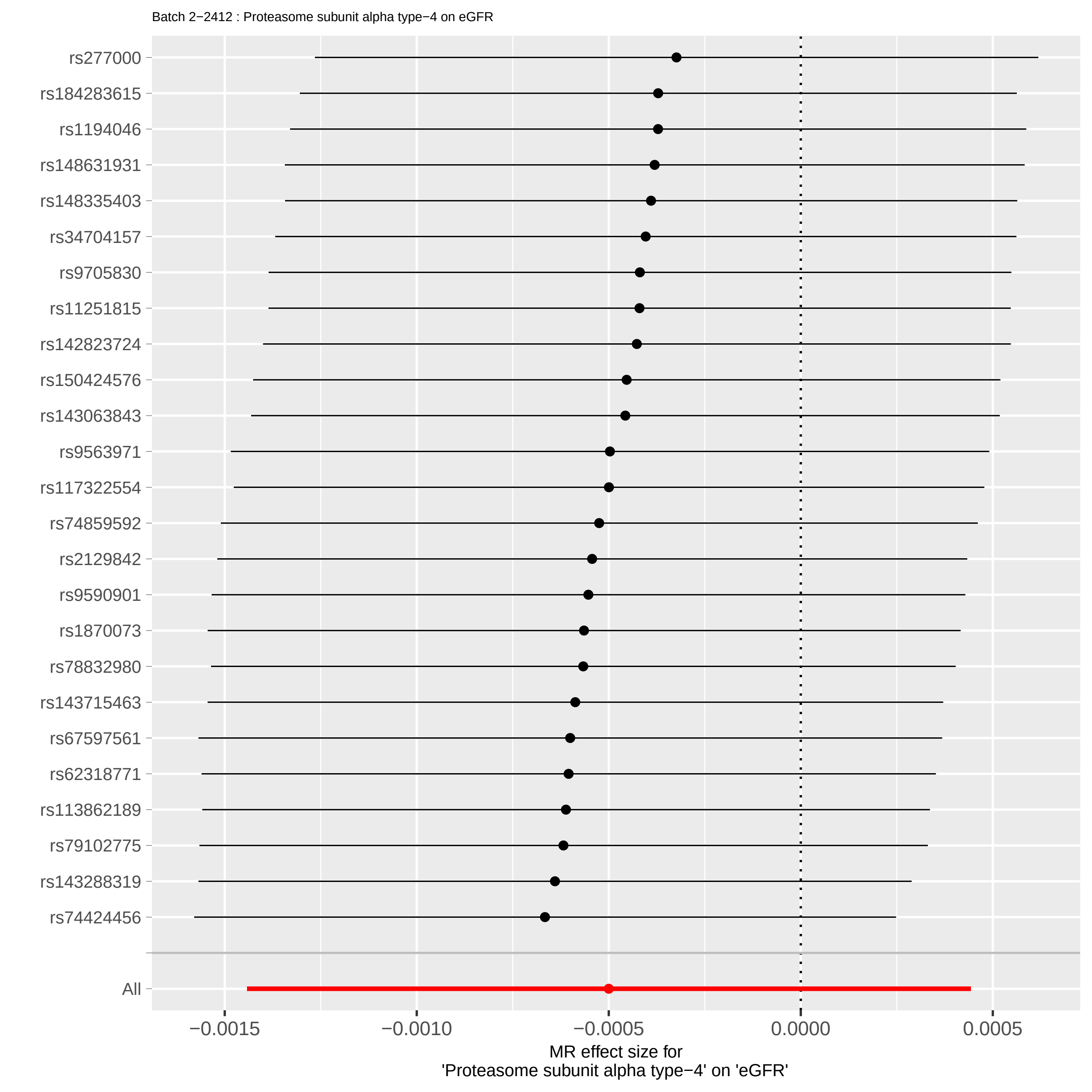

Batch 2−2412 : Proteasome subunit alpha type−4 on eGFR
rs277000
rs184283615
rs1194046
rs148631931
rs148335403
rs34704157
rs9705830
rs11251815
rs142823724
rs150424576
rs143063843
rs9563971
rs117322554
rs74859592
rs2129842
rs9590901
rs1870073
rs78832980
rs143715463
rs67597561
rs62318771
rs113862189
rs79102775
rs143288319
rs74424456
All
−0.0015
−0.0010
−0.0005
0.0000
0.0005
MR effect size for
'Proteasome subunit alpha type−4' on 'eGFR'

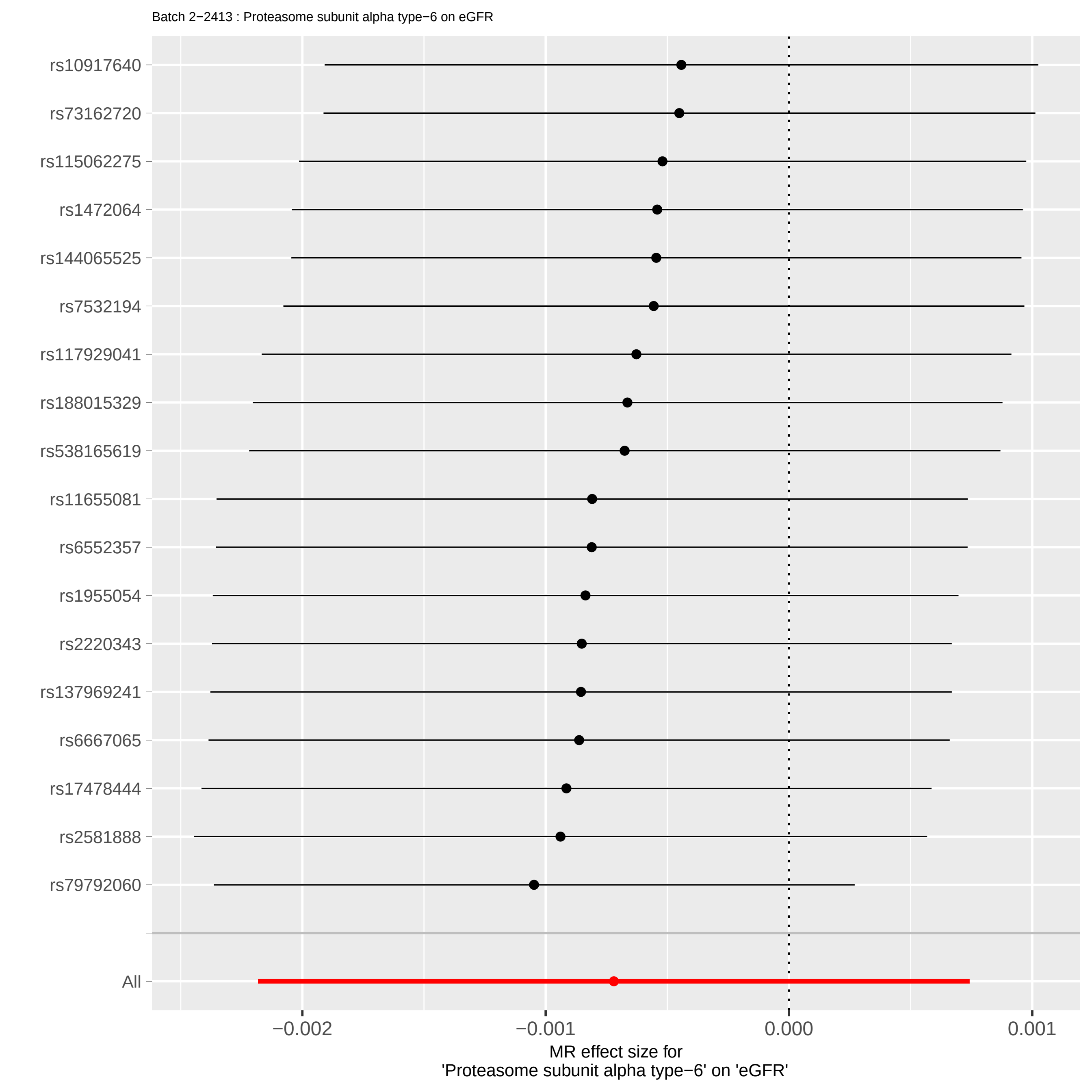

Batch 2−2413 : Proteasome subunit alpha type−6 on eGFR
rs10917640
rs73162720
rs115062275
rs1472064
rs144065525
rs7532194
rs117929041
rs188015329
rs538165619
rs11655081
rs6552357
rs1955054
rs2220343
rs137969241
rs6667065
rs17478444
rs2581888
rs79792060
All
−0.002
−0.001
0.000
0.001
MR effect size for
'Proteasome subunit alpha type−6' on 'eGFR'

Batch 2−2414 : Proteasome subunit beta type−5 on eGFR

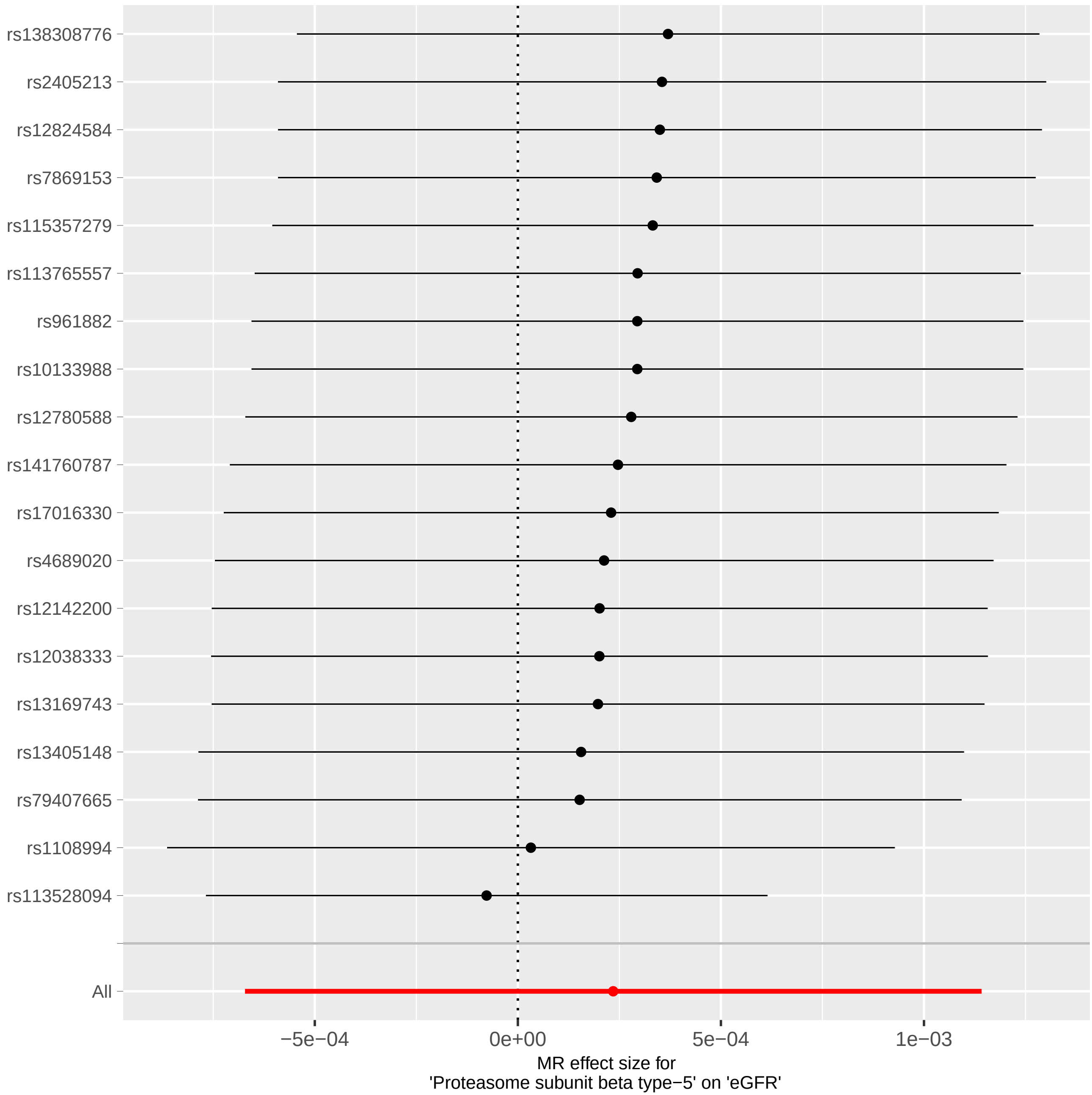

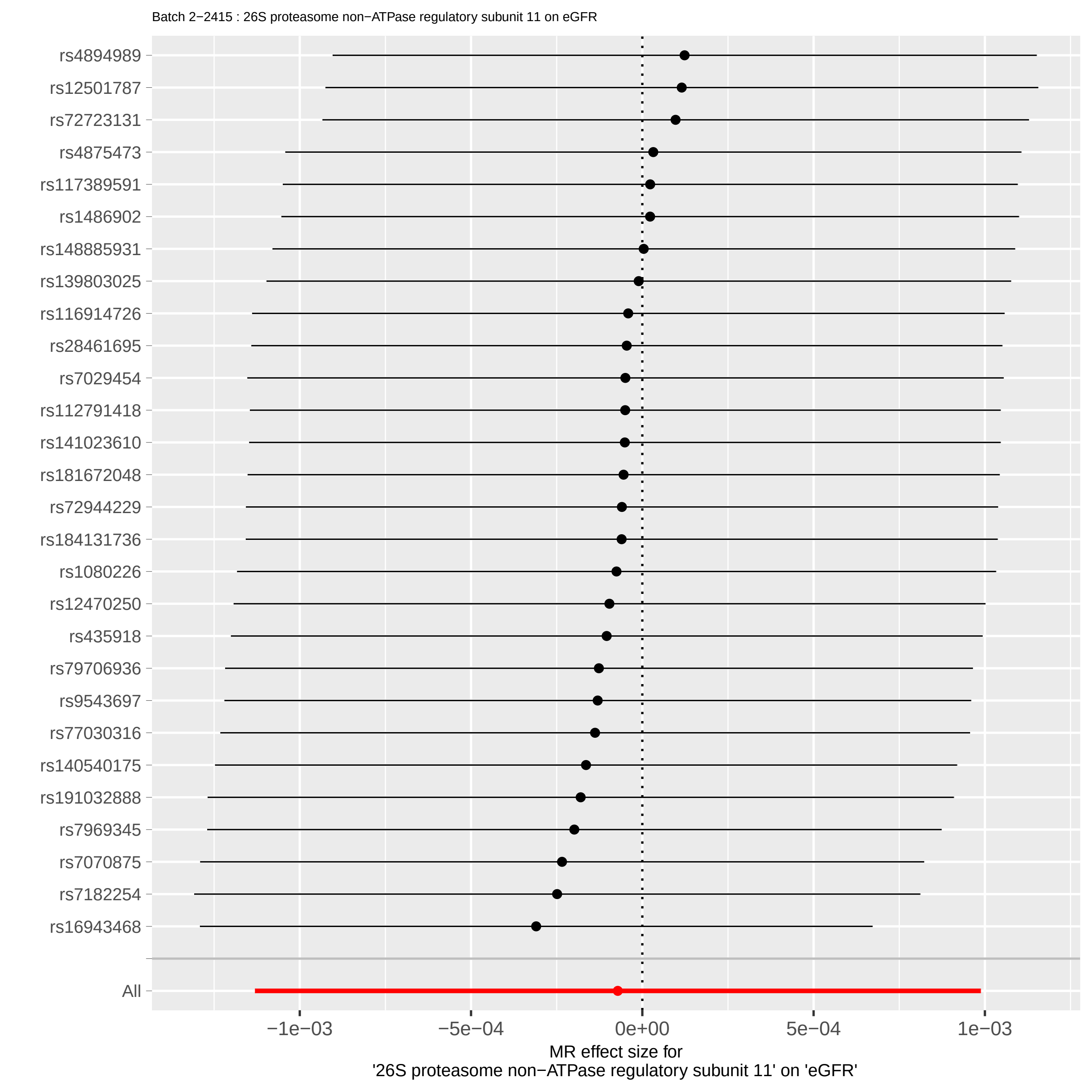

Batch 2−2415 : 26S proteasome non−ATPase regulatory subunit 11 on eGFR
rs4894989
rs12501787
rs72723131
rs4875473
rs117389591
rs1486902
rs148885931
rs139803025
rs116914726
rs28461695
rs7029454
rs112791418
rs141023610
rs181672048
rs72944229
rs184131736
rs1080226
rs12470250
rs435918
rs79706936
rs9543697
rs77030316
rs140540175
rs191032888
rs7969345
rs7070875
rs7182254
rs16943468
All
−1e−03
−5e−04
0e+00
5e−04
1e−03
MR effect size for
'26S proteasome non−ATPase regulatory subunit 11' on 'eGFR'

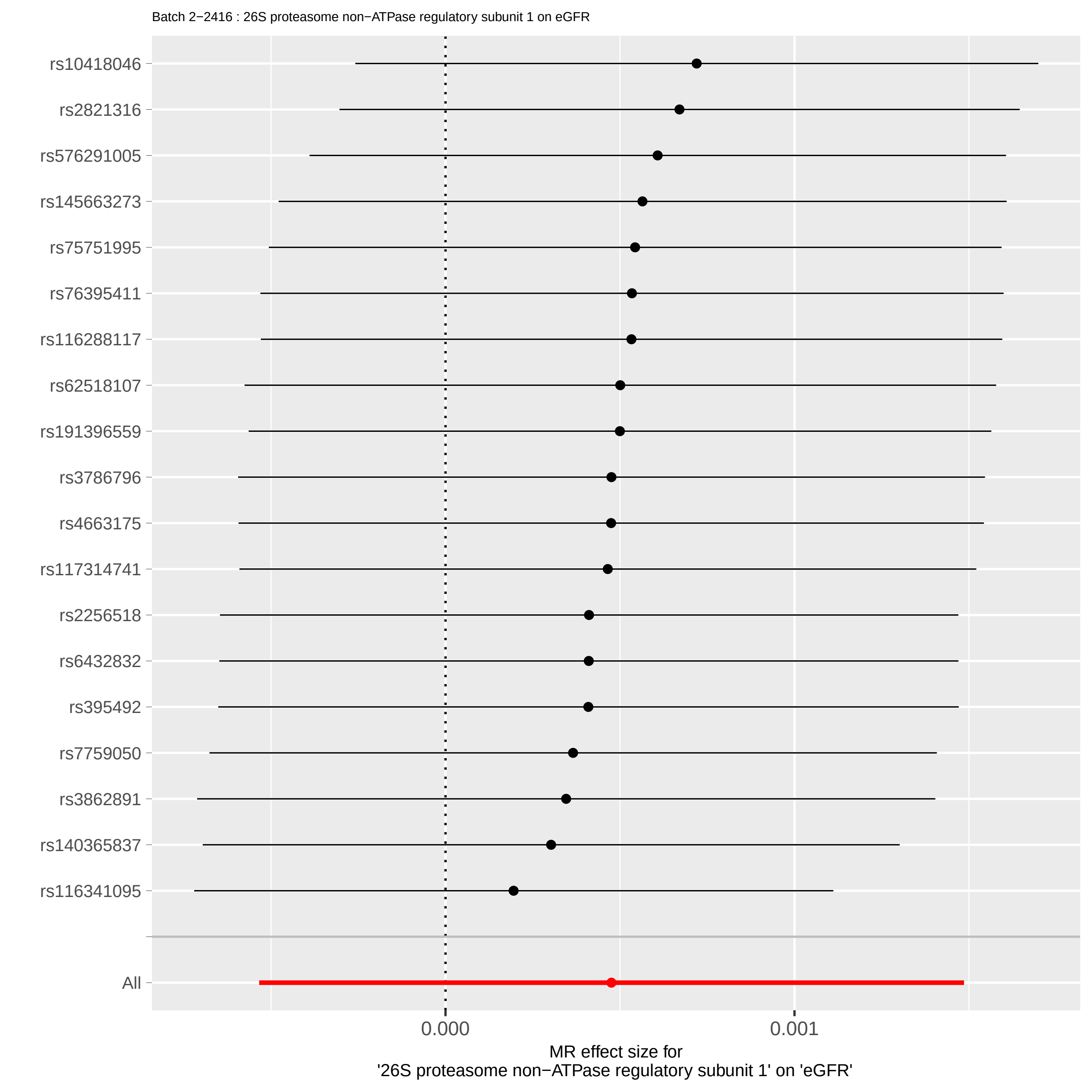

Batch 2−2416 : 26S proteasome non−ATPase regulatory subunit 1 on eGFR
rs10418046
rs2821316
rs576291005
rs145663273
rs75751995
rs76395411
rs116288117
rs62518107
rs191396559
rs3786796
rs4663175
rs117314741
rs2256518
rs6432832
rs395492
rs7759050
rs3862891
rs140365837
rs116341095
All
0.000
0.001
MR effect size for
'26S proteasome non−ATPase regulatory subunit 1' on 'eGFR'

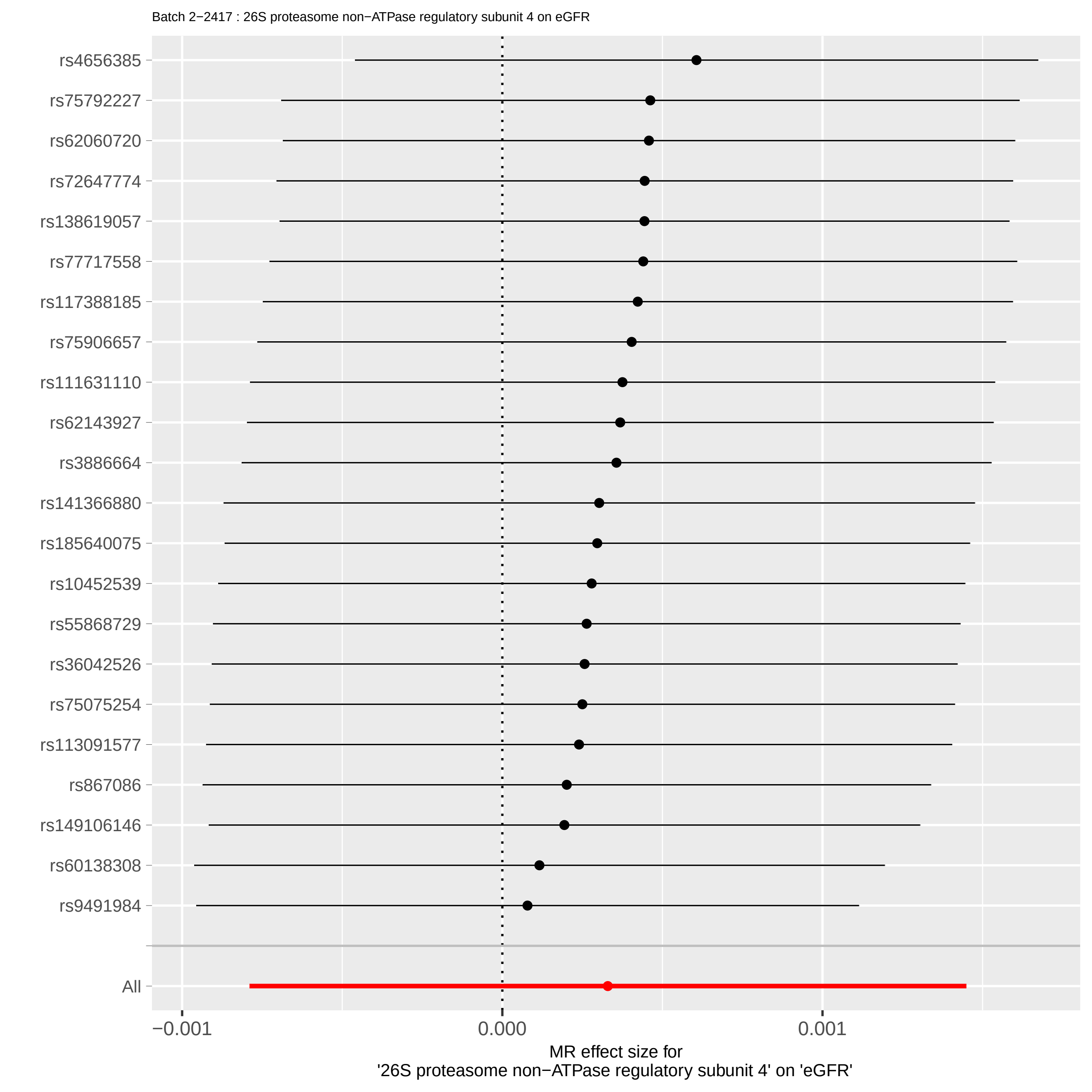

Batch 2−2417 : 26S proteasome non−ATPase regulatory subunit 4 on eGFR
rs4656385
rs75792227
rs62060720
rs72647774
rs138619057
rs77717558
rs117388185
rs75906657
rs111631110
rs62143927
rs3886664
rs141366880
rs185640075
rs10452539
rs55868729
rs36042526
rs75075254
rs113091577
rs867086
rs149106146
rs60138308
rs9491984
All
−0.001
0.000
0.001
MR effect size for
'26S proteasome non−ATPase regulatory subunit 4' on 'eGFR'

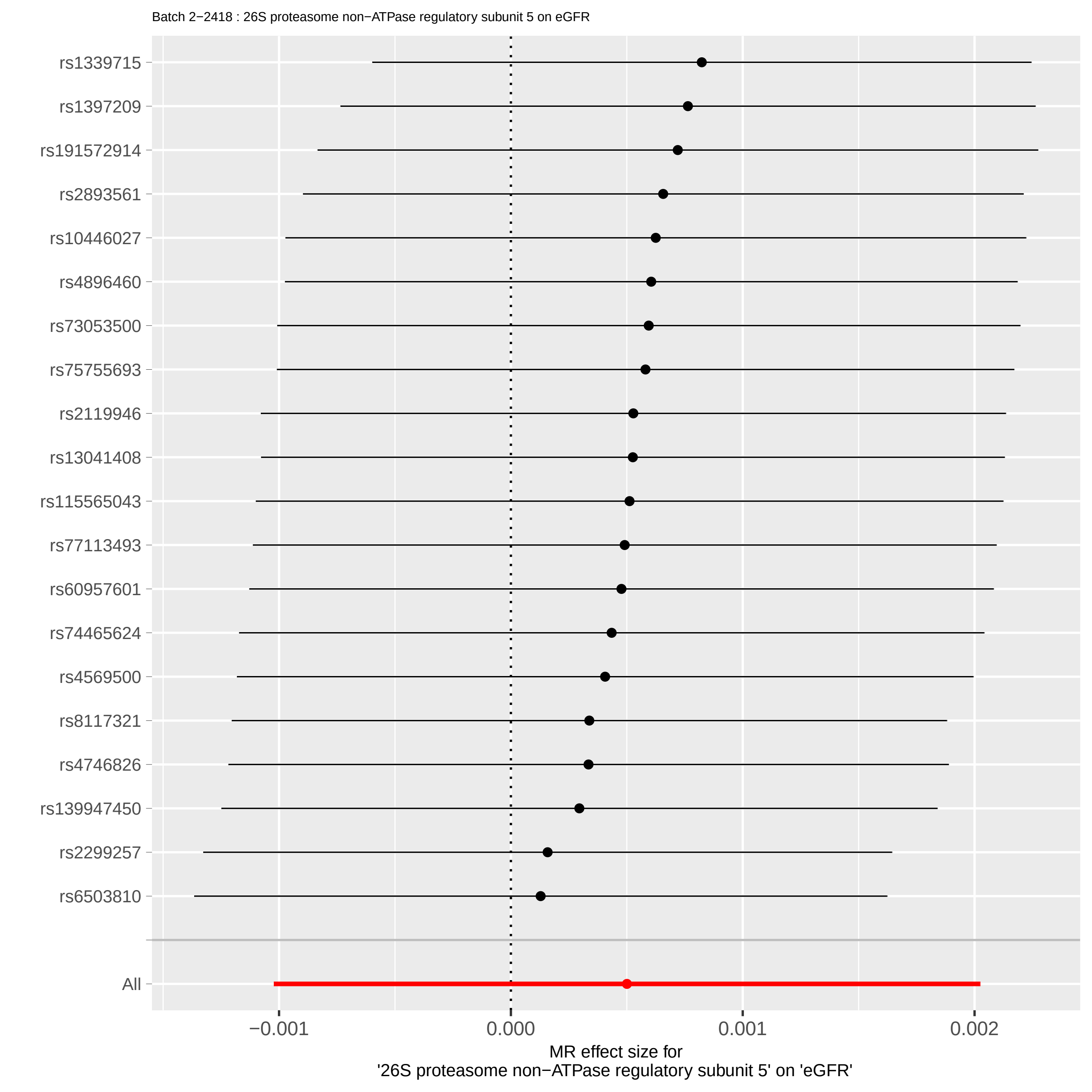

Batch 2−2418 : 26S proteasome non−ATPase regulatory subunit 5 on eGFR
rs1339715
rs1397209
rs191572914
rs2893561
rs10446027
rs4896460
rs73053500
rs75755693
rs2119946
rs13041408
rs115565043
rs77113493
rs60957601
rs74465624
rs4569500
rs8117321
rs4746826
rs139947450
rs2299257
rs6503810
All
−0.001
0.000
0.001
0.002
MR effect size for
'26S proteasome non−ATPase regulatory subunit 5' on 'eGFR'

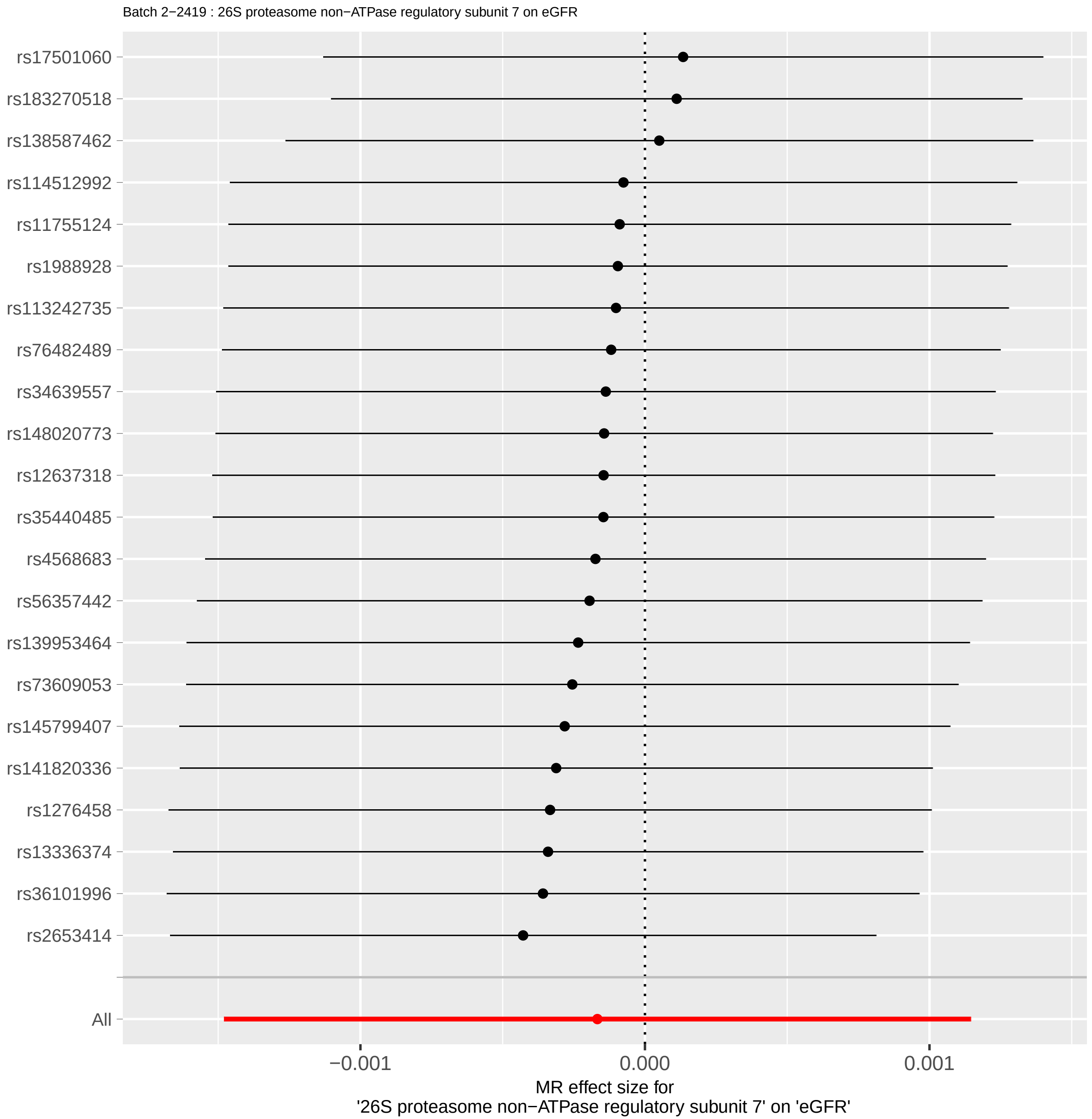

Batch 2−2419 : 26S proteasome non−ATPase regulatory subunit 7 on eGFR
rs17501060
rs183270518
rs138587462
rs114512992
rs11755124
rs1988928
rs113242735
rs76482489
rs34639557
rs148020773
rs12637318
rs35440485
rs4568683
rs56357442
rs139953464
rs73609053
rs145799407
rs141820336
rs1276458
rs13336374
rs36101996
rs2653414
All
−0.001
0.000
0.001
MR effect size for
'26S proteasome non−ATPase regulatory subunit 7' on 'eGFR'

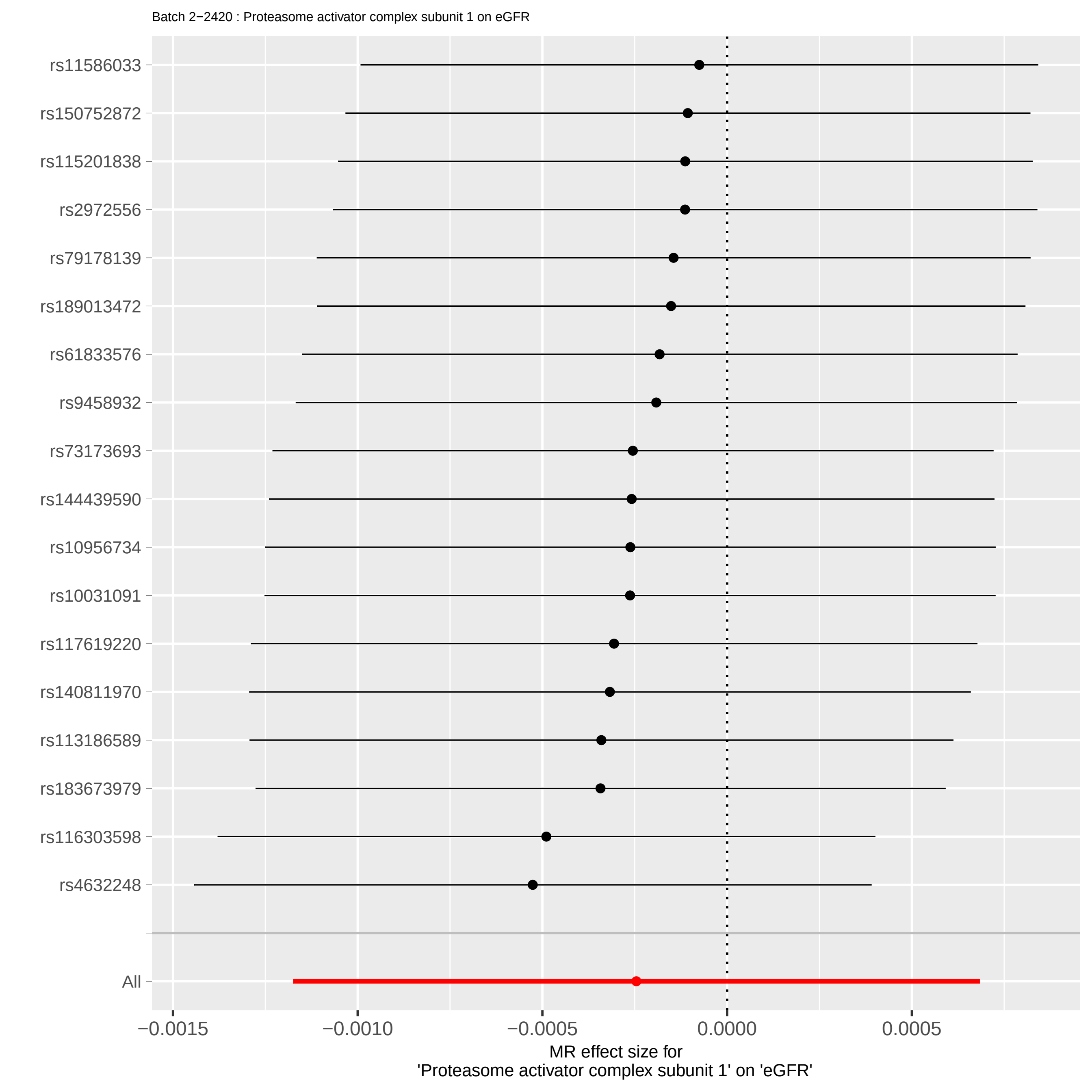

Batch 2−2420 : Proteasome activator complex subunit 1 on eGFR
rs11586033
rs150752872
rs115201838
rs2972556
rs79178139
rs189013472
rs61833576
rs9458932
rs73173693
rs144439590
rs10956734
rs10031091
rs117619220
rs140811970
rs113186589
rs183673979
rs116303598
rs4632248
All
−0.0015
−0.0010
−0.0005
0.0000
0.0005
MR effect size for
'Proteasome activator complex subunit 1' on 'eGFR'

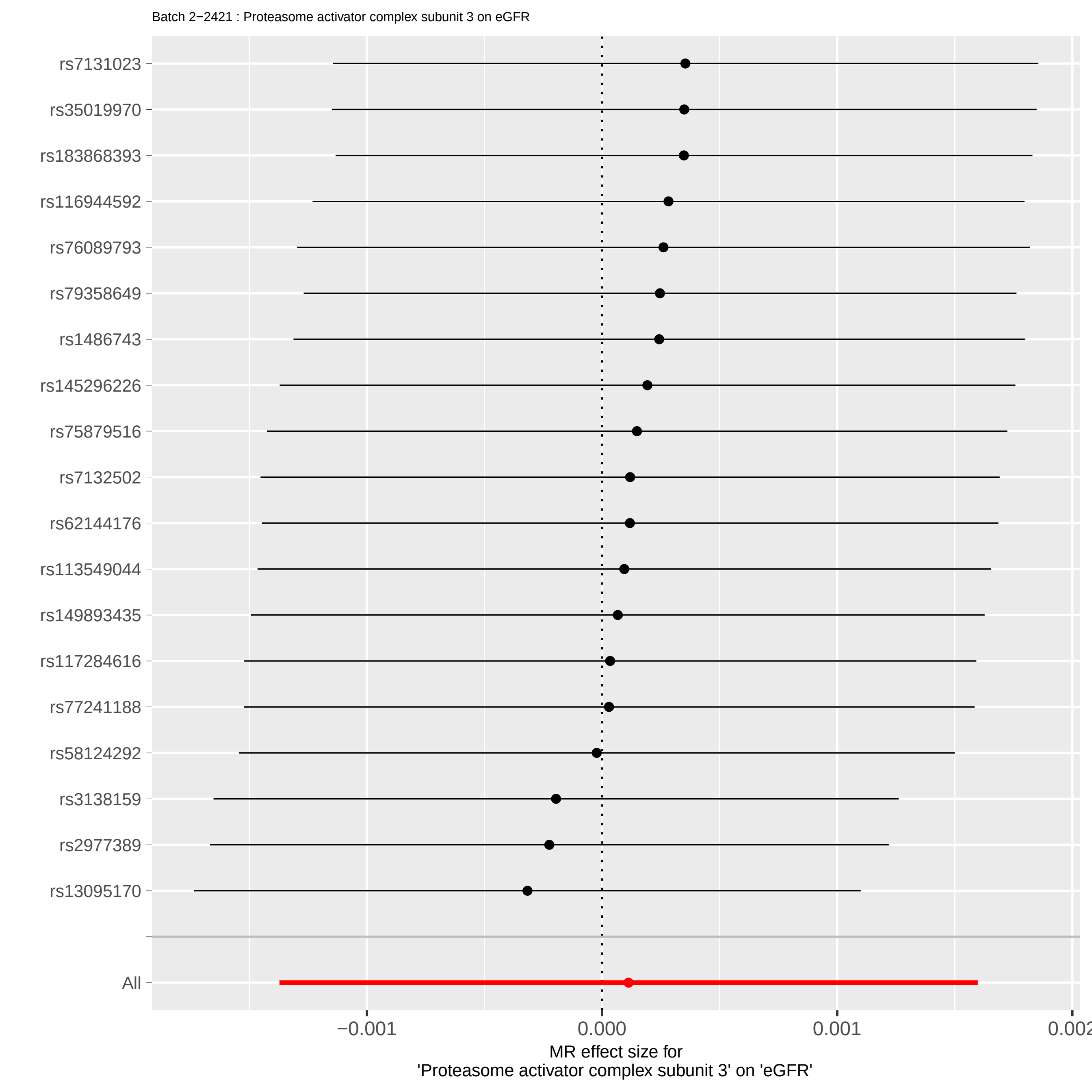
Batch 2−2421 : Proteasome activator complex subunit 3 on eGFR
rs7131023
rs35019970
rs183868393
rs116944592
rs76089793
rs79358649
rs1486743
rs145296226
rs75879516
rs7132502
rs62144176
rs113549044
rs149893435
rs117284616
rs77241188
rs58124292
rs3138159
rs2977389
rs13095170
All
−0.001
0.000
0.001
0.002
MR effect size for
'Proteasome activator complex subunit 3' on 'eGFR'

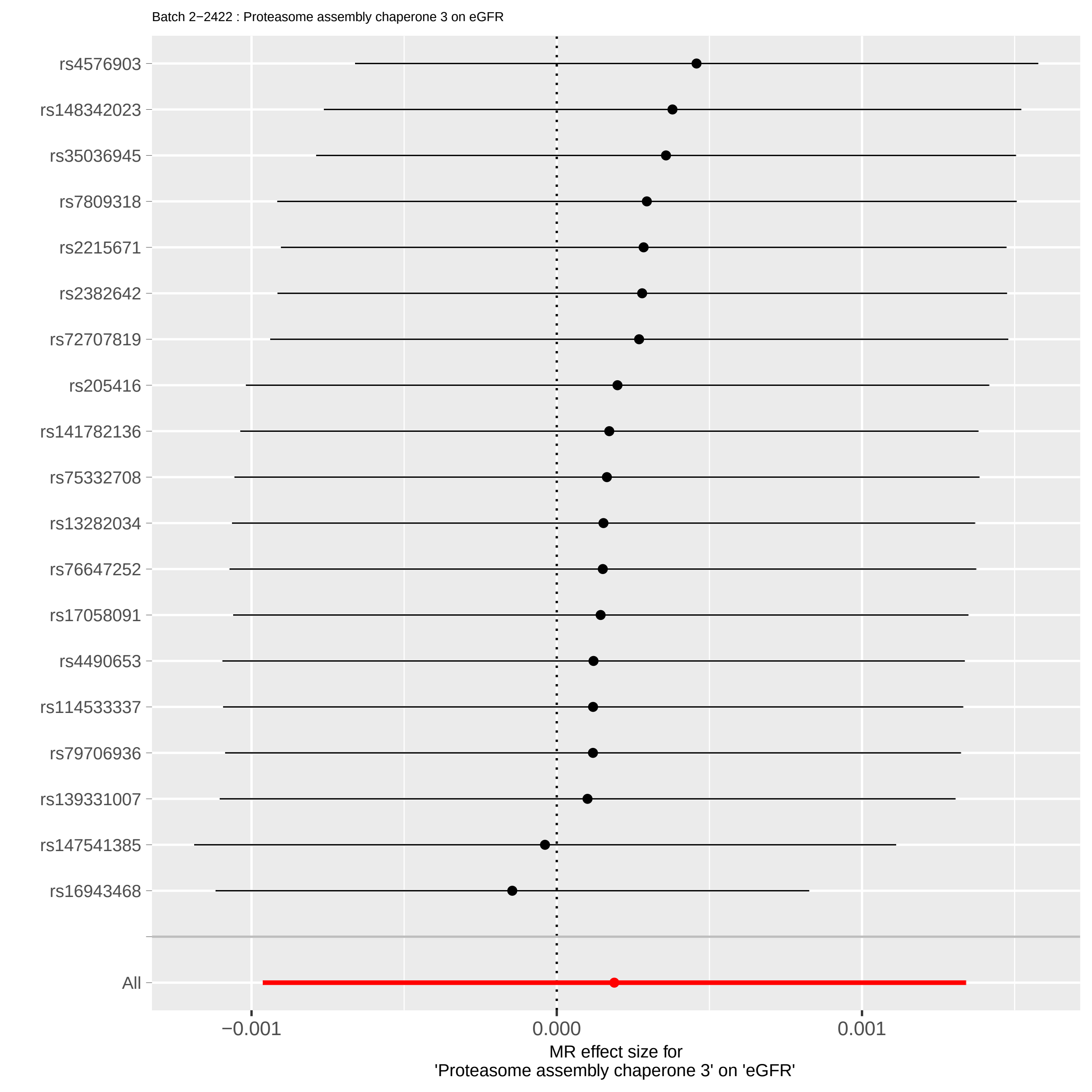

Batch 2−2422 : Proteasome assembly chaperone 3 on eGFR
rs4576903
rs148342023
rs35036945
rs7809318
rs2215671
rs2382642
rs72707819
rs205416
rs141782136
rs75332708
rs13282034
rs76647252
rs17058091
rs4490653
rs114533337
rs79706936
rs139331007
rs147541385
rs16943468
All
−0.001
0.000
0.001
MR effect size for
'Proteasome assembly chaperone 3' on 'eGFR'

Batch 2−2423 : Persephin on eGFR

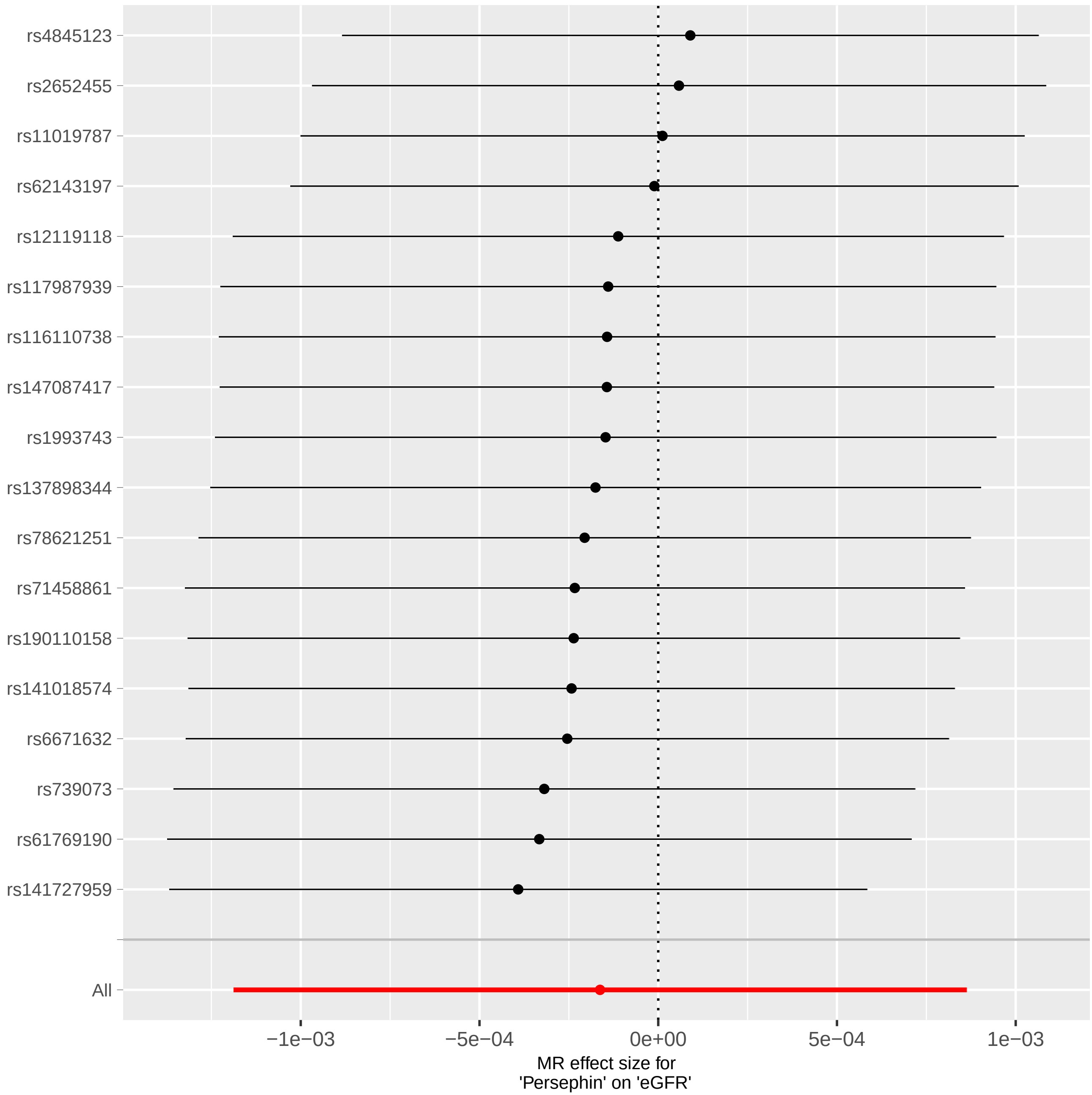

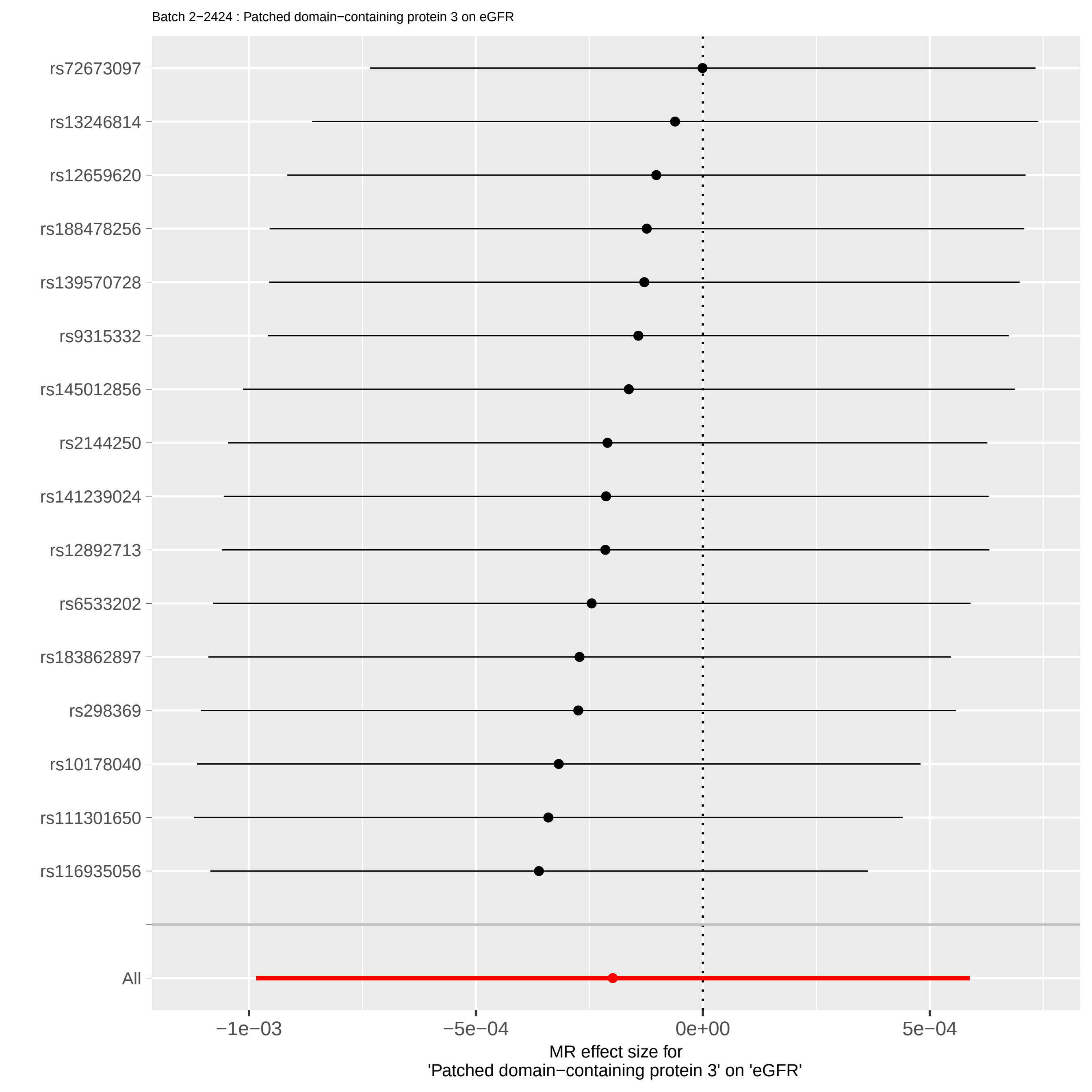
Batch 2−2424 : Patched domain−containing protein 3 on eGFR
rs72673097
rs13246814
rs12659620
rs188478256
rs139570728
rs9315332
rs145012856
rs2144250
rs141239024
rs12892713
rs6533202
rs183862897
rs298369
rs10178040
rs111301650
rs116935056
All
−1e−03
−5e−04
0e+00
5e−04
MR effect size for
'Patched domain−containing protein 3' on 'eGFR'

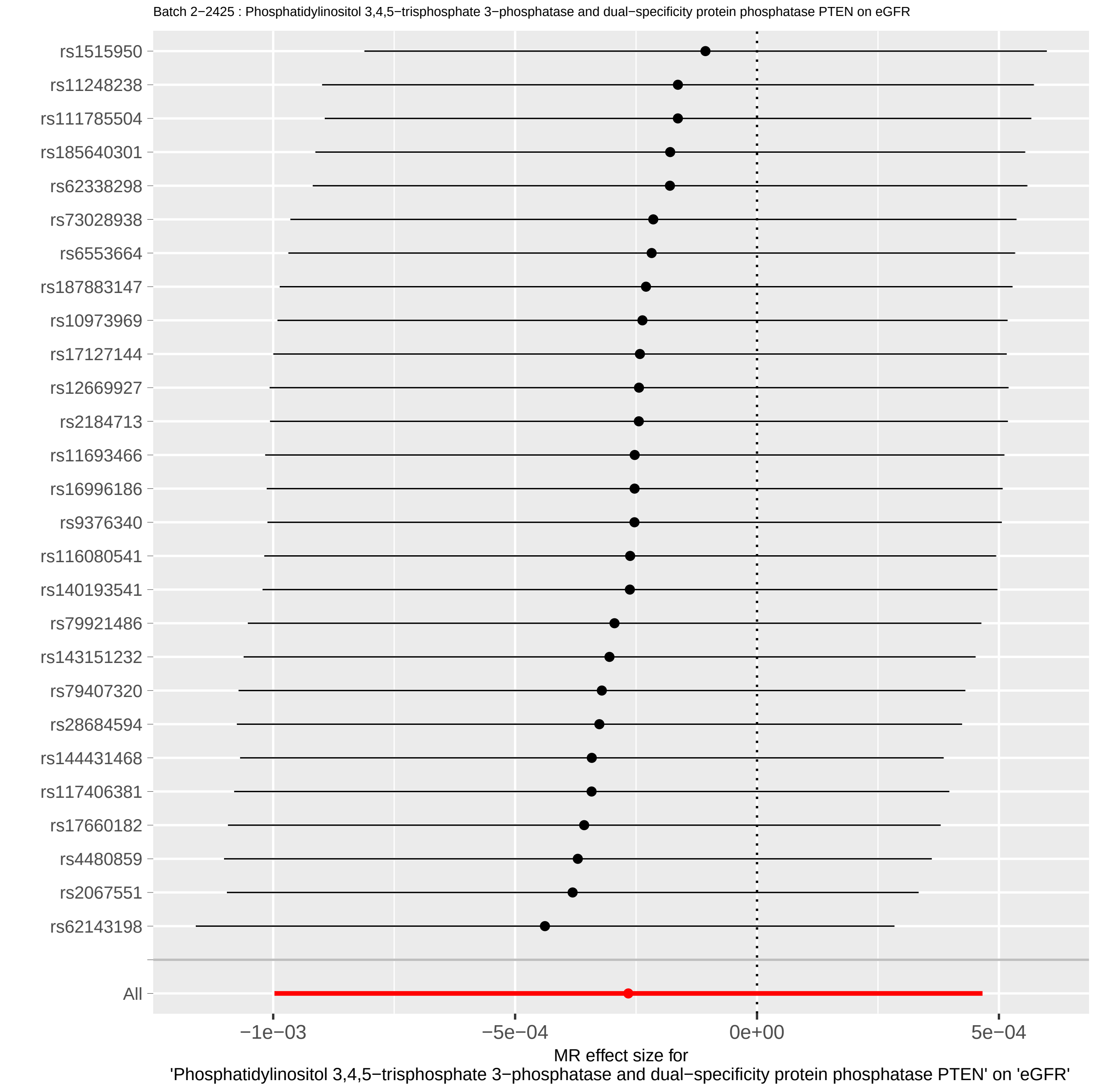

Batch 2−2425 : Phosphatidylinositol 3,4,5−trisphosphate 3−phosphatase and dual−specificity protein phosphatase PTEN on eGFR
rs1515950
rs11248238
rs111785504
rs185640301
rs62338298
rs73028938
rs6553664
rs187883147
rs10973969
rs17127144
rs12669927
rs2184713
rs11693466
rs16996186
rs9376340
rs116080541
rs140193541
rs79921486
rs143151232
rs79407320
rs28684594
rs144431468
rs117406381
rs17660182
rs4480859
rs2067551
rs62143198
All
−1e−03
−5e−04
0e+00
5e−04
MR effect size for
'Phosphatidylinositol 3,4,5−trisphosphate 3−phosphatase and dual−specificity protein phosphatase PTEN' on 'eGFR'

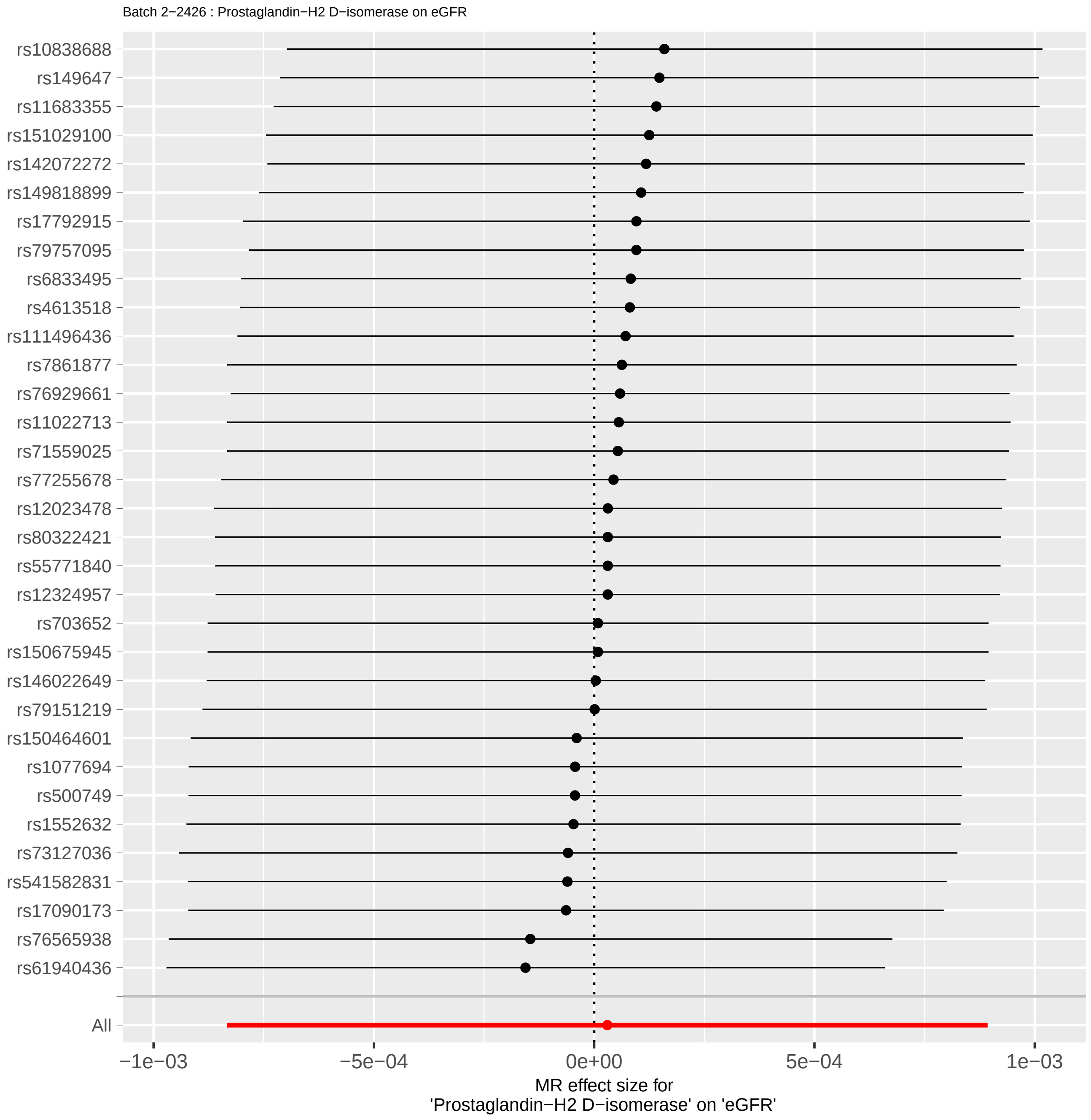

Batch 2−2426 : Prostaglandin−H2 D−isomerase on eGFR
rs10838688
rs149647
rs11683355
rs151029100
rs142072272
rs149818899
rs17792915
rs79757095
rs6833495
rs4613518
rs111496436
rs7861877
rs76929661
rs11022713
rs71559025
rs77255678
rs12023478
rs80322421
rs55771840
rs12324957
rs703652
rs150675945
rs146022649
rs79151219
rs150464601
rs1077694
rs500749
rs1552632
rs73127036
rs541582831
rs17090173
rs76565938
rs61940436
All
−1e−03
−5e−04
0e+00
5e−04
1e−03
MR effect size for
'Prostaglandin−H2 D−isomerase' on 'eGFR'

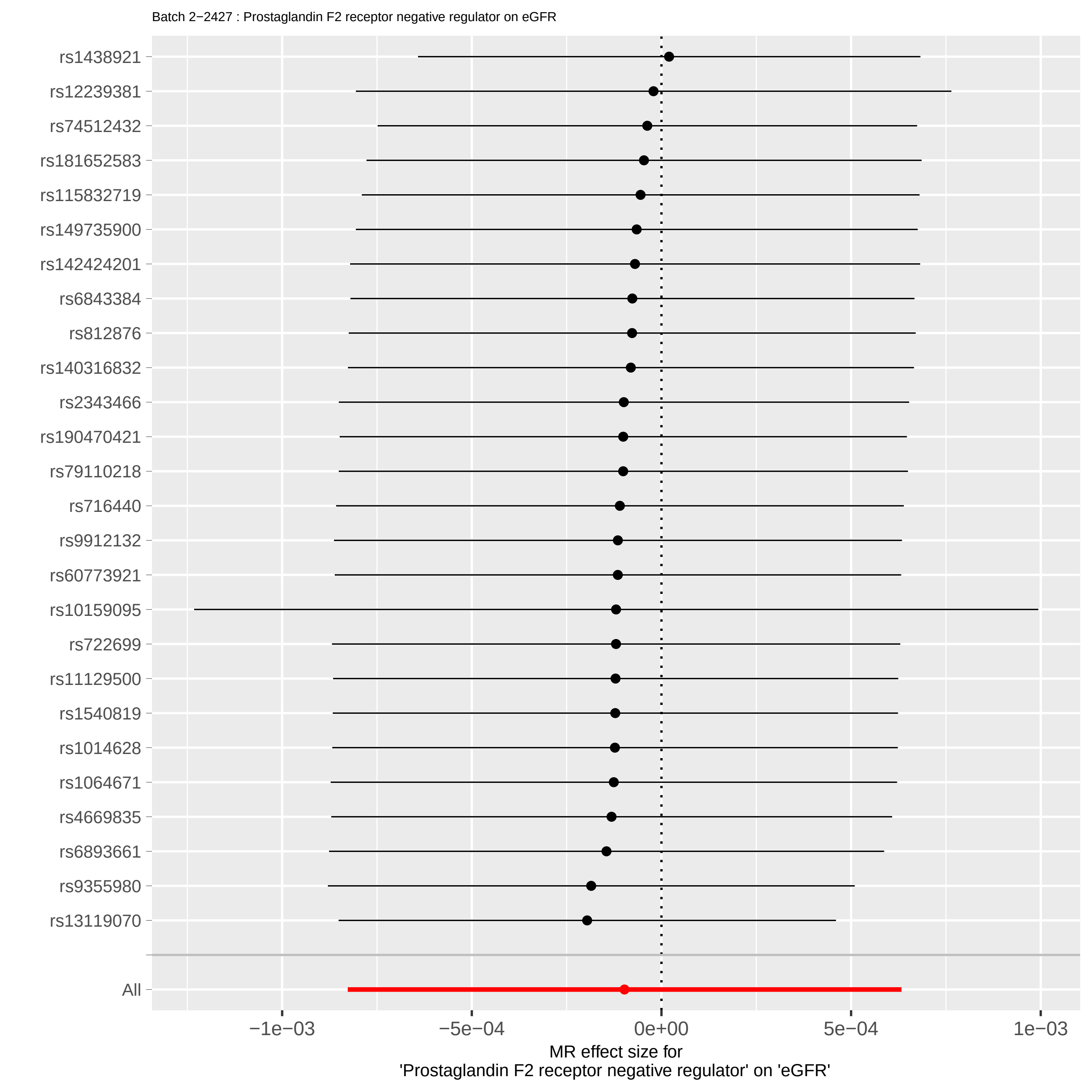

Batch 2−2427 : Prostaglandin F2 receptor negative regulator on eGFR
rs1438921
rs12239381
rs74512432
rs181652583
rs115832719
rs149735900
rs142424201
rs6843384
rs812876
rs140316832
rs2343466
rs190470421
rs79110218
rs716440
rs9912132
rs60773921
rs10159095
rs722699
rs11129500
rs1540819
rs1014628
rs1064671
rs4669835
rs6893661
rs9355980
rs13119070
All
−1e−03
−5e−04
0e+00
5e−04
1e−03
MR effect size for
'Prostaglandin F2 receptor negative regulator' on 'eGFR'

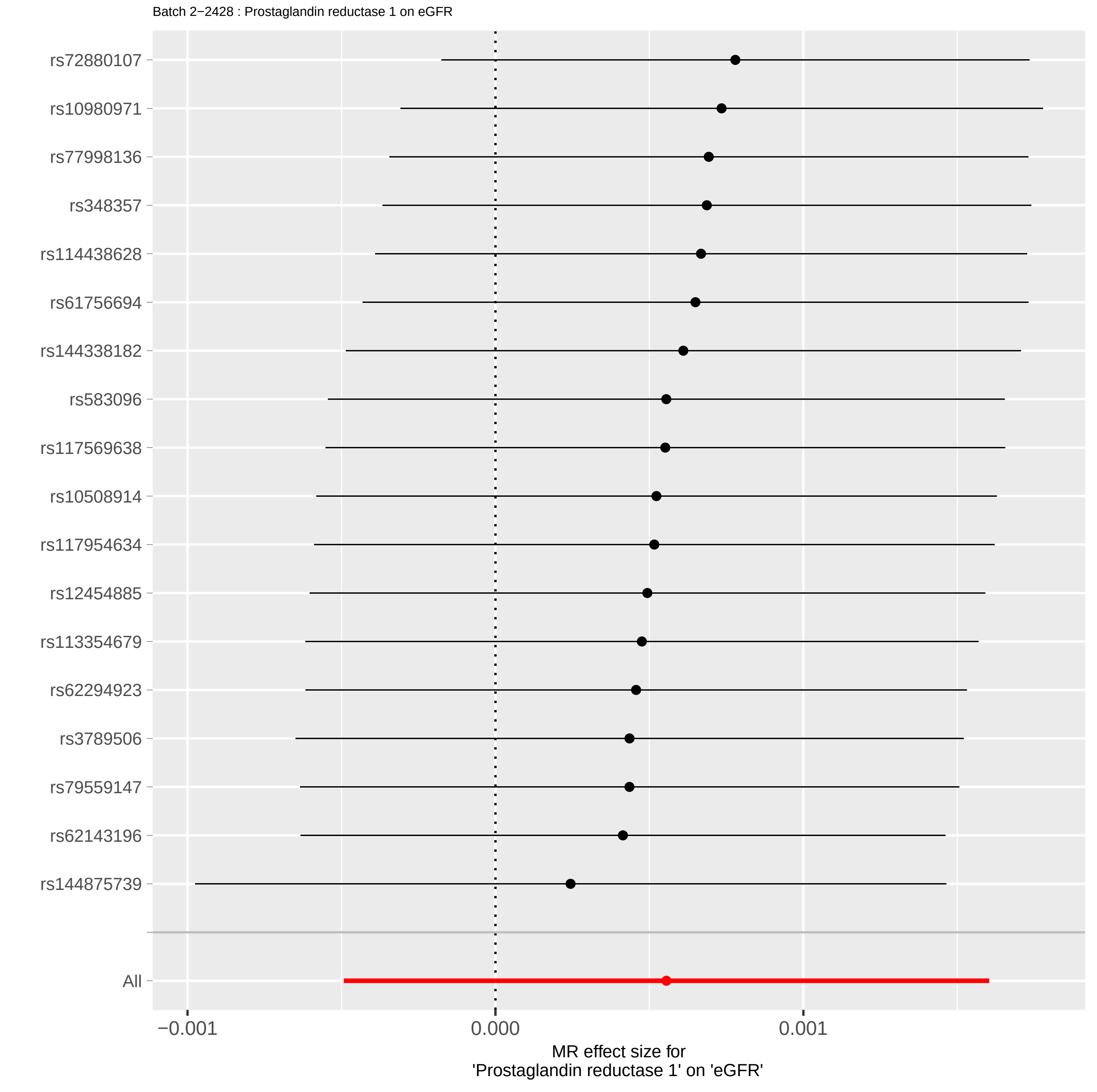

Batch 2−2428 : Prostaglandin reductase 1 on eGFR
rs72880107
rs10980971
rs77998136
rs348357
rs114438628
rs61756694
rs144338182
rs583096
rs117569638
rs10508914
rs117954634
rs12454885
rs113354679
rs62294923
rs3789506
rs79559147
rs62143196
rs144875739
All
−0.001
0.000
0.001
MR effect size for
'Prostaglandin reductase 1' on 'eGFR'

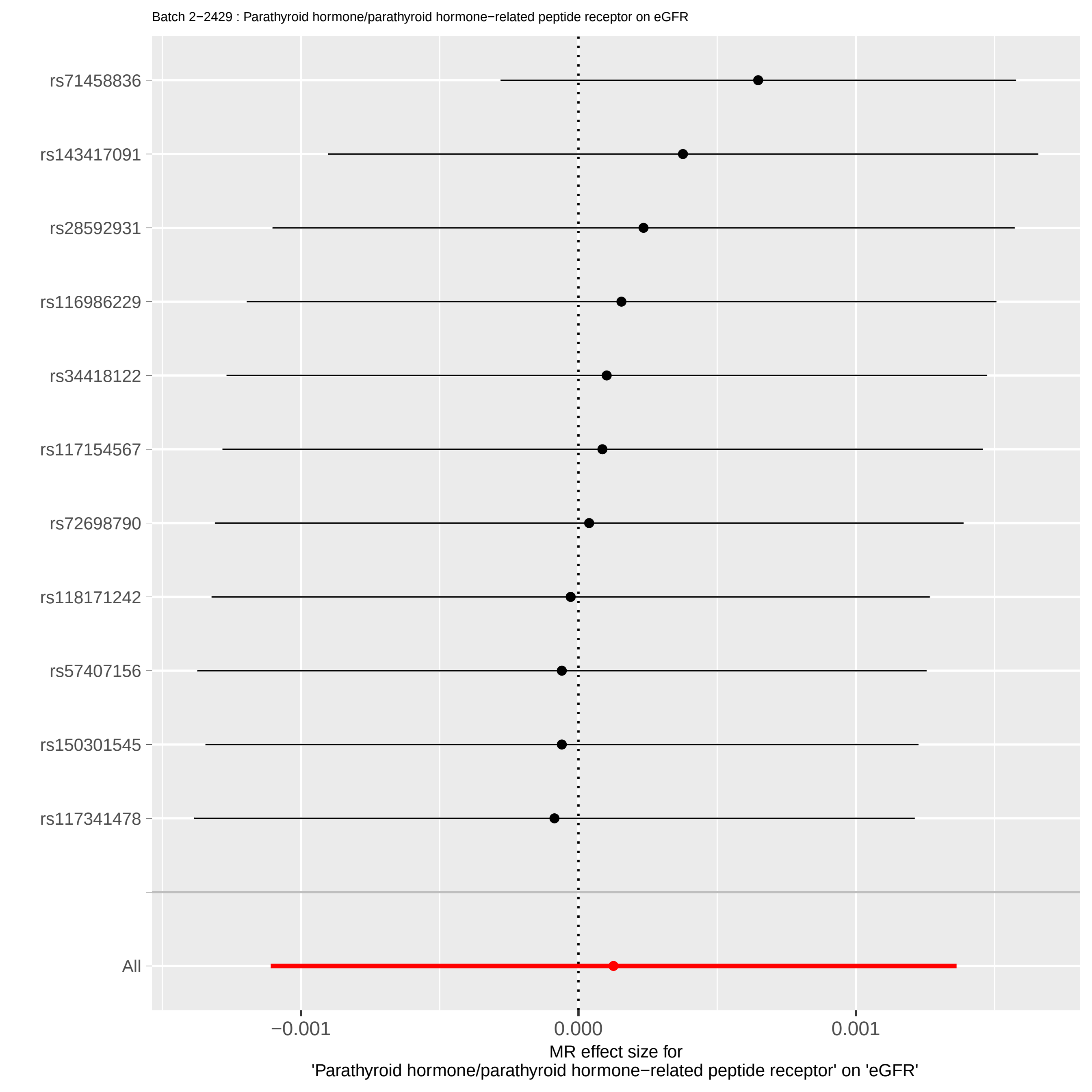

Batch 2−2429 : Parathyroid hormone/parathyroid hormone−related peptide receptor on eGFR
rs71458836
rs143417091
rs28592931
rs116986229
rs34418122
rs117154567
rs72698790
rs118171242
rs57407156
rs150301545
rs117341478
All
−0.001
0.000
0.001
MR effect size for
'Parathyroid hormone/parathyroid hormone−related peptide receptor' on 'eGFR'

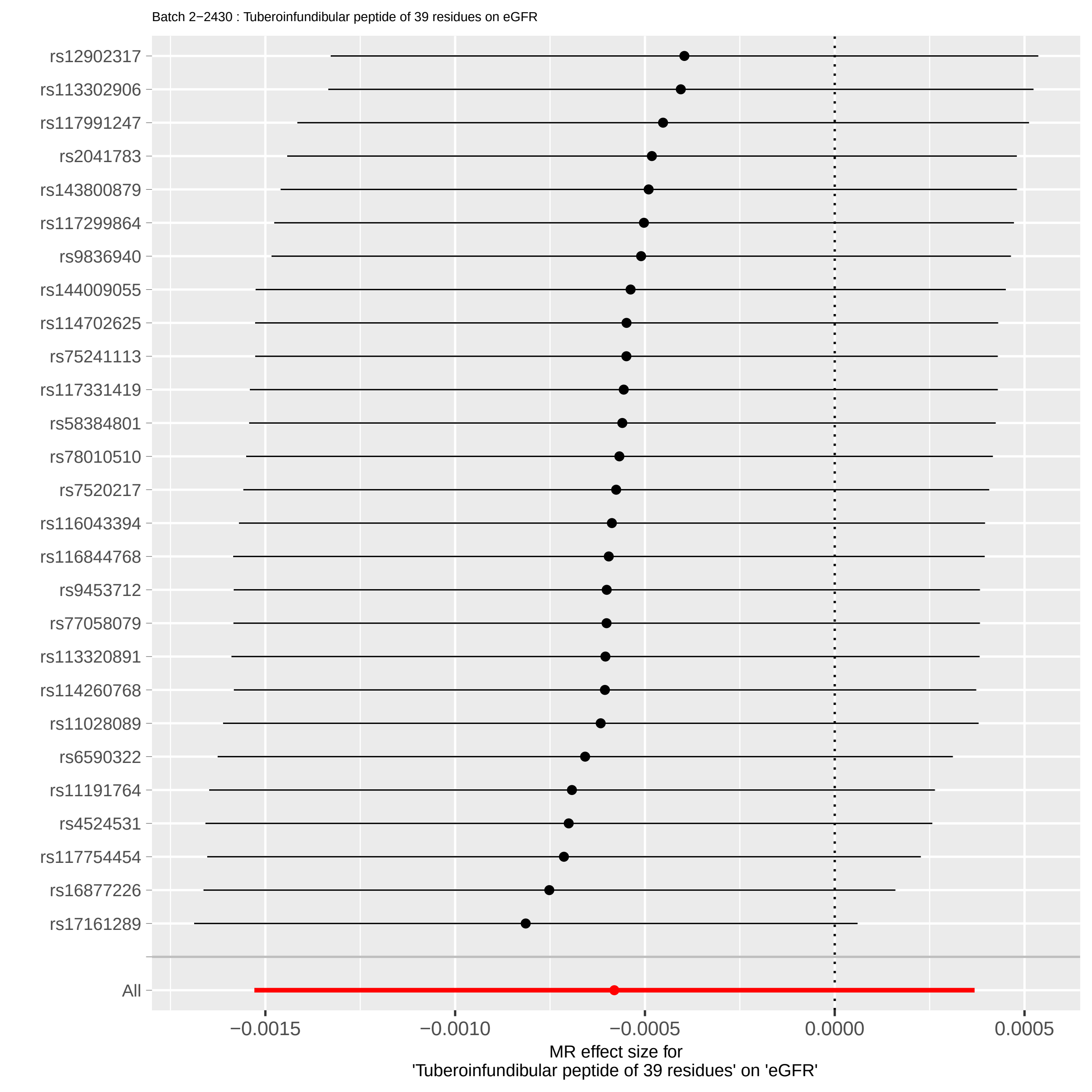

Batch 2−2430 : Tuberoinfundibular peptide of 39 residues on eGFR
rs12902317
rs113302906
rs117991247
rs2041783
rs143800879
rs117299864
rs9836940
rs144009055
rs114702625
rs75241113
rs117331419
rs58384801
rs78010510
rs7520217
rs116043394
rs116844768
rs9453712
rs77058079
rs113320891
rs114260768
rs11028089
rs6590322
rs11191764
rs4524531
rs117754454
rs16877226
rs17161289
All
−0.0015
−0.0010
−0.0005
0.0000
0.0005
MR effect size for
'Tuberoinfundibular peptide of 39 residues' on 'eGFR'

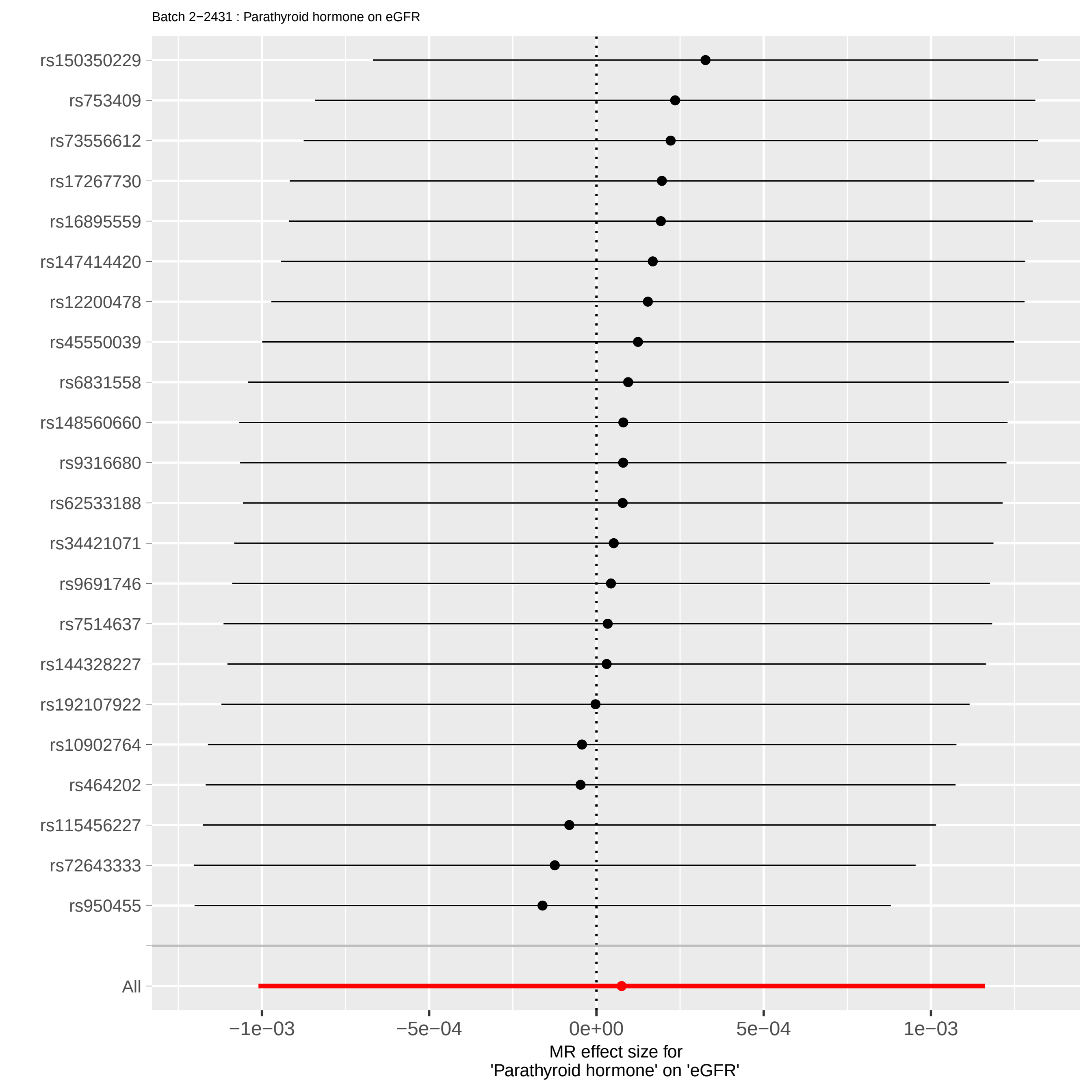

Batch 2−2431 : Parathyroid hormone on eGFR
rs150350229
rs753409
rs73556612
rs17267730
rs16895559
rs147414420
rs12200478
rs45550039
rs6831558
rs148560660
rs9316680
rs62533188
rs34421071
rs9691746
rs7514637
rs144328227
rs192107922
rs10902764
rs464202
rs115456227
rs72643333
rs950455
All
−1e−03
−5e−04
0e+00
5e−04
1e−03
MR effect size for
'Parathyroid hormone' on 'eGFR'

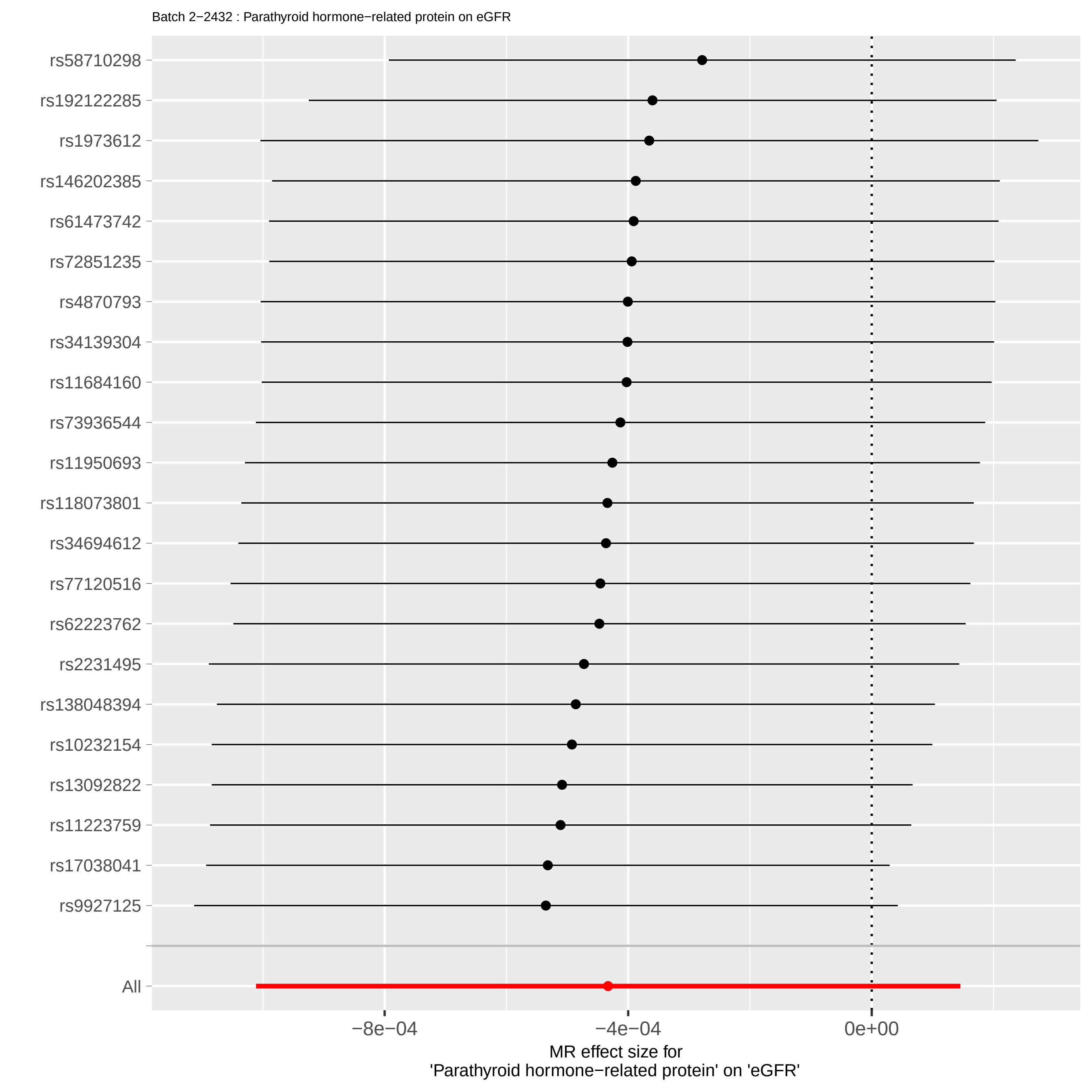

Batch 2−2432 : Parathyroid hormone−related protein on eGFR
rs58710298
rs192122285
rs1973612
rs146202385
rs61473742
rs72851235
rs4870793
rs34139304
rs11684160
rs73936544
rs11950693
rs118073801
rs34694612
rs77120516
rs62223762
rs2231495
rs138048394
rs10232154
rs13092822
rs11223759
rs17038041
rs9927125
All
−8e−04
−4e−04
0e+00
MR effect size for
'Parathyroid hormone−related protein' on 'eGFR'

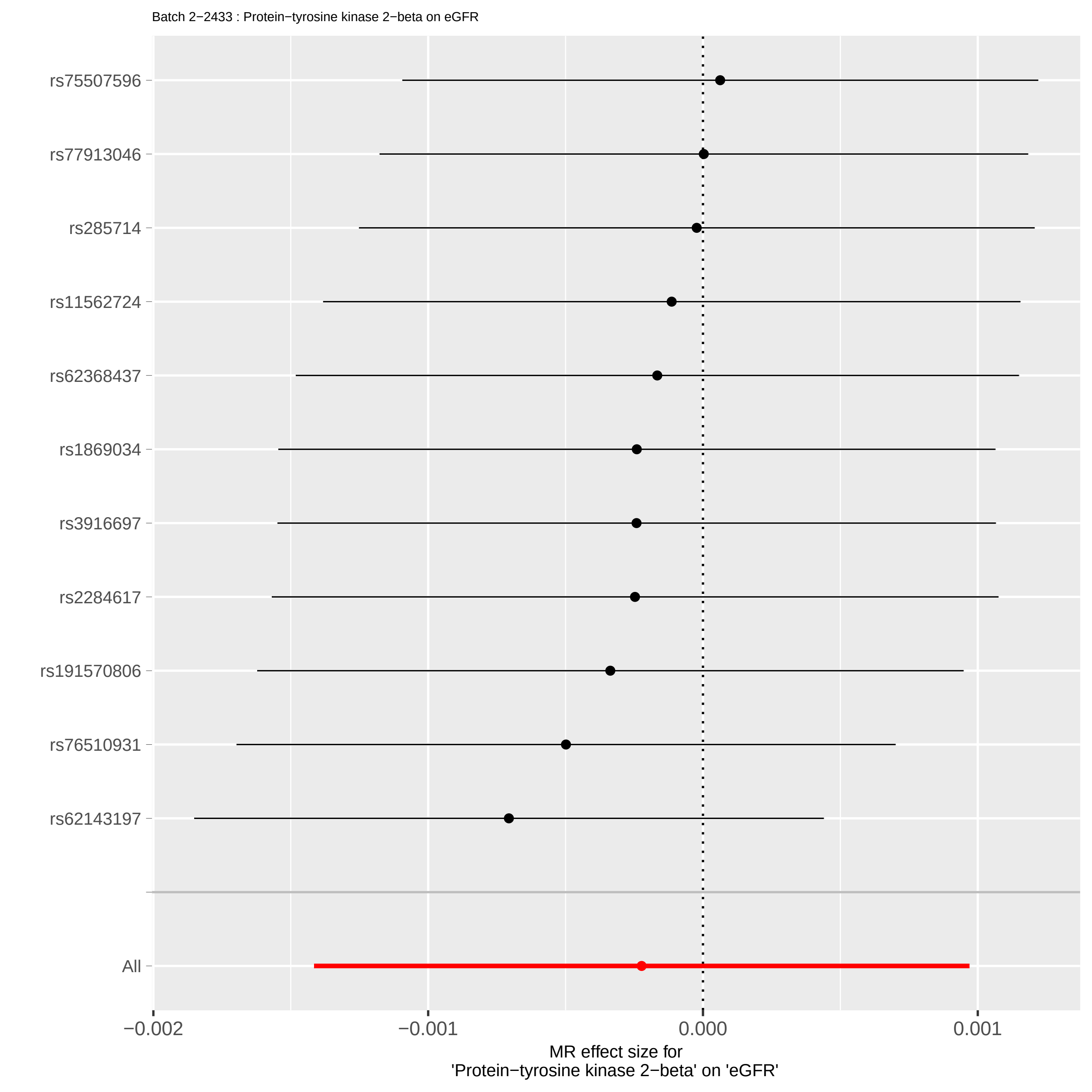
Batch 2−2433 : Protein−tyrosine kinase 2−beta on eGFR
rs75507596
rs77913046
rs285714
rs11562724
rs62368437
rs1869034
rs3916697
rs2284617
rs191570806
rs76510931
rs62143197
All
−0.002
−0.001
0.000
0.001
MR effect size for
'Protein−tyrosine kinase 2−beta' on 'eGFR'

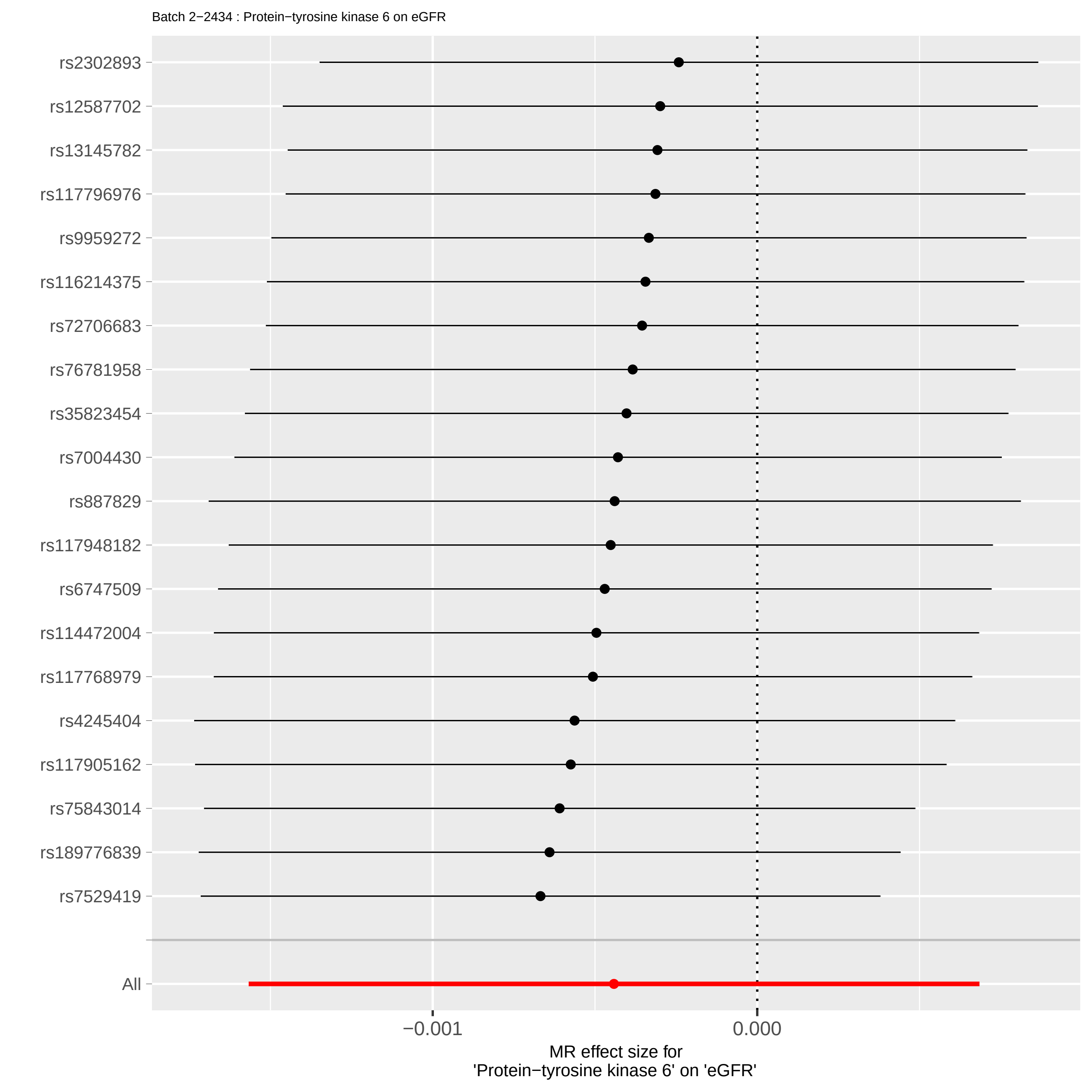

Batch 2−2434 : Protein−tyrosine kinase 6 on eGFR
rs2302893
rs12587702
rs13145782
rs117796976
rs9959272
rs116214375
rs72706683
rs76781958
rs35823454
rs7004430
rs887829
rs117948182
rs6747509
rs114472004
rs117768979
rs4245404
rs117905162
rs75843014
rs189776839
rs7529419
All
−0.001
0.000
MR effect size for
'Protein−tyrosine kinase 6' on 'eGFR'

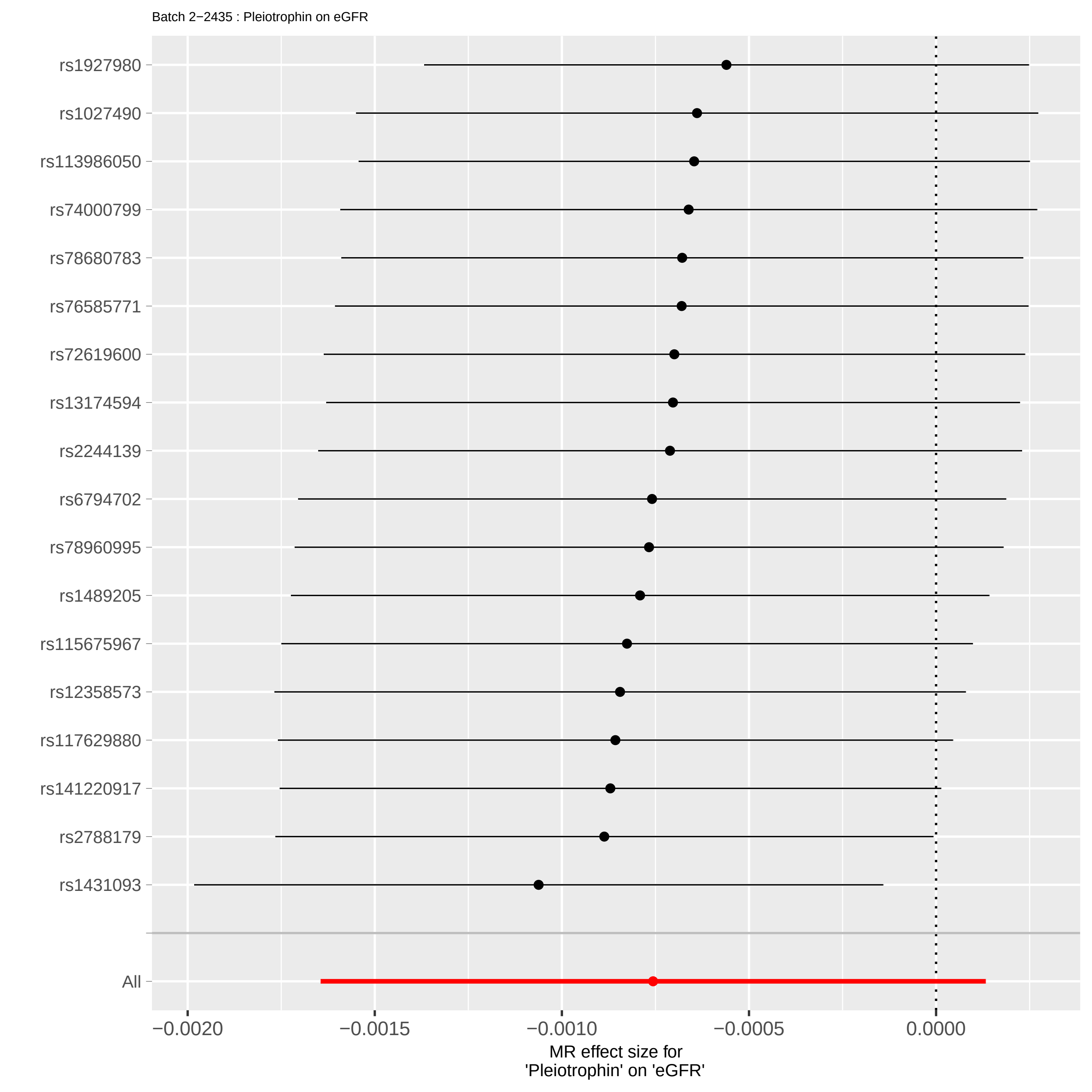

Batch 2−2435 : Pleiotrophin on eGFR
rs1927980
rs1027490
rs113986050
rs74000799
rs78680783
rs76585771
rs72619600
rs13174594
rs2244139
rs6794702
rs78960995
rs1489205
rs115675967
rs12358573
rs117629880
rs141220917
rs2788179
rs1431093
All
−0.0020
−0.0015
−0.0010
−0.0005
0.0000
MR effect size for
'Pleiotrophin' on 'eGFR'

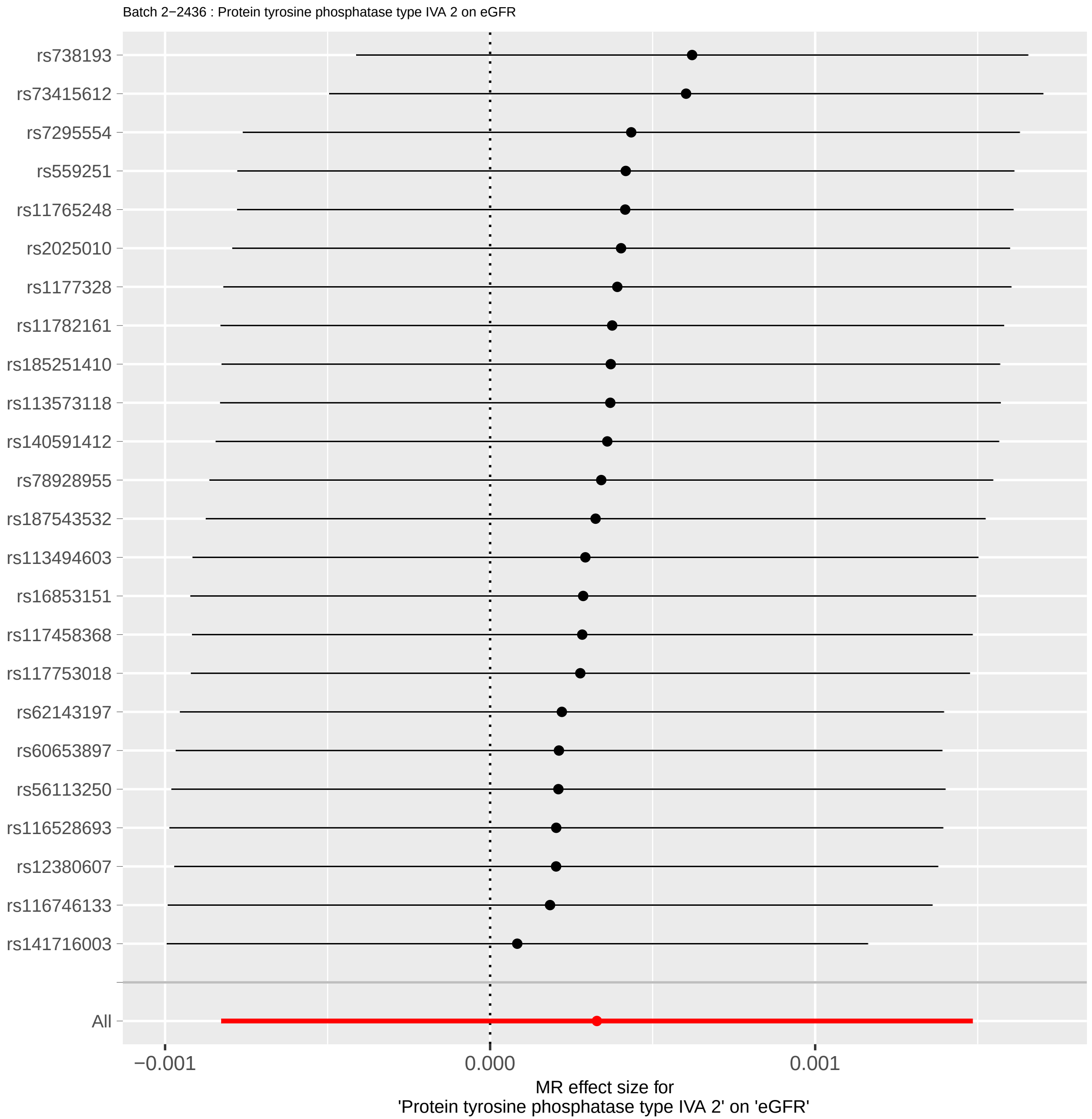

Batch 2−2436 : Protein tyrosine phosphatase type IVA 2 on eGFR
rs738193
rs73415612
rs7295554
rs559251
rs11765248
rs2025010
rs1177328
rs11782161
rs185251410
rs113573118
rs140591412
rs78928955
rs187543532
rs113494603
rs16853151
rs117458368
rs117753018
rs62143197
rs60653897
rs56113250
rs116528693
rs12380607
rs116746133
rs141716003
All
−0.001
0.000
0.001
MR effect size for
'Protein tyrosine phosphatase type IVA 2' on 'eGFR'

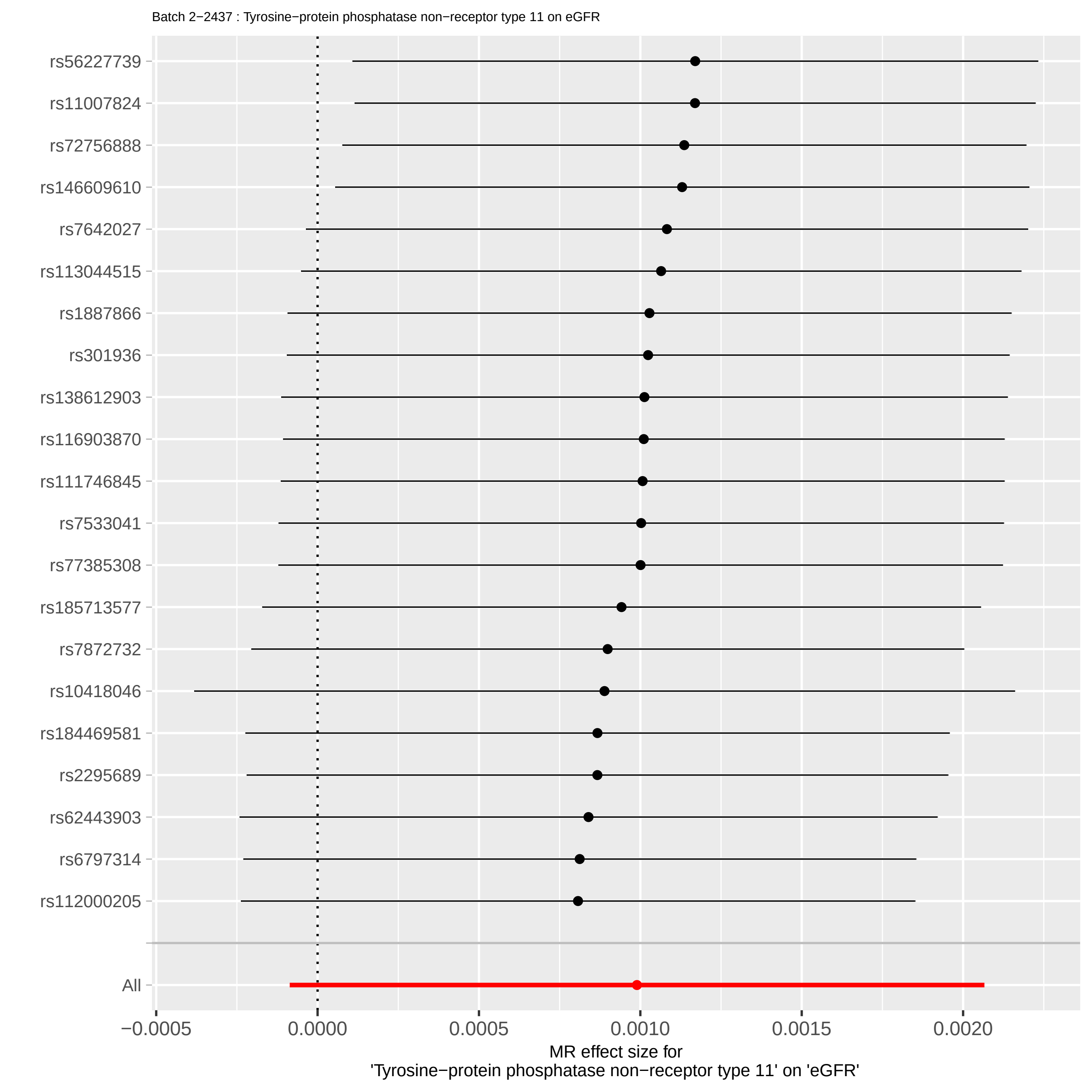
Batch 2−2437 : Tyrosine−protein phosphatase non−receptor type 11 on eGFR
rs56227739
rs11007824
rs72756888
rs146609610
rs7642027
rs113044515
rs1887866
rs301936
rs138612903
rs116903870
rs111746845
rs7533041
rs77385308
rs185713577
rs7872732
rs10418046
rs184469581
rs2295689
rs62443903
rs6797314
rs112000205
All
−0.0005
0.0000
0.0005
0.0010
0.0015
0.0020
MR effect size for
'Tyrosine−protein phosphatase non−receptor type 11' on 'eGFR'

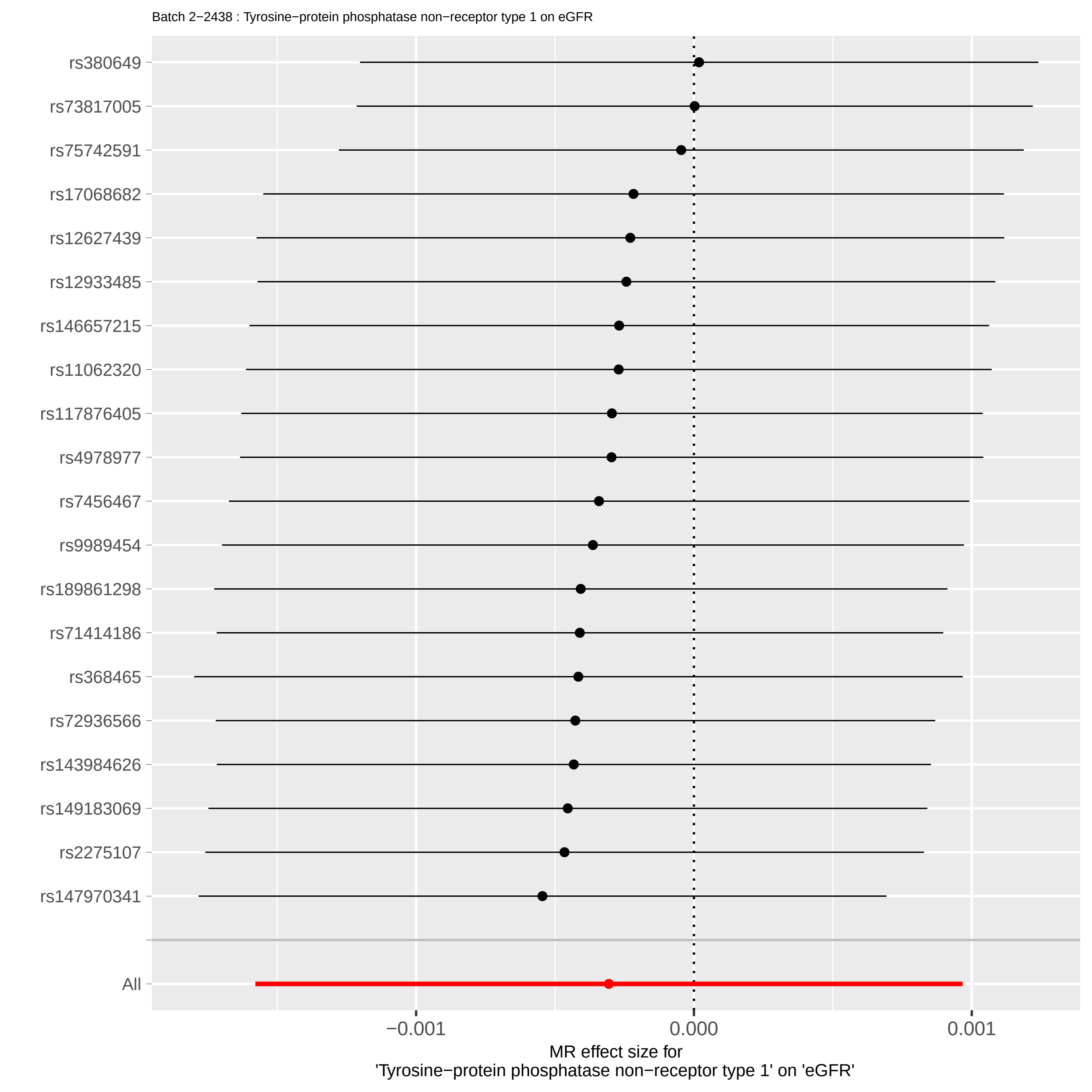

Batch 2−2438 : Tyrosine−protein phosphatase non−receptor type 1 on eGFR
rs380649
rs73817005
rs75742591
rs17068682
rs12627439
rs12933485
rs146657215
rs11062320
rs117876405
rs4978977
rs7456467
rs9989454
rs189861298
rs71414186
rs368465
rs72936566
rs143984626
rs149183069
rs2275107
rs147970341
All
−0.001
0.000
0.001
MR effect size for
'Tyrosine−protein phosphatase non−receptor type 1' on 'eGFR'

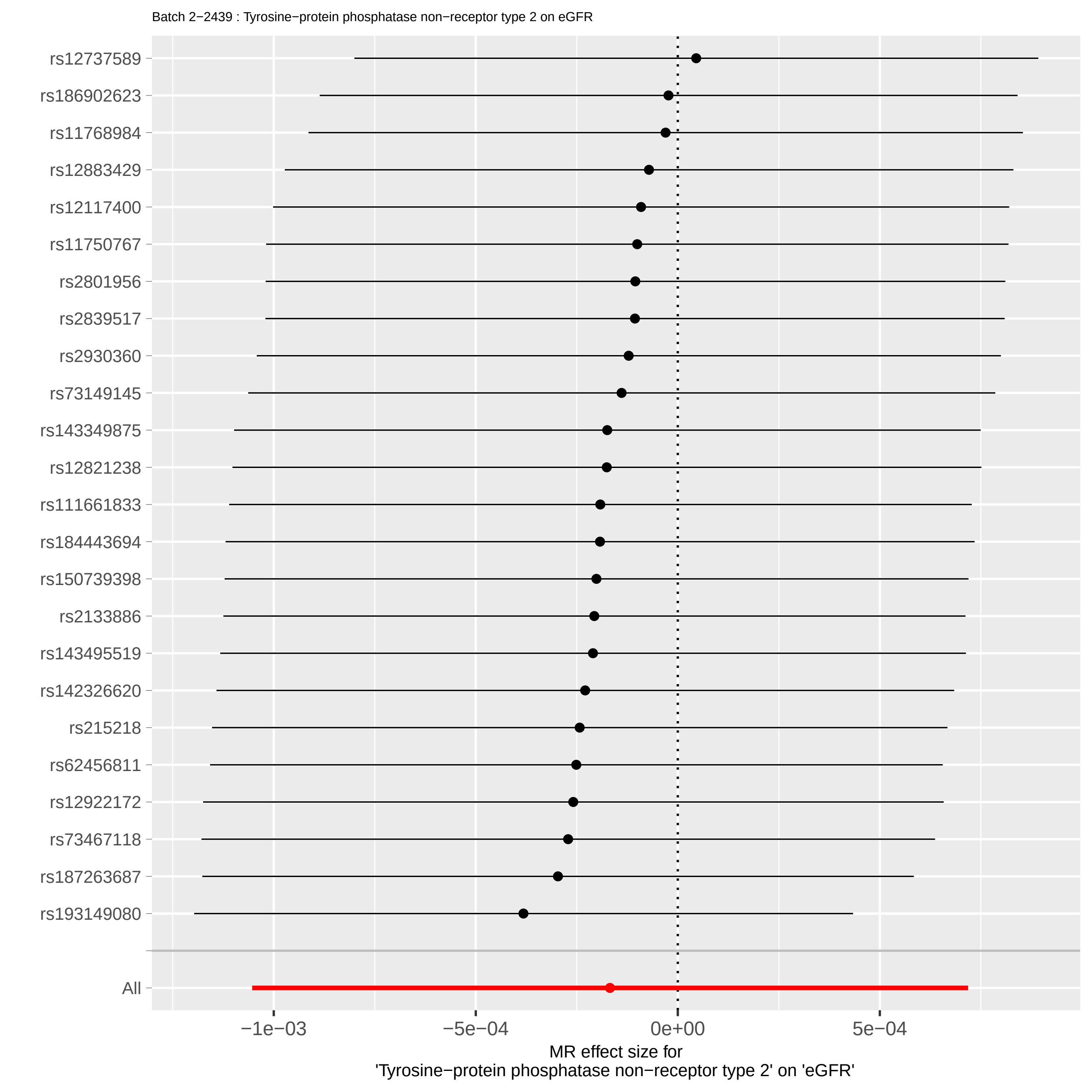

Batch 2−2439 : Tyrosine−protein phosphatase non−receptor type 2 on eGFR
rs12737589
rs186902623
rs11768984
rs12883429
rs12117400
rs11750767
rs2801956
rs2839517
rs2930360
rs73149145
rs143349875
rs12821238
rs111661833
rs184443694
rs150739398
rs2133886
rs143495519
rs142326620
rs215218
rs62456811
rs12922172
rs73467118
rs187263687
rs193149080
All
−1e−03
−5e−04
0e+00
5e−04
MR effect size for
'Tyrosine−protein phosphatase non−receptor type 2' on 'eGFR'

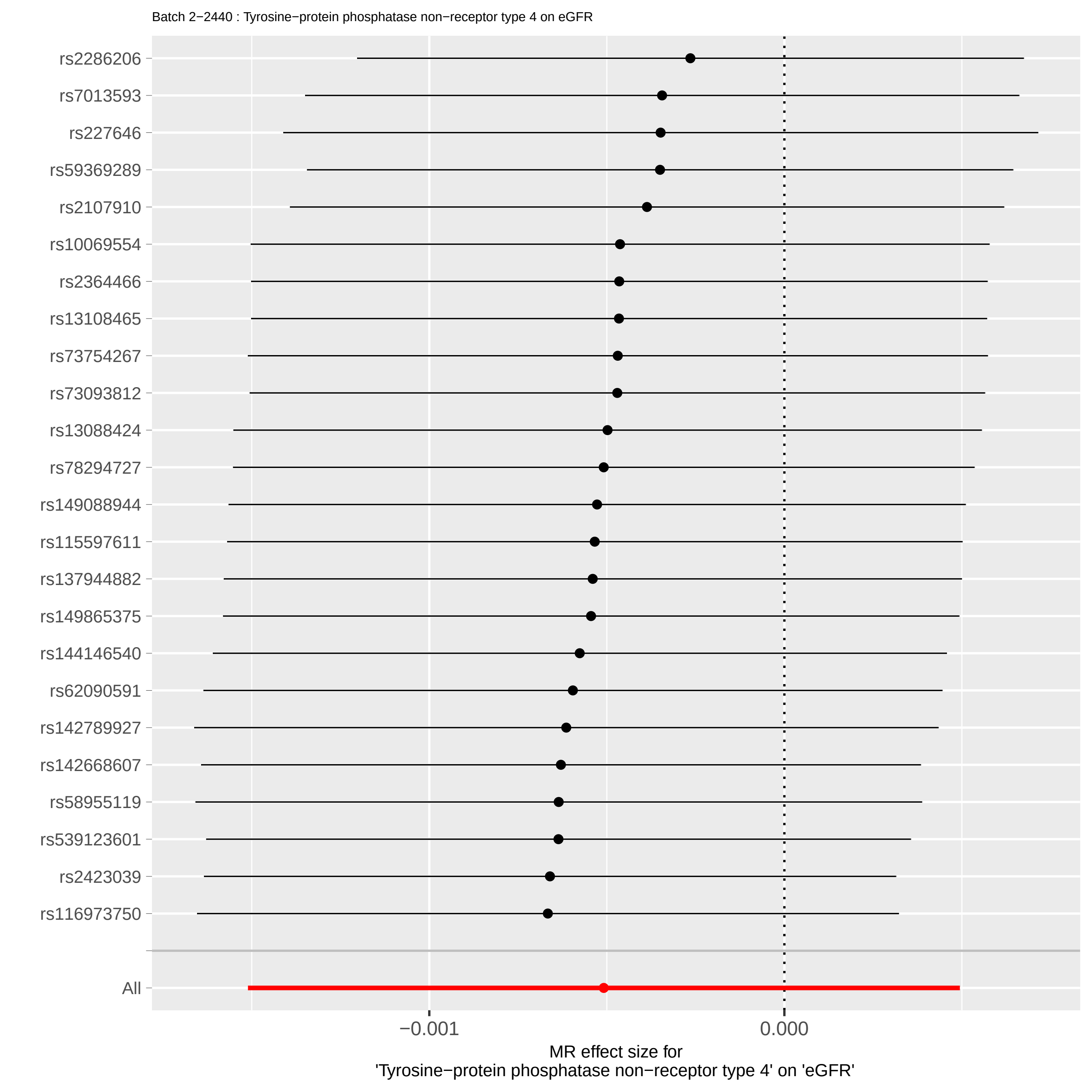
Batch 2−2440 : Tyrosine−protein phosphatase non−receptor type 4 on eGFR
rs2286206
rs7013593
rs227646
rs59369289
rs2107910
rs10069554
rs2364466
rs13108465
rs73754267
rs73093812
rs13088424
rs78294727
rs149088944
rs115597611
rs137944882
rs149865375
rs144146540
rs62090591
rs142789927
rs142668607
rs58955119
rs539123601
rs2423039
rs116973750
All
−0.001
0.000
MR effect size for
'Tyrosine−protein phosphatase non−receptor type 4' on 'eGFR'

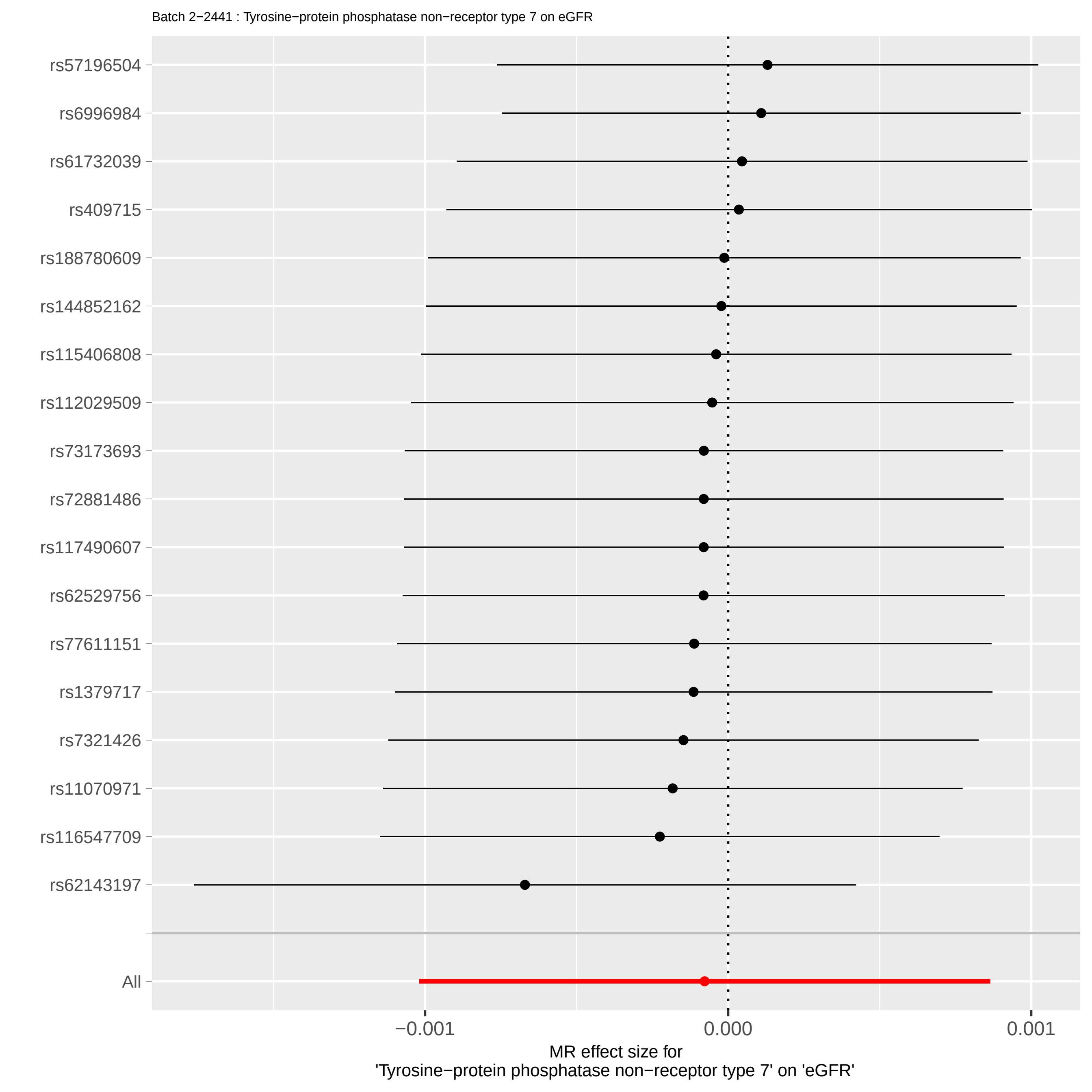

Batch 2−2441 : Tyrosine−protein phosphatase non−receptor type 7 on eGFR
rs57196504
rs6996984
rs61732039
rs409715
rs188780609
rs144852162
rs115406808
rs112029509
rs73173693
rs72881486
rs117490607
rs62529756
rs77611151
rs1379717
rs7321426
rs11070971
rs116547709
rs62143197
All
−0.001
0.000
0.001
MR effect size for
'Tyrosine−protein phosphatase non−receptor type 7' on 'eGFR'

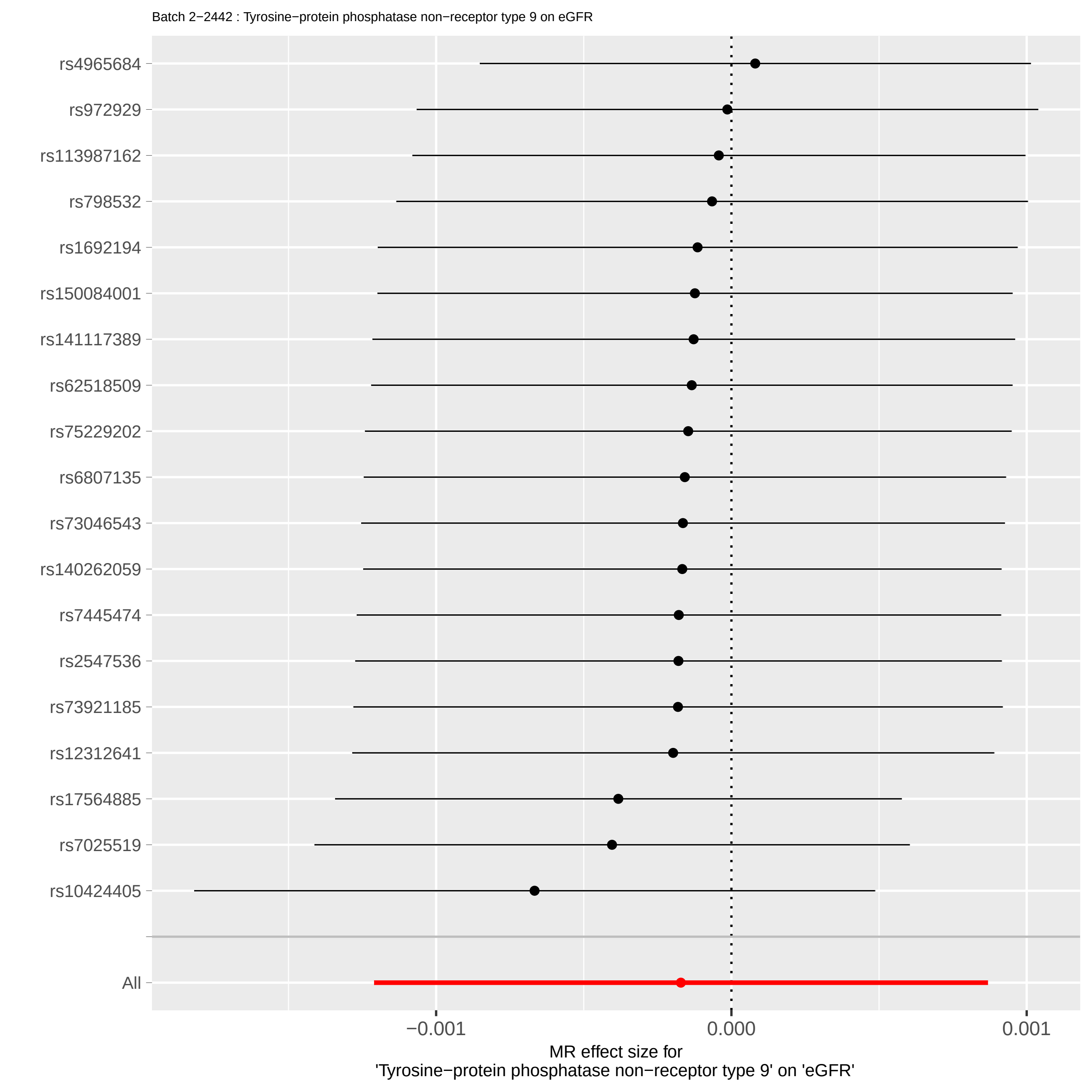
Batch 2−2442 : Tyrosine−protein phosphatase non−receptor type 9 on eGFR
rs4965684
rs972929
rs113987162
rs798532
rs1692194
rs150084001
rs141117389
rs62518509
rs75229202
rs6807135
rs73046543
rs140262059
rs7445474
rs2547536
rs73921185
rs12312641
rs17564885
rs7025519
rs10424405
All
−0.001
0.000
0.001
MR effect size for
'Tyrosine−protein phosphatase non−receptor type 9' on 'eGFR'

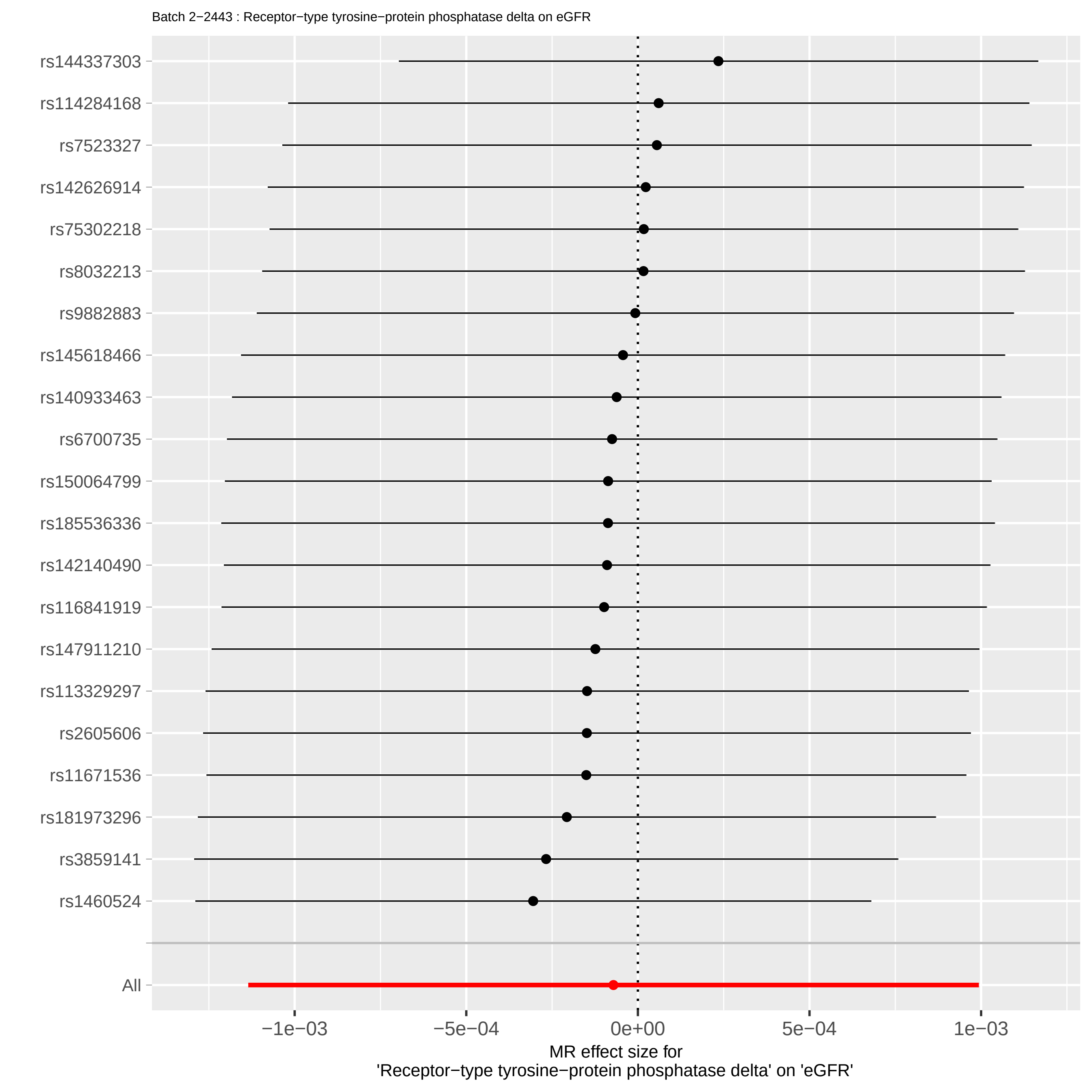

Batch 2−2443 : Receptor−type tyrosine−protein phosphatase delta on eGFR
rs144337303
rs114284168
rs7523327
rs142626914
rs75302218
rs8032213
rs9882883
rs145618466
rs140933463
rs6700735
rs150064799
rs185536336
rs142140490
rs116841919
rs147911210
rs113329297
rs2605606
rs11671536
rs181973296
rs3859141
rs1460524
All
−1e−03
−5e−04
0e+00
5e−04
1e−03
MR effect size for
'Receptor−type tyrosine−protein phosphatase delta' on 'eGFR'

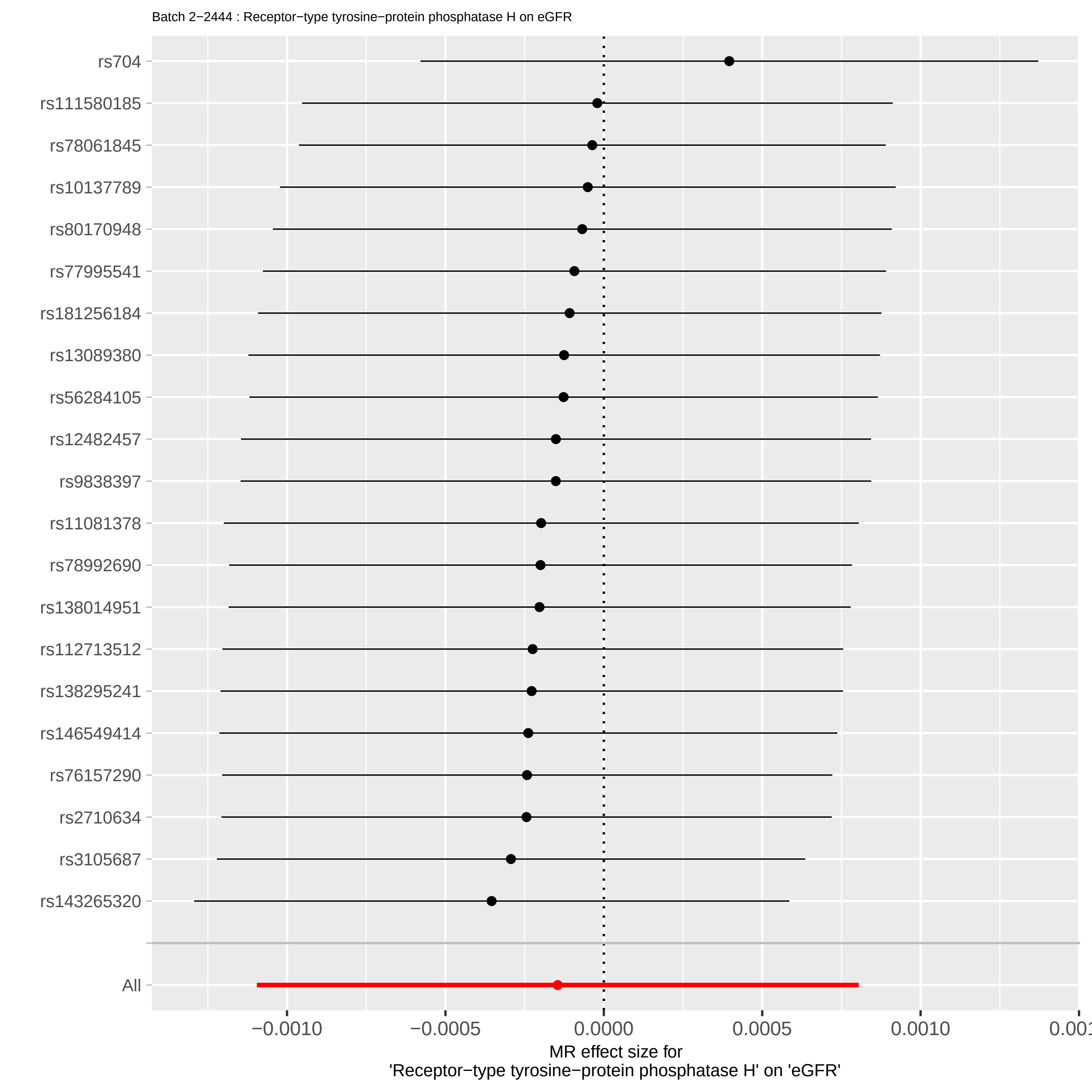

Batch 2−2444 : Receptor−type tyrosine−protein phosphatase H on eGFR
rs704
rs111580185
rs78061845
rs10137789
rs80170948
rs77995541
rs181256184
rs13089380
rs56284105
rs12482457
rs9838397
rs11081378
rs78992690
rs138014951
rs112713512
rs138295241
rs146549414
rs76157290
rs2710634
rs3105687
rs143265320
All
−0.0010
−0.0005
0.0000
0.0005
0.0010
MR effect size for
'Receptor−type tyrosine−protein phosphatase H' on 'eGFR'

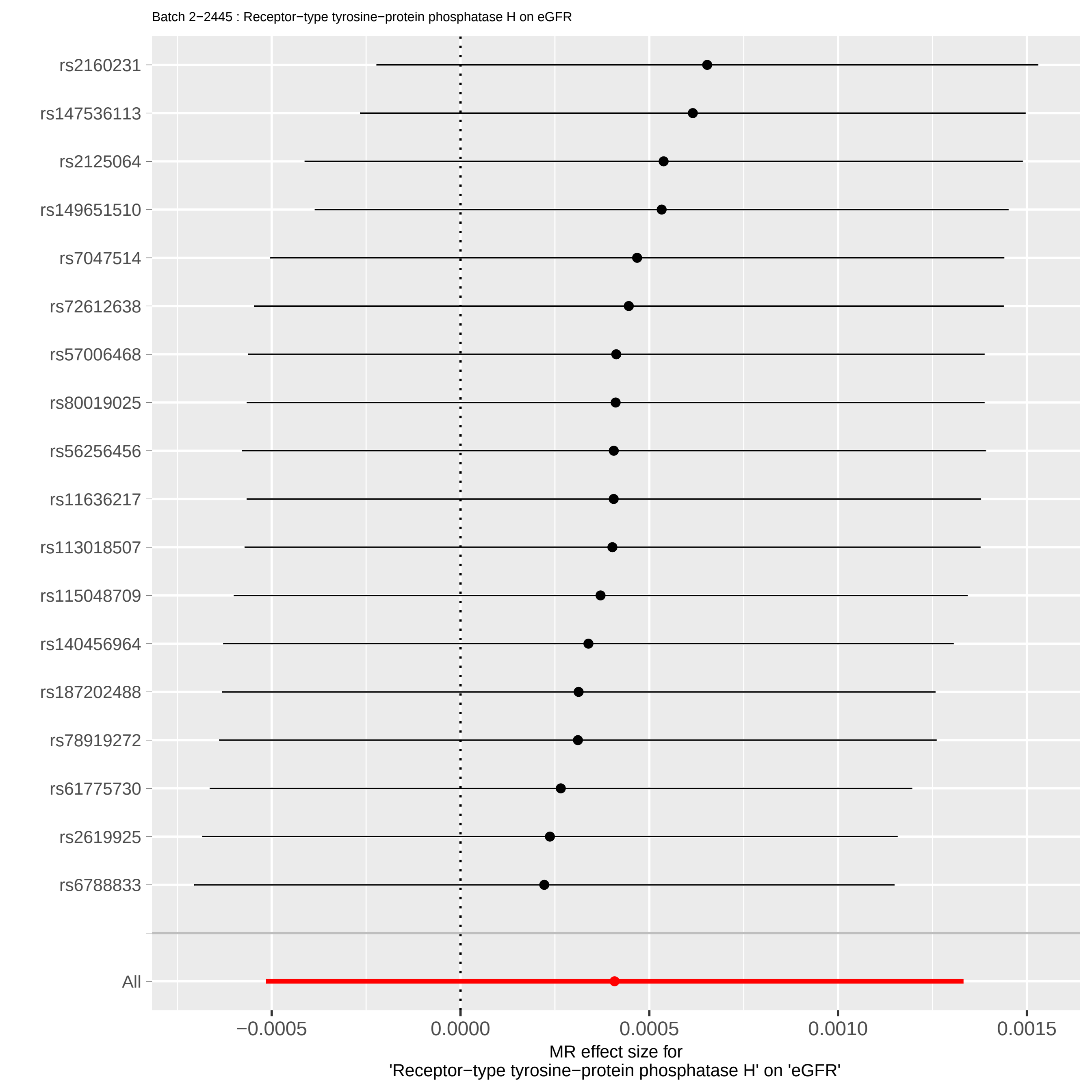

Batch 2−2445 : Receptor−type tyrosine−protein phosphatase H on eGFR
rs2160231
rs147536113
rs2125064
rs149651510
rs7047514
rs72612638
rs57006468
rs80019025
rs56256456
rs11636217
rs113018507
rs115048709
rs140456964
rs187202488
rs78919272
rs61775730
rs2619925
rs6788833
All
−0.0005
0.0000
0.0005
0.0010
0.0015
MR effect size for
'Receptor−type tyrosine−protein phosphatase H' on 'eGFR'

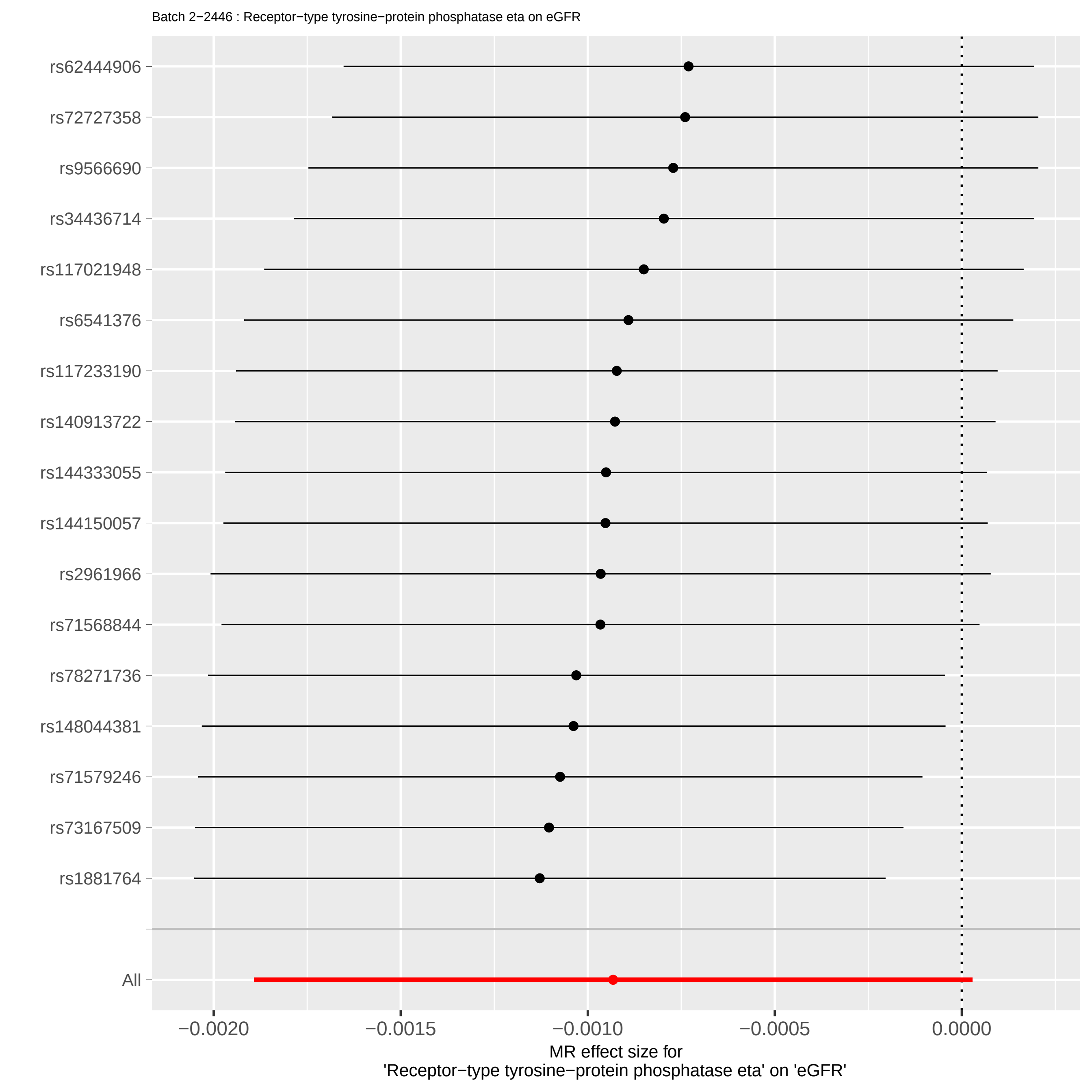
Batch 2−2446 : Receptor−type tyrosine−protein phosphatase eta on eGFR
rs62444906
rs72727358
rs9566690
rs34436714
rs117021948
rs6541376
rs117233190
rs140913722
rs144333055
rs144150057
rs2961966
rs71568844
rs78271736
rs148044381
rs71579246
rs73167509
rs1881764
All
−0.0020
−0.0015
−0.0010
−0.0005
0.0000
MR effect size for
'Receptor−type tyrosine−protein phosphatase eta' on 'eGFR'

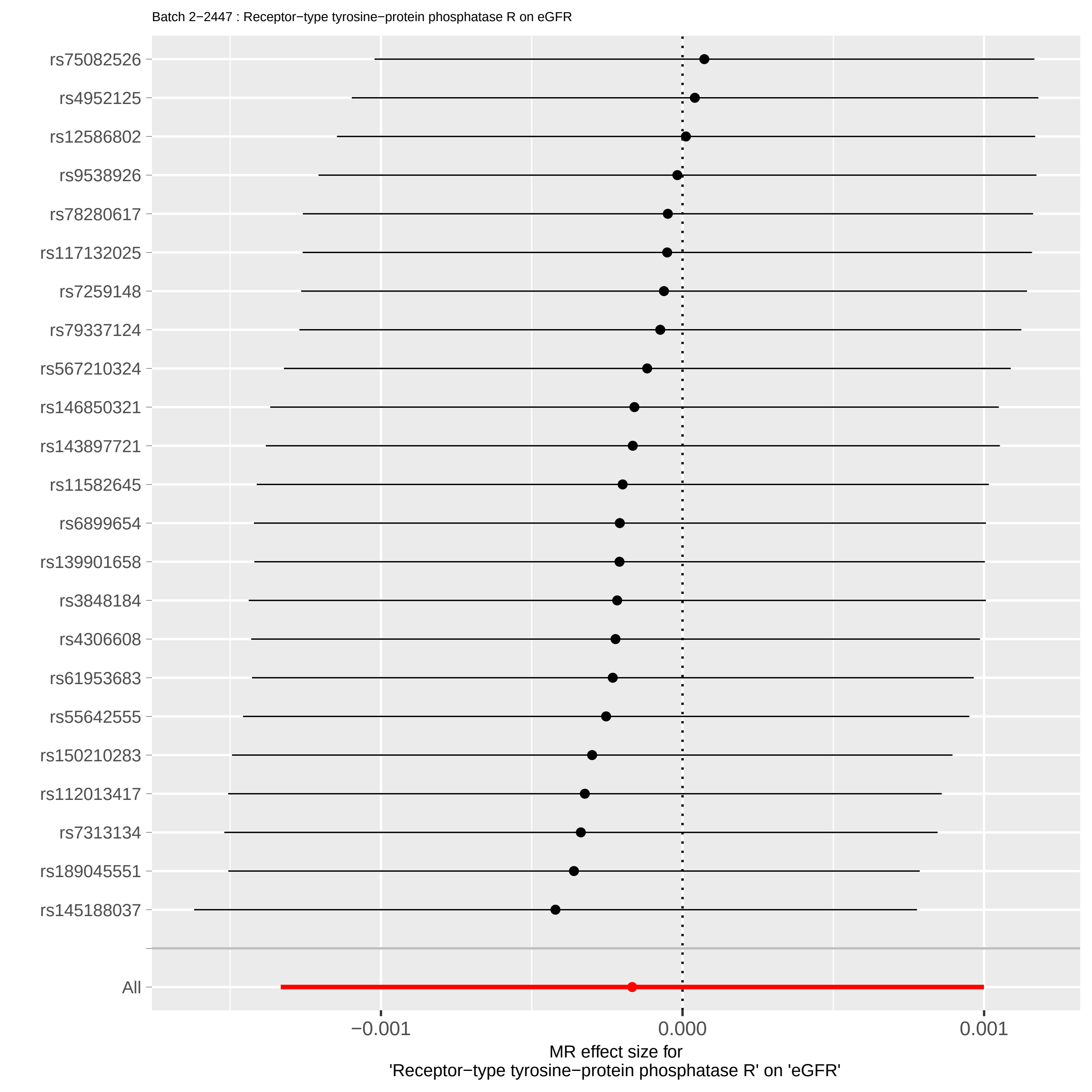
Batch 2−2447 : Receptor−type tyrosine−protein phosphatase R on eGFR
rs75082526
rs4952125
rs12586802
rs9538926
rs78280617
rs117132025
rs7259148
rs79337124
rs567210324
rs146850321
rs143897721
rs11582645
rs6899654
rs139901658
rs3848184
rs4306608
rs61953683
rs55642555
rs150210283
rs112013417
rs7313134
rs189045551
rs145188037
All
−0.001
0.000
0.001
MR effect size for
'Receptor−type tyrosine−protein phosphatase R' on 'eGFR'

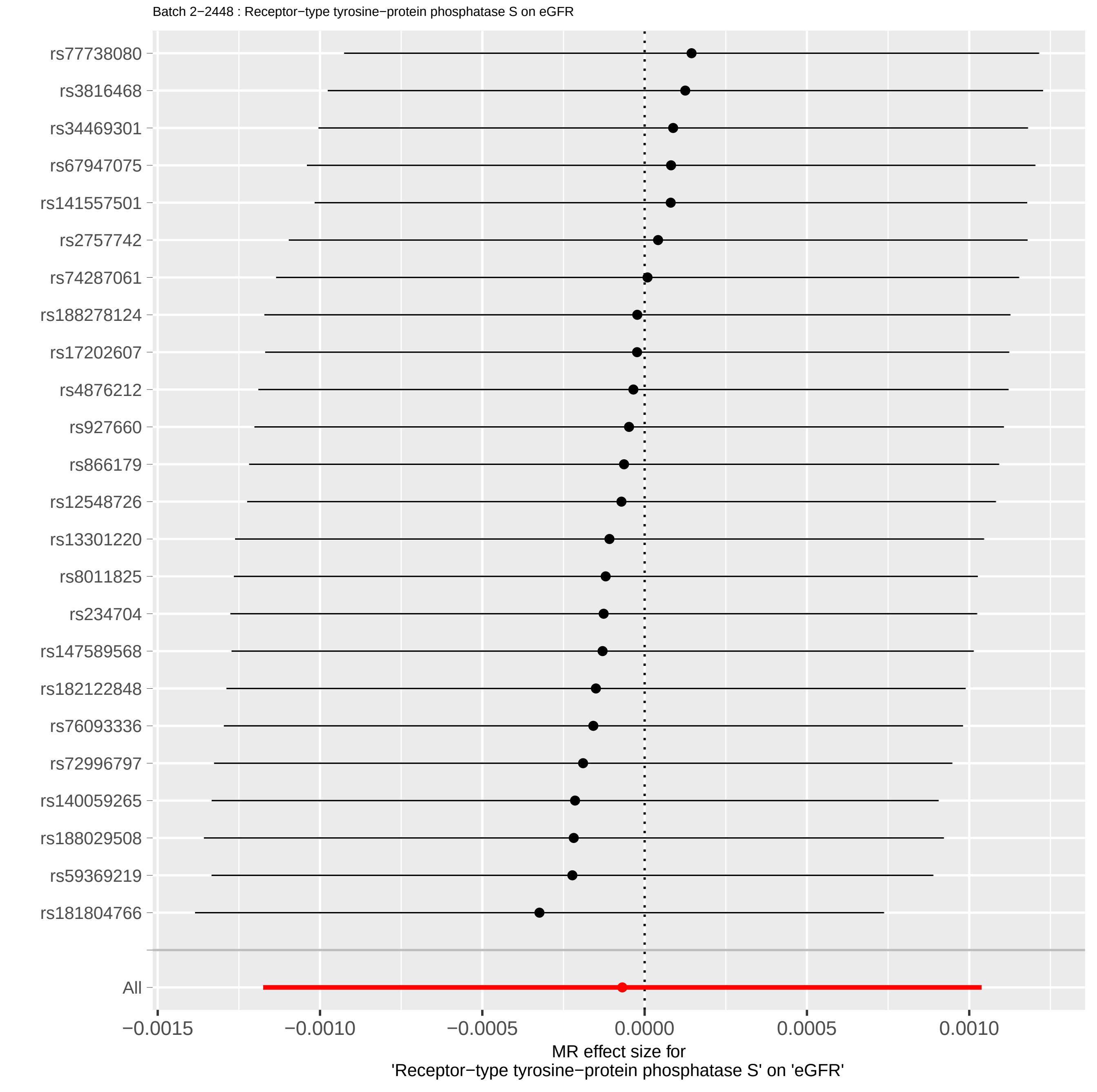
Batch 2−2448 : Receptor−type tyrosine−protein phosphatase S on eGFR
rs77738080
rs3816468
rs34469301
rs67947075
rs141557501
rs2757742
rs74287061
rs188278124
rs17202607
rs4876212
rs927660
rs866179
rs12548726
rs13301220
rs8011825
rs234704
rs147589568
rs182122848
rs76093336
rs72996797
rs140059265
rs188029508
rs59369219
rs181804766
All
−0.0015
−0.0010
−0.0005
0.0000
0.0005
0.0010
MR effect size for
'Receptor−type tyrosine−protein phosphatase S' on 'eGFR'

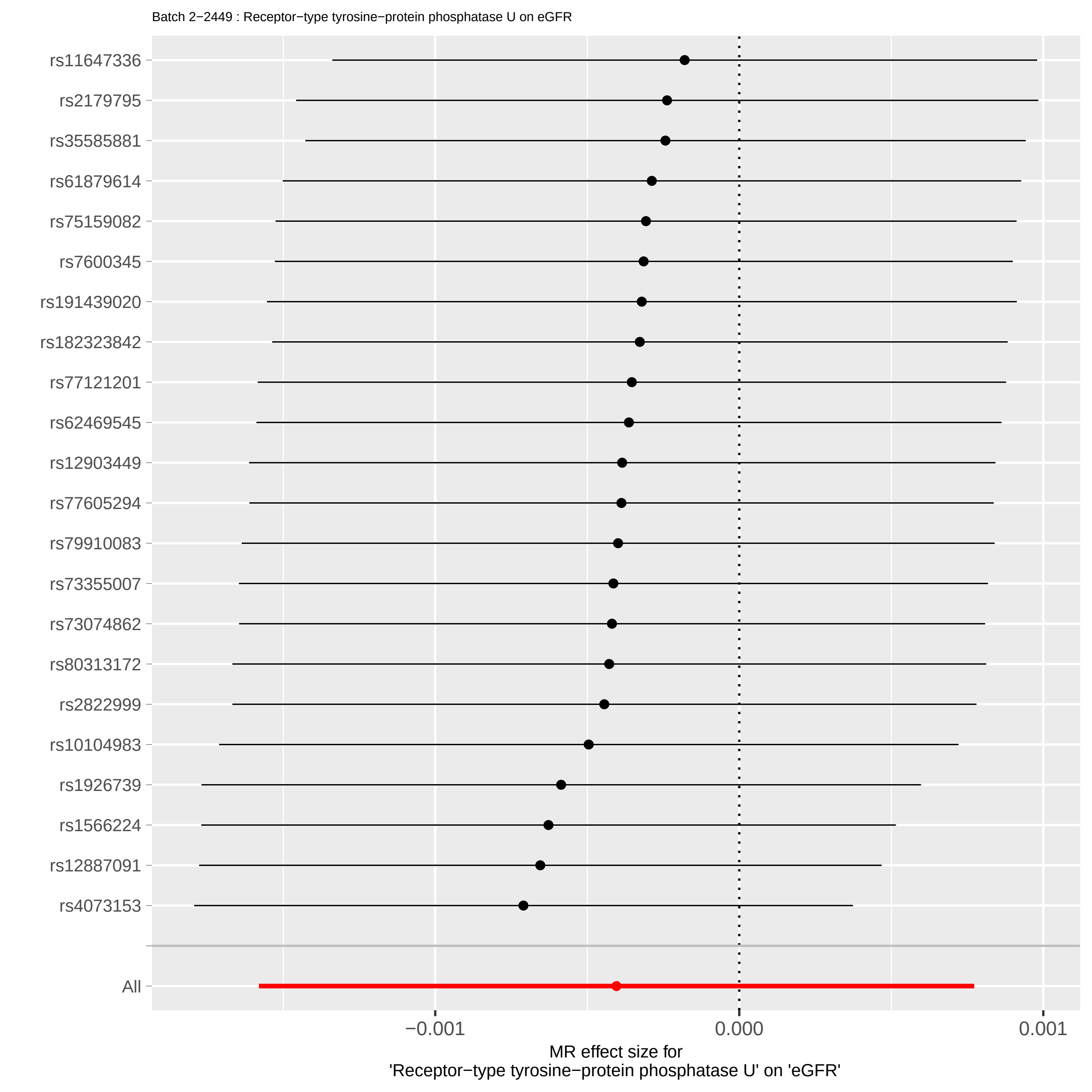

Batch 2−2449 : Receptor−type tyrosine−protein phosphatase U on eGFR
rs11647336
rs2179795
rs35585881
rs61879614
rs75159082
rs7600345
rs191439020
rs182323842
rs77121201
rs62469545
rs12903449
rs77605294
rs79910083
rs73355007
rs73074862
rs80313172
rs2822999
rs10104983
rs1926739
rs1566224
rs12887091
rs4073153
All
−0.001
0.000
0.001
MR effect size for
'Receptor−type tyrosine−protein phosphatase U' on 'eGFR'

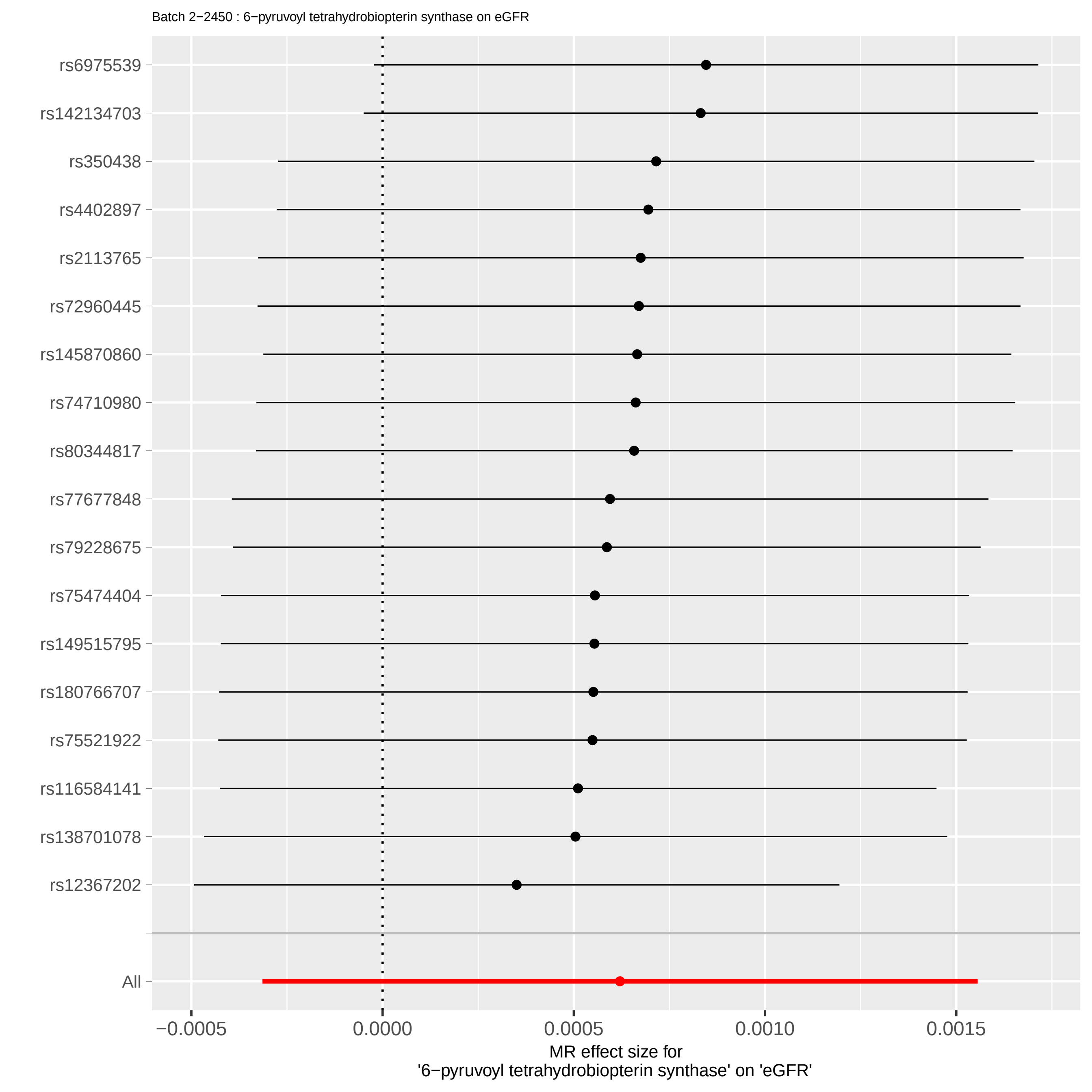
Batch 2−2450 : 6−pyruvoyl tetrahydrobiopterin synthase on eGFR
rs6975539
rs142134703
rs350438
rs4402897
rs2113765
rs72960445
rs145870860
rs74710980
rs80344817
rs77677848
rs79228675
rs75474404
rs149515795
rs180766707
rs75521922
rs116584141
rs138701078
rs12367202
All
−0.0005
0.0000
0.0005
0.0010
0.0015
MR effect size for
'6−pyruvoyl tetrahydrobiopterin synthase' on 'eGFR'

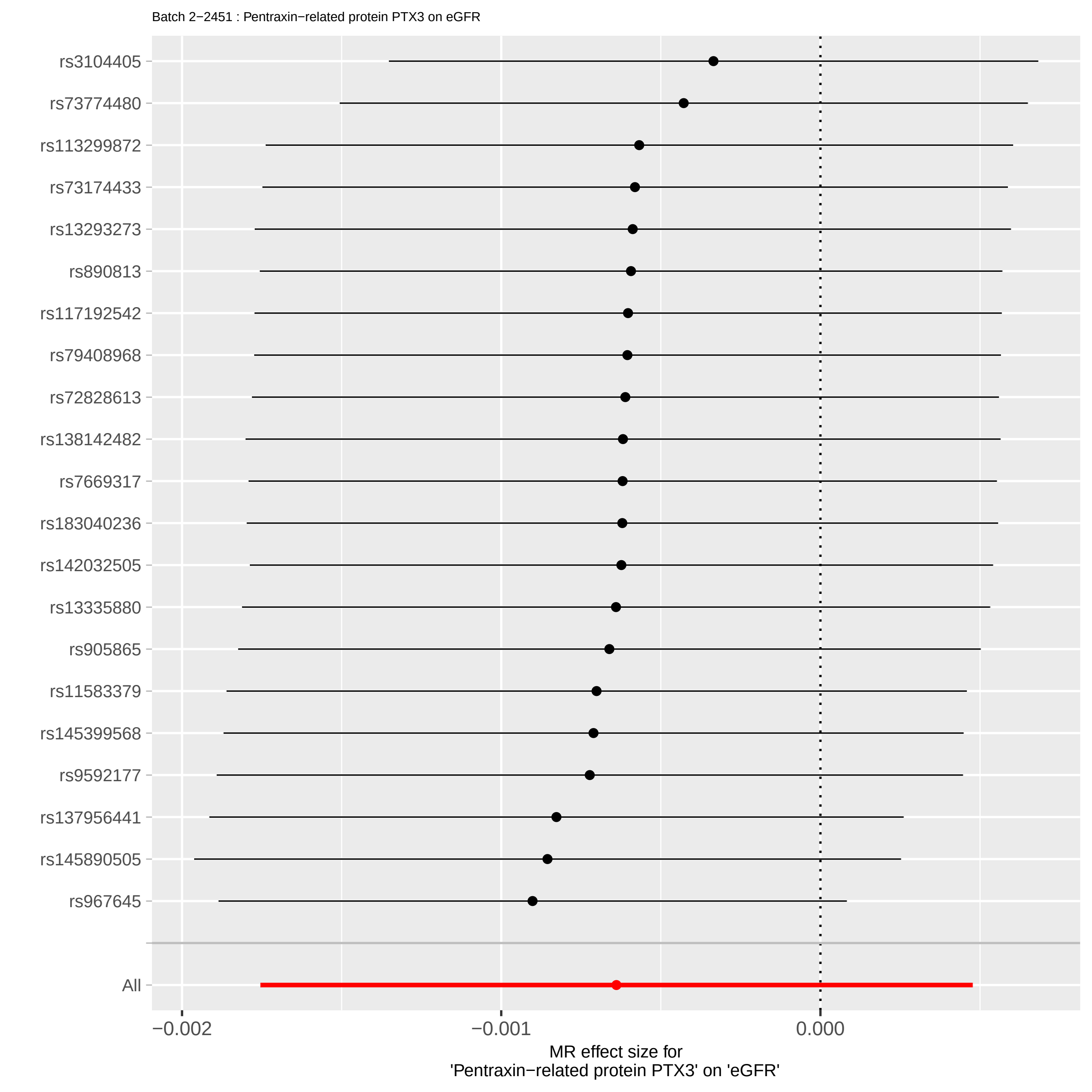

Batch 2−2451 : Pentraxin−related protein PTX3 on eGFR
rs3104405
rs73774480
rs113299872
rs73174433
rs13293273
rs890813
rs117192542
rs79408968
rs72828613
rs138142482
rs7669317
rs183040236
rs142032505
rs13335880
rs905865
rs11583379
rs145399568
rs9592177
rs137956441
rs145890505
rs967645
All
−0.002
−0.001
0.000
MR effect size for
'Pentraxin−related protein PTX3' on 'eGFR'

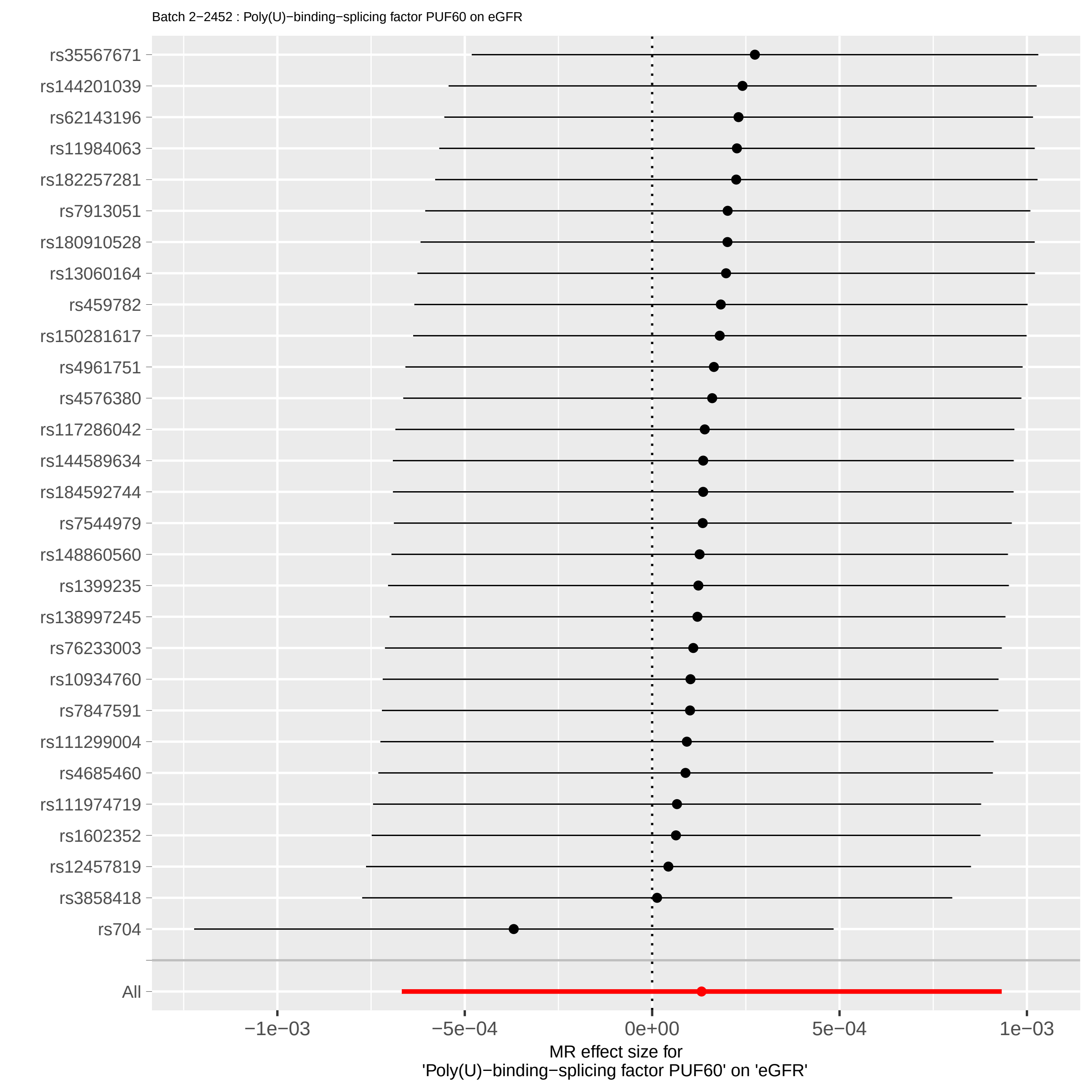

Batch 2−2452 : Poly(U)−binding−splicing factor PUF60 on eGFR
rs35567671
rs144201039
rs62143196
rs11984063
rs182257281
rs7913051
rs180910528
rs13060164
rs459782
rs150281617
rs4961751
rs4576380
rs117286042
rs144589634
rs184592744
rs7544979
rs148860560
rs1399235
rs138997245
rs76233003
rs10934760
rs7847591
rs111299004
rs4685460
rs111974719
rs1602352
rs12457819
rs3858418
rs704
All
−1e−03
−5e−04
0e+00
5e−04
1e−03
MR effect size for
'Poly(U)−binding−splicing factor PUF60' on 'eGFR'

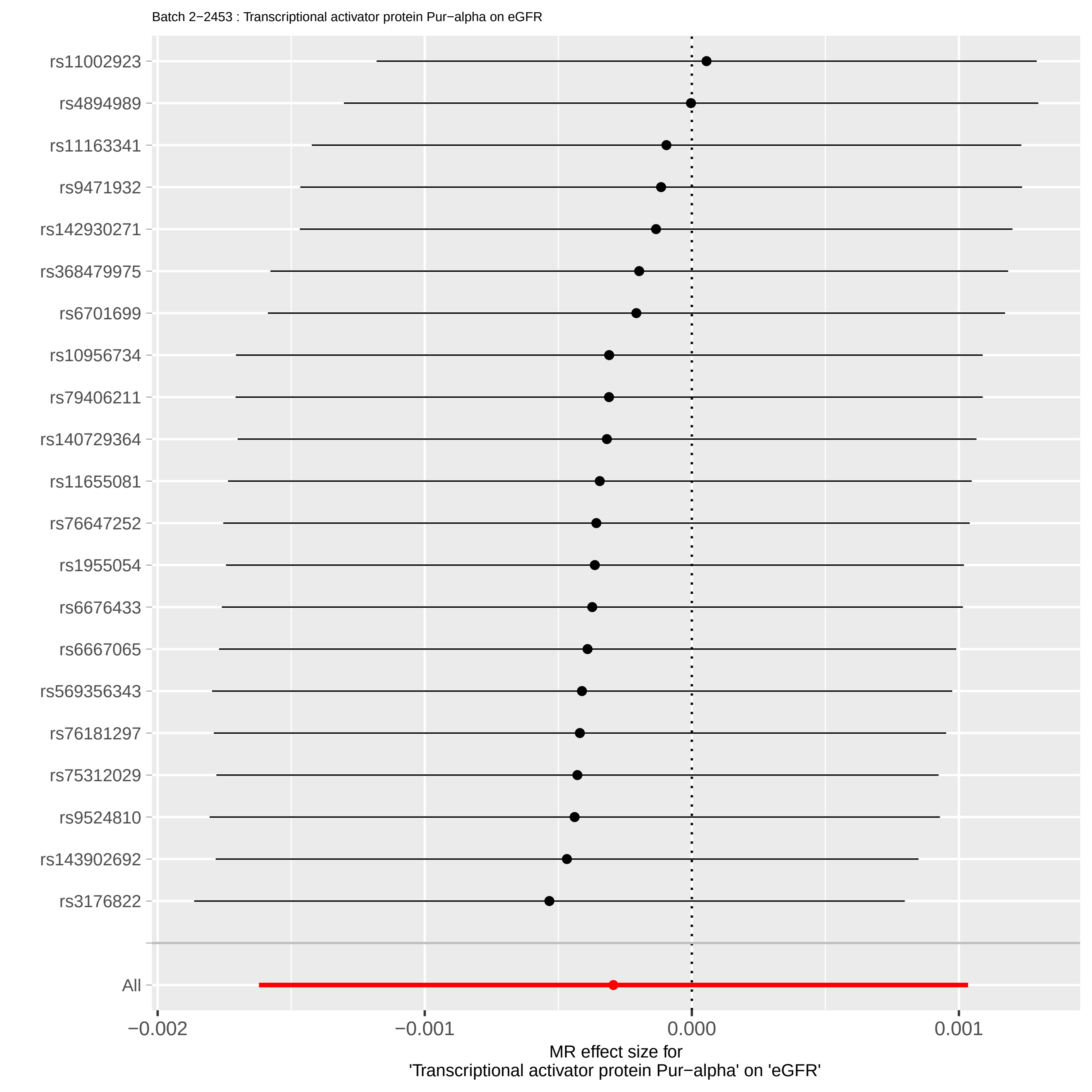

Batch 2−2453 : Transcriptional activator protein Pur−alpha on eGFR
rs11002923
rs4894989
rs11163341
rs9471932
rs142930271
rs368479975
rs6701699
rs10956734
rs79406211
rs140729364
rs11655081
rs76647252
rs1955054
rs6676433
rs6667065
rs569356343
rs76181297
rs75312029
rs9524810
rs143902692
rs3176822
All
−0.002
−0.001
0.000
0.001
MR effect size for
'Transcriptional activator protein Pur−alpha' on 'eGFR'

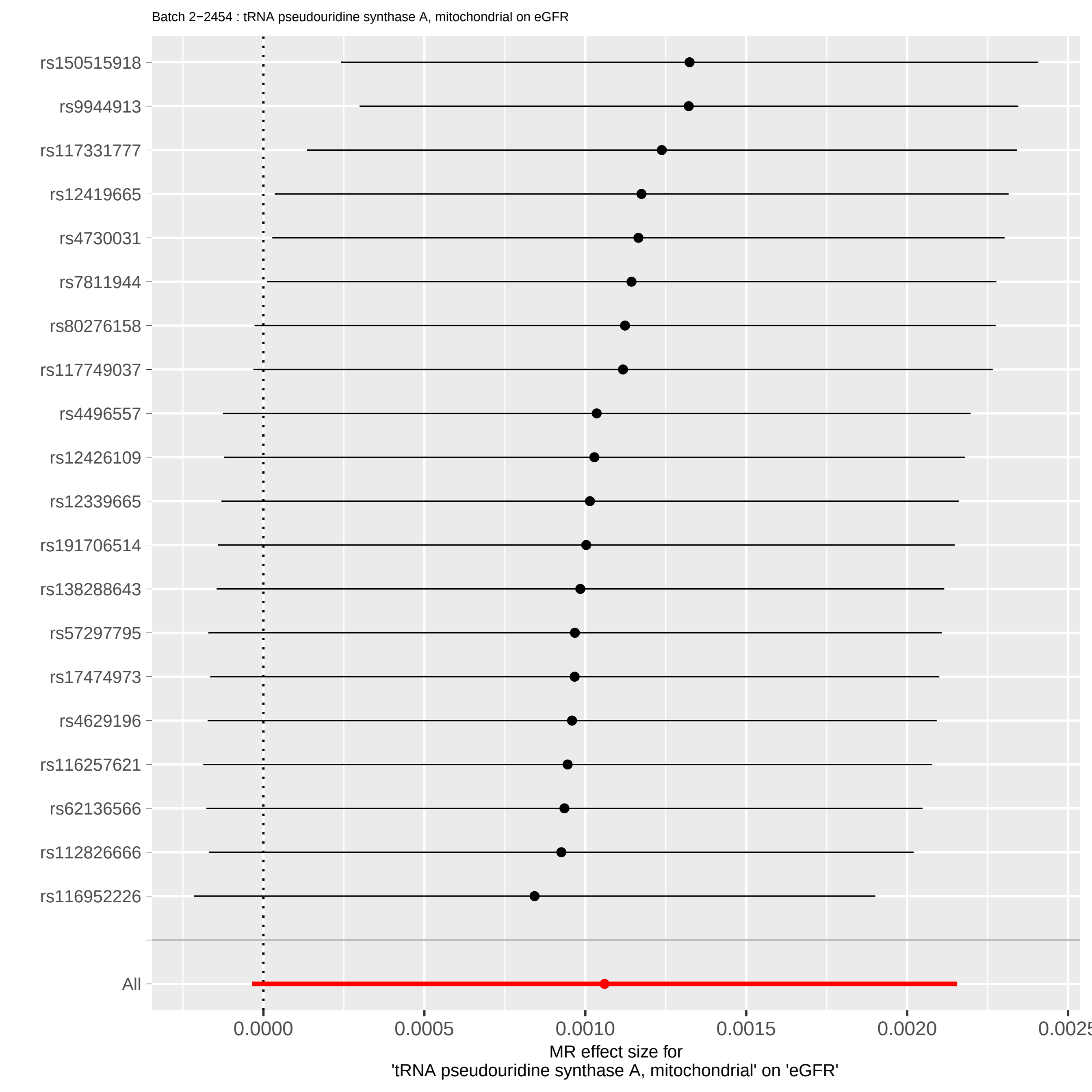
Batch 2−2454 : tRNA pseudouridine synthase A, mitochondrial on eGFR
rs150515918
rs9944913
rs117331777
rs12419665
rs4730031
rs7811944
rs80276158
rs117749037
rs4496557
rs12426109
rs12339665
rs191706514
rs138288643
rs57297795
rs17474973
rs4629196
rs116257621
rs62136566
rs112826666
rs116952226
All
0.0000
0.0005
0.0010
0.0015
0.0020
MR effect size for
'tRNA pseudouridine synthase A, mitochondrial' on 'eGFR'

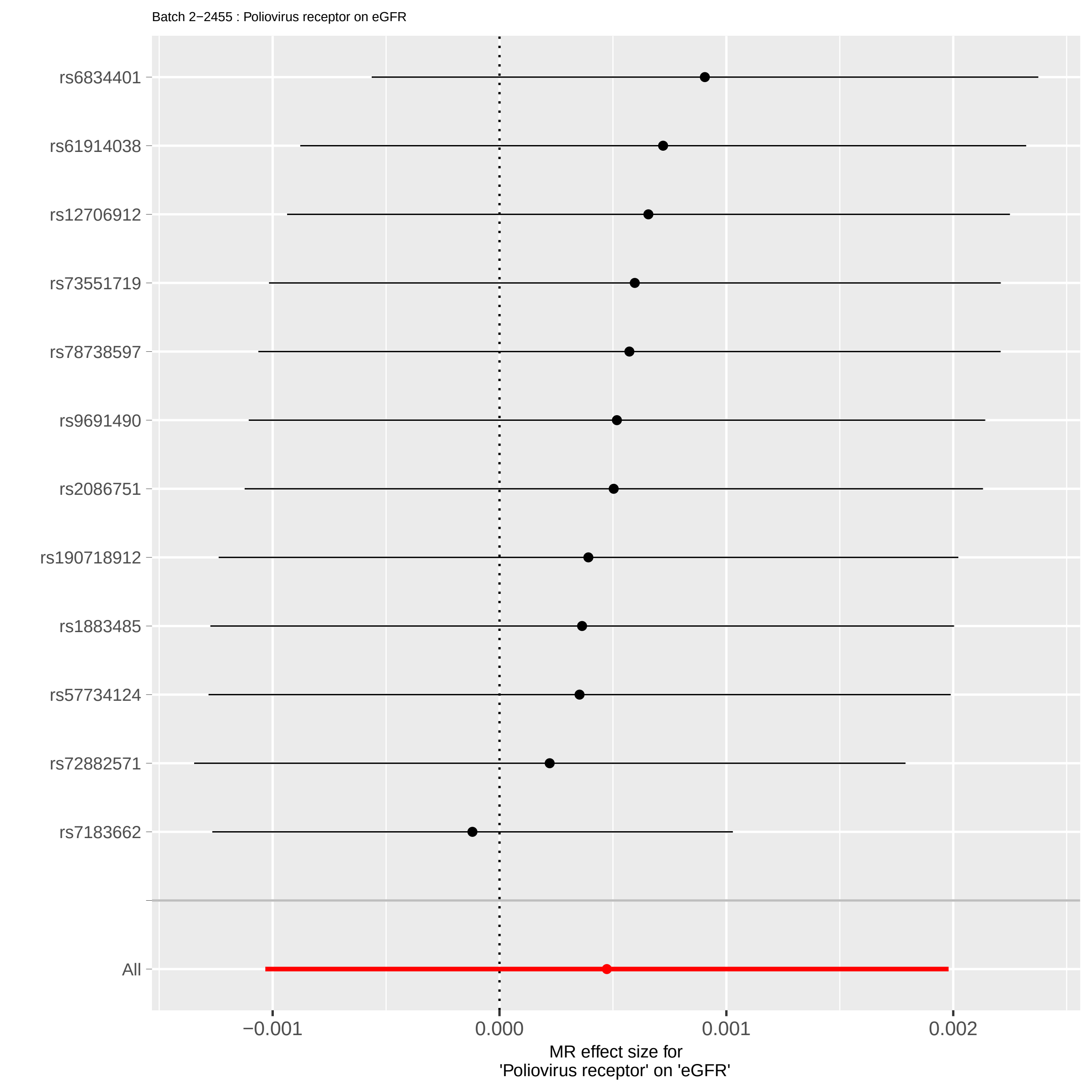
Batch 2−2455 : Poliovirus receptor on eGFR
rs6834401
rs61914038
rs12706912
rs73551719
rs78738597
rs9691490
rs2086751
rs190718912
rs1883485
rs57734124
rs72882571
rs7183662
All
−0.001
0.000
0.001
0.002
MR effect size for
'Poliovirus receptor' on 'eGFR'

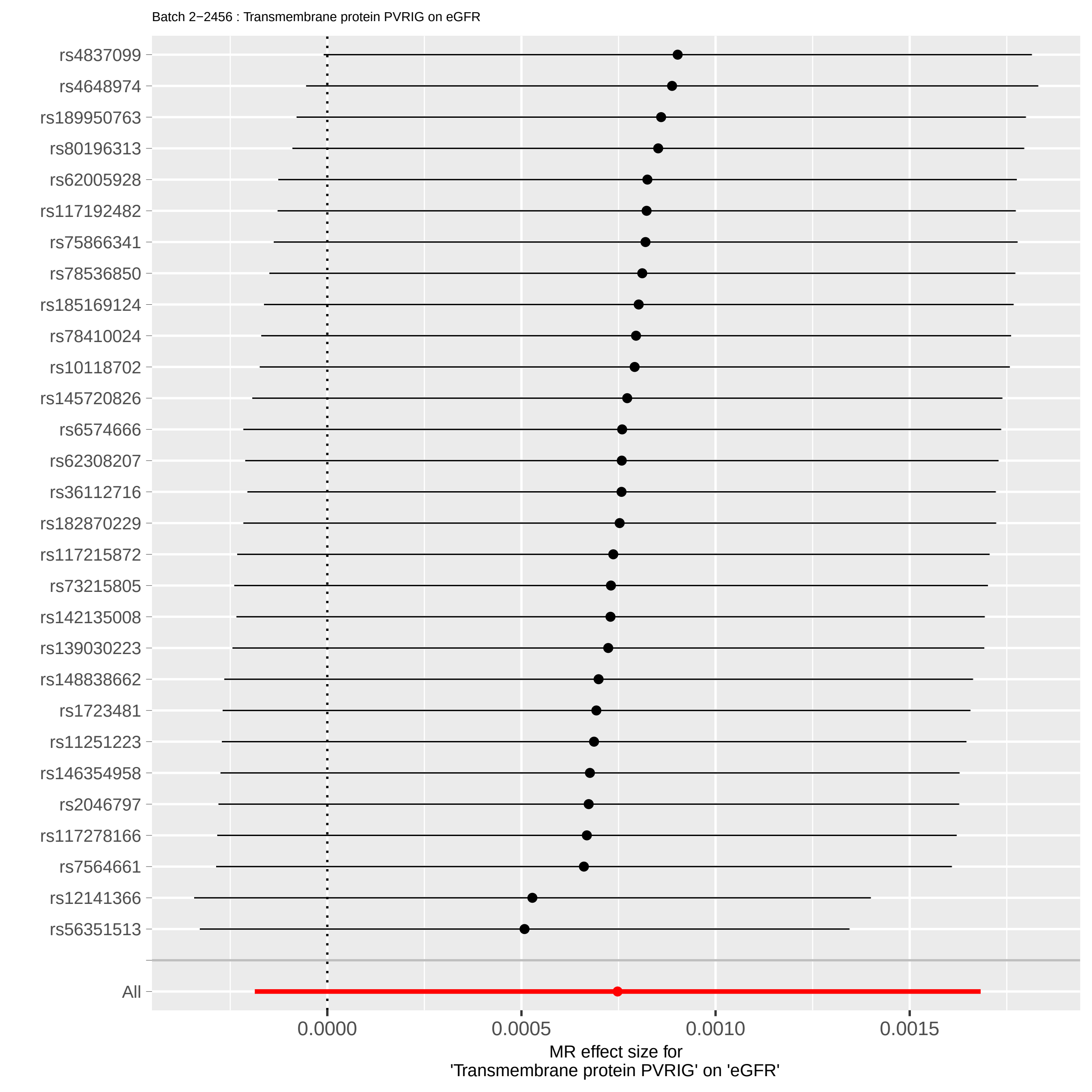

Batch 2−2456 : Transmembrane protein PVRIG on eGFR
rs4837099
rs4648974
rs189950763
rs80196313
rs62005928
rs117192482
rs75866341
rs78536850
rs185169124
rs78410024
rs10118702
rs145720826
rs6574666
rs62308207
rs36112716
rs182870229
rs117215872
rs73215805
rs142135008
rs139030223
rs148838662
rs1723481
rs11251223
rs146354958
rs2046797
rs117278166
rs7564661
rs12141366
rs56351513
All
0.0000
0.0005
0.0010
0.0015
MR effect size for
'Transmembrane protein PVRIG' on 'eGFR'

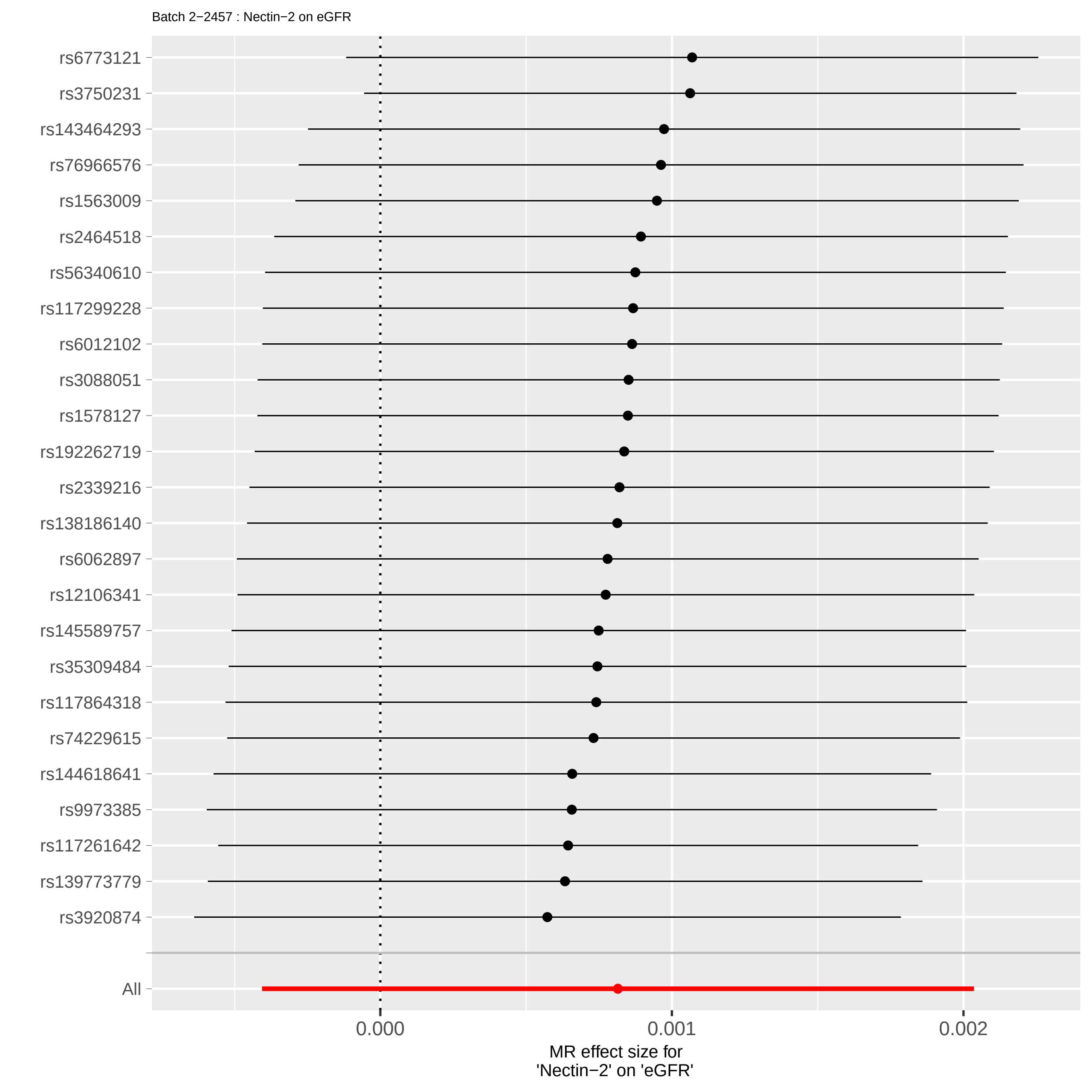

Batch 2−2457 : Nectin−2 on eGFR
rs6773121
rs3750231
rs143464293
rs76966576
rs1563009
rs2464518
rs56340610
rs117299228
rs6012102
rs3088051
rs1578127
rs192262719
rs2339216
rs138186140
rs6062897
rs12106341
rs145589757
rs35309484
rs117864318
rs74229615
rs144618641
rs9973385
rs117261642
rs139773779
rs3920874
All
0.000
0.001
0.002
MR effect size for
'Nectin−2' on 'eGFR'

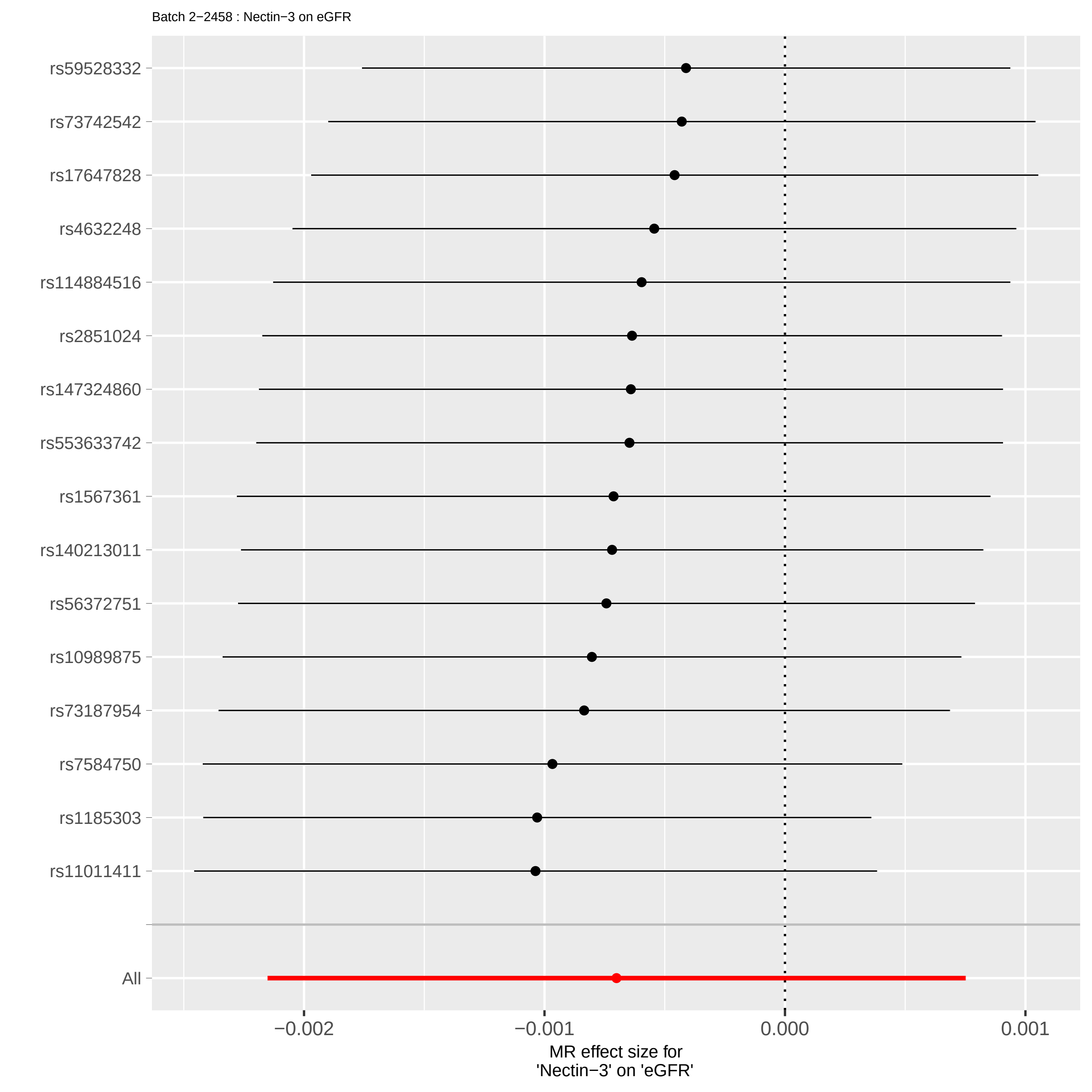

Batch 2−2458 : Nectin−3 on eGFR
rs59528332
rs73742542
rs17647828
rs4632248
rs114884516
rs2851024
rs147324860
rs553633742
rs1567361
rs140213011
rs56372751
rs10989875
rs73187954
rs7584750
rs1185303
rs11011411
All
−0.002
−0.001
0.000
0.001
MR effect size for
'Nectin−3' on 'eGFR'

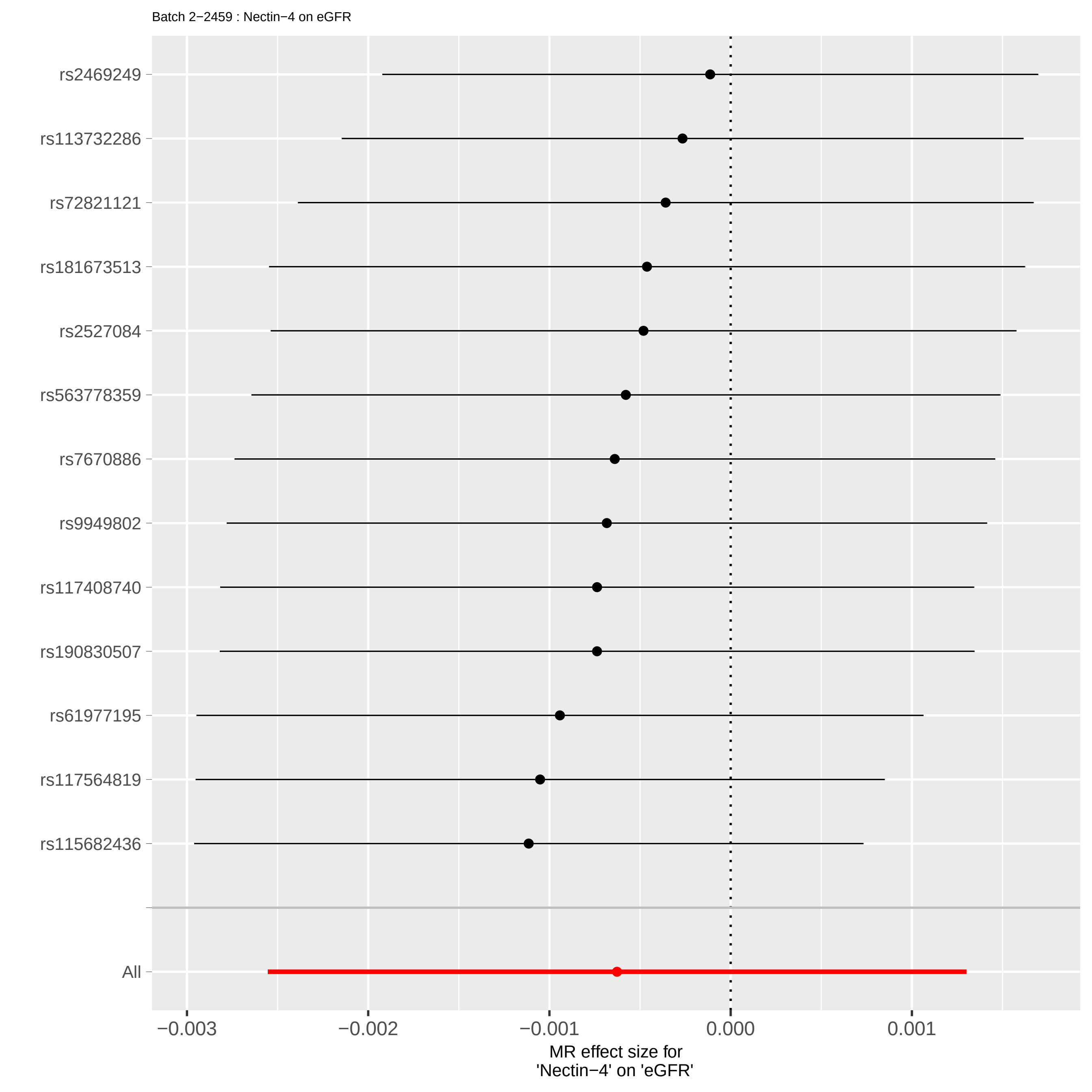
Batch 2−2459 : Nectin−4 on eGFR
rs2469249
rs113732286
rs72821121
rs181673513
rs2527084
rs563778359
rs7670886
rs9949802
rs117408740
rs190830507
rs61977195
rs117564819
rs115682436
All
−0.003
−0.002
−0.001
0.000
0.001
MR effect size for
'Nectin−4' on 'eGFR'

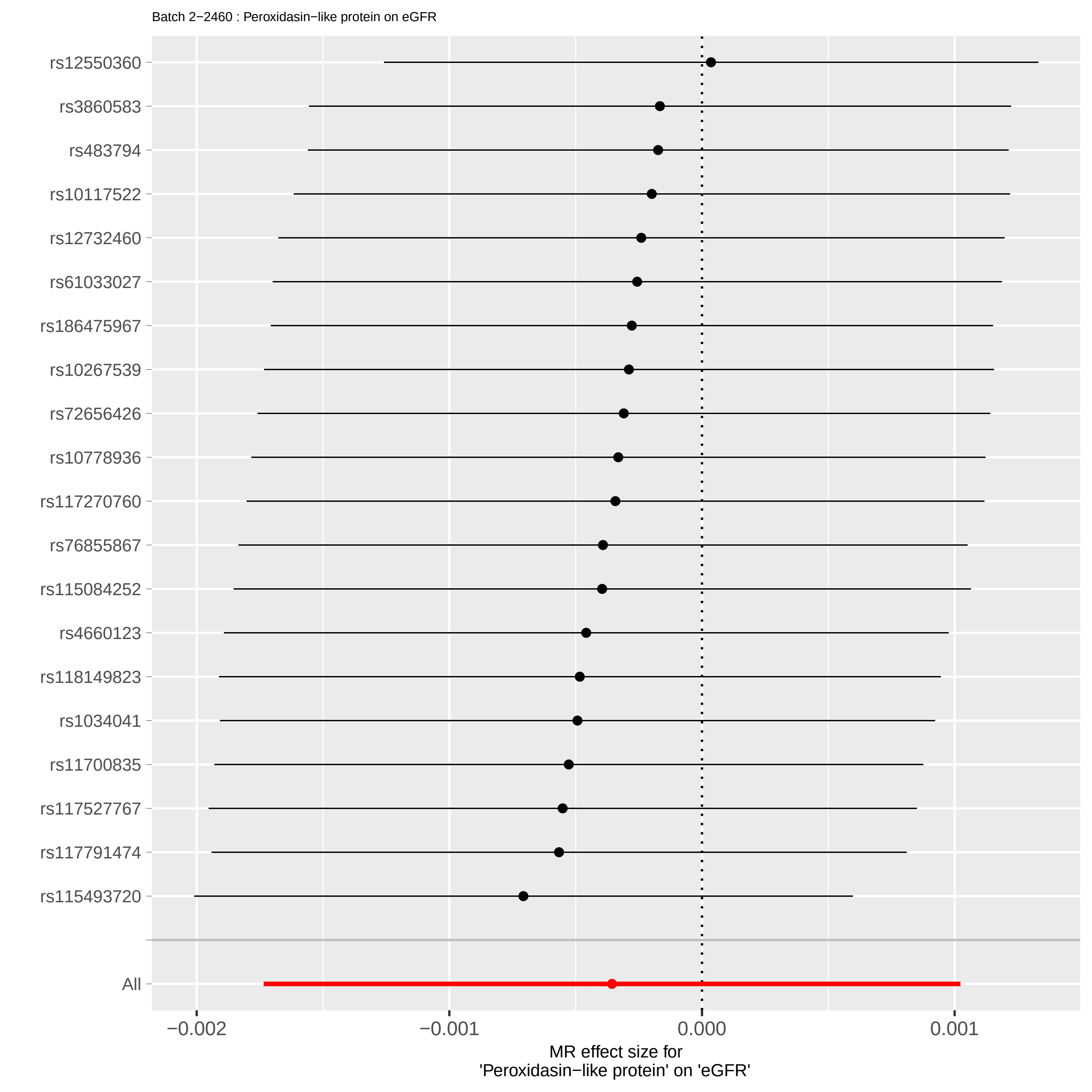
Batch 2−2460 : Peroxidasin−like protein on eGFR
rs12550360
rs3860583
rs483794
rs10117522
rs12732460
rs61033027
rs186475967
rs10267539
rs72656426
rs10778936
rs117270760
rs76855867
rs115084252
rs4660123
rs118149823
rs1034041
rs11700835
rs117527767
rs117791474
rs115493720
All
−0.002
−0.001
0.000
0.001
MR effect size for
'Peroxidasin−like protein' on 'eGFR'

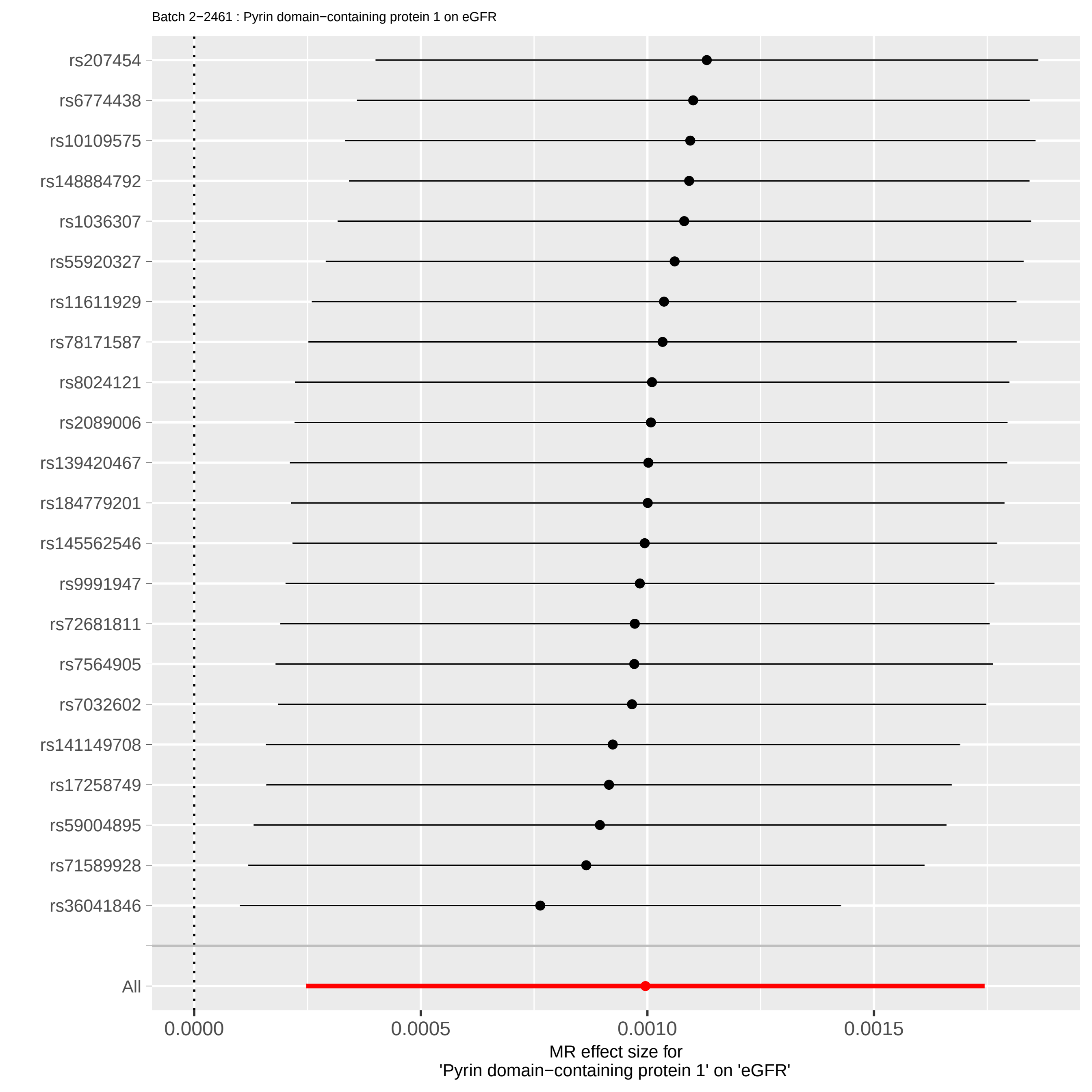

Batch 2−2461 : Pyrin domain−containing protein 1 on eGFR
rs207454
rs6774438
rs10109575
rs148884792
rs1036307
rs55920327
rs11611929
rs78171587
rs8024121
rs2089006
rs139420467
rs184779201
rs145562546
rs9991947
rs72681811
rs7564905
rs7032602
rs141149708
rs17258749
rs59004895
rs71589928
rs36041846
All
0.0000
0.0005
0.0010
0.0015
MR effect size for
'Pyrin domain−containing protein 1' on 'eGFR'

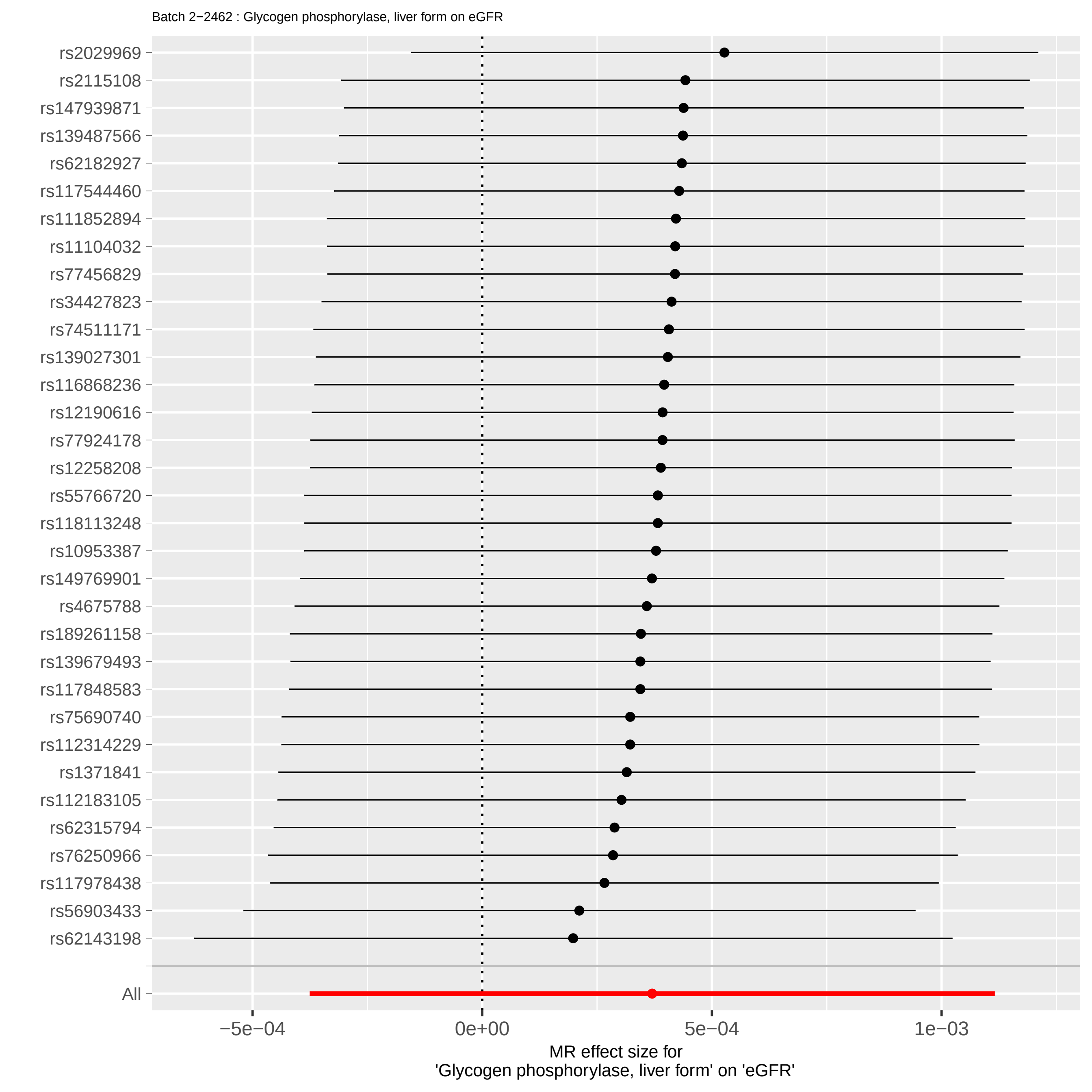

Batch 2−2462 : Glycogen phosphorylase, liver form on eGFR
rs2029969
rs2115108
rs147939871
rs139487566
rs62182927
rs117544460
rs111852894
rs11104032
rs77456829
rs34427823
rs74511171
rs139027301
rs116868236
rs12190616
rs77924178
rs12258208
rs55766720
rs118113248
rs10953387
rs149769901
rs4675788
rs189261158
rs139679493
rs117848583
rs75690740
rs112314229
rs1371841
rs112183105
rs62315794
rs76250966
rs117978438
rs56903433
rs62143198
All
−5e−04
0e+00
5e−04
1e−03
MR effect size for
'Glycogen phosphorylase, liver form' on 'eGFR'

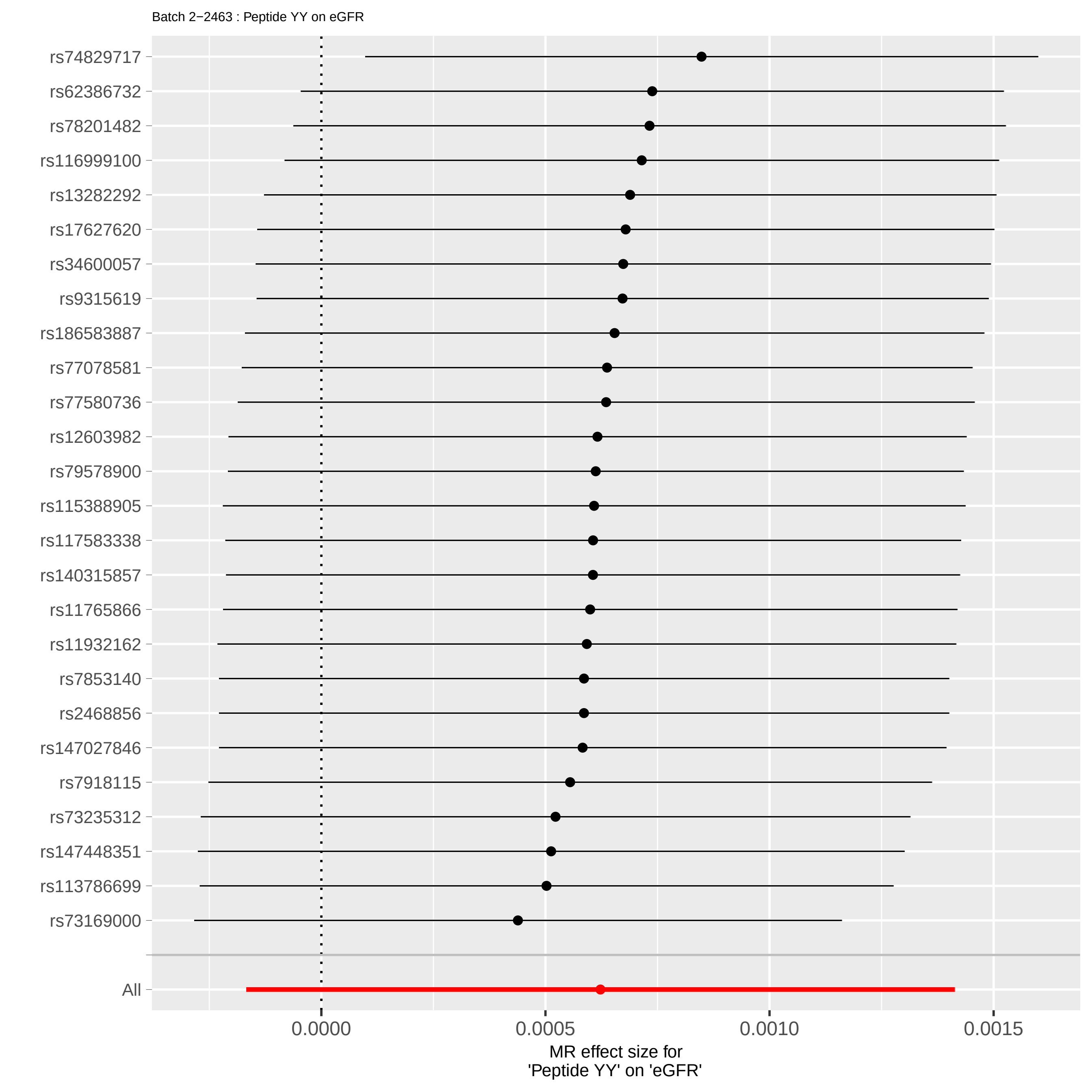

Batch 2−2463 : Peptide YY on eGFR
rs74829717
rs62386732
rs78201482
rs116999100
rs13282292
rs17627620
rs34600057
rs9315619
rs186583887
rs77078581
rs77580736
rs12603982
rs79578900
rs115388905
rs117583338
rs140315857
rs11765866
rs11932162
rs7853140
rs2468856
rs147027846
rs7918115
rs73235312
rs147448351
rs113786699
rs73169000
All
0.0000
0.0005
0.0010
0.0015
MR effect size for
'Peptide YY' on 'eGFR'

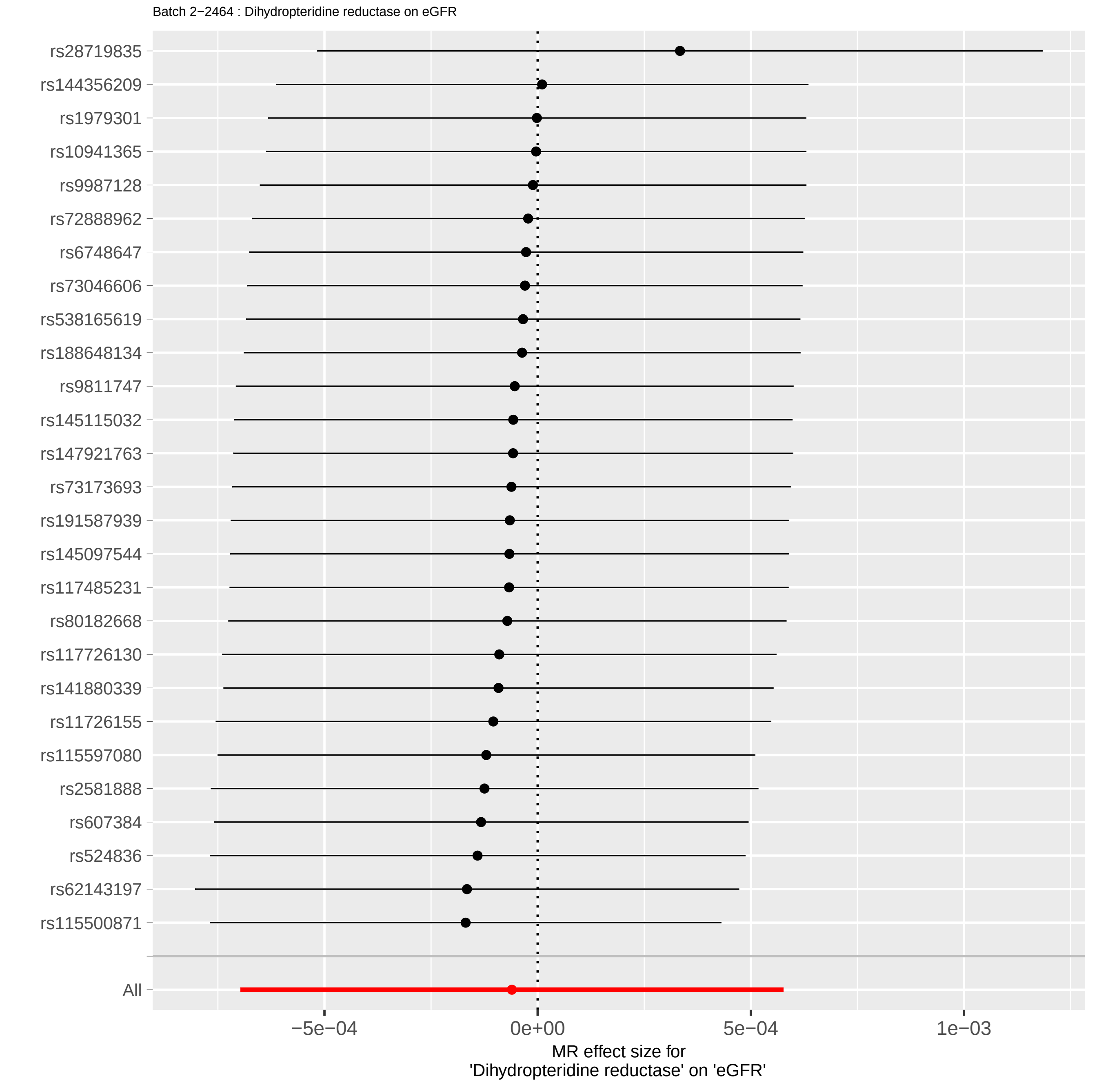

Batch 2−2464 : Dihydropteridine reductase on eGFR
rs28719835
rs144356209
rs1979301
rs10941365
rs9987128
rs72888962
rs6748647
rs73046606
rs538165619
rs188648134
rs9811747
rs145115032
rs147921763
rs73173693
rs191587939
rs145097544
rs117485231
rs80182668
rs117726130
rs141880339
rs11726155
rs115597080
rs2581888
rs607384
rs524836
rs62143197
rs115500871
All
−5e−04
0e+00
5e−04
1e−03
MR effect size for
'Dihydropteridine reductase' on 'eGFR'

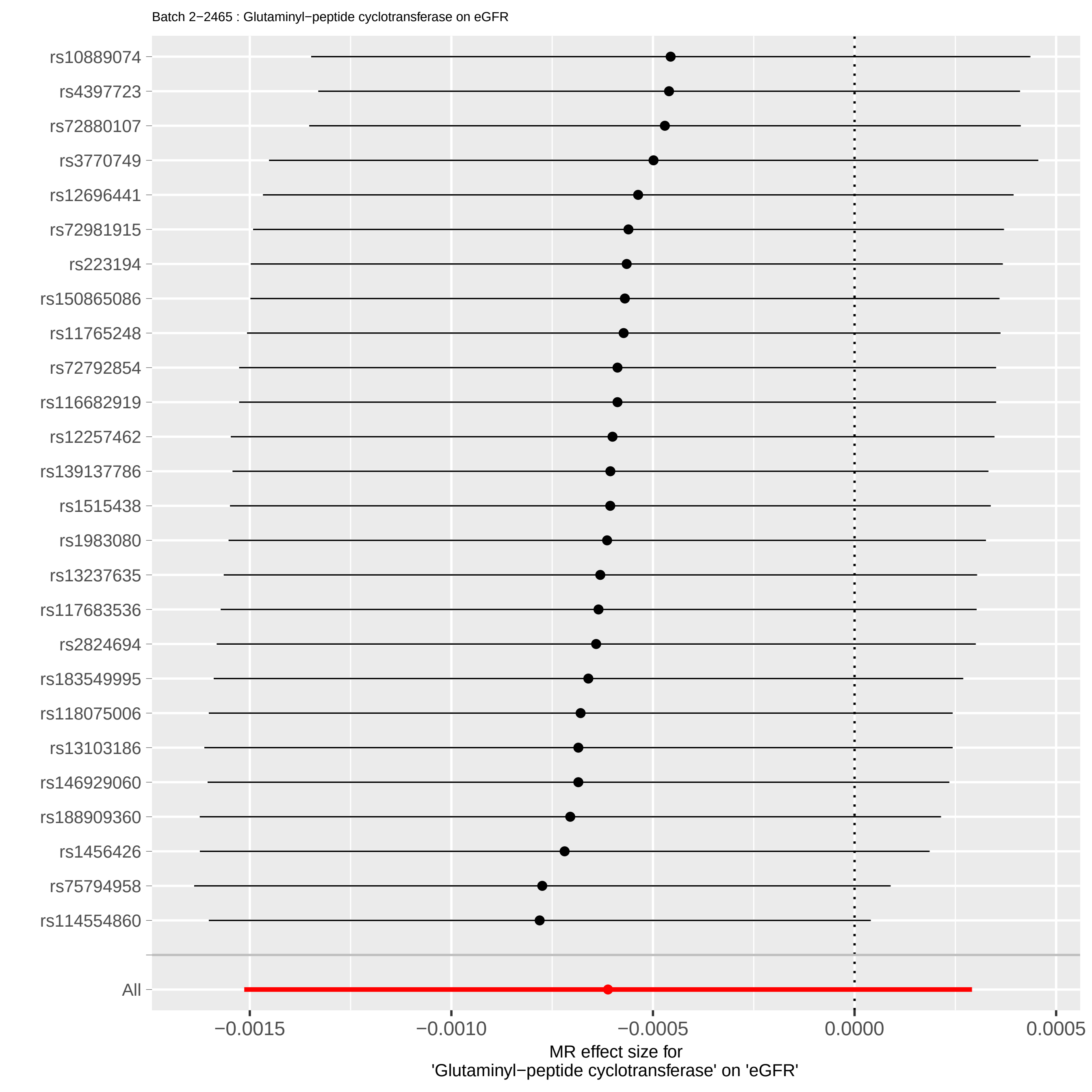
Batch 2−2465 : Glutaminyl−peptide cyclotransferase on eGFR
rs10889074
rs4397723
rs72880107
rs3770749
rs12696441
rs72981915
rs223194
rs150865086
rs11765248
rs72792854
rs116682919
rs12257462
rs139137786
rs1515438
rs1983080
rs13237635
rs117683536
rs2824694
rs183549995
rs118075006
rs13103186
rs146929060
rs188909360
rs1456426
rs75794958
rs114554860
All
−0.0015
−0.0010
−0.0005
0.0000
0.0005
MR effect size for
'Glutaminyl−peptide cyclotransferase' on 'eGFR'

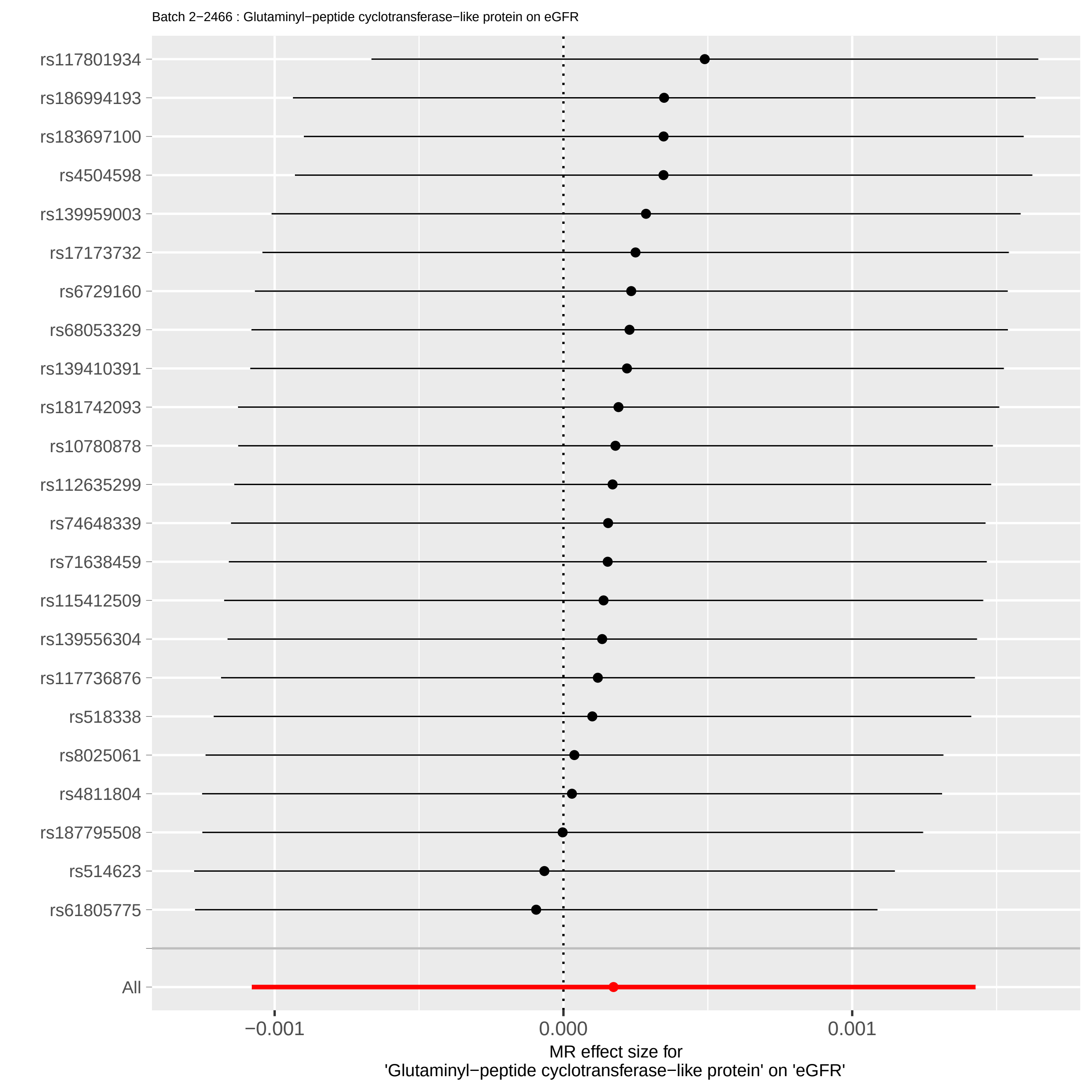
Batch 2−2466 : Glutaminyl−peptide cyclotransferase−like protein on eGFR
rs117801934
rs186994193
rs183697100
rs4504598
rs139959003
rs17173732
rs6729160
rs68053329
rs139410391
rs181742093
rs10780878
rs112635299
rs74648339
rs71638459
rs115412509
rs139556304
rs117736876
rs518338
rs8025061
rs4811804
rs187795508
rs514623
rs61805775
All
−0.001
0.000
0.001
MR effect size for
'Glutaminyl−peptide cyclotransferase−like protein' on 'eGFR'

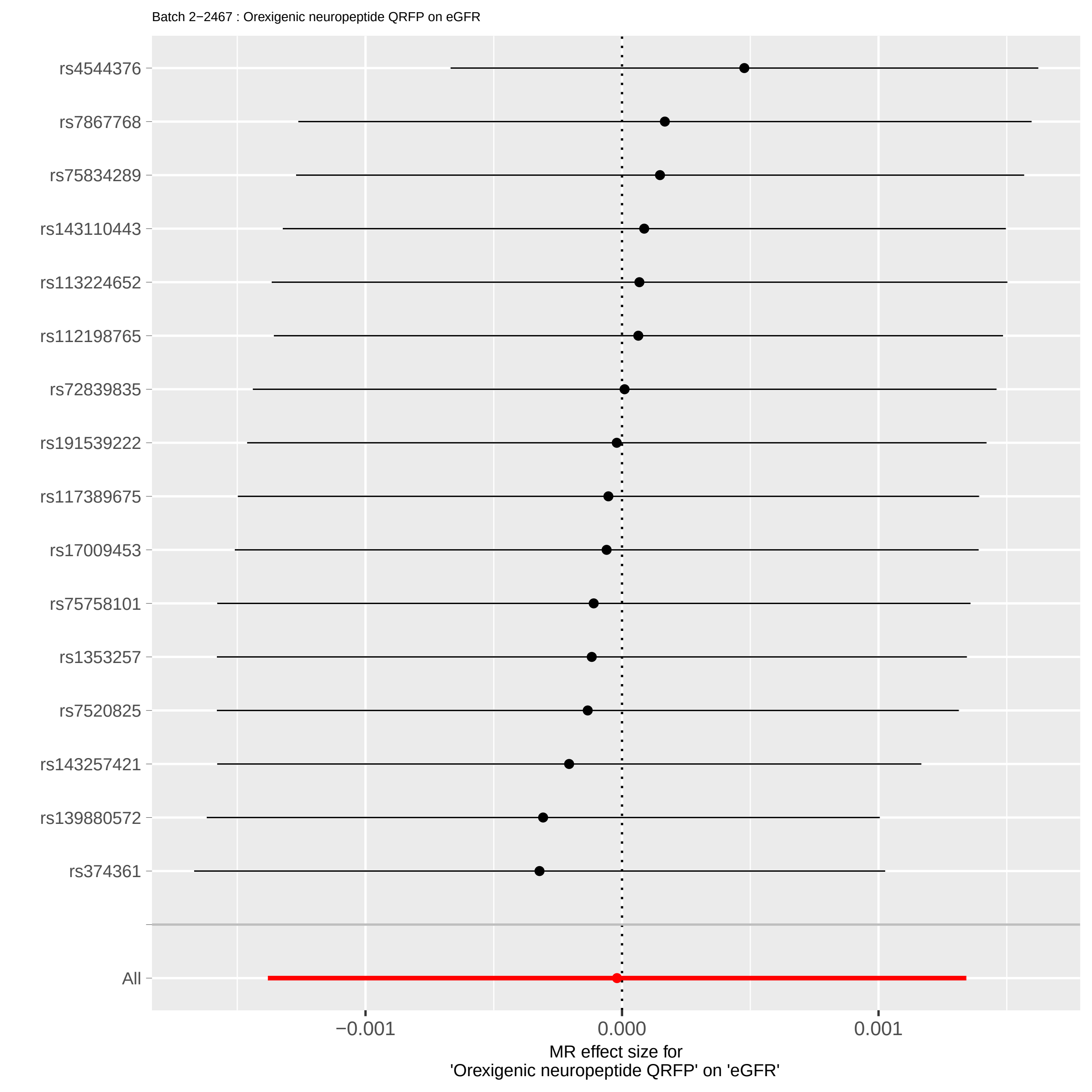

Batch 2−2467 : Orexigenic neuropeptide QRFP on eGFR
rs4544376
rs7867768
rs75834289
rs143110443
rs113224652
rs112198765
rs72839835
rs191539222
rs117389675
rs17009453
rs75758101
rs1353257
rs7520825
rs143257421
rs139880572
rs374361
All
−0.001
0.000
0.001
MR effect size for
'Orexigenic neuropeptide QRFP' on 'eGFR'

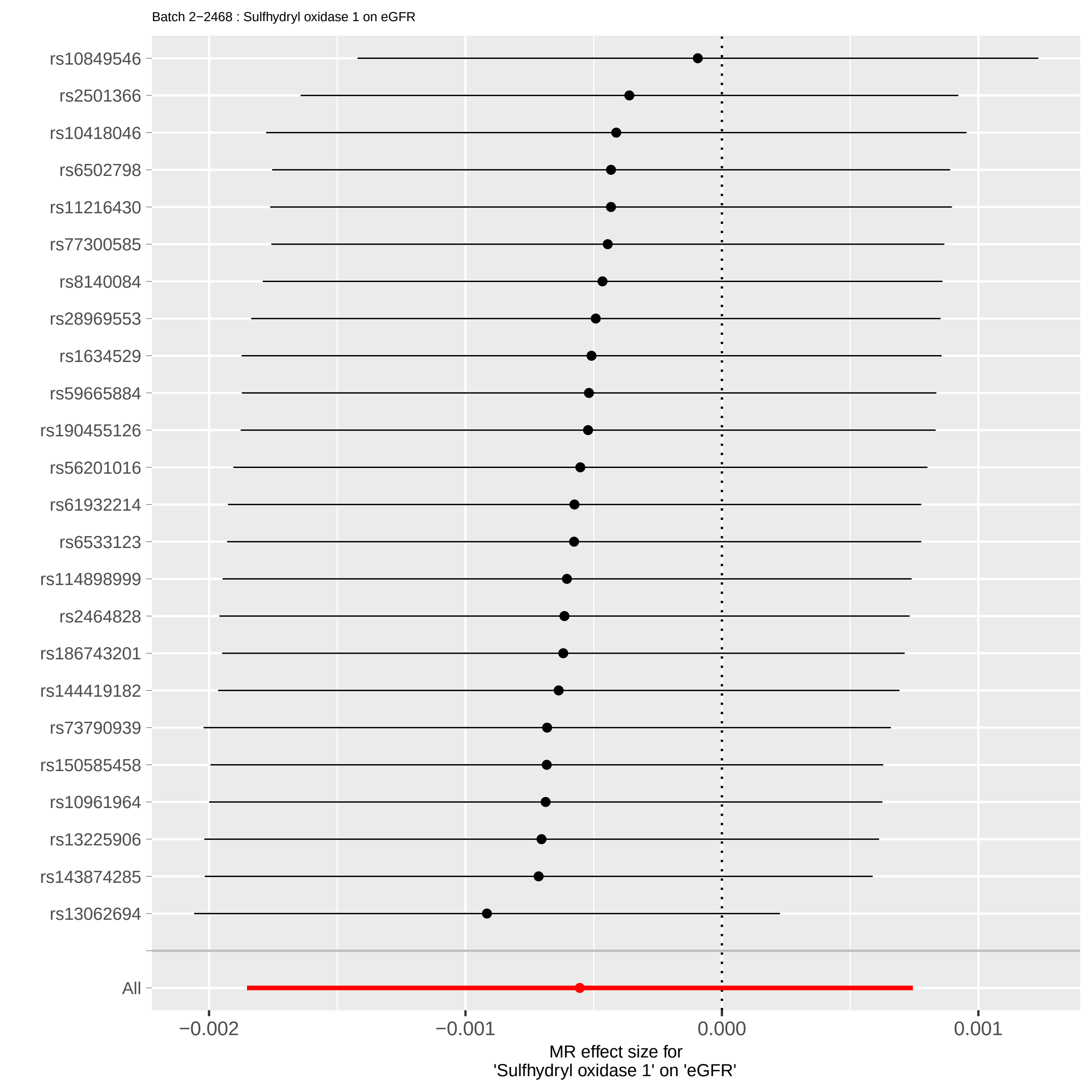
Batch 2−2468 : Sulfhydryl oxidase 1 on eGFR
rs10849546
rs2501366
rs10418046
rs6502798
rs11216430
rs77300585
rs8140084
rs28969553
rs1634529
rs59665884
rs190455126
rs56201016
rs61932214
rs6533123
rs114898999
rs2464828
rs186743201
rs144419182
rs73790939
rs150585458
rs10961964
rs13225906
rs143874285
rs13062694
All
−0.002
−0.001
0.000
0.001
MR effect size for
'Sulfhydryl oxidase 1' on 'eGFR'

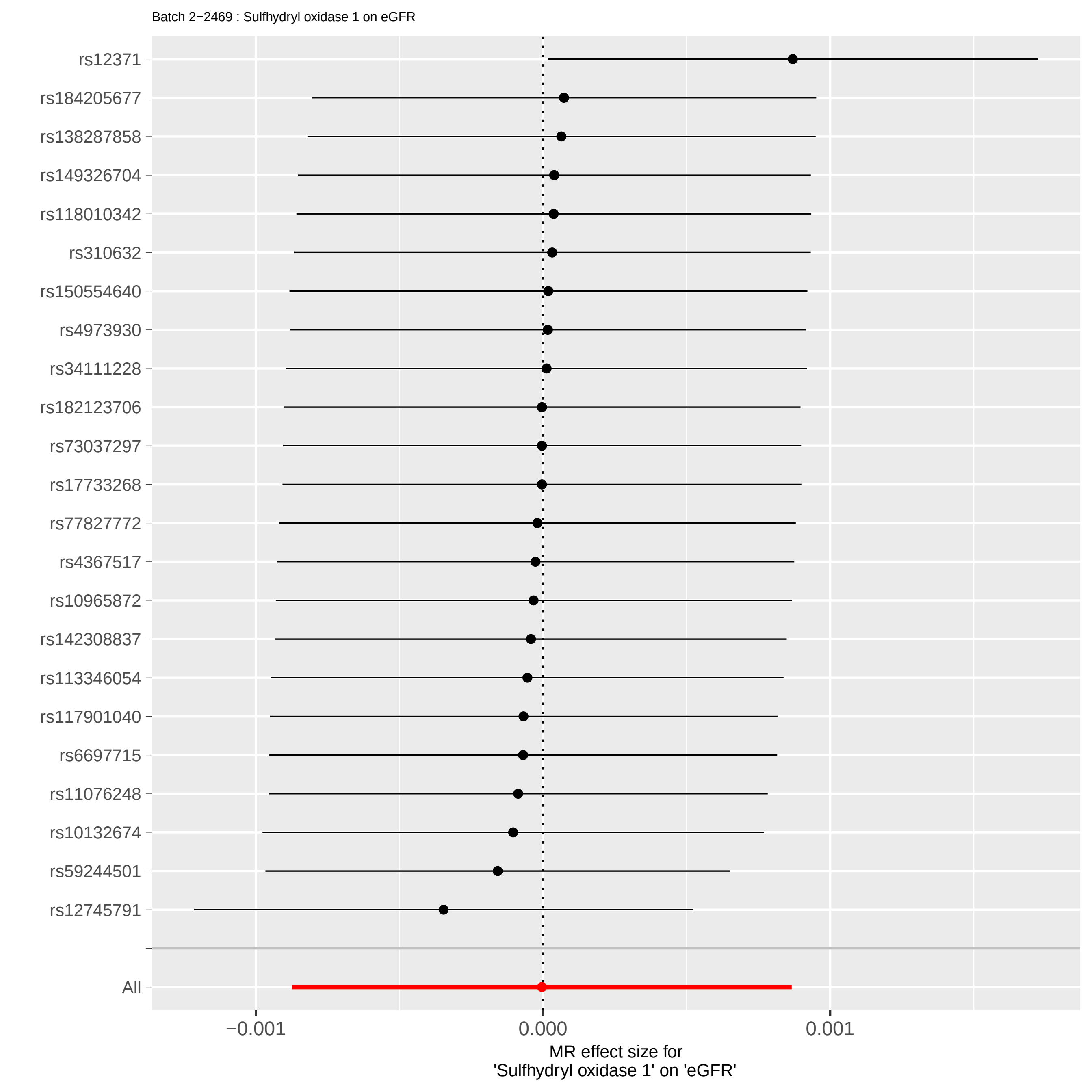

Batch 2−2469 : Sulfhydryl oxidase 1 on eGFR
rs12371
rs184205677
rs138287858
rs149326704
rs118010342
rs310632
rs150554640
rs4973930
rs34111228
rs182123706
rs73037297
rs17733268
rs77827772
rs4367517
rs10965872
rs142308837
rs113346054
rs117901040
rs6697715
rs11076248
rs10132674
rs59244501
rs12745791
All
−0.001
0.000
0.001
MR effect size for
'Sulfhydryl oxidase 1' on 'eGFR'

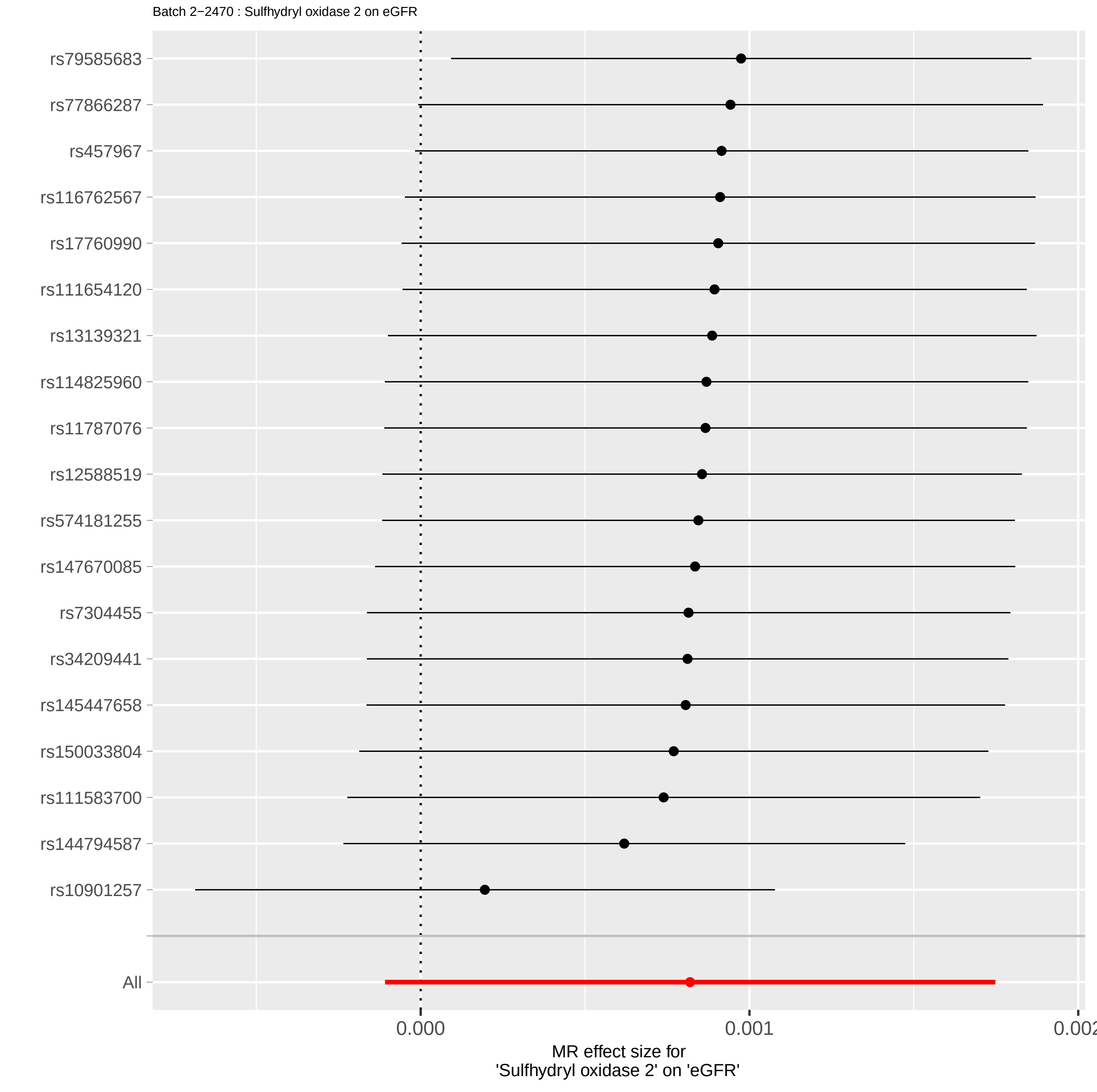

Batch 2−2470 : Sulfhydryl oxidase 2 on eGFR
rs79585683
rs77866287
rs457967
rs116762567
rs17760990
rs111654120
rs13139321
rs114825960
rs11787076
rs12588519
rs574181255
rs147670085
rs7304455
rs34209441
rs145447658
rs150033804
rs111583700
rs144794587
rs10901257
All
0.000
0.001
0.002
MR effect size for
'Sulfhydryl oxidase 2' on 'eGFR'

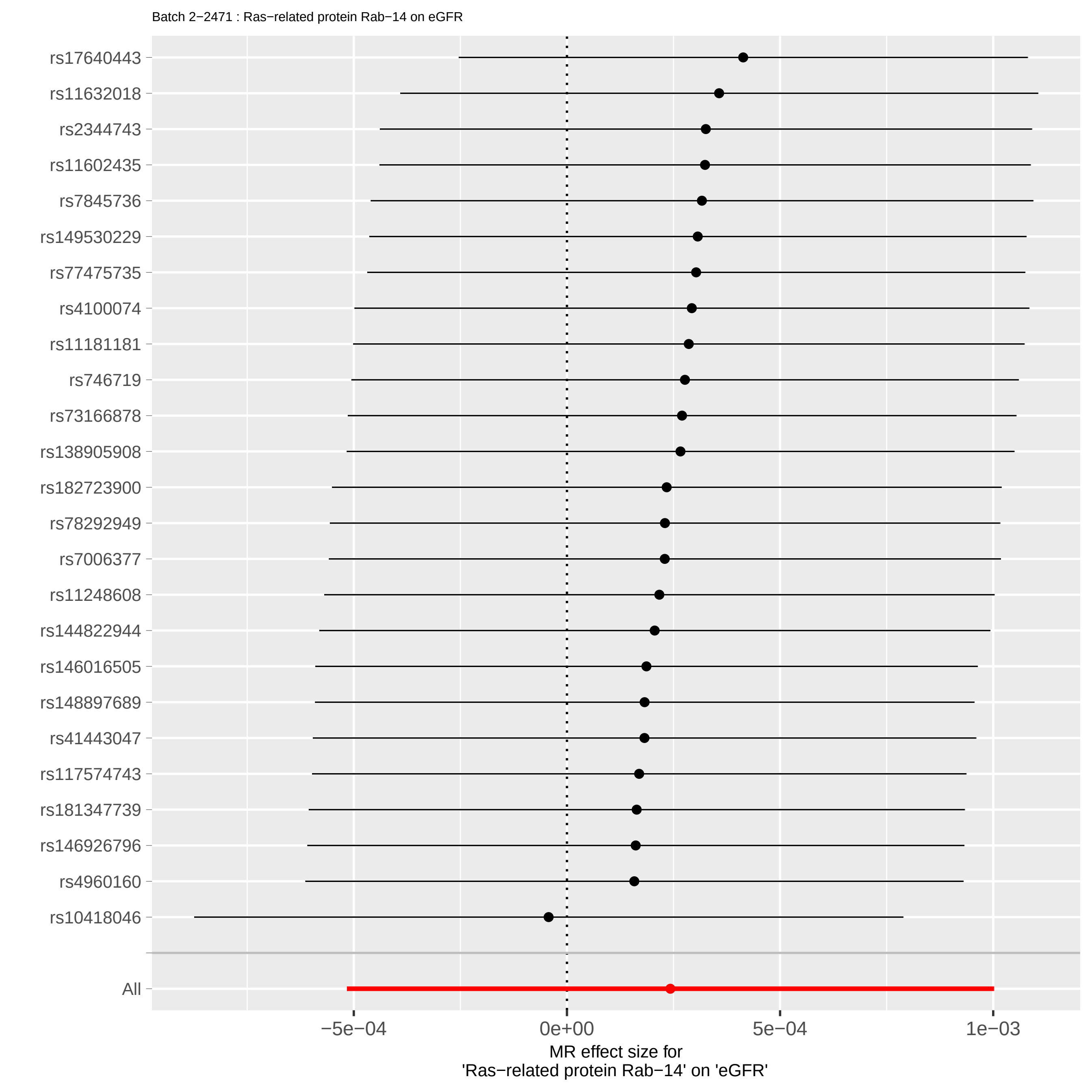

Batch 2−2471 : Ras−related protein Rab−14 on eGFR
rs17640443
rs11632018
rs2344743
rs11602435
rs7845736
rs149530229
rs77475735
rs4100074
rs11181181
rs746719
rs73166878
rs138905908
rs182723900
rs78292949
rs7006377
rs11248608
rs144822944
rs146016505
rs148897689
rs41443047
rs117574743
rs181347739
rs146926796
rs4960160
rs10418046
All
−5e−04
0e+00
5e−04
1e−03
MR effect size for
'Ras−related protein Rab−14' on 'eGFR'

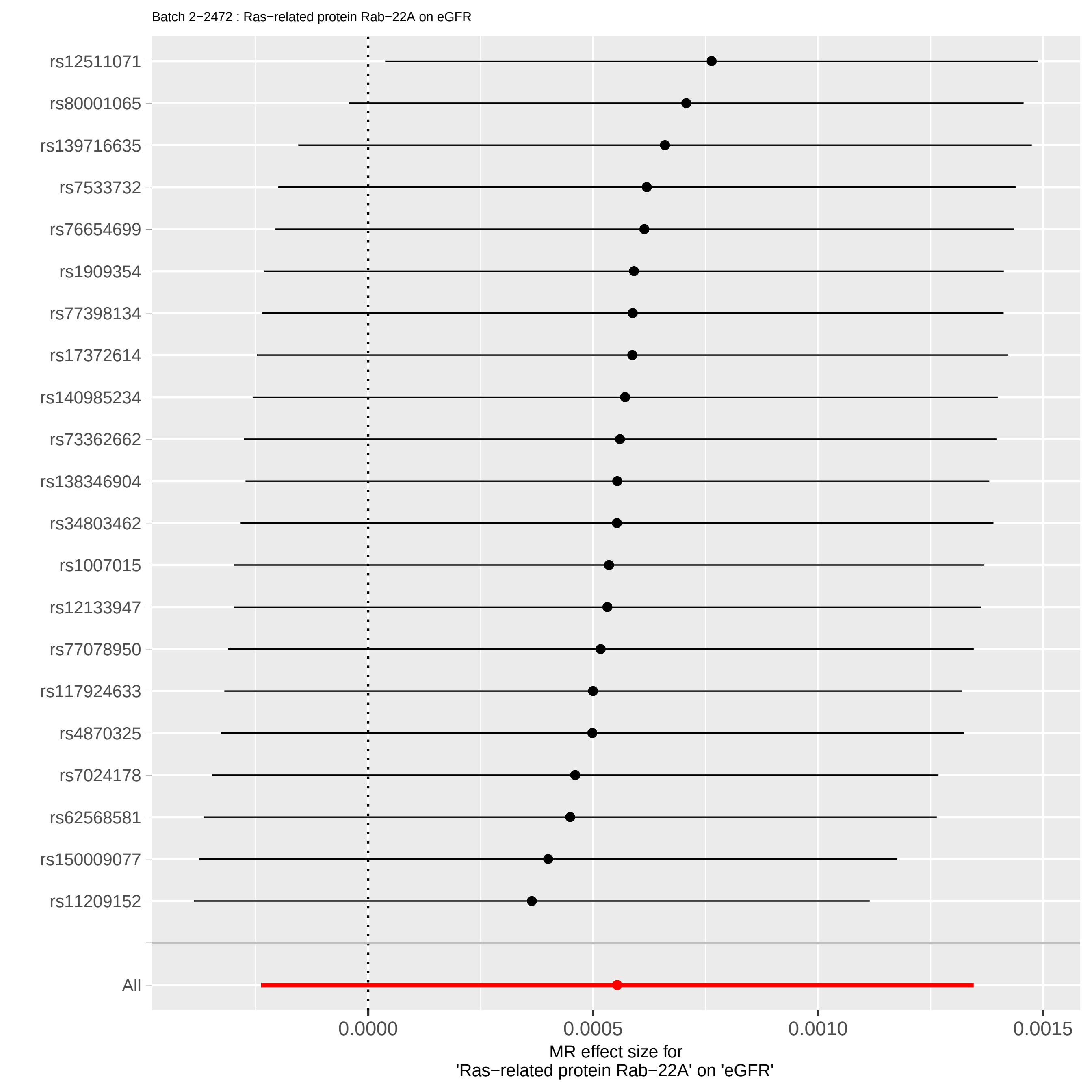

Batch 2−2472 : Ras−related protein Rab−22A on eGFR
rs12511071
rs80001065
rs139716635
rs7533732
rs76654699
rs1909354
rs77398134
rs17372614
rs140985234
rs73362662
rs138346904
rs34803462
rs1007015
rs12133947
rs77078950
rs117924633
rs4870325
rs7024178
rs62568581
rs150009077
rs11209152
All
0.0000
0.0005
0.0010
0.0015
MR effect size for
'Ras−related protein Rab−22A' on 'eGFR'

Batch 2−2473 : Ras−related protein Rab−26 on eGFR

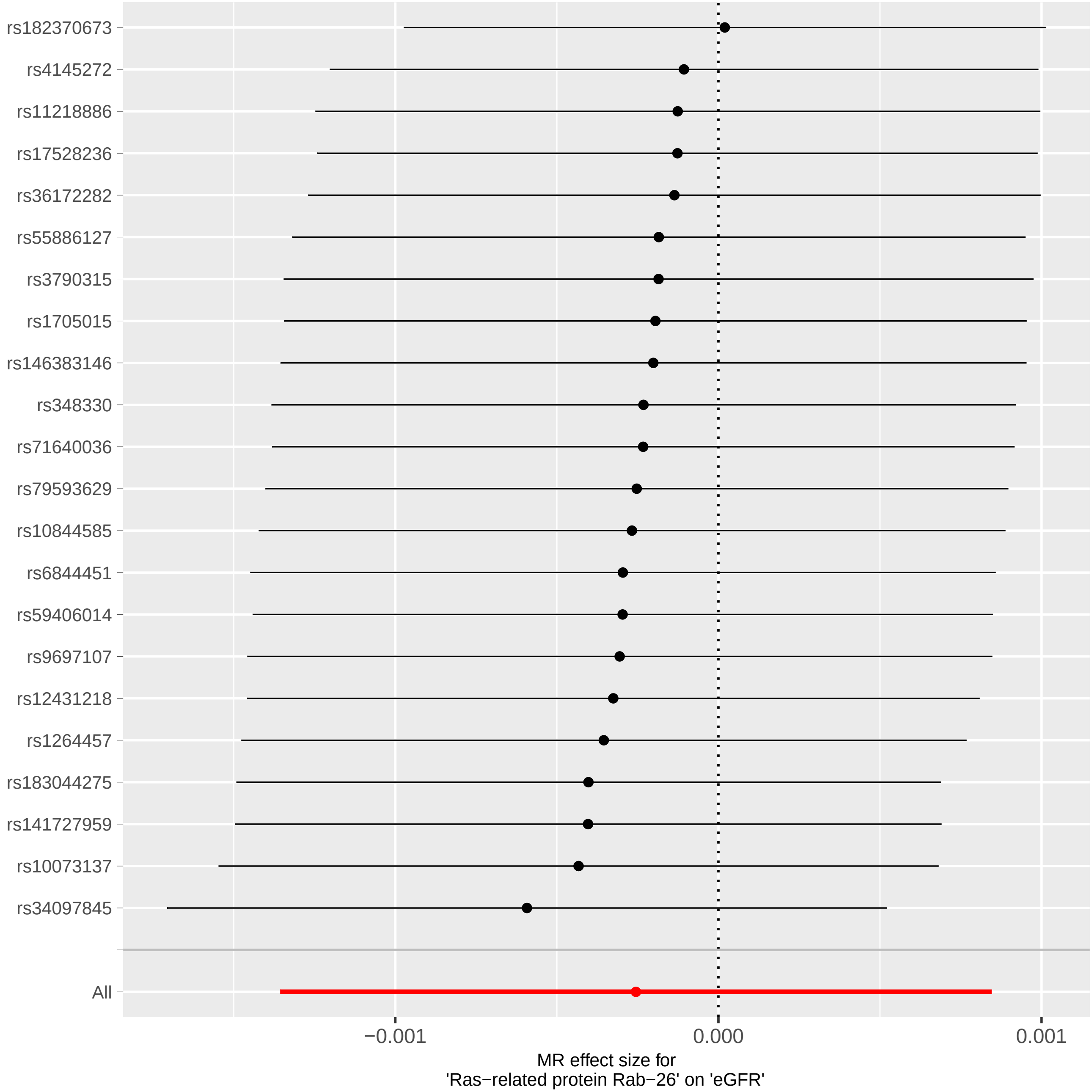

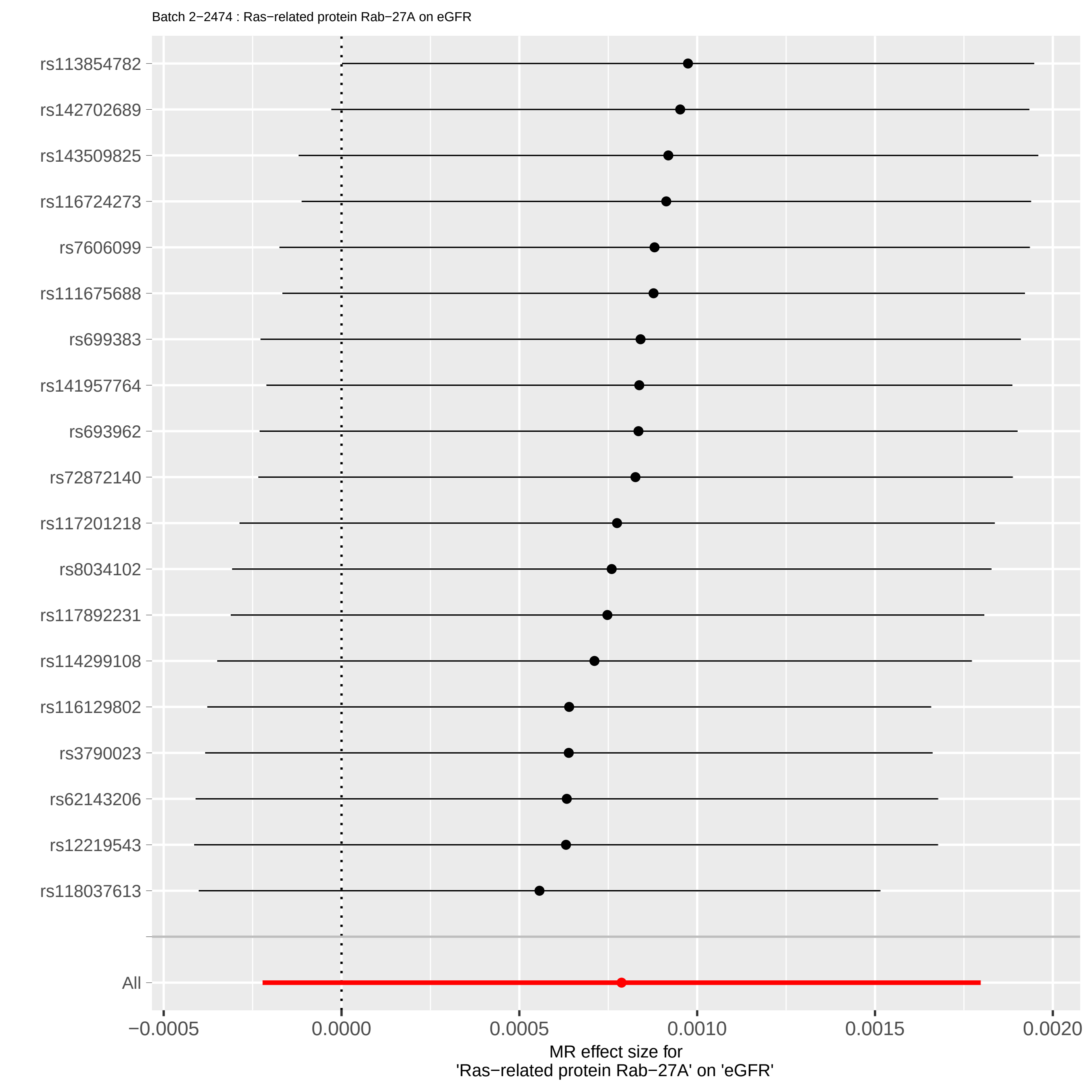

Batch 2−2474 : Ras−related protein Rab−27A on eGFR
rs113854782
rs142702689
rs143509825
rs116724273
rs7606099
rs111675688
rs699383
rs141957764
rs693962
rs72872140
rs117201218
rs8034102
rs117892231
rs114299108
rs116129802
rs3790023
rs62143206
rs12219543
rs118037613
All
−0.0005
0.0000
0.0005
0.0010
0.0015
0.0020
MR effect size for
'Ras−related protein Rab−27A' on 'eGFR'

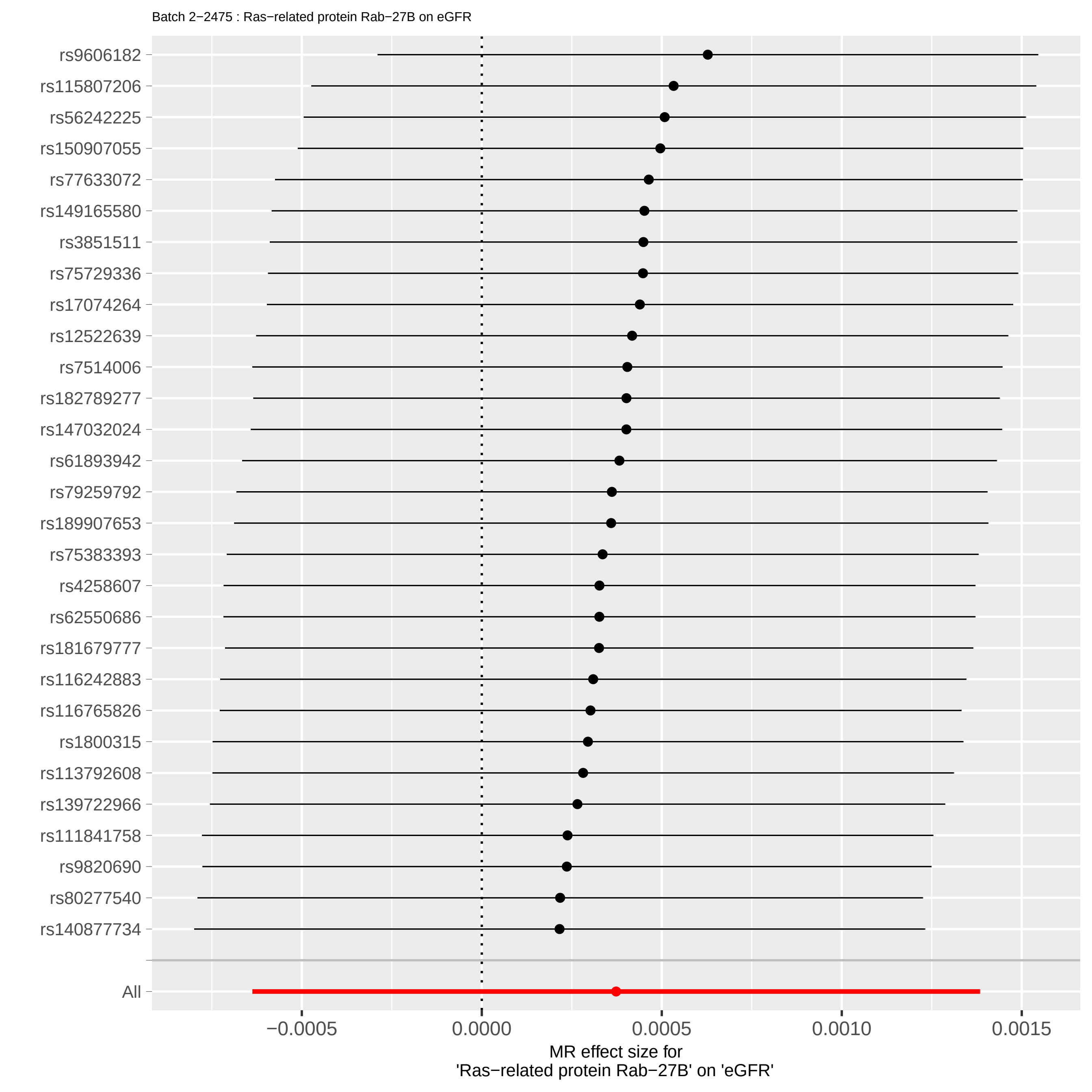

Batch 2−2475 : Ras−related protein Rab−27B on eGFR
rs9606182
rs115807206
rs56242225
rs150907055
rs77633072
rs149165580
rs3851511
rs75729336
rs17074264
rs12522639
rs7514006
rs182789277
rs147032024
rs61893942
rs79259792
rs189907653
rs75383393
rs4258607
rs62550686
rs181679777
rs116242883
rs116765826
rs1800315
rs113792608
rs139722966
rs111841758
rs9820690
rs80277540
rs140877734
All
−0.0005
0.0000
0.0005
0.0010
0.0015
MR effect size for
'Ras−related protein Rab−27B' on 'eGFR'

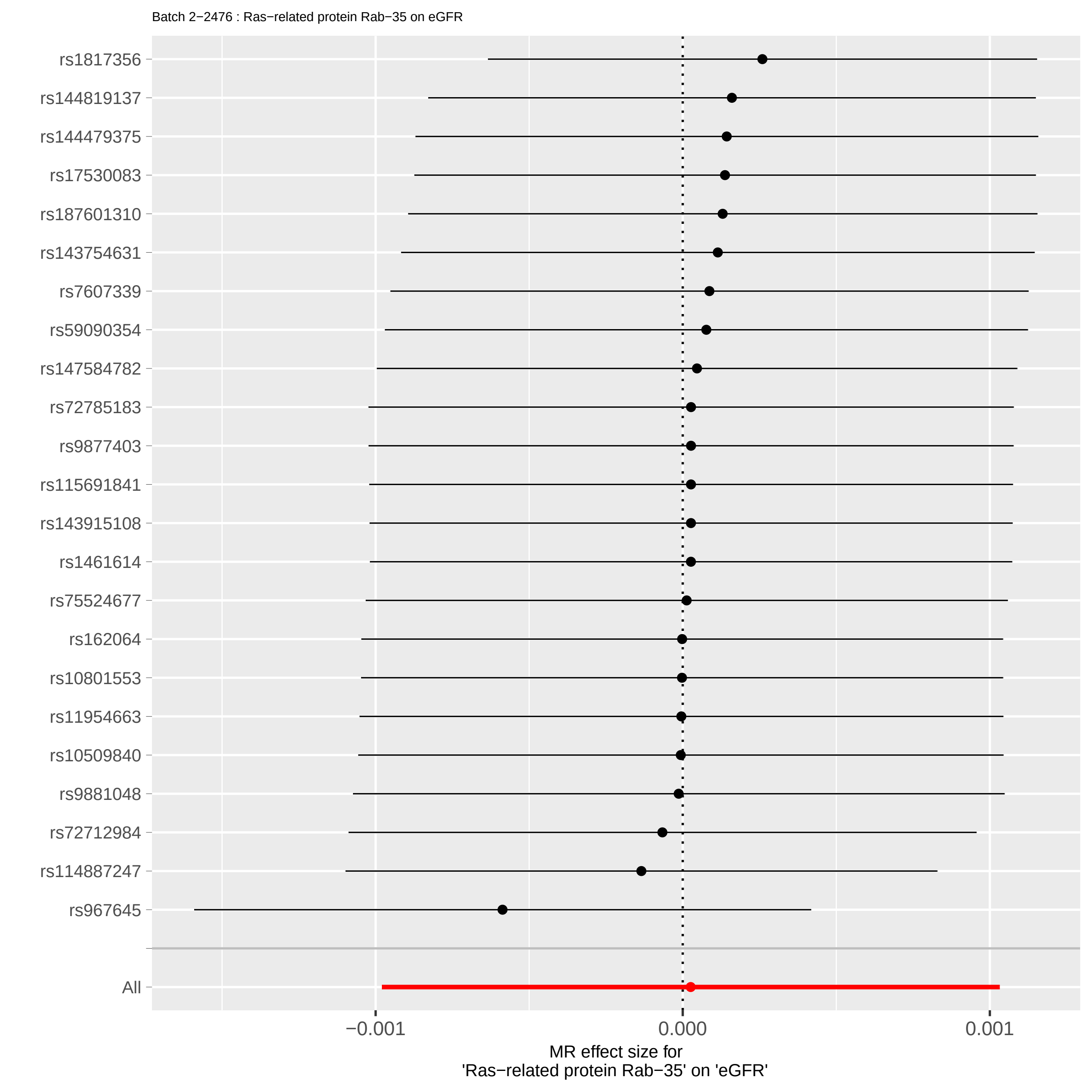

Batch 2−2476 : Ras−related protein Rab−35 on eGFR
rs1817356
rs144819137
rs144479375
rs17530083
rs187601310
rs143754631
rs7607339
rs59090354
rs147584782
rs72785183
rs9877403
rs115691841
rs143915108
rs1461614
rs75524677
rs162064
rs10801553
rs11954663
rs10509840
rs9881048
rs72712984
rs114887247
rs967645
All
−0.001
0.000
0.001
MR effect size for
'Ras−related protein Rab−35' on 'eGFR'

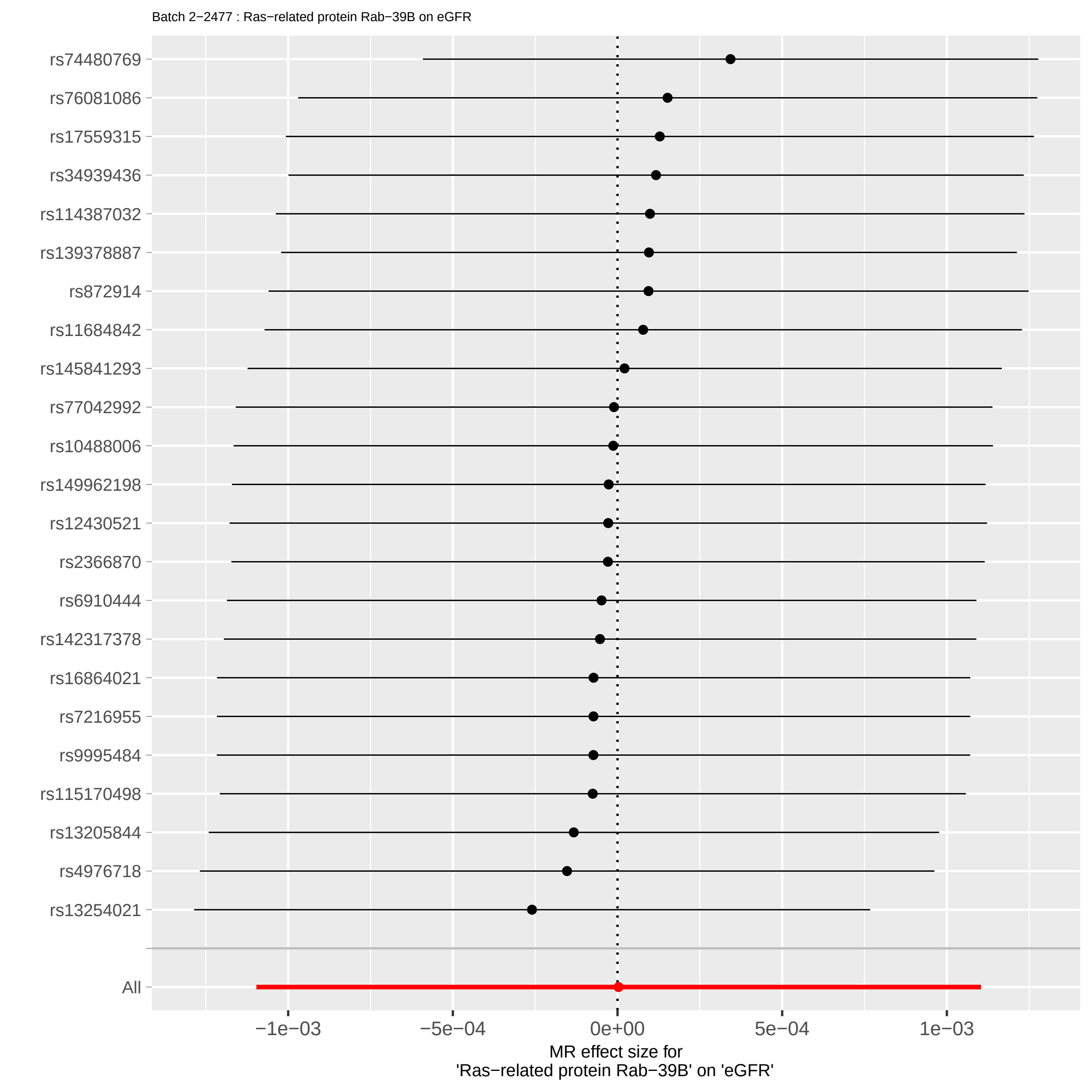

Batch 2−2477 : Ras−related protein Rab−39B on eGFR
rs74480769
rs76081086
rs17559315
rs34939436
rs114387032
rs139378887
rs872914
rs11684842
rs145841293
rs77042992
rs10488006
rs149962198
rs12430521
rs2366870
rs6910444
rs142317378
rs16864021
rs7216955
rs9995484
rs115170498
rs13205844
rs4976718
rs13254021
All
−1e−03
−5e−04
0e+00
5e−04
1e−03
MR effect size for
'Ras−related protein Rab−39B' on 'eGFR'

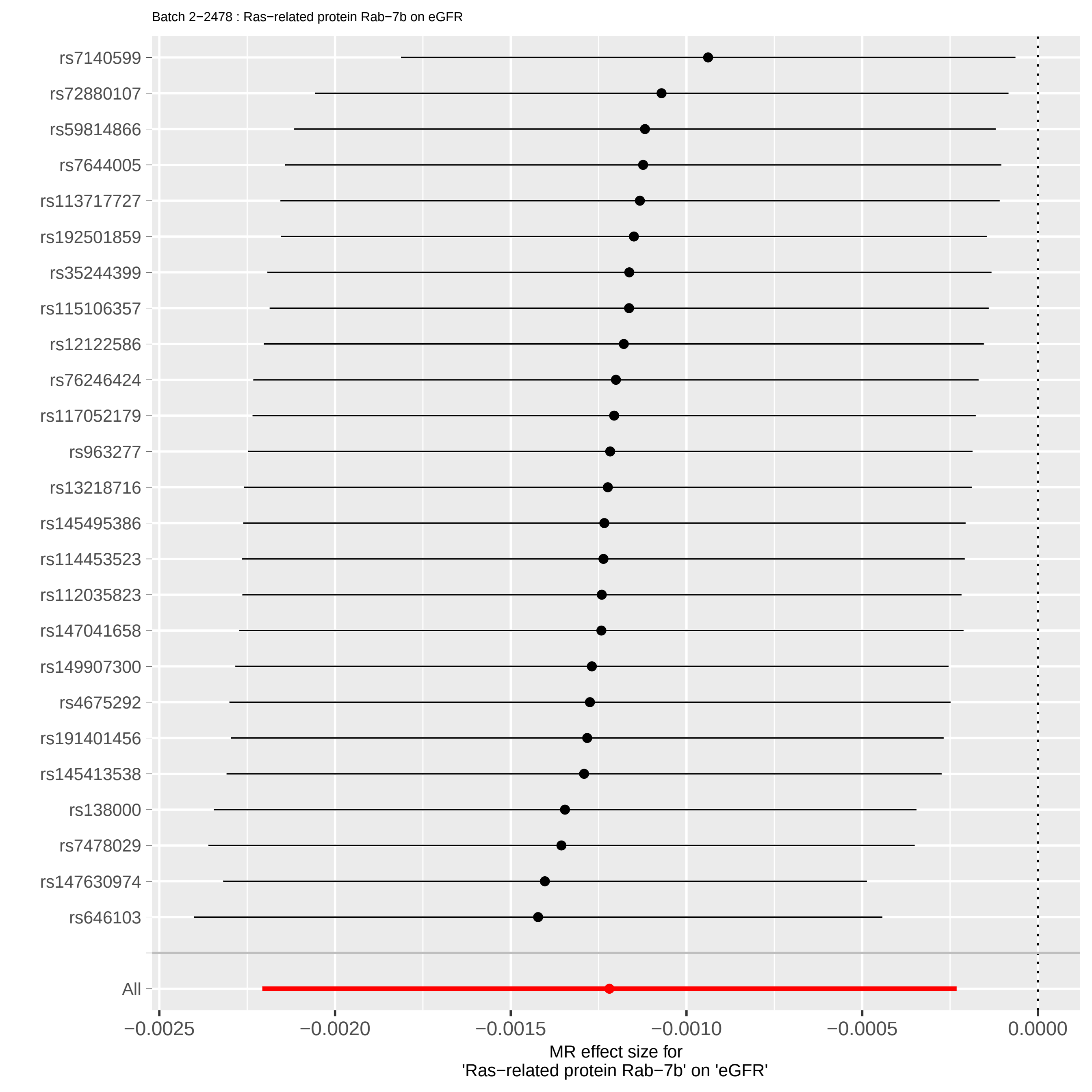

Batch 2−2478 : Ras−related protein Rab−7b on eGFR
rs7140599
rs72880107
rs59814866
rs7644005
rs113717727
rs192501859
rs35244399
rs115106357
rs12122586
rs76246424
rs117052179
rs963277
rs13218716
rs145495386
rs114453523
rs112035823
rs147041658
rs149907300
rs4675292
rs191401456
rs145413538
rs138000
rs7478029
rs147630974
rs646103
All
−0.0025
−0.0020
−0.0015
−0.0010
−0.0005
0.0000
MR effect size for
'Ras−related protein Rab−7b' on 'eGFR'

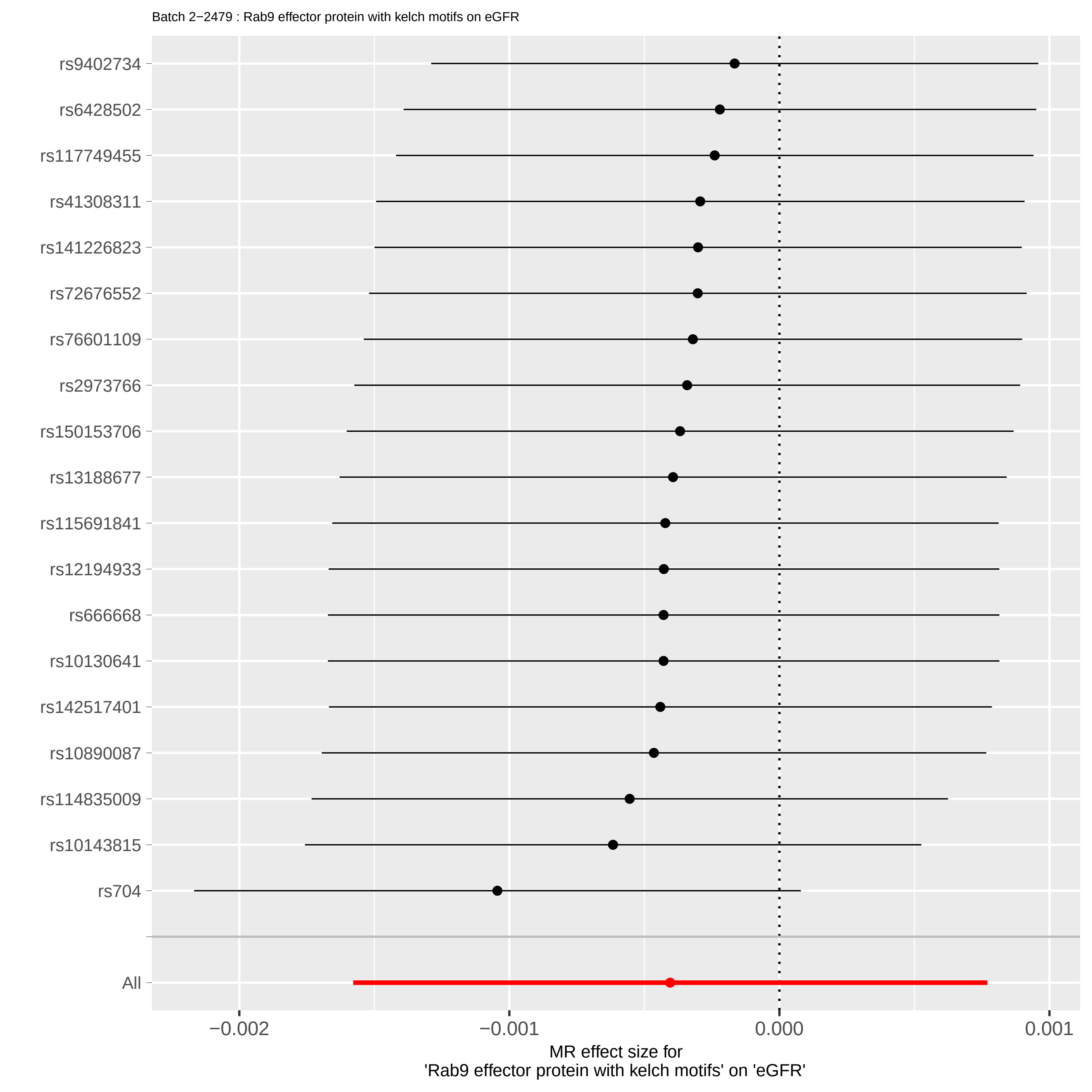

Batch 2−2479 : Rab9 effector protein with kelch motifs on eGFR
rs9402734
rs6428502
rs117749455
rs41308311
rs141226823
rs72676552
rs76601109
rs2973766
rs150153706
rs13188677
rs115691841
rs12194933
rs666668
rs10130641
rs142517401
rs10890087
rs114835009
rs10143815
rs704
All
−0.002
−0.001
0.000
0.001
MR effect size for
'Rab9 effector protein with kelch motifs' on 'eGFR'

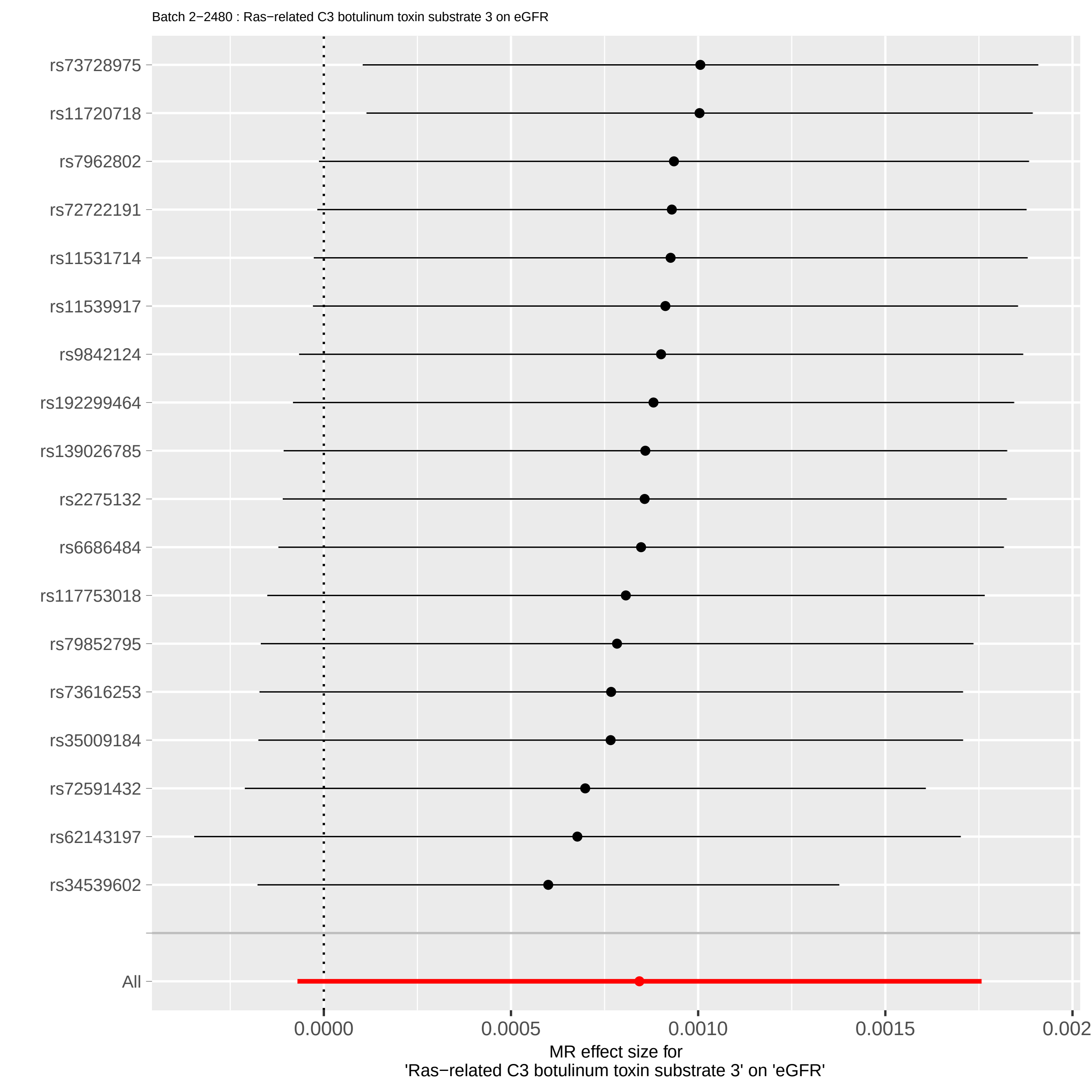

Batch 2−2480 : Ras−related C3 botulinum toxin substrate 3 on eGFR
rs73728975
rs11720718
rs7962802
rs72722191
rs11531714
rs11539917
rs9842124
rs192299464
rs139026785
rs2275132
rs6686484
rs117753018
rs79852795
rs73616253
rs35009184
rs72591432
rs62143197
rs34539602
All
0.0000
0.0005
0.0010
0.0015
0.002
MR effect size for
'Ras−related C3 botulinum toxin substrate 3' on 'eGFR'

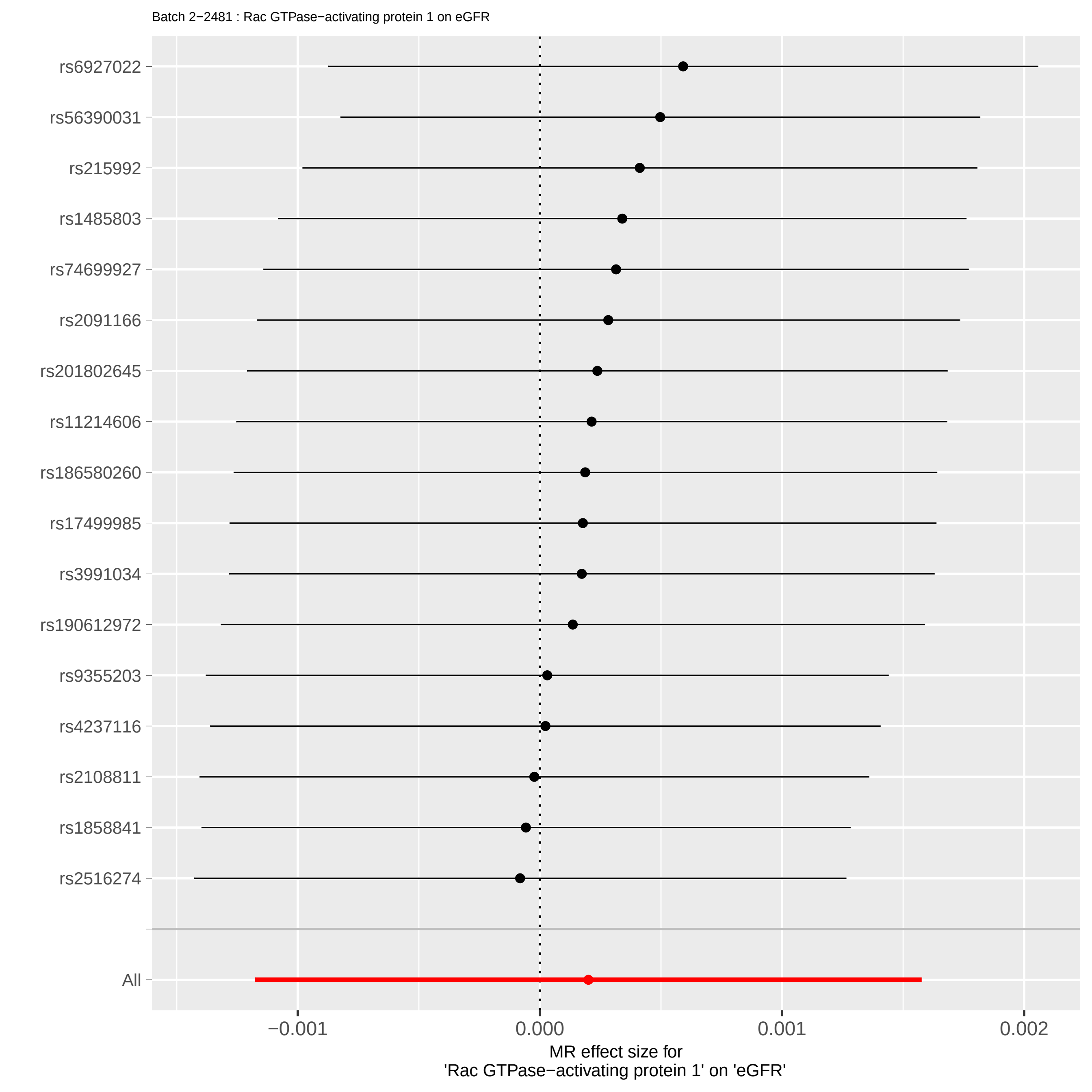

Batch 2−2481 : Rac GTPase−activating protein 1 on eGFR
rs6927022
rs56390031
rs215992
rs1485803
rs74699927
rs2091166
rs201802645
rs11214606
rs186580260
rs17499985
rs3991034
rs190612972
rs9355203
rs4237116
rs2108811
rs1858841
rs2516274
All
−0.001
0.000
0.001
0.002
MR effect size for
'Rac GTPase−activating protein 1' on 'eGFR'

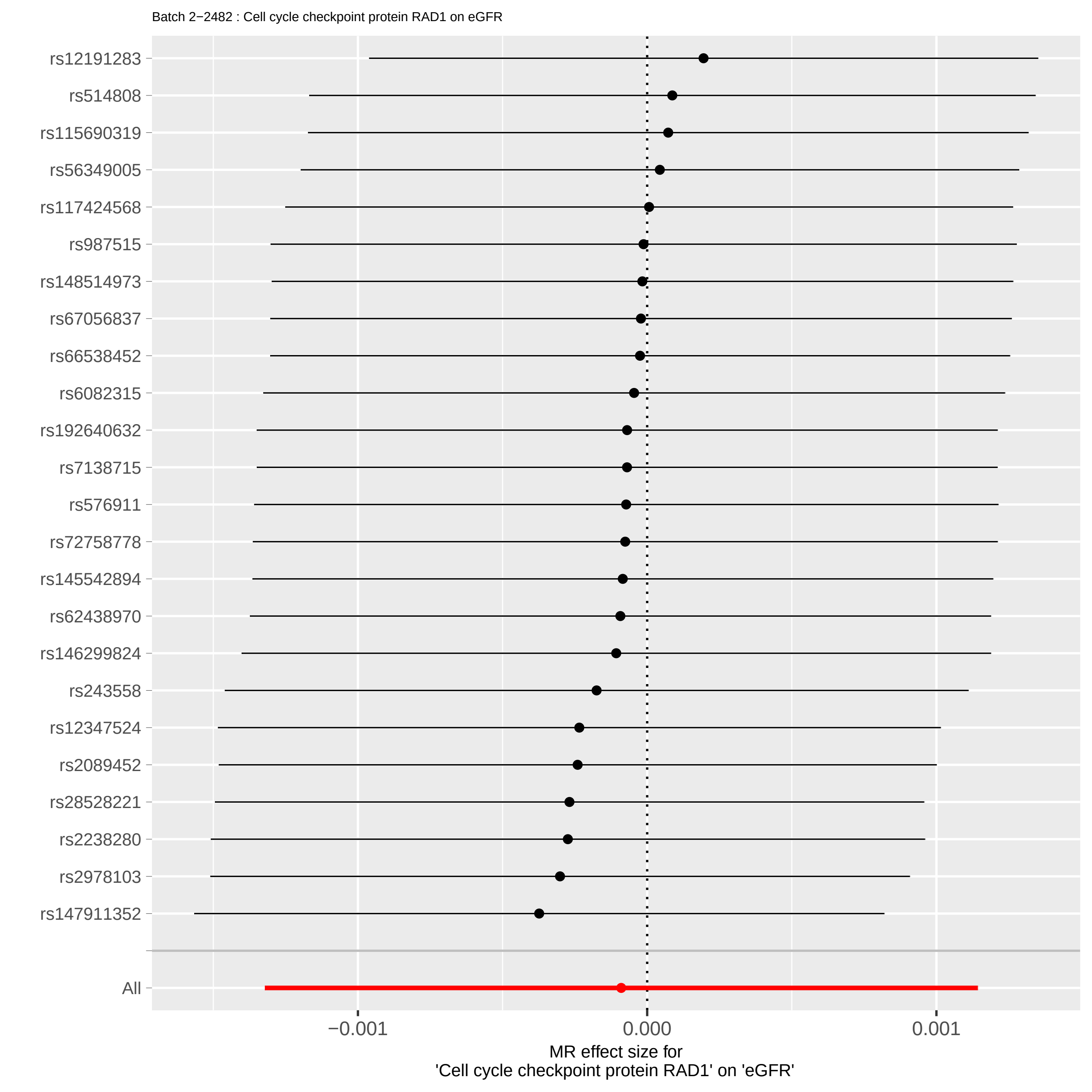

Batch 2−2482 : Cell cycle checkpoint protein RAD1 on eGFR
rs12191283
rs514808
rs115690319
rs56349005
rs117424568
rs987515
rs148514973
rs67056837
rs66538452
rs6082315
rs192640632
rs7138715
rs576911
rs72758778
rs145542894
rs62438970
rs146299824
rs243558
rs12347524
rs2089452
rs28528221
rs2238280
rs2978103
rs147911352
All
−0.001
0.000
0.001
MR effect size for
'Cell cycle checkpoint protein RAD1' on 'eGFR'

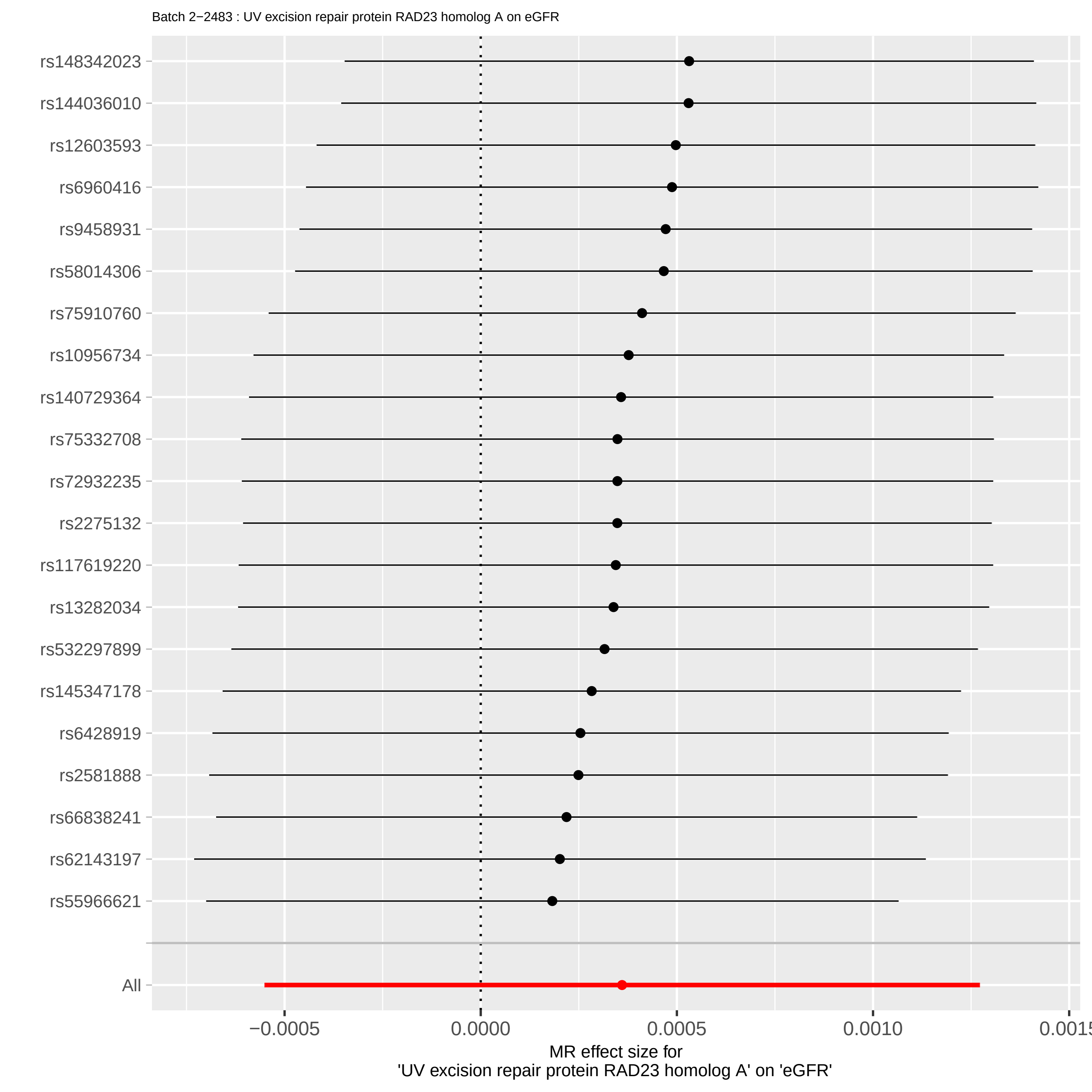

Batch 2−2483 : UV excision repair protein RAD23 homolog A on eGFR
rs148342023
rs144036010
rs12603593
rs6960416
rs9458931
rs58014306
rs75910760
rs10956734
rs140729364
rs75332708
rs72932235
rs2275132
rs117619220
rs13282034
rs532297899
rs145347178
rs6428919
rs2581888
rs66838241
rs62143197
rs55966621
All
−0.0005
0.0000
0.0005
0.0010
0.0015
MR effect size for
'UV excision repair protein RAD23 homolog A' on 'eGFR'

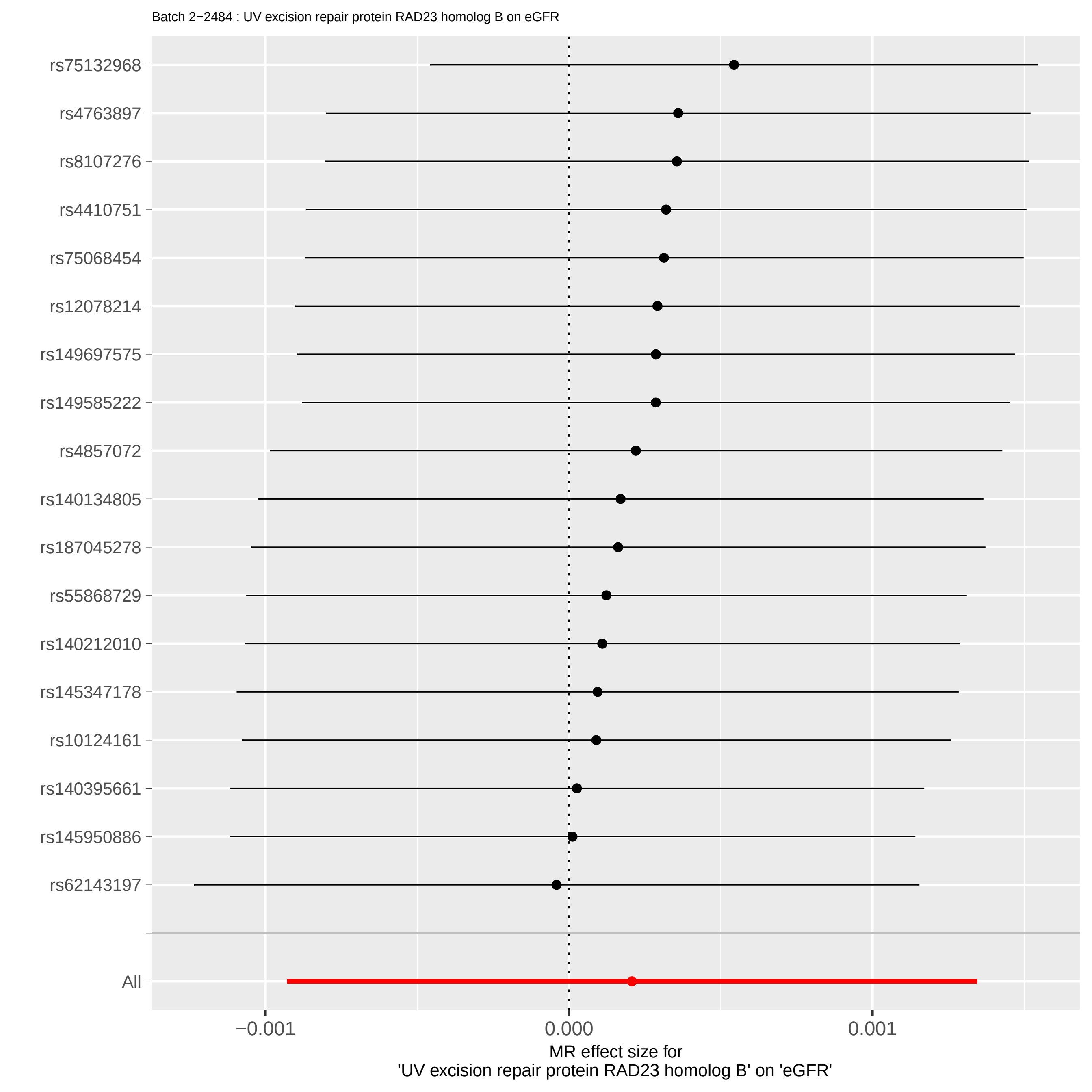

Batch 2−2484 : UV excision repair protein RAD23 homolog B on eGFR
rs75132968
rs4763897
rs8107276
rs4410751
rs75068454
rs12078214
rs149697575
rs149585222
rs4857072
rs140134805
rs187045278
rs55868729
rs140212010
rs145347178
rs10124161
rs140395661
rs145950886
rs62143197
All
−0.001
0.000
0.001
MR effect size for
'UV excision repair protein RAD23 homolog B' on 'eGFR'

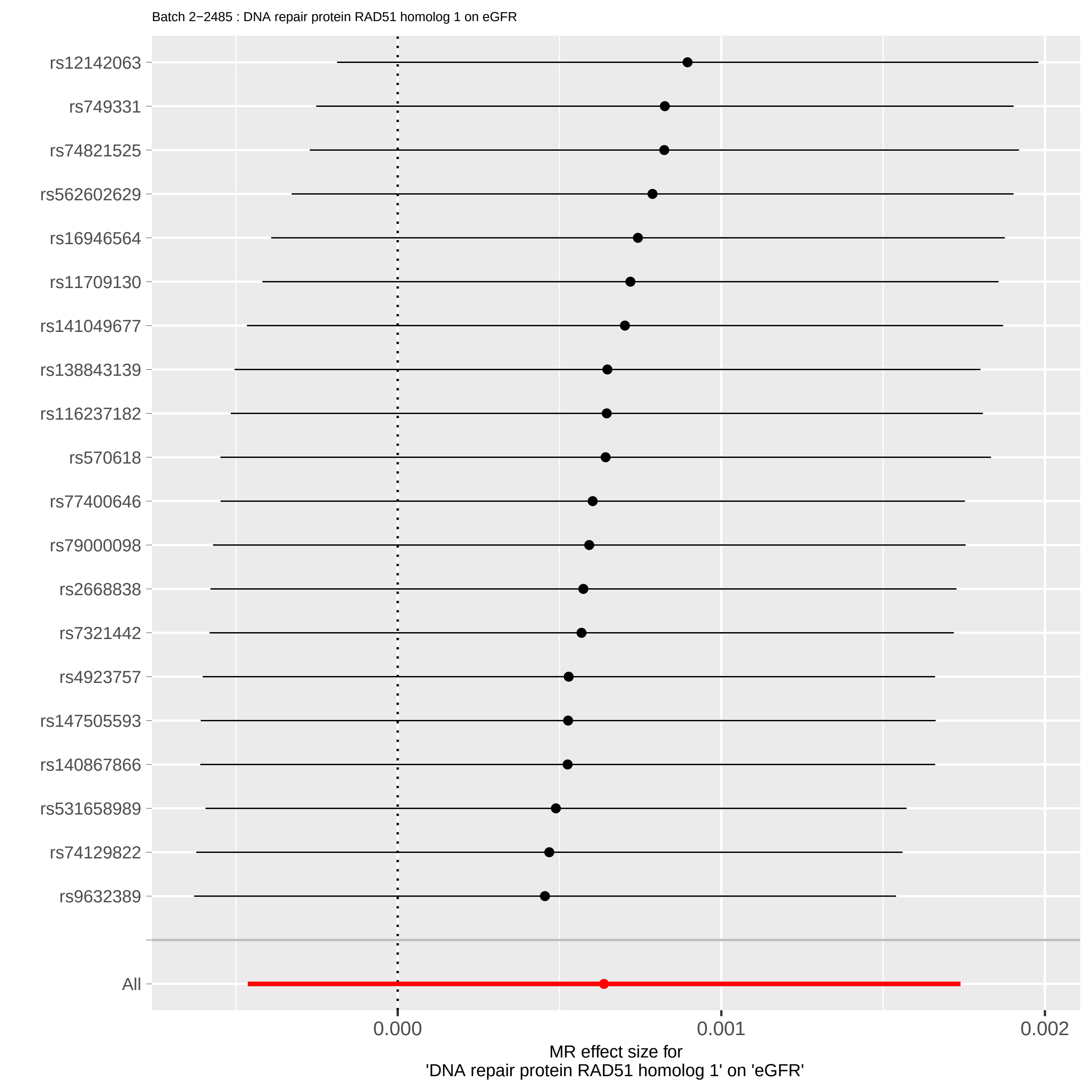

Batch 2−2485 : DNA repair protein RAD51 homolog 1 on eGFR
rs12142063
rs749331
rs74821525
rs562602629
rs16946564
rs11709130
rs141049677
rs138843139
rs116237182
rs570618
rs77400646
rs79000098
rs2668838
rs7321442
rs4923757
rs147505593
rs140867866
rs531658989
rs74129822
rs9632389
All
0.000
0.001
0.002
MR effect size for
'DNA repair protein RAD51 homolog 1' on 'eGFR'

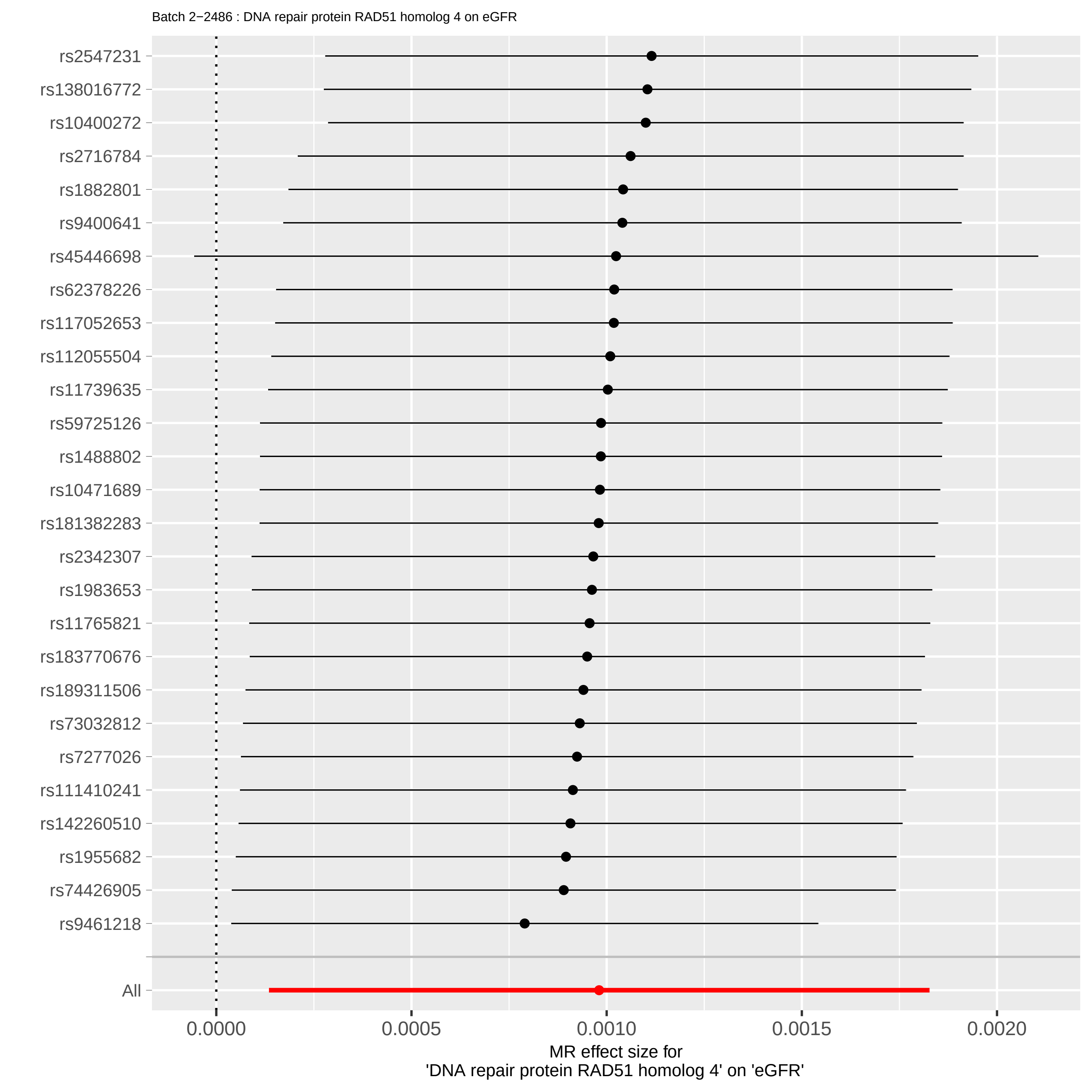

Batch 2−2486 : DNA repair protein RAD51 homolog 4 on eGFR
rs2547231
rs138016772
rs10400272
rs2716784
rs1882801
rs9400641
rs45446698
rs62378226
rs117052653
rs112055504
rs11739635
rs59725126
rs1488802
rs10471689
rs181382283
rs2342307
rs1983653
rs11765821
rs183770676
rs189311506
rs73032812
rs7277026
rs111410241
rs142260510
rs1955682
rs74426905
rs9461218
All
0.0000
0.0005
0.0010
0.0015
0.0020
MR effect size for
'DNA repair protein RAD51 homolog 4' on 'eGFR'

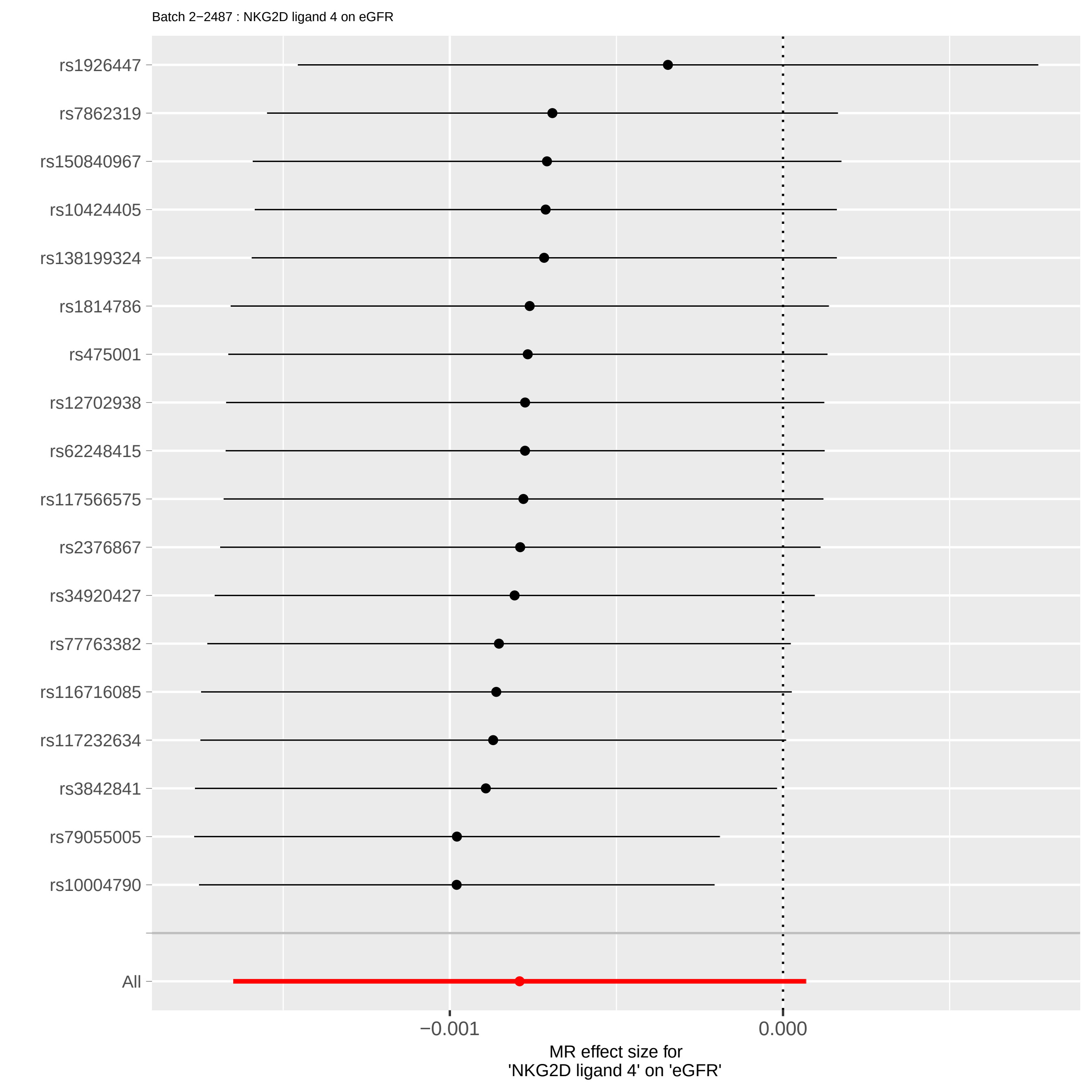

Batch 2−2487 : NKG2D ligand 4 on eGFR
rs1926447
rs7862319
rs150840967
rs10424405
rs138199324
rs1814786
rs475001
rs12702938
rs62248415
rs117566575
rs2376867
rs34920427
rs77763382
rs116716085
rs117232634
rs3842841
rs79055005
rs10004790
All
−0.001
0.000
MR effect size for
'NKG2D ligand 4' on 'eGFR'

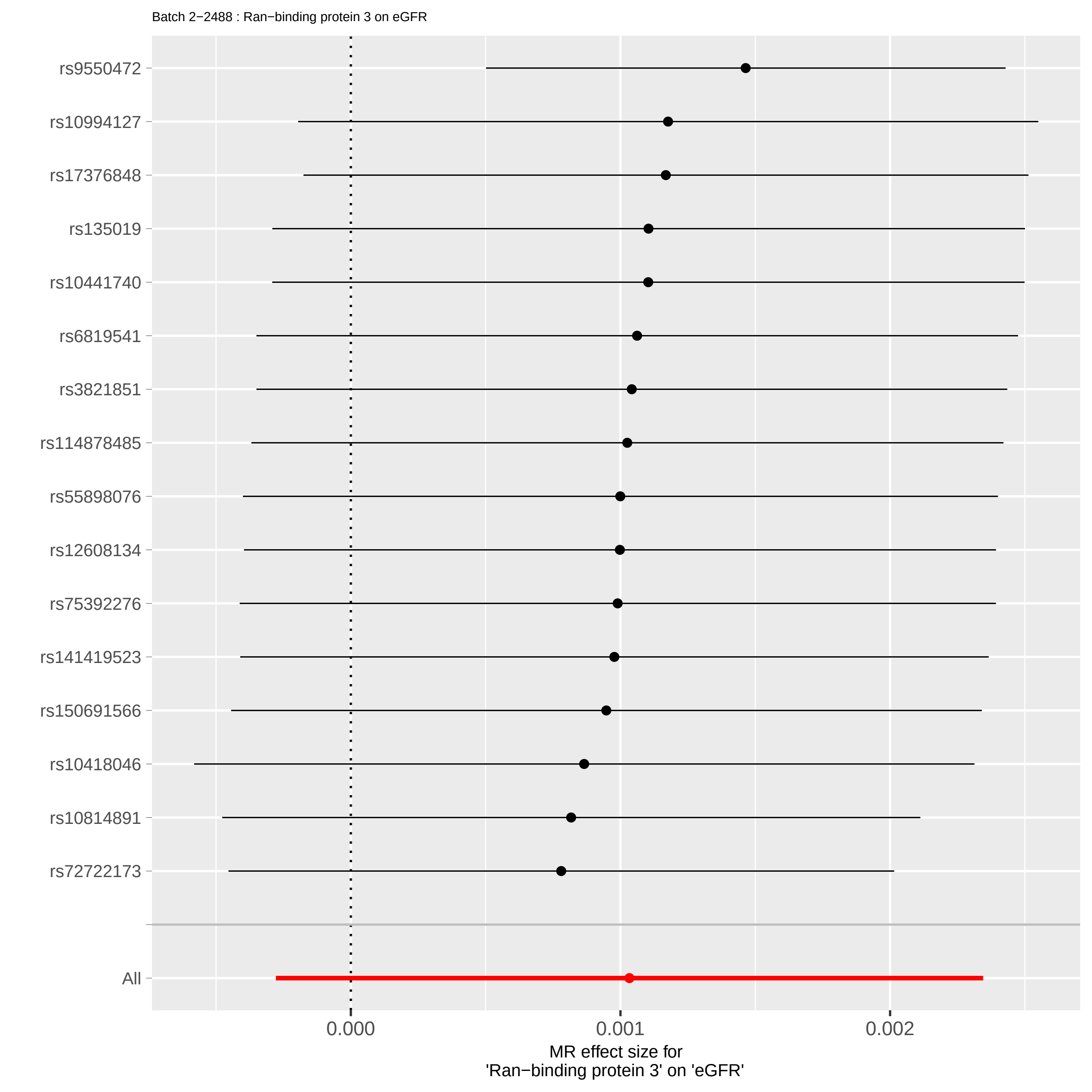

Batch 2−2488 : Ran−binding protein 3 on eGFR
rs9550472
rs10994127
rs17376848
rs135019
rs10441740
rs6819541
rs3821851
rs114878485
rs55898076
rs12608134
rs75392276
rs141419523
rs150691566
rs10418046
rs10814891
rs72722173
All
0.000
0.001
0.002
MR effect size for
'Ran−binding protein 3' on 'eGFR'

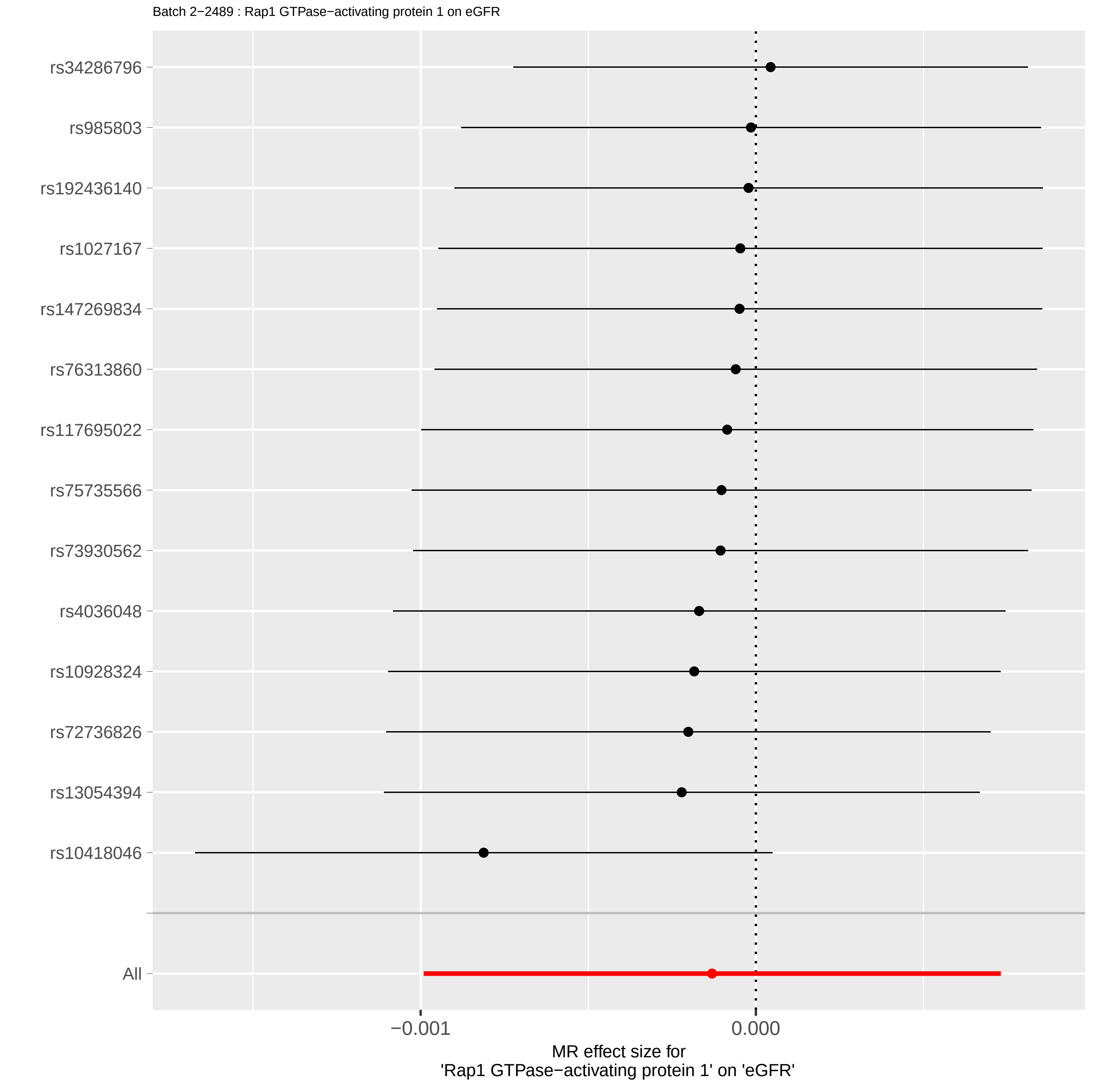

Batch 2−2489 : Rap1 GTPase−activating protein 1 on eGFR
rs34286796
rs985803
rs192436140
rs1027167
rs147269834
rs76313860
rs117695022
rs75735566
rs73930562
rs4036048
rs10928324
rs72736826
rs13054394
rs10418046
All
−0.001
0.000
MR effect size for
'Rap1 GTPase−activating protein 1' on 'eGFR'

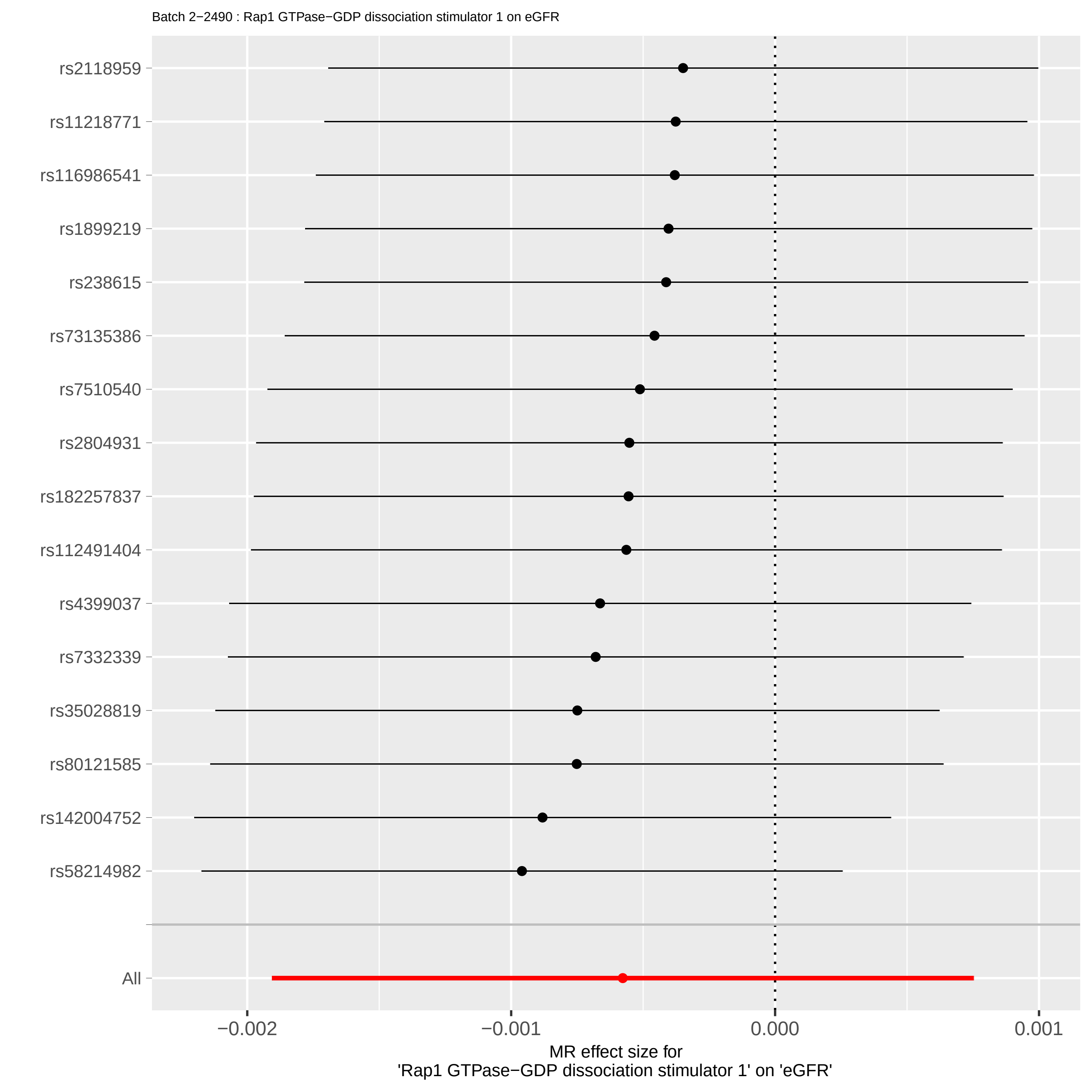

Batch 2−2490 : Rap1 GTPase−GDP dissociation stimulator 1 on eGFR
rs2118959
rs11218771
rs116986541
rs1899219
rs238615
rs73135386
rs7510540
rs2804931
rs182257837
rs112491404
rs4399037
rs7332339
rs35028819
rs80121585
rs142004752
rs58214982
All
−0.002
−0.001
0.000
0.001
MR effect size for
'Rap1 GTPase−GDP dissociation stimulator 1' on 'eGFR'

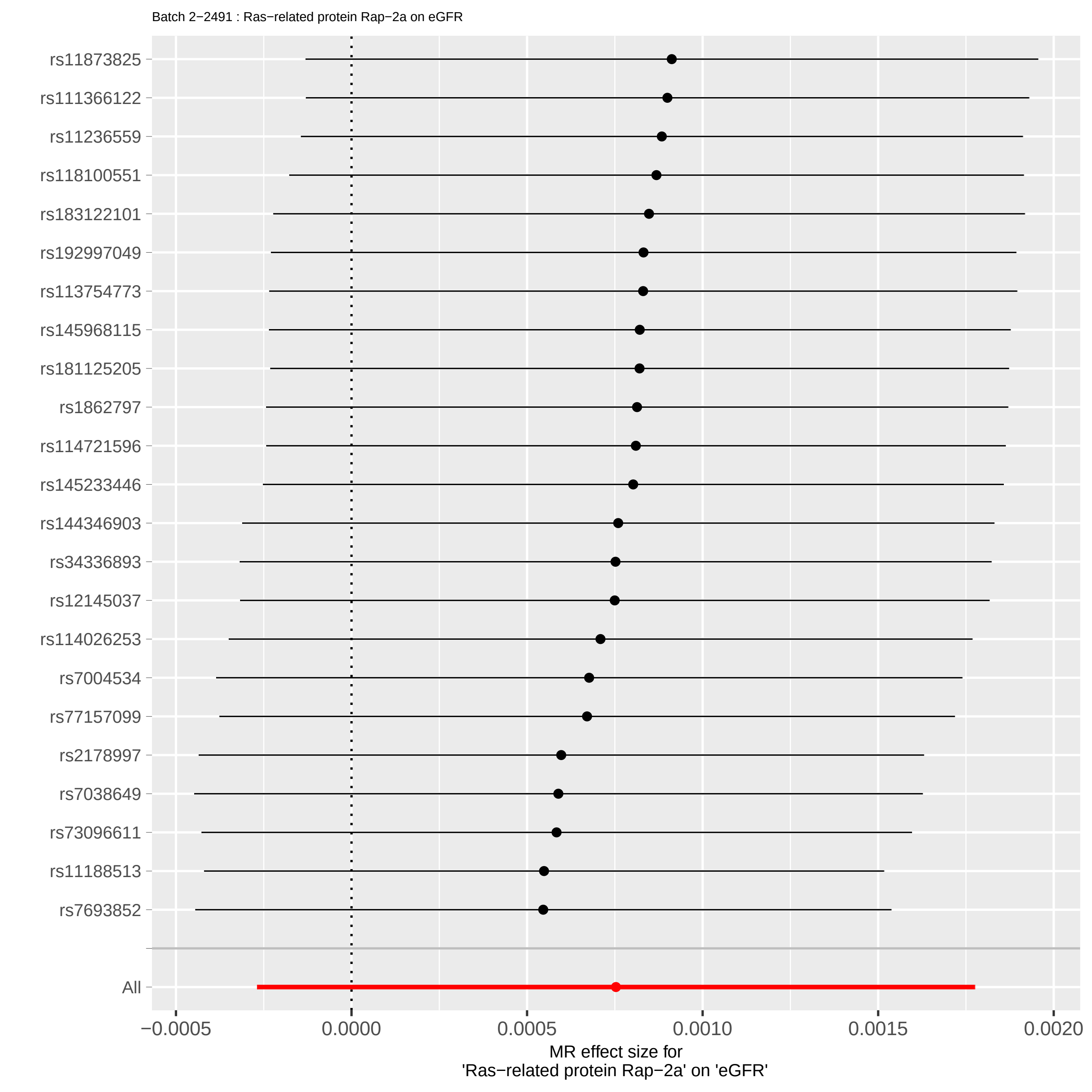

Batch 2−2491 : Ras−related protein Rap−2a on eGFR
rs11873825
rs111366122
rs11236559
rs118100551
rs183122101
rs192997049
rs113754773
rs145968115
rs181125205
rs1862797
rs114721596
rs145233446
rs144346903
rs34336893
rs12145037
rs114026253
rs7004534
rs77157099
rs2178997
rs7038649
rs73096611
rs11188513
rs7693852
All
−0.0005
0.0000
0.0005
0.0010
0.0015
0.0020
MR effect size for
'Ras−related protein Rap−2a' on 'eGFR'

Batch 2−2492 : Rap guanine nucleotide exchange factor 5 on eGFR

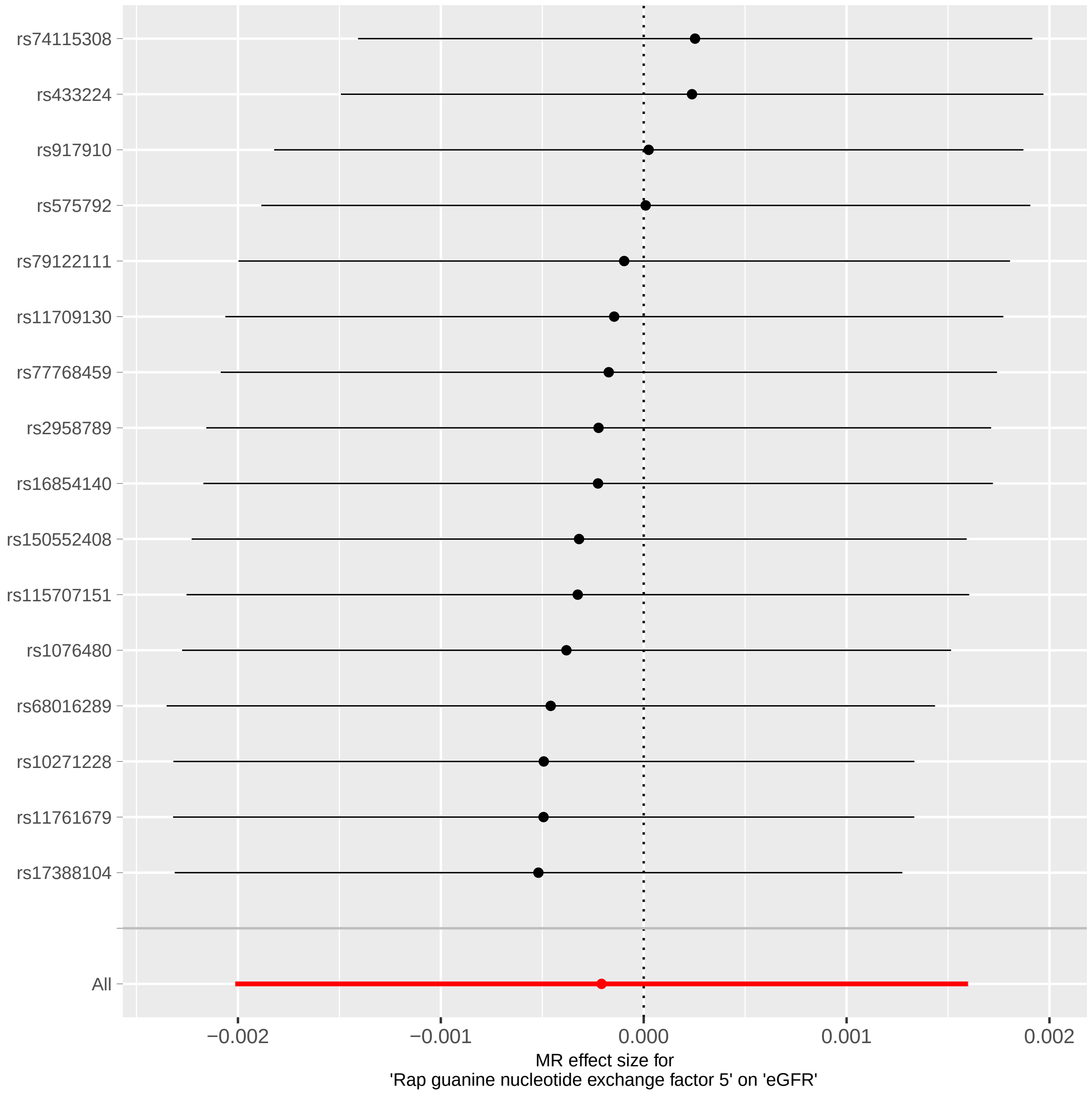

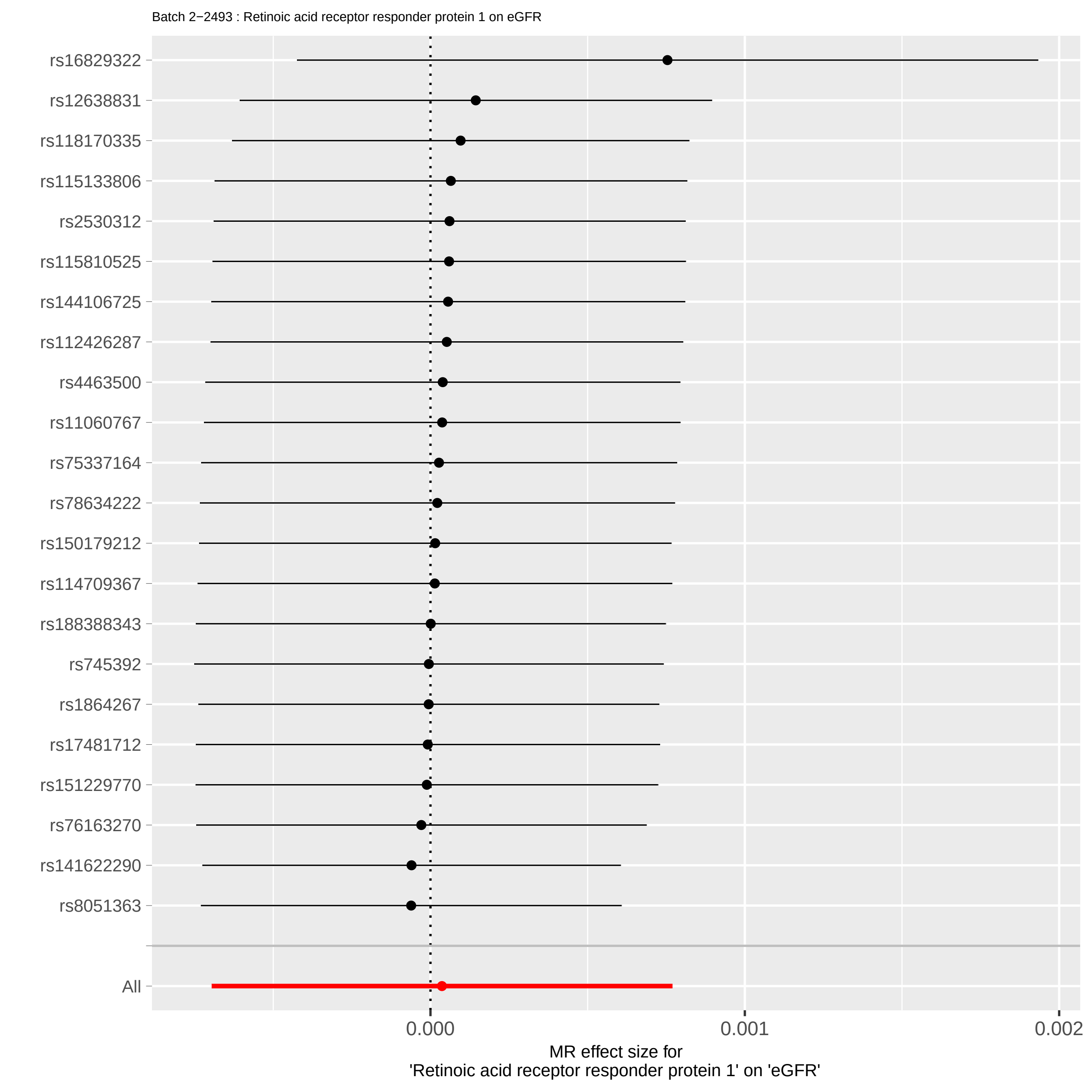
Batch 2−2493 : Retinoic acid receptor responder protein 1 on eGFR
rs16829322
rs12638831
rs118170335
rs115133806
rs2530312
rs115810525
rs144106725
rs112426287
rs4463500
rs11060767
rs75337164
rs78634222
rs150179212
rs114709367
rs188388343
rs745392
rs1864267
rs17481712
rs151229770
rs76163270
rs141622290
rs8051363
All
0.000
0.001
0.002
MR effect size for
'Retinoic acid receptor responder protein 1' on 'eGFR'

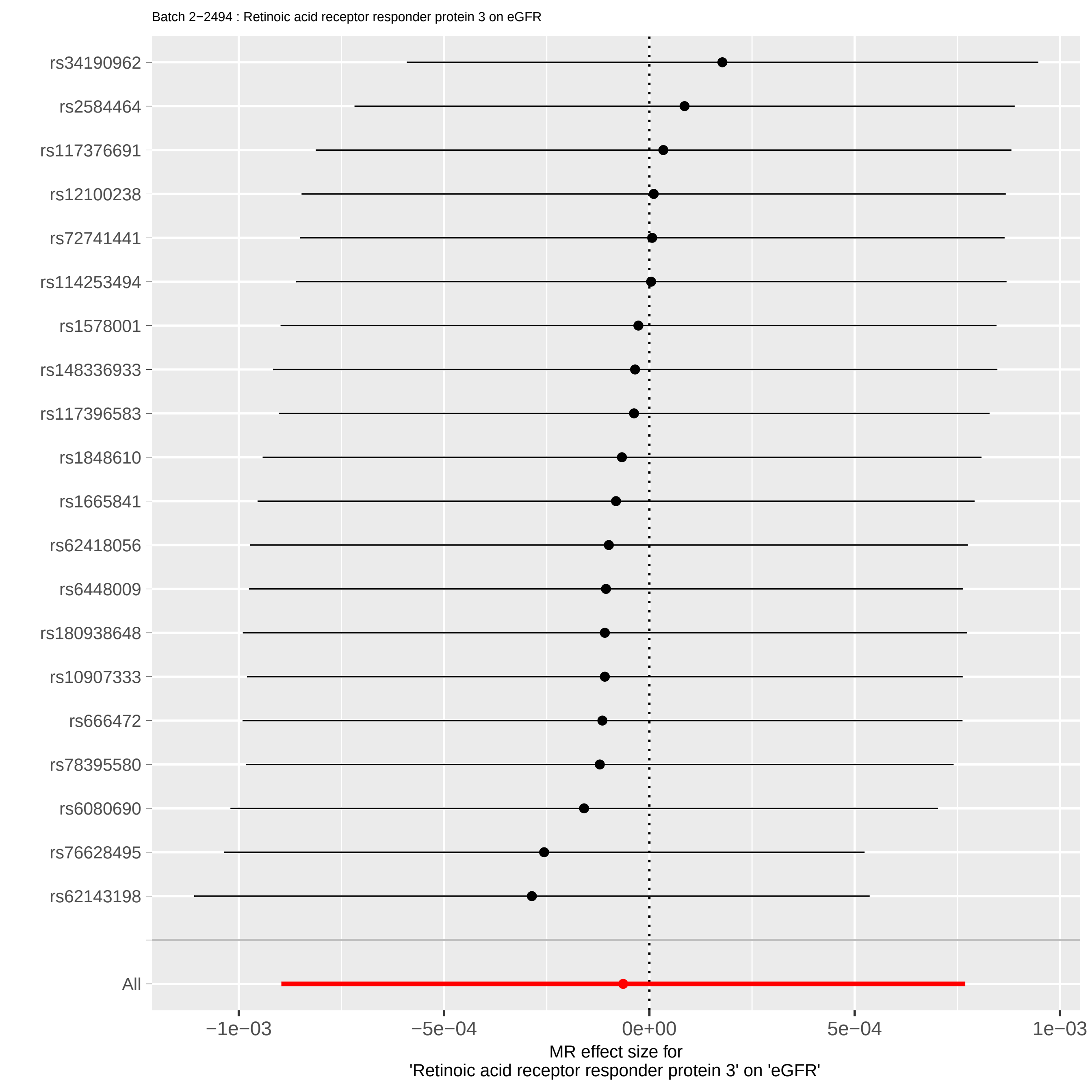

Batch 2−2494 : Retinoic acid receptor responder protein 3 on eGFR
rs34190962
rs2584464
rs117376691
rs12100238
rs72741441
rs114253494
rs1578001
rs148336933
rs117396583
rs1848610
rs1665841
rs62418056
rs6448009
rs180938648
rs10907333
rs666472
rs78395580
rs6080690
rs76628495
rs62143198
All
−1e−03
−5e−04
0e+00
5e−04
1e−03
MR effect size for
'Retinoic acid receptor responder protein 3' on 'eGFR'

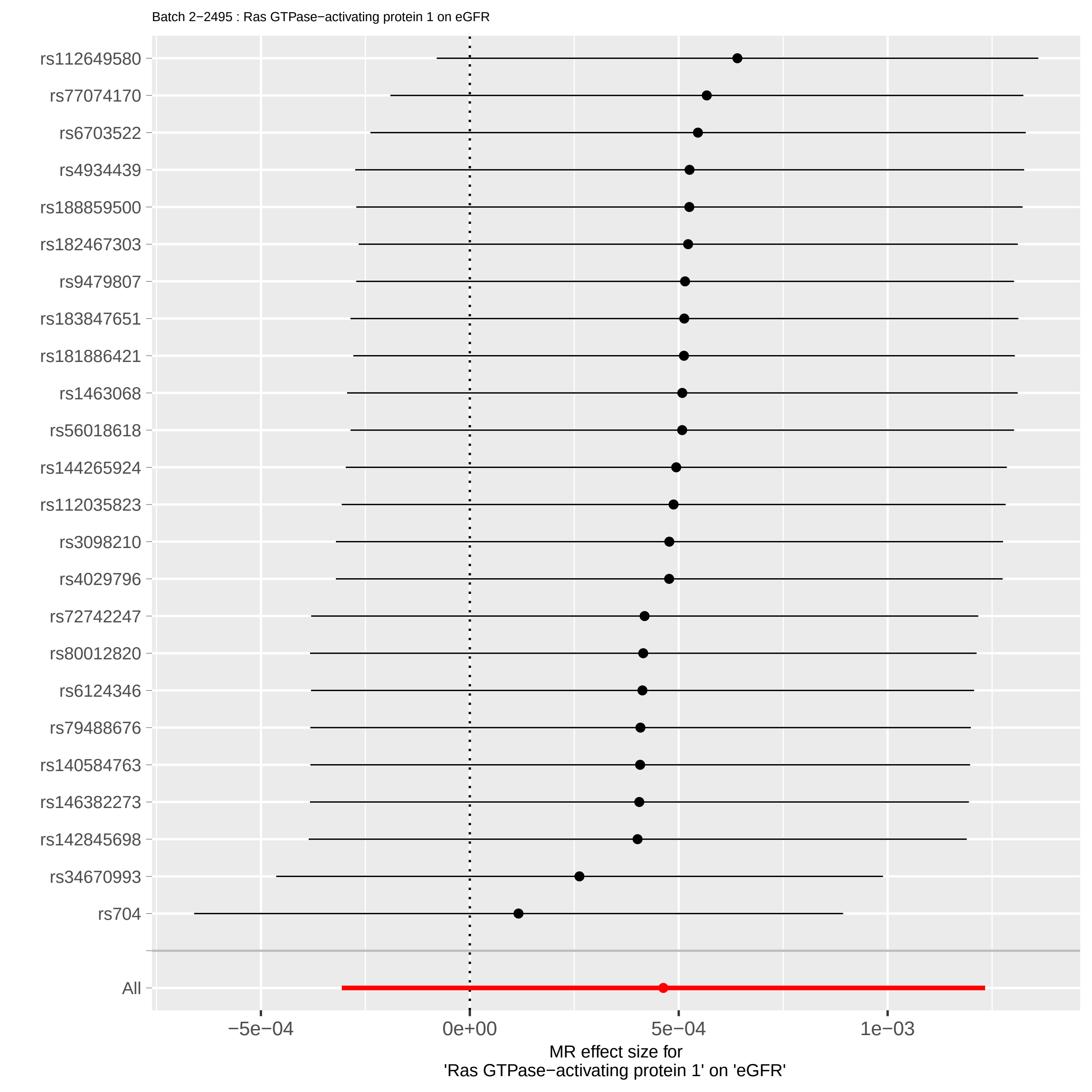
Batch 2−2495 : Ras GTPase−activating protein 1 on eGFR
rs112649580
rs77074170
rs6703522
rs4934439
rs188859500
rs182467303
rs9479807
rs183847651
rs181886421
rs1463068
rs56018618
rs144265924
rs112035823
rs3098210
rs4029796
rs72742247
rs80012820
rs6124346
rs79488676
rs140584763
rs146382273
rs142845698
rs34670993
rs704
All
−5e−04
0e+00
5e−04
1e−03
MR effect size for
'Ras GTPase−activating protein 1' on 'eGFR'

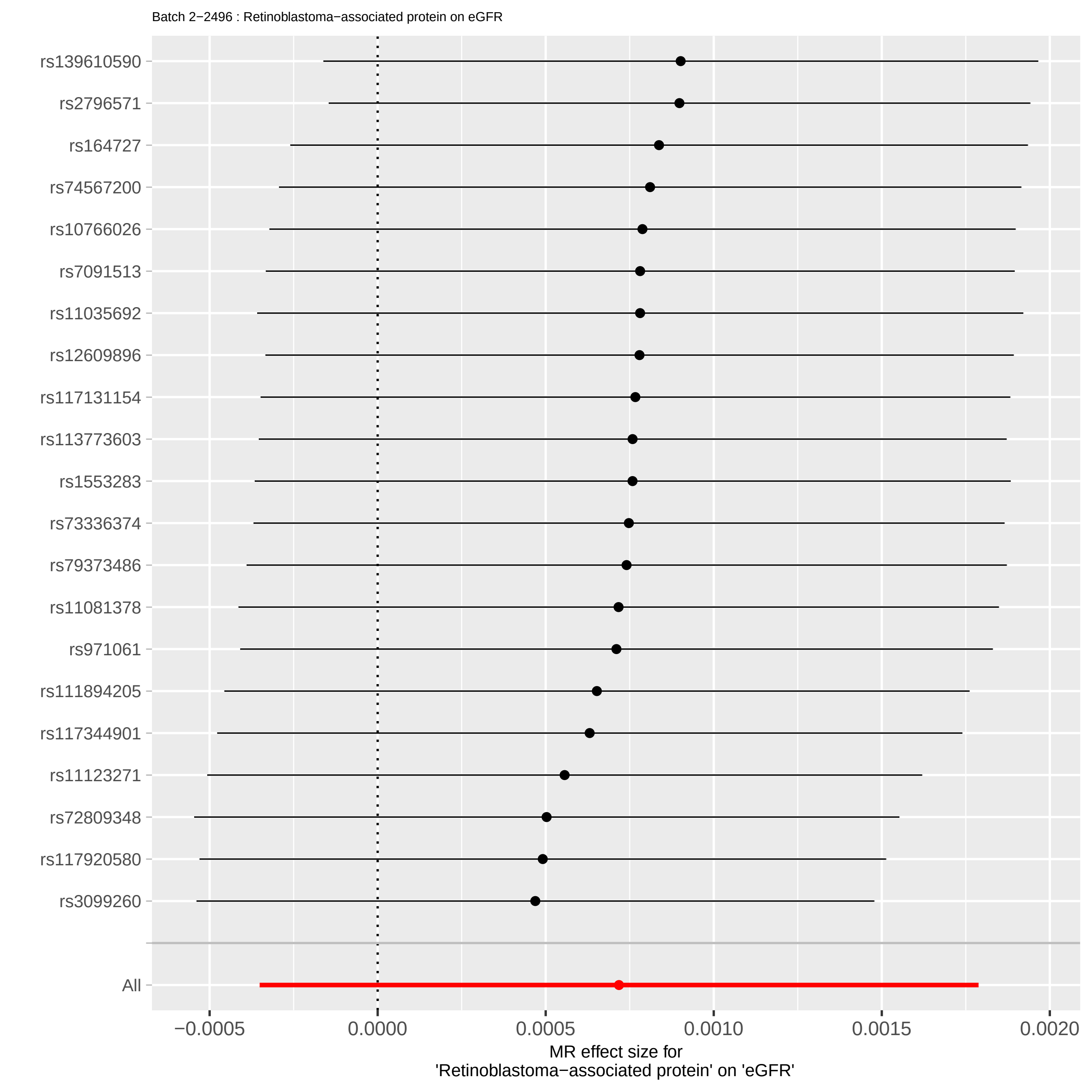

Batch 2−2496 : Retinoblastoma−associated protein on eGFR
rs139610590
rs2796571
rs164727
rs74567200
rs10766026
rs7091513
rs11035692
rs12609896
rs117131154
rs113773603
rs1553283
rs73336374
rs79373486
rs11081378
rs971061
rs111894205
rs117344901
rs11123271
rs72809348
rs117920580
rs3099260
All
−0.0005
0.0000
0.0005
0.0010
0.0015
0.0020
MR effect size for
'Retinoblastoma−associated protein' on 'eGFR'

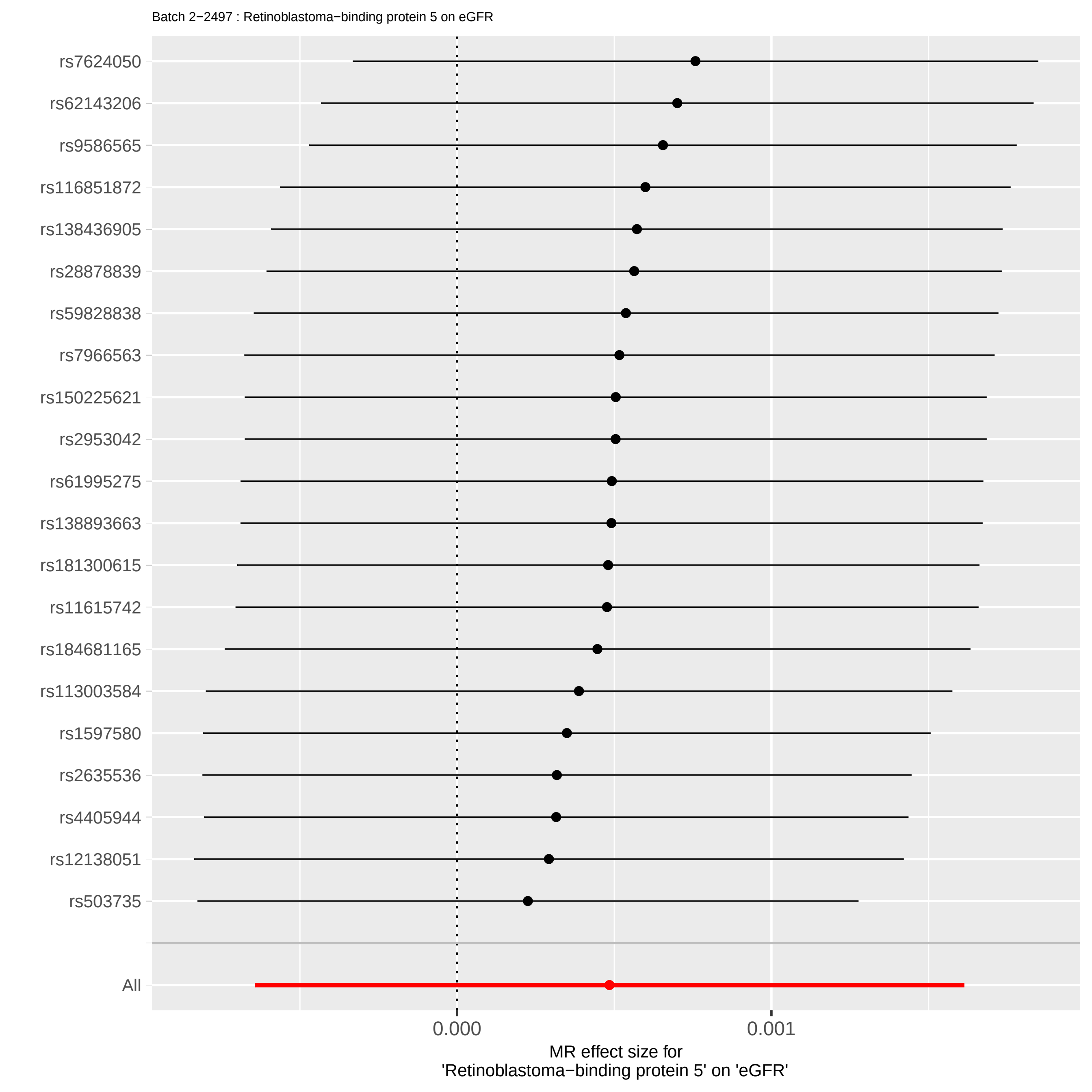

Batch 2−2497 : Retinoblastoma−binding protein 5 on eGFR
rs7624050
rs62143206
rs9586565
rs116851872
rs138436905
rs28878839
rs59828838
rs7966563
rs150225621
rs2953042
rs61995275
rs138893663
rs181300615
rs11615742
rs184681165
rs113003584
rs1597580
rs2635536
rs4405944
rs12138051
rs503735
All
0.000
0.001
MR effect size for
'Retinoblastoma−binding protein 5' on 'eGFR'

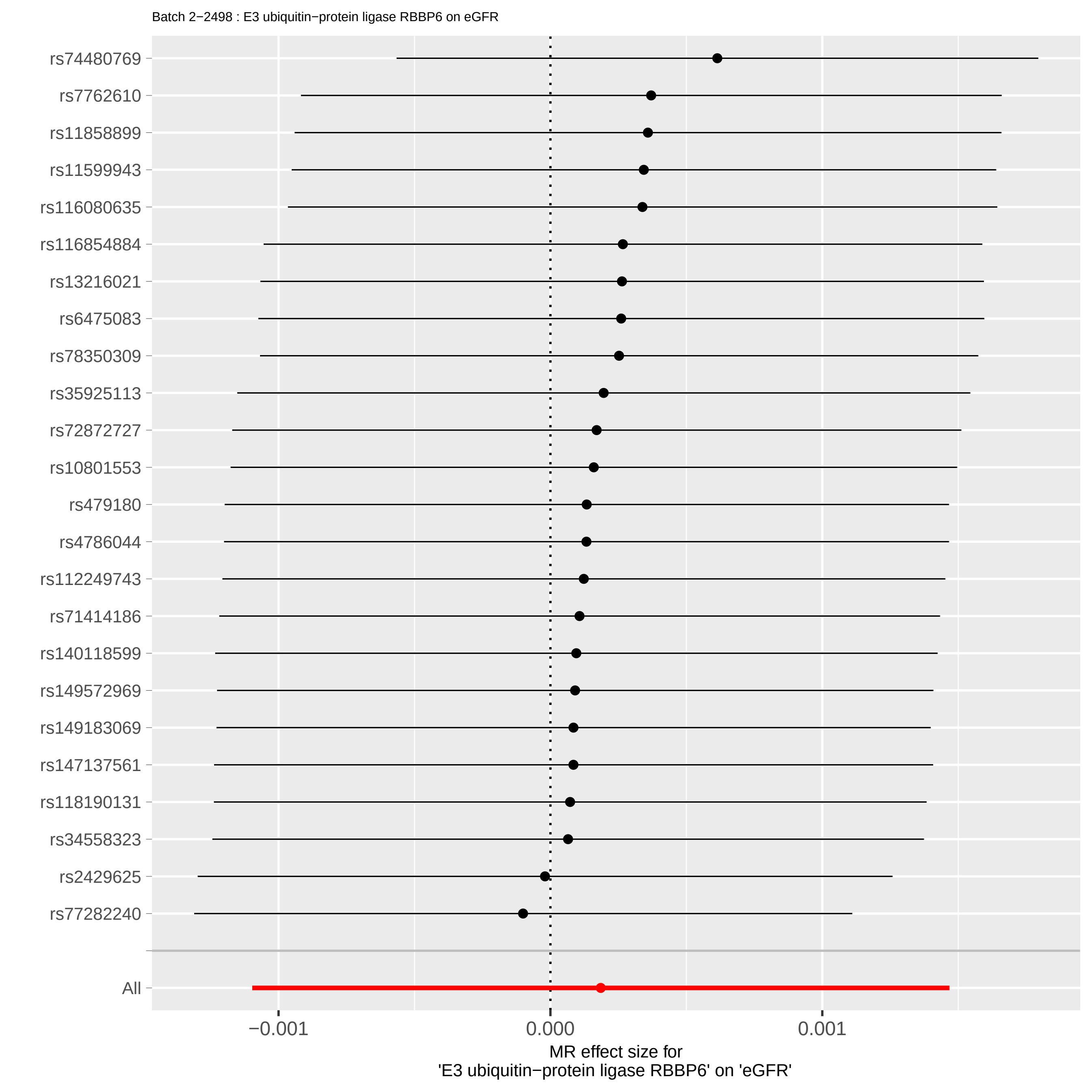

Batch 2−2498 : E3 ubiquitin−protein ligase RBBP6 on eGFR
rs74480769
rs7762610
rs11858899
rs11599943
rs116080635
rs116854884
rs13216021
rs6475083
rs78350309
rs35925113
rs72872727
rs10801553
rs479180
rs4786044
rs112249743
rs71414186
rs140118599
rs149572969
rs149183069
rs147137561
rs118190131
rs34558323
rs2429625
rs77282240
All
−0.001
0.000
0.001
MR effect size for
'E3 ubiquitin−protein ligase RBBP6' on 'eGFR'

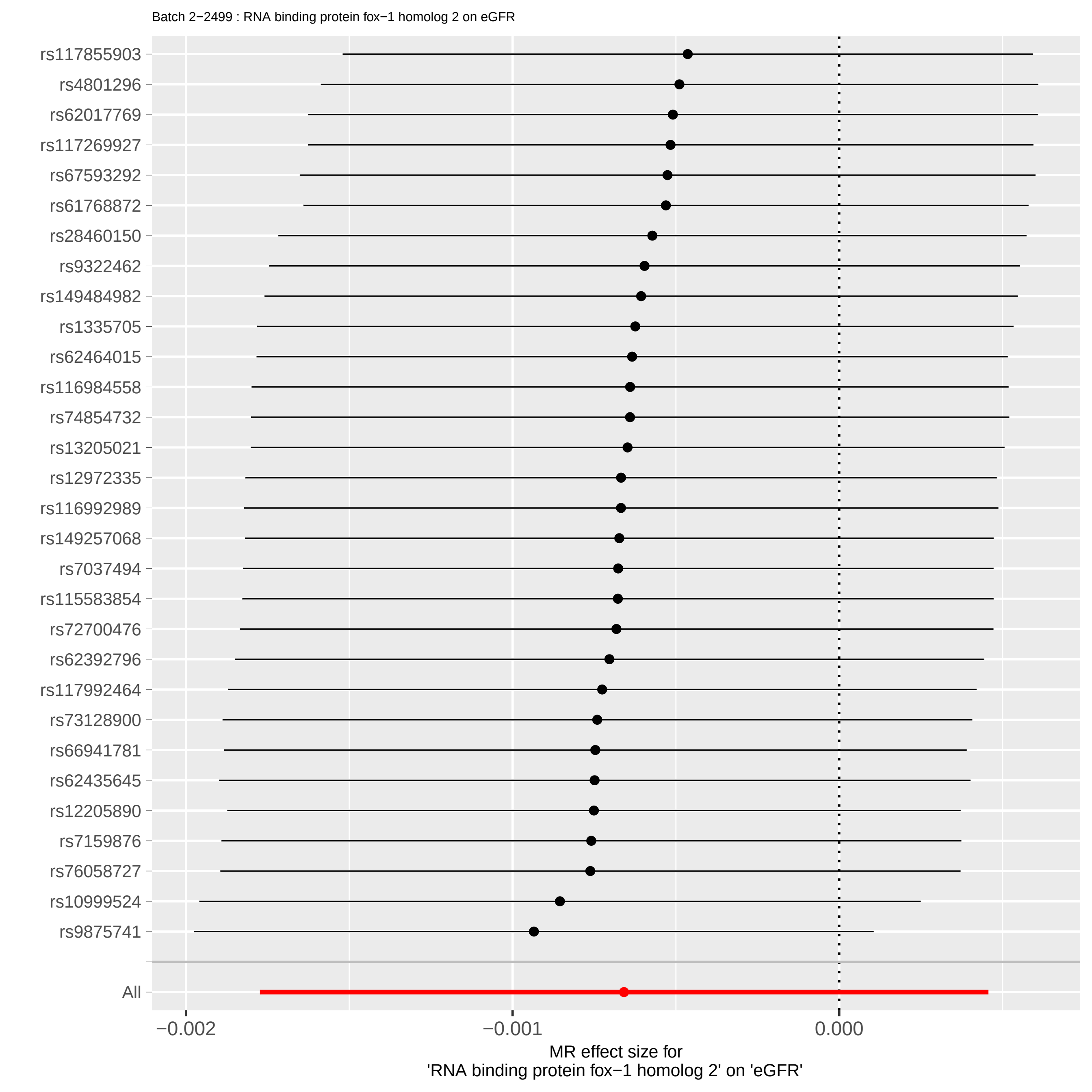

Batch 2−2499 : RNA binding protein fox−1 homolog 2 on eGFR
rs117855903
rs4801296
rs62017769
rs117269927
rs67593292
rs61768872
rs28460150
rs9322462
rs149484982
rs1335705
rs62464015
rs116984558
rs74854732
rs13205021
rs12972335
rs116992989
rs149257068
rs7037494
rs115583854
rs72700476
rs62392796
rs117992464
rs73128900
rs66941781
rs62435645
rs12205890
rs7159876
rs76058727
rs10999524
rs9875741
All
−0.002
−0.001
0.000
MR effect size for
'RNA binding protein fox−1 homolog 2' on 'eGFR'

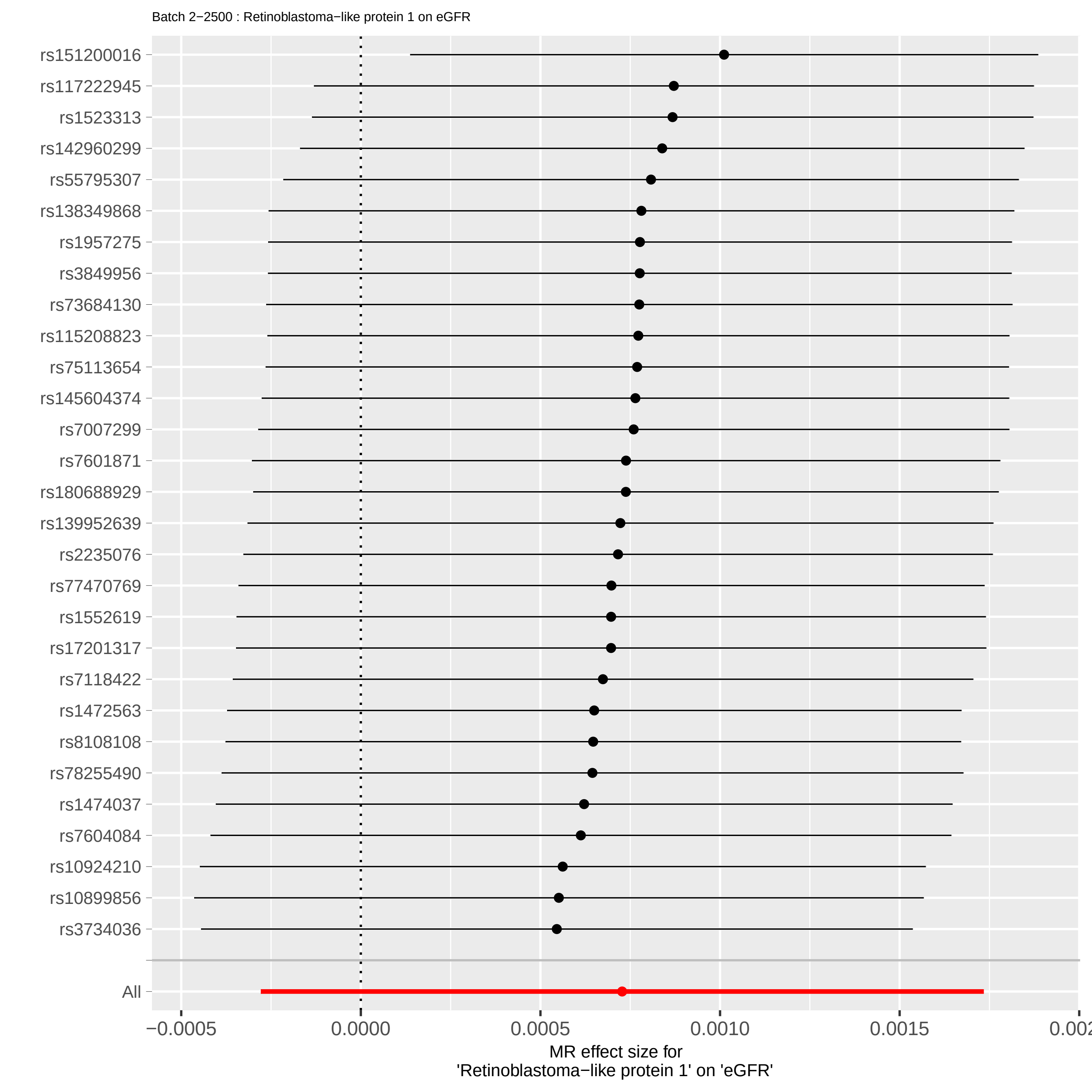

Batch 2−2500 : Retinoblastoma−like protein 1 on eGFR
rs151200016
rs117222945
rs1523313
rs142960299
rs55795307
rs138349868
rs1957275
rs3849956
rs73684130
rs115208823
rs75113654
rs145604374
rs7007299
rs7601871
rs180688929
rs139952639
rs2235076
rs77470769
rs1552619
rs17201317
rs7118422
rs1472563
rs8108108
rs78255490
rs1474037
rs7604084
rs10924210
rs10899856
rs3734036
All
−0.0005
0.0000
0.0005
0.0010
0.0015
MR effect size for
'Retinoblastoma−like protein 1' on 'eGFR'

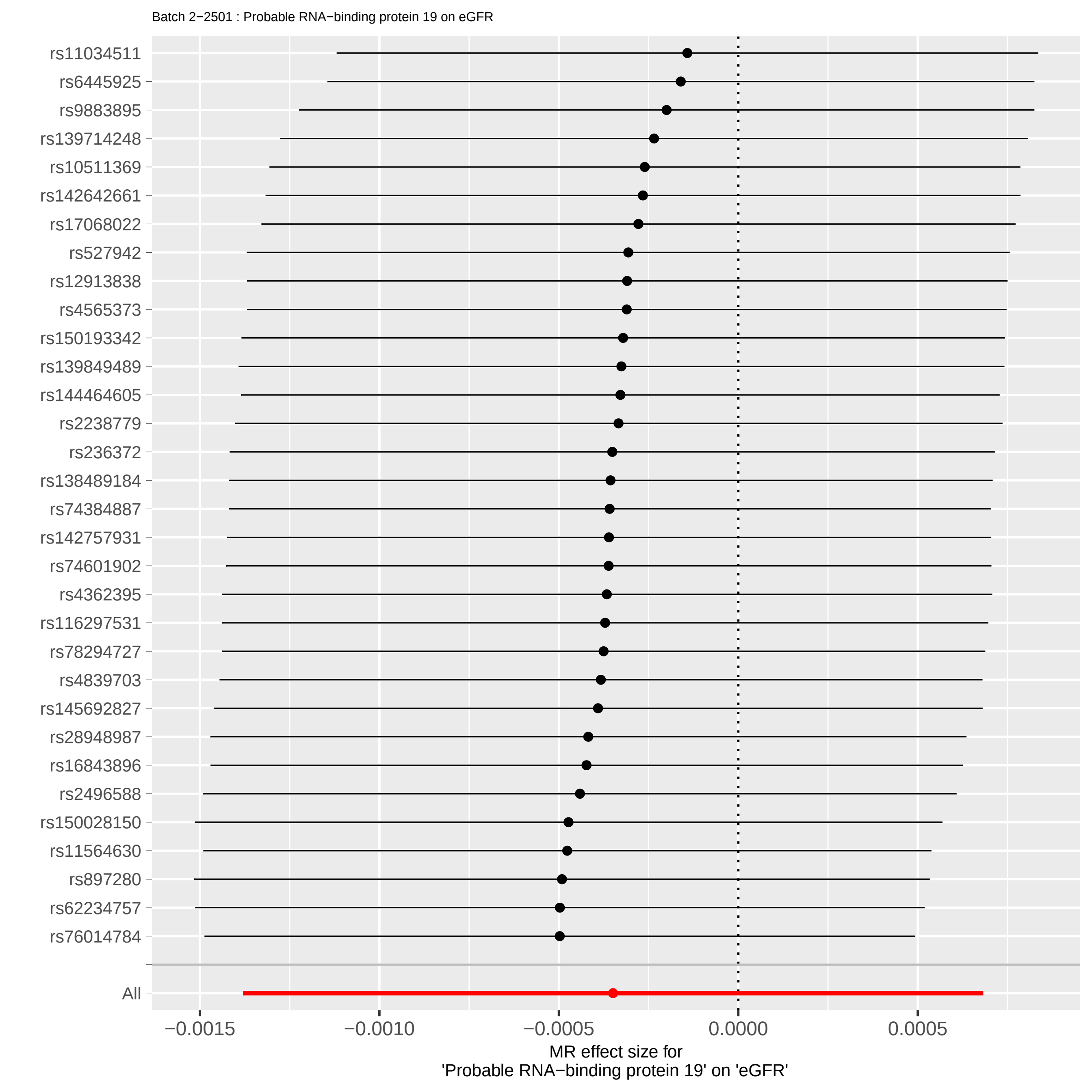

Batch 2−2501 : Probable RNA−binding protein 19 on eGFR
rs11034511
rs6445925
rs9883895
rs139714248
rs10511369
rs142642661
rs17068022
rs527942
rs12913838
rs4565373
rs150193342
rs139849489
rs144464605
rs2238779
rs236372
rs138489184
rs74384887
rs142757931
rs74601902
rs4362395
rs116297531
rs78294727
rs4839703
rs145692827
rs28948987
rs16843896
rs2496588
rs150028150
rs11564630
rs897280
rs62234757
rs76014784
All
−0.0015
−0.0010
−0.0005
0.0000
0.0005
MR effect size for
'Probable RNA−binding protein 19' on 'eGFR'

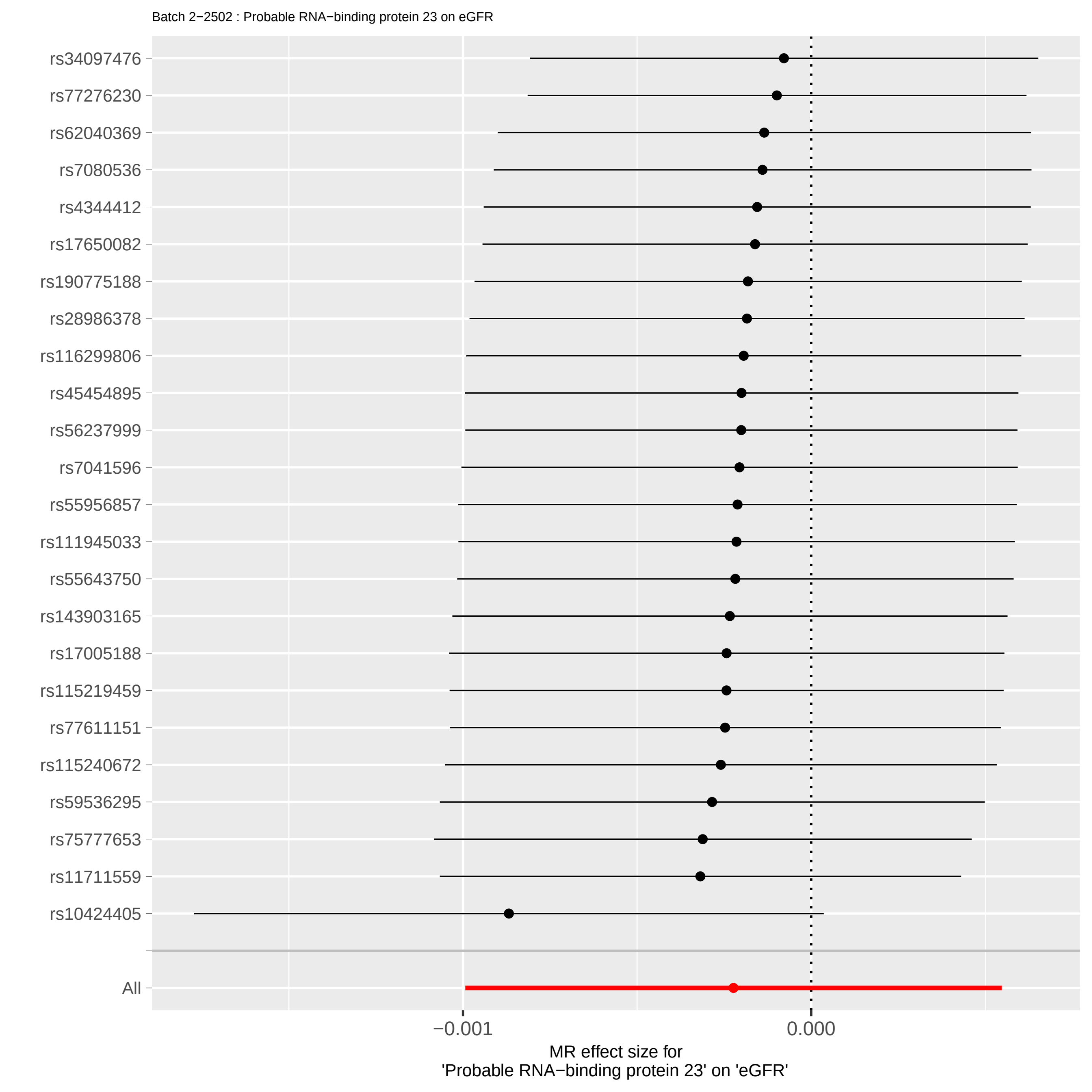

Batch 2−2502 : Probable RNA−binding protein 23 on eGFR
rs34097476
rs77276230
rs62040369
rs7080536
rs4344412
rs17650082
rs190775188
rs28986378
rs116299806
rs45454895
rs56237999
rs7041596
rs55956857
rs111945033
rs55643750
rs143903165
rs17005188
rs115219459
rs77611151
rs115240672
rs59536295
rs75777653
rs11711559
rs10424405
All
−0.001
0.000
MR effect size for
'Probable RNA−binding protein 23' on 'eGFR'

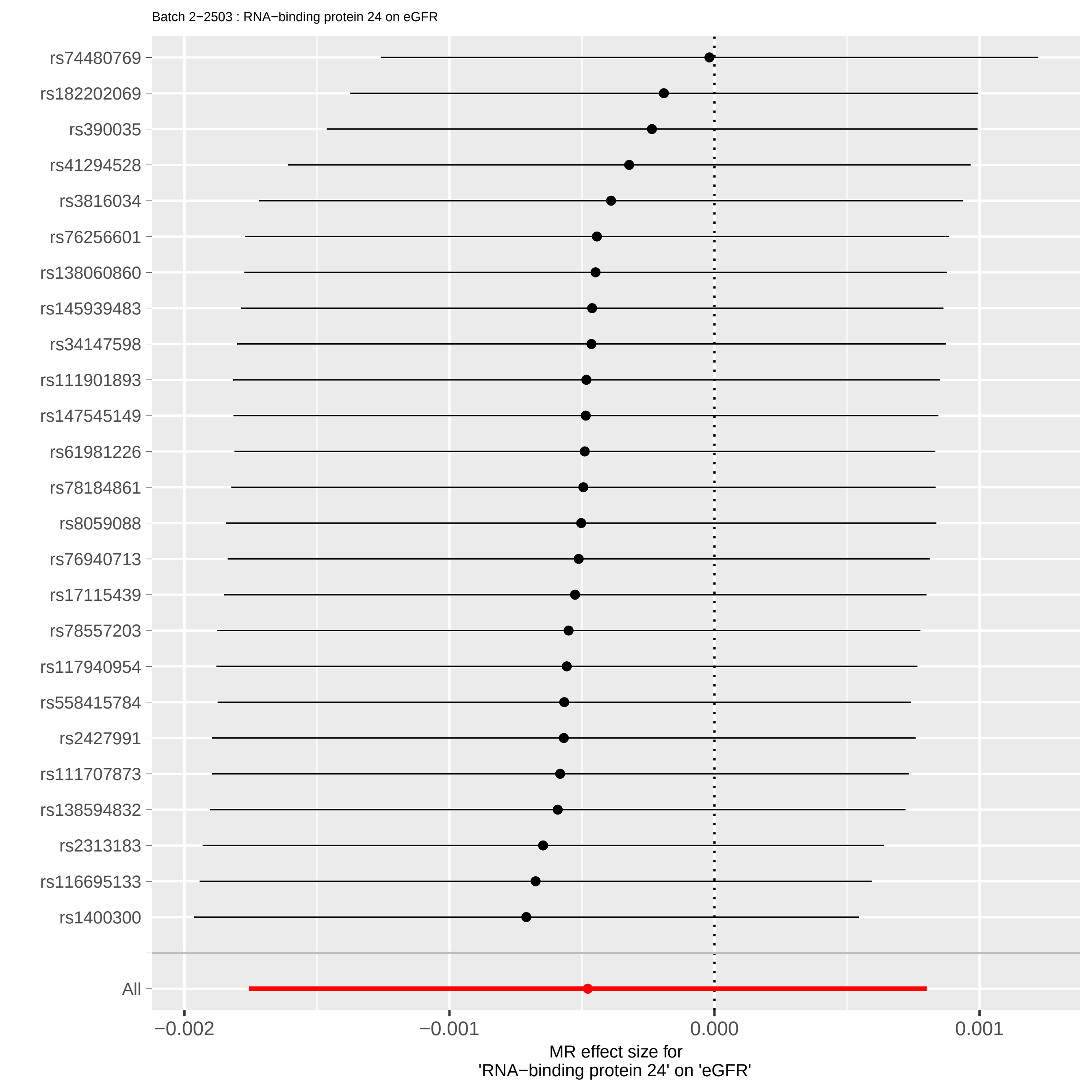

Batch 2−2503 : RNA−binding protein 24 on eGFR
rs74480769
rs182202069
rs390035
rs41294528
rs3816034
rs76256601
rs138060860
rs145939483
rs34147598
rs111901893
rs147545149
rs61981226
rs78184861
rs8059088
rs76940713
rs17115439
rs78557203
rs117940954
rs558415784
rs2427991
rs111707873
rs138594832
rs2313183
rs116695133
rs1400300
All
−0.002
−0.001
0.000
0.001
MR effect size for
'RNA−binding protein 24' on 'eGFR'

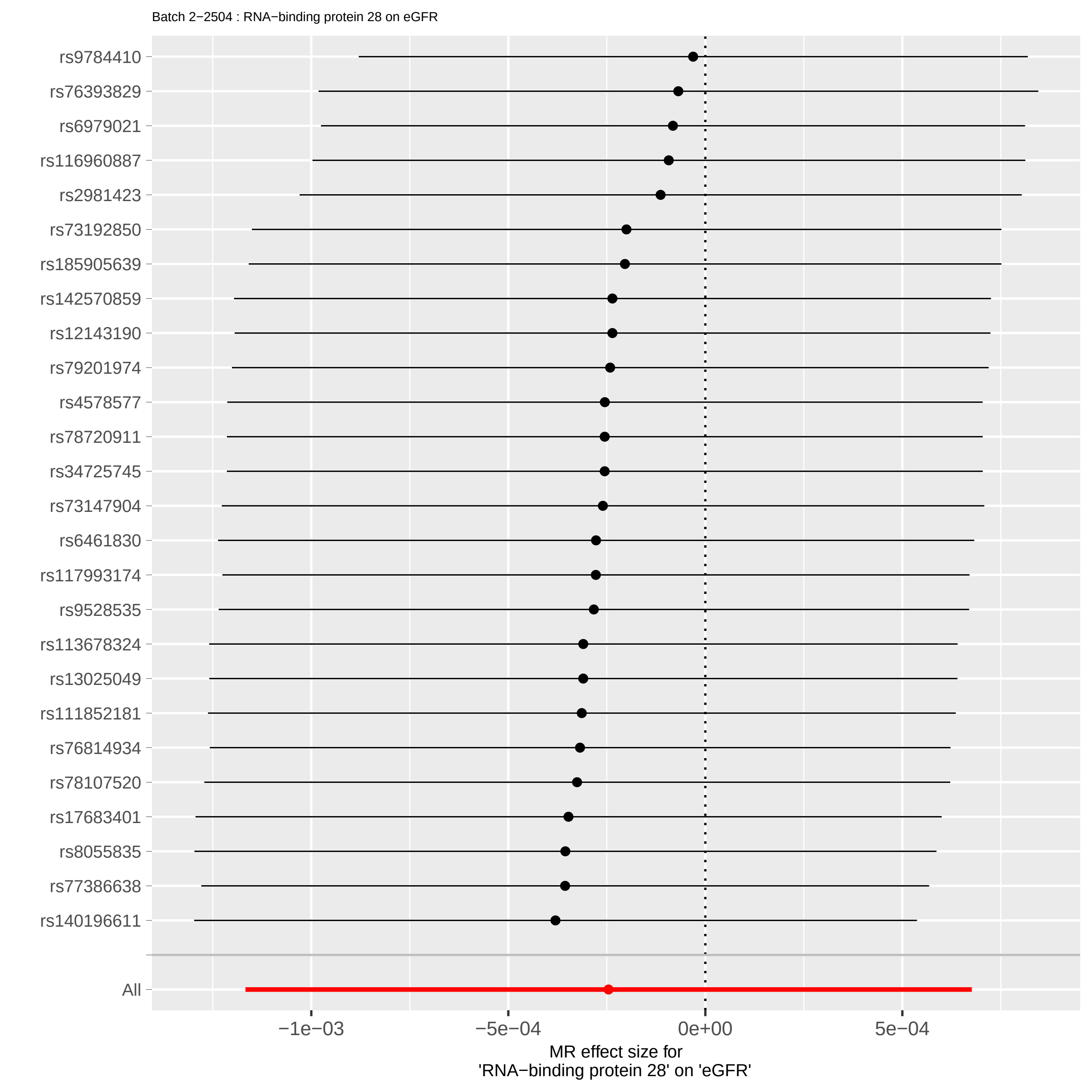

Batch 2−2504 : RNA−binding protein 28 on eGFR
rs9784410
rs76393829
rs6979021
rs116960887
rs2981423
rs73192850
rs185905639
rs142570859
rs12143190
rs79201974
rs4578577
rs78720911
rs34725745
rs73147904
rs6461830
rs117993174
rs9528535
rs113678324
rs13025049
rs111852181
rs76814934
rs78107520
rs17683401
rs8055835
rs77386638
rs140196611
All
−1e−03
−5e−04
0e+00
5e−04
MR effect size for
'RNA−binding protein 28' on 'eGFR'

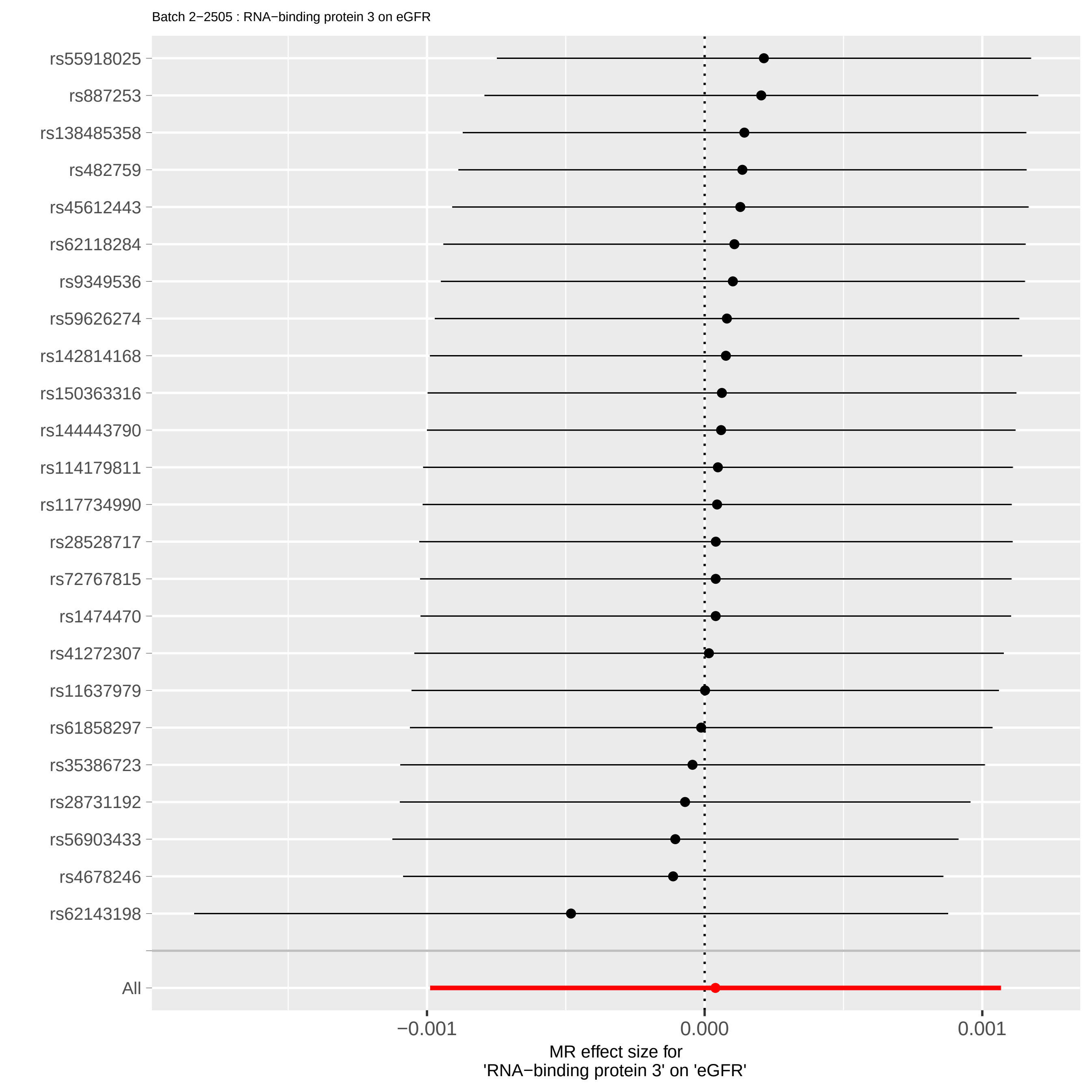

Batch 2−2505 : RNA−binding protein 3 on eGFR
rs55918025
rs887253
rs138485358
rs482759
rs45612443
rs62118284
rs9349536
rs59626274
rs142814168
rs150363316
rs144443790
rs114179811
rs117734990
rs28528717
rs72767815
rs1474470
rs41272307
rs11637979
rs61858297
rs35386723
rs28731192
rs56903433
rs4678246
rs62143198
All
−0.001
0.000
0.001
MR effect size for
'RNA−binding protein 3' on 'eGFR'

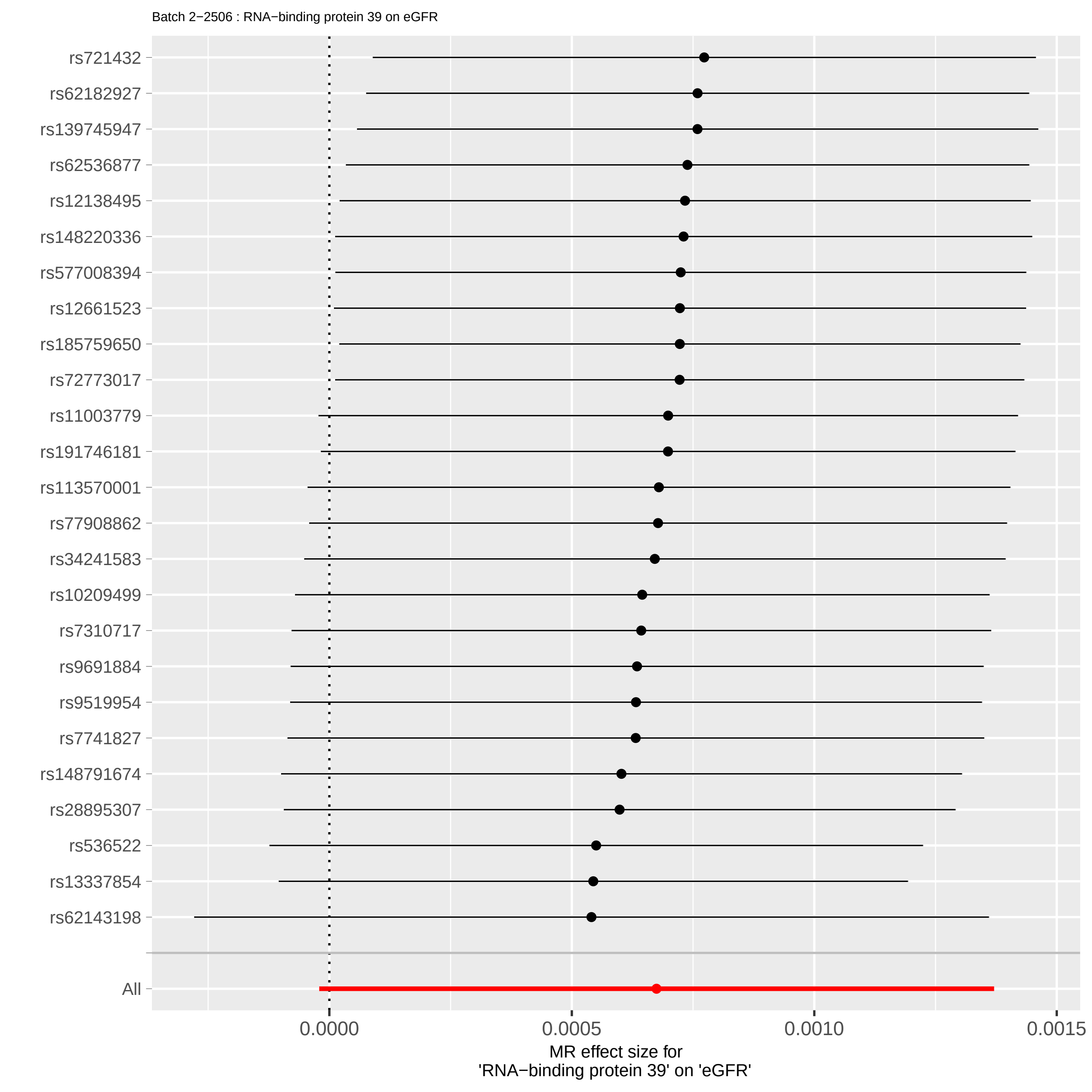
Batch 2−2506 : RNA−binding protein 39 on eGFR
rs721432
rs62182927
rs139745947
rs62536877
rs12138495
rs148220336
rs577008394
rs12661523
rs185759650
rs72773017
rs11003779
rs191746181
rs113570001
rs77908862
rs34241583
rs10209499
rs7310717
rs9691884
rs9519954
rs7741827
rs148791674
rs28895307
rs536522
rs13337854
rs62143198
All
0.0000
0.0005
0.0010
0.0015
MR effect size for
'RNA−binding protein 39' on 'eGFR'

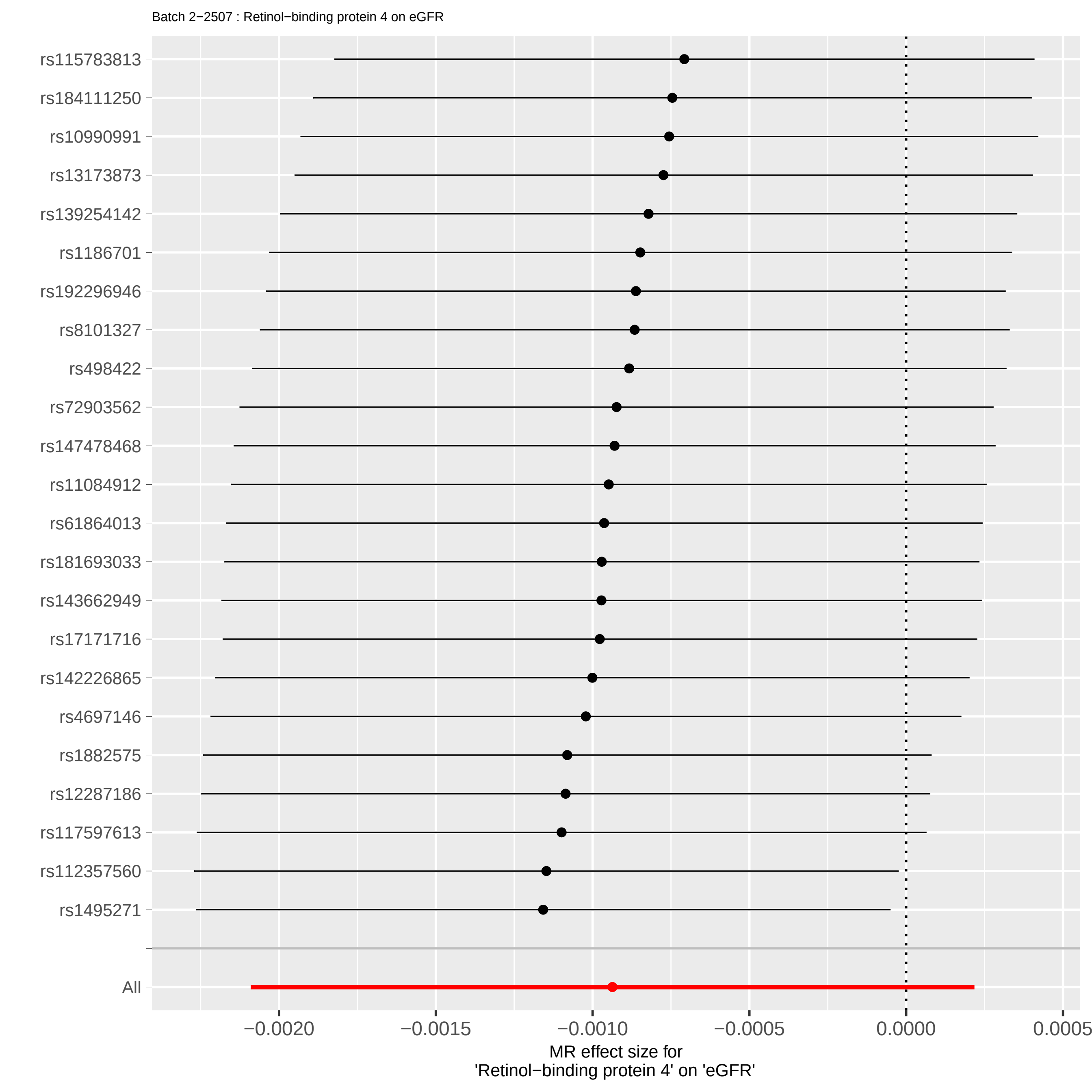
Batch 2−2507 : Retinol−binding protein 4 on eGFR
rs115783813
rs184111250
rs10990991
rs13173873
rs139254142
rs1186701
rs192296946
rs8101327
rs498422
rs72903562
rs147478468
rs11084912
rs61864013
rs181693033
rs143662949
rs17171716
rs142226865
rs4697146
rs1882575
rs12287186
rs117597613
rs112357560
rs1495271
All
−0.0020
−0.0015
−0.0010
−0.0005
0.0000
0.0005
MR effect size for
'Retinol−binding protein 4' on 'eGFR'

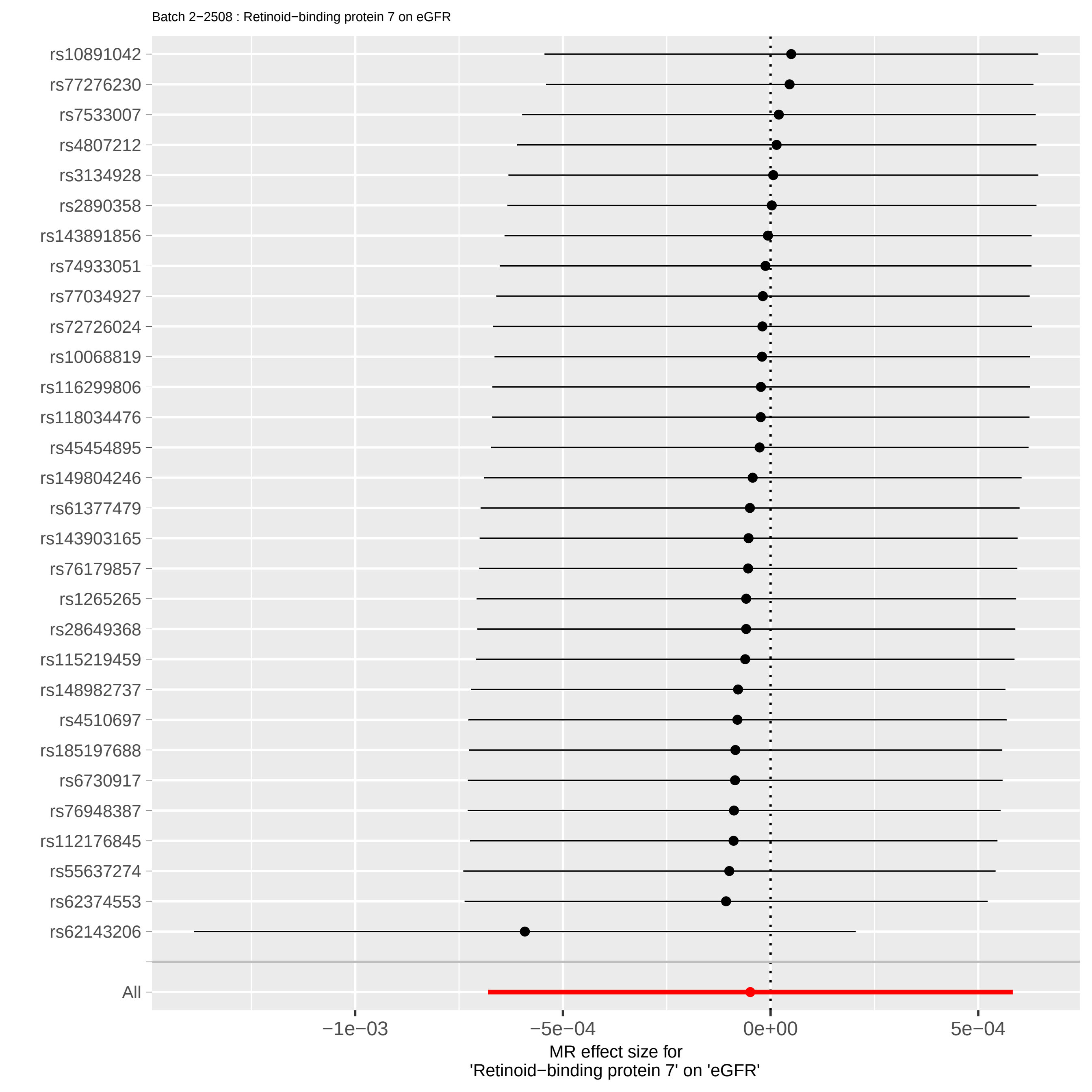

Batch 2−2508 : Retinoid−binding protein 7 on eGFR
rs10891042
rs77276230
rs7533007
rs4807212
rs3134928
rs2890358
rs143891856
rs74933051
rs77034927
rs72726024
rs10068819
rs116299806
rs118034476
rs45454895
rs149804246
rs61377479
rs143903165
rs76179857
rs1265265
rs28649368
rs115219459
rs148982737
rs4510697
rs185197688
rs6730917
rs76948387
rs112176845
rs55637274
rs62374553
rs62143206
All
−1e−03
−5e−04
0e+00
5e−04
MR effect size for
'Retinoid−binding protein 7' on 'eGFR'

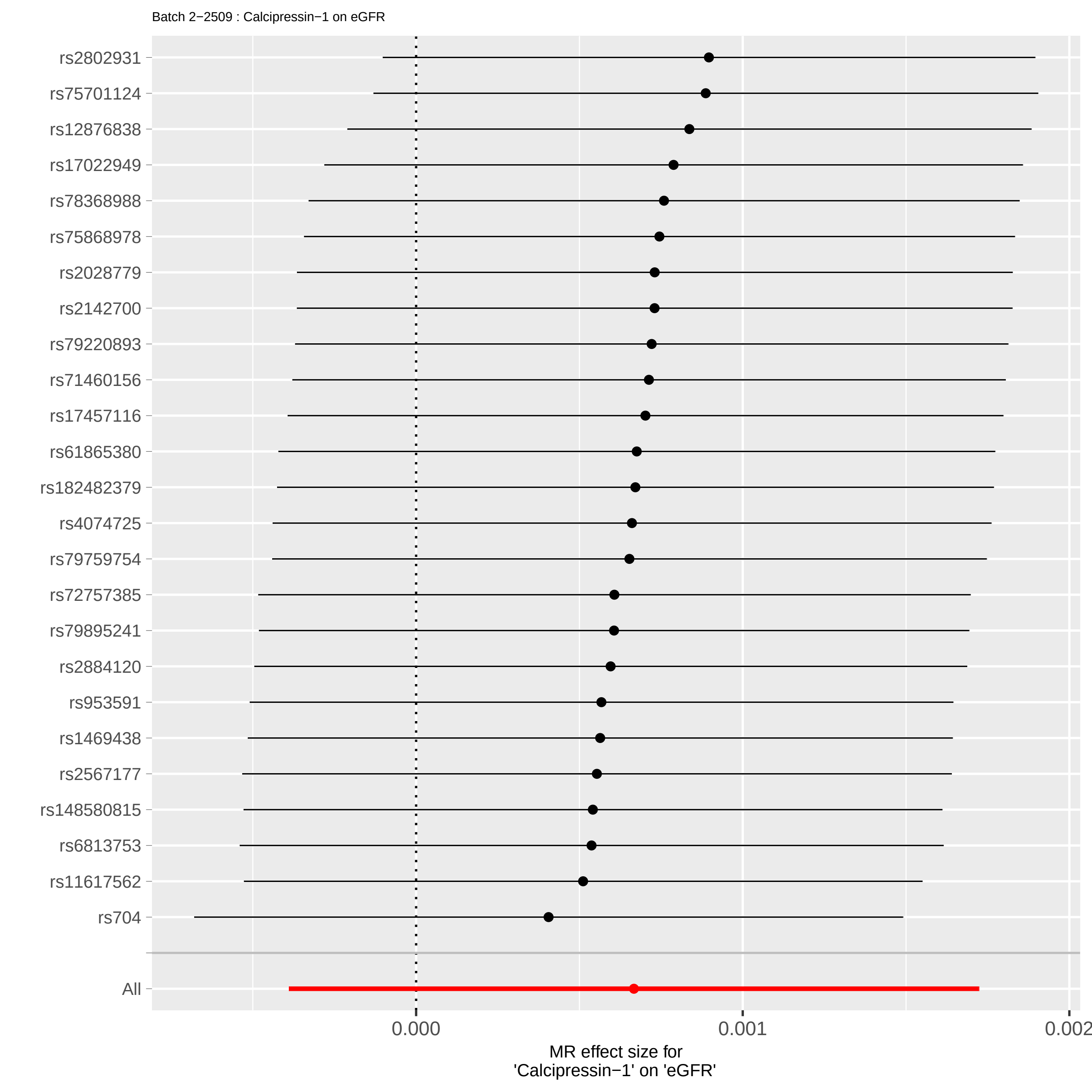

Batch 2−2509 : Calcipressin−1 on eGFR
rs2802931
rs75701124
rs12876838
rs17022949
rs78368988
rs75868978
rs2028779
rs2142700
rs79220893
rs71460156
rs17457116
rs61865380
rs182482379
rs4074725
rs79759754
rs72757385
rs79895241
rs2884120
rs953591
rs1469438
rs2567177
rs148580815
rs6813753
rs11617562
rs704
All
0.000
0.001
0.002
MR effect size for
'Calcipressin−1' on 'eGFR'

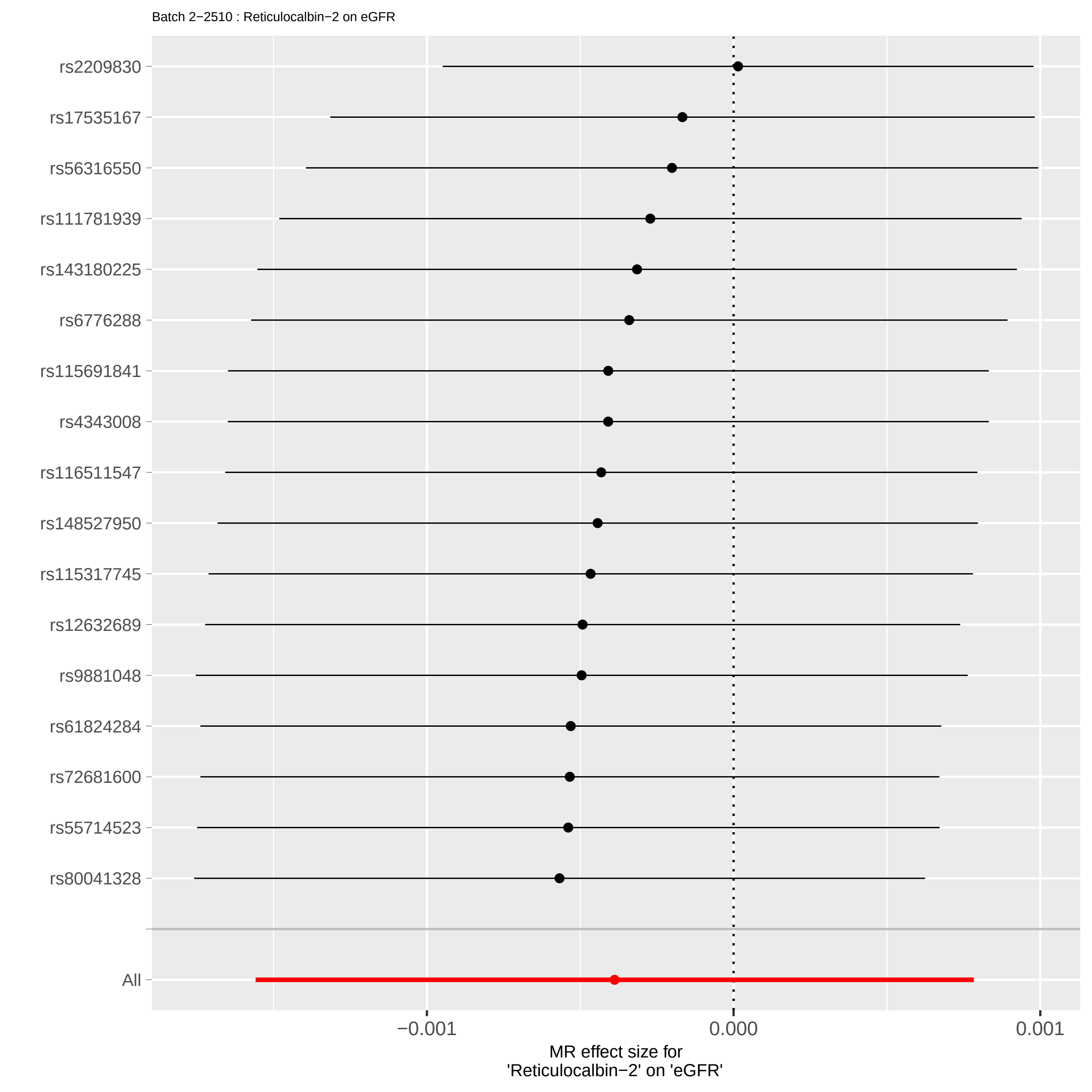

Batch 2−2510 : Reticulocalbin−2 on eGFR
rs2209830
rs17535167
rs56316550
rs111781939
rs143180225
rs6776288
rs115691841
rs4343008
rs116511547
rs148527950
rs115317745
rs12632689
rs9881048
rs61824284
rs72681600
rs55714523
rs80041328
All
−0.001
0.000
0.001
MR effect size for
'Reticulocalbin−2' on 'eGFR'

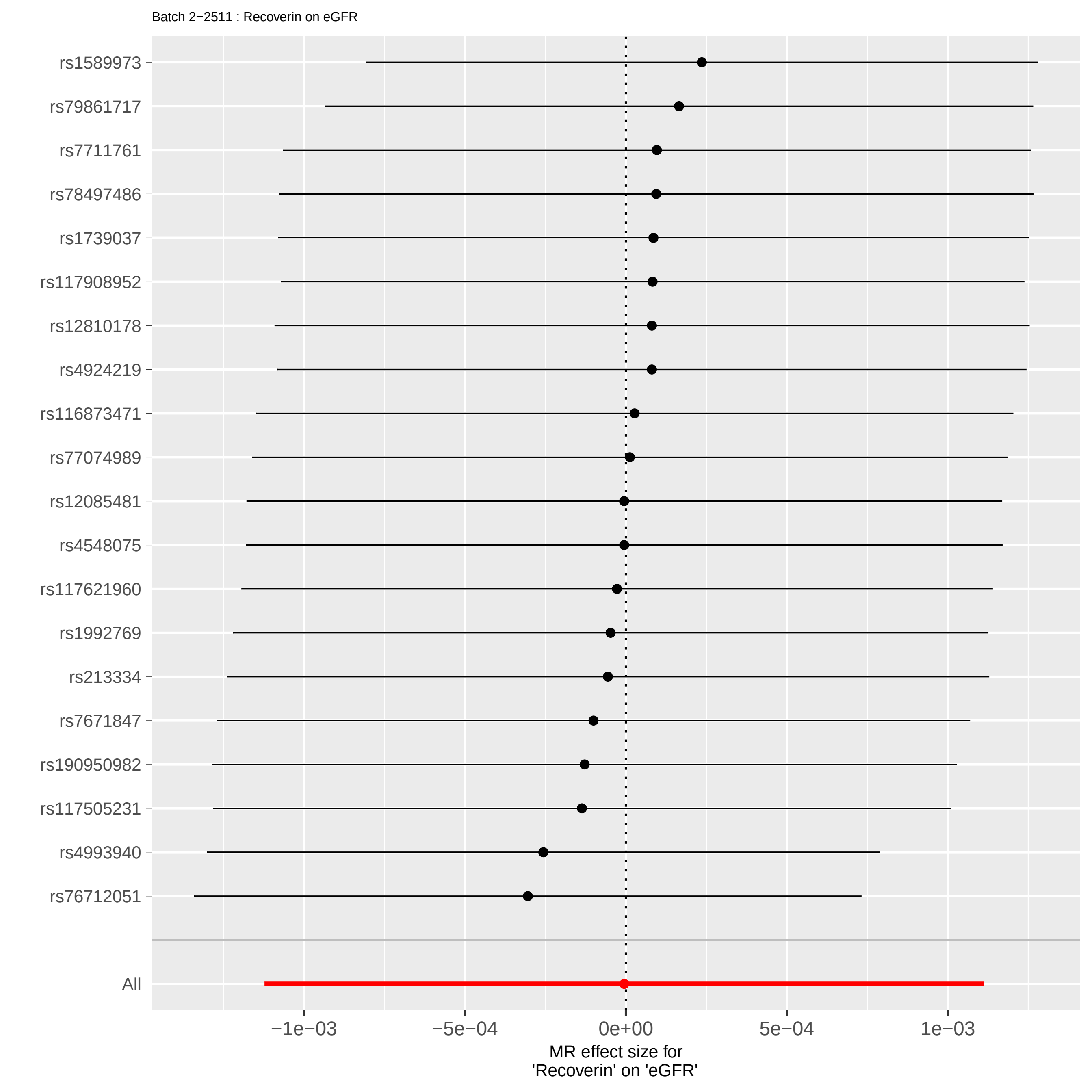

Batch 2−2511 : Recoverin on eGFR
rs1589973
rs79861717
rs7711761
rs78497486
rs1739037
rs117908952
rs12810178
rs4924219
rs116873471
rs77074989
rs12085481
rs4548075
rs117621960
rs1992769
rs213334
rs7671847
rs190950982
rs117505231
rs4993940
rs76712051
All
−1e−03
−5e−04
0e+00
5e−04
1e−03
MR effect size for
'Recoverin' on 'eGFR'

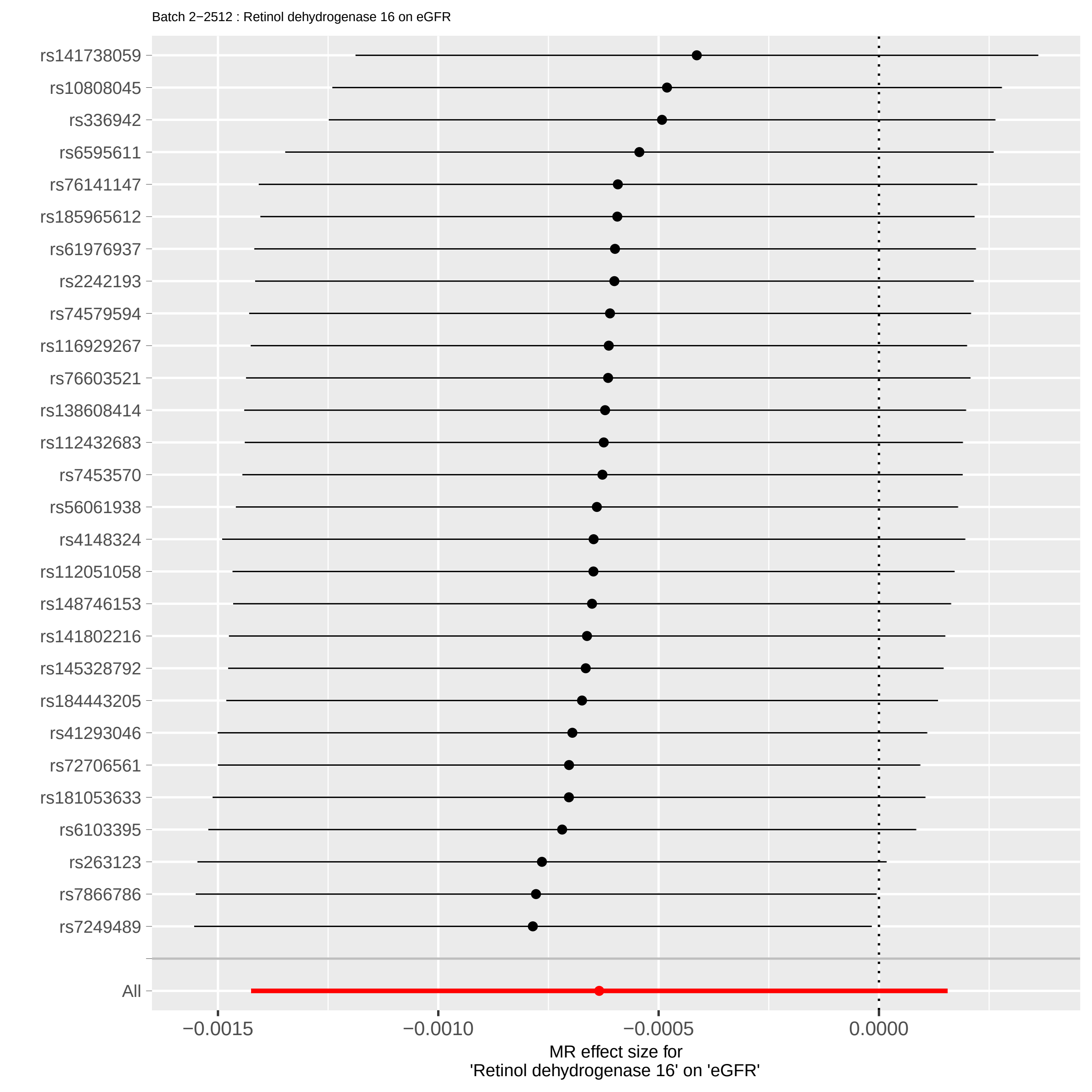

Batch 2−2512 : Retinol dehydrogenase 16 on eGFR
rs141738059
rs10808045
rs336942
rs6595611
rs76141147
rs185965612
rs61976937
rs2242193
rs74579594
rs116929267
rs76603521
rs138608414
rs112432683
rs7453570
rs56061938
rs4148324
rs112051058
rs148746153
rs141802216
rs145328792
rs184443205
rs41293046
rs72706561
rs181053633
rs6103395
rs263123
rs7866786
rs7249489
All
−0.0015
−0.0010
−0.0005
0.0000
MR effect size for
'Retinol dehydrogenase 16' on 'eGFR'

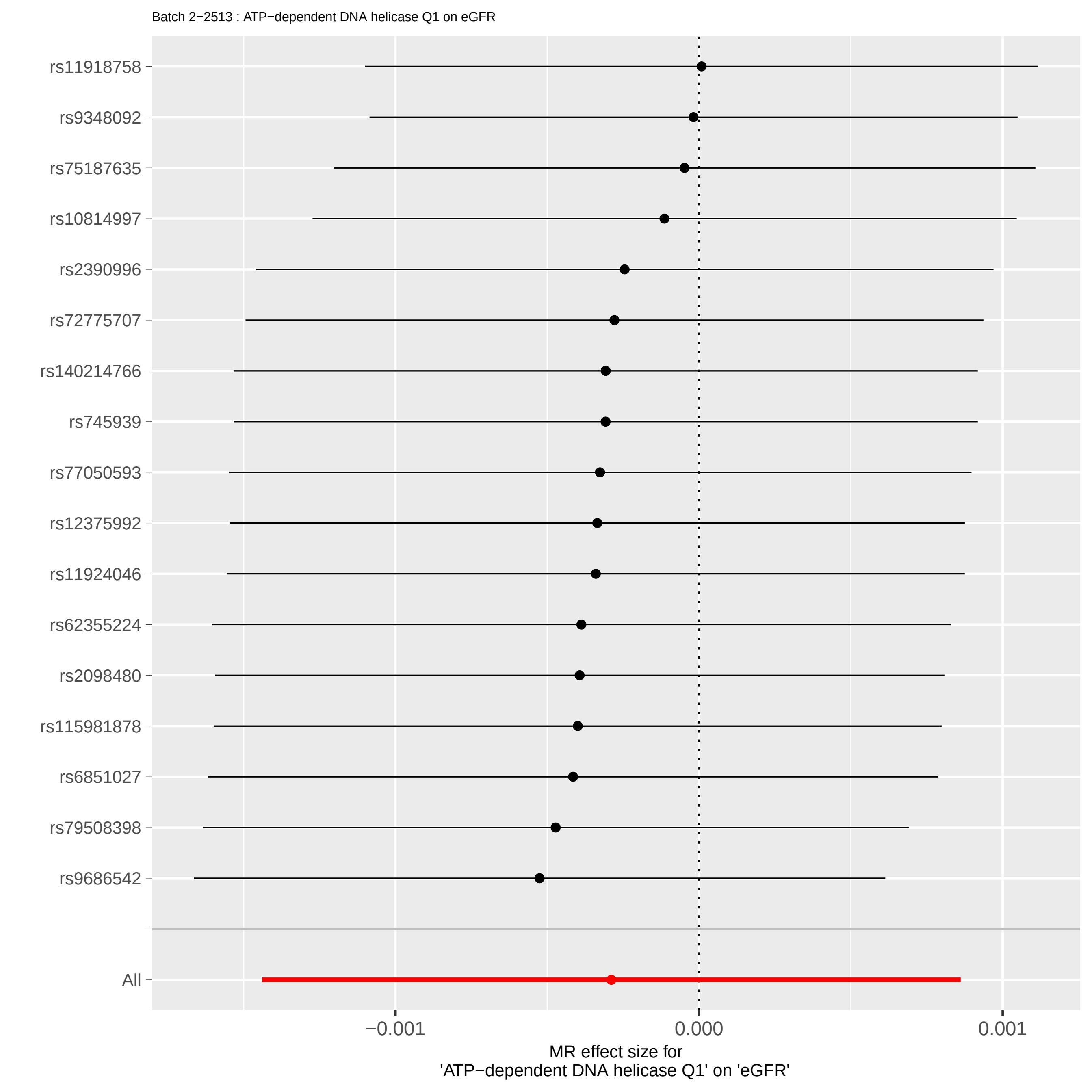

Batch 2−2513 : ATP−dependent DNA helicase Q1 on eGFR
rs11918758
rs9348092
rs75187635
rs10814997
rs2390996
rs72775707
rs140214766
rs745939
rs77050593
rs12375992
rs11924046
rs62355224
rs2098480
rs115981878
rs6851027
rs79508398
rs9686542
All
−0.001
0.000
0.001
MR effect size for
'ATP−dependent DNA helicase Q1' on 'eGFR'

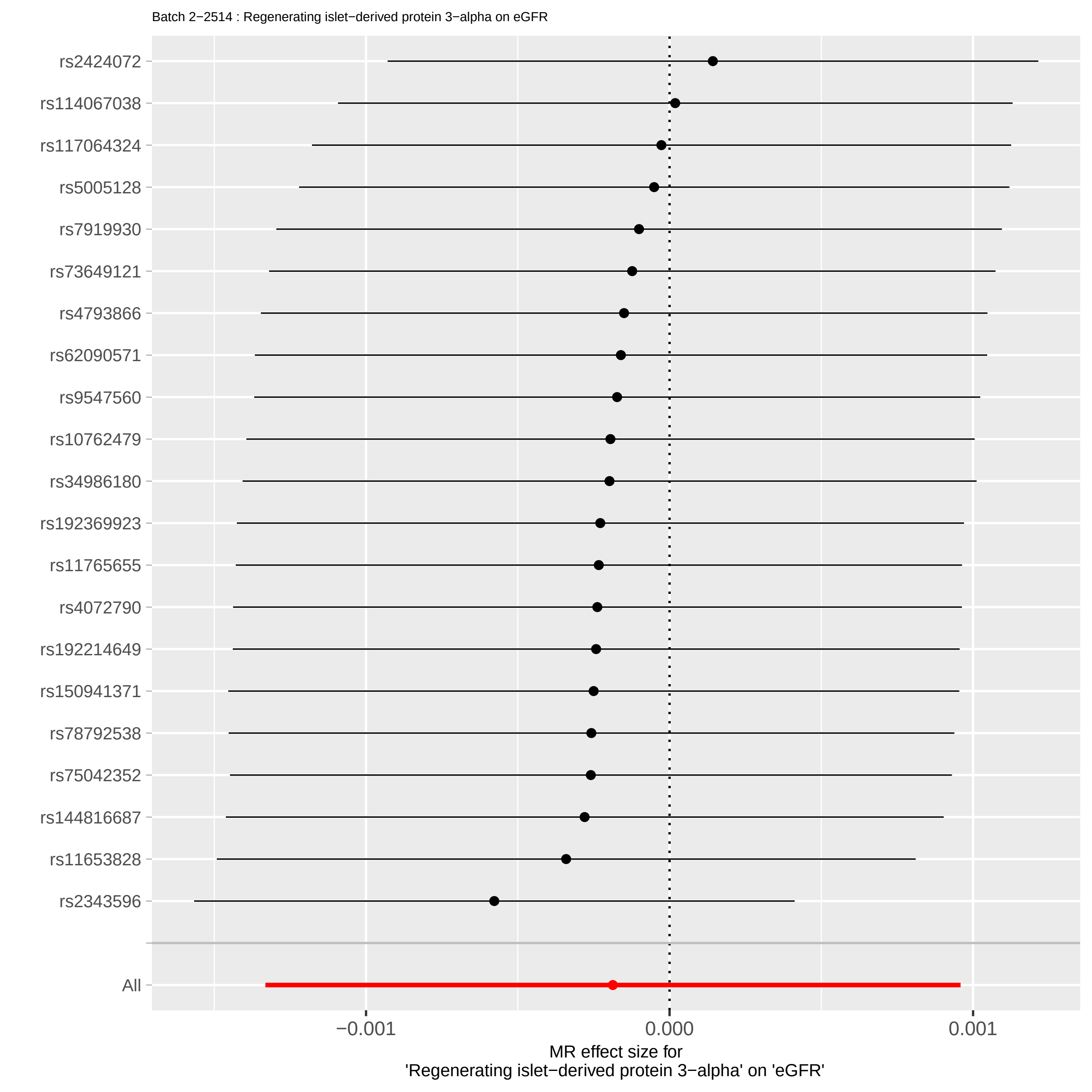
Batch 2−2514 : Regenerating islet−derived protein 3−alpha on eGFR
rs2424072
rs114067038
rs117064324
rs5005128
rs7919930
rs73649121
rs4793866
rs62090571
rs9547560
rs10762479
rs34986180
rs192369923
rs11765655
rs4072790
rs192214649
rs150941371
rs78792538
rs75042352
rs144816687
rs11653828
rs2343596
All
−0.001
0.000
0.001
MR effect size for
'Regenerating islet−derived protein 3−alpha' on 'eGFR'

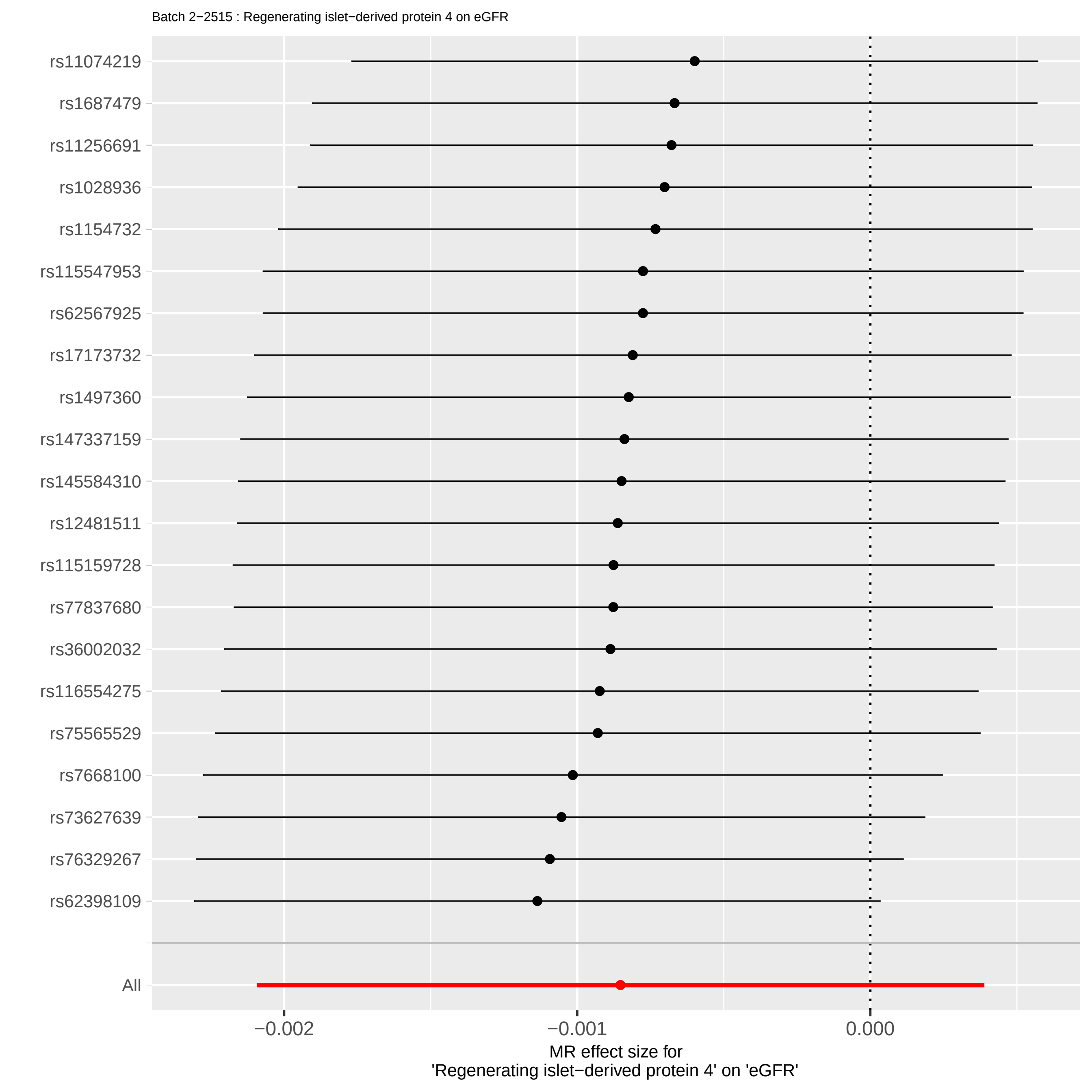
Batch 2−2515 : Regenerating islet−derived protein 4 on eGFR
rs11074219
rs1687479
rs11256691
rs1028936
rs1154732
rs115547953
rs62567925
rs17173732
rs1497360
rs147337159
rs145584310
rs12481511
rs115159728
rs77837680
rs36002032
rs116554275
rs75565529
rs7668100
rs73627639
rs76329267
rs62398109
All
−0.002
−0.001
0.000
MR effect size for
'Regenerating islet−derived protein 4' on 'eGFR'

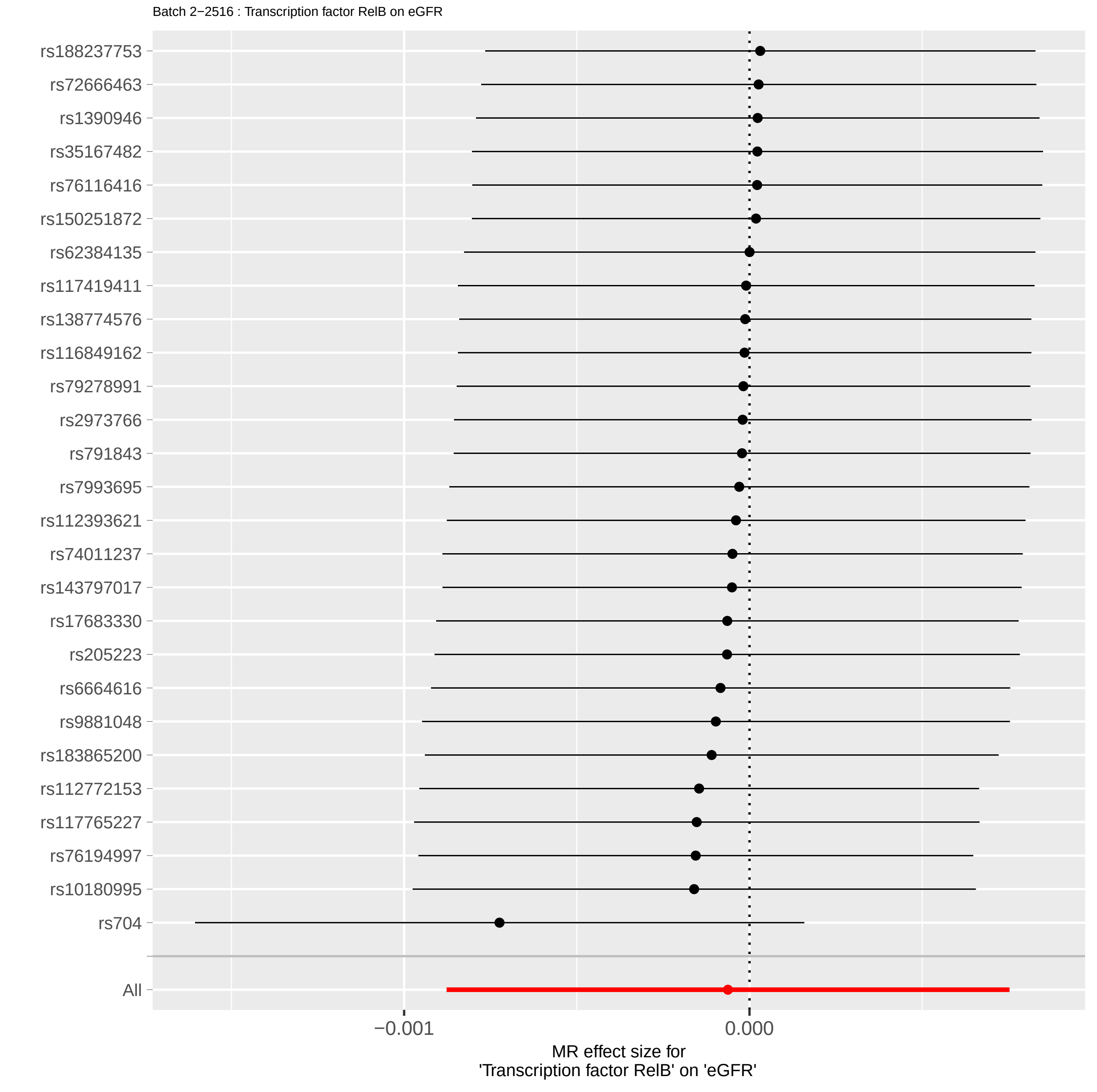

Batch 2−2516 : Transcription factor RelB on eGFR
rs188237753
rs72666463
rs1390946
rs35167482
rs76116416
rs150251872
rs62384135
rs117419411
rs138774576
rs116849162
rs79278991
rs2973766
rs791843
rs7993695
rs112393621
rs74011237
rs143797017
rs17683330
rs205223
rs6664616
rs9881048
rs183865200
rs112772153
rs117765227
rs76194997
rs10180995
rs704
All
−0.001
0.000
MR effect size for
'Transcription factor RelB' on 'eGFR'

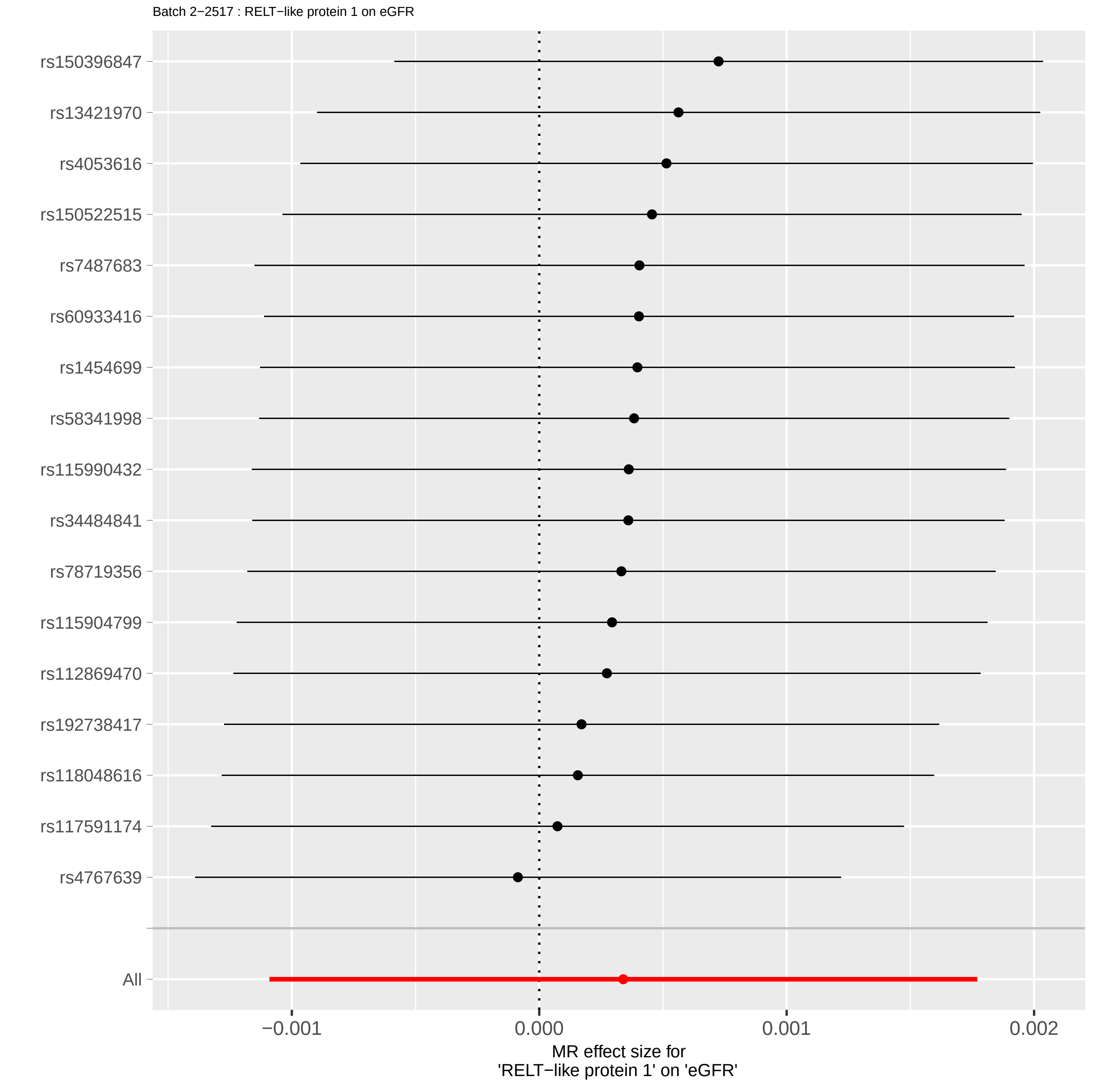

Batch 2−2517 : RELT−like protein 1 on eGFR
rs150396847
rs13421970
rs4053616
rs150522515
rs7487683
rs60933416
rs1454699
rs58341998
rs115990432
rs34484841
rs78719356
rs115904799
rs112869470
rs192738417
rs118048616
rs117591174
rs4767639
All
−0.001
0.000
0.001
0.002
MR effect size for
'RELT−like protein 1' on 'eGFR'

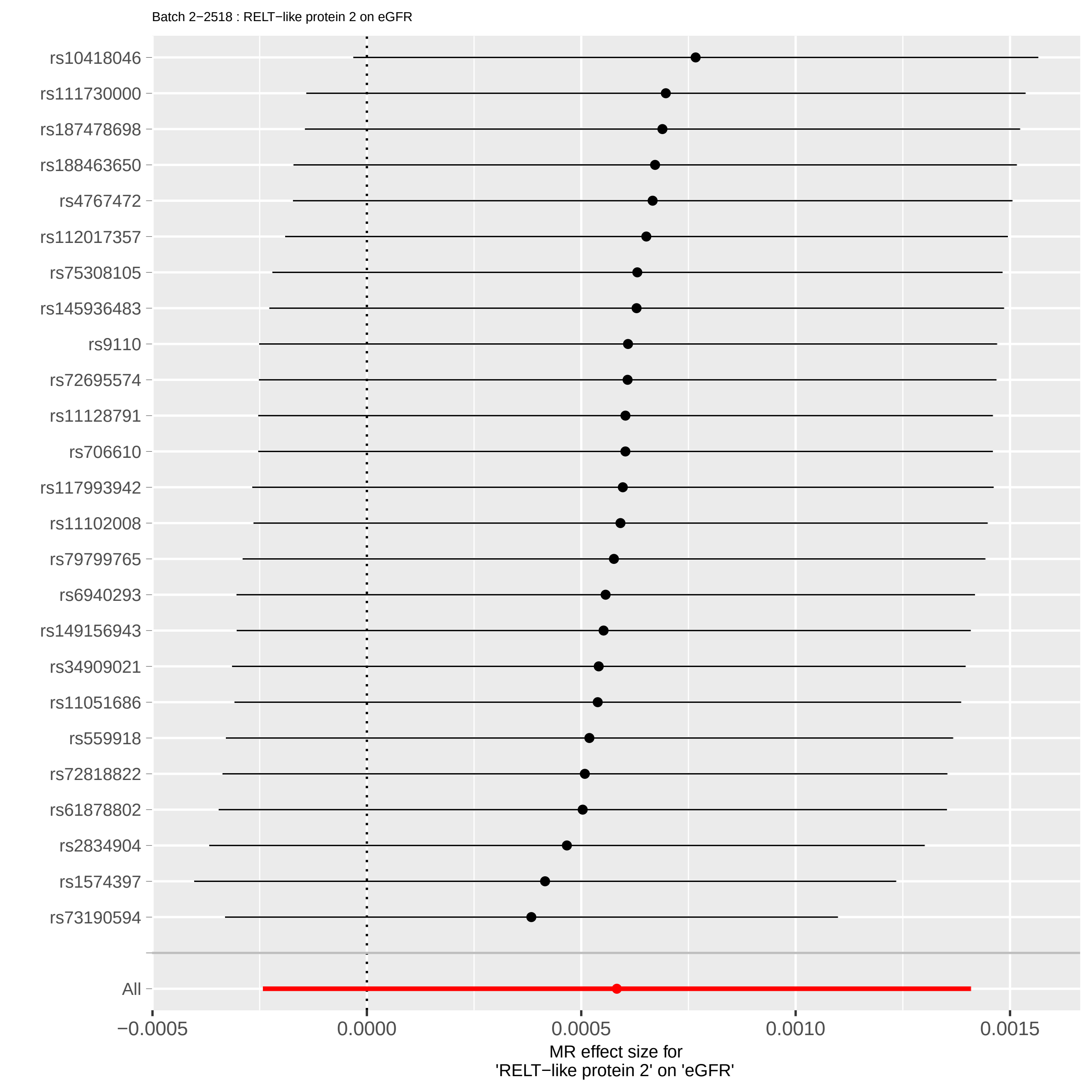

Batch 2−2518 : RELT−like protein 2 on eGFR
rs10418046
rs111730000
rs187478698
rs188463650
rs4767472
rs112017357
rs75308105
rs145936483
rs9110
rs72695574
rs11128791
rs706610
rs117993942
rs11102008
rs79799765
rs6940293
rs149156943
rs34909021
rs11051686
rs559918
rs72818822
rs61878802
rs2834904
rs1574397
rs73190594
All
−0.0005
0.0000
0.0005
0.0010
0.0015
MR effect size for
'RELT−like protein 2' on 'eGFR'

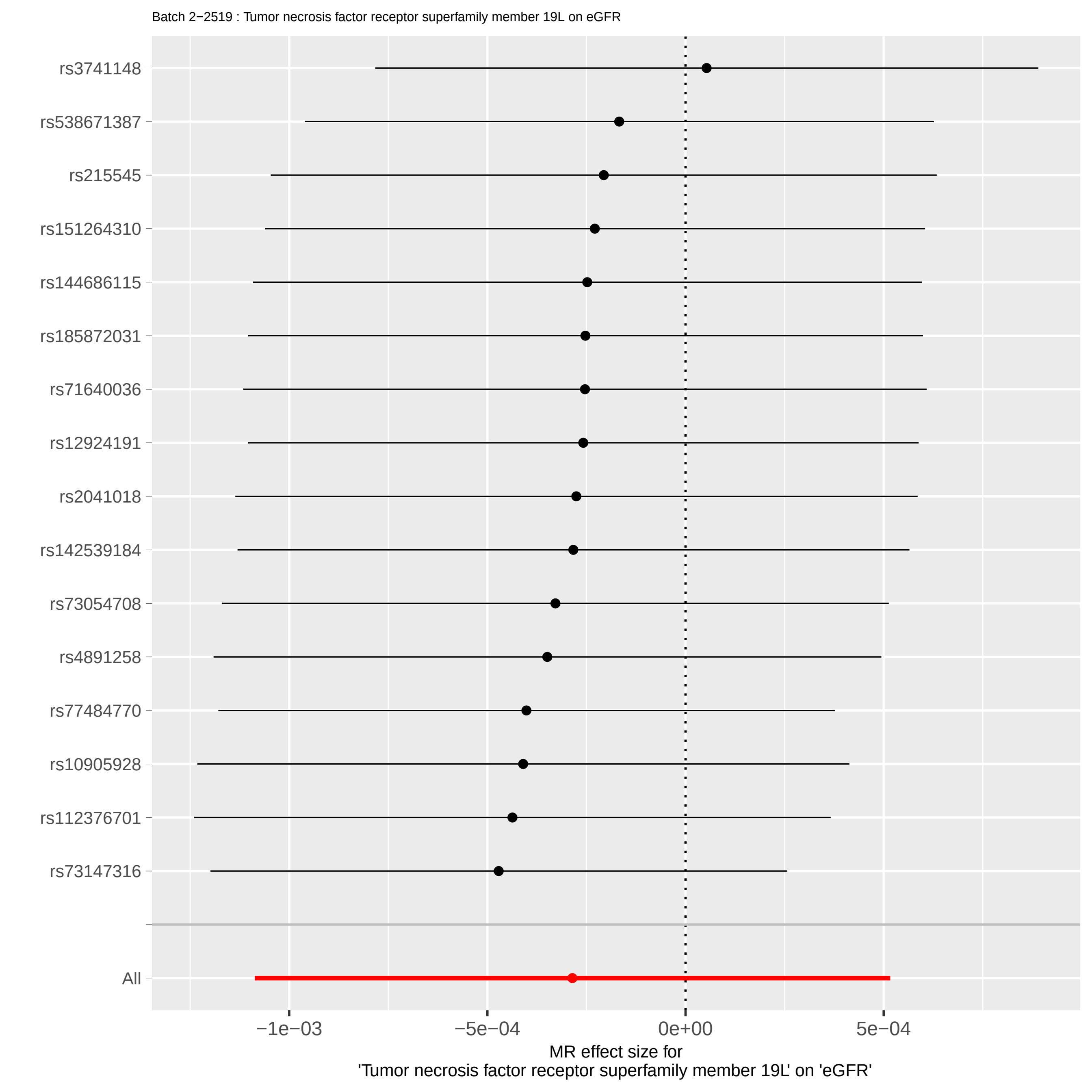

Batch 2−2519 : Tumor necrosis factor receptor superfamily member 19L on eGFR
rs3741148
rs538671387
rs215545
rs151264310
rs144686115
rs185872031
rs71640036
rs12924191
rs2041018
rs142539184
rs73054708
rs4891258
rs77484770
rs10905928
rs112376701
rs73147316
All
−1e−03
−5e−04
0e+00
5e−04
MR effect size for
'Tumor necrosis factor receptor superfamily member 19L' on 'eGFR'

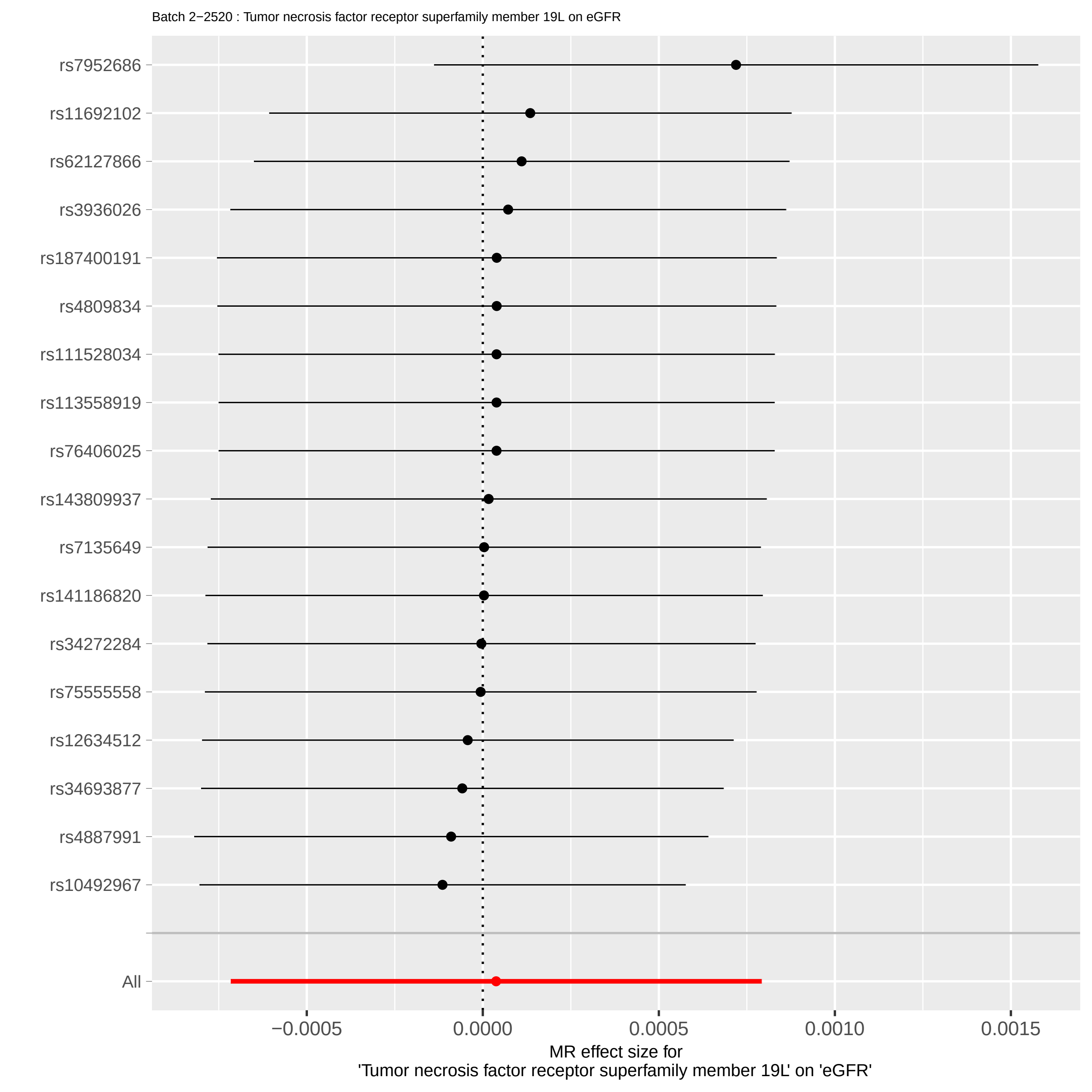

Batch 2−2520 : Tumor necrosis factor receptor superfamily member 19L on eGFR
rs7952686
rs11692102
rs62127866
rs3936026
rs187400191
rs4809834
rs111528034
rs113558919
rs76406025
rs143809937
rs7135649
rs141186820
rs34272284
rs75555558
rs12634512
rs34693877
rs4887991
rs10492967
All
−0.0005
0.0000
0.0005
0.0010
0.0015
MR effect size for
'Tumor necrosis factor receptor superfamily member 19L' on 'eGFR'

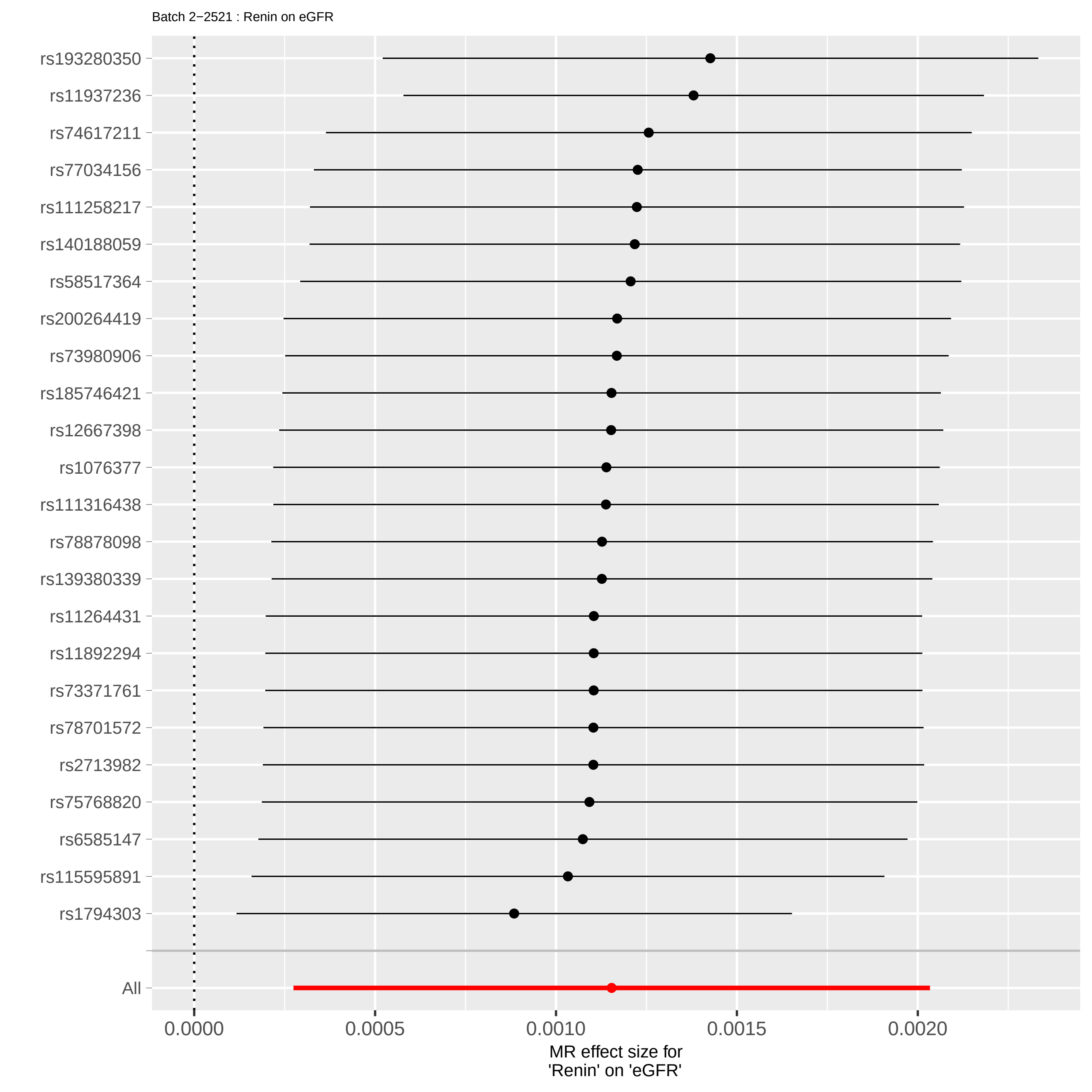

Batch 2−2521 : Renin on eGFR
rs193280350
rs11937236
rs74617211
rs77034156
rs111258217
rs140188059
rs58517364
rs200264419
rs73980906
rs185746421
rs12667398
rs1076377
rs111316438
rs78878098
rs139380339
rs11264431
rs11892294
rs73371761
rs78701572
rs2713982
rs75768820
rs6585147
rs115595891
rs1794303
All
0.0000
0.0005
0.0010
0.0015
0.0020
MR effect size for
'Renin' on 'eGFR'

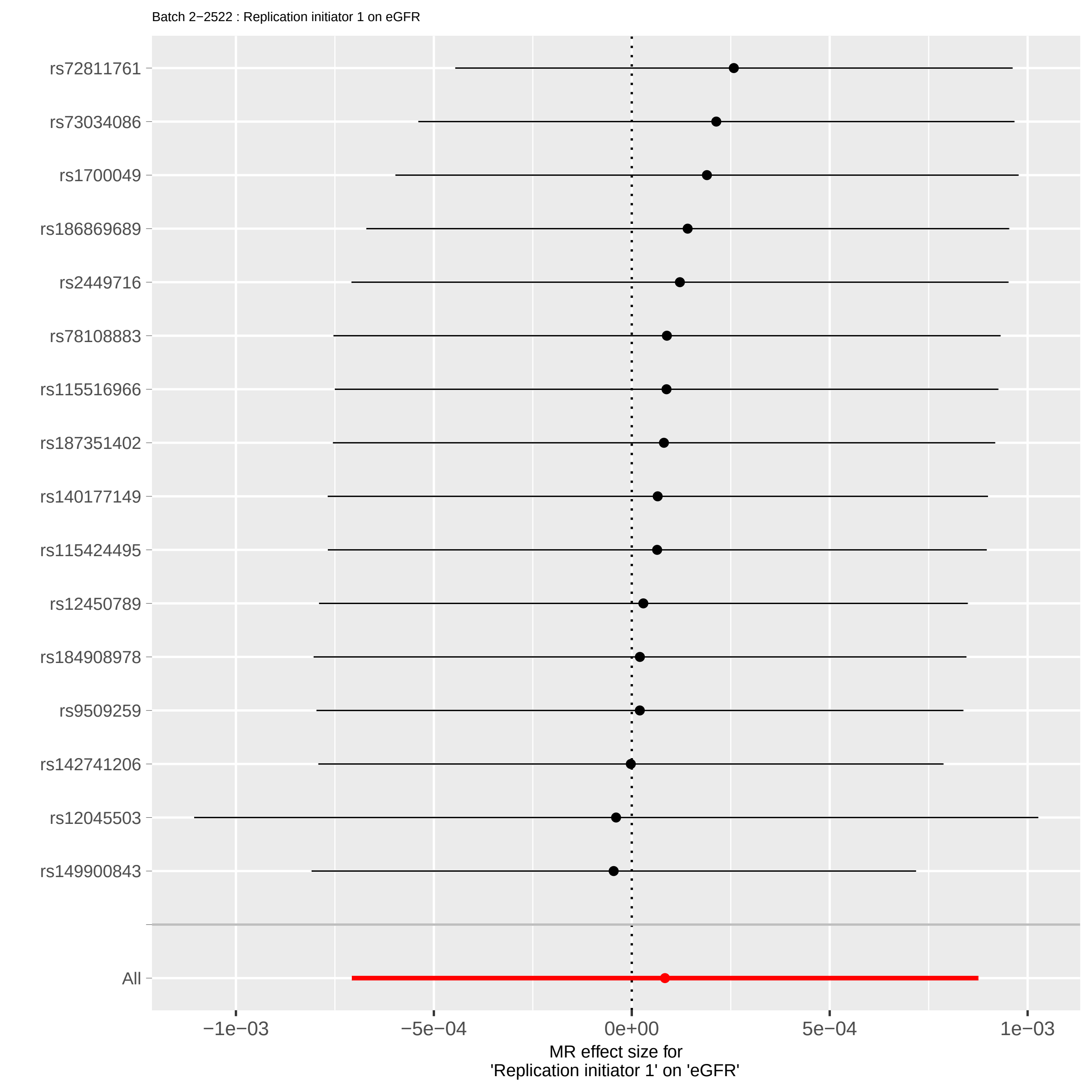

Batch 2−2522 : Replication initiator 1 on eGFR
rs72811761
rs73034086
rs1700049
rs186869689
rs2449716
rs78108883
rs115516966
rs187351402
rs140177149
rs115424495
rs12450789
rs184908978
rs9509259
rs142741206
rs12045503
rs149900843
All
−1e−03
−5e−04
0e+00
5e−04
1e−03
MR effect size for
'Replication initiator 1' on 'eGFR'

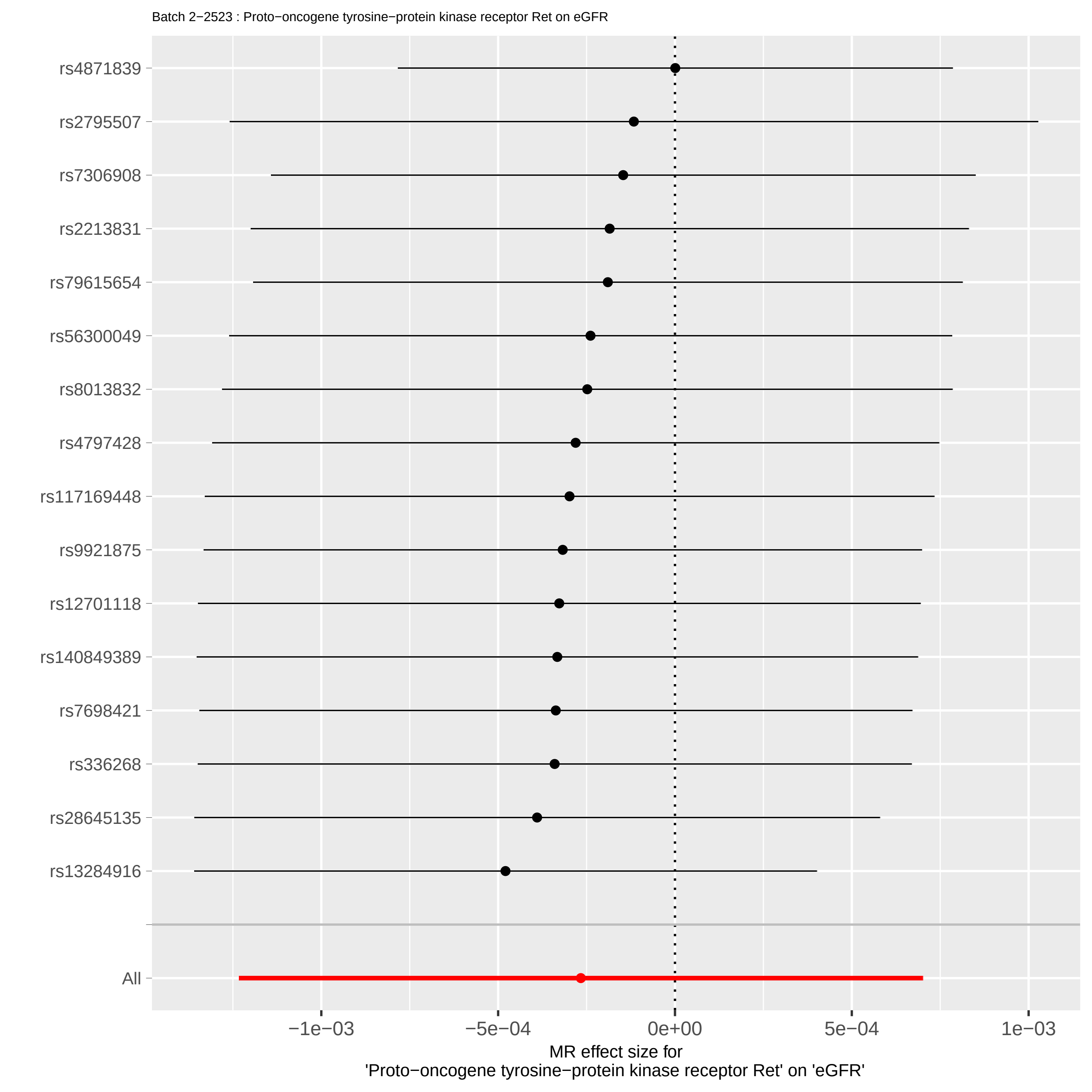

Batch 2−2523 : Proto−oncogene tyrosine−protein kinase receptor Ret on eGFR
rs4871839
rs2795507
rs7306908
rs2213831
rs79615654
rs56300049
rs8013832
rs4797428
rs117169448
rs9921875
rs12701118
rs140849389
rs7698421
rs336268
rs28645135
rs13284916
All
−1e−03
−5e−04
0e+00
5e−04
1e−03
MR effect size for
'Proto−oncogene tyrosine−protein kinase receptor Ret' on 'eGFR'

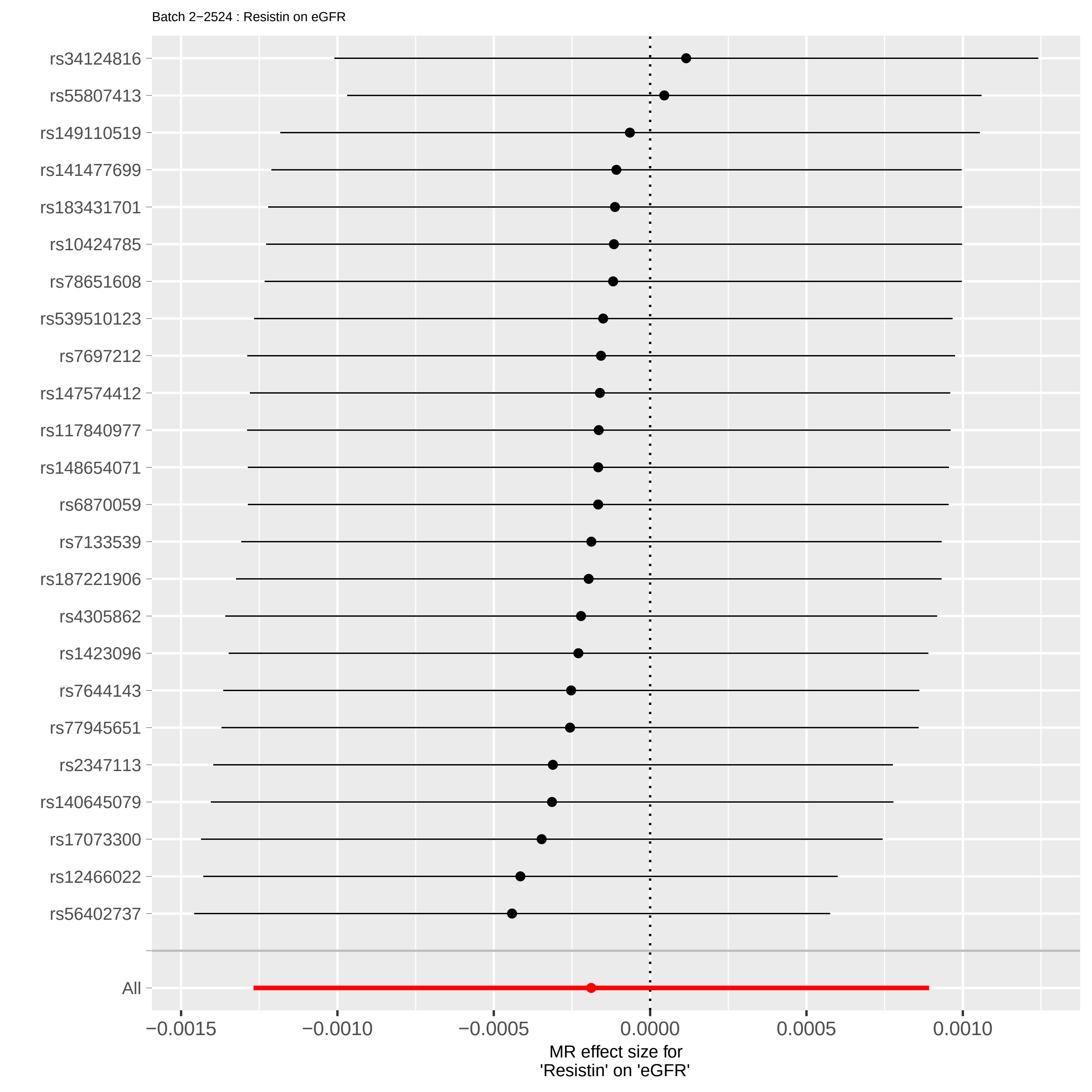

Batch 2−2524 : Resistin on eGFR
rs34124816
rs55807413
rs149110519
rs141477699
rs183431701
rs10424785
rs78651608
rs539510123
rs7697212
rs147574412
rs117840977
rs148654071
rs6870059
rs7133539
rs187221906
rs4305862
rs1423096
rs7644143
rs77945651
rs2347113
rs140645079
rs17073300
rs12466022
rs56402737
All
−0.0015
−0.0010
−0.0005
0.0000
0.0005
0.0010
MR effect size for
'Resistin' on 'eGFR'

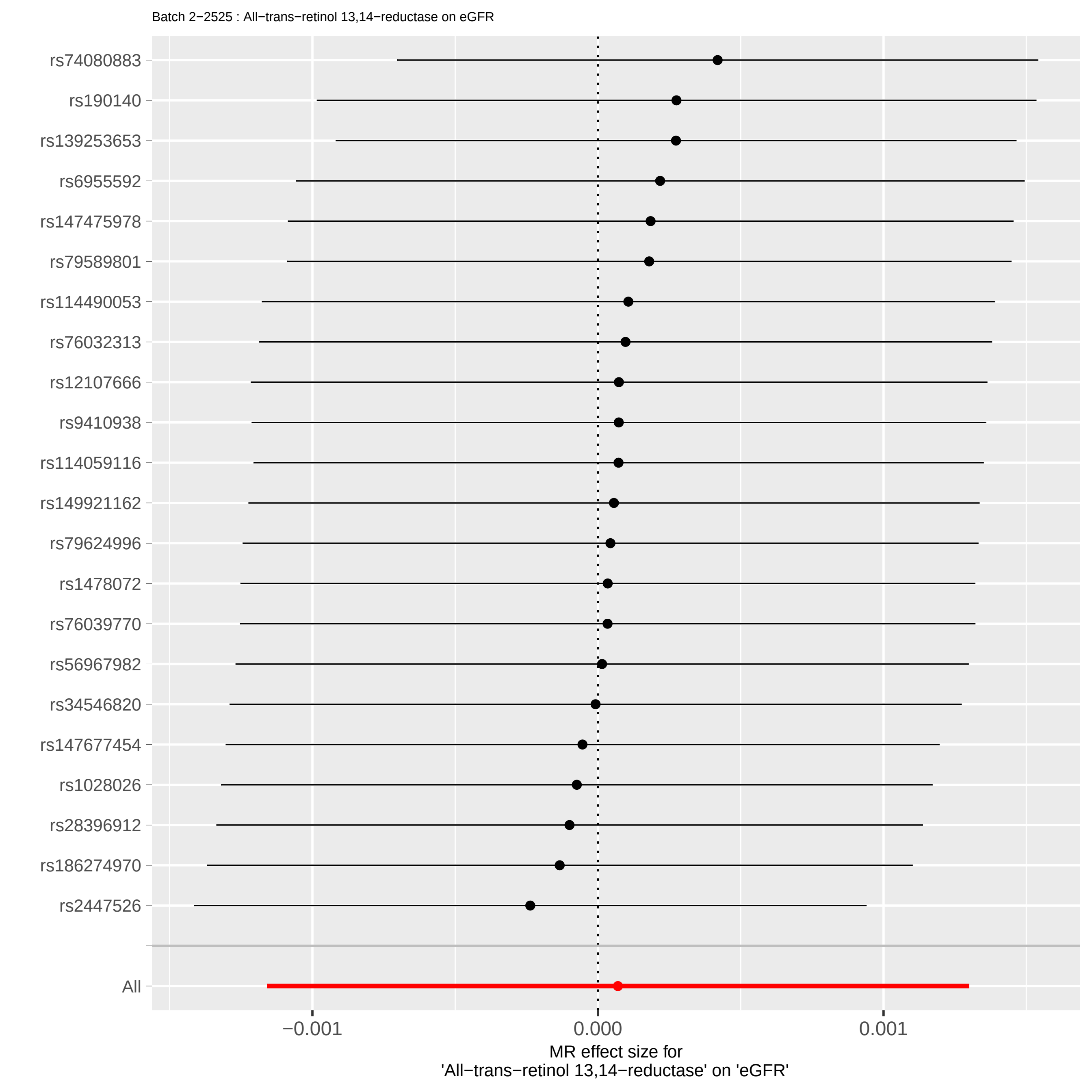
Batch 2−2525 : All−trans−retinol 13,14−reductase on eGFR
rs74080883
rs190140
rs139253653
rs6955592
rs147475978
rs79589801
rs114490053
rs76032313
rs12107666
rs9410938
rs114059116
rs149921162
rs79624996
rs1478072
rs76039770
rs56967982
rs34546820
rs147677454
rs1028026
rs28396912
rs186274970
rs2447526
All
−0.001
0.000
0.001
MR effect size for
'All−trans−retinol 13,14−reductase' on 'eGFR'

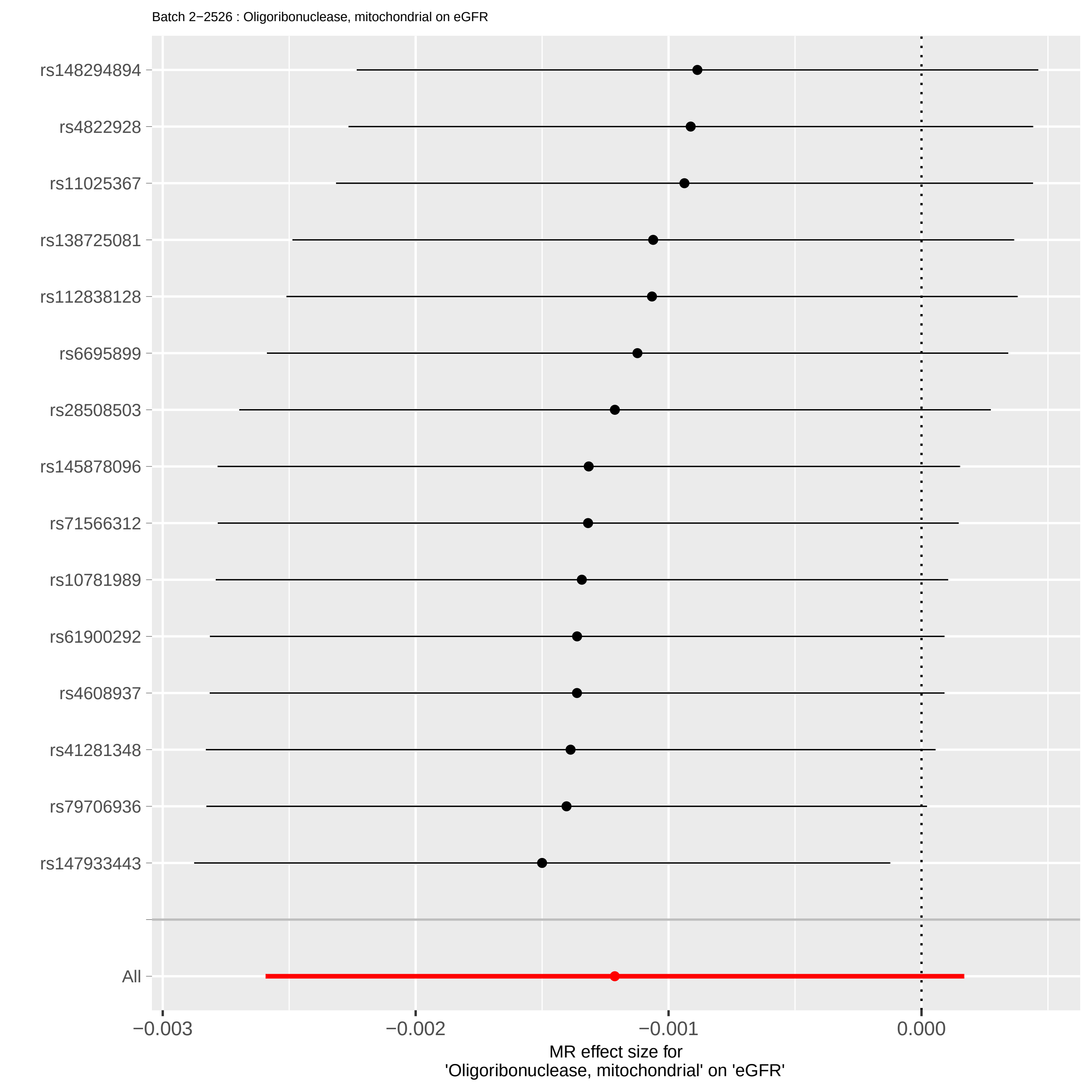

Batch 2−2526 : Oligoribonuclease, mitochondrial on eGFR
rs148294894
rs4822928
rs11025367
rs138725081
rs112838128
rs6695899
rs28508503
rs145878096
rs71566312
rs10781989
rs61900292
rs4608937
rs41281348
rs79706936
rs147933443
All
−0.003
−0.002
−0.001
0.000
MR effect size for
'Oligoribonuclease, mitochondrial' on 'eGFR'

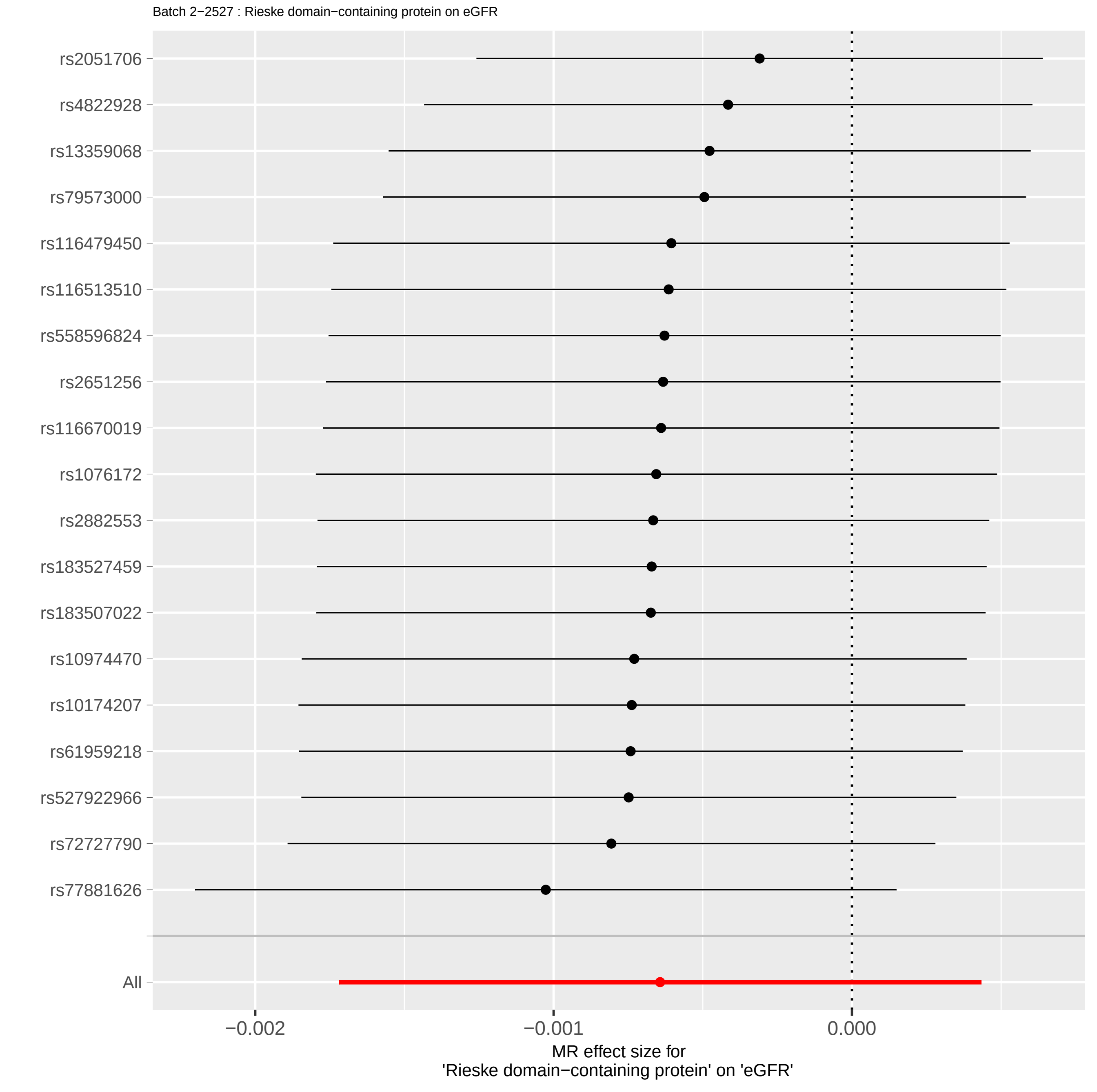
Batch 2−2527 : Rieske domain−containing protein on eGFR
rs2051706
rs4822928
rs13359068
rs79573000
rs116479450
rs116513510
rs558596824
rs2651256
rs116670019
rs1076172
rs2882553
rs183527459
rs183507022
rs10974470
rs10174207
rs61959218
rs527922966
rs72727790
rs77881626
All
−0.002
−0.001
0.000
MR effect size for
'Rieske domain−containing protein' on 'eGFR'

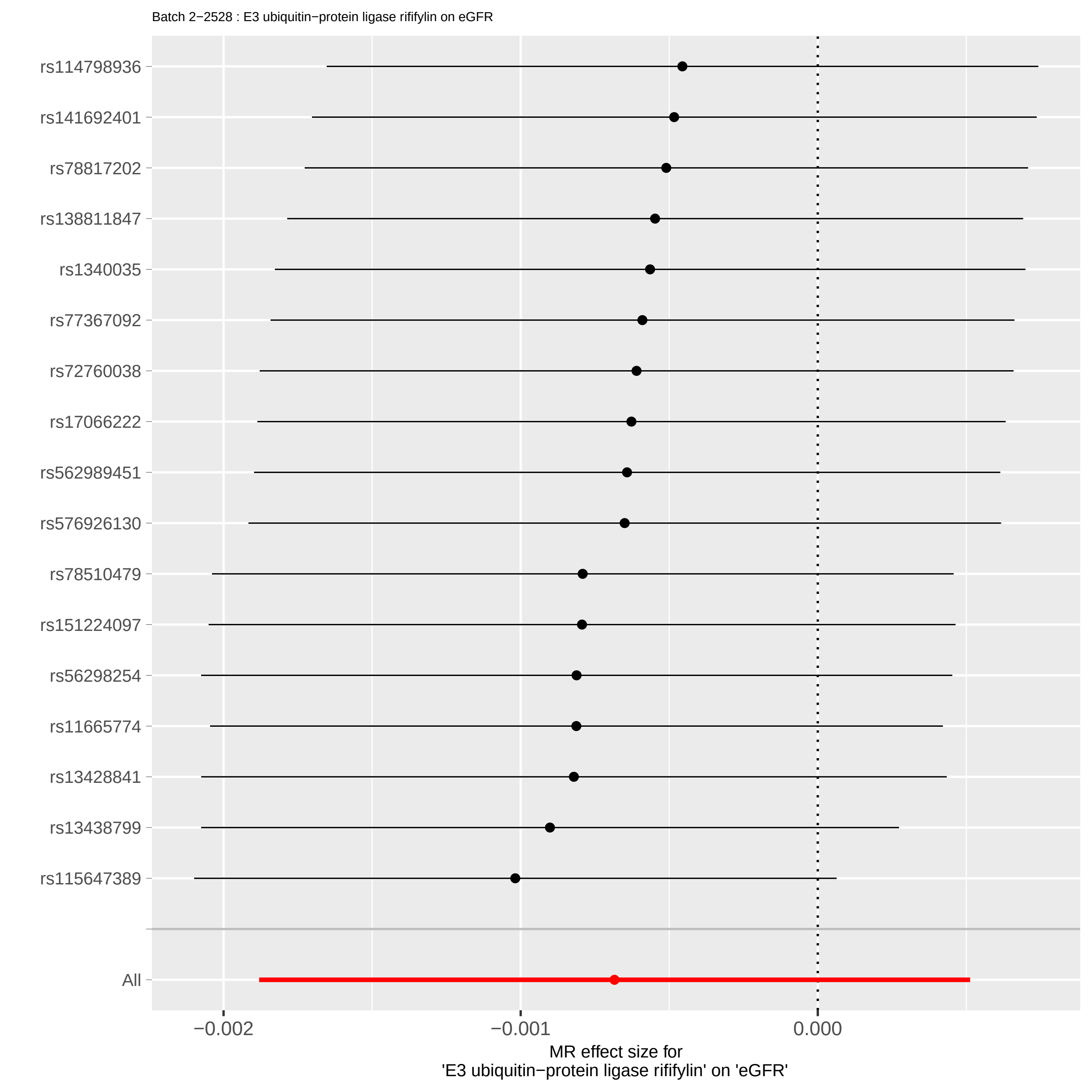

Batch 2−2528 : E3 ubiquitin−protein ligase rififylin on eGFR
rs114798936
rs141692401
rs78817202
rs138811847
rs1340035
rs77367092
rs72760038
rs17066222
rs562989451
rs576926130
rs78510479
rs151224097
rs56298254
rs11665774
rs13428841
rs13438799
rs115647389
All
−0.002
−0.001
0.000
MR effect size for
'E3 ubiquitin−protein ligase rififylin' on 'eGFR'

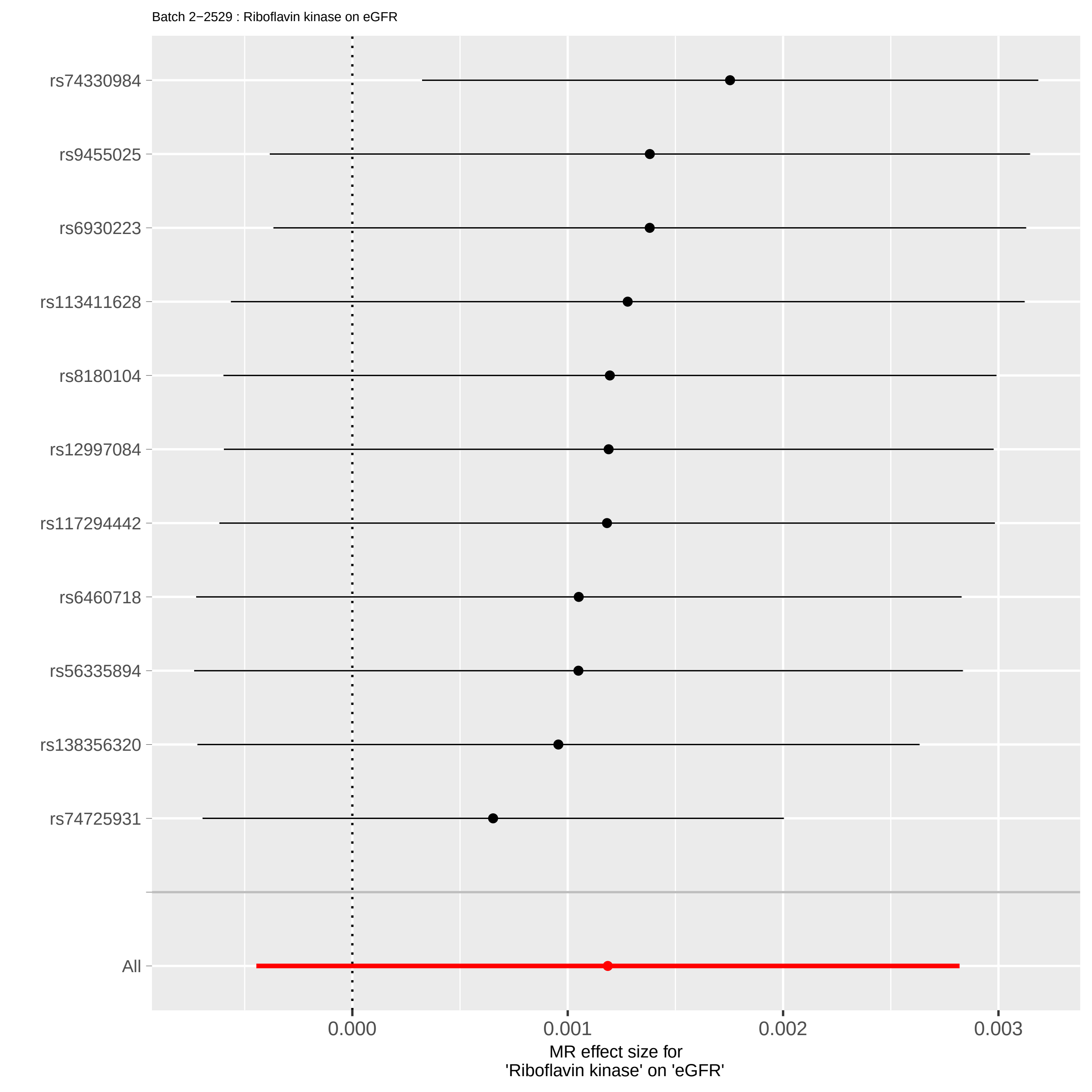
Batch 2−2529 : Riboflavin kinase on eGFR
rs74330984
rs9455025
rs6930223
rs113411628
rs8180104
rs12997084
rs117294442
rs6460718
rs56335894
rs138356320
rs74725931
All
0.000
0.001
0.002
0.003
MR effect size for
'Riboflavin kinase' on 'eGFR'

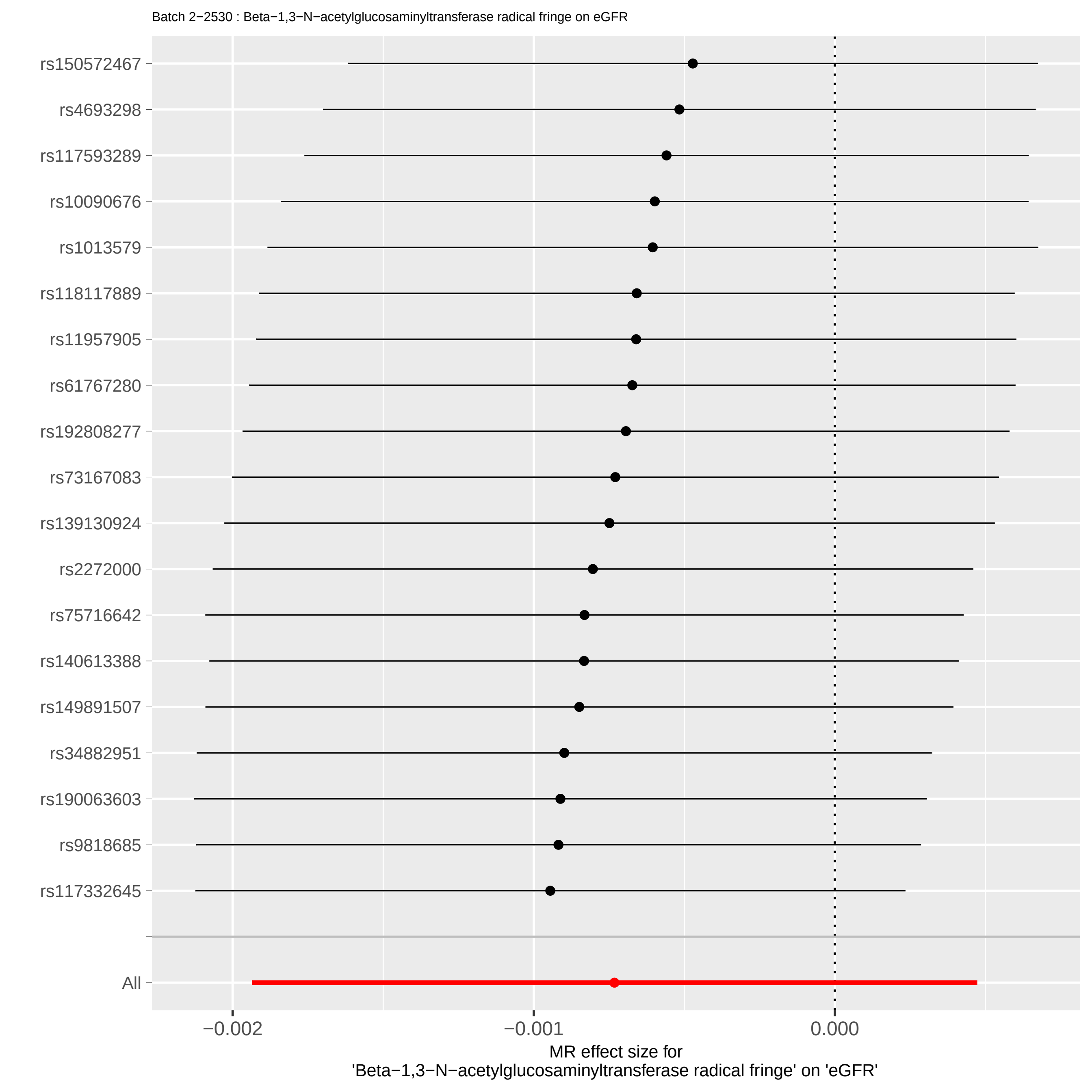

Batch 2−2530 : Beta−1,3−N−acetylglucosaminyltransferase radical fringe on eGFR
rs150572467
rs4693298
rs117593289
rs10090676
rs1013579
rs118117889
rs11957905
rs61767280
rs192808277
rs73167083
rs139130924
rs2272000
rs75716642
rs140613388
rs149891507
rs34882951
rs190063603
rs9818685
rs117332645
All
−0.002
−0.001
0.000
MR effect size for
'Beta−1,3−N−acetylglucosaminyltransferase radical fringe' on 'eGFR'

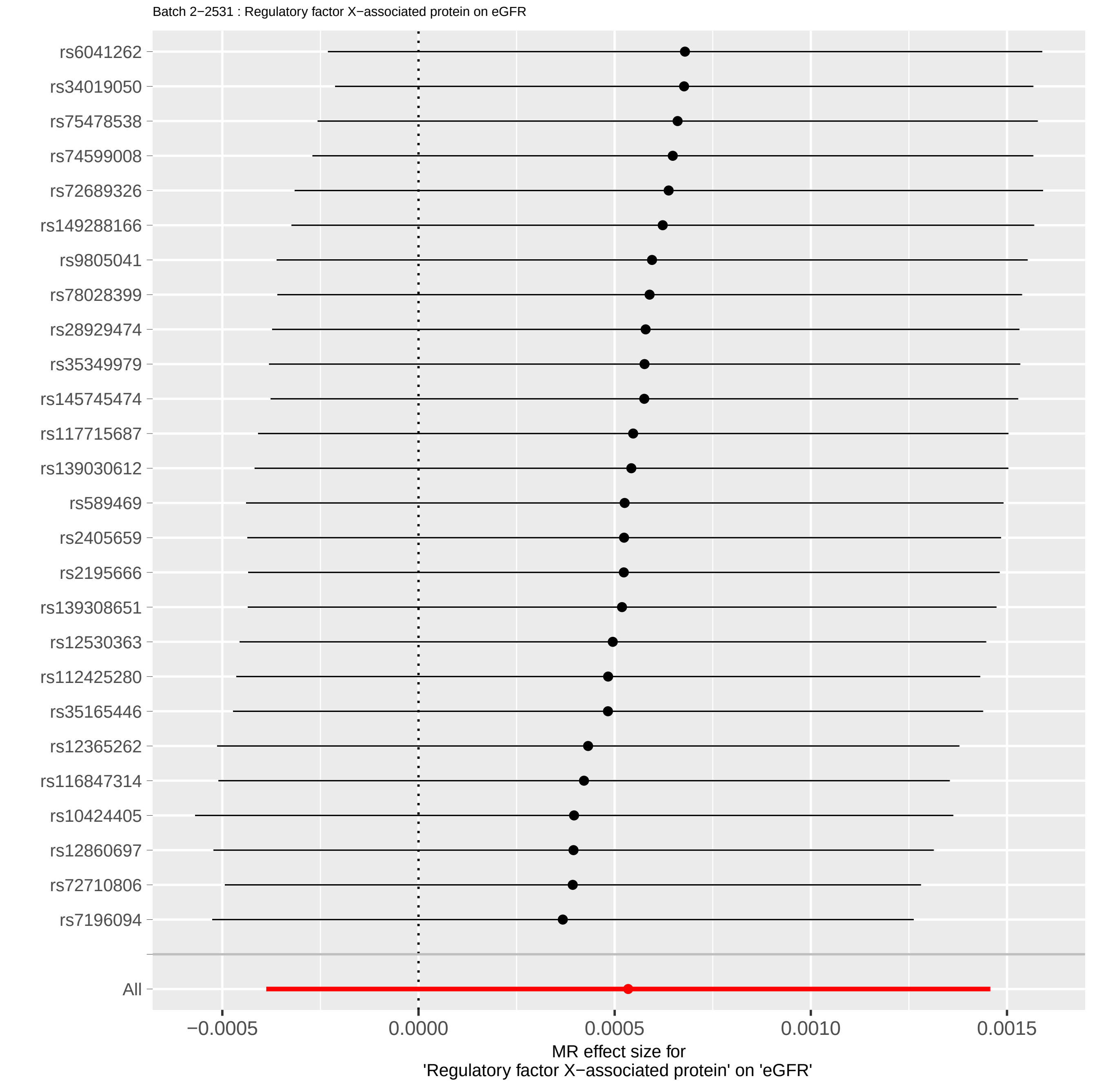

Batch 2−2531 : Regulatory factor X−associated protein on eGFR
rs6041262
rs34019050
rs75478538
rs74599008
rs72689326
rs149288166
rs9805041
rs78028399
rs28929474
rs35349979
rs145745474
rs117715687
rs139030612
rs589469
rs2405659
rs2195666
rs139308651
rs12530363
rs112425280
rs35165446
rs12365262
rs116847314
rs10424405
rs12860697
rs72710806
rs7196094
All
−0.0005
0.0000
0.0005
0.0010
0.0015
MR effect size for
'Regulatory factor X−associated protein' on 'eGFR'

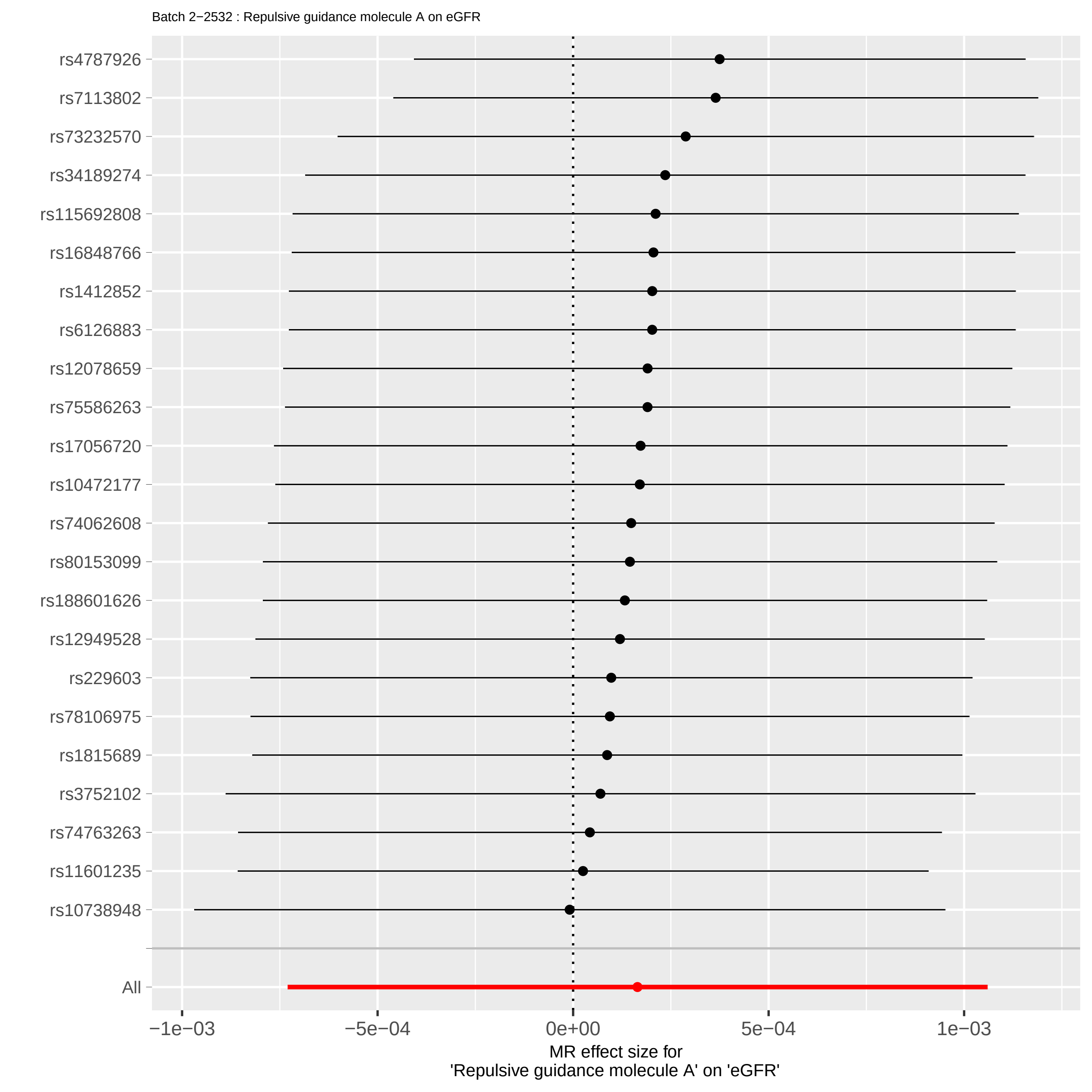

Batch 2−2532 : Repulsive guidance molecule A on eGFR
rs4787926
rs7113802
rs73232570
rs34189274
rs115692808
rs16848766
rs1412852
rs6126883
rs12078659
rs75586263
rs17056720
rs10472177
rs74062608
rs80153099
rs188601626
rs12949528
rs229603
rs78106975
rs1815689
rs3752102
rs74763263
rs11601235
rs10738948
All
−1e−03
−5e−04
0e+00
5e−04
1e−03
MR effect size for
'Repulsive guidance molecule A' on 'eGFR'

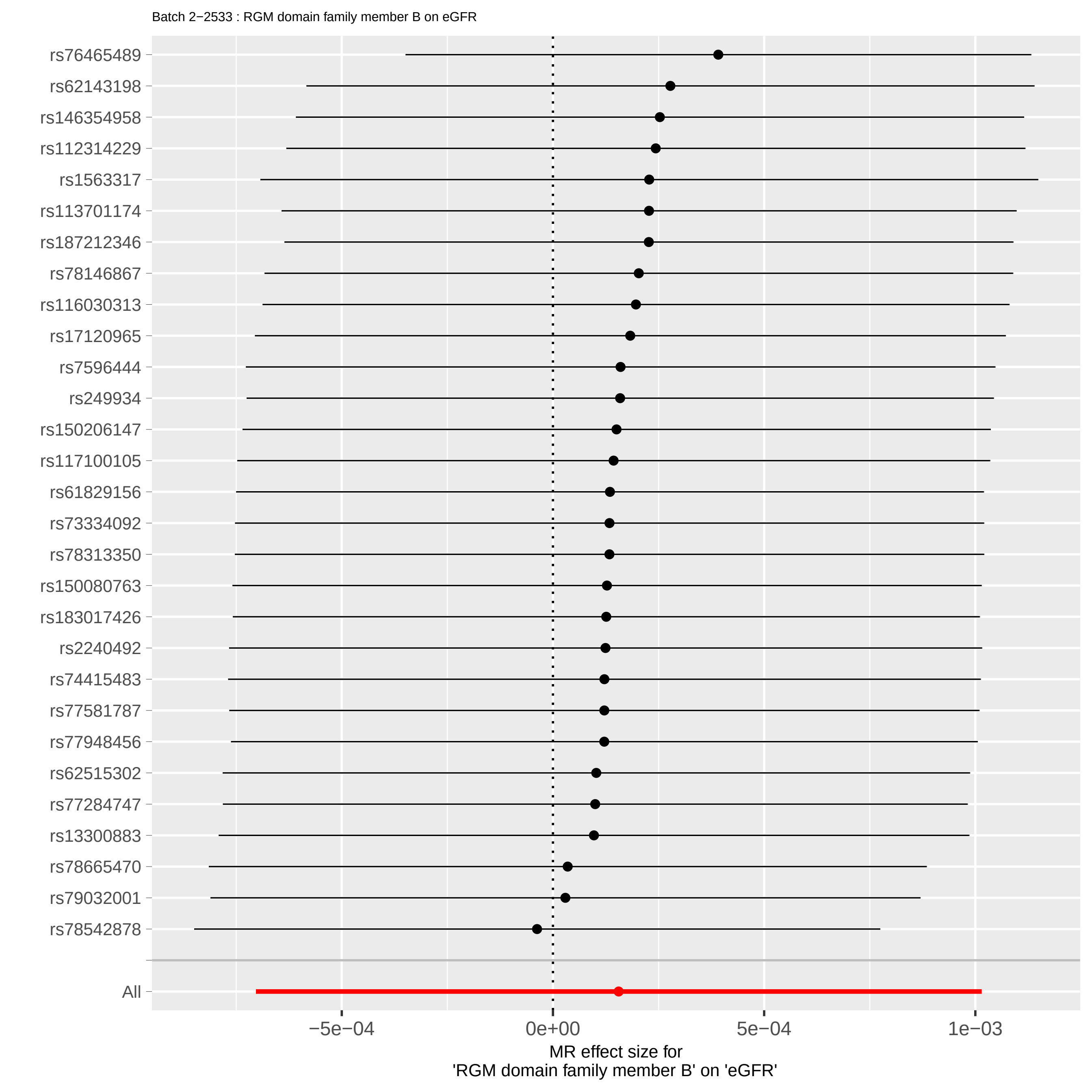

Batch 2−2533 : RGM domain family member B on eGFR
rs76465489
rs62143198
rs146354958
rs112314229
rs1563317
rs113701174
rs187212346
rs78146867
rs116030313
rs17120965
rs7596444
rs249934
rs150206147
rs117100105
rs61829156
rs73334092
rs78313350
rs150080763
rs183017426
rs2240492
rs74415483
rs77581787
rs77948456
rs62515302
rs77284747
rs13300883
rs78665470
rs79032001
rs78542878
All
−5e−04
0e+00
5e−04
1e−03
MR effect size for
'RGM domain family member B' on 'eGFR'

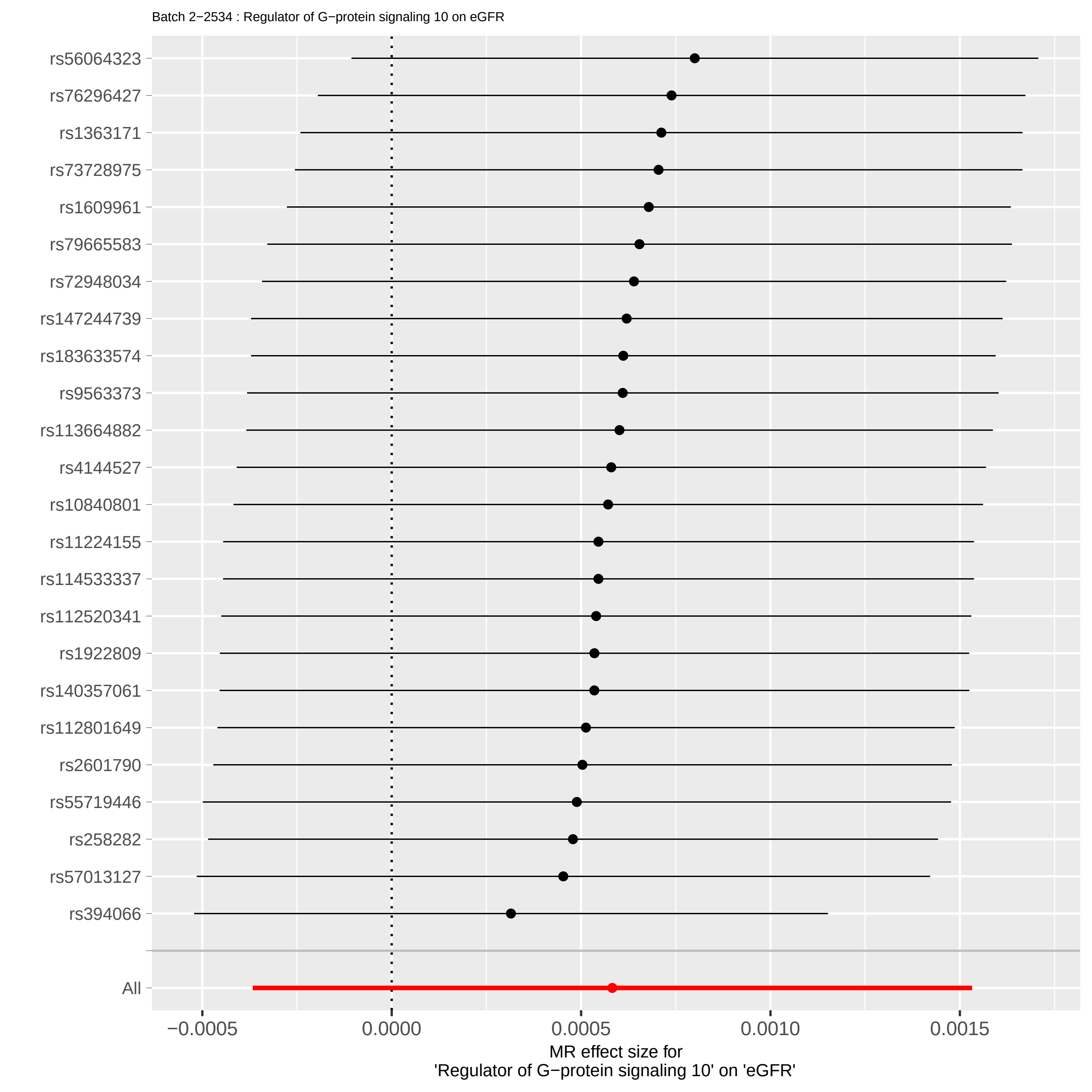

Batch 2−2534 : Regulator of G−protein signaling 10 on eGFR
rs56064323
rs76296427
rs1363171
rs73728975
rs1609961
rs79665583
rs72948034
rs147244739
rs183633574
rs9563373
rs113664882
rs4144527
rs10840801
rs11224155
rs114533337
rs112520341
rs1922809
rs140357061
rs112801649
rs2601790
rs55719446
rs258282
rs57013127
rs394066
All
−0.0005
0.0000
0.0005
0.0010
0.0015
MR effect size for
'Regulator of G−protein signaling 10' on 'eGFR'

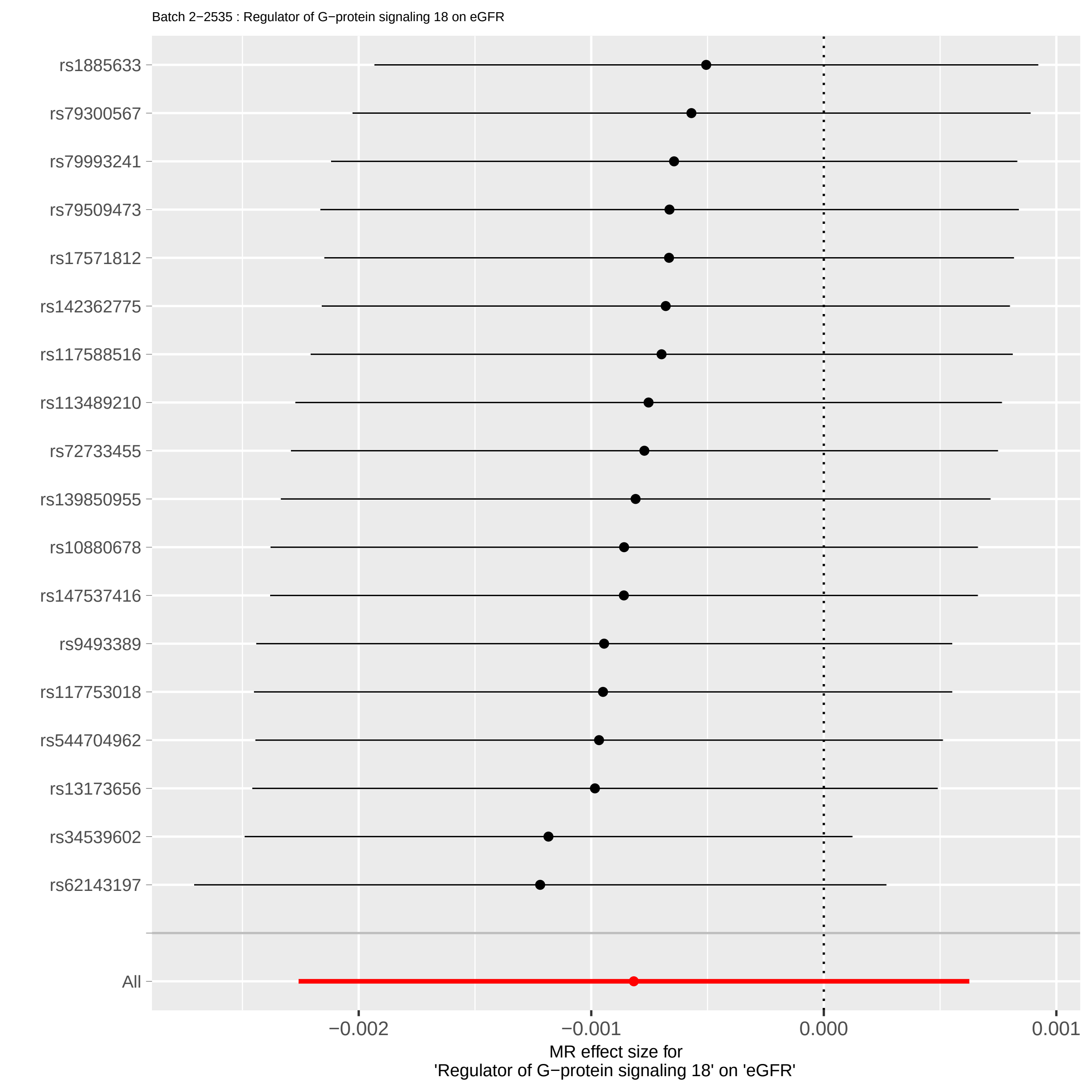

Batch 2−2535 : Regulator of G−protein signaling 18 on eGFR
rs1885633
rs79300567
rs79993241
rs79509473
rs17571812
rs142362775
rs117588516
rs113489210
rs72733455
rs139850955
rs10880678
rs147537416
rs9493389
rs117753018
rs544704962
rs13173656
rs34539602
rs62143197
All
−0.002
−0.001
0.000
0.001
MR effect size for
'Regulator of G−protein signaling 18' on 'eGFR'

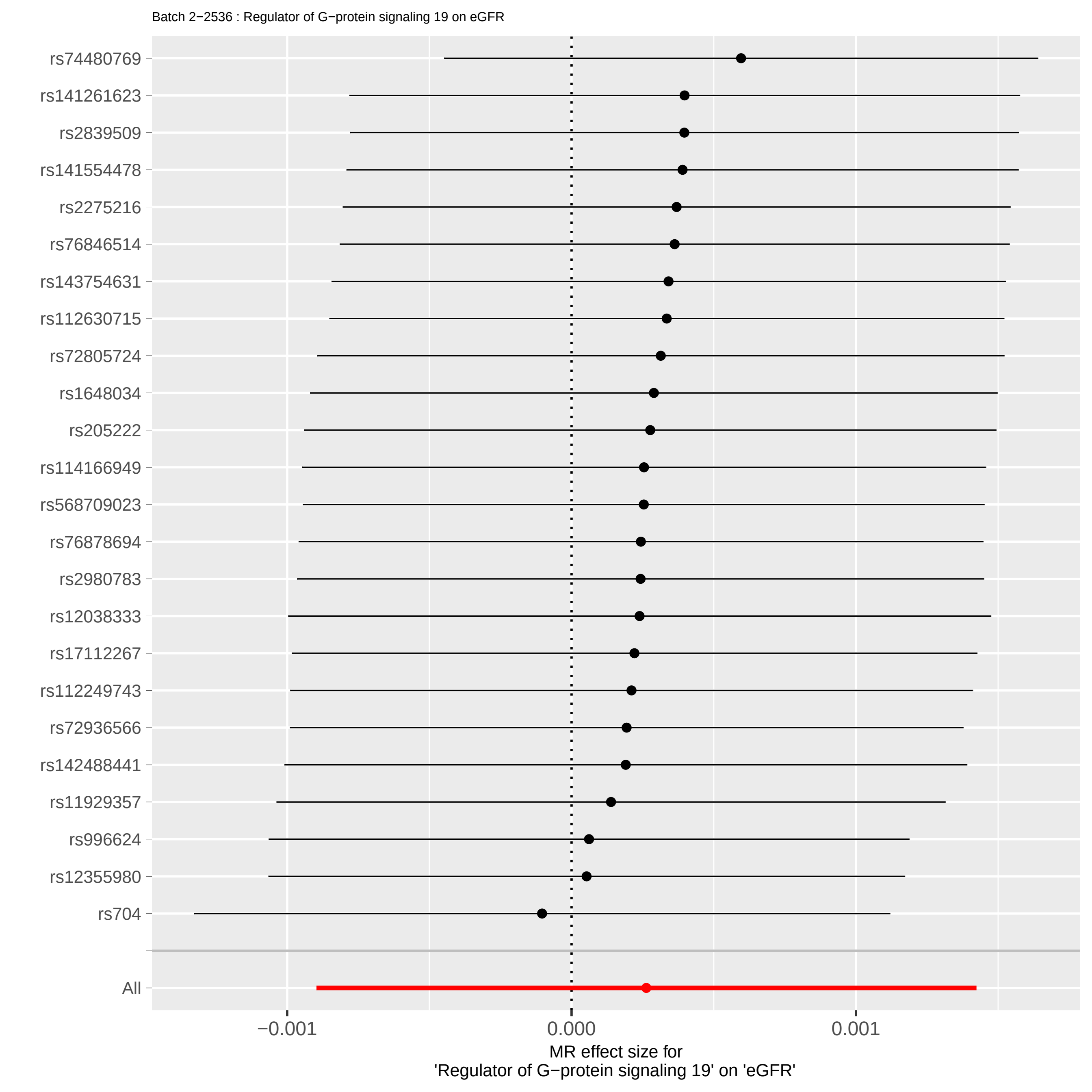

Batch 2−2536 : Regulator of G−protein signaling 19 on eGFR
rs74480769
rs141261623
rs2839509
rs141554478
rs2275216
rs76846514
rs143754631
rs112630715
rs72805724
rs1648034
rs205222
rs114166949
rs568709023
rs76878694
rs2980783
rs12038333
rs17112267
rs112249743
rs72936566
rs142488441
rs11929357
rs996624
rs12355980
rs704
All
−0.001
0.000
0.001
MR effect size for
'Regulator of G−protein signaling 19' on 'eGFR'

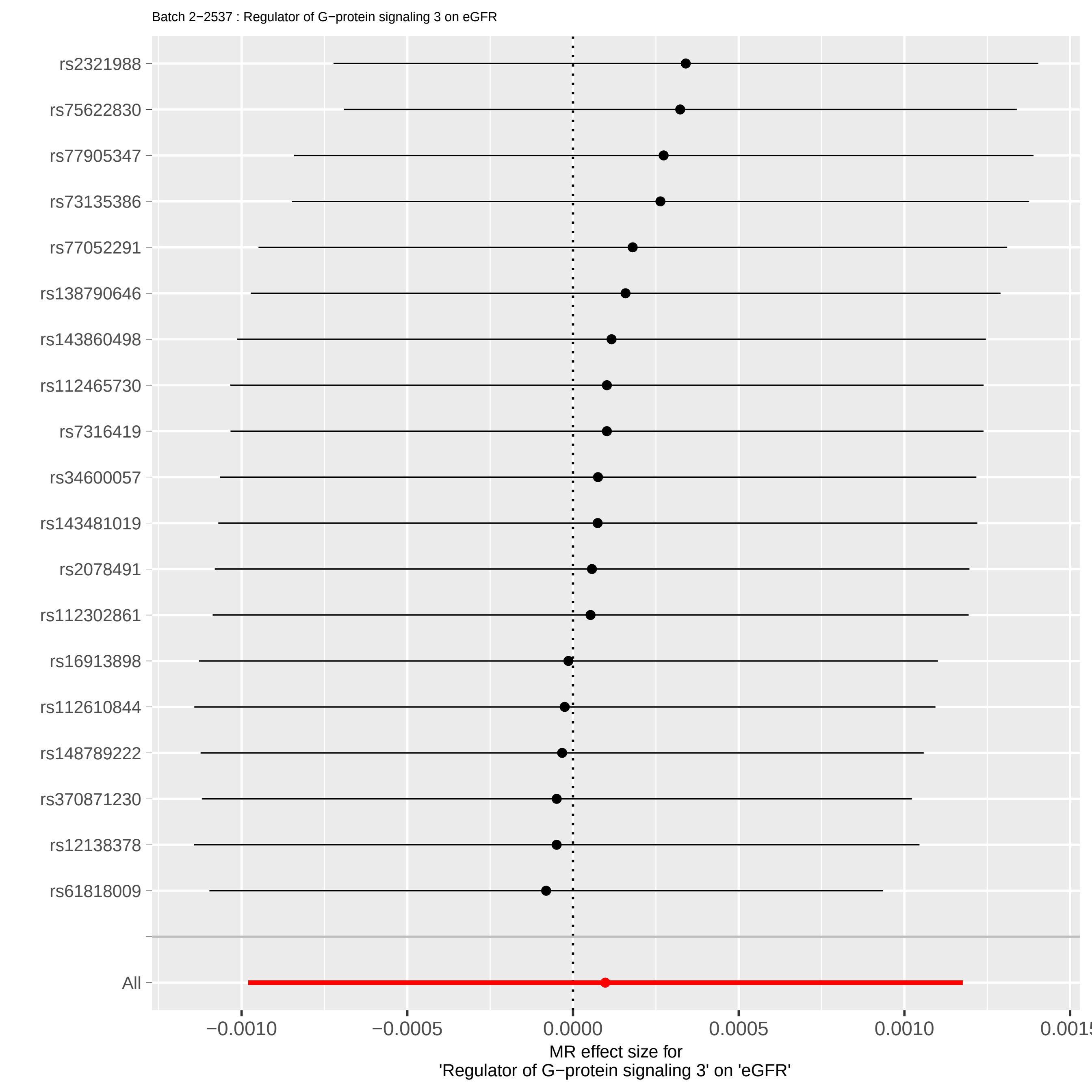

Batch 2−2537 : Regulator of G−protein signaling 3 on eGFR
rs2321988
rs75622830
rs77905347
rs73135386
rs77052291
rs138790646
rs143860498
rs112465730
rs7316419
rs34600057
rs143481019
rs2078491
rs112302861
rs16913898
rs112610844
rs148789222
rs370871230
rs12138378
rs61818009
All
−0.0010
−0.0005
0.0000
0.0005
0.0010
MR effect size for
'Regulator of G−protein signaling 3' on 'eGFR'

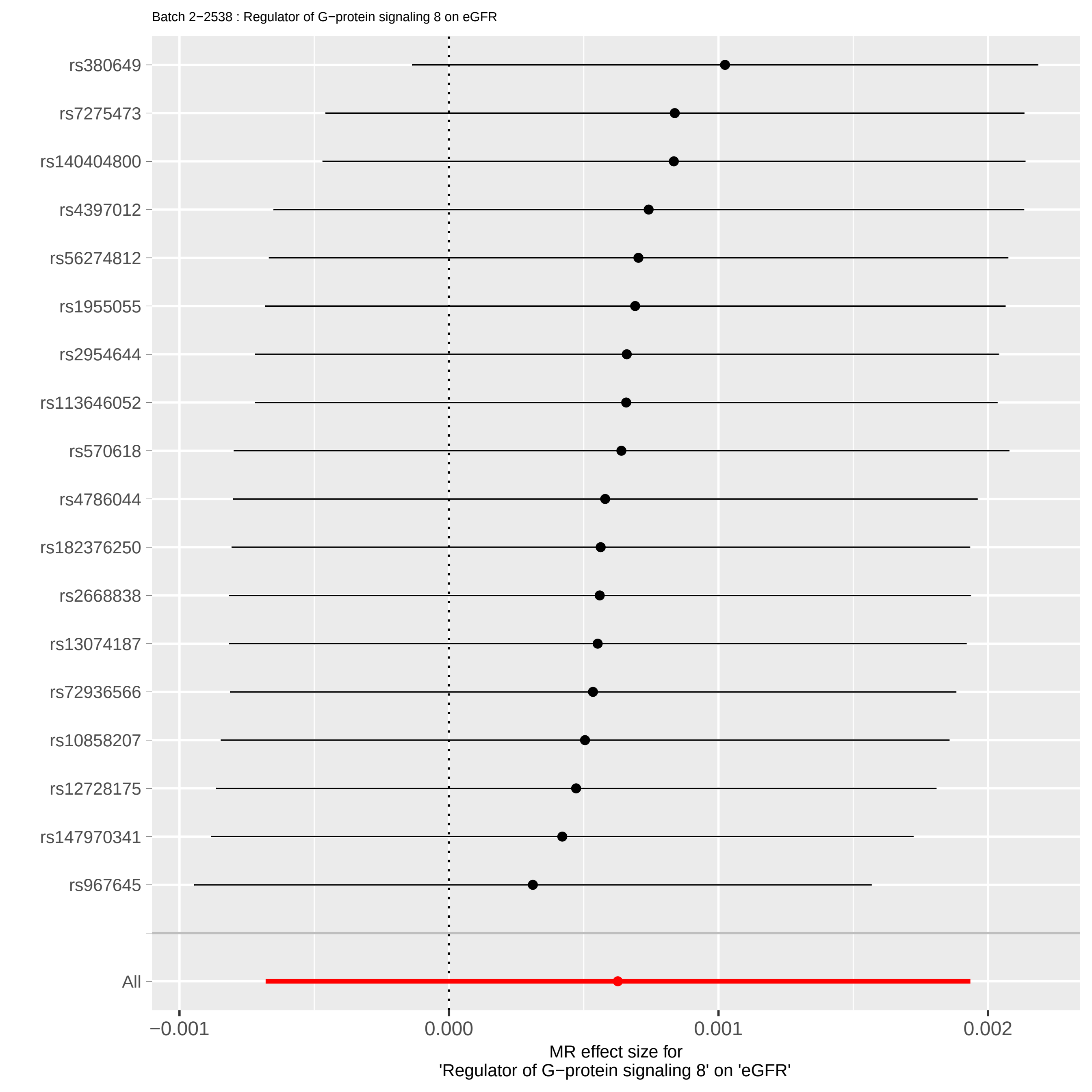
Batch 2−2538 : Regulator of G−protein signaling 8 on eGFR
rs380649
rs7275473
rs140404800
rs4397012
rs56274812
rs1955055
rs2954644
rs113646052
rs570618
rs4786044
rs182376250
rs2668838
rs13074187
rs72936566
rs10858207
rs12728175
rs147970341
rs967645
All
−0.001
0.000
0.001
0.002
MR effect size for
'Regulator of G−protein signaling 8' on 'eGFR'

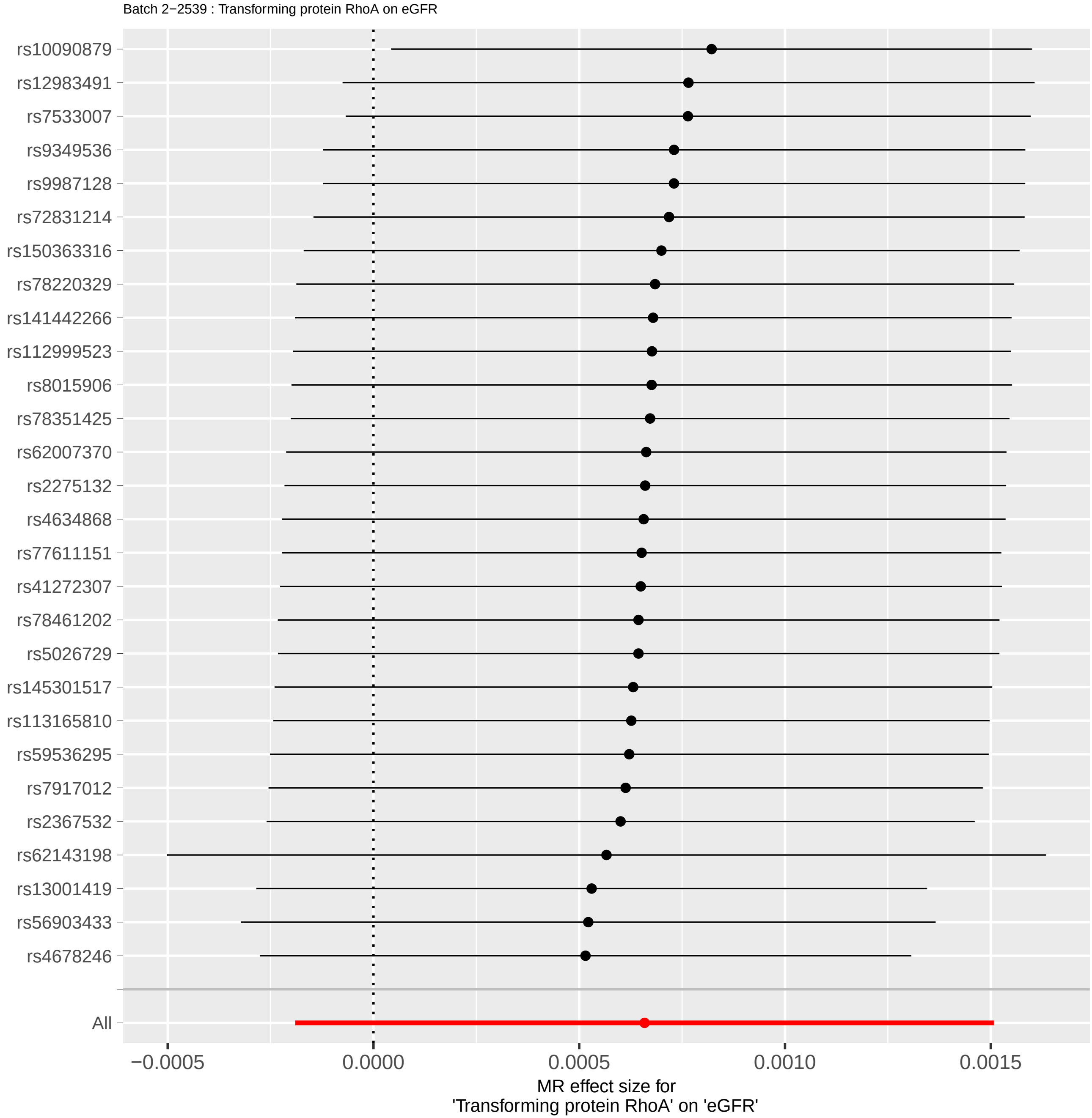
Batch 2−2539 : Transforming protein RhoA on eGFR
rs10090879
rs12983491
rs7533007
rs9349536
rs9987128
rs72831214
rs150363316
rs78220329
rs141442266
rs112999523
rs8015906
rs78351425
rs62007370
rs2275132
rs4634868
rs77611151
rs41272307
rs78461202
rs5026729
rs145301517
rs113165810
rs59536295
rs7917012
rs2367532
rs62143198
rs13001419
rs56903433
rs4678246
All
−0.0005
0.0000
0.0005
0.0010
0.0015
MR effect size for
'Transforming protein RhoA' on 'eGFR'

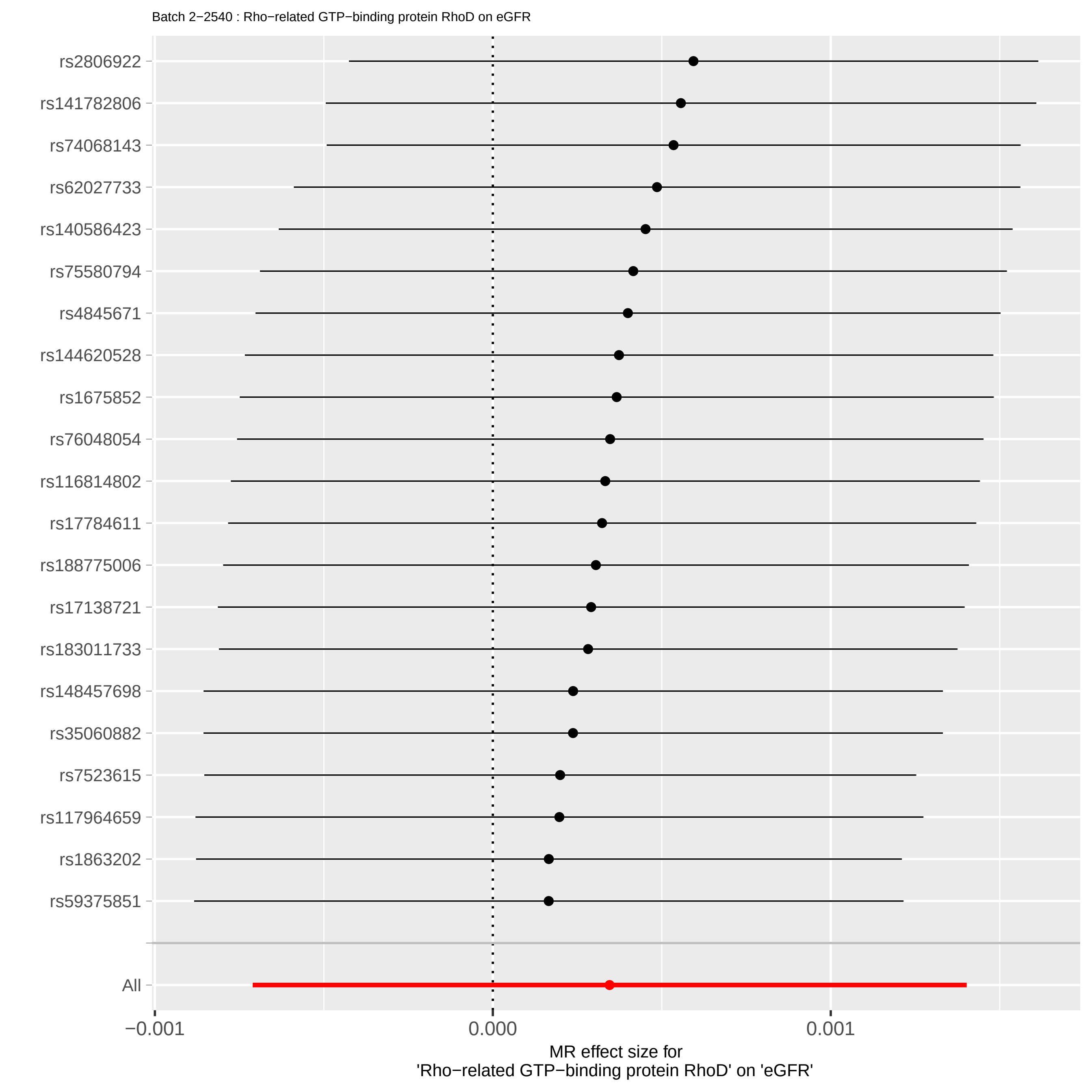

Batch 2−2540 : Rho−related GTP−binding protein RhoD on eGFR
rs2806922
rs141782806
rs74068143
rs62027733
rs140586423
rs75580794
rs4845671
rs144620528
rs1675852
rs76048054
rs116814802
rs17784611
rs188775006
rs17138721
rs183011733
rs148457698
rs35060882
rs7523615
rs117964659
rs1863202
rs59375851
All
−0.001
0.000
0.001
MR effect size for
'Rho−related GTP−binding protein RhoD' on 'eGFR'

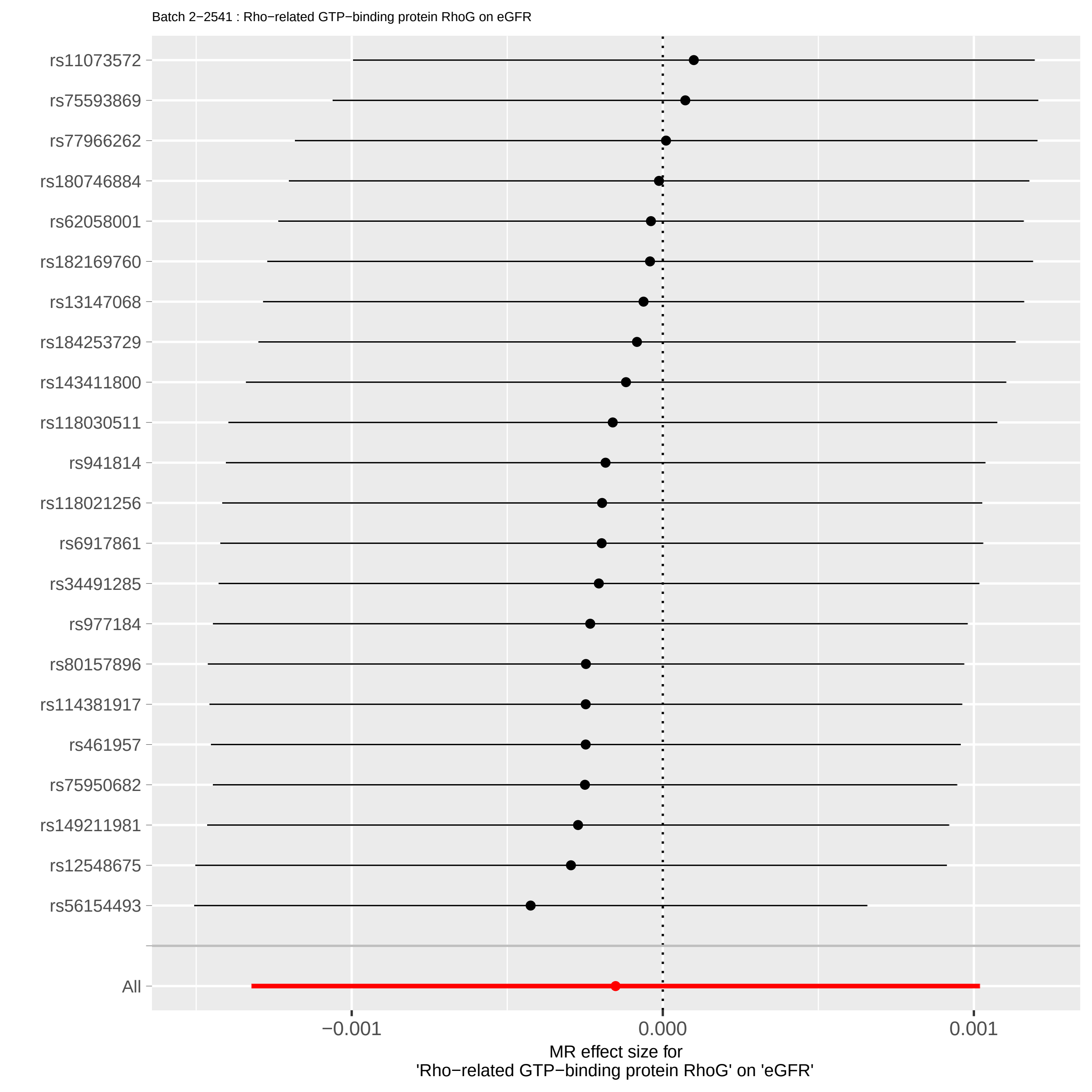

Batch 2−2541 : Rho−related GTP−binding protein RhoG on eGFR
rs11073572
rs75593869
rs77966262
rs180746884
rs62058001
rs182169760
rs13147068
rs184253729
rs143411800
rs118030511
rs941814
rs118021256
rs6917861
rs34491285
rs977184
rs80157896
rs114381917
rs461957
rs75950682
rs149211981
rs12548675
rs56154493
All
−0.001
0.000
0.001
MR effect size for
'Rho−related GTP−binding protein RhoG' on 'eGFR'

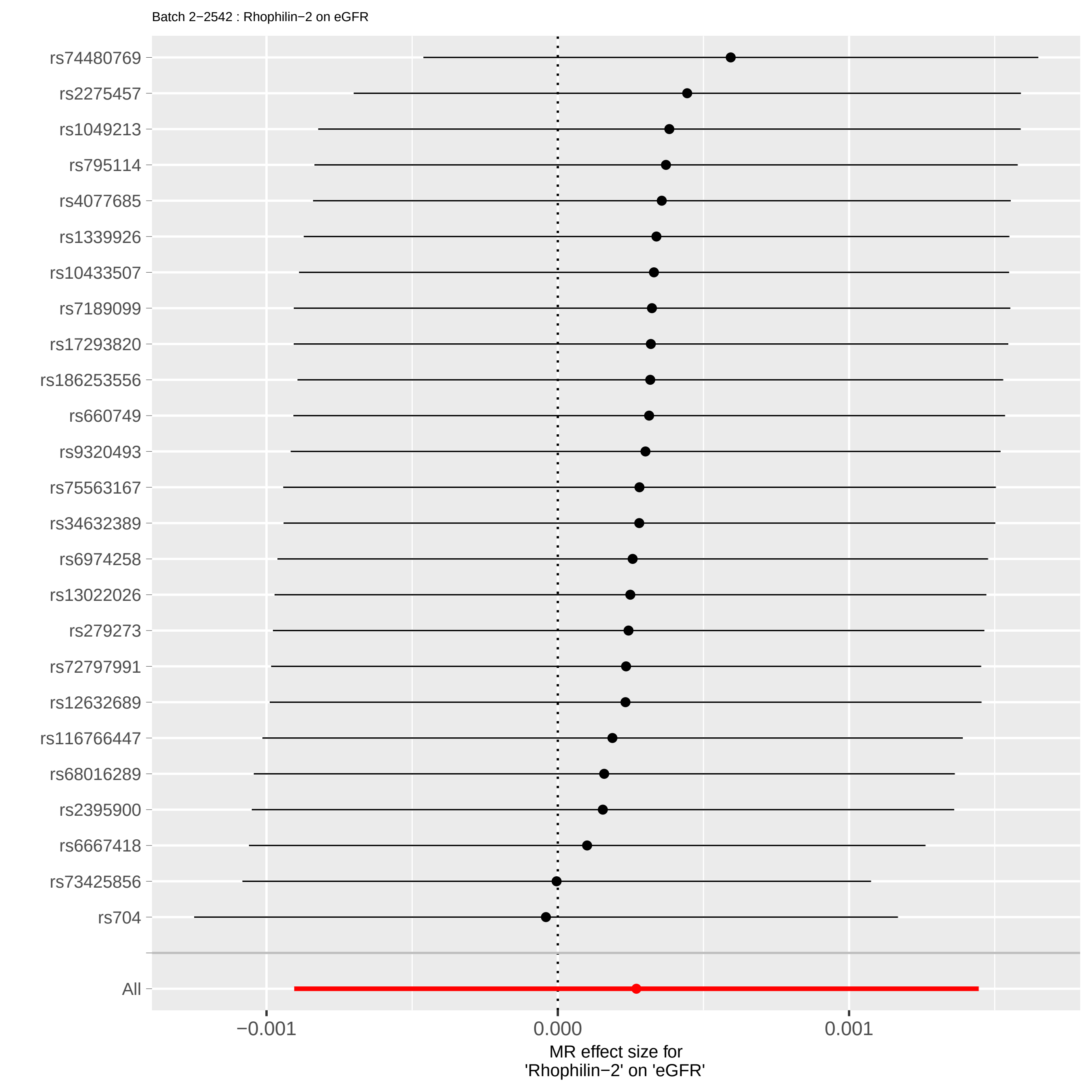
Batch 2−2542 : Rhophilin−2 on eGFR
rs74480769
rs2275457
rs1049213
rs795114
rs4077685
rs1339926
rs10433507
rs7189099
rs17293820
rs186253556
rs660749
rs9320493
rs75563167
rs34632389
rs6974258
rs13022026
rs279273
rs72797991
rs12632689
rs116766447
rs68016289
rs2395900
rs6667418
rs73425856
rs704
All
−0.001
0.000
0.001
MR effect size for
'Rhophilin−2' on 'eGFR'

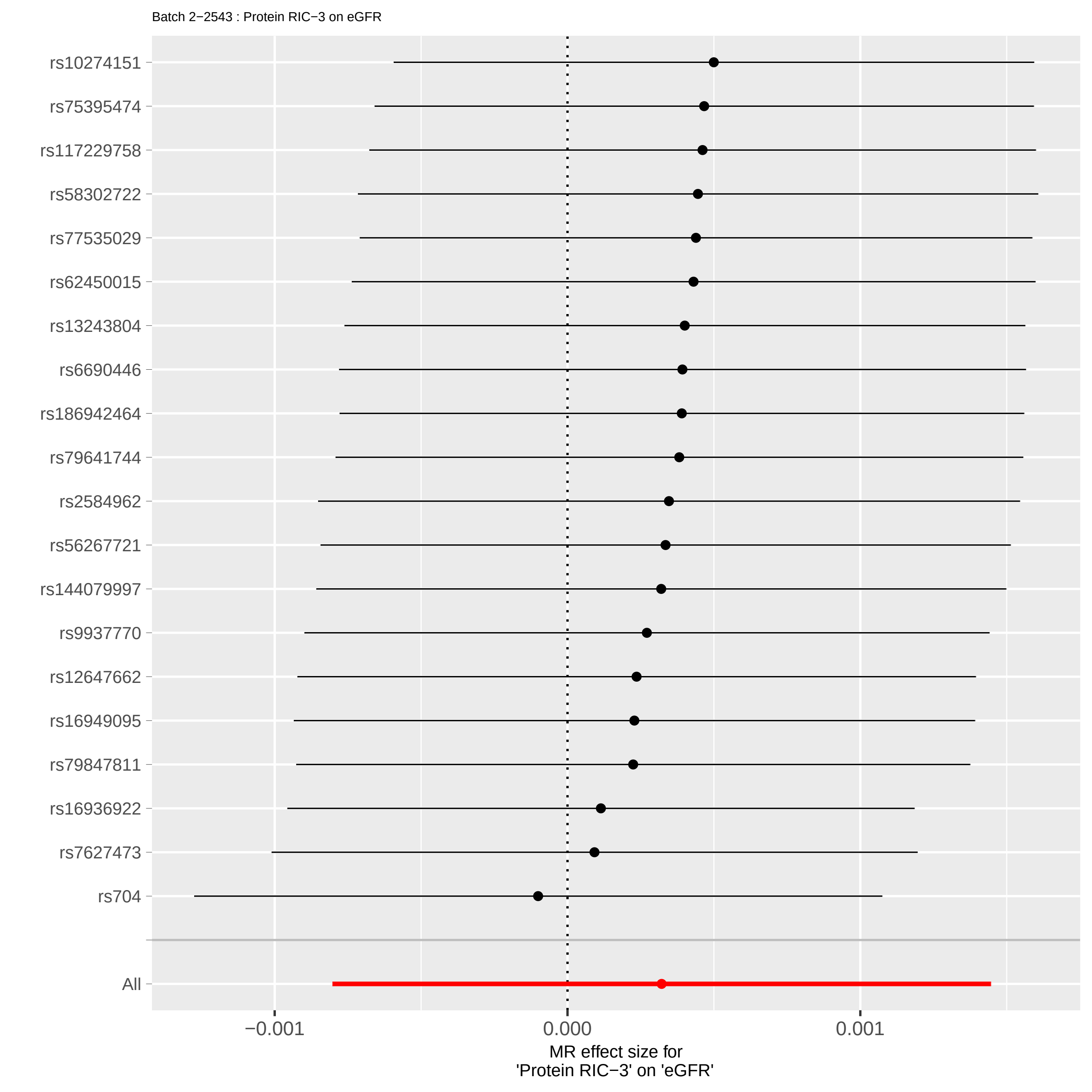

Batch 2−2543 : Protein RIC−3 on eGFR
rs10274151
rs75395474
rs117229758
rs58302722
rs77535029
rs62450015
rs13243804
rs6690446
rs186942464
rs79641744
rs2584962
rs56267721
rs144079997
rs9937770
rs12647662
rs16949095
rs79847811
rs16936922
rs7627473
rs704
All
−0.001
0.000
0.001
MR effect size for
'Protein RIC−3' on 'eGFR'

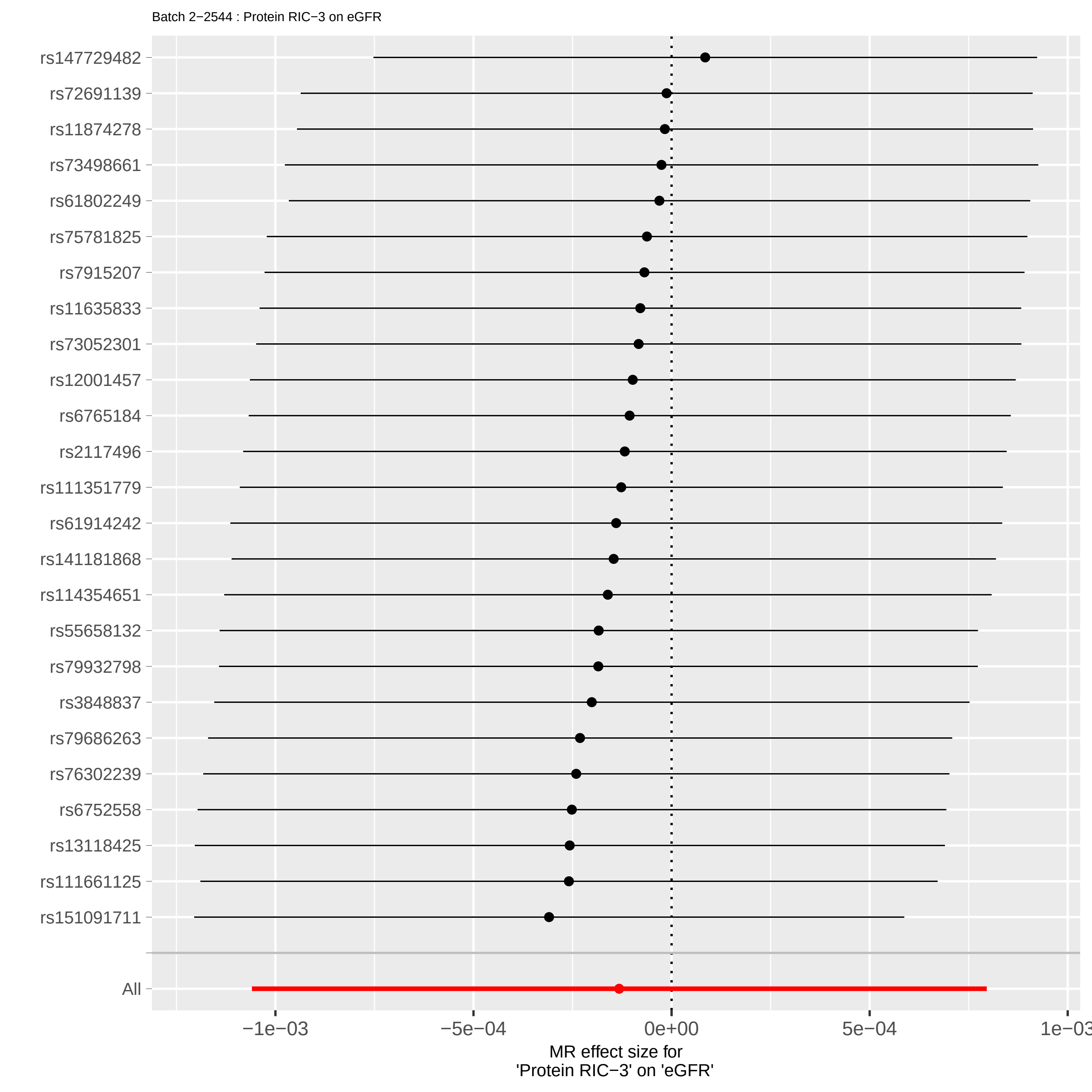
Batch 2−2544 : Protein RIC−3 on eGFR
rs147729482
rs72691139
rs11874278
rs73498661
rs61802249
rs75781825
rs7915207
rs11635833
rs73052301
rs12001457
rs6765184
rs2117496
rs111351779
rs61914242
rs141181868
rs114354651
rs55658132
rs79932798
rs3848837
rs79686263
rs76302239
rs6752558
rs13118425
rs111661125
rs151091711
All
−1e−03
−5e−04
0e+00
5e−04
1e−03
MR effect size for
'Protein RIC−3' on 'eGFR'

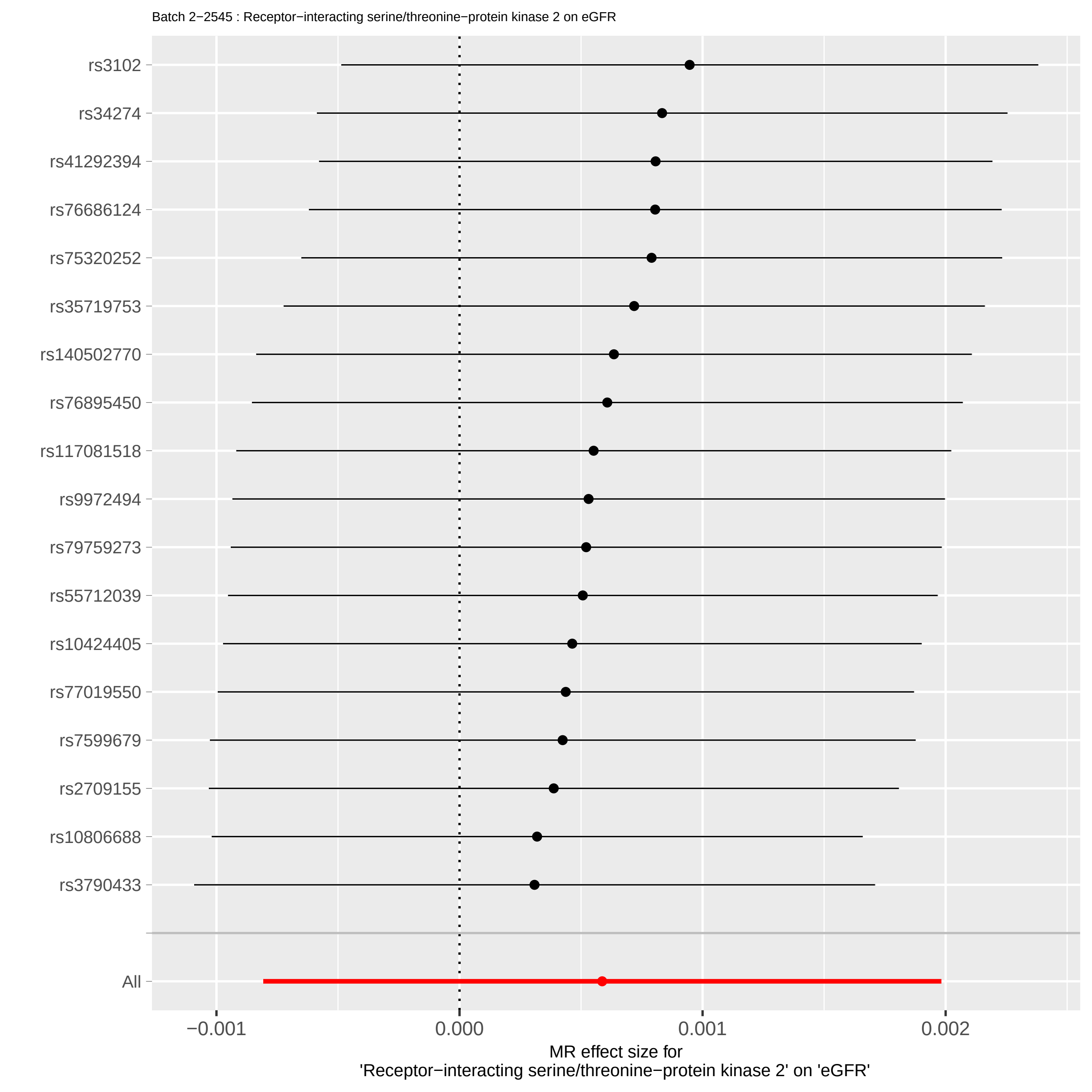
Batch 2−2545 : Receptor−interacting serine/threonine−protein kinase 2 on eGFR
rs3102
rs34274
rs41292394
rs76686124
rs75320252
rs35719753
rs140502770
rs76895450
rs117081518
rs9972494
rs79759273
rs55712039
rs10424405
rs77019550
rs7599679
rs2709155
rs10806688
rs3790433
All
−0.001
0.000
0.001
0.002
MR effect size for
'Receptor−interacting serine/threonine−protein kinase 2' on 'eGFR'

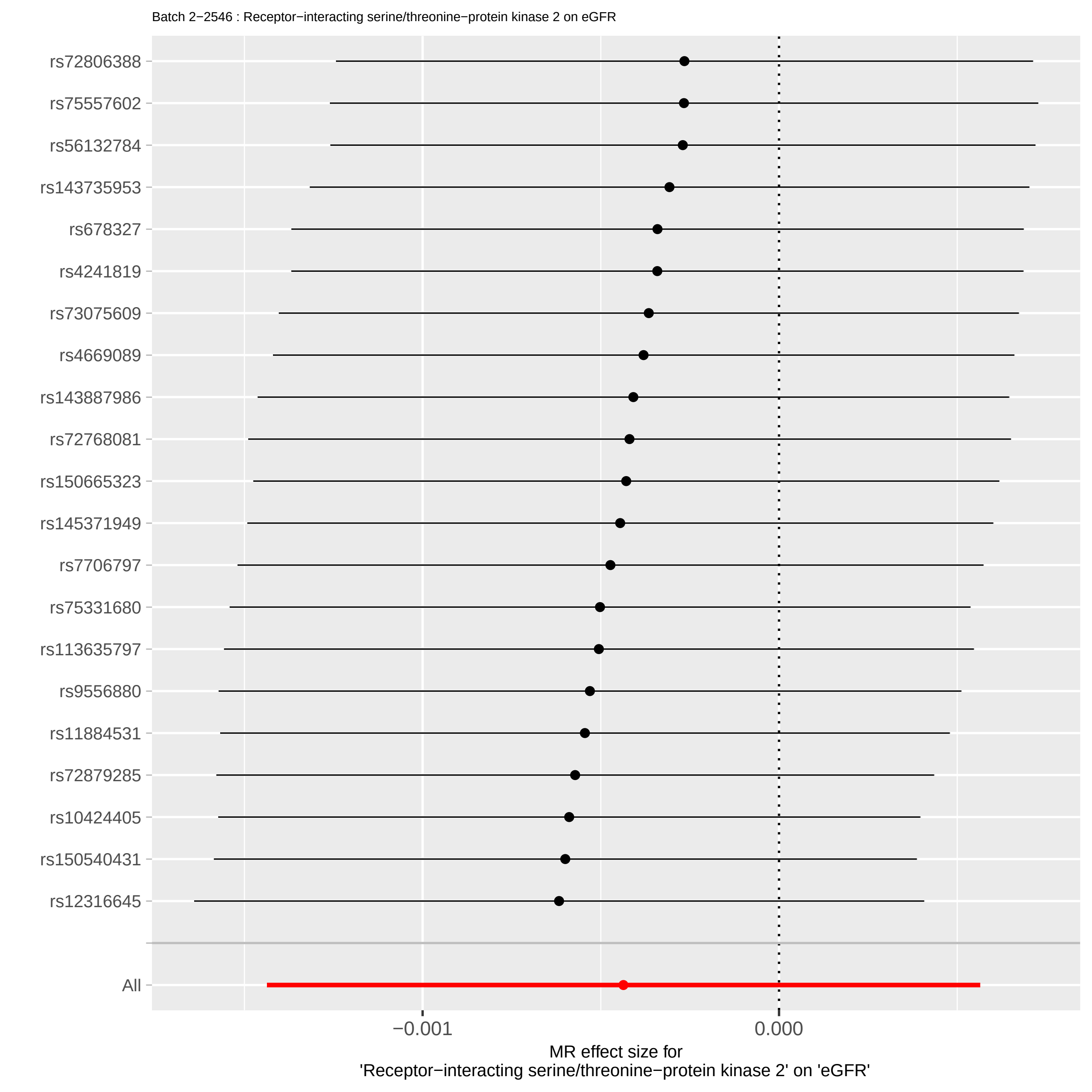

Batch 2−2546 : Receptor−interacting serine/threonine−protein kinase 2 on eGFR
rs72806388
rs75557602
rs56132784
rs143735953
rs678327
rs4241819
rs73075609
rs4669089
rs143887986
rs72768081
rs150665323
rs145371949
rs7706797
rs75331680
rs113635797
rs9556880
rs11884531
rs72879285
rs10424405
rs150540431
rs12316645
All
−0.001
0.000
MR effect size for
'Receptor−interacting serine/threonine−protein kinase 2' on 'eGFR'

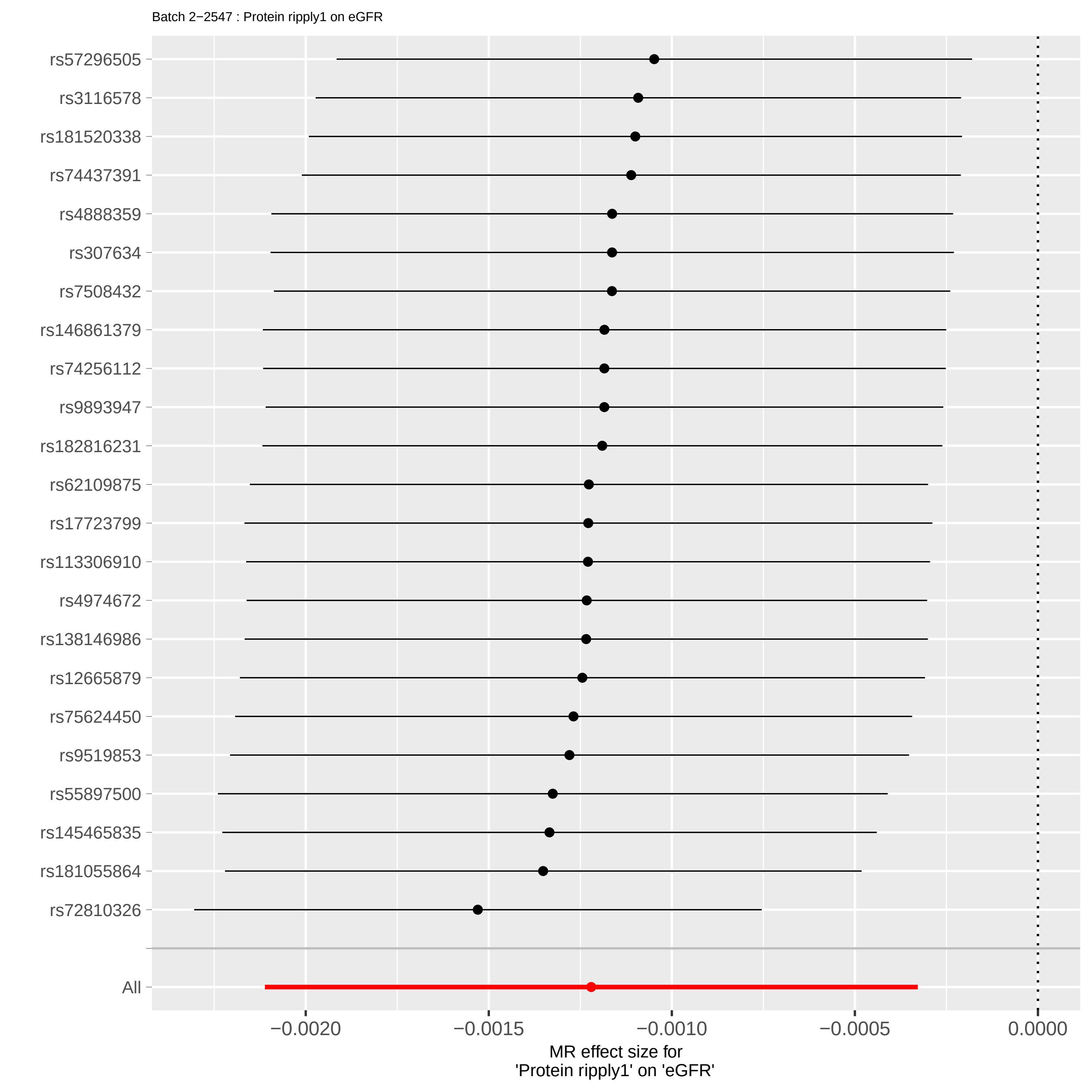

Batch 2−2547 : Protein ripply1 on eGFR
rs57296505
rs3116578
rs181520338
rs74437391
rs4888359
rs307634
rs7508432
rs146861379
rs74256112
rs9893947
rs182816231
rs62109875
rs17723799
rs113306910
rs4974672
rs138146986
rs12665879
rs75624450
rs9519853
rs55897500
rs145465835
rs181055864
rs72810326
All
−0.0020
−0.0015
−0.0010
−0.0005
0.0000
MR effect size for
'Protein ripply1' on 'eGFR'

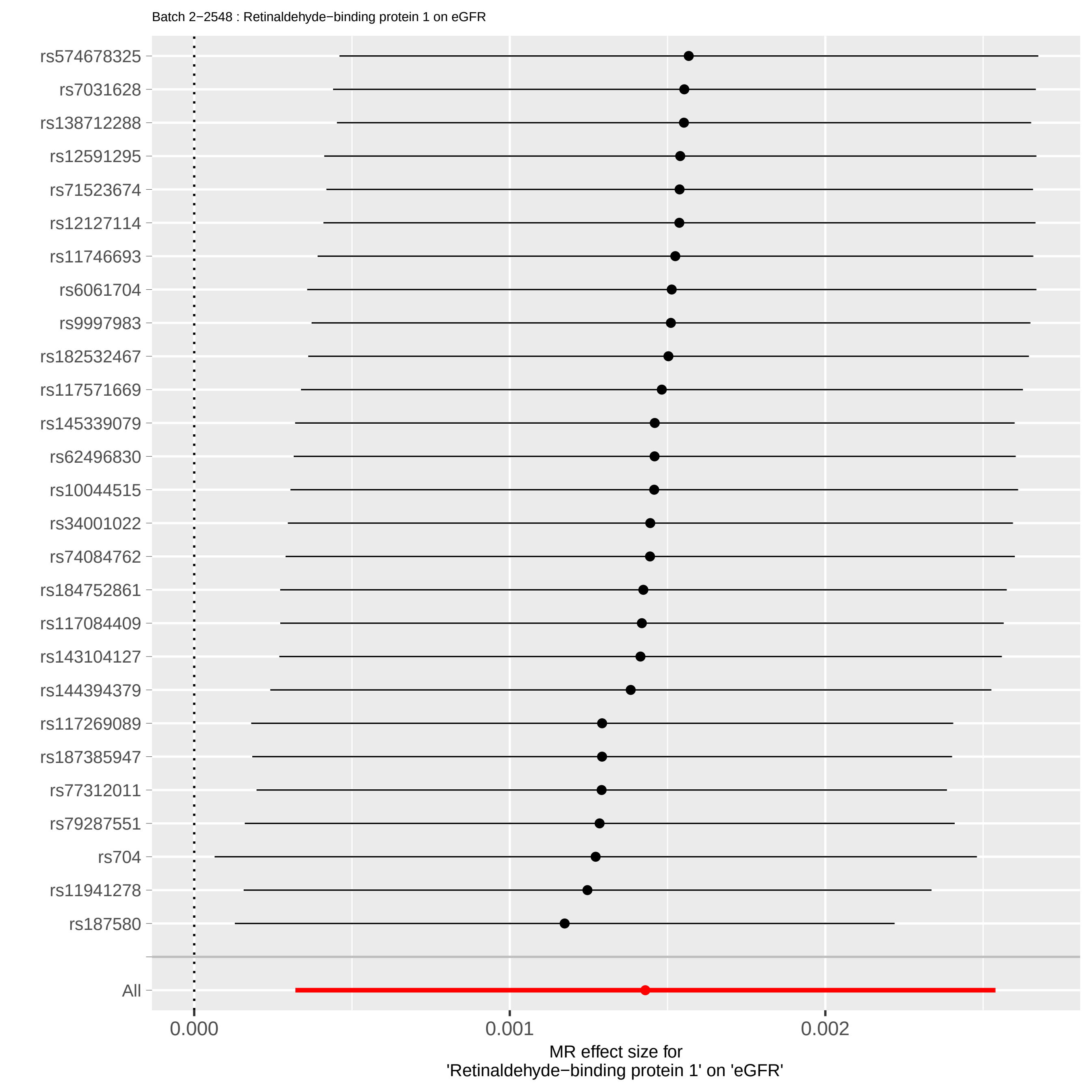

Batch 2−2548 : Retinaldehyde−binding protein 1 on eGFR
rs574678325
rs7031628
rs138712288
rs12591295
rs71523674
rs12127114
rs11746693
rs6061704
rs9997983
rs182532467
rs117571669
rs145339079
rs62496830
rs10044515
rs34001022
rs74084762
rs184752861
rs117084409
rs143104127
rs144394379
rs117269089
rs187385947
rs77312011
rs79287551
rs704
rs11941278
rs187580
All
0.000
0.001
0.002
MR effect size for
'Retinaldehyde−binding protein 1' on 'eGFR'

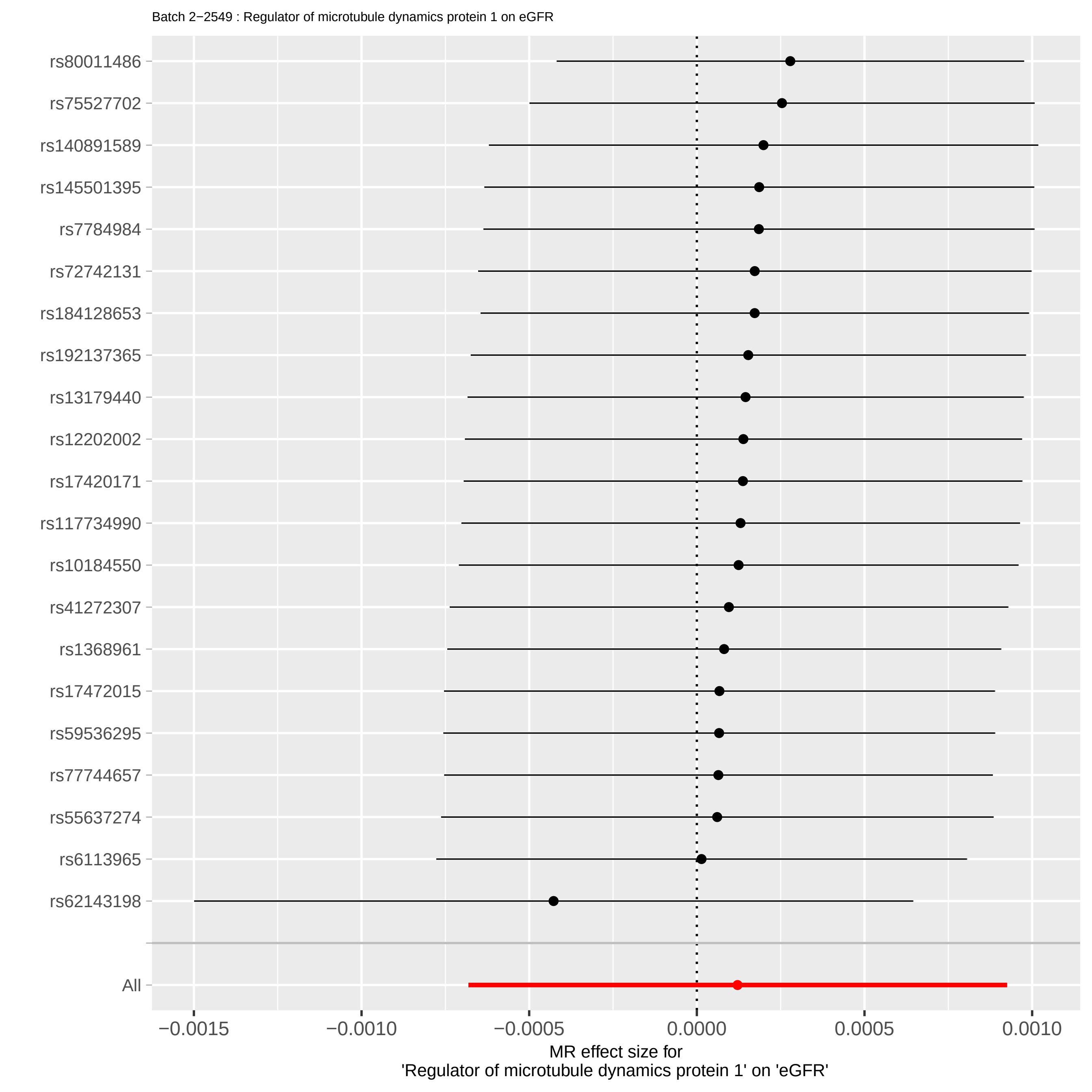

Batch 2−2549 : Regulator of microtubule dynamics protein 1 on eGFR
rs80011486
rs75527702
rs140891589
rs145501395
rs7784984
rs72742131
rs184128653
rs192137365
rs13179440
rs12202002
rs17420171
rs117734990
rs10184550
rs41272307
rs1368961
rs17472015
rs59536295
rs77744657
rs55637274
rs6113965
rs62143198
All
−0.0015
−0.0010
−0.0005
0.0000
0.0005
0.0010
MR effect size for
'Regulator of microtubule dynamics protein 1' on 'eGFR'

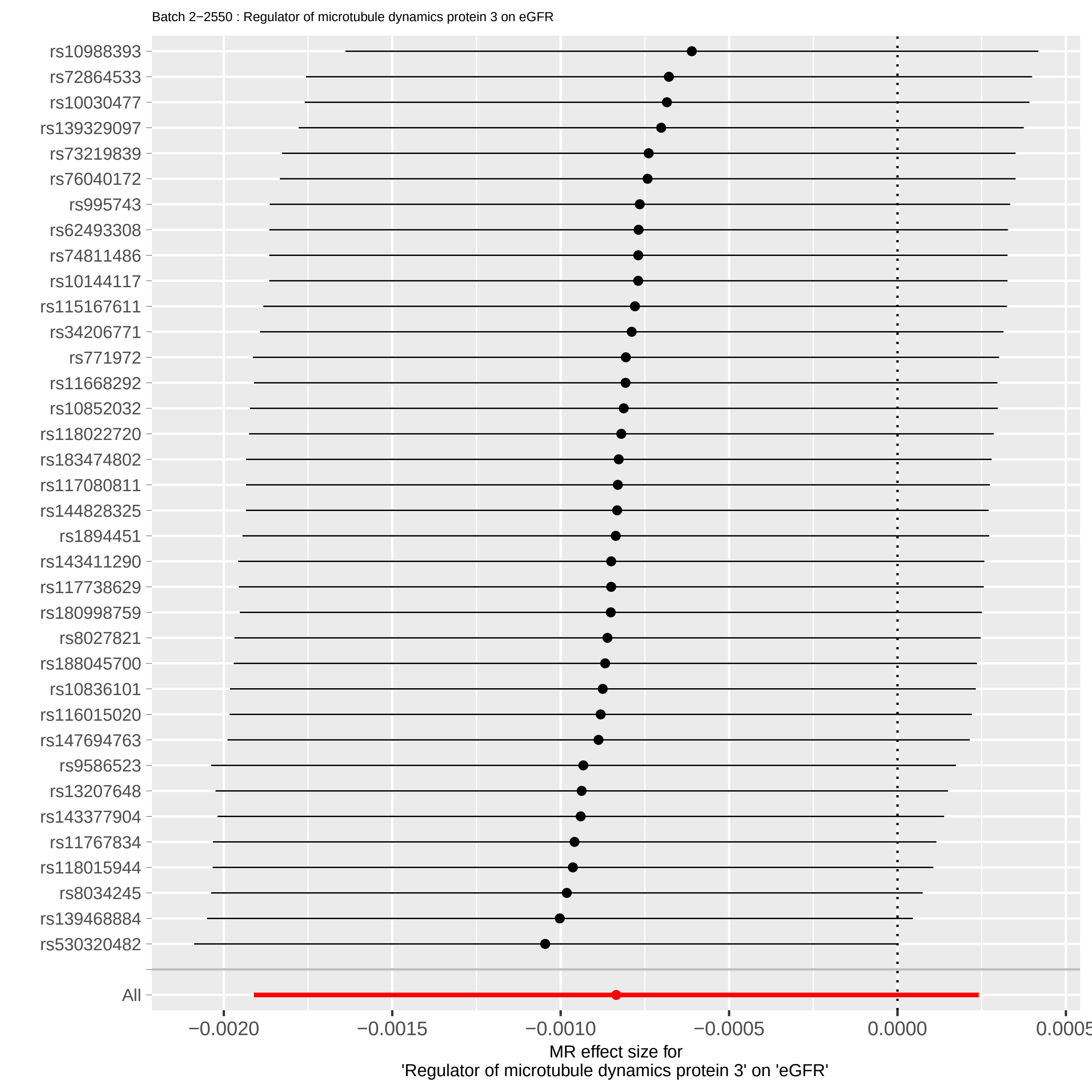

Batch 2−2550 : Regulator of microtubule dynamics protein 3 on eGFR
rs10988393
rs72864533
rs10030477
rs139329097
rs73219839
rs76040172
rs995743
rs62493308
rs74811486
rs10144117
rs115167611
rs34206771
rs771972
rs11668292
rs10852032
rs118022720
rs183474802
rs117080811
rs144828325
rs1894451
rs143411290
rs117738629
rs180998759
rs8027821
rs188045700
rs10836101
rs116015020
rs147694763
rs9586523
rs13207648
rs143377904
rs11767834
rs118015944
rs8034245
rs139468884
rs530320482
All
−0.0020
−0.0015
−0.0010
−0.0005
0.0000
0.0005
MR effect size for
'Regulator of microtubule dynamics protein 3' on 'eGFR'

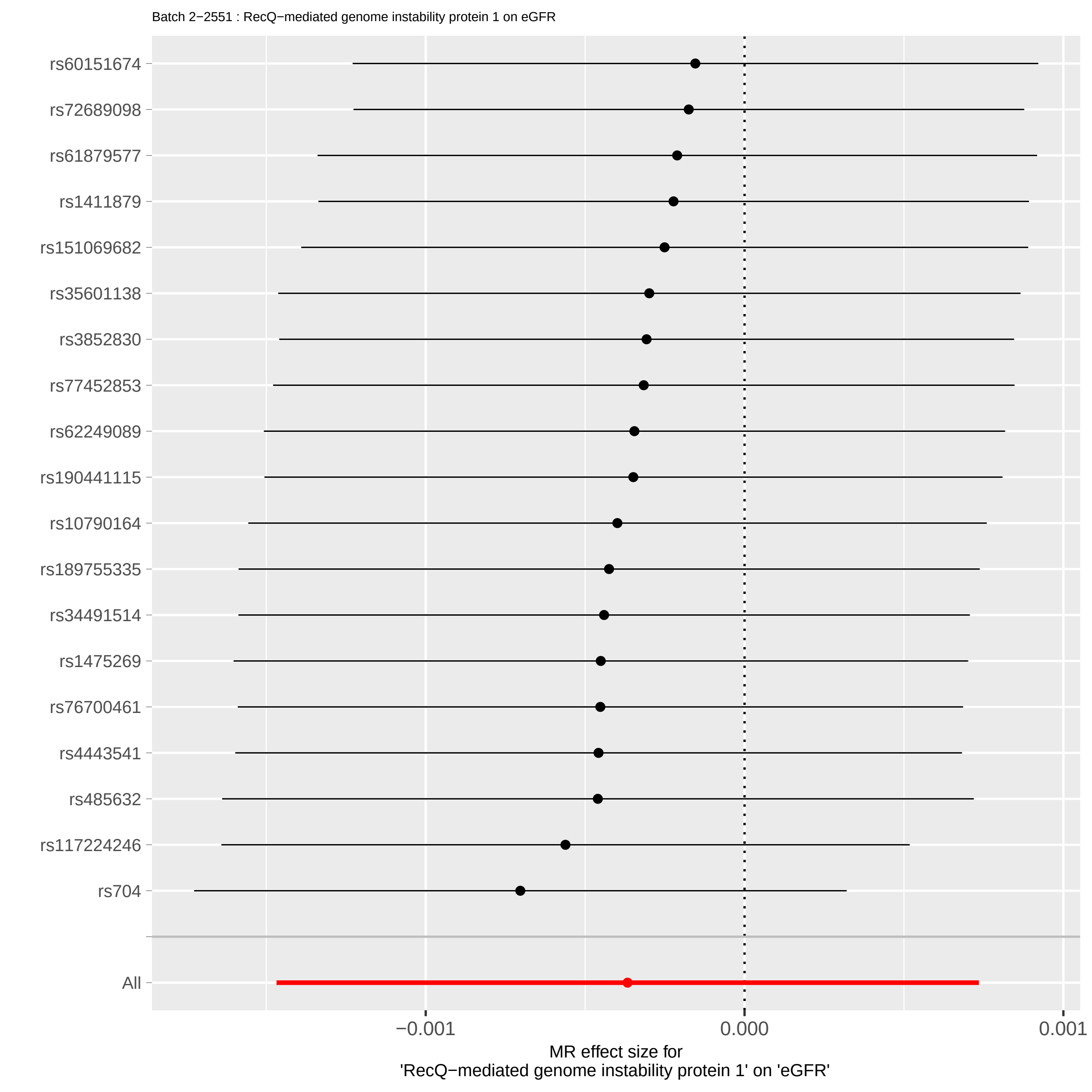
Batch 2−2551 : RecQ−mediated genome instability protein 1 on eGFR
rs60151674
rs72689098
rs61879577
rs1411879
rs151069682
rs35601138
rs3852830
rs77452853
rs62249089
rs190441115
rs10790164
rs189755335
rs34491514
rs1475269
rs76700461
rs4443541
rs485632
rs117224246
rs704
All
−0.001
0.000
0.001
MR effect size for
'RecQ−mediated genome instability protein 1' on 'eGFR'

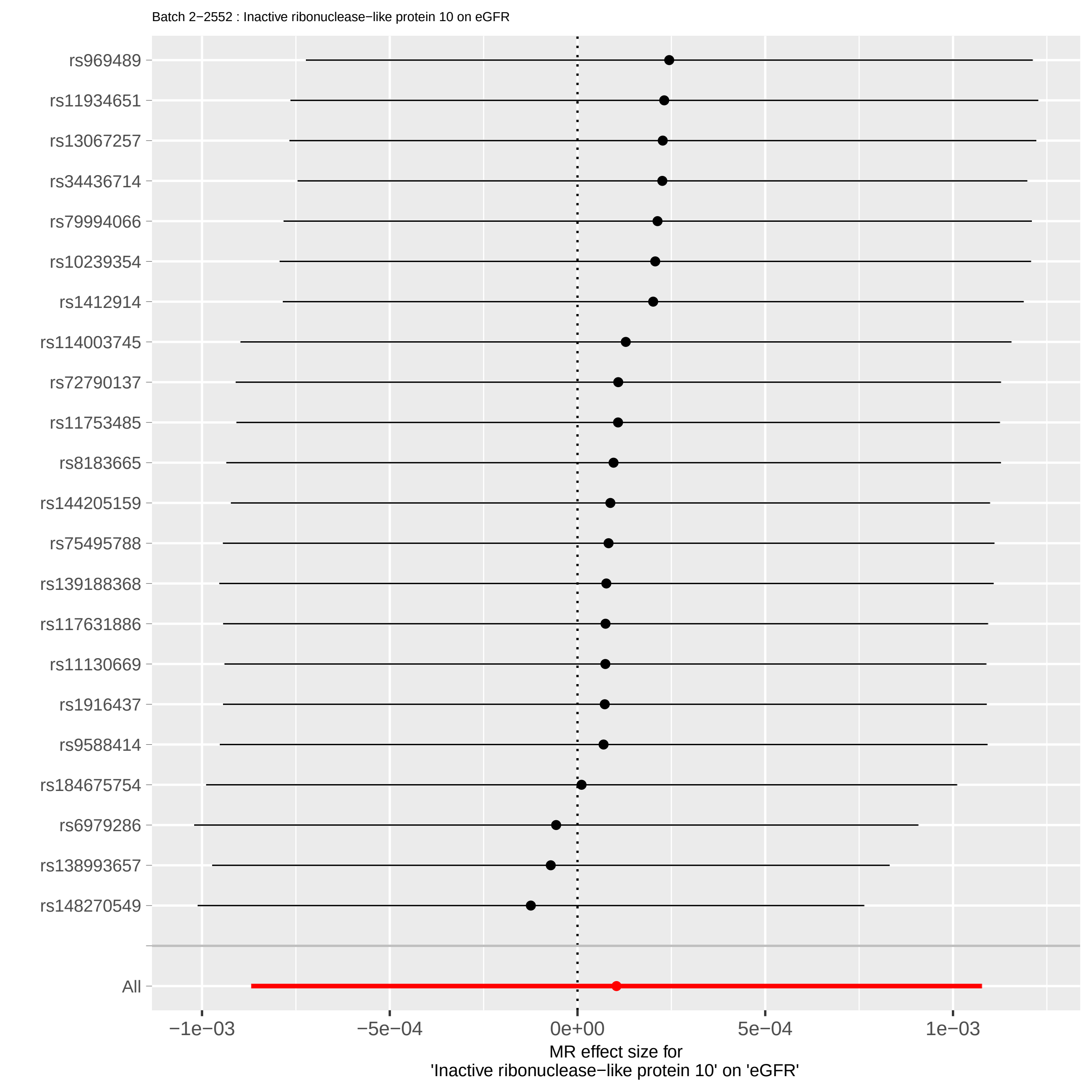

Batch 2−2552 : Inactive ribonuclease−like protein 10 on eGFR
rs969489
rs11934651
rs13067257
rs34436714
rs79994066
rs10239354
rs1412914
rs114003745
rs72790137
rs11753485
rs8183665
rs144205159
rs75495788
rs139188368
rs117631886
rs11130669
rs1916437
rs9588414
rs184675754
rs6979286
rs138993657
rs148270549
All
−1e−03
−5e−04
0e+00
5e−04
1e−03
MR effect size for
'Inactive ribonuclease−like protein 10' on 'eGFR'

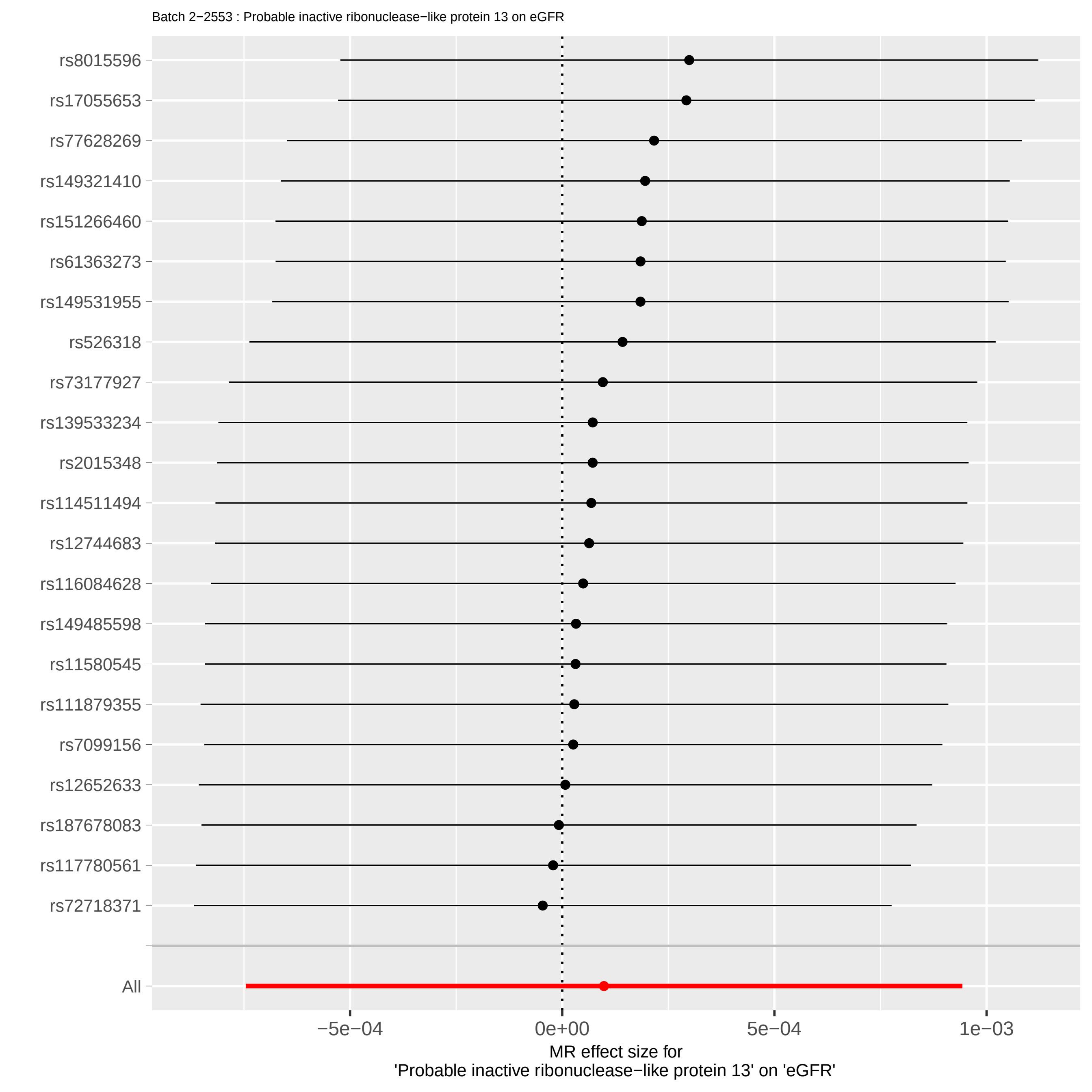

Batch 2−2553 : Probable inactive ribonuclease−like protein 13 on eGFR
rs8015596
rs17055653
rs77628269
rs149321410
rs151266460
rs61363273
rs149531955
rs526318
rs73177927
rs139533234
rs2015348
rs114511494
rs12744683
rs116084628
rs149485598
rs11580545
rs111879355
rs7099156
rs12652633
rs187678083
rs117780561
rs72718371
All
−5e−04
0e+00
5e−04
1e−03
MR effect size for
'Probable inactive ribonuclease−like protein 13' on 'eGFR'

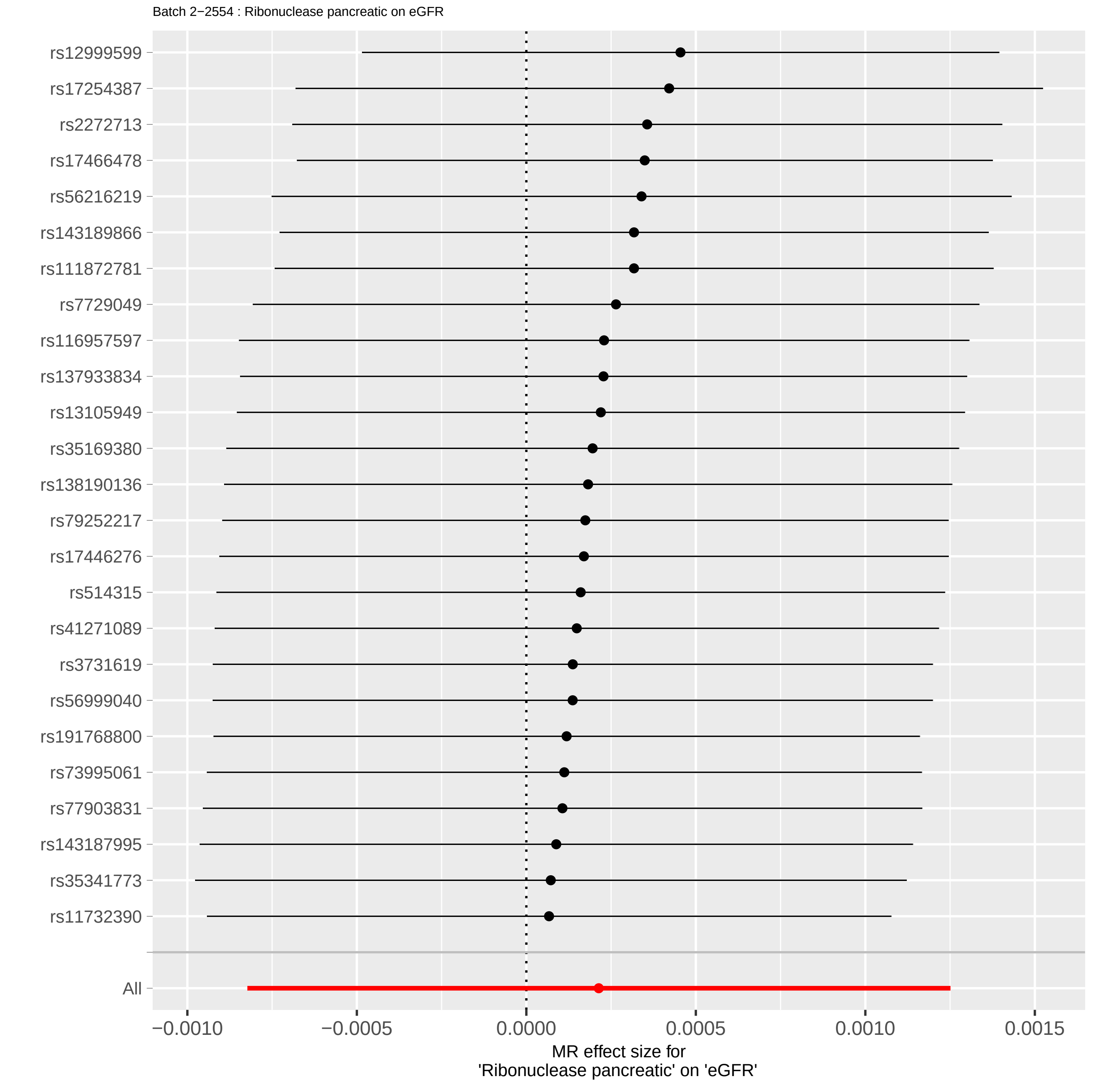
Batch 2−2554 : Ribonuclease pancreatic on eGFR
rs12999599
rs17254387
rs2272713
rs17466478
rs56216219
rs143189866
rs111872781
rs7729049
rs116957597
rs137933834
rs13105949
rs35169380
rs138190136
rs79252217
rs17446276
rs514315
rs41271089
rs3731619
rs56999040
rs191768800
rs73995061
rs77903831
rs143187995
rs35341773
rs11732390
All
−0.0010
−0.0005
0.0000
0.0005
0.0010
0.0015
MR effect size for
'Ribonuclease pancreatic' on 'eGFR'

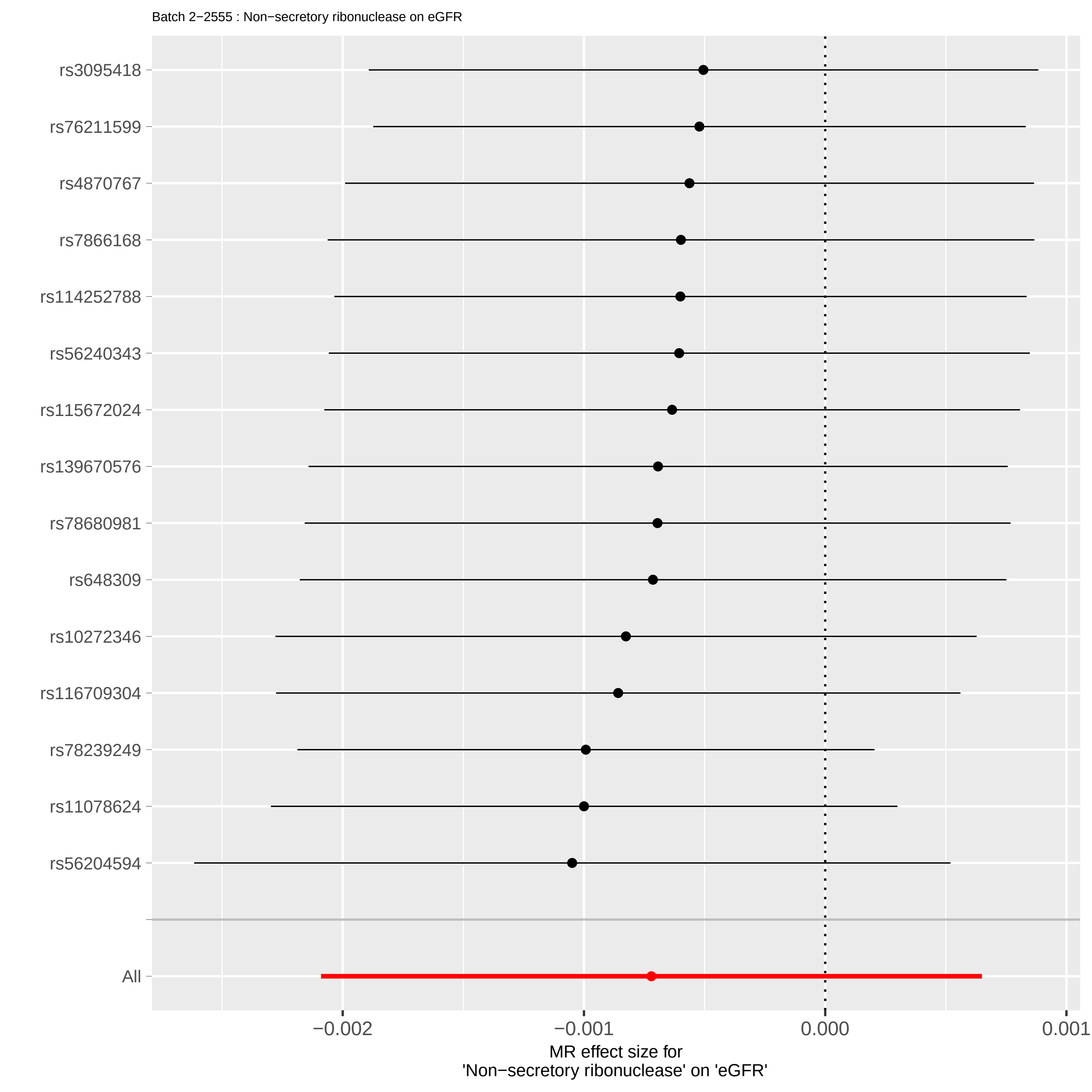

Batch 2−2555 : Non−secretory ribonuclease on eGFR
rs3095418
rs76211599
rs4870767
rs7866168
rs114252788
rs56240343
rs115672024
rs139670576
rs78680981
rs648309
rs10272346
rs116709304
rs78239249
rs11078624
rs56204594
All
−0.002
−0.001
0.000
0.001
MR effect size for
'Non−secretory ribonuclease' on 'eGFR'

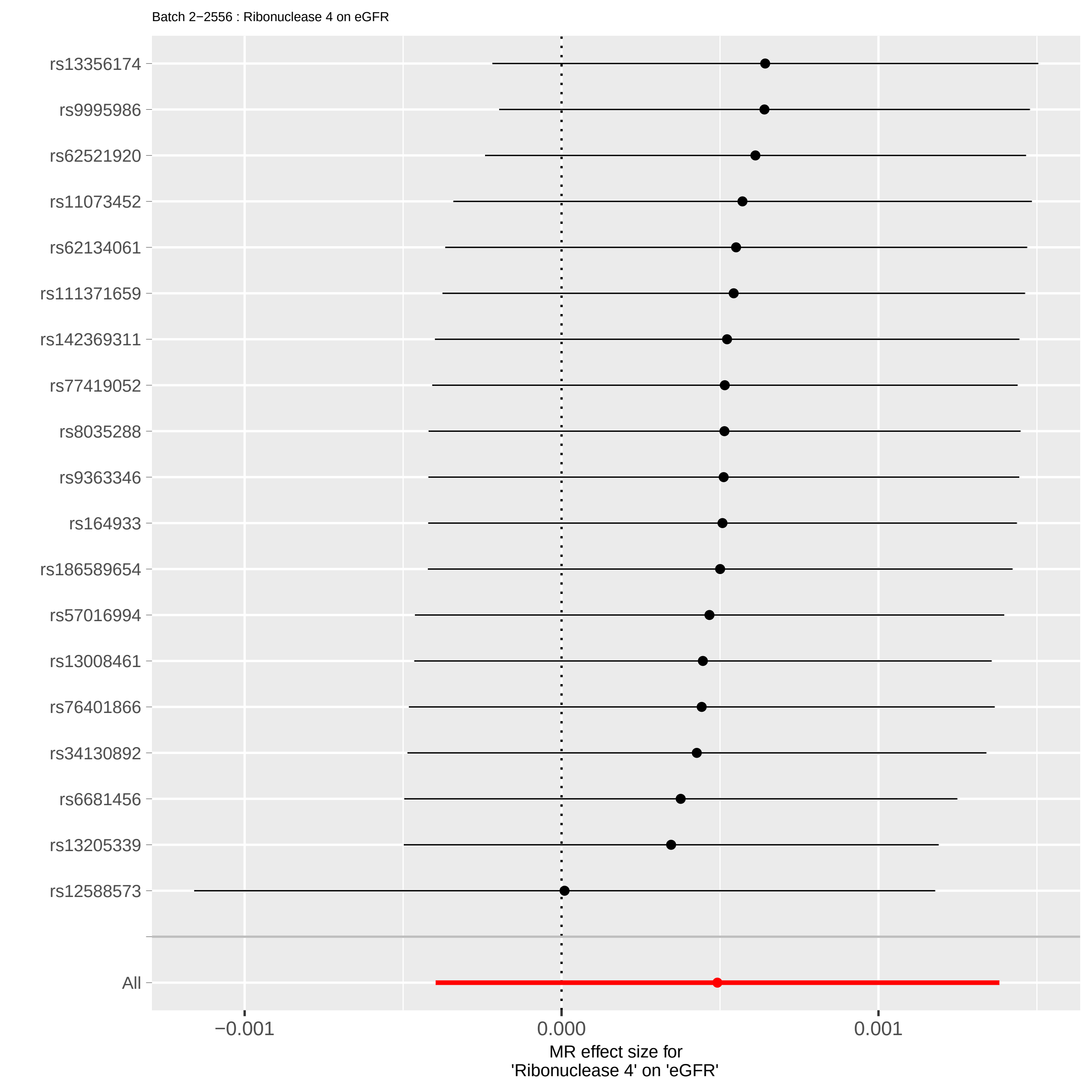

Batch 2−2556 : Ribonuclease 4 on eGFR
rs13356174
rs9995986
rs62521920
rs11073452
rs62134061
rs111371659
rs142369311
rs77419052
rs8035288
rs9363346
rs164933
rs186589654
rs57016994
rs13008461
rs76401866
rs34130892
rs6681456
rs13205339
rs12588573
All
−0.001
0.000
0.001
MR effect size for
'Ribonuclease 4' on 'eGFR'

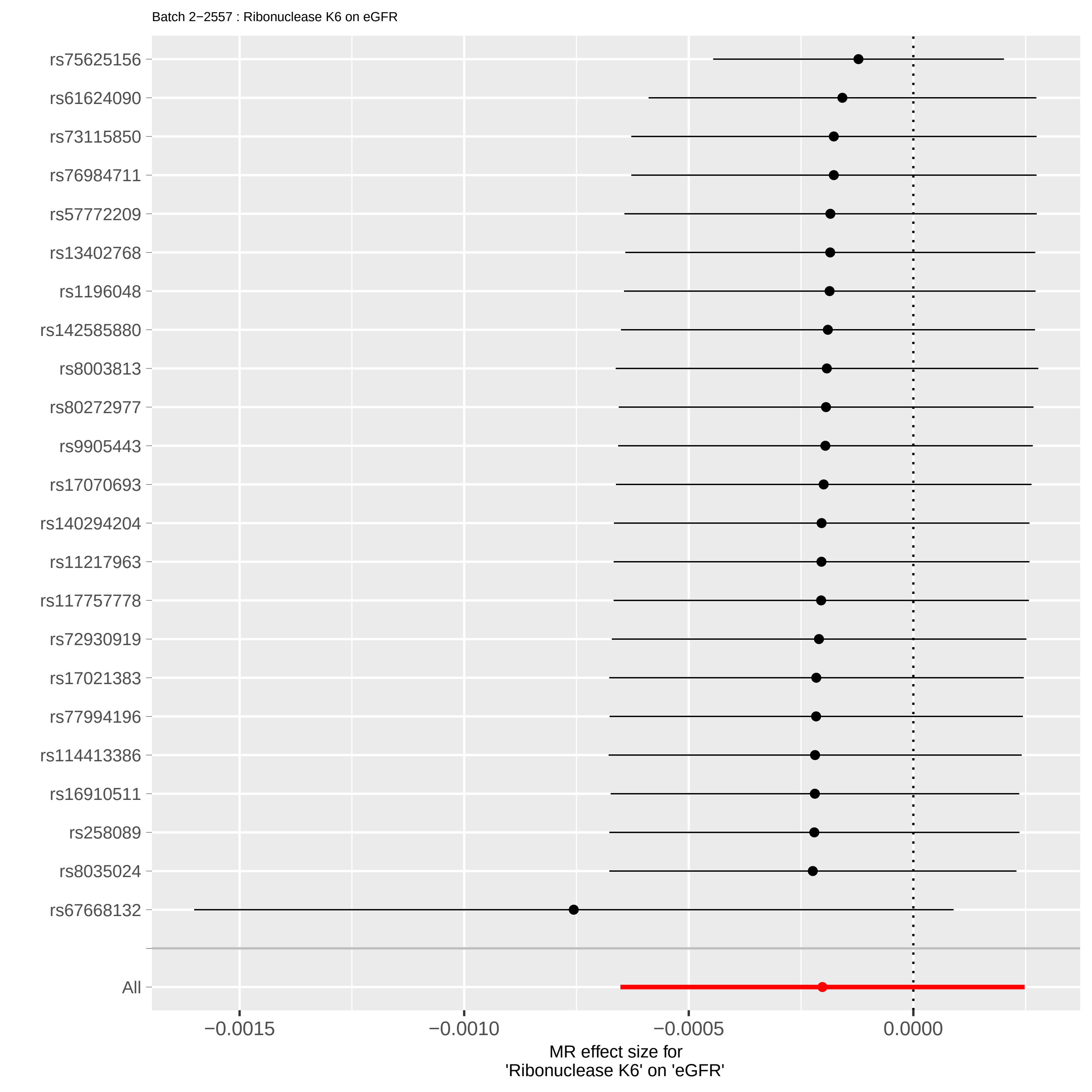

Batch 2−2557 : Ribonuclease K6 on eGFR
rs75625156
rs61624090
rs73115850
rs76984711
rs57772209
rs13402768
rs1196048
rs142585880
rs8003813
rs80272977
rs9905443
rs17070693
rs140294204
rs11217963
rs117757778
rs72930919
rs17021383
rs77994196
rs114413386
rs16910511
rs258089
rs8035024
rs67668132
All
−0.0015
−0.0010
−0.0005
0.0000
MR effect size for
'Ribonuclease K6' on 'eGFR'

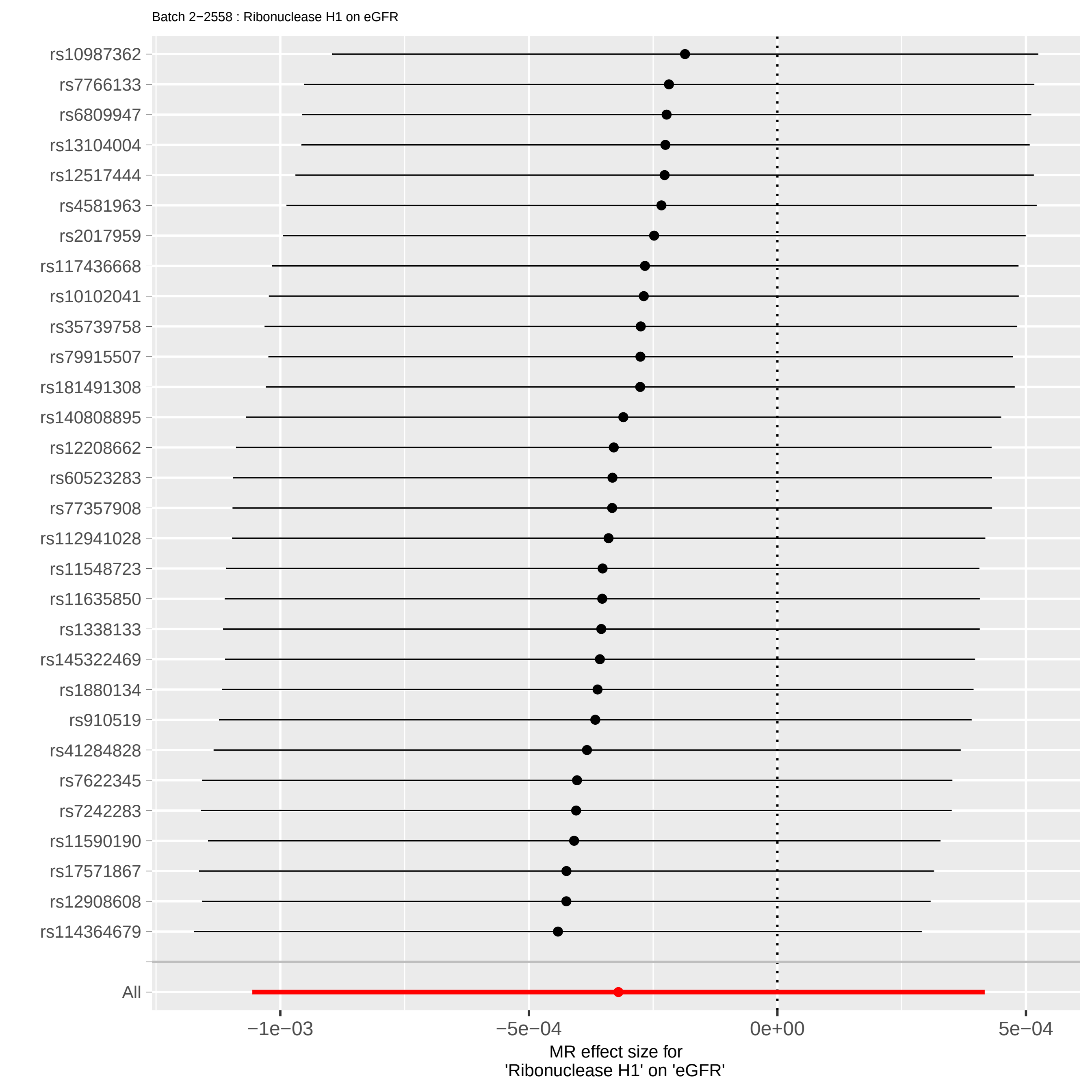

Batch 2−2558 : Ribonuclease H1 on eGFR
rs10987362
rs7766133
rs6809947
rs13104004
rs12517444
rs4581963
rs2017959
rs117436668
rs10102041
rs35739758
rs79915507
rs181491308
rs140808895
rs12208662
rs60523283
rs77357908
rs112941028
rs11548723
rs11635850
rs1338133
rs145322469
rs1880134
rs910519
rs41284828
rs7622345
rs7242283
rs11590190
rs17571867
rs12908608
rs114364679
All
−1e−03
−5e−04
0e+00
5e−04
MR effect size for
'Ribonuclease H1' on 'eGFR'

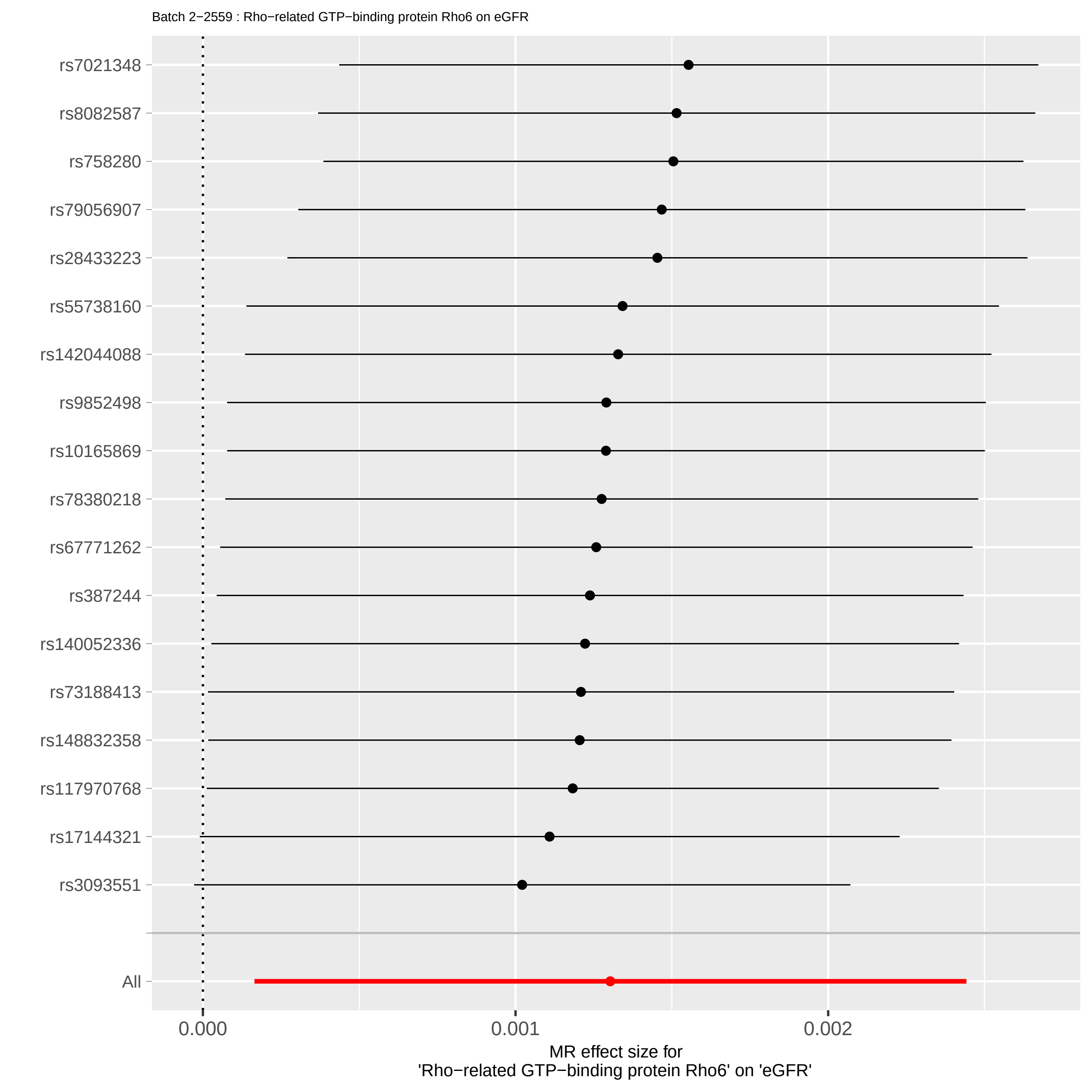

Batch 2−2559 : Rho−related GTP−binding protein Rho6 on eGFR
rs7021348
rs8082587
rs758280
rs79056907
rs28433223
rs55738160
rs142044088
rs9852498
rs10165869
rs78380218
rs67771262
rs387244
rs140052336
rs73188413
rs148832358
rs117970768
rs17144321
rs3093551
All
0.000
0.001
0.002
MR effect size for
'Rho−related GTP−binding protein Rho6' on 'eGFR'

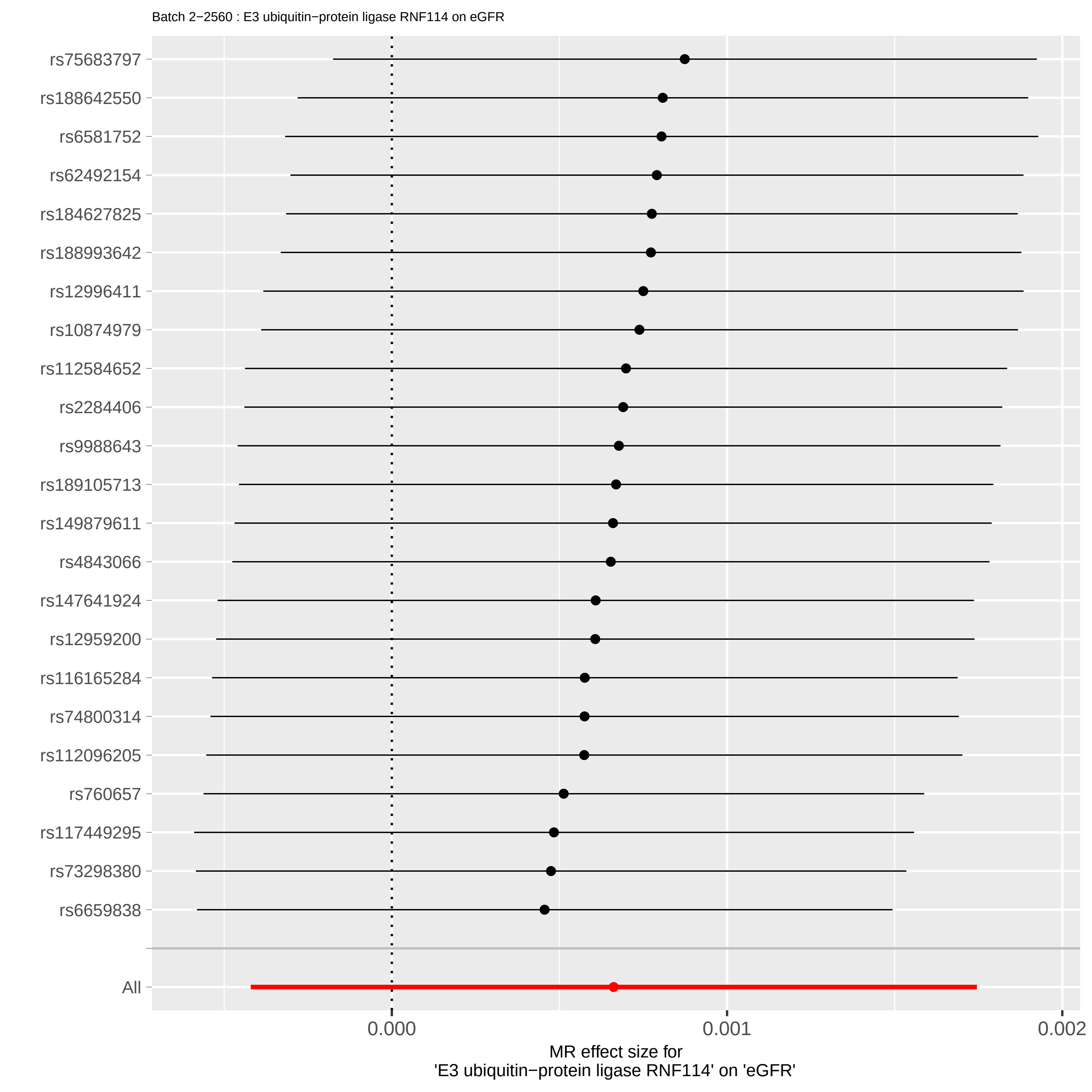
Batch 2−2560 : E3 ubiquitin−protein ligase RNF114 on eGFR
rs75683797
rs188642550
rs6581752
rs62492154
rs184627825
rs188993642
rs12996411
rs10874979
rs112584652
rs2284406
rs9988643
rs189105713
rs149879611
rs4843066
rs147641924
rs12959200
rs116165284
rs74800314
rs112096205
rs760657
rs117449295
rs73298380
rs6659838
All
0.000
0.001
0.002
MR effect size for
'E3 ubiquitin−protein ligase RNF114' on 'eGFR'

Batch 2−2561 : RING finger protein 122 on eGFR

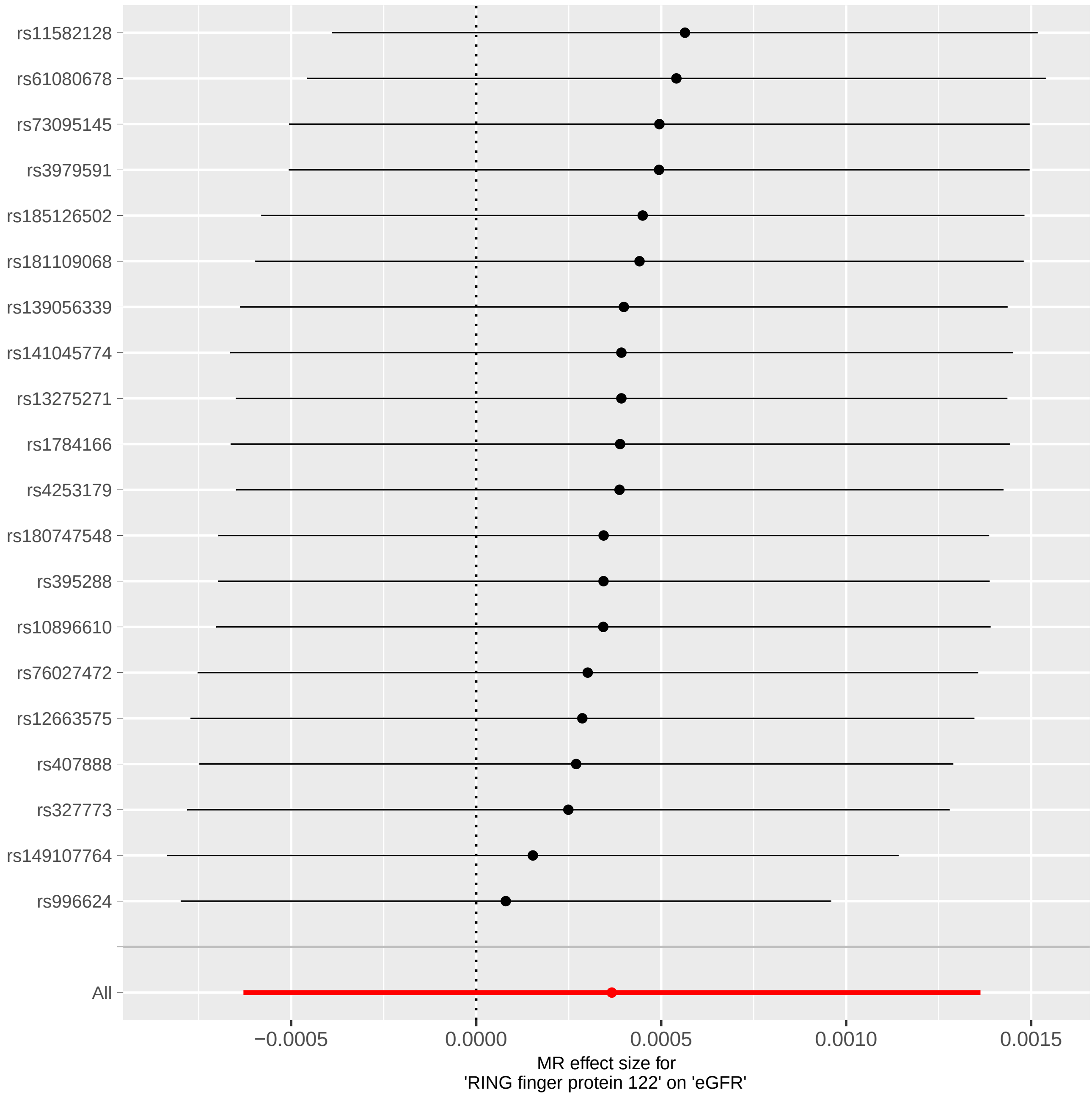

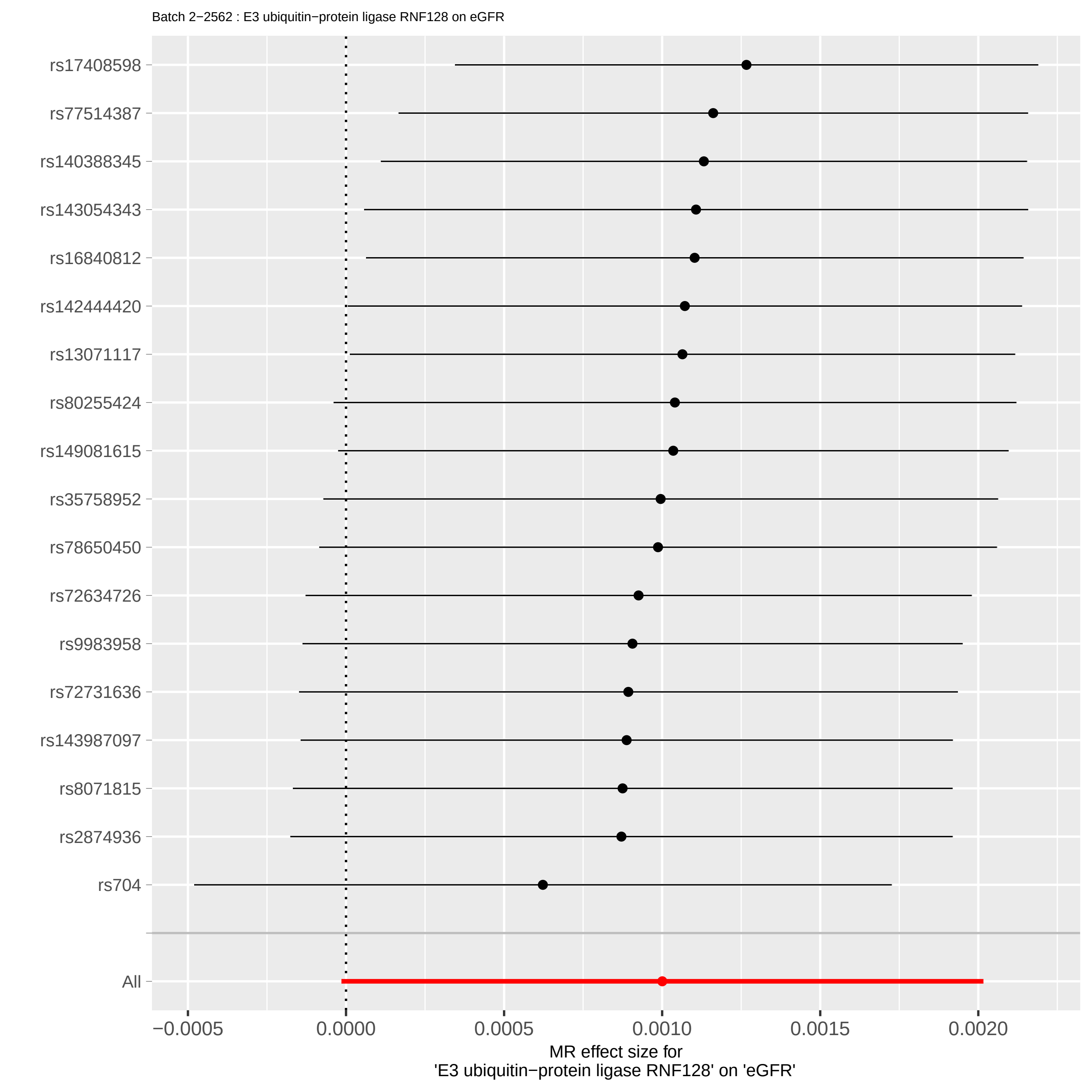

Batch 2−2562 : E3 ubiquitin−protein ligase RNF128 on eGFR
rs17408598
rs77514387
rs140388345
rs143054343
rs16840812
rs142444420
rs13071117
rs80255424
rs149081615
rs35758952
rs78650450
rs72634726
rs9983958
rs72731636
rs143987097
rs8071815
rs2874936
rs704
All
−0.0005
0.0000
0.0005
0.0010
0.0015
0.0020
MR effect size for
'E3 ubiquitin−protein ligase RNF128' on 'eGFR'

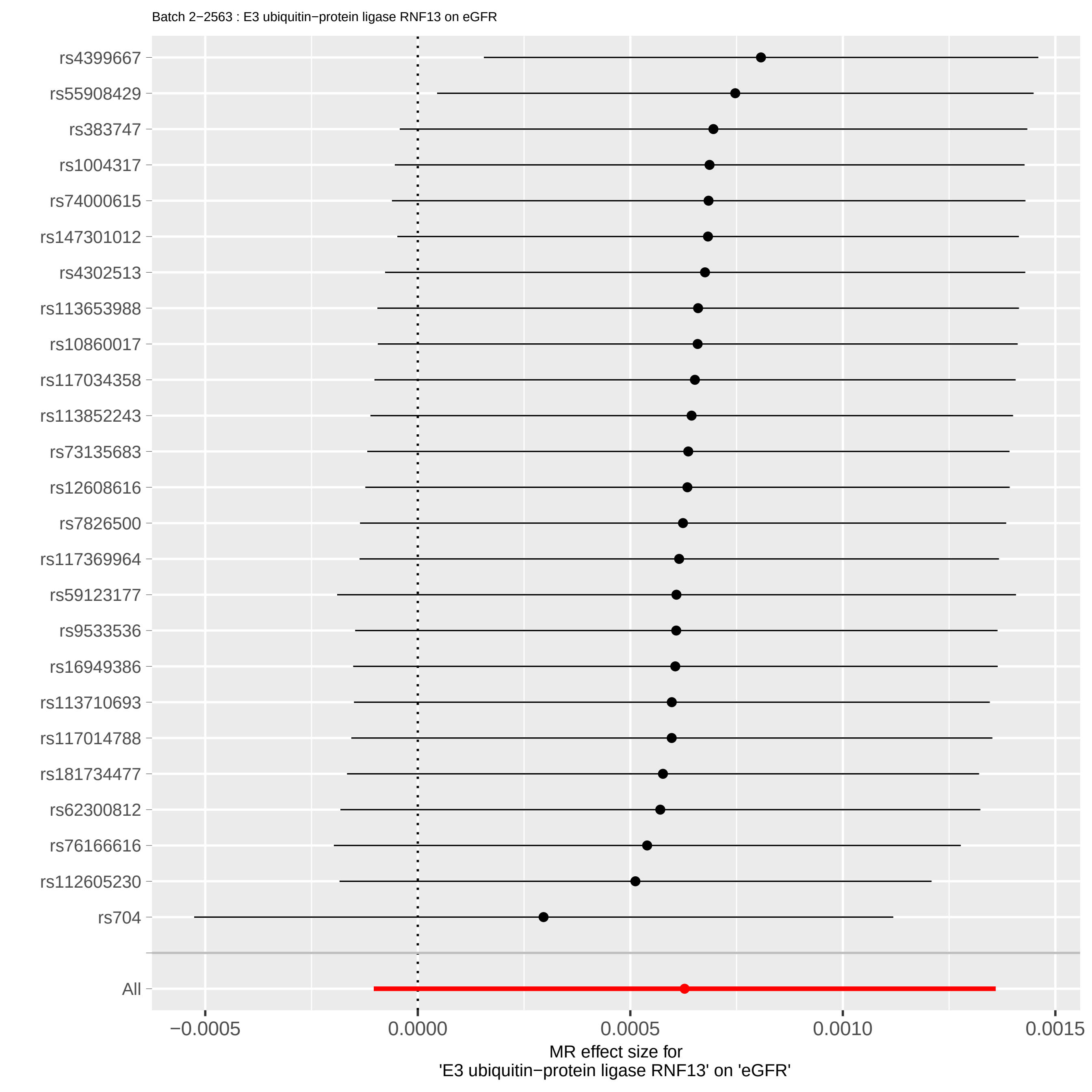

Batch 2−2563 : E3 ubiquitin−protein ligase RNF13 on eGFR
rs4399667
rs55908429
rs383747
rs1004317
rs74000615
rs147301012
rs4302513
rs113653988
rs10860017
rs117034358
rs113852243
rs73135683
rs12608616
rs7826500
rs117369964
rs59123177
rs9533536
rs16949386
rs113710693
rs117014788
rs181734477
rs62300812
rs76166616
rs112605230
rs704
All
−0.0005
0.0000
0.0005
0.0010
0.0015
MR effect size for
'E3 ubiquitin−protein ligase RNF13' on 'eGFR'

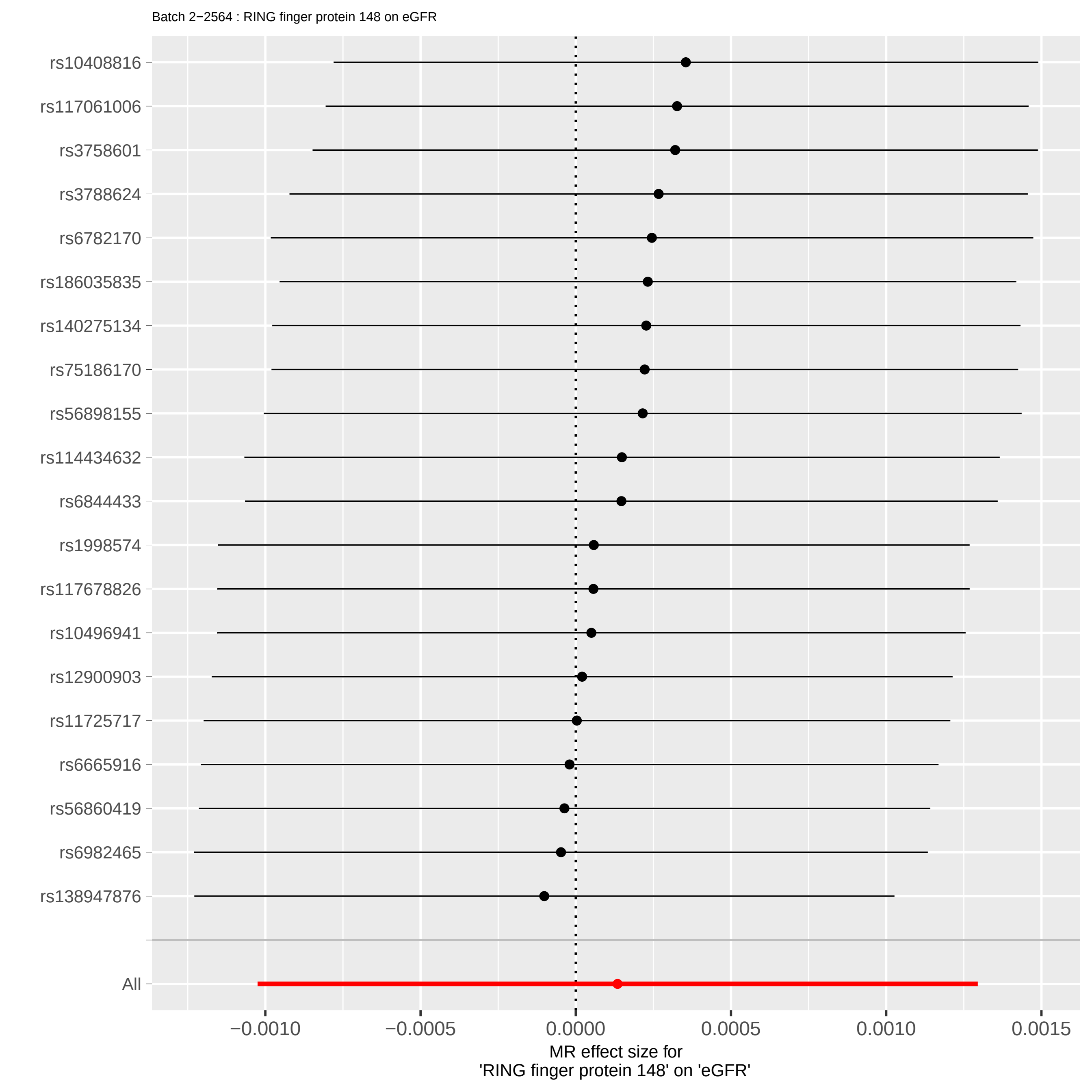

Batch 2−2564 : RING finger protein 148 on eGFR
rs10408816
rs117061006
rs3758601
rs3788624
rs6782170
rs186035835
rs140275134
rs75186170
rs56898155
rs114434632
rs6844433
rs1998574
rs117678826
rs10496941
rs12900903
rs11725717
rs6665916
rs56860419
rs6982465
rs138947876
All
−0.0010
−0.0005
0.0000
0.0005
0.0010
0.0015
MR effect size for
'RING finger protein 148' on 'eGFR'

Batch 2−2565 : RING finger protein 148 on eGFR

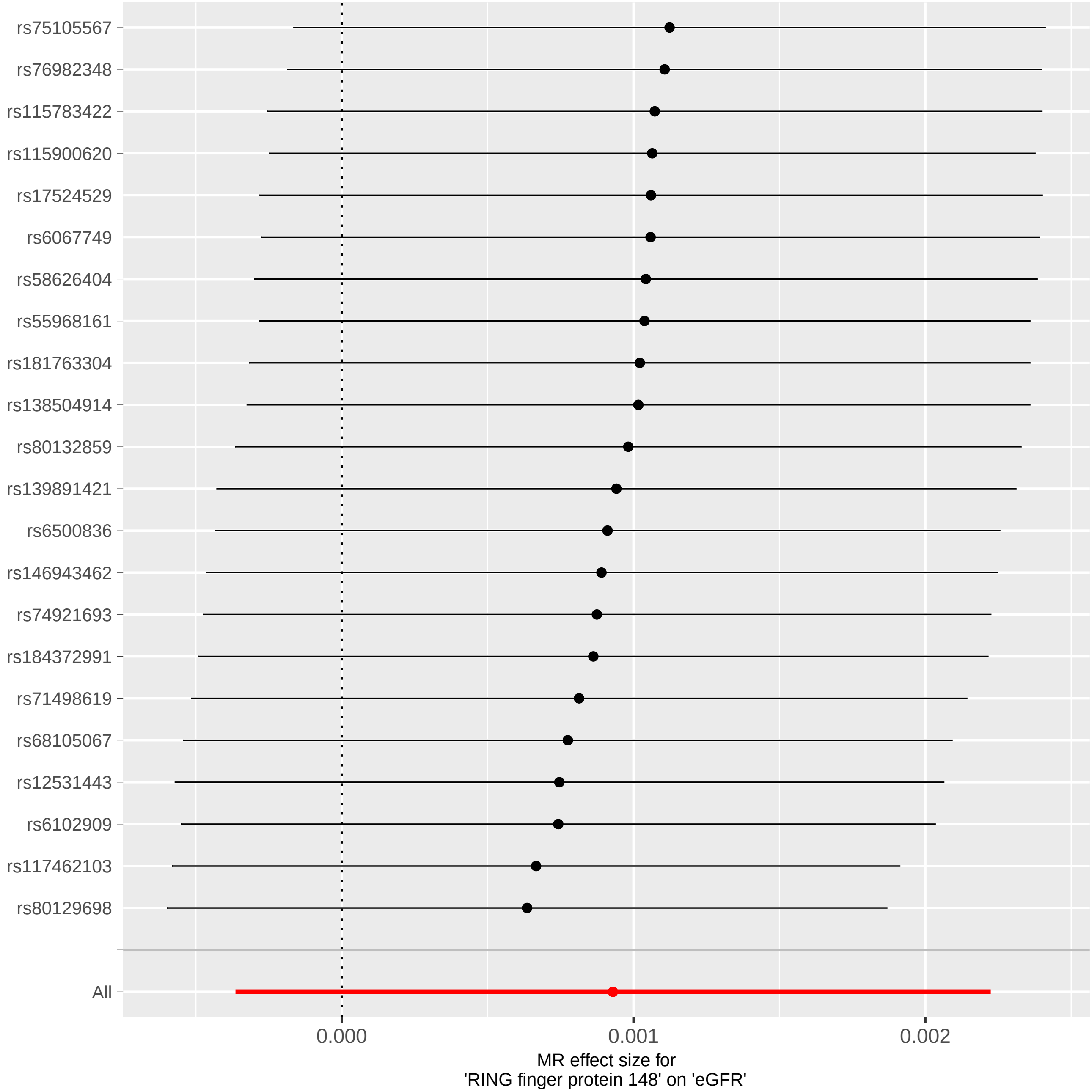

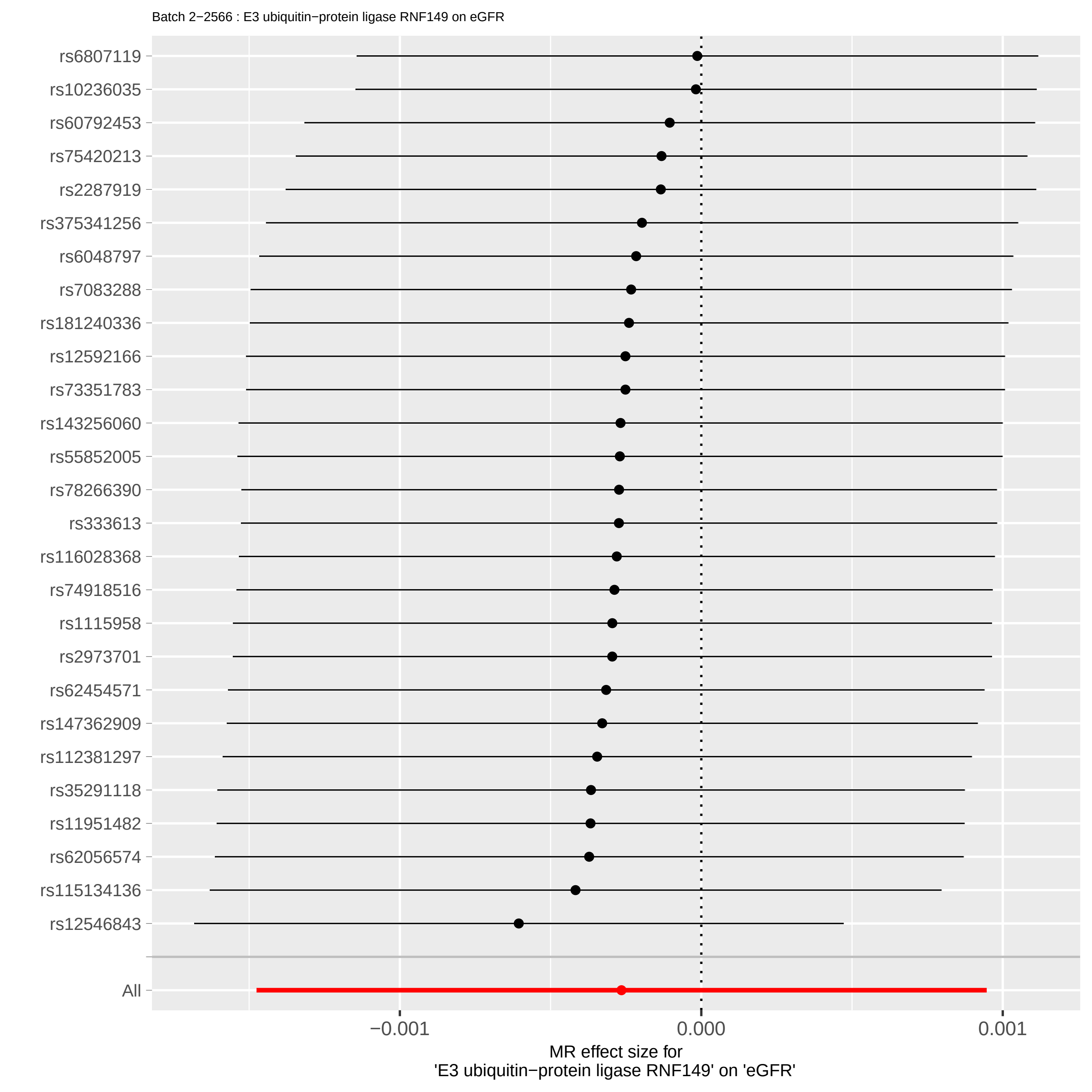

Batch 2−2566 : E3 ubiquitin−protein ligase RNF149 on eGFR
rs6807119
rs10236035
rs60792453
rs75420213
rs2287919
rs375341256
rs6048797
rs7083288
rs181240336
rs12592166
rs73351783
rs143256060
rs55852005
rs78266390
rs333613
rs116028368
rs74918516
rs1115958
rs2973701
rs62454571
rs147362909
rs112381297
rs35291118
rs11951482
rs62056574
rs115134136
rs12546843
All
−0.001
0.000
0.001
MR effect size for
'E3 ubiquitin−protein ligase RNF149' on 'eGFR'

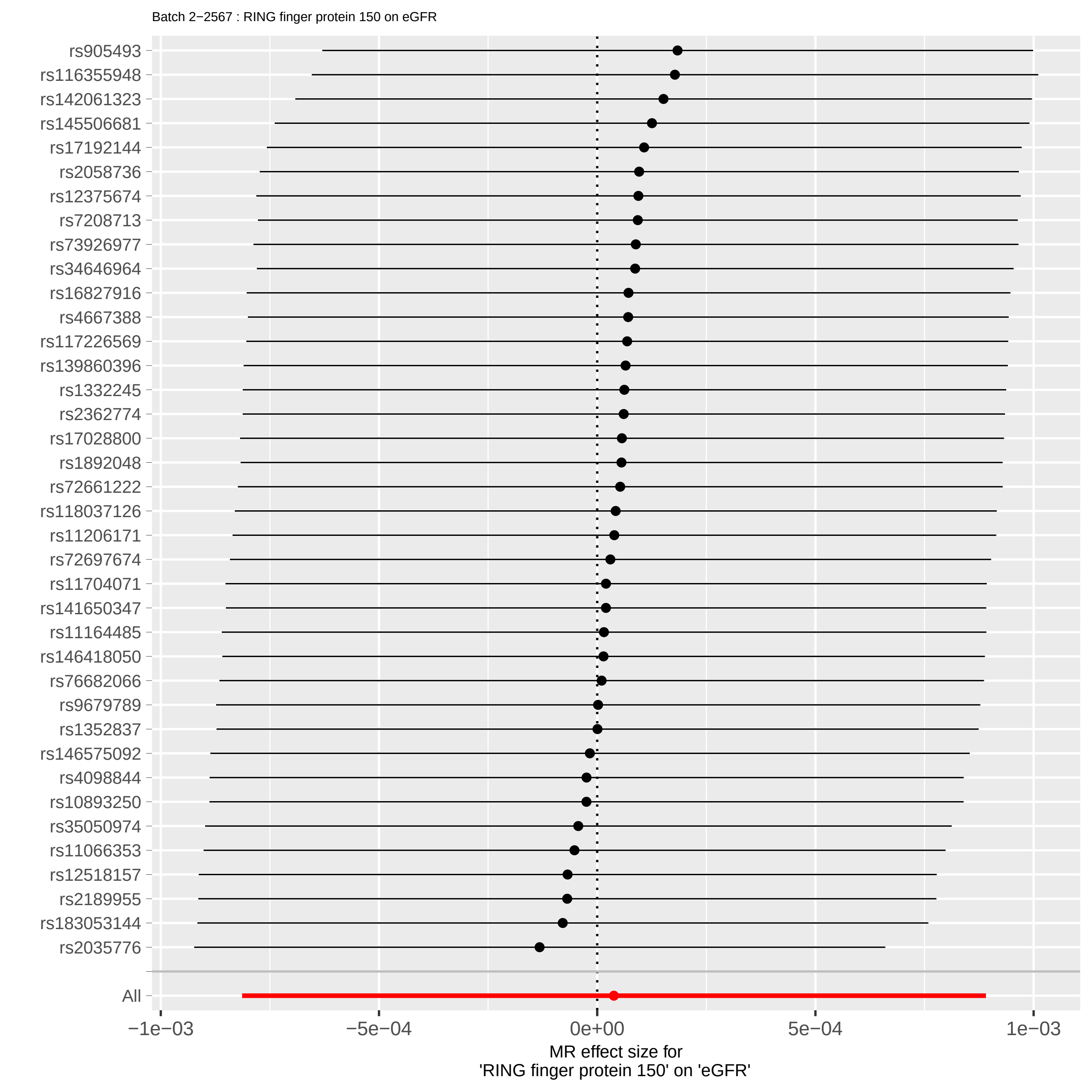
Batch 2−2567 : RING finger protein 150 on eGFR
rs905493
rs116355948
rs142061323
rs145506681
rs17192144
rs2058736
rs12375674
rs7208713
rs73926977
rs34646964
rs16827916
rs4667388
rs117226569
rs139860396
rs1332245
rs2362774
rs17028800
rs1892048
rs72661222
rs118037126
rs11206171
rs72697674
rs11704071
rs141650347
rs11164485
rs146418050
rs76682066
rs9679789
rs1352837
rs146575092
rs4098844
rs10893250
rs35050974
rs11066353
rs12518157
rs2189955
rs183053144
rs2035776
All
−1e−03
−5e−04
0e+00
5e−04
1e−03
MR effect size for
'RING finger protein 150' on 'eGFR'

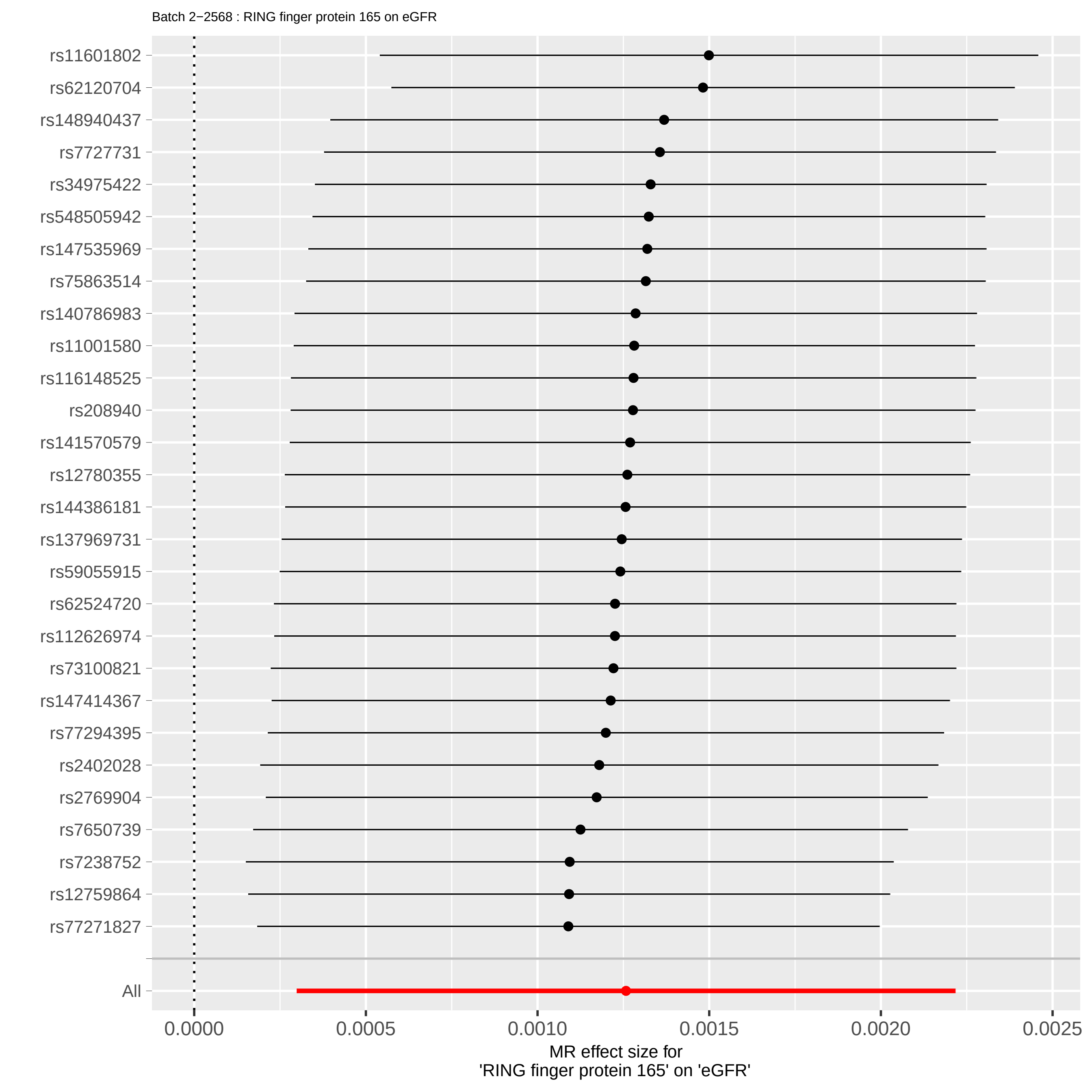

Batch 2−2568 : RING finger protein 165 on eGFR
rs11601802
rs62120704
rs148940437
rs7727731
rs34975422
rs548505942
rs147535969
rs75863514
rs140786983
rs11001580
rs116148525
rs208940
rs141570579
rs12780355
rs144386181
rs137969731
rs59055915
rs62524720
rs112626974
rs73100821
rs147414367
rs77294395
rs2402028
rs2769904
rs7650739
rs7238752
rs12759864
rs77271827
All
0.0000
0.0005
0.0010
0.0015
0.0020
0.0025
MR effect size for
'RING finger protein 165' on 'eGFR'

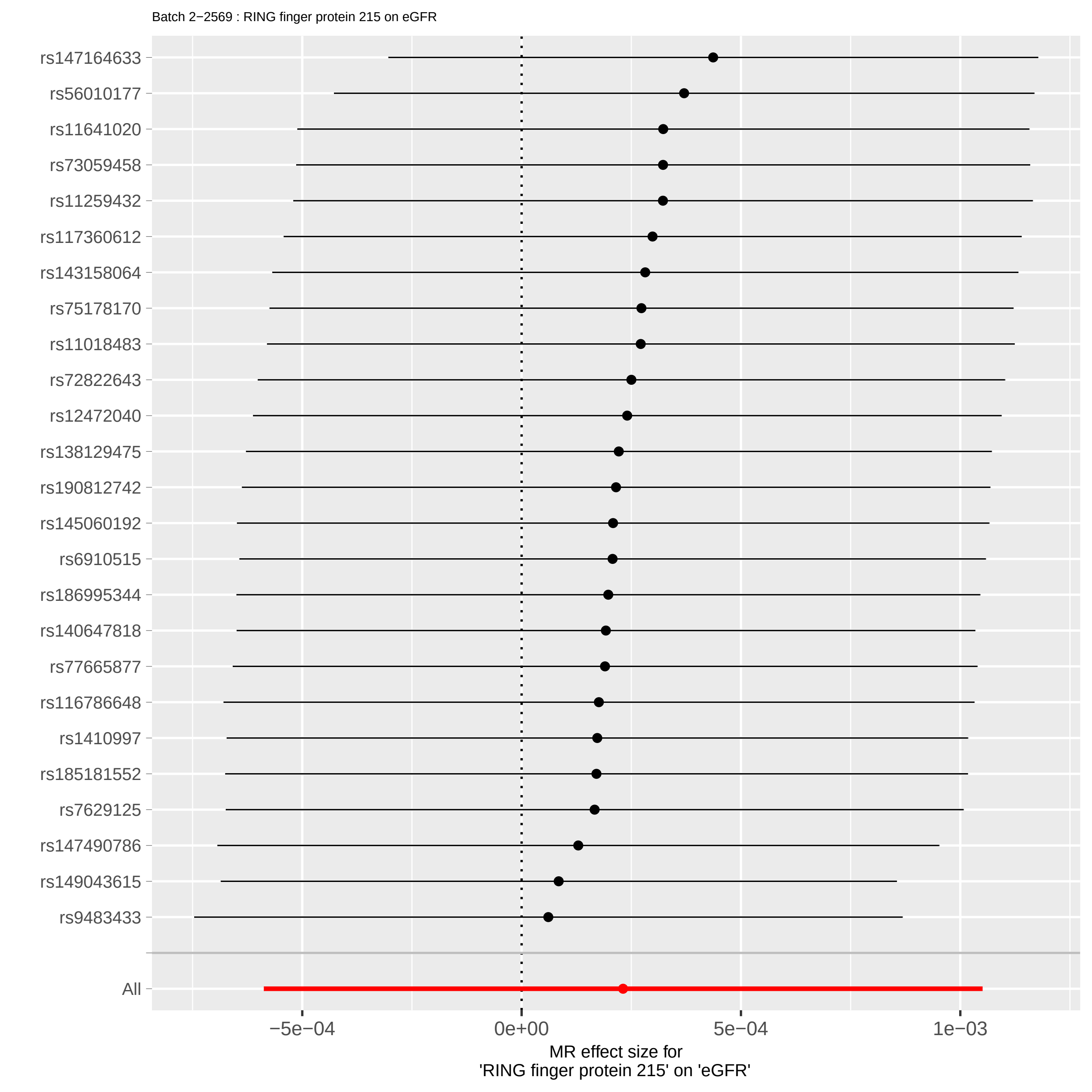

Batch 2−2569 : RING finger protein 215 on eGFR
rs147164633
rs56010177
rs11641020
rs73059458
rs11259432
rs117360612
rs143158064
rs75178170
rs11018483
rs72822643
rs12472040
rs138129475
rs190812742
rs145060192
rs6910515
rs186995344
rs140647818
rs77665877
rs116786648
rs1410997
rs185181552
rs7629125
rs147490786
rs149043615
rs9483433
All
−5e−04
0e+00
5e−04
1e−03
MR effect size for
'RING finger protein 215' on 'eGFR'

Batch 2−2570 : RING finger protein 219 on eGFR

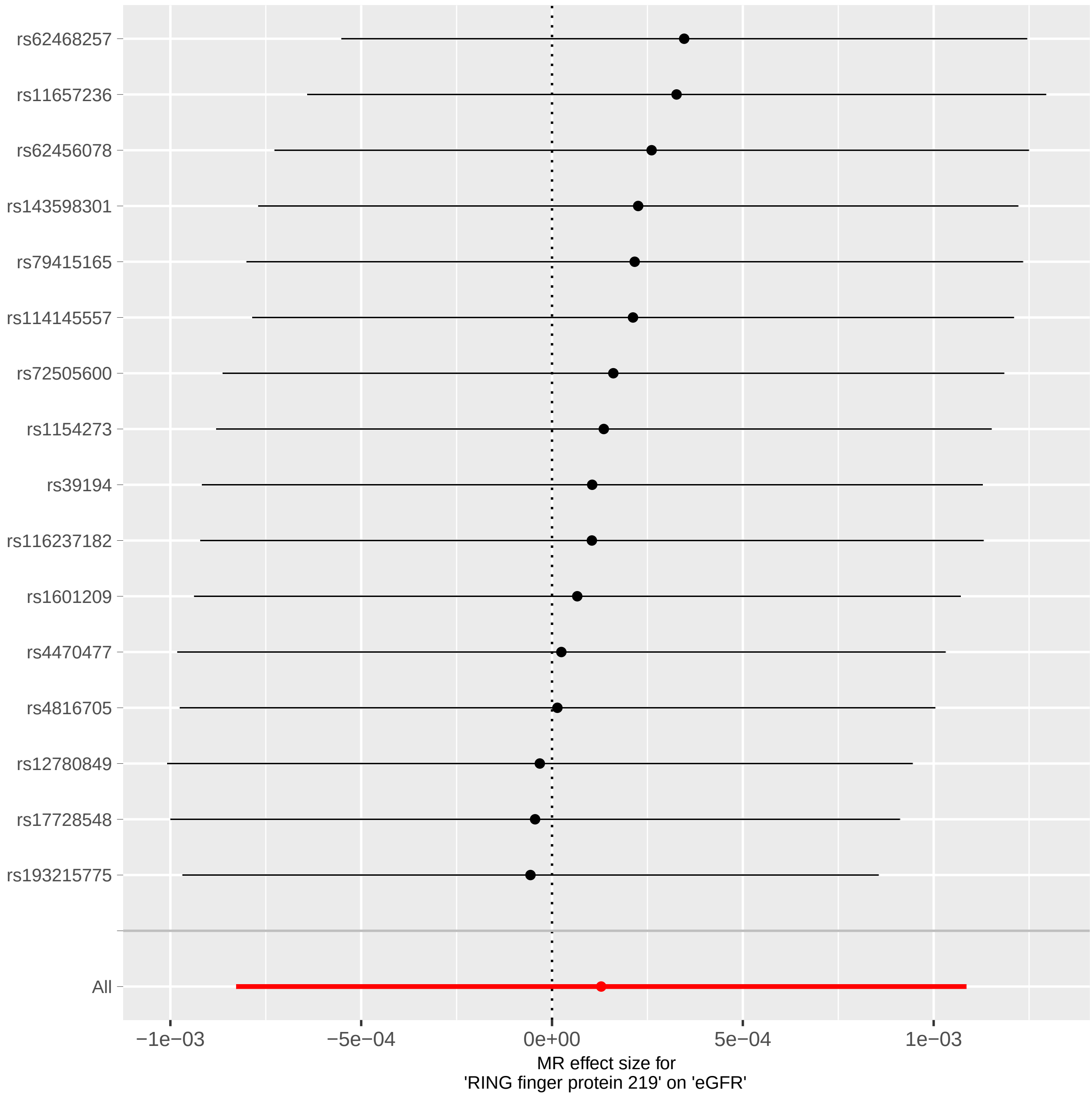

Batch 2−2571 : E3 ubiquitin−protein ligase RNF34 on eGFR

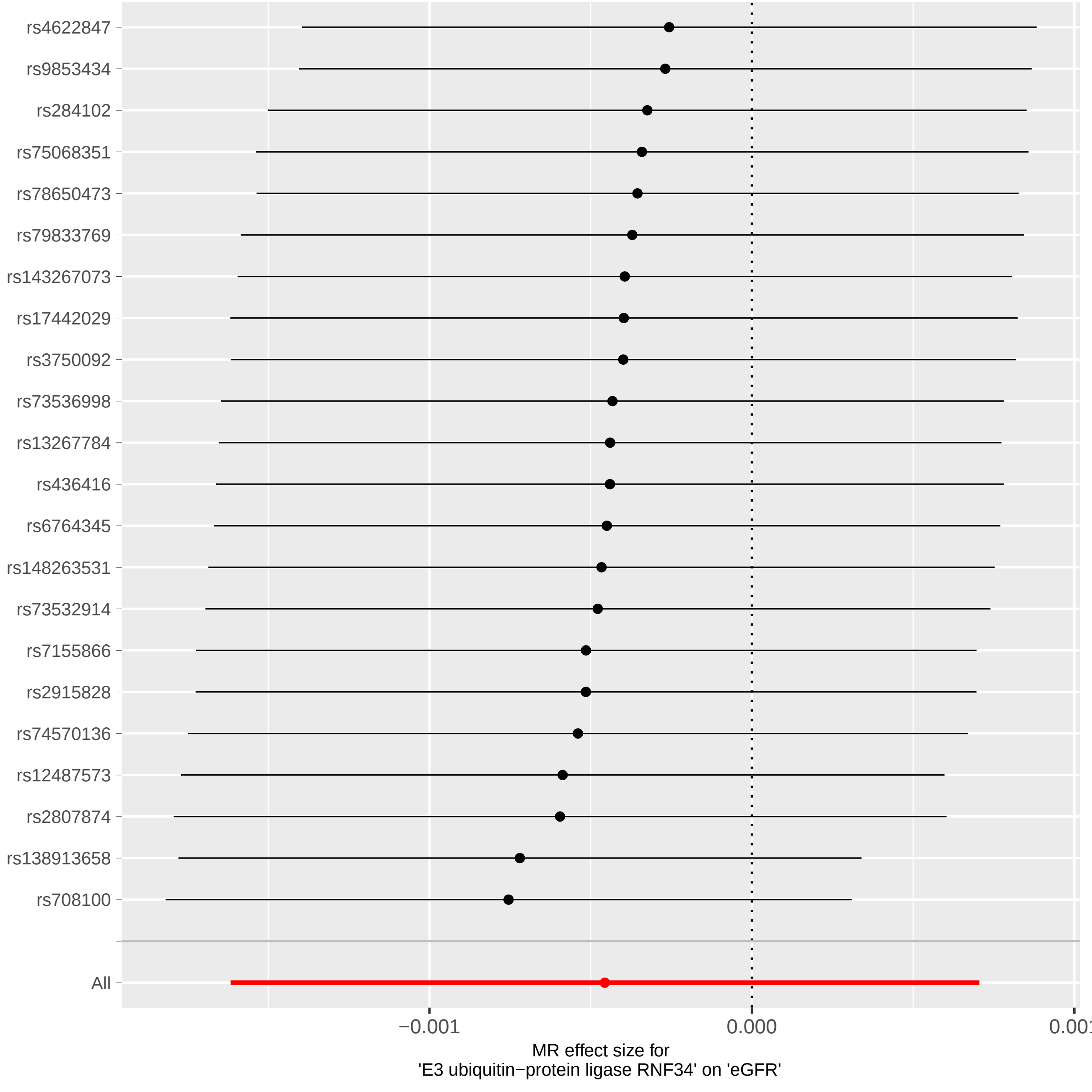

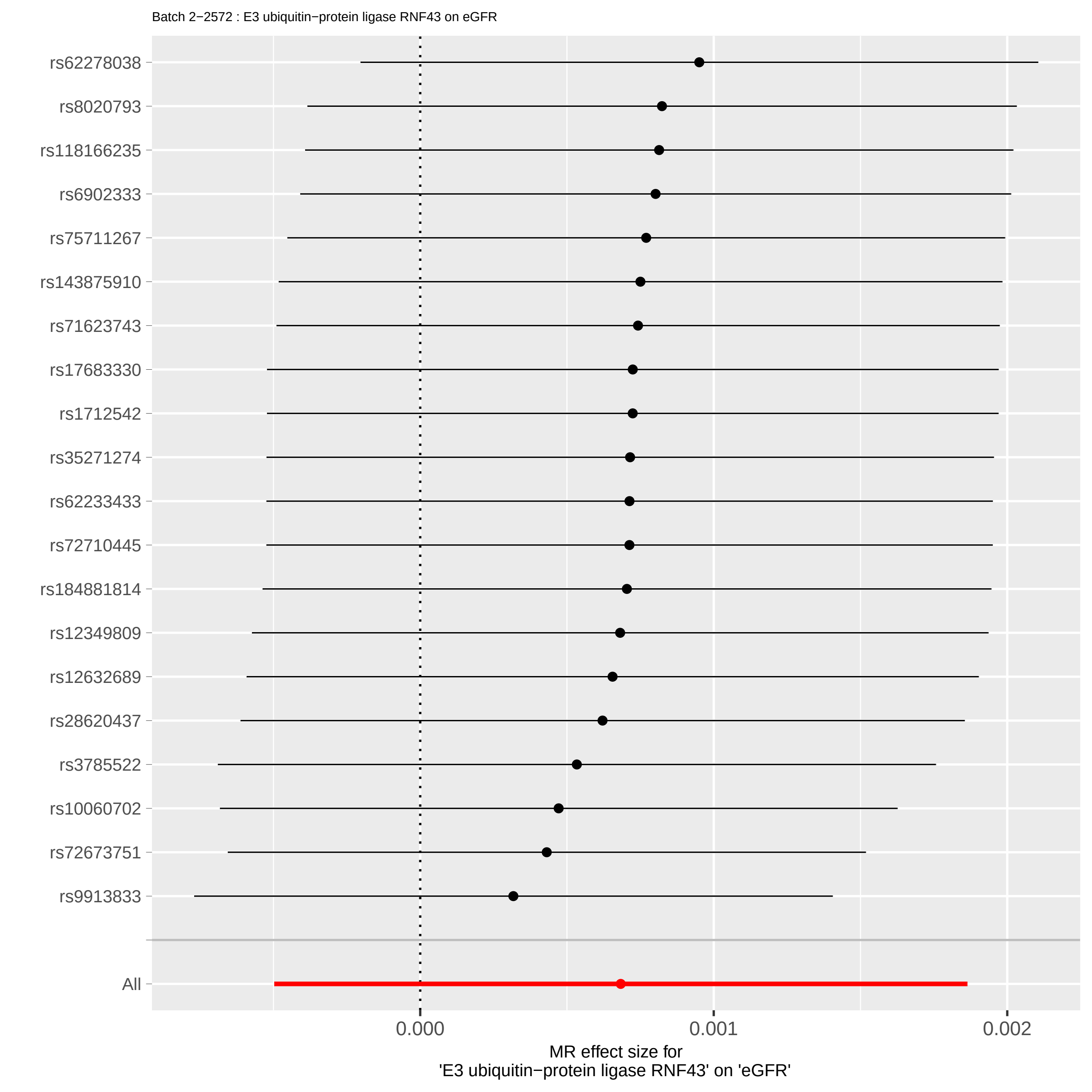

Batch 2−2572 : E3 ubiquitin−protein ligase RNF43 on eGFR
rs62278038
rs8020793
rs118166235
rs6902333
rs75711267
rs143875910
rs71623743
rs17683330
rs1712542
rs35271274
rs62233433
rs72710445
rs184881814
rs12349809
rs12632689
rs28620437
rs3785522
rs10060702
rs72673751
rs9913833
All
0.000
0.001
0.002
MR effect size for
'E3 ubiquitin−protein ligase RNF43' on 'eGFR'

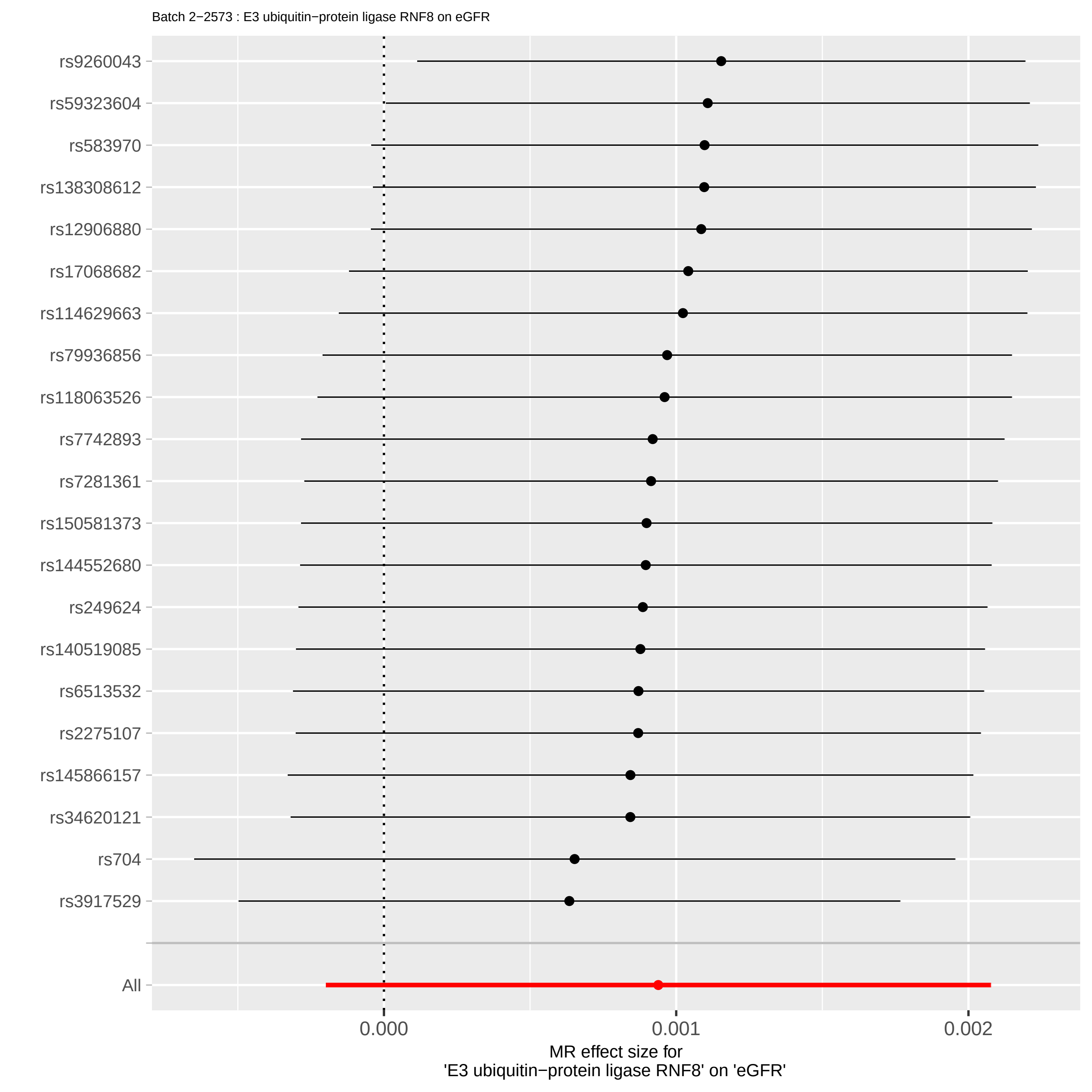

Batch 2−2573 : E3 ubiquitin−protein ligase RNF8 on eGFR
rs9260043
rs59323604
rs583970
rs138308612
rs12906880
rs17068682
rs114629663
rs79936856
rs118063526
rs7742893
rs7281361
rs150581373
rs144552680
rs249624
rs140519085
rs6513532
rs2275107
rs145866157
rs34620121
rs704
rs3917529
All
0.000
0.001
0.002
MR effect size for
'E3 ubiquitin−protein ligase RNF8' on 'eGFR'

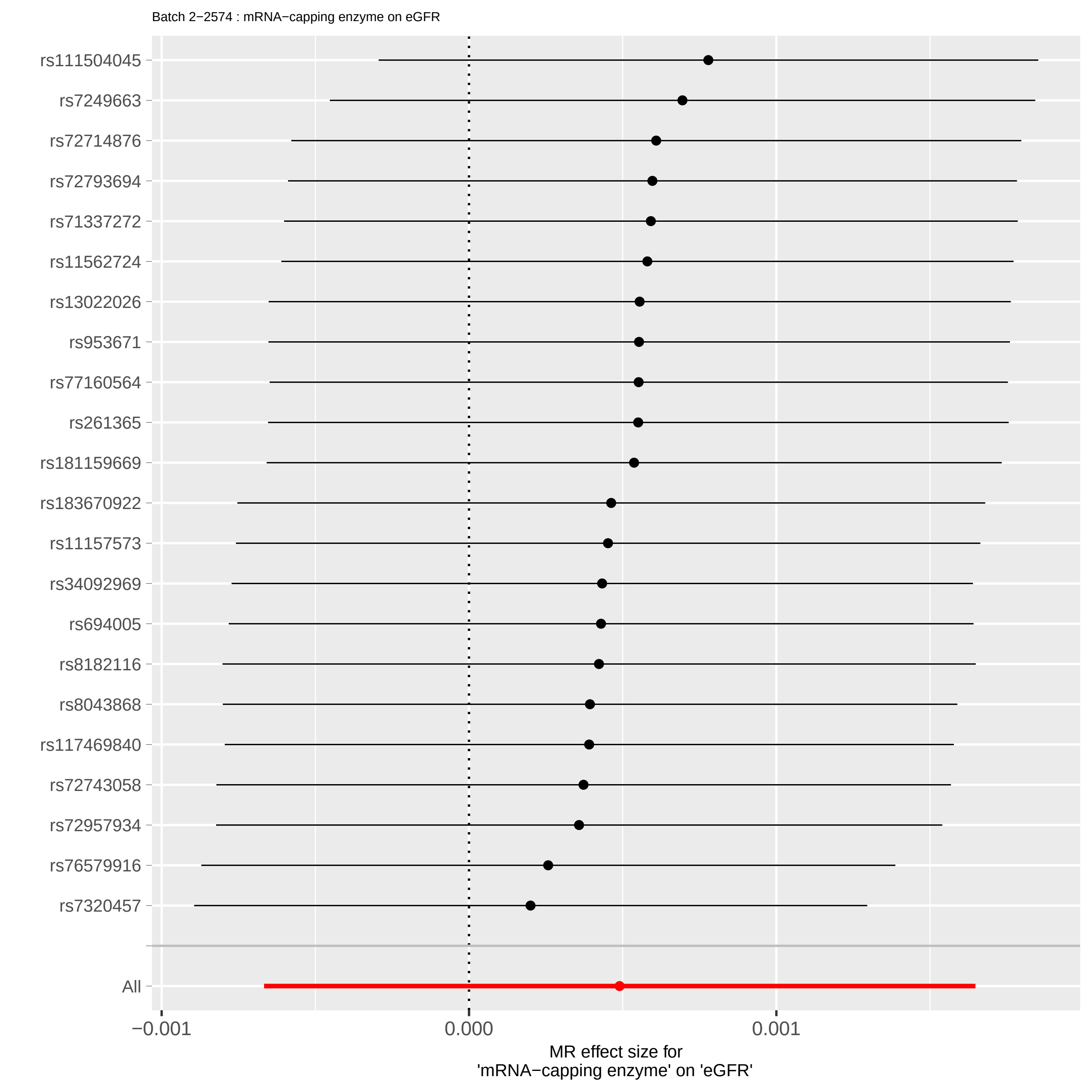

Batch 2−2574 : mRNA−capping enzyme on eGFR
rs111504045
rs7249663
rs72714876
rs72793694
rs71337272
rs11562724
rs13022026
rs953671
rs77160564
rs261365
rs181159669
rs183670922
rs11157573
rs34092969
rs694005
rs8182116
rs8043868
rs117469840
rs72743058
rs72957934
rs76579916
rs7320457
All
−0.001
0.000
0.001
MR effect size for
'mRNA−capping enzyme' on 'eGFR'

Batch 2−2575 : rRNA methyltransferase 3, mitochondrial on eGFR

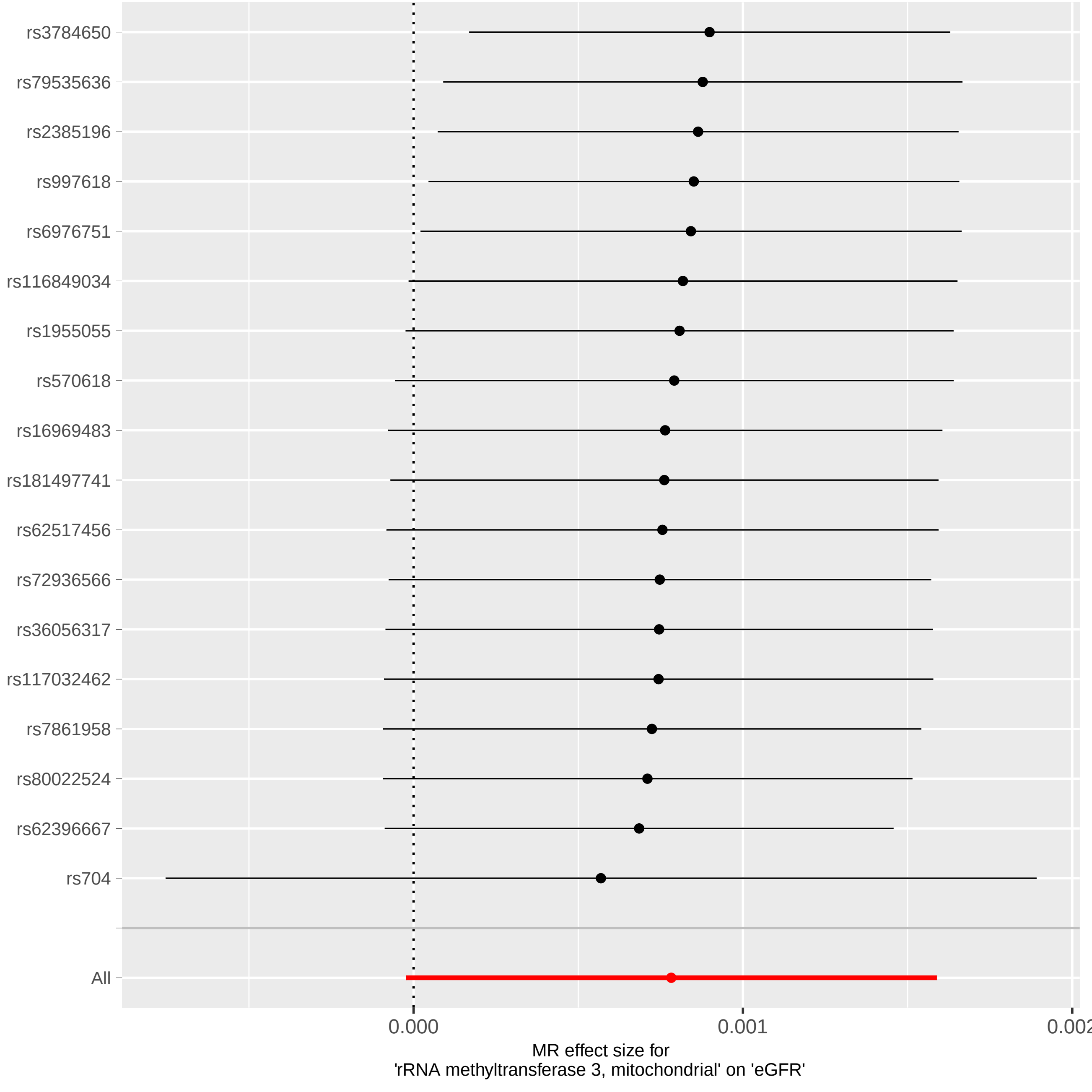

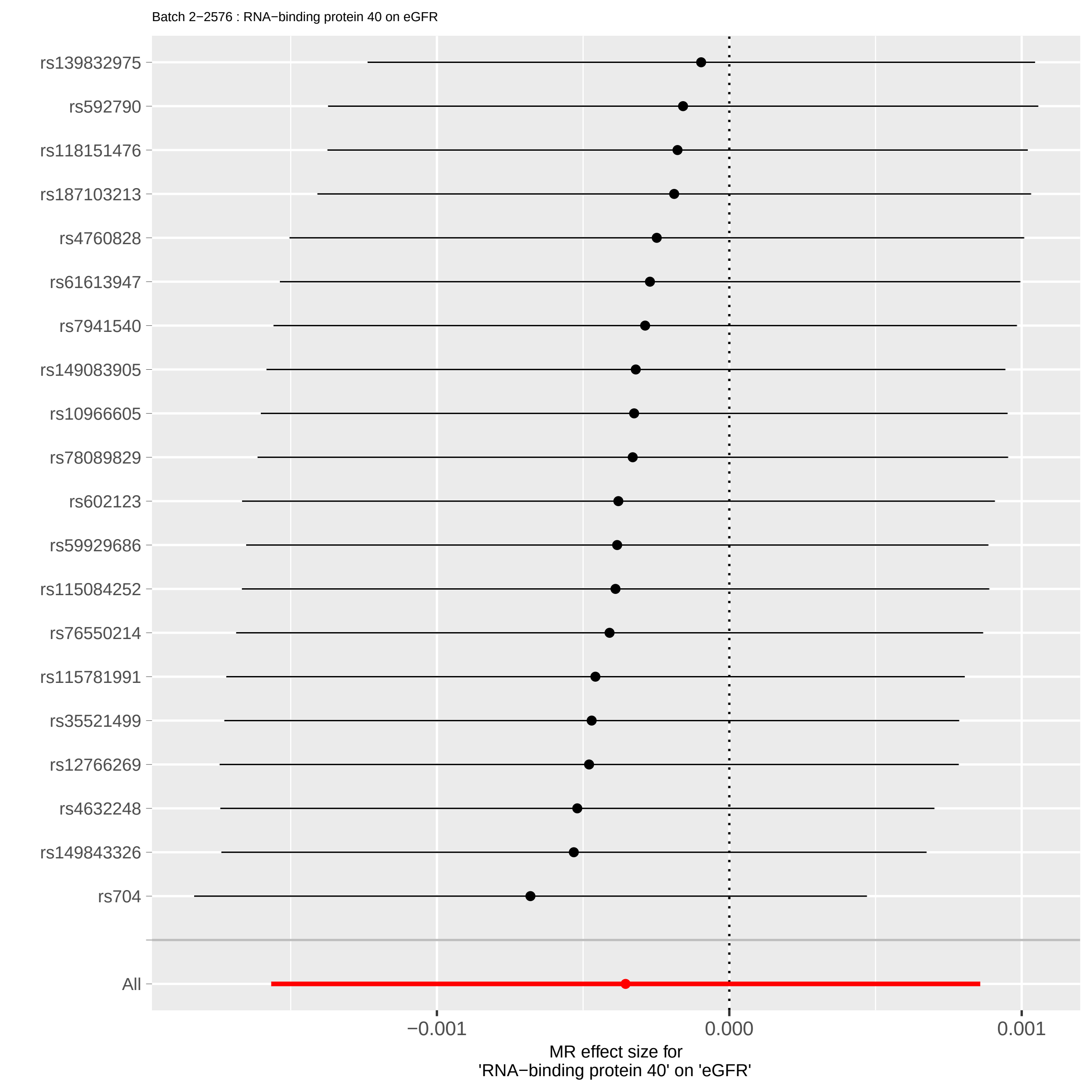

Batch 2−2576 : RNA−binding protein 40 on eGFR
rs139832975
rs592790
rs118151476
rs187103213
rs4760828
rs61613947
rs7941540
rs149083905
rs10966605
rs78089829
rs602123
rs59929686
rs115084252
rs76550214
rs115781991
rs35521499
rs12766269
rs4632248
rs149843326
rs704
All
−0.001
0.000
0.001
MR effect size for
'RNA−binding protein 40' on 'eGFR'

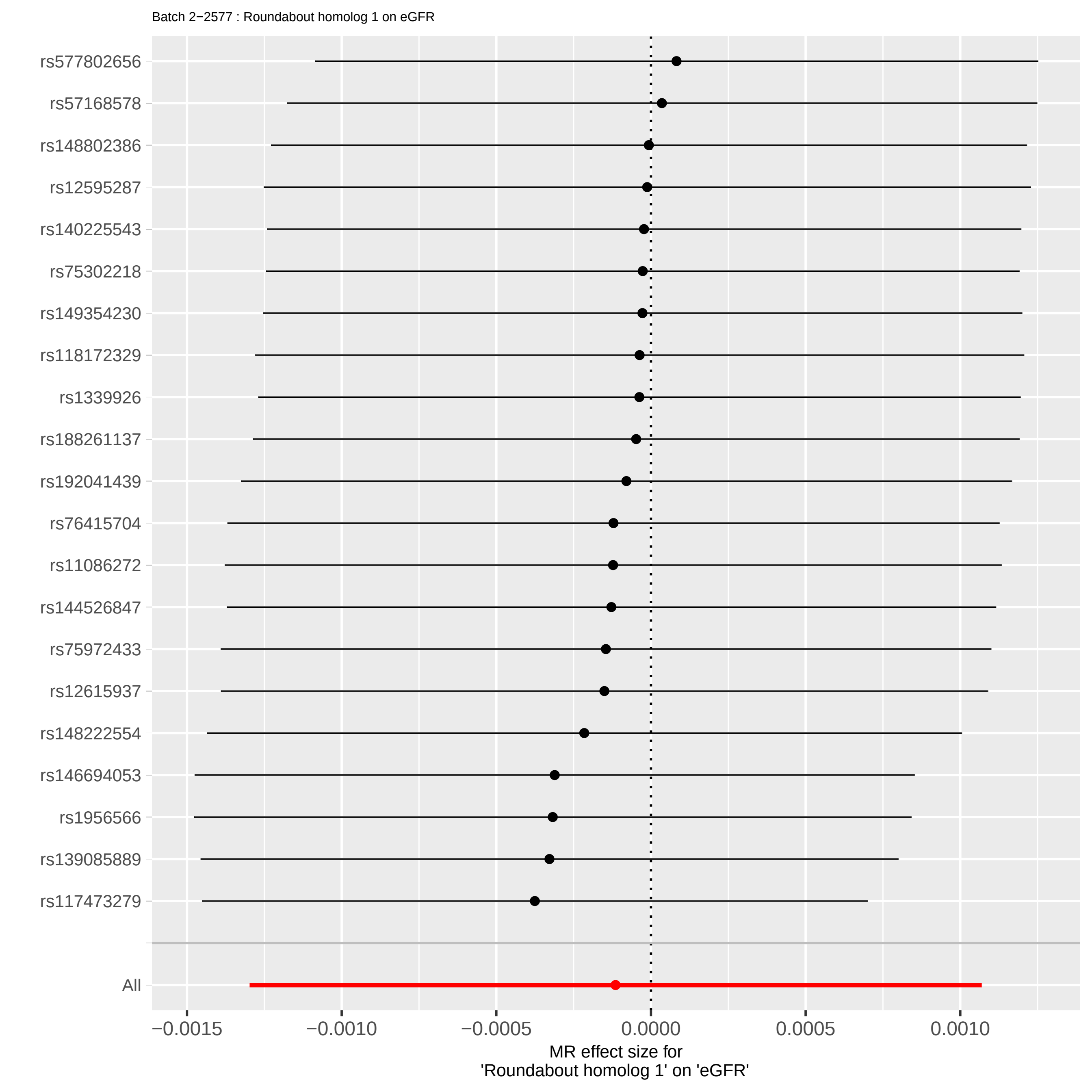

Batch 2−2577 : Roundabout homolog 1 on eGFR
rs577802656
rs57168578
rs148802386
rs12595287
rs140225543
rs75302218
rs149354230
rs118172329
rs1339926
rs188261137
rs192041439
rs76415704
rs11086272
rs144526847
rs75972433
rs12615937
rs148222554
rs146694053
rs1956566
rs139085889
rs117473279
All
−0.0015
−0.0010
−0.0005
0.0000
0.0005
0.0010
MR effect size for
'Roundabout homolog 1' on 'eGFR'

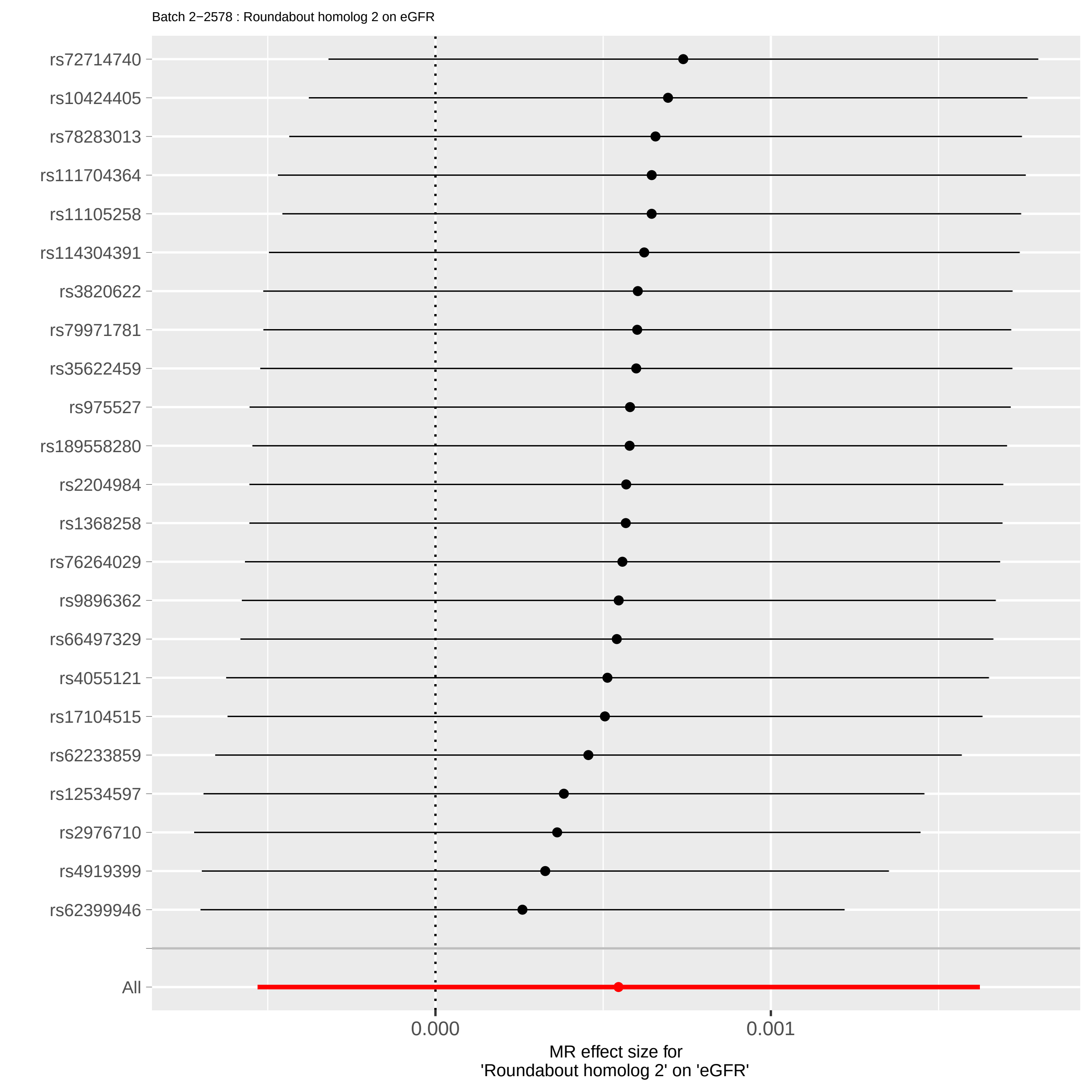
Batch 2−2578 : Roundabout homolog 2 on eGFR
rs72714740
rs10424405
rs78283013
rs111704364
rs11105258
rs114304391
rs3820622
rs79971781
rs35622459
rs975527
rs189558280
rs2204984
rs1368258
rs76264029
rs9896362
rs66497329
rs4055121
rs17104515
rs62233859
rs12534597
rs2976710
rs4919399
rs62399946
All
0.000
0.001
MR effect size for
'Roundabout homolog 2' on 'eGFR'

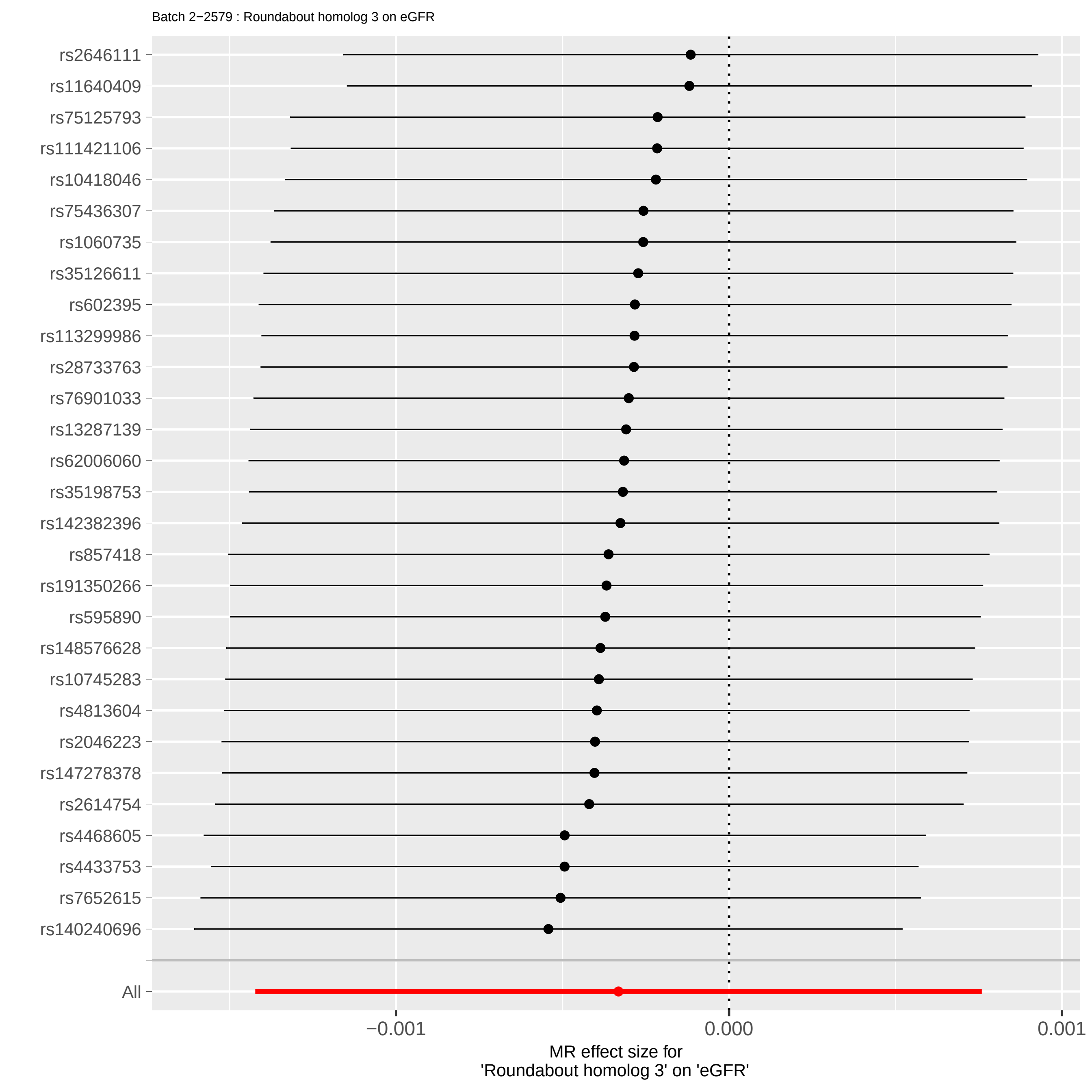

Batch 2−2579 : Roundabout homolog 3 on eGFR
rs2646111
rs11640409
rs75125793
rs111421106
rs10418046
rs75436307
rs1060735
rs35126611
rs602395
rs113299986
rs28733763
rs76901033
rs13287139
rs62006060
rs35198753
rs142382396
rs857418
rs191350266
rs595890
rs148576628
rs10745283
rs4813604
rs2046223
rs147278378
rs2614754
rs4468605
rs4433753
rs7652615
rs140240696
All
−0.001
0.000
0.001
MR effect size for
'Roundabout homolog 3' on 'eGFR'

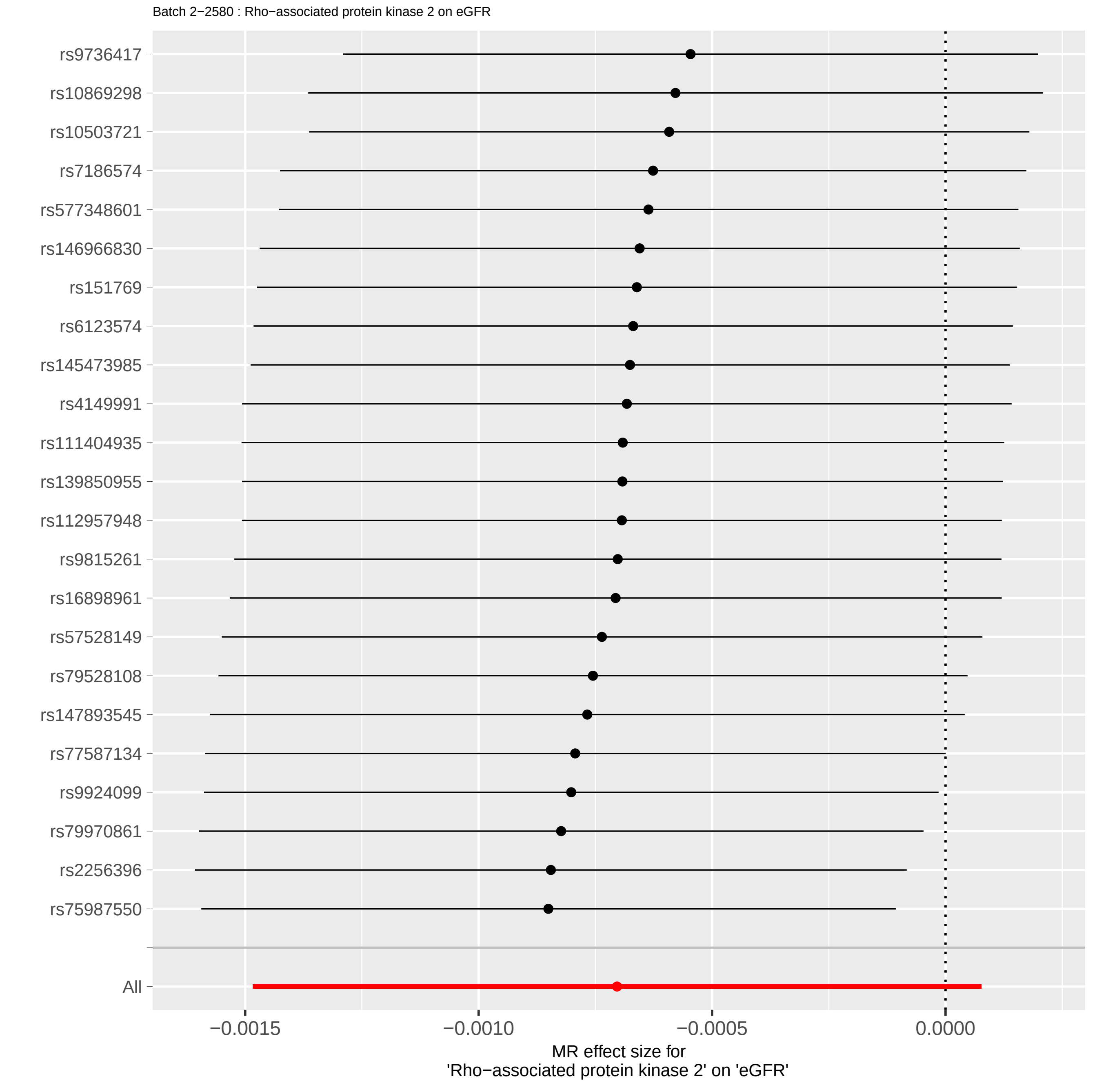
Batch 2−2580 : Rho−associated protein kinase 2 on eGFR
rs9736417
rs10869298
rs10503721
rs7186574
rs577348601
rs146966830
rs151769
rs6123574
rs145473985
rs4149991
rs111404935
rs139850955
rs112957948
rs9815261
rs16898961
rs57528149
rs79528108
rs147893545
rs77587134
rs9924099
rs79970861
rs2256396
rs75987550
All
−0.0015
−0.0010
−0.0005
0.0000
MR effect size for
'Rho−associated protein kinase 2' on 'eGFR'

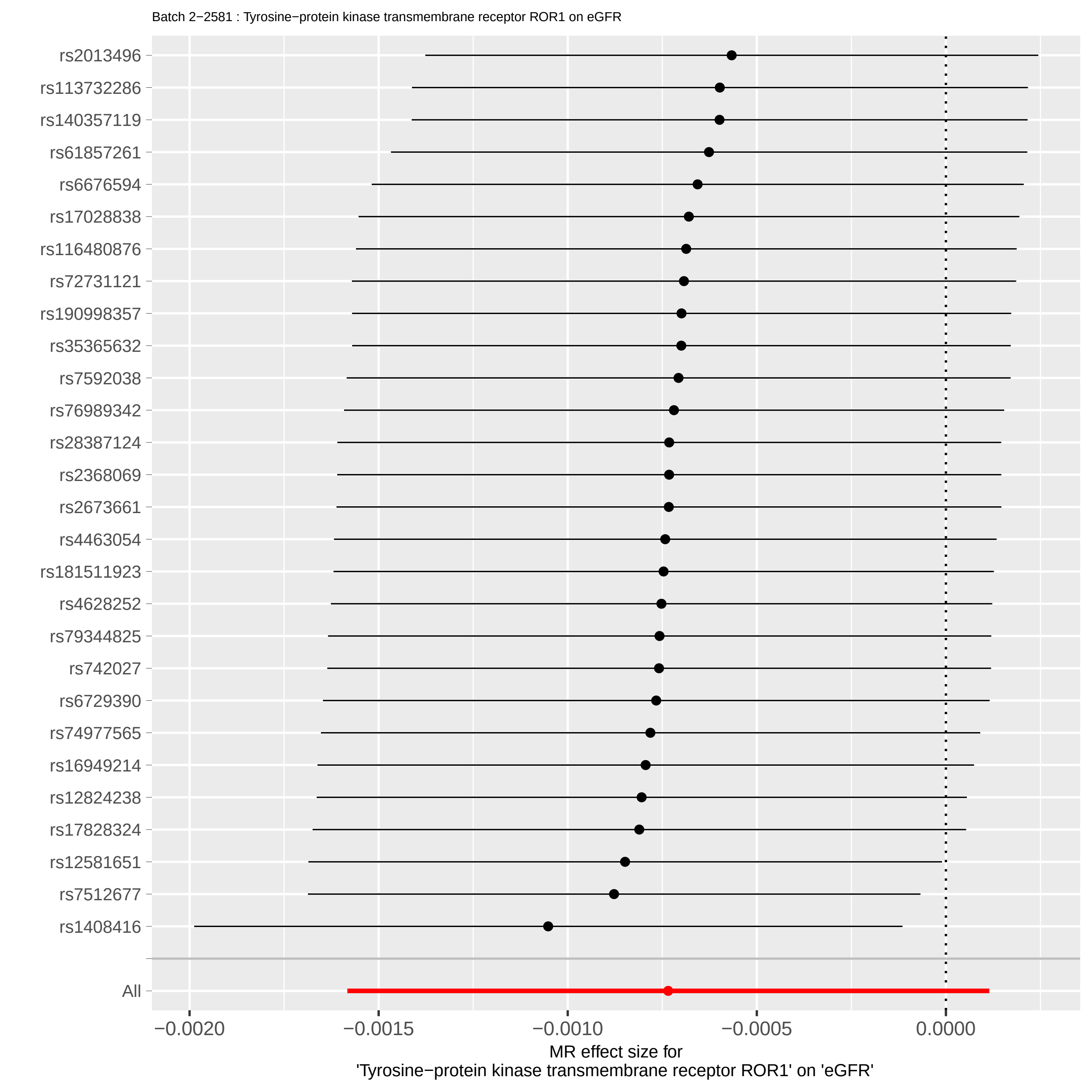

Batch 2−2581 : Tyrosine−protein kinase transmembrane receptor ROR1 on eGFR
rs2013496
rs113732286
rs140357119
rs61857261
rs6676594
rs17028838
rs116480876
rs72731121
rs190998357
rs35365632
rs7592038
rs76989342
rs28387124
rs2368069
rs2673661
rs4463054
rs181511923
rs4628252
rs79344825
rs742027
rs6729390
rs74977565
rs16949214
rs12824238
rs17828324
rs12581651
rs7512677
rs1408416
All
−0.0020
−0.0015
−0.0010
−0.0005
0.0000
MR effect size for
'Tyrosine−protein kinase transmembrane receptor ROR1' on 'eGFR'

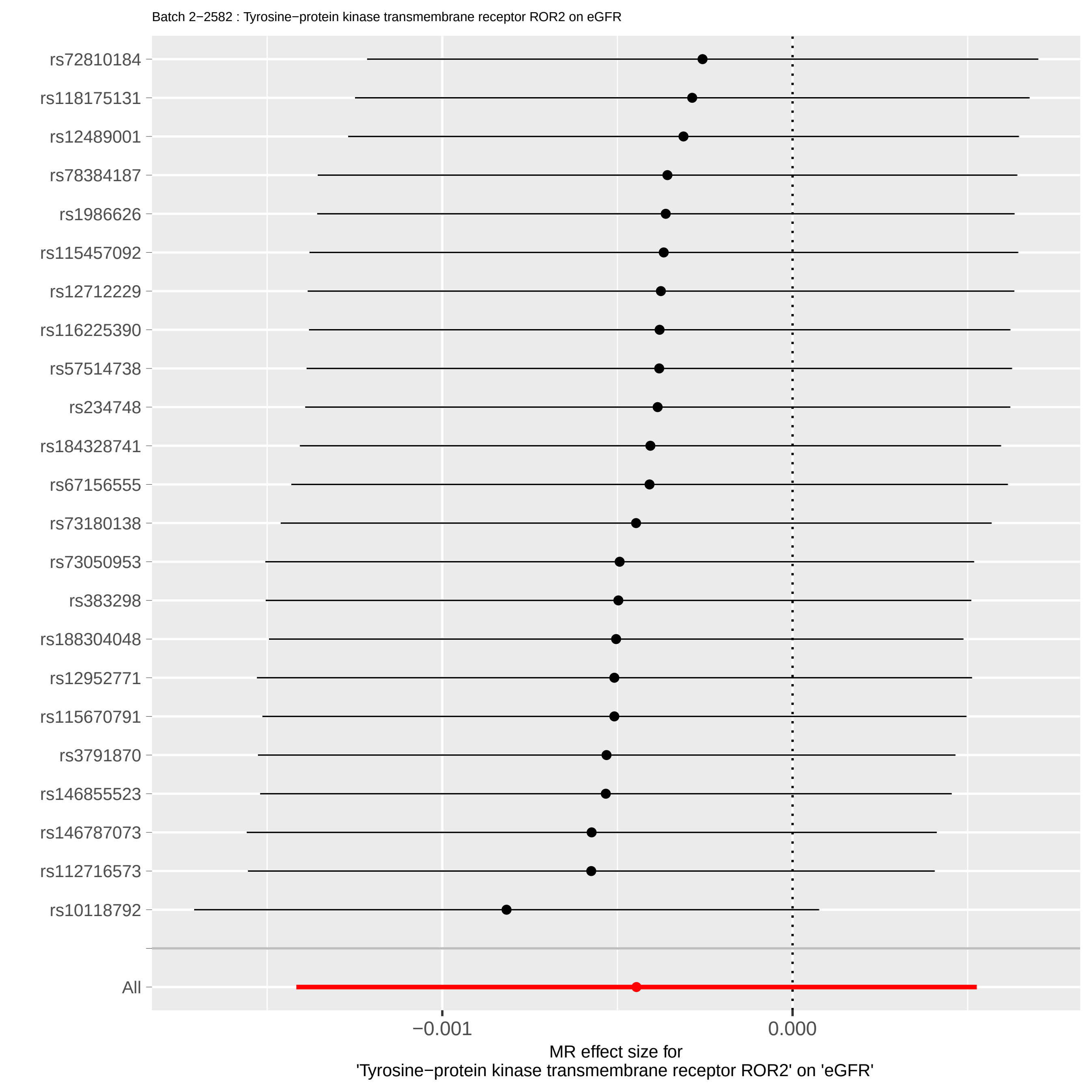

Batch 2−2582 : Tyrosine−protein kinase transmembrane receptor ROR2 on eGFR
rs72810184
rs118175131
rs12489001
rs78384187
rs1986626
rs115457092
rs12712229
rs116225390
rs57514738
rs234748
rs184328741
rs67156555
rs73180138
rs73050953
rs383298
rs188304048
rs12952771
rs115670791
rs3791870
rs146855523
rs146787073
rs112716573
rs10118792
All
−0.001
0.000
MR effect size for
'Tyrosine−protein kinase transmembrane receptor ROR2' on 'eGFR'

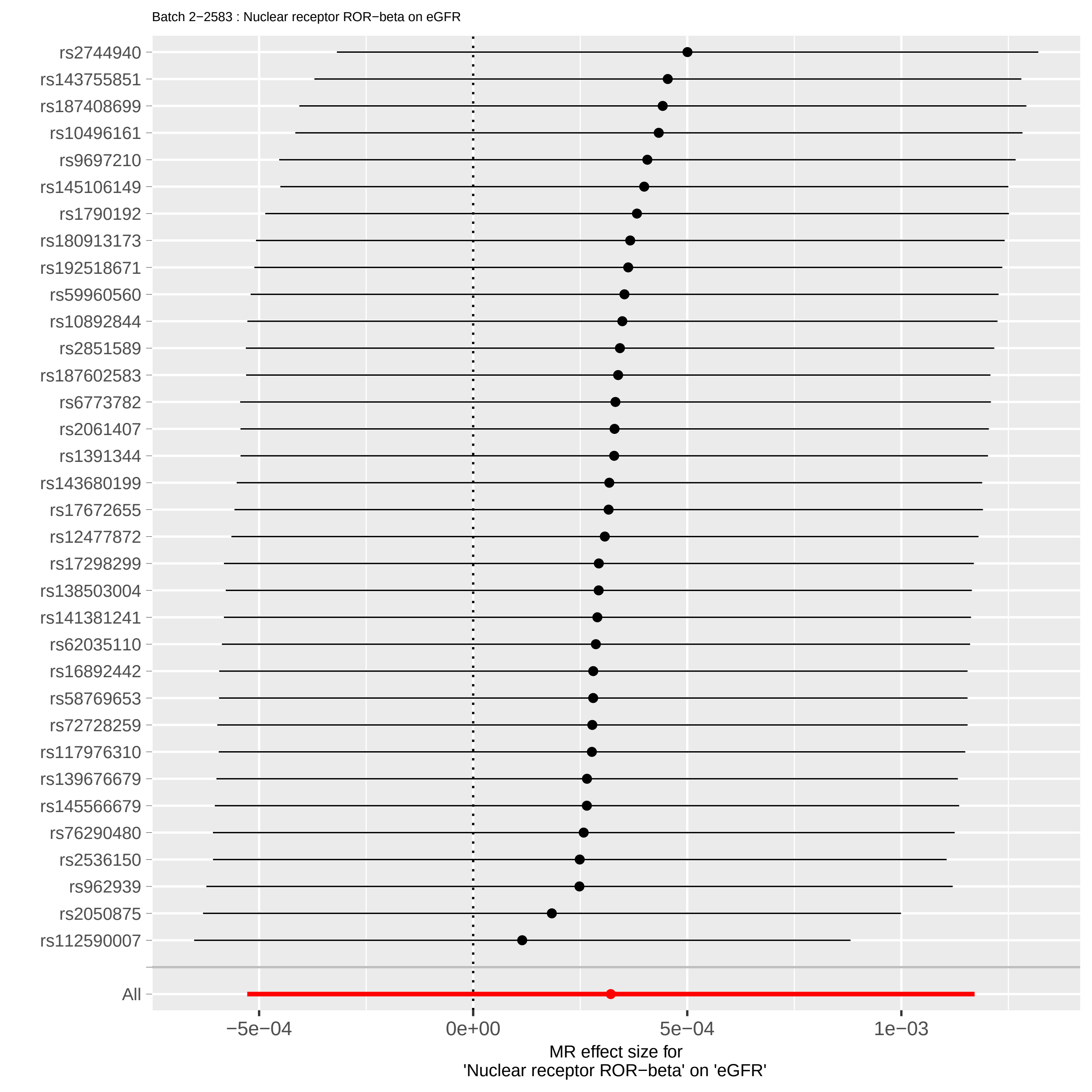
Batch 2−2583 : Nuclear receptor ROR−beta on eGFR
rs2744940
rs143755851
rs187408699
rs10496161
rs9697210
rs145106149
rs1790192
rs180913173
rs192518671
rs59960560
rs10892844
rs2851589
rs187602583
rs6773782
rs2061407
rs1391344
rs143680199
rs17672655
rs12477872
rs17298299
rs138503004
rs141381241
rs62035110
rs16892442
rs58769653
rs72728259
rs117976310
rs139676679
rs145566679
rs76290480
rs2536150
rs962939
rs2050875
rs112590007
All
−5e−04
0e+00
5e−04
1e−03
MR effect size for
'Nuclear receptor ROR−beta' on 'eGFR'

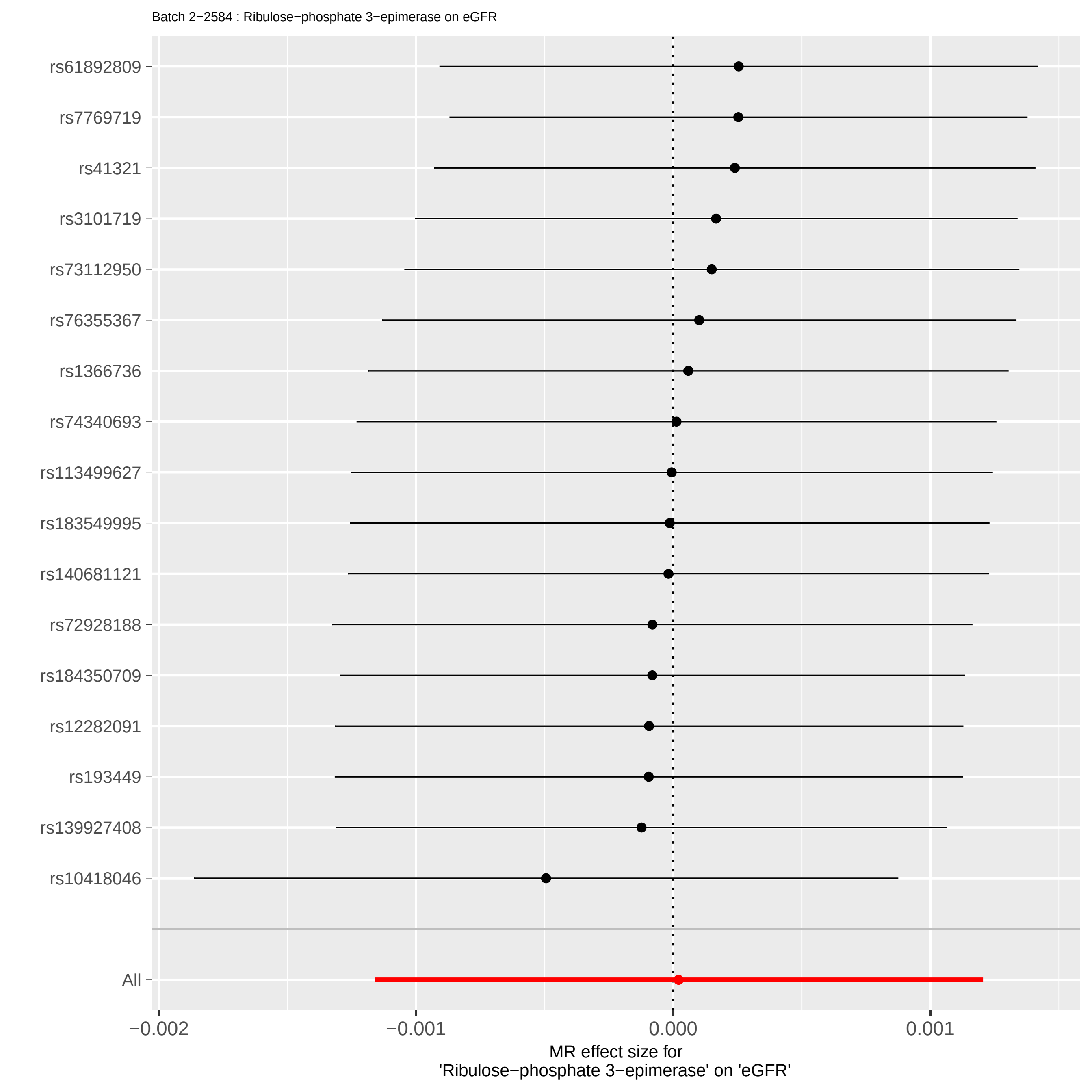
Batch 2−2584 : Ribulose−phosphate 3−epimerase on eGFR
rs61892809
rs7769719
rs41321
rs3101719
rs73112950
rs76355367
rs1366736
rs74340693
rs113499627
rs183549995
rs140681121
rs72928188
rs184350709
rs12282091
rs193449
rs139927408
rs10418046
All
−0.002
−0.001
0.000
0.001
MR effect size for
'Ribulose−phosphate 3−epimerase' on 'eGFR'

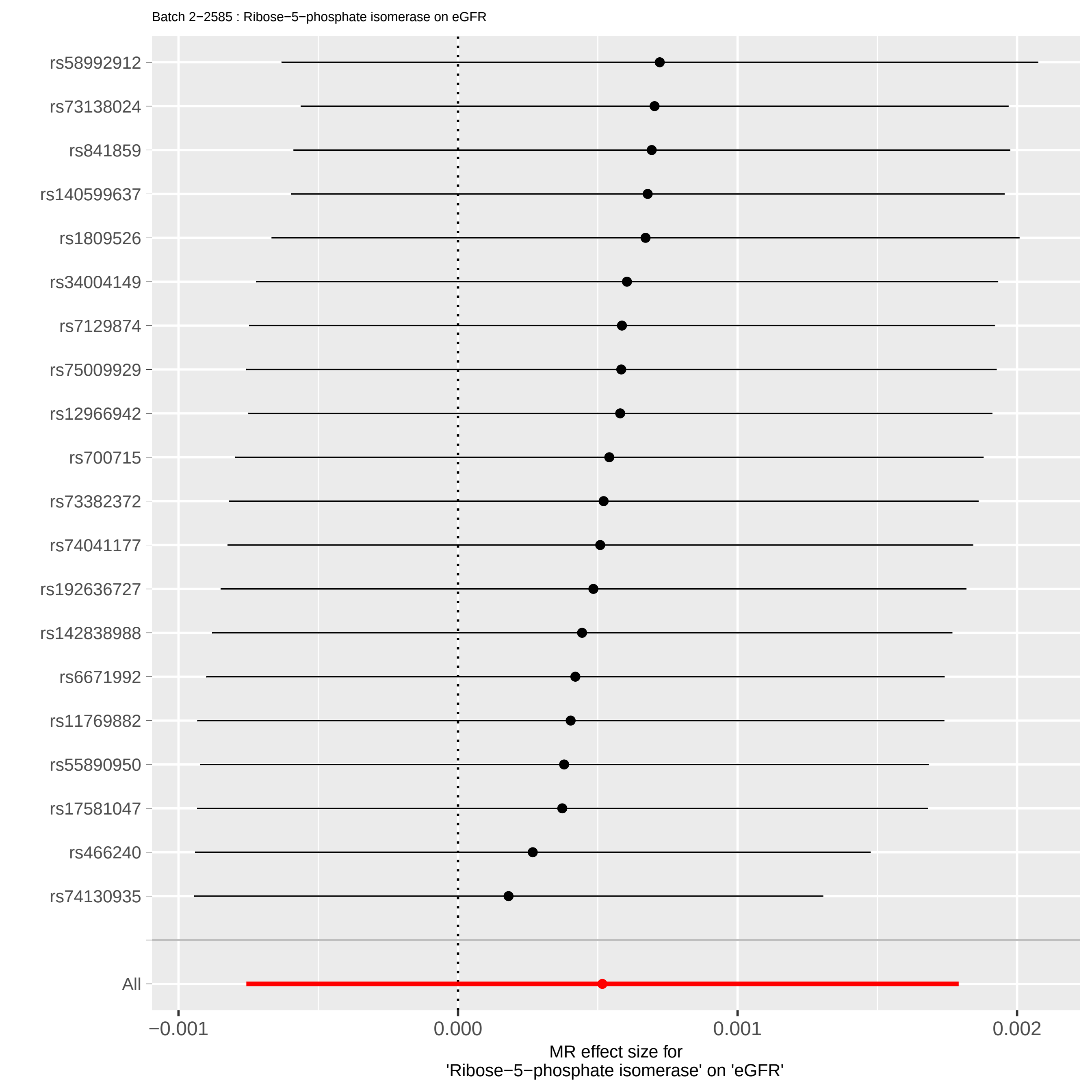
Batch 2−2585 : Ribose−5−phosphate isomerase on eGFR
rs58992912
rs73138024
rs841859
rs140599637
rs1809526
rs34004149
rs7129874
rs75009929
rs12966942
rs700715
rs73382372
rs74041177
rs192636727
rs142838988
rs6671992
rs11769882
rs55890950
rs17581047
rs466240
rs74130935
All
−0.001
0.000
0.001
0.002
MR effect size for
'Ribose−5−phosphate isomerase' on 'eGFR'

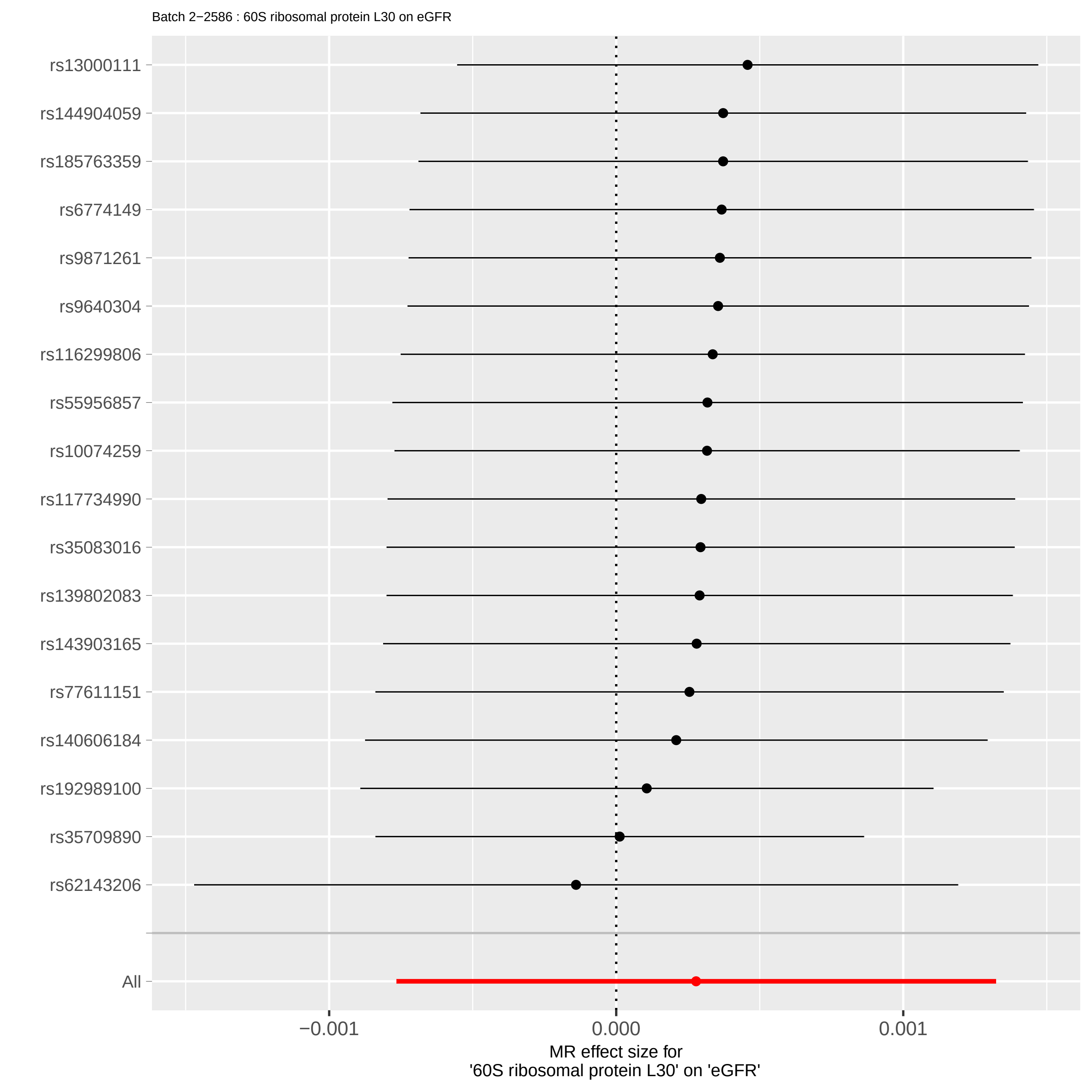
Batch 2−2586 : 60S ribosomal protein L30 on eGFR
rs13000111
rs144904059
rs185763359
rs6774149
rs9871261
rs9640304
rs116299806
rs55956857
rs10074259
rs117734990
rs35083016
rs139802083
rs143903165
rs77611151
rs140606184
rs192989100
rs35709890
rs62143206
All
−0.001
0.000
0.001
MR effect size for
'60S ribosomal protein L30' on 'eGFR'

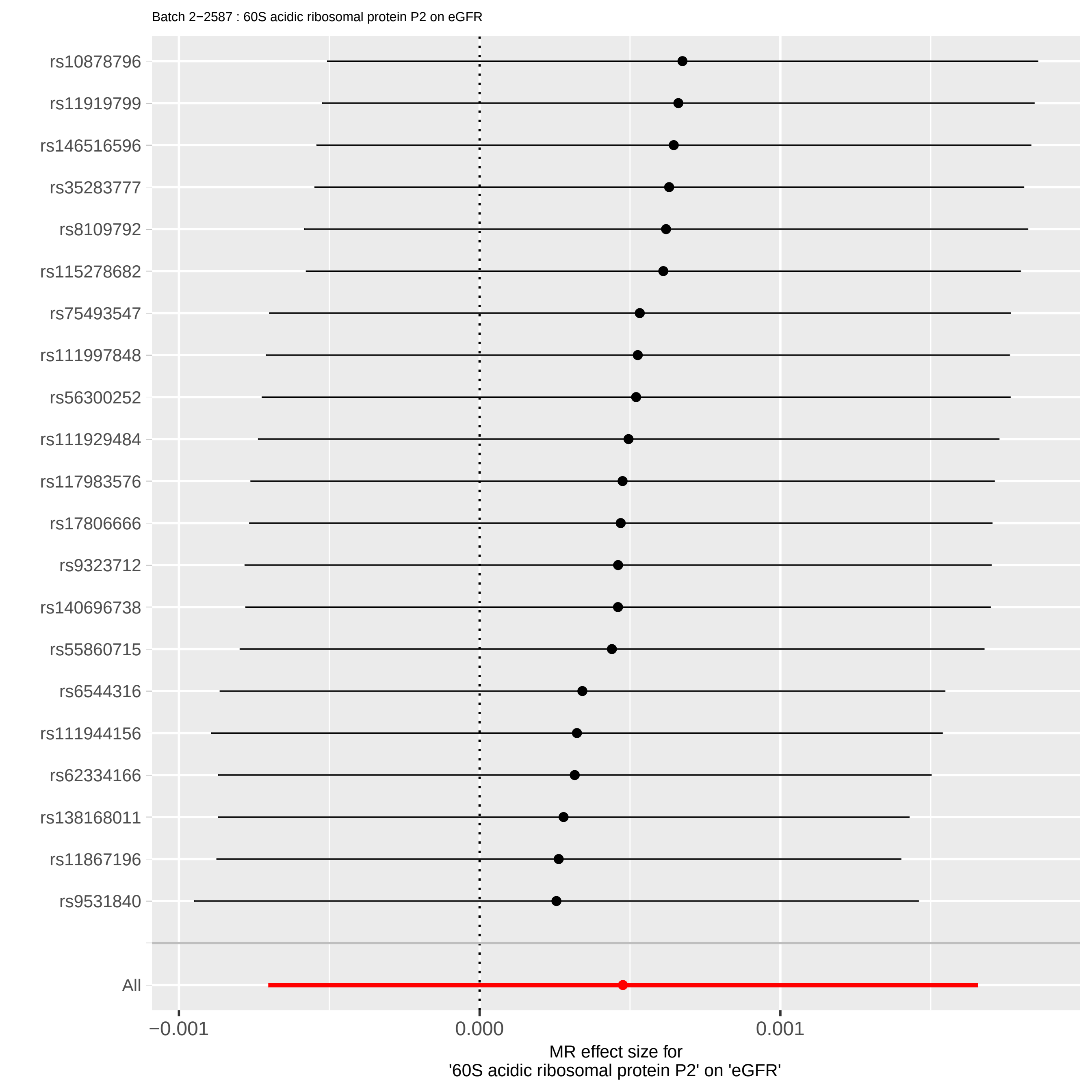

Batch 2−2587 : 60S acidic ribosomal protein P2 on eGFR
rs10878796
rs11919799
rs146516596
rs35283777
rs8109792
rs115278682
rs75493547
rs111997848
rs56300252
rs111929484
rs117983576
rs17806666
rs9323712
rs140696738
rs55860715
rs6544316
rs111944156
rs62334166
rs138168011
rs11867196
rs9531840
All
−0.001
0.000
0.001
MR effect size for
'60S acidic ribosomal protein P2' on 'eGFR'

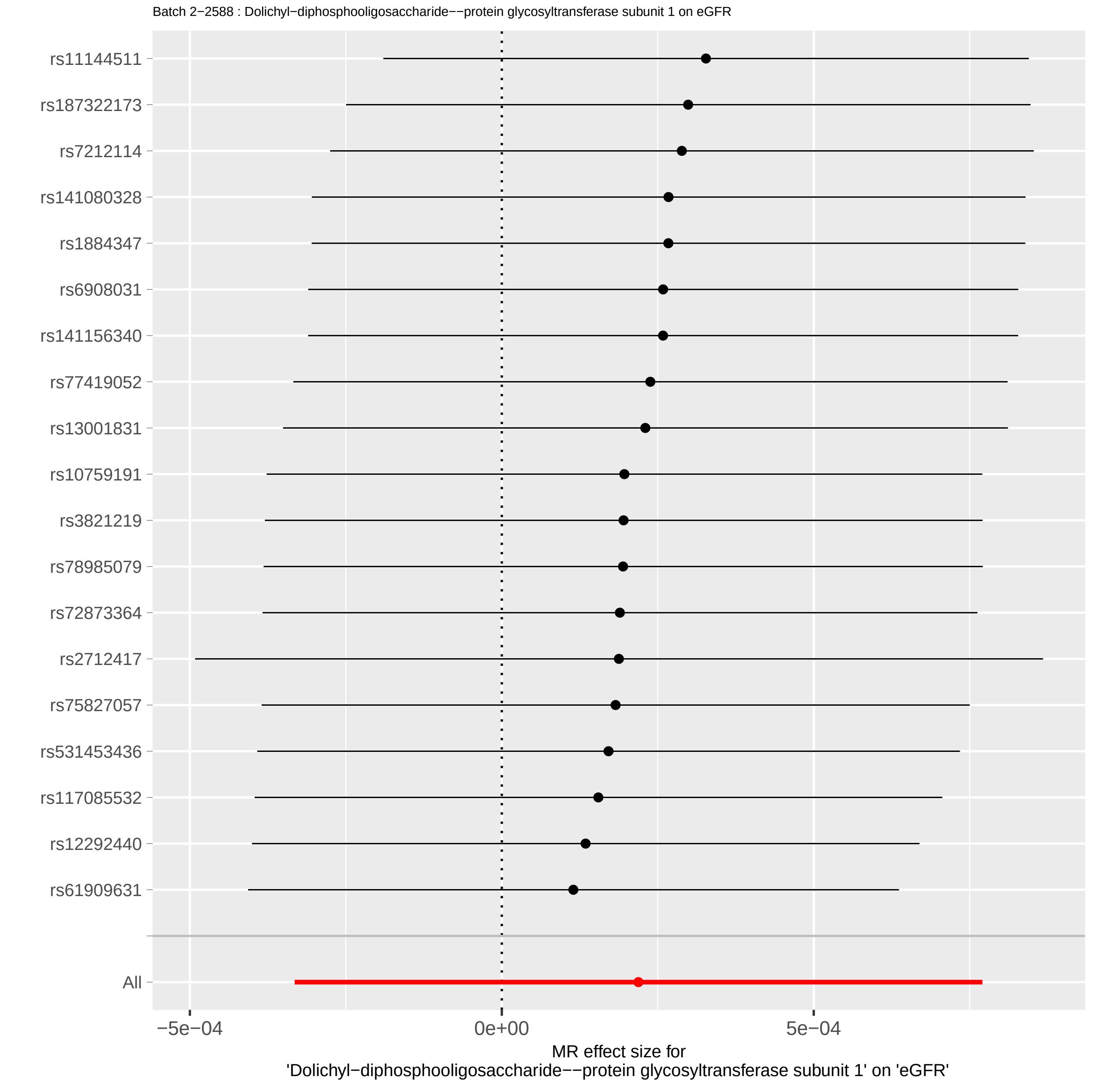

Batch 2−2588 : Dolichyl−diphosphooligosaccharide−−protein glycosyltransferase subunit 1 on eGFR
rs11144511
rs187322173
rs7212114
rs141080328
rs1884347
rs6908031
rs141156340
rs77419052
rs13001831
rs10759191
rs3821219
rs78985079
rs72873364
rs2712417
rs75827057
rs531453436
rs117085532
rs12292440
rs61909631
All
−5e−04
0e+00
5e−04
MR effect size for
'Dolichyl−diphosphooligosaccharide−−protein glycosyltransferase subunit 1' on 'eGFR'

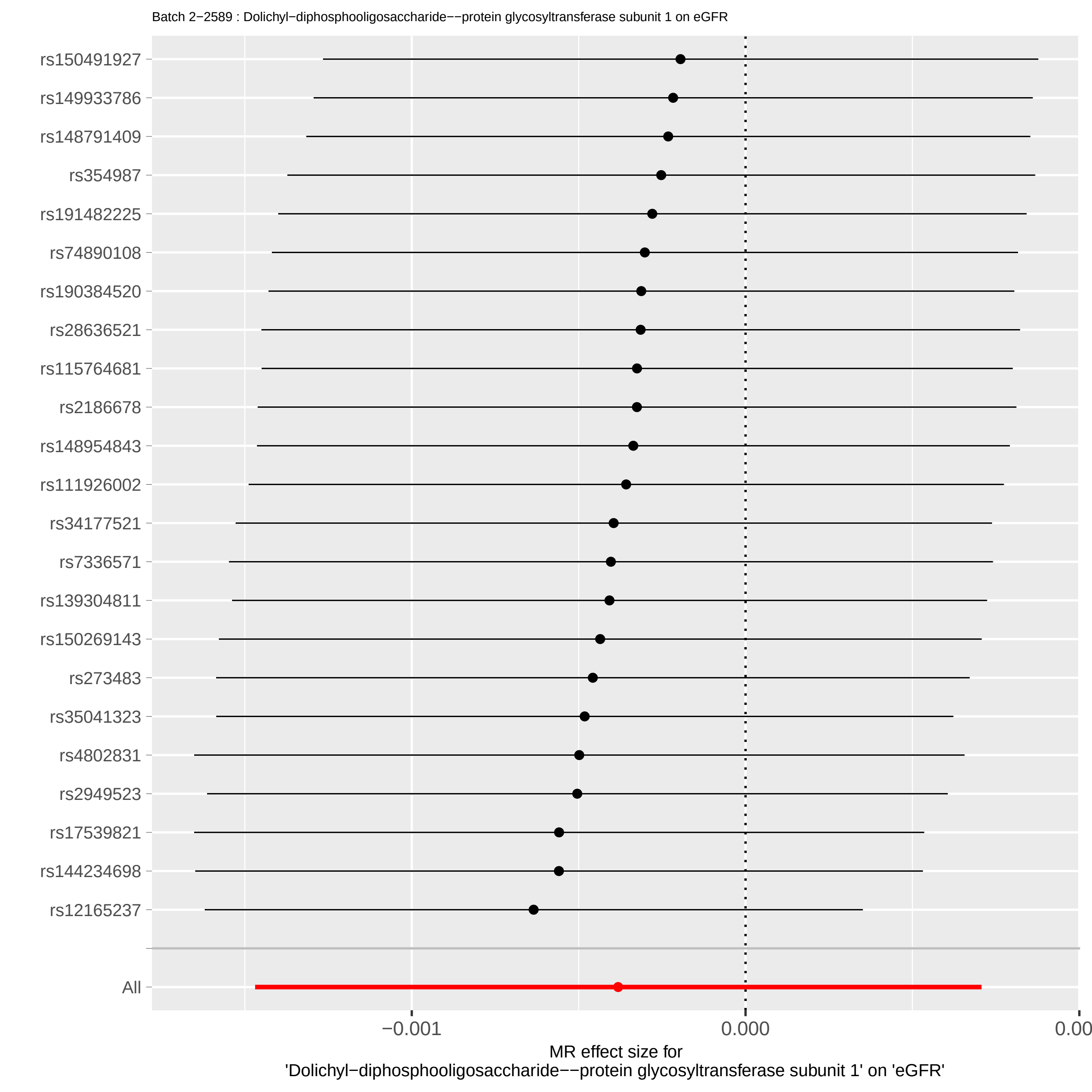

Batch 2−2589 : Dolichyl−diphosphooligosaccharide−−protein glycosyltransferase subunit 1 on eGFR
rs150491927
rs149933786
rs148791409
rs354987
rs191482225
rs74890108
rs190384520
rs28636521
rs115764681
rs2186678
rs148954843
rs111926002
rs34177521
rs7336571
rs139304811
rs150269143
rs273483
rs35041323
rs4802831
rs2949523
rs17539821
rs144234698
rs12165237
All
−0.001
0.000
0.00
MR effect size for
'Dolichyl−diphosphooligosaccharide−−protein glycosyltransferase subunit 1' on 'eGFR'

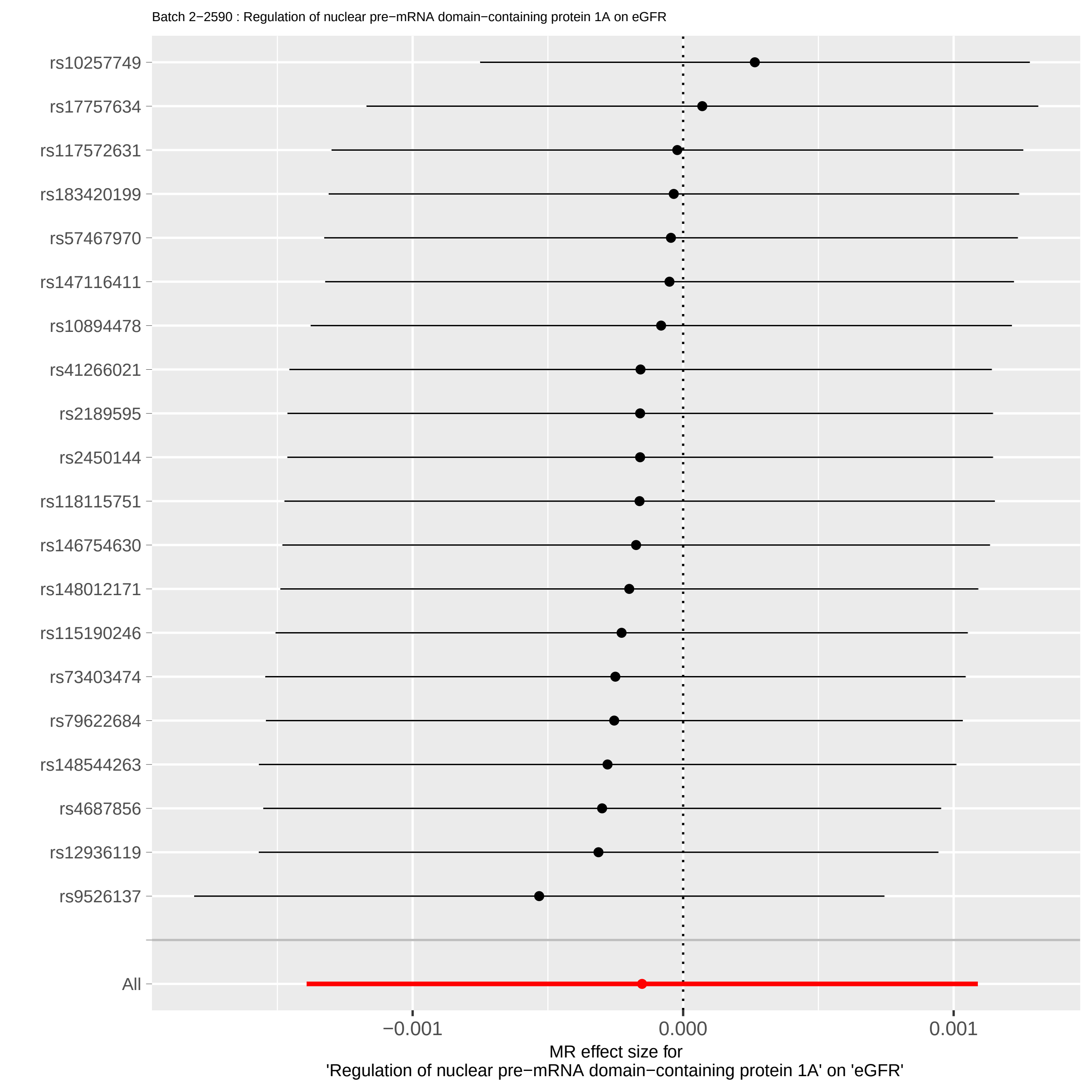
Batch 2−2590 : Regulation of nuclear pre−mRNA domain−containing protein 1A on eGFR
rs10257749
rs17757634
rs117572631
rs183420199
rs57467970
rs147116411
rs10894478
rs41266021
rs2189595
rs2450144
rs118115751
rs146754630
rs148012171
rs115190246
rs73403474
rs79622684
rs148544263
rs4687856
rs12936119
rs9526137
All
−0.001
0.000
0.001
MR effect size for
'Regulation of nuclear pre−mRNA domain−containing protein 1A' on 'eGFR'

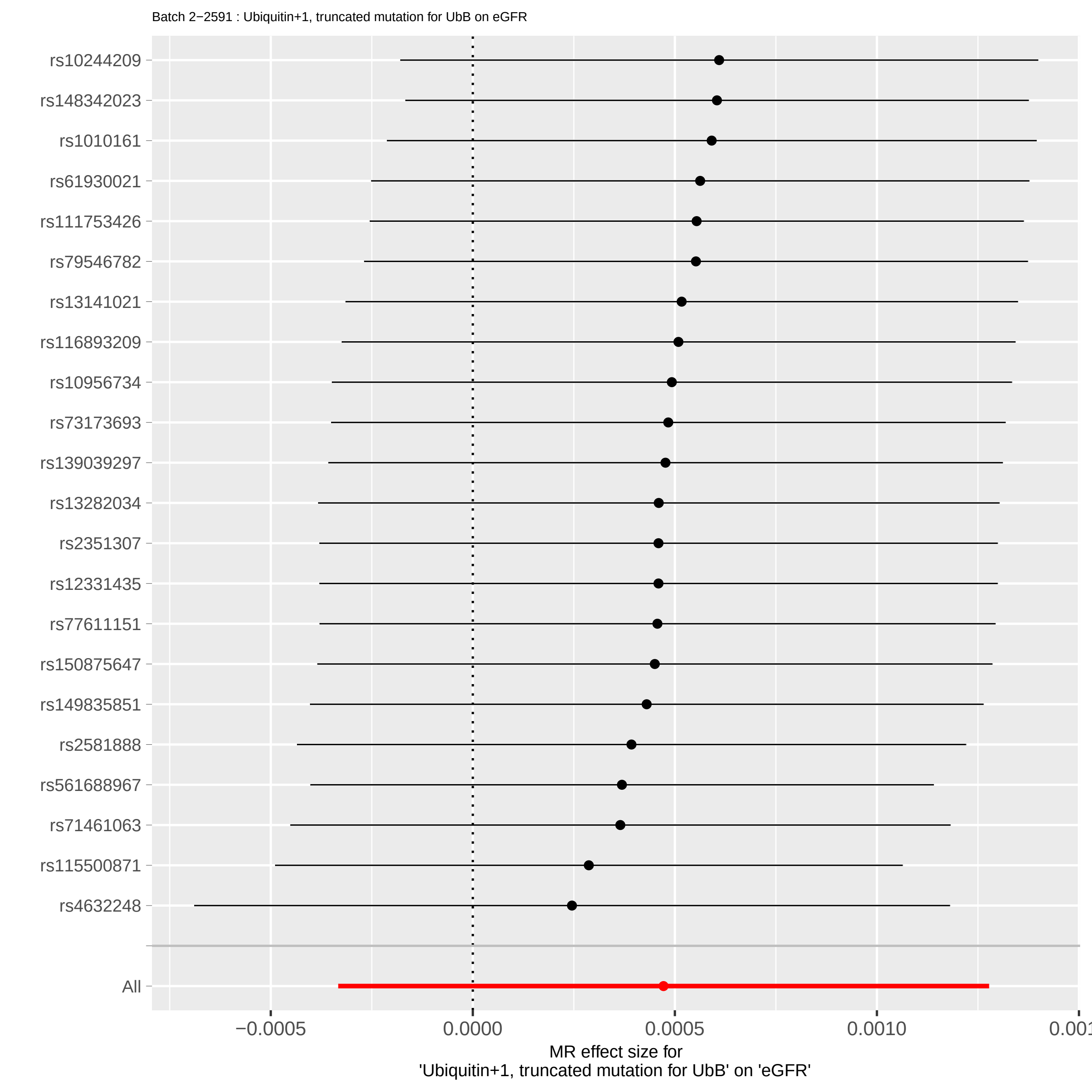

Batch 2−2591 : Ubiquitin+1, truncated mutation for UbB on eGFR
rs10244209
rs148342023
rs1010161
rs61930021
rs111753426
rs79546782
rs13141021
rs116893209
rs10956734
rs73173693
rs139039297
rs13282034
rs2351307
rs12331435
rs77611151
rs150875647
rs149835851
rs2581888
rs561688967
rs71461063
rs115500871
rs4632248
All
−0.0005
0.0000
0.0005
0.0010
MR effect size for
'Ubiquitin+1, truncated mutation for UbB' on 'eGFR'

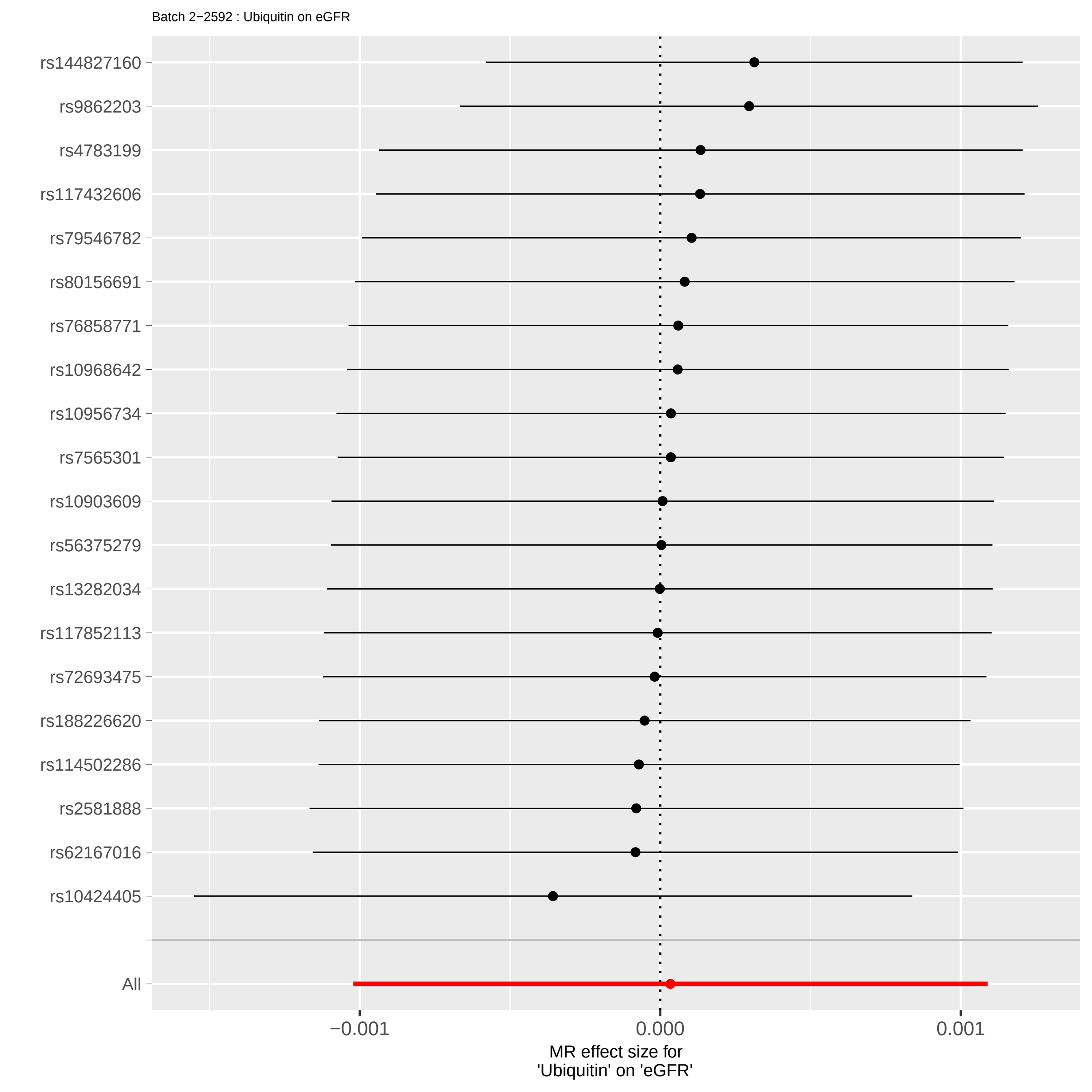
Batch 2−2592 : Ubiquitin on eGFR
rs144827160
rs9862203
rs4783199
rs117432606
rs79546782
rs80156691
rs76858771
rs10968642
rs10956734
rs7565301
rs10903609
rs56375279
rs13282034
rs117852113
rs72693475
rs188226620
rs114502286
rs2581888
rs62167016
rs10424405
All
−0.001
0.000
0.001
MR effect size for
'Ubiquitin' on 'eGFR'

Batch 2−2593 : 40S ribosomal protein S3 on eGFR

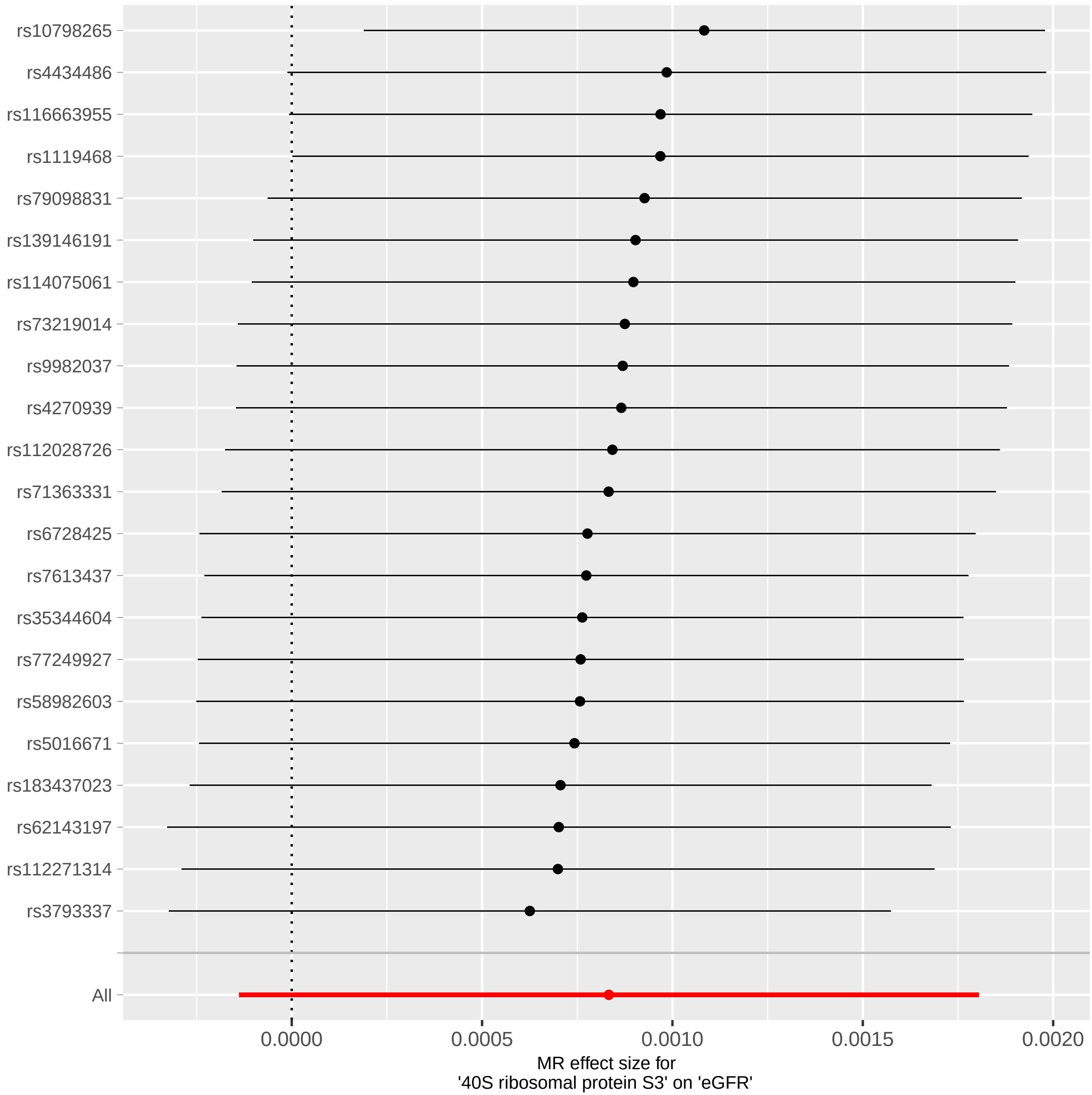

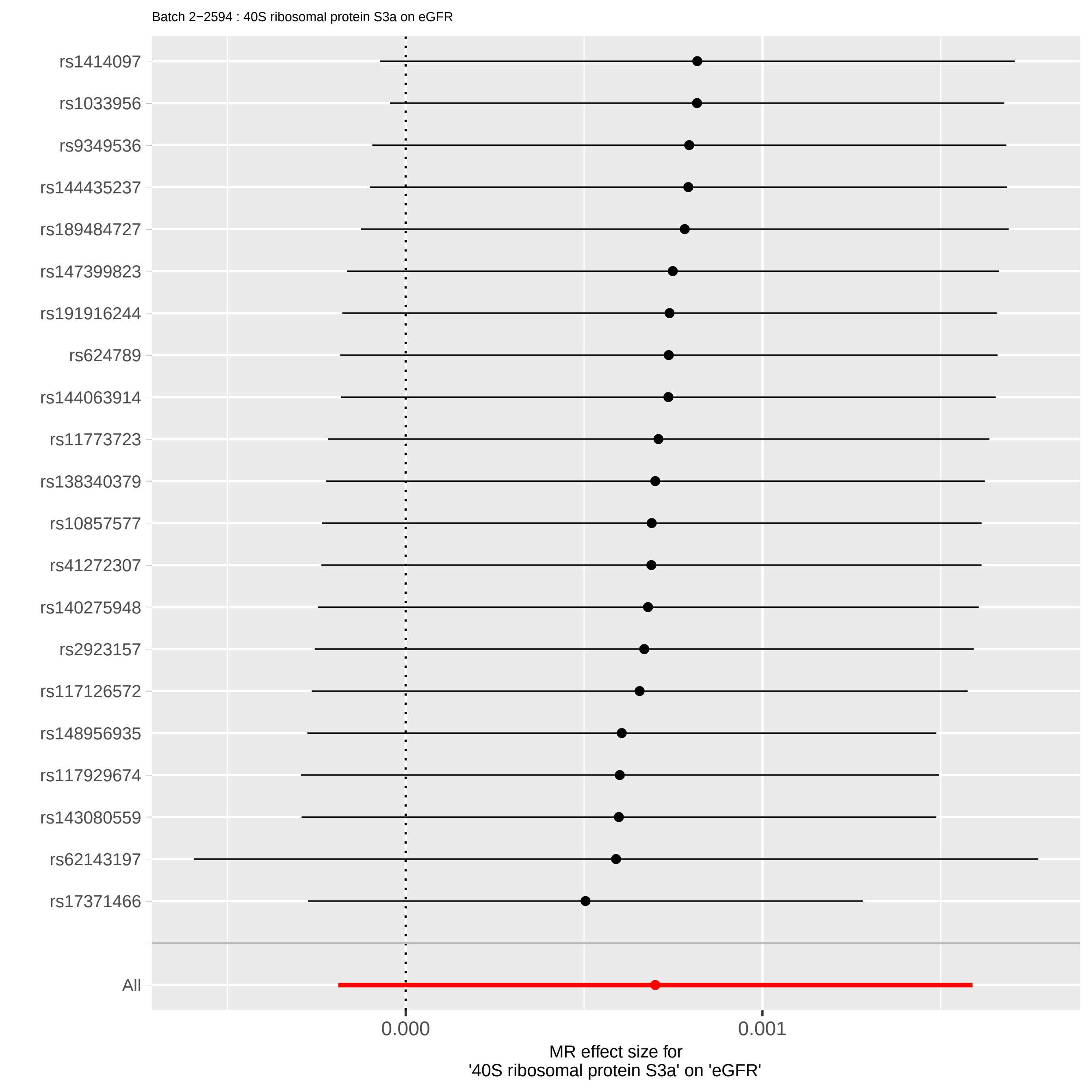

Batch 2−2594 : 40S ribosomal protein S3a on eGFR
rs1414097
rs1033956
rs9349536
rs144435237
rs189484727
rs147399823
rs191916244
rs624789
rs144063914
rs11773723
rs138340379
rs10857577
rs41272307
rs140275948
rs2923157
rs117126572
rs148956935
rs117929674
rs143080559
rs62143197
rs17371466
All
0.000
0.001
MR effect size for
'40S ribosomal protein S3a' on 'eGFR'

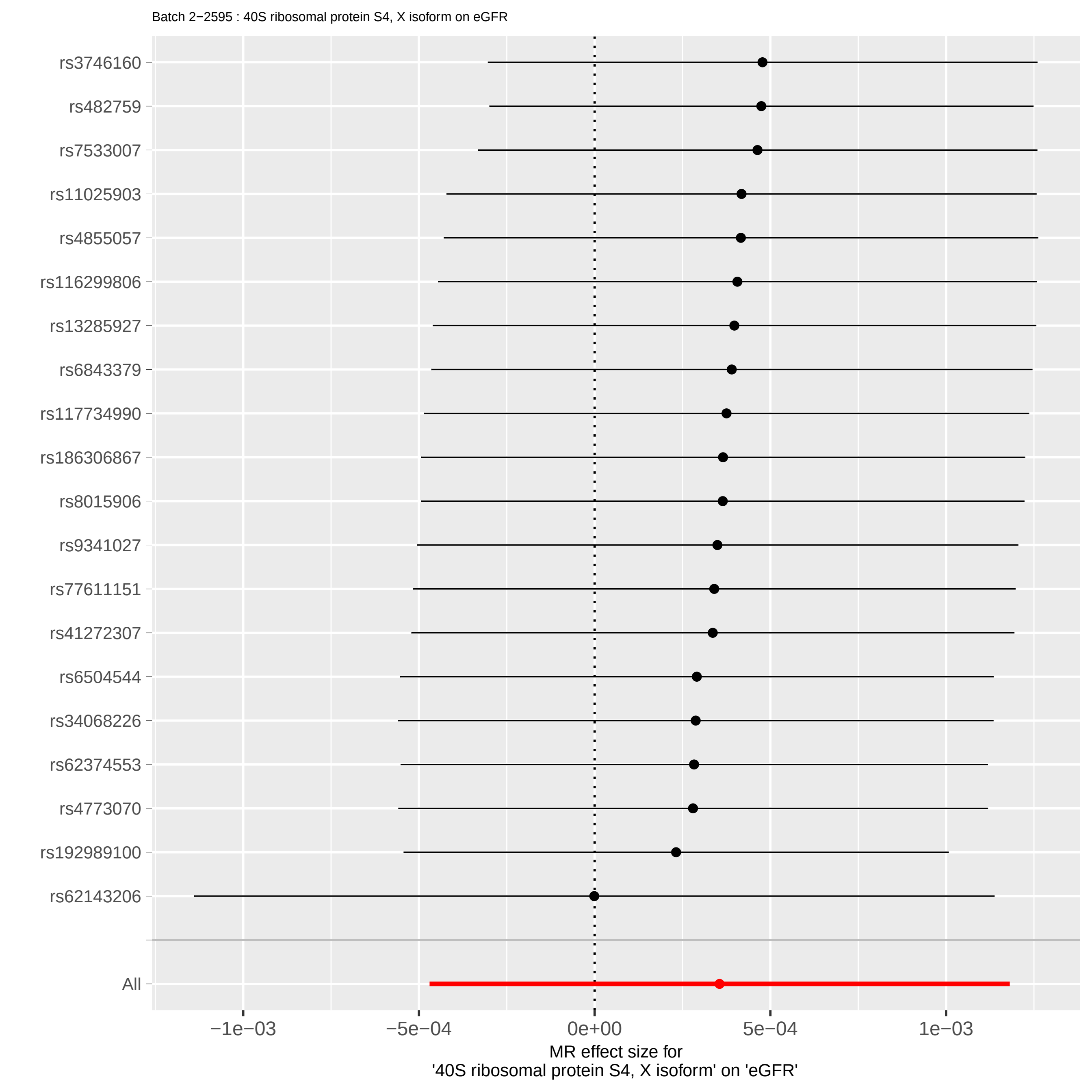

Batch 2−2595 : 40S ribosomal protein S4, X isoform on eGFR
rs3746160
rs482759
rs7533007
rs11025903
rs4855057
rs116299806
rs13285927
rs6843379
rs117734990
rs186306867
rs8015906
rs9341027
rs77611151
rs41272307
rs6504544
rs34068226
rs62374553
rs4773070
rs192989100
rs62143206
All
−1e−03
−5e−04
0e+00
5e−04
1e−03
MR effect size for
'40S ribosomal protein S4, X isoform' on 'eGFR'

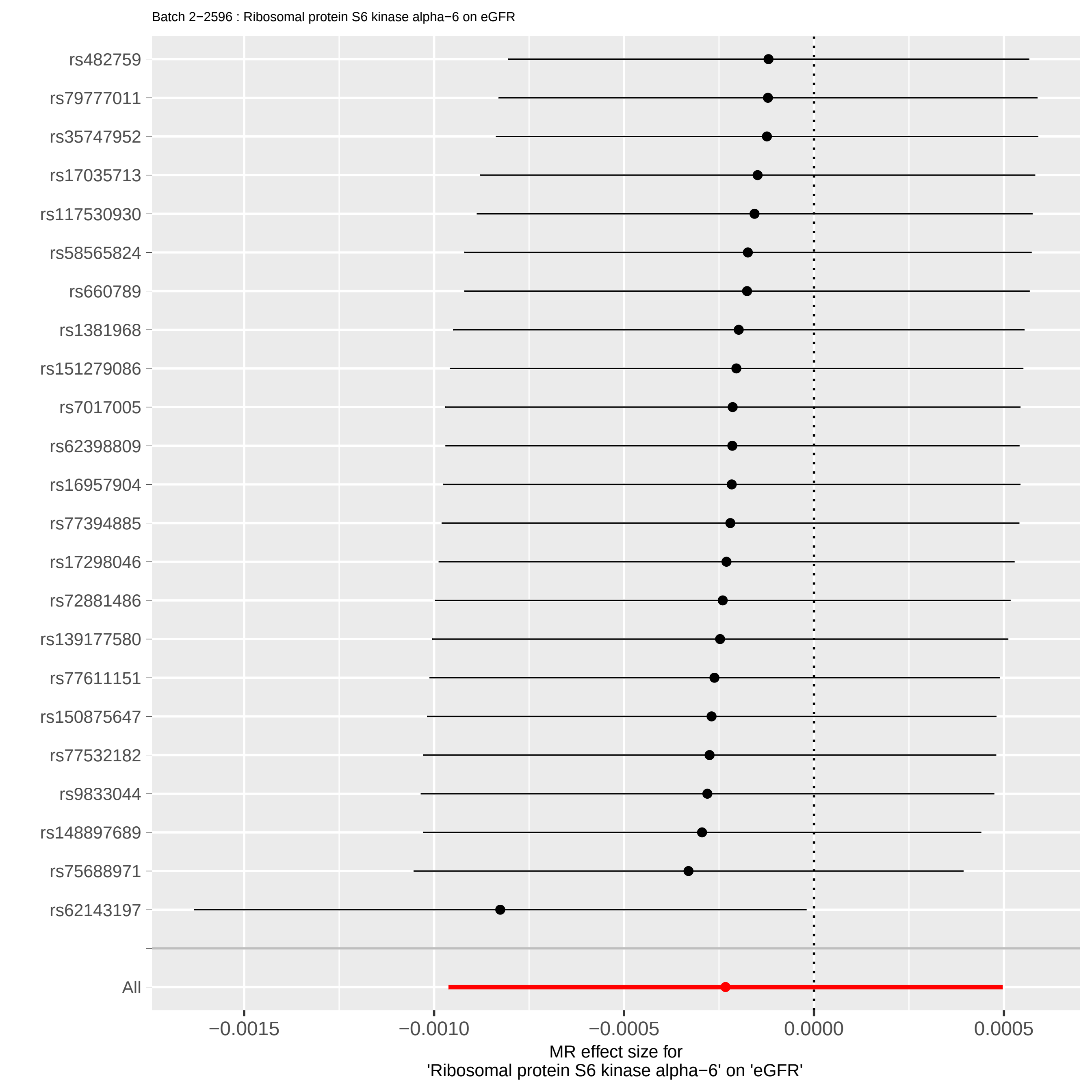

Batch 2−2596 : Ribosomal protein S6 kinase alpha−6 on eGFR
rs482759
rs79777011
rs35747952
rs17035713
rs117530930
rs58565824
rs660789
rs1381968
rs151279086
rs7017005
rs62398809
rs16957904
rs77394885
rs17298046
rs72881486
rs139177580
rs77611151
rs150875647
rs77532182
rs9833044
rs148897689
rs75688971
rs62143197
All
−0.0015
−0.0010
−0.0005
0.0000
0.0005
MR effect size for
'Ribosomal protein S6 kinase alpha−6' on 'eGFR'

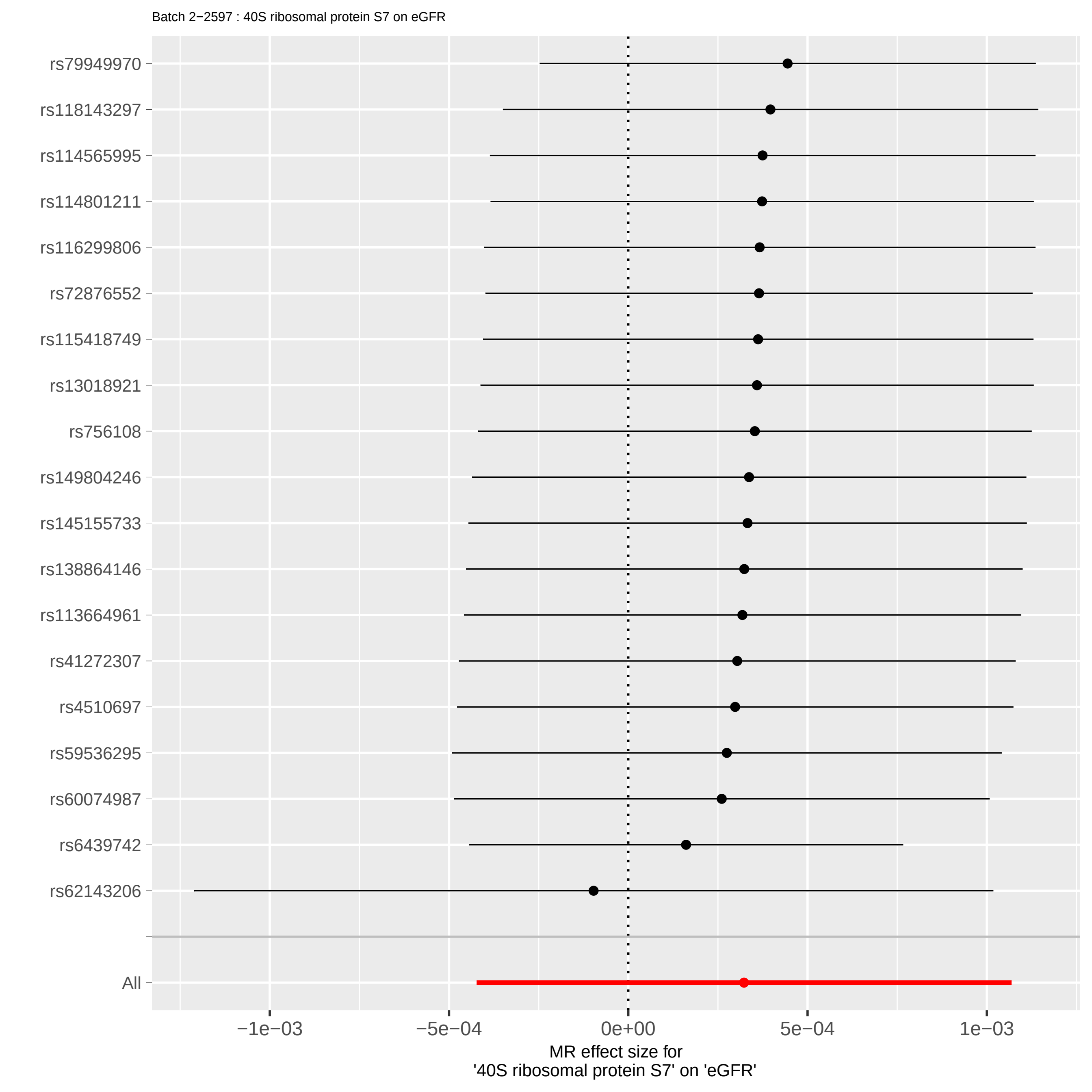
Batch 2−2597 : 40S ribosomal protein S7 on eGFR
rs79949970
rs118143297
rs114565995
rs114801211
rs116299806
rs72876552
rs115418749
rs13018921
rs756108
rs149804246
rs145155733
rs138864146
rs113664961
rs41272307
rs4510697
rs59536295
rs60074987
rs6439742
rs62143206
All
−1e−03
−5e−04
0e+00
5e−04
1e−03
MR effect size for
'40S ribosomal protein S7' on 'eGFR'

Batch 2−2598 : 40S ribosomal protein SA on eGFR

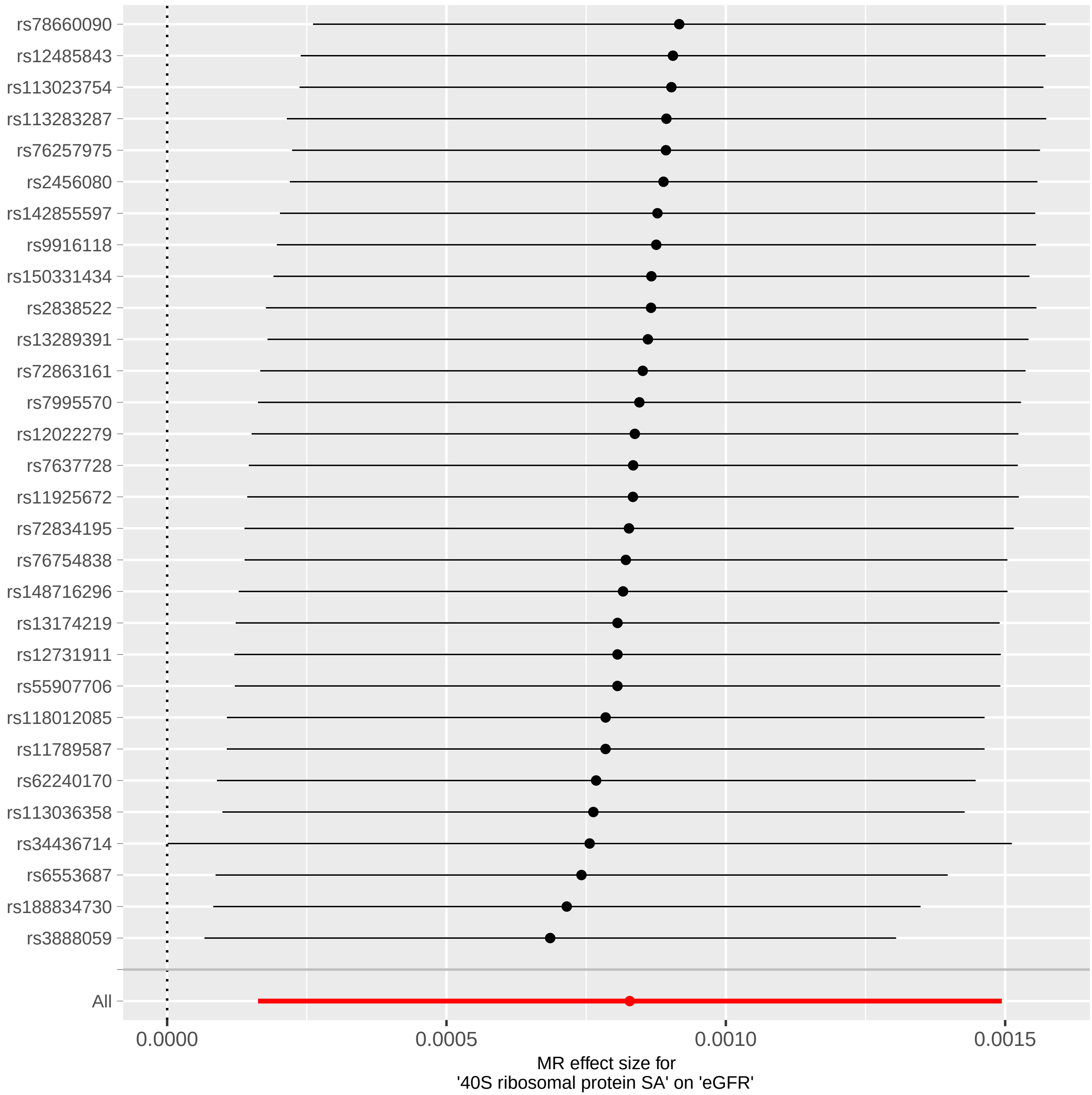

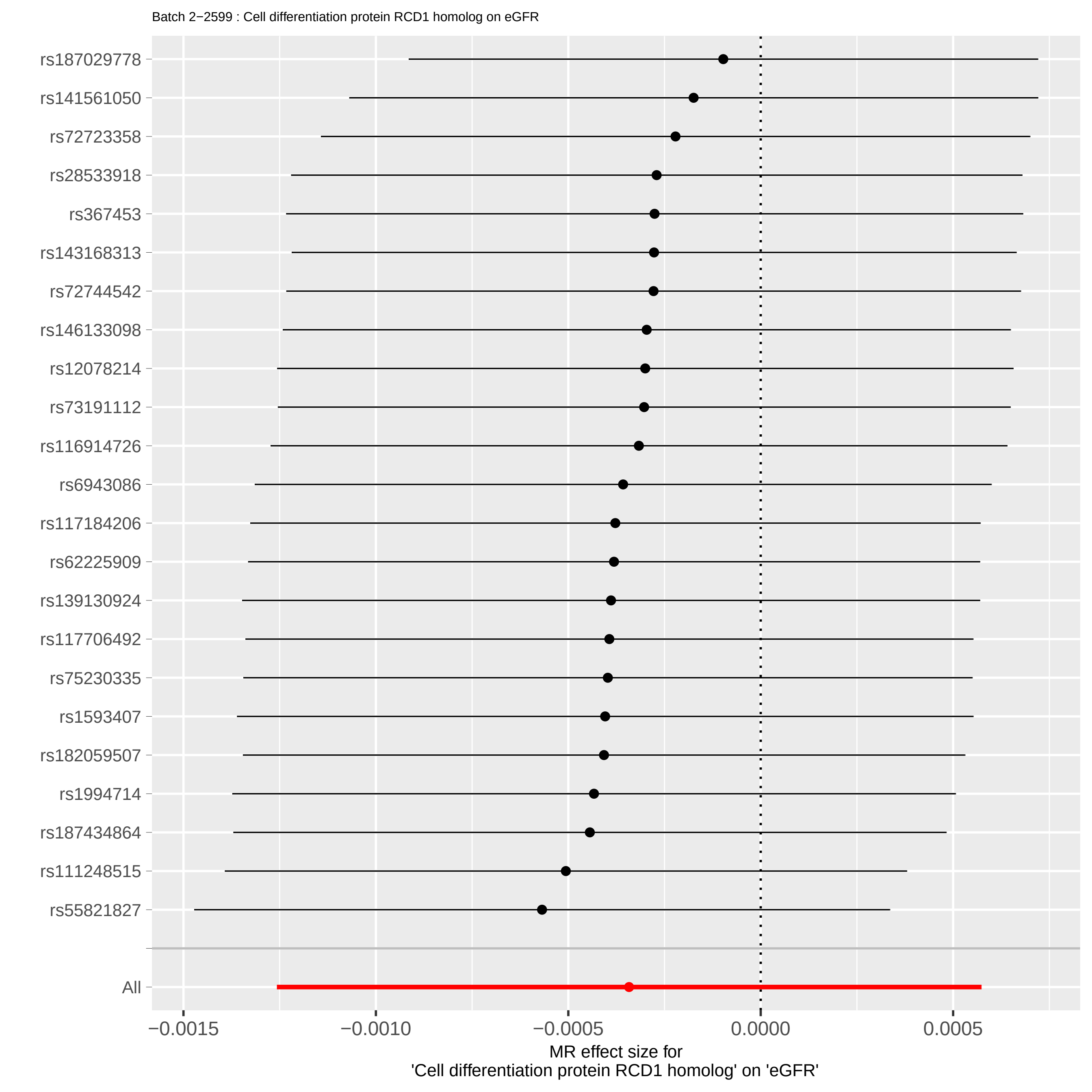
Batch 2−2599 : Cell differentiation protein RCD1 homolog on eGFR
rs187029778
rs141561050
rs72723358
rs28533918
rs367453
rs143168313
rs72744542
rs146133098
rs12078214
rs73191112
rs116914726
rs6943086
rs117184206
rs62225909
rs139130924
rs117706492
rs75230335
rs1593407
rs182059507
rs1994714
rs187434864
rs111248515
rs55821827
All
−0.0015
−0.0010
−0.0005
0.0000
0.0005
MR effect size for
'Cell differentiation protein RCD1 homolog' on 'eGFR'

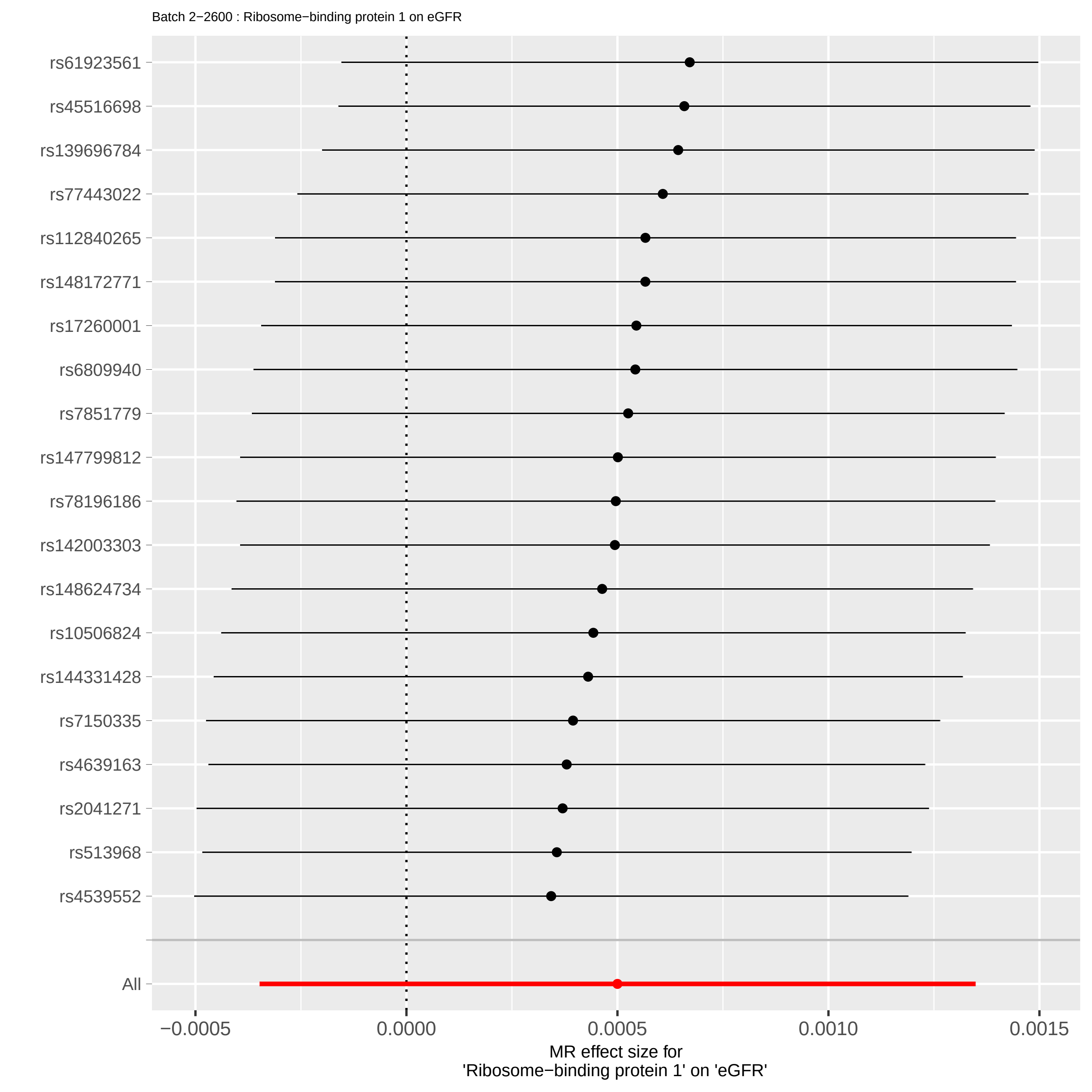
Batch 2−2600 : Ribosome−binding protein 1 on eGFR
rs61923561
rs45516698
rs139696784
rs77443022
rs112840265
rs148172771
rs17260001
rs6809940
rs7851779
rs147799812
rs78196186
rs142003303
rs148624734
rs10506824
rs144331428
rs7150335
rs4639163
rs2041271
rs513968
rs4539552
All
−0.0005
0.0000
0.0005
0.0010
0.0015
MR effect size for
'Ribosome−binding protein 1' on 'eGFR'

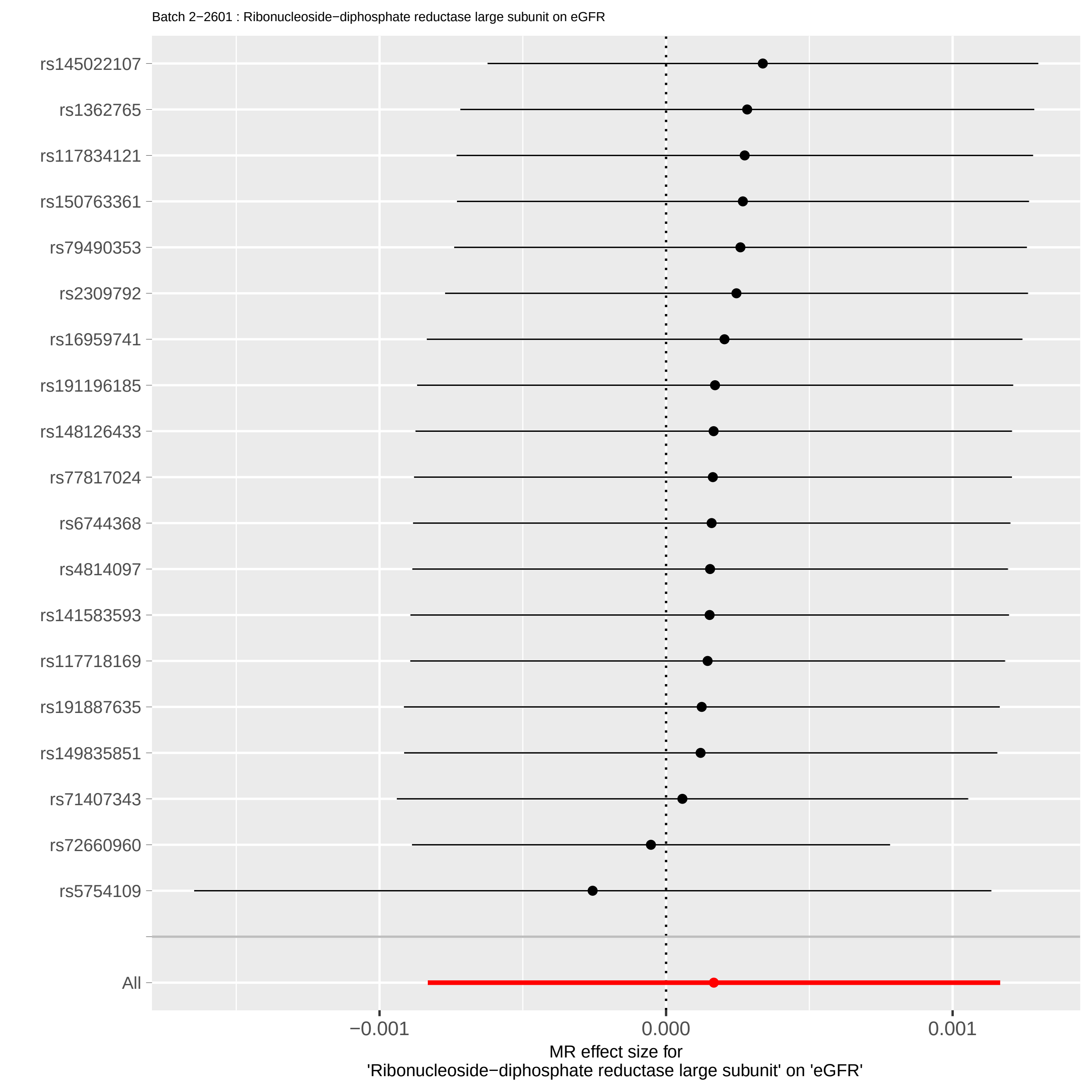

Batch 2−2601 : Ribonucleoside−diphosphate reductase large subunit on eGFR
rs145022107
rs1362765
rs117834121
rs150763361
rs79490353
rs2309792
rs16959741
rs191196185
rs148126433
rs77817024
rs6744368
rs4814097
rs141583593
rs117718169
rs191887635
rs149835851
rs71407343
rs72660960
rs5754109
All
−0.001
0.000
0.001
MR effect size for
'Ribonucleoside−diphosphate reductase large subunit' on 'eGFR'

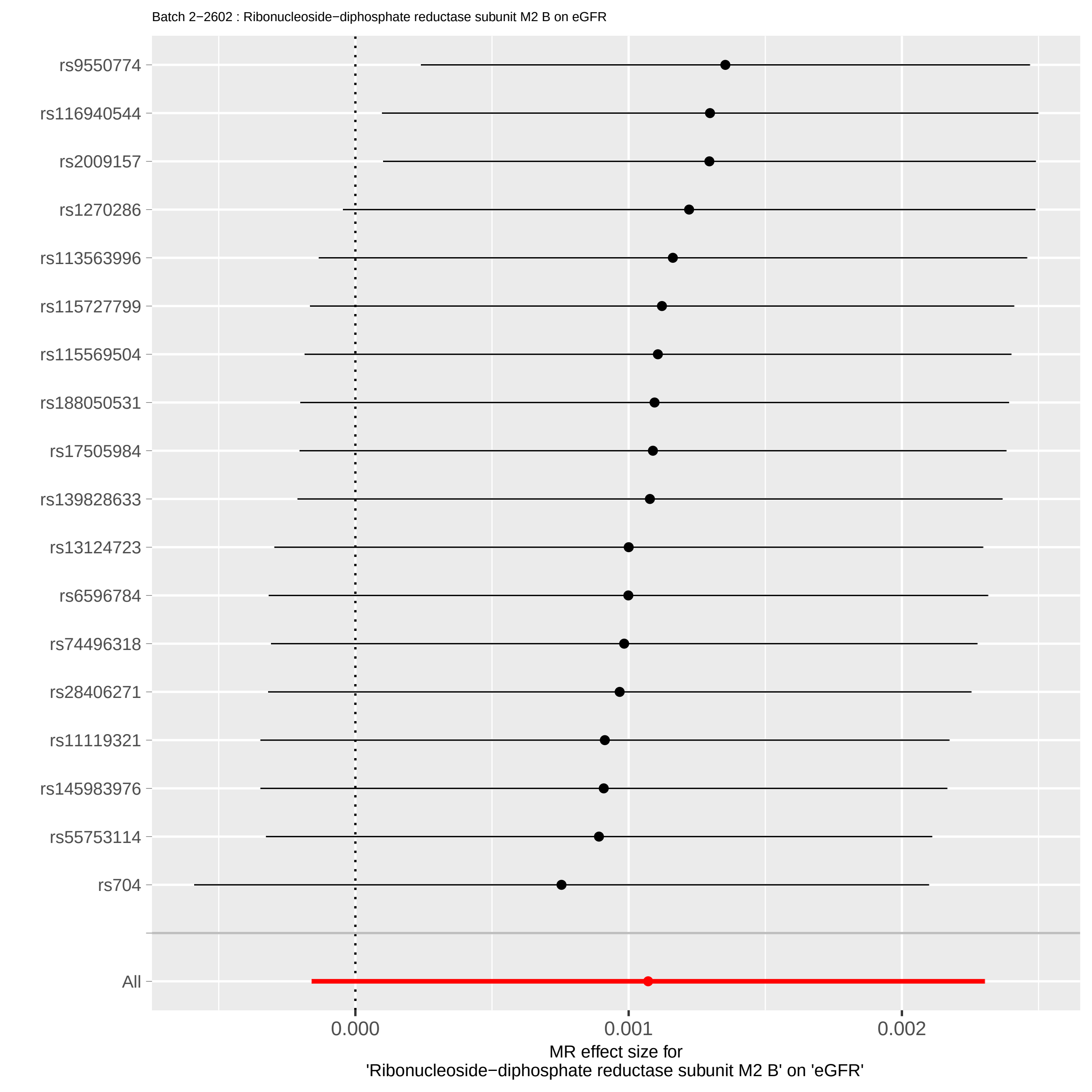

Batch 2−2602 : Ribonucleoside−diphosphate reductase subunit M2 B on eGFR
rs9550774
rs116940544
rs2009157
rs1270286
rs113563996
rs115727799
rs115569504
rs188050531
rs17505984
rs139828633
rs13124723
rs6596784
rs74496318
rs28406271
rs11119321
rs145983976
rs55753114
rs704
All
0.000
0.001
0.002
MR effect size for
'Ribonucleoside−diphosphate reductase subunit M2 B' on 'eGFR'

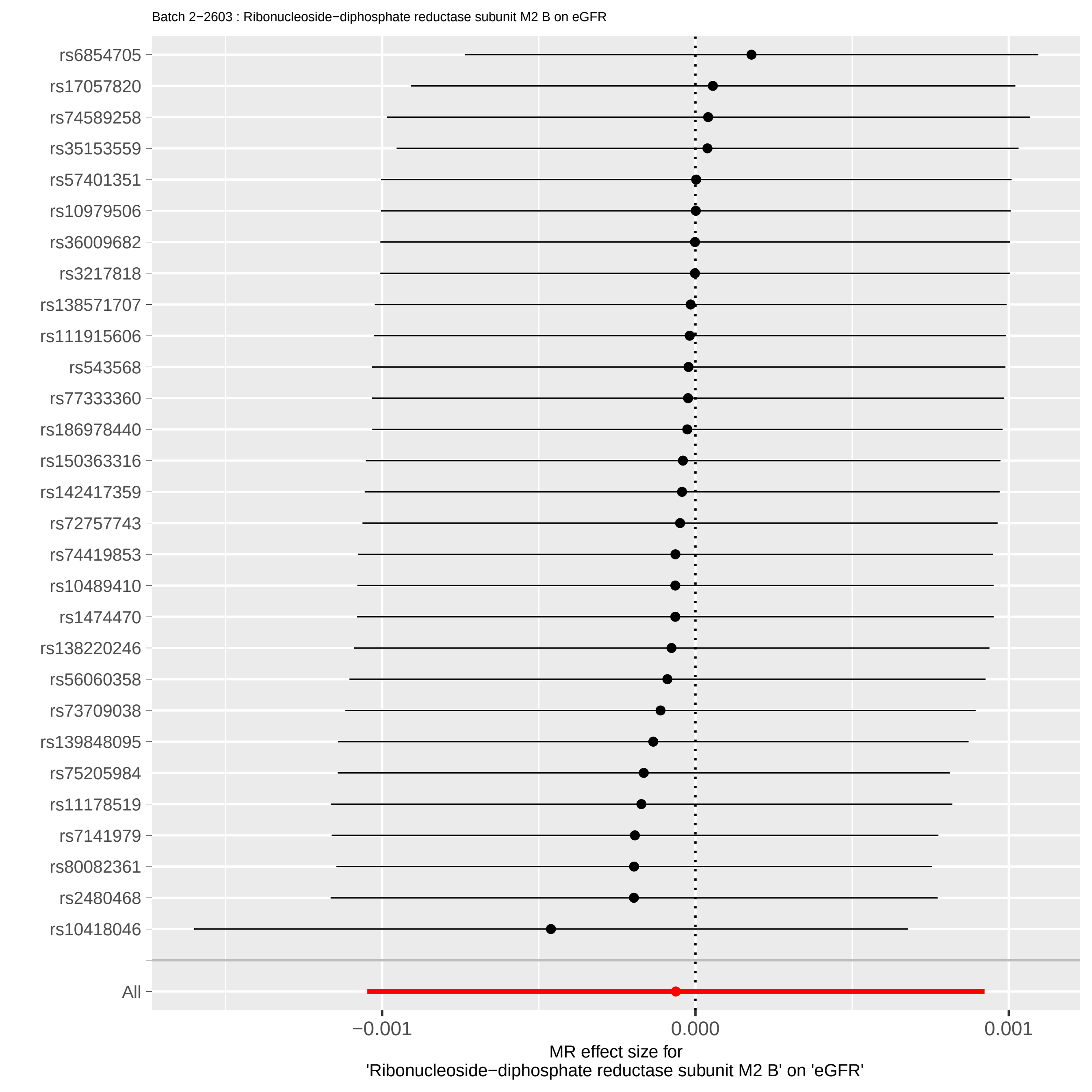
Batch 2−2603 : Ribonucleoside−diphosphate reductase subunit M2 B on eGFR
rs6854705
rs17057820
rs74589258
rs35153559
rs57401351
rs10979506
rs36009682
rs3217818
rs138571707
rs111915606
rs543568
rs77333360
rs186978440
rs150363316
rs142417359
rs72757743
rs74419853
rs10489410
rs1474470
rs138220246
rs56060358
rs73709038
rs139848095
rs75205984
rs11178519
rs7141979
rs80082361
rs2480468
rs10418046
All
−0.001
0.000
0.001
MR effect size for
'Ribonucleoside−diphosphate reductase subunit M2 B' on 'eGFR'

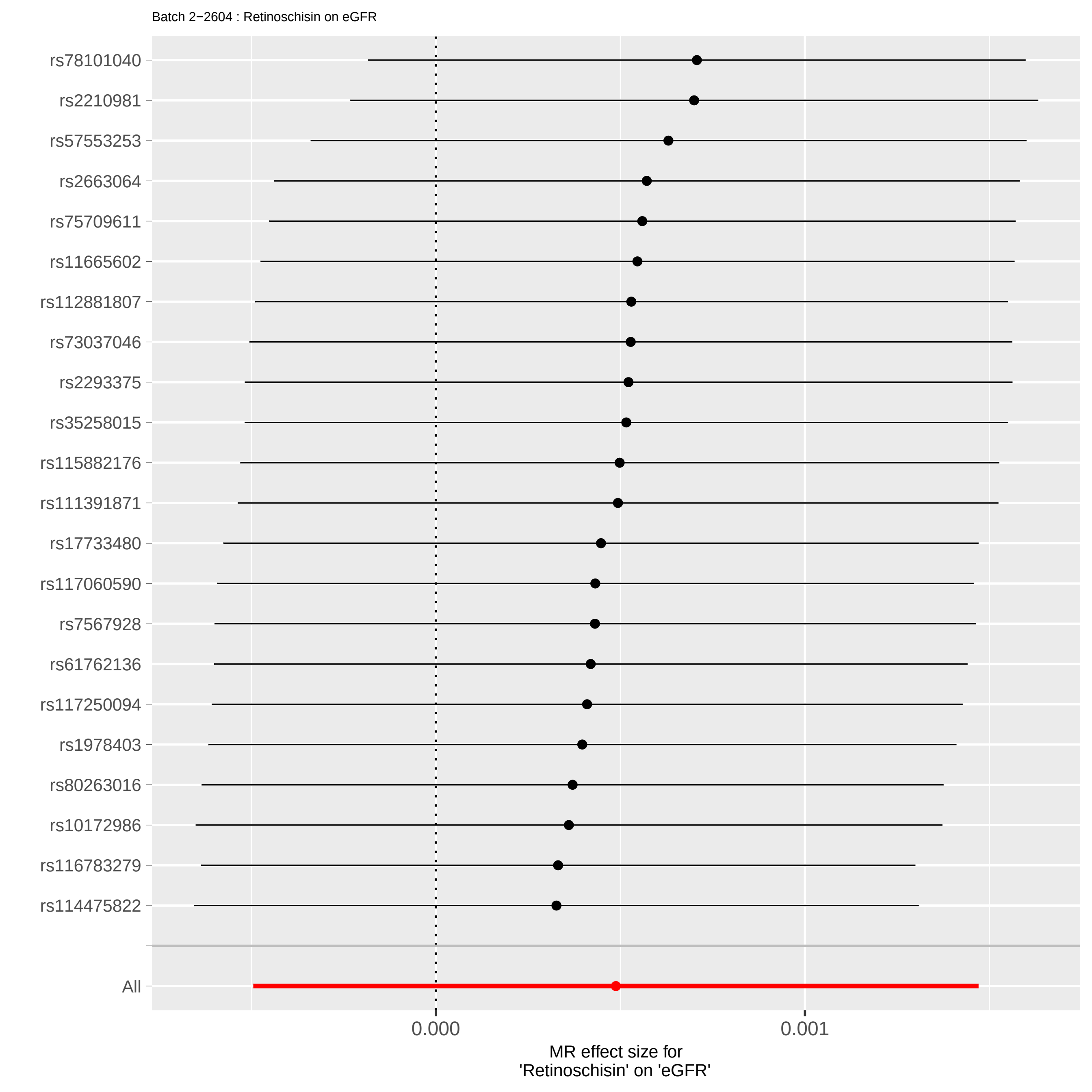

Batch 2−2604 : Retinoschisin on eGFR
rs78101040
rs2210981
rs57553253
rs2663064
rs75709611
rs11665602
rs112881807
rs73037046
rs2293375
rs35258015
rs115882176
rs111391871
rs17733480
rs117060590
rs7567928
rs61762136
rs117250094
rs1978403
rs80263016
rs10172986
rs116783279
rs114475822
All
0.000
0.001
MR effect size for
'Retinoschisin' on 'eGFR'

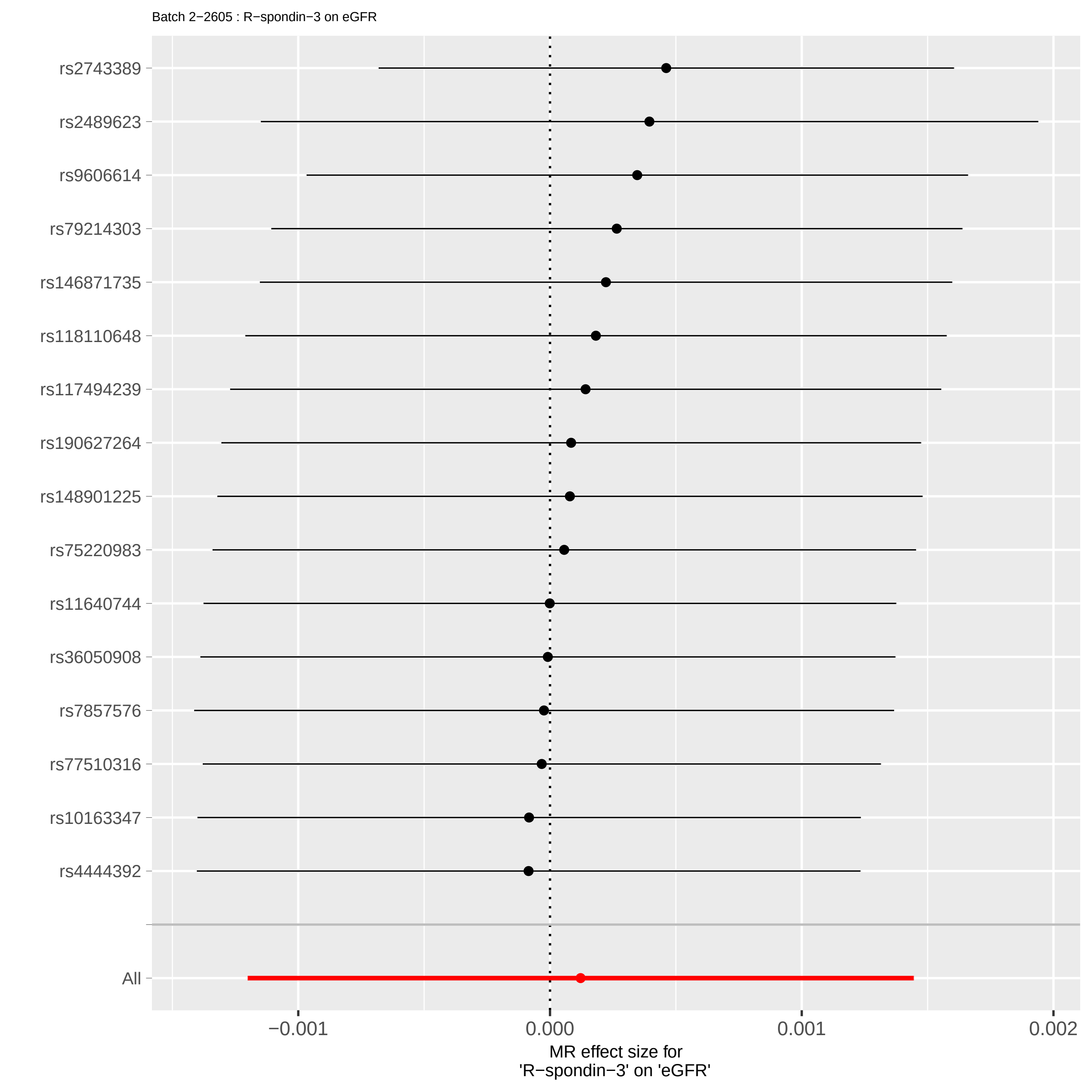

Batch 2−2605 : R−spondin−3 on eGFR
rs2743389
rs2489623
rs9606614
rs79214303
rs146871735
rs118110648
rs117494239
rs190627264
rs148901225
rs75220983
rs11640744
rs36050908
rs7857576
rs77510316
rs10163347
rs4444392
All
−0.001
0.000
0.001
0.002
MR effect size for
'R−spondin−3' on 'eGFR'

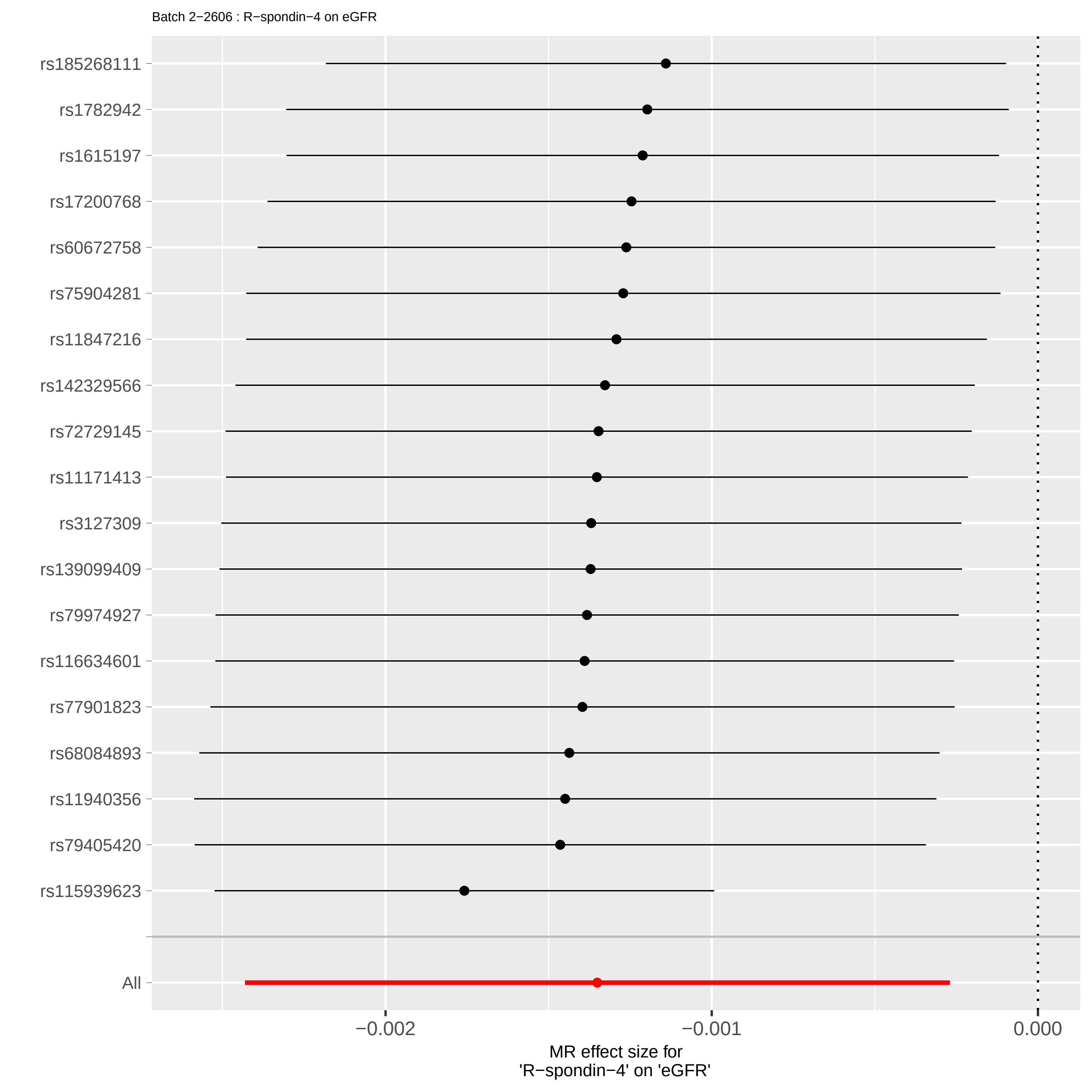

Batch 2−2606 : R−spondin−4 on eGFR
rs185268111
rs1782942
rs1615197
rs17200768
rs60672758
rs75904281
rs11847216
rs142329566
rs72729145
rs11171413
rs3127309
rs139099409
rs79974927
rs116634601
rs77901823
rs68084893
rs11940356
rs79405420
rs115939623
All
−0.002
−0.001
0.000
MR effect size for
'R−spondin−4' on 'eGFR'

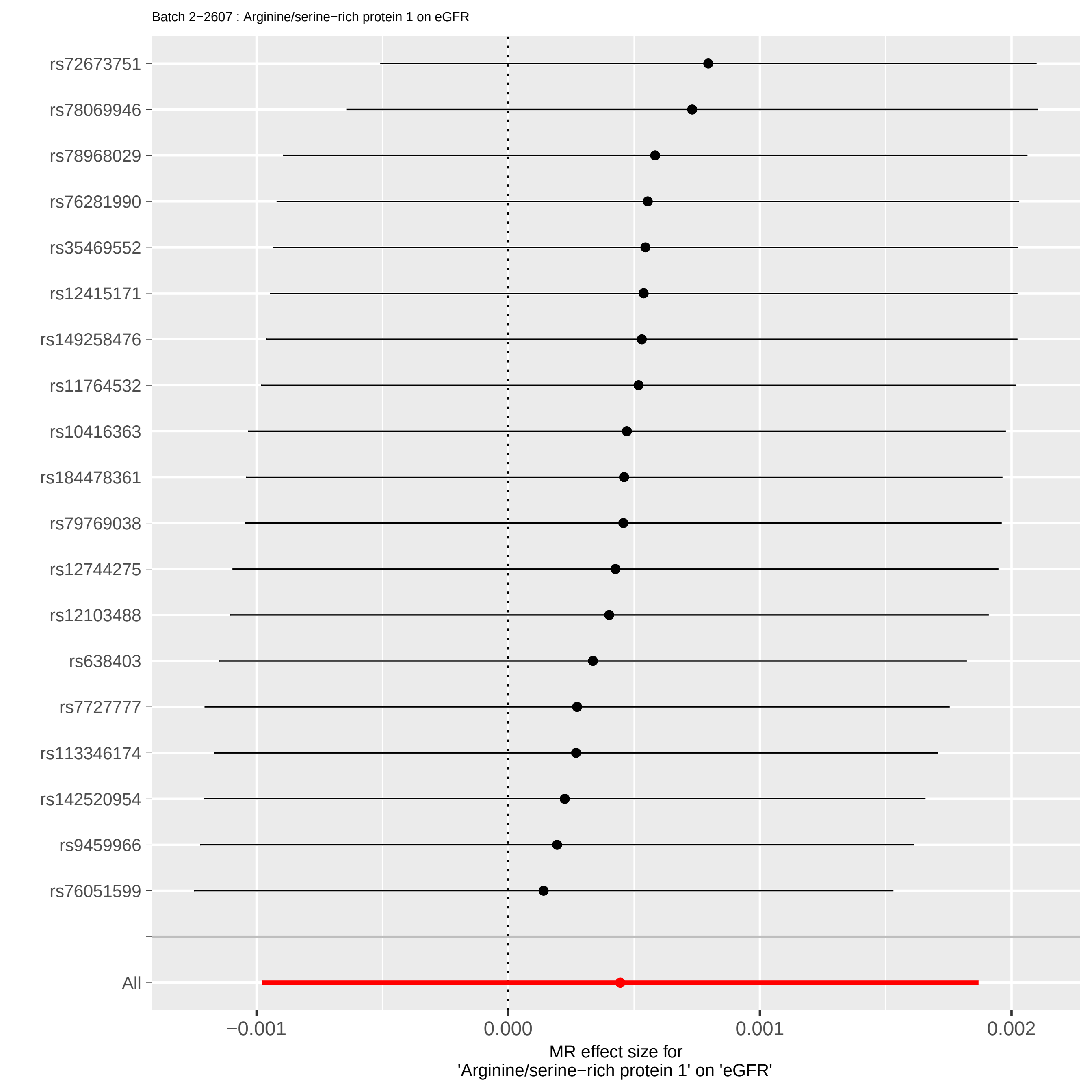

Batch 2−2607 : Arginine/serine−rich protein 1 on eGFR
rs72673751
rs78069946
rs78968029
rs76281990
rs35469552
rs12415171
rs149258476
rs11764532
rs10416363
rs184478361
rs79769038
rs12744275
rs12103488
rs638403
rs7727777
rs113346174
rs142520954
rs9459966
rs76051599
All
−0.001
0.000
0.001
0.002
MR effect size for
'Arginine/serine−rich protein 1' on 'eGFR'

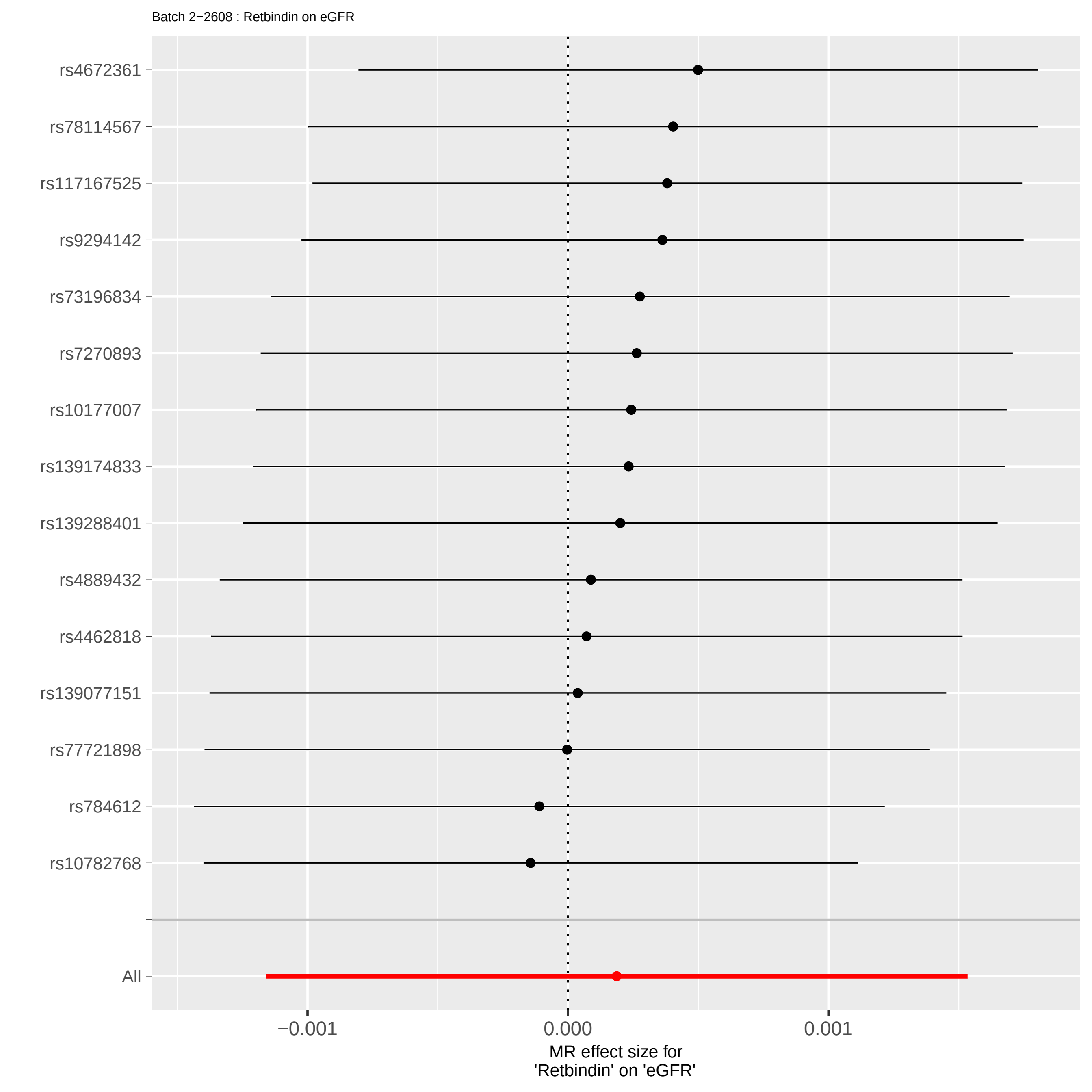

Batch 2−2608 : Retbindin on eGFR
rs4672361
rs78114567
rs117167525
rs9294142
rs73196834
rs7270893
rs10177007
rs139174833
rs139288401
rs4889432
rs4462818
rs139077151
rs77721898
rs784612
rs10782768
All
−0.001
0.000
0.001
MR effect size for
'Retbindin' on 'eGFR'

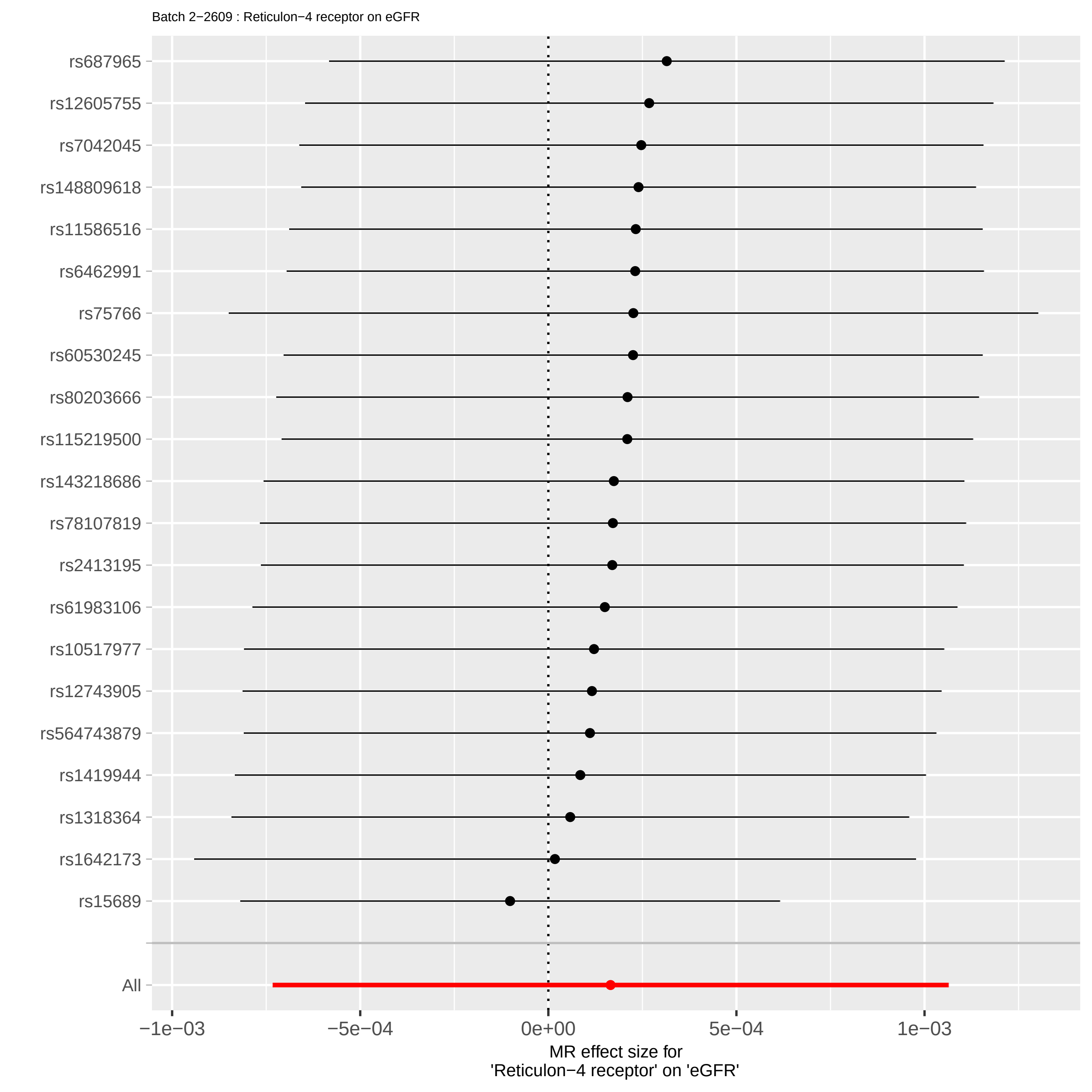

Batch 2−2609 : Reticulon−4 receptor on eGFR
rs687965
rs12605755
rs7042045
rs148809618
rs11586516
rs6462991
rs75766
rs60530245
rs80203666
rs115219500
rs143218686
rs78107819
rs2413195
rs61983106
rs10517977
rs12743905
rs564743879
rs1419944
rs1318364
rs1642173
rs15689
All
−1e−03
−5e−04
0e+00
5e−04
1e−03
MR effect size for
'Reticulon−4 receptor' on 'eGFR'

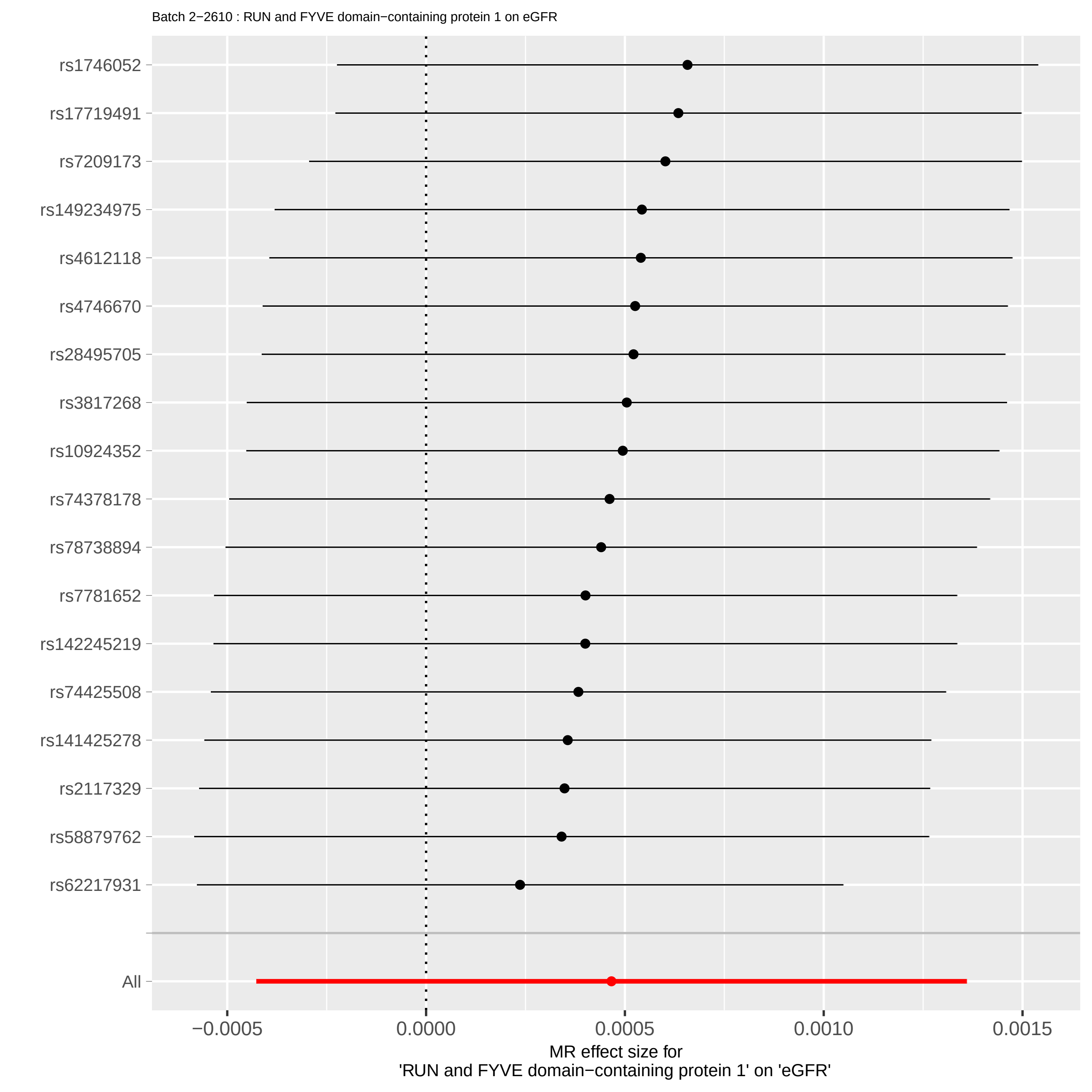

Batch 2−2610 : RUN and FYVE domain−containing protein 1 on eGFR
rs1746052
rs17719491
rs7209173
rs149234975
rs4612118
rs4746670
rs28495705
rs3817268
rs10924352
rs74378178
rs78738894
rs7781652
rs142245219
rs74425508
rs141425278
rs2117329
rs58879762
rs62217931
All
−0.0005
0.0000
0.0005
0.0010
0.0015
MR effect size for
'RUN and FYVE domain−containing protein 1' on 'eGFR'

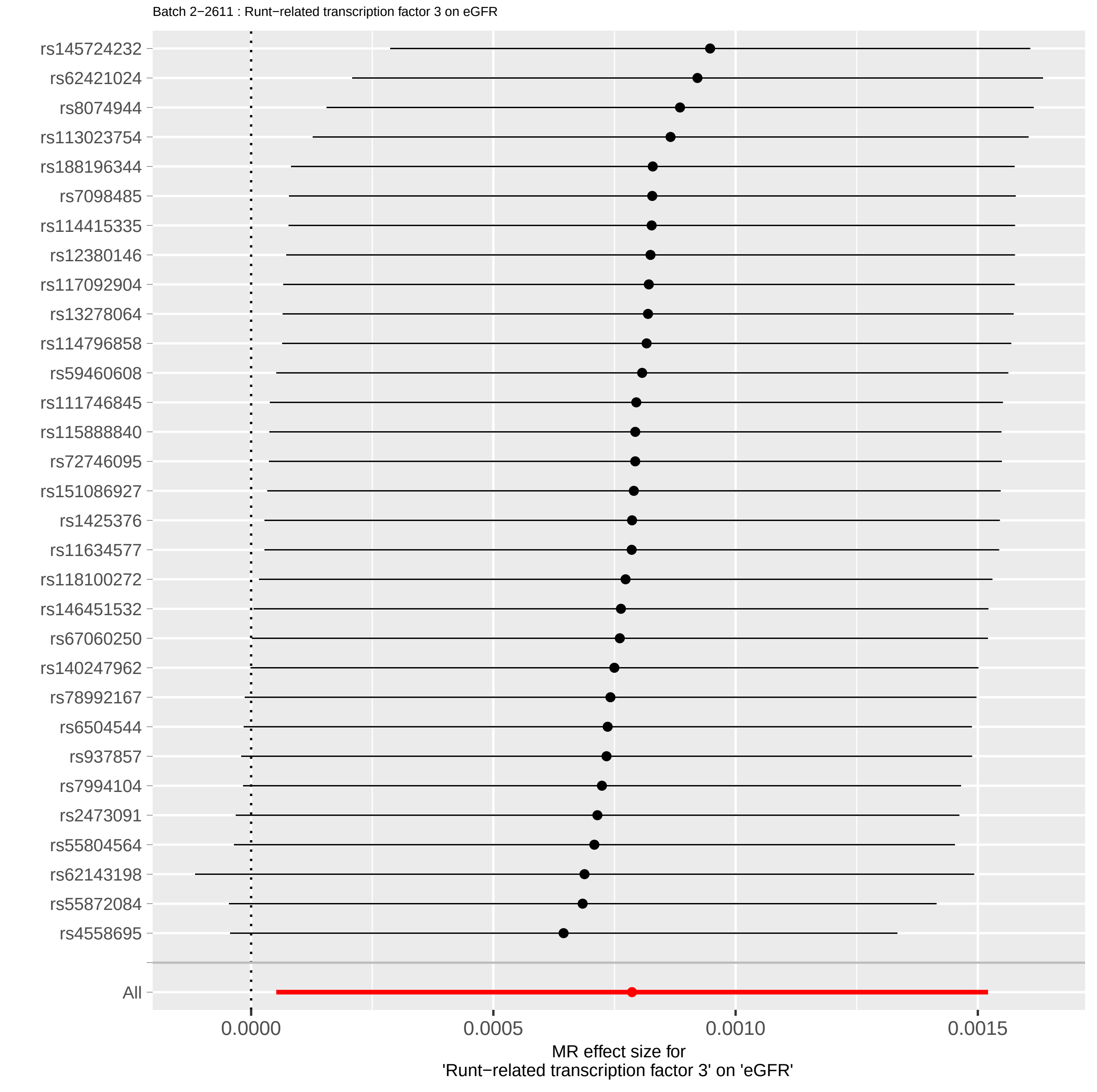
Batch 2−2611 : Runt−related transcription factor 3 on eGFR
rs145724232
rs62421024
rs8074944
rs113023754
rs188196344
rs7098485
rs114415335
rs12380146
rs117092904
rs13278064
rs114796858
rs59460608
rs111746845
rs115888840
rs72746095
rs151086927
rs1425376
rs11634577
rs118100272
rs146451532
rs67060250
rs140247962
rs78992167
rs6504544
rs937857
rs7994104
rs2473091
rs55804564
rs62143198
rs55872084
rs4558695
All
0.0000
0.0005
0.0010
0.0015
MR effect size for
'Runt−related transcription factor 3' on 'eGFR'

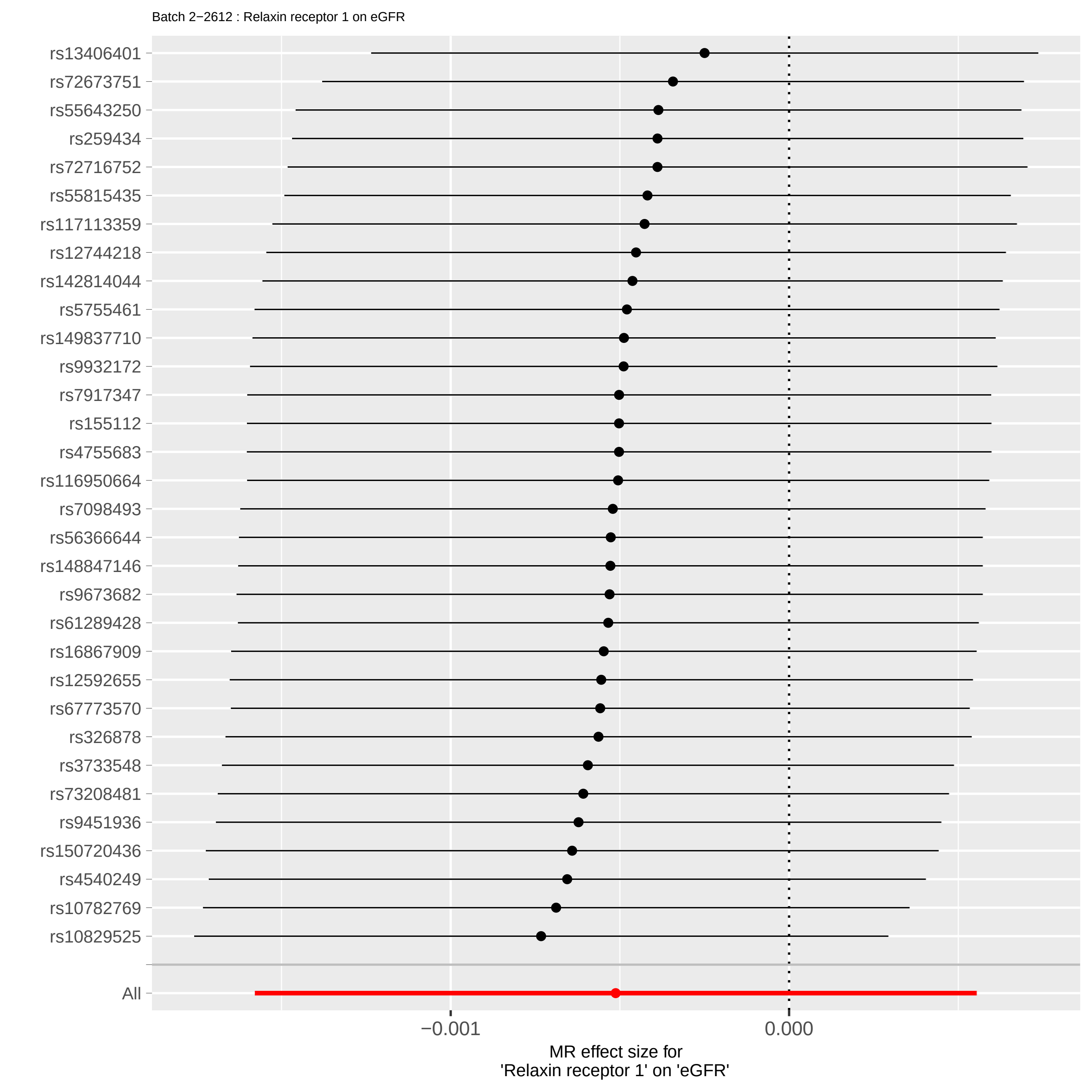

Batch 2−2612 : Relaxin receptor 1 on eGFR
rs13406401
rs72673751
rs55643250
rs259434
rs72716752
rs55815435
rs117113359
rs12744218
rs142814044
rs5755461
rs149837710
rs9932172
rs7917347
rs155112
rs4755683
rs116950664
rs7098493
rs56366644
rs148847146
rs9673682
rs61289428
rs16867909
rs12592655
rs67773570
rs326878
rs3733548
rs73208481
rs9451936
rs150720436
rs4540249
rs10782769
rs10829525
All
−0.001
0.000
MR effect size for
'Relaxin receptor 1' on 'eGFR'

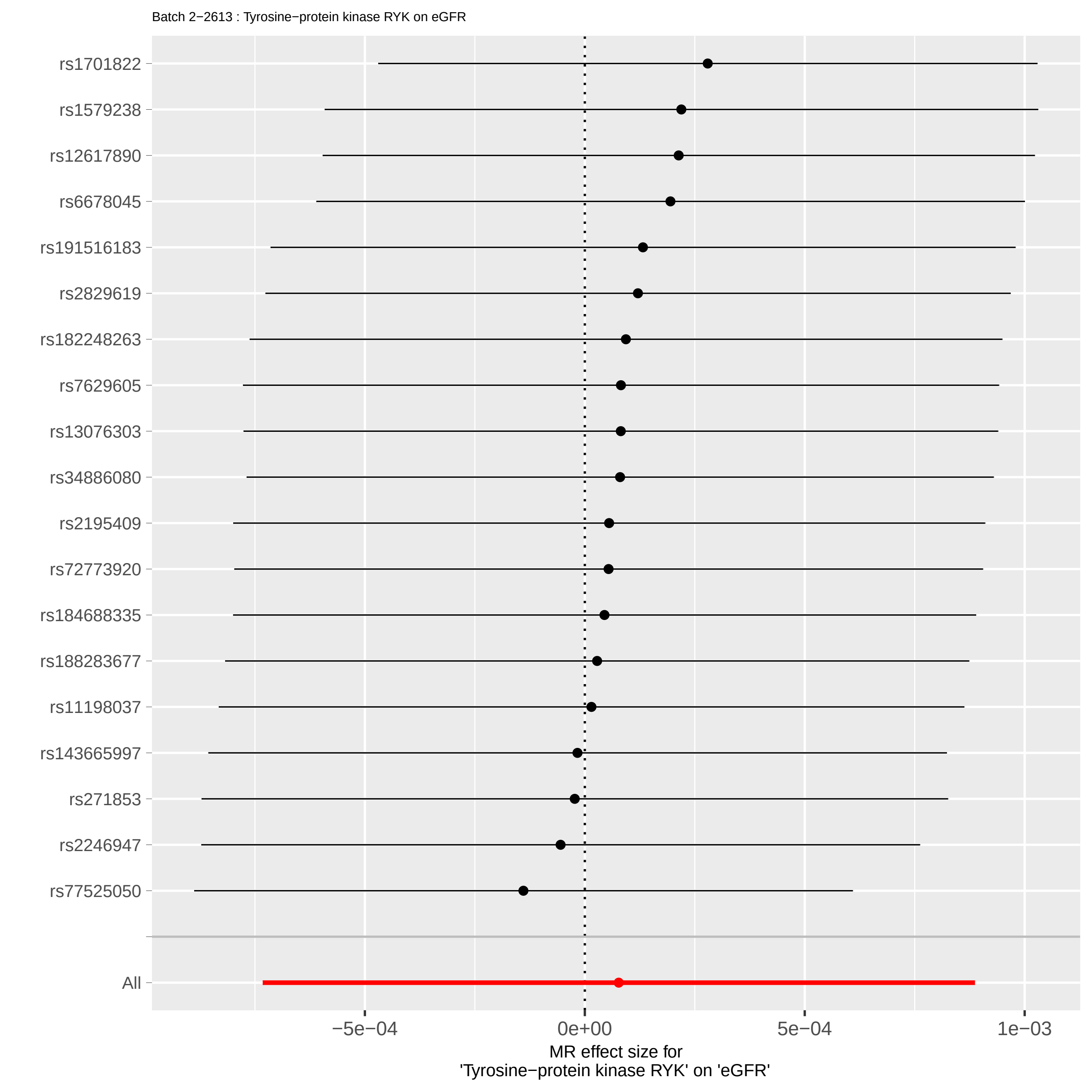

Batch 2−2613 : Tyrosine−protein kinase RYK on eGFR
rs1701822
rs1579238
rs12617890
rs6678045
rs191516183
rs2829619
rs182248263
rs7629605
rs13076303
rs34886080
rs2195409
rs72773920
rs184688335
rs188283677
rs11198037
rs143665997
rs271853
rs2246947
rs77525050
All
−5e−04
0e+00
5e−04
1e−03
MR effect size for
'Tyrosine−protein kinase RYK' on 'eGFR'

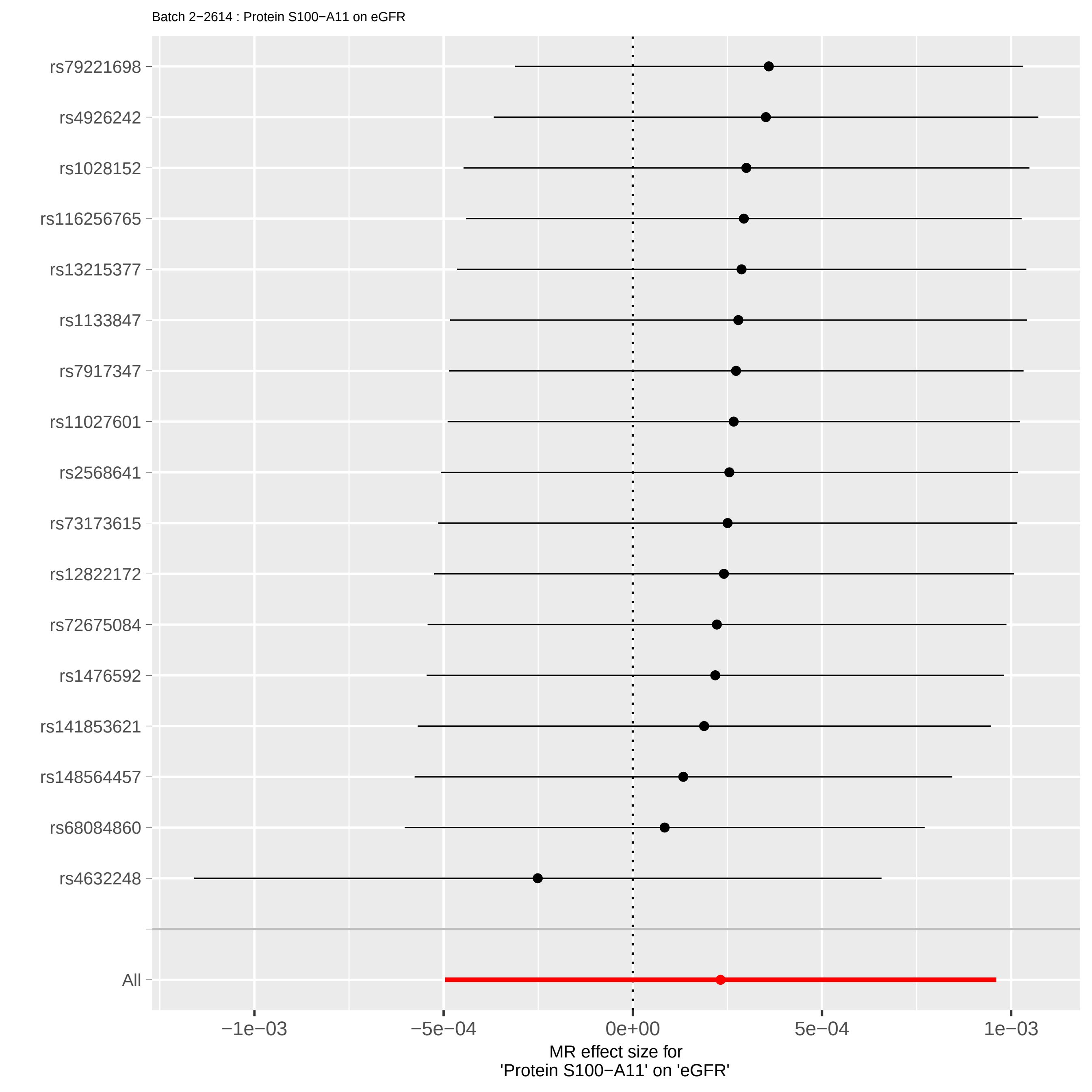

Batch 2−2614 : Protein S100−A11 on eGFR
rs79221698
rs4926242
rs1028152
rs116256765
rs13215377
rs1133847
rs7917347
rs11027601
rs2568641
rs73173615
rs12822172
rs72675084
rs1476592
rs141853621
rs148564457
rs68084860
rs4632248
All
−1e−03
−5e−04
0e+00
5e−04
1e−03
MR effect size for
'Protein S100−A11' on 'eGFR'

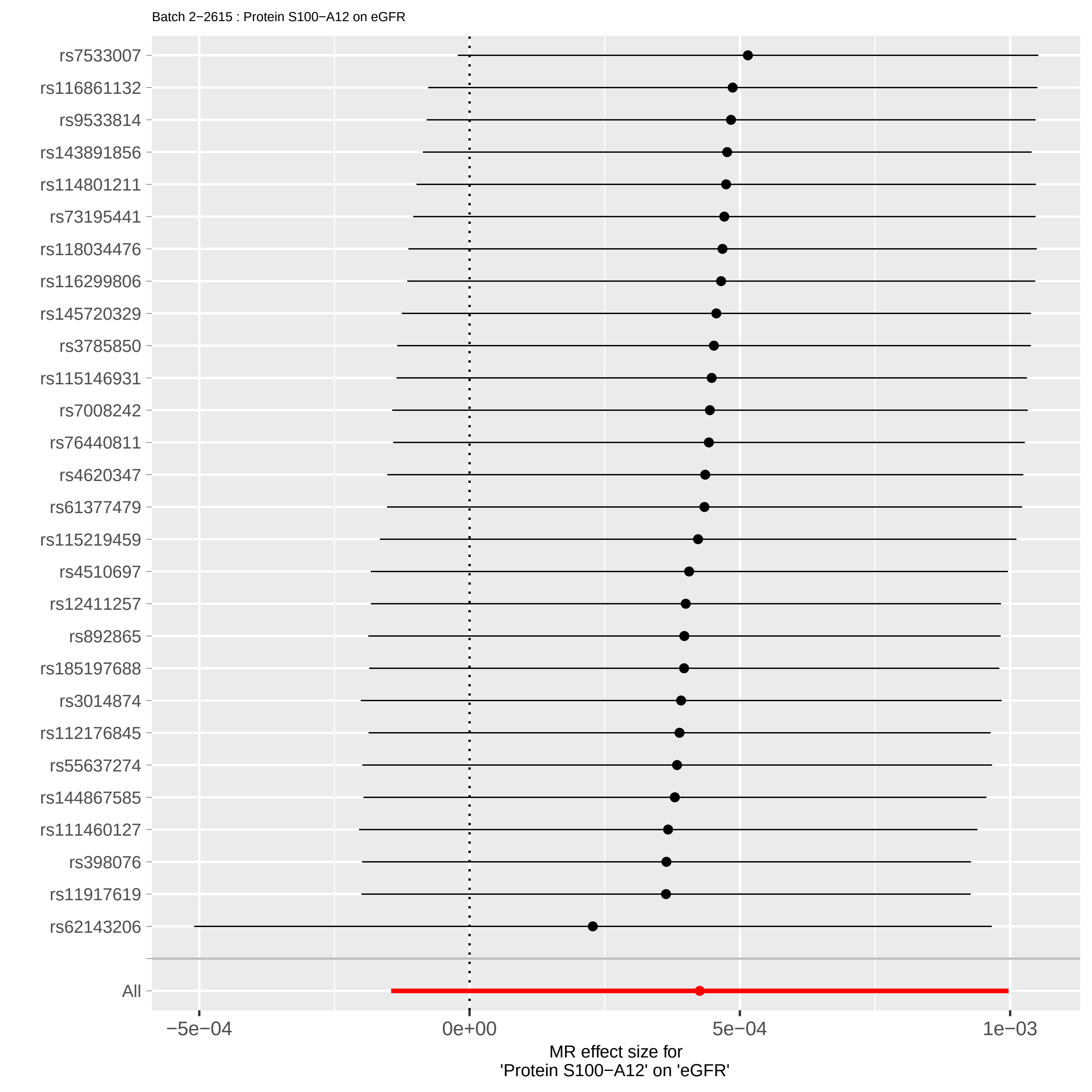

Batch 2−2615 : Protein S100−A12 on eGFR
rs7533007
rs116861132
rs9533814
rs143891856
rs114801211
rs73195441
rs118034476
rs116299806
rs145720329
rs3785850
rs115146931
rs7008242
rs76440811
rs4620347
rs61377479
rs115219459
rs4510697
rs12411257
rs892865
rs185197688
rs3014874
rs112176845
rs55637274
rs144867585
rs111460127
rs398076
rs11917619
rs62143206
All
−5e−04
0e+00
5e−04
1e−03
MR effect size for
'Protein S100−A12' on 'eGFR'

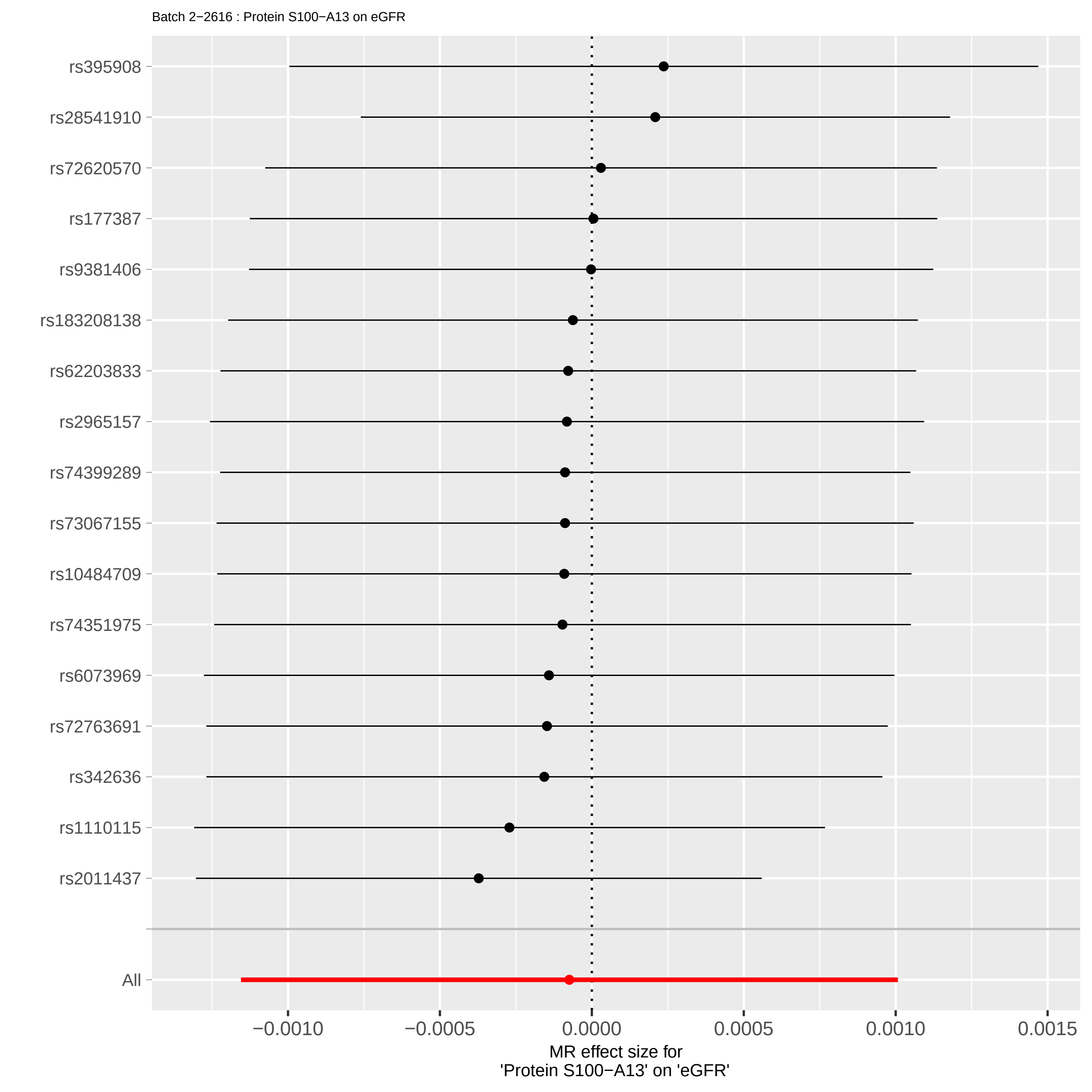

Batch 2−2616 : Protein S100−A13 on eGFR
rs395908
rs28541910
rs72620570
rs177387
rs9381406
rs183208138
rs62203833
rs2965157
rs74399289
rs73067155
rs10484709
rs74351975
rs6073969
rs72763691
rs342636
rs1110115
rs2011437
All
−0.0010
−0.0005
0.0000
0.0005
0.0010
0.0015
MR effect size for
'Protein S100−A13' on 'eGFR'

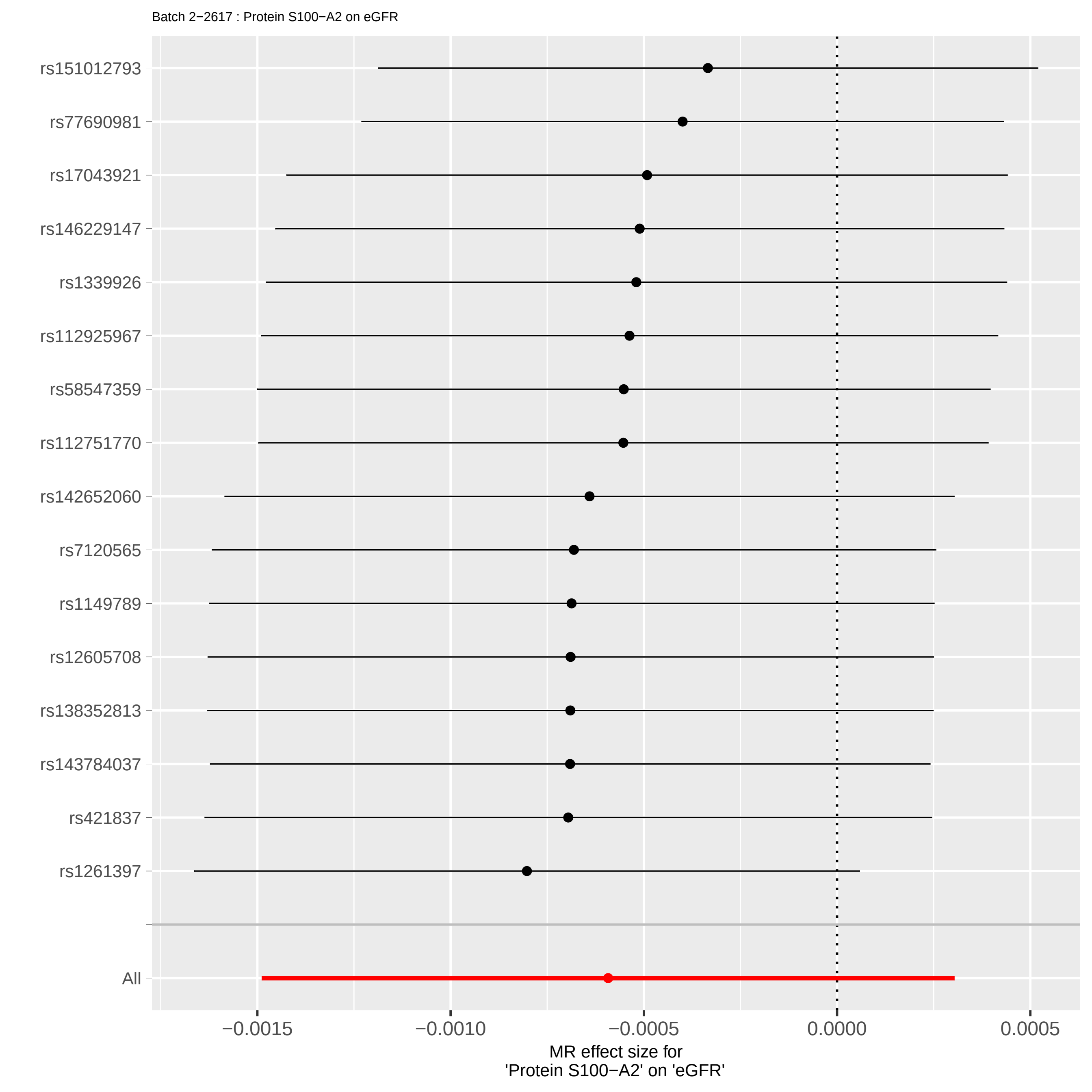

Batch 2−2617 : Protein S100−A2 on eGFR
rs151012793
rs77690981
rs17043921
rs146229147
rs1339926
rs112925967
rs58547359
rs112751770
rs142652060
rs7120565
rs1149789
rs12605708
rs138352813
rs143784037
rs421837
rs1261397
All
−0.0015
−0.0010
−0.0005
0.0000
0.0005
MR effect size for
'Protein S100−A2' on 'eGFR'

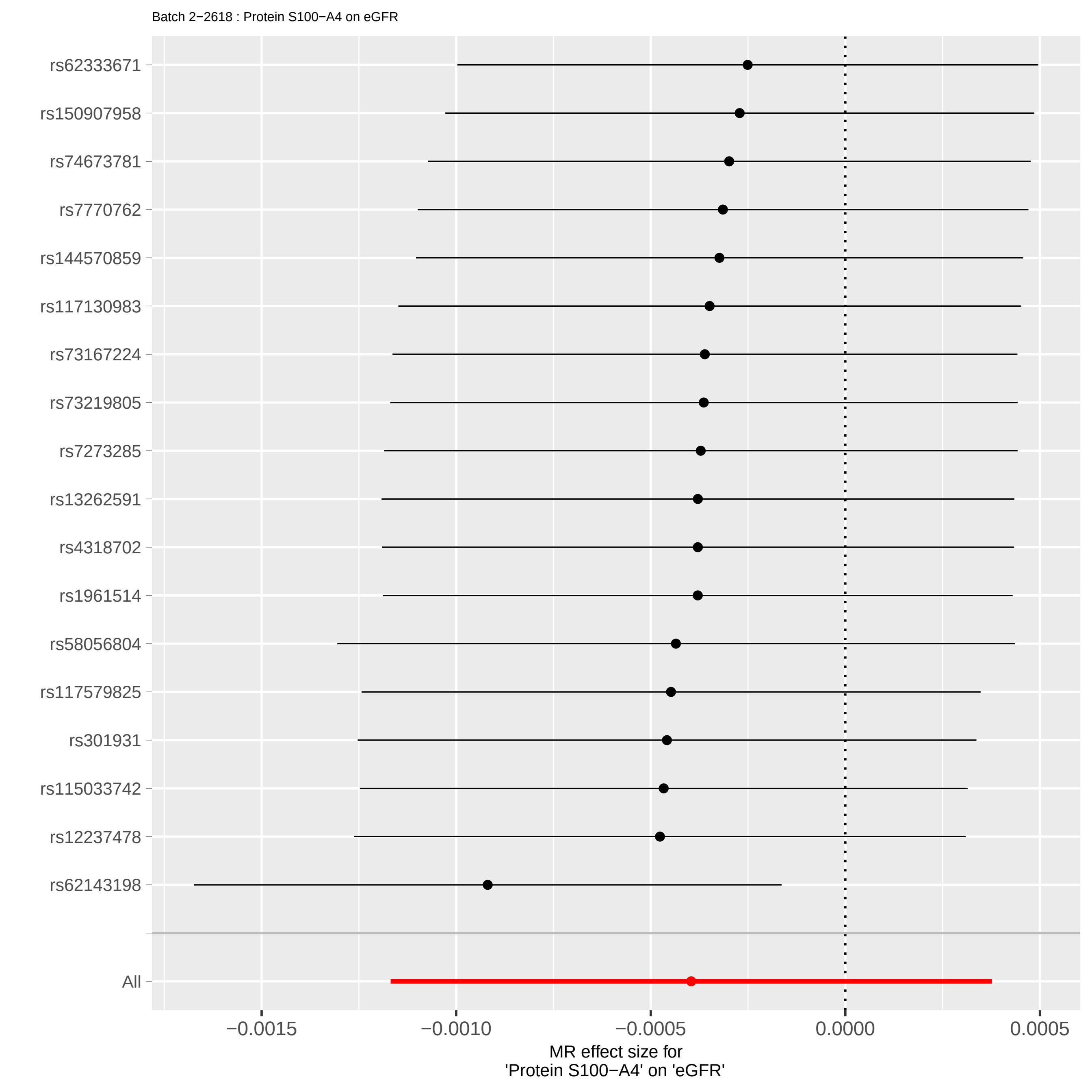

Batch 2−2618 : Protein S100−A4 on eGFR
rs62333671
rs150907958
rs74673781
rs7770762
rs144570859
rs117130983
rs73167224
rs73219805
rs7273285
rs13262591
rs4318702
rs1961514
rs58056804
rs117579825
rs301931
rs115033742
rs12237478
rs62143198
All
−0.0015
−0.0010
−0.0005
0.0000
0.0005
MR effect size for
'Protein S100−A4' on 'eGFR'

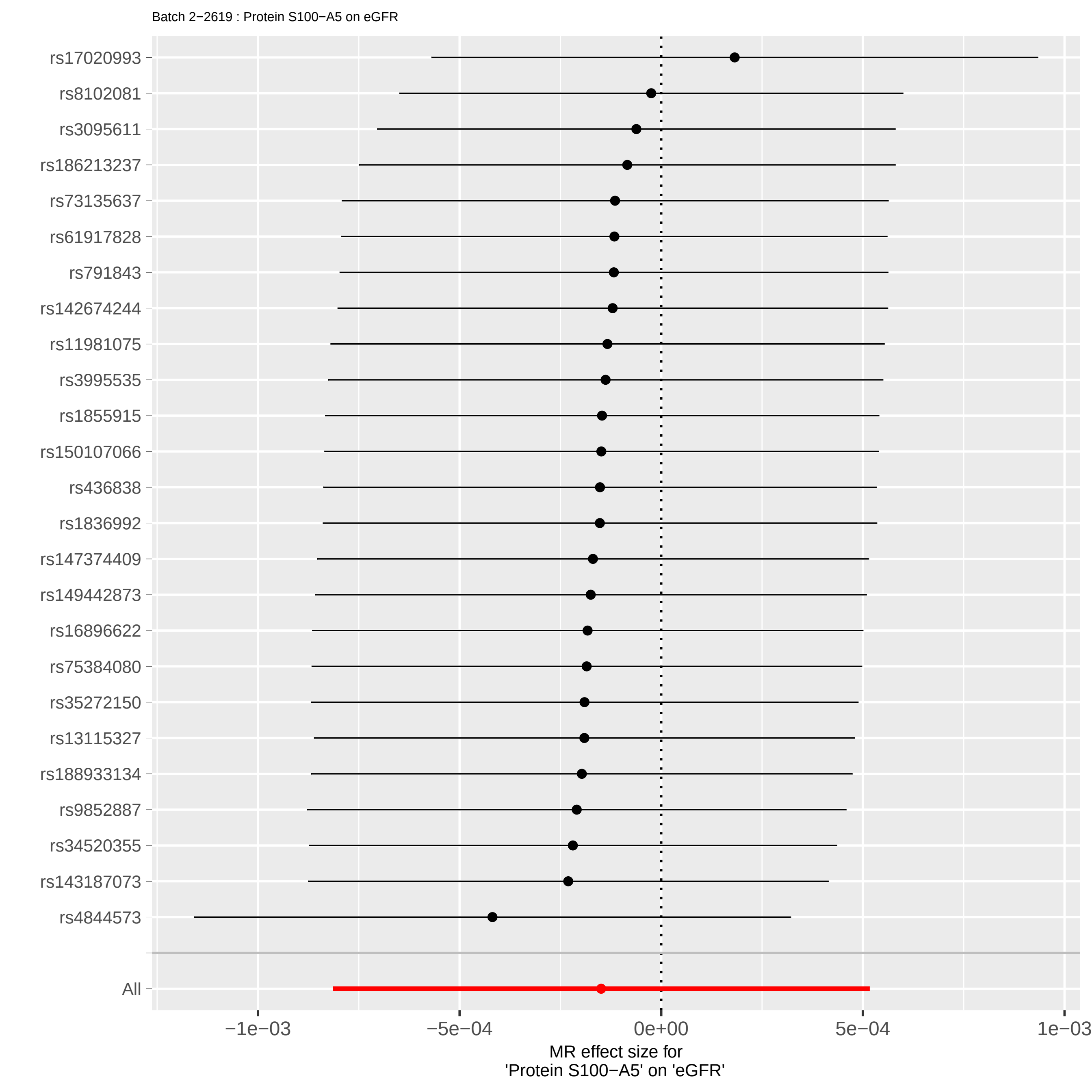

Batch 2−2619 : Protein S100−A5 on eGFR
rs17020993
rs8102081
rs3095611
rs186213237
rs73135637
rs61917828
rs791843
rs142674244
rs11981075
rs3995535
rs1855915
rs150107066
rs436838
rs1836992
rs147374409
rs149442873
rs16896622
rs75384080
rs35272150
rs13115327
rs188933134
rs9852887
rs34520355
rs143187073
rs4844573
All
−1e−03
−5e−04
0e+00
5e−04
1e−03
MR effect size for
'Protein S100−A5' on 'eGFR'

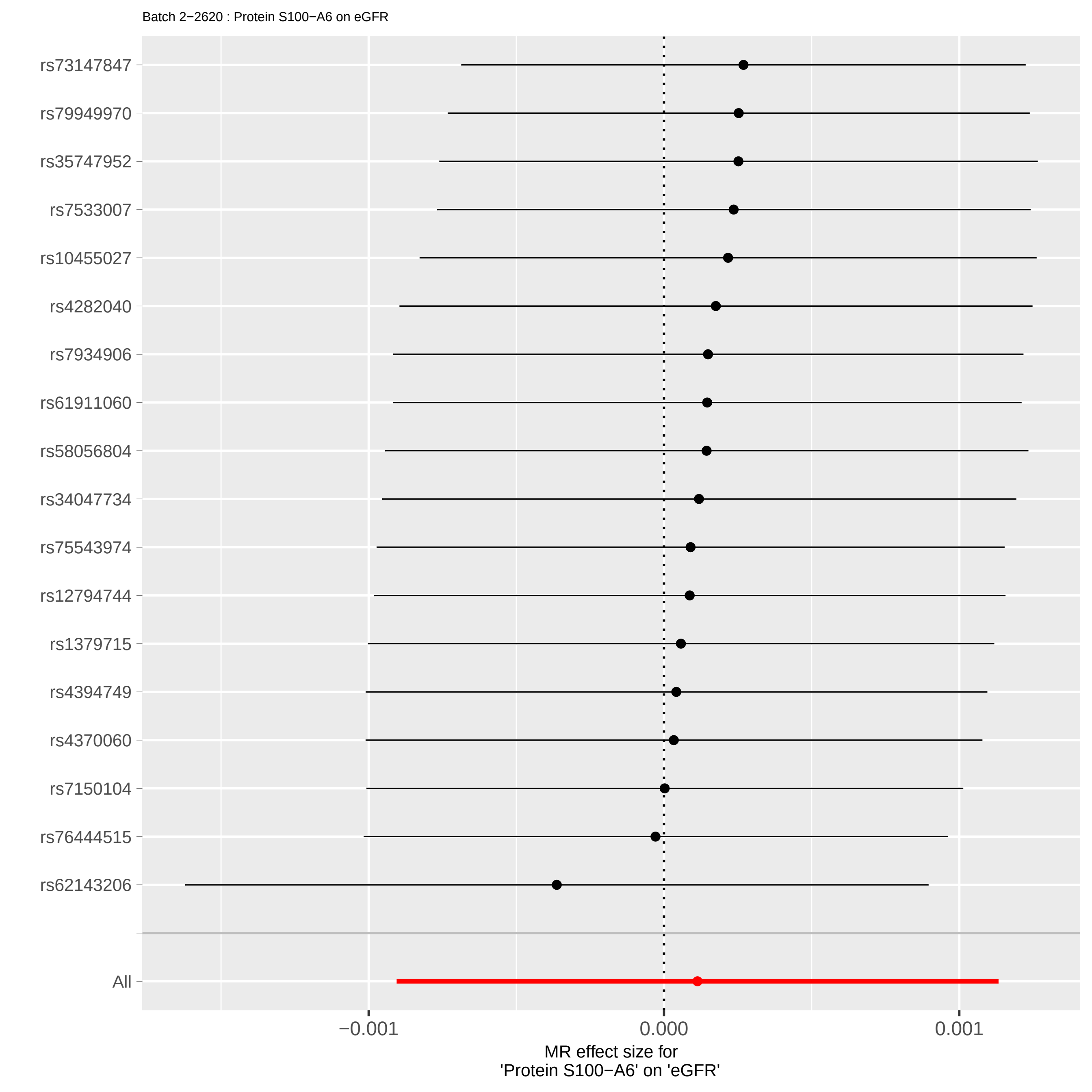

Batch 2−2620 : Protein S100−A6 on eGFR
rs73147847
rs79949970
rs35747952
rs7533007
rs10455027
rs4282040
rs7934906
rs61911060
rs58056804
rs34047734
rs75543974
rs12794744
rs1379715
rs4394749
rs4370060
rs7150104
rs76444515
rs62143206
All
−0.001
0.000
0.001
MR effect size for
'Protein S100−A6' on 'eGFR'

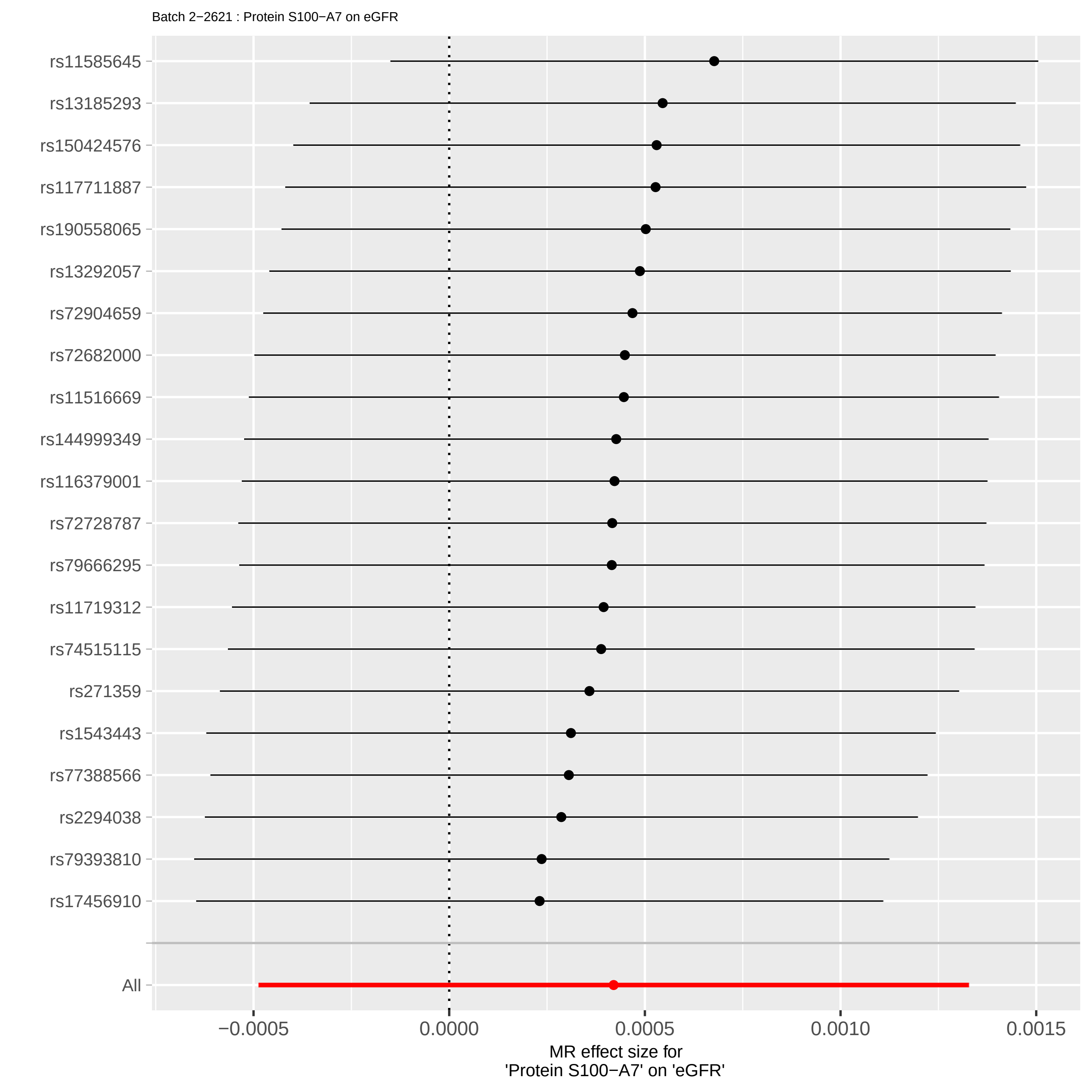

Batch 2−2621 : Protein S100−A7 on eGFR
rs11585645
rs13185293
rs150424576
rs117711887
rs190558065
rs13292057
rs72904659
rs72682000
rs11516669
rs144999349
rs116379001
rs72728787
rs79666295
rs11719312
rs74515115
rs271359
rs1543443
rs77388566
rs2294038
rs79393810
rs17456910
All
−0.0005
0.0000
0.0005
0.0010
0.0015
MR effect size for
'Protein S100−A7' on 'eGFR'

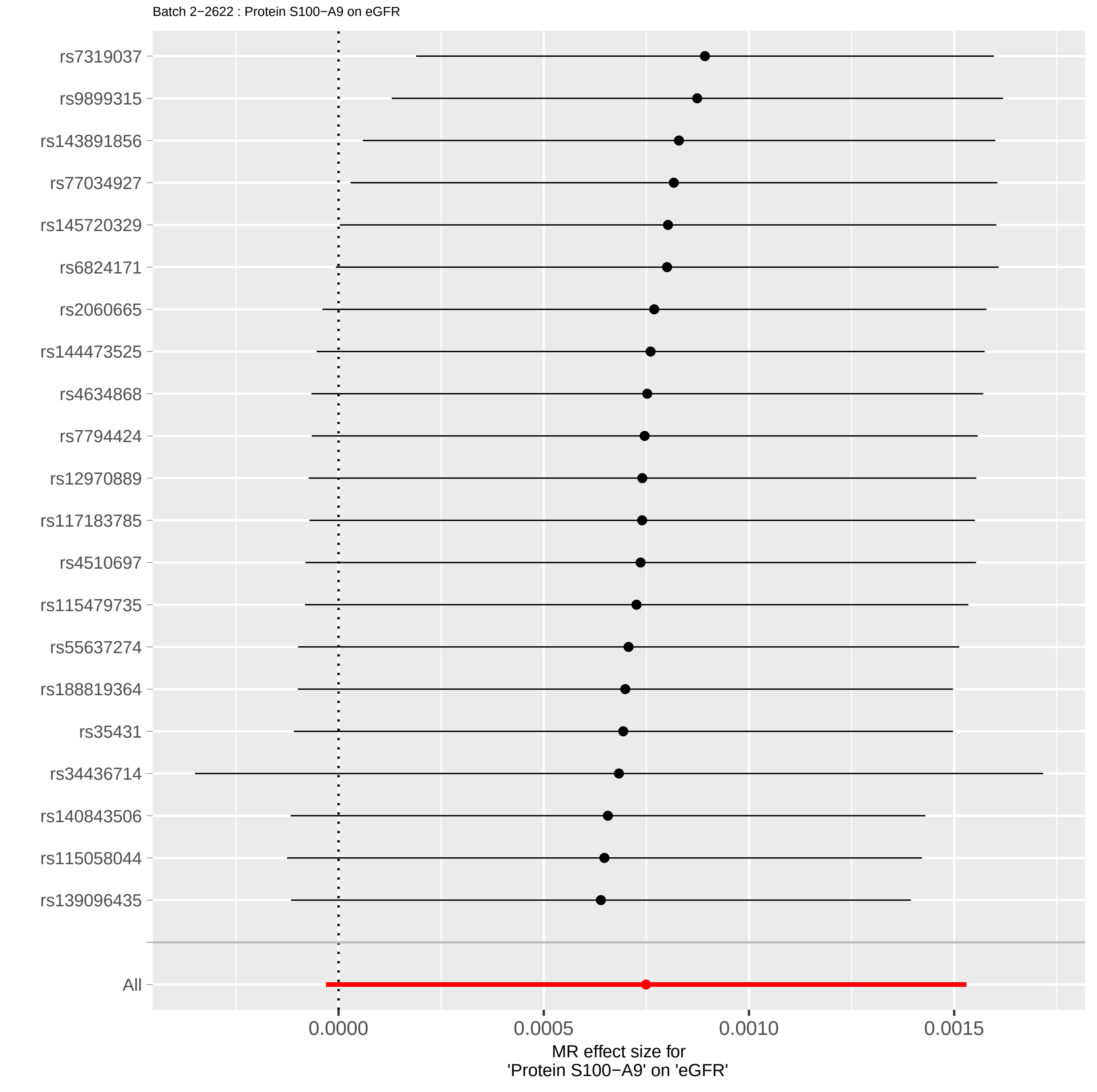

Batch 2−2622 : Protein S100−A9 on eGFR
rs7319037
rs9899315
rs143891856
rs77034927
rs145720329
rs6824171
rs2060665
rs144473525
rs4634868
rs7794424
rs12970889
rs117183785
rs4510697
rs115479735
rs55637274
rs188819364
rs35431
rs34436714
rs140843506
rs115058044
rs139096435
All
0.0000
0.0005
0.0010
0.0015
MR effect size for
'Protein S100−A9' on 'eGFR'

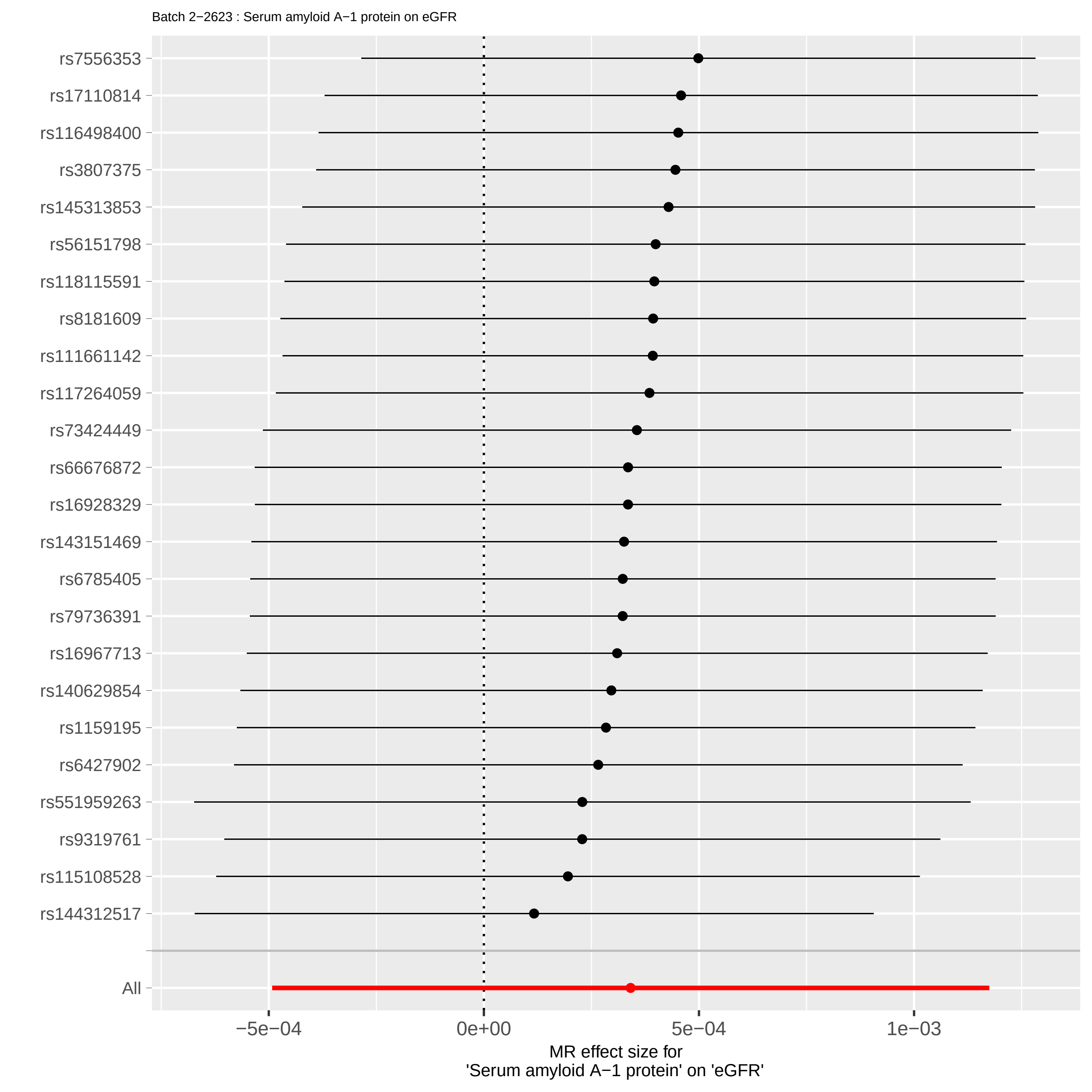

Batch 2−2623 : Serum amyloid A−1 protein on eGFR
rs7556353
rs17110814
rs116498400
rs3807375
rs145313853
rs56151798
rs118115591
rs8181609
rs111661142
rs117264059
rs73424449
rs66676872
rs16928329
rs143151469
rs6785405
rs79736391
rs16967713
rs140629854
rs1159195
rs6427902
rs551959263
rs9319761
rs115108528
rs144312517
All
−5e−04
0e+00
5e−04
1e−03
MR effect size for
'Serum amyloid A−1 protein' on 'eGFR'

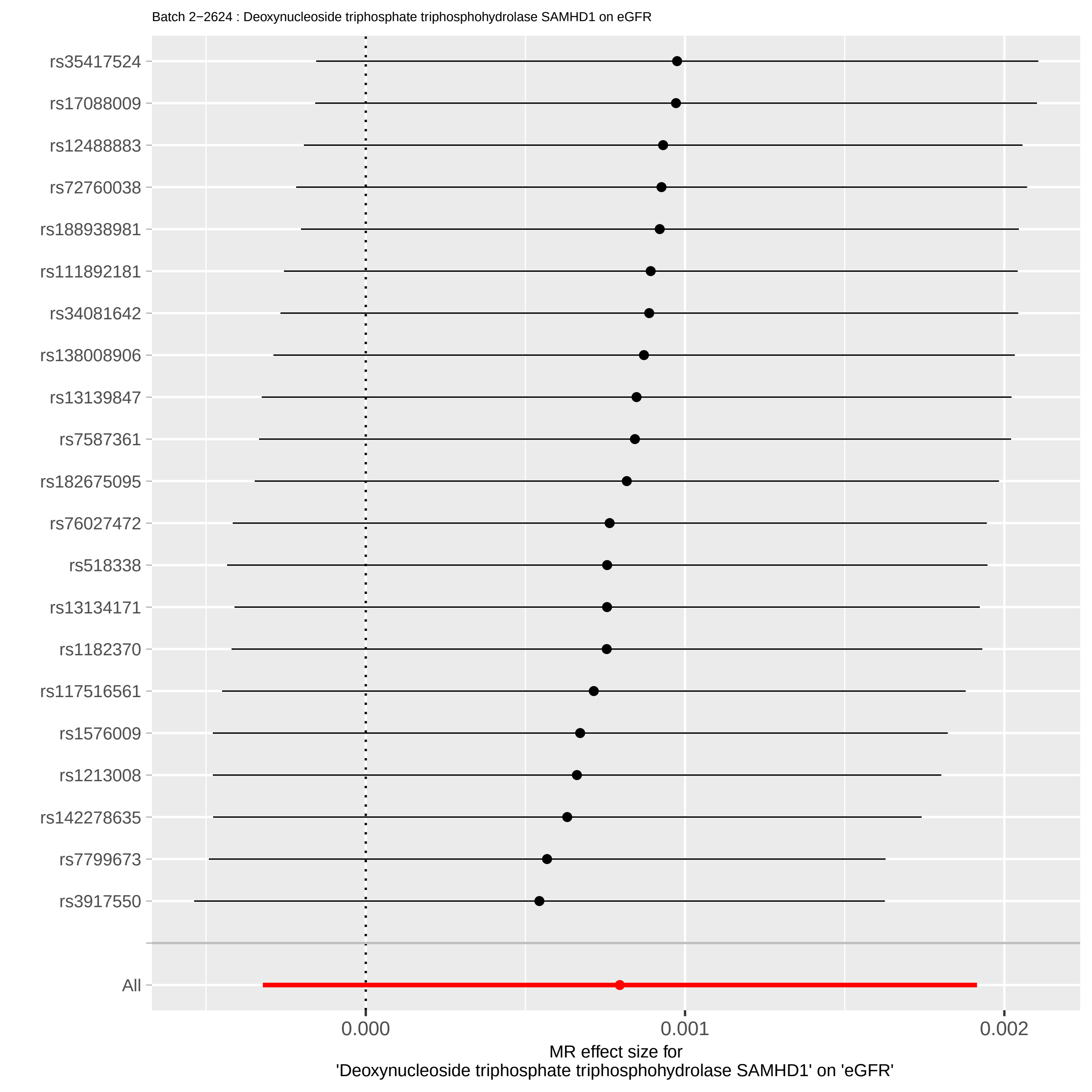

Batch 2−2624 : Deoxynucleoside triphosphate triphosphohydrolase SAMHD1 on eGFR
rs35417524
rs17088009
rs12488883
rs72760038
rs188938981
rs111892181
rs34081642
rs138008906
rs13139847
rs7587361
rs182675095
rs76027472
rs518338
rs13134171
rs1182370
rs117516561
rs1576009
rs1213008
rs142278635
rs7799673
rs3917550
All
0.000
0.001
0.002
MR effect size for
'Deoxynucleoside triphosphate triphosphohydrolase SAMHD1' on 'eGFR'

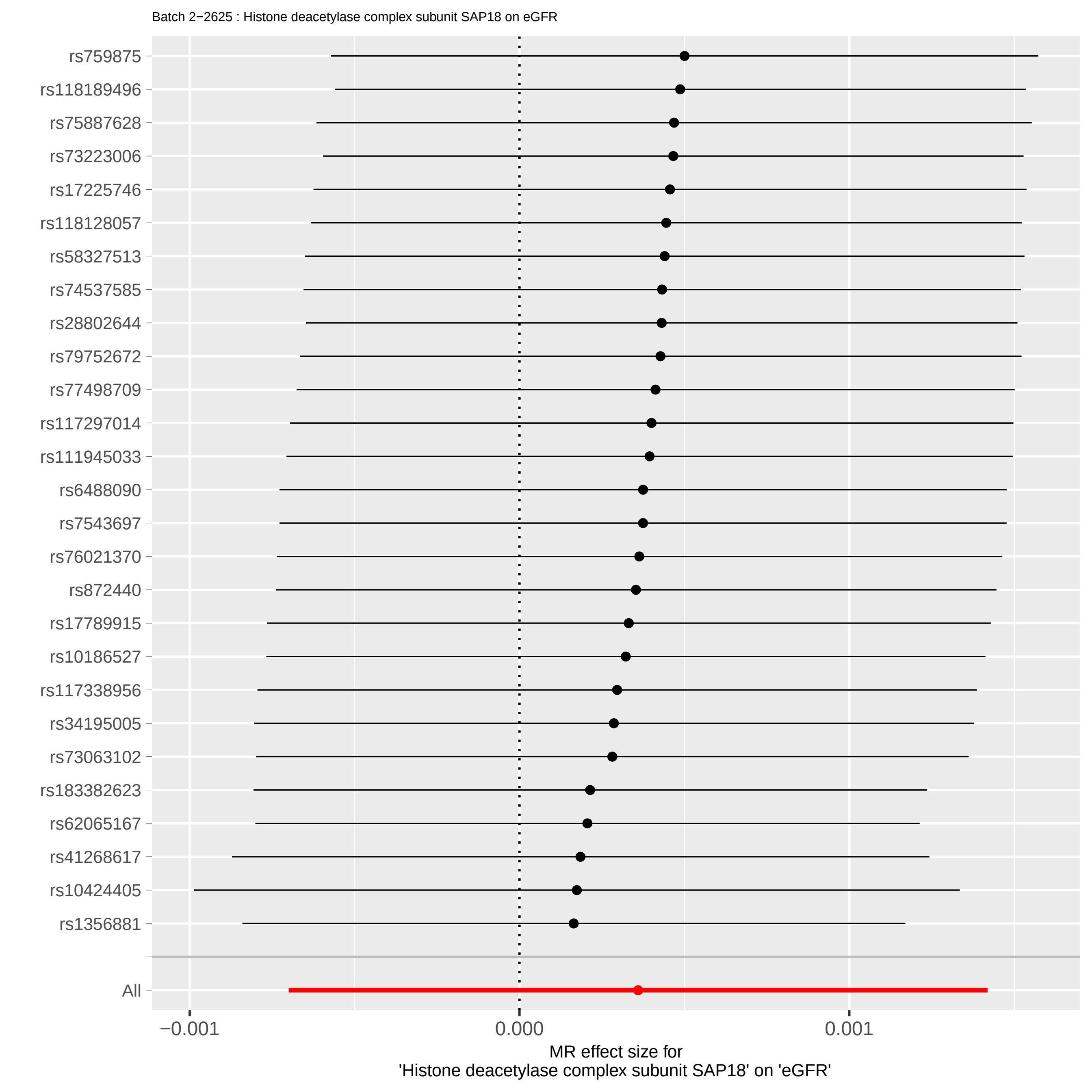
Batch 2−2625 : Histone deacetylase complex subunit SAP18 on eGFR
rs759875
rs118189496
rs75887628
rs73223006
rs17225746
rs118128057
rs58327513
rs74537585
rs28802644
rs79752672
rs77498709
rs117297014
rs111945033
rs6488090
rs7543697
rs76021370
rs872440
rs17789915
rs10186527
rs117338956
rs34195005
rs73063102
rs183382623
rs62065167
rs41268617
rs10424405
rs1356881
All
−0.001
0.000
0.001
MR effect size for
'Histone deacetylase complex subunit SAP18' on 'eGFR'

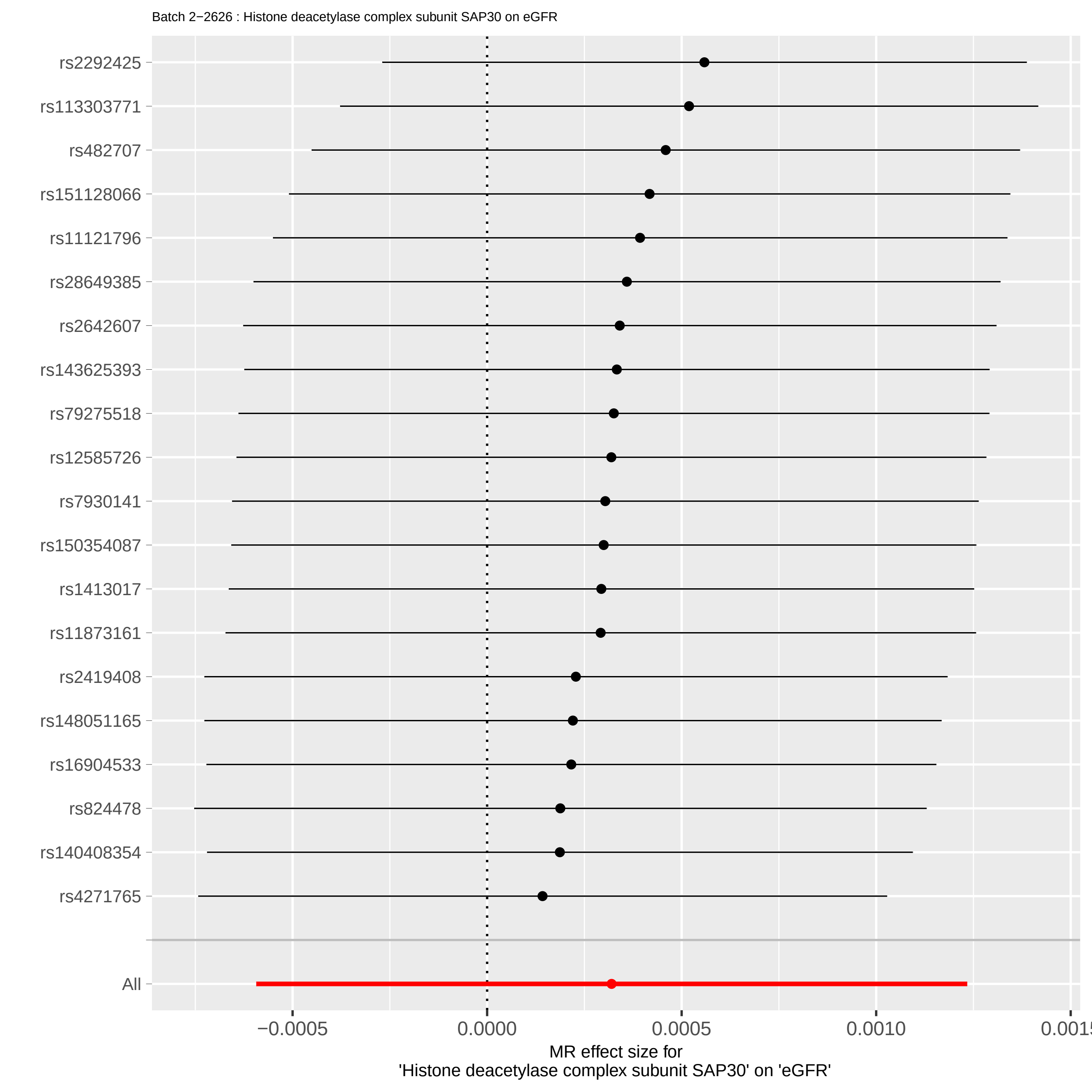
Batch 2−2626 : Histone deacetylase complex subunit SAP30 on eGFR
rs2292425
rs113303771
rs482707
rs151128066
rs11121796
rs28649385
rs2642607
rs143625393
rs79275518
rs12585726
rs7930141
rs150354087
rs1413017
rs11873161
rs2419408
rs148051165
rs16904533
rs824478
rs140408354
rs4271765
All
−0.0005
0.0000
0.0005
0.0010
MR effect size for
'Histone deacetylase complex subunit SAP30' on 'eGFR'

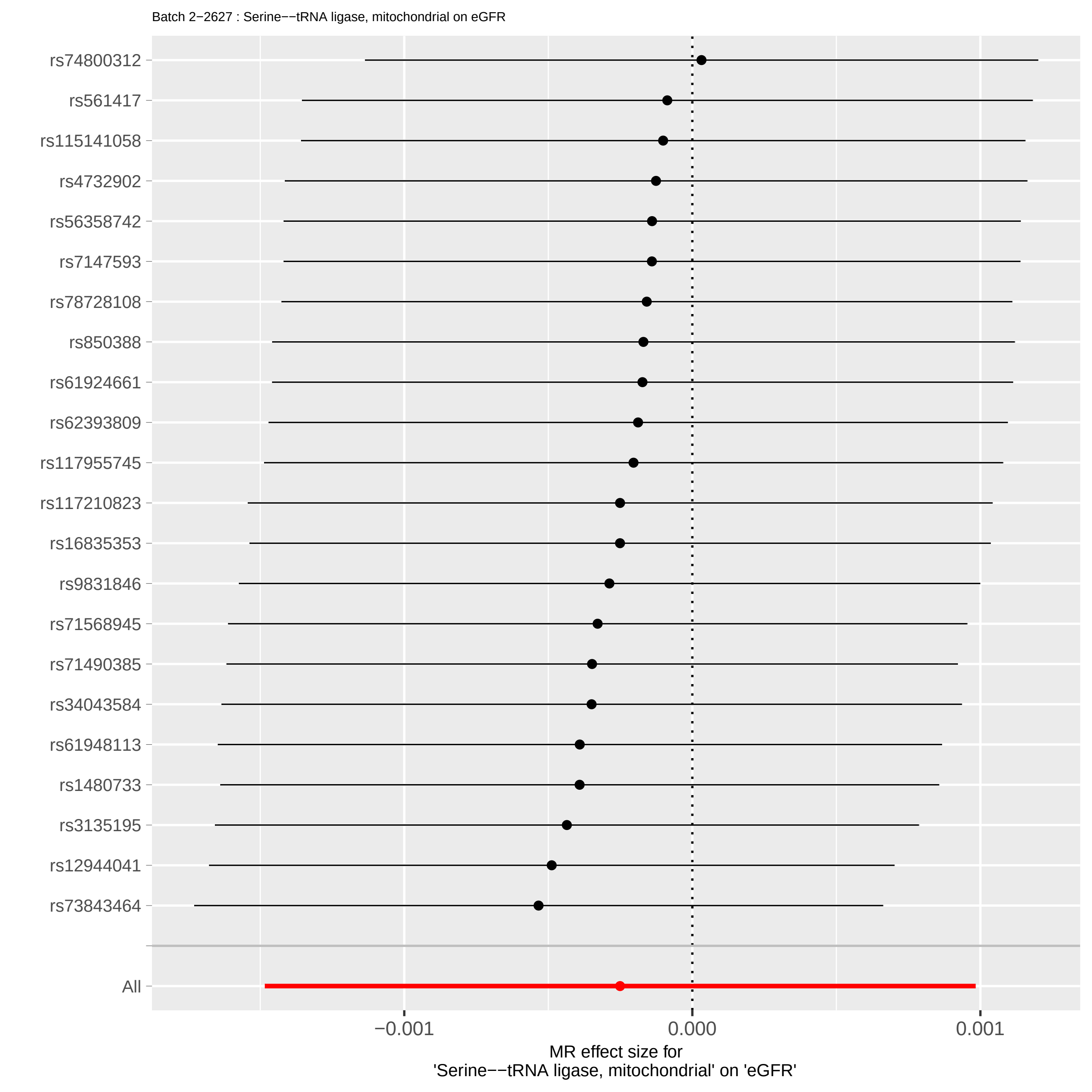

Batch 2−2627 : Serine−−tRNA ligase, mitochondrial on eGFR
rs74800312
rs561417
rs115141058
rs4732902
rs56358742
rs7147593
rs78728108
rs850388
rs61924661
rs62393809
rs117955745
rs117210823
rs16835353
rs9831846
rs71568945
rs71490385
rs34043584
rs61948113
rs1480733
rs3135195
rs12944041
rs73843464
All
−0.001
0.000
0.001
MR effect size for
'Serine−−tRNA ligase, mitochondrial' on 'eGFR'

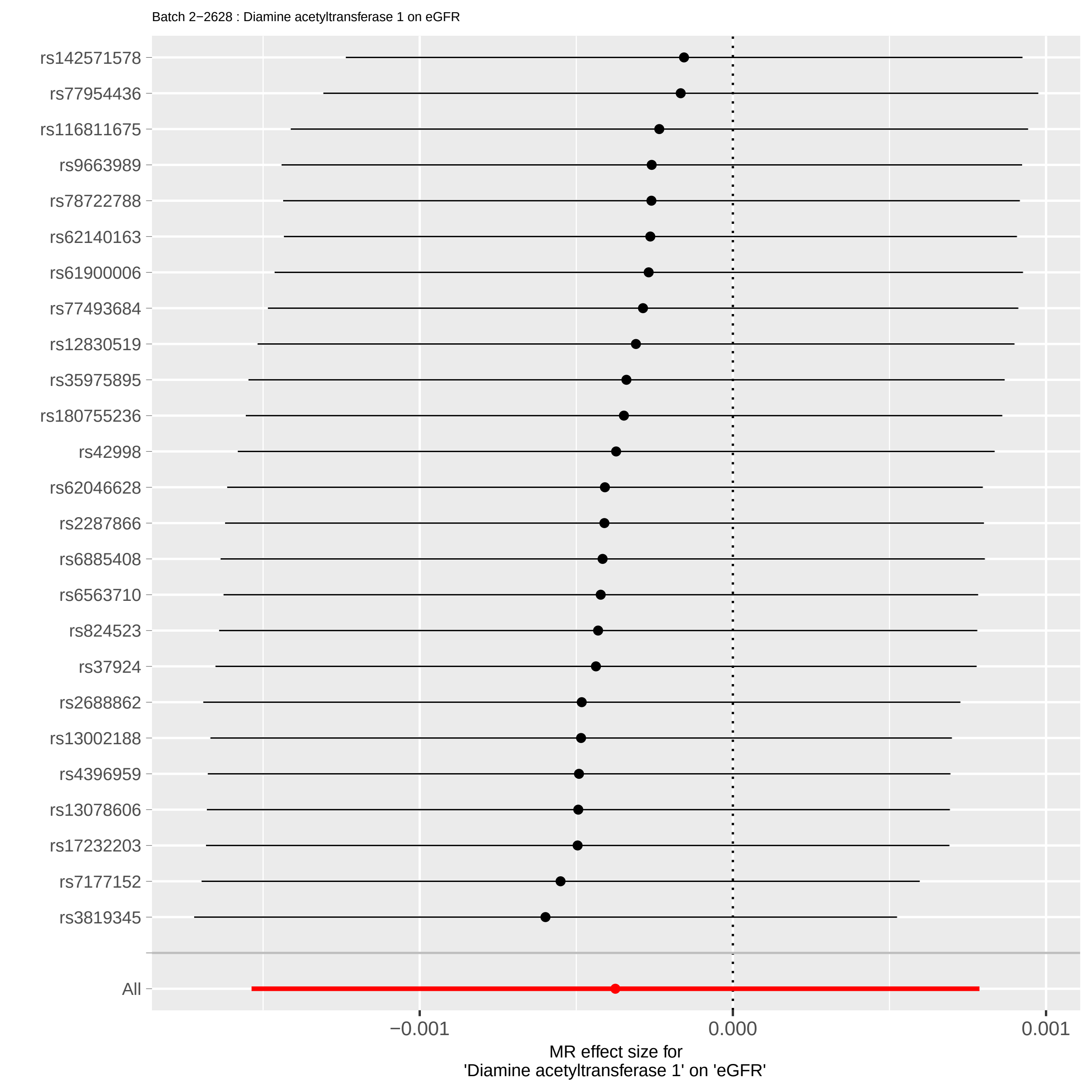

Batch 2−2628 : Diamine acetyltransferase 1 on eGFR
rs142571578
rs77954436
rs116811675
rs9663989
rs78722788
rs62140163
rs61900006
rs77493684
rs12830519
rs35975895
rs180755236
rs42998
rs62046628
rs2287866
rs6885408
rs6563710
rs824523
rs37924
rs2688862
rs13002188
rs4396959
rs13078606
rs17232203
rs7177152
rs3819345
All
−0.001
0.000
0.001
MR effect size for
'Diamine acetyltransferase 1' on 'eGFR'

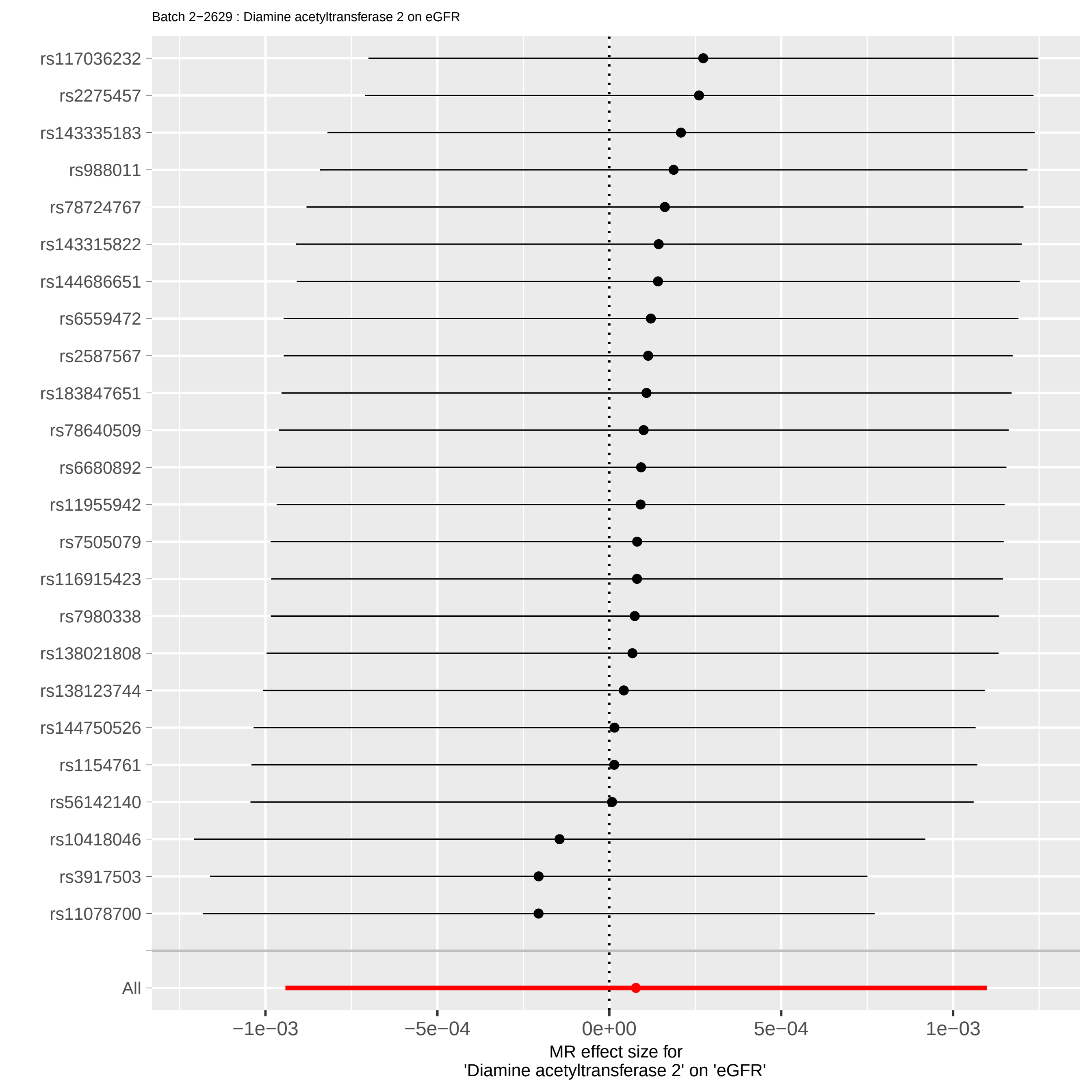

Batch 2−2629 : Diamine acetyltransferase 2 on eGFR
rs117036232
rs2275457
rs143335183
rs988011
rs78724767
rs143315822
rs144686651
rs6559472
rs2587567
rs183847651
rs78640509
rs6680892
rs11955942
rs7505079
rs116915423
rs7980338
rs138021808
rs138123744
rs144750526
rs1154761
rs56142140
rs10418046
rs3917503
rs11078700
All
−1e−03
−5e−04
0e+00
5e−04
1e−03
MR effect size for
'Diamine acetyltransferase 2' on 'eGFR'

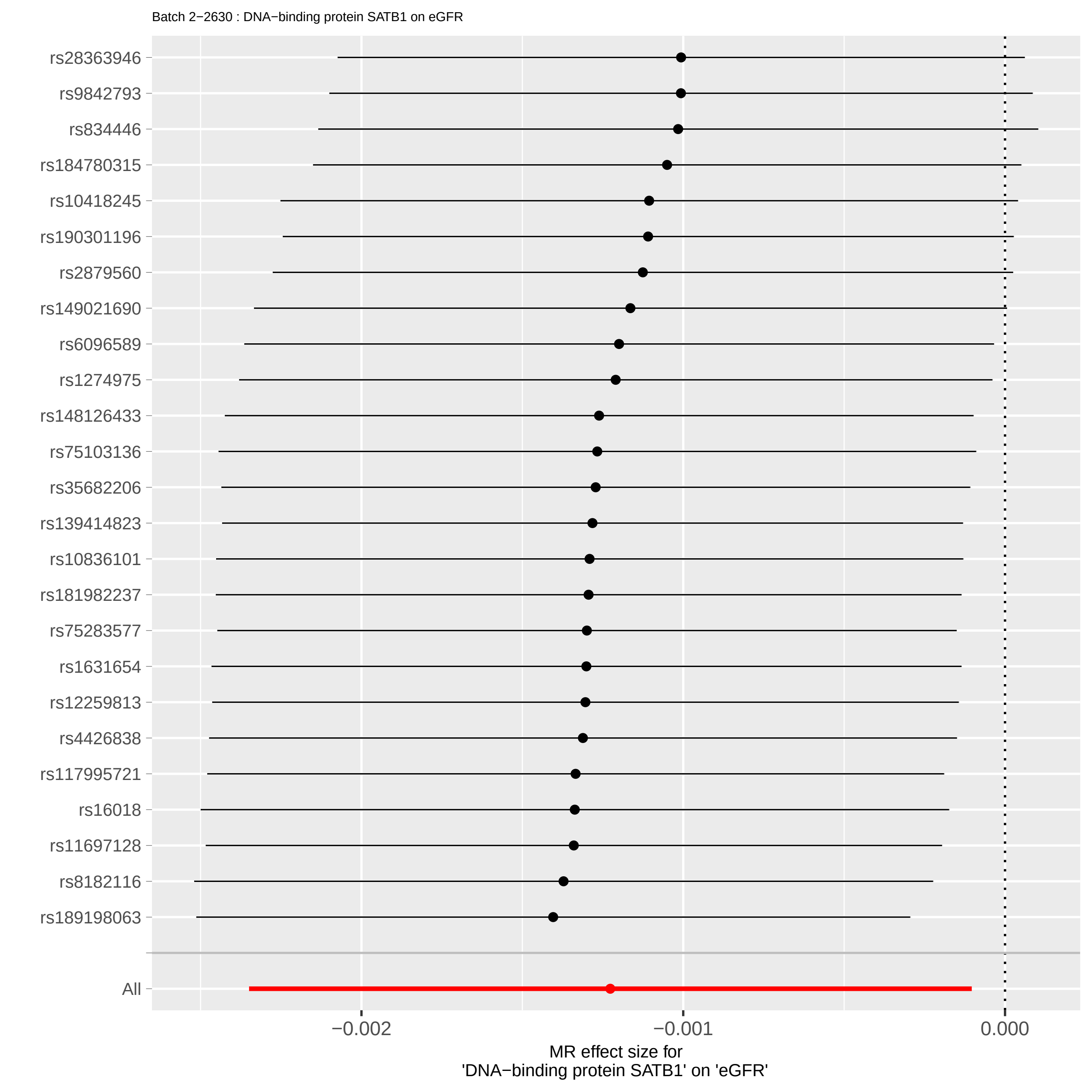

Batch 2−2630 : DNA−binding protein SATB1 on eGFR
rs28363946
rs9842793
rs834446
rs184780315
rs10418245
rs190301196
rs2879560
rs149021690
rs6096589
rs1274975
rs148126433
rs75103136
rs35682206
rs139414823
rs10836101
rs181982237
rs75283577
rs1631654
rs12259813
rs4426838
rs117995721
rs16018
rs11697128
rs8182116
rs189198063
All
−0.002
−0.001
0.000
MR effect size for
'DNA−binding protein SATB1' on 'eGFR'

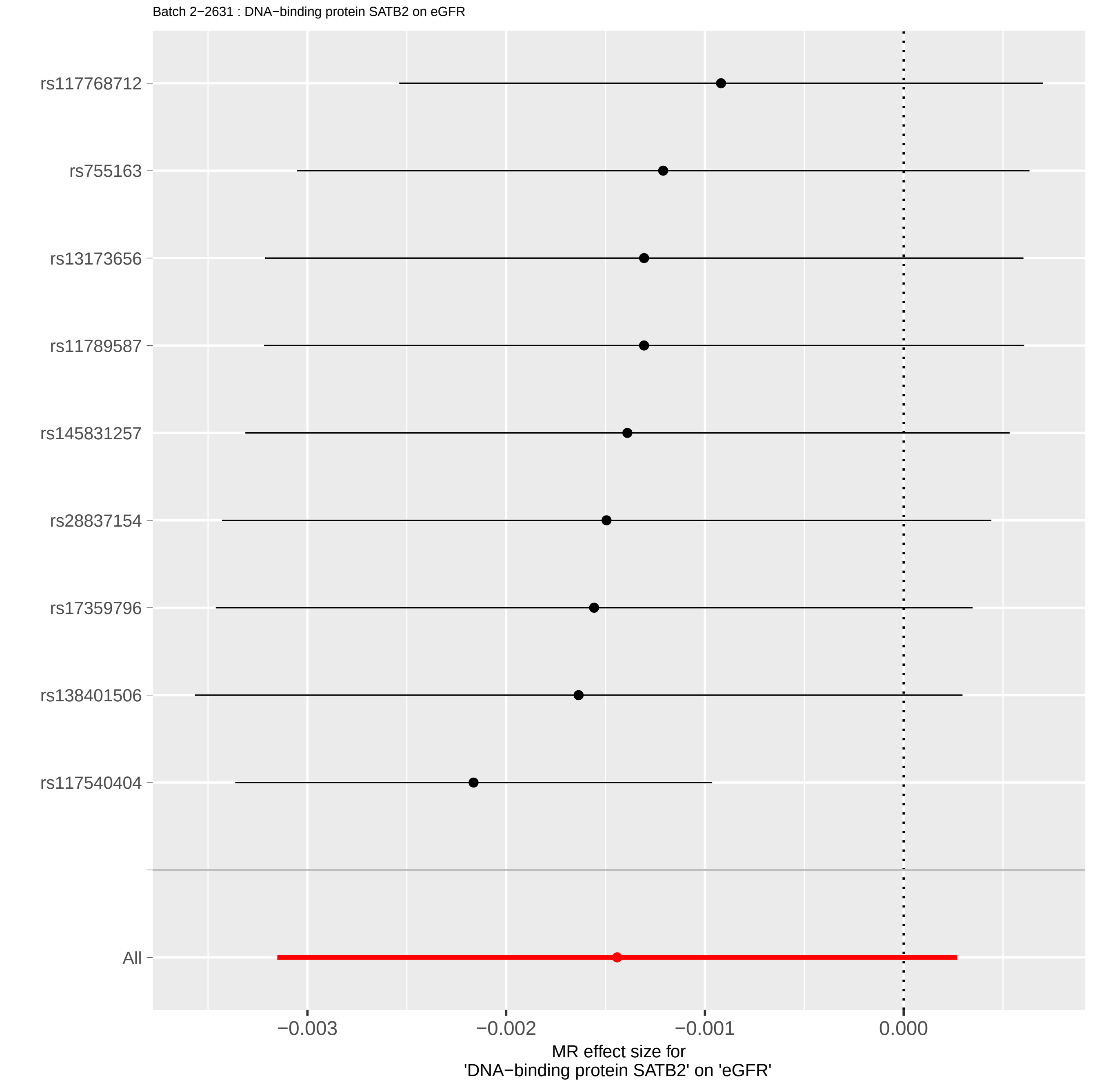

Batch 2−2631 : DNA−binding protein SATB2 on eGFR
rs117768712
rs755163
rs13173656
rs11789587
rs145831257
rs28837154
rs17359796
rs138401506
rs117540404
All
−0.003
−0.002
−0.001
0.000
MR effect size for
'DNA−binding protein SATB2' on 'eGFR'

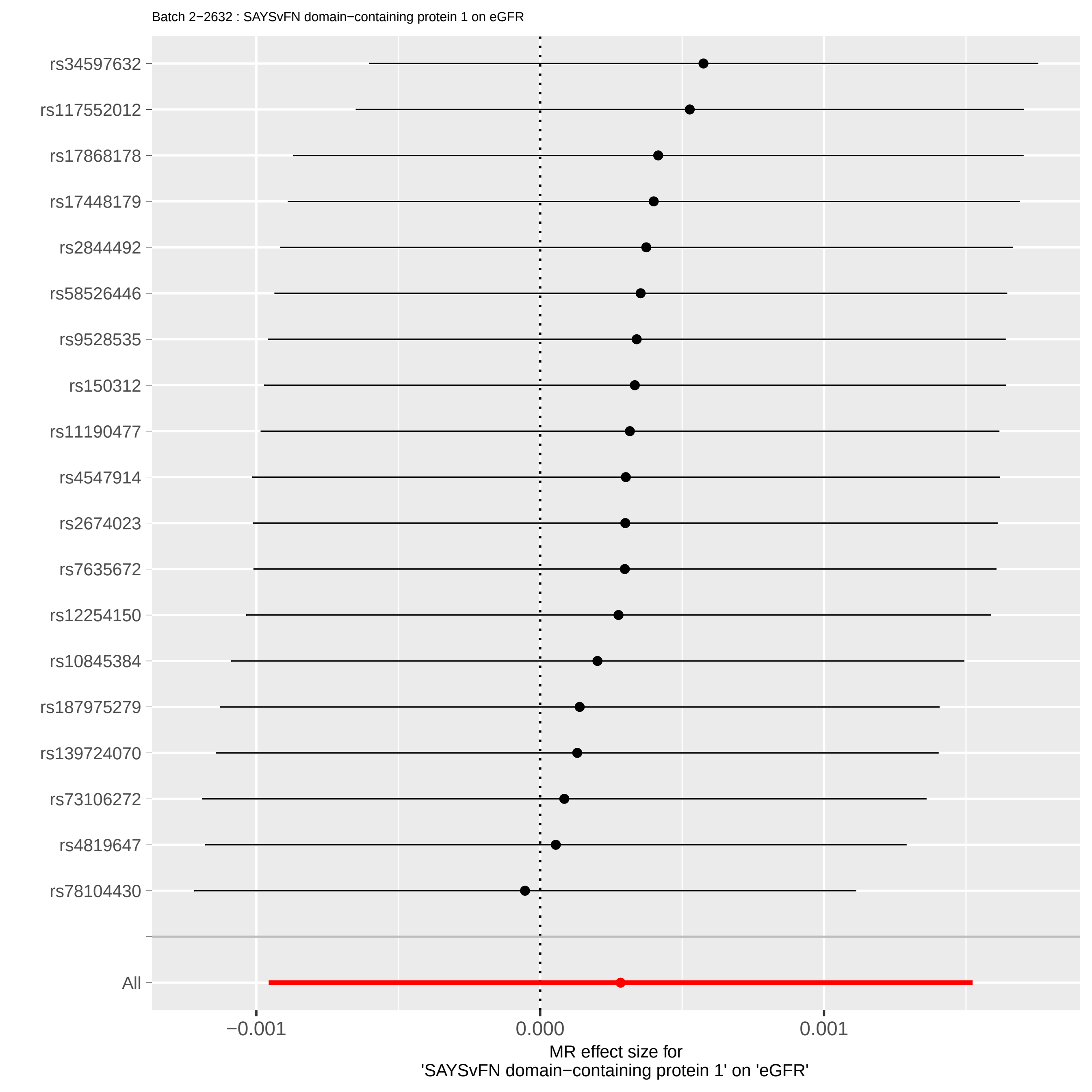
Batch 2−2632 : SAYSvFN domain−containing protein 1 on eGFR
rs34597632
rs117552012
rs17868178
rs17448179
rs2844492
rs58526446
rs9528535
rs150312
rs11190477
rs4547914
rs2674023
rs7635672
rs12254150
rs10845384
rs187975279
rs139724070
rs73106272
rs4819647
rs78104430
All
−0.001
0.000
0.001
MR effect size for
'SAYSvFN domain−containing protein 1' on 'eGFR'

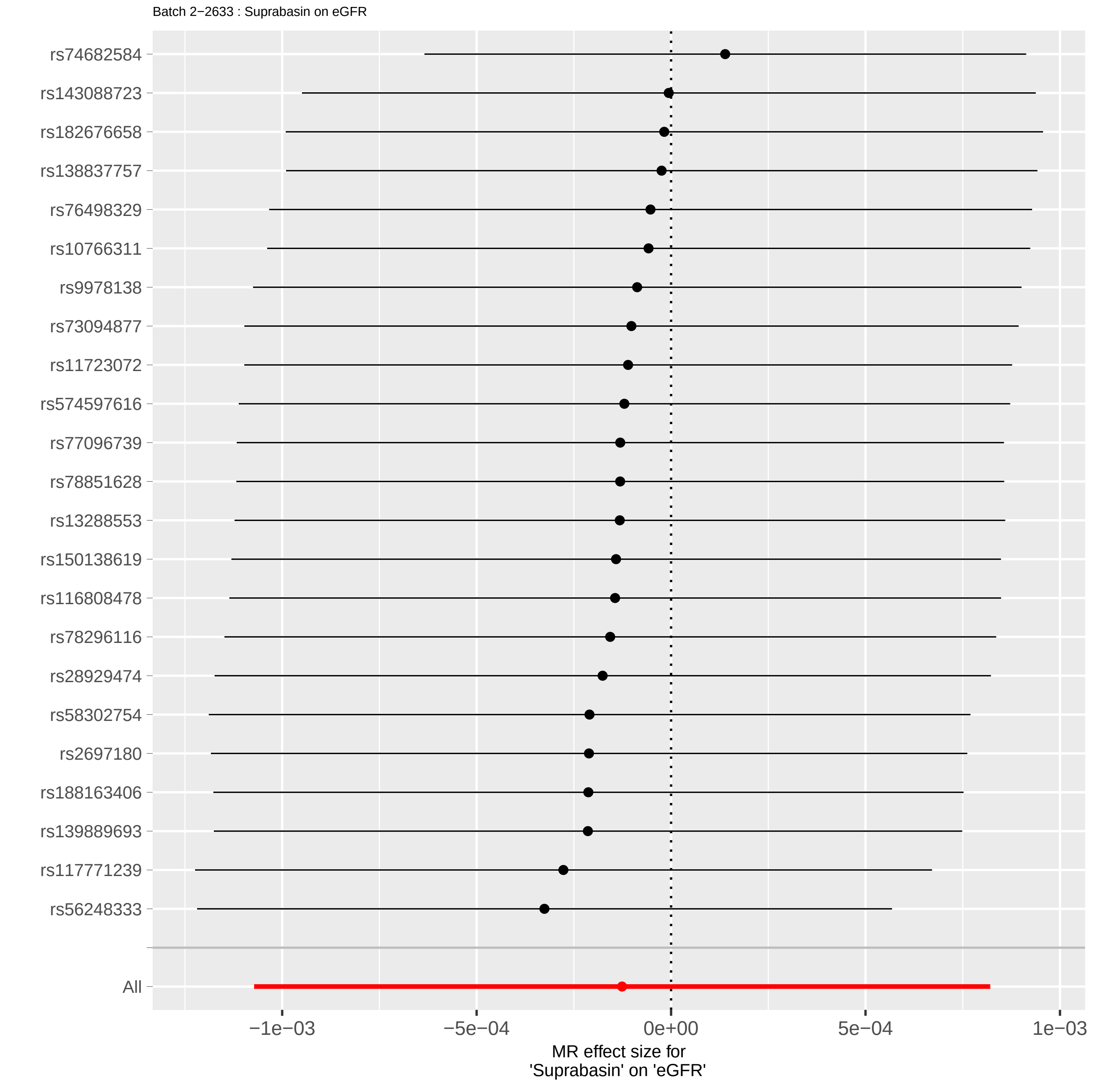

Batch 2−2633 : Suprabasin on eGFR
rs74682584
rs143088723
rs182676658
rs138837757
rs76498329
rs10766311
rs9978138
rs73094877
rs11723072
rs574597616
rs77096739
rs78851628
rs13288553
rs150138619
rs116808478
rs78296116
rs28929474
rs58302754
rs2697180
rs188163406
rs139889693
rs117771239
rs56248333
All
−1e−03
−5e−04
0e+00
5e−04
1e−03
MR effect size for
'Suprabasin' on 'eGFR'

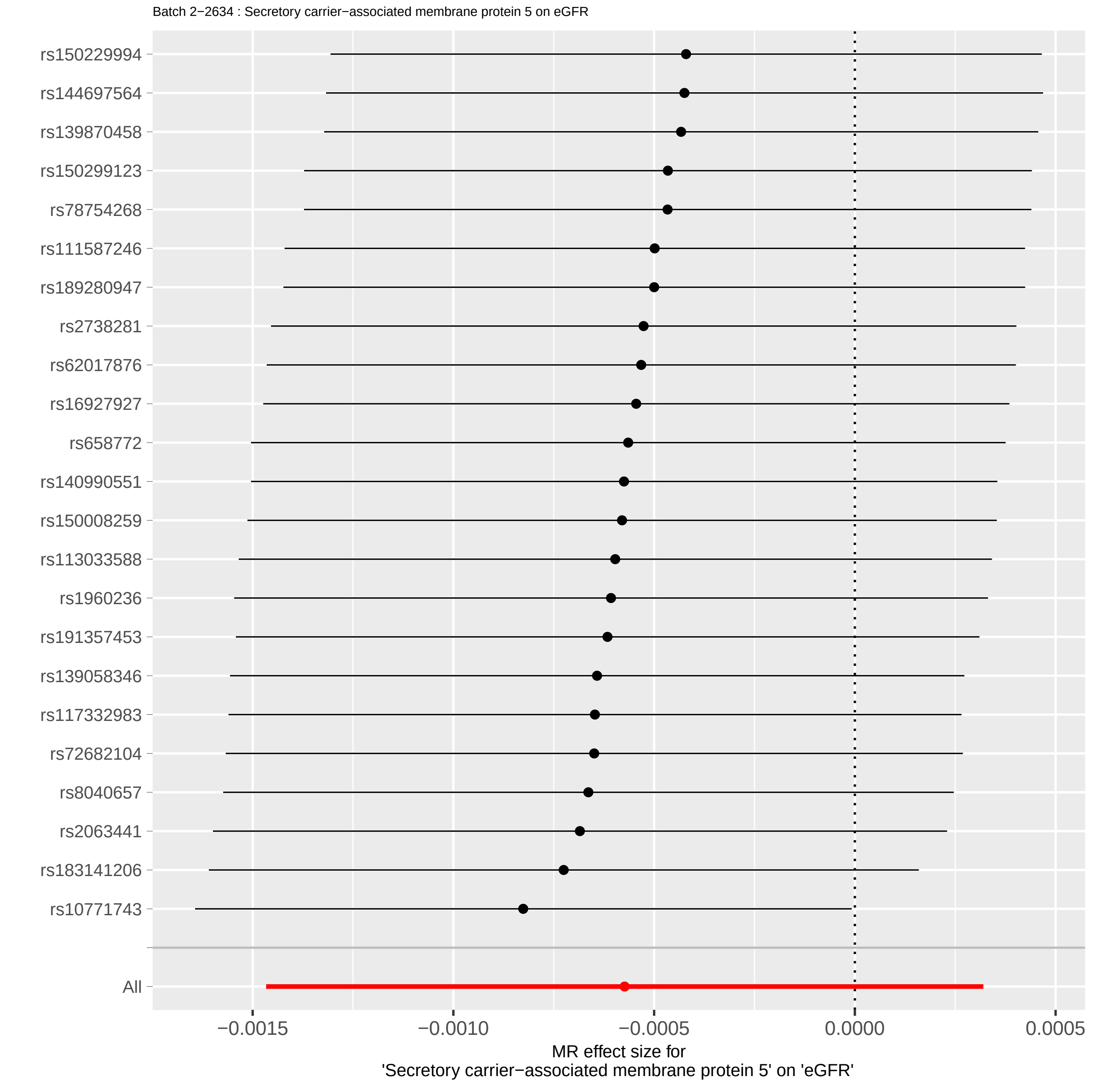
Batch 2−2634 : Secretory carrier−associated membrane protein 5 on eGFR
rs150229994
rs144697564
rs139870458
rs150299123
rs78754268
rs111587246
rs189280947
rs2738281
rs62017876
rs16927927
rs658772
rs140990551
rs150008259
rs113033588
rs1960236
rs191357453
rs139058346
rs117332983
rs72682104
rs8040657
rs2063441
rs183141206
rs10771743
All
−0.0015
−0.0010
−0.0005
0.0000
0.0005
MR effect size for
'Secretory carrier−associated membrane protein 5' on 'eGFR'

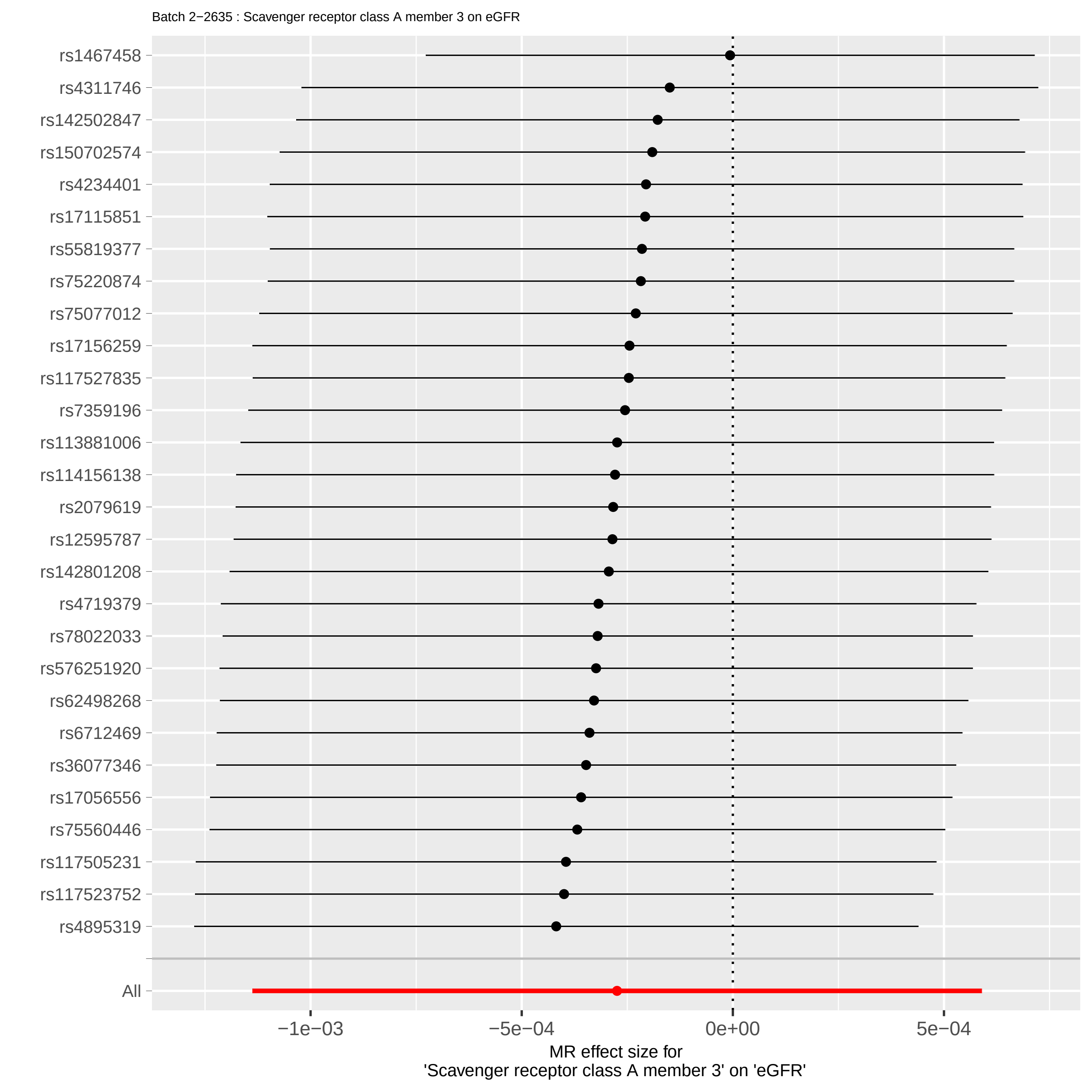

Batch 2−2635 : Scavenger receptor class A member 3 on eGFR
rs1467458
rs4311746
rs142502847
rs150702574
rs4234401
rs17115851
rs55819377
rs75220874
rs75077012
rs17156259
rs117527835
rs7359196
rs113881006
rs114156138
rs2079619
rs12595787
rs142801208
rs4719379
rs78022033
rs576251920
rs62498268
rs6712469
rs36077346
rs17056556
rs75560446
rs117505231
rs117523752
rs4895319
All
−1e−03
−5e−04
0e+00
5e−04
MR effect size for
'Scavenger receptor class A member 3' on 'eGFR'

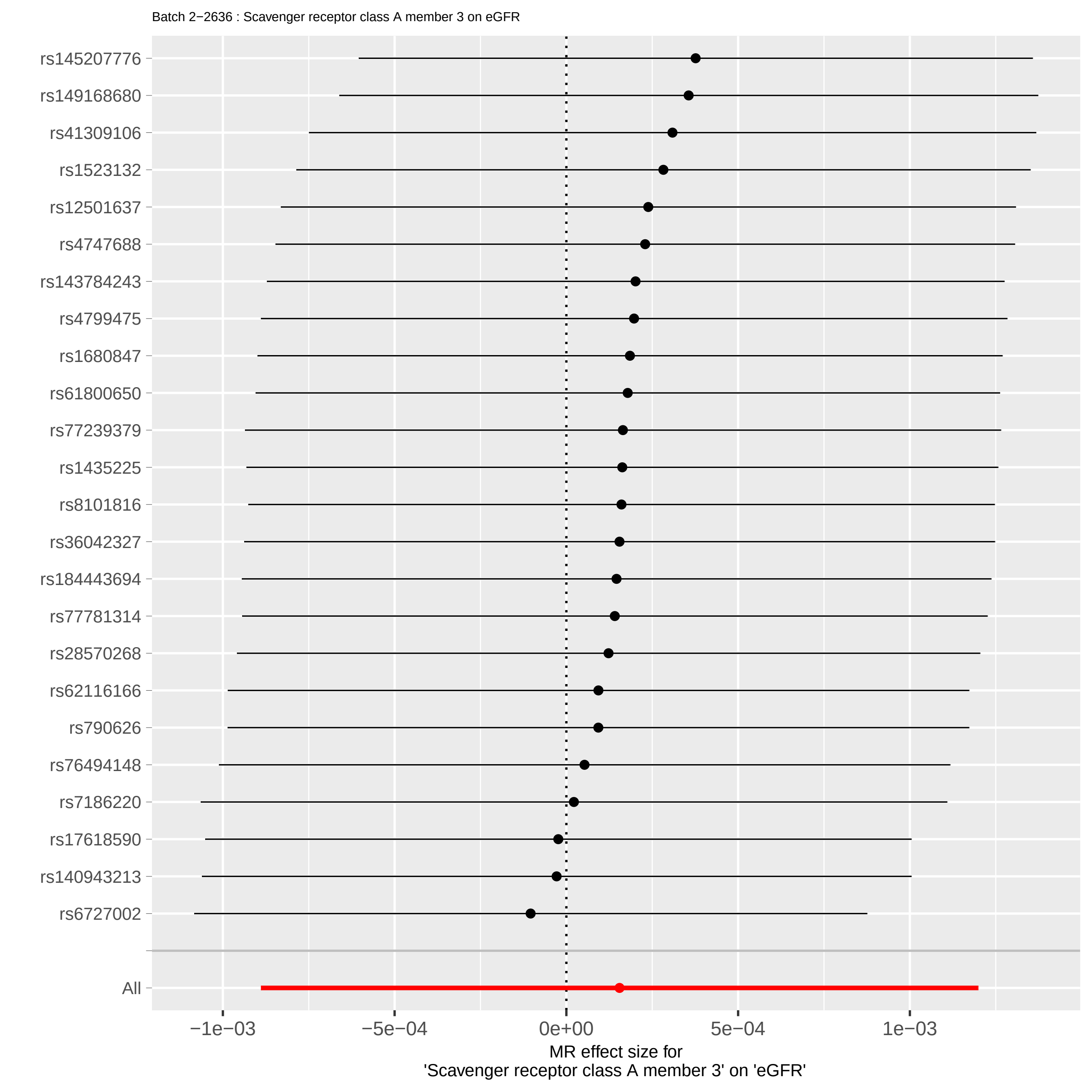
Batch 2−2636 : Scavenger receptor class A member 3 on eGFR
rs145207776
rs149168680
rs41309106
rs1523132
rs12501637
rs4747688
rs143784243
rs4799475
rs1680847
rs61800650
rs77239379
rs1435225
rs8101816
rs36042327
rs184443694
rs77781314
rs28570268
rs62116166
rs790626
rs76494148
rs7186220
rs17618590
rs140943213
rs6727002
All
−1e−03
−5e−04
0e+00
5e−04
1e−03
MR effect size for
'Scavenger receptor class A member 3' on 'eGFR'

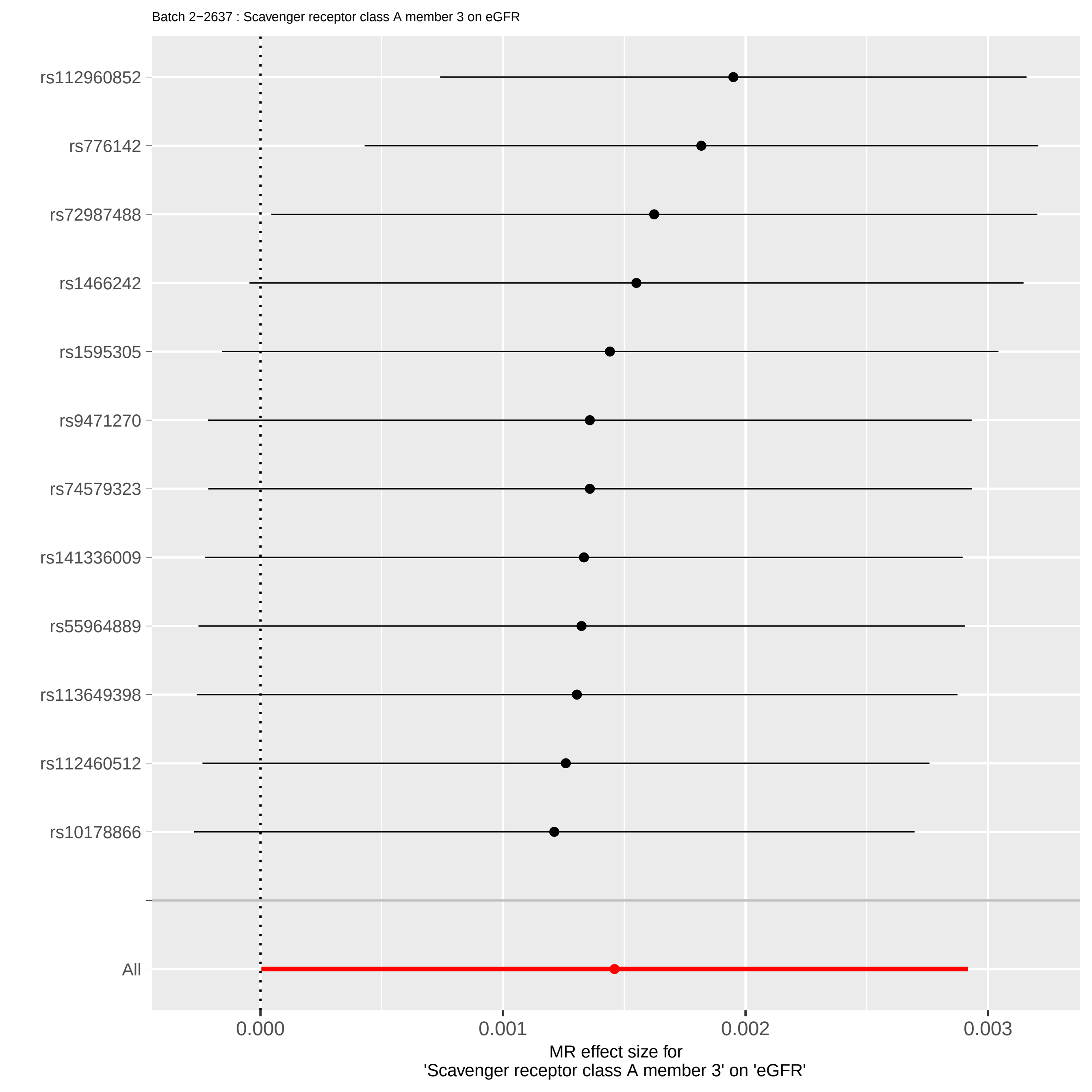
Batch 2−2637 : Scavenger receptor class A member 3 on eGFR
rs112960852
rs776142
rs72987488
rs1466242
rs1595305
rs9471270
rs74579323
rs141336009
rs55964889
rs113649398
rs112460512
rs10178866
All
0.000
0.001
0.002
0.003
MR effect size for
'Scavenger receptor class A member 3' on 'eGFR'

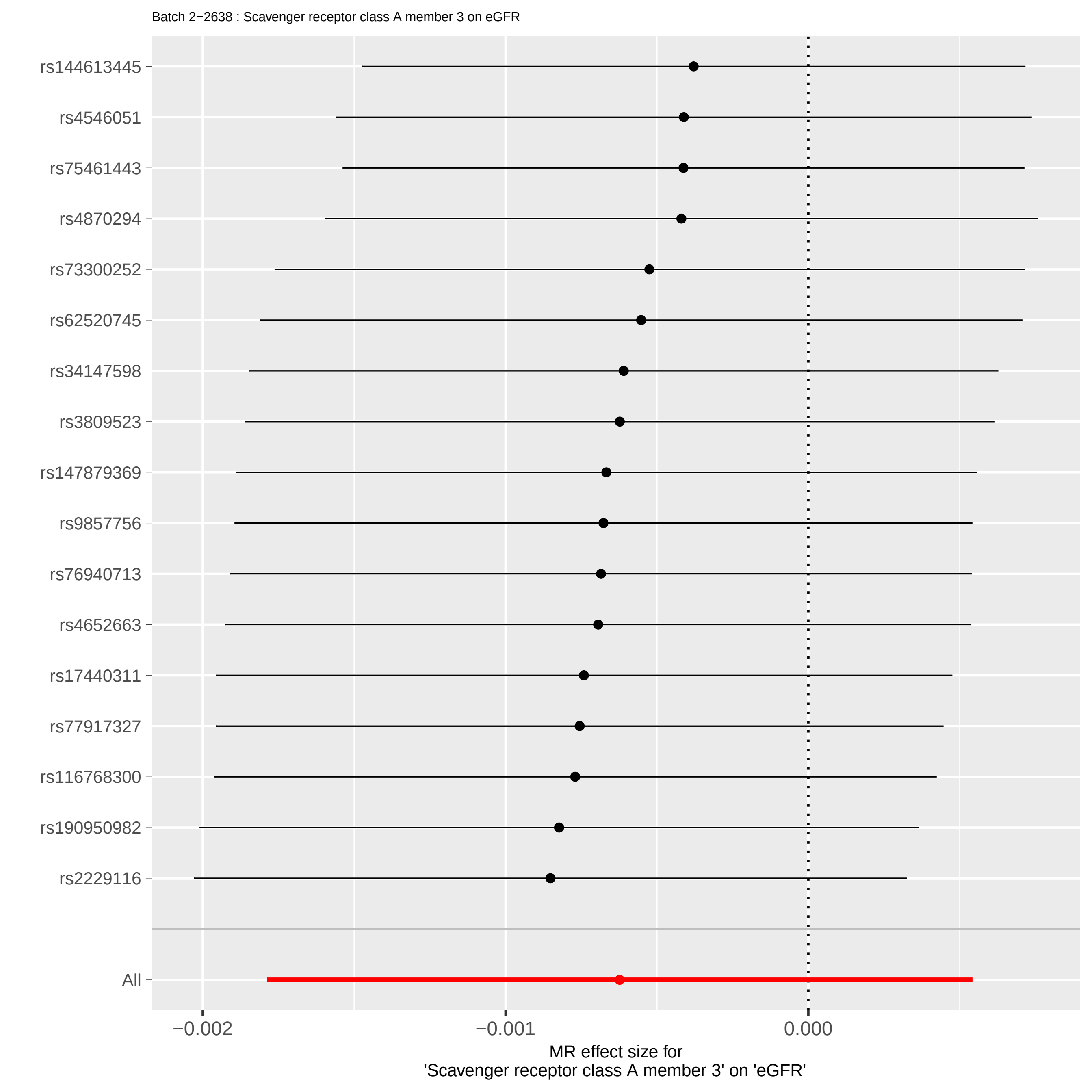

Batch 2−2638 : Scavenger receptor class A member 3 on eGFR
rs144613445
rs4546051
rs75461443
rs4870294
rs73300252
rs62520745
rs34147598
rs3809523
rs147879369
rs9857756
rs76940713
rs4652663
rs17440311
rs77917327
rs116768300
rs190950982
rs2229116
All
−0.002
−0.001
0.000
MR effect size for
'Scavenger receptor class A member 3' on 'eGFR'

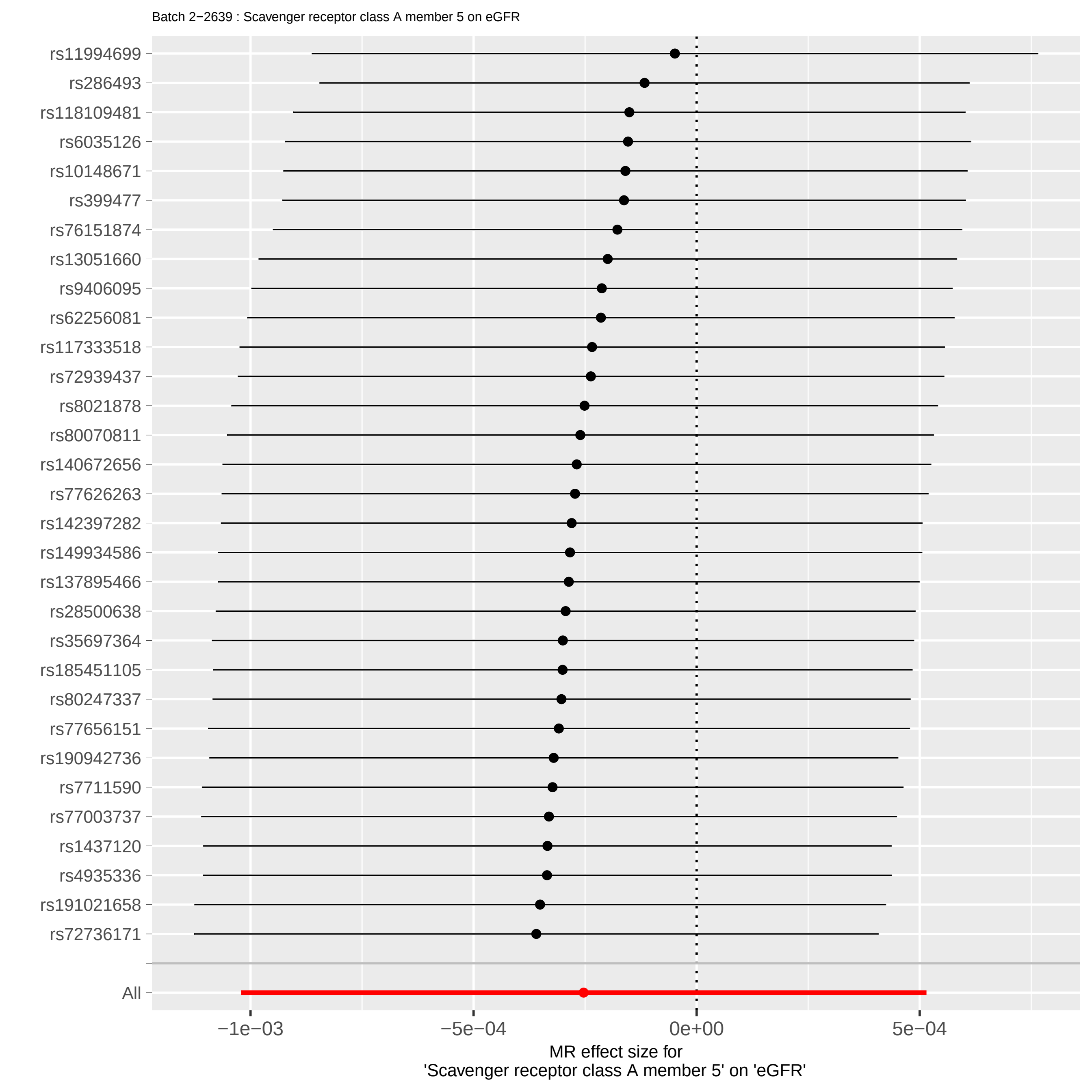

Batch 2−2639 : Scavenger receptor class A member 5 on eGFR
rs11994699
rs286493
rs118109481
rs6035126
rs10148671
rs399477
rs76151874
rs13051660
rs9406095
rs62256081
rs117333518
rs72939437
rs8021878
rs80070811
rs140672656
rs77626263
rs142397282
rs149934586
rs137895466
rs28500638
rs35697364
rs185451105
rs80247337
rs77656151
rs190942736
rs7711590
rs77003737
rs1437120
rs4935336
rs191021658
rs72736171
All
−1e−03
−5e−04
0e+00
5e−04
MR effect size for
'Scavenger receptor class A member 5' on 'eGFR'

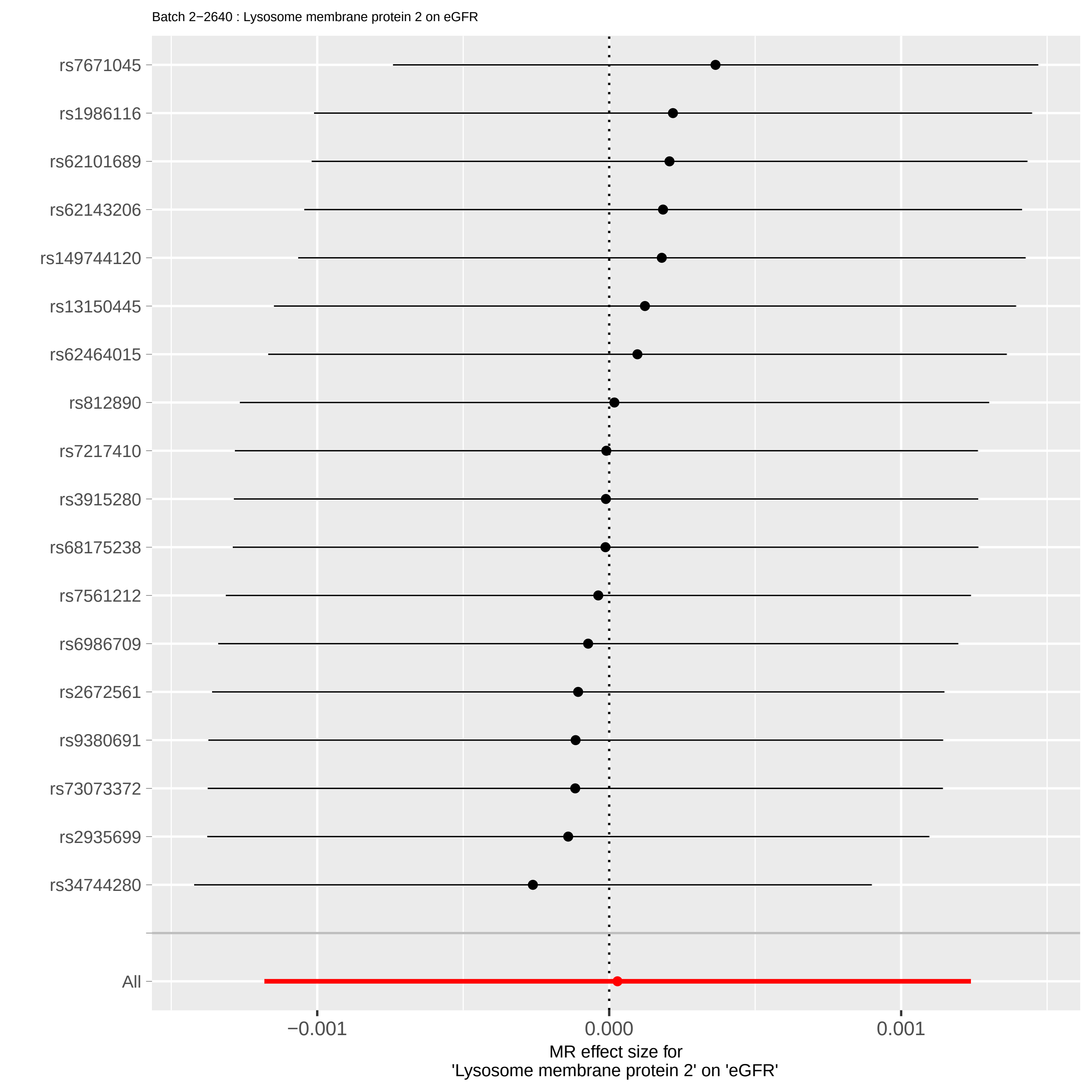

Batch 2−2640 : Lysosome membrane protein 2 on eGFR
rs7671045
rs1986116
rs62101689
rs62143206
rs149744120
rs13150445
rs62464015
rs812890
rs7217410
rs3915280
rs68175238
rs7561212
rs6986709
rs2672561
rs9380691
rs73073372
rs2935699
rs34744280
All
−0.001
0.000
0.001
MR effect size for
'Lysosome membrane protein 2' on 'eGFR'

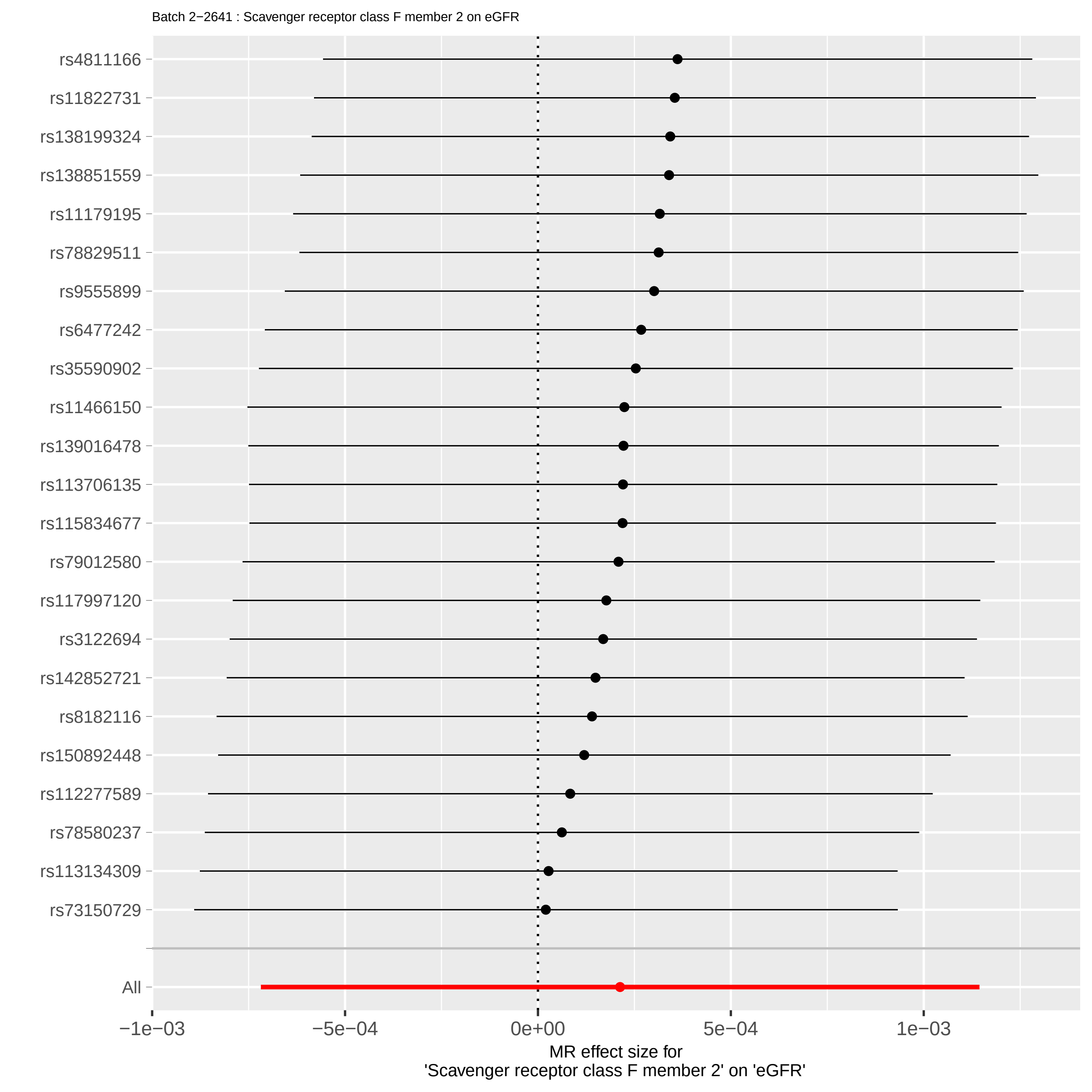
Batch 2−2641 : Scavenger receptor class F member 2 on eGFR
rs4811166
rs11822731
rs138199324
rs138851559
rs11179195
rs78829511
rs9555899
rs6477242
rs35590902
rs11466150
rs139016478
rs113706135
rs115834677
rs79012580
rs117997120
rs3122694
rs142852721
rs8182116
rs150892448
rs112277589
rs78580237
rs113134309
rs73150729
All
−1e−03
−5e−04
0e+00
5e−04
1e−03
MR effect size for
'Scavenger receptor class F member 2' on 'eGFR'

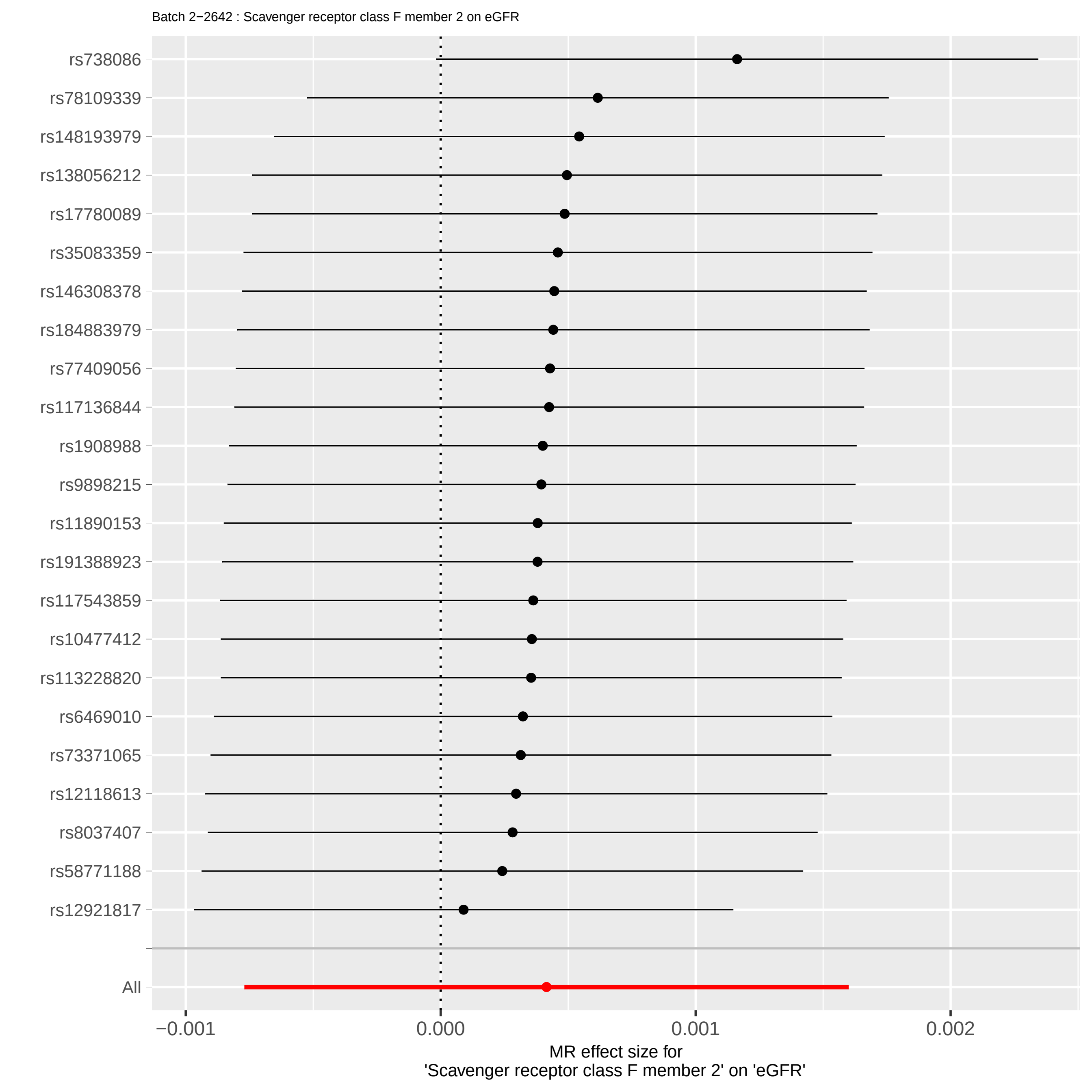
Batch 2−2642 : Scavenger receptor class F member 2 on eGFR
rs738086
rs78109339
rs148193979
rs138056212
rs17780089
rs35083359
rs146308378
rs184883979
rs77409056
rs117136844
rs1908988
rs9898215
rs11890153
rs191388923
rs117543859
rs10477412
rs113228820
rs6469010
rs73371065
rs12118613
rs8037407
rs58771188
rs12921817
All
−0.001
0.000
0.001
0.002
MR effect size for
'Scavenger receptor class F member 2' on 'eGFR'

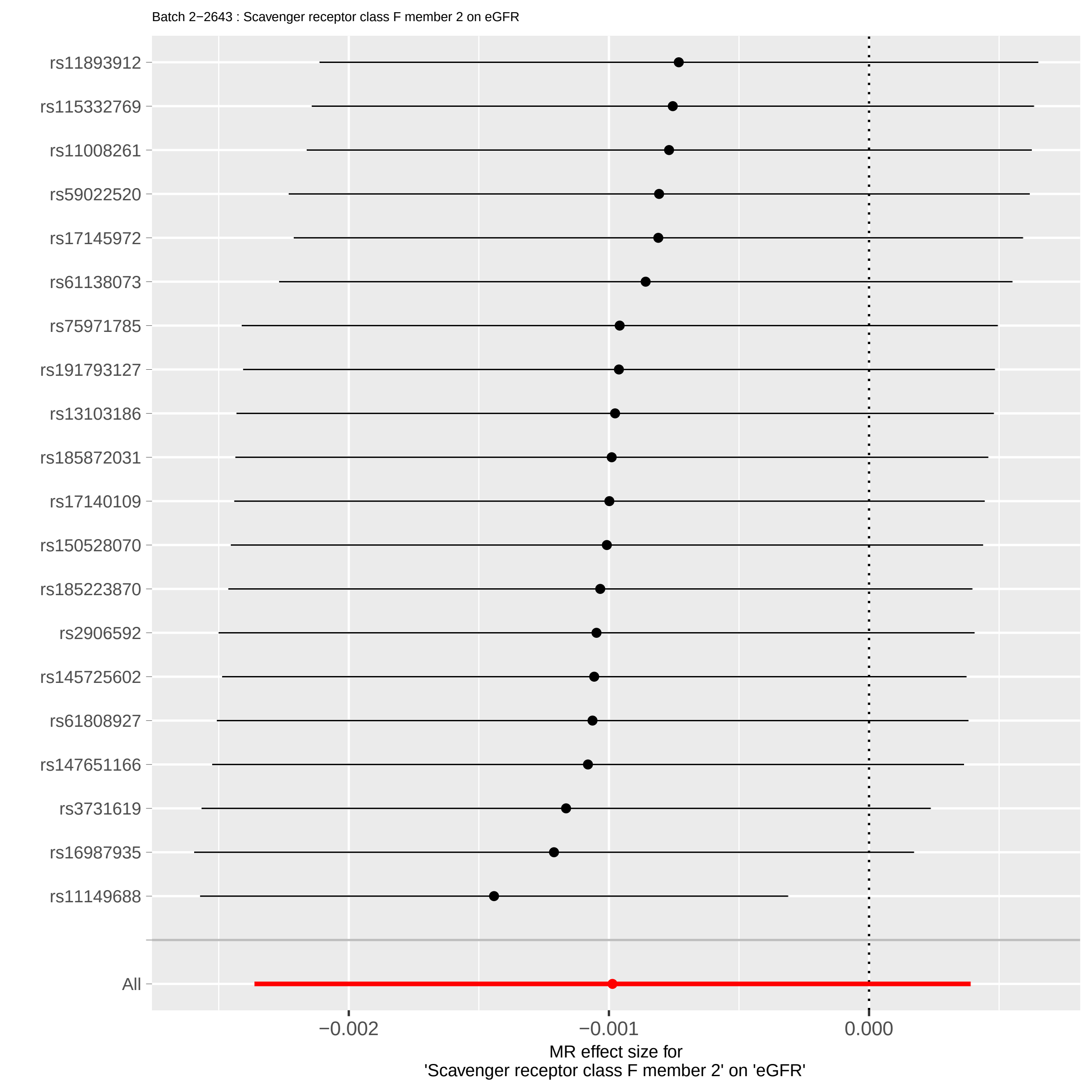
Batch 2−2643 : Scavenger receptor class F member 2 on eGFR
rs11893912
rs115332769
rs11008261
rs59022520
rs17145972
rs61138073
rs75971785
rs191793127
rs13103186
rs185872031
rs17140109
rs150528070
rs185223870
rs2906592
rs145725602
rs61808927
rs147651166
rs3731619
rs16987935
rs11149688
All
−0.002
−0.001
0.000
MR effect size for
'Scavenger receptor class F member 2' on 'eGFR'

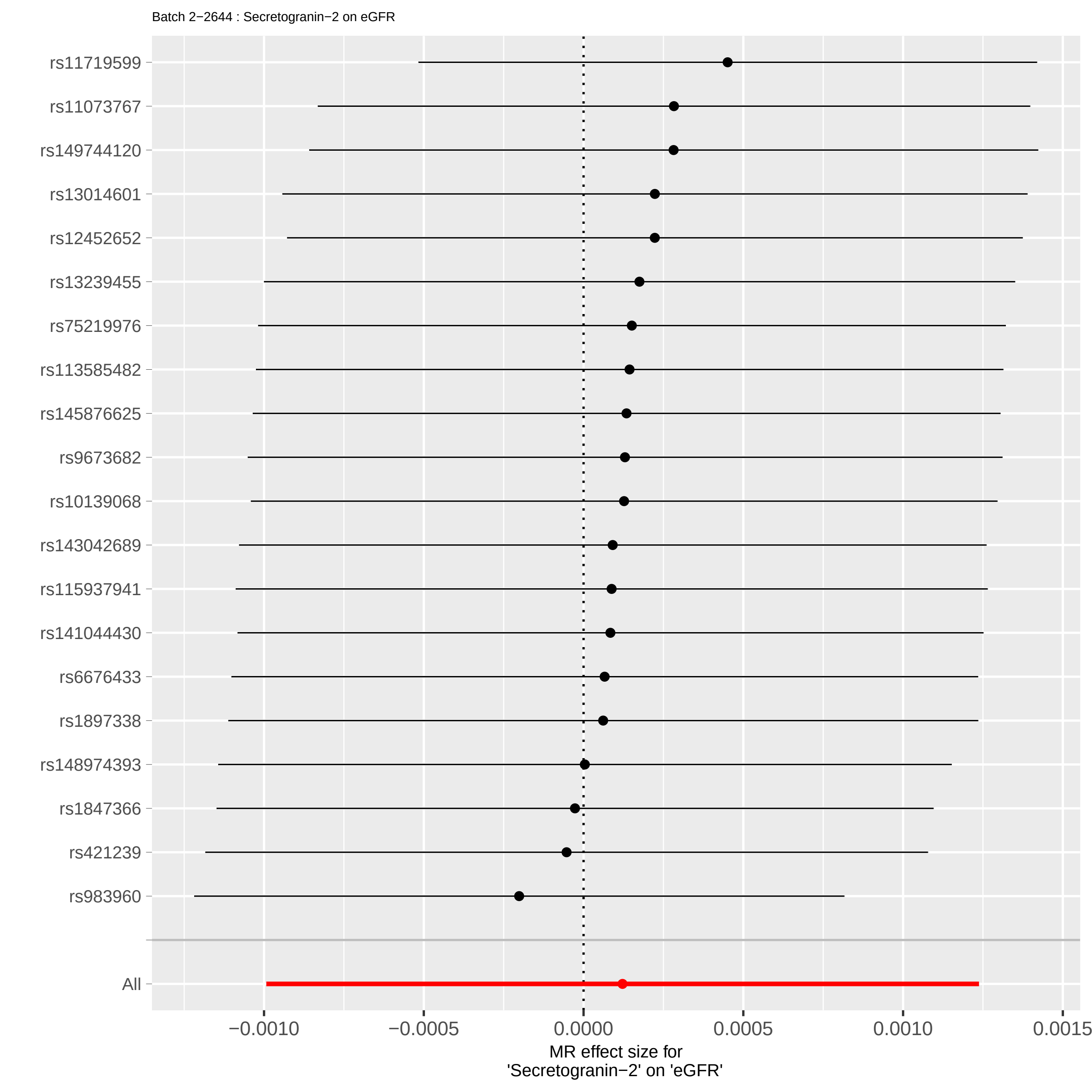

Batch 2−2644 : Secretogranin−2 on eGFR
rs11719599
rs11073767
rs149744120
rs13014601
rs12452652
rs13239455
rs75219976
rs113585482
rs145876625
rs9673682
rs10139068
rs143042689
rs115937941
rs141044430
rs6676433
rs1897338
rs148974393
rs1847366
rs421239
rs983960
All
−0.0010
−0.0005
0.0000
0.0005
0.0010
0.0015
MR effect size for
'Secretogranin−2' on 'eGFR'

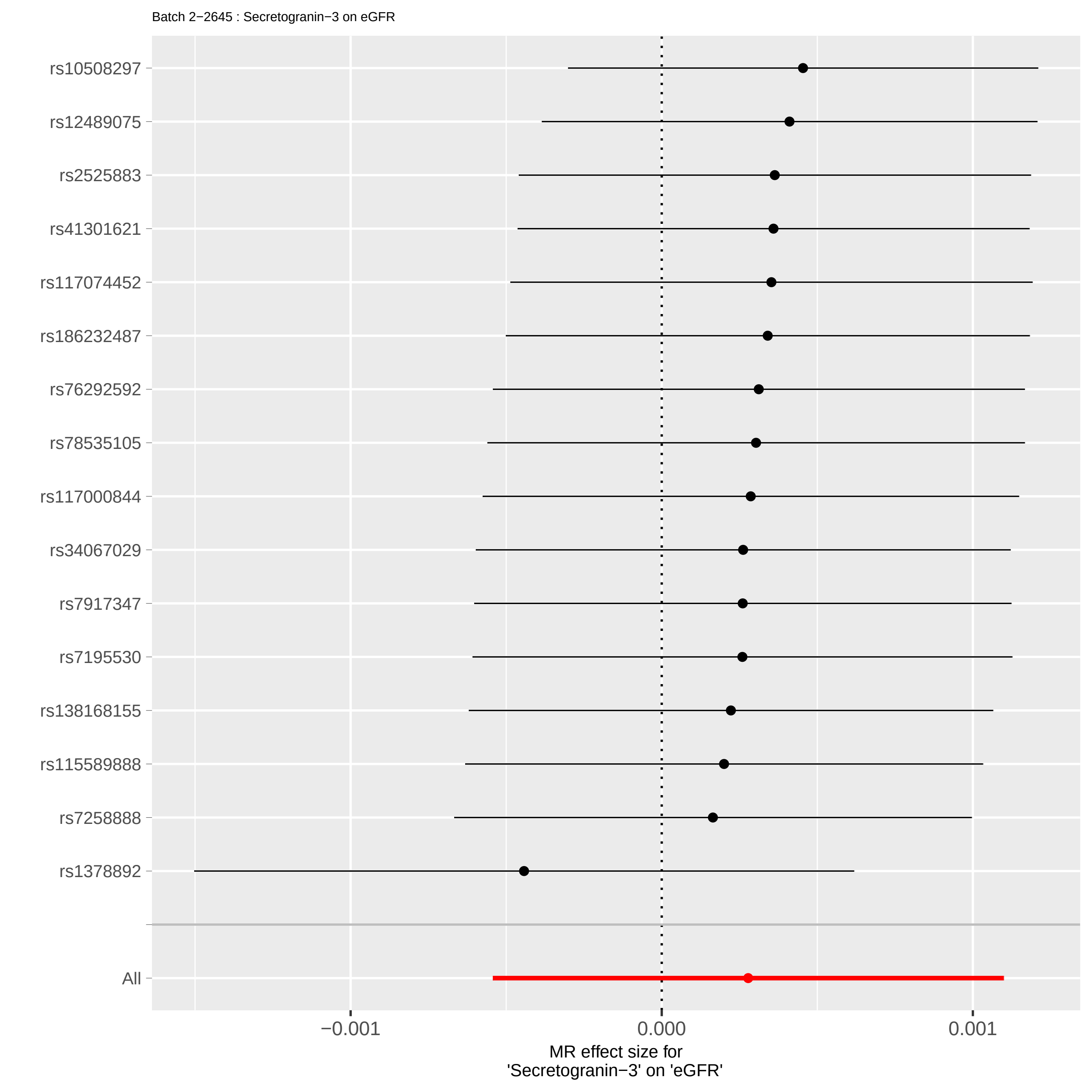

Batch 2−2645 : Secretogranin−3 on eGFR
rs10508297
rs12489075
rs2525883
rs41301621
rs117074452
rs186232487
rs76292592
rs78535105
rs117000844
rs34067029
rs7917347
rs7195530
rs138168155
rs115589888
rs7258888
rs1378892
All
−0.001
0.000
0.001
MR effect size for
'Secretogranin−3' on 'eGFR'

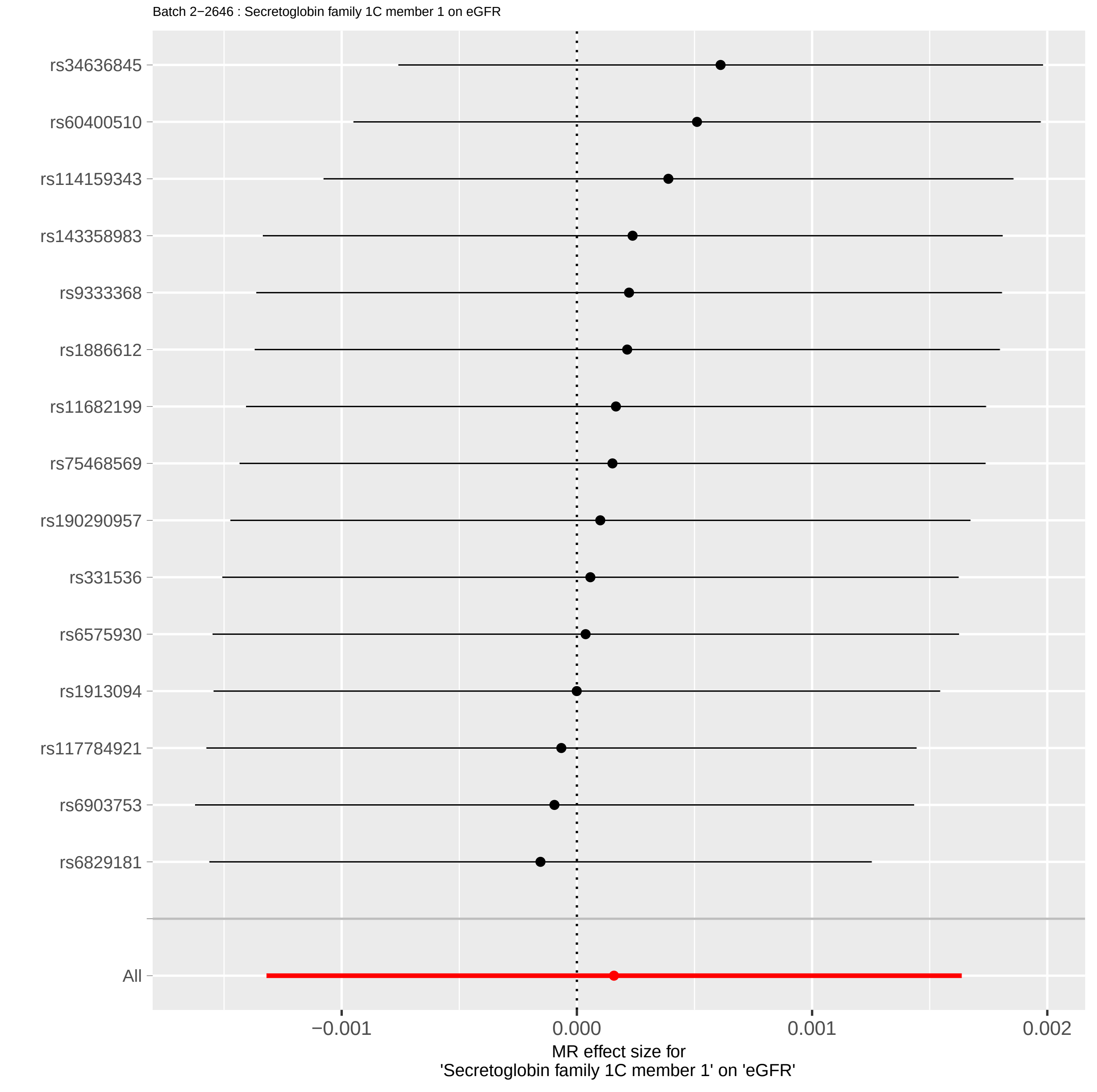
Batch 2−2646 : Secretoglobin family 1C member 1 on eGFR
rs34636845
rs60400510
rs114159343
rs143358983
rs9333368
rs1886612
rs11682199
rs75468569
rs190290957
rs331536
rs6575930
rs1913094
rs117784921
rs6903753
rs6829181
All
−0.001
0.000
0.001
0.002
MR effect size for
'Secretoglobin family 1C member 1' on 'eGFR'

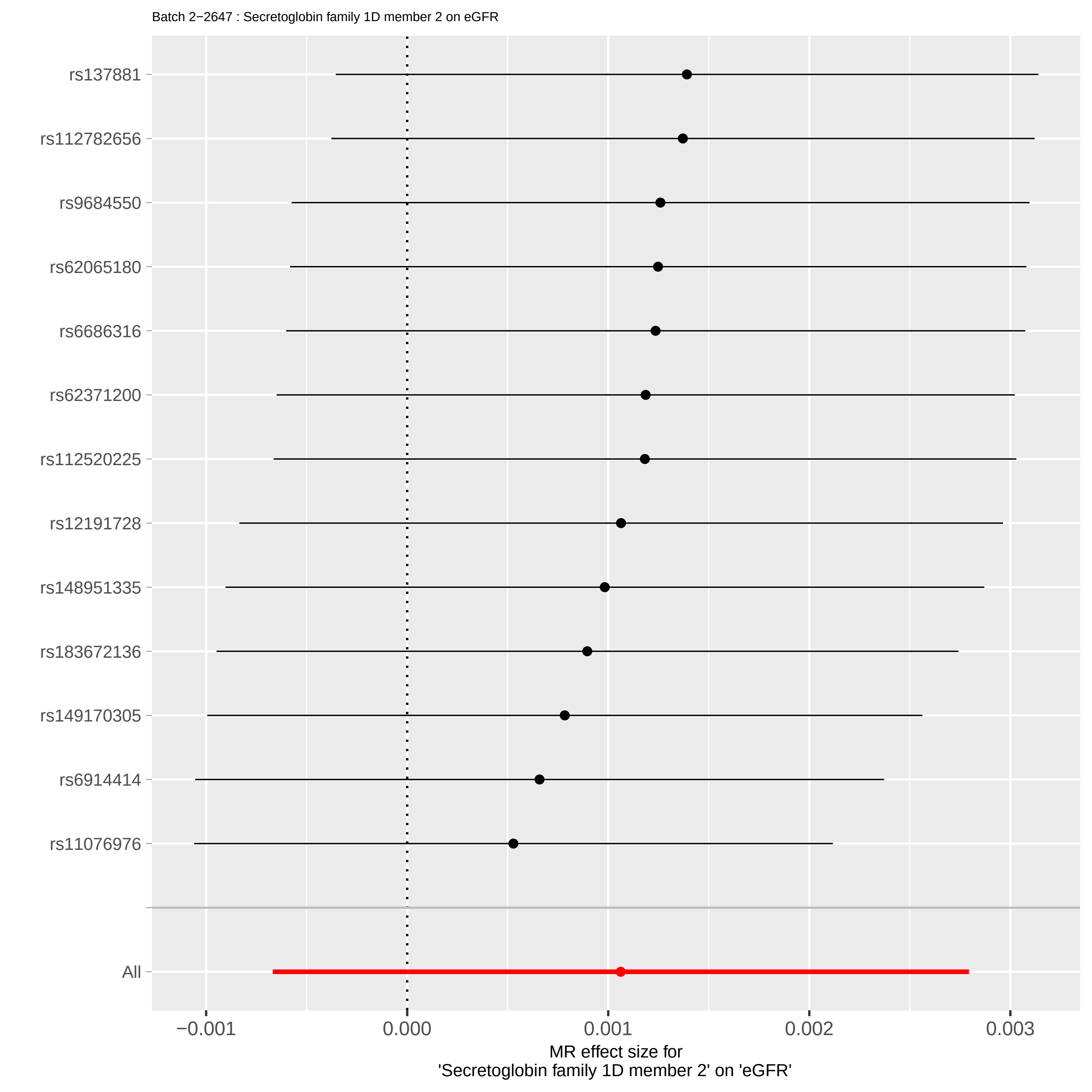
Batch 2−2647 : Secretoglobin family 1D member 2 on eGFR
rs137881
rs112782656
rs9684550
rs62065180
rs6686316
rs62371200
rs112520225
rs12191728
rs148951335
rs183672136
rs149170305
rs6914414
rs11076976
All
−0.001
0.000
0.001
0.002
0.003
MR effect size for
'Secretoglobin family 1D member 2' on 'eGFR'

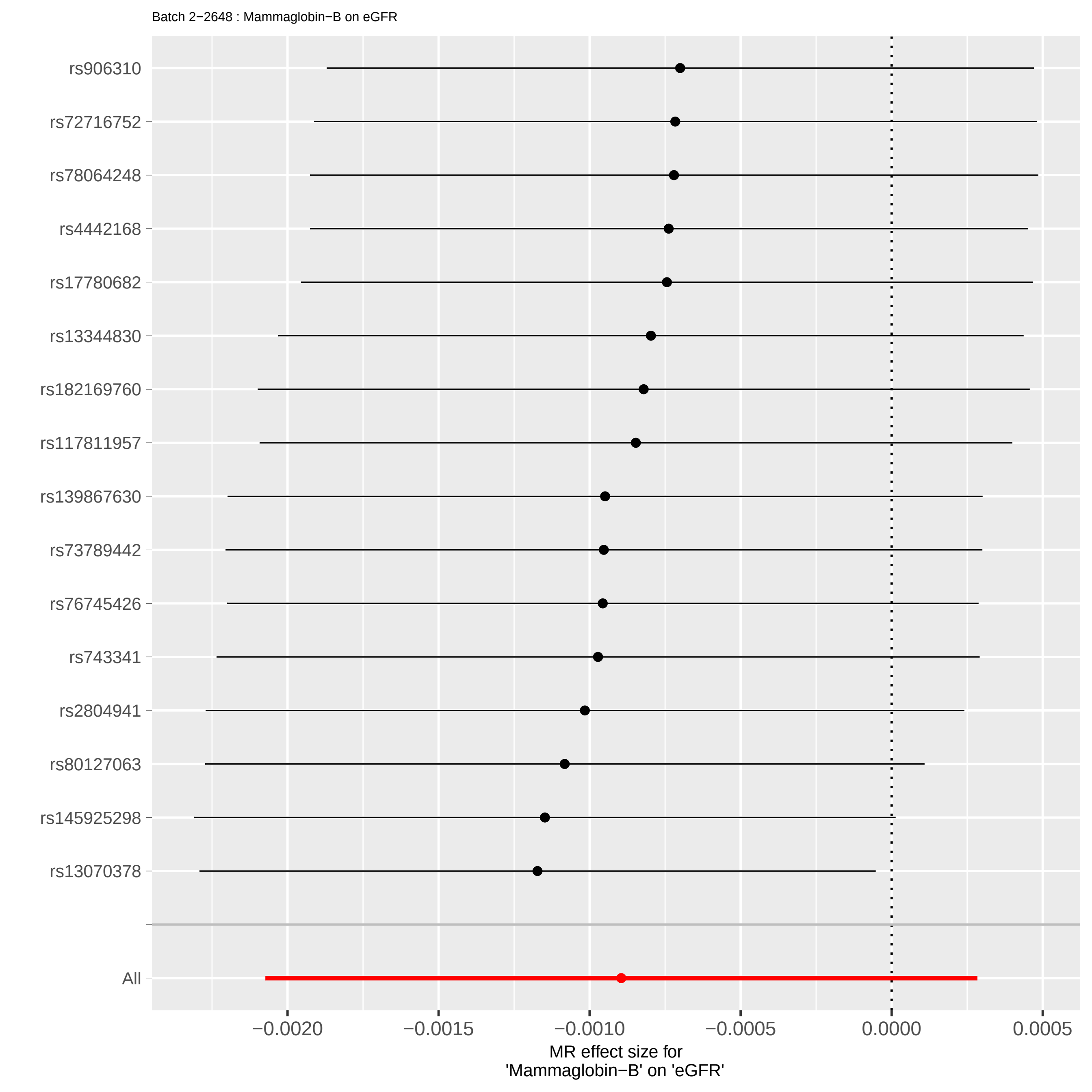
Batch 2−2648 : Mammaglobin−B on eGFR
rs906310
rs72716752
rs78064248
rs4442168
rs17780682
rs13344830
rs182169760
rs117811957
rs139867630
rs73789442
rs76745426
rs743341
rs2804941
rs80127063
rs145925298
rs13070378
All
−0.0020
−0.0015
−0.0010
−0.0005
0.0000
0.0005
MR effect size for
'Mammaglobin−B' on 'eGFR'

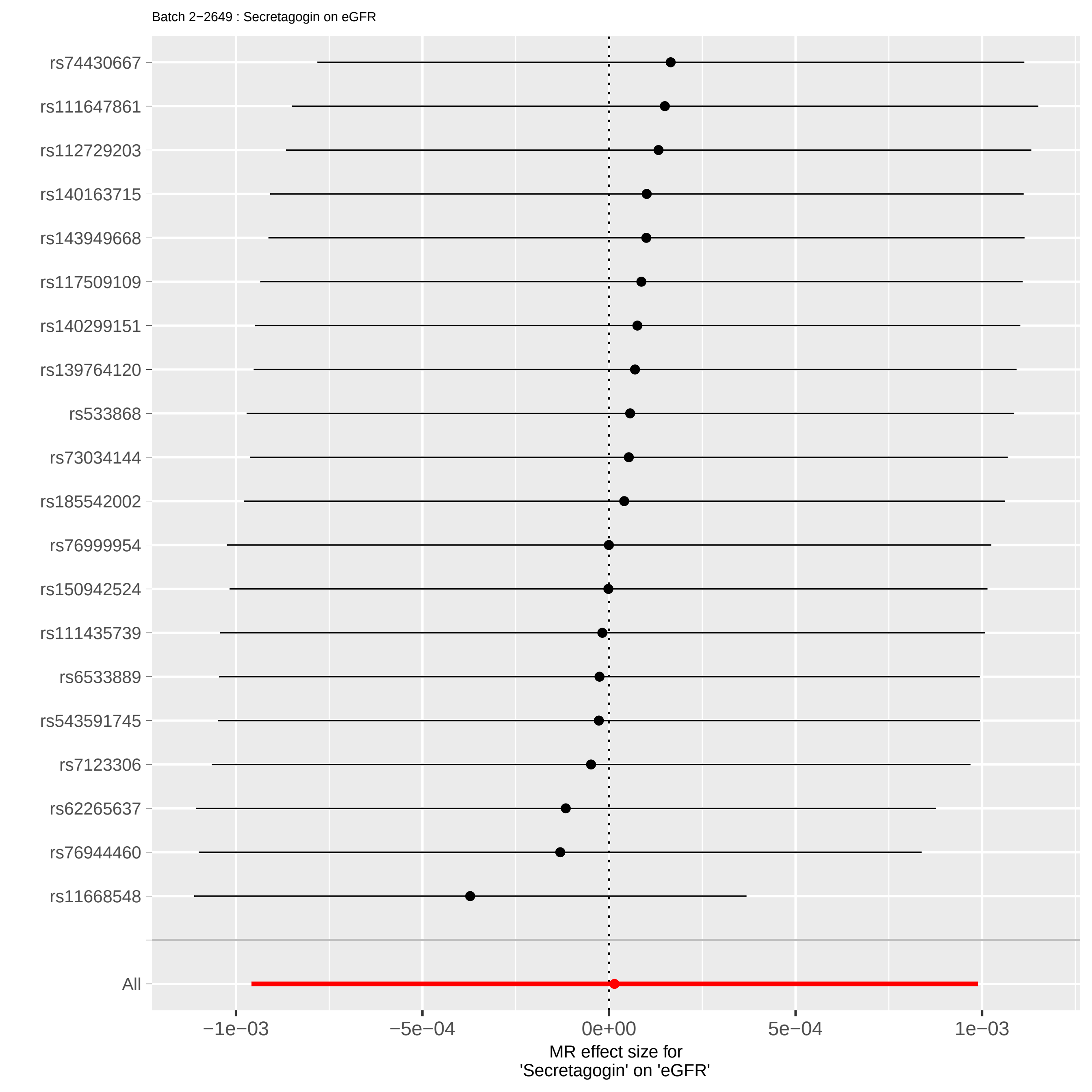
Batch 2−2649 : Secretagogin on eGFR
rs74430667
rs111647861
rs112729203
rs140163715
rs143949668
rs117509109
rs140299151
rs139764120
rs533868
rs73034144
rs185542002
rs76999954
rs150942524
rs111435739
rs6533889
rs543591745
rs7123306
rs62265637
rs76944460
rs11668548
All
−1e−03
−5e−04
0e+00
5e−04
1e−03
MR effect size for
'Secretagogin' on 'eGFR'

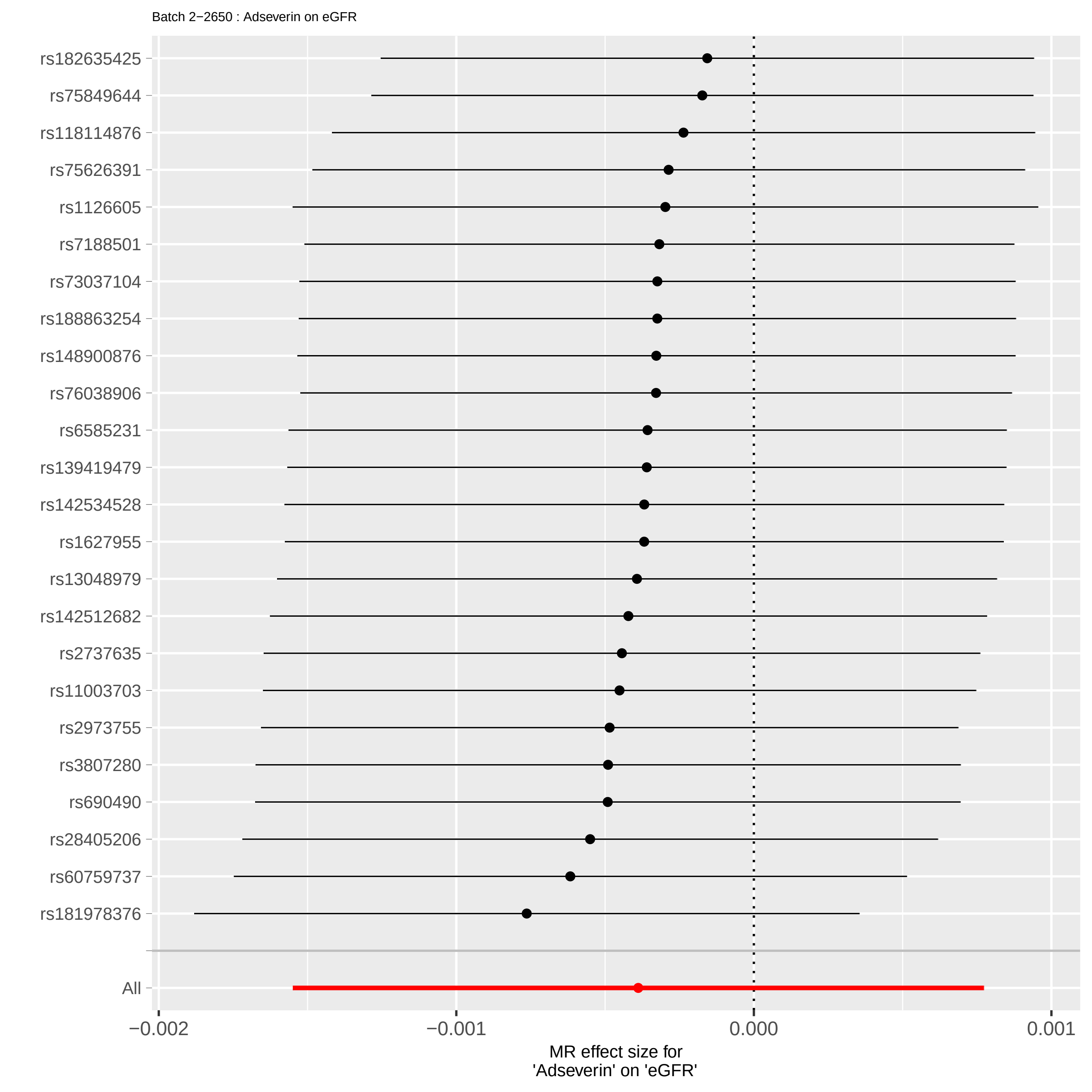

Batch 2−2650 : Adseverin on eGFR
rs182635425
rs75849644
rs118114876
rs75626391
rs1126605
rs7188501
rs73037104
rs188863254
rs148900876
rs76038906
rs6585231
rs139419479
rs142534528
rs1627955
rs13048979
rs142512682
rs2737635
rs11003703
rs2973755
rs3807280
rs690490
rs28405206
rs60759737
rs181978376
All
−0.002
−0.001
0.000
0.001
MR effect size for
'Adseverin' on 'eGFR'

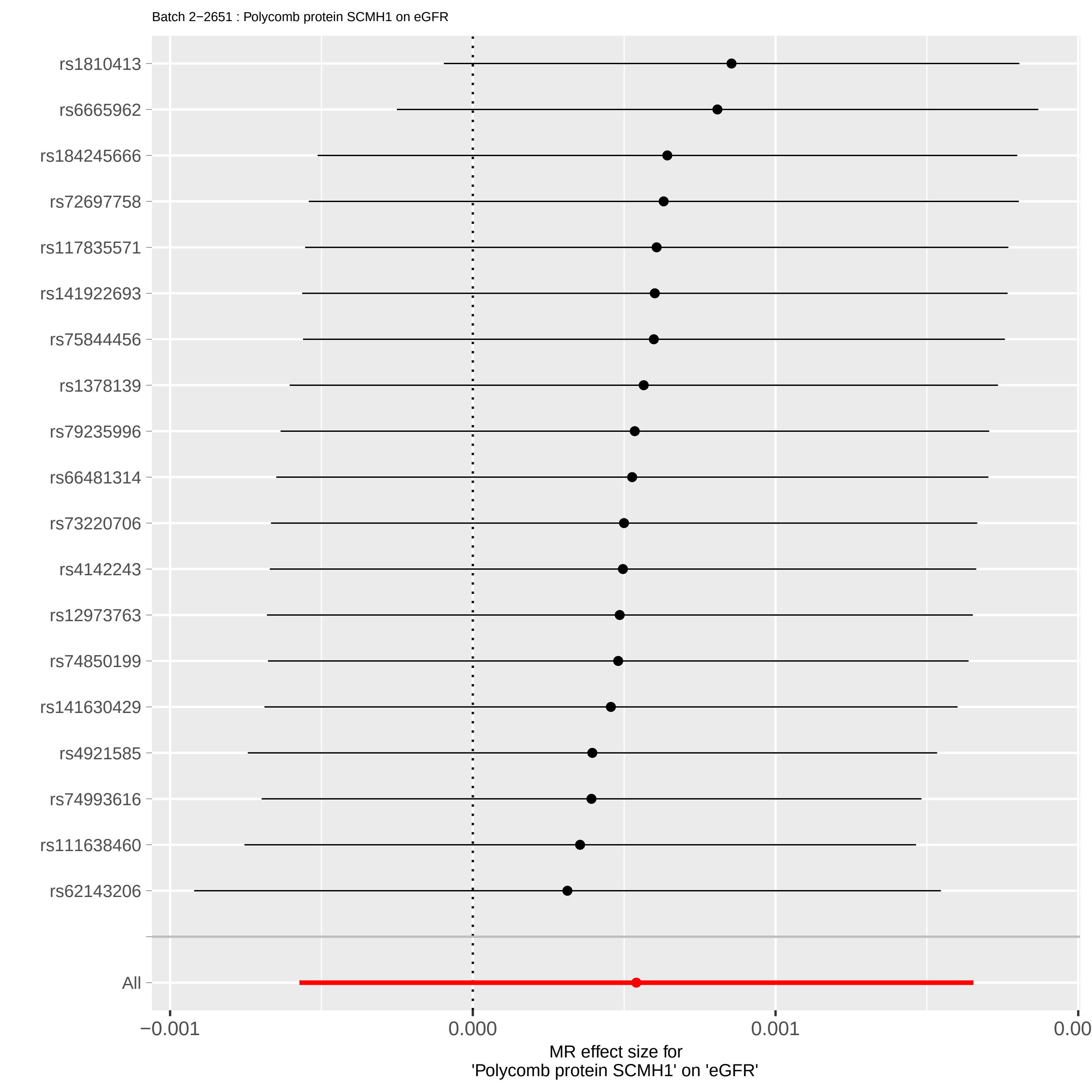
Batch 2−2651 : Polycomb protein SCMH1 on eGFR
rs1810413
rs6665962
rs184245666
rs72697758
rs117835571
rs141922693
rs75844456
rs1378139
rs79235996
rs66481314
rs73220706
rs4142243
rs12973763
rs74850199
rs141630429
rs4921585
rs74993616
rs111638460
rs62143206
All
−0.001
0.000
0.001
0.00
MR effect size for
'Polycomb protein SCMH1' on 'eGFR'

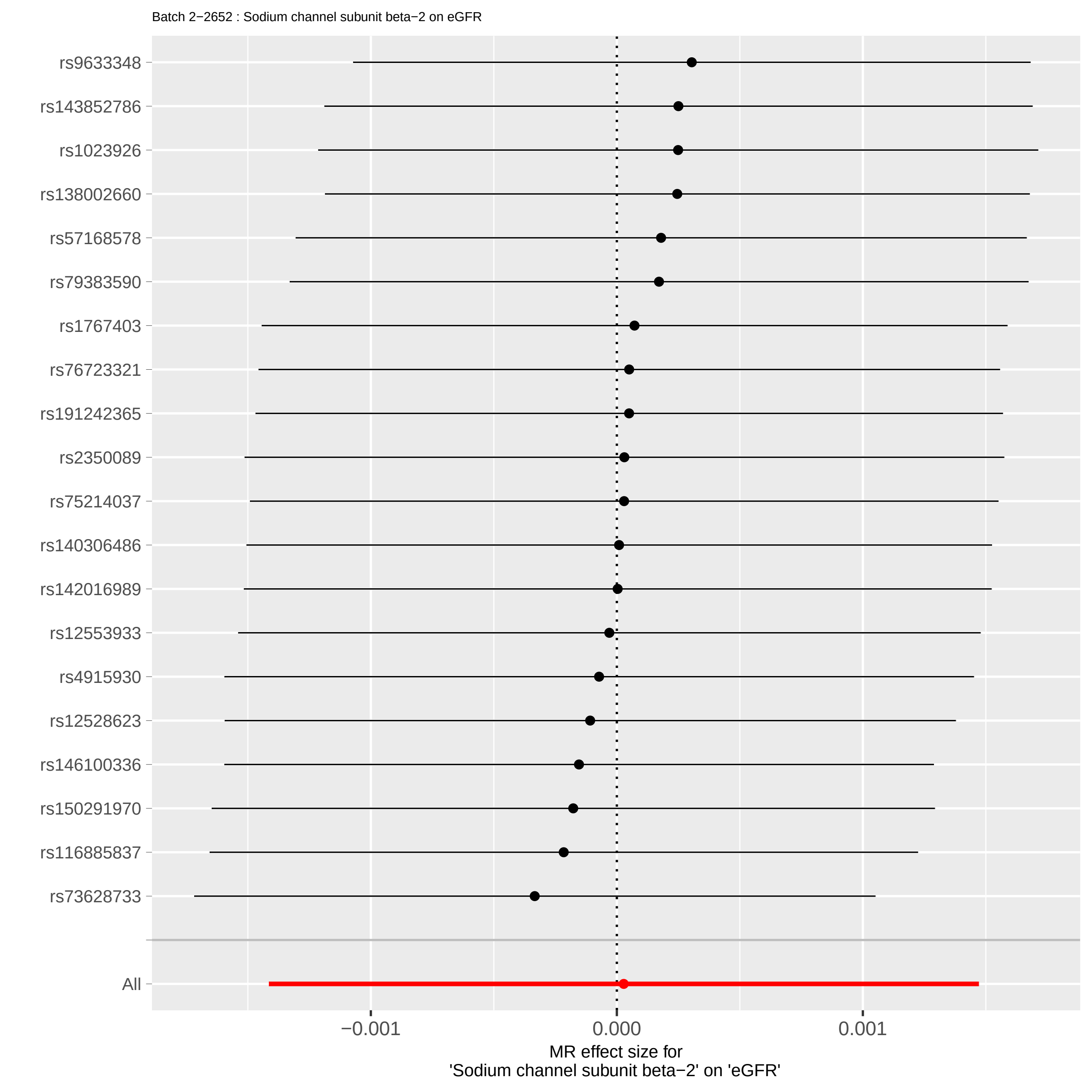

Batch 2−2652 : Sodium channel subunit beta−2 on eGFR
rs9633348
rs143852786
rs1023926
rs138002660
rs57168578
rs79383590
rs1767403
rs76723321
rs191242365
rs2350089
rs75214037
rs140306486
rs142016989
rs12553933
rs4915930
rs12528623
rs146100336
rs150291970
rs116885837
rs73628733
All
−0.001
0.000
0.001
MR effect size for
'Sodium channel subunit beta−2' on 'eGFR'

Batch 2−2653 : Protein SCO1 homolog, mitochondrial on eGFR

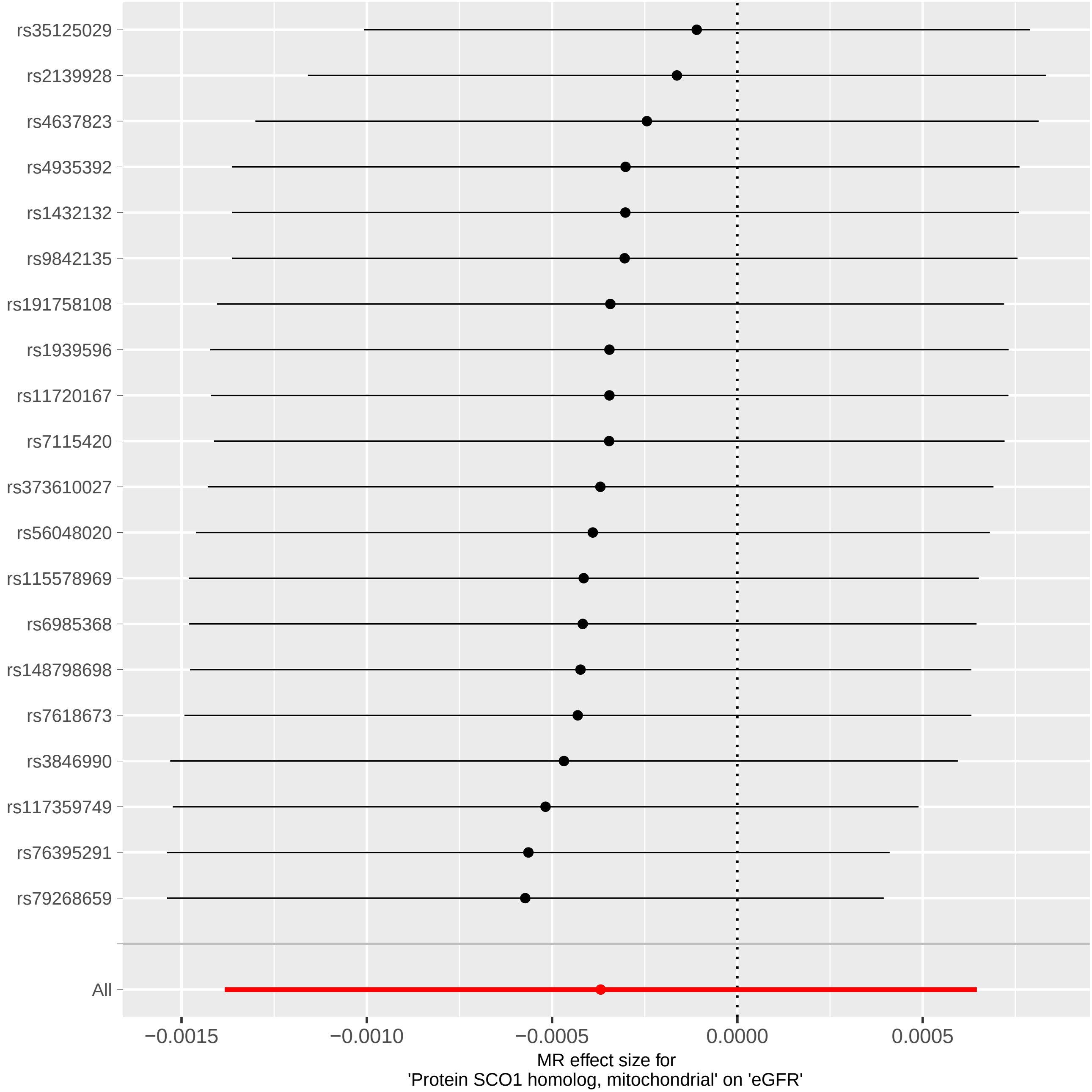

Batch 2−2654 : SCP2 sterol−binding domain−containing protein 1 on eGFR

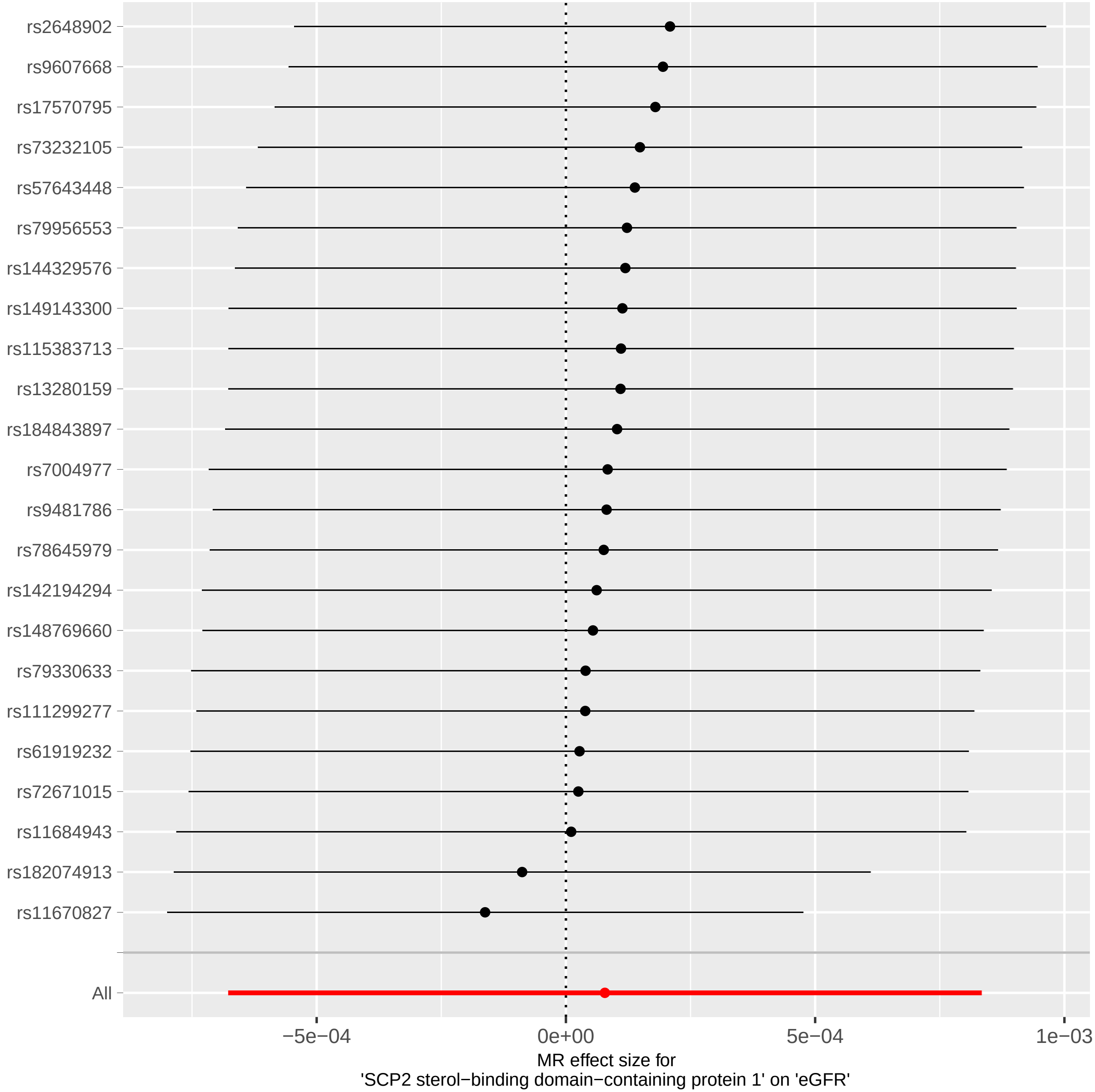

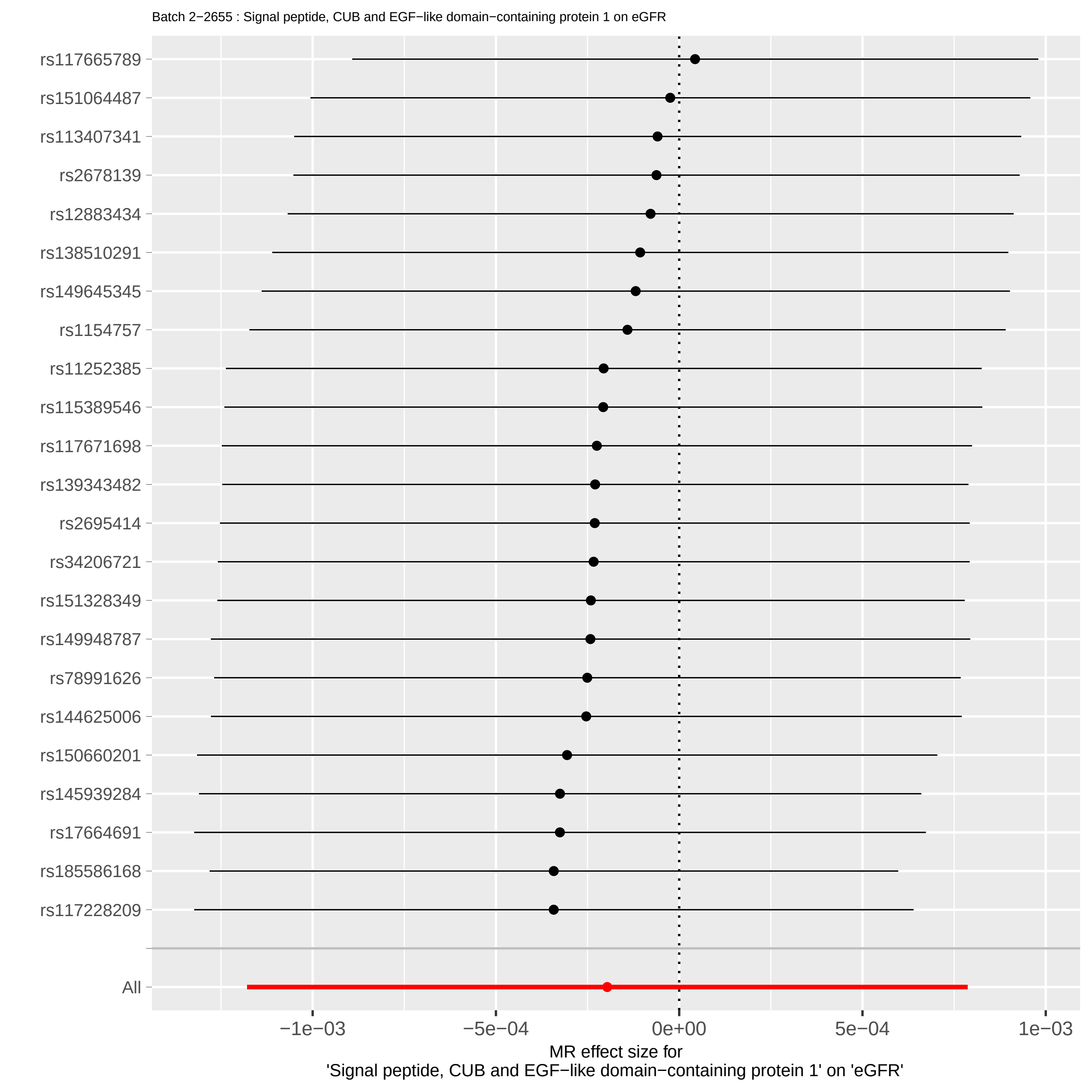
Batch 2−2655 : Signal peptide, CUB and EGF−like domain−containing protein 1 on eGFR
rs117665789
rs151064487
rs113407341
rs2678139
rs12883434
rs138510291
rs149645345
rs1154757
rs11252385
rs115389546
rs117671698
rs139343482
rs2695414
rs34206721
rs151328349
rs149948787
rs78991626
rs144625006
rs150660201
rs145939284
rs17664691
rs185586168
rs117228209
All
−1e−03
−5e−04
0e+00
5e−04
1e−03
MR effect size for
'Signal peptide, CUB and EGF−like domain−containing protein 1' on 'eGFR'

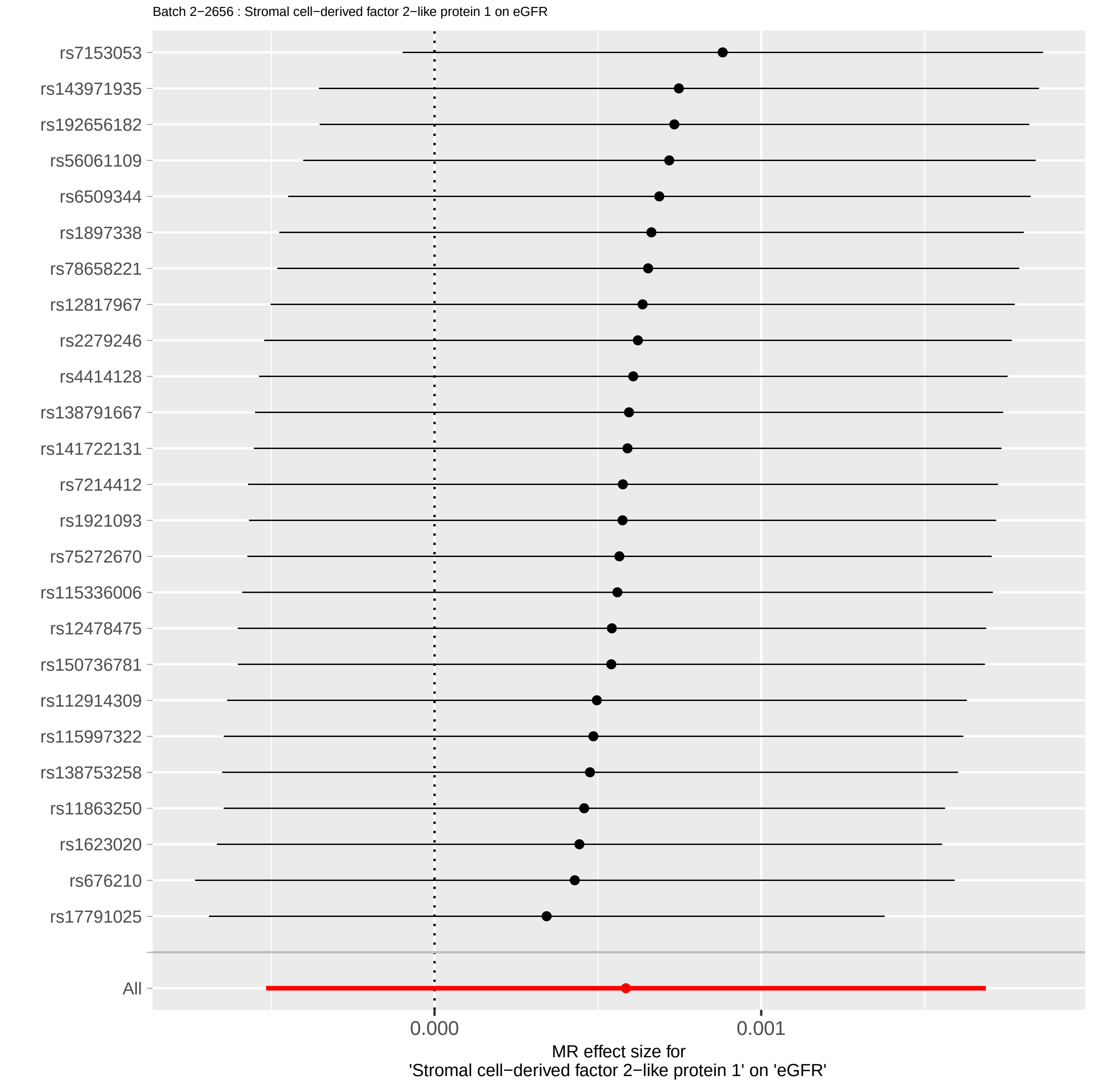

Batch 2−2656 : Stromal cell−derived factor 2−like protein 1 on eGFR
rs7153053
rs143971935
rs192656182
rs56061109
rs6509344
rs1897338
rs78658221
rs12817967
rs2279246
rs4414128
rs138791667
rs141722131
rs7214412
rs1921093
rs75272670
rs115336006
rs12478475
rs150736781
rs112914309
rs115997322
rs138753258
rs11863250
rs1623020
rs676210
rs17791025
All
0.000
0.001
MR effect size for
'Stromal cell−derived factor 2−like protein 1' on 'eGFR'

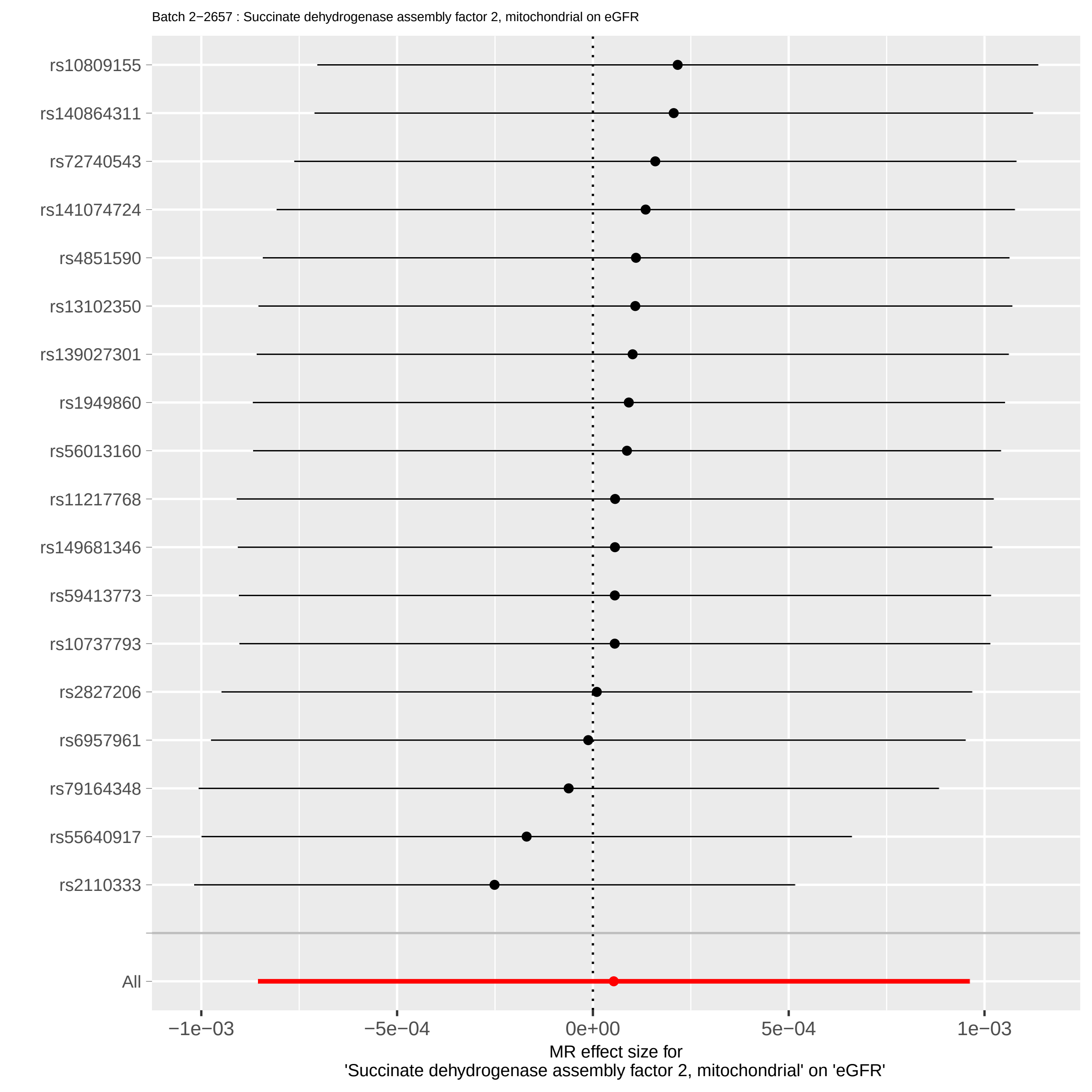

Batch 2−2657 : Succinate dehydrogenase assembly factor 2, mitochondrial on eGFR
rs10809155
rs140864311
rs72740543
rs141074724
rs4851590
rs13102350
rs139027301
rs1949860
rs56013160
rs11217768
rs149681346
rs59413773
rs10737793
rs2827206
rs6957961
rs79164348
rs55640917
rs2110333
All
−1e−03
−5e−04
0e+00
5e−04
1e−03
MR effect size for
'Succinate dehydrogenase assembly factor 2, mitochondrial' on 'eGFR'

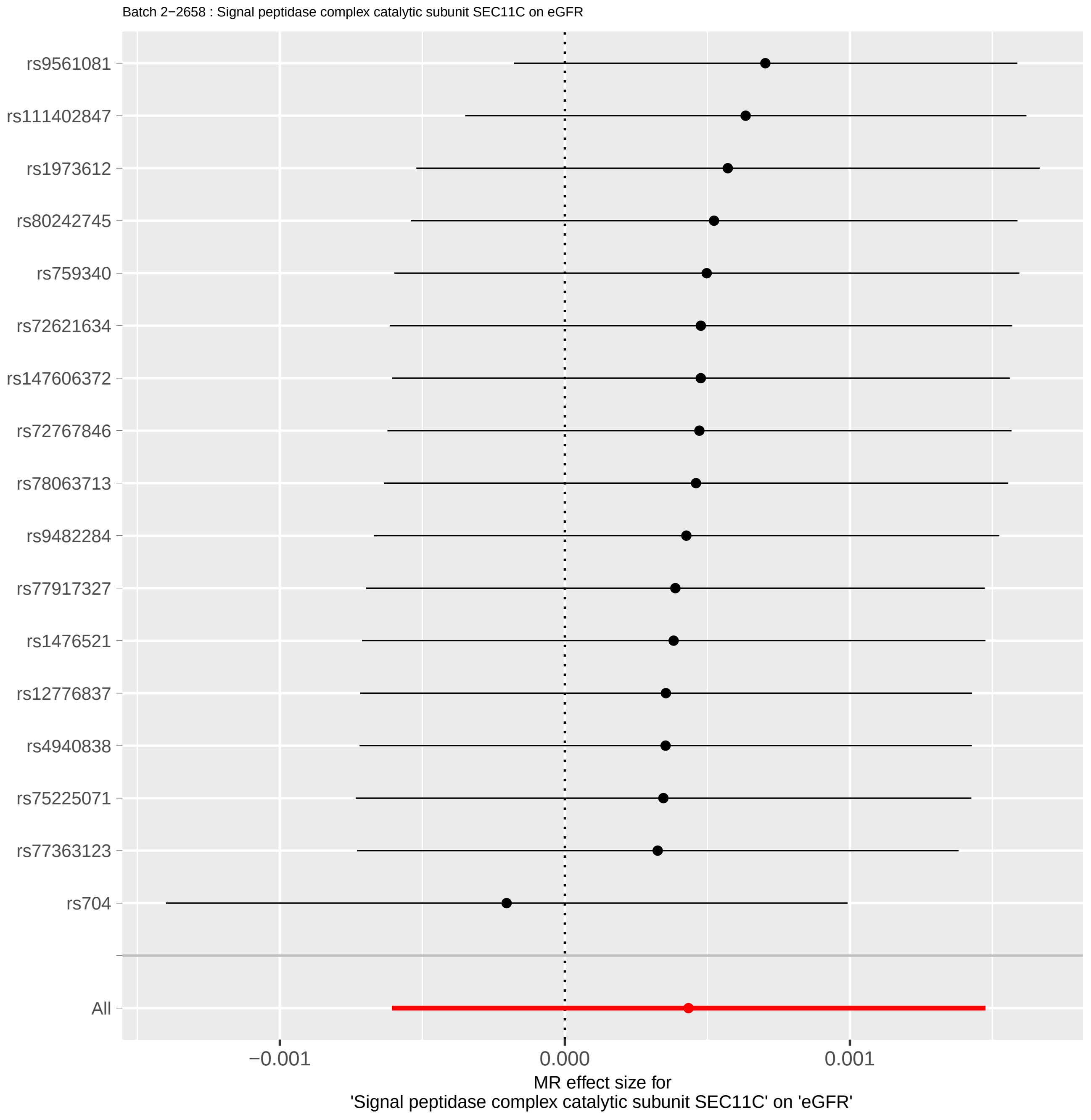
Batch 2−2658 : Signal peptidase complex catalytic subunit SEC11C on eGFR
rs9561081
rs111402847
rs1973612
rs80242745
rs759340
rs72621634
rs147606372
rs72767846
rs78063713
rs9482284
rs77917327
rs1476521
rs12776837
rs4940838
rs75225071
rs77363123
rs704
All
−0.001
0.000
0.001
MR effect size for
'Signal peptidase complex catalytic subunit SEC11C' on 'eGFR'

Batch 2−2659 : Protein SEC13 homolog on eGFR

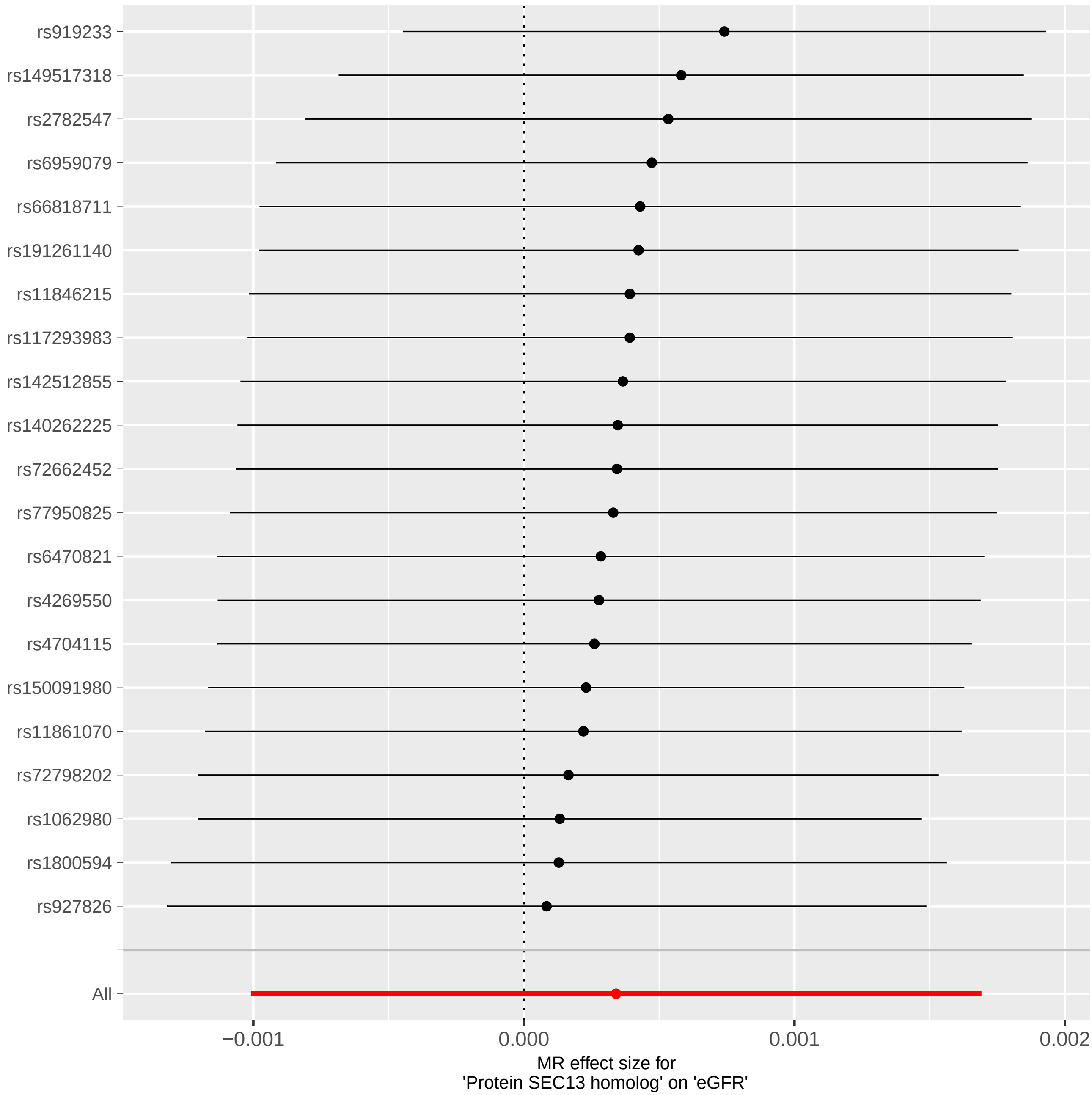

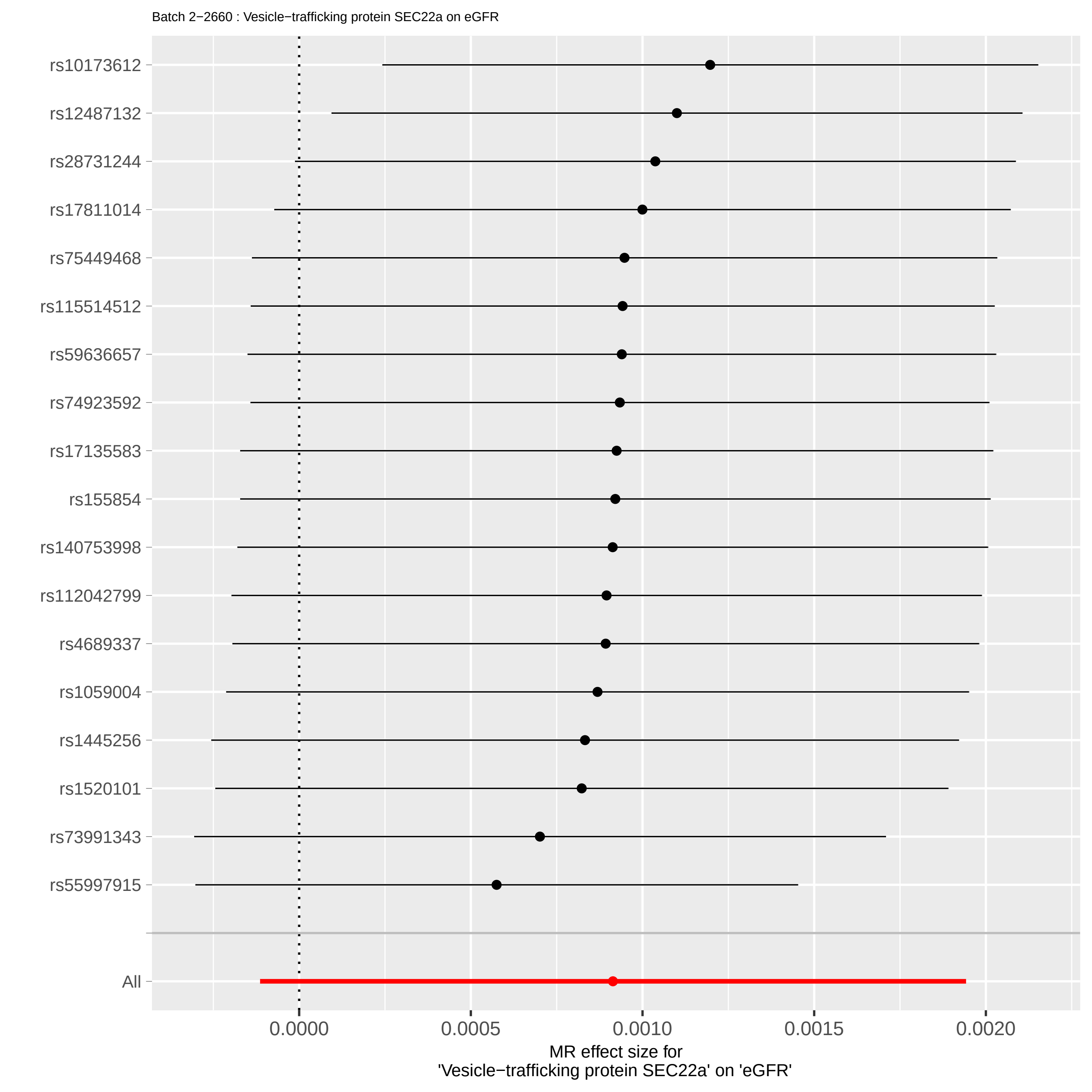

Batch 2−2660 : Vesicle−trafficking protein SEC22a on eGFR
rs10173612
rs12487132
rs28731244
rs17811014
rs75449468
rs115514512
rs59636657
rs74923592
rs17135583
rs155854
rs140753998
rs112042799
rs4689337
rs1059004
rs1445256
rs1520101
rs73991343
rs55997915
All
0.0000
0.0005
0.0010
0.0015
0.0020
MR effect size for
'Vesicle−trafficking protein SEC22a' on 'eGFR'

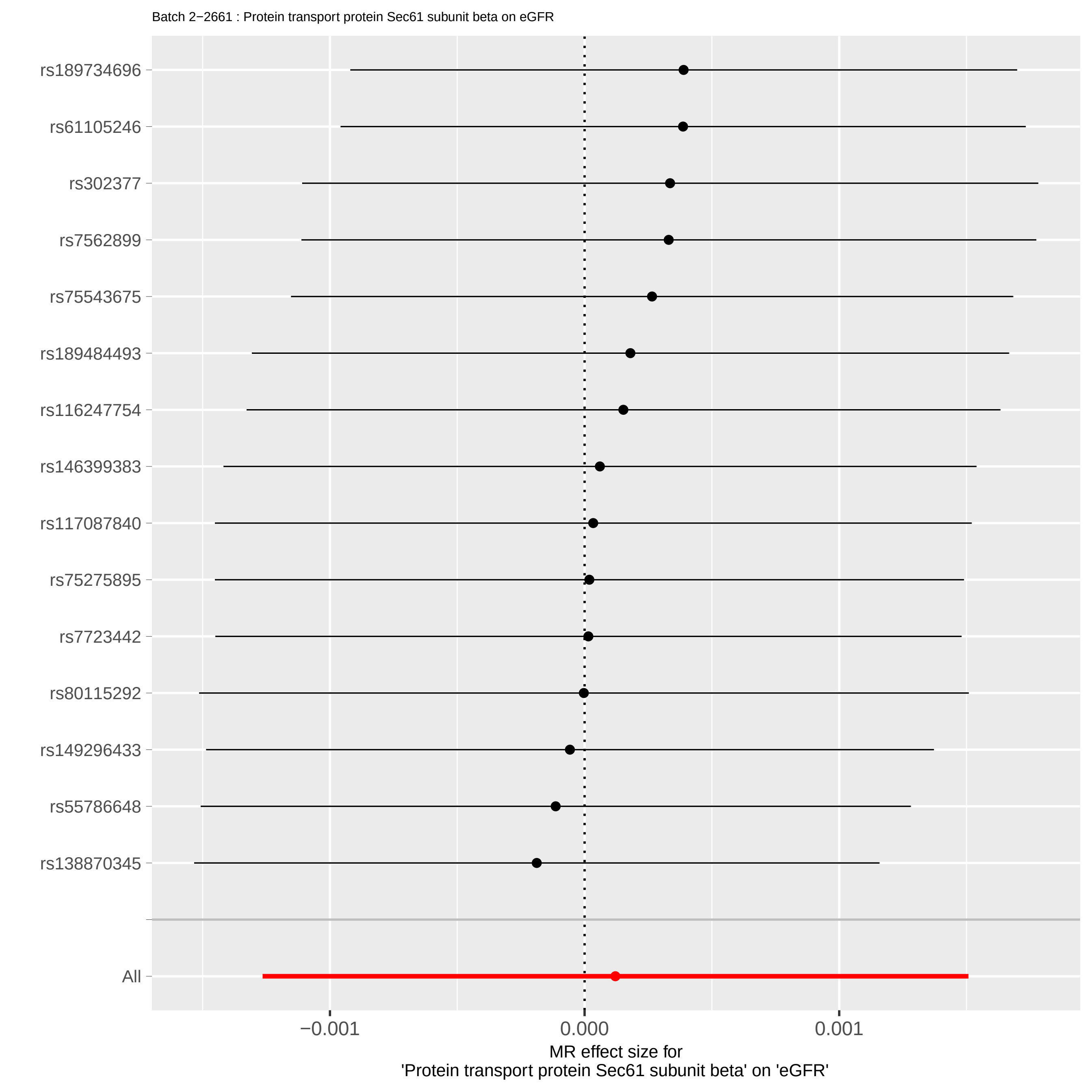
Batch 2−2661 : Protein transport protein Sec61 subunit beta on eGFR
rs189734696
rs61105246
rs302377
rs7562899
rs75543675
rs189484493
rs116247754
rs146399383
rs117087840
rs75275895
rs7723442
rs80115292
rs149296433
rs55786648
rs138870345
All
−0.001
0.000
0.001
MR effect size for
'Protein transport protein Sec61 subunit beta' on 'eGFR'

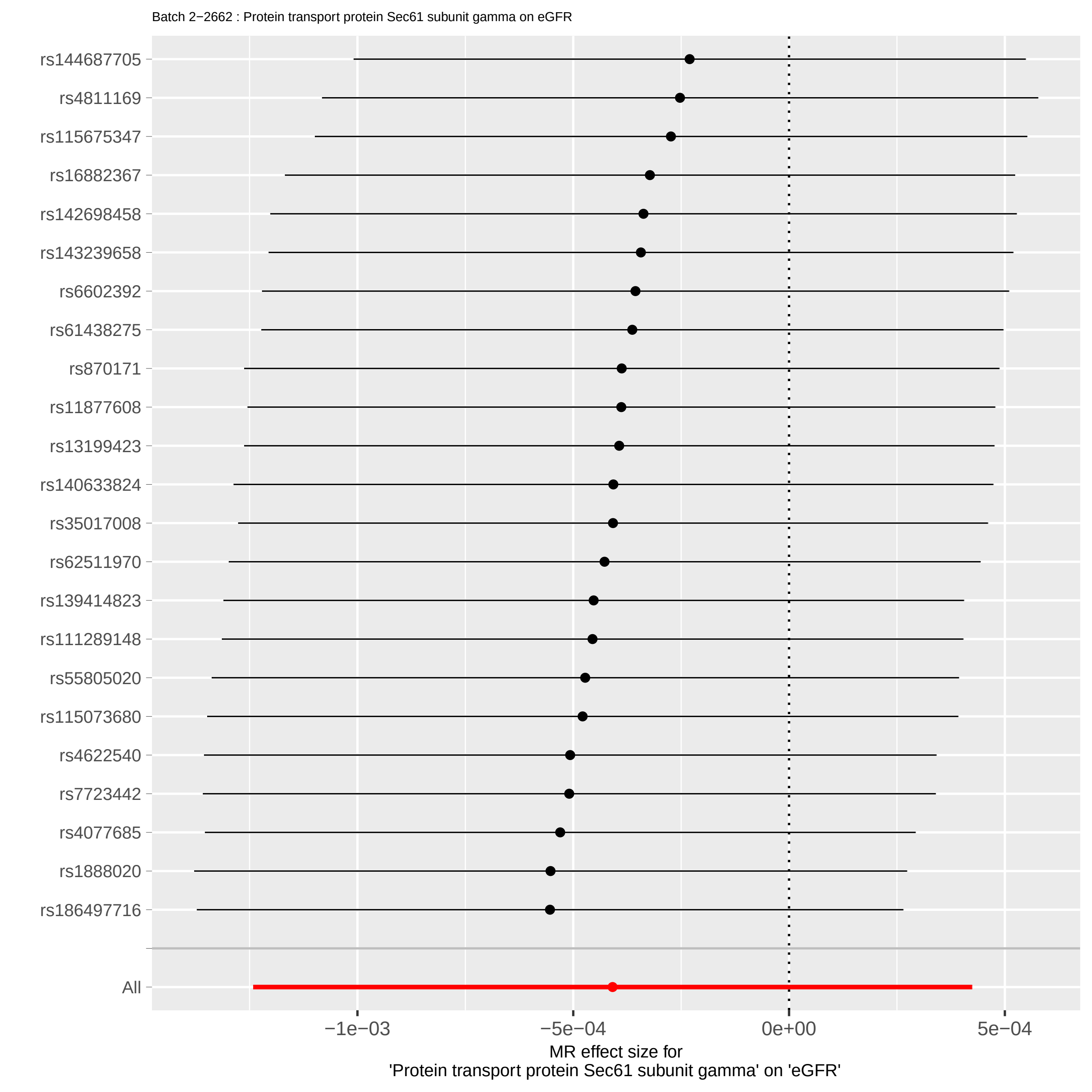
Batch 2−2662 : Protein transport protein Sec61 subunit gamma on eGFR
rs144687705
rs4811169
rs115675347
rs16882367
rs142698458
rs143239658
rs6602392
rs61438275
rs870171
rs11877608
rs13199423
rs140633824
rs35017008
rs62511970
rs139414823
rs111289148
rs55805020
rs115073680
rs4622540
rs7723442
rs4077685
rs1888020
rs186497716
All
−1e−03
−5e−04
0e+00
5e−04
MR effect size for
'Protein transport protein Sec61 subunit gamma' on 'eGFR'

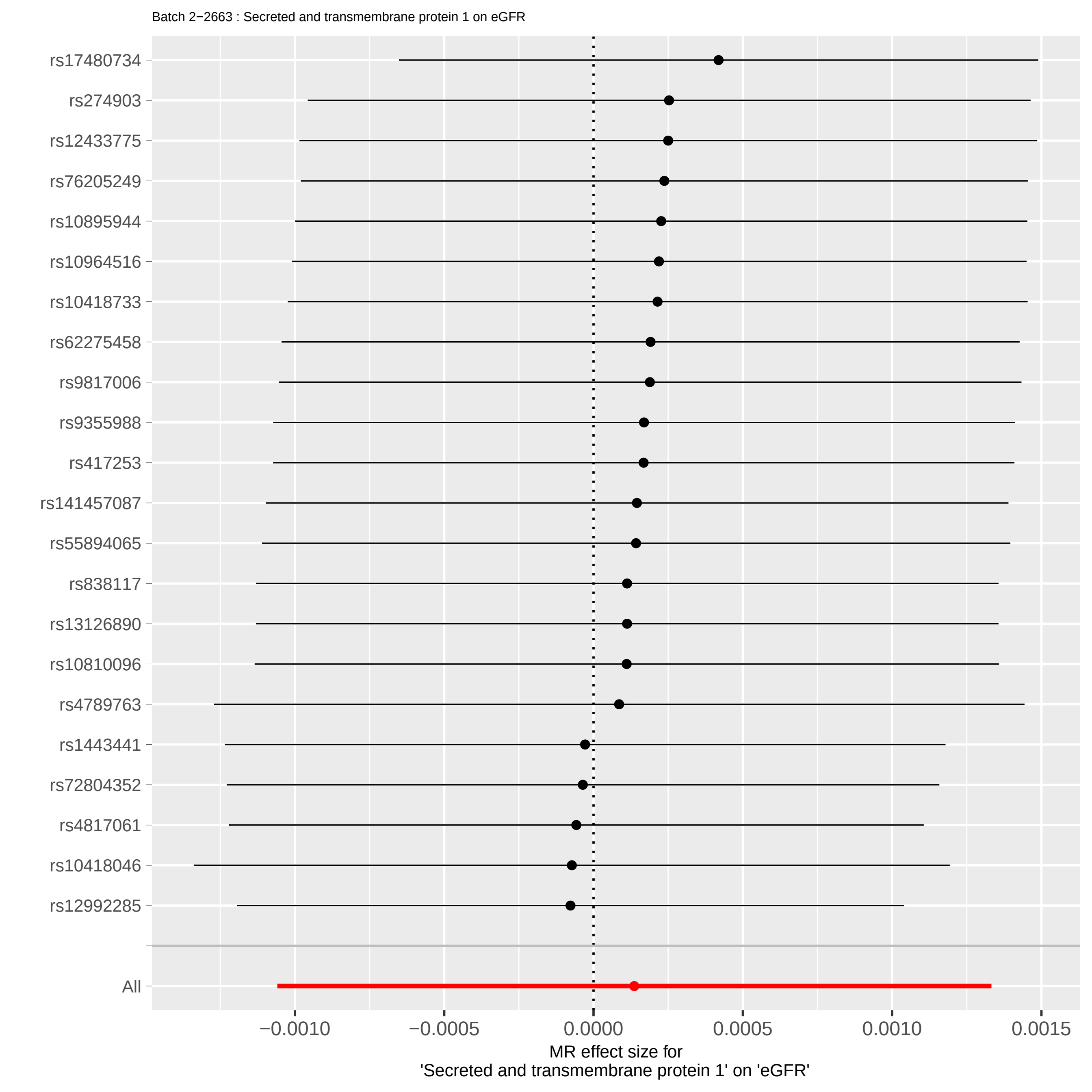

Batch 2−2663 : Secreted and transmembrane protein 1 on eGFR
rs17480734
rs274903
rs12433775
rs76205249
rs10895944
rs10964516
rs10418733
rs62275458
rs9817006
rs9355988
rs417253
rs141457087
rs55894065
rs838117
rs13126890
rs10810096
rs4789763
rs1443441
rs72804352
rs4817061
rs10418046
rs12992285
All
−0.0010
−0.0005
0.0000
0.0005
0.0010
0.0015
MR effect size for
'Secreted and transmembrane protein 1' on 'eGFR'

Batch 2−2664 : Protein sel−1 homolog 2 on eGFR

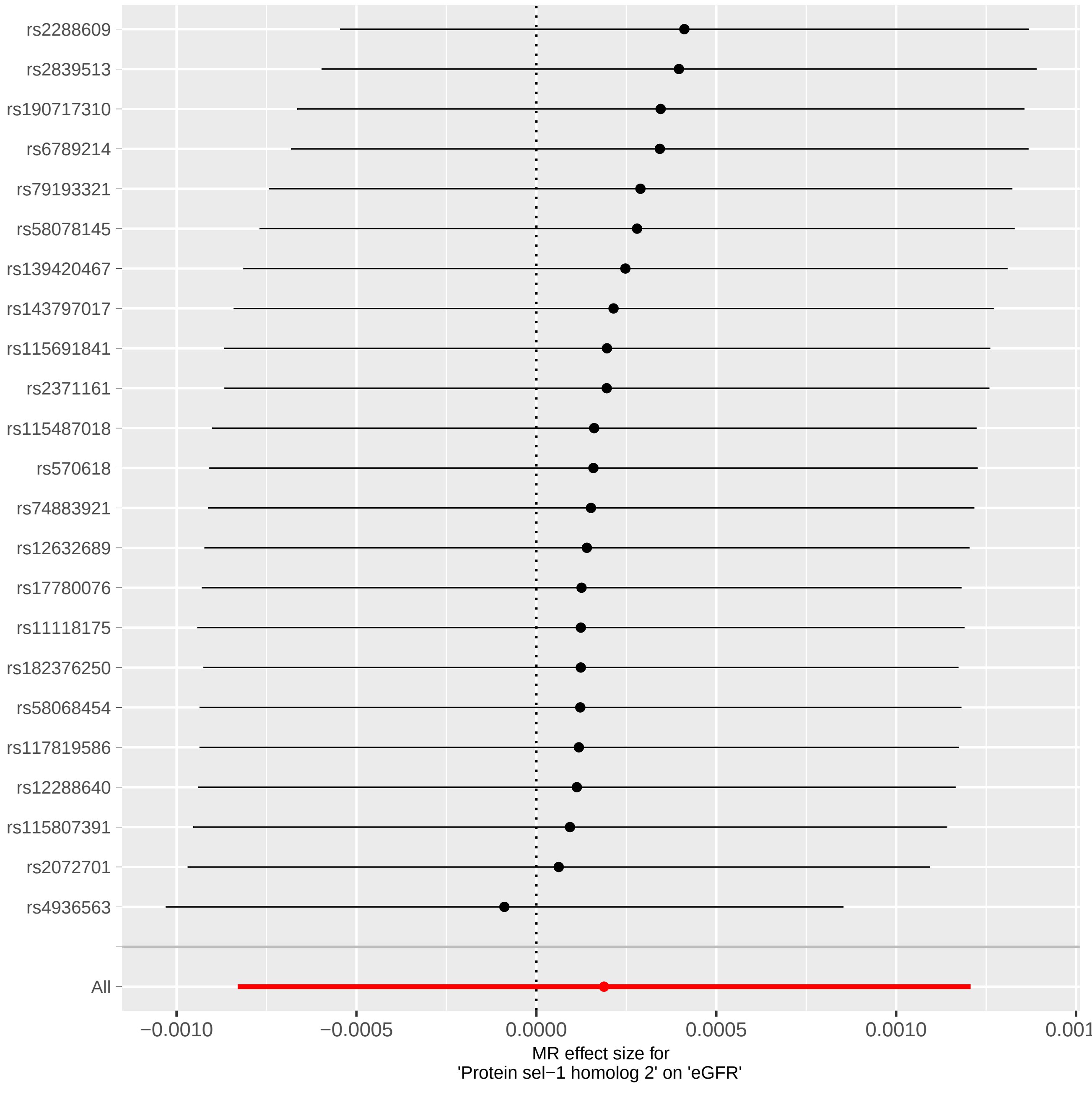

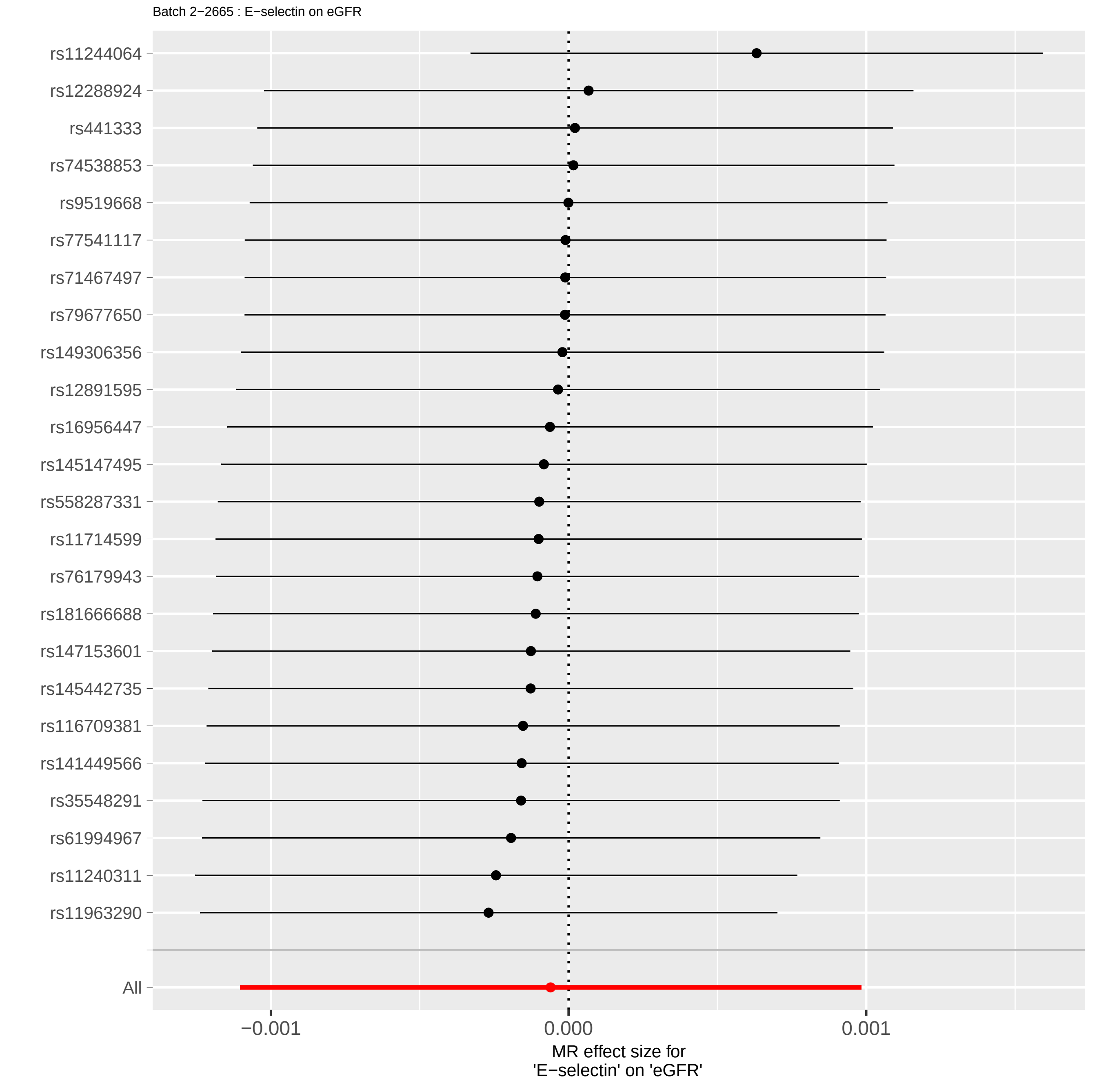

Batch 2−2665 : E−selectin on eGFR
rs11244064
rs12288924
rs441333
rs74538853
rs9519668
rs77541117
rs71467497
rs79677650
rs149306356
rs12891595
rs16956447
rs145147495
rs558287331
rs11714599
rs76179943
rs181666688
rs147153601
rs145442735
rs116709381
rs141449566
rs35548291
rs61994967
rs11240311
rs11963290
All
−0.001
0.000
0.001
MR effect size for
'E−selectin' on 'eGFR'

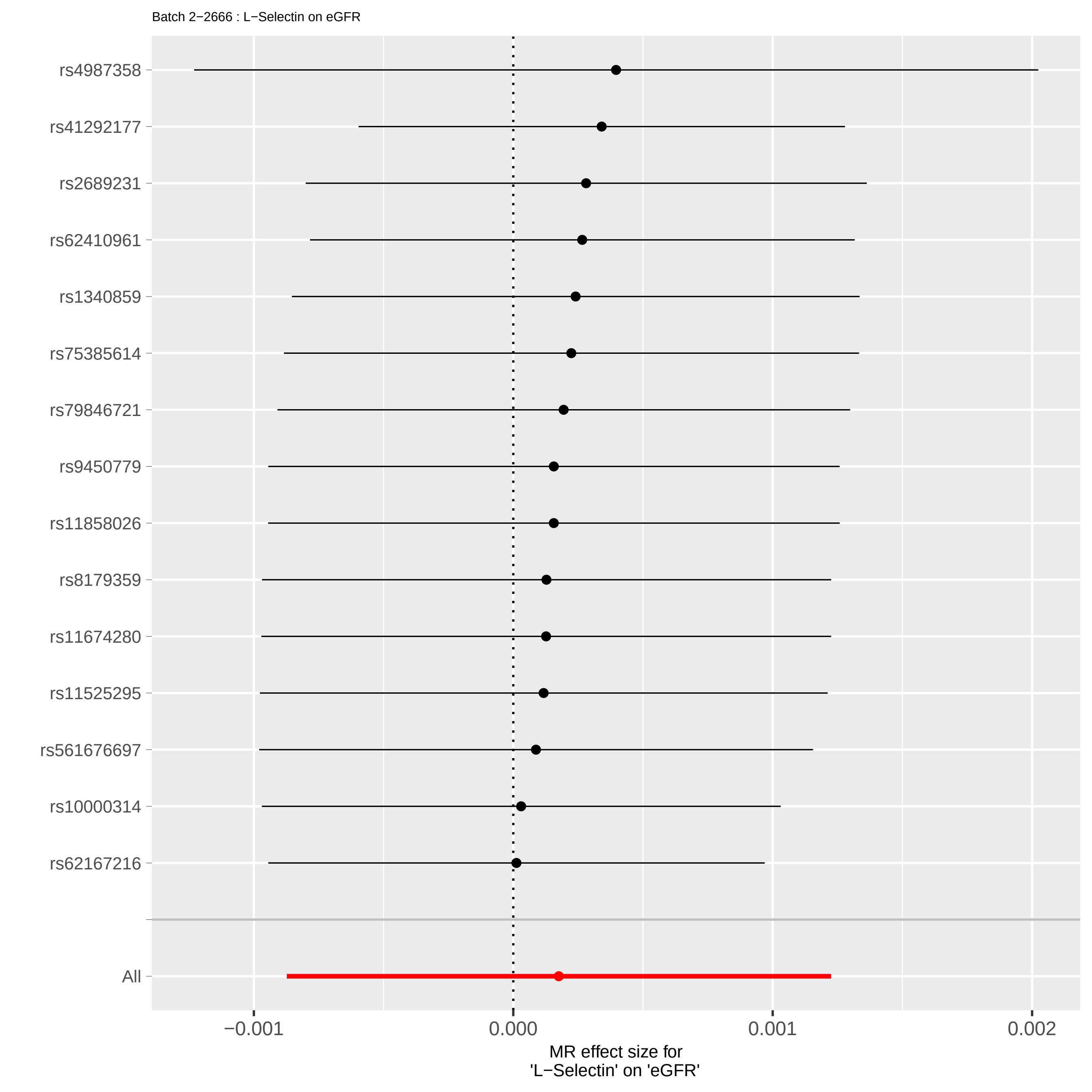

Batch 2−2666 : L−Selectin on eGFR
rs4987358
rs41292177
rs2689231
rs62410961
rs1340859
rs75385614
rs79846721
rs9450779
rs11858026
rs8179359
rs11674280
rs11525295
rs561676697
rs10000314
rs62167216
All
−0.001
0.000
0.001
0.002
MR effect size for
'L−Selectin' on 'eGFR'

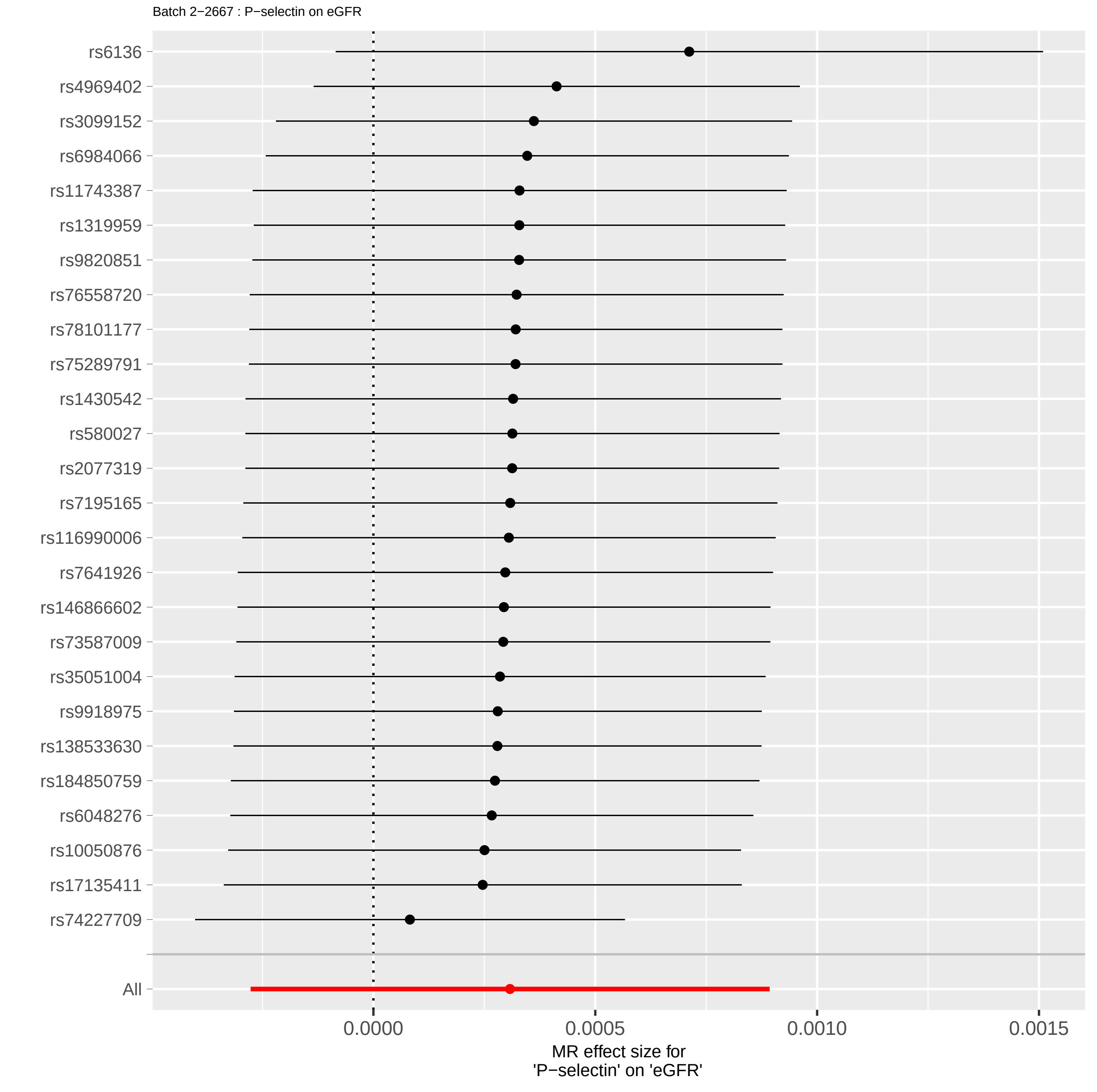
Batch 2−2667 : P−selectin on eGFR
rs6136
rs4969402
rs3099152
rs6984066
rs11743387
rs1319959
rs9820851
rs76558720
rs78101177
rs75289791
rs1430542
rs580027
rs2077319
rs7195165
rs116990006
rs7641926
rs146866602
rs73587009
rs35051004
rs9918975
rs138533630
rs184850759
rs6048276
rs10050876
rs17135411
rs74227709
All
0.0000
0.0005
0.0010
0.0015
MR effect size for
'P−selectin' on 'eGFR'

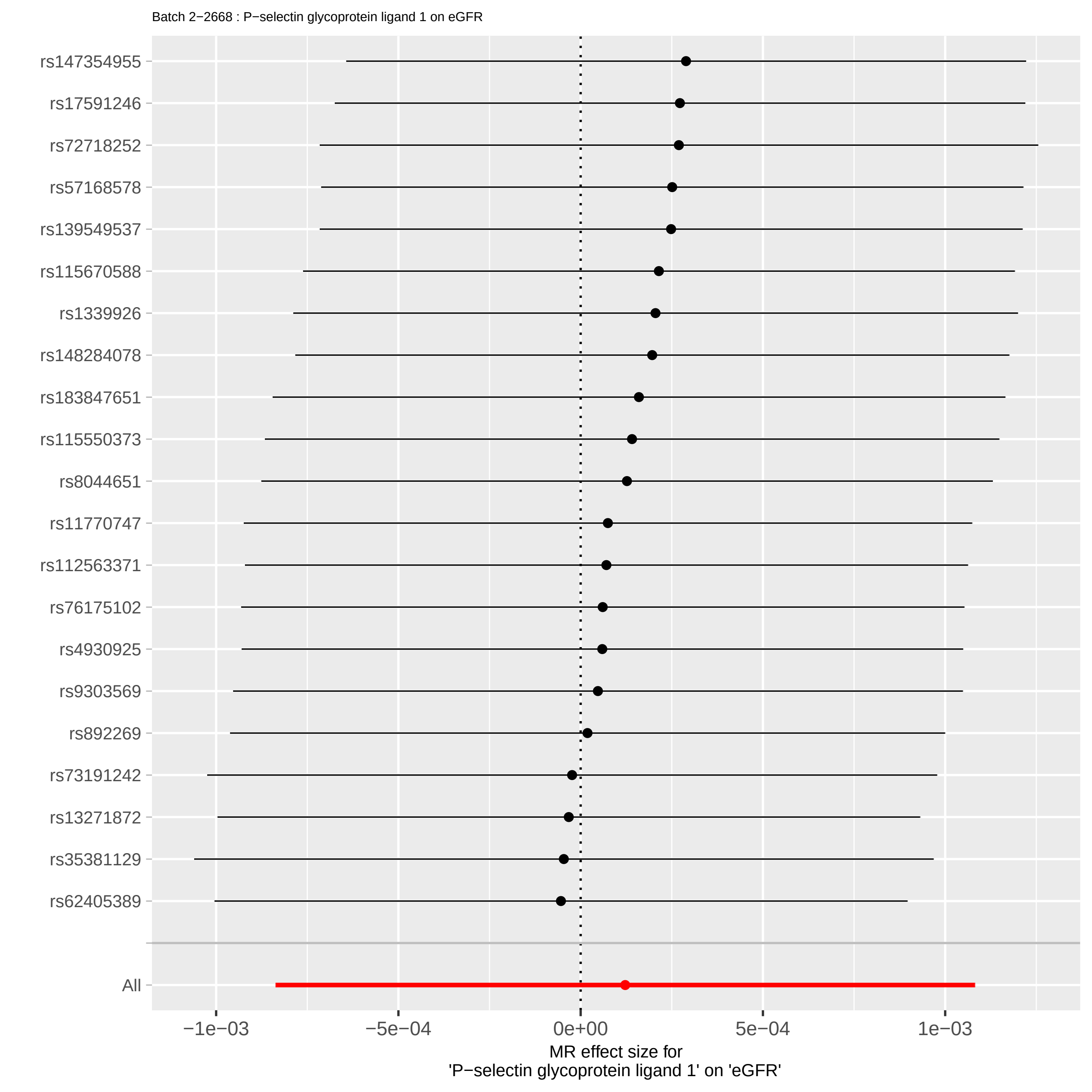
Batch 2−2668 : P−selectin glycoprotein ligand 1 on eGFR
rs147354955
rs17591246
rs72718252
rs57168578
rs139549537
rs115670588
rs1339926
rs148284078
rs183847651
rs115550373
rs8044651
rs11770747
rs112563371
rs76175102
rs4930925
rs9303569
rs892269
rs73191242
rs13271872
rs35381129
rs62405389
All
−1e−03
−5e−04
0e+00
5e−04
1e−03
MR effect size for
'P−selectin glycoprotein ligand 1' on 'eGFR'

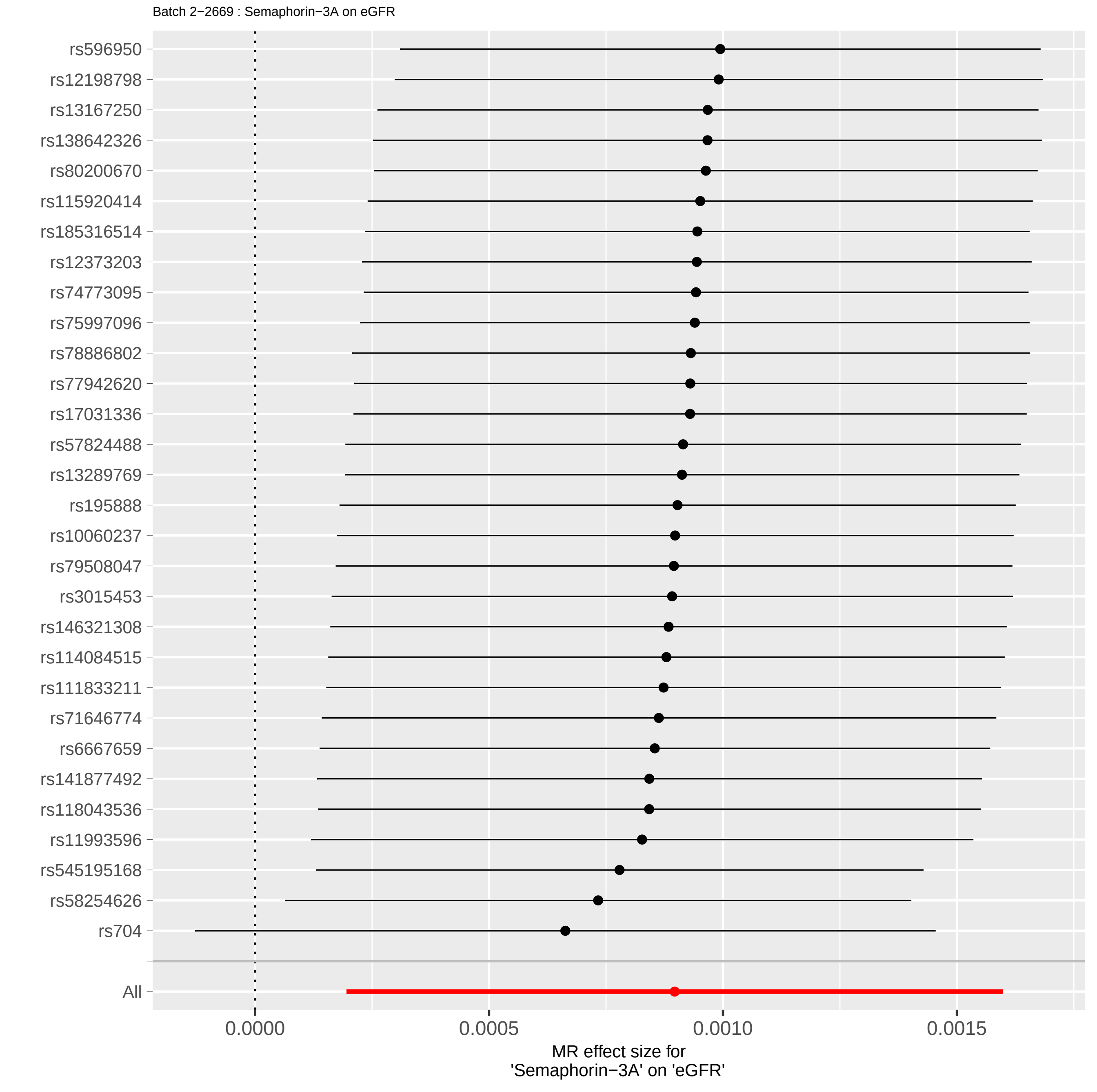

Batch 2−2669 : Semaphorin−3A on eGFR
rs596950
rs12198798
rs13167250
rs138642326
rs80200670
rs115920414
rs185316514
rs12373203
rs74773095
rs75997096
rs78886802
rs77942620
rs17031336
rs57824488
rs13289769
rs195888
rs10060237
rs79508047
rs3015453
rs146321308
rs114084515
rs111833211
rs71646774
rs6667659
rs141877492
rs118043536
rs11993596
rs545195168
rs58254626
rs704
All
0.0000
0.0005
0.0010
0.0015
MR effect size for
'Semaphorin−3A' on 'eGFR'

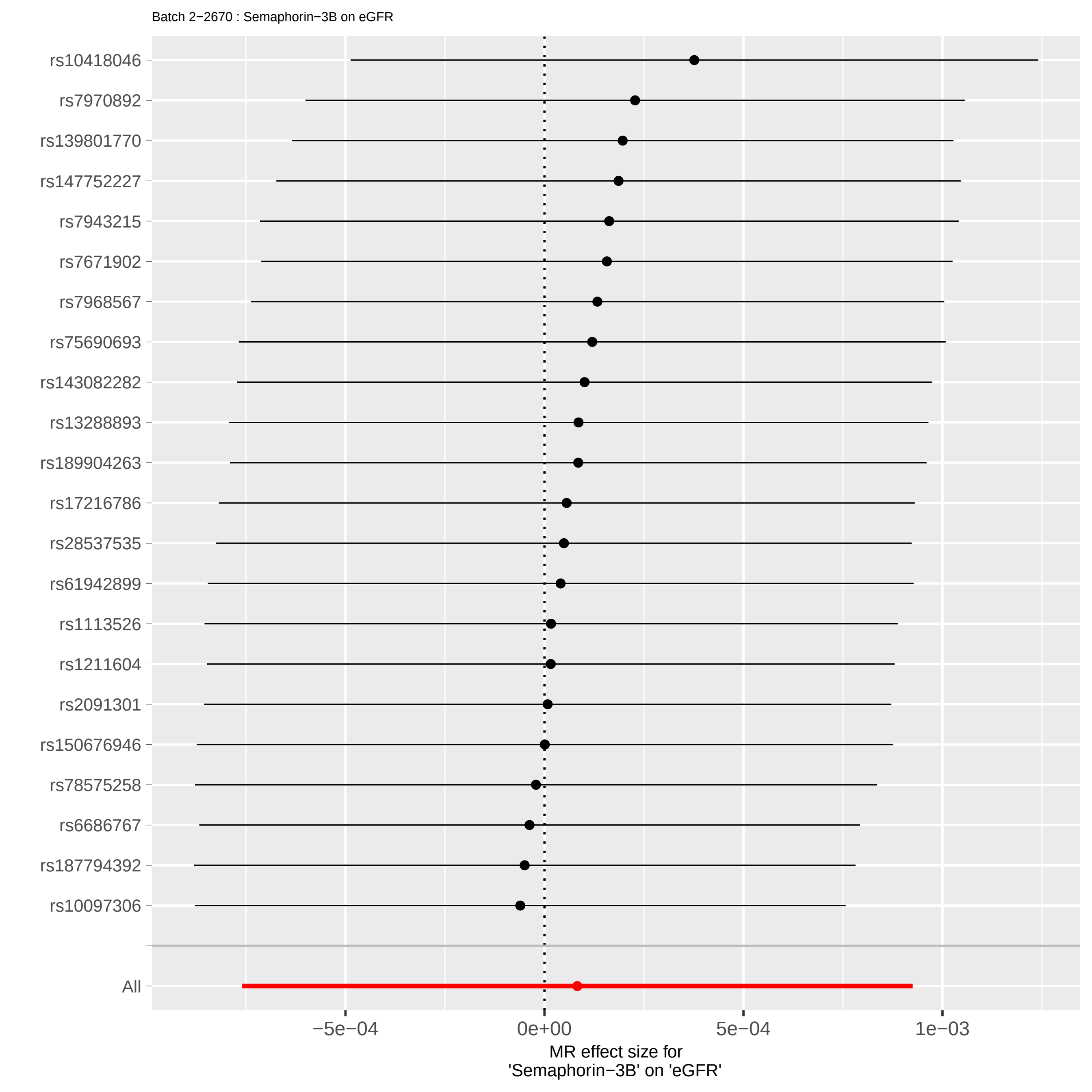
Batch 2−2670 : Semaphorin−3B on eGFR
rs10418046
rs7970892
rs139801770
rs147752227
rs7943215
rs7671902
rs7968567
rs75690693
rs143082282
rs13288893
rs189904263
rs17216786
rs28537535
rs61942899
rs1113526
rs1211604
rs2091301
rs150676946
rs78575258
rs6686767
rs187794392
rs10097306
All
−5e−04
0e+00
5e−04
1e−03
MR effect size for
'Semaphorin−3B' on 'eGFR'

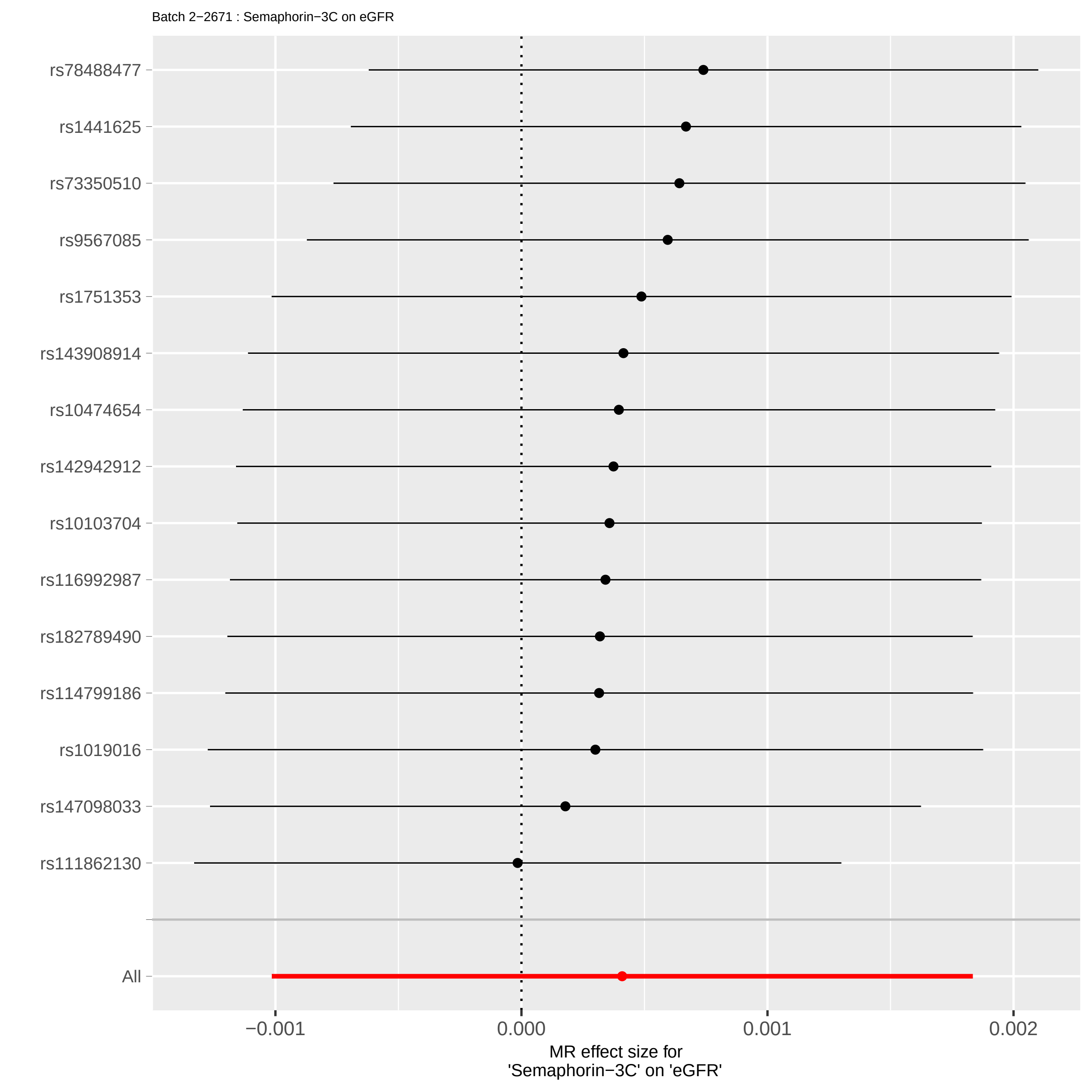

Batch 2−2671 : Semaphorin−3C on eGFR
rs78488477
rs1441625
rs73350510
rs9567085
rs1751353
rs143908914
rs10474654
rs142942912
rs10103704
rs116992987
rs182789490
rs114799186
rs1019016
rs147098033
rs111862130
All
−0.001
0.000
0.001
0.002
MR effect size for
'Semaphorin−3C' on 'eGFR'

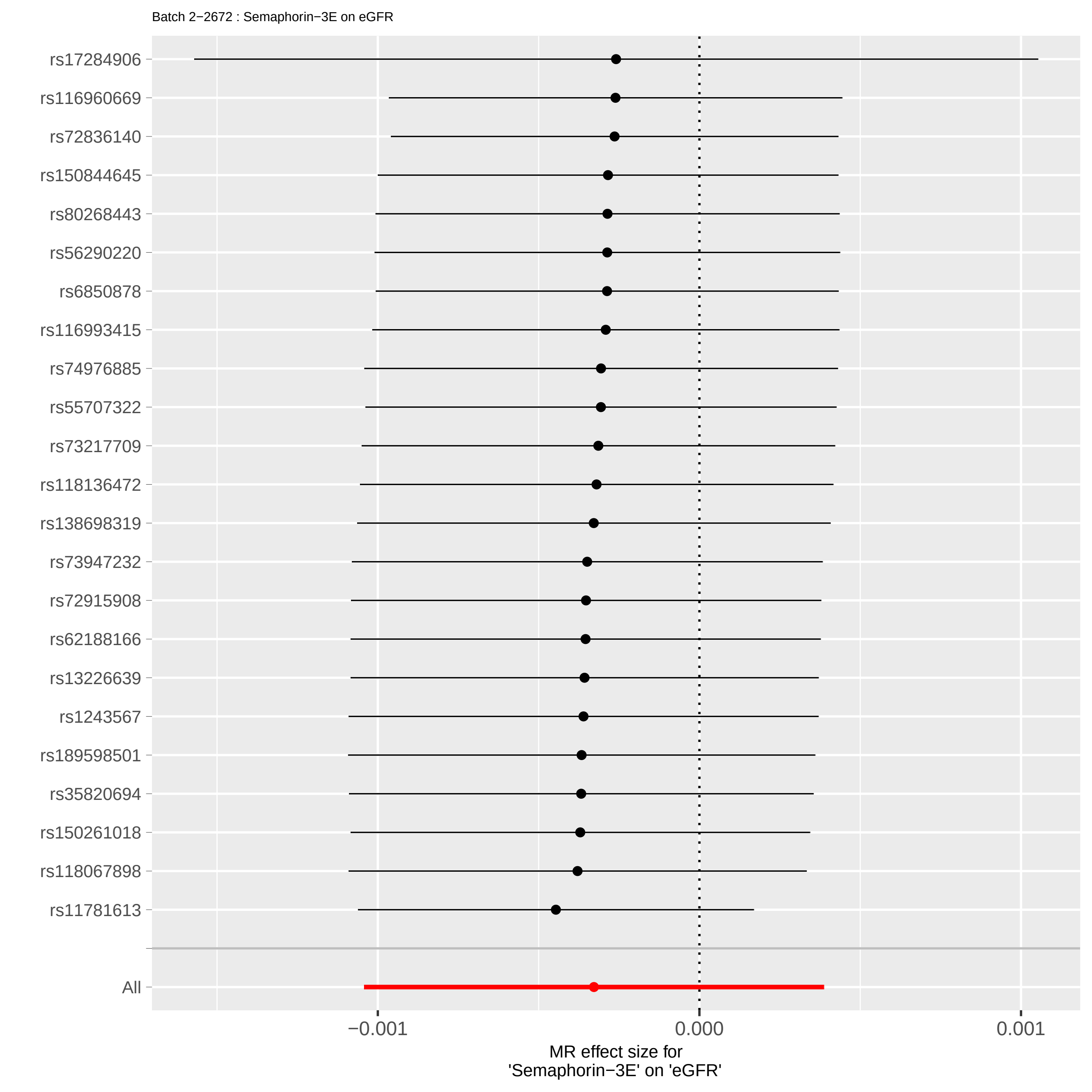

Batch 2−2672 : Semaphorin−3E on eGFR
rs17284906
rs116960669
rs72836140
rs150844645
rs80268443
rs56290220
rs6850878
rs116993415
rs74976885
rs55707322
rs73217709
rs118136472
rs138698319
rs73947232
rs72915908
rs62188166
rs13226639
rs1243567
rs189598501
rs35820694
rs150261018
rs118067898
rs11781613
All
−0.001
0.000
0.001
MR effect size for
'Semaphorin−3E' on 'eGFR'

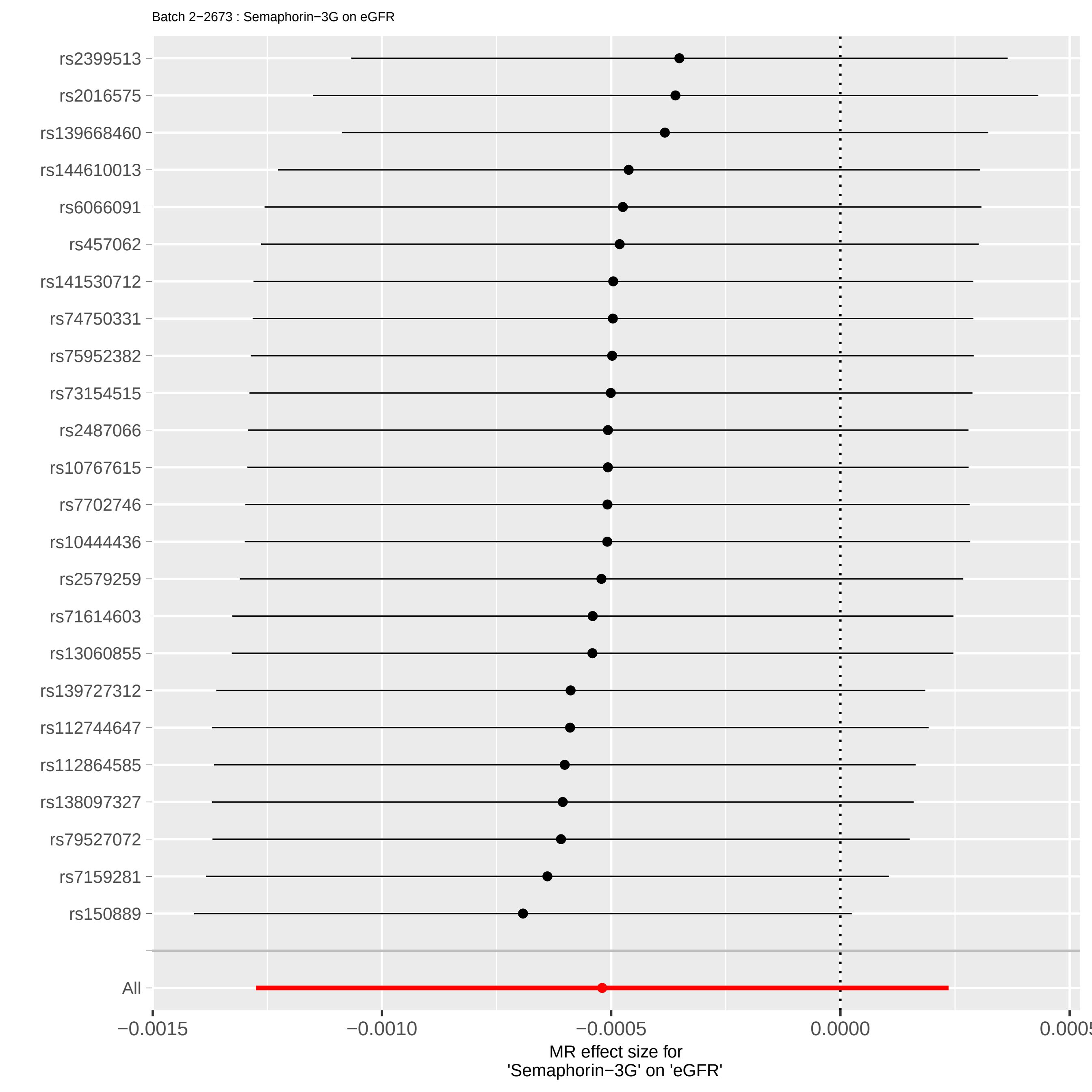
Batch 2−2673 : Semaphorin−3G on eGFR
rs2399513
rs2016575
rs139668460
rs144610013
rs6066091
rs457062
rs141530712
rs74750331
rs75952382
rs73154515
rs2487066
rs10767615
rs7702746
rs10444436
rs2579259
rs71614603
rs13060855
rs139727312
rs112744647
rs112864585
rs138097327
rs79527072
rs7159281
rs150889
All
−0.0015
−0.0010
−0.0005
0.0000
MR effect size for
'Semaphorin−3G' on 'eGFR'

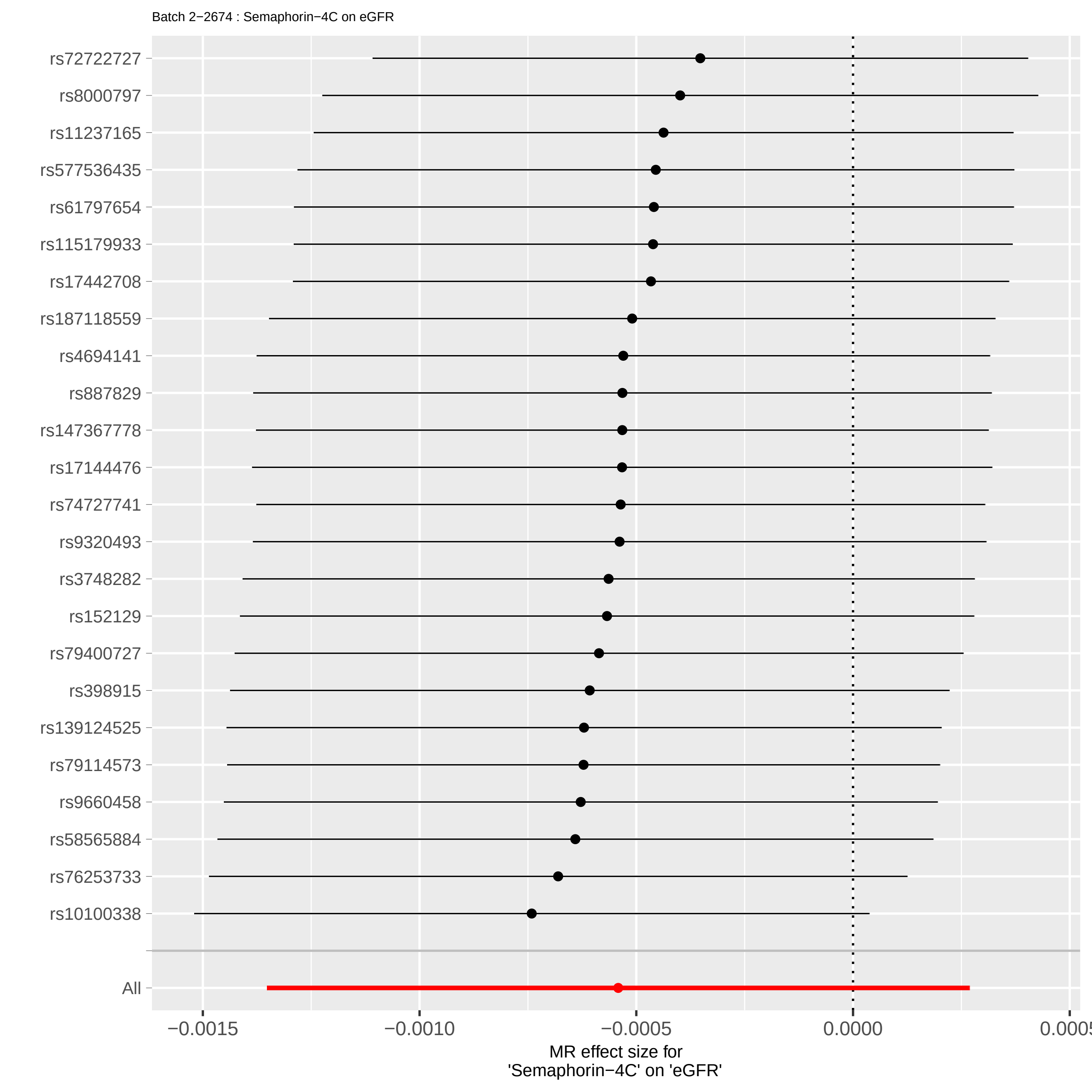

Batch 2−2674 : Semaphorin−4C on eGFR
rs72722727
rs8000797
rs11237165
rs577536435
rs61797654
rs115179933
rs17442708
rs187118559
rs4694141
rs887829
rs147367778
rs17144476
rs74727741
rs9320493
rs3748282
rs152129
rs79400727
rs398915
rs139124525
rs79114573
rs9660458
rs58565884
rs76253733
rs10100338
All
−0.0015
−0.0010
−0.0005
0.0000
MR effect size for
'Semaphorin−4C' on 'eGFR'

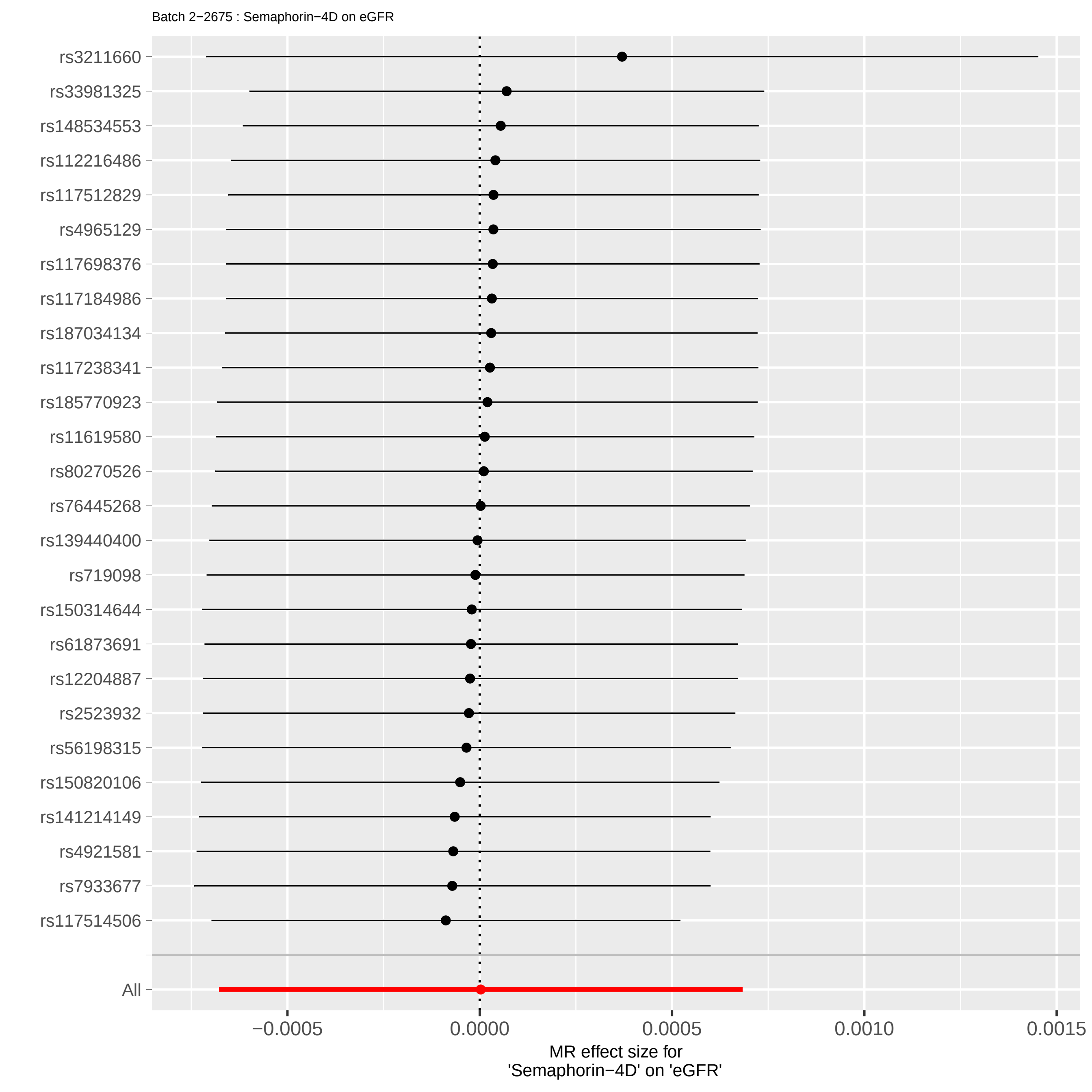

Batch 2−2675 : Semaphorin−4D on eGFR
rs3211660
rs33981325
rs148534553
rs112216486
rs117512829
rs4965129
rs117698376
rs117184986
rs187034134
rs117238341
rs185770923
rs11619580
rs80270526
rs76445268
rs139440400
rs719098
rs150314644
rs61873691
rs12204887
rs2523932
rs56198315
rs150820106
rs141214149
rs4921581
rs7933677
rs117514506
All
−0.0005
0.0000
0.0005
0.0010
0.0015
MR effect size for
'Semaphorin−4D' on 'eGFR'

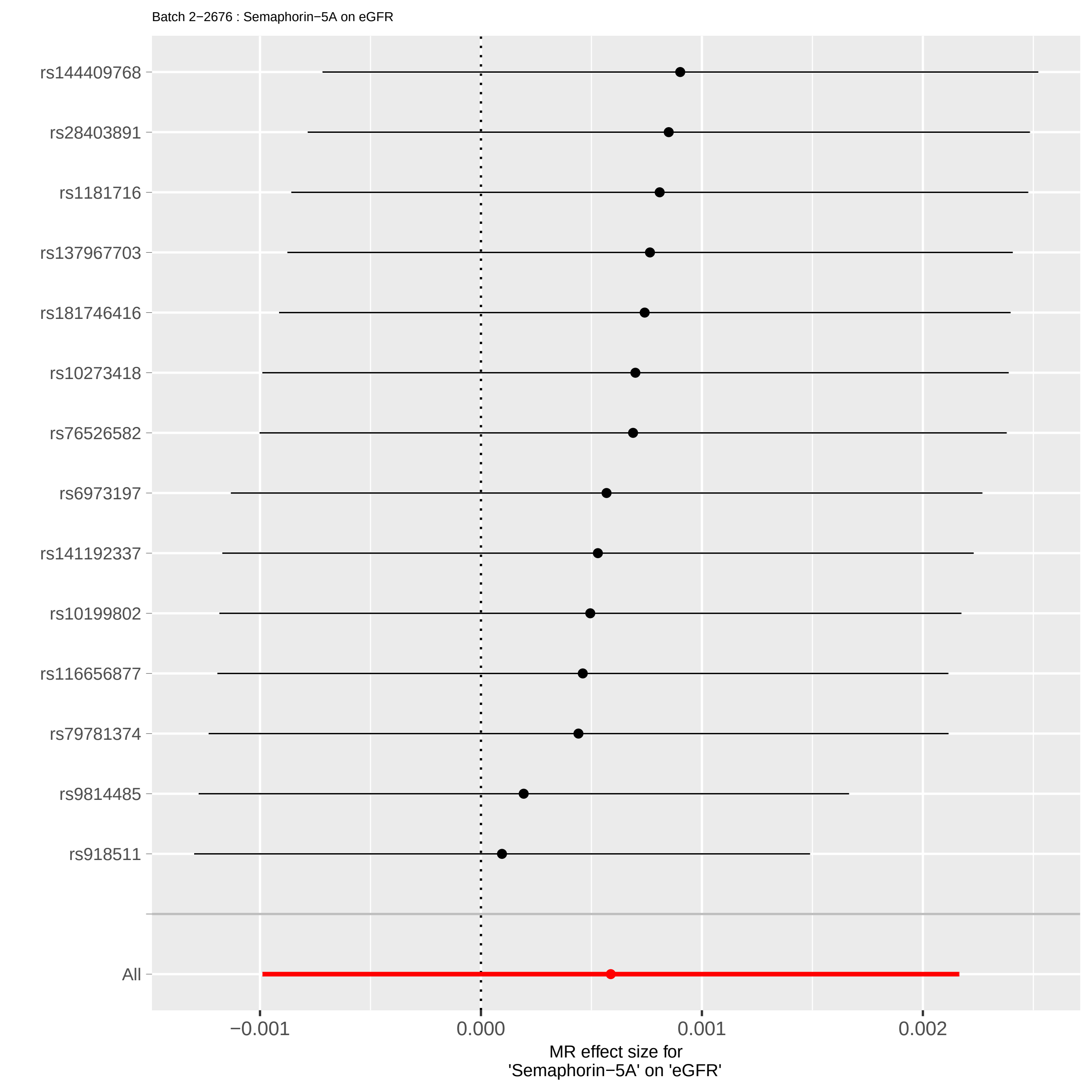

Batch 2−2676 : Semaphorin−5A on eGFR
rs144409768
rs28403891
rs1181716
rs137967703
rs181746416
rs10273418
rs76526582
rs6973197
rs141192337
rs10199802
rs116656877
rs79781374
rs9814485
rs918511
All
−0.001
0.000
0.001
0.002
MR effect size for
'Semaphorin−5A' on 'eGFR'

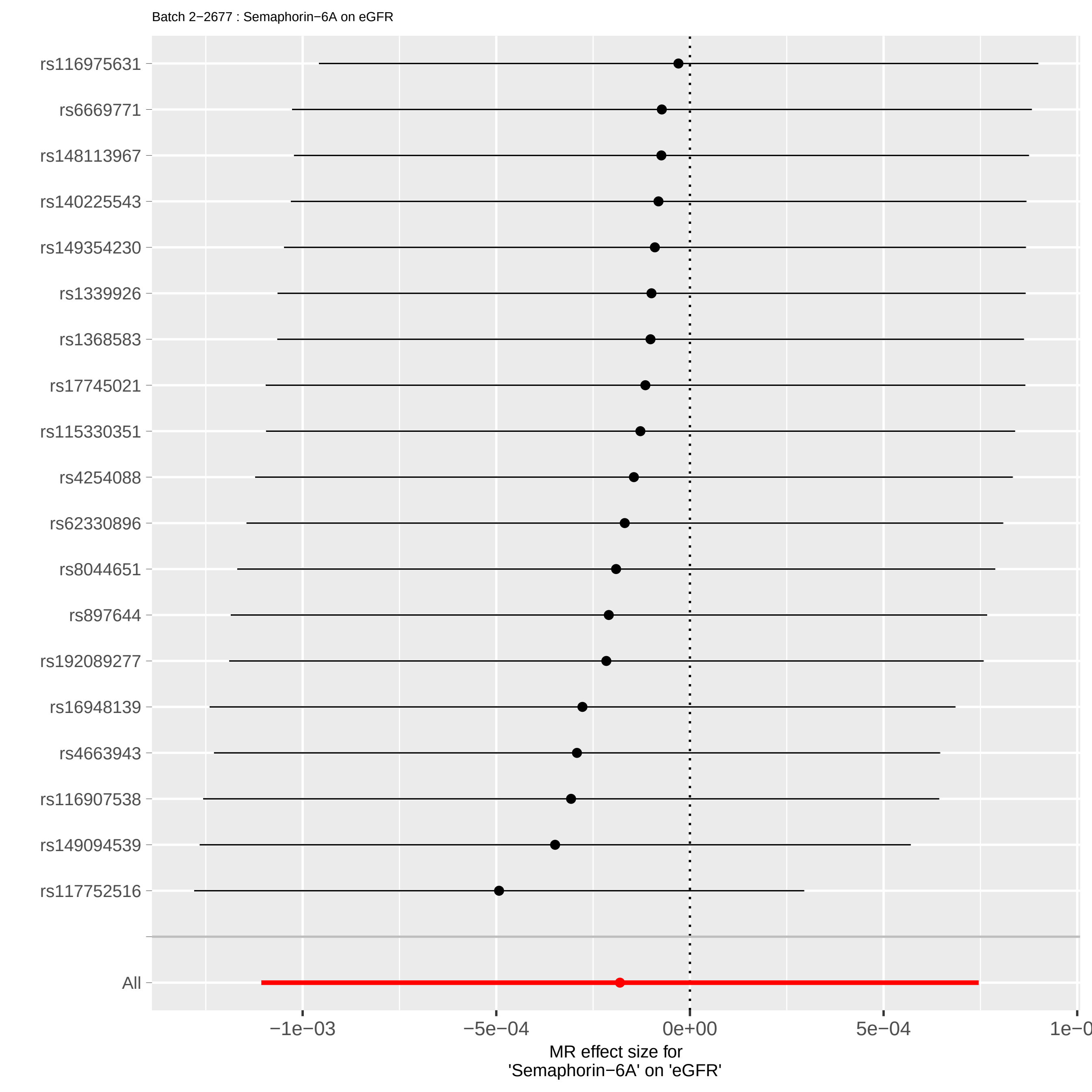

Batch 2−2677 : Semaphorin−6A on eGFR
rs116975631
rs6669771
rs148113967
rs140225543
rs149354230
rs1339926
rs1368583
rs17745021
rs115330351
rs4254088
rs62330896
rs8044651
rs897644
rs192089277
rs16948139
rs4663943
rs116907538
rs149094539
rs117752516
All
−1e−03
−5e−04
0e+00
5e−04
MR effect size for
'Semaphorin−6A' on 'eGFR'

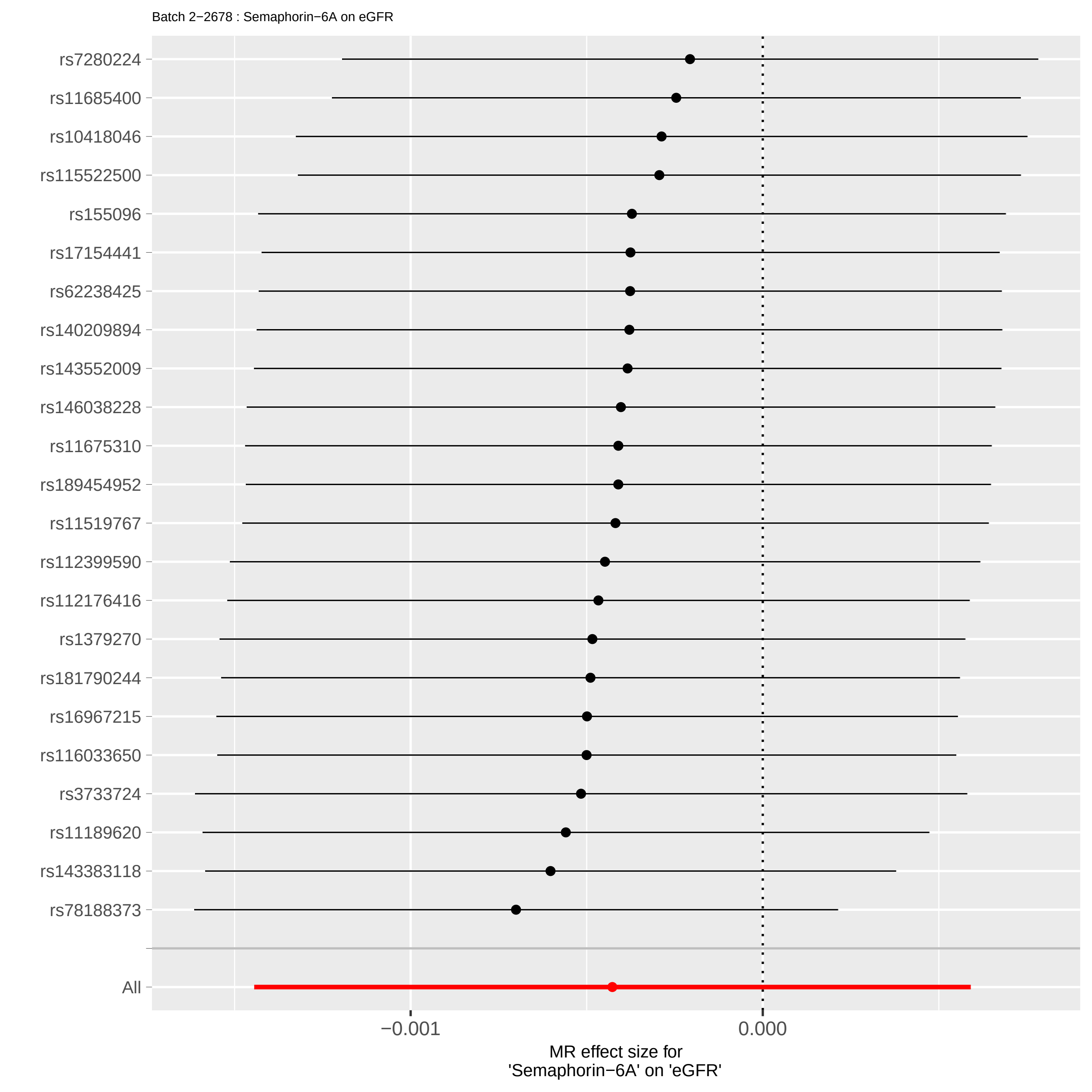
Batch 2−2678 : Semaphorin−6A on eGFR
rs7280224
rs11685400
rs10418046
rs115522500
rs155096
rs17154441
rs62238425
rs140209894
rs143552009
rs146038228
rs11675310
rs189454952
rs11519767
rs112399590
rs112176416
rs1379270
rs181790244
rs16967215
rs116033650
rs3733724
rs11189620
rs143383118
rs78188373
All
−0.001
0.000
MR effect size for
'Semaphorin−6A' on 'eGFR'

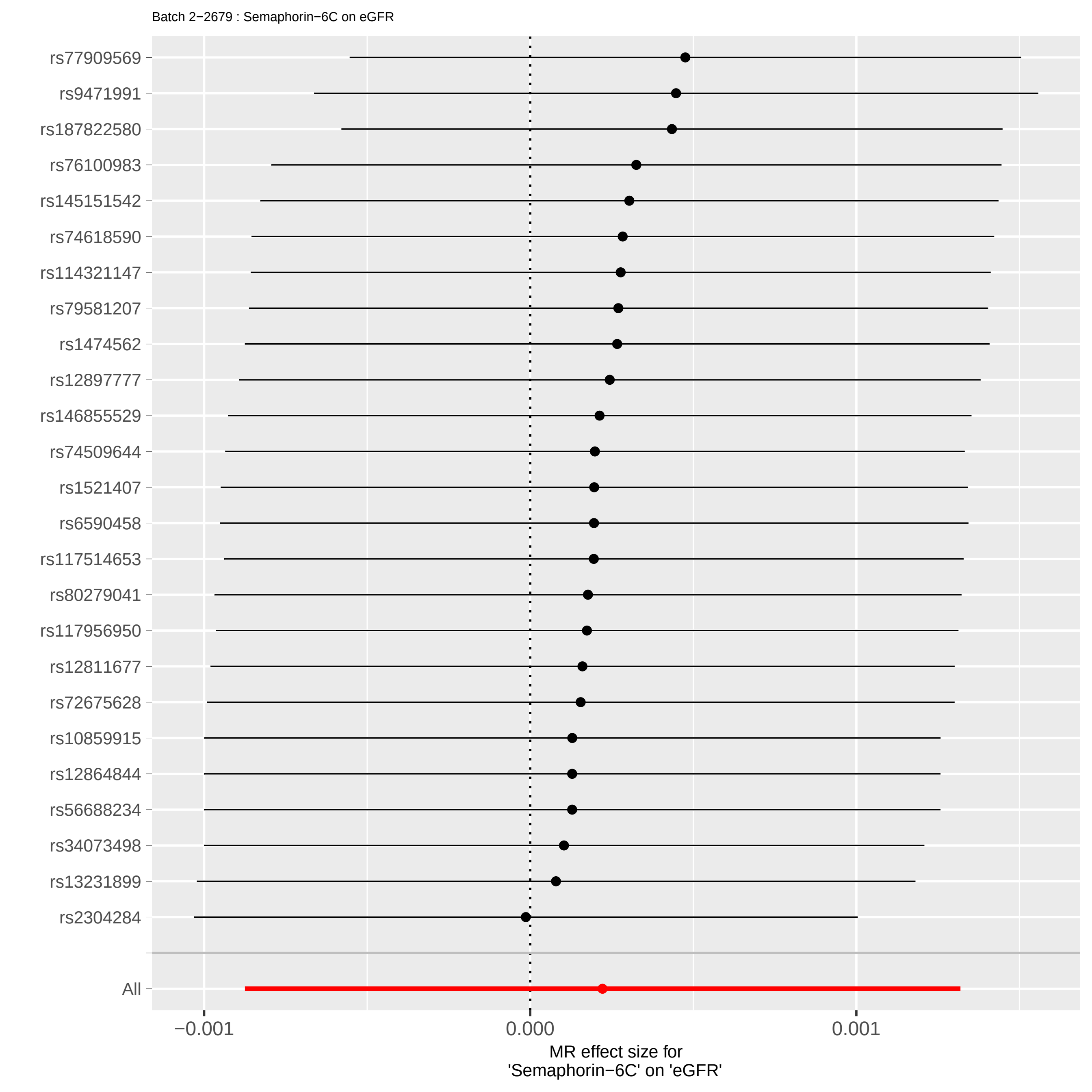
Batch 2−2679 : Semaphorin−6C on eGFR
rs77909569
rs9471991
rs187822580
rs76100983
rs145151542
rs74618590
rs114321147
rs79581207
rs1474562
rs12897777
rs146855529
rs74509644
rs1521407
rs6590458
rs117514653
rs80279041
rs117956950
rs12811677
rs72675628
rs10859915
rs12864844
rs56688234
rs34073498
rs13231899
rs2304284
All
−0.001
0.000
0.001
MR effect size for
'Semaphorin−6C' on 'eGFR'

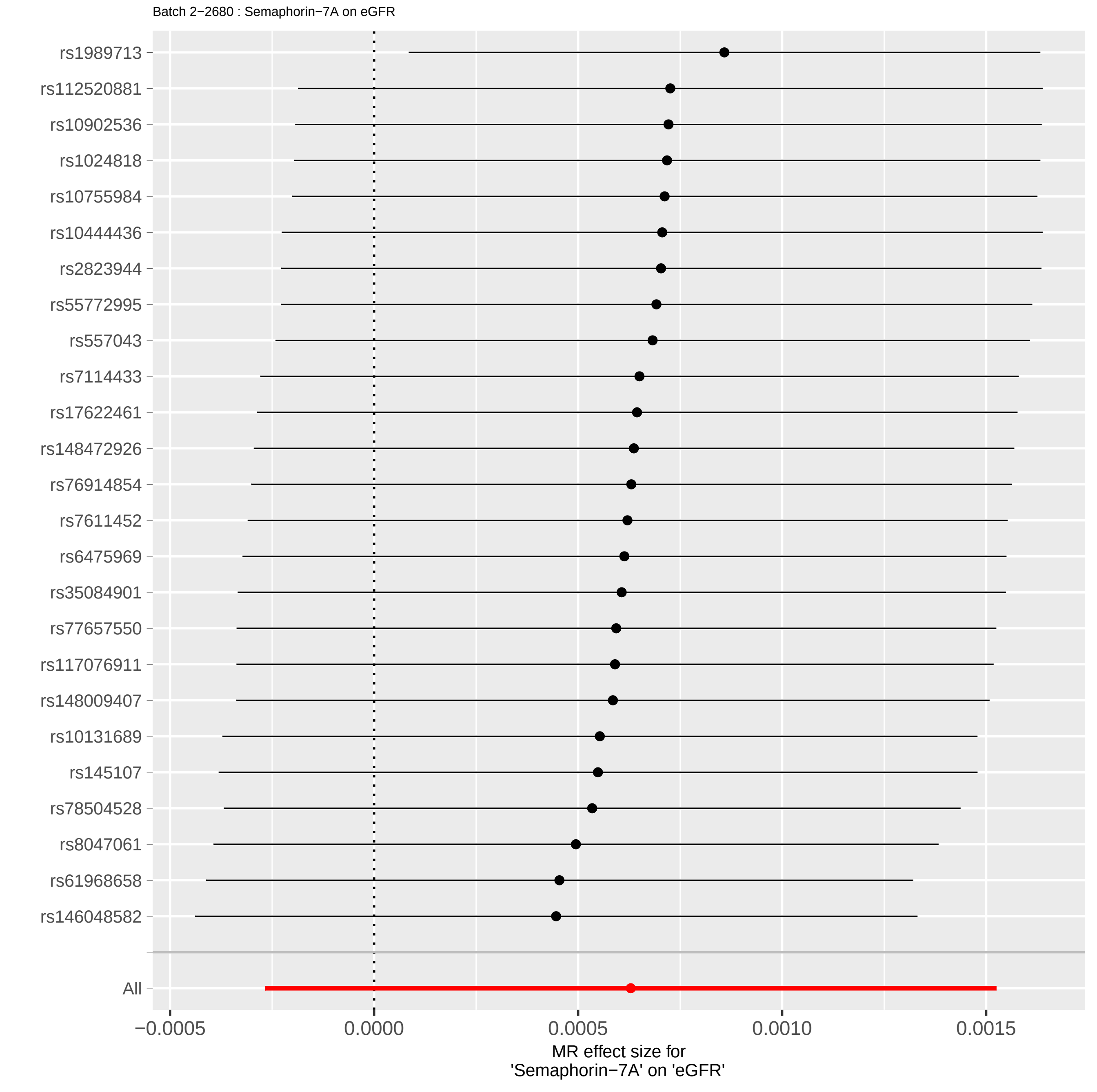

Batch 2−2680 : Semaphorin−7A on eGFR
rs1989713
rs112520881
rs10902536
rs1024818
rs10755984
rs10444436
rs2823944
rs55772995
rs557043
rs7114433
rs17622461
rs148472926
rs76914854
rs7611452
rs6475969
rs35084901
rs77657550
rs117076911
rs148009407
rs10131689
rs145107
rs78504528
rs8047061
rs61968658
rs146048582
All
−0.0005
0.0000
0.0005
0.0010
0.0015
MR effect size for
'Semaphorin−7A' on 'eGFR'

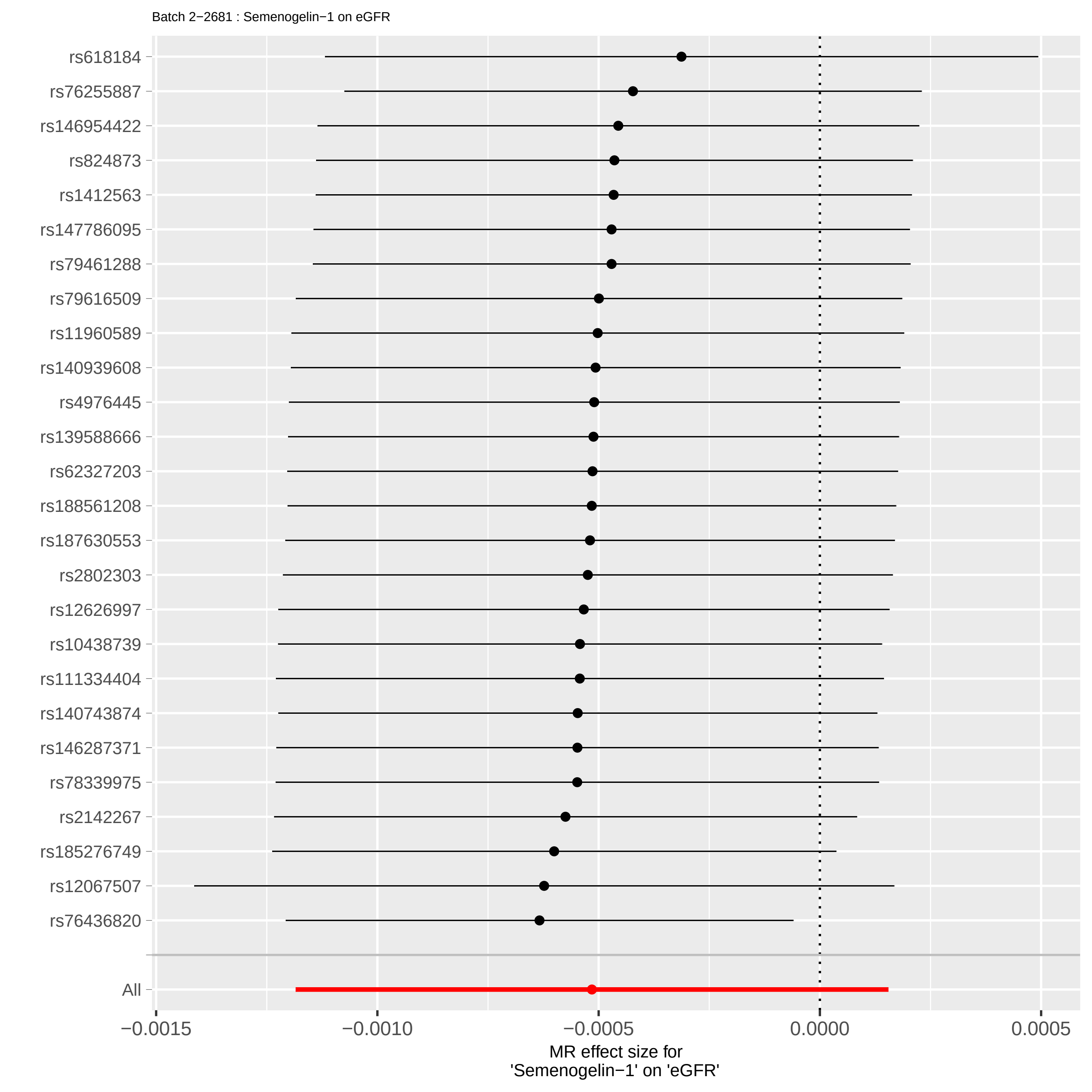

Batch 2−2681 : Semenogelin−1 on eGFR
rs618184
rs76255887
rs146954422
rs824873
rs1412563
rs147786095
rs79461288
rs79616509
rs11960589
rs140939608
rs4976445
rs139588666
rs62327203
rs188561208
rs187630553
rs2802303
rs12626997
rs10438739
rs111334404
rs140743874
rs146287371
rs78339975
rs2142267
rs185276749
rs12067507
rs76436820
All
−0.0015
−0.0010
−0.0005
0.0000
0.0005
MR effect size for
'Semenogelin−1' on 'eGFR'

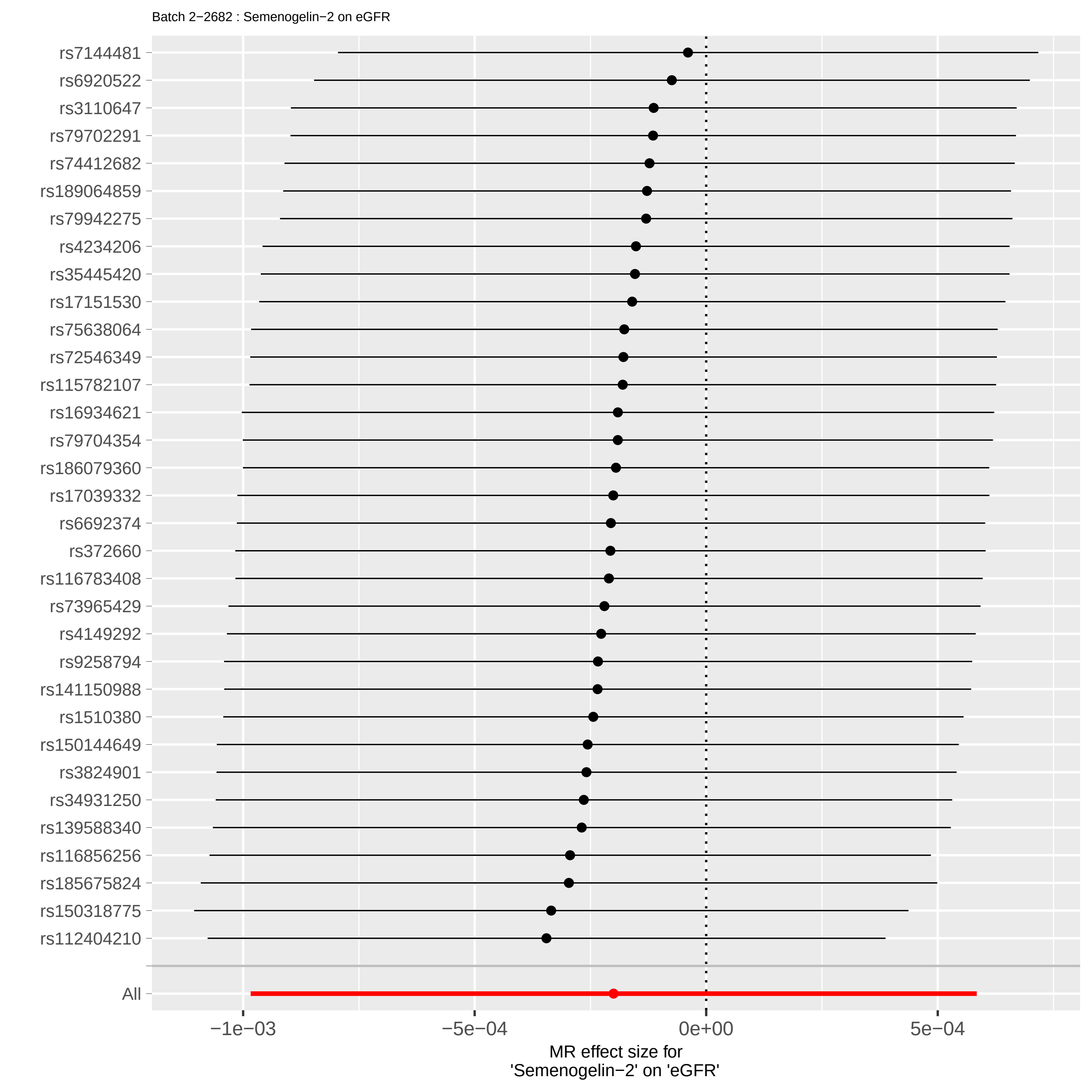

Batch 2−2682 : Semenogelin−2 on eGFR
rs7144481
rs6920522
rs3110647
rs79702291
rs74412682
rs189064859
rs79942275
rs4234206
rs35445420
rs17151530
rs75638064
rs72546349
rs115782107
rs16934621
rs79704354
rs186079360
rs17039332
rs6692374
rs372660
rs116783408
rs73965429
rs4149292
rs9258794
rs141150988
rs1510380
rs150144649
rs3824901
rs34931250
rs139588340
rs116856256
rs185675824
rs150318775
rs112404210
All
−1e−03
−5e−04
0e+00
5e−04
MR effect size for
'Semenogelin−2' on 'eGFR'

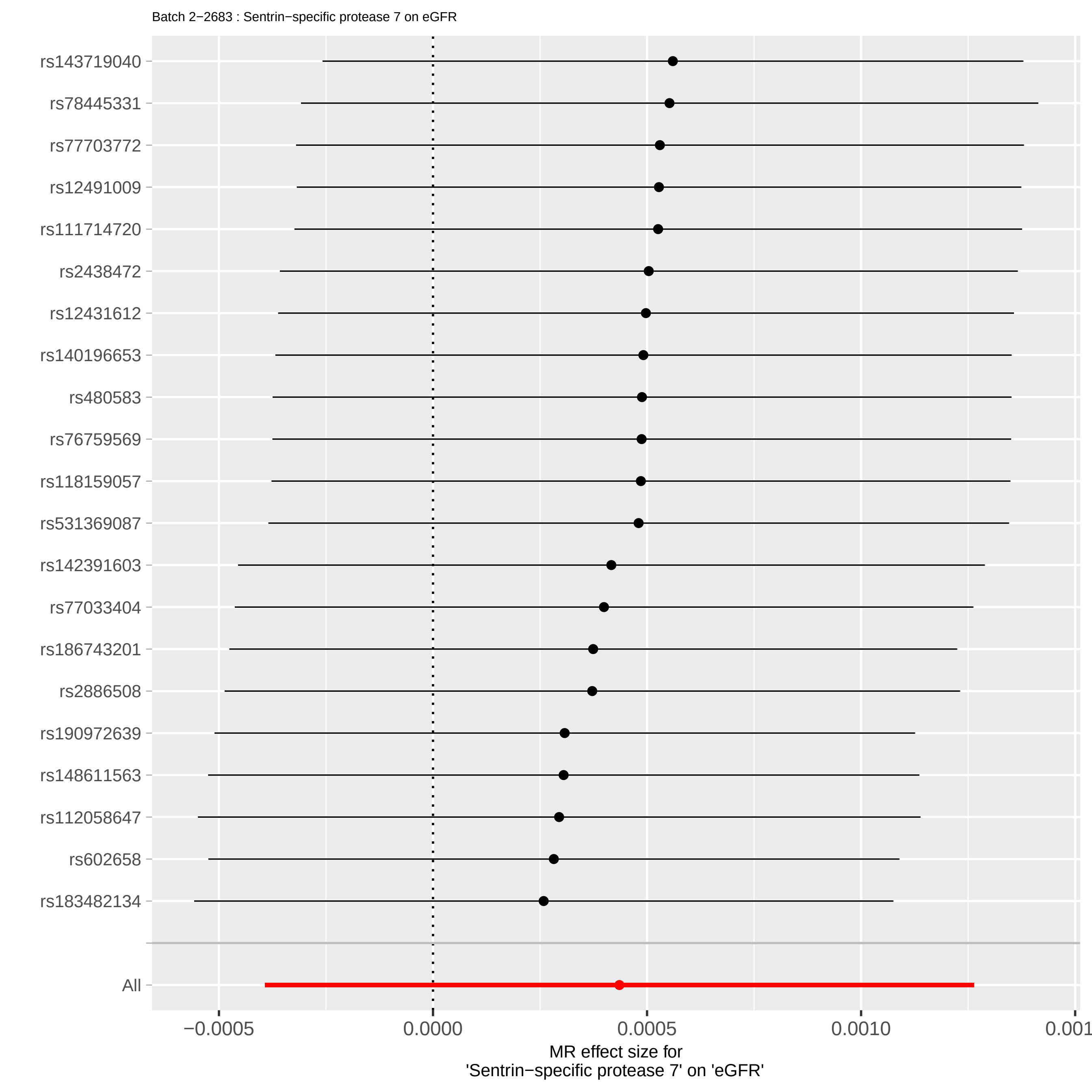

Batch 2−2683 : Sentrin−specific protease 7 on eGFR
rs143719040
rs78445331
rs77703772
rs12491009
rs111714720
rs2438472
rs12431612
rs140196653
rs480583
rs76759569
rs118159057
rs531369087
rs142391603
rs77033404
rs186743201
rs2886508
rs190972639
rs148611563
rs112058647
rs602658
rs183482134
All
−0.0005
0.0000
0.0005
0.0010
MR effect size for
'Sentrin−specific protease 7' on 'eGFR'

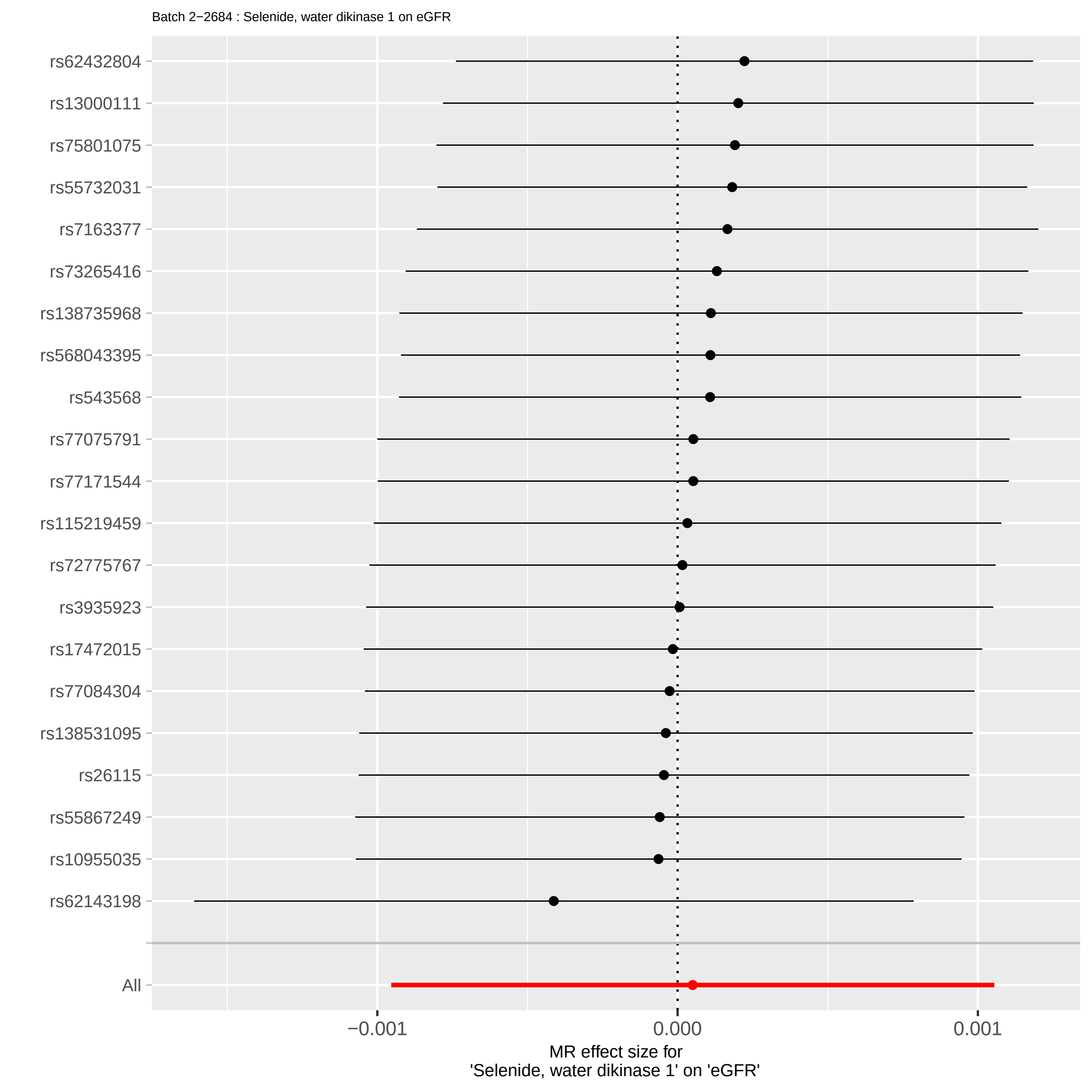

Batch 2−2684 : Selenide, water dikinase 1 on eGFR
rs62432804
rs13000111
rs75801075
rs55732031
rs7163377
rs73265416
rs138735968
rs568043395
rs543568
rs77075791
rs77171544
rs115219459
rs72775767
rs3935923
rs17472015
rs77084304
rs138531095
rs26115
rs55867249
rs10955035
rs62143198
All
−0.001
0.000
0.001
MR effect size for
'Selenide, water dikinase 1' on 'eGFR'

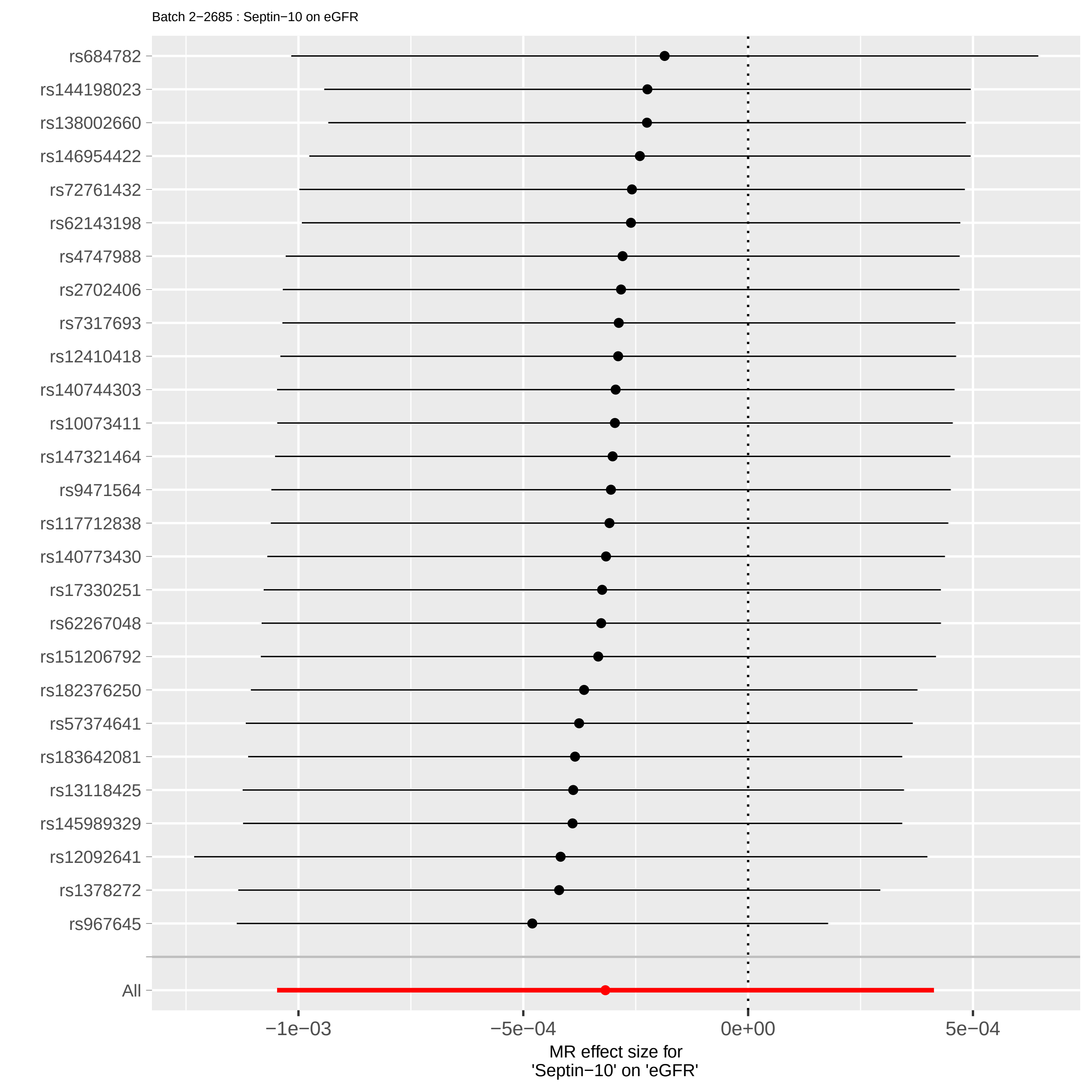

Batch 2−2685 : Septin−10 on eGFR
rs684782
rs144198023
rs138002660
rs146954422
rs72761432
rs62143198
rs4747988
rs2702406
rs7317693
rs12410418
rs140744303
rs10073411
rs147321464
rs9471564
rs117712838
rs140773430
rs17330251
rs62267048
rs151206792
rs182376250
rs57374641
rs183642081
rs13118425
rs145989329
rs12092641
rs1378272
rs967645
All
−1e−03
−5e−04
0e+00
5e−04
MR effect size for
'Septin−10' on 'eGFR'

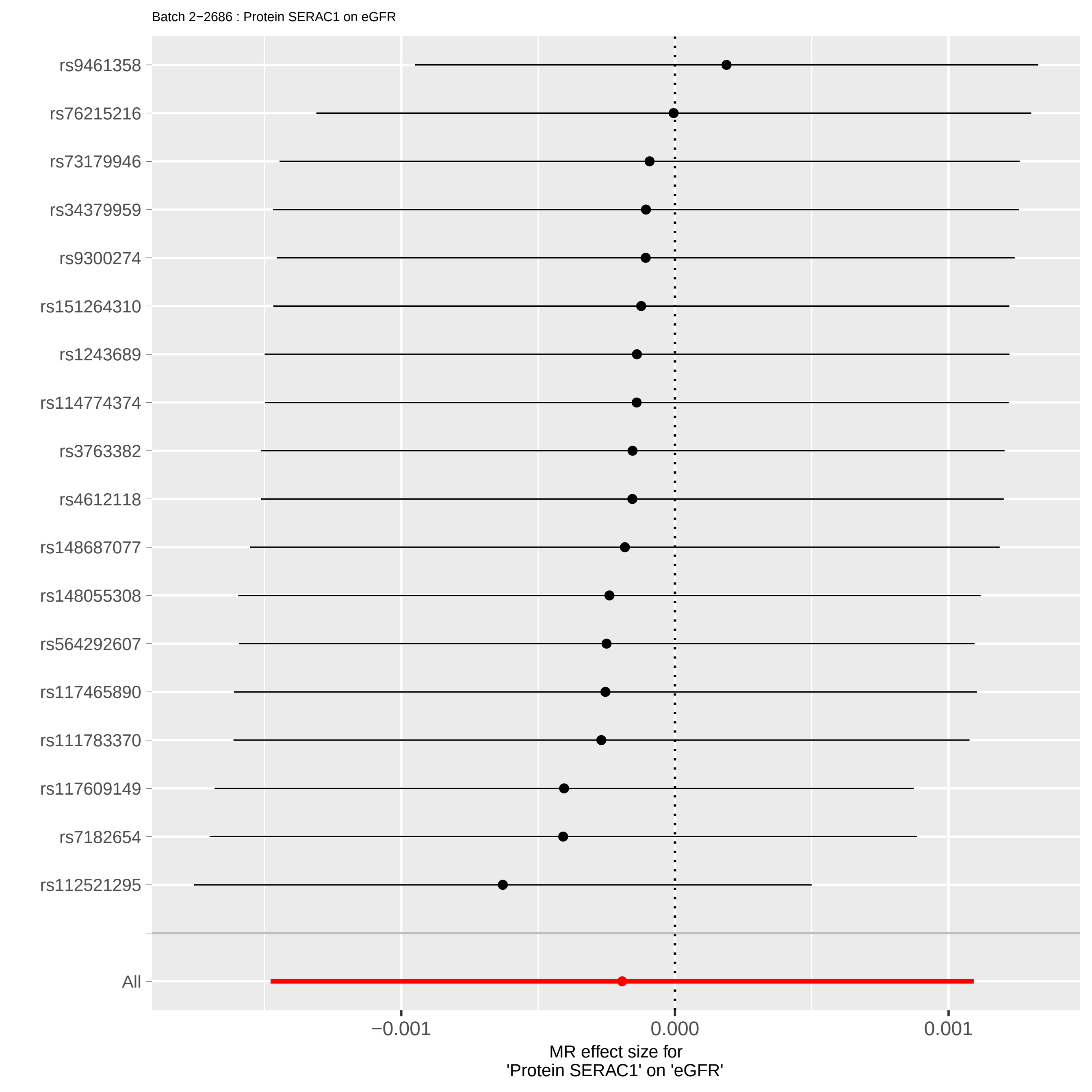

Batch 2−2686 : Protein SERAC1 on eGFR
rs9461358
rs76215216
rs73179946
rs34379959
rs9300274
rs151264310
rs1243689
rs114774374
rs3763382
rs4612118
rs148687077
rs148055308
rs564292607
rs117465890
rs111783370
rs117609149
rs7182654
rs112521295
All
−0.001
0.000
0.001
MR effect size for
'Protein SERAC1' on 'eGFR'

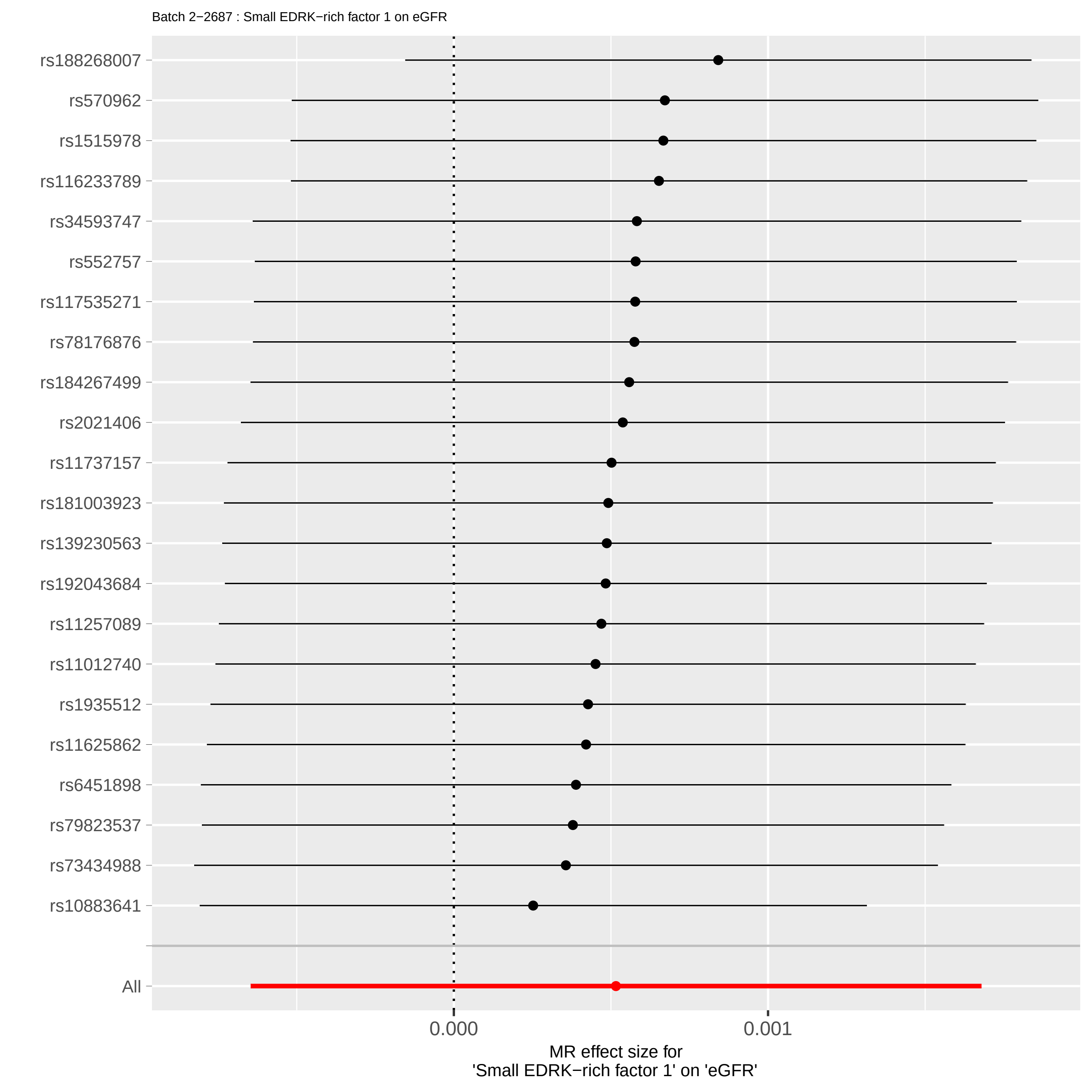

Batch 2−2687 : Small EDRK−rich factor 1 on eGFR
rs188268007
rs570962
rs1515978
rs116233789
rs34593747
rs552757
rs117535271
rs78176876
rs184267499
rs2021406
rs11737157
rs181003923
rs139230563
rs192043684
rs11257089
rs11012740
rs1935512
rs11625862
rs6451898
rs79823537
rs73434988
rs10883641
All
0.000
0.001
MR effect size for
'Small EDRK−rich factor 1' on 'eGFR'

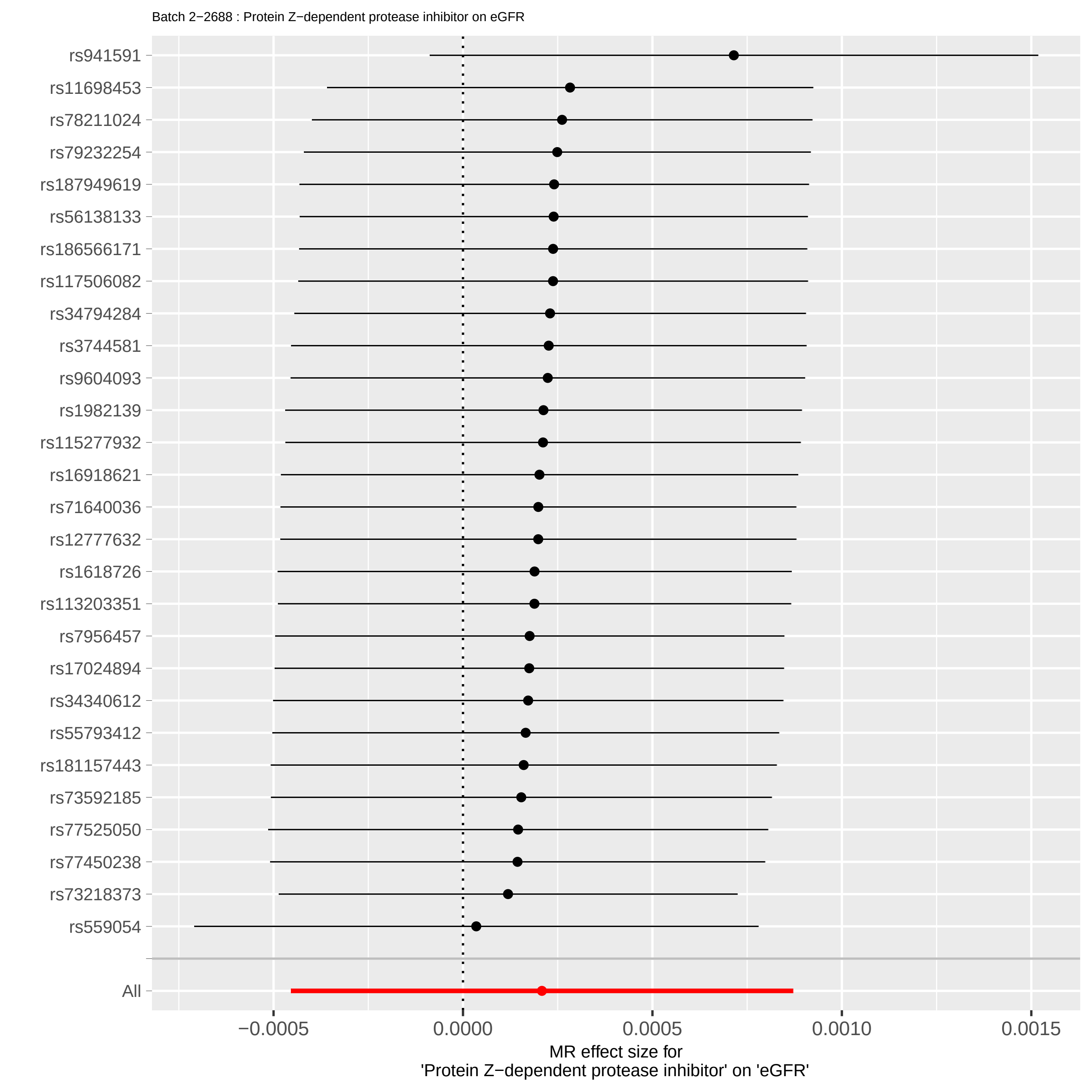
Batch 2−2688 : Protein Z−dependent protease inhibitor on eGFR
rs941591
rs11698453
rs78211024
rs79232254
rs187949619
rs56138133
rs186566171
rs117506082
rs34794284
rs3744581
rs9604093
rs1982139
rs115277932
rs16918621
rs71640036
rs12777632
rs1618726
rs113203351
rs7956457
rs17024894
rs34340612
rs55793412
rs181157443
rs73592185
rs77525050
rs77450238
rs73218373
rs559054
All
−0.0005
0.0000
0.0005
0.0010
0.0015
MR effect size for
'Protein Z−dependent protease inhibitor' on 'eGFR'

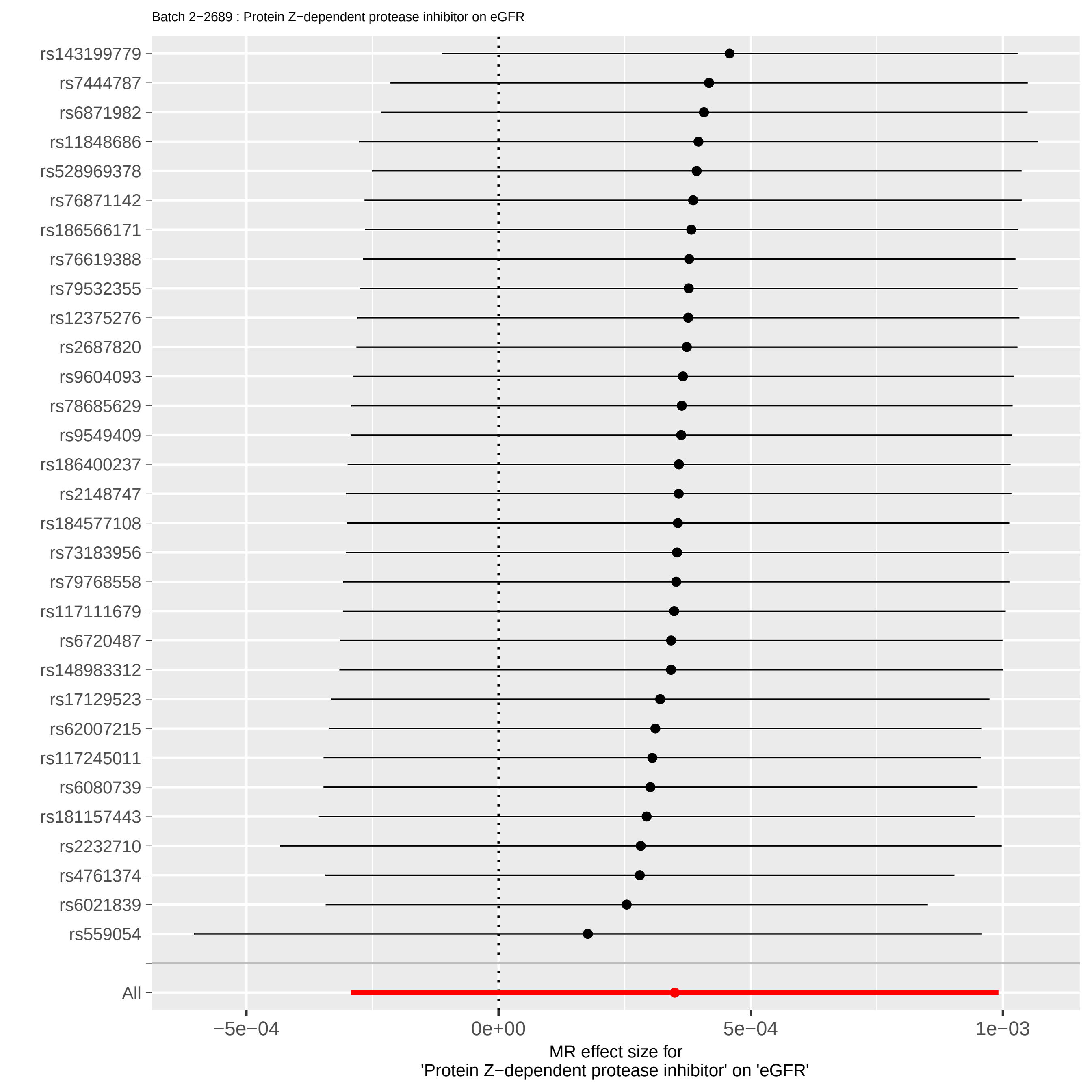

Batch 2−2689 : Protein Z−dependent protease inhibitor on eGFR
rs143199779
rs7444787
rs6871982
rs11848686
rs528969378
rs76871142
rs186566171
rs76619388
rs79532355
rs12375276
rs2687820
rs9604093
rs78685629
rs9549409
rs186400237
rs2148747
rs184577108
rs73183956
rs79768558
rs117111679
rs6720487
rs148983312
rs17129523
rs62007215
rs117245011
rs6080739
rs181157443
rs2232710
rs4761374
rs6021839
rs559054
All
−5e−04
0e+00
5e−04
1e−03
MR effect size for
'Protein Z−dependent protease inhibitor' on 'eGFR'

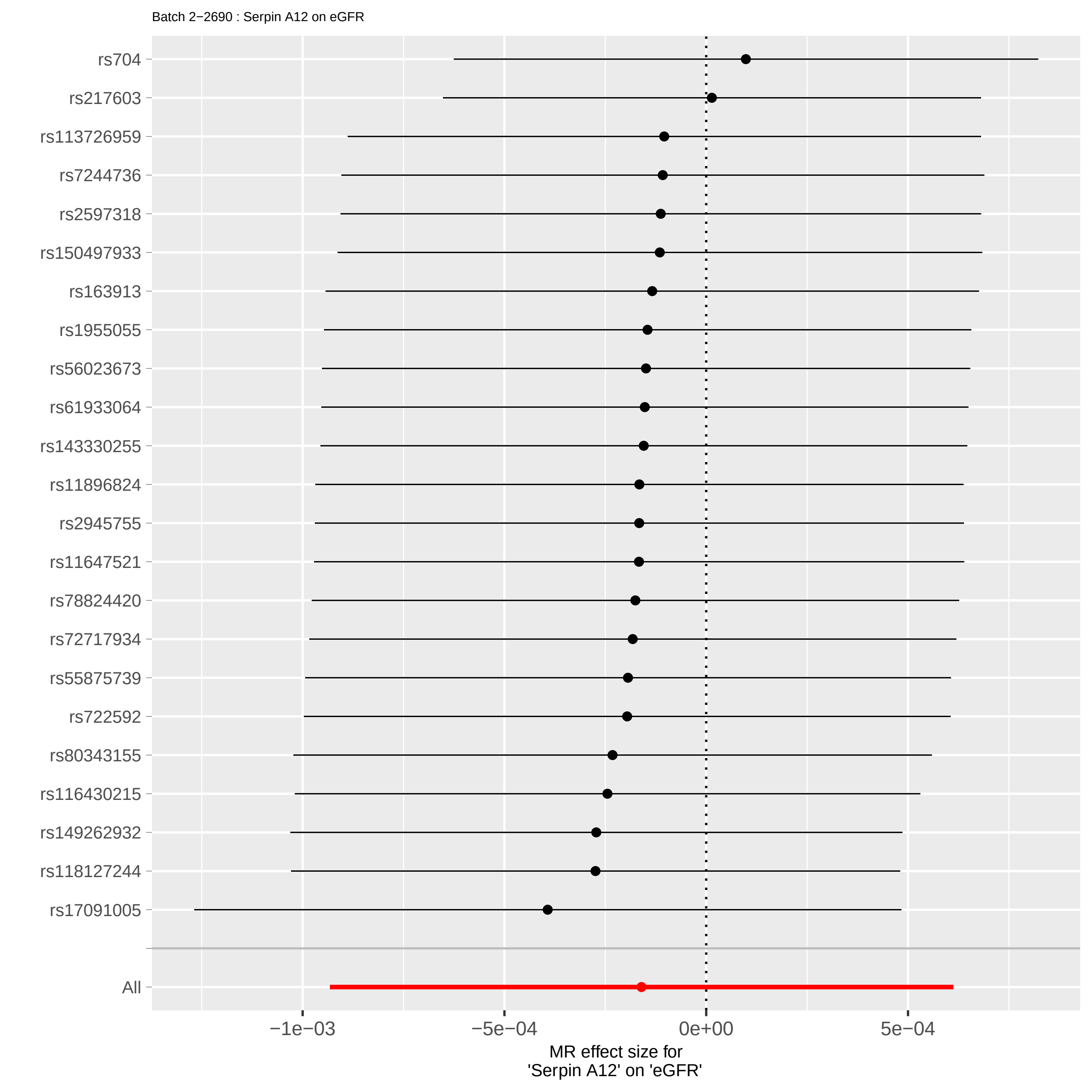
Batch 2−2690 : Serpin A12 on eGFR
rs704
rs217603
rs113726959
rs7244736
rs2597318
rs150497933
rs163913
rs1955055
rs56023673
rs61933064
rs143330255
rs11896824
rs2945755
rs11647521
rs78824420
rs72717934
rs55875739
rs722592
rs80343155
rs116430215
rs149262932
rs118127244
rs17091005
All
−1e−03
−5e−04
0e+00
5e−04
MR effect size for
'Serpin A12' on 'eGFR'

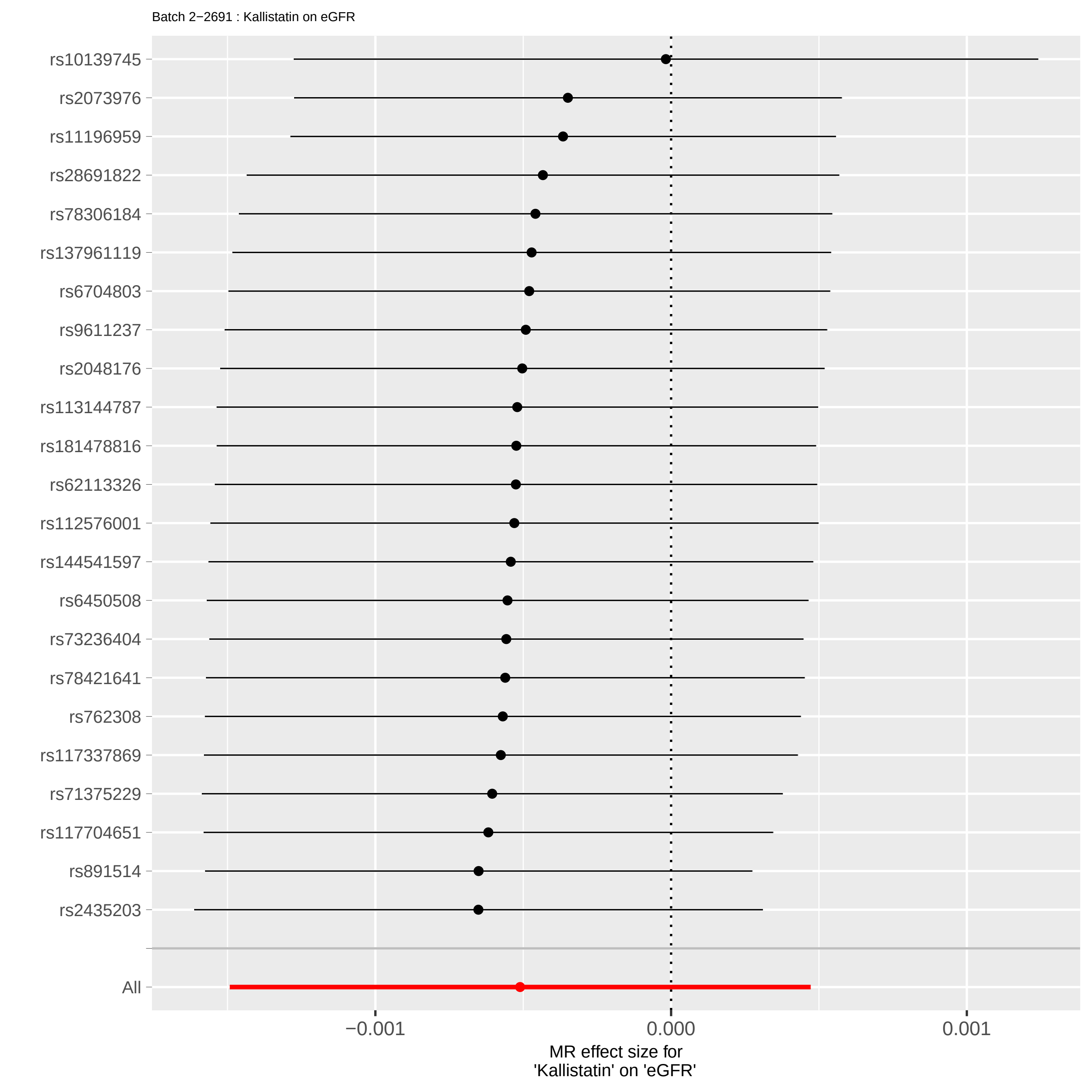

Batch 2−2691 : Kallistatin on eGFR
rs10139745
rs2073976
rs11196959
rs28691822
rs78306184
rs137961119
rs6704803
rs9611237
rs2048176
rs113144787
rs181478816
rs62113326
rs112576001
rs144541597
rs6450508
rs73236404
rs78421641
rs762308
rs117337869
rs71375229
rs117704651
rs891514
rs2435203
All
−0.001
0.000
0.001
MR effect size for
'Kallistatin' on 'eGFR'

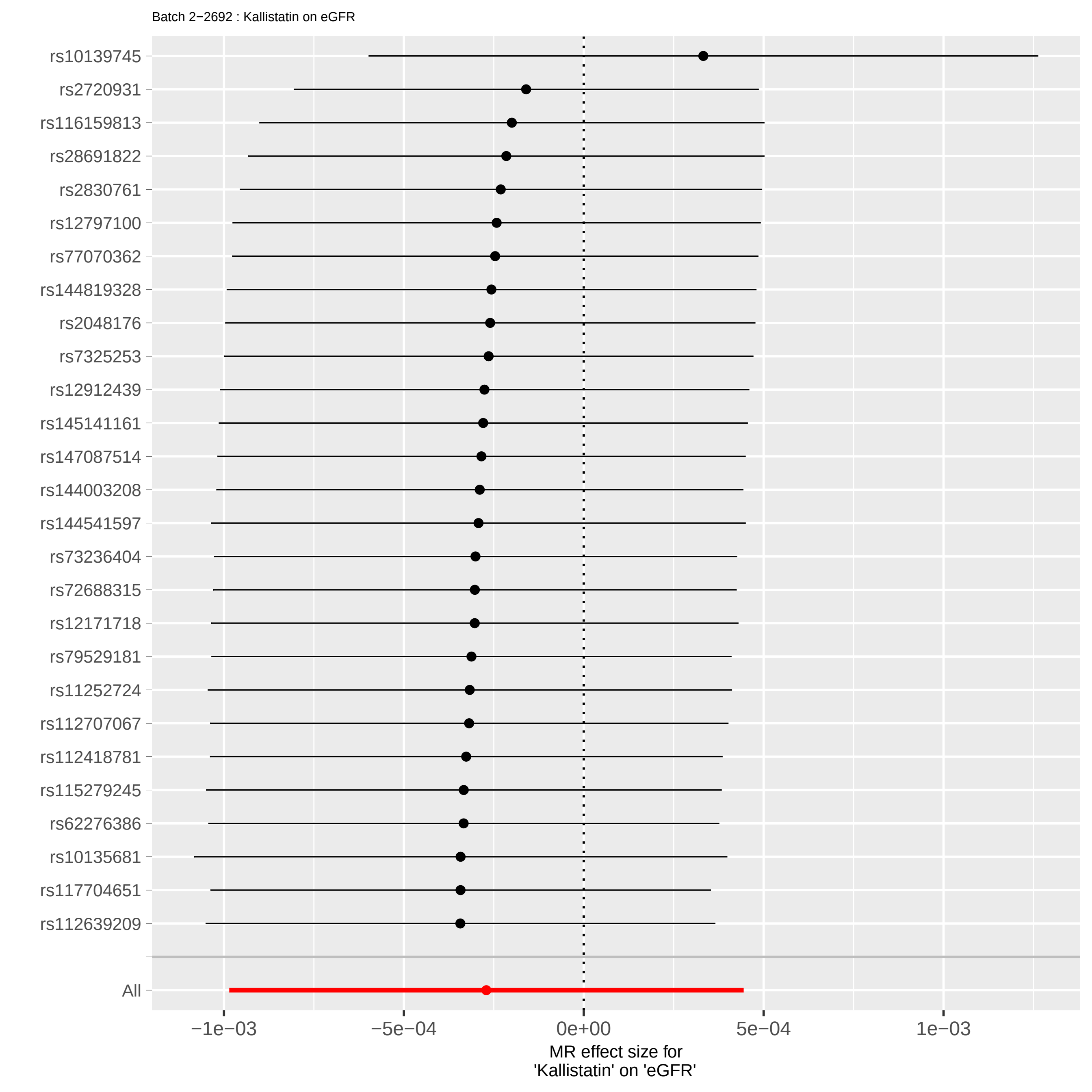

Batch 2−2692 : Kallistatin on eGFR
rs10139745
rs2720931
rs116159813
rs28691822
rs2830761
rs12797100
rs77070362
rs144819328
rs2048176
rs7325253
rs12912439
rs145141161
rs147087514
rs144003208
rs144541597
rs73236404
rs72688315
rs12171718
rs79529181
rs11252724
rs112707067
rs112418781
rs115279245
rs62276386
rs10135681
rs117704651
rs112639209
All
−1e−03
−5e−04
0e+00
5e−04
1e−03
MR effect size for
'Kallistatin' on 'eGFR'

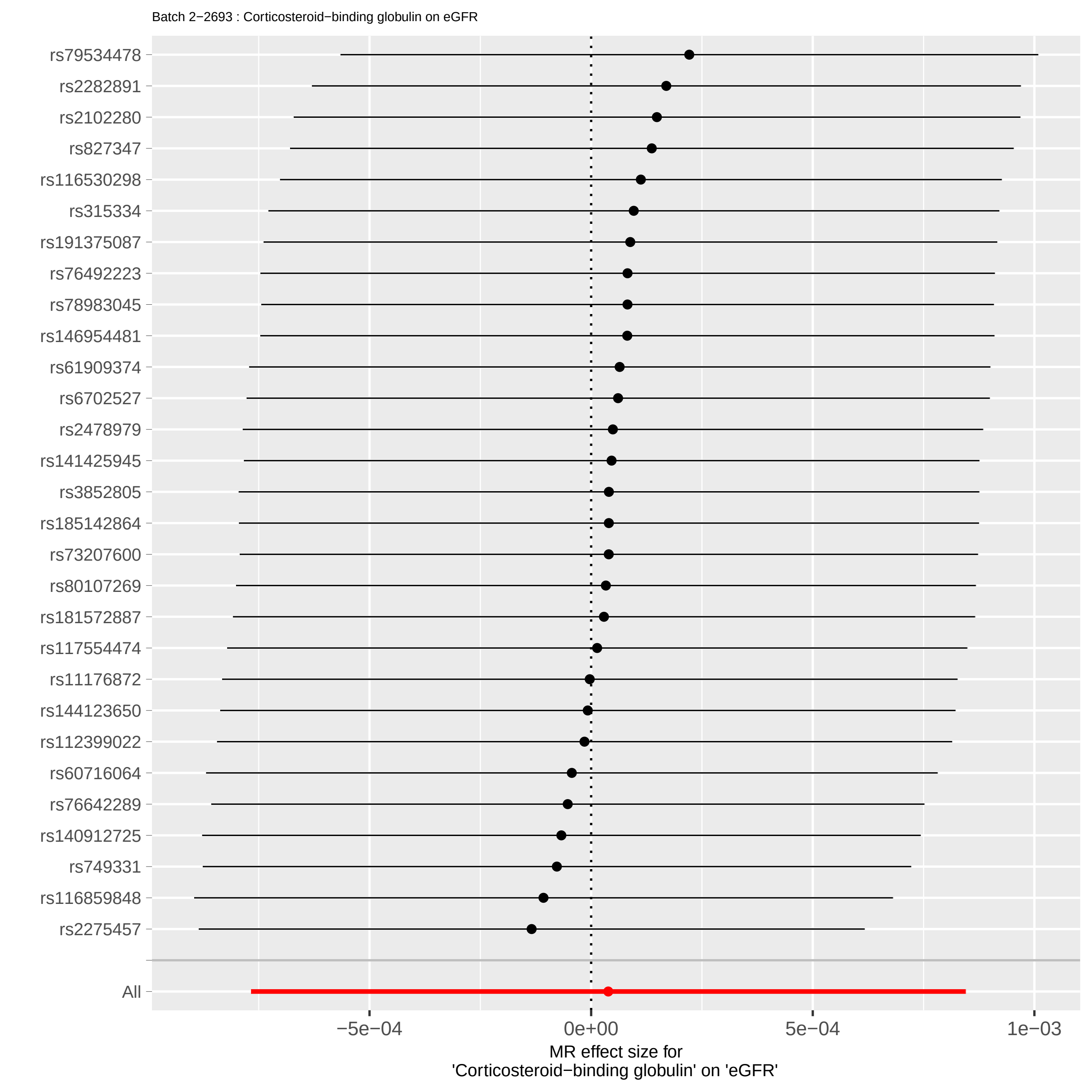

Batch 2−2693 : Corticosteroid−binding globulin on eGFR
rs79534478
rs2282891
rs2102280
rs827347
rs116530298
rs315334
rs191375087
rs76492223
rs78983045
rs146954481
rs61909374
rs6702527
rs2478979
rs141425945
rs3852805
rs185142864
rs73207600
rs80107269
rs181572887
rs117554474
rs11176872
rs144123650
rs112399022
rs60716064
rs76642289
rs140912725
rs749331
rs116859848
rs2275457
All
−5e−04
0e+00
5e−04
1e−03
MR effect size for
'Corticosteroid−binding globulin' on 'eGFR'

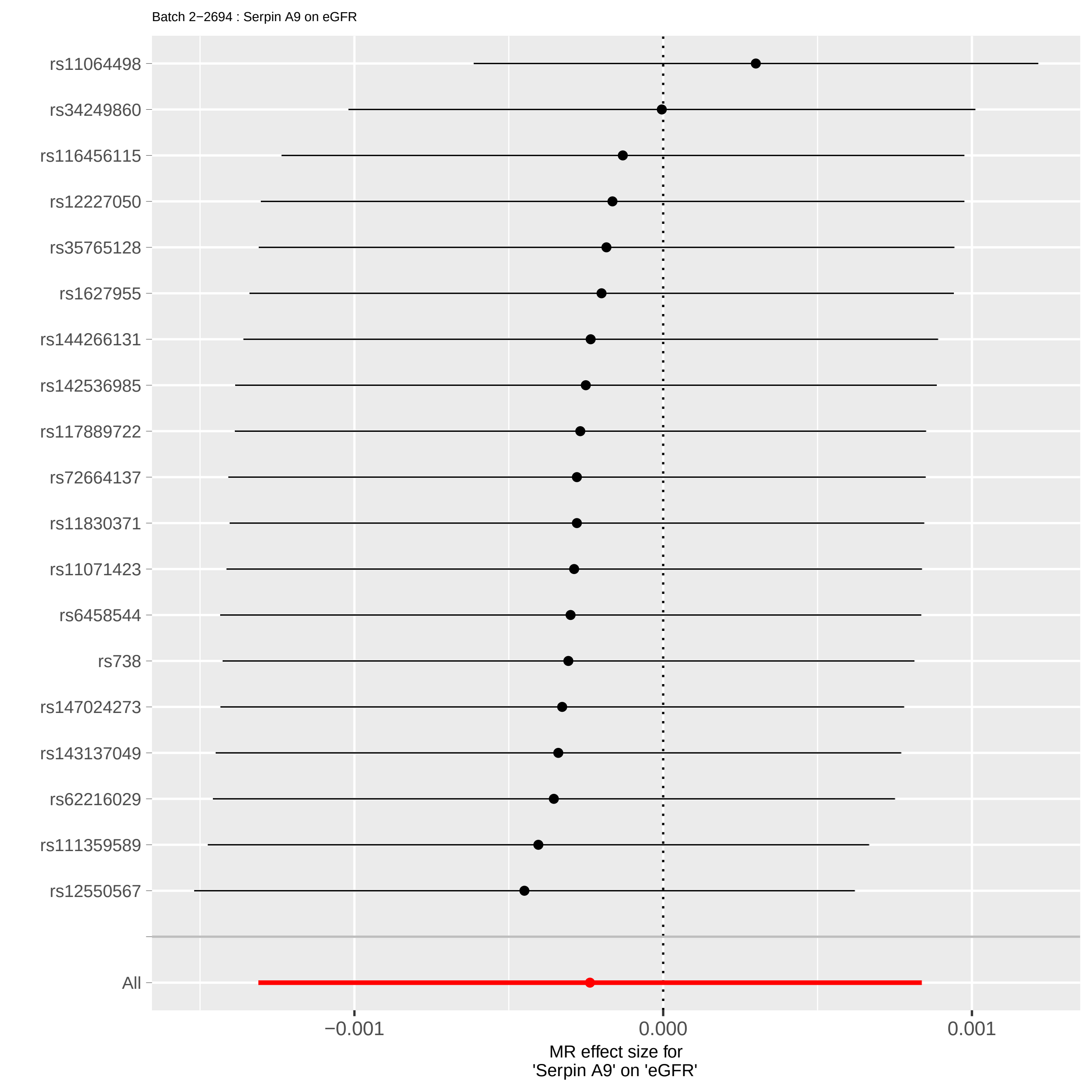

Batch 2−2694 : Serpin A9 on eGFR
rs11064498
rs34249860
rs116456115
rs12227050
rs35765128
rs1627955
rs144266131
rs142536985
rs117889722
rs72664137
rs11830371
rs11071423
rs6458544
rs738
rs147024273
rs143137049
rs62216029
rs111359589
rs12550567
All
−0.001
0.000
0.001
MR effect size for
'Serpin A9' on 'eGFR'

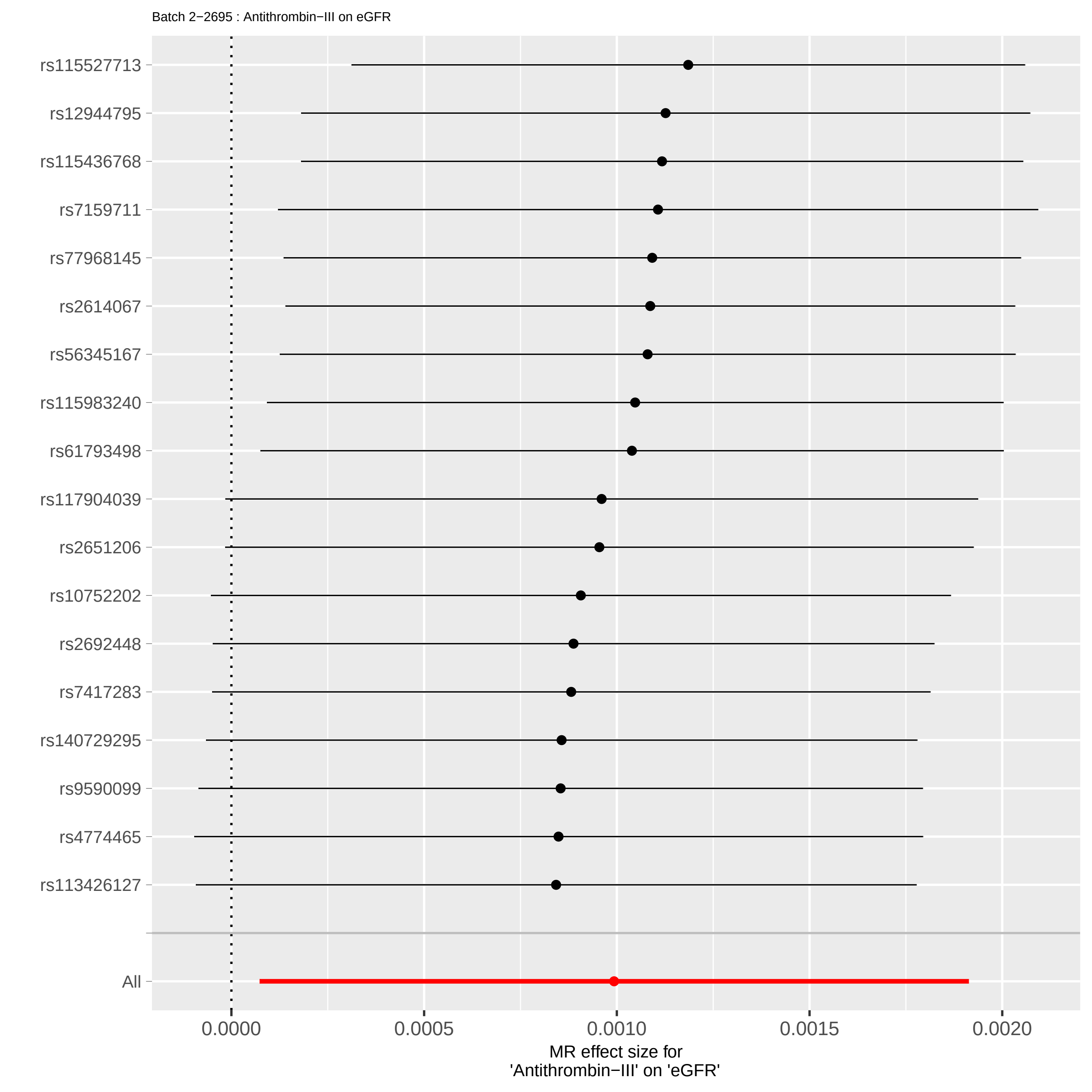

Batch 2−2695 : Antithrombin−III on eGFR
rs115527713
rs12944795
rs115436768
rs7159711
rs77968145
rs2614067
rs56345167
rs115983240
rs61793498
rs117904039
rs2651206
rs10752202
rs2692448
rs7417283
rs140729295
rs9590099
rs4774465
rs113426127
All
0.0000
0.0005
0.0010
0.0015
0.0020
MR effect size for
'Antithrombin−III' on 'eGFR'

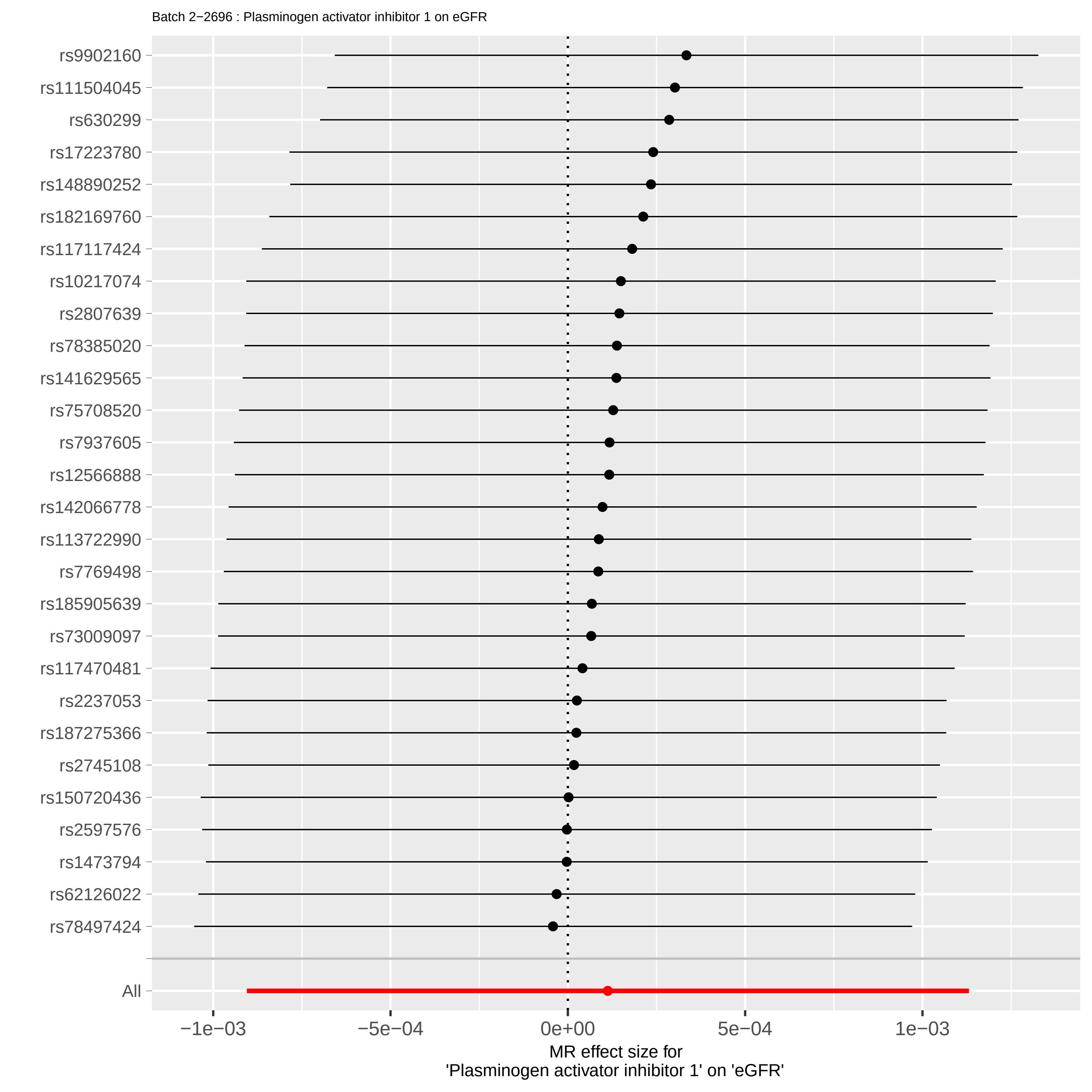
Batch 2−2696 : Plasminogen activator inhibitor 1 on eGFR
rs9902160
rs111504045
rs630299
rs17223780
rs148890252
rs182169760
rs117117424
rs10217074
rs2807639
rs78385020
rs141629565
rs75708520
rs7937605
rs12566888
rs142066778
rs113722990
rs7769498
rs185905639
rs73009097
rs117470481
rs2237053
rs187275366
rs2745108
rs150720436
rs2597576
rs1473794
rs62126022
rs78497424
All
−1e−03
−5e−04
0e+00
5e−04
1e−03
MR effect size for
'Plasminogen activator inhibitor 1' on 'eGFR'

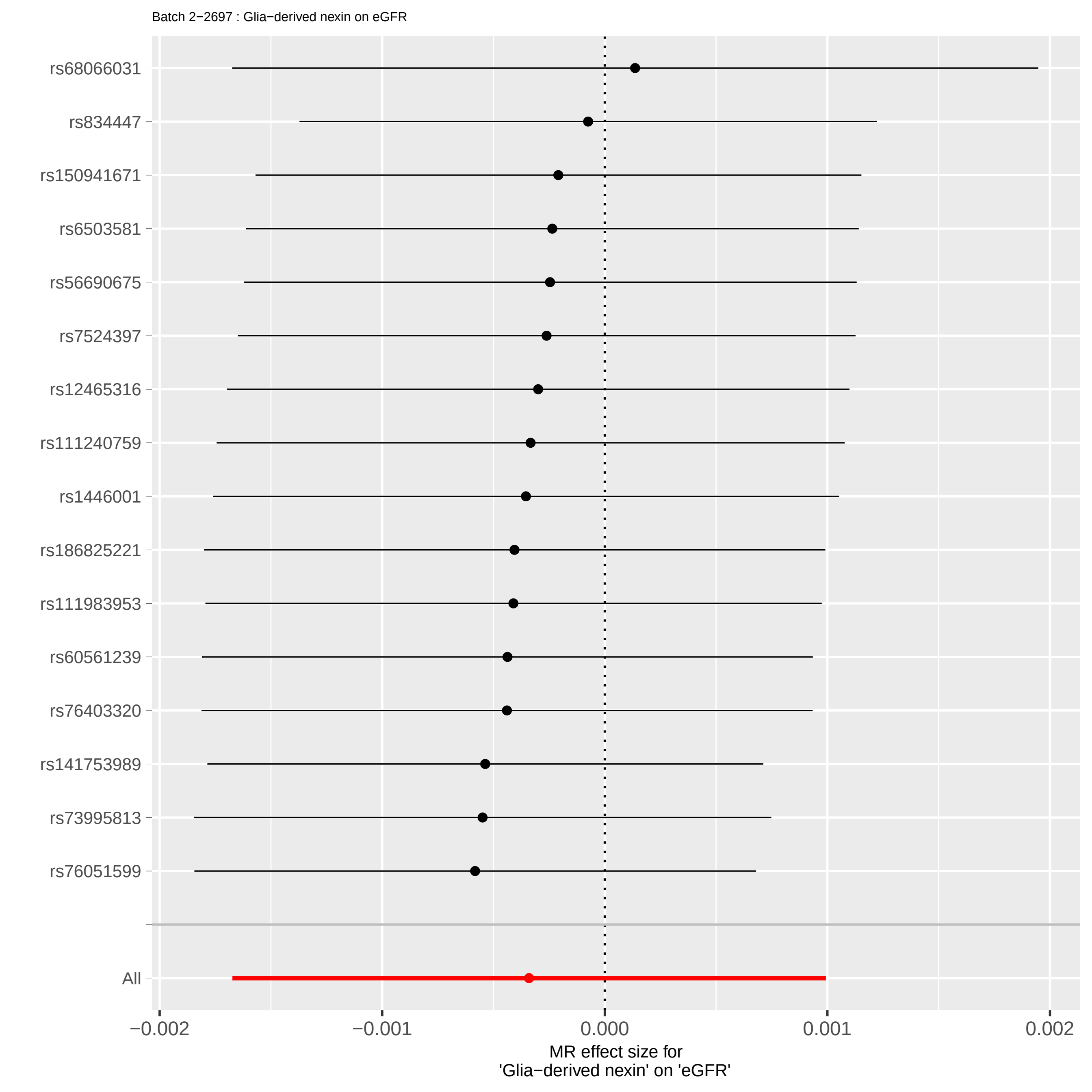

Batch 2−2697 : Glia−derived nexin on eGFR
rs68066031
rs834447
rs150941671
rs6503581
rs56690675
rs7524397
rs12465316
rs111240759
rs1446001
rs186825221
rs111983953
rs60561239
rs76403320
rs141753989
rs73995813
rs76051599
All
−0.002
−0.001
0.000
0.001
0.002
MR effect size for
'Glia−derived nexin' on 'eGFR'

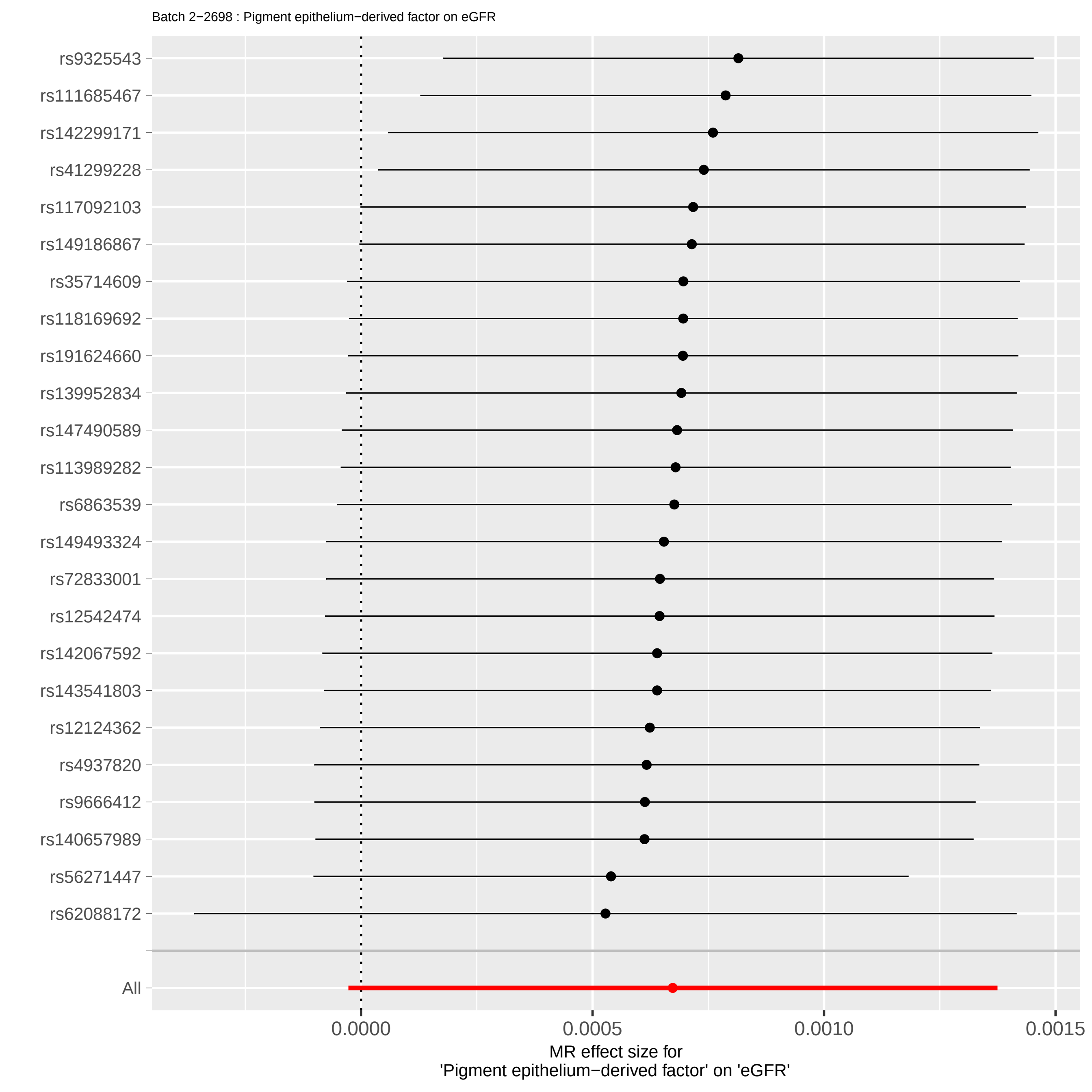

Batch 2−2698 : Pigment epithelium−derived factor on eGFR
rs9325543
rs111685467
rs142299171
rs41299228
rs117092103
rs149186867
rs35714609
rs118169692
rs191624660
rs139952834
rs147490589
rs113989282
rs6863539
rs149493324
rs72833001
rs12542474
rs142067592
rs143541803
rs12124362
rs4937820
rs9666412
rs140657989
rs56271447
rs62088172
All
0.0000
0.0005
0.0010
0.0015
MR effect size for
'Pigment epithelium−derived factor' on 'eGFR'

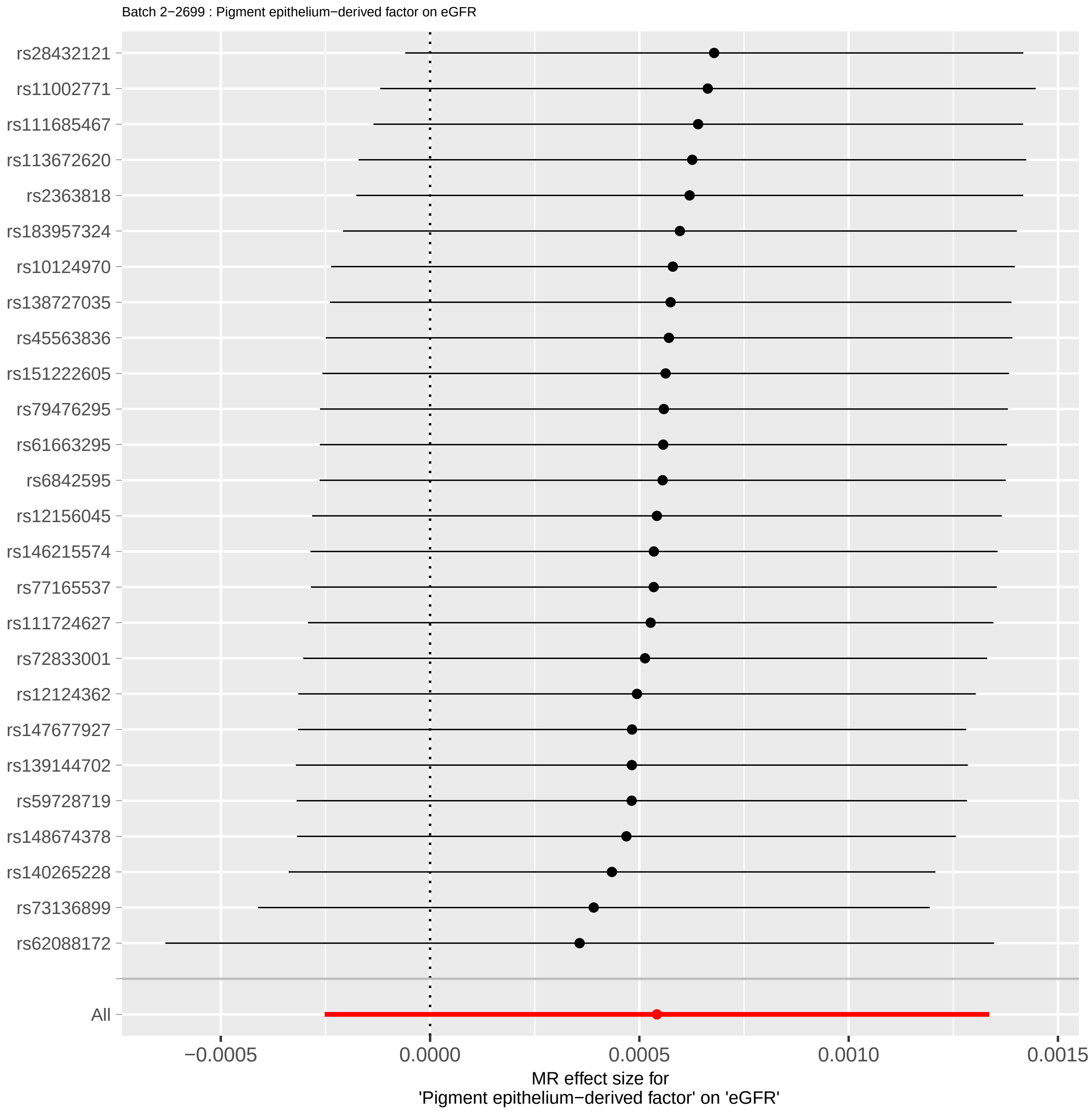

Batch 2−2699 : Pigment epithelium−derived factor on eGFR
rs28432121
rs11002771
rs111685467
rs113672620
rs2363818
rs183957324
rs10124970
rs138727035
rs45563836
rs151222605
rs79476295
rs61663295
rs6842595
rs12156045
rs146215574
rs77165537
rs111724627
rs72833001
rs12124362
rs147677927
rs139144702
rs59728719
rs148674378
rs140265228
rs73136899
rs62088172
All
−0.0005
0.0000
0.0005
0.0010
0.0015
MR effect size for
'Pigment epithelium−derived factor' on 'eGFR'

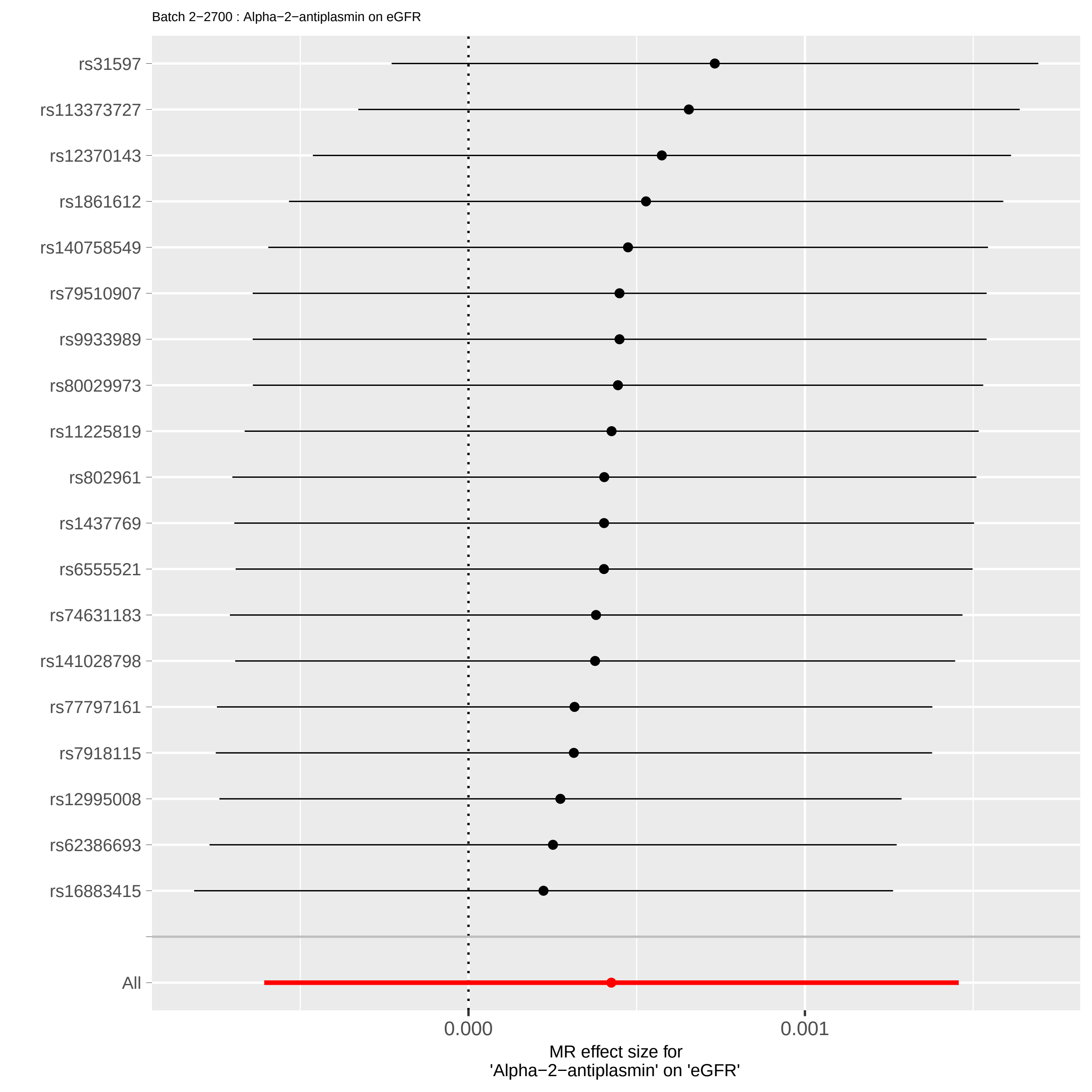

Batch 2−2700 : Alpha−2−antiplasmin on eGFR
rs31597
rs113373727
rs12370143
rs1861612
rs140758549
rs79510907
rs9933989
rs80029973
rs11225819
rs802961
rs1437769
rs6555521
rs74631183
rs141028798
rs77797161
rs7918115
rs12995008
rs62386693
rs16883415
All
0.000
0.001
MR effect size for
'Alpha−2−antiplasmin' on 'eGFR'

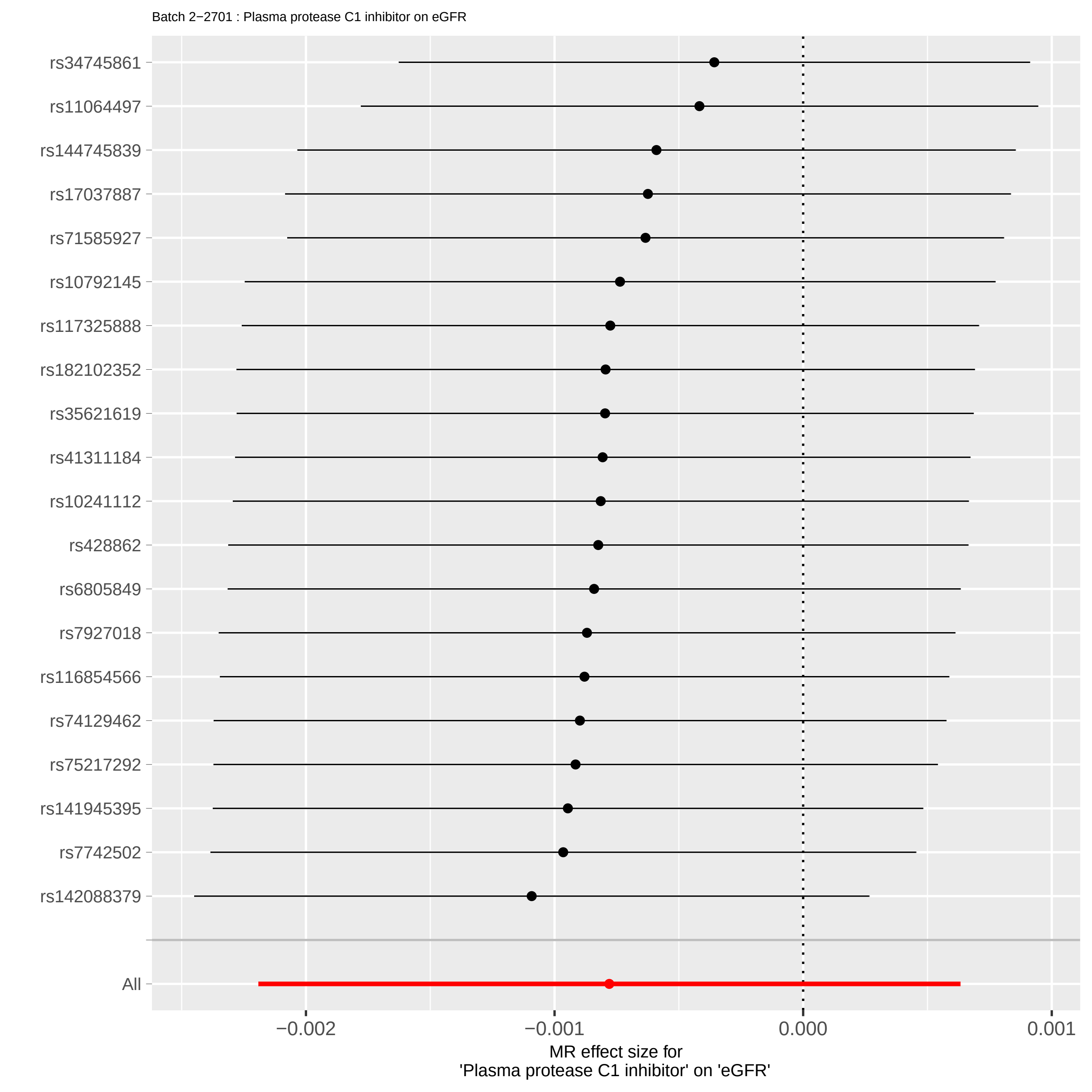

Batch 2−2701 : Plasma protease C1 inhibitor on eGFR
rs34745861
rs11064497
rs144745839
rs17037887
rs71585927
rs10792145
rs117325888
rs182102352
rs35621619
rs41311184
rs10241112
rs428862
rs6805849
rs7927018
rs116854566
rs74129462
rs75217292
rs141945395
rs7742502
rs142088379
All
−0.002
−0.001
0.000
0.001
MR effect size for
'Plasma protease C1 inhibitor' on 'eGFR'

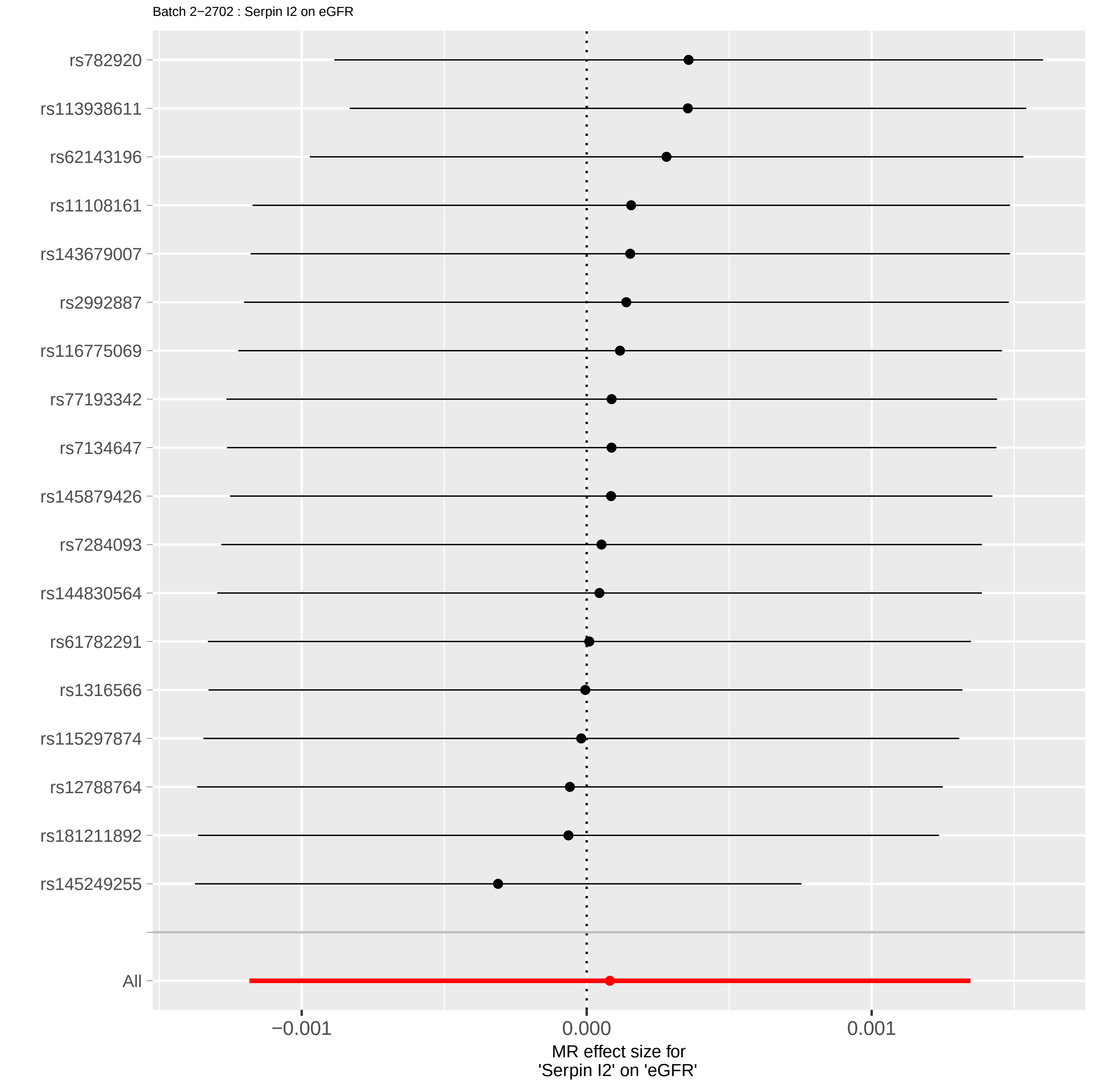

Batch 2−2702 : Serpin I2 on eGFR
rs782920
rs113938611
rs62143196
rs11108161
rs143679007
rs2992887
rs116775069
rs77193342
rs7134647
rs145879426
rs7284093
rs144830564
rs61782291
rs1316566
rs115297874
rs12788764
rs181211892
rs145249255
All
−0.001
0.000
0.001
MR effect size for
'Serpin I2' on 'eGFR'

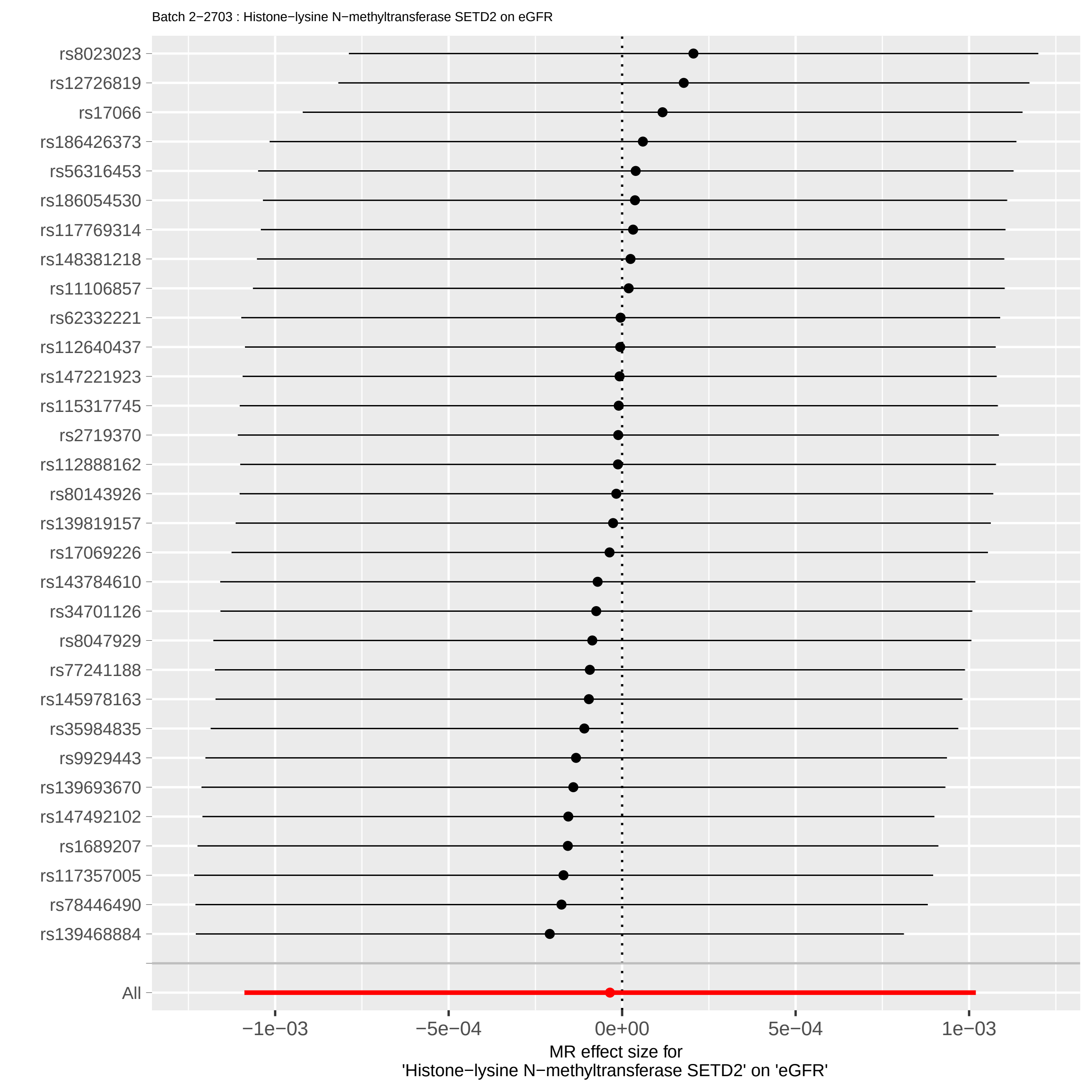

Batch 2−2703 : Histone−lysine N−methyltransferase SETD2 on eGFR
rs8023023
rs12726819
rs17066
rs186426373
rs56316453
rs186054530
rs117769314
rs148381218
rs11106857
rs62332221
rs112640437
rs147221923
rs115317745
rs2719370
rs112888162
rs80143926
rs139819157
rs17069226
rs143784610
rs34701126
rs8047929
rs77241188
rs145978163
rs35984835
rs9929443
rs139693670
rs147492102
rs1689207
rs117357005
rs78446490
rs139468884
All
−1e−03
−5e−04
0e+00
5e−04
1e−03
MR effect size for
'Histone−lysine N−methyltransferase SETD2' on 'eGFR'

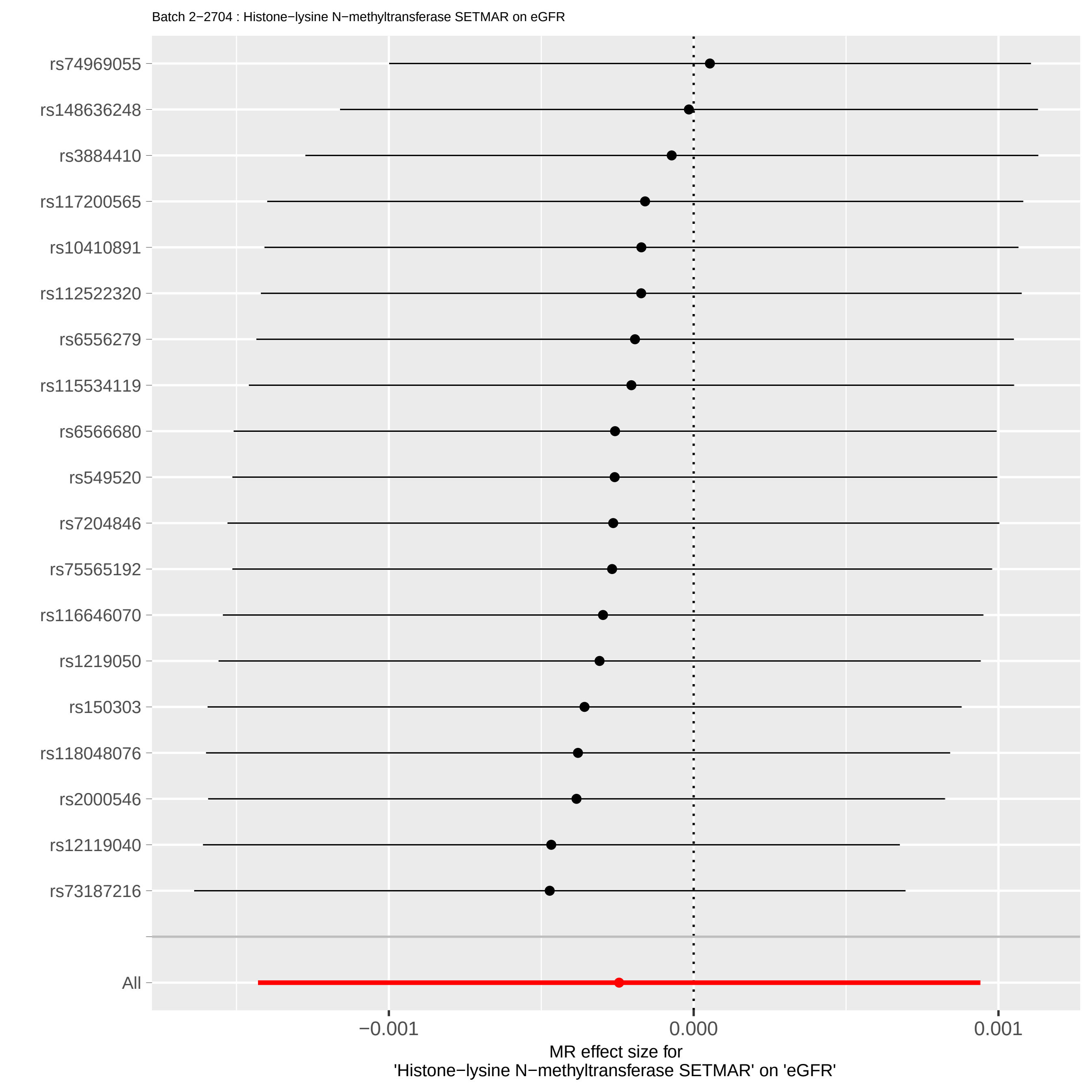

Batch 2−2704 : Histone−lysine N−methyltransferase SETMAR on eGFR
rs74969055
rs148636248
rs3884410
rs117200565
rs10410891
rs112522320
rs6556279
rs115534119
rs6566680
rs549520
rs7204846
rs75565192
rs116646070
rs1219050
rs150303
rs118048076
rs2000546
rs12119040
rs73187216
All
−0.001
0.000
0.001
MR effect size for
'Histone−lysine N−methyltransferase SETMAR' on 'eGFR'

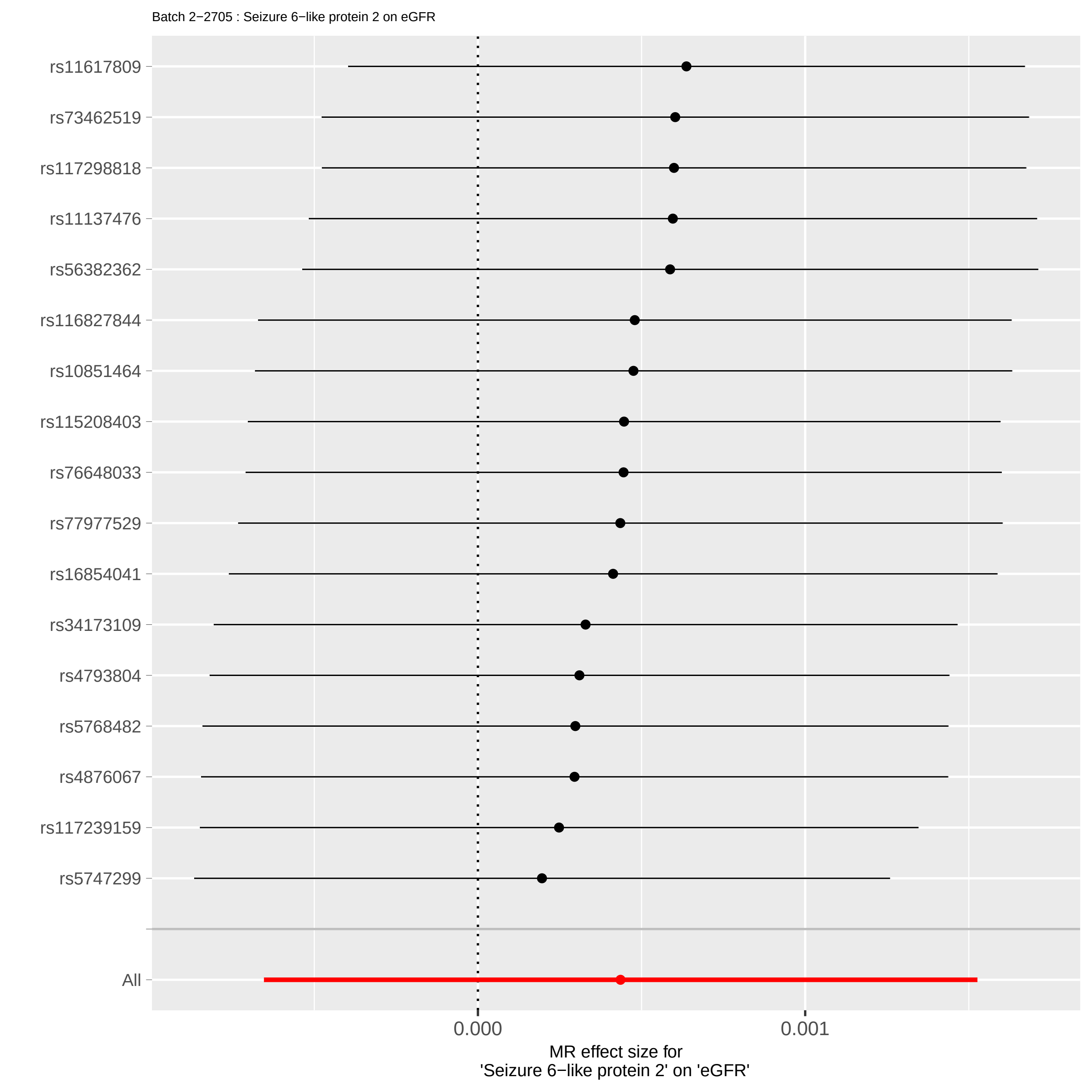

Batch 2−2705 : Seizure 6−like protein 2 on eGFR
rs11617809
rs73462519
rs117298818
rs11137476
rs56382362
rs116827844
rs10851464
rs115208403
rs76648033
rs77977529
rs16854041
rs34173109
rs4793804
rs5768482
rs4876067
rs117239159
rs5747299
All
0.000
0.001
MR effect size for
'Seizure 6−like protein 2' on 'eGFR'

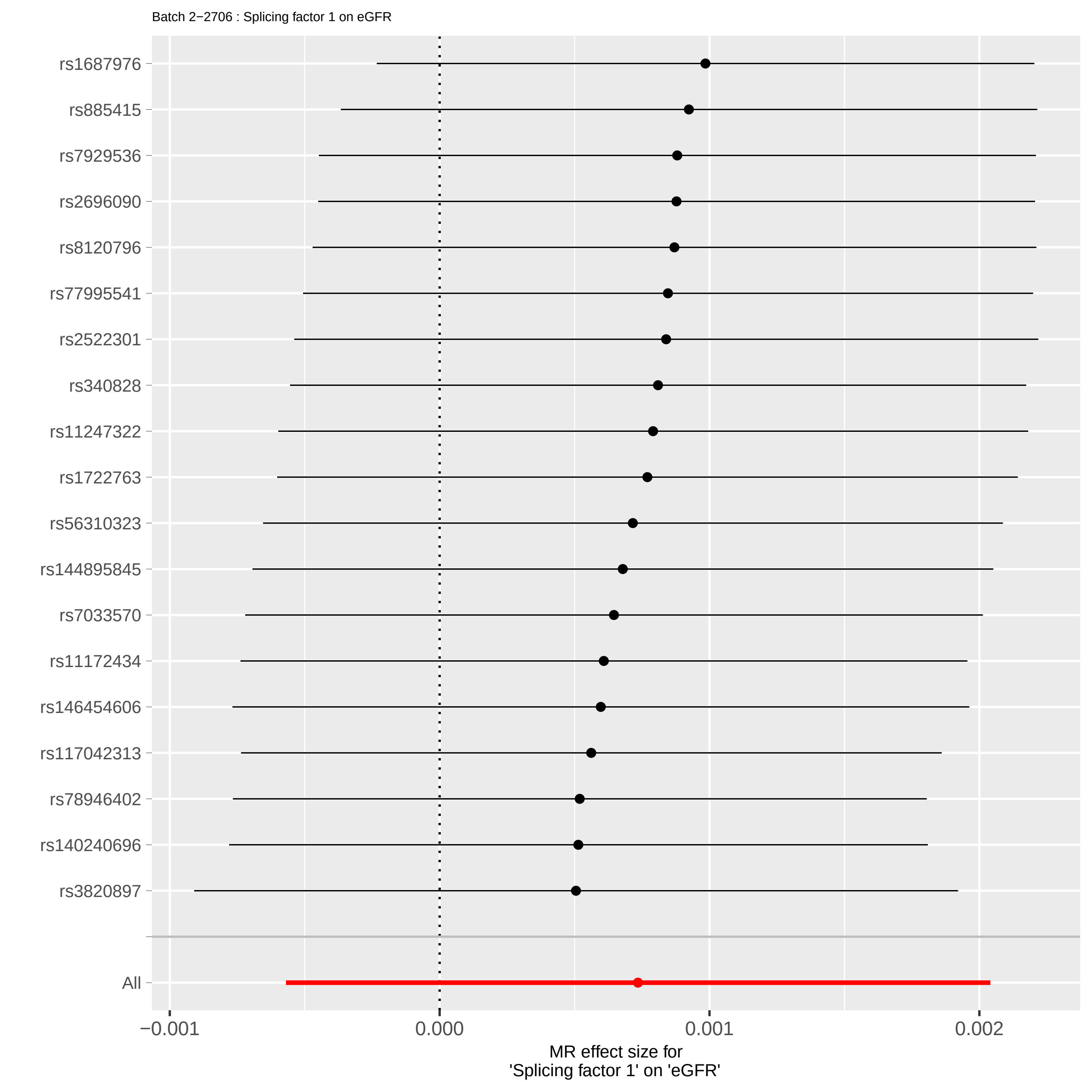

Batch 2−2706 : Splicing factor 1 on eGFR
rs1687976
rs885415
rs7929536
rs2696090
rs8120796
rs77995541
rs2522301
rs340828
rs11247322
rs1722763
rs56310323
rs144895845
rs7033570
rs11172434
rs146454606
rs117042313
rs78946402
rs140240696
rs3820897
All
−0.001
0.000
0.001
0.002
MR effect size for
'Splicing factor 1' on 'eGFR'

Batch 2−2707 : Splicing factor 3B subunit 4 on eGFR

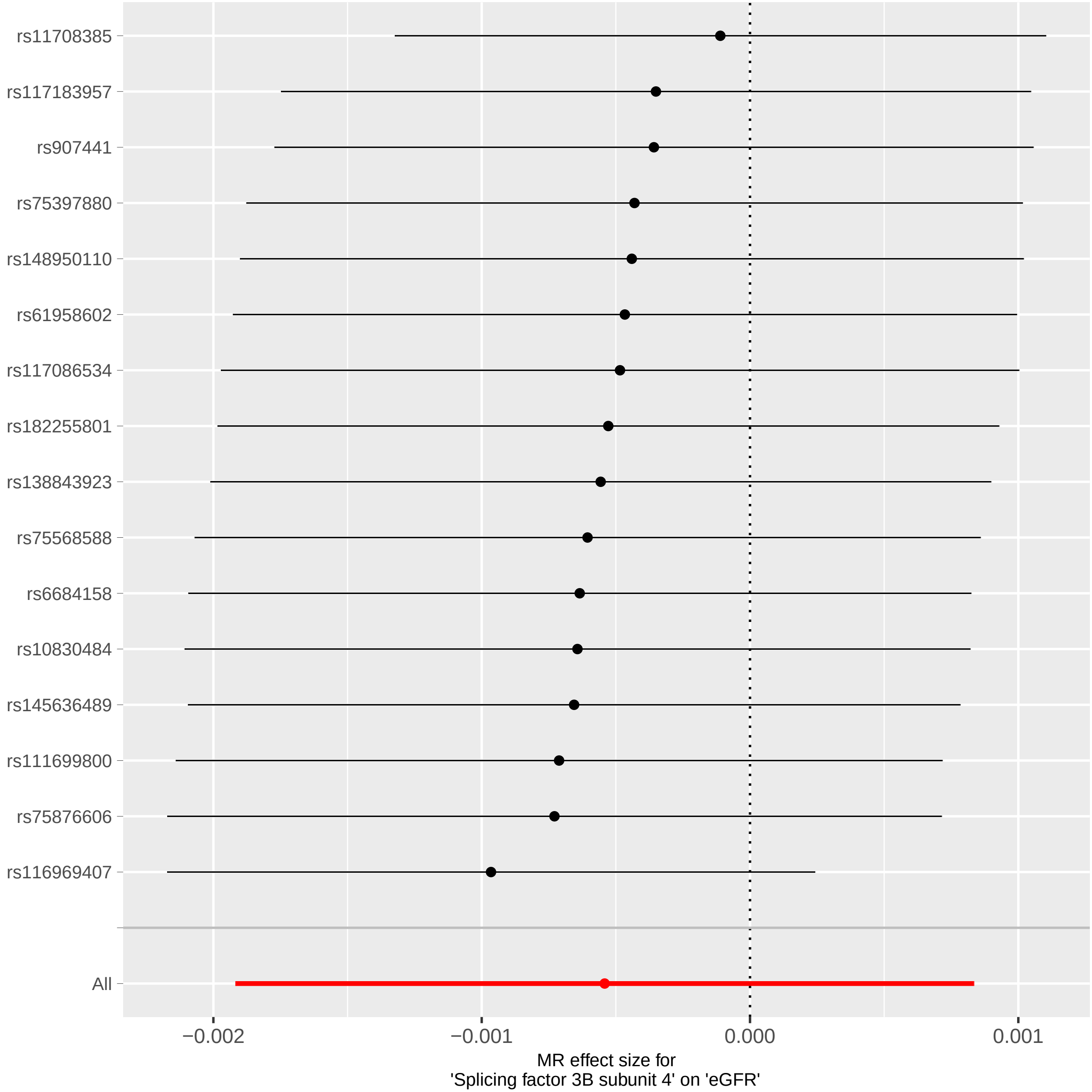

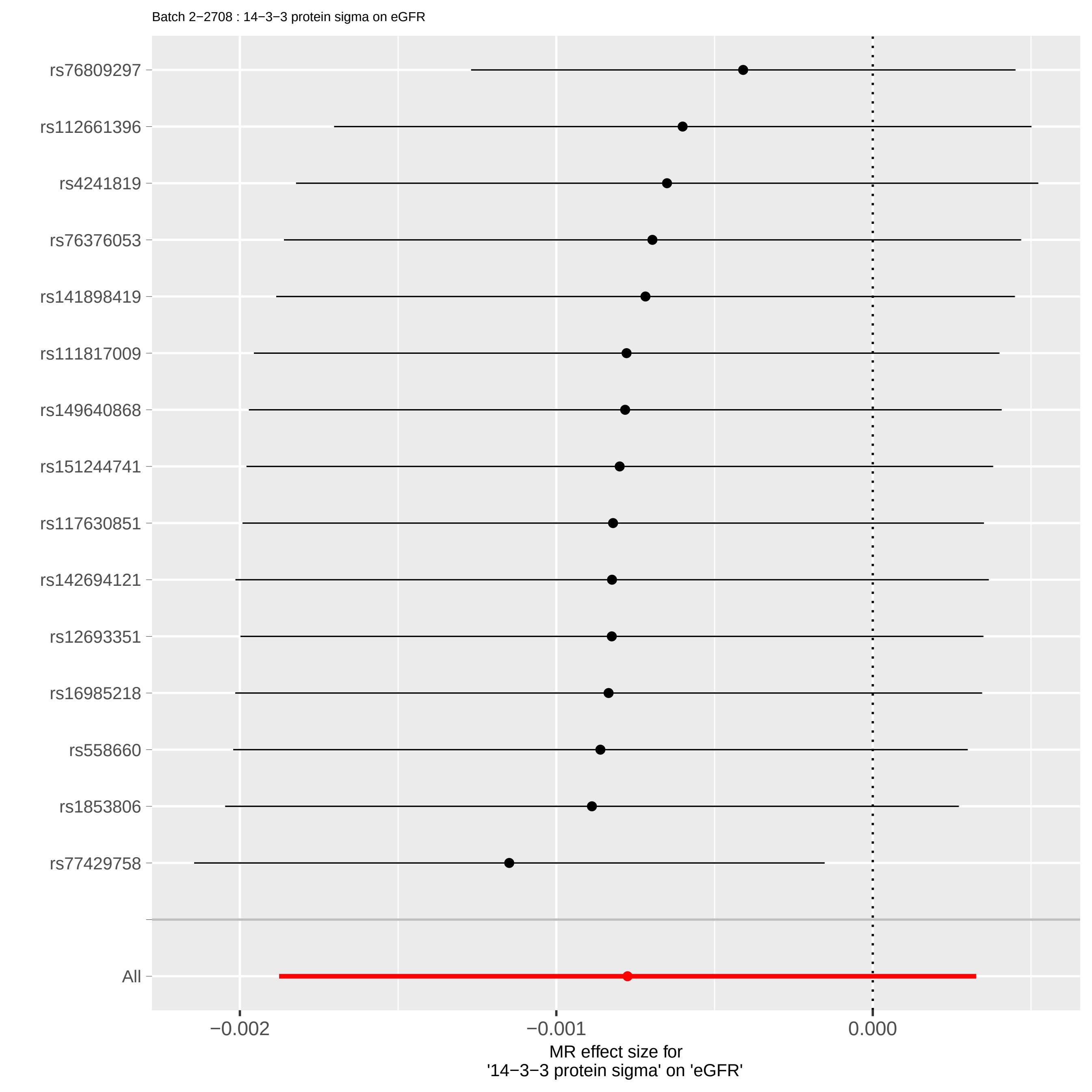

Batch 2−2708 : 14−3−3 protein sigma on eGFR
rs76809297
rs112661396
rs4241819
rs76376053
rs141898419
rs111817009
rs149640868
rs151244741
rs117630851
rs142694121
rs12693351
rs16985218
rs558660
rs1853806
rs77429758
All
−0.002
−0.001
0.000
MR effect size for
'14−3−3 protein sigma' on 'eGFR'

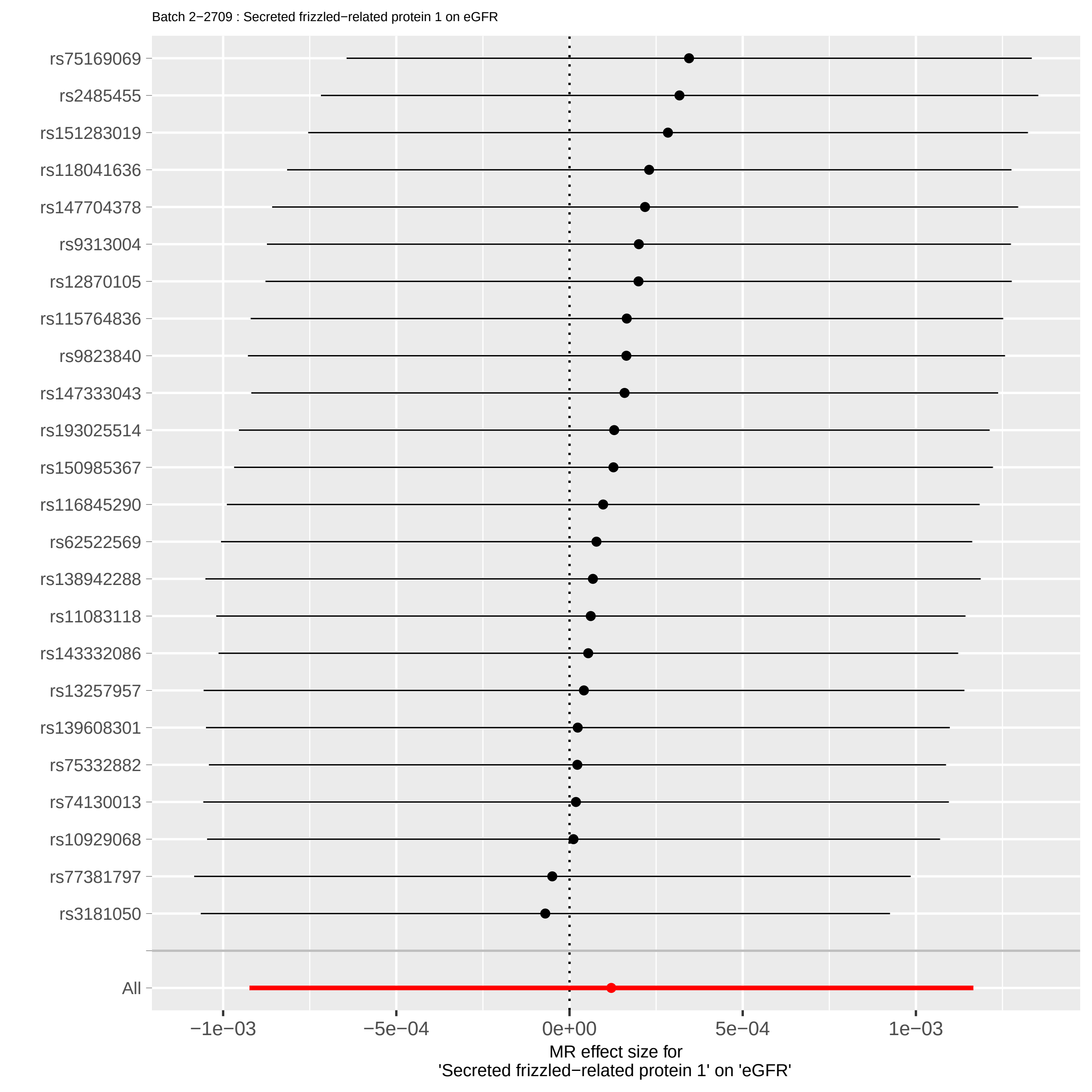

Batch 2−2709 : Secreted frizzled−related protein 1 on eGFR
rs75169069
rs2485455
rs151283019
rs118041636
rs147704378
rs9313004
rs12870105
rs115764836
rs9823840
rs147333043
rs193025514
rs150985367
rs116845290
rs62522569
rs138942288
rs11083118
rs143332086
rs13257957
rs139608301
rs75332882
rs74130013
rs10929068
rs77381797
rs3181050
All
−1e−03
−5e−04
0e+00
5e−04
1e−03
MR effect size for
'Secreted frizzled−related protein 1' on 'eGFR'

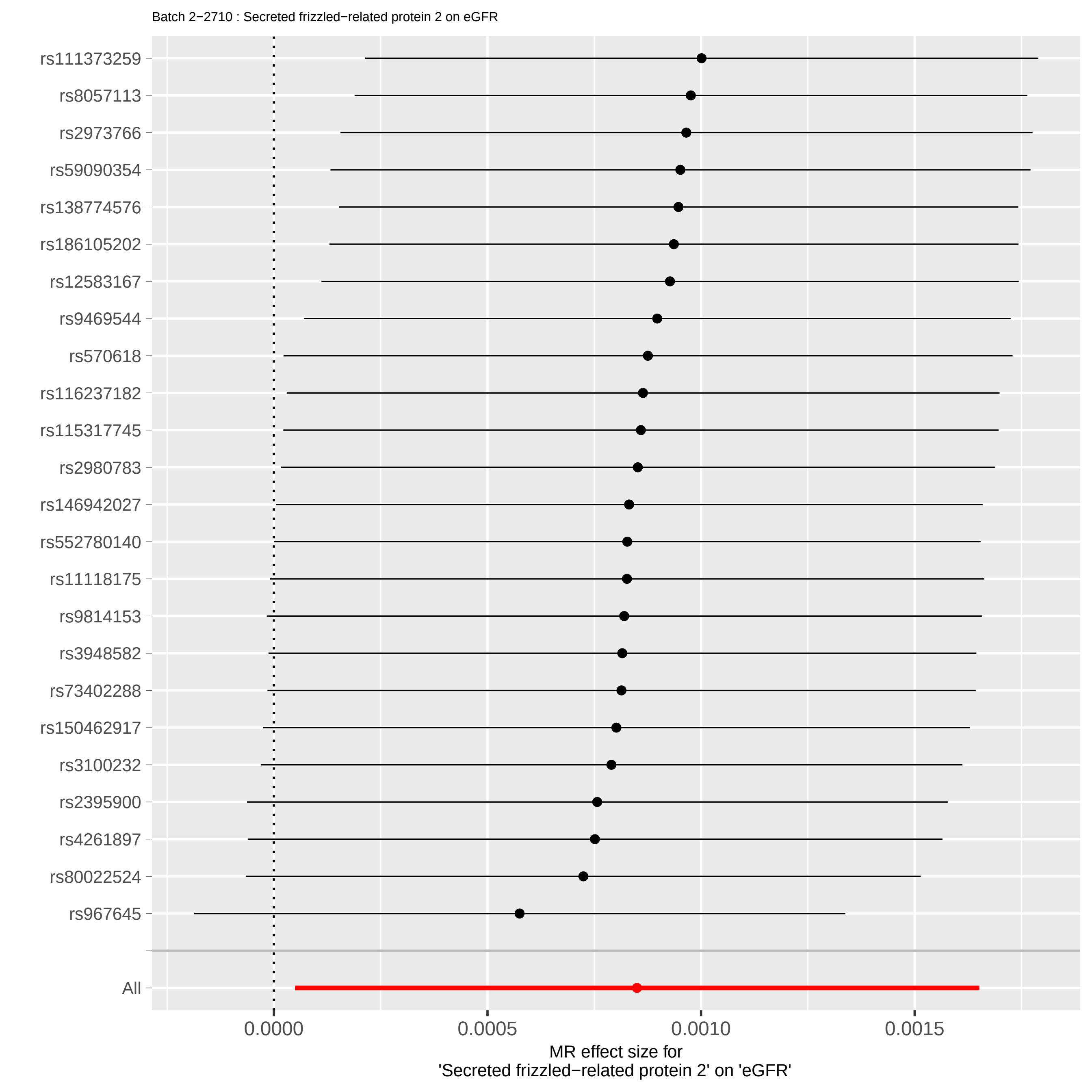

Batch 2−2710 : Secreted frizzled−related protein 2 on eGFR
rs111373259
rs8057113
rs2973766
rs59090354
rs138774576
rs186105202
rs12583167
rs9469544
rs570618
rs116237182
rs115317745
rs2980783
rs146942027
rs552780140
rs11118175
rs9814153
rs3948582
rs73402288
rs150462917
rs3100232
rs2395900
rs4261897
rs80022524
rs967645
All
0.0000
0.0005
0.0010
0.0015
MR effect size for
'Secreted frizzled−related protein 2' on 'eGFR'

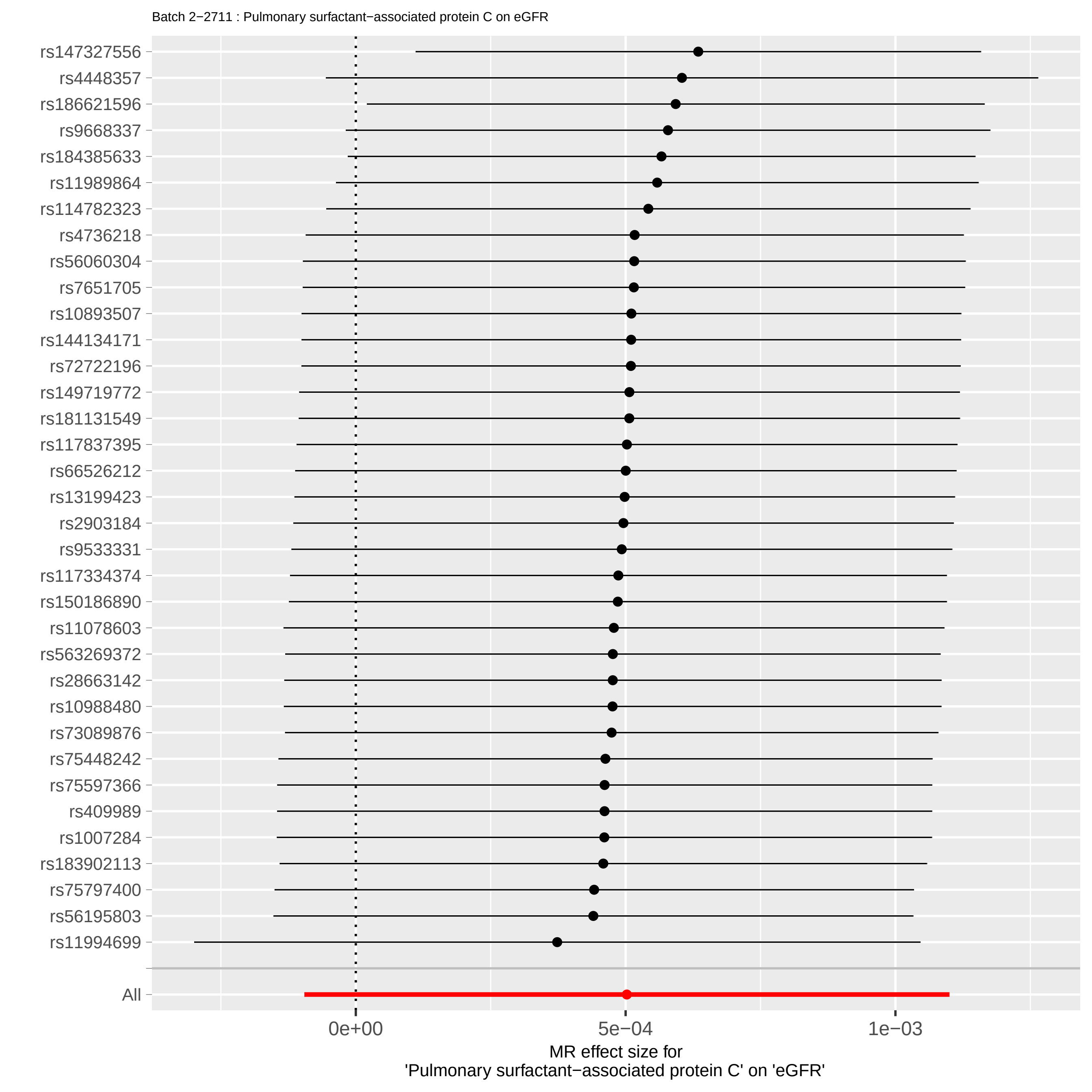

Batch 2−2711 : Pulmonary surfactant−associated protein C on eGFR
rs147327556
rs4448357
rs186621596
rs9668337
rs184385633
rs11989864
rs114782323
rs4736218
rs56060304
rs7651705
rs10893507
rs144134171
rs72722196
rs149719772
rs181131549
rs117837395
rs66526212
rs13199423
rs2903184
rs9533331
rs117334374
rs150186890
rs11078603
rs563269372
rs28663142
rs10988480
rs73089876
rs75448242
rs75597366
rs409989
rs1007284
rs183902113
rs75797400
rs56195803
rs11994699
All
0e+00
5e−04
1e−03
MR effect size for
'Pulmonary surfactant−associated protein C' on 'eGFR'

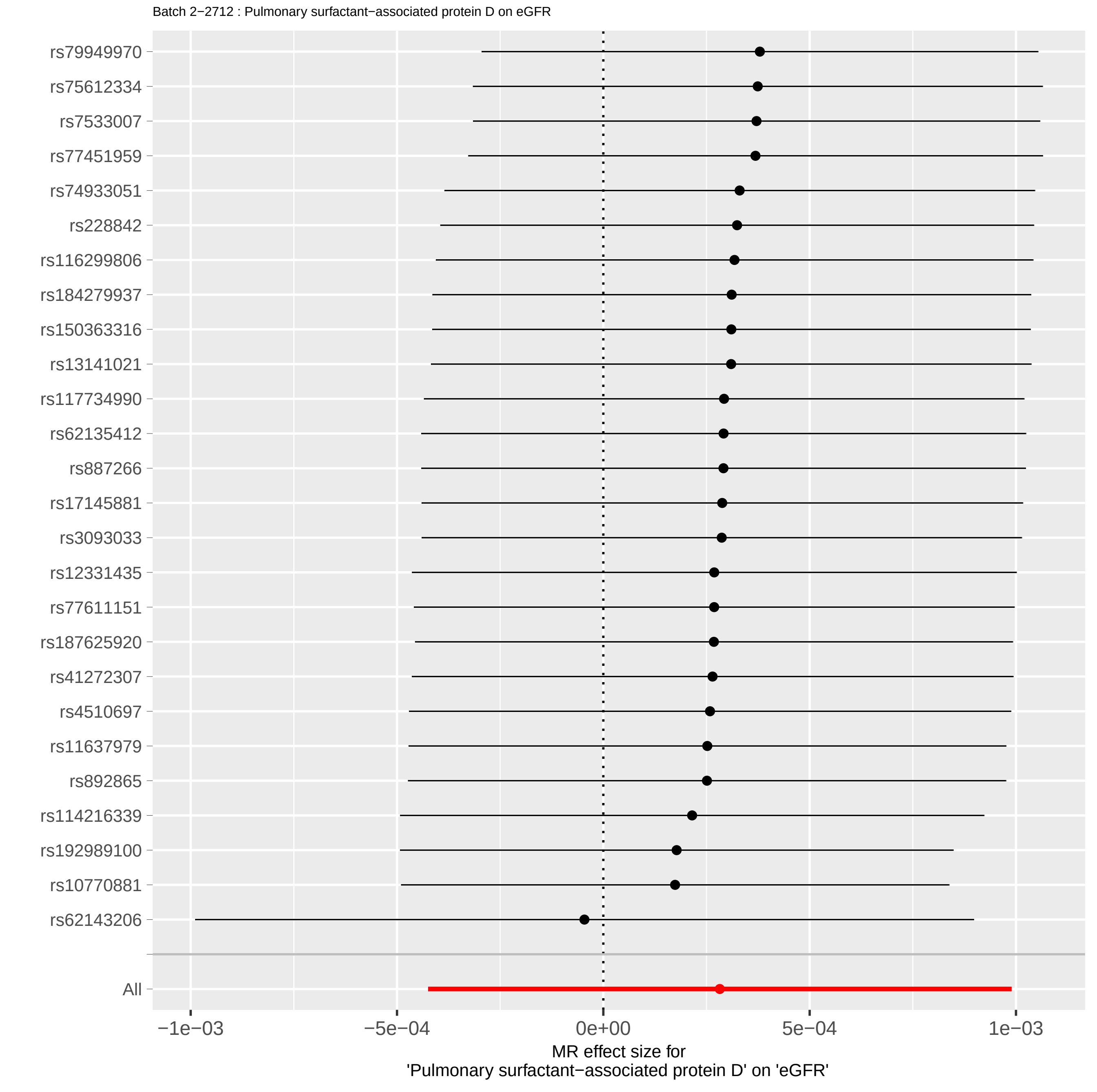
Batch 2−2712 : Pulmonary surfactant−associated protein D on eGFR
rs79949970
rs75612334
rs7533007
rs77451959
rs74933051
rs228842
rs116299806
rs184279937
rs150363316
rs13141021
rs117734990
rs62135412
rs887266
rs17145881
rs3093033
rs12331435
rs77611151
rs187625920
rs41272307
rs4510697
rs11637979
rs892865
rs114216339
rs192989100
rs10770881
rs62143206
All
−1e−03
−5e−04
0e+00
5e−04
1e−03
MR effect size for
'Pulmonary surfactant−associated protein D' on 'eGFR'

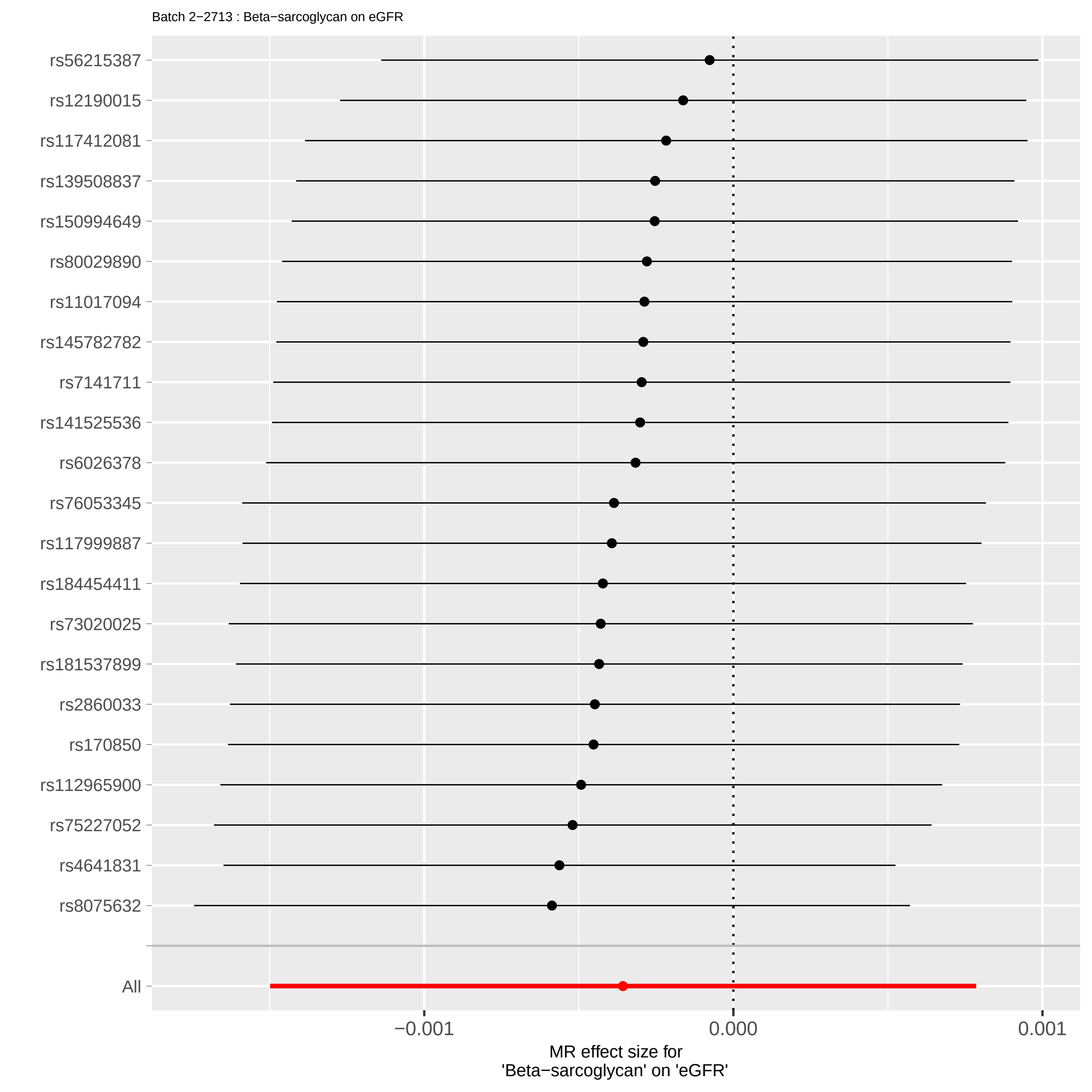
Batch 2−2713 : Beta−sarcoglycan on eGFR
rs56215387
rs12190015
rs117412081
rs139508837
rs150994649
rs80029890
rs11017094
rs145782782
rs7141711
rs141525536
rs6026378
rs76053345
rs117999887
rs184454411
rs73020025
rs181537899
rs2860033
rs170850
rs112965900
rs75227052
rs4641831
rs8075632
All
−0.001
0.000
0.001
MR effect size for
'Beta−sarcoglycan' on 'eGFR'

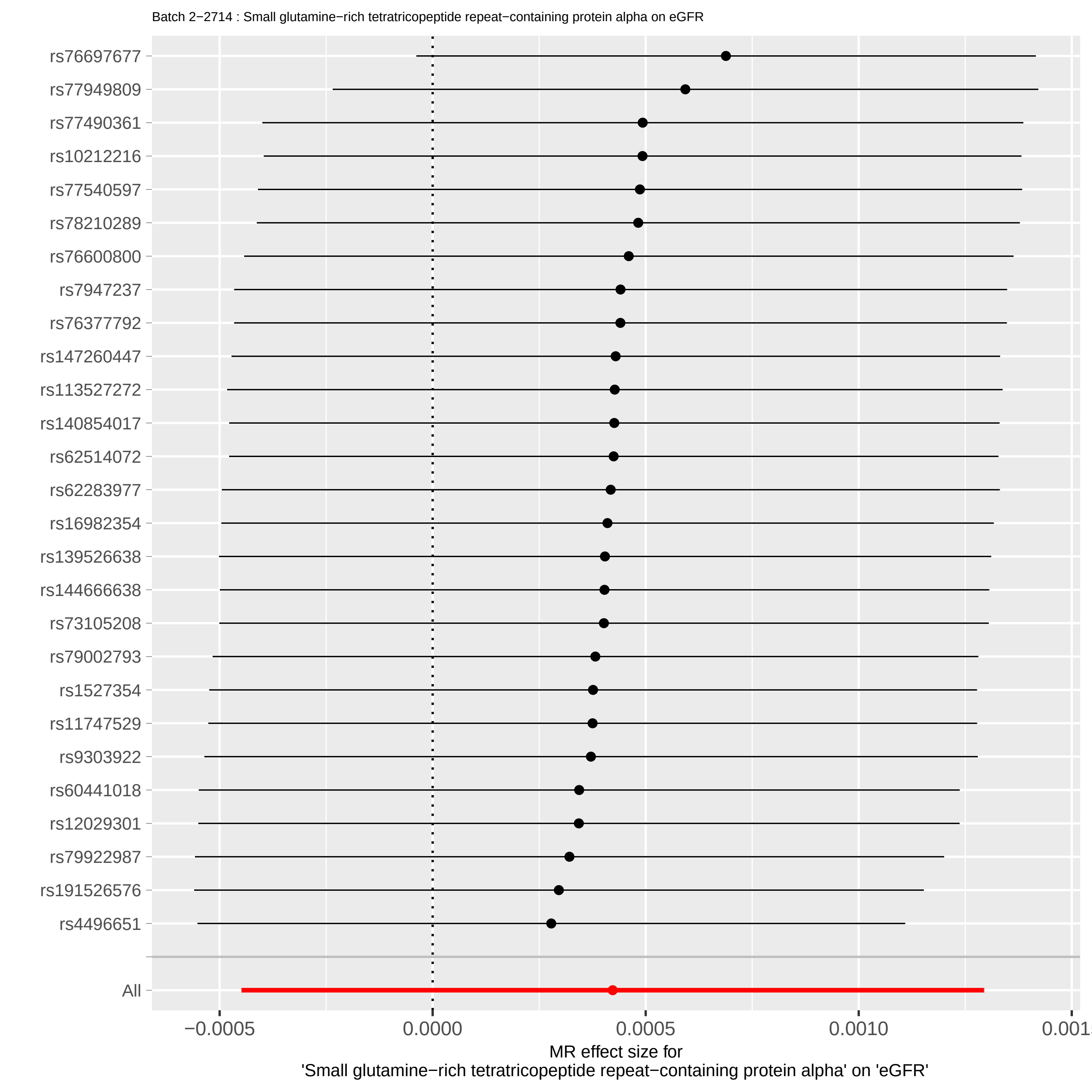
Batch 2−2714 : Small glutamine−rich tetratricopeptide repeat−containing protein alpha on eGFR
rs76697677
rs77949809
rs77490361
rs10212216
rs77540597
rs78210289
rs76600800
rs7947237
rs76377792
rs147260447
rs113527272
rs140854017
rs62514072
rs62283977
rs16982354
rs139526638
rs144666638
rs73105208
rs79002793
rs1527354
rs11747529
rs9303922
rs60441018
rs12029301
rs79922987
rs191526576
rs4496651
All
−0.0005
0.0000
0.0005
0.0010
MR effect size for
'Small glutamine−rich tetratricopeptide repeat−containing protein alpha' on 'eGFR'

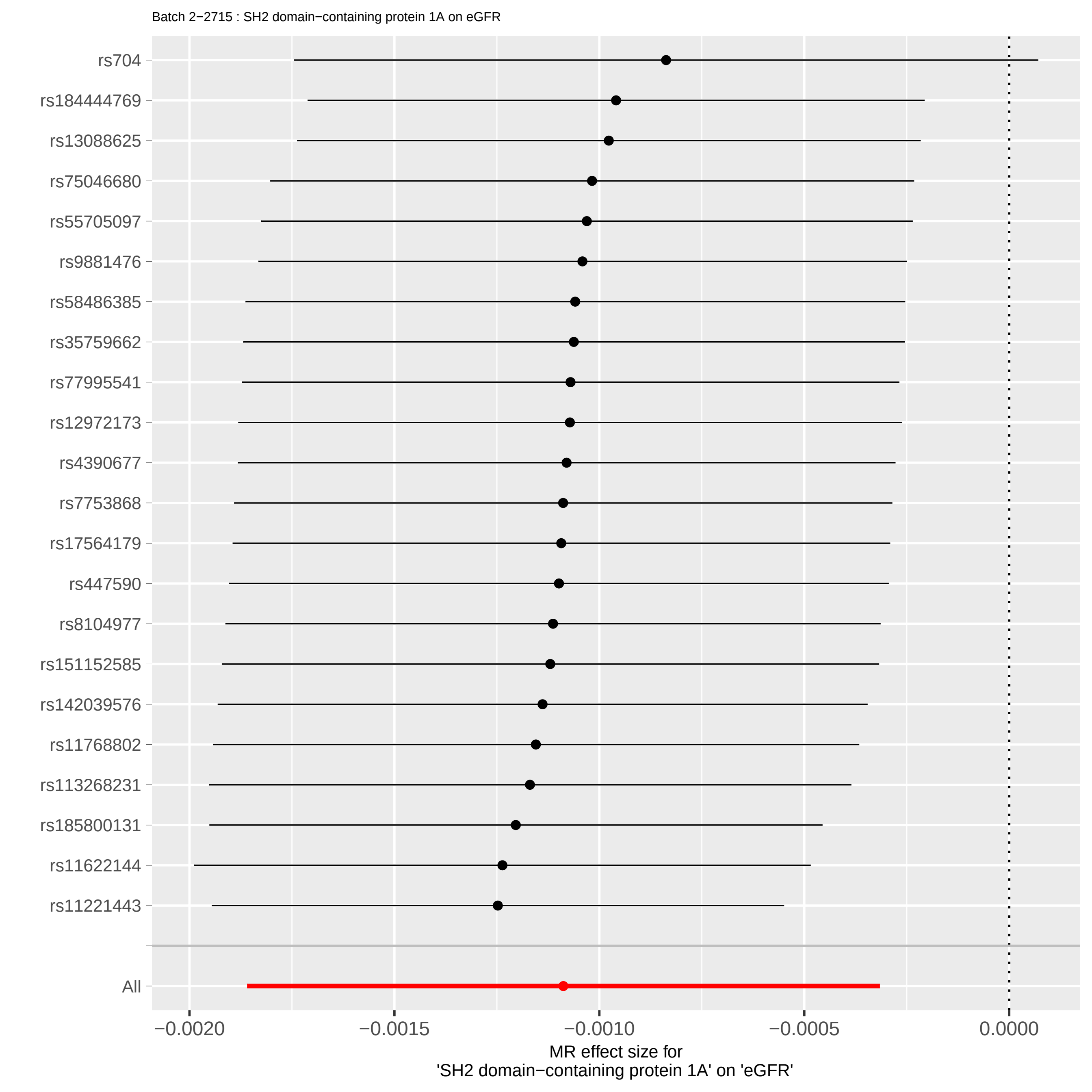
Batch 2−2715 : SH2 domain−containing protein 1A on eGFR
rs704
rs184444769
rs13088625
rs75046680
rs55705097
rs9881476
rs58486385
rs35759662
rs77995541
rs12972173
rs4390677
rs7753868
rs17564179
rs447590
rs8104977
rs151152585
rs142039576
rs11768802
rs113268231
rs185800131
rs11622144
rs11221443
All
−0.0020
−0.0015
−0.0010
−0.0005
0.0000
MR effect size for
'SH2 domain−containing protein 1A' on 'eGFR'

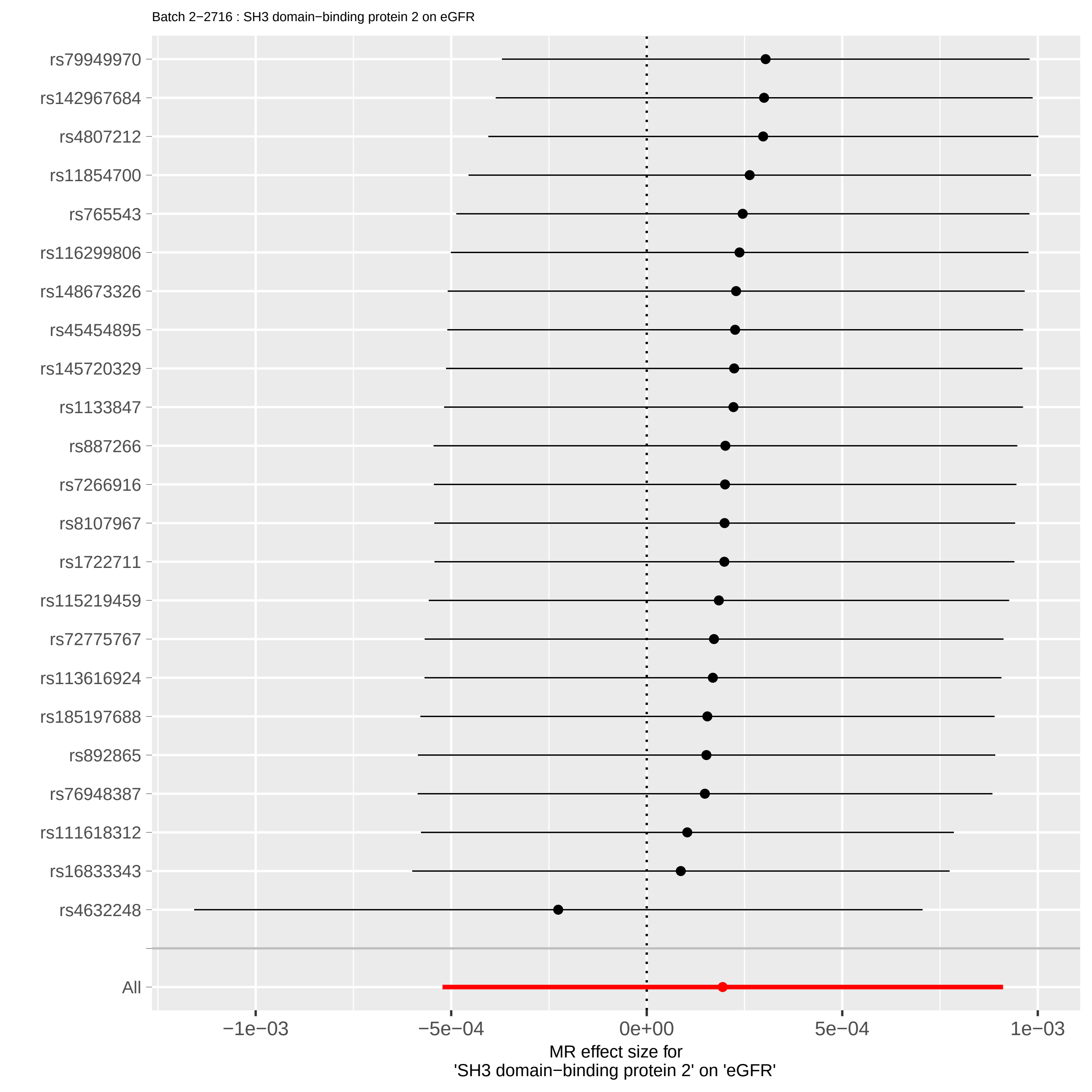

Batch 2−2716 : SH3 domain−binding protein 2 on eGFR
rs79949970
rs142967684
rs4807212
rs11854700
rs765543
rs116299806
rs148673326
rs45454895
rs145720329
rs1133847
rs887266
rs7266916
rs8107967
rs1722711
rs115219459
rs72775767
rs113616924
rs185197688
rs892865
rs76948387
rs111618312
rs16833343
rs4632248
All
−1e−03
−5e−04
0e+00
5e−04
1e−03
MR effect size for
'SH3 domain−binding protein 2' on 'eGFR'

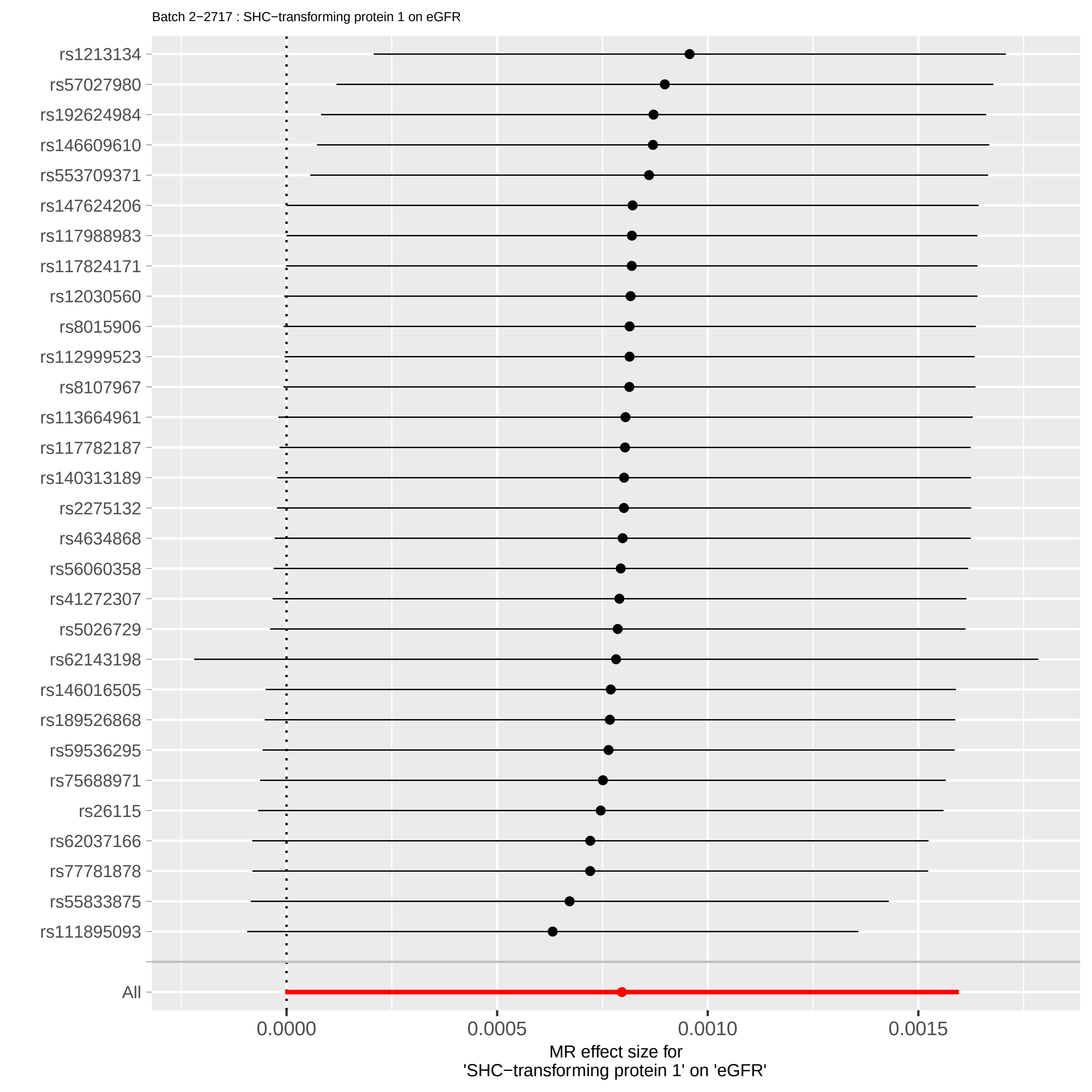

Batch 2−2717 : SHC−transforming protein 1 on eGFR
rs1213134
rs57027980
rs192624984
rs146609610
rs553709371
rs147624206
rs117988983
rs117824171
rs12030560
rs8015906
rs112999523
rs8107967
rs113664961
rs117782187
rs140313189
rs2275132
rs4634868
rs56060358
rs41272307
rs5026729
rs62143198
rs146016505
rs189526868
rs59536295
rs75688971
rs26115
rs62037166
rs77781878
rs55833875
rs111895093
All
0.0000
0.0005
0.0010
0.0015
MR effect size for
'SHC−transforming protein 1' on 'eGFR'

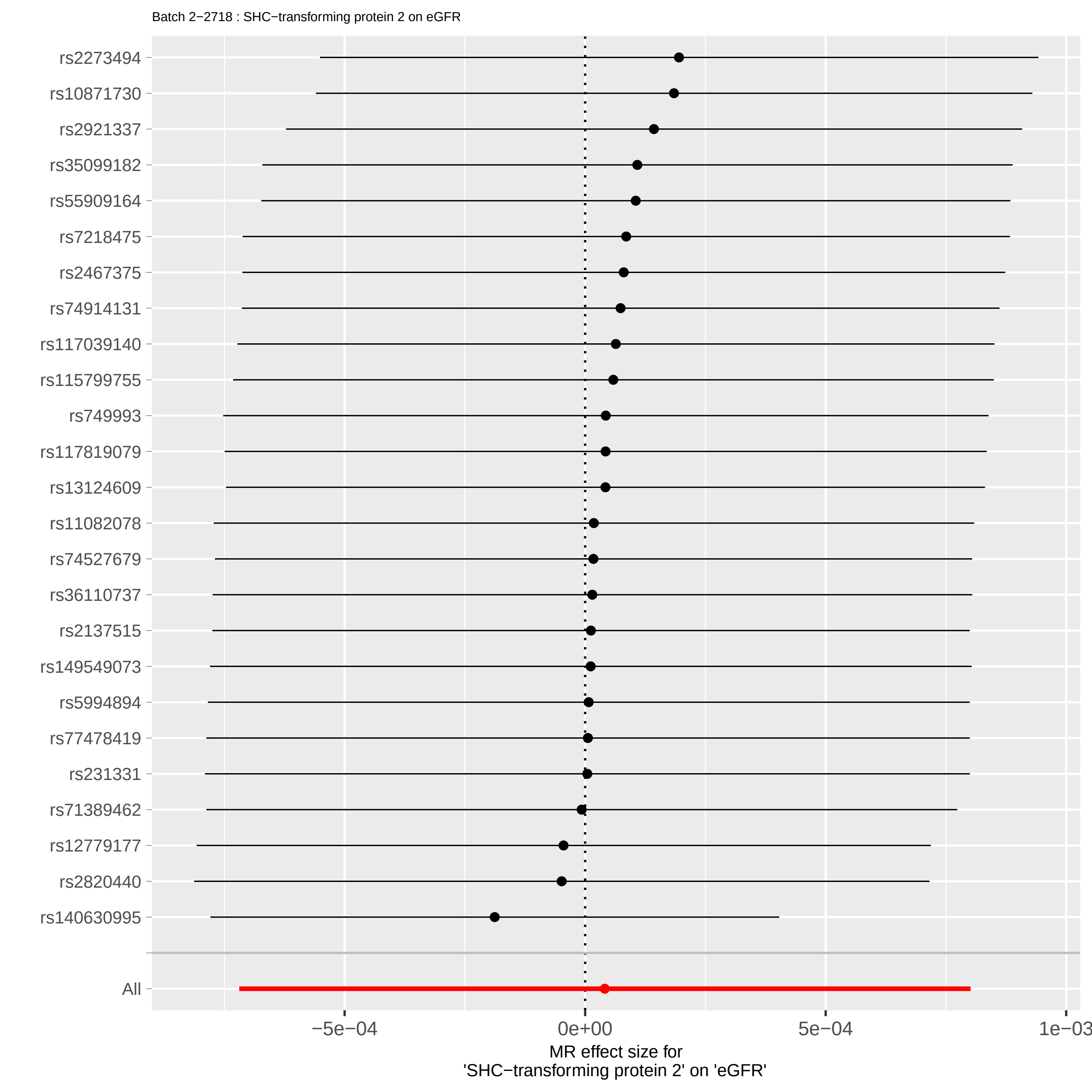
Batch 2−2718 : SHC−transforming protein 2 on eGFR
rs2273494
rs10871730
rs2921337
rs35099182
rs55909164
rs7218475
rs2467375
rs74914131
rs117039140
rs115799755
rs749993
rs117819079
rs13124609
rs11082078
rs74527679
rs36110737
rs2137515
rs149549073
rs5994894
rs77478419
rs231331
rs71389462
rs12779177
rs2820440
rs140630995
All
−5e−04
0e+00
5e−04
1e−03
MR effect size for
'SHC−transforming protein 2' on 'eGFR'

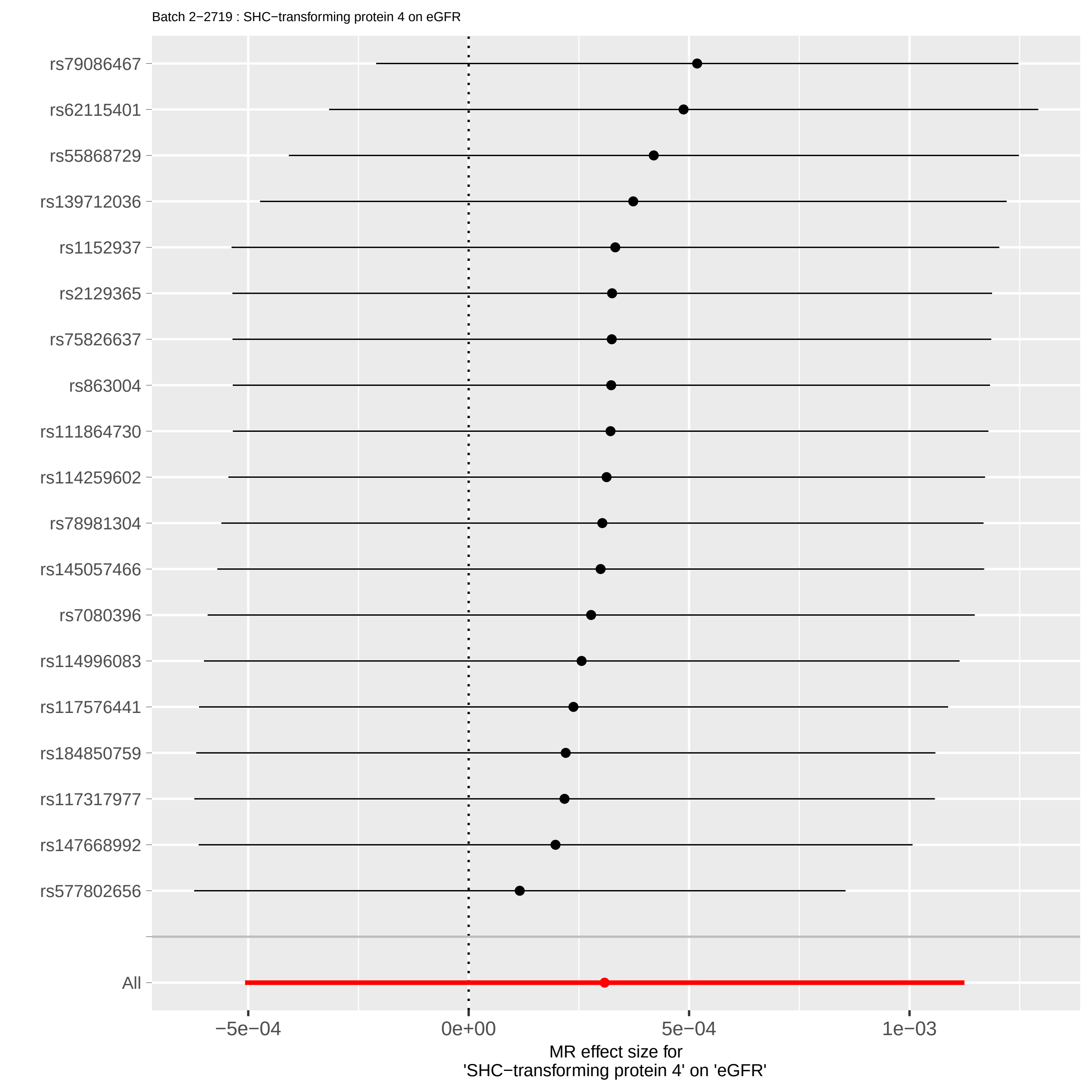

Batch 2−2719 : SHC−transforming protein 4 on eGFR
rs79086467
rs62115401
rs55868729
rs139712036
rs1152937
rs2129365
rs75826637
rs863004
rs111864730
rs114259602
rs78981304
rs145057466
rs7080396
rs114996083
rs117576441
rs184850759
rs117317977
rs147668992
rs577802656
All
−5e−04
0e+00
5e−04
1e−03
MR effect size for
'SHC−transforming protein 4' on 'eGFR'

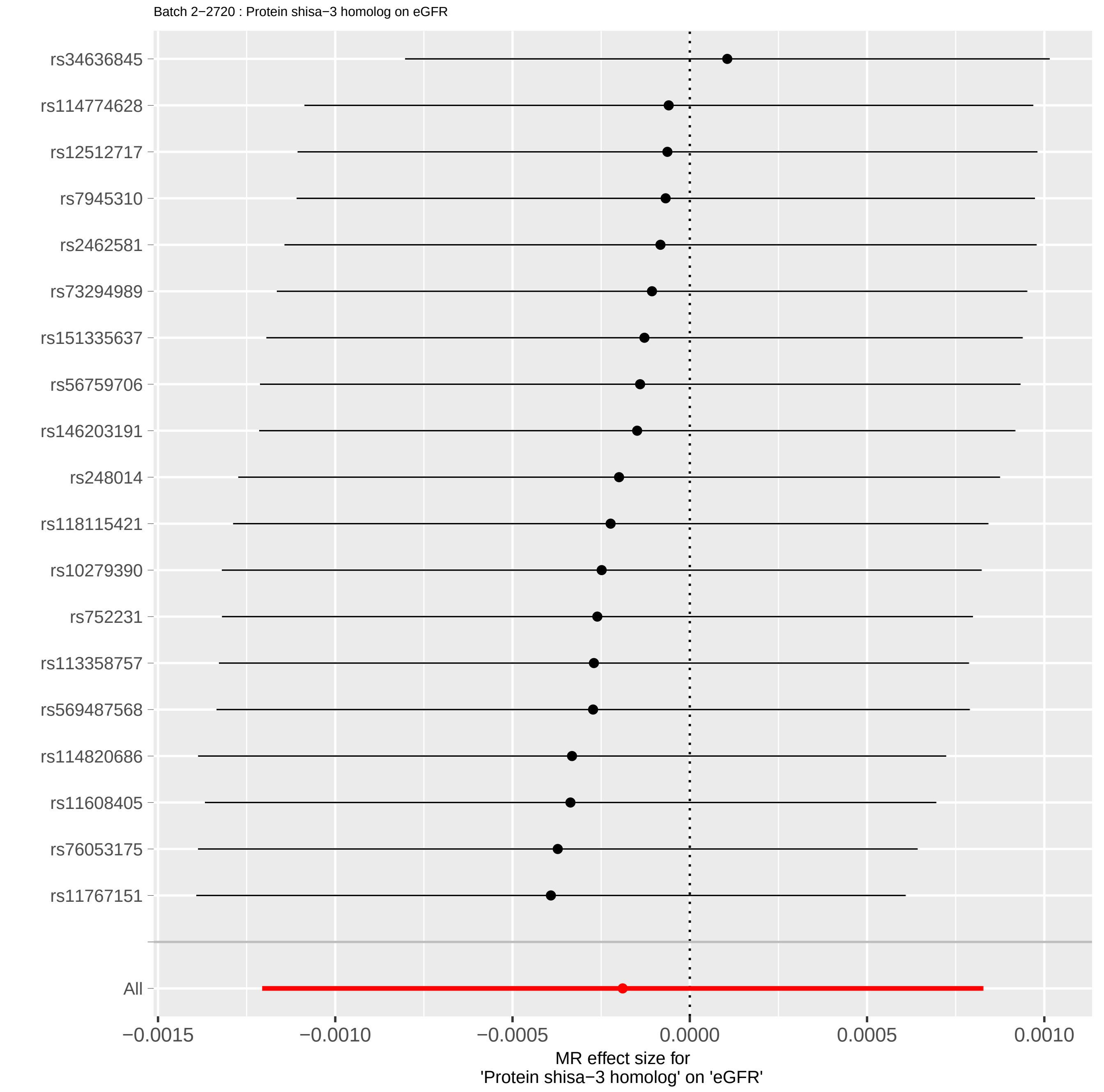

Batch 2−2720 : Protein shisa−3 homolog on eGFR
rs34636845
rs114774628
rs12512717
rs7945310
rs2462581
rs73294989
rs151335637
rs56759706
rs146203191
rs248014
rs118115421
rs10279390
rs752231
rs113358757
rs569487568
rs114820686
rs11608405
rs76053175
rs11767151
All
−0.0015
−0.0010
−0.0005
0.0000
0.0005
0.0010
MR effect size for
'Protein shisa−3 homolog' on 'eGFR'

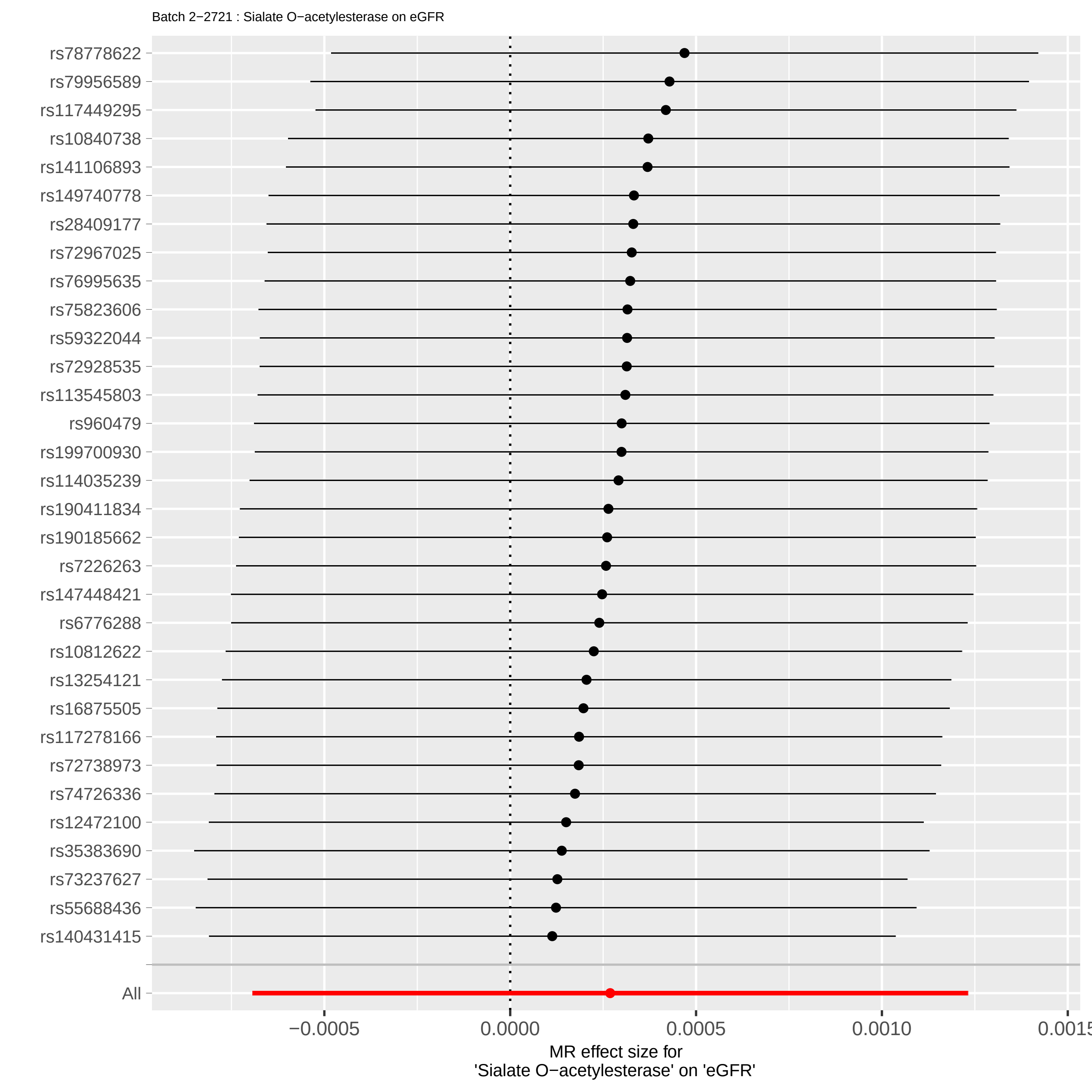

Batch 2−2721 : Sialate O−acetylesterase on eGFR
rs78778622
rs79956589
rs117449295
rs10840738
rs141106893
rs149740778
rs28409177
rs72967025
rs76995635
rs75823606
rs59322044
rs72928535
rs113545803
rs960479
rs199700930
rs114035239
rs190411834
rs190185662
rs7226263
rs147448421
rs6776288
rs10812622
rs13254121
rs16875505
rs117278166
rs72738973
rs74726336
rs12472100
rs35383690
rs73237627
rs55688436
rs140431415
All
−0.0005
0.0000
0.0005
0.0010
0.0015
MR effect size for
'Sialate O−acetylesterase' on 'eGFR'

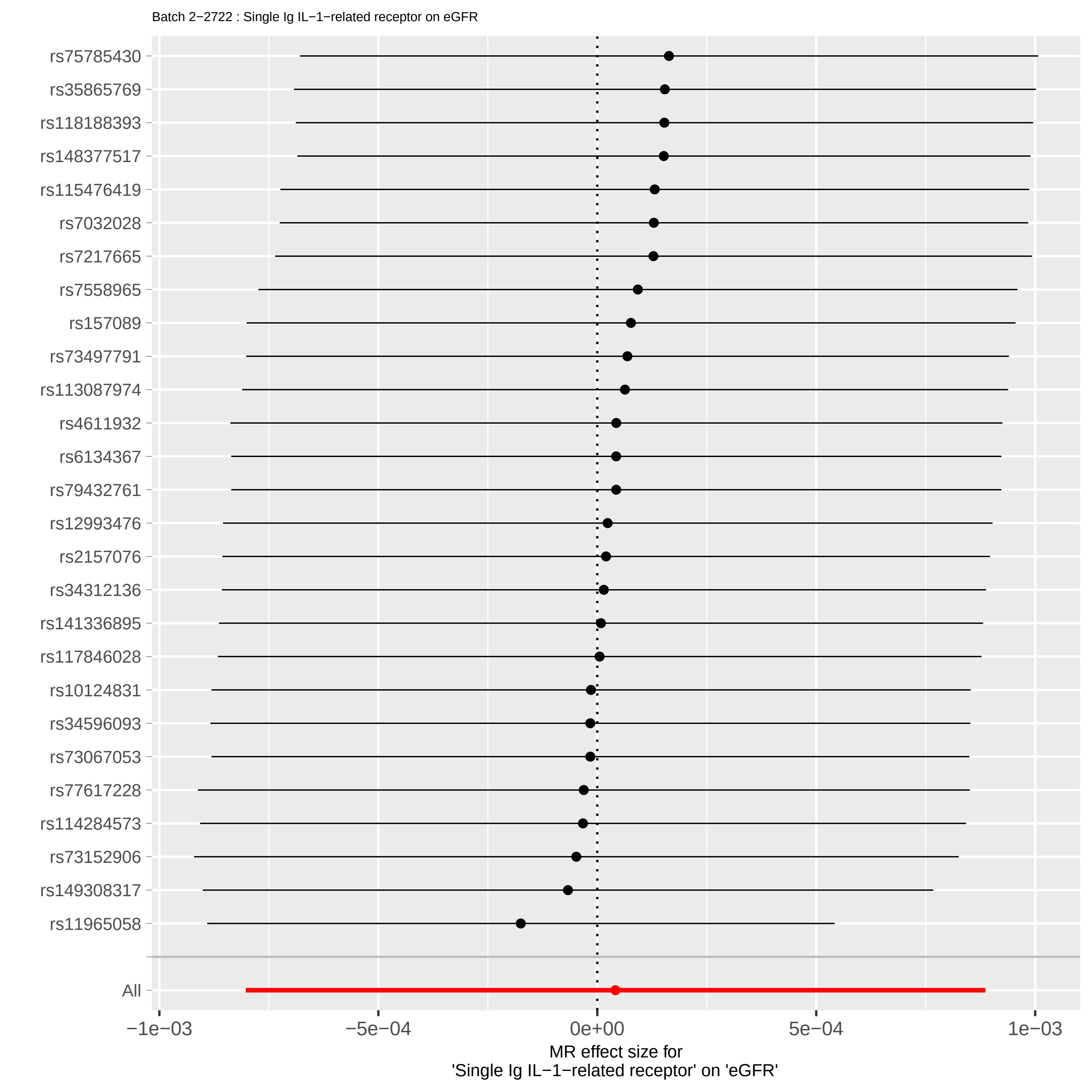

Batch 2−2722 : Single Ig IL−1−related receptor on eGFR
rs75785430
rs35865769
rs118188393
rs148377517
rs115476419
rs7032028
rs7217665
rs7558965
rs157089
rs73497791
rs113087974
rs4611932
rs6134367
rs79432761
rs12993476
rs2157076
rs34312136
rs141336895
rs117846028
rs10124831
rs34596093
rs73067053
rs77617228
rs114284573
rs73152906
rs149308317
rs11965058
All
−1e−03
−5e−04
0e+00
5e−04
1e−03
MR effect size for
'Single Ig IL−1−related receptor' on 'eGFR'

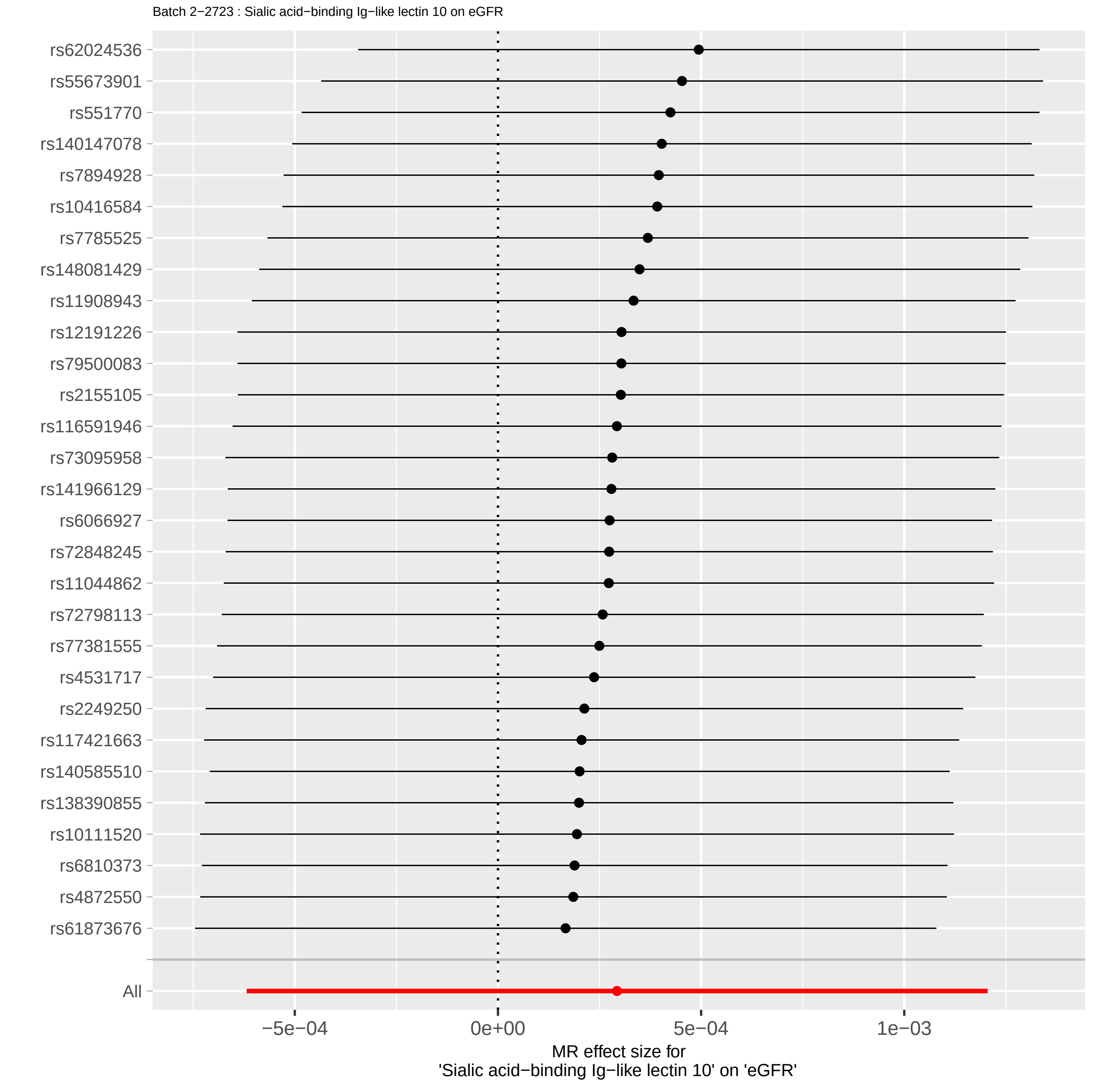
Batch 2−2723 : Sialic acid−binding Ig−like lectin 10 on eGFR
rs62024536
rs55673901
rs551770
rs140147078
rs7894928
rs10416584
rs7785525
rs148081429
rs11908943
rs12191226
rs79500083
rs2155105
rs116591946
rs73095958
rs141966129
rs6066927
rs72848245
rs11044862
rs72798113
rs77381555
rs4531717
rs2249250
rs117421663
rs140585510
rs138390855
rs10111520
rs6810373
rs4872550
rs61873676
All
−5e−04
0e+00
5e−04
1e−03
MR effect size for
'Sialic acid−binding Ig−like lectin 10' on 'eGFR'

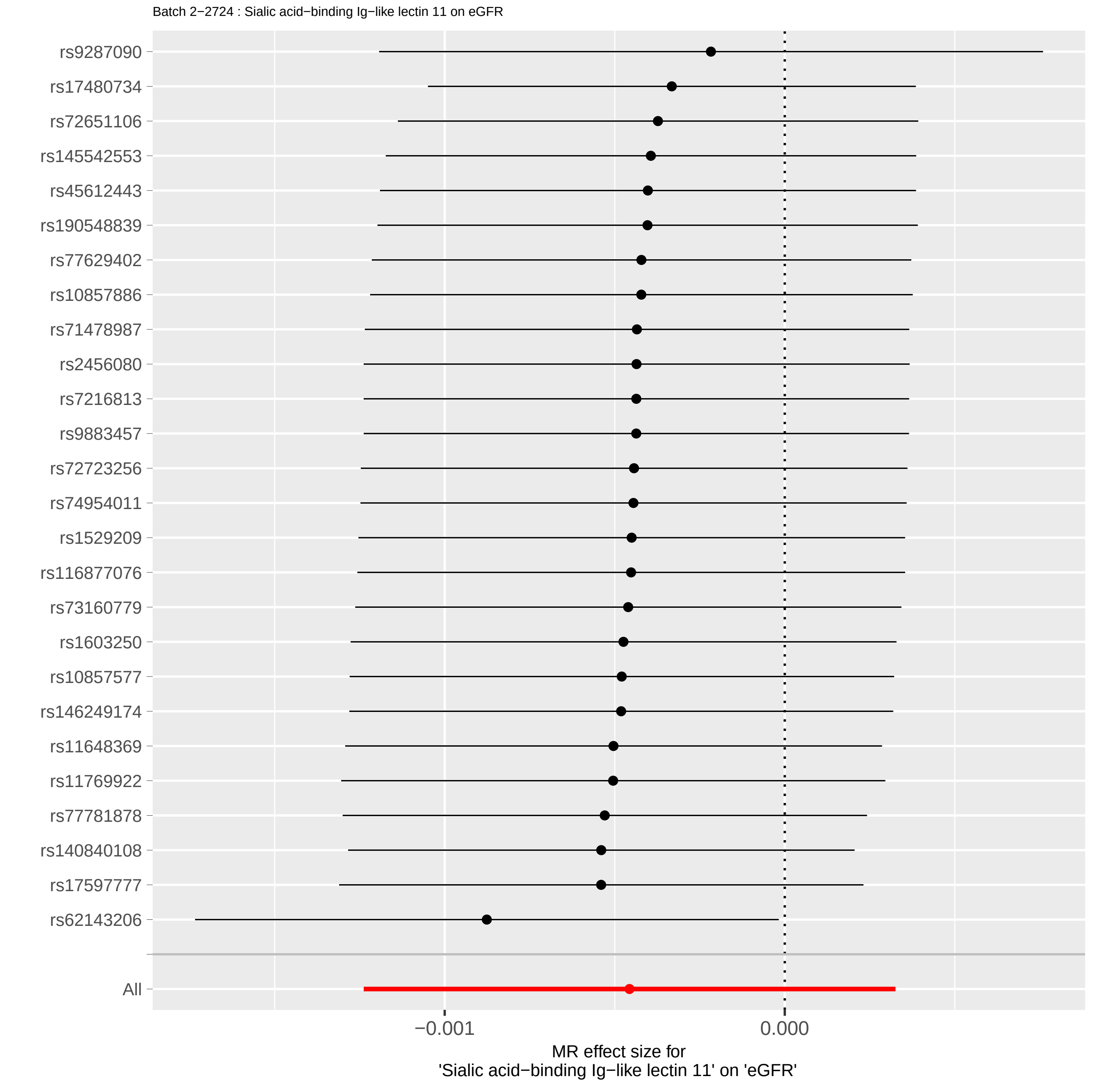

Batch 2−2724 : Sialic acid−binding Ig−like lectin 11 on eGFR
rs9287090
rs17480734
rs72651106
rs145542553
rs45612443
rs190548839
rs77629402
rs10857886
rs71478987
rs2456080
rs7216813
rs9883457
rs72723256
rs74954011
rs1529209
rs116877076
rs73160779
rs1603250
rs10857577
rs146249174
rs11648369
rs11769922
rs77781878
rs140840108
rs17597777
rs62143206
All
−0.001
0.000
MR effect size for
'Sialic acid−binding Ig−like lectin 11' on 'eGFR'

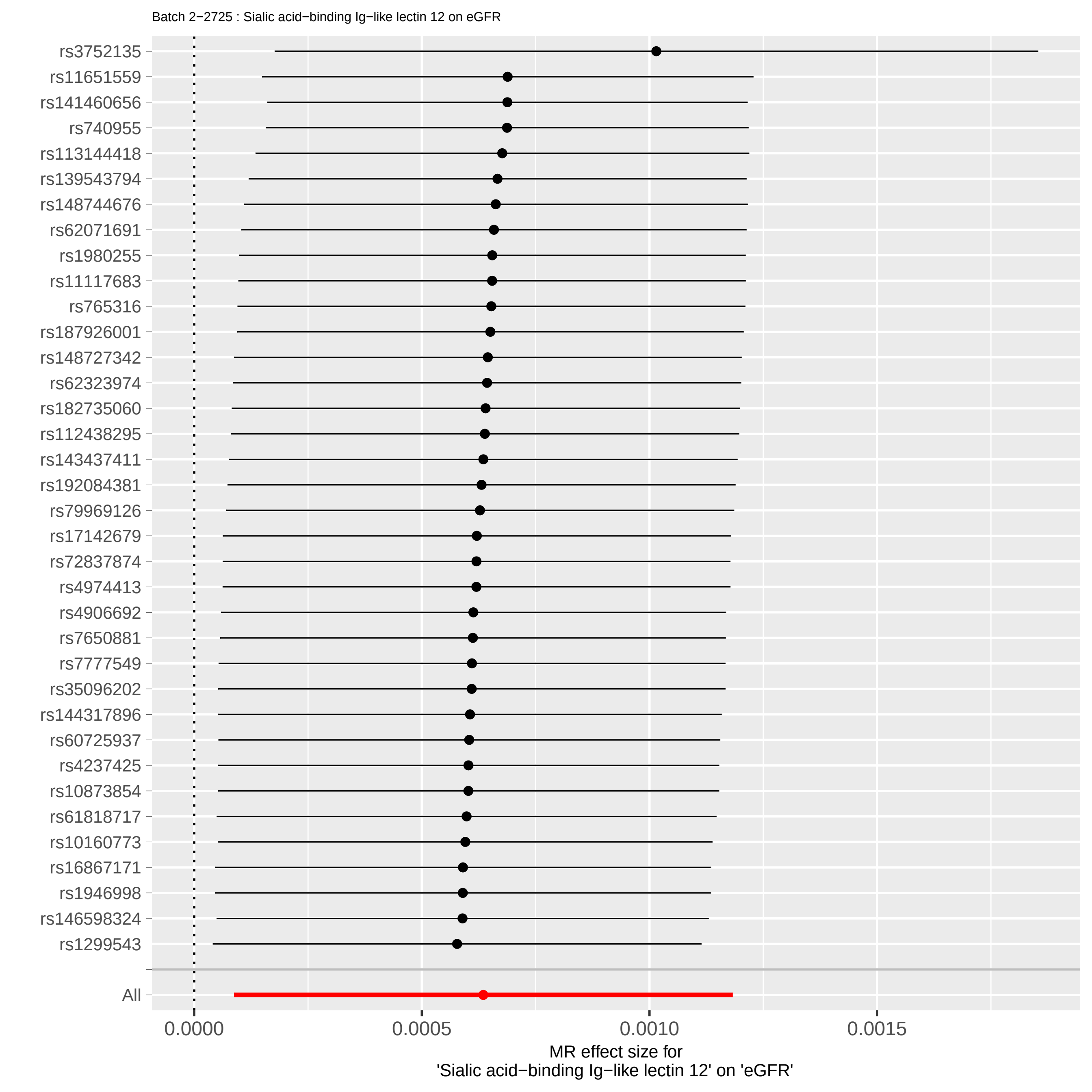
Batch 2−2725 : Sialic acid−binding Ig−like lectin 12 on eGFR
rs3752135
rs11651559
rs141460656
rs740955
rs113144418
rs139543794
rs148744676
rs62071691
rs1980255
rs11117683
rs765316
rs187926001
rs148727342
rs62323974
rs182735060
rs112438295
rs143437411
rs192084381
rs79969126
rs17142679
rs72837874
rs4974413
rs4906692
rs7650881
rs7777549
rs35096202
rs144317896
rs60725937
rs4237425
rs10873854
rs61818717
rs10160773
rs16867171
rs1946998
rs146598324
rs1299543
All
0.0000
0.0005
0.0010
0.0015
MR effect size for
'Sialic acid−binding Ig−like lectin 12' on 'eGFR'

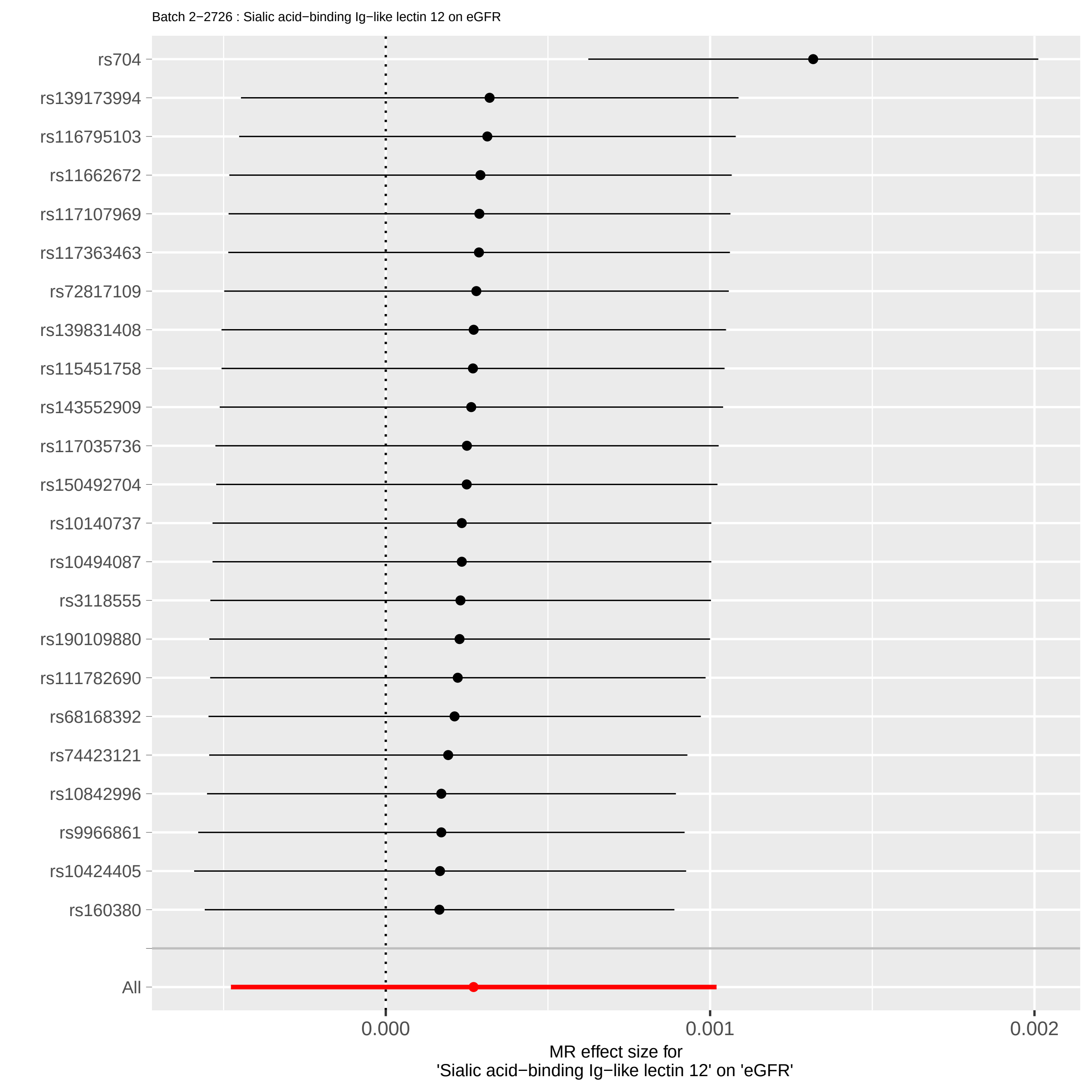

Batch 2−2726 : Sialic acid−binding Ig−like lectin 12 on eGFR
rs704
rs139173994
rs116795103
rs11662672
rs117107969
rs117363463
rs72817109
rs139831408
rs115451758
rs143552909
rs117035736
rs150492704
rs10140737
rs10494087
rs3118555
rs190109880
rs111782690
rs68168392
rs74423121
rs10842996
rs9966861
rs10424405
rs160380
All
0.000
0.001
0.002
MR effect size for
'Sialic acid−binding Ig−like lectin 12' on 'eGFR'

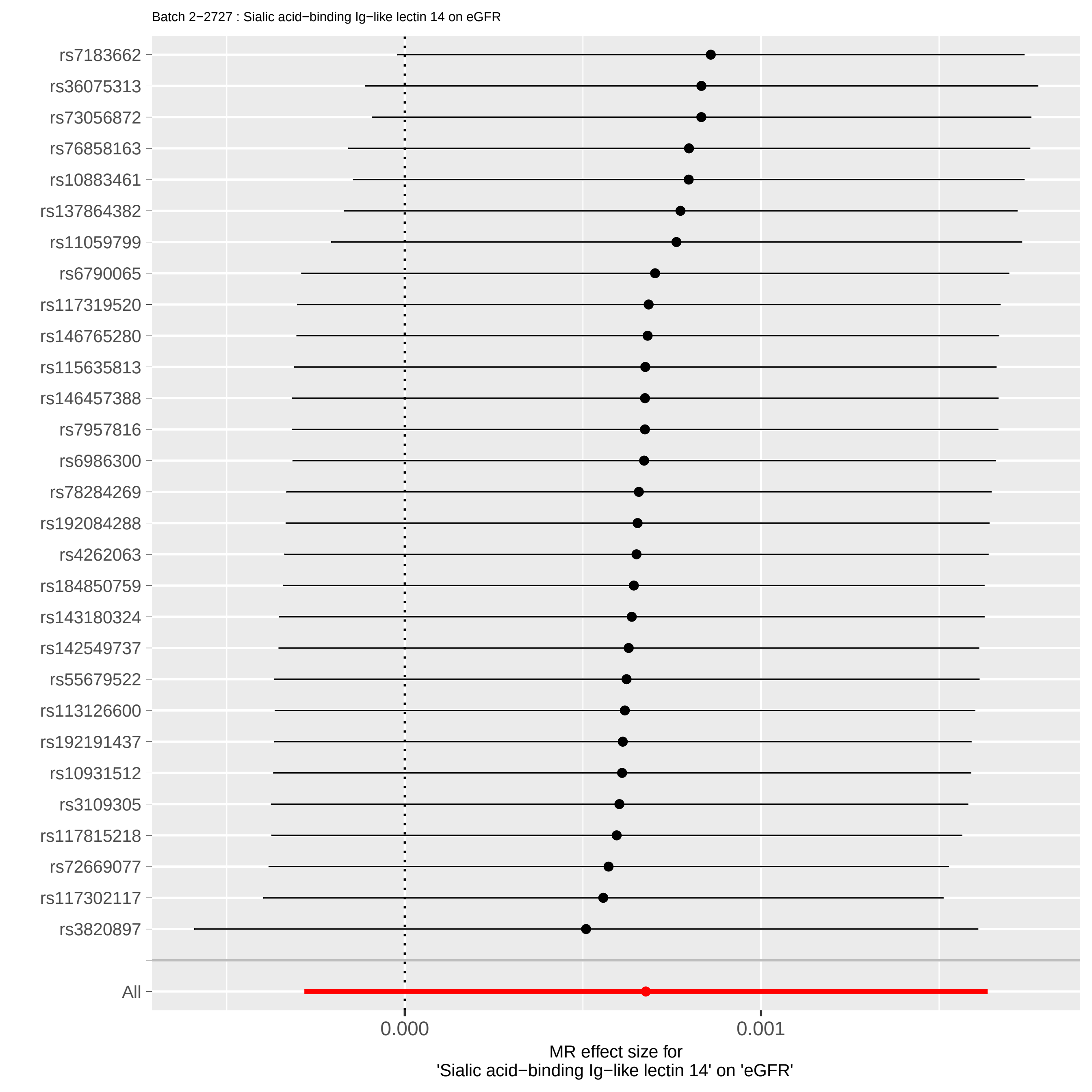

Batch 2−2727 : Sialic acid−binding Ig−like lectin 14 on eGFR
rs7183662
rs36075313
rs73056872
rs76858163
rs10883461
rs137864382
rs11059799
rs6790065
rs117319520
rs146765280
rs115635813
rs146457388
rs7957816
rs6986300
rs78284269
rs192084288
rs4262063
rs184850759
rs143180324
rs142549737
rs55679522
rs113126600
rs192191437
rs10931512
rs3109305
rs117815218
rs72669077
rs117302117
rs3820897
All
0.000
0.001
MR effect size for
'Sialic acid−binding Ig−like lectin 14' on 'eGFR'

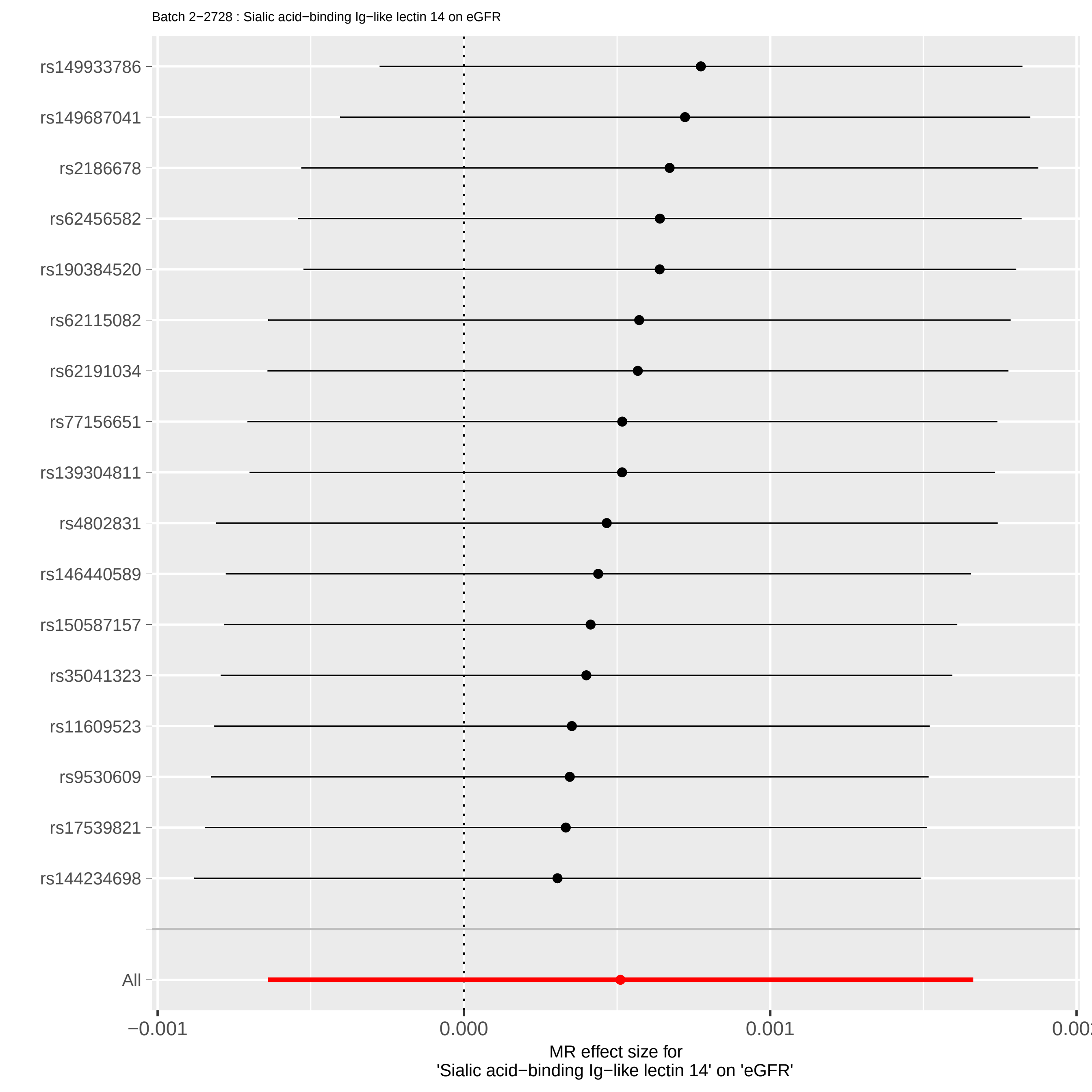

Batch 2−2728 : Sialic acid−binding Ig−like lectin 14 on eGFR
rs149933786
rs149687041
rs2186678
rs62456582
rs190384520
rs62115082
rs62191034
rs77156651
rs139304811
rs4802831
rs146440589
rs150587157
rs35041323
rs11609523
rs9530609
rs17539821
rs144234698
All
−0.001
0.000
0.001
MR effect size for
'Sialic acid−binding Ig−like lectin 14' on 'eGFR'

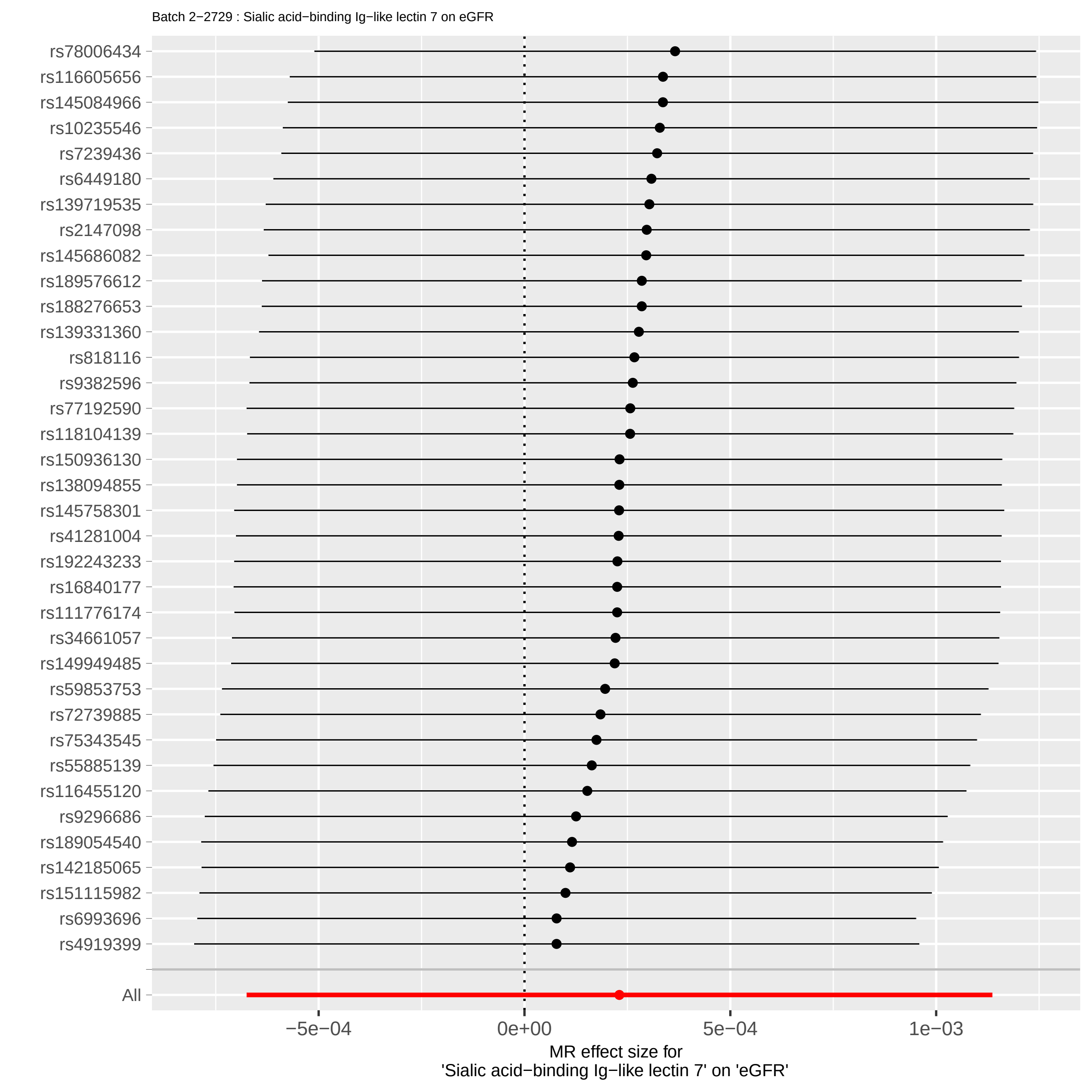

Batch 2−2729 : Sialic acid−binding Ig−like lectin 7 on eGFR
rs78006434
rs116605656
rs145084966
rs10235546
rs7239436
rs6449180
rs139719535
rs2147098
rs145686082
rs189576612
rs188276653
rs139331360
rs818116
rs9382596
rs77192590
rs118104139
rs150936130
rs138094855
rs145758301
rs41281004
rs192243233
rs16840177
rs111776174
rs34661057
rs149949485
rs59853753
rs72739885
rs75343545
rs55885139
rs116455120
rs9296686
rs189054540
rs142185065
rs151115982
rs6993696
rs4919399
All
−5e−04
0e+00
5e−04
1e−03
MR effect size for
'Sialic acid−binding Ig−like lectin 7' on 'eGFR'

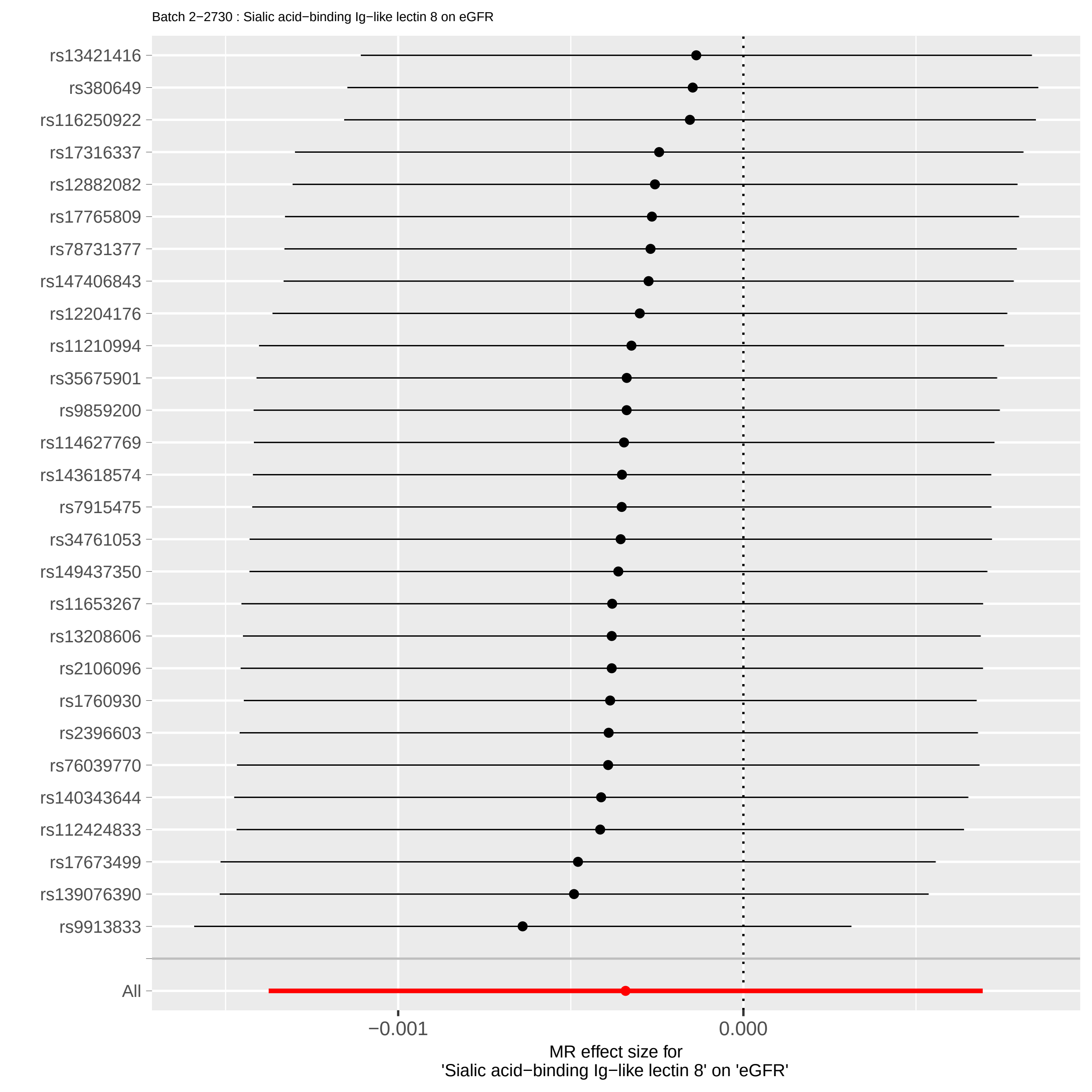

Batch 2−2730 : Sialic acid−binding Ig−like lectin 8 on eGFR
rs13421416
rs380649
rs116250922
rs17316337
rs12882082
rs17765809
rs78731377
rs147406843
rs12204176
rs11210994
rs35675901
rs9859200
rs114627769
rs143618574
rs7915475
rs34761053
rs149437350
rs11653267
rs13208606
rs2106096
rs1760930
rs2396603
rs76039770
rs140343644
rs112424833
rs17673499
rs139076390
rs9913833
All
−0.001
0.000
MR effect size for
'Sialic acid−binding Ig−like lectin 8' on 'eGFR'

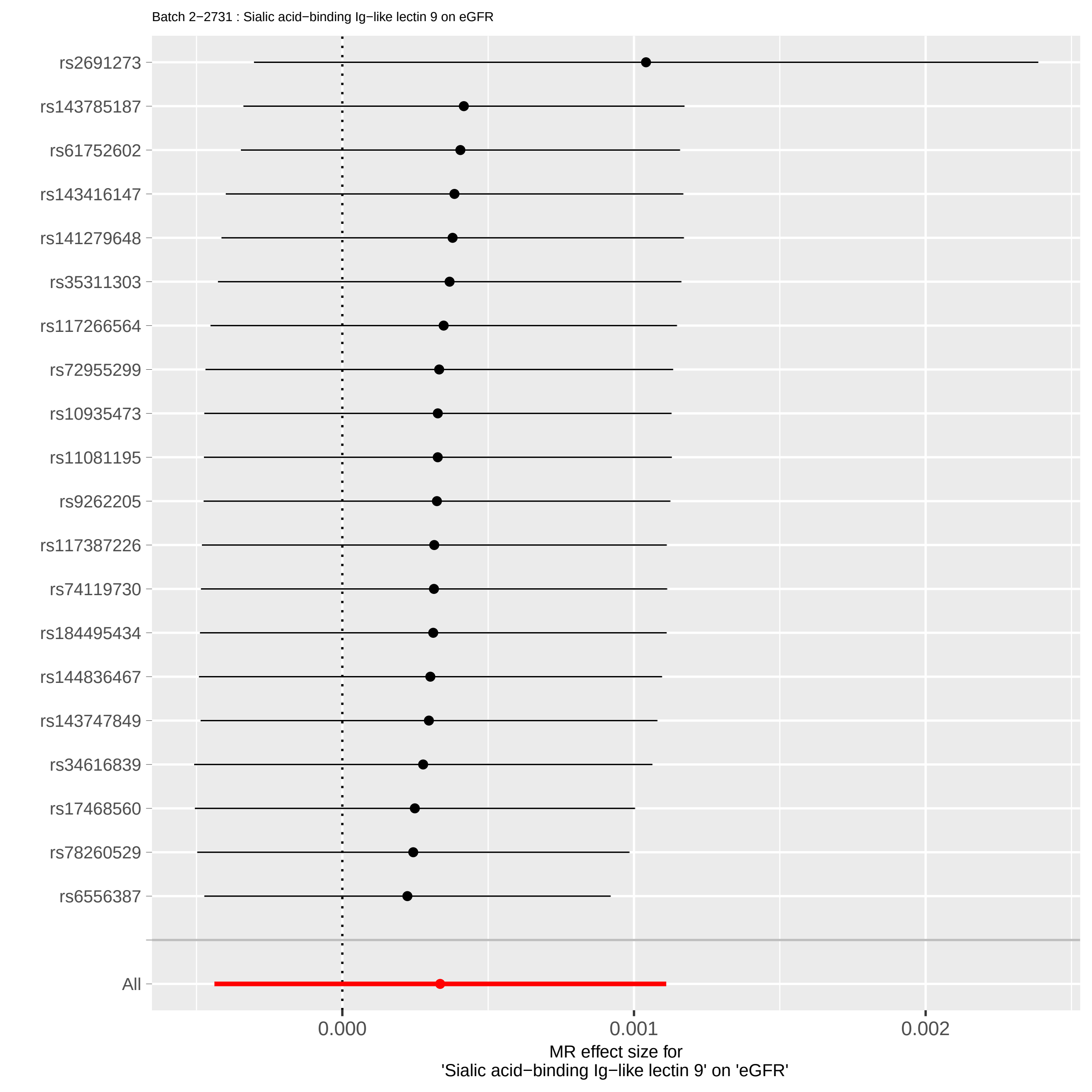
Batch 2−2731 : Sialic acid−binding Ig−like lectin 9 on eGFR
rs2691273
rs143785187
rs61752602
rs143416147
rs141279648
rs35311303
rs117266564
rs72955299
rs10935473
rs11081195
rs9262205
rs117387226
rs74119730
rs184495434
rs144836467
rs143747849
rs34616839
rs17468560
rs78260529
rs6556387
All
0.000
0.001
0.002
MR effect size for
'Sialic acid−binding Ig−like lectin 9' on 'eGFR'

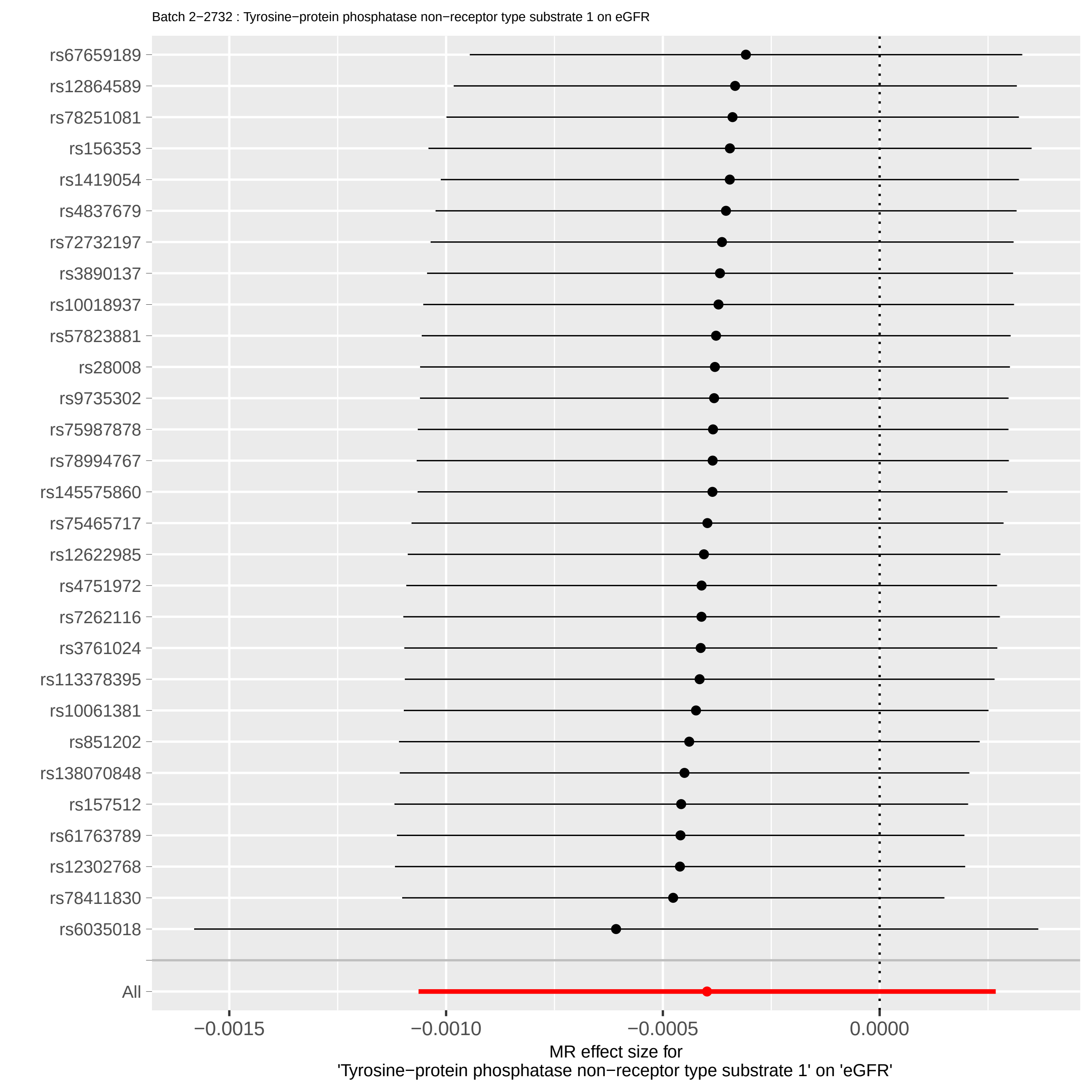

Batch 2−2732 : Tyrosine−protein phosphatase non−receptor type substrate 1 on eGFR
rs67659189
rs12864589
rs78251081
rs156353
rs1419054
rs4837679
rs72732197
rs3890137
rs10018937
rs57823881
rs28008
rs9735302
rs75987878
rs78994767
rs145575860
rs75465717
rs12622985
rs4751972
rs7262116
rs3761024
rs113378395
rs10061381
rs851202
rs138070848
rs157512
rs61763789
rs12302768
rs78411830
rs6035018
All
−0.0015
−0.0010
−0.0005
0.0000
MR effect size for
'Tyrosine−protein phosphatase non−receptor type substrate 1' on 'eGFR'

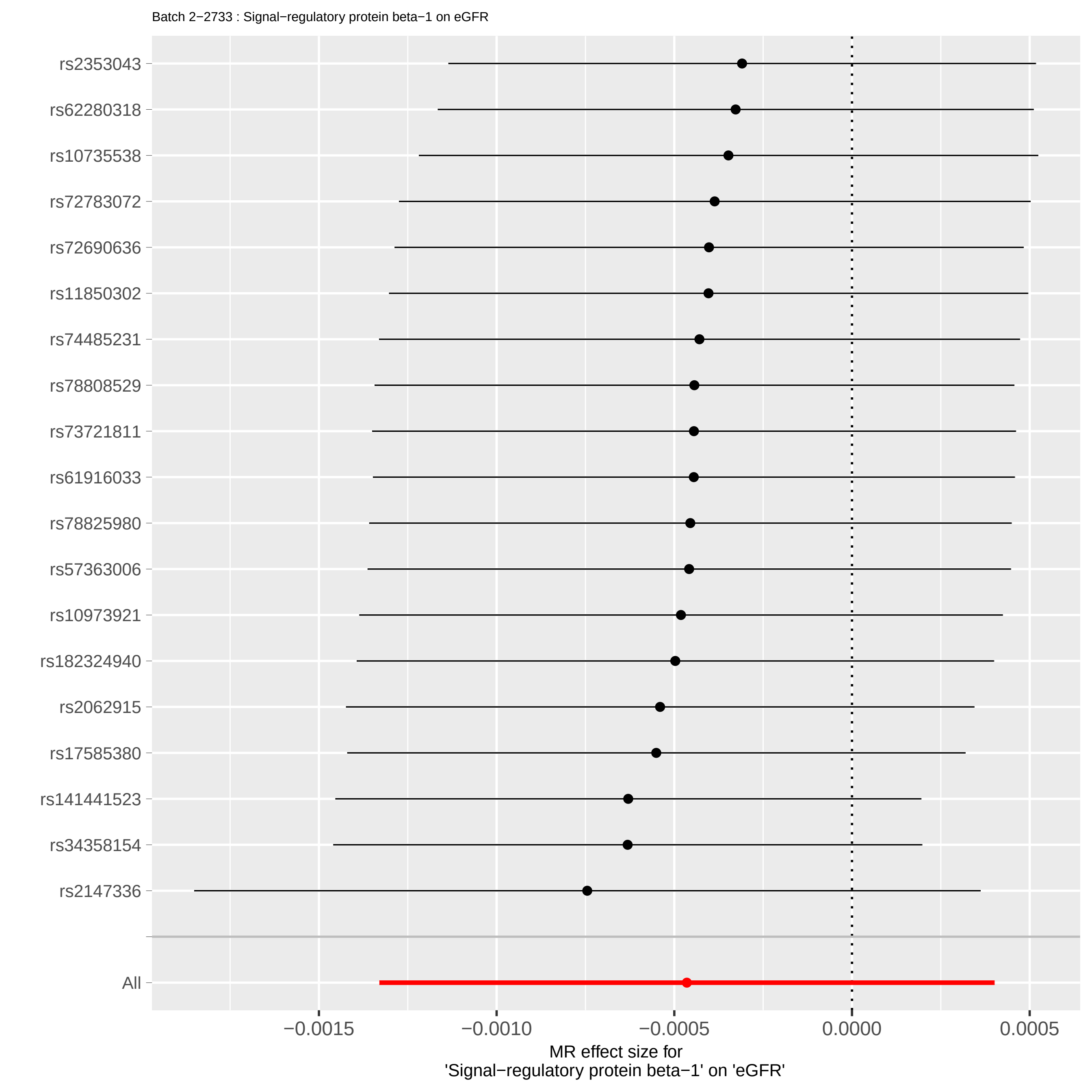

Batch 2−2733 : Signal−regulatory protein beta−1 on eGFR
rs2353043
rs62280318
rs10735538
rs72783072
rs72690636
rs11850302
rs74485231
rs78808529
rs73721811
rs61916033
rs78825980
rs57363006
rs10973921
rs182324940
rs2062915
rs17585380
rs141441523
rs34358154
rs2147336
All
−0.0015
−0.0010
−0.0005
0.0000
0.0005
MR effect size for
'Signal−regulatory protein beta−1' on 'eGFR'

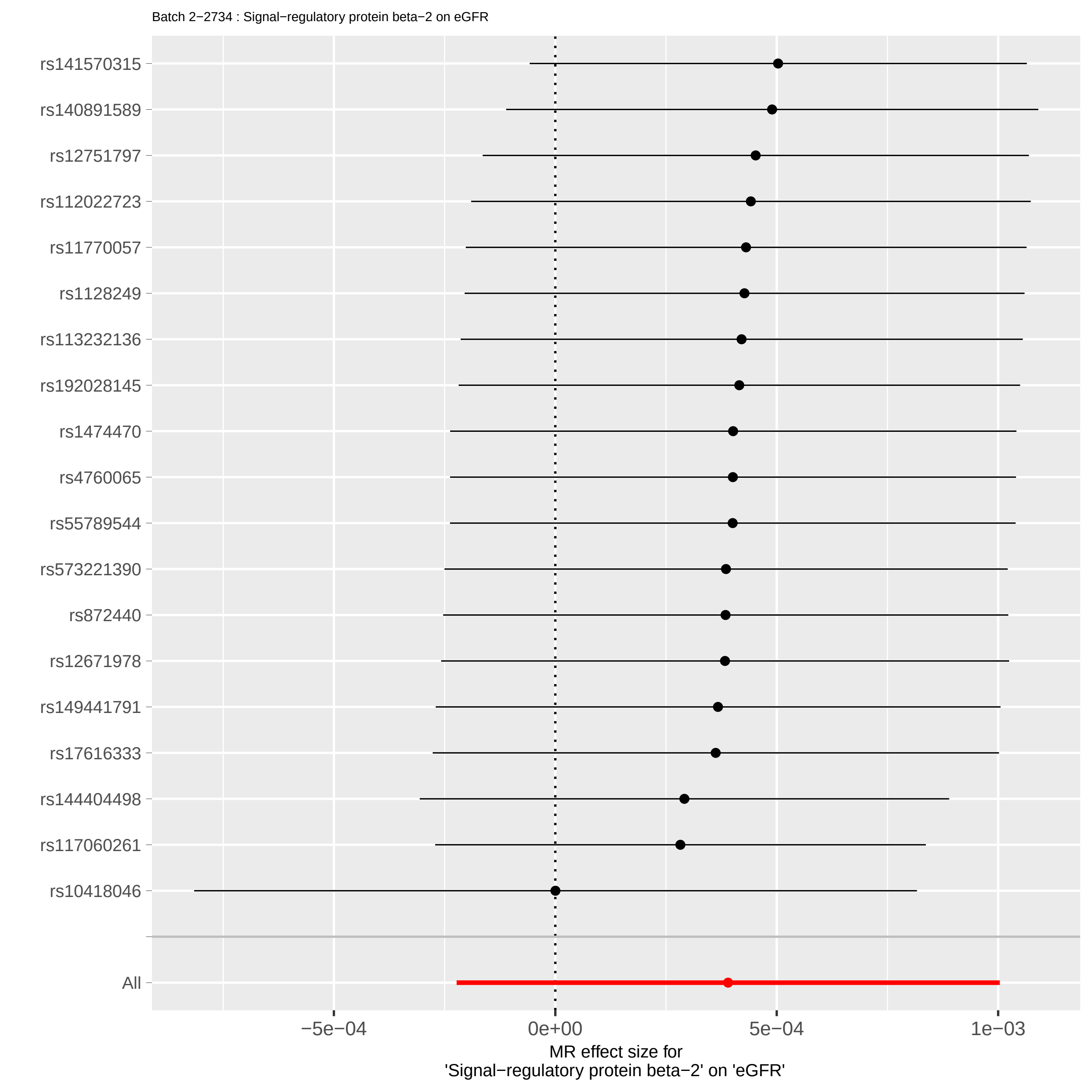

Batch 2−2734 : Signal−regulatory protein beta−2 on eGFR
rs141570315
rs140891589
rs12751797
rs112022723
rs11770057
rs1128249
rs113232136
rs192028145
rs1474470
rs4760065
rs55789544
rs573221390
rs872440
rs12671978
rs149441791
rs17616333
rs144404498
rs117060261
rs10418046
All
−5e−04
0e+00
5e−04
1e−03
MR effect size for
'Signal−regulatory protein beta−2' on 'eGFR'

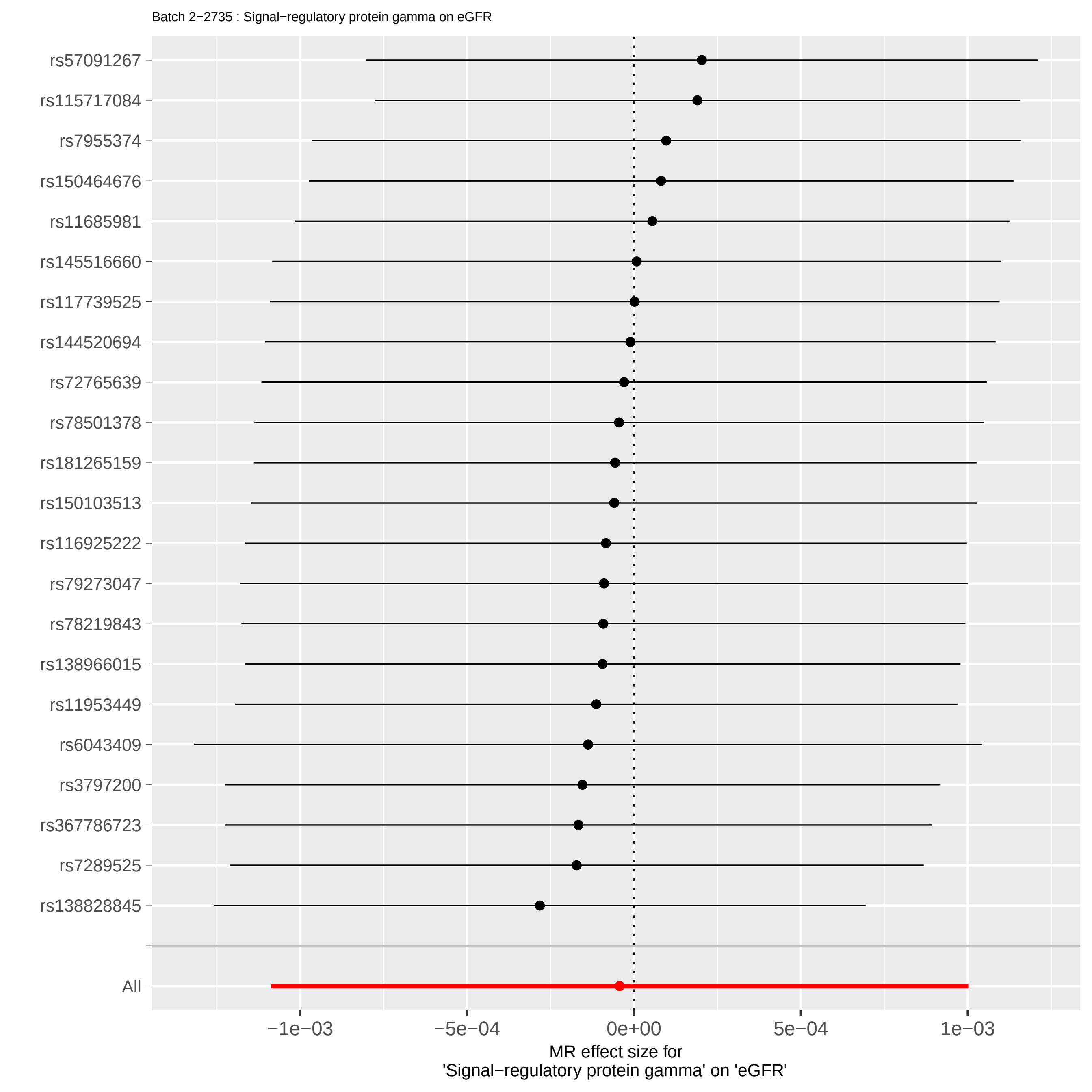

Batch 2−2735 : Signal−regulatory protein gamma on eGFR
rs57091267
rs115717084
rs7955374
rs150464676
rs11685981
rs145516660
rs117739525
rs144520694
rs72765639
rs78501378
rs181265159
rs150103513
rs116925222
rs79273047
rs78219843
rs138966015
rs11953449
rs6043409
rs3797200
rs367786723
rs7289525
rs138828845
All
−1e−03
−5e−04
0e+00
5e−04
1e−03
MR effect size for
'Signal−regulatory protein gamma' on 'eGFR'

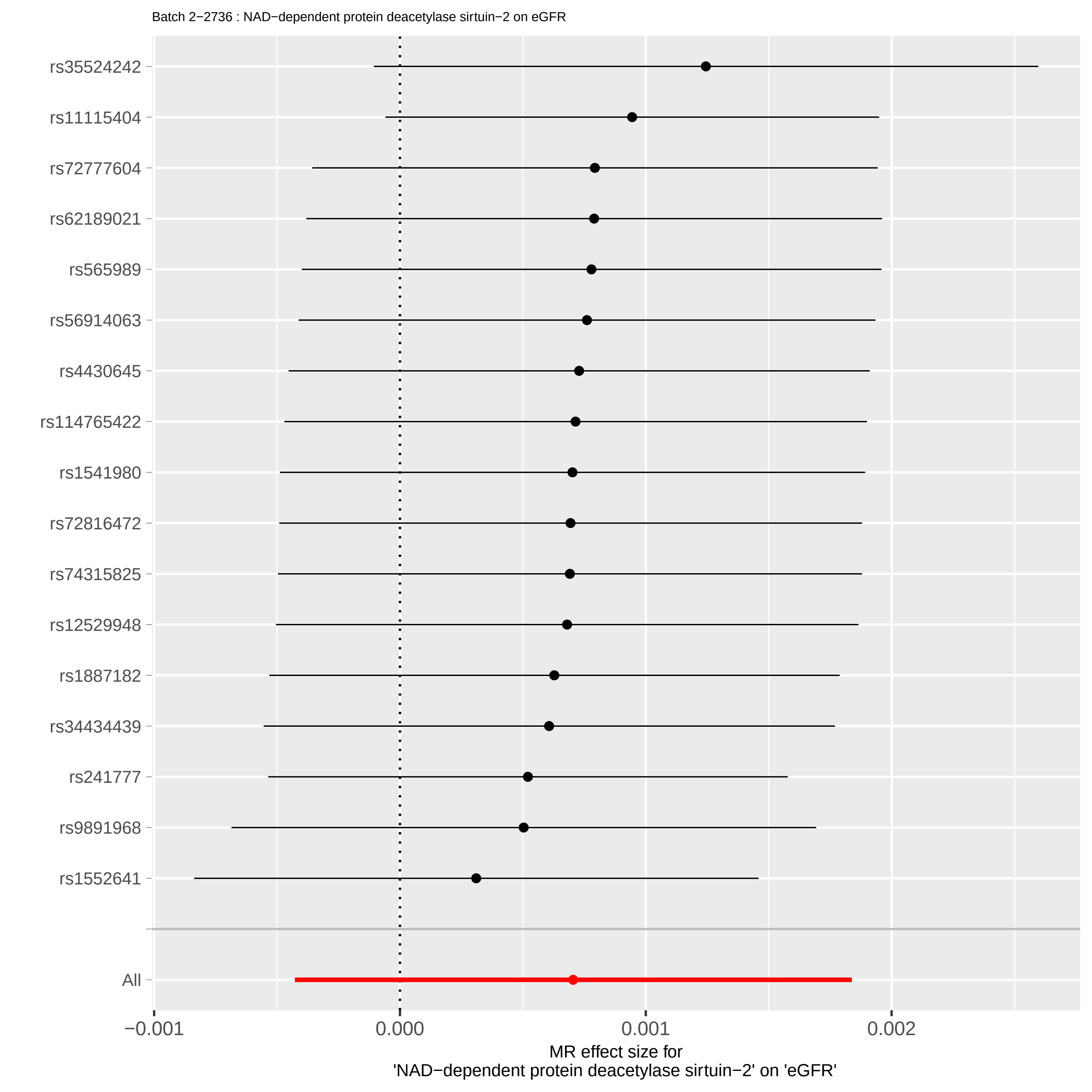

Batch 2−2736 : NAD−dependent protein deacetylase sirtuin−2 on eGFR
rs35524242
rs11115404
rs72777604
rs62189021
rs565989
rs56914063
rs4430645
rs114765422
rs1541980
rs72816472
rs74315825
rs12529948
rs1887182
rs34434439
rs241777
rs9891968
rs1552641
All
−0.001
0.000
0.001
0.002
MR effect size for
'NAD−dependent protein deacetylase sirtuin−2' on 'eGFR'

Batch 2−2737 : NAD−dependent protein deacylase sirtuin−5, mitochondrial on eGFR

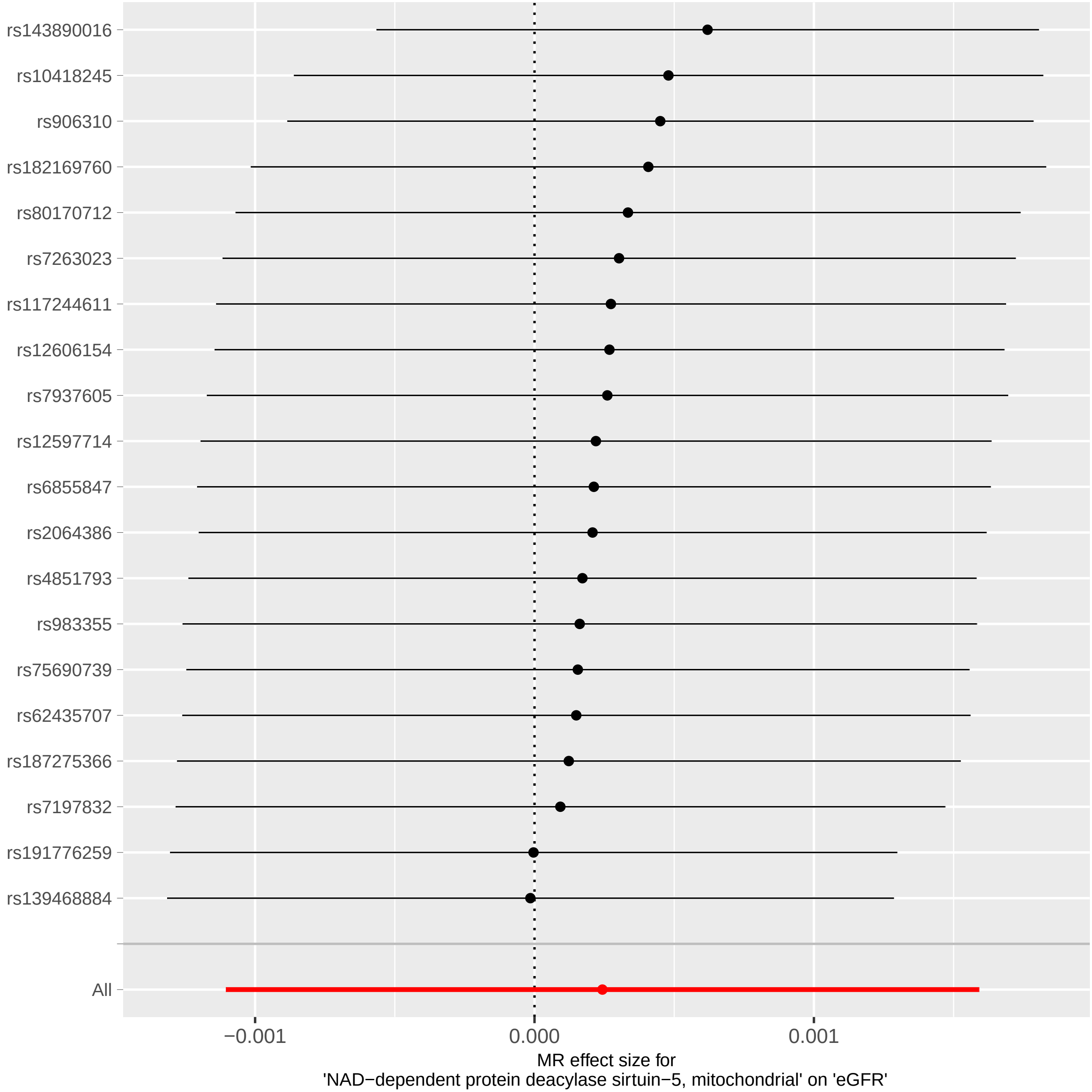

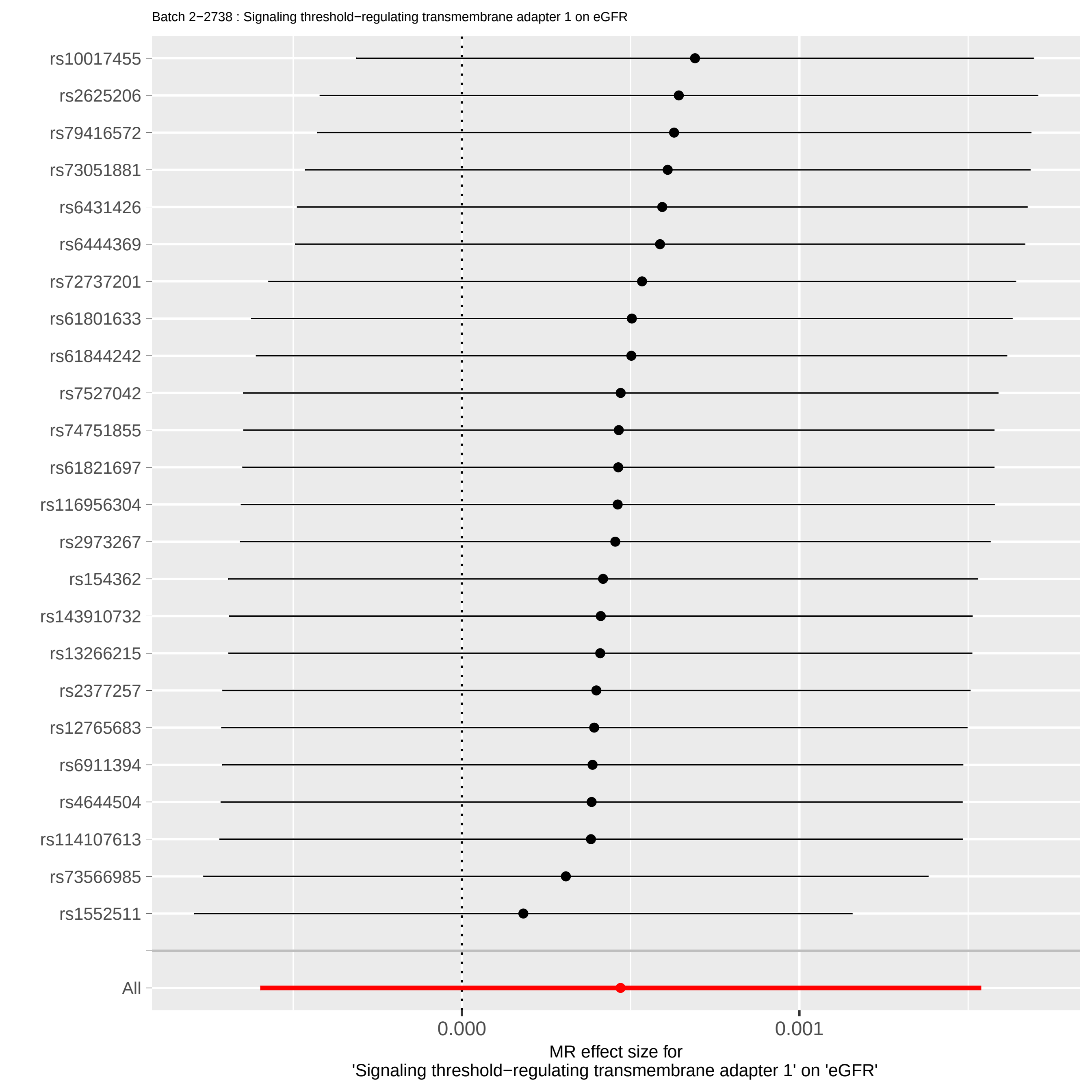

Batch 2−2738 : Signaling threshold−regulating transmembrane adapter 1 on eGFR
rs10017455
rs2625206
rs79416572
rs73051881
rs6431426
rs6444369
rs72737201
rs61801633
rs61844242
rs7527042
rs74751855
rs61821697
rs116956304
rs2973267
rs154362
rs143910732
rs13266215
rs2377257
rs12765683
rs6911394
rs4644504
rs114107613
rs73566985
rs1552511
All
0.000
0.001
MR effect size for
'Signaling threshold−regulating transmembrane adapter 1' on 'eGFR'

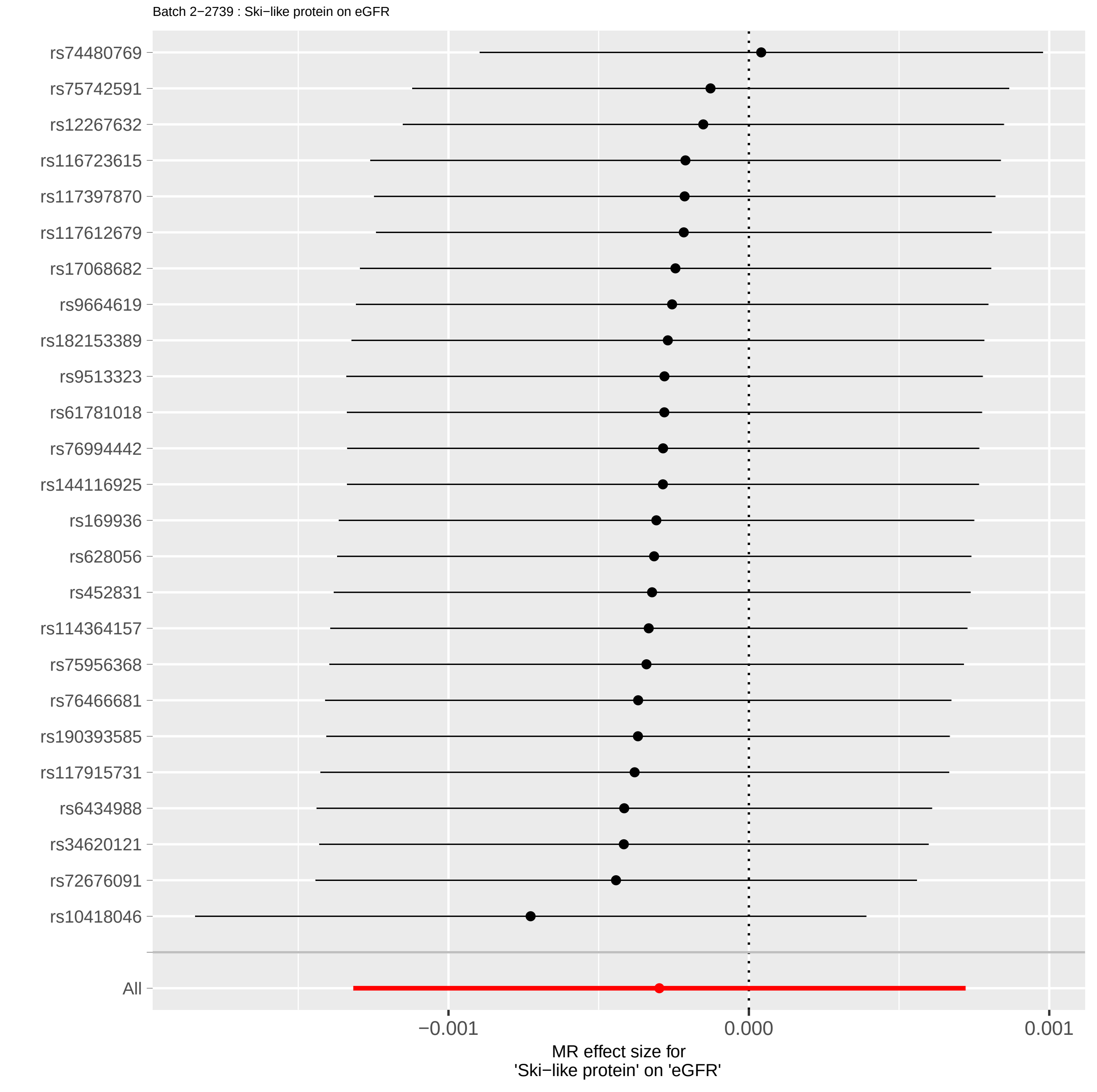

Batch 2−2739 : Ski−like protein on eGFR
rs74480769
rs75742591
rs12267632
rs116723615
rs117397870
rs117612679
rs17068682
rs9664619
rs182153389
rs9513323
rs61781018
rs76994442
rs144116925
rs169936
rs628056
rs452831
rs114364157
rs75956368
rs76466681
rs190393585
rs117915731
rs6434988
rs34620121
rs72676091
rs10418046
All
−0.001
0.000
0.001
MR effect size for
'Ski−like protein' on 'eGFR'

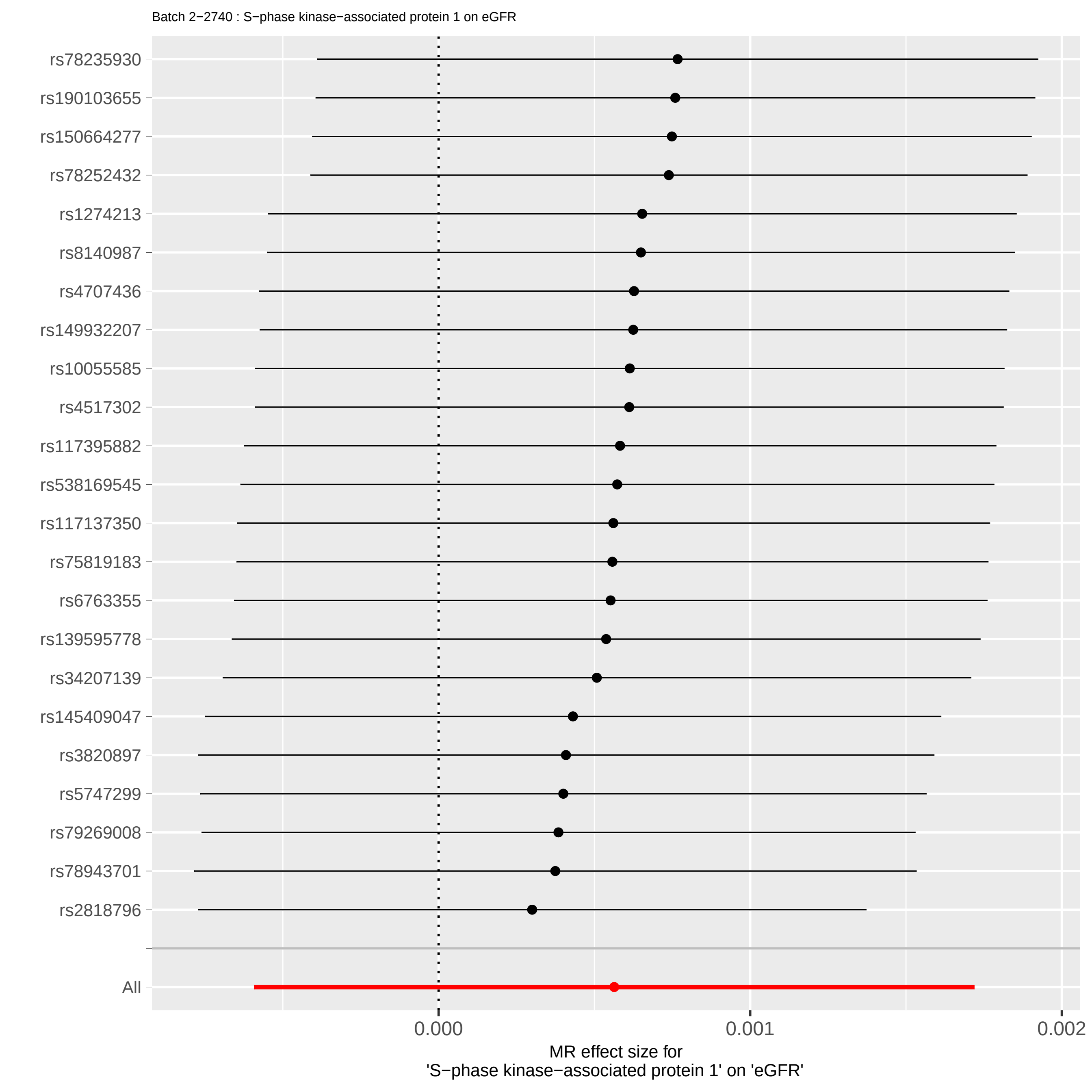

Batch 2−2740 : S−phase kinase−associated protein 1 on eGFR
rs78235930
rs190103655
rs150664277
rs78252432
rs1274213
rs8140987
rs4707436
rs149932207
rs10055585
rs4517302
rs117395882
rs538169545
rs117137350
rs75819183
rs6763355
rs139595778
rs34207139
rs145409047
rs3820897
rs5747299
rs79269008
rs78943701
rs2818796
All
0.000
0.001
0.002
MR effect size for
'S−phase kinase−associated protein 1' on 'eGFR'

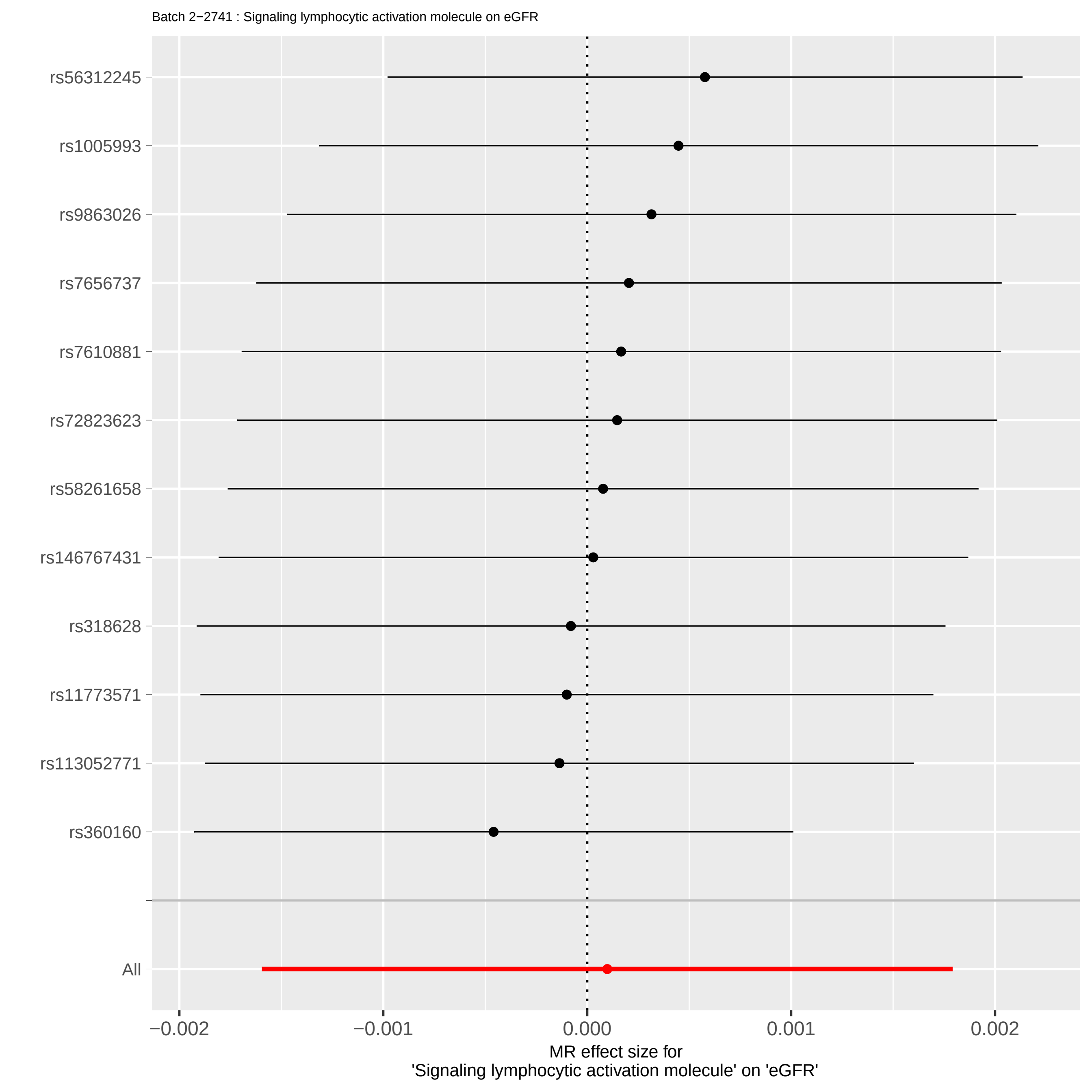

Batch 2−2741 : Signaling lymphocytic activation molecule on eGFR
rs56312245
rs1005993
rs9863026
rs7656737
rs7610881
rs72823623
rs58261658
rs146767431
rs318628
rs11773571
rs113052771
rs360160
All
−0.002
−0.001
0.000
0.001
0.002
MR effect size for
'Signaling lymphocytic activation molecule' on 'eGFR'

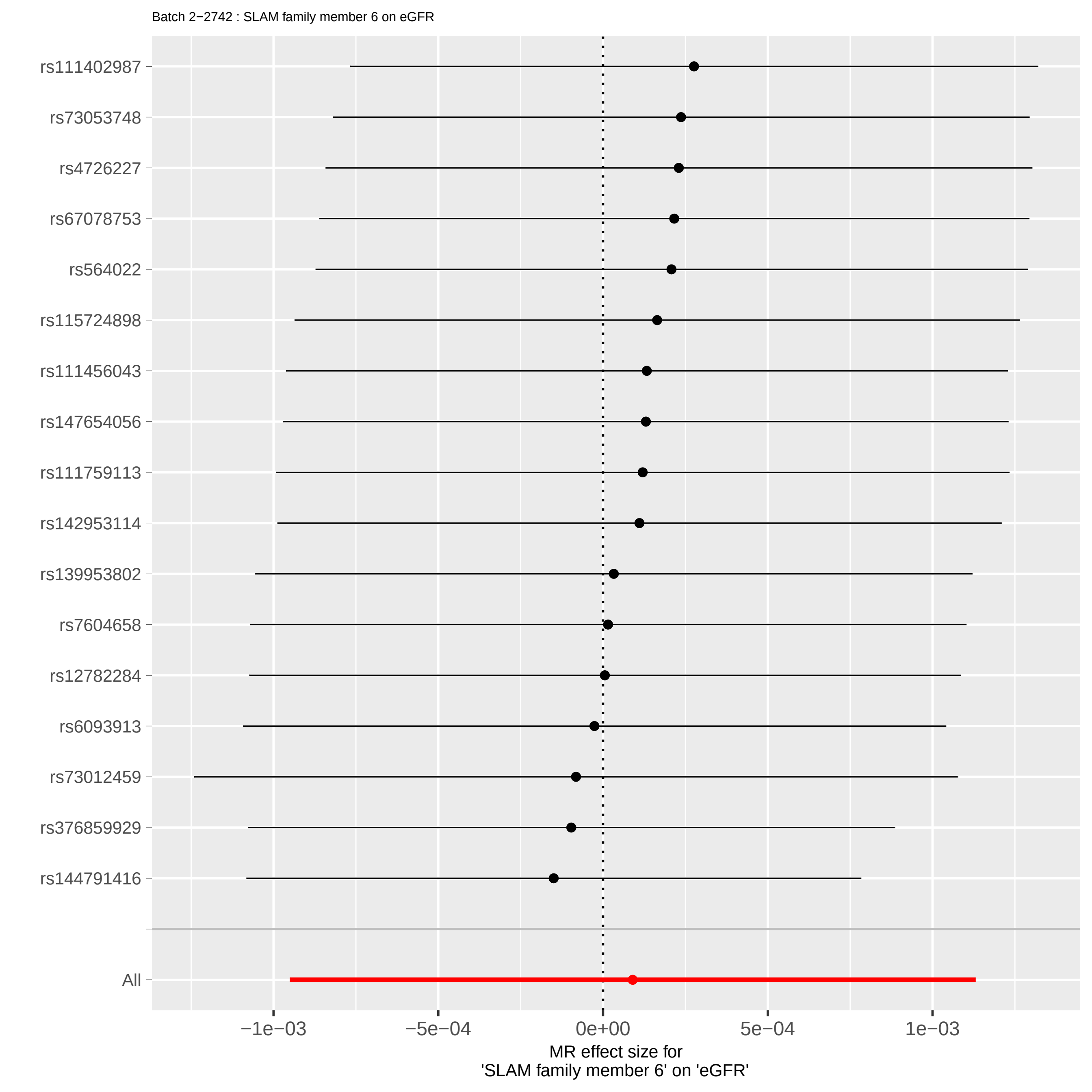

Batch 2−2742 : SLAM family member 6 on eGFR
rs111402987
rs73053748
rs4726227
rs67078753
rs564022
rs115724898
rs111456043
rs147654056
rs111759113
rs142953114
rs139953802
rs7604658
rs12782284
rs6093913
rs73012459
rs376859929
rs144791416
All
−1e−03
−5e−04
0e+00
5e−04
1e−03
MR effect size for
'SLAM family member 6' on 'eGFR'

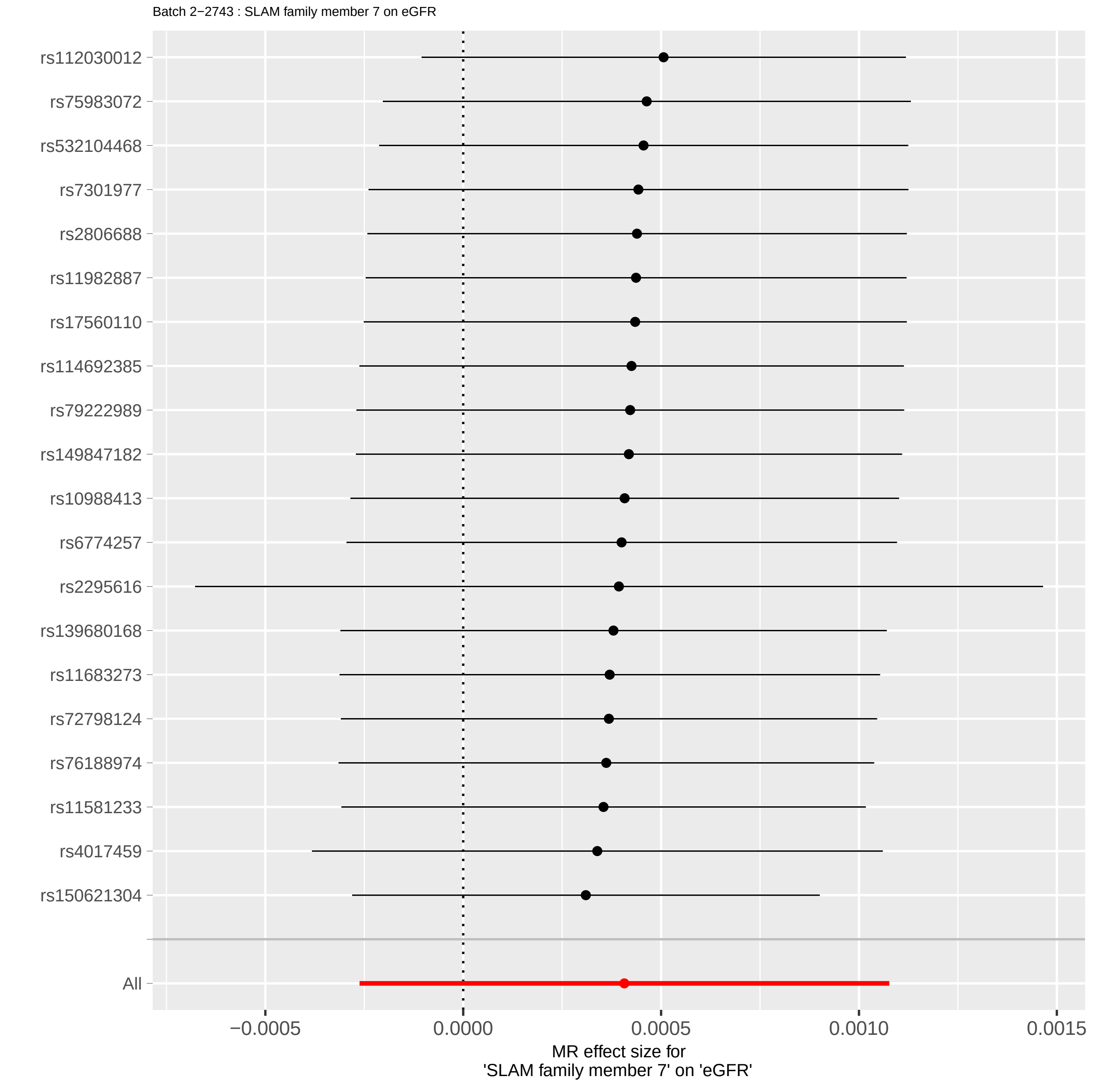

Batch 2−2743 : SLAM family member 7 on eGFR
rs112030012
rs75983072
rs532104468
rs7301977
rs2806688
rs11982887
rs17560110
rs114692385
rs79222989
rs149847182
rs10988413
rs6774257
rs2295616
rs139680168
rs11683273
rs72798124
rs76188974
rs11581233
rs4017459
rs150621304
All
−0.0005
0.0000
0.0005
0.0010
0.0015
MR effect size for
'SLAM family member 7' on 'eGFR'

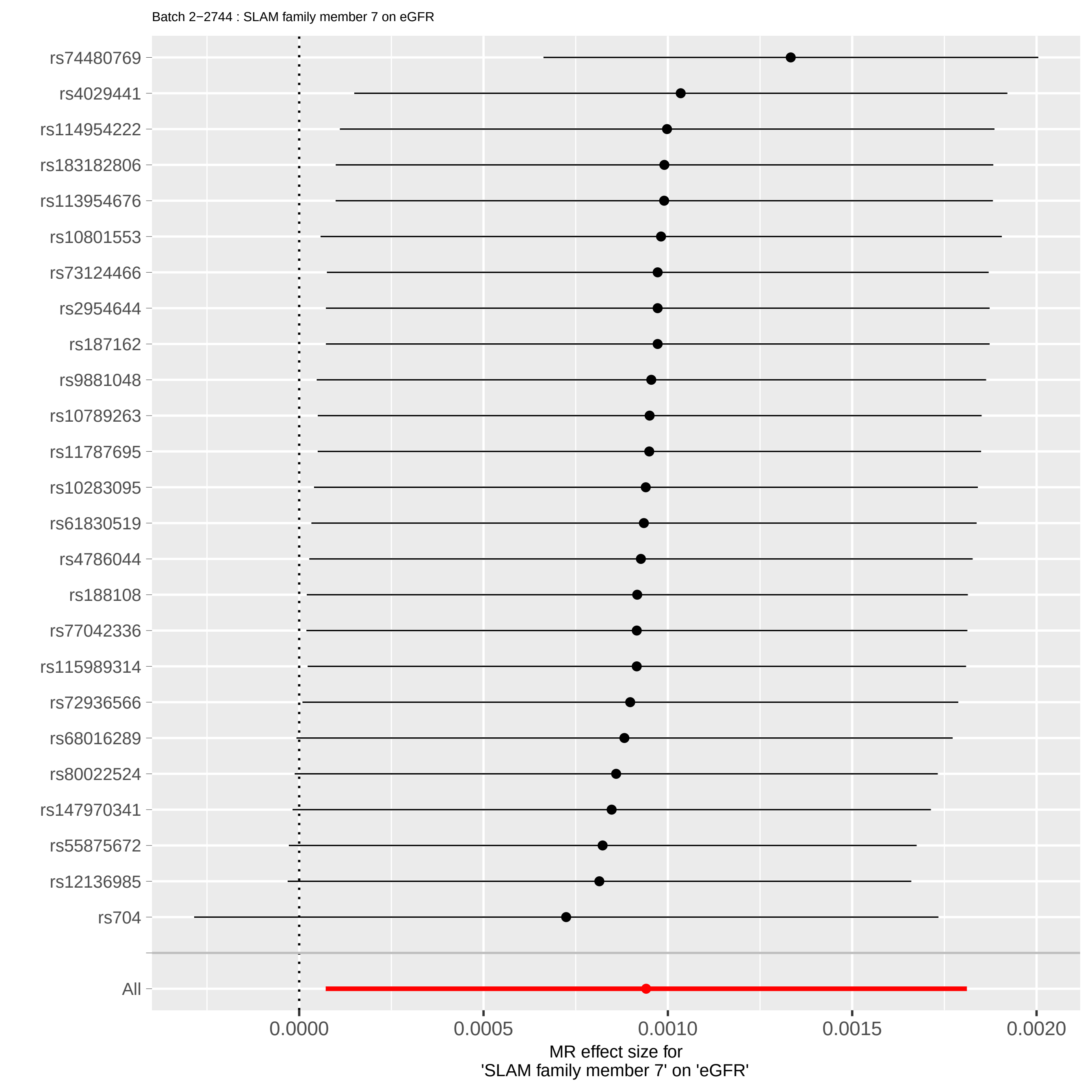

Batch 2−2744 : SLAM family member 7 on eGFR
rs74480769
rs4029441
rs114954222
rs183182806
rs113954676
rs10801553
rs73124466
rs2954644
rs187162
rs9881048
rs10789263
rs11787695
rs10283095
rs61830519
rs4786044
rs188108
rs77042336
rs115989314
rs72936566
rs68016289
rs80022524
rs147970341
rs55875672
rs12136985
rs704
All
0.0000
0.0005
0.0010
0.0015
0.0020
MR effect size for
'SLAM family member 7' on 'eGFR'

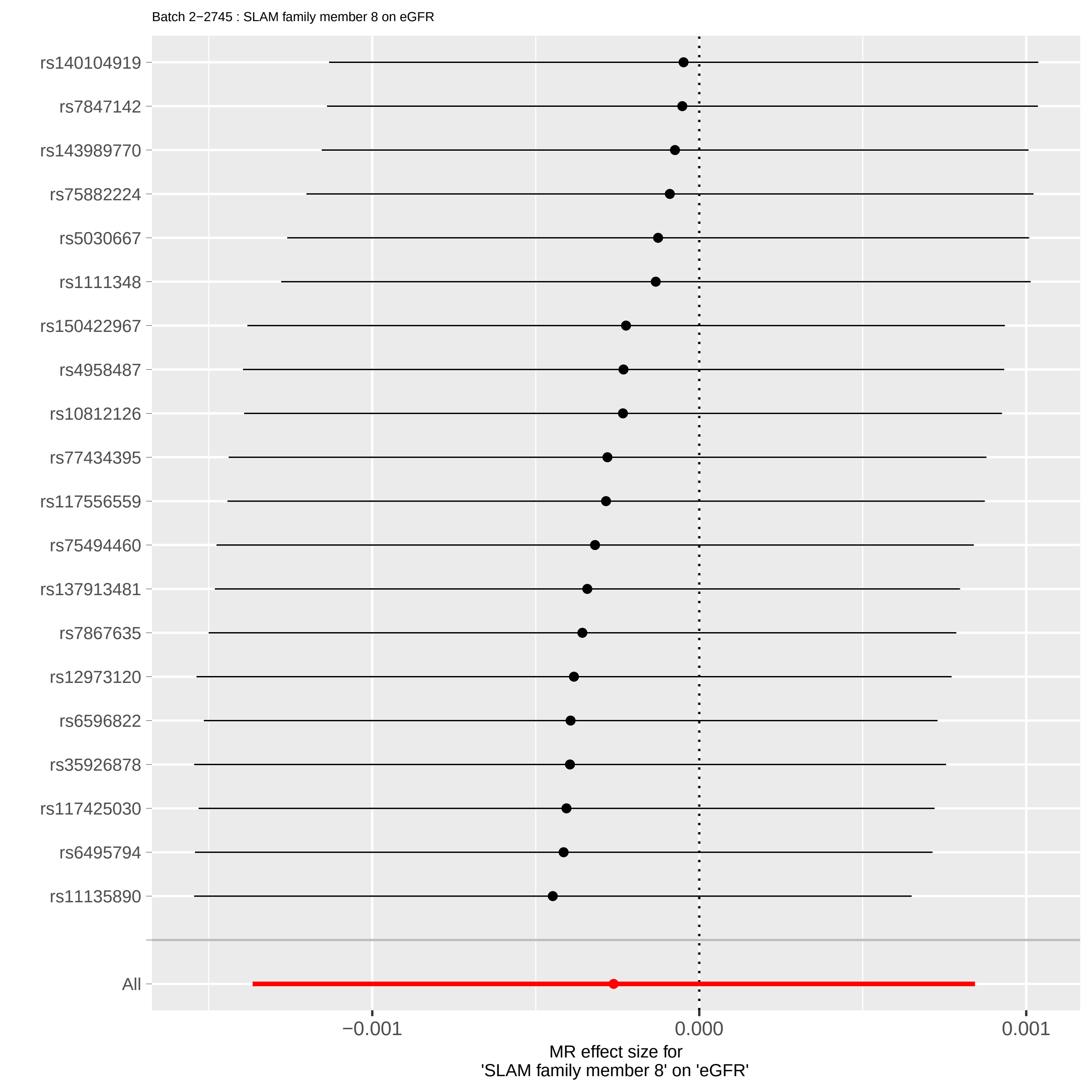

Batch 2−2745 : SLAM family member 8 on eGFR
rs140104919
rs7847142
rs143989770
rs75882224
rs5030667
rs1111348
rs150422967
rs4958487
rs10812126
rs77434395
rs117556559
rs75494460
rs137913481
rs7867635
rs12973120
rs6596822
rs35926878
rs117425030
rs6495794
rs11135890
All
−0.001
0.000
0.001
MR effect size for
'SLAM family member 8' on 'eGFR'

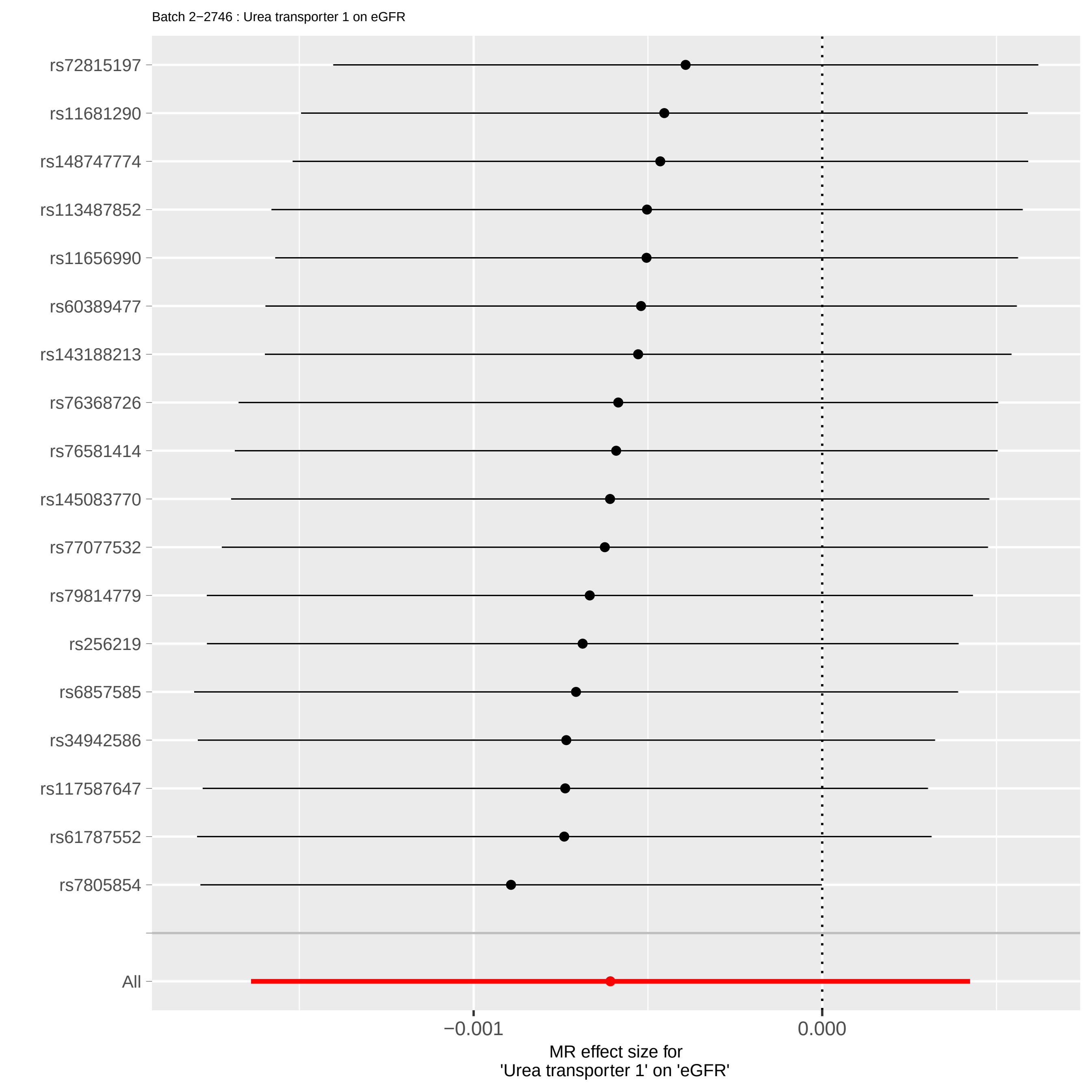

Batch 2−2746 : Urea transporter 1 on eGFR
rs72815197
rs11681290
rs148747774
rs113487852
rs11656990
rs60389477
rs143188213
rs76368726
rs76581414
rs145083770
rs77077532
rs79814779
rs256219
rs6857585
rs34942586
rs117587647
rs61787552
rs7805854
All
−0.001
0.000
MR effect size for
'Urea transporter 1' on 'eGFR'

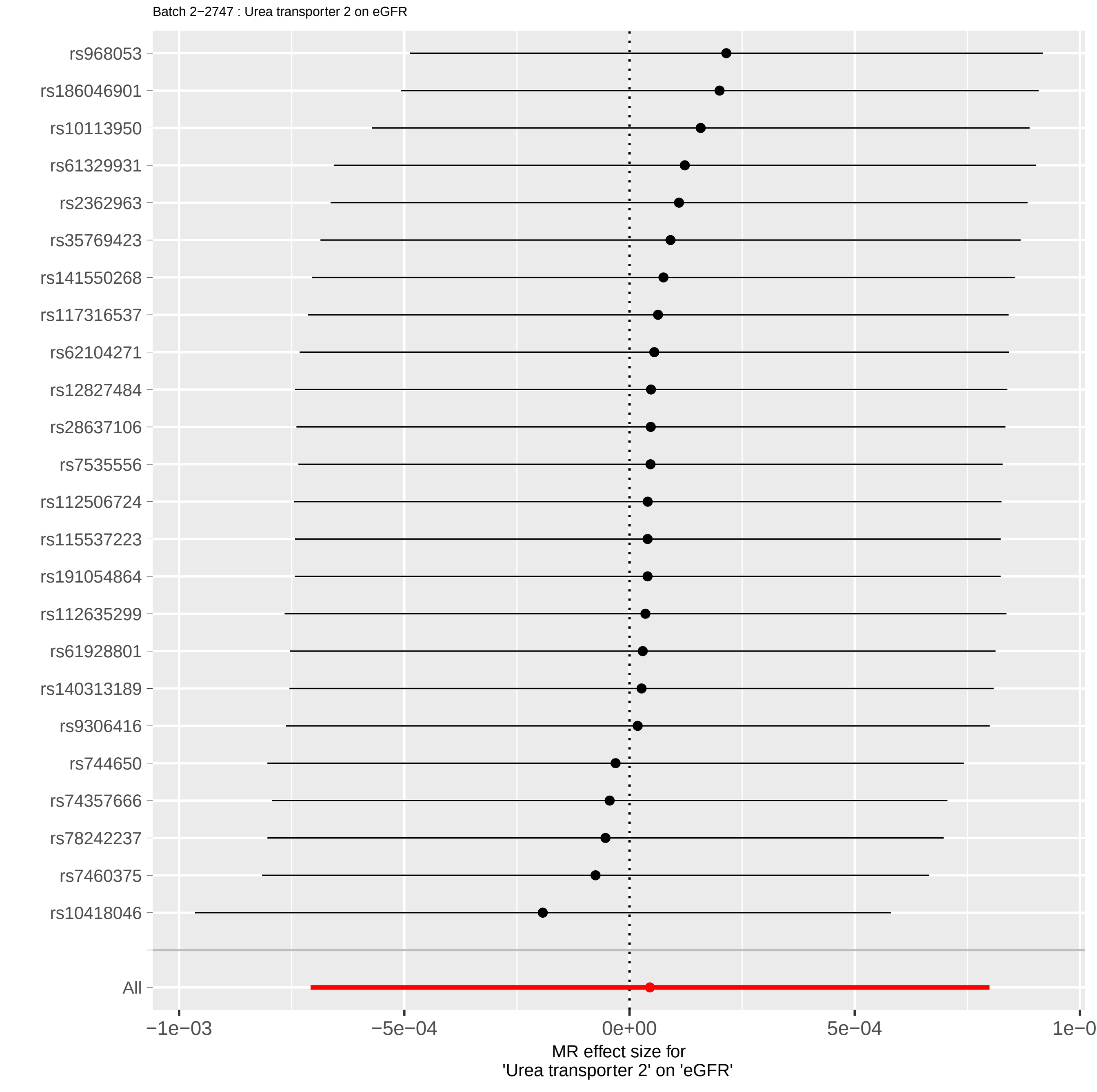

Batch 2−2747 : Urea transporter 2 on eGFR
rs968053
rs186046901
rs10113950
rs61329931
rs2362963
rs35769423
rs141550268
rs117316537
rs62104271
rs12827484
rs28637106
rs7535556
rs112506724
rs115537223
rs191054864
rs112635299
rs61928801
rs140313189
rs9306416
rs744650
rs74357666
rs78242237
rs7460375
rs10418046
All
−1e−03
−5e−04
0e+00
5e−04
MR effect size for
'Urea transporter 2' on 'eGFR'

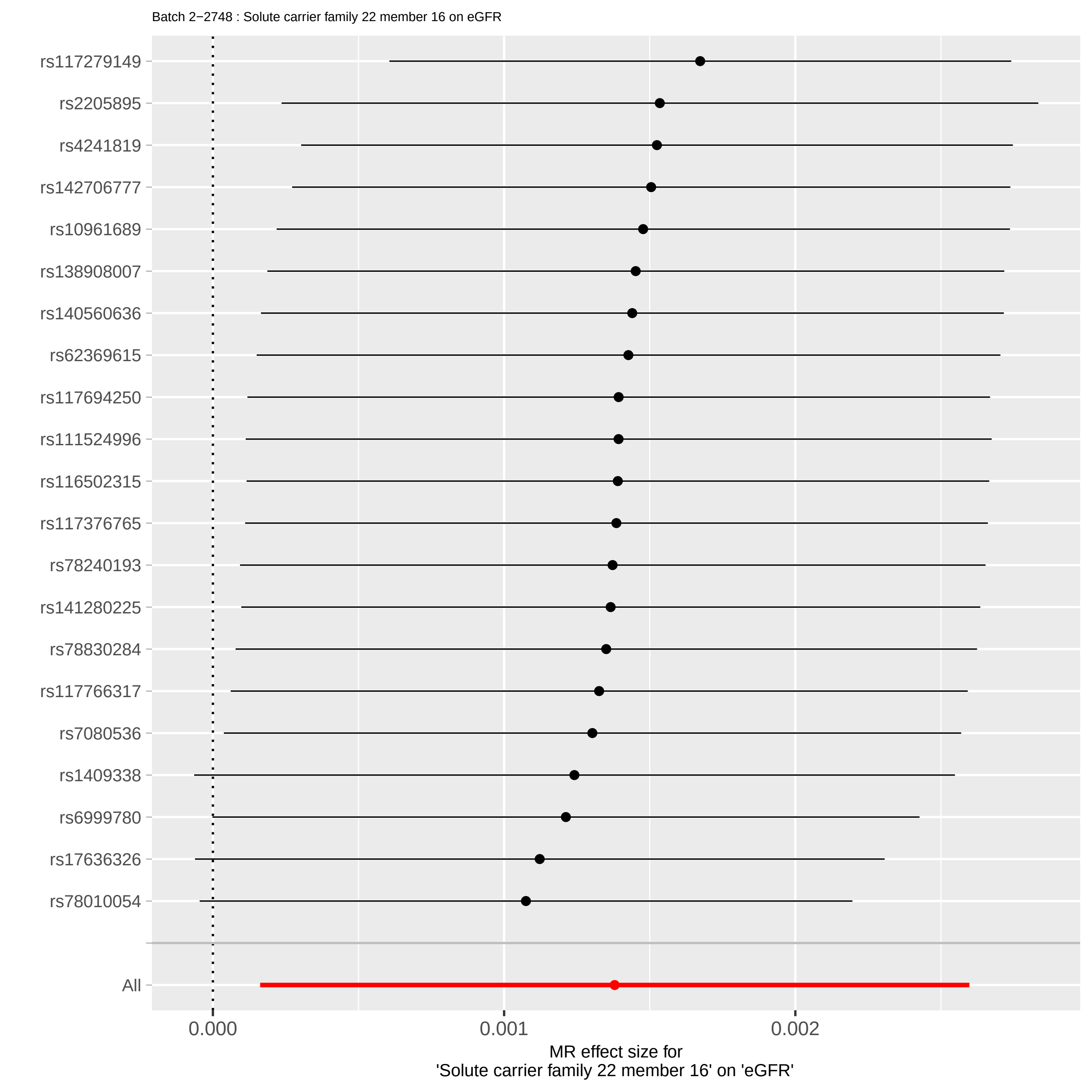

Batch 2−2748 : Solute carrier family 22 member 16 on eGFR
rs117279149
rs2205895
rs4241819
rs142706777
rs10961689
rs138908007
rs140560636
rs62369615
rs117694250
rs111524996
rs116502315
rs117376765
rs78240193
rs141280225
rs78830284
rs117766317
rs7080536
rs1409338
rs6999780
rs17636326
rs78010054
All
0.000
0.001
0.002
MR effect size for
'Solute carrier family 22 member 16' on 'eGFR'

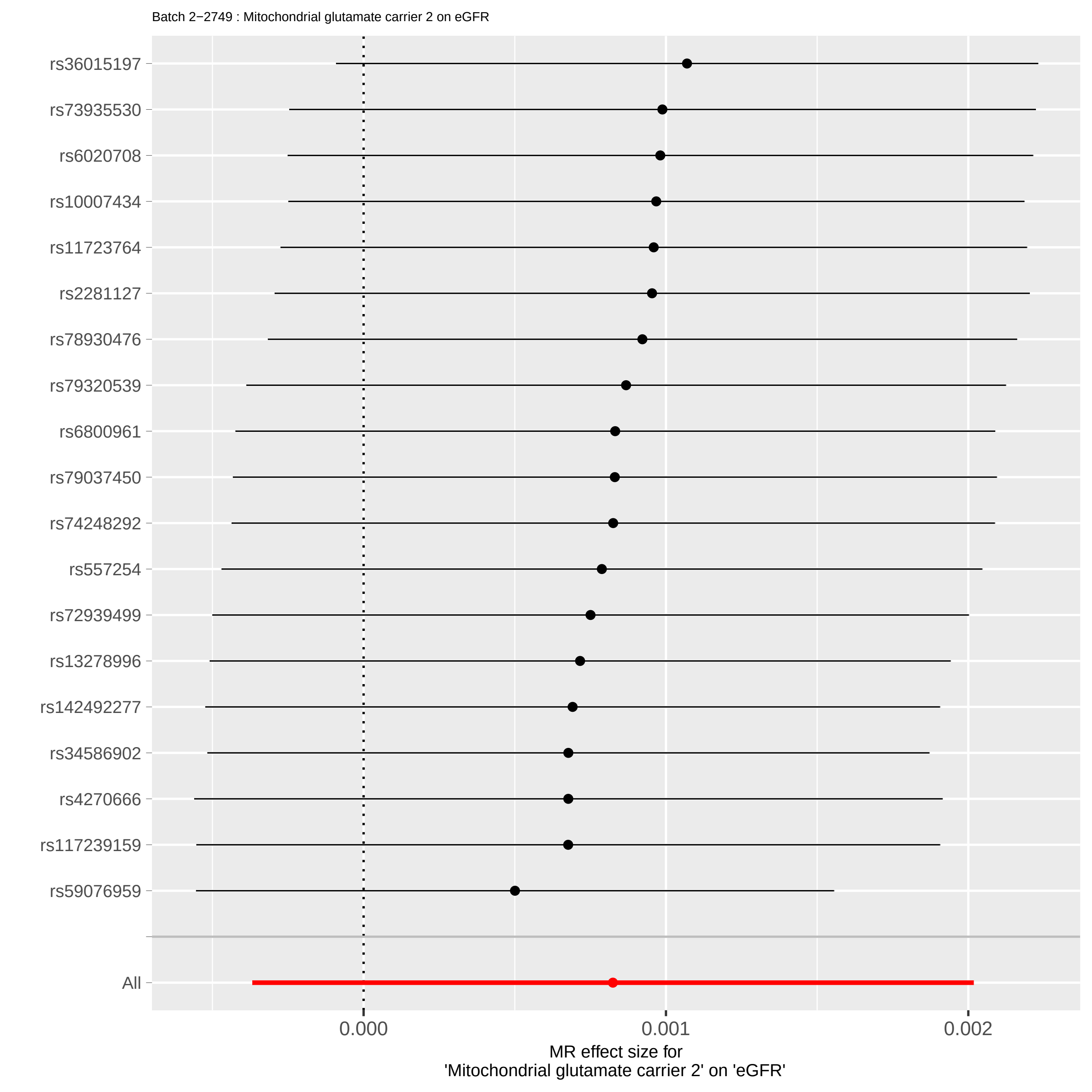

Batch 2−2749 : Mitochondrial glutamate carrier 2 on eGFR
rs36015197
rs73935530
rs6020708
rs10007434
rs11723764
rs2281127
rs78930476
rs79320539
rs6800961
rs79037450
rs74248292
rs557254
rs72939499
rs13278996
rs142492277
rs34586902
rs4270666
rs117239159
rs59076959
All
0.000
0.001
0.002
MR effect size for
'Mitochondrial glutamate carrier 2' on 'eGFR'

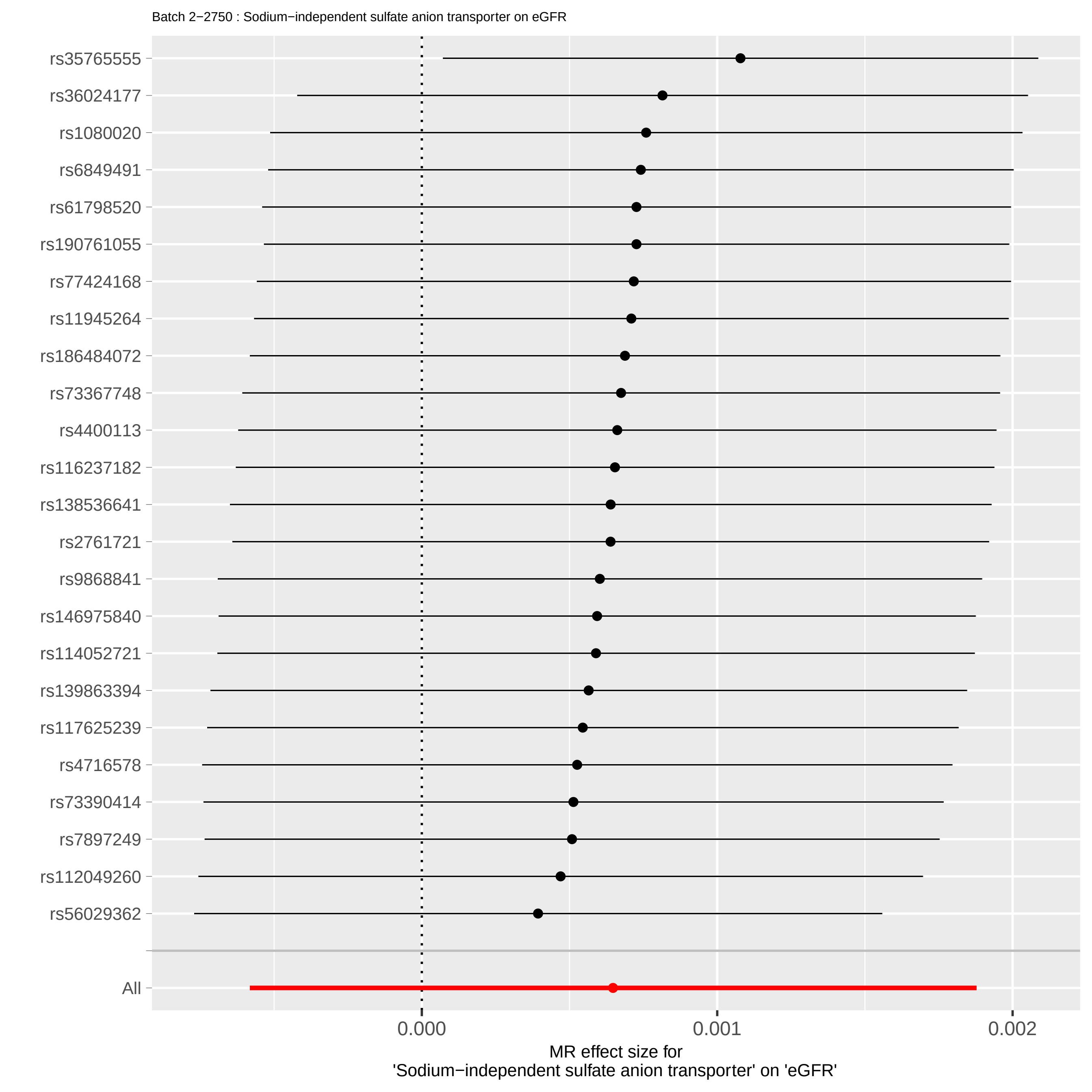

Batch 2−2750 : Sodium−independent sulfate anion transporter on eGFR
rs35765555
rs36024177
rs1080020
rs6849491
rs61798520
rs190761055
rs77424168
rs11945264
rs186484072
rs73367748
rs4400113
rs116237182
rs138536641
rs2761721
rs9868841
rs146975840
rs114052721
rs139863394
rs117625239
rs4716578
rs73390414
rs7897249
rs112049260
rs56029362
All
0.000
0.001
0.002
MR effect size for
'Sodium−independent sulfate anion transporter' on 'eGFR'

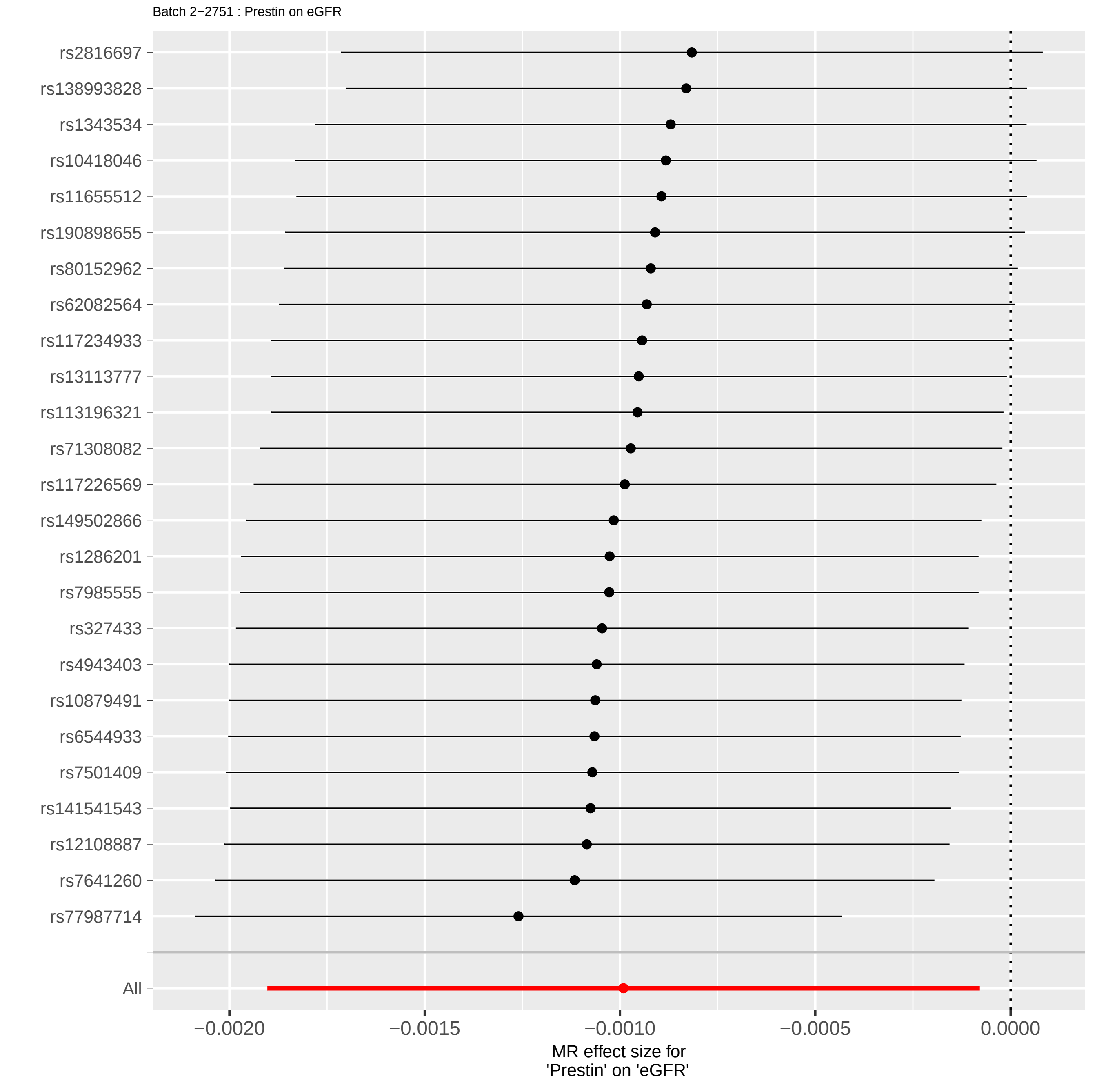

Batch 2−2751 : Prestin on eGFR
rs2816697
rs138993828
rs1343534
rs10418046
rs11655512
rs190898655
rs80152962
rs62082564
rs117234933
rs13113777
rs113196321
rs71308082
rs117226569
rs149502866
rs1286201
rs7985555
rs327433
rs4943403
rs10879491
rs6544933
rs7501409
rs141541543
rs12108887
rs7641260
rs77987714
All
−0.0020
−0.0015
−0.0010
−0.0005
0.0000
MR effect size for
'Prestin' on 'eGFR'

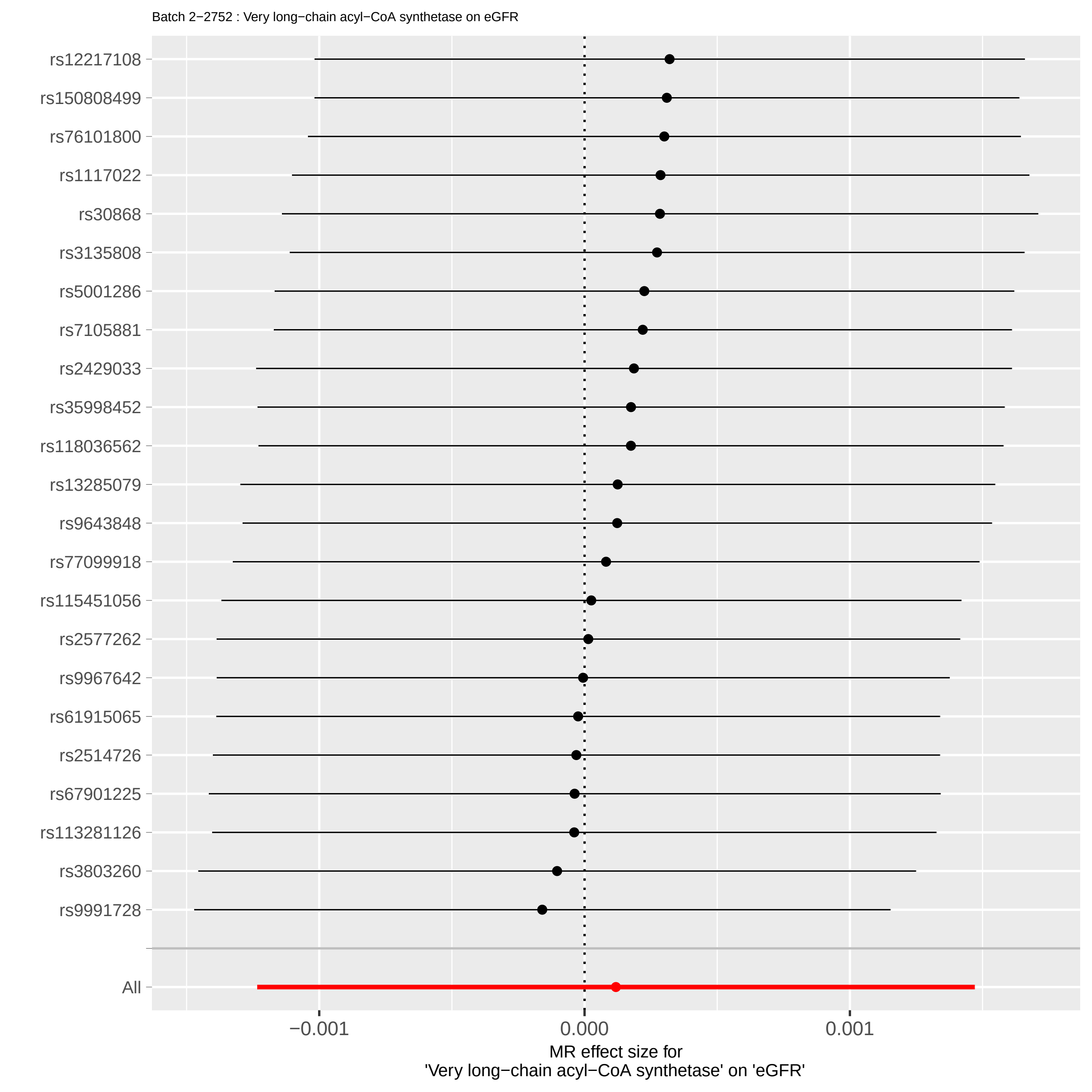

Batch 2−2752 : Very long−chain acyl−CoA synthetase on eGFR
rs12217108
rs150808499
rs76101800
rs1117022
rs30868
rs3135808
rs5001286
rs7105881
rs2429033
rs35998452
rs118036562
rs13285079
rs9643848
rs77099918
rs115451056
rs2577262
rs9967642
rs61915065
rs2514726
rs67901225
rs113281126
rs3803260
rs9991728
All
−0.001
0.000
0.001
MR effect size for
'Very long−chain acyl−CoA synthetase' on 'eGFR'

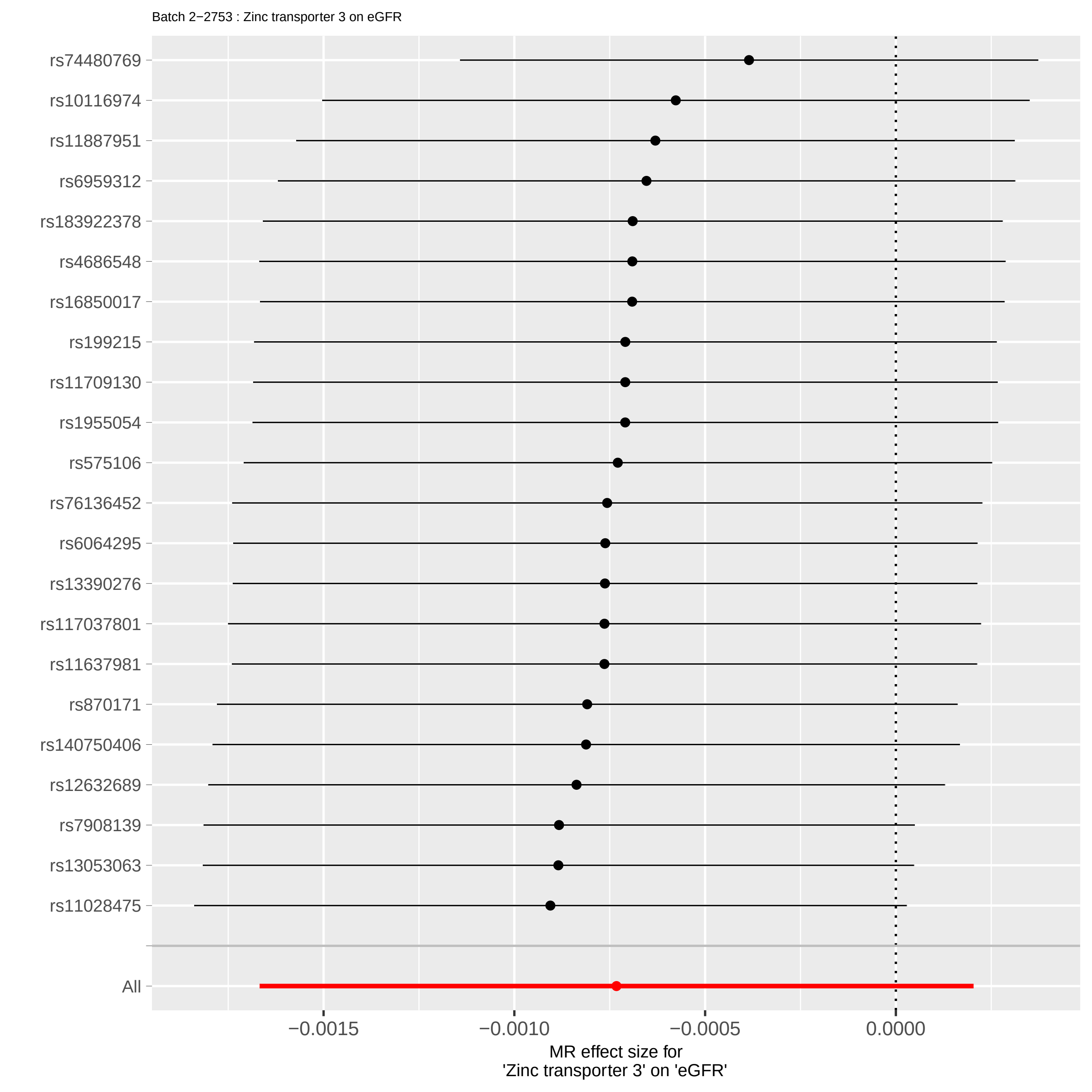

Batch 2−2753 : Zinc transporter 3 on eGFR
rs74480769
rs10116974
rs11887951
rs6959312
rs183922378
rs4686548
rs16850017
rs199215
rs11709130
rs1955054
rs575106
rs76136452
rs6064295
rs13390276
rs117037801
rs11637981
rs870171
rs140750406
rs12632689
rs7908139
rs13053063
rs11028475
All
−0.0015
−0.0010
−0.0005
0.0000
MR effect size for
'Zinc transporter 3' on 'eGFR'

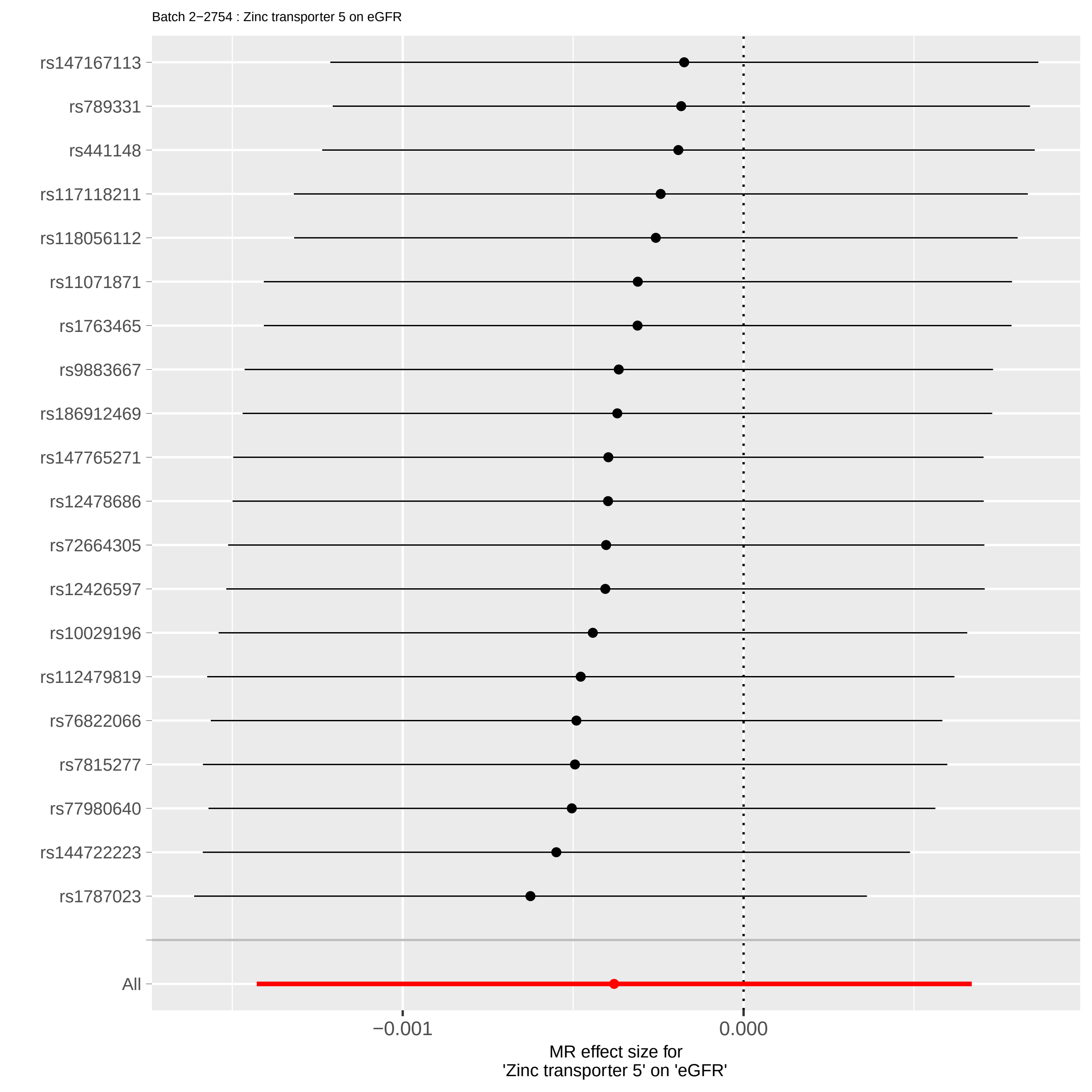

Batch 2−2754 : Zinc transporter 5 on eGFR
rs147167113
rs789331
rs441148
rs117118211
rs118056112
rs11071871
rs1763465
rs9883667
rs186912469
rs147765271
rs12478686
rs72664305
rs12426597
rs10029196
rs112479819
rs76822066
rs7815277
rs77980640
rs144722223
rs1787023
All
−0.001
0.000
MR effect size for
'Zinc transporter 5' on 'eGFR'

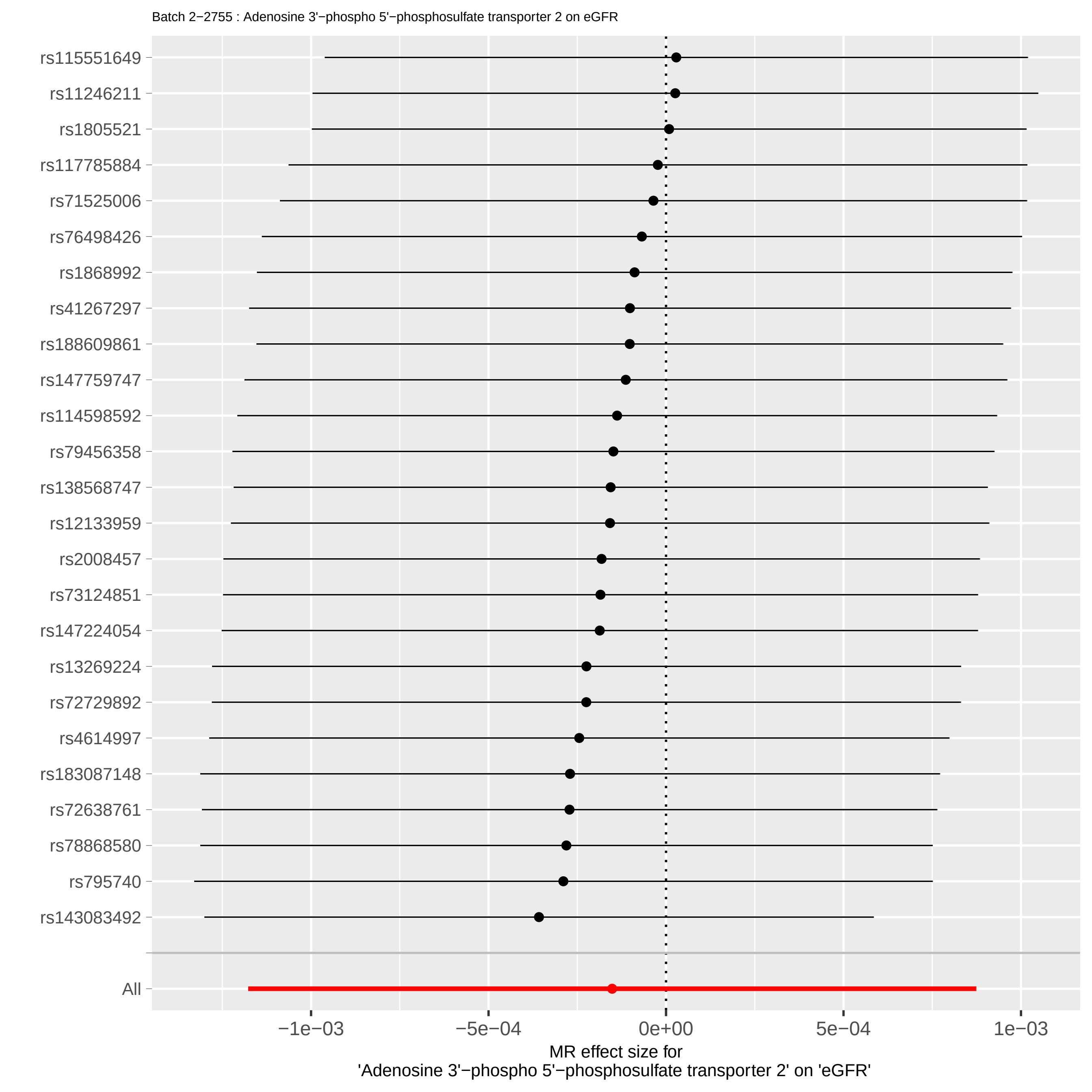

Batch 2−2755 : Adenosine 3'−phospho 5'−phosphosulfate transporter 2 on eGFR
rs115551649
rs11246211
rs1805521
rs117785884
rs71525006
rs76498426
rs1868992
rs41267297
rs188609861
rs147759747
rs114598592
rs79456358
rs138568747
rs12133959
rs2008457
rs73124851
rs147224054
rs13269224
rs72729892
rs4614997
rs183087148
rs72638761
rs78868580
rs795740
rs143083492
All
−1e−03
−5e−04
0e+00
5e−04
1e−03
MR effect size for
'Adenosine 3'−phospho 5'−phosphosulfate transporter 2' on 'eGFR'

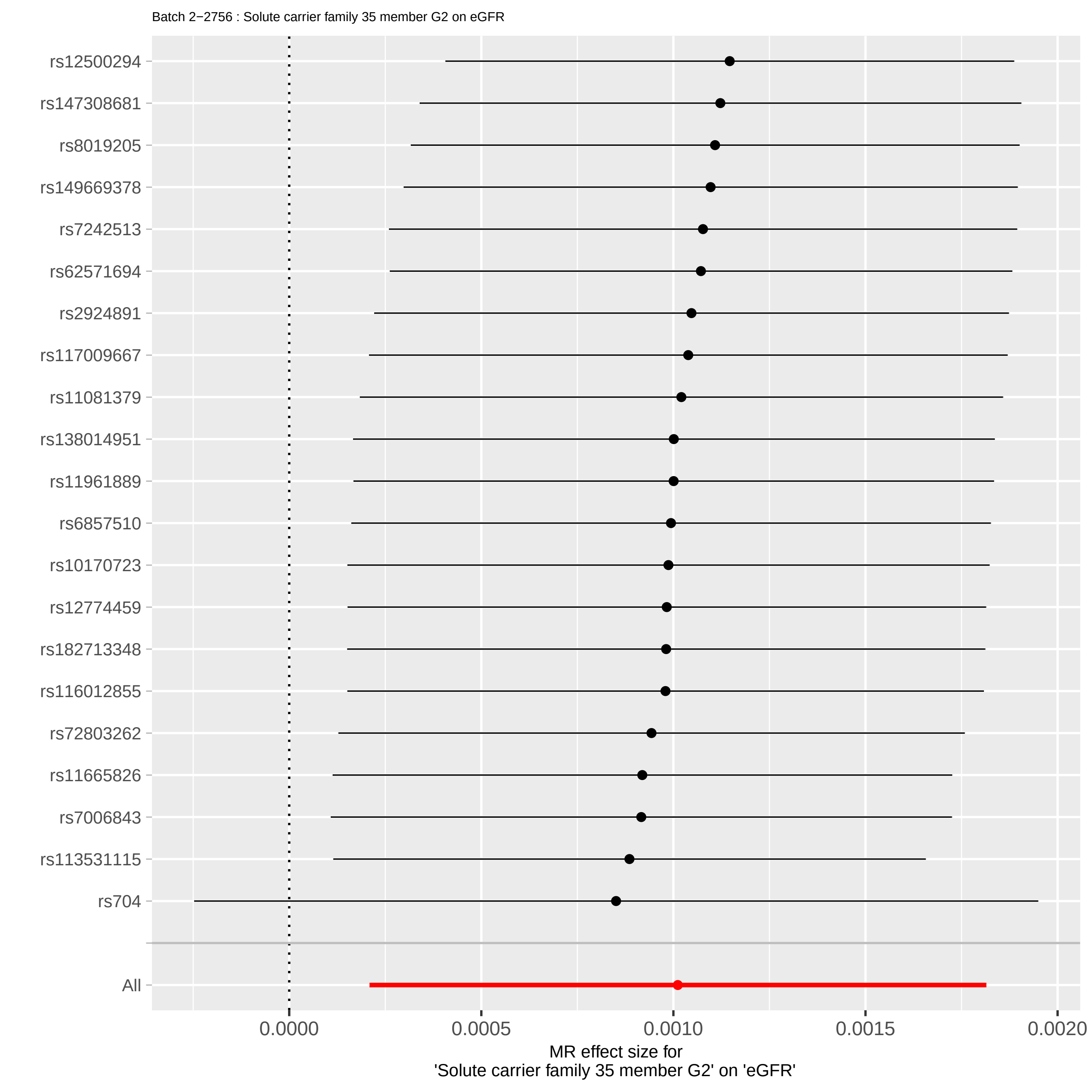

Batch 2−2756 : Solute carrier family 35 member G2 on eGFR
rs12500294
rs147308681
rs8019205
rs149669378
rs7242513
rs62571694
rs2924891
rs117009667
rs11081379
rs138014951
rs11961889
rs6857510
rs10170723
rs12774459
rs182713348
rs116012855
rs72803262
rs11665826
rs7006843
rs113531115
rs704
All
0.0000
0.0005
0.0010
0.0015
0.0020
MR effect size for
'Solute carrier family 35 member G2' on 'eGFR'

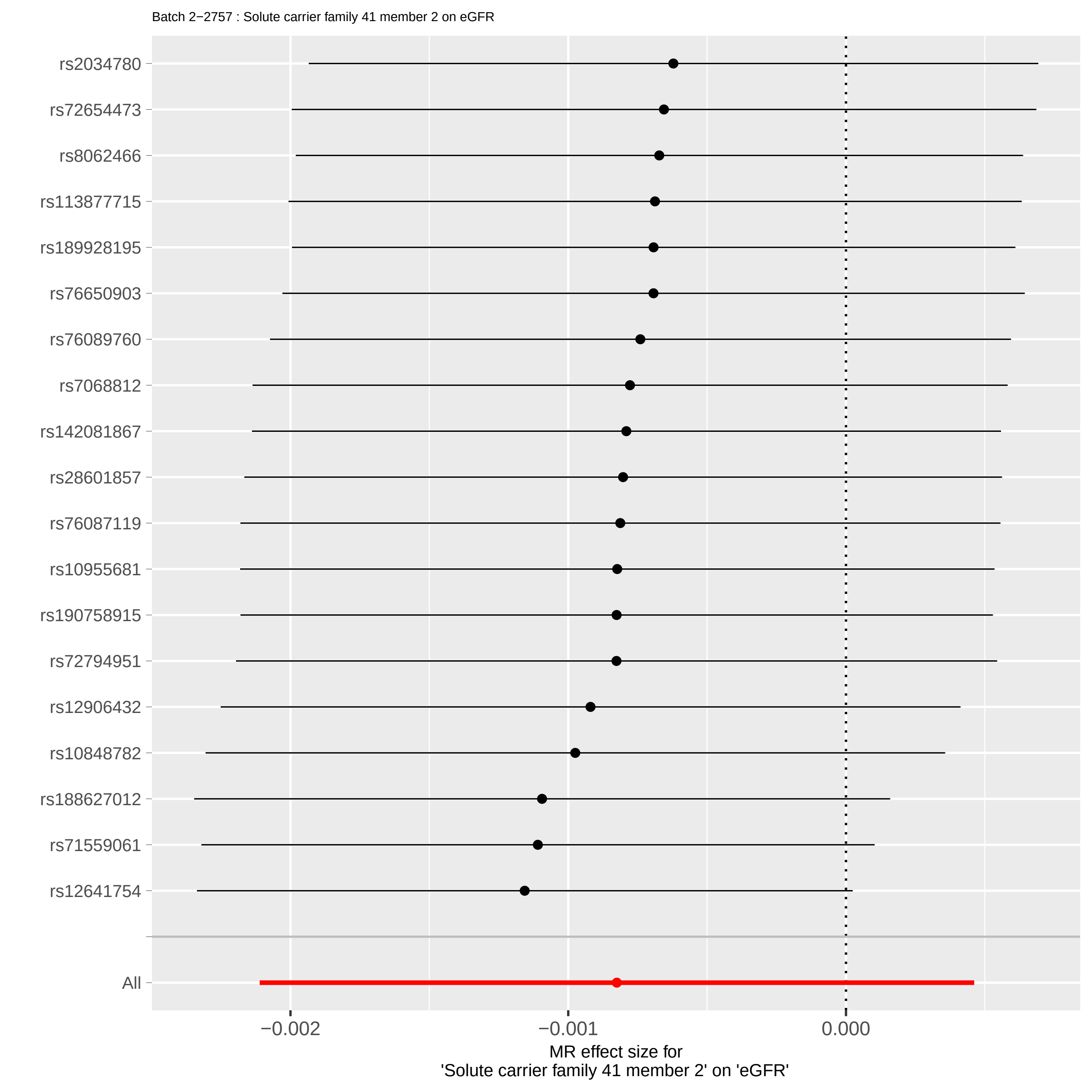

Batch 2−2757 : Solute carrier family 41 member 2 on eGFR
rs2034780
rs72654473
rs8062466
rs113877715
rs189928195
rs76650903
rs76089760
rs7068812
rs142081867
rs28601857
rs76087119
rs10955681
rs190758915
rs72794951
rs12906432
rs10848782
rs188627012
rs71559061
rs12641754
All
−0.002
−0.001
0.000
MR effect size for
'Solute carrier family 41 member 2' on 'eGFR'

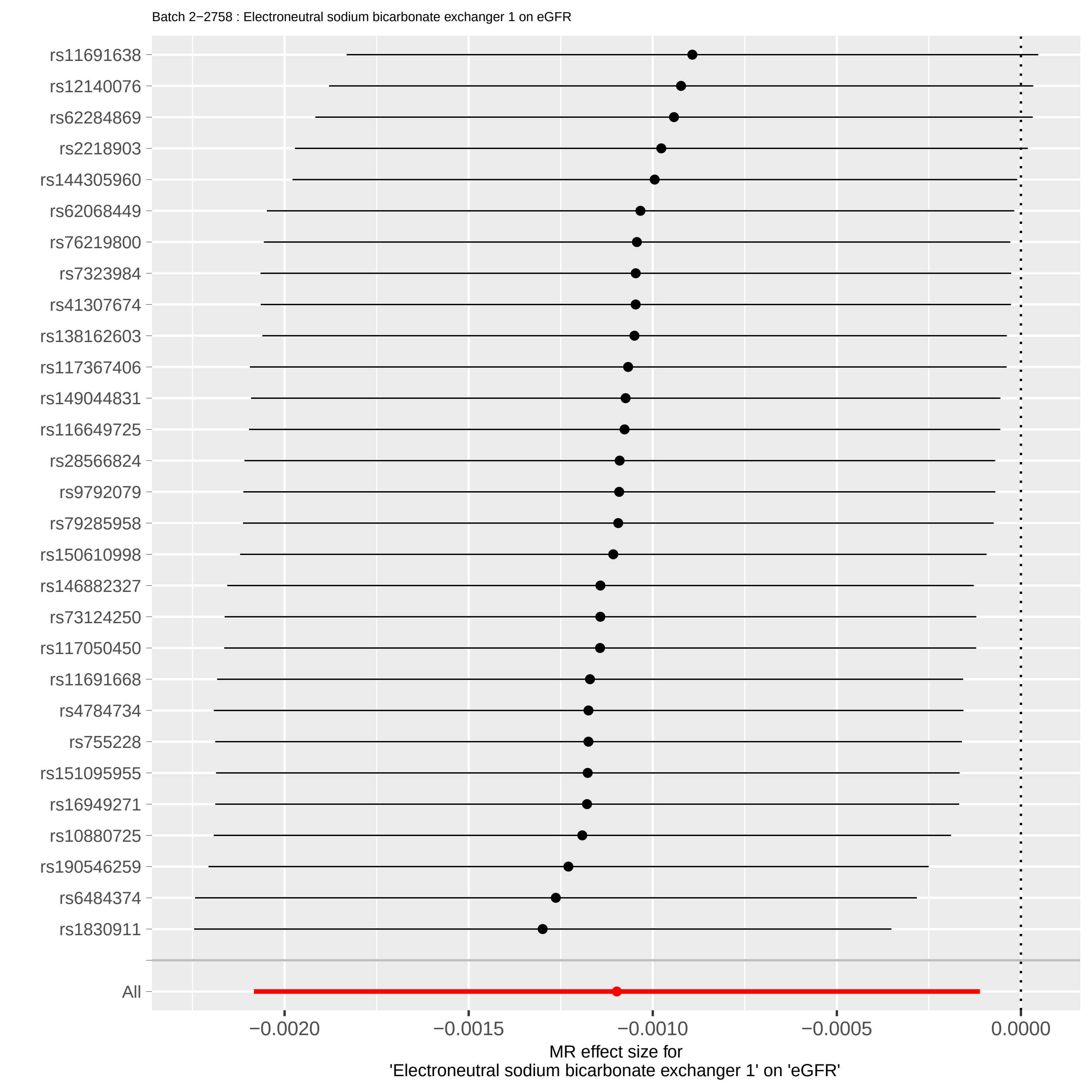
Batch 2−2758 : Electroneutral sodium bicarbonate exchanger 1 on eGFR
rs11691638
rs12140076
rs62284869
rs2218903
rs144305960
rs62068449
rs76219800
rs7323984
rs41307674
rs138162603
rs117367406
rs149044831
rs116649725
rs28566824
rs9792079
rs79285958
rs150610998
rs146882327
rs73124250
rs117050450
rs11691668
rs4784734
rs755228
rs151095955
rs16949271
rs10880725
rs190546259
rs6484374
rs1830911
All
−0.0020
−0.0015
−0.0010
−0.0005
0.0000
MR effect size for
'Electroneutral sodium bicarbonate exchanger 1' on 'eGFR'

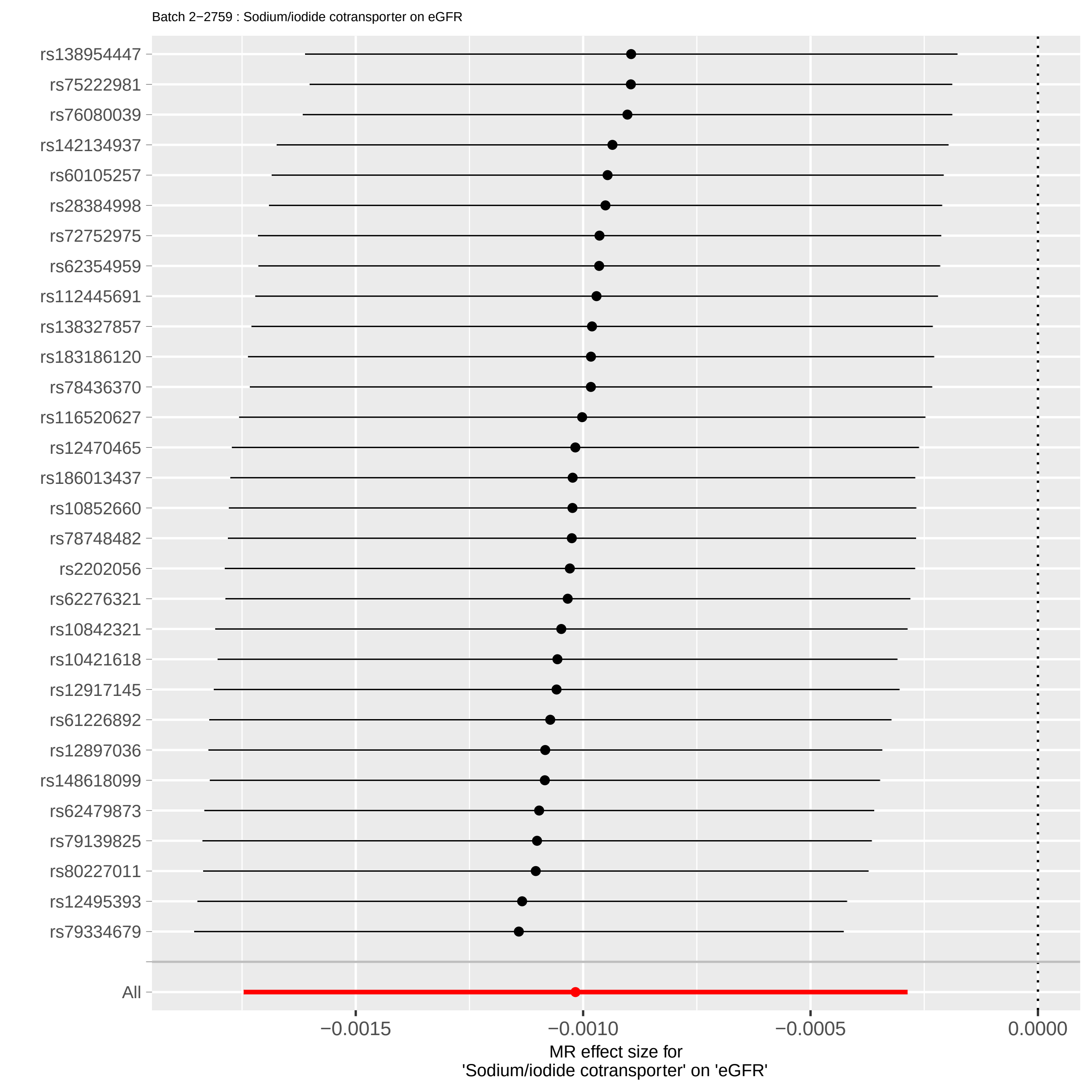

Batch 2−2759 : Sodium/iodide cotransporter on eGFR
rs138954447
rs75222981
rs76080039
rs142134937
rs60105257
rs28384998
rs72752975
rs62354959
rs112445691
rs138327857
rs183186120
rs78436370
rs116520627
rs12470465
rs186013437
rs10852660
rs78748482
rs2202056
rs62276321
rs10842321
rs10421618
rs12917145
rs61226892
rs12897036
rs148618099
rs62479873
rs79139825
rs80227011
rs12495393
rs79334679
All
−0.0015
−0.0010
−0.0005
0.0000
MR effect size for
'Sodium/iodide cotransporter' on 'eGFR'

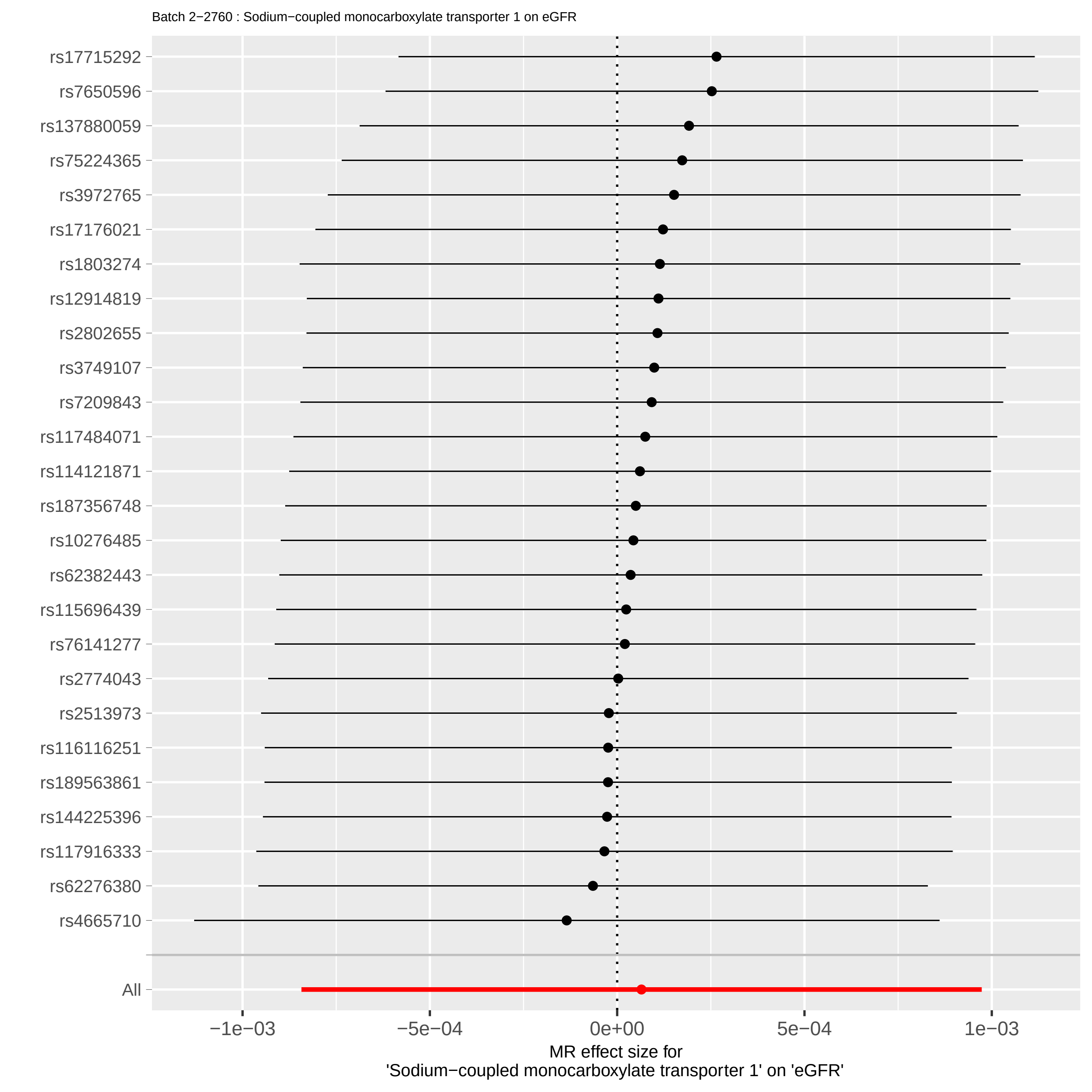

Batch 2−2760 : Sodium−coupled monocarboxylate transporter 1 on eGFR
rs17715292
rs7650596
rs137880059
rs75224365
rs3972765
rs17176021
rs1803274
rs12914819
rs2802655
rs3749107
rs7209843
rs117484071
rs114121871
rs187356748
rs10276485
rs62382443
rs115696439
rs76141277
rs2774043
rs2513973
rs116116251
rs189563861
rs144225396
rs117916333
rs62276380
rs4665710
All
−1e−03
−5e−04
0e+00
5e−04
1e−03
MR effect size for
'Sodium−coupled monocarboxylate transporter 1' on 'eGFR'

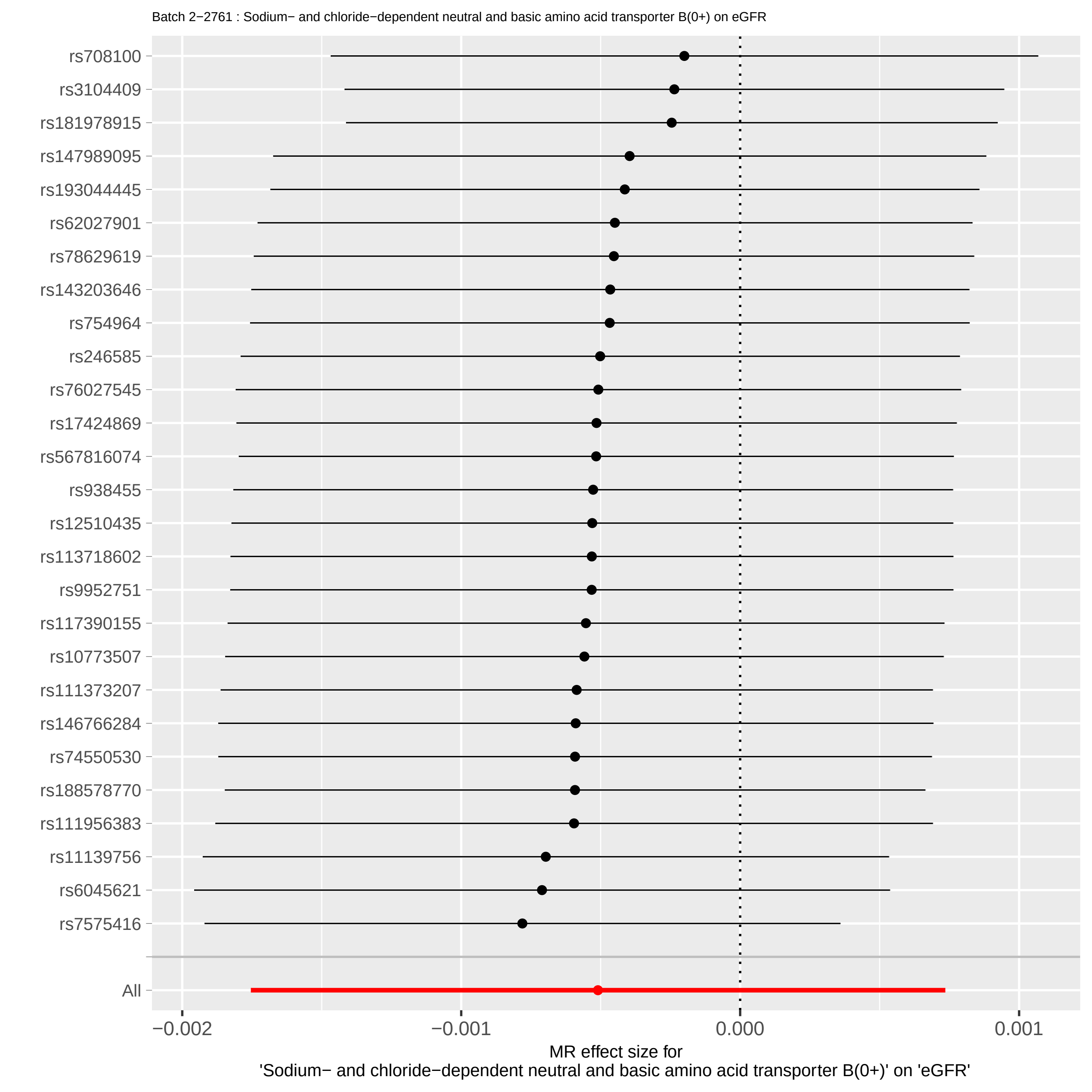

Batch 2−2761 : Sodium− and chloride−dependent neutral and basic amino acid transporter B(0+) on eGFR
rs708100
rs3104409
rs181978915
rs147989095
rs193044445
rs62027901
rs78629619
rs143203646
rs754964
rs246585
rs76027545
rs17424869
rs567816074
rs938455
rs12510435
rs113718602
rs9952751
rs117390155
rs10773507
rs111373207
rs146766284
rs74550530
rs188578770
rs111956383
rs11139756
rs6045621
rs7575416
All
−0.002
−0.001
0.000
0.001
MR effect size for
'Sodium− and chloride−dependent neutral and basic amino acid transporter B(0+)' on 'eGFR'

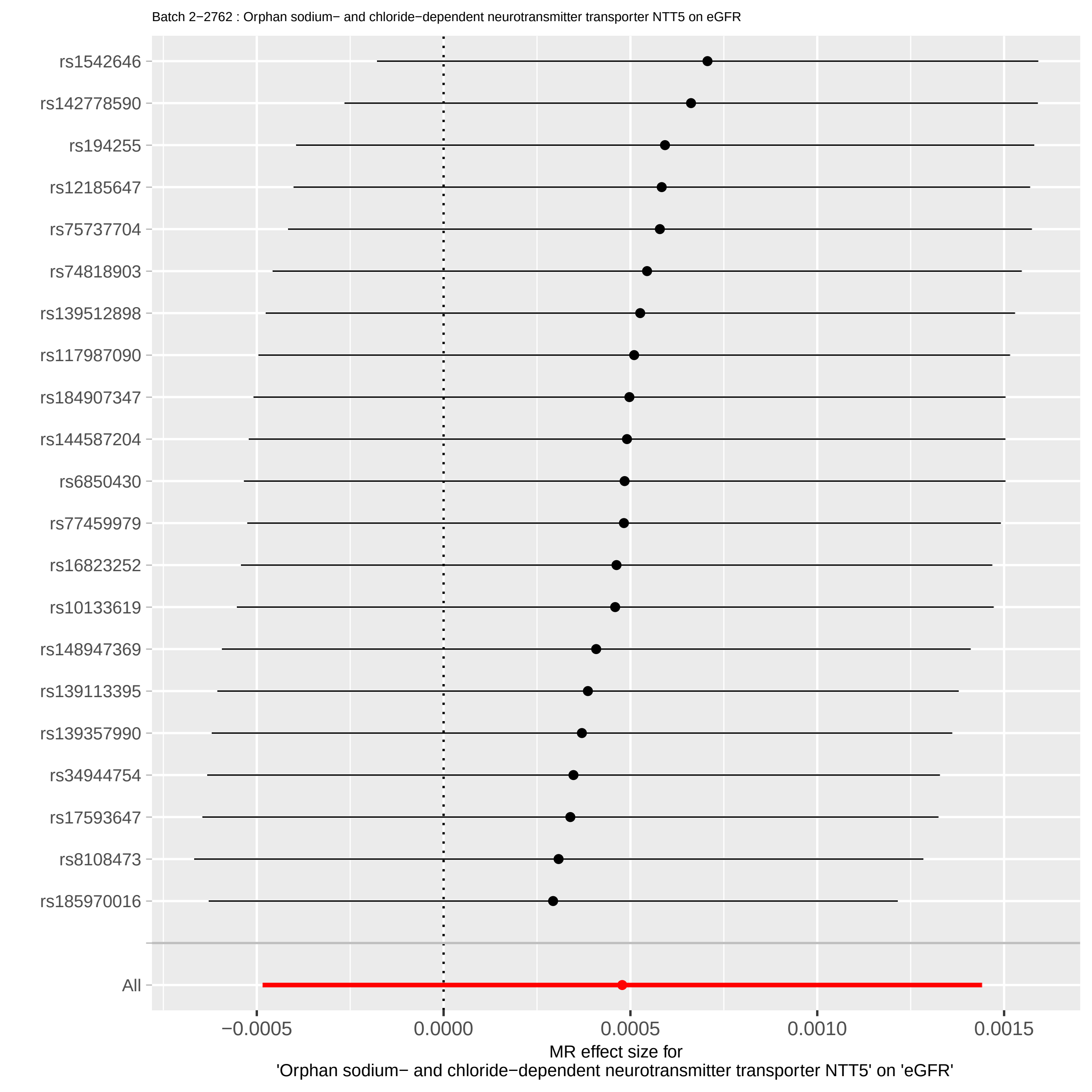

Batch 2−2762 : Orphan sodium− and chloride−dependent neurotransmitter transporter NTT5 on eGFR
rs1542646
rs142778590
rs194255
rs12185647
rs75737704
rs74818903
rs139512898
rs117987090
rs184907347
rs144587204
rs6850430
rs77459979
rs16823252
rs10133619
rs148947369
rs139113395
rs139357990
rs34944754
rs17593647
rs8108473
rs185970016
All
−0.0005
0.0000
0.0005
0.0010
0.0015
MR effect size for
'Orphan sodium− and chloride−dependent neurotransmitter transporter NTT5' on 'eGFR'

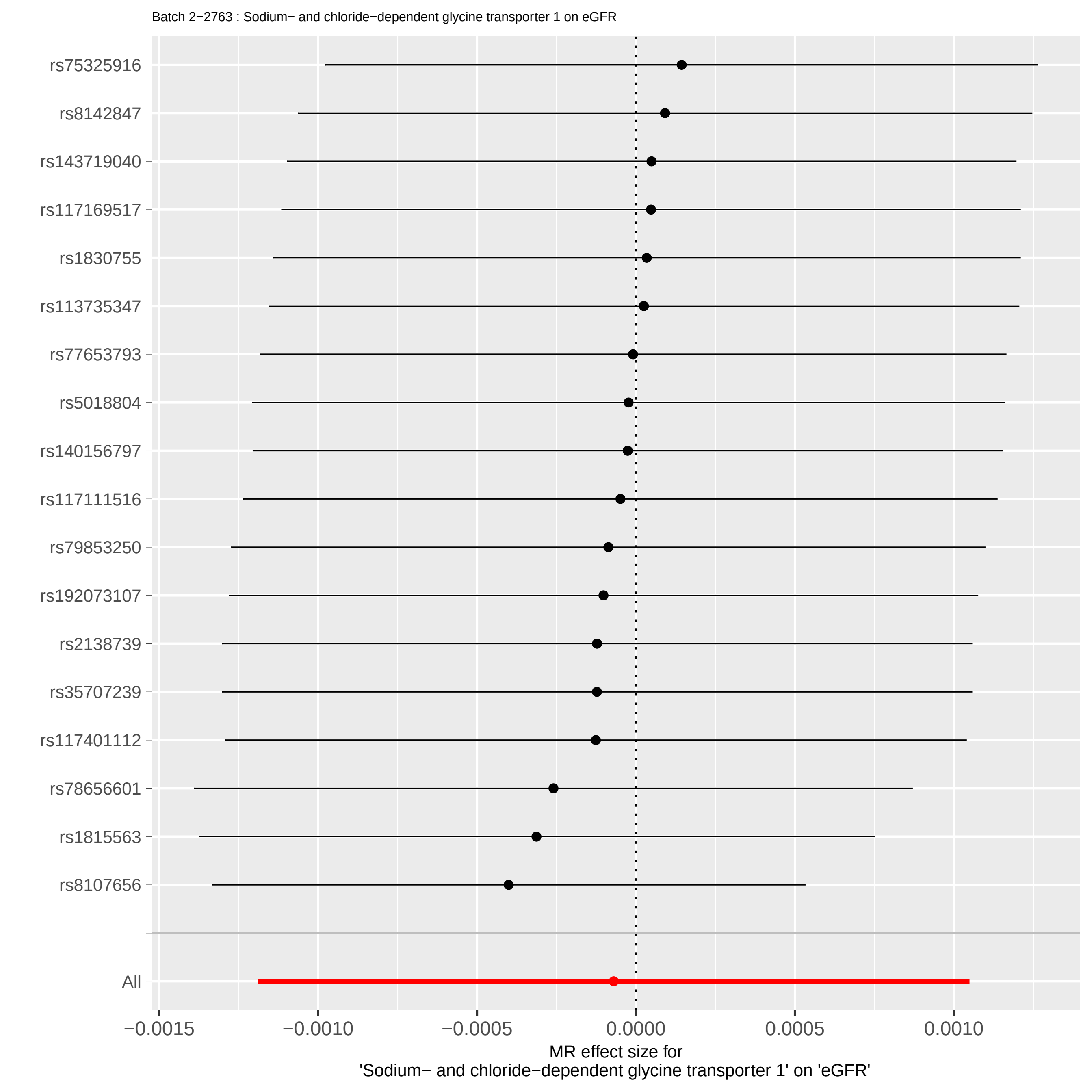

Batch 2−2763 : Sodium− and chloride−dependent glycine transporter 1 on eGFR
rs75325916
rs8142847
rs143719040
rs117169517
rs1830755
rs113735347
rs77653793
rs5018804
rs140156797
rs117111516
rs79853250
rs192073107
rs2138739
rs35707239
rs117401112
rs78656601
rs1815563
rs8107656
All
−0.0015
−0.0010
−0.0005
0.0000
0.0005
0.0010
MR effect size for
'Sodium− and chloride−dependent glycine transporter 1' on 'eGFR'

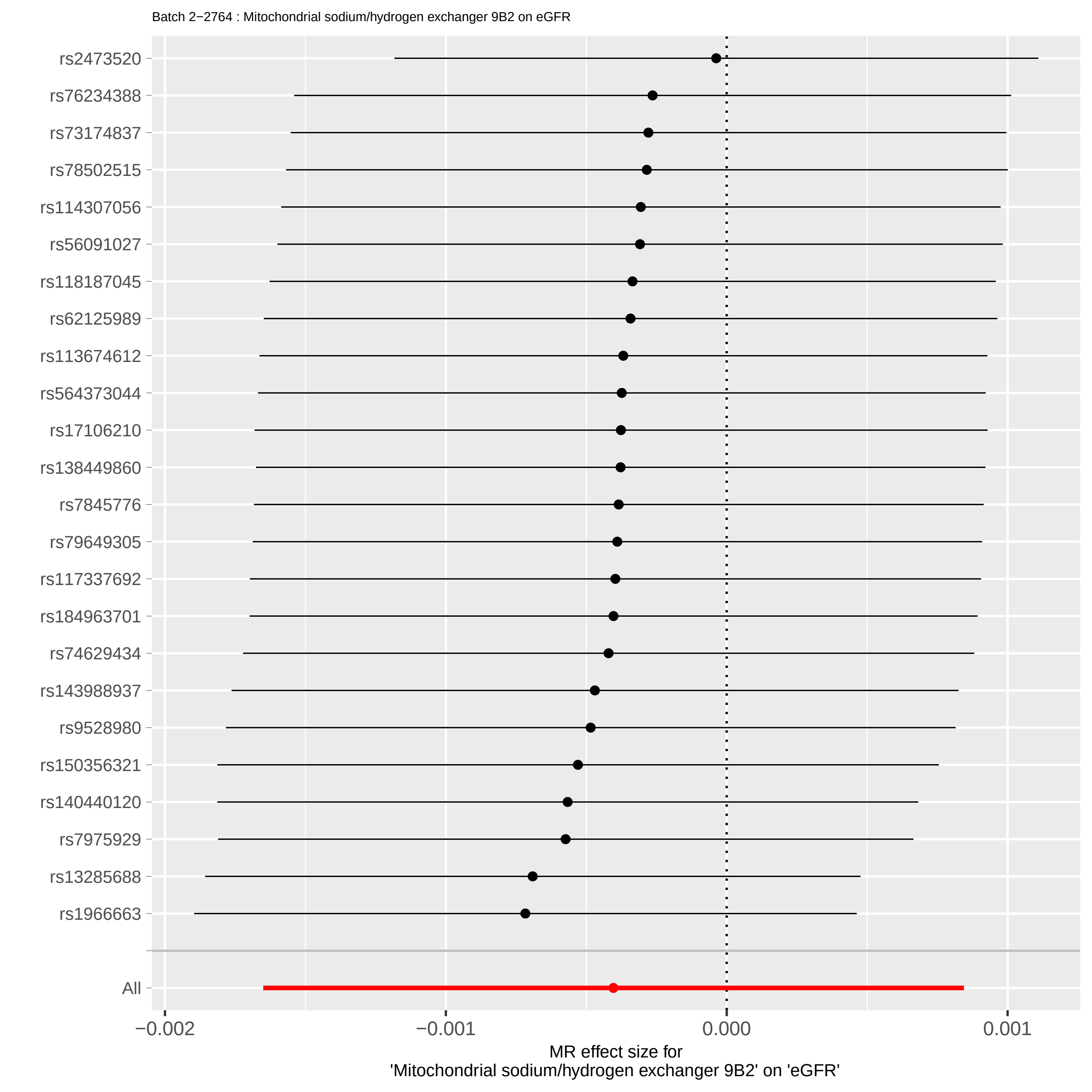

Batch 2−2764 : Mitochondrial sodium/hydrogen exchanger 9B2 on eGFR
rs2473520
rs76234388
rs73174837
rs78502515
rs114307056
rs56091027
rs118187045
rs62125989
rs113674612
rs564373044
rs17106210
rs138449860
rs7845776
rs79649305
rs117337692
rs184963701
rs74629434
rs143988937
rs9528980
rs150356321
rs140440120
rs7975929
rs13285688
rs1966663
All
−0.002
−0.001
0.000
0.001
MR effect size for
'Mitochondrial sodium/hydrogen exchanger 9B2' on 'eGFR'

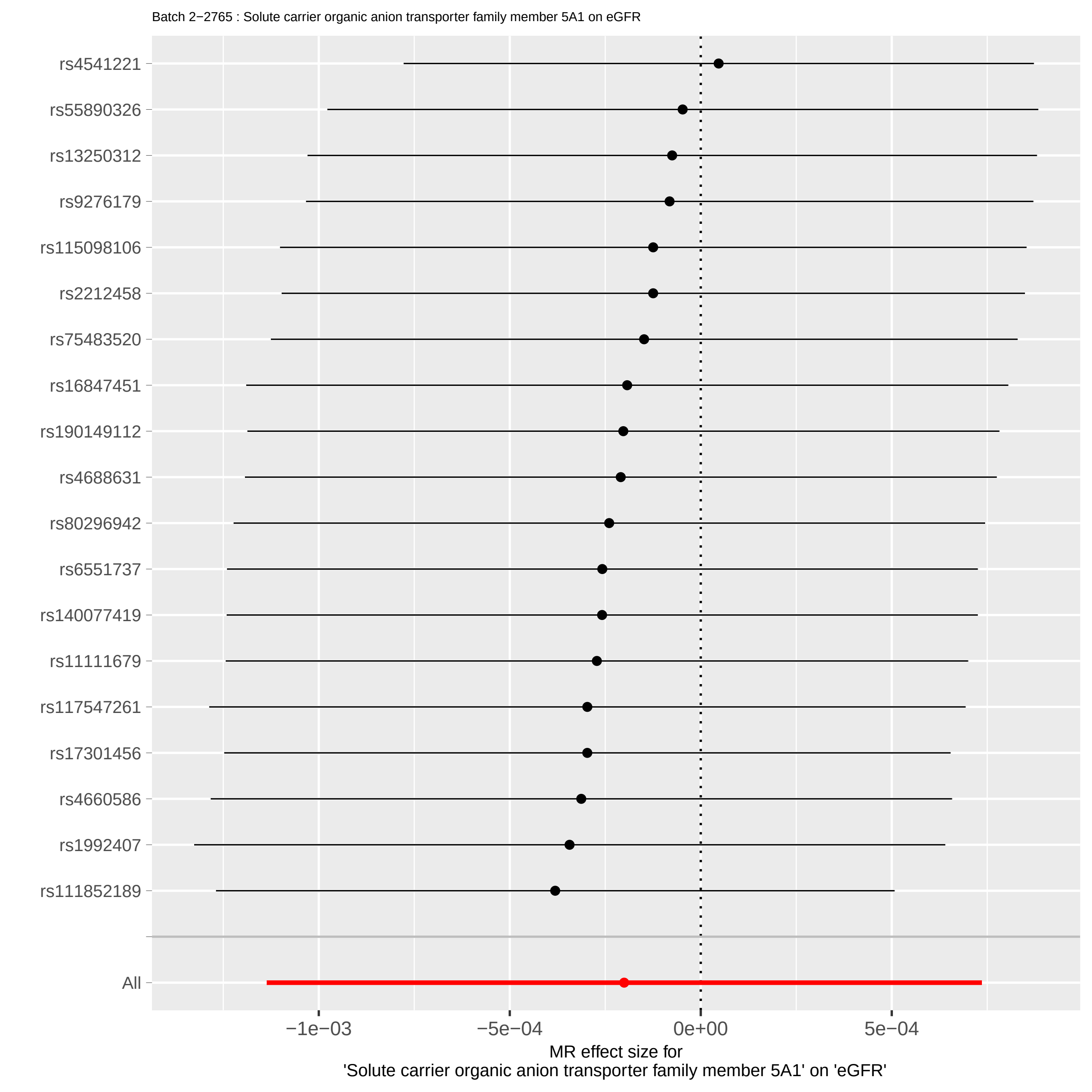

Batch 2−2765 : Solute carrier organic anion transporter family member 5A1 on eGFR
rs4541221
rs55890326
rs13250312
rs9276179
rs115098106
rs2212458
rs75483520
rs16847451
rs190149112
rs4688631
rs80296942
rs6551737
rs140077419
rs11111679
rs117547261
rs17301456
rs4660586
rs1992407
rs111852189
All
−1e−03
−5e−04
0e+00
5e−04
MR effect size for
'Solute carrier organic anion transporter family member 5A1' on 'eGFR'

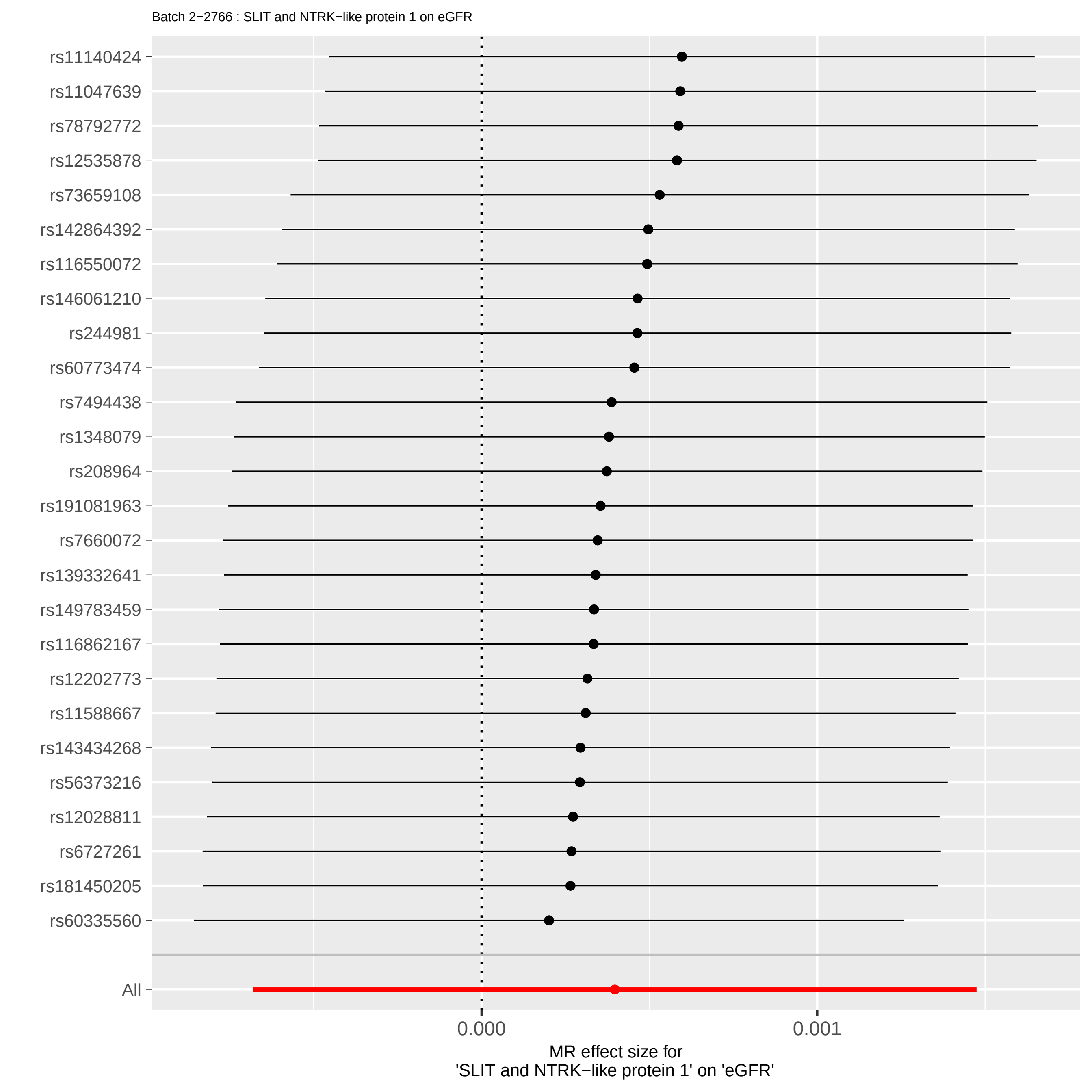
Batch 2−2766 : SLIT and NTRK−like protein 1 on eGFR
rs11140424
rs11047639
rs78792772
rs12535878
rs73659108
rs142864392
rs116550072
rs146061210
rs244981
rs60773474
rs7494438
rs1348079
rs208964
rs191081963
rs7660072
rs139332641
rs149783459
rs116862167
rs12202773
rs11588667
rs143434268
rs56373216
rs12028811
rs6727261
rs181450205
rs60335560
All
0.000
0.001
MR effect size for
'SLIT and NTRK−like protein 1' on 'eGFR'

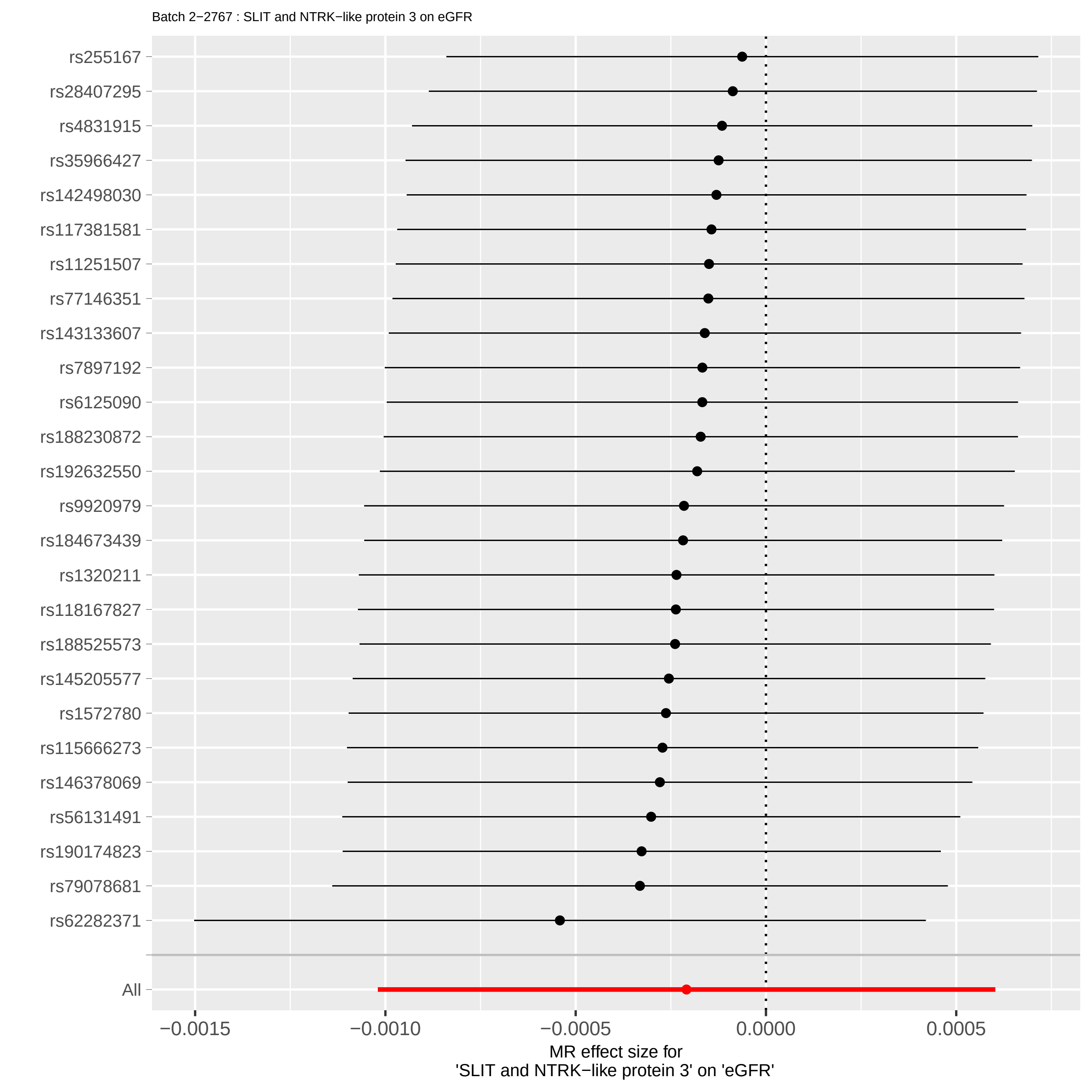
Batch 2−2767 : SLIT and NTRK−like protein 3 on eGFR
rs255167
rs28407295
rs4831915
rs35966427
rs142498030
rs117381581
rs11251507
rs77146351
rs143133607
rs7897192
rs6125090
rs188230872
rs192632550
rs9920979
rs184673439
rs1320211
rs118167827
rs188525573
rs145205577
rs1572780
rs115666273
rs146378069
rs56131491
rs190174823
rs79078681
rs62282371
All
−0.0015
−0.0010
−0.0005
0.0000
0.0005
MR effect size for
'SLIT and NTRK−like protein 3' on 'eGFR'

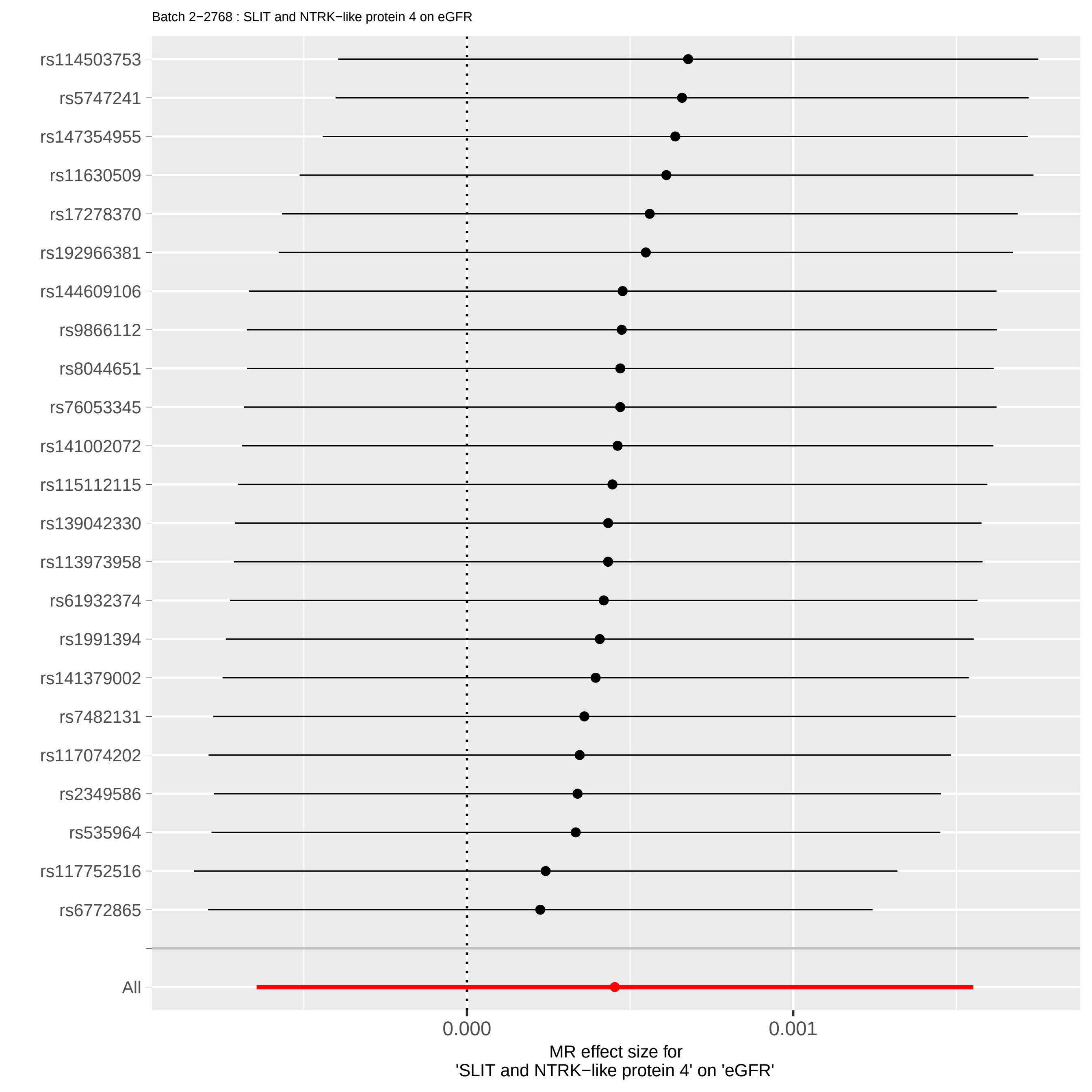
Batch 2−2768 : SLIT and NTRK−like protein 4 on eGFR
rs114503753
rs5747241
rs147354955
rs11630509
rs17278370
rs192966381
rs144609106
rs9866112
rs8044651
rs76053345
rs141002072
rs115112115
rs139042330
rs113973958
rs61932374
rs1991394
rs141379002
rs7482131
rs117074202
rs2349586
rs535964
rs117752516
rs6772865
All
0.000
0.001
MR effect size for
'SLIT and NTRK−like protein 4' on 'eGFR'

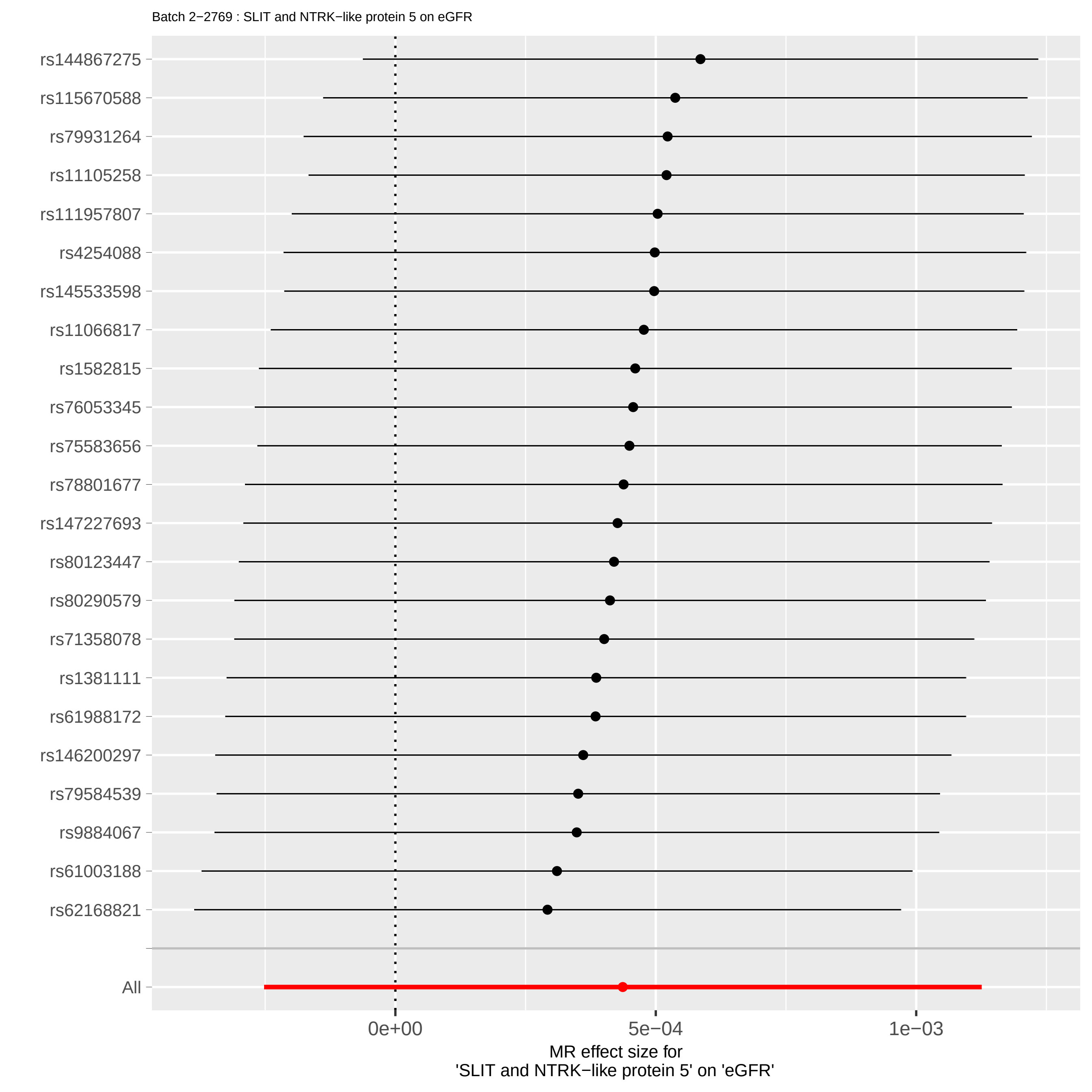

Batch 2−2769 : SLIT and NTRK−like protein 5 on eGFR
rs144867275
rs115670588
rs79931264
rs11105258
rs111957807
rs4254088
rs145533598
rs11066817
rs1582815
rs76053345
rs75583656
rs78801677
rs147227693
rs80123447
rs80290579
rs71358078
rs1381111
rs61988172
rs146200297
rs79584539
rs9884067
rs61003188
rs62168821
All
0e+00
5e−04
1e−03
MR effect size for
'SLIT and NTRK−like protein 5' on 'eGFR'

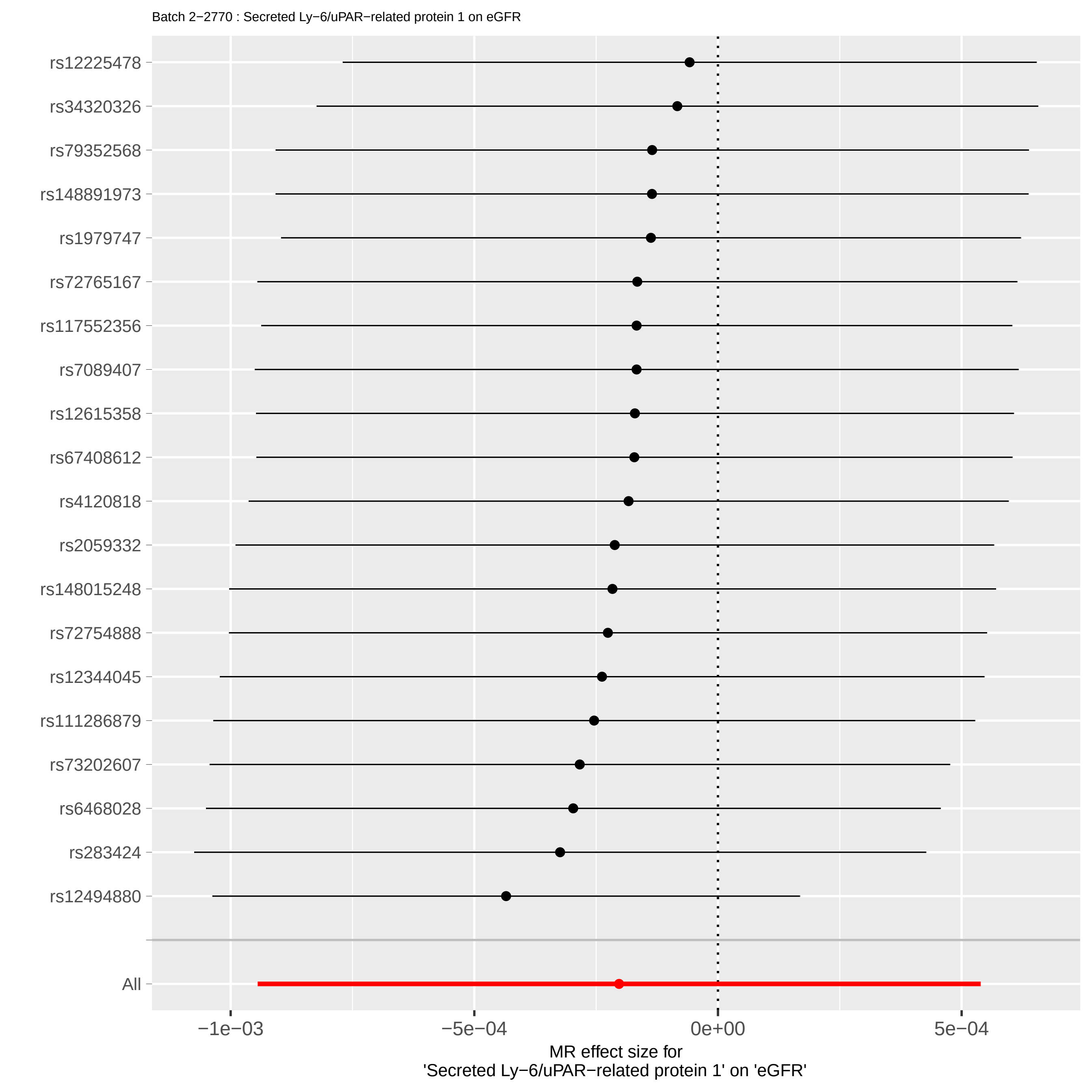

Batch 2−2770 : Secreted Ly−6/uPAR−related protein 1 on eGFR
rs12225478
rs34320326
rs79352568
rs148891973
rs1979747
rs72765167
rs117552356
rs7089407
rs12615358
rs67408612
rs4120818
rs2059332
rs148015248
rs72754888
rs12344045
rs111286879
rs73202607
rs6468028
rs283424
rs12494880
All
−1e−03
−5e−04
0e+00
5e−04
MR effect size for
'Secreted Ly−6/uPAR−related protein 1' on 'eGFR'

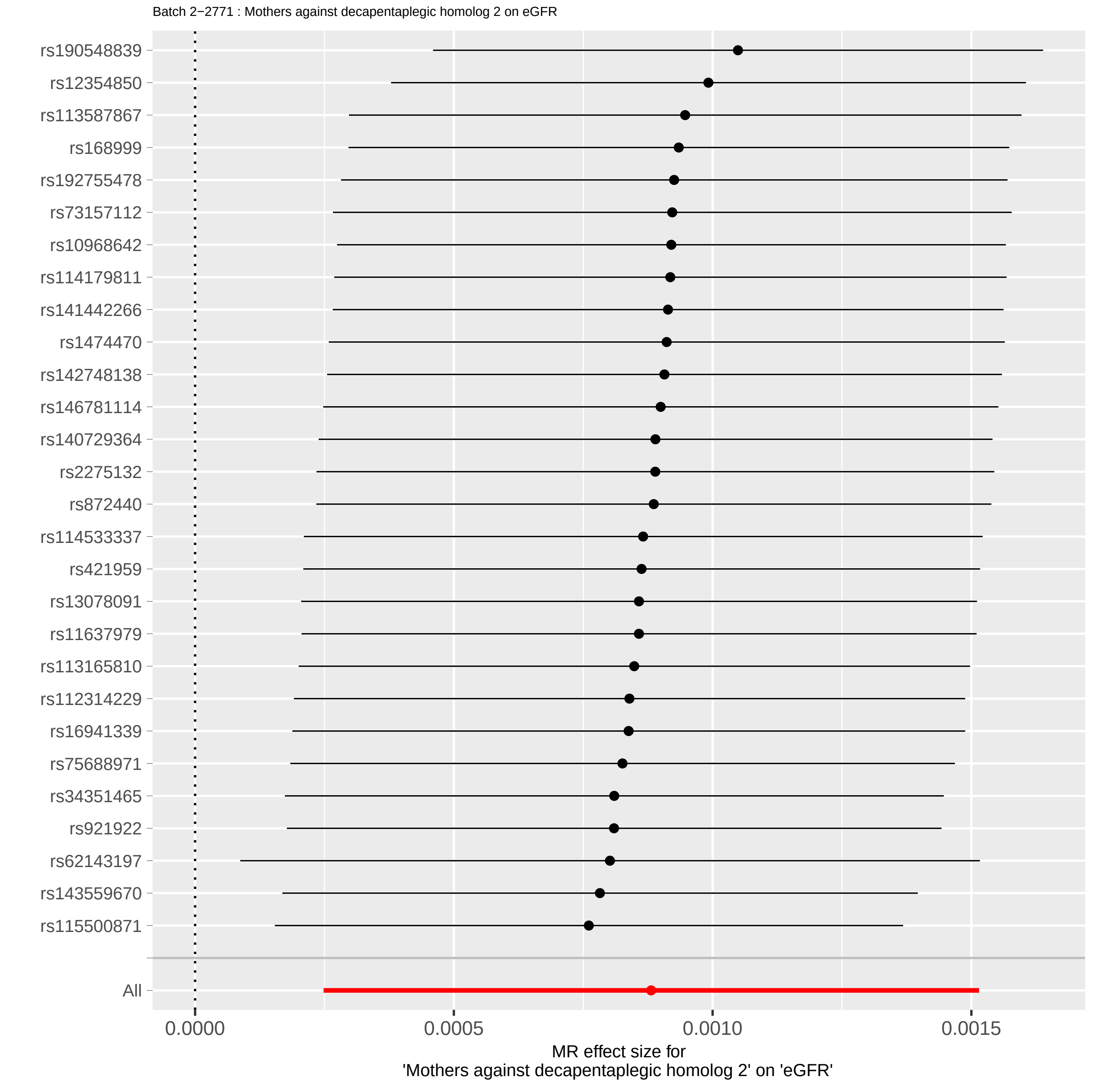
Batch 2−2771 : Mothers against decapentaplegic homolog 2 on eGFR
rs190548839
rs12354850
rs113587867
rs168999
rs192755478
rs73157112
rs10968642
rs114179811
rs141442266
rs1474470
rs142748138
rs146781114
rs140729364
rs2275132
rs872440
rs114533337
rs421959
rs13078091
rs11637979
rs113165810
rs112314229
rs16941339
rs75688971
rs34351465
rs921922
rs62143197
rs143559670
rs115500871
All
0.0000
0.0005
0.0010
0.0015
MR effect size for
'Mothers against decapentaplegic homolog 2' on 'eGFR'

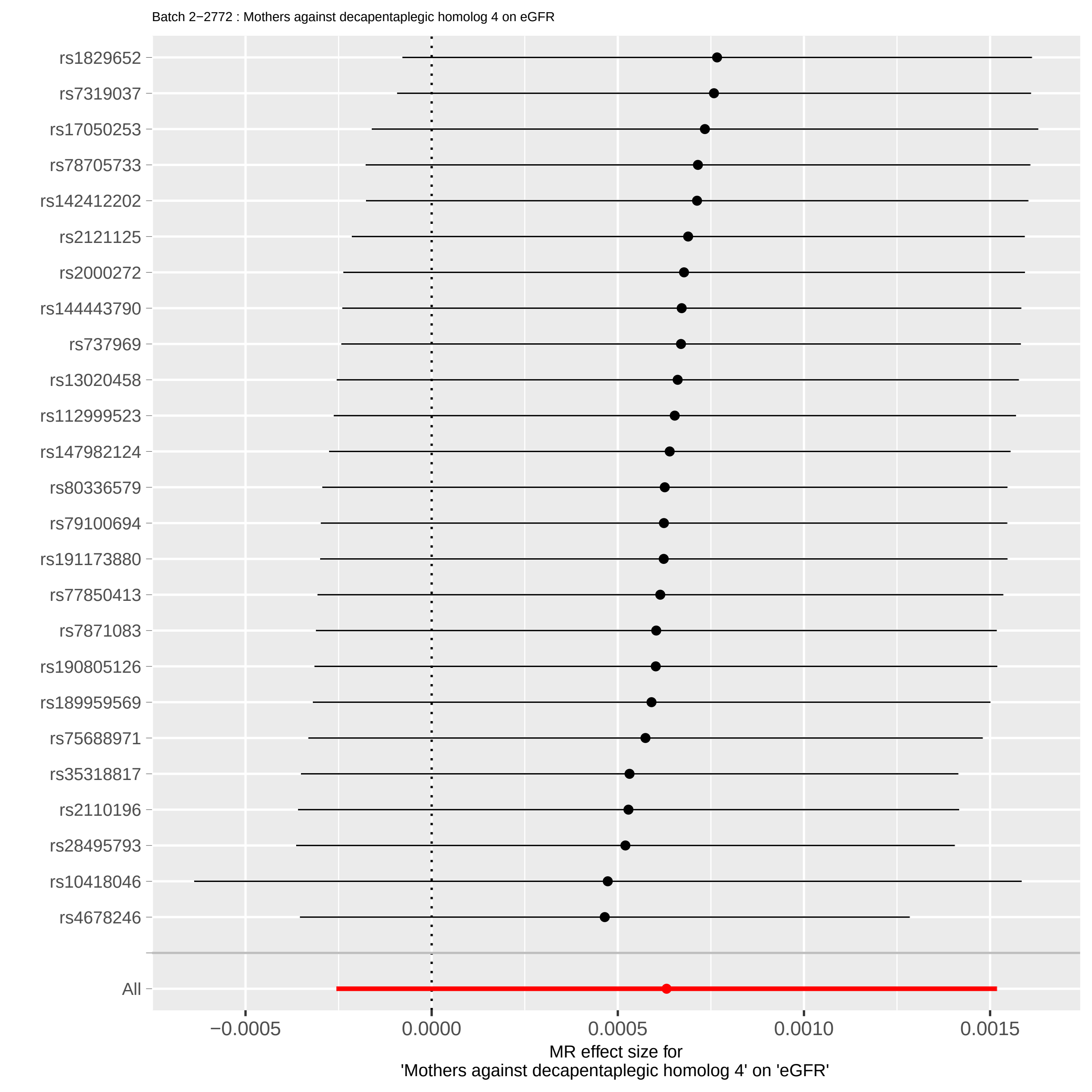

Batch 2−2772 : Mothers against decapentaplegic homolog 4 on eGFR
rs1829652
rs7319037
rs17050253
rs78705733
rs142412202
rs2121125
rs2000272
rs144443790
rs737969
rs13020458
rs112999523
rs147982124
rs80336579
rs79100694
rs191173880
rs77850413
rs7871083
rs190805126
rs189959569
rs75688971
rs35318817
rs2110196
rs28495793
rs10418046
rs4678246
All
−0.0005
0.0000
0.0005
0.0010
0.0015
MR effect size for
'Mothers against decapentaplegic homolog 4' on 'eGFR'

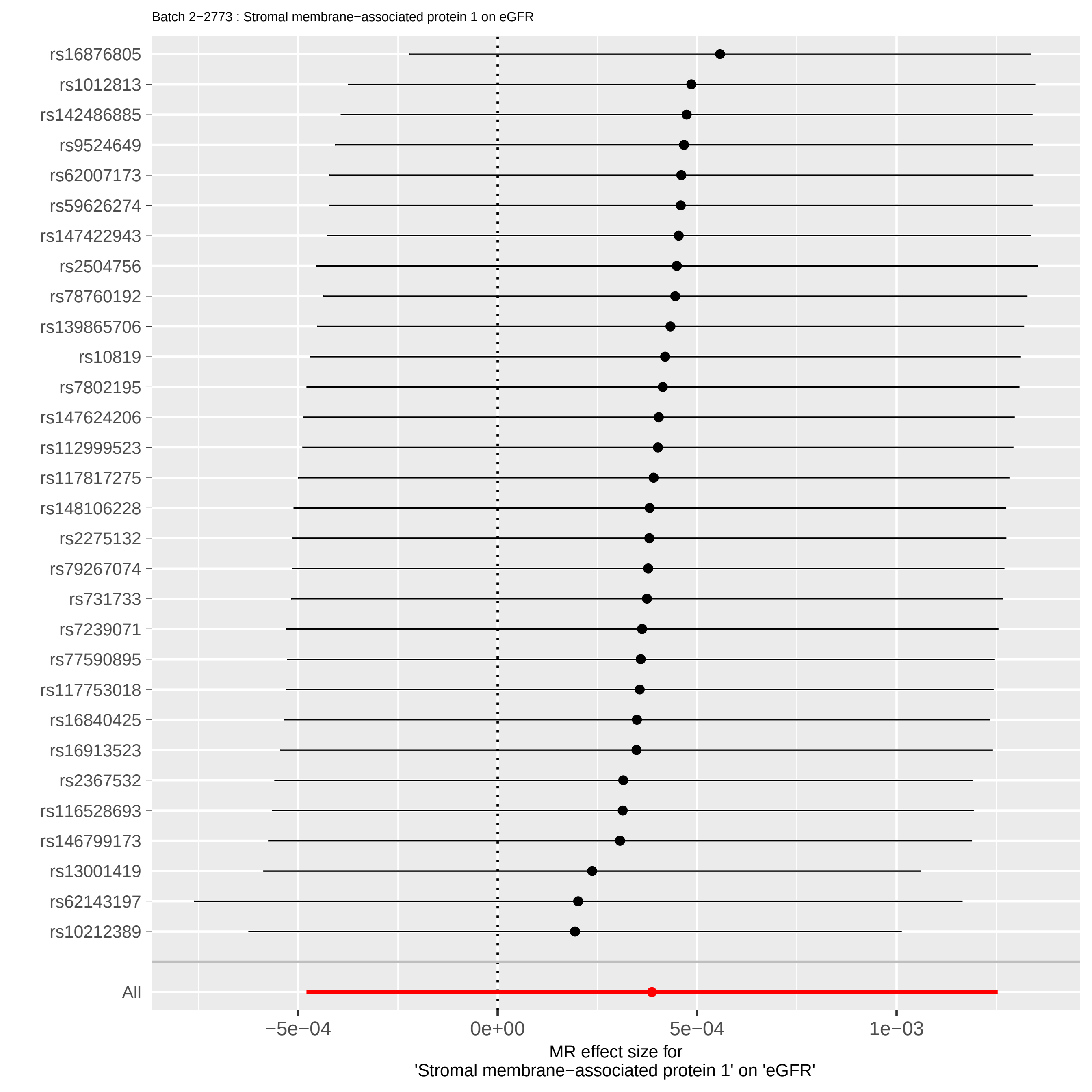

Batch 2−2773 : Stromal membrane−associated protein 1 on eGFR
rs16876805
rs1012813
rs142486885
rs9524649
rs62007173
rs59626274
rs147422943
rs2504756
rs78760192
rs139865706
rs10819
rs7802195
rs147624206
rs112999523
rs117817275
rs148106228
rs2275132
rs79267074
rs731733
rs7239071
rs77590895
rs117753018
rs16840425
rs16913523
rs2367532
rs116528693
rs146799173
rs13001419
rs62143197
rs10212389
All
−5e−04
0e+00
5e−04
1e−03
MR effect size for
'Stromal membrane−associated protein 1' on 'eGFR'

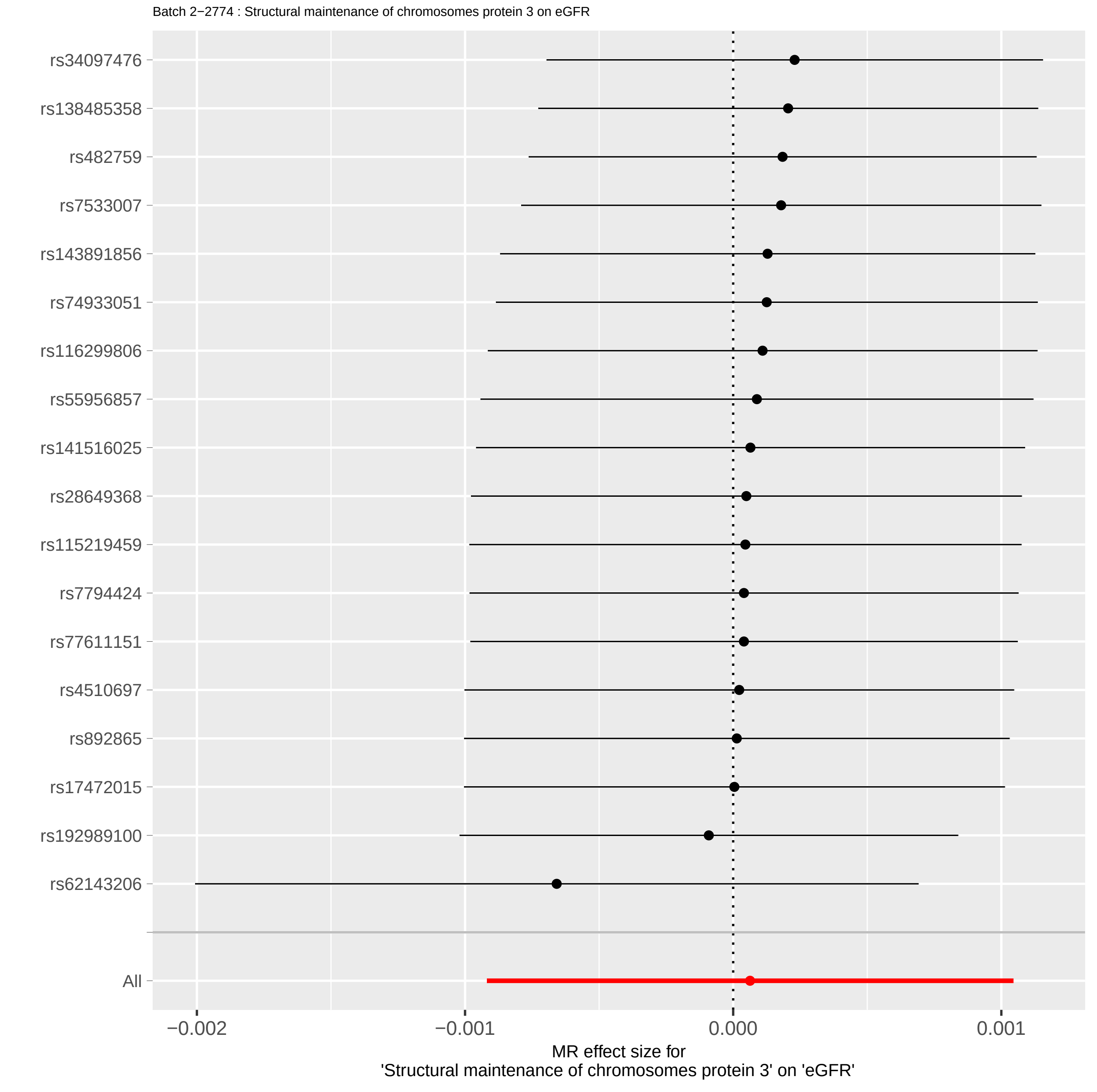
Batch 2−2774 : Structural maintenance of chromosomes protein 3 on eGFR
rs34097476
rs138485358
rs482759
rs7533007
rs143891856
rs74933051
rs116299806
rs55956857
rs141516025
rs28649368
rs115219459
rs7794424
rs77611151
rs4510697
rs892865
rs17472015
rs192989100
rs62143206
All
−0.002
−0.001
0.000
0.001
MR effect size for
'Structural maintenance of chromosomes protein 3' on 'eGFR'

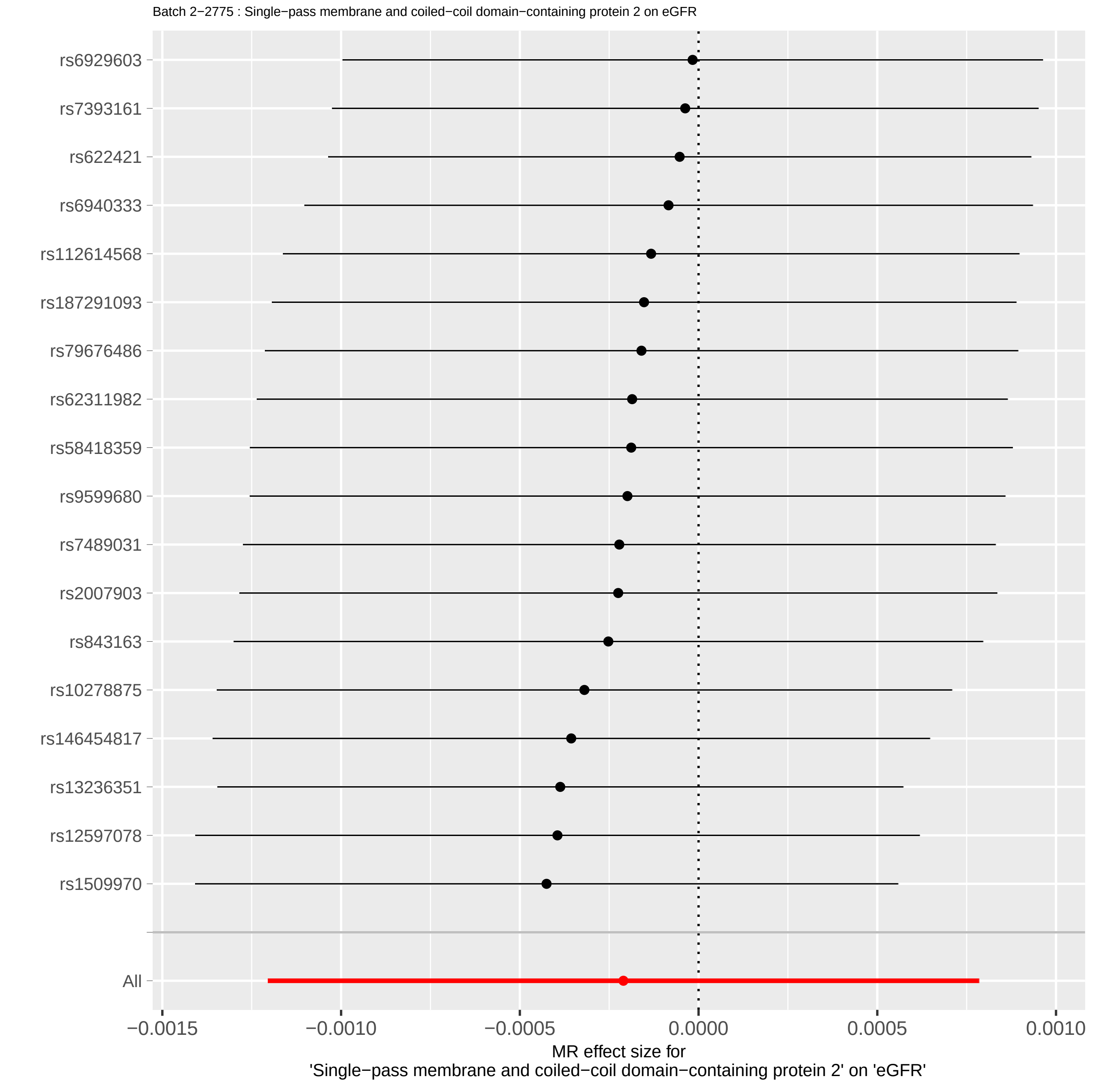
Batch 2−2775 : Single−pass membrane and coiled−coil domain−containing protein 2 on eGFR
rs6929603
rs7393161
rs622421
rs6940333
rs112614568
rs187291093
rs79676486
rs62311982
rs58418359
rs9599680
rs7489031
rs2007903
rs843163
rs10278875
rs146454817
rs13236351
rs12597078
rs1509970
All
−0.0015
−0.0010
−0.0005
0.0000
0.0005
0.0010
MR effect size for
'Single−pass membrane and coiled−coil domain−containing protein 2' on 'eGFR'

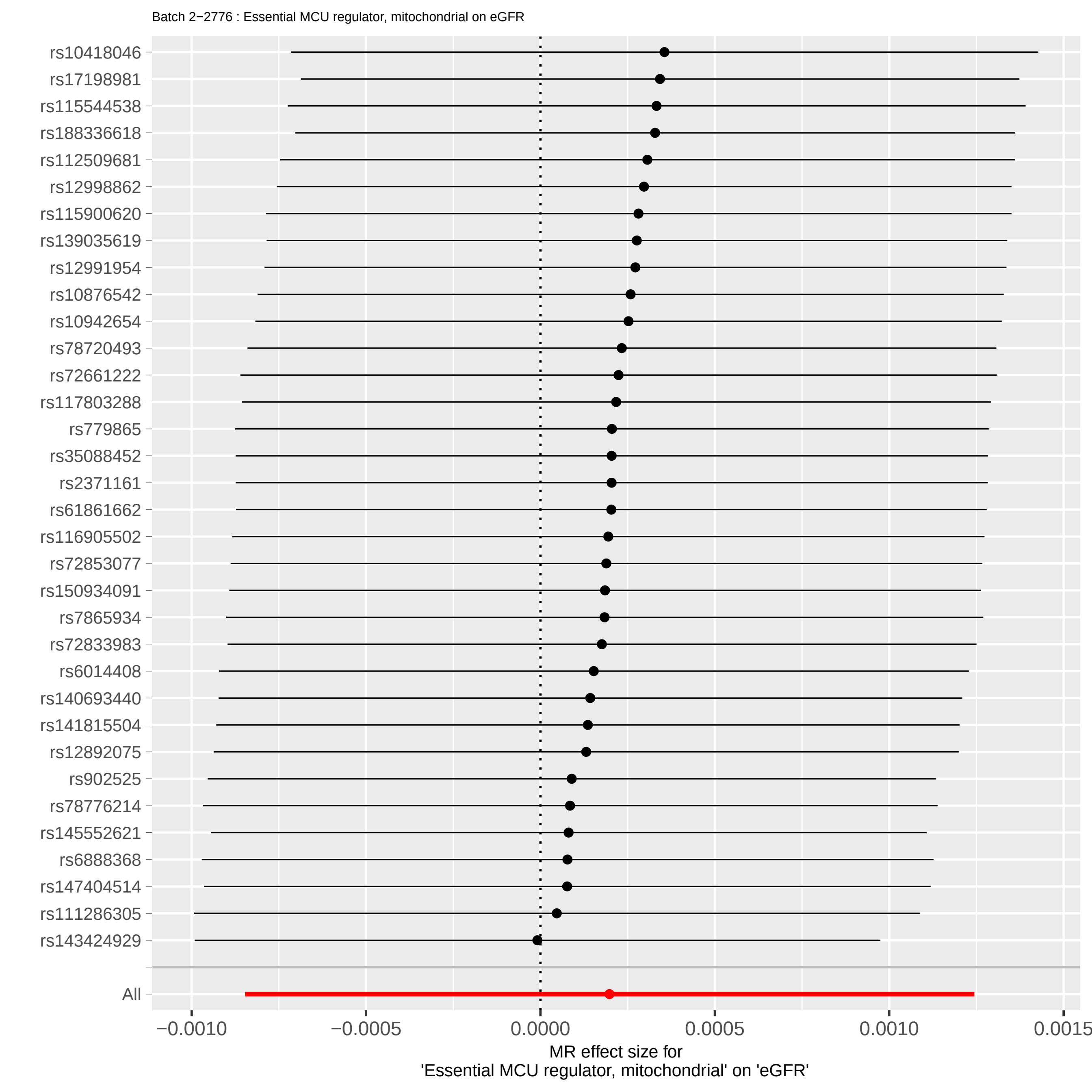

Batch 2−2776 : Essential MCU regulator, mitochondrial on eGFR
rs10418046
rs17198981
rs115544538
rs188336618
rs112509681
rs12998862
rs115900620
rs139035619
rs12991954
rs10876542
rs10942654
rs78720493
rs72661222
rs117803288
rs779865
rs35088452
rs2371161
rs61861662
rs116905502
rs72853077
rs150934091
rs7865934
rs72833983
rs6014408
rs140693440
rs141815504
rs12892075
rs902525
rs78776214
rs145552621
rs6888368
rs147404514
rs111286305
rs143424929
All
−0.0010
−0.0005
0.0000
0.0005
0.0010
0.0015
MR effect size for
'Essential MCU regulator, mitochondrial' on 'eGFR'

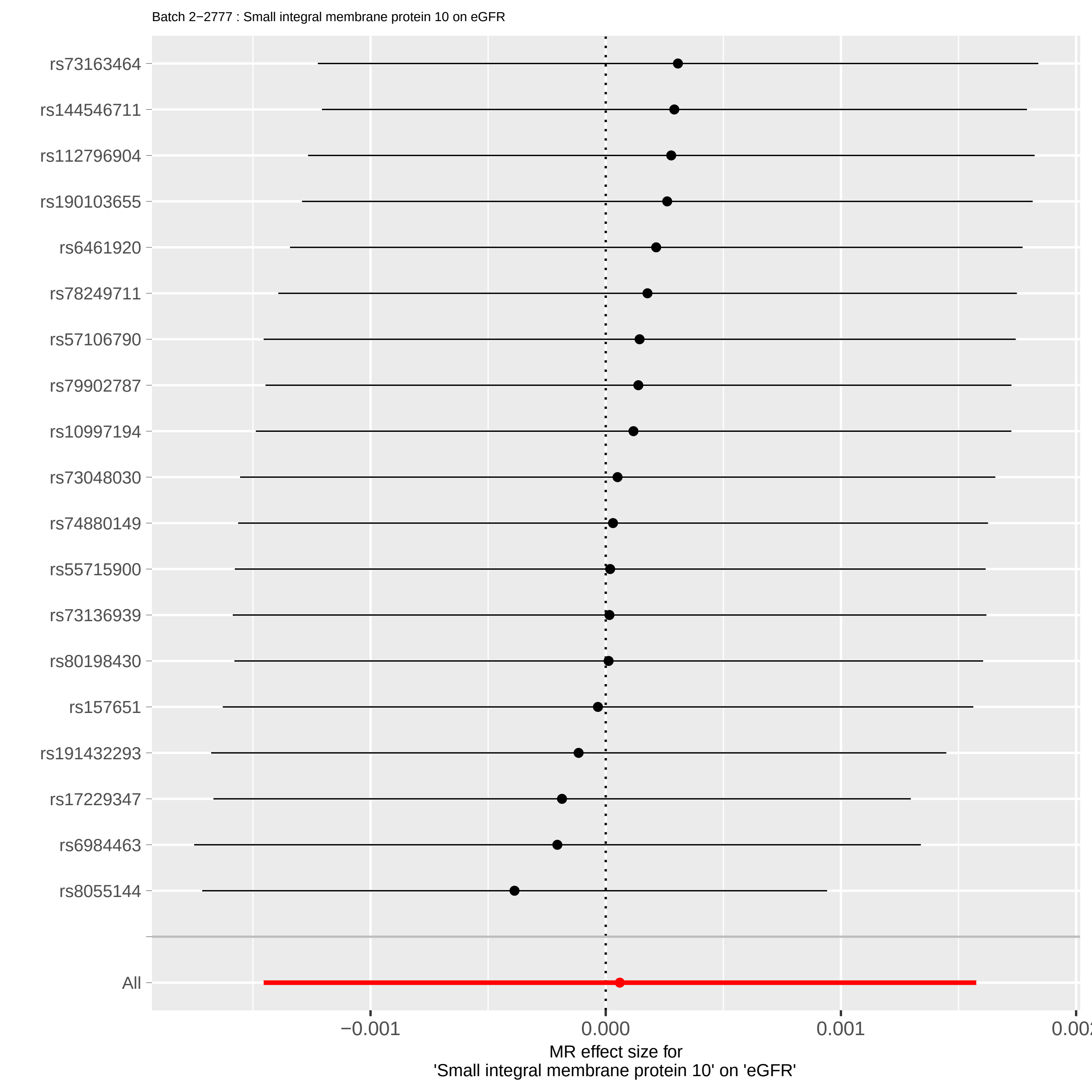
Batch 2−2777 : Small integral membrane protein 10 on eGFR
rs73163464
rs144546711
rs112796904
rs190103655
rs6461920
rs78249711
rs57106790
rs79902787
rs10997194
rs73048030
rs74880149
rs55715900
rs73136939
rs80198430
rs157651
rs191432293
rs17229347
rs6984463
rs8055144
All
−0.001
0.000
0.001
MR effect size for
'Small integral membrane protein 10' on 'eGFR'

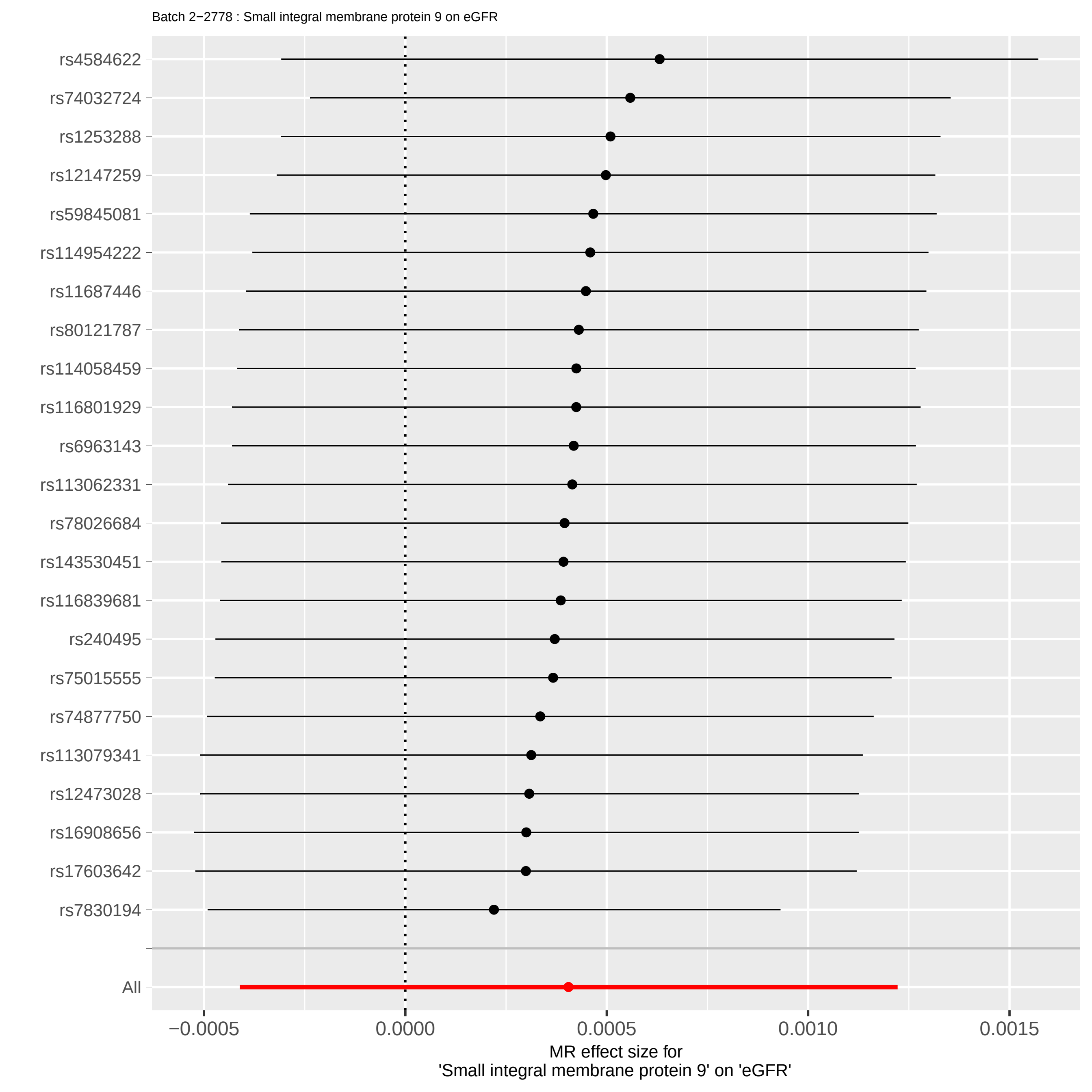

Batch 2−2778 : Small integral membrane protein 9 on eGFR
rs4584622
rs74032724
rs1253288
rs12147259
rs59845081
rs114954222
rs11687446
rs80121787
rs114058459
rs116801929
rs6963143
rs113062331
rs78026684
rs143530451
rs116839681
rs240495
rs75015555
rs74877750
rs113079341
rs12473028
rs16908656
rs17603642
rs7830194
All
−0.0005
0.0000
0.0005
0.0010
0.0015
MR effect size for
'Small integral membrane protein 9' on 'eGFR'

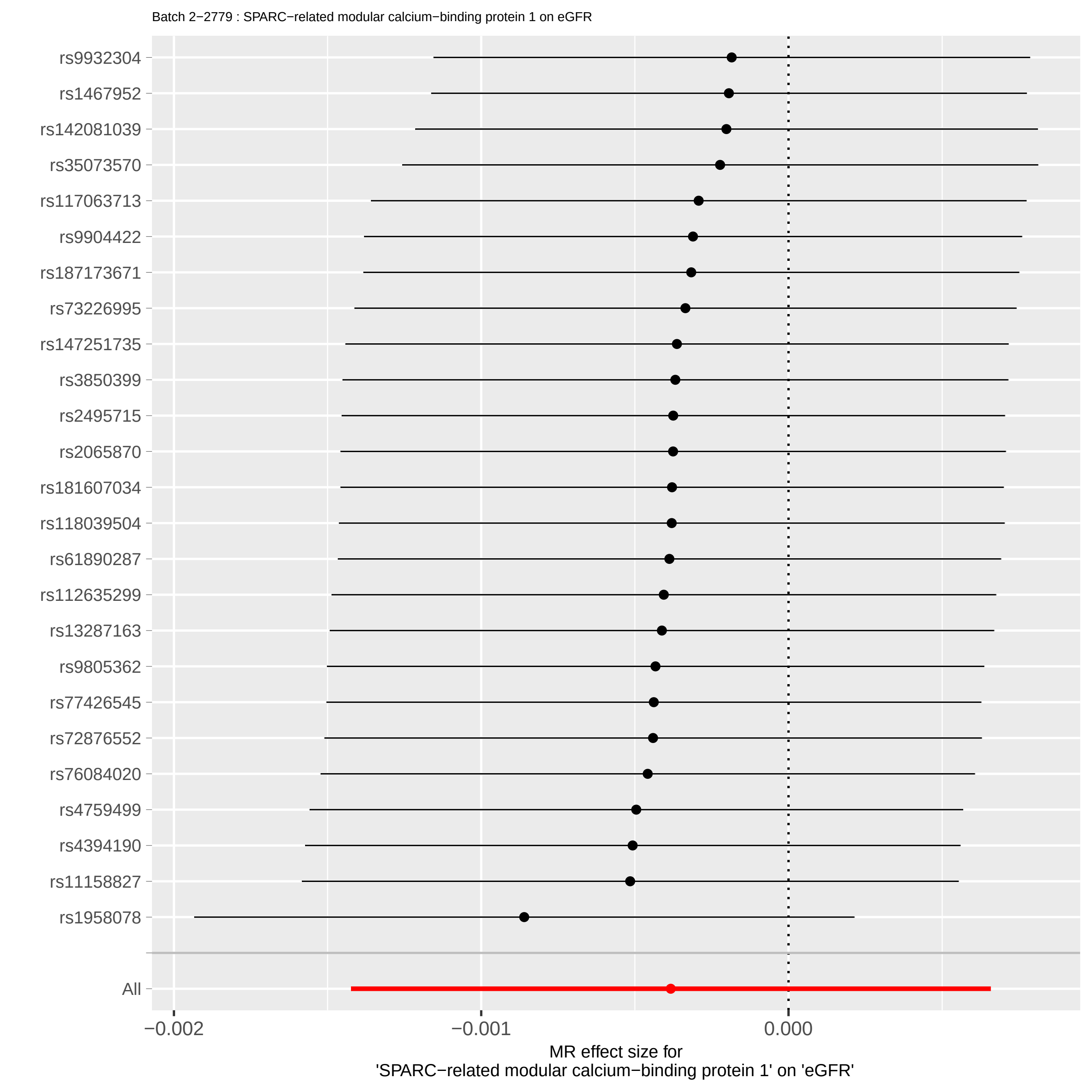
Batch 2−2779 : SPARC−related modular calcium−binding protein 1 on eGFR
rs9932304
rs1467952
rs142081039
rs35073570
rs117063713
rs9904422
rs187173671
rs73226995
rs147251735
rs3850399
rs2495715
rs2065870
rs181607034
rs118039504
rs61890287
rs112635299
rs13287163
rs9805362
rs77426545
rs72876552
rs76084020
rs4759499
rs4394190
rs11158827
rs1958078
All
−0.002
−0.001
0.000
MR effect size for
'SPARC−related modular calcium−binding protein 1' on 'eGFR'

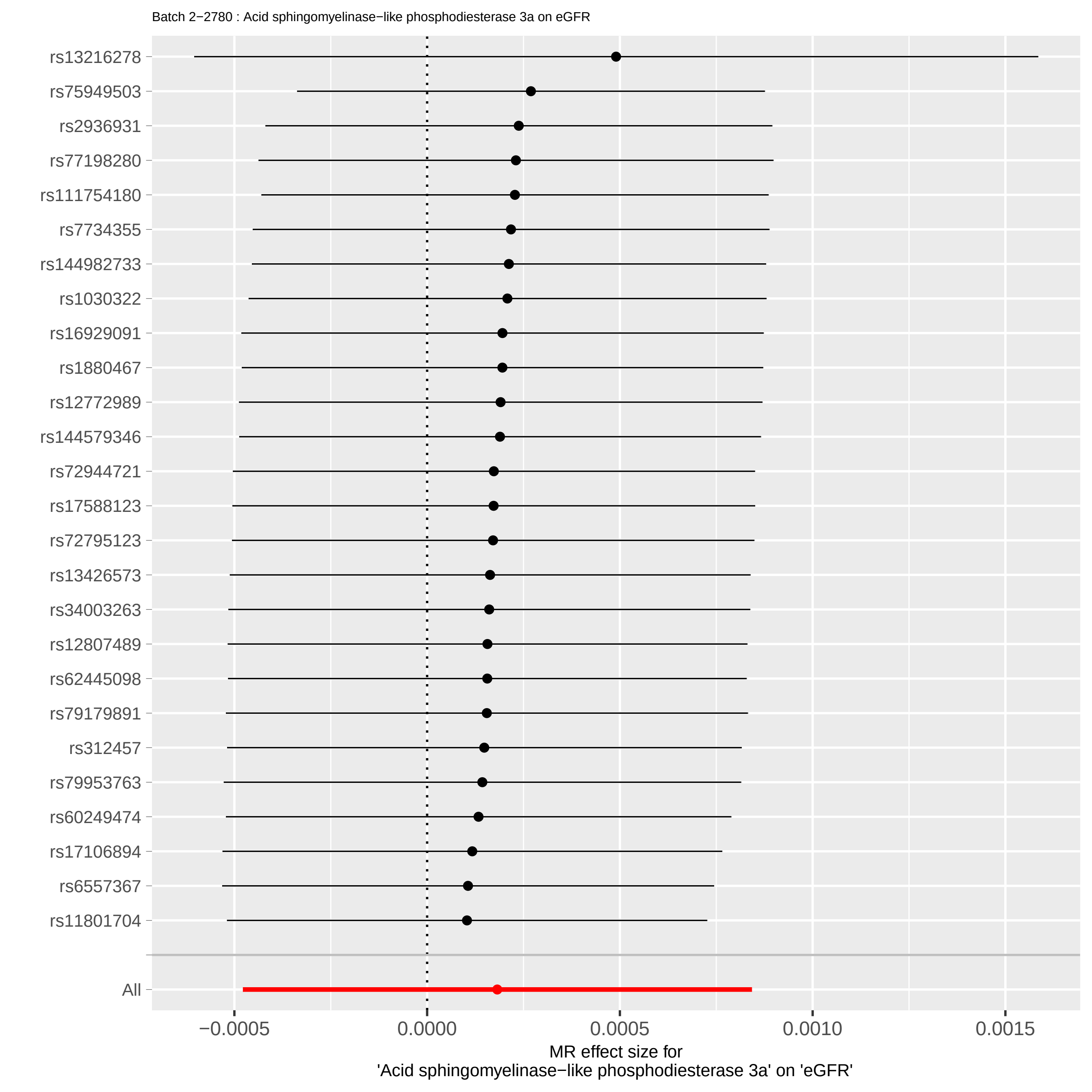
Batch 2−2780 : Acid sphingomyelinase−like phosphodiesterase 3a on eGFR
rs13216278
rs75949503
rs2936931
rs77198280
rs111754180
rs7734355
rs144982733
rs1030322
rs16929091
rs1880467
rs12772989
rs144579346
rs72944721
rs17588123
rs72795123
rs13426573
rs34003263
rs12807489
rs62445098
rs79179891
rs312457
rs79953763
rs60249474
rs17106894
rs6557367
rs11801704
All
−0.0005
0.0000
0.0005
0.0010
0.0015
MR effect size for
'Acid sphingomyelinase−like phosphodiesterase 3a' on 'eGFR'

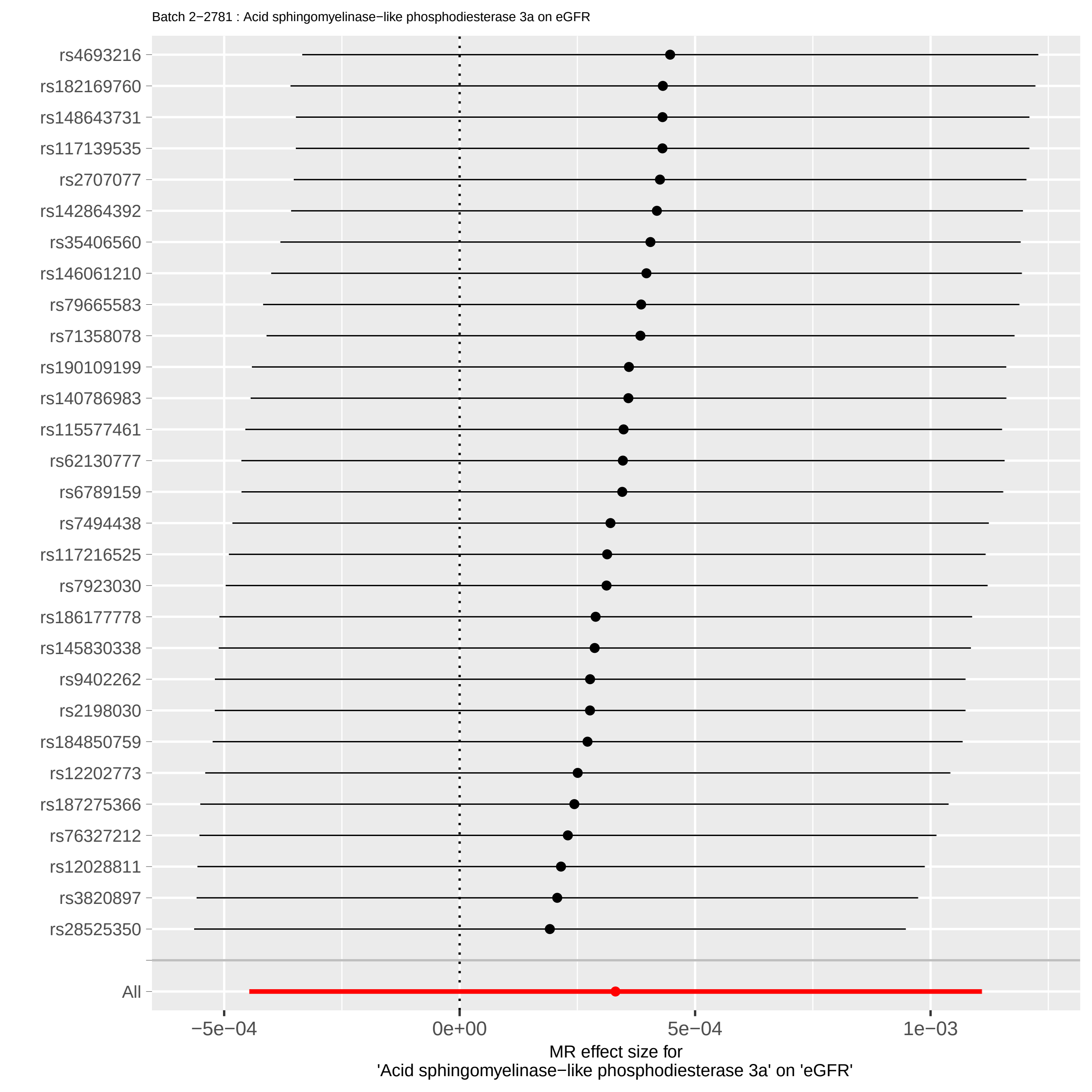

Batch 2−2781 : Acid sphingomyelinase−like phosphodiesterase 3a on eGFR
rs4693216
rs182169760
rs148643731
rs117139535
rs2707077
rs142864392
rs35406560
rs146061210
rs79665583
rs71358078
rs190109199
rs140786983
rs115577461
rs62130777
rs6789159
rs7494438
rs117216525
rs7923030
rs186177778
rs145830338
rs9402262
rs2198030
rs184850759
rs12202773
rs187275366
rs76327212
rs12028811
rs3820897
rs28525350
All
−5e−04
0e+00
5e−04
1e−03
MR effect size for
'Acid sphingomyelinase−like phosphodiesterase 3a' on 'eGFR'

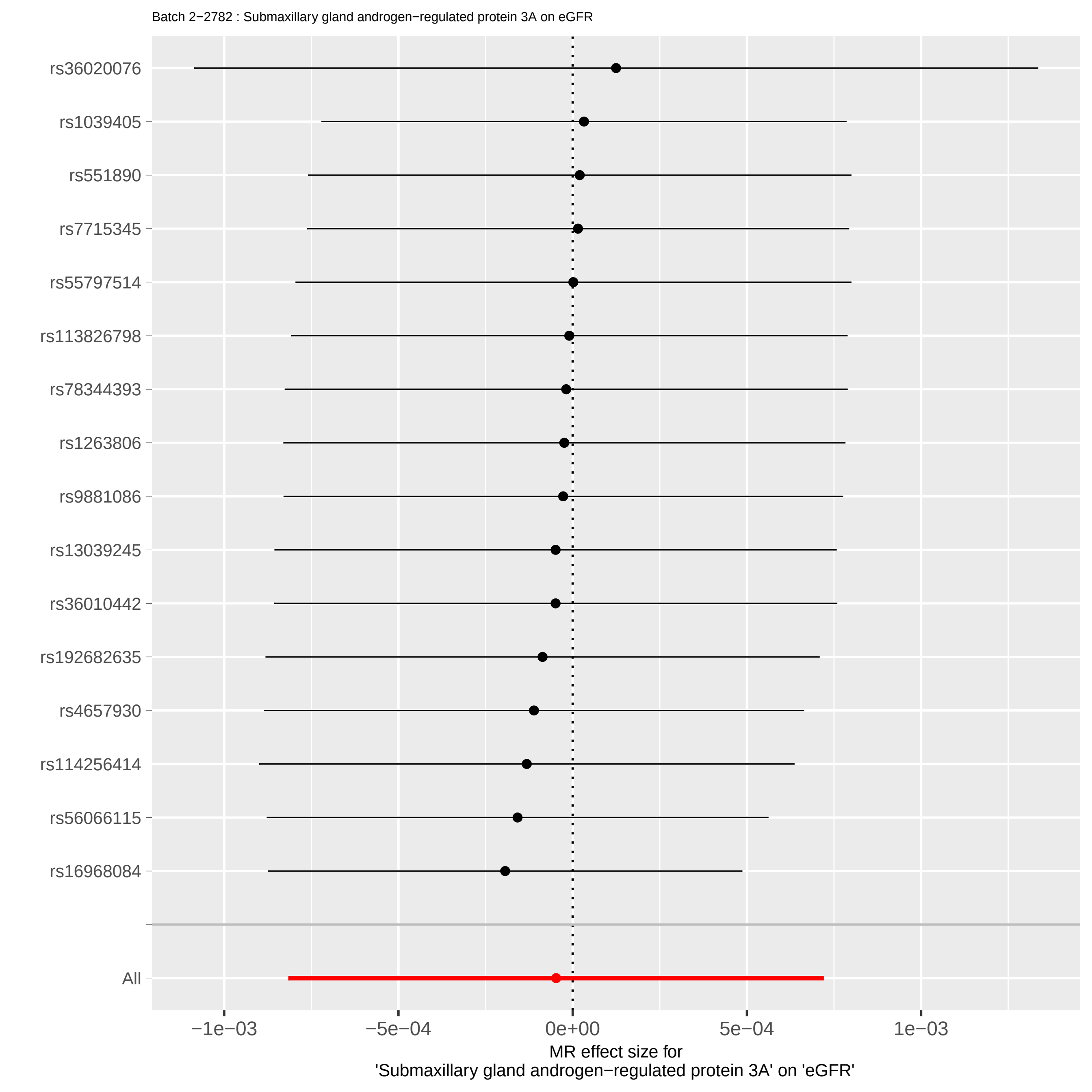

Batch 2−2782 : Submaxillary gland androgen−regulated protein 3A on eGFR
rs36020076
rs1039405
rs551890
rs7715345
rs55797514
rs113826798
rs78344393
rs1263806
rs9881086
rs13039245
rs36010442
rs192682635
rs4657930
rs114256414
rs56066115
rs16968084
All
−1e−03
−5e−04
0e+00
5e−04
1e−03
MR effect size for
'Submaxillary gland androgen−regulated protein 3A' on 'eGFR'

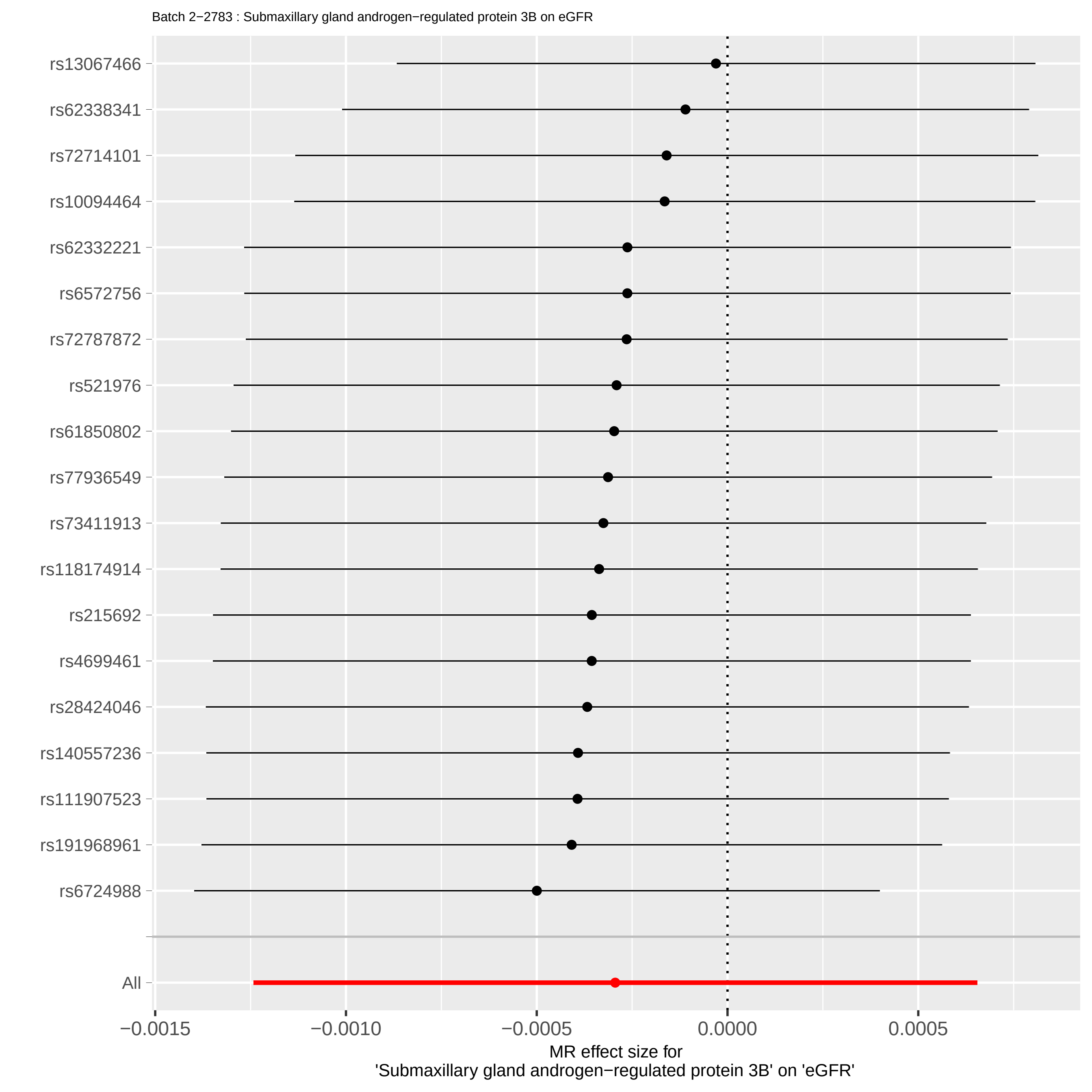
Batch 2−2783 : Submaxillary gland androgen−regulated protein 3B on eGFR
rs13067466
rs62338341
rs72714101
rs10094464
rs62332221
rs6572756
rs72787872
rs521976
rs61850802
rs77936549
rs73411913
rs118174914
rs215692
rs4699461
rs28424046
rs140557236
rs111907523
rs191968961
rs6724988
All
−0.0015
−0.0010
−0.0005
0.0000
0.0005
MR effect size for
'Submaxillary gland androgen−regulated protein 3B' on 'eGFR'

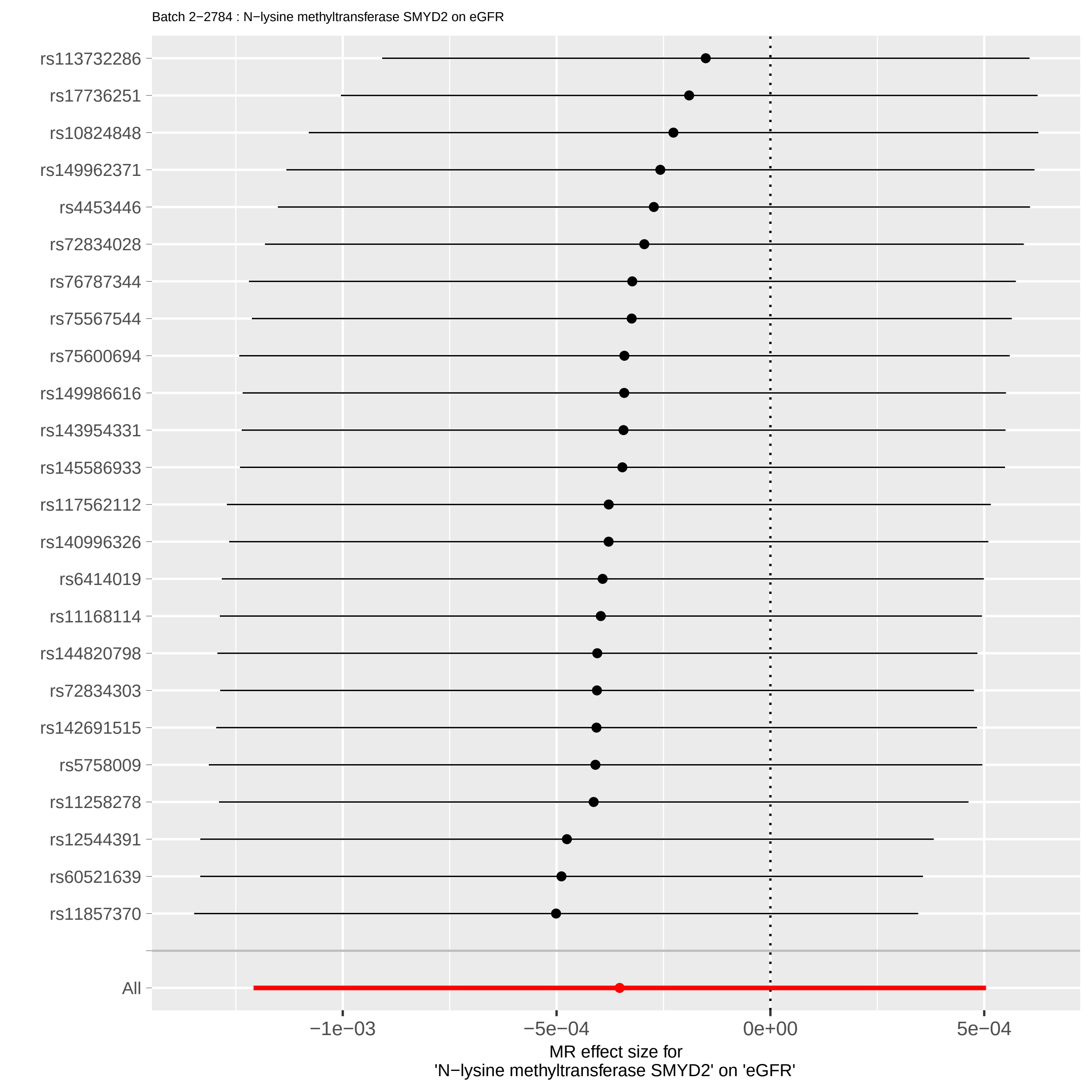
Batch 2−2784 : N−lysine methyltransferase SMYD2 on eGFR
rs113732286
rs17736251
rs10824848
rs149962371
rs4453446
rs72834028
rs76787344
rs75567544
rs75600694
rs149986616
rs143954331
rs145586933
rs117562112
rs140996326
rs6414019
rs11168114
rs144820798
rs72834303
rs142691515
rs5758009
rs11258278
rs12544391
rs60521639
rs11857370
All
−1e−03
−5e−04
0e+00
5e−04
MR effect size for
'N−lysine methyltransferase SMYD2' on 'eGFR'

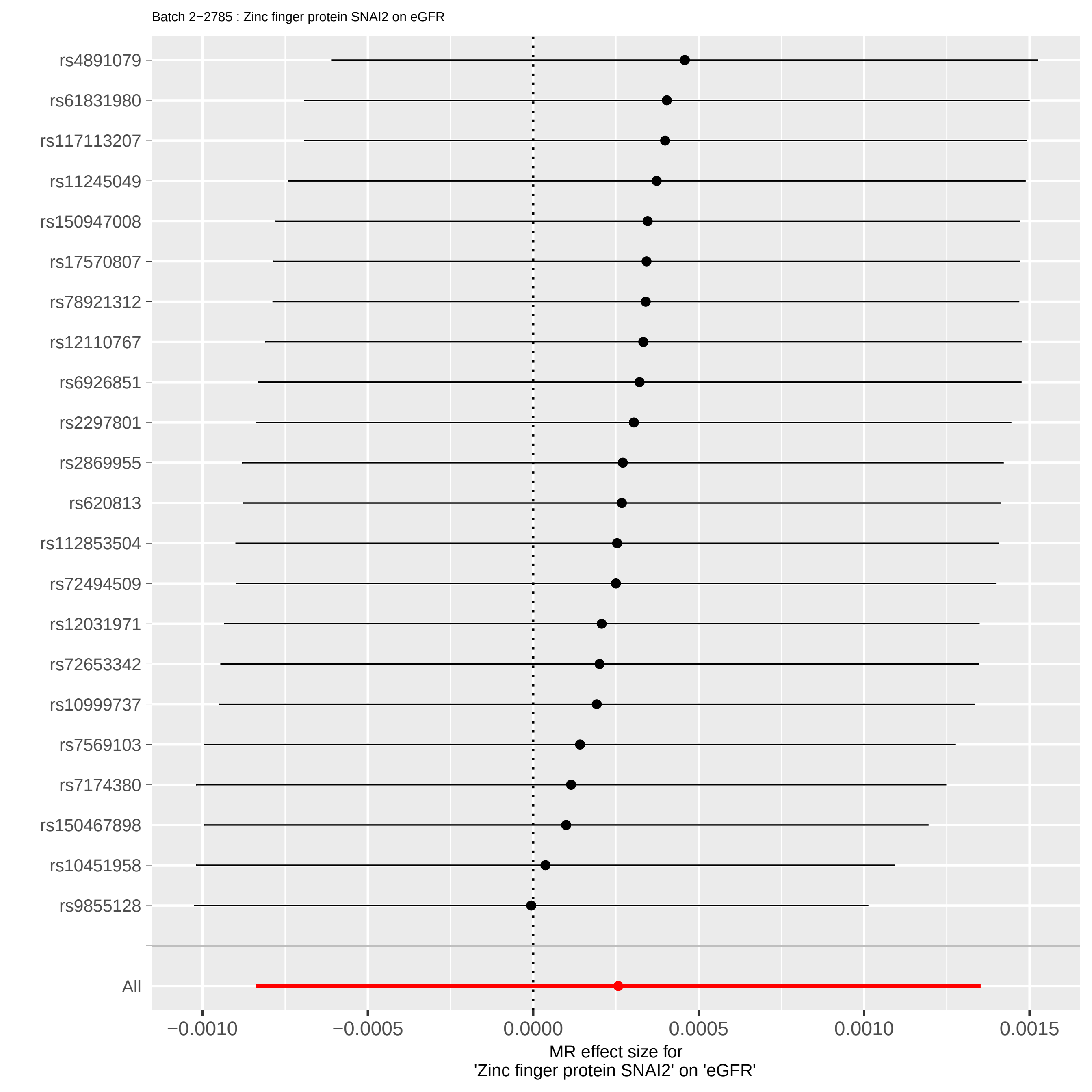
Batch 2−2785 : Zinc finger protein SNAI2 on eGFR
rs4891079
rs61831980
rs117113207
rs11245049
rs150947008
rs17570807
rs78921312
rs12110767
rs6926851
rs2297801
rs2869955
rs620813
rs112853504
rs72494509
rs12031971
rs72653342
rs10999737
rs7569103
rs7174380
rs150467898
rs10451958
rs9855128
All
−0.0010
−0.0005
0.0000
0.0005
0.0010
0.0015
MR effect size for
'Zinc finger protein SNAI2' on 'eGFR'

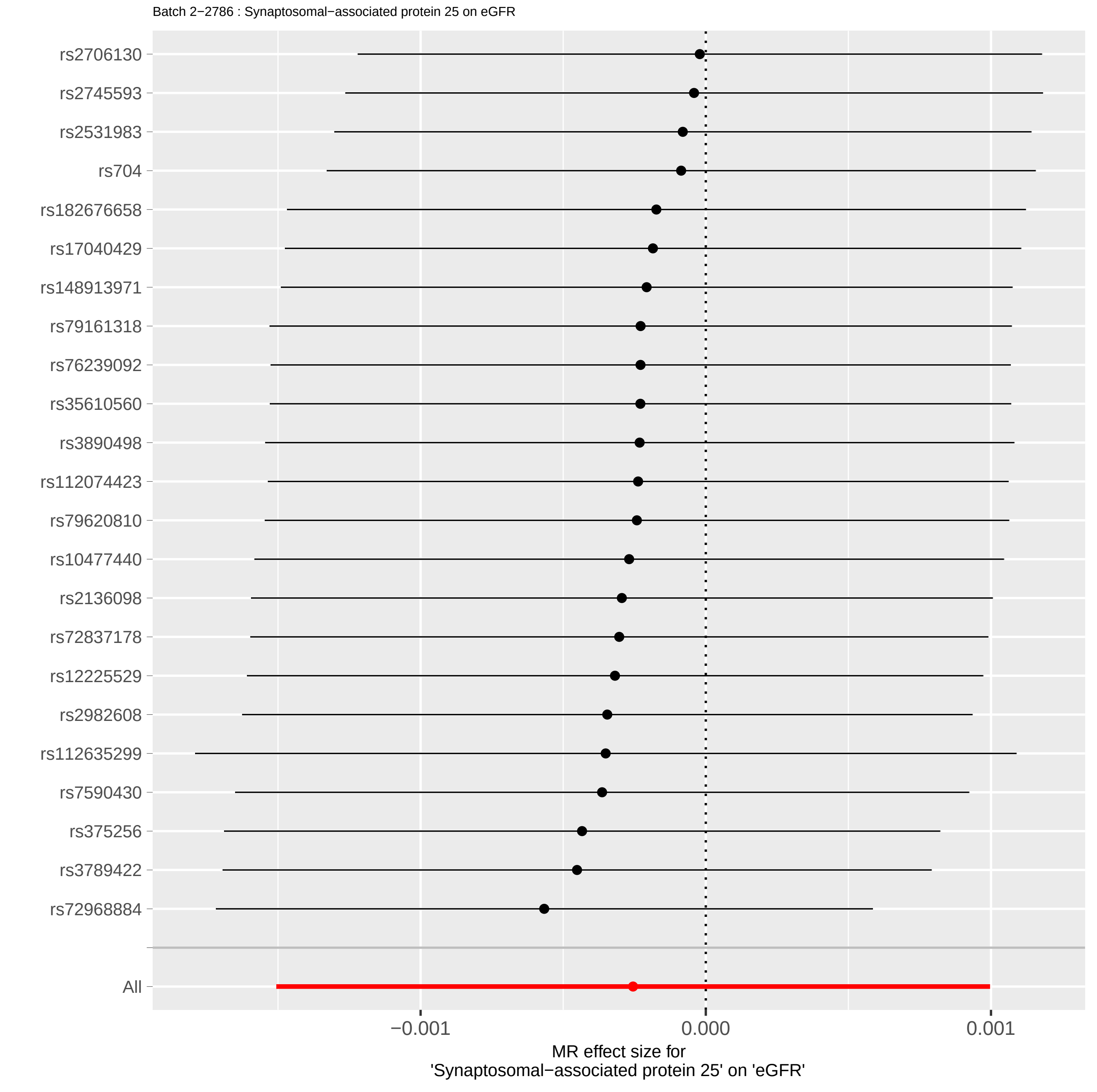

Batch 2−2786 : Synaptosomal−associated protein 25 on eGFR
rs2706130
rs2745593
rs2531983
rs704
rs182676658
rs17040429
rs148913971
rs79161318
rs76239092
rs35610560
rs3890498
rs112074423
rs79620810
rs10477440
rs2136098
rs72837178
rs12225529
rs2982608
rs112635299
rs7590430
rs375256
rs3789422
rs72968884
All
−0.001
0.000
0.001
MR effect size for
'Synaptosomal−associated protein 25' on 'eGFR'

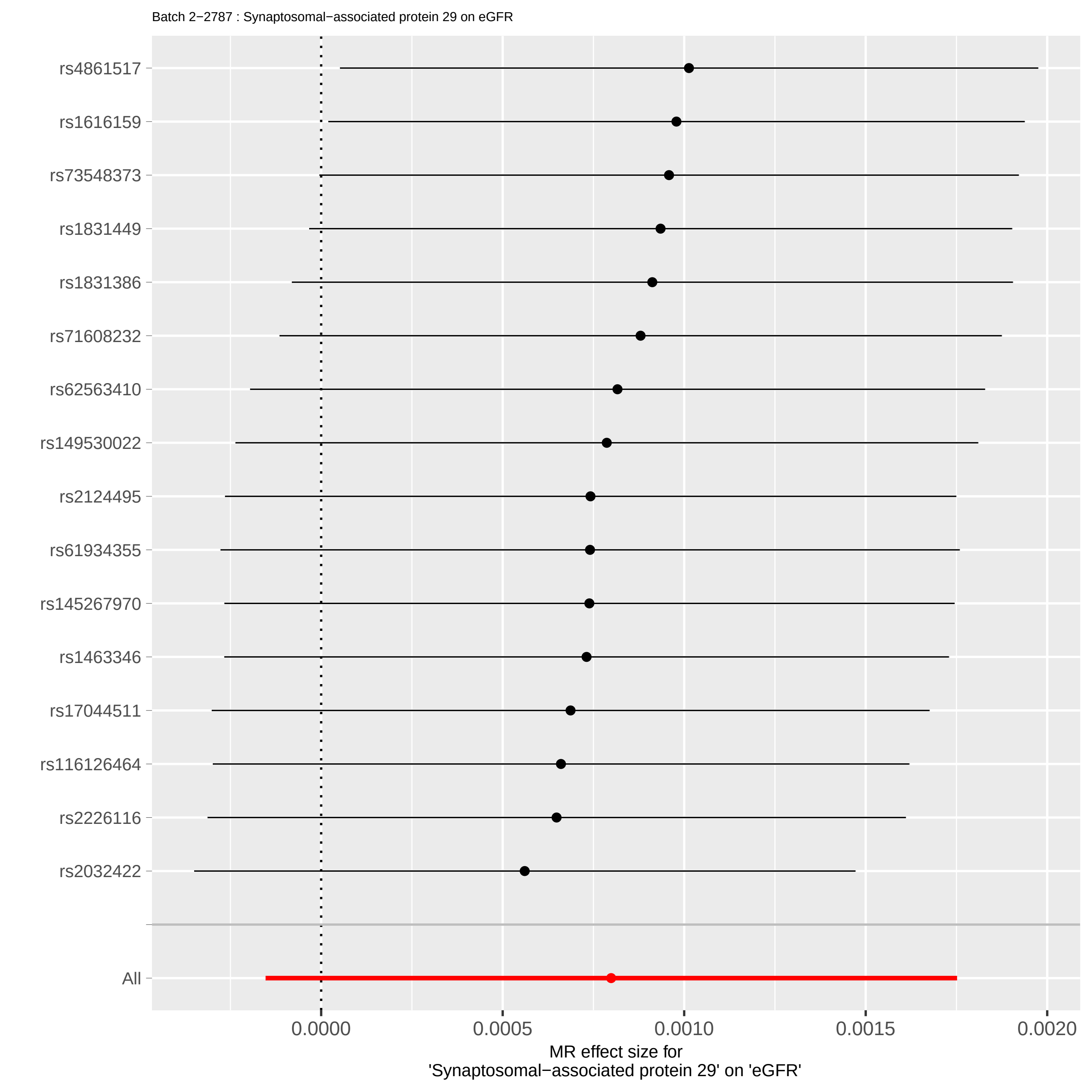

Batch 2−2787 : Synaptosomal−associated protein 29 on eGFR
rs4861517
rs1616159
rs73548373
rs1831449
rs1831386
rs71608232
rs62563410
rs149530022
rs2124495
rs61934355
rs145267970
rs1463346
rs17044511
rs116126464
rs2226116
rs2032422
All
0.0000
0.0005
0.0010
0.0015
0.0020
MR effect size for
'Synaptosomal−associated protein 29' on 'eGFR'

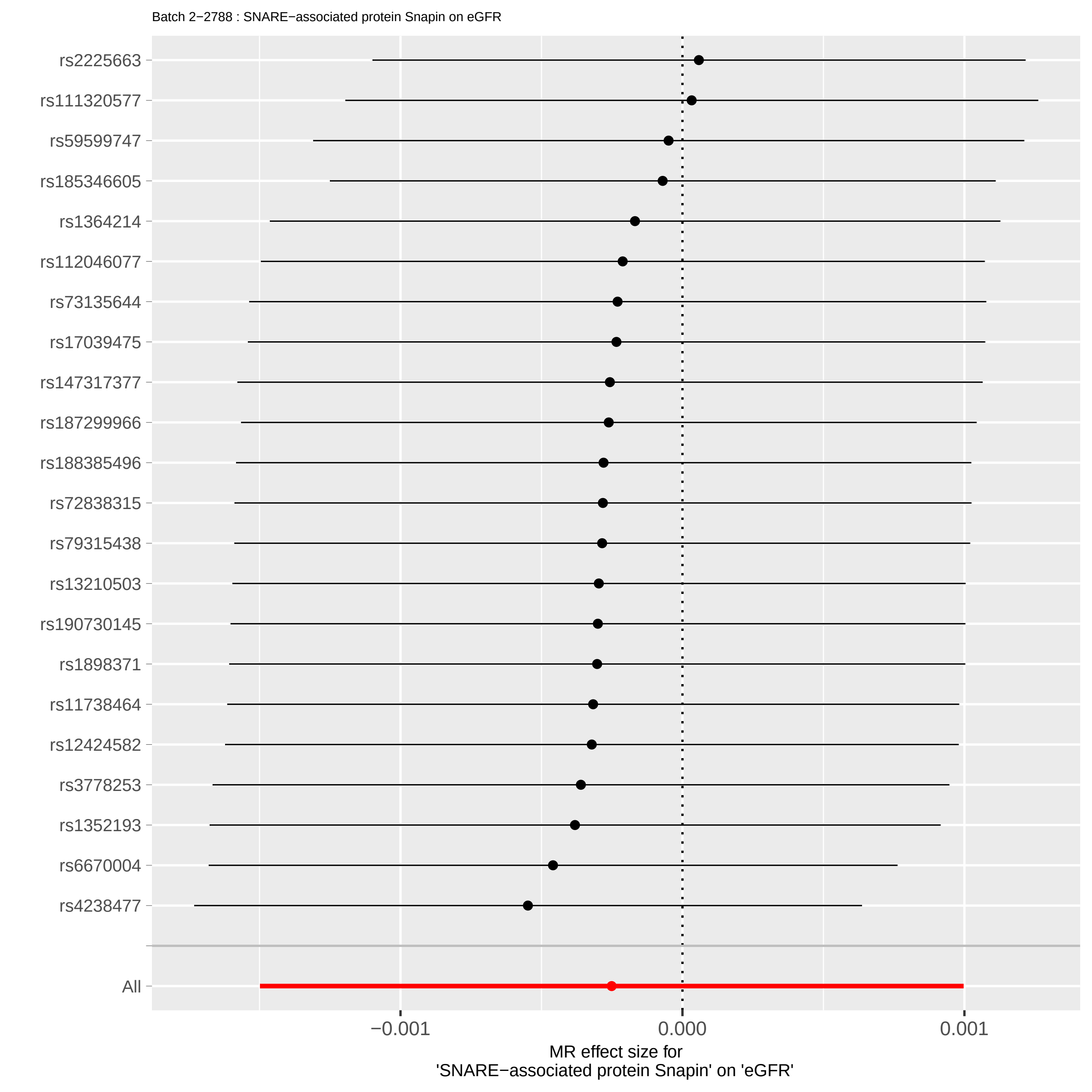

Batch 2−2788 : SNARE−associated protein Snapin on eGFR
rs2225663
rs111320577
rs59599747
rs185346605
rs1364214
rs112046077
rs73135644
rs17039475
rs147317377
rs187299966
rs188385496
rs72838315
rs79315438
rs13210503
rs190730145
rs1898371
rs11738464
rs12424582
rs3778253
rs1352193
rs6670004
rs4238477
All
−0.001
0.000
0.001
MR effect size for
'SNARE−associated protein Snapin' on 'eGFR'

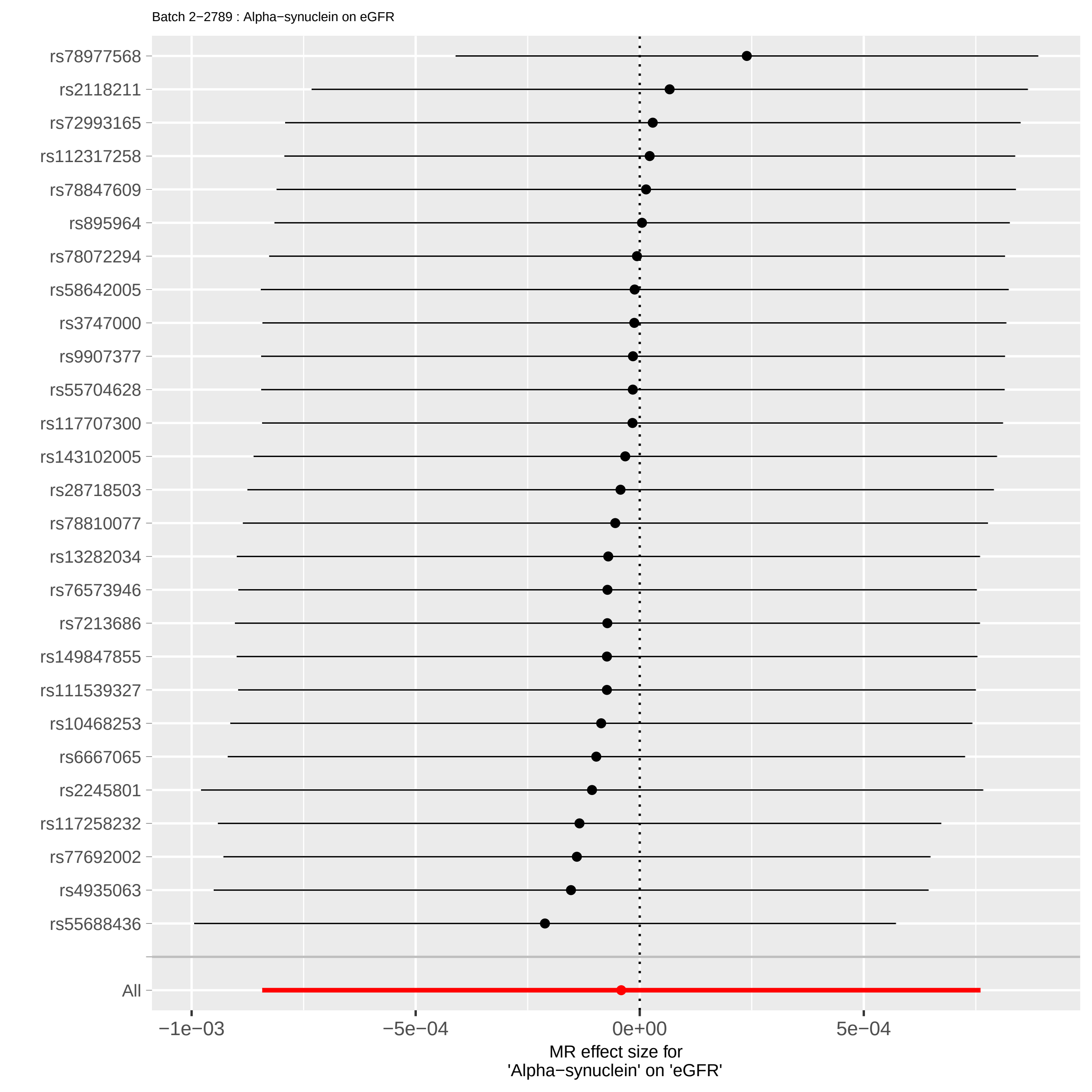

Batch 2−2789 : Alpha−synuclein on eGFR
rs78977568
rs2118211
rs72993165
rs112317258
rs78847609
rs895964
rs78072294
rs58642005
rs3747000
rs9907377
rs55704628
rs117707300
rs143102005
rs28718503
rs78810077
rs13282034
rs76573946
rs7213686
rs149847855
rs111539327
rs10468253
rs6667065
rs2245801
rs117258232
rs77692002
rs4935063
rs55688436
All
−1e−03
−5e−04
0e+00
5e−04
MR effect size for
'Alpha−synuclein' on 'eGFR'

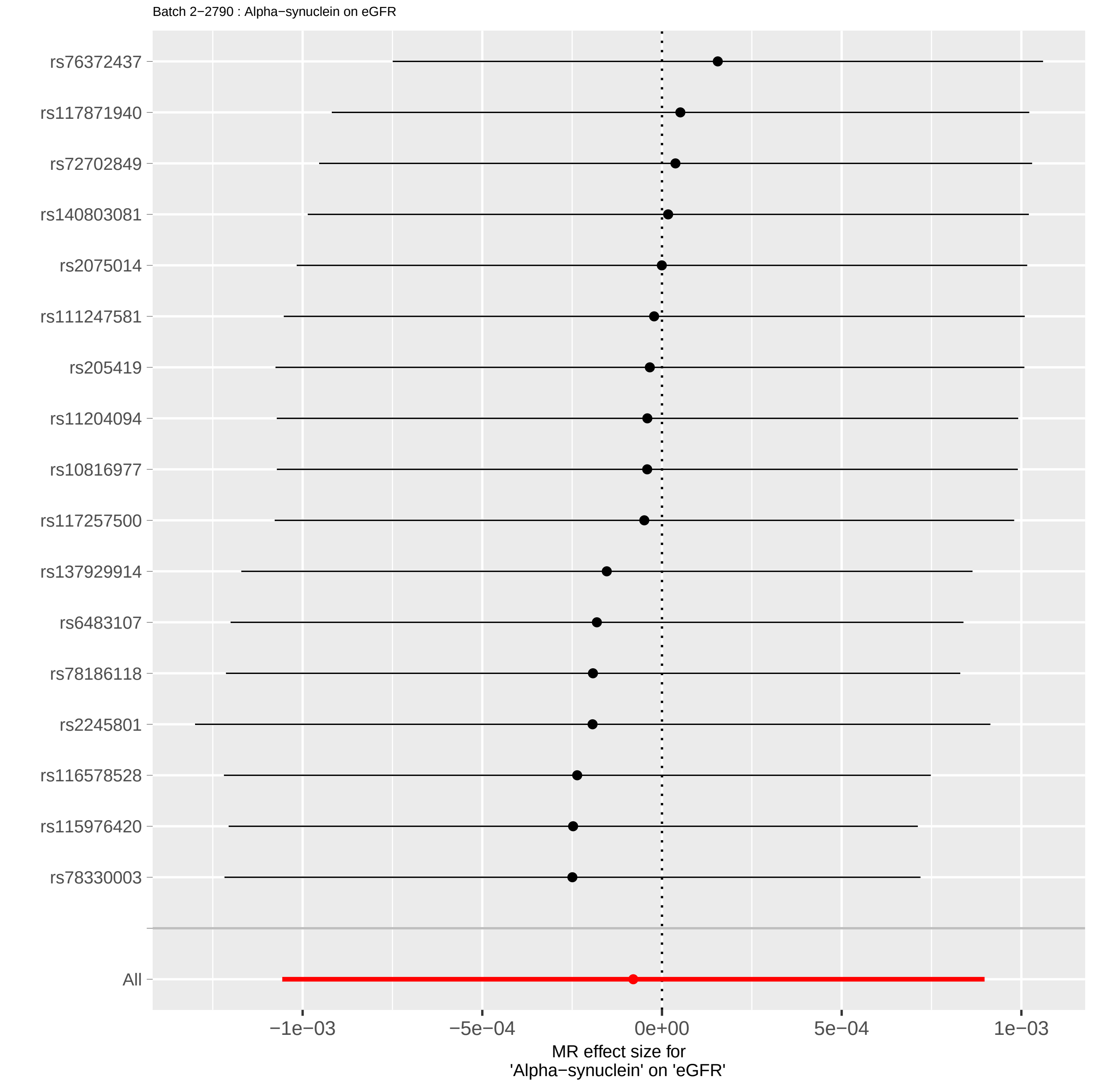

Batch 2−2790 : Alpha−synuclein on eGFR
rs76372437
rs117871940
rs72702849
rs140803081
rs2075014
rs111247581
rs205419
rs11204094
rs10816977
rs117257500
rs137929914
rs6483107
rs78186118
rs2245801
rs116578528
rs115976420
rs78330003
All
−1e−03
−5e−04
0e+00
5e−04
1e−03
MR effect size for
'Alpha−synuclein' on 'eGFR'

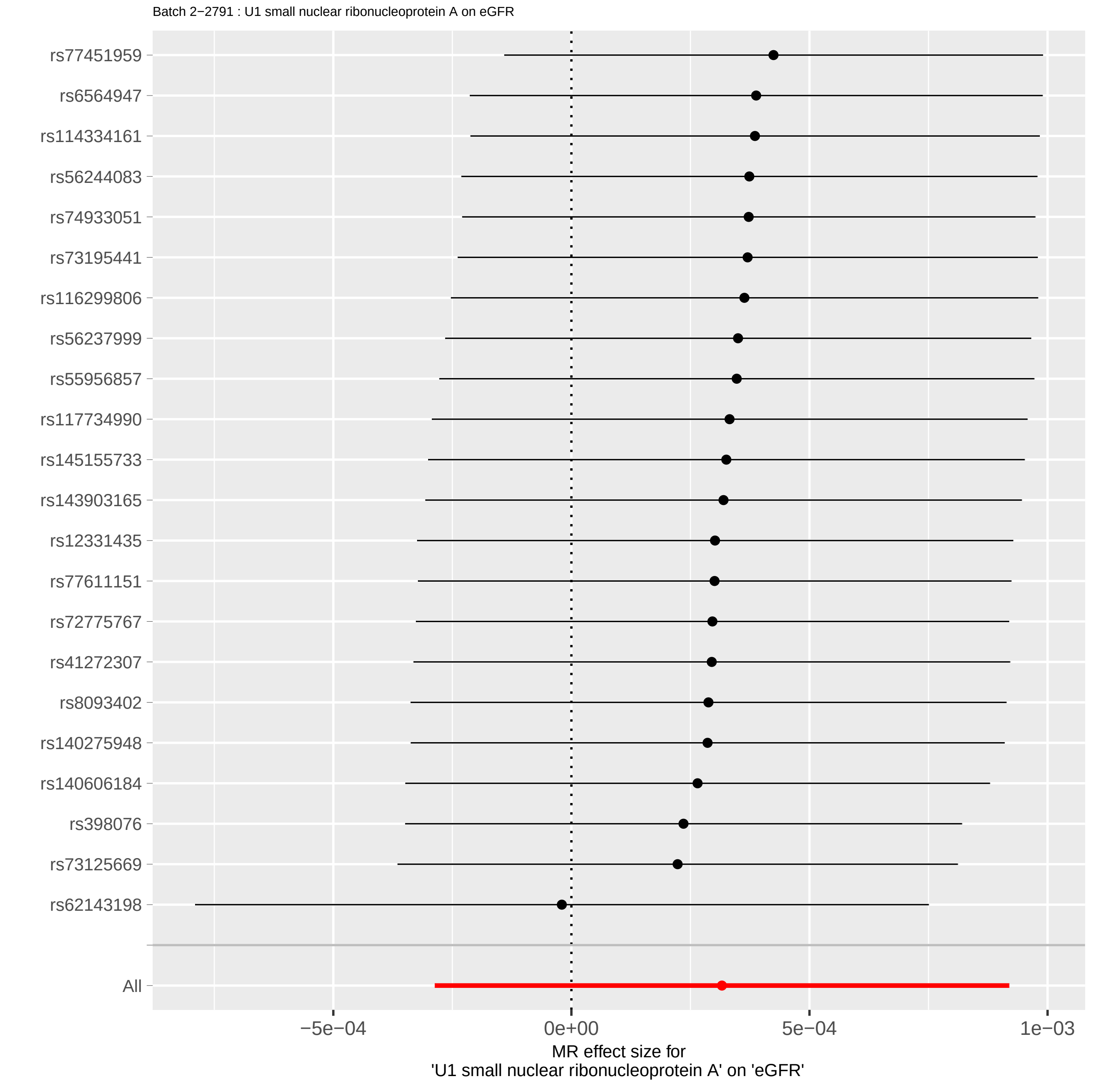

Batch 2−2791 : U1 small nuclear ribonucleoprotein A on eGFR
rs77451959
rs6564947
rs114334161
rs56244083
rs74933051
rs73195441
rs116299806
rs56237999
rs55956857
rs117734990
rs145155733
rs143903165
rs12331435
rs77611151
rs72775767
rs41272307
rs8093402
rs140275948
rs140606184
rs398076
rs73125669
rs62143198
All
−5e−04
0e+00
5e−04
1e−03
MR effect size for
'U1 small nuclear ribonucleoprotein A' on 'eGFR'

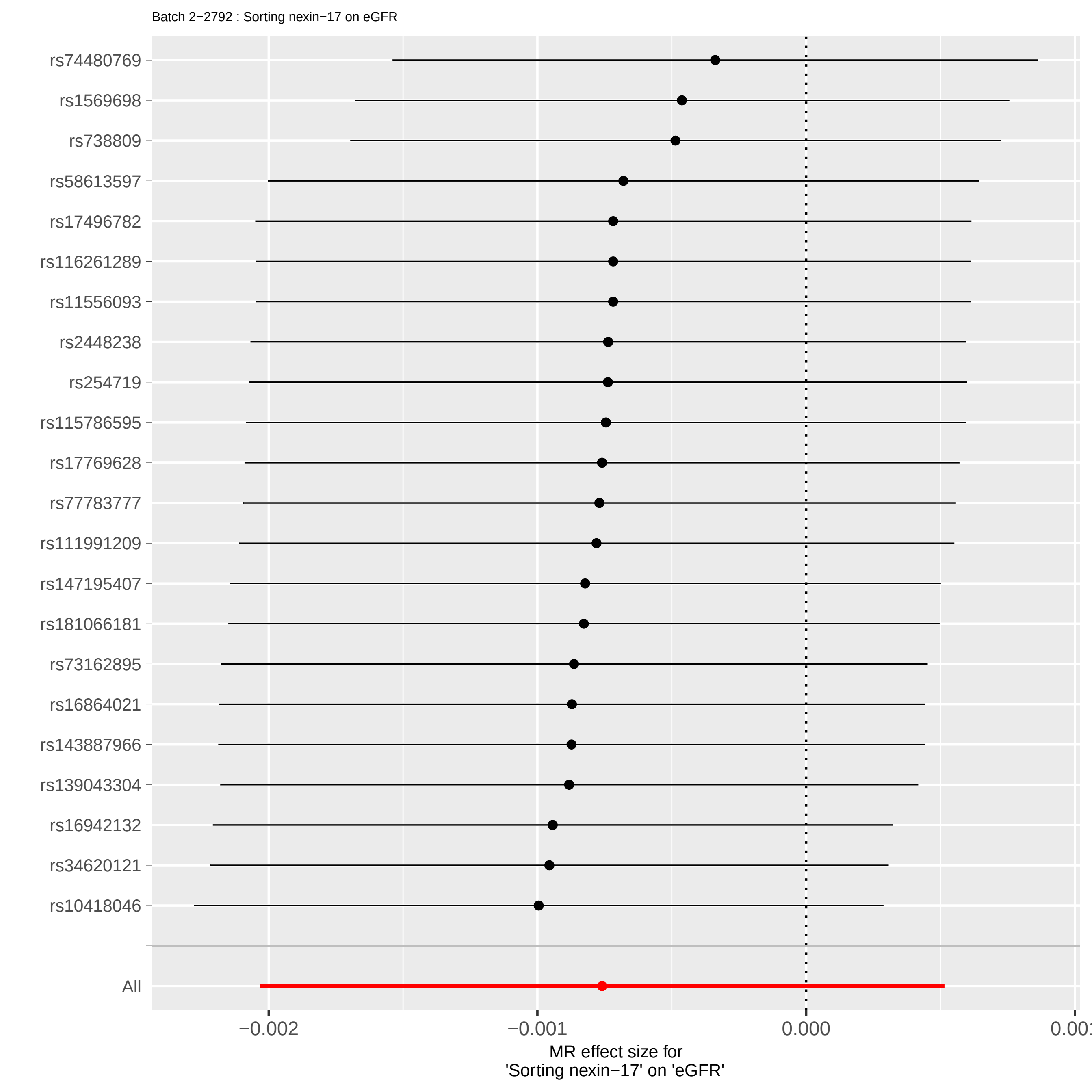

Batch 2−2792 : Sorting nexin−17 on eGFR
rs74480769
rs1569698
rs738809
rs58613597
rs17496782
rs116261289
rs11556093
rs2448238
rs254719
rs115786595
rs17769628
rs77783777
rs111991209
rs147195407
rs181066181
rs73162895
rs16864021
rs143887966
rs139043304
rs16942132
rs34620121
rs10418046
All
−0.002
−0.001
0.000
MR effect size for
'Sorting nexin−17' on 'eGFR'

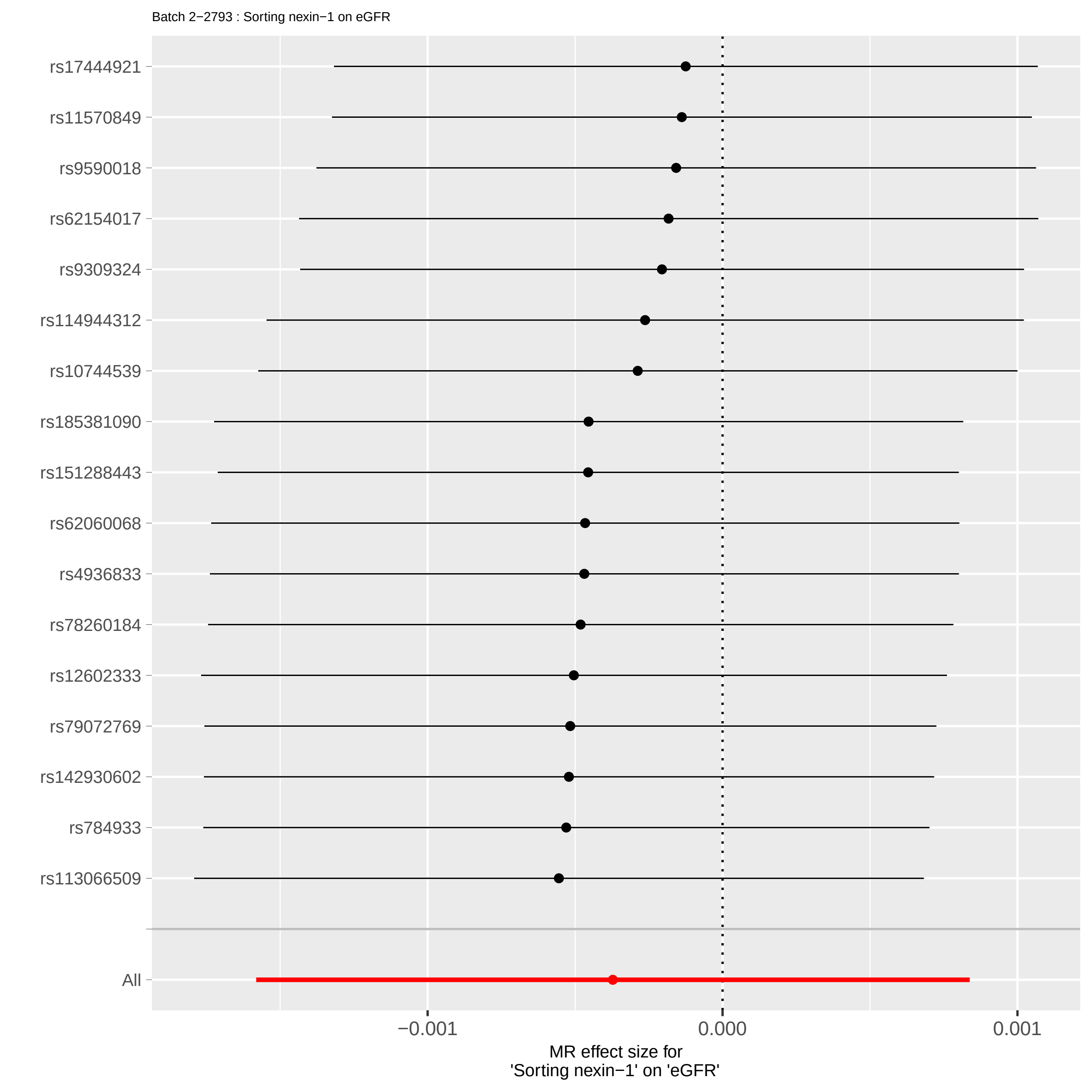

Batch 2−2793 : Sorting nexin−1 on eGFR
rs17444921
rs11570849
rs9590018
rs62154017
rs9309324
rs114944312
rs10744539
rs185381090
rs151288443
rs62060068
rs4936833
rs78260184
rs12602333
rs79072769
rs142930602
rs784933
rs113066509
All
−0.001
0.000
0.001
MR effect size for
'Sorting nexin−1' on 'eGFR'

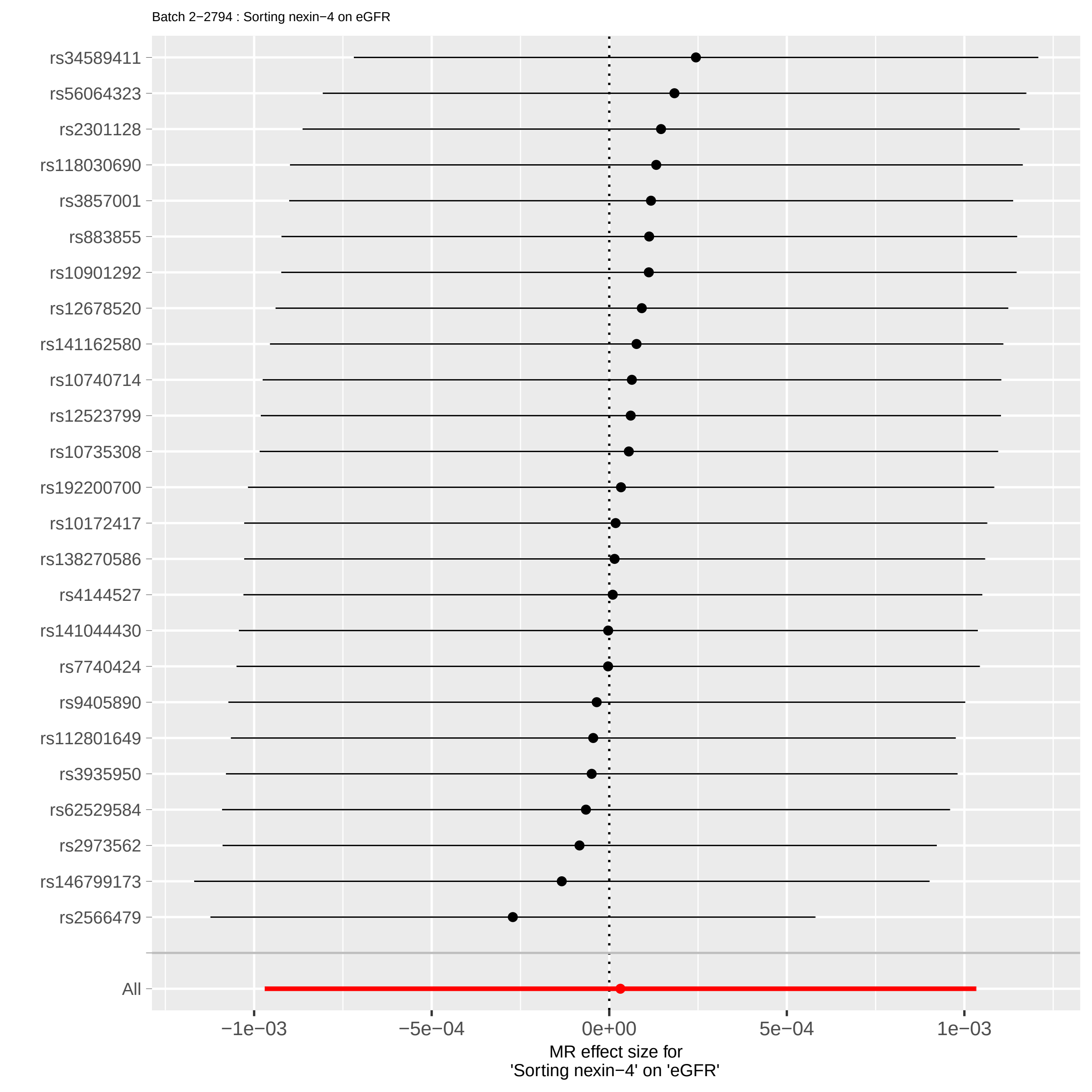

Batch 2−2794 : Sorting nexin−4 on eGFR
rs34589411
rs56064323
rs2301128
rs118030690
rs3857001
rs883855
rs10901292
rs12678520
rs141162580
rs10740714
rs12523799
rs10735308
rs192200700
rs10172417
rs138270586
rs4144527
rs141044430
rs7740424
rs9405890
rs112801649
rs3935950
rs62529584
rs2973562
rs146799173
rs2566479
All
−1e−03
−5e−04
0e+00
5e−04
1e−03
MR effect size for
'Sorting nexin−4' on 'eGFR'

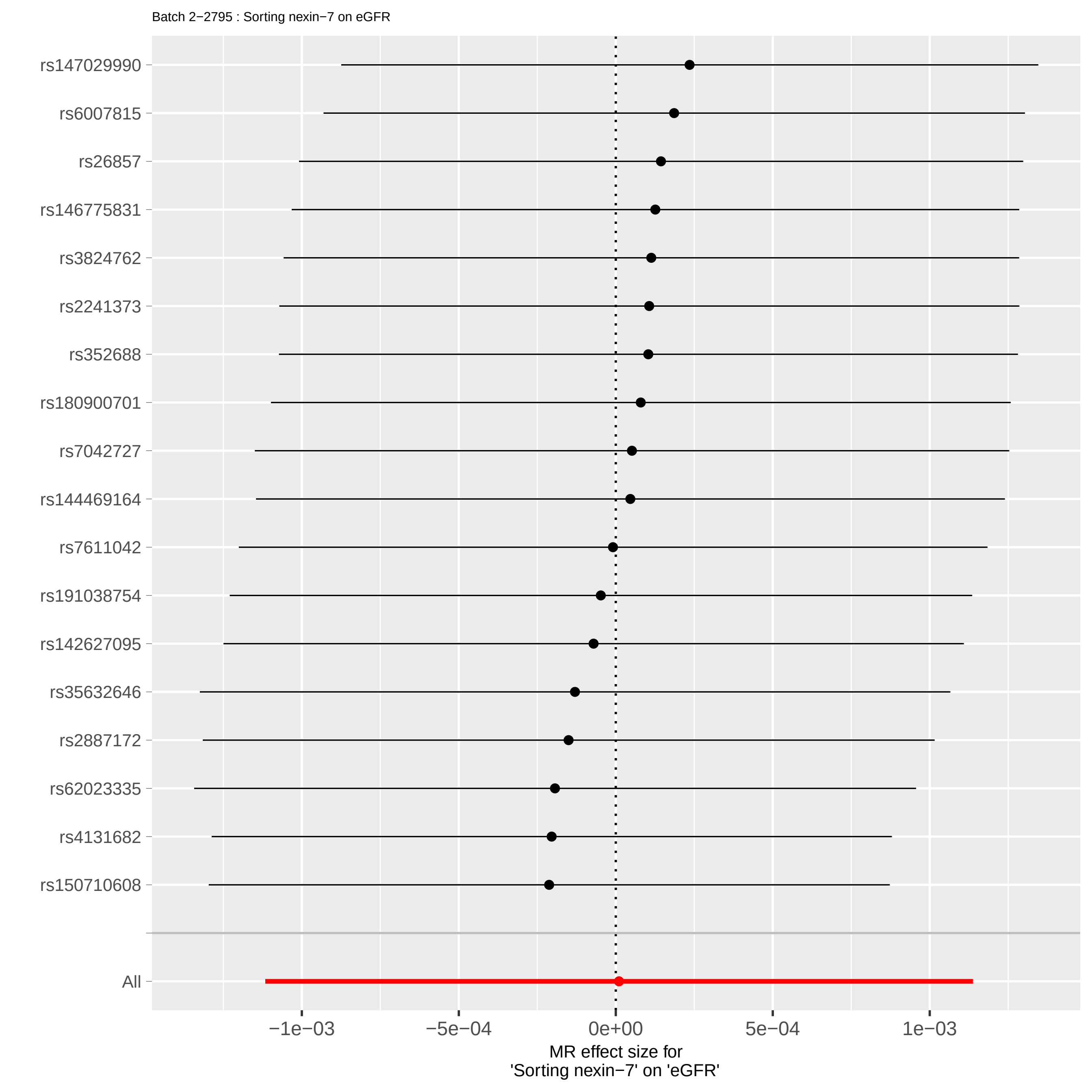

Batch 2−2795 : Sorting nexin−7 on eGFR
rs147029990
rs6007815
rs26857
rs146775831
rs3824762
rs2241373
rs352688
rs180900701
rs7042727
rs144469164
rs7611042
rs191038754
rs142627095
rs35632646
rs2887172
rs62023335
rs4131682
rs150710608
All
−1e−03
−5e−04
0e+00
5e−04
1e−03
MR effect size for
'Sorting nexin−7' on 'eGFR'

Batch 2−2796 : Sorting nexin−8 on eGFR

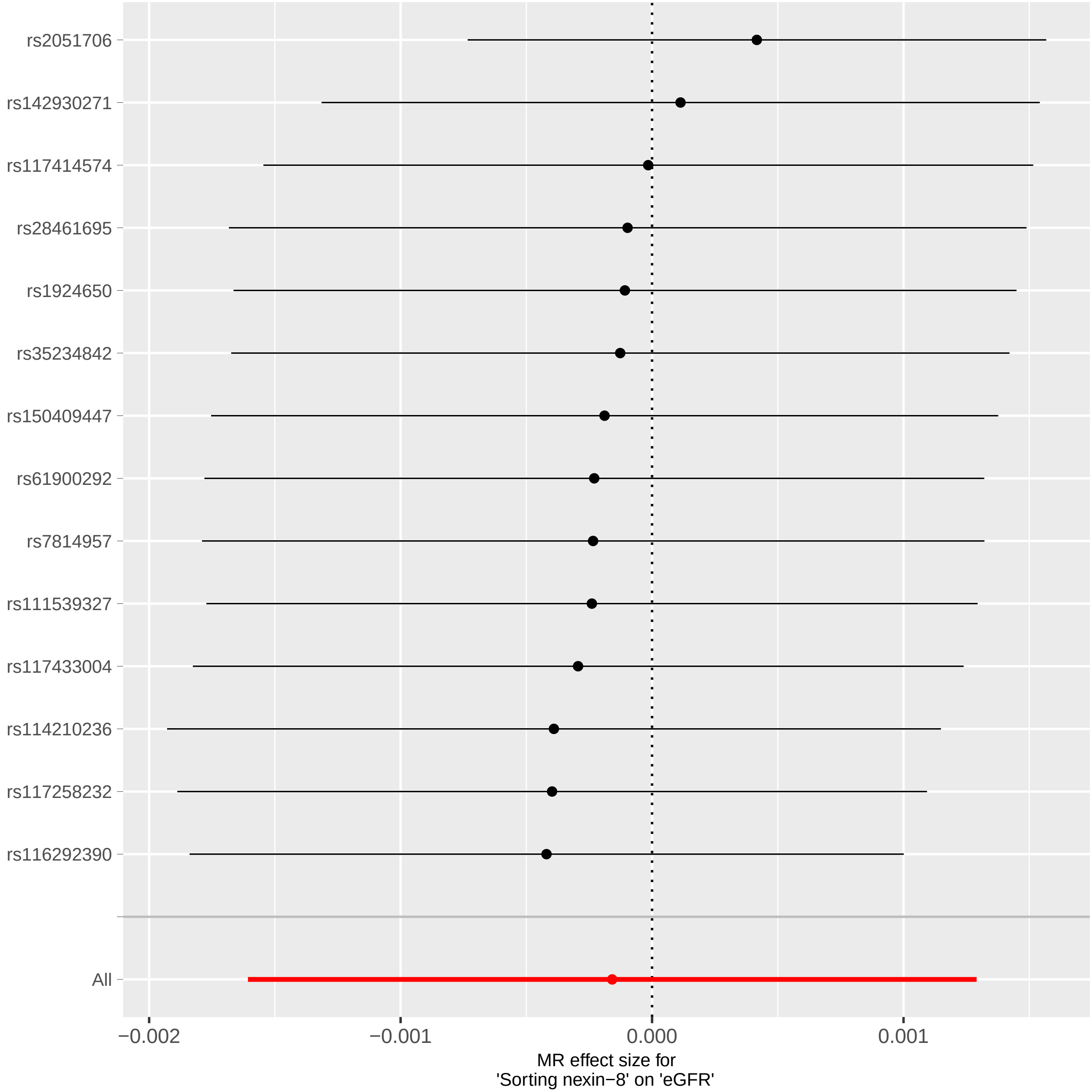

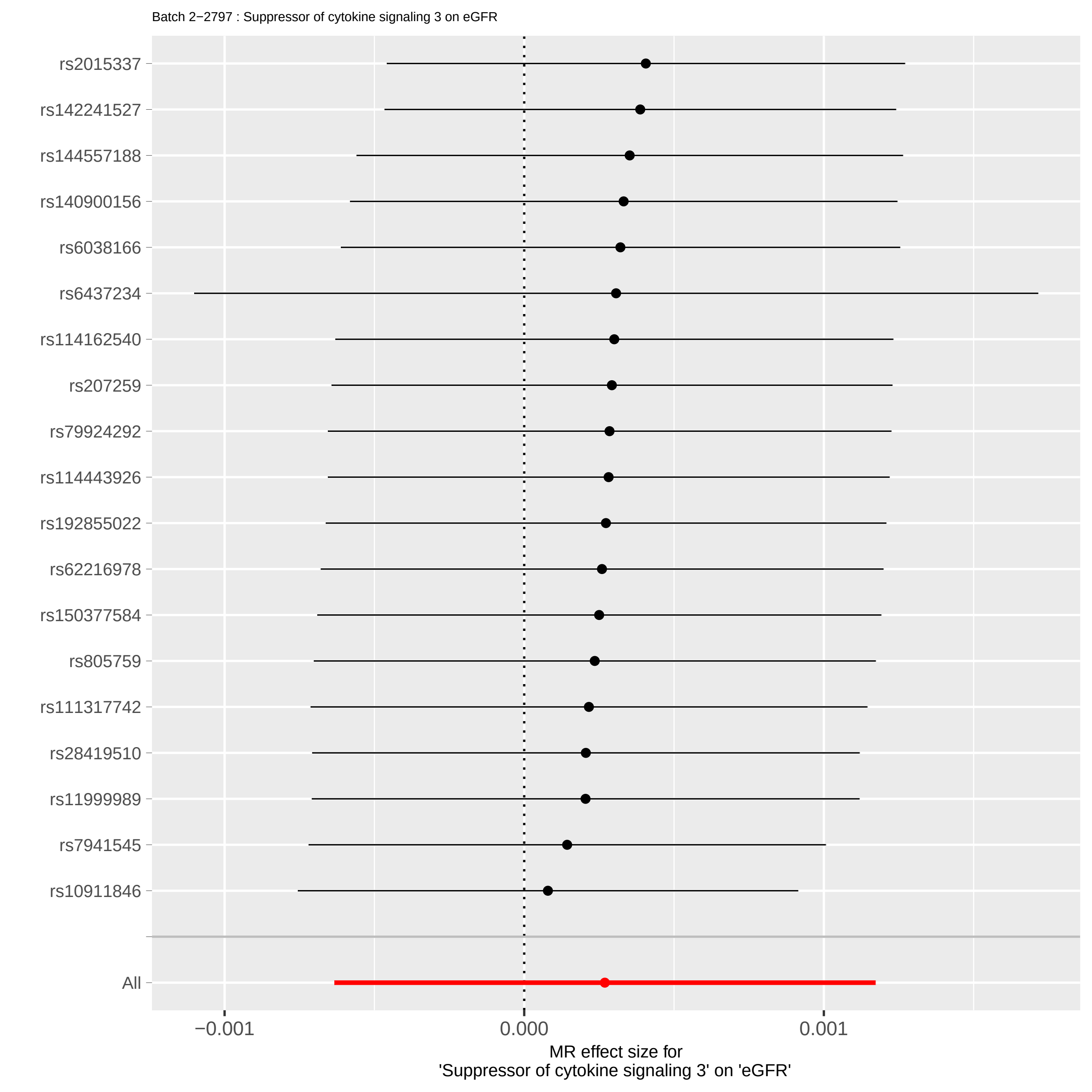
Batch 2−2797 : Suppressor of cytokine signaling 3 on eGFR
rs2015337
rs142241527
rs144557188
rs140900156
rs6038166
rs6437234
rs114162540
rs207259
rs79924292
rs114443926
rs192855022
rs62216978
rs150377584
rs805759
rs111317742
rs28419510
rs11999989
rs7941545
rs10911846
All
−0.001
0.000
0.001
MR effect size for
'Suppressor of cytokine signaling 3' on 'eGFR'

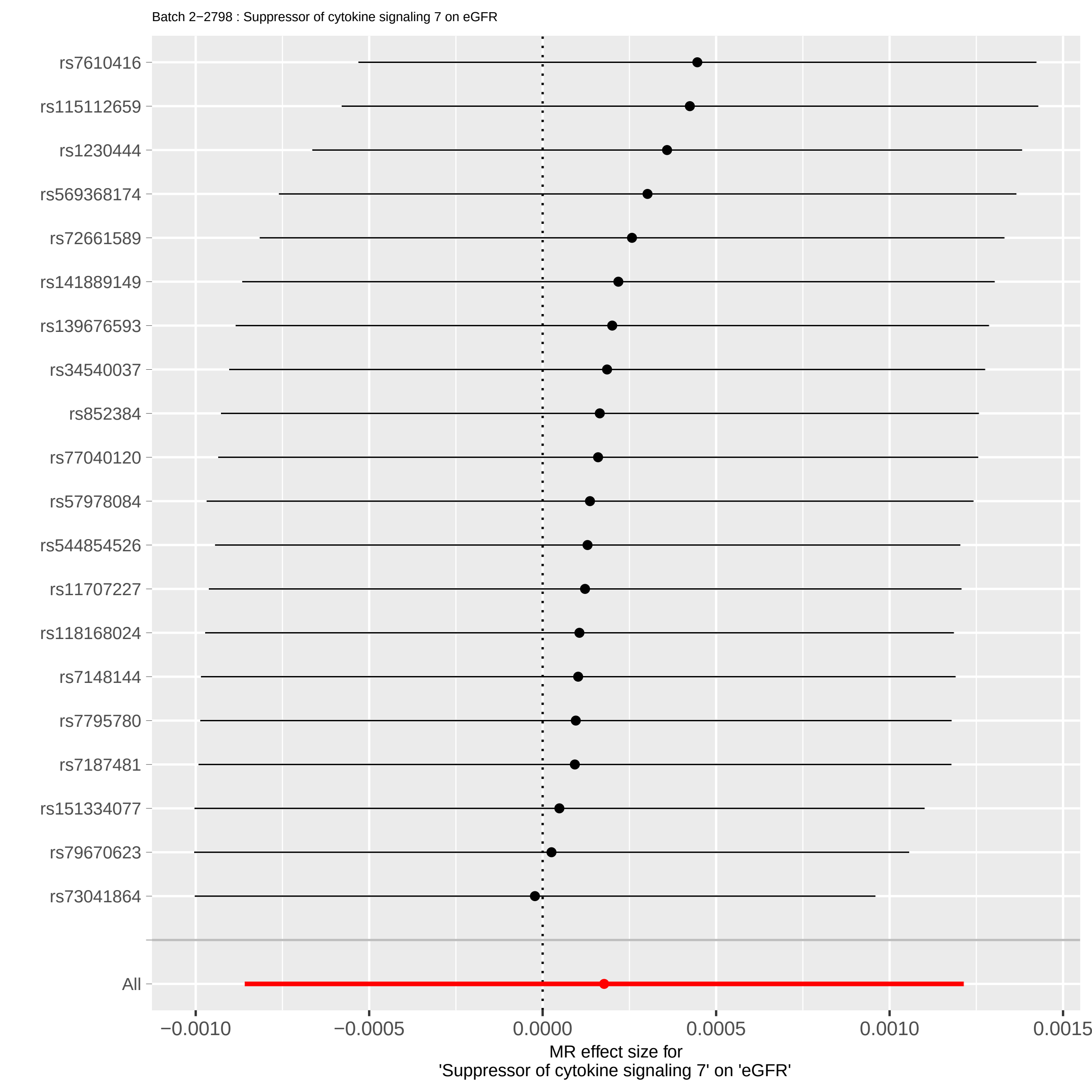

Batch 2−2798 : Suppressor of cytokine signaling 7 on eGFR
rs7610416
rs115112659
rs1230444
rs569368174
rs72661589
rs141889149
rs139676593
rs34540037
rs852384
rs77040120
rs57978084
rs544854526
rs11707227
rs118168024
rs7148144
rs7795780
rs7187481
rs151334077
rs79670623
rs73041864
All
−0.0010
−0.0005
0.0000
0.0005
0.0010
0.0015
MR effect size for
'Suppressor of cytokine signaling 7' on 'eGFR'

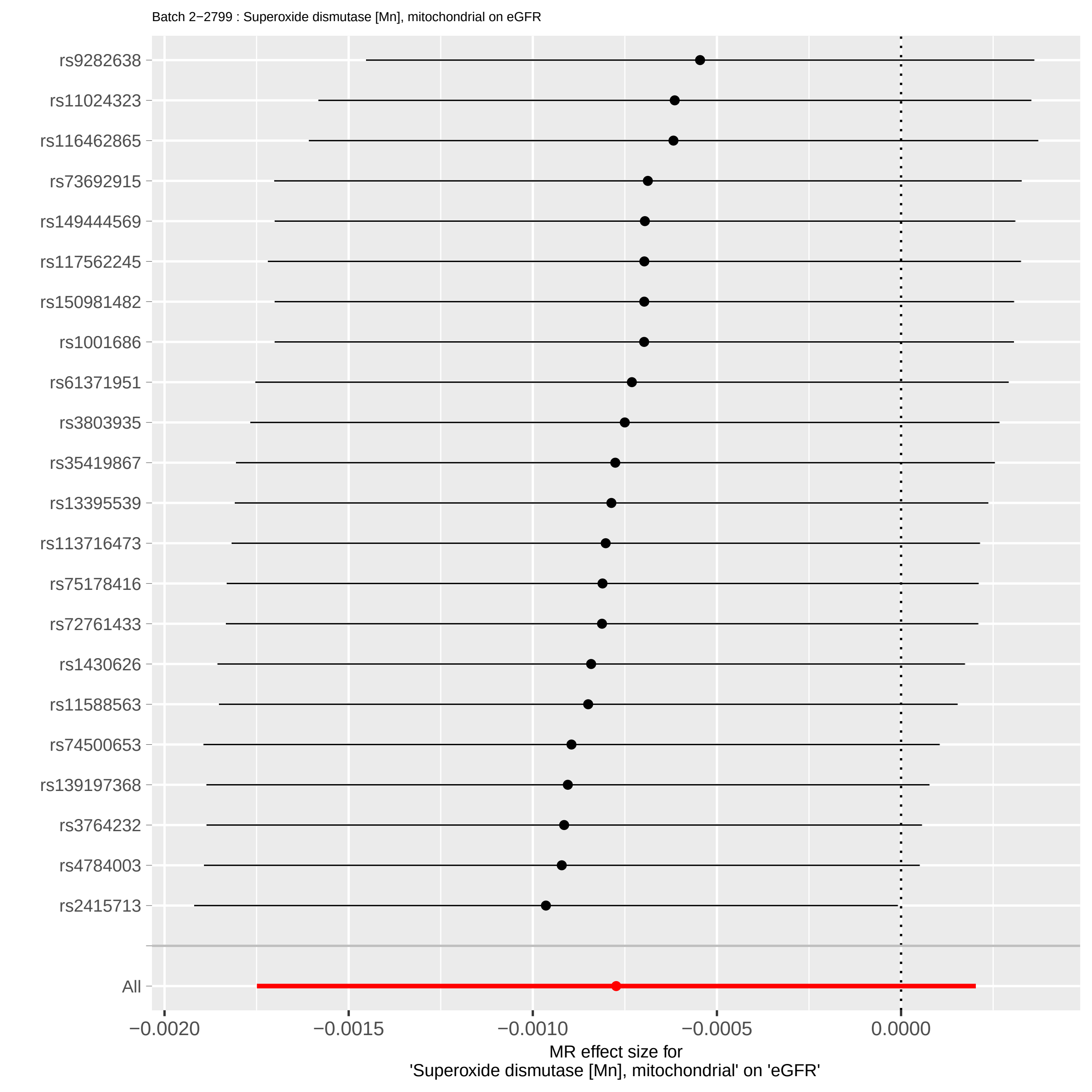

Batch 2−2799 : Superoxide dismutase [Mn], mitochondrial on eGFR
rs9282638
rs11024323
rs116462865
rs73692915
rs149444569
rs117562245
rs150981482
rs1001686
rs61371951
rs3803935
rs35419867
rs13395539
rs113716473
rs75178416
rs72761433
rs1430626
rs11588563
rs74500653
rs139197368
rs3764232
rs4784003
rs2415713
All
−0.0020
−0.0015
−0.0010
−0.0005
0.0000
MR effect size for
'Superoxide dismutase [Mn], mitochondrial' on 'eGFR'

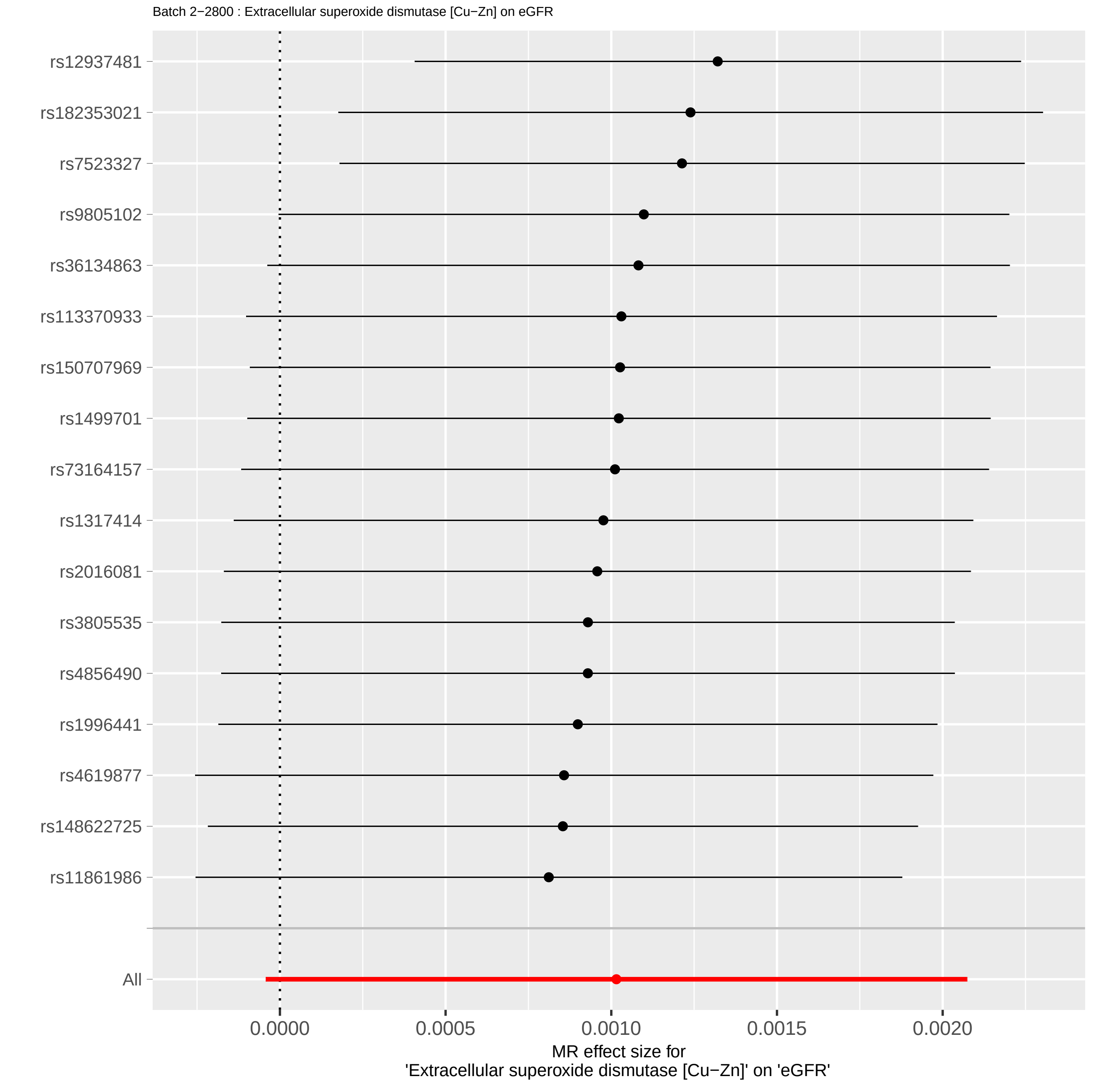
Batch 2−2800 : Extracellular superoxide dismutase [Cu−Zn] on eGFR
rs12937481
rs182353021
rs7523327
rs9805102
rs36134863
rs113370933
rs150707969
rs1499701
rs73164157
rs1317414
rs2016081
rs3805535
rs4856490
rs1996441
rs4619877
rs148622725
rs11861986
All
0.0000
0.0005
0.0010
0.0015
0.0020
MR effect size for
'Extracellular superoxide dismutase [Cu−Zn]' on 'eGFR'

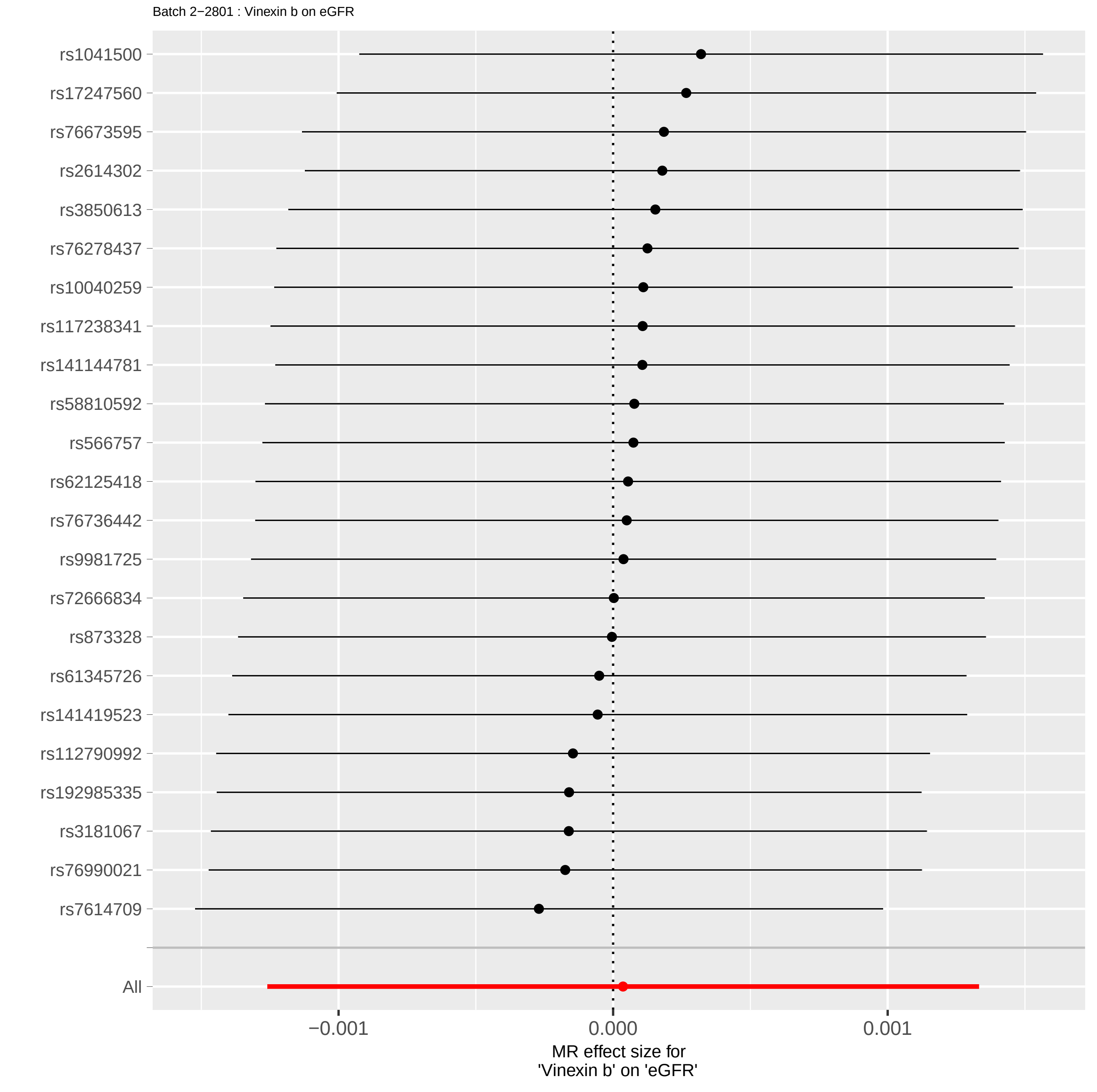

Batch 2−2801 : Vinexin b on eGFR
rs1041500
rs17247560
rs76673595
rs2614302
rs3850613
rs76278437
rs10040259
rs117238341
rs141144781
rs58810592
rs566757
rs62125418
rs76736442
rs9981725
rs72666834
rs873328
rs61345726
rs141419523
rs112790992
rs192985335
rs3181067
rs76990021
rs7614709
All
−0.001
0.000
0.001
MR effect size for
'Vinexin b' on 'eGFR'

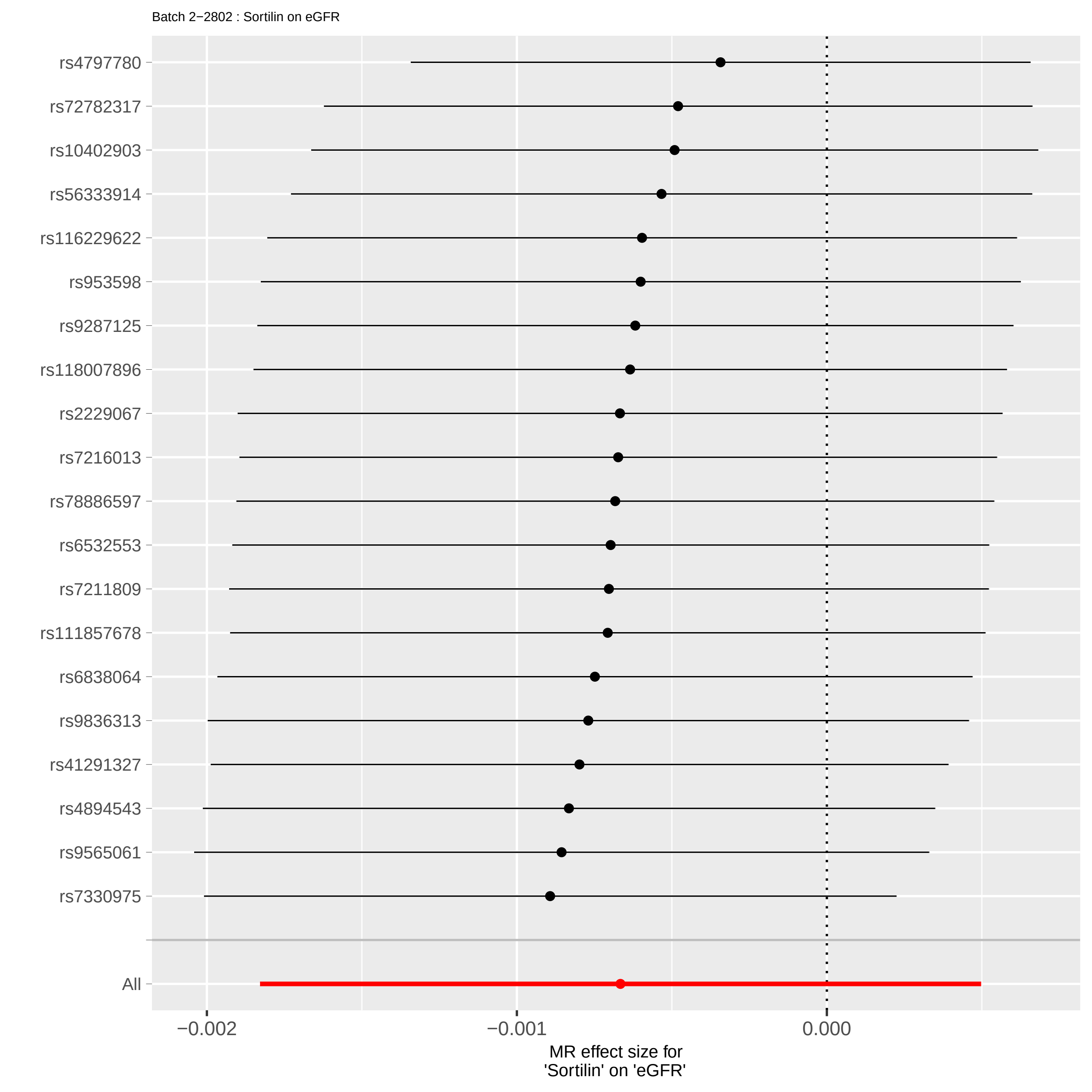

Batch 2−2802 : Sortilin on eGFR
rs4797780
rs72782317
rs10402903
rs56333914
rs116229622
rs953598
rs9287125
rs118007896
rs2229067
rs7216013
rs78886597
rs6532553
rs7211809
rs111857678
rs6838064
rs9836313
rs41291327
rs4894543
rs9565061
rs7330975
All
−0.002
−0.001
0.000
MR effect size for
'Sortilin' on 'eGFR'

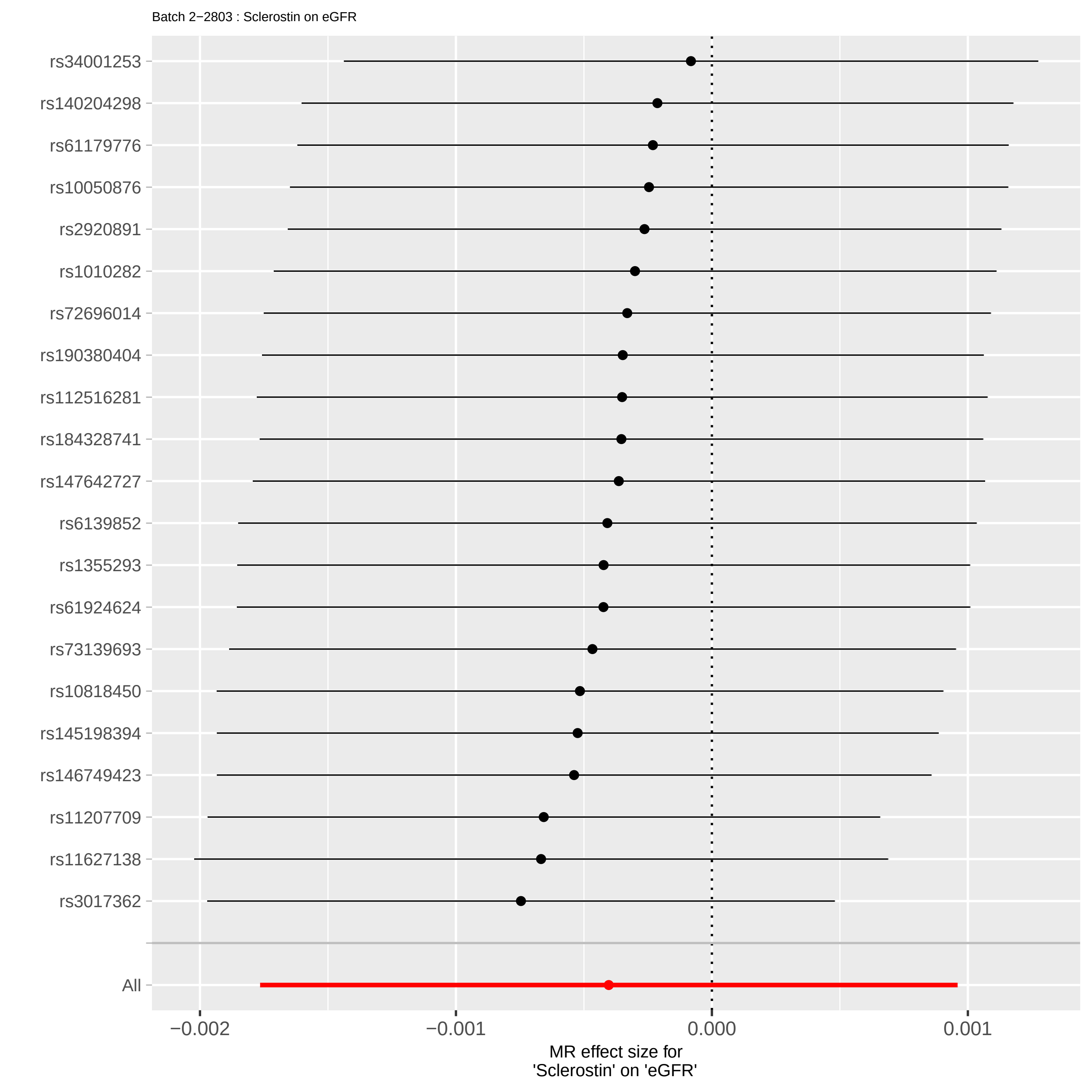

Batch 2−2803 : Sclerostin on eGFR
rs34001253
rs140204298
rs61179776
rs10050876
rs2920891
rs1010282
rs72696014
rs190380404
rs112516281
rs184328741
rs147642727
rs6139852
rs1355293
rs61924624
rs73139693
rs10818450
rs145198394
rs146749423
rs11207709
rs11627138
rs3017362
All
−0.002
−0.001
0.000
0.001
MR effect size for
'Sclerostin' on 'eGFR'

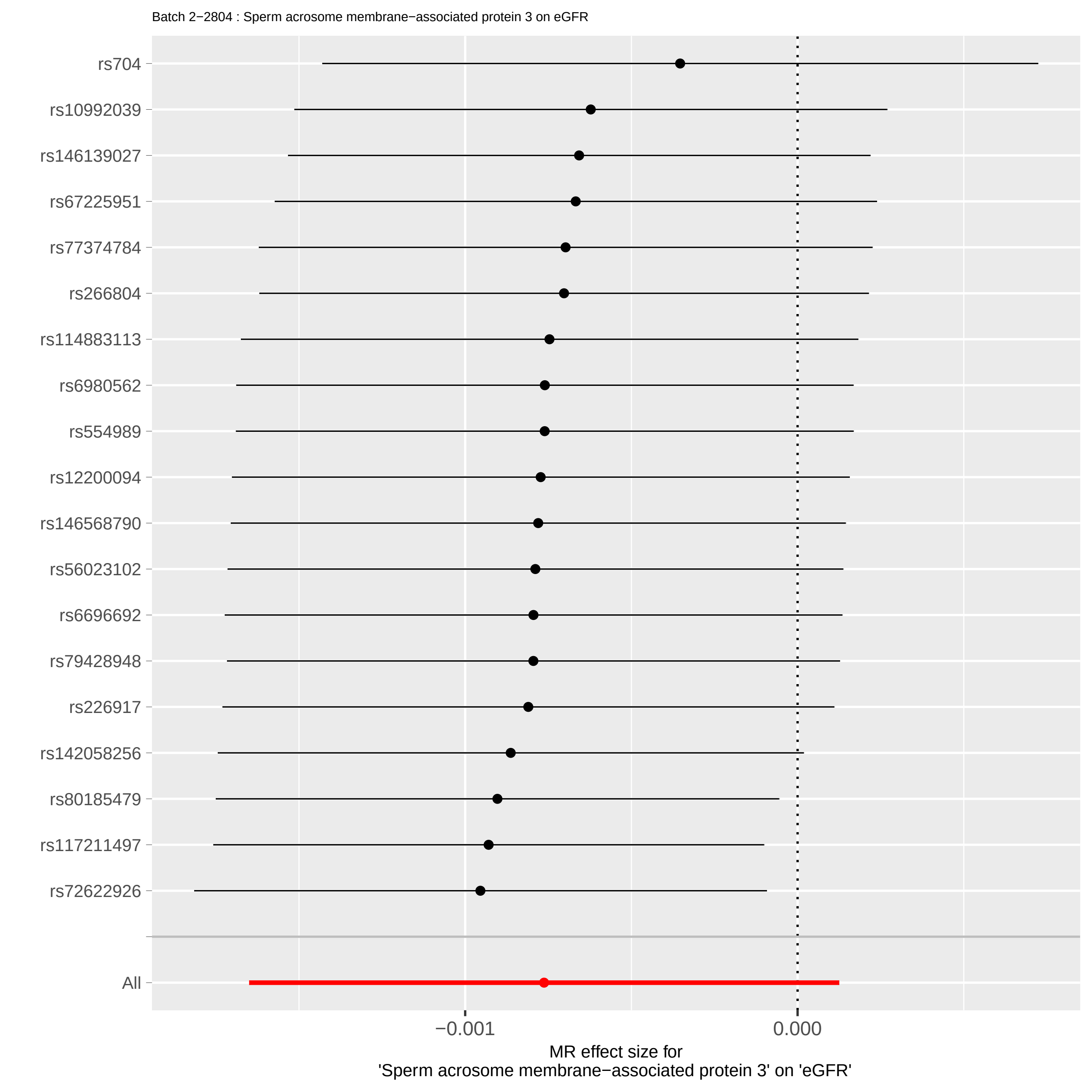

Batch 2−2804 : Sperm acrosome membrane−associated protein 3 on eGFR
rs704
rs10992039
rs146139027
rs67225951
rs77374784
rs266804
rs114883113
rs6980562
rs554989
rs12200094
rs146568790
rs56023102
rs6696692
rs79428948
rs226917
rs142058256
rs80185479
rs117211497
rs72622926
All
−0.001
0.000
MR effect size for
'Sperm acrosome membrane−associated protein 3' on 'eGFR'

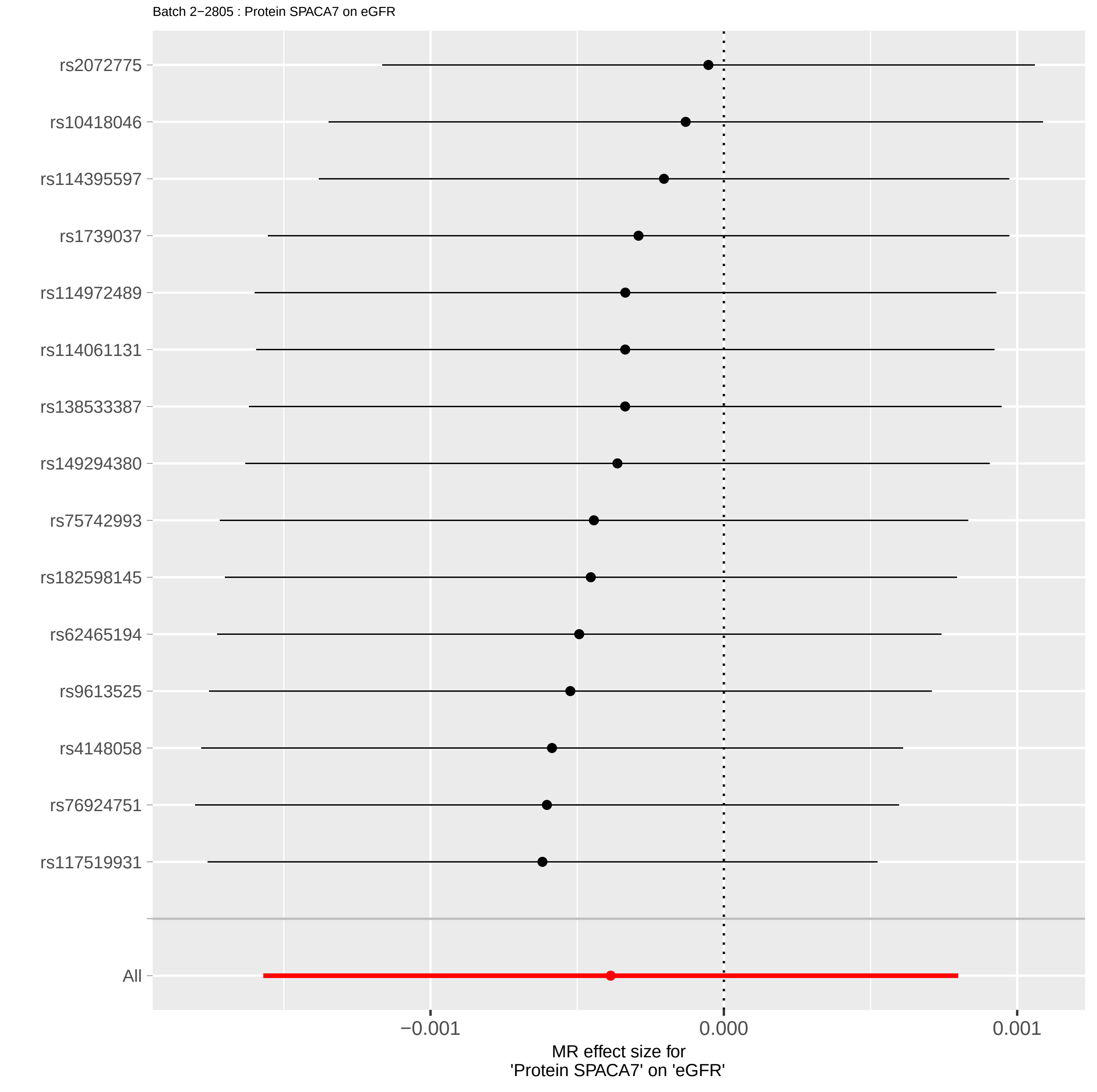

Batch 2−2805 : Protein SPACA7 on eGFR
rs2072775
rs10418046
rs114395597
rs1739037
rs114972489
rs114061131
rs138533387
rs149294380
rs75742993
rs182598145
rs62465194
rs9613525
rs4148058
rs76924751
rs117519931
All
−0.001
0.000
0.001
MR effect size for
'Protein SPACA7' on 'eGFR'

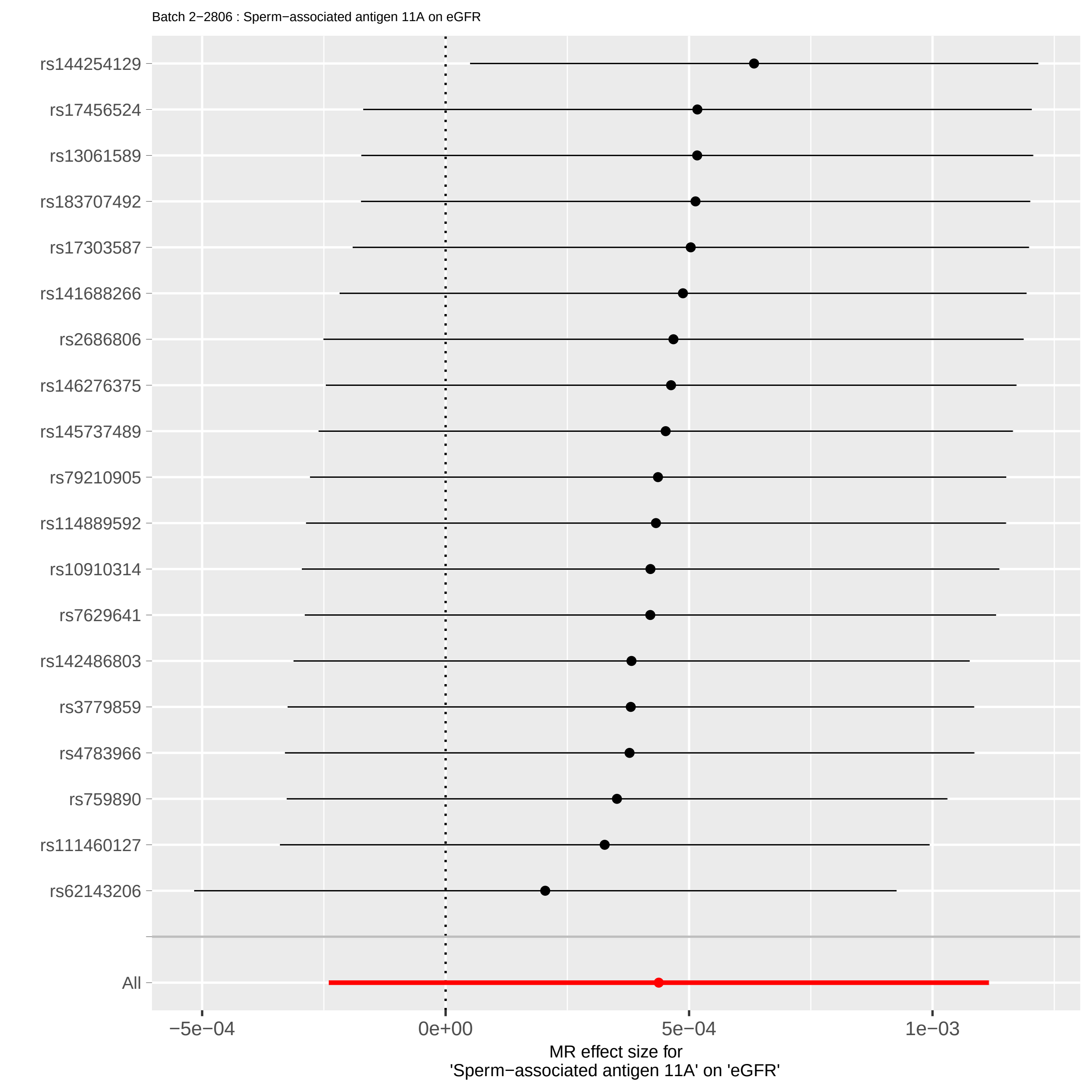

Batch 2−2806 : Sperm−associated antigen 11A on eGFR
rs144254129
rs17456524
rs13061589
rs183707492
rs17303587
rs141688266
rs2686806
rs146276375
rs145737489
rs79210905
rs114889592
rs10910314
rs7629641
rs142486803
rs3779859
rs4783966
rs759890
rs111460127
rs62143206
All
−5e−04
0e+00
5e−04
1e−03
MR effect size for
'Sperm−associated antigen 11A' on 'eGFR'

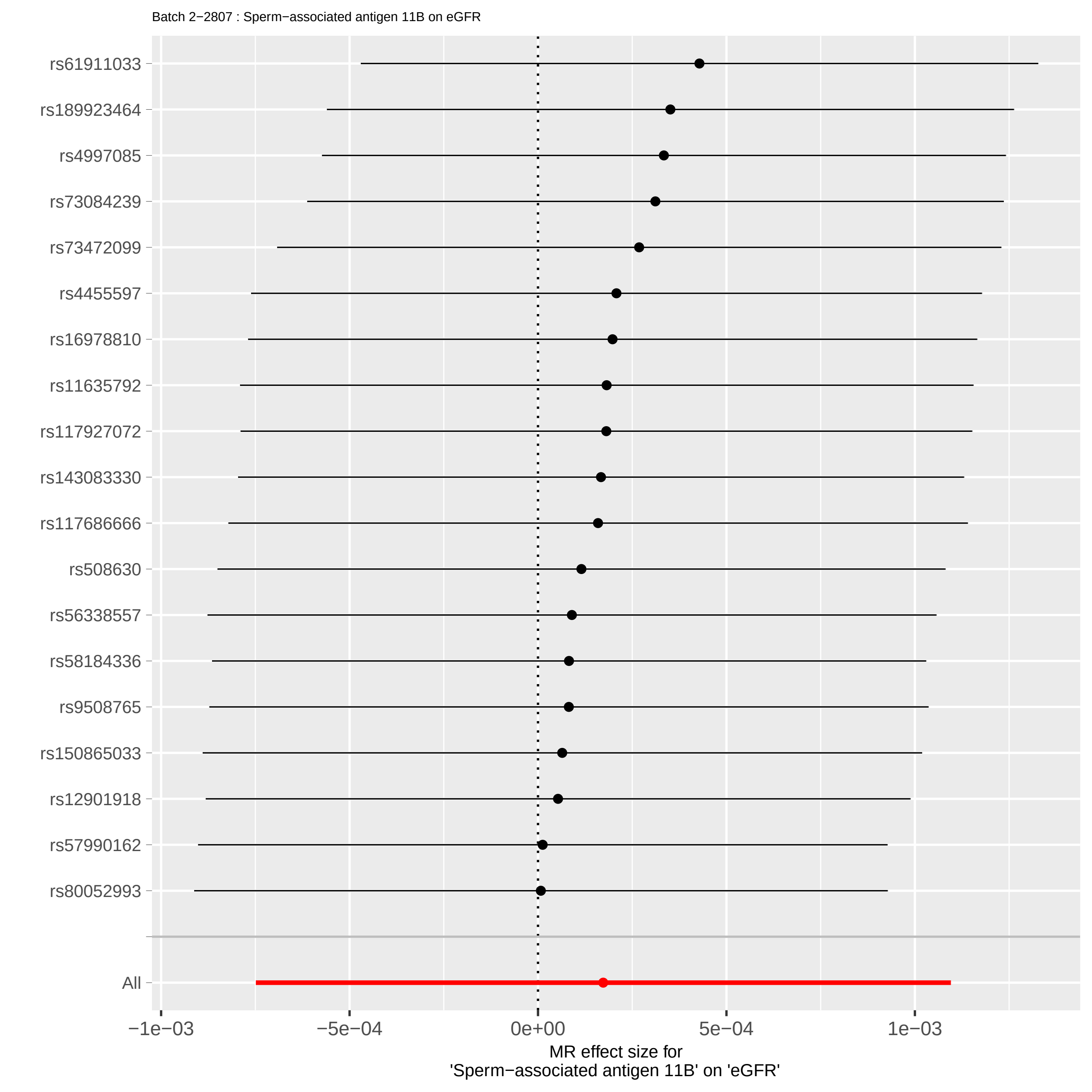

Batch 2−2807 : Sperm−associated antigen 11B on eGFR
rs61911033
rs189923464
rs4997085
rs73084239
rs73472099
rs4455597
rs16978810
rs11635792
rs117927072
rs143083330
rs117686666
rs508630
rs56338557
rs58184336
rs9508765
rs150865033
rs12901918
rs57990162
rs80052993
All
−1e−03
−5e−04
0e+00
5e−04
1e−03
MR effect size for
'Sperm−associated antigen 11B' on 'eGFR'

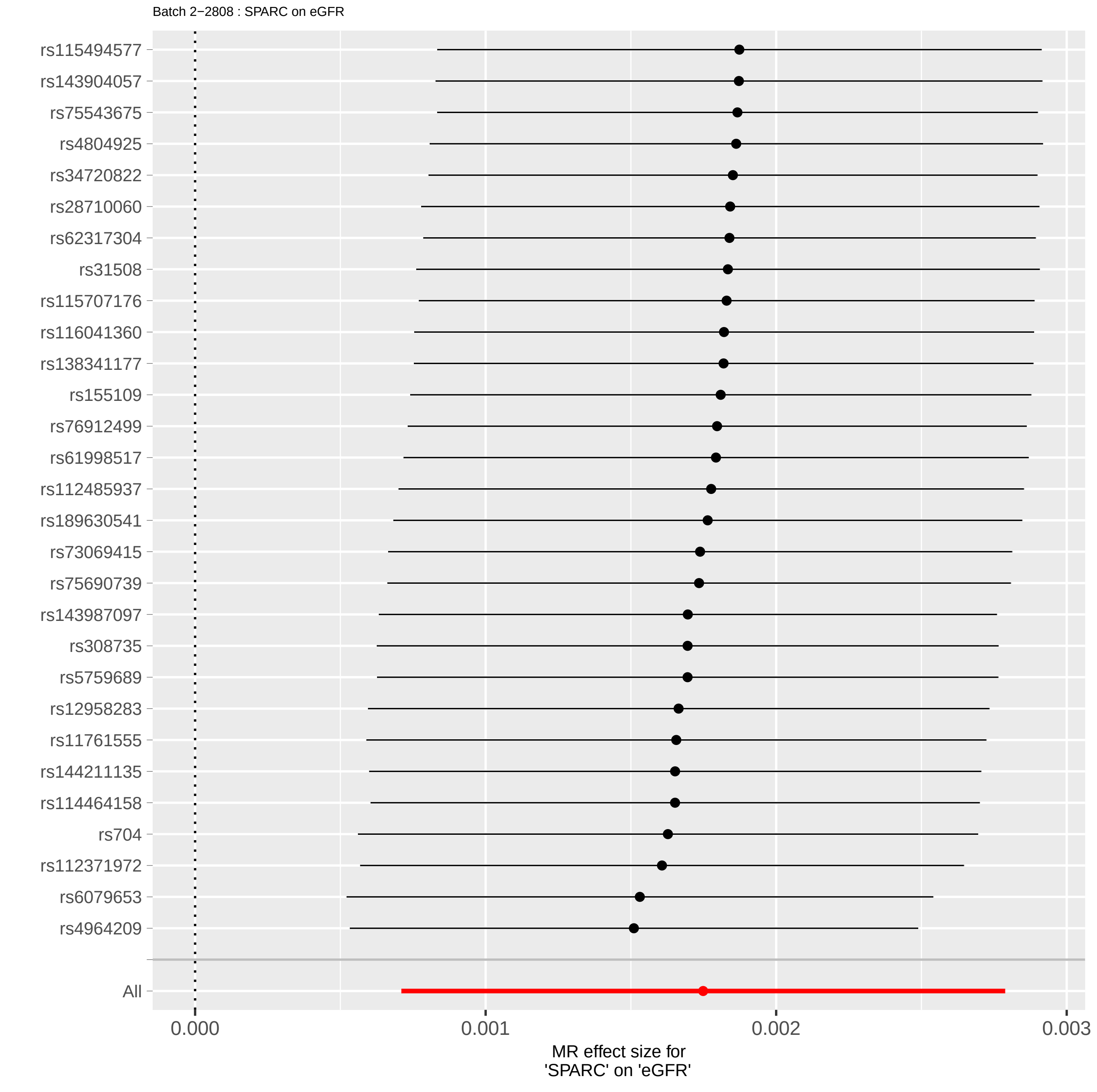

Batch 2−2808 : SPARC on eGFR
rs115494577
rs143904057
rs75543675
rs4804925
rs34720822
rs28710060
rs62317304
rs31508
rs115707176
rs116041360
rs138341177
rs155109
rs76912499
rs61998517
rs112485937
rs189630541
rs73069415
rs75690739
rs143987097
rs308735
rs5759689
rs12958283
rs11761555
rs144211135
rs114464158
rs704
rs112371972
rs6079653
rs4964209
All
0.000
0.001
0.002
0.003
MR effect size for
'SPARC' on 'eGFR'

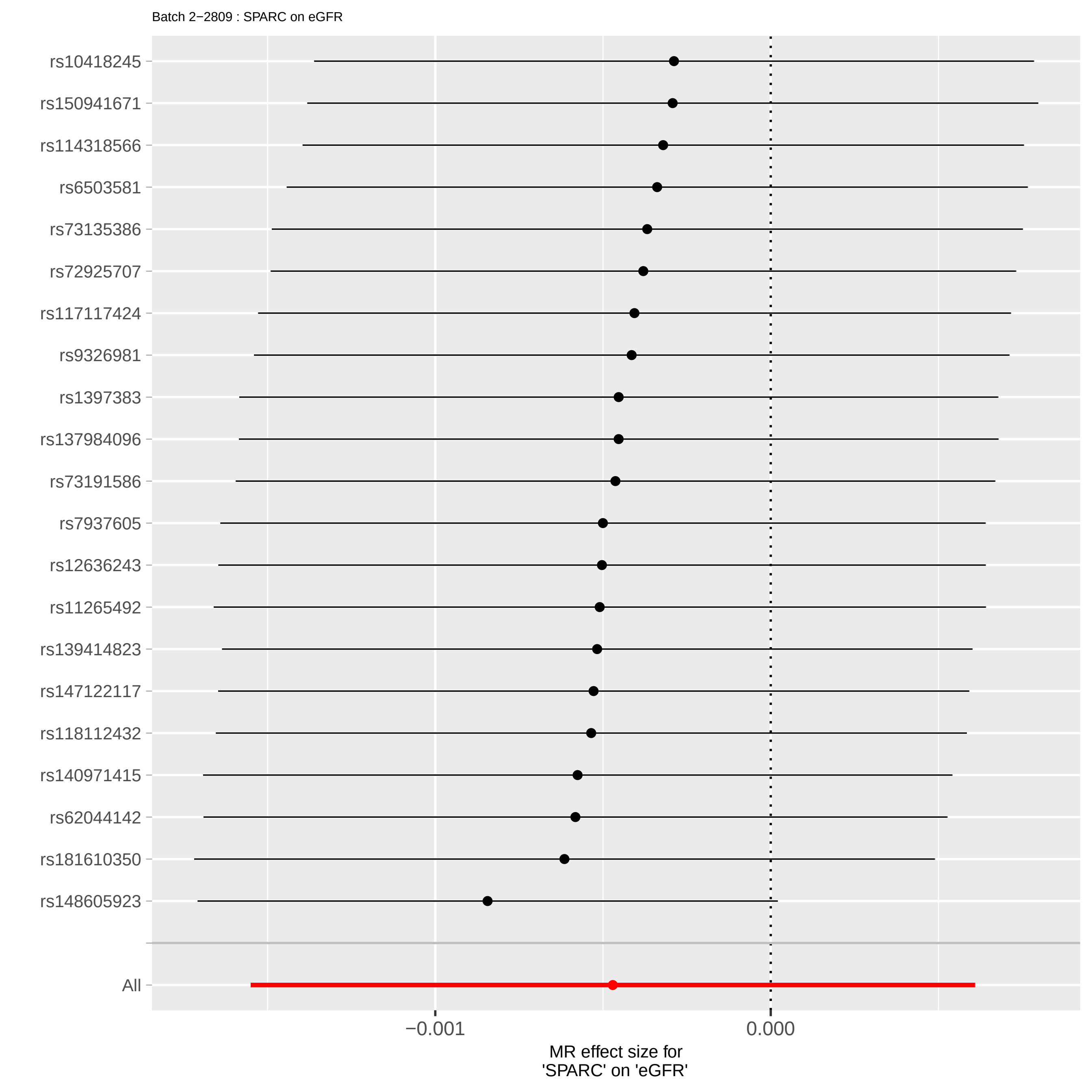
Batch 2−2809 : SPARC on eGFR
rs10418245
rs150941671
rs114318566
rs6503581
rs73135386
rs72925707
rs117117424
rs9326981
rs1397383
rs137984096
rs73191586
rs7937605
rs12636243
rs11265492
rs139414823
rs147122117
rs118112432
rs140971415
rs62044142
rs181610350
rs148605923
All
−0.001
0.000
MR effect size for
'SPARC' on 'eGFR'

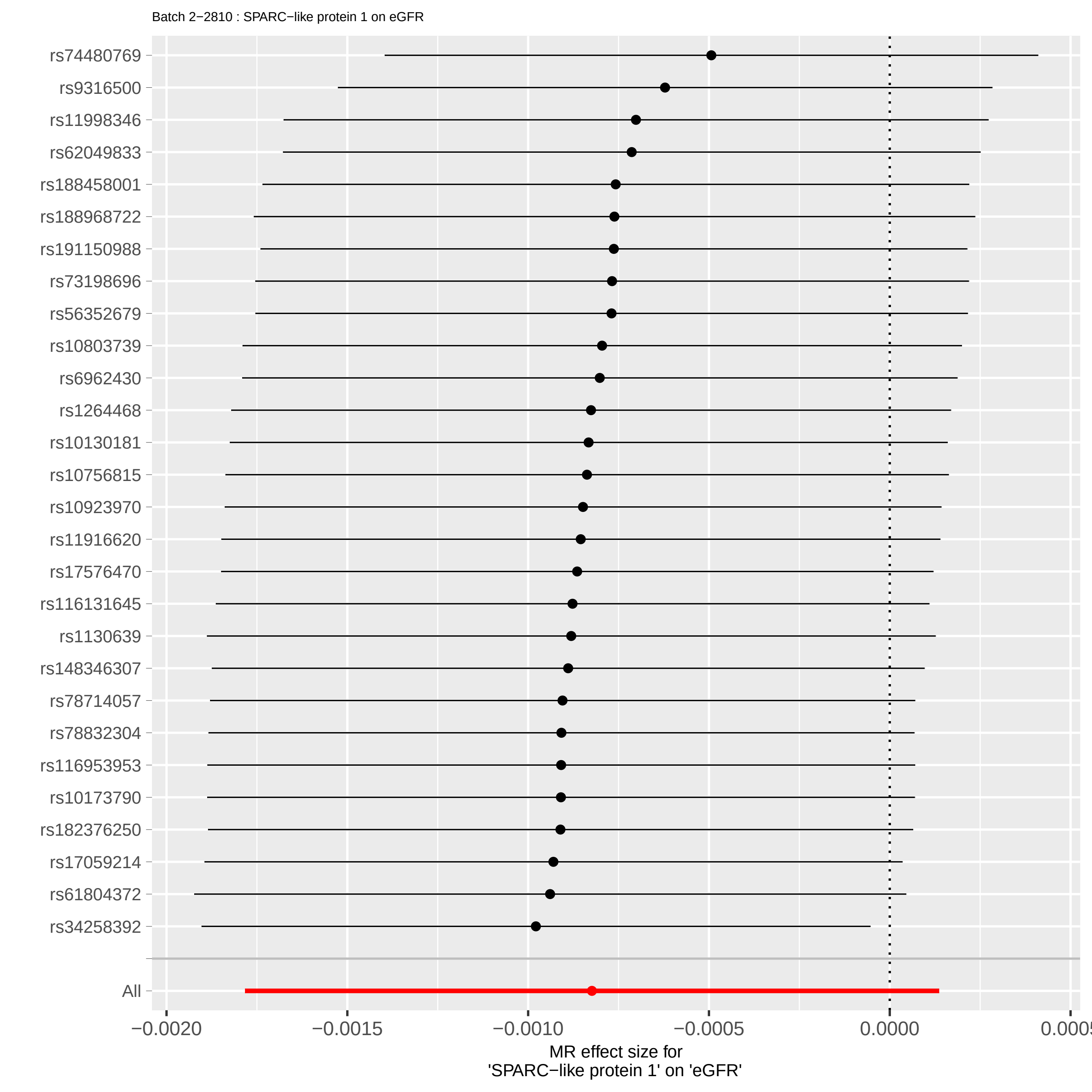

Batch 2−2810 : SPARC−like protein 1 on eGFR
rs74480769
rs9316500
rs11998346
rs62049833
rs188458001
rs188968722
rs191150988
rs73198696
rs56352679
rs10803739
rs6962430
rs1264468
rs10130181
rs10756815
rs10923970
rs11916620
rs17576470
rs116131645
rs1130639
rs148346307
rs78714057
rs78832304
rs116953953
rs10173790
rs182376250
rs17059214
rs61804372
rs34258392
All
−0.0020
−0.0015
−0.0010
−0.0005
0.0000
MR effect size for
'SPARC−like protein 1' on 'eGFR'

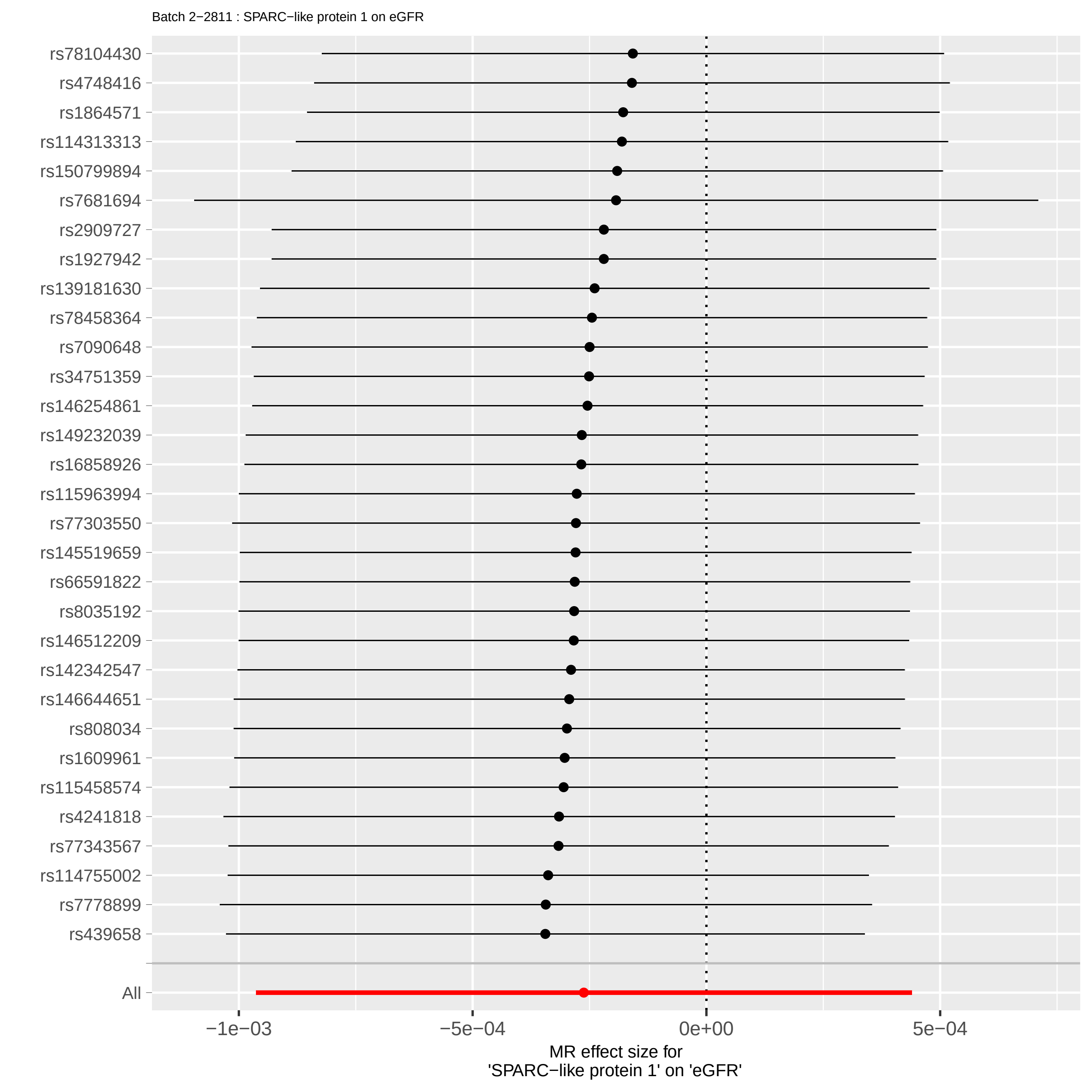
Batch 2−2811 : SPARC−like protein 1 on eGFR
rs78104430
rs4748416
rs1864571
rs114313313
rs150799894
rs7681694
rs2909727
rs1927942
rs139181630
rs78458364
rs7090648
rs34751359
rs146254861
rs149232039
rs16858926
rs115963994
rs77303550
rs145519659
rs66591822
rs8035192
rs146512209
rs142342547
rs146644651
rs808034
rs1609961
rs115458574
rs4241818
rs77343567
rs114755002
rs7778899
rs439658
All
−1e−03
−5e−04
0e+00
5e−04
MR effect size for
'SPARC−like protein 1' on 'eGFR'

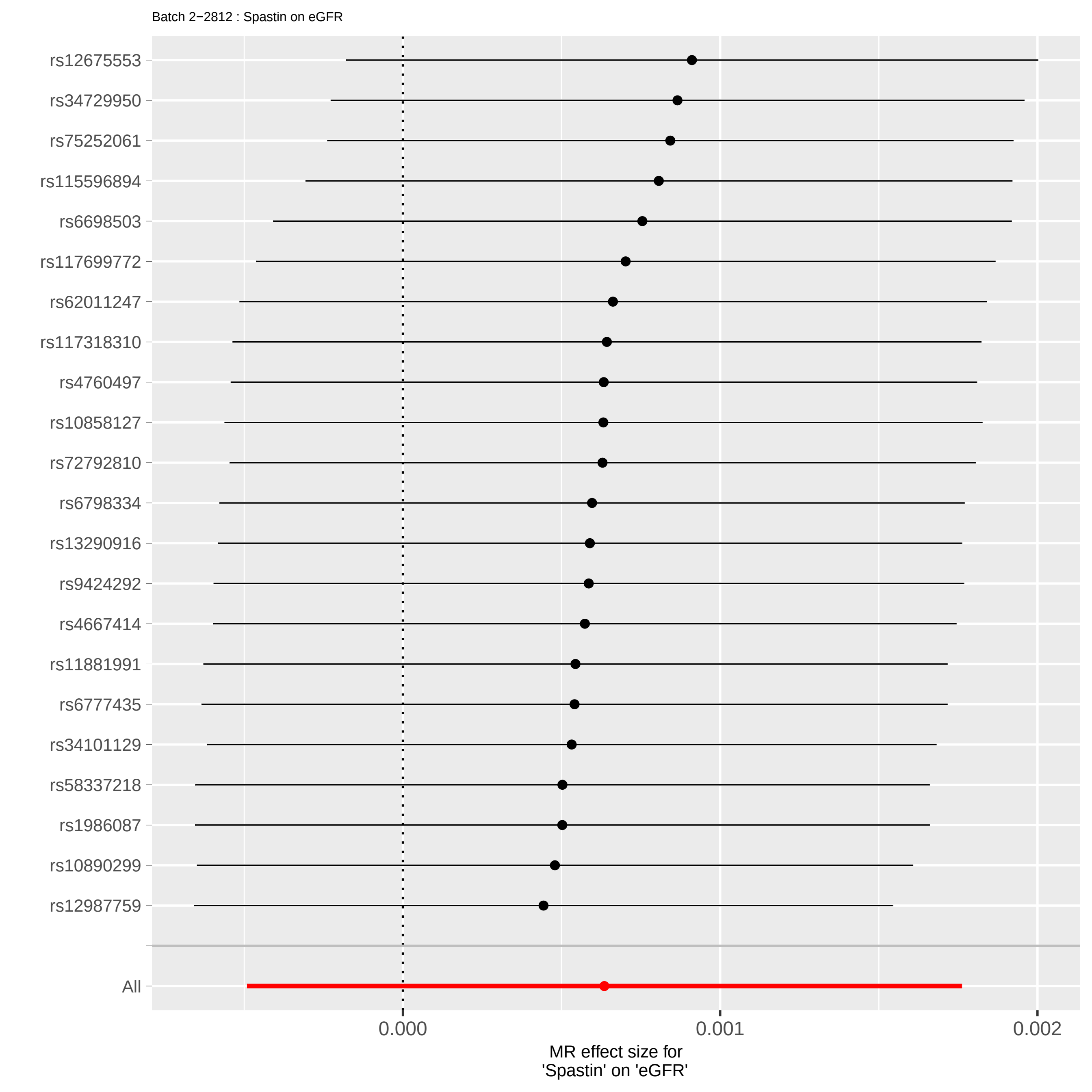

Batch 2−2812 : Spastin on eGFR
rs12675553
rs34729950
rs75252061
rs115596894
rs6698503
rs117699772
rs62011247
rs117318310
rs4760497
rs10858127
rs72792810
rs6798334
rs13290916
rs9424292
rs4667414
rs11881991
rs6777435
rs34101129
rs58337218
rs1986087
rs10890299
rs12987759
All
0.000
0.001
0.002
MR effect size for
'Spastin' on 'eGFR'

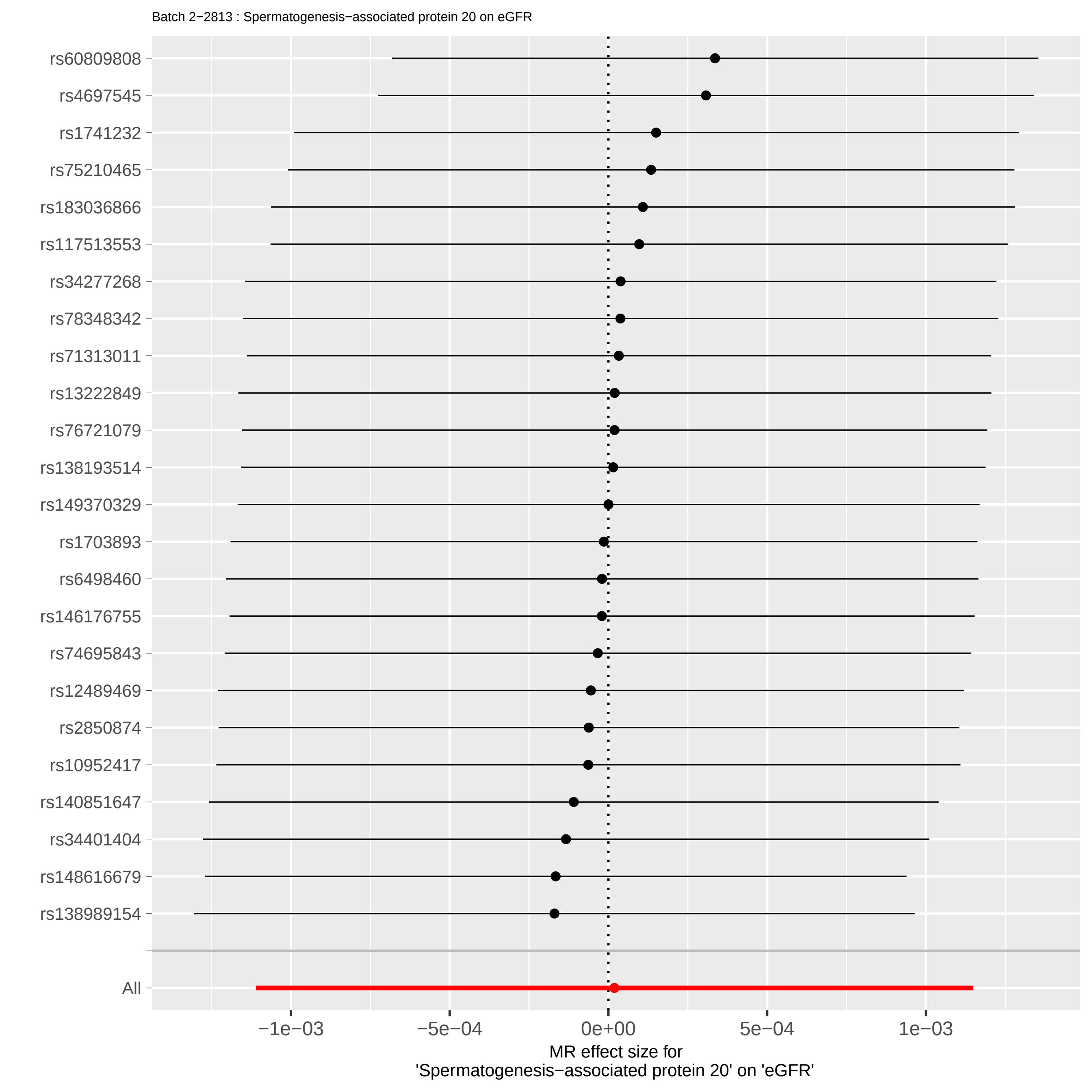

Batch 2−2813 : Spermatogenesis−associated protein 20 on eGFR
rs60809808
rs4697545
rs1741232
rs75210465
rs183036866
rs117513553
rs34277268
rs78348342
rs71313011
rs13222849
rs76721079
rs138193514
rs149370329
rs1703893
rs6498460
rs146176755
rs74695843
rs12489469
rs2850874
rs10952417
rs140851647
rs34401404
rs148616679
rs138989154
All
−1e−03
−5e−04
0e+00
5e−04
1e−03
MR effect size for
'Spermatogenesis−associated protein 20' on 'eGFR'

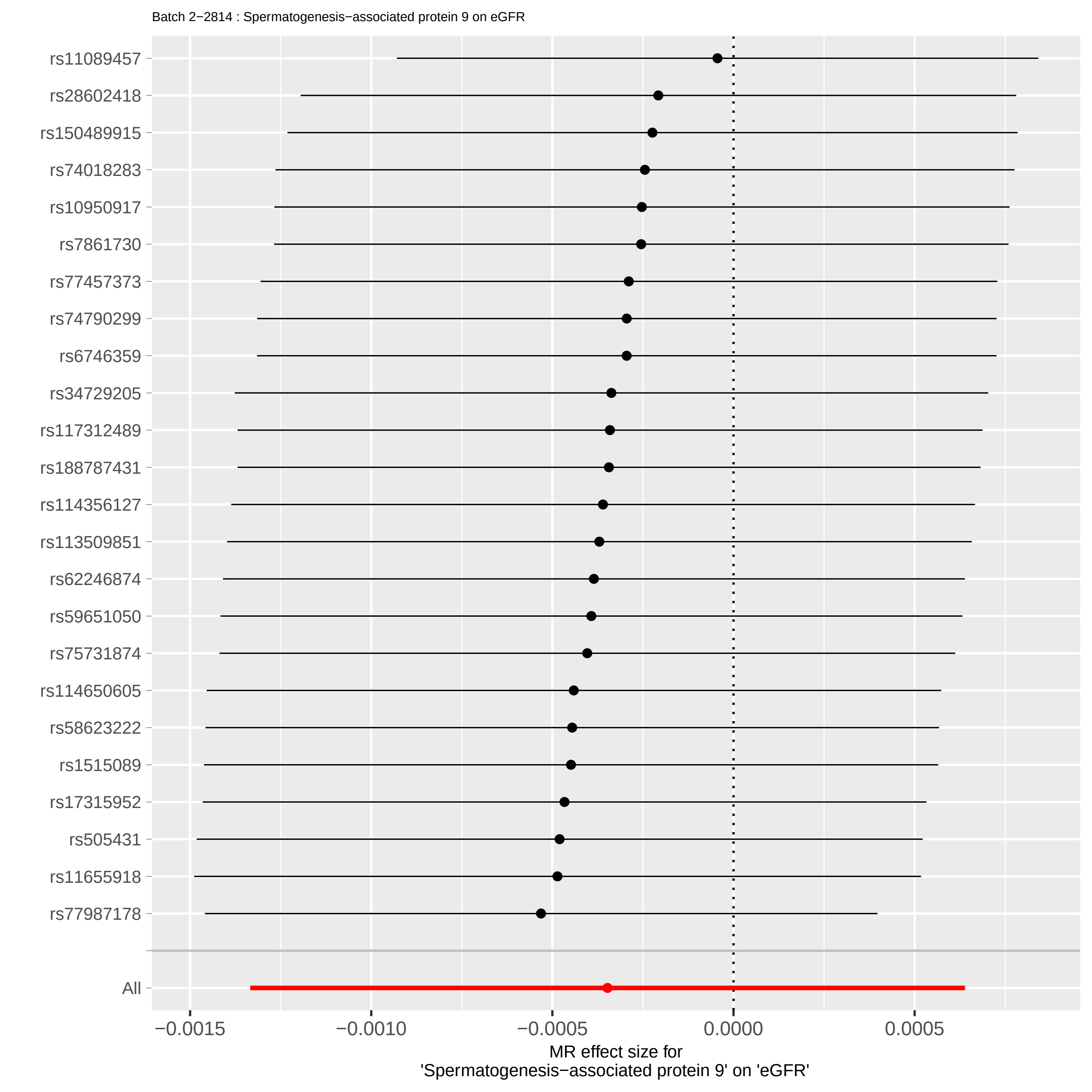

Batch 2−2814 : Spermatogenesis−associated protein 9 on eGFR
rs11089457
rs28602418
rs150489915
rs74018283
rs10950917
rs7861730
rs77457373
rs74790299
rs6746359
rs34729205
rs117312489
rs188787431
rs114356127
rs113509851
rs62246874
rs59651050
rs75731874
rs114650605
rs58623222
rs1515089
rs17315952
rs505431
rs11655918
rs77987178
All
−0.0015
−0.0010
−0.0005
0.0000
0.0005
MR effect size for
'Spermatogenesis−associated protein 9' on 'eGFR'

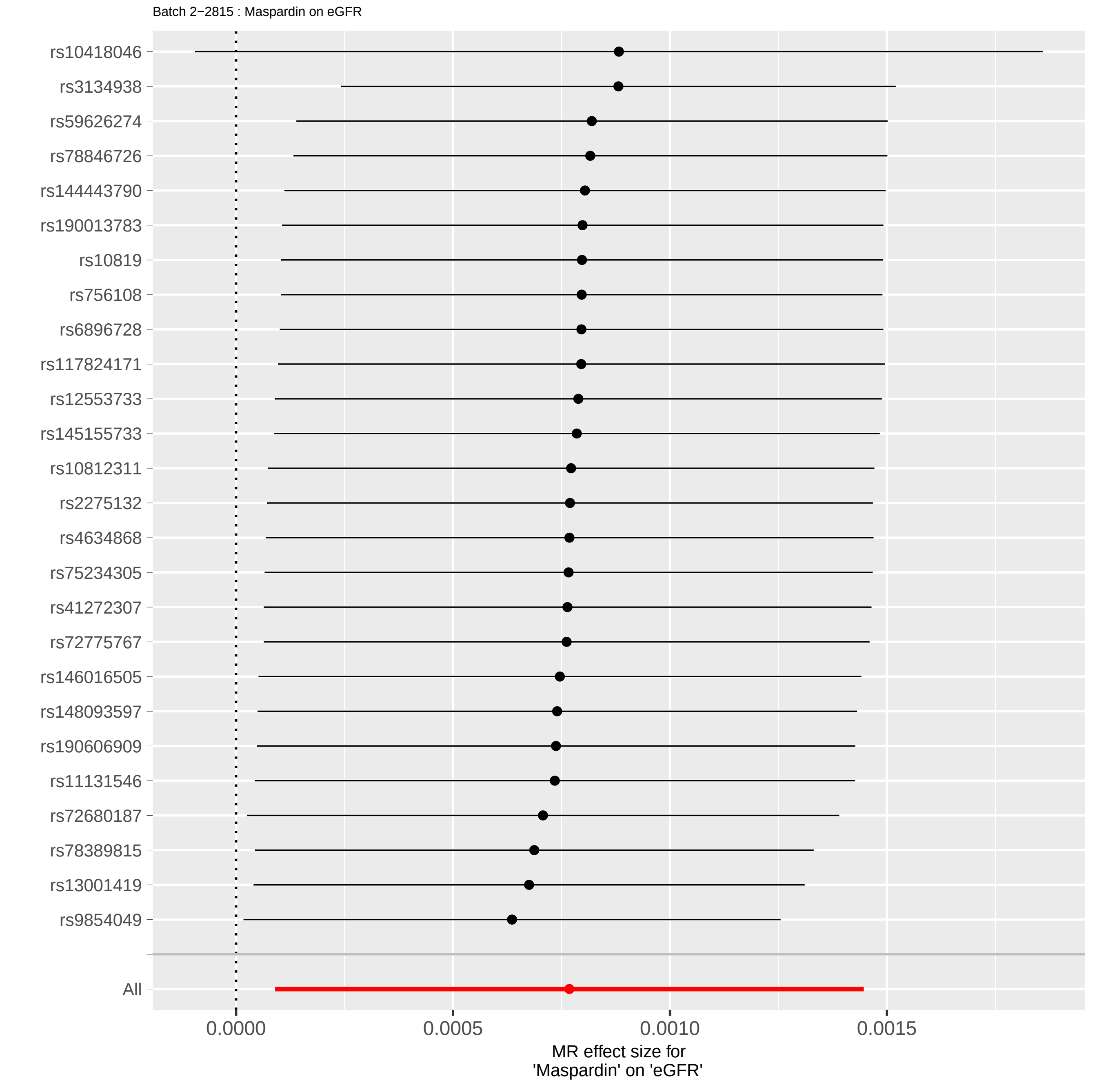

Batch 2−2815 : Maspardin on eGFR
rs10418046
rs3134938
rs59626274
rs78846726
rs144443790
rs190013783
rs10819
rs756108
rs6896728
rs117824171
rs12553733
rs145155733
rs10812311
rs2275132
rs4634868
rs75234305
rs41272307
rs72775767
rs146016505
rs148093597
rs190606909
rs11131546
rs72680187
rs78389815
rs13001419
rs9854049
All
0.0000
0.0005
0.0010
0.0015
MR effect size for
'Maspardin' on 'eGFR'

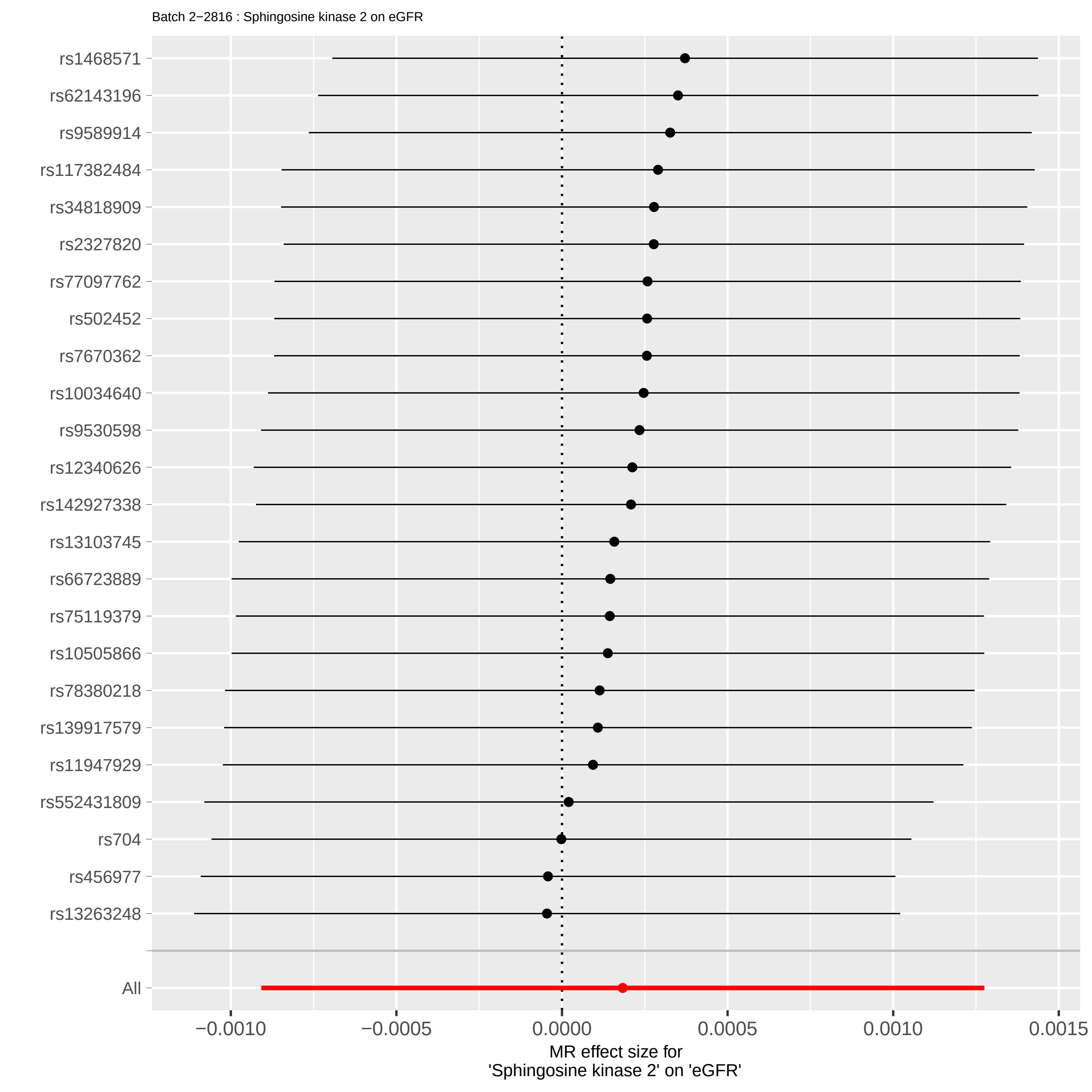

Batch 2−2816 : Sphingosine kinase 2 on eGFR
rs1468571
rs62143196
rs9589914
rs117382484
rs34818909
rs2327820
rs77097762
rs502452
rs7670362
rs10034640
rs9530598
rs12340626
rs142927338
rs13103745
rs66723889
rs75119379
rs10505866
rs78380218
rs139917579
rs11947929
rs552431809
rs704
rs456977
rs13263248
All
−0.0010
−0.0005
0.0000
0.0005
0.0010
0.0015
MR effect size for
'Sphingosine kinase 2' on 'eGFR'

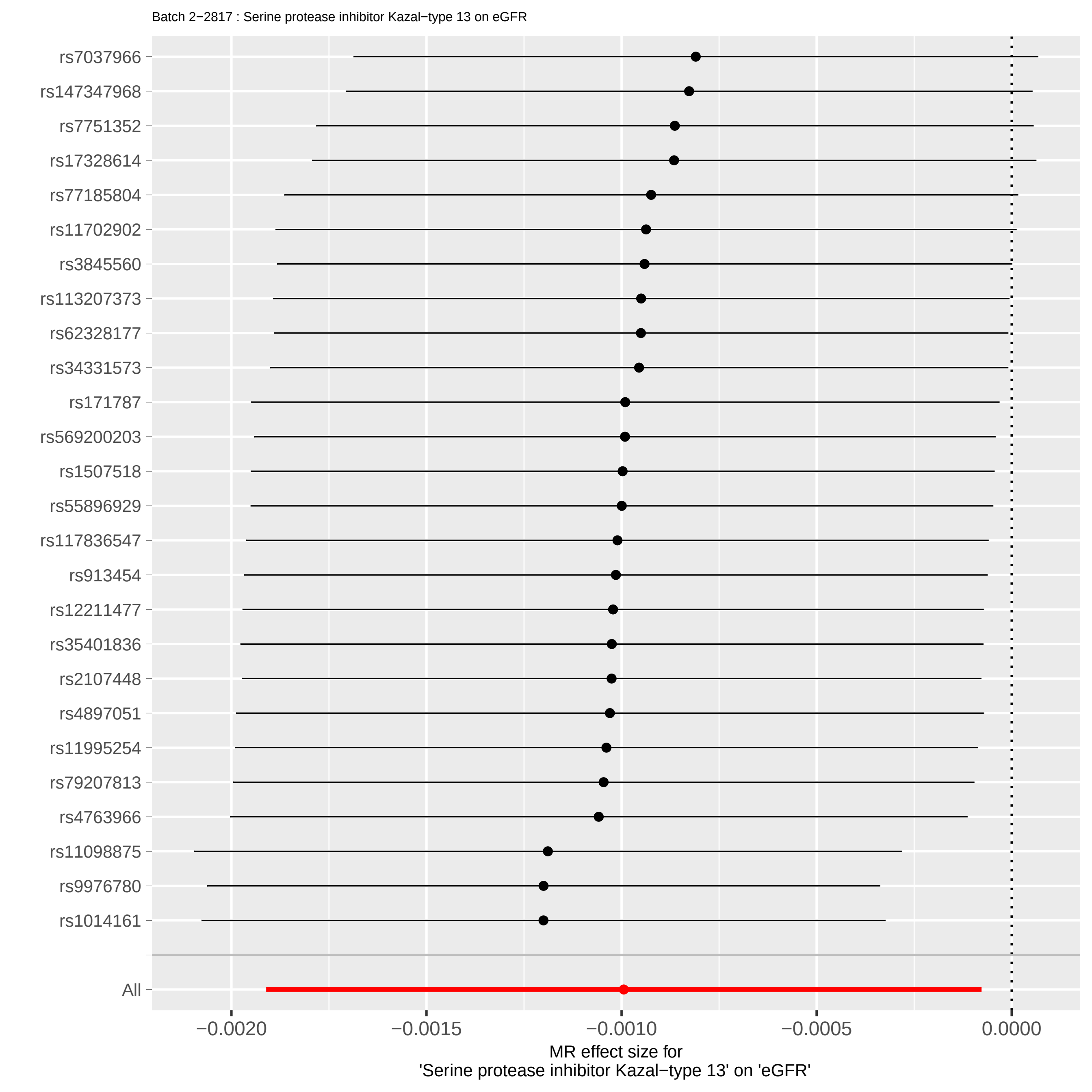

Batch 2−2817 : Serine protease inhibitor Kazal−type 13 on eGFR
rs7037966
rs147347968
rs7751352
rs17328614
rs77185804
rs11702902
rs3845560
rs113207373
rs62328177
rs34331573
rs171787
rs569200203
rs1507518
rs55896929
rs117836547
rs913454
rs12211477
rs35401836
rs2107448
rs4897051
rs11995254
rs79207813
rs4763966
rs11098875
rs9976780
rs1014161
All
−0.0020
−0.0015
−0.0010
−0.0005
0.0000
MR effect size for
'Serine protease inhibitor Kazal−type 13' on 'eGFR'

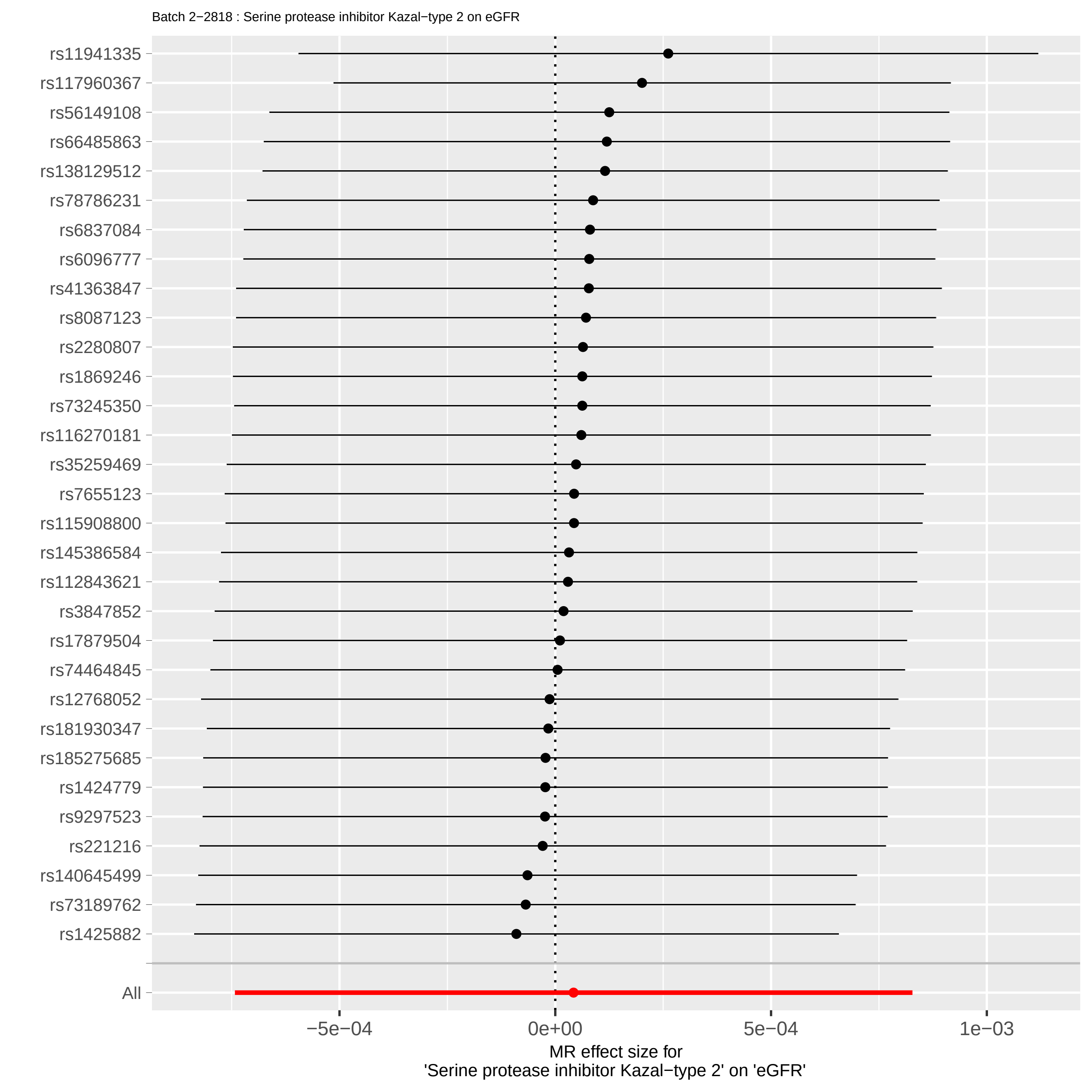
Batch 2−2818 : Serine protease inhibitor Kazal−type 2 on eGFR
rs11941335
rs117960367
rs56149108
rs66485863
rs138129512
rs78786231
rs6837084
rs6096777
rs41363847
rs8087123
rs2280807
rs1869246
rs73245350
rs116270181
rs35259469
rs7655123
rs115908800
rs145386584
rs112843621
rs3847852
rs17879504
rs74464845
rs12768052
rs181930347
rs185275685
rs1424779
rs9297523
rs221216
rs140645499
rs73189762
rs1425882
All
−5e−04
0e+00
5e−04
1e−03
MR effect size for
'Serine protease inhibitor Kazal−type 2' on 'eGFR'

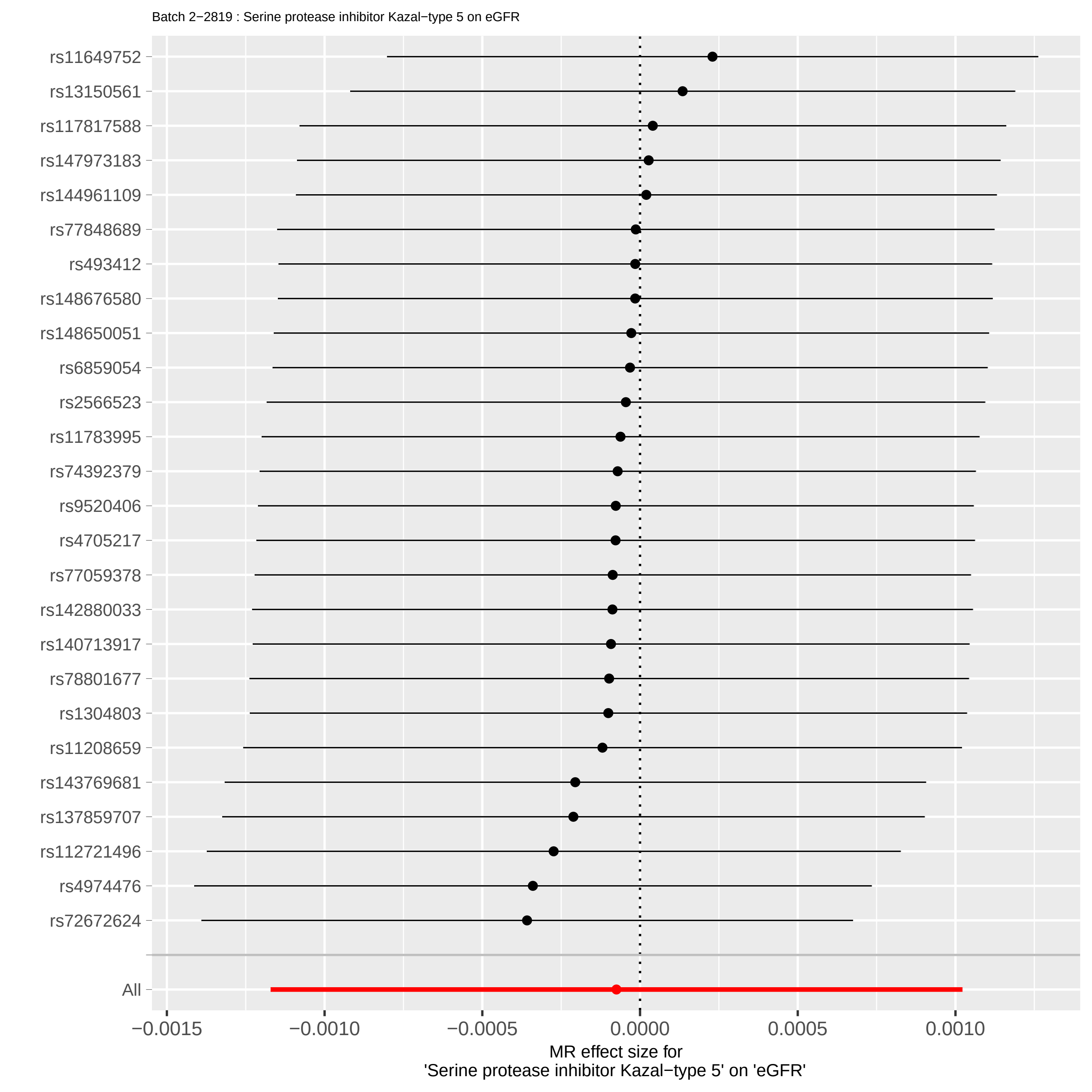

Batch 2−2819 : Serine protease inhibitor Kazal−type 5 on eGFR
rs11649752
rs13150561
rs117817588
rs147973183
rs144961109
rs77848689
rs493412
rs148676580
rs148650051
rs6859054
rs2566523
rs11783995
rs74392379
rs9520406
rs4705217
rs77059378
rs142880033
rs140713917
rs78801677
rs1304803
rs11208659
rs143769681
rs137859707
rs112721496
rs4974476
rs72672624
All
−0.0015
−0.0010
−0.0005
0.0000
0.0005
0.0010
MR effect size for
'Serine protease inhibitor Kazal−type 5' on 'eGFR'

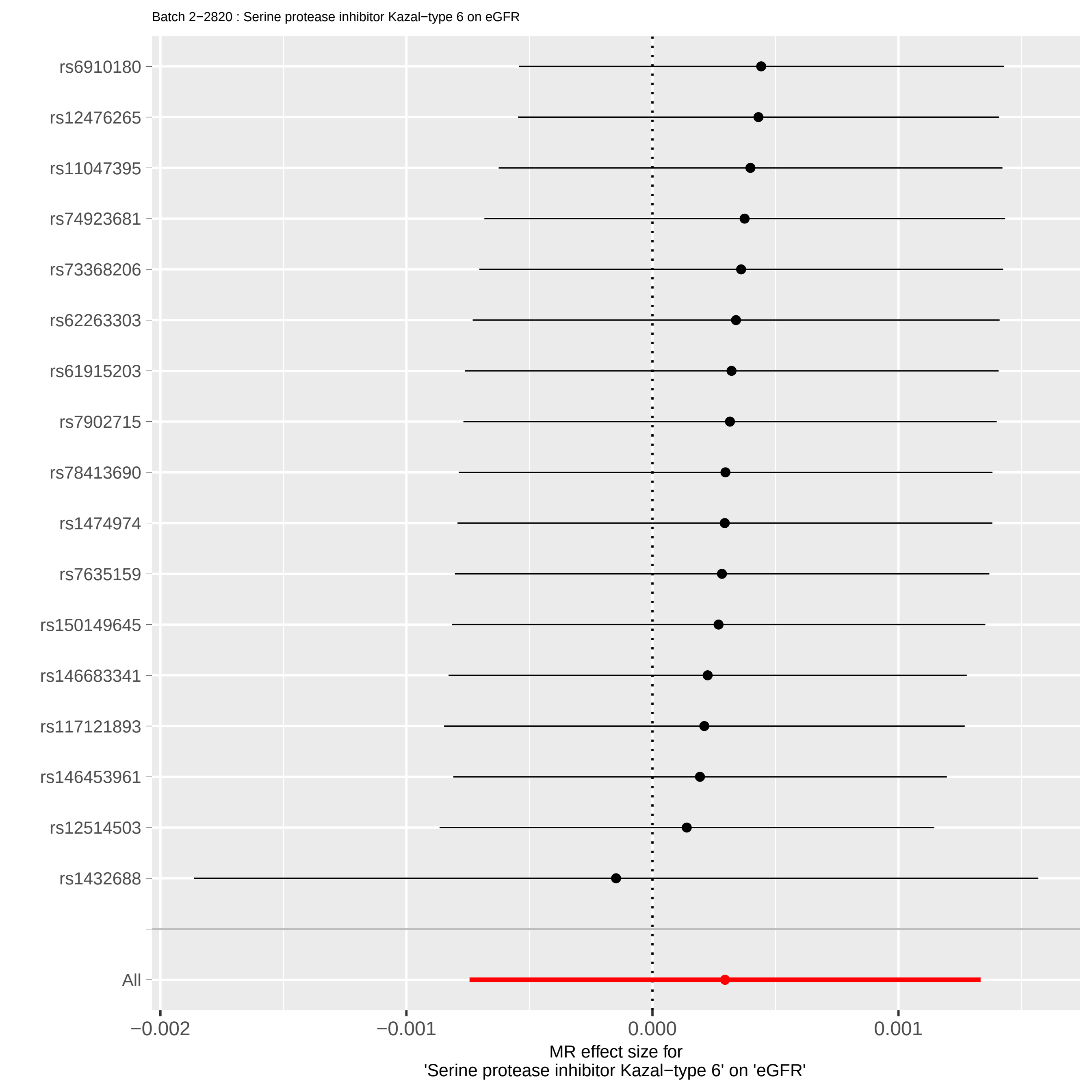

Batch 2−2820 : Serine protease inhibitor Kazal−type 6 on eGFR
rs6910180
rs12476265
rs11047395
rs74923681
rs73368206
rs62263303
rs61915203
rs7902715
rs78413690
rs1474974
rs7635159
rs150149645
rs146683341
rs117121893
rs146453961
rs12514503
rs1432688
All
−0.002
−0.001
0.000
0.001
MR effect size for
'Serine protease inhibitor Kazal−type 6' on 'eGFR'

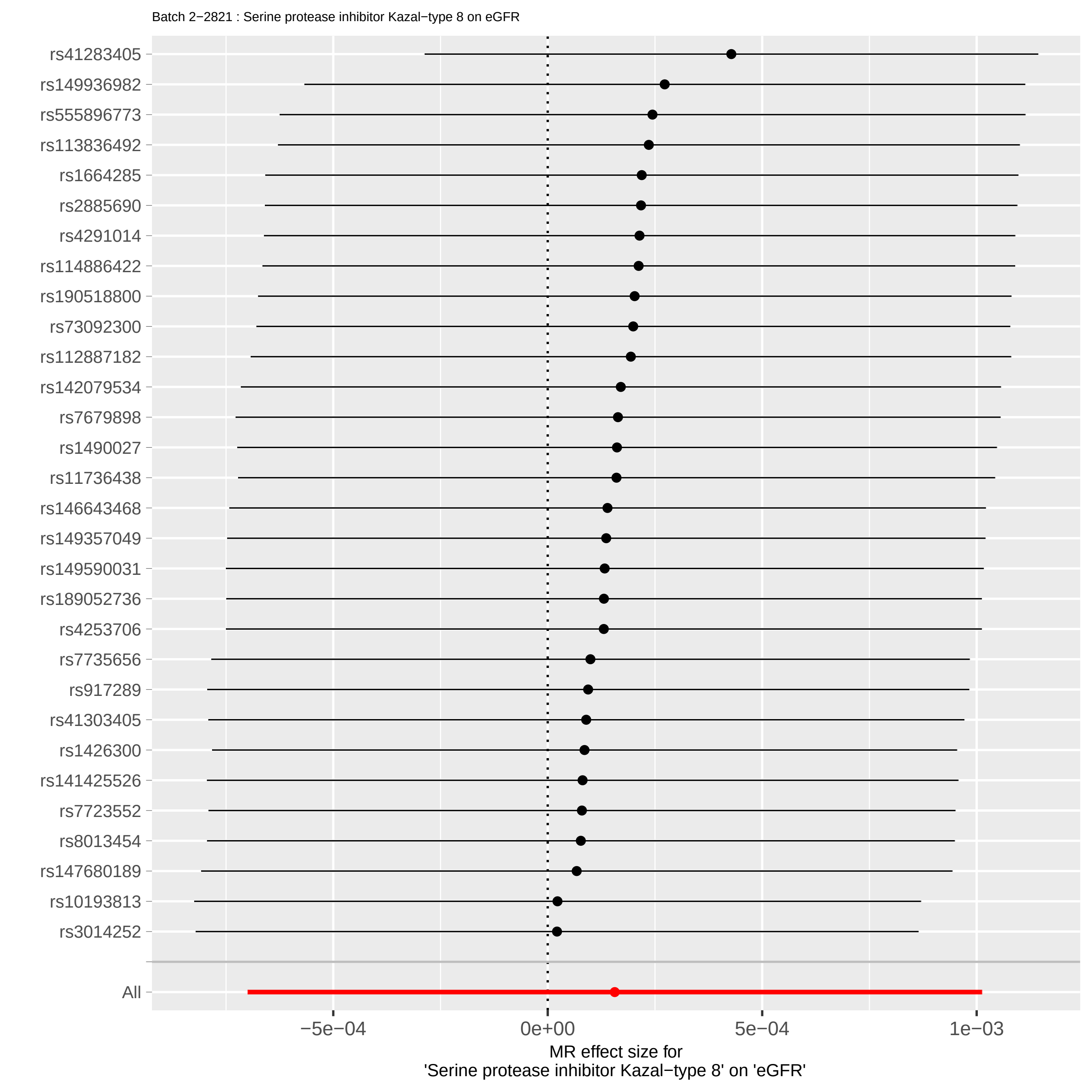

Batch 2−2821 : Serine protease inhibitor Kazal−type 8 on eGFR
rs41283405
rs149936982
rs555896773
rs113836492
rs1664285
rs2885690
rs4291014
rs114886422
rs190518800
rs73092300
rs112887182
rs142079534
rs7679898
rs1490027
rs11736438
rs146643468
rs149357049
rs149590031
rs189052736
rs4253706
rs7735656
rs917289
rs41303405
rs1426300
rs141425526
rs7723552
rs8013454
rs147680189
rs10193813
rs3014252
All
−5e−04
0e+00
5e−04
1e−03
MR effect size for
'Serine protease inhibitor Kazal−type 8' on 'eGFR'

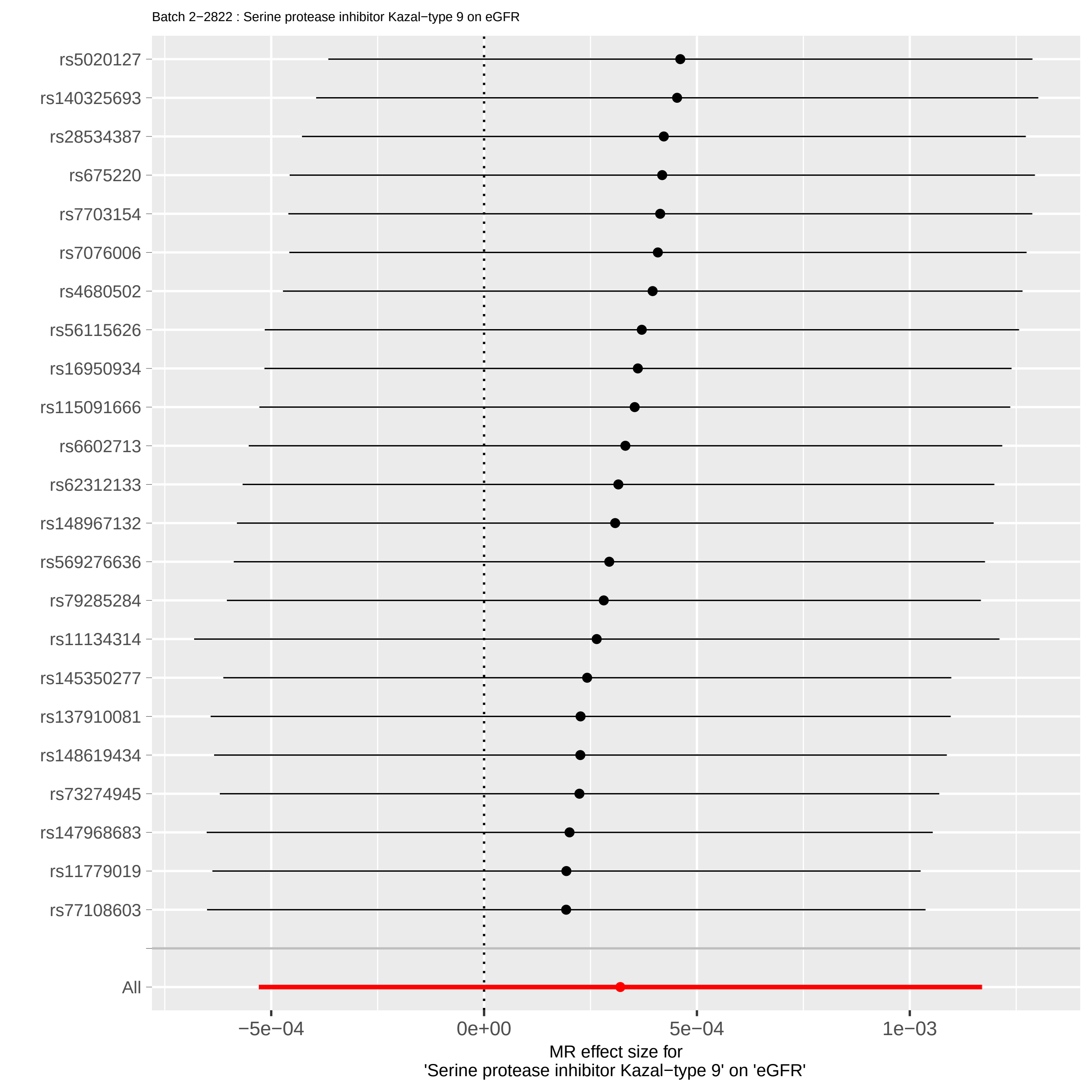

Batch 2−2822 : Serine protease inhibitor Kazal−type 9 on eGFR
rs5020127
rs140325693
rs28534387
rs675220
rs7703154
rs7076006
rs4680502
rs56115626
rs16950934
rs115091666
rs6602713
rs62312133
rs148967132
rs569276636
rs79285284
rs11134314
rs145350277
rs137910081
rs148619434
rs73274945
rs147968683
rs11779019
rs77108603
All
−5e−04
0e+00
5e−04
1e−03
MR effect size for
'Serine protease inhibitor Kazal−type 9' on 'eGFR'

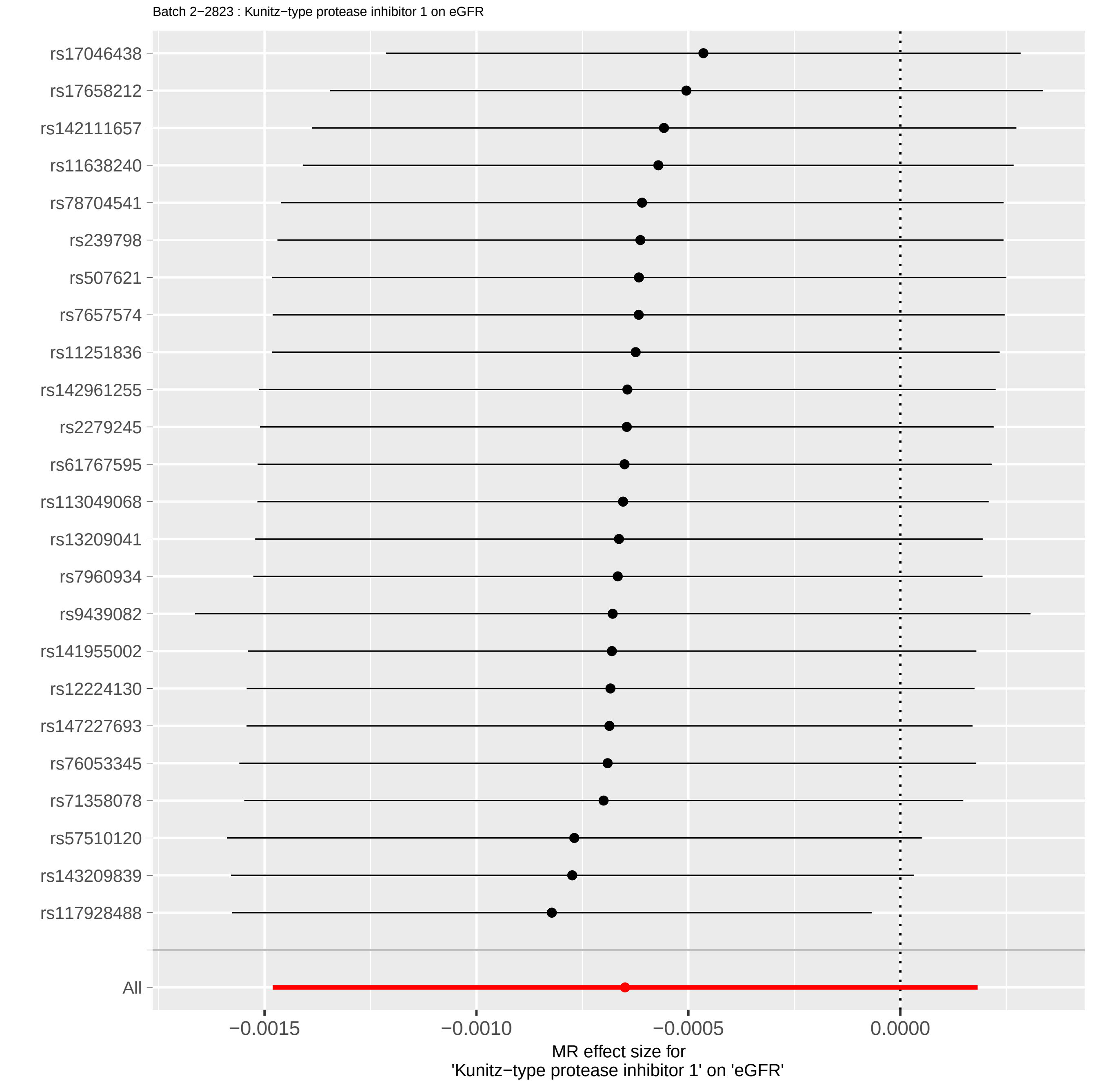
Batch 2−2823 : Kunitz−type protease inhibitor 1 on eGFR
rs17046438
rs17658212
rs142111657
rs11638240
rs78704541
rs239798
rs507621
rs7657574
rs11251836
rs142961255
rs2279245
rs61767595
rs113049068
rs13209041
rs7960934
rs9439082
rs141955002
rs12224130
rs147227693
rs76053345
rs71358078
rs57510120
rs143209839
rs117928488
All
−0.0015
−0.0010
−0.0005
0.0000
MR effect size for
'Kunitz−type protease inhibitor 1' on 'eGFR'

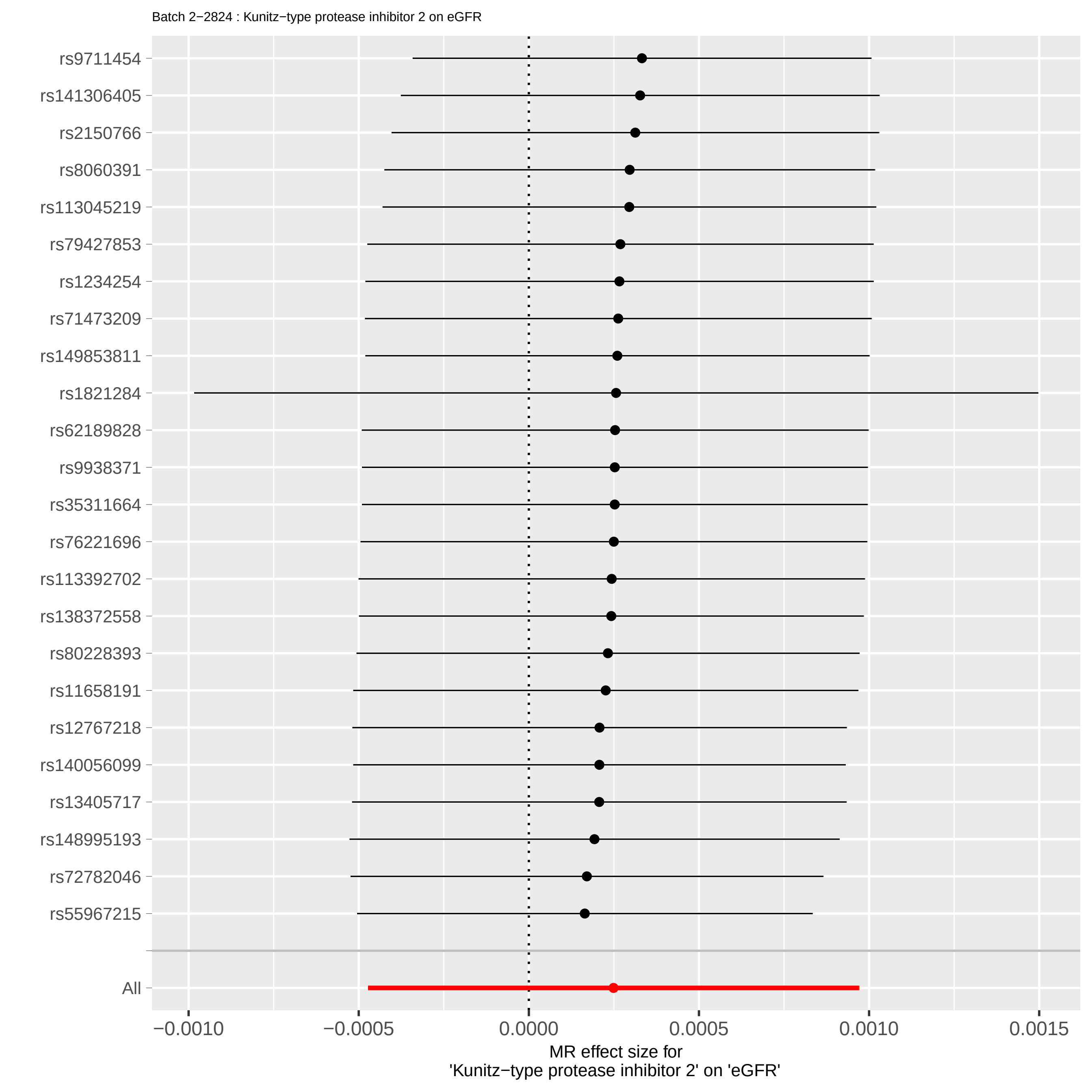

Batch 2−2824 : Kunitz−type protease inhibitor 2 on eGFR
rs9711454
rs141306405
rs2150766
rs8060391
rs113045219
rs79427853
rs1234254
rs71473209
rs149853811
rs1821284
rs62189828
rs9938371
rs35311664
rs76221696
rs113392702
rs138372558
rs80228393
rs11658191
rs12767218
rs140056099
rs13405717
rs148995193
rs72782046
rs55967215
All
−0.0010
−0.0005
0.0000
0.0005
0.0010
0.0015
MR effect size for
'Kunitz−type protease inhibitor 2' on 'eGFR'

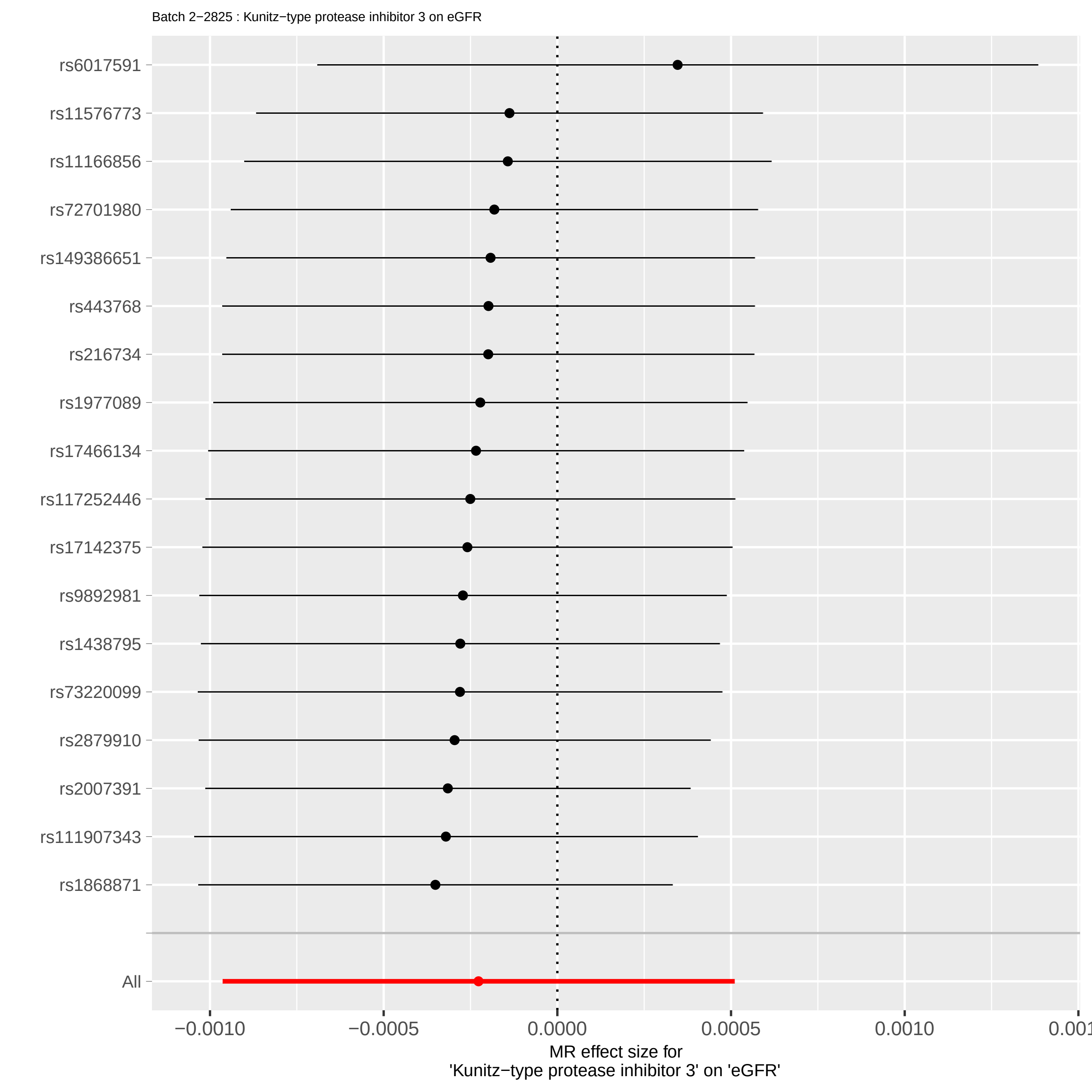

Batch 2−2825 : Kunitz−type protease inhibitor 3 on eGFR
rs6017591
rs11576773
rs11166856
rs72701980
rs149386651
rs443768
rs216734
rs1977089
rs17466134
rs117252446
rs17142375
rs9892981
rs1438795
rs73220099
rs2879910
rs2007391
rs111907343
rs1868871
All
−0.0010
−0.0005
0.0000
0.0005
0.0010
MR effect size for
'Kunitz−type protease inhibitor 3' on 'eGFR'

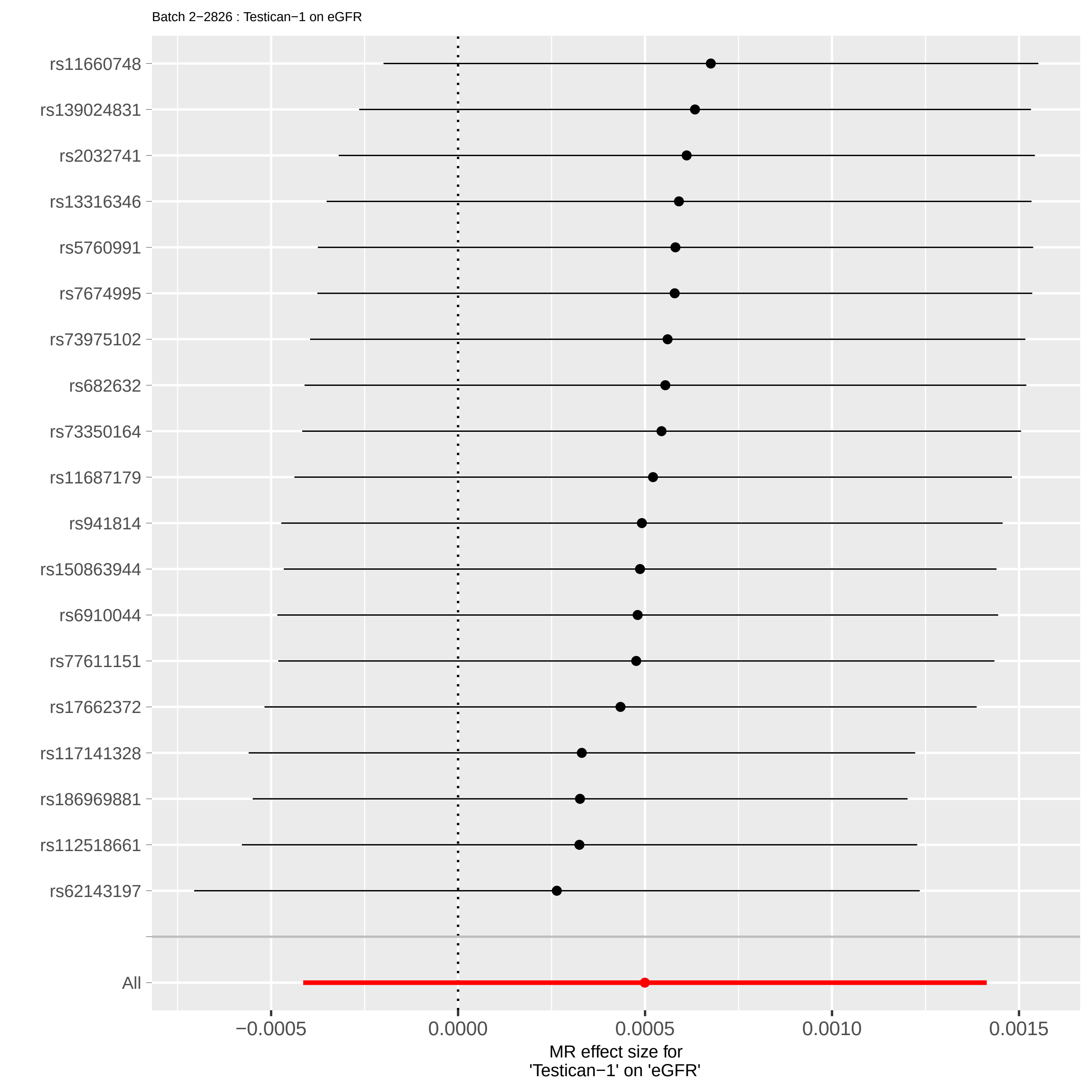

Batch 2−2826 : Testican−1 on eGFR
rs11660748
rs139024831
rs2032741
rs13316346
rs5760991
rs7674995
rs73975102
rs682632
rs73350164
rs11687179
rs941814
rs150863944
rs6910044
rs77611151
rs17662372
rs117141328
rs186969881
rs112518661
rs62143197
All
−0.0005
0.0000
0.0005
0.0010
0.0015
MR effect size for
'Testican−1' on 'eGFR'

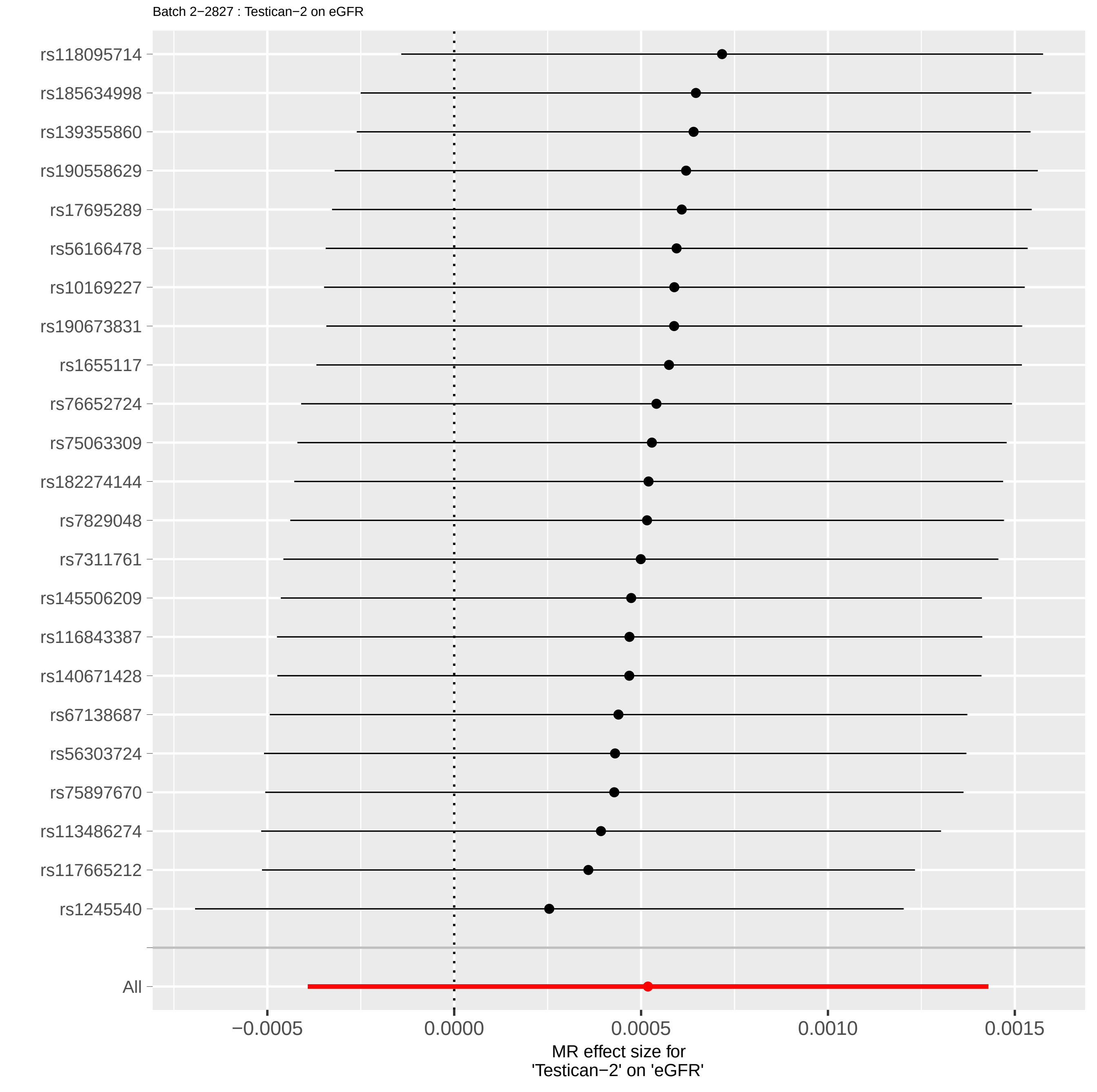

Batch 2−2827 : Testican−2 on eGFR
rs118095714
rs185634998
rs139355860
rs190558629
rs17695289
rs56166478
rs10169227
rs190673831
rs1655117
rs76652724
rs75063309
rs182274144
rs7829048
rs7311761
rs145506209
rs116843387
rs140671428
rs67138687
rs56303724
rs75897670
rs113486274
rs117665212
rs1245540
All
−0.0005
0.0000
0.0005
0.0010
0.0015
MR effect size for
'Testican−2' on 'eGFR'

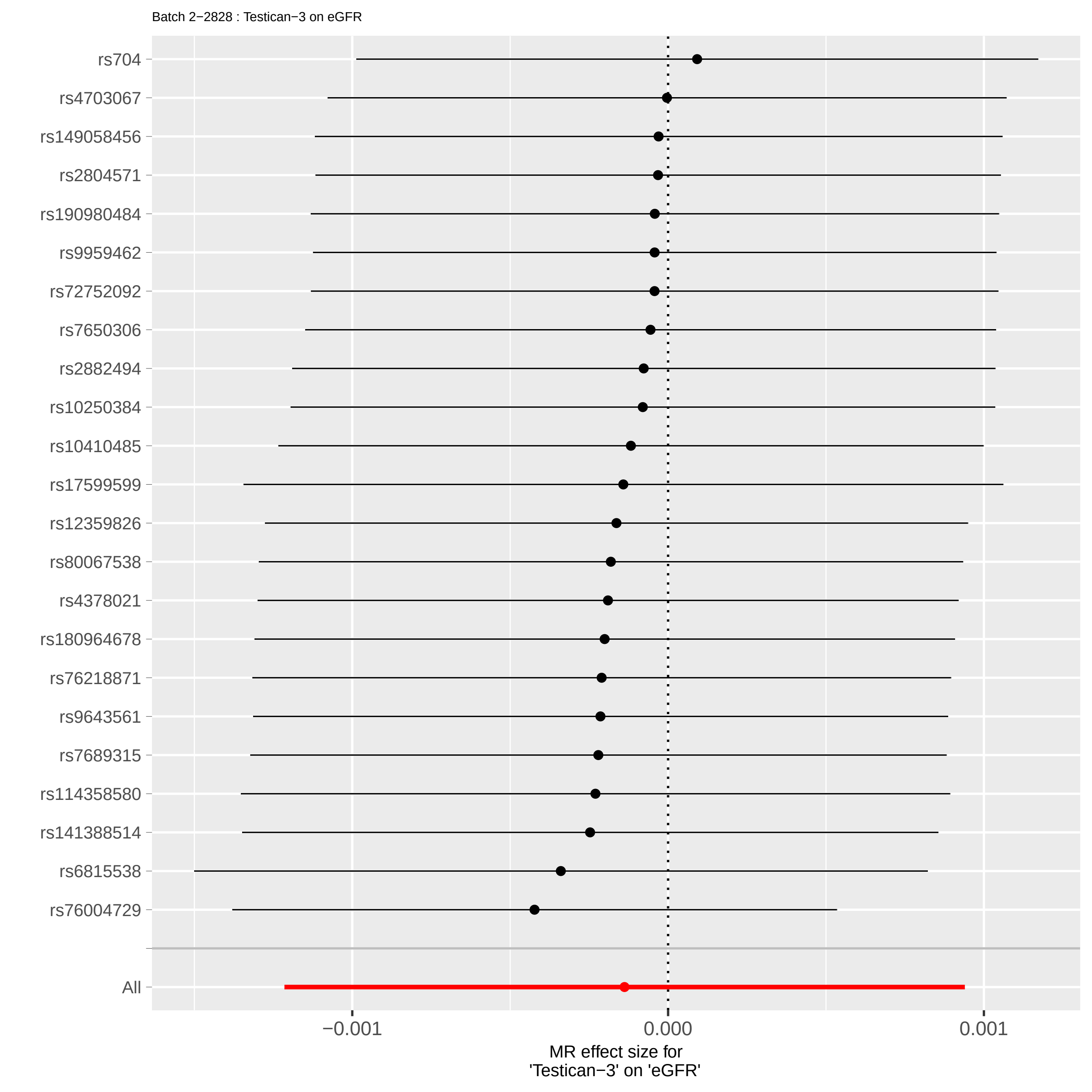

Batch 2−2828 : Testican−3 on eGFR
rs704
rs4703067
rs149058456
rs2804571
rs190980484
rs9959462
rs72752092
rs7650306
rs2882494
rs10250384
rs10410485
rs17599599
rs12359826
rs80067538
rs4378021
rs180964678
rs76218871
rs9643561
rs7689315
rs114358580
rs141388514
rs6815538
rs76004729
All
−0.001
0.000
0.001
MR effect size for
'Testican−3' on 'eGFR'

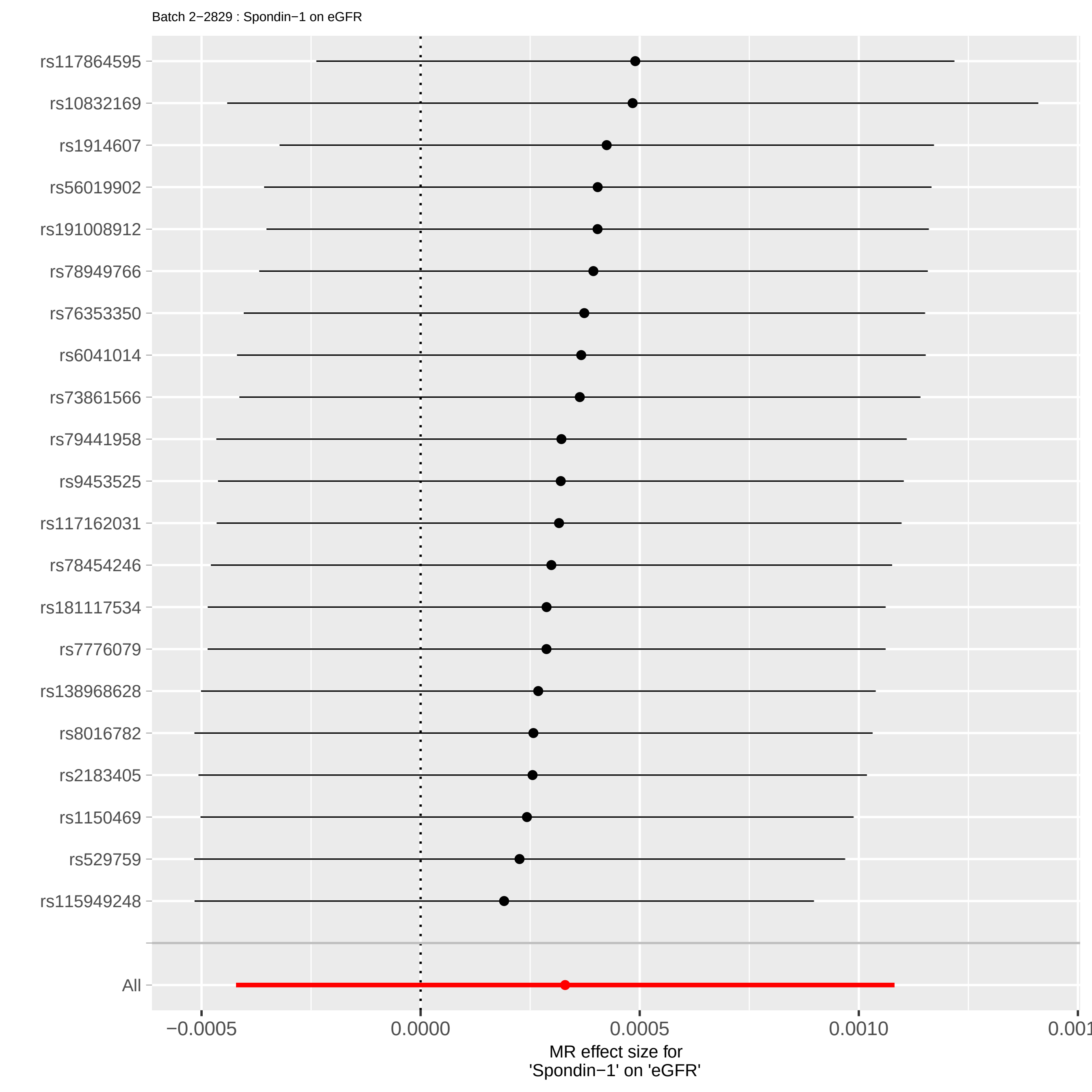

Batch 2−2829 : Spondin−1 on eGFR
rs117864595
rs10832169
rs1914607
rs56019902
rs191008912
rs78949766
rs76353350
rs6041014
rs73861566
rs79441958
rs9453525
rs117162031
rs78454246
rs181117534
rs7776079
rs138968628
rs8016782
rs2183405
rs1150469
rs529759
rs115949248
All
−0.0005
0.0000
0.0005
0.0010
MR effect size for
'Spondin−1' on 'eGFR'

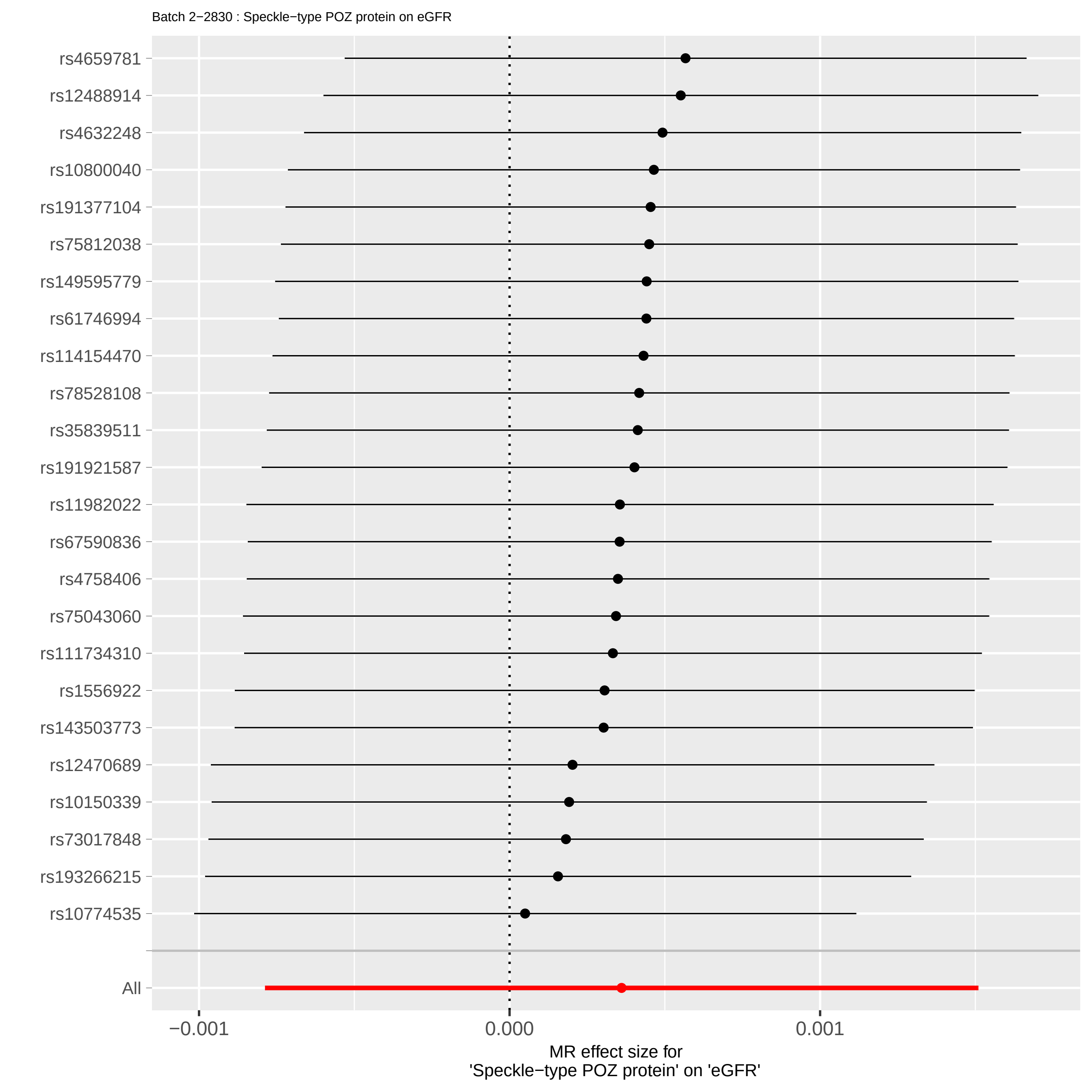

Batch 2−2830 : Speckle−type POZ protein on eGFR
rs4659781
rs12488914
rs4632248
rs10800040
rs191377104
rs75812038
rs149595779
rs61746994
rs114154470
rs78528108
rs35839511
rs191921587
rs11982022
rs67590836
rs4758406
rs75043060
rs111734310
rs1556922
rs143503773
rs12470689
rs10150339
rs73017848
rs193266215
rs10774535
All
−0.001
0.000
0.001
MR effect size for
'Speckle−type POZ protein' on 'eGFR'

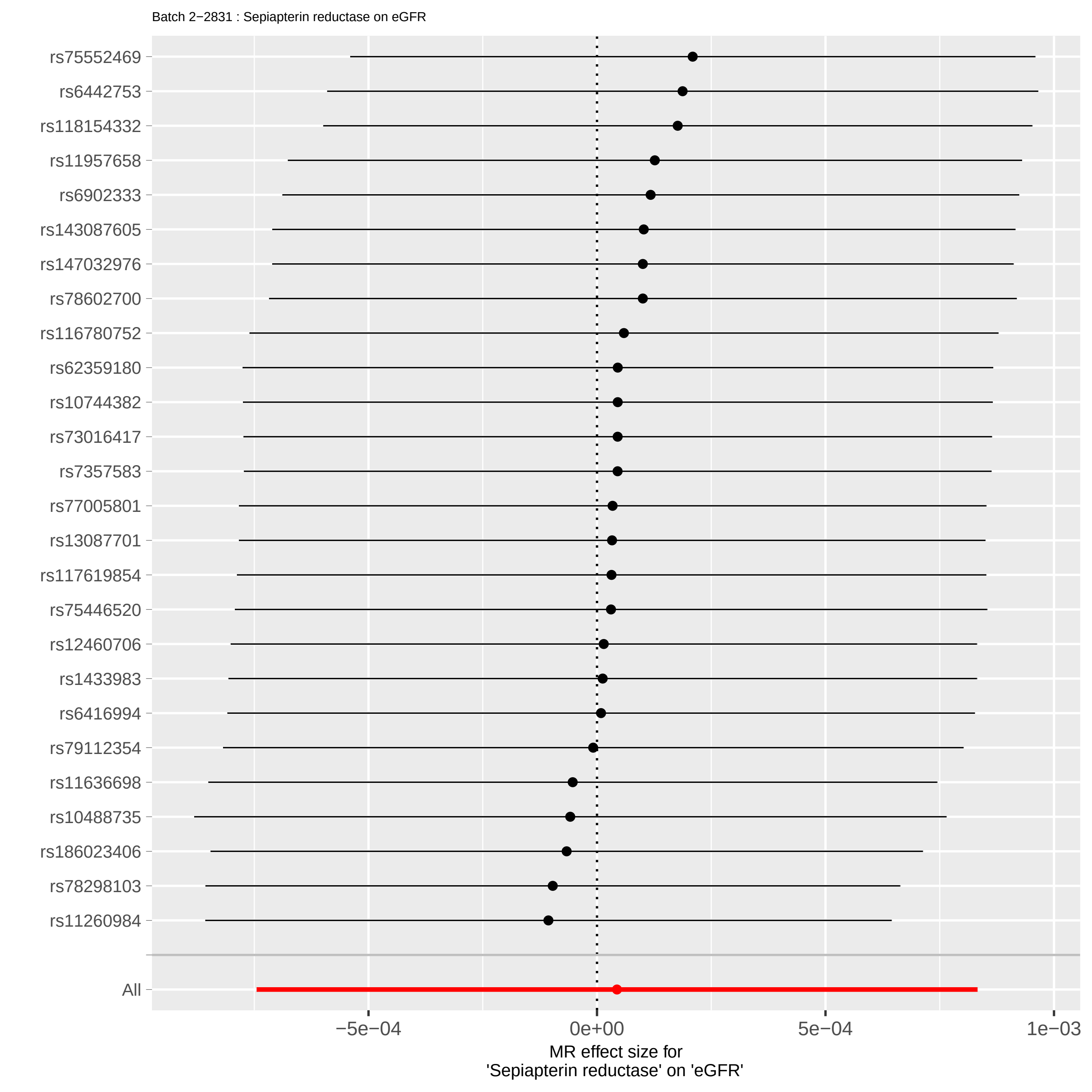

Batch 2−2831 : Sepiapterin reductase on eGFR
rs75552469
rs6442753
rs118154332
rs11957658
rs6902333
rs143087605
rs147032976
rs78602700
rs116780752
rs62359180
rs10744382
rs73016417
rs7357583
rs77005801
rs13087701
rs117619854
rs75446520
rs12460706
rs1433983
rs6416994
rs79112354
rs11636698
rs10488735
rs186023406
rs78298103
rs11260984
All
−5e−04
0e+00
5e−04
1e−03
MR effect size for
'Sepiapterin reductase' on 'eGFR'

Batch 2−2832 : Sprouty−related, EVH1 domain−containing protein 1 on eGFR

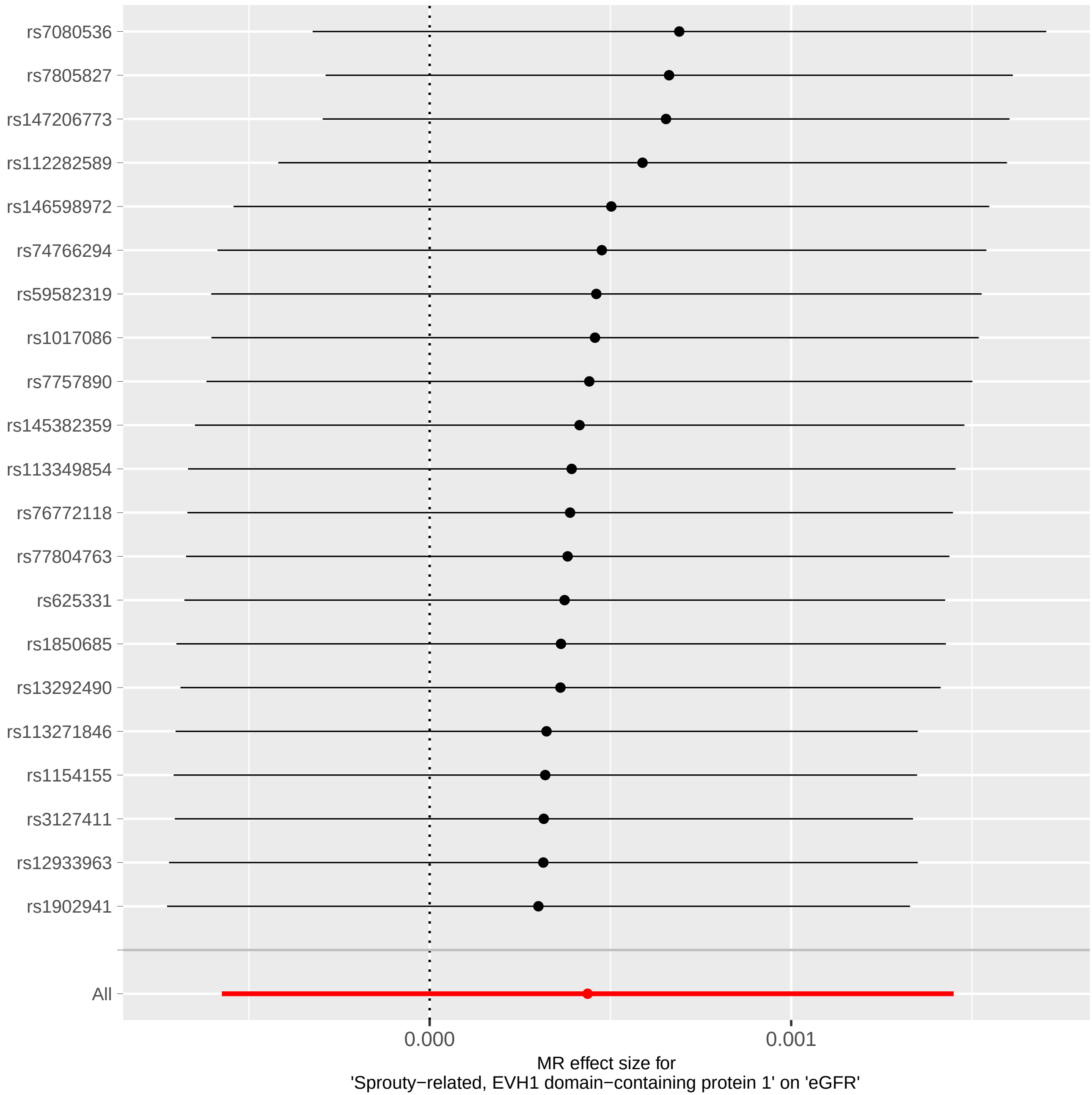

Batch 2−2833 : Protein sprouty homolog 1 on eGFR

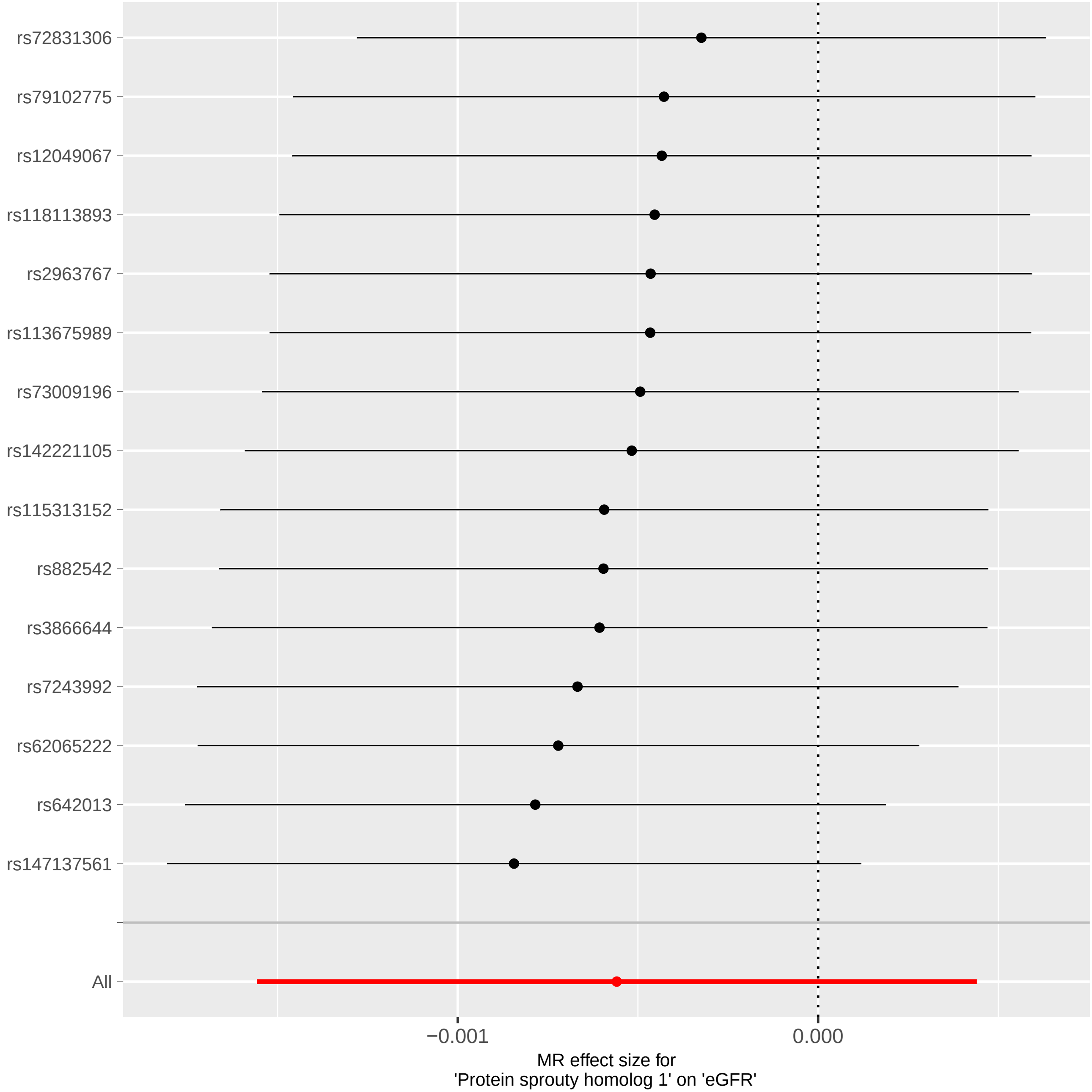

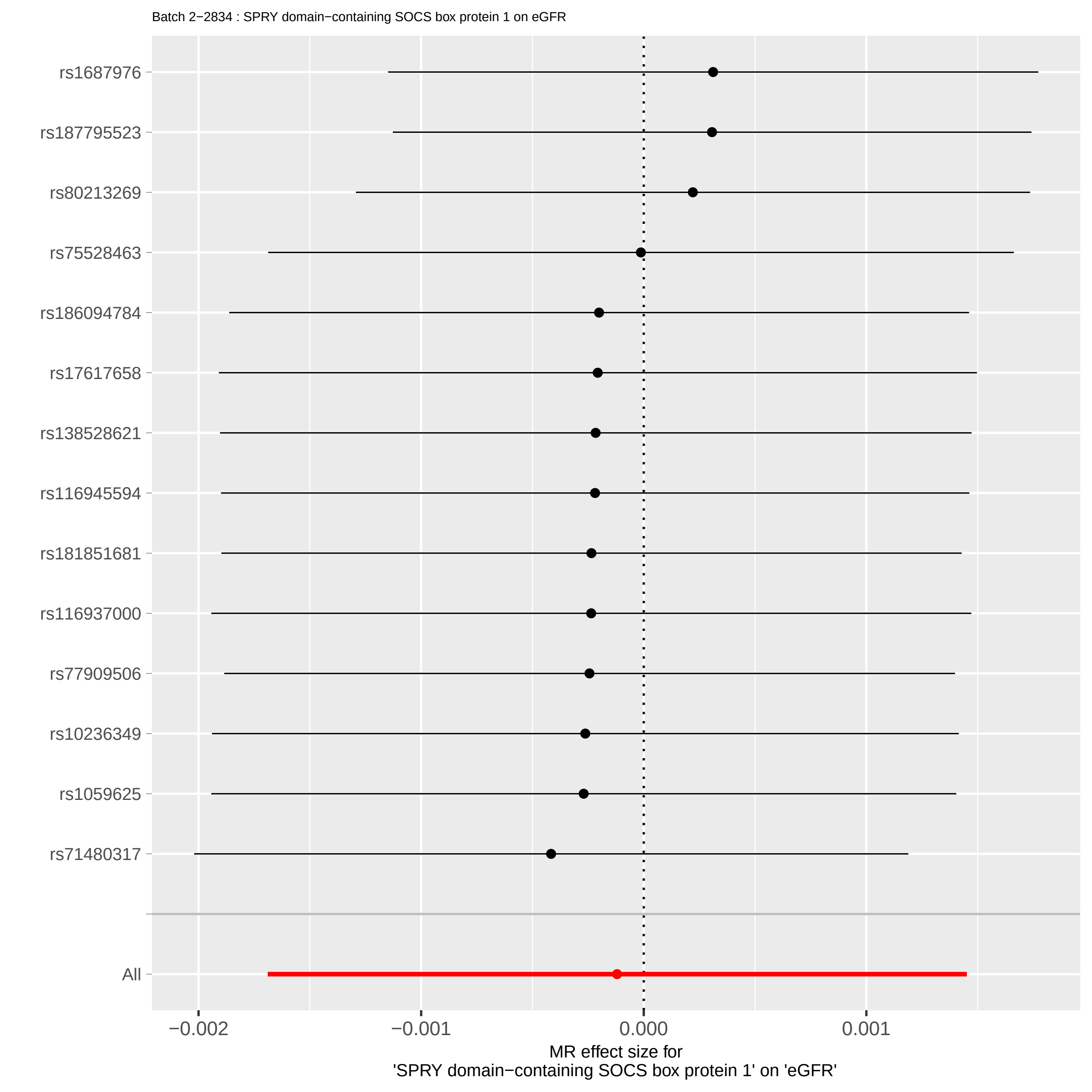

Batch 2−2834 : SPRY domain−containing SOCS box protein 1 on eGFR
rs1687976
rs187795523
rs80213269
rs75528463
rs186094784
rs17617658
rs138528621
rs116945594
rs181851681
rs116937000
rs77909506
rs10236349
rs1059625
rs71480317
All
−0.002
−0.001
0.000
0.001
MR effect size for
'SPRY domain−containing SOCS box protein 1' on 'eGFR'

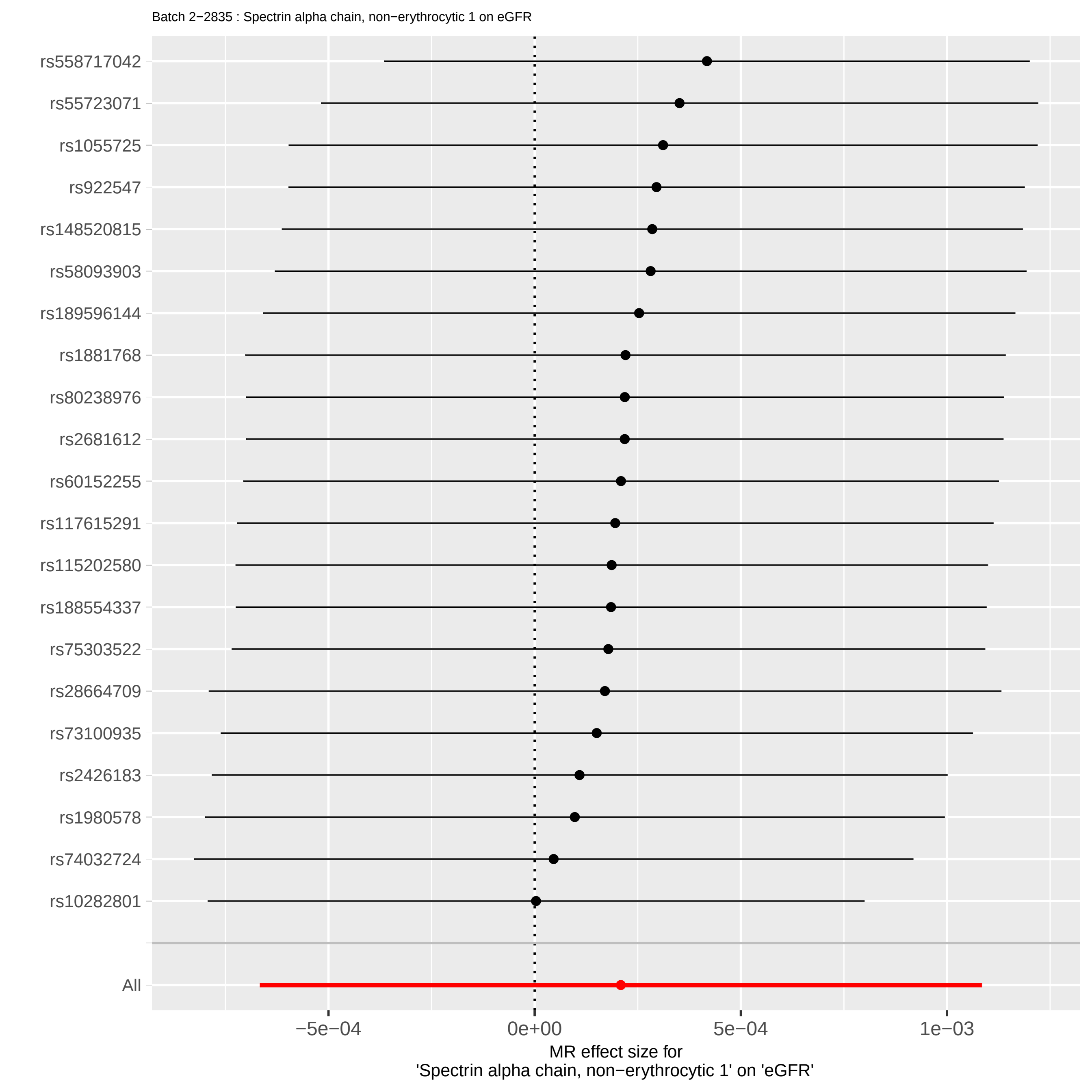

Batch 2−2835 : Spectrin alpha chain, non−erythrocytic 1 on eGFR
rs558717042
rs55723071
rs1055725
rs922547
rs148520815
rs58093903
rs189596144
rs1881768
rs80238976
rs2681612
rs60152255
rs117615291
rs115202580
rs188554337
rs75303522
rs28664709
rs73100935
rs2426183
rs1980578
rs74032724
rs10282801
All
−5e−04
0e+00
5e−04
1e−03
MR effect size for
'Spectrin alpha chain, non−erythrocytic 1' on 'eGFR'

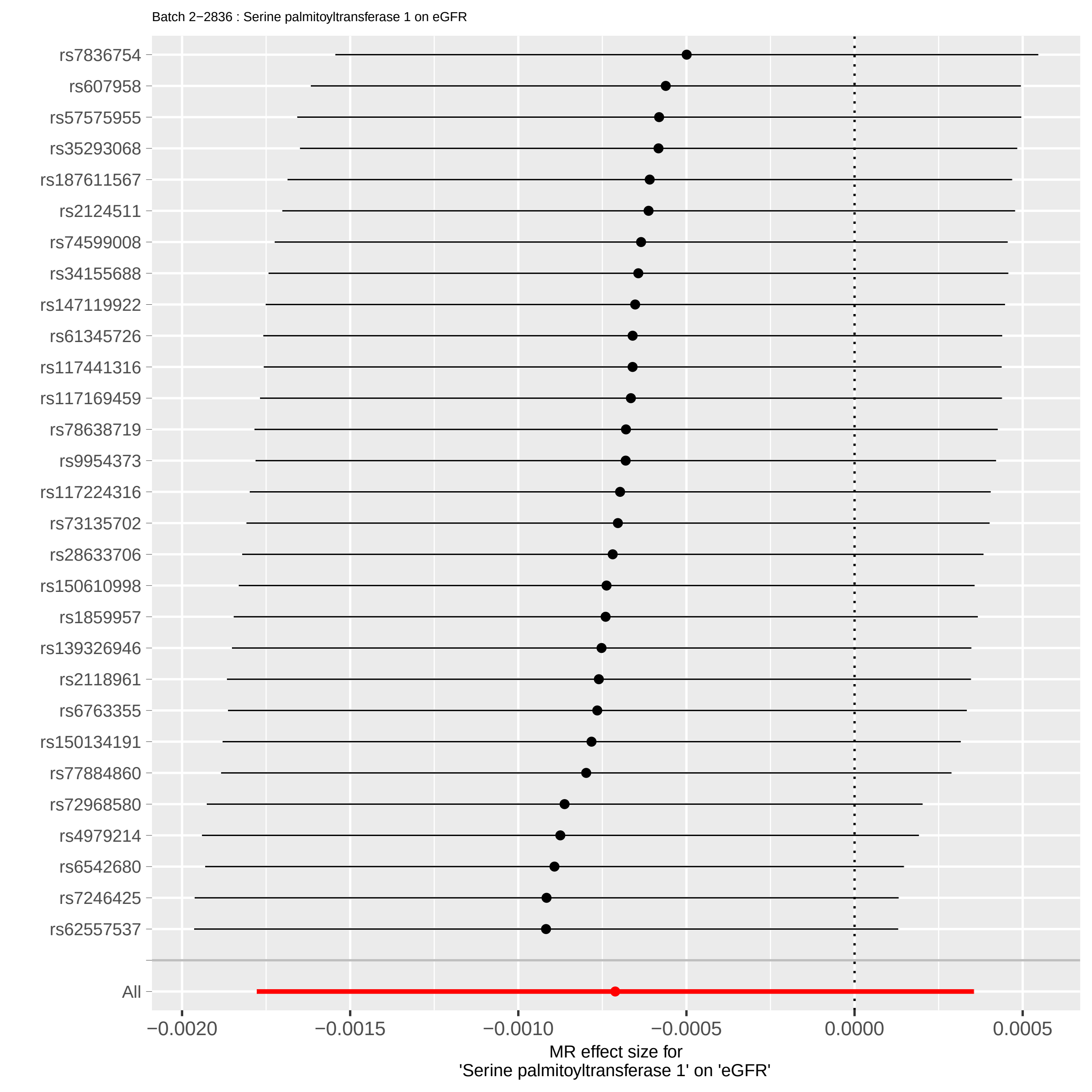
Batch 2−2836 : Serine palmitoyltransferase 1 on eGFR
rs7836754
rs607958
rs57575955
rs35293068
rs187611567
rs2124511
rs74599008
rs34155688
rs147119922
rs61345726
rs117441316
rs117169459
rs78638719
rs9954373
rs117224316
rs73135702
rs28633706
rs150610998
rs1859957
rs139326946
rs2118961
rs6763355
rs150134191
rs77884860
rs72968580
rs4979214
rs6542680
rs7246425
rs62557537
All
−0.0020
−0.0015
−0.0010
−0.0005
0.0000
0.0005
MR effect size for
'Serine palmitoyltransferase 1' on 'eGFR'

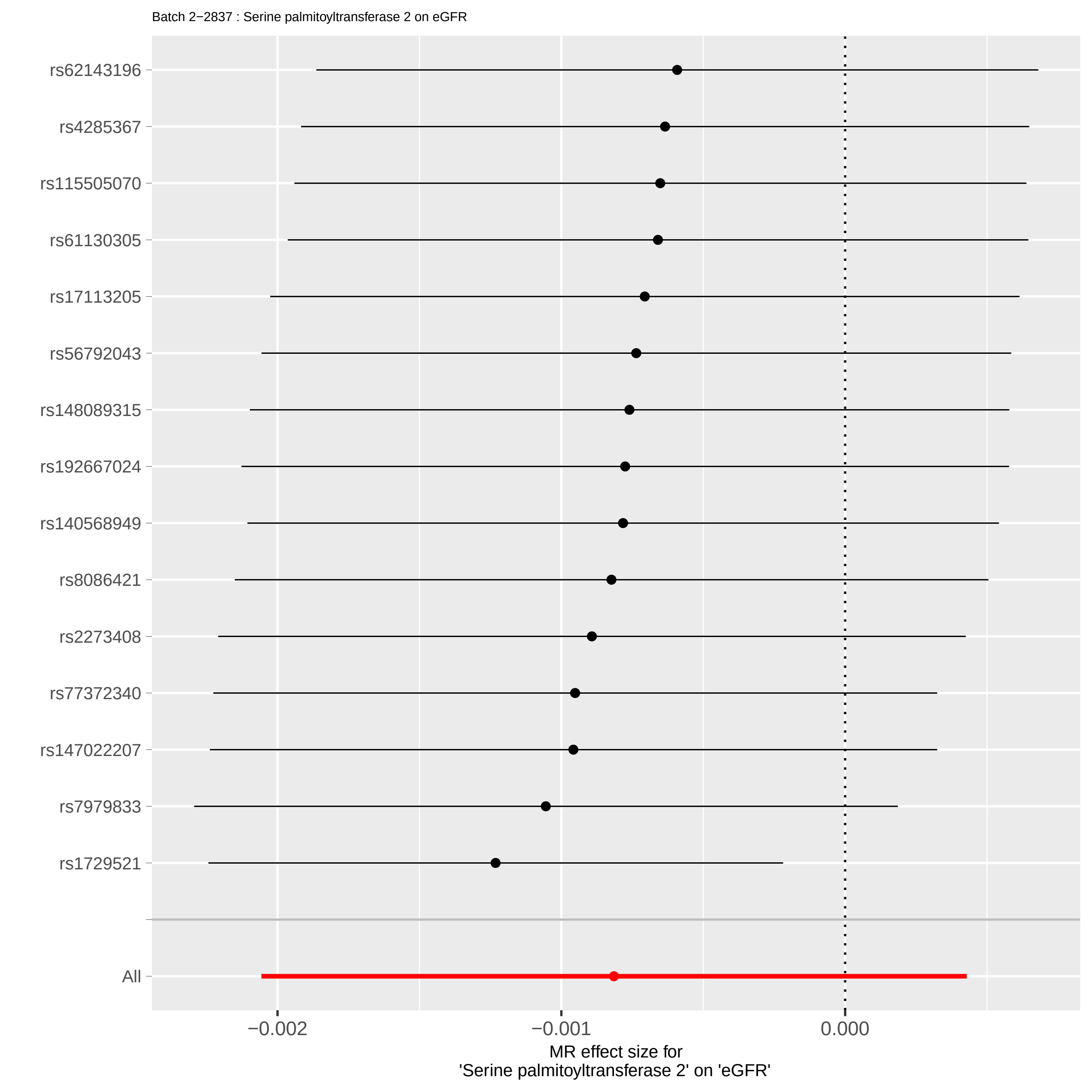
Batch 2−2837 : Serine palmitoyltransferase 2 on eGFR
rs62143196
rs4285367
rs115505070
rs61130305
rs17113205
rs56792043
rs148089315
rs192667024
rs140568949
rs8086421
rs2273408
rs77372340
rs147022207
rs7979833
rs1729521
All
−0.002
−0.001
0.000
MR effect size for
'Serine palmitoyltransferase 2' on 'eGFR'

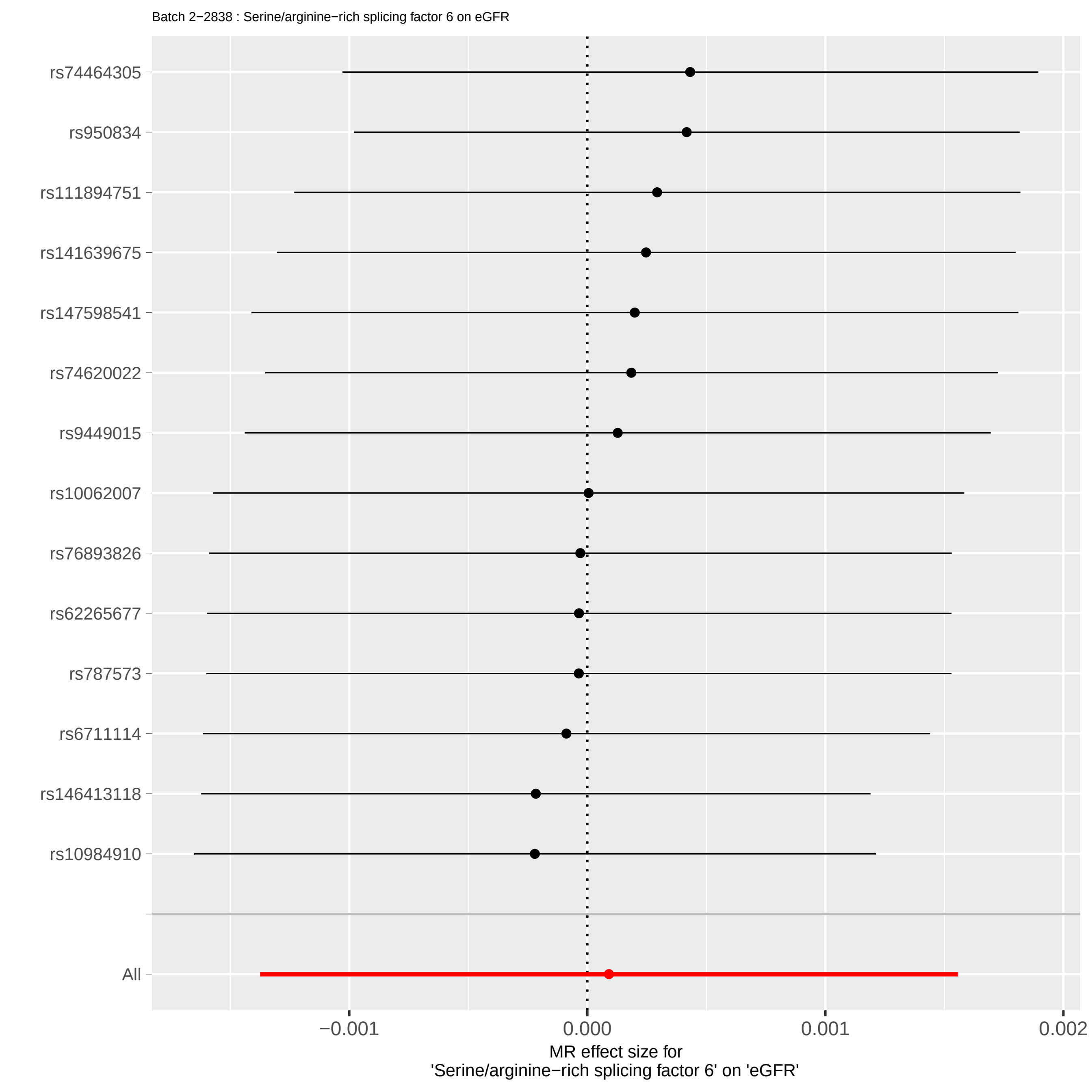

Batch 2−2838 : Serine/arginine−rich splicing factor 6 on eGFR
rs74464305
rs950834
rs111894751
rs141639675
rs147598541
rs74620022
rs9449015
rs10062007
rs76893826
rs62265677
rs787573
rs6711114
rs146413118
rs10984910
All
−0.001
0.000
0.001
0.002
MR effect size for
'Serine/arginine−rich splicing factor 6' on 'eGFR'

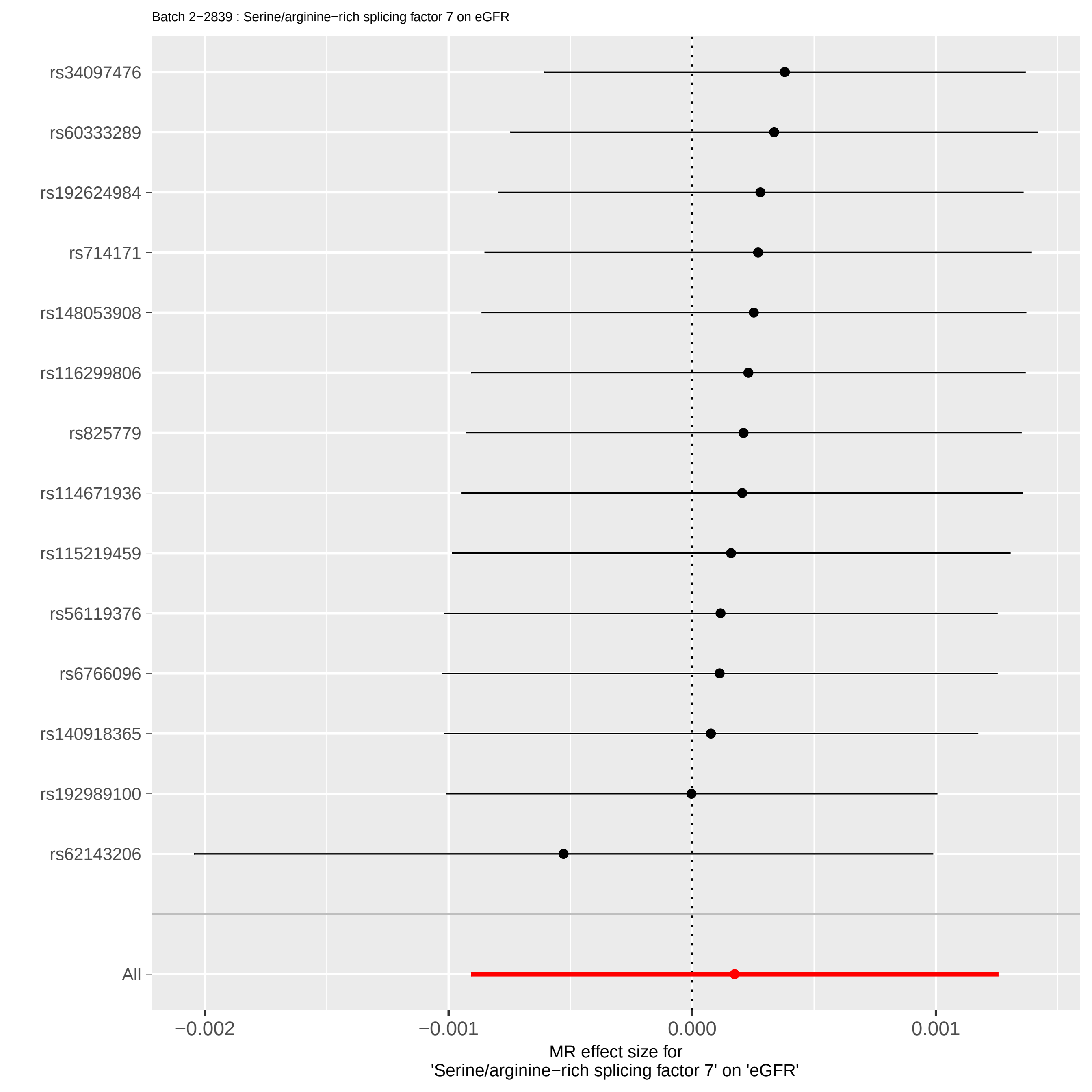

Batch 2−2839 : Serine/arginine−rich splicing factor 7 on eGFR
rs34097476
rs60333289
rs192624984
rs714171
rs148053908
rs116299806
rs825779
rs114671936
rs115219459
rs56119376
rs6766096
rs140918365
rs192989100
rs62143206
All
−0.002
−0.001
0.000
0.001
MR effect size for
'Serine/arginine−rich splicing factor 7' on 'eGFR'

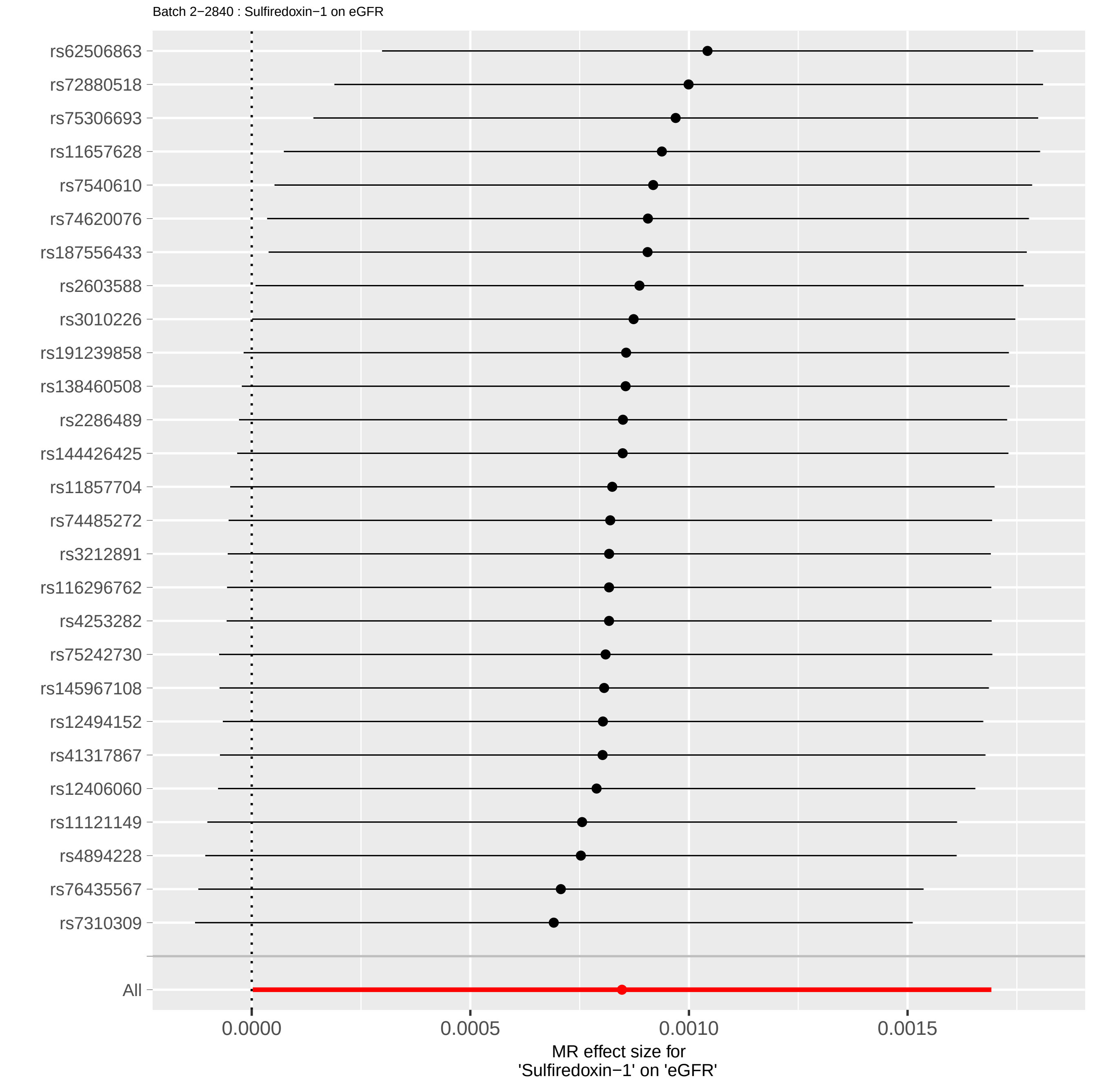
Batch 2−2840 : Sulfiredoxin−1 on eGFR
rs62506863
rs72880518
rs75306693
rs11657628
rs7540610
rs74620076
rs187556433
rs2603588
rs3010226
rs191239858
rs138460508
rs2286489
rs144426425
rs11857704
rs74485272
rs3212891
rs116296762
rs4253282
rs75242730
rs145967108
rs12494152
rs41317867
rs12406060
rs11121149
rs4894228
rs76435567
rs7310309
All
0.0000
0.0005
0.0010
0.0015
MR effect size for
'Sulfiredoxin−1' on 'eGFR'

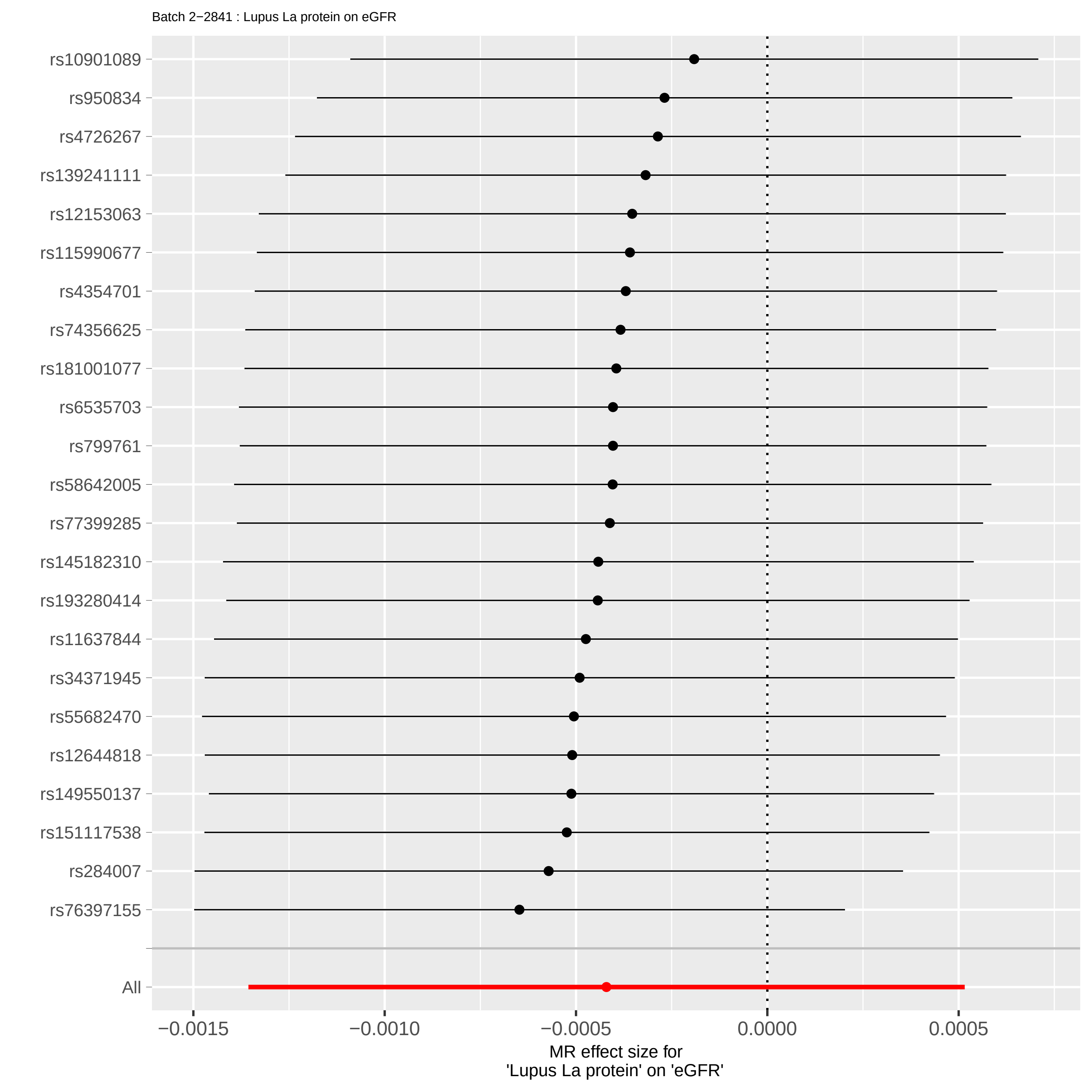
Batch 2−2841 : Lupus La protein on eGFR
rs10901089
rs950834
rs4726267
rs139241111
rs12153063
rs115990677
rs4354701
rs74356625
rs181001077
rs6535703
rs799761
rs58642005
rs77399285
rs145182310
rs193280414
rs11637844
rs34371945
rs55682470
rs12644818
rs149550137
rs151117538
rs284007
rs76397155
All
−0.0015
−0.0010
−0.0005
0.0000
0.0005
MR effect size for
'Lupus La protein' on 'eGFR'

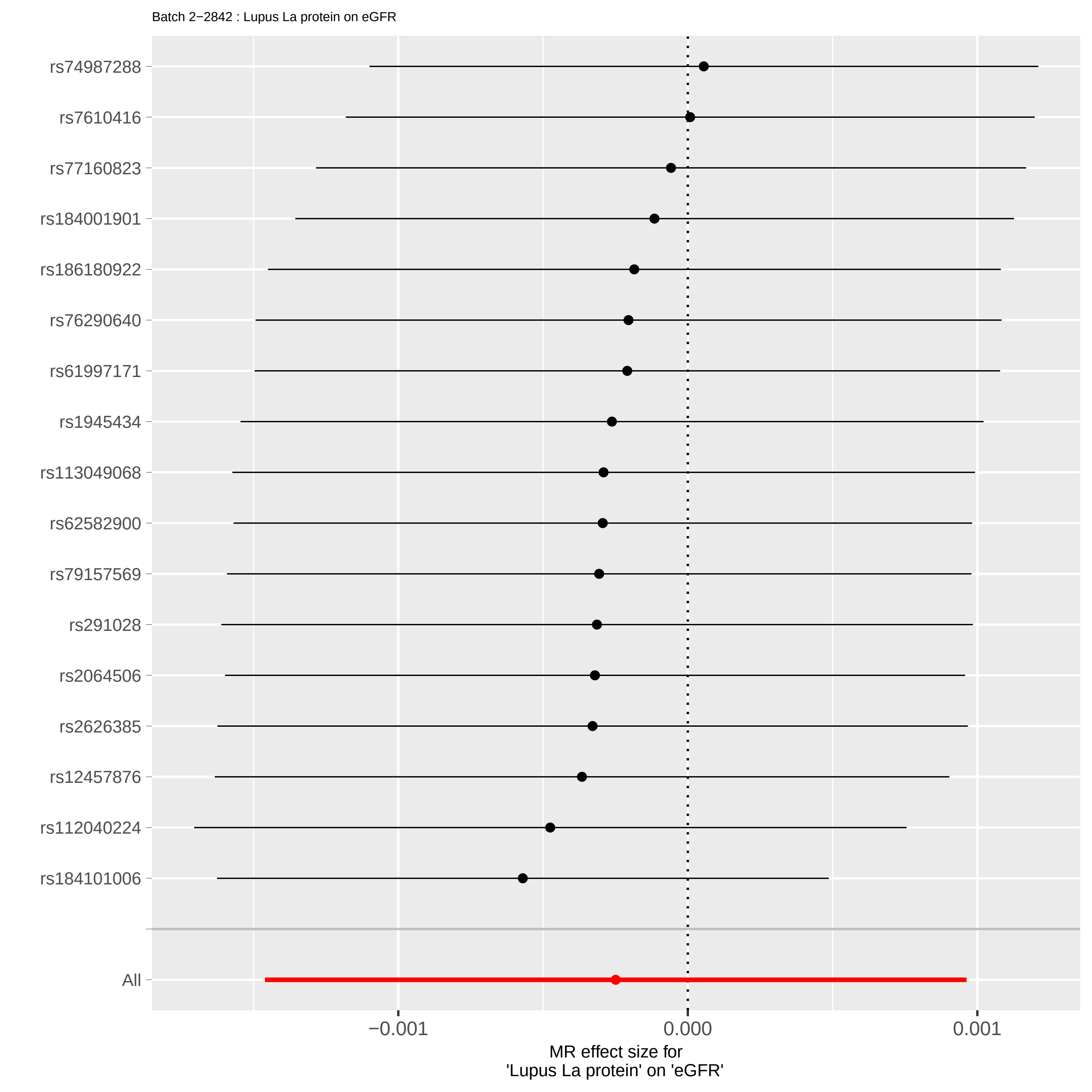

Batch 2−2842 : Lupus La protein on eGFR
rs74987288
rs7610416
rs77160823
rs184001901
rs186180922
rs76290640
rs61997171
rs1945434
rs113049068
rs62582900
rs79157569
rs291028
rs2064506
rs2626385
rs12457876
rs112040224
rs184101006
All
−0.001
0.000
0.001
MR effect size for
'Lupus La protein' on 'eGFR'

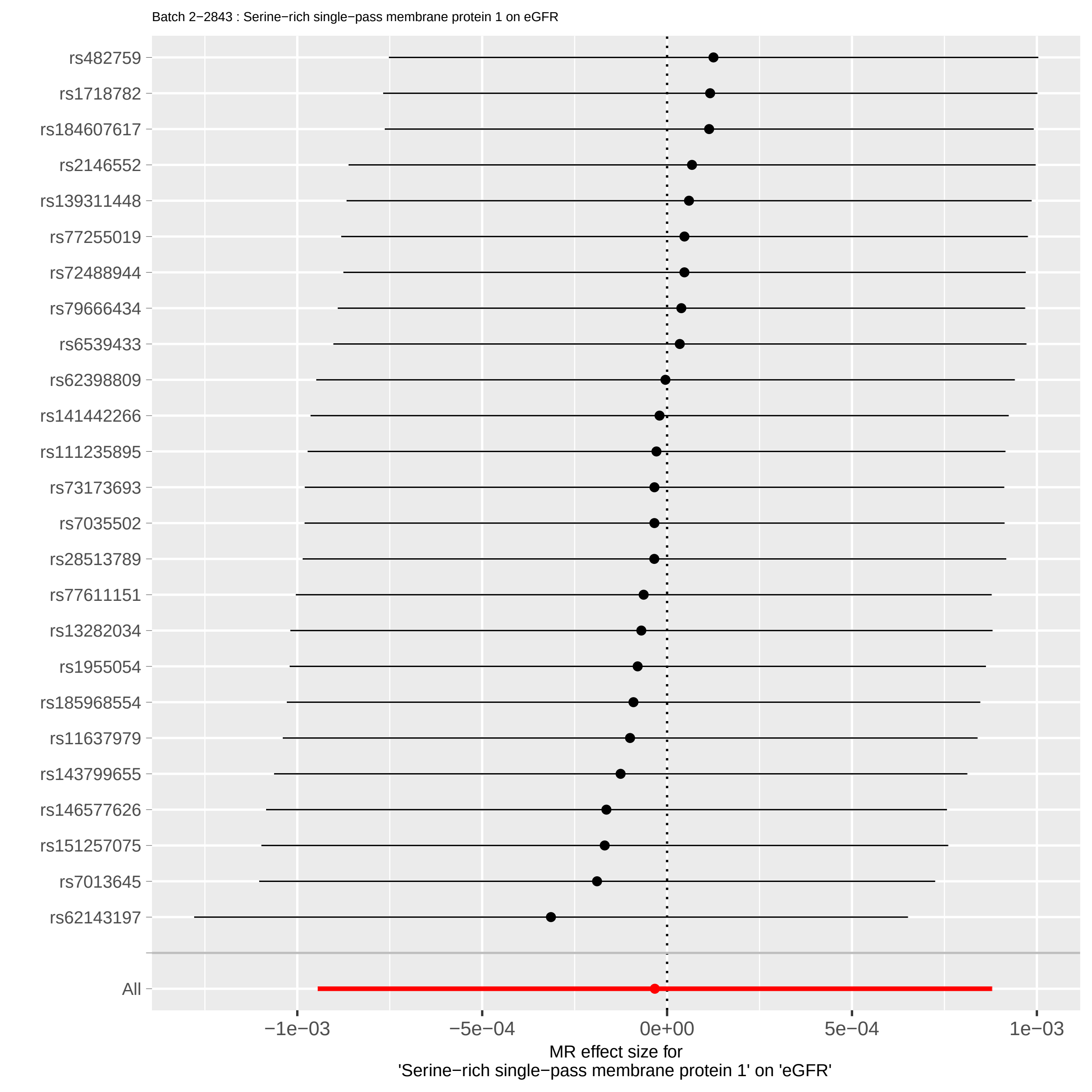

Batch 2−2843 : Serine−rich single−pass membrane protein 1 on eGFR
rs482759
rs1718782
rs184607617
rs2146552
rs139311448
rs77255019
rs72488944
rs79666434
rs6539433
rs62398809
rs141442266
rs111235895
rs73173693
rs7035502
rs28513789
rs77611151
rs13282034
rs1955054
rs185968554
rs11637979
rs143799655
rs146577626
rs151257075
rs7013645
rs62143197
All
−1e−03
−5e−04
0e+00
5e−04
1e−03
MR effect size for
'Serine−rich single−pass membrane protein 1' on 'eGFR'

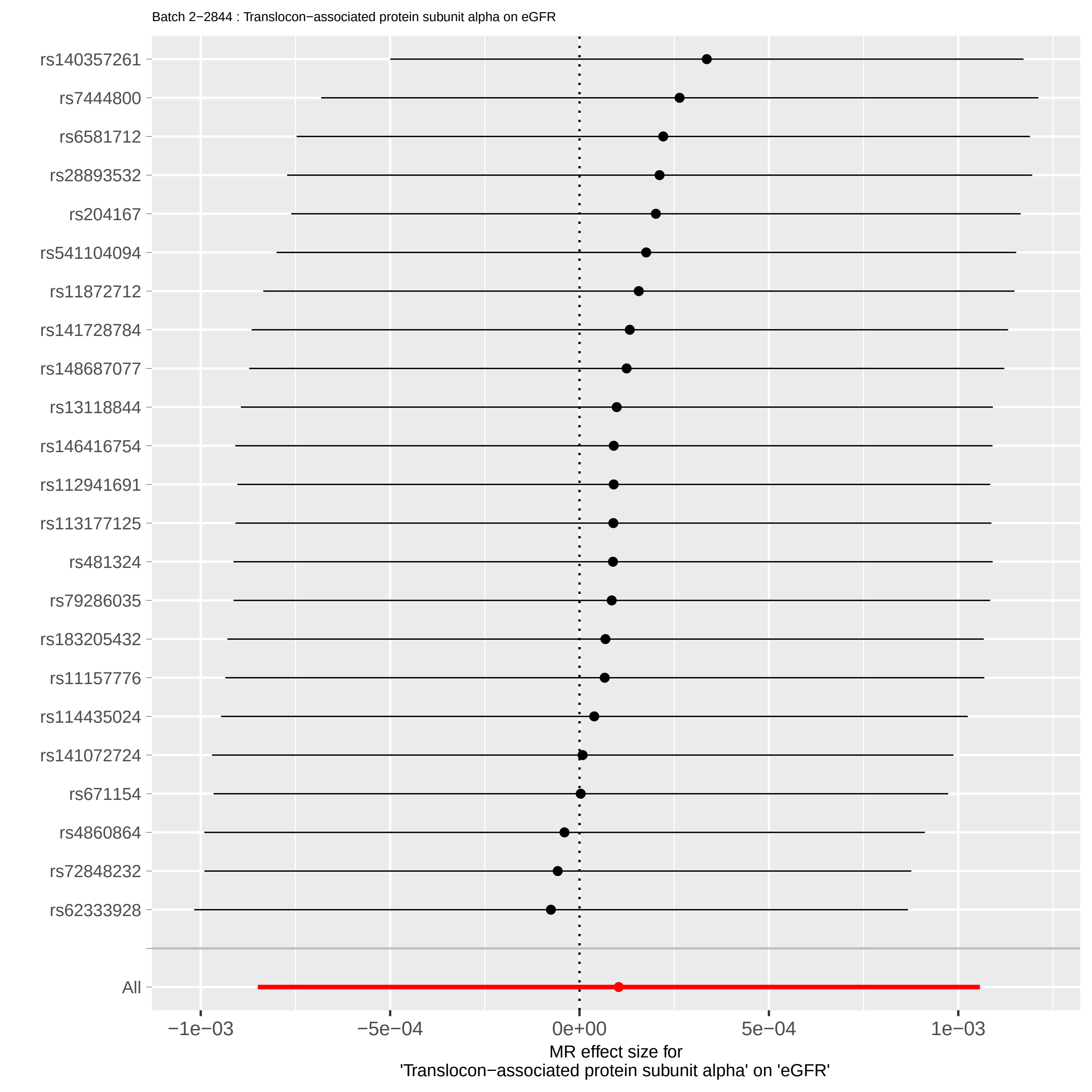
Batch 2−2844 : Translocon−associated protein subunit alpha on eGFR
rs140357261
rs7444800
rs6581712
rs28893532
rs204167
rs541104094
rs11872712
rs141728784
rs148687077
rs13118844
rs146416754
rs112941691
rs113177125
rs481324
rs79286035
rs183205432
rs11157776
rs114435024
rs141072724
rs671154
rs4860864
rs72848232
rs62333928
All
−1e−03
−5e−04
0e+00
5e−04
1e−03
MR effect size for
'Translocon−associated protein subunit alpha' on 'eGFR'

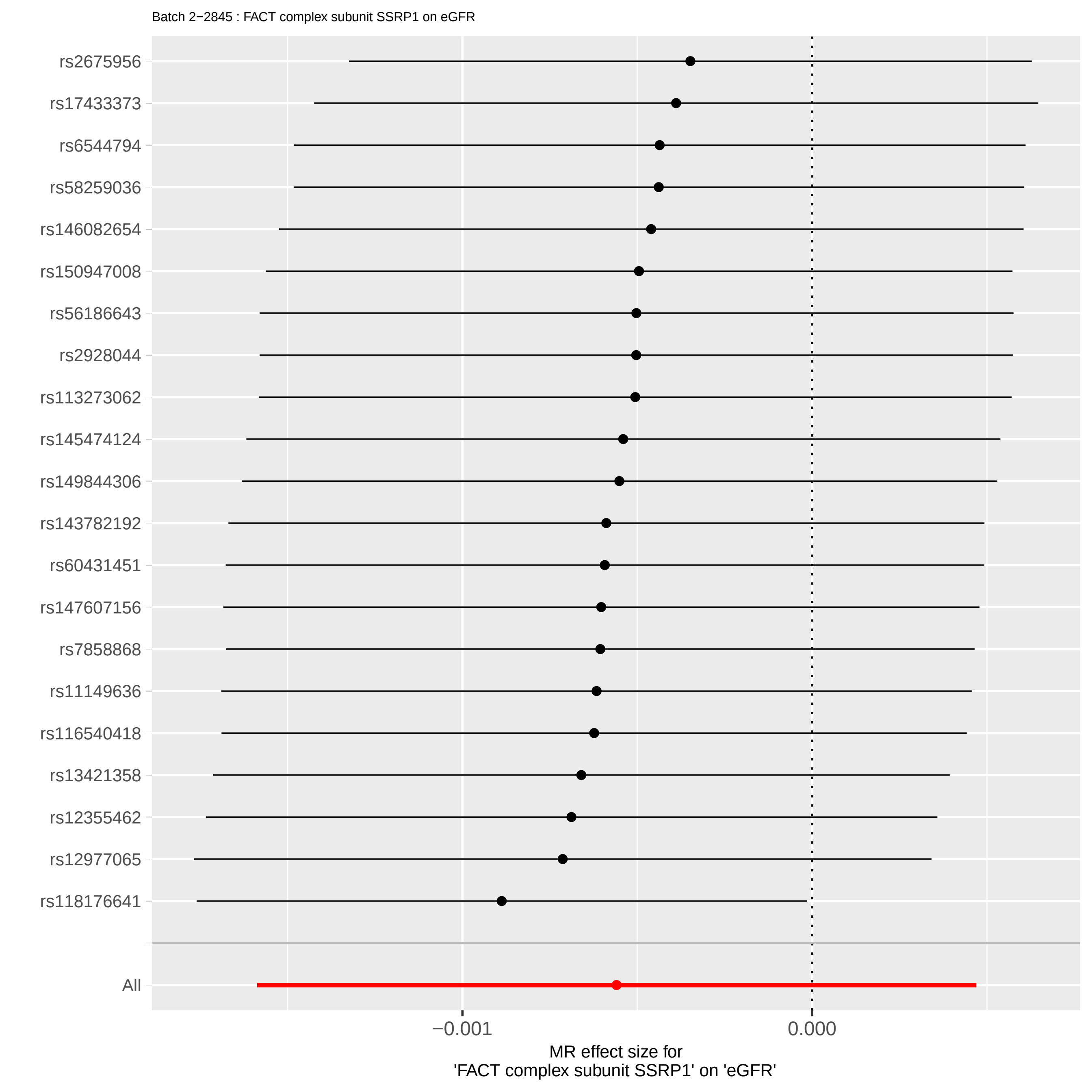
Batch 2−2845 : FACT complex subunit SSRP1 on eGFR
rs2675956
rs17433373
rs6544794
rs58259036
rs146082654
rs150947008
rs56186643
rs2928044
rs113273062
rs145474124
rs149844306
rs143782192
rs60431451
rs147607156
rs7858868
rs11149636
rs116540418
rs13421358
rs12355462
rs12977065
rs118176641
All
−0.001
0.000
MR effect size for
'FACT complex subunit SSRP1' on 'eGFR'

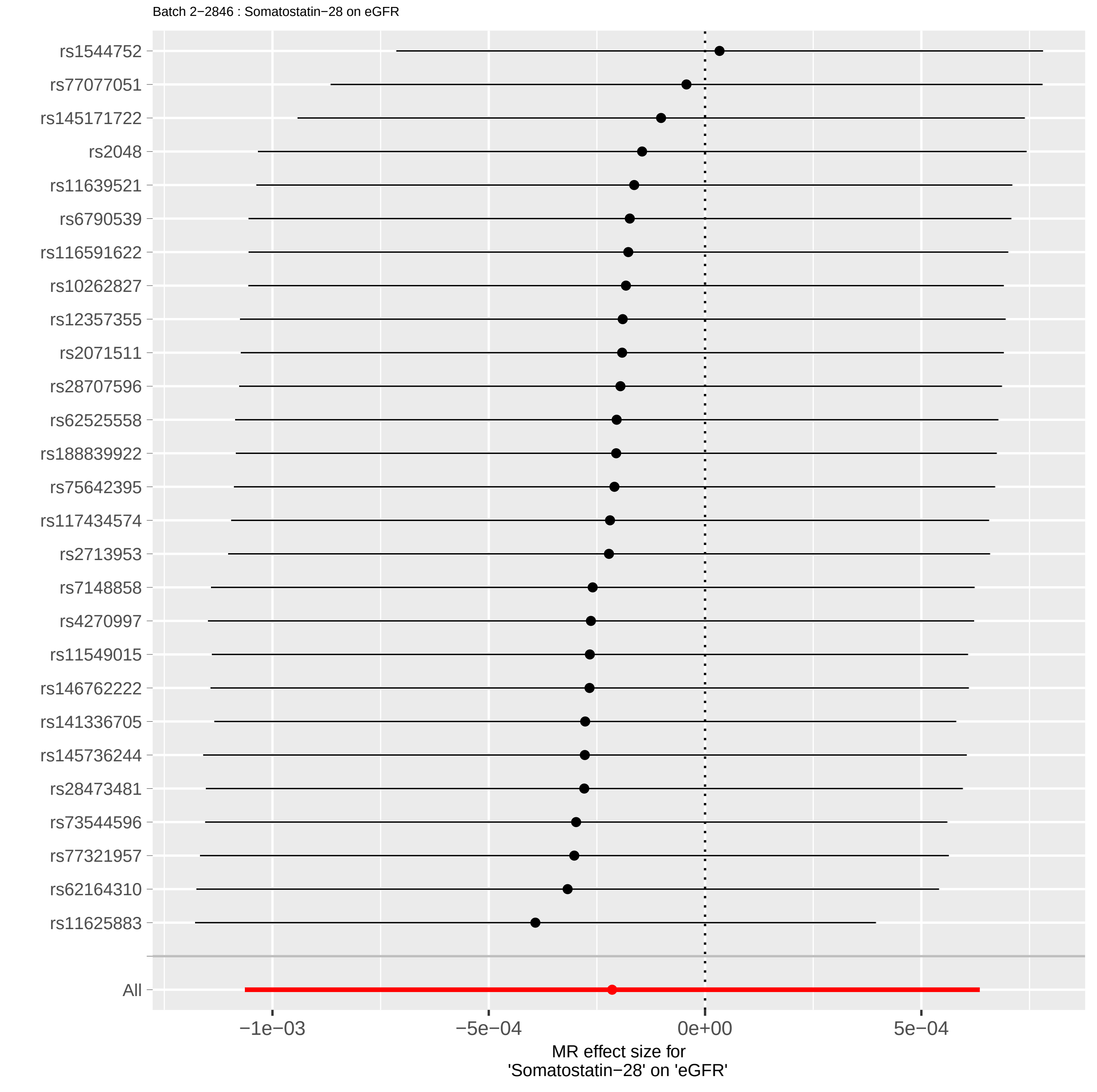

Batch 2−2846 : Somatostatin−28 on eGFR
rs1544752
rs77077051
rs145171722
rs2048
rs11639521
rs6790539
rs116591622
rs10262827
rs12357355
rs2071511
rs28707596
rs62525558
rs188839922
rs75642395
rs117434574
rs2713953
rs7148858
rs4270997
rs11549015
rs146762222
rs141336705
rs145736244
rs28473481
rs73544596
rs77321957
rs62164310
rs11625883
All
−1e−03
−5e−04
0e+00
5e−04
MR effect size for
'Somatostatin−28' on 'eGFR'

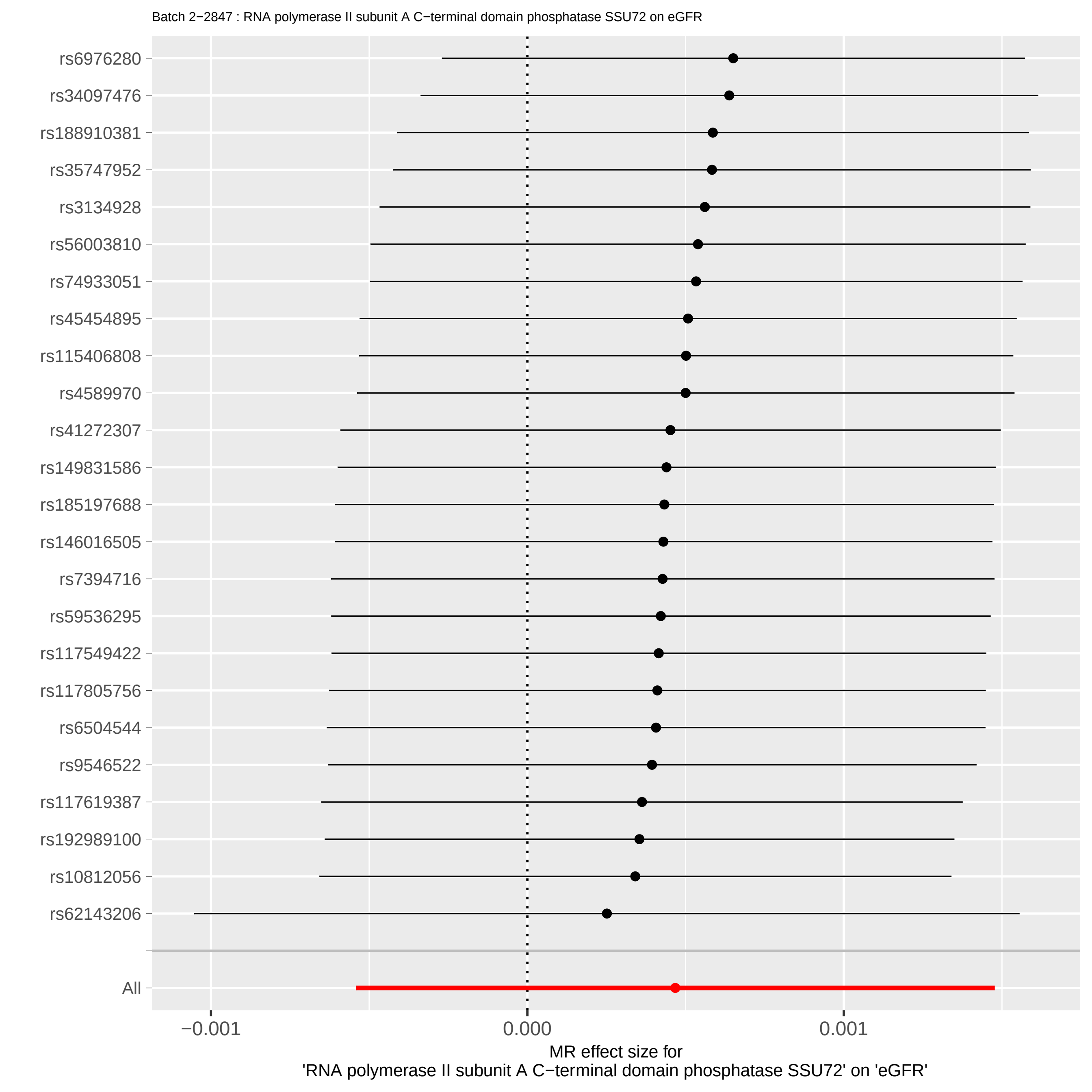

Batch 2−2847 : RNA polymerase II subunit A C−terminal domain phosphatase SSU72 on eGFR
rs6976280
rs34097476
rs188910381
rs35747952
rs3134928
rs56003810
rs74933051
rs45454895
rs115406808
rs4589970
rs41272307
rs149831586
rs185197688
rs146016505
rs7394716
rs59536295
rs117549422
rs117805756
rs6504544
rs9546522
rs117619387
rs192989100
rs10812056
rs62143206
All
−0.001
0.000
0.001
MR effect size for
'RNA polymerase II subunit A C−terminal domain phosphatase SSU72' on 'eGFR'

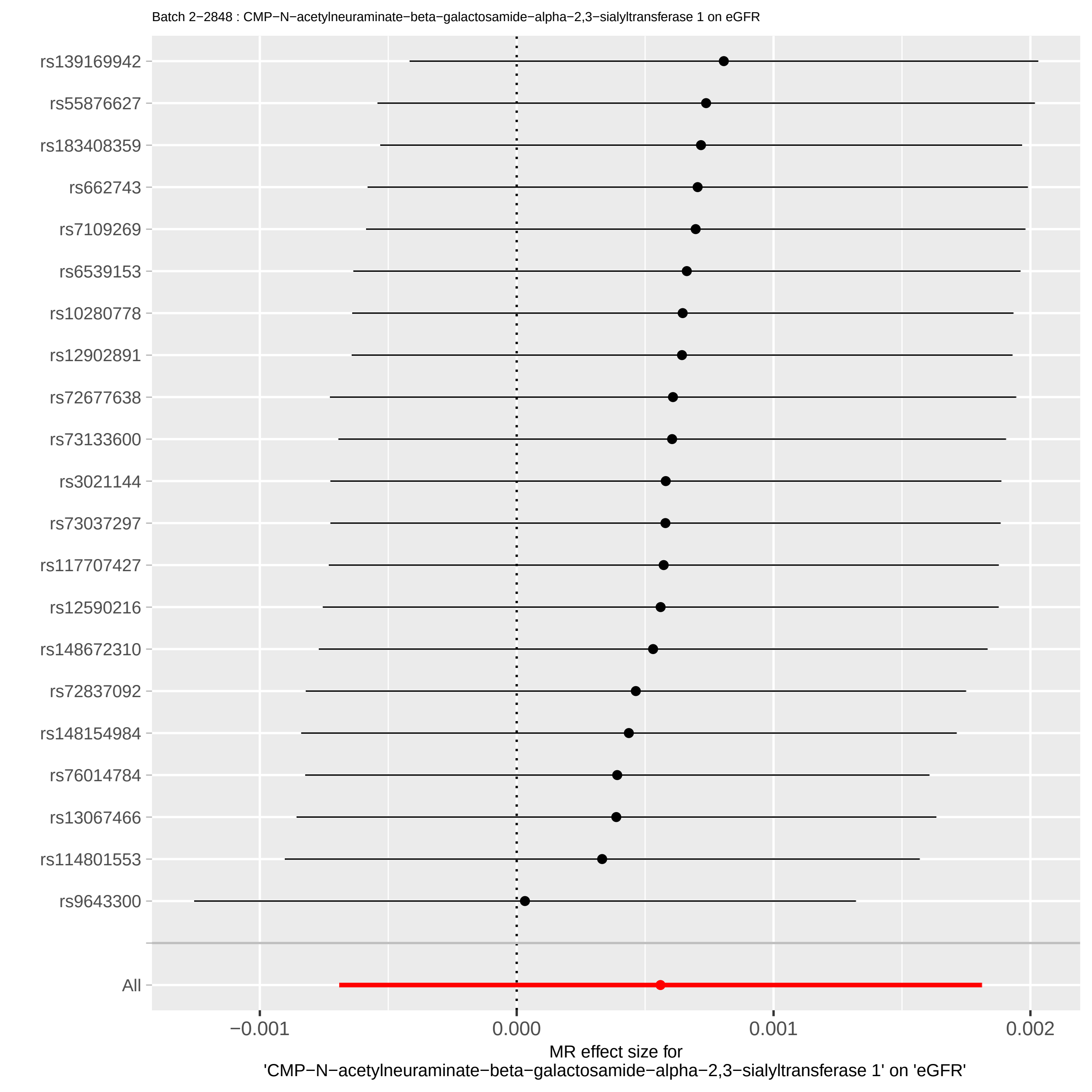

Batch 2−2848 : CMP−N−acetylneuraminate−beta−galactosamide−alpha−2,3−sialyltransferase 1 on eGFR
rs139169942
rs55876627
rs183408359
rs662743
rs7109269
rs6539153
rs10280778
rs12902891
rs72677638
rs73133600
rs3021144
rs73037297
rs117707427
rs12590216
rs148672310
rs72837092
rs148154984
rs76014784
rs13067466
rs114801553
rs9643300
All
−0.001
0.000
0.001
0.002
MR effect size for
'CMP−N−acetylneuraminate−beta−galactosamide−alpha−2,3−sialyltransferase 1' on 'eGFR'

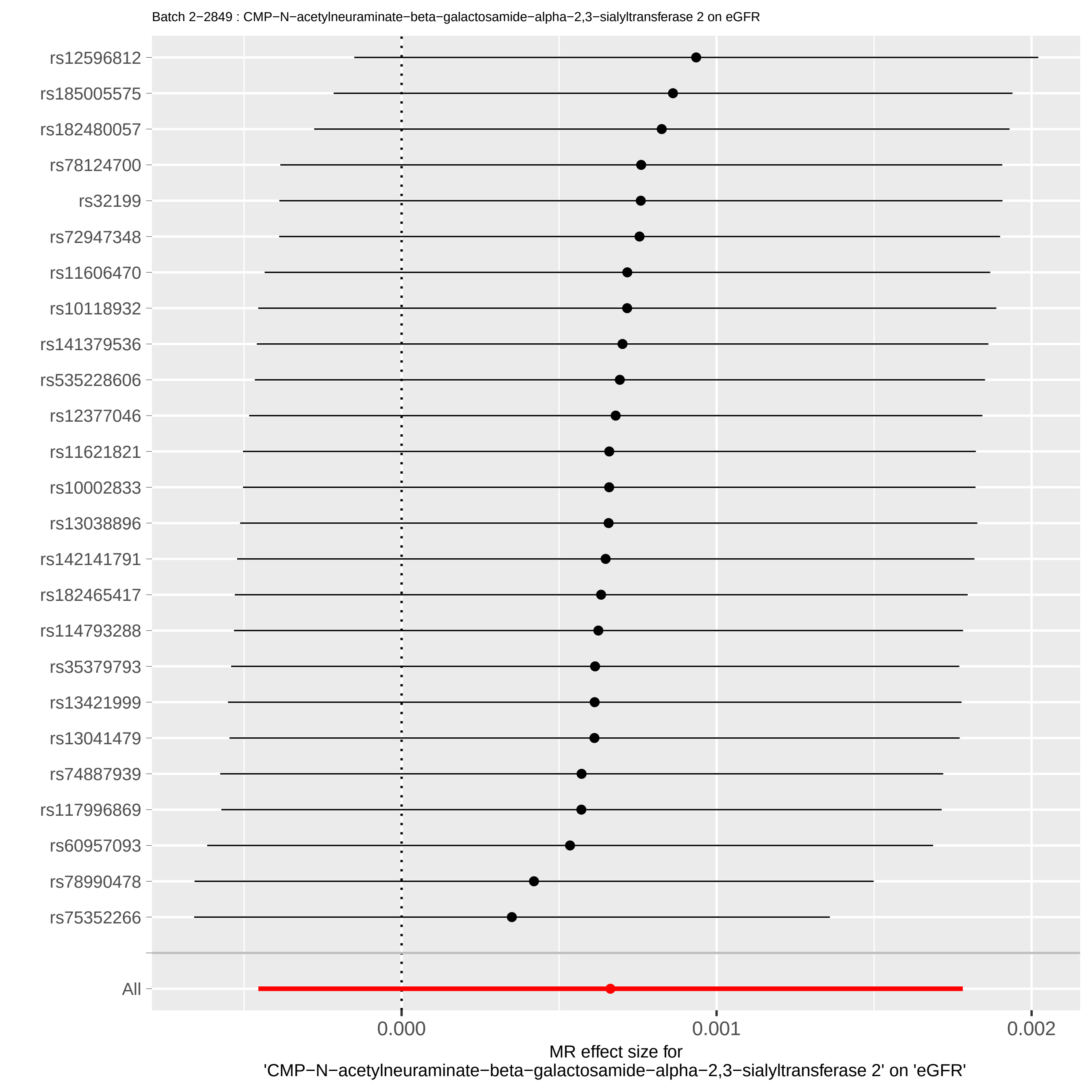
Batch 2−2849 : CMP−N−acetylneuraminate−beta−galactosamide−alpha−2,3−sialyltransferase 2 on eGFR
rs12596812
rs185005575
rs182480057
rs78124700
rs32199
rs72947348
rs11606470
rs10118932
rs141379536
rs535228606
rs12377046
rs11621821
rs10002833
rs13038896
rs142141791
rs182465417
rs114793288
rs35379793
rs13421999
rs13041479
rs74887939
rs117996869
rs60957093
rs78990478
rs75352266
All
0.000
0.001
0.002
MR effect size for
'CMP−N−acetylneuraminate−beta−galactosamide−alpha−2,3−sialyltransferase 2' on 'eGFR'

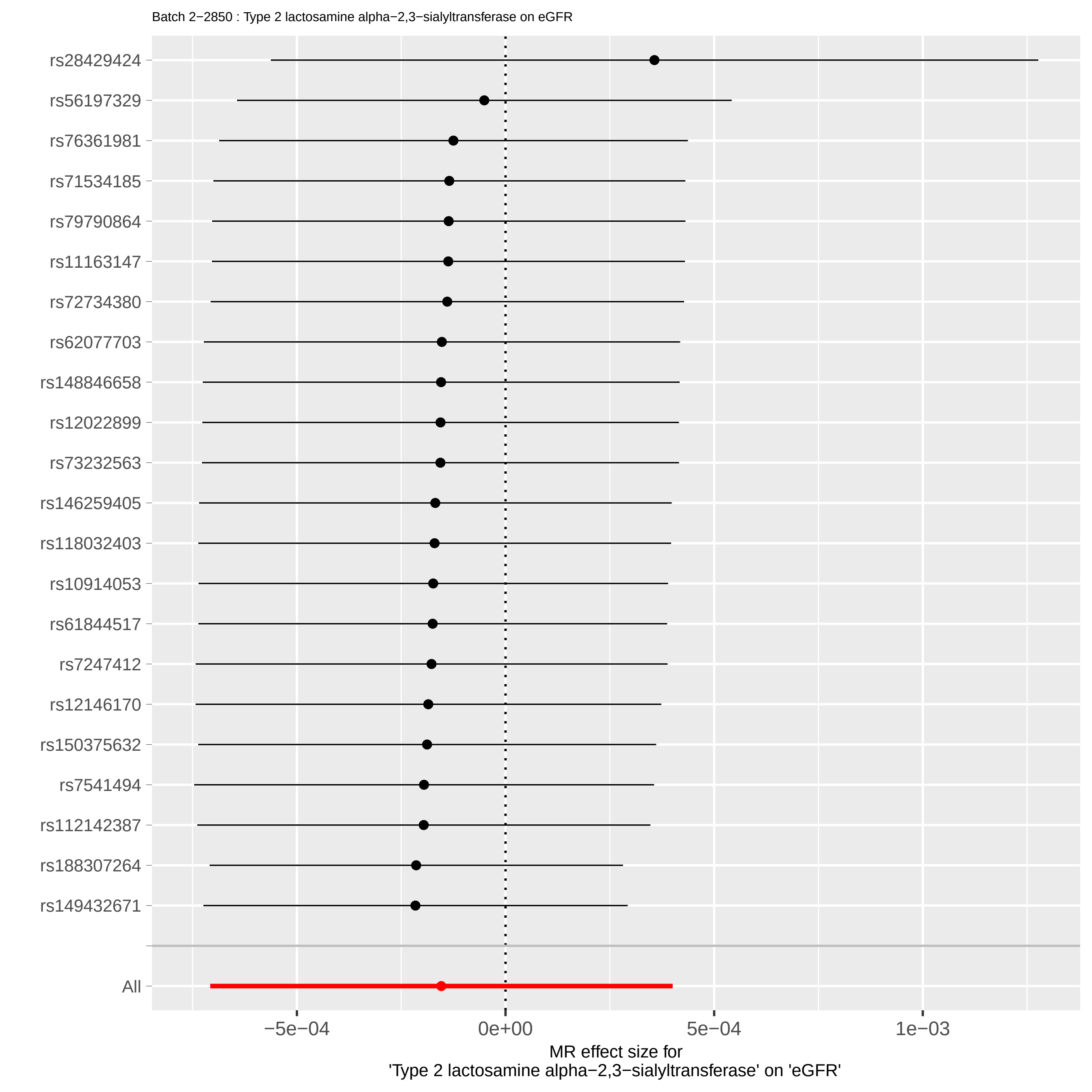

Batch 2−2850 : Type 2 lactosamine alpha−2,3−sialyltransferase on eGFR
rs28429424
rs56197329
rs76361981
rs71534185
rs79790864
rs11163147
rs72734380
rs62077703
rs148846658
rs12022899
rs73232563
rs146259405
rs118032403
rs10914053
rs61844517
rs7247412
rs12146170
rs150375632
rs7541494
rs112142387
rs188307264
rs149432671
All
−5e−04
0e+00
5e−04
1e−03
MR effect size for
'Type 2 lactosamine alpha−2,3−sialyltransferase' on 'eGFR'

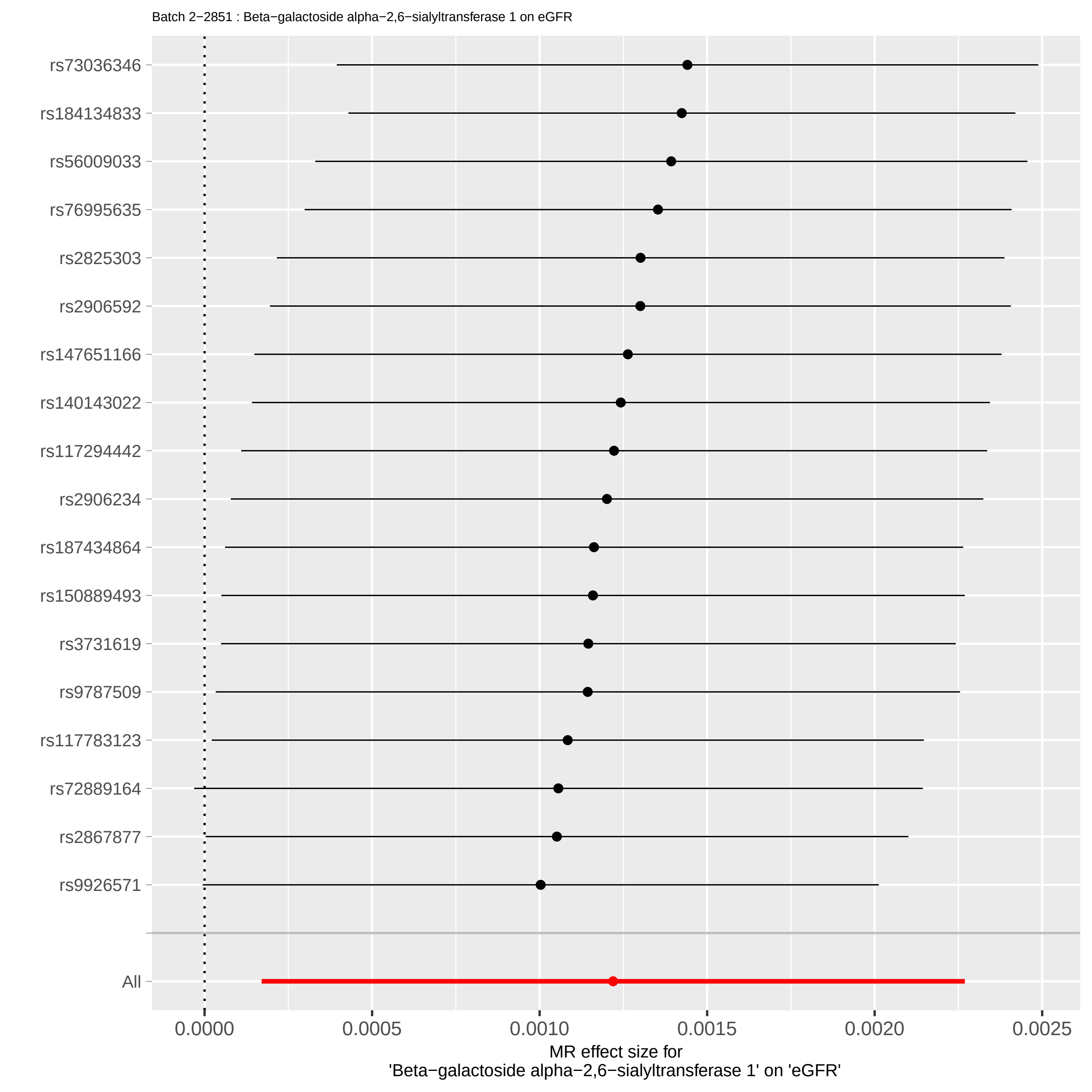

Batch 2−2851 : Beta−galactoside alpha−2,6−sialyltransferase 1 on eGFR
rs73036346
rs184134833
rs56009033
rs76995635
rs2825303
rs2906592
rs147651166
rs140143022
rs117294442
rs2906234
rs187434864
rs150889493
rs3731619
rs9787509
rs117783123
rs72889164
rs2867877
rs9926571
All
0.0000
0.0005
0.0010
0.0015
0.0020
0.0025
MR effect size for
'Beta−galactoside alpha−2,6−sialyltransferase 1' on 'eGFR'

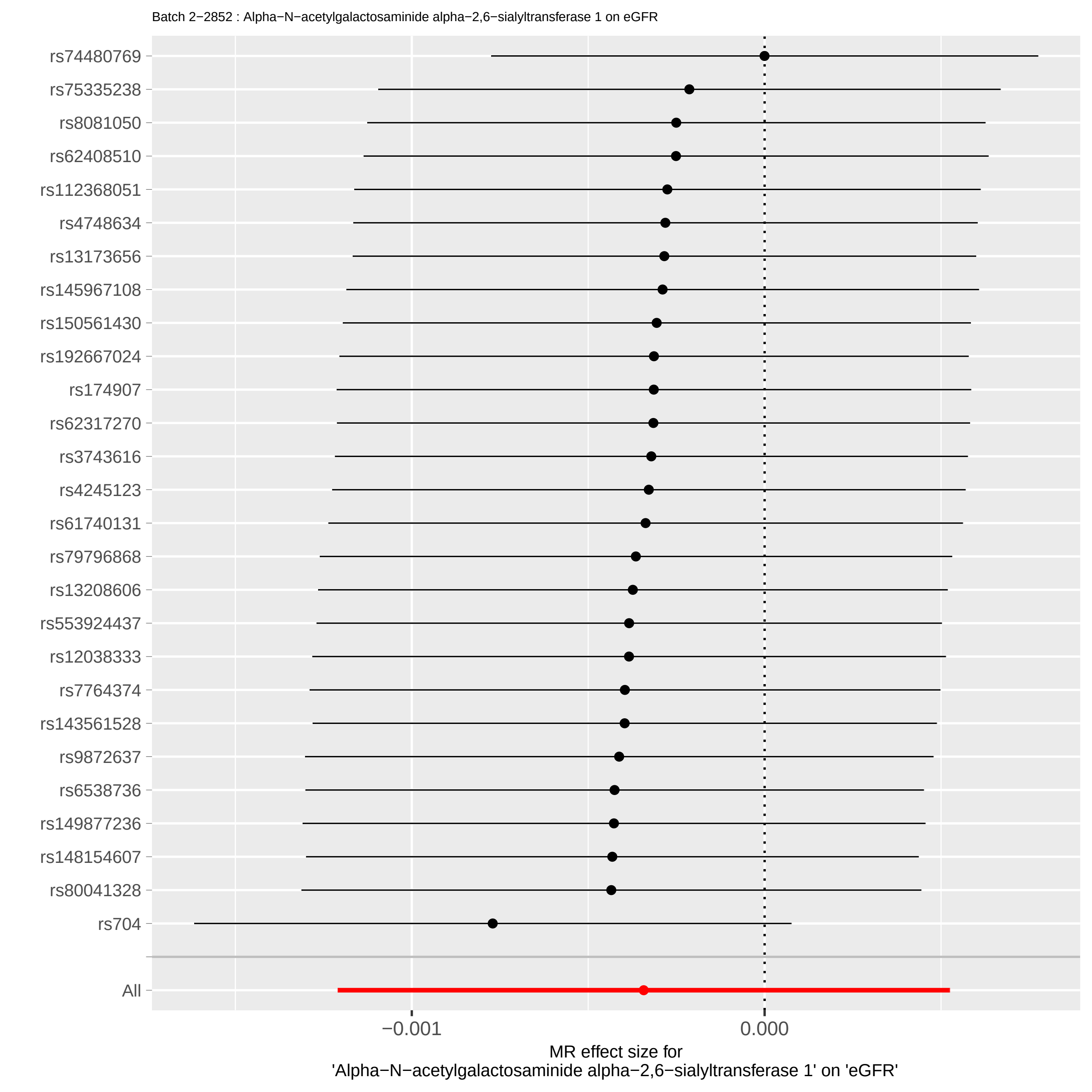

Batch 2−2852 : Alpha−N−acetylgalactosaminide alpha−2,6−sialyltransferase 1 on eGFR
rs74480769
rs75335238
rs8081050
rs62408510
rs112368051
rs4748634
rs13173656
rs145967108
rs150561430
rs192667024
rs174907
rs62317270
rs3743616
rs4245123
rs61740131
rs79796868
rs13208606
rs553924437
rs12038333
rs7764374
rs143561528
rs9872637
rs6538736
rs149877236
rs148154607
rs80041328
rs704
All
−0.001
0.000
MR effect size for
'Alpha−N−acetylgalactosaminide alpha−2,6−sialyltransferase 1' on 'eGFR'

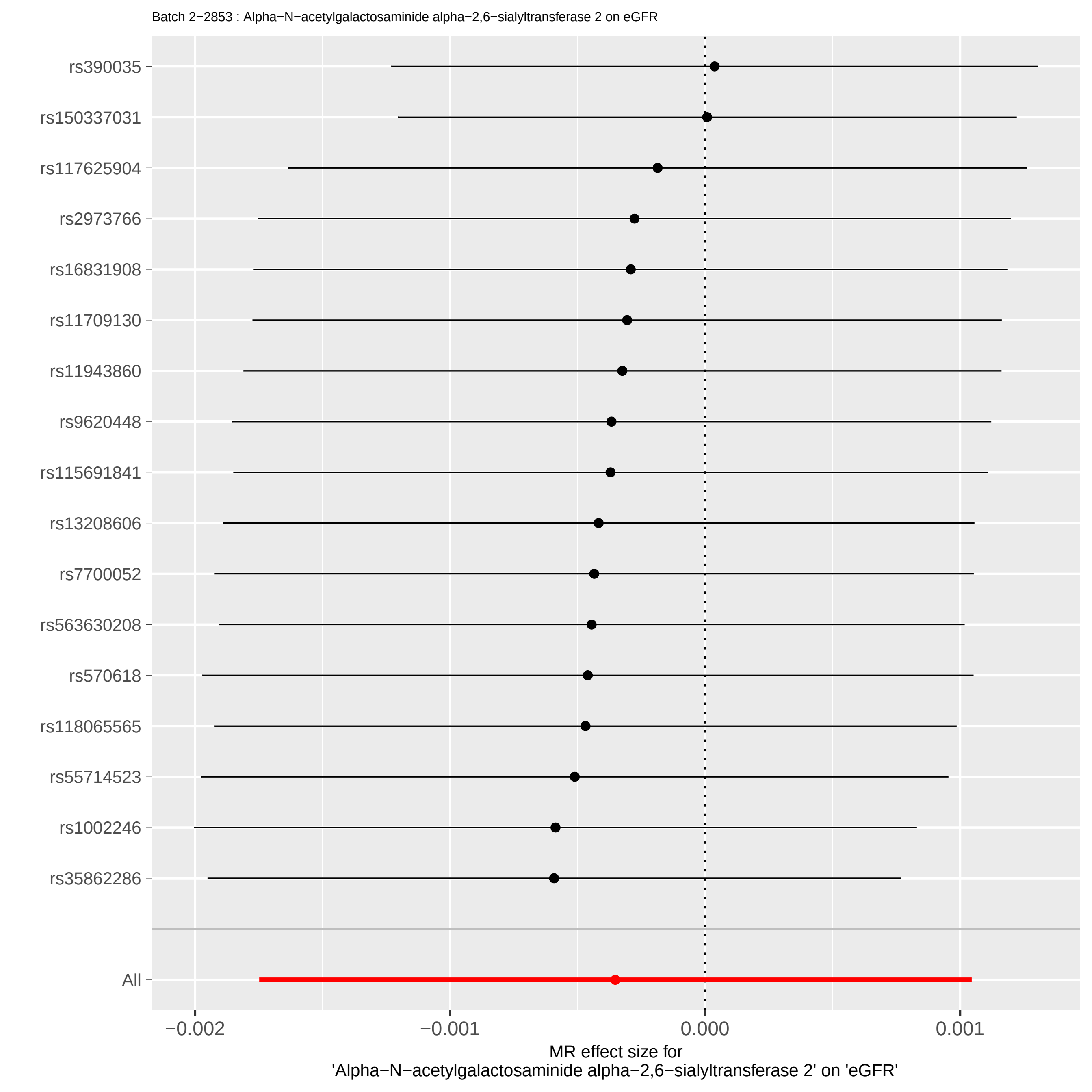

Batch 2−2853 : Alpha−N−acetylgalactosaminide alpha−2,6−sialyltransferase 2 on eGFR
rs390035
rs150337031
rs117625904
rs2973766
rs16831908
rs11709130
rs11943860
rs9620448
rs115691841
rs13208606
rs7700052
rs563630208
rs570618
rs118065565
rs55714523
rs1002246
rs35862286
All
−0.002
−0.001
0.000
0.001
MR effect size for
'Alpha−N−acetylgalactosaminide alpha−2,6−sialyltransferase 2' on 'eGFR'

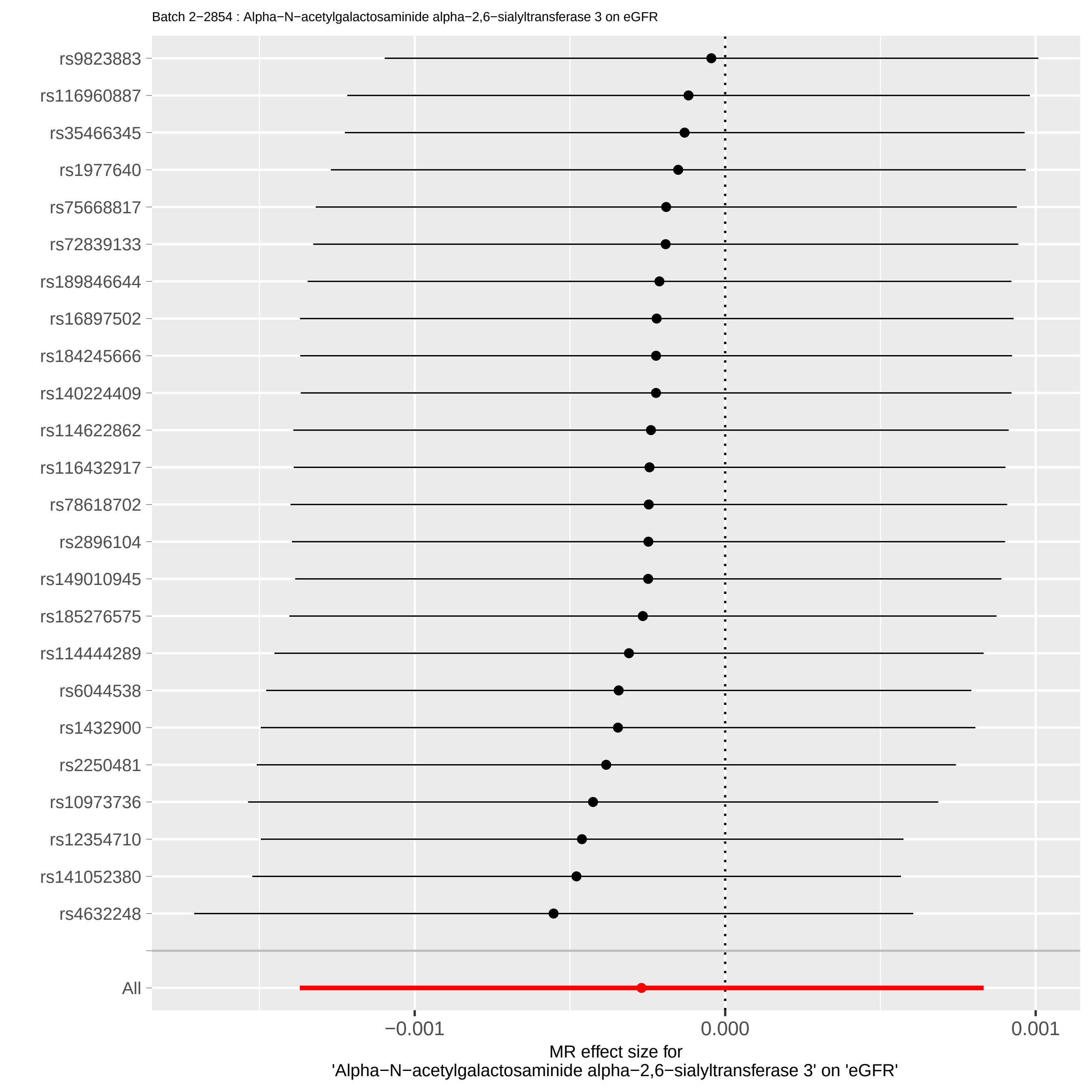

Batch 2−2854 : Alpha−N−acetylgalactosaminide alpha−2,6−sialyltransferase 3 on eGFR
rs9823883
rs116960887
rs35466345
rs1977640
rs75668817
rs72839133
rs189846644
rs16897502
rs184245666
rs140224409
rs114622862
rs116432917
rs78618702
rs2896104
rs149010945
rs185276575
rs114444289
rs6044538
rs1432900
rs2250481
rs10973736
rs12354710
rs141052380
rs4632248
All
−0.001
0.000
0.001
MR effect size for
'Alpha−N−acetylgalactosaminide alpha−2,6−sialyltransferase 3' on 'eGFR'

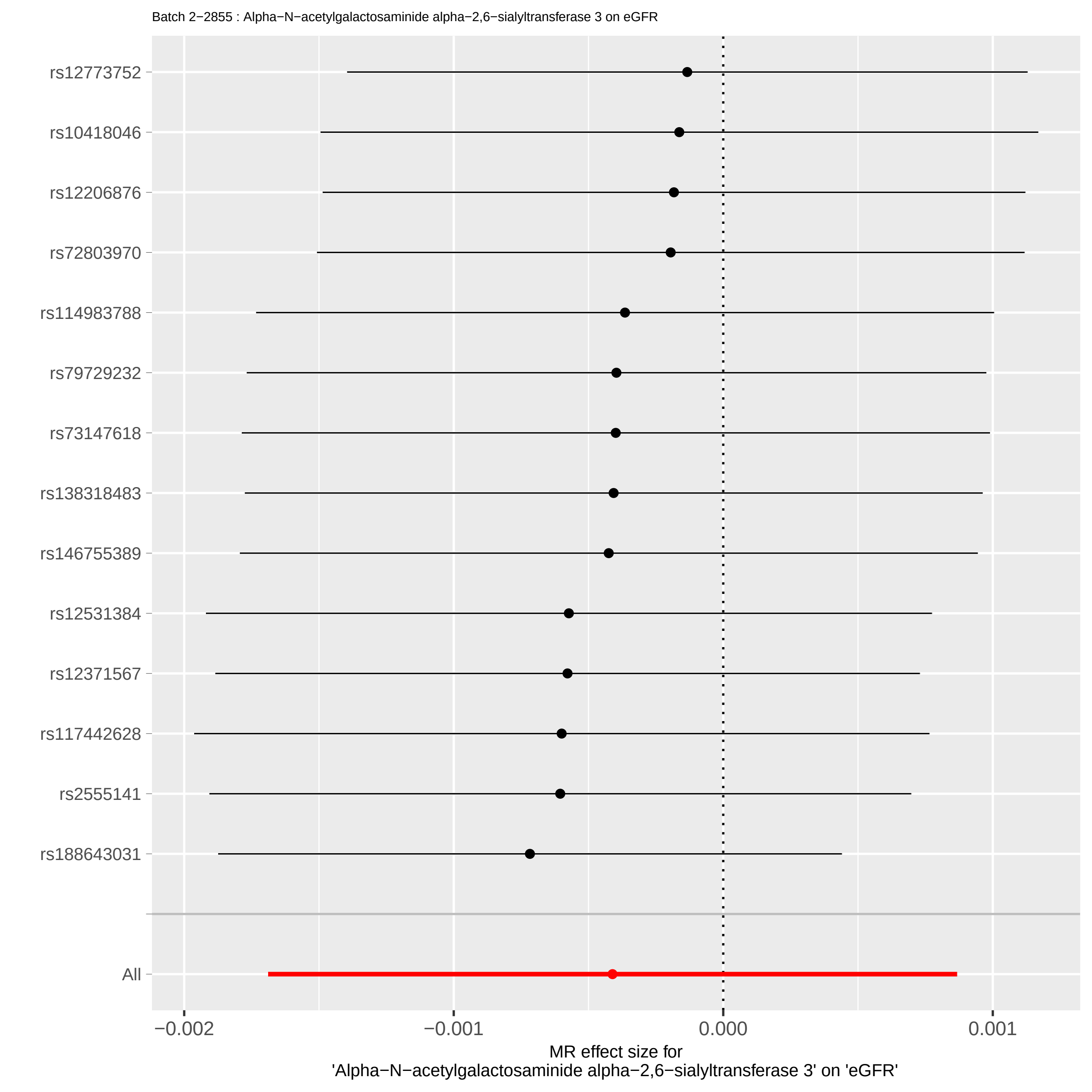

Batch 2−2855 : Alpha−N−acetylgalactosaminide alpha−2,6−sialyltransferase 3 on eGFR
rs12773752
rs10418046
rs12206876
rs72803970
rs114983788
rs79729232
rs73147618
rs138318483
rs146755389
rs12531384
rs12371567
rs117442628
rs2555141
rs188643031
All
−0.002
−0.001
0.000
0.001
MR effect size for
'Alpha−N−acetylgalactosaminide alpha−2,6−sialyltransferase 3' on 'eGFR'

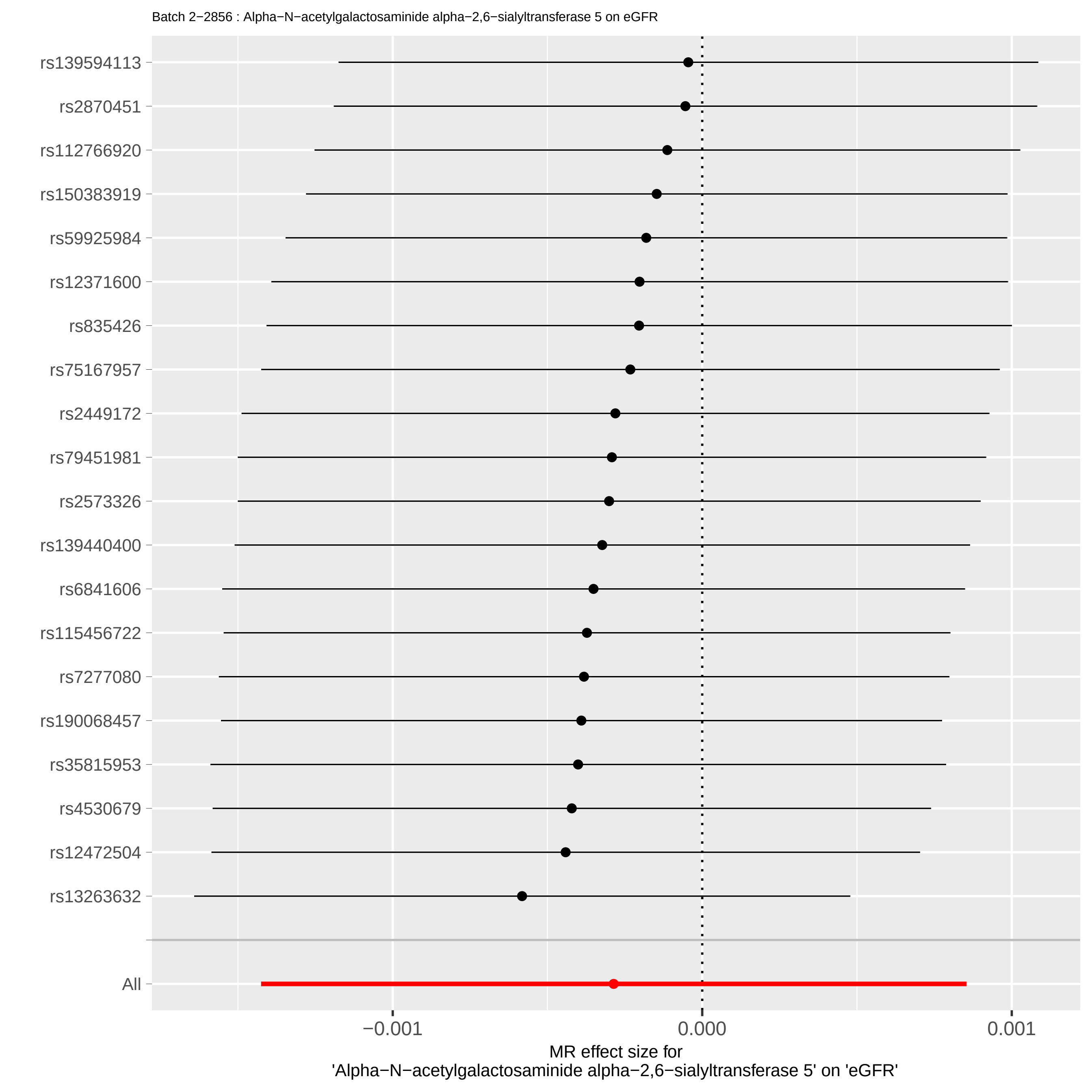
Batch 2−2856 : Alpha−N−acetylgalactosaminide alpha−2,6−sialyltransferase 5 on eGFR
rs139594113
rs2870451
rs112766920
rs150383919
rs59925984
rs12371600
rs835426
rs75167957
rs2449172
rs79451981
rs2573326
rs139440400
rs6841606
rs115456722
rs7277080
rs190068457
rs35815953
rs4530679
rs12472504
rs13263632
All
−0.001
0.000
0.001
MR effect size for
'Alpha−N−acetylgalactosaminide alpha−2,6−sialyltransferase 5' on 'eGFR'

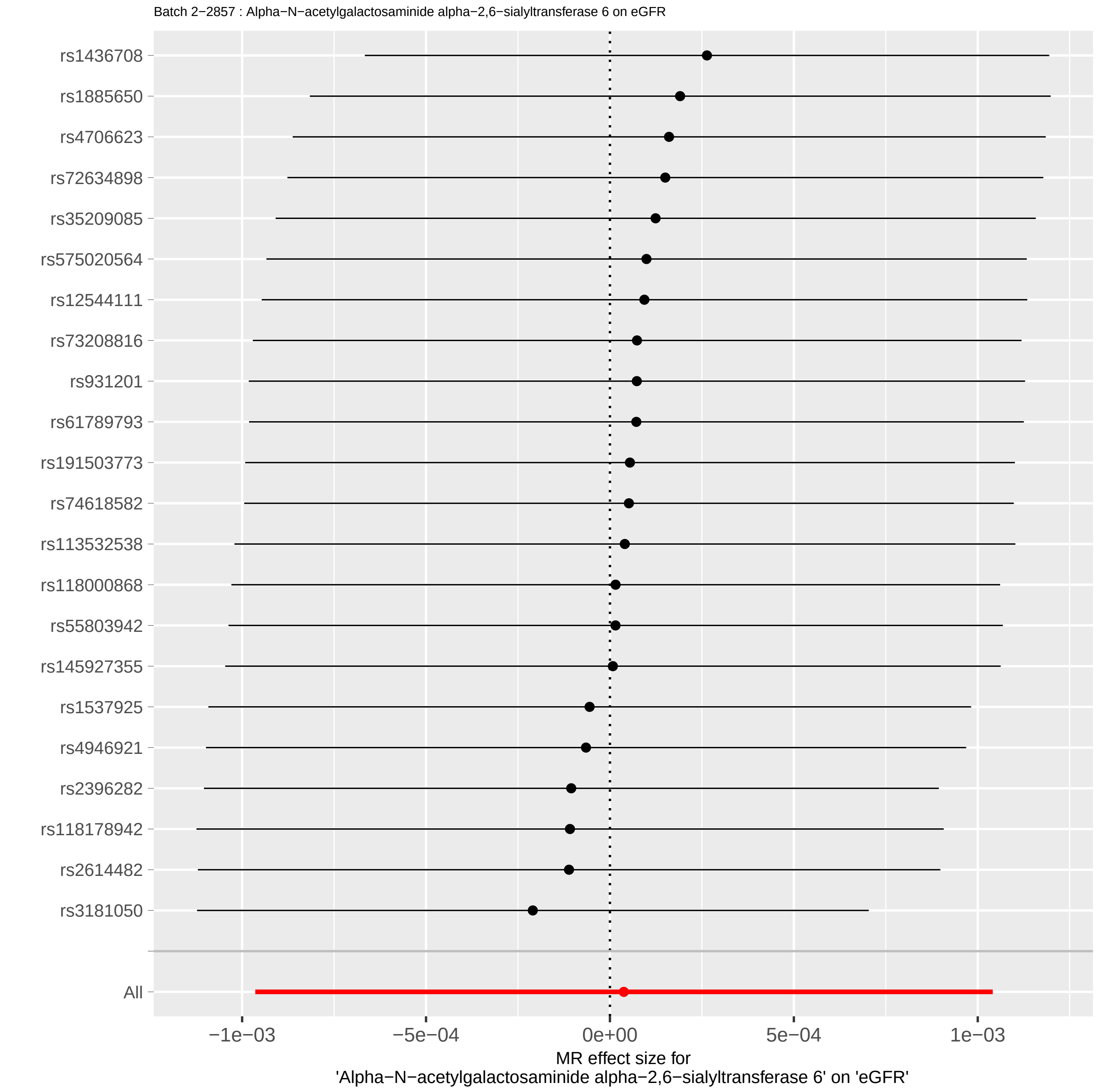
Batch 2−2857 : Alpha−N−acetylgalactosaminide alpha−2,6−sialyltransferase 6 on eGFR
rs1436708
rs1885650
rs4706623
rs72634898
rs35209085
rs575020564
rs12544111
rs73208816
rs931201
rs61789793
rs191503773
rs74618582
rs113532538
rs118000868
rs55803942
rs145927355
rs1537925
rs4946921
rs2396282
rs118178942
rs2614482
rs3181050
All
−1e−03
−5e−04
0e+00
5e−04
1e−03
MR effect size for
'Alpha−N−acetylgalactosaminide alpha−2,6−sialyltransferase 6' on 'eGFR'

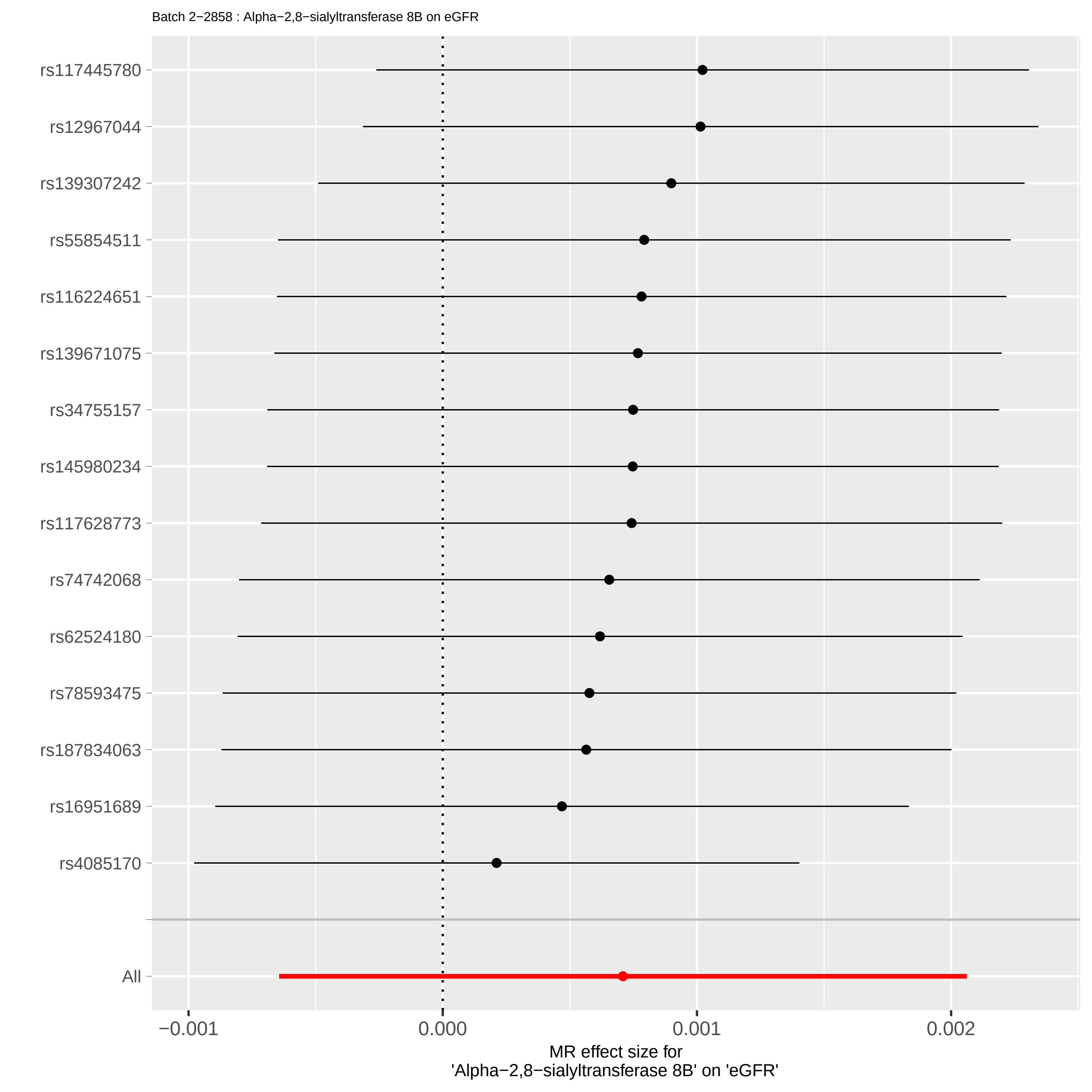

Batch 2−2858 : Alpha−2,8−sialyltransferase 8B on eGFR
rs117445780
rs12967044
rs139307242
rs55854511
rs116224651
rs139671075
rs34755157
rs145980234
rs117628773
rs74742068
rs62524180
rs78593475
rs187834063
rs16951689
rs4085170
All
−0.001
0.000
0.001
0.002
MR effect size for
'Alpha−2,8−sialyltransferase 8B' on 'eGFR'

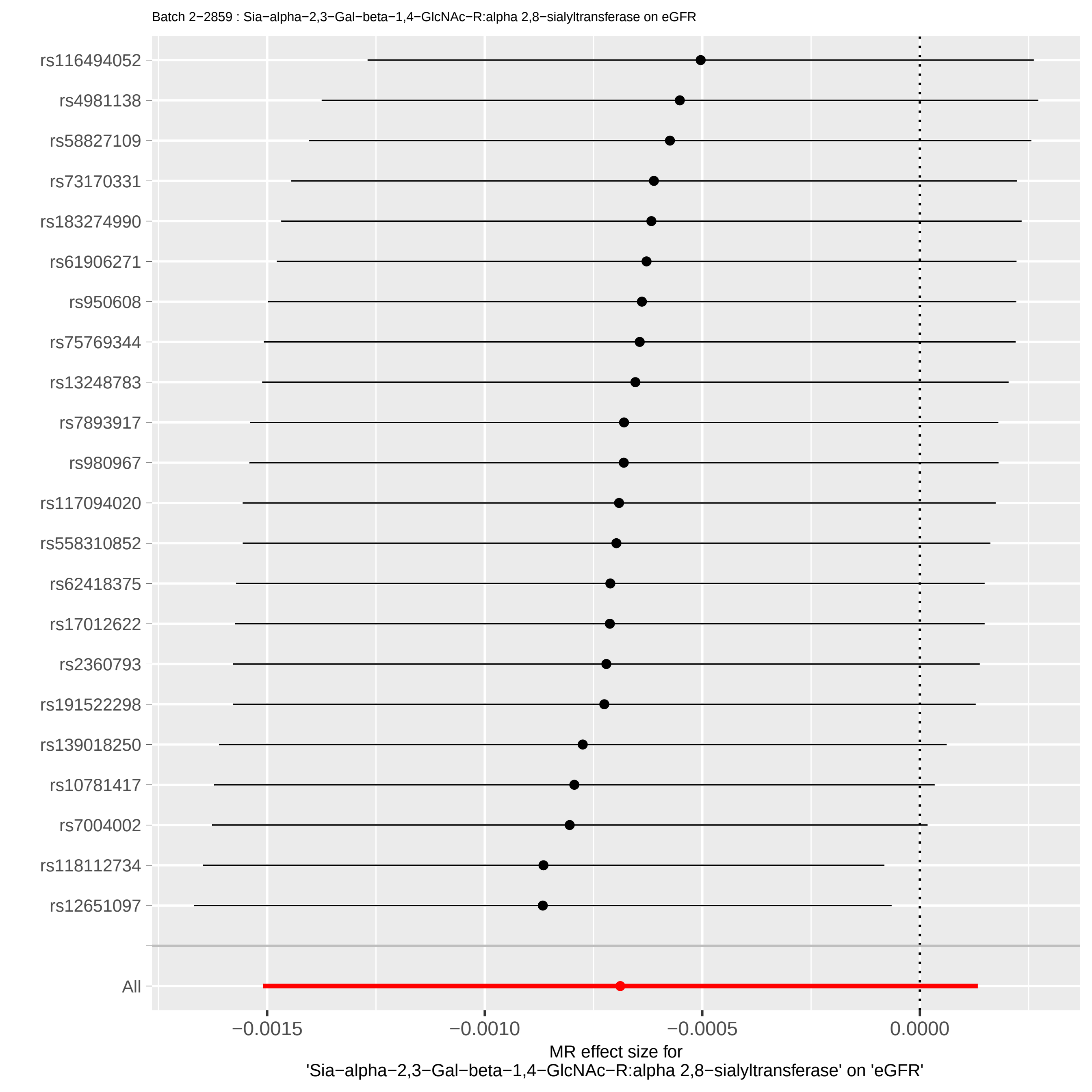

Batch 2−2859 : Sia−alpha−2,3−Gal−beta−1,4−GlcNAc−R:alpha 2,8−sialyltransferase on eGFR
rs116494052
rs4981138
rs58827109
rs73170331
rs183274990
rs61906271
rs950608
rs75769344
rs13248783
rs7893917
rs980967
rs117094020
rs558310852
rs62418375
rs17012622
rs2360793
rs191522298
rs139018250
rs10781417
rs7004002
rs118112734
rs12651097
All
−0.0015
−0.0010
−0.0005
0.0000
MR effect size for
'Sia−alpha−2,3−Gal−beta−1,4−GlcNAc−R:alpha 2,8−sialyltransferase' on 'eGFR'

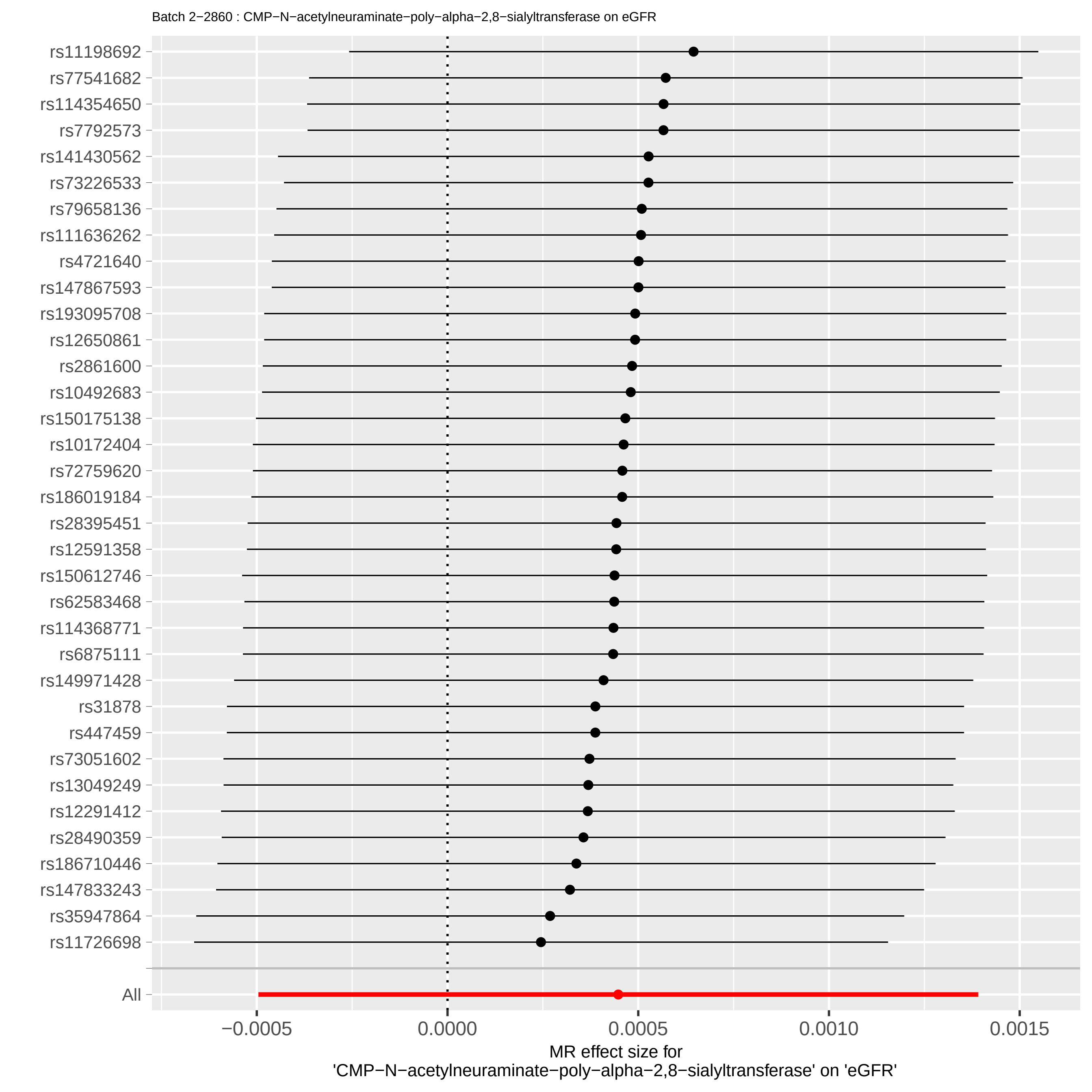

Batch 2−2860 : CMP−N−acetylneuraminate−poly−alpha−2,8−sialyltransferase on eGFR
rs11198692
rs77541682
rs114354650
rs7792573
rs141430562
rs73226533
rs79658136
rs111636262
rs4721640
rs147867593
rs193095708
rs12650861
rs2861600
rs10492683
rs150175138
rs10172404
rs72759620
rs186019184
rs28395451
rs12591358
rs150612746
rs62583468
rs114368771
rs6875111
rs149971428
rs31878
rs447459
rs73051602
rs13049249
rs12291412
rs28490359
rs186710446
rs147833243
rs35947864
rs11726698
All
−0.0005
0.0000
0.0005
0.0010
0.0015
MR effect size for
'CMP−N−acetylneuraminate−poly−alpha−2,8−sialyltransferase' on 'eGFR'

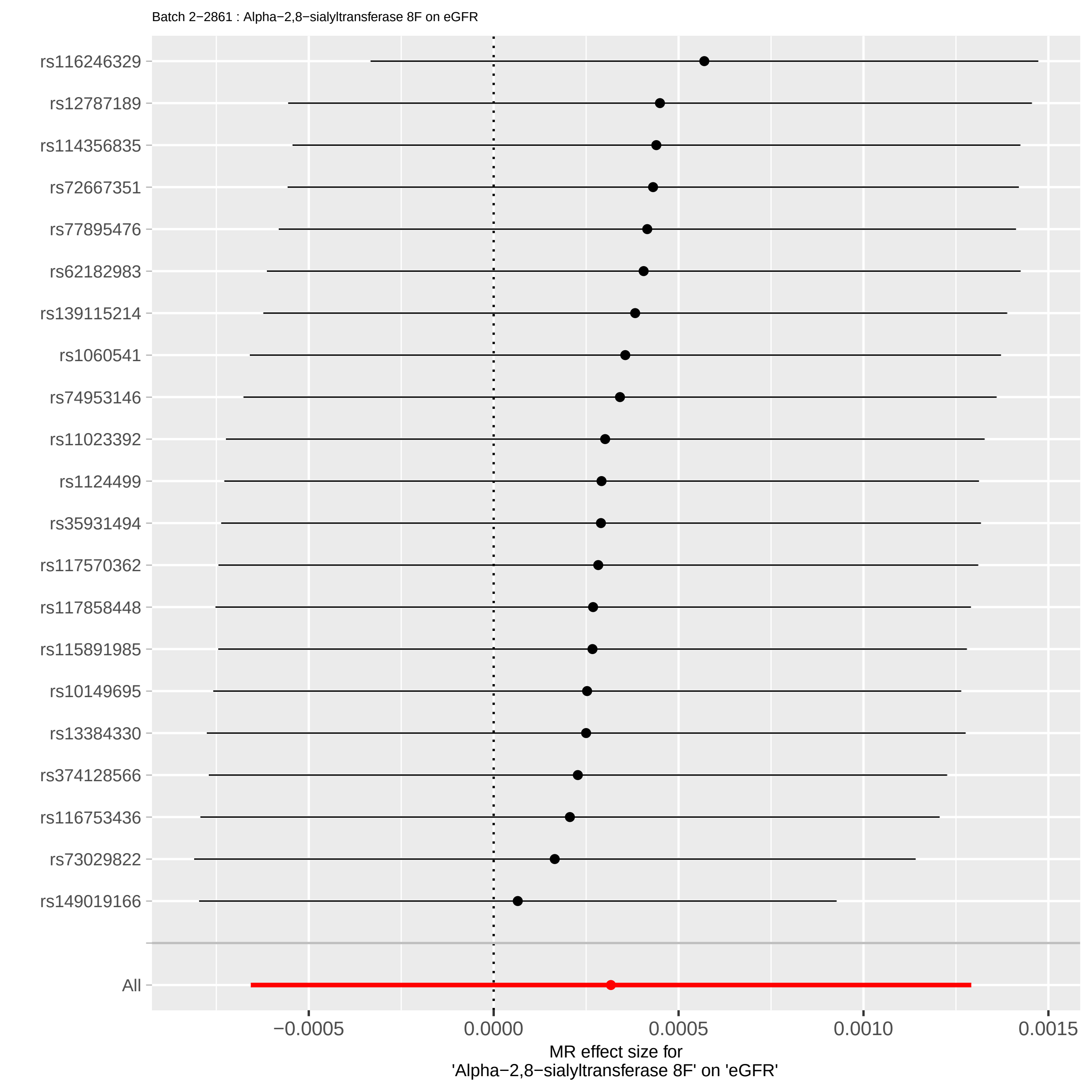

Batch 2−2861 : Alpha−2,8−sialyltransferase 8F on eGFR
rs116246329
rs12787189
rs114356835
rs72667351
rs77895476
rs62182983
rs139115214
rs1060541
rs74953146
rs11023392
rs1124499
rs35931494
rs117570362
rs117858448
rs115891985
rs10149695
rs13384330
rs374128566
rs116753436
rs73029822
rs149019166
All
−0.0005
0.0000
0.0005
0.0010
0.0015
MR effect size for
'Alpha−2,8−sialyltransferase 8F' on 'eGFR'

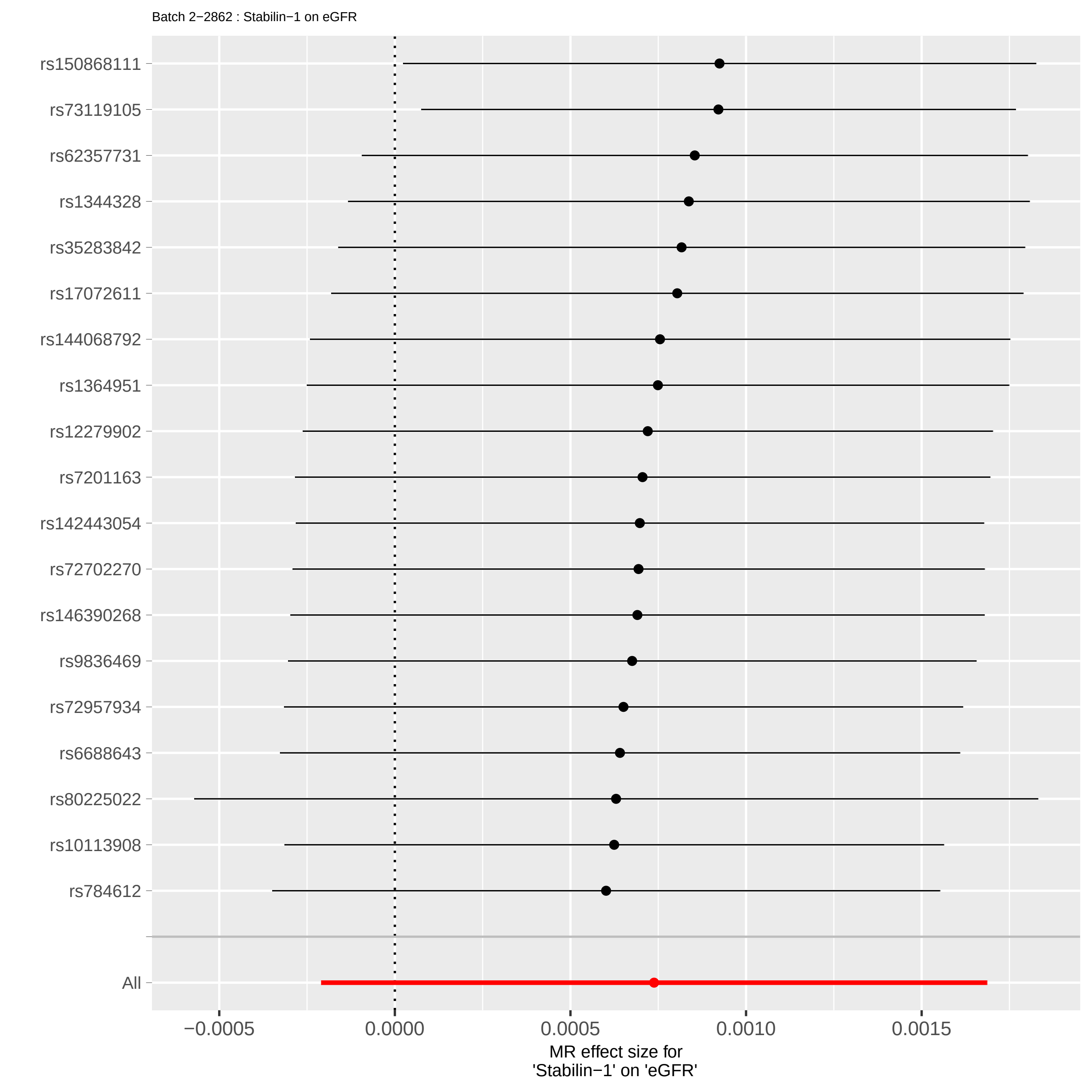
Batch 2−2862 : Stabilin−1 on eGFR
rs150868111
rs73119105
rs62357731
rs1344328
rs35283842
rs17072611
rs144068792
rs1364951
rs12279902
rs7201163
rs142443054
rs72702270
rs146390268
rs9836469
rs72957934
rs6688643
rs80225022
rs10113908
rs784612
All
−0.0005
0.0000
0.0005
0.0010
0.0015
MR effect size for
'Stabilin−1' on 'eGFR'

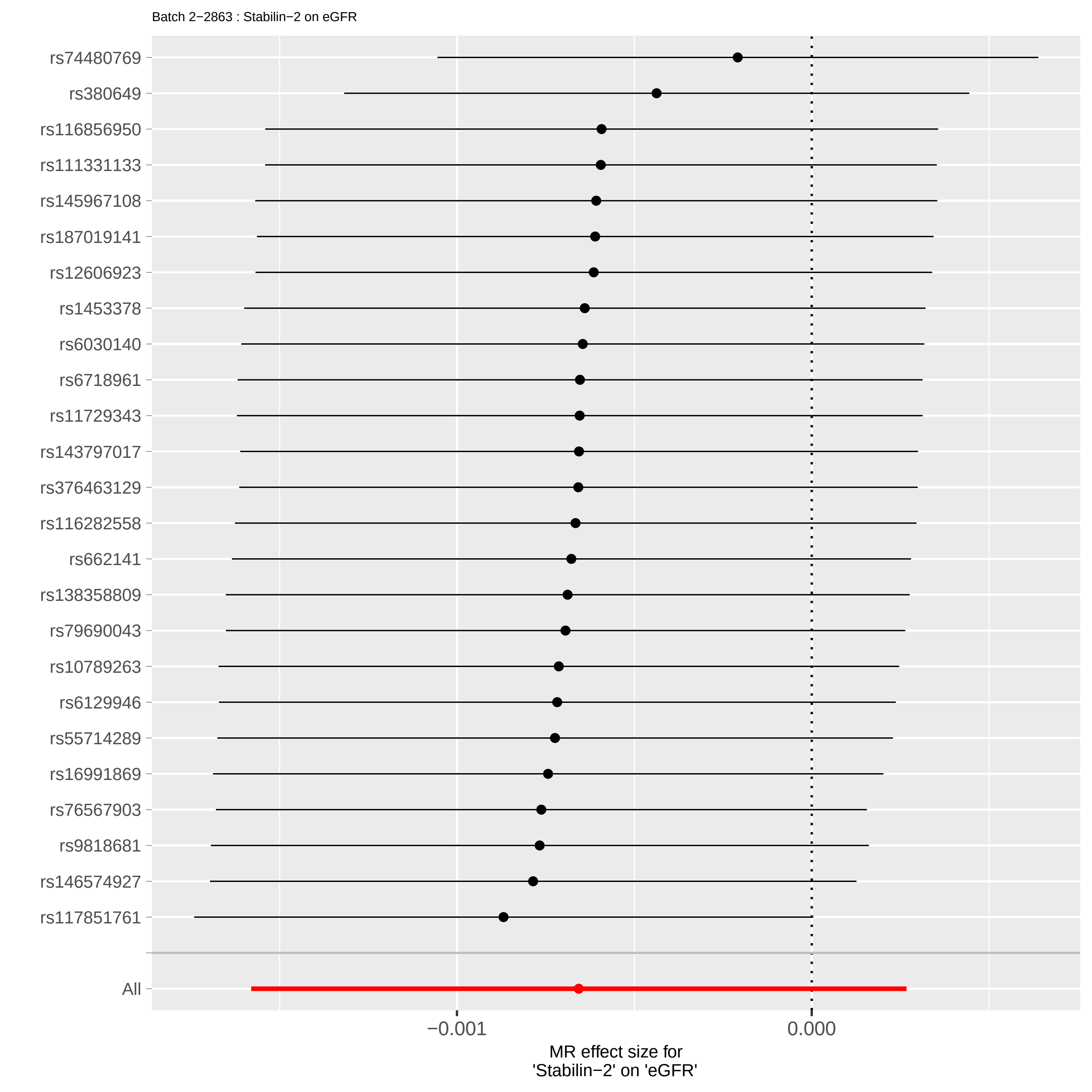

Batch 2−2863 : Stabilin−2 on eGFR
rs74480769
rs380649
rs116856950
rs111331133
rs145967108
rs187019141
rs12606923
rs1453378
rs6030140
rs6718961
rs11729343
rs143797017
rs376463129
rs116282558
rs662141
rs138358809
rs79690043
rs10789263
rs6129946
rs55714289
rs16991869
rs76567903
rs9818681
rs146574927
rs117851761
All
−0.001
0.000
MR effect size for
'Stabilin−2' on 'eGFR'

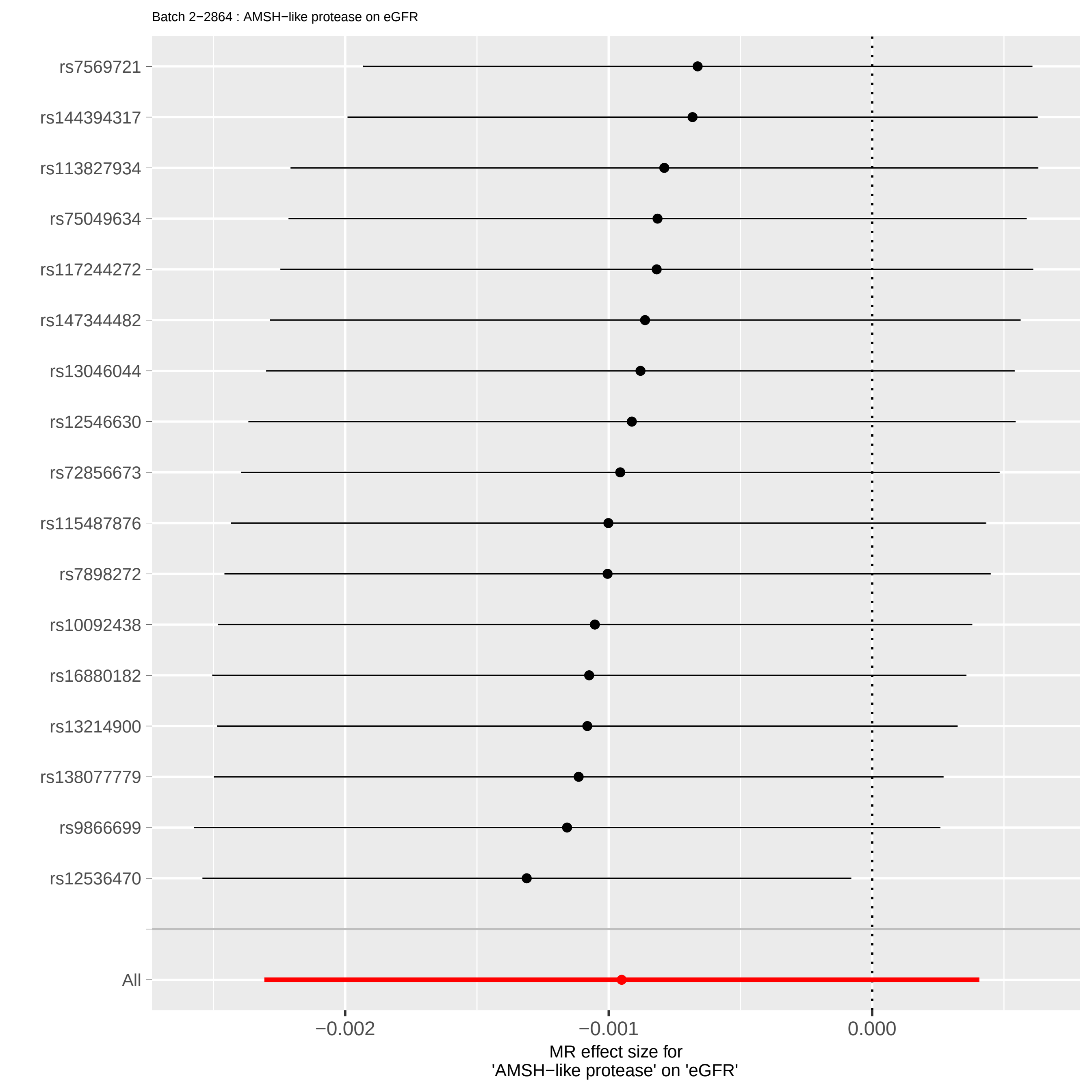
Batch 2−2864 : AMSH−like protease on eGFR
rs7569721
rs144394317
rs113827934
rs75049634
rs117244272
rs147344482
rs13046044
rs12546630
rs72856673
rs115487876
rs7898272
rs10092438
rs16880182
rs13214900
rs138077779
rs9866699
rs12536470
All
−0.002
−0.001
0.000
MR effect size for
'AMSH−like protease' on 'eGFR'

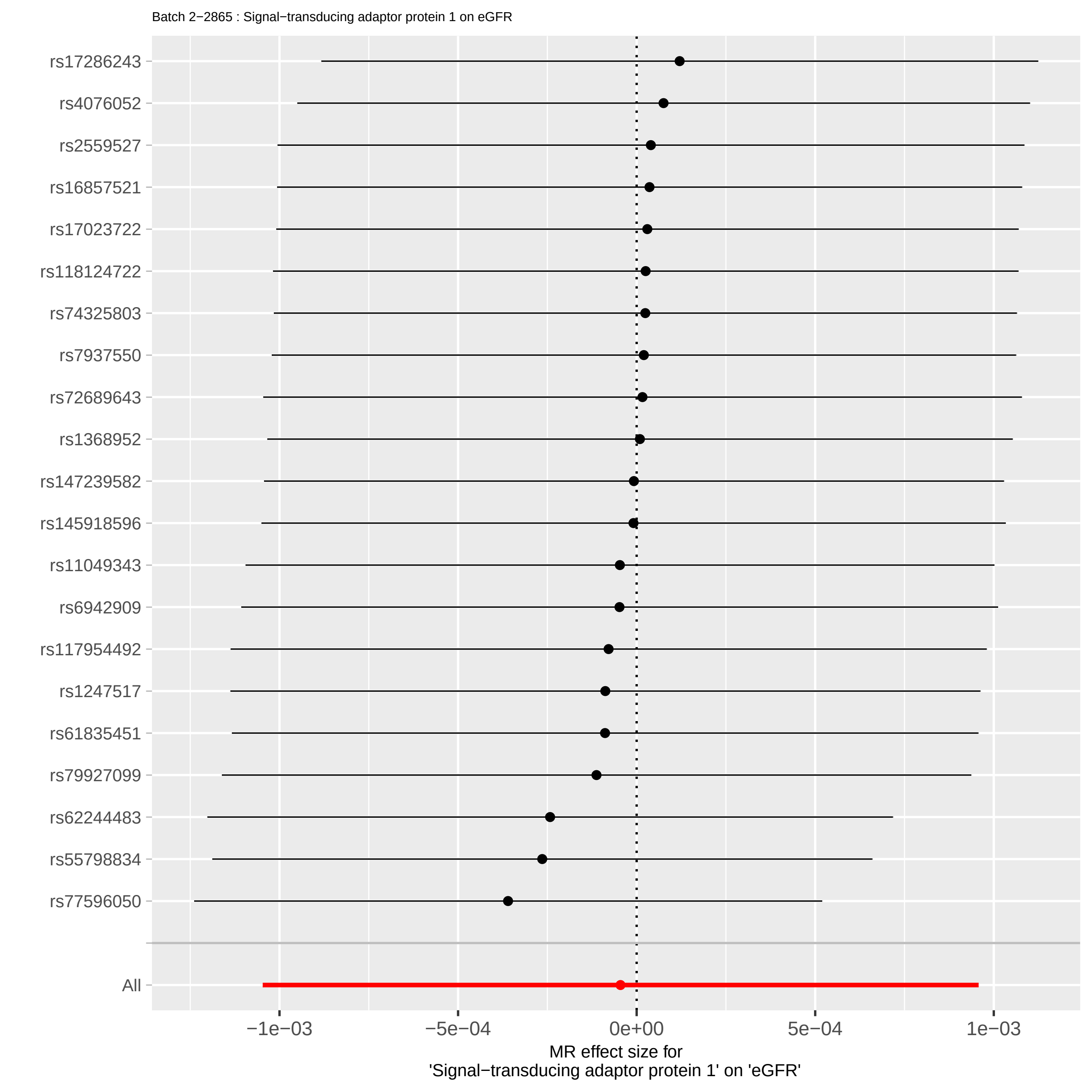

Batch 2−2865 : Signal−transducing adaptor protein 1 on eGFR
rs17286243
rs4076052
rs2559527
rs16857521
rs17023722
rs118124722
rs74325803
rs7937550
rs72689643
rs1368952
rs147239582
rs145918596
rs11049343
rs6942909
rs117954492
rs1247517
rs61835451
rs79927099
rs62244483
rs55798834
rs77596050
All
−1e−03
−5e−04
0e+00
5e−04
1e−03
MR effect size for
'Signal−transducing adaptor protein 1' on 'eGFR'

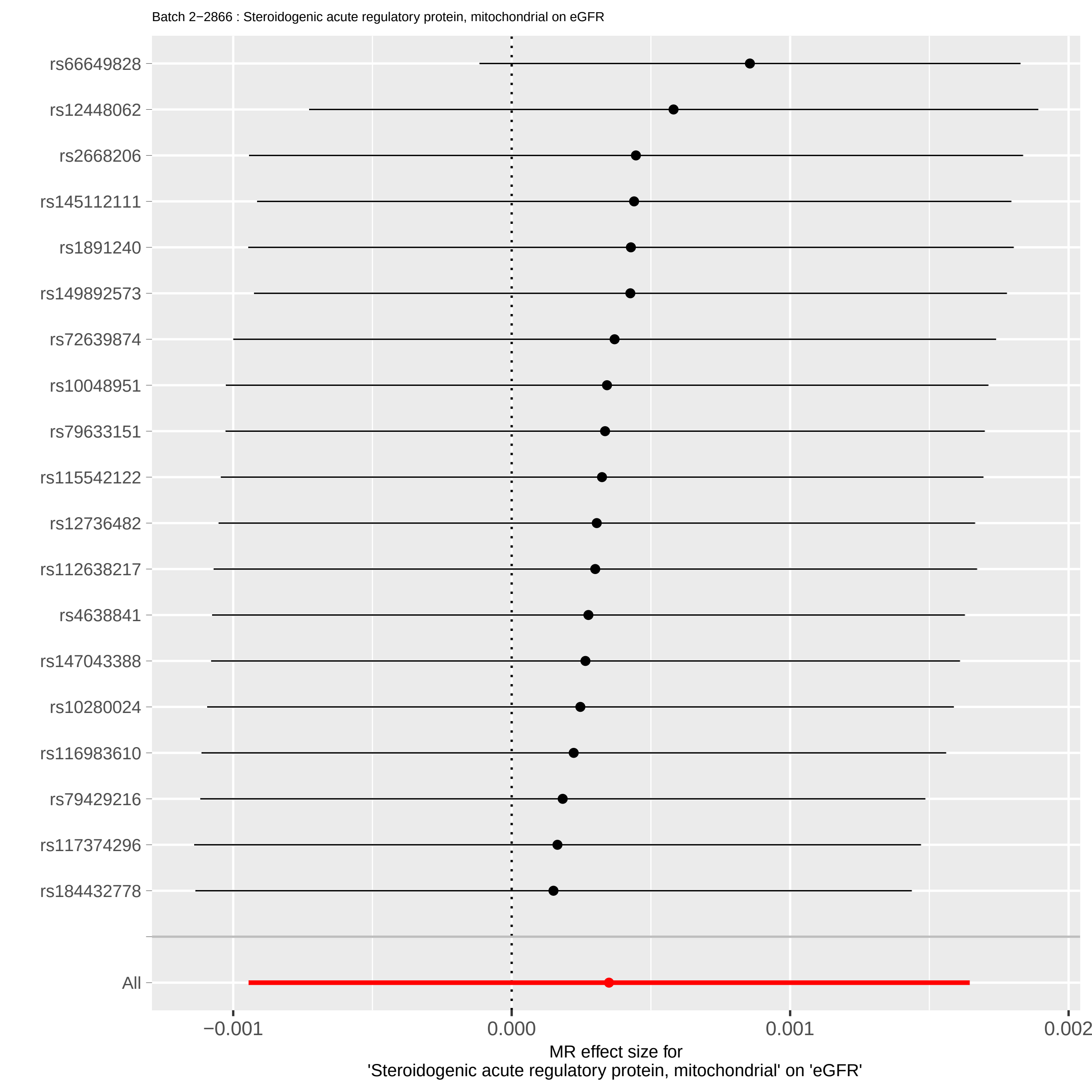

Batch 2−2866 : Steroidogenic acute regulatory protein, mitochondrial on eGFR
rs66649828
rs12448062
rs2668206
rs145112111
rs1891240
rs149892573
rs72639874
rs10048951
rs79633151
rs115542122
rs12736482
rs112638217
rs4638841
rs147043388
rs10280024
rs116983610
rs79429216
rs117374296
rs184432778
All
−0.001
0.000
0.001
0.002
MR effect size for
'Steroidogenic acute regulatory protein, mitochondrial' on 'eGFR'

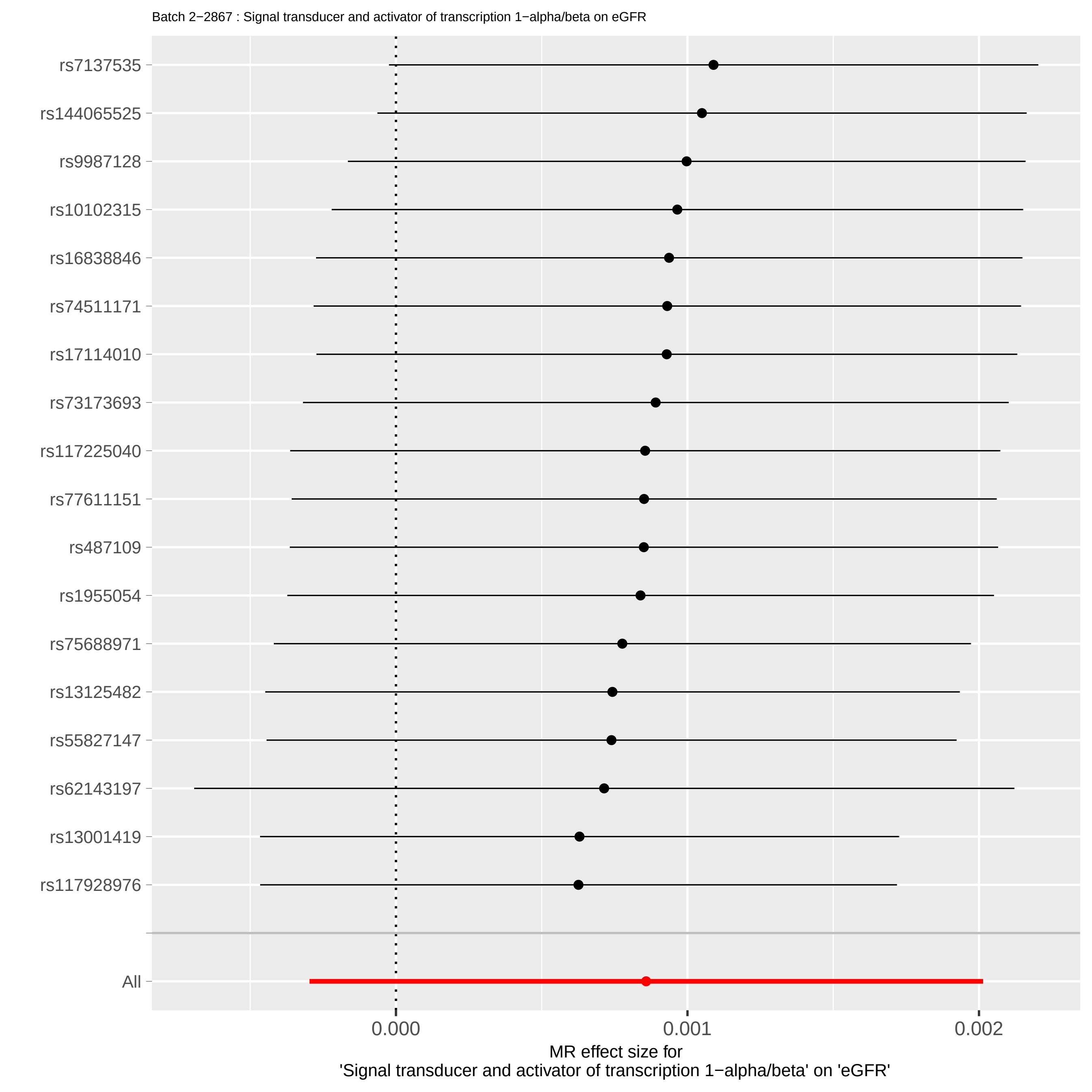

Batch 2−2867 : Signal transducer and activator of transcription 1−alpha/beta on eGFR
rs7137535
rs144065525
rs9987128
rs10102315
rs16838846
rs74511171
rs17114010
rs73173693
rs117225040
rs77611151
rs487109
rs1955054
rs75688971
rs13125482
rs55827147
rs62143197
rs13001419
rs117928976
All
0.000
0.001
0.002
MR effect size for
'Signal transducer and activator of transcription 1−alpha/beta' on 'eGFR'

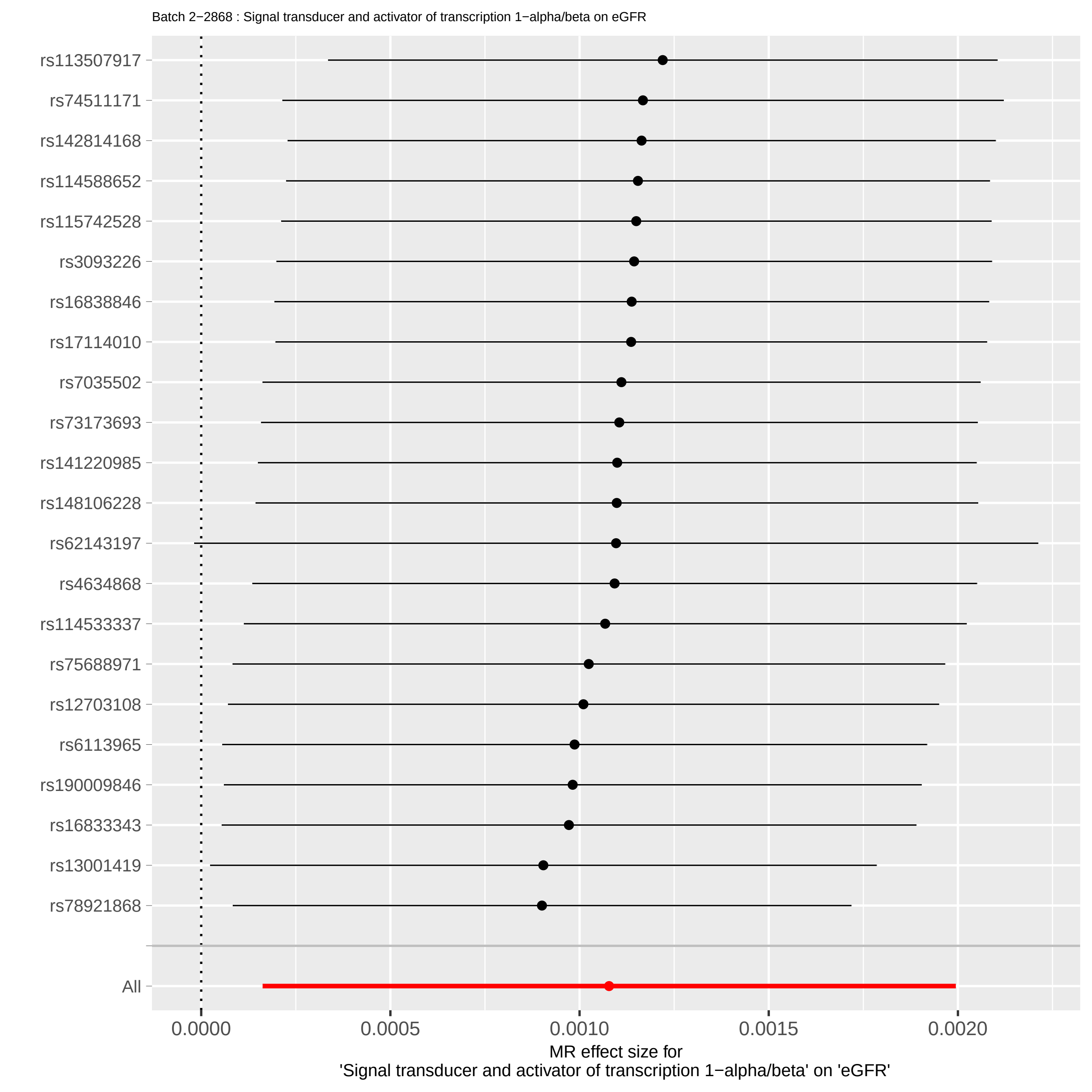

Batch 2−2868 : Signal transducer and activator of transcription 1−alpha/beta on eGFR
rs113507917
rs74511171
rs142814168
rs114588652
rs115742528
rs3093226
rs16838846
rs17114010
rs7035502
rs73173693
rs141220985
rs148106228
rs62143197
rs4634868
rs114533337
rs75688971
rs12703108
rs6113965
rs190009846
rs16833343
rs13001419
rs78921868
All
0.0000
0.0005
0.0010
0.0015
0.0020
MR effect size for
'Signal transducer and activator of transcription 1−alpha/beta' on 'eGFR'

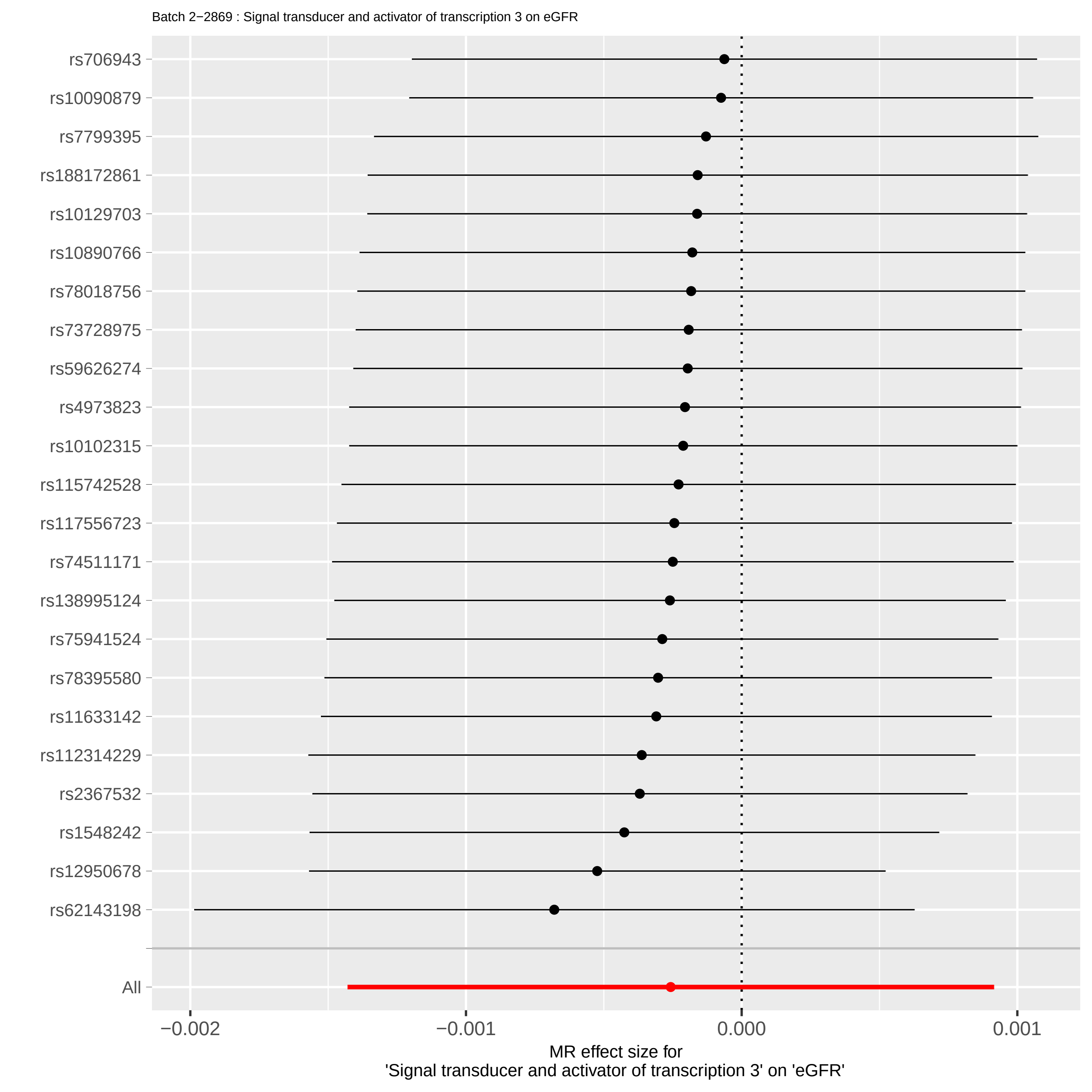

Batch 2−2869 : Signal transducer and activator of transcription 3 on eGFR
rs706943
rs10090879
rs7799395
rs188172861
rs10129703
rs10890766
rs78018756
rs73728975
rs59626274
rs4973823
rs10102315
rs115742528
rs117556723
rs74511171
rs138995124
rs75941524
rs78395580
rs11633142
rs112314229
rs2367532
rs1548242
rs12950678
rs62143198
All
−0.002
−0.001
0.000
0.001
MR effect size for
'Signal transducer and activator of transcription 3' on 'eGFR'

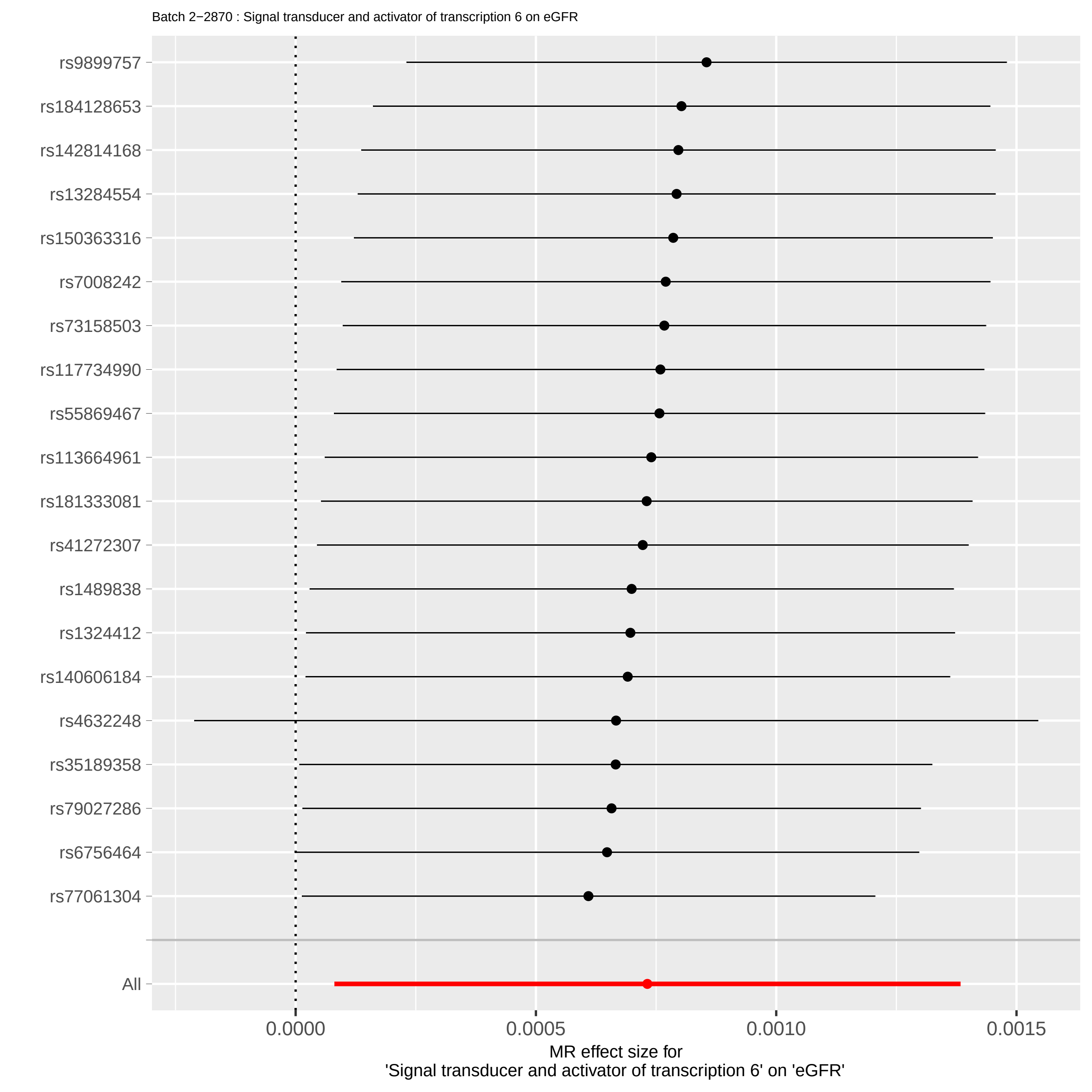
Batch 2−2870 : Signal transducer and activator of transcription 6 on eGFR
rs9899757
rs184128653
rs142814168
rs13284554
rs150363316
rs7008242
rs73158503
rs117734990
rs55869467
rs113664961
rs181333081
rs41272307
rs1489838
rs1324412
rs140606184
rs4632248
rs35189358
rs79027286
rs6756464
rs77061304
All
0.0000
0.0005
0.0010
0.0015
MR effect size for
'Signal transducer and activator of transcription 6' on 'eGFR'

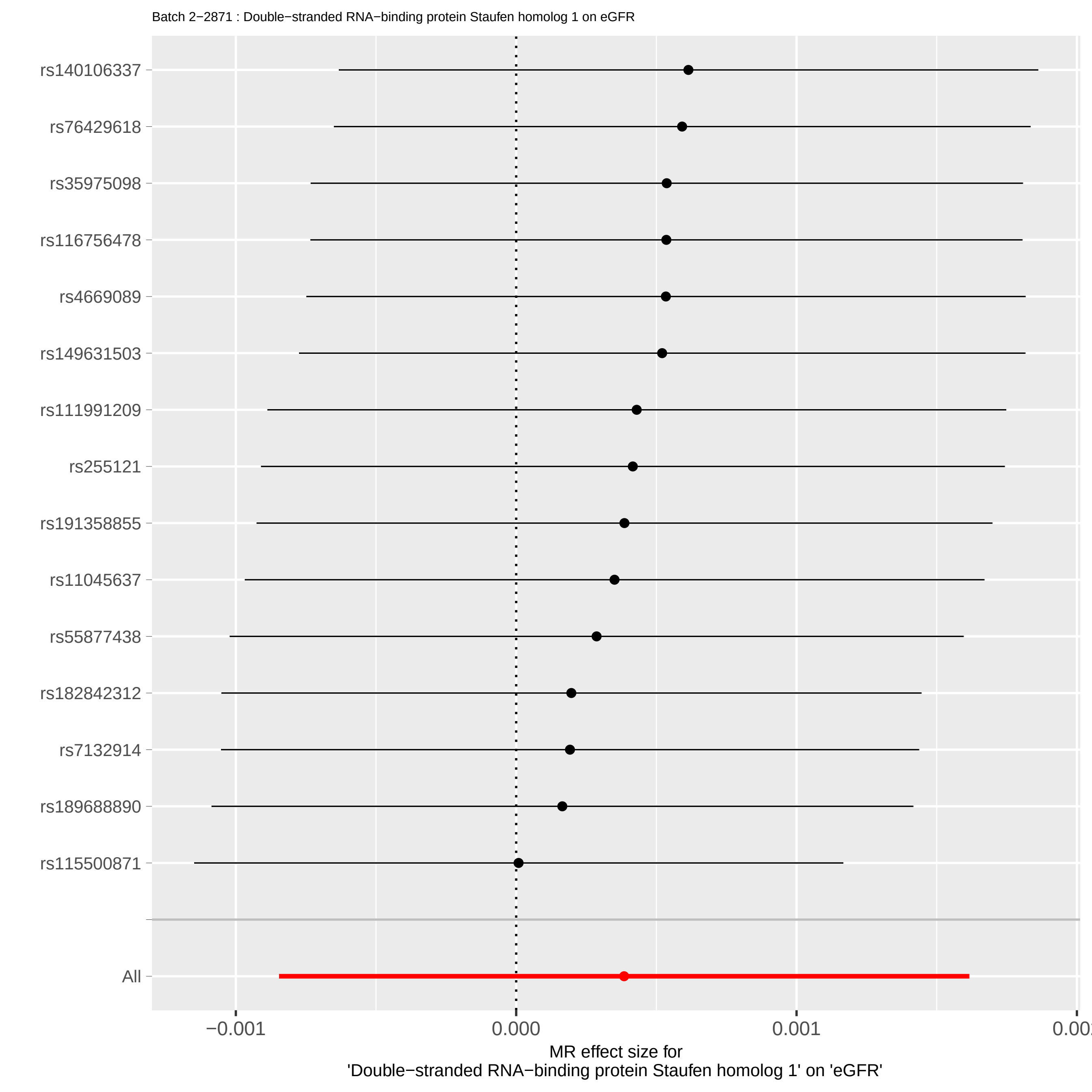
Batch 2−2871 : Double−stranded RNA−binding protein Staufen homolog 1 on eGFR
rs140106337
rs76429618
rs35975098
rs116756478
rs4669089
rs149631503
rs111991209
rs255121
rs191358855
rs11045637
rs55877438
rs182842312
rs7132914
rs189688890
rs115500871
All
−0.001
0.000
0.001
MR effect size for
'Double−stranded RNA−binding protein Staufen homolog 1' on 'eGFR'

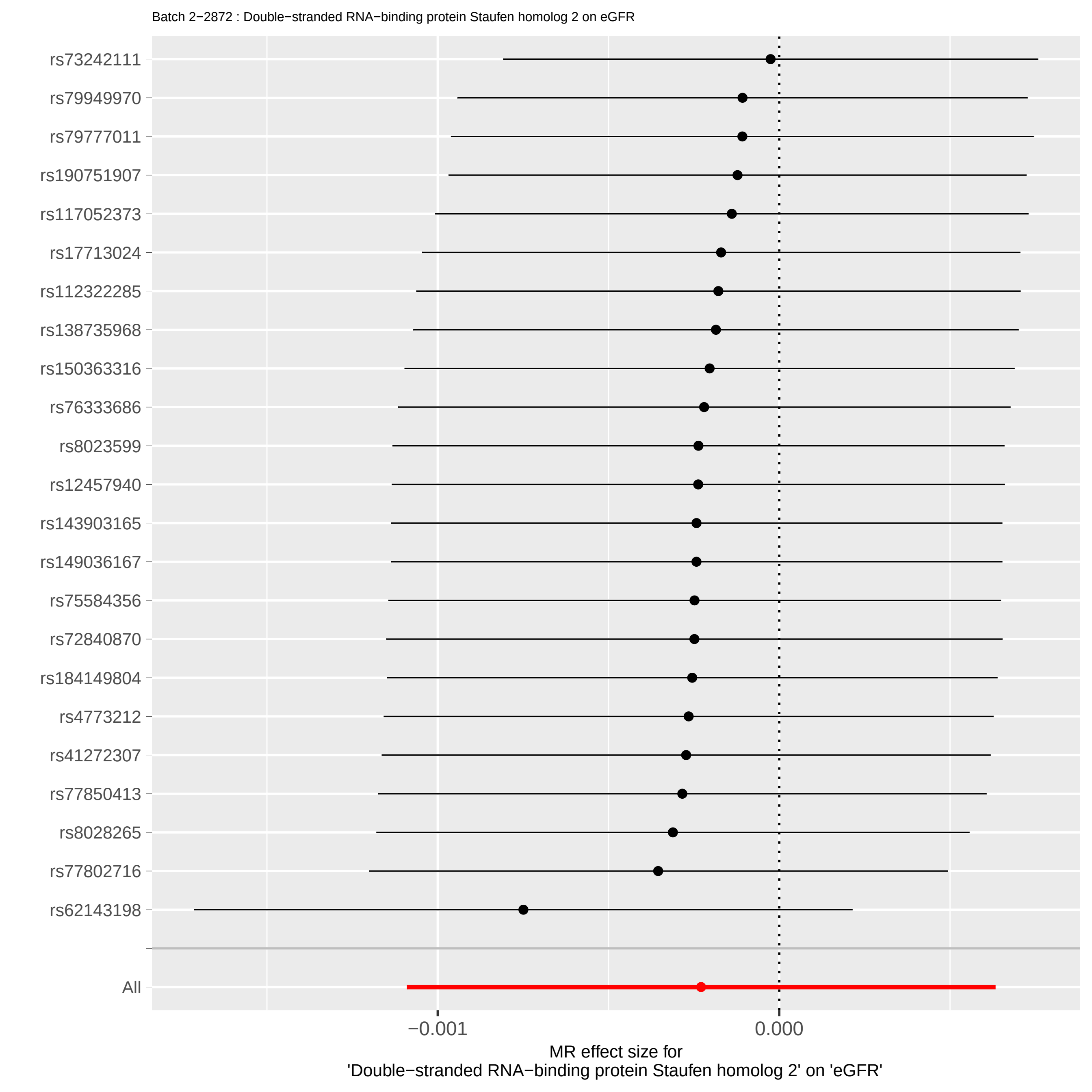

Batch 2−2872 : Double−stranded RNA−binding protein Staufen homolog 2 on eGFR
rs73242111
rs79949970
rs79777011
rs190751907
rs117052373
rs17713024
rs112322285
rs138735968
rs150363316
rs76333686
rs8023599
rs12457940
rs143903165
rs149036167
rs75584356
rs72840870
rs184149804
rs4773212
rs41272307
rs77850413
rs8028265
rs77802716
rs62143198
All
−0.001
0.000
MR effect size for
'Double−stranded RNA−binding protein Staufen homolog 2' on 'eGFR'

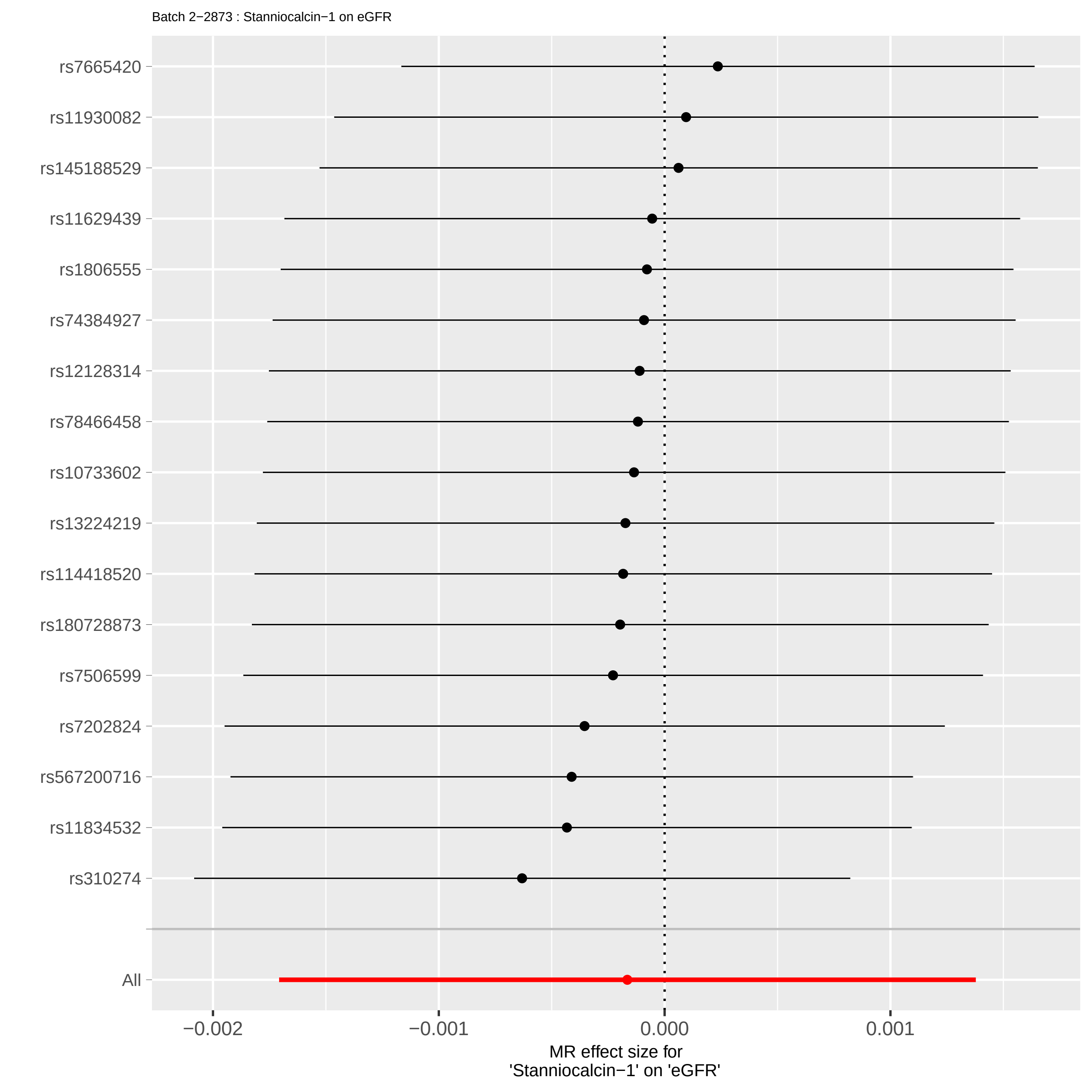

Batch 2−2873 : Stanniocalcin−1 on eGFR
rs7665420
rs11930082
rs145188529
rs11629439
rs1806555
rs74384927
rs12128314
rs78466458
rs10733602
rs13224219
rs114418520
rs180728873
rs7506599
rs7202824
rs567200716
rs11834532
rs310274
All
−0.002
−0.001
0.000
0.001
MR effect size for
'Stanniocalcin−1' on 'eGFR'

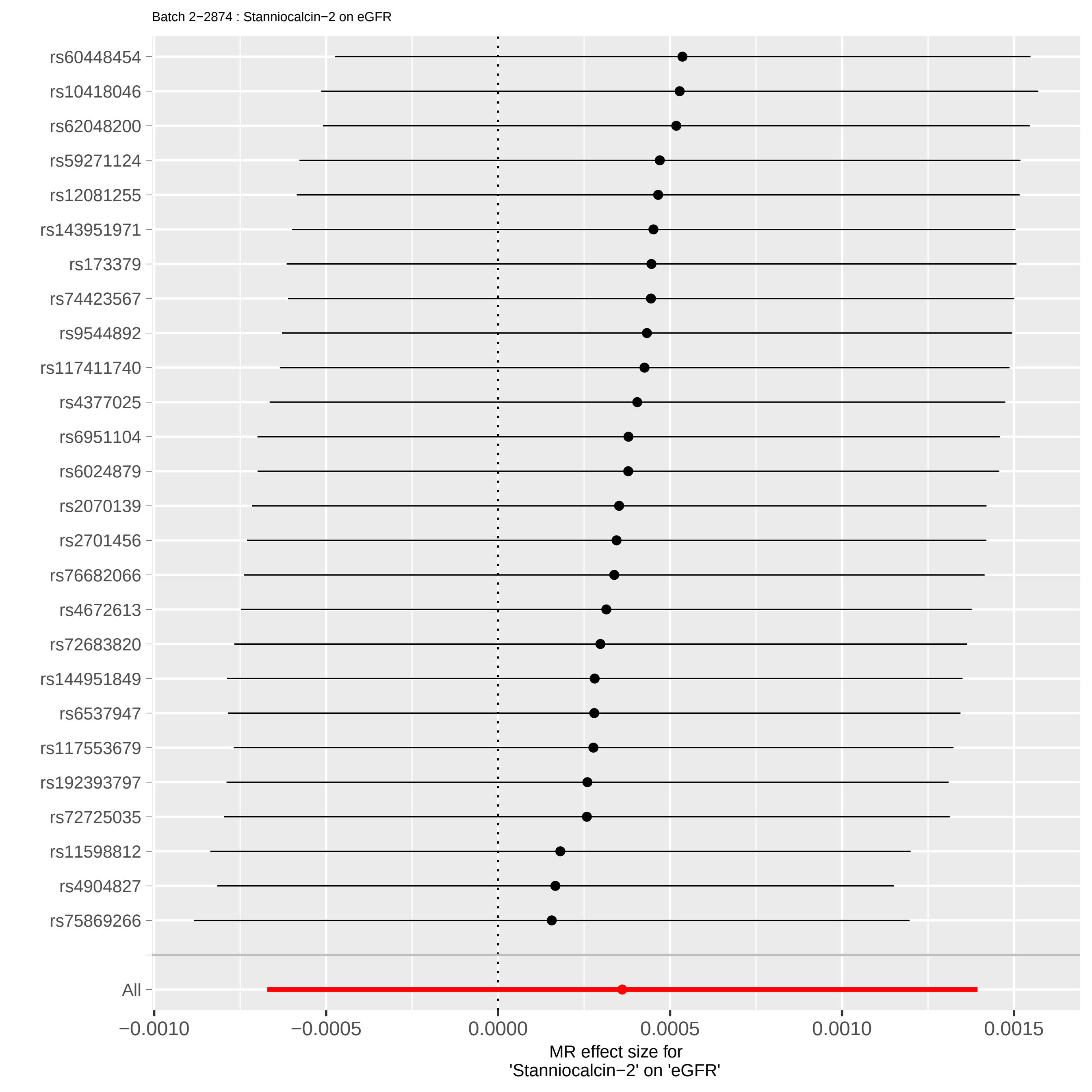
Batch 2−2874 : Stanniocalcin−2 on eGFR
rs60448454
rs10418046
rs62048200
rs59271124
rs12081255
rs143951971
rs173379
rs74423567
rs9544892
rs117411740
rs4377025
rs6951104
rs6024879
rs2070139
rs2701456
rs76682066
rs4672613
rs72683820
rs144951849
rs6537947
rs117553679
rs192393797
rs72725035
rs11598812
rs4904827
rs75869266
All
−0.0010
−0.0005
0.0000
0.0005
0.0010
0.0015
MR effect size for
'Stanniocalcin−2' on 'eGFR'

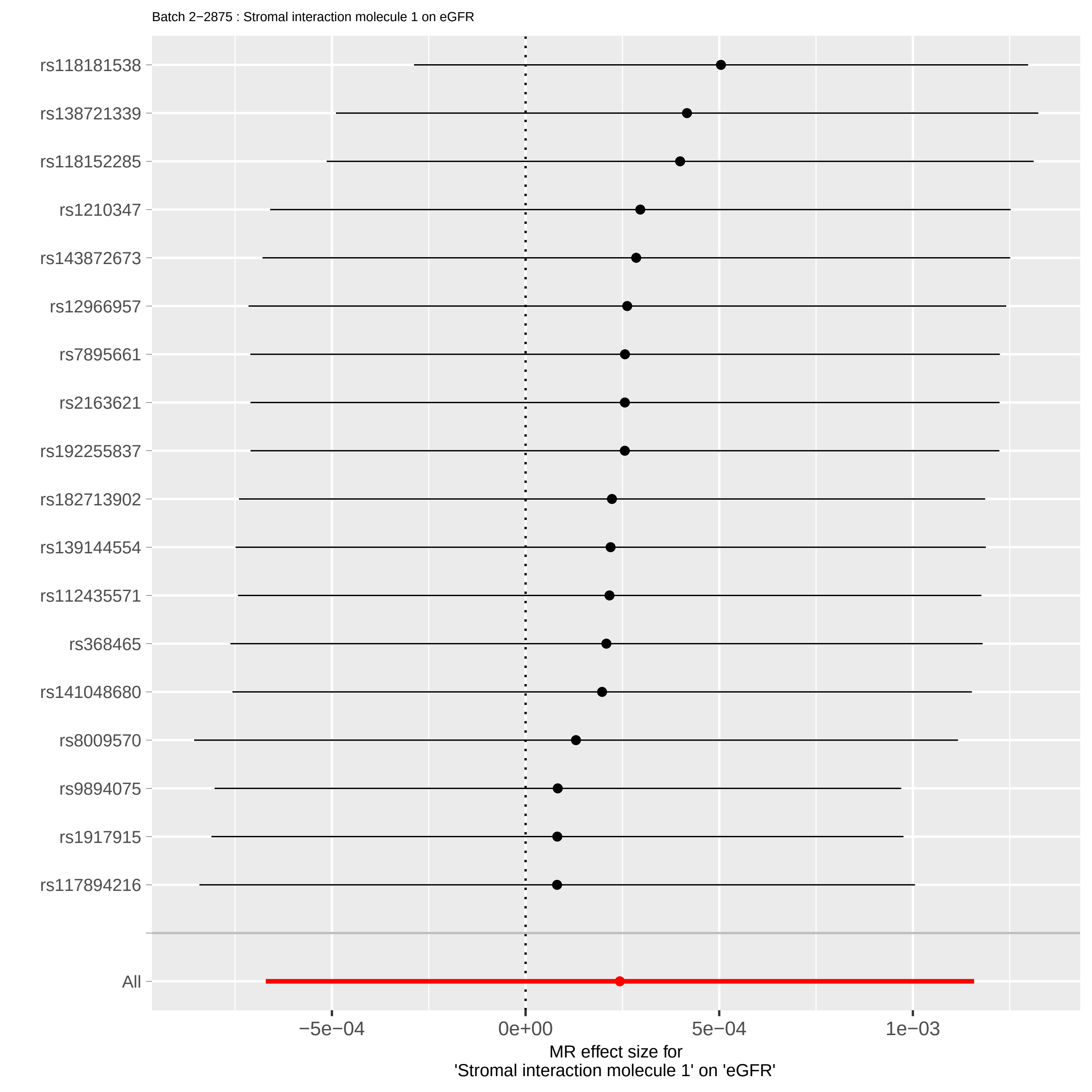

Batch 2−2875 : Stromal interaction molecule 1 on eGFR
rs118181538
rs138721339
rs118152285
rs1210347
rs143872673
rs12966957
rs7895661
rs2163621
rs192255837
rs182713902
rs139144554
rs112435571
rs368465
rs141048680
rs8009570
rs9894075
rs1917915
rs117894216
All
−5e−04
0e+00
5e−04
1e−03
MR effect size for
'Stromal interaction molecule 1' on 'eGFR'

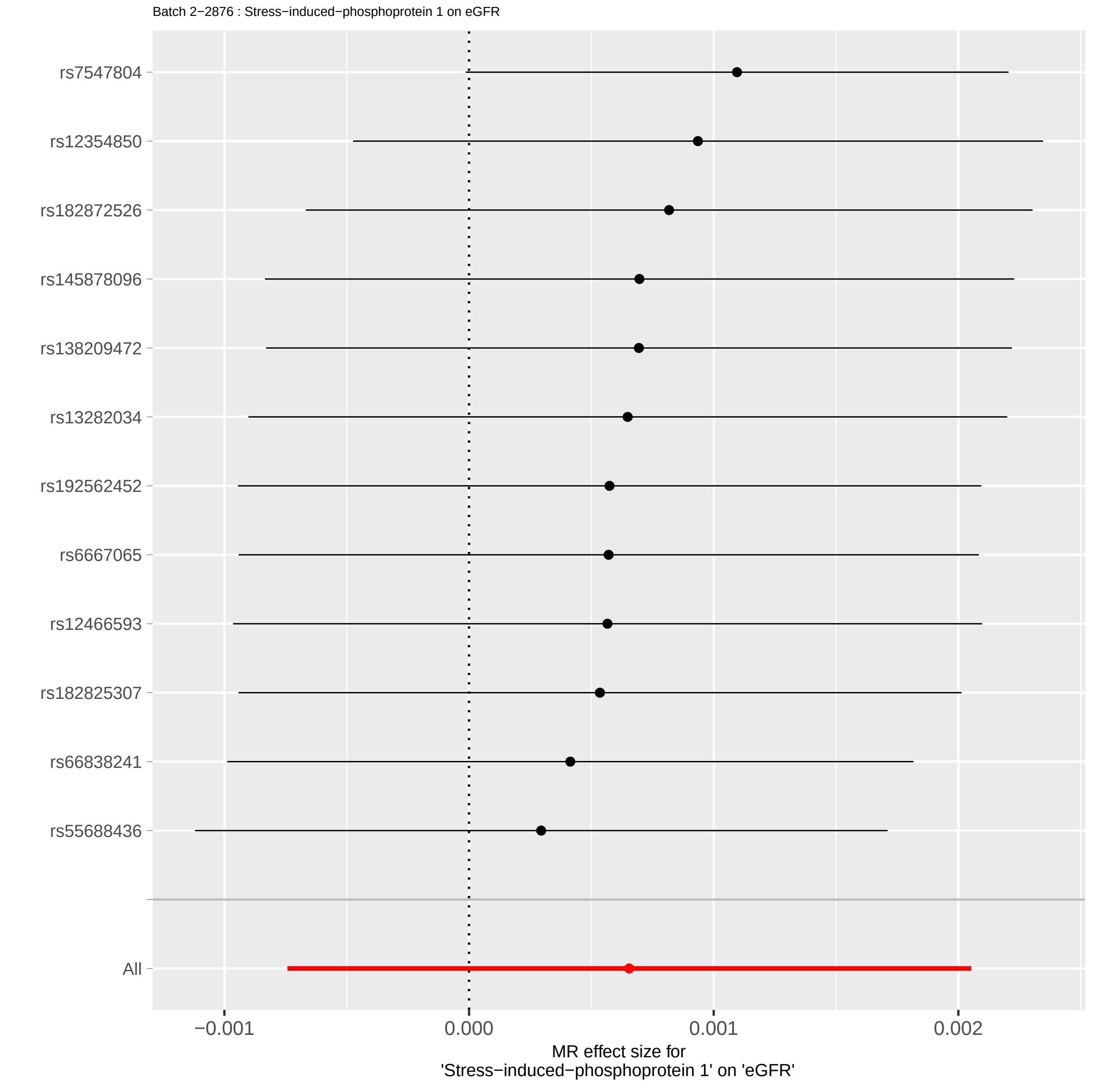

Batch 2−2876 : Stress−induced−phosphoprotein 1 on eGFR
rs7547804
rs12354850
rs182872526
rs145878096
rs138209472
rs13282034
rs192562452
rs6667065
rs12466593
rs182825307
rs66838241
rs55688436
All
−0.001
0.000
0.001
0.002
MR effect size for
'Stress−induced−phosphoprotein 1' on 'eGFR'

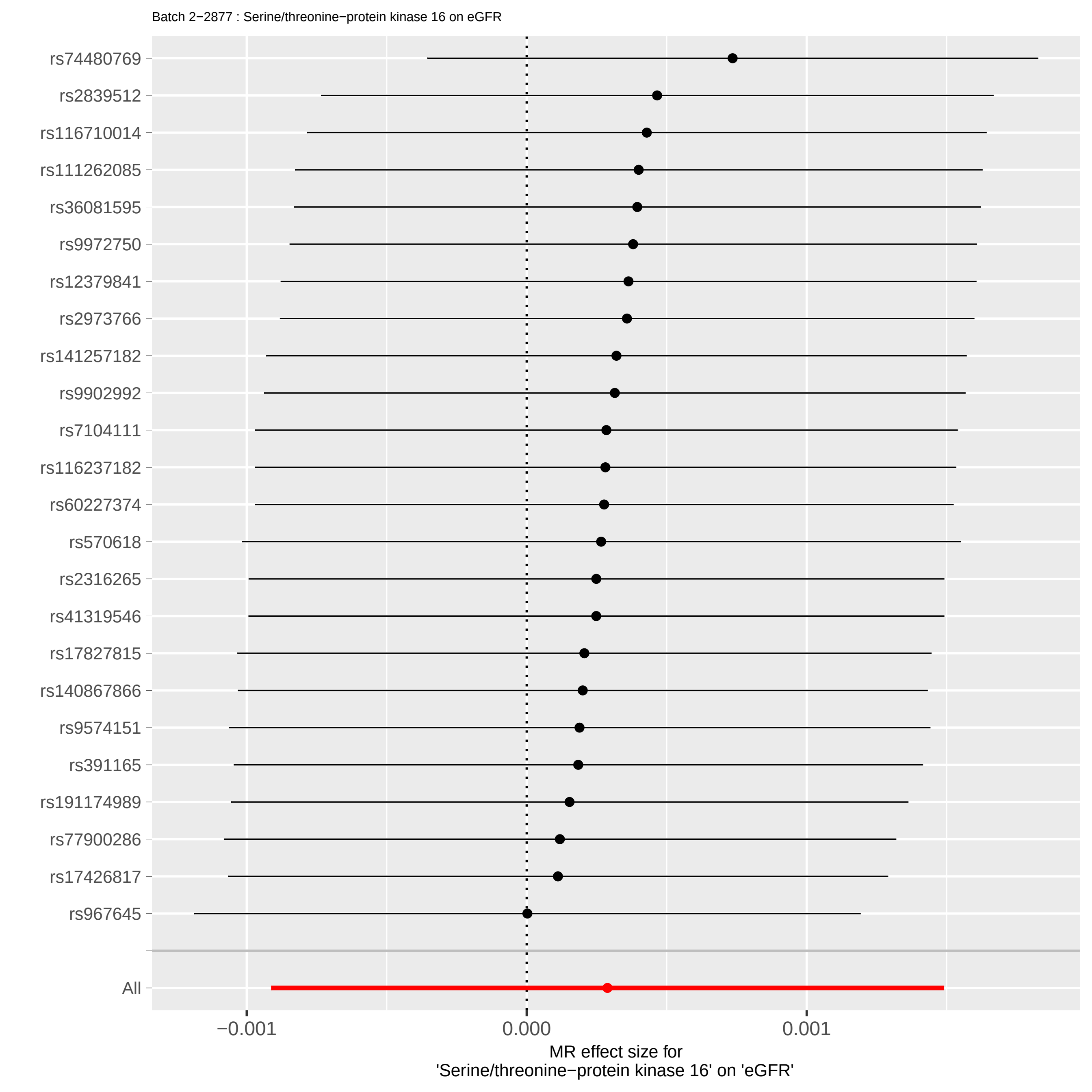

Batch 2−2877 : Serine/threonine−protein kinase 16 on eGFR
rs74480769
rs2839512
rs116710014
rs111262085
rs36081595
rs9972750
rs12379841
rs2973766
rs141257182
rs9902992
rs7104111
rs116237182
rs60227374
rs570618
rs2316265
rs41319546
rs17827815
rs140867866
rs9574151
rs391165
rs191174989
rs77900286
rs17426817
rs967645
All
−0.001
0.000
0.001
MR effect size for
'Serine/threonine−protein kinase 16' on 'eGFR'

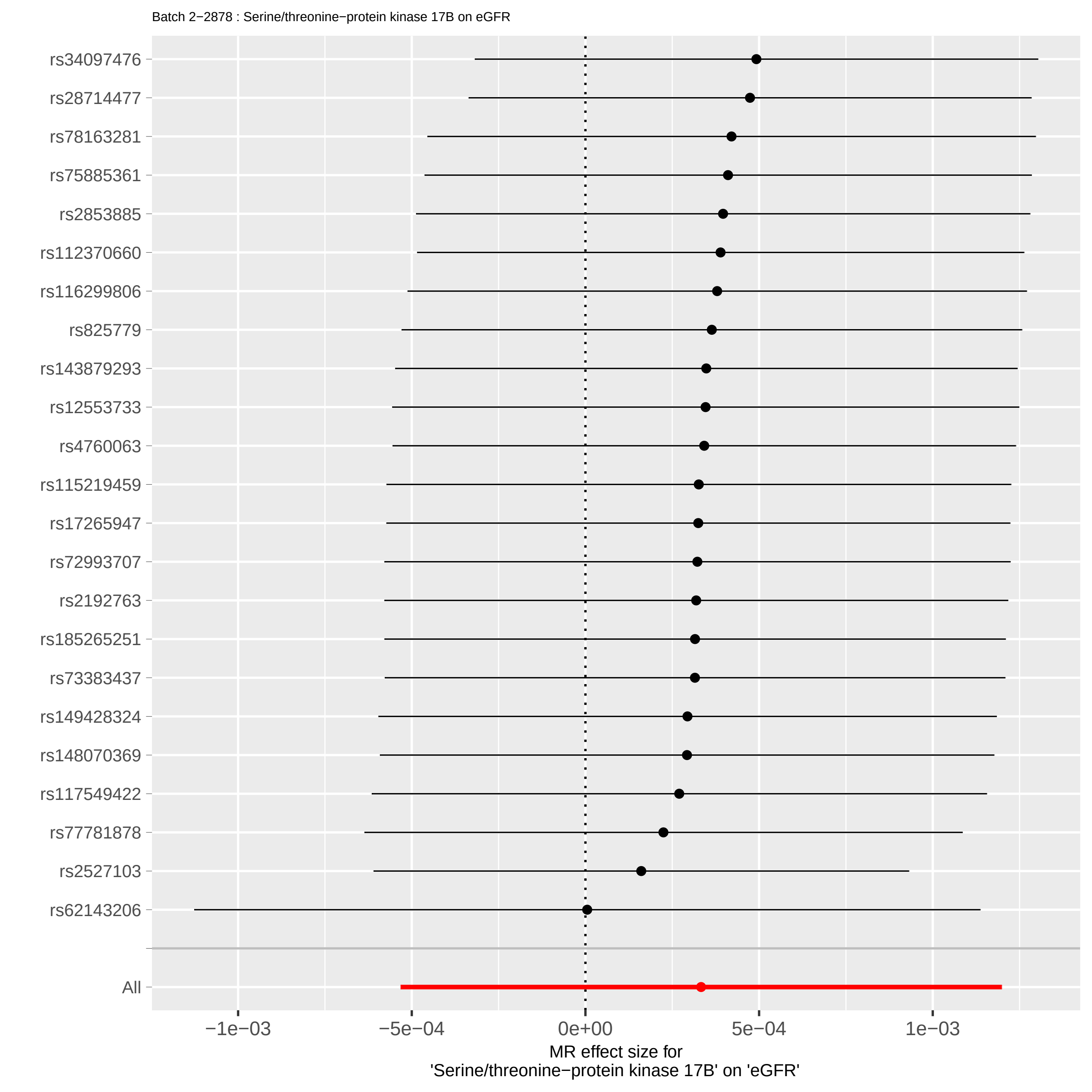

Batch 2−2878 : Serine/threonine−protein kinase 17B on eGFR
rs34097476
rs28714477
rs78163281
rs75885361
rs2853885
rs112370660
rs116299806
rs825779
rs143879293
rs12553733
rs4760063
rs115219459
rs17265947
rs72993707
rs2192763
rs185265251
rs73383437
rs149428324
rs148070369
rs117549422
rs77781878
rs2527103
rs62143206
All
−1e−03
−5e−04
0e+00
5e−04
1e−03
MR effect size for
'Serine/threonine−protein kinase 17B' on 'eGFR'

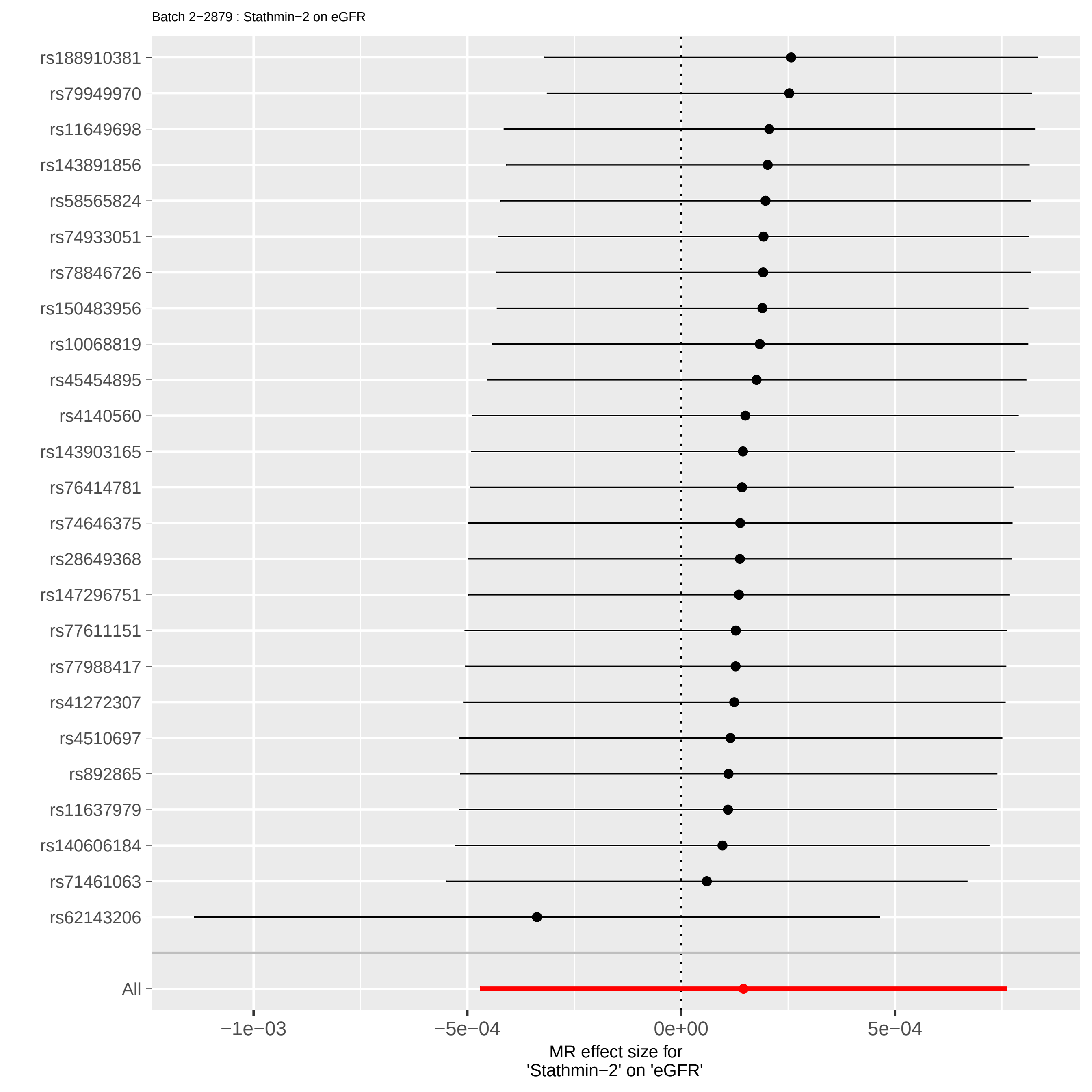

Batch 2−2879 : Stathmin−2 on eGFR
rs188910381
rs79949970
rs11649698
rs143891856
rs58565824
rs74933051
rs78846726
rs150483956
rs10068819
rs45454895
rs4140560
rs143903165
rs76414781
rs74646375
rs28649368
rs147296751
rs77611151
rs77988417
rs41272307
rs4510697
rs892865
rs11637979
rs140606184
rs71461063
rs62143206
All
−1e−03
−5e−04
0e+00
5e−04
MR effect size for
'Stathmin−2' on 'eGFR'

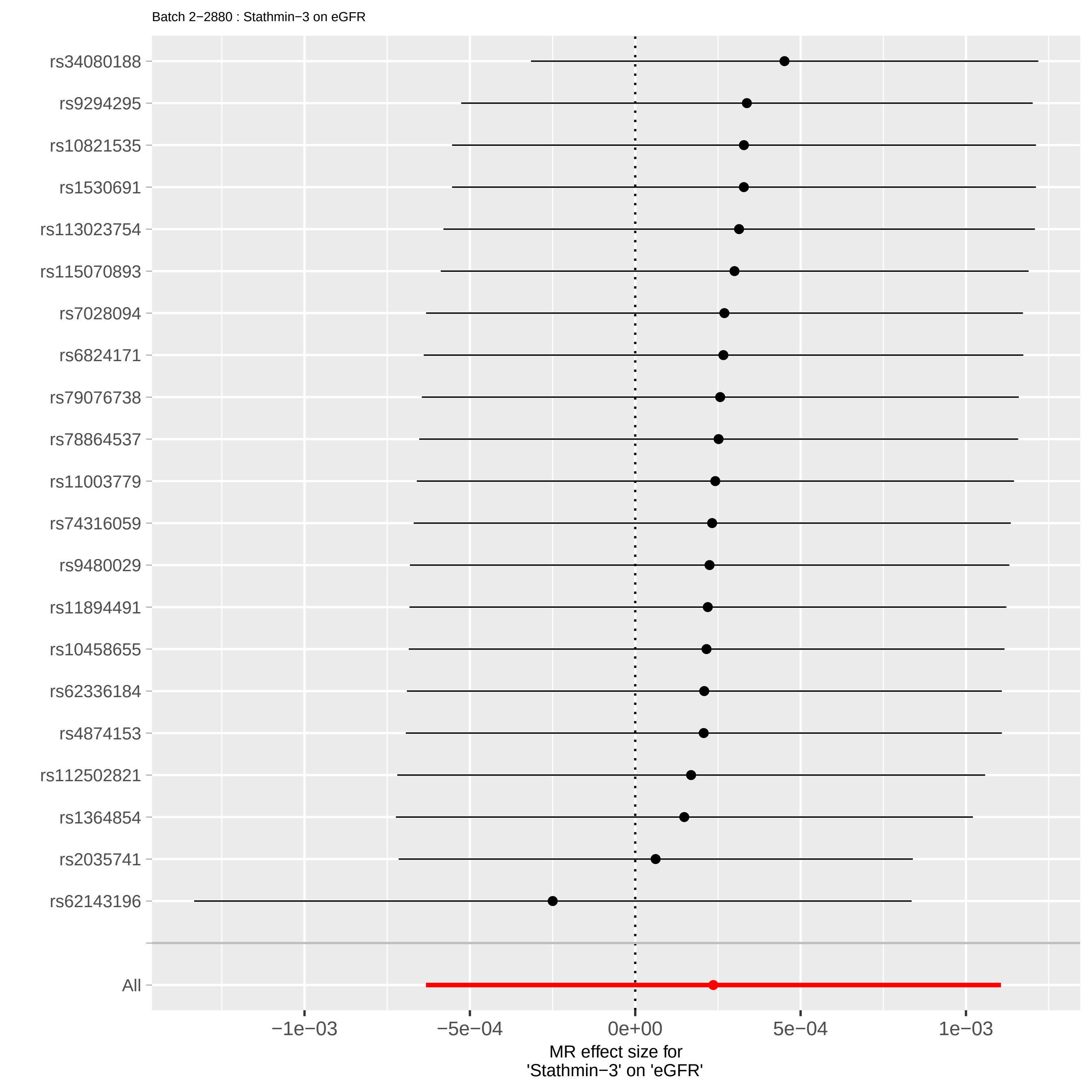

Batch 2−2880 : Stathmin−3 on eGFR
rs34080188
rs9294295
rs10821535
rs1530691
rs113023754
rs115070893
rs7028094
rs6824171
rs79076738
rs78864537
rs11003779
rs74316059
rs9480029
rs11894491
rs10458655
rs62336184
rs4874153
rs112502821
rs1364854
rs2035741
rs62143196
All
−1e−03
−5e−04
0e+00
5e−04
1e−03
MR effect size for
'Stathmin−3' on 'eGFR'

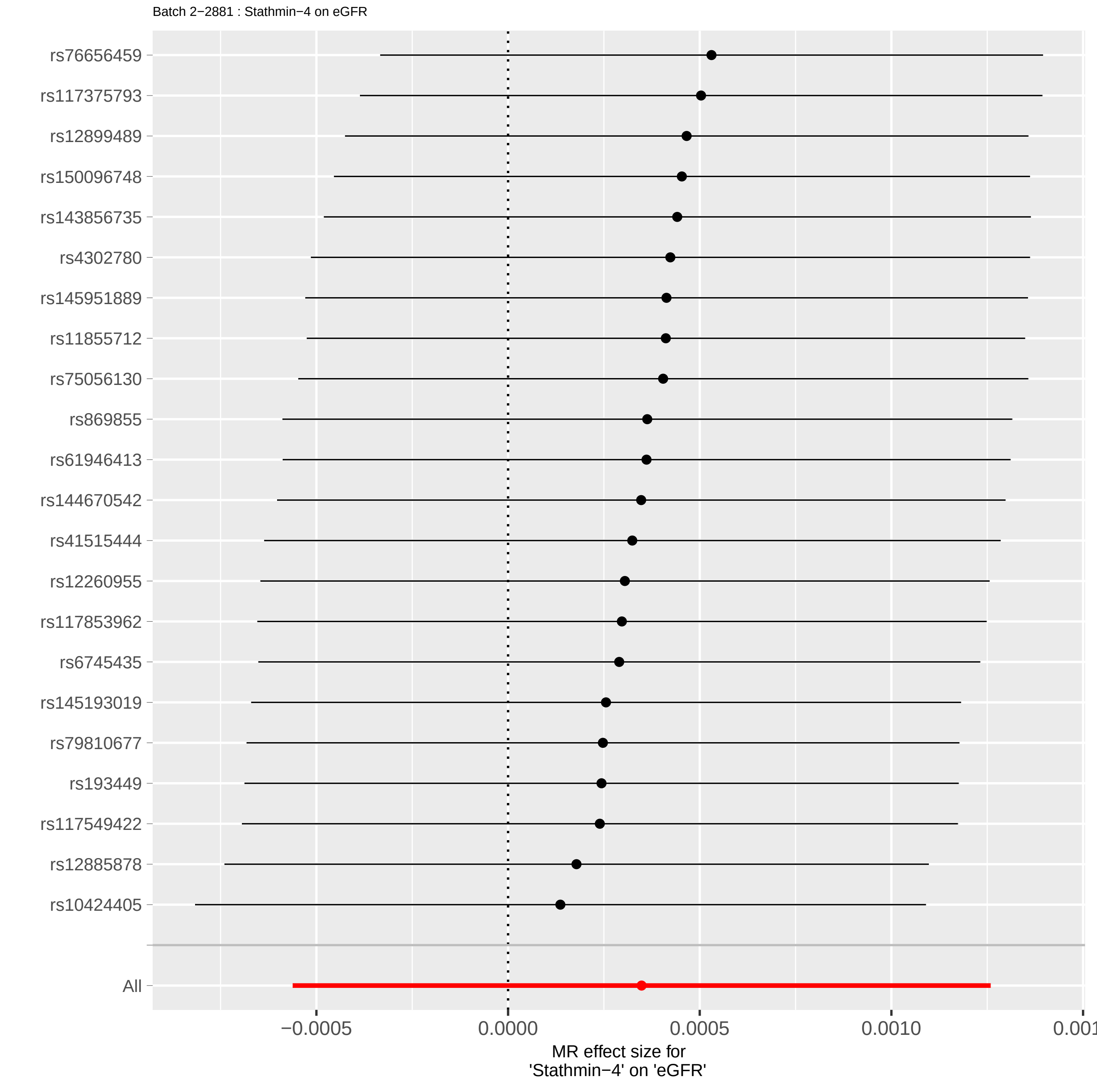
Batch 2−2881 : Stathmin−4 on eGFR
rs76656459
rs117375793
rs12899489
rs150096748
rs143856735
rs4302780
rs145951889
rs11855712
rs75056130
rs869855
rs61946413
rs144670542
rs41515444
rs12260955
rs117853962
rs6745435
rs145193019
rs79810677
rs193449
rs117549422
rs12885878
rs10424405
All
−0.0005
0.0000
0.0005
0.0010
MR effect size for
'Stathmin−4' on 'eGFR'

Batch 2−2882 : Erythrocyte band 7 integral membrane protein on eGFR

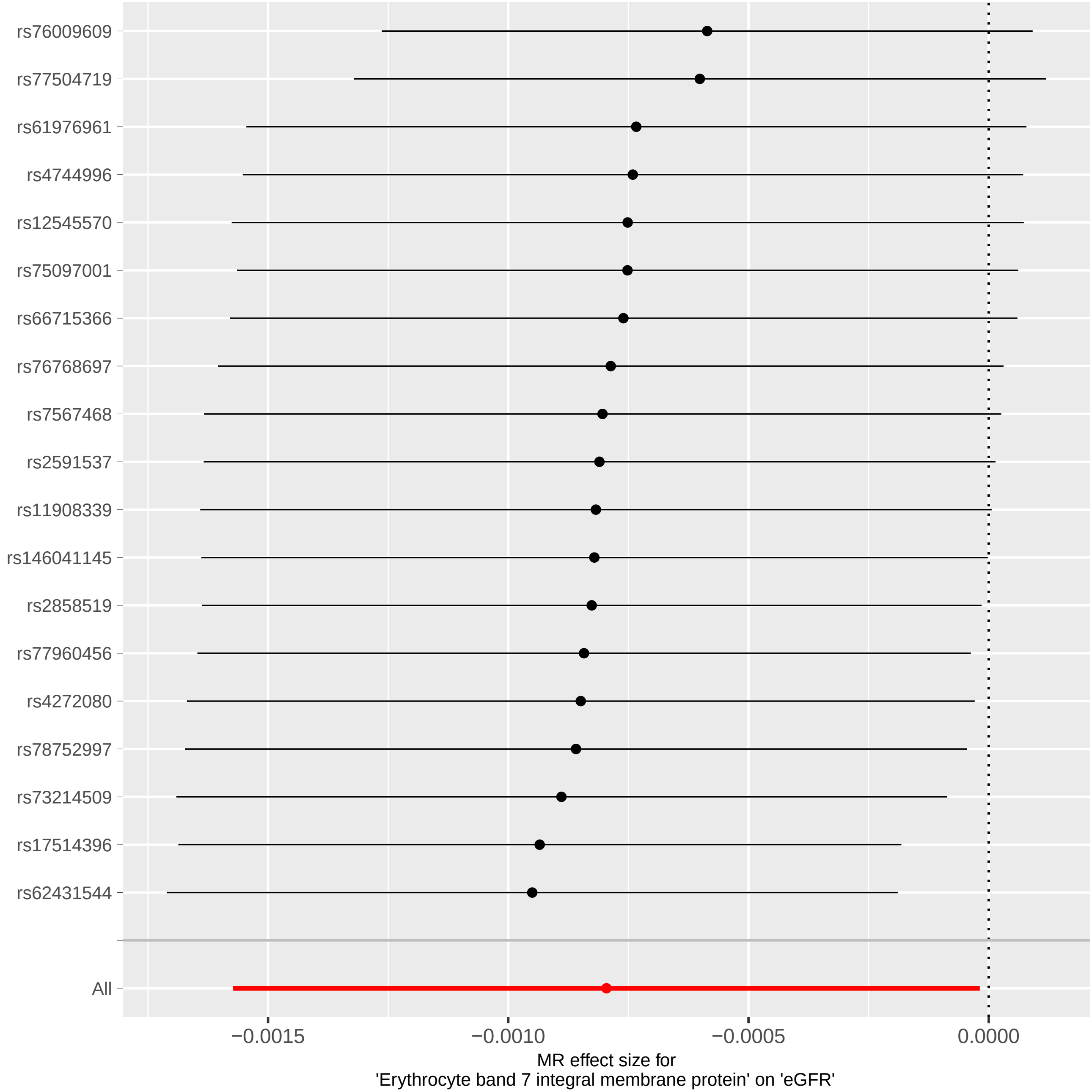

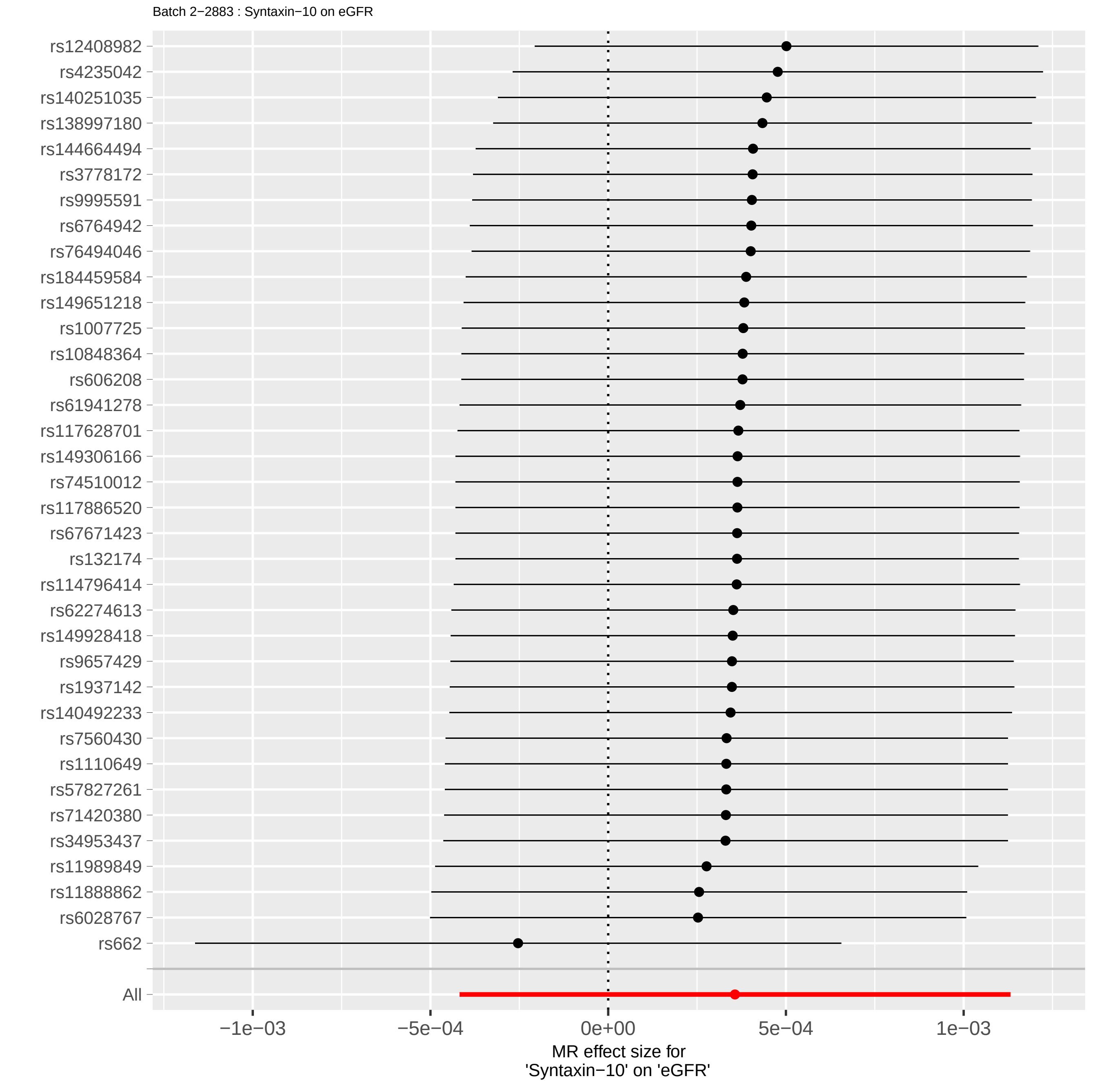
Batch 2−2883 : Syntaxin−10 on eGFR
rs12408982
rs4235042
rs140251035
rs138997180
rs144664494
rs3778172
rs9995591
rs6764942
rs76494046
rs184459584
rs149651218
rs1007725
rs10848364
rs606208
rs61941278
rs117628701
rs149306166
rs74510012
rs117886520
rs67671423
rs132174
rs114796414
rs62274613
rs149928418
rs9657429
rs1937142
rs140492233
rs7560430
rs1110649
rs57827261
rs71420380
rs34953437
rs11989849
rs11888862
rs6028767
rs662
All
−1e−03
−5e−04
0e+00
5e−04
1e−03
MR effect size for
'Syntaxin−10' on 'eGFR'

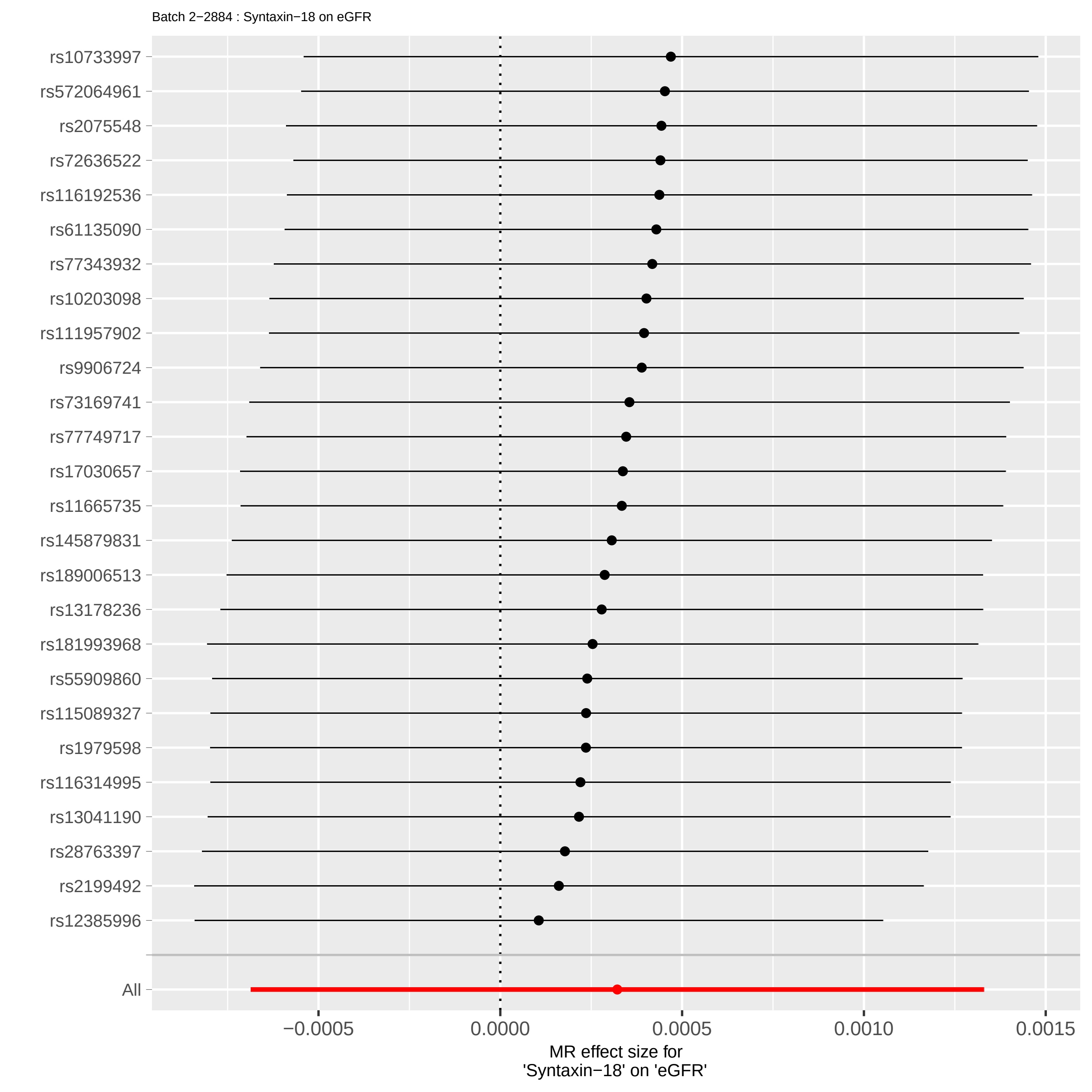
Batch 2−2884 : Syntaxin−18 on eGFR
rs10733997
rs572064961
rs2075548
rs72636522
rs116192536
rs61135090
rs77343932
rs10203098
rs111957902
rs9906724
rs73169741
rs77749717
rs17030657
rs11665735
rs145879831
rs189006513
rs13178236
rs181993968
rs55909860
rs115089327
rs1979598
rs116314995
rs13041190
rs28763397
rs2199492
rs12385996
All
−0.0005
0.0000
0.0005
0.0010
0.0015
MR effect size for
'Syntaxin−18' on 'eGFR'

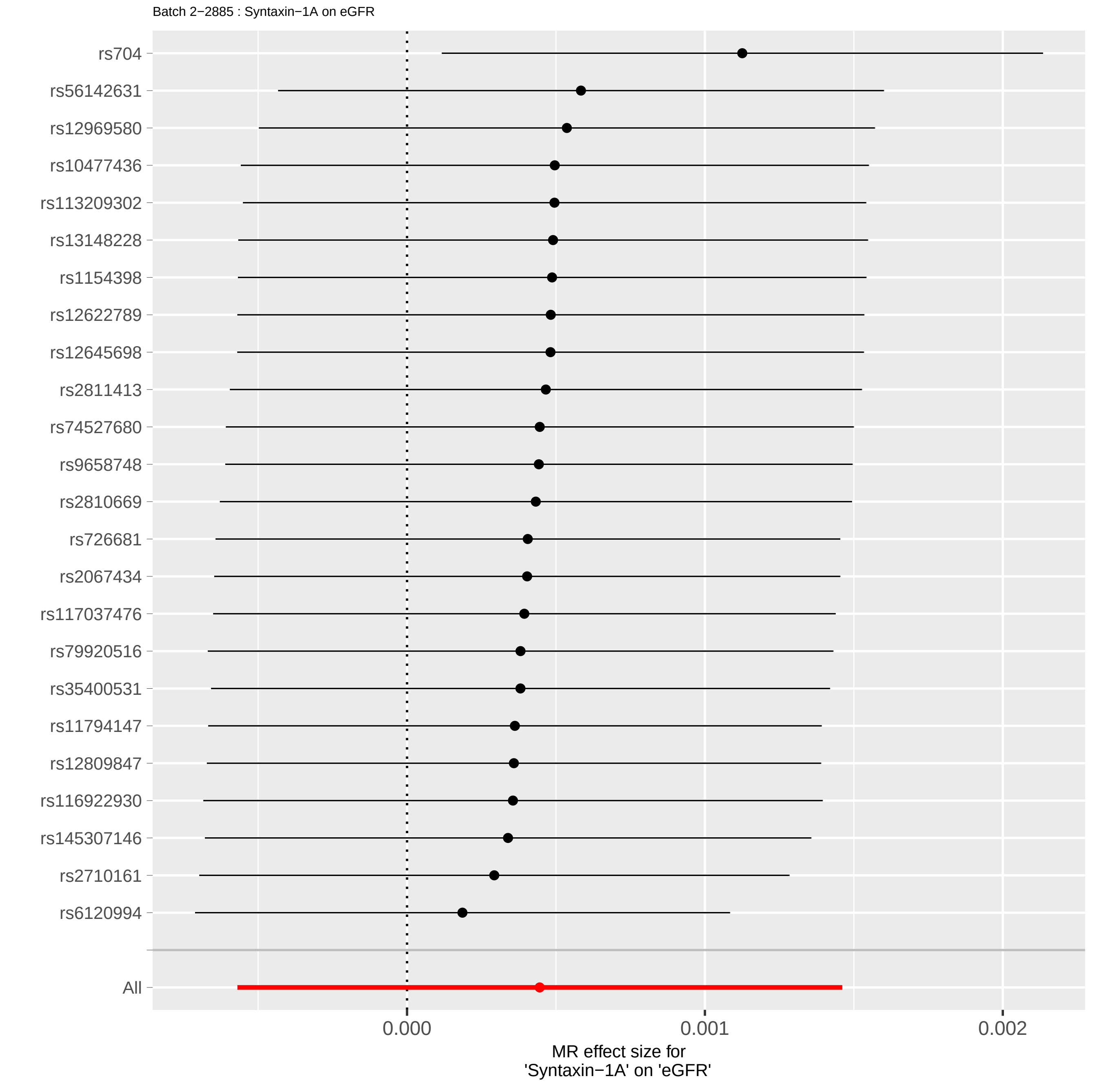

Batch 2−2885 : Syntaxin−1A on eGFR
rs704
rs56142631
rs12969580
rs10477436
rs113209302
rs13148228
rs1154398
rs12622789
rs12645698
rs2811413
rs74527680
rs9658748
rs2810669
rs726681
rs2067434
rs117037476
rs79920516
rs35400531
rs11794147
rs12809847
rs116922930
rs145307146
rs2710161
rs6120994
All
0.000
0.001
0.002
MR effect size for
'Syntaxin−1A' on 'eGFR'

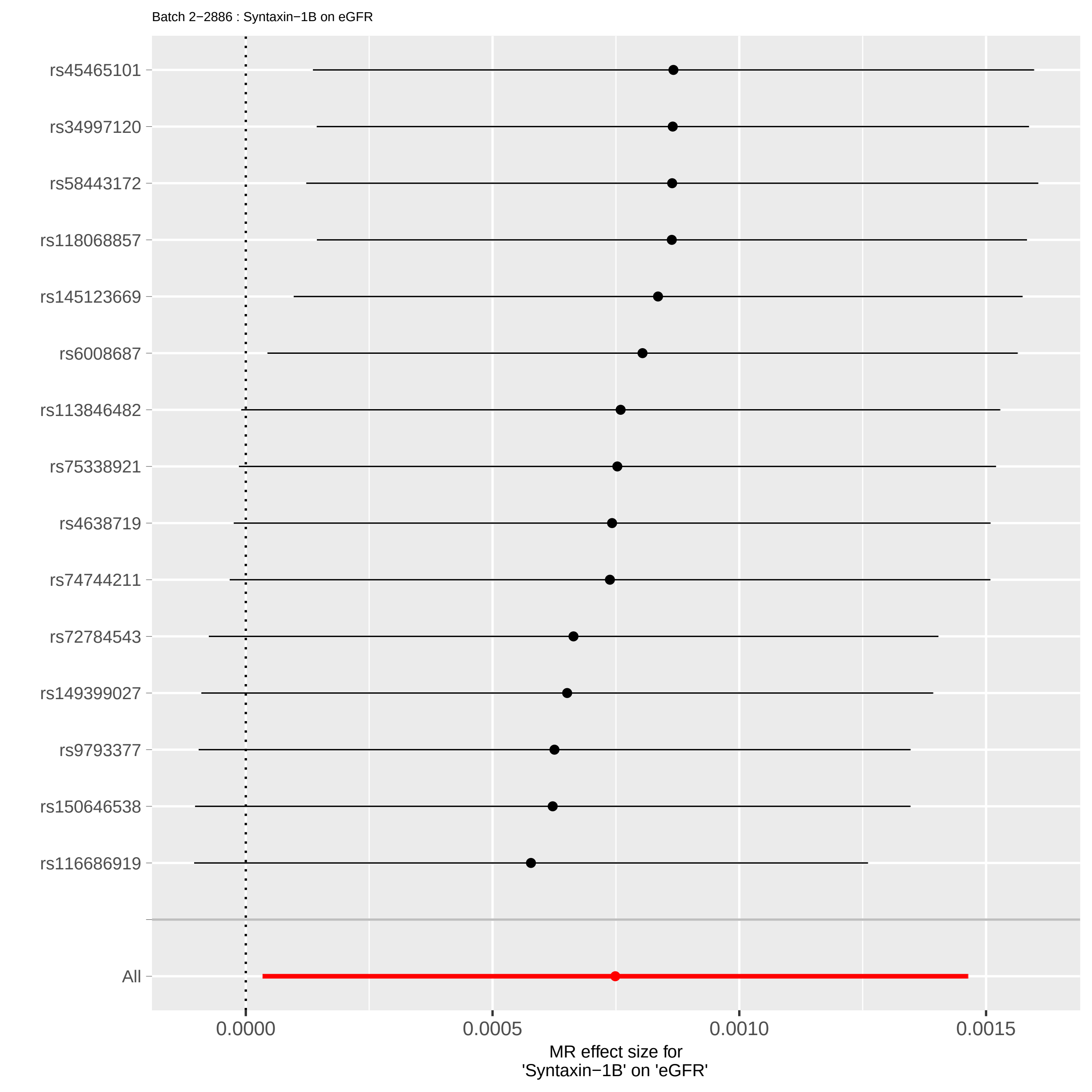

Batch 2−2886 : Syntaxin−1B on eGFR
rs45465101
rs34997120
rs58443172
rs118068857
rs145123669
rs6008687
rs113846482
rs75338921
rs4638719
rs74744211
rs72784543
rs149399027
rs9793377
rs150646538
rs116686919
All
0.0000
0.0005
0.0010
0.0015
MR effect size for
'Syntaxin−1B' on 'eGFR'

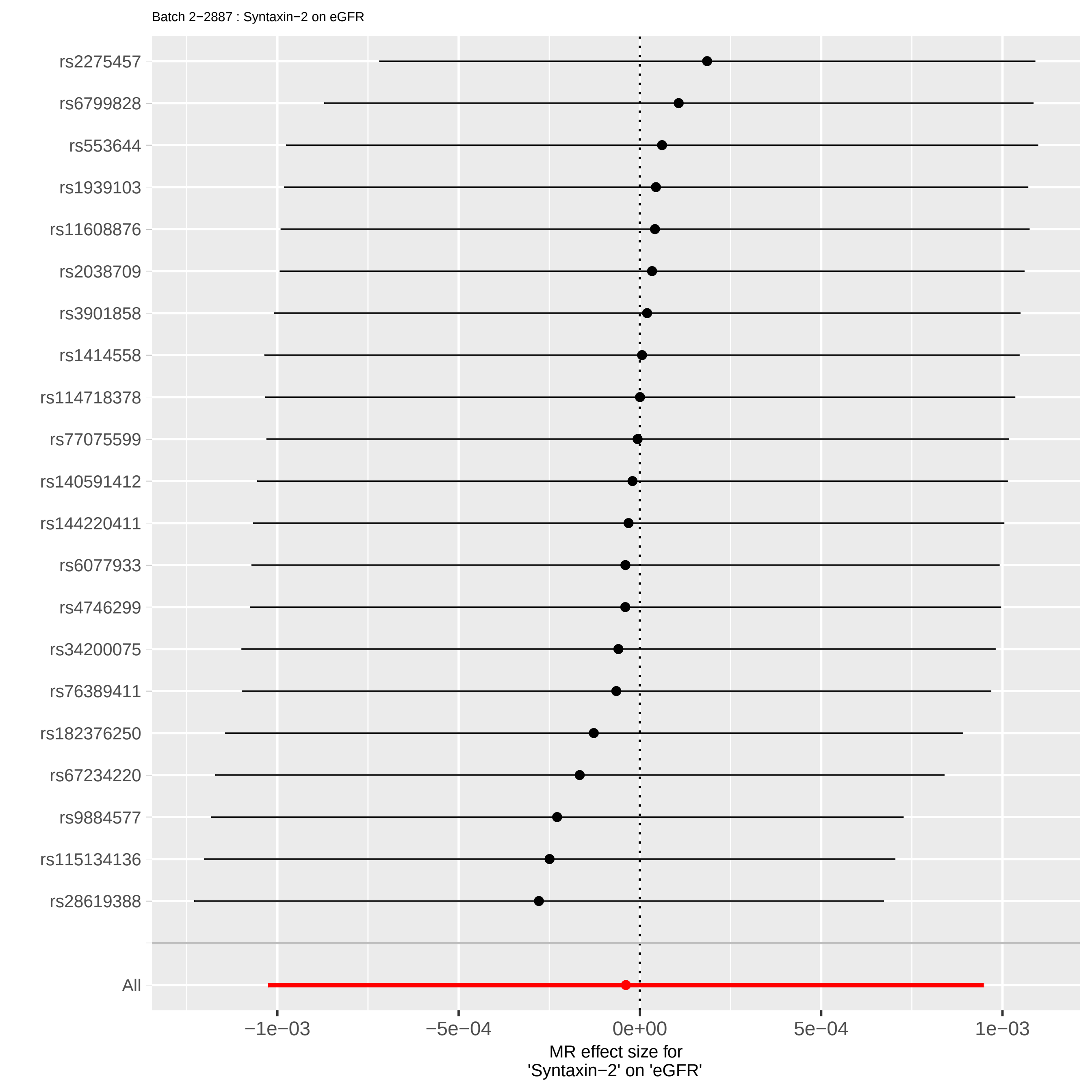

Batch 2−2887 : Syntaxin−2 on eGFR
rs2275457
rs6799828
rs553644
rs1939103
rs11608876
rs2038709
rs3901858
rs1414558
rs114718378
rs77075599
rs140591412
rs144220411
rs6077933
rs4746299
rs34200075
rs76389411
rs182376250
rs67234220
rs9884577
rs115134136
rs28619388
All
−1e−03
−5e−04
0e+00
5e−04
1e−03
MR effect size for
'Syntaxin−2' on 'eGFR'

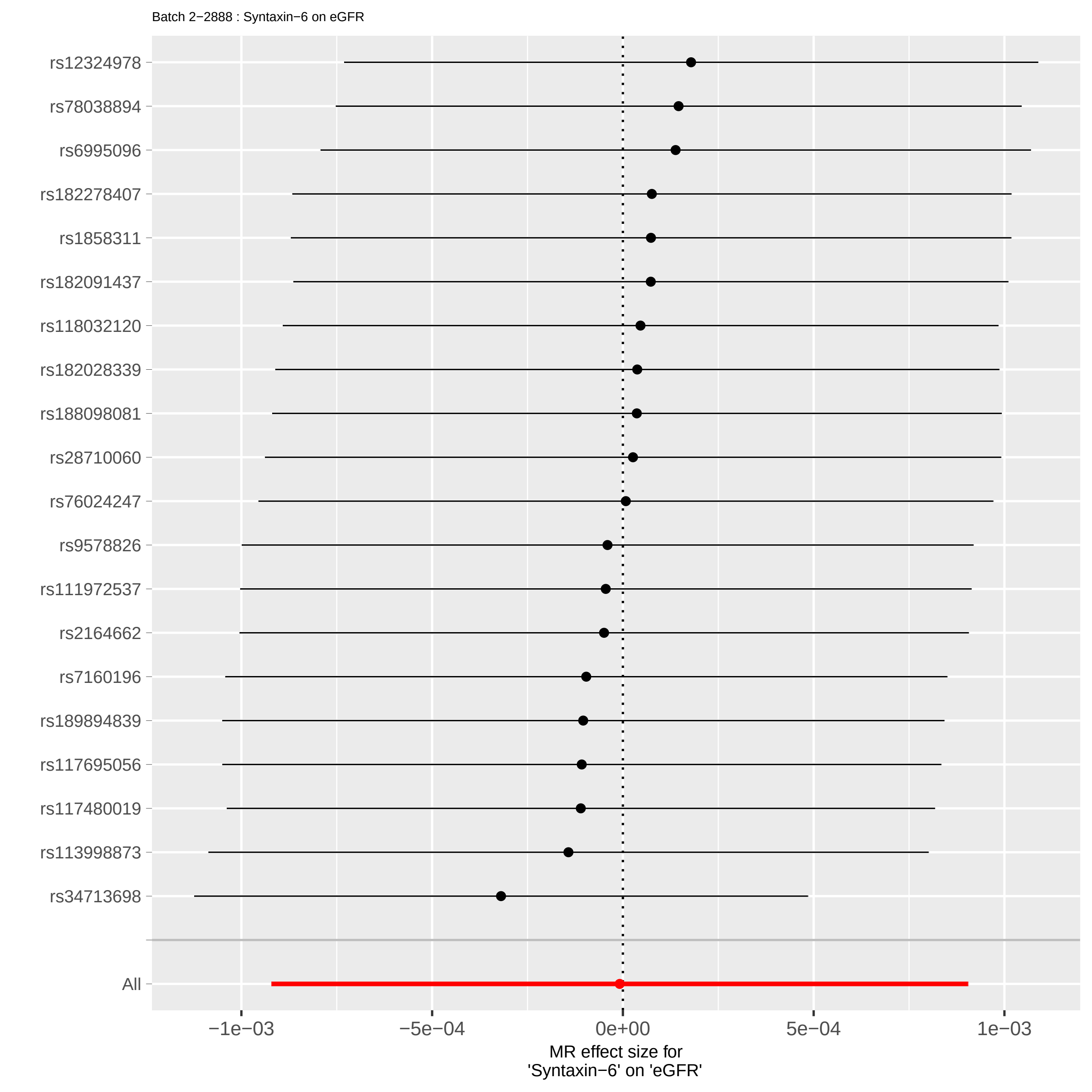

Batch 2−2888 : Syntaxin−6 on eGFR
rs12324978
rs78038894
rs6995096
rs182278407
rs1858311
rs182091437
rs118032120
rs182028339
rs188098081
rs28710060
rs76024247
rs9578826
rs111972537
rs2164662
rs7160196
rs189894839
rs117695056
rs117480019
rs113998873
rs34713698
All
−1e−03
−5e−04
0e+00
5e−04
1e−03
MR effect size for
'Syntaxin−6' on 'eGFR'

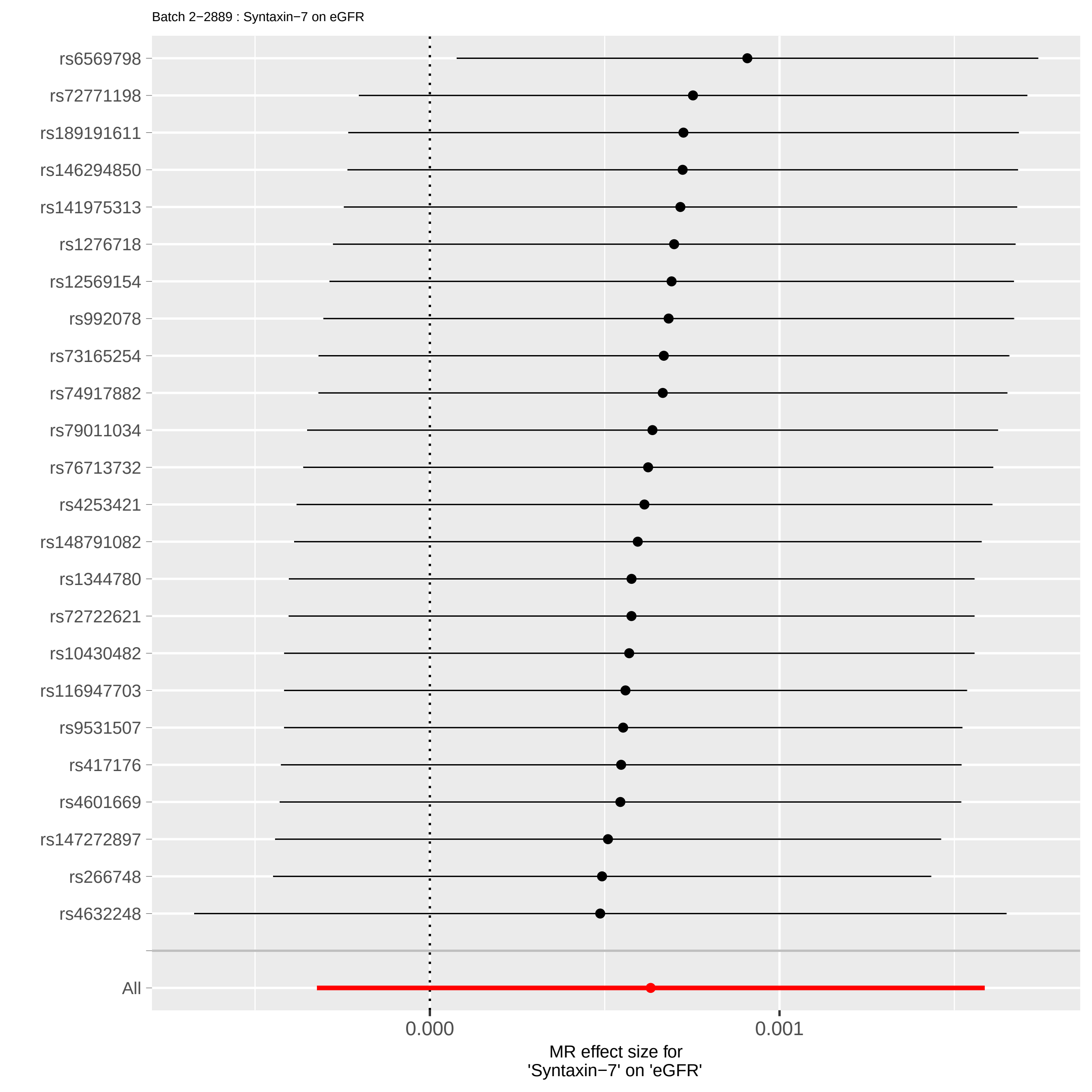

Batch 2−2889 : Syntaxin−7 on eGFR
rs6569798
rs72771198
rs189191611
rs146294850
rs141975313
rs1276718
rs12569154
rs992078
rs73165254
rs74917882
rs79011034
rs76713732
rs4253421
rs148791082
rs1344780
rs72722621
rs10430482
rs116947703
rs9531507
rs417176
rs4601669
rs147272897
rs266748
rs4632248
All
0.000
0.001
MR effect size for
'Syntaxin−7' on 'eGFR'

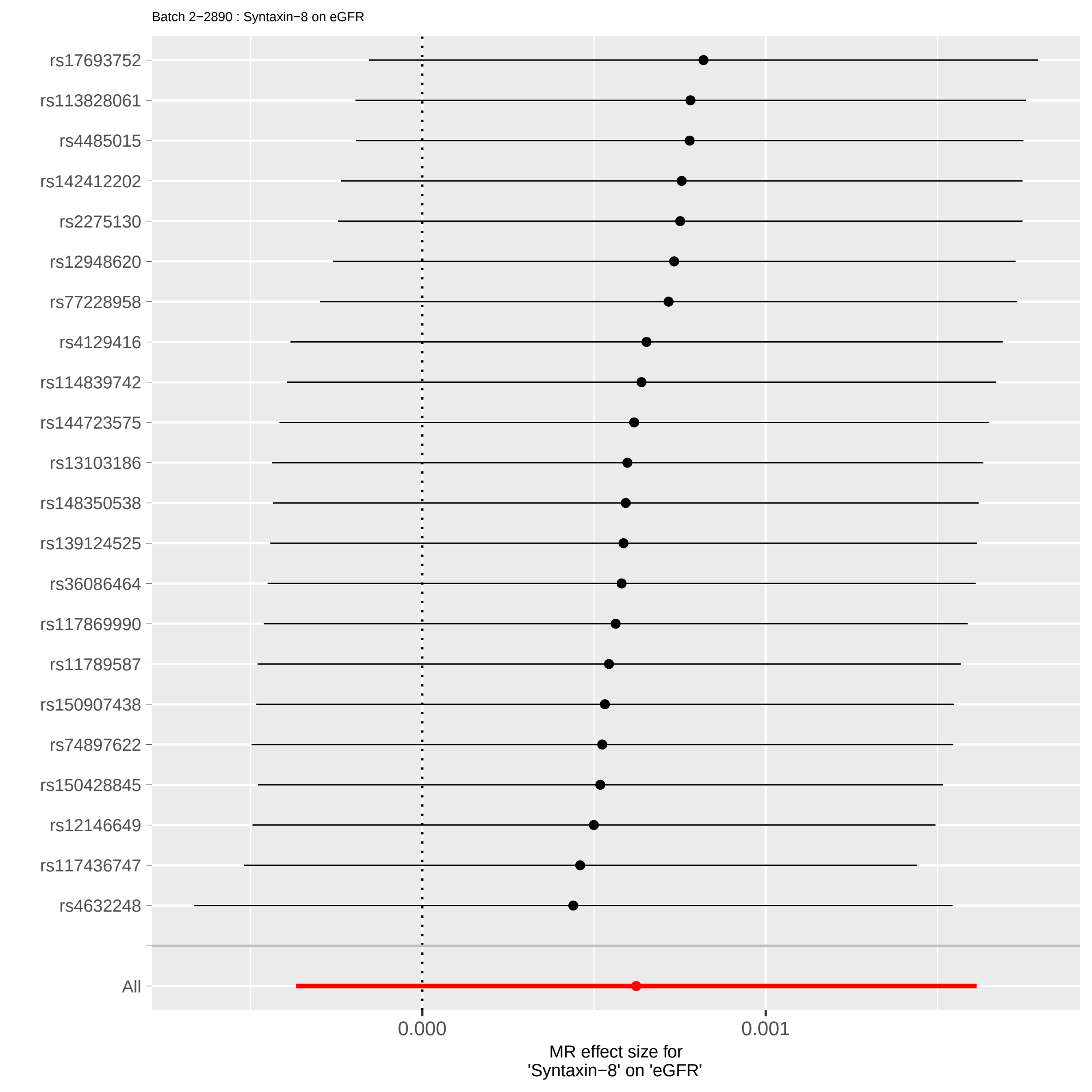

Batch 2−2890 : Syntaxin−8 on eGFR
rs17693752
rs113828061
rs4485015
rs142412202
rs2275130
rs12948620
rs77228958
rs4129416
rs114839742
rs144723575
rs13103186
rs148350538
rs139124525
rs36086464
rs117869990
rs11789587
rs150907438
rs74897622
rs150428845
rs12146649
rs117436747
rs4632248
All
0.000
0.001
MR effect size for
'Syntaxin−8' on 'eGFR'

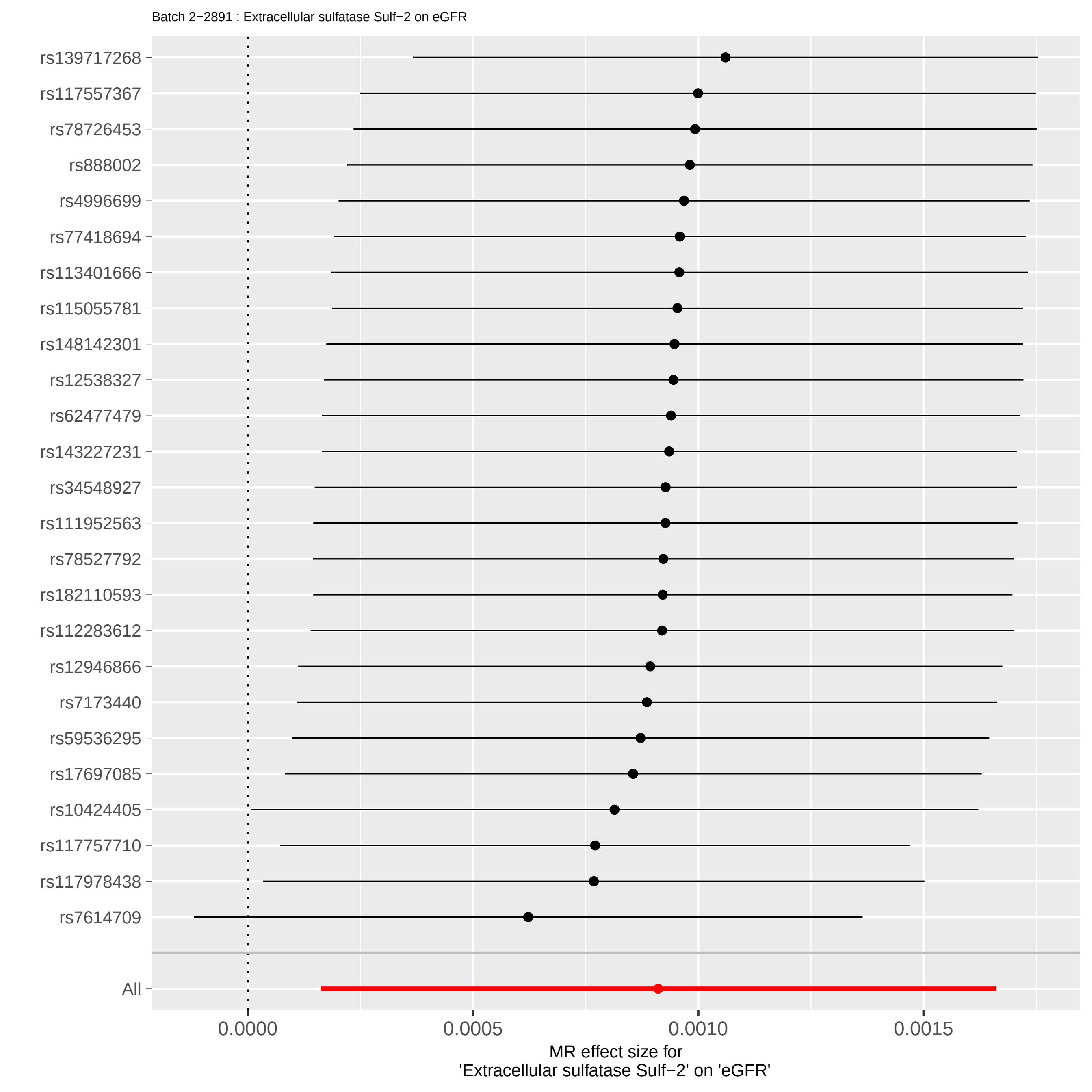

Batch 2−2891 : Extracellular sulfatase Sulf−2 on eGFR
rs139717268
rs117557367
rs78726453
rs888002
rs4996699
rs77418694
rs113401666
rs115055781
rs148142301
rs12538327
rs62477479
rs143227231
rs34548927
rs111952563
rs78527792
rs182110593
rs112283612
rs12946866
rs7173440
rs59536295
rs17697085
rs10424405
rs117757710
rs117978438
rs7614709
All
0.0000
0.0005
0.0010
0.0015
MR effect size for
'Extracellular sulfatase Sulf−2' on 'eGFR'

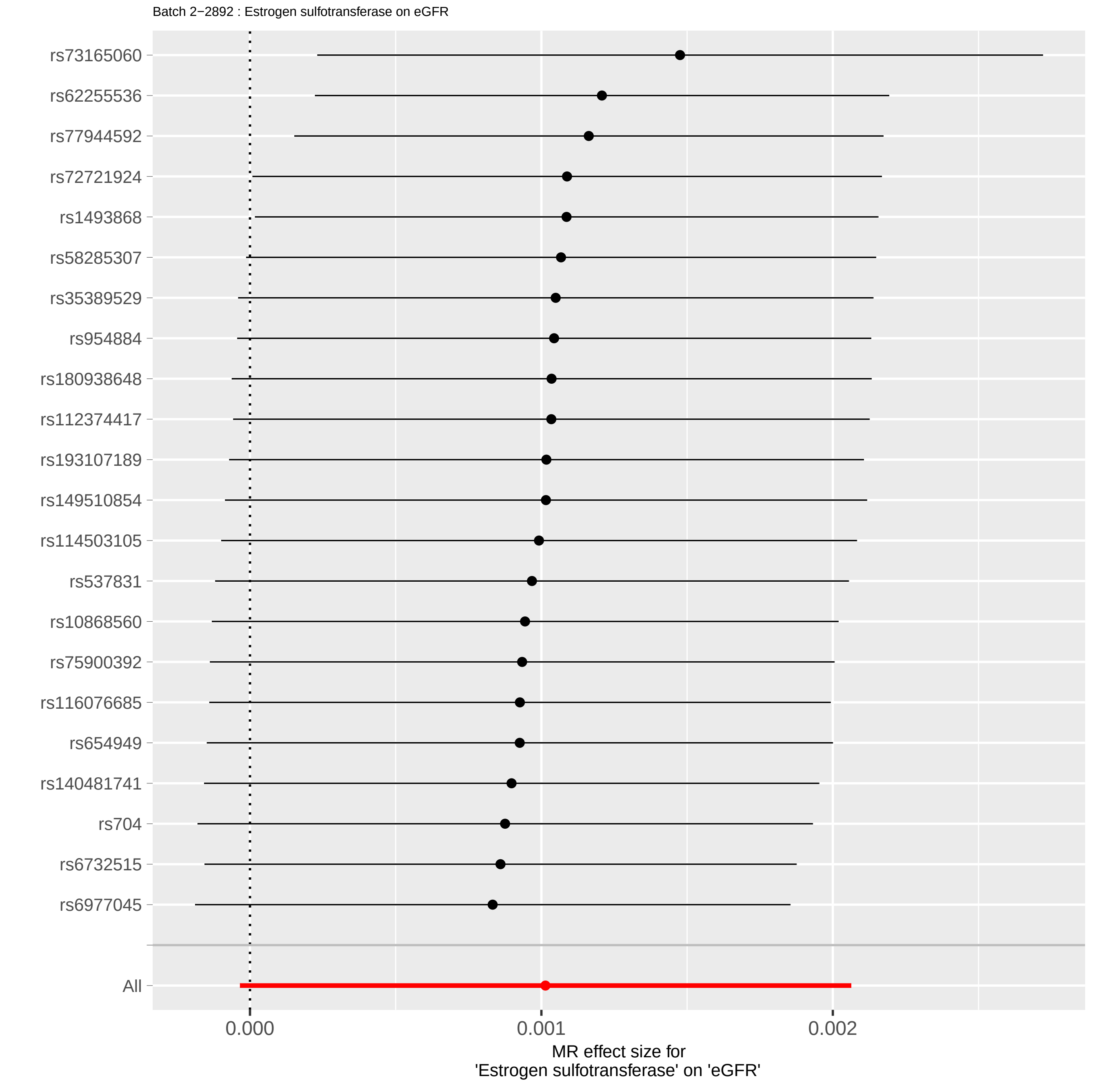

Batch 2−2892 : Estrogen sulfotransferase on eGFR
rs73165060
rs62255536
rs77944592
rs72721924
rs1493868
rs58285307
rs35389529
rs954884
rs180938648
rs112374417
rs193107189
rs149510854
rs114503105
rs537831
rs10868560
rs75900392
rs116076685
rs654949
rs140481741
rs704
rs6732515
rs6977045
All
0.000
0.001
0.002
MR effect size for
'Estrogen sulfotransferase' on 'eGFR'

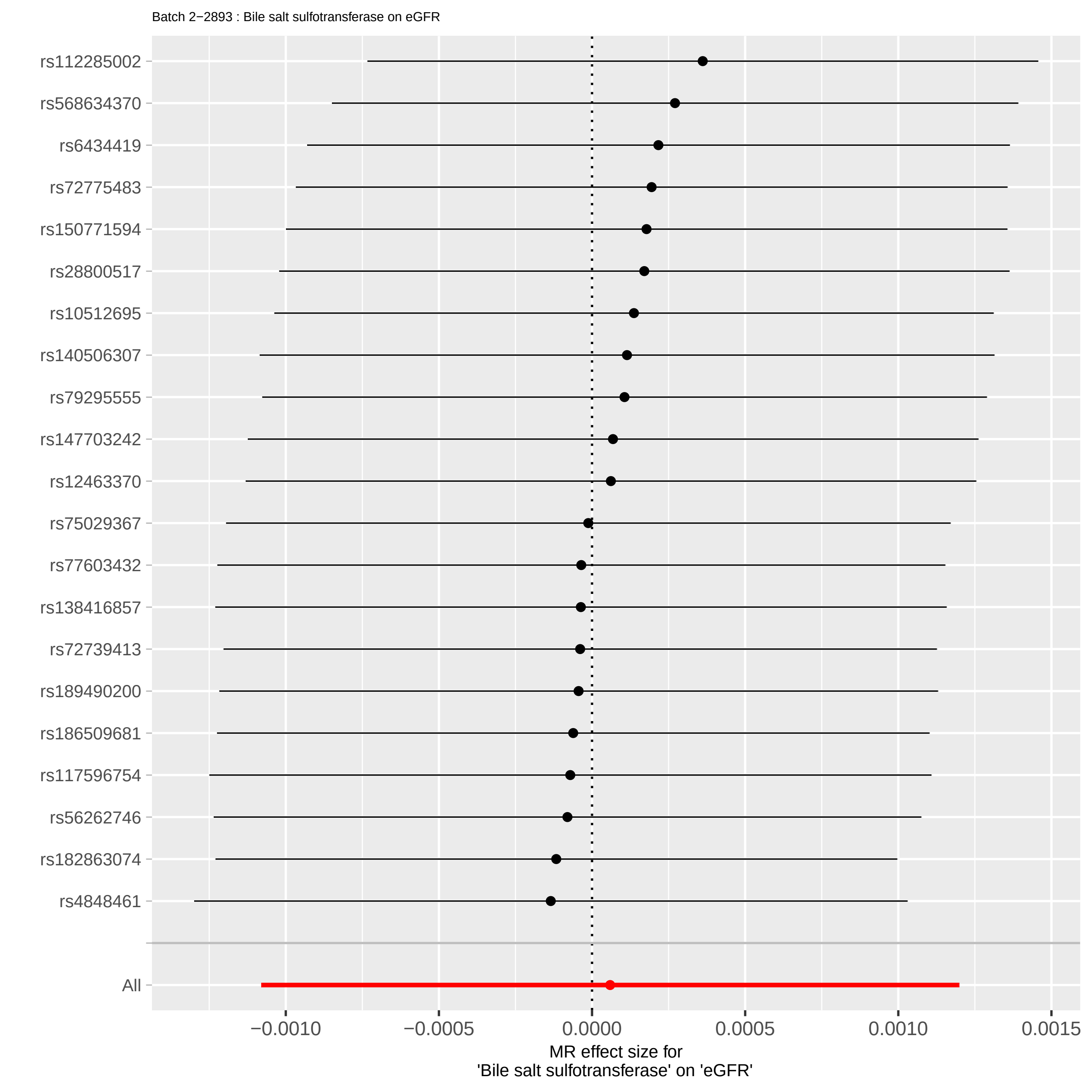
Batch 2−2893 : Bile salt sulfotransferase on eGFR
rs112285002
rs568634370
rs6434419
rs72775483
rs150771594
rs28800517
rs10512695
rs140506307
rs79295555
rs147703242
rs12463370
rs75029367
rs77603432
rs138416857
rs72739413
rs189490200
rs186509681
rs117596754
rs56262746
rs182863074
rs4848461
All
−0.0010
−0.0005
0.0000
0.0005
0.0010
0.0015
MR effect size for
'Bile salt sulfotransferase' on 'eGFR'

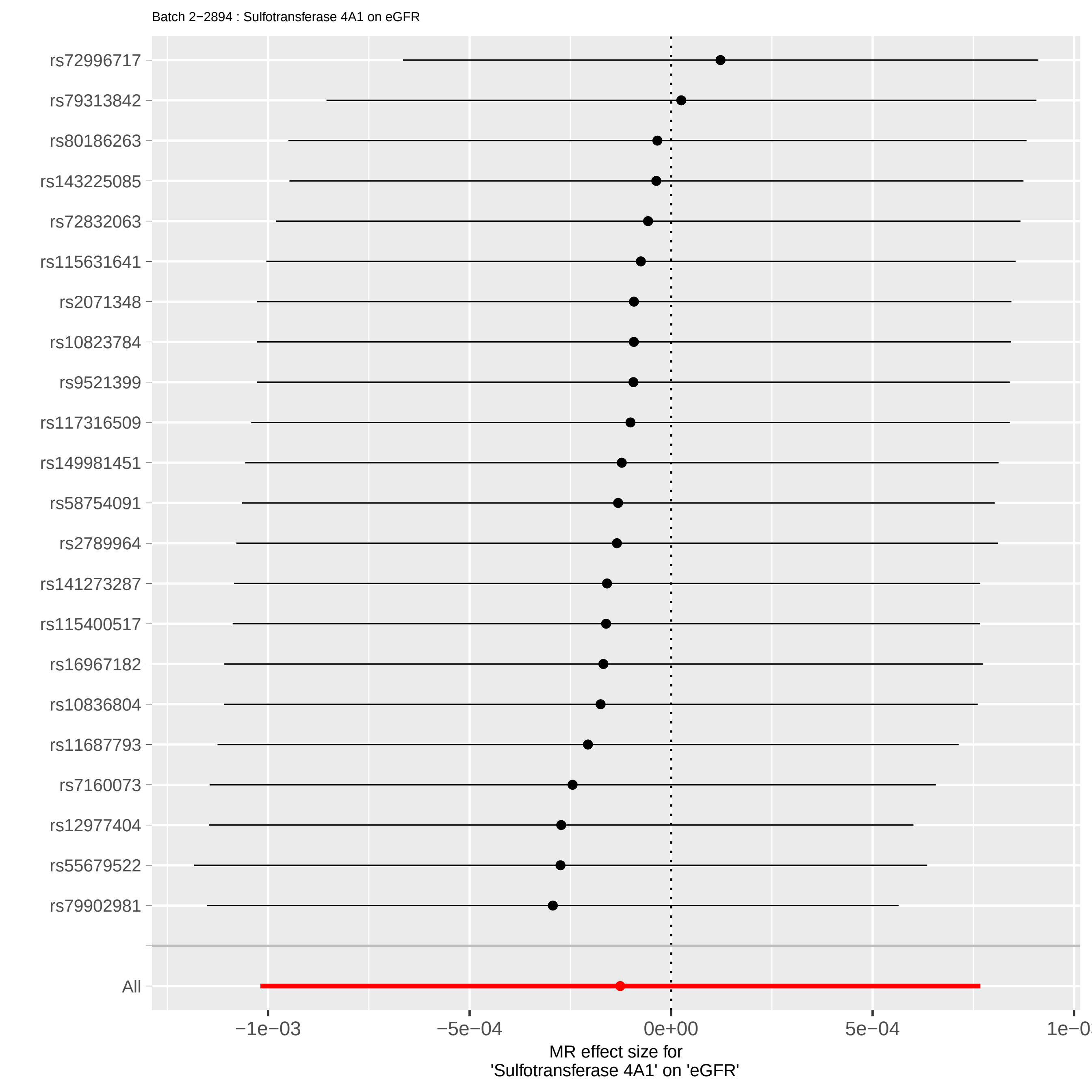
Batch 2−2894 : Sulfotransferase 4A1 on eGFR
rs72996717
rs79313842
rs80186263
rs143225085
rs72832063
rs115631641
rs2071348
rs10823784
rs9521399
rs117316509
rs149981451
rs58754091
rs2789964
rs141273287
rs115400517
rs16967182
rs10836804
rs11687793
rs7160073
rs12977404
rs55679522
rs79902981
All
−1e−03
−5e−04
0e+00
5e−04
MR effect size for
'Sulfotransferase 4A1' on 'eGFR'

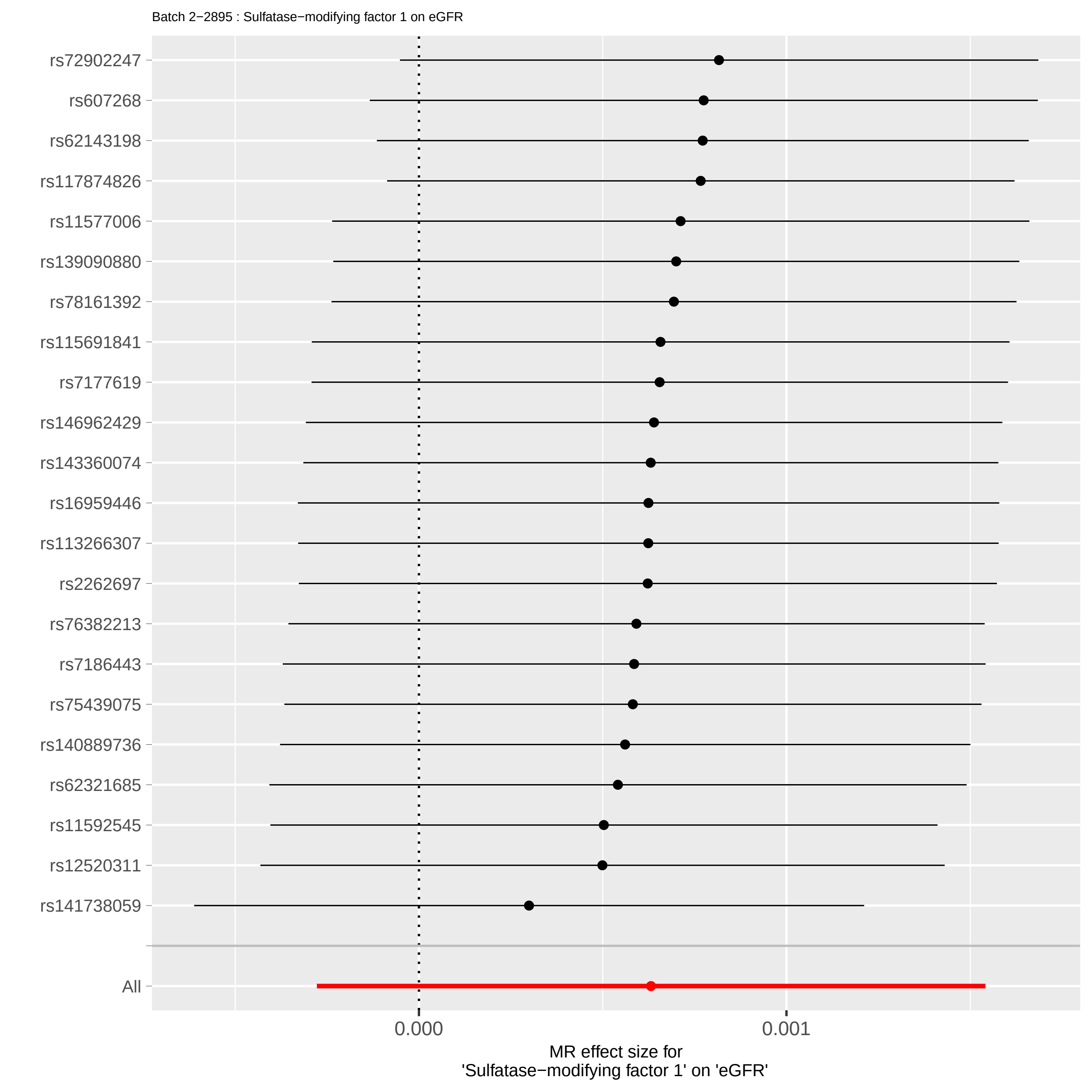
Batch 2−2895 : Sulfatase−modifying factor 1 on eGFR
rs72902247
rs607268
rs62143198
rs117874826
rs11577006
rs139090880
rs78161392
rs115691841
rs7177619
rs146962429
rs143360074
rs16959446
rs113266307
rs2262697
rs76382213
rs7186443
rs75439075
rs140889736
rs62321685
rs11592545
rs12520311
rs141738059
All
0.000
0.001
MR effect size for
'Sulfatase−modifying factor 1' on 'eGFR'

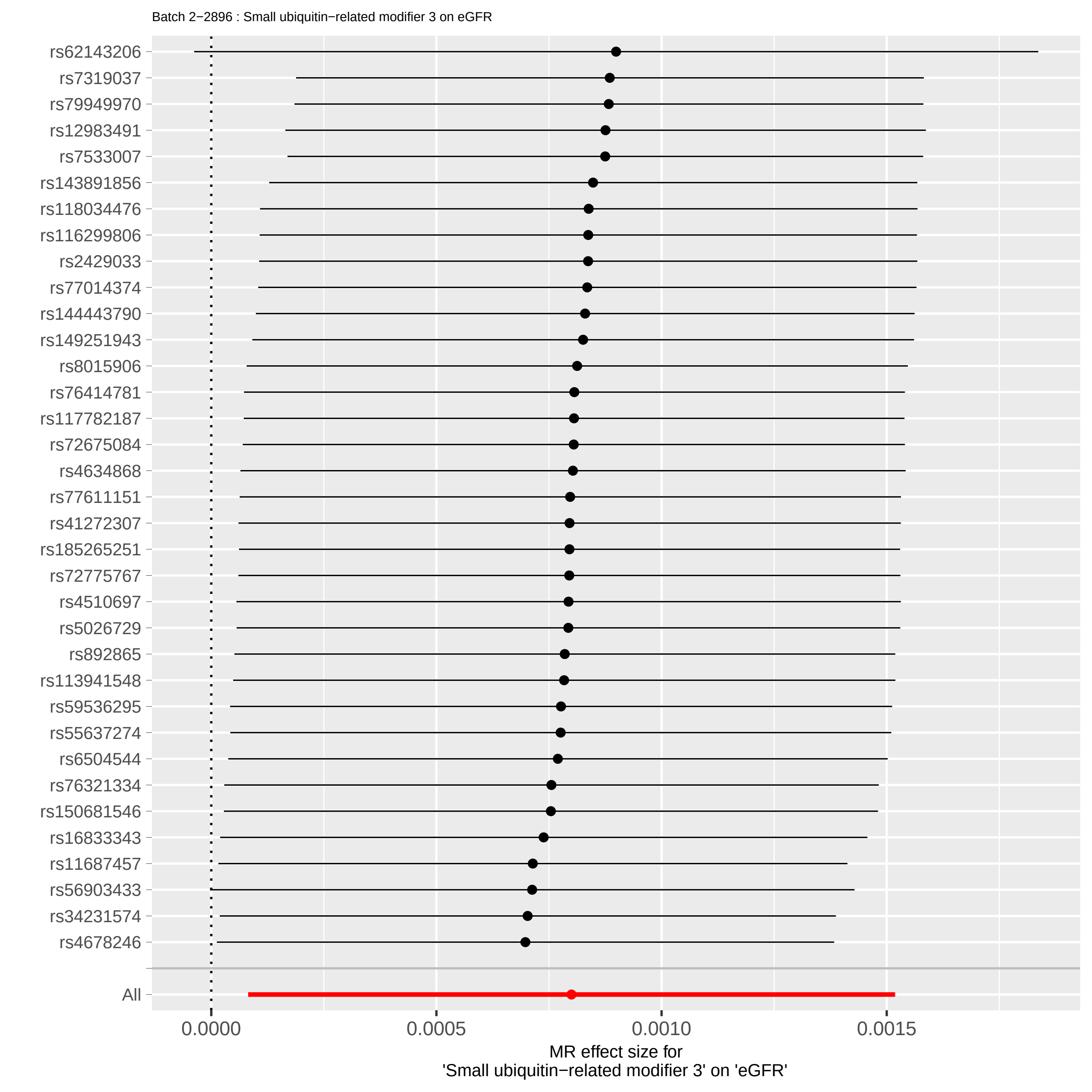
Batch 2−2896 : Small ubiquitin−related modifier 3 on eGFR
rs62143206
rs7319037
rs79949970
rs12983491
rs7533007
rs143891856
rs118034476
rs116299806
rs2429033
rs77014374
rs144443790
rs149251943
rs8015906
rs76414781
rs117782187
rs72675084
rs4634868
rs77611151
rs41272307
rs185265251
rs72775767
rs4510697
rs5026729
rs892865
rs113941548
rs59536295
rs55637274
rs6504544
rs76321334
rs150681546
rs16833343
rs11687457
rs56903433
rs34231574
rs4678246
All
0.0000
0.0005
0.0010
0.0015
MR effect size for
'Small ubiquitin−related modifier 3' on 'eGFR'

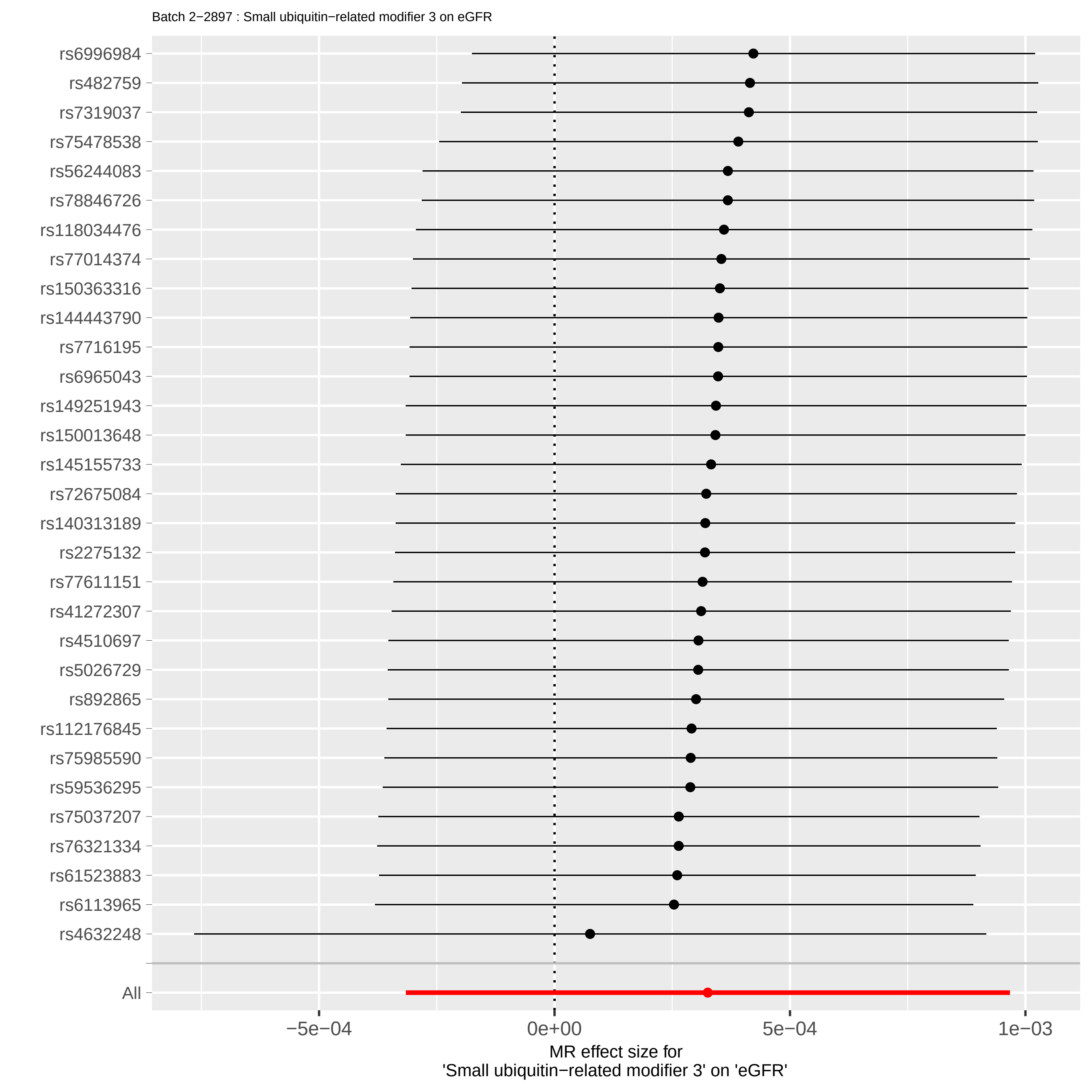
Batch 2−2897 : Small ubiquitin−related modifier 3 on eGFR
rs6996984
rs482759
rs7319037
rs75478538
rs56244083
rs78846726
rs118034476
rs77014374
rs150363316
rs144443790
rs7716195
rs6965043
rs149251943
rs150013648
rs145155733
rs72675084
rs140313189
rs2275132
rs77611151
rs41272307
rs4510697
rs5026729
rs892865
rs112176845
rs75985590
rs59536295
rs75037207
rs76321334
rs61523883
rs6113965
rs4632248
All
−5e−04
0e+00
5e−04
1e−03
MR effect size for
'Small ubiquitin−related modifier 3' on 'eGFR'

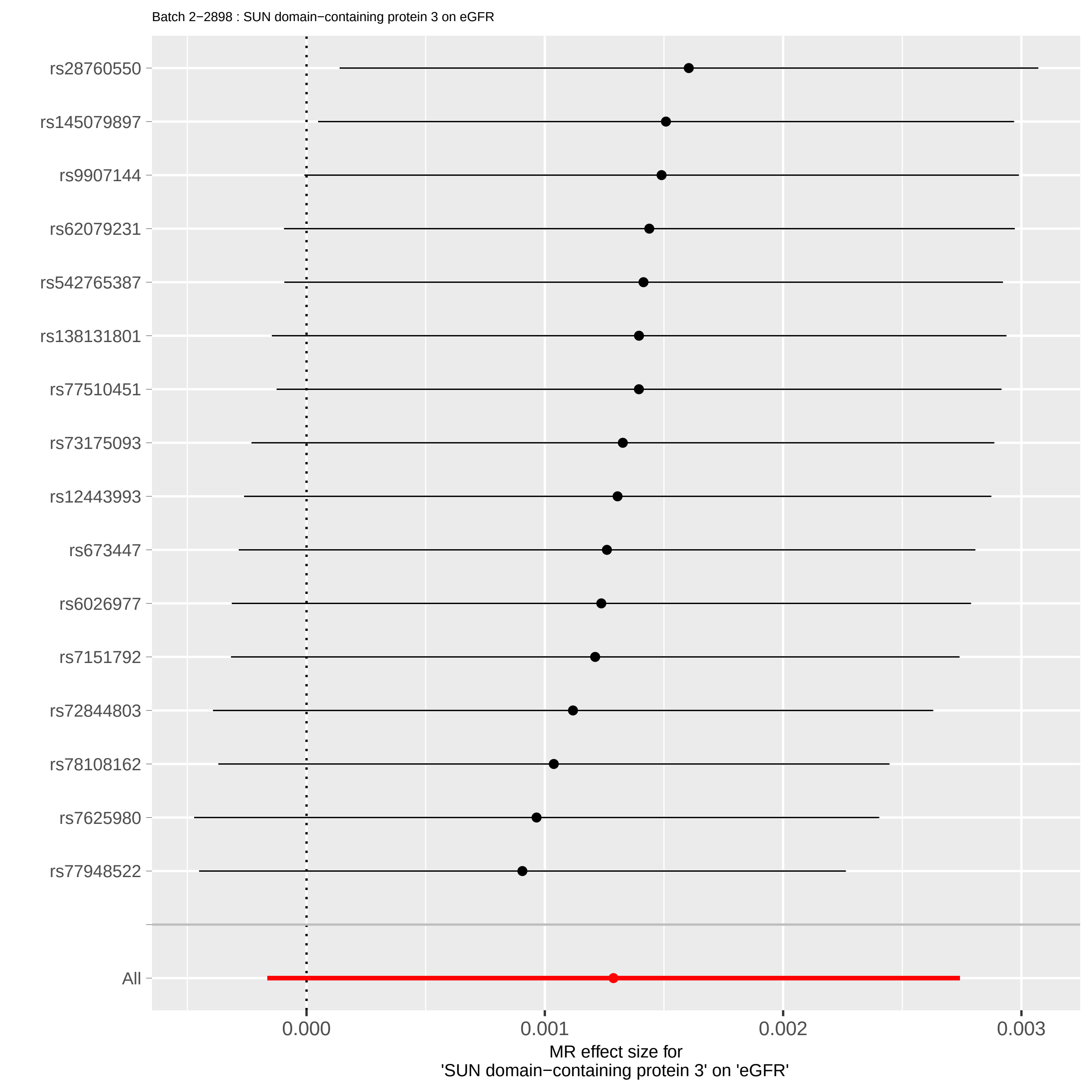

Batch 2−2898 : SUN domain−containing protein 3 on eGFR
rs28760550
rs145079897
rs9907144
rs62079231
rs542765387
rs138131801
rs77510451
rs73175093
rs12443993
rs673447
rs6026977
rs7151792
rs72844803
rs78108162
rs7625980
rs77948522
All
0.000
0.001
0.002
0.003
MR effect size for
'SUN domain−containing protein 3' on 'eGFR'

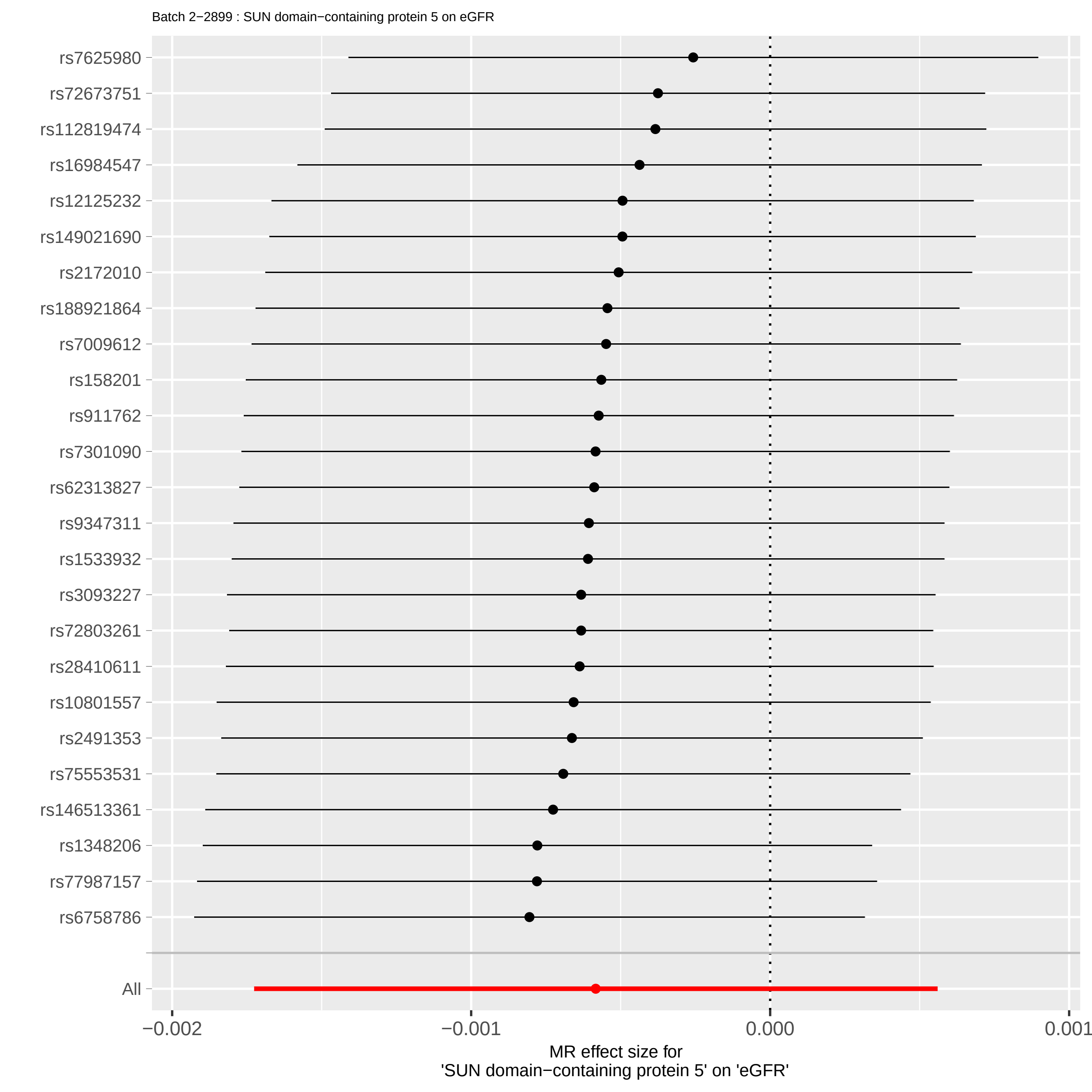
Batch 2−2899 : SUN domain−containing protein 5 on eGFR
rs7625980
rs72673751
rs112819474
rs16984547
rs12125232
rs149021690
rs2172010
rs188921864
rs7009612
rs158201
rs911762
rs7301090
rs62313827
rs9347311
rs1533932
rs3093227
rs72803261
rs28410611
rs10801557
rs2491353
rs75553531
rs146513361
rs1348206
rs77987157
rs6758786
All
−0.002
−0.001
0.000
0.001
MR effect size for
'SUN domain−containing protein 5' on 'eGFR'

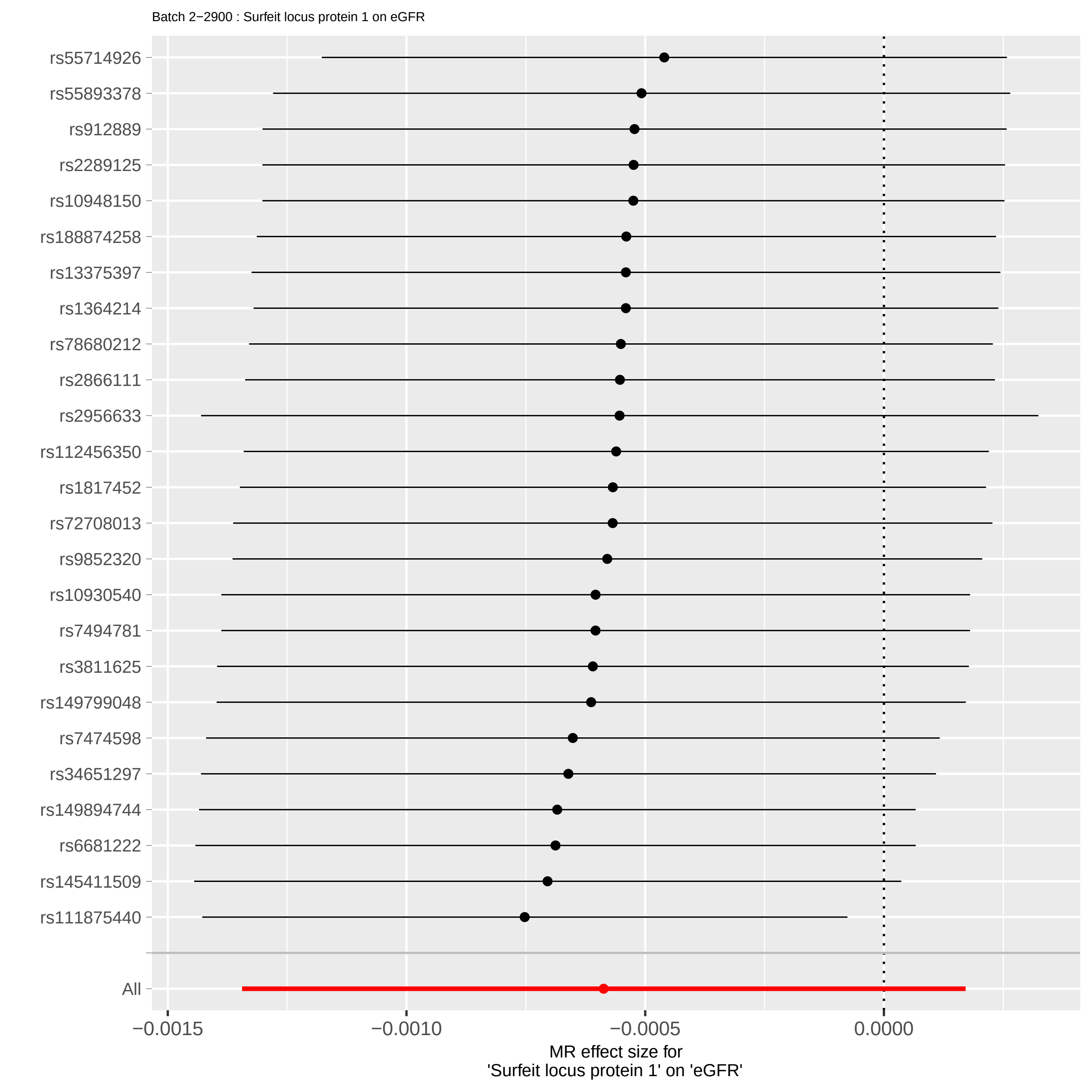

Batch 2−2900 : Surfeit locus protein 1 on eGFR
rs55714926
rs55893378
rs912889
rs2289125
rs10948150
rs188874258
rs13375397
rs1364214
rs78680212
rs2866111
rs2956633
rs112456350
rs1817452
rs72708013
rs9852320
rs10930540
rs7494781
rs3811625
rs149799048
rs7474598
rs34651297
rs149894744
rs6681222
rs145411509
rs111875440
All
−0.0015
−0.0010
−0.0005
0.0000
MR effect size for
'Surfeit locus protein 1' on 'eGFR'

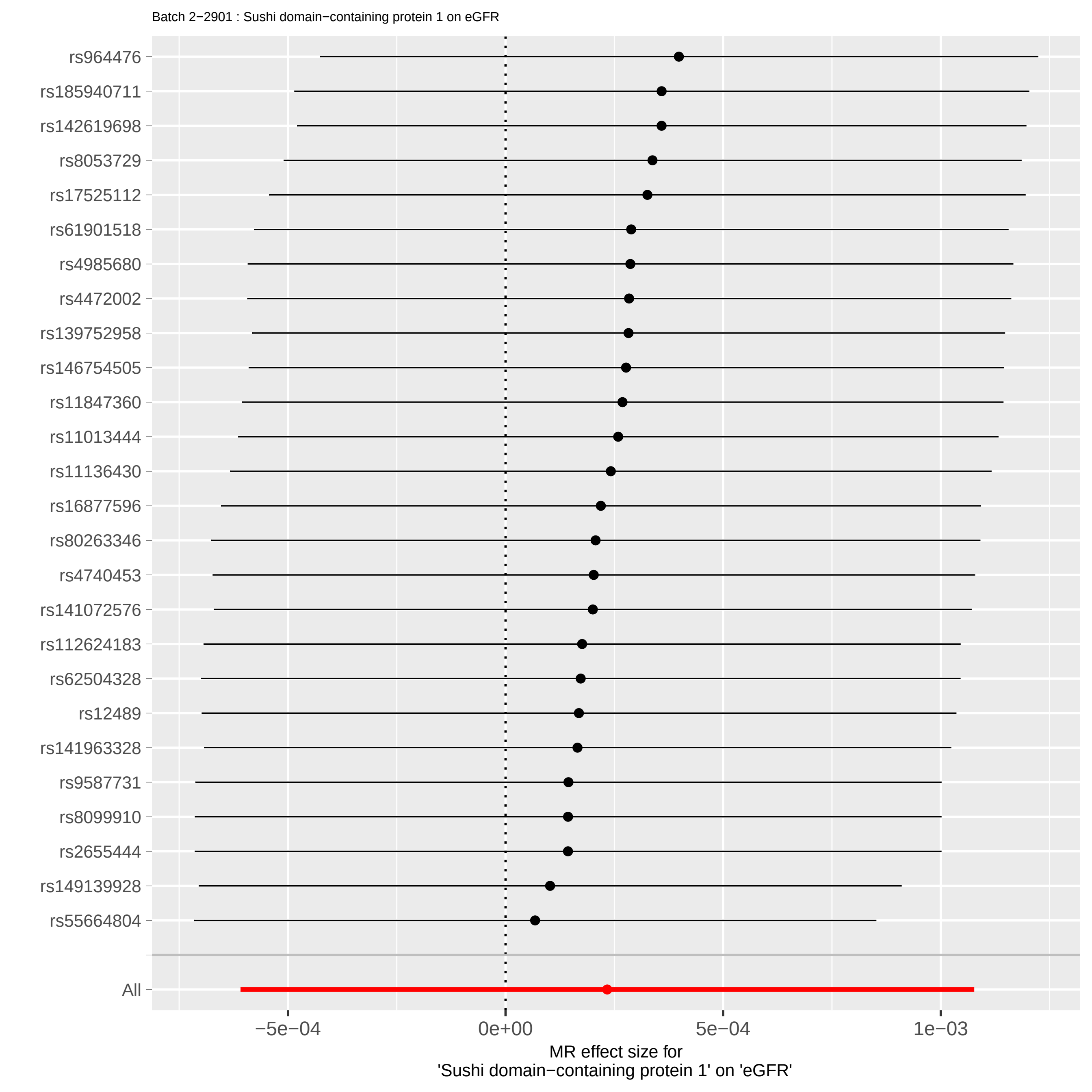
Batch 2−2901 : Sushi domain−containing protein 1 on eGFR
rs964476
rs185940711
rs142619698
rs8053729
rs17525112
rs61901518
rs4985680
rs4472002
rs139752958
rs146754505
rs11847360
rs11013444
rs11136430
rs16877596
rs80263346
rs4740453
rs141072576
rs112624183
rs62504328
rs12489
rs141963328
rs9587731
rs8099910
rs2655444
rs149139928
rs55664804
All
−5e−04
0e+00
5e−04
1e−03
MR effect size for
'Sushi domain−containing protein 1' on 'eGFR'

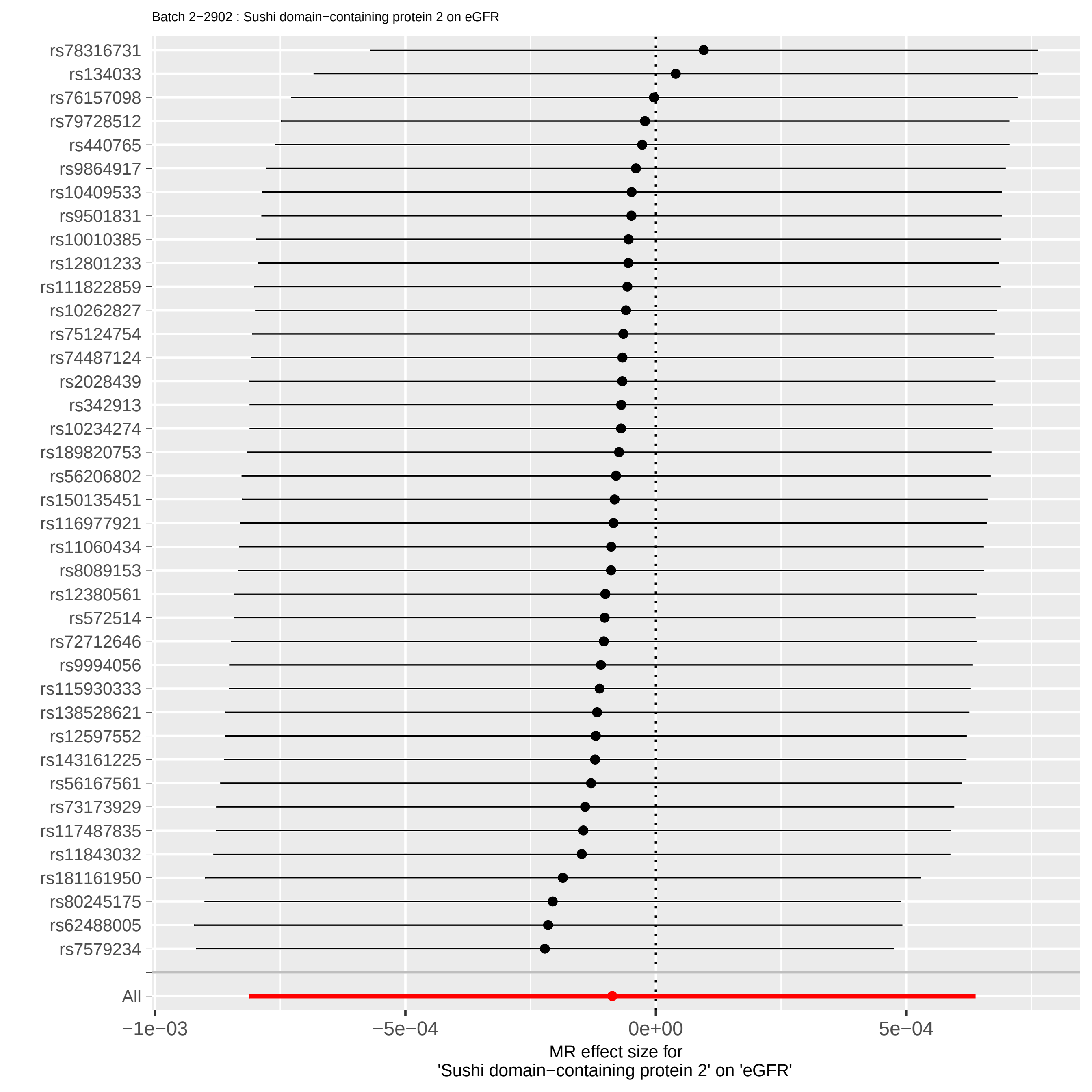

Batch 2−2902 : Sushi domain−containing protein 2 on eGFR
rs78316731
rs134033
rs76157098
rs79728512
rs440765
rs9864917
rs10409533
rs9501831
rs10010385
rs12801233
rs111822859
rs10262827
rs75124754
rs74487124
rs2028439
rs342913
rs10234274
rs189820753
rs56206802
rs150135451
rs116977921
rs11060434
rs8089153
rs12380561
rs572514
rs72712646
rs9994056
rs115930333
rs138528621
rs12597552
rs143161225
rs56167561
rs73173929
rs117487835
rs11843032
rs181161950
rs80245175
rs62488005
rs7579234
All
−1e−03
−5e−04
0e+00
5e−04
MR effect size for
'Sushi domain−containing protein 2' on 'eGFR'

Batch 2−2903 : Sushi domain−containing protein 3 on eGFR

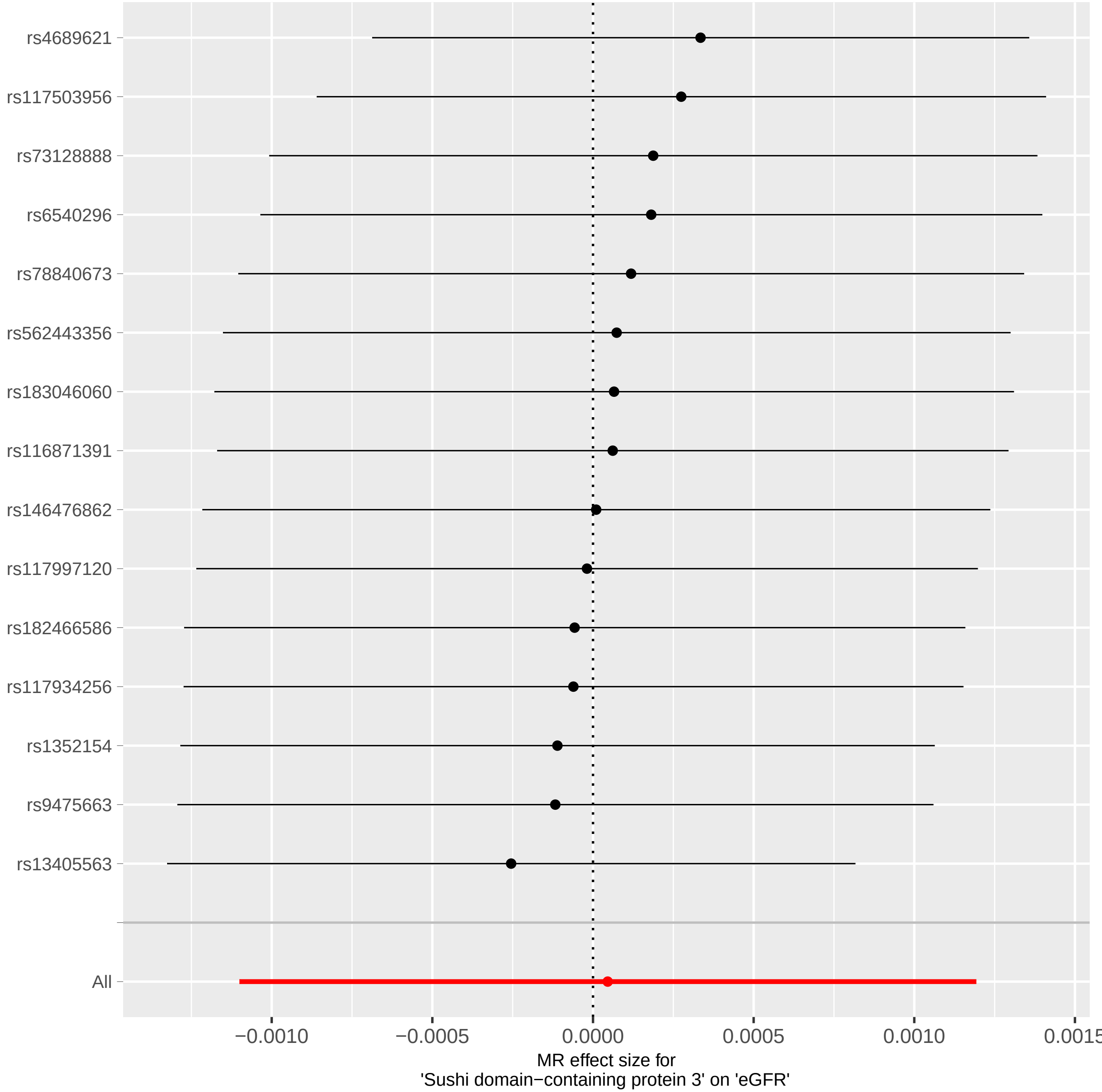

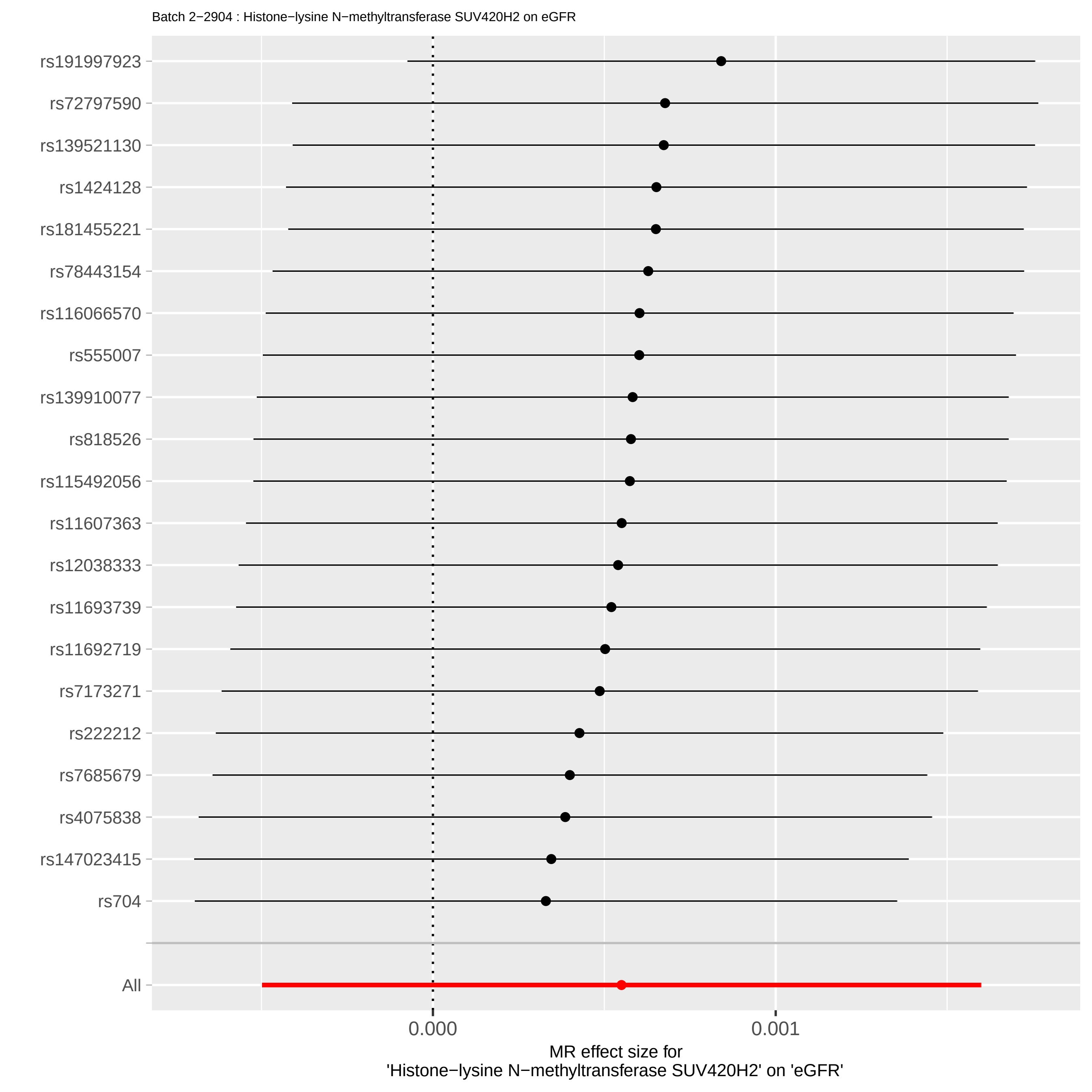
Batch 2−2904 : Histone−lysine N−methyltransferase SUV420H2 on eGFR
rs191997923
rs72797590
rs139521130
rs1424128
rs181455221
rs78443154
rs116066570
rs555007
rs139910077
rs818526
rs115492056
rs11607363
rs12038333
rs11693739
rs11692719
rs7173271
rs222212
rs7685679
rs4075838
rs147023415
rs704
All
0.000
0.001
MR effect size for
'Histone−lysine N−methyltransferase SUV420H2' on 'eGFR'

Batch 2−2905 : Synaptic vesicle glycoprotein 2A on eGFR

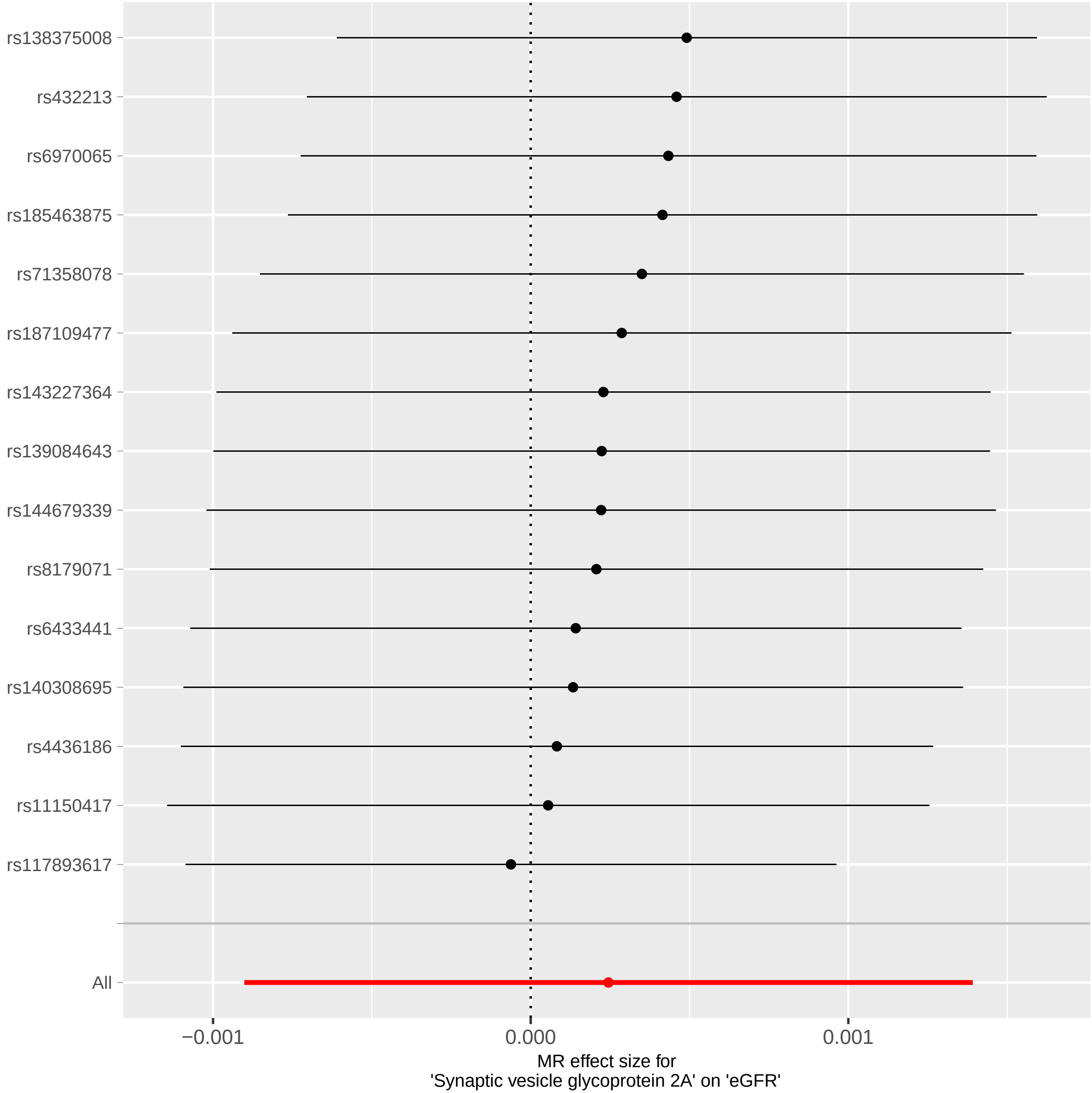

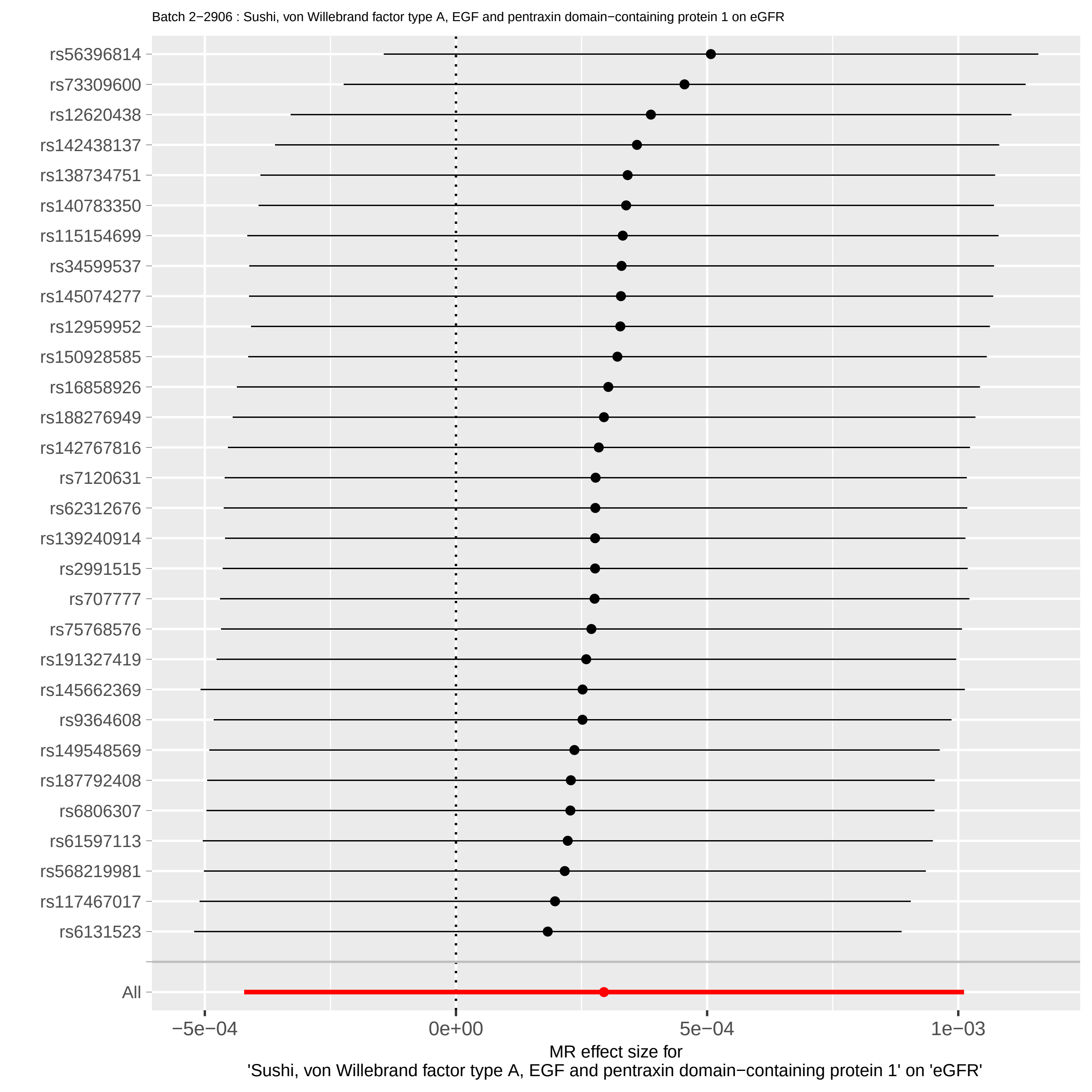

Batch 2−2906 : Sushi, von Willebrand factor type A, EGF and pentraxin domain−containing protein 1 on eGFR
rs56396814
rs73309600
rs12620438
rs142438137
rs138734751
rs140783350
rs115154699
rs34599537
rs145074277
rs12959952
rs150928585
rs16858926
rs188276949
rs142767816
rs7120631
rs62312676
rs139240914
rs2991515
rs707777
rs75768576
rs191327419
rs145662369
rs9364608
rs149548569
rs187792408
rs6806307
rs61597113
rs568219981
rs117467017
rs6131523
All
−5e−04
0e+00
5e−04
1e−03
MR effect size for
'Sushi, von Willebrand factor type A, EGF and pentraxin domain−containing protein 1' on 'eGFR'

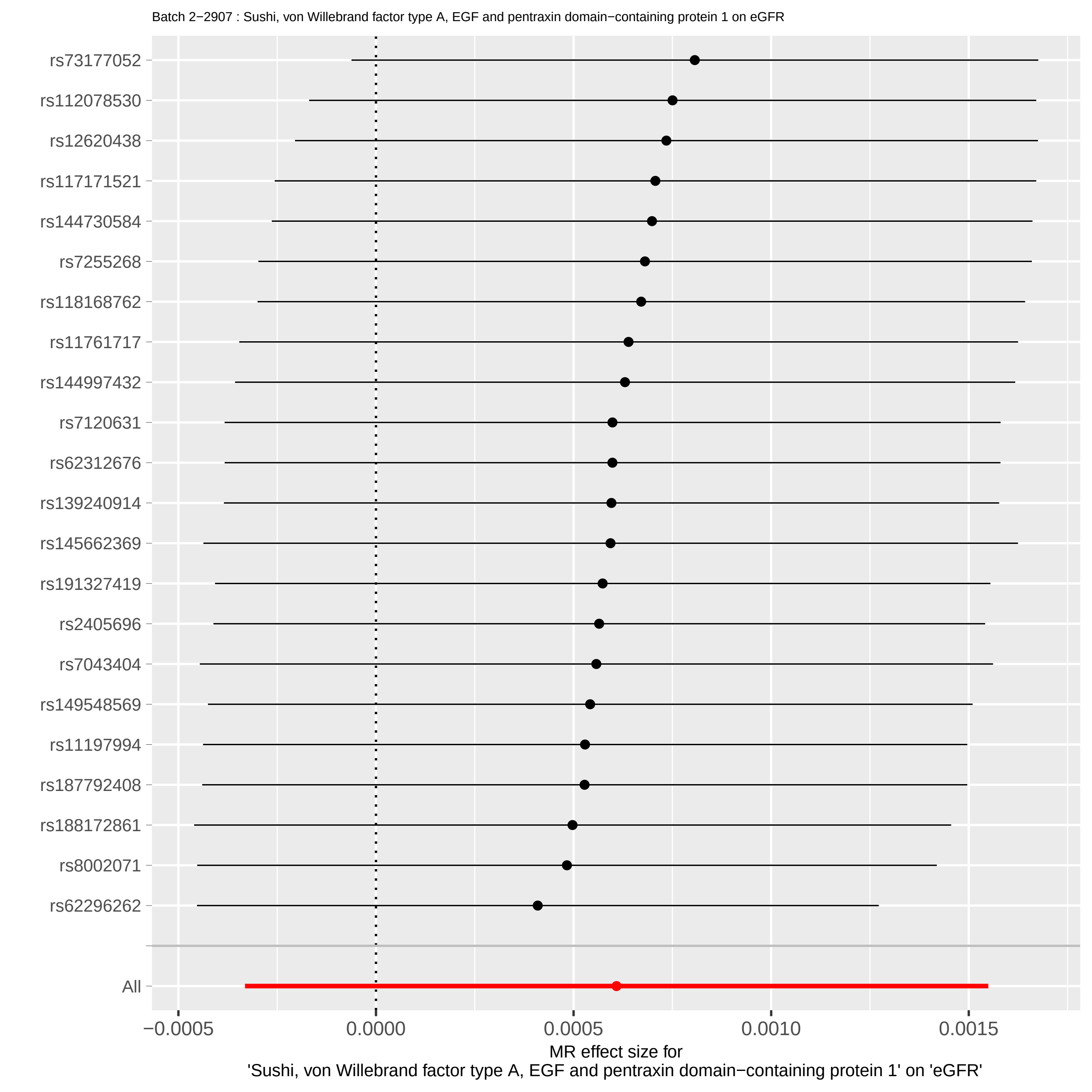

Batch 2−2907 : Sushi, von Willebrand factor type A, EGF and pentraxin domain−containing protein 1 on eGFR
rs73177052
rs112078530
rs12620438
rs117171521
rs144730584
rs7255268
rs118168762
rs11761717
rs144997432
rs7120631
rs62312676
rs139240914
rs145662369
rs191327419
rs2405696
rs7043404
rs149548569
rs11197994
rs187792408
rs188172861
rs8002071
rs62296262
All
−0.0005
0.0000
0.0005
0.0010
0.0015
MR effect size for
'Sushi, von Willebrand factor type A, EGF and pentraxin domain−containing protein 1' on 'eGFR'

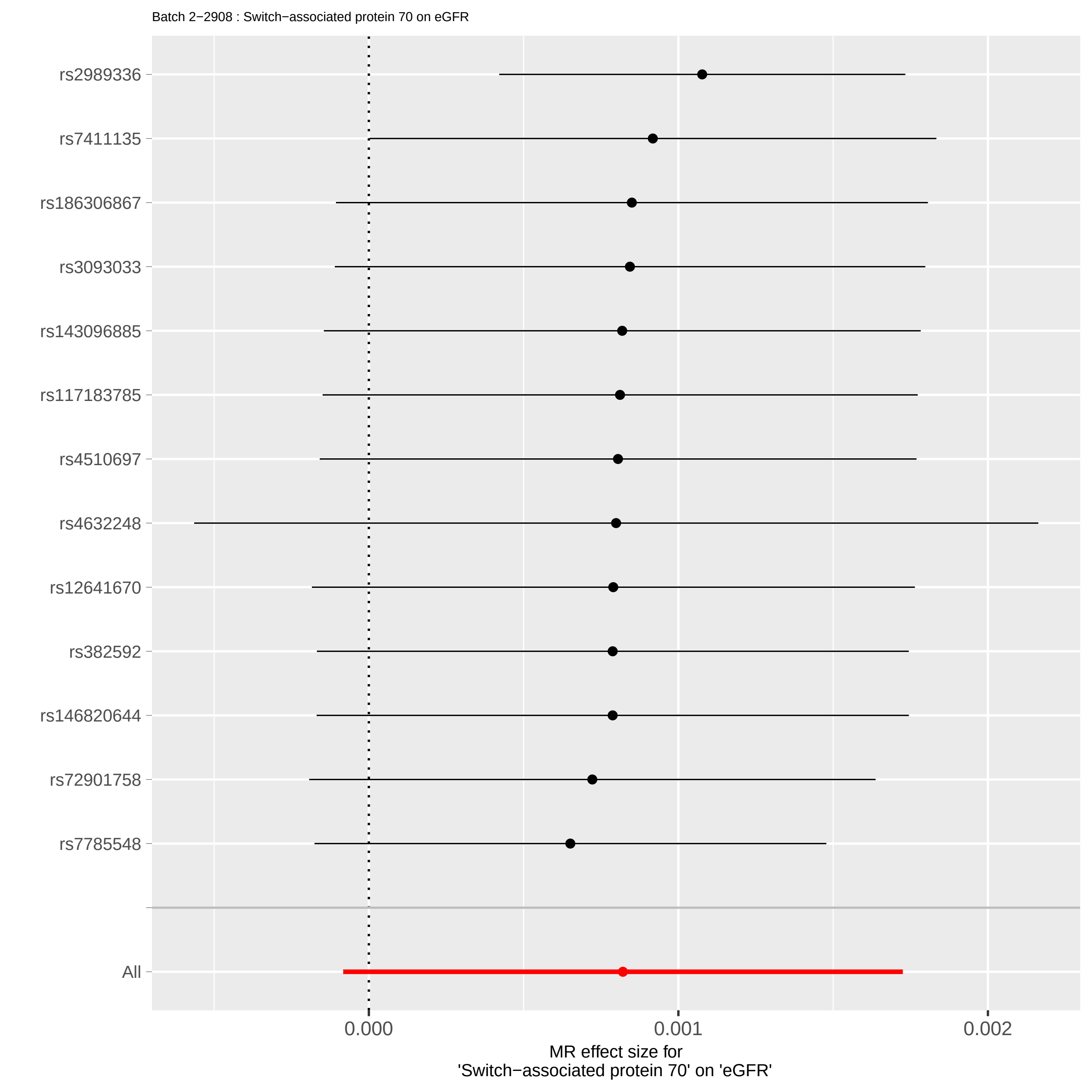
Batch 2−2908 : Switch−associated protein 70 on eGFR
rs2989336
rs7411135
rs186306867
rs3093033
rs143096885
rs117183785
rs4510697
rs4632248
rs12641670
rs382592
rs146820644
rs72901758
rs7785548
All
0.000
0.001
0.002
MR effect size for
'Switch−associated protein 70' on 'eGFR'

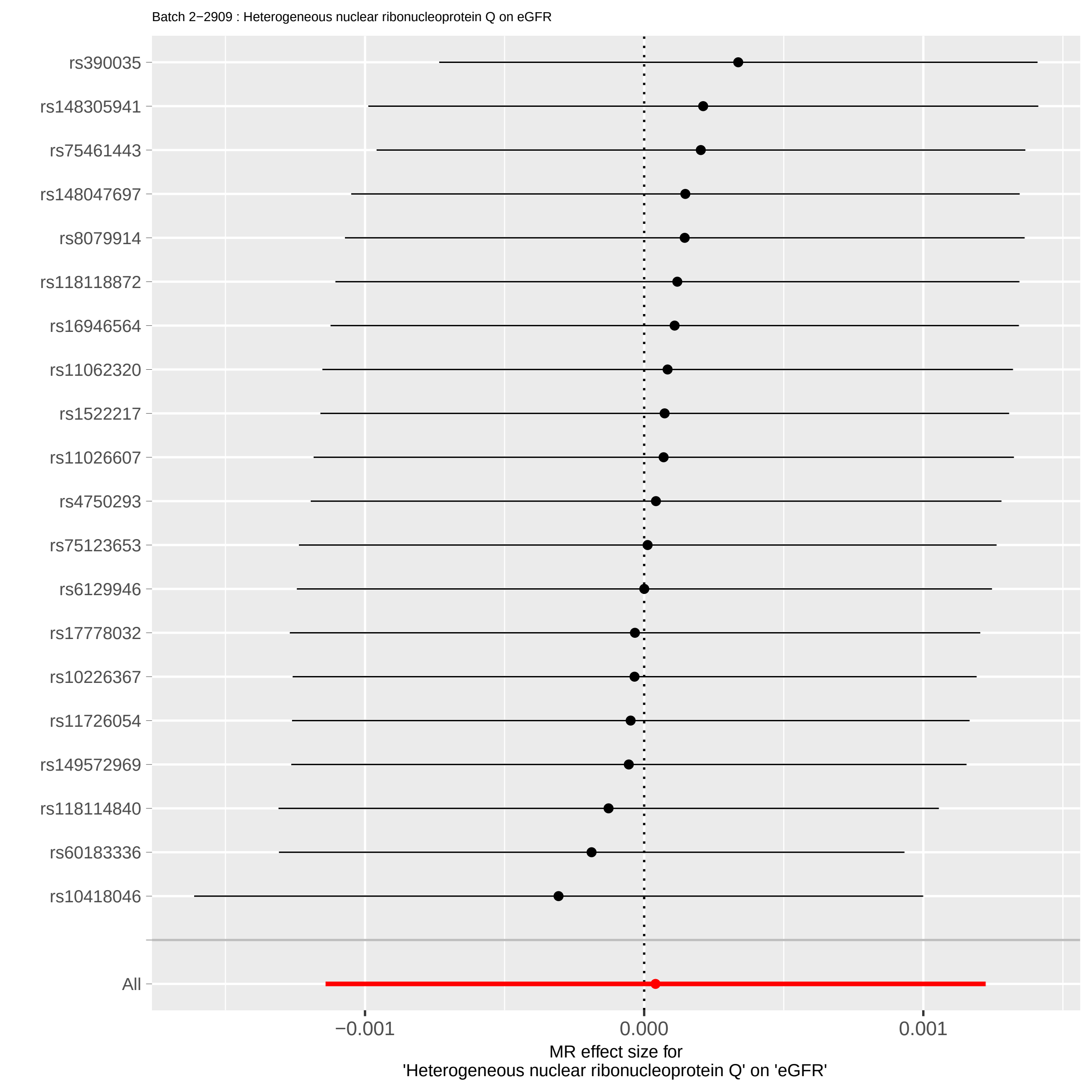

Batch 2−2909 : Heterogeneous nuclear ribonucleoprotein Q on eGFR
rs390035
rs148305941
rs75461443
rs148047697
rs8079914
rs118118872
rs16946564
rs11062320
rs1522217
rs11026607
rs4750293
rs75123653
rs6129946
rs17778032
rs10226367
rs11726054
rs149572969
rs118114840
rs60183336
rs10418046
All
−0.001
0.000
0.001
MR effect size for
'Heterogeneous nuclear ribonucleoprotein Q' on 'eGFR'

Batch 2−2910 : Nesprin−2 on eGFR

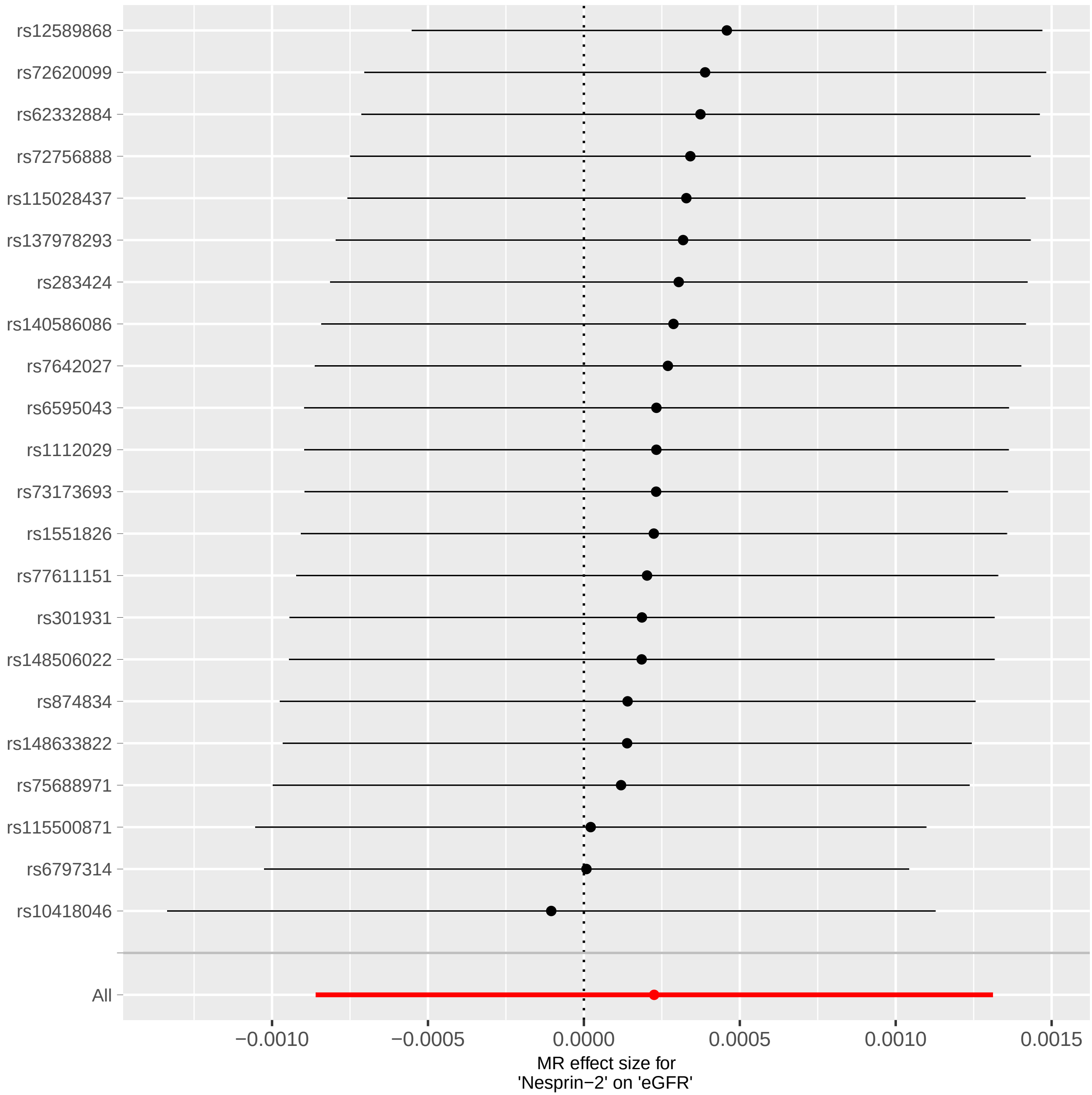

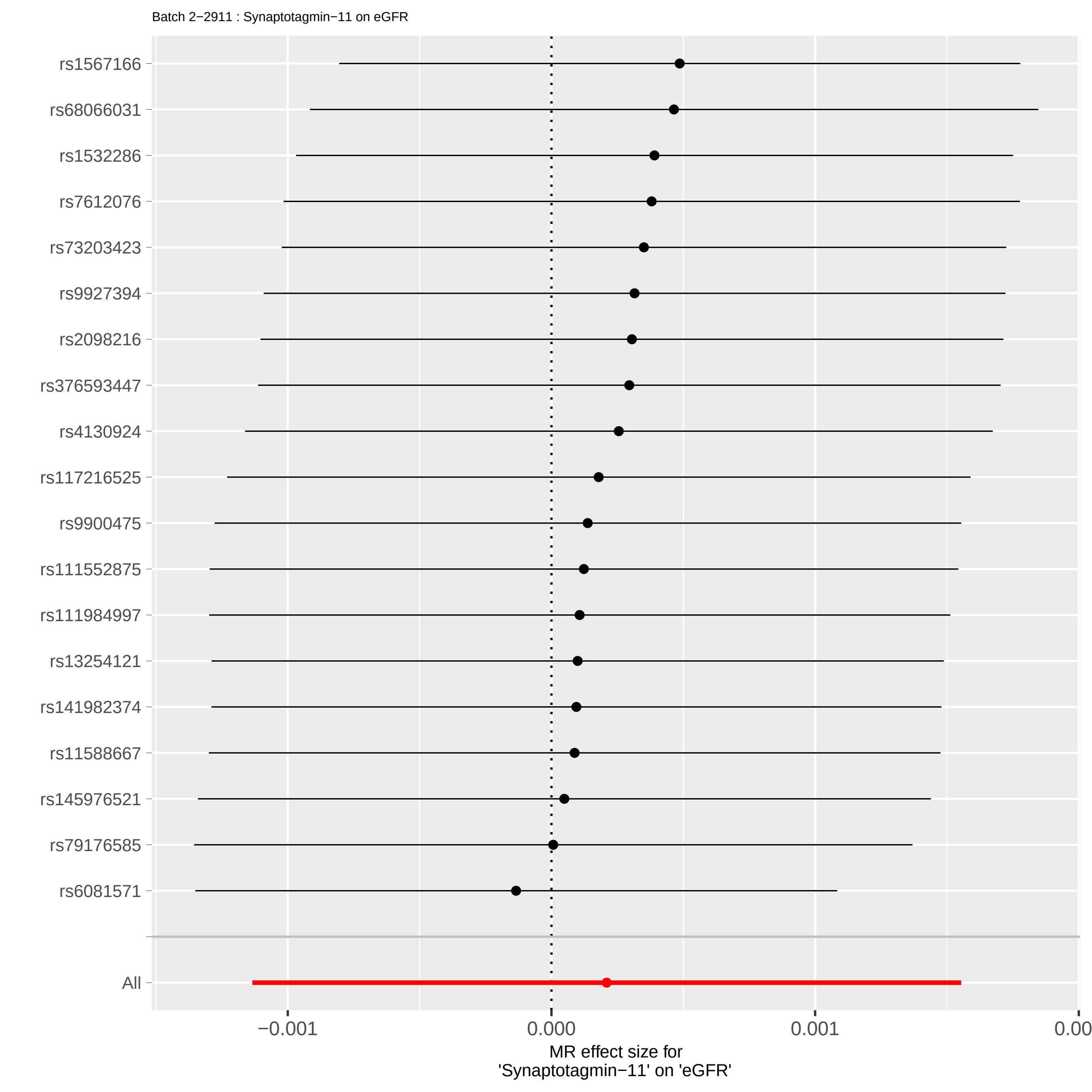

Batch 2−2911 : Synaptotagmin−11 on eGFR
rs1567166
rs68066031
rs1532286
rs7612076
rs73203423
rs9927394
rs2098216
rs376593447
rs4130924
rs117216525
rs9900475
rs111552875
rs111984997
rs13254121
rs141982374
rs11588667
rs145976521
rs79176585
rs6081571
All
−0.001
0.000
0.001
0.00
MR effect size for
'Synaptotagmin−11' on 'eGFR'

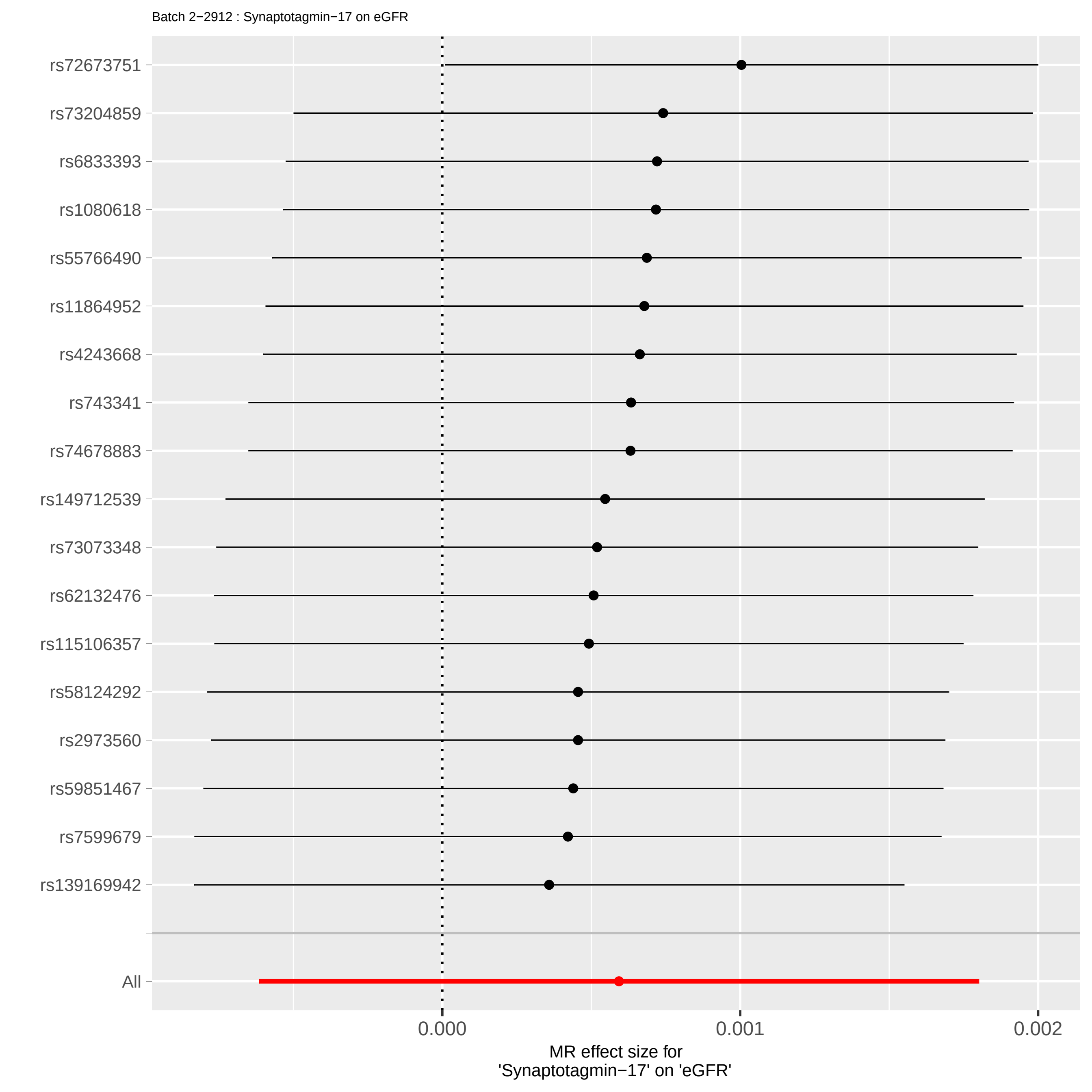
Batch 2−2912 : Synaptotagmin−17 on eGFR
rs72673751
rs73204859
rs6833393
rs1080618
rs55766490
rs11864952
rs4243668
rs743341
rs74678883
rs149712539
rs73073348
rs62132476
rs115106357
rs58124292
rs2973560
rs59851467
rs7599679
rs139169942
All
0.000
0.001
0.002
MR effect size for
'Synaptotagmin−17' on 'eGFR'

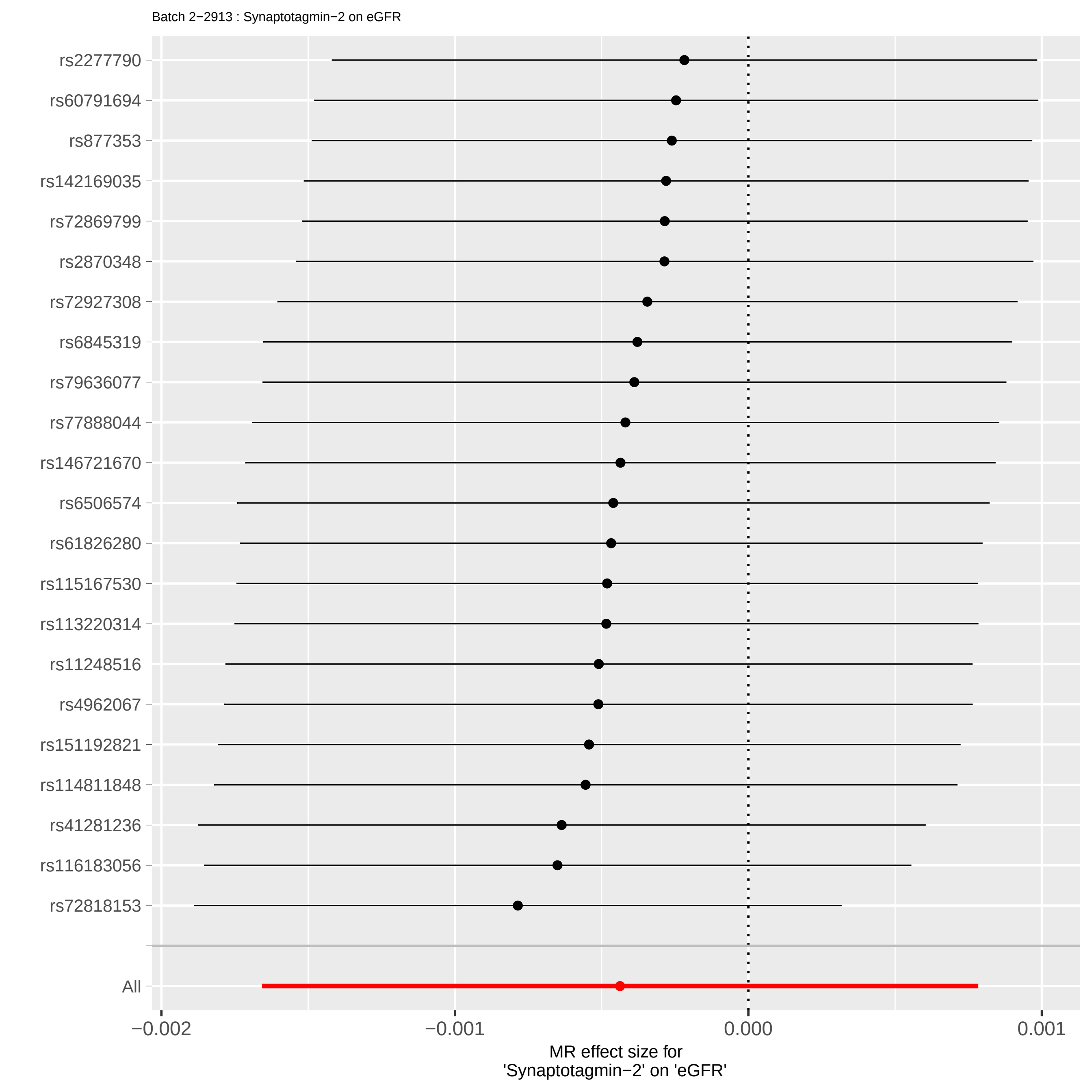

Batch 2−2913 : Synaptotagmin−2 on eGFR
rs2277790
rs60791694
rs877353
rs142169035
rs72869799
rs2870348
rs72927308
rs6845319
rs79636077
rs77888044
rs146721670
rs6506574
rs61826280
rs115167530
rs113220314
rs11248516
rs4962067
rs151192821
rs114811848
rs41281236
rs116183056
rs72818153
All
−0.002
−0.001
0.000
0.001
MR effect size for
'Synaptotagmin−2' on 'eGFR'

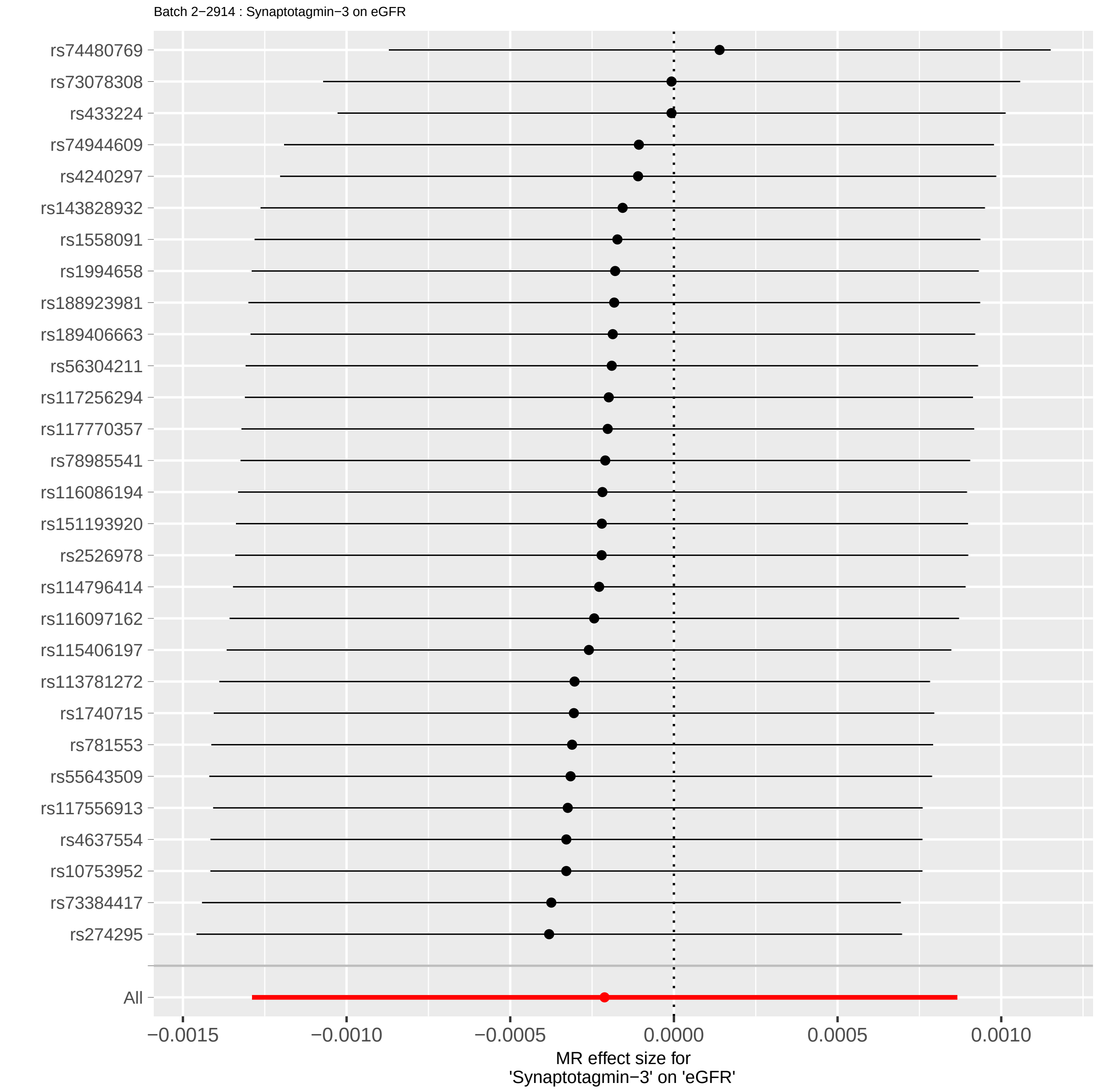

Batch 2−2914 : Synaptotagmin−3 on eGFR
rs74480769
rs73078308
rs433224
rs74944609
rs4240297
rs143828932
rs1558091
rs1994658
rs188923981
rs189406663
rs56304211
rs117256294
rs117770357
rs78985541
rs116086194
rs151193920
rs2526978
rs114796414
rs116097162
rs115406197
rs113781272
rs1740715
rs781553
rs55643509
rs117556913
rs4637554
rs10753952
rs73384417
rs274295
All
−0.0015
−0.0010
−0.0005
0.0000
0.0005
0.0010
MR effect size for
'Synaptotagmin−3' on 'eGFR'

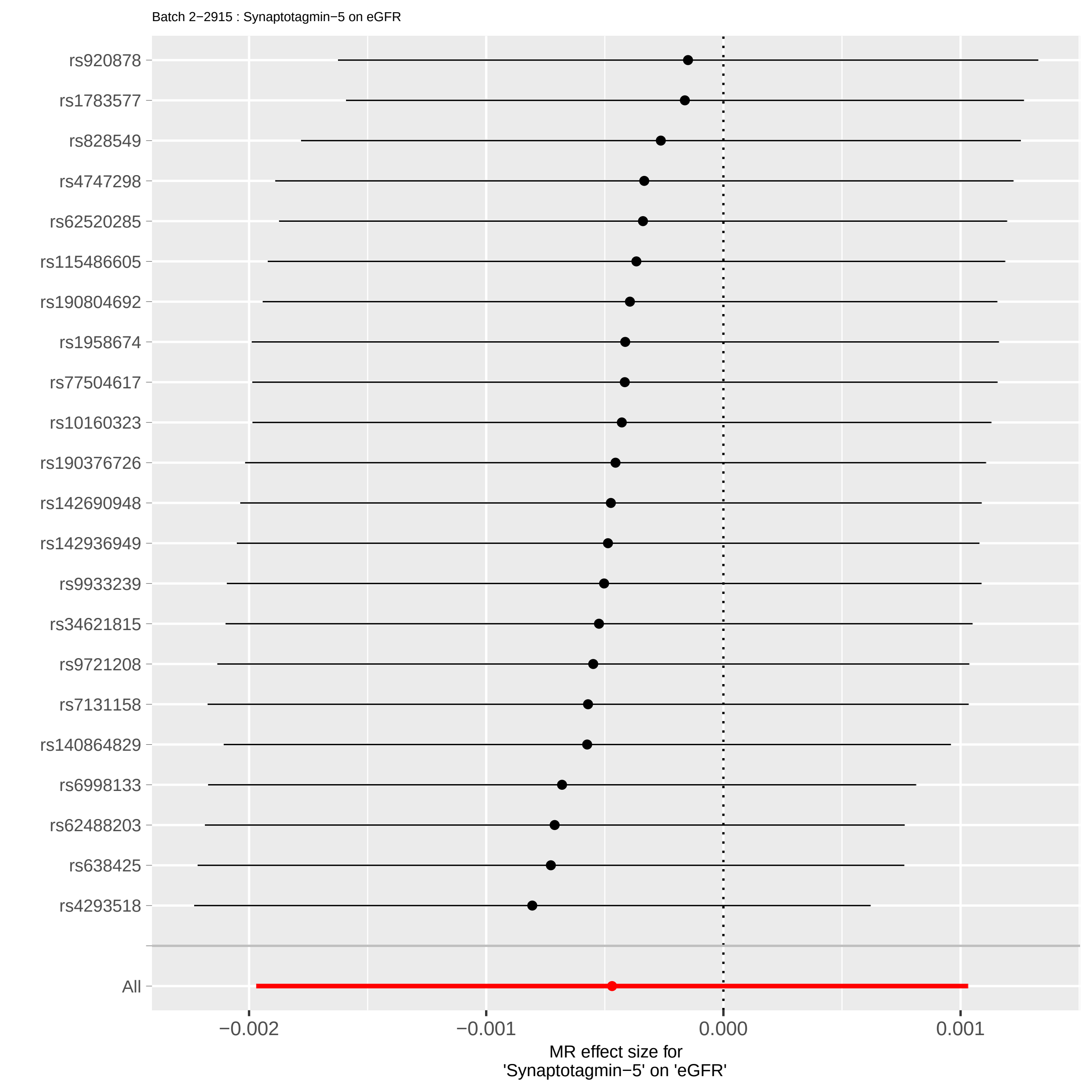

Batch 2−2915 : Synaptotagmin−5 on eGFR
rs920878
rs1783577
rs828549
rs4747298
rs62520285
rs115486605
rs190804692
rs1958674
rs77504617
rs10160323
rs190376726
rs142690948
rs142936949
rs9933239
rs34621815
rs9721208
rs7131158
rs140864829
rs6998133
rs62488203
rs638425
rs4293518
All
−0.002
−0.001
0.000
0.001
MR effect size for
'Synaptotagmin−5' on 'eGFR'

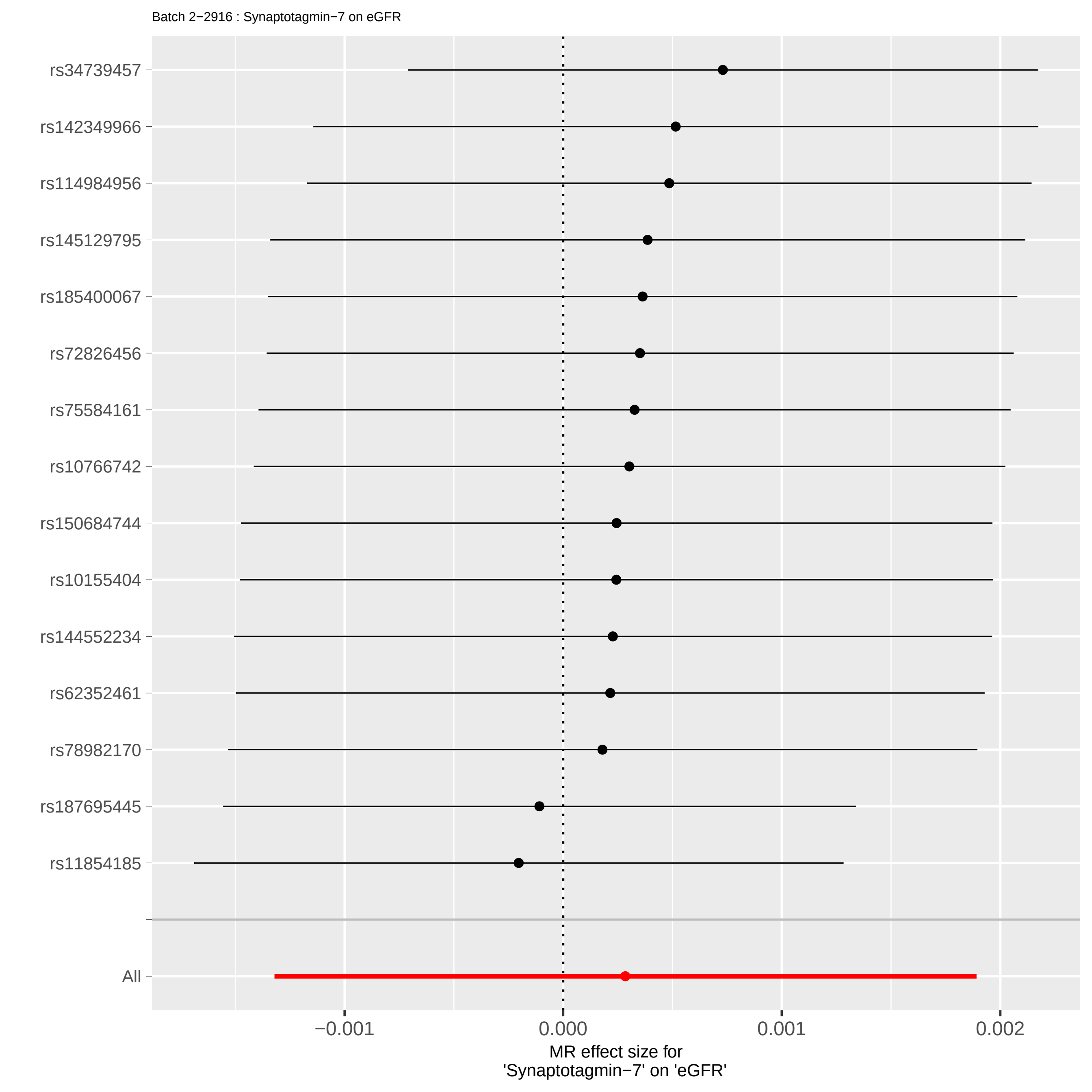

Batch 2−2916 : Synaptotagmin−7 on eGFR
rs34739457
rs142349966
rs114984956
rs145129795
rs185400067
rs72826456
rs75584161
rs10766742
rs150684744
rs10155404
rs144552234
rs62352461
rs78982170
rs187695445
rs11854185
All
−0.001
0.000
0.001
0.002
MR effect size for
'Synaptotagmin−7' on 'eGFR'

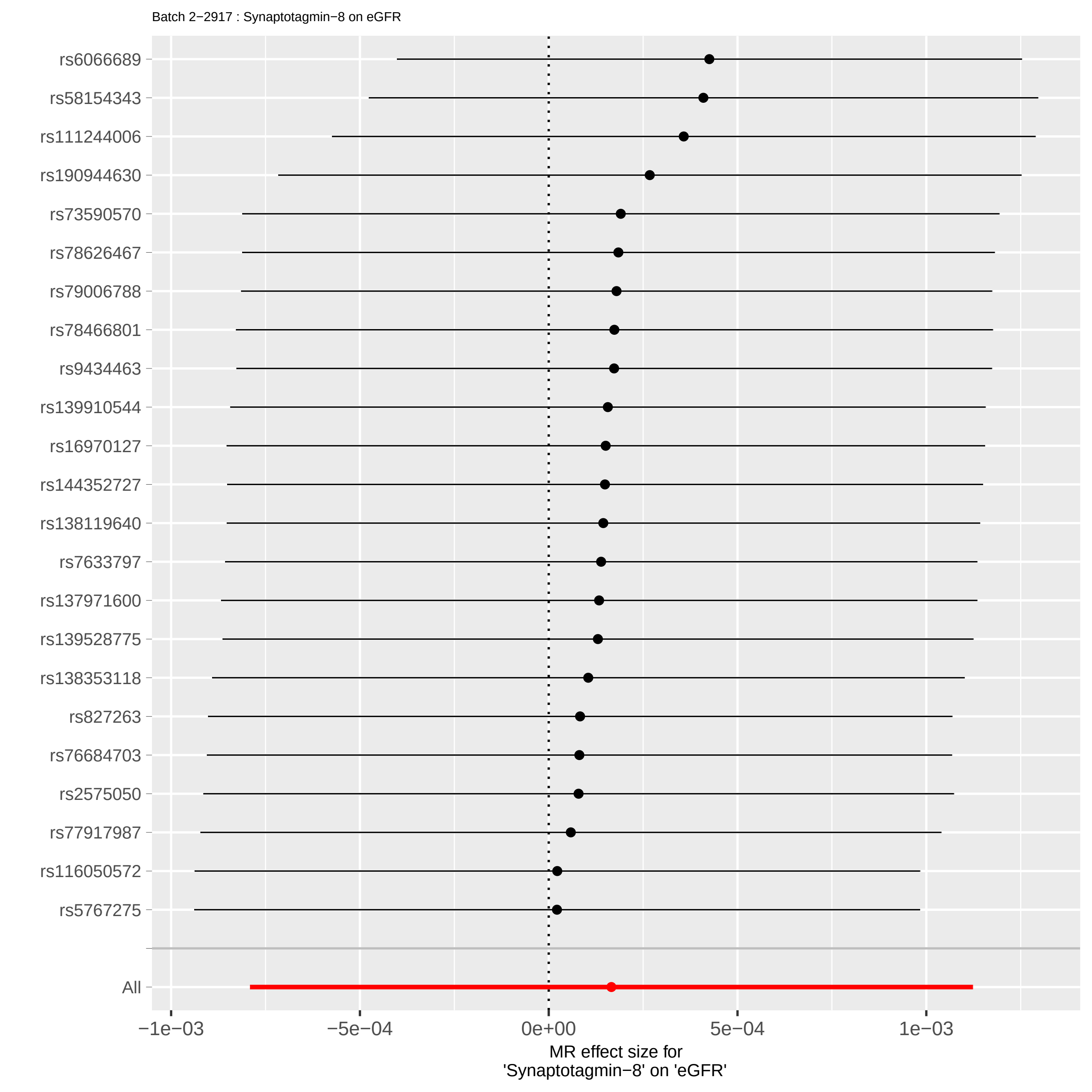
Batch 2−2917 : Synaptotagmin−8 on eGFR
rs6066689
rs58154343
rs111244006
rs190944630
rs73590570
rs78626467
rs79006788
rs78466801
rs9434463
rs139910544
rs16970127
rs144352727
rs138119640
rs7633797
rs137971600
rs139528775
rs138353118
rs827263
rs76684703
rs2575050
rs77917987
rs116050572
rs5767275
All
−1e−03
−5e−04
0e+00
5e−04
1e−03
MR effect size for
'Synaptotagmin−8' on 'eGFR'

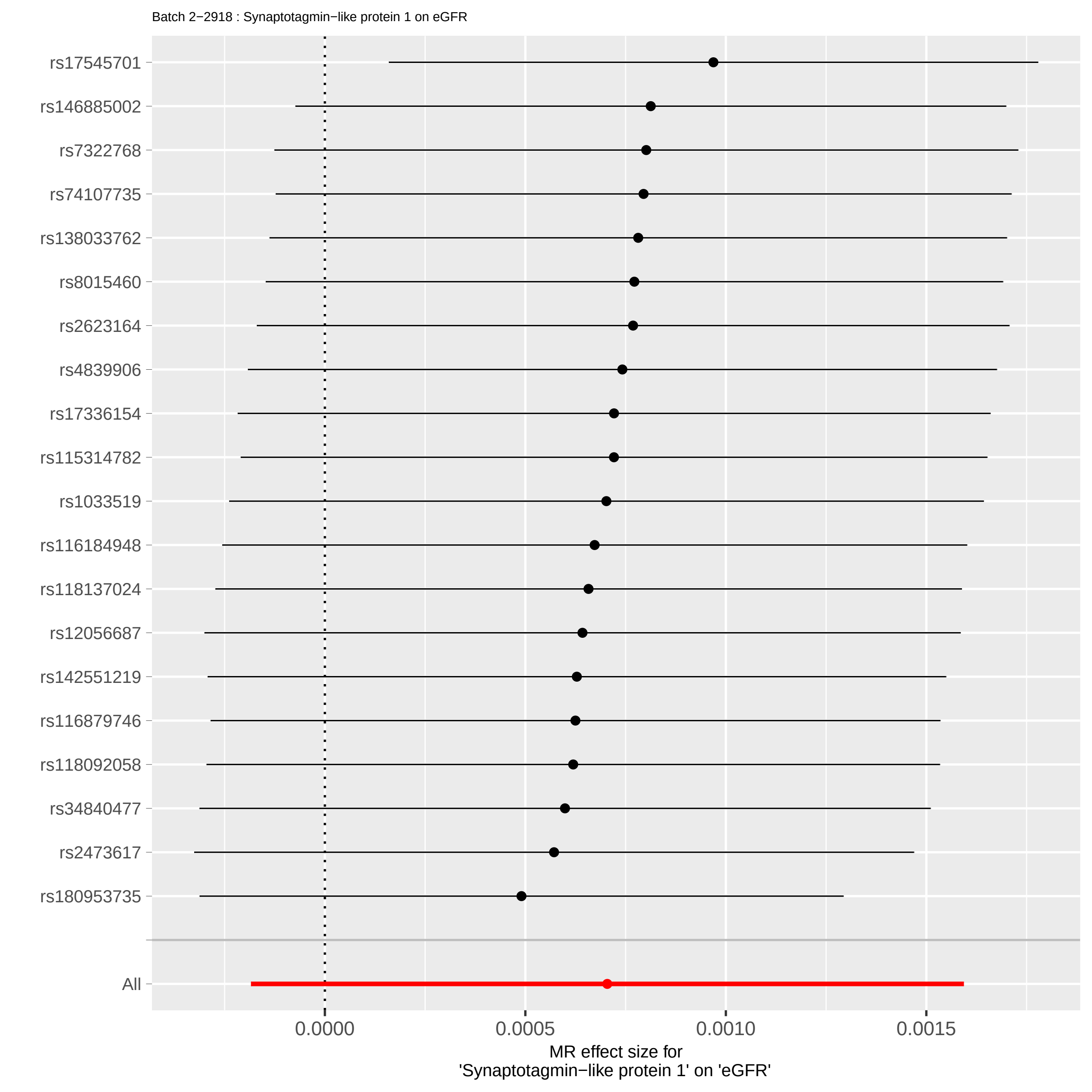
Batch 2−2918 : Synaptotagmin−like protein 1 on eGFR
rs17545701
rs146885002
rs7322768
rs74107735
rs138033762
rs8015460
rs2623164
rs4839906
rs17336154
rs115314782
rs1033519
rs116184948
rs118137024
rs12056687
rs142551219
rs116879746
rs118092058
rs34840477
rs2473617
rs180953735
All
0.0000
0.0005
0.0010
0.0015
MR effect size for
'Synaptotagmin−like protein 1' on 'eGFR'

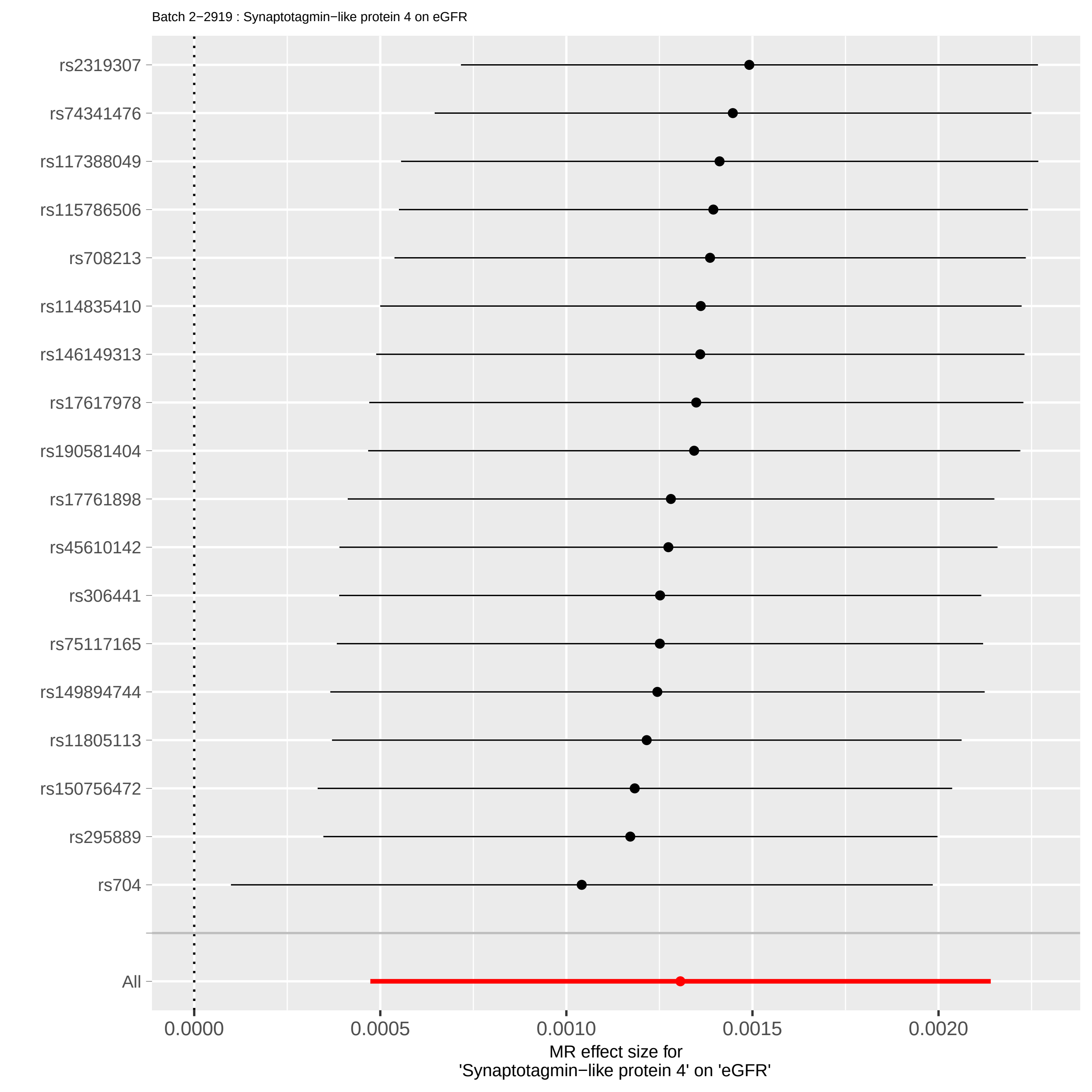

Batch 2−2919 : Synaptotagmin−like protein 4 on eGFR
rs2319307
rs74341476
rs117388049
rs115786506
rs708213
rs114835410
rs146149313
rs17617978
rs190581404
rs17761898
rs45610142
rs306441
rs75117165
rs149894744
rs11805113
rs150756472
rs295889
rs704
All
0.0000
0.0005
0.0010
0.0015
0.0020
MR effect size for
'Synaptotagmin−like protein 4' on 'eGFR'

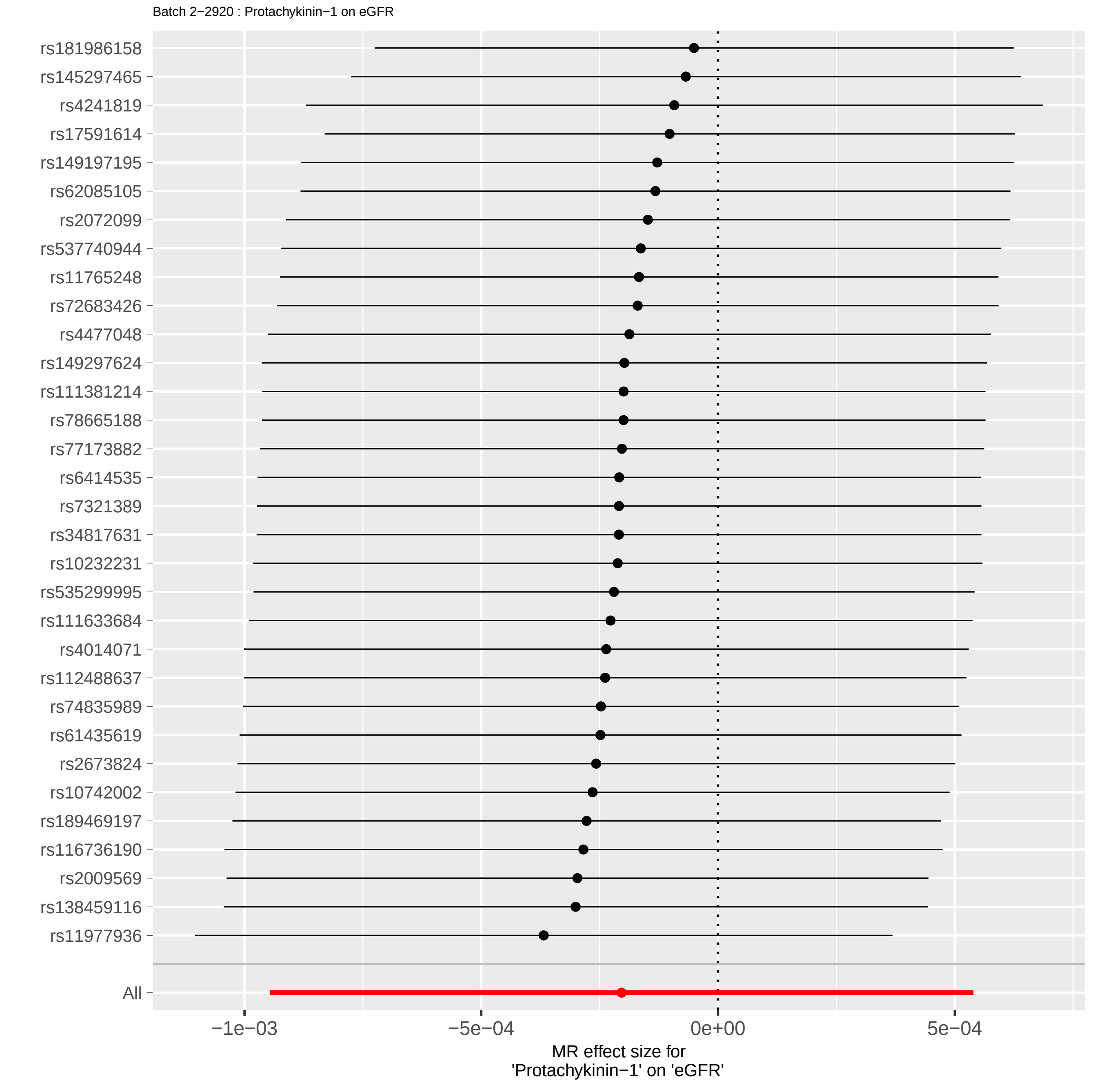
Batch 2−2920 : Protachykinin−1 on eGFR
rs181986158
rs145297465
rs4241819
rs17591614
rs149197195
rs62085105
rs2072099
rs537740944
rs11765248
rs72683426
rs4477048
rs149297624
rs111381214
rs78665188
rs77173882
rs6414535
rs7321389
rs34817631
rs10232231
rs535299995
rs111633684
rs4014071
rs112488637
rs74835989
rs61435619
rs2673824
rs10742002
rs189469197
rs116736190
rs2009569
rs138459116
rs11977936
All
−1e−03
−5e−04
0e+00
5e−04
MR effect size for
'Protachykinin−1' on 'eGFR'

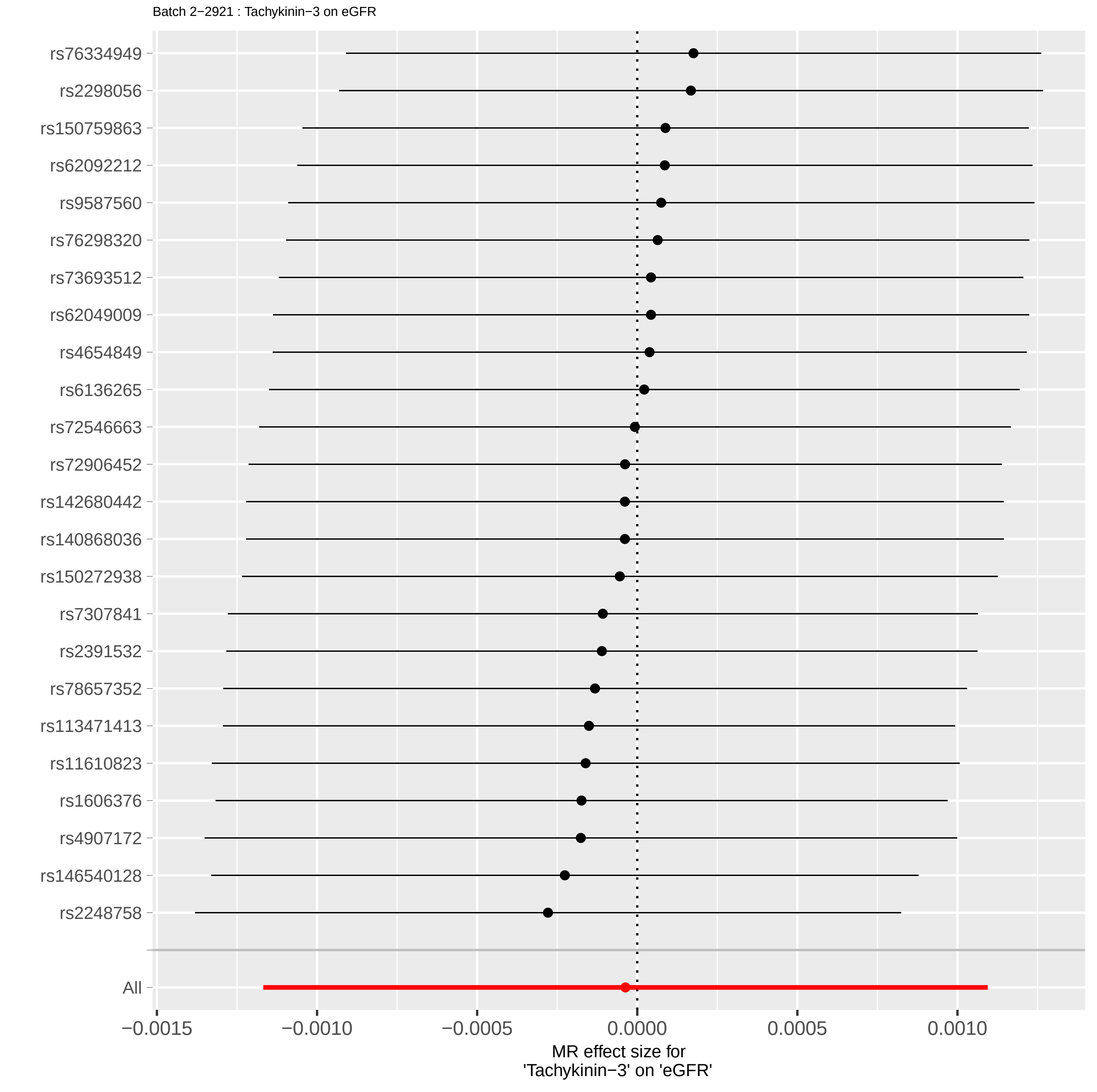

Batch 2−2921 : Tachykinin−3 on eGFR
rs76334949
rs2298056
rs150759863
rs62092212
rs9587560
rs76298320
rs73693512
rs62049009
rs4654849
rs6136265
rs72546663
rs72906452
rs142680442
rs140868036
rs150272938
rs7307841
rs2391532
rs78657352
rs113471413
rs11610823
rs1606376
rs4907172
rs146540128
rs2248758
All
−0.0015
−0.0010
−0.0005
0.0000
0.0005
0.0010
MR effect size for
'Tachykinin−3' on 'eGFR'

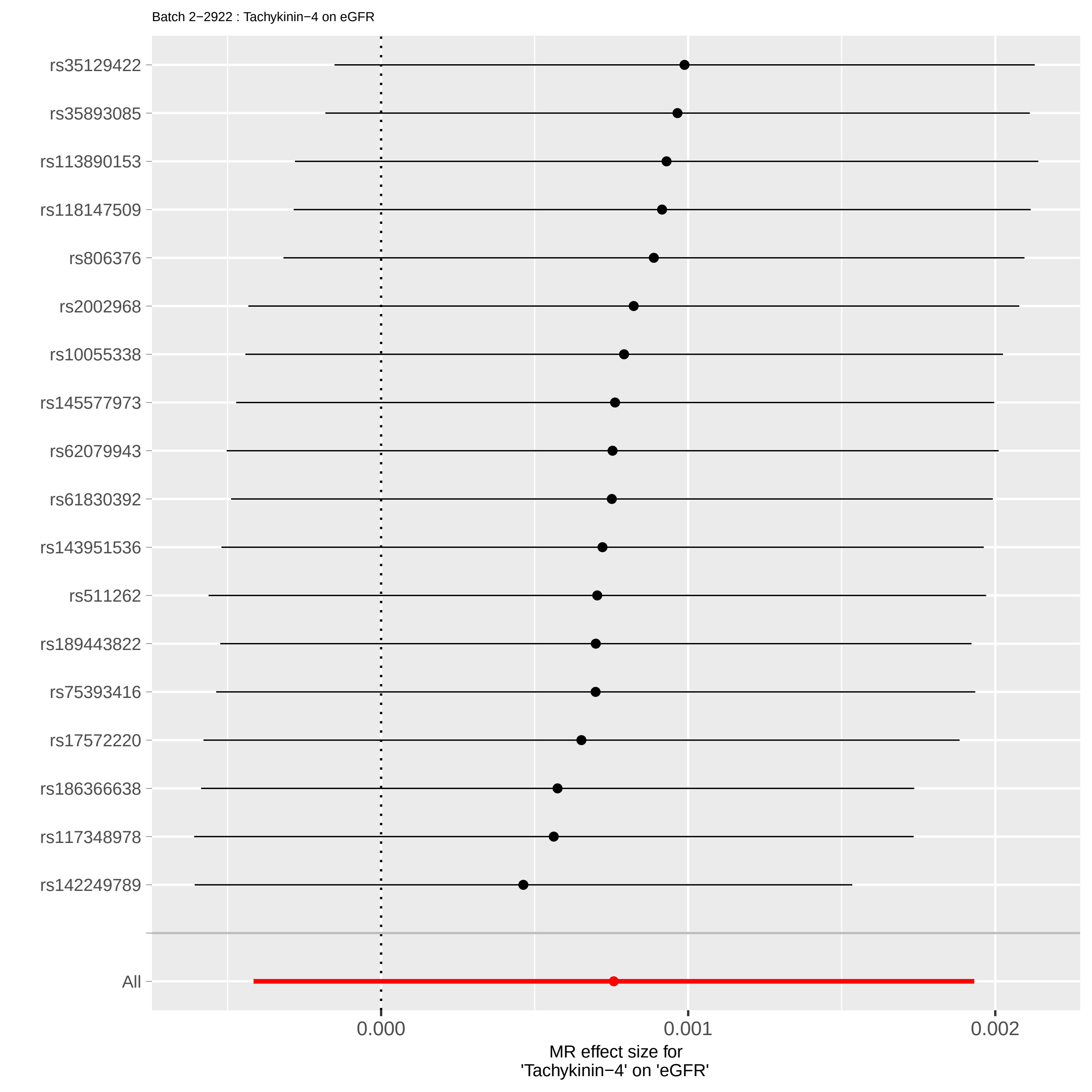

Batch 2−2922 : Tachykinin−4 on eGFR
rs35129422
rs35893085
rs113890153
rs118147509
rs806376
rs2002968
rs10055338
rs145577973
rs62079943
rs61830392
rs143951536
rs511262
rs189443822
rs75393416
rs17572220
rs186366638
rs117348978
rs142249789
All
0.000
0.001
0.002
MR effect size for
'Tachykinin−4' on 'eGFR'

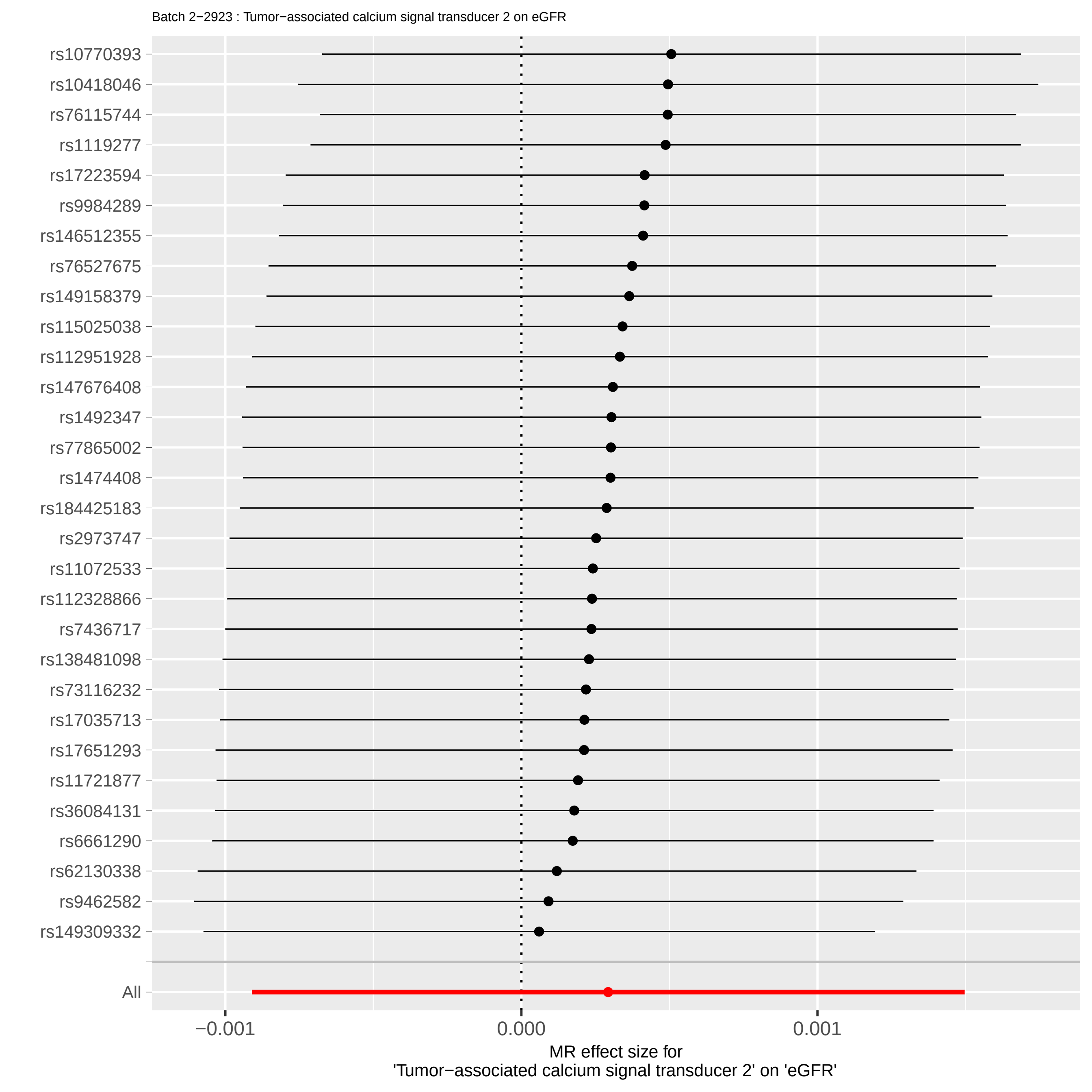
Batch 2−2923 : Tumor−associated calcium signal transducer 2 on eGFR
rs10770393
rs10418046
rs76115744
rs1119277
rs17223594
rs9984289
rs146512355
rs76527675
rs149158379
rs115025038
rs112951928
rs147676408
rs1492347
rs77865002
rs1474408
rs184425183
rs2973747
rs11072533
rs112328866
rs7436717
rs138481098
rs73116232
rs17035713
rs17651293
rs11721877
rs36084131
rs6661290
rs62130338
rs9462582
rs149309332
All
−0.001
0.000
0.001
MR effect size for
'Tumor−associated calcium signal transducer 2' on 'eGFR'

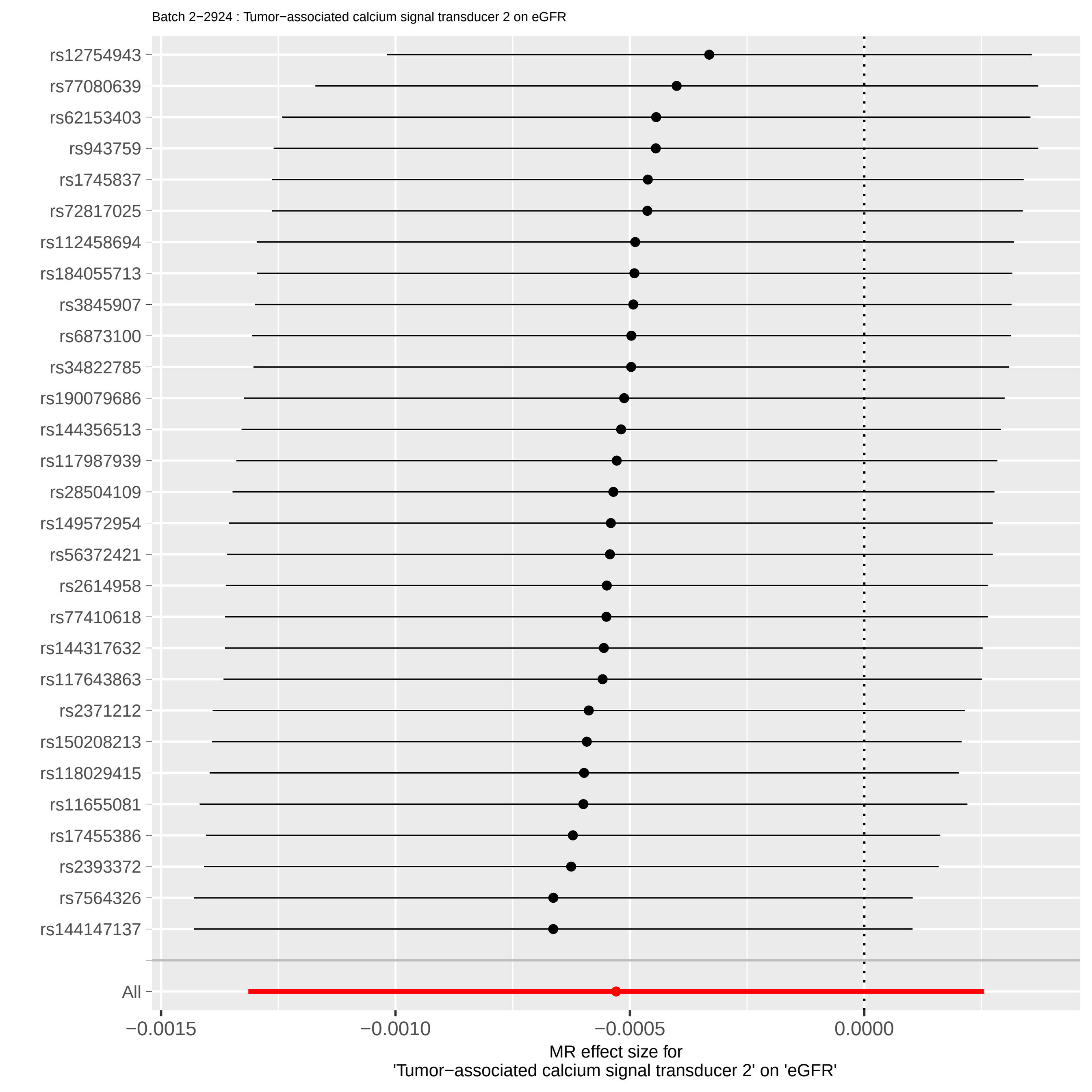
Batch 2−2924 : Tumor−associated calcium signal transducer 2 on eGFR
rs12754943
rs77080639
rs62153403
rs943759
rs1745837
rs72817025
rs112458694
rs184055713
rs3845907
rs6873100
rs34822785
rs190079686
rs144356513
rs117987939
rs28504109
rs149572954
rs56372421
rs2614958
rs77410618
rs144317632
rs117643863
rs2371212
rs150208213
rs118029415
rs11655081
rs17455386
rs2393372
rs7564326
rs144147137
All
−0.0015
−0.0010
−0.0005
0.0000
MR effect size for
'Tumor−associated calcium signal transducer 2' on 'eGFR'

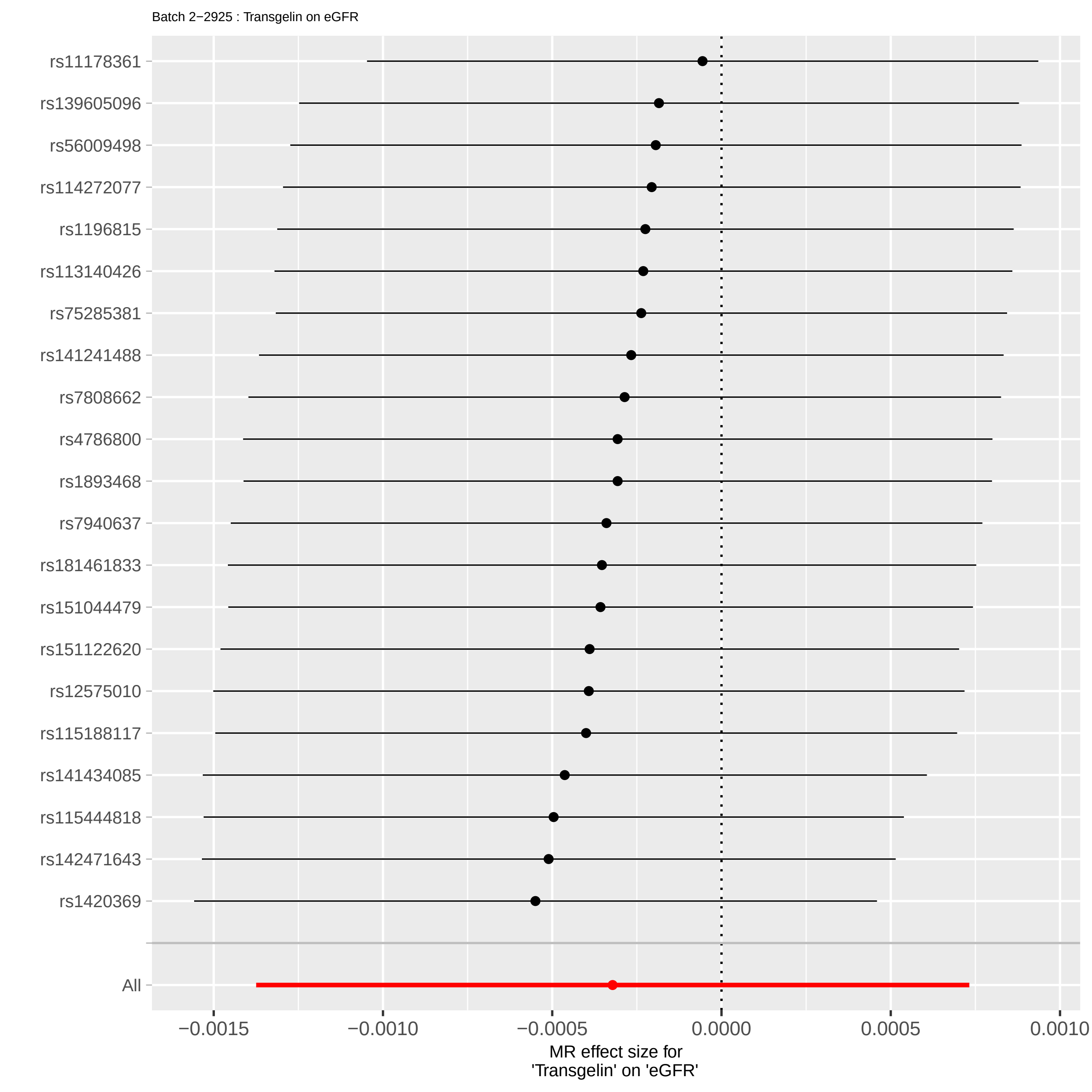

Batch 2−2925 : Transgelin on eGFR
rs11178361
rs139605096
rs56009498
rs114272077
rs1196815
rs113140426
rs75285381
rs141241488
rs7808662
rs4786800
rs1893468
rs7940637
rs181461833
rs151044479
rs151122620
rs12575010
rs115188117
rs141434085
rs115444818
rs142471643
rs1420369
All
−0.0015
−0.0010
−0.0005
0.0000
0.0005
0.0010
MR effect size for
'Transgelin' on 'eGFR'

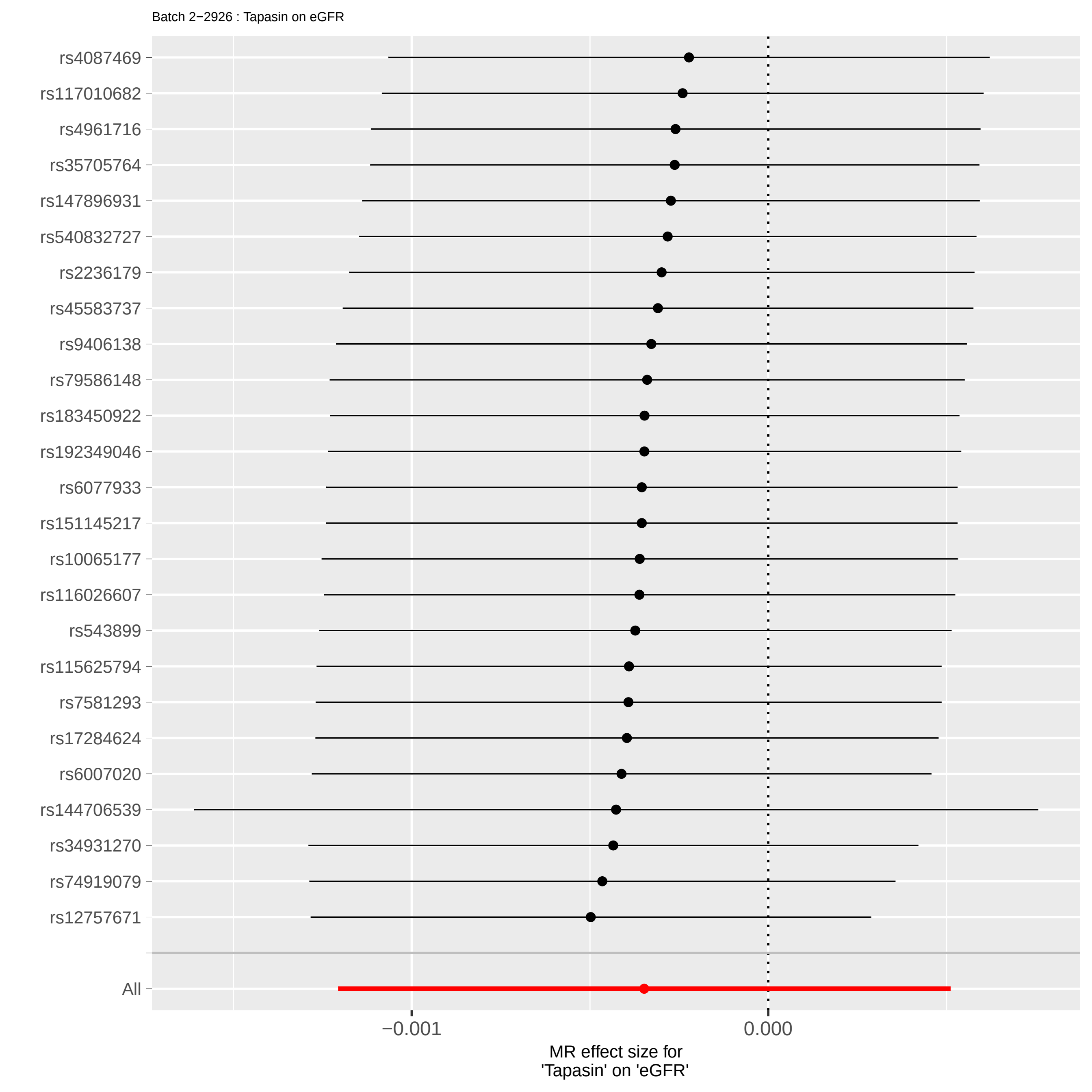
Batch 2−2926 : Tapasin on eGFR
rs4087469
rs117010682
rs4961716
rs35705764
rs147896931
rs540832727
rs2236179
rs45583737
rs9406138
rs79586148
rs183450922
rs192349046
rs6077933
rs151145217
rs10065177
rs116026607
rs543899
rs115625794
rs7581293
rs17284624
rs6007020
rs144706539
rs34931270
rs74919079
rs12757671
All
−0.001
0.000
MR effect size for
'Tapasin' on 'eGFR'

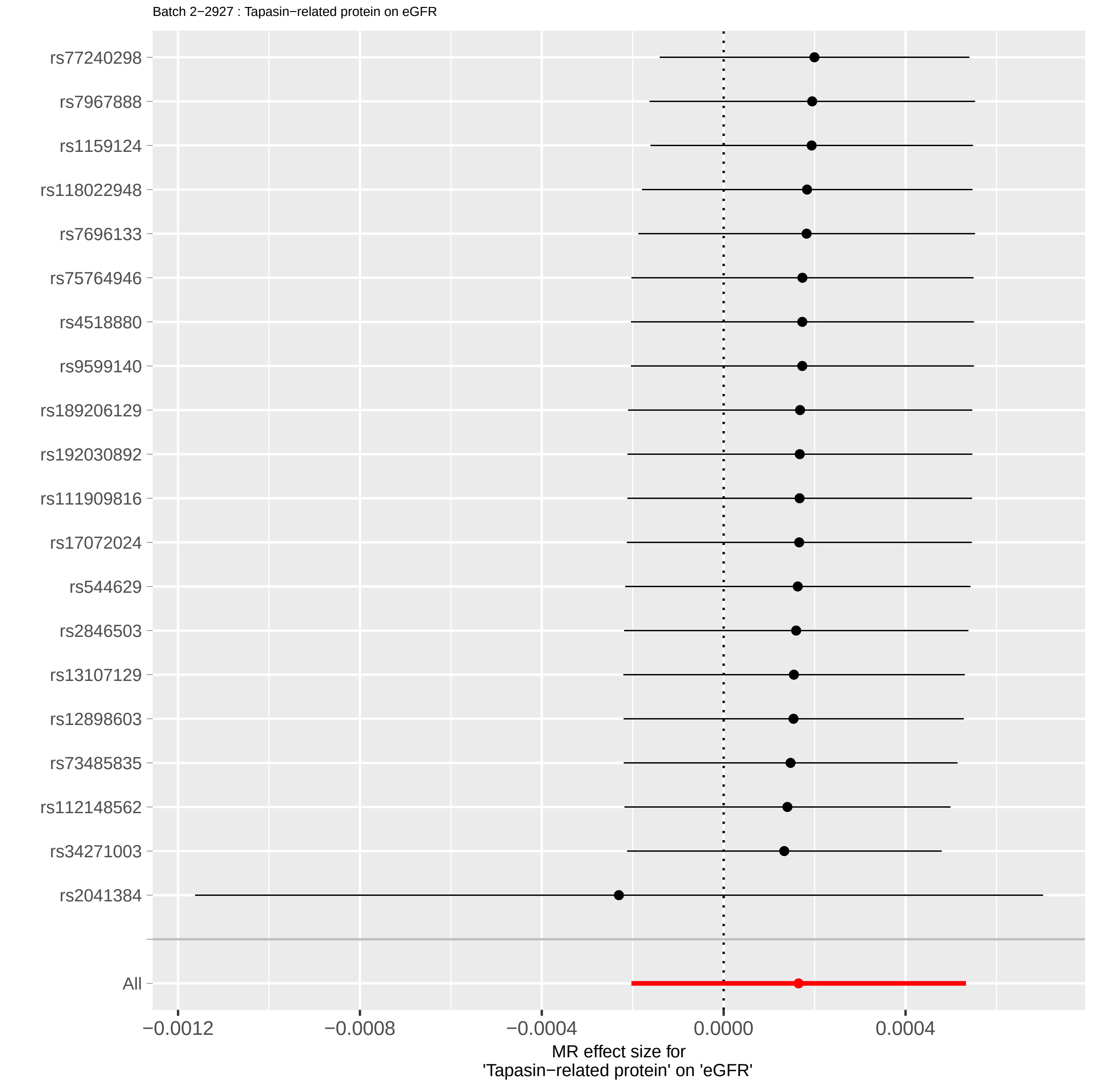

Batch 2−2927 : Tapasin−related protein on eGFR
rs77240298
rs7967888
rs1159124
rs118022948
rs7696133
rs75764946
rs4518880
rs9599140
rs189206129
rs192030892
rs111909816
rs17072024
rs544629
rs2846503
rs13107129
rs12898603
rs73485835
rs112148562
rs34271003
rs2041384
All
−0.0012
−0.0008
−0.0004
0.0000
0.0004
MR effect size for
'Tapasin−related protein' on 'eGFR'

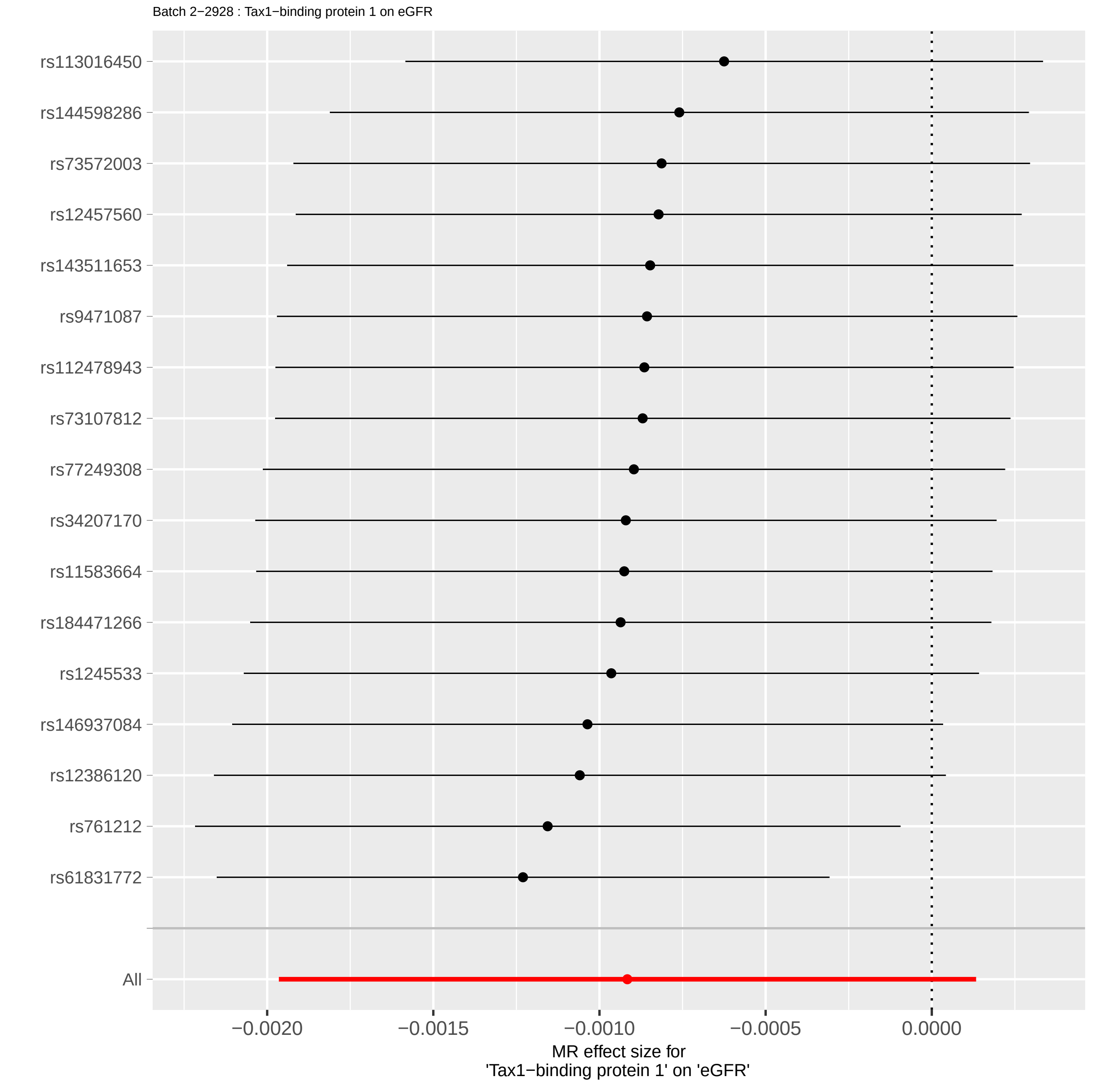

Batch 2−2928 : Tax1−binding protein 1 on eGFR
rs113016450
rs144598286
rs73572003
rs12457560
rs143511653
rs9471087
rs112478943
rs73107812
rs77249308
rs34207170
rs11583664
rs184471266
rs1245533
rs146937084
rs12386120
rs761212
rs61831772
All
−0.0020
−0.0015
−0.0010
−0.0005
0.0000
MR effect size for
'Tax1−binding protein 1' on 'eGFR'

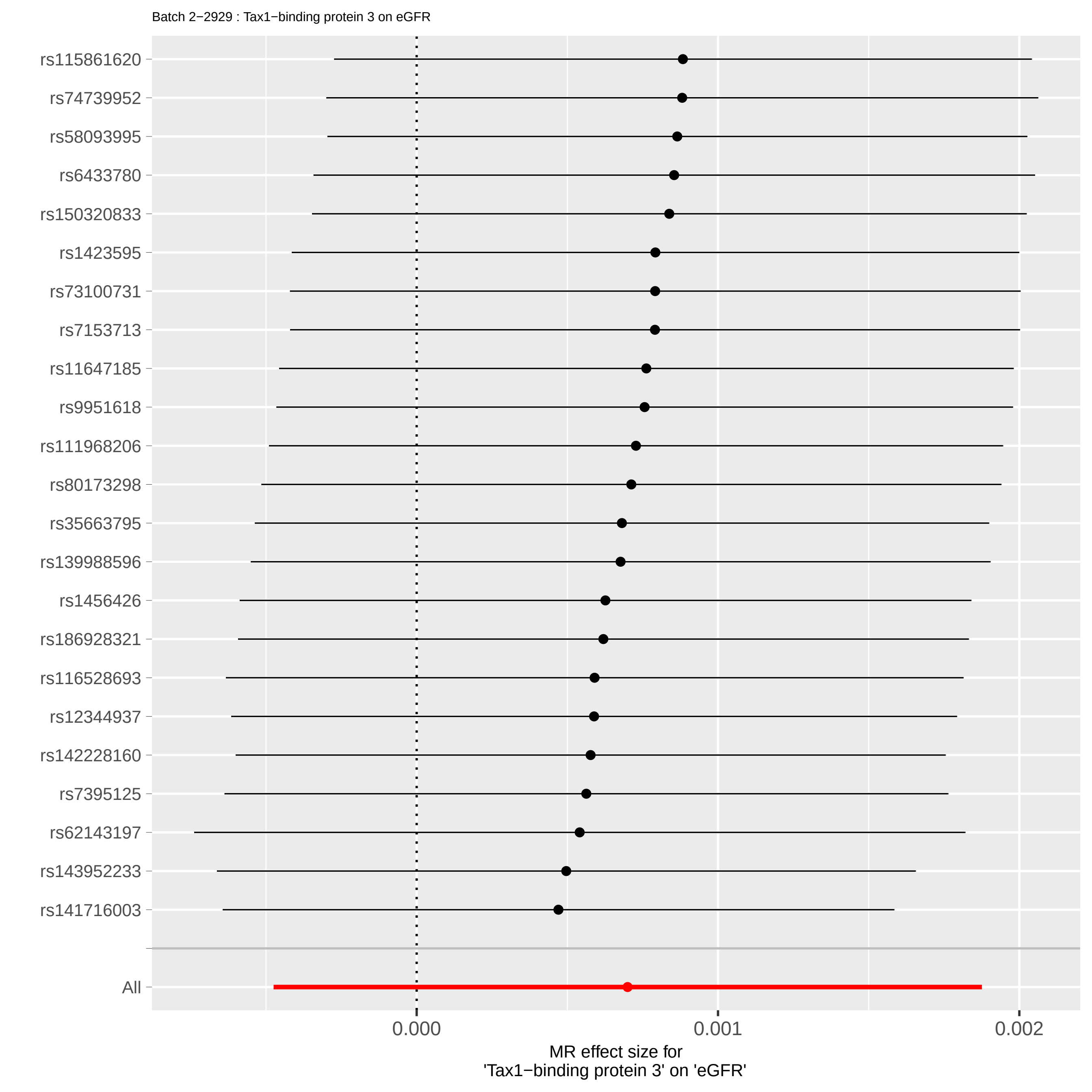

Batch 2−2929 : Tax1−binding protein 3 on eGFR
rs115861620
rs74739952
rs58093995
rs6433780
rs150320833
rs1423595
rs73100731
rs7153713
rs11647185
rs9951618
rs111968206
rs80173298
rs35663795
rs139988596
rs1456426
rs186928321
rs116528693
rs12344937
rs142228160
rs7395125
rs62143197
rs143952233
rs141716003
All
0.000
0.001
0.002
MR effect size for
'Tax1−binding protein 3' on 'eGFR'

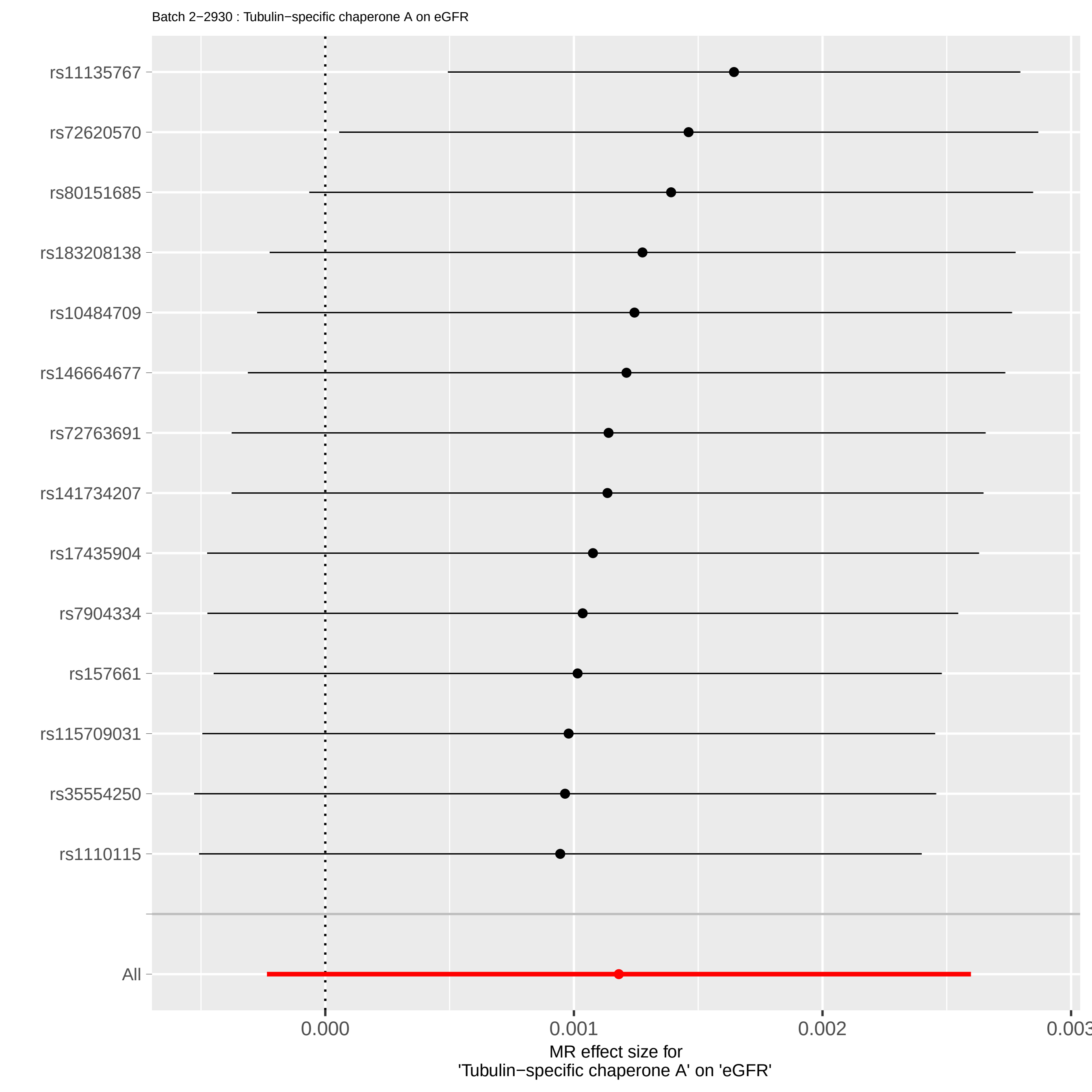

Batch 2−2930 : Tubulin−specific chaperone A on eGFR
rs11135767
rs72620570
rs80151685
rs183208138
rs10484709
rs146664677
rs72763691
rs141734207
rs17435904
rs7904334
rs157661
rs115709031
rs35554250
rs1110115
All
0.000
0.001
0.002
MR effect size for
'Tubulin−specific chaperone A' on 'eGFR'

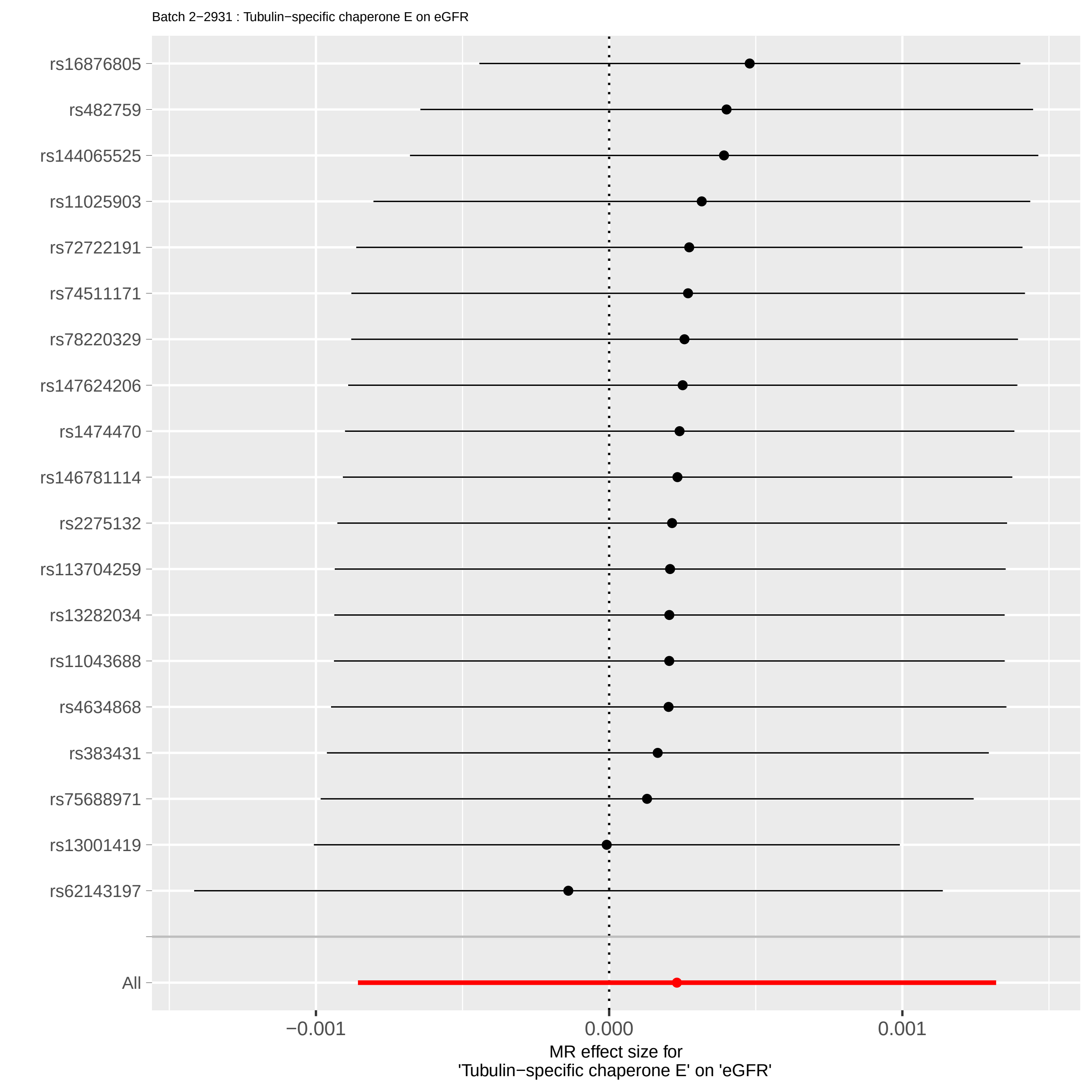

Batch 2−2931 : Tubulin−specific chaperone E on eGFR
rs16876805
rs482759
rs144065525
rs11025903
rs72722191
rs74511171
rs78220329
rs147624206
rs1474470
rs146781114
rs2275132
rs113704259
rs13282034
rs11043688
rs4634868
rs383431
rs75688971
rs13001419
rs62143197
All
−0.001
0.000
0.001
MR effect size for
'Tubulin−specific chaperone E' on 'eGFR'

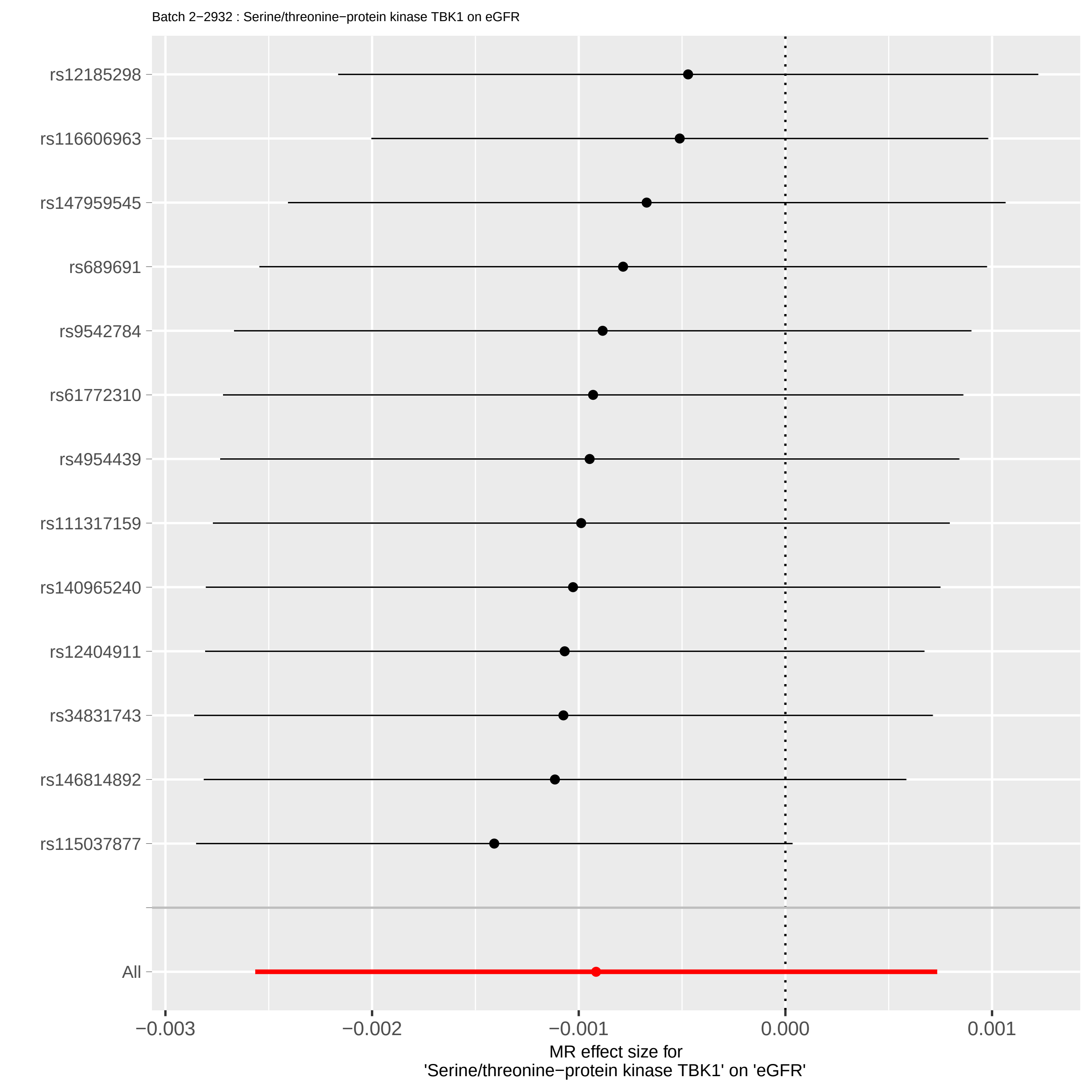

Batch 2−2932 : Serine/threonine−protein kinase TBK1 on eGFR
rs12185298
rs116606963
rs147959545
rs689691
rs9542784
rs61772310
rs4954439
rs111317159
rs140965240
rs12404911
rs34831743
rs146814892
rs115037877
All
−0.003
−0.002
−0.001
0.000
0.001
MR effect size for
'Serine/threonine−protein kinase TBK1' on 'eGFR'

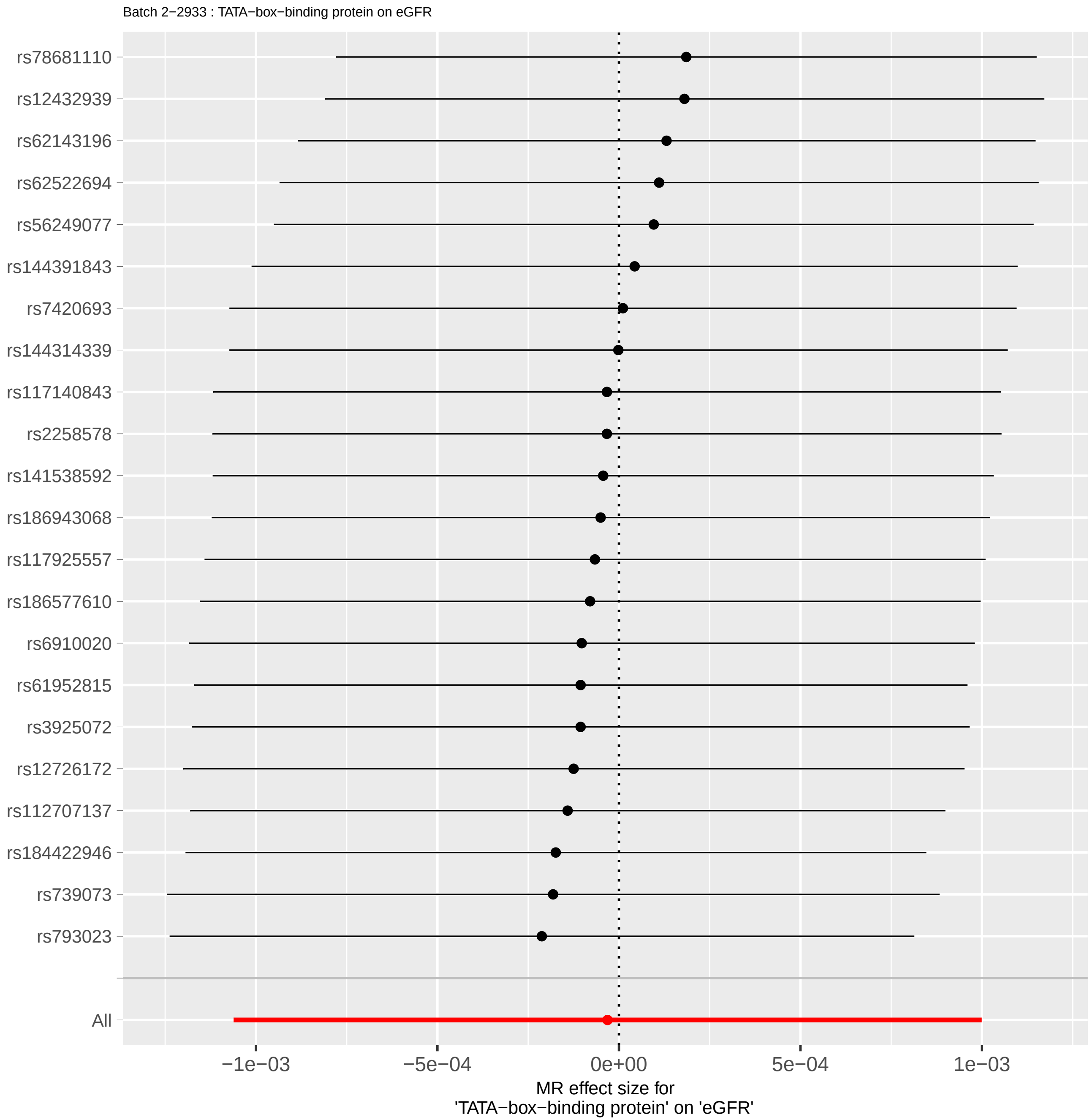
Batch 2−2933 : TATA−box−binding protein on eGFR
rs78681110
rs12432939
rs62143196
rs62522694
rs56249077
rs144391843
rs7420693
rs144314339
rs117140843
rs2258578
rs141538592
rs186943068
rs117925557
rs186577610
rs6910020
rs61952815
rs3925072
rs12726172
rs112707137
rs184422946
rs739073
rs793023
All
−1e−03
−5e−04
0e+00
5e−04
1e−03
MR effect size for
'TATA−box−binding protein' on 'eGFR'

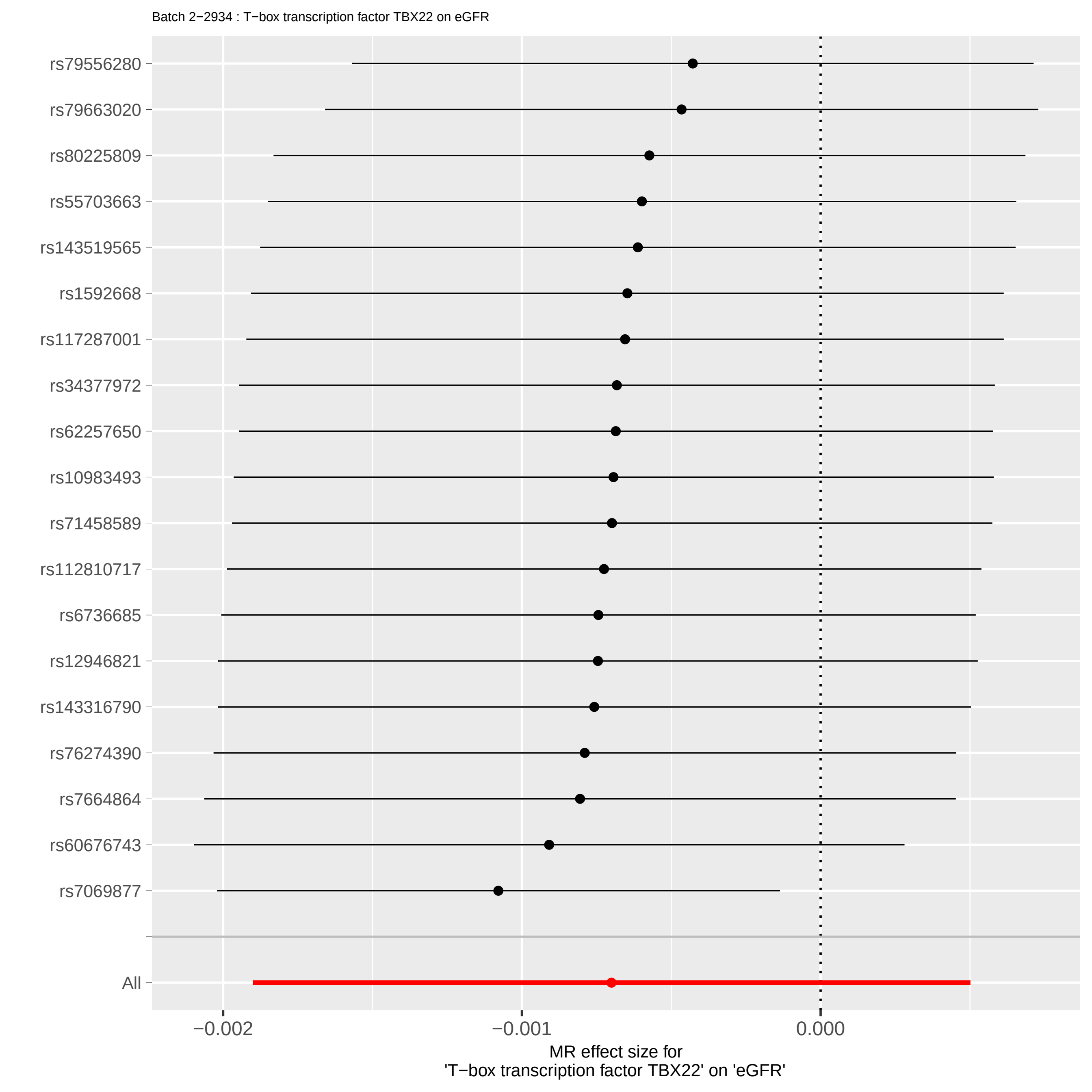

Batch 2−2934 : T−box transcription factor TBX22 on eGFR
rs79556280
rs79663020
rs80225809
rs55703663
rs143519565
rs1592668
rs117287001
rs34377972
rs62257650
rs10983493
rs71458589
rs112810717
rs6736685
rs12946821
rs143316790
rs76274390
rs7664864
rs60676743
rs7069877
All
−0.002
−0.001
0.000
MR effect size for
'T−box transcription factor TBX22' on 'eGFR'

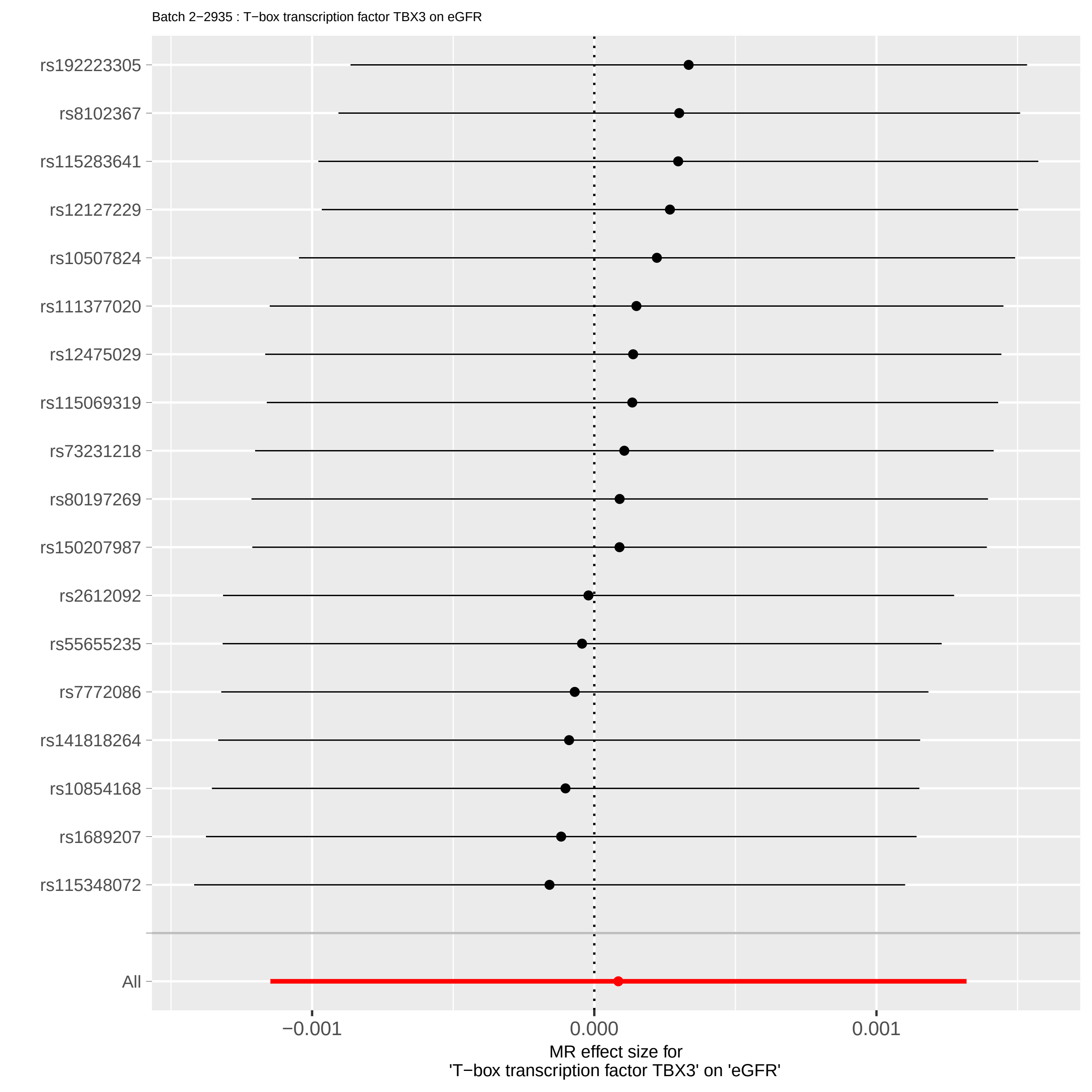

Batch 2−2935 : T−box transcription factor TBX3 on eGFR
rs192223305
rs8102367
rs115283641
rs12127229
rs10507824
rs111377020
rs12475029
rs115069319
rs73231218
rs80197269
rs150207987
rs2612092
rs55655235
rs7772086
rs141818264
rs10854168
rs1689207
rs115348072
All
−0.001
0.000
0.001
MR effect size for
'T−box transcription factor TBX3' on 'eGFR'

Batch 2−2936 : T−box transcription factor TBX5 on eGFR

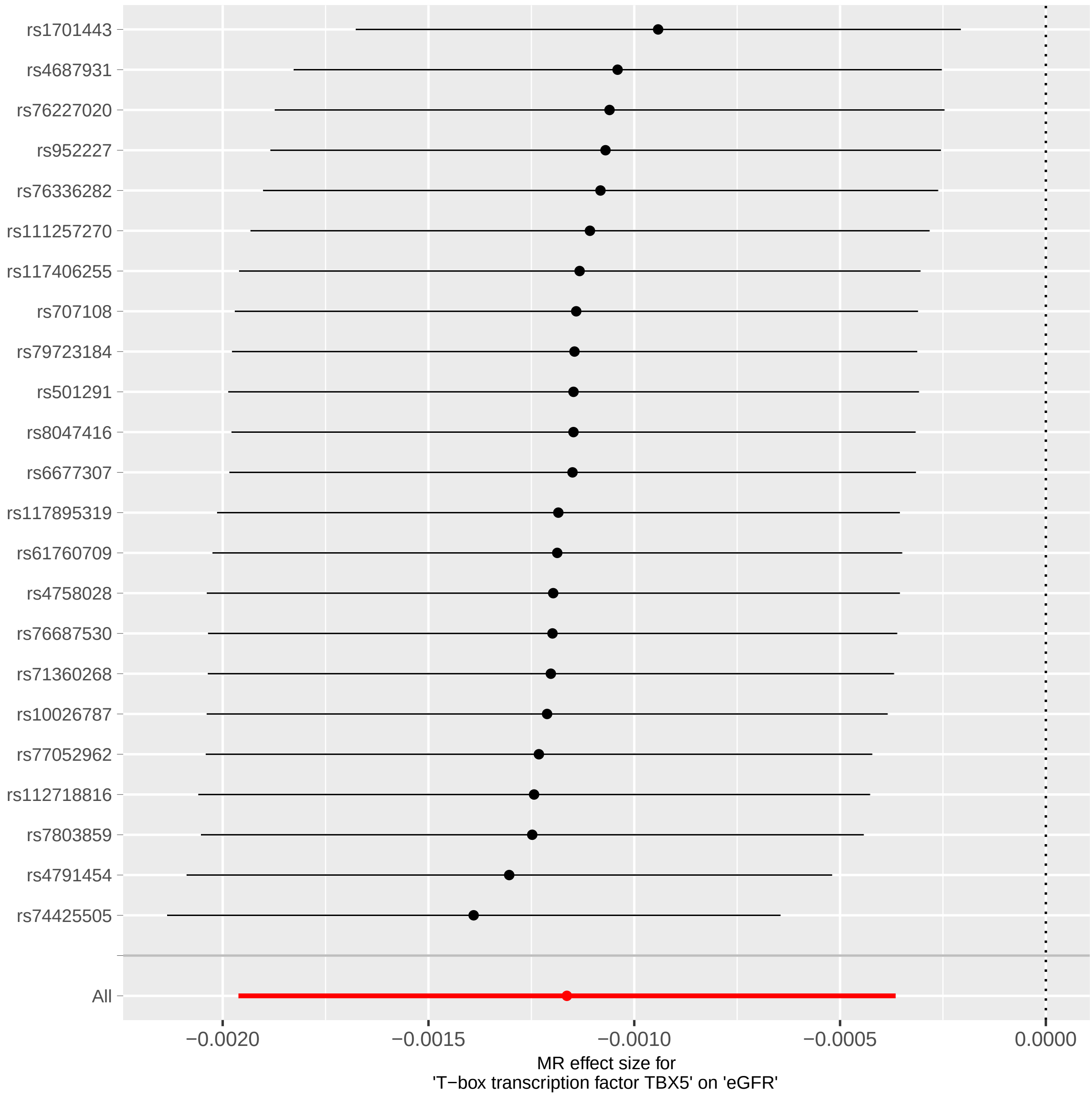

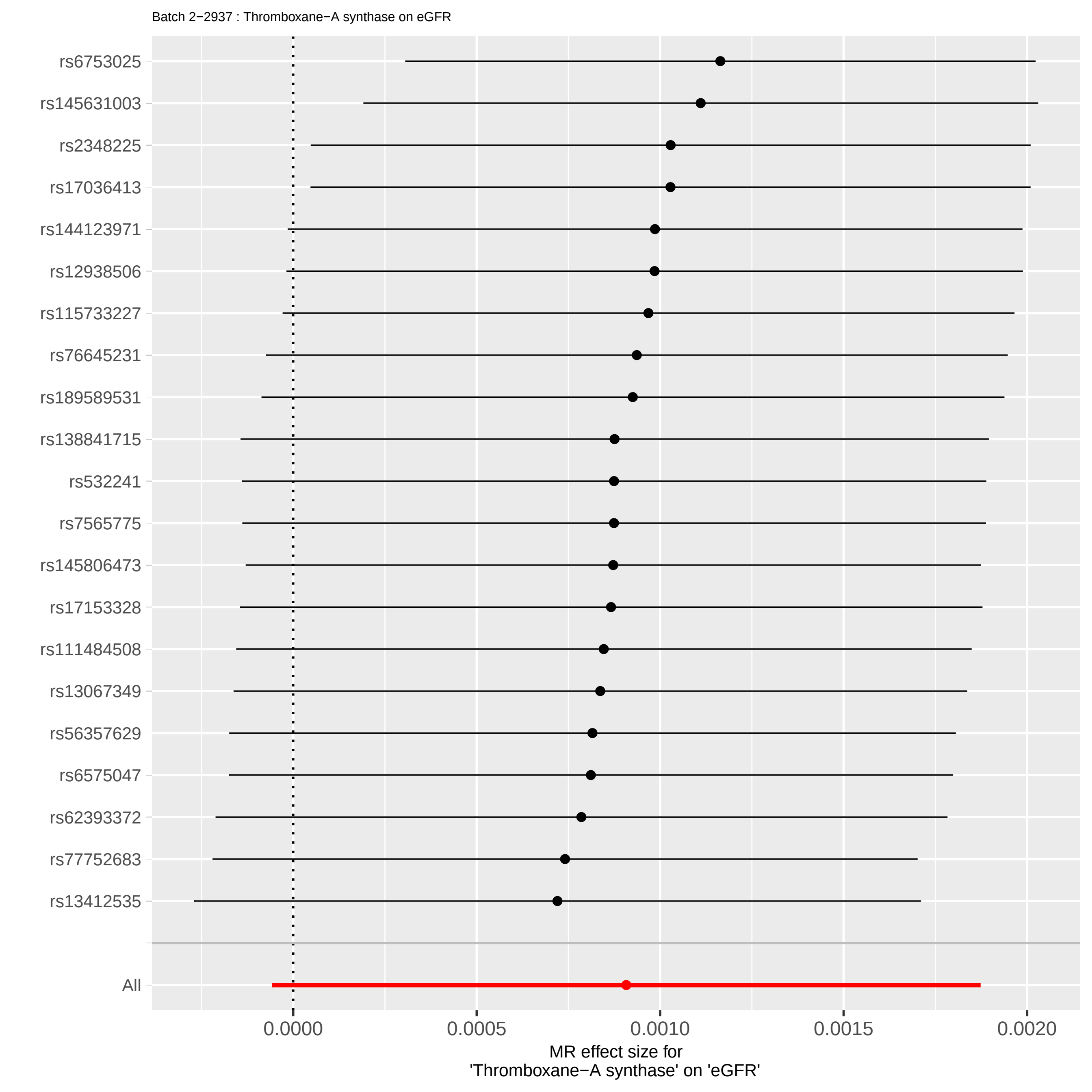

Batch 2−2937 : Thromboxane−A synthase on eGFR
rs6753025
rs145631003
rs2348225
rs17036413
rs144123971
rs12938506
rs115733227
rs76645231
rs189589531
rs138841715
rs532241
rs7565775
rs145806473
rs17153328
rs111484508
rs13067349
rs56357629
rs6575047
rs62393372
rs77752683
rs13412535
All
0.0000
0.0005
0.0010
0.0015
0.0020
MR effect size for
'Thromboxane−A synthase' on 'eGFR'

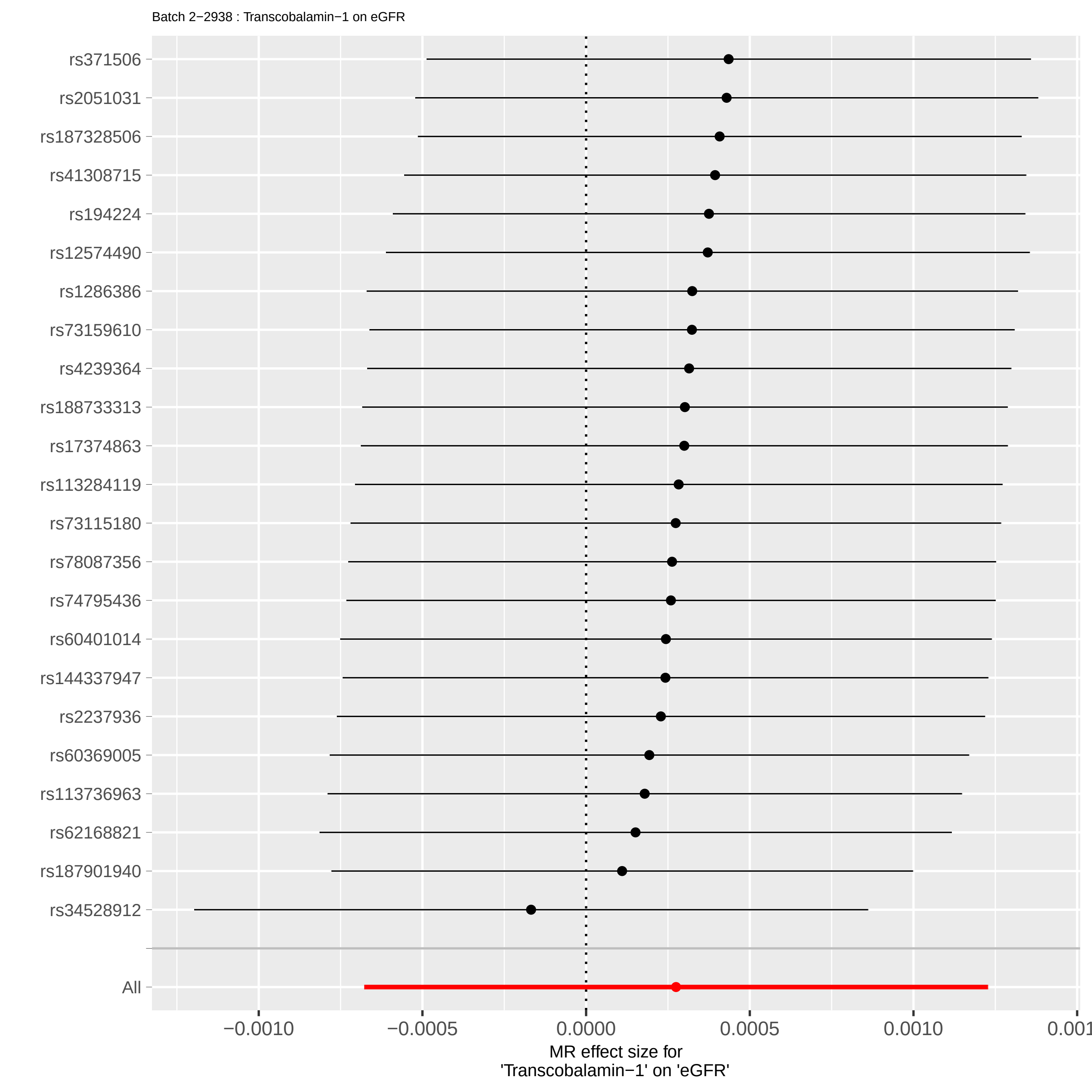

Batch 2−2938 : Transcobalamin−1 on eGFR
rs371506
rs2051031
rs187328506
rs41308715
rs194224
rs12574490
rs1286386
rs73159610
rs4239364
rs188733313
rs17374863
rs113284119
rs73115180
rs78087356
rs74795436
rs60401014
rs144337947
rs2237936
rs60369005
rs113736963
rs62168821
rs187901940
rs34528912
All
−0.0010
−0.0005
0.0000
0.0005
0.0010
MR effect size for
'Transcobalamin−1' on 'eGFR'

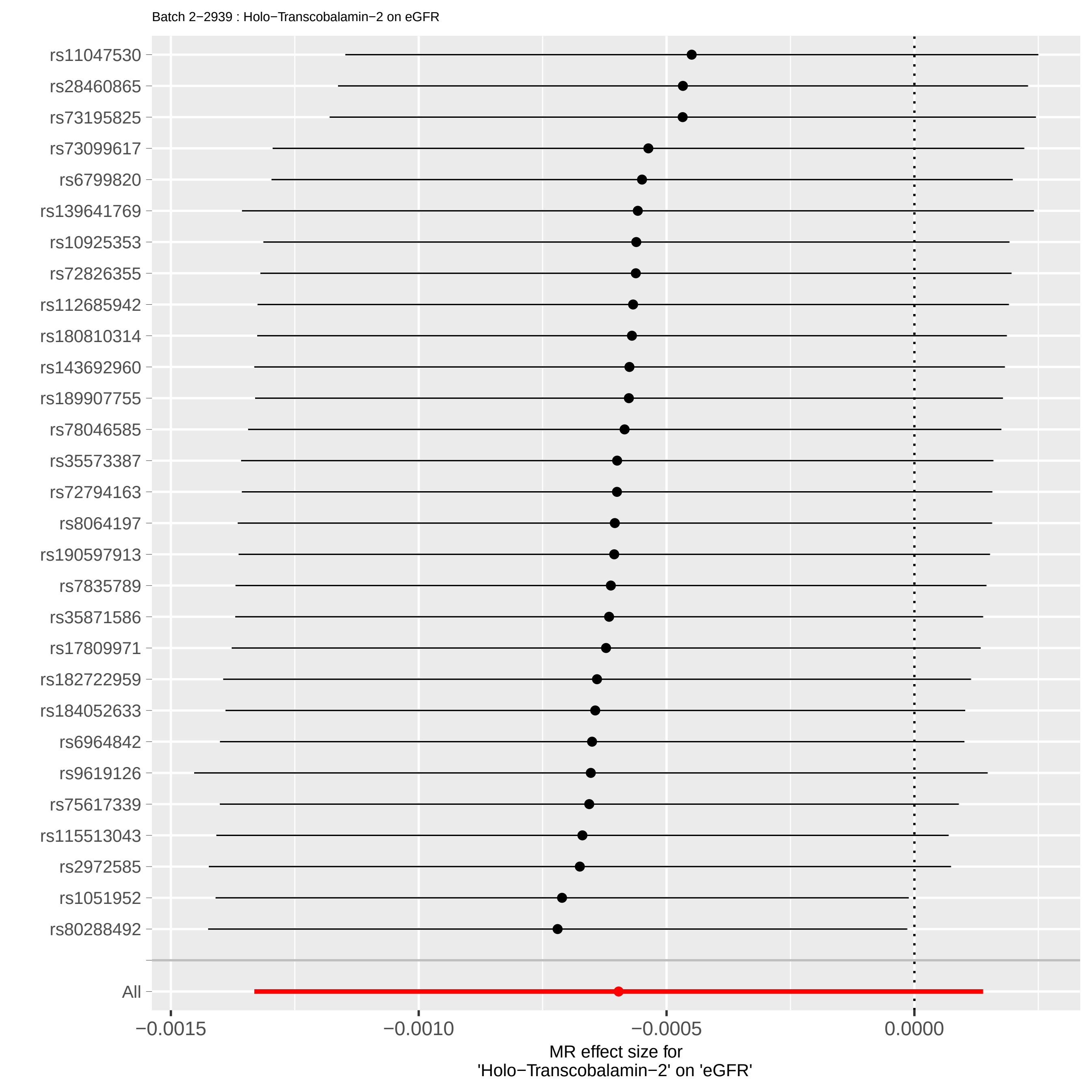

Batch 2−2939 : Holo−Transcobalamin−2 on eGFR
rs11047530
rs28460865
rs73195825
rs73099617
rs6799820
rs139641769
rs10925353
rs72826355
rs112685942
rs180810314
rs143692960
rs189907755
rs78046585
rs35573387
rs72794163
rs8064197
rs190597913
rs7835789
rs35871586
rs17809971
rs182722959
rs184052633
rs6964842
rs9619126
rs75617339
rs115513043
rs2972585
rs1051952
rs80288492
All
−0.0015
−0.0010
−0.0005
0.0000
MR effect size for
'Holo−Transcobalamin−2' on 'eGFR'

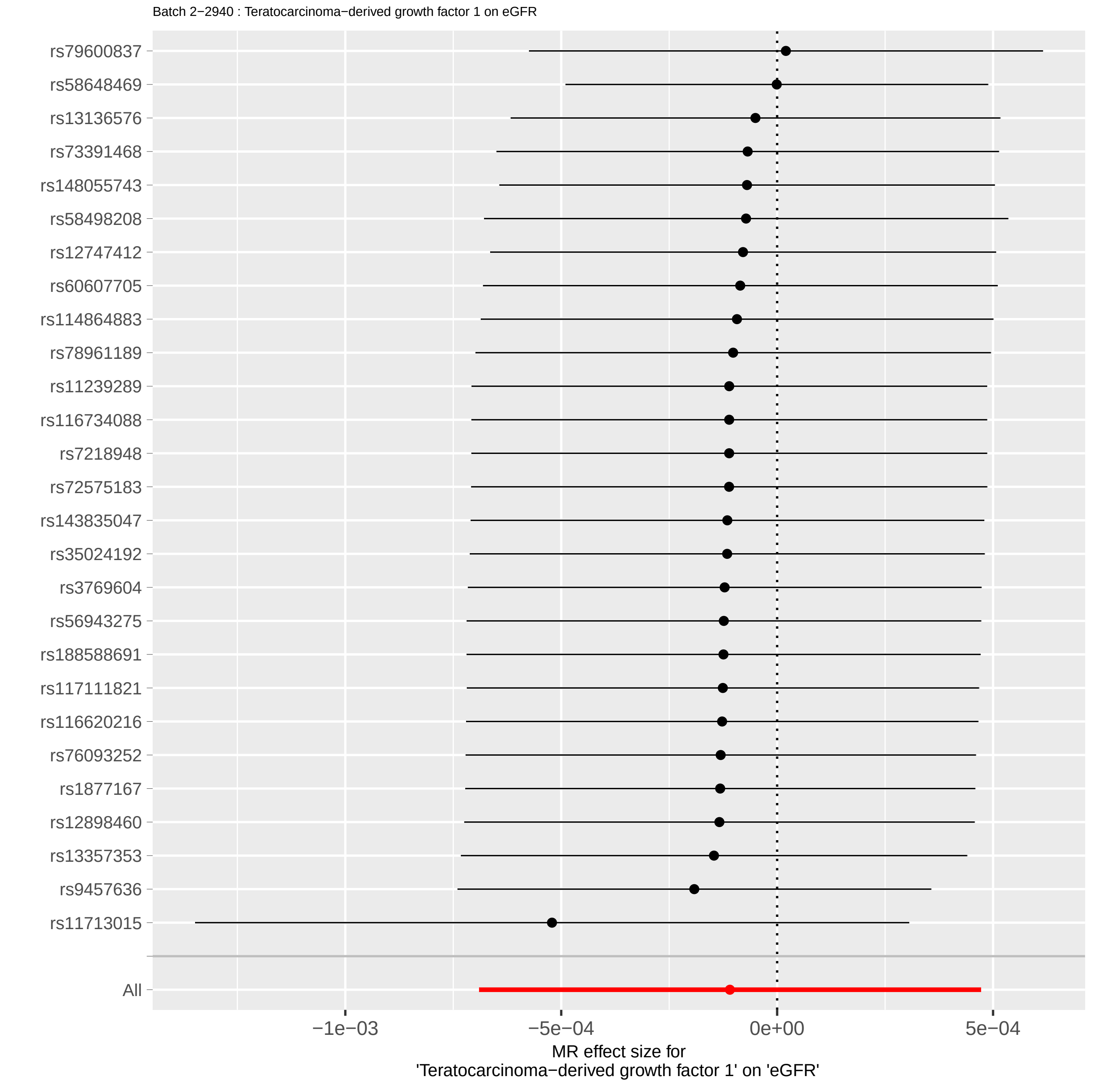

Batch 2−2940 : Teratocarcinoma−derived growth factor 1 on eGFR
rs79600837
rs58648469
rs13136576
rs73391468
rs148055743
rs58498208
rs12747412
rs60607705
rs114864883
rs78961189
rs11239289
rs116734088
rs7218948
rs72575183
rs143835047
rs35024192
rs3769604
rs56943275
rs188588691
rs117111821
rs116620216
rs76093252
rs1877167
rs12898460
rs13357353
rs9457636
rs11713015
All
−1e−03
−5e−04
0e+00
5e−04
MR effect size for
'Teratocarcinoma−derived growth factor 1' on 'eGFR'

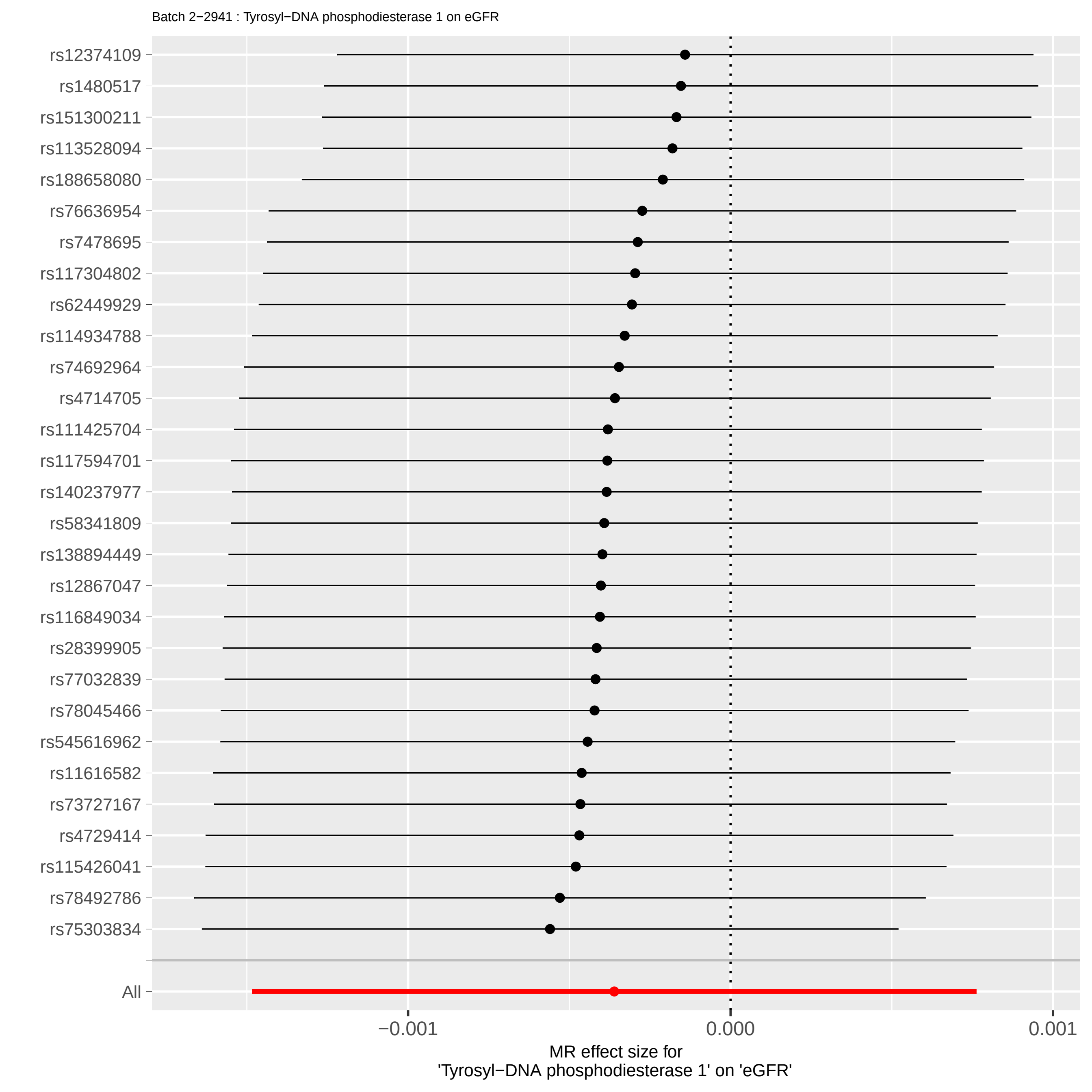

Batch 2−2941 : Tyrosyl−DNA phosphodiesterase 1 on eGFR
rs12374109
rs1480517
rs151300211
rs113528094
rs188658080
rs76636954
rs7478695
rs117304802
rs62449929
rs114934788
rs74692964
rs4714705
rs111425704
rs117594701
rs140237977
rs58341809
rs138894449
rs12867047
rs116849034
rs28399905
rs77032839
rs78045466
rs545616962
rs11616582
rs73727167
rs4729414
rs115426041
rs78492786
rs75303834
All
−0.001
0.000
0.001
MR effect size for
'Tyrosyl−DNA phosphodiesterase 1' on 'eGFR'

Batch 2−2942 : Transcriptional enhancer factor TEF−5 on eGFR

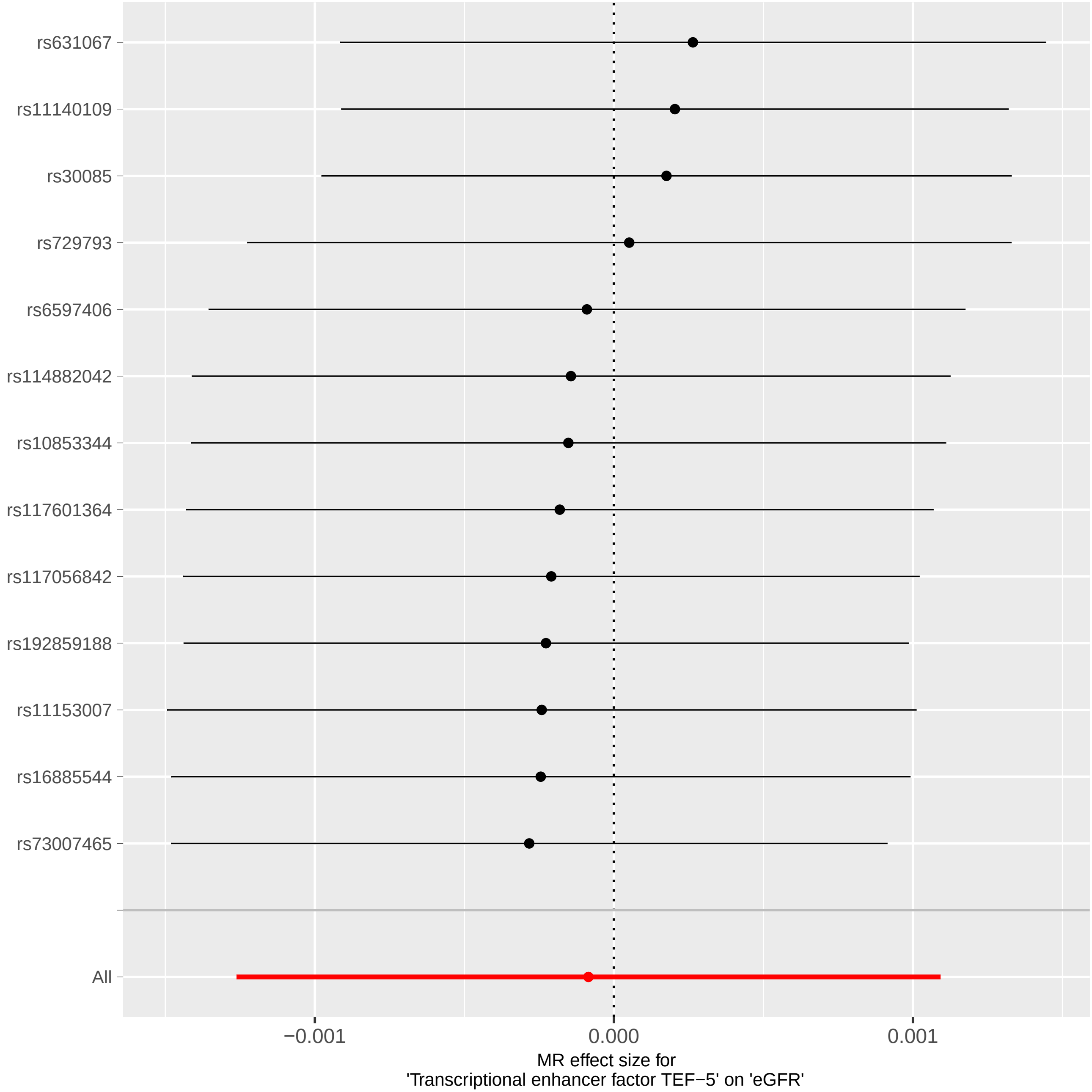

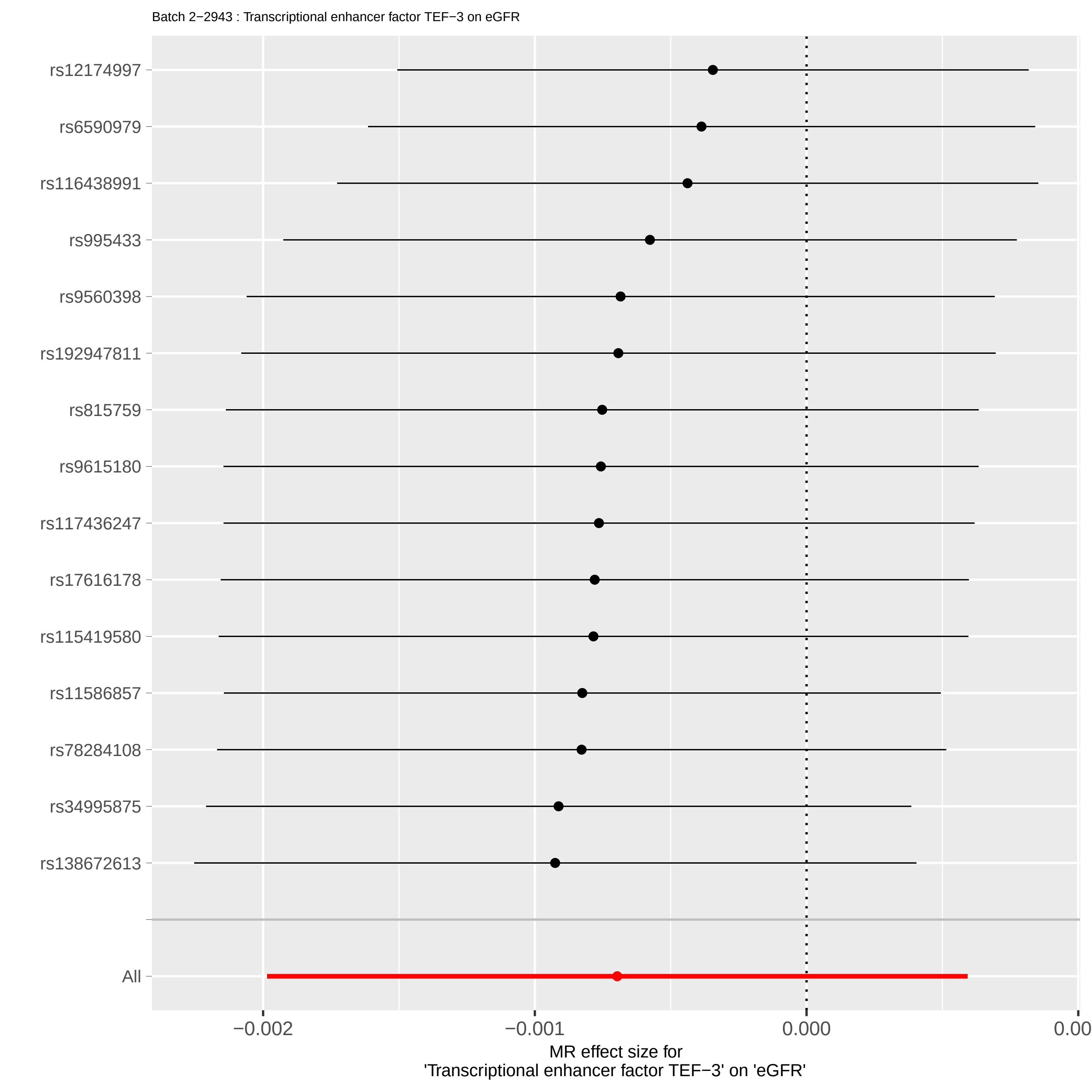

Batch 2−2943 : Transcriptional enhancer factor TEF−3 on eGFR
rs12174997
rs6590979
rs116438991
rs995433
rs9560398
rs192947811
rs815759
rs9615180
rs117436247
rs17616178
rs115419580
rs11586857
rs78284108
rs34995875
rs138672613
All
−0.002
−0.001
0.000
0.00
MR effect size for
'Transcriptional enhancer factor TEF−3' on 'eGFR'

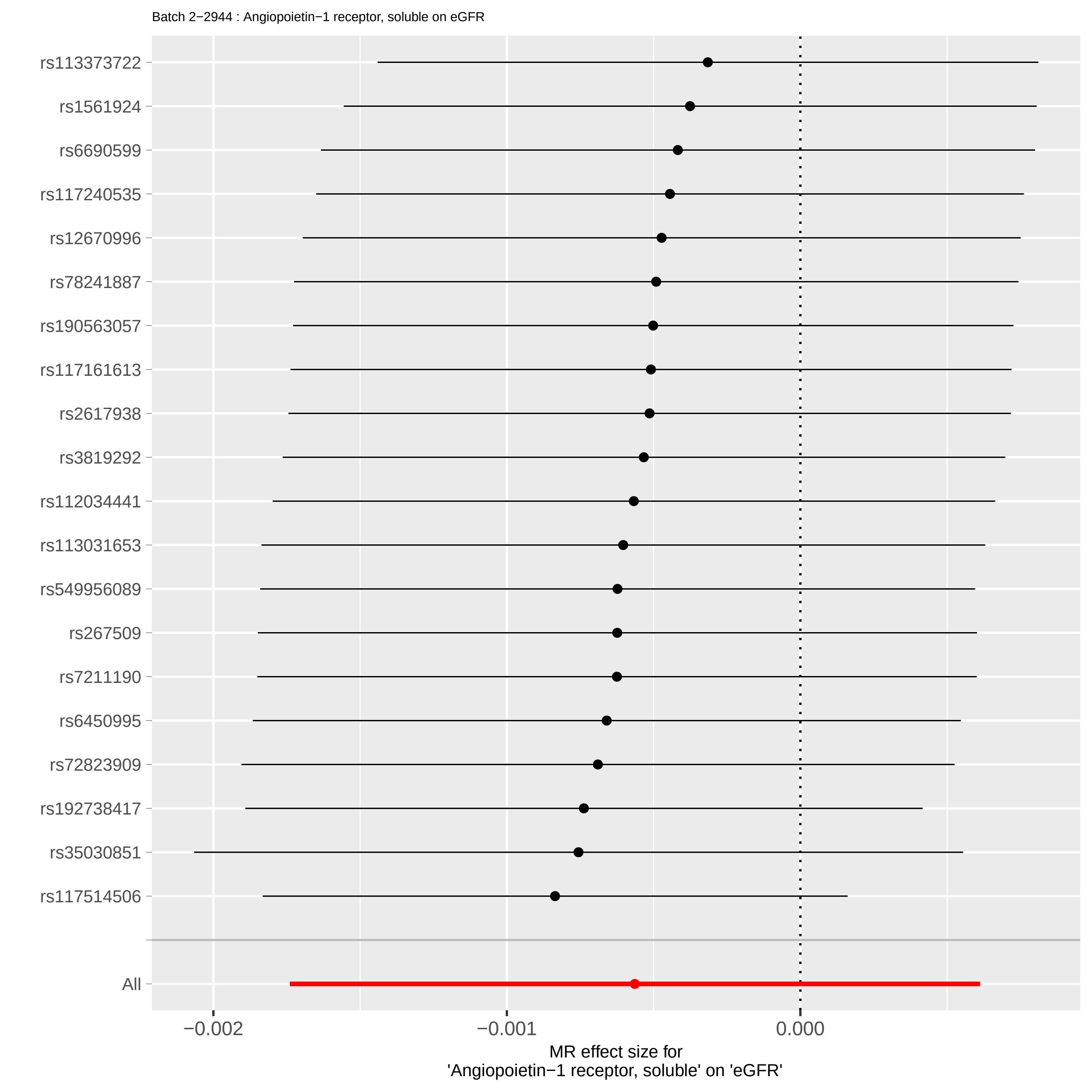

Batch 2−2944 : Angiopoietin−1 receptor, soluble on eGFR
rs113373722
rs1561924
rs6690599
rs117240535
rs12670996
rs78241887
rs190563057
rs117161613
rs2617938
rs3819292
rs112034441
rs113031653
rs549956089
rs267509
rs7211190
rs6450995
rs72823909
rs192738417
rs35030851
rs117514506
All
−0.002
−0.001
0.000
MR effect size for
'Angiopoietin−1 receptor, soluble' on 'eGFR'

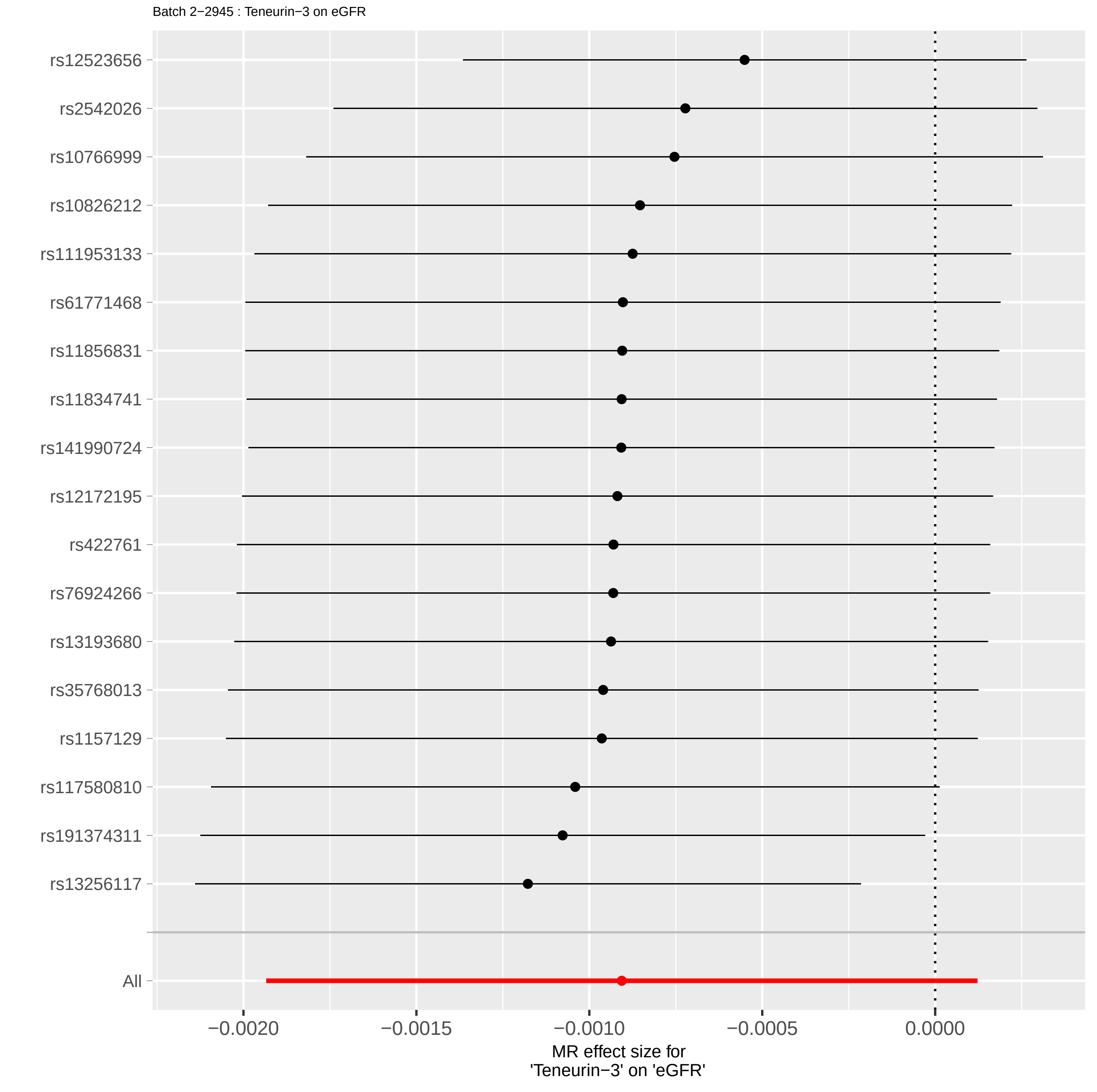

Batch 2−2945 : Teneurin−3 on eGFR
rs12523656
rs2542026
rs10766999
rs10826212
rs111953133
rs61771468
rs11856831
rs11834741
rs141990724
rs12172195
rs422761
rs76924266
rs13193680
rs35768013
rs1157129
rs117580810
rs191374311
rs13256117
All
−0.0020
−0.0015
−0.0010
−0.0005
0.0000
MR effect size for
'Teneurin−3' on 'eGFR'

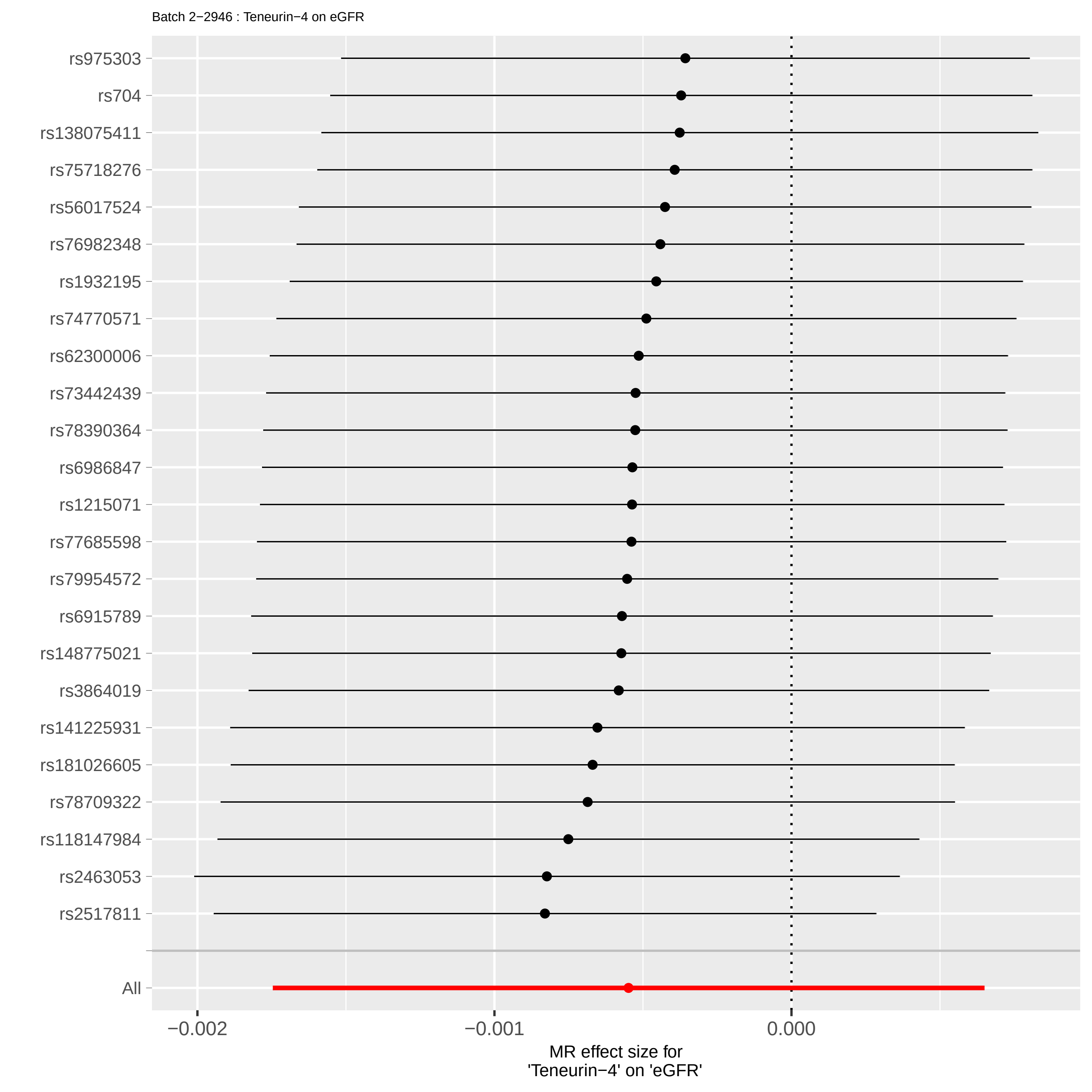
Batch 2−2946 : Teneurin−4 on eGFR
rs975303
rs704
rs138075411
rs75718276
rs56017524
rs76982348
rs1932195
rs74770571
rs62300006
rs73442439
rs78390364
rs6986847
rs1215071
rs77685598
rs79954572
rs6915789
rs148775021
rs3864019
rs141225931
rs181026605
rs78709322
rs118147984
rs2463053
rs2517811
All
−0.002
−0.001
0.000
MR effect size for
'Teneurin−4' on 'eGFR'

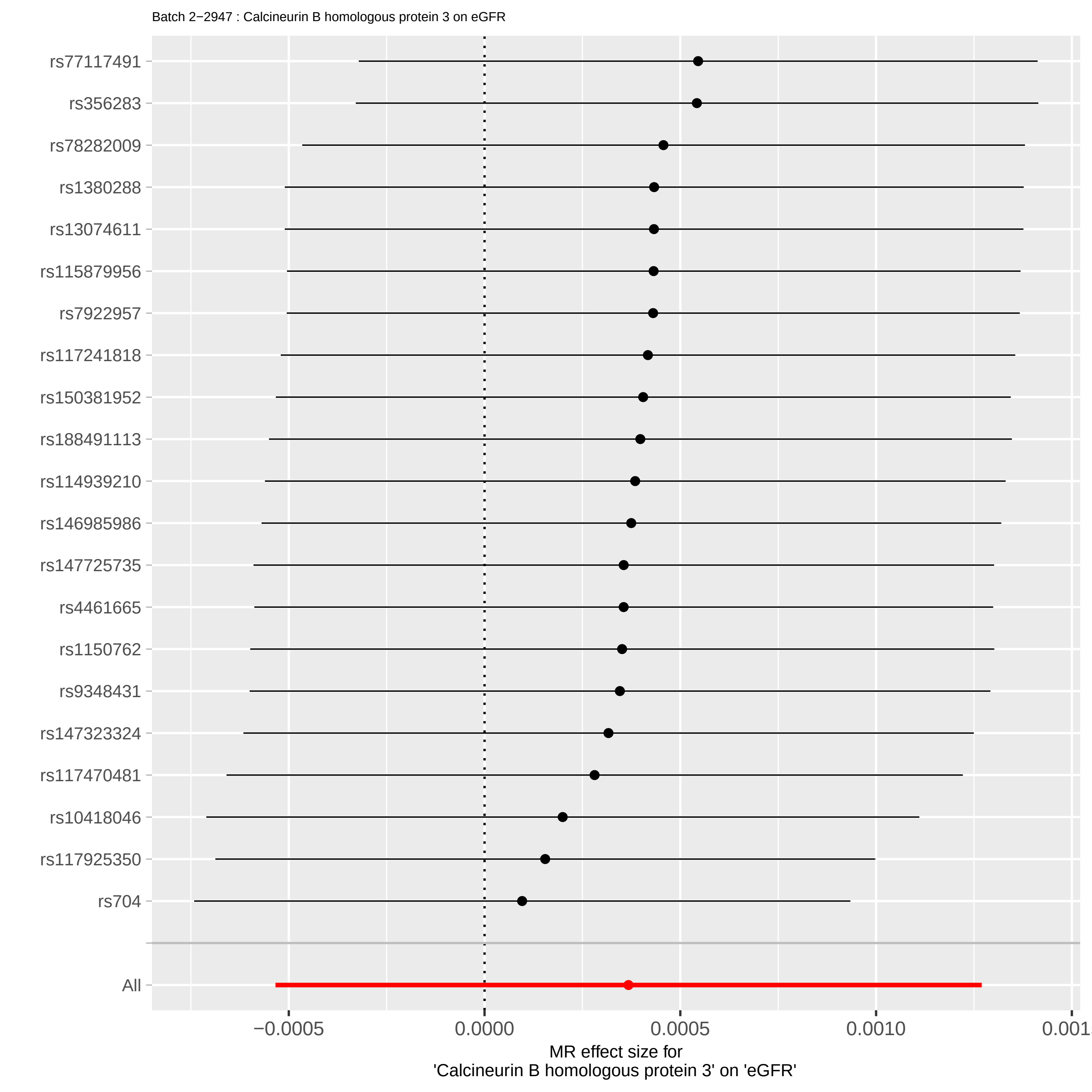
Batch 2−2947 : Calcineurin B homologous protein 3 on eGFR
rs77117491
rs356283
rs78282009
rs1380288
rs13074611
rs115879956
rs7922957
rs117241818
rs150381952
rs188491113
rs114939210
rs146985986
rs147725735
rs4461665
rs1150762
rs9348431
rs147323324
rs117470481
rs10418046
rs117925350
rs704
All
−0.0005
0.0000
0.0005
0.0010
MR effect size for
'Calcineurin B homologous protein 3' on 'eGFR'

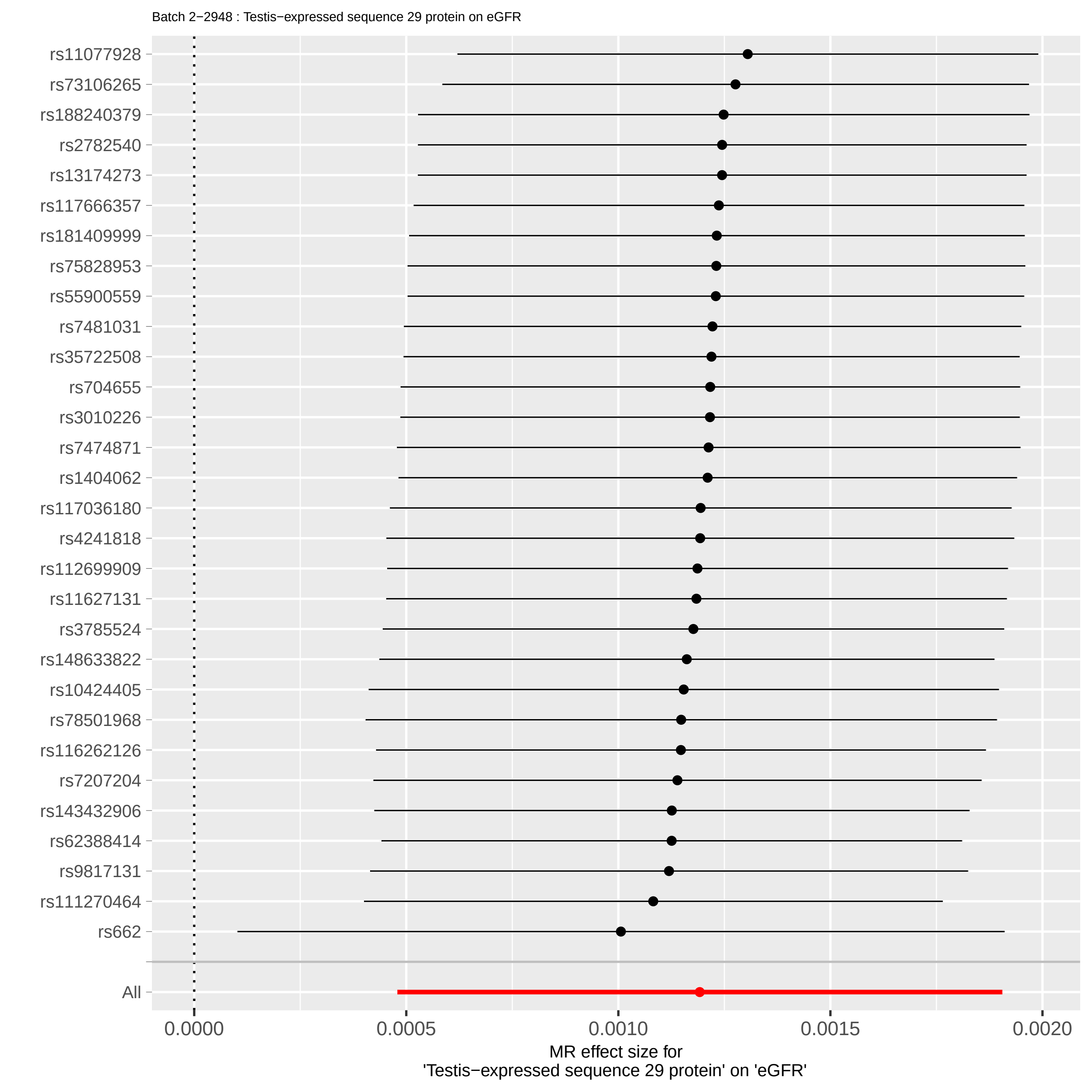

Batch 2−2948 : Testis−expressed sequence 29 protein on eGFR
rs11077928
rs73106265
rs188240379
rs2782540
rs13174273
rs117666357
rs181409999
rs75828953
rs55900559
rs7481031
rs35722508
rs704655
rs3010226
rs7474871
rs1404062
rs117036180
rs4241818
rs112699909
rs11627131
rs3785524
rs148633822
rs10424405
rs78501968
rs116262126
rs7207204
rs143432906
rs62388414
rs9817131
rs111270464
rs662
All
0.0000
0.0005
0.0010
0.0015
0.0020
MR effect size for
'Testis−expressed sequence 29 protein' on 'eGFR'

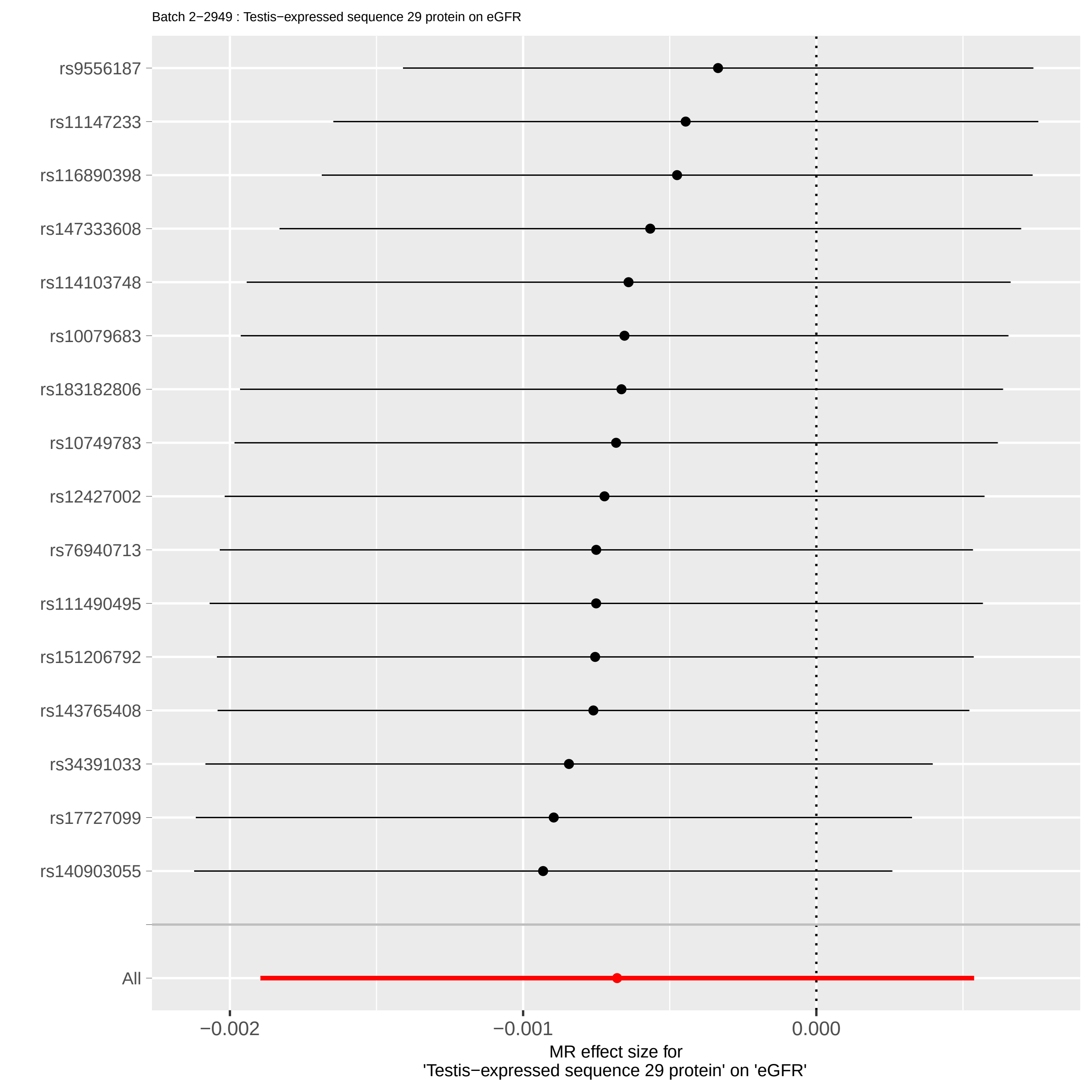
Batch 2−2949 : Testis−expressed sequence 29 protein on eGFR
rs9556187
rs11147233
rs116890398
rs147333608
rs114103748
rs10079683
rs183182806
rs10749783
rs12427002
rs76940713
rs111490495
rs151206792
rs143765408
rs34391033
rs17727099
rs140903055
All
−0.002
−0.001
0.000
MR effect size for
'Testis−expressed sequence 29 protein' on 'eGFR'

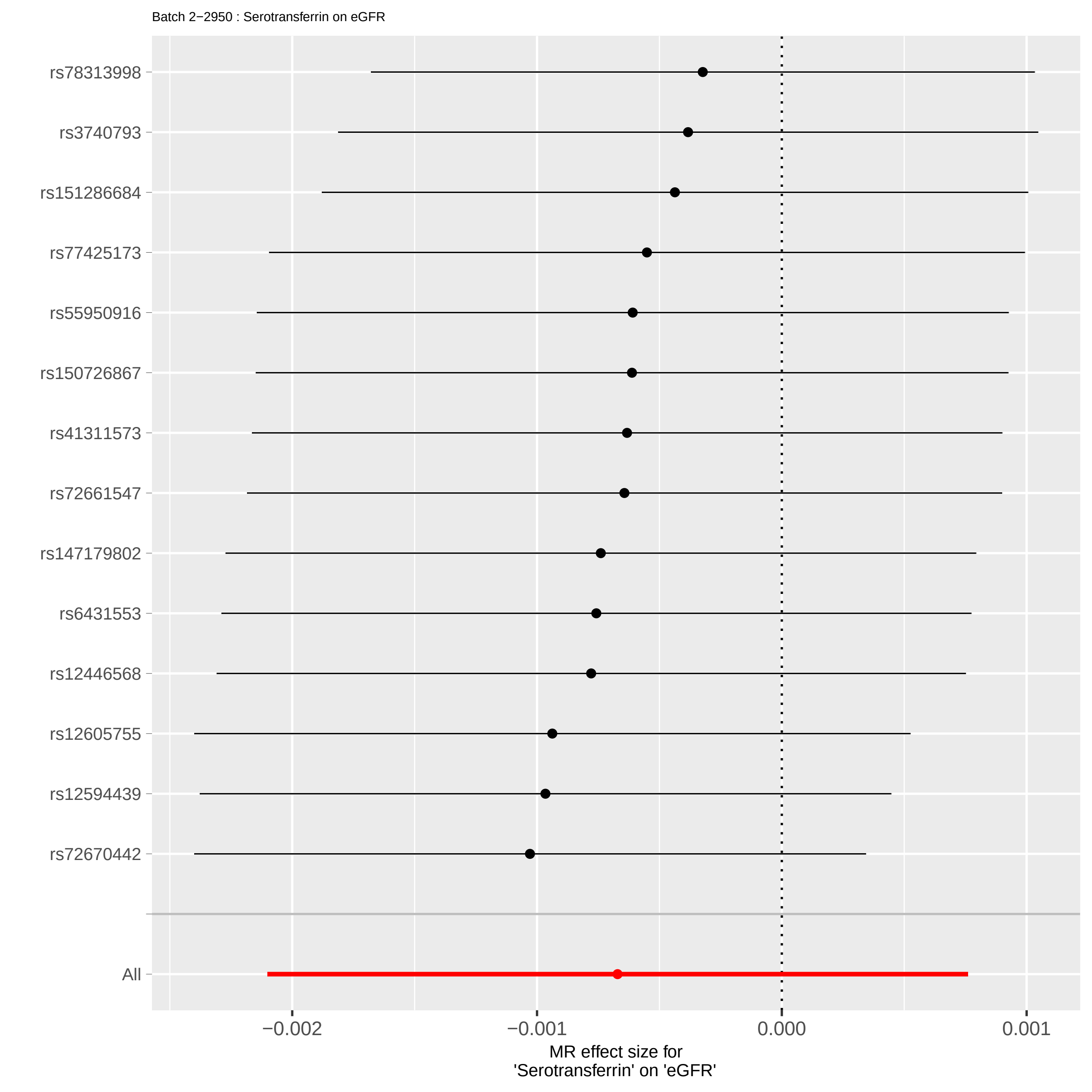

Batch 2−2950 : Serotransferrin on eGFR
rs78313998
rs3740793
rs151286684
rs77425173
rs55950916
rs150726867
rs41311573
rs72661547
rs147179802
rs6431553
rs12446568
rs12605755
rs12594439
rs72670442
All
−0.002
−0.001
0.000
0.001
MR effect size for
'Serotransferrin' on 'eGFR'

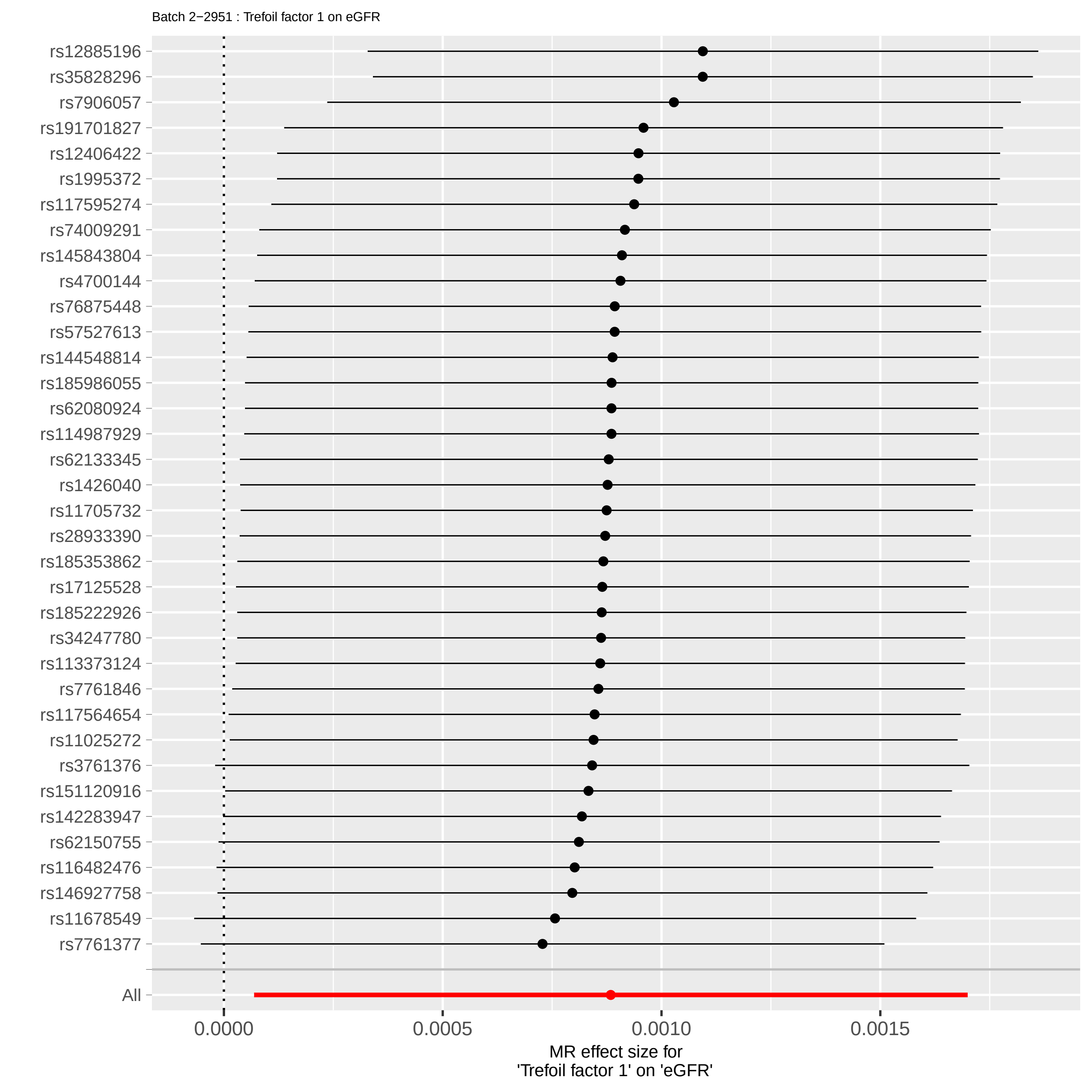

Batch 2−2951 : Trefoil factor 1 on eGFR
rs12885196
rs35828296
rs7906057
rs191701827
rs12406422
rs1995372
rs117595274
rs74009291
rs145843804
rs4700144
rs76875448
rs57527613
rs144548814
rs185986055
rs62080924
rs114987929
rs62133345
rs1426040
rs11705732
rs28933390
rs185353862
rs17125528
rs185222926
rs34247780
rs113373124
rs7761846
rs117564654
rs11025272
rs3761376
rs151120916
rs142283947
rs62150755
rs116482476
rs146927758
rs11678549
rs7761377
All
0.0000
0.0005
0.0010
0.0015
MR effect size for
'Trefoil factor 1' on 'eGFR'

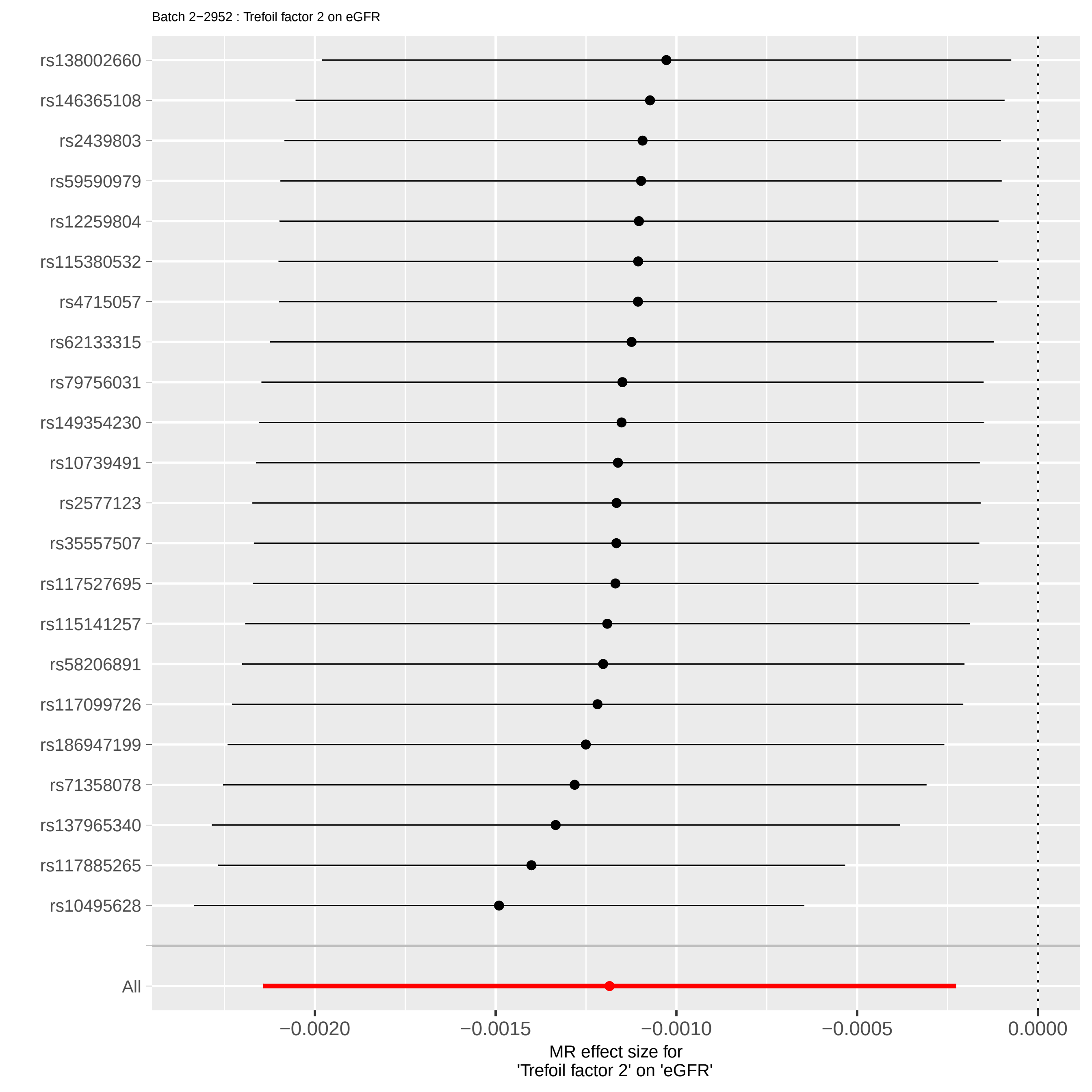
Batch 2−2952 : Trefoil factor 2 on eGFR
rs138002660
rs146365108
rs2439803
rs59590979
rs12259804
rs115380532
rs4715057
rs62133315
rs79756031
rs149354230
rs10739491
rs2577123
rs35557507
rs117527695
rs115141257
rs58206891
rs117099726
rs186947199
rs71358078
rs137965340
rs117885265
rs10495628
All
−0.0020
−0.0015
−0.0010
−0.0005
0.0000
MR effect size for
'Trefoil factor 2' on 'eGFR'

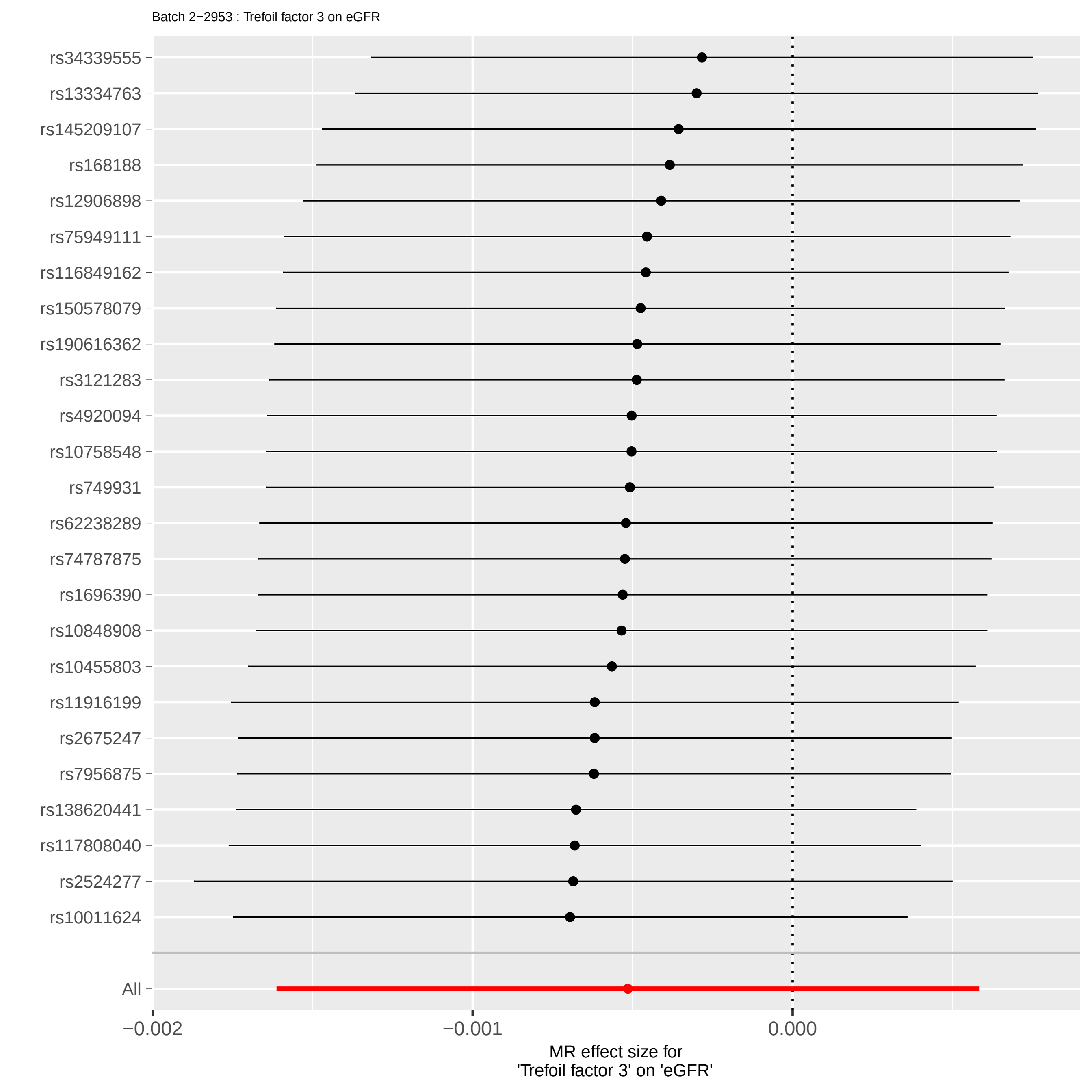

Batch 2−2953 : Trefoil factor 3 on eGFR
rs34339555
rs13334763
rs145209107
rs168188
rs12906898
rs75949111
rs116849162
rs150578079
rs190616362
rs3121283
rs4920094
rs10758548
rs749931
rs62238289
rs74787875
rs1696390
rs10848908
rs10455803
rs11916199
rs2675247
rs7956875
rs138620441
rs117808040
rs2524277
rs10011624
All
−0.002
−0.001
0.000
MR effect size for
'Trefoil factor 3' on 'eGFR'

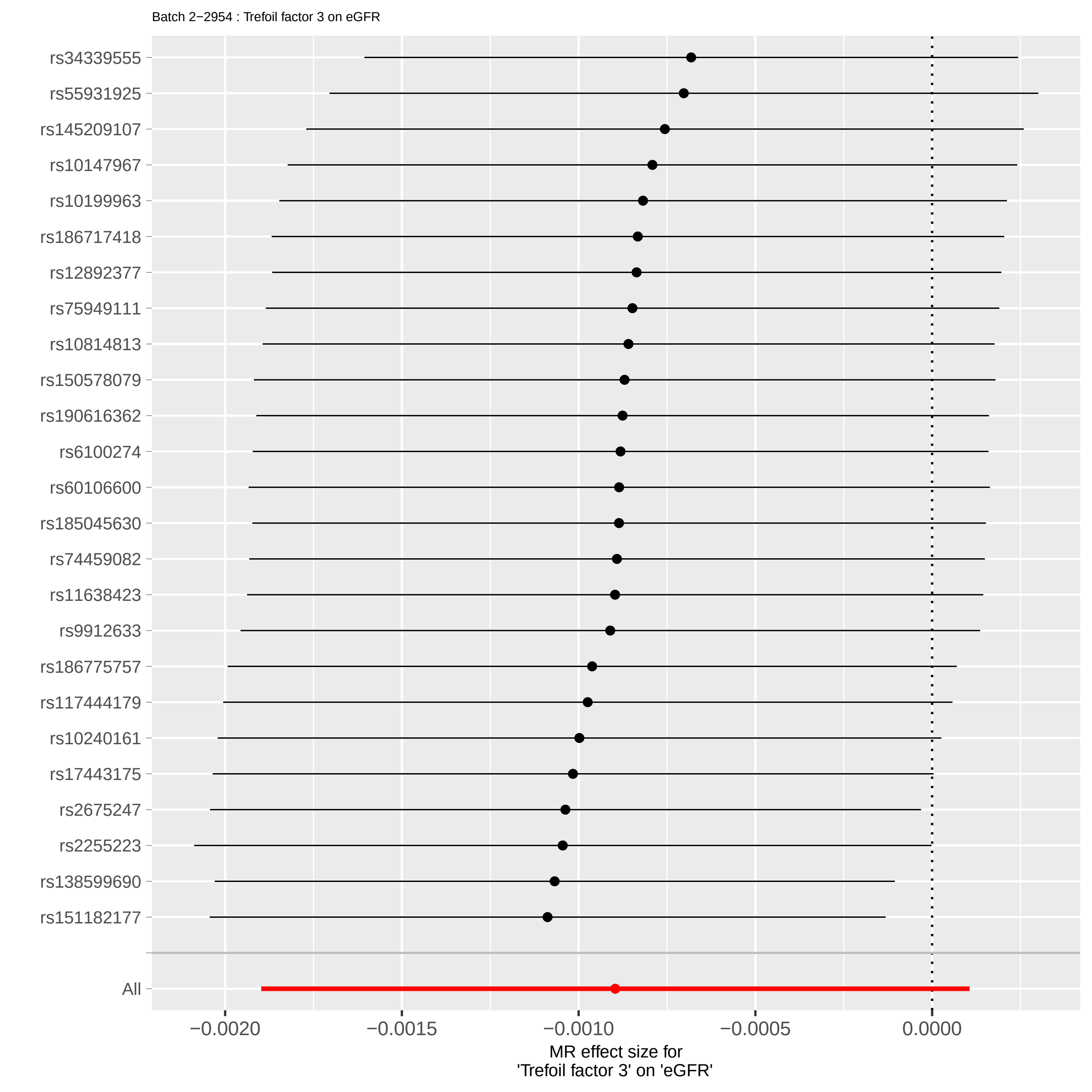
Batch 2−2954 : Trefoil factor 3 on eGFR
rs34339555
rs55931925
rs145209107
rs10147967
rs10199963
rs186717418
rs12892377
rs75949111
rs10814813
rs150578079
rs190616362
rs6100274
rs60106600
rs185045630
rs74459082
rs11638423
rs9912633
rs186775757
rs117444179
rs10240161
rs17443175
rs2675247
rs2255223
rs138599690
rs151182177
All
−0.0020
−0.0015
−0.0010
−0.0005
0.0000
MR effect size for
'Trefoil factor 3' on 'eGFR'

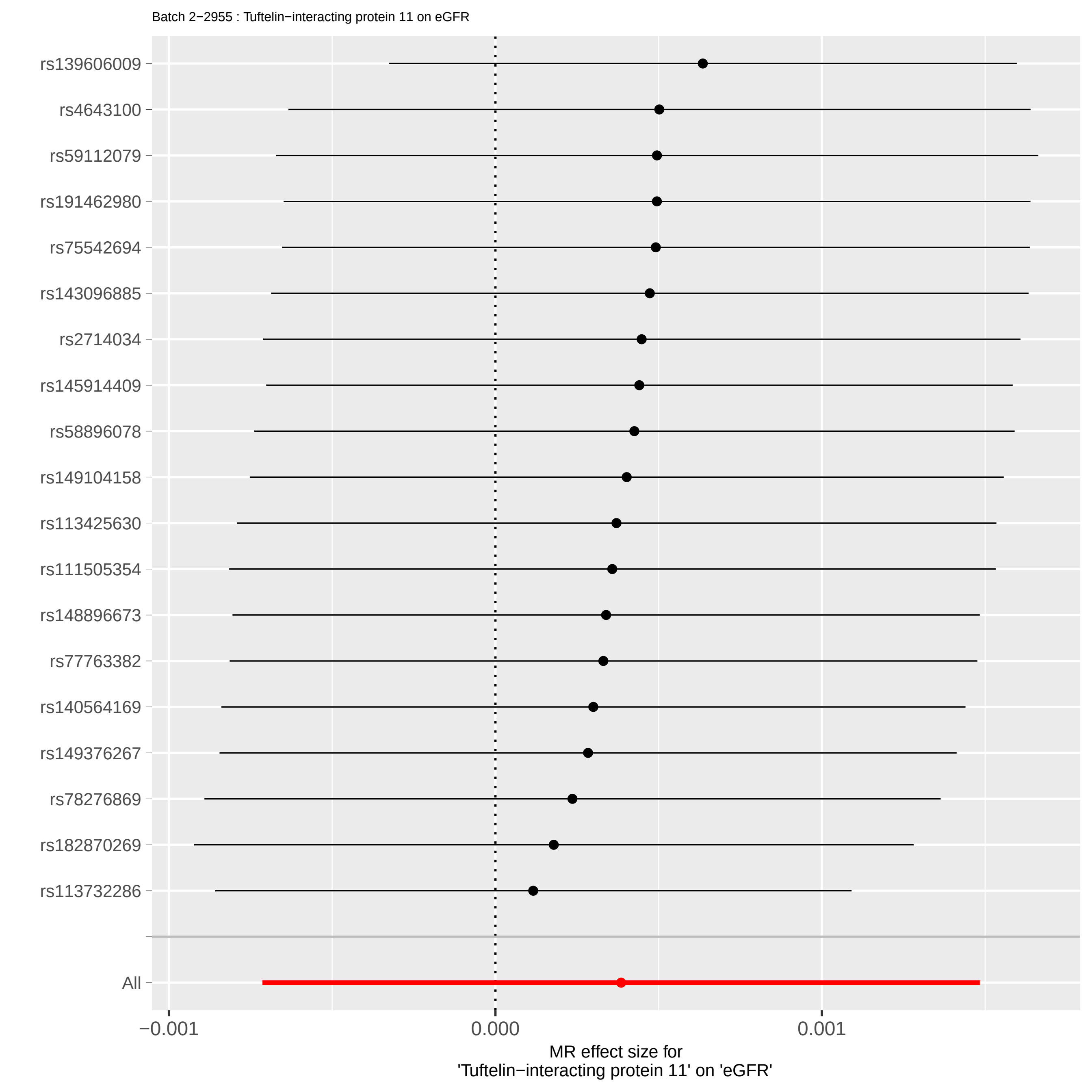

Batch 2−2955 : Tuftelin−interacting protein 11 on eGFR
rs139606009
rs4643100
rs59112079
rs191462980
rs75542694
rs143096885
rs2714034
rs145914409
rs58896078
rs149104158
rs113425630
rs111505354
rs148896673
rs77763382
rs140564169
rs149376267
rs78276869
rs182870269
rs113732286
All
−0.001
0.000
0.001
MR effect size for
'Tuftelin−interacting protein 11' on 'eGFR'

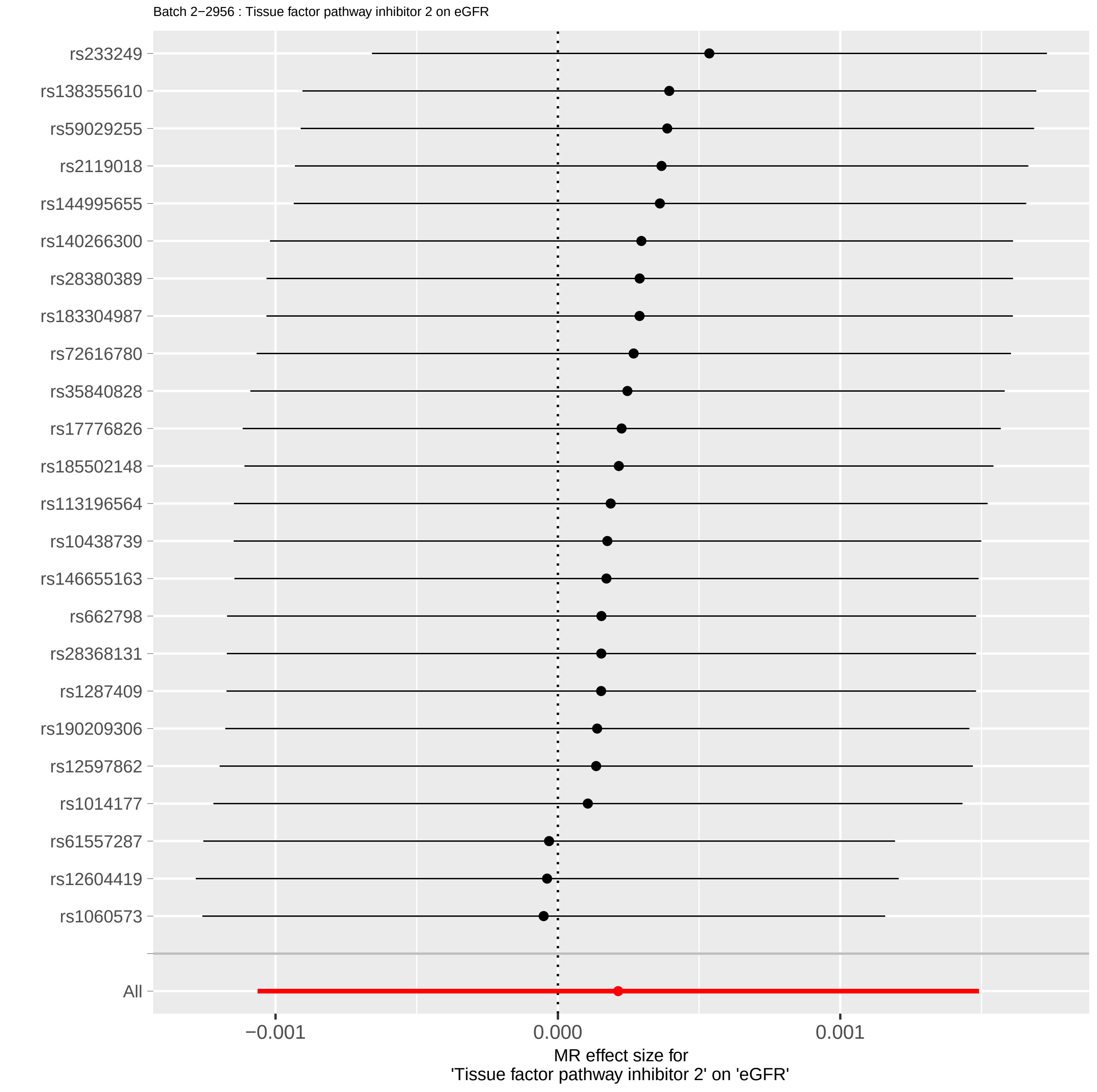

Batch 2−2956 : Tissue factor pathway inhibitor 2 on eGFR
rs233249
rs138355610
rs59029255
rs2119018
rs144995655
rs140266300
rs28380389
rs183304987
rs72616780
rs35840828
rs17776826
rs185502148
rs113196564
rs10438739
rs146655163
rs662798
rs28368131
rs1287409
rs190209306
rs12597862
rs1014177
rs61557287
rs12604419
rs1060573
All
−0.001
0.000
0.001
MR effect size for
'Tissue factor pathway inhibitor 2' on 'eGFR'

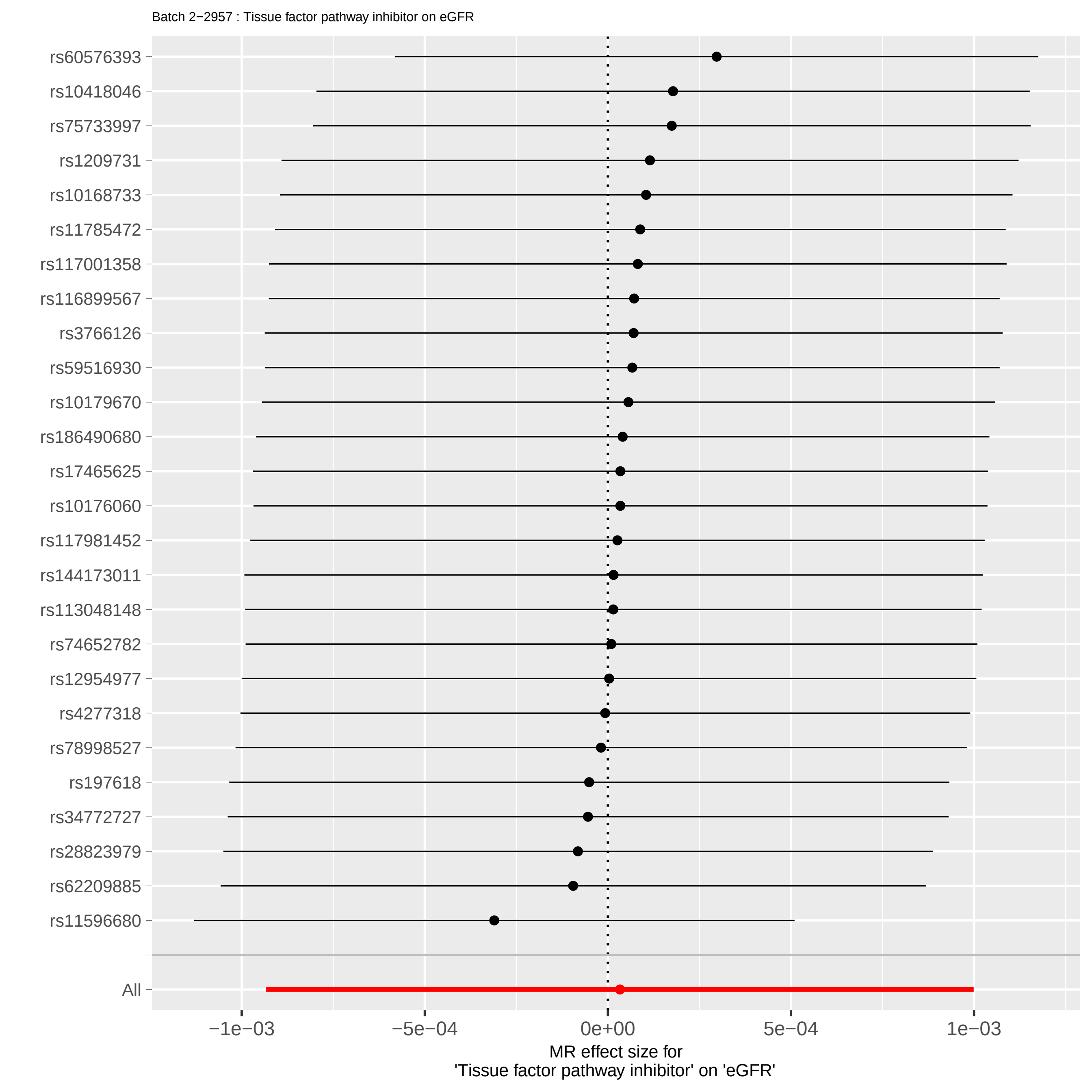
Batch 2−2957 : Tissue factor pathway inhibitor on eGFR
rs60576393
rs10418046
rs75733997
rs1209731
rs10168733
rs11785472
rs117001358
rs116899567
rs3766126
rs59516930
rs10179670
rs186490680
rs17465625
rs10176060
rs117981452
rs144173011
rs113048148
rs74652782
rs12954977
rs4277318
rs78998527
rs197618
rs34772727
rs28823979
rs62209885
rs11596680
All
−1e−03
−5e−04
0e+00
5e−04
1e−03
MR effect size for
'Tissue factor pathway inhibitor' on 'eGFR'

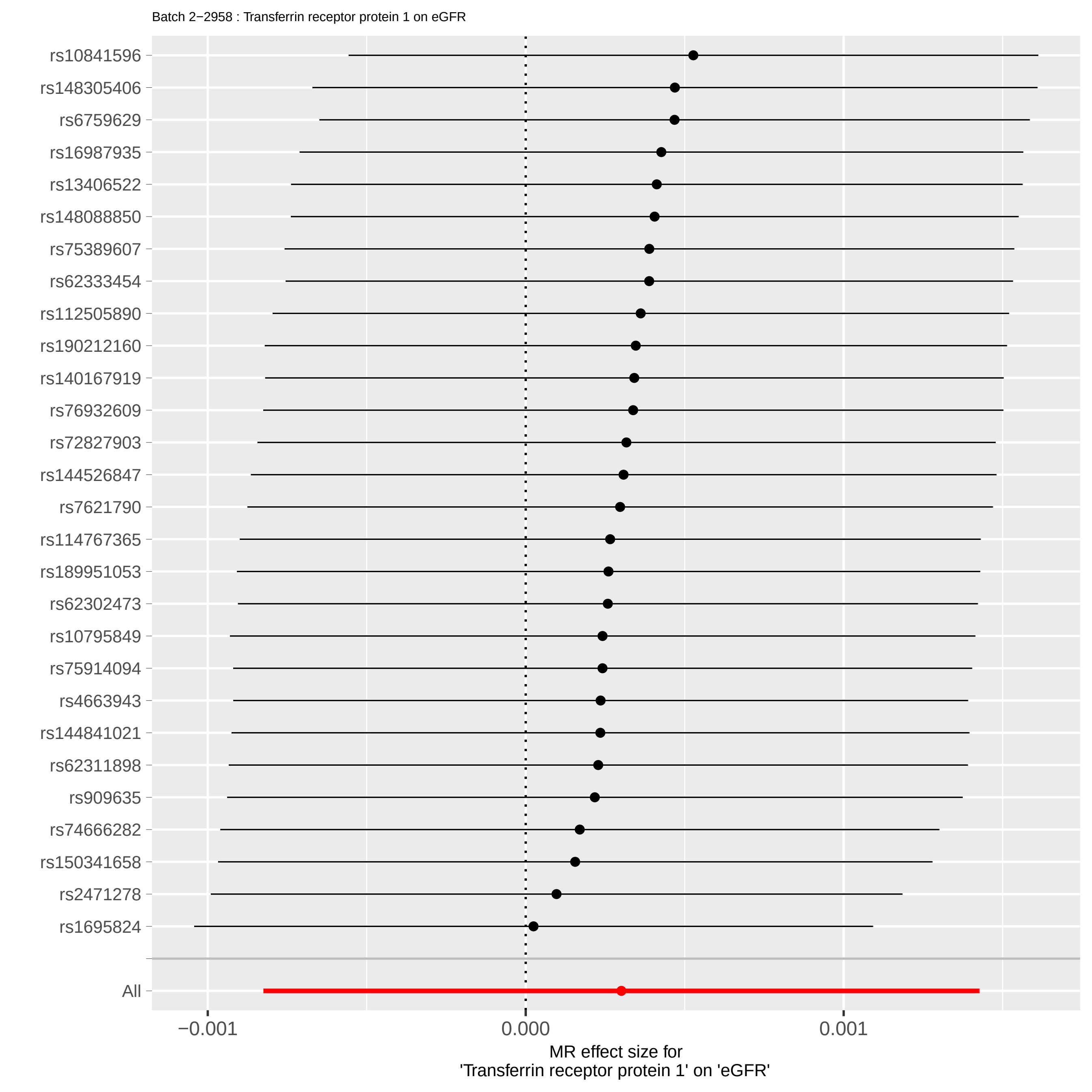

Batch 2−2958 : Transferrin receptor protein 1 on eGFR
rs10841596
rs148305406
rs6759629
rs16987935
rs13406522
rs148088850
rs75389607
rs62333454
rs112505890
rs190212160
rs140167919
rs76932609
rs72827903
rs144526847
rs7621790
rs114767365
rs189951053
rs62302473
rs10795849
rs75914094
rs4663943
rs144841021
rs62311898
rs909635
rs74666282
rs150341658
rs2471278
rs1695824
All
−0.001
0.000
0.001
MR effect size for
'Transferrin receptor protein 1' on 'eGFR'

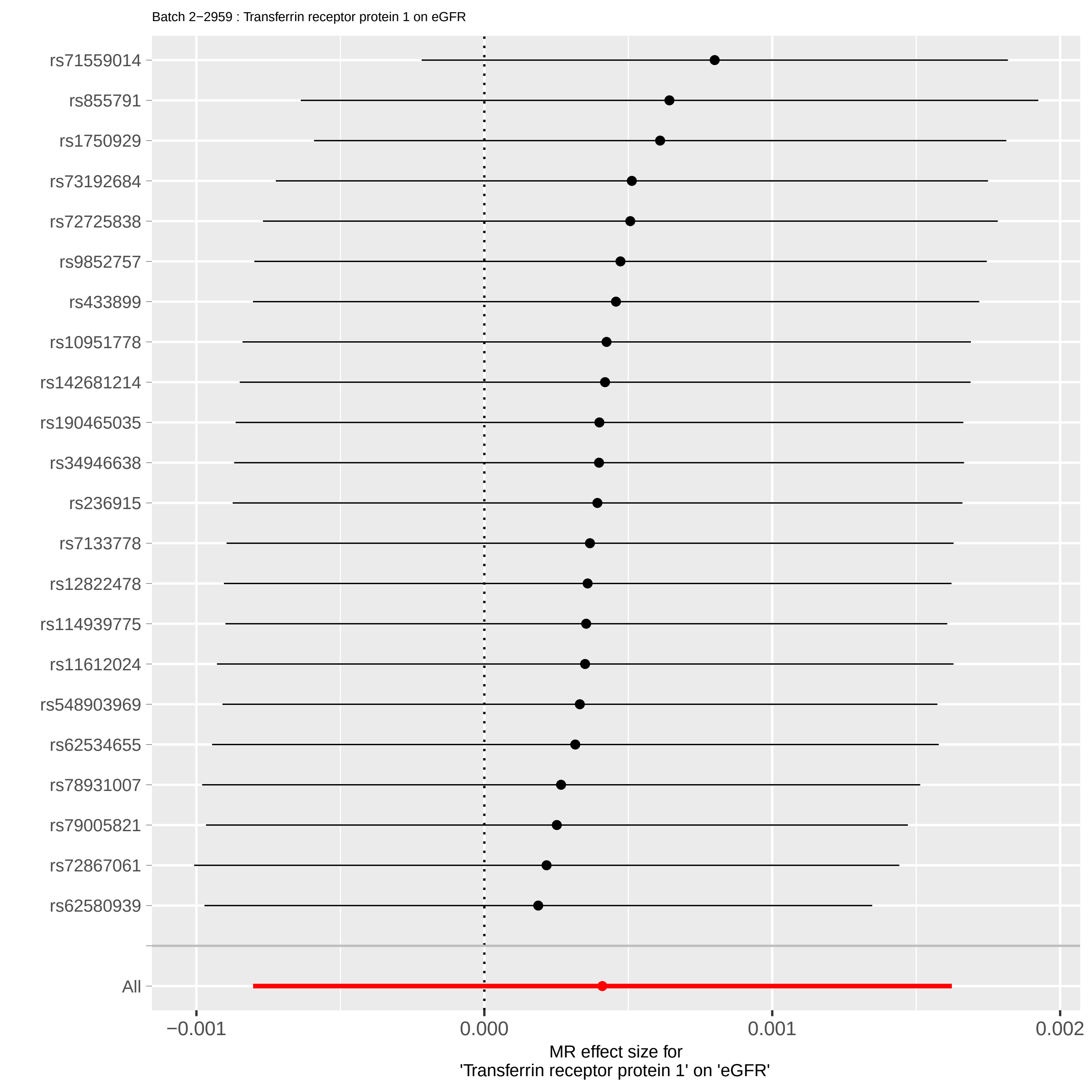

Batch 2−2959 : Transferrin receptor protein 1 on eGFR
rs71559014
rs855791
rs1750929
rs73192684
rs72725838
rs9852757
rs433899
rs10951778
rs142681214
rs190465035
rs34946638
rs236915
rs7133778
rs12822478
rs114939775
rs11612024
rs548903969
rs62534655
rs78931007
rs79005821
rs72867061
rs62580939
All
−0.001
0.000
0.001
0.002
MR effect size for
'Transferrin receptor protein 1' on 'eGFR'

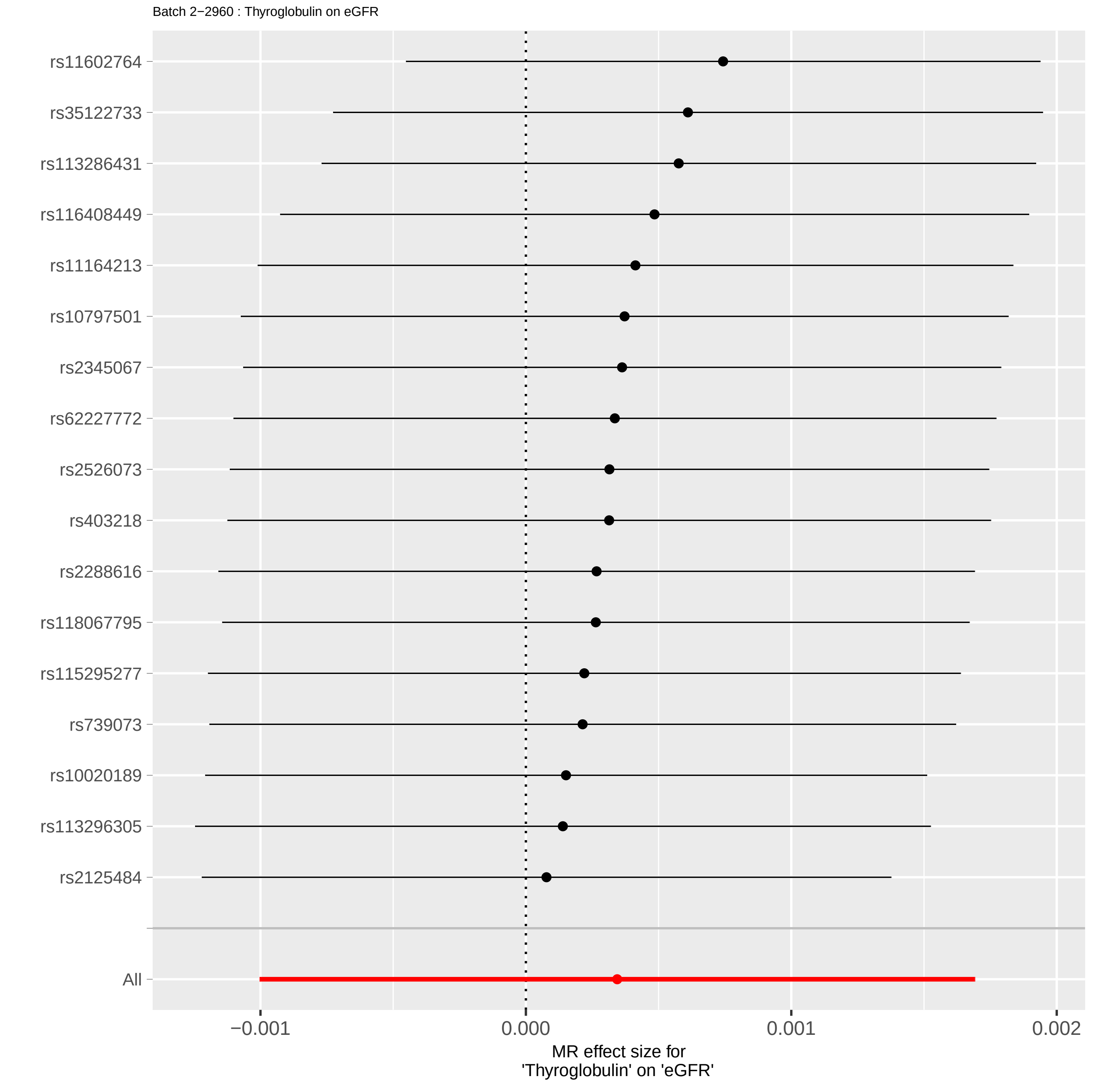
Batch 2−2960 : Thyroglobulin on eGFR
rs11602764
rs35122733
rs113286431
rs116408449
rs11164213
rs10797501
rs2345067
rs62227772
rs2526073
rs403218
rs2288616
rs118067795
rs115295277
rs739073
rs10020189
rs113296305
rs2125484
All
−0.001
0.000
0.001
0.002
MR effect size for
'Thyroglobulin' on 'eGFR'

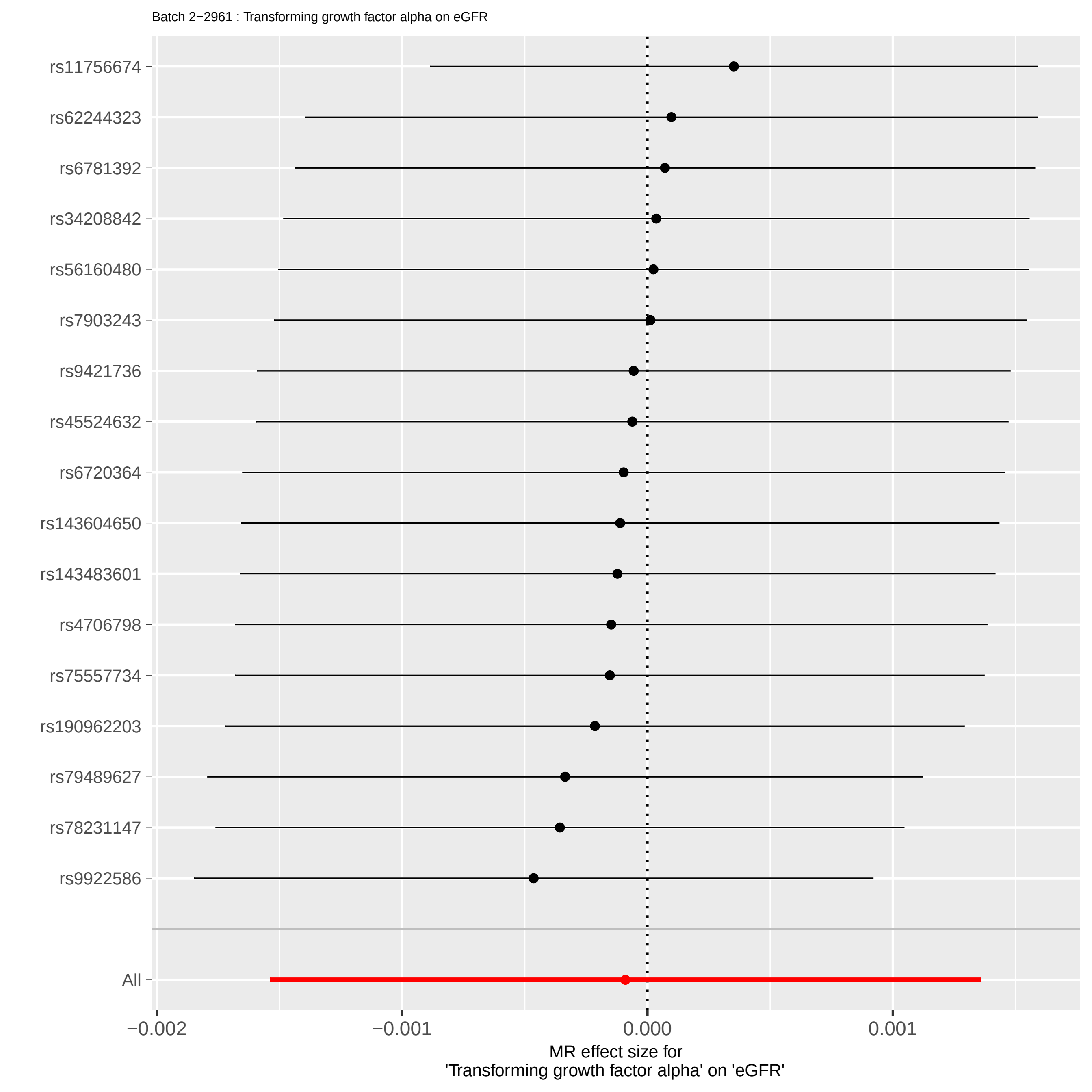
Batch 2−2961 : Transforming growth factor alpha on eGFR
rs11756674
rs62244323
rs6781392
rs34208842
rs56160480
rs7903243
rs9421736
rs45524632
rs6720364
rs143604650
rs143483601
rs4706798
rs75557734
rs190962203
rs79489627
rs78231147
rs9922586
All
−0.002
−0.001
0.000
0.001
MR effect size for
'Transforming growth factor alpha' on 'eGFR'

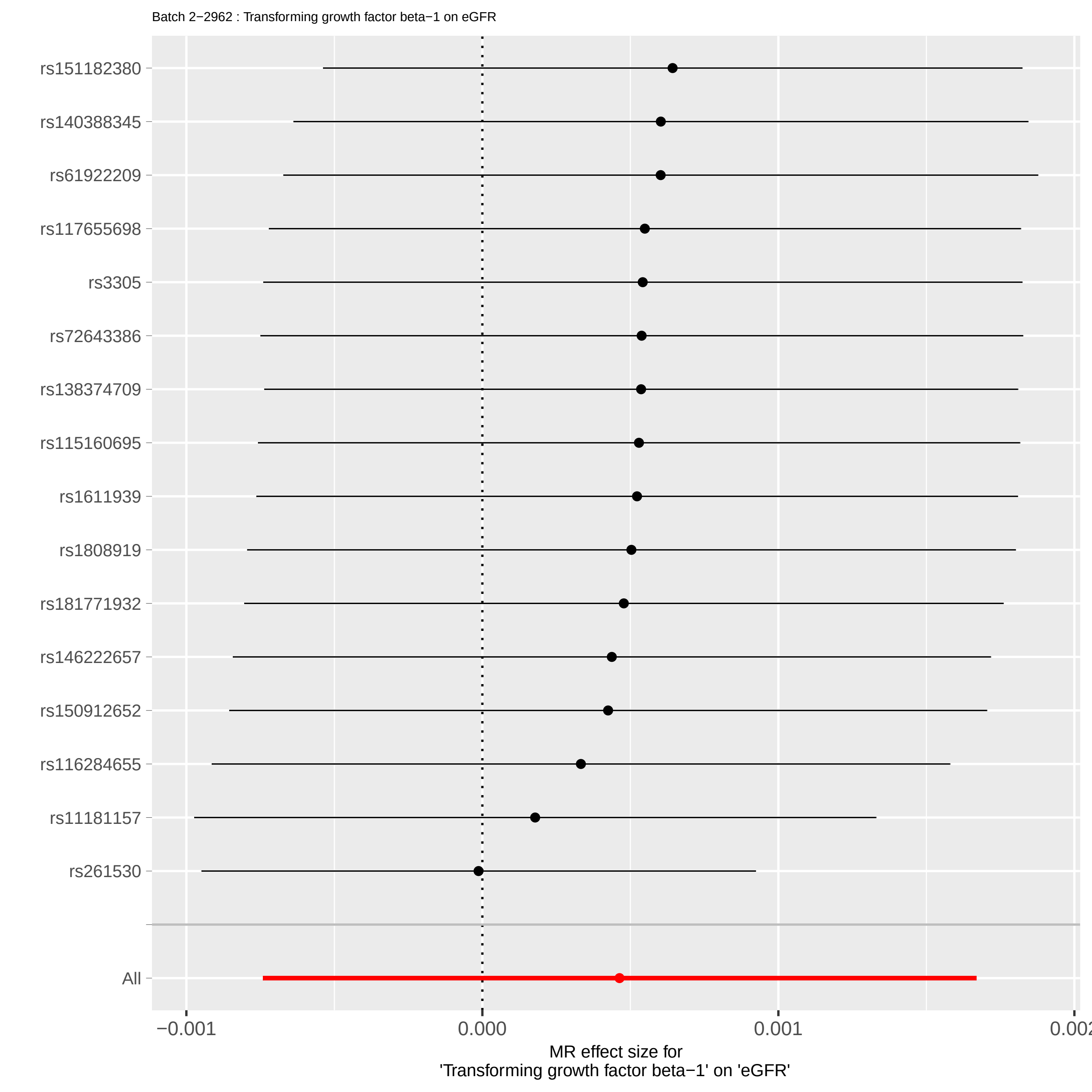

Batch 2−2962 : Transforming growth factor beta−1 on eGFR
rs151182380
rs140388345
rs61922209
rs117655698
rs3305
rs72643386
rs138374709
rs115160695
rs1611939
rs1808919
rs181771932
rs146222657
rs150912652
rs116284655
rs11181157
rs261530
All
−0.001
0.000
0.001
MR effect size for
'Transforming growth factor beta−1' on 'eGFR'

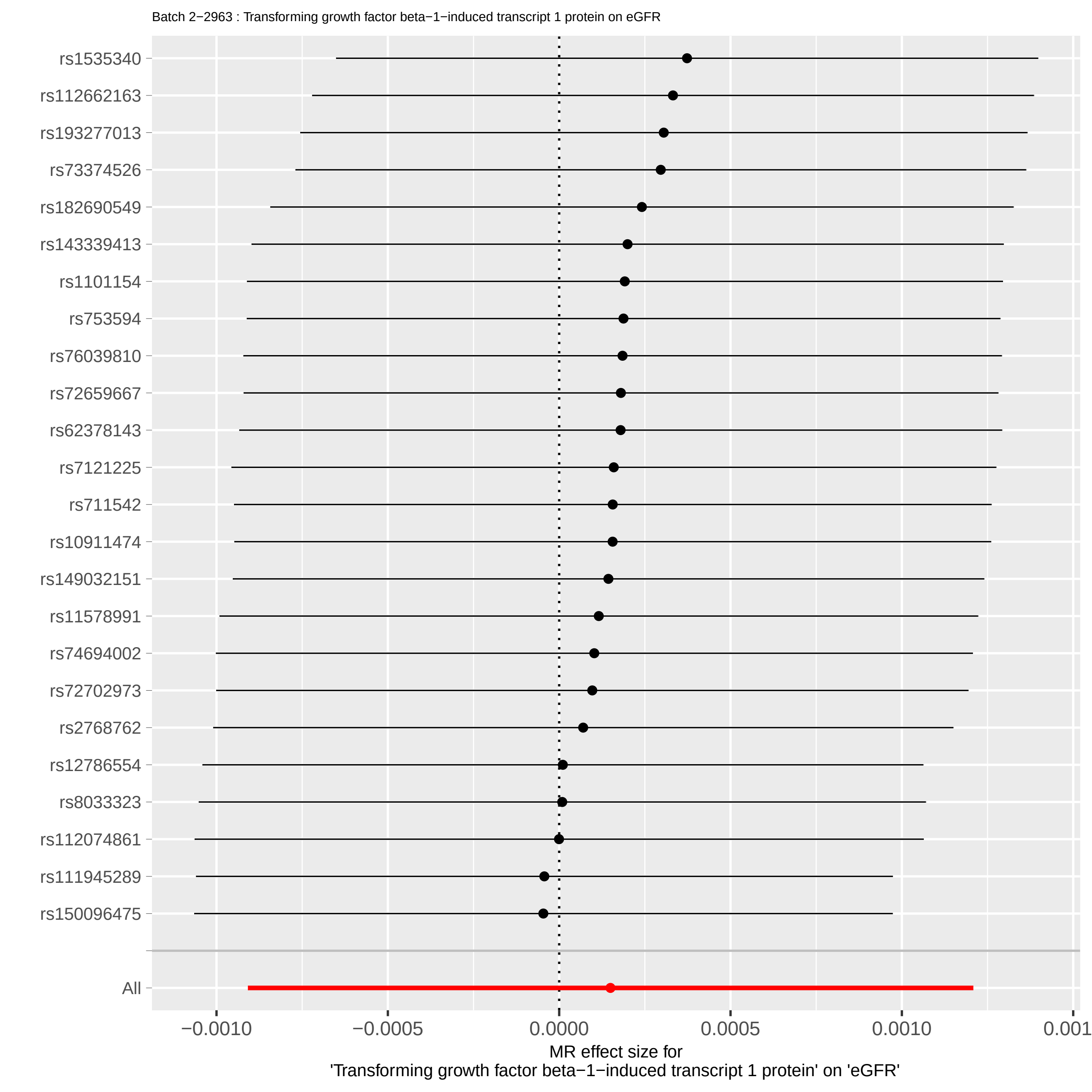

Batch 2−2963 : Transforming growth factor beta−1−induced transcript 1 protein on eGFR
rs1535340
rs112662163
rs193277013
rs73374526
rs182690549
rs143339413
rs1101154
rs753594
rs76039810
rs72659667
rs62378143
rs7121225
rs711542
rs10911474
rs149032151
rs11578991
rs74694002
rs72702973
rs2768762
rs12786554
rs8033323
rs112074861
rs111945289
rs150096475
All
−0.0010
−0.0005
0.0000
0.0005
0.0010
0.001
MR effect size for
'Transforming growth factor beta−1−induced transcript 1 protein' on 'eGFR'

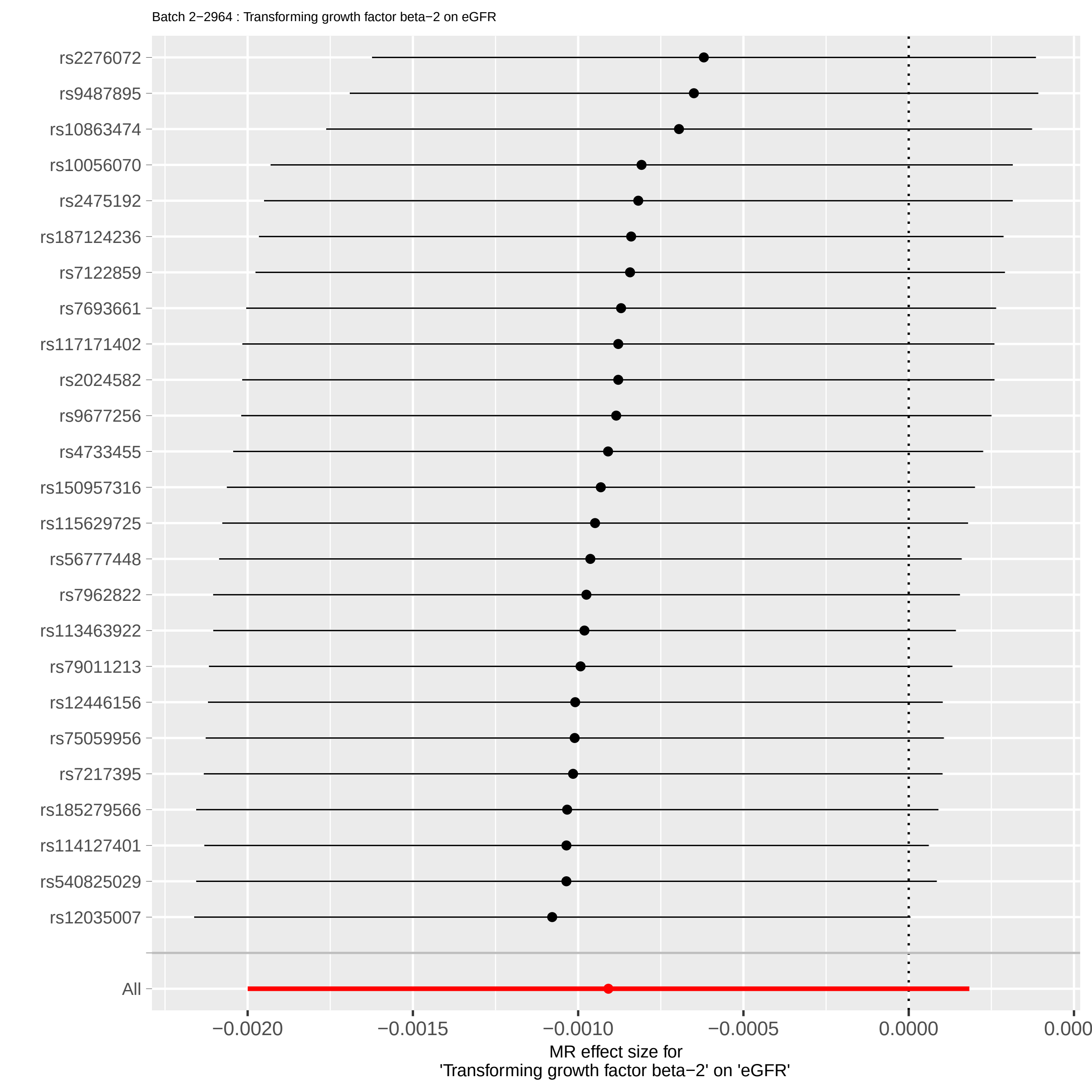
Batch 2−2964 : Transforming growth factor beta−2 on eGFR
rs2276072
rs9487895
rs10863474
rs10056070
rs2475192
rs187124236
rs7122859
rs7693661
rs117171402
rs2024582
rs9677256
rs4733455
rs150957316
rs115629725
rs56777448
rs7962822
rs113463922
rs79011213
rs12446156
rs75059956
rs7217395
rs185279566
rs114127401
rs540825029
rs12035007
All
−0.0020
−0.0015
−0.0010
−0.0005
0.0000
0.000
MR effect size for
'Transforming growth factor beta−2' on 'eGFR'

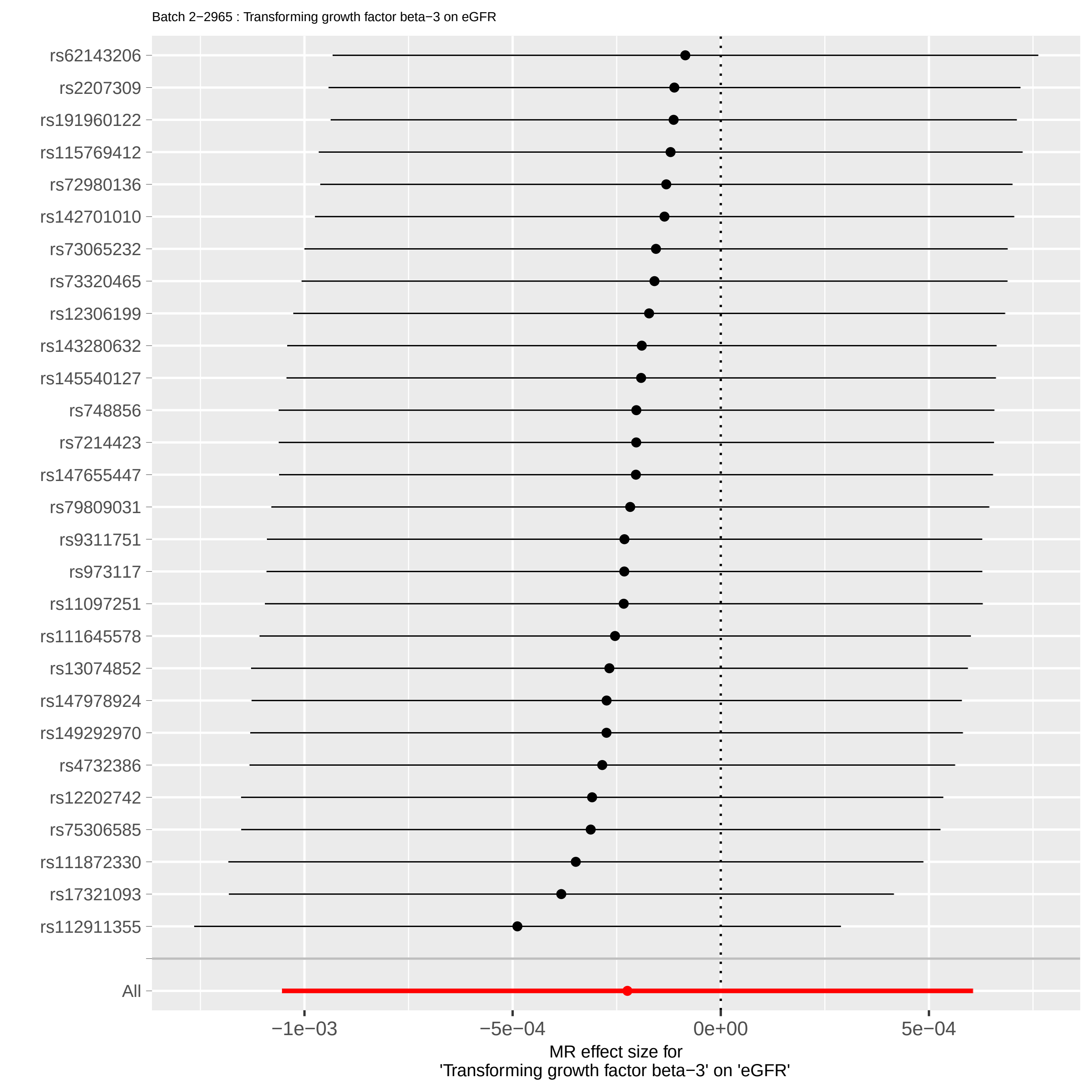

Batch 2−2965 : Transforming growth factor beta−3 on eGFR
rs62143206
rs2207309
rs191960122
rs115769412
rs72980136
rs142701010
rs73065232
rs73320465
rs12306199
rs143280632
rs145540127
rs748856
rs7214423
rs147655447
rs79809031
rs9311751
rs973117
rs11097251
rs111645578
rs13074852
rs147978924
rs149292970
rs4732386
rs12202742
rs75306585
rs111872330
rs17321093
rs112911355
All
−1e−03
−5e−04
0e+00
5e−04
MR effect size for
'Transforming growth factor beta−3' on 'eGFR'

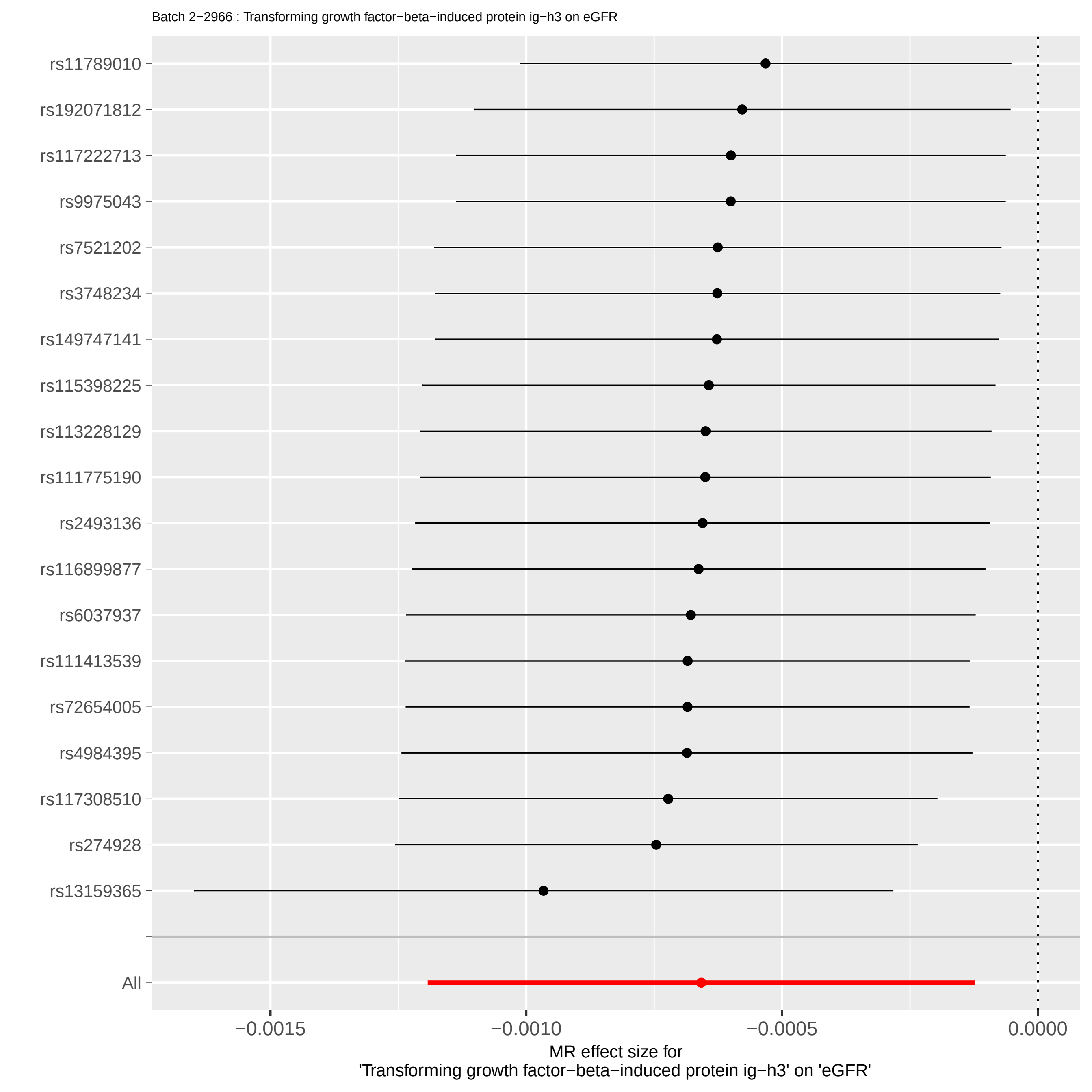

Batch 2−2966 : Transforming growth factor−beta−induced protein ig−h3 on eGFR
rs11789010
rs192071812
rs117222713
rs9975043
rs7521202
rs3748234
rs149747141
rs115398225
rs113228129
rs111775190
rs2493136
rs116899877
rs6037937
rs111413539
rs72654005
rs4984395
rs117308510
rs274928
rs13159365
All
−0.0015
−0.0010
−0.0005
0.0000
MR effect size for
'Transforming growth factor−beta−induced protein ig−h3' on 'eGFR'

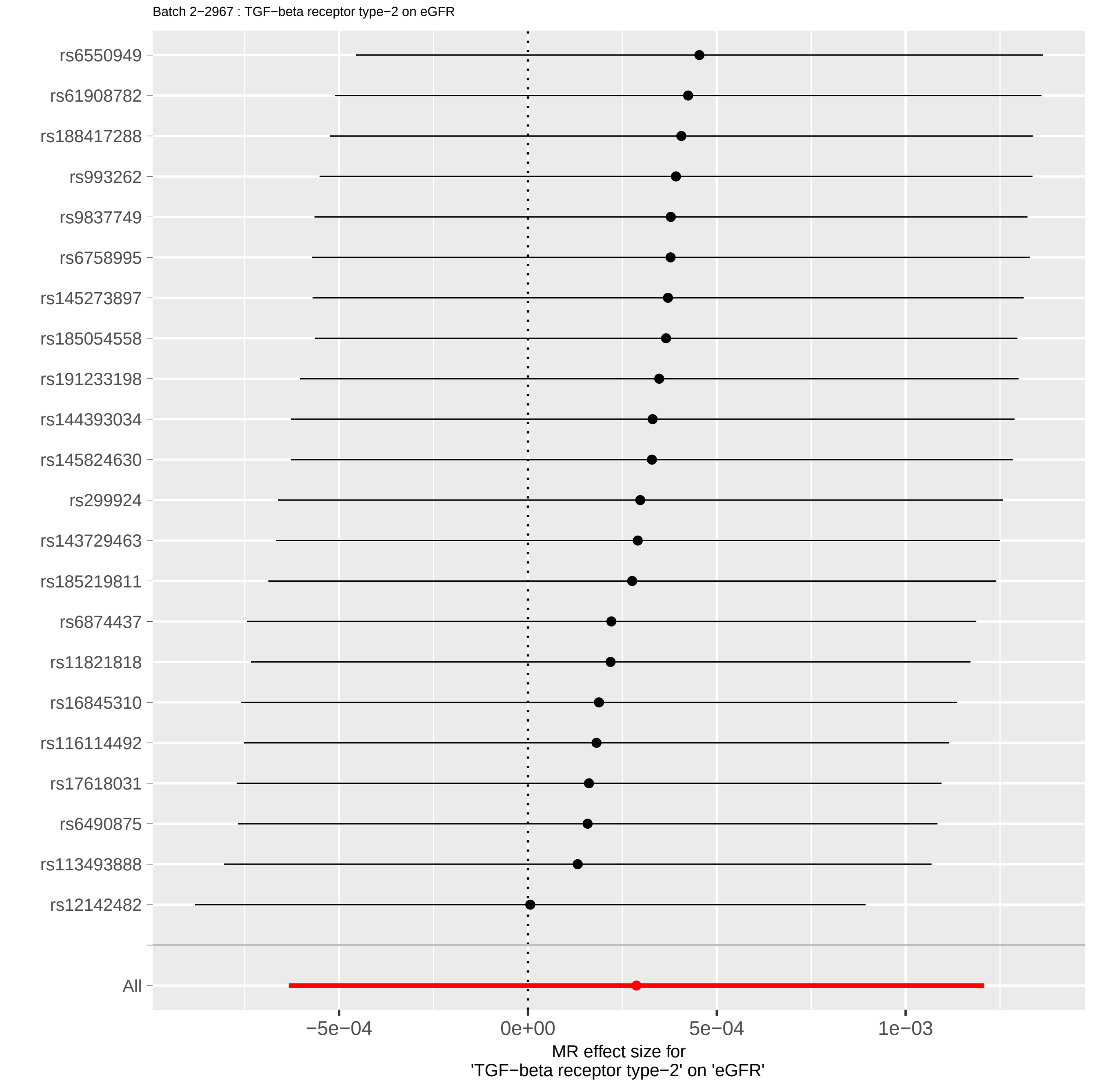
Batch 2−2967 : TGF−beta receptor type−2 on eGFR
rs6550949
rs61908782
rs188417288
rs993262
rs9837749
rs6758995
rs145273897
rs185054558
rs191233198
rs144393034
rs145824630
rs299924
rs143729463
rs185219811
rs6874437
rs11821818
rs16845310
rs116114492
rs17618031
rs6490875
rs113493888
rs12142482
All
−5e−04
0e+00
5e−04
1e−03
MR effect size for
'TGF−beta receptor type−2' on 'eGFR'

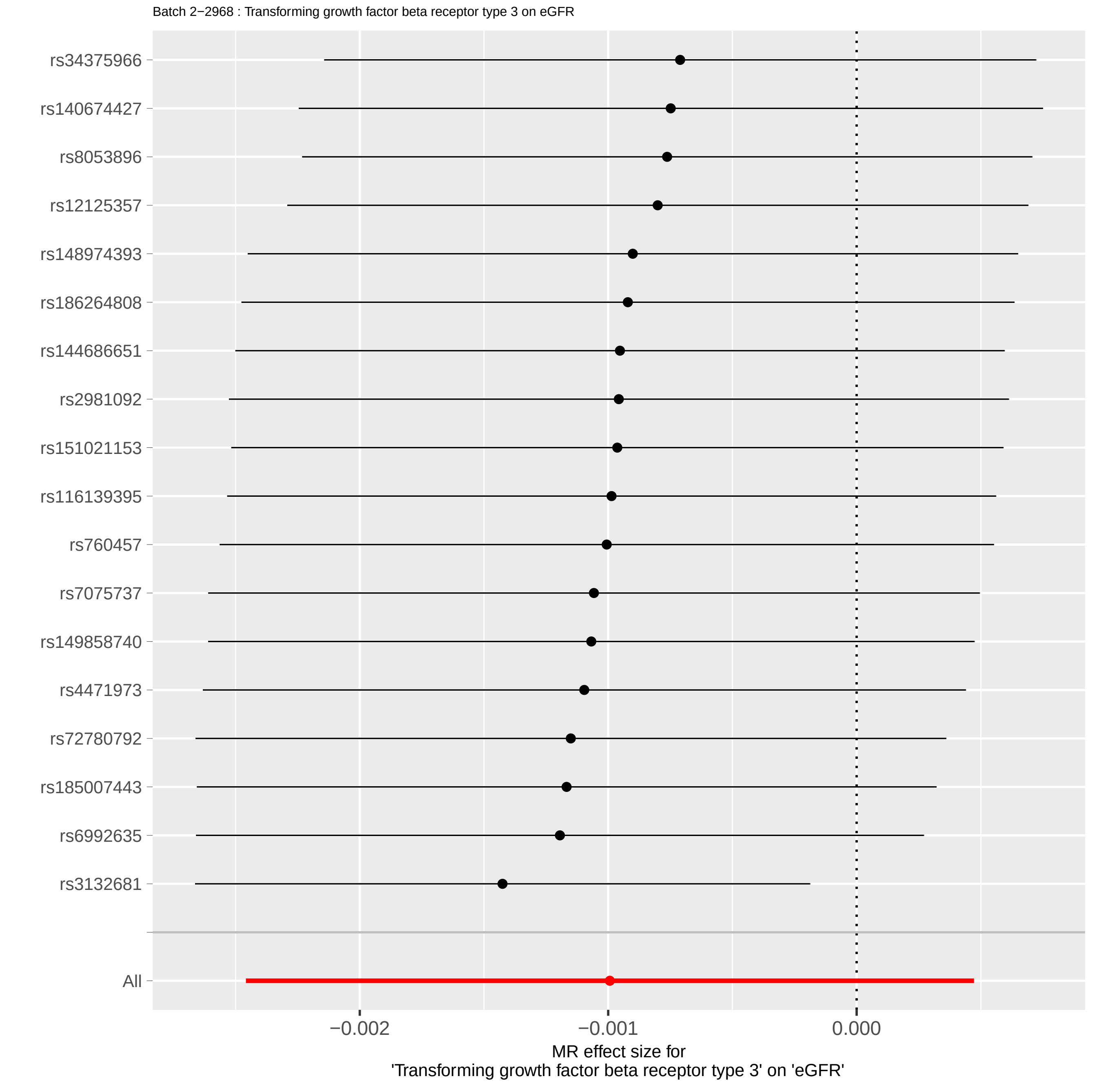

Batch 2−2968 : Transforming growth factor beta receptor type 3 on eGFR
rs34375966
rs140674427
rs8053896
rs12125357
rs148974393
rs186264808
rs144686651
rs2981092
rs151021153
rs116139395
rs760457
rs7075737
rs149858740
rs4471973
rs72780792
rs185007443
rs6992635
rs3132681
All
−0.002
−0.001
0.000
MR effect size for
'Transforming growth factor beta receptor type 3' on 'eGFR'

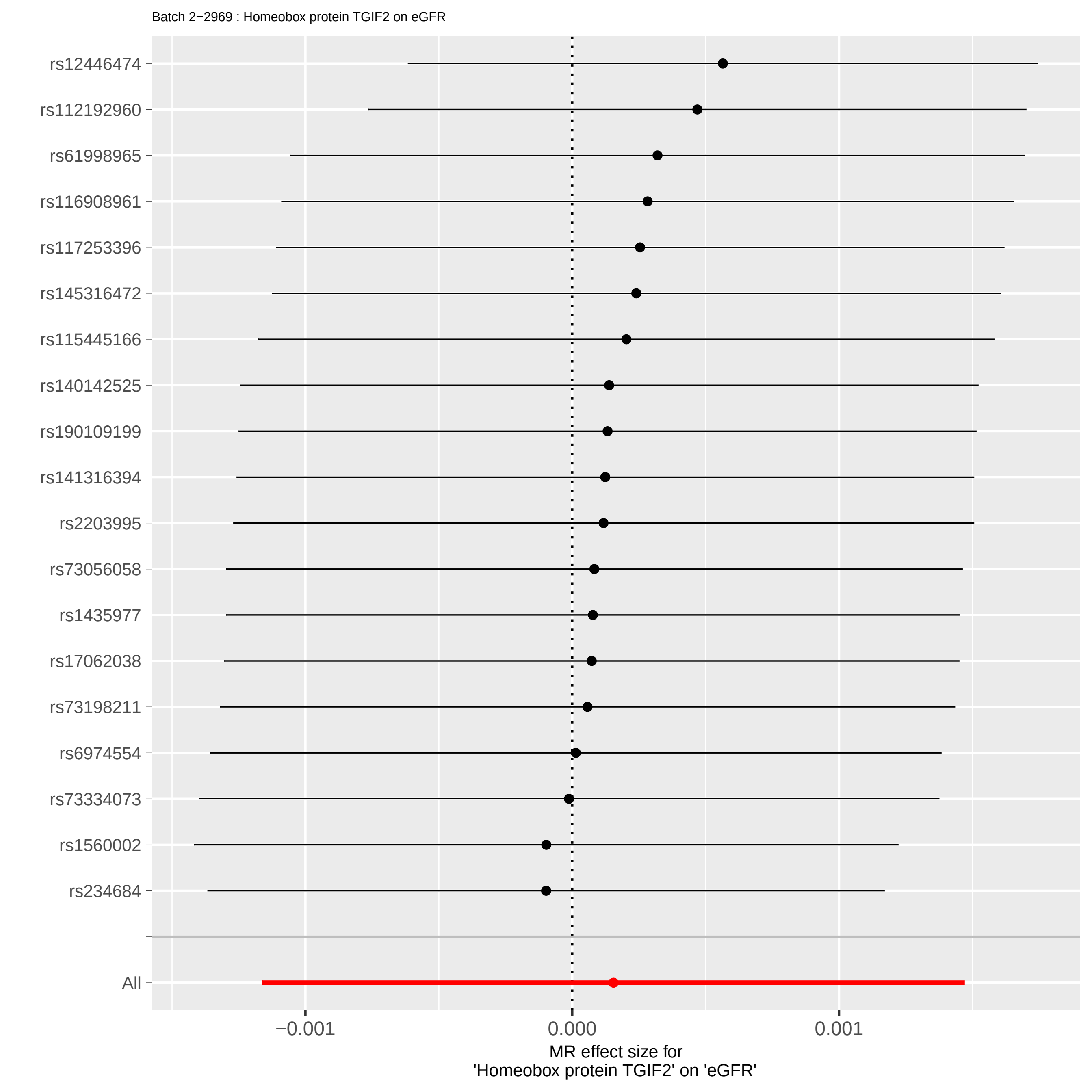

Batch 2−2969 : Homeobox protein TGIF2 on eGFR
rs12446474
rs112192960
rs61998965
rs116908961
rs117253396
rs145316472
rs115445166
rs140142525
rs190109199
rs141316394
rs2203995
rs73056058
rs1435977
rs17062038
rs73198211
rs6974554
rs73334073
rs1560002
rs234684
All
−0.001
0.000
0.001
MR effect size for
'Homeobox protein TGIF2' on 'eGFR'

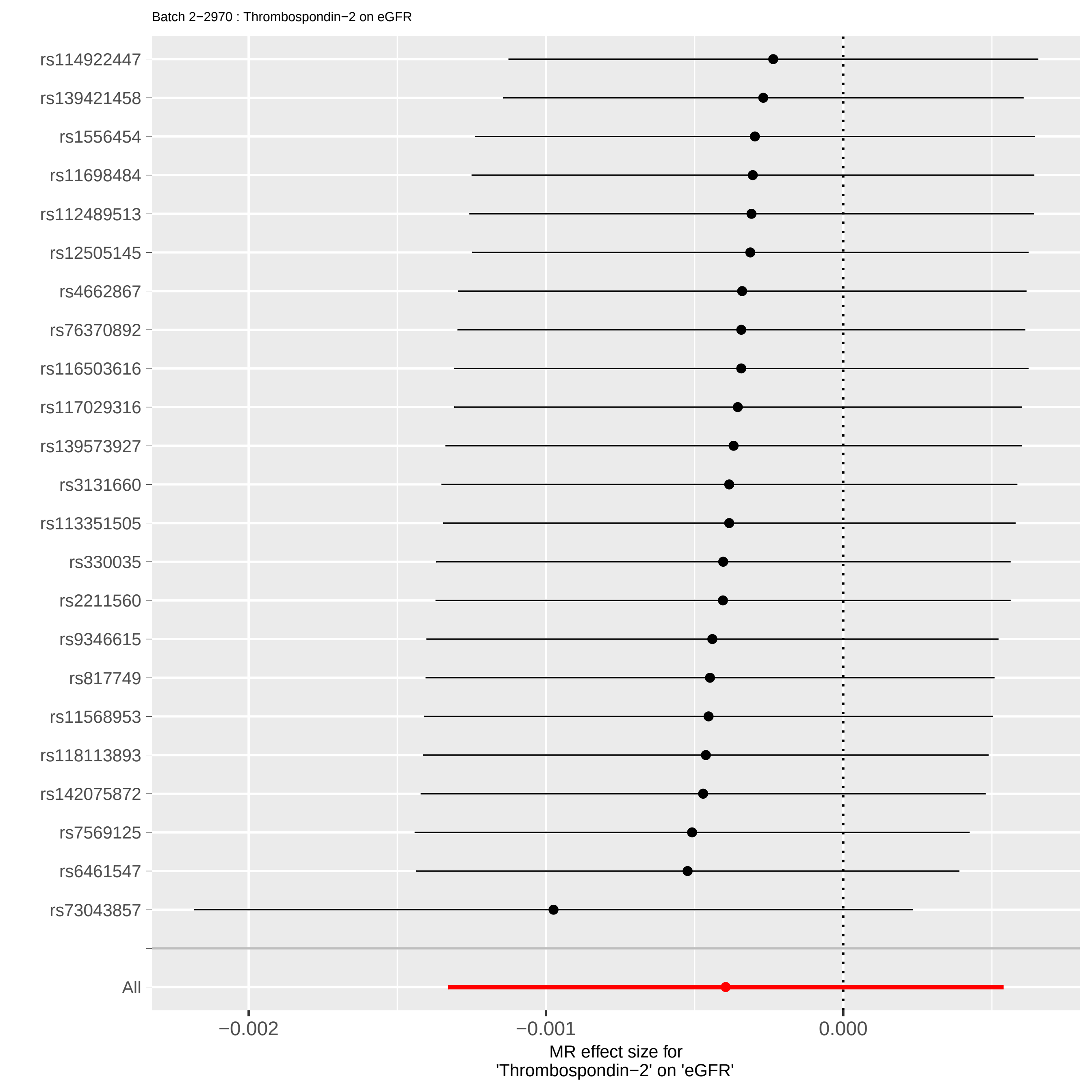

Batch 2−2970 : Thrombospondin−2 on eGFR
rs114922447
rs139421458
rs1556454
rs11698484
rs112489513
rs12505145
rs4662867
rs76370892
rs116503616
rs117029316
rs139573927
rs3131660
rs113351505
rs330035
rs2211560
rs9346615
rs817749
rs11568953
rs118113893
rs142075872
rs7569125
rs6461547
rs73043857
All
−0.002
−0.001
0.000
MR effect size for
'Thrombospondin−2' on 'eGFR'

Batch 2−2971 : Thrombospondin−3 on eGFR

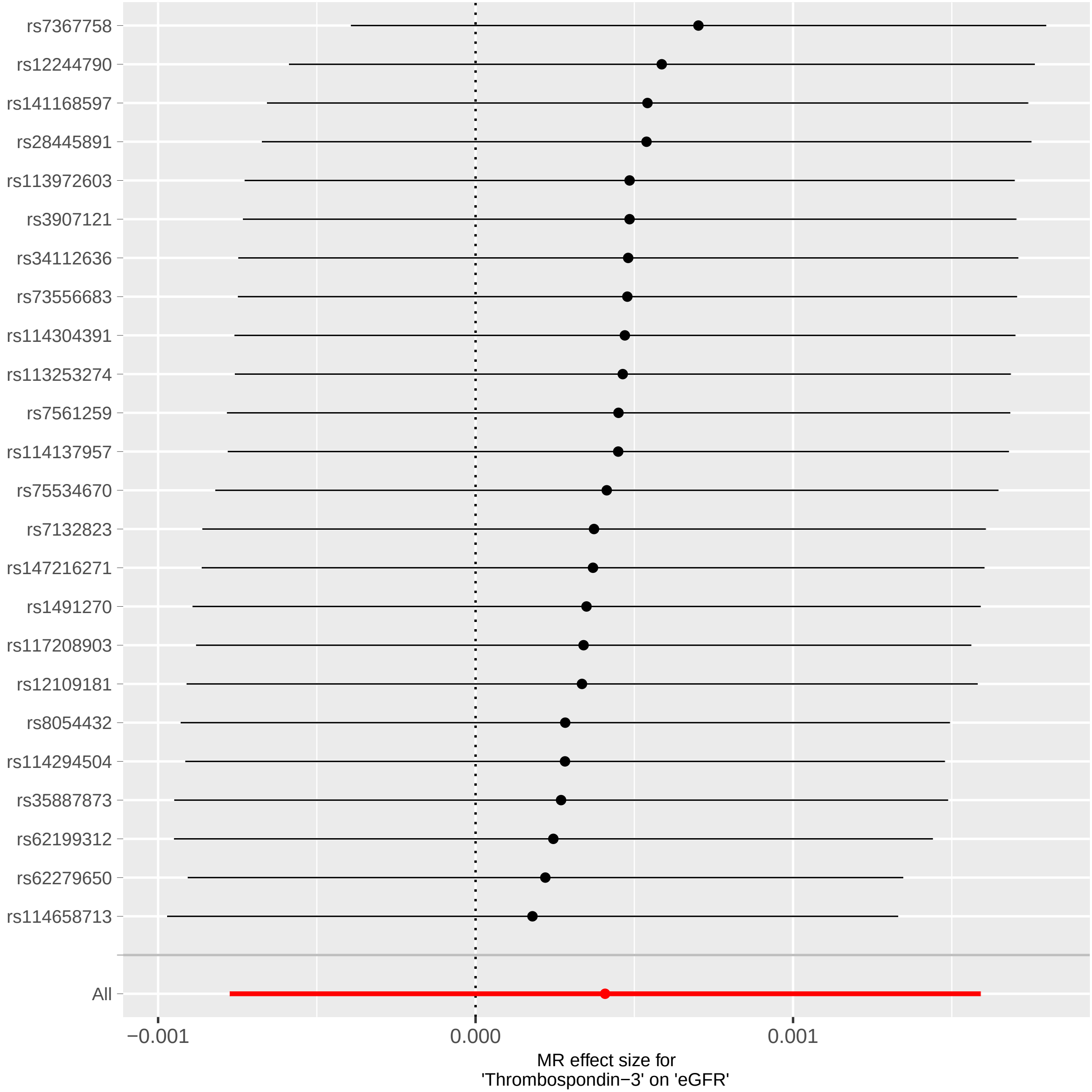

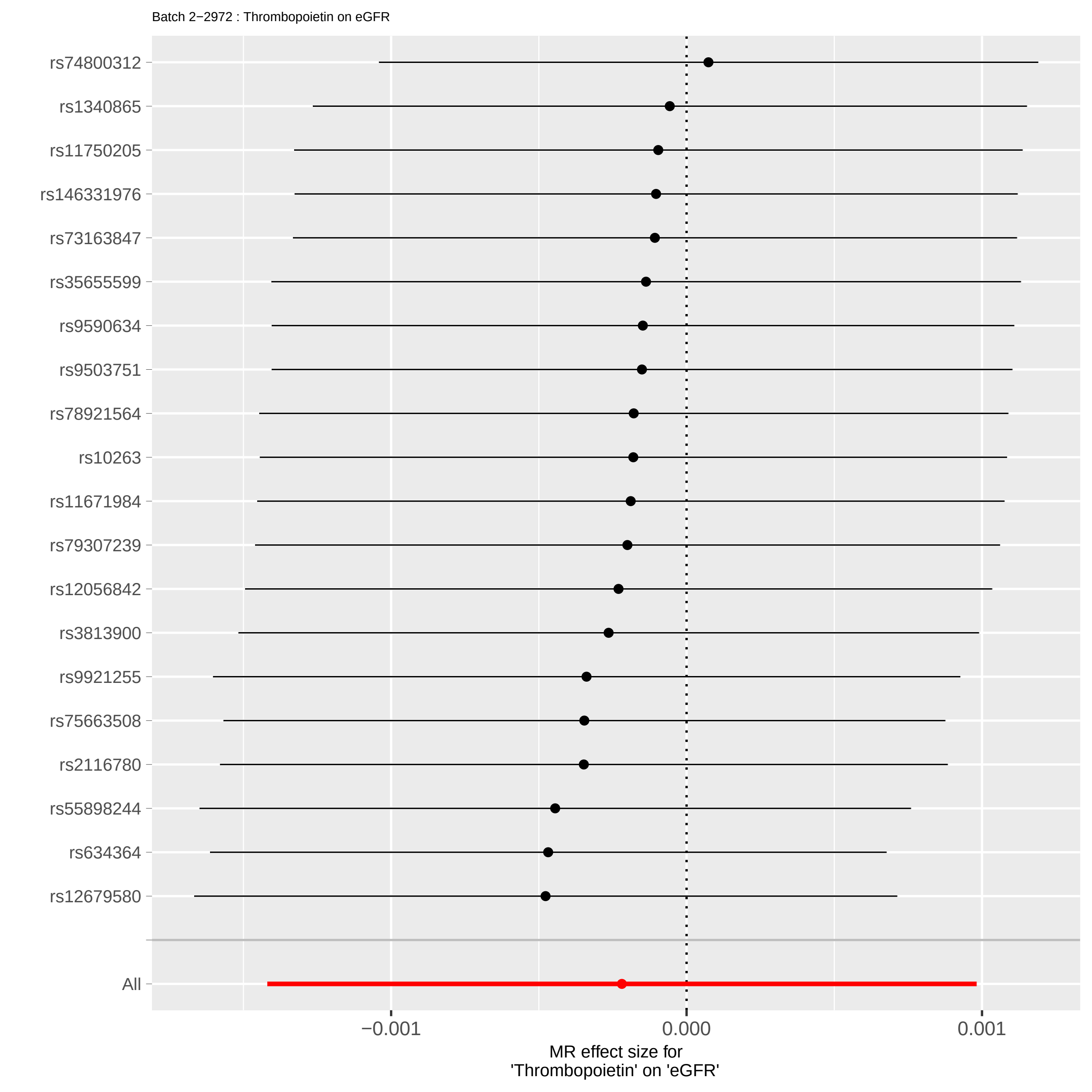

Batch 2−2972 : Thrombopoietin on eGFR
rs74800312
rs1340865
rs11750205
rs146331976
rs73163847
rs35655599
rs9590634
rs9503751
rs78921564
rs10263
rs11671984
rs79307239
rs12056842
rs3813900
rs9921255
rs75663508
rs2116780
rs55898244
rs634364
rs12679580
All
−0.001
0.000
0.001
MR effect size for
'Thrombopoietin' on 'eGFR'

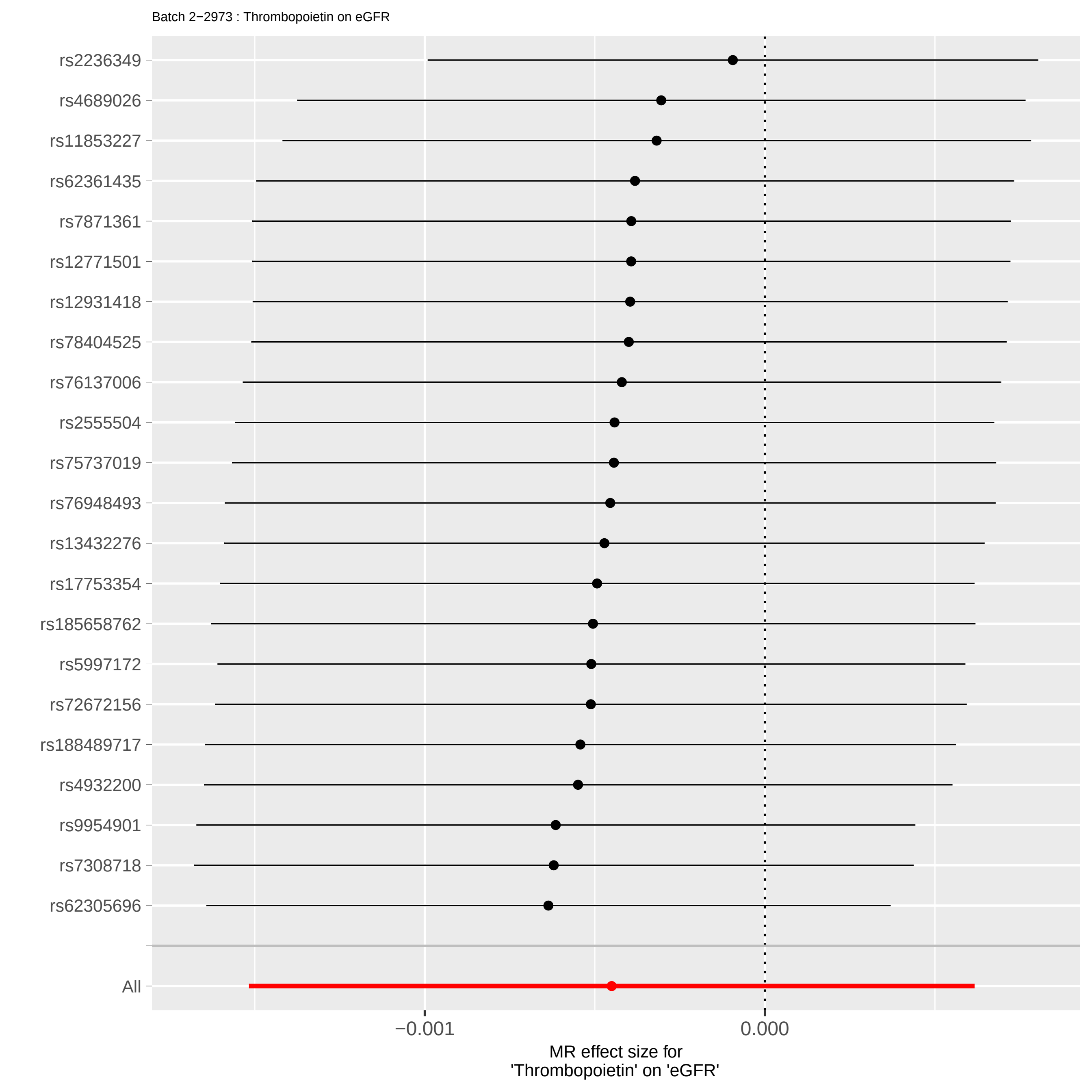

Batch 2−2973 : Thrombopoietin on eGFR
rs2236349
rs4689026
rs11853227
rs62361435
rs7871361
rs12771501
rs12931418
rs78404525
rs76137006
rs2555504
rs75737019
rs76948493
rs13432276
rs17753354
rs185658762
rs5997172
rs72672156
rs188489717
rs4932200
rs9954901
rs7308718
rs62305696
All
−0.001
0.000
MR effect size for
'Thrombopoietin' on 'eGFR'

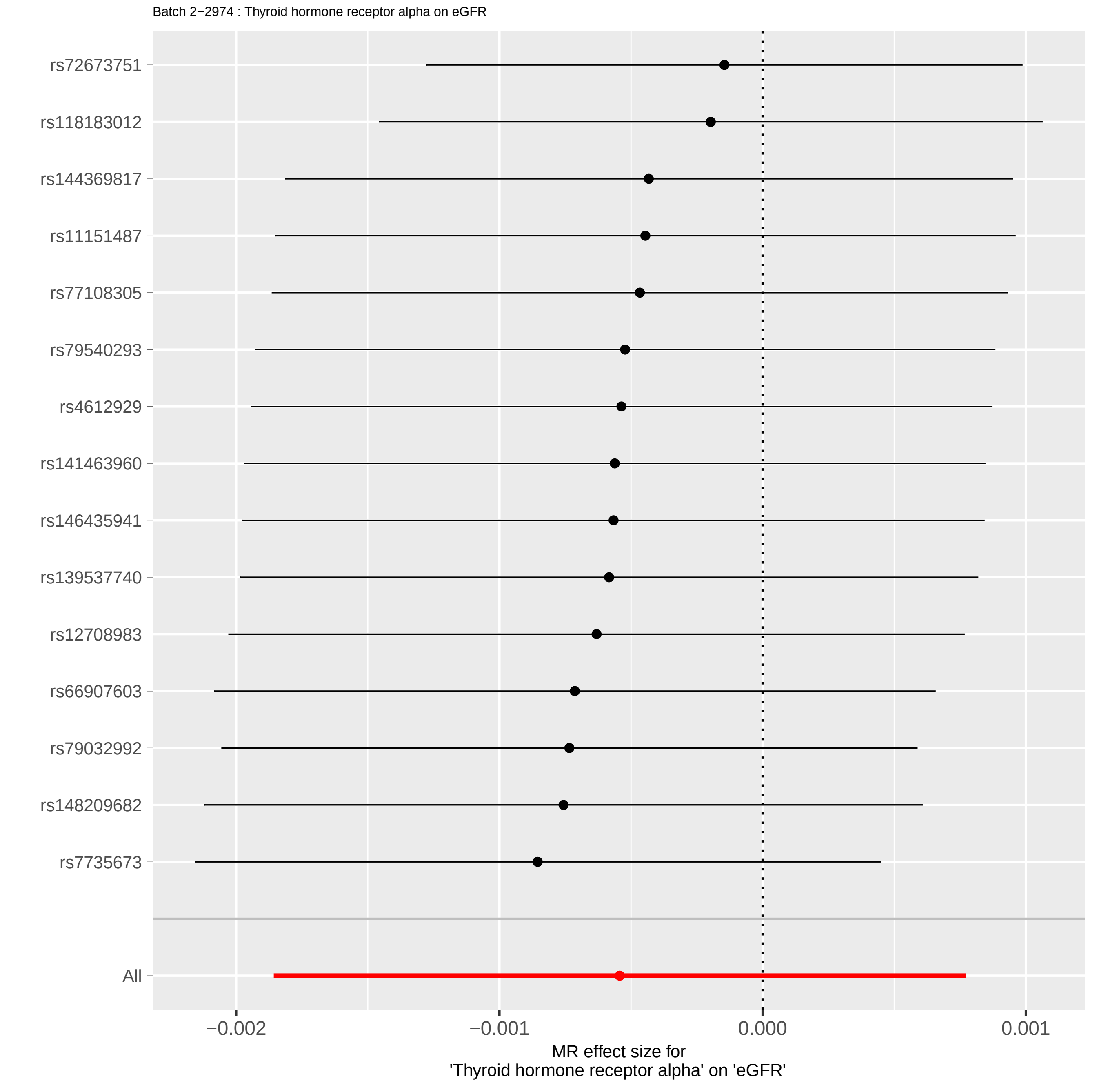

Batch 2−2974 : Thyroid hormone receptor alpha on eGFR
rs72673751
rs118183012
rs144369817
rs11151487
rs77108305
rs79540293
rs4612929
rs141463960
rs146435941
rs139537740
rs12708983
rs66907603
rs79032992
rs148209682
rs7735673
All
−0.002
−0.001
0.000
0.001
MR effect size for
'Thyroid hormone receptor alpha' on 'eGFR'

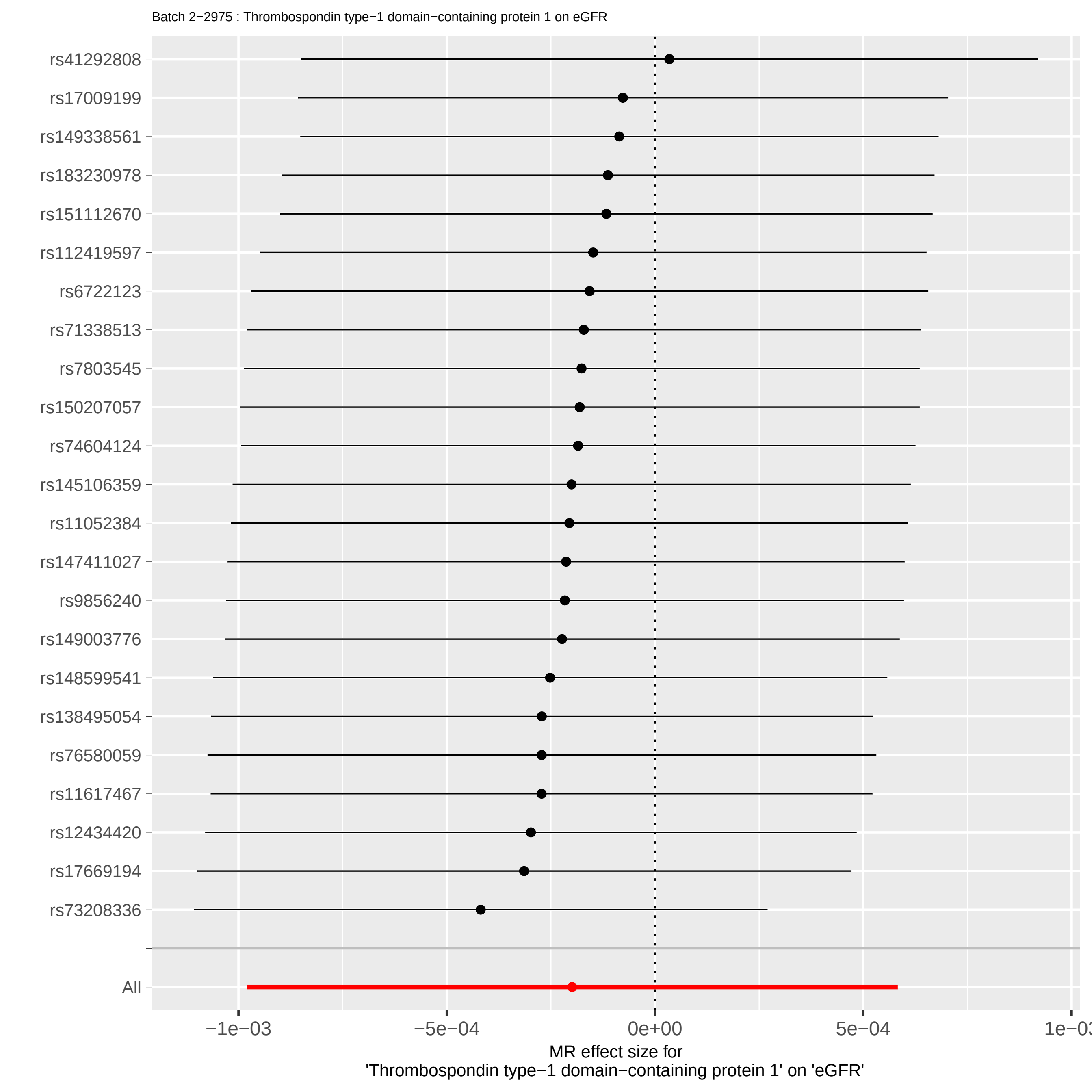
Batch 2−2975 : Thrombospondin type−1 domain−containing protein 1 on eGFR
rs41292808
rs17009199
rs149338561
rs183230978
rs151112670
rs112419597
rs6722123
rs71338513
rs7803545
rs150207057
rs74604124
rs145106359
rs11052384
rs147411027
rs9856240
rs149003776
rs148599541
rs138495054
rs76580059
rs11617467
rs12434420
rs17669194
rs73208336
All
−1e−03
−5e−04
0e+00
5e−04
1e−03
MR effect size for
'Thrombospondin type−1 domain−containing protein 1' on 'eGFR'

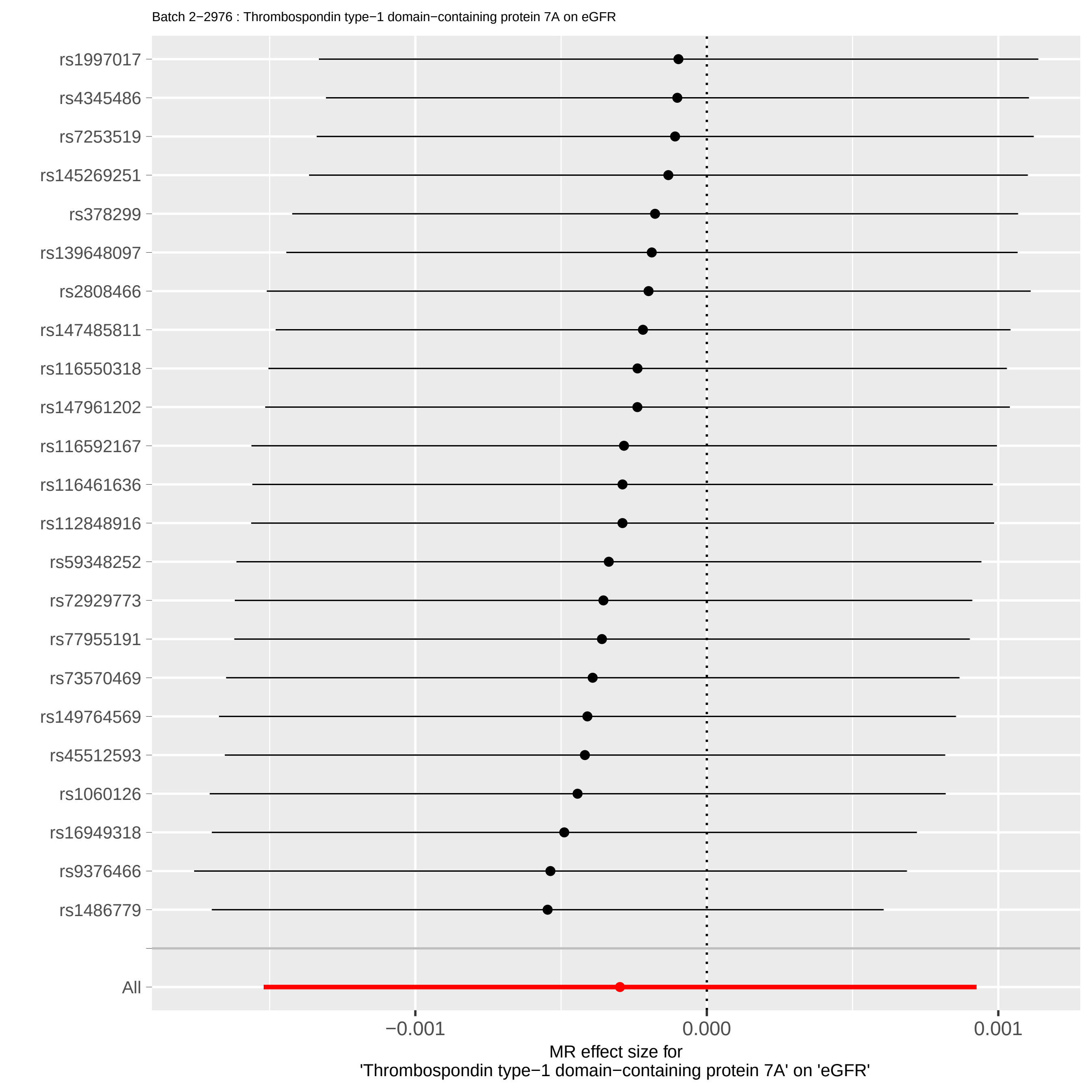

Batch 2−2976 : Thrombospondin type−1 domain−containing protein 7A on eGFR
rs1997017
rs4345486
rs7253519
rs145269251
rs378299
rs139648097
rs2808466
rs147485811
rs116550318
rs147961202
rs116592167
rs116461636
rs112848916
rs59348252
rs72929773
rs77955191
rs73570469
rs149764569
rs45512593
rs1060126
rs16949318
rs9376466
rs1486779
All
−0.001
0.000
0.001
MR effect size for
'Thrombospondin type−1 domain−containing protein 7A' on 'eGFR'

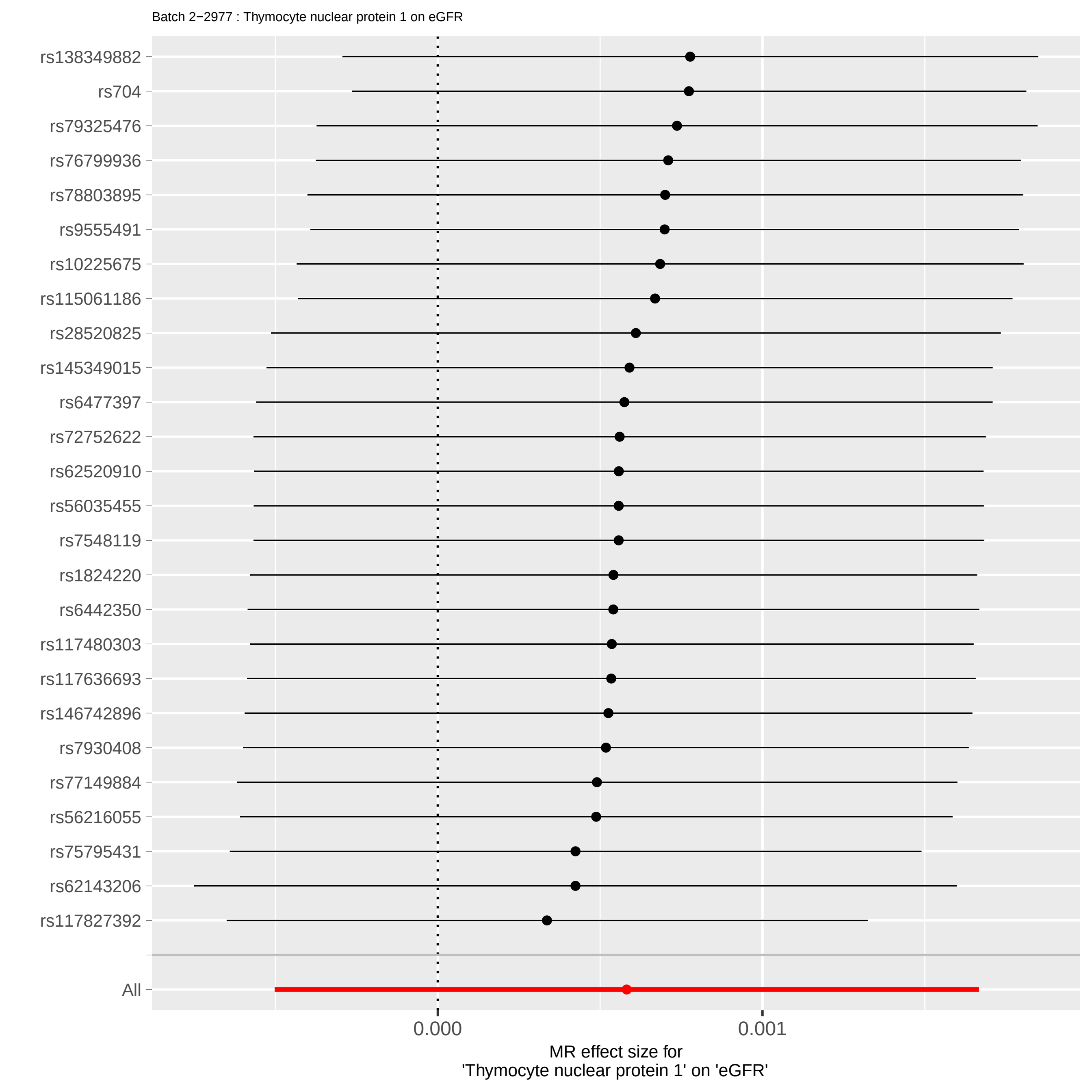

Batch 2−2977 : Thymocyte nuclear protein 1 on eGFR
rs138349882
rs704
rs79325476
rs76799936
rs78803895
rs9555491
rs10225675
rs115061186
rs28520825
rs145349015
rs6477397
rs72752622
rs62520910
rs56035455
rs7548119
rs1824220
rs6442350
rs117480303
rs117636693
rs146742896
rs7930408
rs77149884
rs56216055
rs75795431
rs62143206
rs117827392
All
0.000
0.001
MR effect size for
'Thymocyte nuclear protein 1' on 'eGFR'

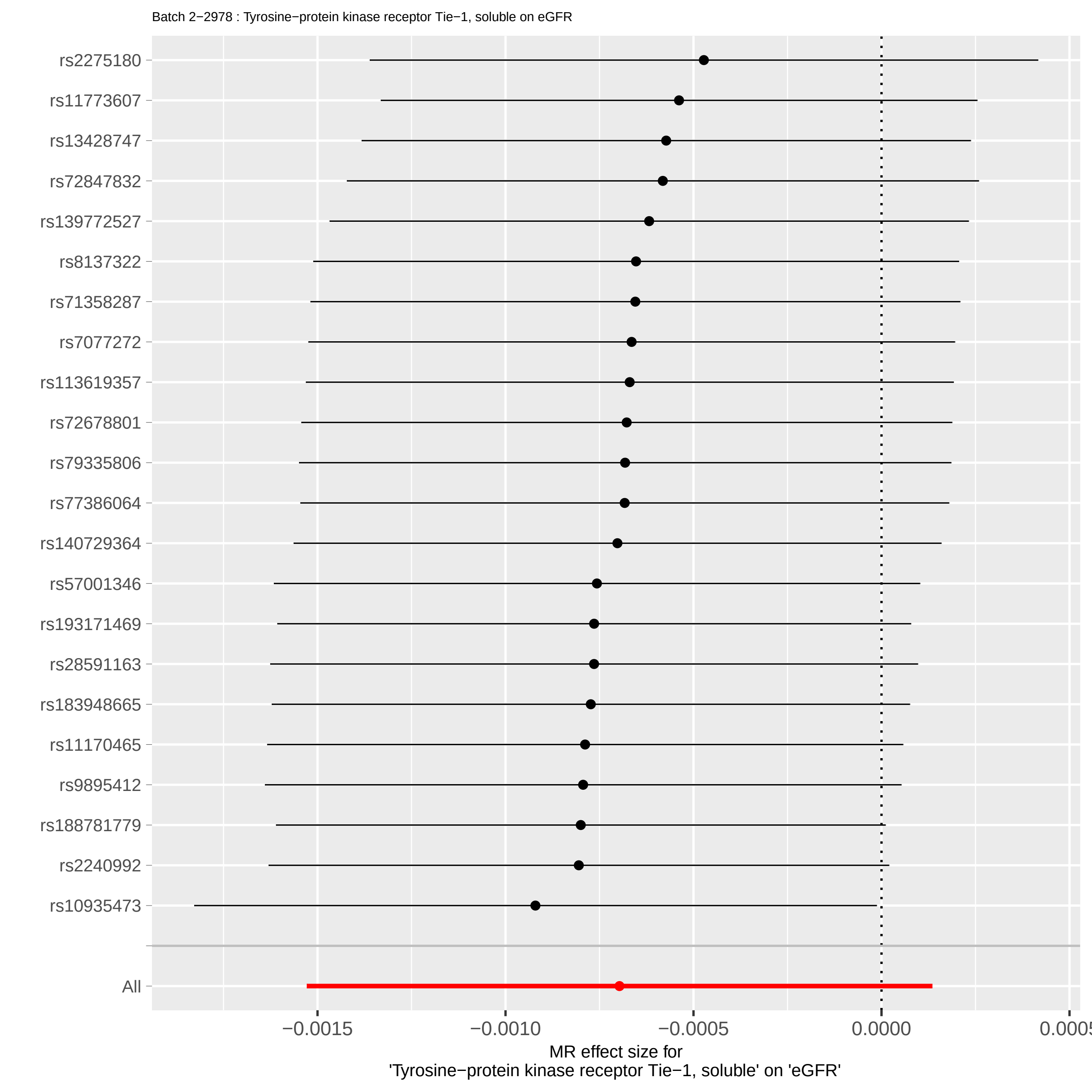

Batch 2−2978 : Tyrosine−protein kinase receptor Tie−1, soluble on eGFR
rs2275180
rs11773607
rs13428747
rs72847832
rs139772527
rs8137322
rs71358287
rs7077272
rs113619357
rs72678801
rs79335806
rs77386064
rs140729364
rs57001346
rs193171469
rs28591163
rs183948665
rs11170465
rs9895412
rs188781779
rs2240992
rs10935473
All
−0.0015
−0.0010
−0.0005
0.0000
MR effect size for
'Tyrosine−protein kinase receptor Tie−1, soluble' on 'eGFR'

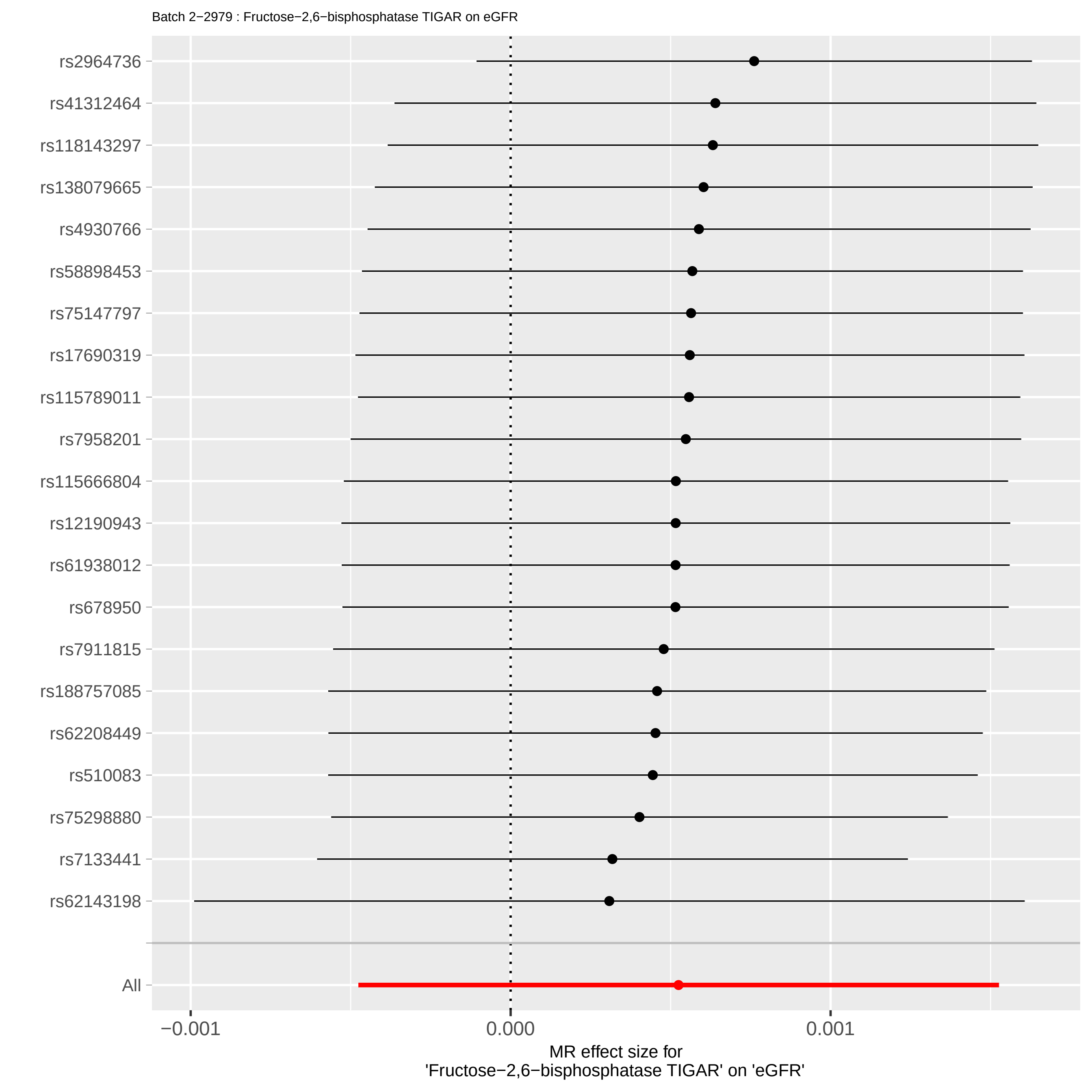

Batch 2−2979 : Fructose−2,6−bisphosphatase TIGAR on eGFR
rs2964736
rs41312464
rs118143297
rs138079665
rs4930766
rs58898453
rs75147797
rs17690319
rs115789011
rs7958201
rs115666804
rs12190943
rs61938012
rs678950
rs7911815
rs188757085
rs62208449
rs510083
rs75298880
rs7133441
rs62143198
All
−0.001
0.000
0.001
MR effect size for
'Fructose−2,6−bisphosphatase TIGAR' on 'eGFR'

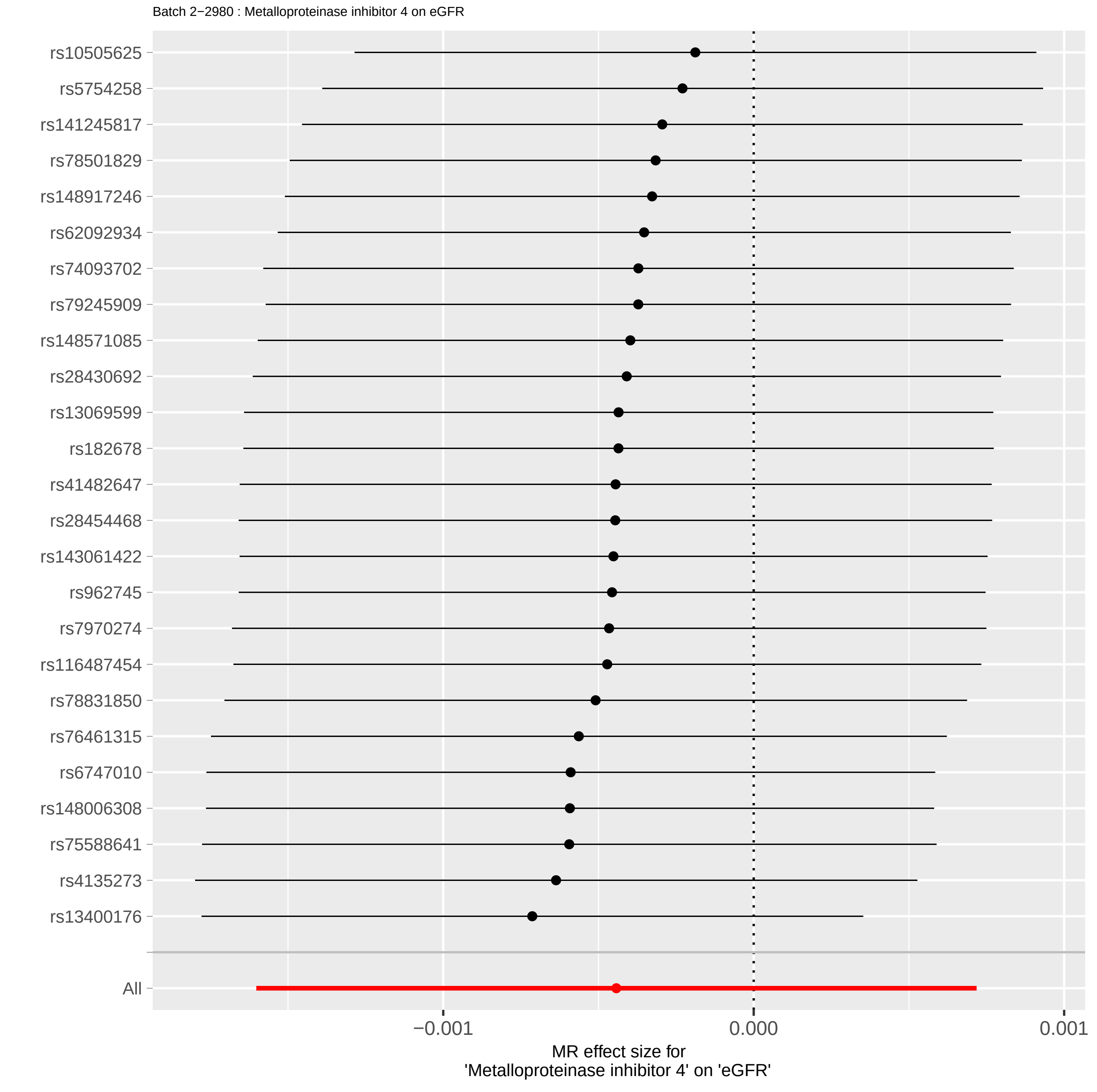

Batch 2−2980 : Metalloproteinase inhibitor 4 on eGFR
rs10505625
rs5754258
rs141245817
rs78501829
rs148917246
rs62092934
rs74093702
rs79245909
rs148571085
rs28430692
rs13069599
rs182678
rs41482647
rs28454468
rs143061422
rs962745
rs7970274
rs116487454
rs78831850
rs76461315
rs6747010
rs148006308
rs75588641
rs4135273
rs13400176
All
−0.001
0.000
0.001
MR effect size for
'Metalloproteinase inhibitor 4' on 'eGFR'

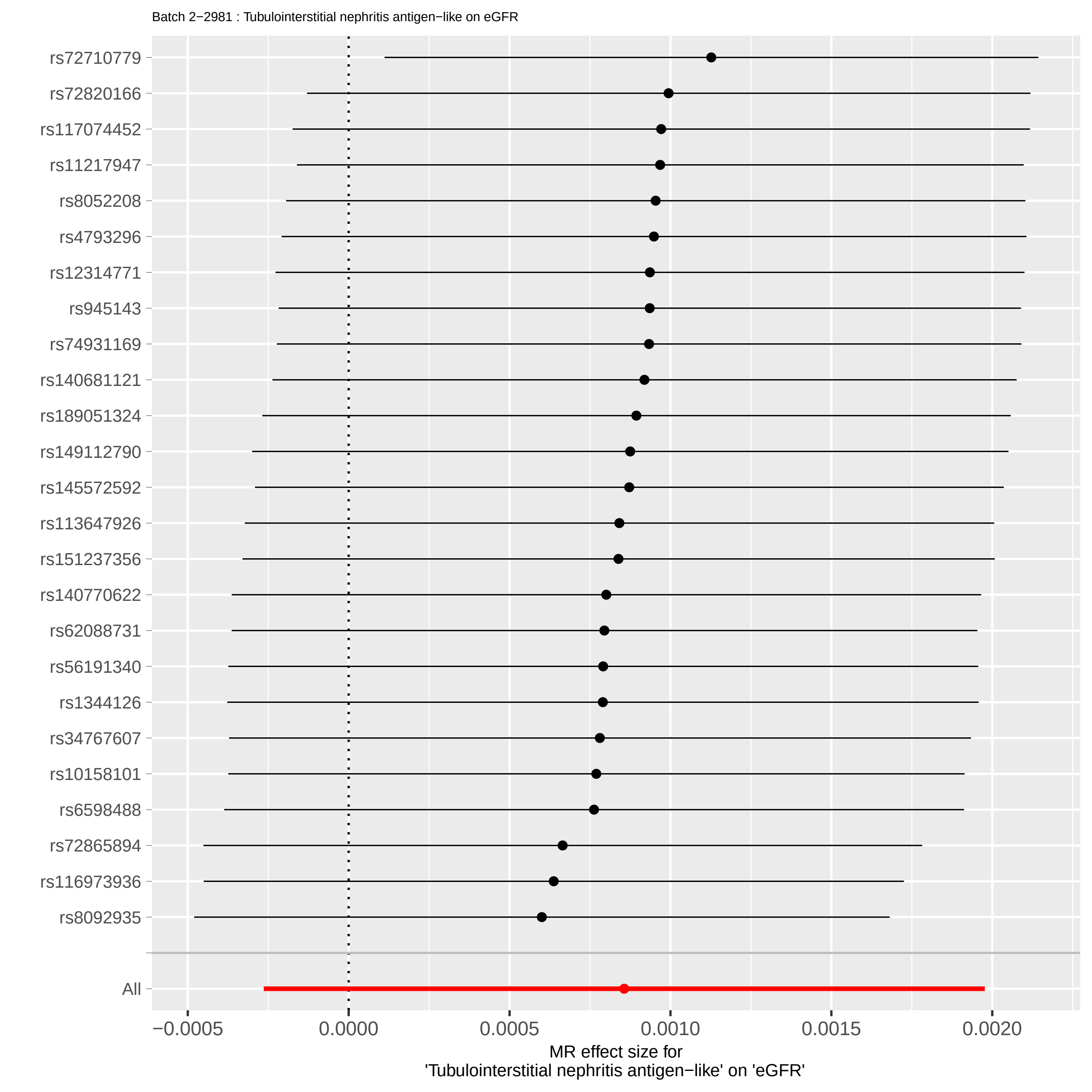

Batch 2−2981 : Tubulointerstitial nephritis antigen−like on eGFR
rs72710779
rs72820166
rs117074452
rs11217947
rs8052208
rs4793296
rs12314771
rs945143
rs74931169
rs140681121
rs189051324
rs149112790
rs145572592
rs113647926
rs151237356
rs140770622
rs62088731
rs56191340
rs1344126
rs34767607
rs10158101
rs6598488
rs72865894
rs116973936
rs8092935
All
−0.0005
0.0000
0.0005
0.0010
0.0015
0.0020
MR effect size for
'Tubulointerstitial nephritis antigen−like' on 'eGFR'

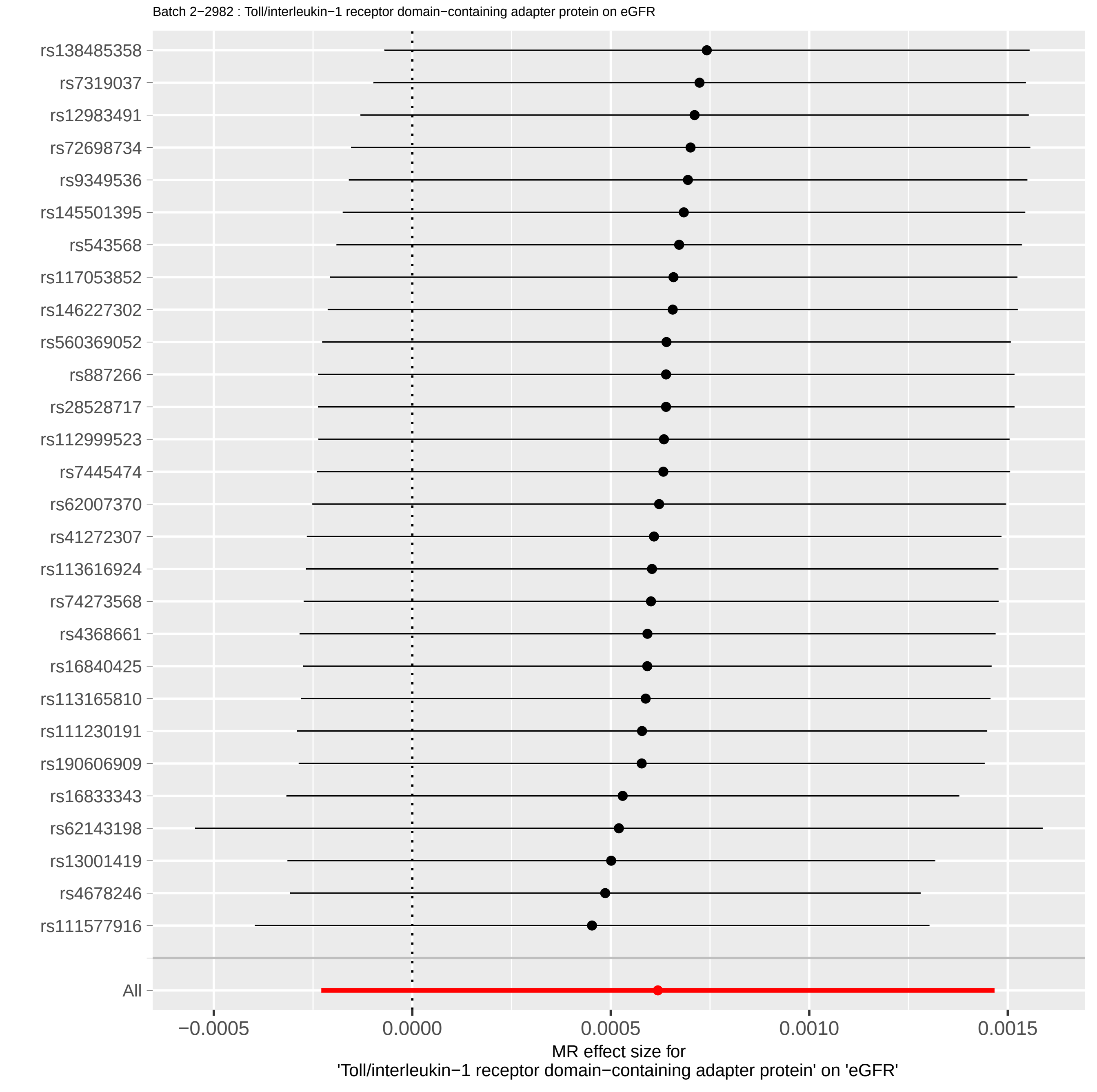

Batch 2−2982 : Toll/interleukin−1 receptor domain−containing adapter protein on eGFR
rs138485358
rs7319037
rs12983491
rs72698734
rs9349536
rs145501395
rs543568
rs117053852
rs146227302
rs560369052
rs887266
rs28528717
rs112999523
rs7445474
rs62007370
rs41272307
rs113616924
rs74273568
rs4368661
rs16840425
rs113165810
rs111230191
rs190606909
rs16833343
rs62143198
rs13001419
rs4678246
rs111577916
All
−0.0005
0.0000
0.0005
0.0010
0.0015
MR effect size for
'Toll/interleukin−1 receptor domain−containing adapter protein' on 'eGFR'

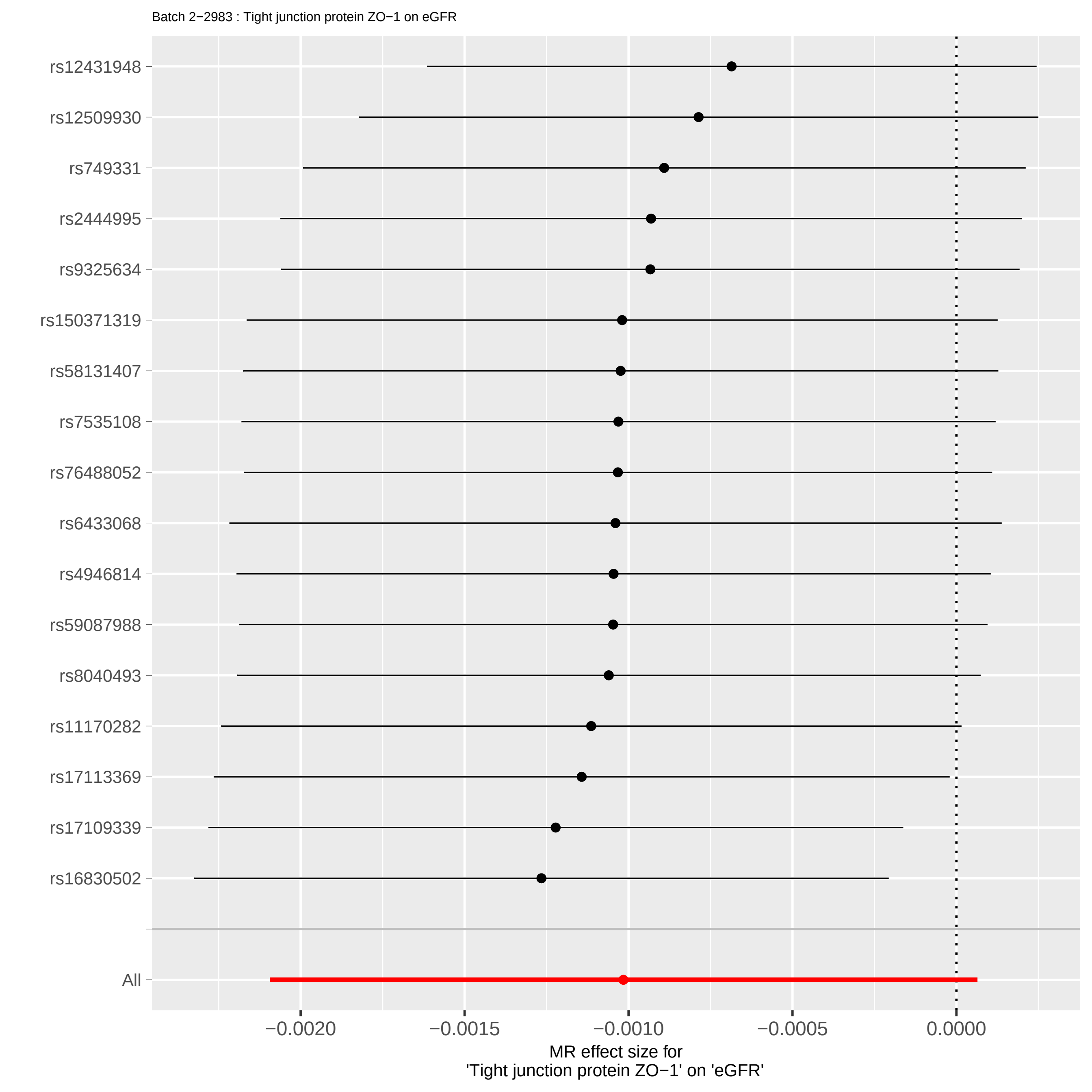

Batch 2−2983 : Tight junction protein ZO−1 on eGFR
rs12431948
rs12509930
rs749331
rs2444995
rs9325634
rs150371319
rs58131407
rs7535108
rs76488052
rs6433068
rs4946814
rs59087988
rs8040493
rs11170282
rs17113369
rs17109339
rs16830502
All
−0.0020
−0.0015
−0.0010
−0.0005
0.0000
MR effect size for
'Tight junction protein ZO−1' on 'eGFR'

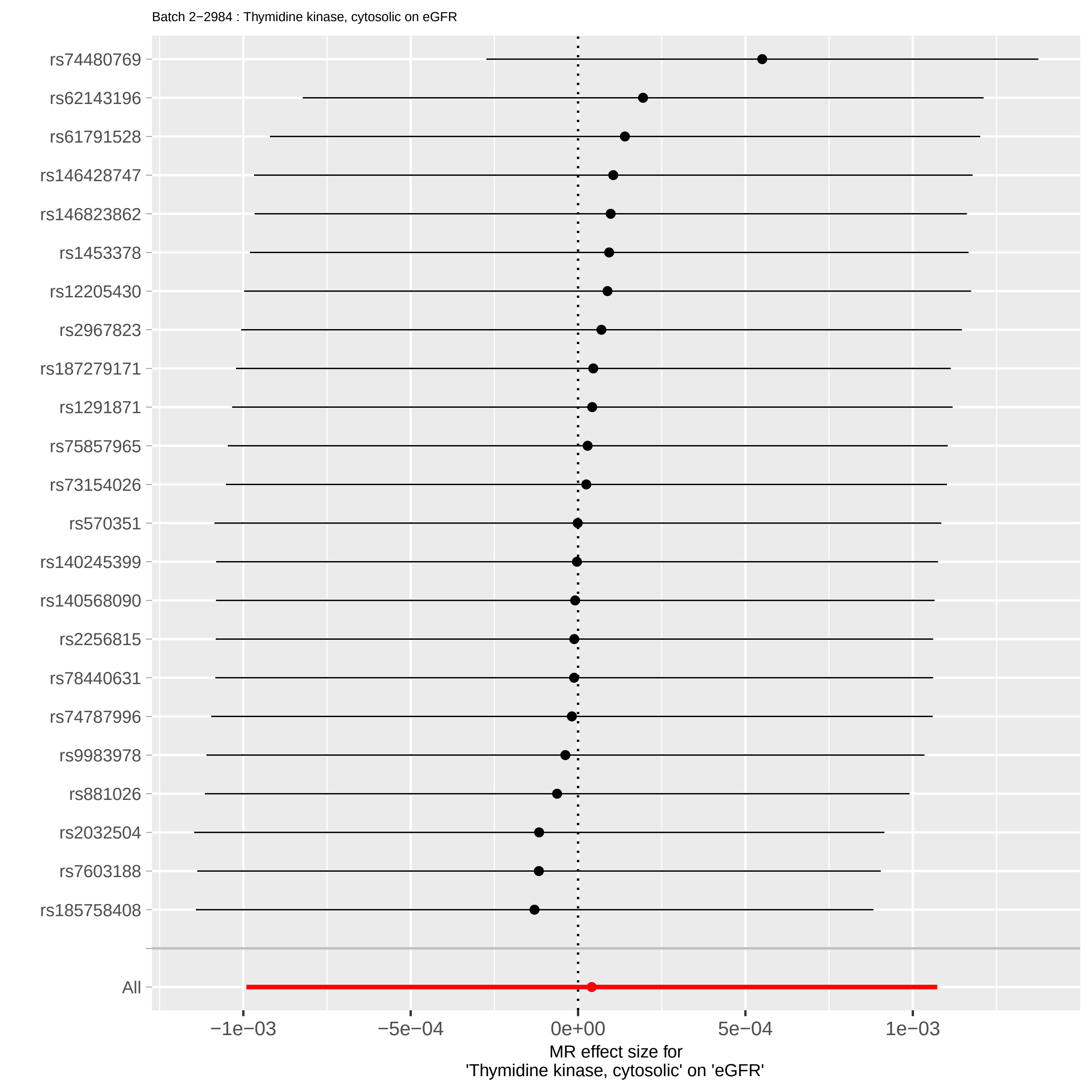
Batch 2−2984 : Thymidine kinase, cytosolic on eGFR
rs74480769
rs62143196
rs61791528
rs146428747
rs146823862
rs1453378
rs12205430
rs2967823
rs187279171
rs1291871
rs75857965
rs73154026
rs570351
rs140245399
rs140568090
rs2256815
rs78440631
rs74787996
rs9983978
rs881026
rs2032504
rs7603188
rs185758408
All
−1e−03
−5e−04
0e+00
5e−04
1e−03
MR effect size for
'Thymidine kinase, cytosolic' on 'eGFR'

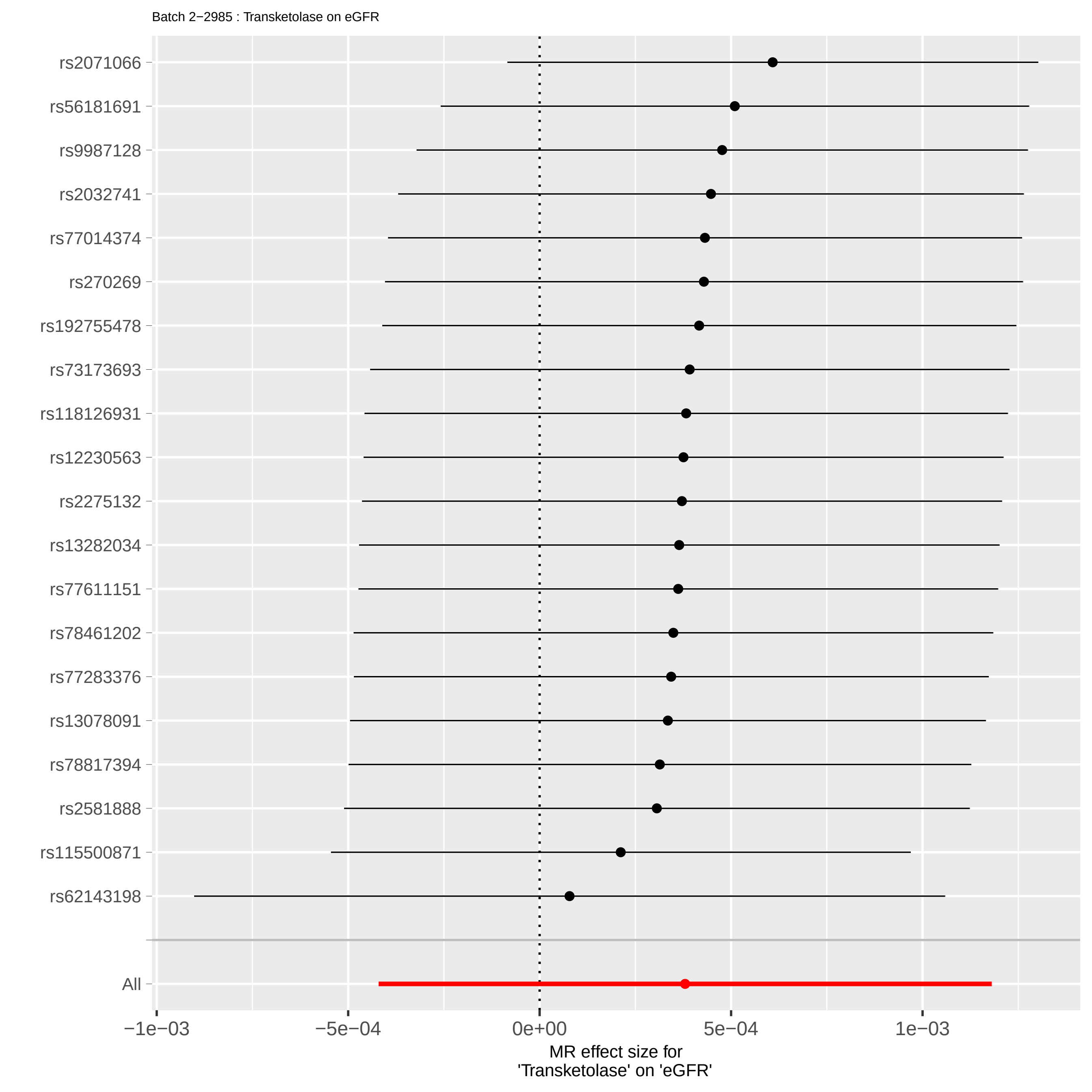

Batch 2−2985 : Transketolase on eGFR
rs2071066
rs56181691
rs9987128
rs2032741
rs77014374
rs270269
rs192755478
rs73173693
rs118126931
rs12230563
rs2275132
rs13282034
rs77611151
rs78461202
rs77283376
rs13078091
rs78817394
rs2581888
rs115500871
rs62143198
All
−1e−03
−5e−04
0e+00
5e−04
1e−03
MR effect size for
'Transketolase' on 'eGFR'

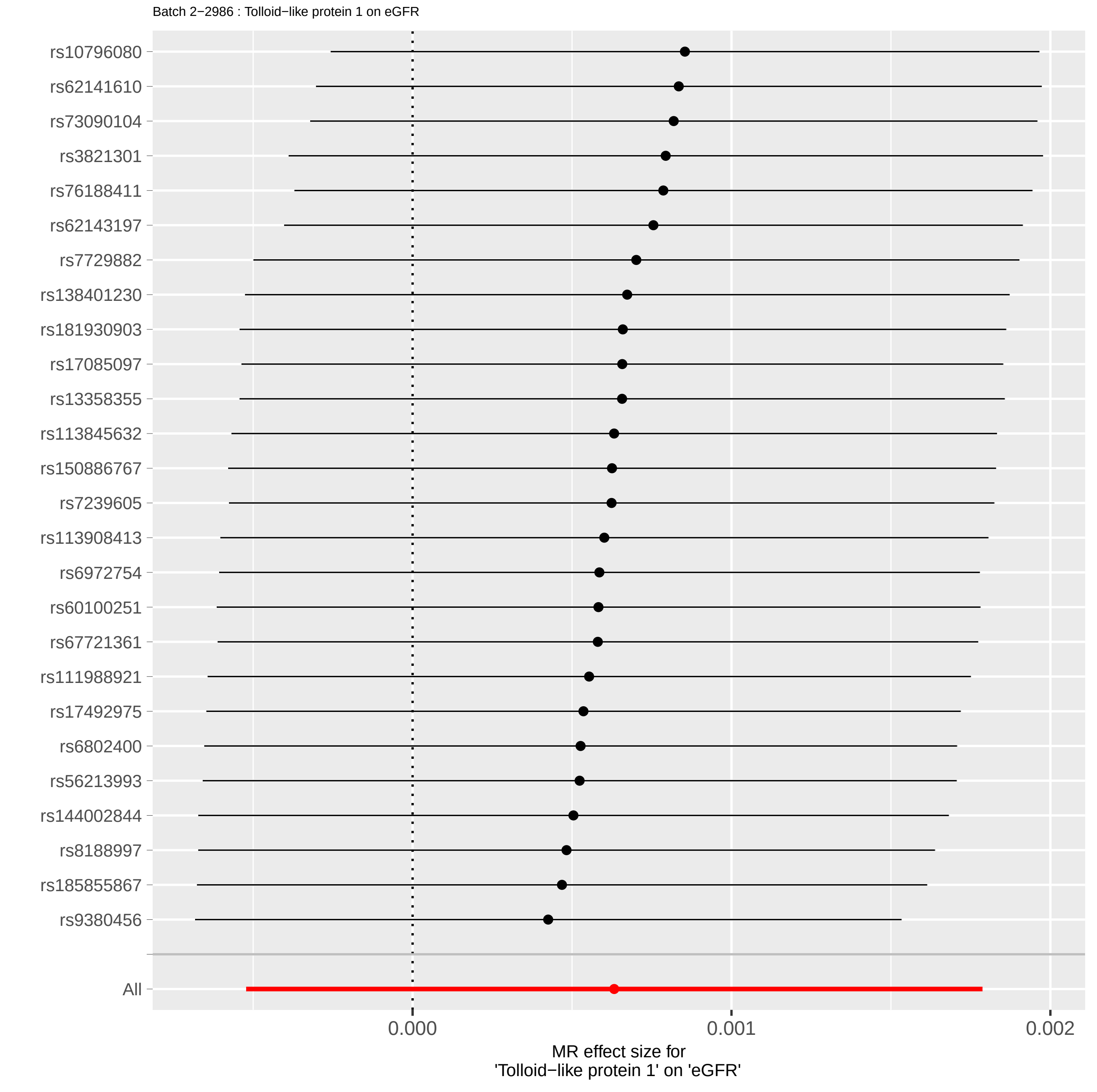

Batch 2−2986 : Tolloid−like protein 1 on eGFR
rs10796080
rs62141610
rs73090104
rs3821301
rs76188411
rs62143197
rs7729882
rs138401230
rs181930903
rs17085097
rs13358355
rs113845632
rs150886767
rs7239605
rs113908413
rs6972754
rs60100251
rs67721361
rs111988921
rs17492975
rs6802400
rs56213993
rs144002844
rs8188997
rs185855867
rs9380456
All
0.000
0.001
0.002
MR effect size for
'Tolloid−like protein 1' on 'eGFR'

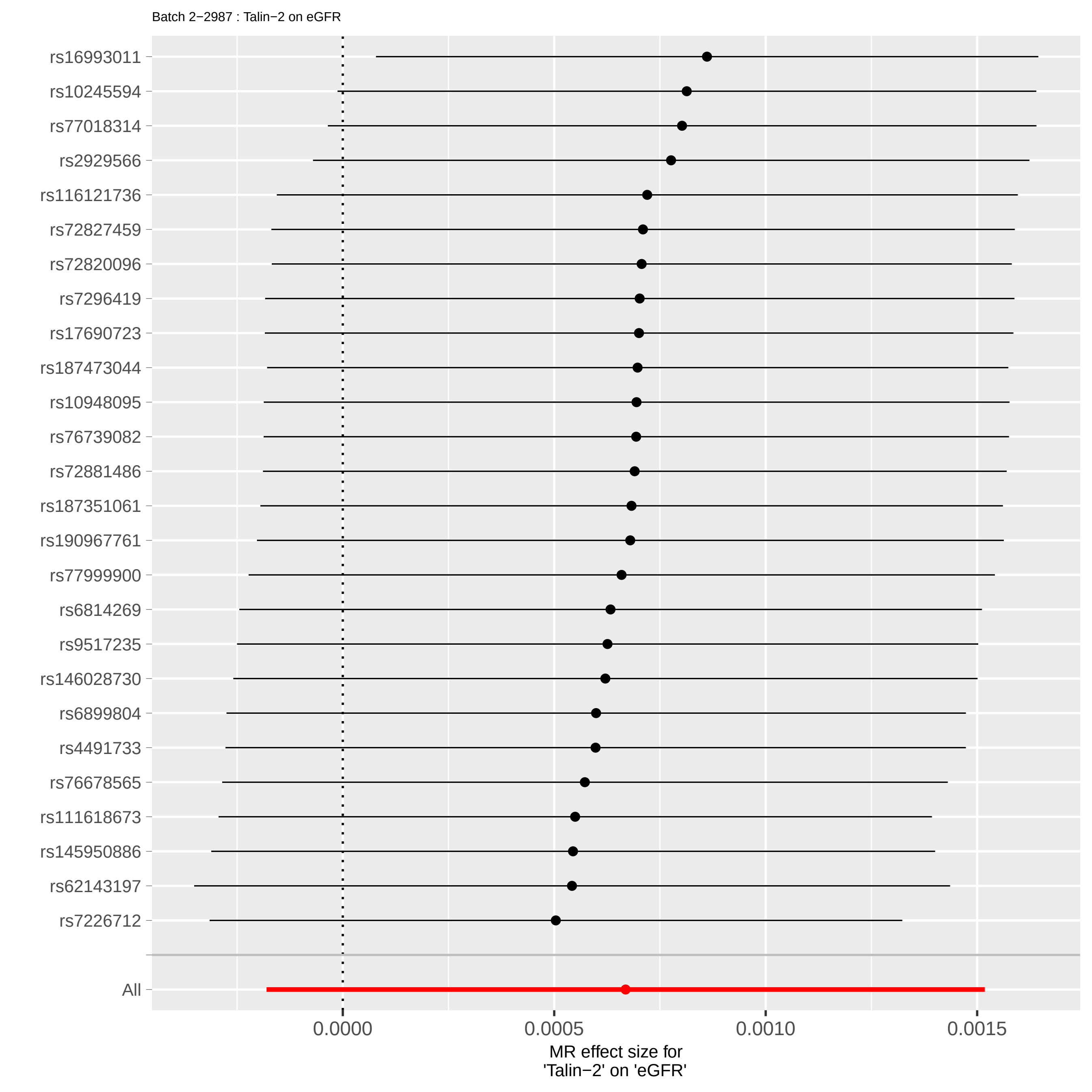

Batch 2−2987 : Talin−2 on eGFR
rs16993011
rs10245594
rs77018314
rs2929566
rs116121736
rs72827459
rs72820096
rs7296419
rs17690723
rs187473044
rs10948095
rs76739082
rs72881486
rs187351061
rs190967761
rs77999900
rs6814269
rs9517235
rs146028730
rs6899804
rs4491733
rs76678565
rs111618673
rs145950886
rs62143197
rs7226712
All
0.0000
0.0005
0.0010
0.0015
MR effect size for
'Talin−2' on 'eGFR'

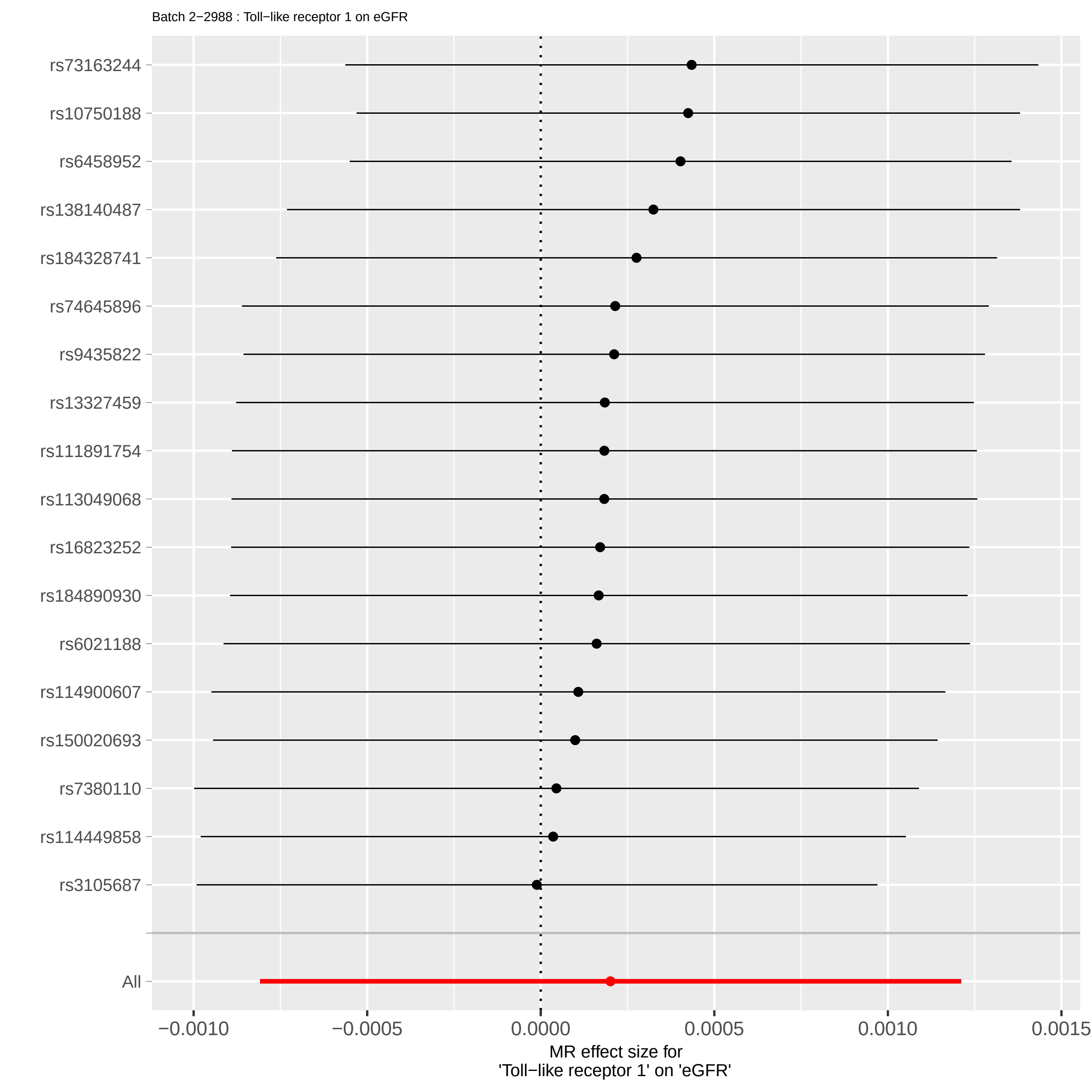

Batch 2−2988 : Toll−like receptor 1 on eGFR
rs73163244
rs10750188
rs6458952
rs138140487
rs184328741
rs74645896
rs9435822
rs13327459
rs111891754
rs113049068
rs16823252
rs184890930
rs6021188
rs114900607
rs150020693
rs7380110
rs114449858
rs3105687
All
−0.0010
−0.0005
0.0000
0.0005
0.0010
0.0015
MR effect size for
'Toll−like receptor 1' on 'eGFR'

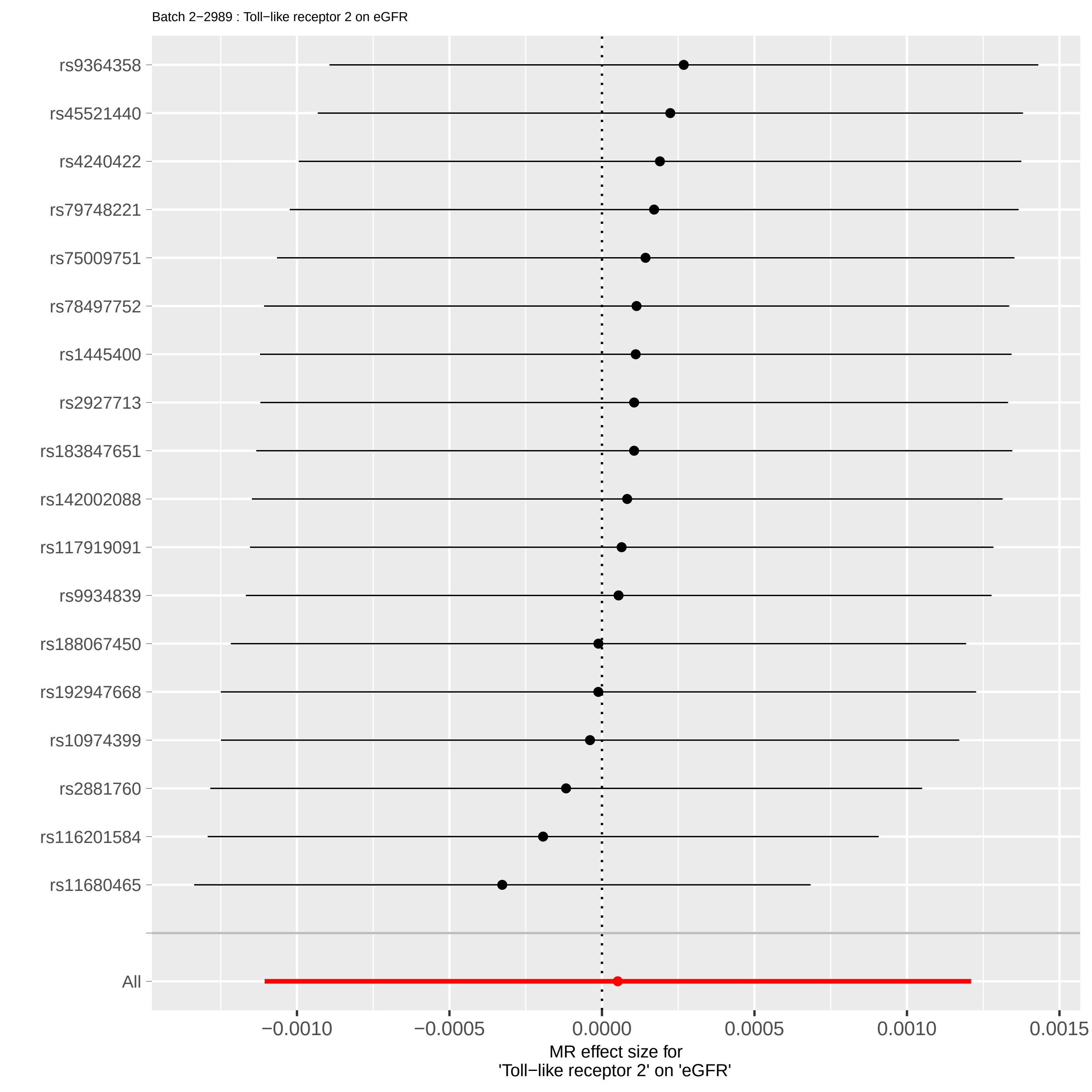

Batch 2−2989 : Toll−like receptor 2 on eGFR
rs9364358
rs45521440
rs4240422
rs79748221
rs75009751
rs78497752
rs1445400
rs2927713
rs183847651
rs142002088
rs117919091
rs9934839
rs188067450
rs192947668
rs10974399
rs2881760
rs116201584
rs11680465
All
−0.0010
−0.0005
0.0000
0.0005
0.0010
0.0015
MR effect size for
'Toll−like receptor 2' on 'eGFR'

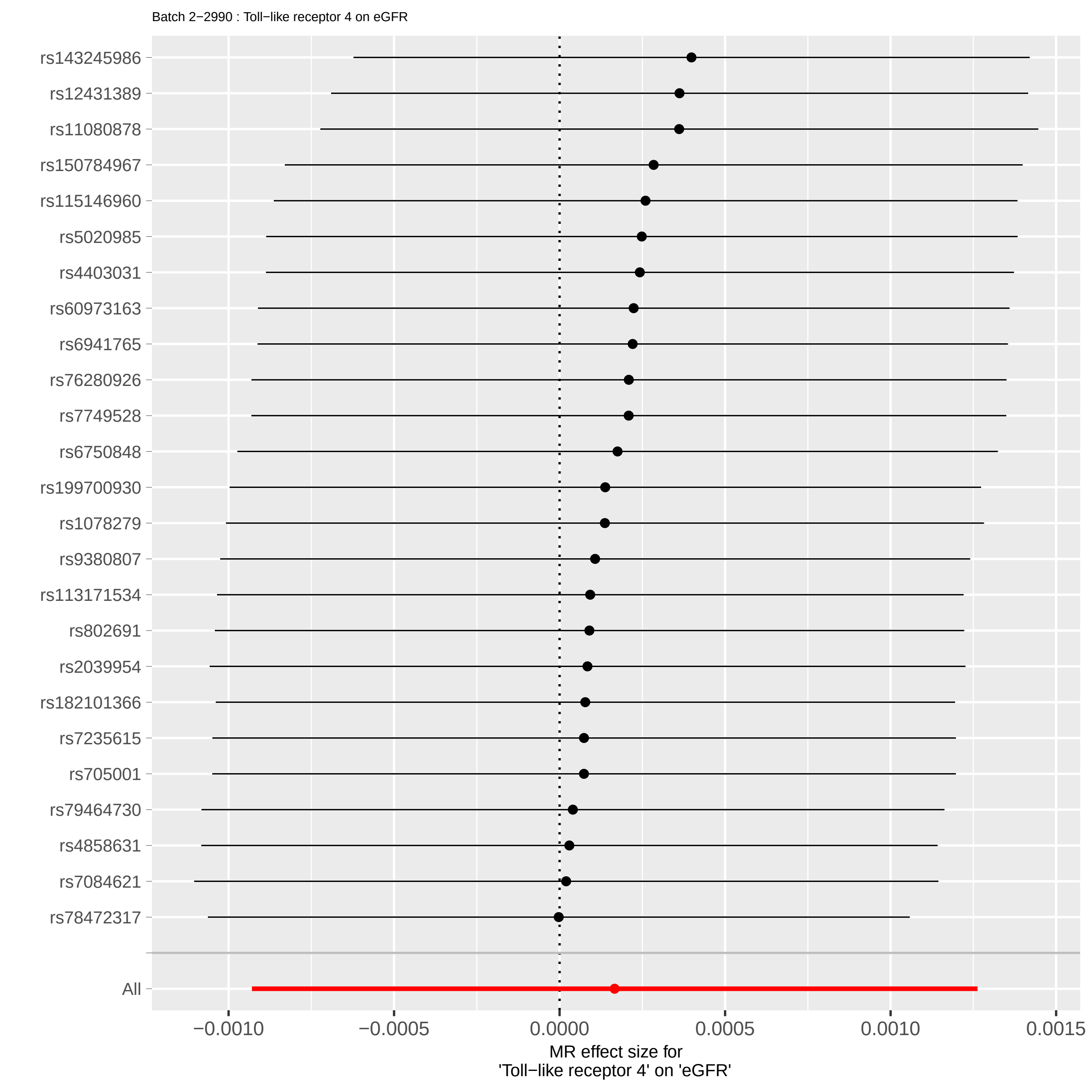

Batch 2−2990 : Toll−like receptor 4 on eGFR
rs143245986
rs12431389
rs11080878
rs150784967
rs115146960
rs5020985
rs4403031
rs60973163
rs6941765
rs76280926
rs7749528
rs6750848
rs199700930
rs1078279
rs9380807
rs113171534
rs802691
rs2039954
rs182101366
rs7235615
rs705001
rs79464730
rs4858631
rs7084621
rs78472317
All
−0.0010
−0.0005
0.0000
0.0005
0.0010
0.0015
MR effect size for
'Toll−like receptor 4' on 'eGFR'

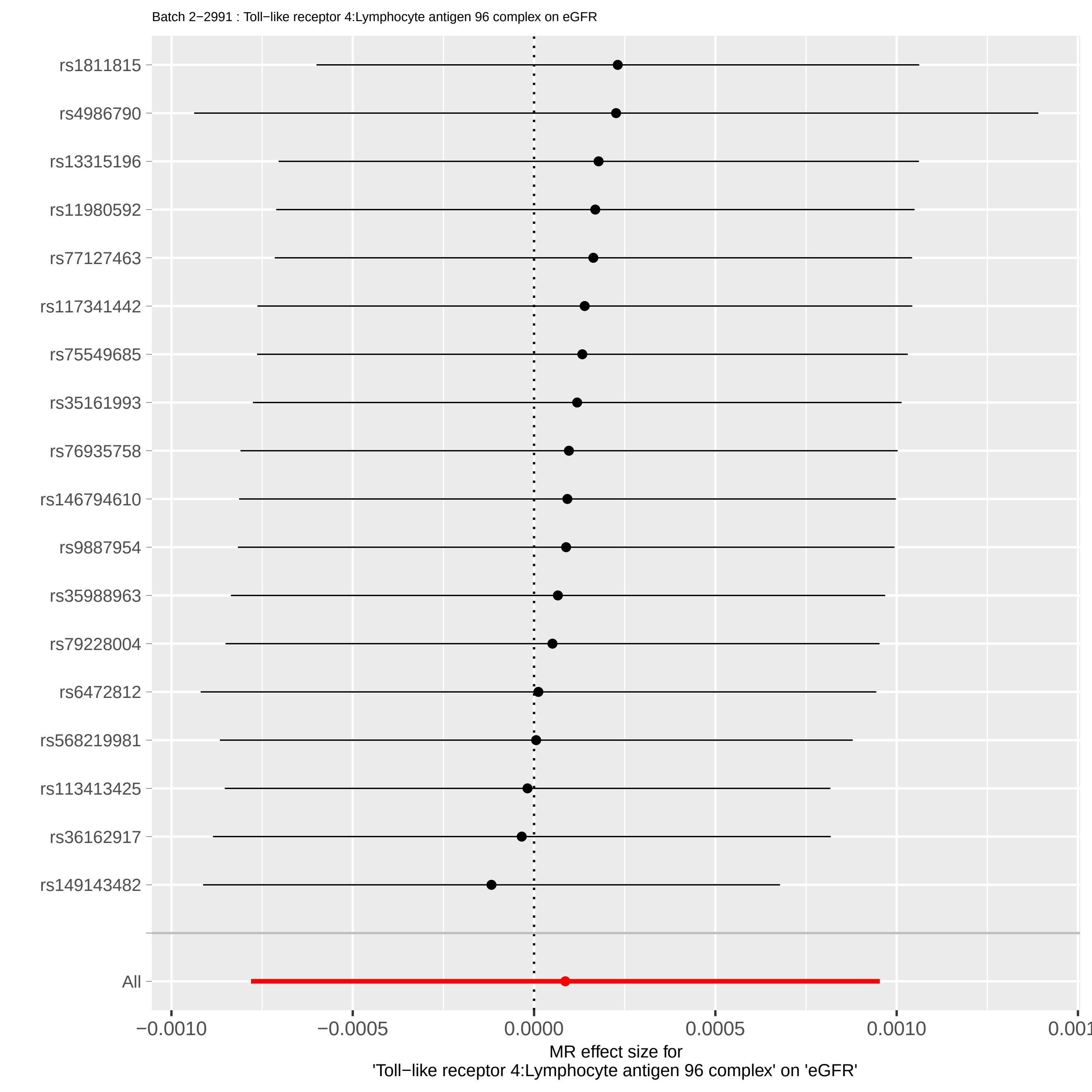

Batch 2−2991 : Toll−like receptor 4:Lymphocyte antigen 96 complex on eGFR
rs1811815
rs4986790
rs13315196
rs11980592
rs77127463
rs117341442
rs75549685
rs35161993
rs76935758
rs146794610
rs9887954
rs35988963
rs79228004
rs6472812
rs568219981
rs113413425
rs36162917
rs149143482
All
−0.0010
−0.0005
0.0000
0.0005
0.0010
MR effect size for
'Toll−like receptor 4:Lymphocyte antigen 96 complex' on 'eGFR'

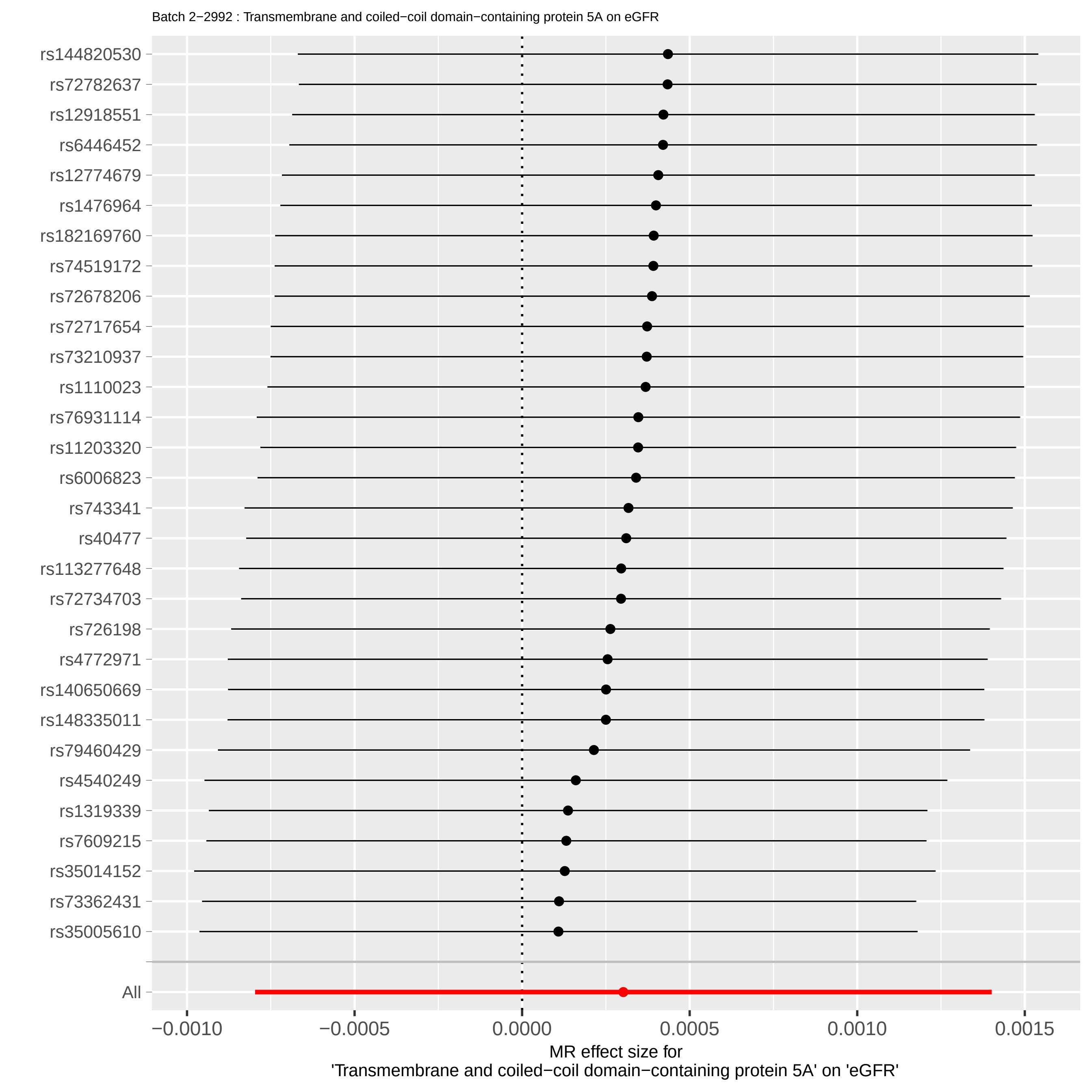

Batch 2−2992 : Transmembrane and coiled−coil domain−containing protein 5A on eGFR
rs144820530
rs72782637
rs12918551
rs6446452
rs12774679
rs1476964
rs182169760
rs74519172
rs72678206
rs72717654
rs73210937
rs1110023
rs76931114
rs11203320
rs6006823
rs743341
rs40477
rs113277648
rs72734703
rs726198
rs4772971
rs140650669
rs148335011
rs79460429
rs4540249
rs1319339
rs7609215
rs35014152
rs73362431
rs35005610
All
−0.0010
−0.0005
0.0000
0.0005
0.0010
0.0015
MR effect size for
'Transmembrane and coiled−coil domain−containing protein 5A' on 'eGFR'

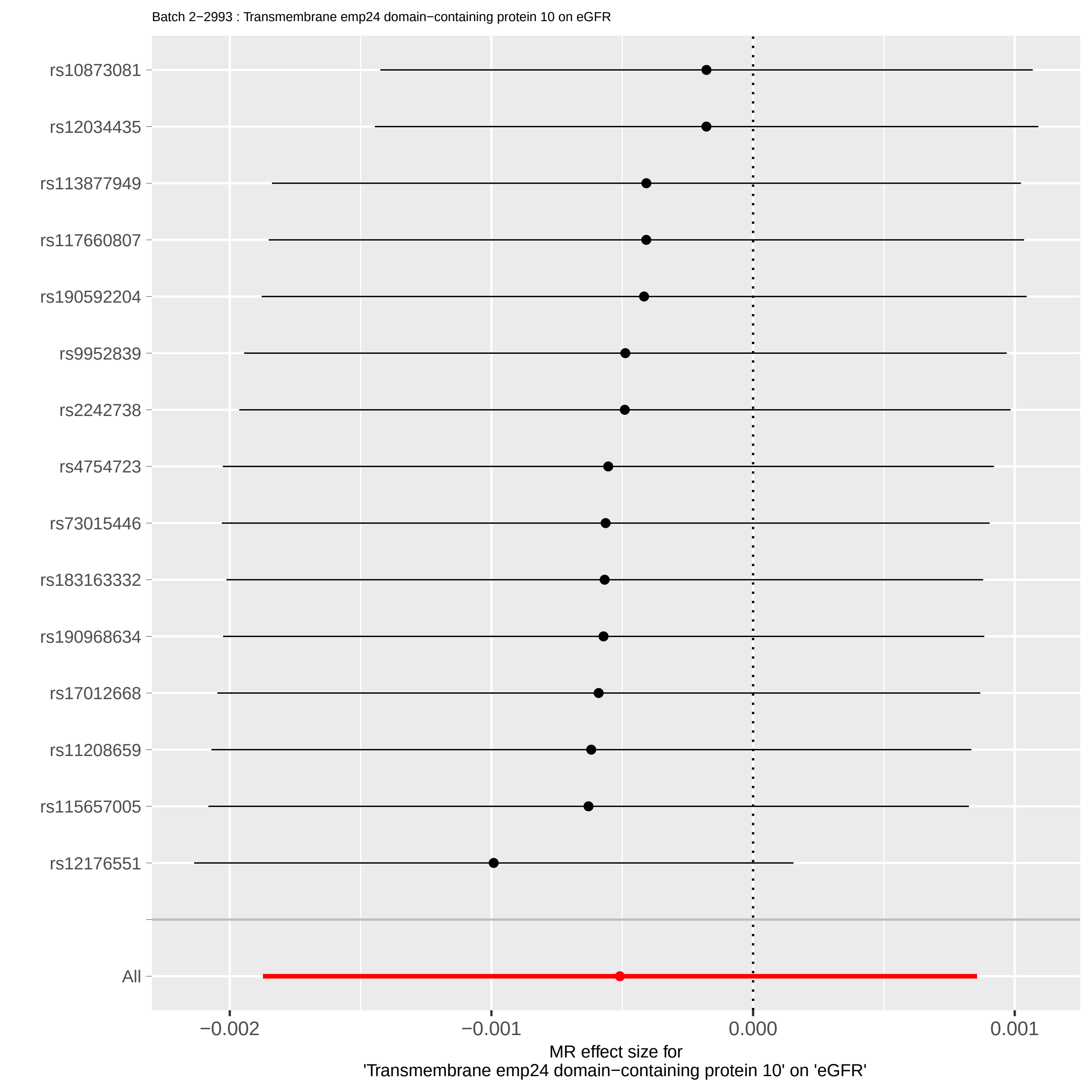

Batch 2−2993 : Transmembrane emp24 domain−containing protein 10 on eGFR
rs10873081
rs12034435
rs113877949
rs117660807
rs190592204
rs9952839
rs2242738
rs4754723
rs73015446
rs183163332
rs190968634
rs17012668
rs11208659
rs115657005
rs12176551
All
−0.002
−0.001
0.000
0.001
MR effect size for
'Transmembrane emp24 domain−containing protein 10' on 'eGFR'

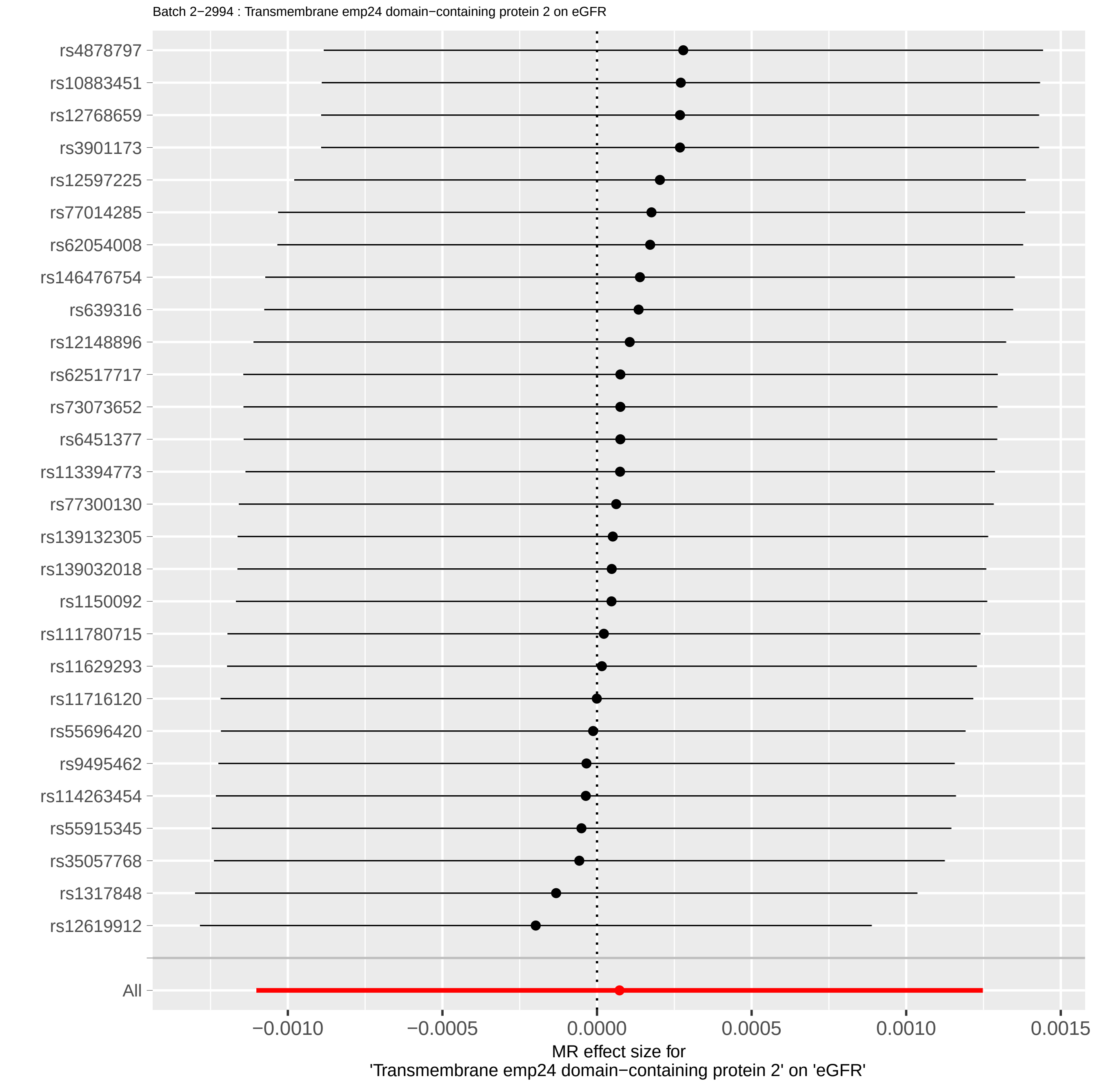

Batch 2−2994 : Transmembrane emp24 domain−containing protein 2 on eGFR
rs4878797
rs10883451
rs12768659
rs3901173
rs12597225
rs77014285
rs62054008
rs146476754
rs639316
rs12148896
rs62517717
rs73073652
rs6451377
rs113394773
rs77300130
rs139132305
rs139032018
rs1150092
rs111780715
rs11629293
rs11716120
rs55696420
rs9495462
rs114263454
rs55915345
rs35057768
rs1317848
rs12619912
All
−0.0010
−0.0005
0.0000
0.0005
0.0010
0.0015
MR effect size for
'Transmembrane emp24 domain−containing protein 2' on 'eGFR'

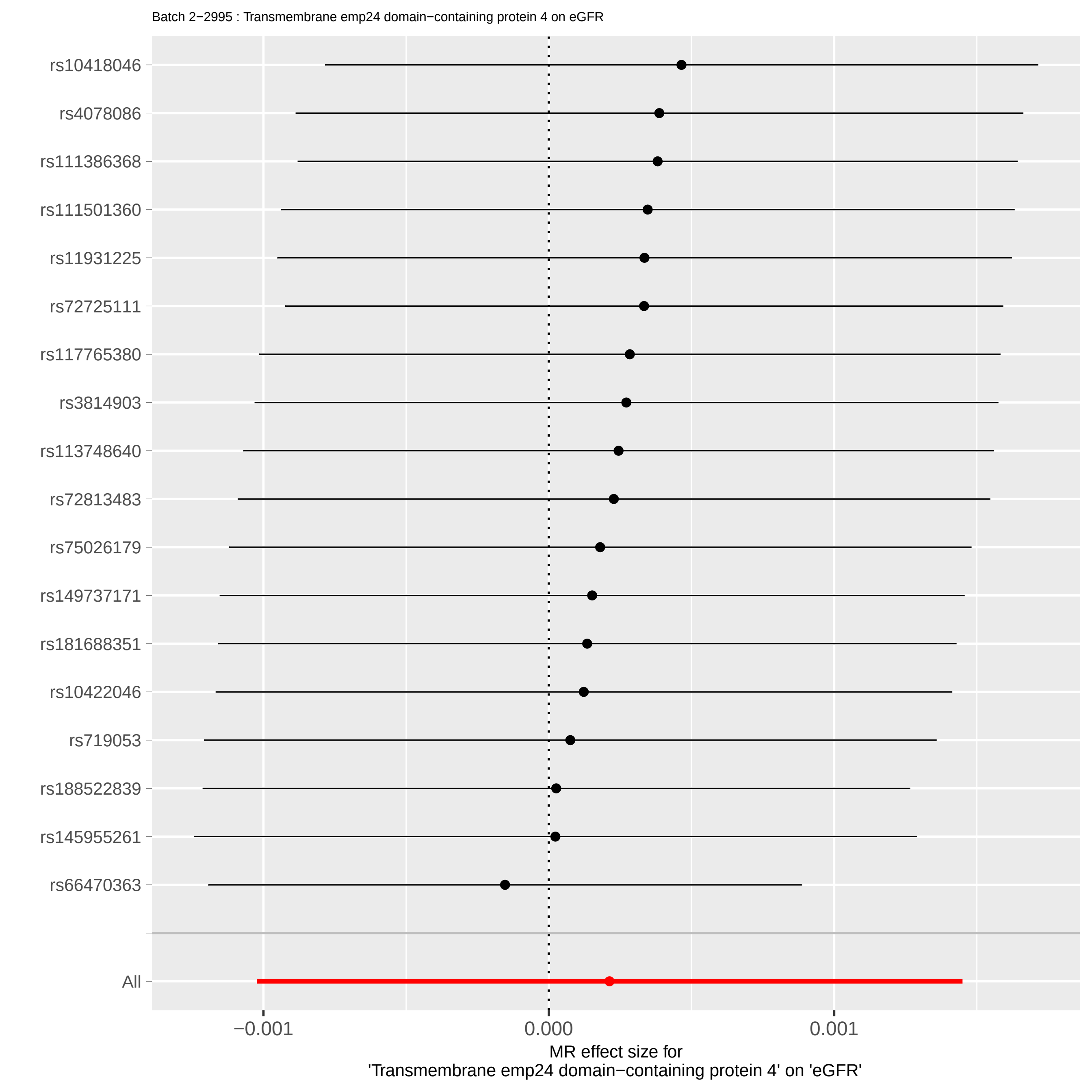

Batch 2−2995 : Transmembrane emp24 domain−containing protein 4 on eGFR
rs10418046
rs4078086
rs111386368
rs111501360
rs11931225
rs72725111
rs117765380
rs3814903
rs113748640
rs72813483
rs75026179
rs149737171
rs181688351
rs10422046
rs719053
rs188522839
rs145955261
rs66470363
All
−0.001
0.000
0.001
MR effect size for
'Transmembrane emp24 domain−containing protein 4' on 'eGFR'

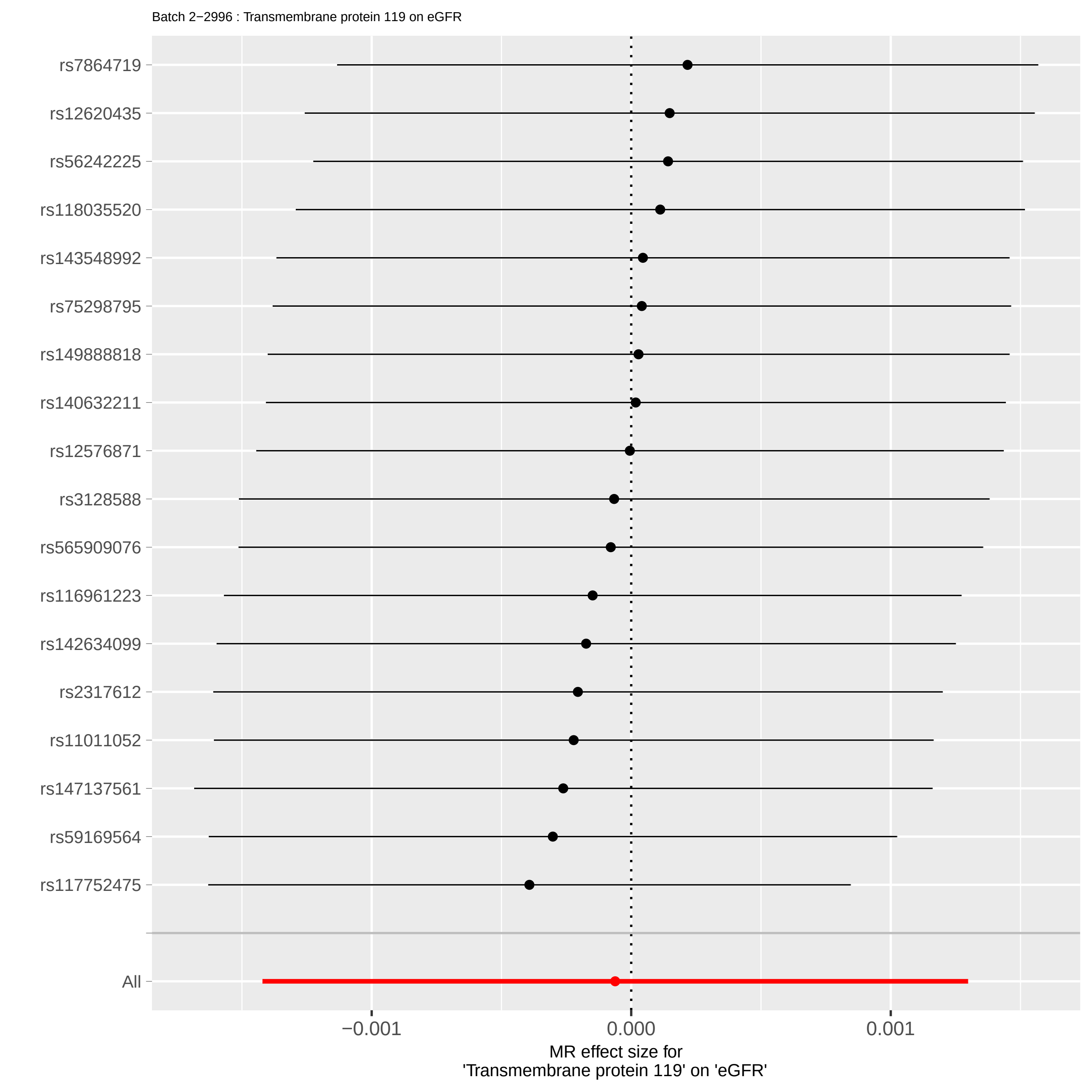

Batch 2−2996 : Transmembrane protein 119 on eGFR
rs7864719
rs12620435
rs56242225
rs118035520
rs143548992
rs75298795
rs149888818
rs140632211
rs12576871
rs3128588
rs565909076
rs116961223
rs142634099
rs2317612
rs11011052
rs147137561
rs59169564
rs117752475
All
−0.001
0.000
0.001
MR effect size for
'Transmembrane protein 119' on 'eGFR'

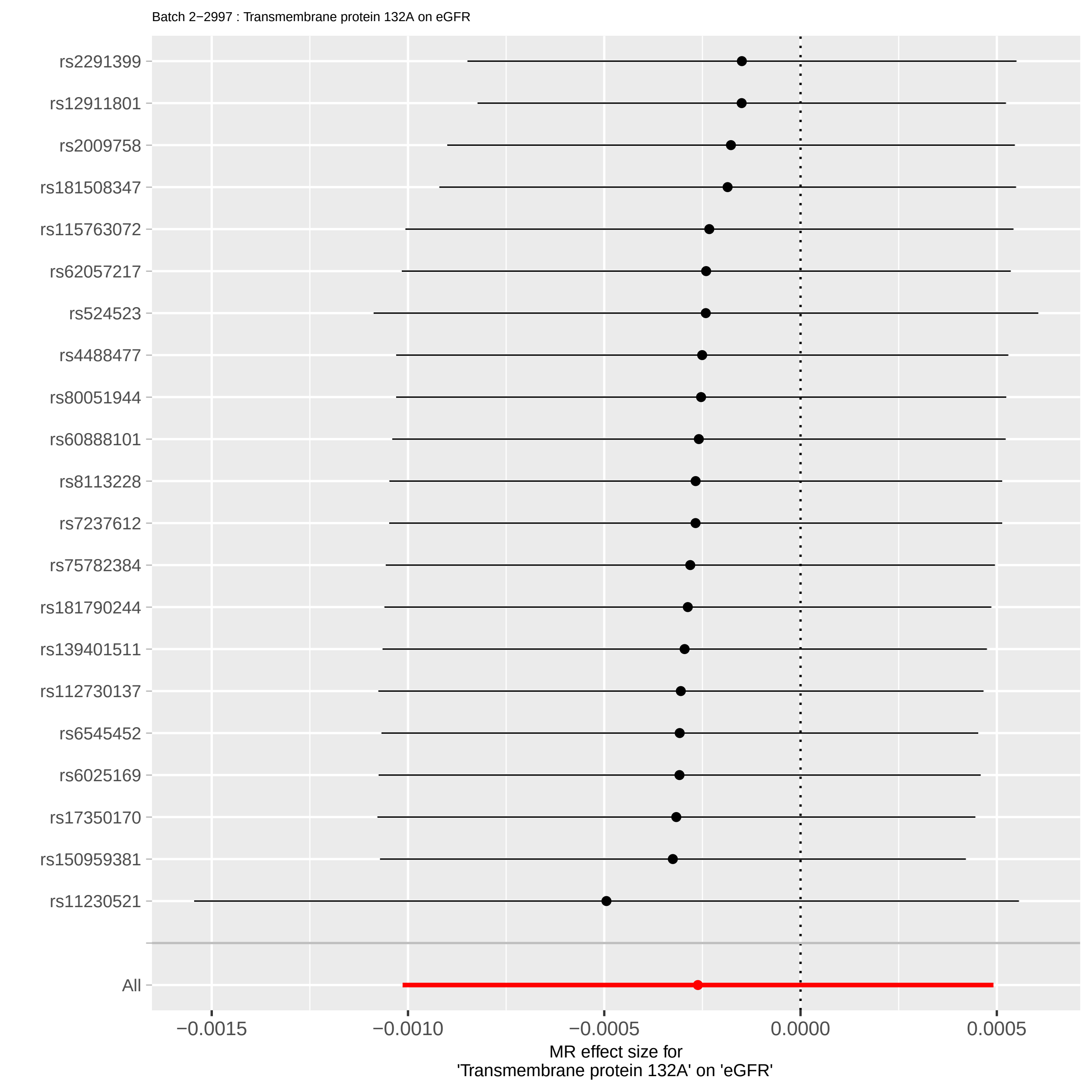
Batch 2−2997 : Transmembrane protein 132A on eGFR
rs2291399
rs12911801
rs2009758
rs181508347
rs115763072
rs62057217
rs524523
rs4488477
rs80051944
rs60888101
rs8113228
rs7237612
rs75782384
rs181790244
rs139401511
rs112730137
rs6545452
rs6025169
rs17350170
rs150959381
rs11230521
All
−0.0015
−0.0010
−0.0005
0.0000
0.0005
MR effect size for
'Transmembrane protein 132A' on 'eGFR'

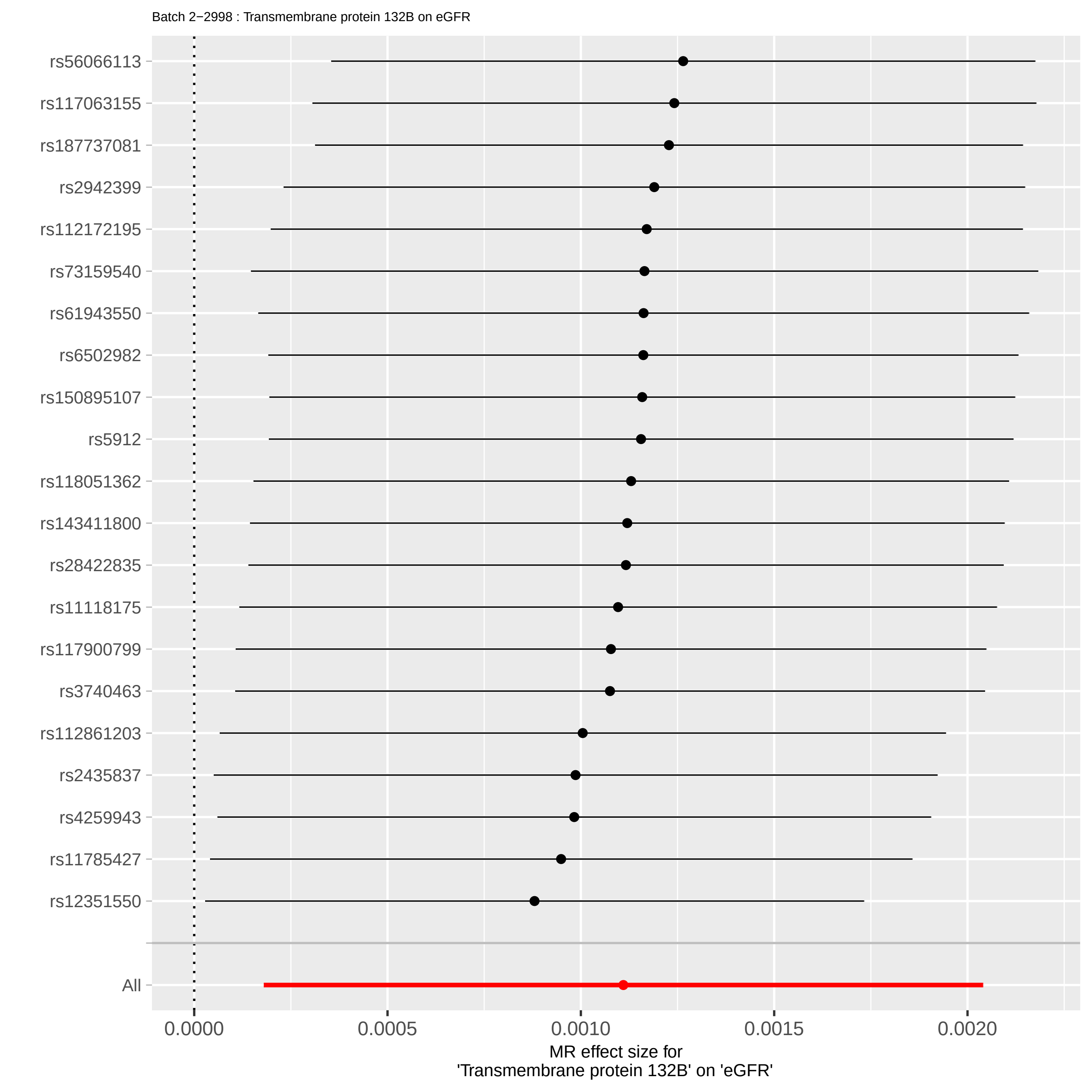

Batch 2−2998 : Transmembrane protein 132B on eGFR
rs56066113
rs117063155
rs187737081
rs2942399
rs112172195
rs73159540
rs61943550
rs6502982
rs150895107
rs5912
rs118051362
rs143411800
rs28422835
rs11118175
rs117900799
rs3740463
rs112861203
rs2435837
rs4259943
rs11785427
rs12351550
All
0.0000
0.0005
0.0010
0.0015
0.0020
MR effect size for
'Transmembrane protein 132B' on 'eGFR'

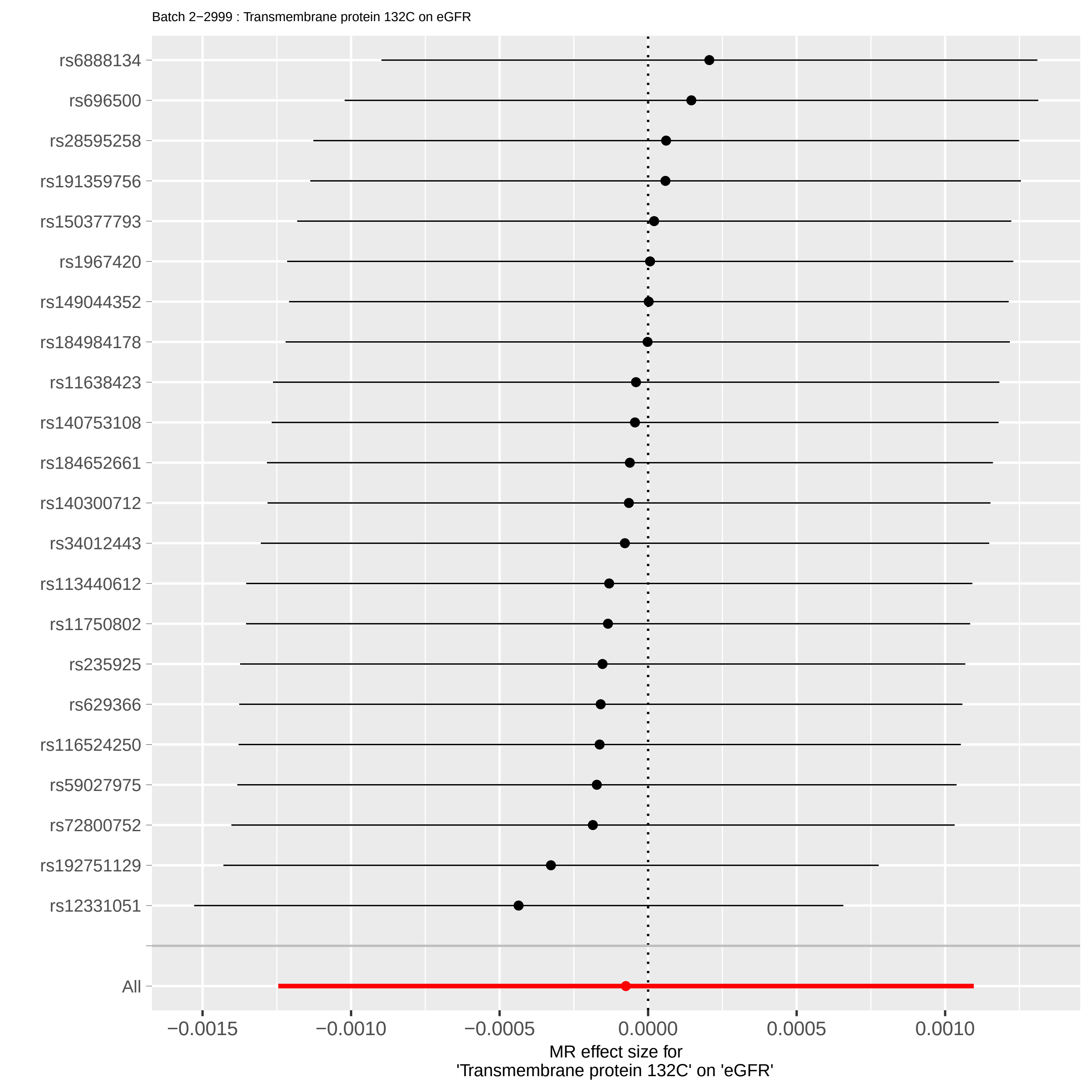

Batch 2−2999 : Transmembrane protein 132C on eGFR
rs6888134
rs696500
rs28595258
rs191359756
rs150377793
rs1967420
rs149044352
rs184984178
rs11638423
rs140753108
rs184652661
rs140300712
rs34012443
rs113440612
rs11750802
rs235925
rs629366
rs116524250
rs59027975
rs72800752
rs192751129
rs12331051
All
−0.0015
−0.0010
−0.0005
0.0000
0.0005
0.0010
MR effect size for
'Transmembrane protein 132C' on 'eGFR'

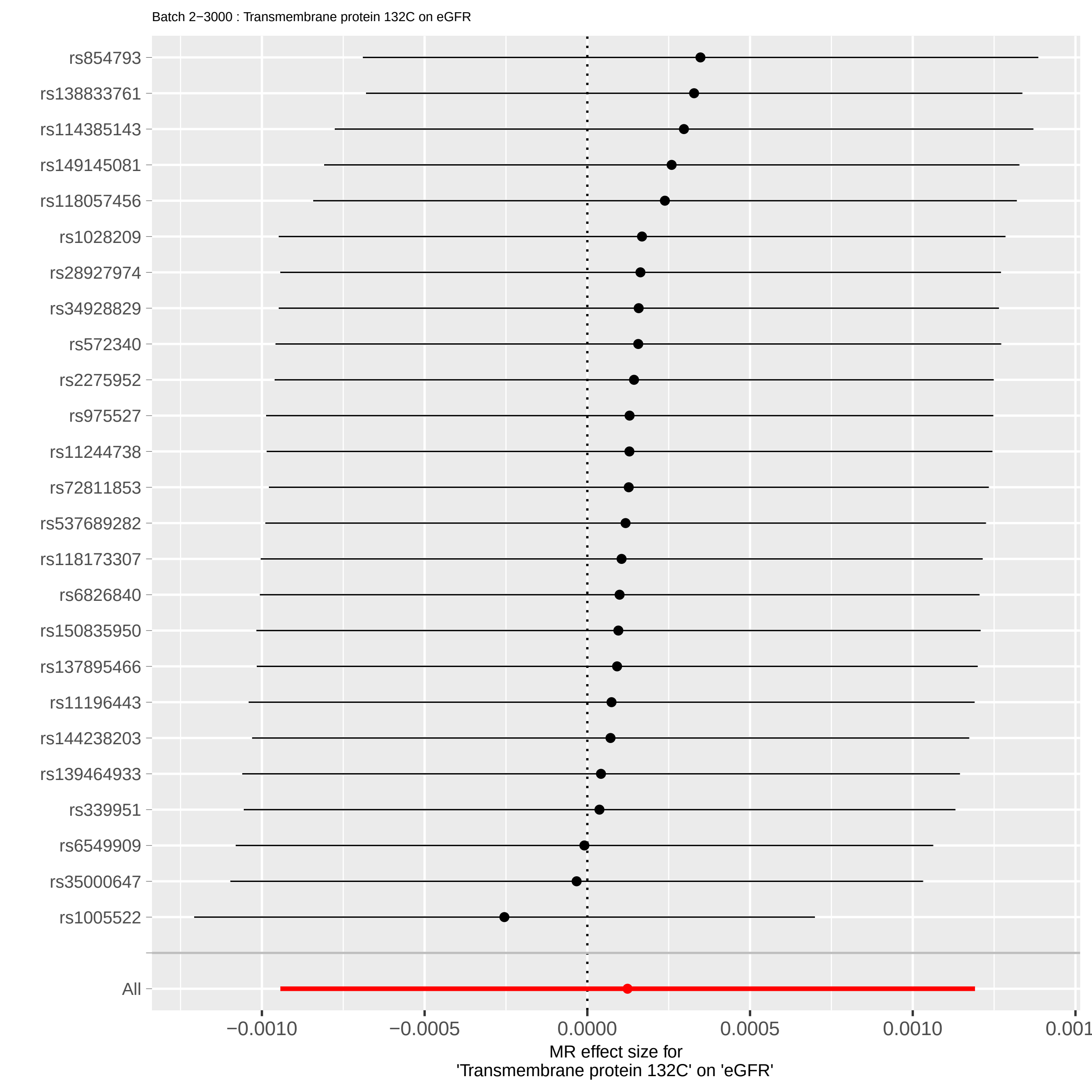

Batch 2−3000 : Transmembrane protein 132C on eGFR
rs854793
rs138833761
rs114385143
rs149145081
rs118057456
rs1028209
rs28927974
rs34928829
rs572340
rs2275952
rs975527
rs11244738
rs72811853
rs537689282
rs118173307
rs6826840
rs150835950
rs137895466
rs11196443
rs144238203
rs139464933
rs339951
rs6549909
rs35000647
rs1005522
All
−0.0010
−0.0005
0.0000
0.0005
0.0010
MR effect size for
'Transmembrane protein 132C' on 'eGFR'

Batch 2−3001 : Transmembrane protein 132D on eGFR

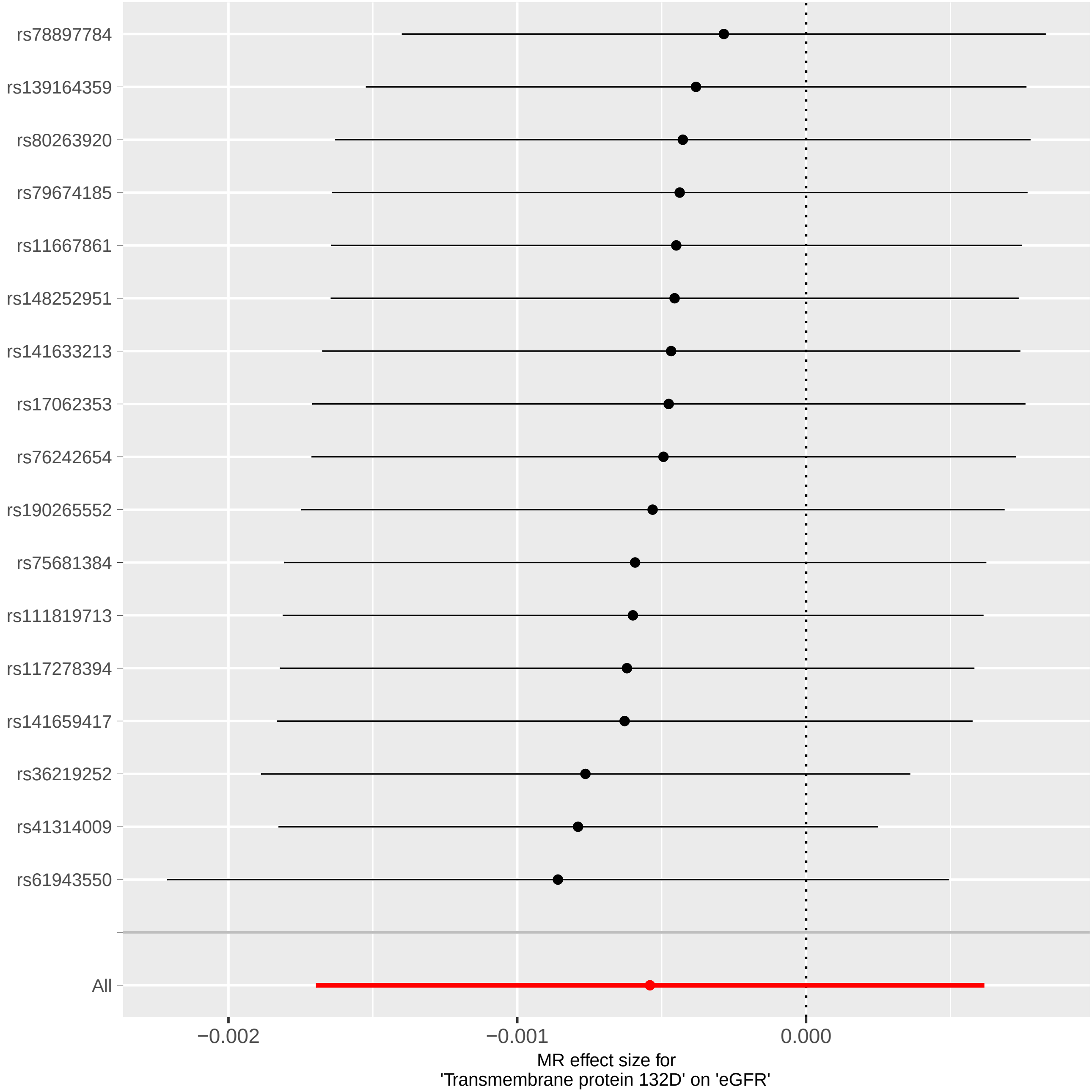

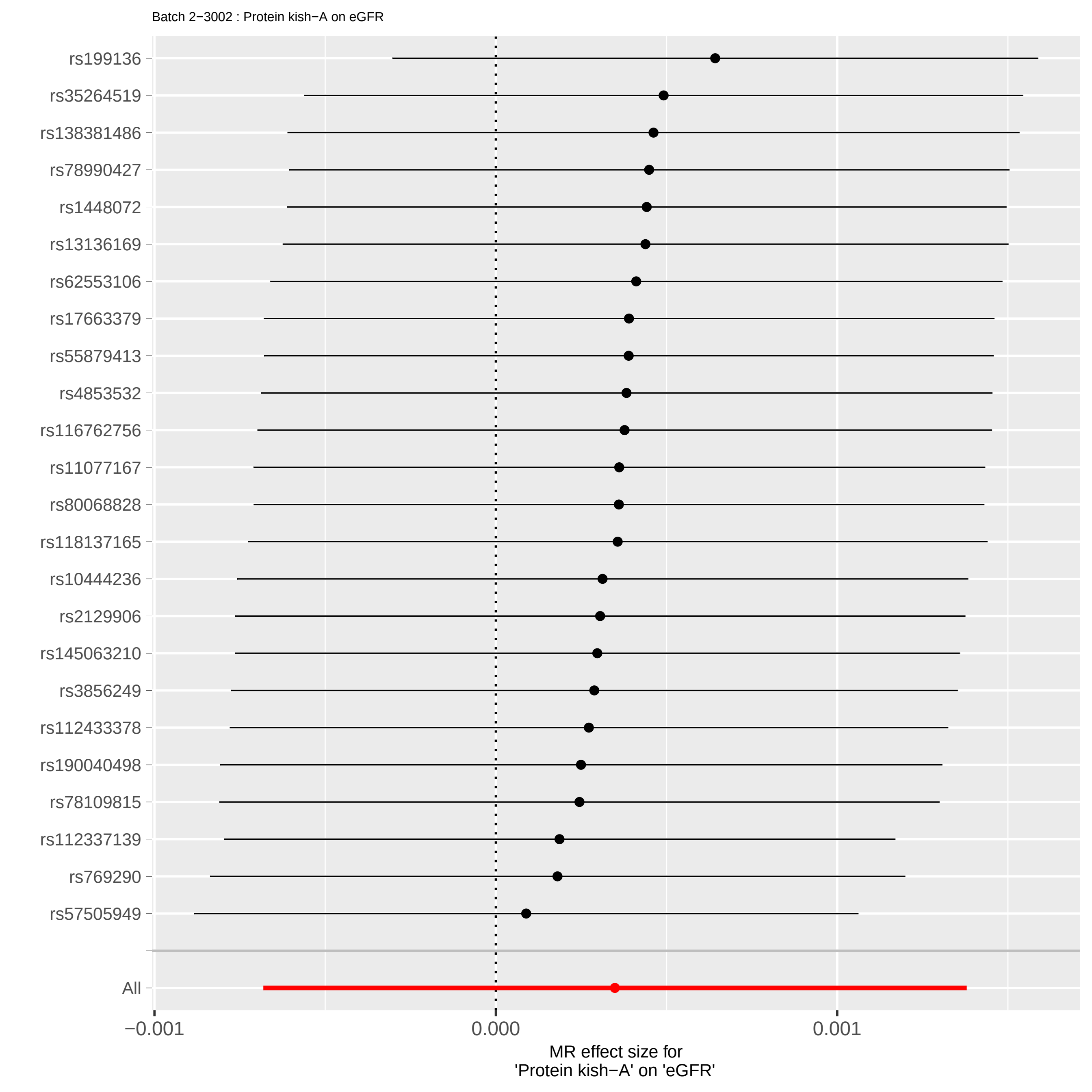
Batch 2−3002 : Protein kish−A on eGFR
rs199136
rs35264519
rs138381486
rs78990427
rs1448072
rs13136169
rs62553106
rs17663379
rs55879413
rs4853532
rs116762756
rs11077167
rs80068828
rs118137165
rs10444236
rs2129906
rs145063210
rs3856249
rs112433378
rs190040498
rs78109815
rs112337139
rs769290
rs57505949
All
−0.001
0.000
0.001
MR effect size for
'Protein kish−A' on 'eGFR'

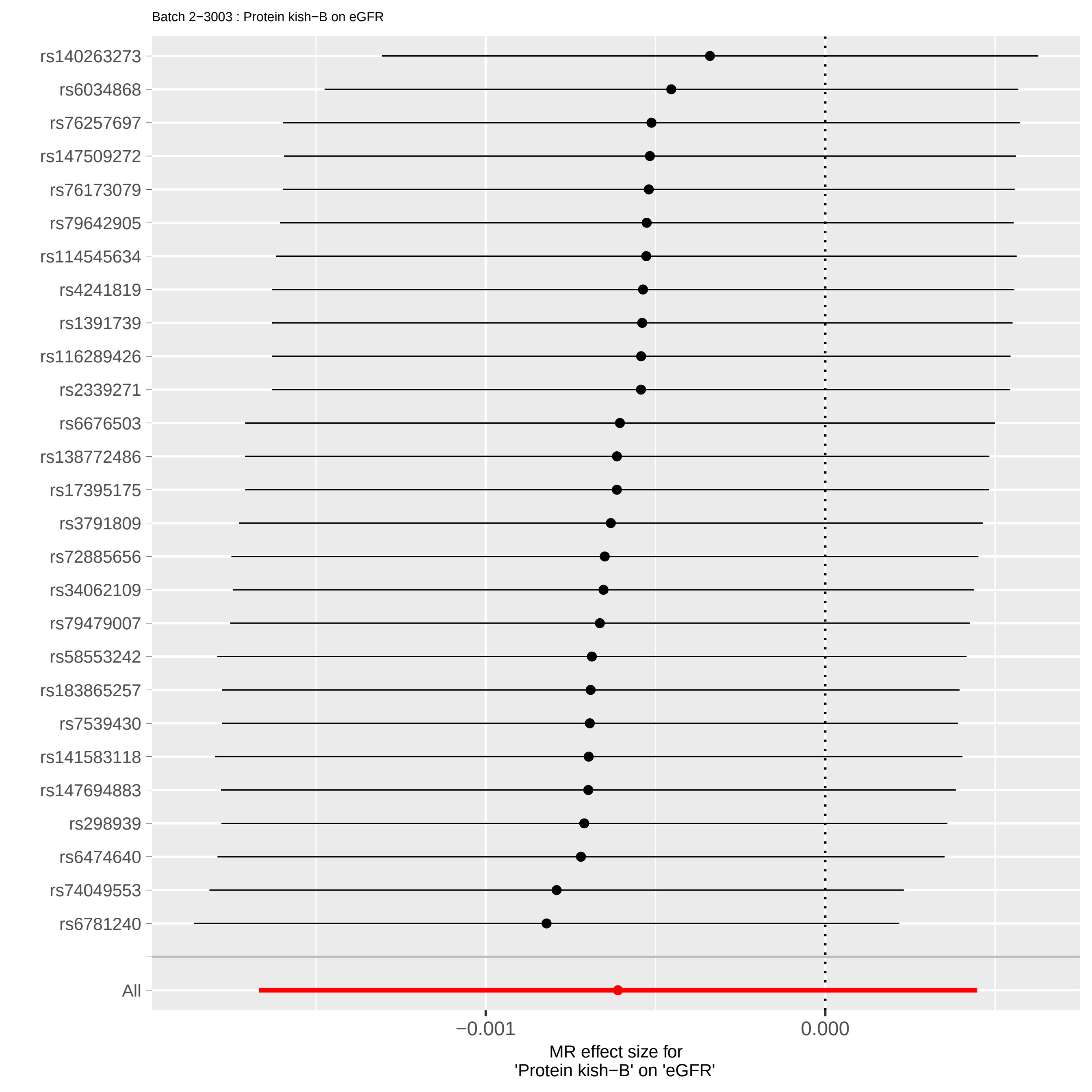
Batch 2−3003 : Protein kish−B on eGFR
rs140263273
rs6034868
rs76257697
rs147509272
rs76173079
rs79642905
rs114545634
rs4241819
rs1391739
rs116289426
rs2339271
rs6676503
rs138772486
rs17395175
rs3791809
rs72885656
rs34062109
rs79479007
rs58553242
rs183865257
rs7539430
rs141583118
rs147694883
rs298939
rs6474640
rs74049553
rs6781240
All
−0.001
0.000
MR effect size for
'Protein kish−B' on 'eGFR'

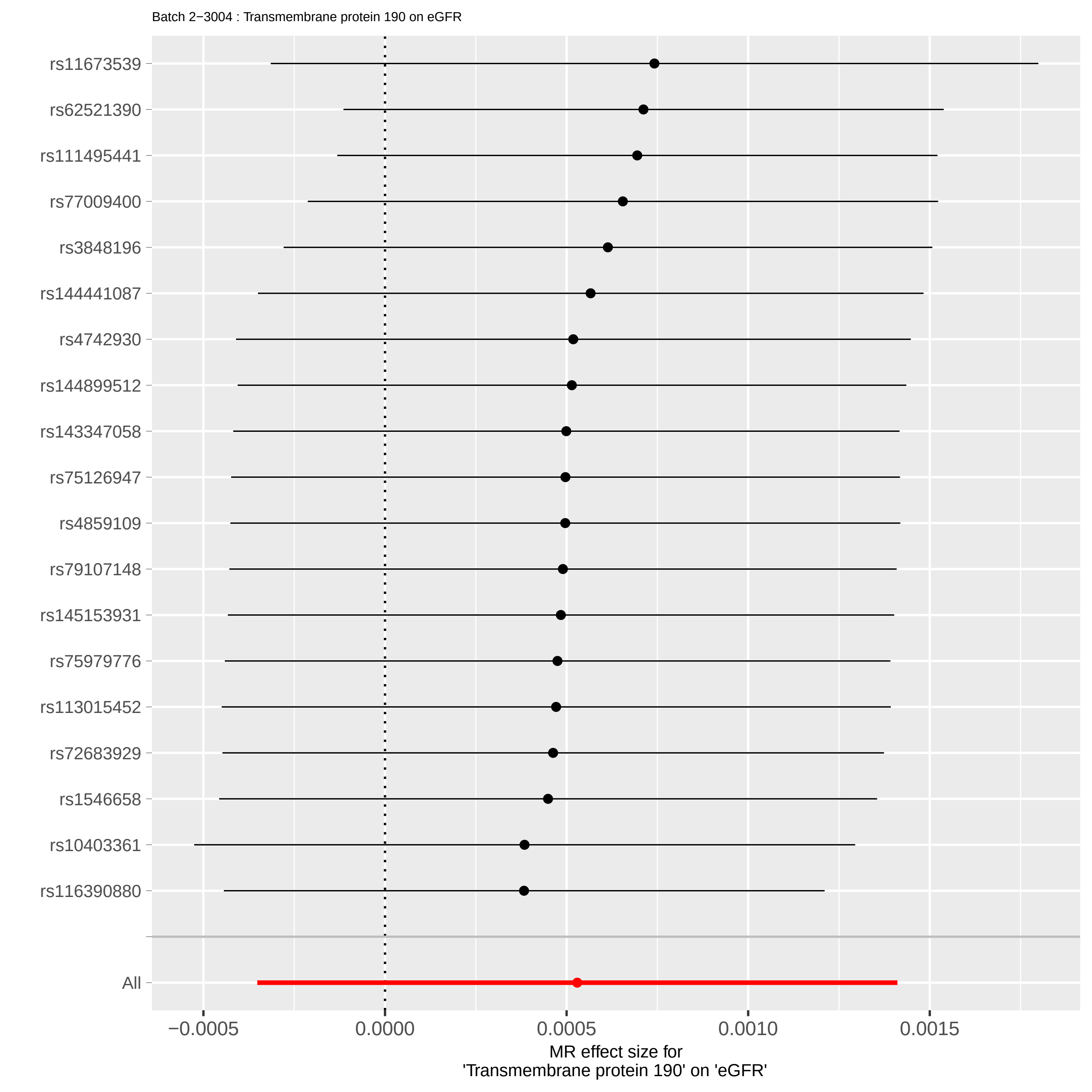

Batch 2−3004 : Transmembrane protein 190 on eGFR
rs11673539
rs62521390
rs111495441
rs77009400
rs3848196
rs144441087
rs4742930
rs144899512
rs143347058
rs75126947
rs4859109
rs79107148
rs145153931
rs75979776
rs113015452
rs72683929
rs1546658
rs10403361
rs116390880
All
−0.0005
0.0000
0.0005
0.0010
0.0015
MR effect size for
'Transmembrane protein 190' on 'eGFR'

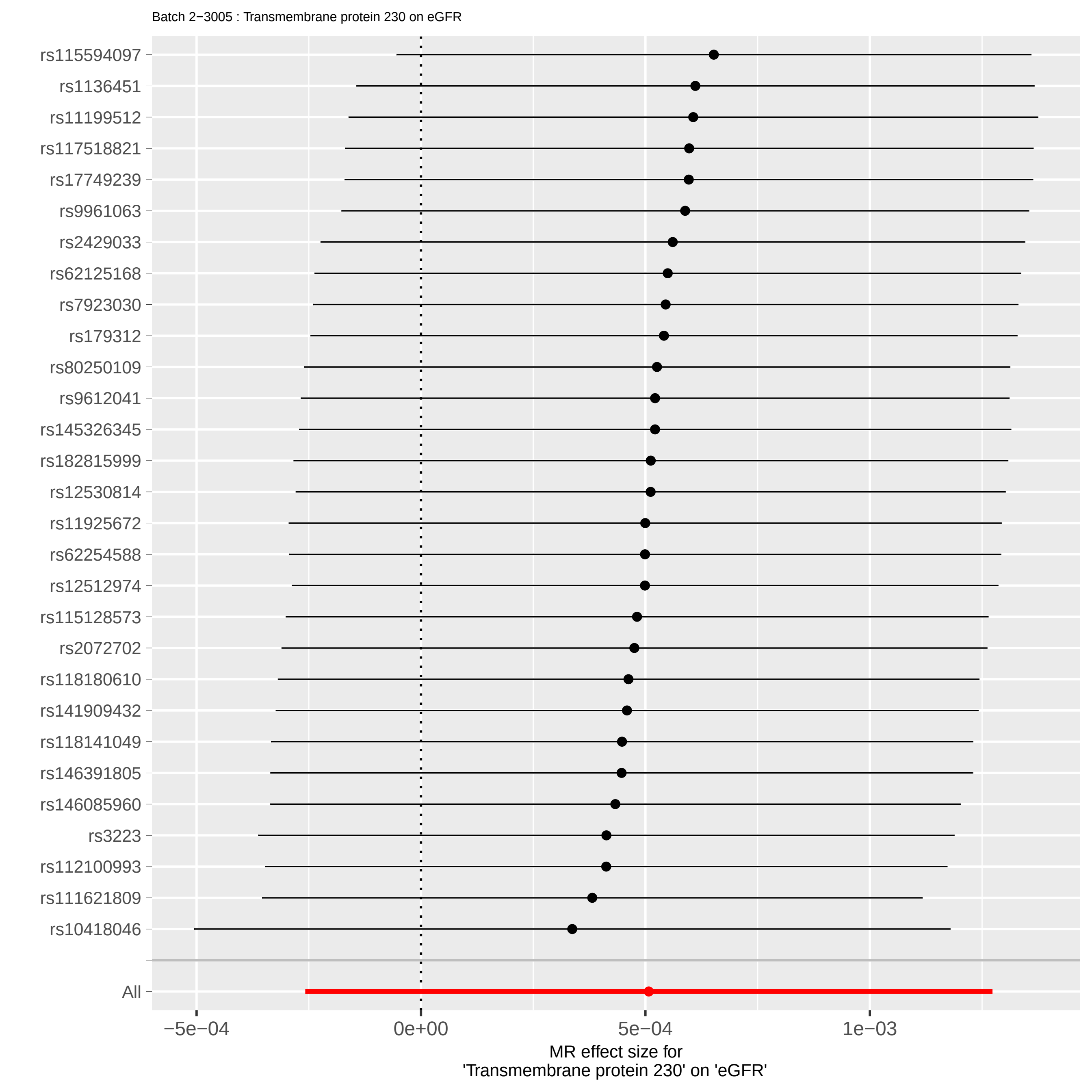
Batch 2−3005 : Transmembrane protein 230 on eGFR
rs115594097
rs1136451
rs11199512
rs117518821
rs17749239
rs9961063
rs2429033
rs62125168
rs7923030
rs179312
rs80250109
rs9612041
rs145326345
rs182815999
rs12530814
rs11925672
rs62254588
rs12512974
rs115128573
rs2072702
rs118180610
rs141909432
rs118141049
rs146391805
rs146085960
rs3223
rs112100993
rs111621809
rs10418046
All
−5e−04
0e+00
5e−04
1e−03
MR effect size for
'Transmembrane protein 230' on 'eGFR'

Batch 2−3006 : Transmembrane protein 234 on eGFR

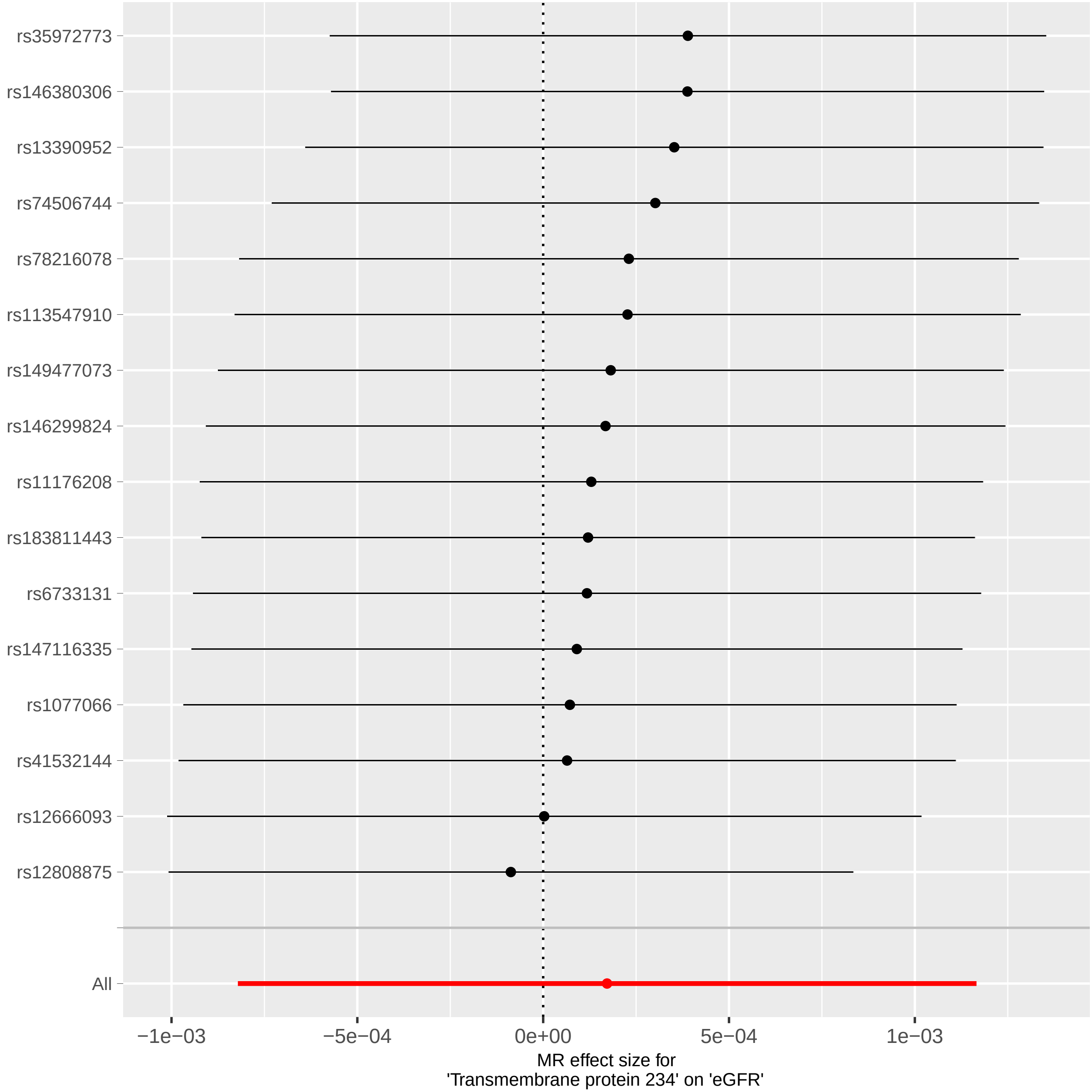

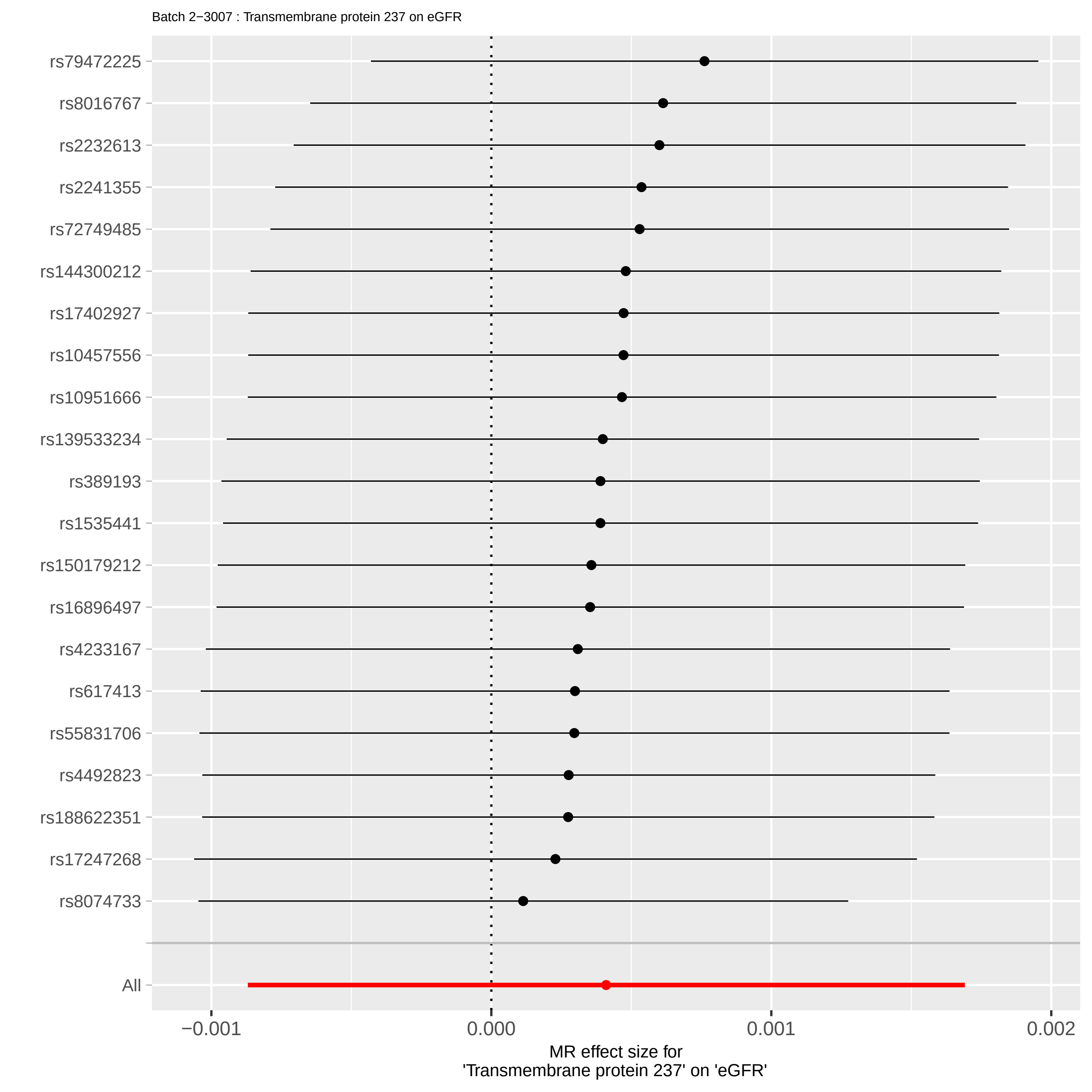

Batch 2−3007 : Transmembrane protein 237 on eGFR
rs79472225
rs8016767
rs2232613
rs2241355
rs72749485
rs144300212
rs17402927
rs10457556
rs10951666
rs139533234
rs389193
rs1535441
rs150179212
rs16896497
rs4233167
rs617413
rs55831706
rs4492823
rs188622351
rs17247268
rs8074733
All
−0.001
0.000
0.001
0.002
MR effect size for
'Transmembrane protein 237' on 'eGFR'

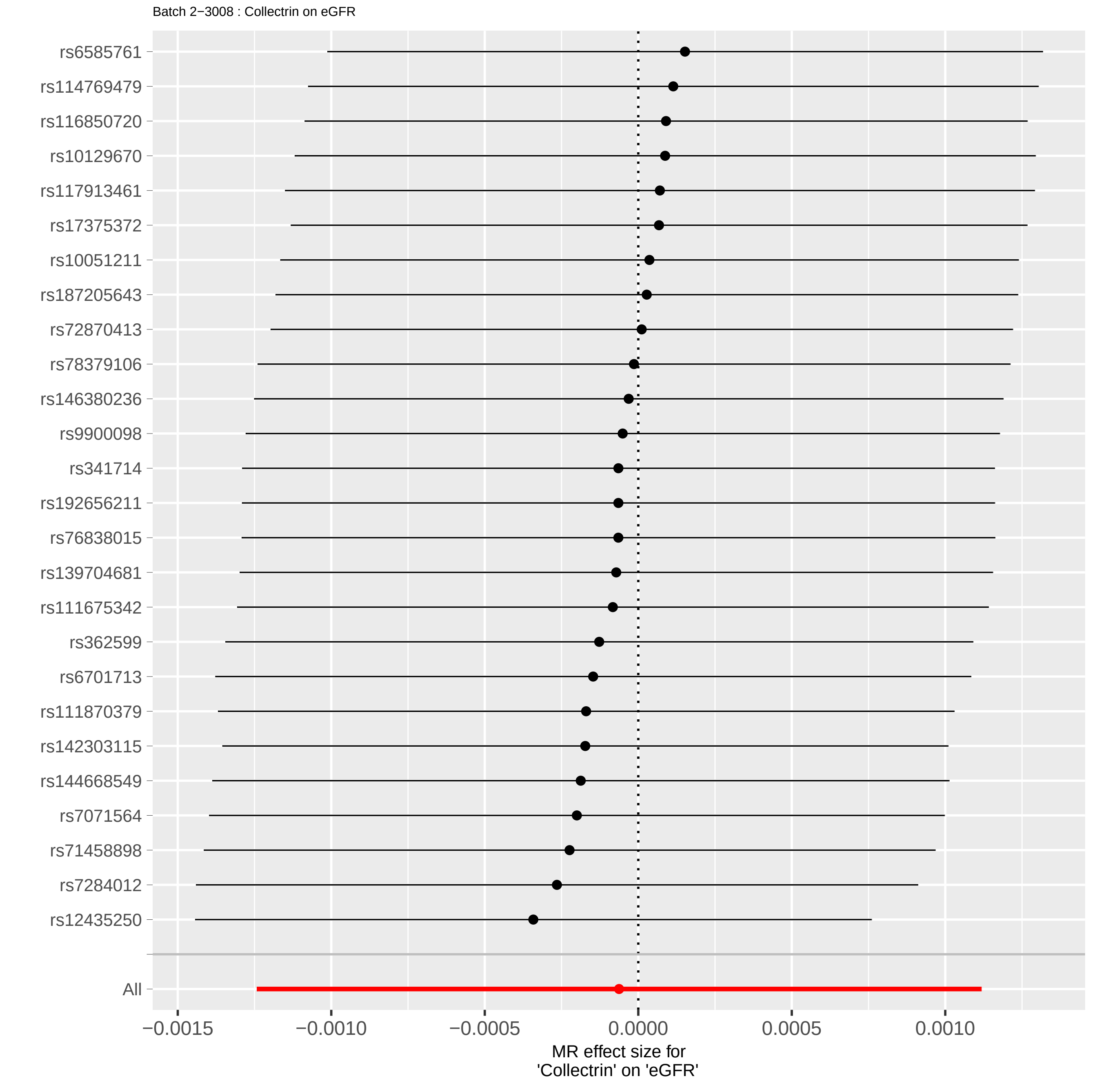

Batch 2−3008 : Collectrin on eGFR
rs6585761
rs114769479
rs116850720
rs10129670
rs117913461
rs17375372
rs10051211
rs187205643
rs72870413
rs78379106
rs146380236
rs9900098
rs341714
rs192656211
rs76838015
rs139704681
rs111675342
rs362599
rs6701713
rs111870379
rs142303115
rs144668549
rs7071564
rs71458898
rs7284012
rs12435250
All
−0.0015
−0.0010
−0.0005
0.0000
0.0005
0.0010
MR effect size for
'Collectrin' on 'eGFR'

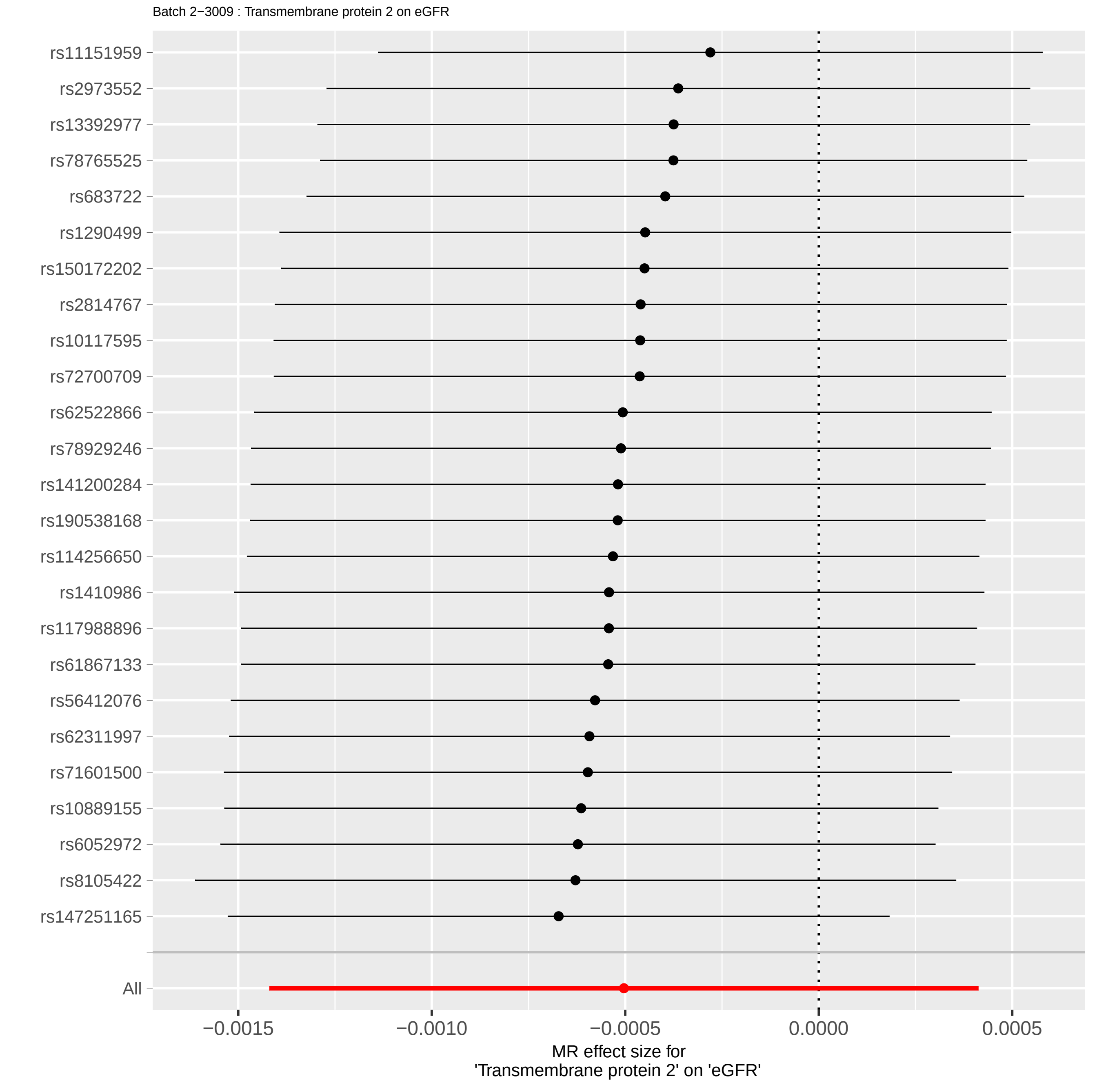

Batch 2−3009 : Transmembrane protein 2 on eGFR
rs11151959
rs2973552
rs13392977
rs78765525
rs683722
rs1290499
rs150172202
rs2814767
rs10117595
rs72700709
rs62522866
rs78929246
rs141200284
rs190538168
rs114256650
rs1410986
rs117988896
rs61867133
rs56412076
rs62311997
rs71601500
rs10889155
rs6052972
rs8105422
rs147251165
All
−0.0015
−0.0010
−0.0005
0.0000
0.0005
MR effect size for
'Transmembrane protein 2' on 'eGFR'

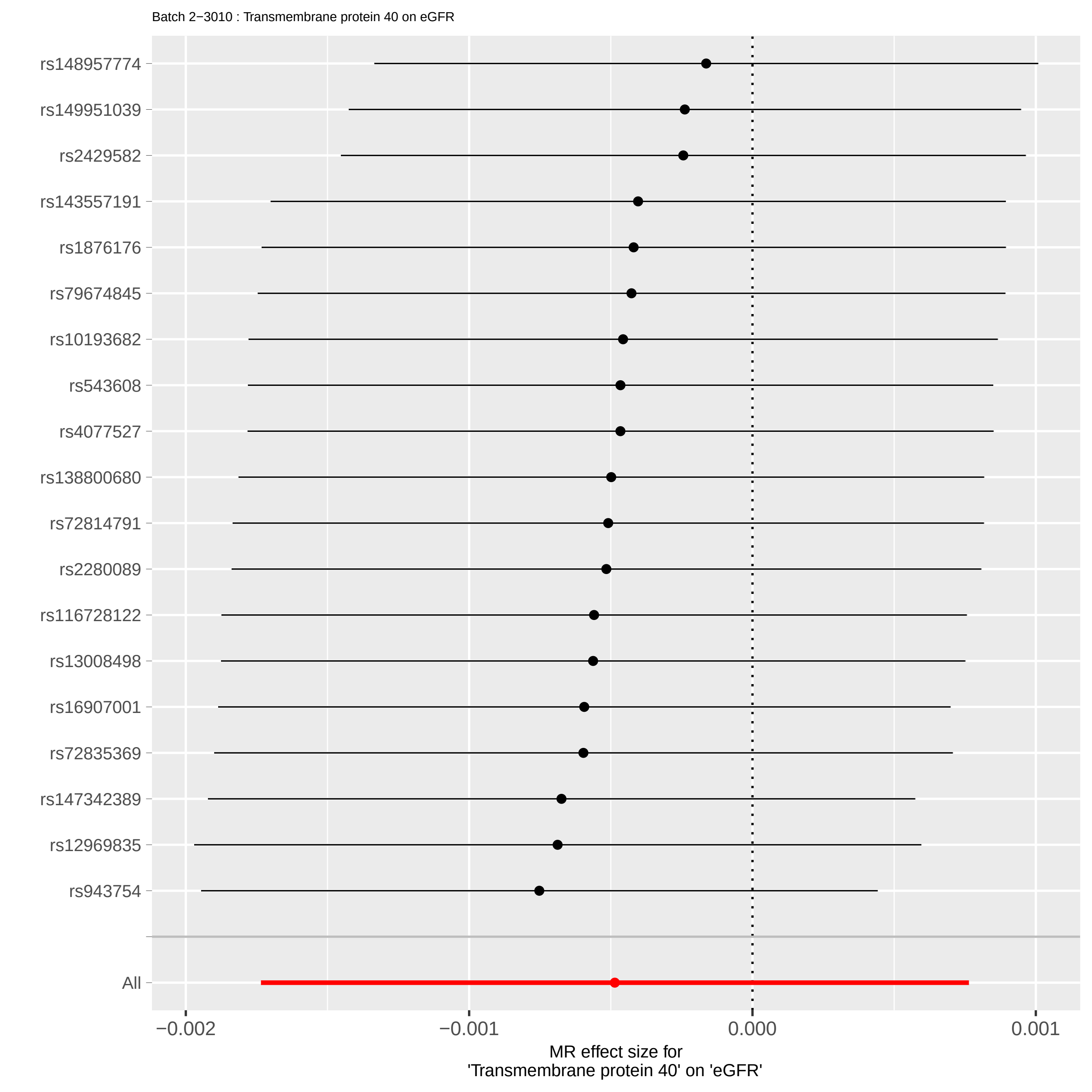

Batch 2−3010 : Transmembrane protein 40 on eGFR
rs148957774
rs149951039
rs2429582
rs143557191
rs1876176
rs79674845
rs10193682
rs543608
rs4077527
rs138800680
rs72814791
rs2280089
rs116728122
rs13008498
rs16907001
rs72835369
rs147342389
rs12969835
rs943754
All
−0.002
−0.001
0.000
0.001
MR effect size for
'Transmembrane protein 40' on 'eGFR'

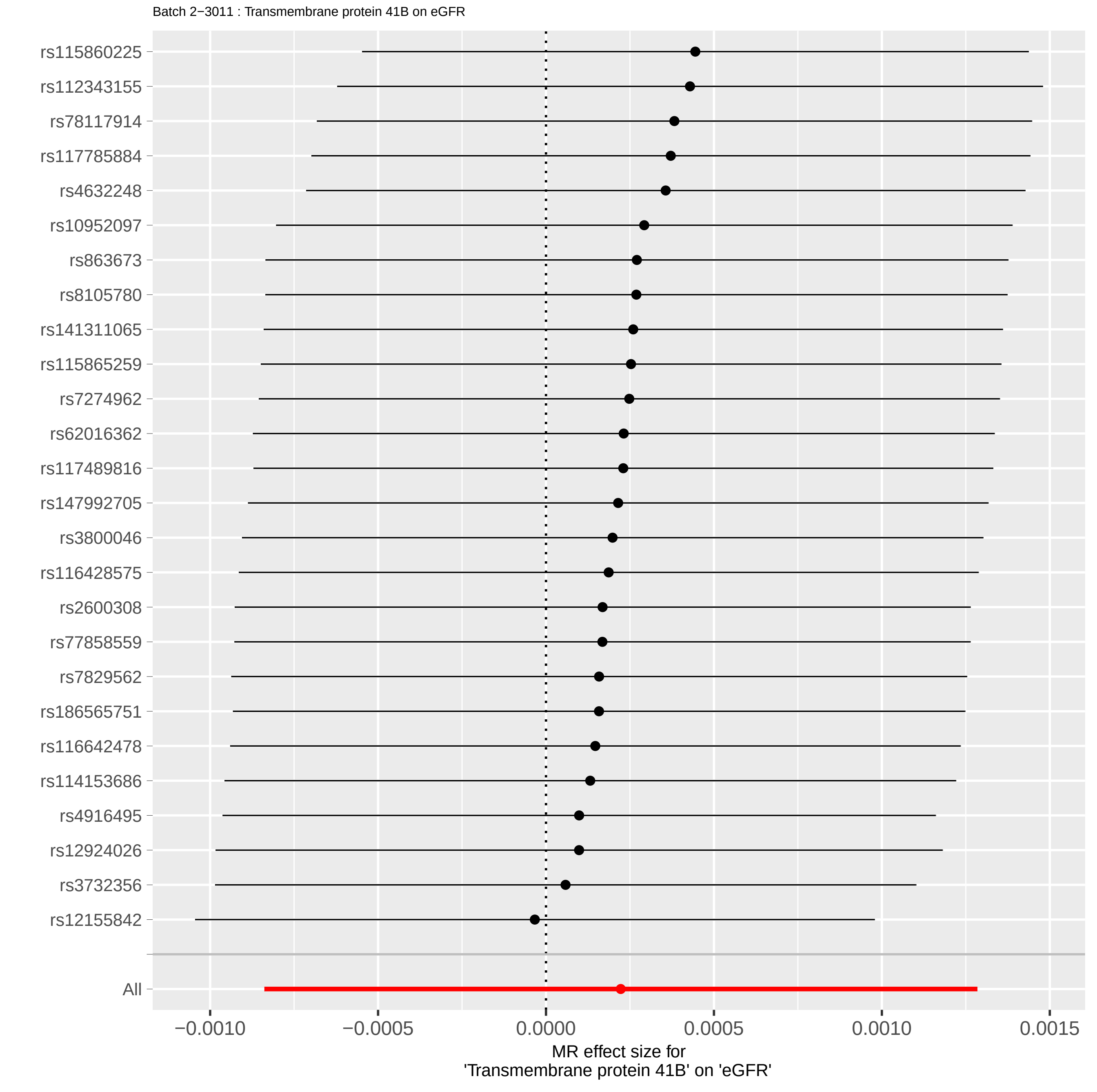

Batch 2−3011 : Transmembrane protein 41B on eGFR
rs115860225
rs112343155
rs78117914
rs117785884
rs4632248
rs10952097
rs863673
rs8105780
rs141311065
rs115865259
rs7274962
rs62016362
rs117489816
rs147992705
rs3800046
rs116428575
rs2600308
rs77858559
rs7829562
rs186565751
rs116642478
rs114153686
rs4916495
rs12924026
rs3732356
rs12155842
All
−0.0010
−0.0005
0.0000
0.0005
0.0010
0.0015
MR effect size for
'Transmembrane protein 41B' on 'eGFR'

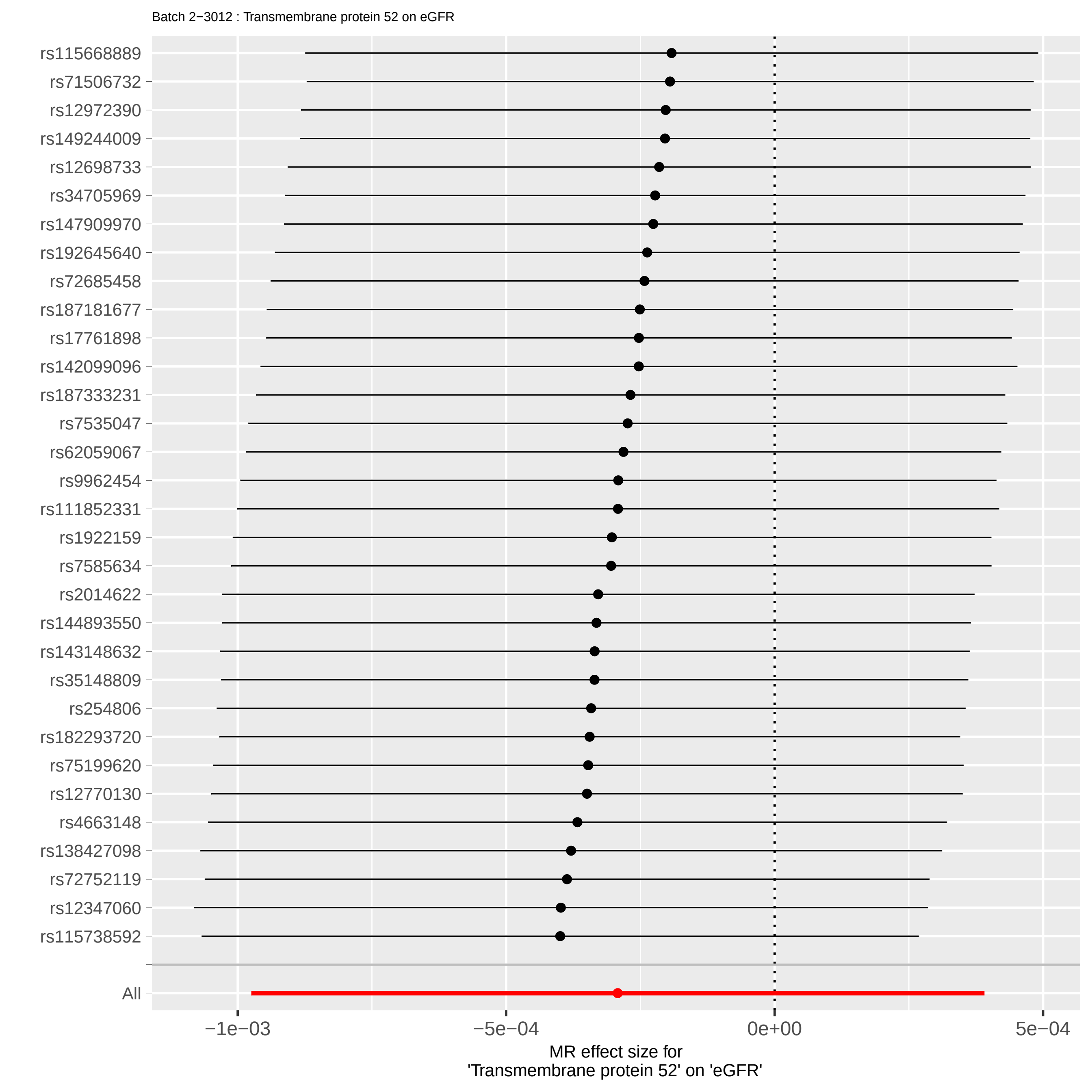

Batch 2−3012 : Transmembrane protein 52 on eGFR
rs115668889
rs71506732
rs12972390
rs149244009
rs12698733
rs34705969
rs147909970
rs192645640
rs72685458
rs187181677
rs17761898
rs142099096
rs187333231
rs7535047
rs62059067
rs9962454
rs111852331
rs1922159
rs7585634
rs2014622
rs144893550
rs143148632
rs35148809
rs254806
rs182293720
rs75199620
rs12770130
rs4663148
rs138427098
rs72752119
rs12347060
rs115738592
All
−1e−03
−5e−04
0e+00
5e−04
MR effect size for
'Transmembrane protein 52' on 'eGFR'

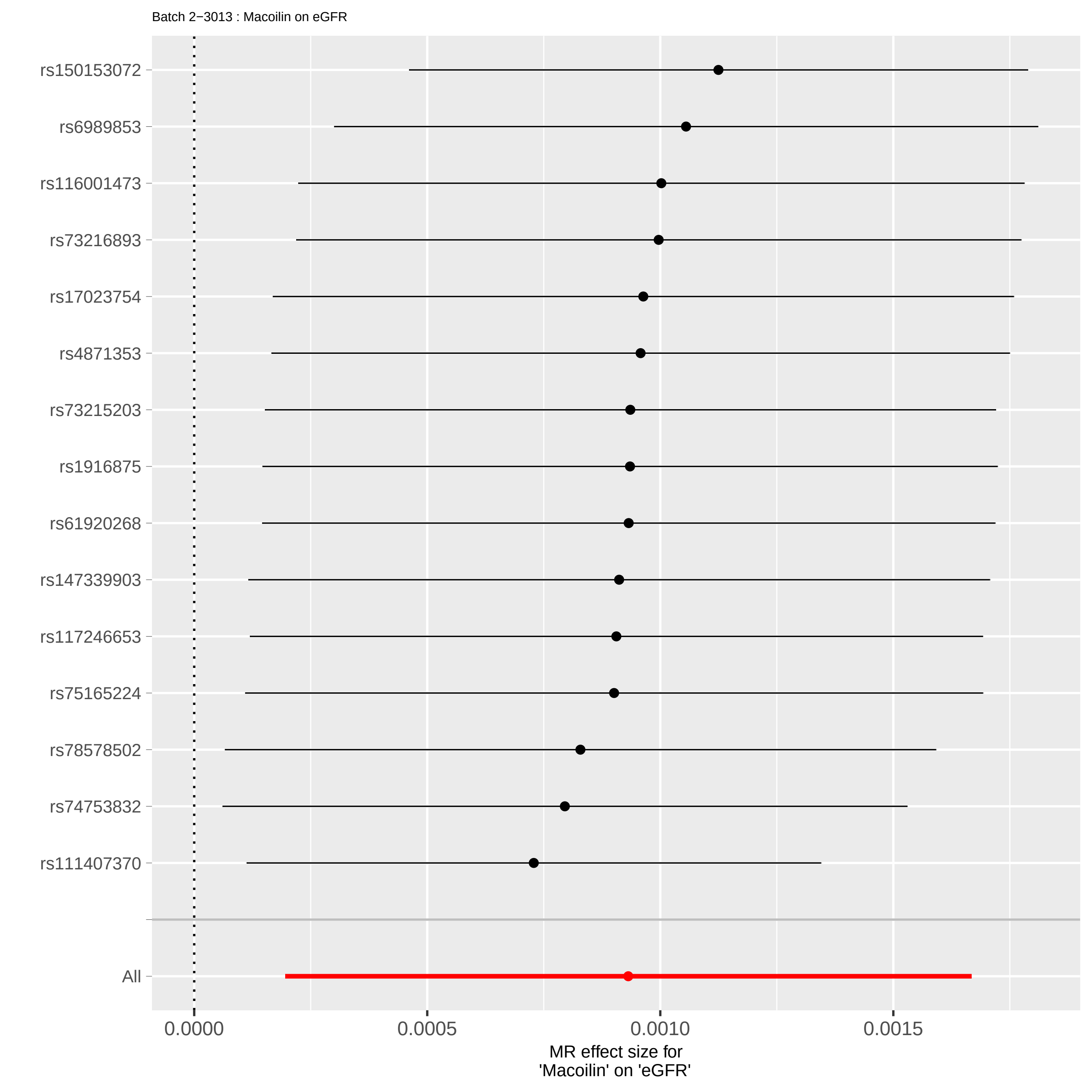

Batch 2−3013 : Macoilin on eGFR
rs150153072
rs6989853
rs116001473
rs73216893
rs17023754
rs4871353
rs73215203
rs1916875
rs61920268
rs147339903
rs117246653
rs75165224
rs78578502
rs74753832
rs111407370
All
0.0000
0.0005
0.0010
0.0015
MR effect size for
'Macoilin' on 'eGFR'

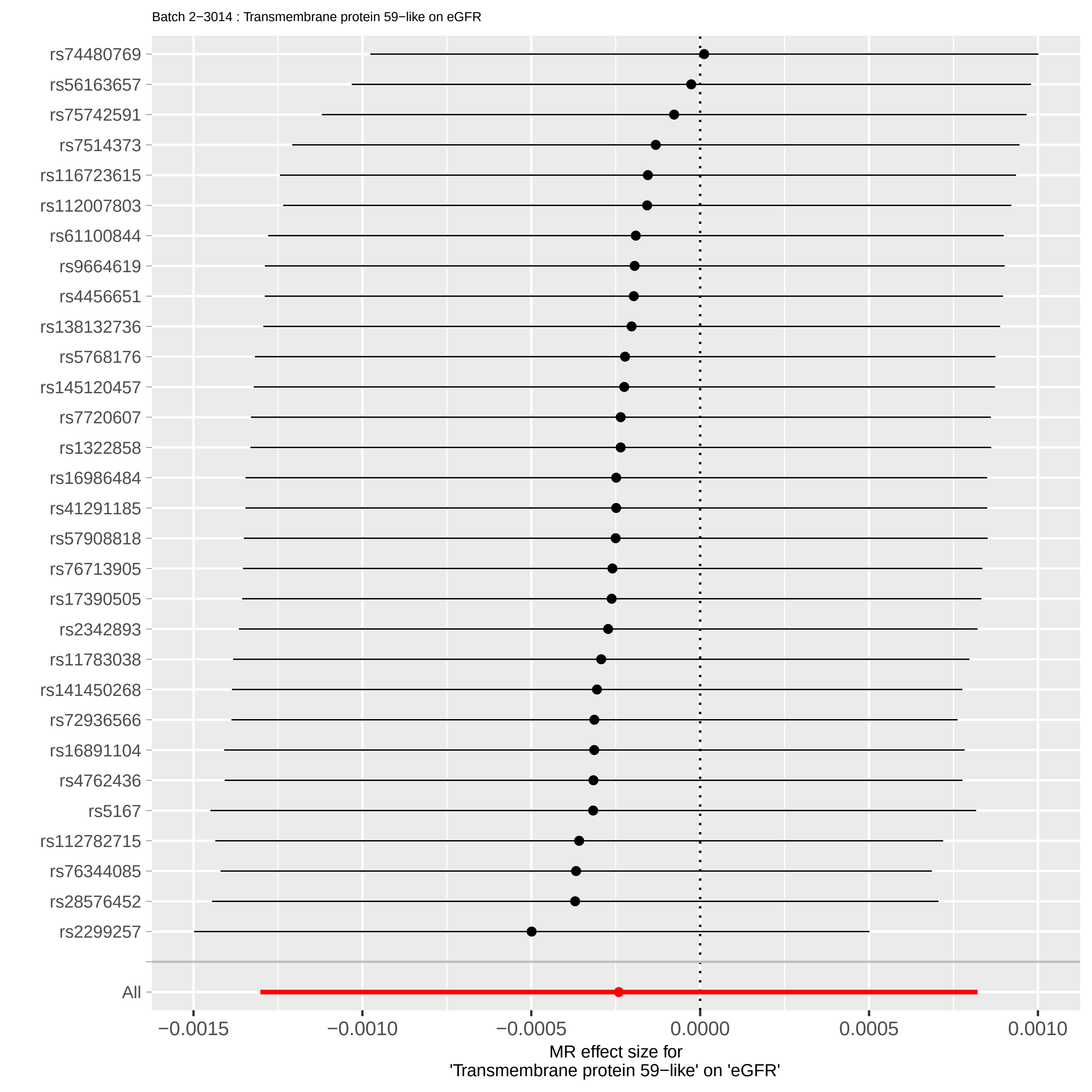

Batch 2−3014 : Transmembrane protein 59−like on eGFR
rs74480769
rs56163657
rs75742591
rs7514373
rs116723615
rs112007803
rs61100844
rs9664619
rs4456651
rs138132736
rs5768176
rs145120457
rs7720607
rs1322858
rs16986484
rs41291185
rs57908818
rs76713905
rs17390505
rs2342893
rs11783038
rs141450268
rs72936566
rs16891104
rs4762436
rs5167
rs112782715
rs76344085
rs28576452
rs2299257
All
−0.0015
−0.0010
−0.0005
0.0000
0.0005
0.0010
MR effect size for
'Transmembrane protein 59−like' on 'eGFR'

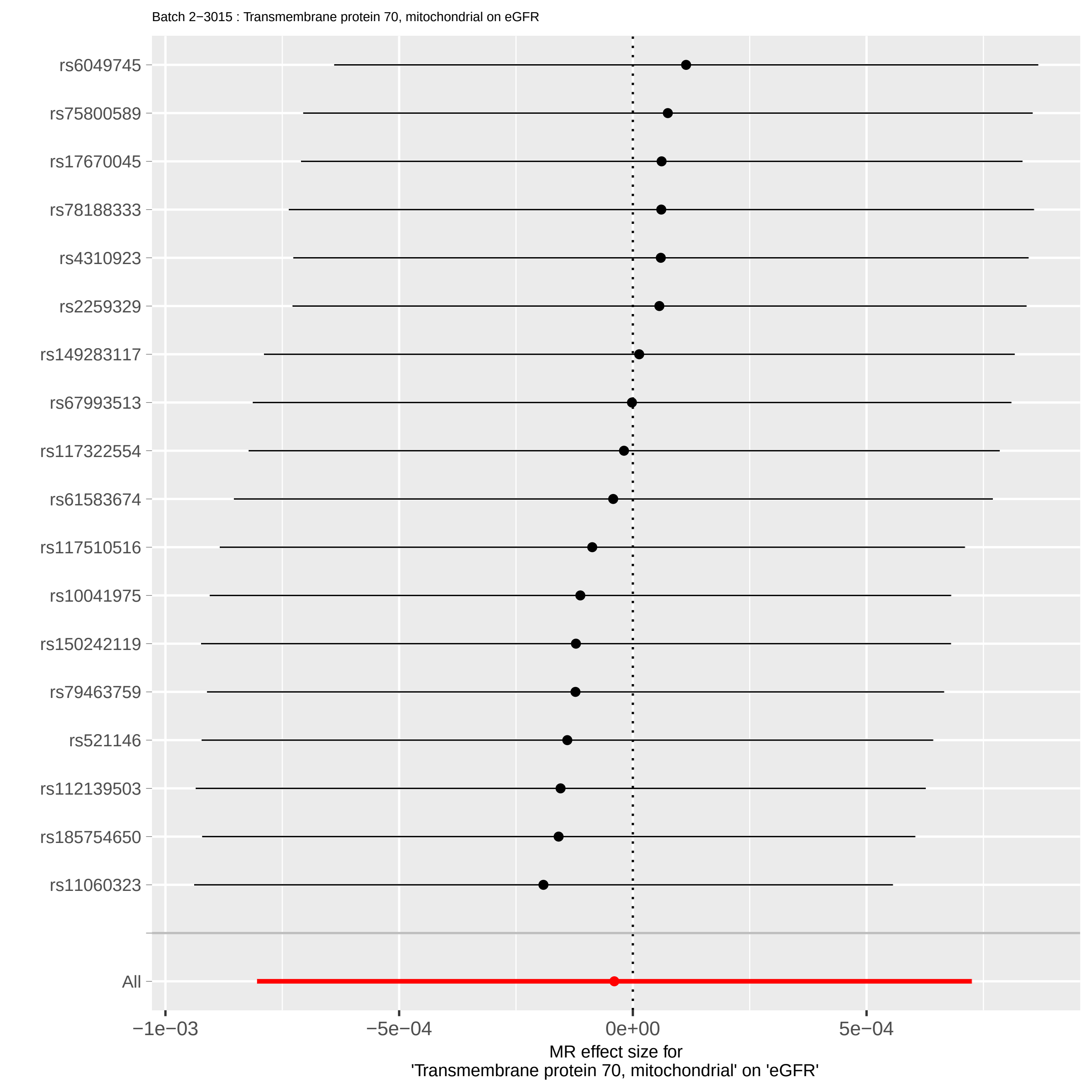

Batch 2−3015 : Transmembrane protein 70, mitochondrial on eGFR
rs6049745
rs75800589
rs17670045
rs78188333
rs4310923
rs2259329
rs149283117
rs67993513
rs117322554
rs61583674
rs117510516
rs10041975
rs150242119
rs79463759
rs521146
rs112139503
rs185754650
rs11060323
All
−1e−03
−5e−04
0e+00
5e−04
MR effect size for
'Transmembrane protein 70, mitochondrial' on 'eGFR'

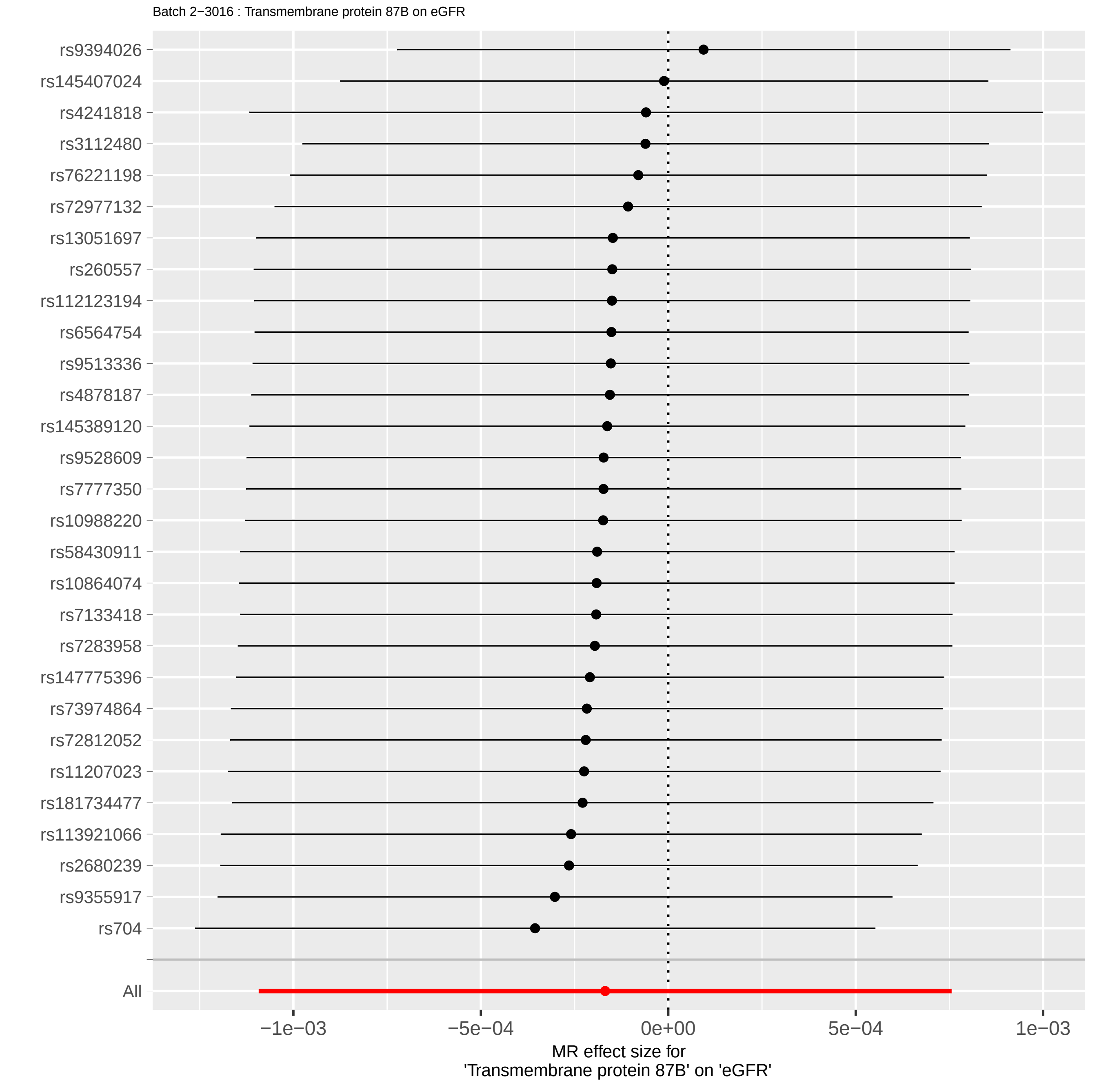

Batch 2−3016 : Transmembrane protein 87B on eGFR
rs9394026
rs145407024
rs4241818
rs3112480
rs76221198
rs72977132
rs13051697
rs260557
rs112123194
rs6564754
rs9513336
rs4878187
rs145389120
rs9528609
rs7777350
rs10988220
rs58430911
rs10864074
rs7133418
rs7283958
rs147775396
rs73974864
rs72812052
rs11207023
rs181734477
rs113921066
rs2680239
rs9355917
rs704
All
−1e−03
−5e−04
0e+00
5e−04
1e−03
MR effect size for
'Transmembrane protein 87B' on 'eGFR'

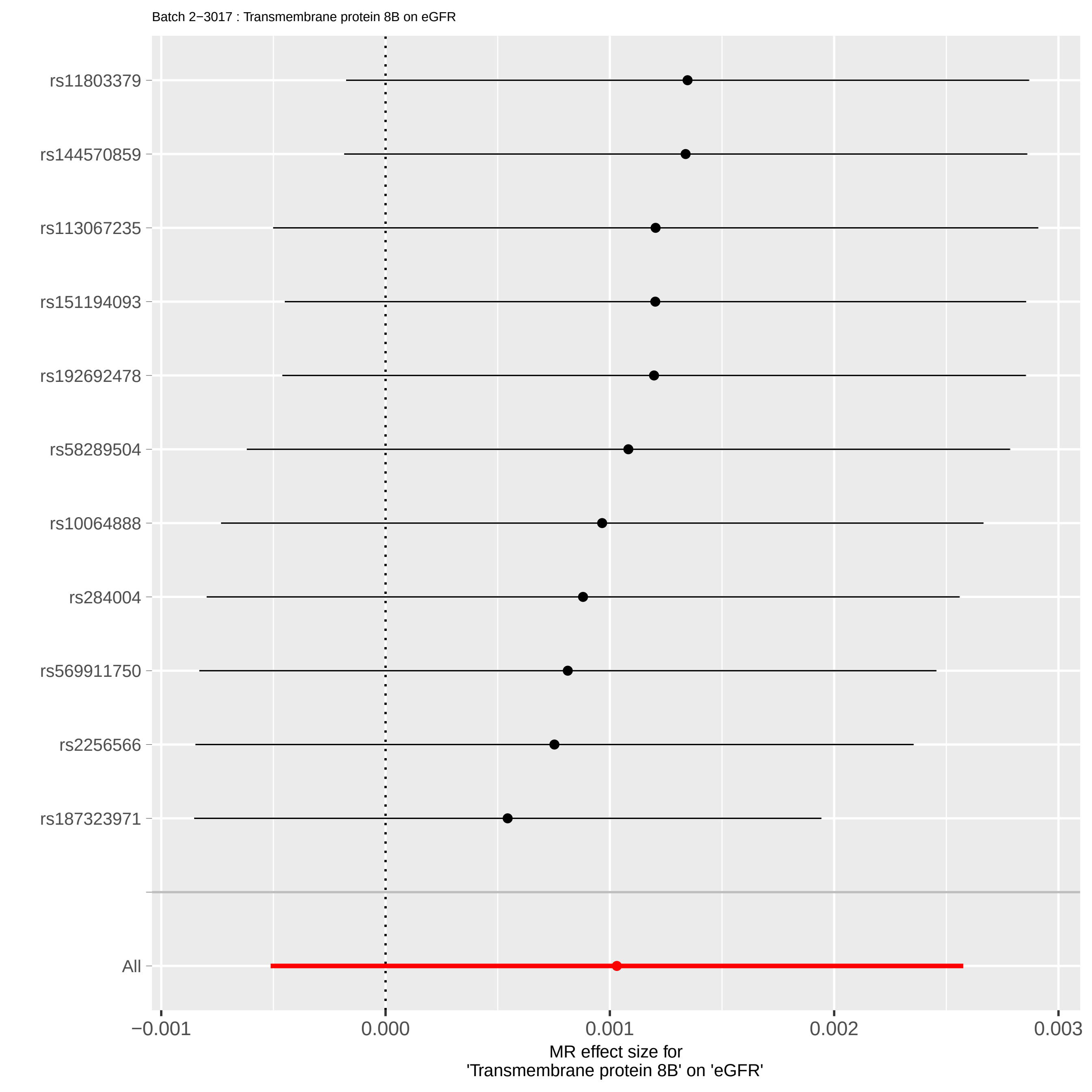

Batch 2−3017 : Transmembrane protein 8B on eGFR
rs11803379
rs144570859
rs113067235
rs151194093
rs192692478
rs58289504
rs10064888
rs284004
rs569911750
rs2256566
rs187323971
All
−0.001
0.000
0.001
0.002
0.003
MR effect size for
'Transmembrane protein 8B' on 'eGFR'

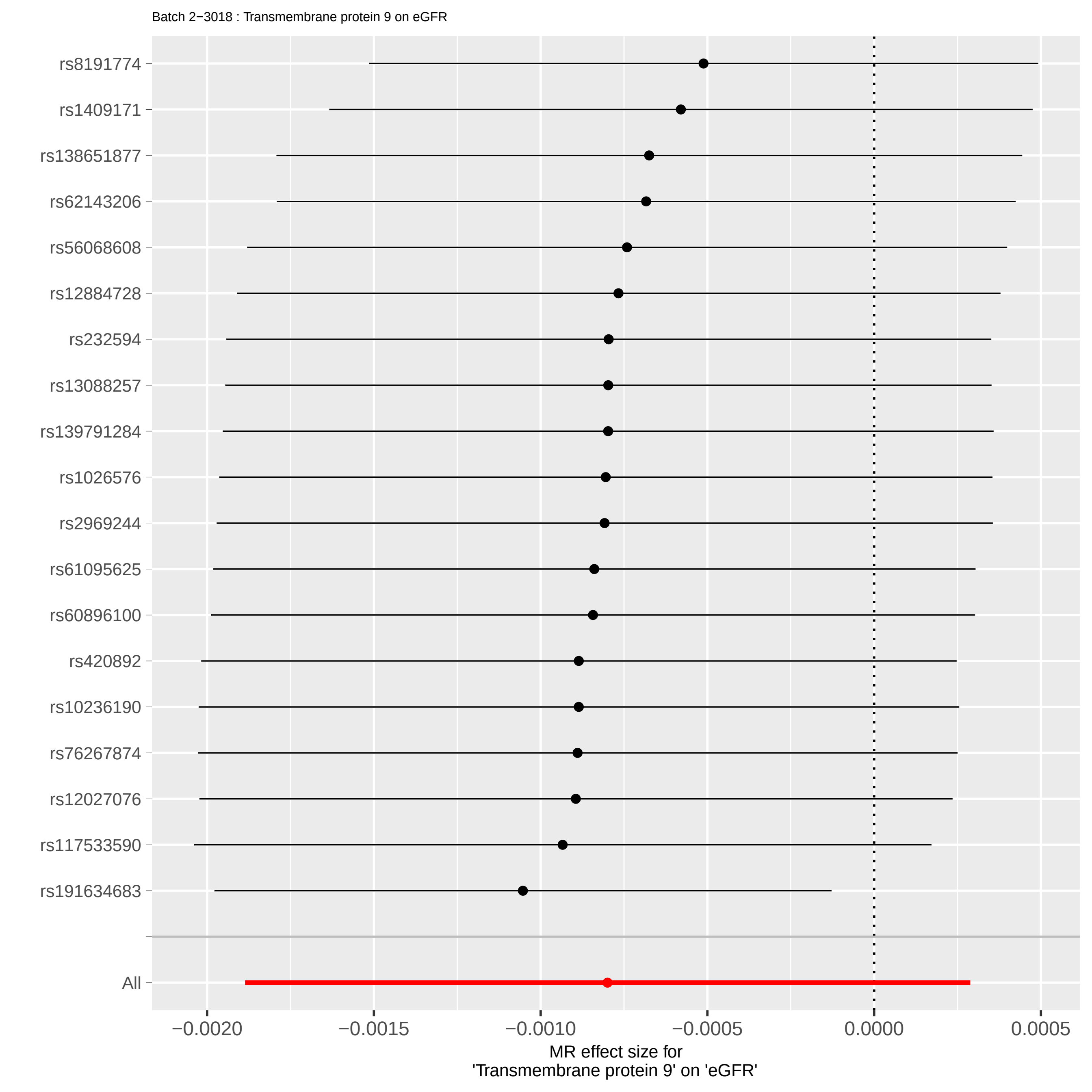

Batch 2−3018 : Transmembrane protein 9 on eGFR
rs8191774
rs1409171
rs138651877
rs62143206
rs56068608
rs12884728
rs232594
rs13088257
rs139791284
rs1026576
rs2969244
rs61095625
rs60896100
rs420892
rs10236190
rs76267874
rs12027076
rs117533590
rs191634683
All
−0.0020
−0.0015
−0.0010
−0.0005
0.0000
0.0005
MR effect size for
'Transmembrane protein 9' on 'eGFR'

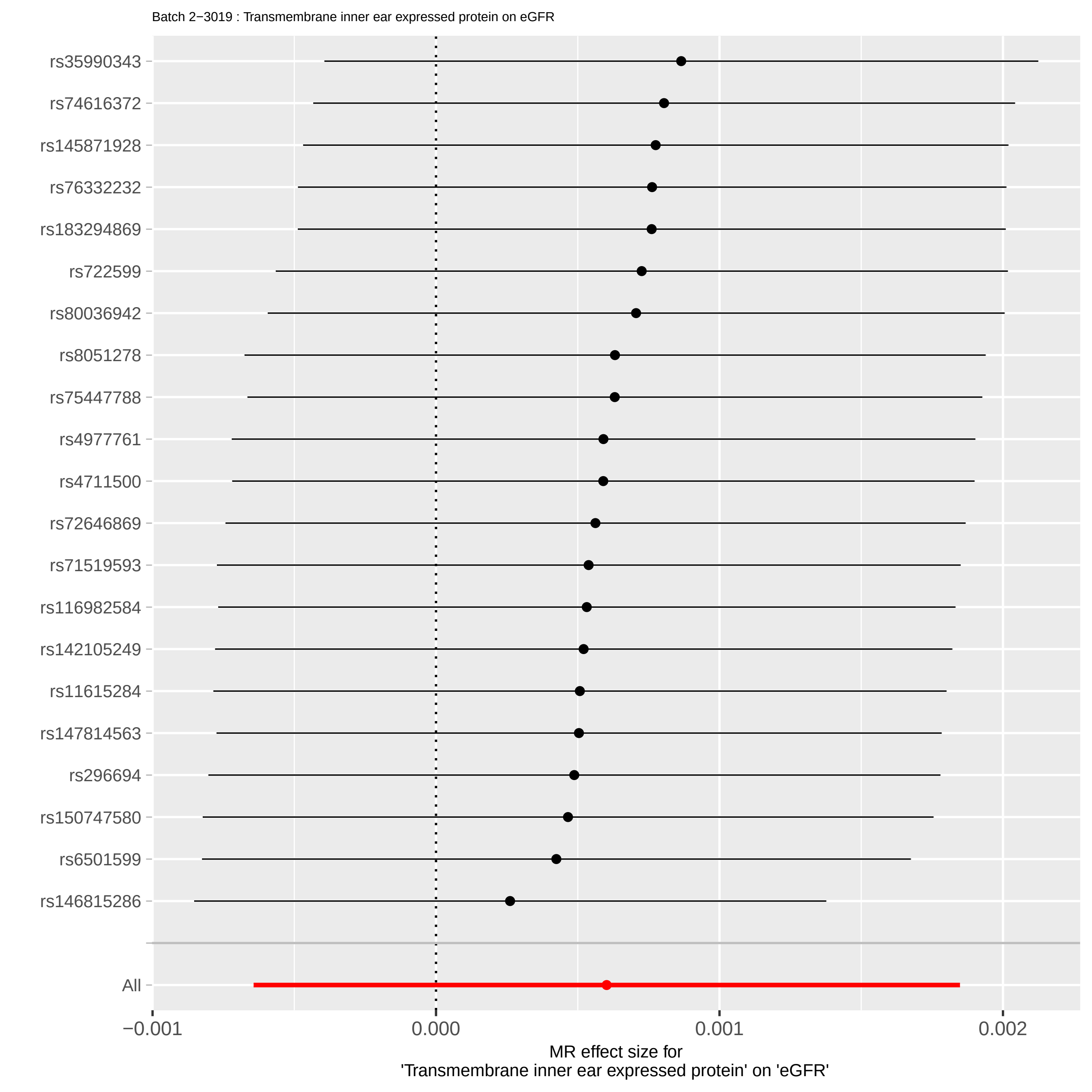
Batch 2−3019 : Transmembrane inner ear expressed protein on eGFR
rs35990343
rs74616372
rs145871928
rs76332232
rs183294869
rs722599
rs80036942
rs8051278
rs75447788
rs4977761
rs4711500
rs72646869
rs71519593
rs116982584
rs142105249
rs11615284
rs147814563
rs296694
rs150747580
rs6501599
rs146815286
All
−0.001
0.000
0.001
0.002
MR effect size for
'Transmembrane inner ear expressed protein' on 'eGFR'

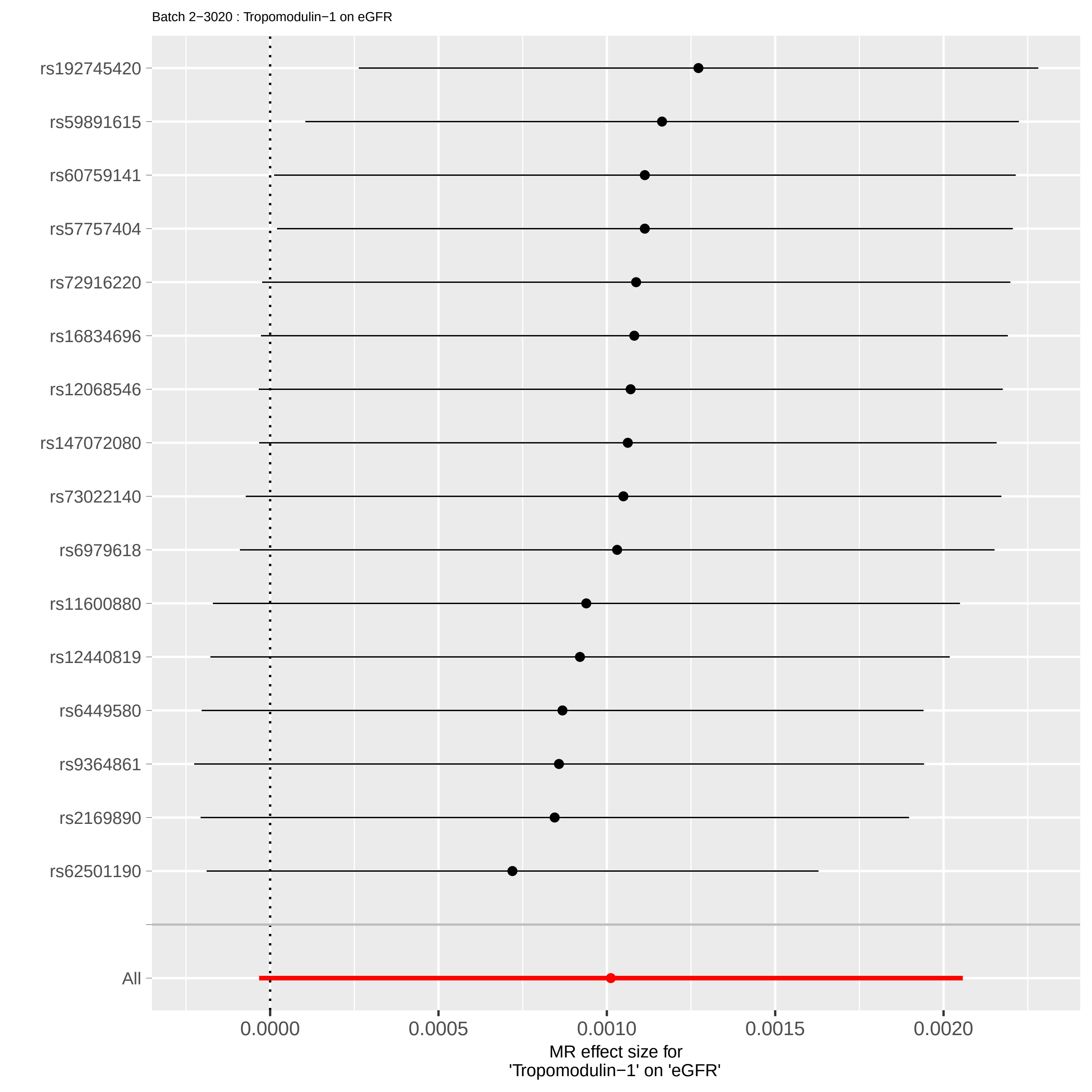

Batch 2−3020 : Tropomodulin−1 on eGFR
rs192745420
rs59891615
rs60759141
rs57757404
rs72916220
rs16834696
rs12068546
rs147072080
rs73022140
rs6979618
rs11600880
rs12440819
rs6449580
rs9364861
rs2169890
rs62501190
All
0.0000
0.0005
0.0010
0.0015
0.0020
MR effect size for
'Tropomodulin−1' on 'eGFR'

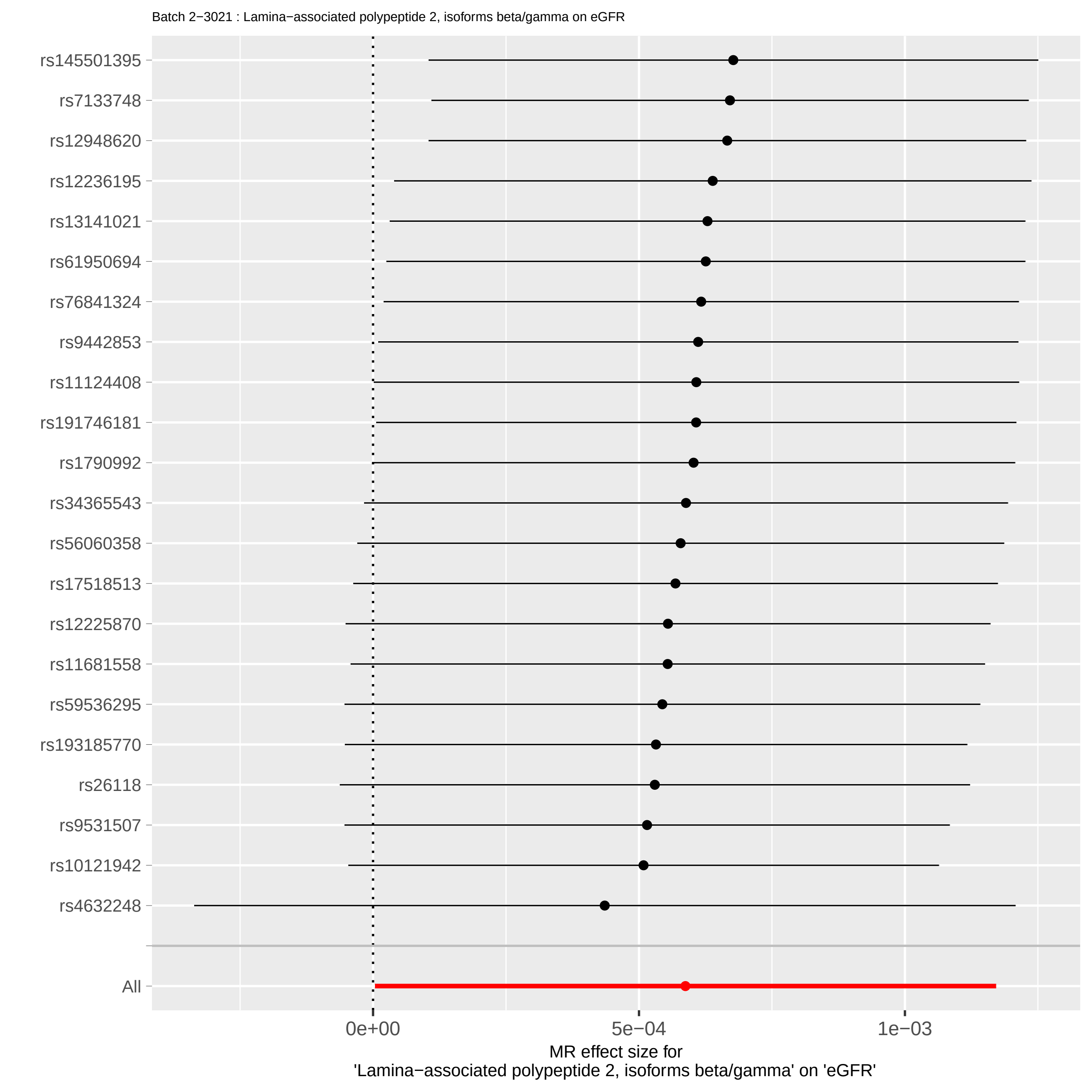

Batch 2−3021 : Lamina−associated polypeptide 2, isoforms beta/gamma on eGFR
rs145501395
rs7133748
rs12948620
rs12236195
rs13141021
rs61950694
rs76841324
rs9442853
rs11124408
rs191746181
rs1790992
rs34365543
rs56060358
rs17518513
rs12225870
rs11681558
rs59536295
rs193185770
rs26118
rs9531507
rs10121942
rs4632248
All
0e+00
5e−04
1e−03
MR effect size for
'Lamina−associated polypeptide 2, isoforms beta/gamma' on 'eGFR'

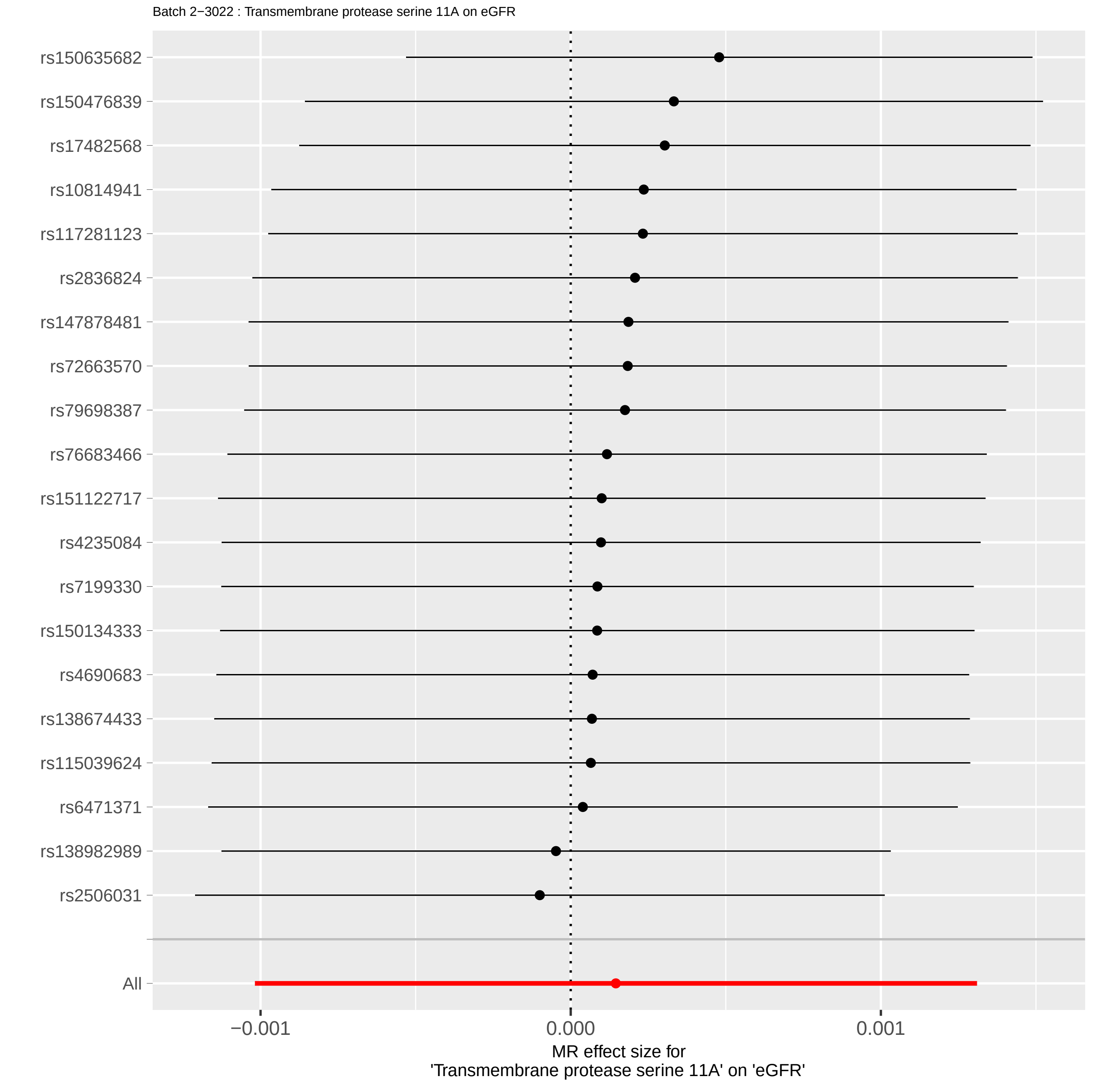

Batch 2−3022 : Transmembrane protease serine 11A on eGFR
rs150635682
rs150476839
rs17482568
rs10814941
rs117281123
rs2836824
rs147878481
rs72663570
rs79698387
rs76683466
rs151122717
rs4235084
rs7199330
rs150134333
rs4690683
rs138674433
rs115039624
rs6471371
rs138982989
rs2506031
All
−0.001
0.000
0.001
MR effect size for
'Transmembrane protease serine 11A' on 'eGFR'

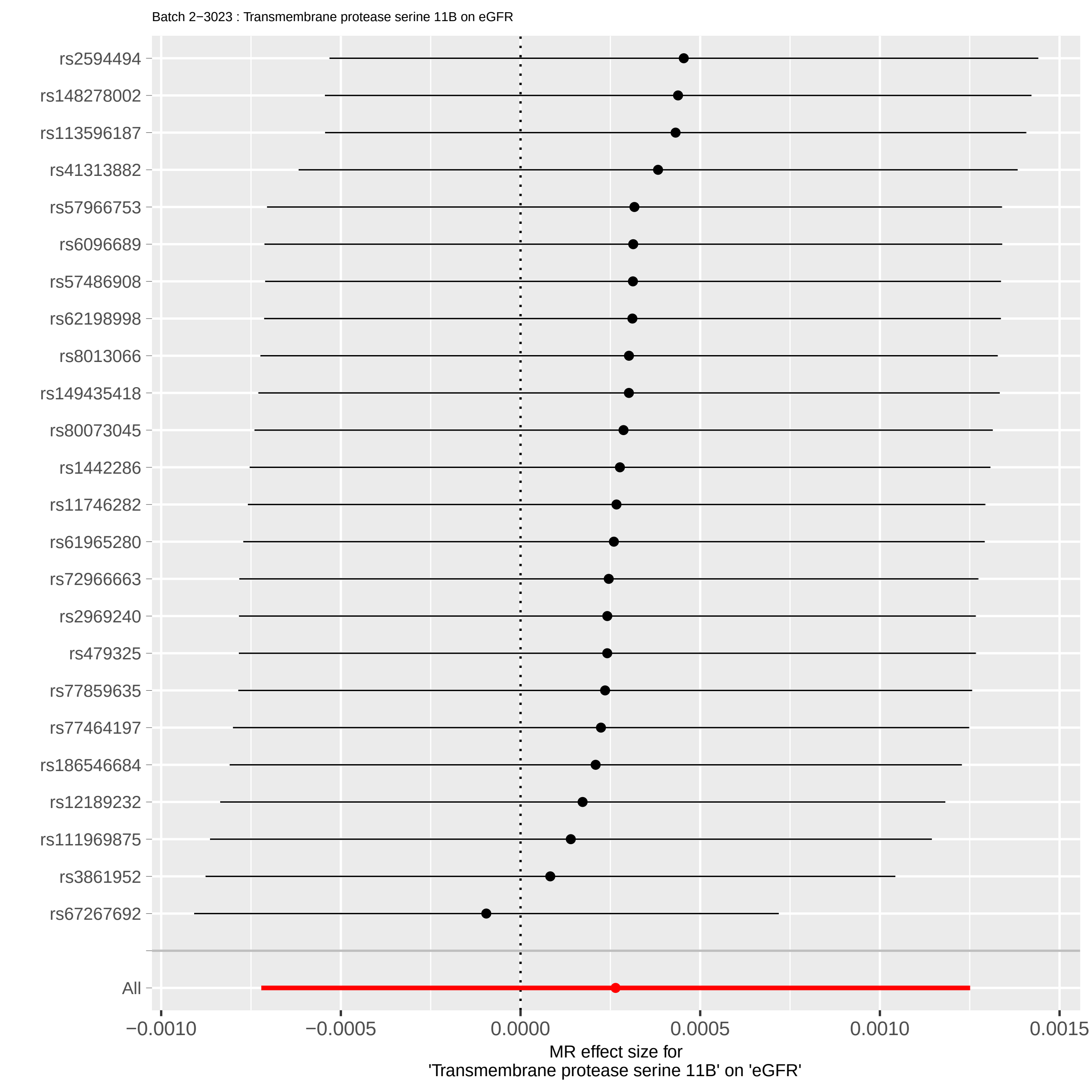

Batch 2−3023 : Transmembrane protease serine 11B on eGFR
rs2594494
rs148278002
rs113596187
rs41313882
rs57966753
rs6096689
rs57486908
rs62198998
rs8013066
rs149435418
rs80073045
rs1442286
rs11746282
rs61965280
rs72966663
rs2969240
rs479325
rs77859635
rs77464197
rs186546684
rs12189232
rs111969875
rs3861952
rs67267692
All
−0.0010
−0.0005
0.0000
0.0005
0.0010
0.0015
MR effect size for
'Transmembrane protease serine 11B' on 'eGFR'

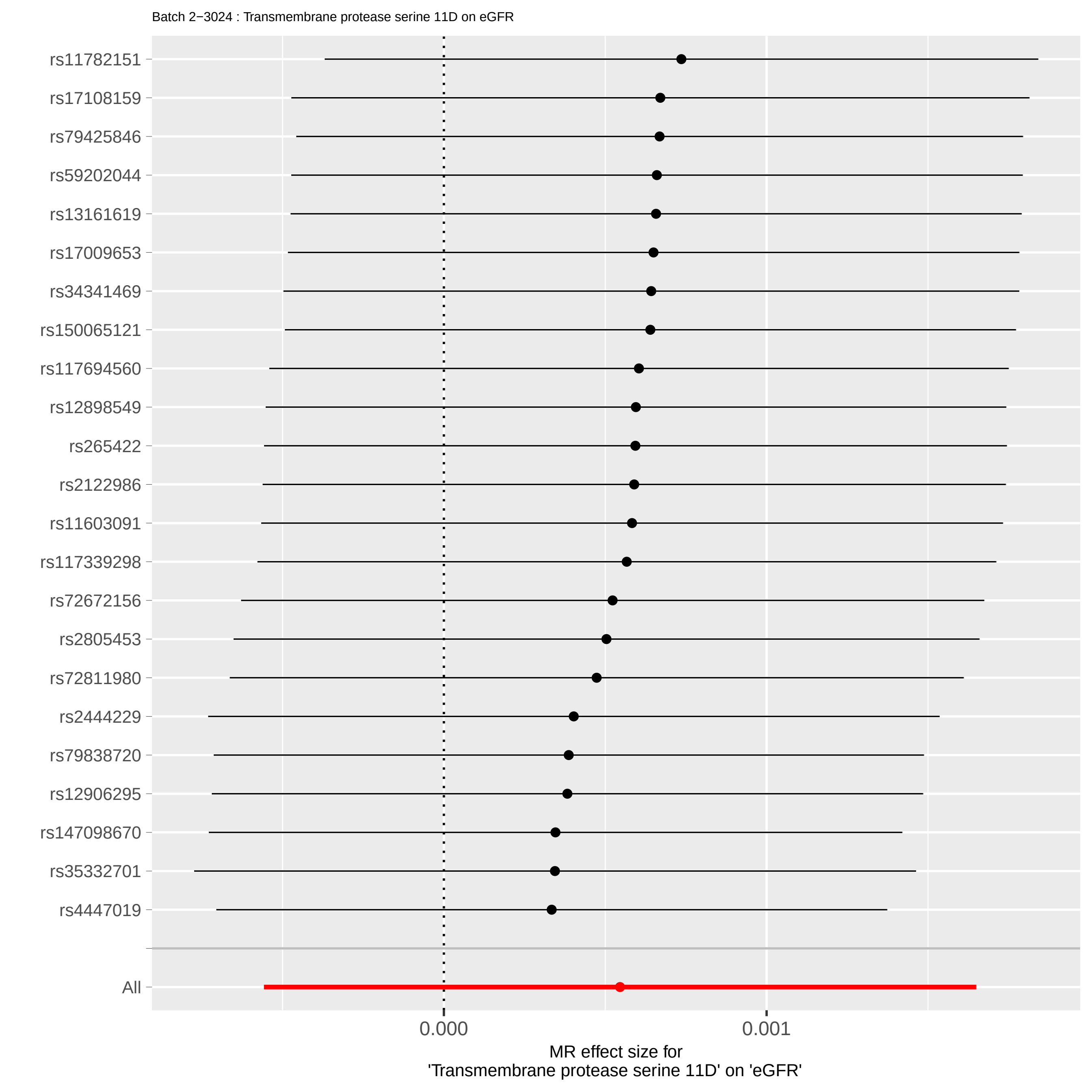

Batch 2−3024 : Transmembrane protease serine 11D on eGFR
rs11782151
rs17108159
rs79425846
rs59202044
rs13161619
rs17009653
rs34341469
rs150065121
rs117694560
rs12898549
rs265422
rs2122986
rs11603091
rs117339298
rs72672156
rs2805453
rs72811980
rs2444229
rs79838720
rs12906295
rs147098670
rs35332701
rs4447019
All
0.000
0.001
MR effect size for
'Transmembrane protease serine 11D' on 'eGFR'

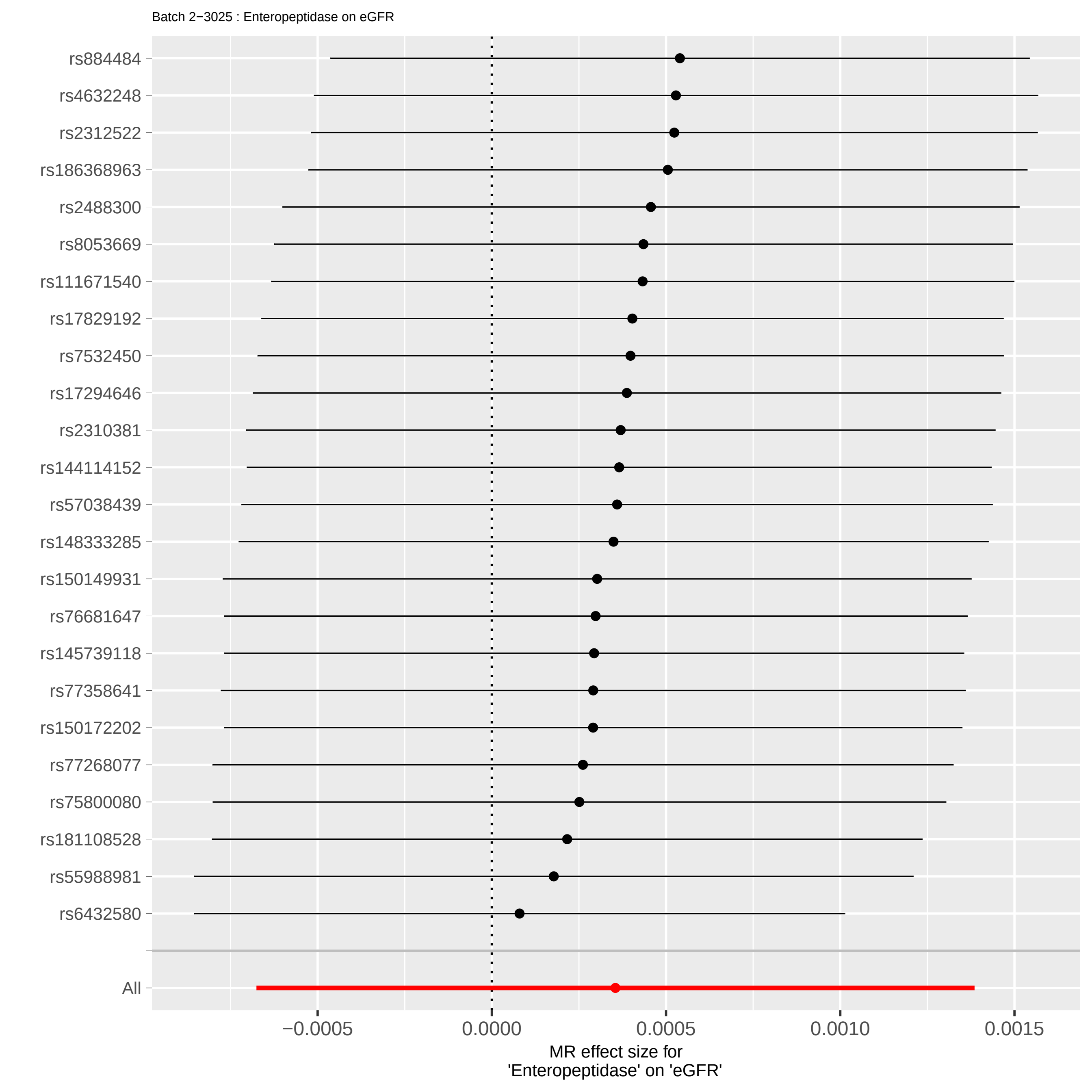
Batch 2−3025 : Enteropeptidase on eGFR
rs884484
rs4632248
rs2312522
rs186368963
rs2488300
rs8053669
rs111671540
rs17829192
rs7532450
rs17294646
rs2310381
rs144114152
rs57038439
rs148333285
rs150149931
rs76681647
rs145739118
rs77358641
rs150172202
rs77268077
rs75800080
rs181108528
rs55988981
rs6432580
All
−0.0005
0.0000
0.0005
0.0010
0.0015
MR effect size for
'Enteropeptidase' on 'eGFR'

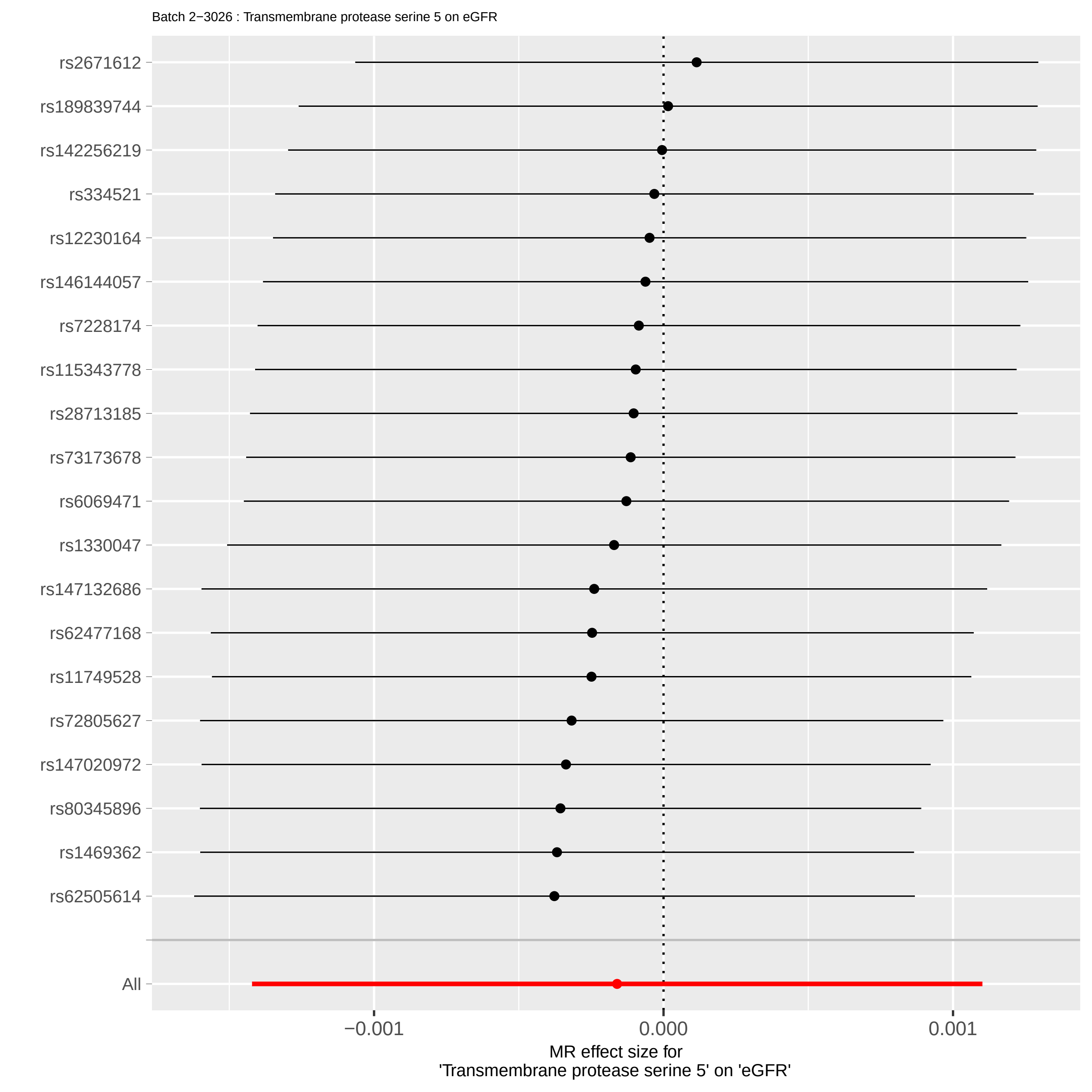

Batch 2−3026 : Transmembrane protease serine 5 on eGFR
rs2671612
rs189839744
rs142256219
rs334521
rs12230164
rs146144057
rs7228174
rs115343778
rs28713185
rs73173678
rs6069471
rs1330047
rs147132686
rs62477168
rs11749528
rs72805627
rs147020972
rs80345896
rs1469362
rs62505614
All
−0.001
0.000
0.001
MR effect size for
'Transmembrane protease serine 5' on 'eGFR'

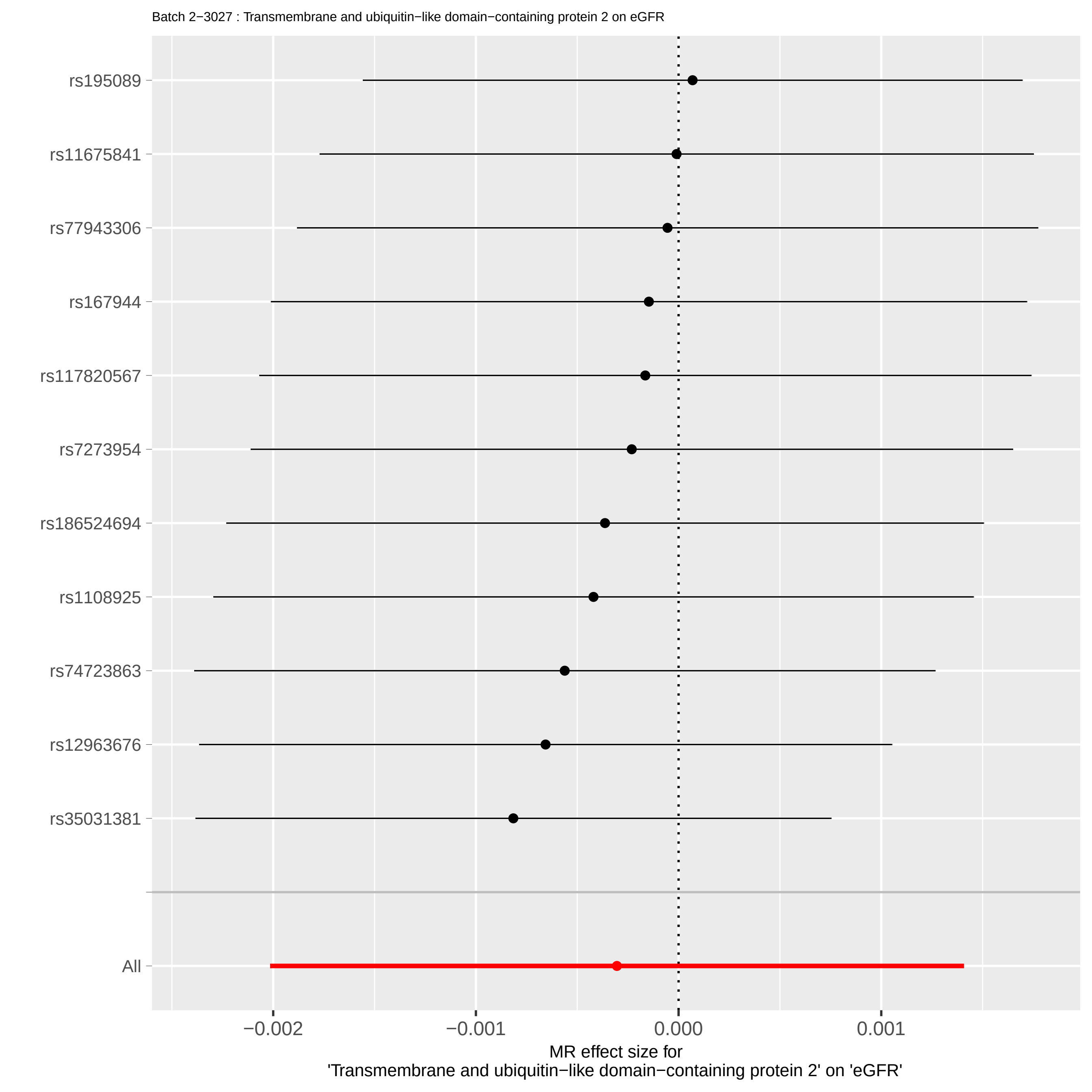

Batch 2−3027 : Transmembrane and ubiquitin−like domain−containing protein 2 on eGFR
rs195089
rs11675841
rs77943306
rs167944
rs117820567
rs7273954
rs186524694
rs1108925
rs74723863
rs12963676
rs35031381
All
−0.002
−0.001
0.000
0.001
MR effect size for
'Transmembrane and ubiquitin−like domain−containing protein 2' on 'eGFR'

Batch 2−3028 : Tenascin on eGFR

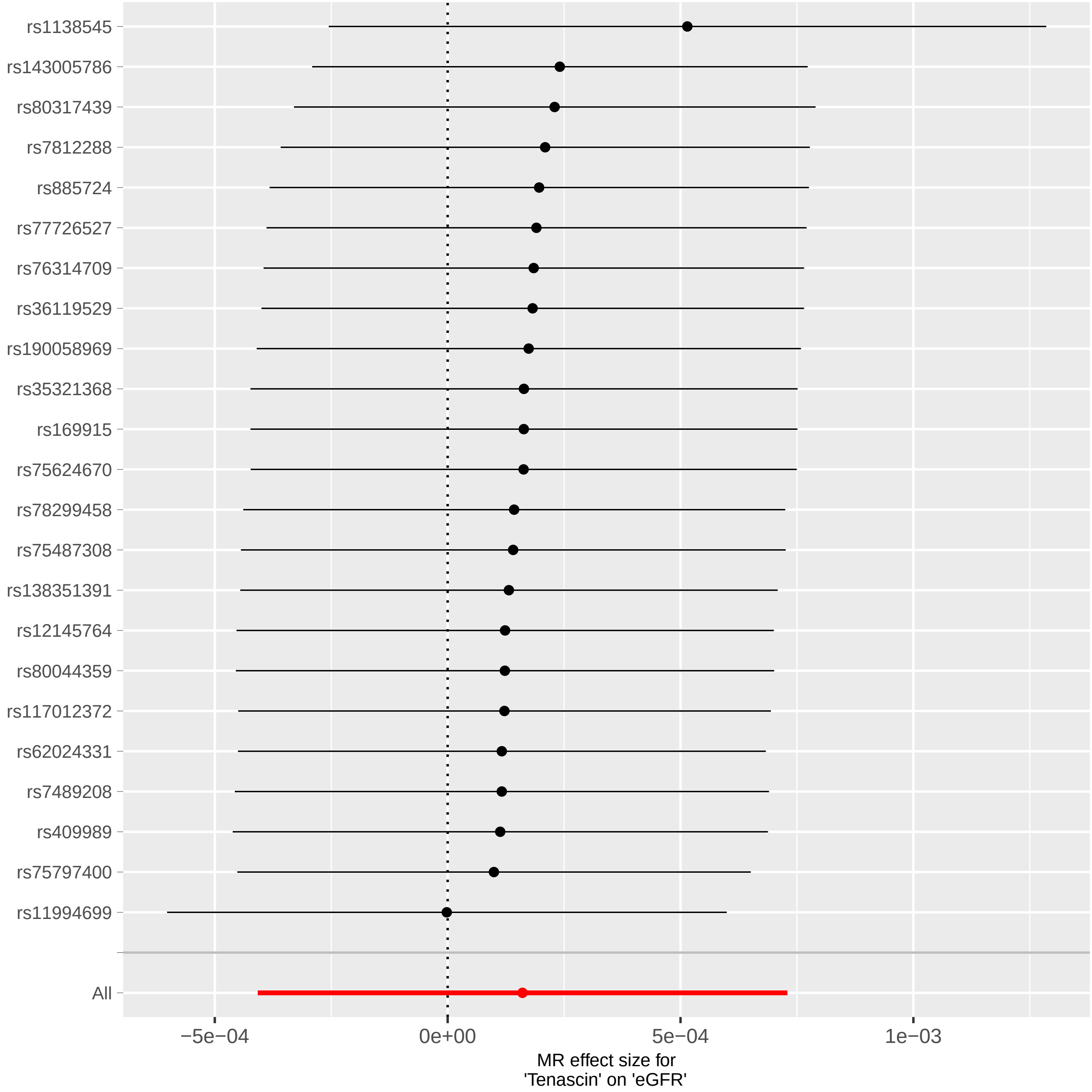

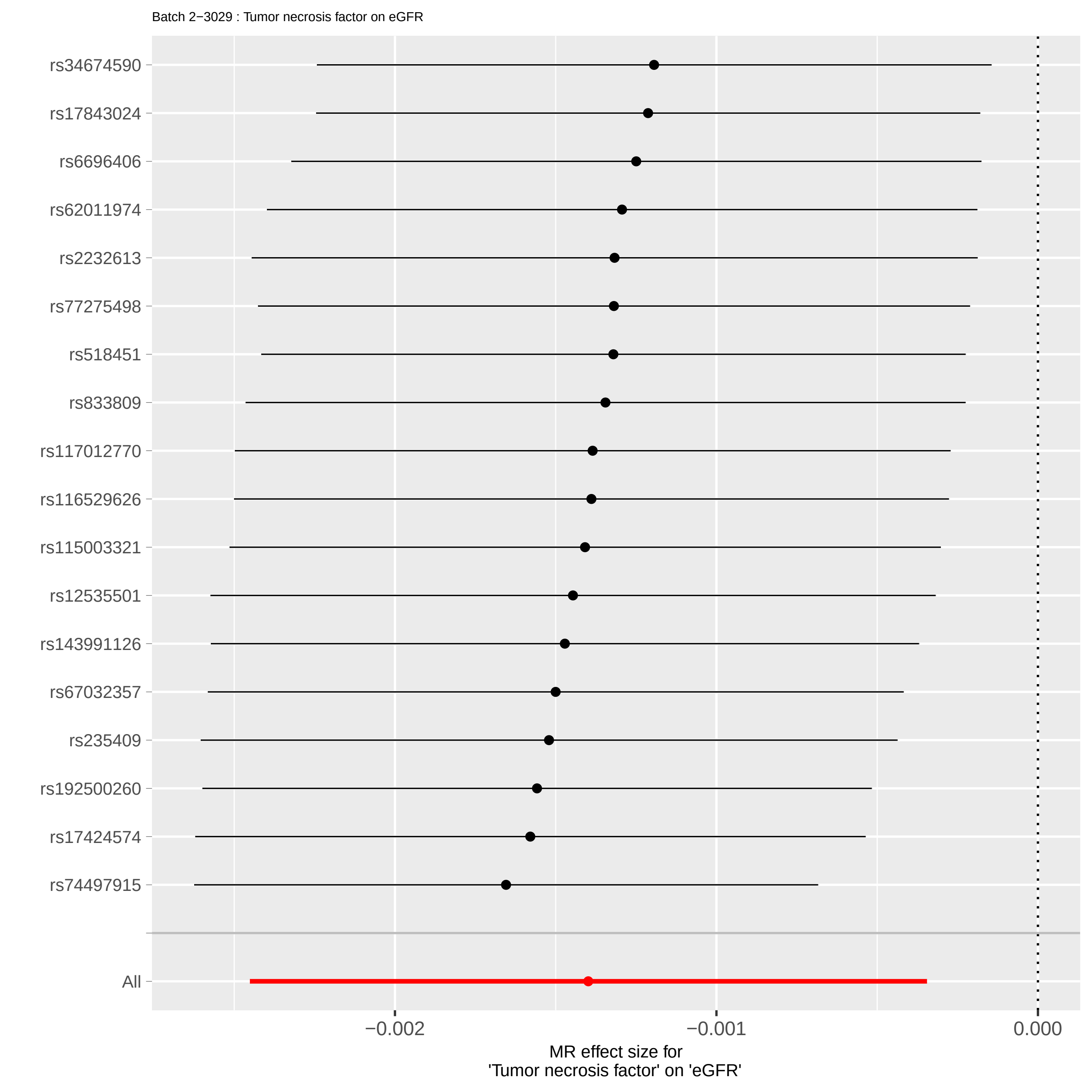
Batch 2−3029 : Tumor necrosis factor on eGFR
rs34674590
rs17843024
rs6696406
rs62011974
rs2232613
rs77275498
rs518451
rs833809
rs117012770
rs116529626
rs115003321
rs12535501
rs143991126
rs67032357
rs235409
rs192500260
rs17424574
rs74497915
All
−0.002
−0.001
0.000
MR effect size for
'Tumor necrosis factor' on 'eGFR'

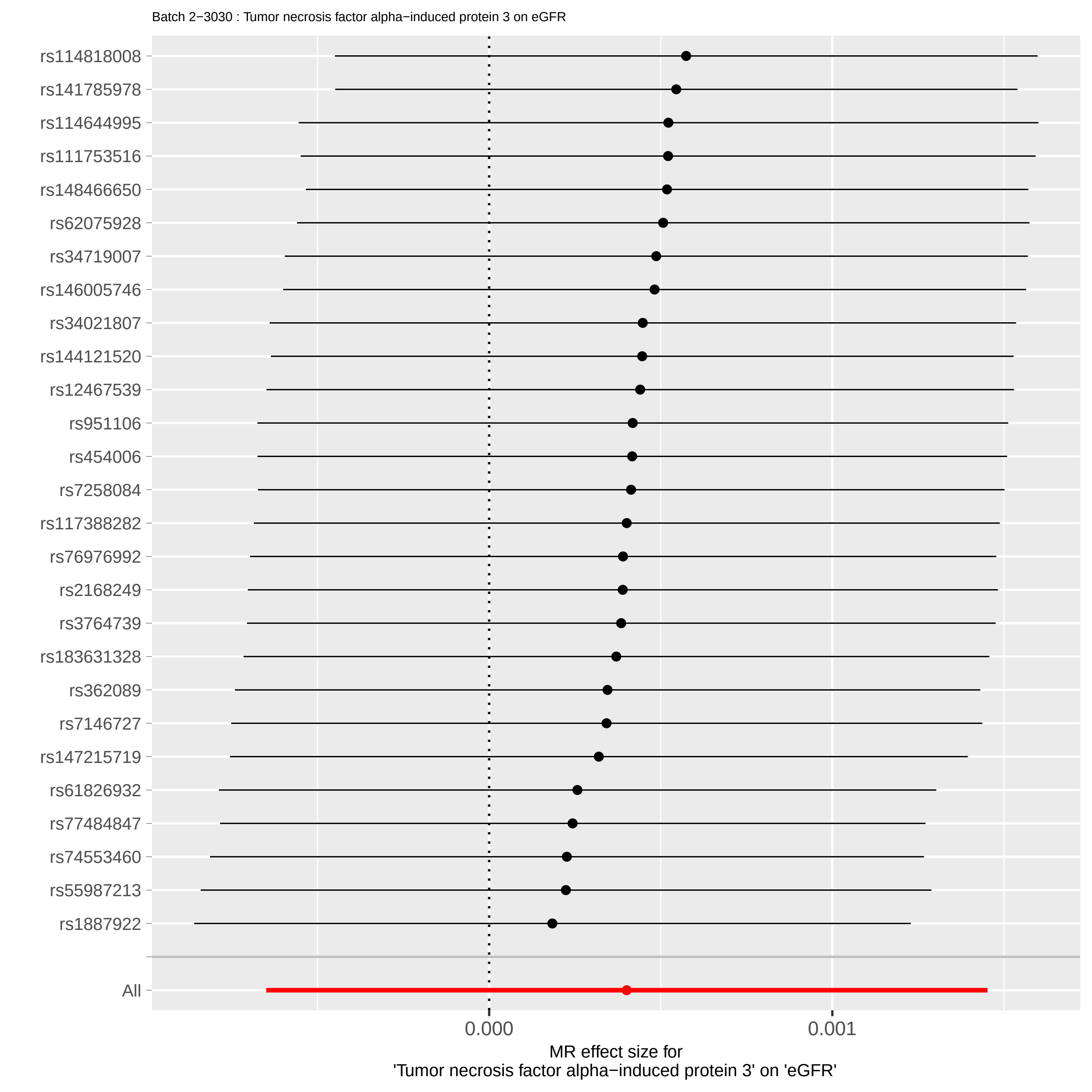

Batch 2−3030 : Tumor necrosis factor alpha−induced protein 3 on eGFR
rs114818008
rs141785978
rs114644995
rs111753516
rs148466650
rs62075928
rs34719007
rs146005746
rs34021807
rs144121520
rs12467539
rs951106
rs454006
rs7258084
rs117388282
rs76976992
rs2168249
rs3764739
rs183631328
rs362089
rs7146727
rs147215719
rs61826932
rs77484847
rs74553460
rs55987213
rs1887922
All
0.000
0.001
MR effect size for
'Tumor necrosis factor alpha−induced protein 3' on 'eGFR'

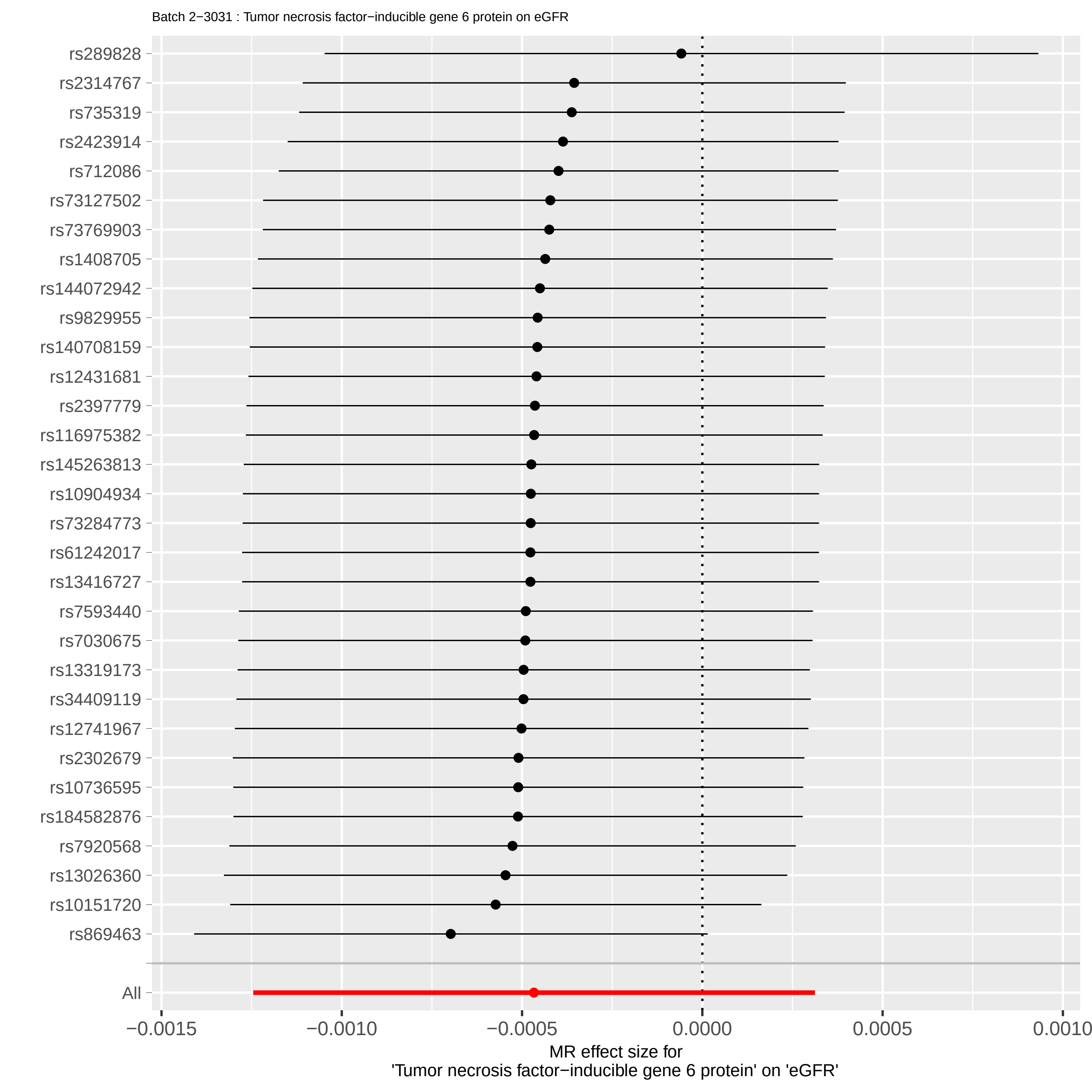

Batch 2−3031 : Tumor necrosis factor−inducible gene 6 protein on eGFR
rs289828
rs2314767
rs735319
rs2423914
rs712086
rs73127502
rs73769903
rs1408705
rs144072942
rs9829955
rs140708159
rs12431681
rs2397779
rs116975382
rs145263813
rs10904934
rs73284773
rs61242017
rs13416727
rs7593440
rs7030675
rs13319173
rs34409119
rs12741967
rs2302679
rs10736595
rs184582876
rs7920568
rs13026360
rs10151720
rs869463
All
−0.0015
−0.0010
−0.0005
0.0000
0.0005
0.0010
MR effect size for
'Tumor necrosis factor−inducible gene 6 protein' on 'eGFR'

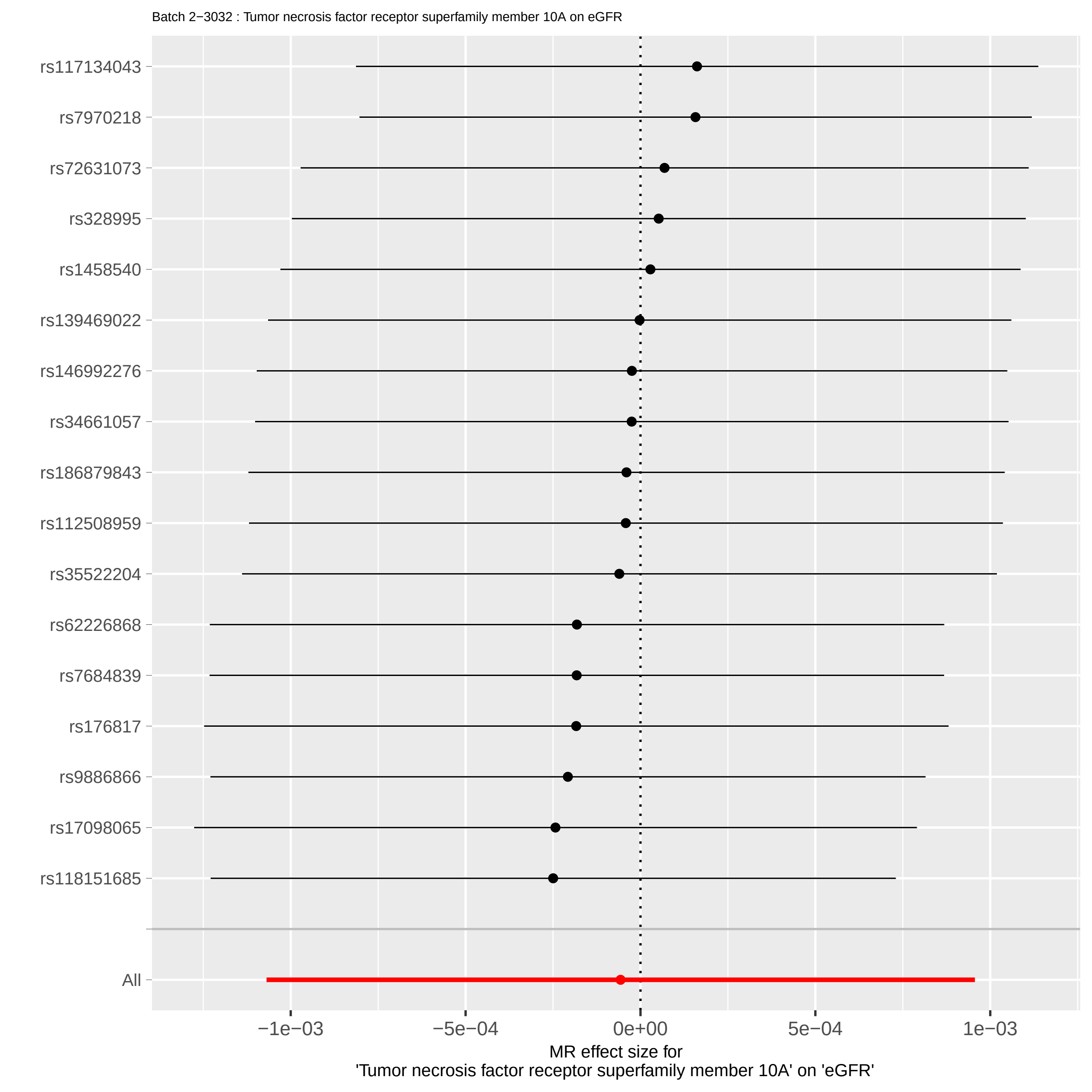
Batch 2−3032 : Tumor necrosis factor receptor superfamily member 10A on eGFR
rs117134043
rs7970218
rs72631073
rs328995
rs1458540
rs139469022
rs146992276
rs34661057
rs186879843
rs112508959
rs35522204
rs62226868
rs7684839
rs176817
rs9886866
rs17098065
rs118151685
All
−1e−03
−5e−04
0e+00
5e−04
1e−03
MR effect size for
'Tumor necrosis factor receptor superfamily member 10A' on 'eGFR'

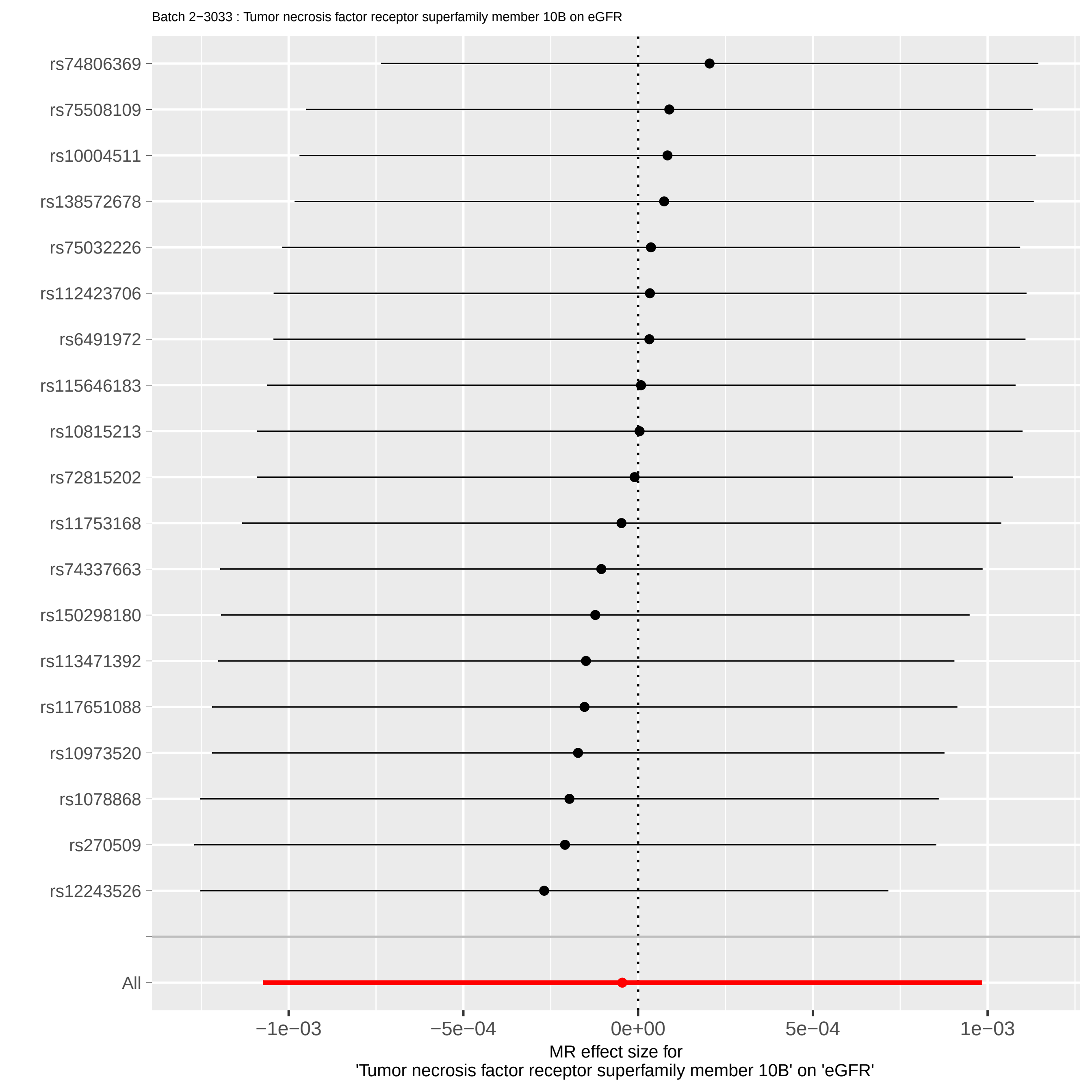
Batch 2−3033 : Tumor necrosis factor receptor superfamily member 10B on eGFR
rs74806369
rs75508109
rs10004511
rs138572678
rs75032226
rs112423706
rs6491972
rs115646183
rs10815213
rs72815202
rs11753168
rs74337663
rs150298180
rs113471392
rs117651088
rs10973520
rs1078868
rs270509
rs12243526
All
−1e−03
−5e−04
0e+00
5e−04
1e−03
MR effect size for
'Tumor necrosis factor receptor superfamily member 10B' on 'eGFR'

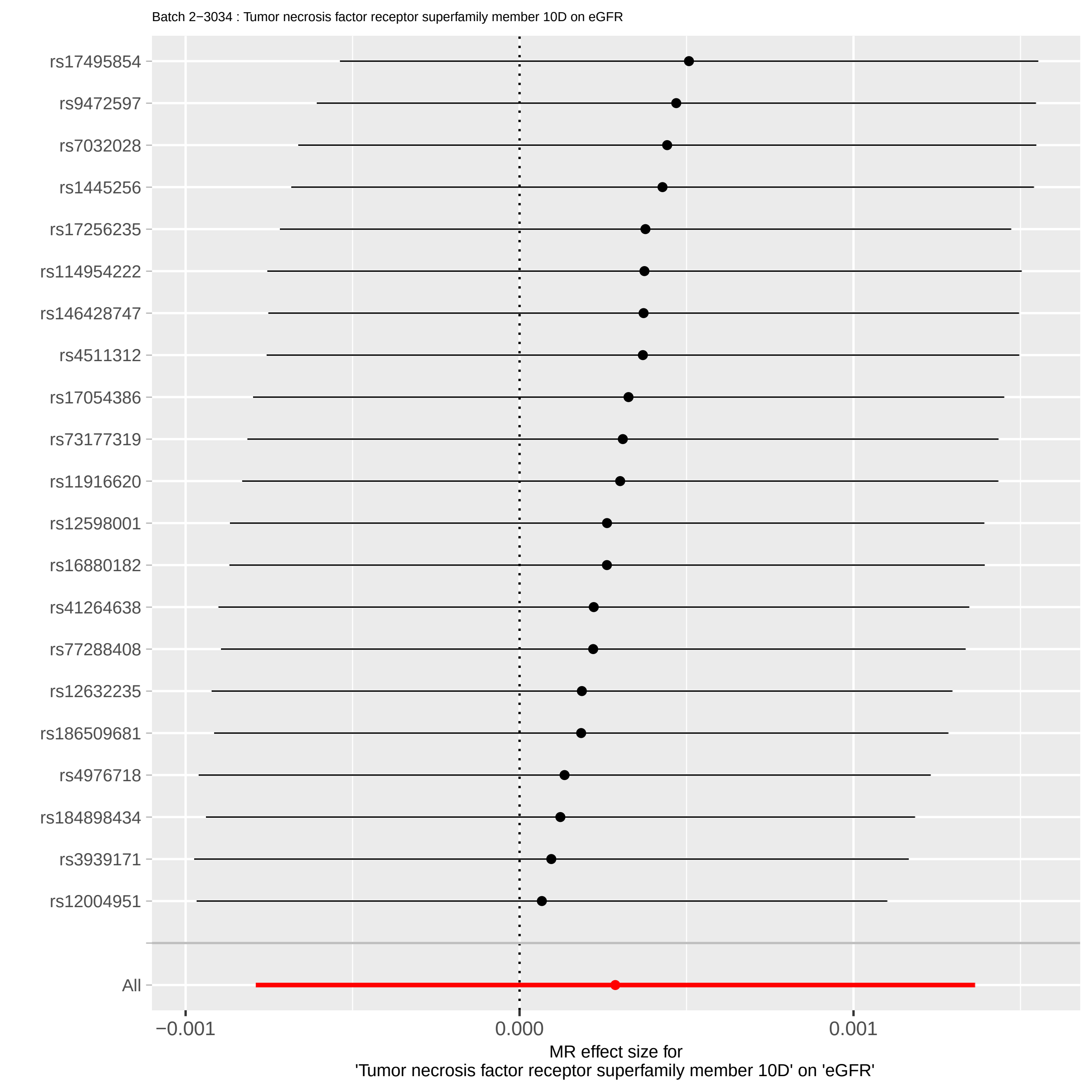

Batch 2−3034 : Tumor necrosis factor receptor superfamily member 10D on eGFR
rs17495854
rs9472597
rs7032028
rs1445256
rs17256235
rs114954222
rs146428747
rs4511312
rs17054386
rs73177319
rs11916620
rs12598001
rs16880182
rs41264638
rs77288408
rs12632235
rs186509681
rs4976718
rs184898434
rs3939171
rs12004951
All
−0.001
0.000
0.001
MR effect size for
'Tumor necrosis factor receptor superfamily member 10D' on 'eGFR'

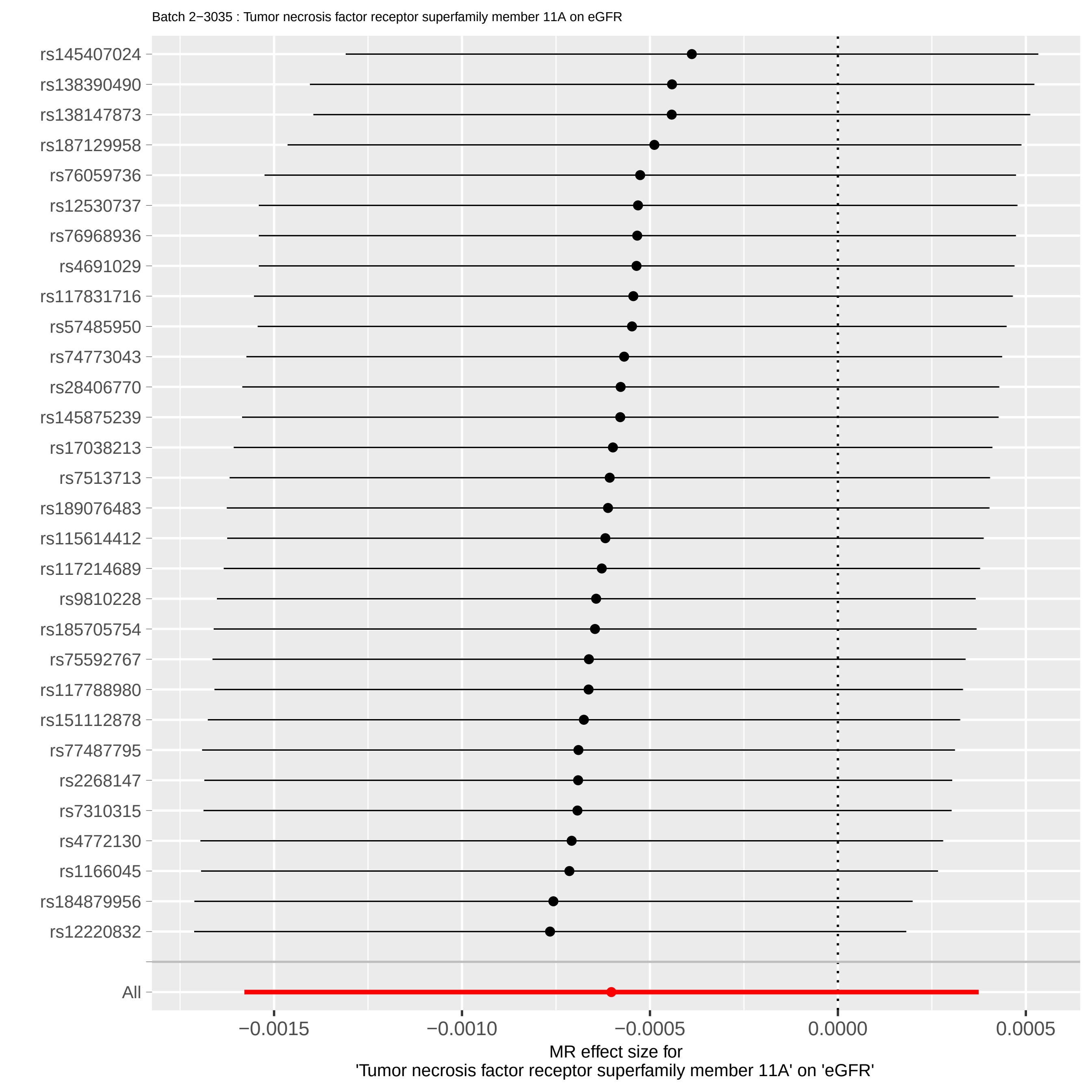

Batch 2−3035 : Tumor necrosis factor receptor superfamily member 11A on eGFR
rs145407024
rs138390490
rs138147873
rs187129958
rs76059736
rs12530737
rs76968936
rs4691029
rs117831716
rs57485950
rs74773043
rs28406770
rs145875239
rs17038213
rs7513713
rs189076483
rs115614412
rs117214689
rs9810228
rs185705754
rs75592767
rs117788980
rs151112878
rs77487795
rs2268147
rs7310315
rs4772130
rs1166045
rs184879956
rs12220832
All
−0.0015
−0.0010
−0.0005
0.0000
0.0005
MR effect size for
'Tumor necrosis factor receptor superfamily member 11A' on 'eGFR'

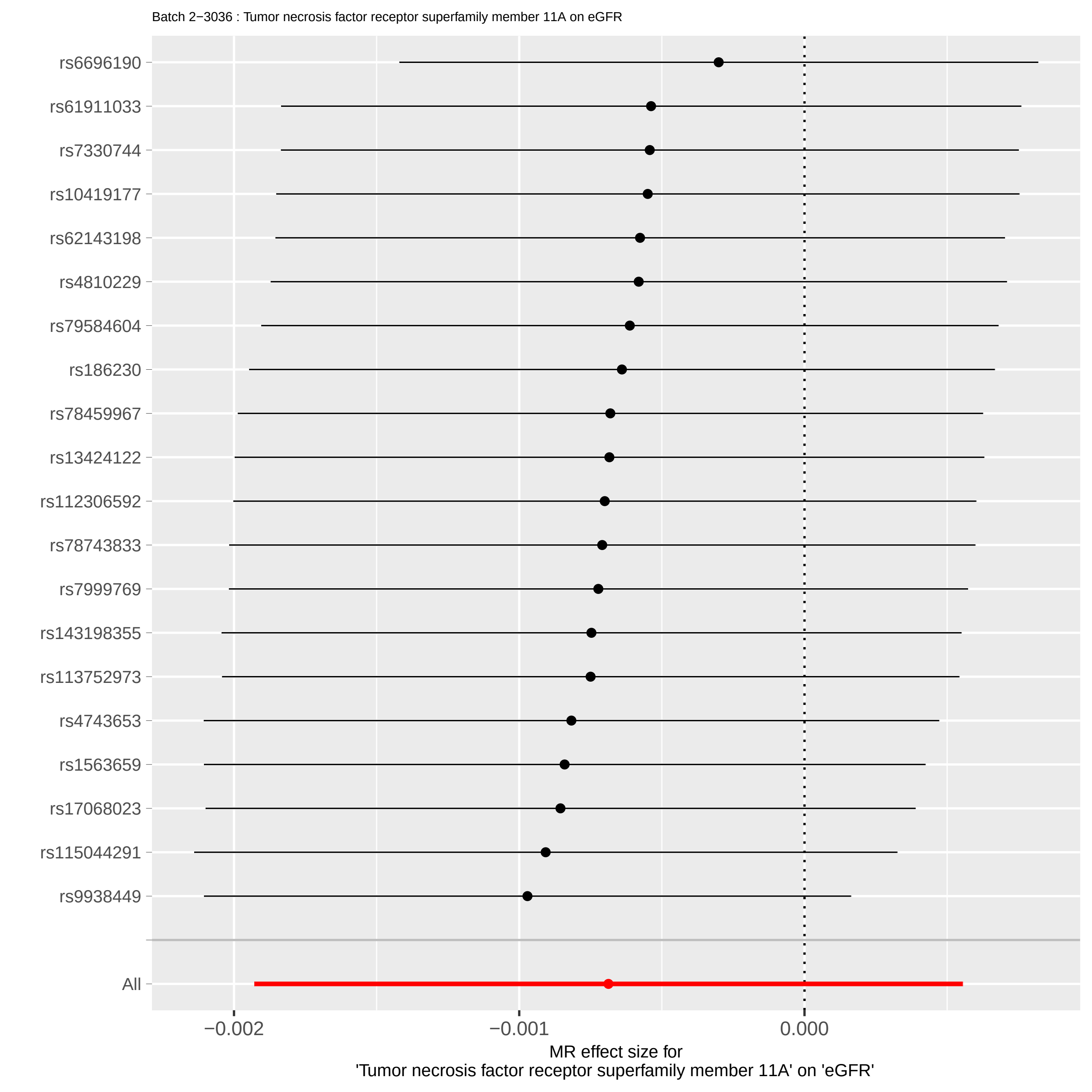

Batch 2−3036 : Tumor necrosis factor receptor superfamily member 11A on eGFR
rs6696190
rs61911033
rs7330744
rs10419177
rs62143198
rs4810229
rs79584604
rs186230
rs78459967
rs13424122
rs112306592
rs78743833
rs7999769
rs143198355
rs113752973
rs4743653
rs1563659
rs17068023
rs115044291
rs9938449
All
−0.002
−0.001
0.000
MR effect size for
'Tumor necrosis factor receptor superfamily member 11A' on 'eGFR'

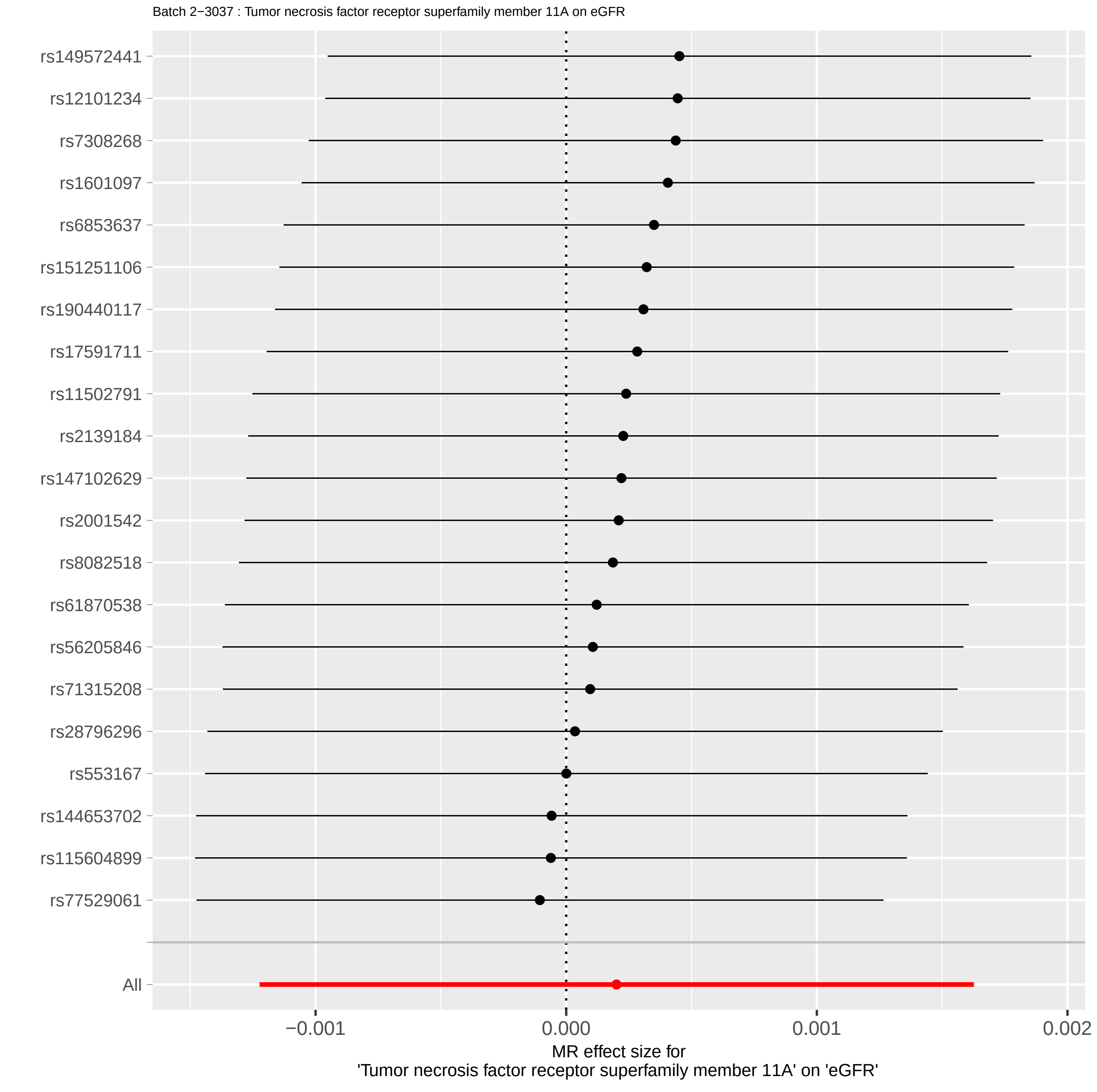

Batch 2−3037 : Tumor necrosis factor receptor superfamily member 11A on eGFR
rs149572441
rs12101234
rs7308268
rs1601097
rs6853637
rs151251106
rs190440117
rs17591711
rs11502791
rs2139184
rs147102629
rs2001542
rs8082518
rs61870538
rs56205846
rs71315208
rs28796296
rs553167
rs144653702
rs115604899
rs77529061
All
−0.001
0.000
0.001
0.002
MR effect size for
'Tumor necrosis factor receptor superfamily member 11A' on 'eGFR'

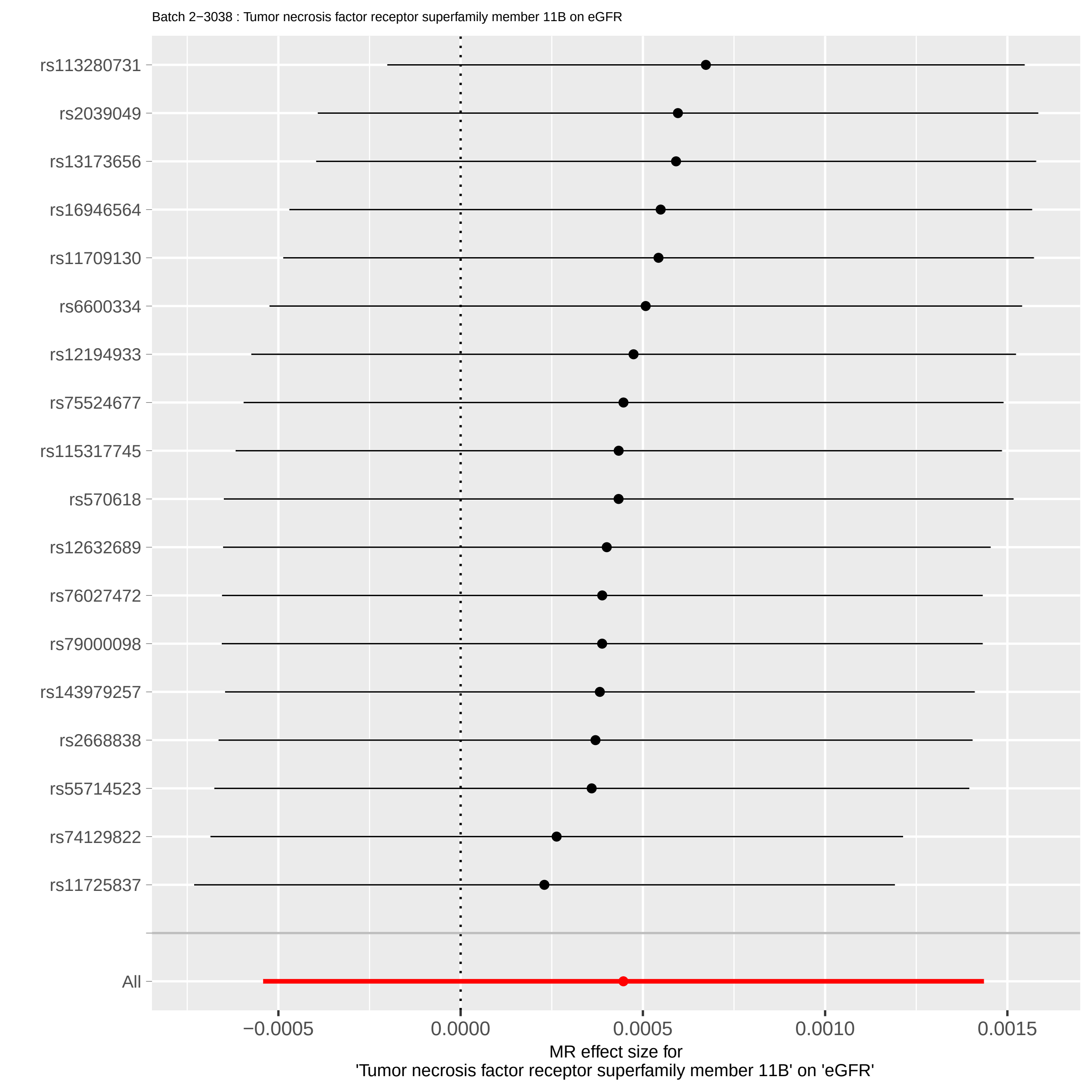
Batch 2−3038 : Tumor necrosis factor receptor superfamily member 11B on eGFR
rs113280731
rs2039049
rs13173656
rs16946564
rs11709130
rs6600334
rs12194933
rs75524677
rs115317745
rs570618
rs12632689
rs76027472
rs79000098
rs143979257
rs2668838
rs55714523
rs74129822
rs11725837
All
−0.0005
0.0000
0.0005
0.0010
0.0015
MR effect size for
'Tumor necrosis factor receptor superfamily member 11B' on 'eGFR'

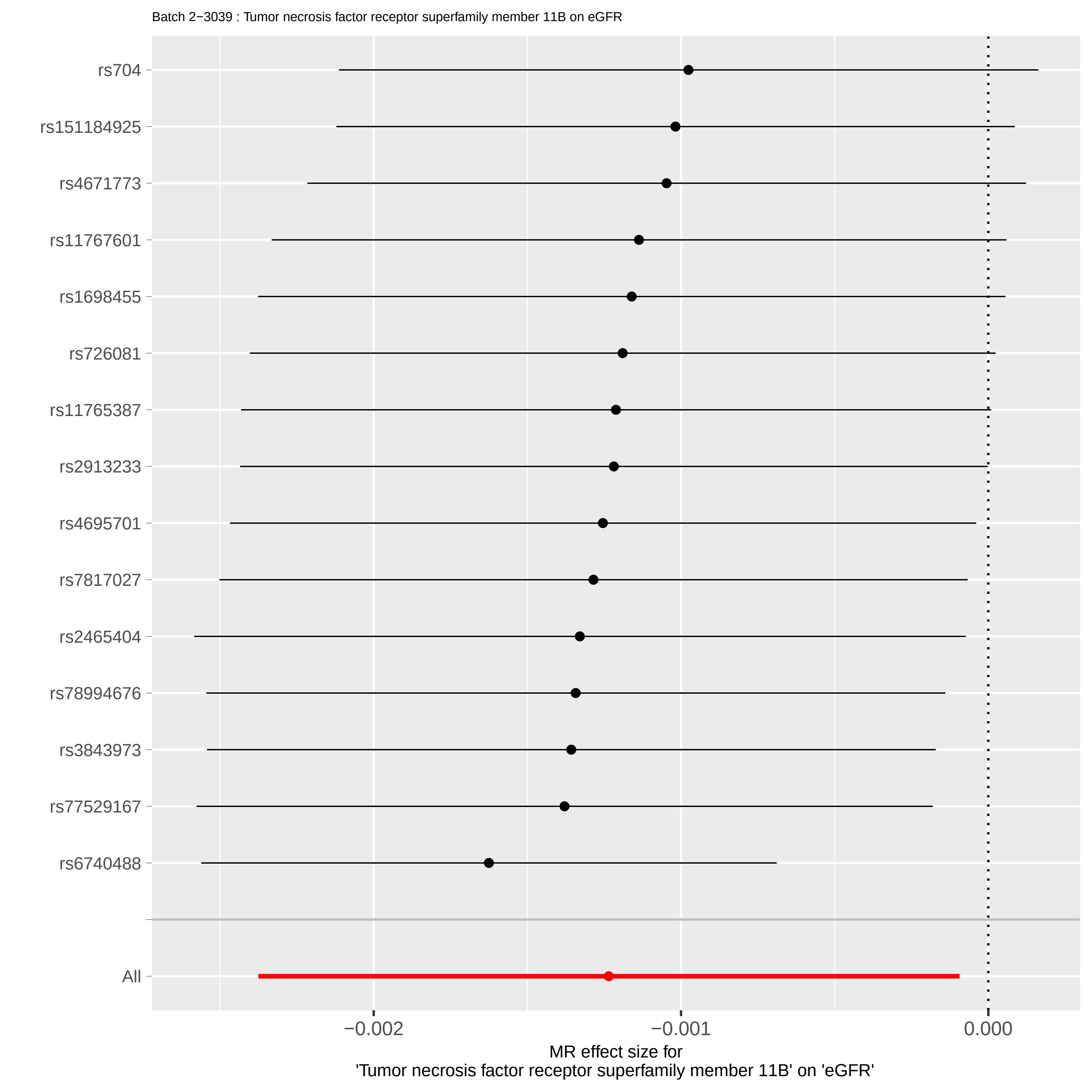

Batch 2−3039 : Tumor necrosis factor receptor superfamily member 11B on eGFR
rs704
rs151184925
rs4671773
rs11767601
rs1698455
rs726081
rs11765387
rs2913233
rs4695701
rs7817027
rs2465404
rs78994676
rs3843973
rs77529167
rs6740488
All
−0.002
−0.001
0.000
MR effect size for
'Tumor necrosis factor receptor superfamily member 11B' on 'eGFR'

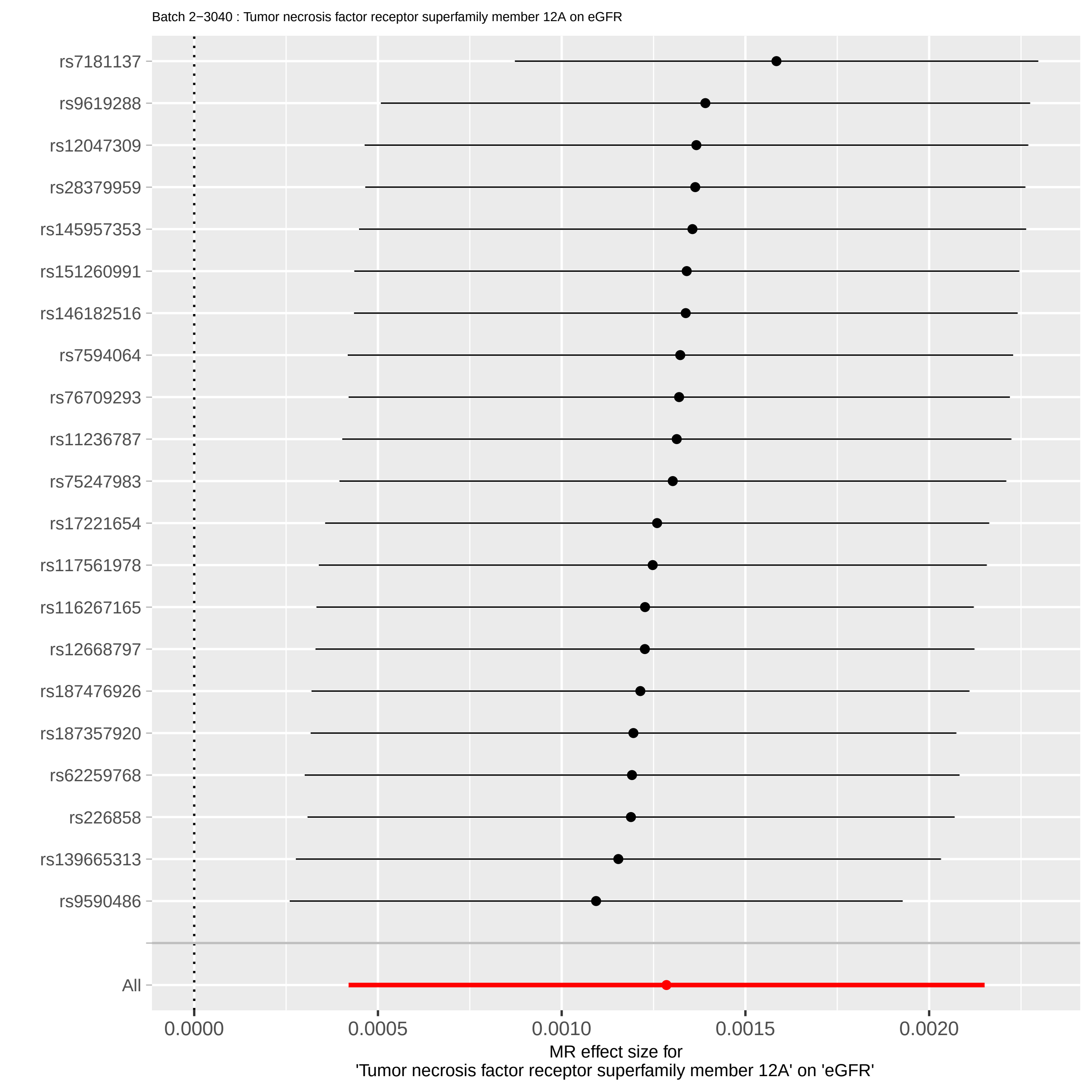

Batch 2−3040 : Tumor necrosis factor receptor superfamily member 12A on eGFR
rs7181137
rs9619288
rs12047309
rs28379959
rs145957353
rs151260991
rs146182516
rs7594064
rs76709293
rs11236787
rs75247983
rs17221654
rs117561978
rs116267165
rs12668797
rs187476926
rs187357920
rs62259768
rs226858
rs139665313
rs9590486
All
0.0000
0.0005
0.0010
0.0015
0.0020
MR effect size for
'Tumor necrosis factor receptor superfamily member 12A' on 'eGFR'

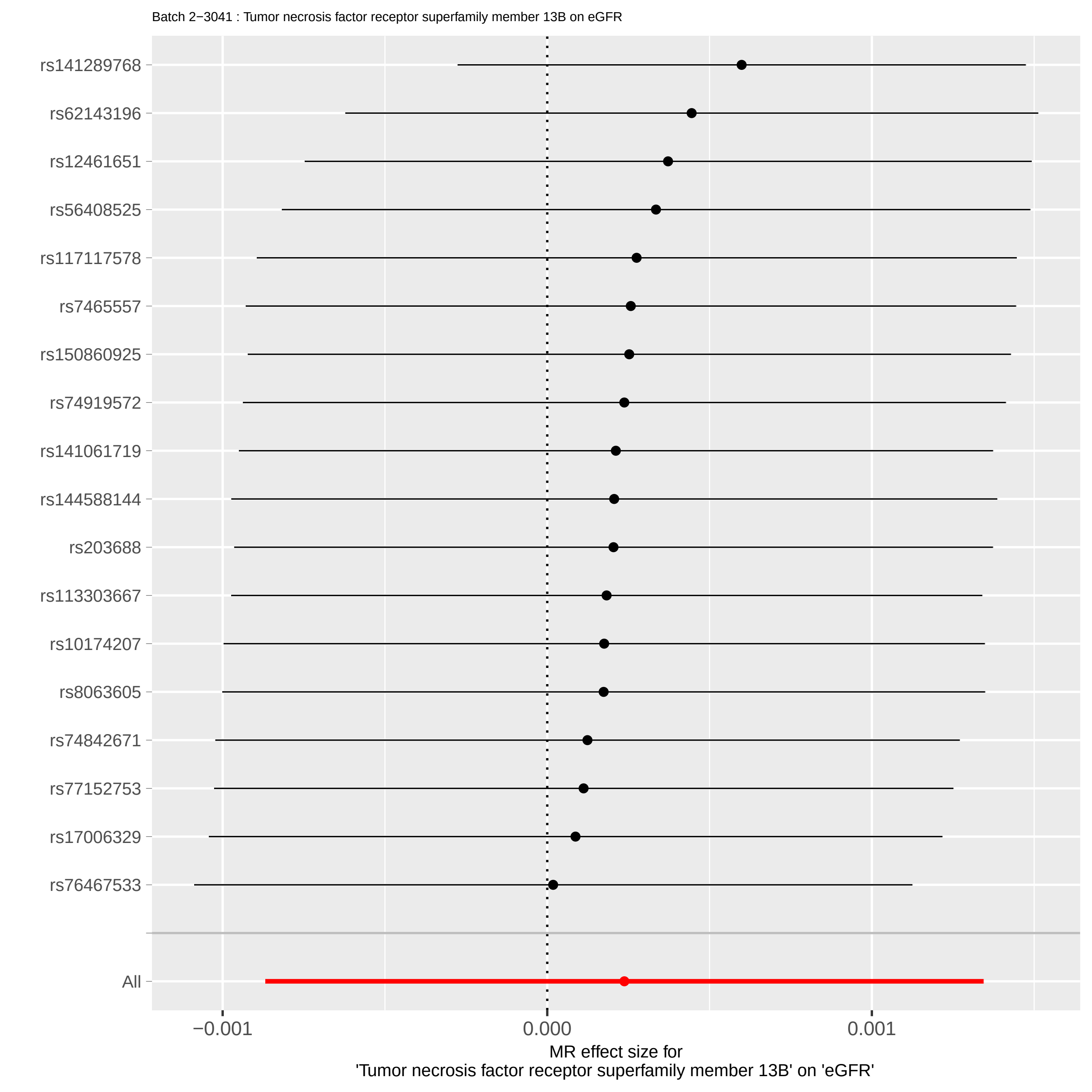

Batch 2−3041 : Tumor necrosis factor receptor superfamily member 13B on eGFR
rs141289768
rs62143196
rs12461651
rs56408525
rs117117578
rs7465557
rs150860925
rs74919572
rs141061719
rs144588144
rs203688
rs113303667
rs10174207
rs8063605
rs74842671
rs77152753
rs17006329
rs76467533
All
−0.001
0.000
0.001
MR effect size for
'Tumor necrosis factor receptor superfamily member 13B' on 'eGFR'

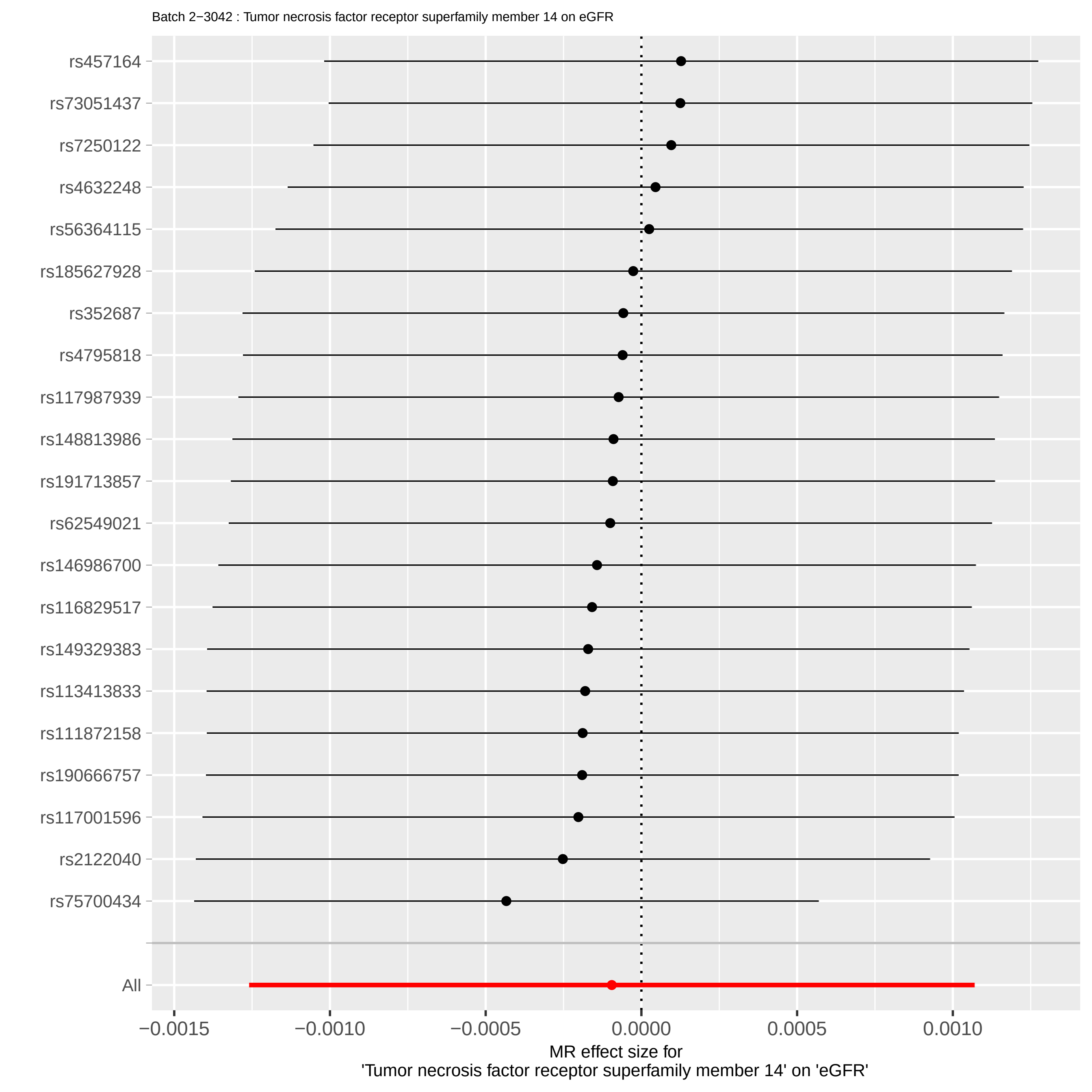

Batch 2−3042 : Tumor necrosis factor receptor superfamily member 14 on eGFR
rs457164
rs73051437
rs7250122
rs4632248
rs56364115
rs185627928
rs352687
rs4795818
rs117987939
rs148813986
rs191713857
rs62549021
rs146986700
rs116829517
rs149329383
rs113413833
rs111872158
rs190666757
rs117001596
rs2122040
rs75700434
All
−0.0015
−0.0010
−0.0005
0.0000
0.0005
0.0010
MR effect size for
'Tumor necrosis factor receptor superfamily member 14' on 'eGFR'

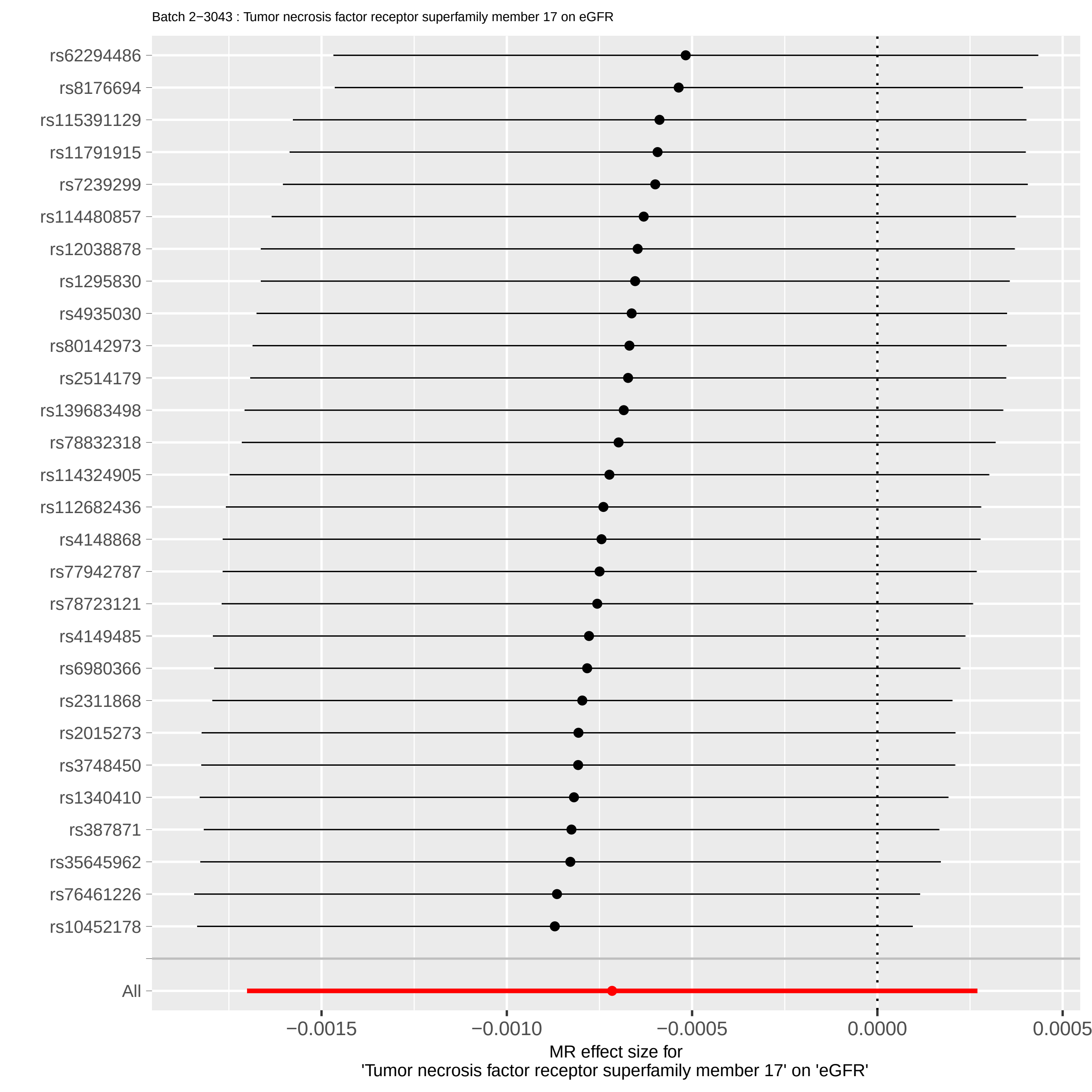

Batch 2−3043 : Tumor necrosis factor receptor superfamily member 17 on eGFR
rs62294486
rs8176694
rs115391129
rs11791915
rs7239299
rs114480857
rs12038878
rs1295830
rs4935030
rs80142973
rs2514179
rs139683498
rs78832318
rs114324905
rs112682436
rs4148868
rs77942787
rs78723121
rs4149485
rs6980366
rs2311868
rs2015273
rs3748450
rs1340410
rs387871
rs35645962
rs76461226
rs10452178
All
−0.0015
−0.0010
−0.0005
0.0000
0.0005
MR effect size for
'Tumor necrosis factor receptor superfamily member 17' on 'eGFR'

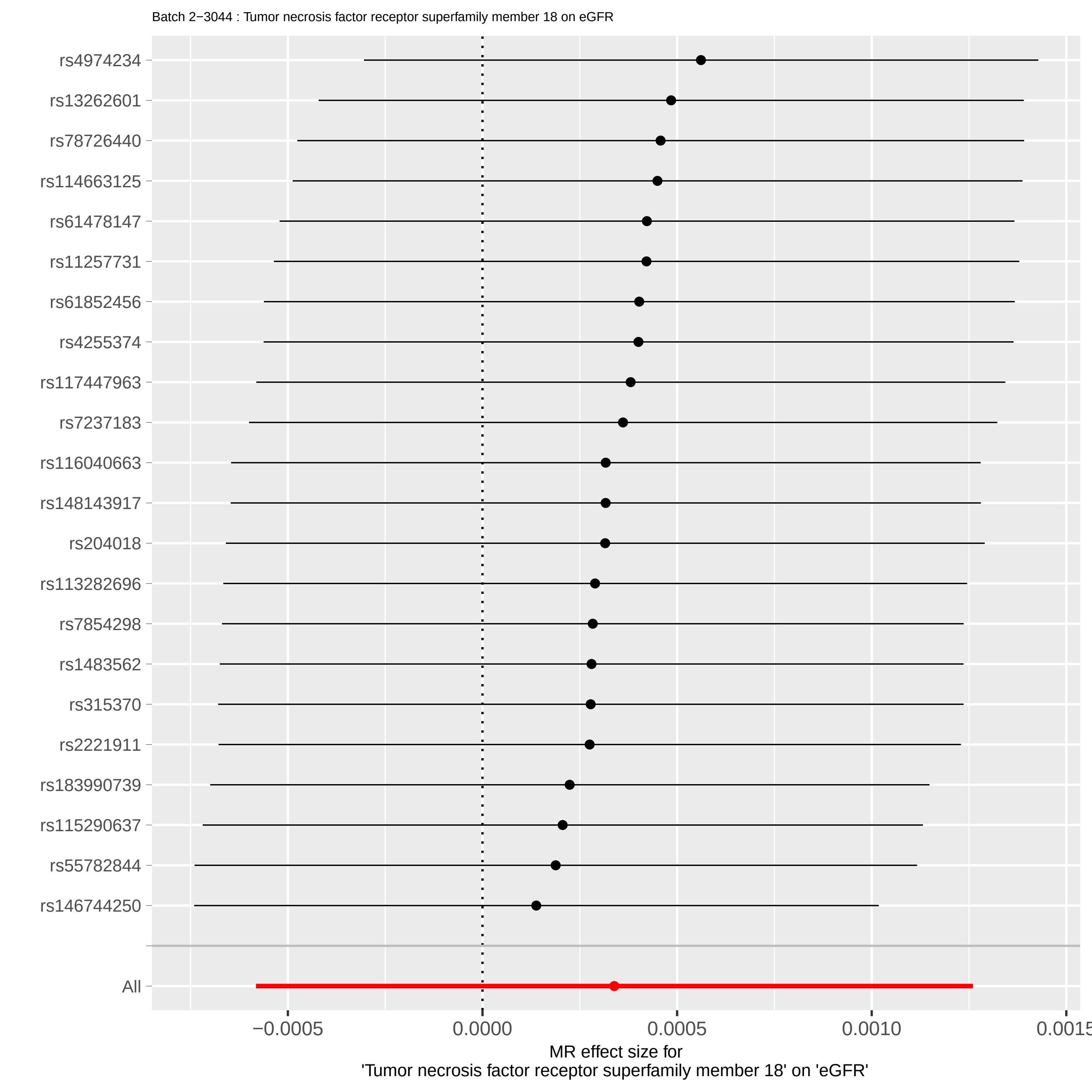
Batch 2−3044 : Tumor necrosis factor receptor superfamily member 18 on eGFR
rs4974234
rs13262601
rs78726440
rs114663125
rs61478147
rs11257731
rs61852456
rs4255374
rs117447963
rs7237183
rs116040663
rs148143917
rs204018
rs113282696
rs7854298
rs1483562
rs315370
rs2221911
rs183990739
rs115290637
rs55782844
rs146744250
All
−0.0005
0.0000
0.0005
0.0010
0.0015
MR effect size for
'Tumor necrosis factor receptor superfamily member 18' on 'eGFR'

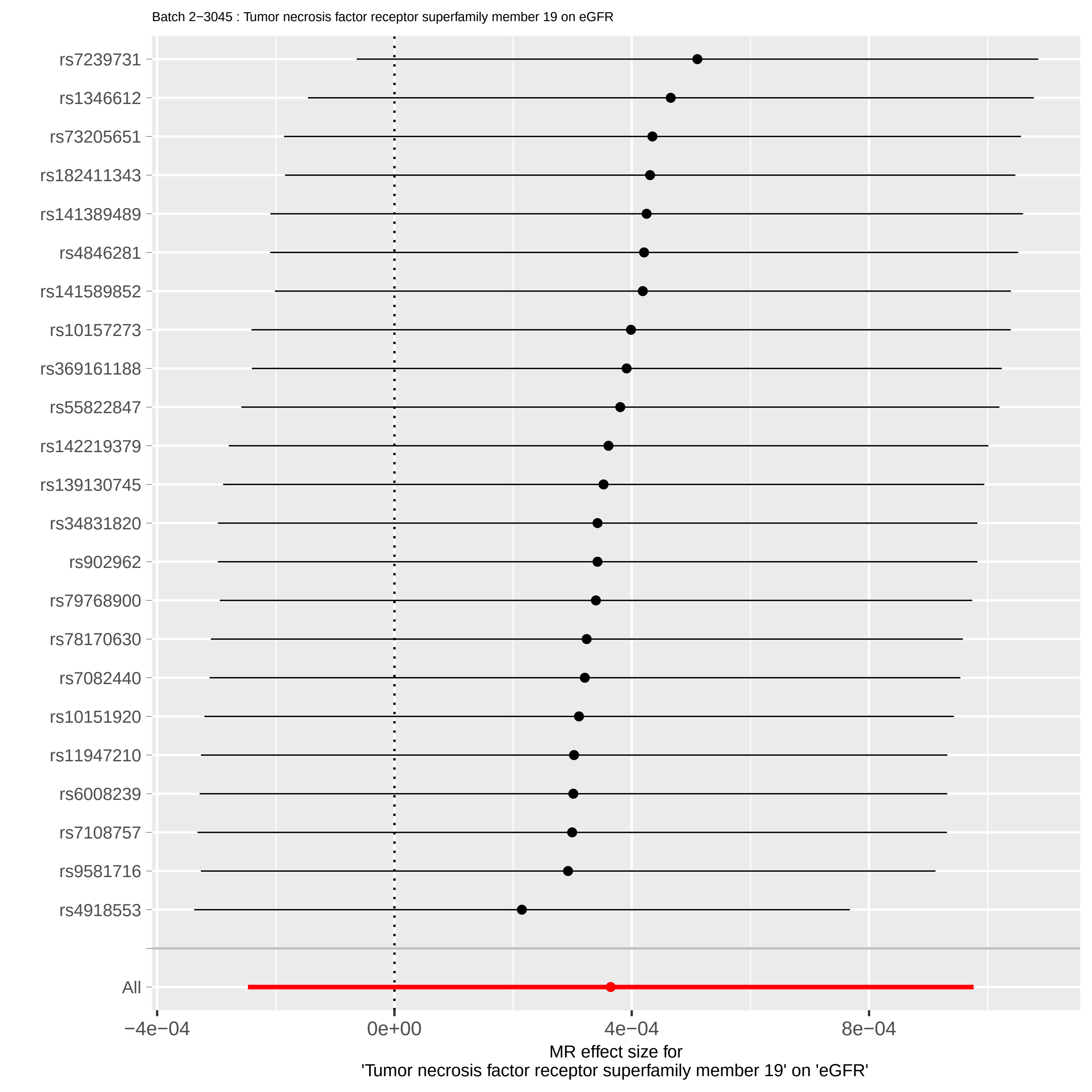

Batch 2−3045 : Tumor necrosis factor receptor superfamily member 19 on eGFR
rs7239731
rs1346612
rs73205651
rs182411343
rs141389489
rs4846281
rs141589852
rs10157273
rs369161188
rs55822847
rs142219379
rs139130745
rs34831820
rs902962
rs79768900
rs78170630
rs7082440
rs10151920
rs11947210
rs6008239
rs7108757
rs9581716
rs4918553
All
−4e−04
0e+00
4e−04
8e−04
MR effect size for
'Tumor necrosis factor receptor superfamily member 19' on 'eGFR'

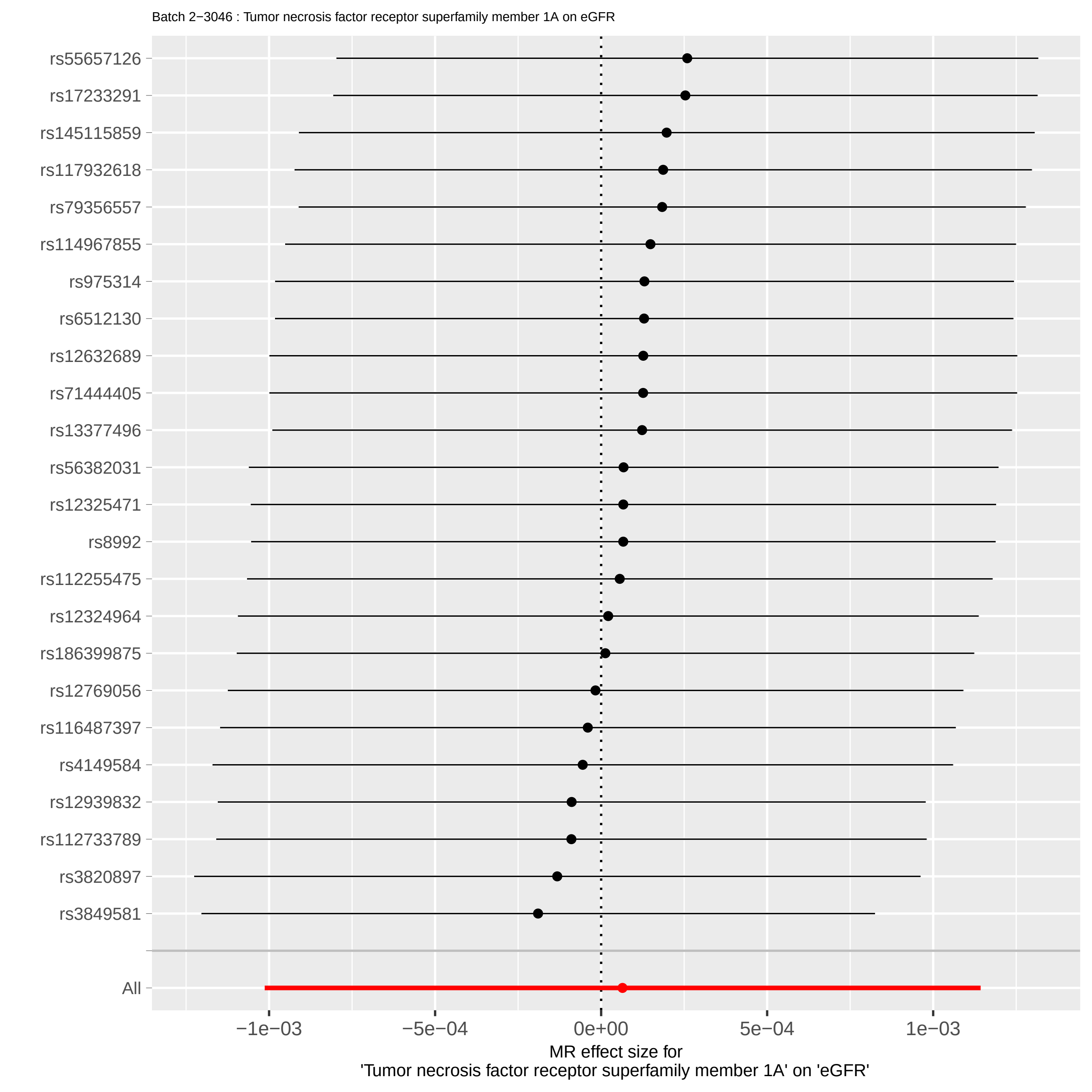

Batch 2−3046 : Tumor necrosis factor receptor superfamily member 1A on eGFR
rs55657126
rs17233291
rs145115859
rs117932618
rs79356557
rs114967855
rs975314
rs6512130
rs12632689
rs71444405
rs13377496
rs56382031
rs12325471
rs8992
rs112255475
rs12324964
rs186399875
rs12769056
rs116487397
rs4149584
rs12939832
rs112733789
rs3820897
rs3849581
All
−1e−03
−5e−04
0e+00
5e−04
1e−03
MR effect size for
'Tumor necrosis factor receptor superfamily member 1A' on 'eGFR'

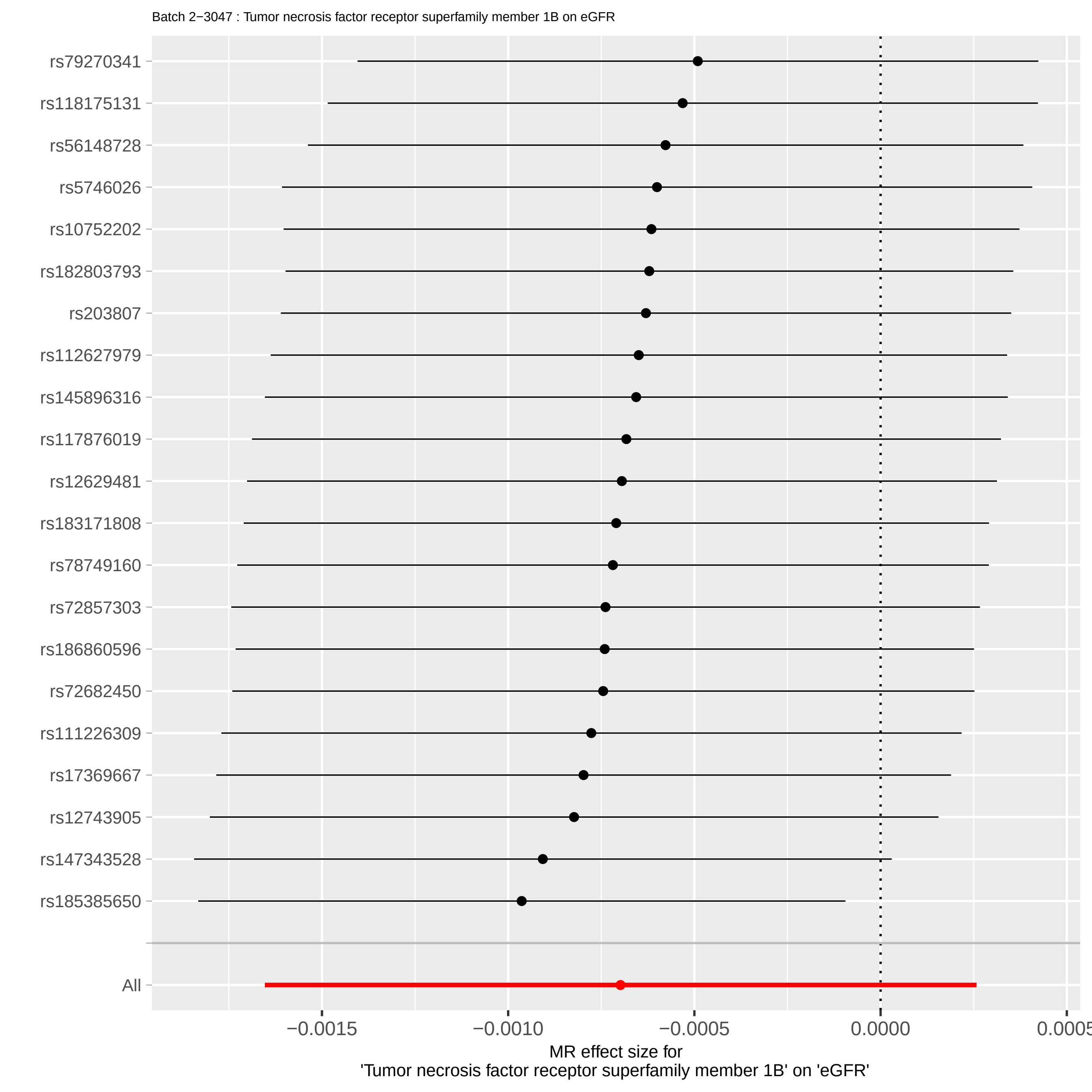
Batch 2−3047 : Tumor necrosis factor receptor superfamily member 1B on eGFR
rs79270341
rs118175131
rs56148728
rs5746026
rs10752202
rs182803793
rs203807
rs112627979
rs145896316
rs117876019
rs12629481
rs183171808
rs78749160
rs72857303
rs186860596
rs72682450
rs111226309
rs17369667
rs12743905
rs147343528
rs185385650
All
−0.0015
−0.0010
−0.0005
0.0000
0.0005
MR effect size for
'Tumor necrosis factor receptor superfamily member 1B' on 'eGFR'

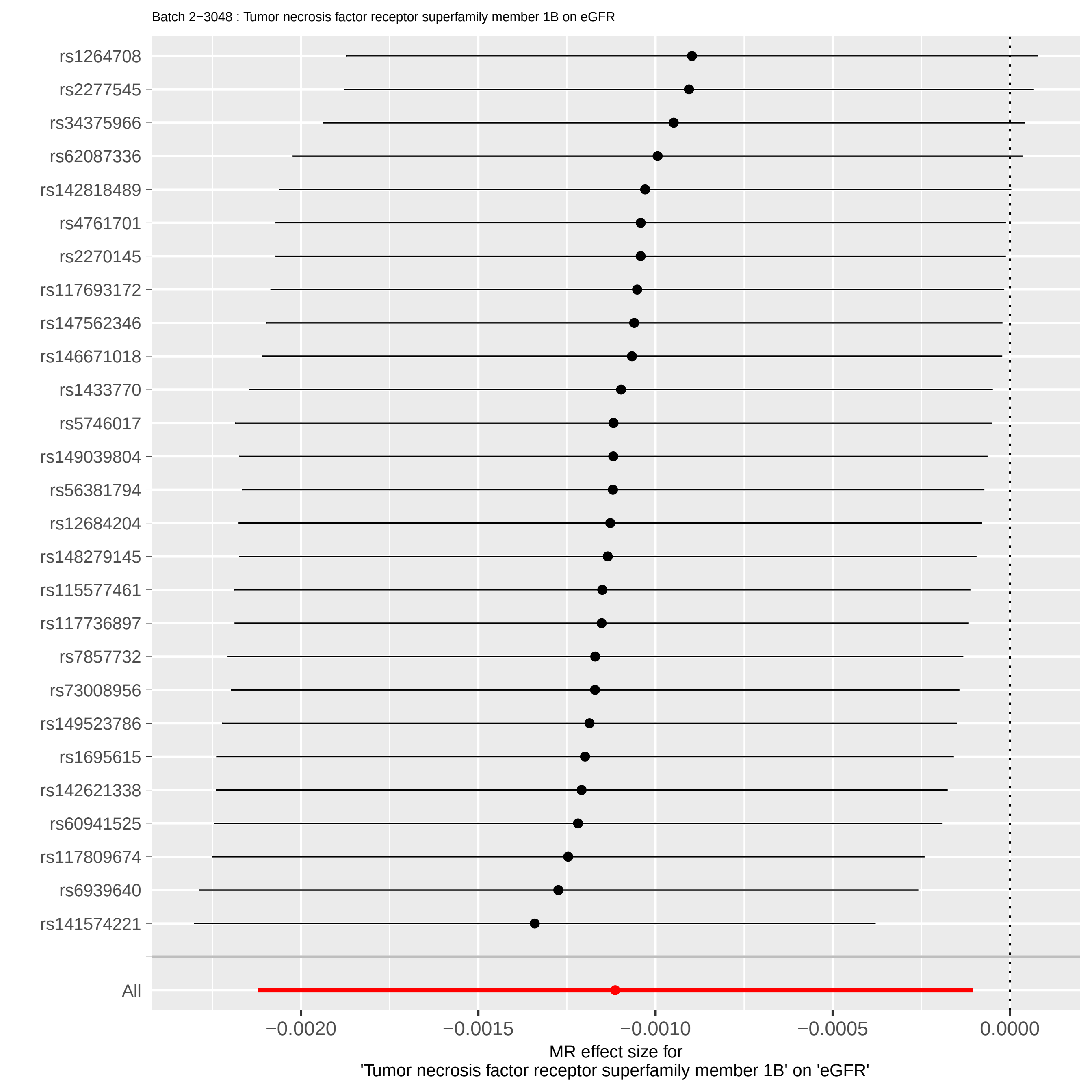

Batch 2−3048 : Tumor necrosis factor receptor superfamily member 1B on eGFR
rs1264708
rs2277545
rs34375966
rs62087336
rs142818489
rs4761701
rs2270145
rs117693172
rs147562346
rs146671018
rs1433770
rs5746017
rs149039804
rs56381794
rs12684204
rs148279145
rs115577461
rs117736897
rs7857732
rs73008956
rs149523786
rs1695615
rs142621338
rs60941525
rs117809674
rs6939640
rs141574221
All
−0.0020
−0.0015
−0.0010
−0.0005
0.0000
MR effect size for
'Tumor necrosis factor receptor superfamily member 1B' on 'eGFR'

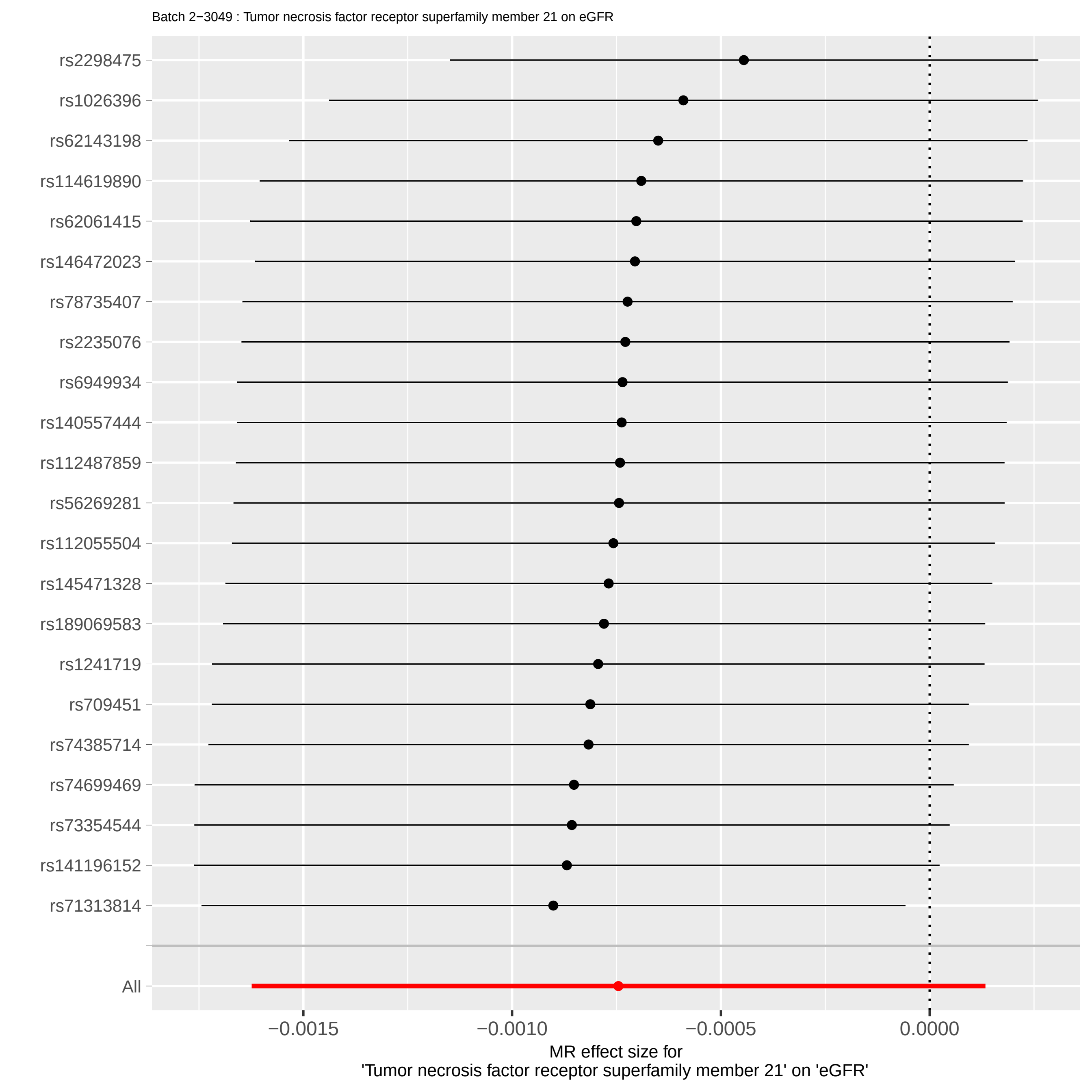

Batch 2−3049 : Tumor necrosis factor receptor superfamily member 21 on eGFR
rs2298475
rs1026396
rs62143198
rs114619890
rs62061415
rs146472023
rs78735407
rs2235076
rs6949934
rs140557444
rs112487859
rs56269281
rs112055504
rs145471328
rs189069583
rs1241719
rs709451
rs74385714
rs74699469
rs73354544
rs141196152
rs71313814
All
−0.0015
−0.0010
−0.0005
0.0000
MR effect size for
'Tumor necrosis factor receptor superfamily member 21' on 'eGFR'

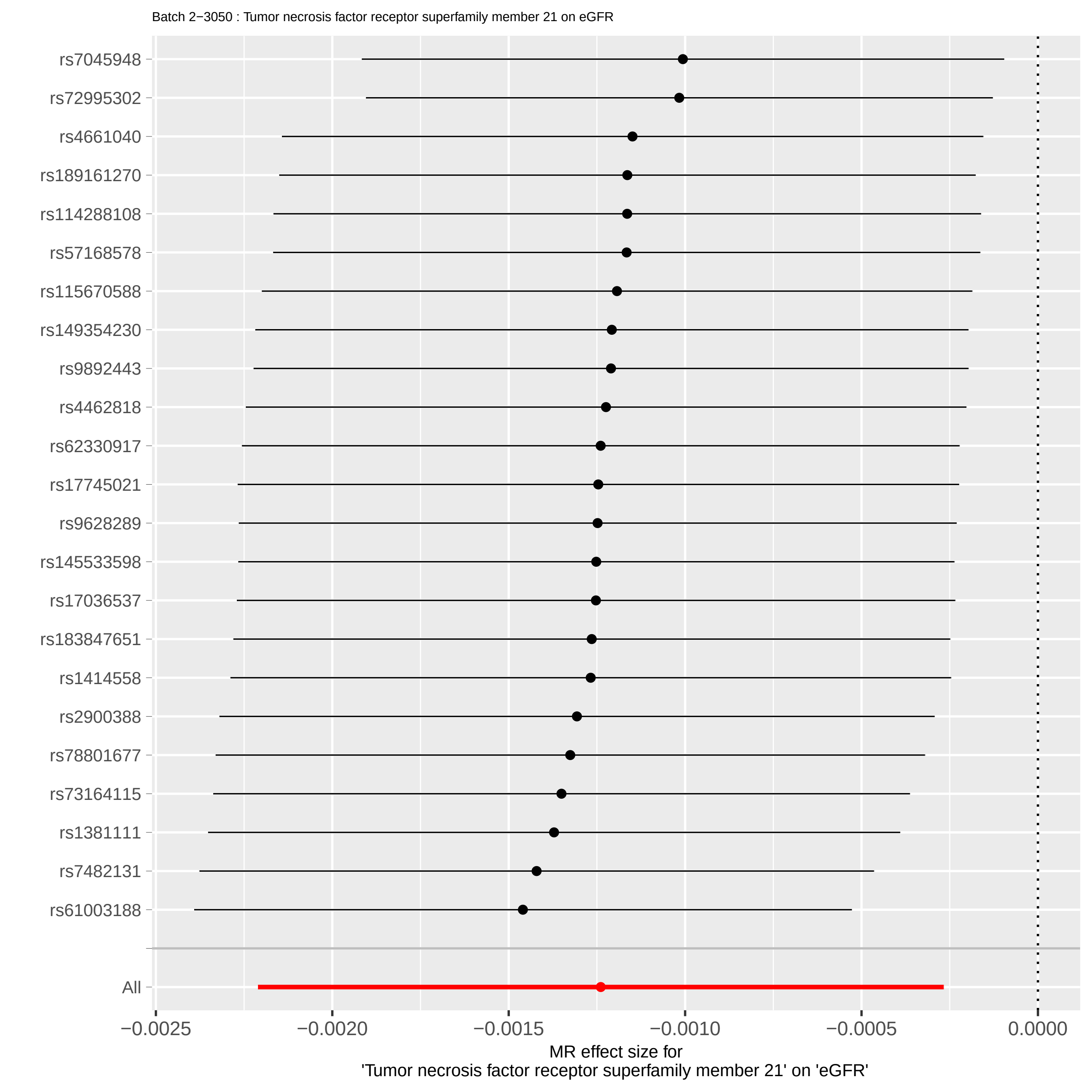

Batch 2−3050 : Tumor necrosis factor receptor superfamily member 21 on eGFR
rs7045948
rs72995302
rs4661040
rs189161270
rs114288108
rs57168578
rs115670588
rs149354230
rs9892443
rs4462818
rs62330917
rs17745021
rs9628289
rs145533598
rs17036537
rs183847651
rs1414558
rs2900388
rs78801677
rs73164115
rs1381111
rs7482131
rs61003188
All
−0.0025
−0.0020
−0.0015
−0.0010
−0.0005
0.0000
MR effect size for
'Tumor necrosis factor receptor superfamily member 21' on 'eGFR'

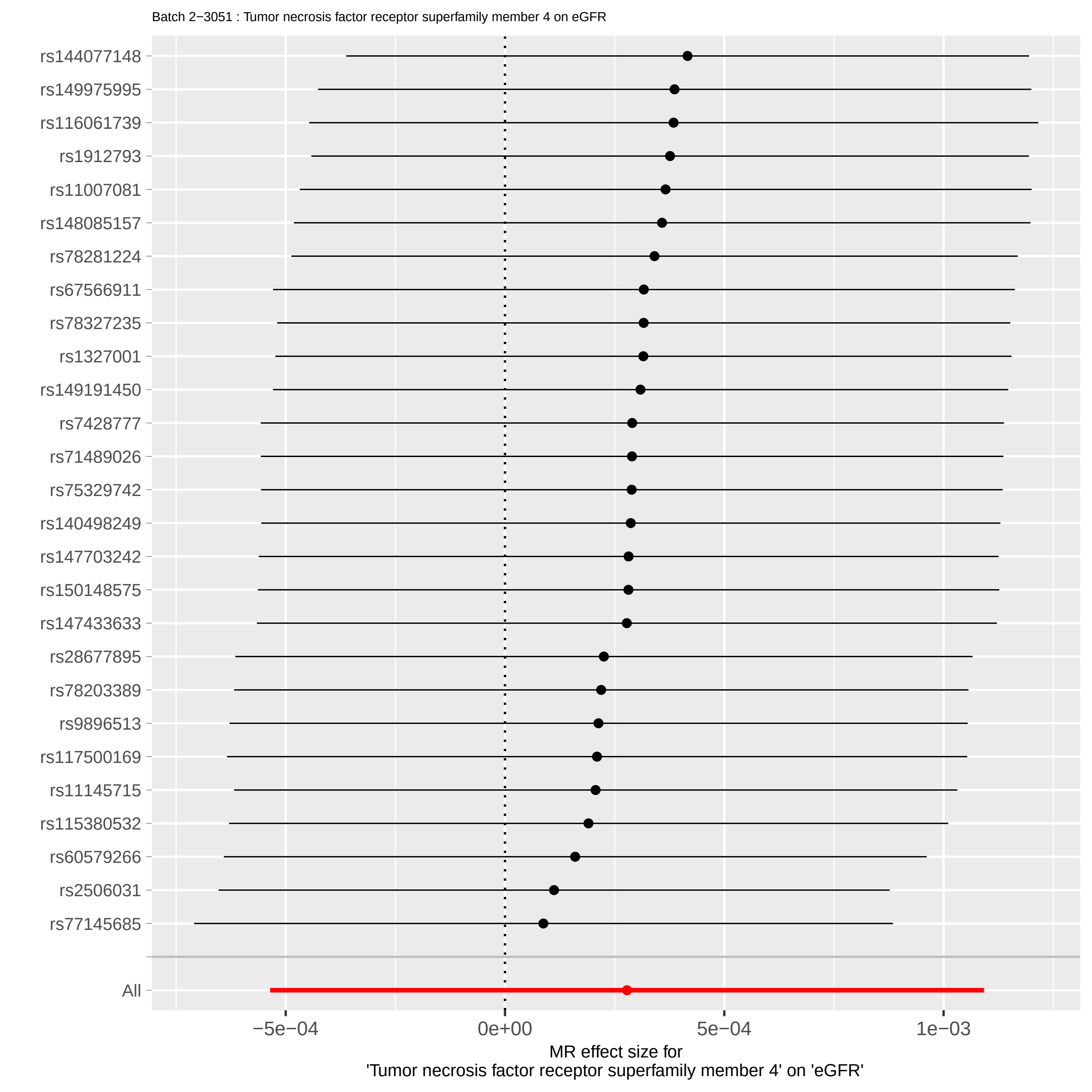

Batch 2−3051 : Tumor necrosis factor receptor superfamily member 4 on eGFR
rs144077148
rs149975995
rs116061739
rs1912793
rs11007081
rs148085157
rs78281224
rs67566911
rs78327235
rs1327001
rs149191450
rs7428777
rs71489026
rs75329742
rs140498249
rs147703242
rs150148575
rs147433633
rs28677895
rs78203389
rs9896513
rs117500169
rs11145715
rs115380532
rs60579266
rs2506031
rs77145685
All
−5e−04
0e+00
5e−04
1e−03
MR effect size for
'Tumor necrosis factor receptor superfamily member 4' on 'eGFR'

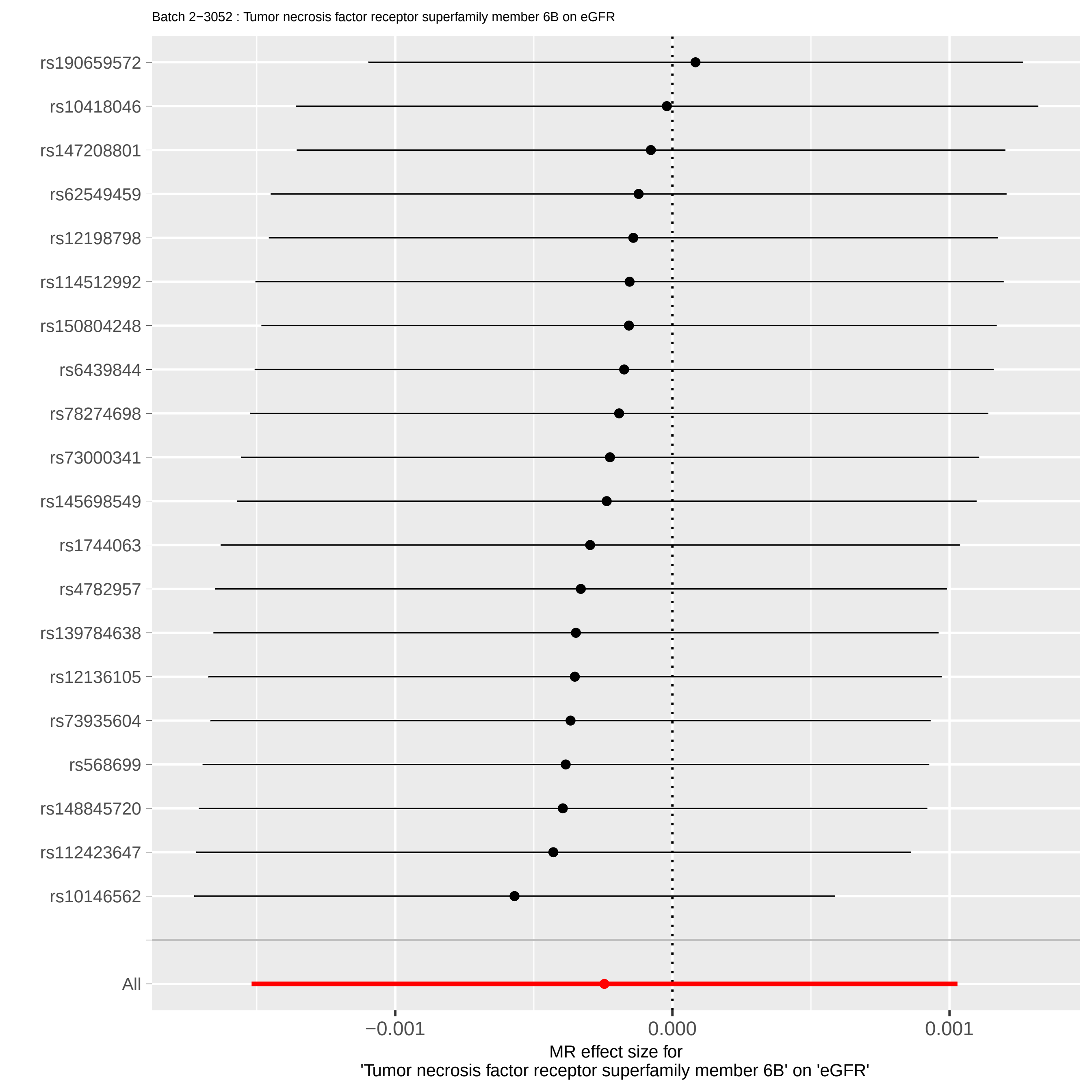

Batch 2−3052 : Tumor necrosis factor receptor superfamily member 6B on eGFR
rs190659572
rs10418046
rs147208801
rs62549459
rs12198798
rs114512992
rs150804248
rs6439844
rs78274698
rs73000341
rs145698549
rs1744063
rs4782957
rs139784638
rs12136105
rs73935604
rs568699
rs148845720
rs112423647
rs10146562
All
−0.001
0.000
0.001
MR effect size for
'Tumor necrosis factor receptor superfamily member 6B' on 'eGFR'

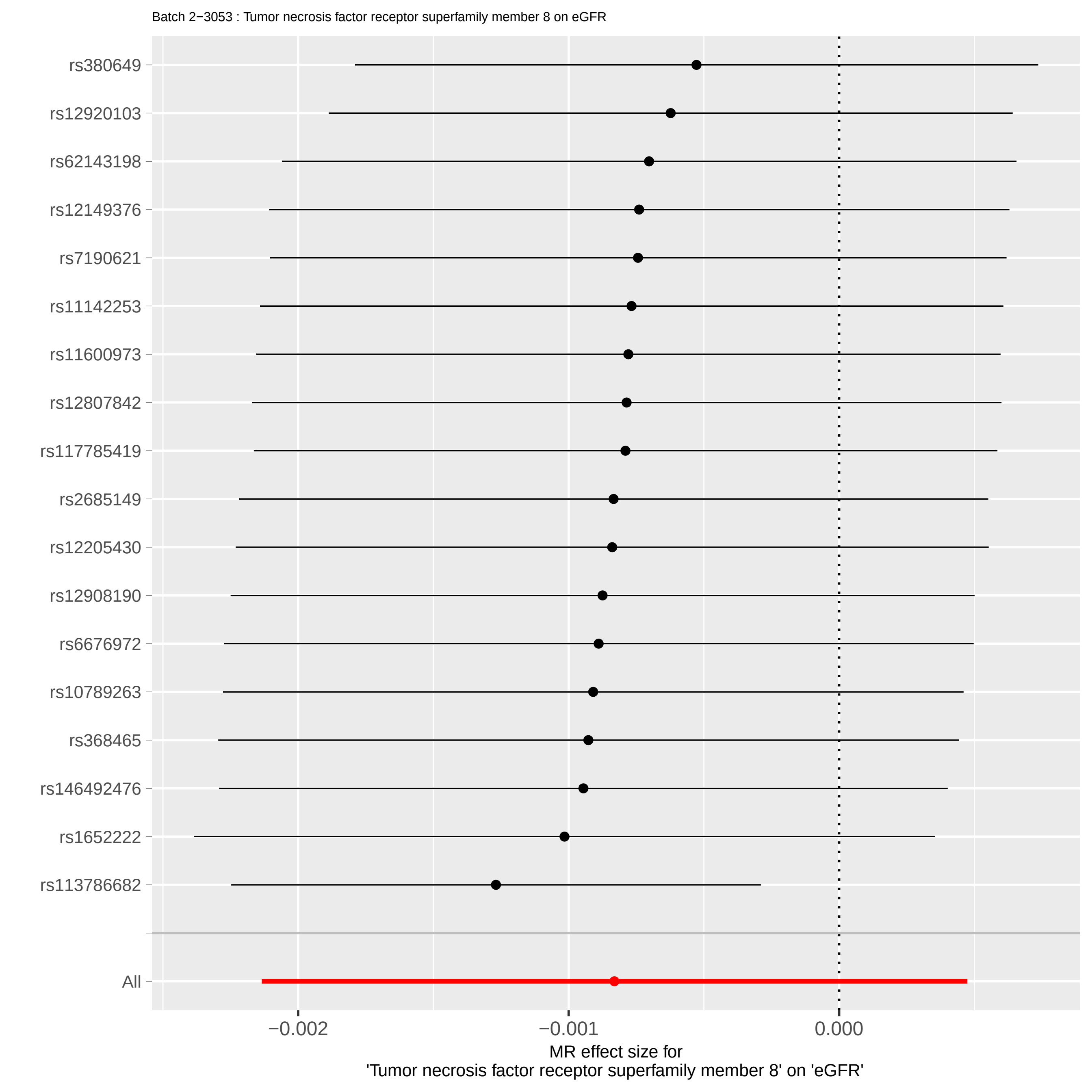

Batch 2−3053 : Tumor necrosis factor receptor superfamily member 8 on eGFR
rs380649
rs12920103
rs62143198
rs12149376
rs7190621
rs11142253
rs11600973
rs12807842
rs117785419
rs2685149
rs12205430
rs12908190
rs6676972
rs10789263
rs368465
rs146492476
rs1652222
rs113786682
All
−0.002
−0.001
0.000
MR effect size for
'Tumor necrosis factor receptor superfamily member 8' on 'eGFR'

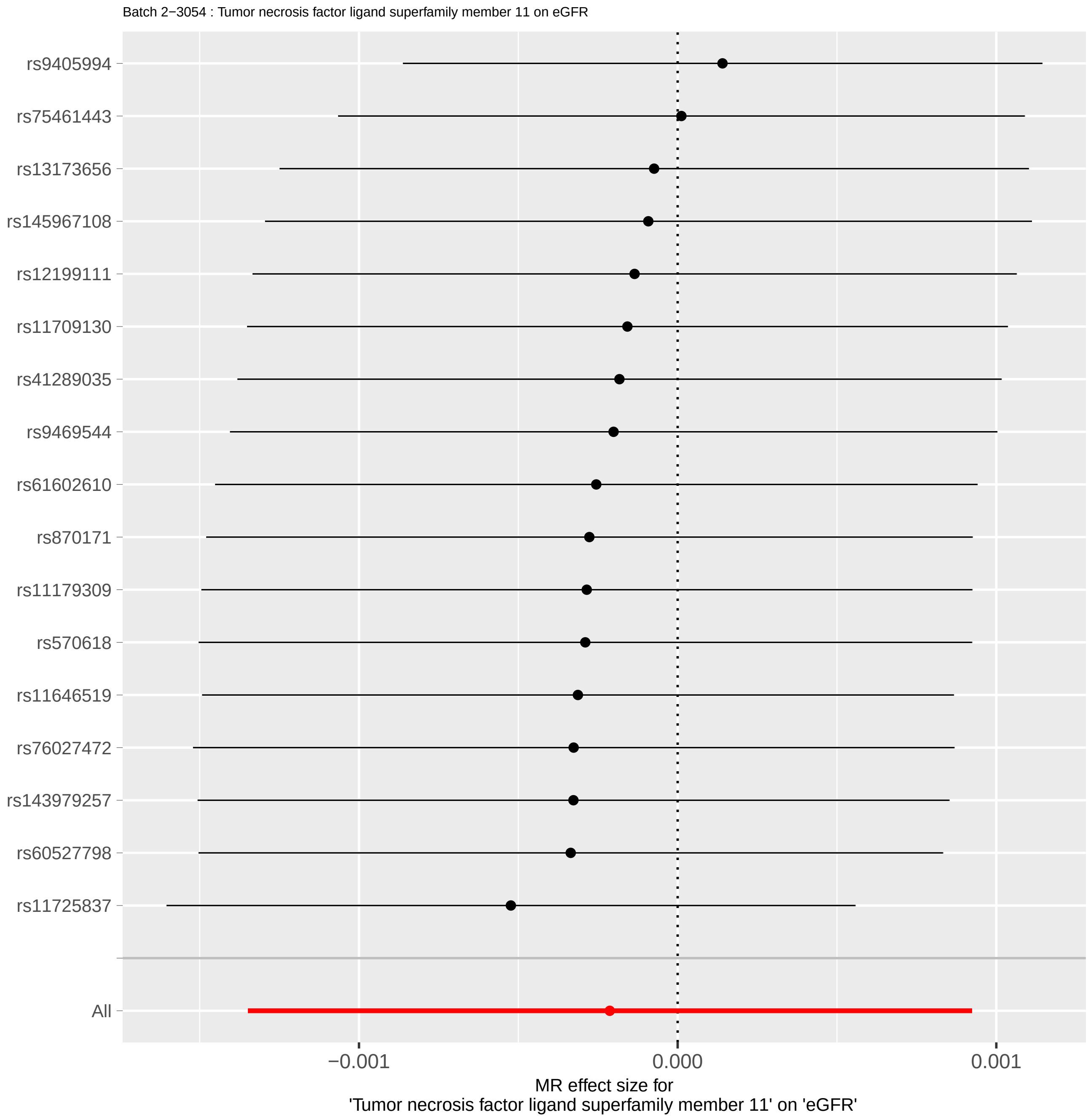
Batch 2−3054 : Tumor necrosis factor ligand superfamily member 11 on eGFR
rs9405994
rs75461443
rs13173656
rs145967108
rs12199111
rs11709130
rs41289035
rs9469544
rs61602610
rs870171
rs11179309
rs570618
rs11646519
rs76027472
rs143979257
rs60527798
rs11725837
All
−0.001
0.000
0.001
MR effect size for
'Tumor necrosis factor ligand superfamily member 11' on 'eGFR'

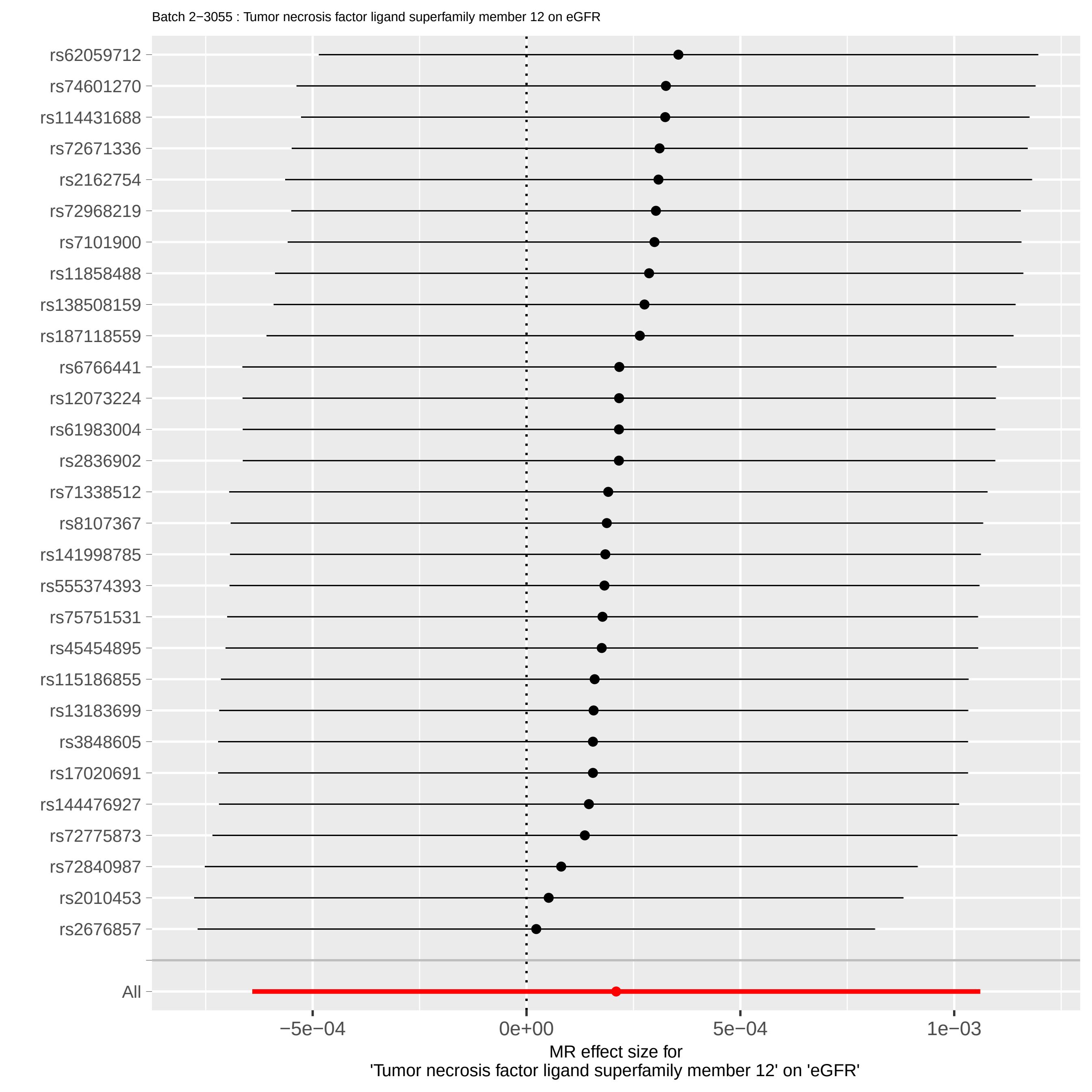
Batch 2−3055 : Tumor necrosis factor ligand superfamily member 12 on eGFR
rs62059712
rs74601270
rs114431688
rs72671336
rs2162754
rs72968219
rs7101900
rs11858488
rs138508159
rs187118559
rs6766441
rs12073224
rs61983004
rs2836902
rs71338512
rs8107367
rs141998785
rs555374393
rs75751531
rs45454895
rs115186855
rs13183699
rs3848605
rs17020691
rs144476927
rs72775873
rs72840987
rs2010453
rs2676857
All
−5e−04
0e+00
5e−04
1e−03
MR effect size for
'Tumor necrosis factor ligand superfamily member 12' on 'eGFR'

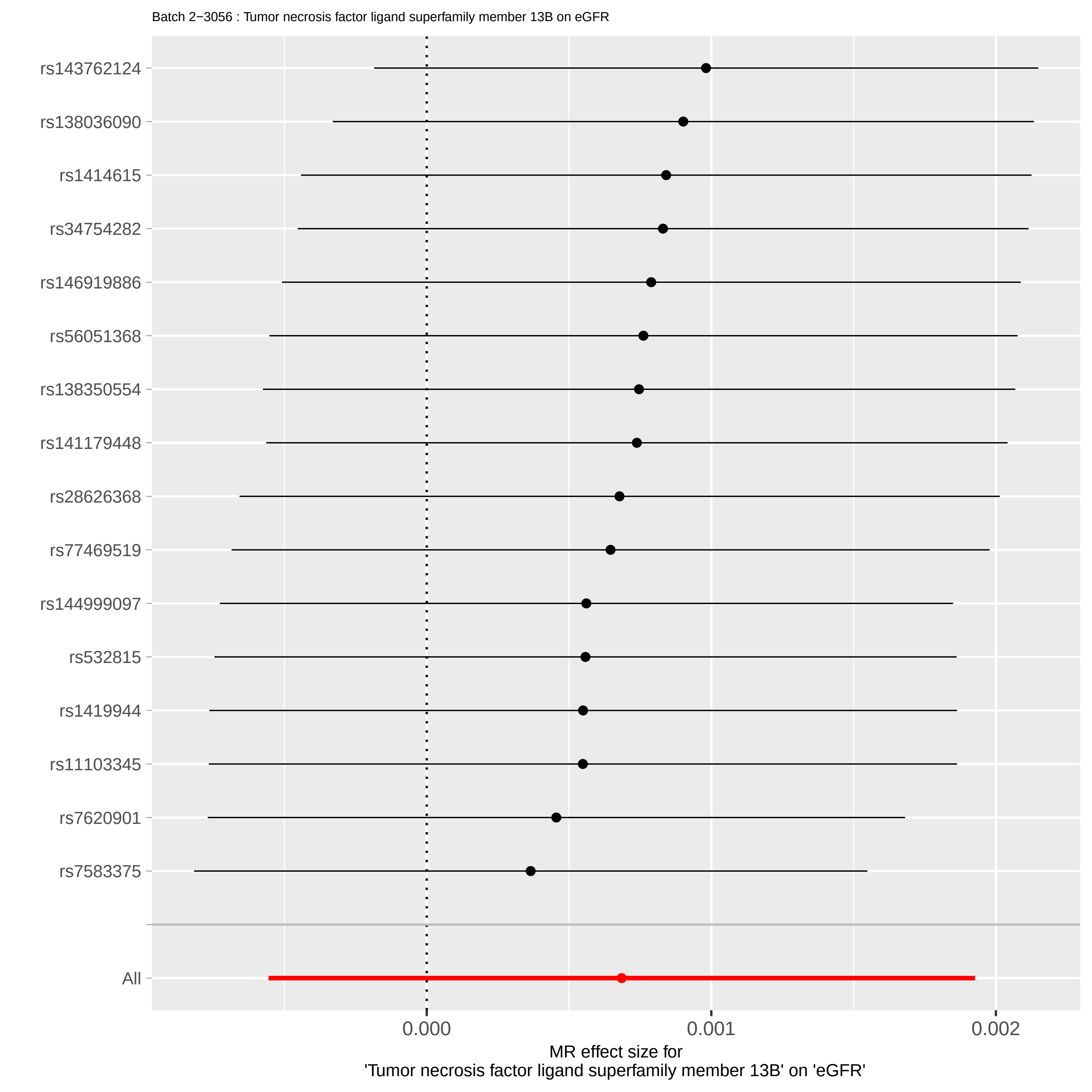

Batch 2−3056 : Tumor necrosis factor ligand superfamily member 13B on eGFR
rs143762124
rs138036090
rs1414615
rs34754282
rs146919886
rs56051368
rs138350554
rs141179448
rs28626368
rs77469519
rs144999097
rs532815
rs1419944
rs11103345
rs7620901
rs7583375
All
0.000
0.001
0.002
MR effect size for
'Tumor necrosis factor ligand superfamily member 13B' on 'eGFR'

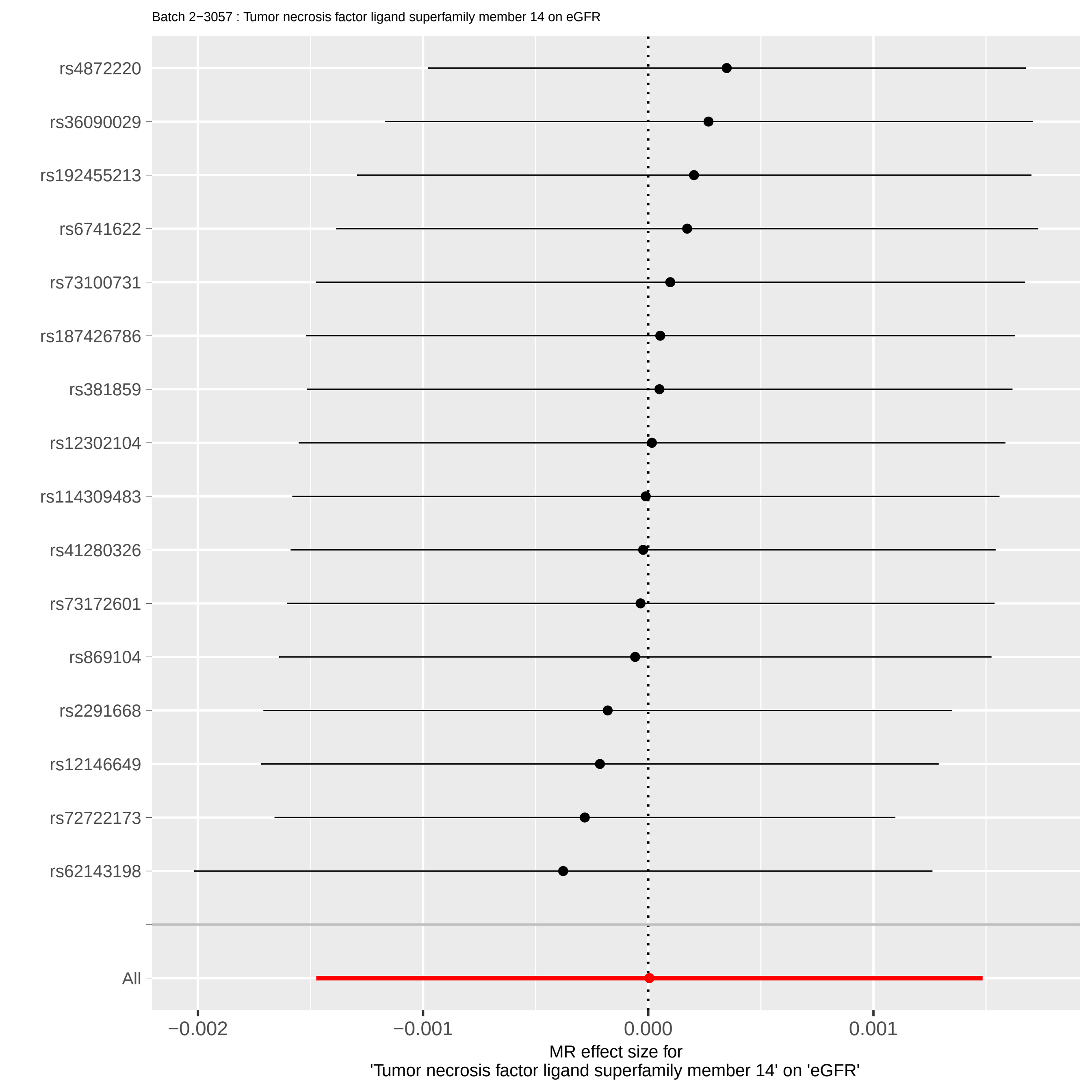

Batch 2−3057 : Tumor necrosis factor ligand superfamily member 14 on eGFR
rs4872220
rs36090029
rs192455213
rs6741622
rs73100731
rs187426786
rs381859
rs12302104
rs114309483
rs41280326
rs73172601
rs869104
rs2291668
rs12146649
rs72722173
rs62143198
All
−0.002
−0.001
0.000
0.001
MR effect size for
'Tumor necrosis factor ligand superfamily member 14' on 'eGFR'

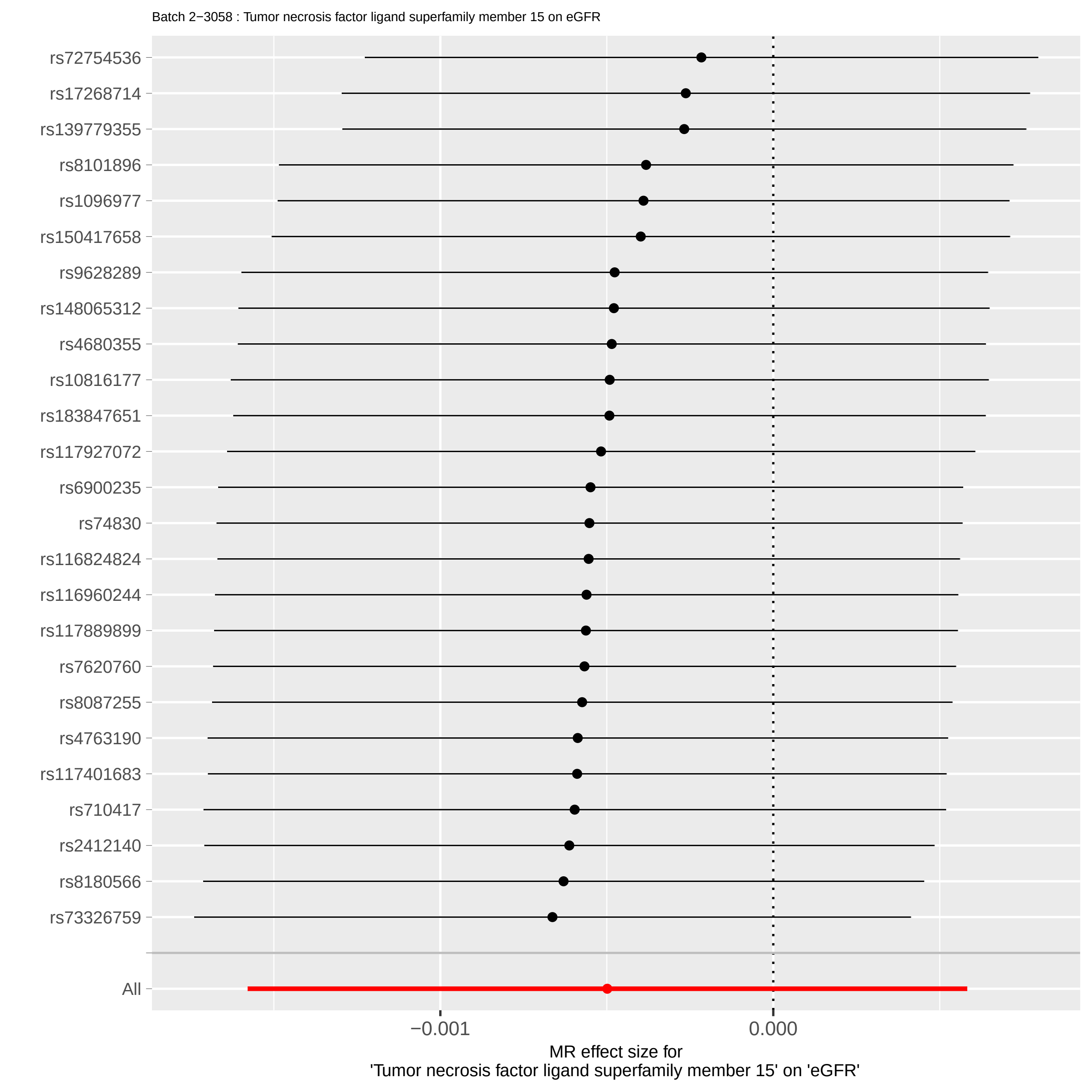

Batch 2−3058 : Tumor necrosis factor ligand superfamily member 15 on eGFR
rs72754536
rs17268714
rs139779355
rs8101896
rs1096977
rs150417658
rs9628289
rs148065312
rs4680355
rs10816177
rs183847651
rs117927072
rs6900235
rs74830
rs116824824
rs116960244
rs117889899
rs7620760
rs8087255
rs4763190
rs117401683
rs710417
rs2412140
rs8180566
rs73326759
All
−0.001
0.000
MR effect size for
'Tumor necrosis factor ligand superfamily member 15' on 'eGFR'

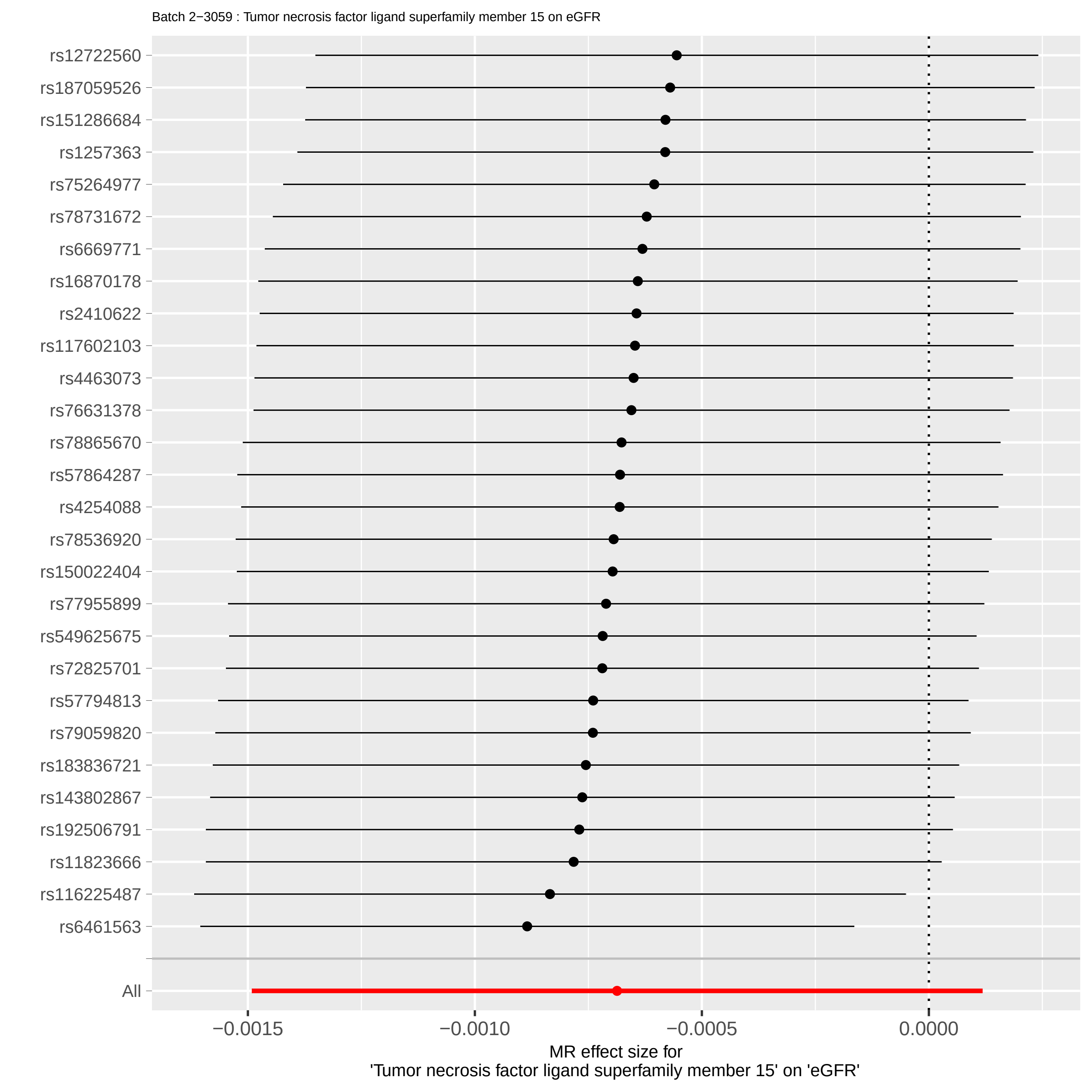

Batch 2−3059 : Tumor necrosis factor ligand superfamily member 15 on eGFR
rs12722560
rs187059526
rs151286684
rs1257363
rs75264977
rs78731672
rs6669771
rs16870178
rs2410622
rs117602103
rs4463073
rs76631378
rs78865670
rs57864287
rs4254088
rs78536920
rs150022404
rs77955899
rs549625675
rs72825701
rs57794813
rs79059820
rs183836721
rs143802867
rs192506791
rs11823666
rs116225487
rs6461563
All
−0.0015
−0.0010
−0.0005
0.0000
MR effect size for
'Tumor necrosis factor ligand superfamily member 15' on 'eGFR'

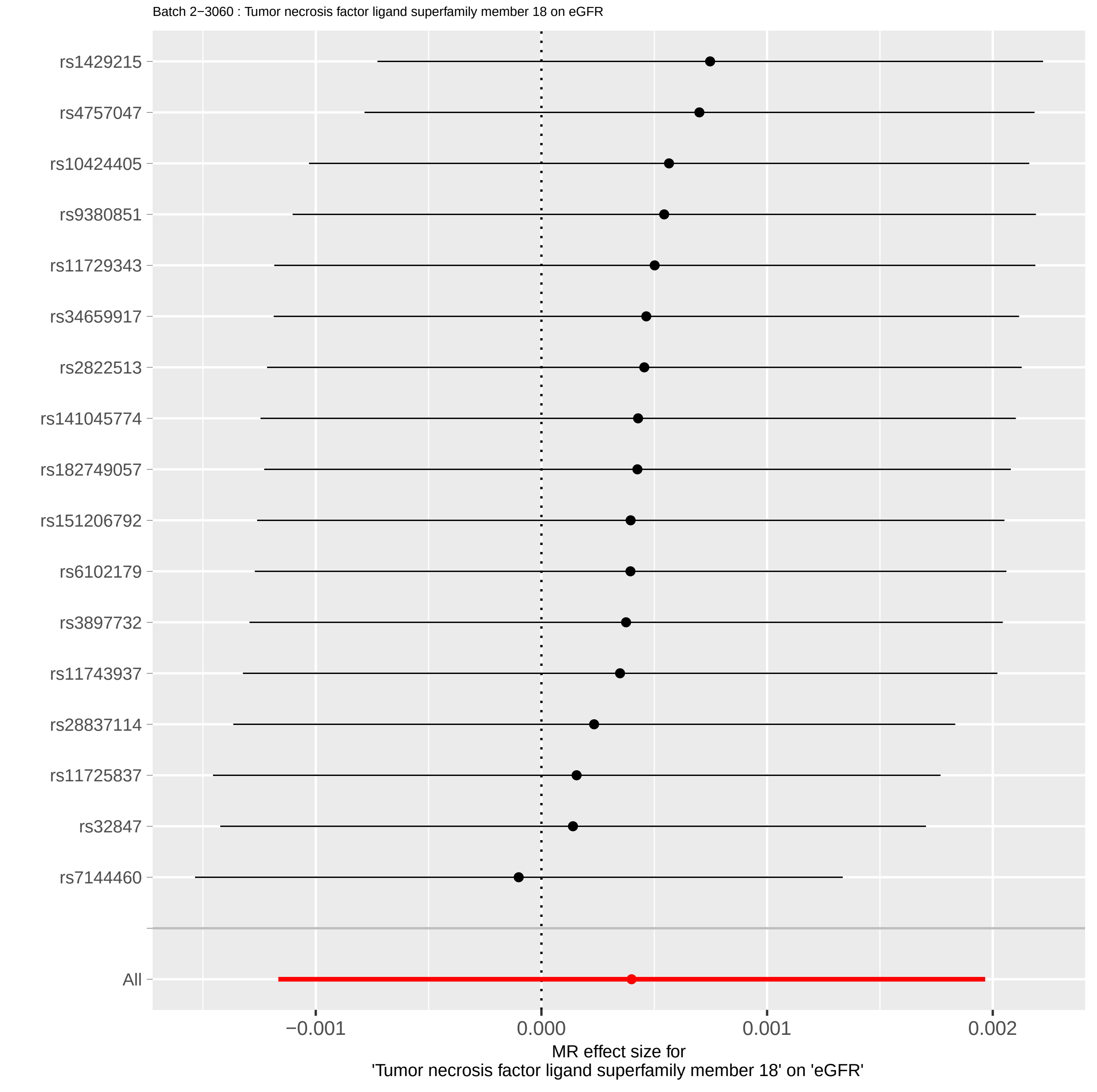

Batch 2−3060 : Tumor necrosis factor ligand superfamily member 18 on eGFR
rs1429215
rs4757047
rs10424405
rs9380851
rs11729343
rs34659917
rs2822513
rs141045774
rs182749057
rs151206792
rs6102179
rs3897732
rs11743937
rs28837114
rs11725837
rs32847
rs7144460
All
−0.001
0.000
0.001
0.002
MR effect size for
'Tumor necrosis factor ligand superfamily member 18' on 'eGFR'

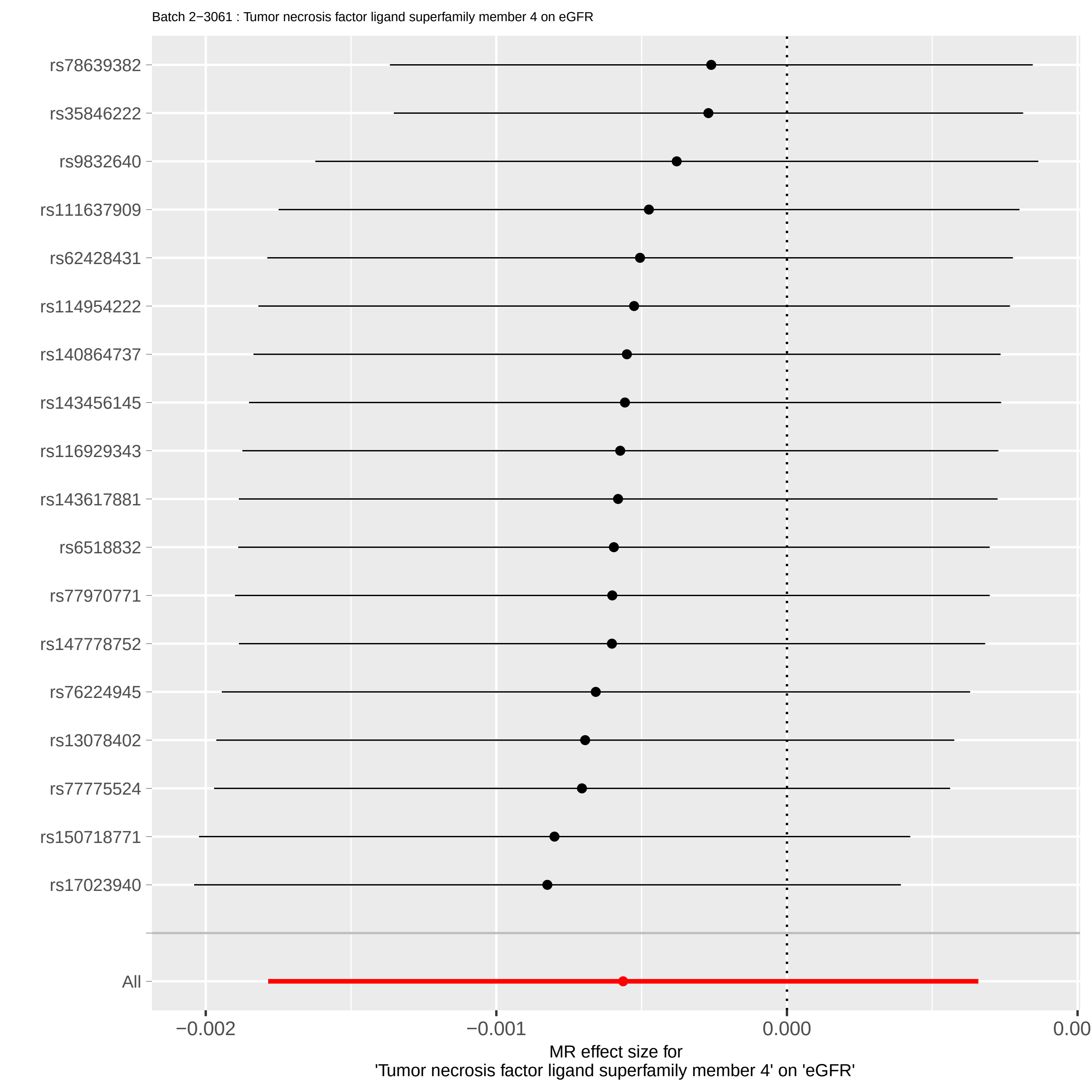

Batch 2−3061 : Tumor necrosis factor ligand superfamily member 4 on eGFR
rs78639382
rs35846222
rs9832640
rs111637909
rs62428431
rs114954222
rs140864737
rs143456145
rs116929343
rs143617881
rs6518832
rs77970771
rs147778752
rs76224945
rs13078402
rs77775524
rs150718771
rs17023940
All
−0.002
−0.001
0.000
0.00
MR effect size for
'Tumor necrosis factor ligand superfamily member 4' on 'eGFR'

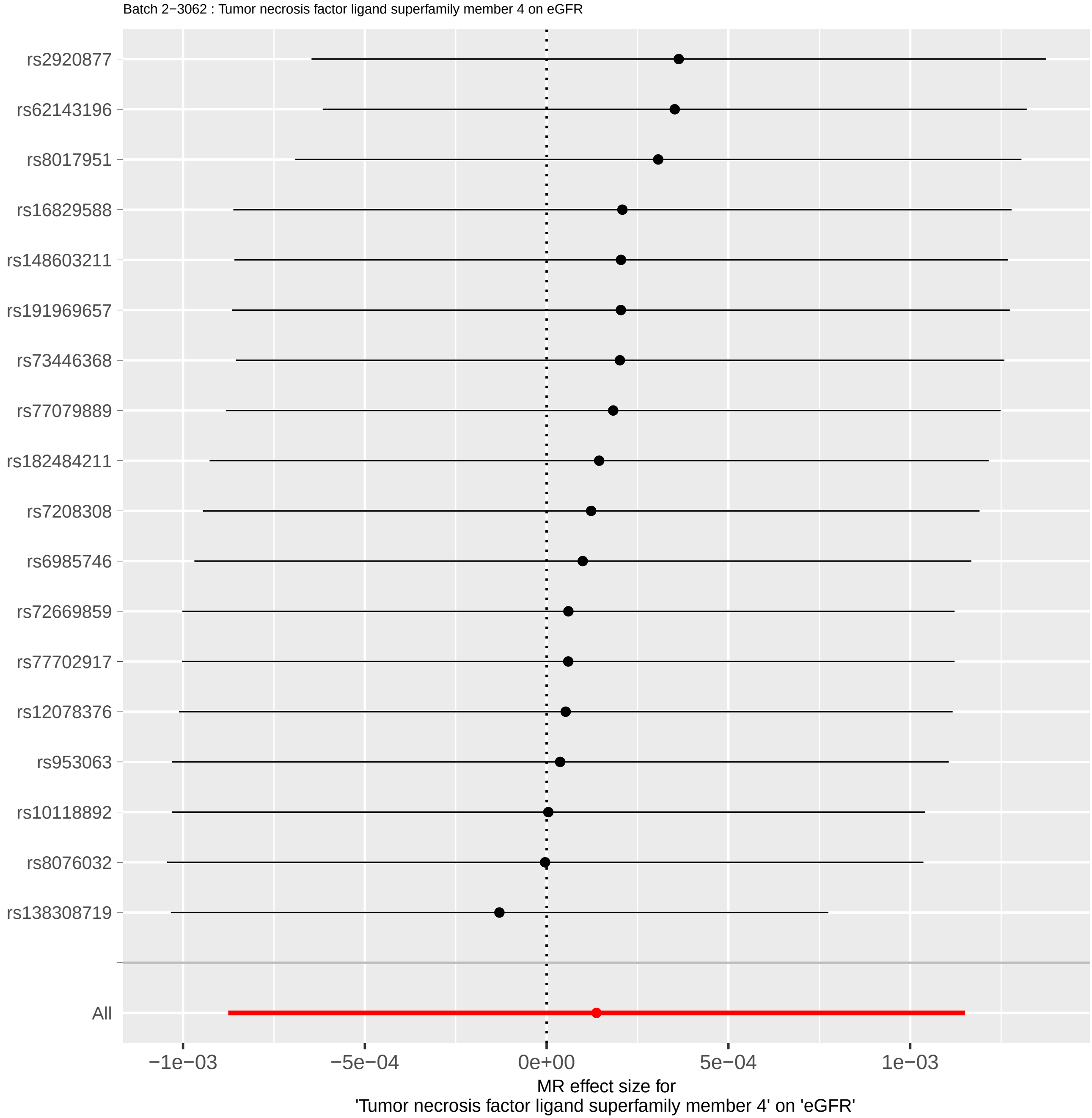

Batch 2−3062 : Tumor necrosis factor ligand superfamily member 4 on eGFR
rs2920877
rs62143196
rs8017951
rs16829588
rs148603211
rs191969657
rs73446368
rs77079889
rs182484211
rs7208308
rs6985746
rs72669859
rs77702917
rs12078376
rs953063
rs10118892
rs8076032
rs138308719
All
−1e−03
−5e−04
0e+00
5e−04
1e−03
MR effect size for
'Tumor necrosis factor ligand superfamily member 4' on 'eGFR'

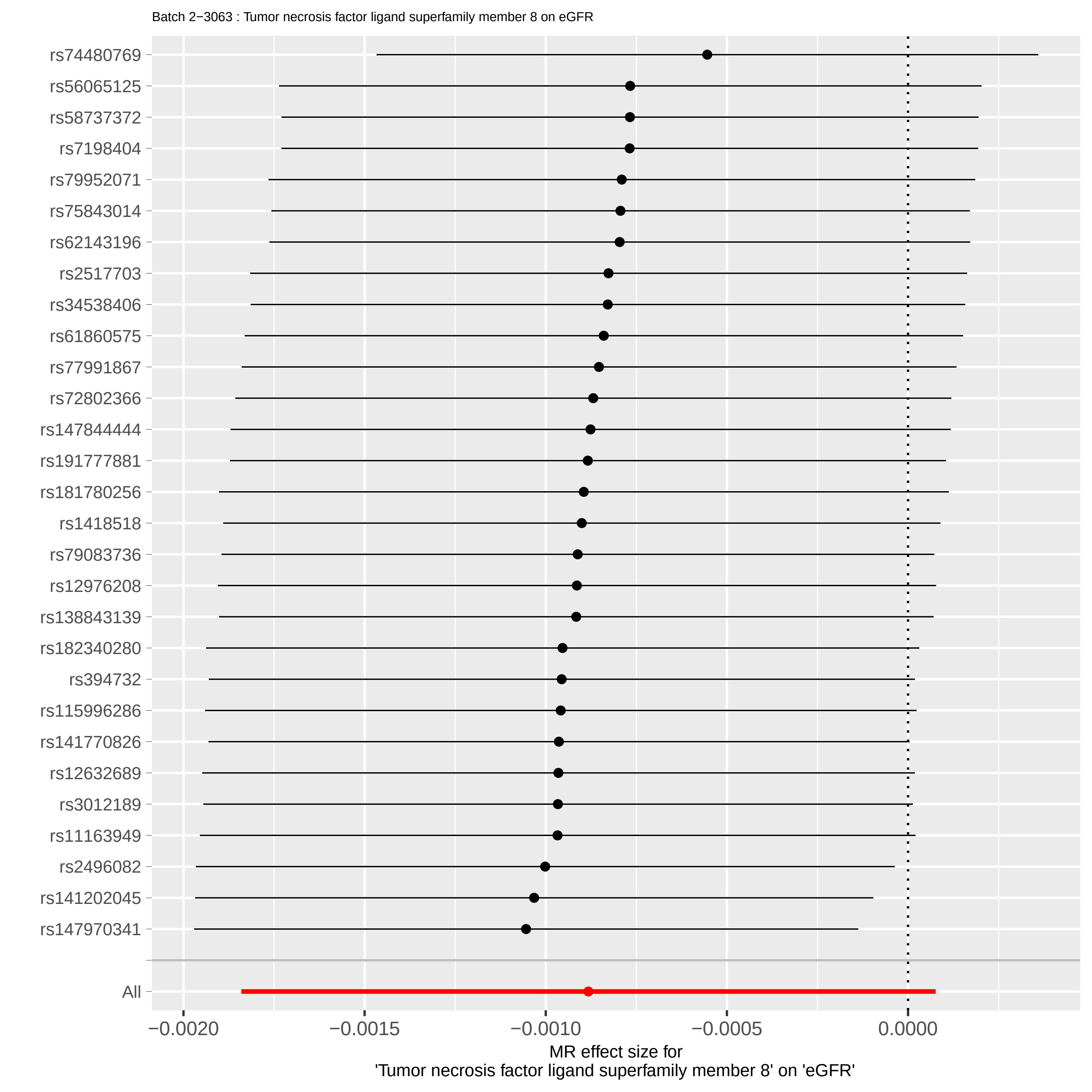

Batch 2−3063 : Tumor necrosis factor ligand superfamily member 8 on eGFR
rs74480769
rs56065125
rs58737372
rs7198404
rs79952071
rs75843014
rs62143196
rs2517703
rs34538406
rs61860575
rs77991867
rs72802366
rs147844444
rs191777881
rs181780256
rs1418518
rs79083736
rs12976208
rs138843139
rs182340280
rs394732
rs115996286
rs141770826
rs12632689
rs3012189
rs11163949
rs2496082
rs141202045
rs147970341
All
−0.0020
−0.0015
−0.0010
−0.0005
0.0000
MR effect size for
'Tumor necrosis factor ligand superfamily member 8' on 'eGFR'

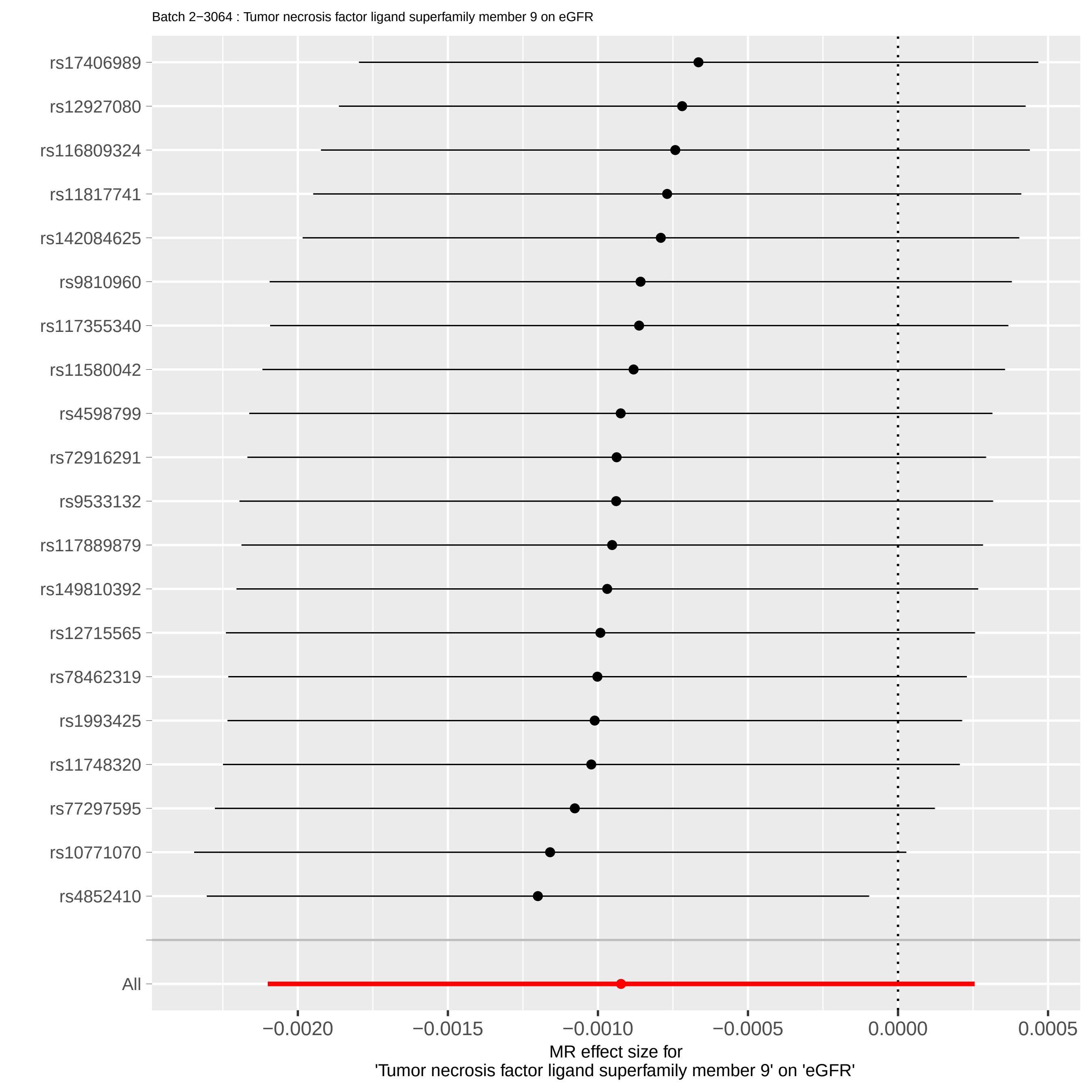

Batch 2−3064 : Tumor necrosis factor ligand superfamily member 9 on eGFR
rs17406989
rs12927080
rs116809324
rs11817741
rs142084625
rs9810960
rs117355340
rs11580042
rs4598799
rs72916291
rs9533132
rs117889879
rs149810392
rs12715565
rs78462319
rs1993425
rs11748320
rs77297595
rs10771070
rs4852410
All
−0.0020
−0.0015
−0.0010
−0.0005
0.0000
0.0005
MR effect size for
'Tumor necrosis factor ligand superfamily member 9' on 'eGFR'

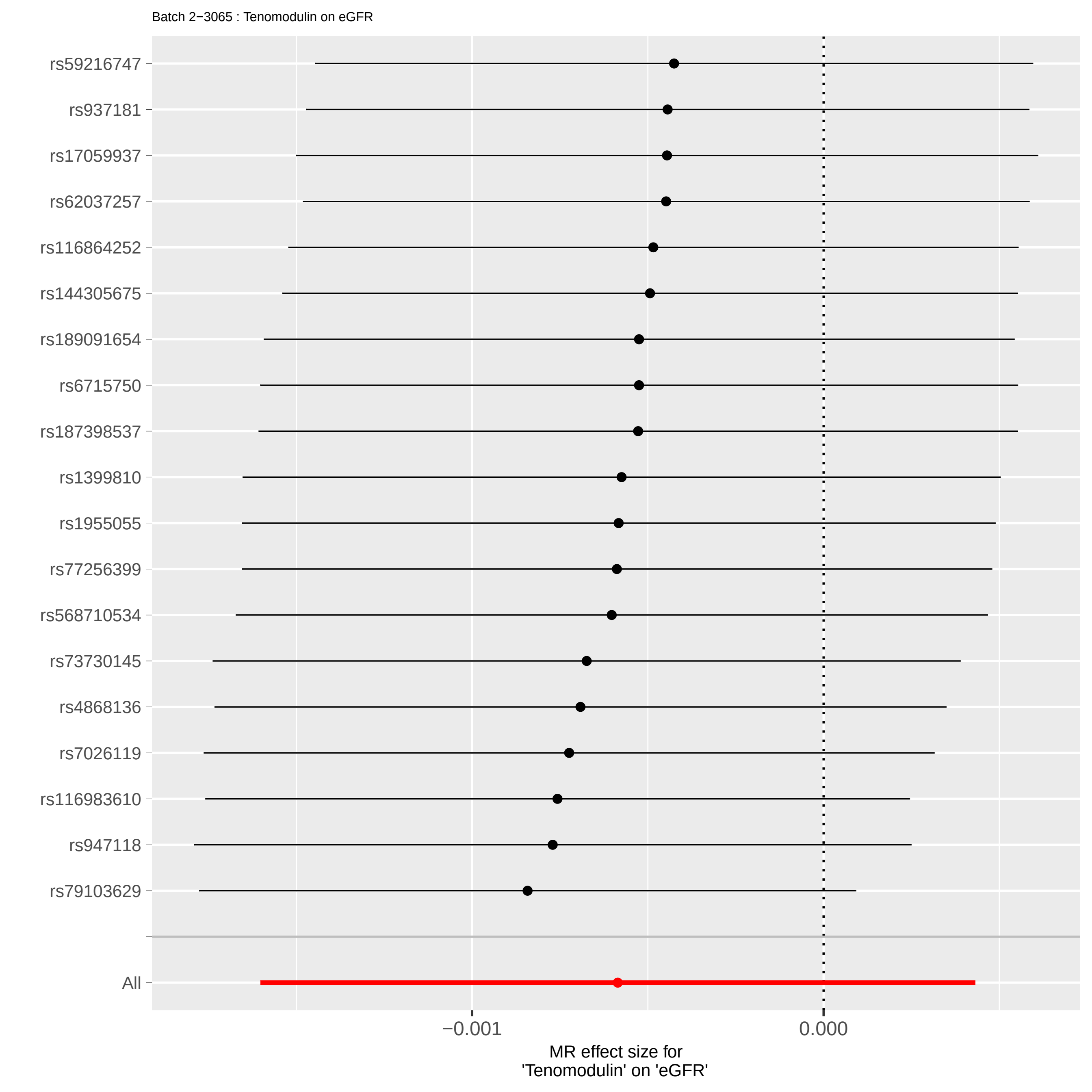

Batch 2−3065 : Tenomodulin on eGFR
rs59216747
rs937181
rs17059937
rs62037257
rs116864252
rs144305675
rs189091654
rs6715750
rs187398537
rs1399810
rs1955055
rs77256399
rs568710534
rs73730145
rs4868136
rs7026119
rs116983610
rs947118
rs79103629
All
−0.001
0.000
MR effect size for
'Tenomodulin' on 'eGFR'

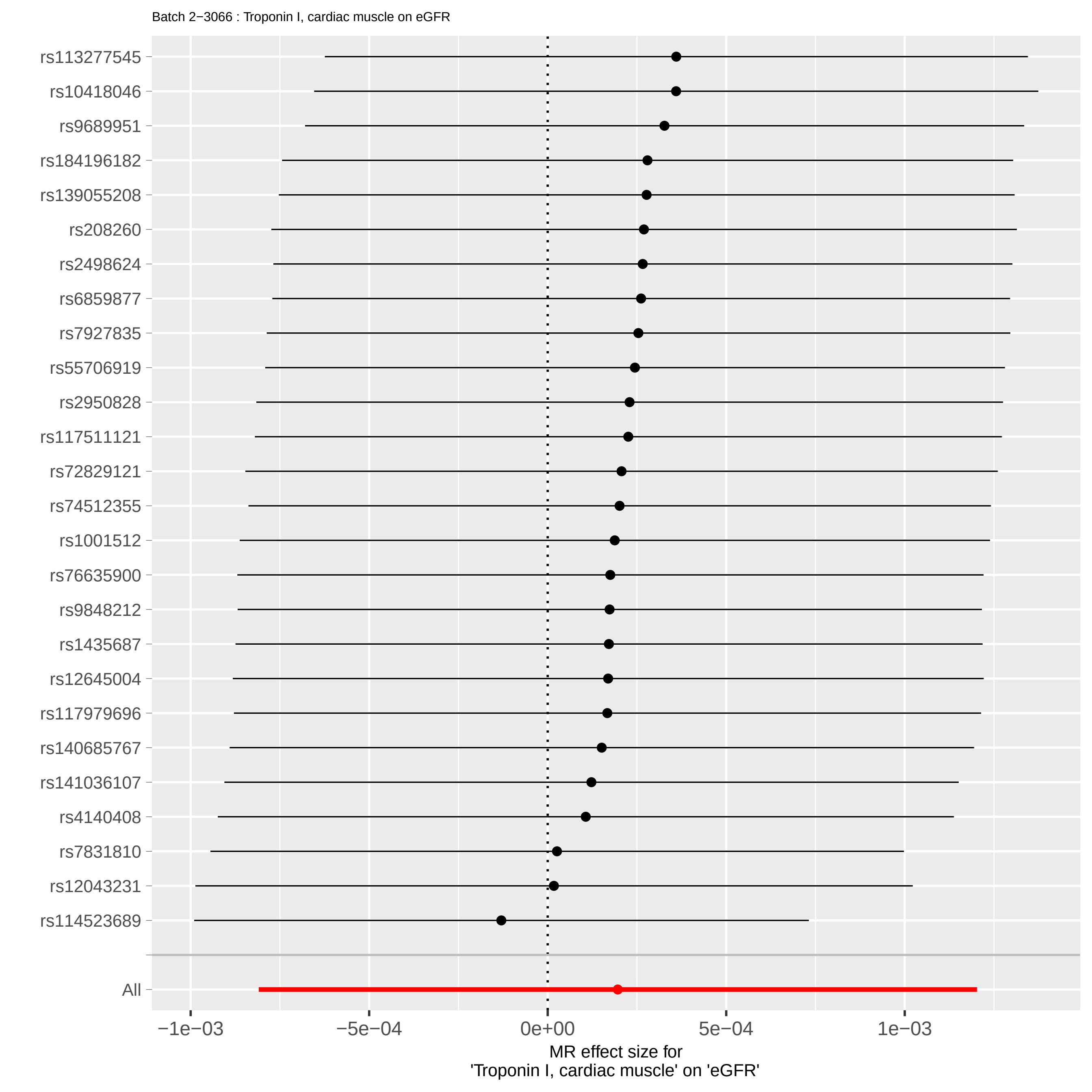

Batch 2−3066 : Troponin I, cardiac muscle on eGFR
rs113277545
rs10418046
rs9689951
rs184196182
rs139055208
rs208260
rs2498624
rs6859877
rs7927835
rs55706919
rs2950828
rs117511121
rs72829121
rs74512355
rs1001512
rs76635900
rs9848212
rs1435687
rs12645004
rs117979696
rs140685767
rs141036107
rs4140408
rs7831810
rs12043231
rs114523689
All
−1e−03
−5e−04
0e+00
5e−04
1e−03
MR effect size for
'Troponin I, cardiac muscle' on 'eGFR'

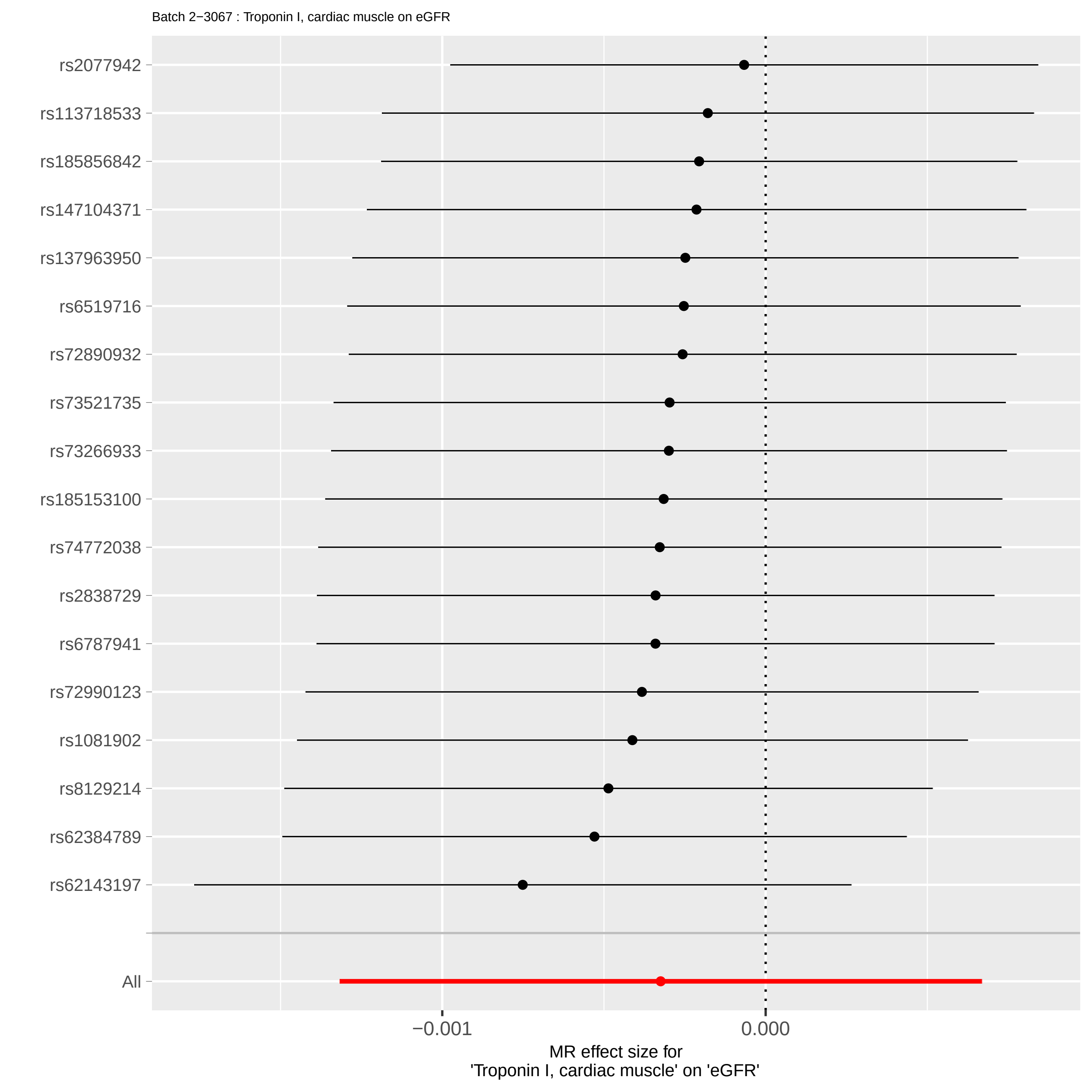

Batch 2−3067 : Troponin I, cardiac muscle on eGFR
rs2077942
rs113718533
rs185856842
rs147104371
rs137963950
rs6519716
rs72890932
rs73521735
rs73266933
rs185153100
rs74772038
rs2838729
rs6787941
rs72990123
rs1081902
rs8129214
rs62384789
rs62143197
All
−0.001
0.000
MR effect size for
'Troponin I, cardiac muscle' on 'eGFR'

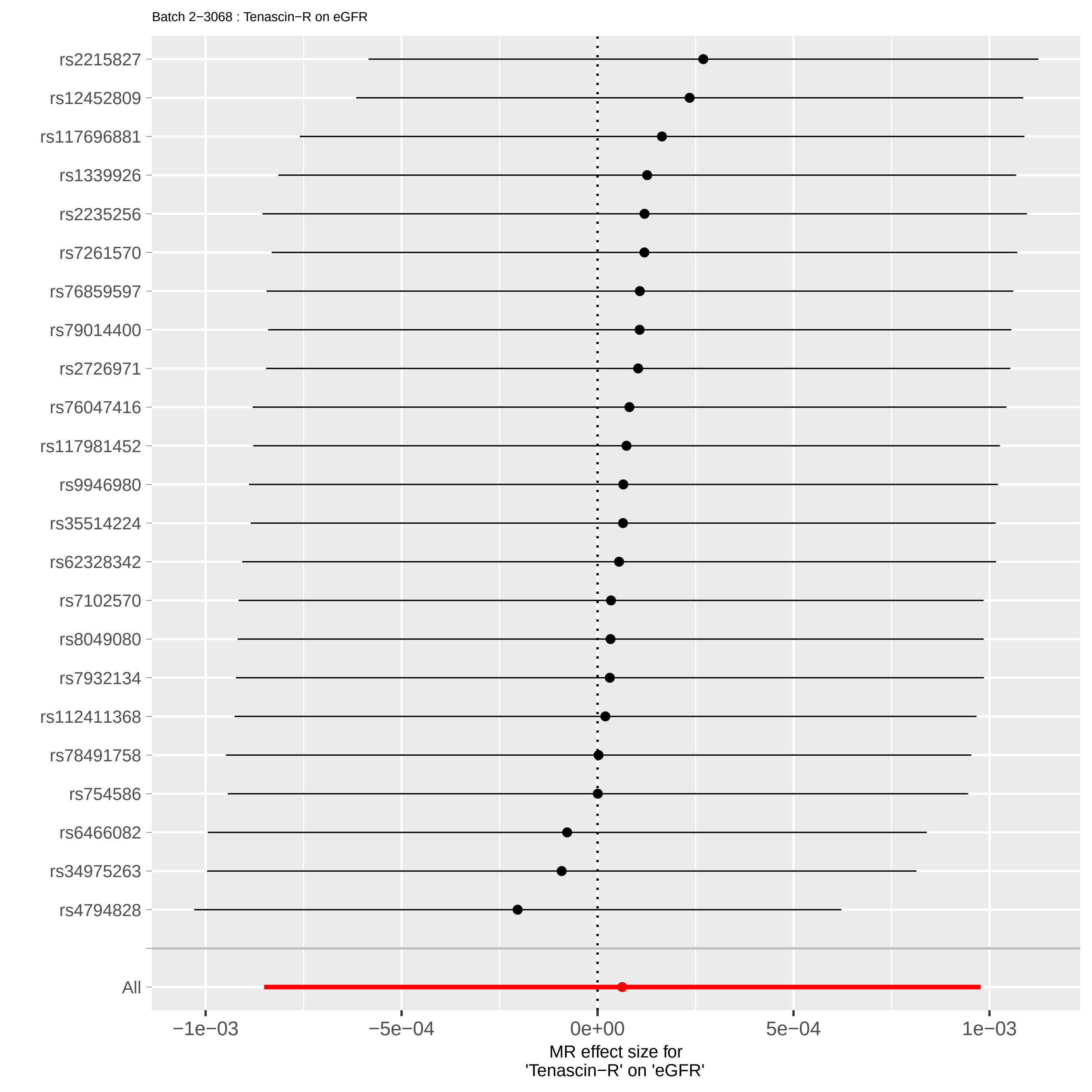
Batch 2−3068 : Tenascin−R on eGFR
rs2215827
rs12452809
rs117696881
rs1339926
rs2235256
rs7261570
rs76859597
rs79014400
rs2726971
rs76047416
rs117981452
rs9946980
rs35514224
rs62328342
rs7102570
rs8049080
rs7932134
rs112411368
rs78491758
rs754586
rs6466082
rs34975263
rs4794828
All
−1e−03
−5e−04
0e+00
5e−04
1e−03
MR effect size for
'Tenascin−R' on 'eGFR'

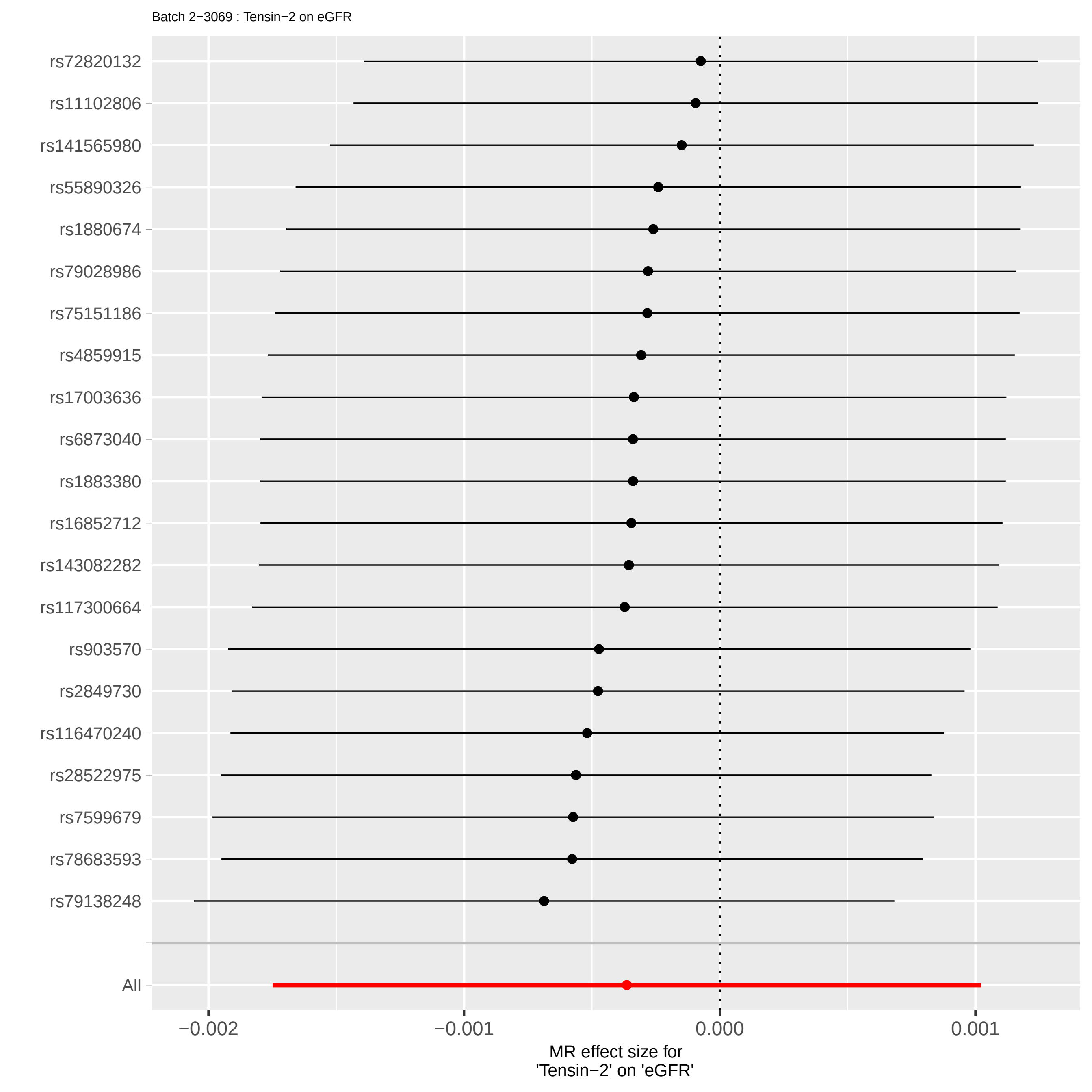
Batch 2−3069 : Tensin−2 on eGFR
rs72820132
rs11102806
rs141565980
rs55890326
rs1880674
rs79028986
rs75151186
rs4859915
rs17003636
rs6873040
rs1883380
rs16852712
rs143082282
rs117300664
rs903570
rs2849730
rs116470240
rs28522975
rs7599679
rs78683593
rs79138248
All
−0.002
−0.001
0.000
0.001
MR effect size for
'Tensin−2' on 'eGFR'

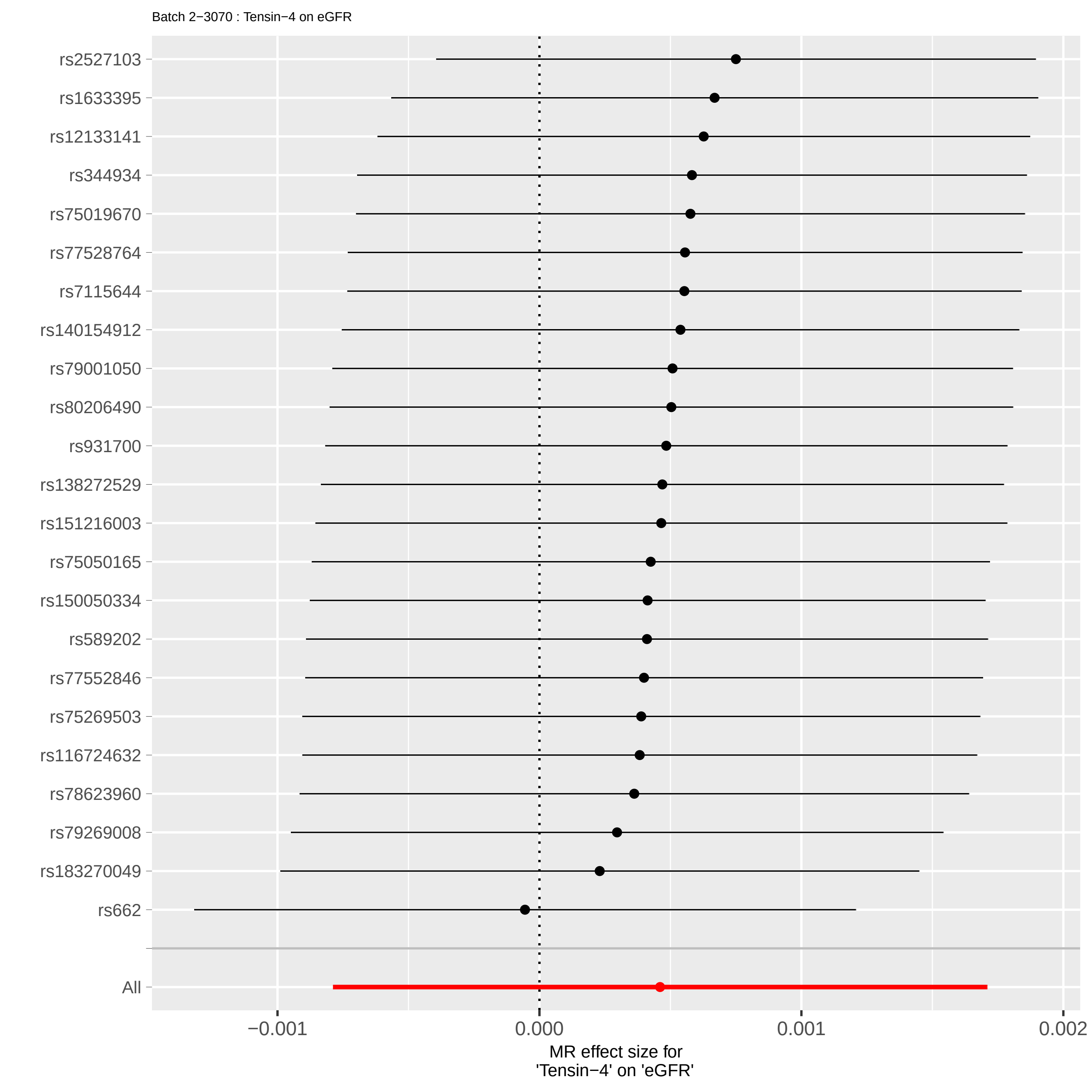

Batch 2−3070 : Tensin−4 on eGFR
rs2527103
rs1633395
rs12133141
rs344934
rs75019670
rs77528764
rs7115644
rs140154912
rs79001050
rs80206490
rs931700
rs138272529
rs151216003
rs75050165
rs150050334
rs589202
rs77552846
rs75269503
rs116724632
rs78623960
rs79269008
rs183270049
rs662
All
−0.001
0.000
0.001
0.002
MR effect size for
'Tensin−4' on 'eGFR'

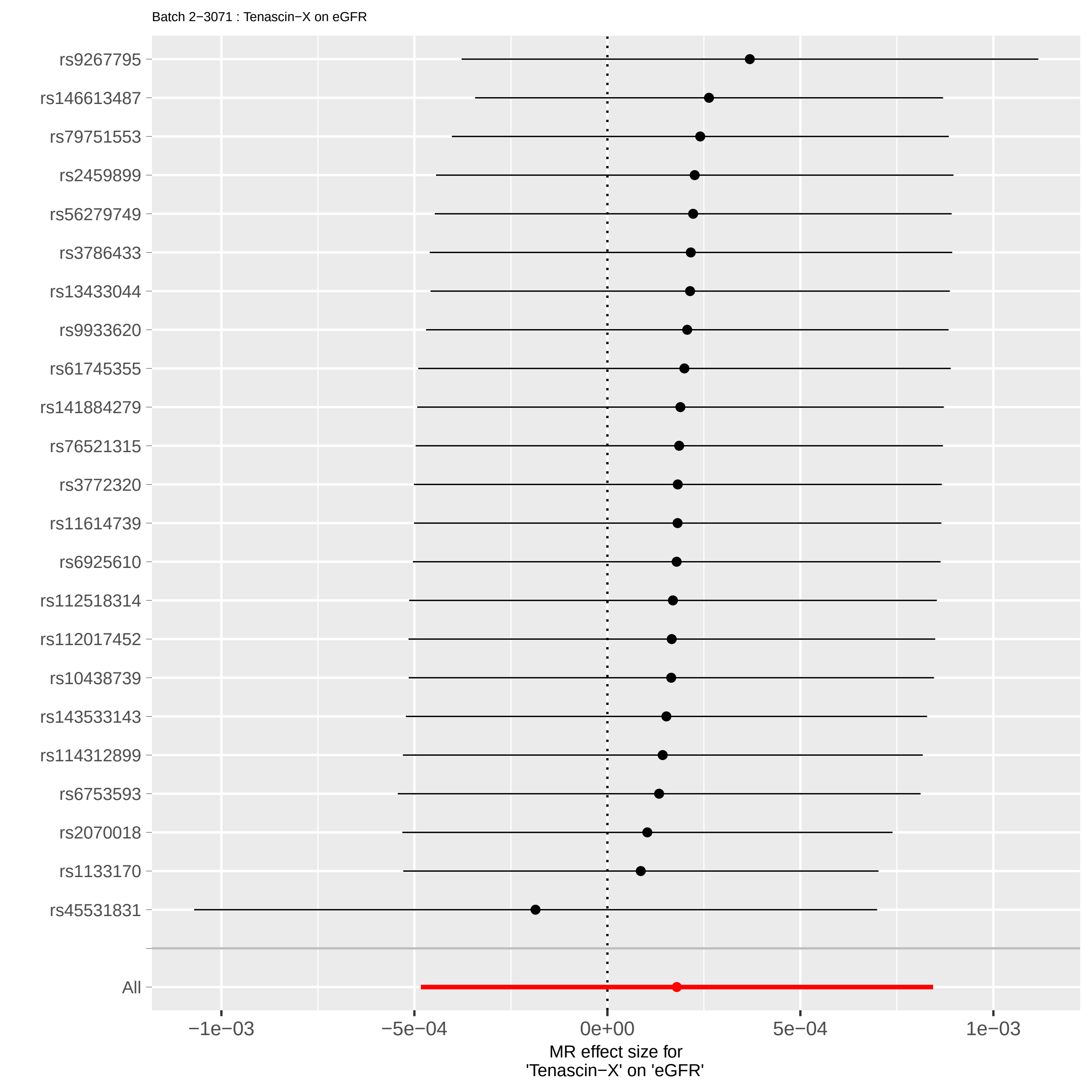

Batch 2−3071 : Tenascin−X on eGFR
rs9267795
rs146613487
rs79751553
rs2459899
rs56279749
rs3786433
rs13433044
rs9933620
rs61745355
rs141884279
rs76521315
rs3772320
rs11614739
rs6925610
rs112518314
rs112017452
rs10438739
rs143533143
rs114312899
rs6753593
rs2070018
rs1133170
rs45531831
All
−1e−03
−5e−04
0e+00
5e−04
1e−03
MR effect size for
'Tenascin−X' on 'eGFR'

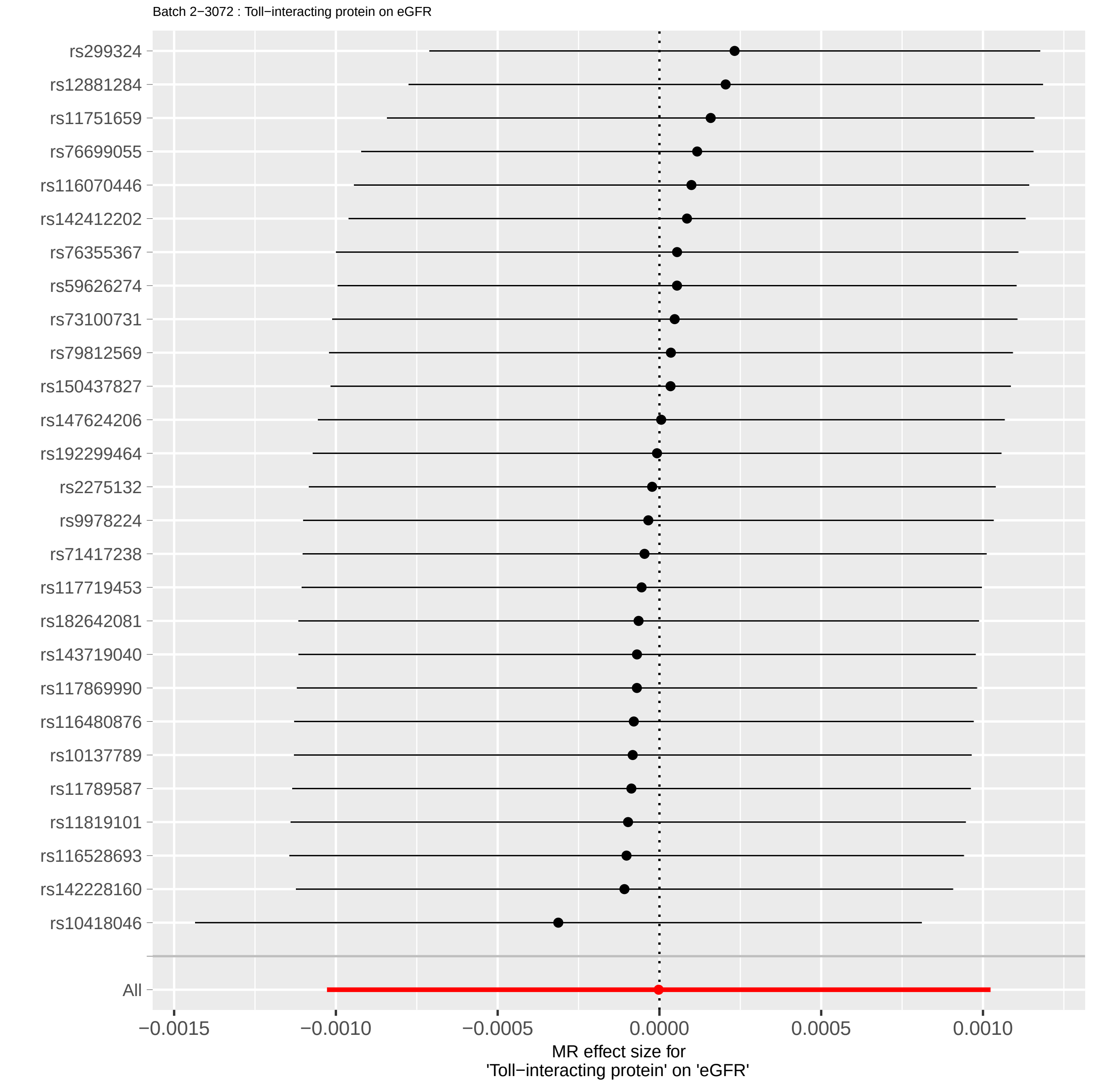
Batch 2−3072 : Toll−interacting protein on eGFR
rs299324
rs12881284
rs11751659
rs76699055
rs116070446
rs142412202
rs76355367
rs59626274
rs73100731
rs79812569
rs150437827
rs147624206
rs192299464
rs2275132
rs9978224
rs71417238
rs117719453
rs182642081
rs143719040
rs117869990
rs116480876
rs10137789
rs11789587
rs11819101
rs116528693
rs142228160
rs10418046
All
−0.0015
−0.0010
−0.0005
0.0000
0.0005
0.0010
MR effect size for
'Toll−interacting protein' on 'eGFR'

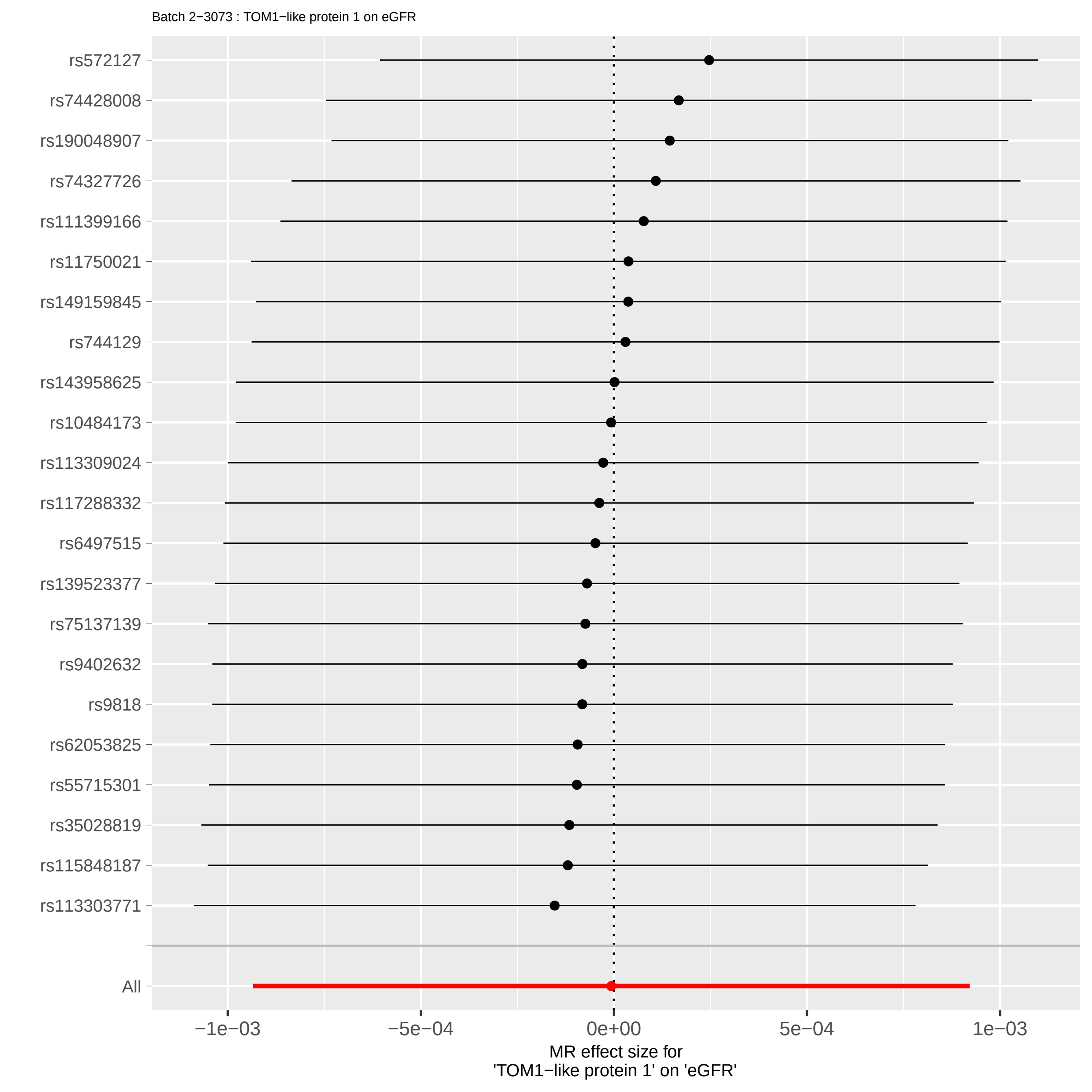
Batch 2−3073 : TOM1−like protein 1 on eGFR
rs572127
rs74428008
rs190048907
rs74327726
rs111399166
rs11750021
rs149159845
rs744129
rs143958625
rs10484173
rs113309024
rs117288332
rs6497515
rs139523377
rs75137139
rs9402632
rs9818
rs62053825
rs55715301
rs35028819
rs115848187
rs113303771
All
−1e−03
−5e−04
0e+00
5e−04
1e−03
MR effect size for
'TOM1−like protein 1' on 'eGFR'

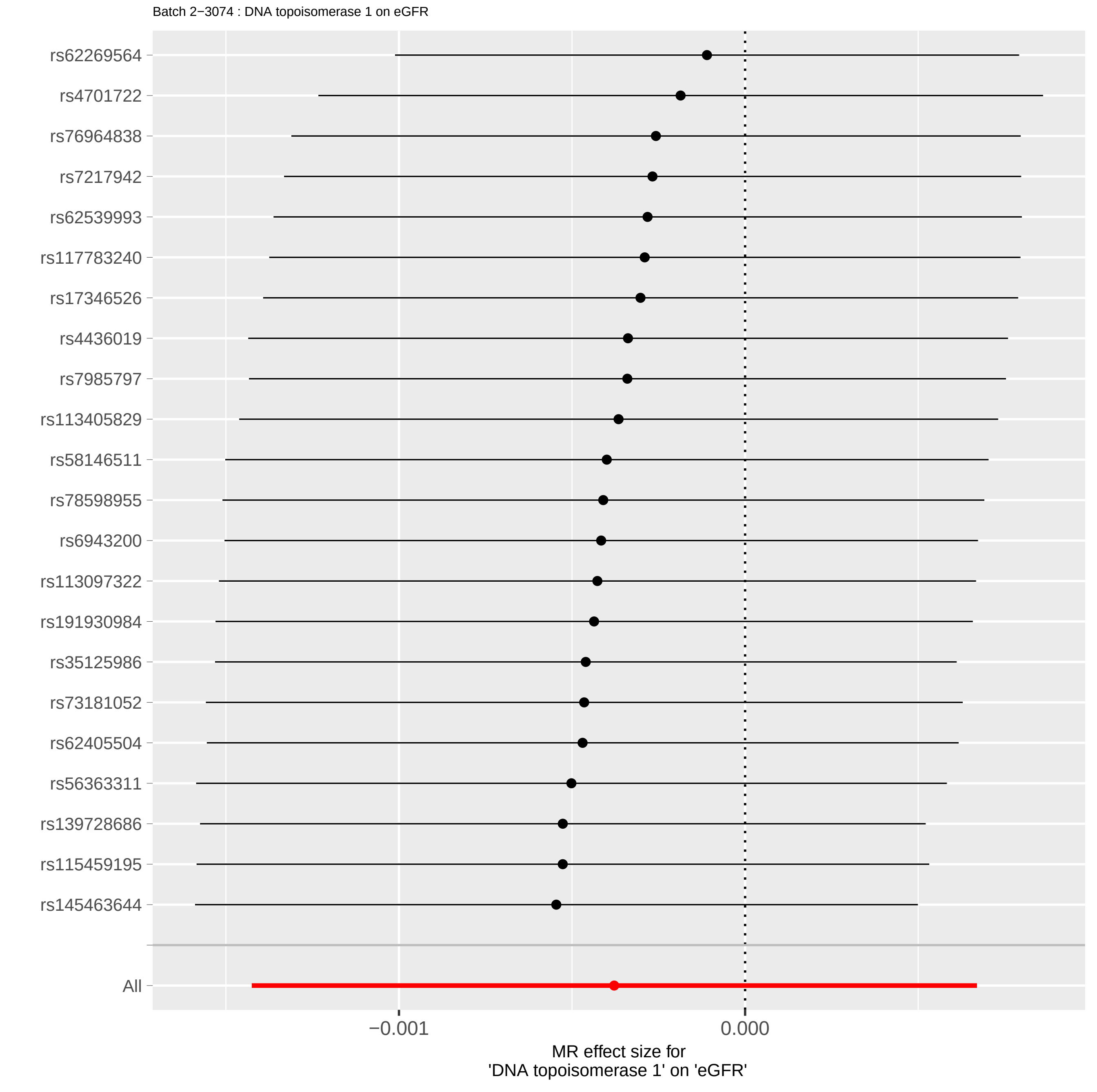

Batch 2−3074 : DNA topoisomerase 1 on eGFR
rs62269564
rs4701722
rs76964838
rs7217942
rs62539993
rs117783240
rs17346526
rs4436019
rs7985797
rs113405829
rs58146511
rs78598955
rs6943200
rs113097322
rs191930984
rs35125986
rs73181052
rs62405504
rs56363311
rs139728686
rs115459195
rs145463644
All
−0.001
0.000
MR effect size for
'DNA topoisomerase 1' on 'eGFR'

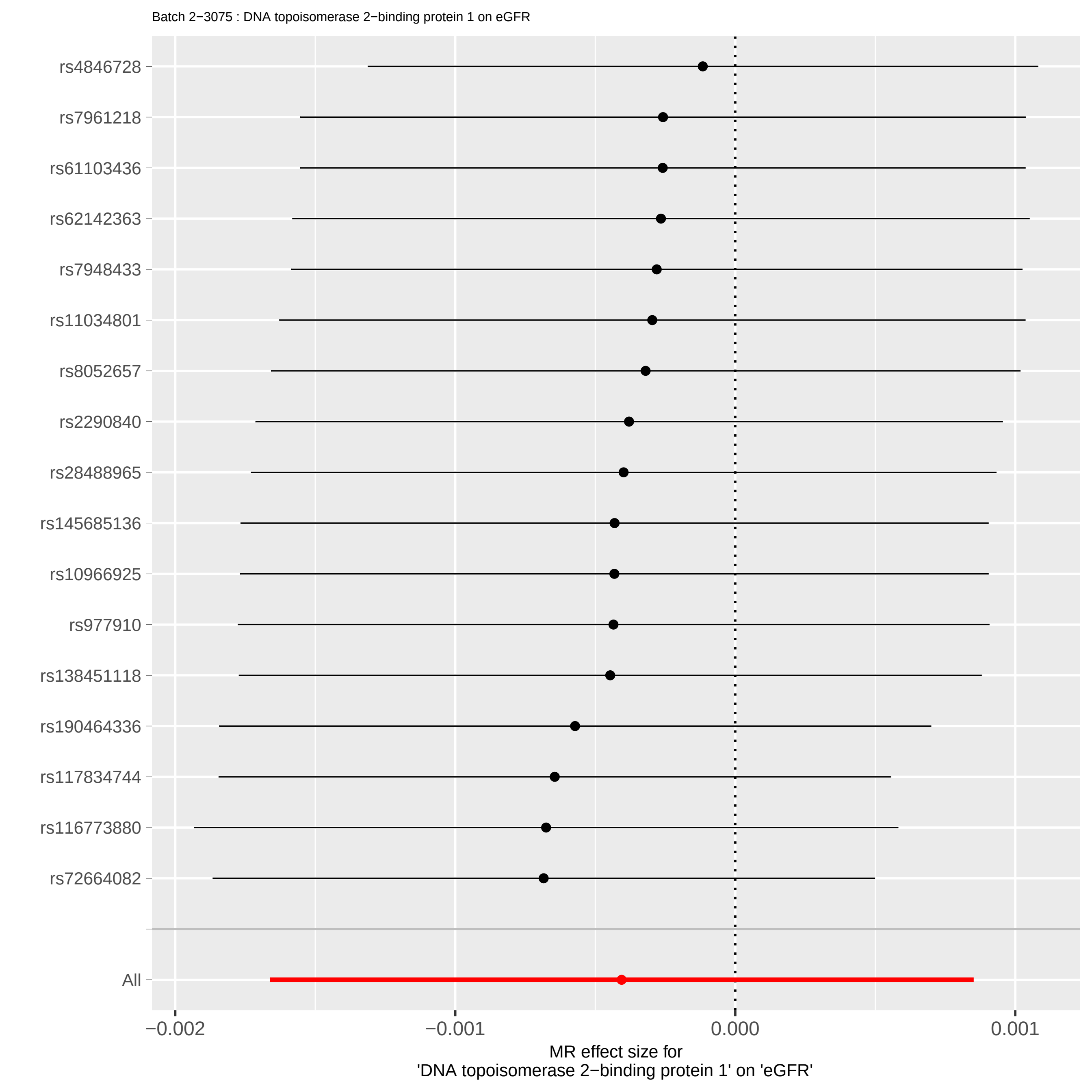
Batch 2−3075 : DNA topoisomerase 2−binding protein 1 on eGFR
rs4846728
rs7961218
rs61103436
rs62142363
rs7948433
rs11034801
rs8052657
rs2290840
rs28488965
rs145685136
rs10966925
rs977910
rs138451118
rs190464336
rs117834744
rs116773880
rs72664082
All
−0.002
−0.001
0.000
0.001
MR effect size for
'DNA topoisomerase 2−binding protein 1' on 'eGFR'

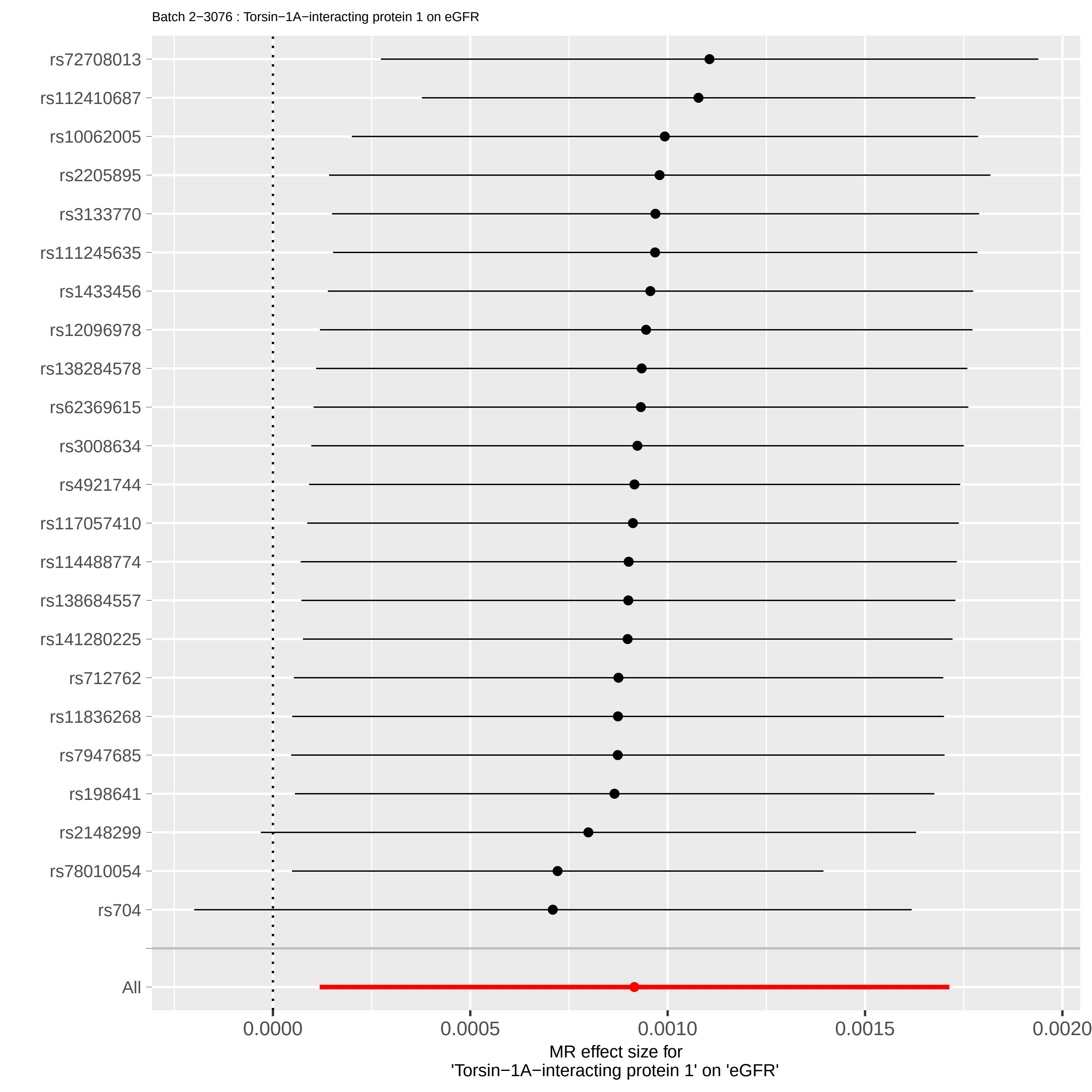

Batch 2−3076 : Torsin−1A−interacting protein 1 on eGFR
rs72708013
rs112410687
rs10062005
rs2205895
rs3133770
rs111245635
rs1433456
rs12096978
rs138284578
rs62369615
rs3008634
rs4921744
rs117057410
rs114488774
rs138684557
rs141280225
rs712762
rs11836268
rs7947685
rs198641
rs2148299
rs78010054
rs704
All
0.0000
0.0005
0.0010
0.0015
0.0020
MR effect size for
'Torsin−1A−interacting protein 1' on 'eGFR'

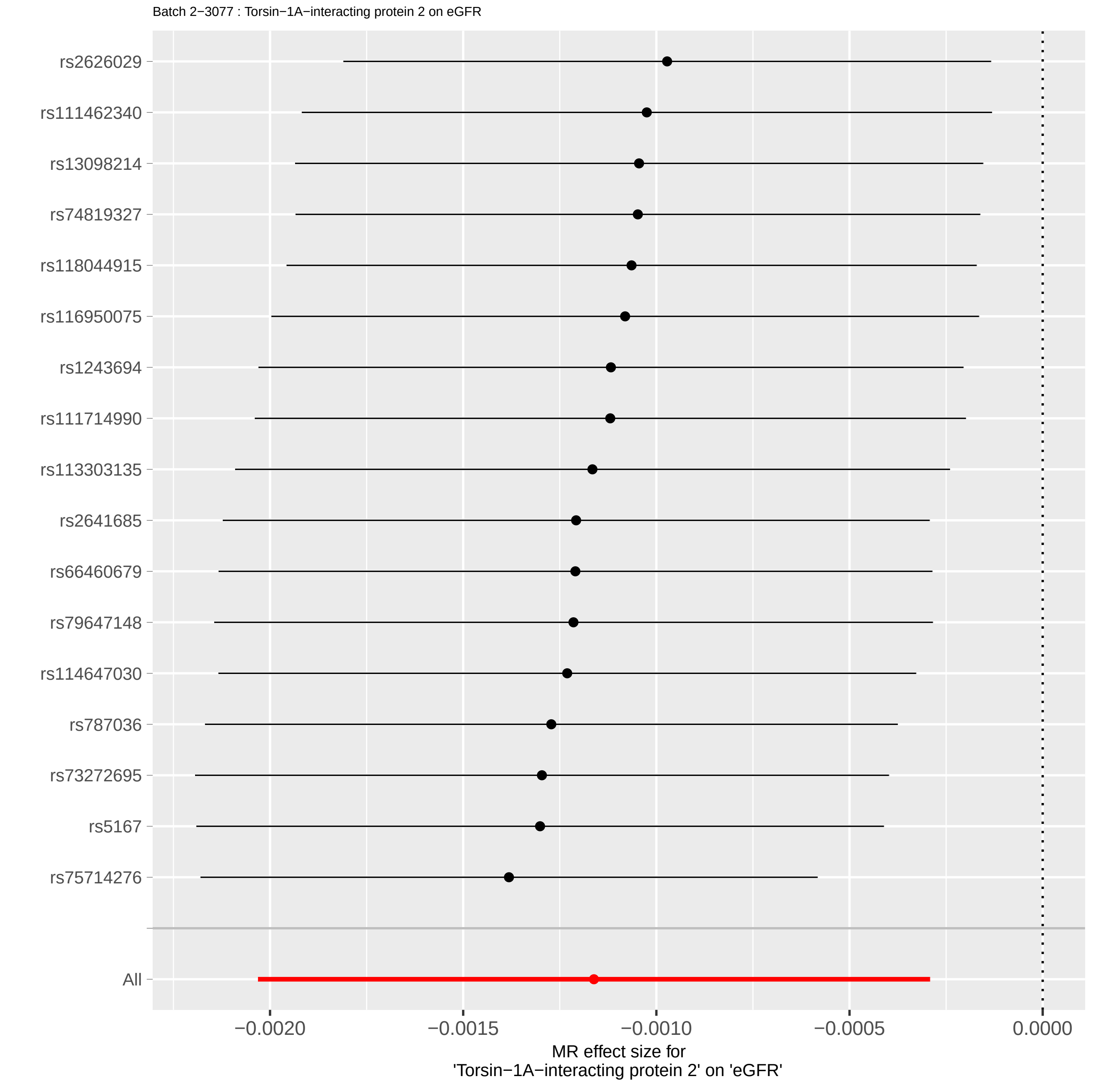

Batch 2−3077 : Torsin−1A−interacting protein 2 on eGFR
rs2626029
rs111462340
rs13098214
rs74819327
rs118044915
rs116950075
rs1243694
rs111714990
rs113303135
rs2641685
rs66460679
rs79647148
rs114647030
rs787036
rs73272695
rs5167
rs75714276
All
−0.0020
−0.0015
−0.0010
−0.0005
0.0000
MR effect size for
'Torsin−1A−interacting protein 2' on 'eGFR'

Batch 2−3078 : Torsin−1A−interacting protein 2 on eGFR

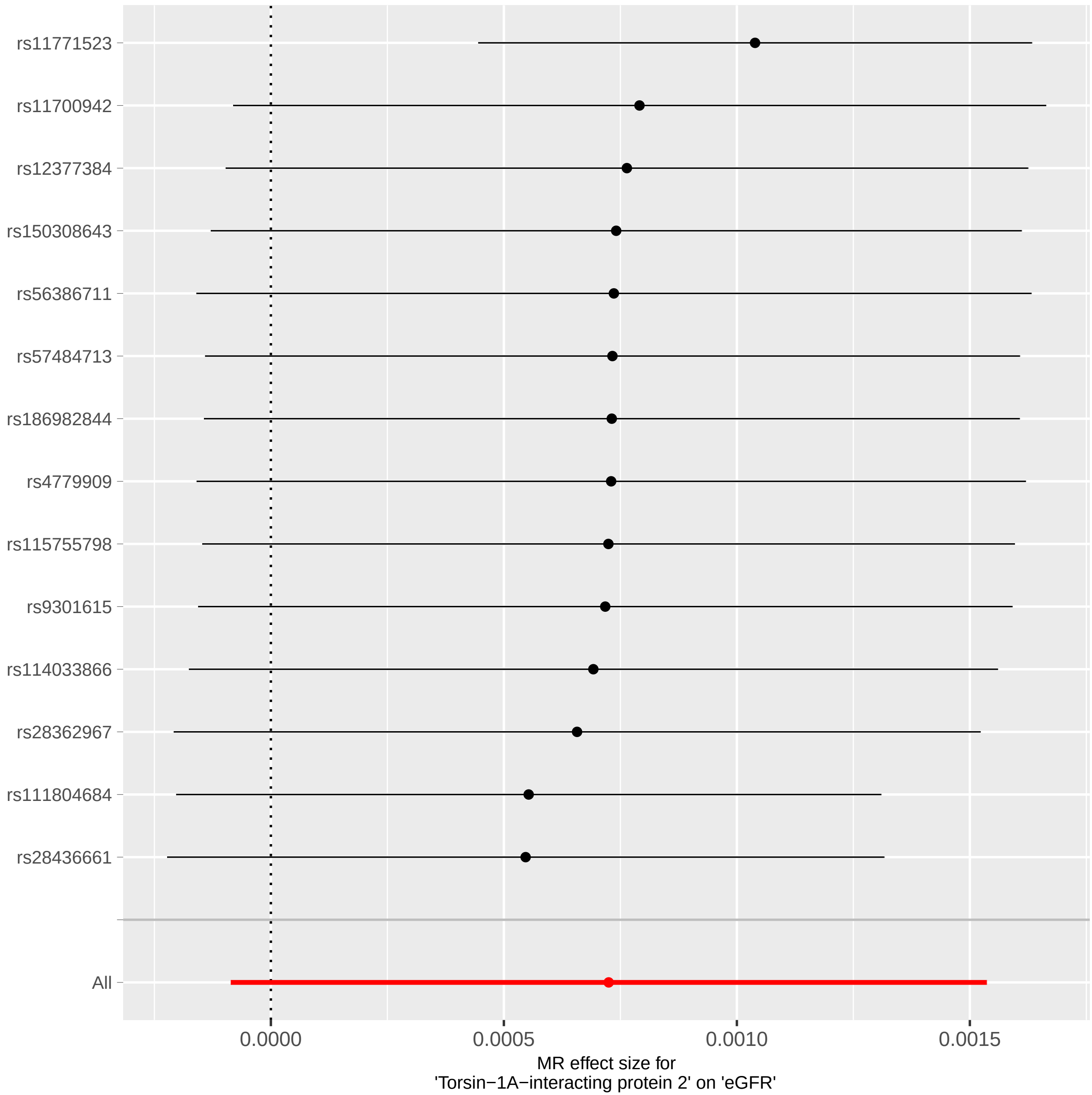

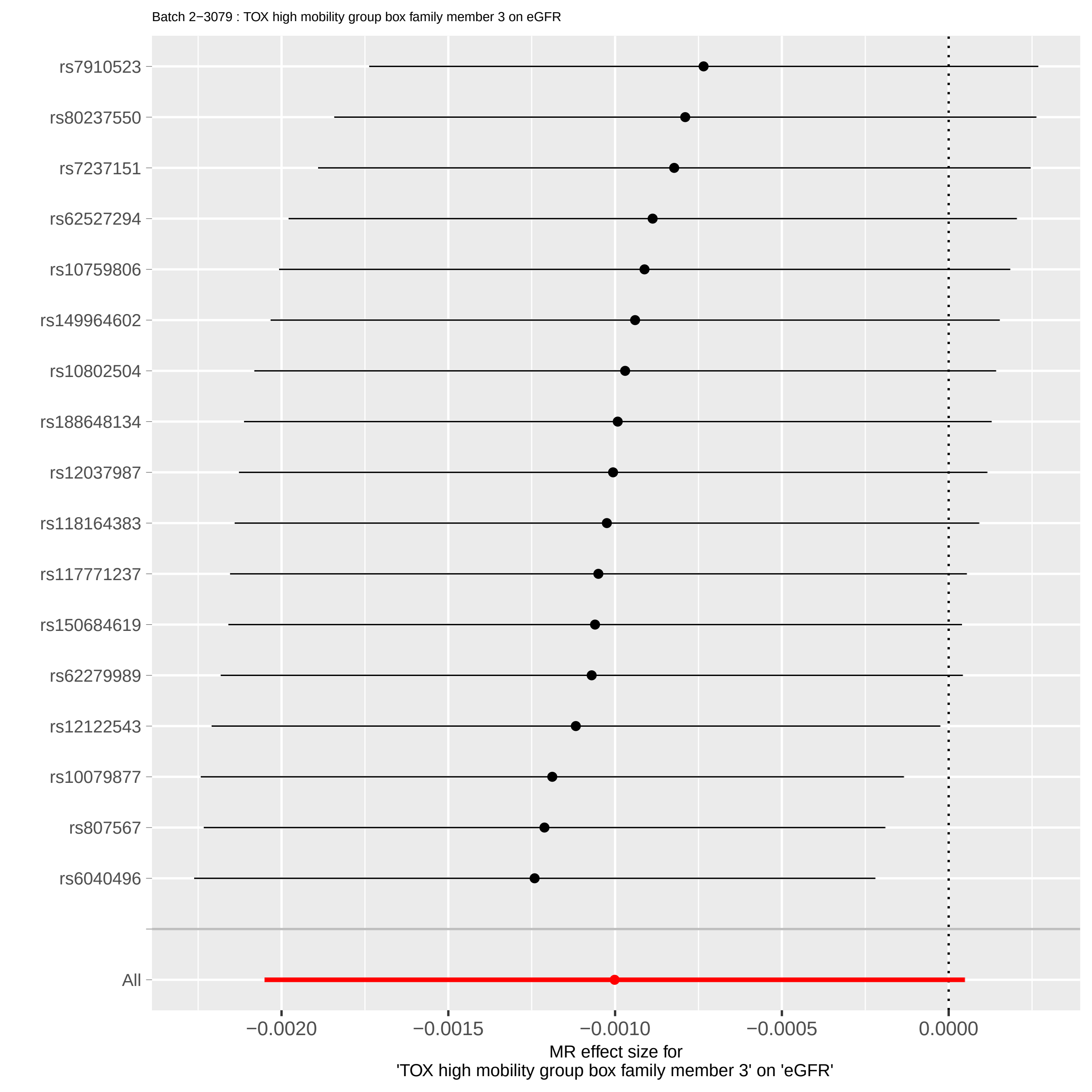

Batch 2−3079 : TOX high mobility group box family member 3 on eGFR
rs7910523
rs80237550
rs7237151
rs62527294
rs10759806
rs149964602
rs10802504
rs188648134
rs12037987
rs118164383
rs117771237
rs150684619
rs62279989
rs12122543
rs10079877
rs807567
rs6040496
All
−0.0020
−0.0015
−0.0010
−0.0005
0.0000
MR effect size for
'TOX high mobility group box family member 3' on 'eGFR'

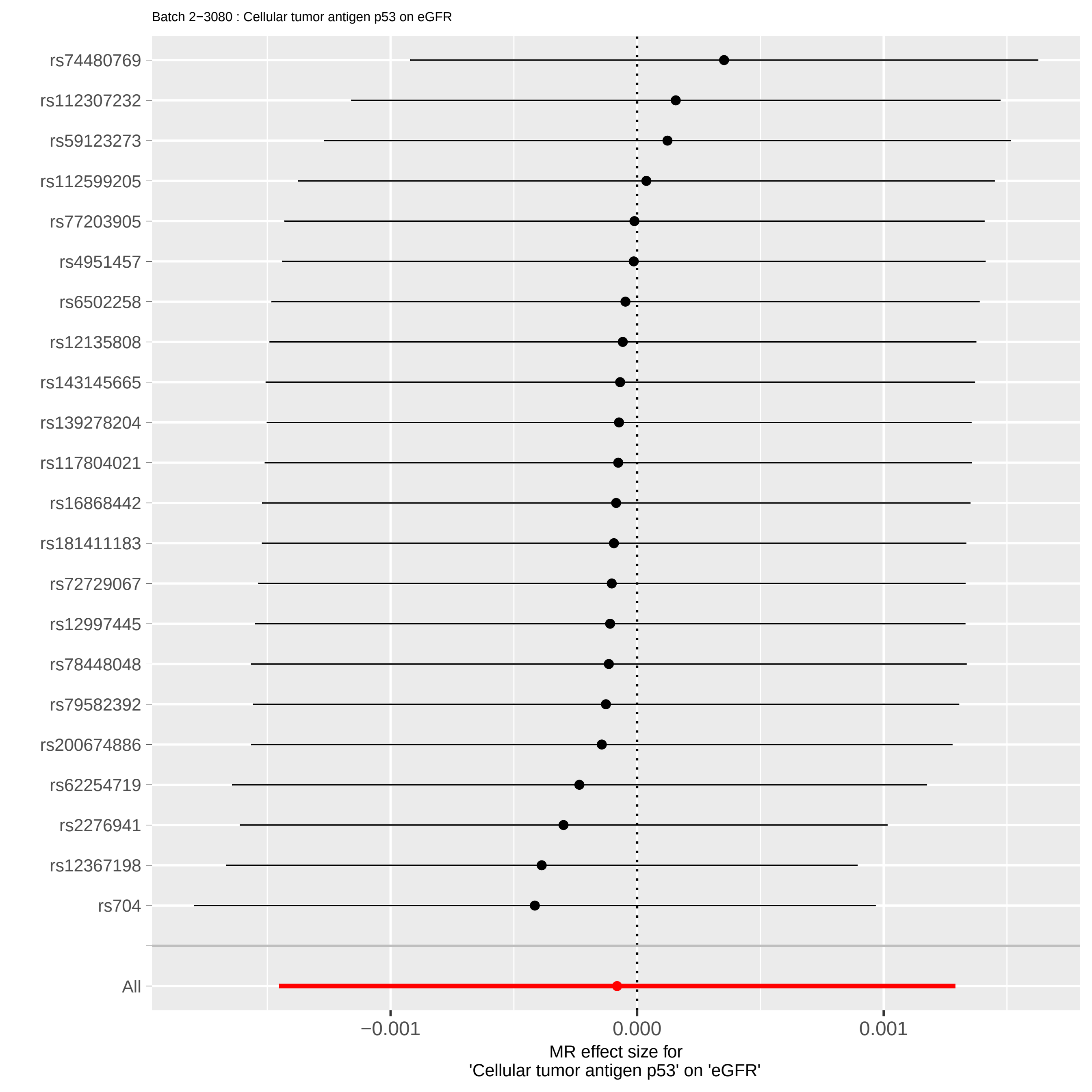

Batch 2−3080 : Cellular tumor antigen p53 on eGFR
rs74480769
rs112307232
rs59123273
rs112599205
rs77203905
rs4951457
rs6502258
rs12135808
rs143145665
rs139278204
rs117804021
rs16868442
rs181411183
rs72729067
rs12997445
rs78448048
rs79582392
rs200674886
rs62254719
rs2276941
rs12367198
rs704
All
−0.001
0.000
0.001
MR effect size for
'Cellular tumor antigen p53' on 'eGFR'

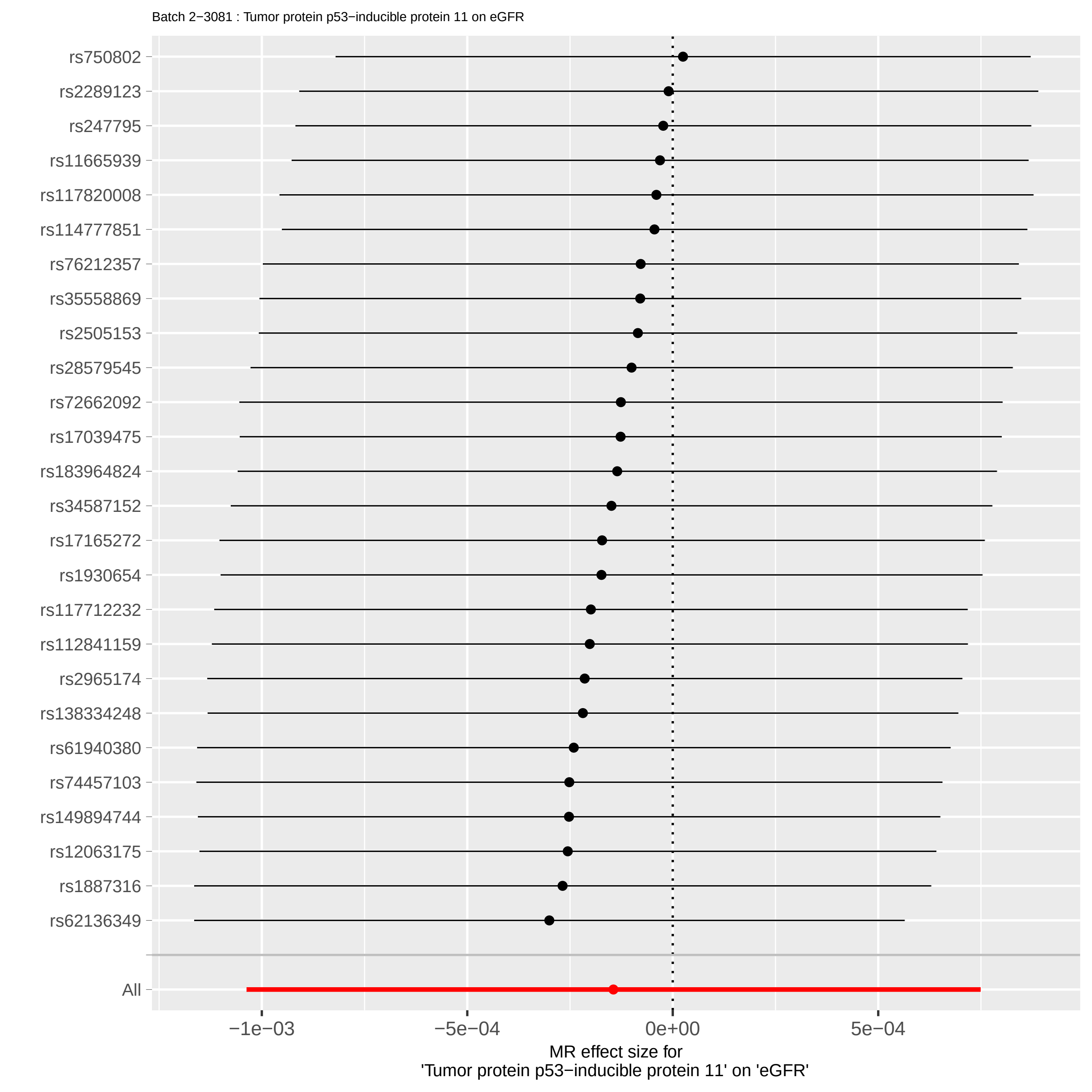

Batch 2−3081 : Tumor protein p53−inducible protein 11 on eGFR
rs750802
rs2289123
rs247795
rs11665939
rs117820008
rs114777851
rs76212357
rs35558869
rs2505153
rs28579545
rs72662092
rs17039475
rs183964824
rs34587152
rs17165272
rs1930654
rs117712232
rs112841159
rs2965174
rs138334248
rs61940380
rs74457103
rs149894744
rs12063175
rs1887316
rs62136349
All
−1e−03
−5e−04
0e+00
5e−04
MR effect size for
'Tumor protein p53−inducible protein 11' on 'eGFR'

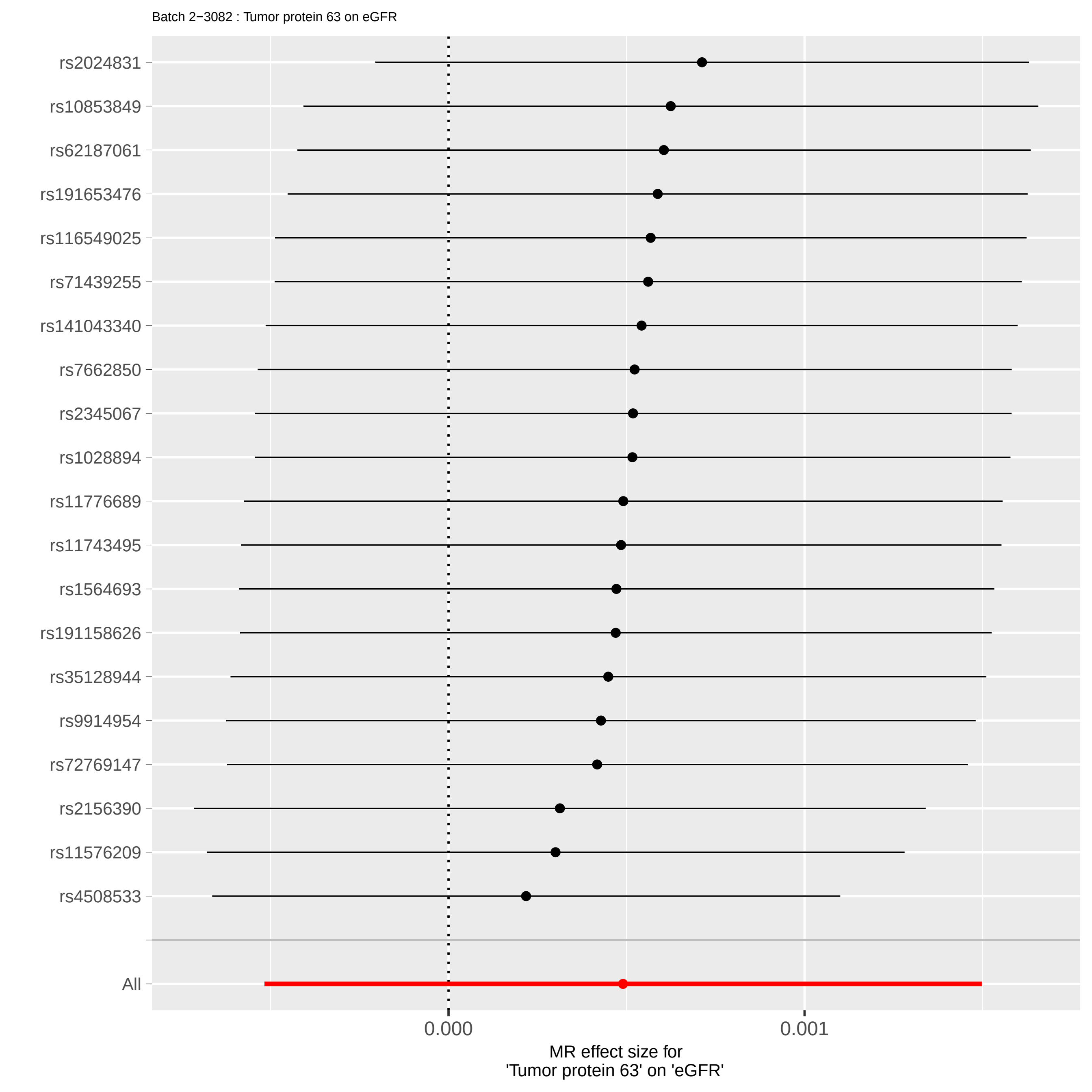
Batch 2−3082 : Tumor protein 63 on eGFR
rs2024831
rs10853849
rs62187061
rs191653476
rs116549025
rs71439255
rs141043340
rs7662850
rs2345067
rs1028894
rs11776689
rs11743495
rs1564693
rs191158626
rs35128944
rs9914954
rs72769147
rs2156390
rs11576209
rs4508533
All
0.000
0.001
MR effect size for
'Tumor protein 63' on 'eGFR'

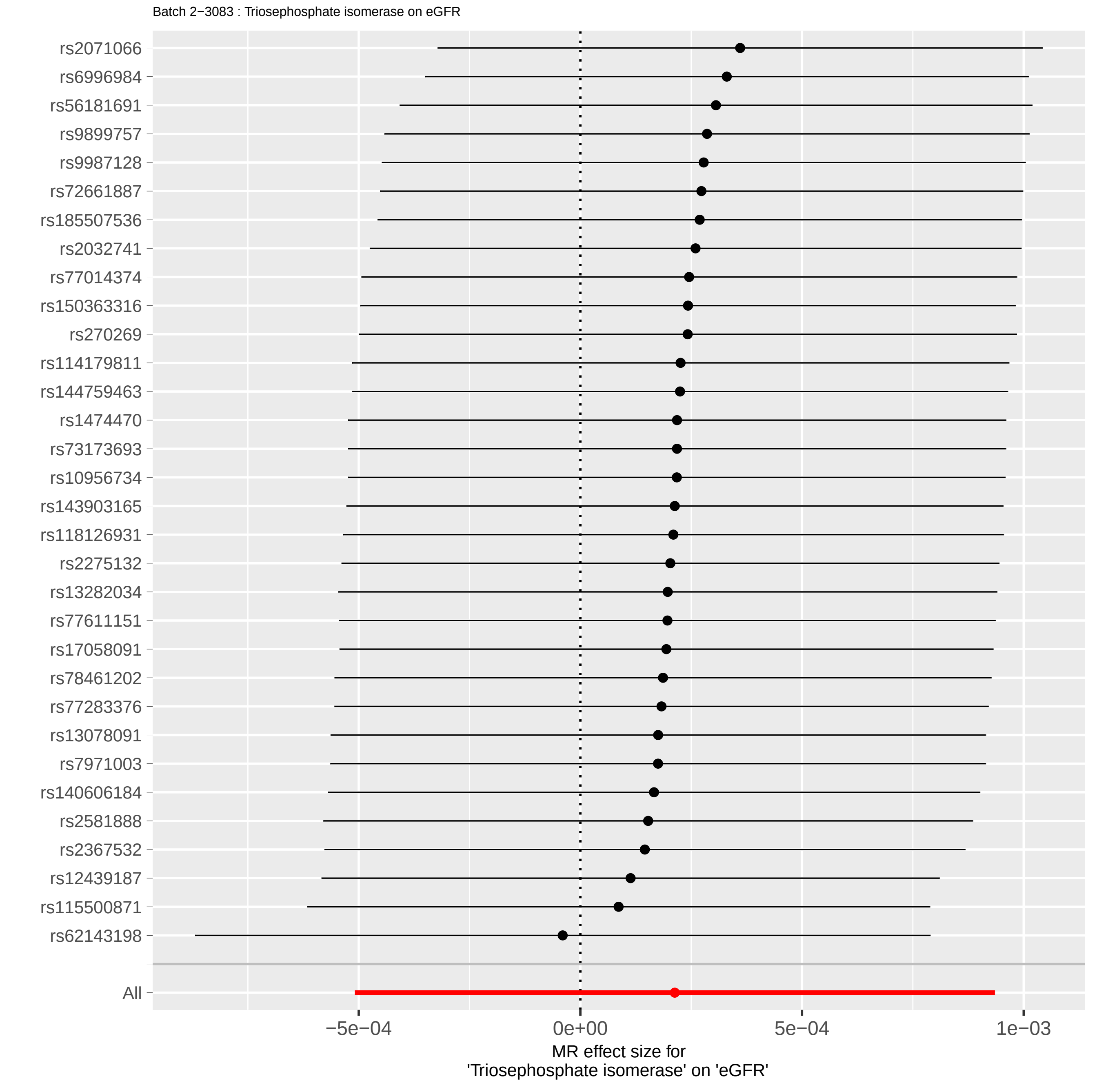

Batch 2−3083 : Triosephosphate isomerase on eGFR
rs2071066
rs6996984
rs56181691
rs9899757
rs9987128
rs72661887
rs185507536
rs2032741
rs77014374
rs150363316
rs270269
rs114179811
rs144759463
rs1474470
rs73173693
rs10956734
rs143903165
rs118126931
rs2275132
rs13282034
rs77611151
rs17058091
rs78461202
rs77283376
rs13078091
rs7971003
rs140606184
rs2581888
rs2367532
rs12439187
rs115500871
rs62143198
All
−5e−04
0e+00
5e−04
1e−03
MR effect size for
'Triosephosphate isomerase' on 'eGFR'

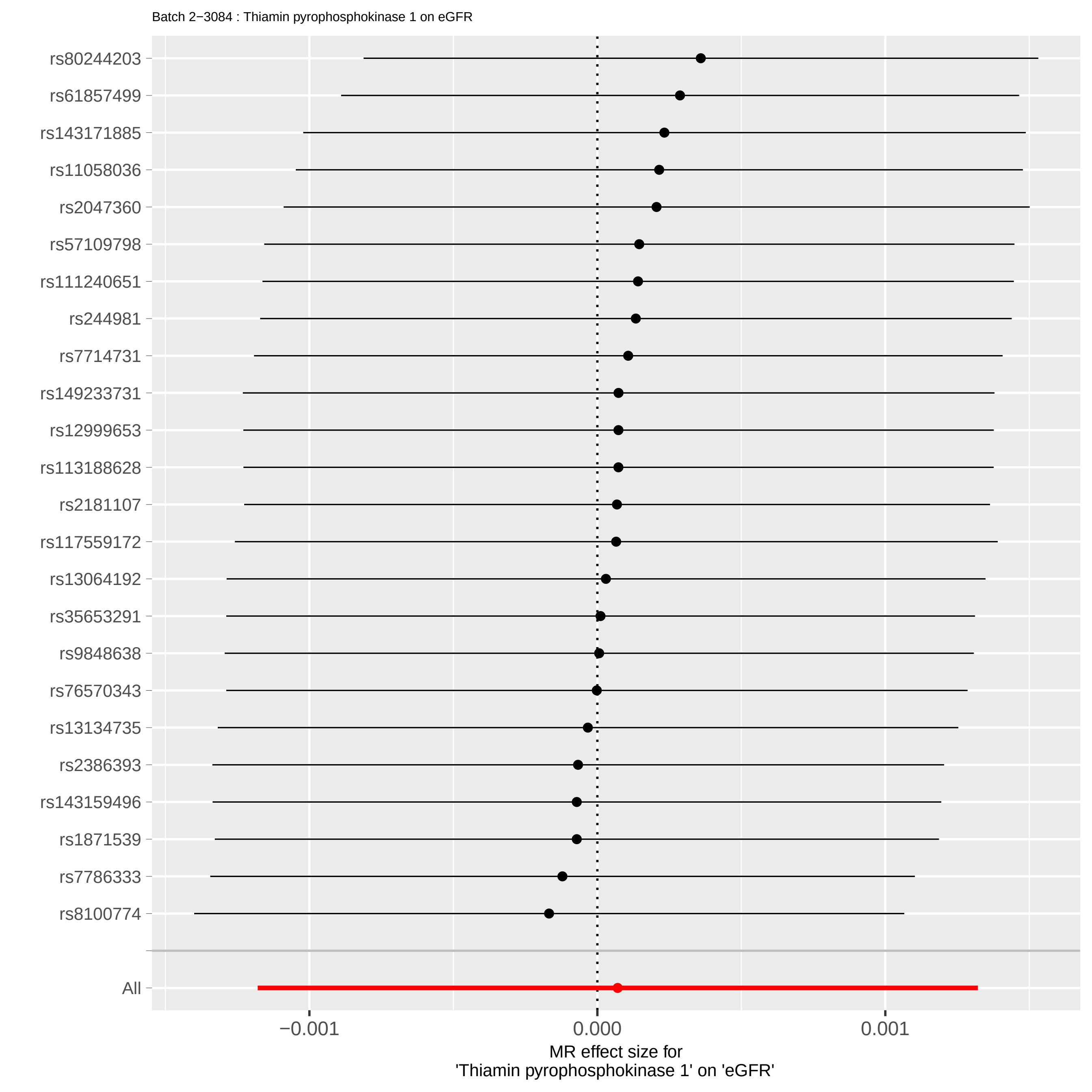
Batch 2−3084 : Thiamin pyrophosphokinase 1 on eGFR
rs80244203
rs61857499
rs143171885
rs11058036
rs2047360
rs57109798
rs111240651
rs244981
rs7714731
rs149233731
rs12999653
rs113188628
rs2181107
rs117559172
rs13064192
rs35653291
rs9848638
rs76570343
rs13134735
rs2386393
rs143159496
rs1871539
rs7786333
rs8100774
All
−0.001
0.000
0.001
MR effect size for
'Thiamin pyrophosphokinase 1' on 'eGFR'

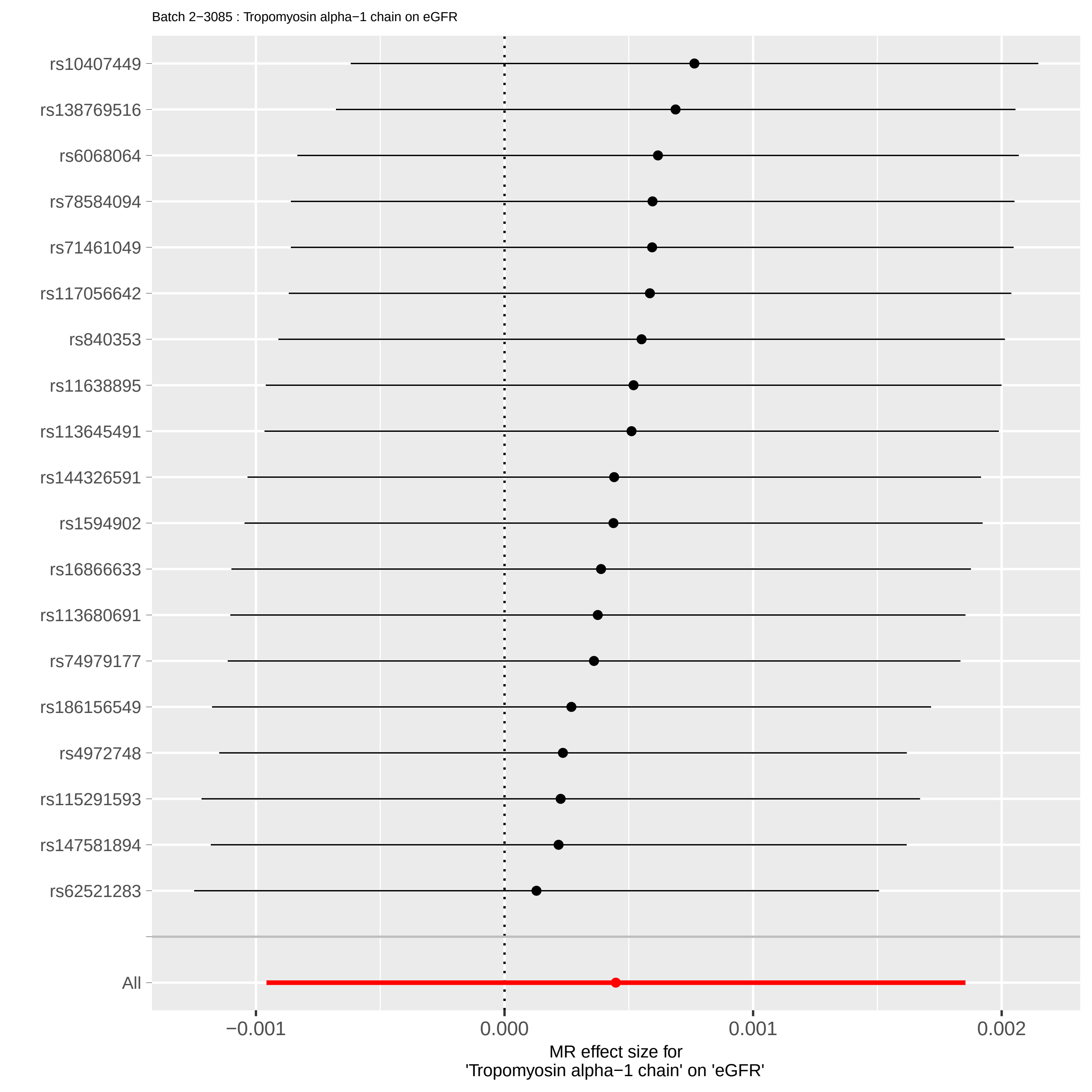
Batch 2−3085 : Tropomyosin alpha−1 chain on eGFR
rs10407449
rs138769516
rs6068064
rs78584094
rs71461049
rs117056642
rs840353
rs11638895
rs113645491
rs144326591
rs1594902
rs16866633
rs113680691
rs74979177
rs186156549
rs4972748
rs115291593
rs147581894
rs62521283
All
−0.001
0.000
0.001
0.002
MR effect size for
'Tropomyosin alpha−1 chain' on 'eGFR'

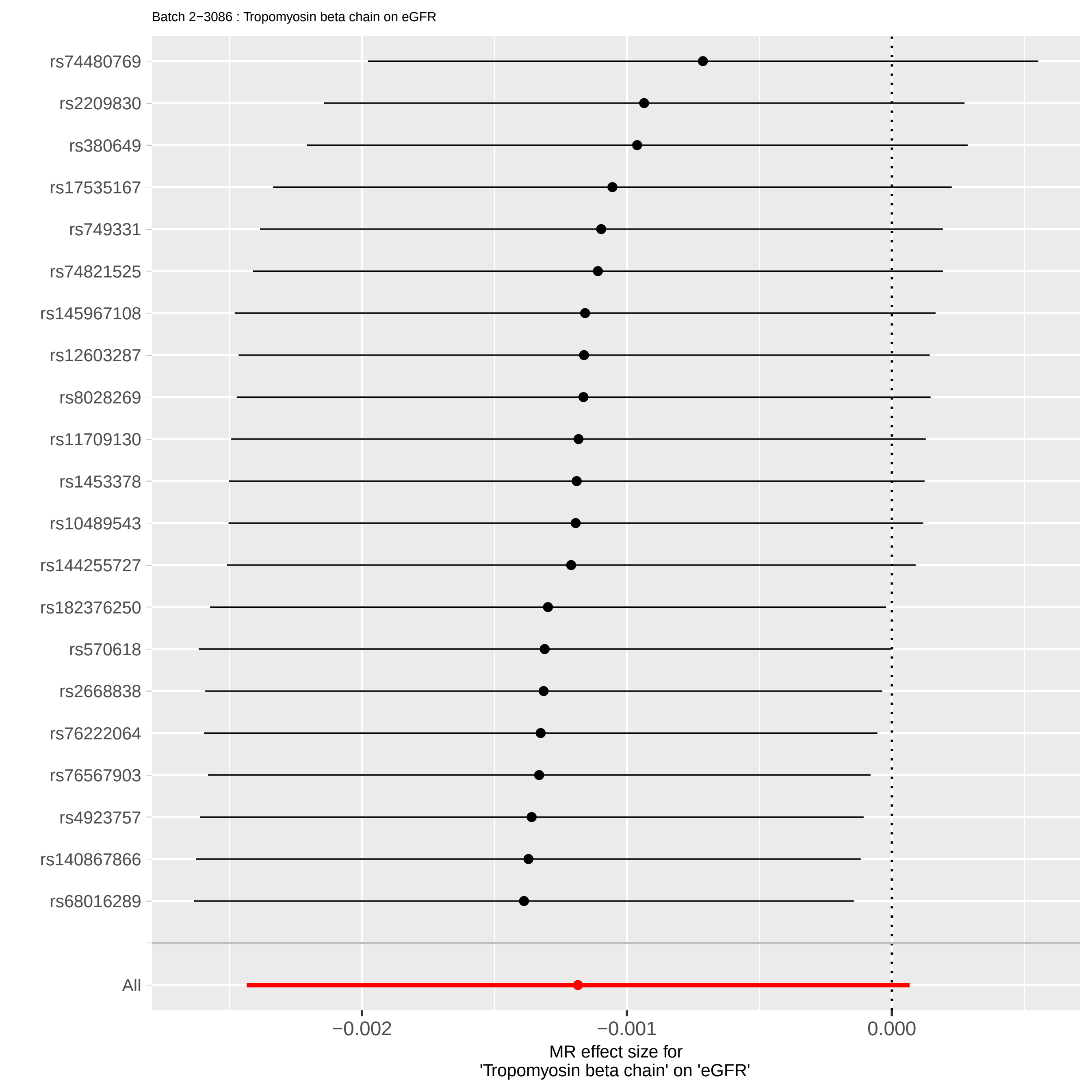

Batch 2−3086 : Tropomyosin beta chain on eGFR
rs74480769
rs2209830
rs380649
rs17535167
rs749331
rs74821525
rs145967108
rs12603287
rs8028269
rs11709130
rs1453378
rs10489543
rs144255727
rs182376250
rs570618
rs2668838
rs76222064
rs76567903
rs4923757
rs140867866
rs68016289
All
−0.002
−0.001
0.000
MR effect size for
'Tropomyosin beta chain' on 'eGFR'

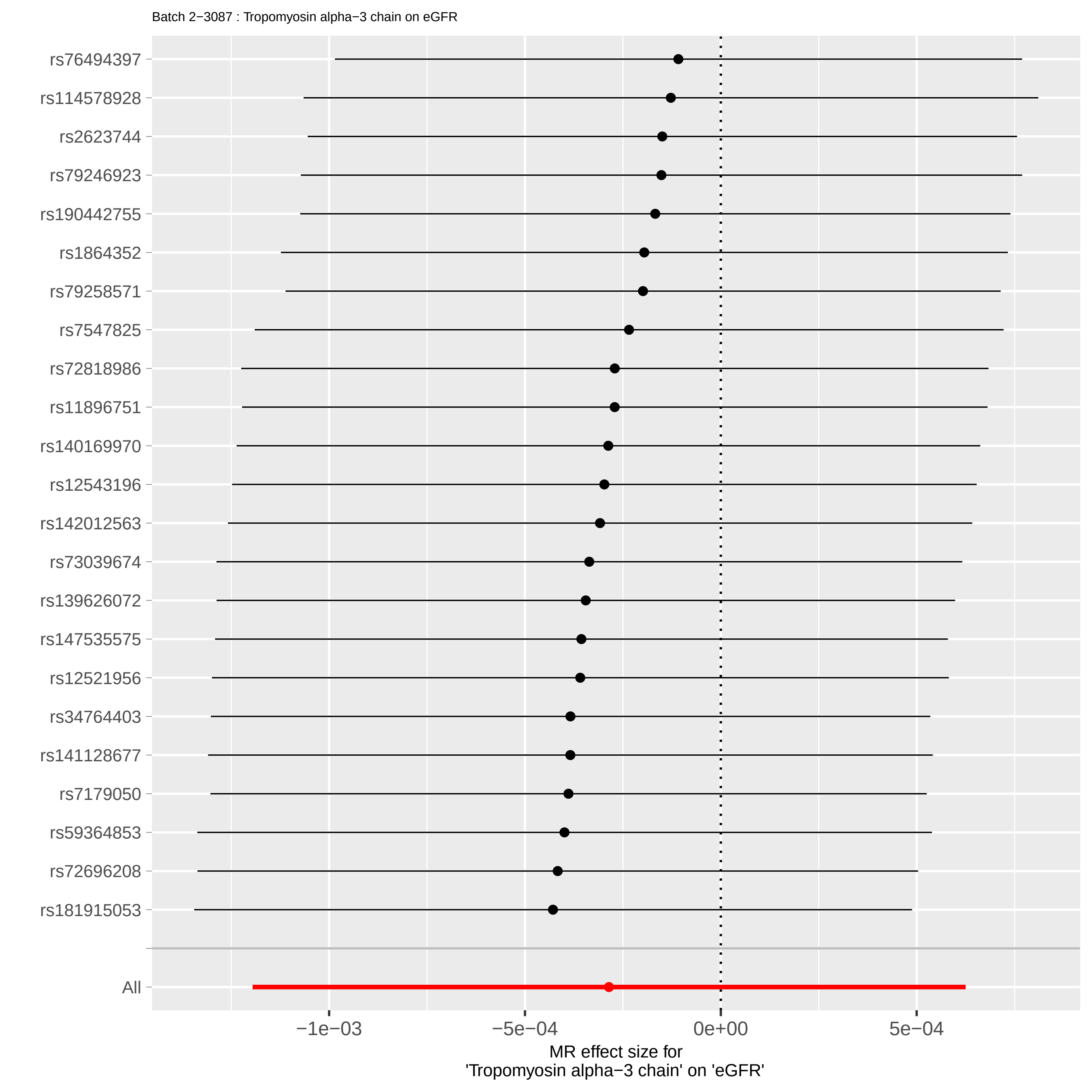

Batch 2−3087 : Tropomyosin alpha−3 chain on eGFR
rs76494397
rs114578928
rs2623744
rs79246923
rs190442755
rs1864352
rs79258571
rs7547825
rs72818986
rs11896751
rs140169970
rs12543196
rs142012563
rs73039674
rs139626072
rs147535575
rs12521956
rs34764403
rs141128677
rs7179050
rs59364853
rs72696208
rs181915053
All
−1e−03
−5e−04
0e+00
5e−04
MR effect size for
'Tropomyosin alpha−3 chain' on 'eGFR'
[truncated: 114,223 more chars]
