# Supplementary material for: A new perspective on selenium’s impact on renal function: European population-based analysis of plasma proteome-mediated Mendelian randomization study
Source: Front Endocrinol (Lausanne). 2024 Sep 12;15:1410463. doi: 10.3389/fendo.2024.1410463 (PMC11424436; doi:10.3389/fendo.2024.1410463)

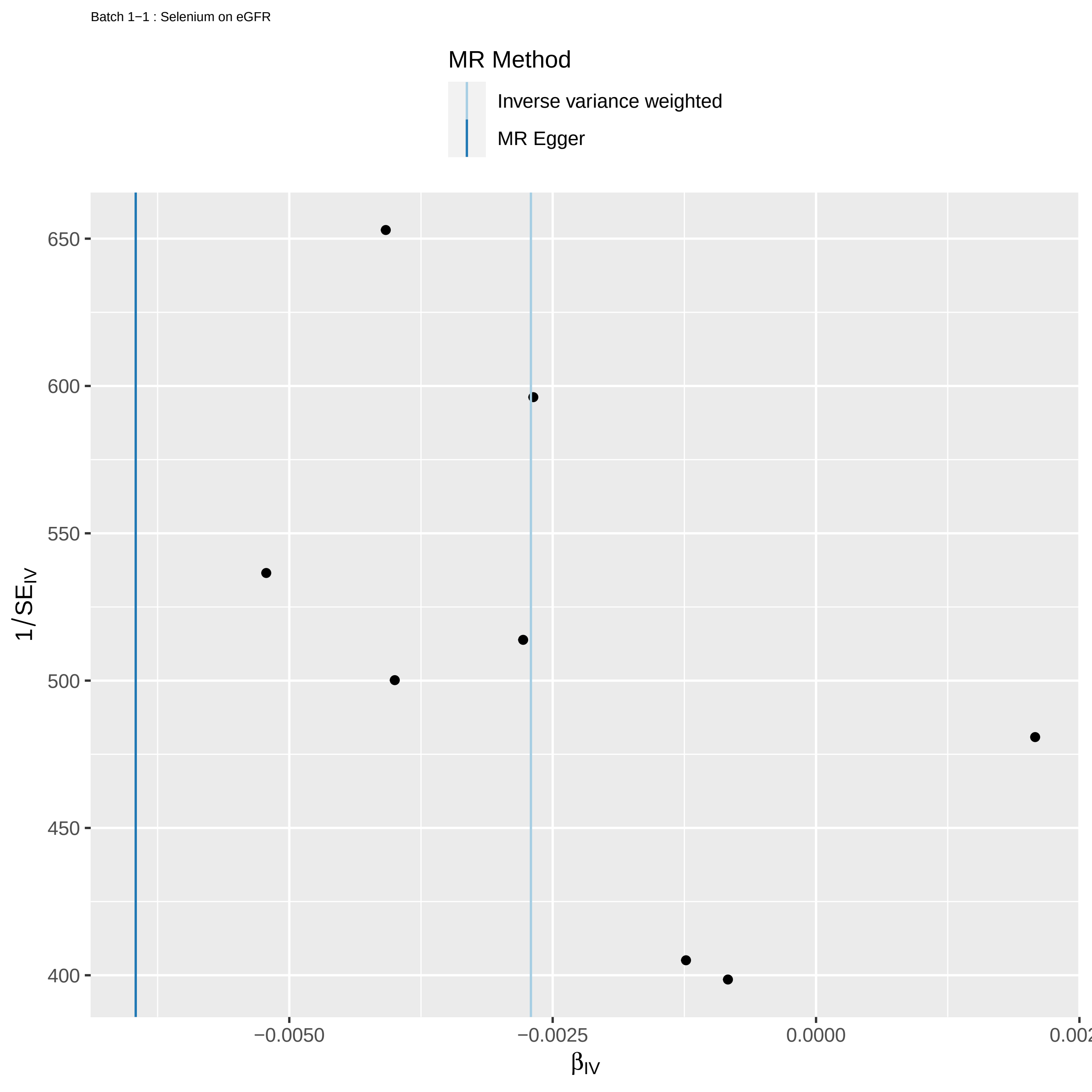

Batch 1−1 : Selenium on eGFR
MR Method
Inverse variance weighted
MR Egger
650
600
550
500
450
400
1/SE$_{IV}$
−0.0050
−0.0025
0.0000
0.002
β$_{IV}$

Batch 3−1 : Selenium on APOBEC1 complementation factor

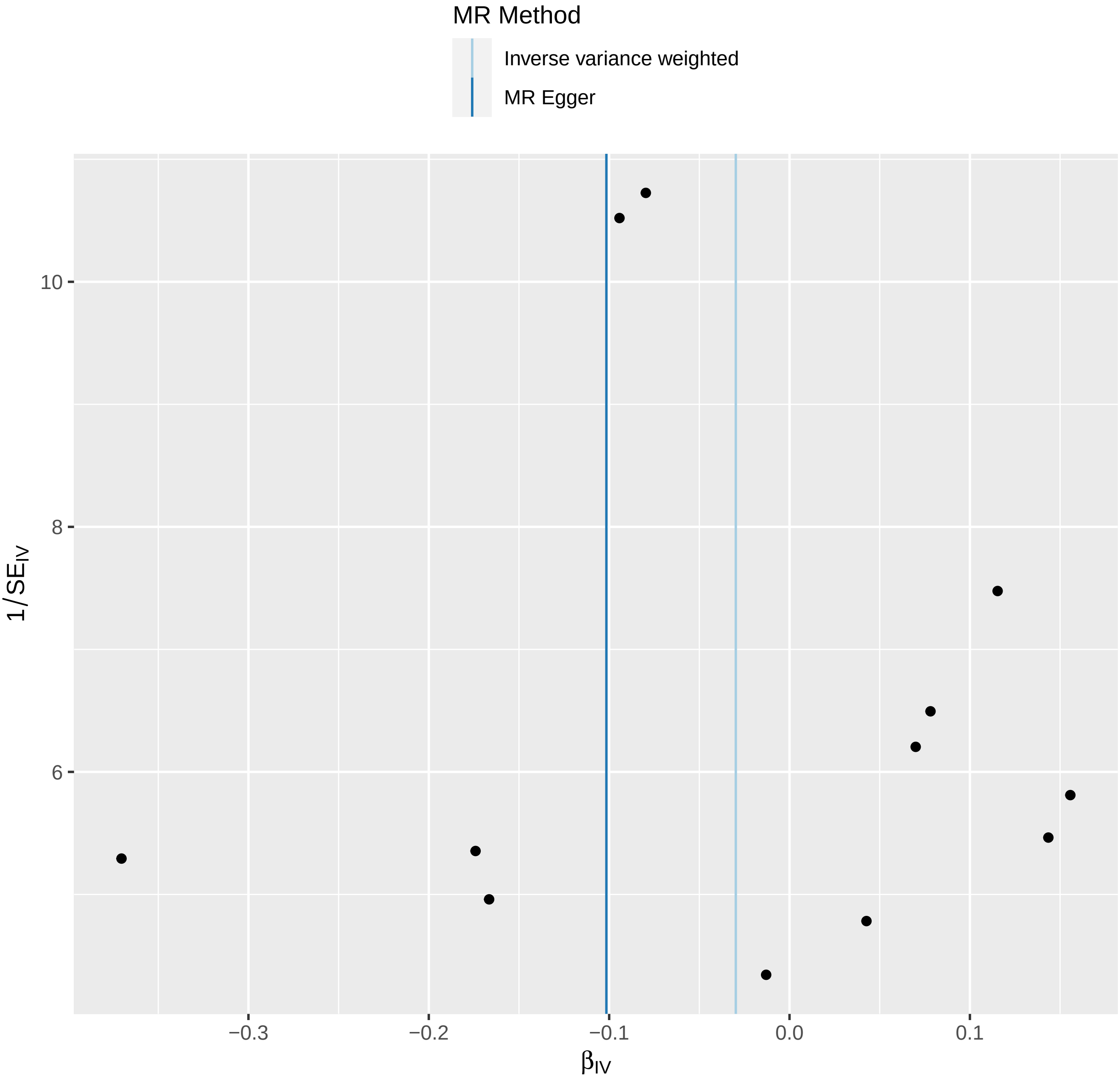

Batch 3−2 : Selenium on Lactosylceramide 4−alpha−galactosyltransferase

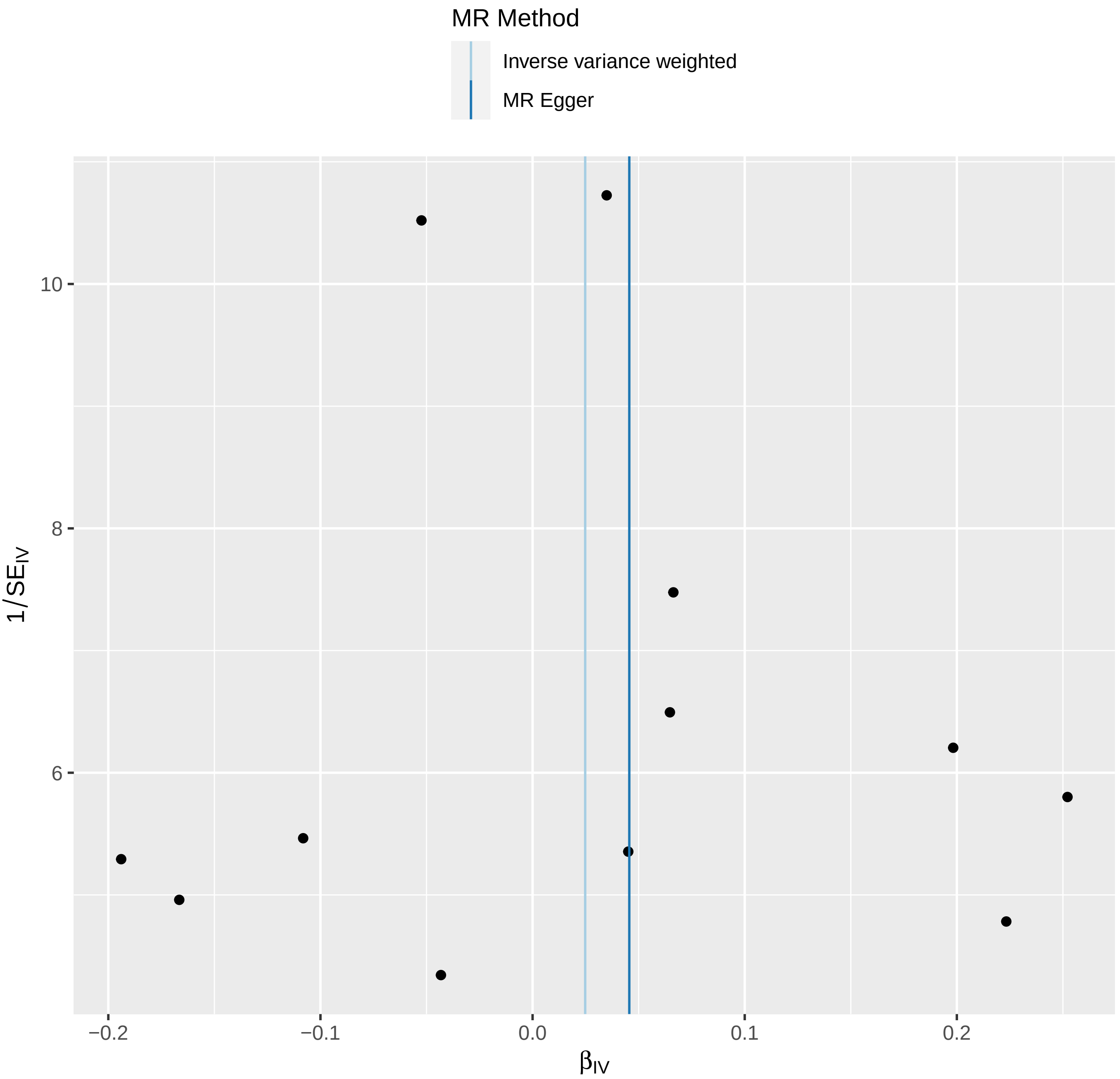

Batch 3−3 : Selenium on Alanine−−tRNA ligase, cytoplasmic

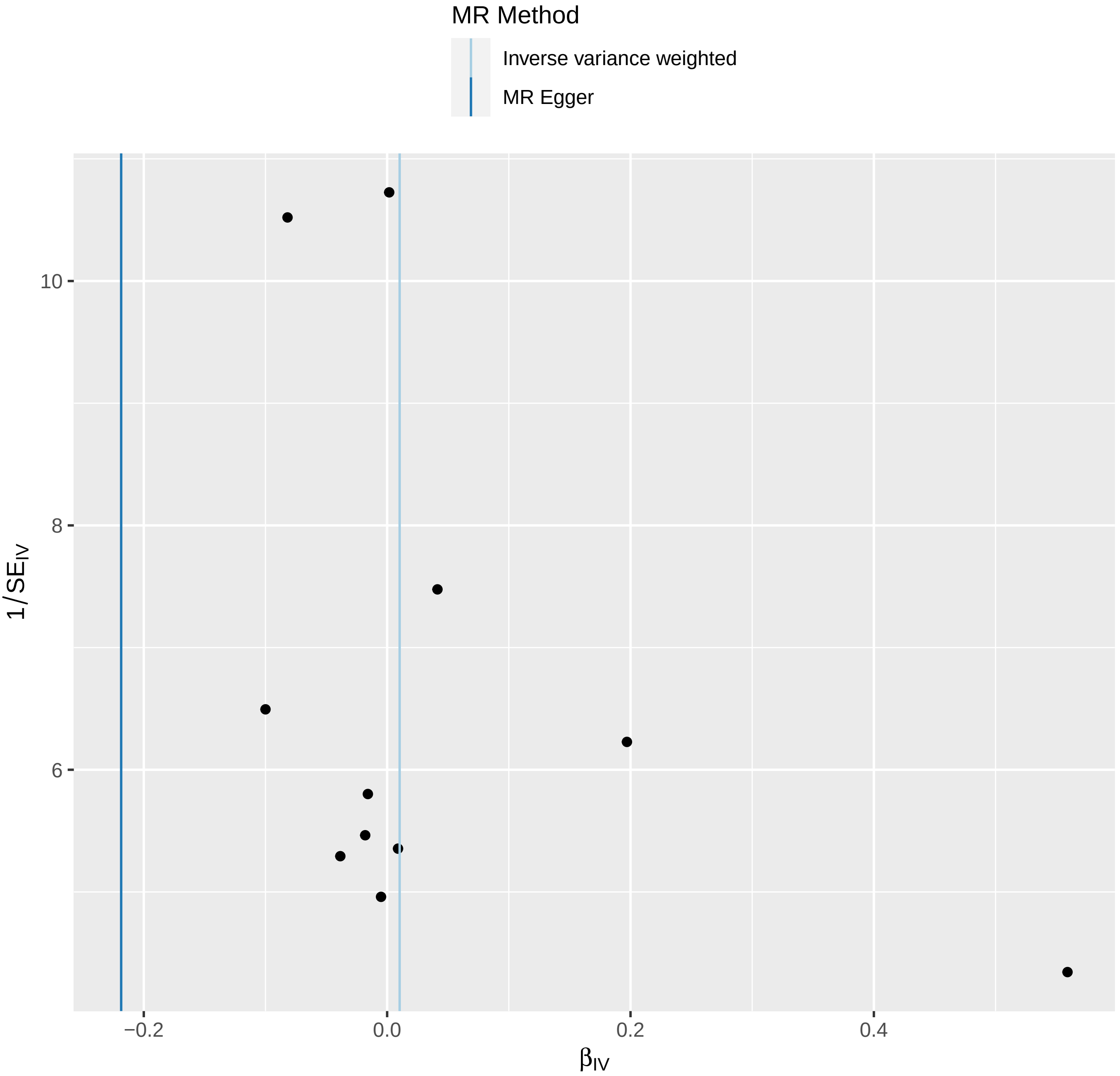

Batch 3−4 : Selenium on Multidrug resistance−associated protein 6

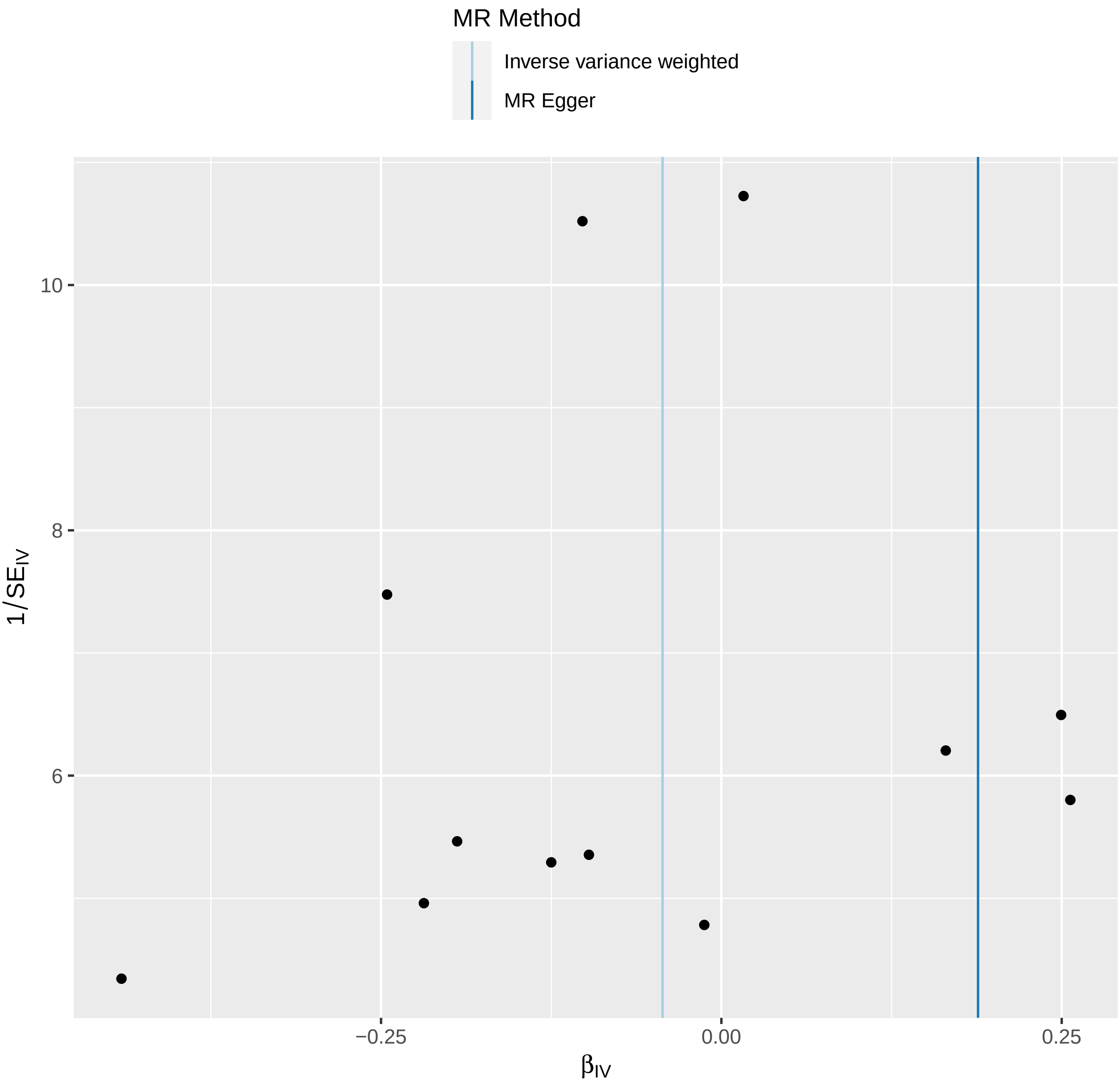

Batch 3−5 : Selenium on Monoacylglycerol lipase ABHD12

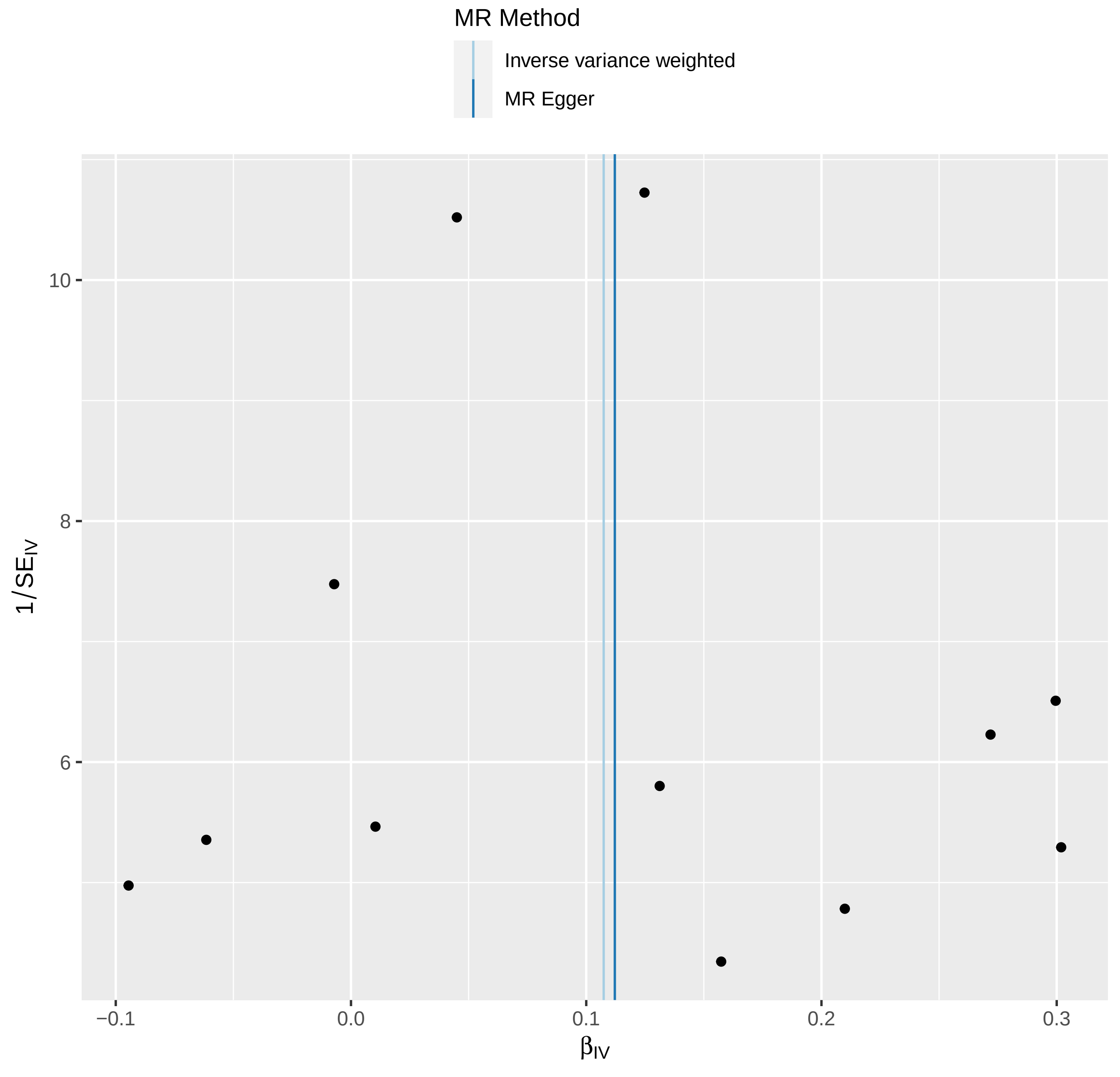

Batch 3−6 : Selenium on Alpha/beta hydrolase domain−containing protein 14A

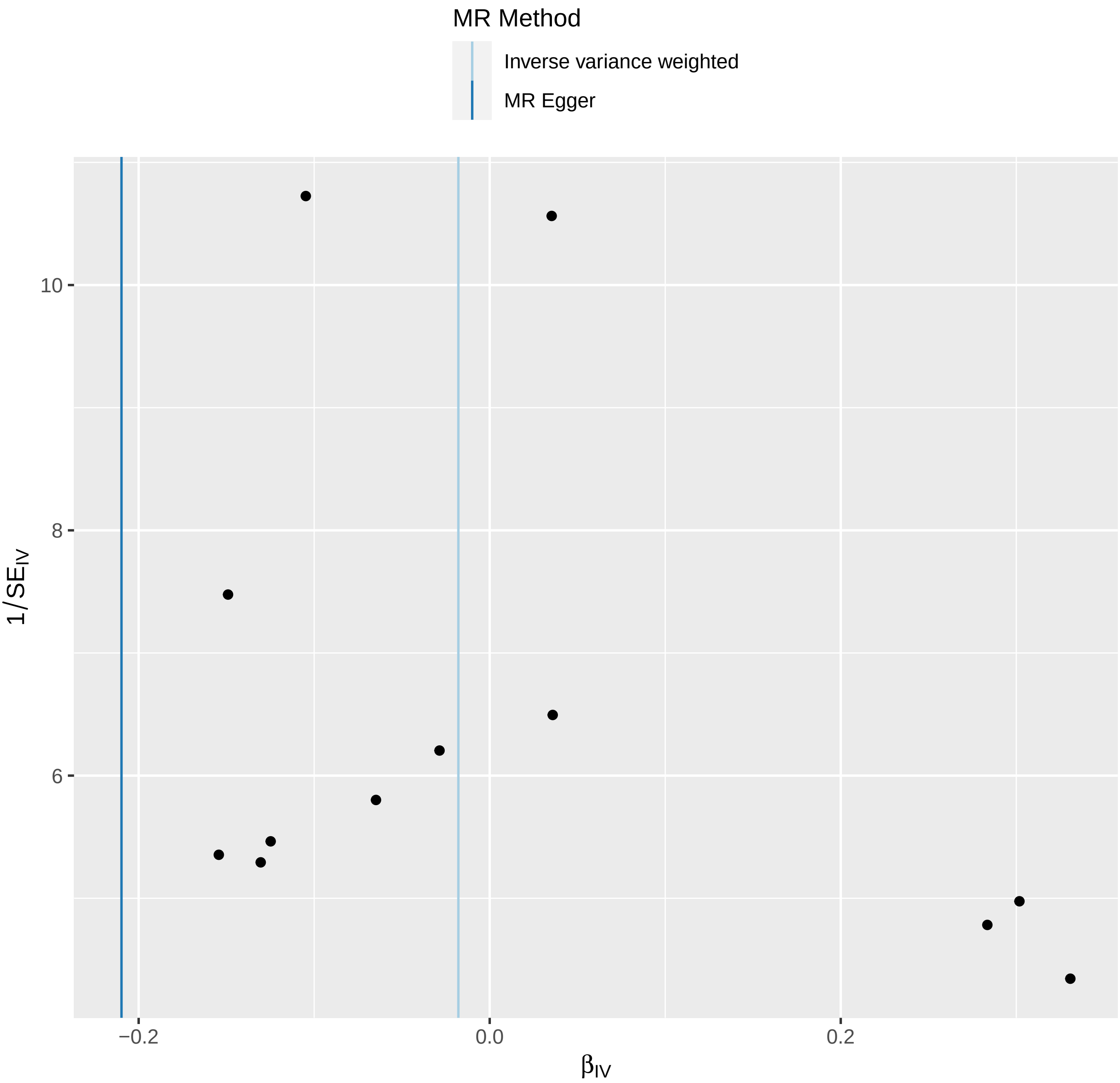

Batch 3−7 : Selenium on Tyrosine−protein kinase ABL1

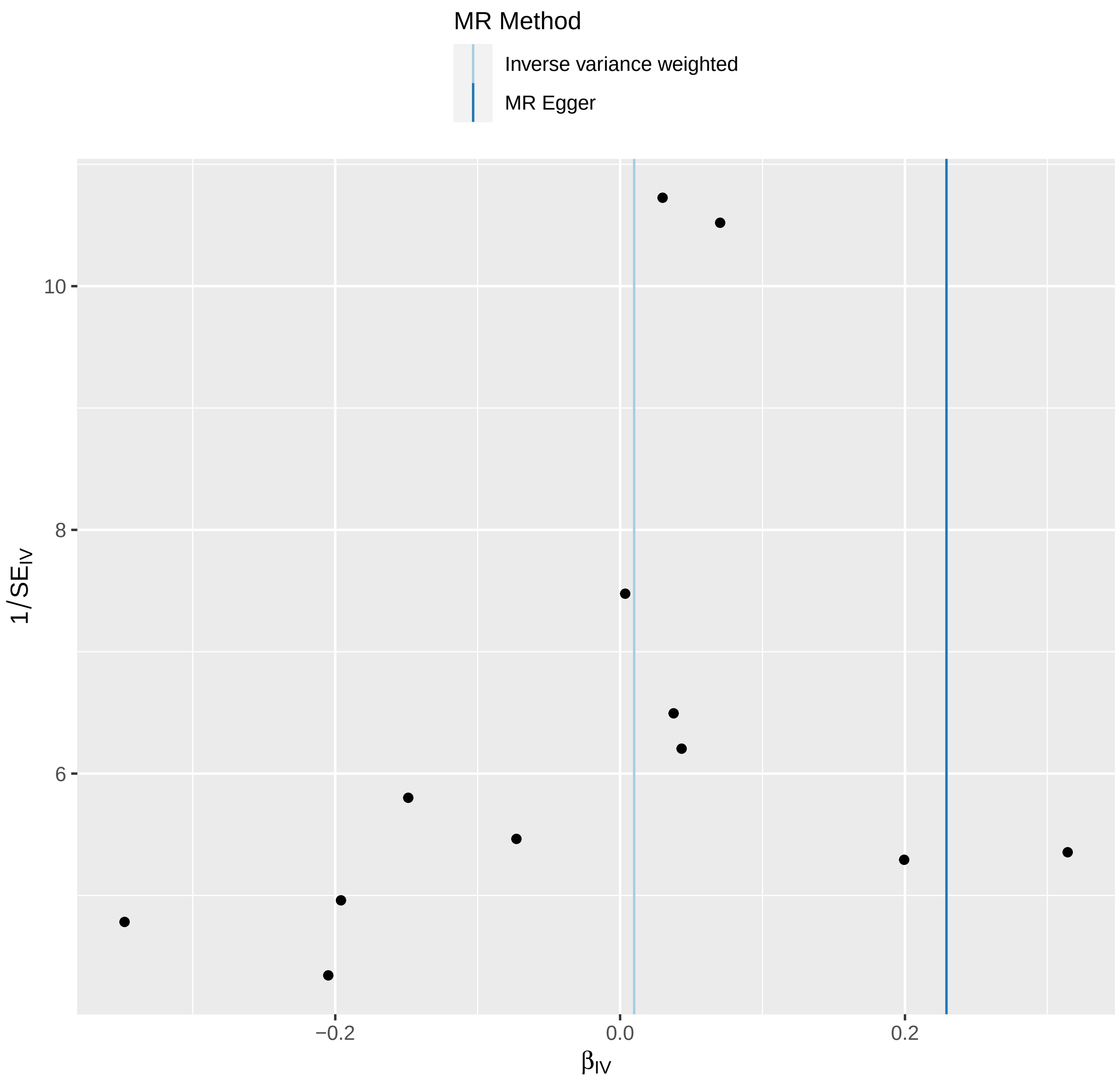

Batch 3−8 : Selenium on Abelson tyrosine−protein kinase 2

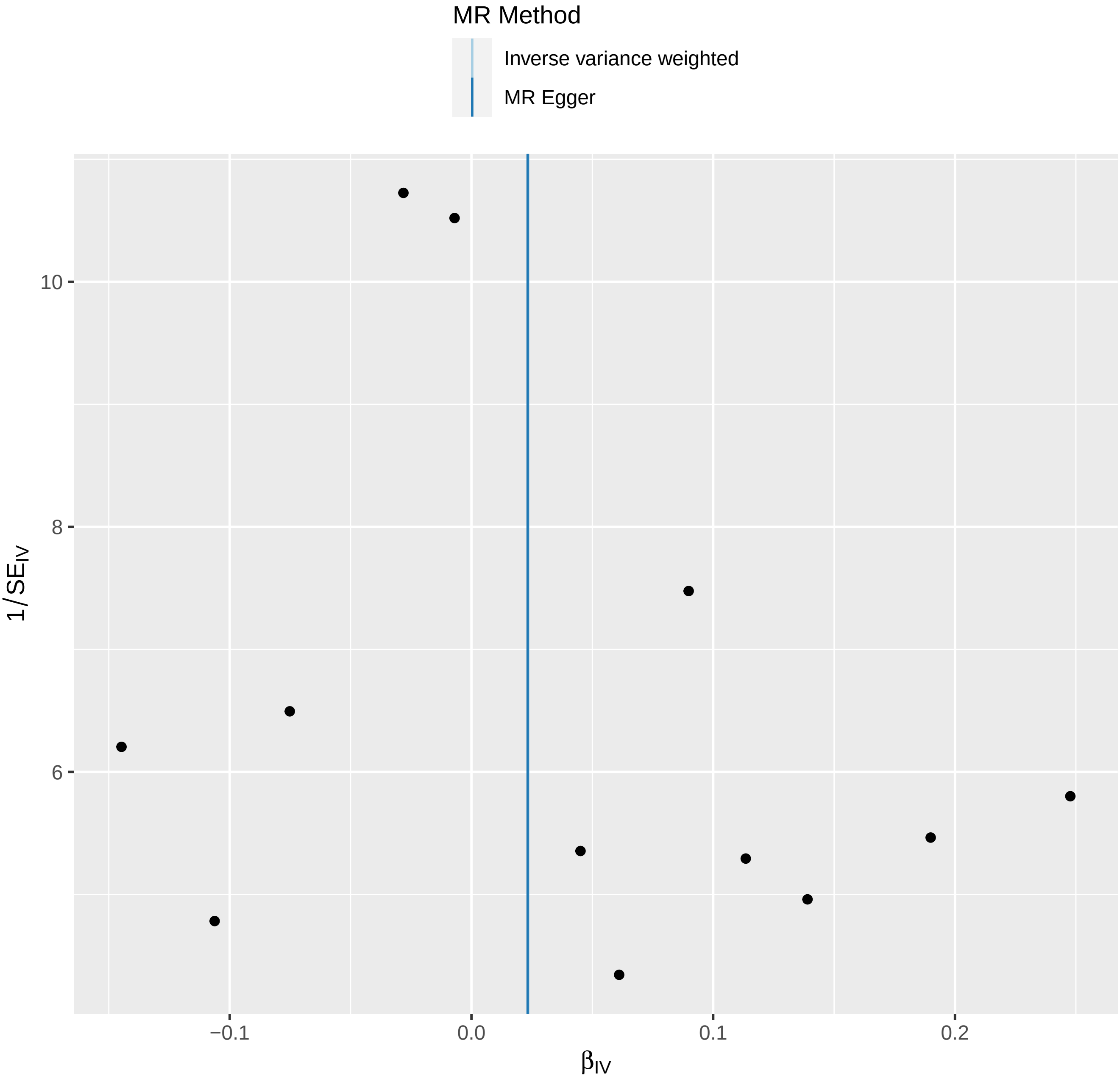

Batch 3−9 : Selenium on Actin−binding LIM protein 3

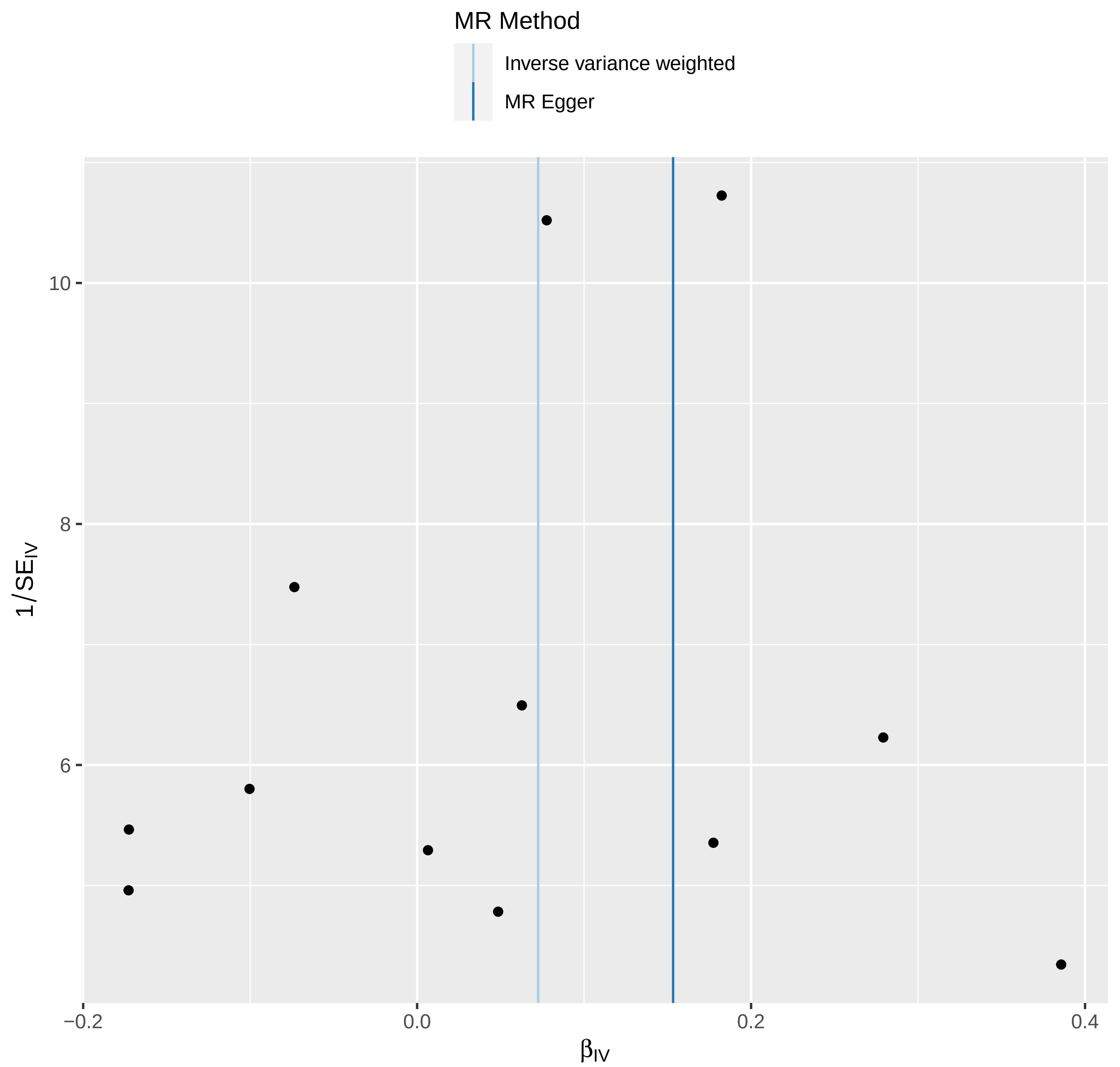

Batch 3−10 : Selenium on Histo−blood group ABO system transferase

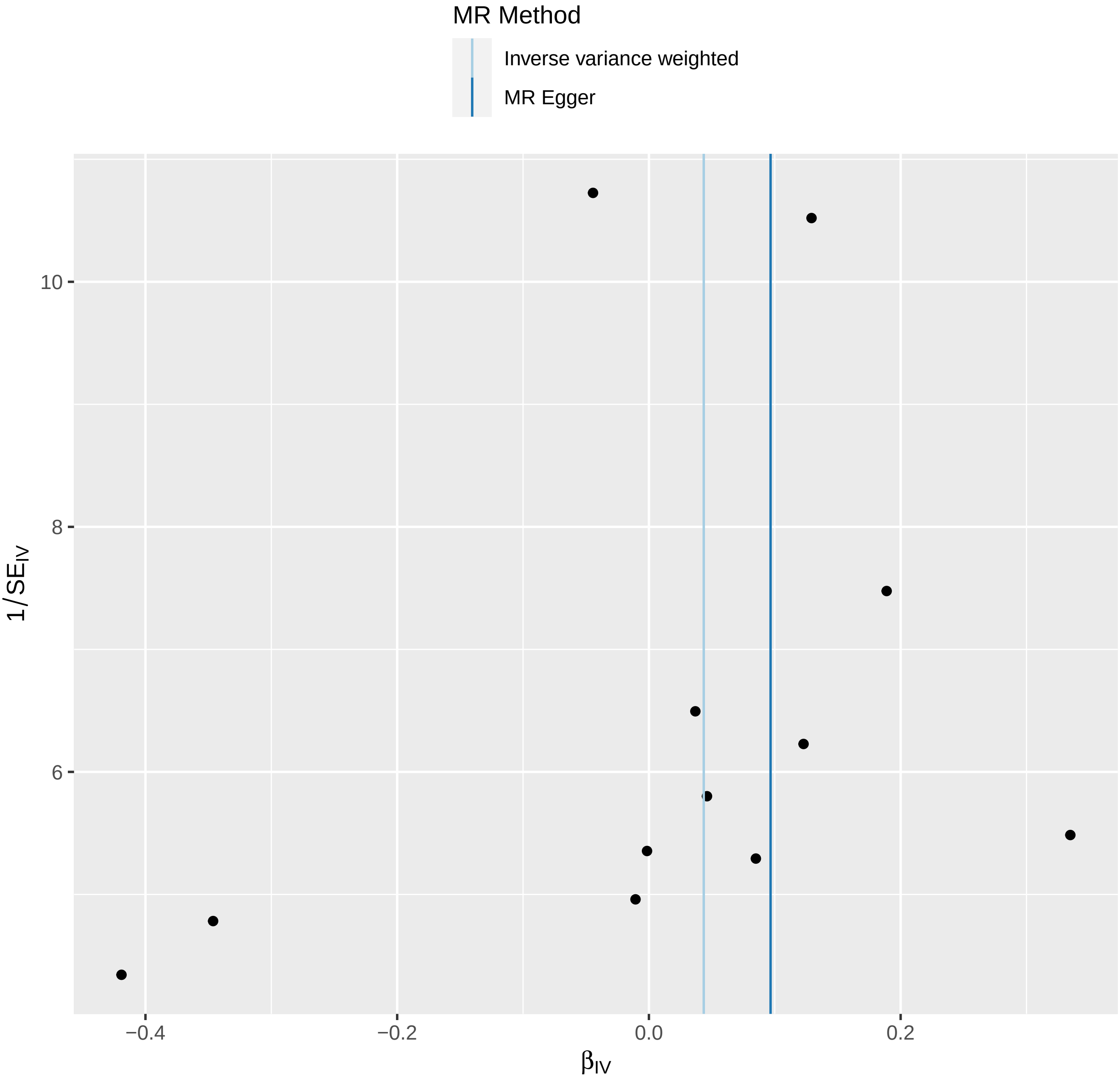

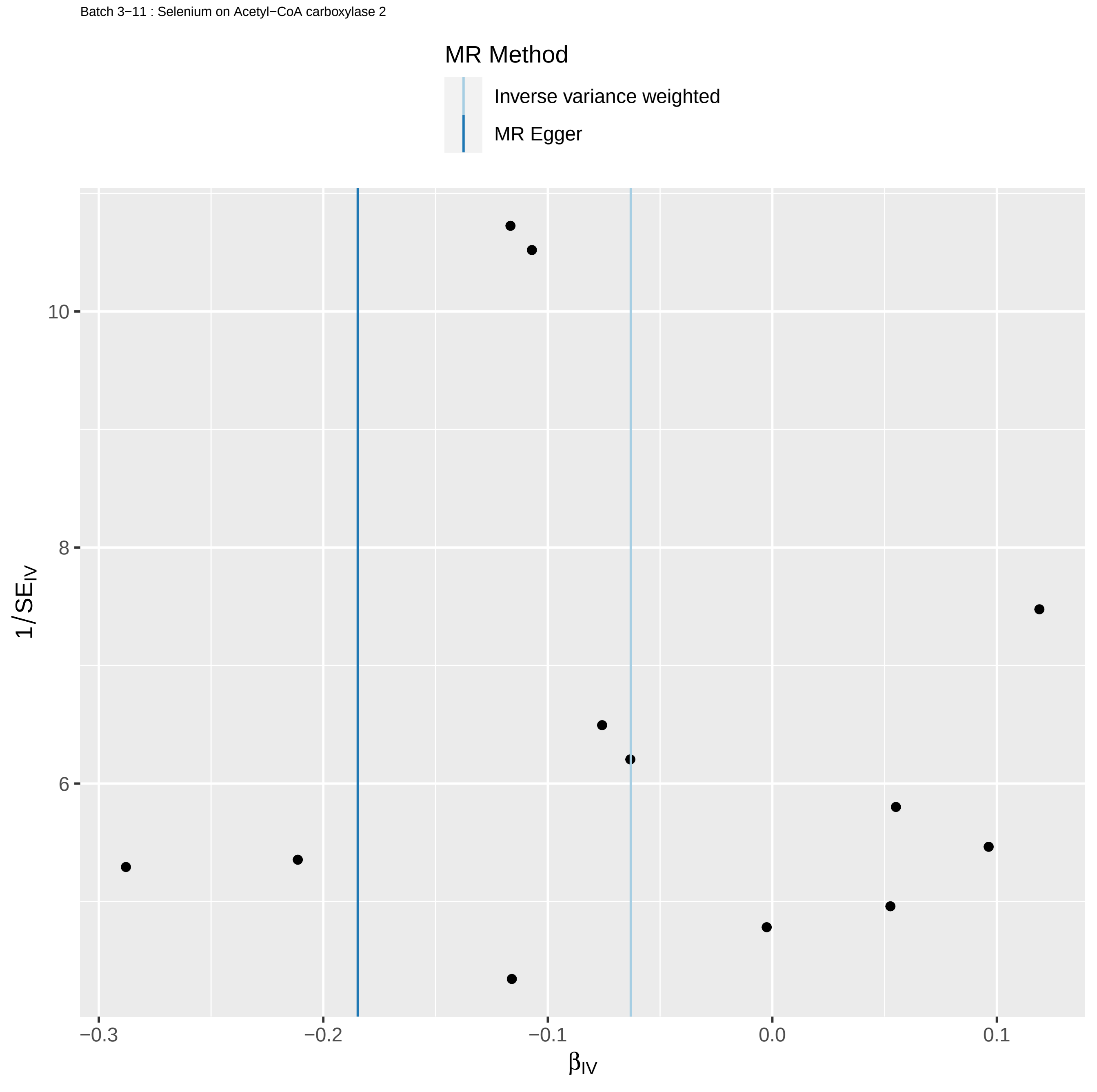
Batch 3−11 : Selenium on Acetyl−CoA carboxylase 2
MR Method
Inverse variance weighted
MR Egger
10
8
6
1/SE_IV
−0.3
−0.2
−0.1
0.0
0.1
β_IV

Batch 3−12 : Selenium on Aggrecan core protein

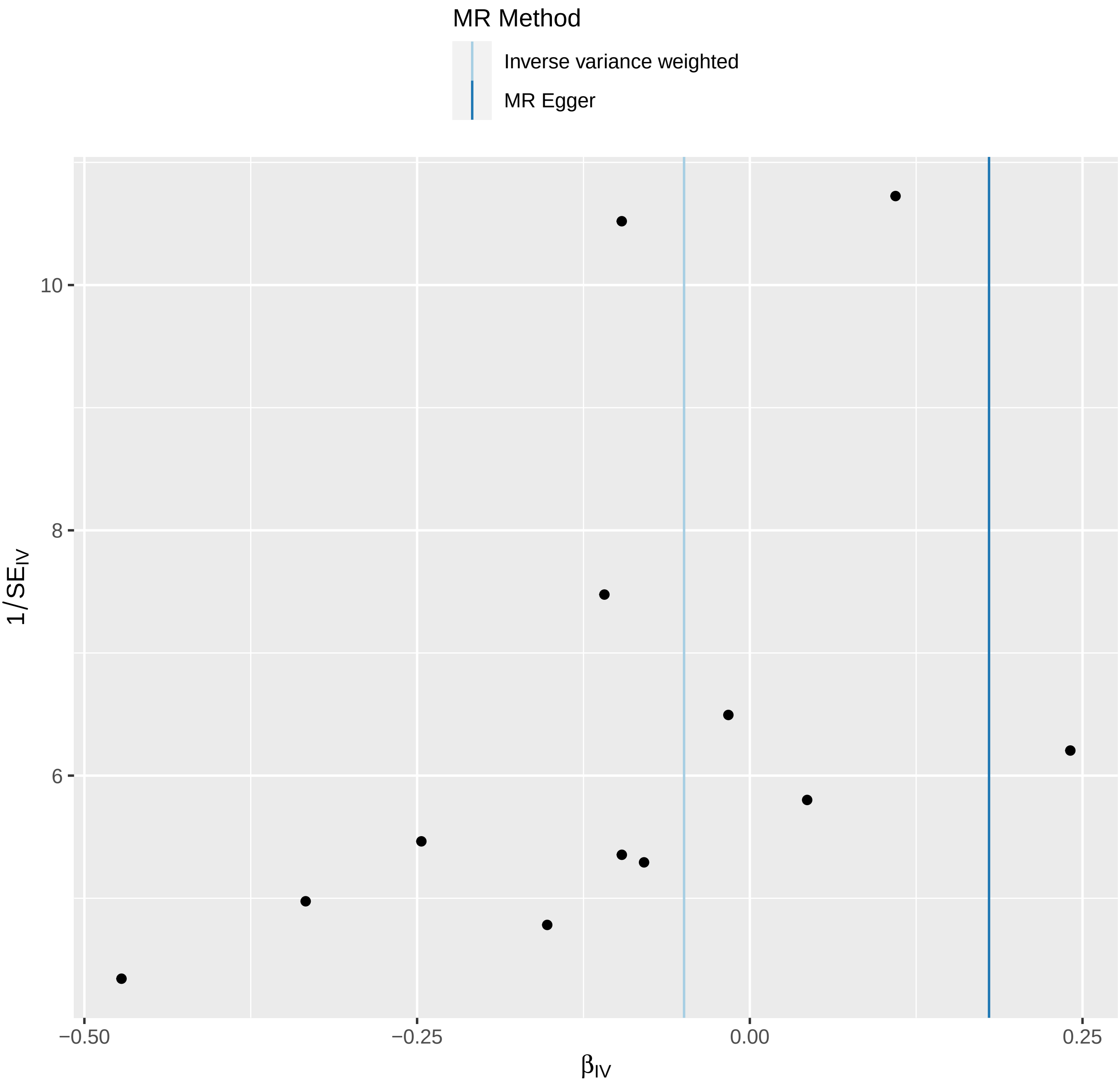

Batch 3−13 : Selenium on Arf−GAP with coiled−coil, ANK repeat and PH domain−containing protein 2

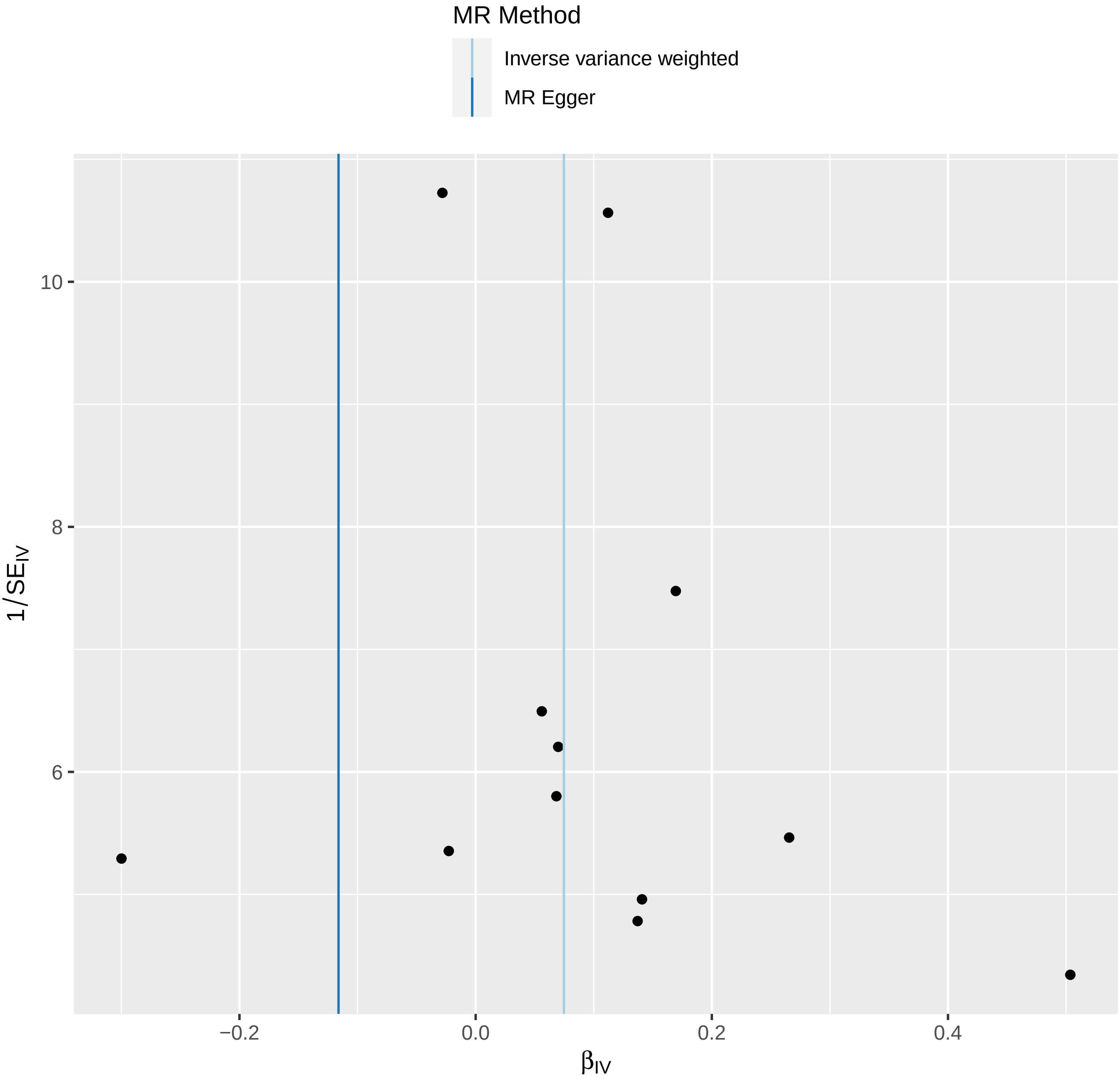

Batch 3−14 : Selenium on Acyl−CoA−binding domain−containing protein 6

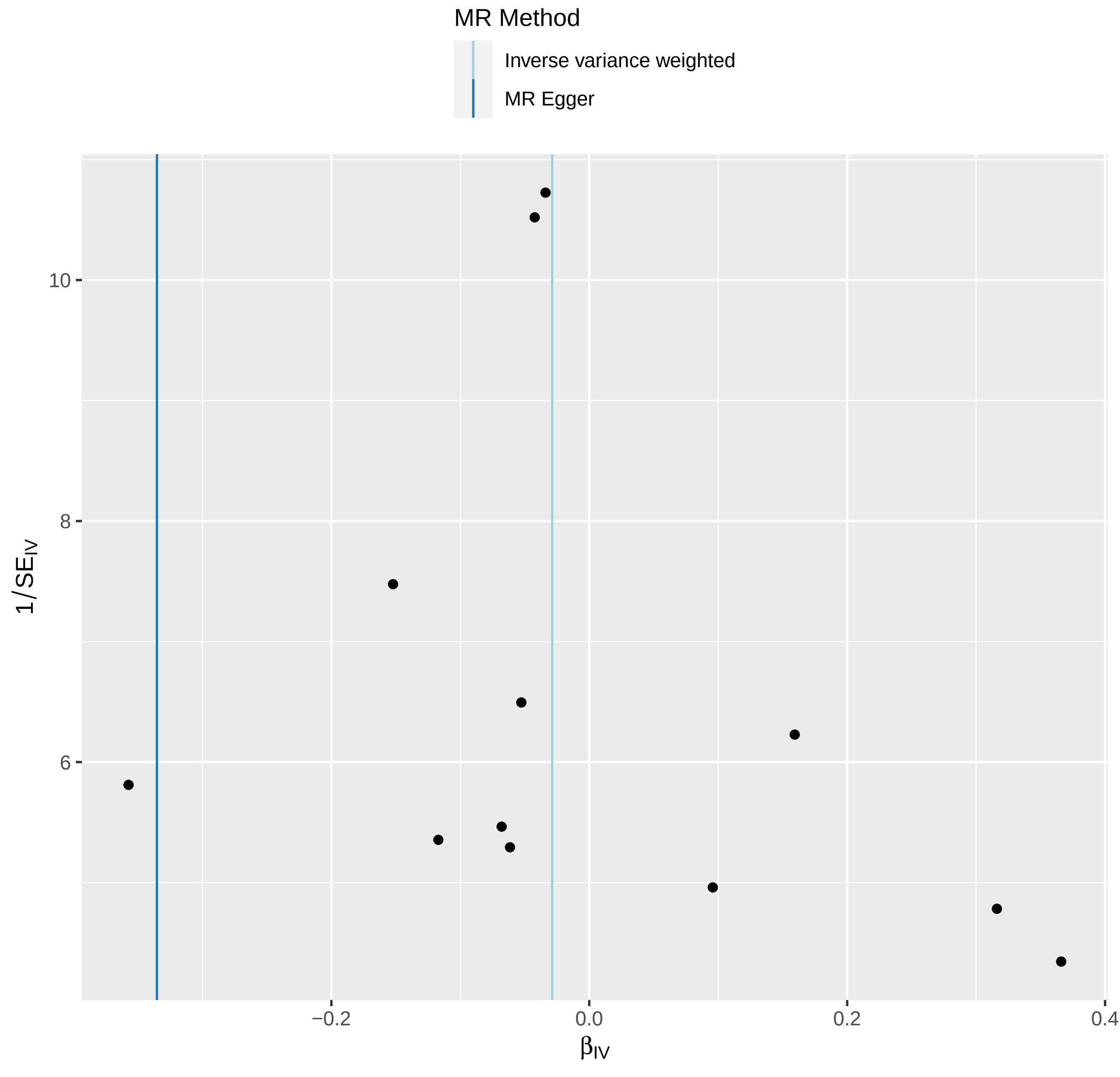

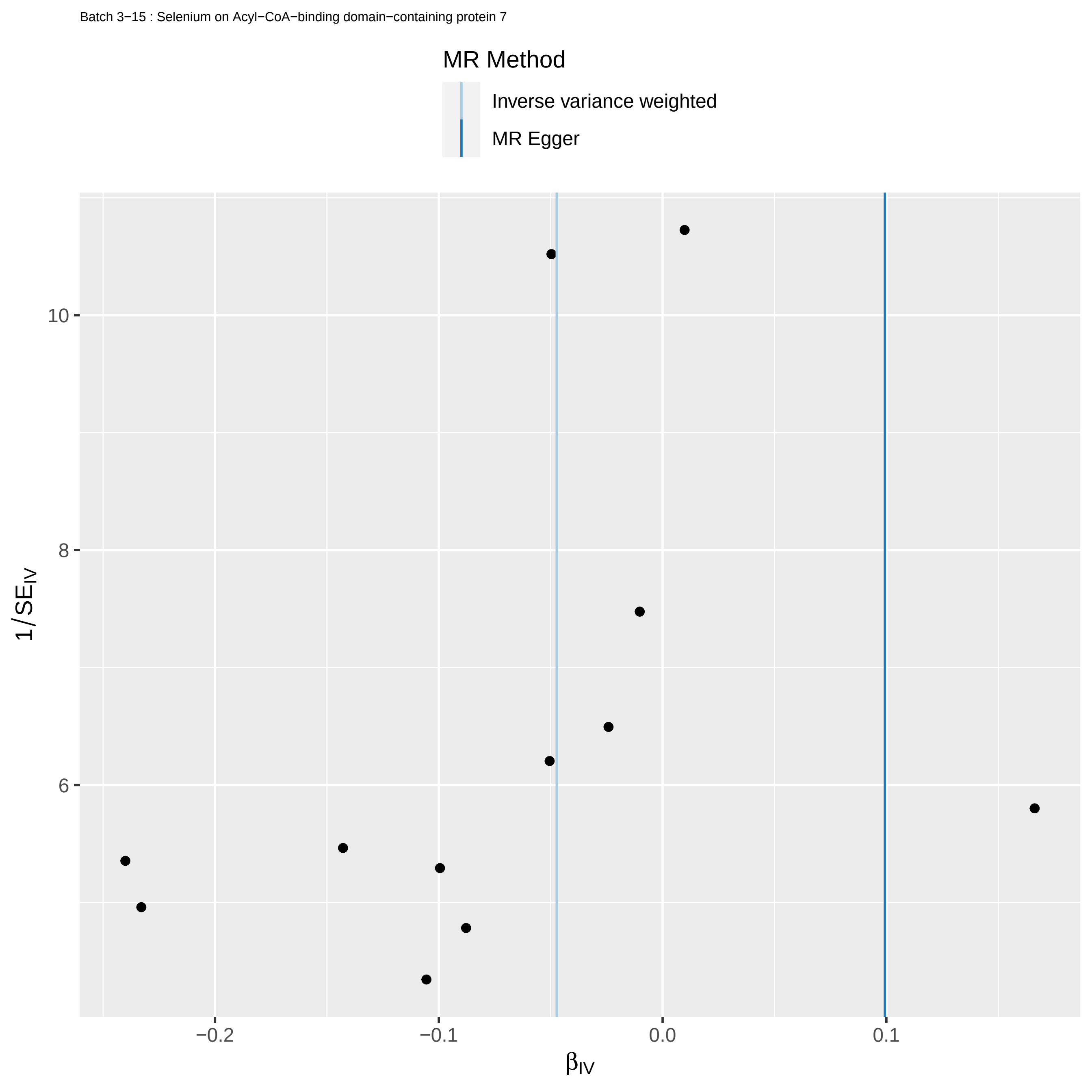

Batch 3−15 : Selenium on Acyl−CoA−binding domain−containing protein 7
MR Method
Inverse variance weighted
MR Egger
10
8
6
1/SE$_{IV}$
−0.2
−0.1
0.0
0.1
β$_{IV}$

Batch 3−16 : Selenium on Angiotensin−converting enzyme 2

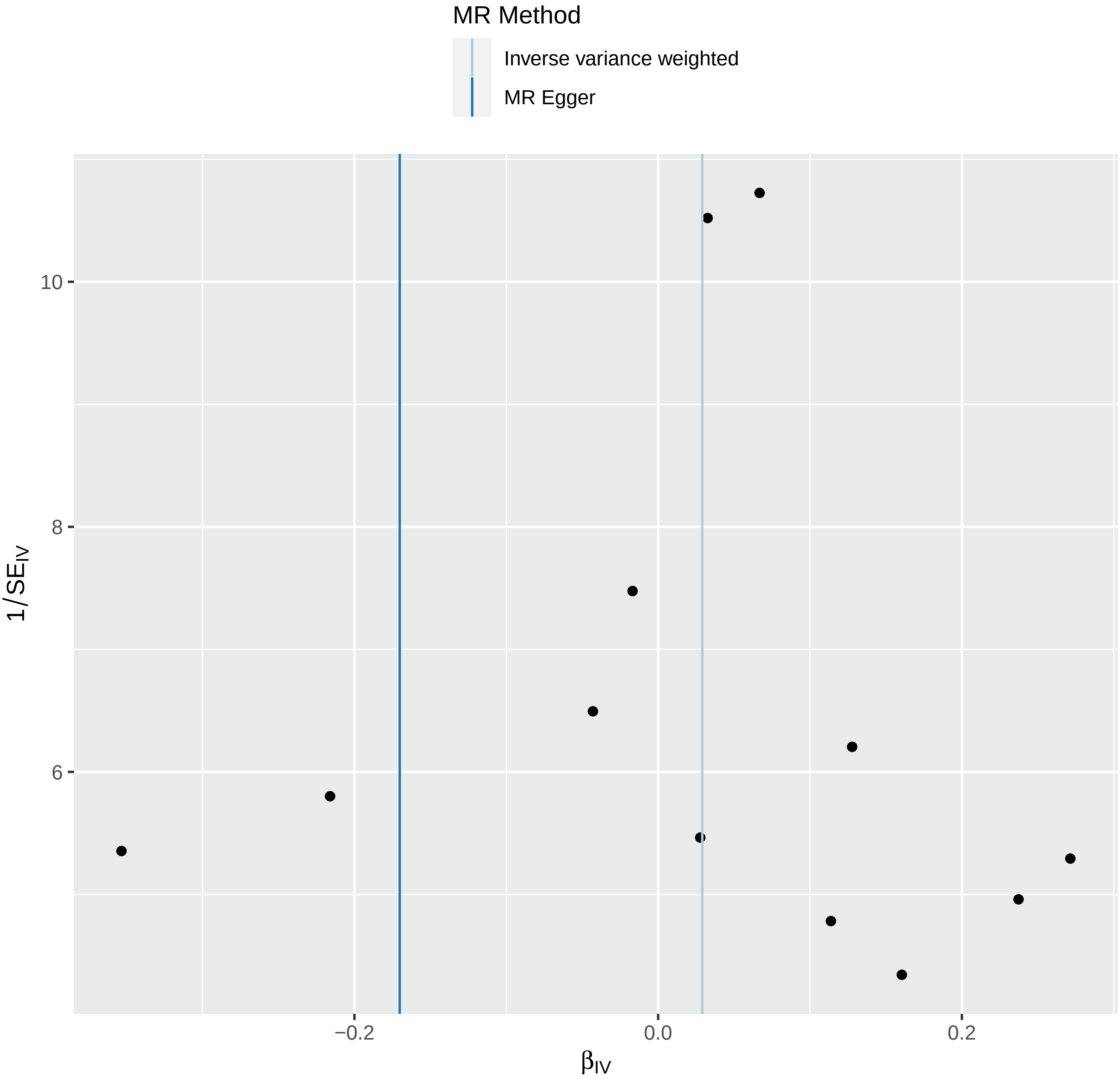

Batch 3−17 : Selenium on ATP−citrate synthase

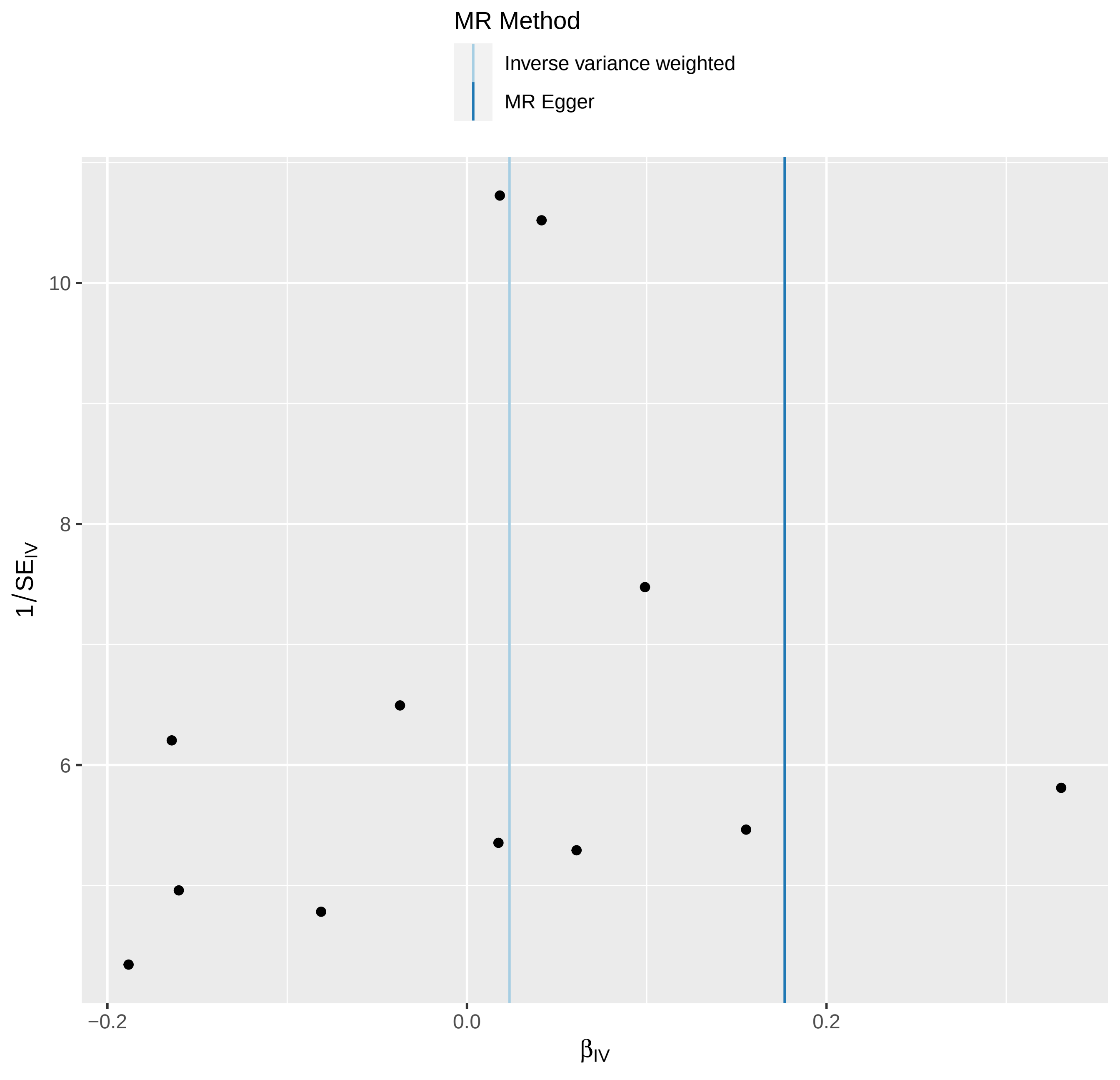

Batch 3−18 : Selenium on Low molecular weight phosphotyrosine protein phosphatase

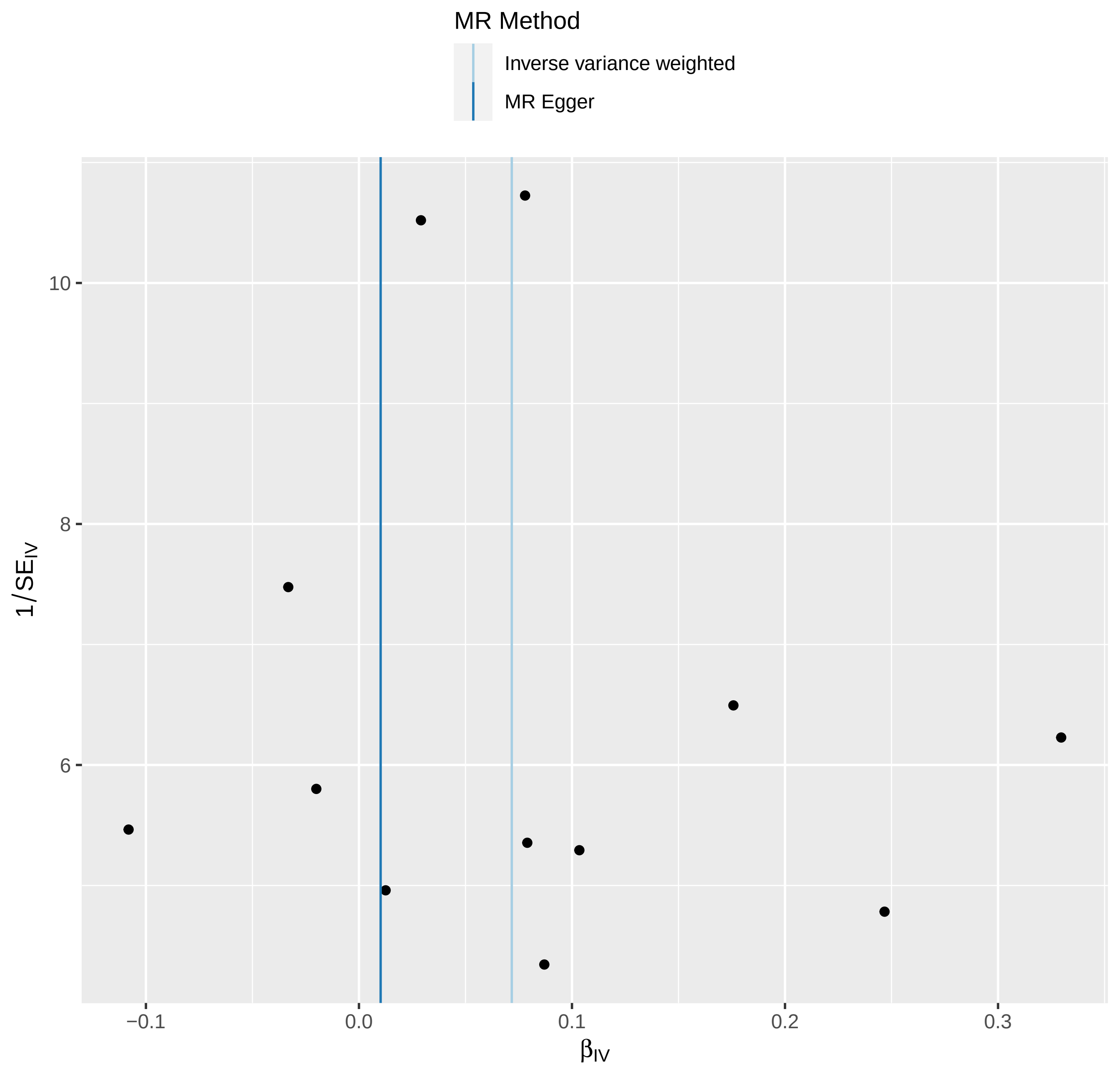

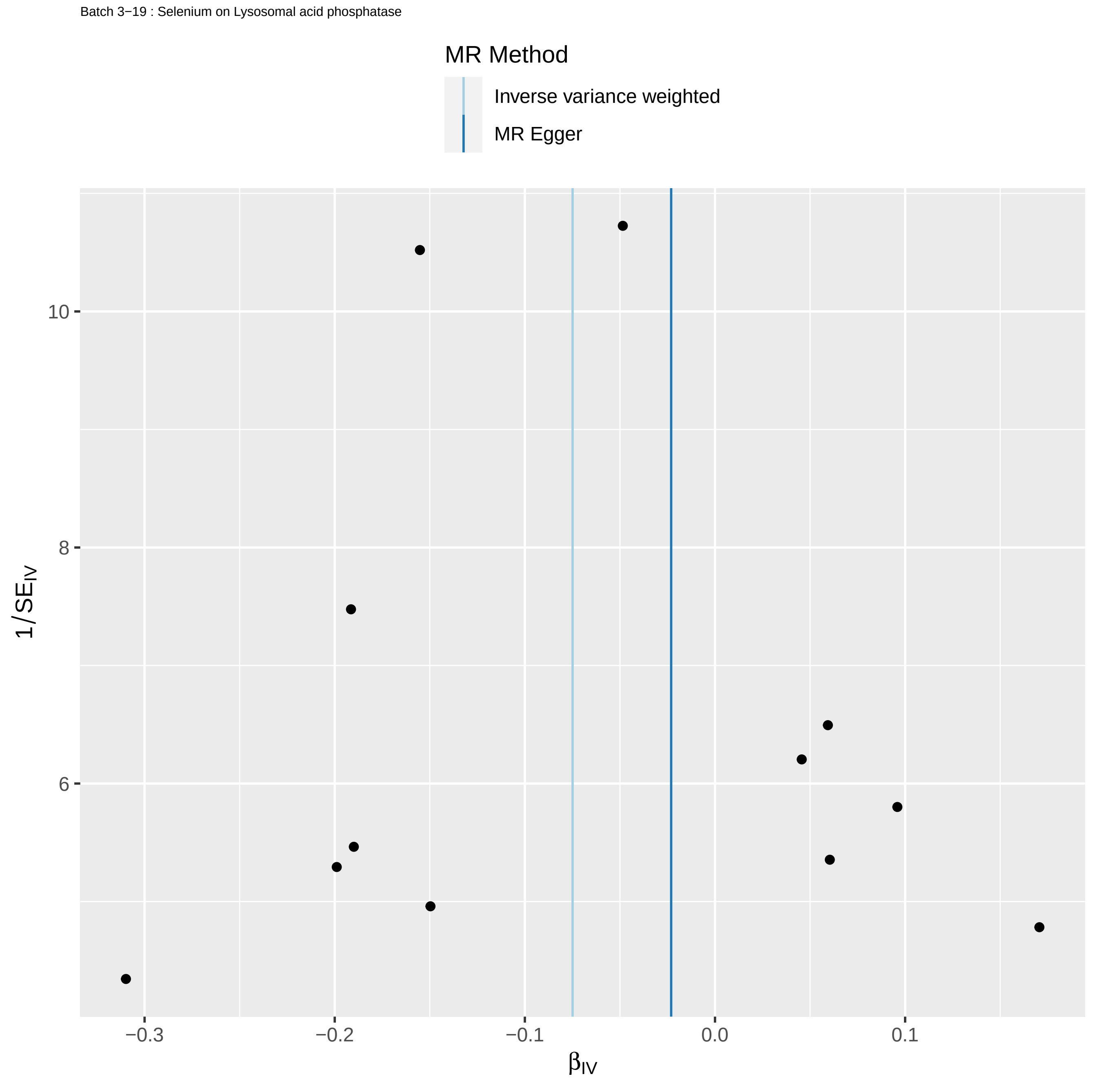
Batch 3−19 : Selenium on Lysosomal acid phosphatase
MR Method
Inverse variance weighted
MR Egger
10
8
6
1/SE_IV
−0.3
−0.2
−0.1
0.0
0.1
β_IV

Batch 3−20 : Selenium on Tartrate−resistant acid phosphatase type 5

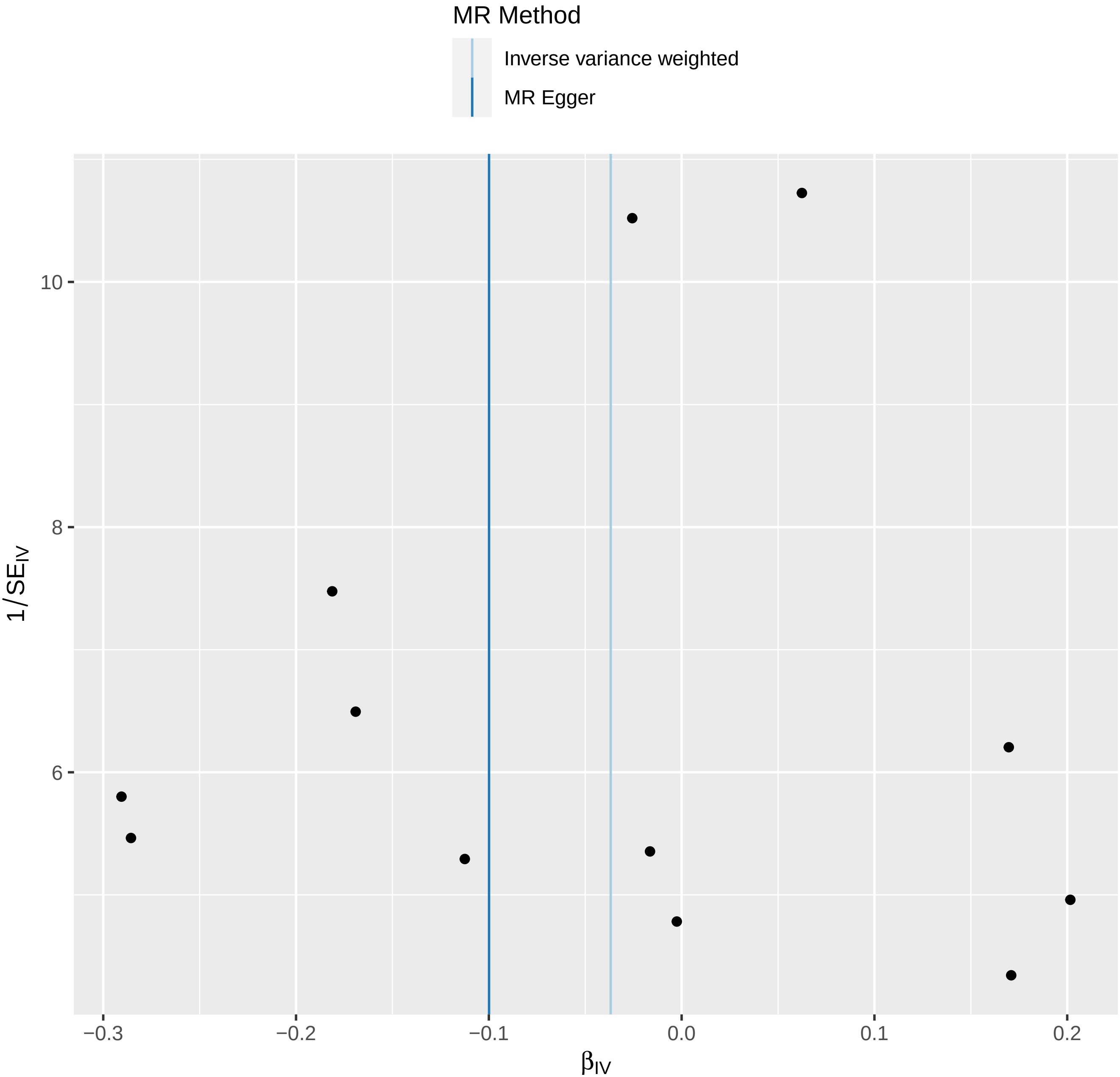

Batch 3−21 : Selenium on 2−phosphoxylose phosphatase 1

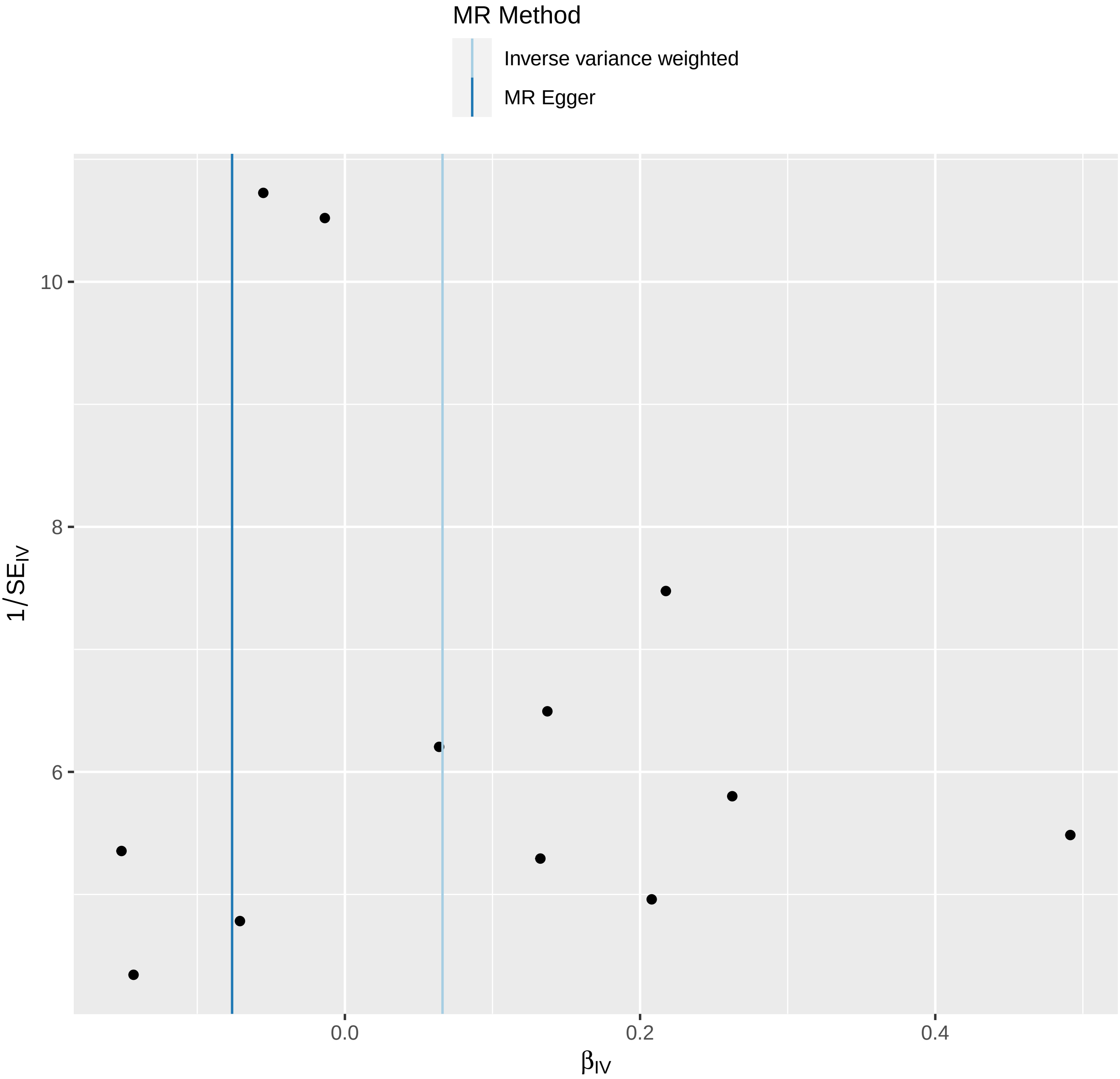

Batch 3−22 : Selenium on Activin receptor type−1B

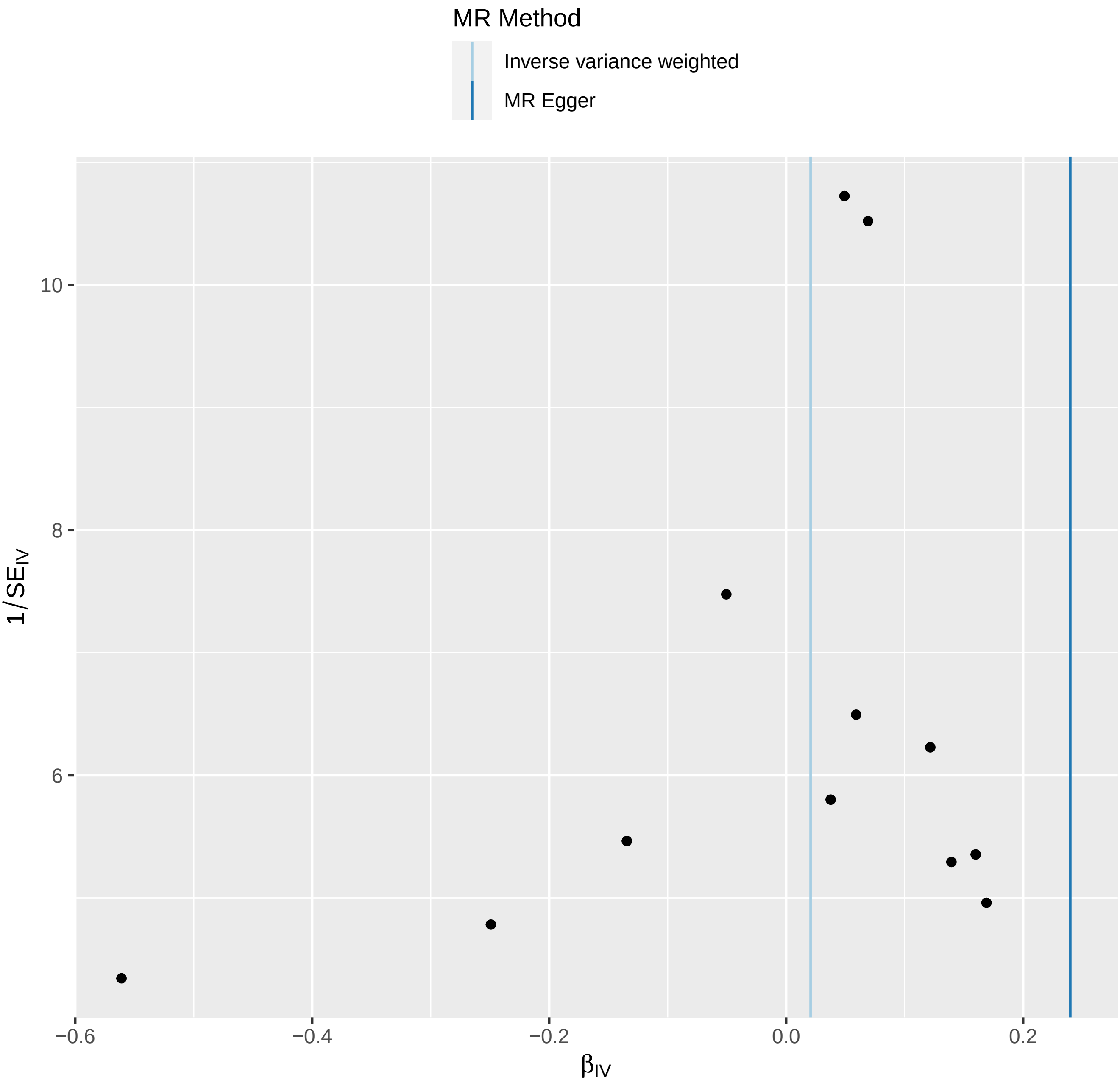

Batch 3−23 : Selenium on Serine/threonine−protein kinase receptor R3

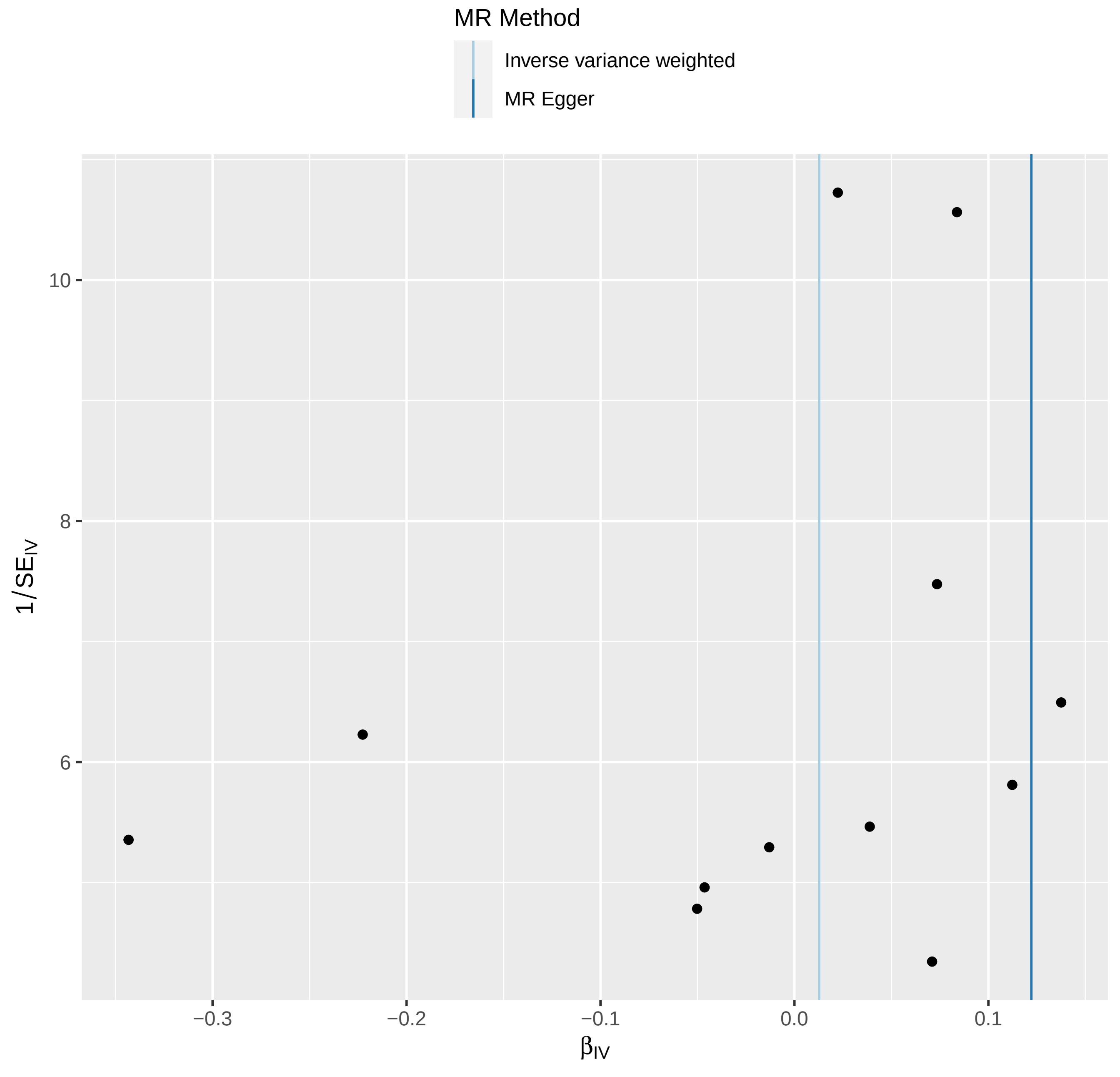

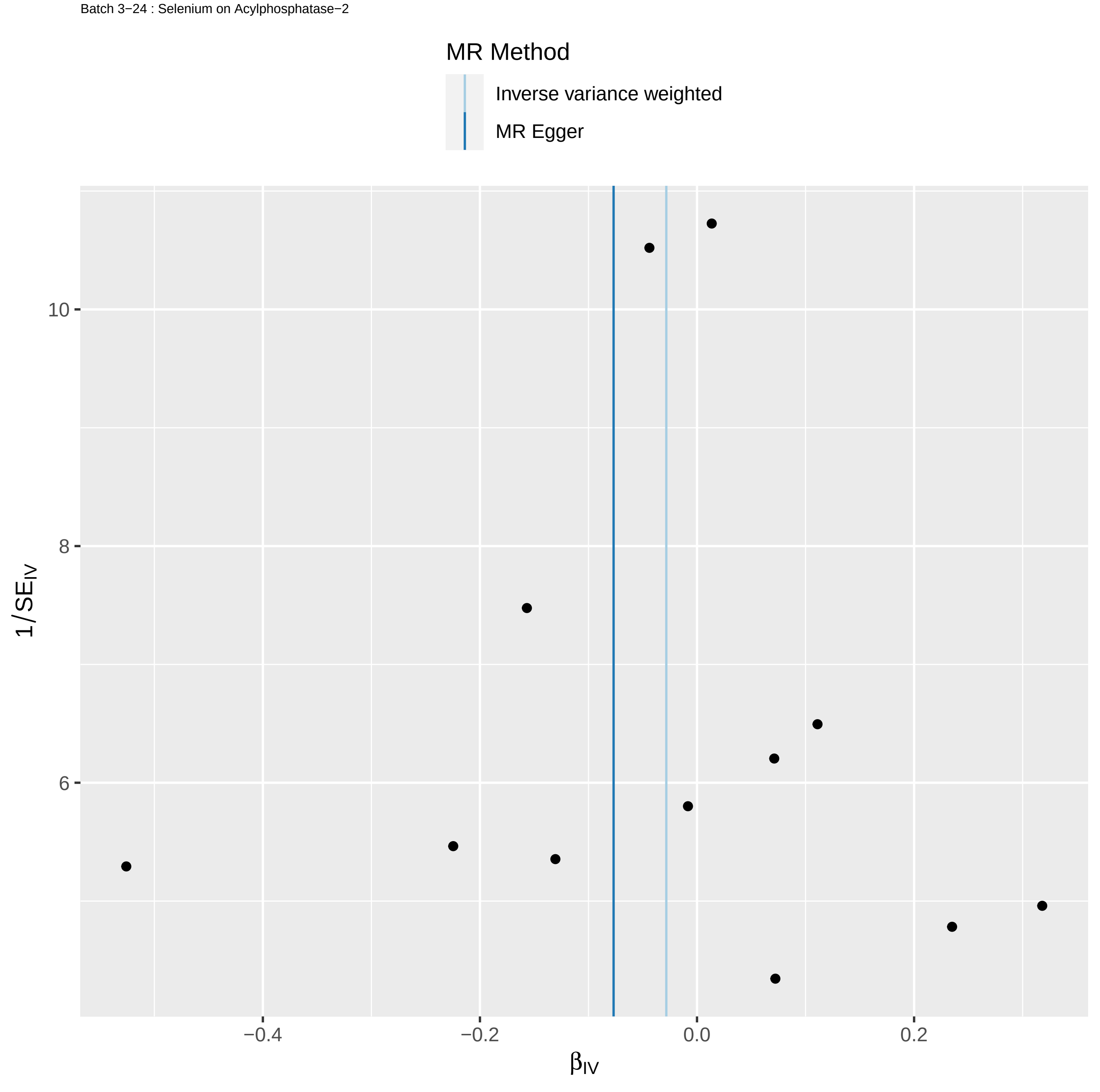

Batch 3−24 : Selenium on Acylphosphatase−2
MR Method
Inverse variance weighted
MR Egger
10
8
6
1/SE$_{IV}$
−0.4
−0.2
0.0
0.2
β$_{IV}$

Batch 3−25 : Selenium on Disintegrin and metalloproteinase domain−containing protein 11

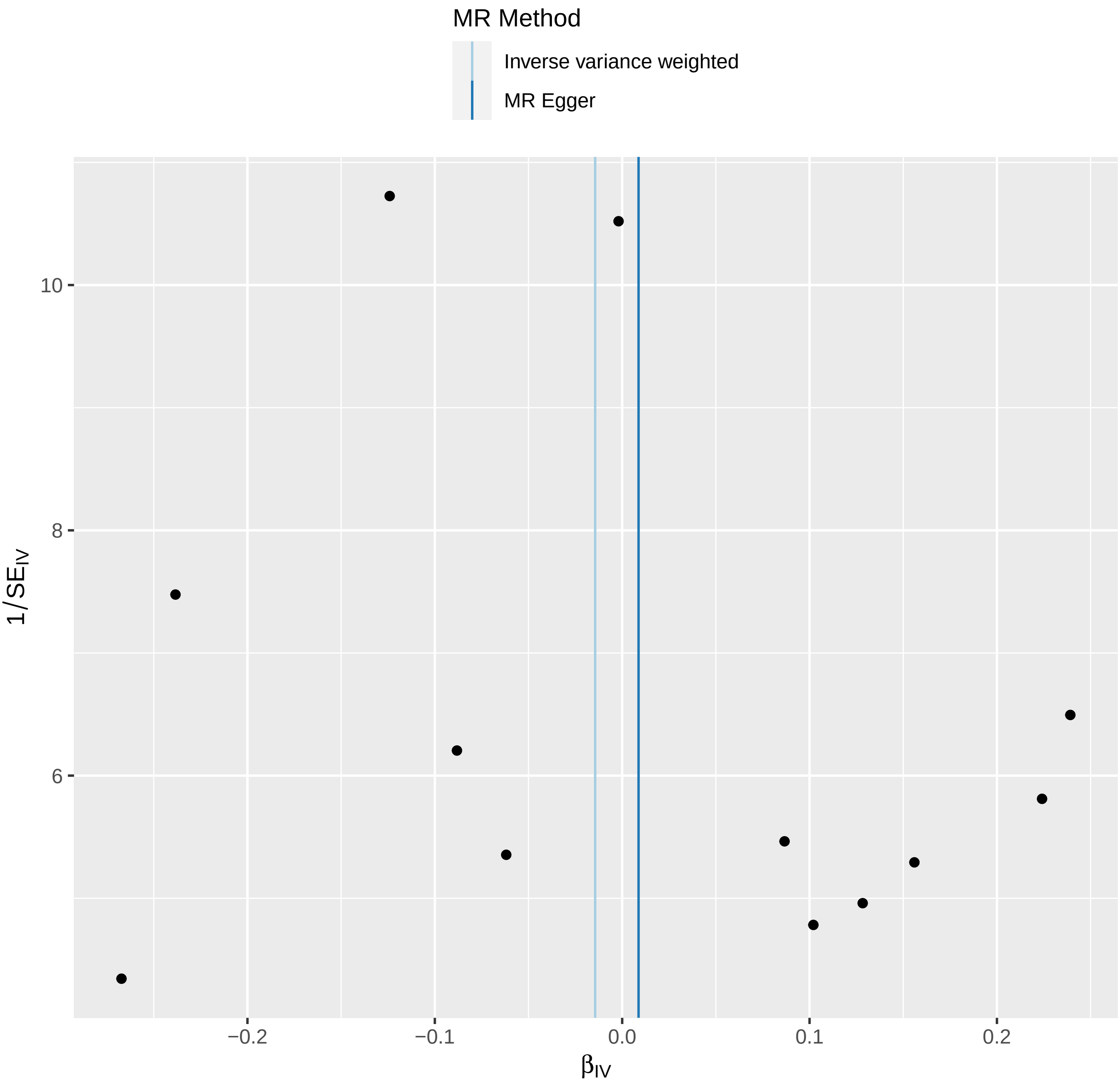

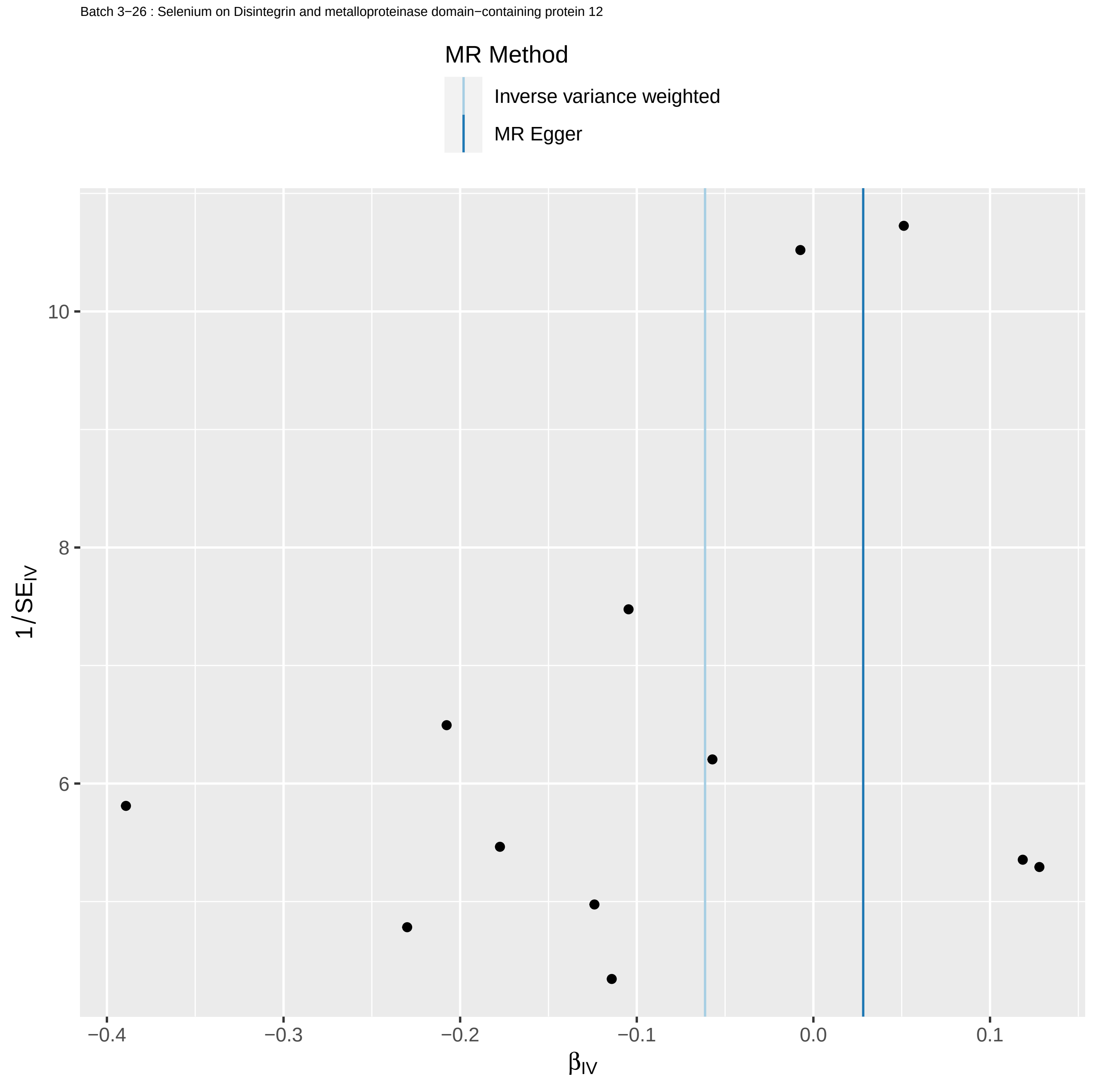

Batch 3−26 : Selenium on Disintegrin and metalloproteinase domain−containing protein 12
MR Method
Inverse variance weighted
MR Egger
10
8
6
1/SE_IV
−0.4
−0.3
−0.2
−0.1
0.0
0.1
β_IV

Batch 3−27 : Selenium on Disintegrin and metalloproteinase domain−containing protein 19

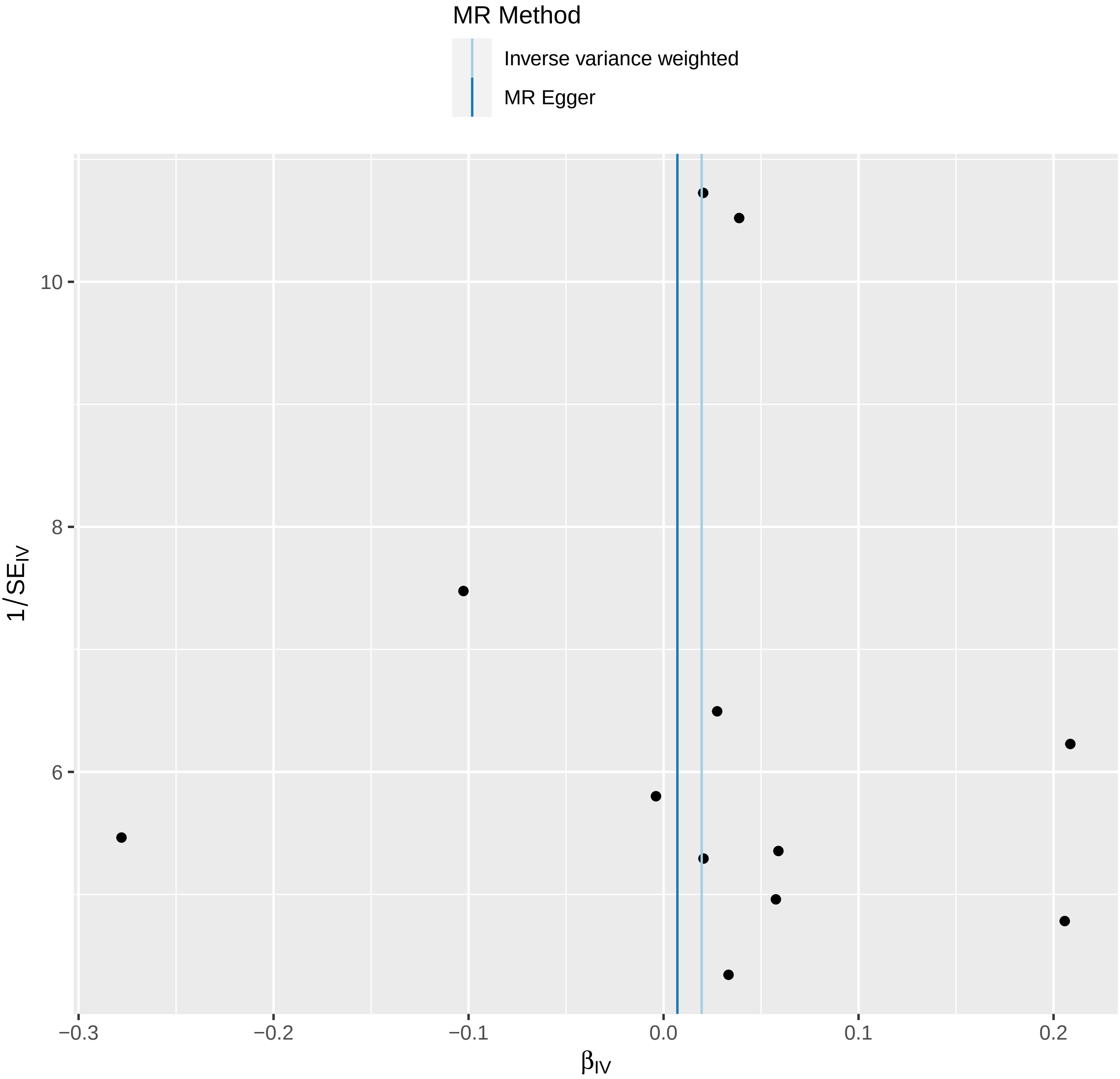

Batch 3−28 : Selenium on Disintegrin and metalloproteinase domain−containing protein 23

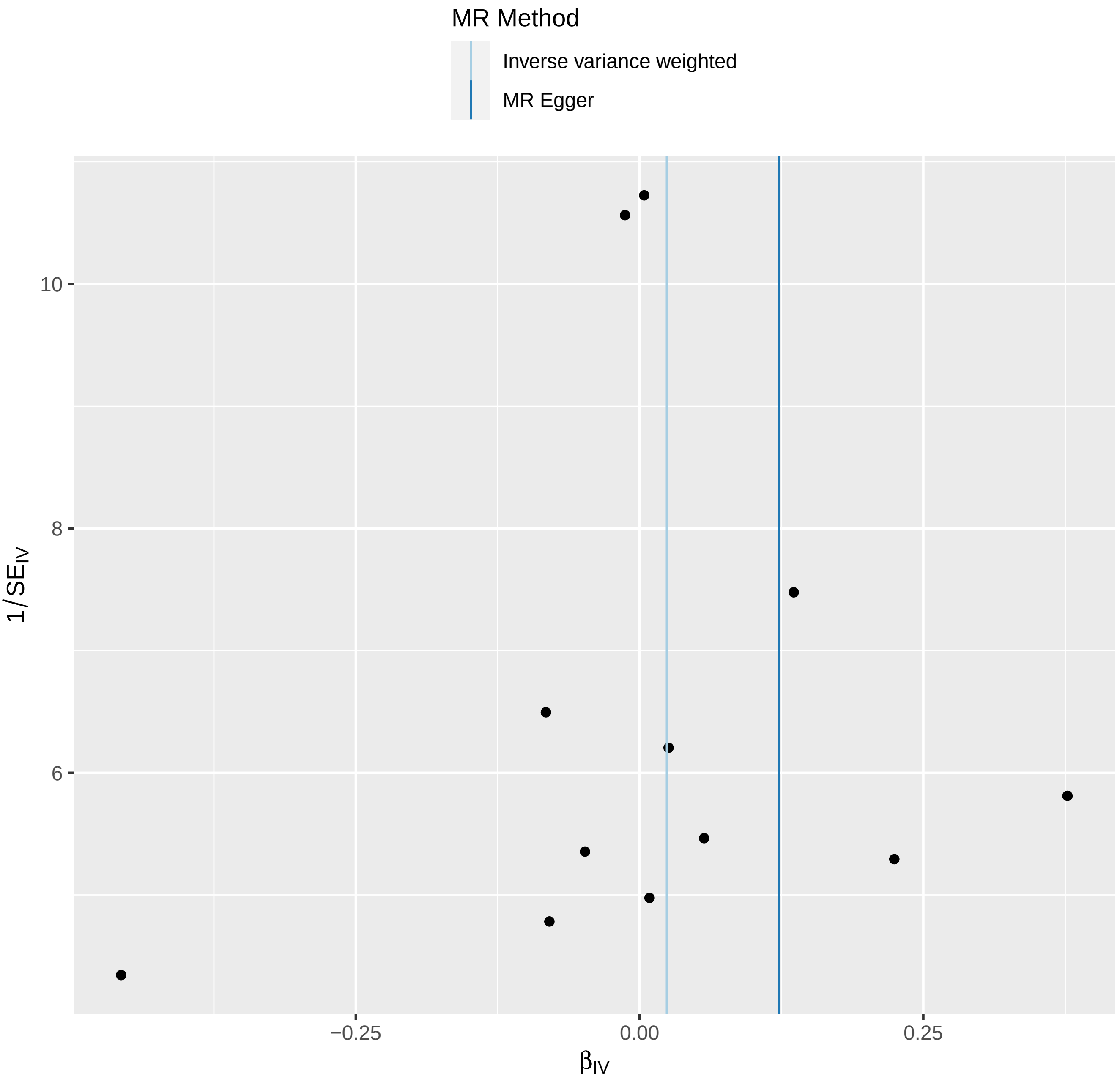

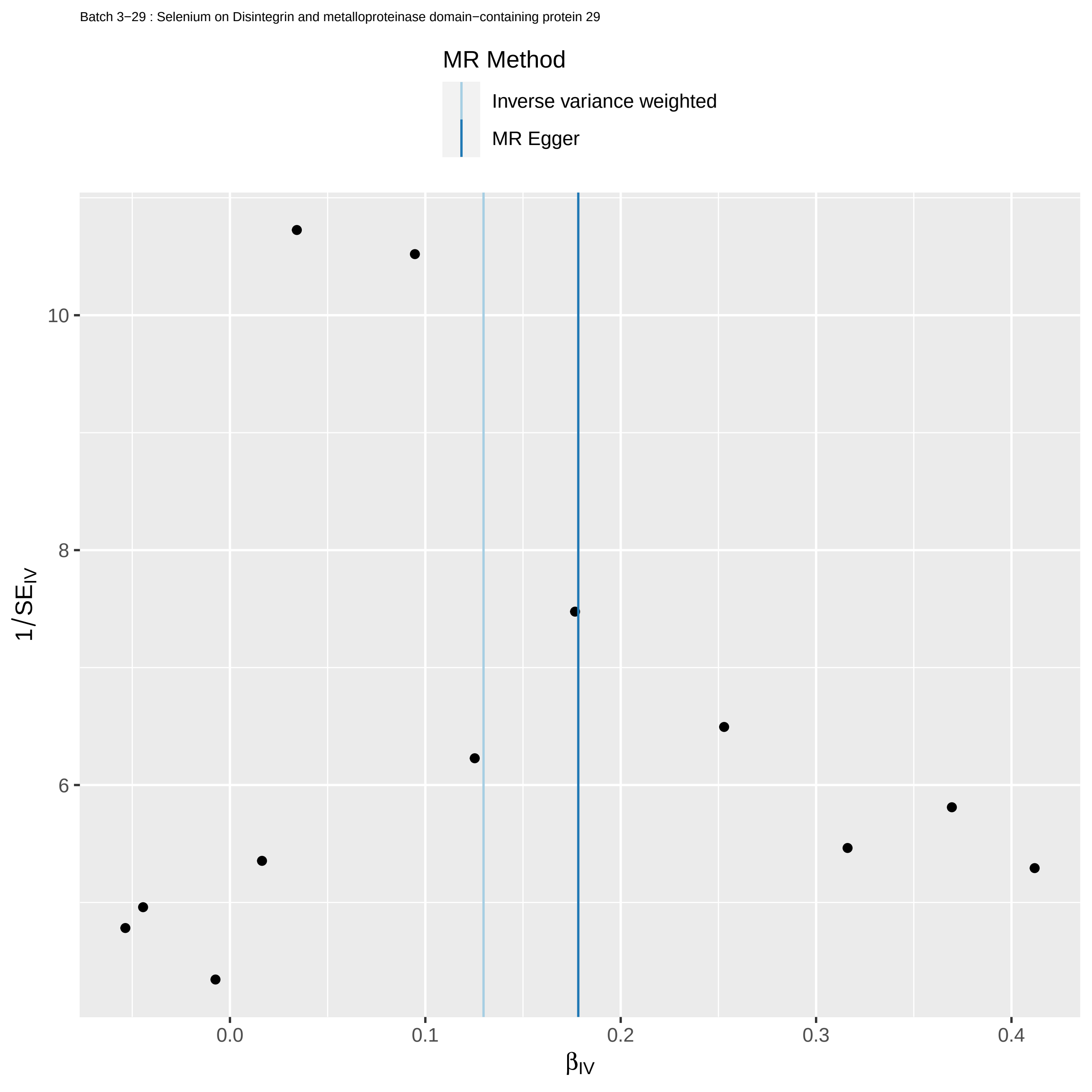

Batch 3−29 : Selenium on Disintegrin and metalloproteinase domain−containing protein 29
MR Method
Inverse variance weighted
MR Egger
10
8
6
1/SE_IV
0.0
0.1
0.2
0.3
0.4
β_IV

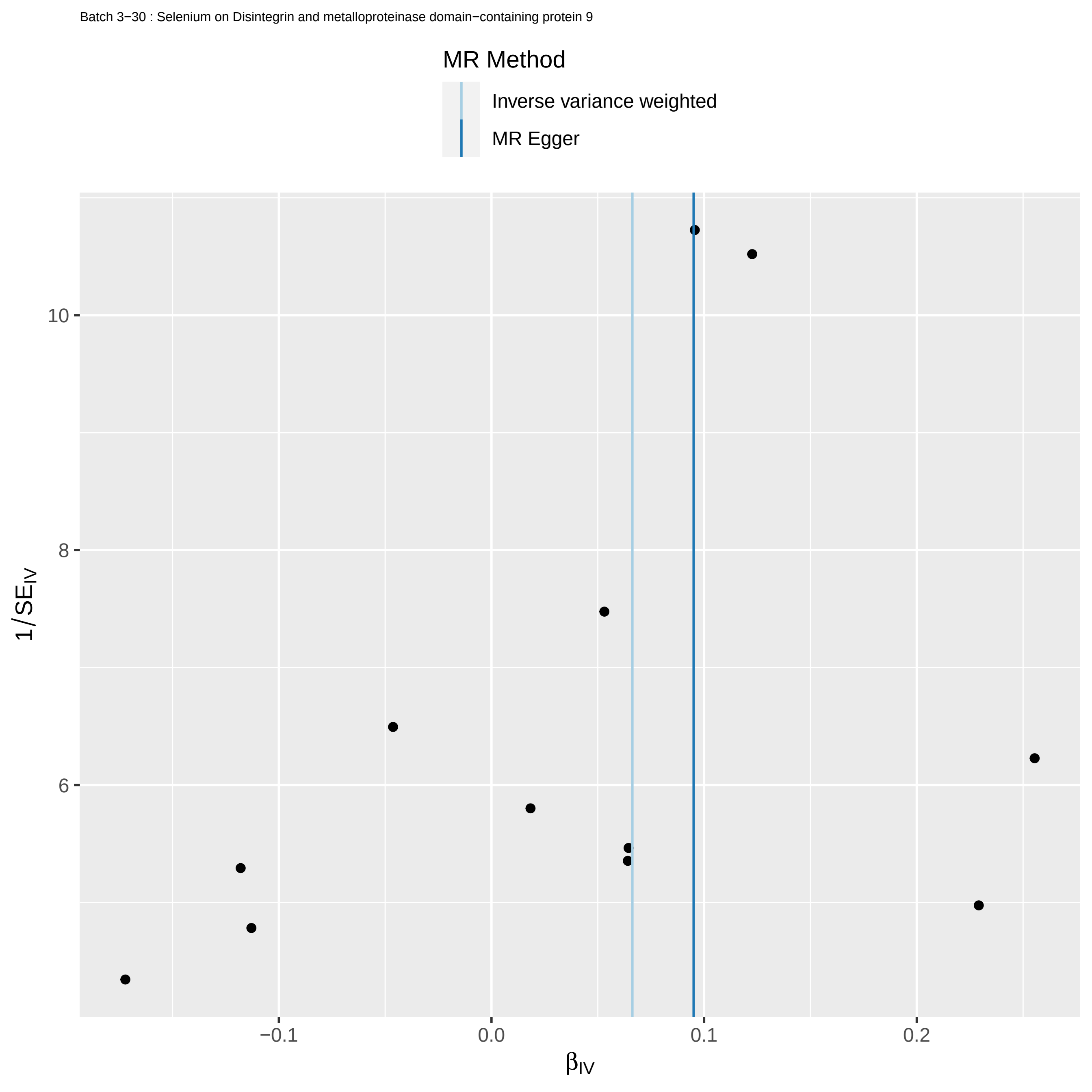
Batch 3−30 : Selenium on Disintegrin and metalloproteinase domain−containing protein 9
MR Method
Inverse variance weighted
MR Egger
10
8
6
1/SE_IV
−0.1
0.0
0.1
0.2
β_IV

Batch 3−31 : Selenium on A disintegrin and metalloproteinase with thrombospondin motifs 1

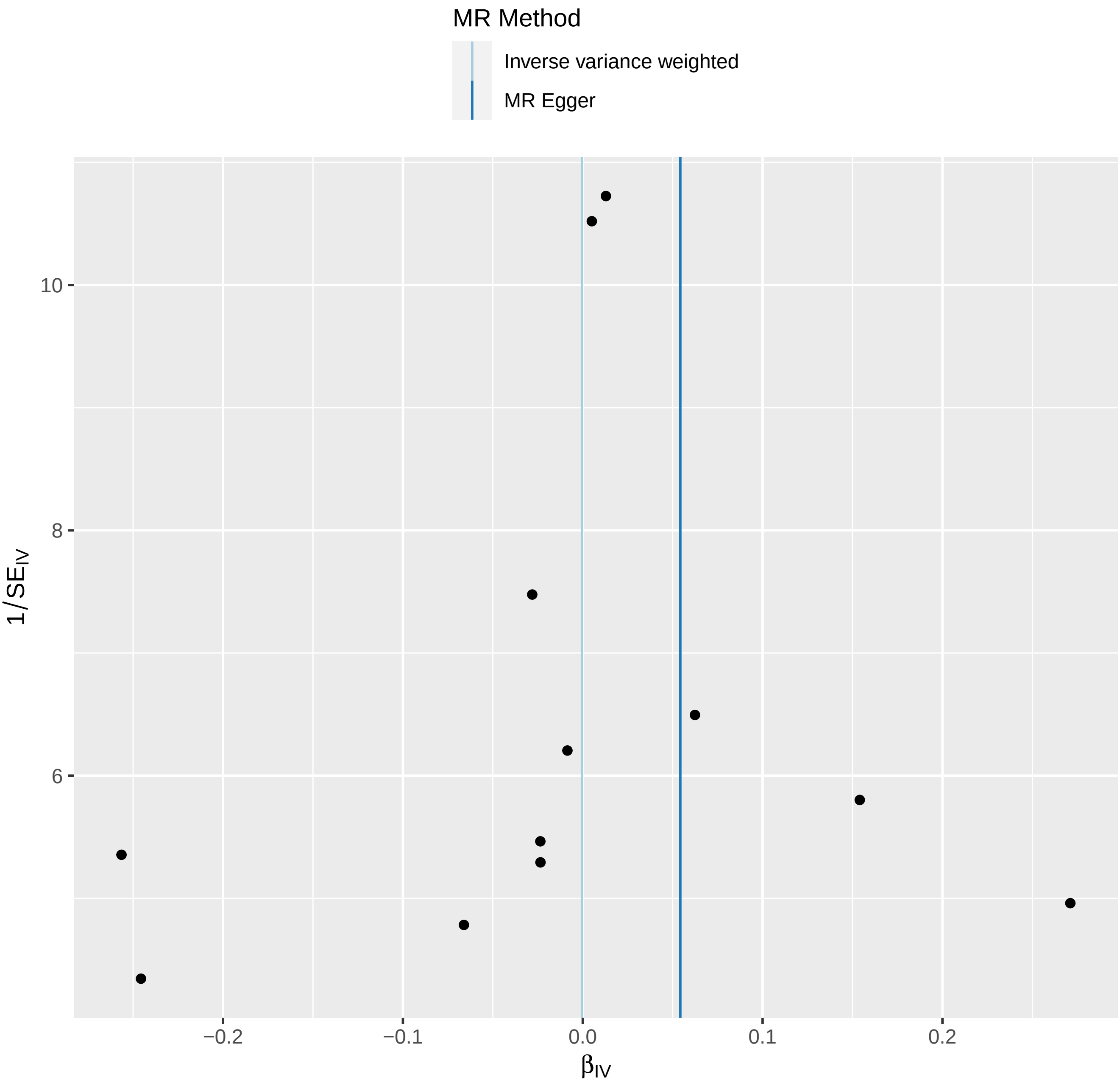

Batch 3−32 : Selenium on A disintegrin and metalloproteinase with thrombospondin motifs 13

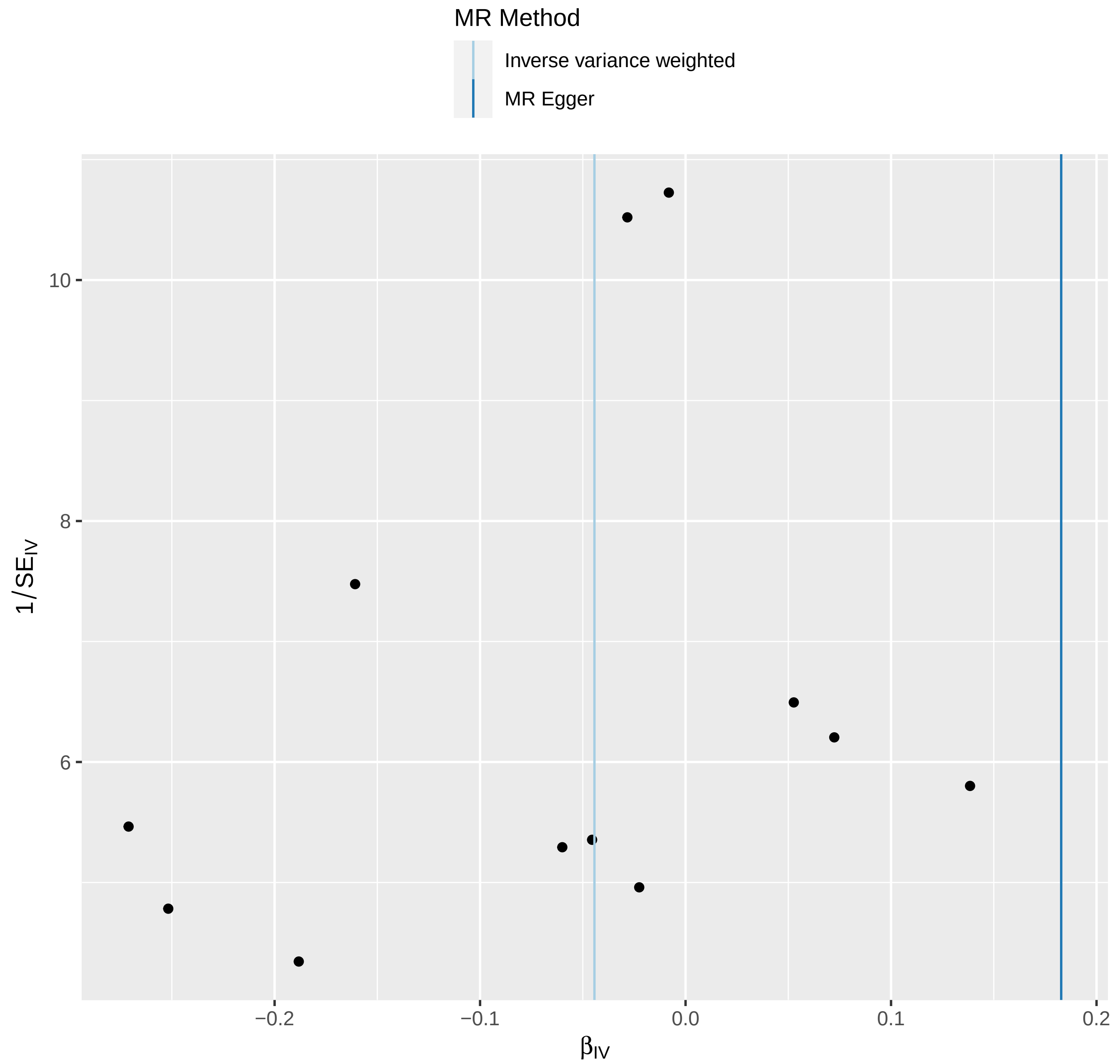

Batch 3−33 : Selenium on A disintegrin and metalloproteinase with thrombospondin motifs 15

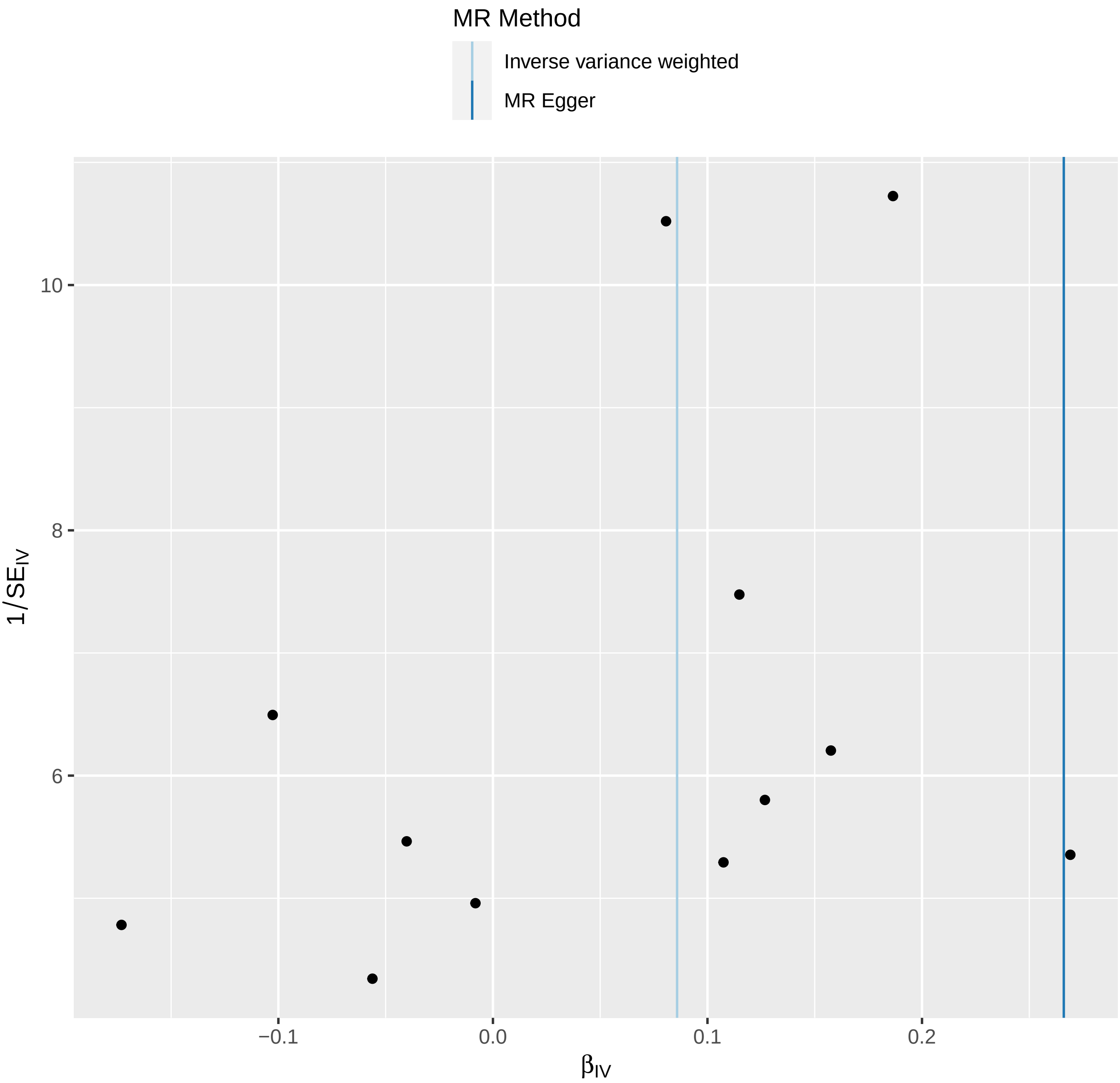

Batch 3−34 : Selenium on A disintegrin and metalloproteinase with thrombospondin motifs 3

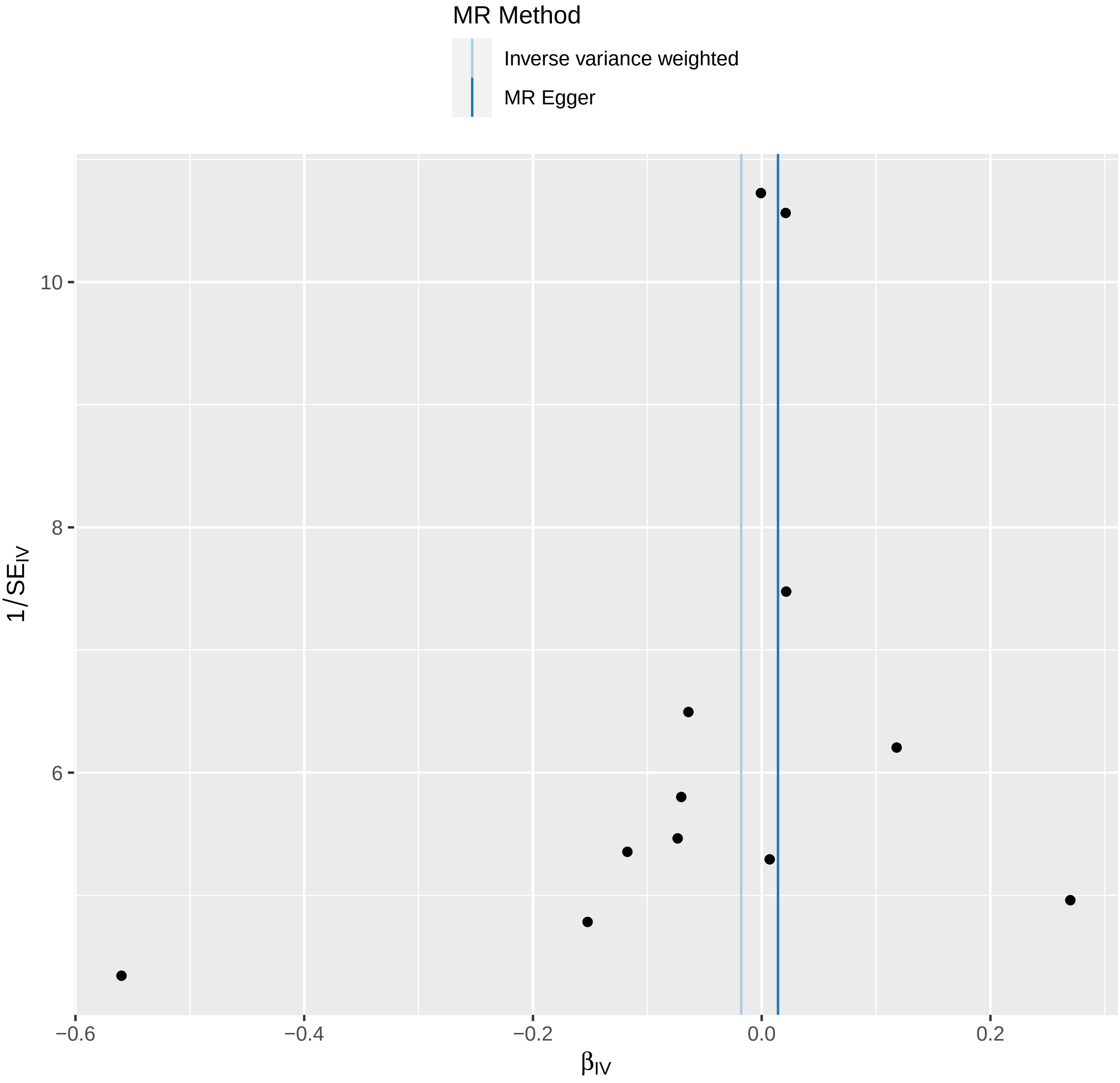

Batch 3−35 : Selenium on A disintegrin and metalloproteinase with thrombospondin motifs 4

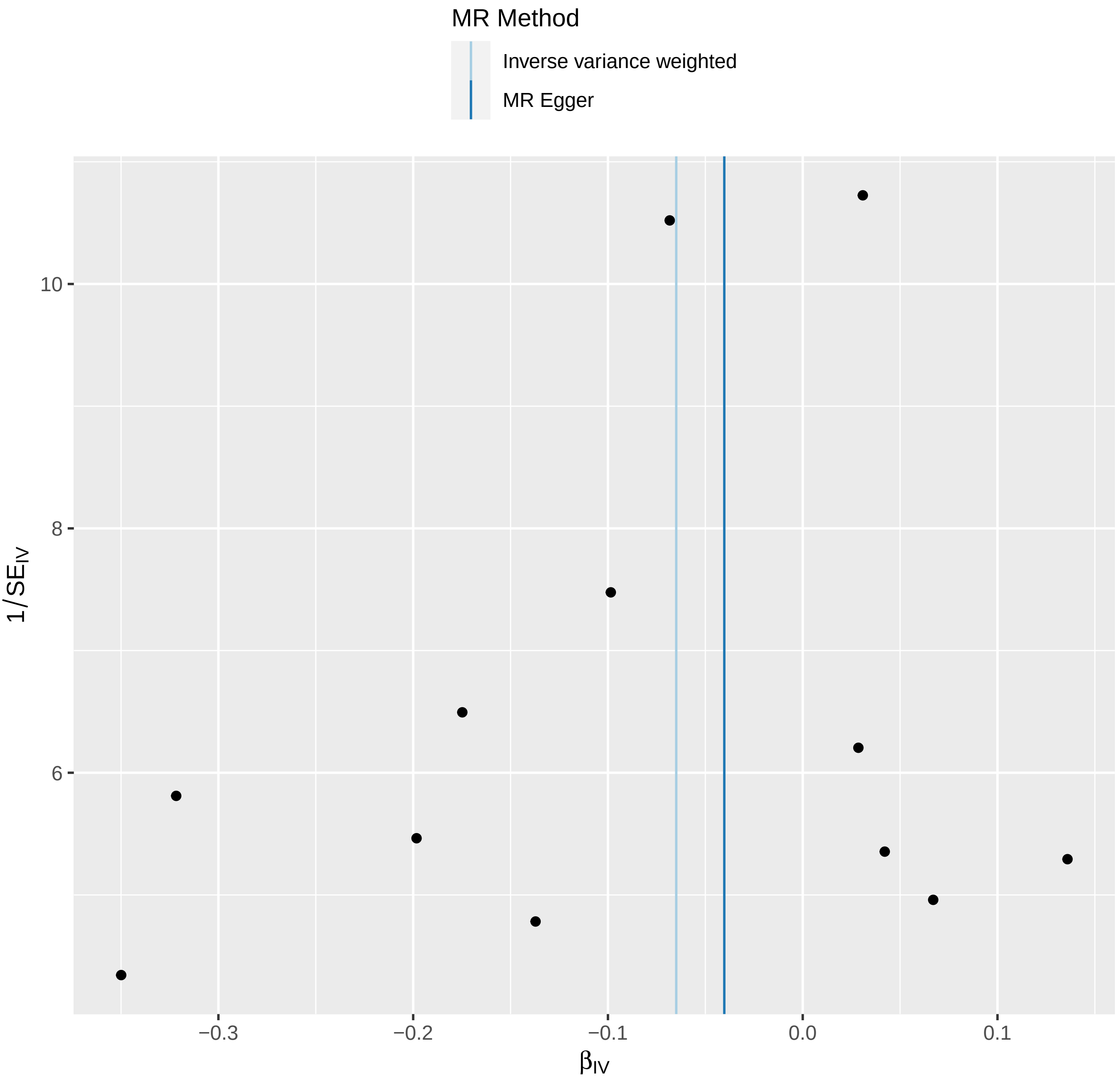

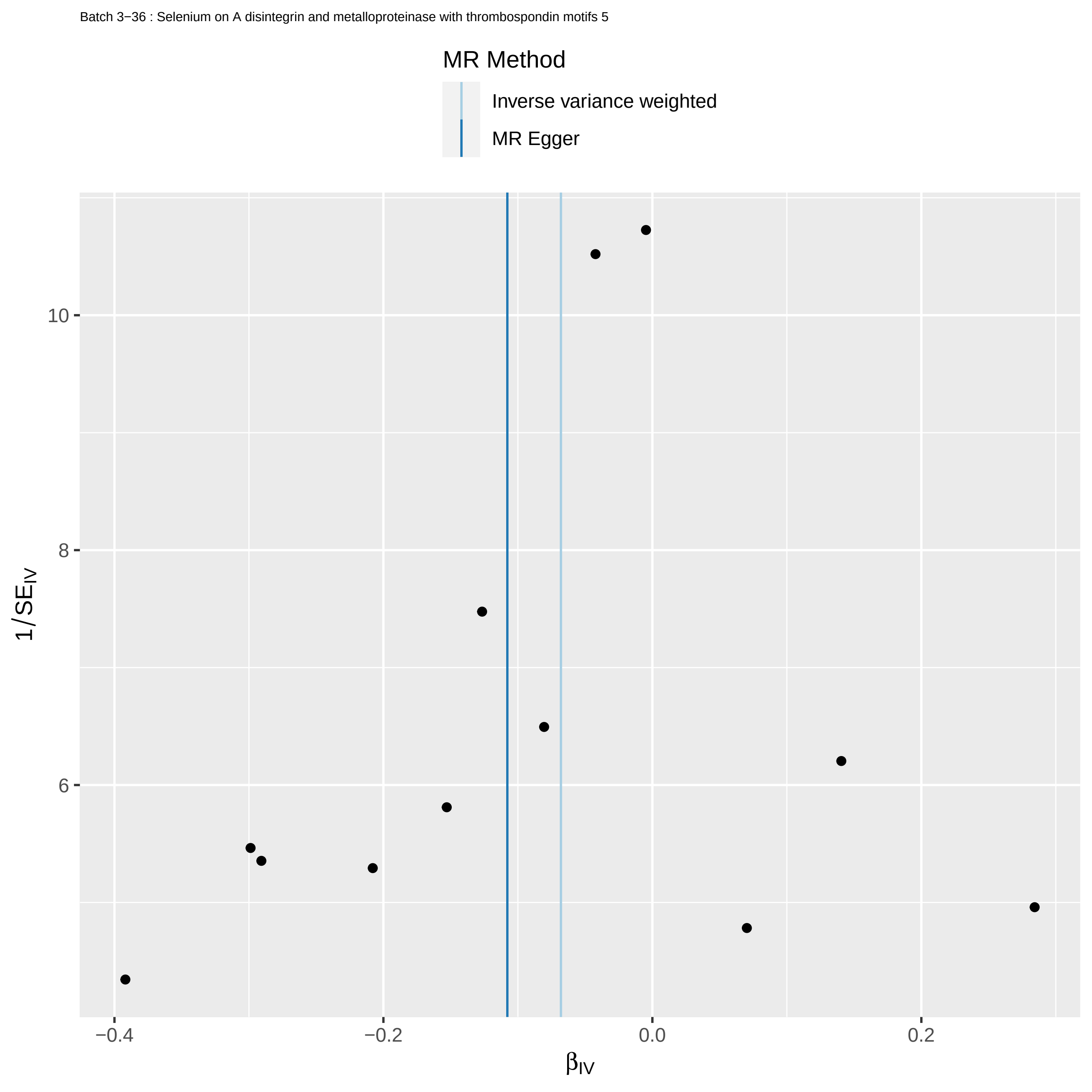
Batch 3−36 : Selenium on A disintegrin and metalloproteinase with thrombospondin motifs 5
MR Method
Inverse variance weighted
MR Egger
10
8
6
1/SE_IV
−0.4
−0.2
0.0
0.2
β_IV

Batch 3−37 : Selenium on A disintegrin and metalloproteinase with thrombospondin motifs 6

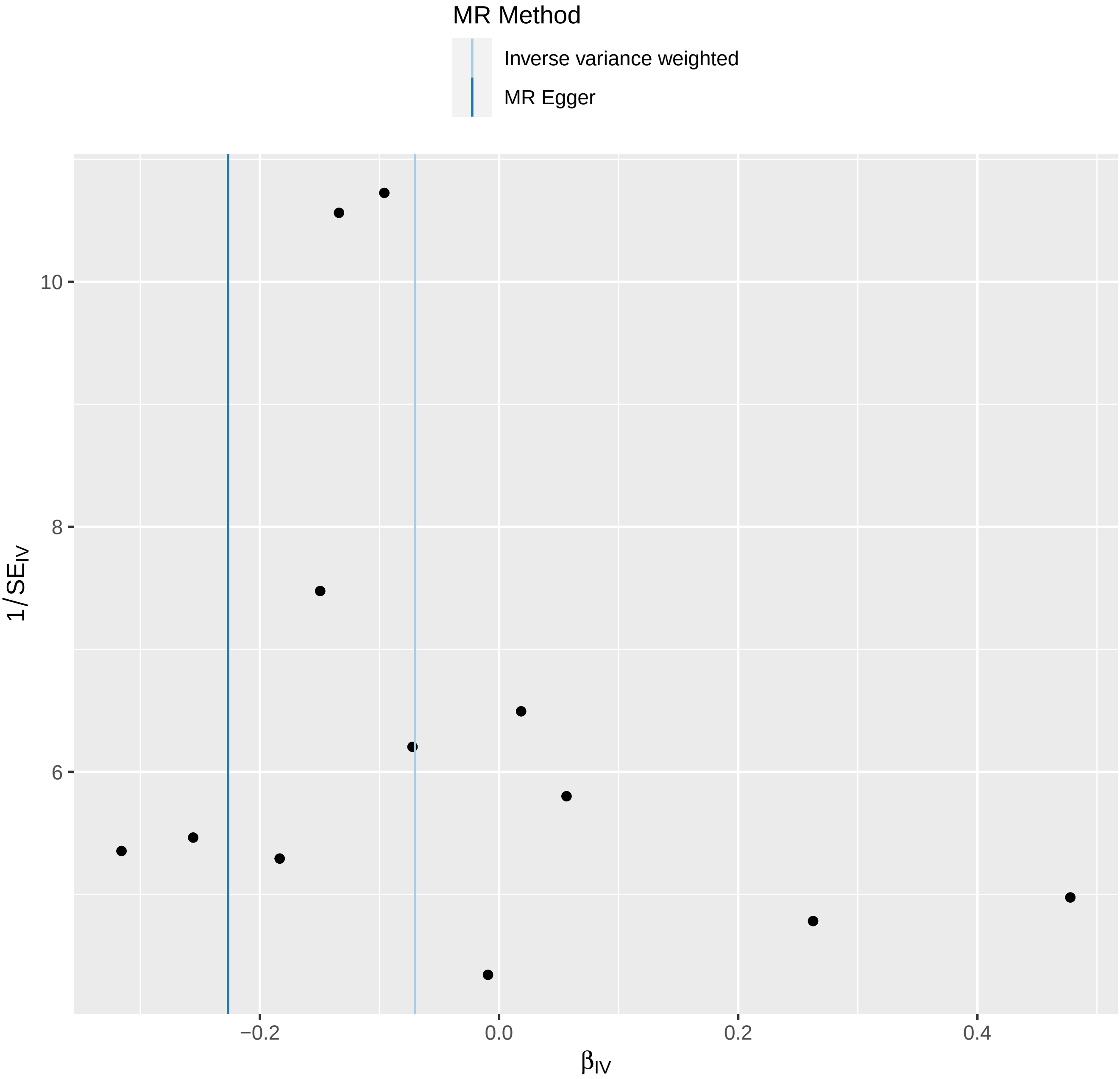

Batch 3−38 : Selenium on ADAMTS−like protein 1

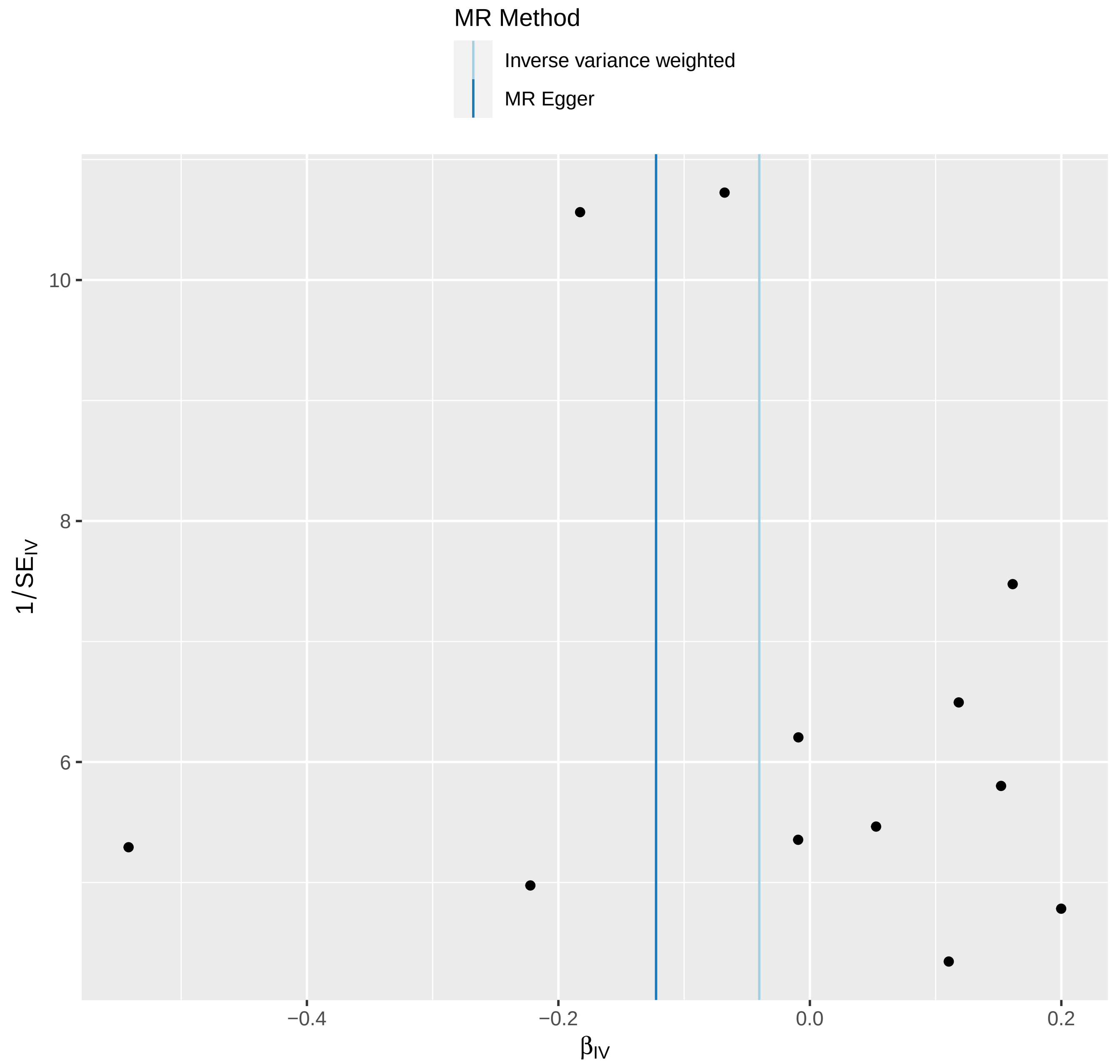

Batch 3−39 : Selenium on AarF domain−containing protein kinase 4

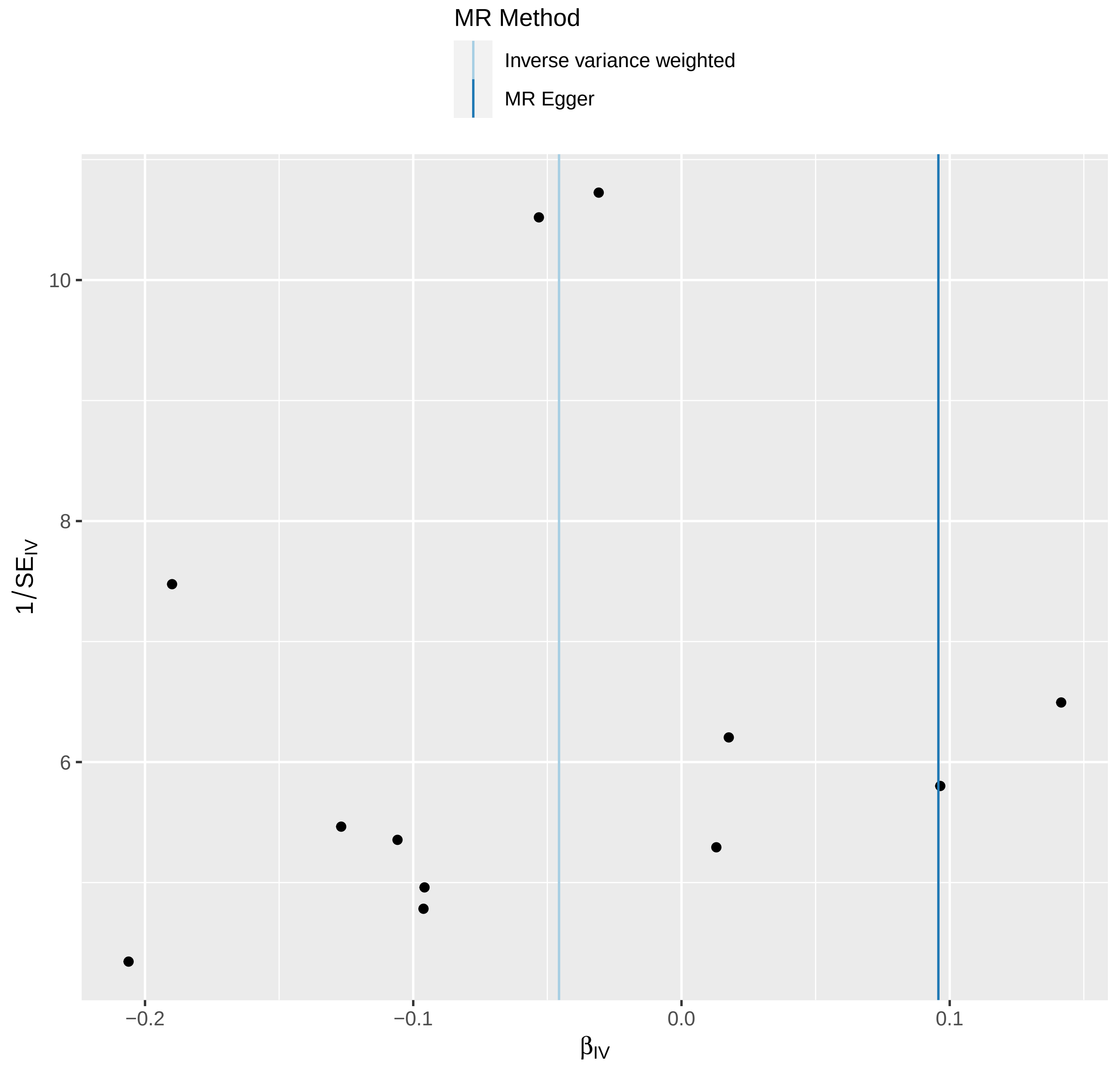

Batch 3−40 : Selenium on Pituitary adenylate cyclase−activating polypeptide 27

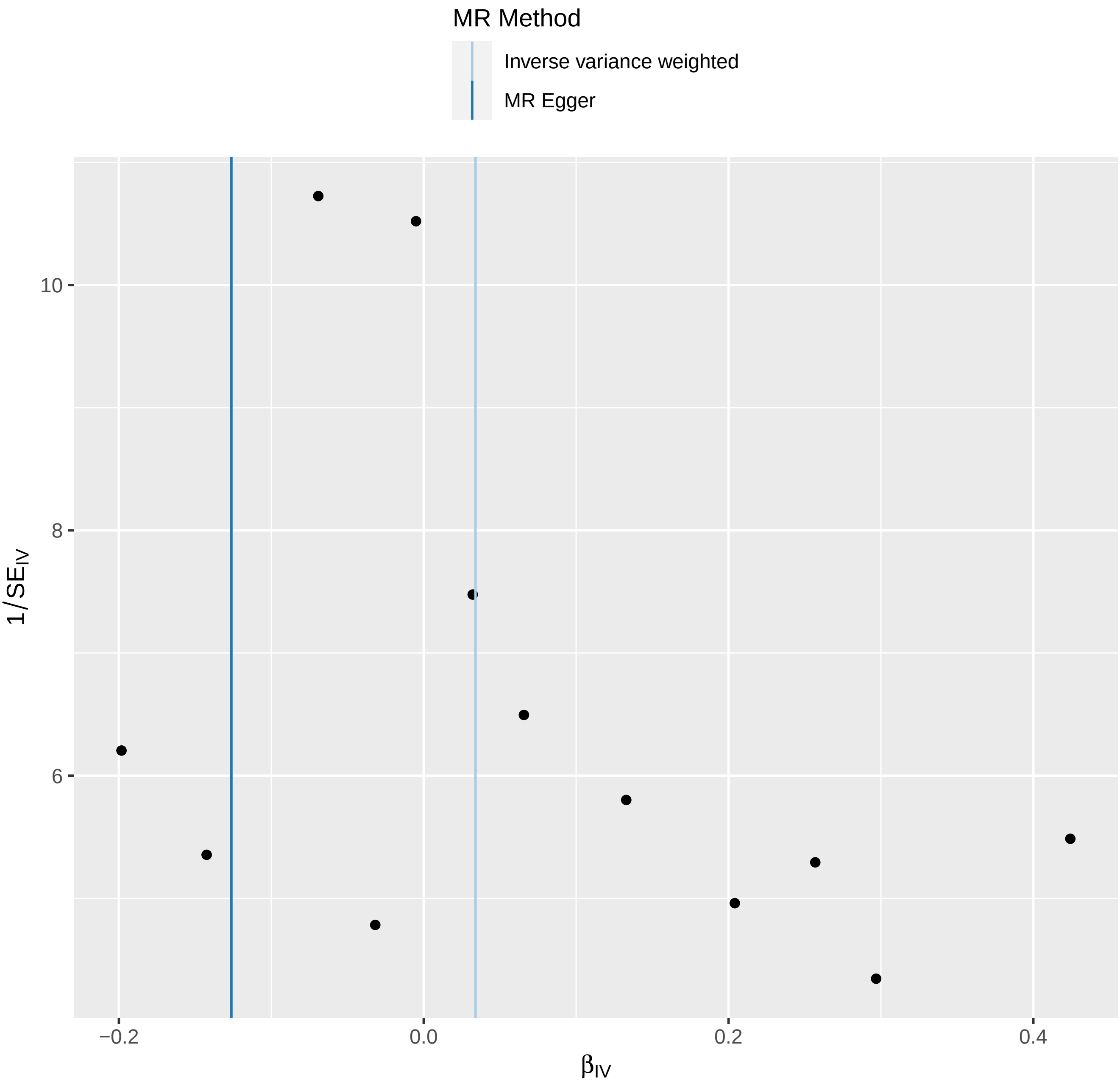

Batch 3−41 : Selenium on Pituitary adenylate cyclase−activating polypeptide 38

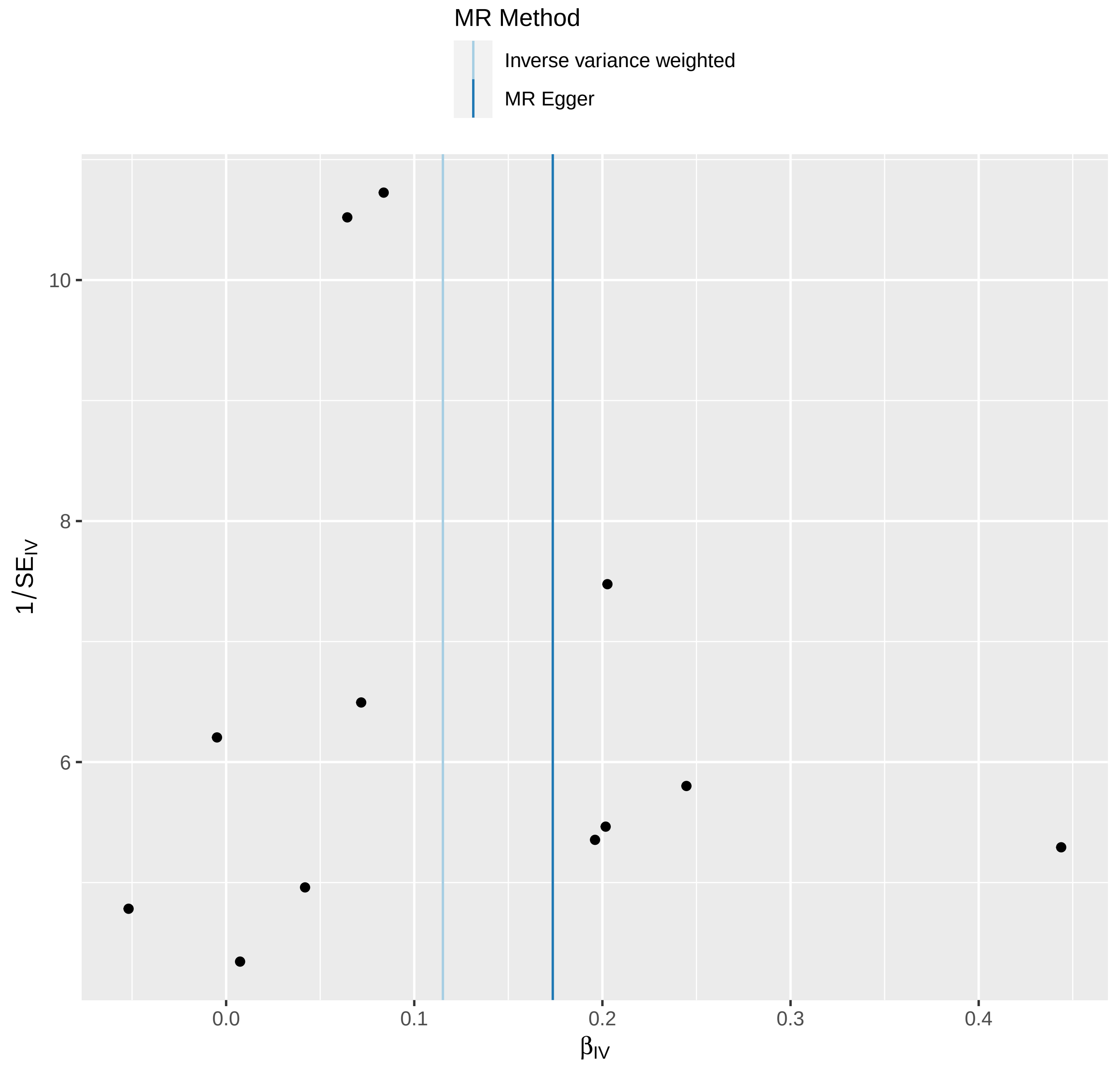

Batch 3−42 : Selenium on Pituitary adenylate cyclase−activating polypeptide

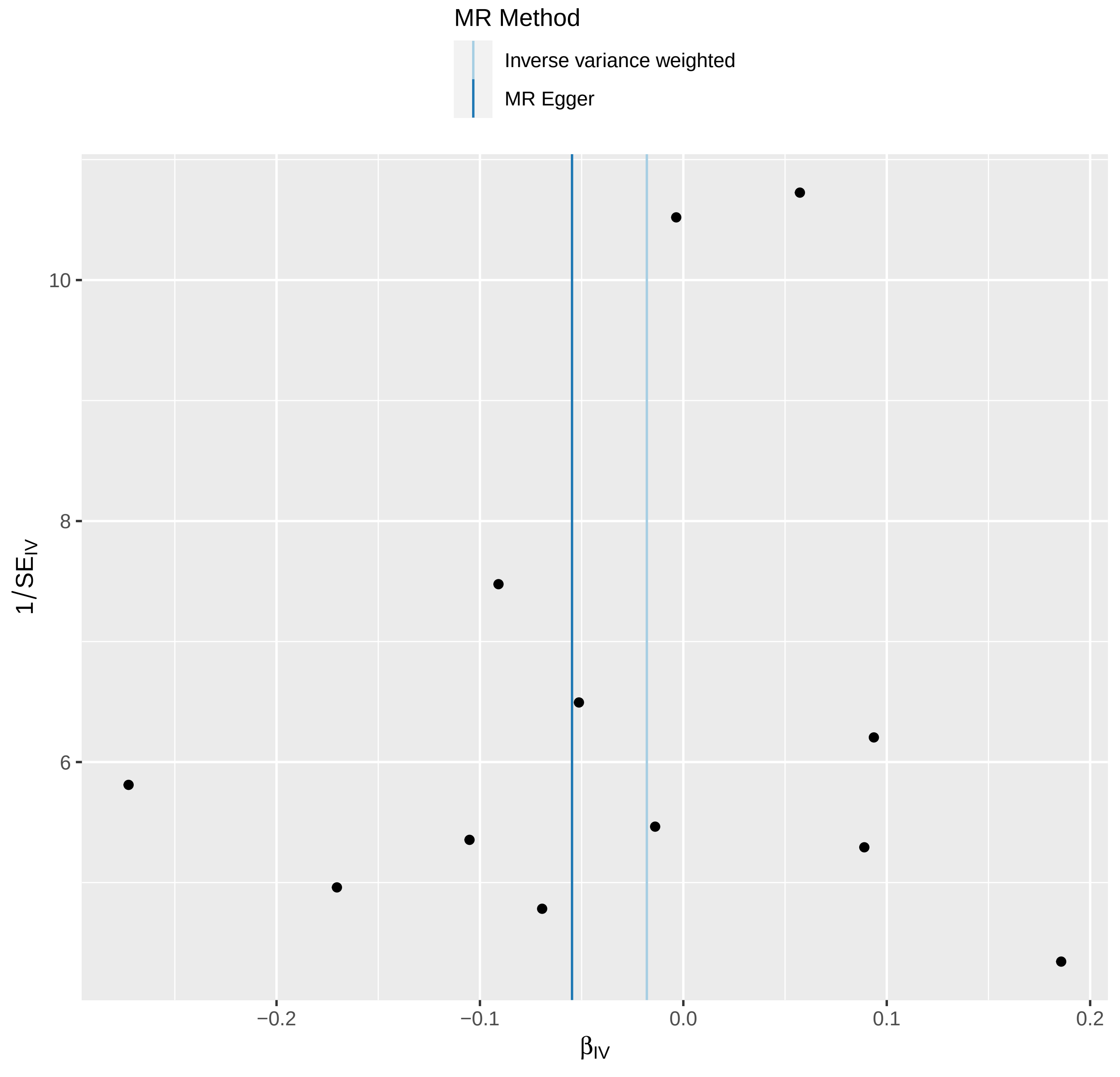

Batch 3−43 : Selenium on Adhesion G−protein coupled receptor F1

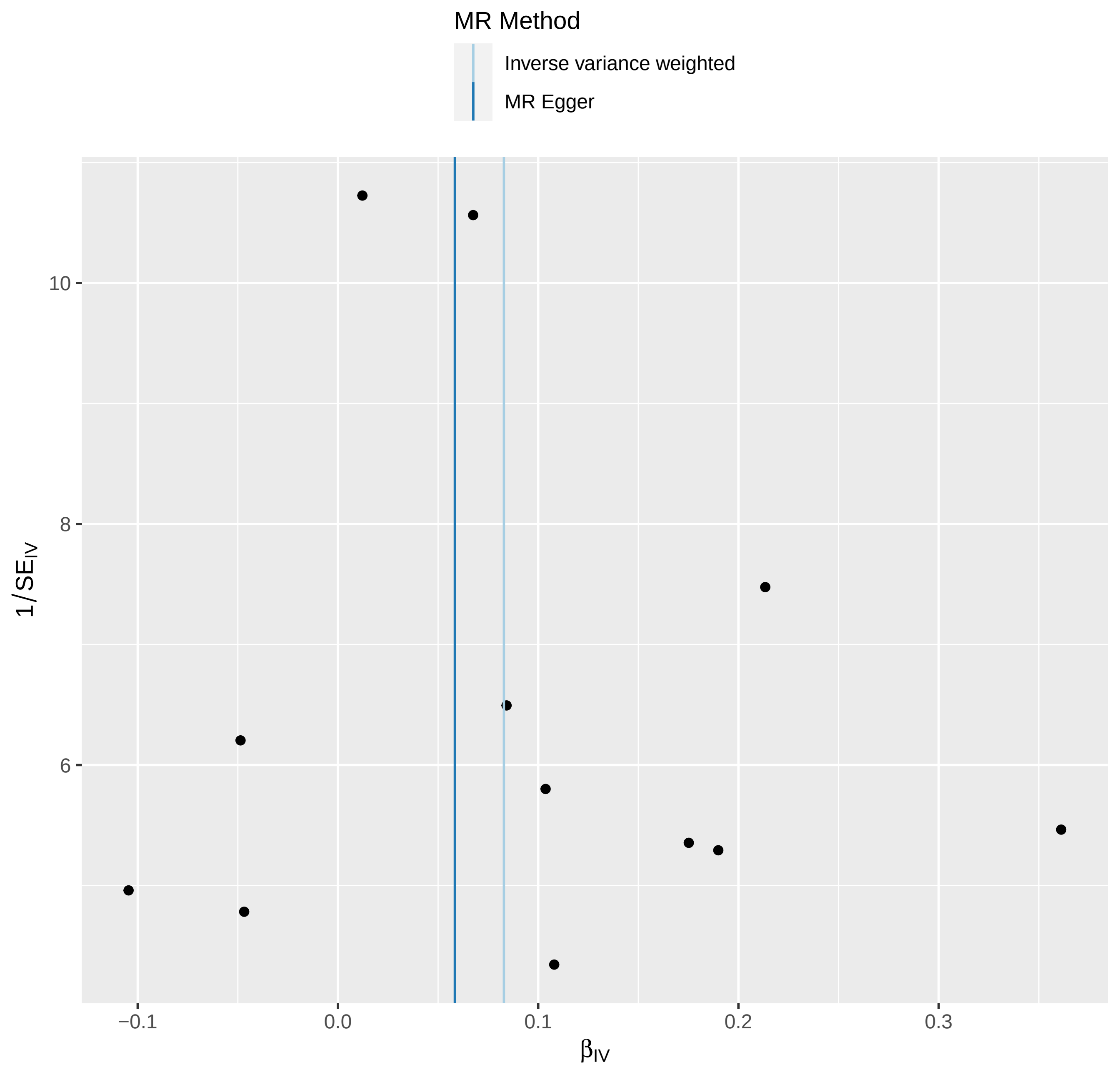

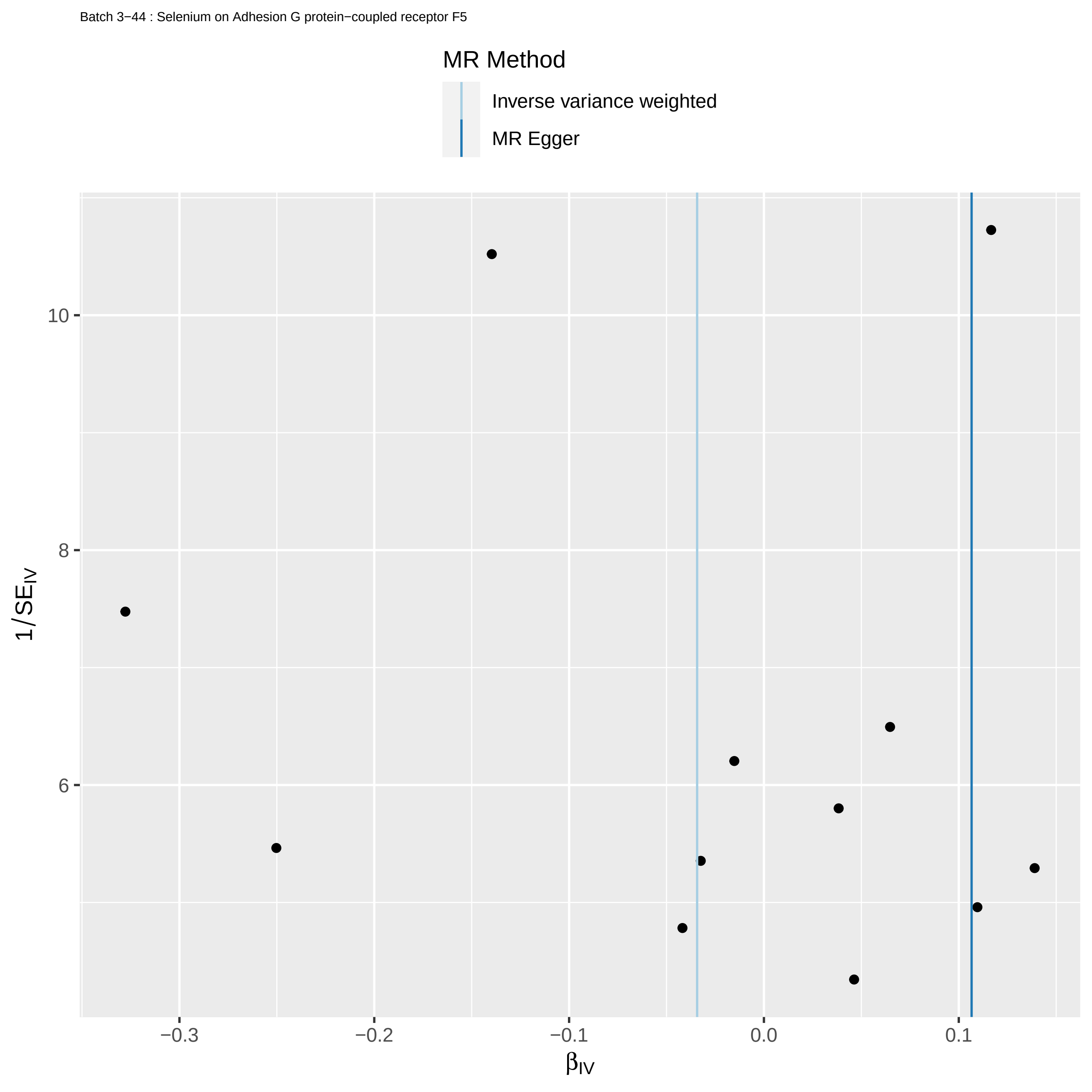
Batch 3−44 : Selenium on Adhesion G protein−coupled receptor F5
MR Method
Inverse variance weighted
MR Egger
10
8
6
1/SE_IV
−0.3
−0.2
−0.1
0.0
0.1
β_IV

Batch 3−45 : Selenium on Adhesion G−protein coupled receptor G5

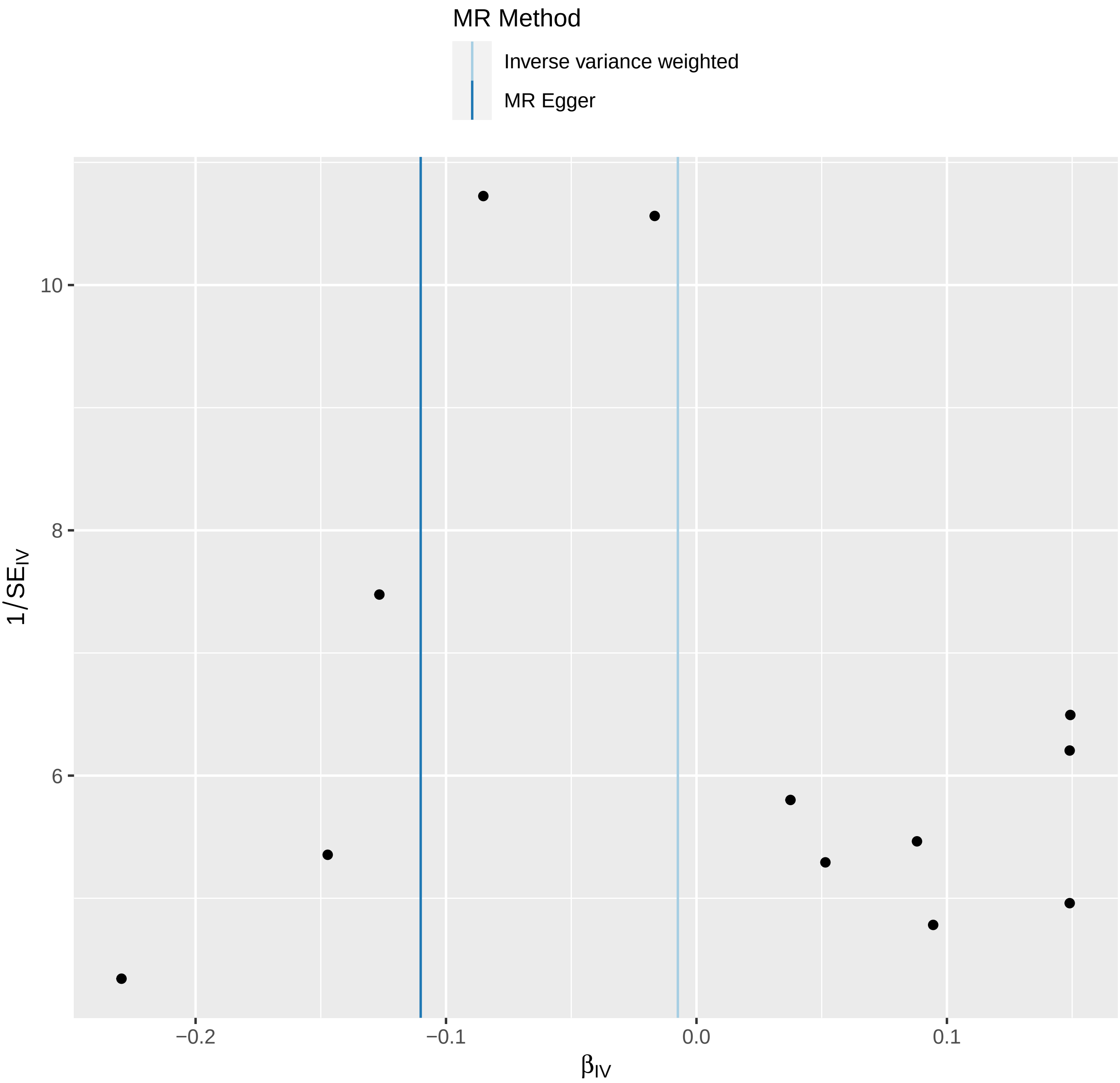

Batch 3−46 : Selenium on Alcohol dehydrogenase 1B

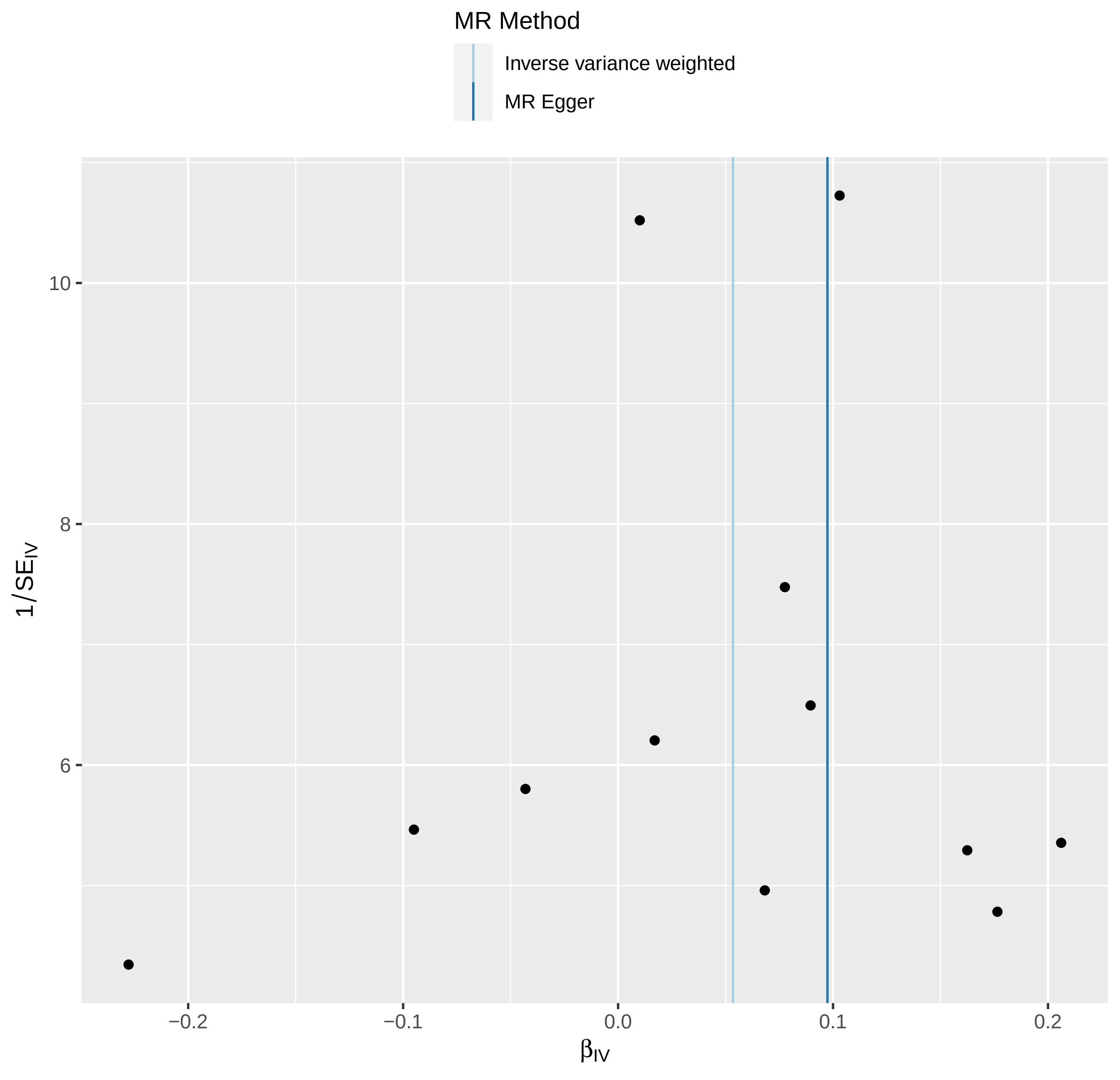

Batch 3−47 : Selenium on Alcohol dehydrogenase 4

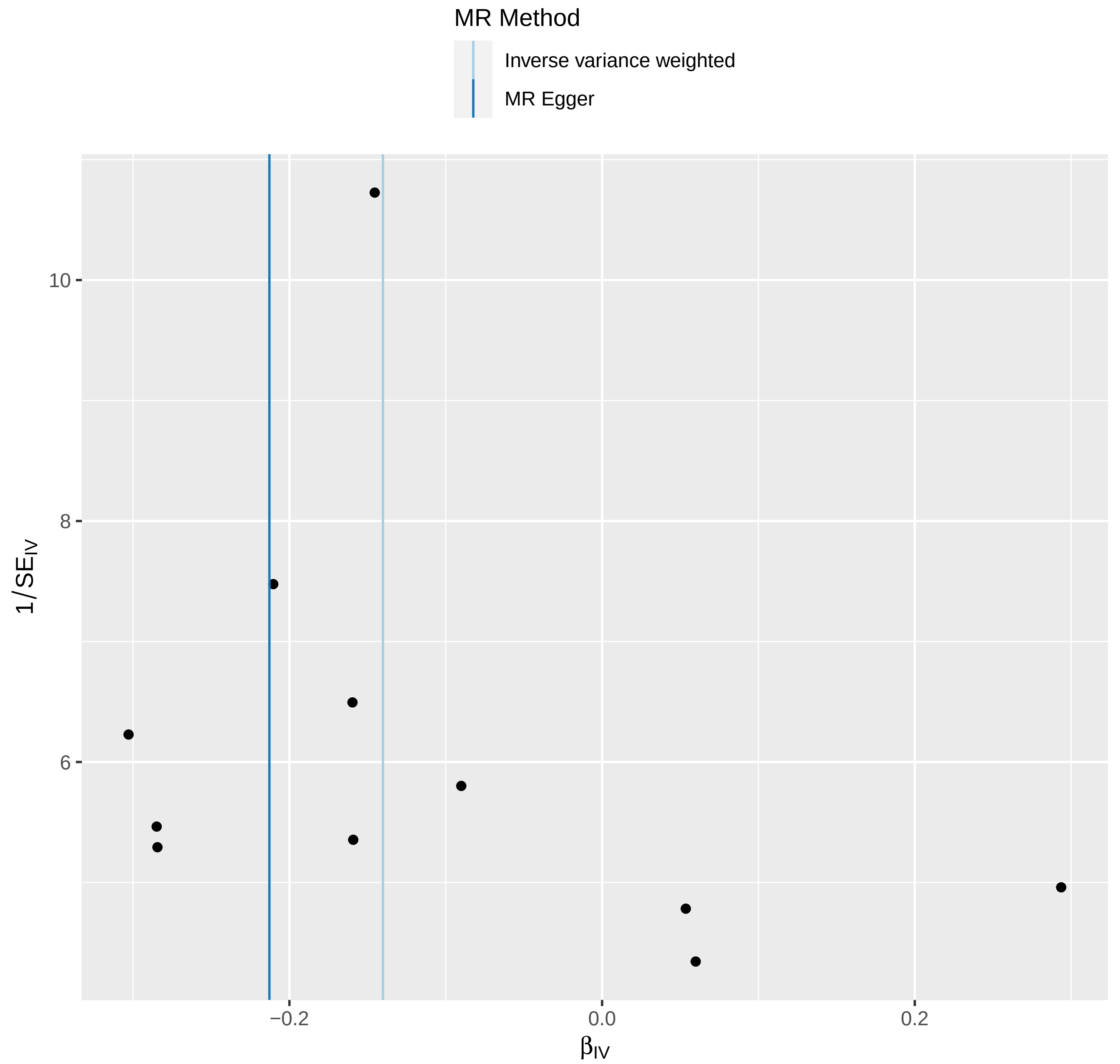

Batch 3−48 : Selenium on Adrenomedullin

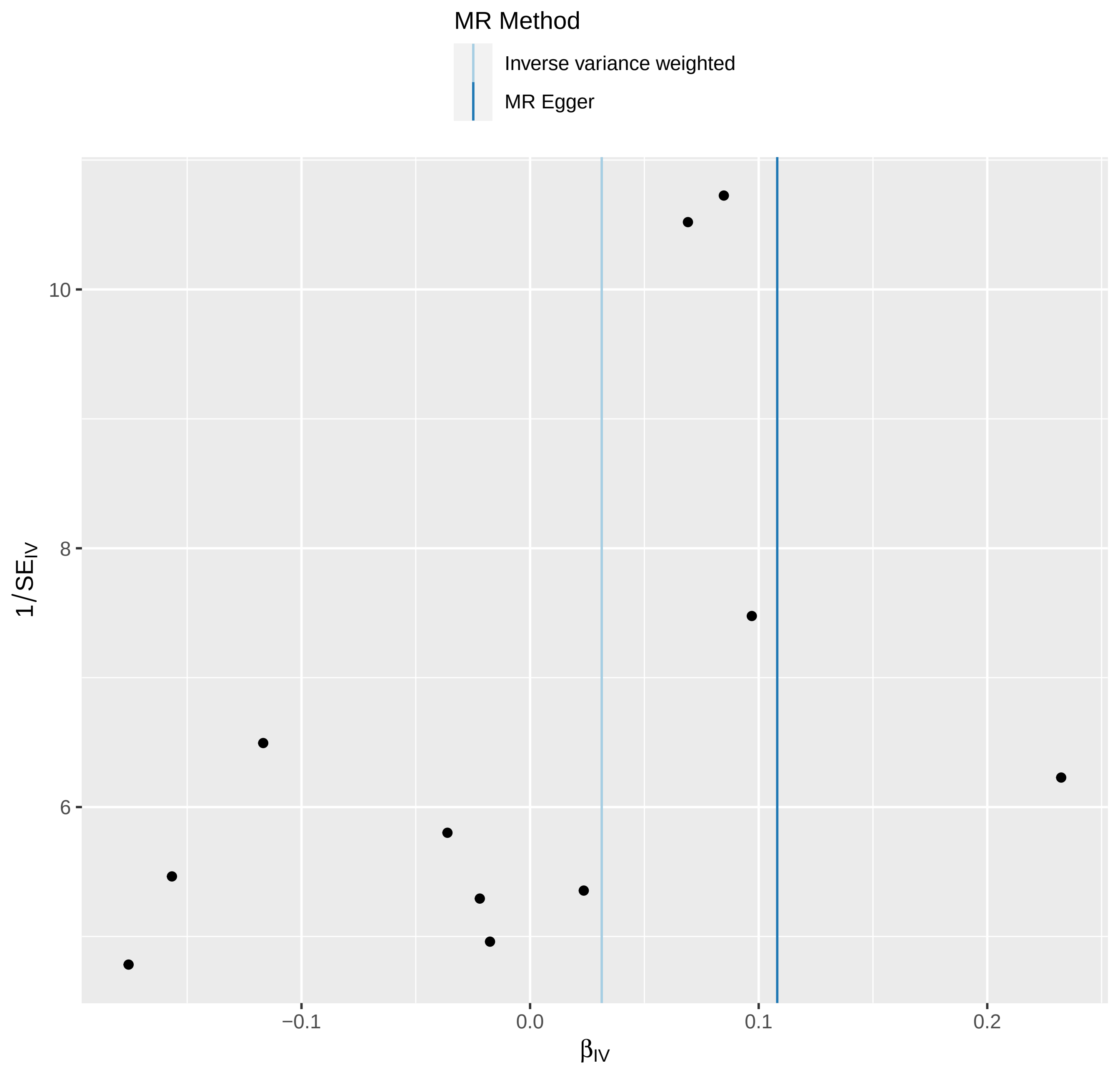

Batch 3−49 : Selenium on ADP−dependent glucokinase

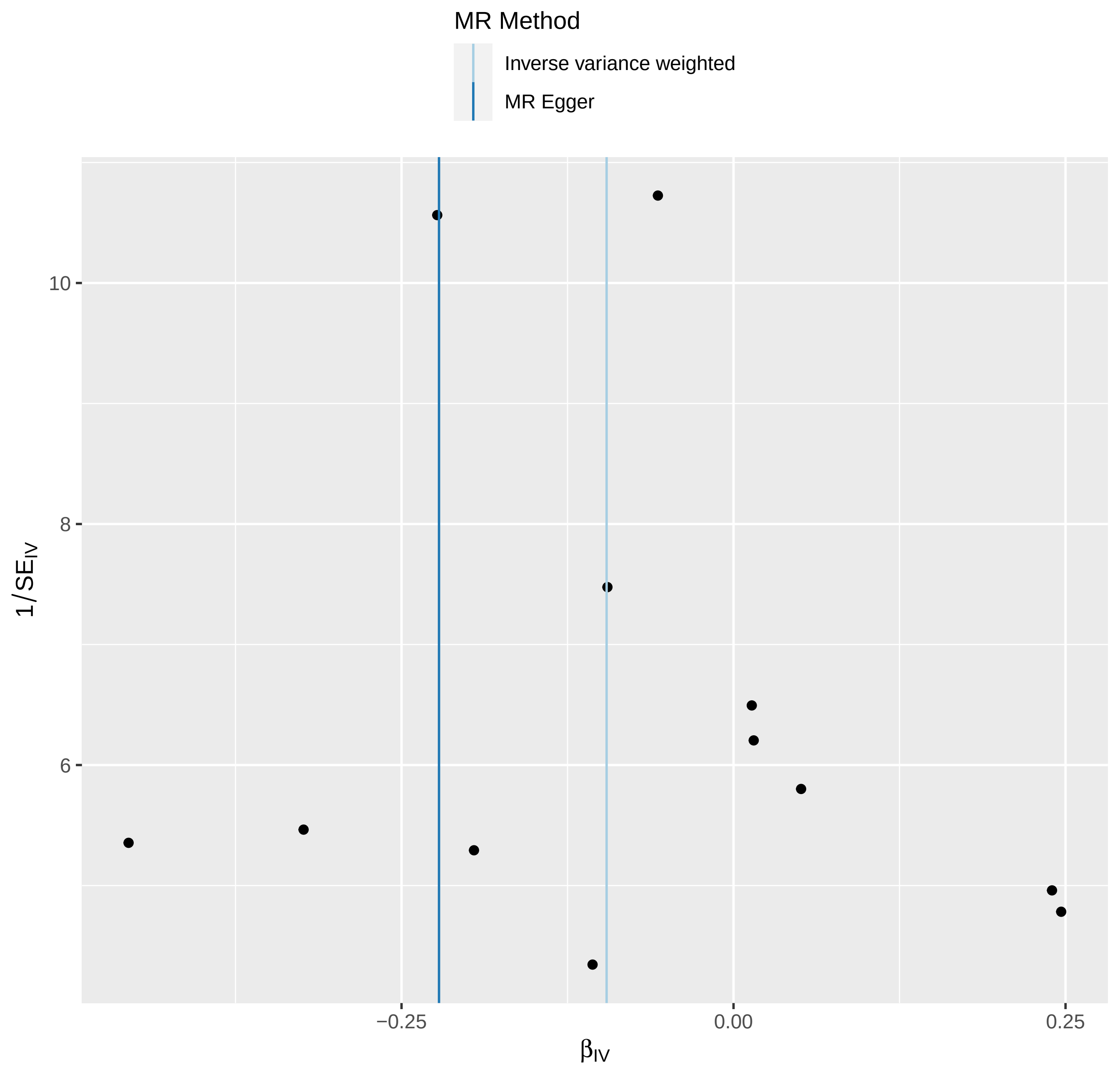

Batch 3−50 : Selenium on beta−adrenergic receptor kinase 1

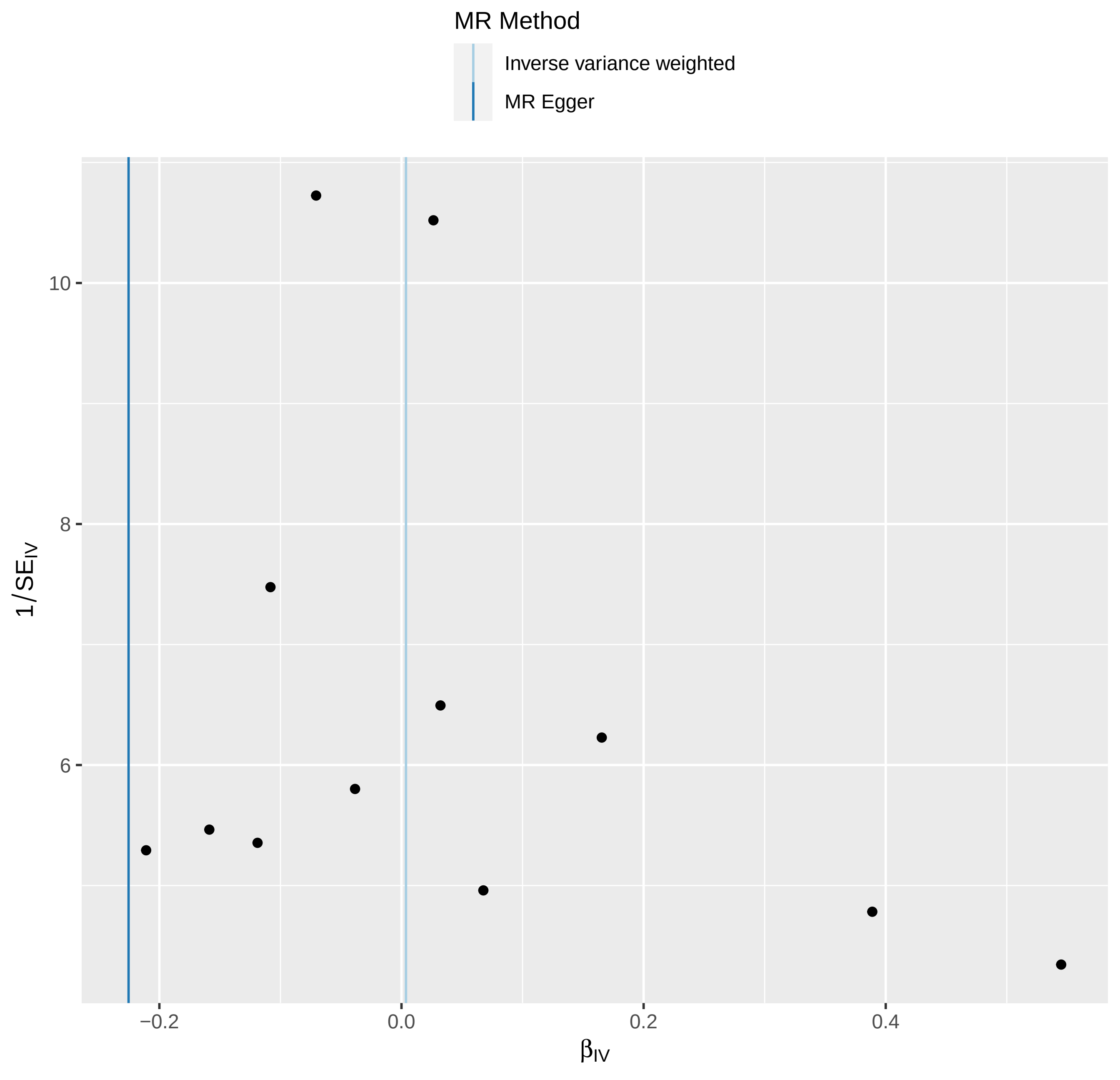

Batch 3−51 : Selenium on Adenylosuccinate synthetase isozyme 2

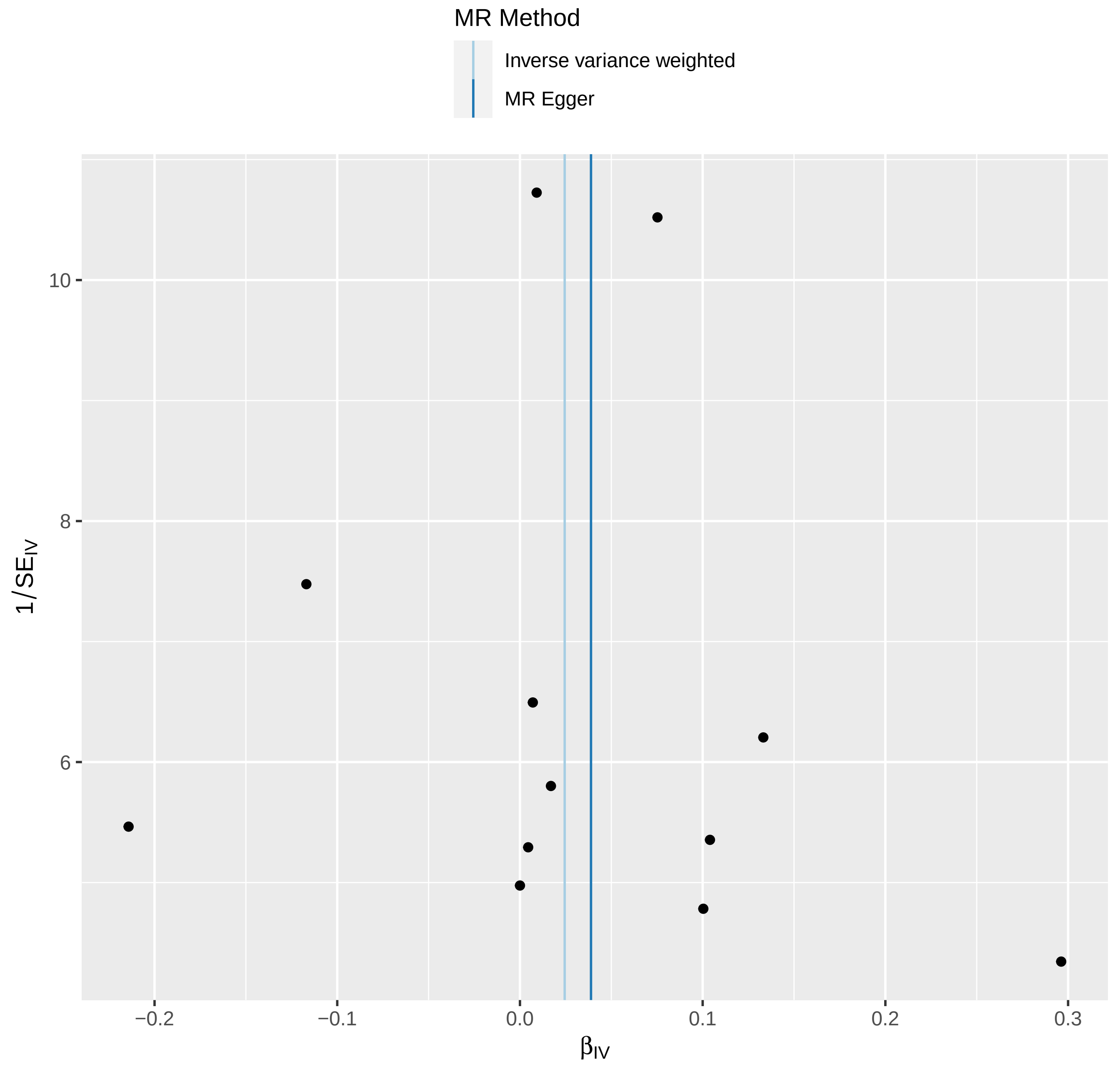

Batch 3−52 : Selenium on Adenylosuccinate synthetase isozyme 1

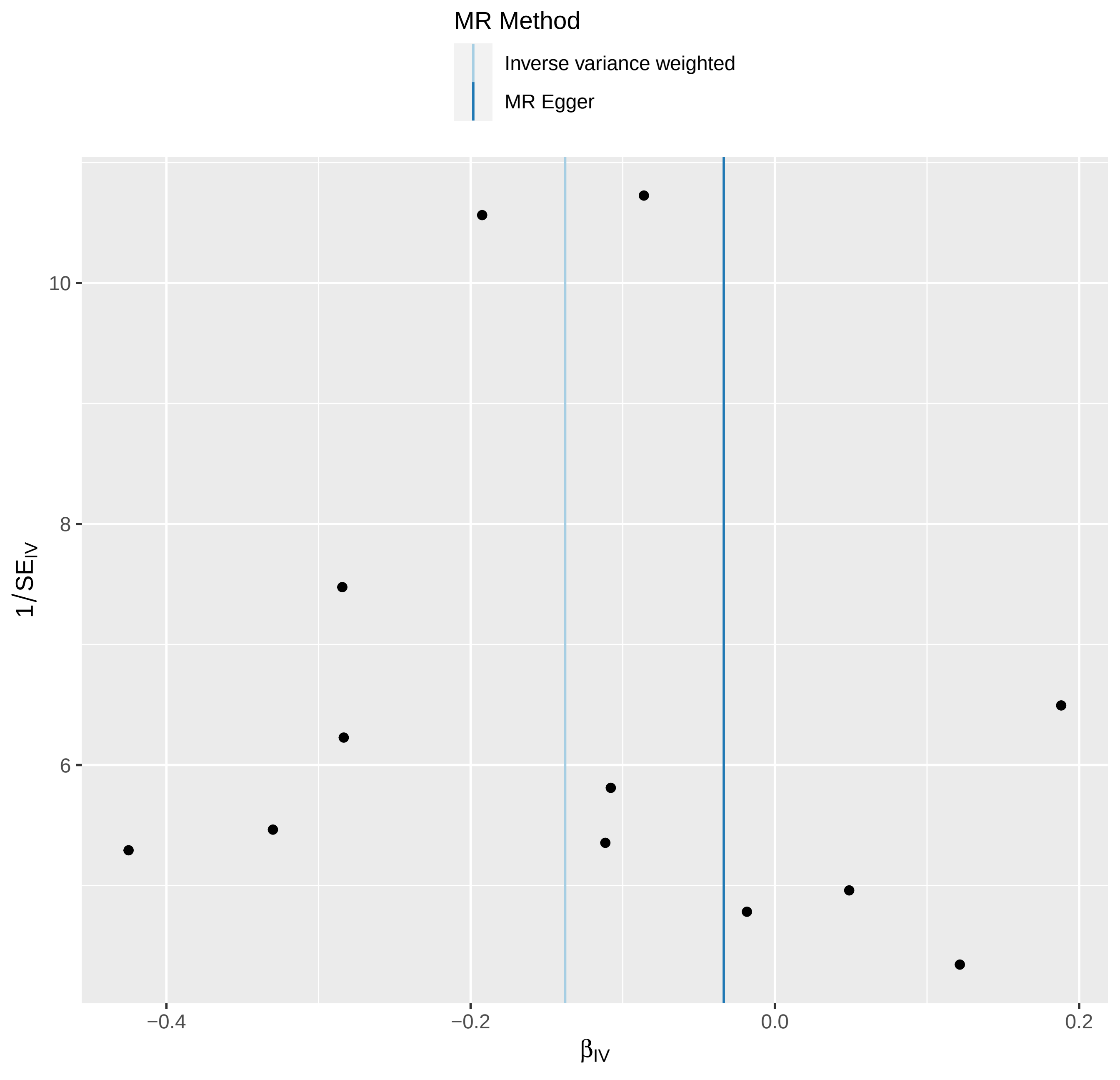

Batch 3−53 : Selenium on alpha−Fetoprotein

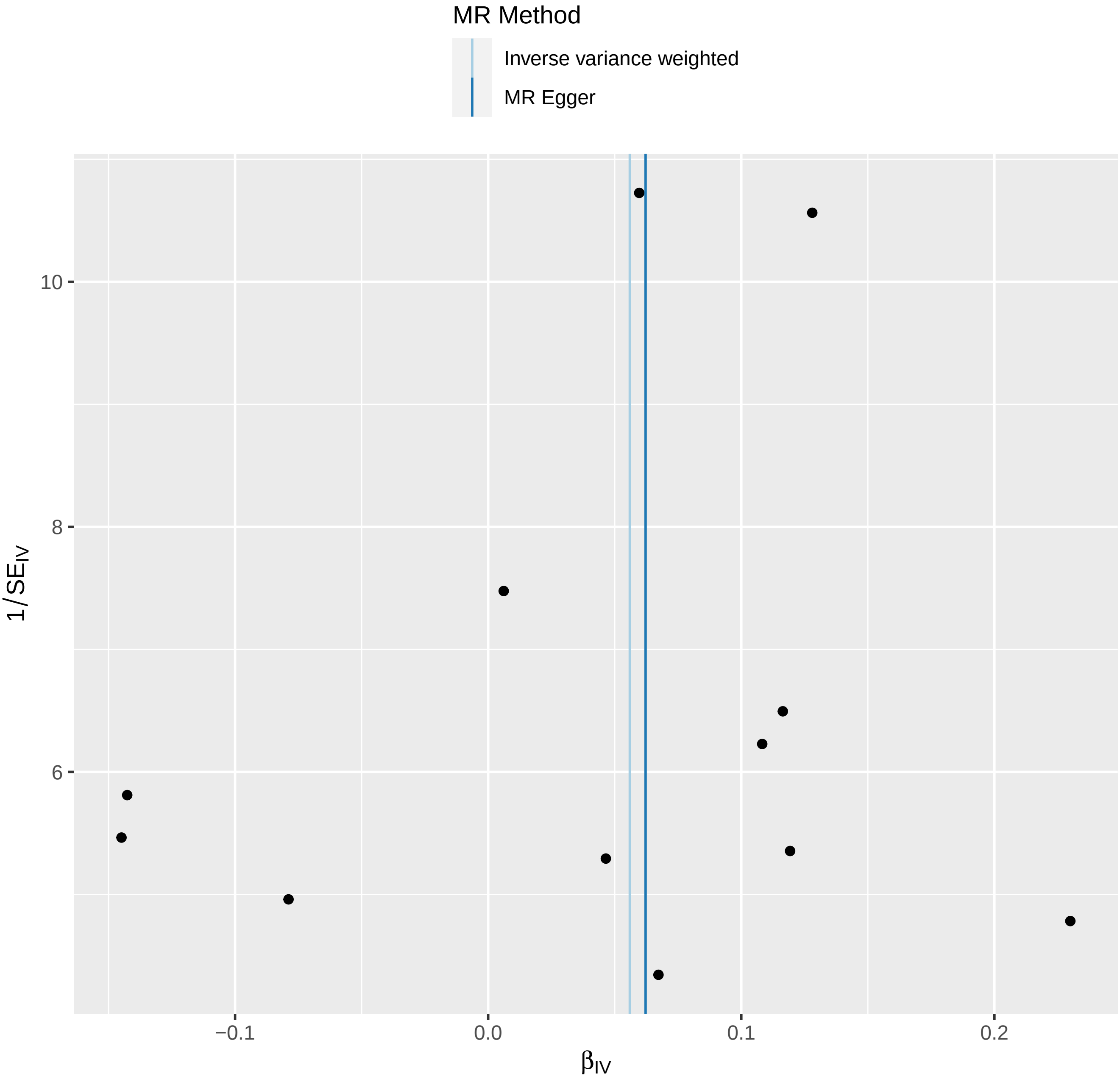

Batch 3−54 : Selenium on Advanced glycosylation end product−specific receptor, soluble

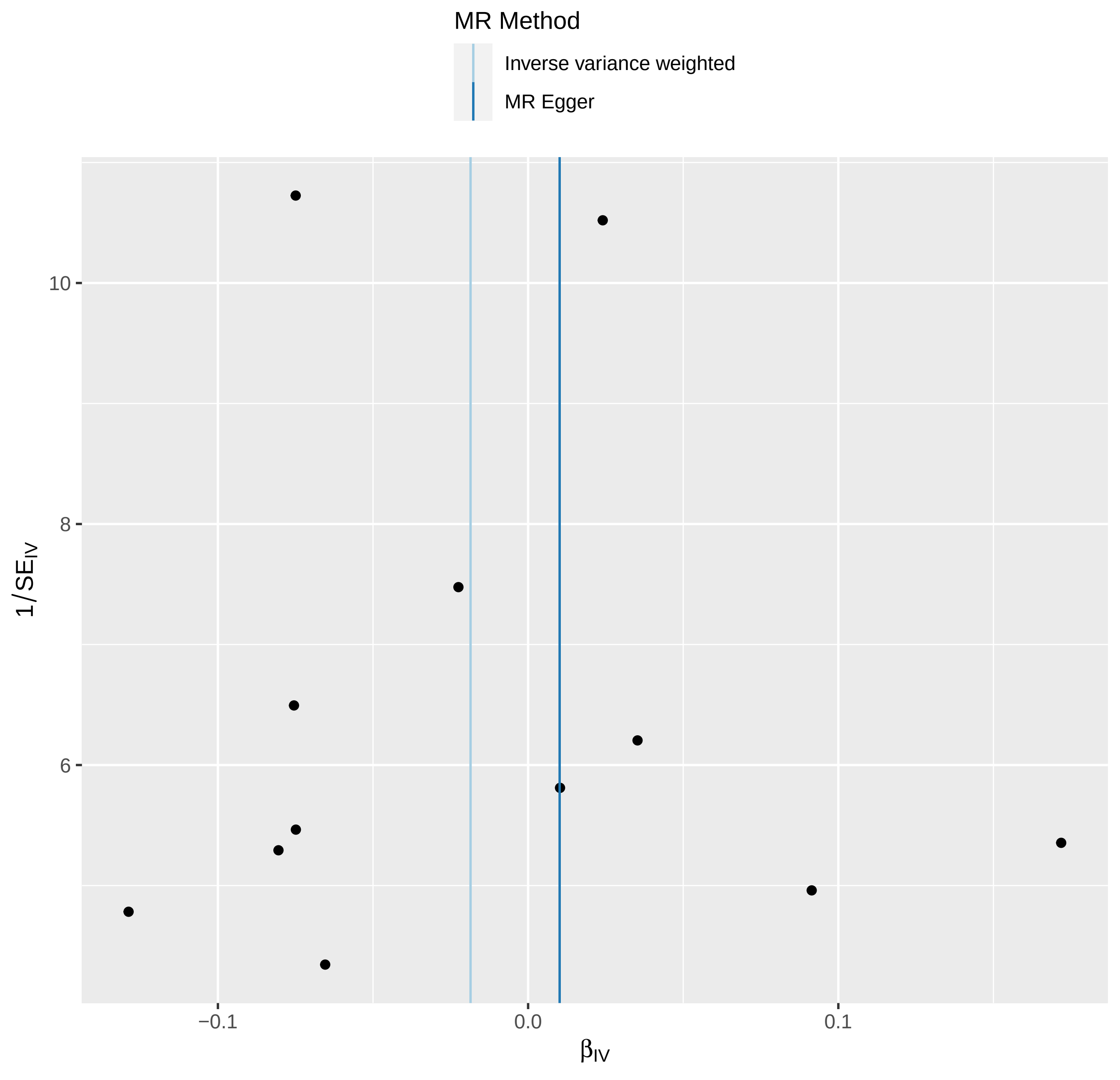

Batch 3−55 : Selenium on Arf−GAP domain and FG repeat−containing protein 1

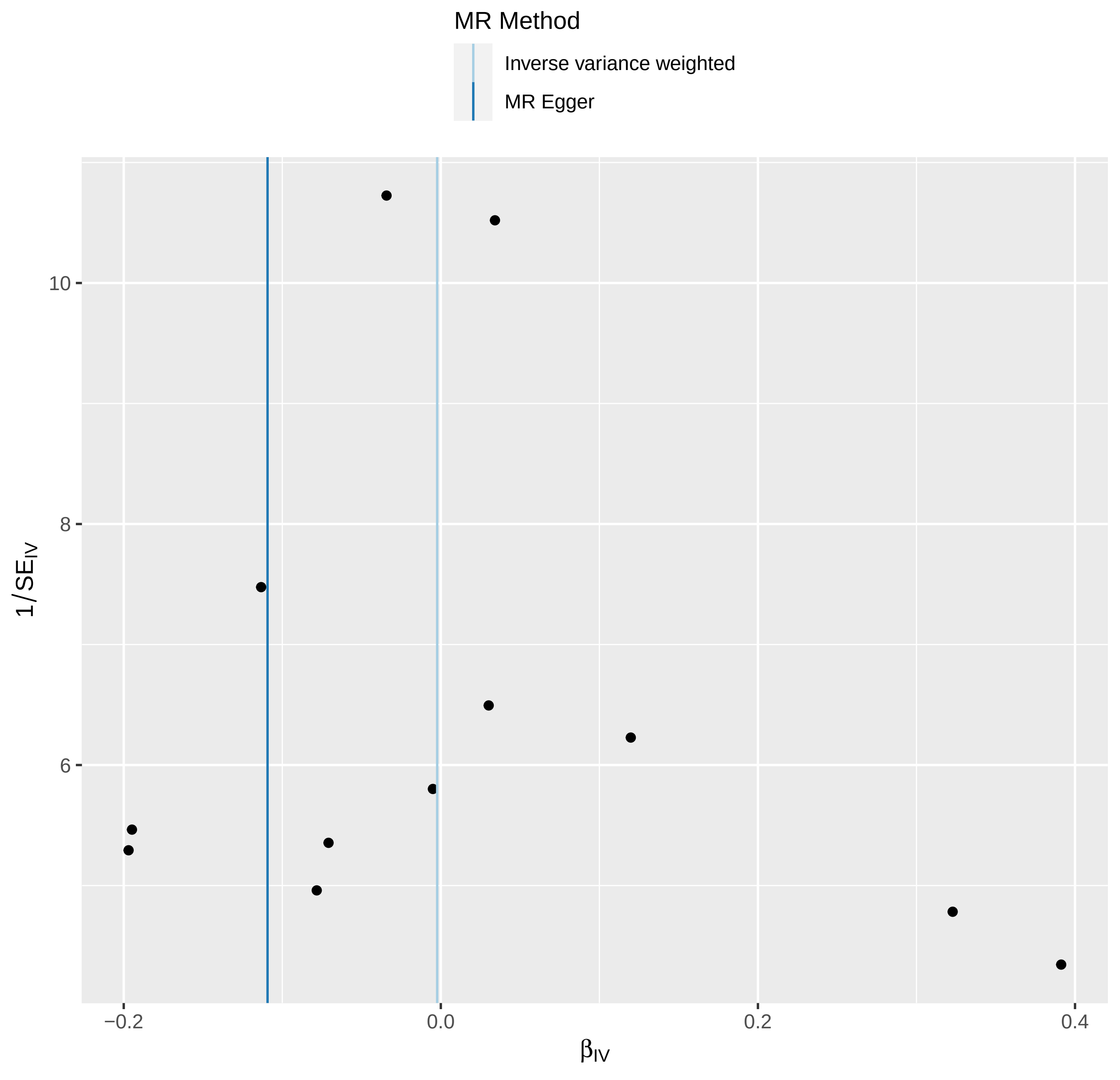

Batch 3−56 : Selenium on Angiogenic factor with G patch and FHA domains 1

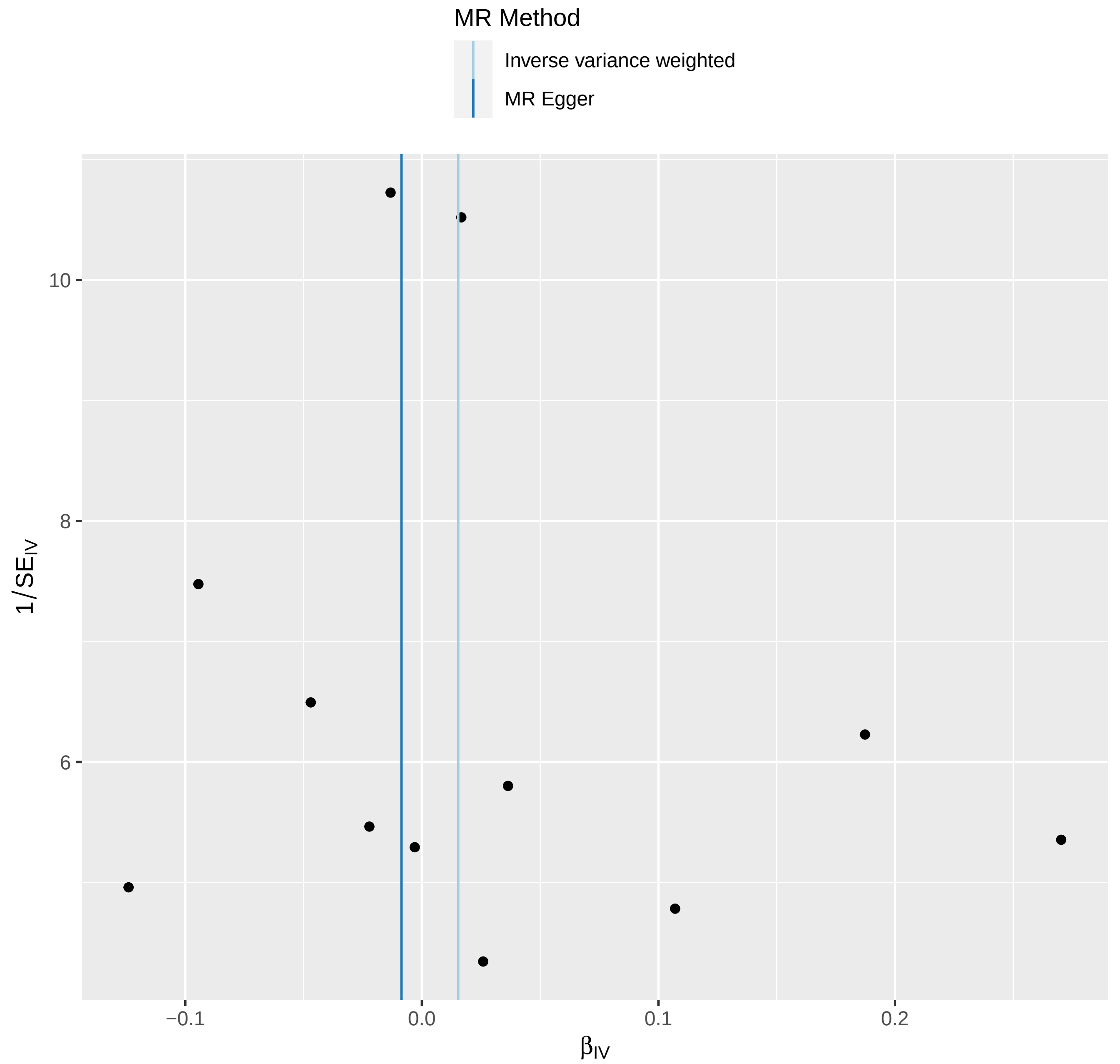

Batch 3−57 : Selenium on Protein argonaute−1

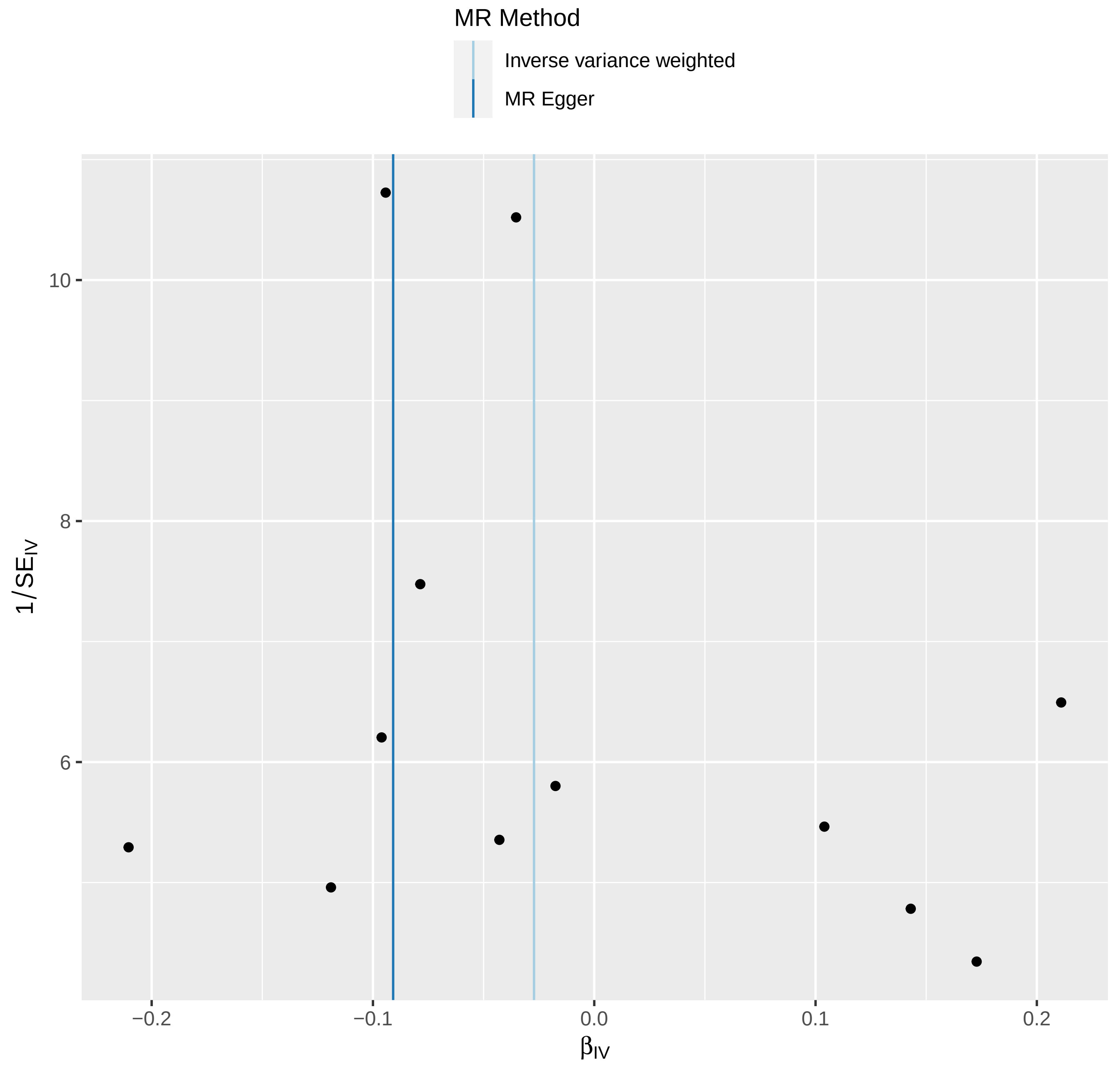

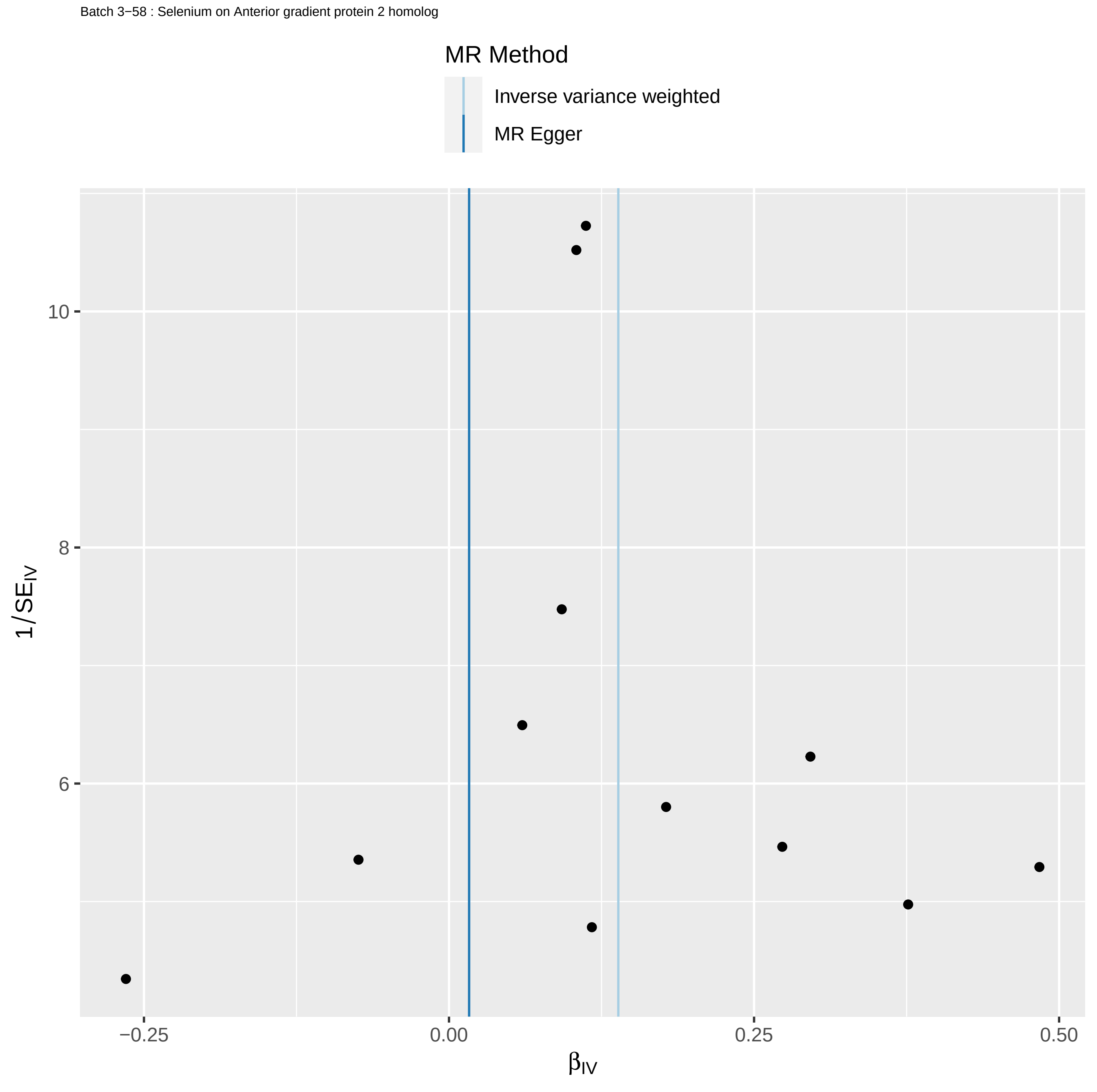
Batch 3−58 : Selenium on Anterior gradient protein 2 homolog
MR Method
Inverse variance weighted
MR Egger
10
8
6
1/SE$_{IV}$
−0.25
0.00
0.25
0.50
β$_{IV}$

Batch 3−59 : Selenium on Agouti−related protein

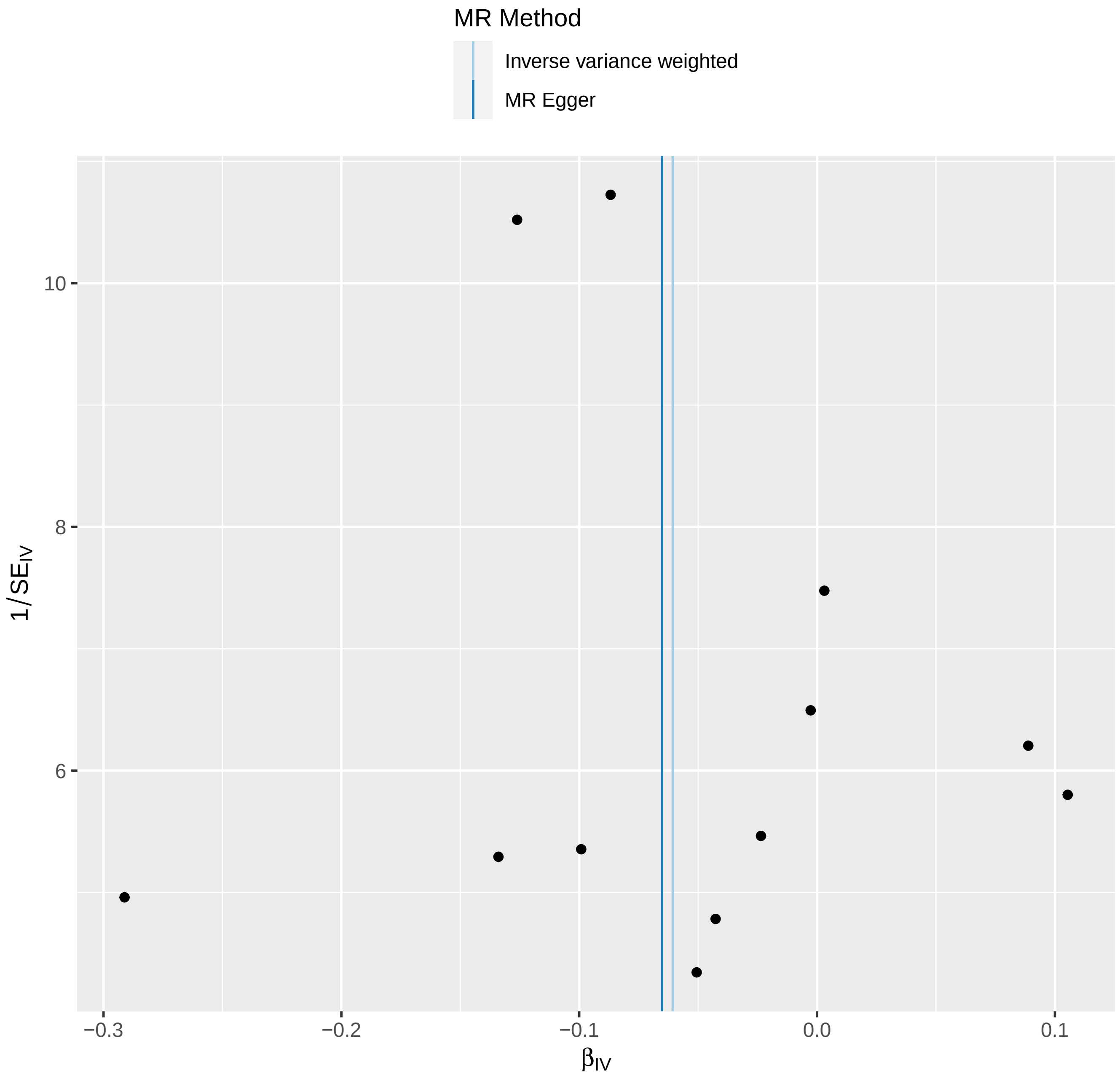

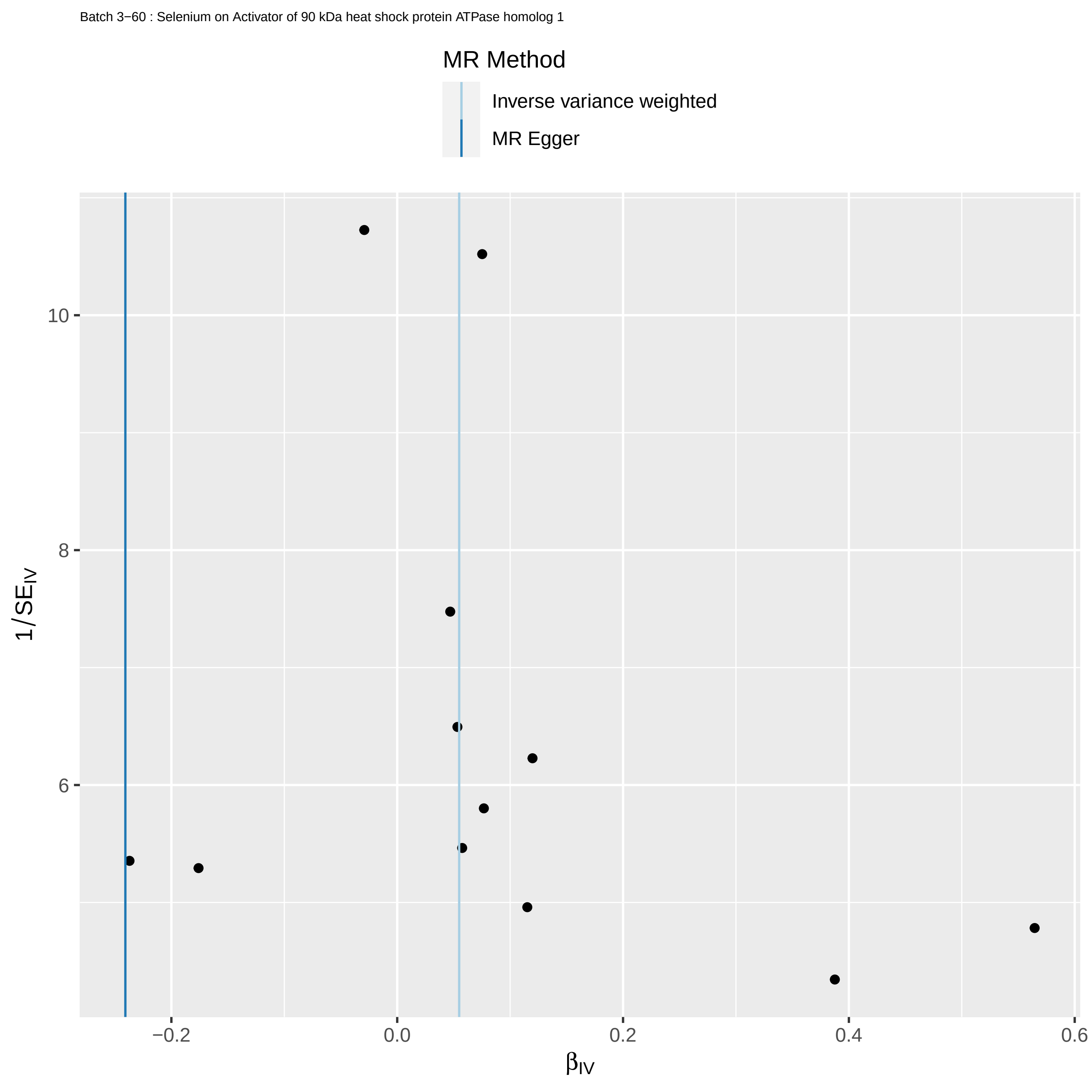

Batch 3−60 : Selenium on Activator of 90 kDa heat shock protein ATPase homolog 1
MR Method
Inverse variance weighted
MR Egger
10
8
6
1/SE_IV
−0.2
0.0
0.2
0.4
0.6
β_IV

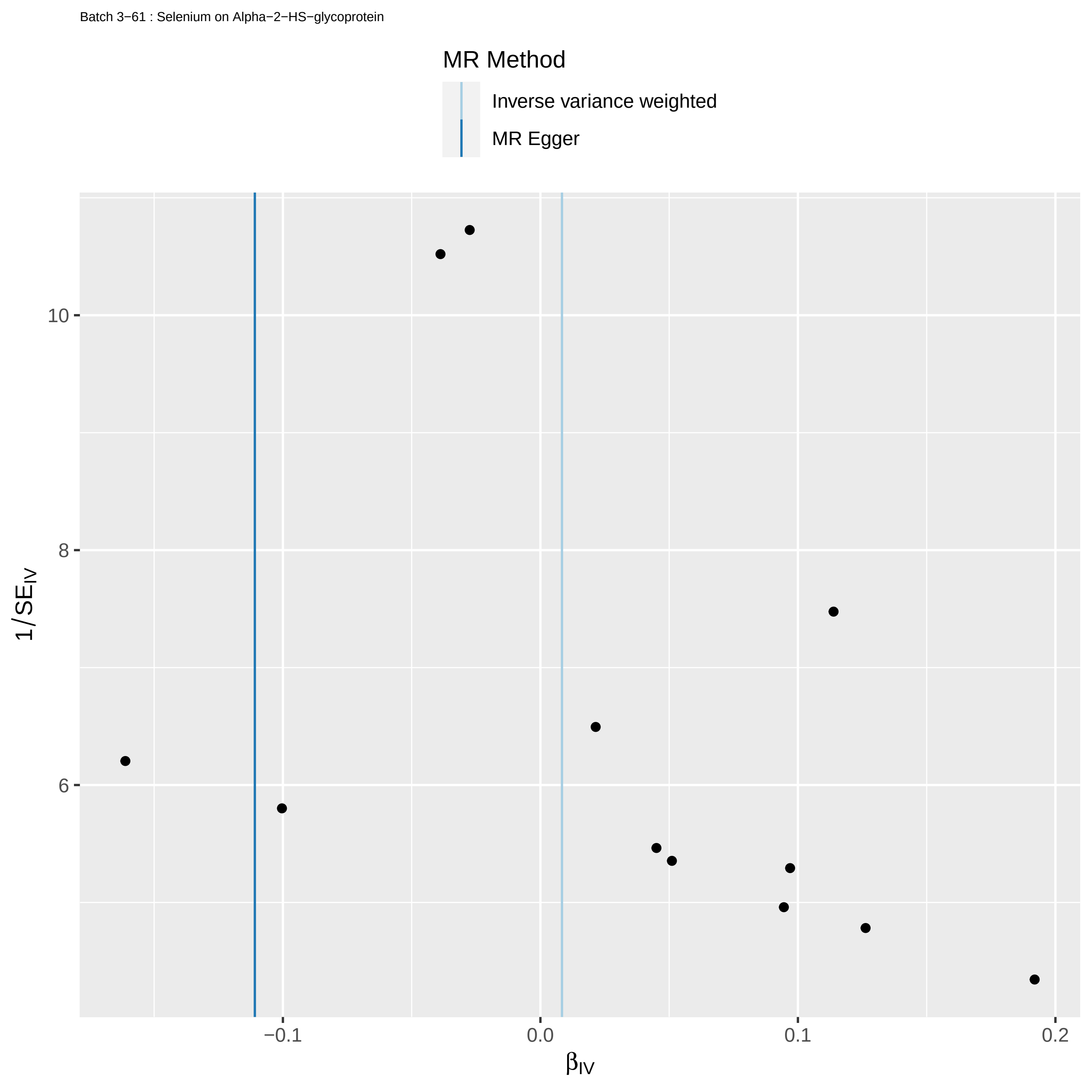

Batch 3−61 : Selenium on Alpha−2−HS−glycoprotein
MR Method
Inverse variance weighted
MR Egger
10
8
6
1/SE_IV
−0.1
0.0
0.1
0.2
β_IV

Batch 3−62 : Selenium on Allograft inflammatory factor 1

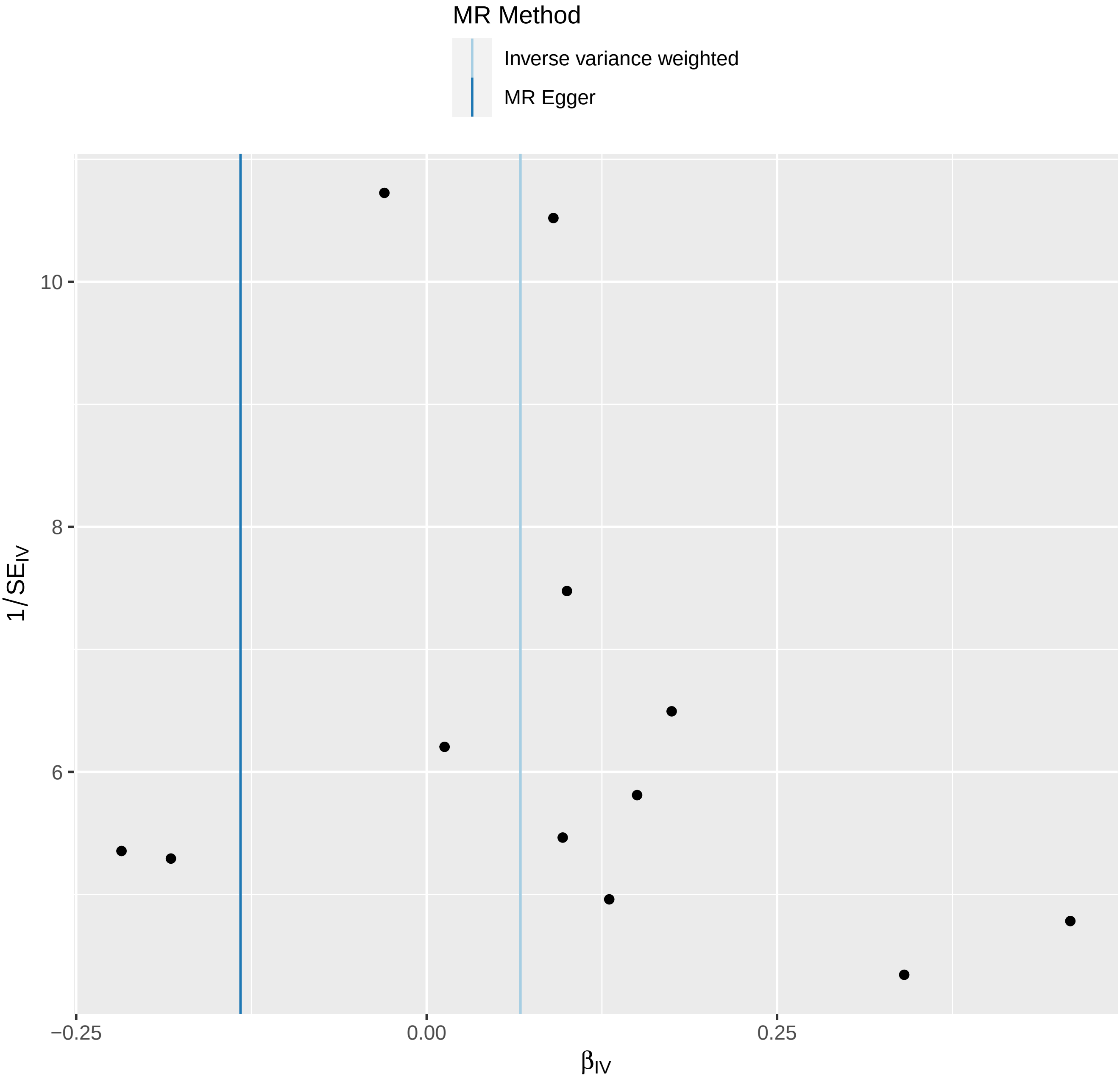

Batch 3−63 : Selenium on Apoptosis−inducing factor 1, mitochondrial

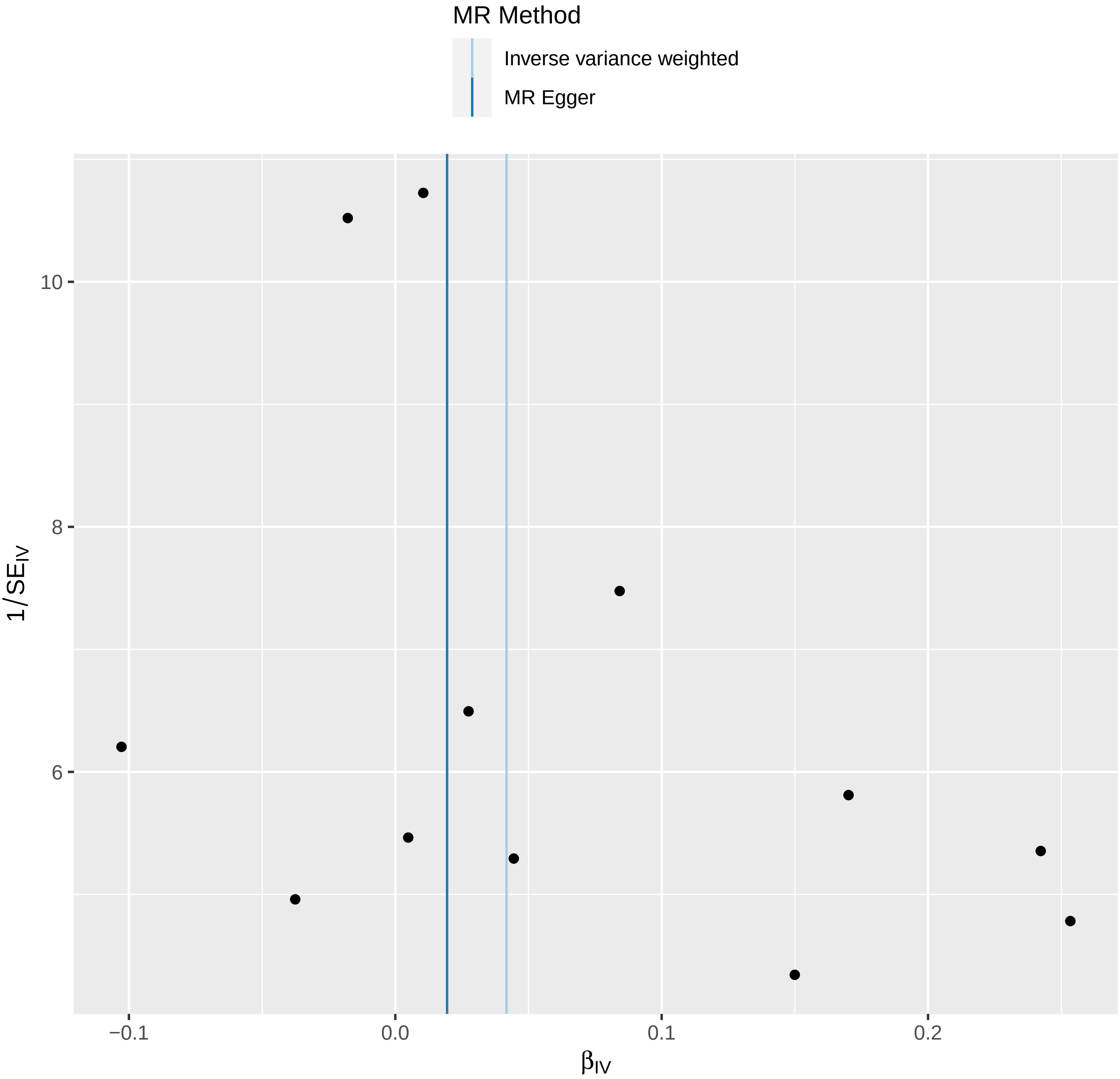

Batch 3−64 : Selenium on Apoptosis−inducing factor 1, mitochondrial

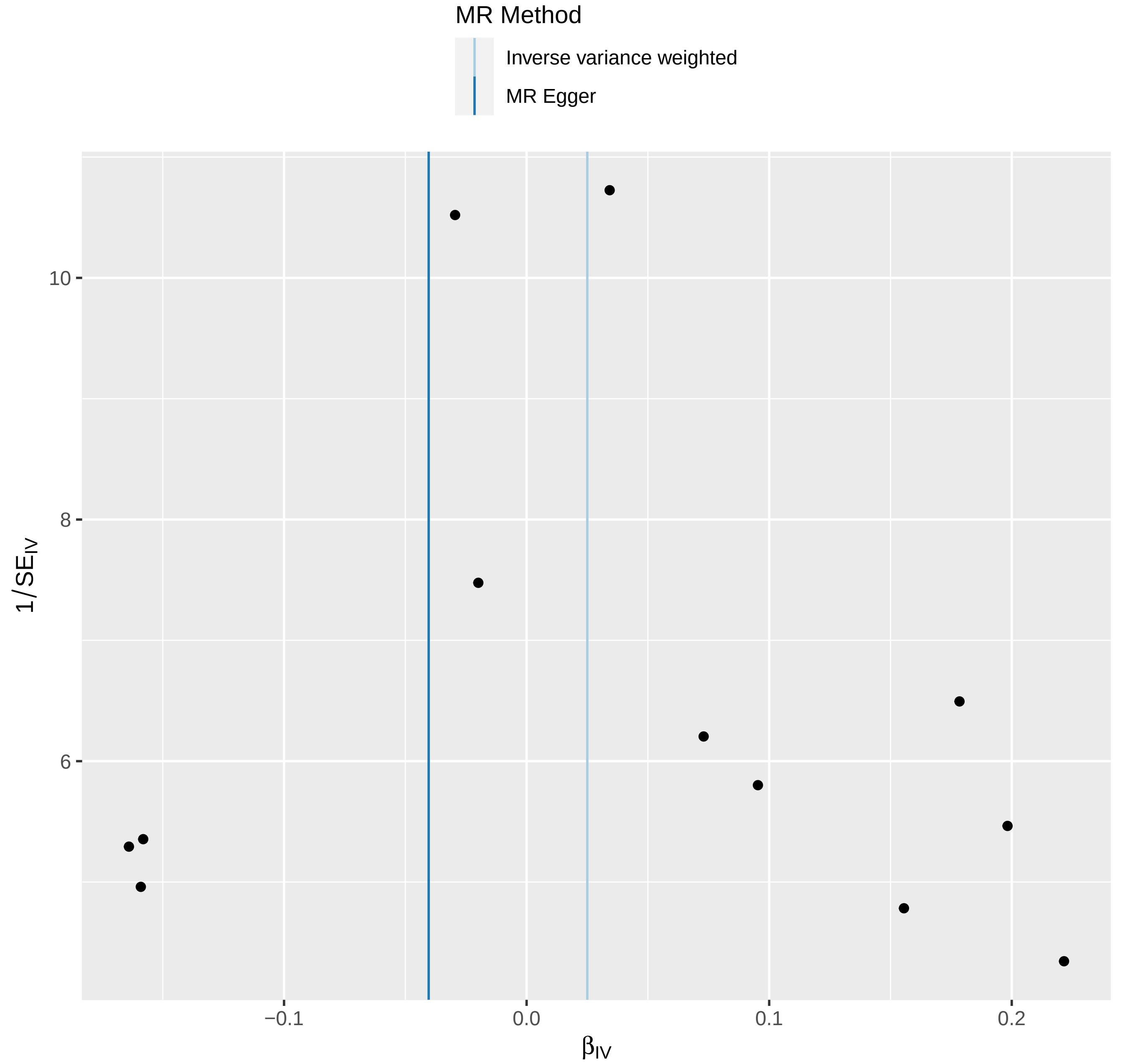

Batch 3−65 : Selenium on Endothelial monocyte−activating polypeptide 2

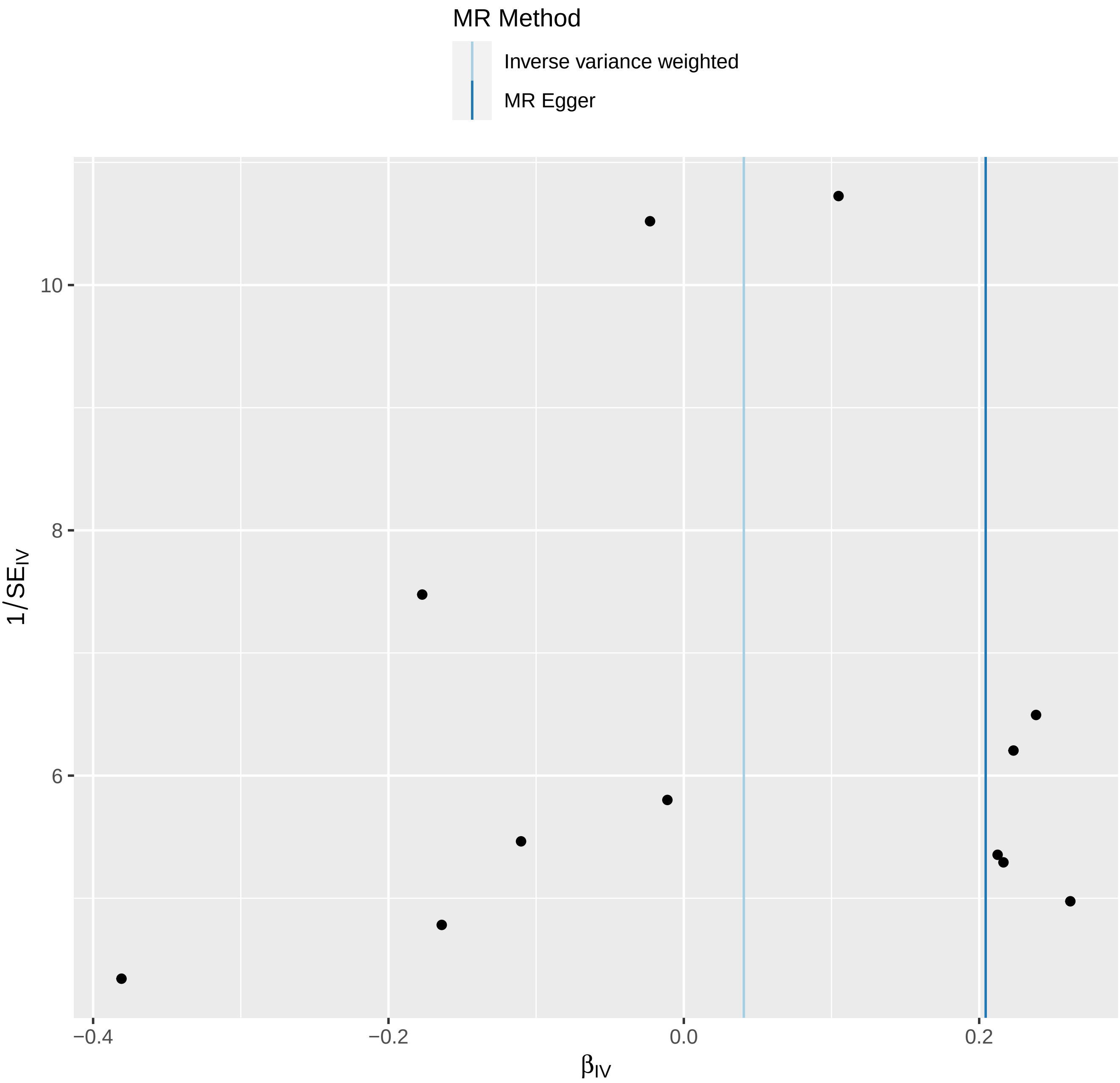

Batch 3−66 : Selenium on Adenylate kinase isoenzyme 1

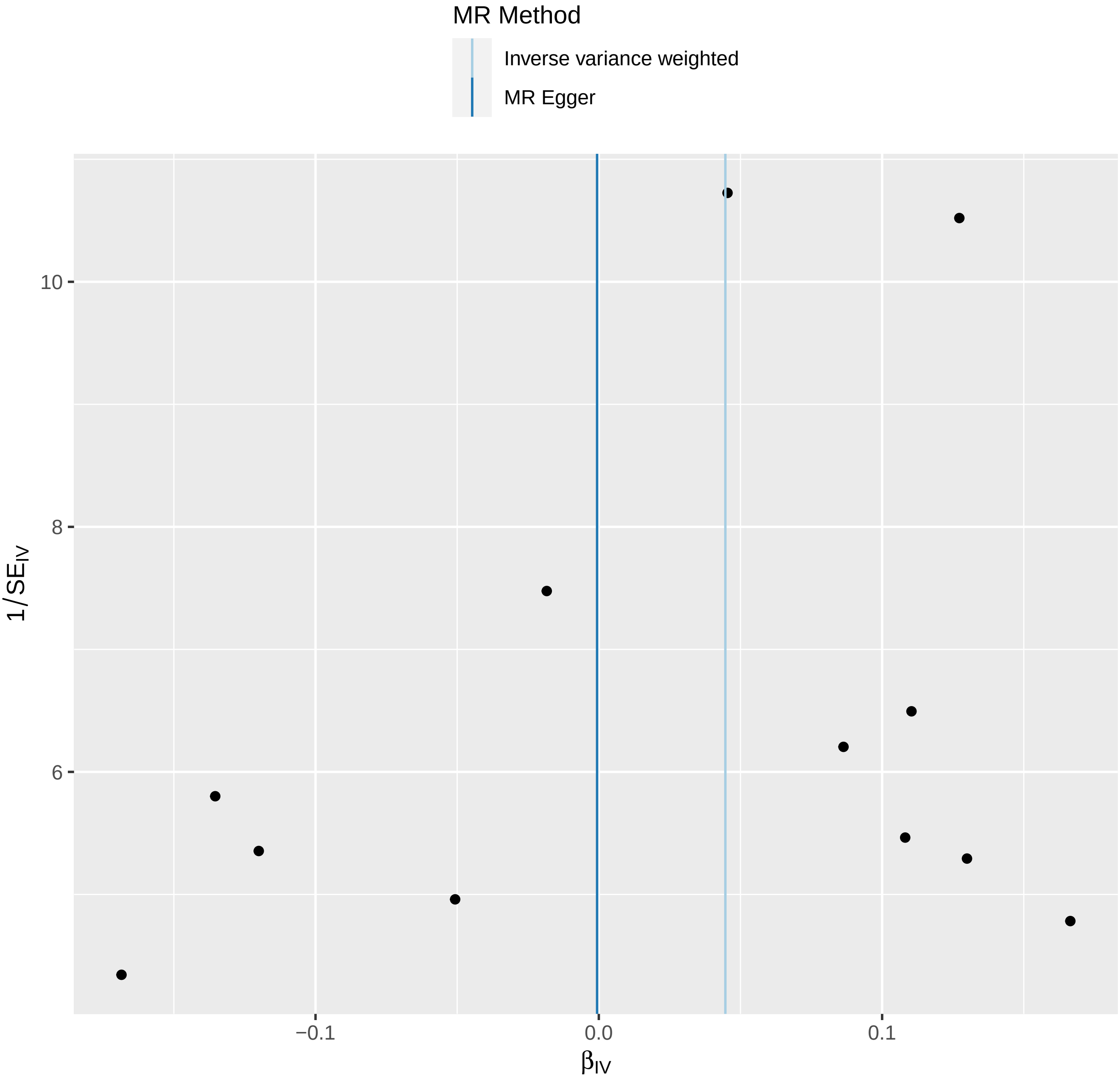

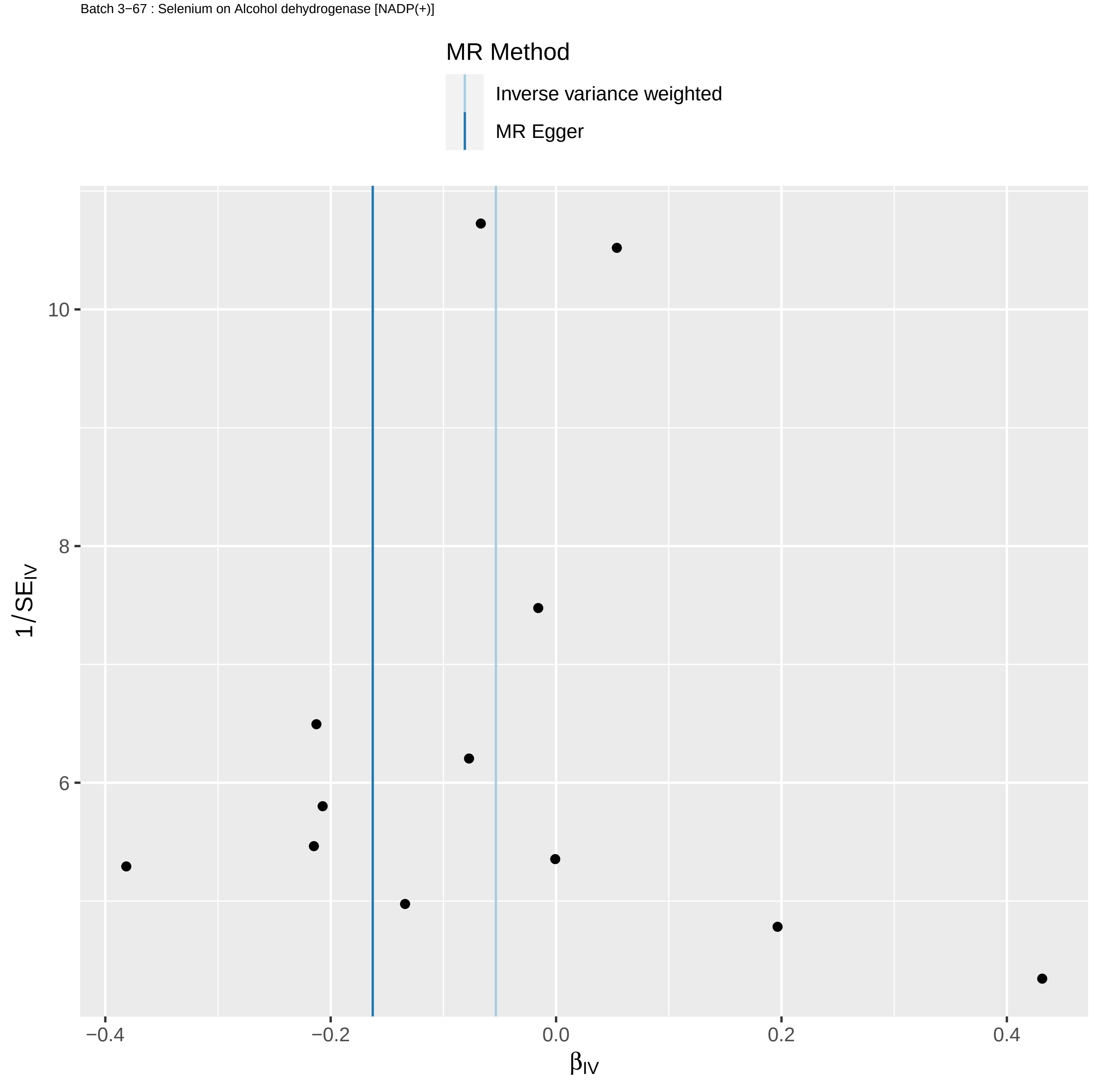
Batch 3−67 : Selenium on Alcohol dehydrogenase [NADP(+)]
MR Method
Inverse variance weighted
MR Egger
10
8
6
1/SE_IV
−0.4
−0.2
0.0
0.2
0.4
β_IV

Batch 3−68 : Selenium on Aldose reductase

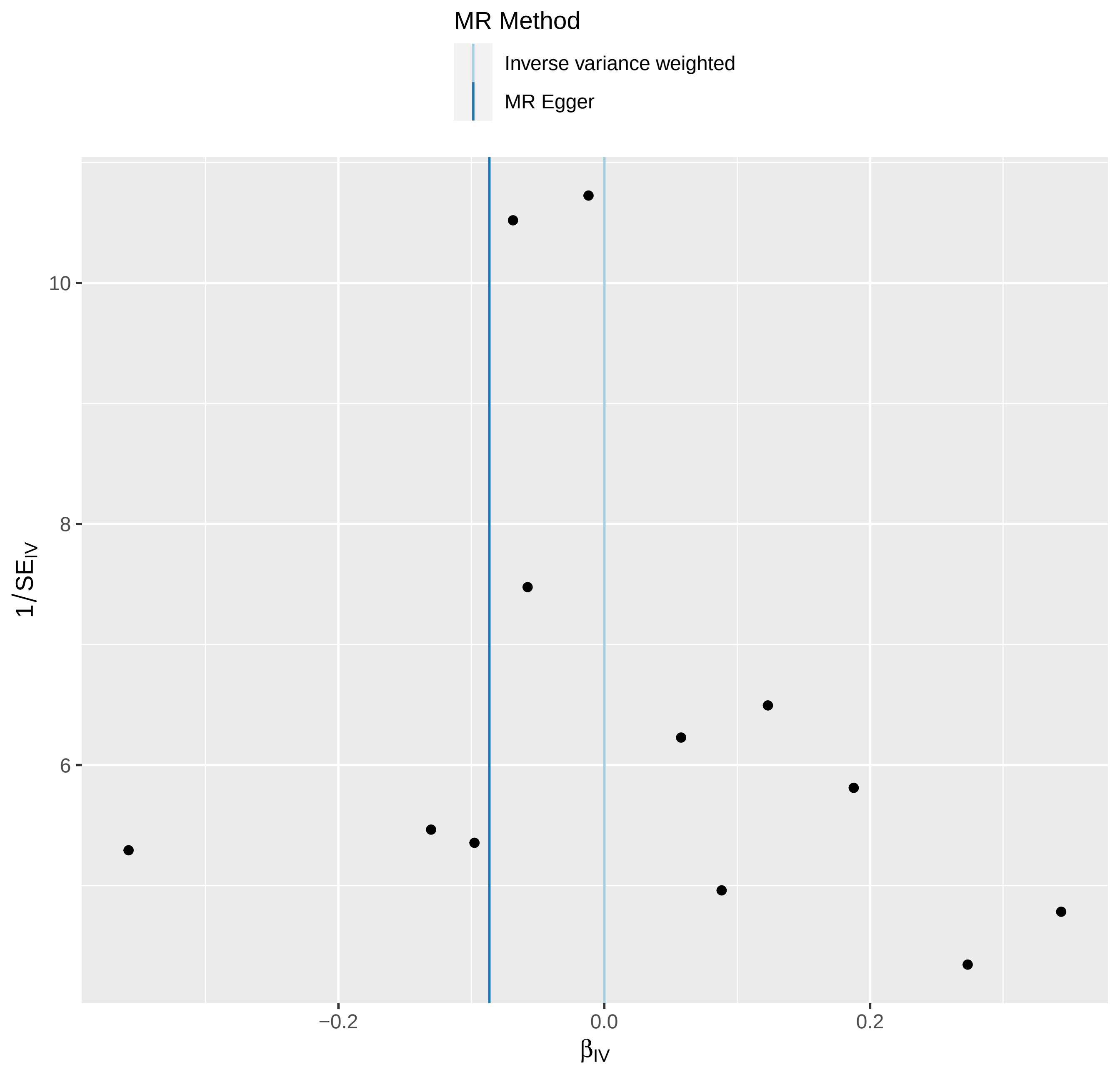

Batch 3−69 : Selenium on Aldo−keto reductase family 1 member C1

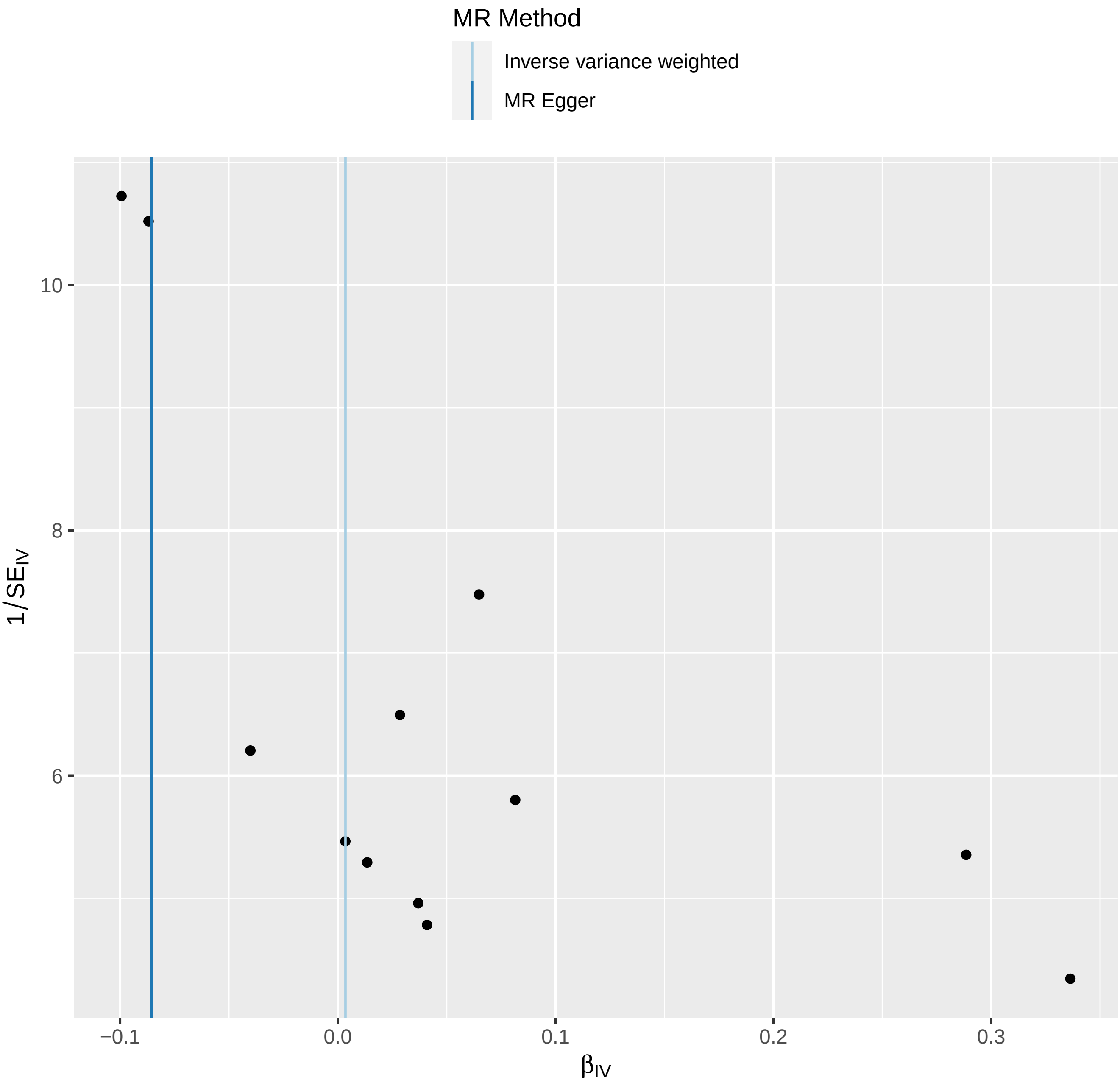

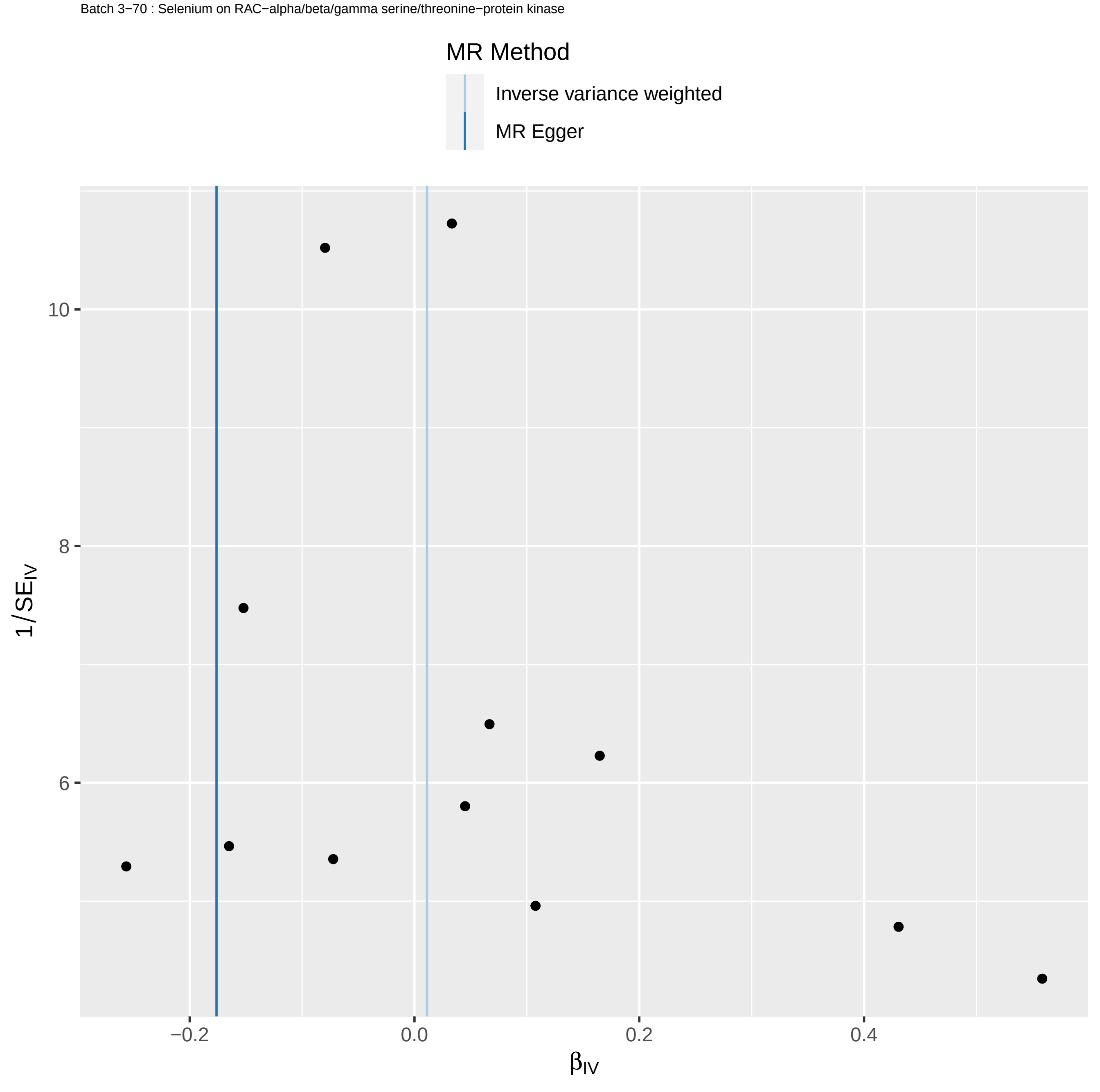

Batch 3−70 : Selenium on RAC−alpha/beta/gamma serine/threonine−protein kinase
MR Method
Inverse variance weighted
MR Egger
10
8
6
1/SE_IV
−0.2
0.0
0.2
0.4
β_IV

Batch 3−71 : Selenium on RAC−alpha/beta/gamma serine/threonine−protein kinase

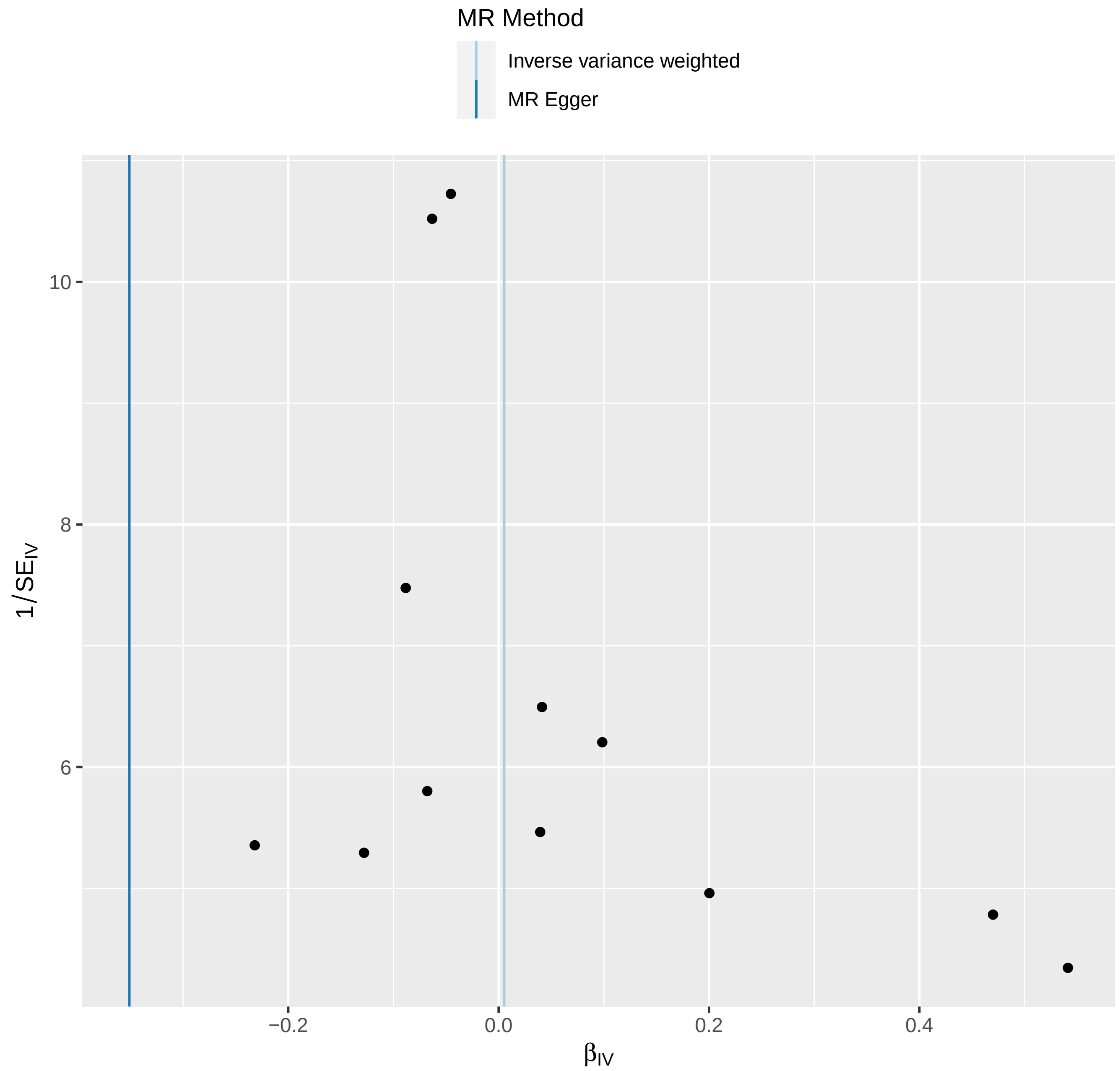

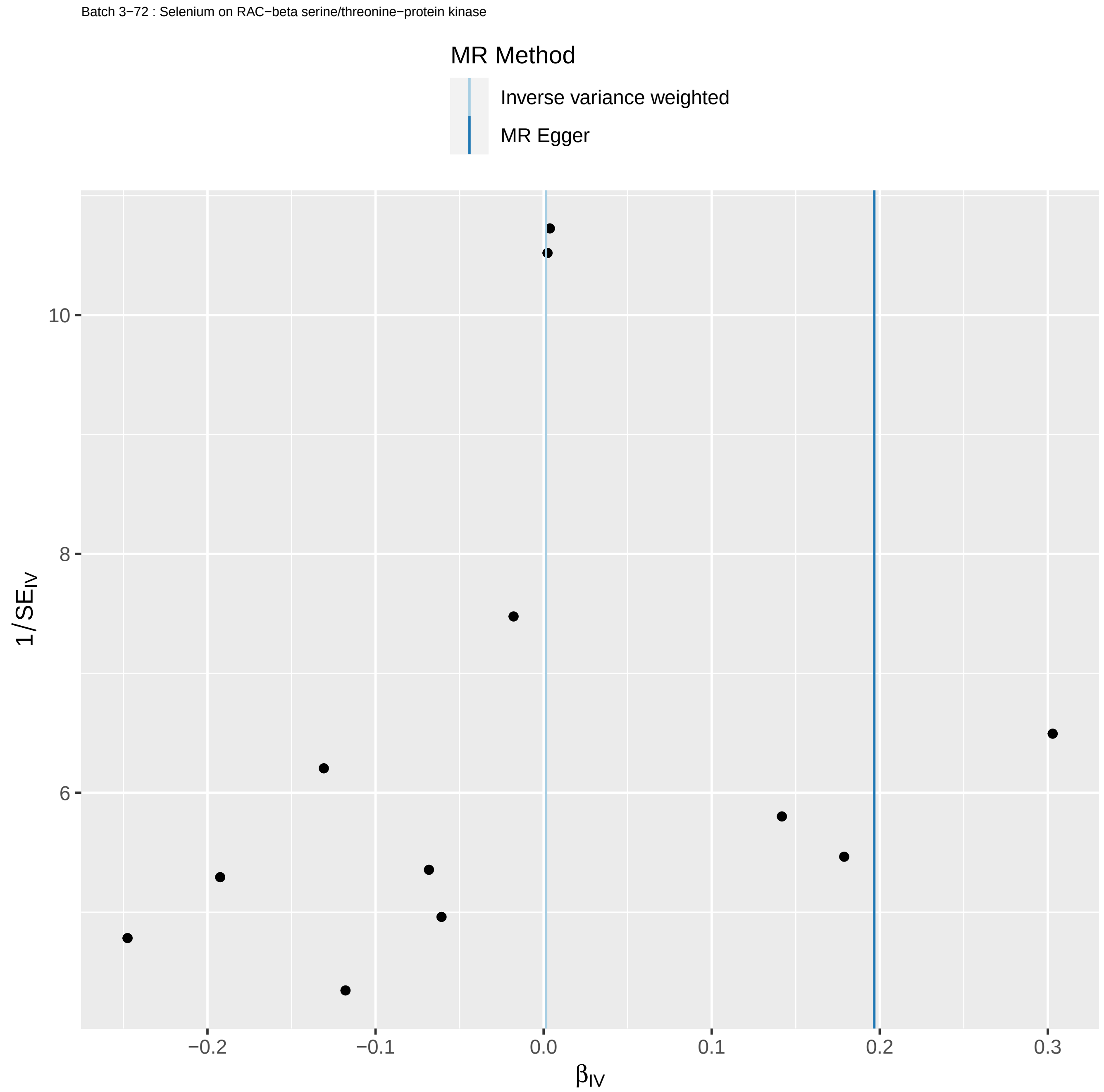

Batch 3−72 : Selenium on RAC−beta serine/threonine−protein kinase
MR Method
Inverse variance weighted
MR Egger
10
8
6
1/SE_IV
−0.2
−0.1
0.0
0.1
0.2
0.3
β_IV

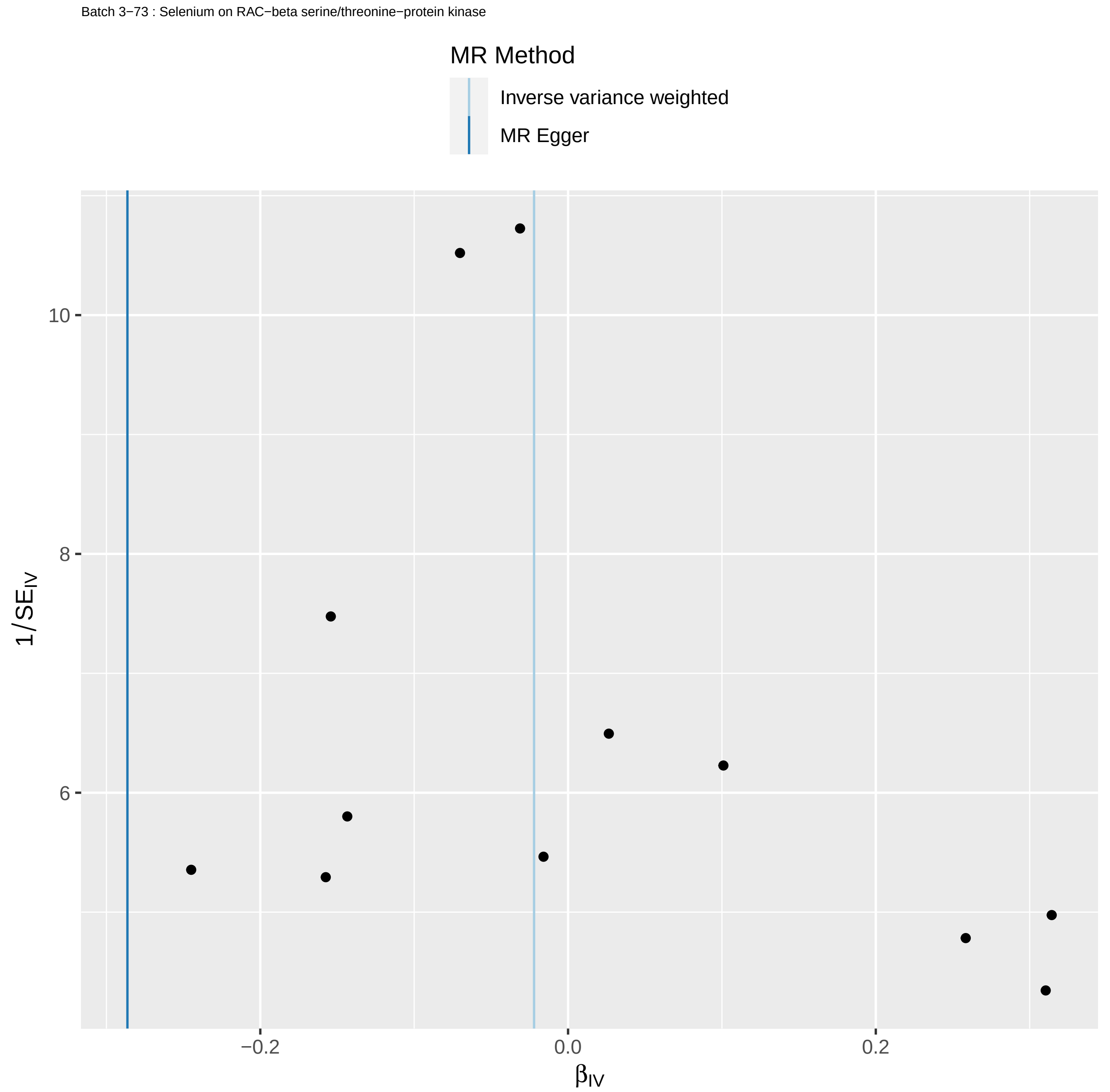

Batch 3−73 : Selenium on RAC−beta serine/threonine−protein kinase
MR Method
Inverse variance weighted
MR Egger
10
8
6
1/SE_IV
−0.2
0.0
0.2
β_IV

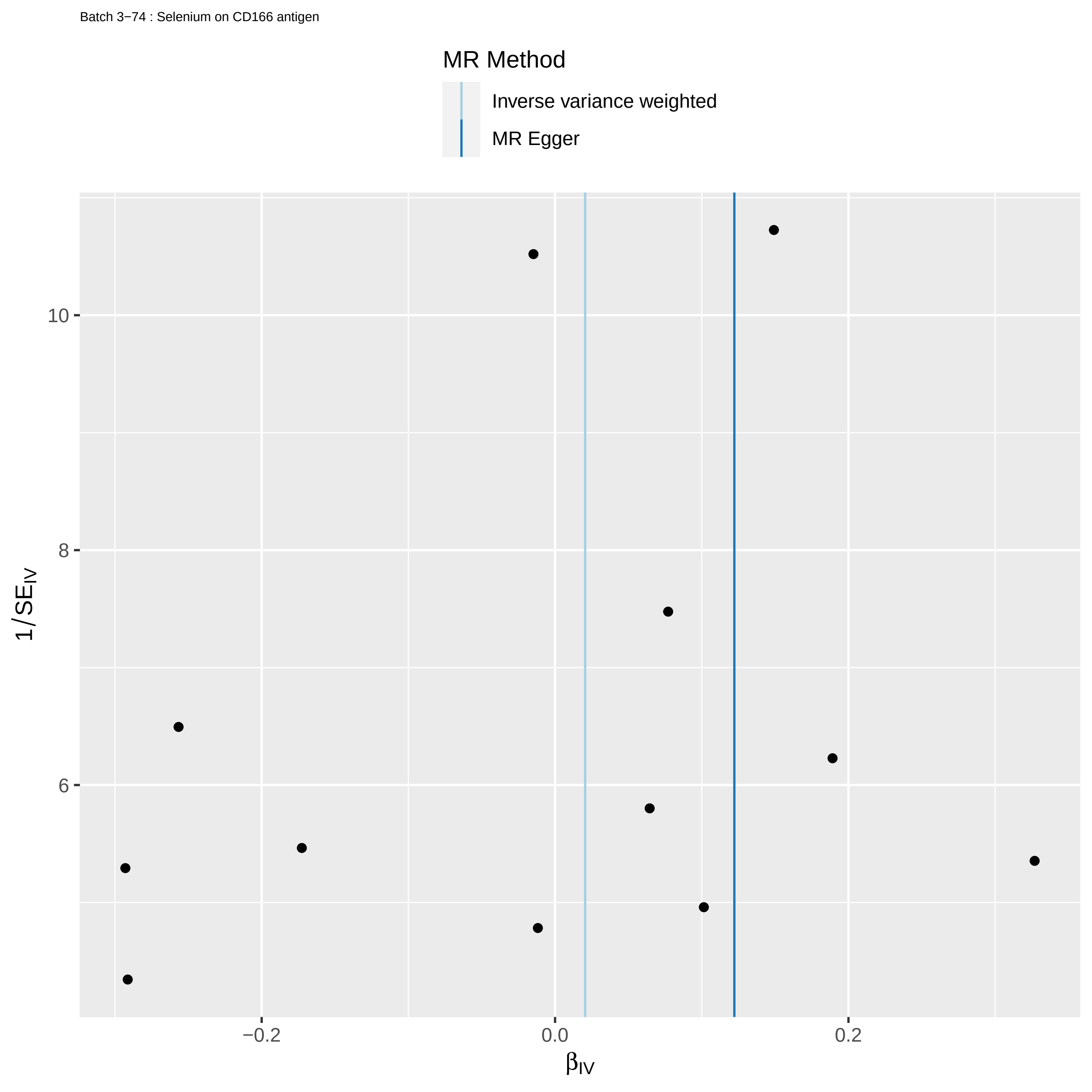

Batch 3−74 : Selenium on CD166 antigen
MR Method
Inverse variance weighted
MR Egger
10
8
6
1/SE$_{IV}$
−0.2
0.0
0.2
β$_{IV}$

Batch 3−75 : Selenium on Aldehyde dehydrogenase, dimeric NADP−preferring

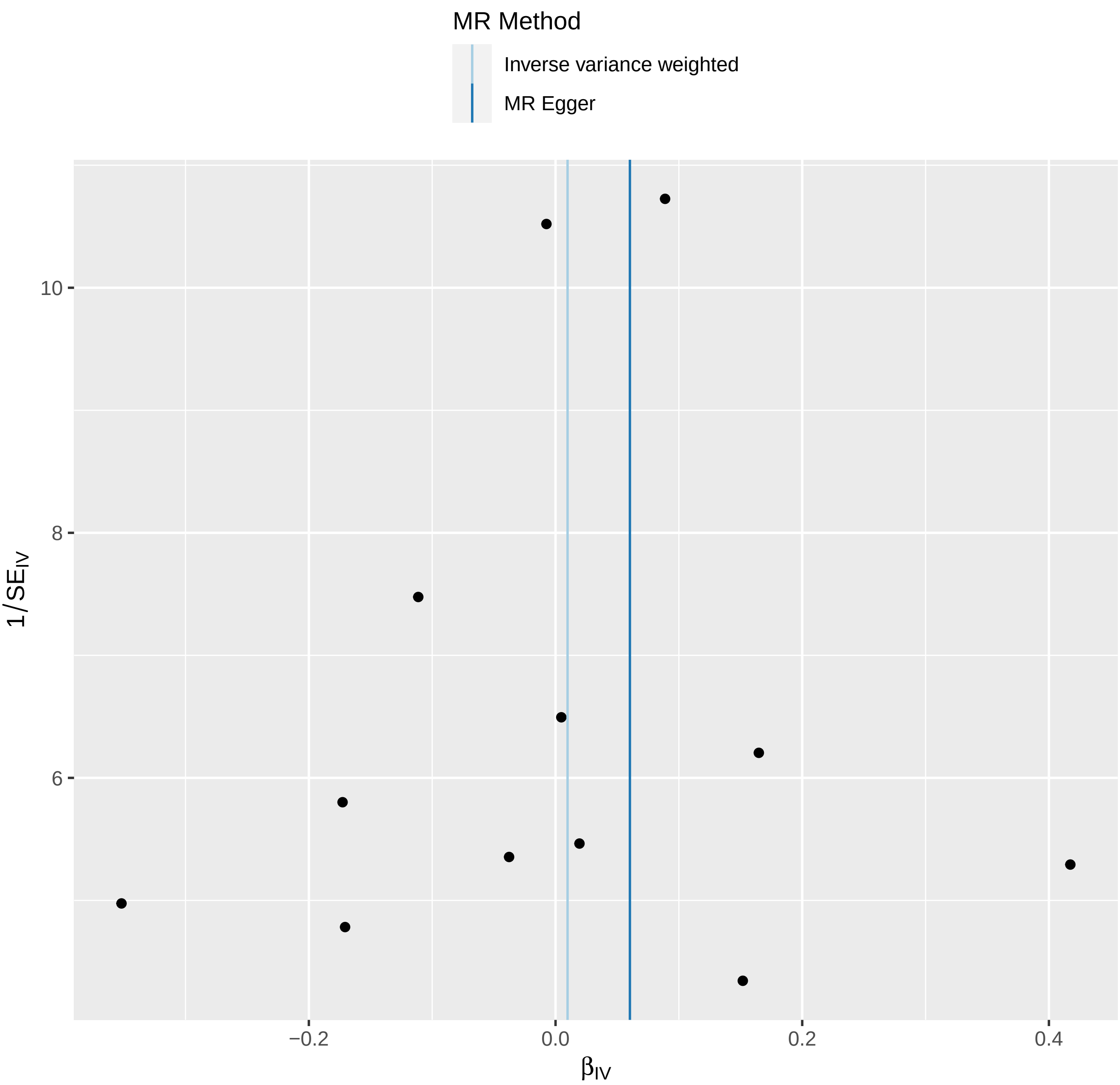

Batch 3−76 : Selenium on Aldehyde dehydrogenase family 3 member B1

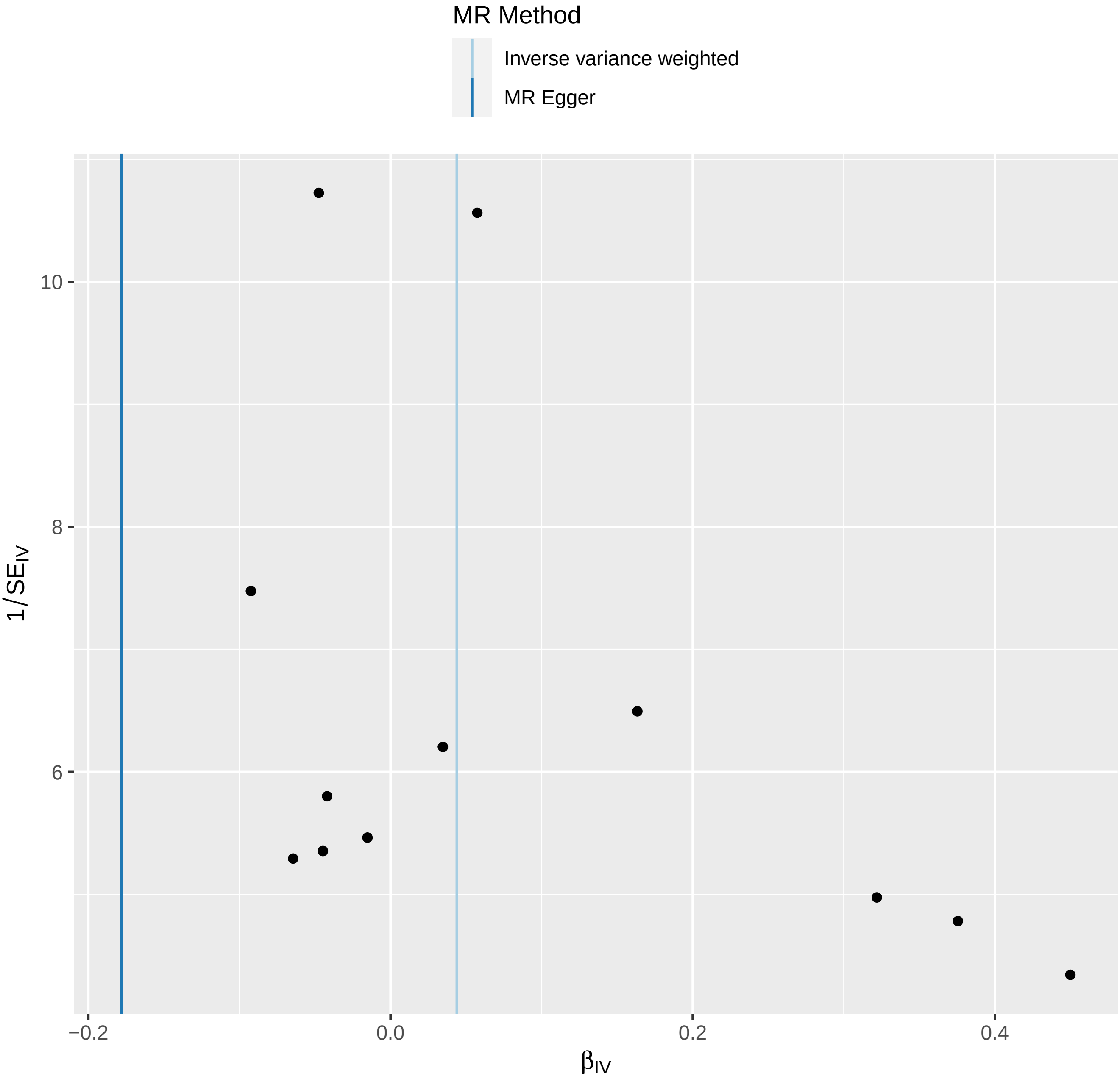

Batch 3−77 : Selenium on Fructose−bisphosphate aldolase A

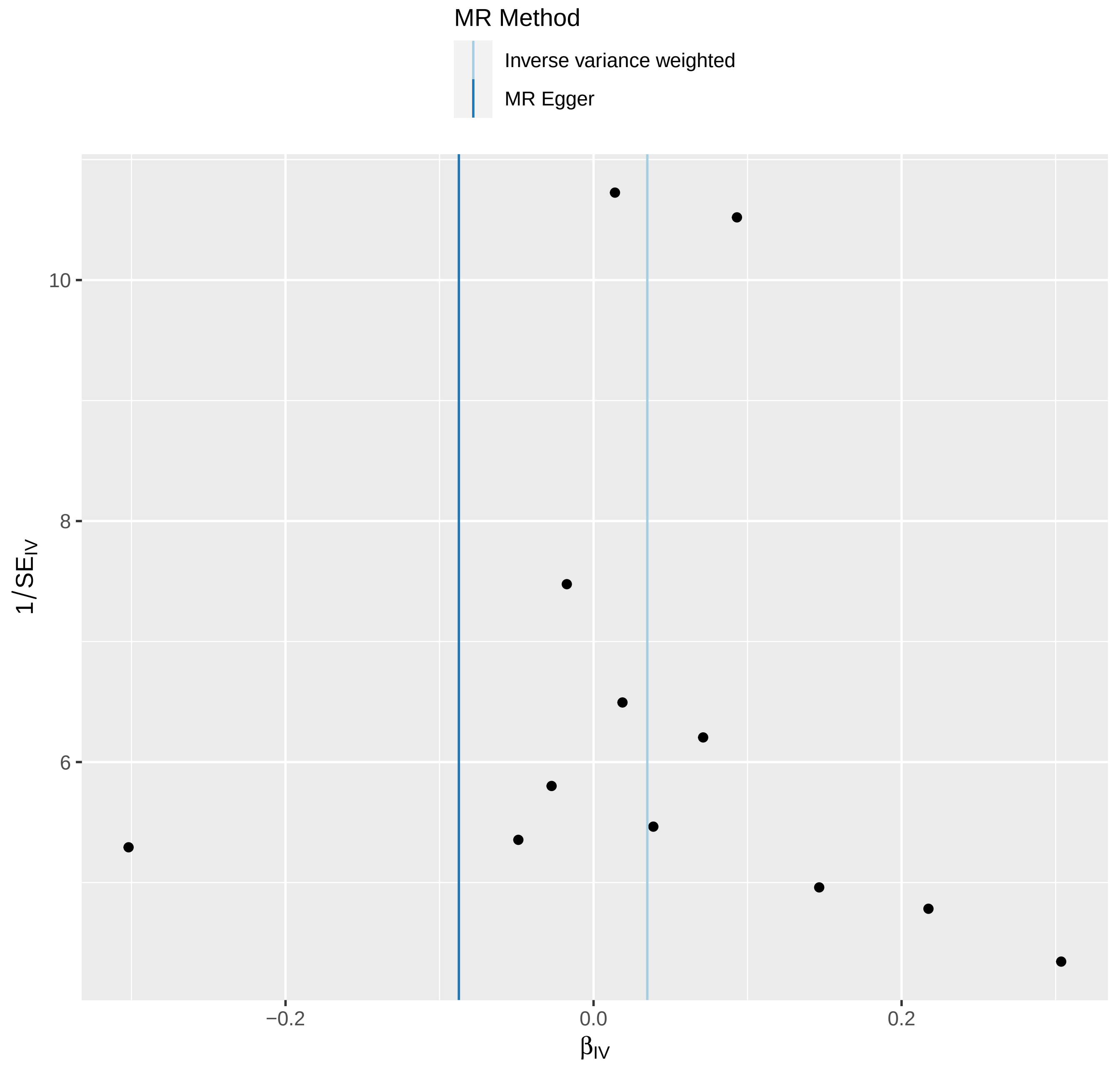

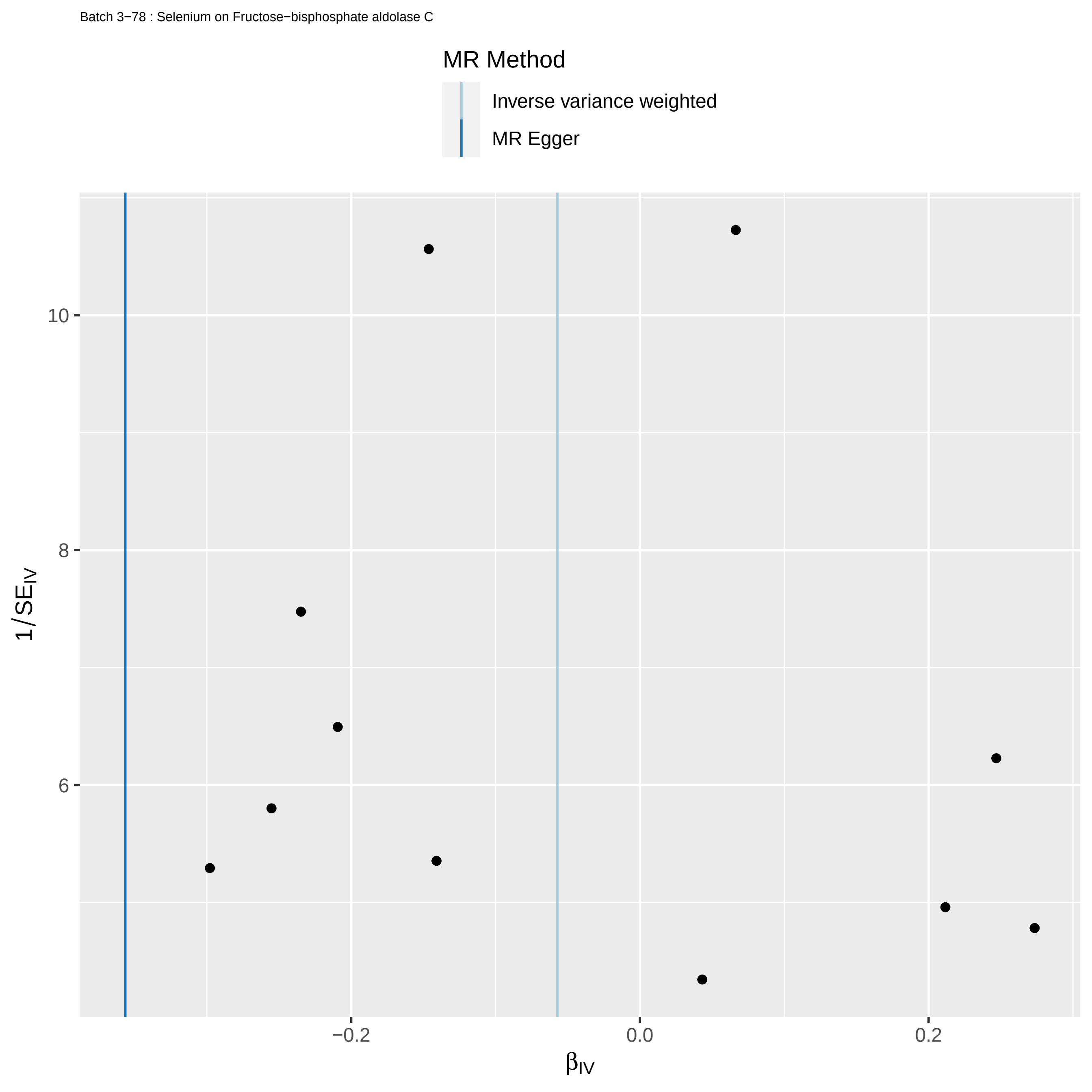

Batch 3−78 : Selenium on Fructose−bisphosphate aldolase C
MR Method
Inverse variance weighted
MR Egger
10
8
6
1/SE_IV
−0.2
0.0
0.2
β_IV

Batch 3−79 : Selenium on RNA demethylase ALKBH5

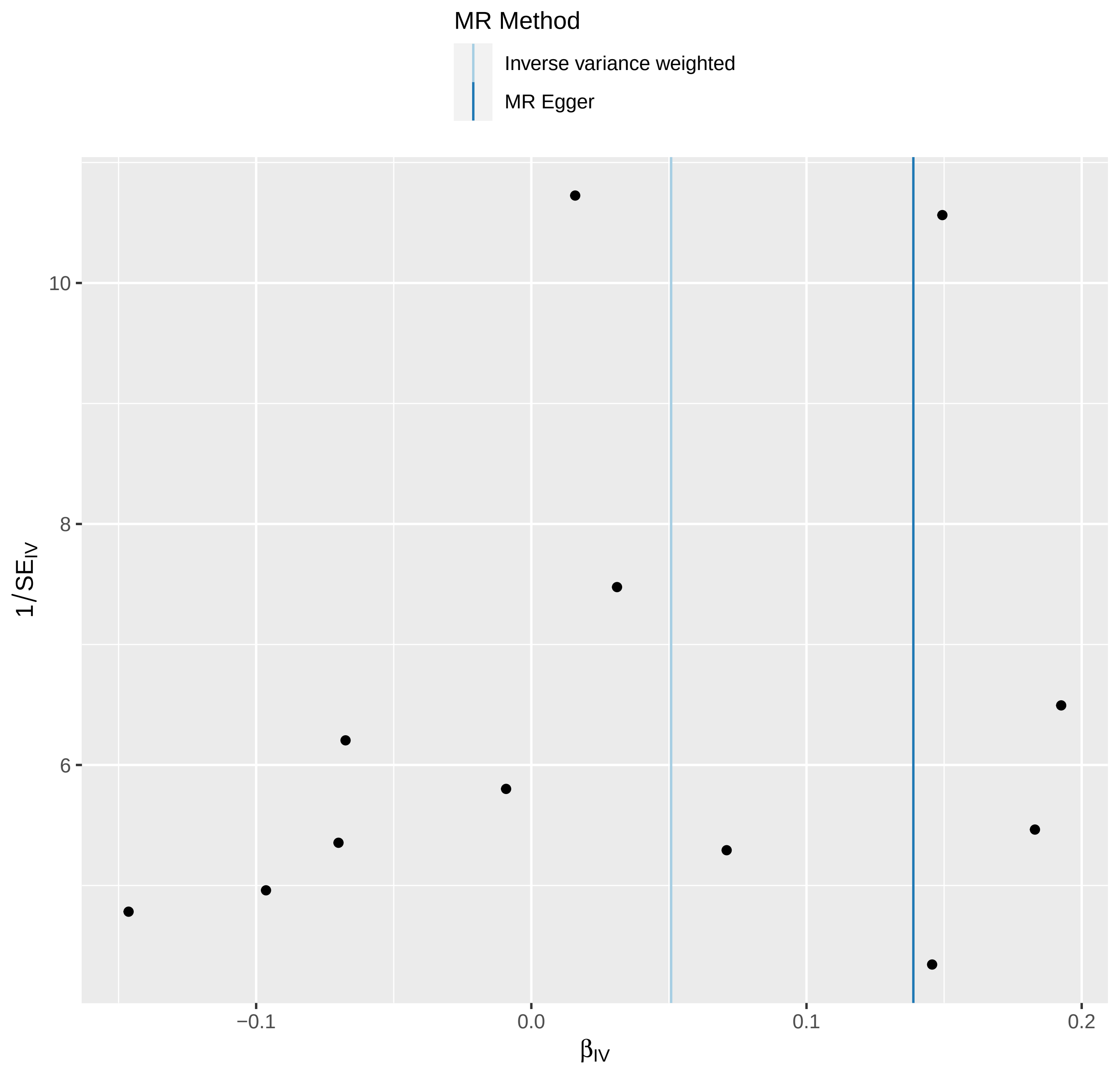

Batch 3−80 : Selenium on Arachidonate 15−lipoxygenase B

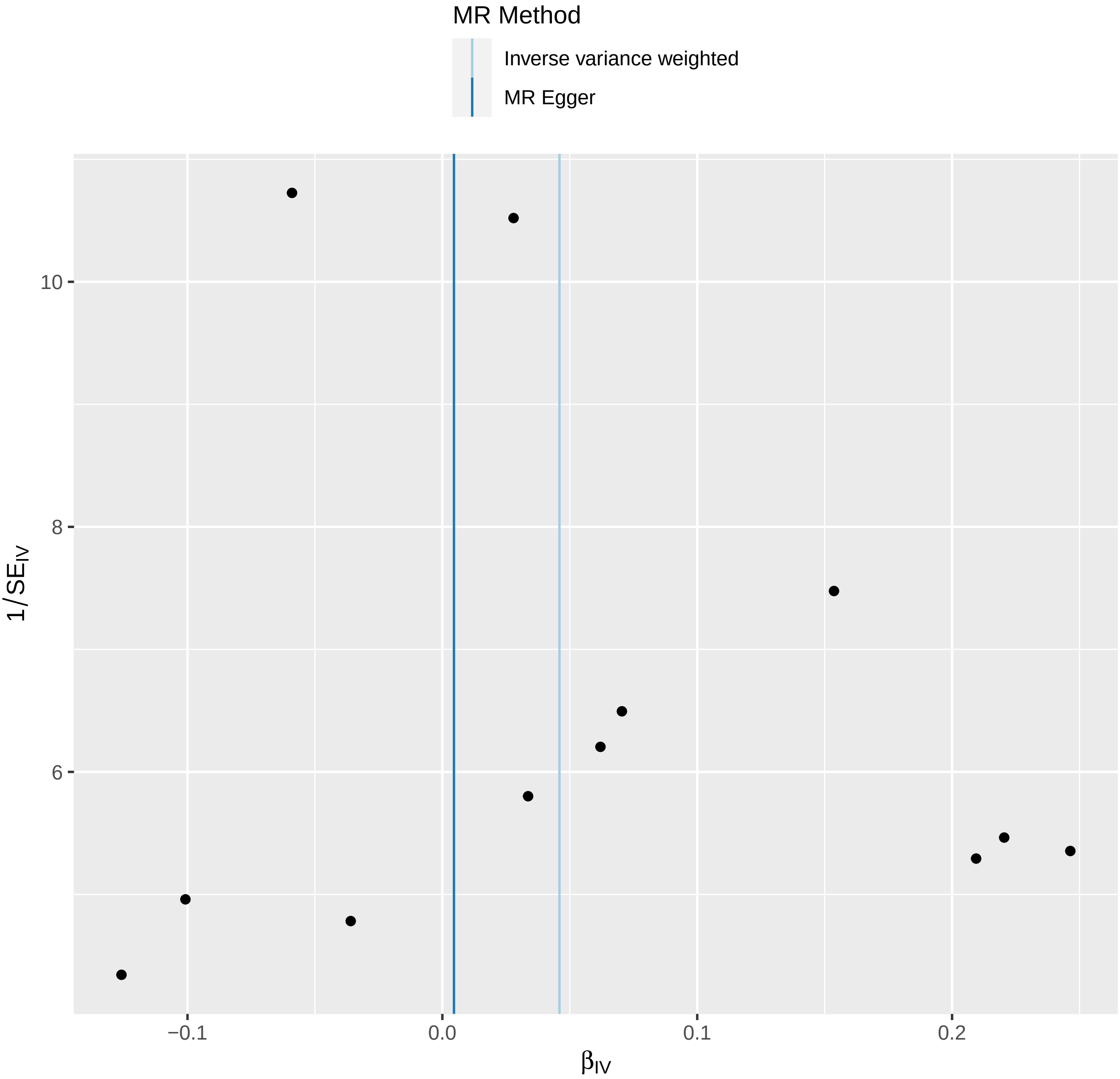

Batch 3−81 : Selenium on Intestinal−type alkaline phosphatase

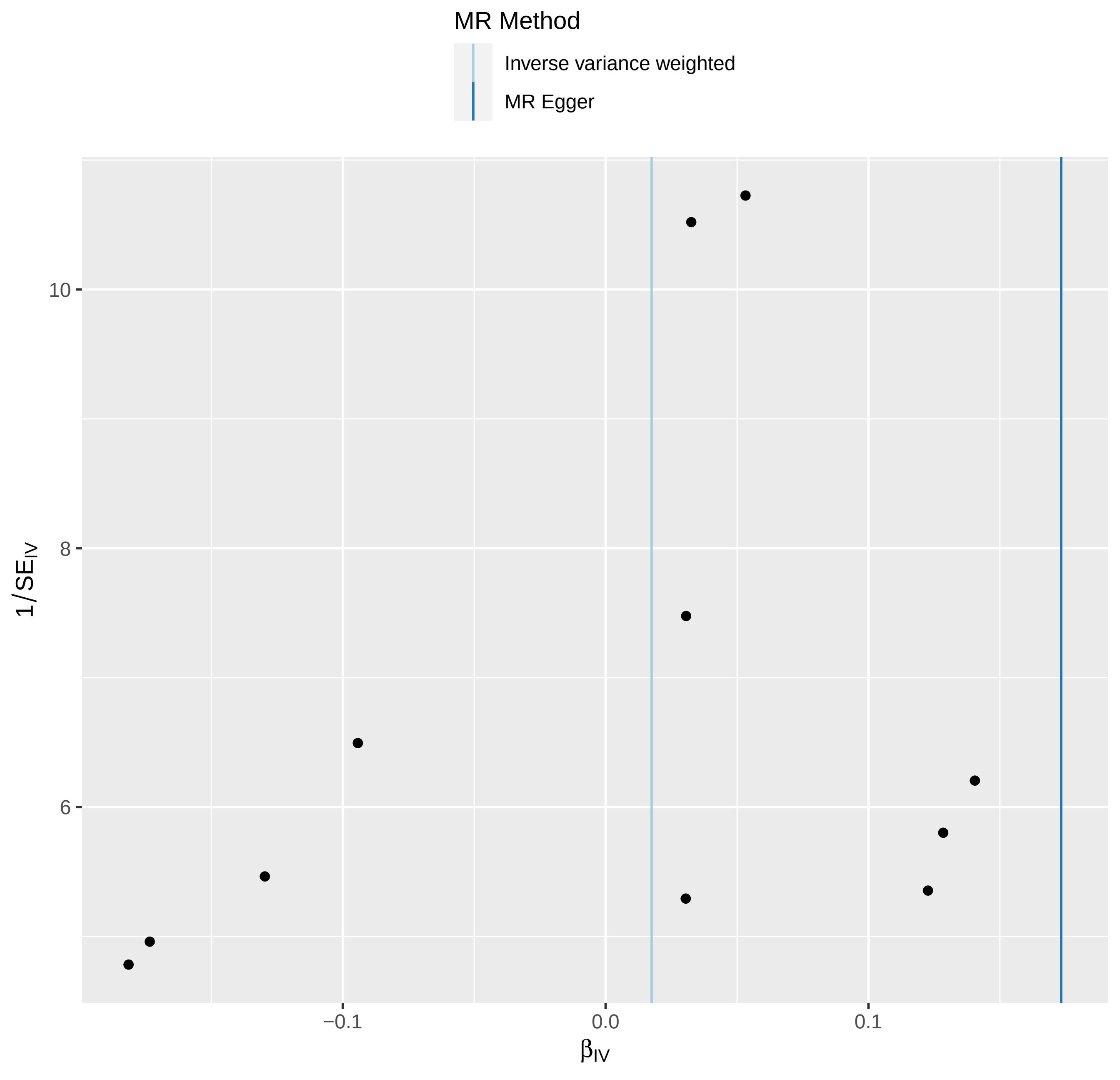

Batch 3−82 : Selenium on Ameloblastin

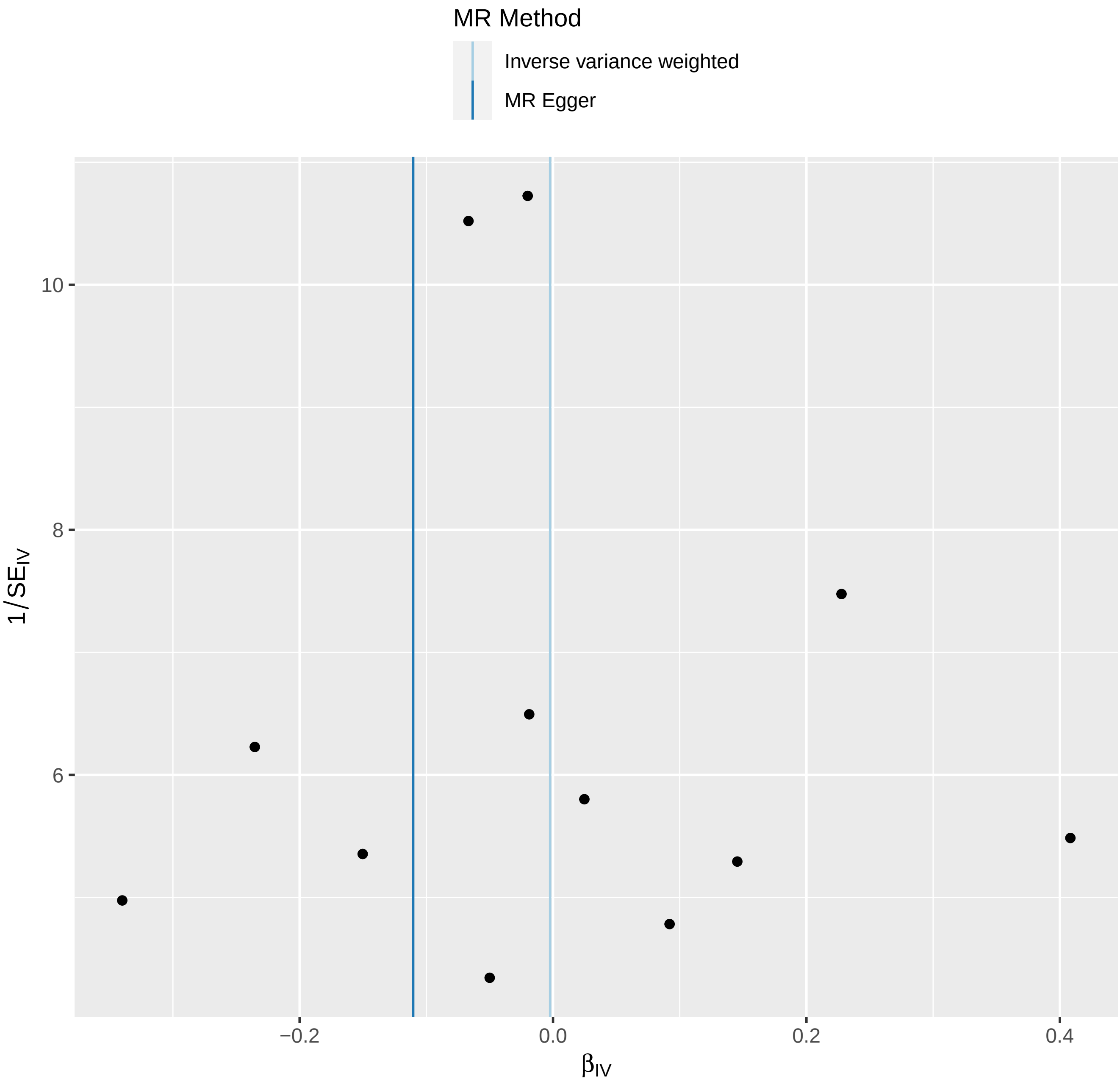

Batch 3−83 : Selenium on Amelogenin, X isoform

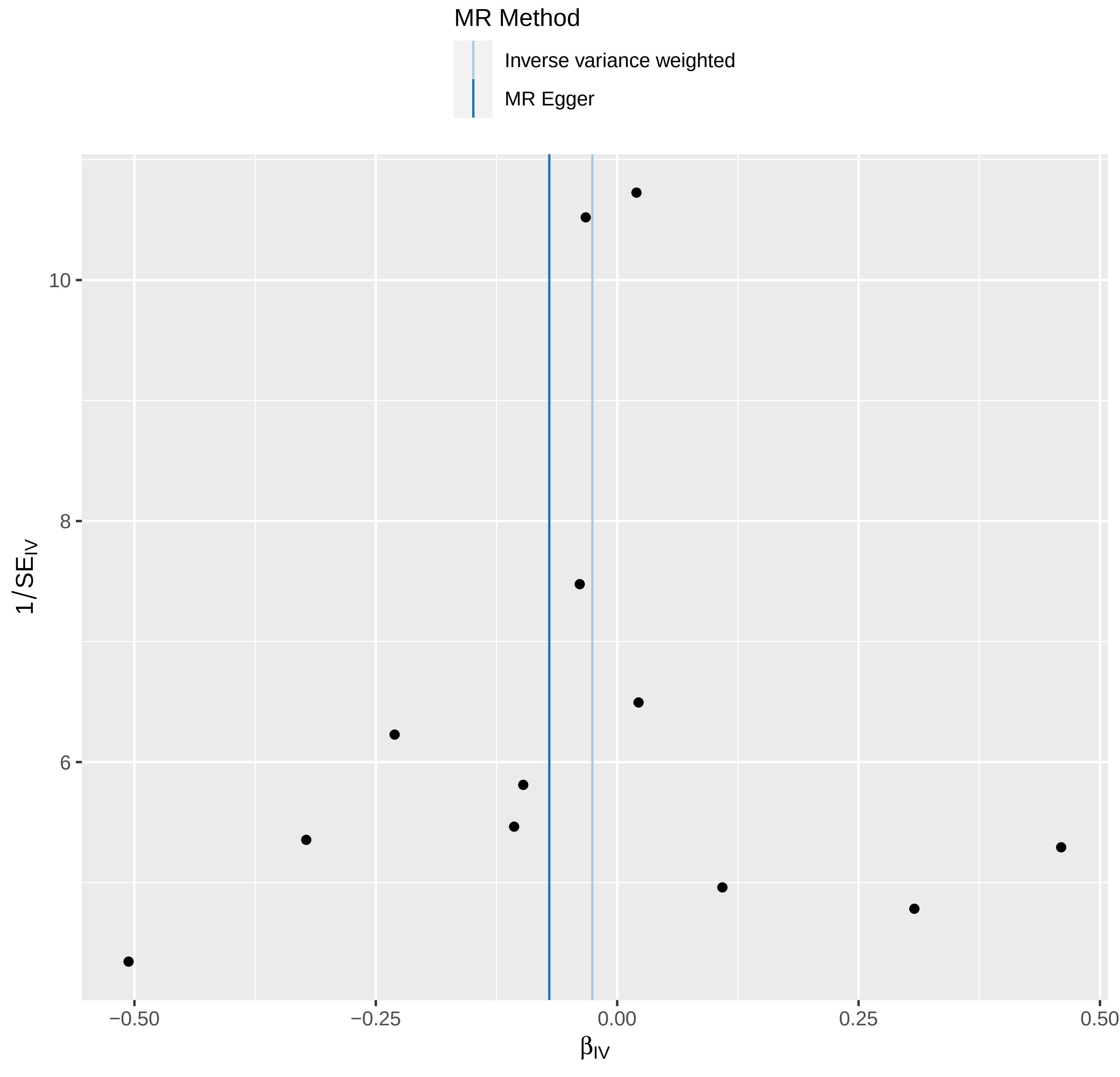

Batch 3−84 : Selenium on Muellerian−inhibiting factor

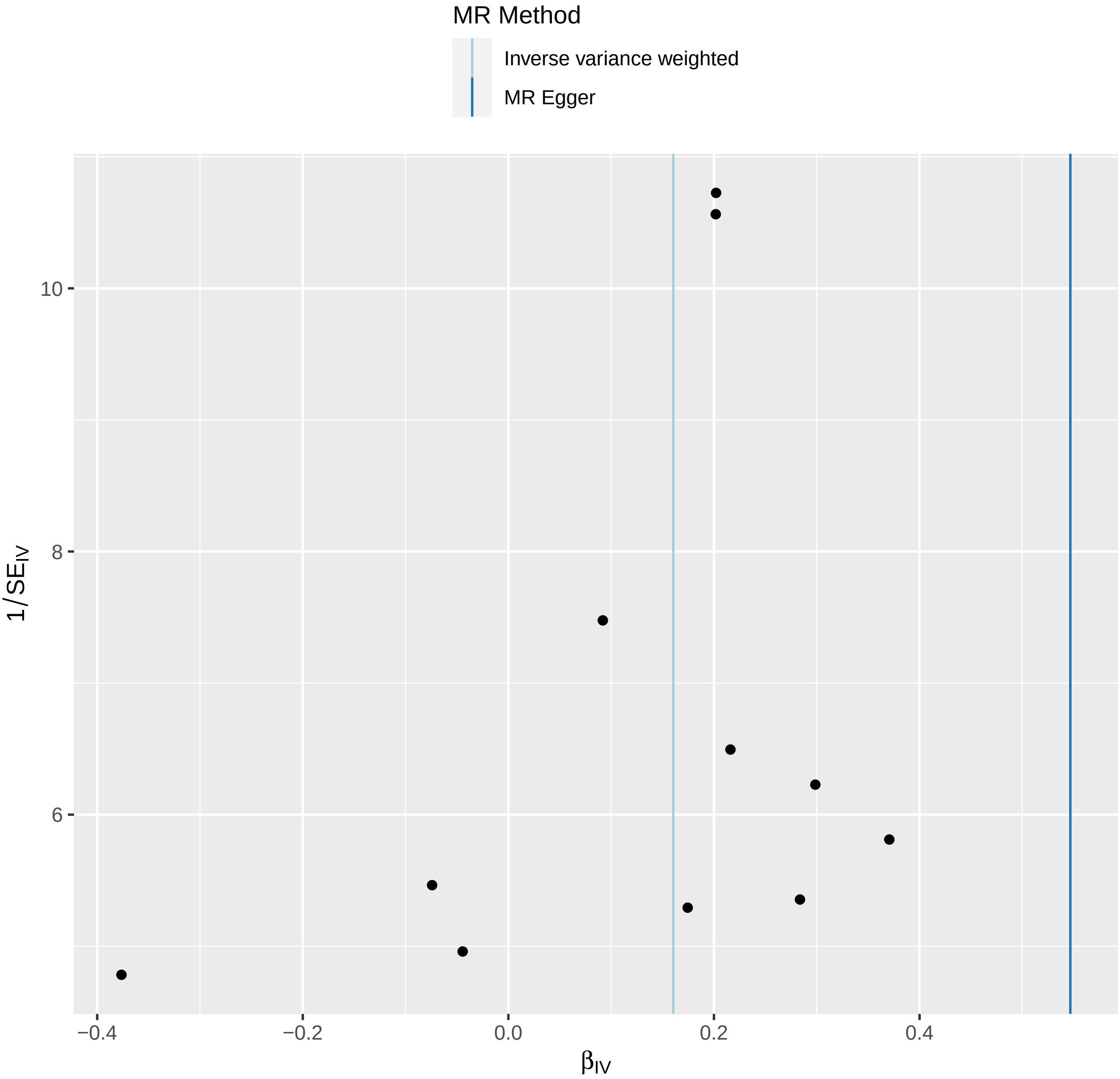

Batch 3−85 : Selenium on Junctional adhesion molecule−like

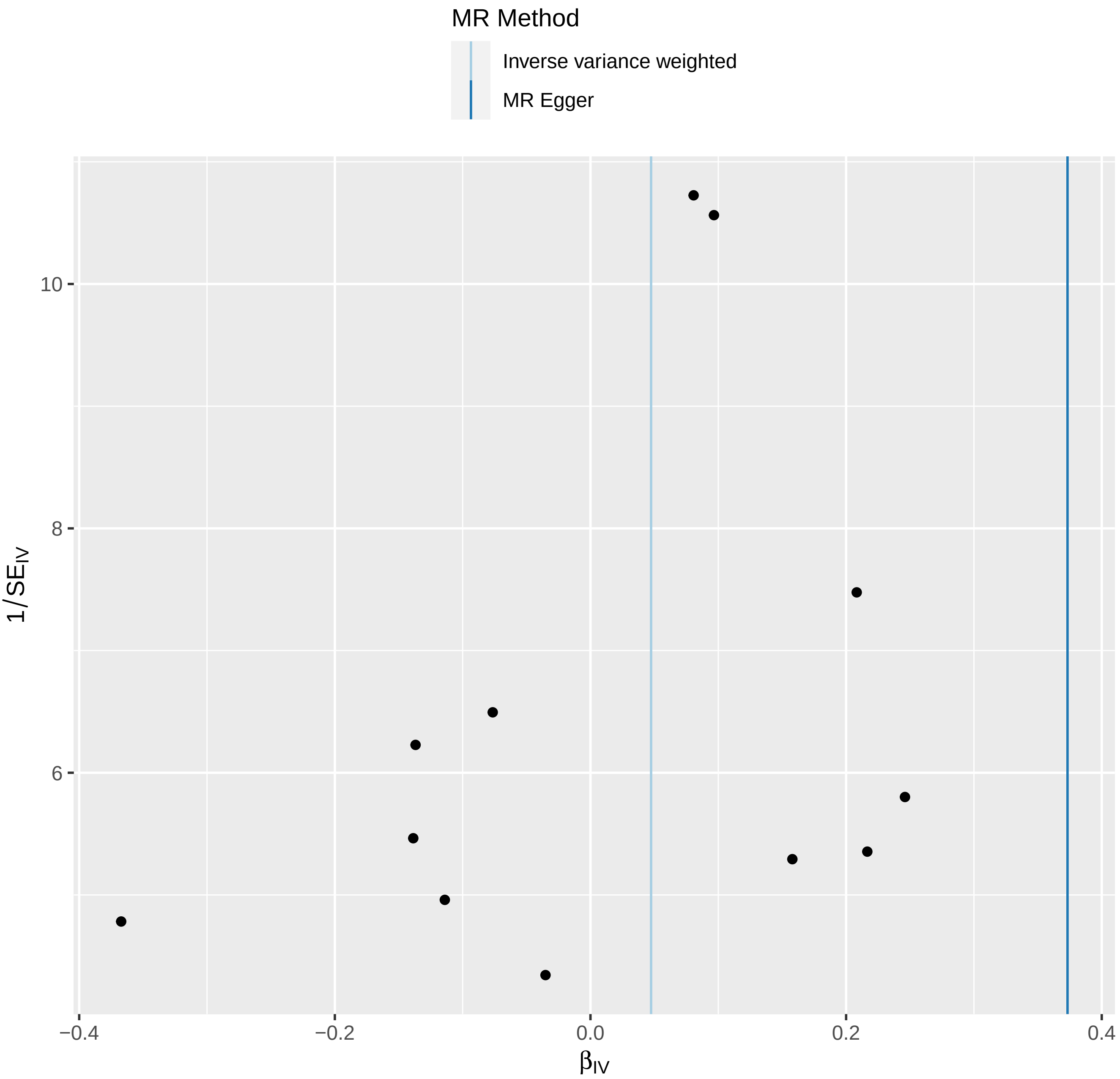

Batch 3−86 : Selenium on Junctional adhesion molecule−like

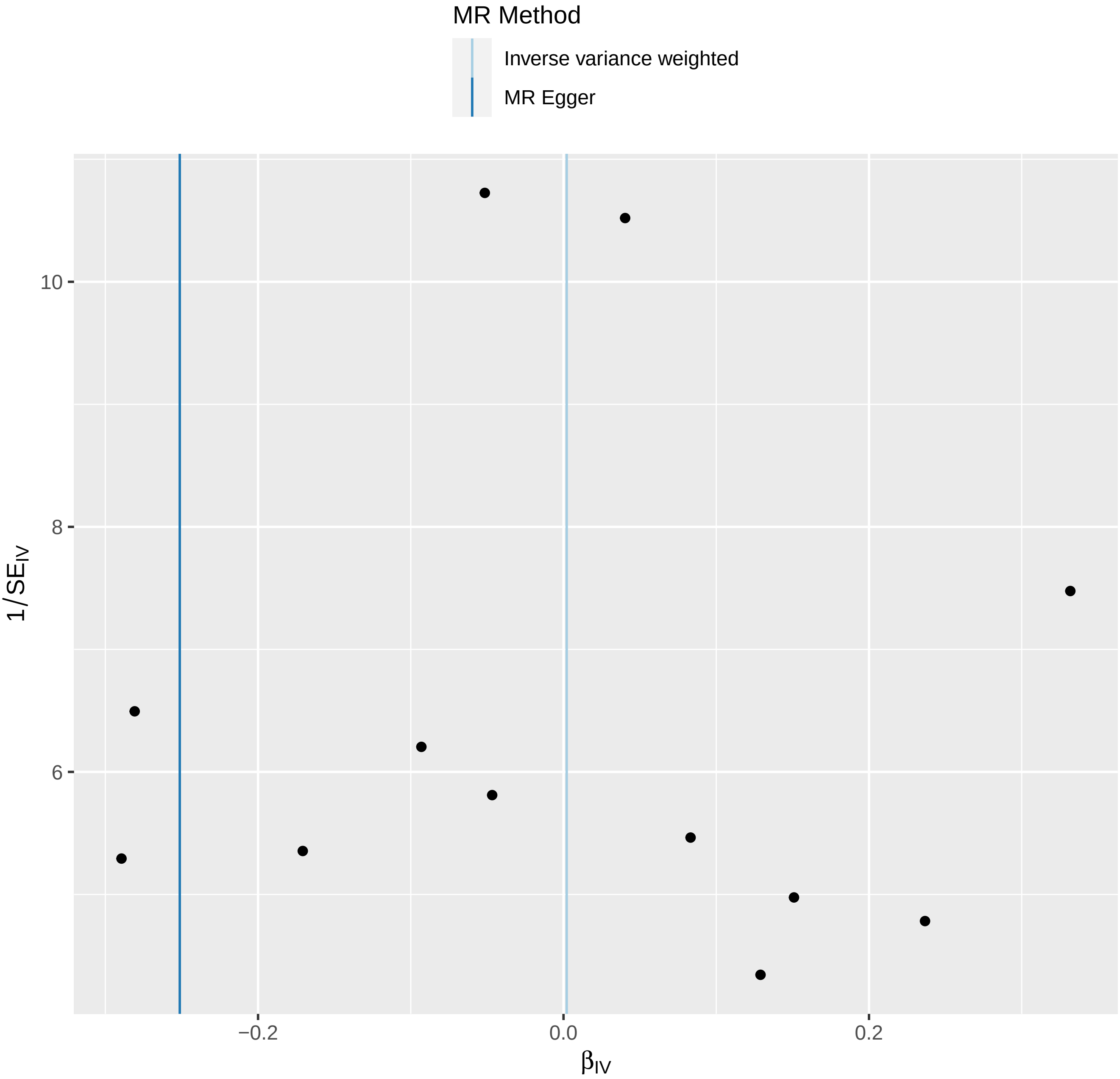

Batch 3−87 : Selenium on Amphoterin−induced protein 1

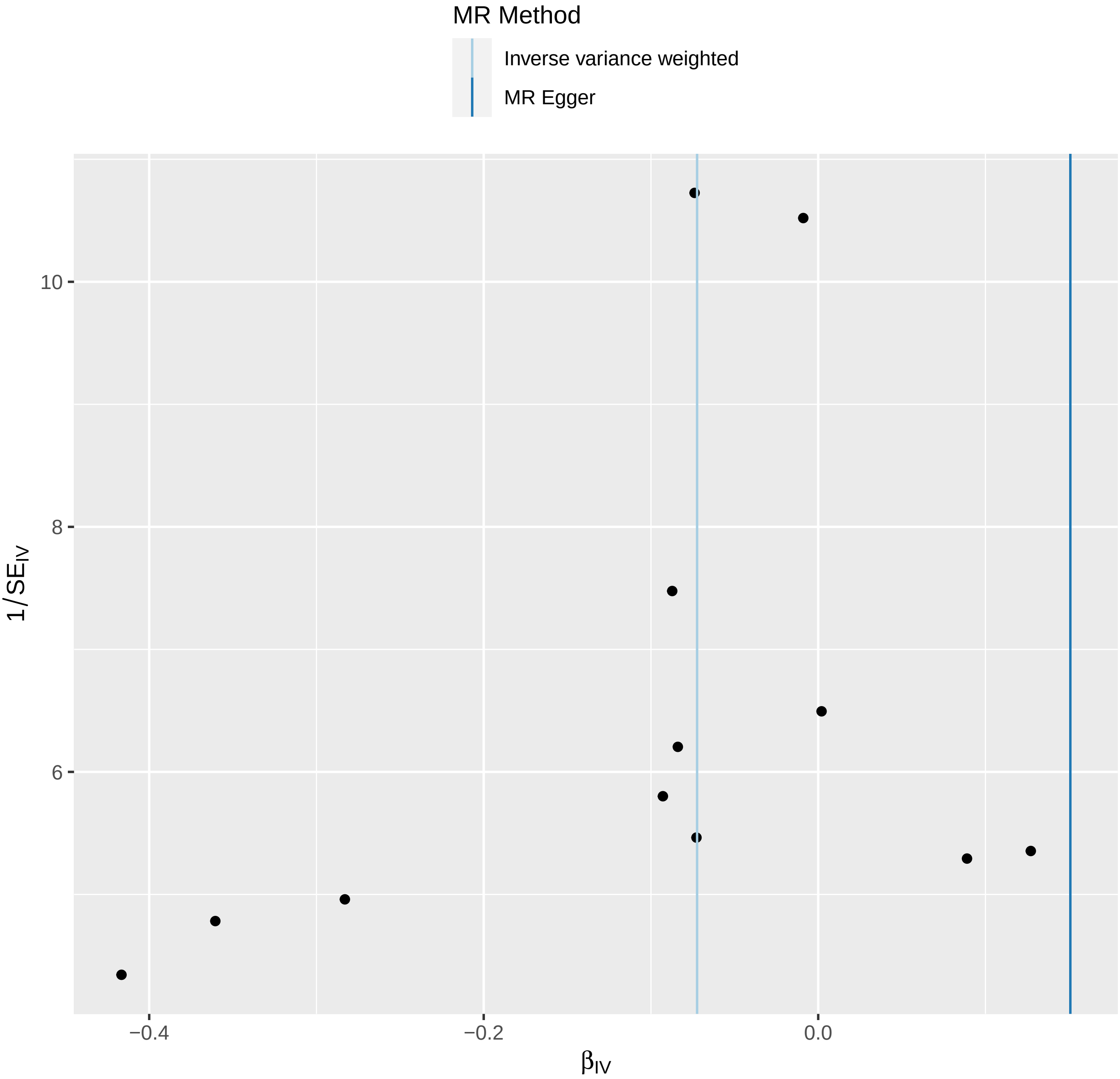

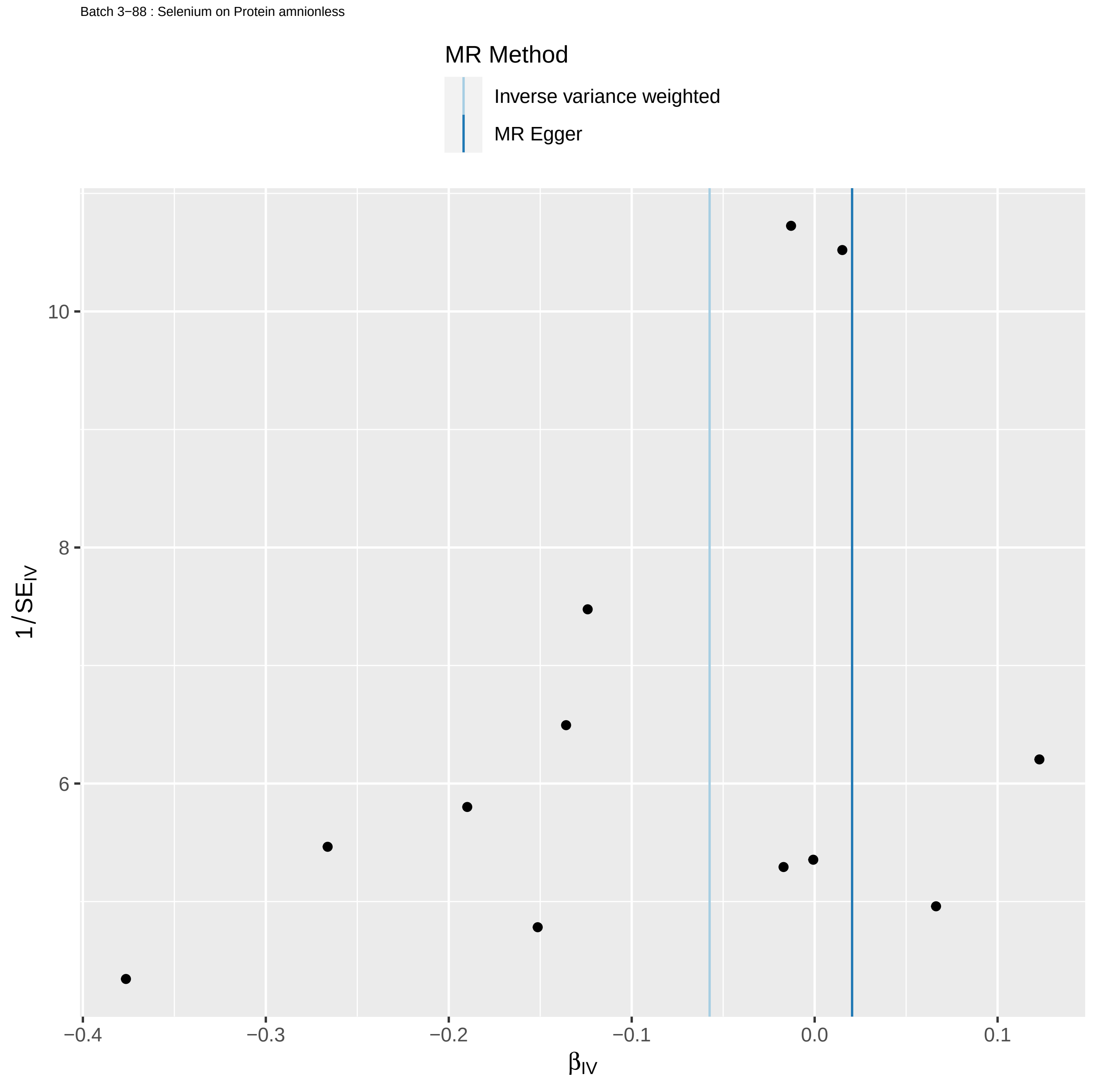

Batch 3−88 : Selenium on Protein amnionless
MR Method
Inverse variance weighted
MR Egger
10
8
6
1/SE_IV
−0.4
−0.3
−0.2
−0.1
0.0
0.1
β_IV

Batch 3−89 : Selenium on Alpha−amylase 1

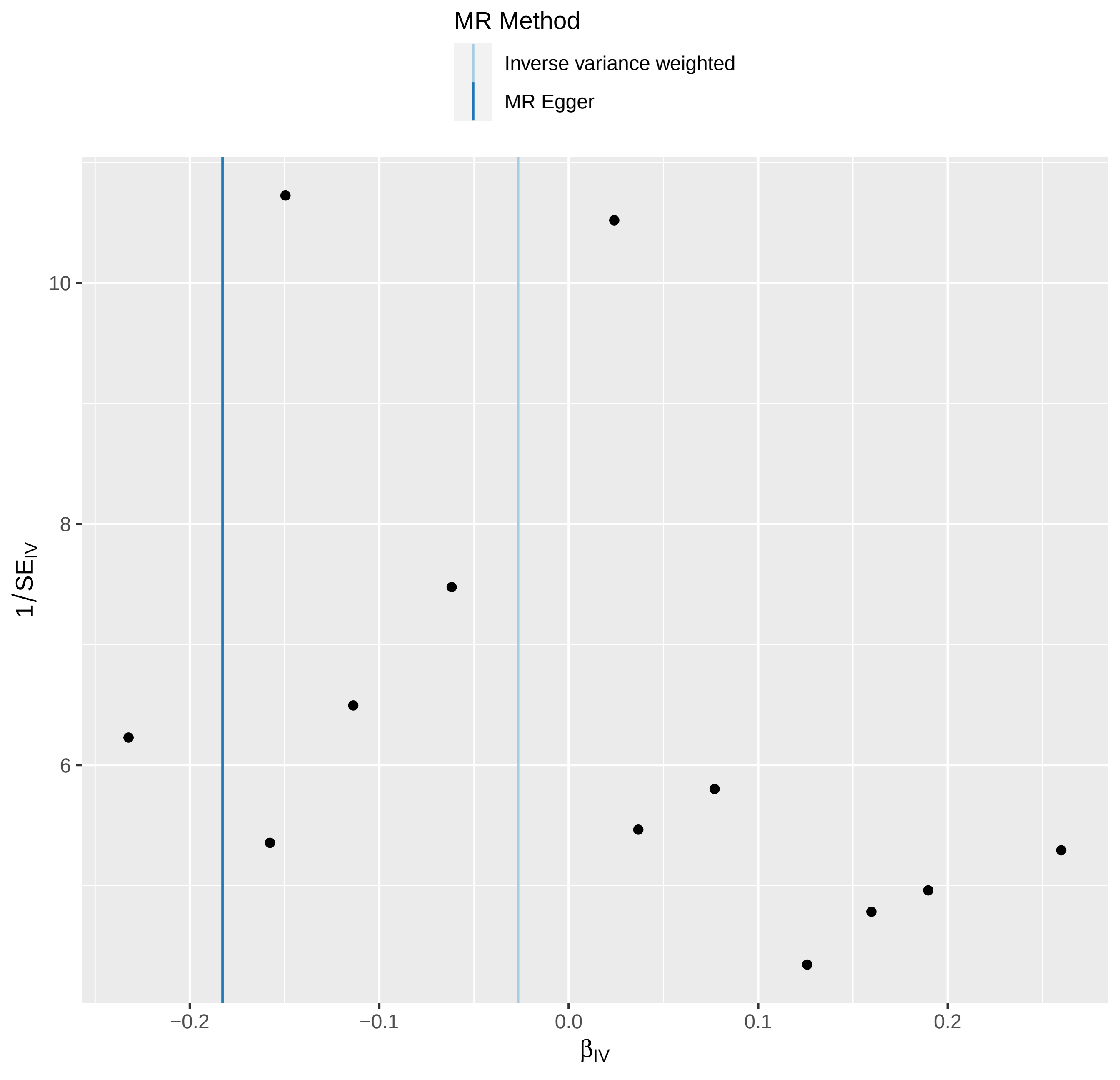

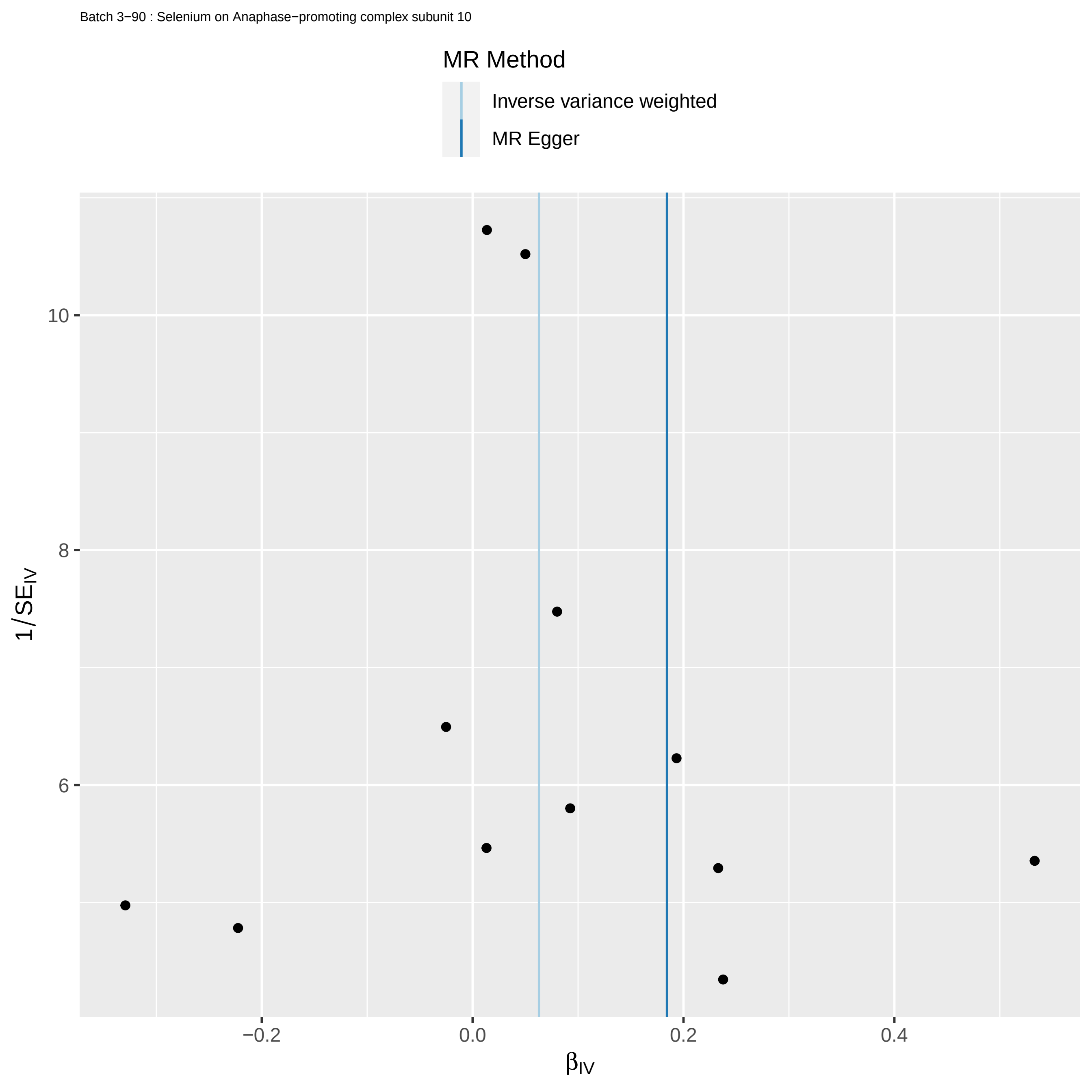

Batch 3−90 : Selenium on Anaphase−promoting complex subunit 10
MR Method
Inverse variance weighted
MR Egger
10
8
6
−0.2
0.0
0.2
0.4
$1/SE_{IV}$
β_{IV}

Batch 3−91 : Selenium on Anaphase−promoting complex subunit 7

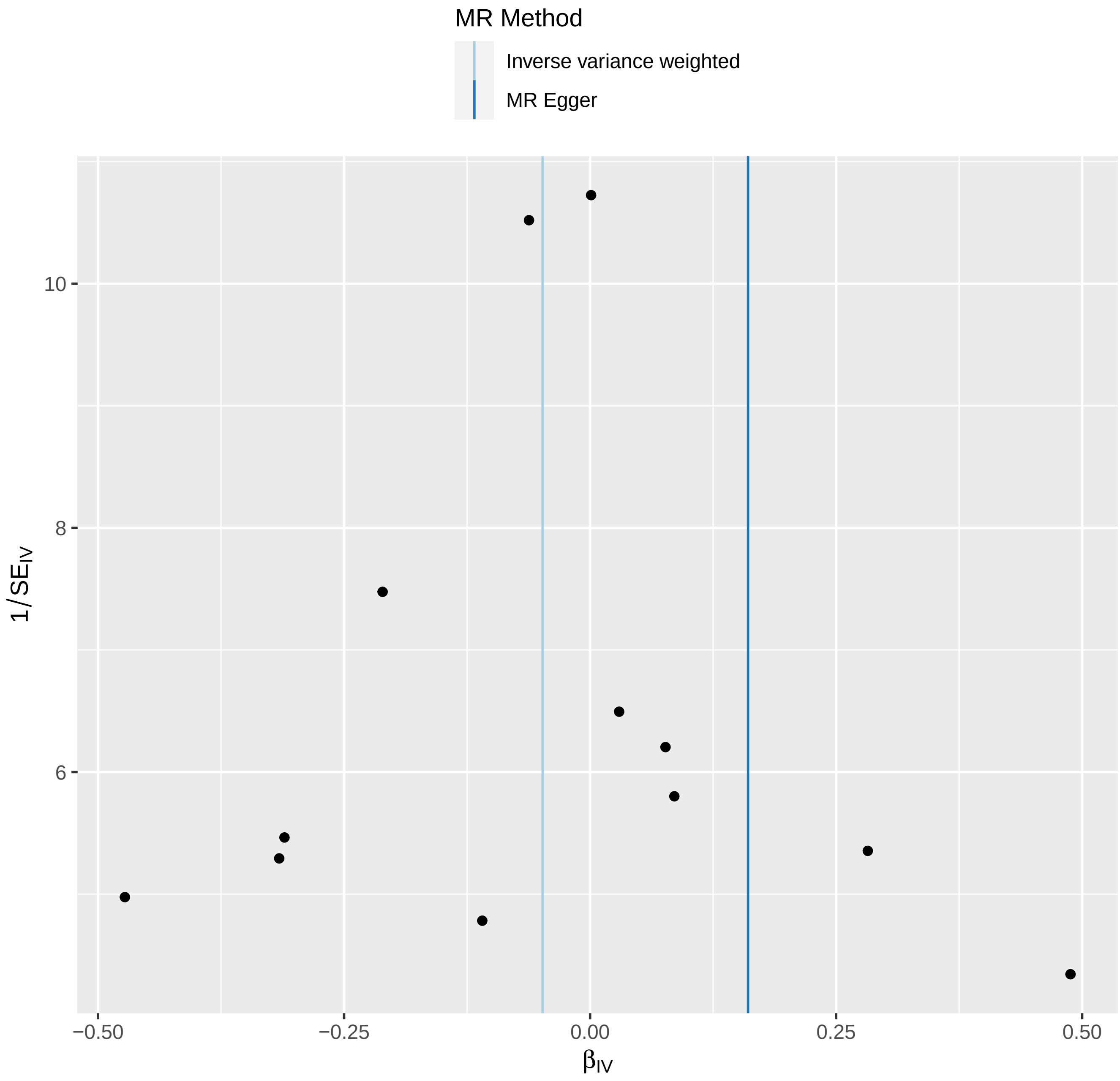

Batch 3−92 : Selenium on Angiopoietin−1

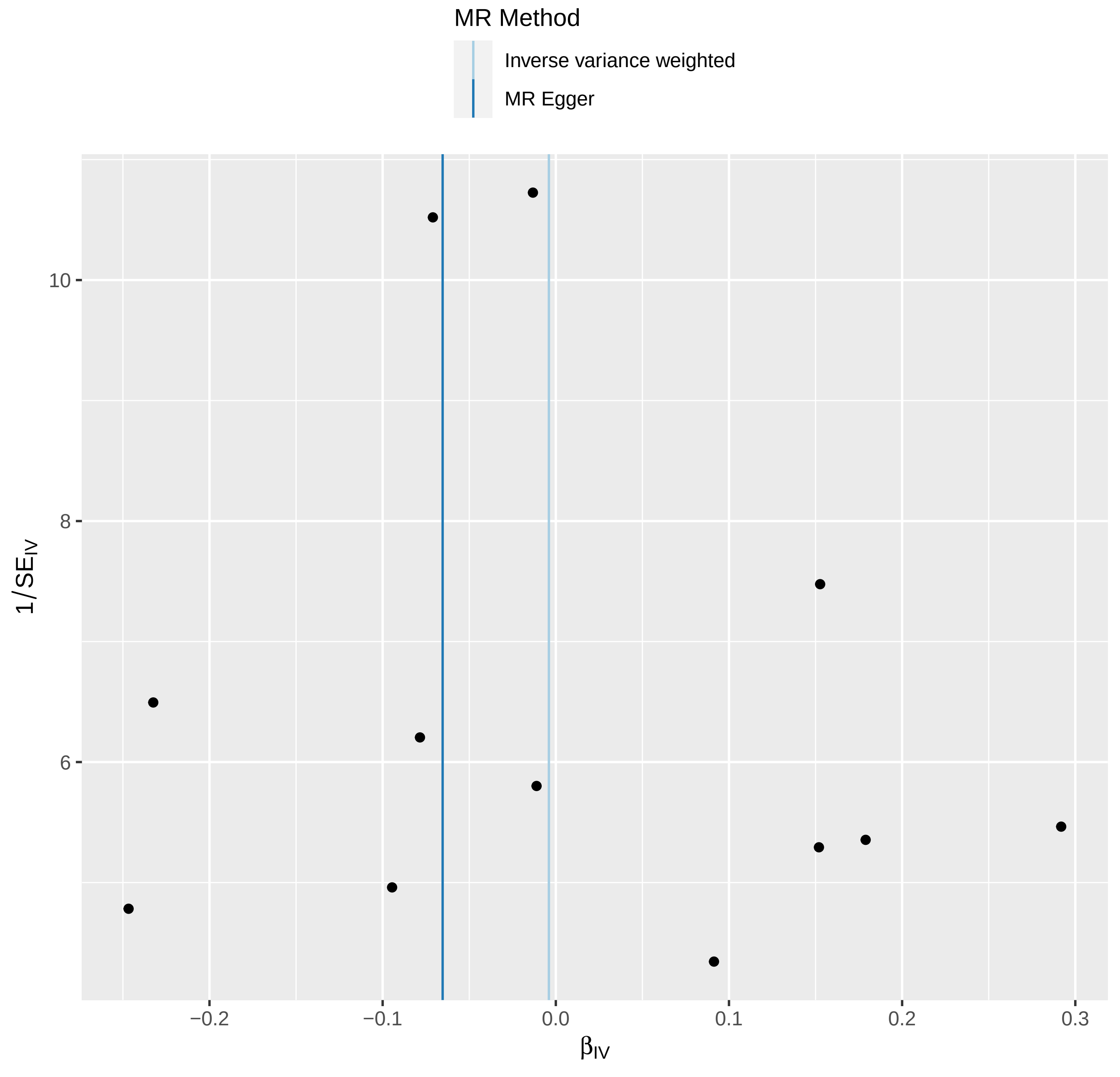

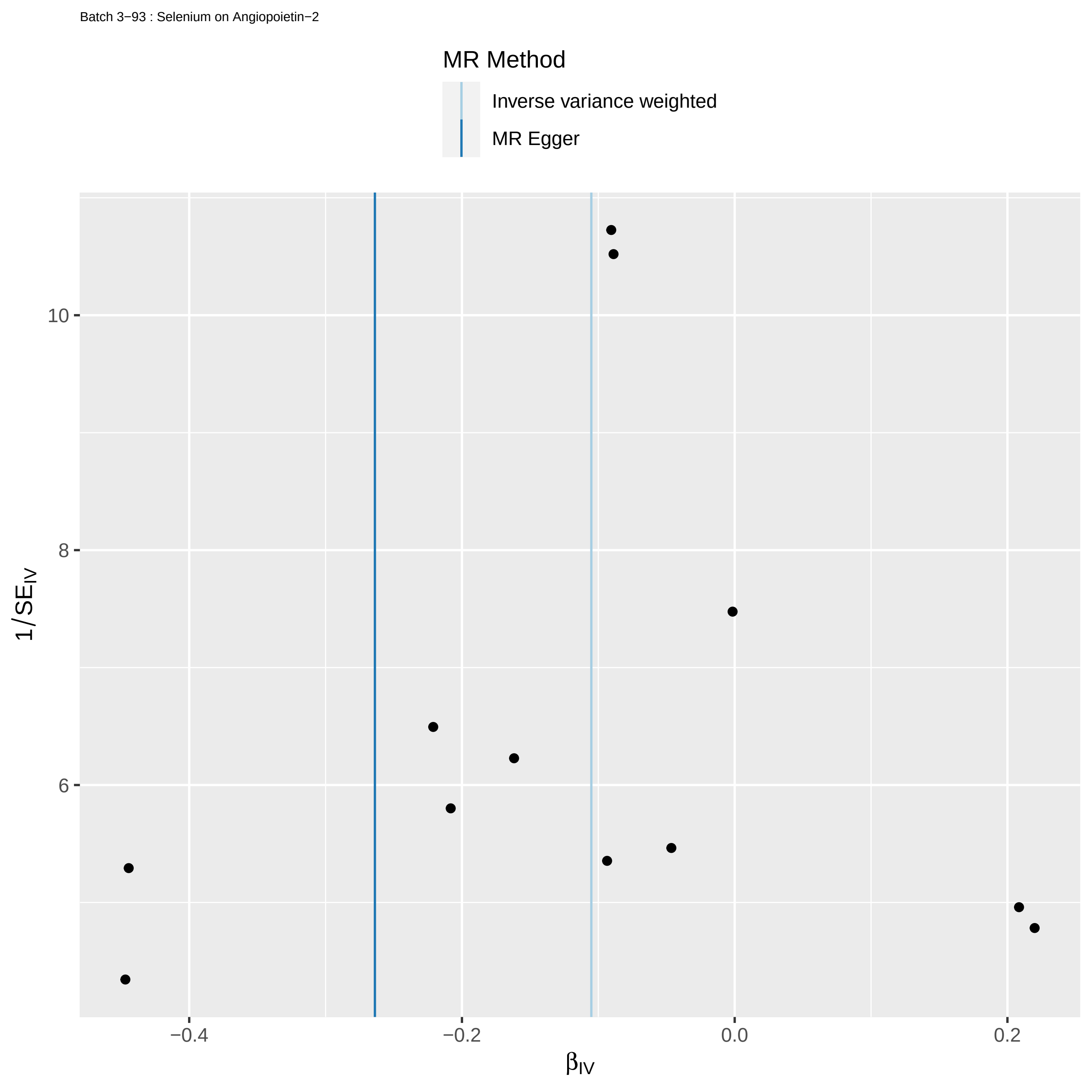

Batch 3−93 : Selenium on Angiopoietin−2
MR Method
Inverse variance weighted
MR Egger
10
8
6
1/SE$_{IV}$
−0.4
−0.2
0.0
0.2
β$_{IV}$

Batch 3−94 : Selenium on Angiopoietin−2

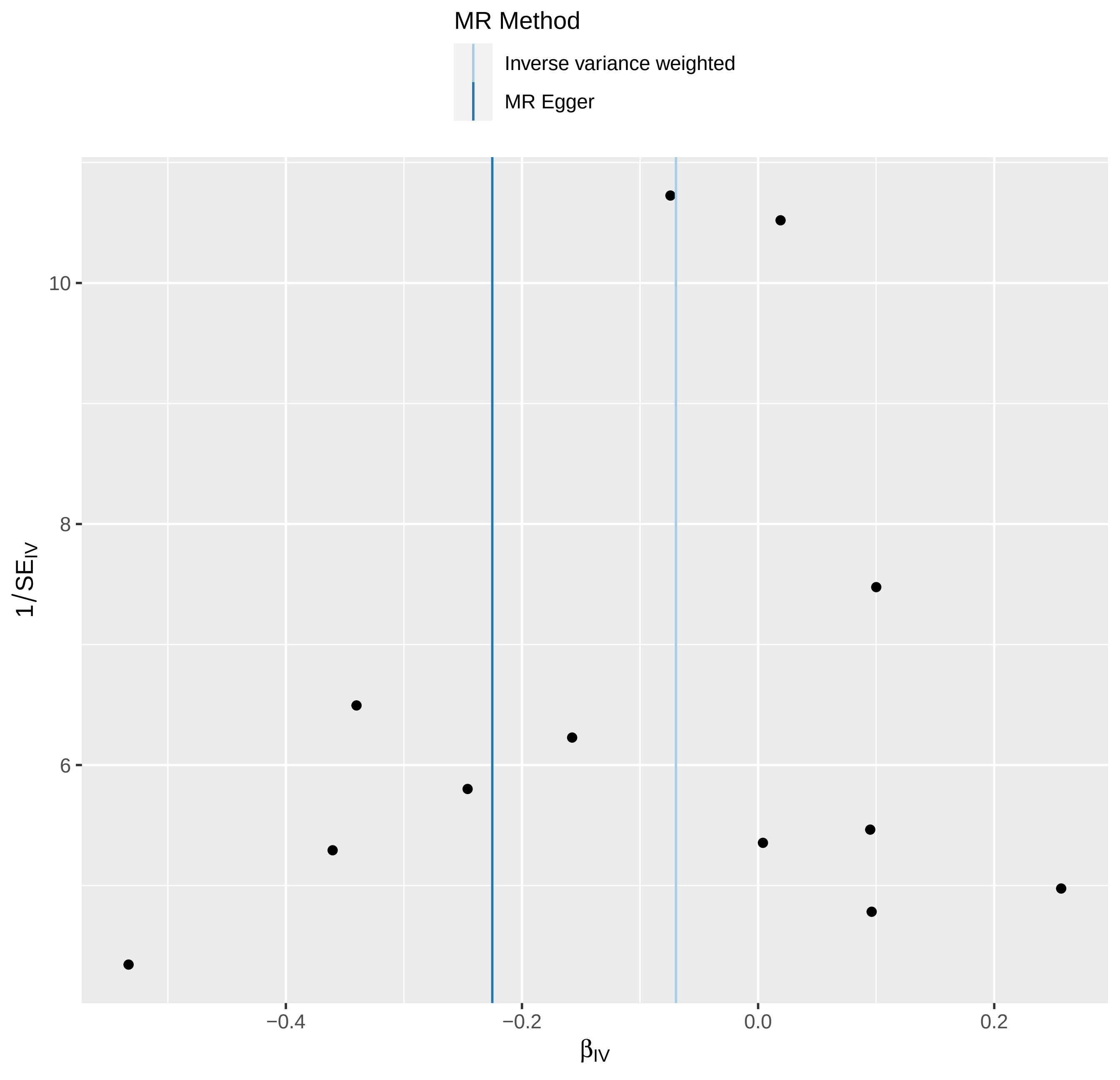

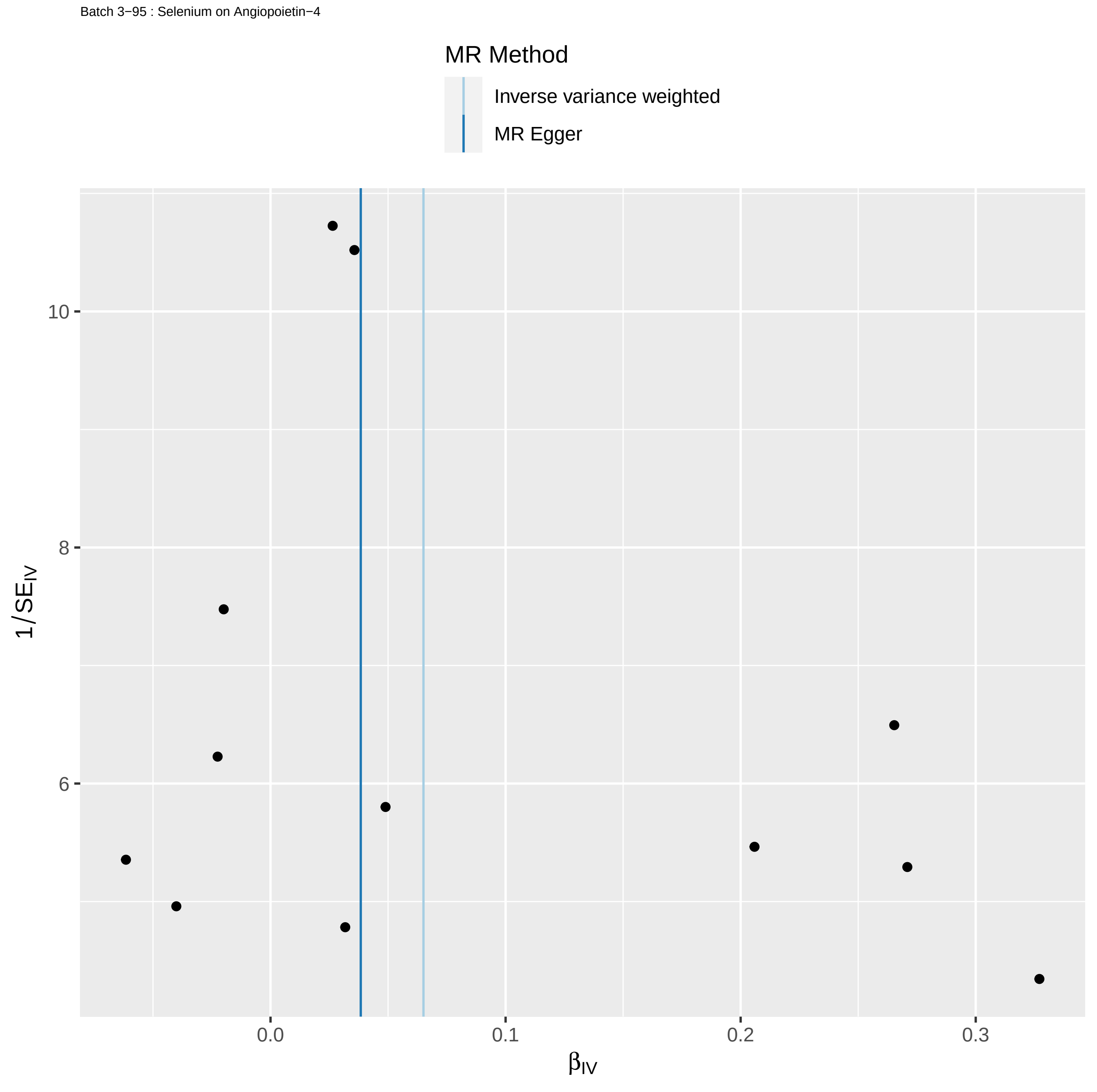

Batch 3−95 : Selenium on Angiopoietin−4
MR Method
Inverse variance weighted
MR Egger
10
8
6
1/SE_IV
0.0
0.1
0.2
0.3
β_IV

Batch 3−96 : Selenium on Angiopoietin−related protein 1

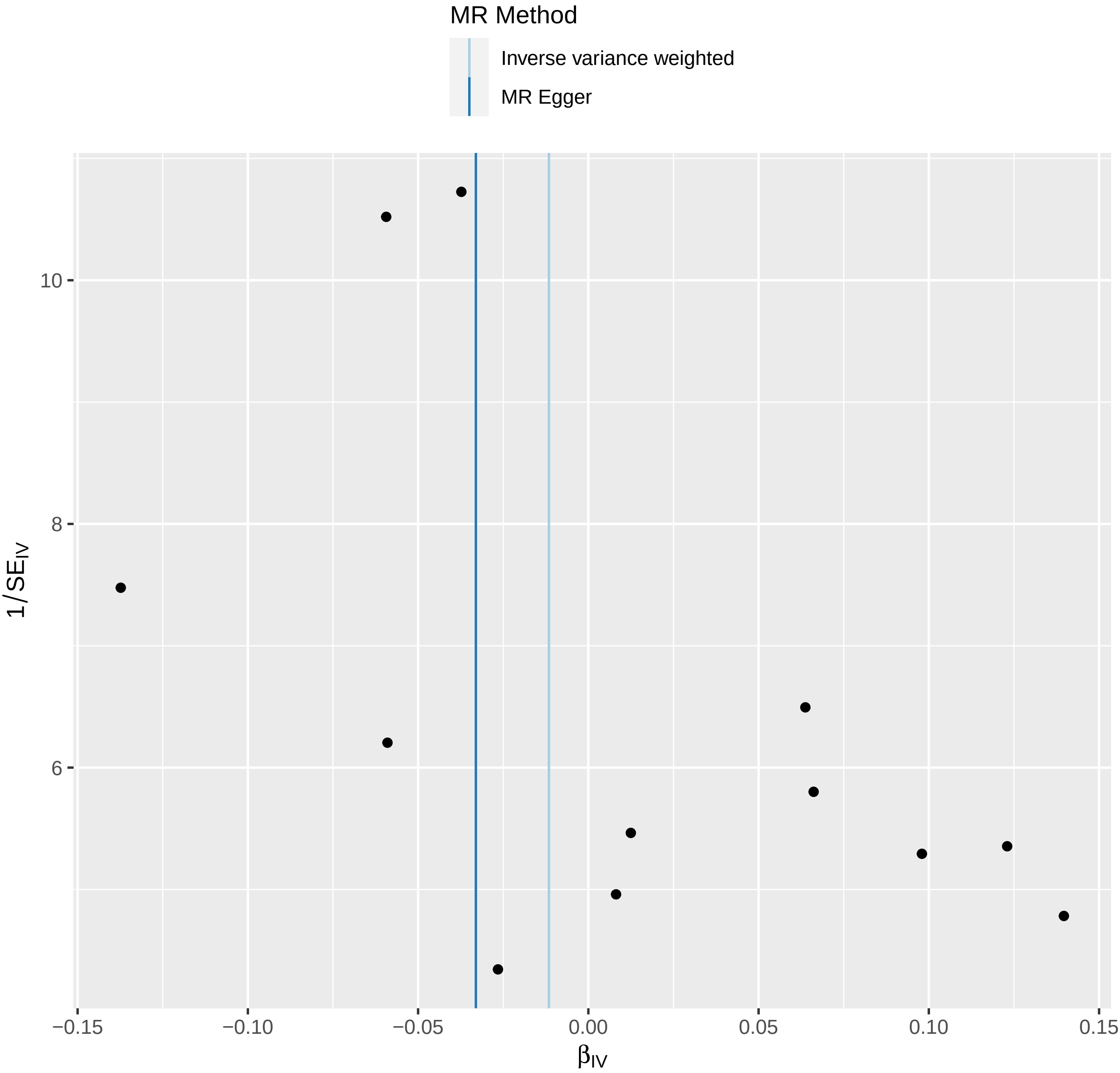

Batch 3−97 : Selenium on Angiopoietin−related protein 1

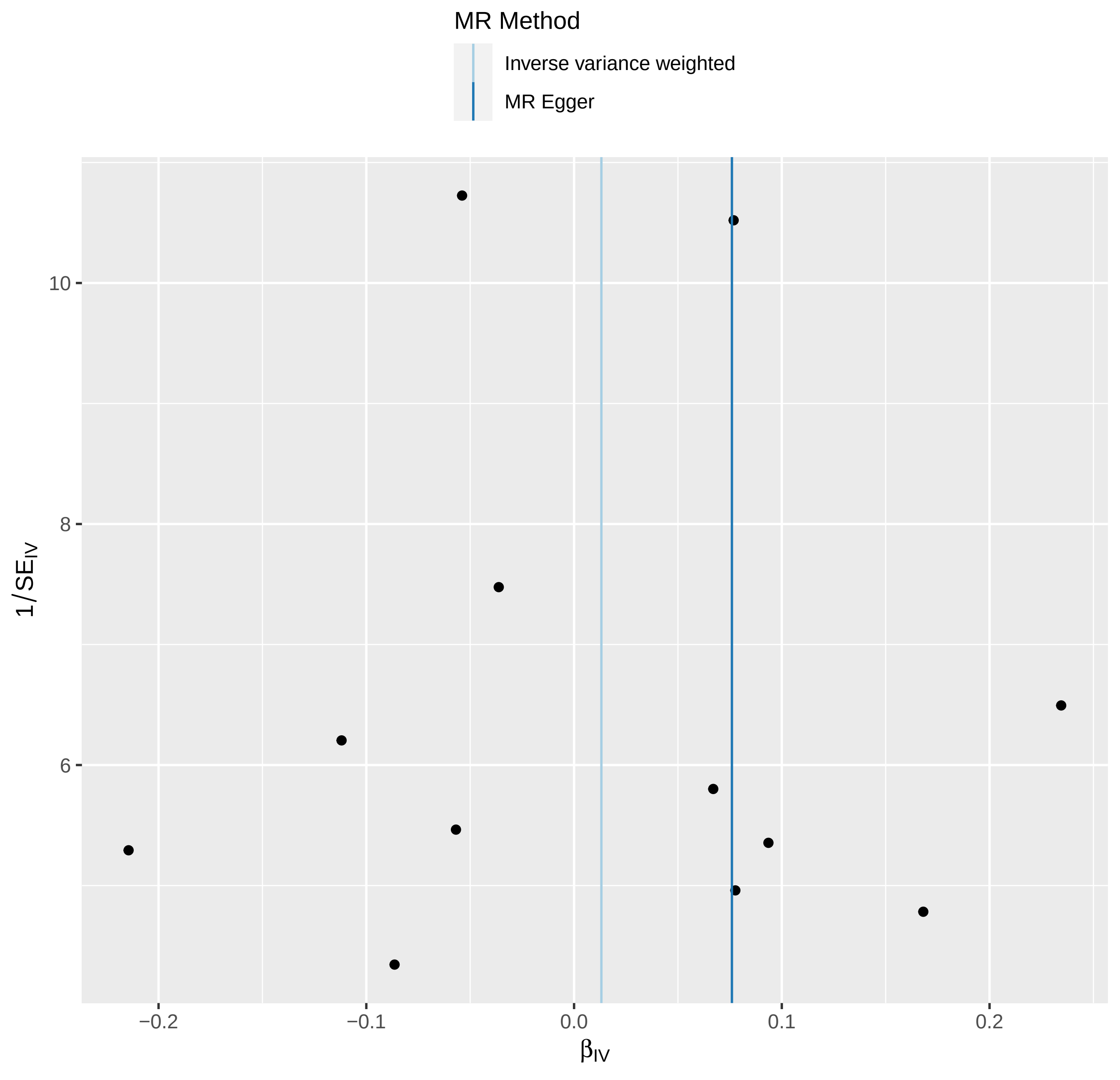

Batch 3−98 : Selenium on Angiopoietin−related protein 3

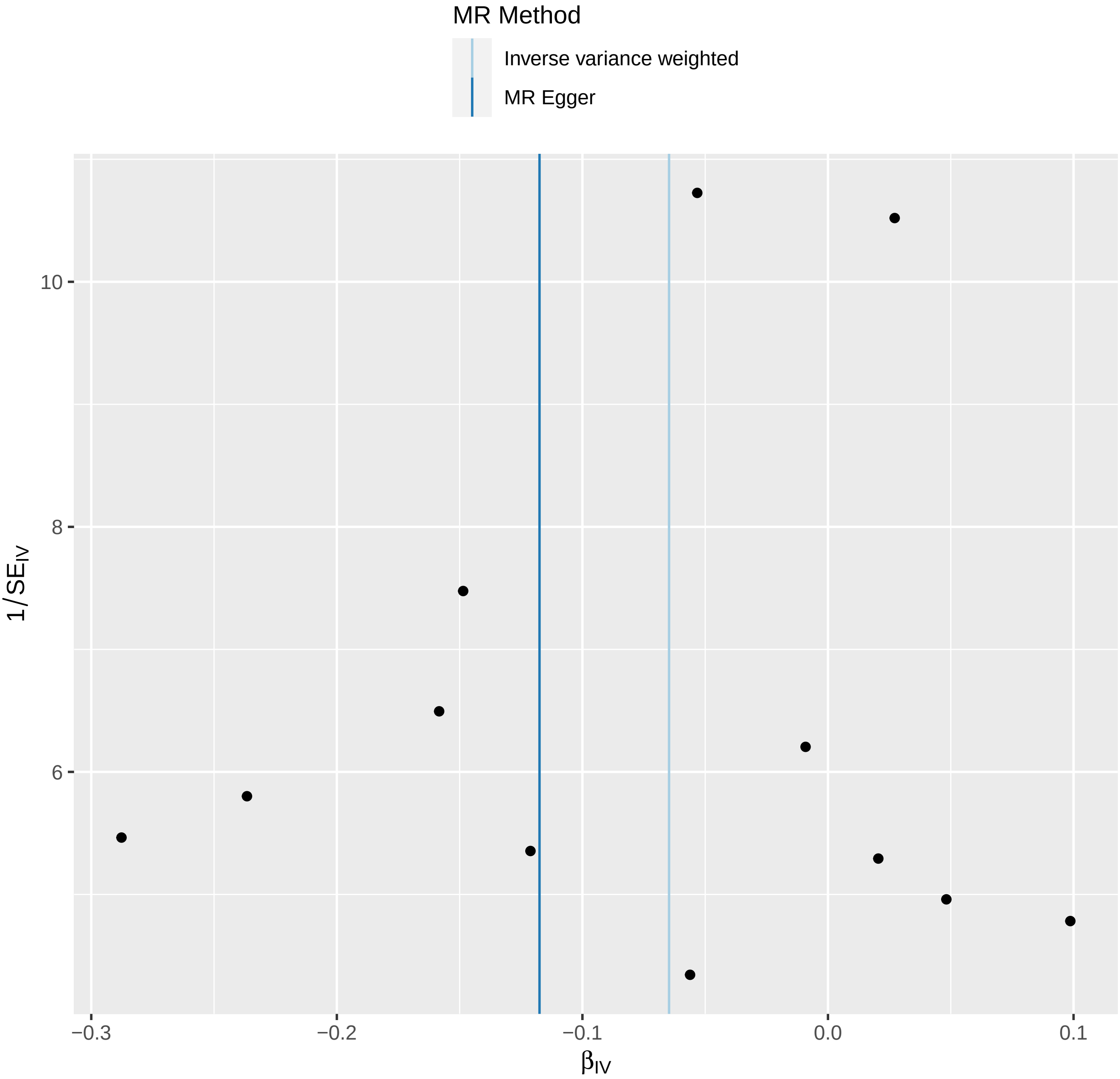

Batch 3−99 : Selenium on Angiopoietin−related protein 4

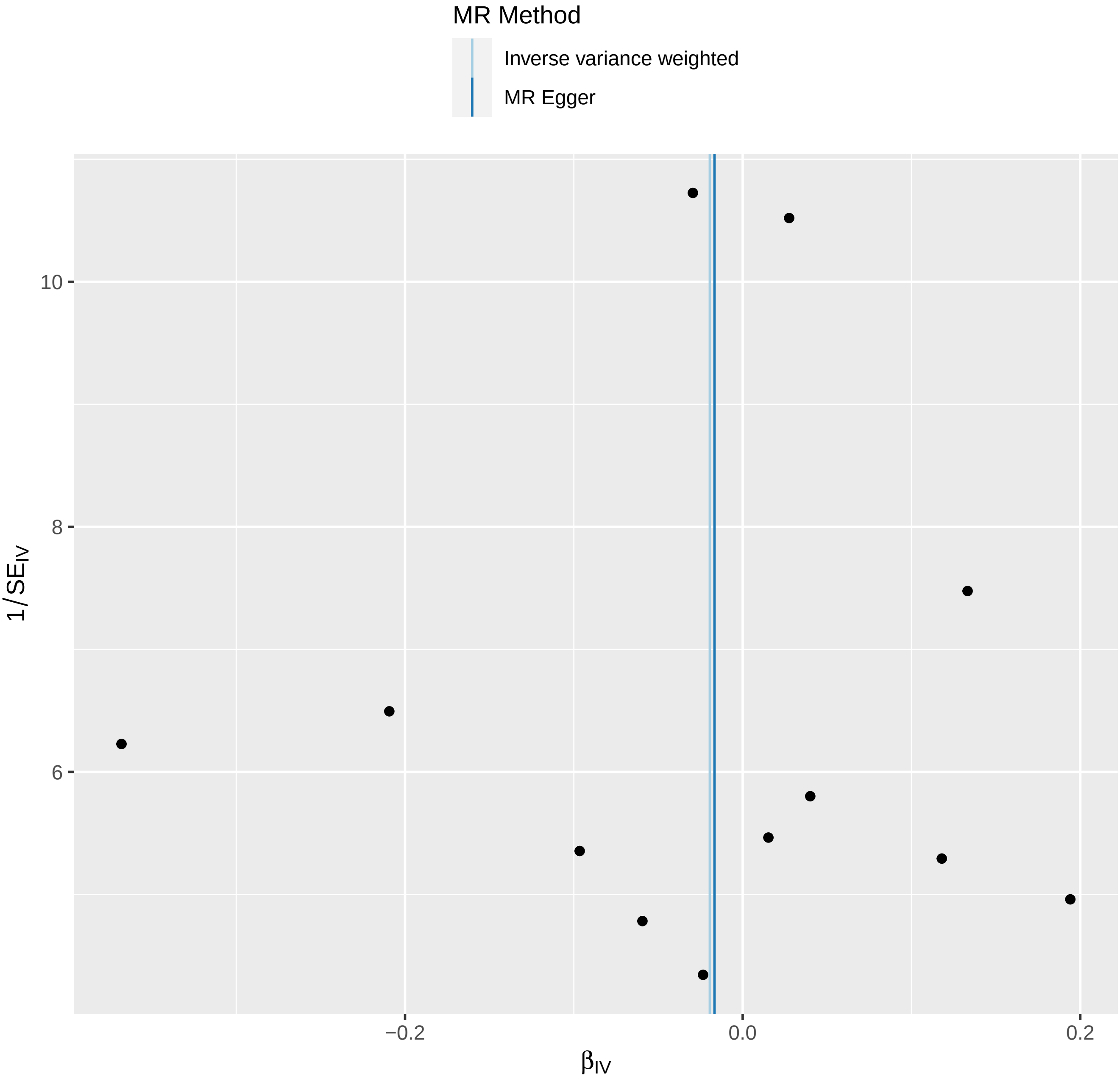

Batch 3−100 : Selenium on Angiopoietin−related protein 7

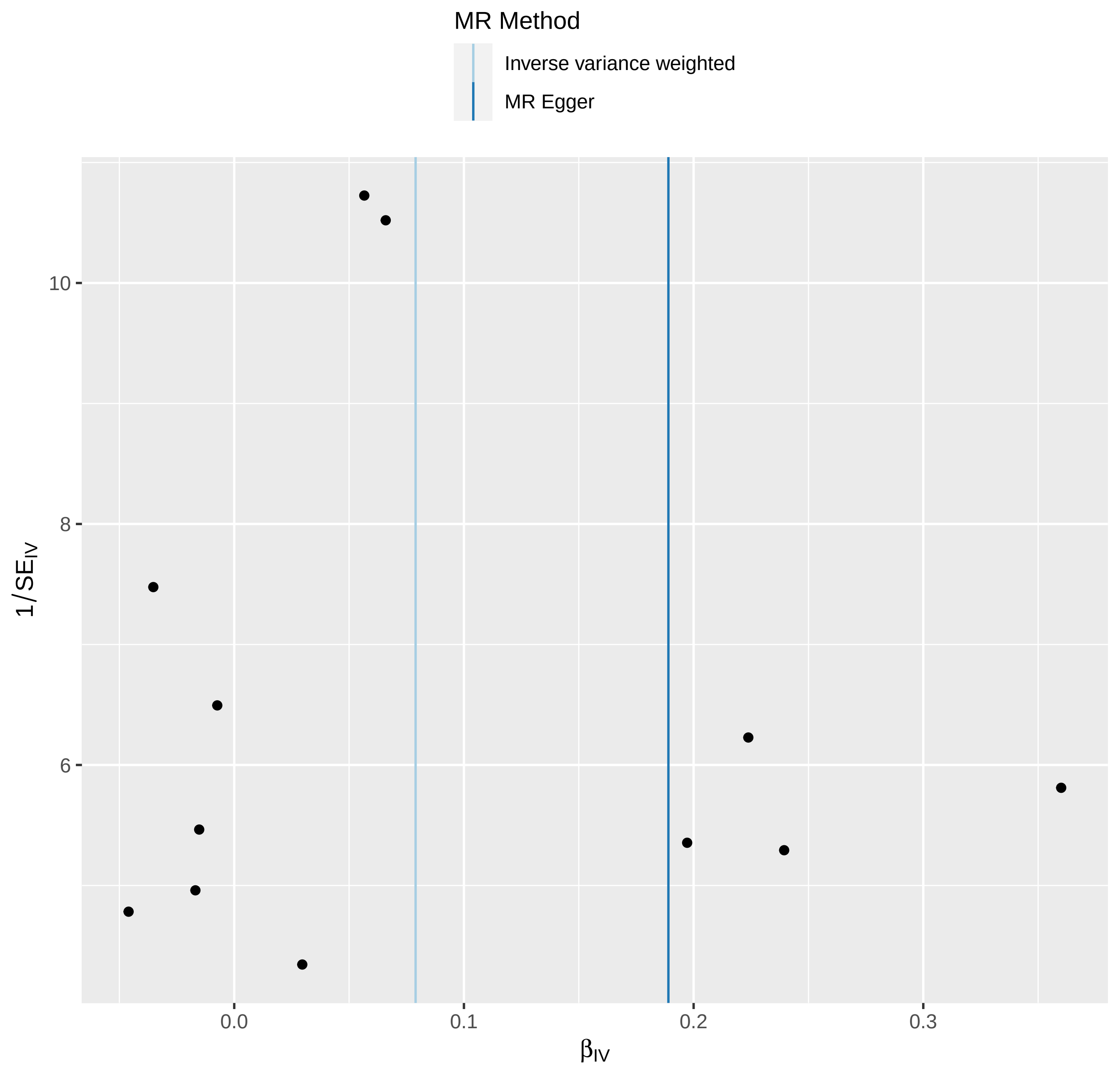

Batch 3−101 : Selenium on Ankyrin−2

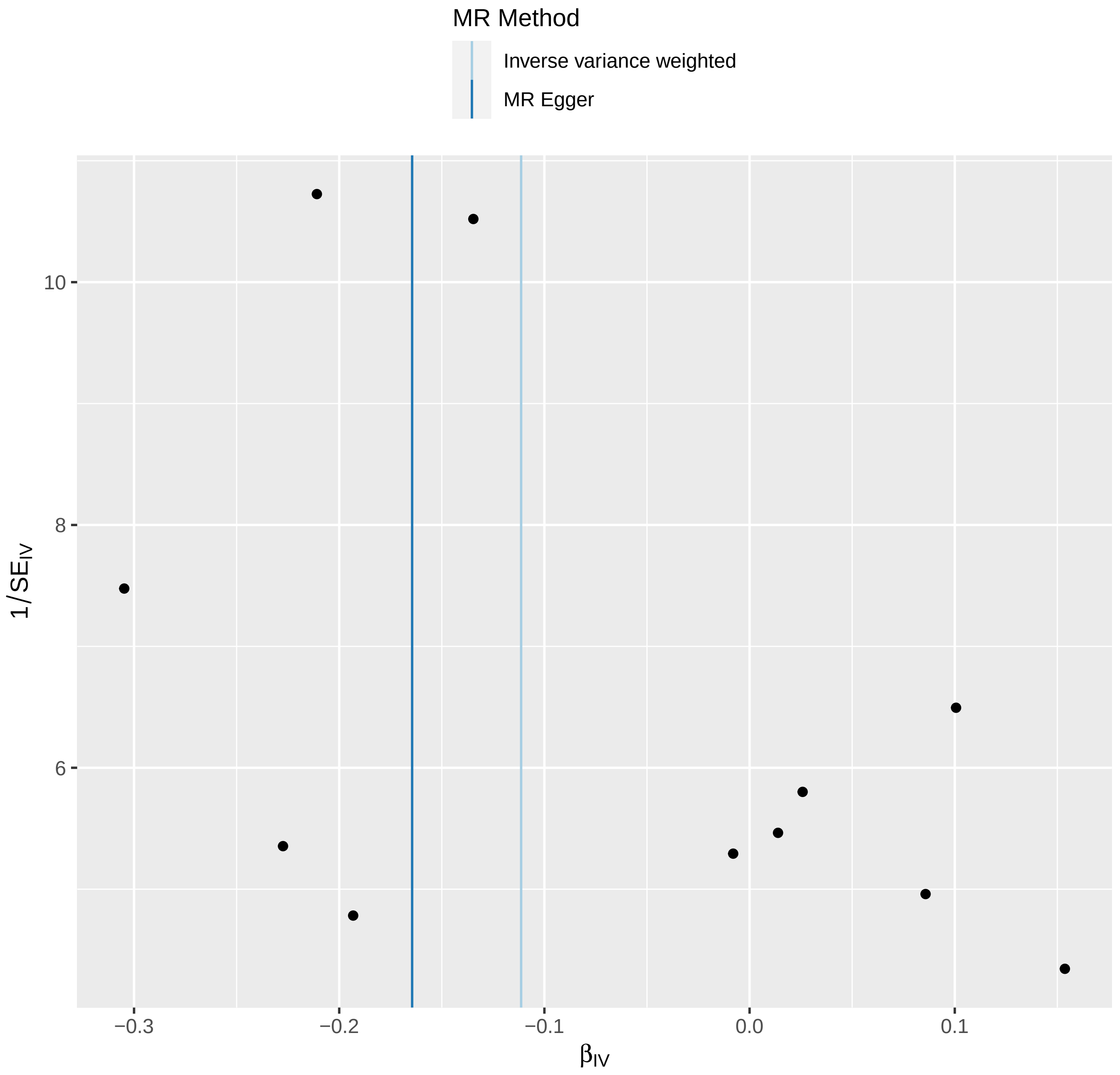

Batch 3−102 : Selenium on Ankyrin repeat domain−containing protein 27

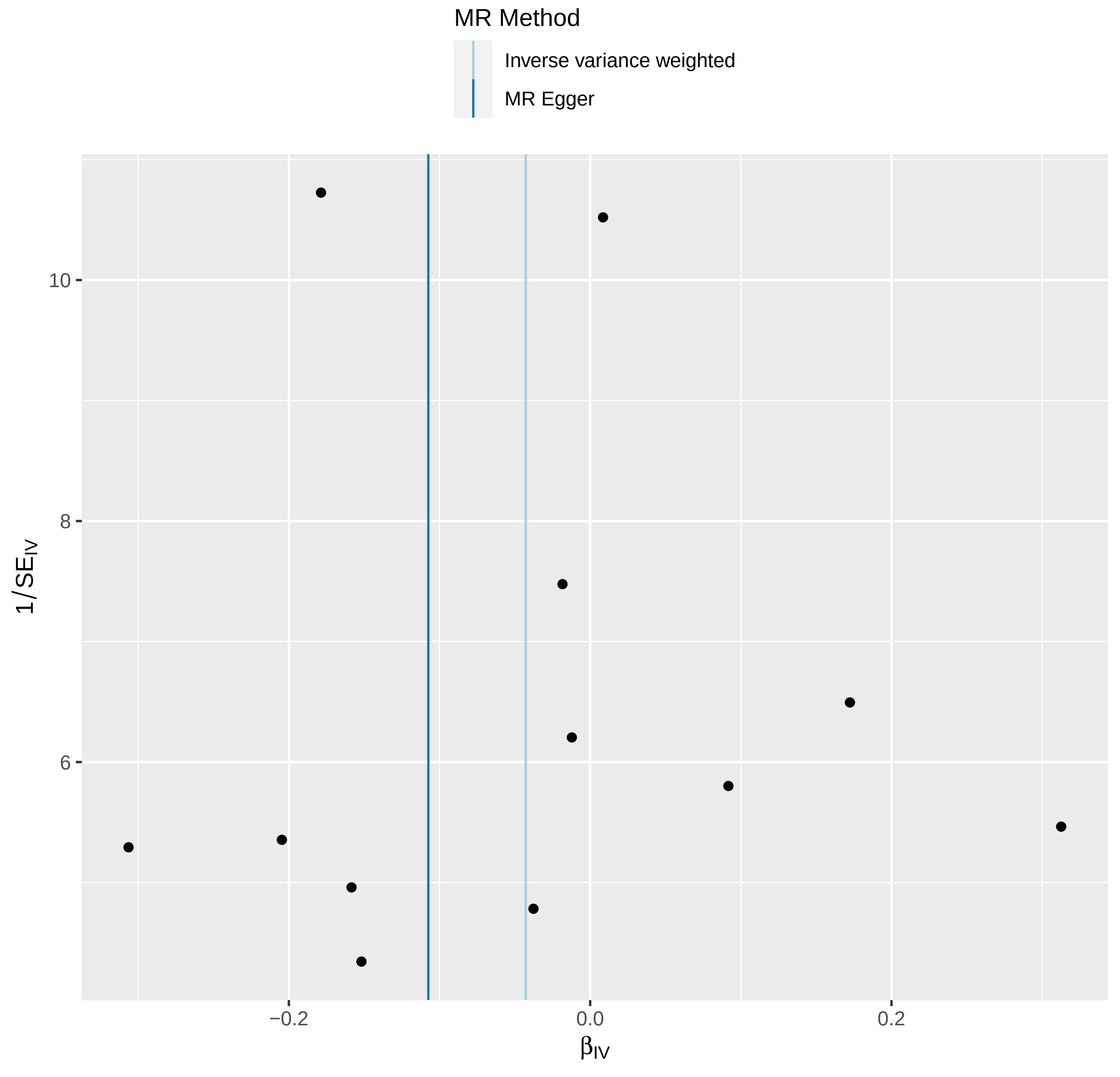

Batch 3−103 : Selenium on Ankyrin repeat domain−containing protein 46

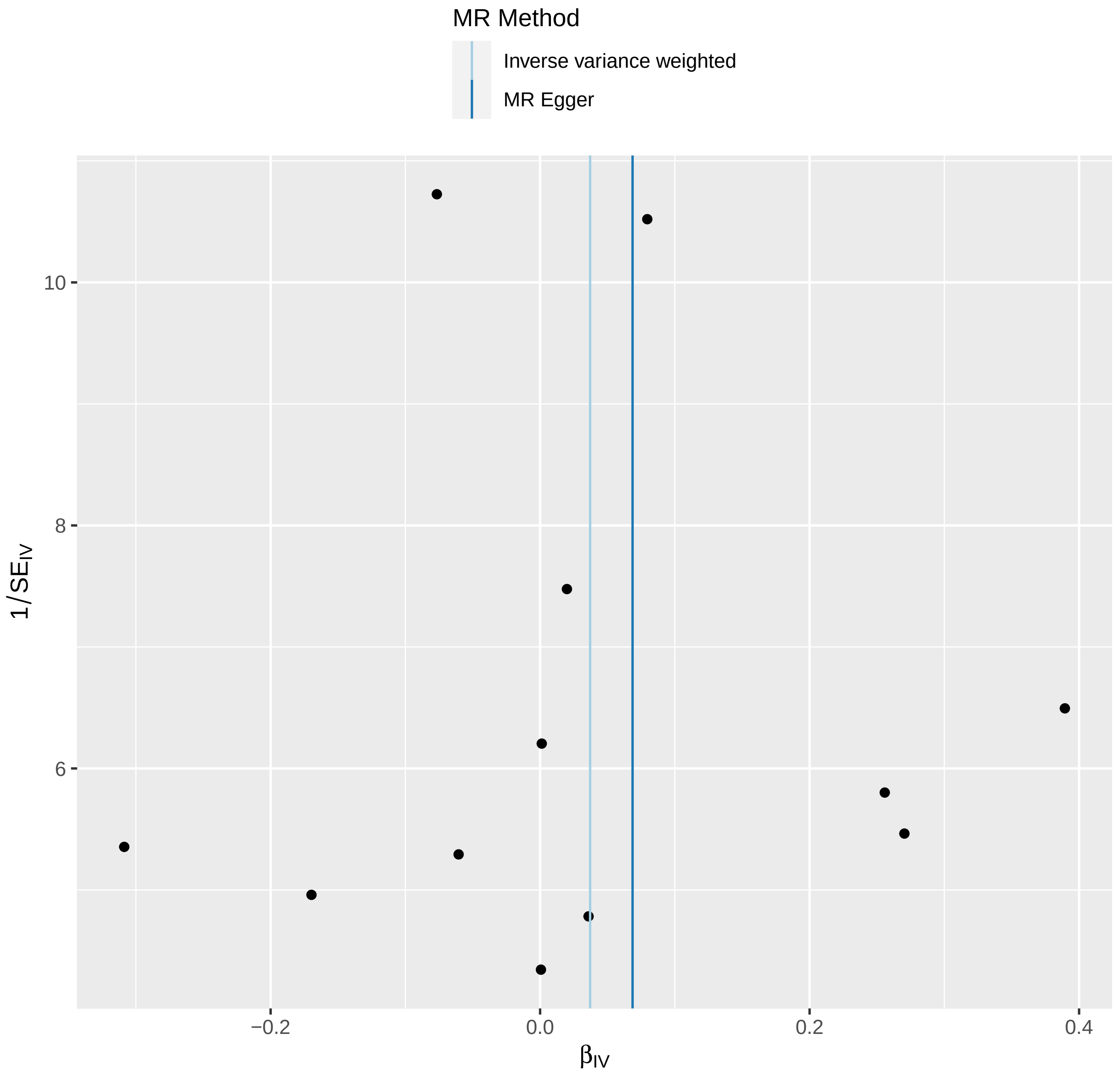

Batch 3−104 : Selenium on Acidic leucine−rich nuclear phosphoprotein 32 family member A

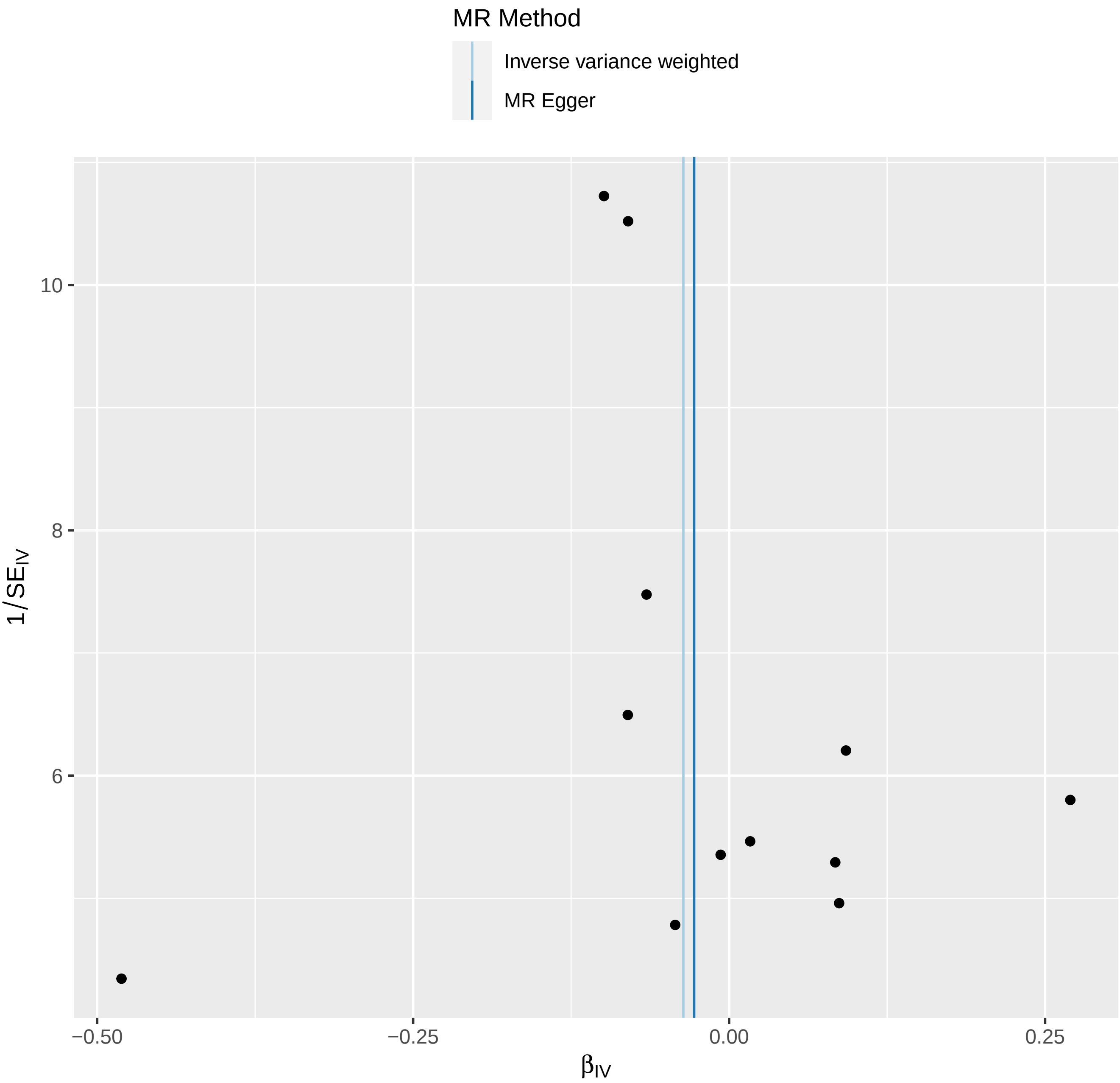

Batch 3−105 : Selenium on Acidic leucine−rich nuclear phosphoprotein 32 family member B

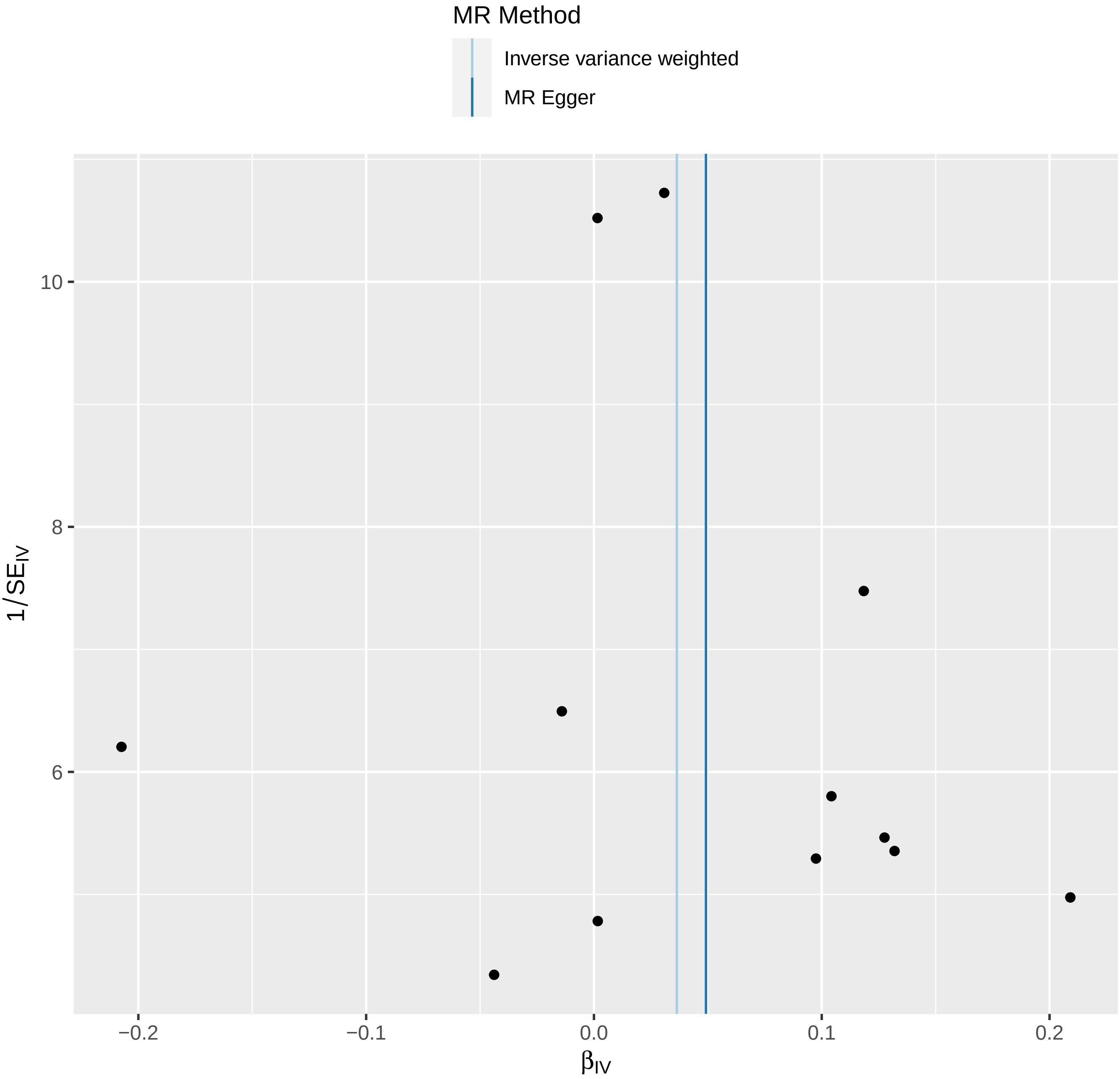

Batch 3−106 : Selenium on Annexin A10

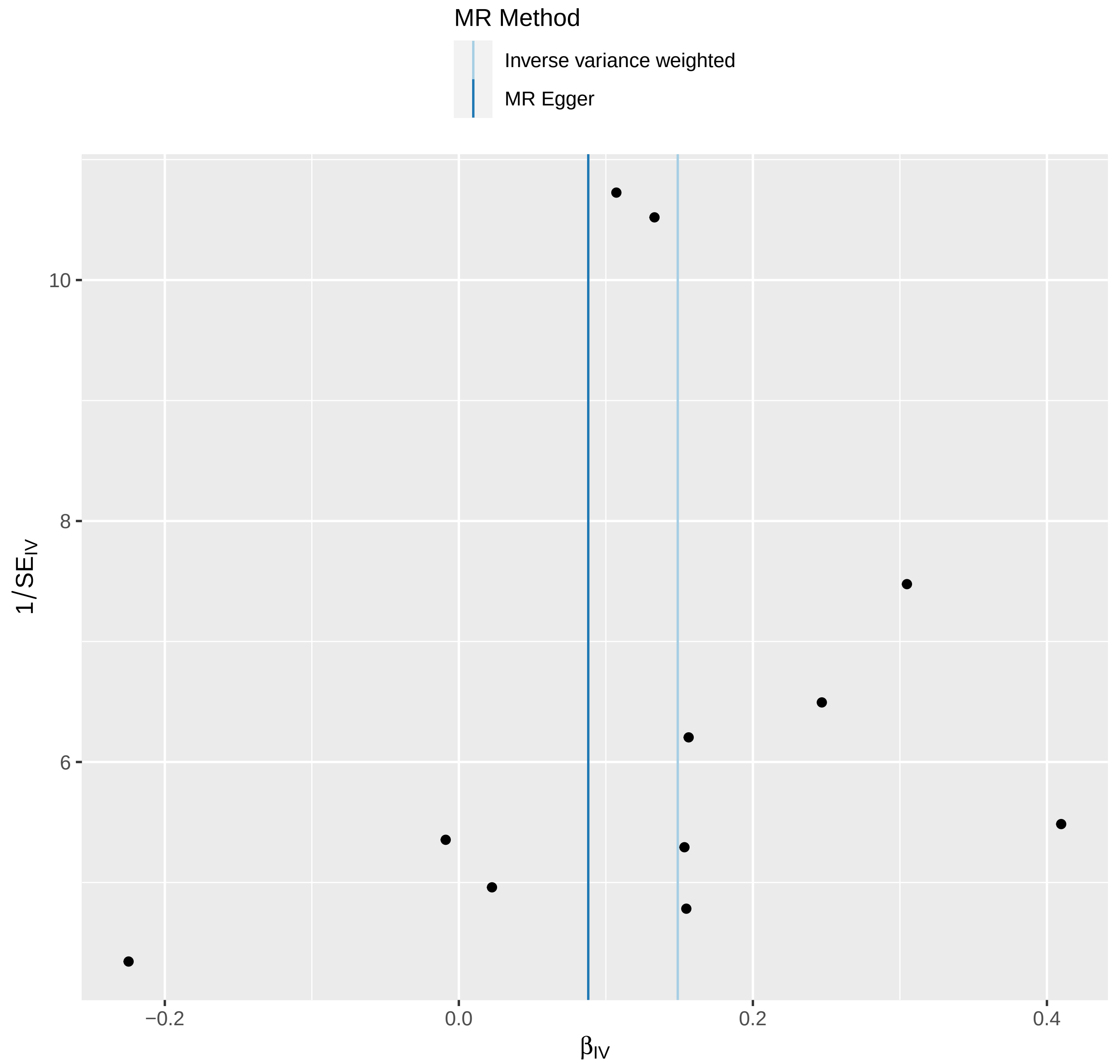

Batch 3−107 : Selenium on Annexin A1

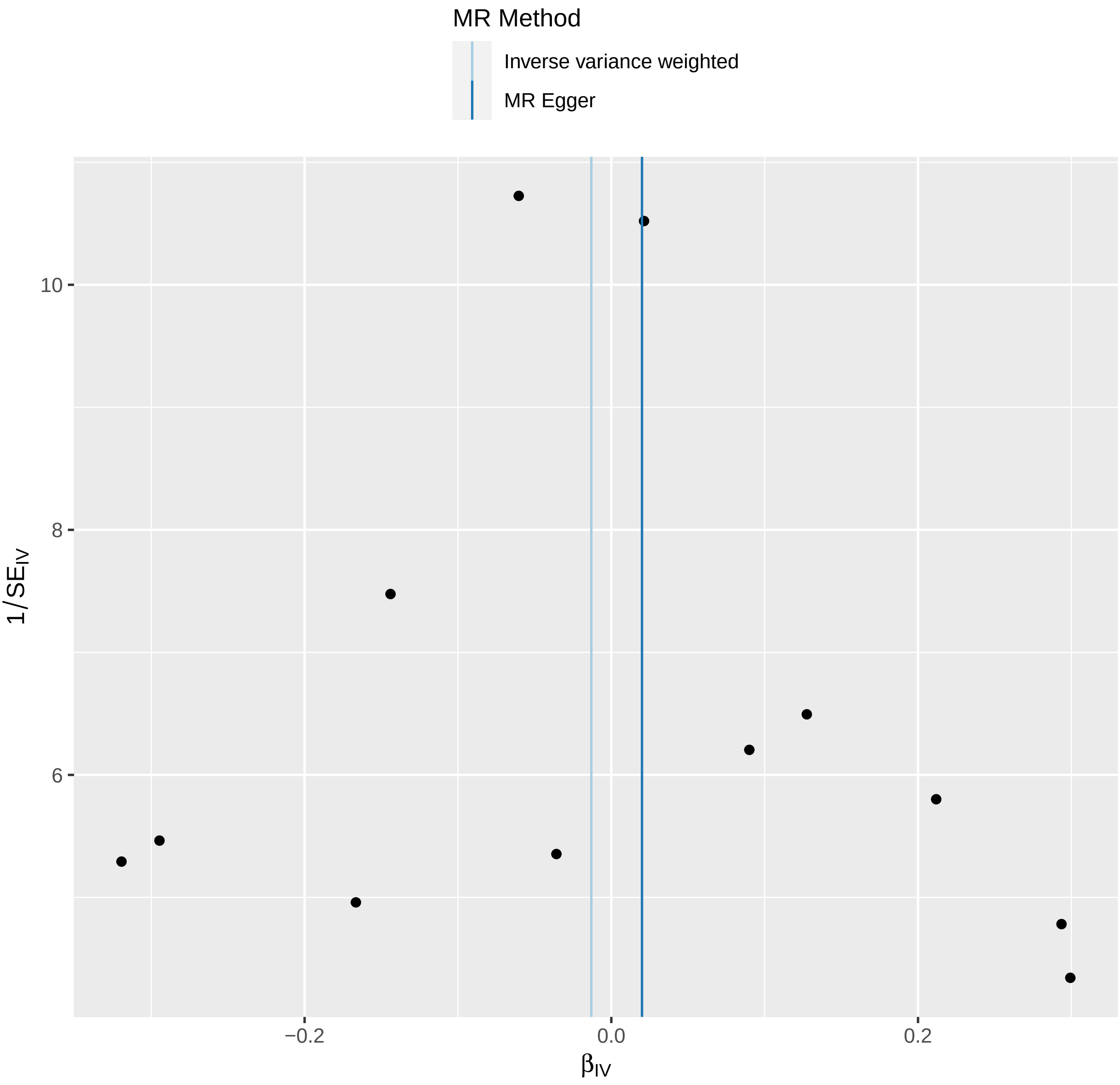

Batch 3−108 : Selenium on Annexin A2

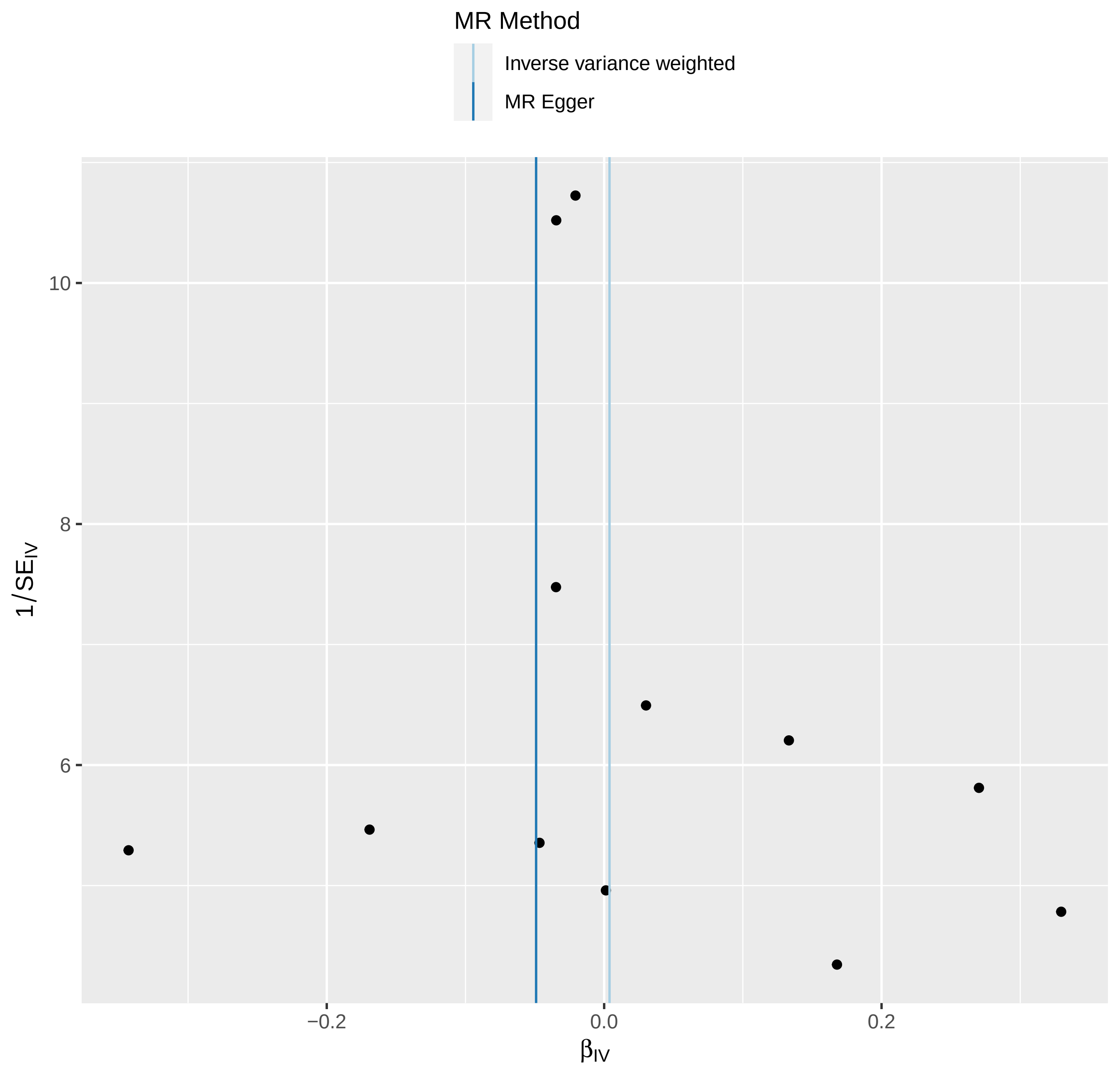

Batch 3−109 : Selenium on Annexin A2

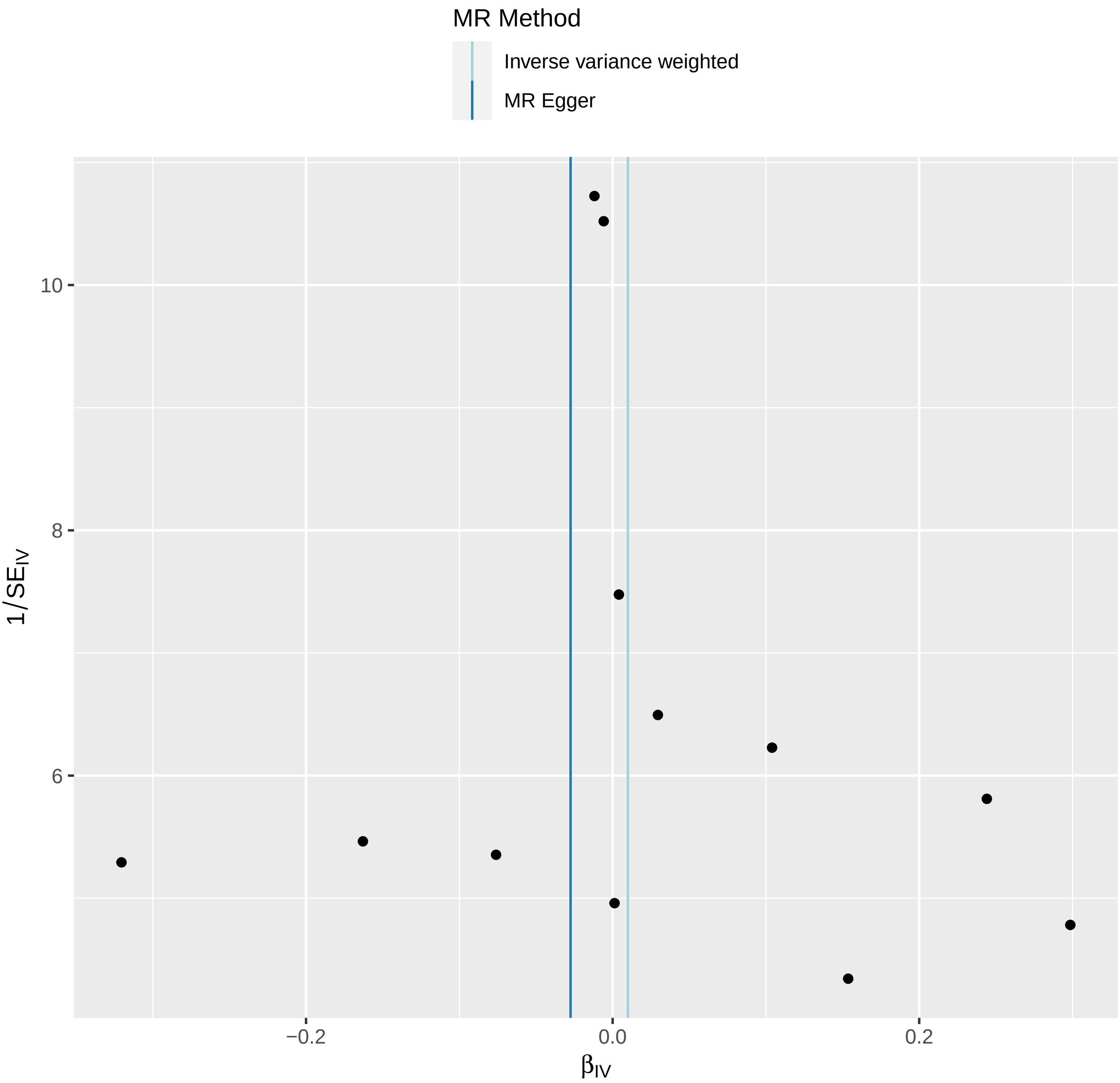

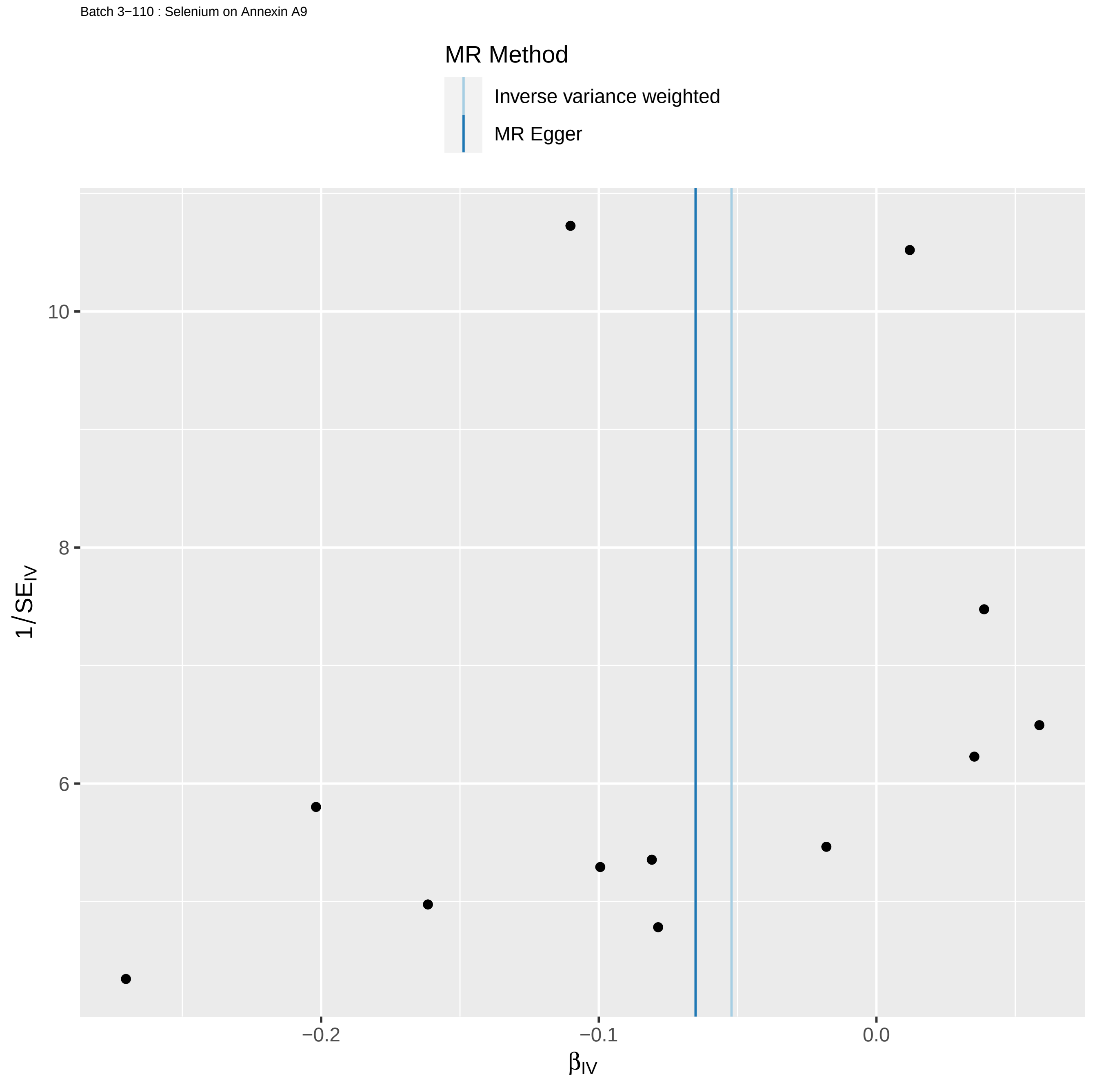
Batch 3−110 : Selenium on Annexin A9
MR Method
Inverse variance weighted
MR Egger
10
8
6
1/SE_IV
−0.2
−0.1
0.0
β_IV

Batch 3−111 : Selenium on Retina−specific copper amine oxidase

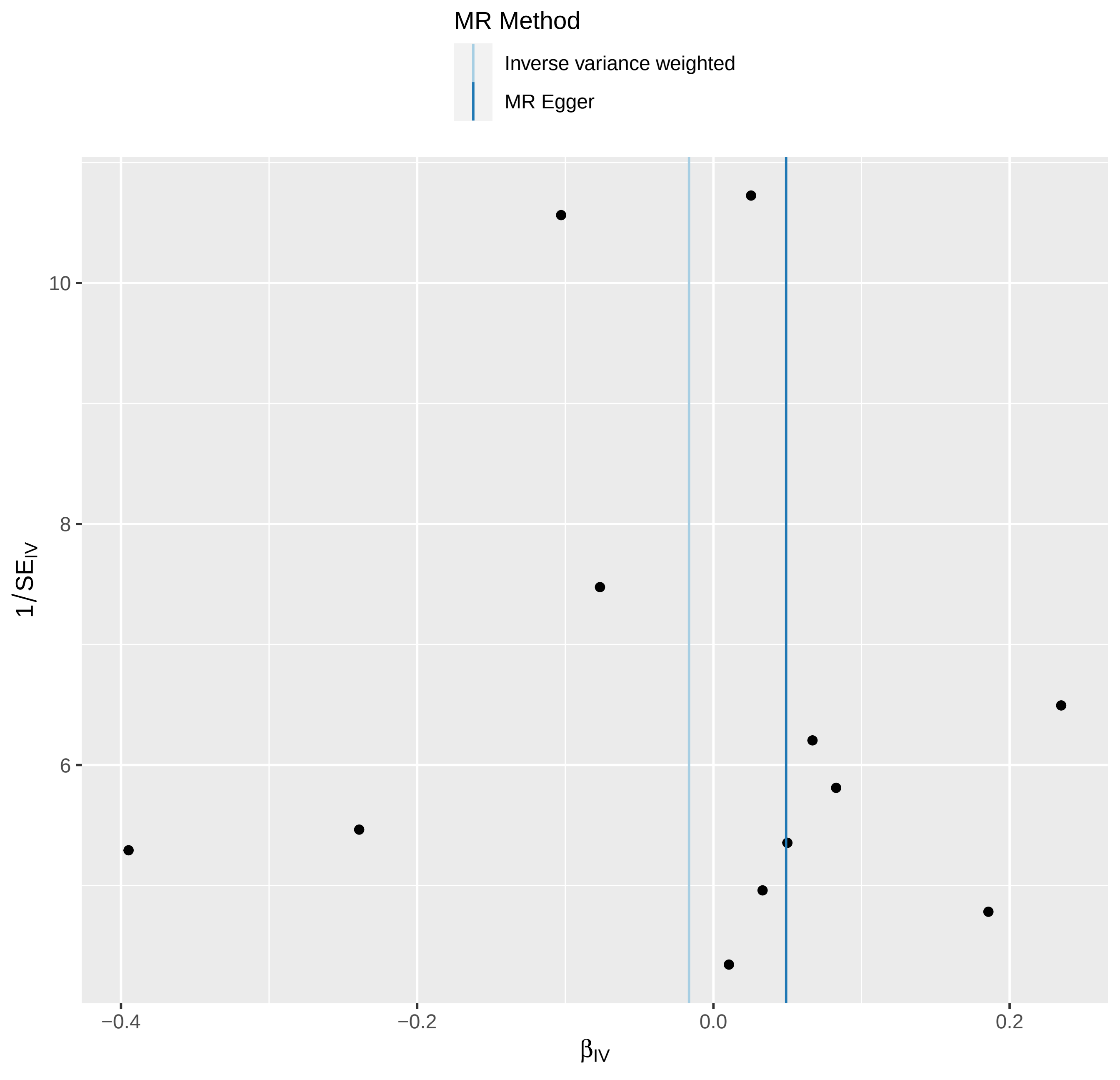

Batch 3−112 : Selenium on AP−4 complex subunit mu−1

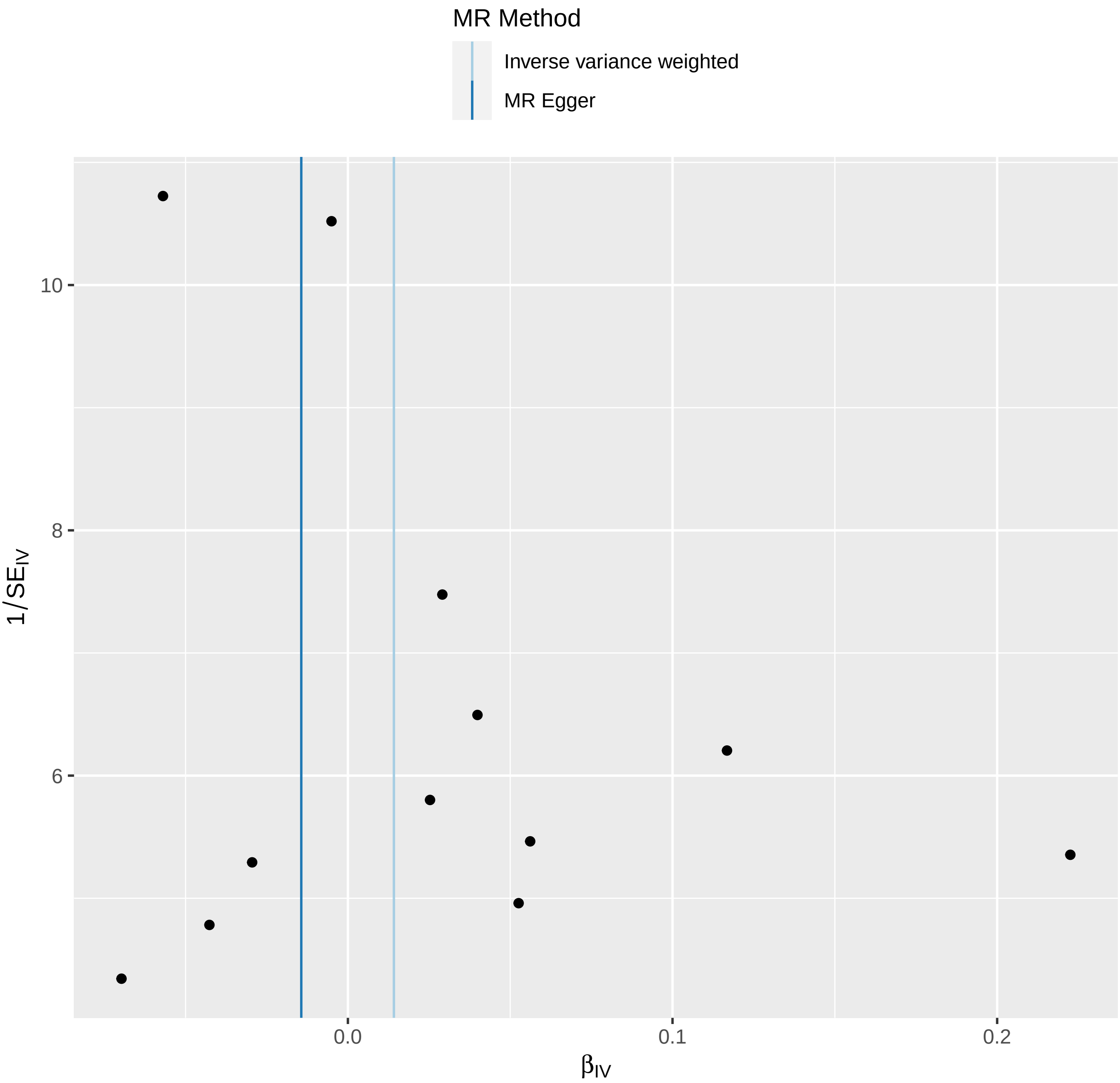

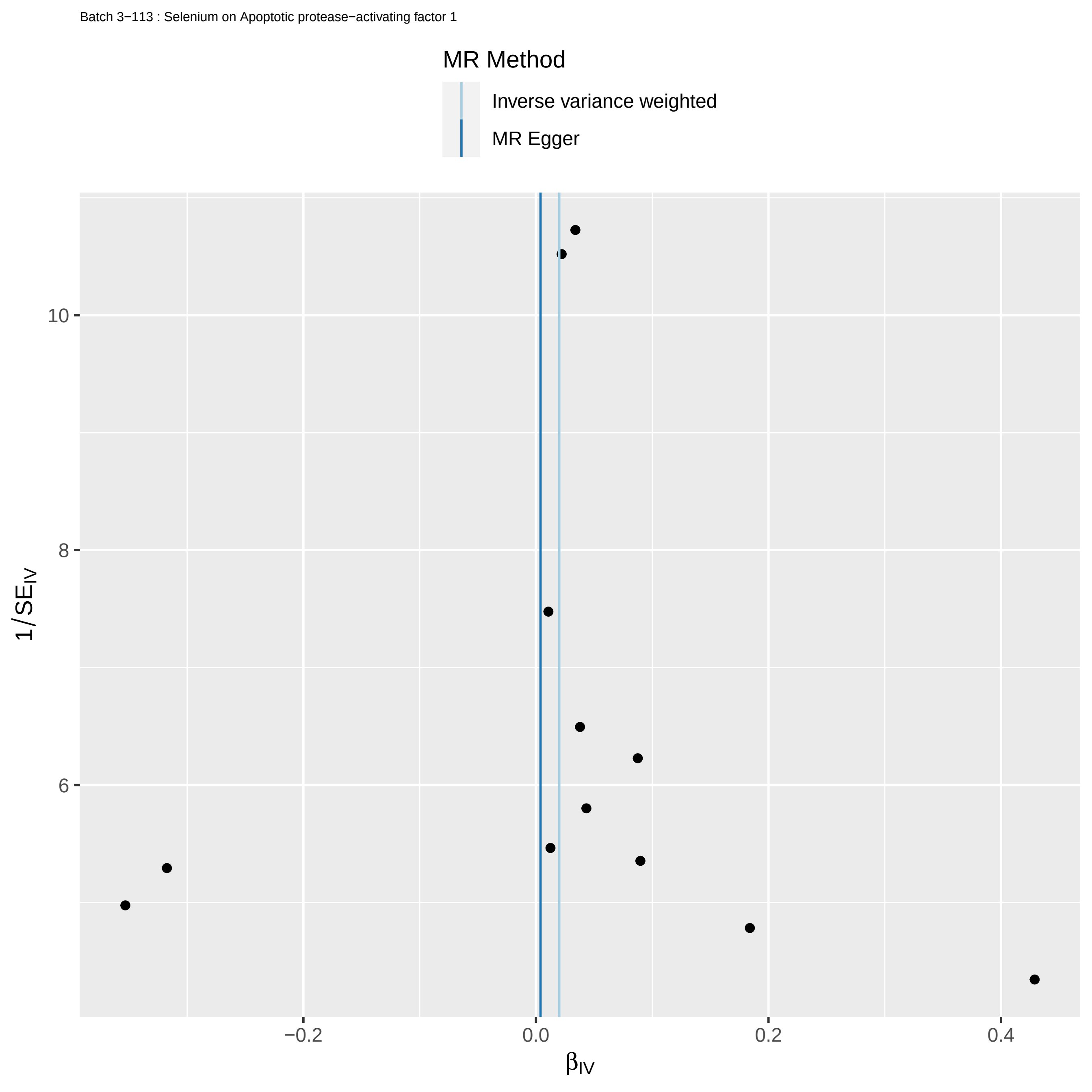

Batch 3−113 : Selenium on Apoptotic protease−activating factor 1
MR Method
Inverse variance weighted
MR Egger
1/SE_IV
β_IV
10
8
6
−0.2
0.0
0.2
0.4

Batch 3−114 : Selenium on Amyloid beta A4 precursor protein−binding family B member 1

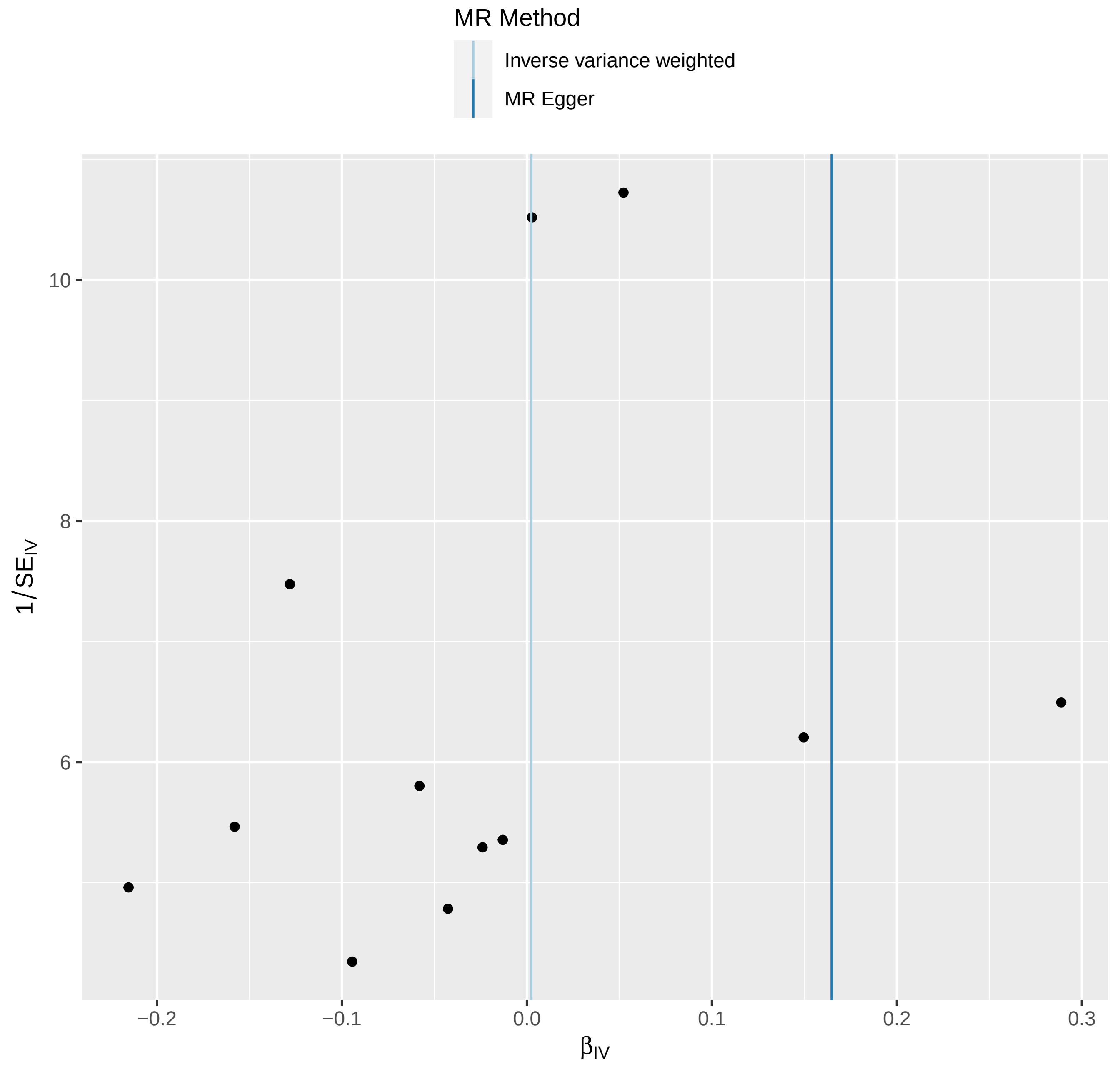

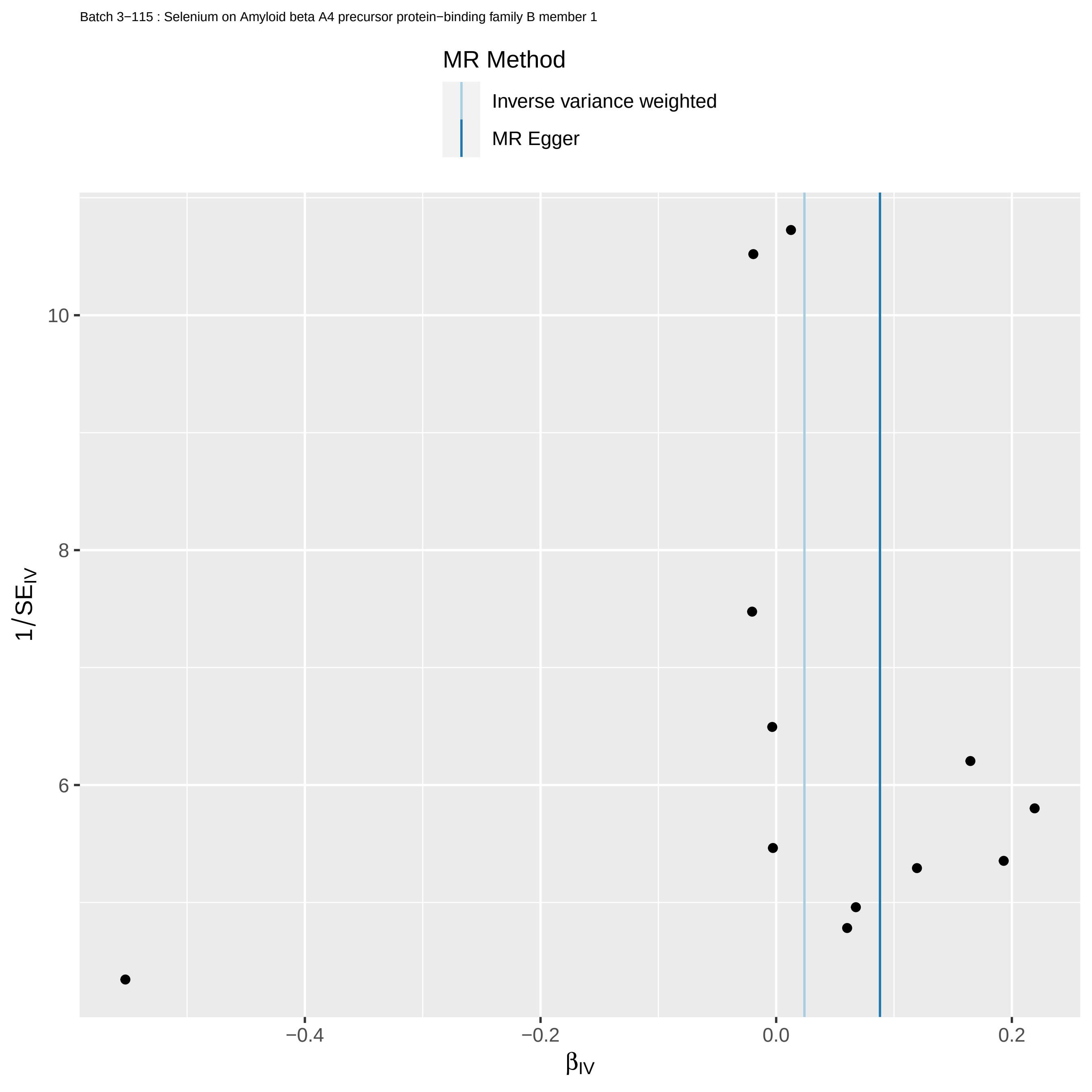
Batch 3−115 : Selenium on Amyloid beta A4 precursor protein−binding family B member 1
MR Method
Inverse variance weighted
MR Egger
10
8
6
1/SE_IV
−0.4
−0.2
0.0
0.2
β_IV

Batch 3−116 : Selenium on Amyloid beta A4 precursor protein−binding family B member 2

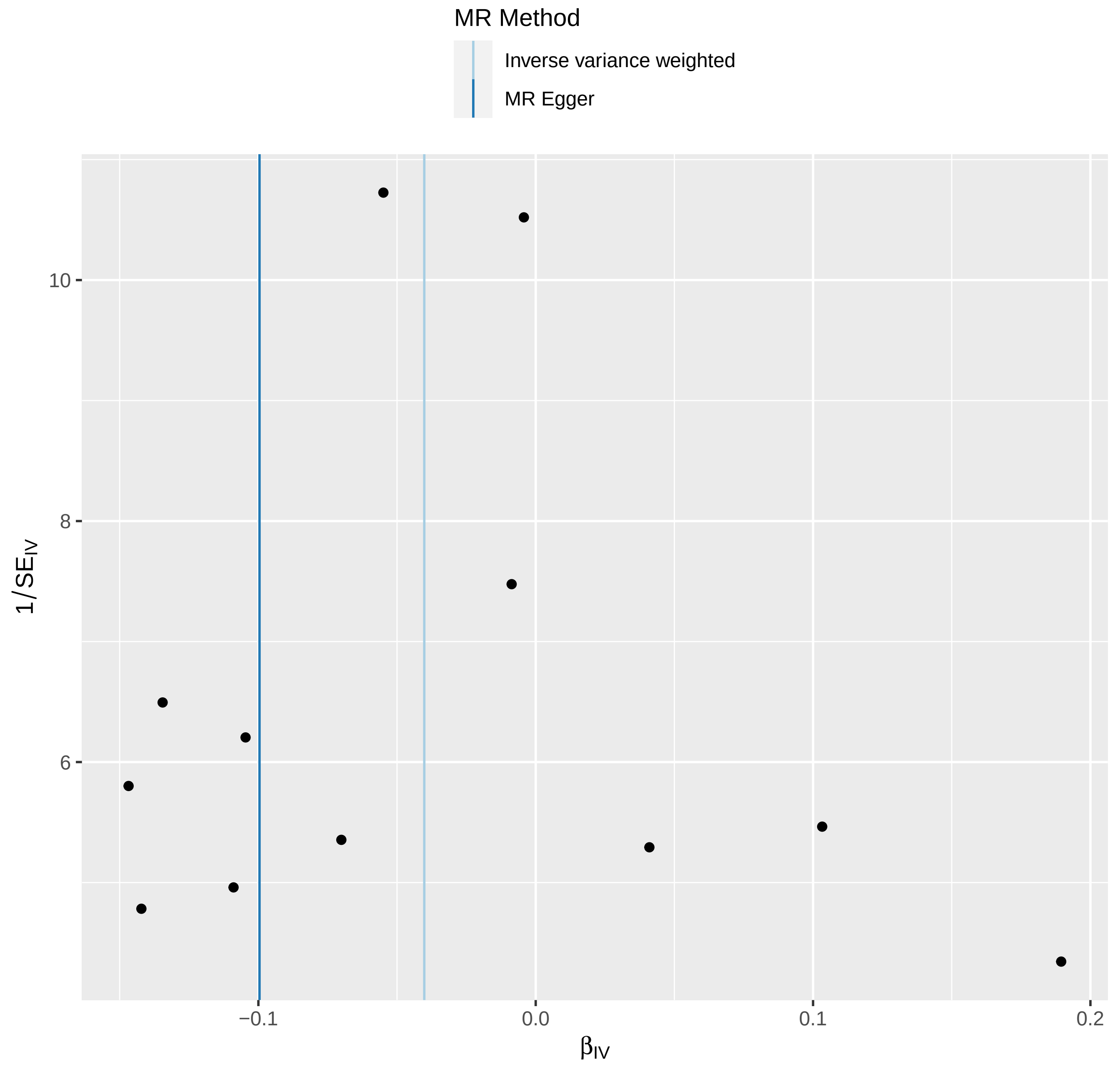

Batch 3−117 : Selenium on Amyloid beta A4 precursor protein−binding family B member 2

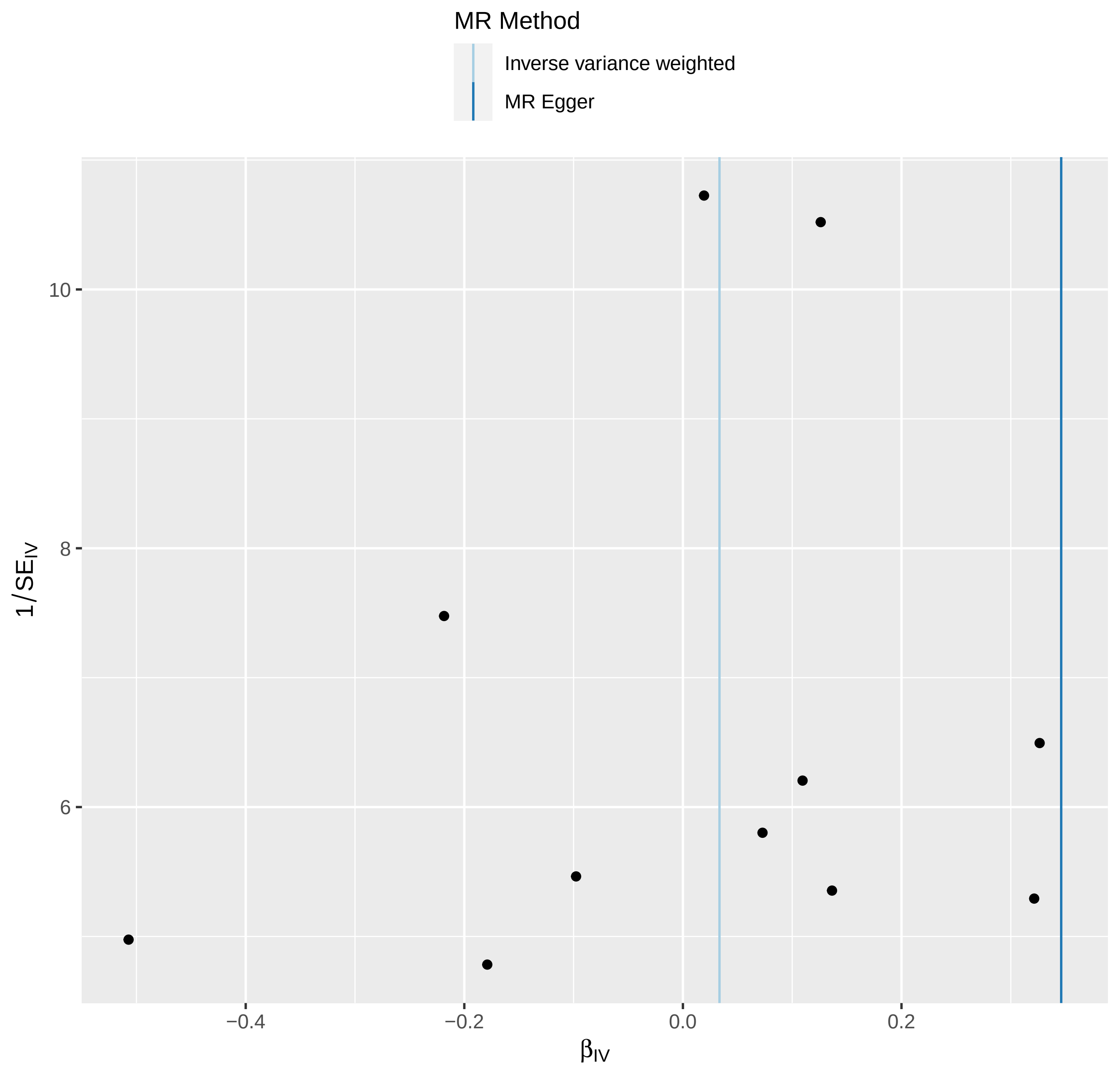

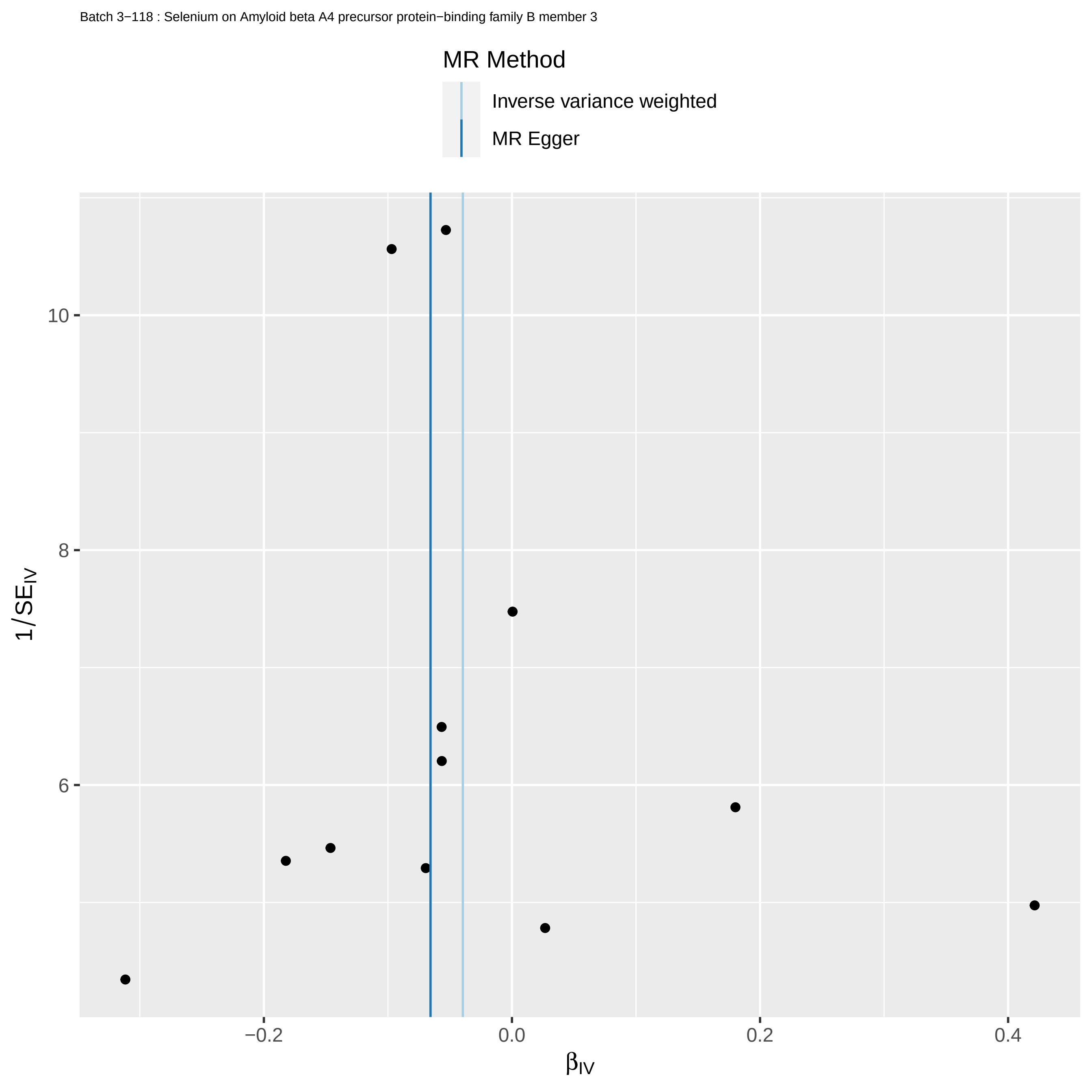

Batch 3−118 : Selenium on Amyloid beta A4 precursor protein−binding family B member 3
MR Method
Inverse variance weighted
MR Egger
10
8
6
1/SE_IV
−0.2
0.0
0.2
0.4
β_IV

Batch 3−119 : Selenium on Protein APCDD1

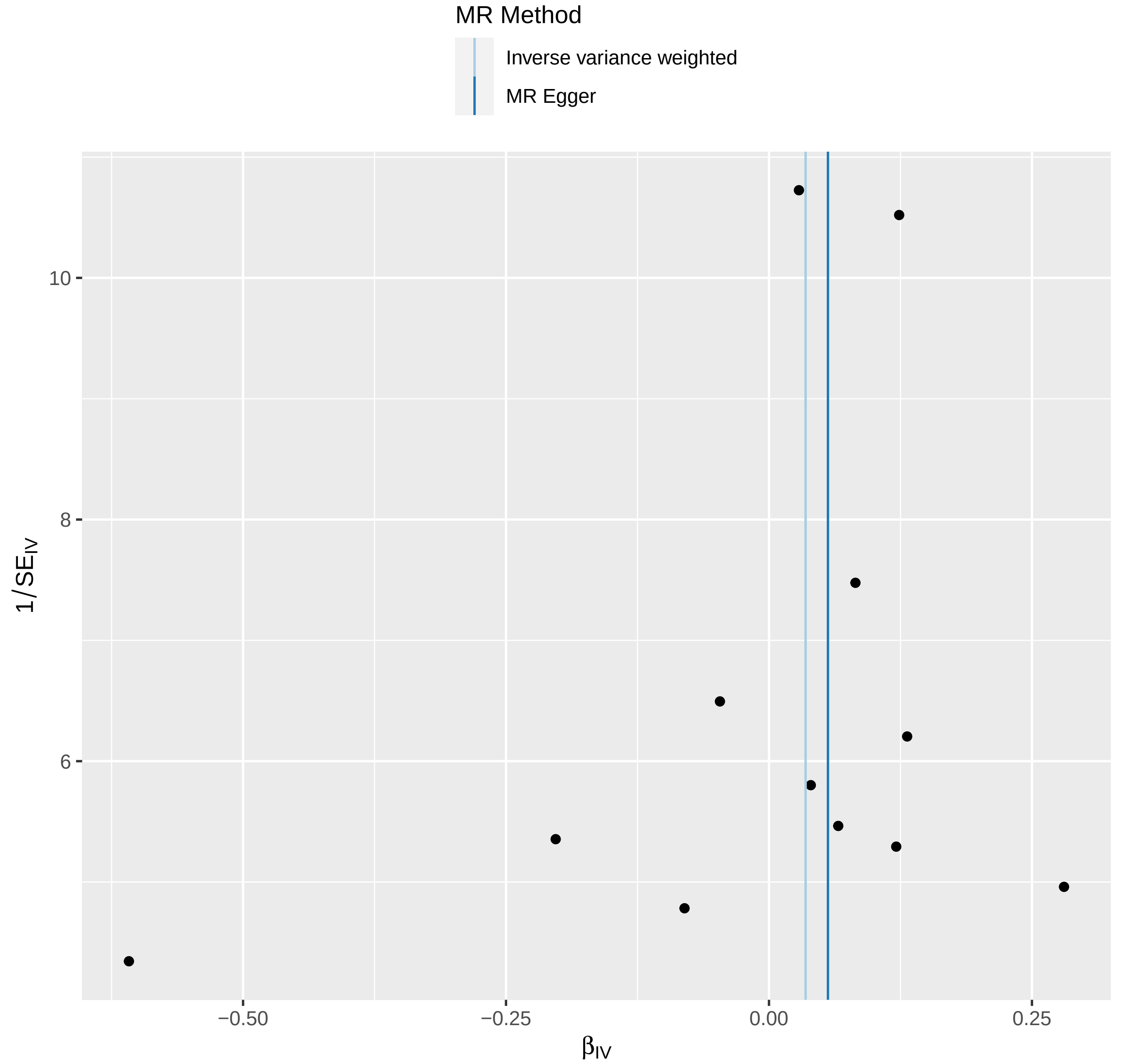

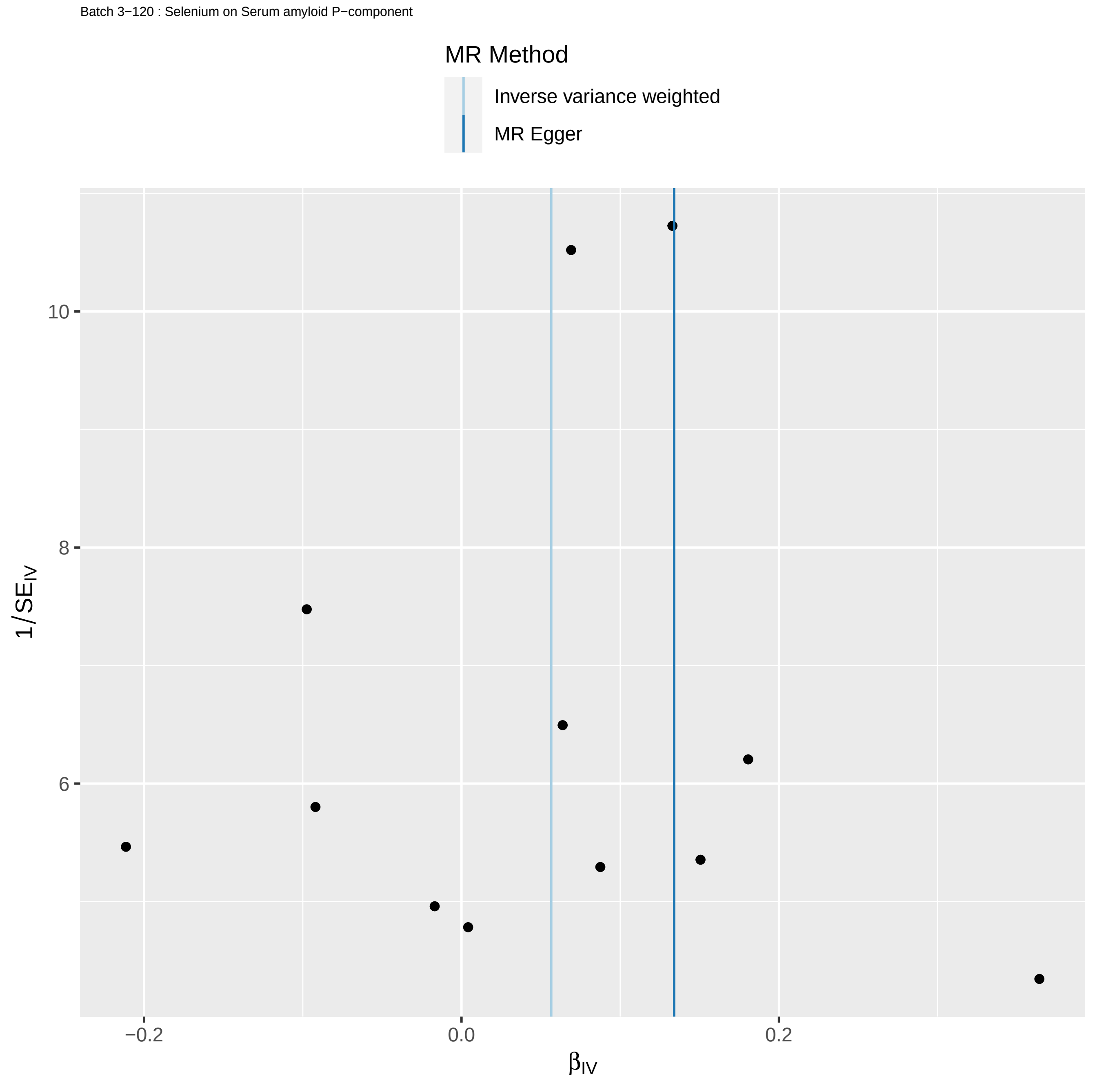

Batch 3−120 : Selenium on Serum amyloid P−component
MR Method
Inverse variance weighted
MR Egger
10
8
6
1/SE_IV
−0.2
0.0
0.2
β_IV

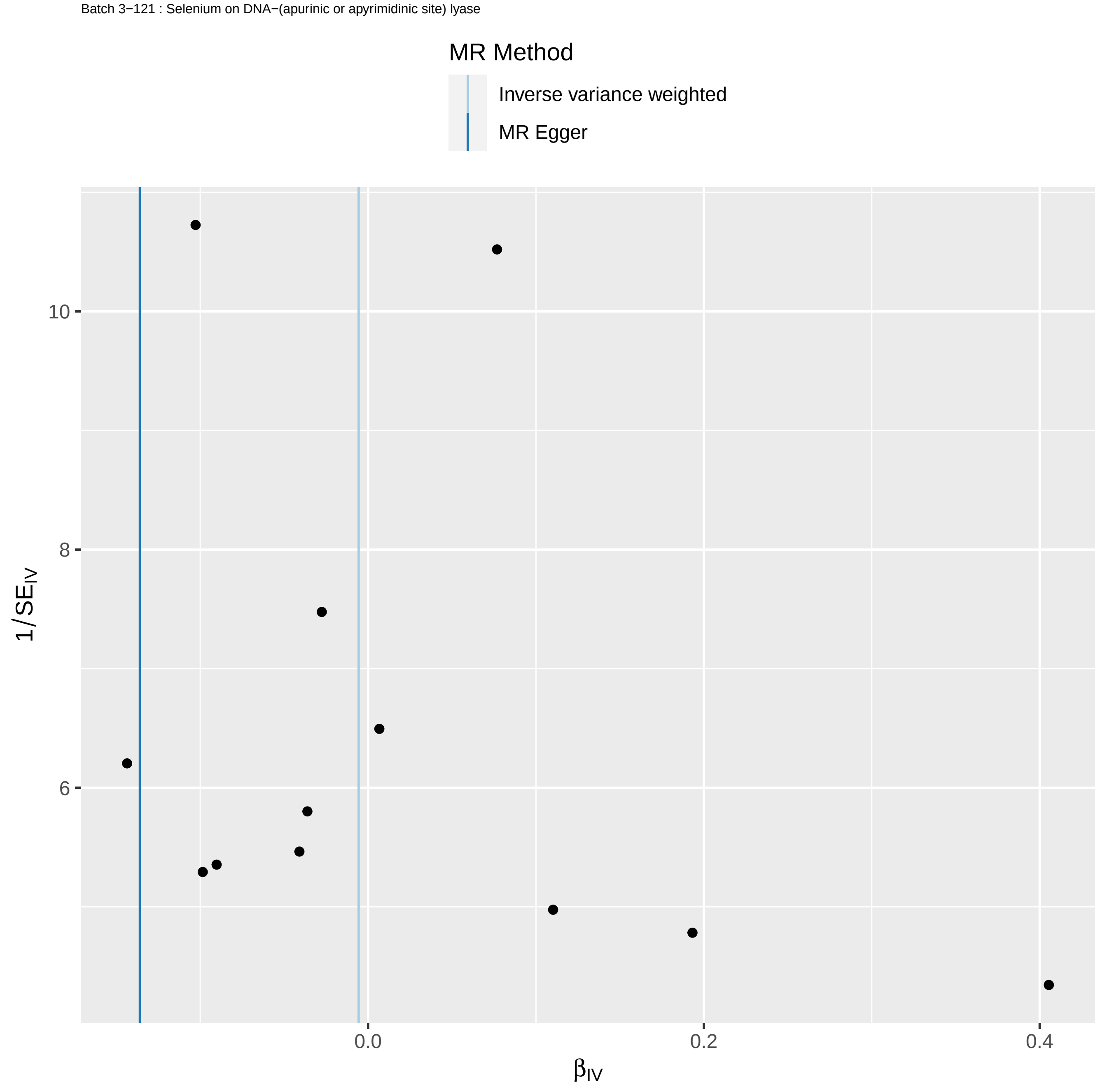

Batch 3−121 : Selenium on DNA−(apurinic or apyrimidinic site) lyase
MR Method
Inverse variance weighted
MR Egger
10
8
6
1/SE$_{IV}$
0.0
0.2
0.4
β$_{IV}$

Batch 3−122 : Selenium on Amyloid−like protein 2

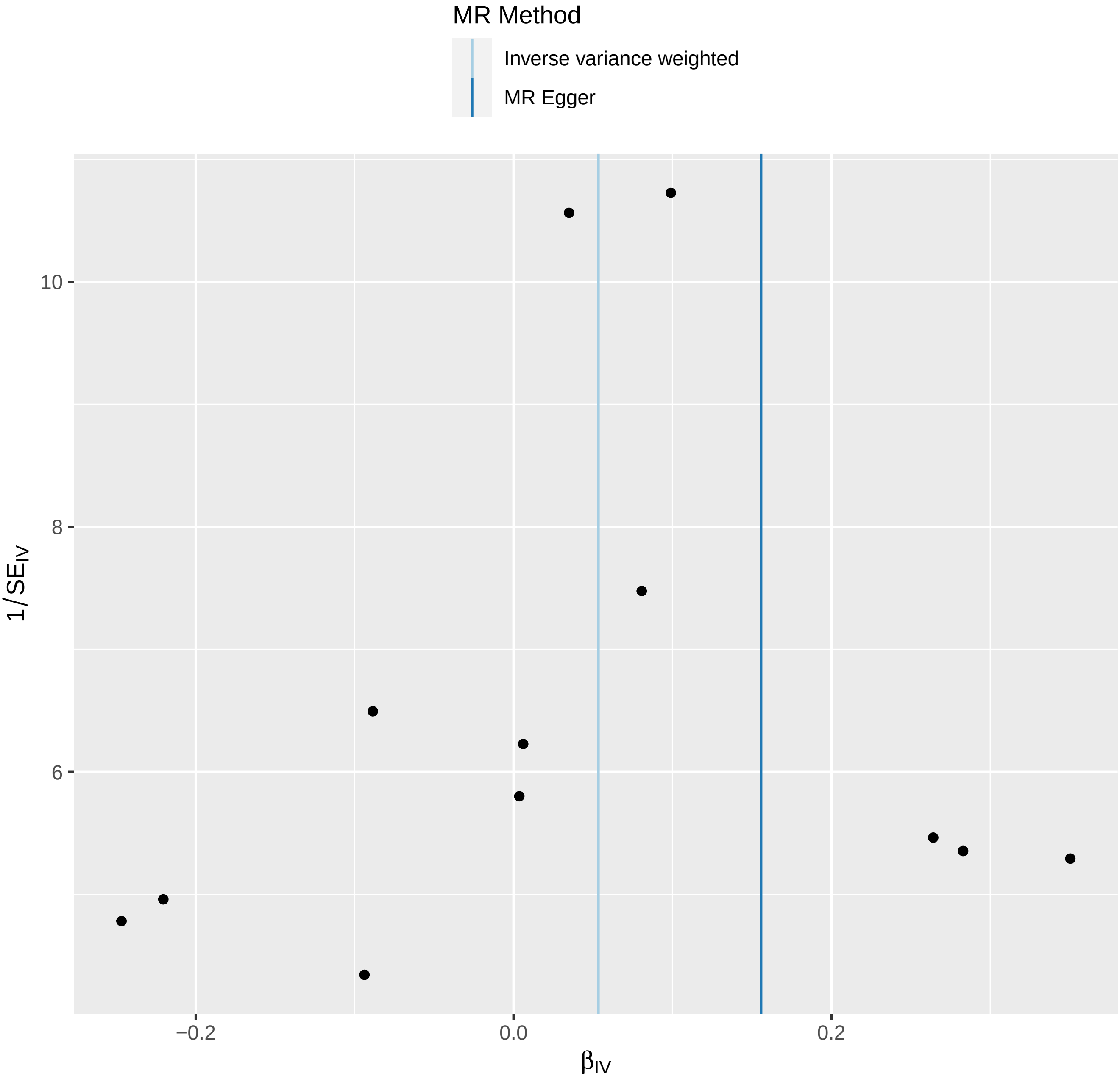

Batch 3−123 : Selenium on Amyloid−like protein 2

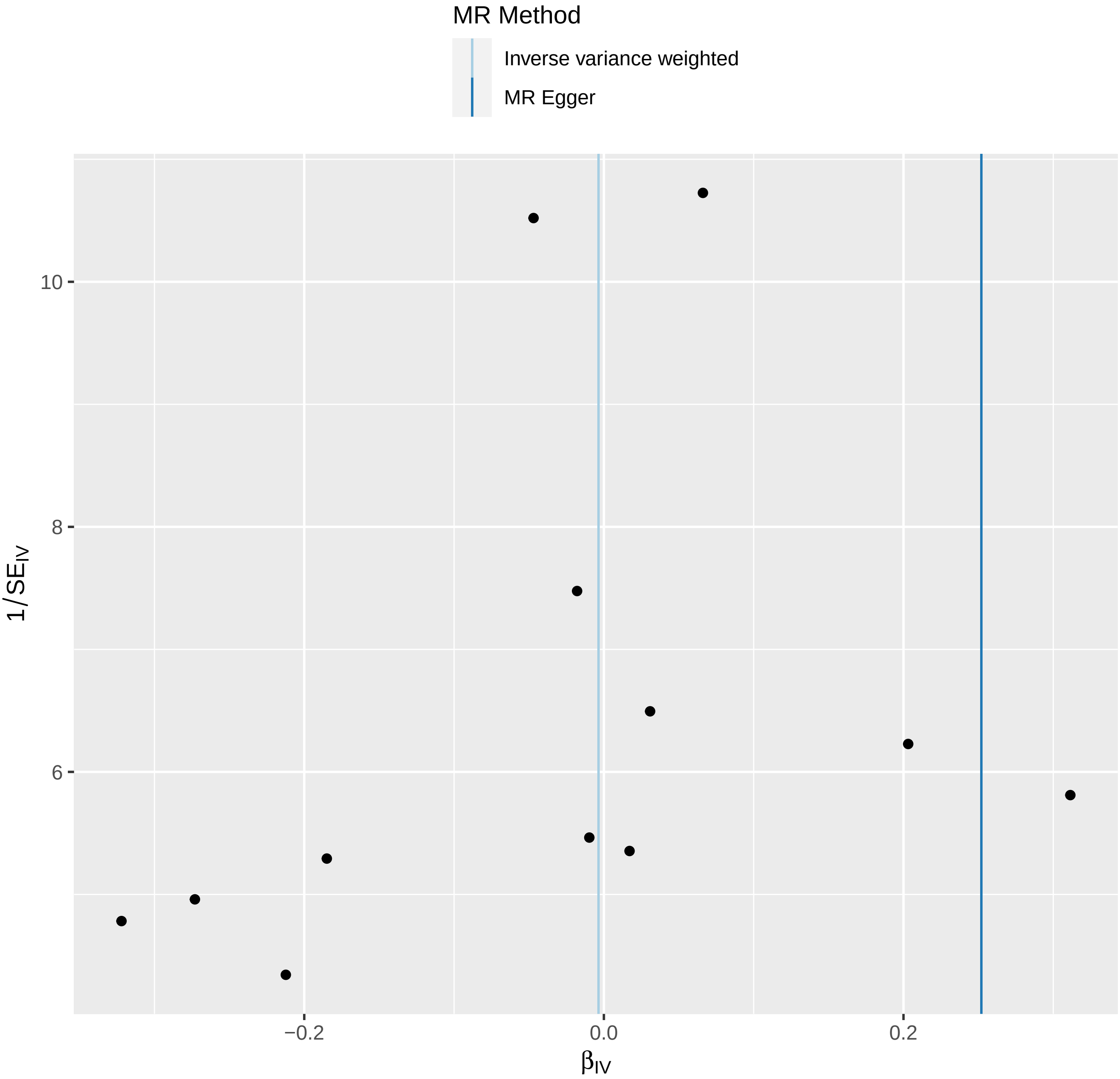

Batch 3−124 : Selenium on Adipocyte plasma membrane−associated protein

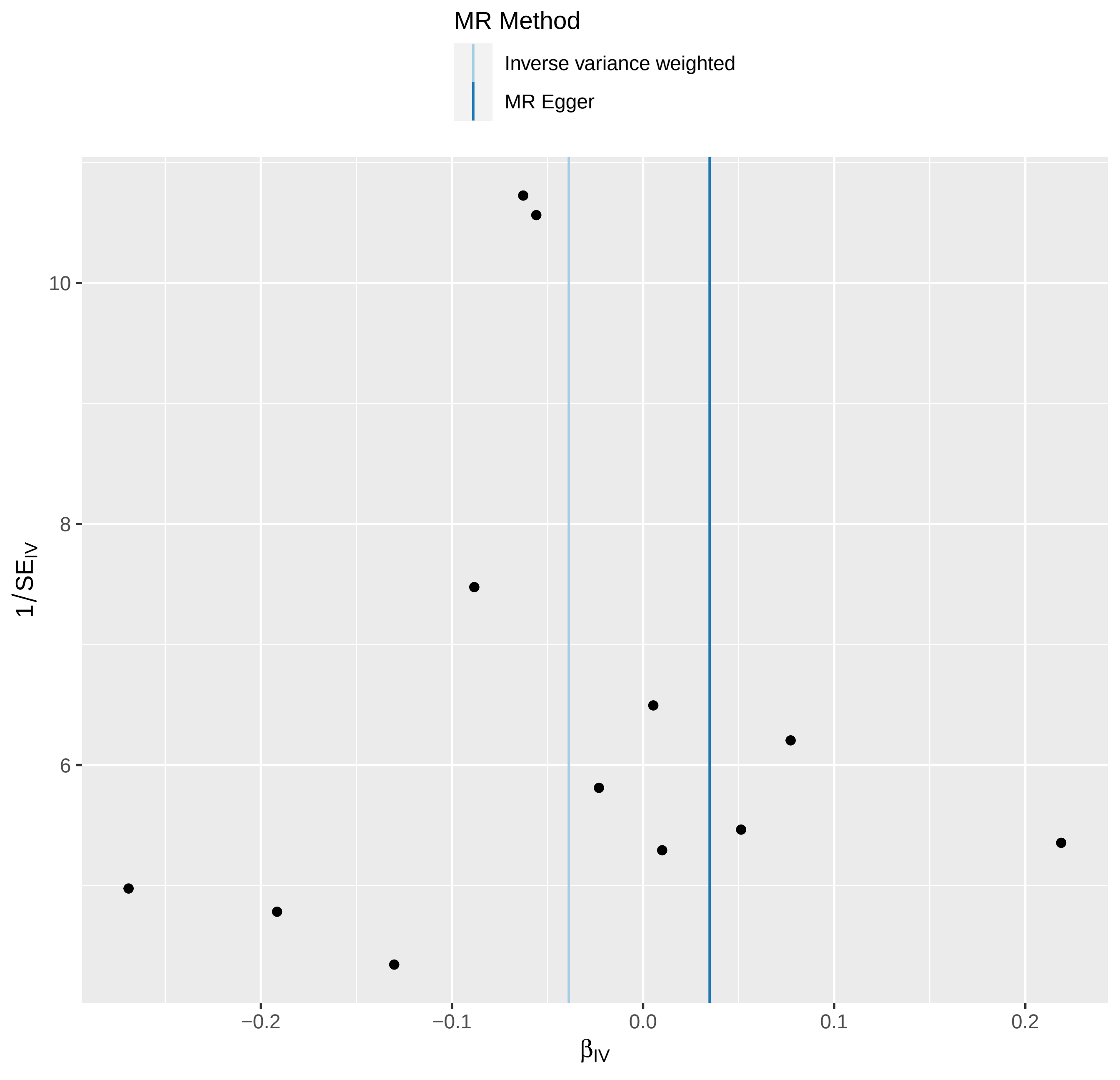

Batch 3−125 : Selenium on Apolipoprotein A−V

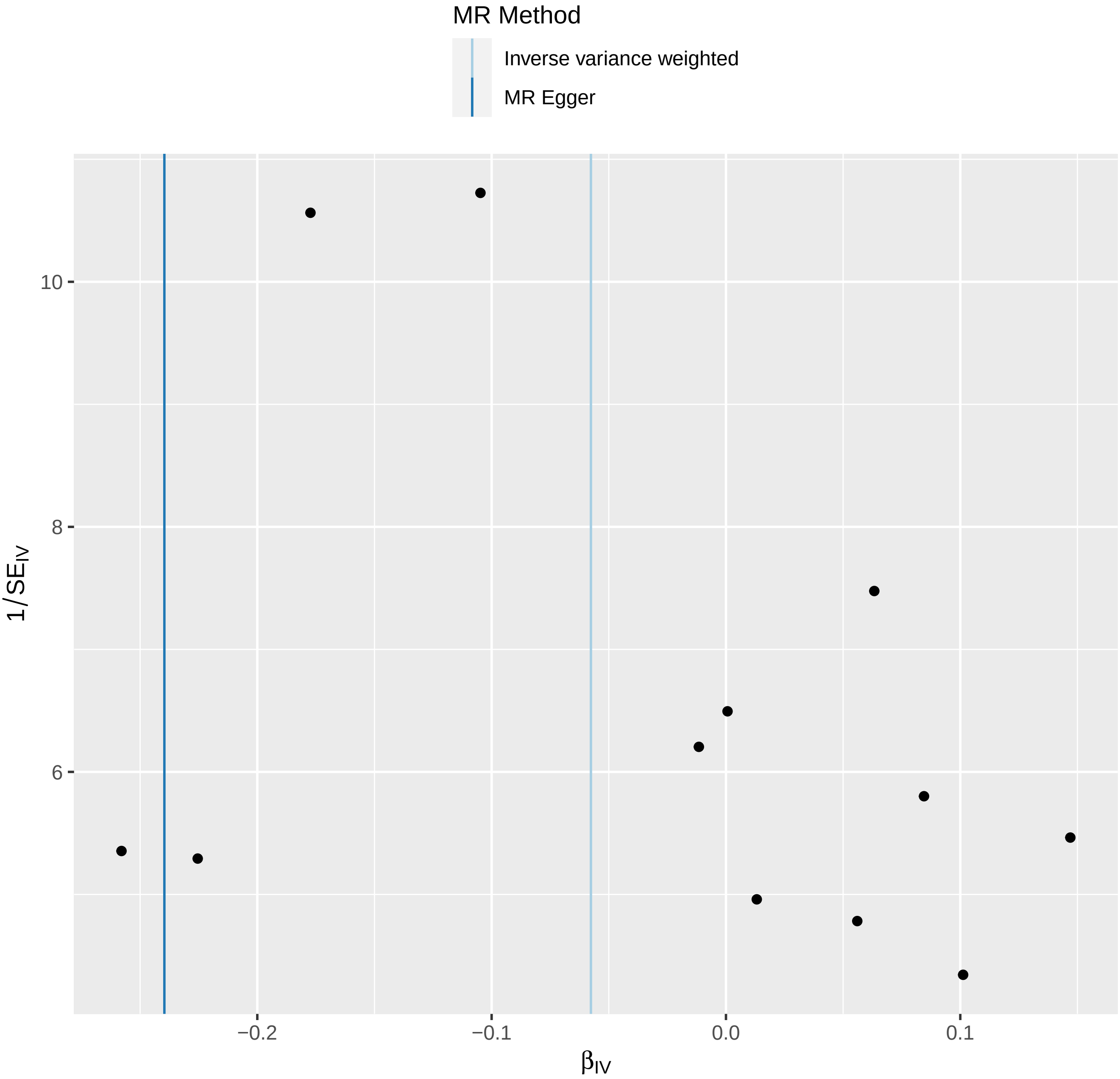

Batch 3−126 : Selenium on Apolipoprotein A−V

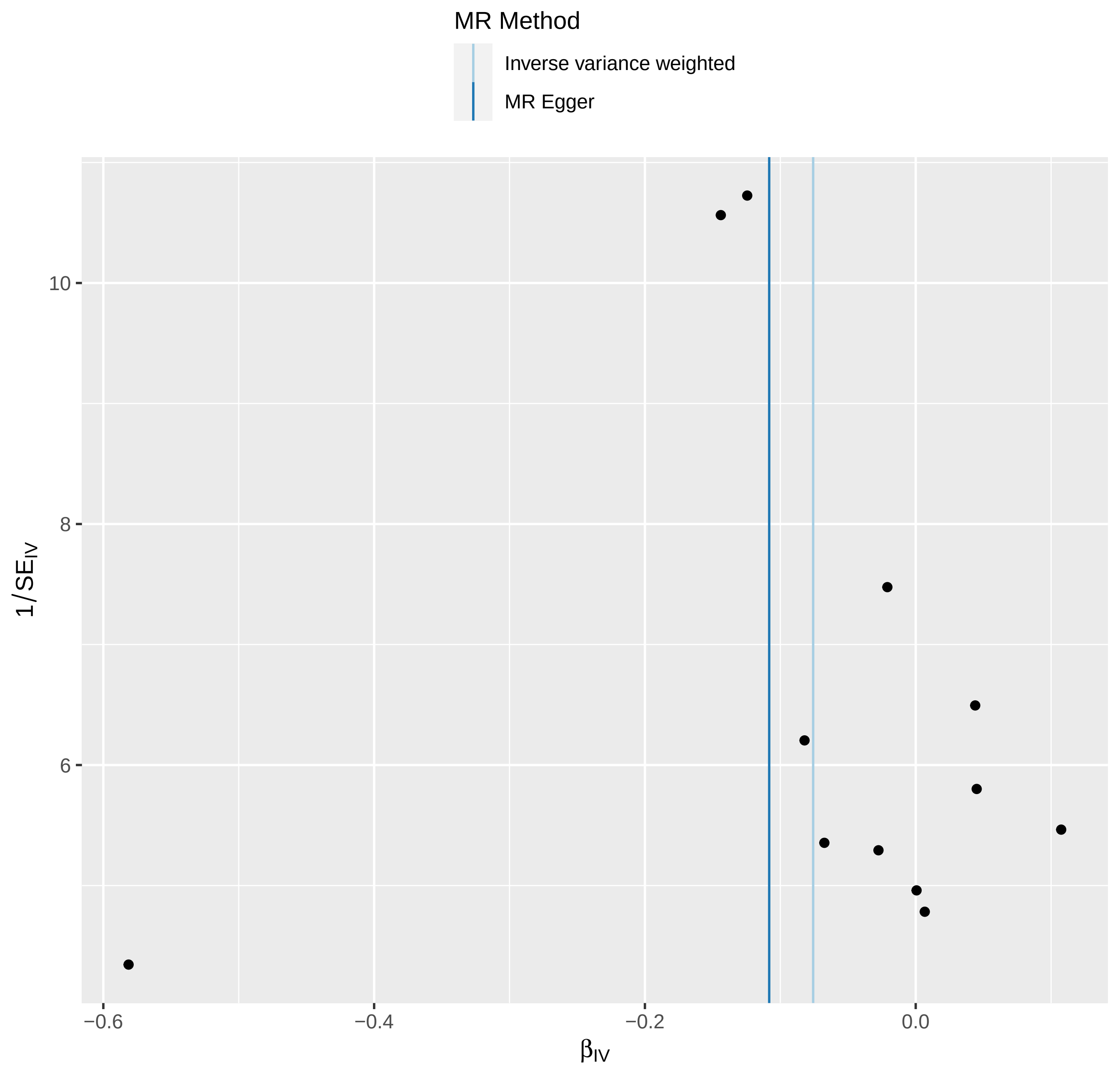

Batch 3−127 : Selenium on Apolipoprotein B

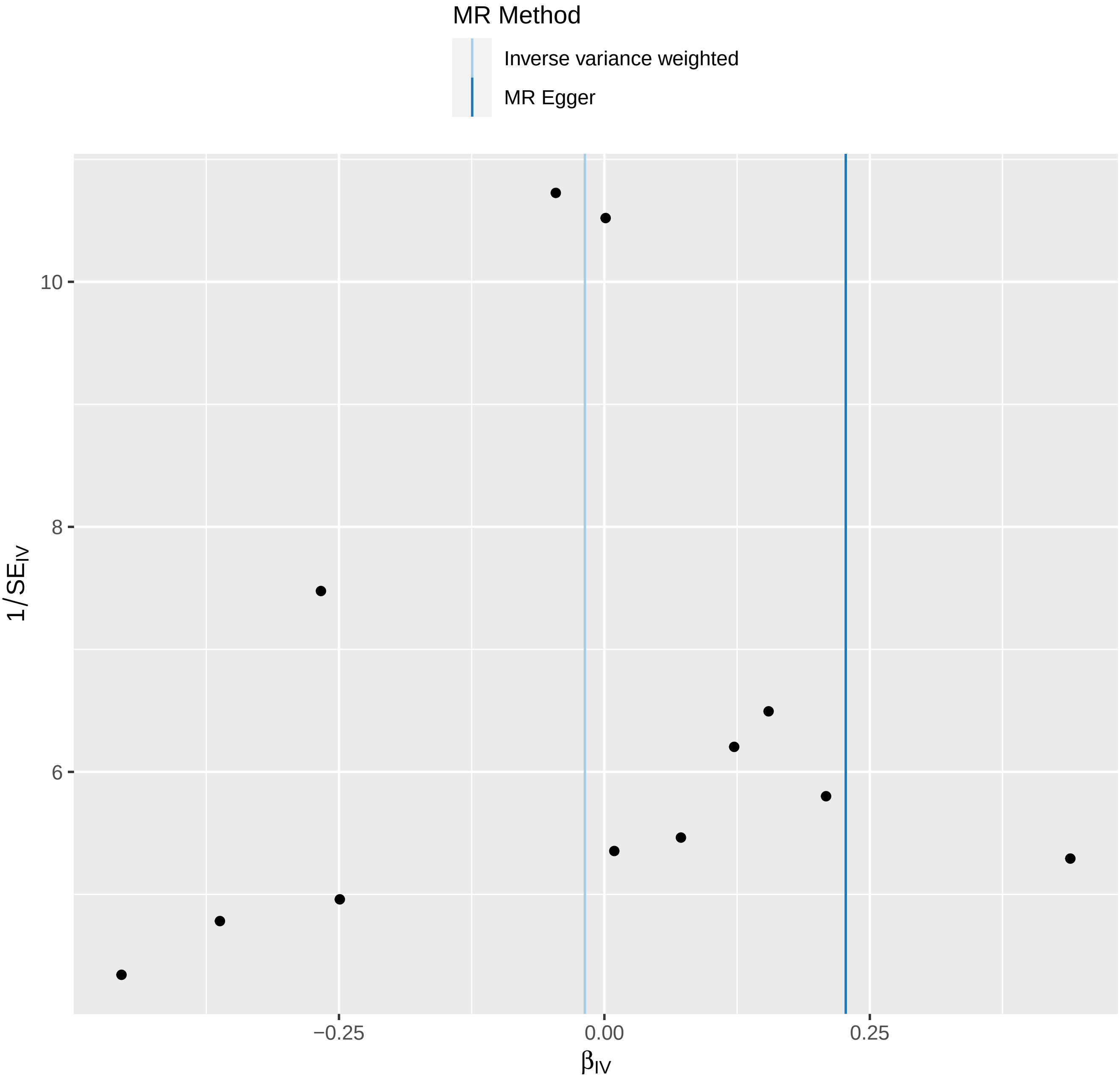

Batch 3−128 : Selenium on DNA dC−>dU−editing enzyme APOBEC−3G

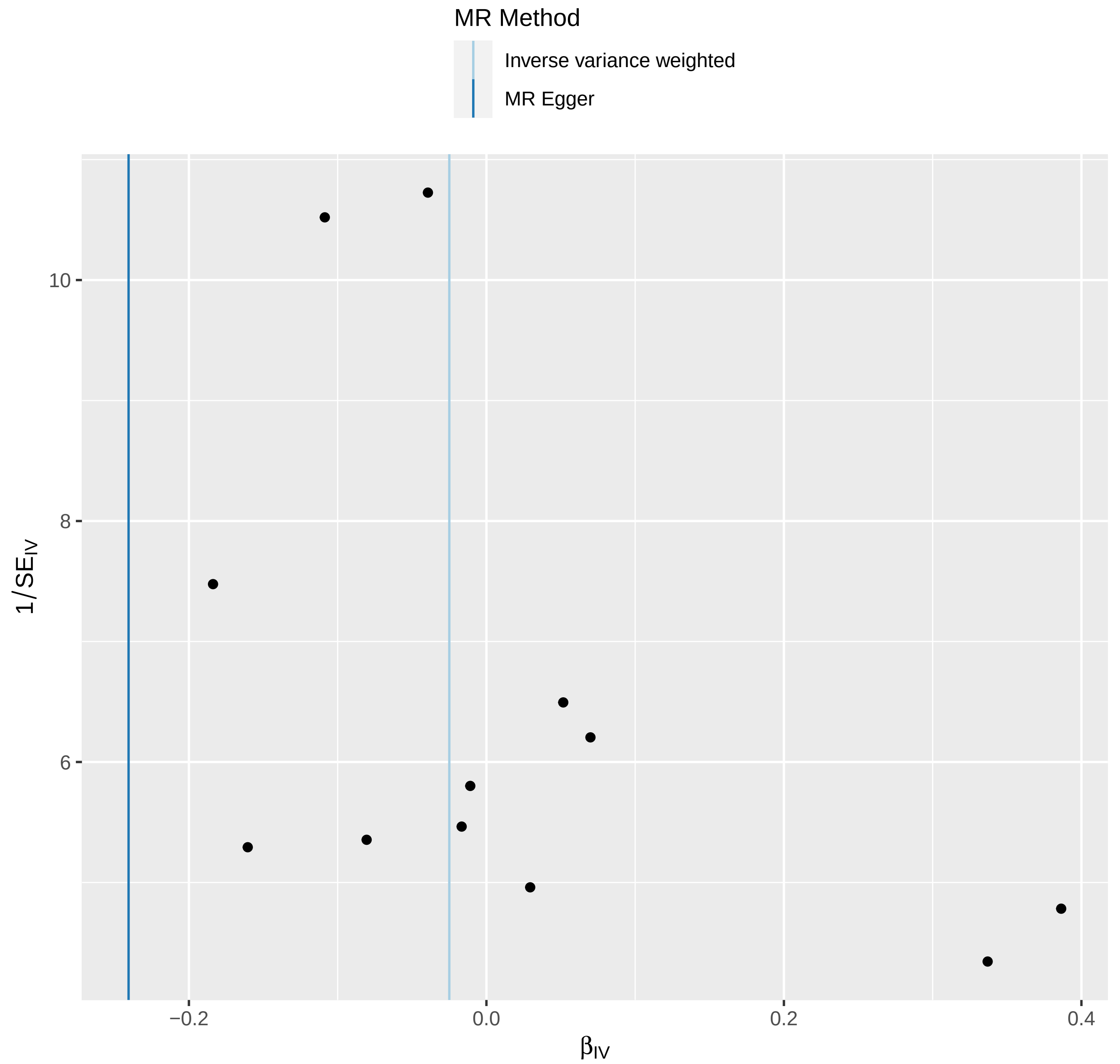

Batch 3−129 : Selenium on Apolipoprotein D

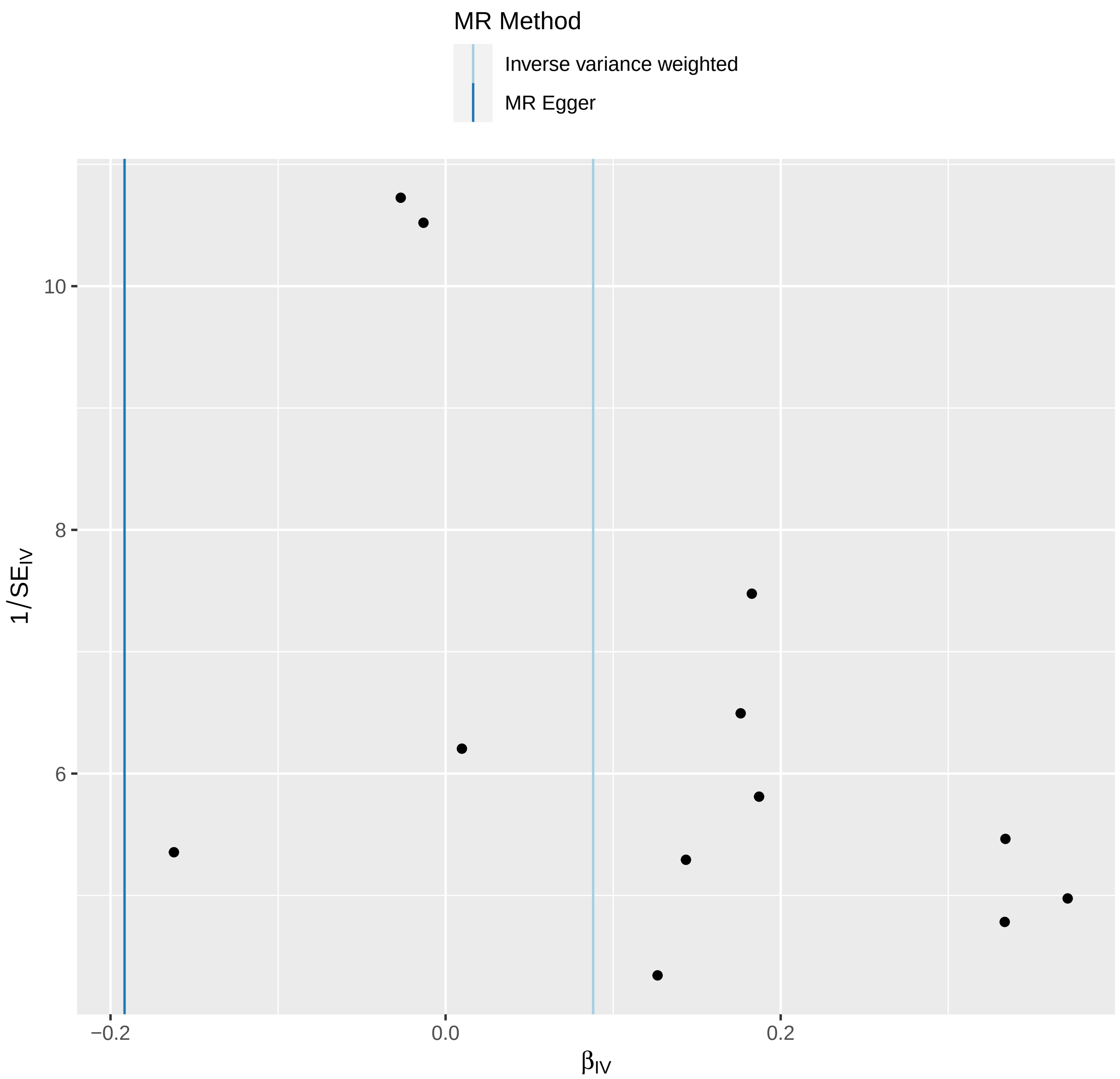

Batch 3−130 : Selenium on Apolipoprotein D

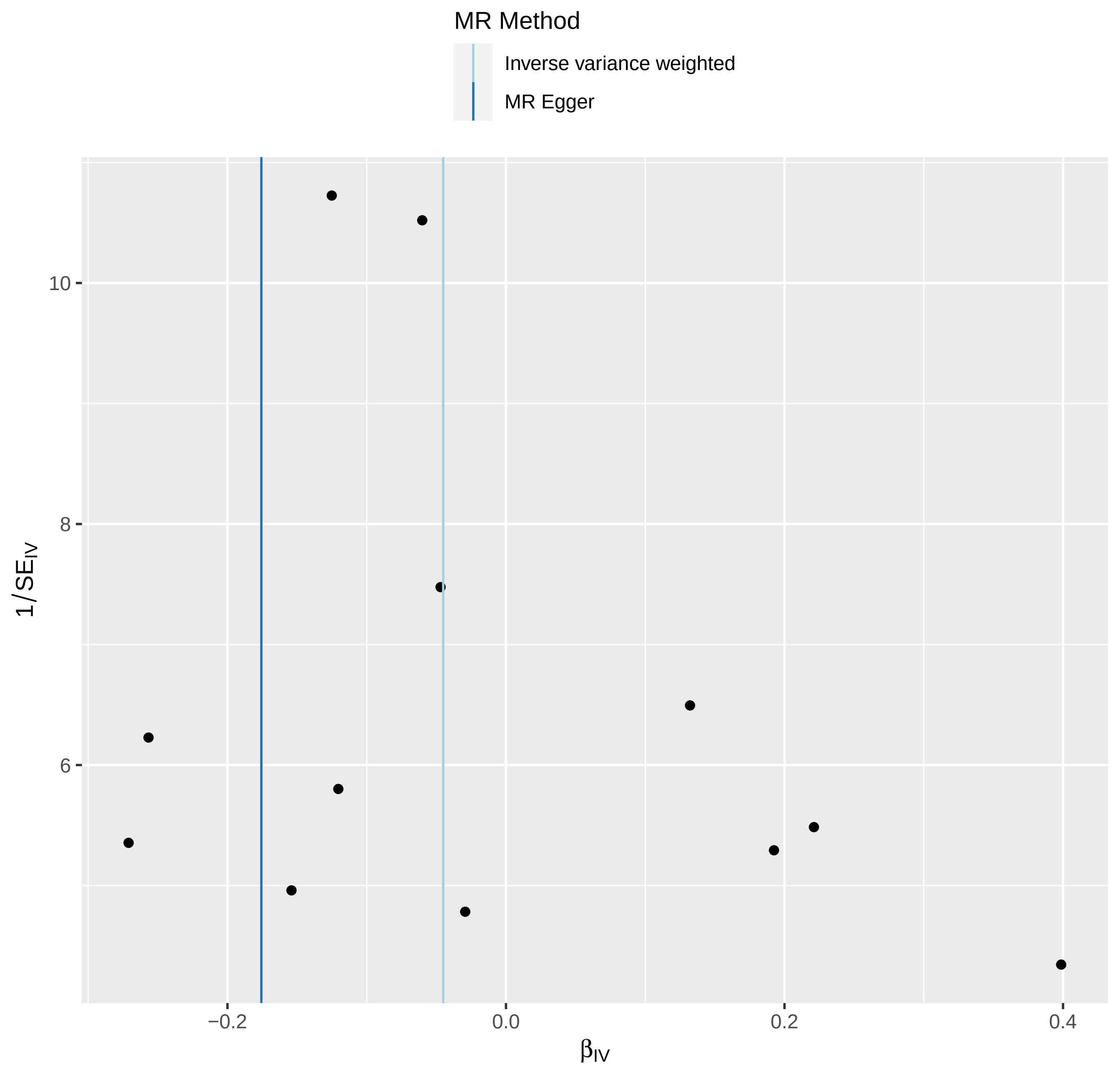

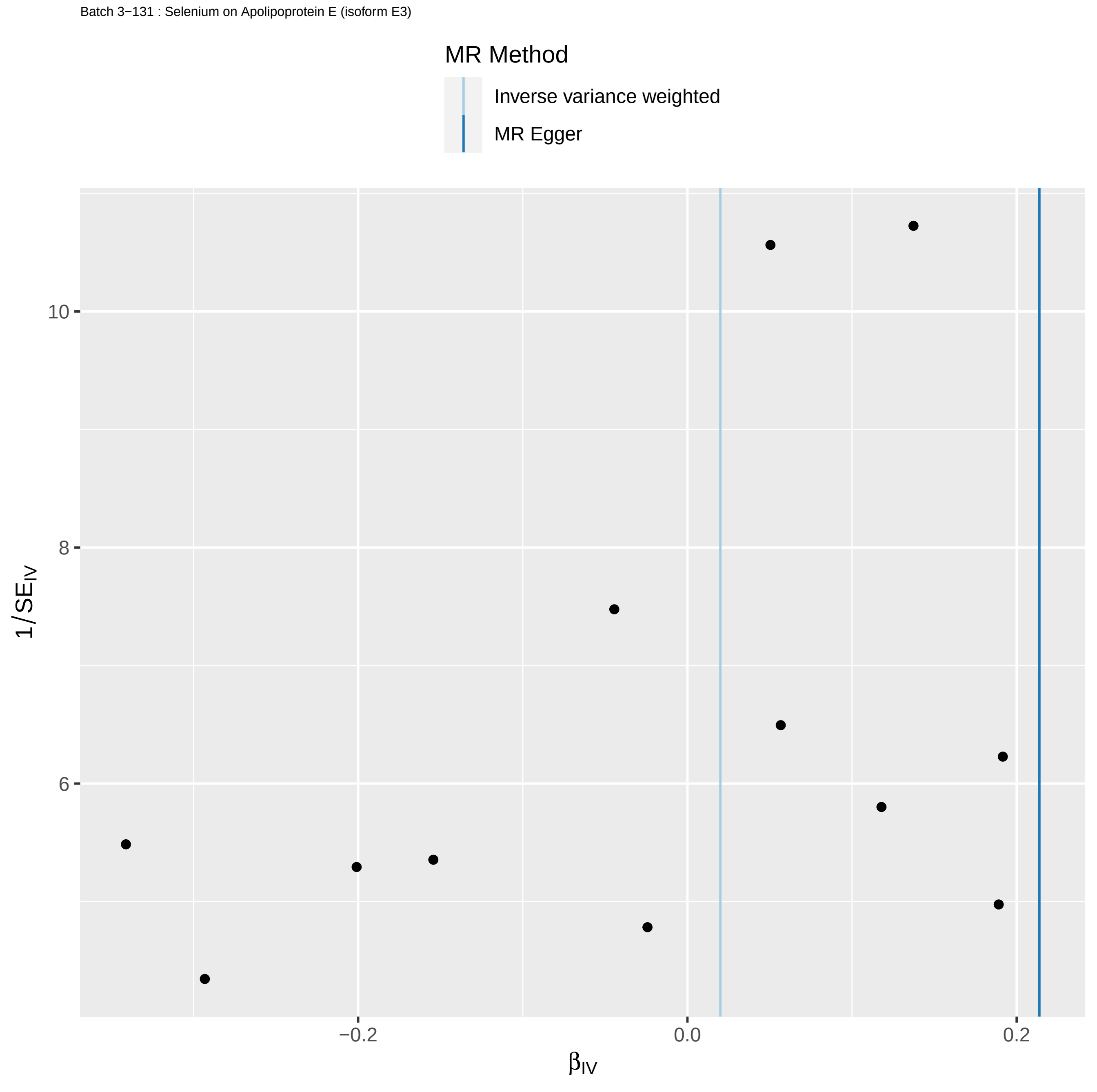

Batch 3−131 : Selenium on Apolipoprotein E (isoform E3)
MR Method
Inverse variance weighted
MR Egger
10
8
6
1/SE_IV
−0.2
0.0
0.2
β_IV

Batch 3−132 : Selenium on Apolipoprotein E (isoform E2)

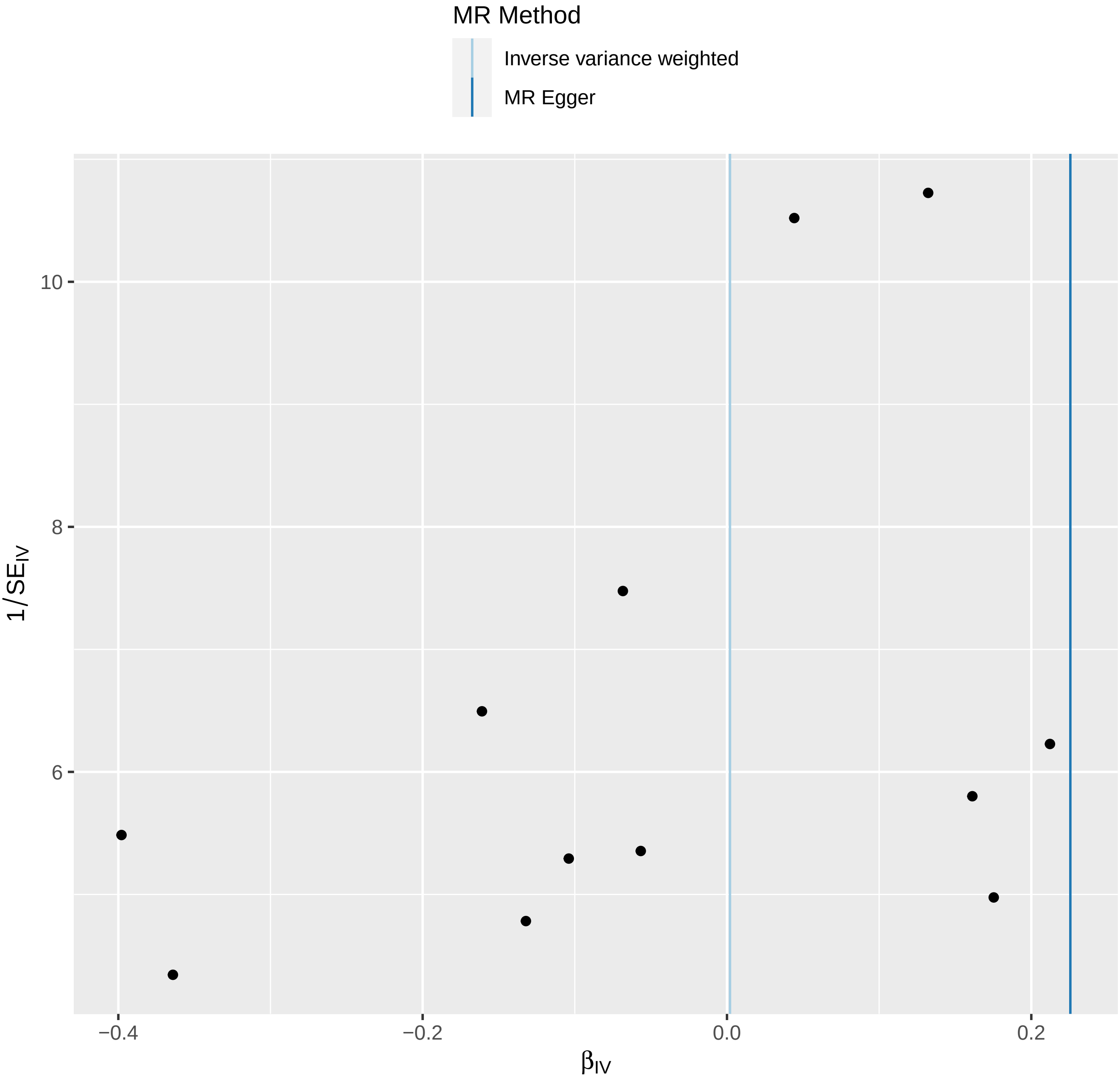

Batch 3−133 : Selenium on Apolipoprotein F

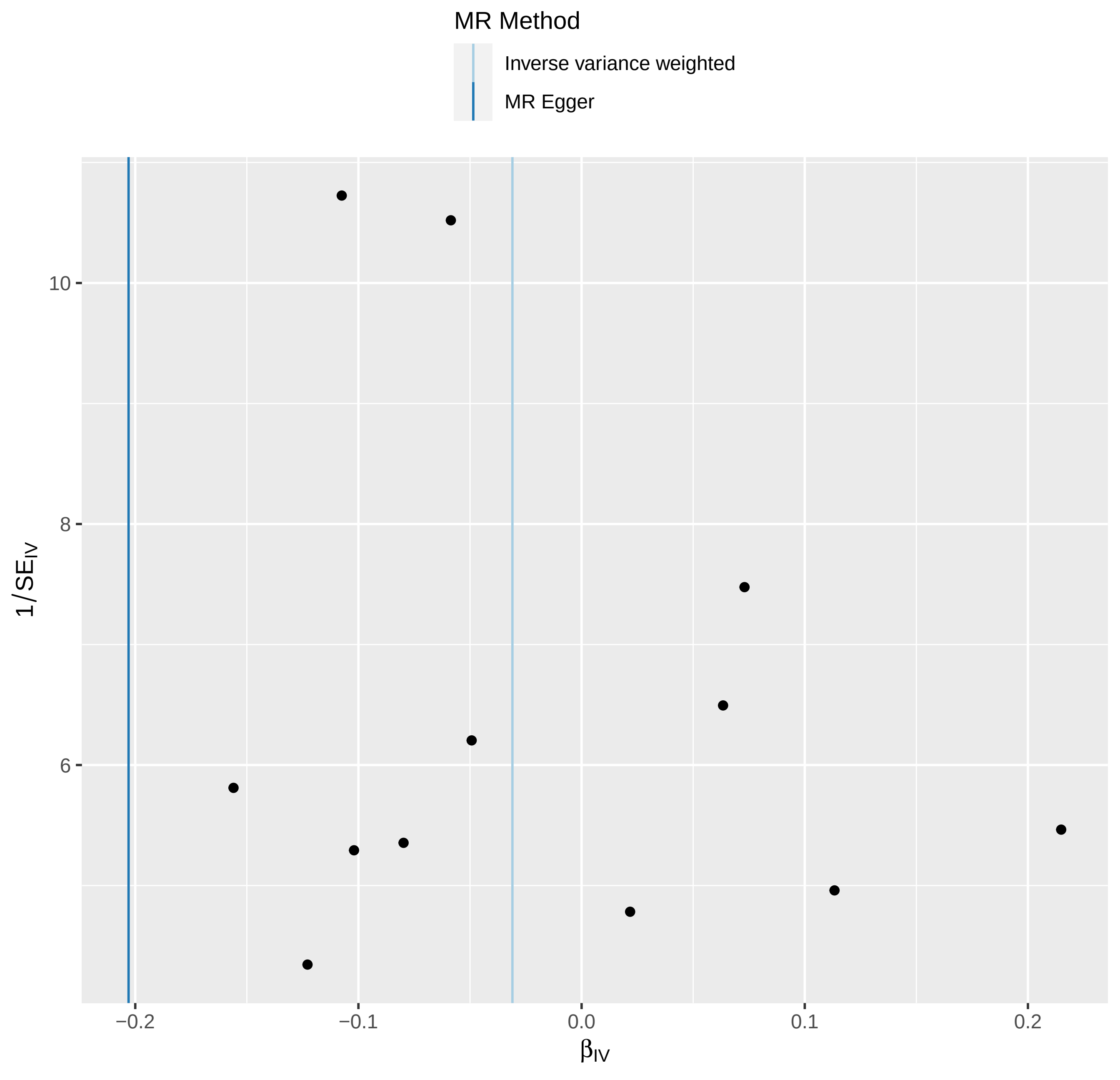

Batch 3−134 : Selenium on Apolipoprotein L1

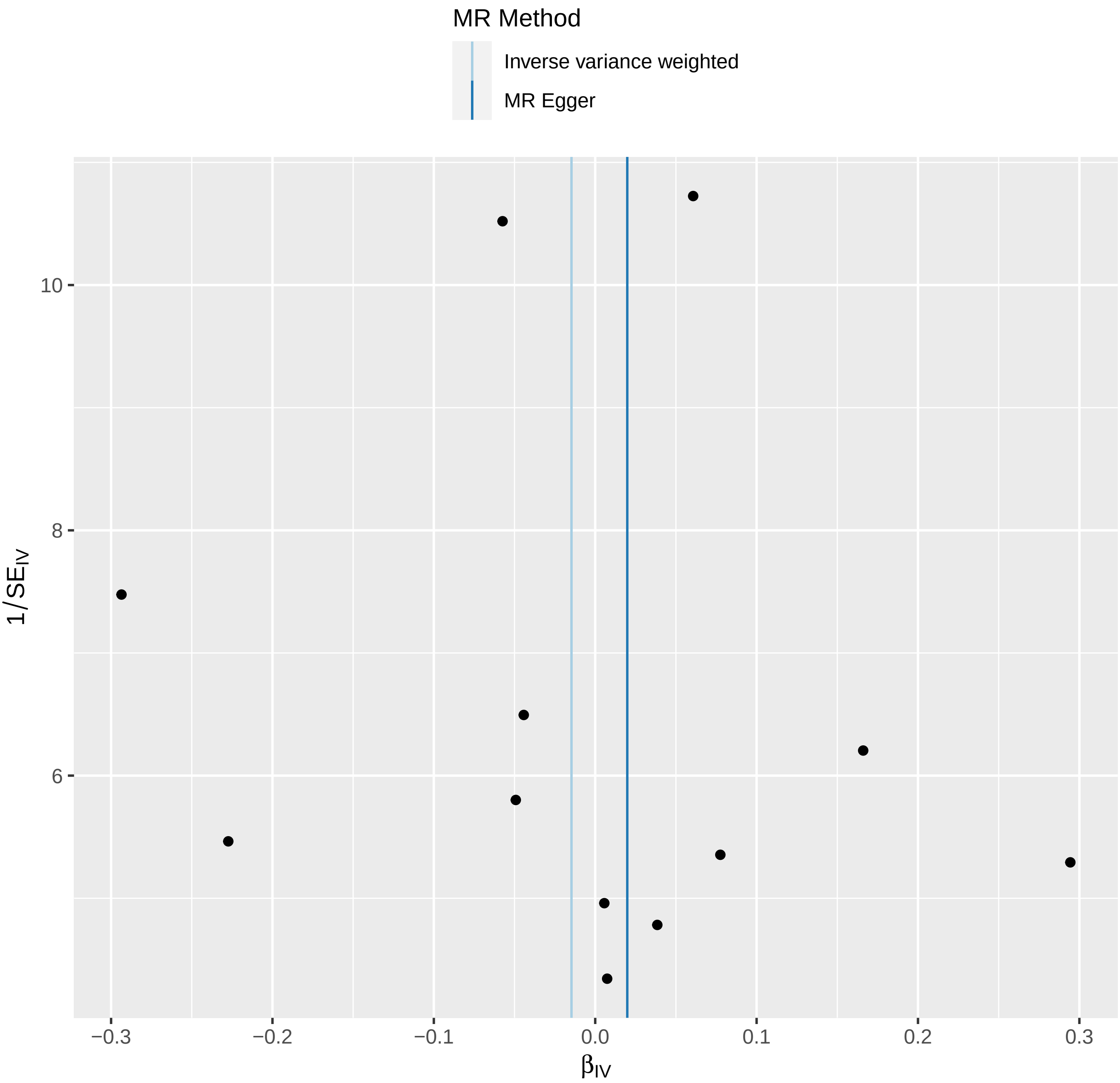

Batch 3−135 : Selenium on Apolipoprotein L1

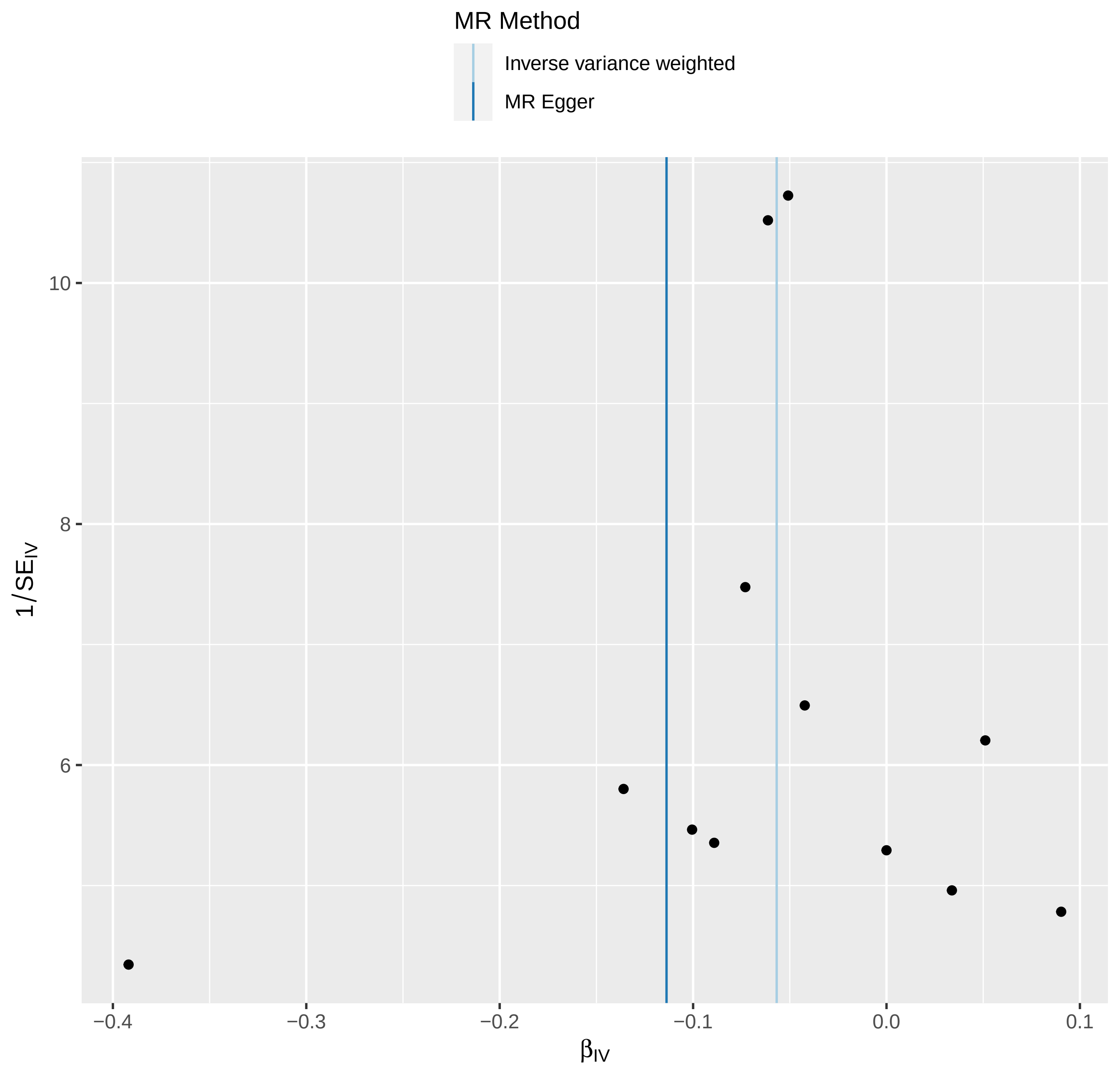

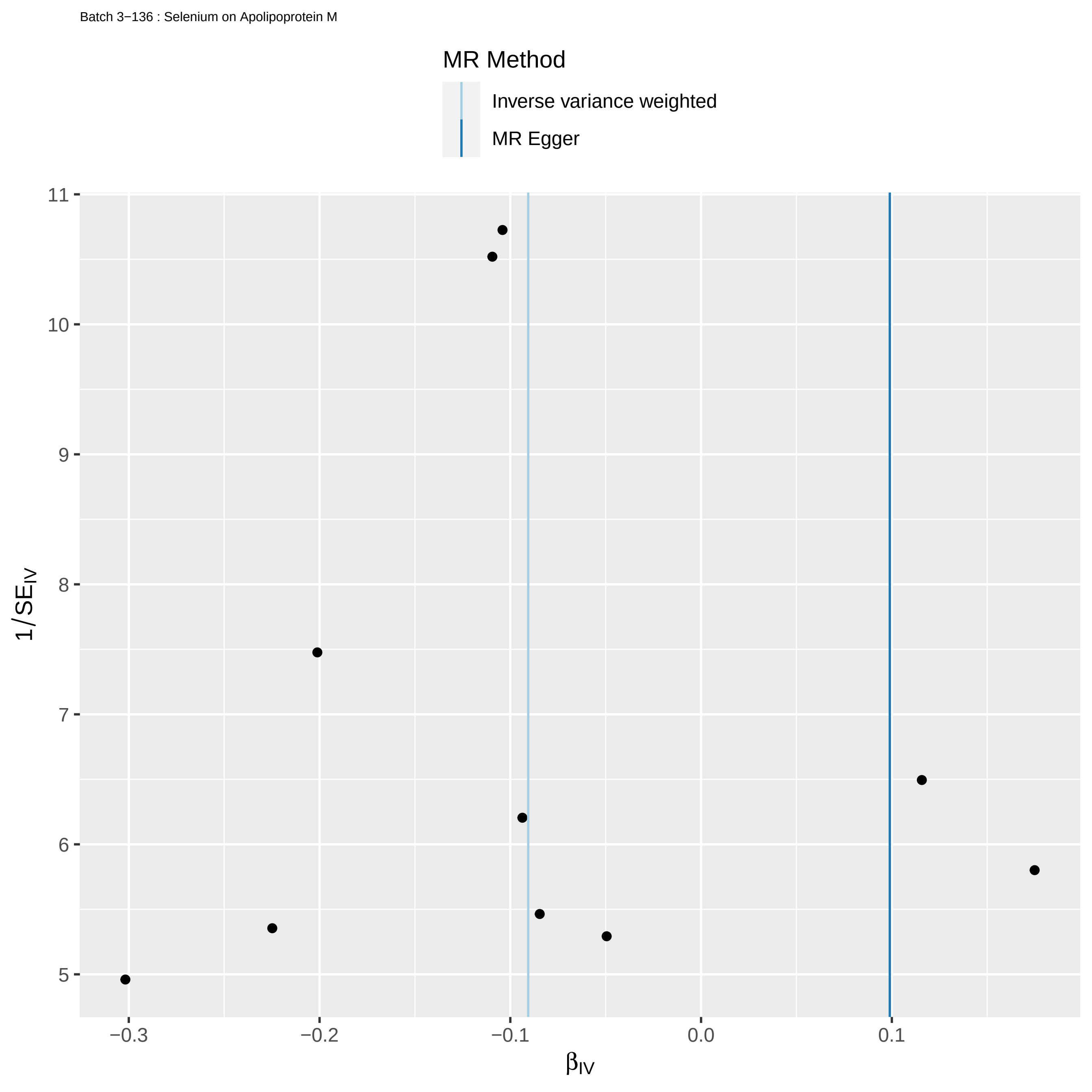
Batch 3−136 : Selenium on Apolipoprotein M
MR Method
Inverse variance weighted
MR Egger
1/SE_IV
β_IV
11
10
9
8
7
6
5
−0.3
−0.2
−0.1
0.0
0.1

Batch 3−137 : Selenium on Apolipoprotein M

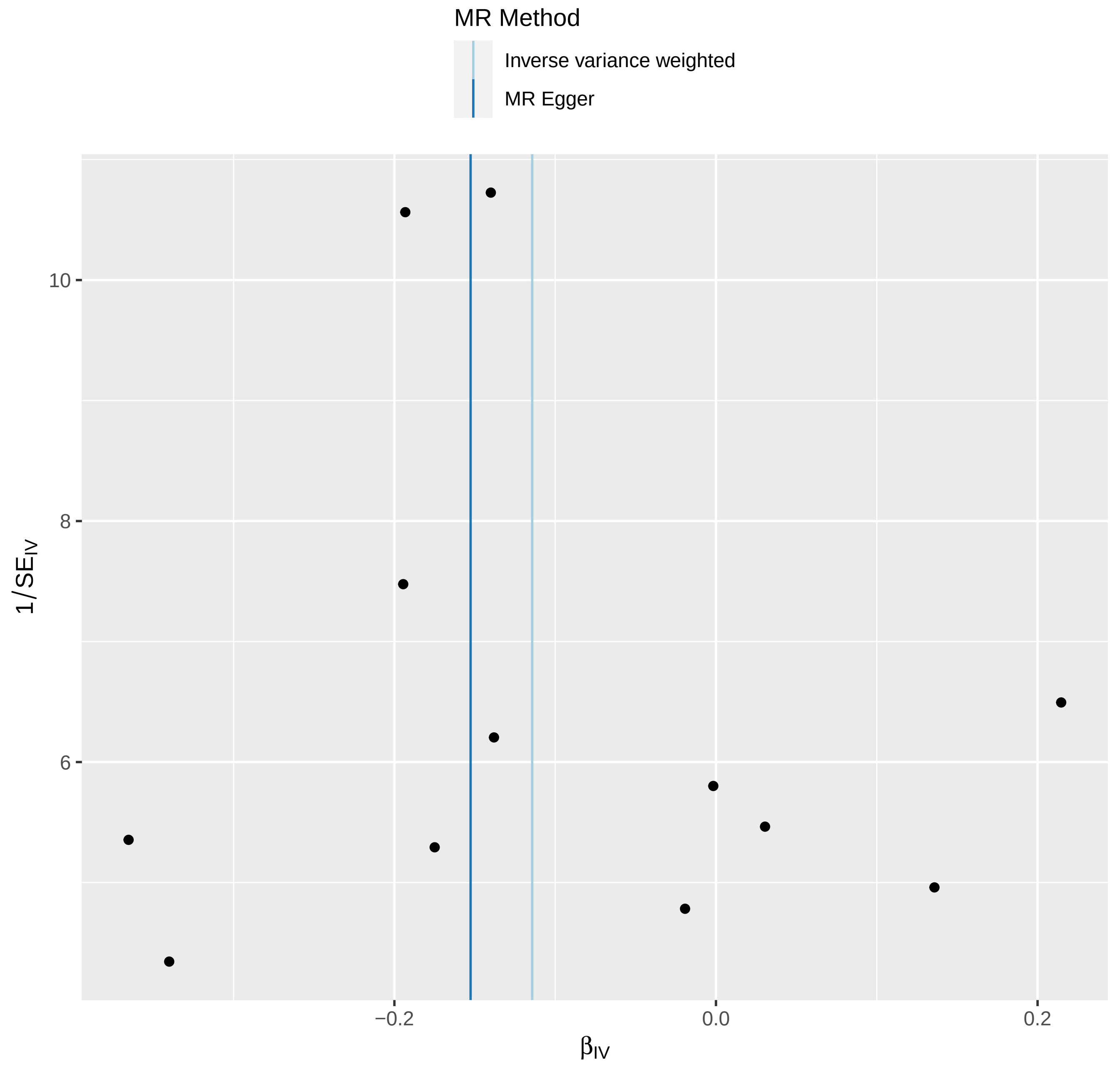

Batch 3−138 : Selenium on MICOS complex subunit MIC26

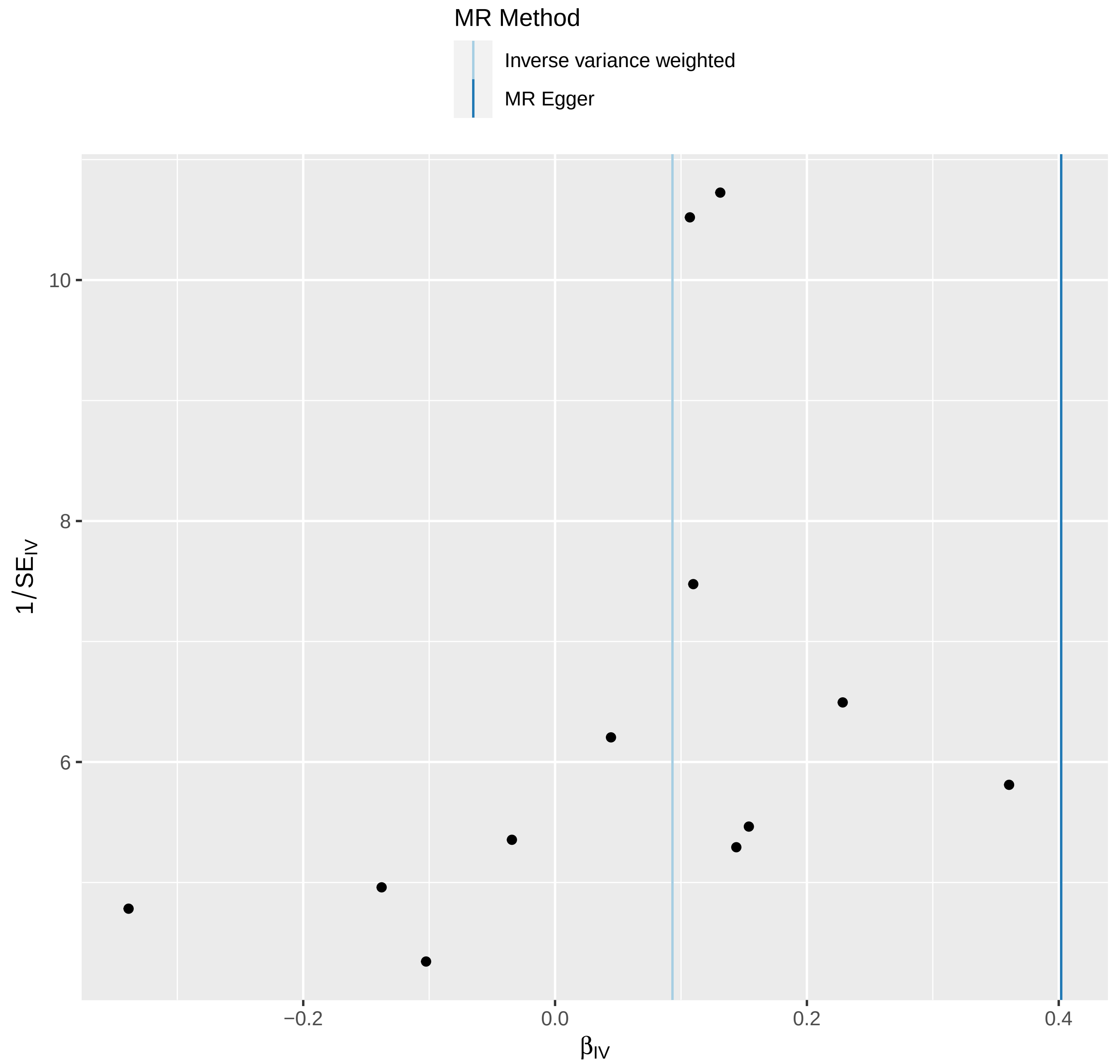

Batch 3−139 : Selenium on Amyloid beta A4 protein

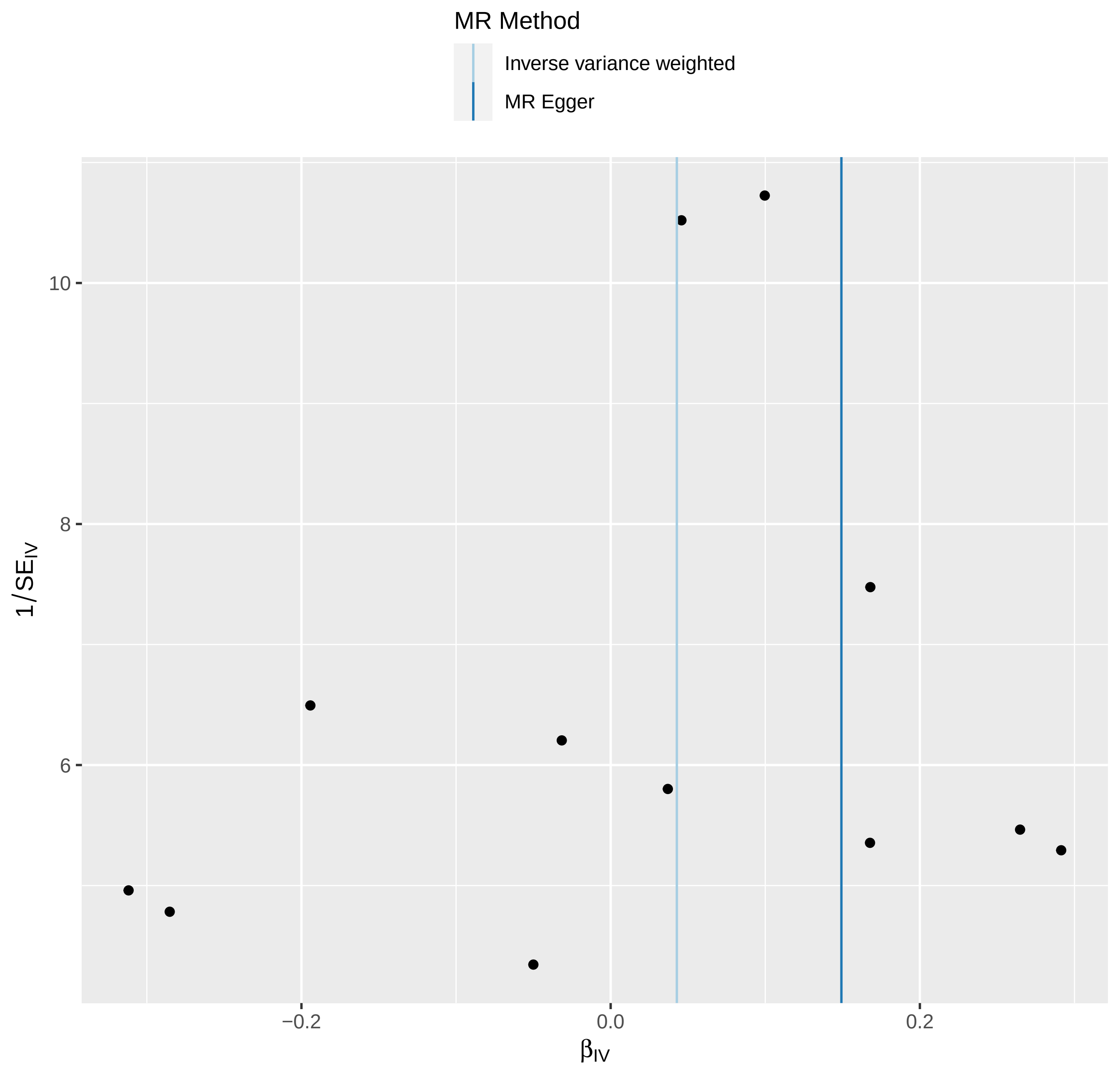

Batch 3−140 : Selenium on DCC−interacting protein 13−alpha

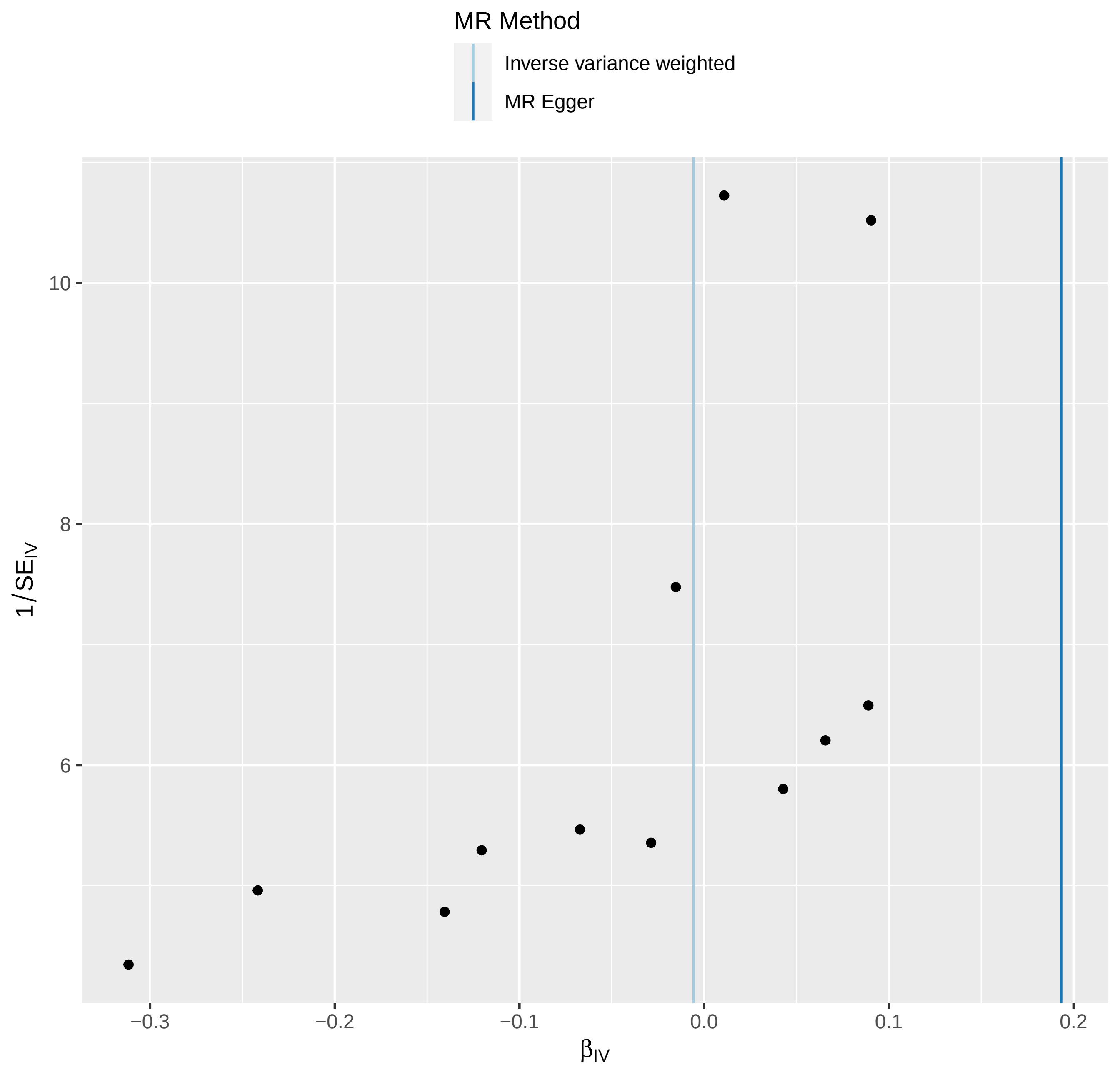

Batch 3−141 : Selenium on Amphiregulin

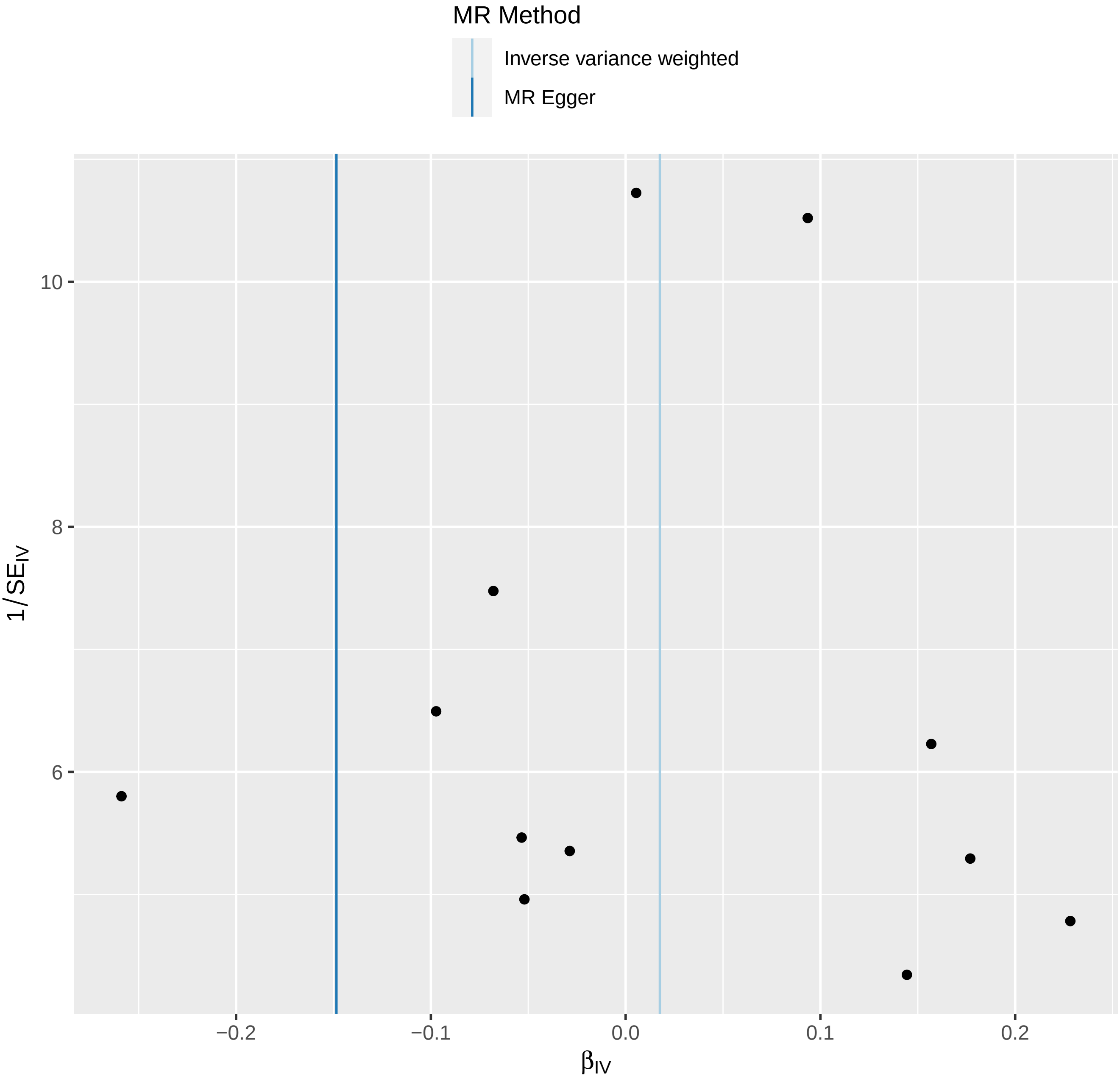

Batch 3−142 : Selenium on ADP−ribosylation factor 3

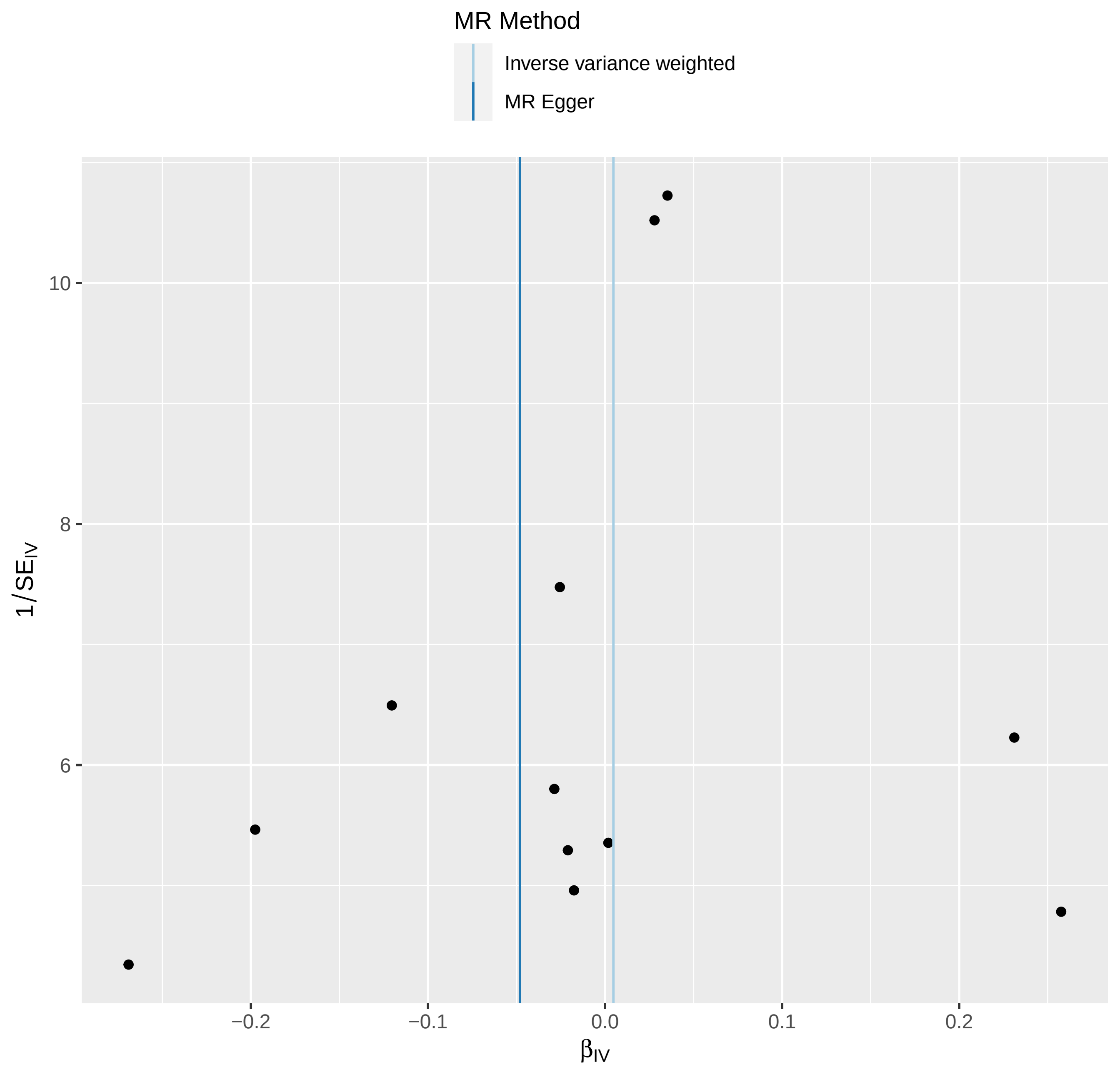

Batch 3−143 : Selenium on ADP−ribosylation factor 6

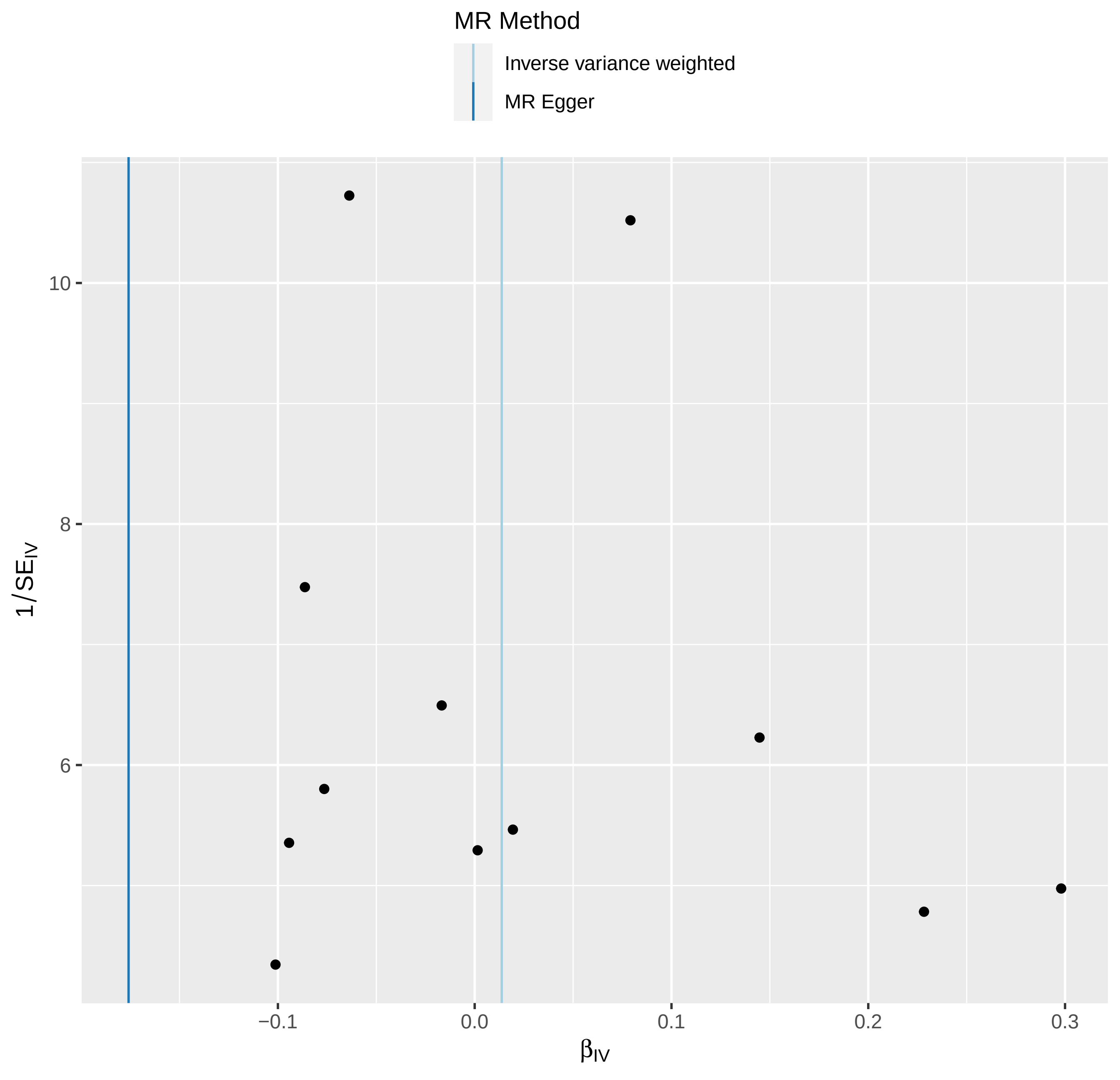

Batch 3−144 : Selenium on ADP−ribosylation factor GTPase−activating protein 2

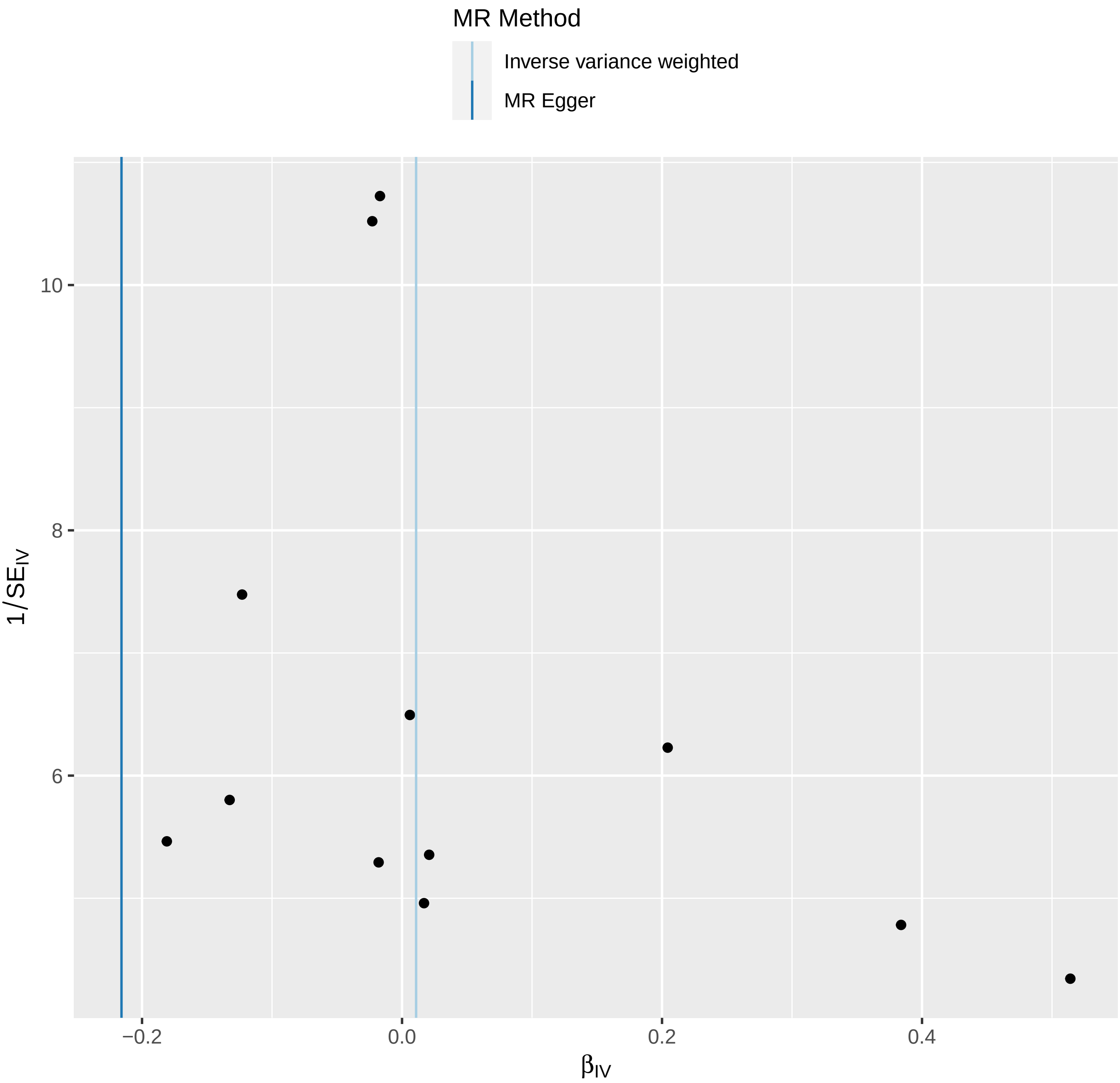

Batch 3−145 : Selenium on Arfaptin−1

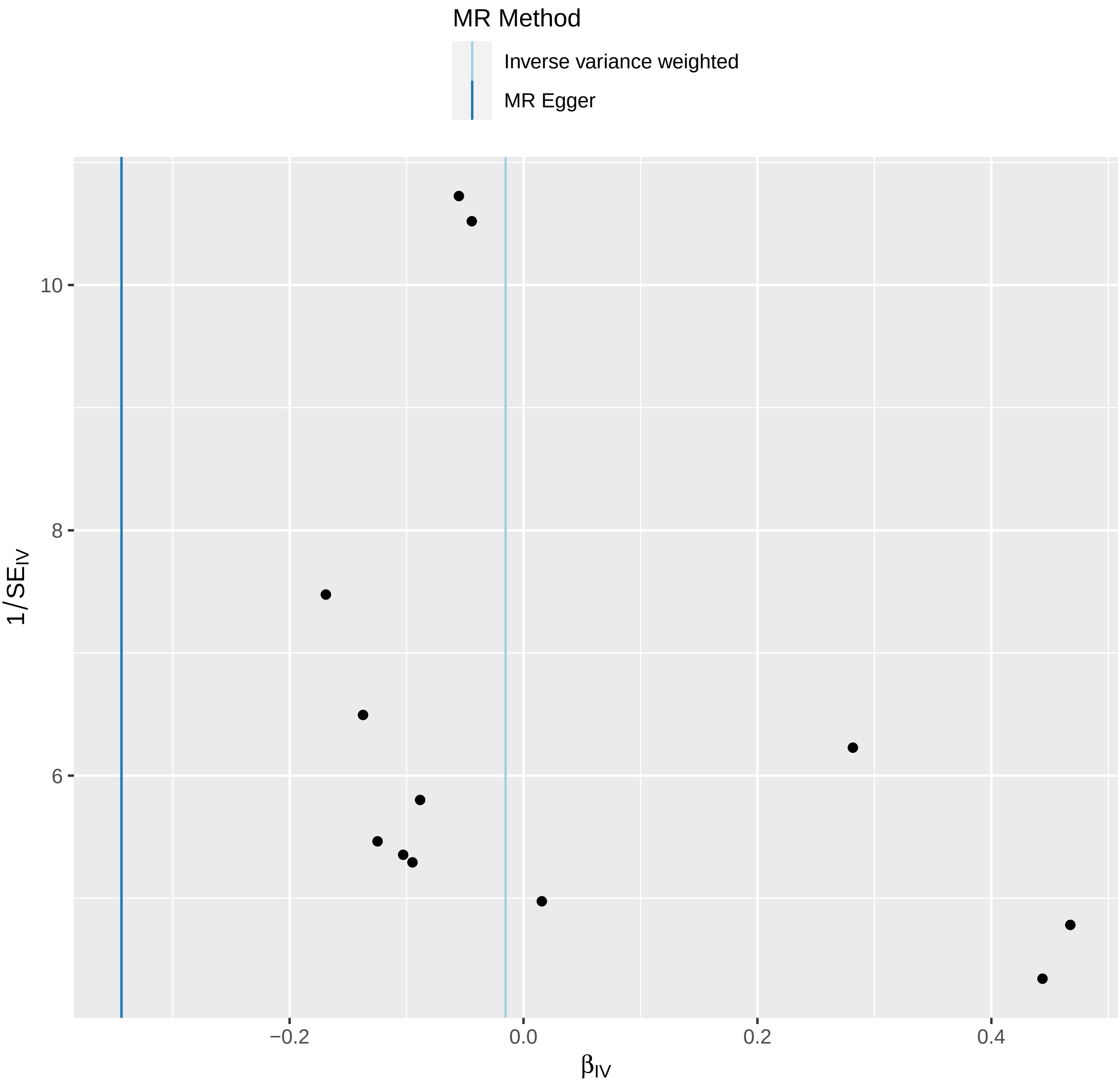

Batch 3−146 : Selenium on Arfaptin−2

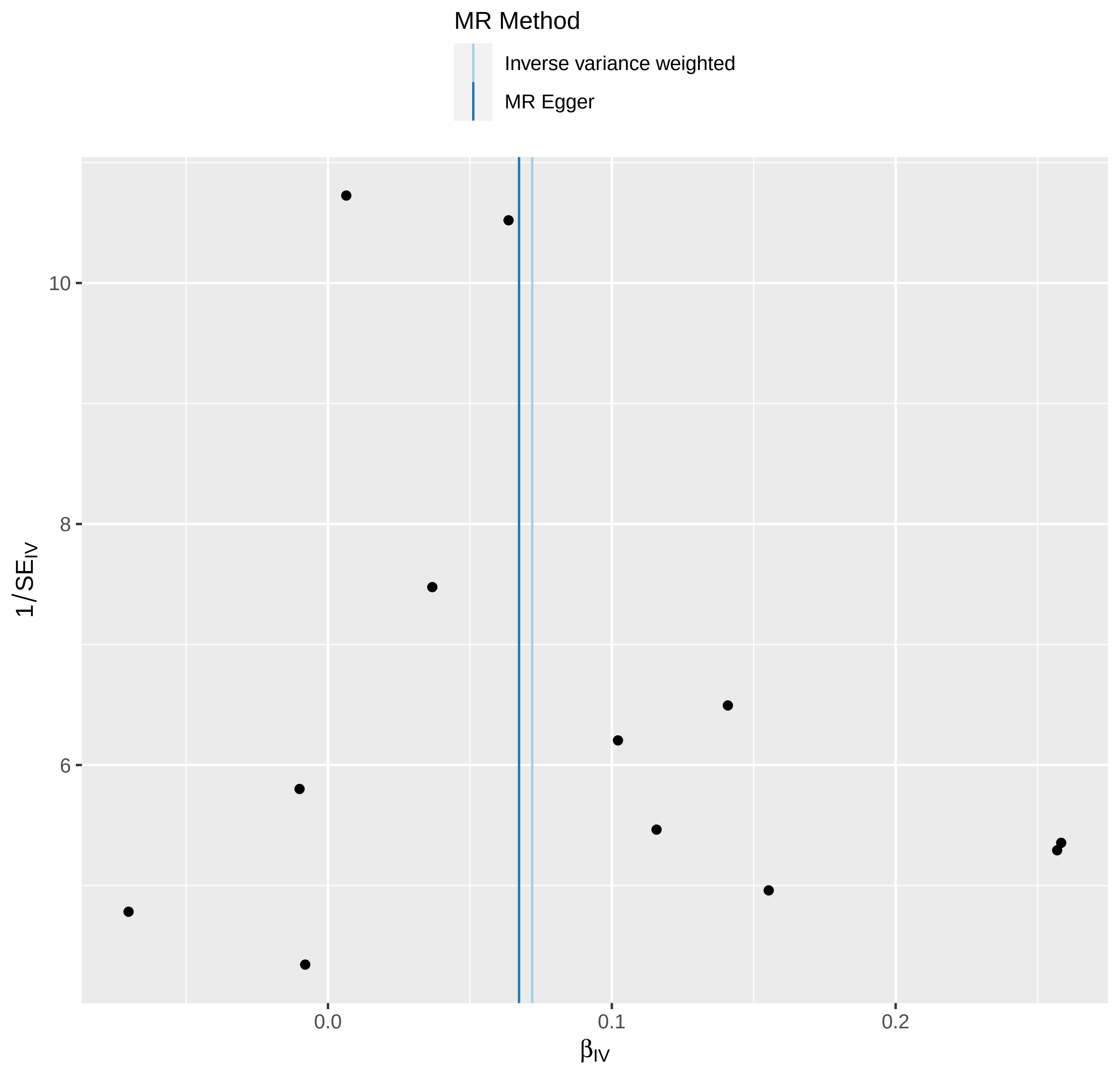

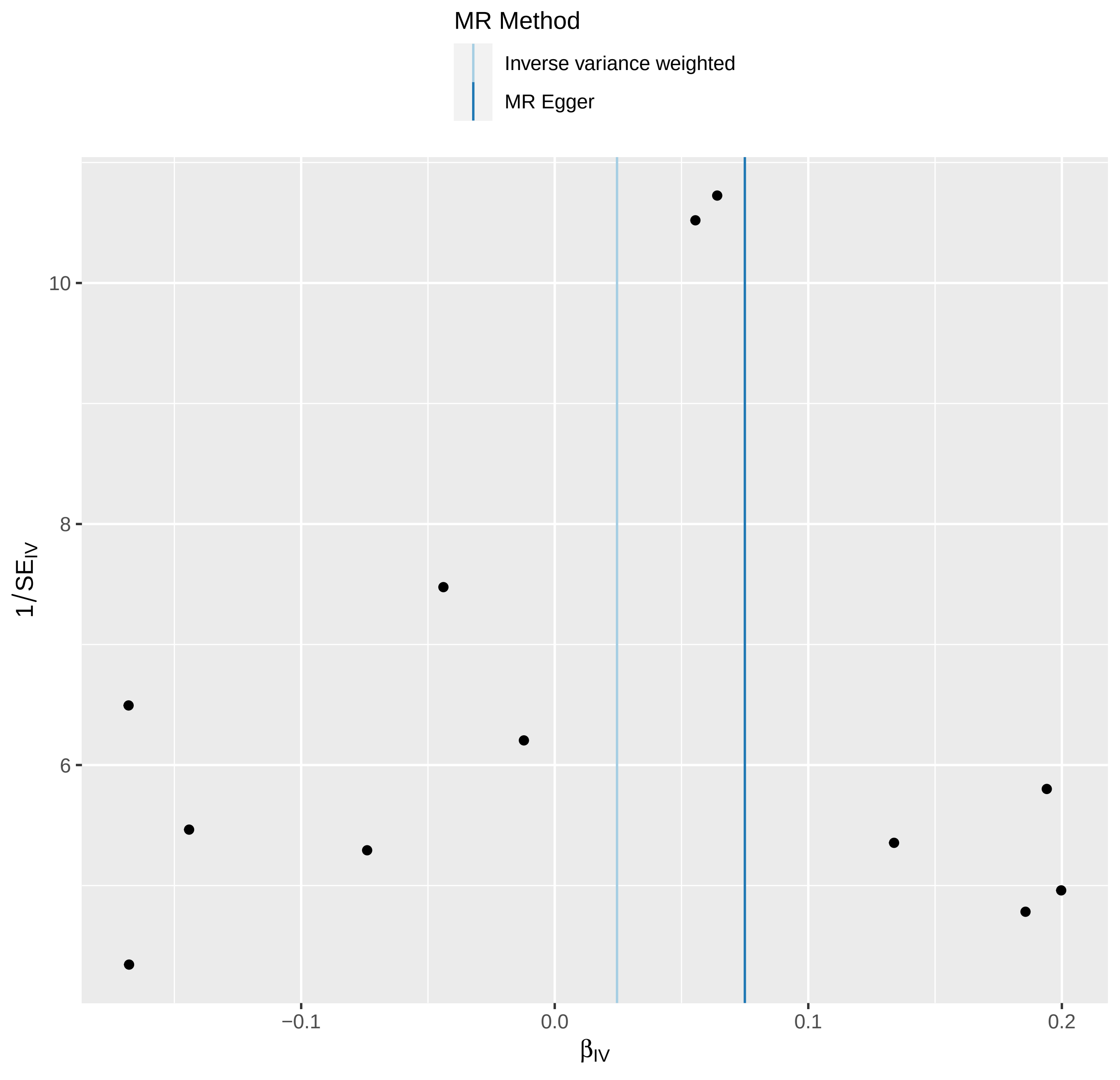
MR Method
Inverse variance weighted
MR Egger
10
8
6
1/SE$_{IV}$
−0.1
0.0
0.1
0.2
β$_{IV}$

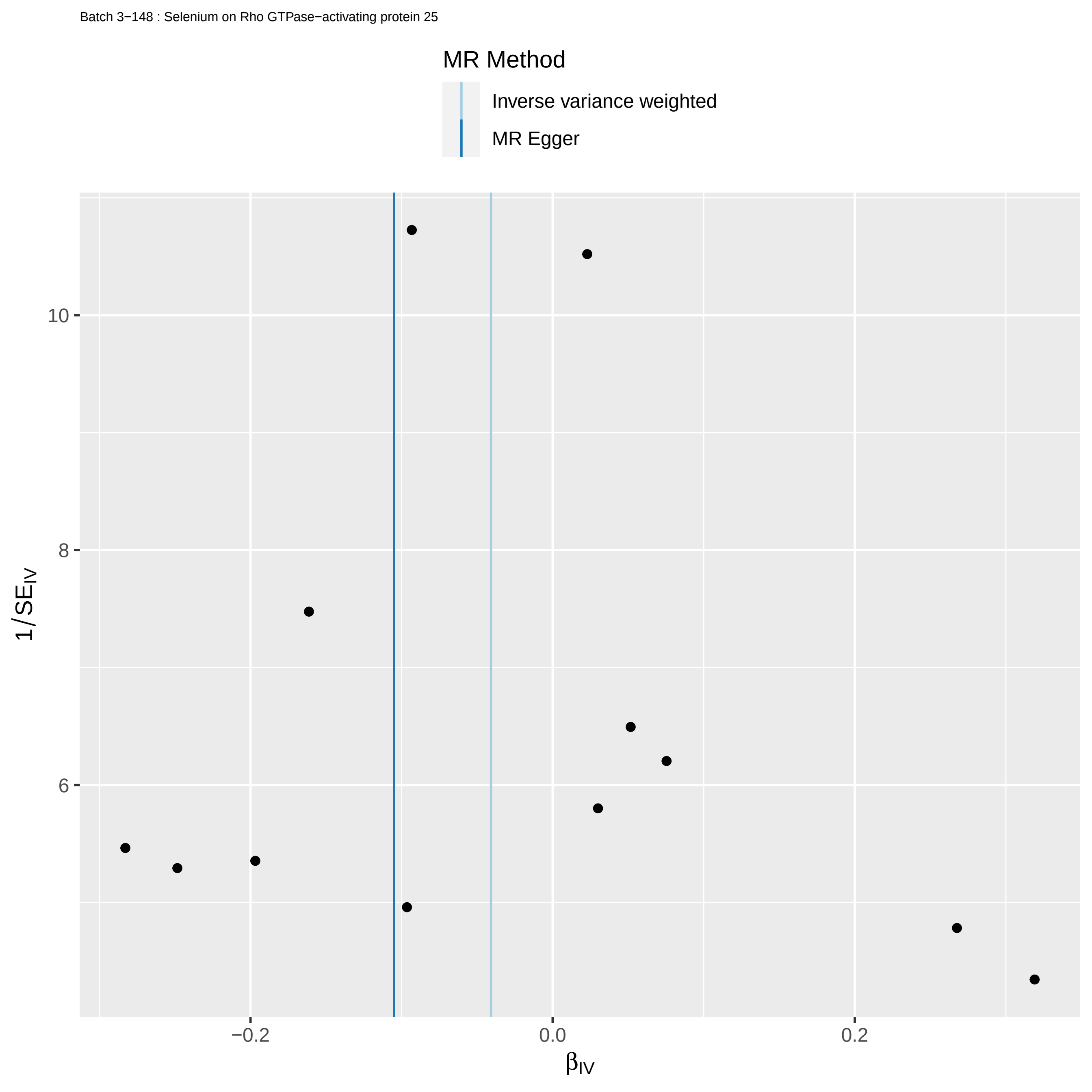

Batch 3−148 : Selenium on Rho GTPase−activating protein 25
MR Method
Inverse variance weighted
MR Egger
10
8
6
1/SE_IV
−0.2
0.0
0.2
β_IV

Batch 3−149 : Selenium on Rho GTPase−activating protein 30

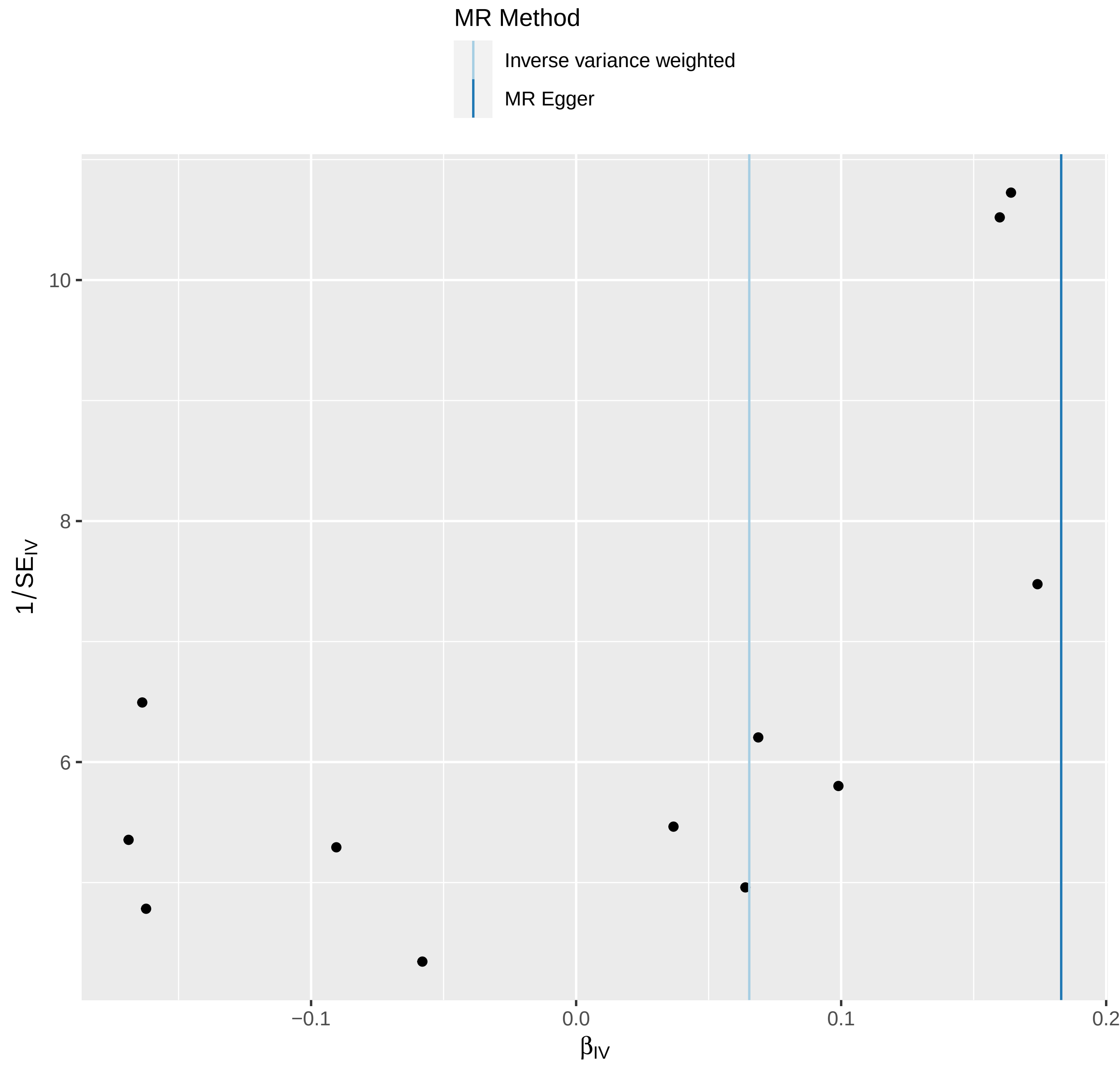

Batch 3−150 : Selenium on Rho GTPase−activating protein 36

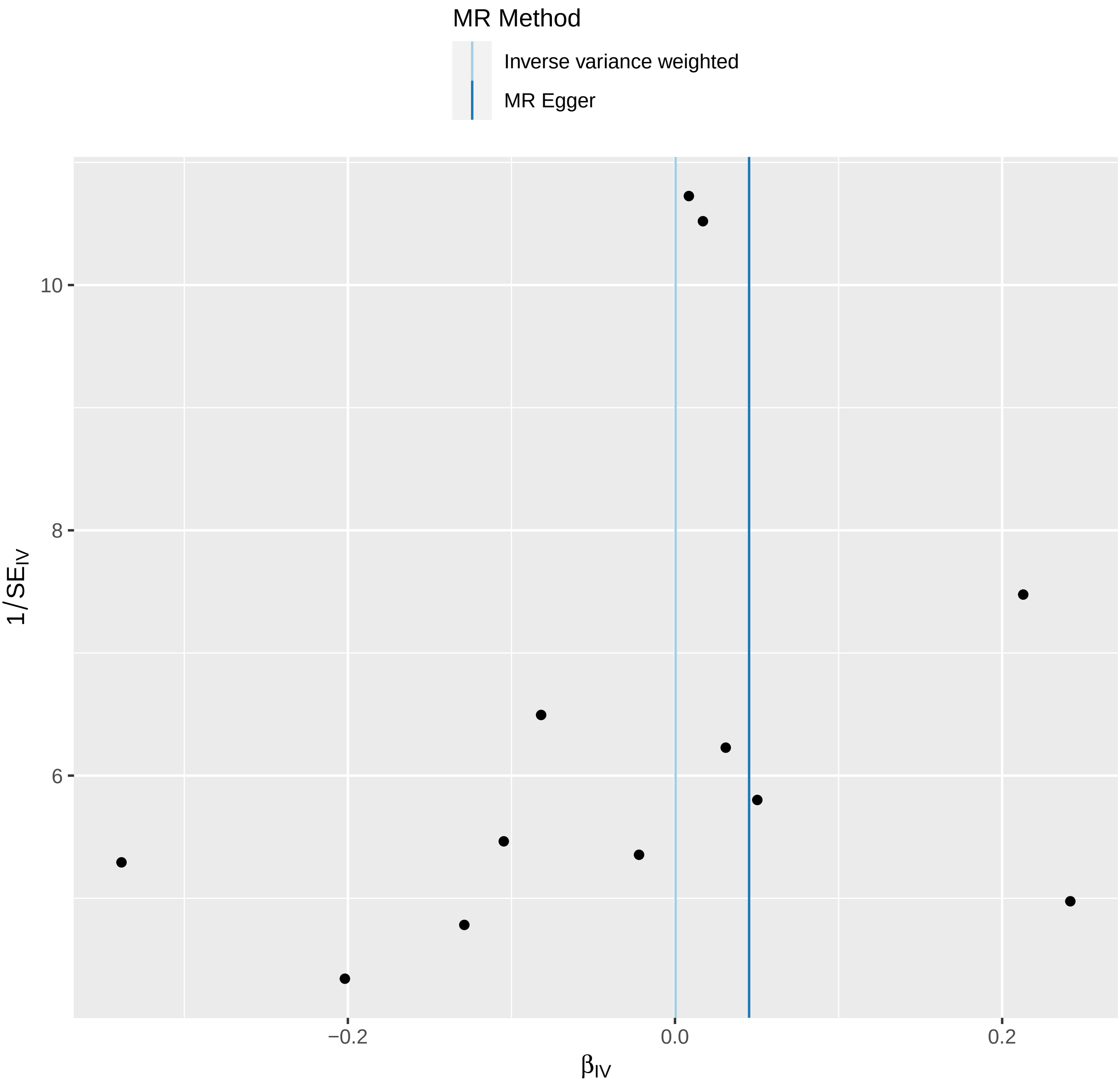

Batch 3−151 : Selenium on Rho GTPase−activating protein 5

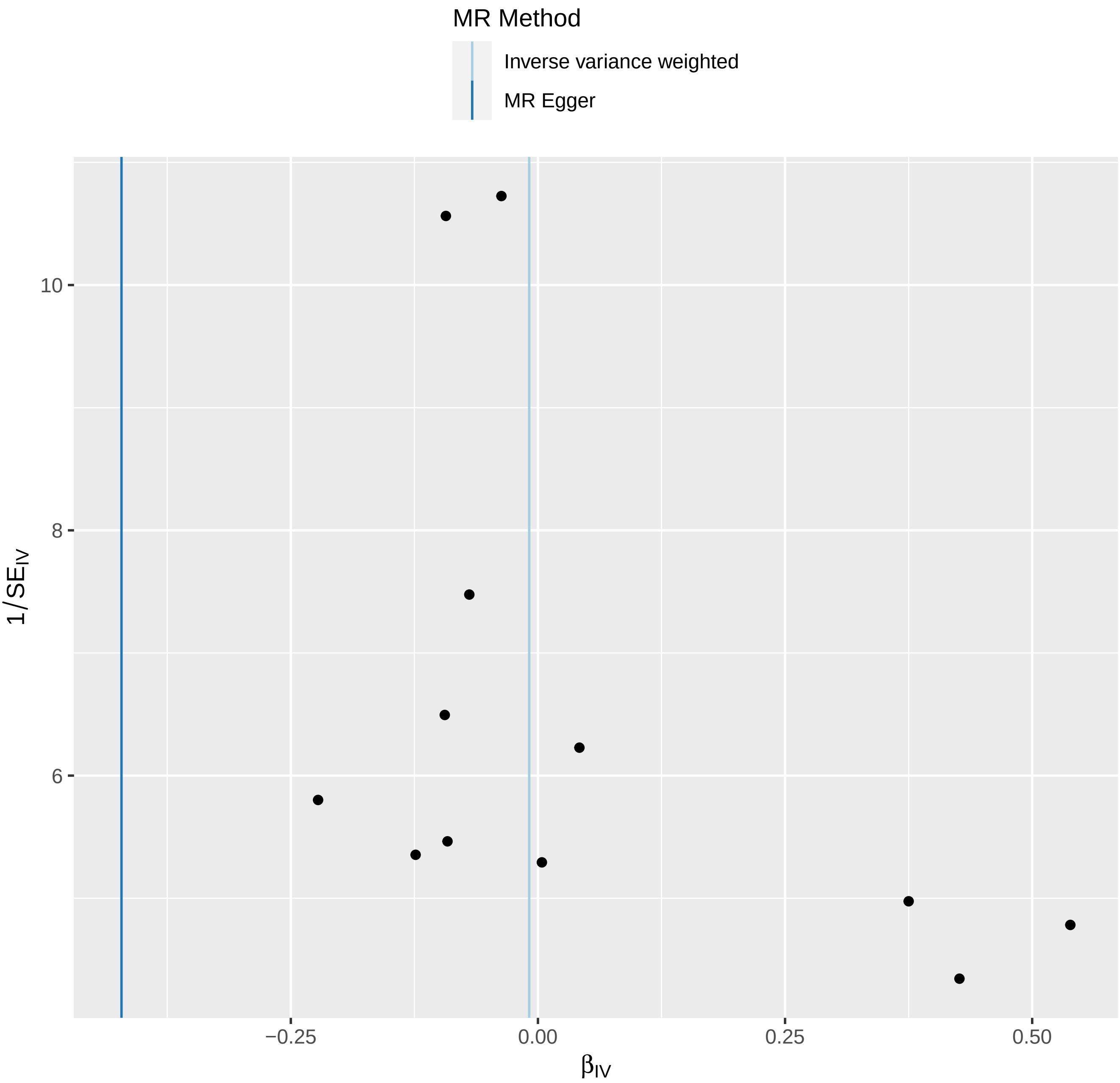

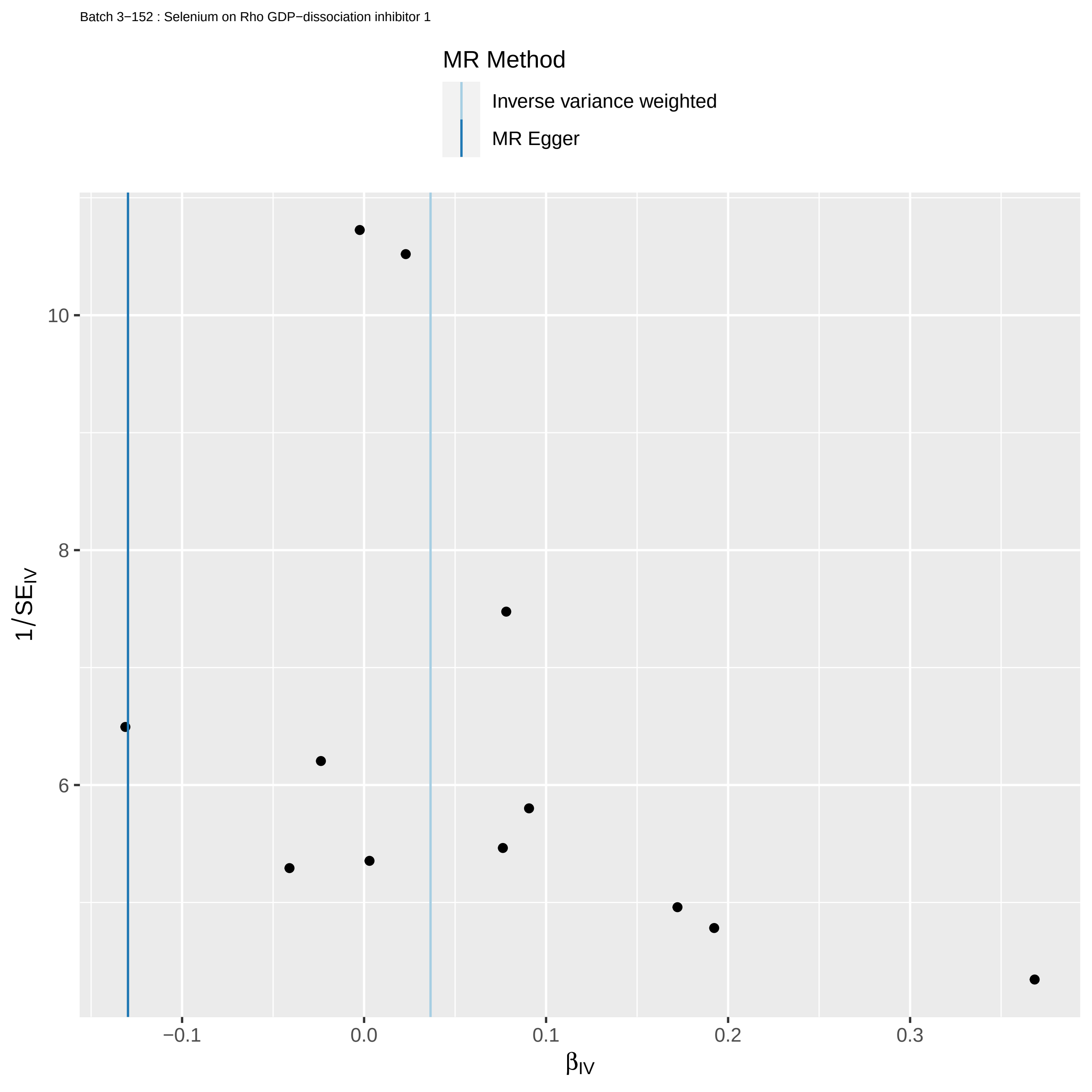

Batch 3−152 : Selenium on Rho GDP−dissociation inhibitor 1
MR Method
Inverse variance weighted
MR Egger
10
8
6
1/SE$_{IV}$
−0.1
0.0
0.1
0.2
0.3
β$_{IV}$

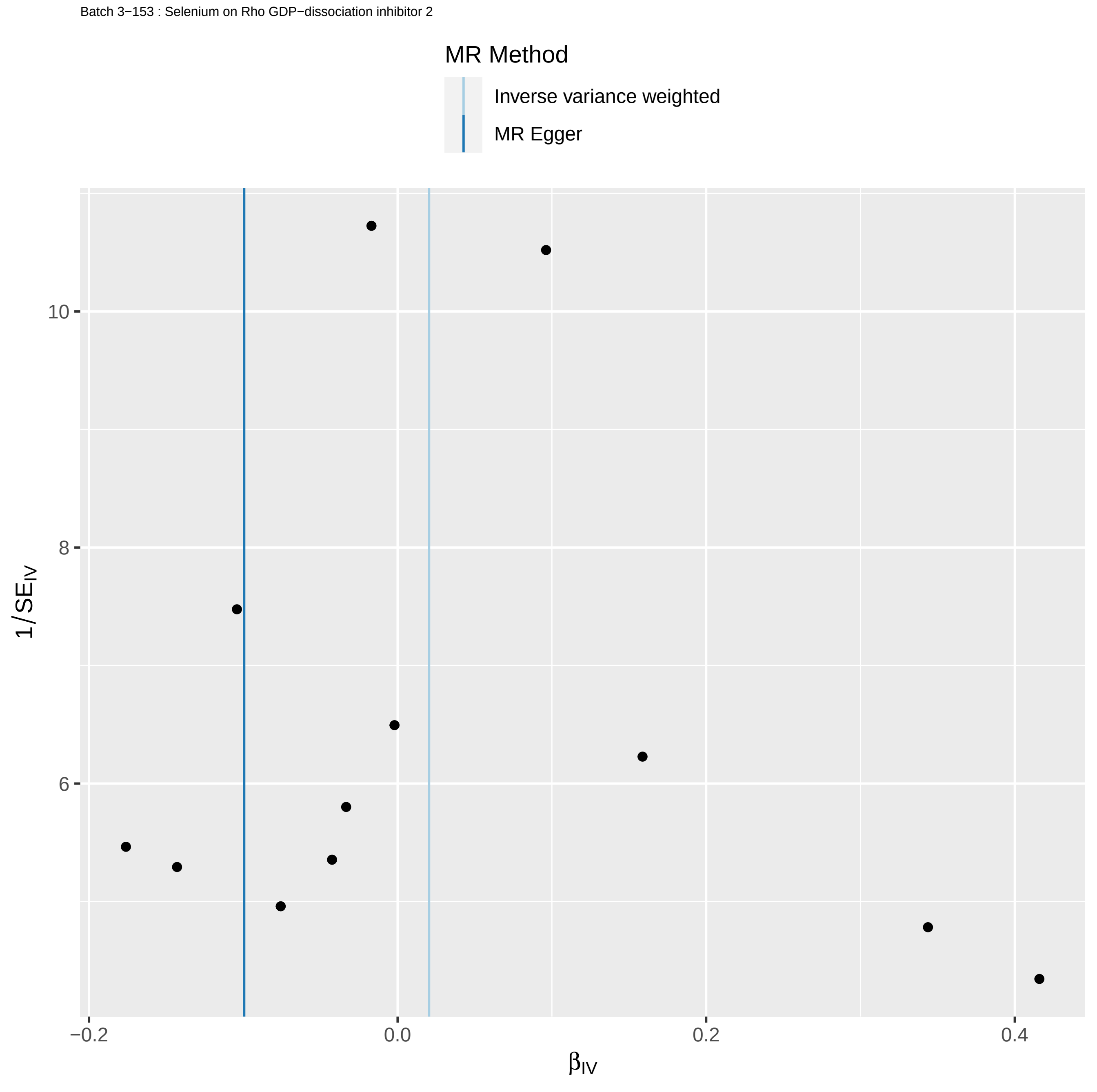

Batch 3−153 : Selenium on Rho GDP−dissociation inhibitor 2
MR Method
Inverse variance weighted
MR Egger
10
8
6
1/SE$_{IV}$
−0.2
0.0
0.2
0.4
β$_{IV}$

Batch 3−154 : Selenium on Rho guanine nucleotide exchange factor 10

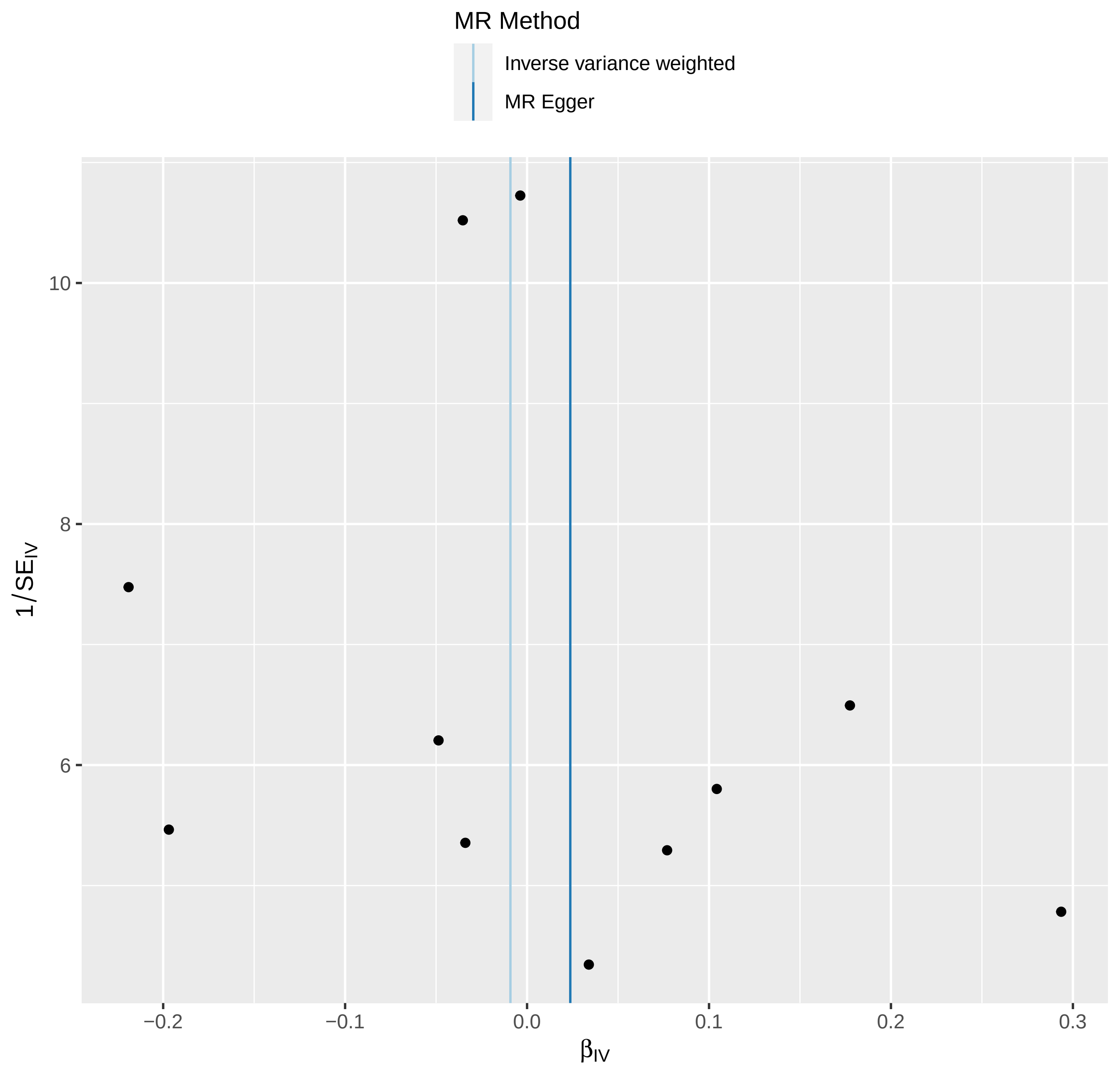

Batch 3−155 : Selenium on Rho guanine nucleotide exchange factor 1

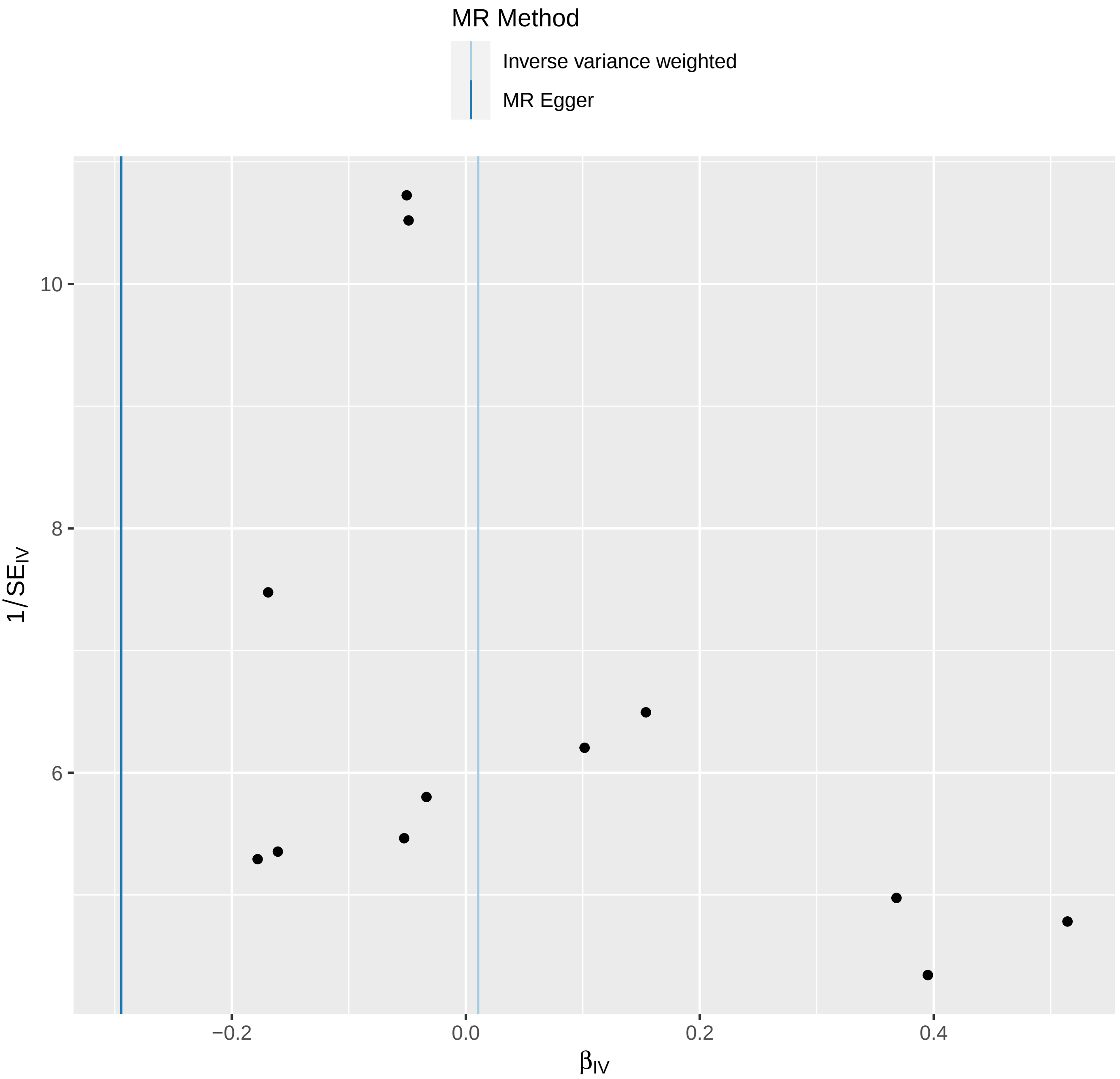

Batch 3−156 : Selenium on Rho guanine nucleotide exchange factor 2

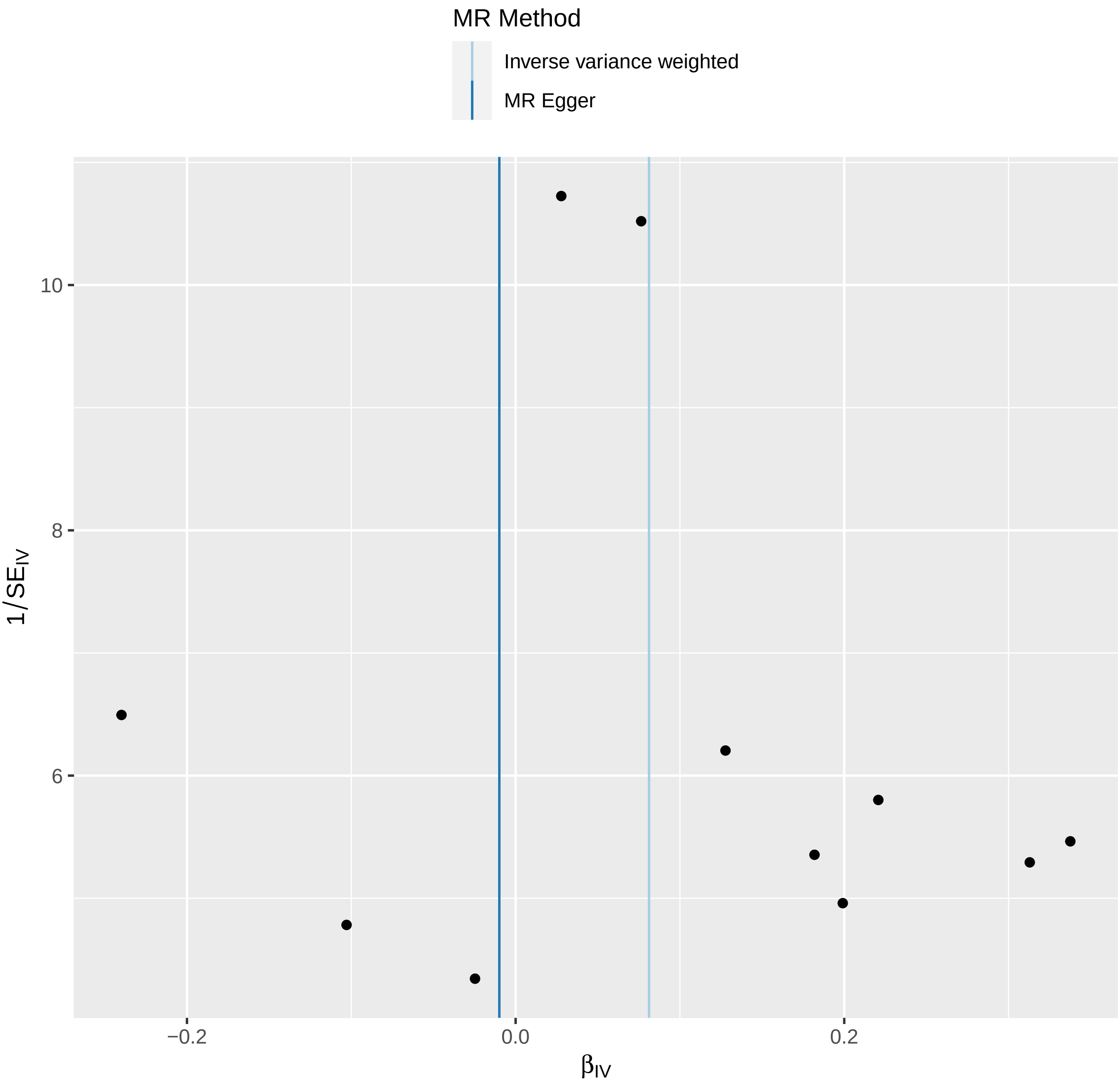

Batch 3−157 : Selenium on Rho guanine nucleotide exchange factor 25

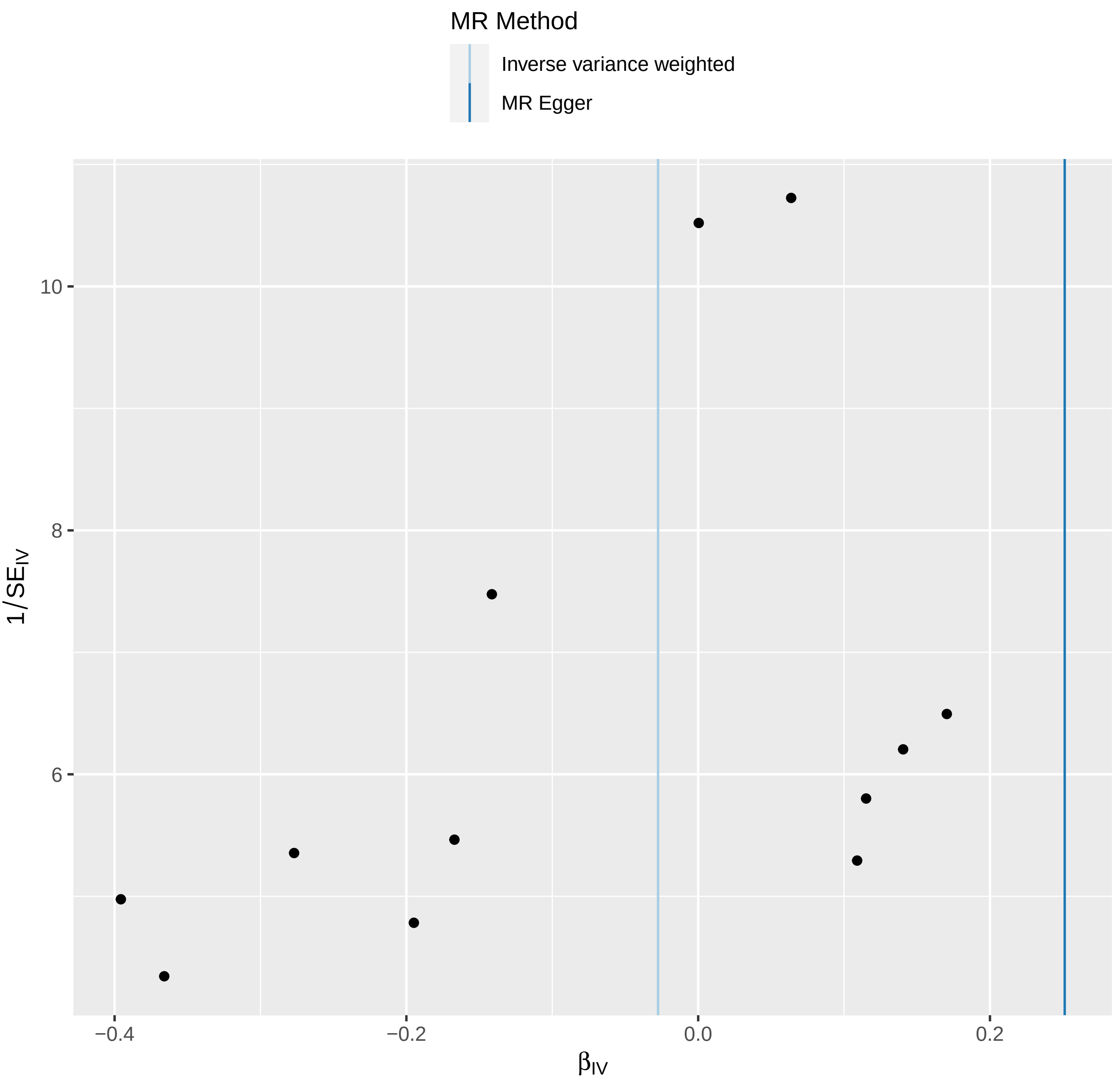

Batch 3−158 : Selenium on Rho guanine nucleotide exchange factor 7

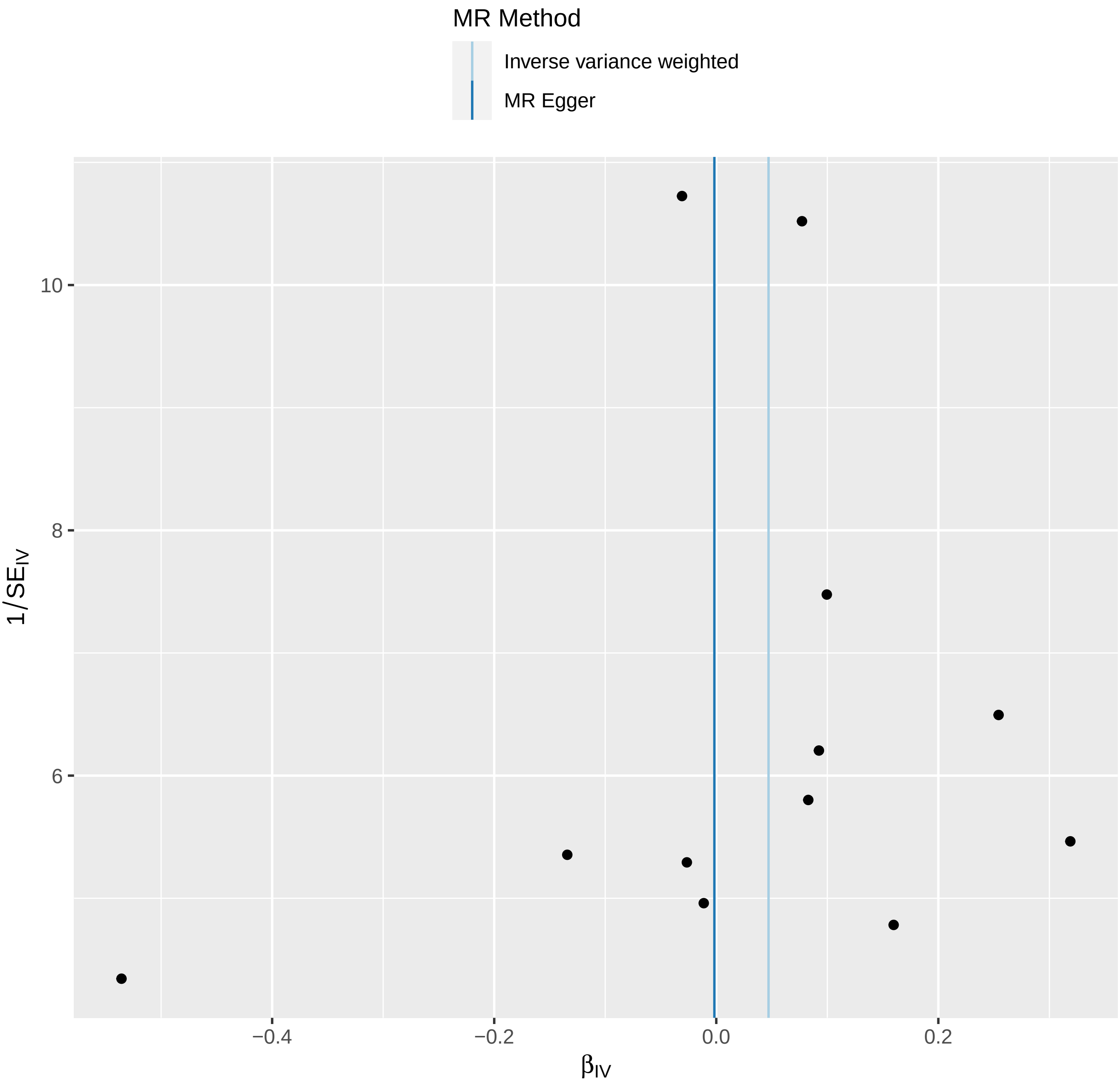

Batch 3−159 : Selenium on AT−rich interactive domain−containing protein 3A

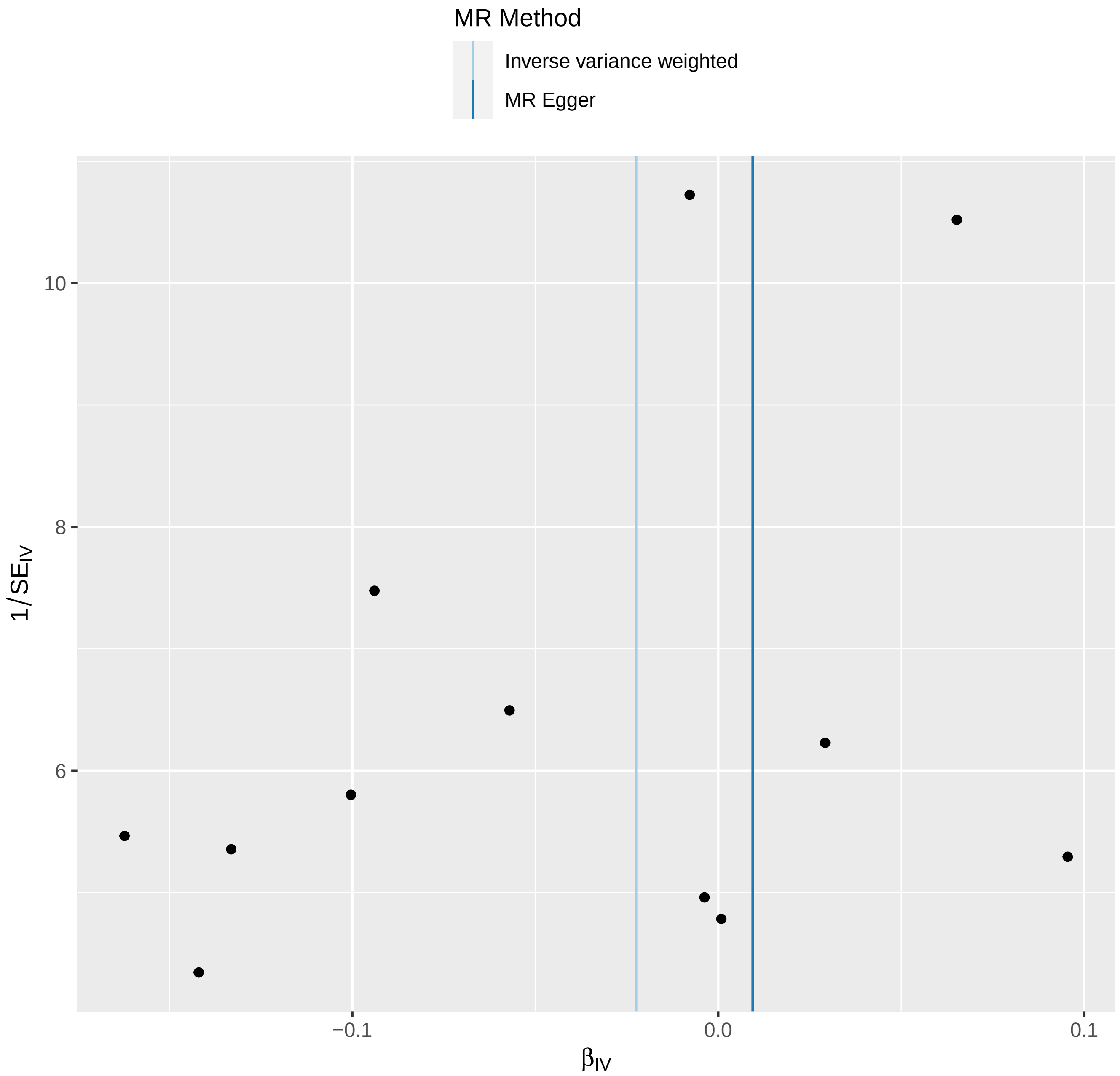

Batch 3−160 : Selenium on ADP−ribosylation factor−like protein 11

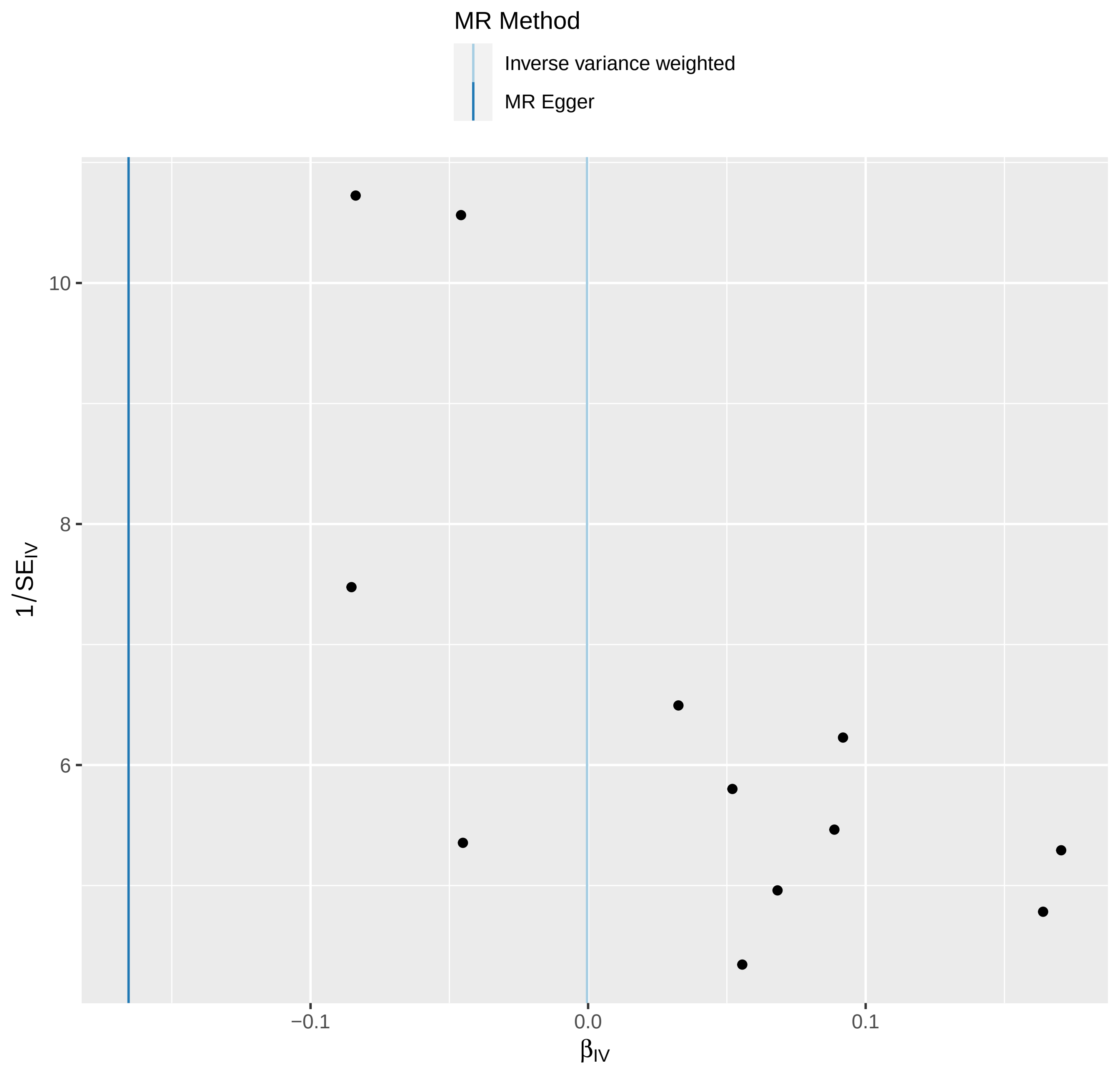

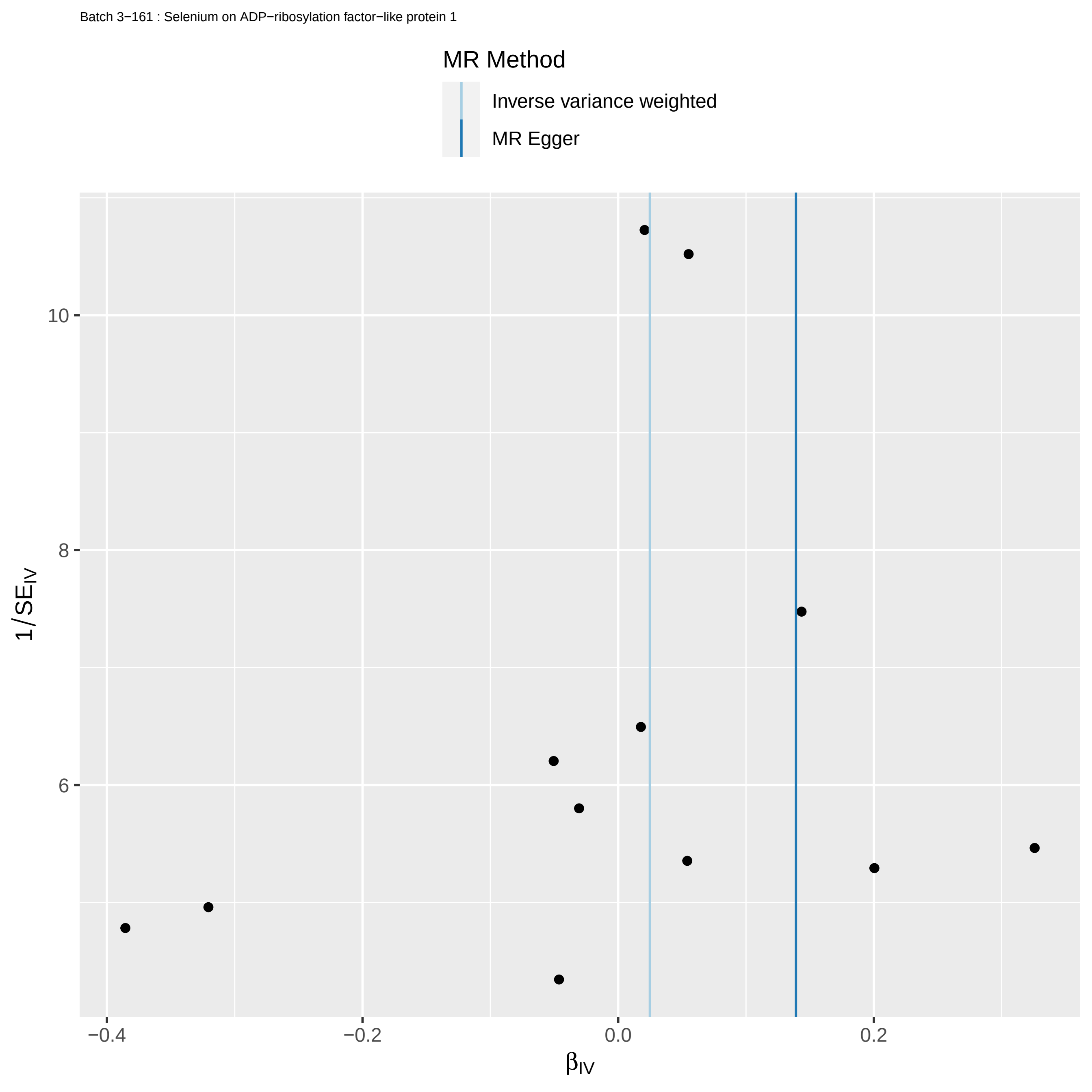
Batch 3−161 : Selenium on ADP−ribosylation factor−like protein 1
MR Method
Inverse variance weighted
MR Egger
10
8
6
1/SE_IV
−0.4
−0.2
0.0
0.2
β_IV

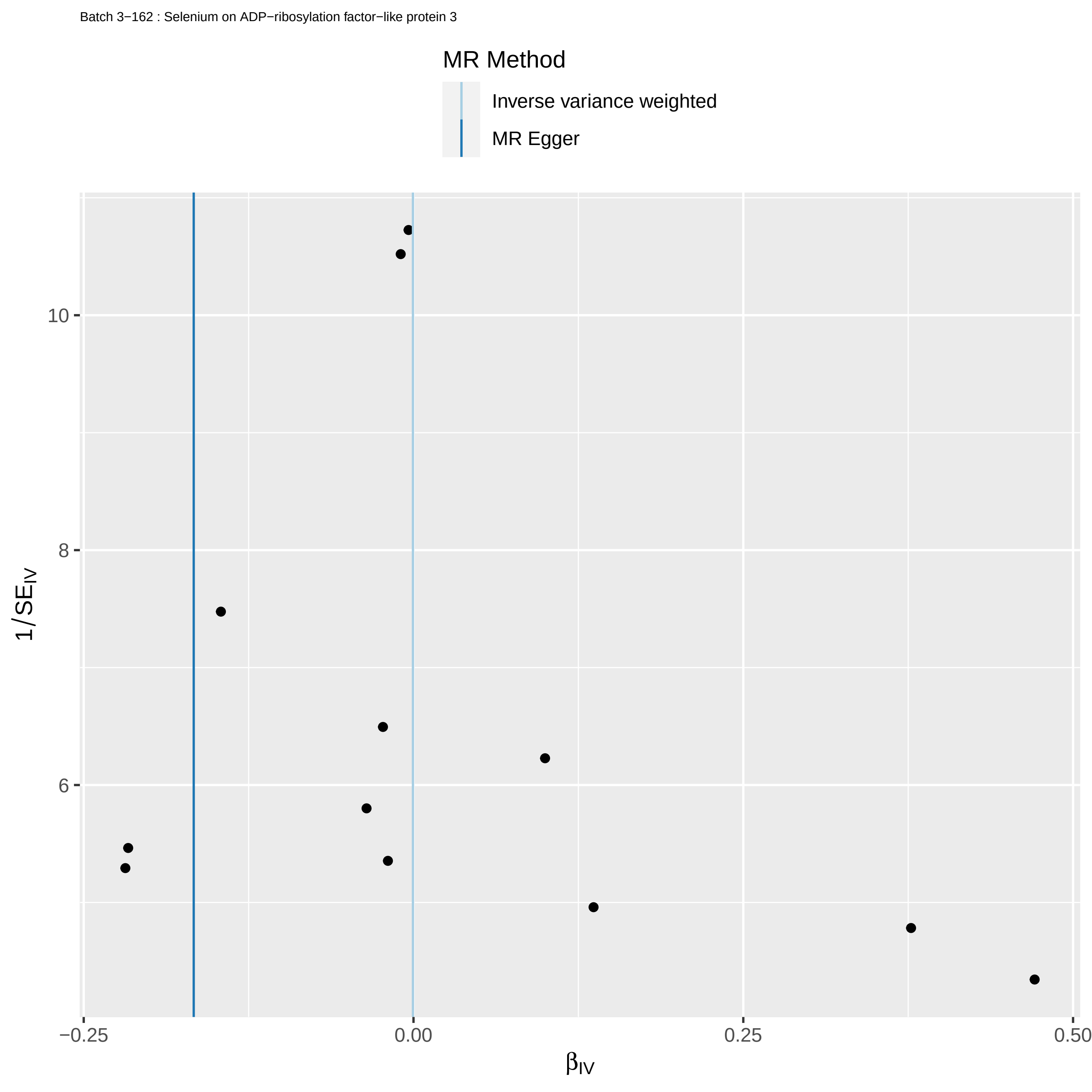
Batch 3−162 : Selenium on ADP−ribosylation factor−like protein 3
MR Method
Inverse variance weighted
MR Egger
10
8
6
1/SE_IV
−0.25
0.00
0.25
0.50
β_IV

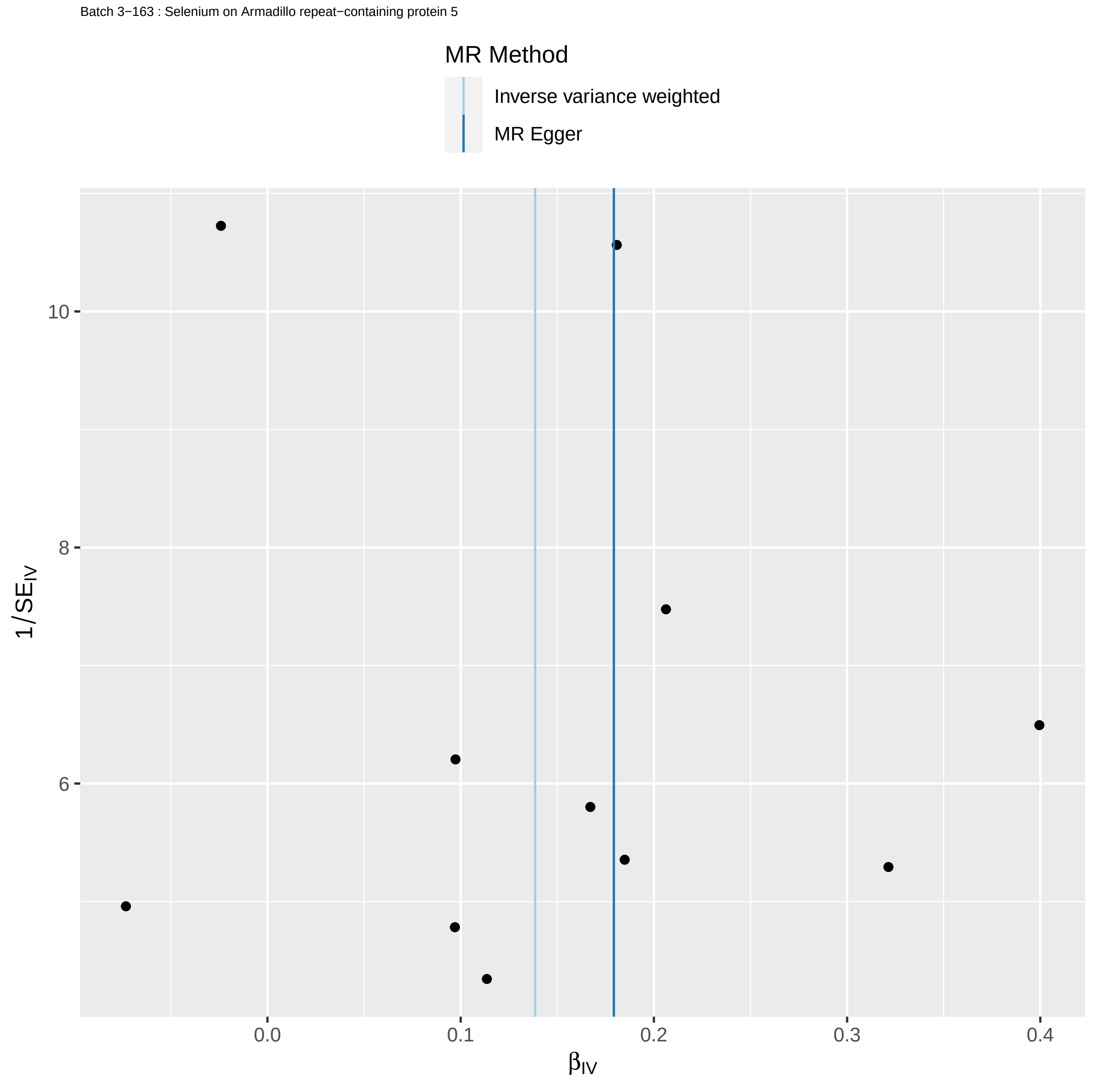
Batch 3−163 : Selenium on Armadillo repeat−containing protein 5
MR Method
Inverse variance weighted
MR Egger
10
8
6
1/SE_IV
0.0
0.1
0.2
0.3
0.4
β_IV

Batch 3−164 : Selenium on Armadillo repeat−containing protein 5

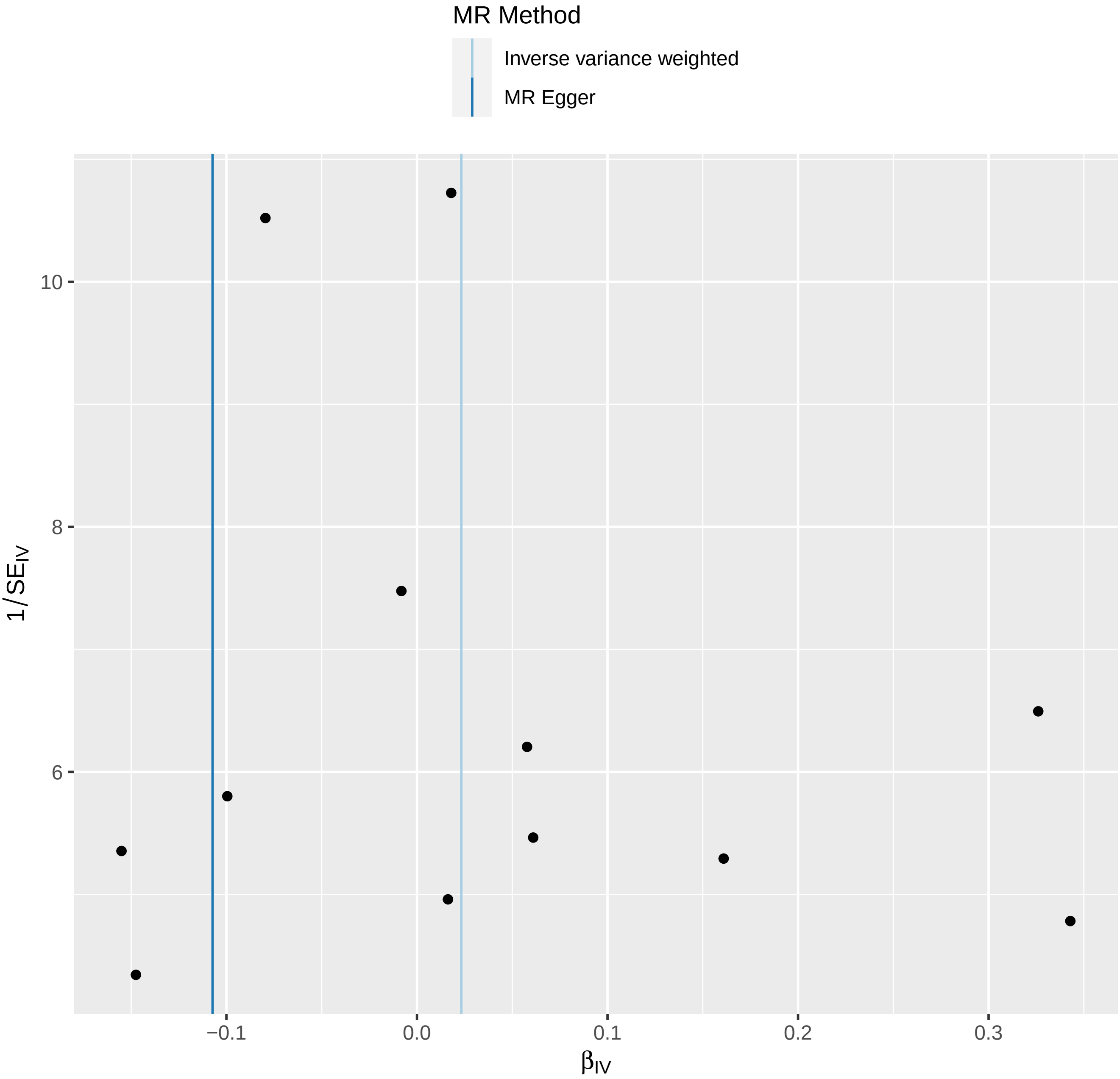

Batch 3−165 : Selenium on Armadillo repeat−containing protein 5

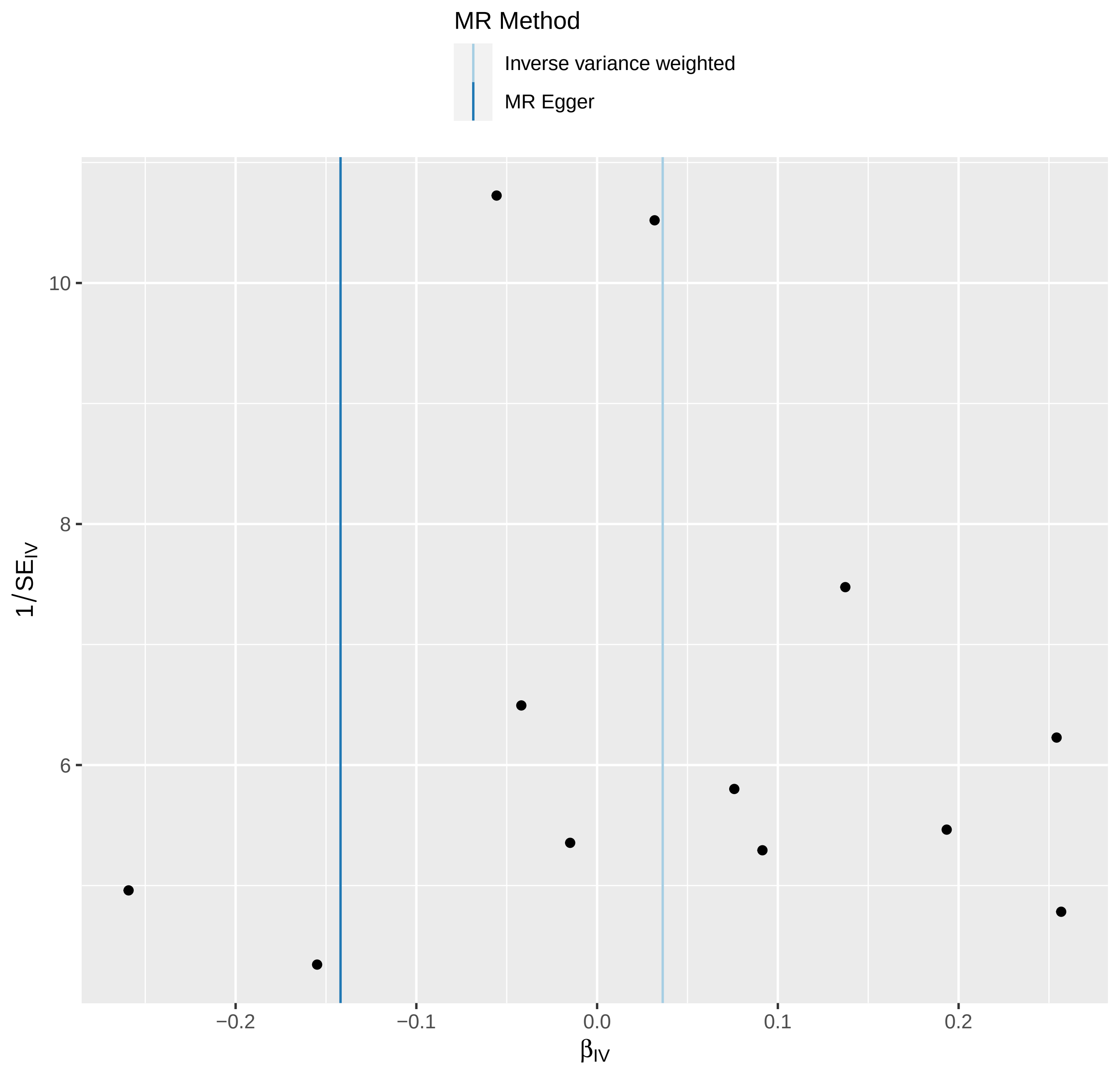

Batch 3−166 : Selenium on Actin−related protein 2/3 complex subunit 3

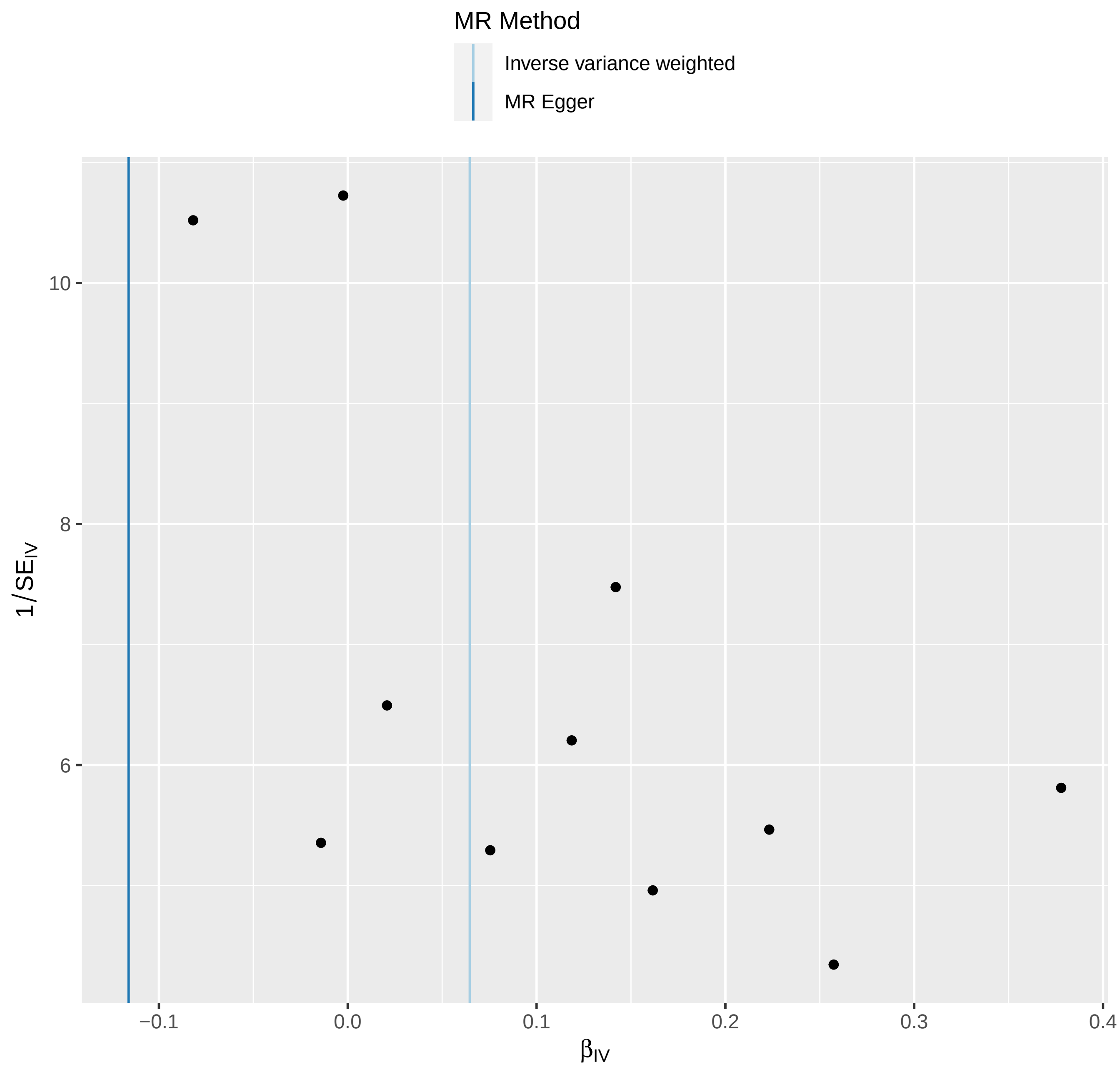

Batch 3−167 : Selenium on cAMP−regulated phosphoprotein 19

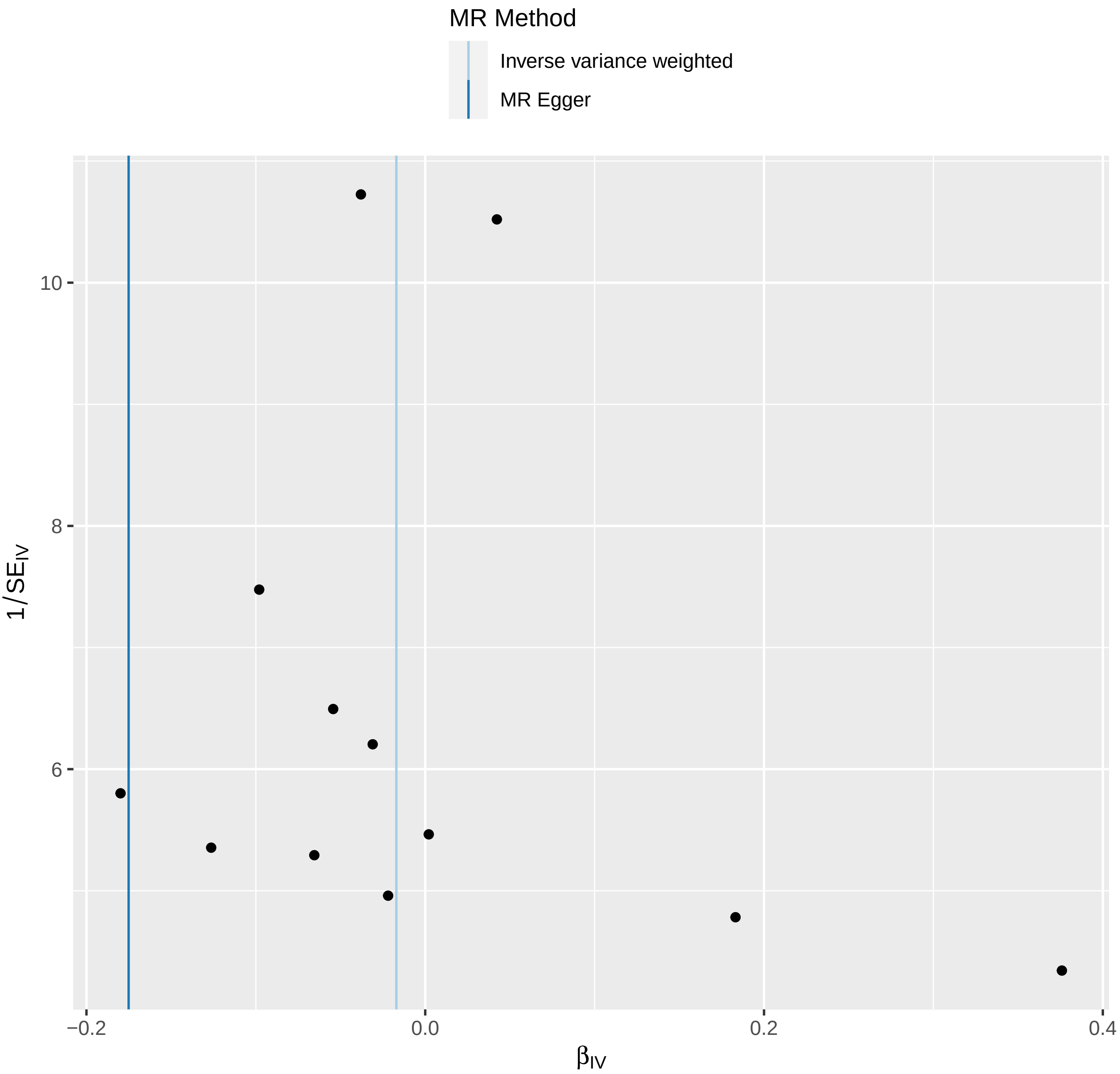

Batch 3−168 : Selenium on cAMP−regulated phosphoprotein 21

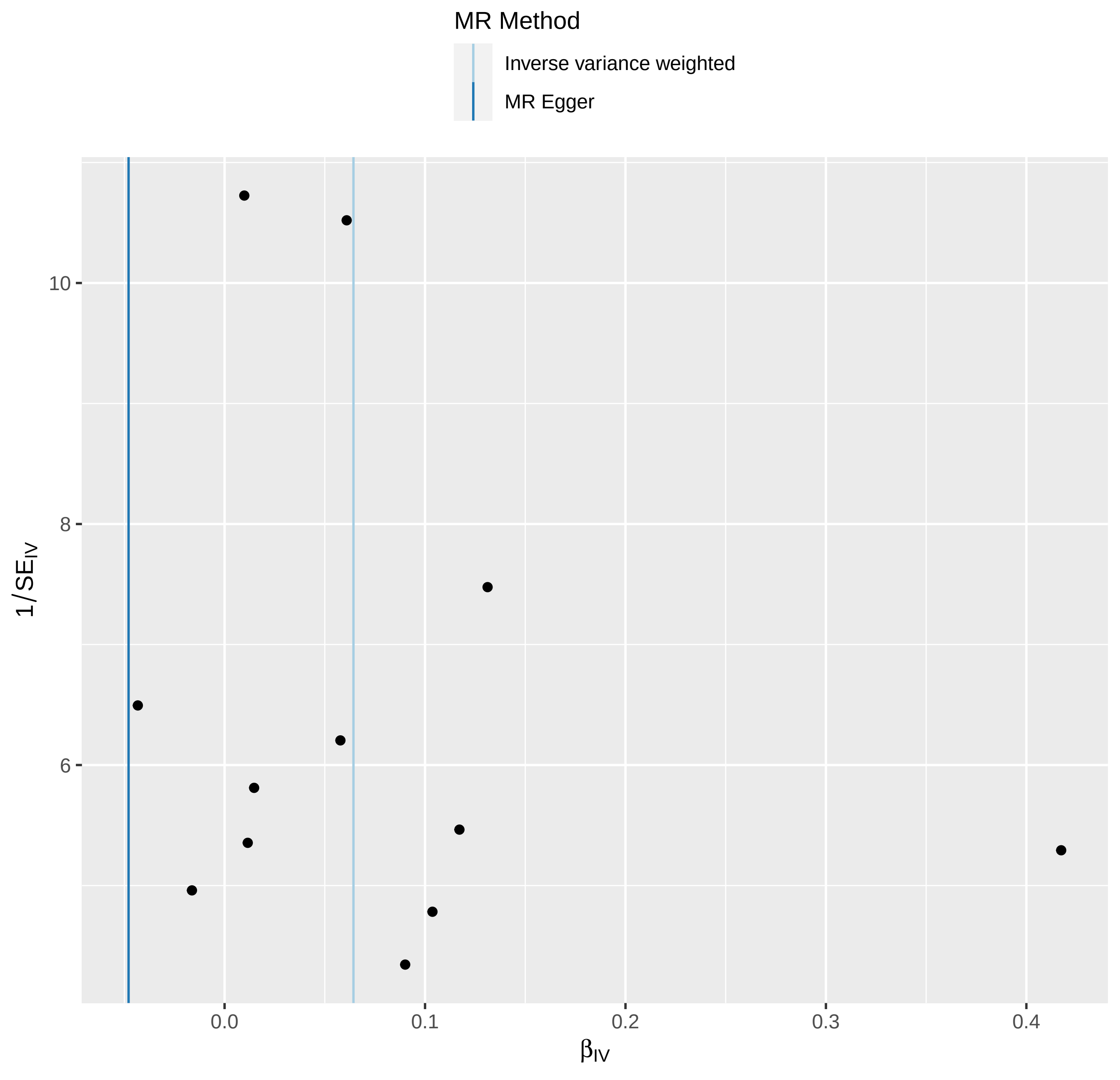

Batch 3−169 : Selenium on Beta−arrestin−1

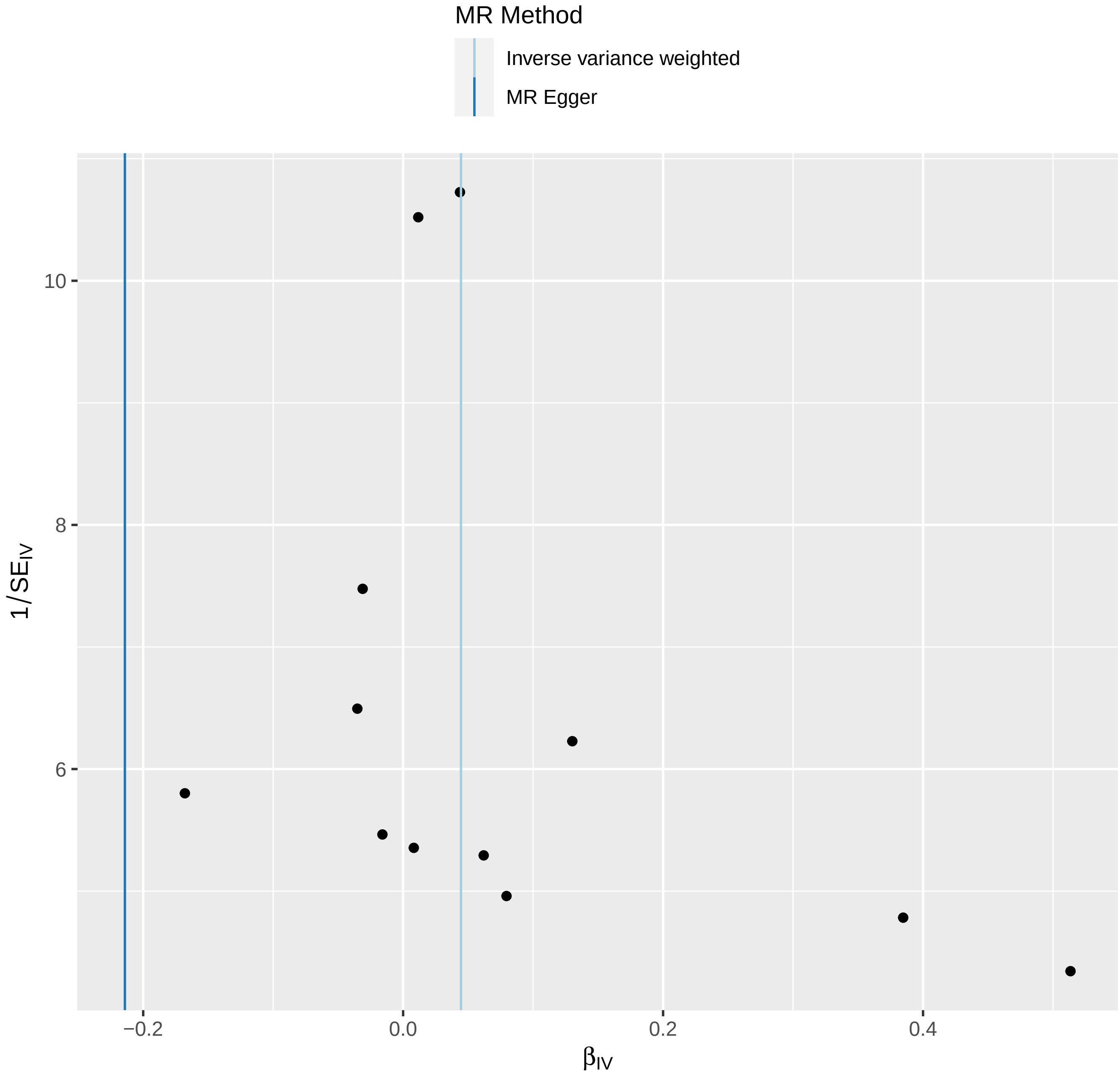

Batch 3−170 : Selenium on Arrestin domain−containing protein 3

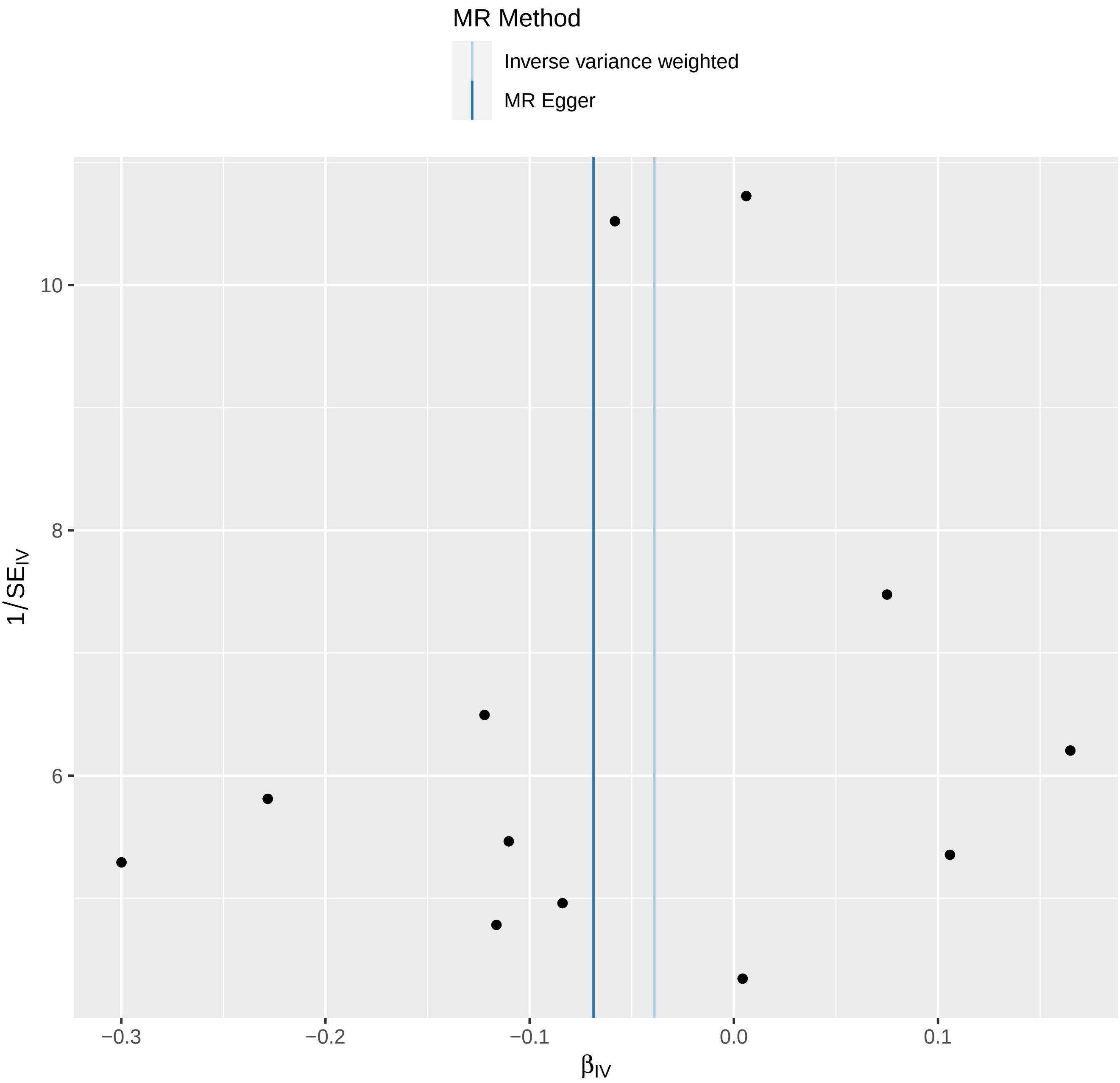

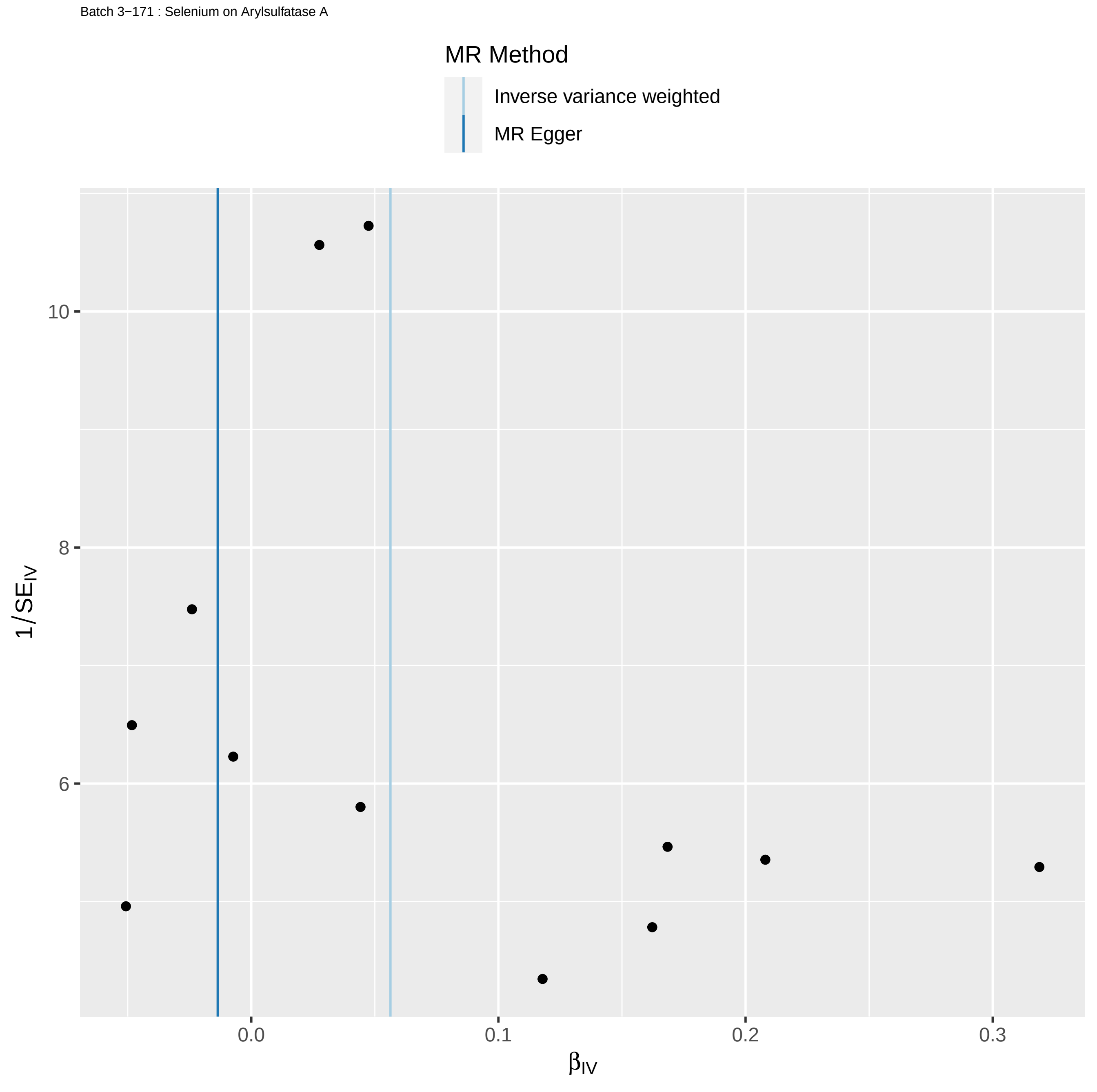

Batch 3−171 : Selenium on Arylsulfatase A
MR Method
Inverse variance weighted
MR Egger
10
8
6
1/SE$_{IV}$
0.0
0.1
0.2
0.3
β$_{IV}$

Batch 3−172 : Selenium on Arylsulfatase B

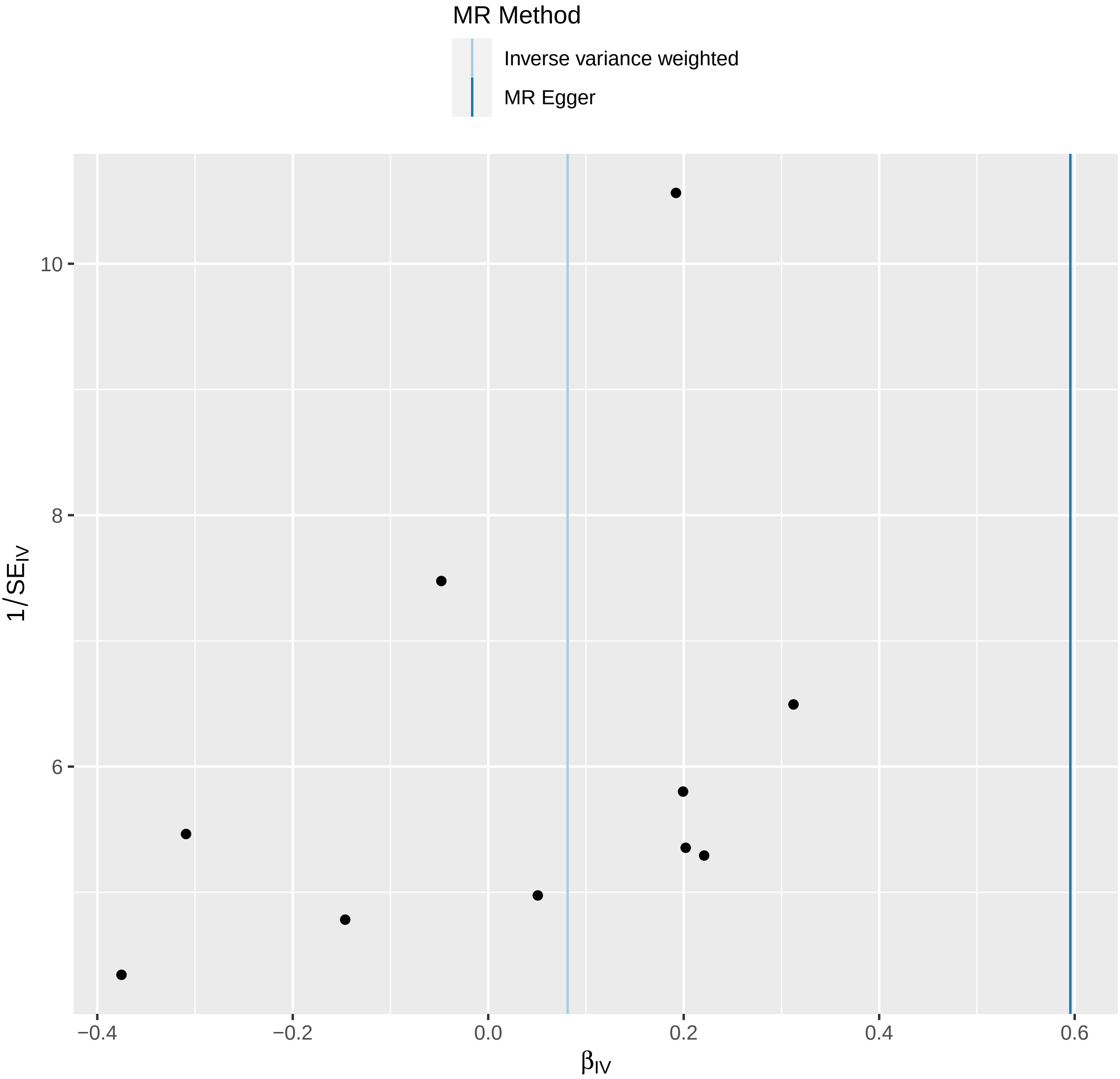

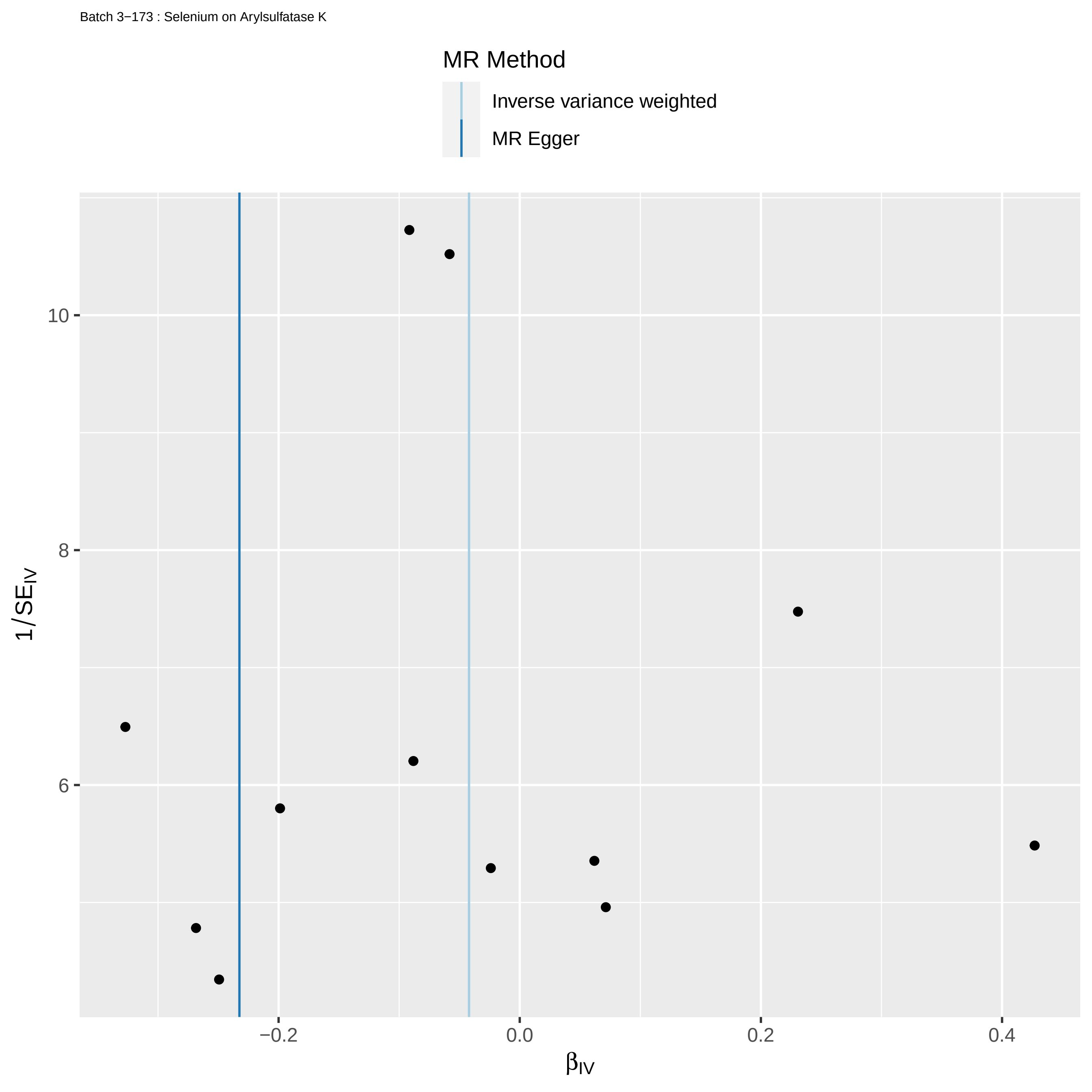

Batch 3−173 : Selenium on Arylsulfatase K
MR Method
Inverse variance weighted
MR Egger
10
8
6
1/SE_IV
−0.2
0.0
0.2
0.4
β_IV

Batch 3−174 : Selenium on Ecto−ADP−ribosyltransferase 3

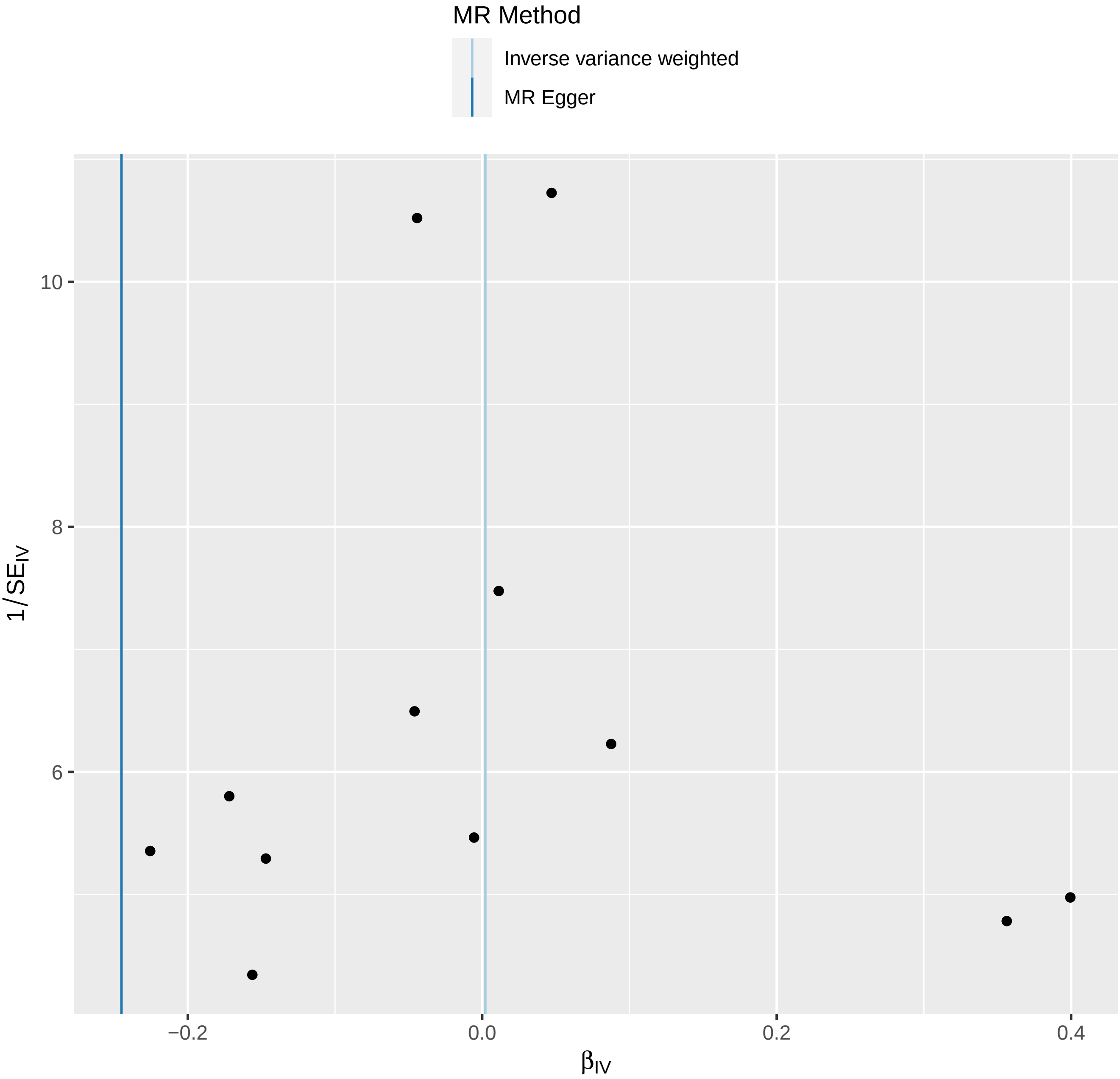

Batch 3−175 : Selenium on Ecto−ADP−ribosyltransferase 3

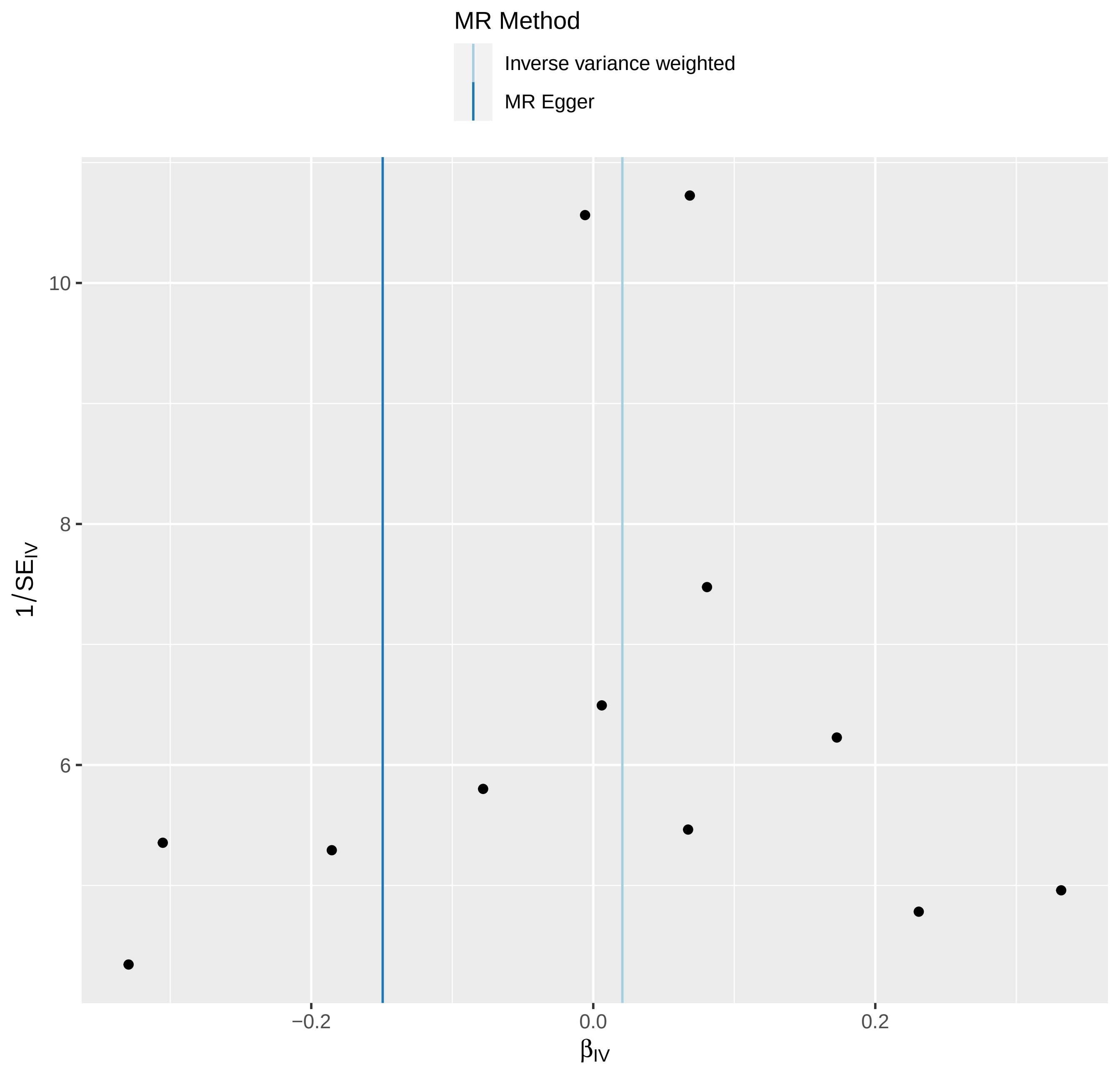

Batch 3−176 : Selenium on Ecto−ADP−ribosyltransferase 4

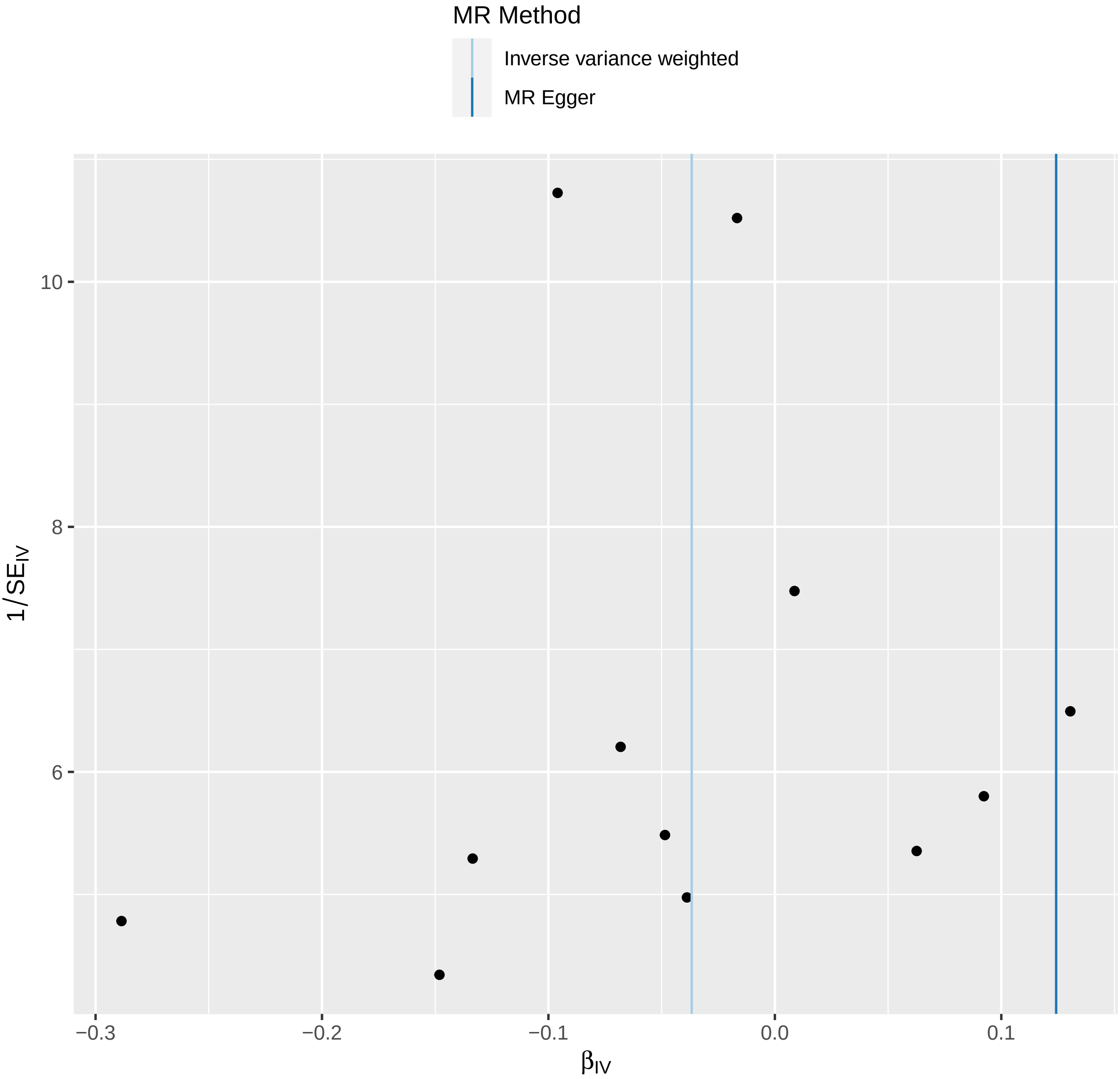

Batch 3−177 : Selenium on Artemin

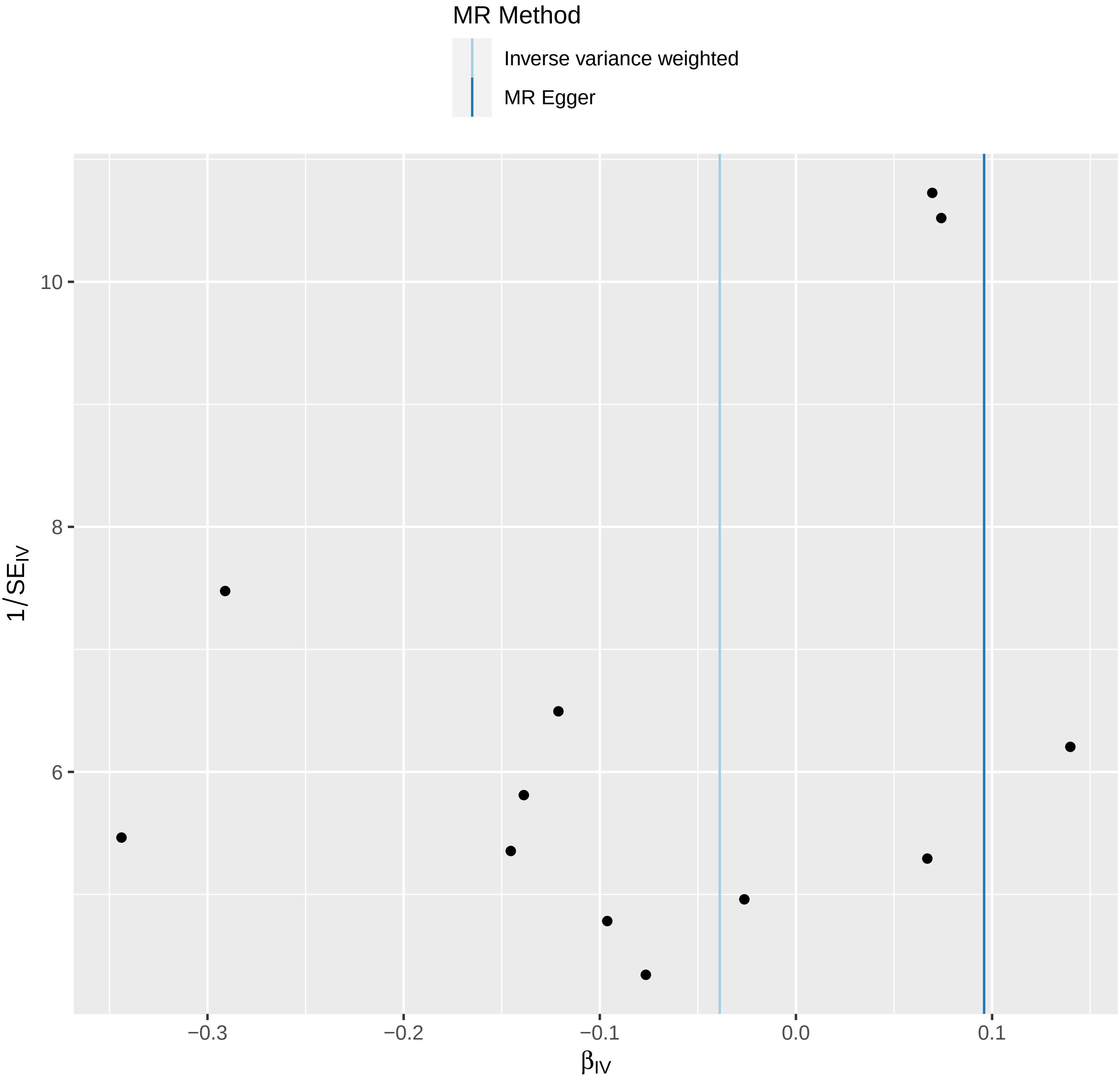

Batch 3−178 : Selenium on Acid ceramidase

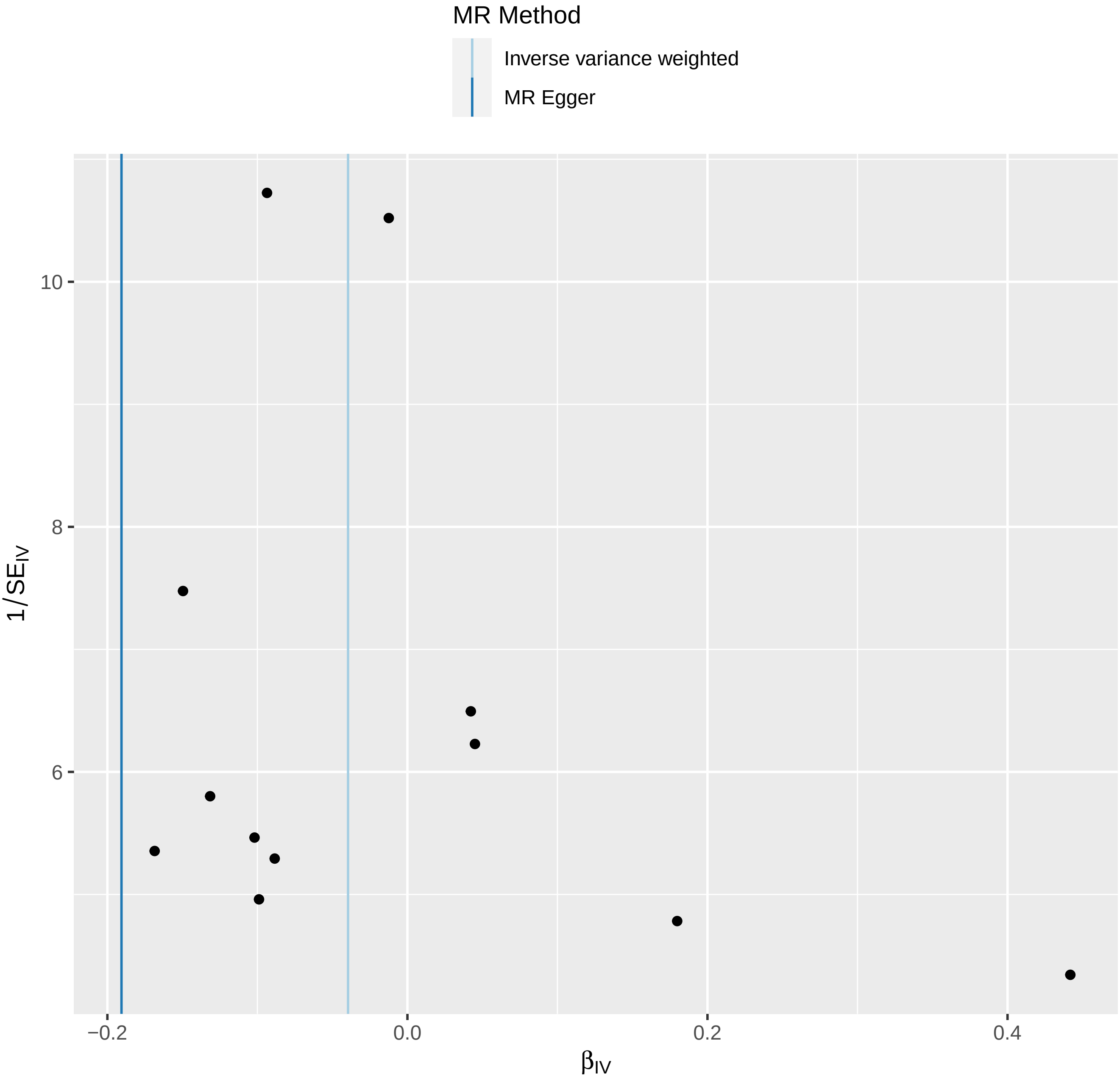

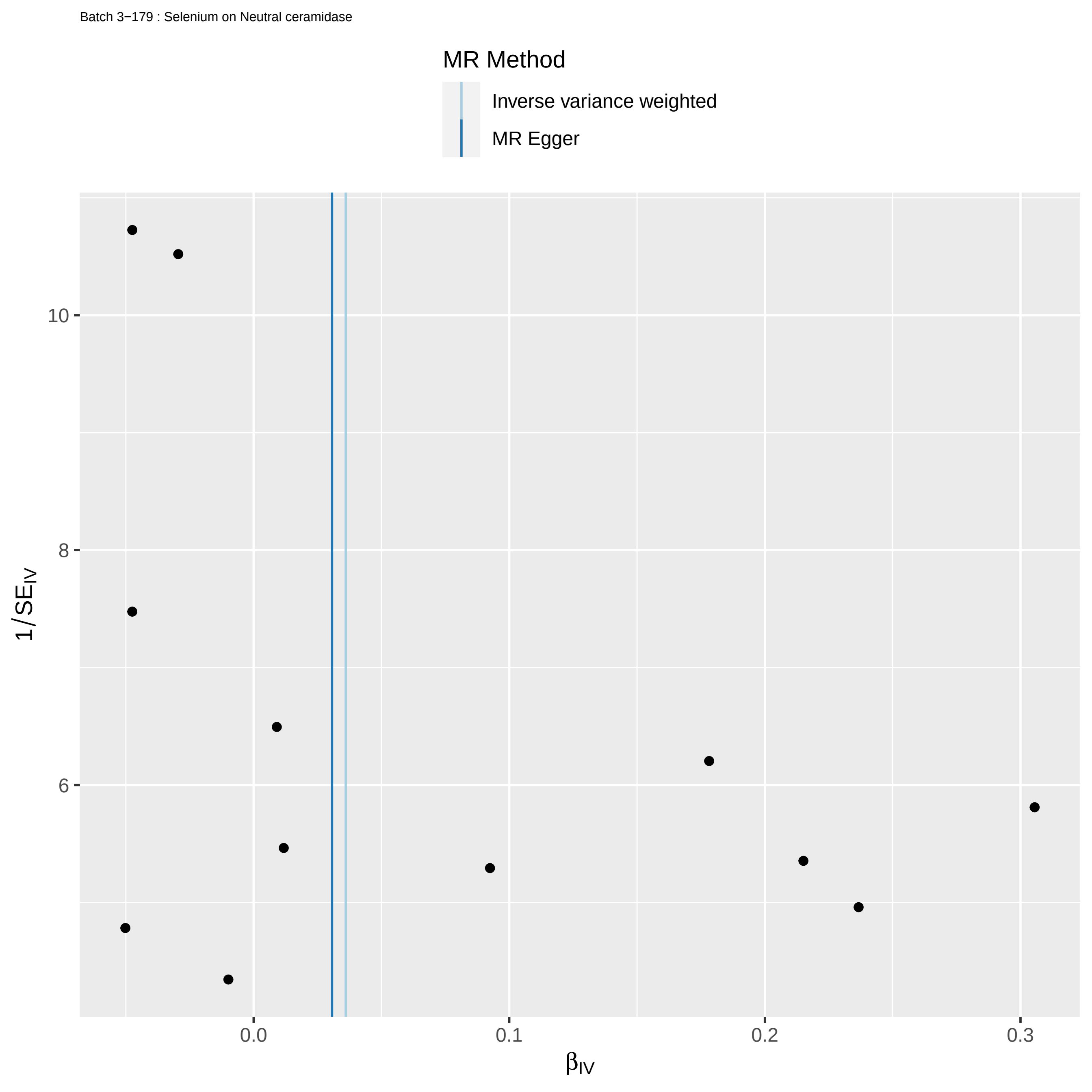

Batch 3−179 : Selenium on Neutral ceramidase
MR Method
Inverse variance weighted
MR Egger
10
8
6
$1/SE_{IV}$
0.0
0.1
0.2
0.3
β_{IV}

Batch 3−180 : Selenium on Arf−GAP with SH3 domain, ANK repeat and PH domain−containing protein 2

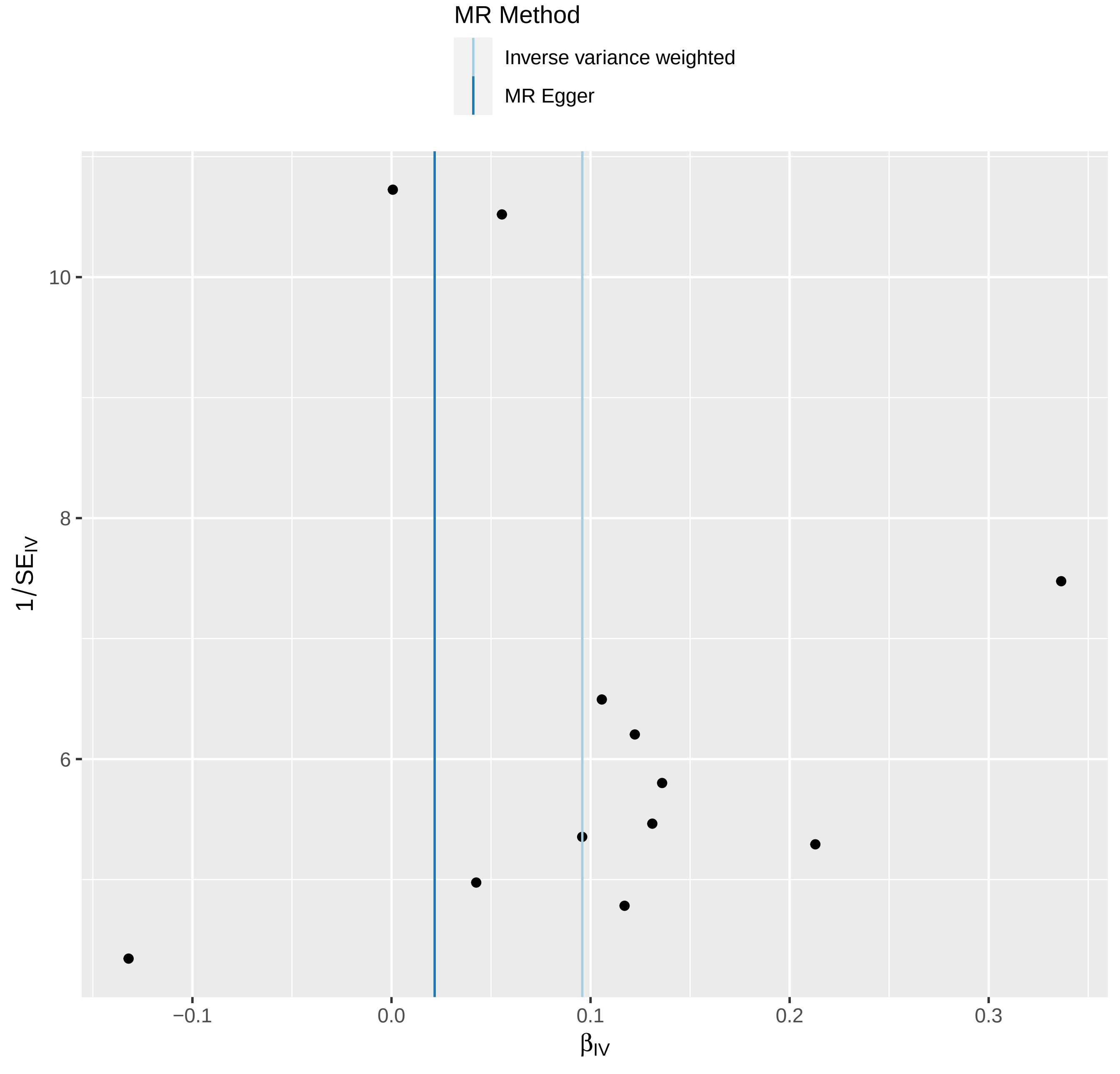

Batch 3−181 : Selenium on Activating signal cointegrator 1 complex subunit 1

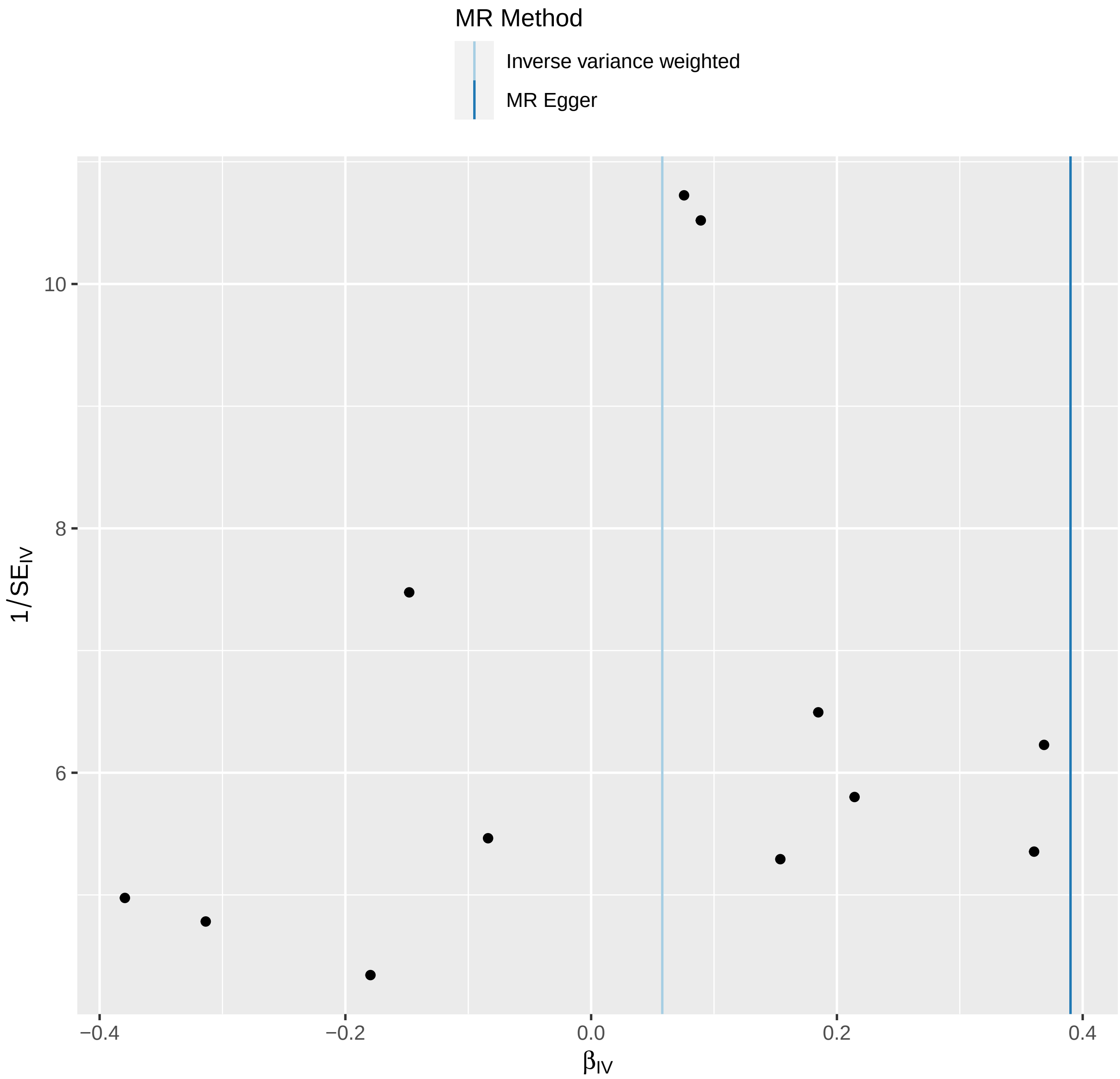

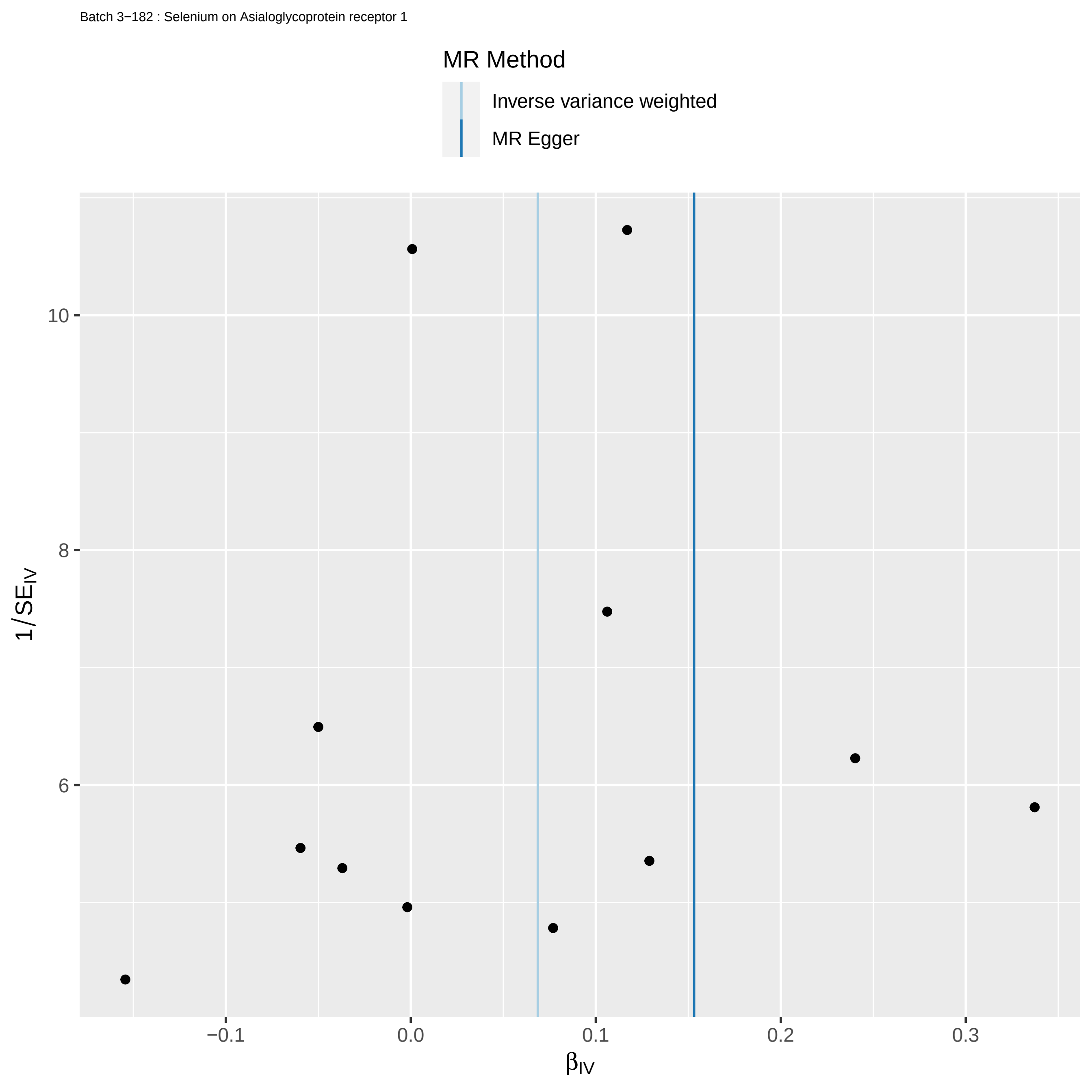

Batch 3−182 : Selenium on Asialoglycoprotein receptor 1
MR Method
Inverse variance weighted
MR Egger
10
8
6
1/SE_IV
−0.1
0.0
0.1
0.2
0.3
β_IV

Batch 3−183 : Selenium on Asialoglycoprotein receptor 2

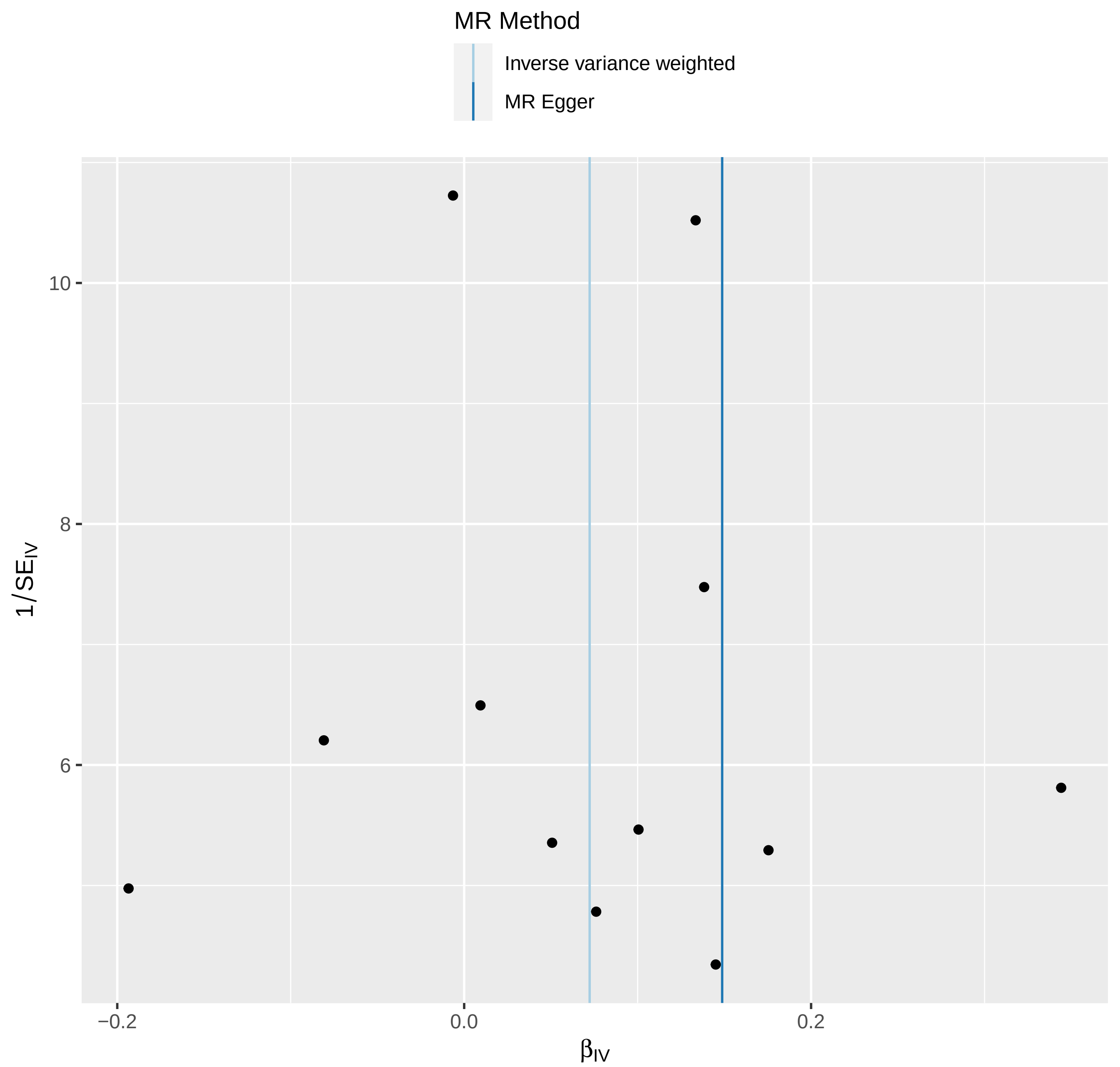

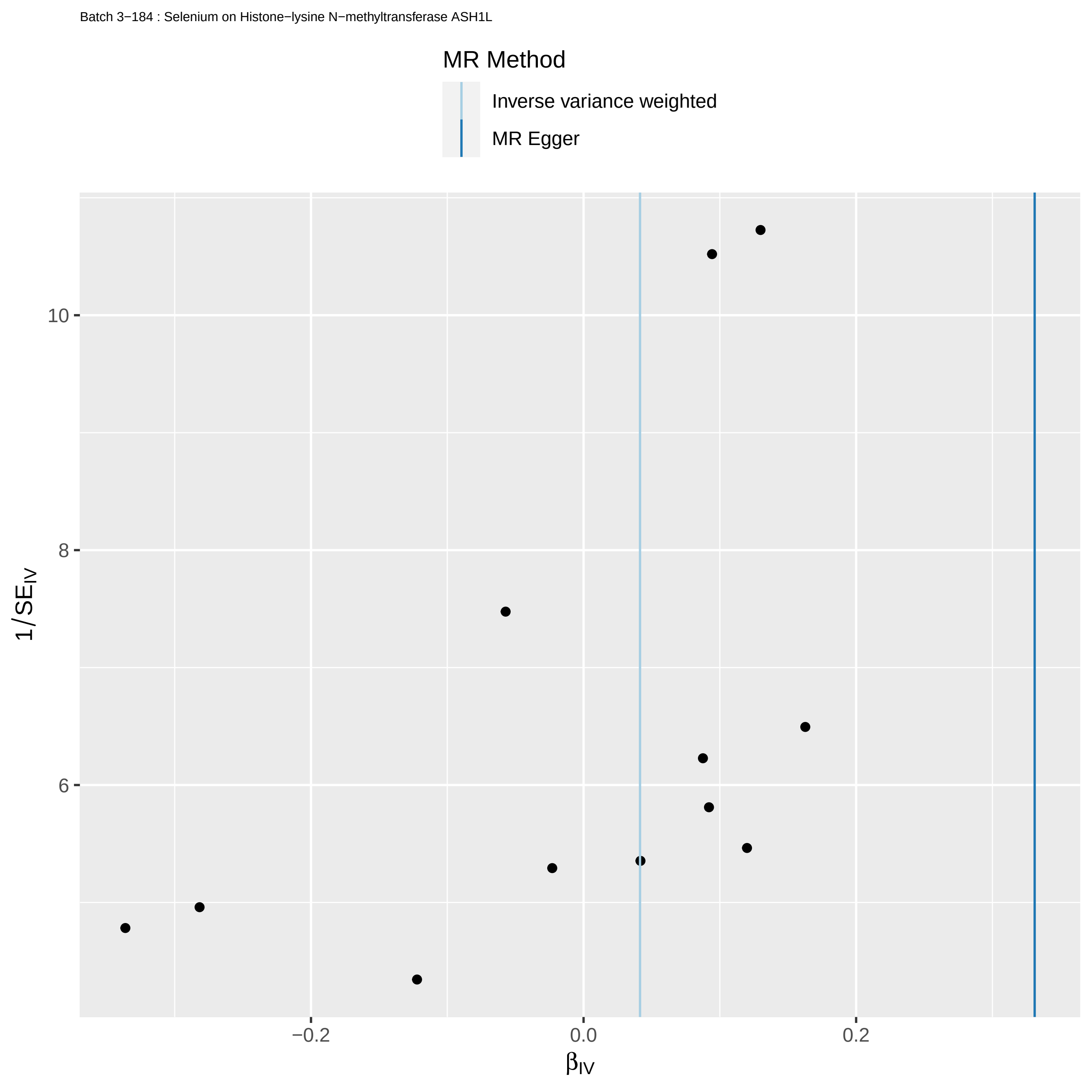
Batch 3−184 : Selenium on Histone−lysine N−methyltransferase ASH1L
MR Method
Inverse variance weighted
MR Egger
10
8
6
1/SE_IV
−0.2
0.0
0.2
β_IV

Batch 3−185 : Selenium on Set1/Ash2 histone methyltransferase complex subunit ASH2

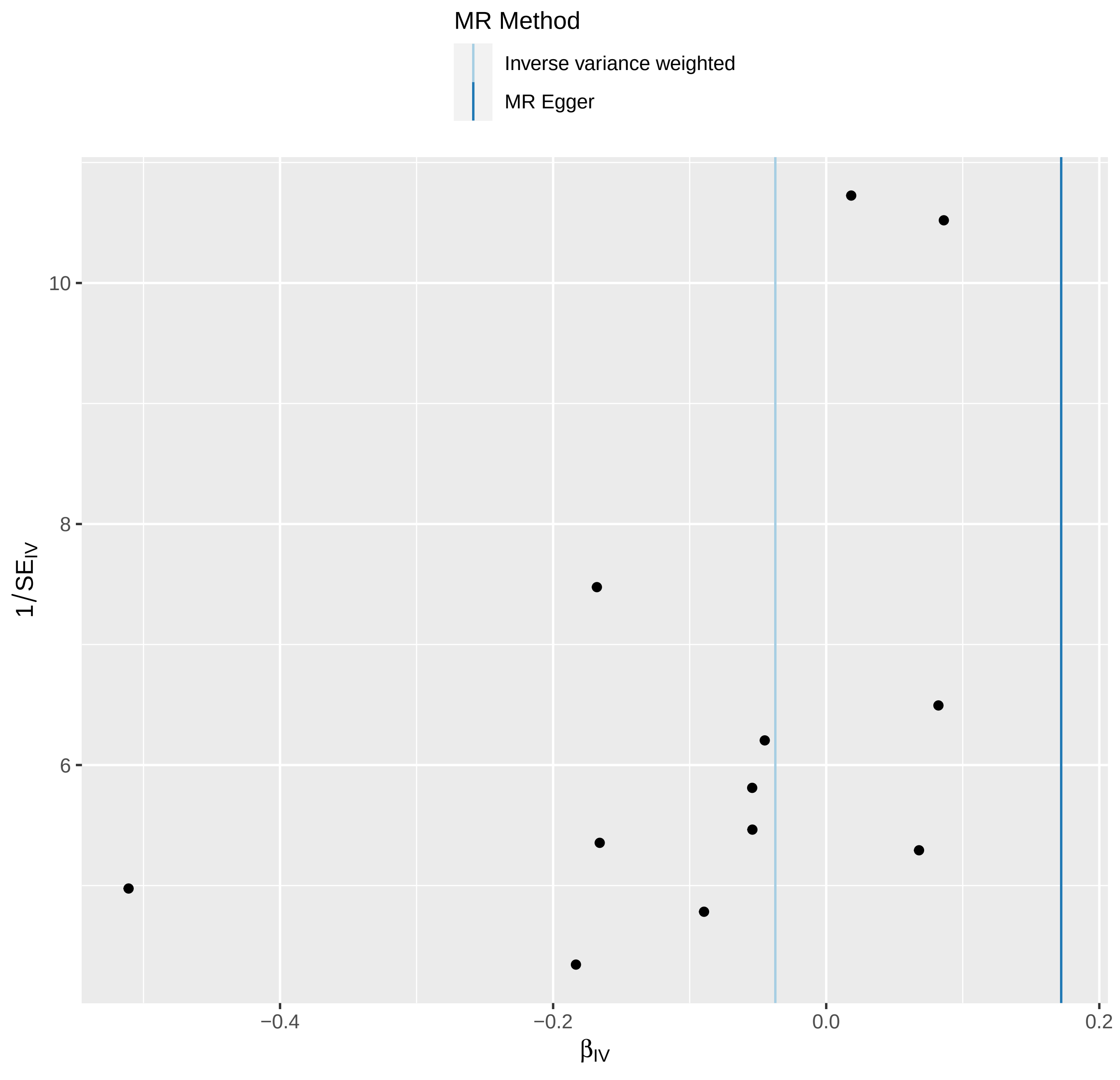

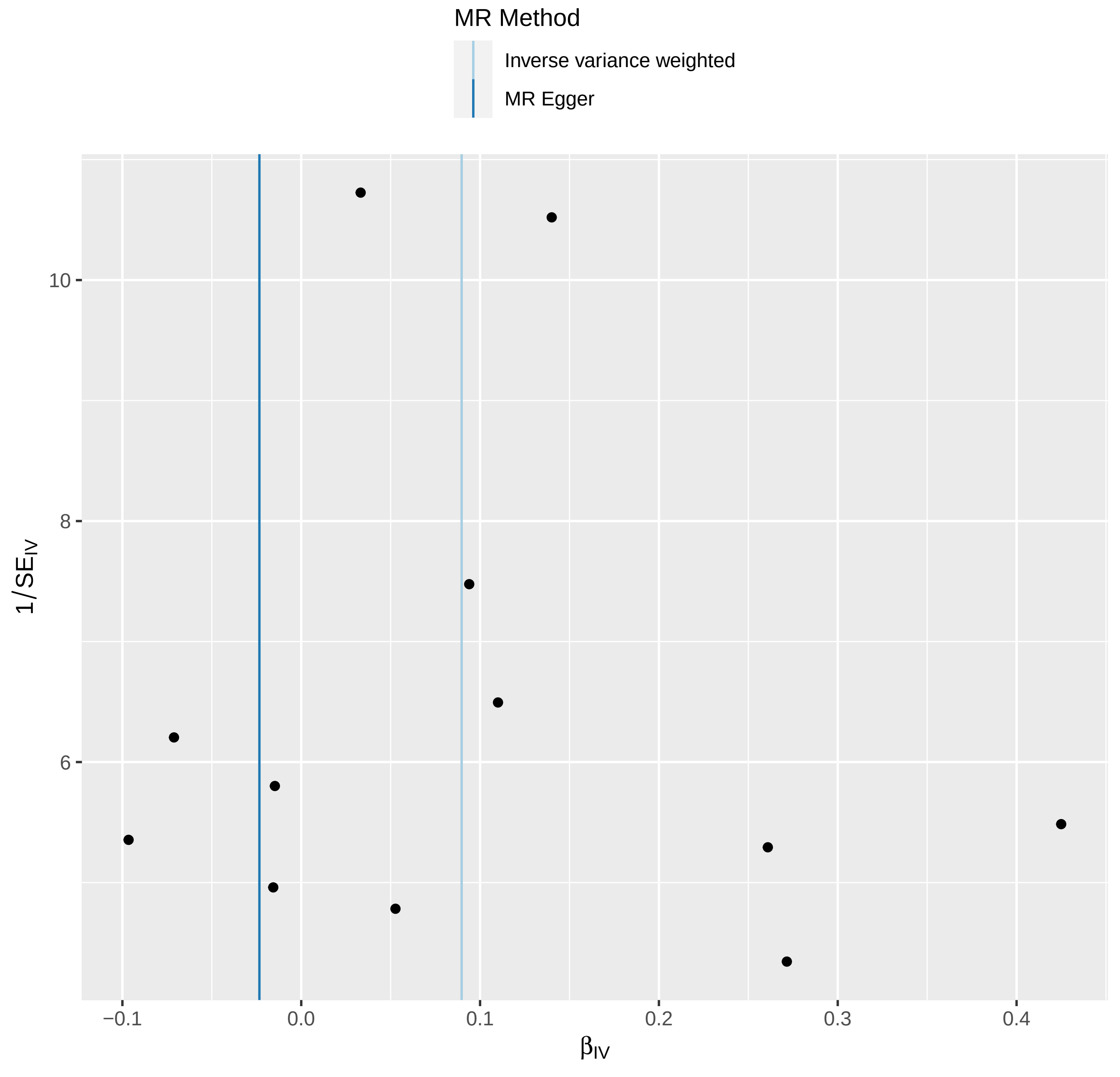
MR Method
Inverse variance weighted
MR Egger
10
8
6
1/SE$_{IV}$
−0.1
0.0
0.1
0.2
0.3
0.4
β$_{IV}$

Batch 3−187 : Selenium on Agouti−signaling protein

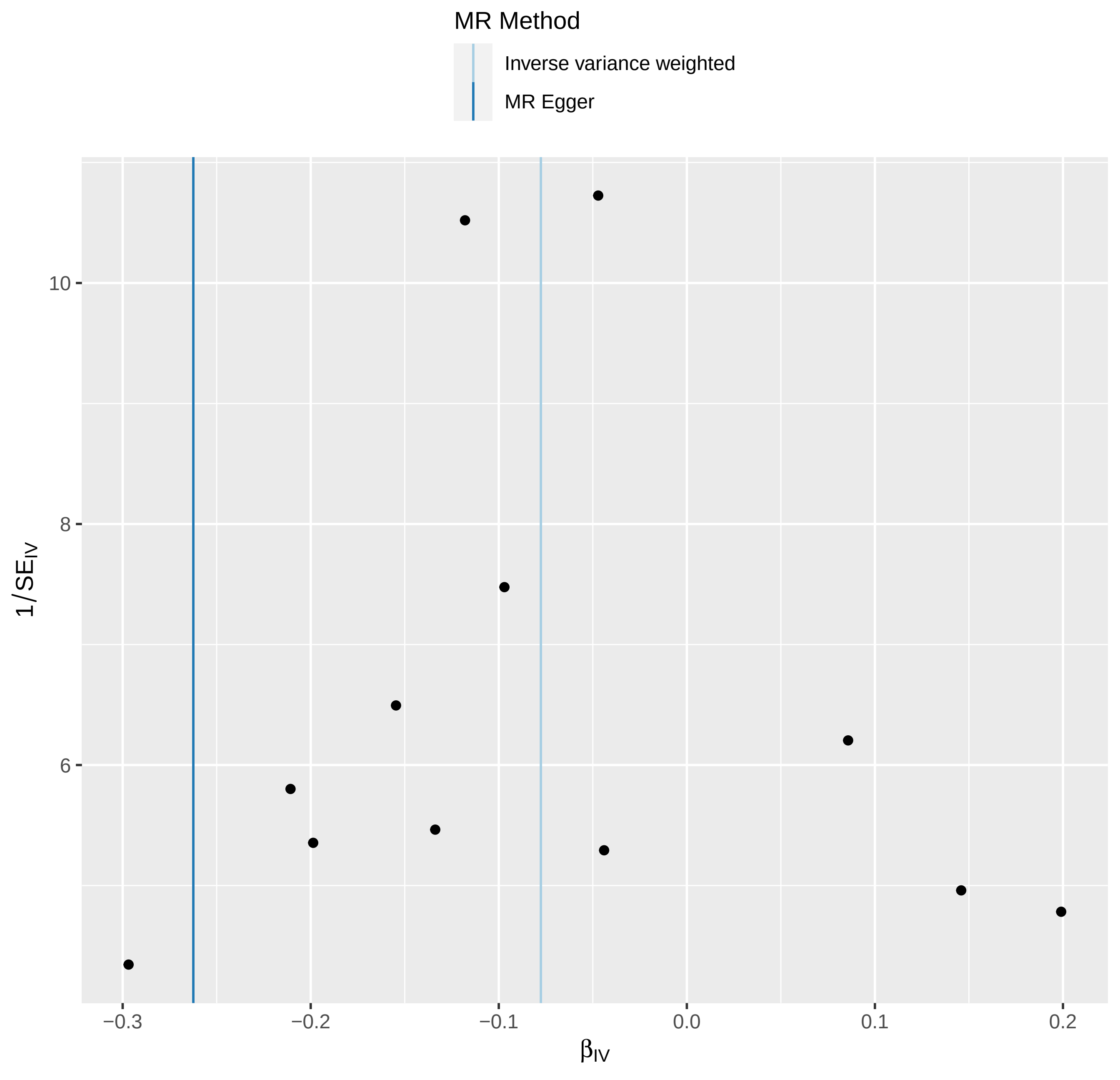

Batch 3−188 : Selenium on Argininosuccinate lyase

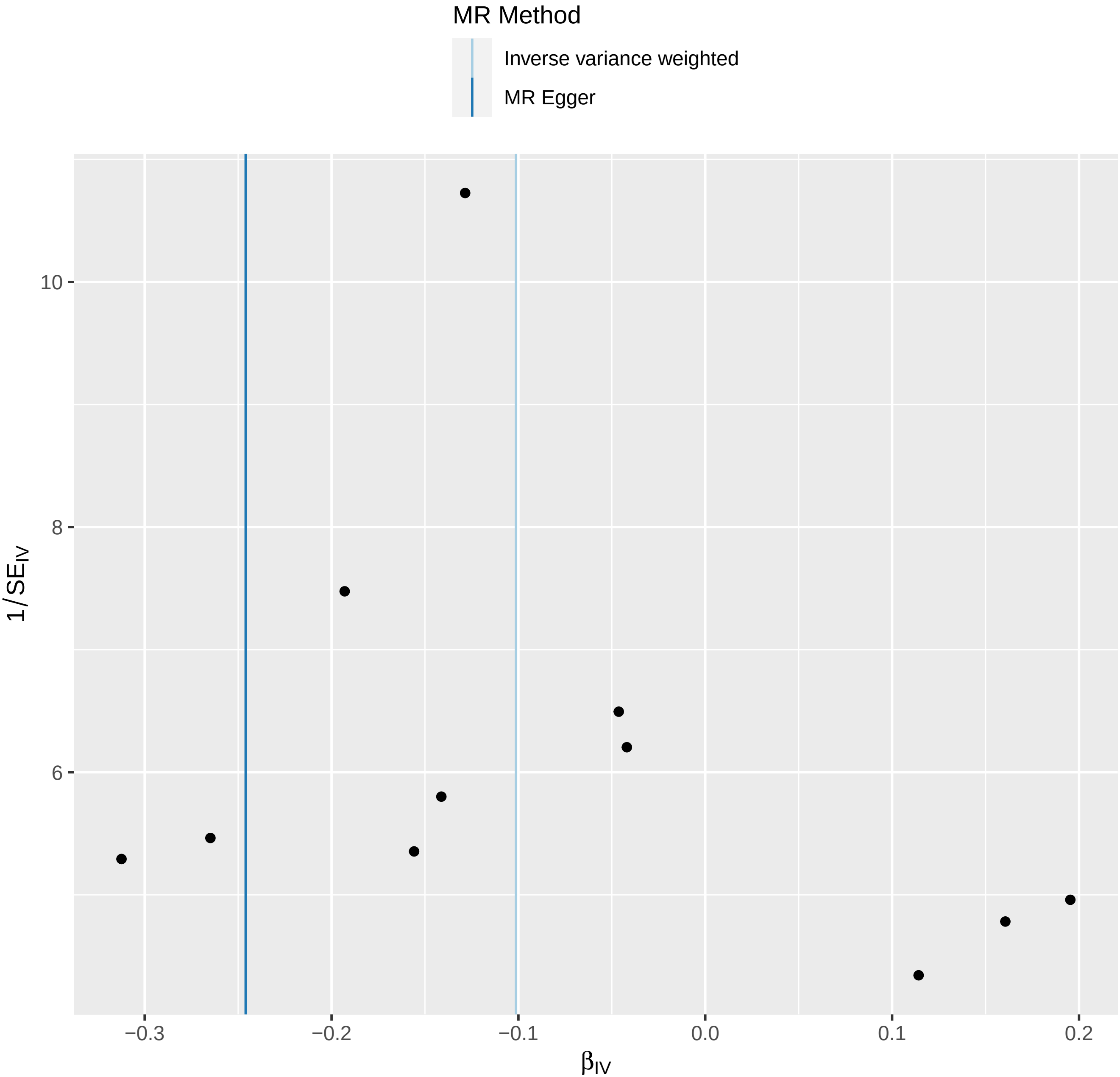

Batch 3−189 : Selenium on N−acetylserotonin O−methyltransferase−like protein

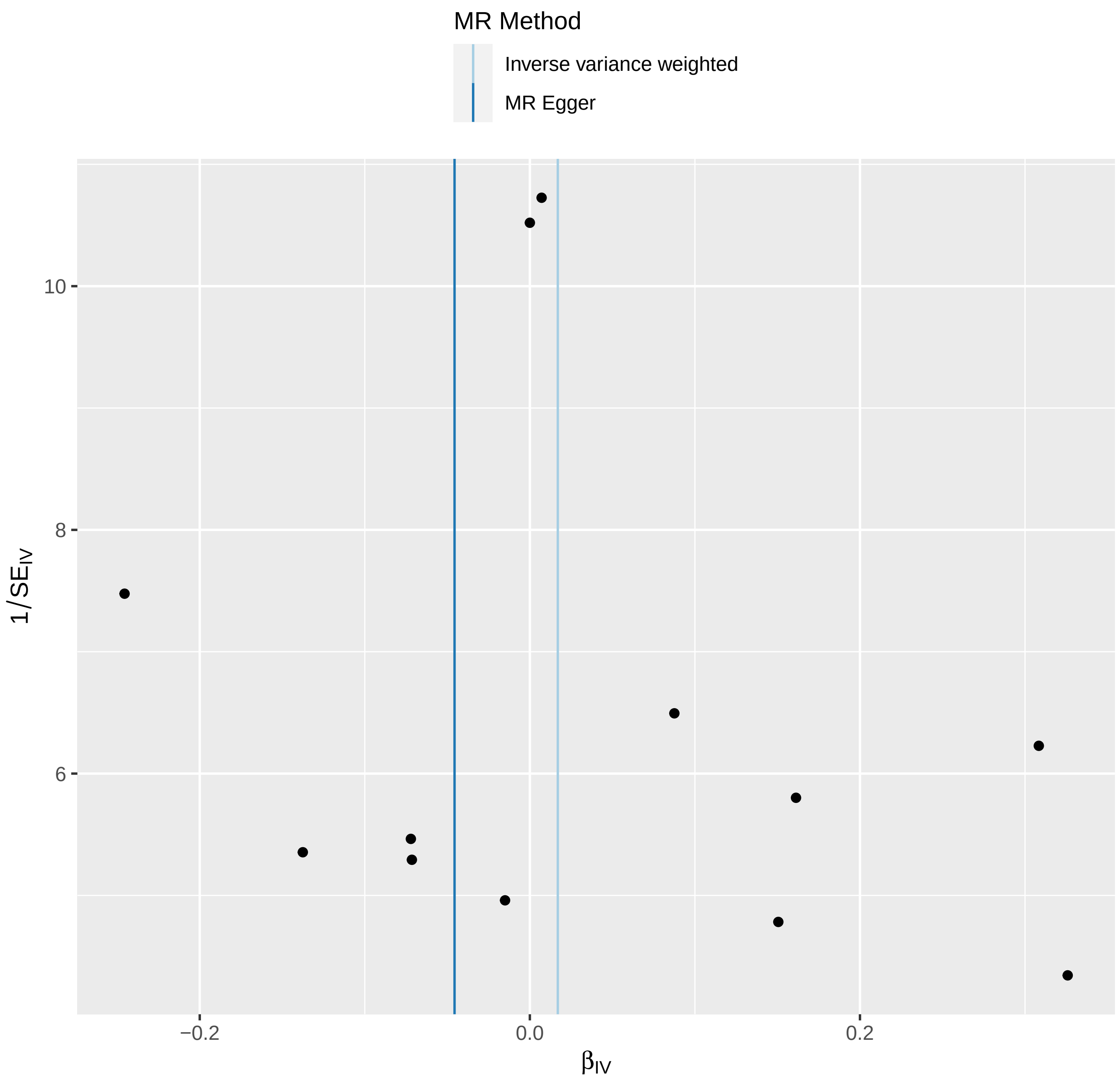

Batch 3−190 : Selenium on ATPase ASNA1

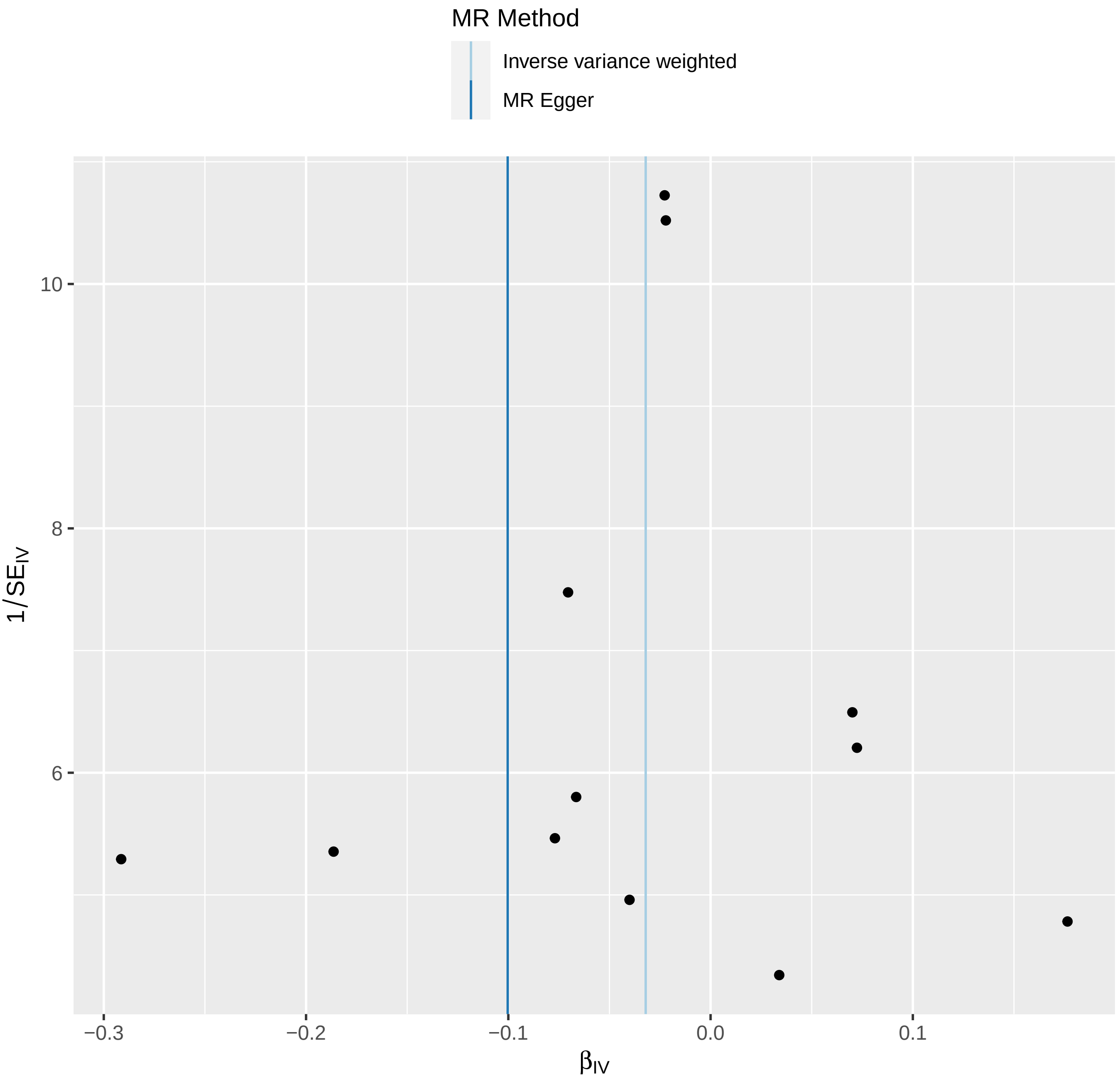

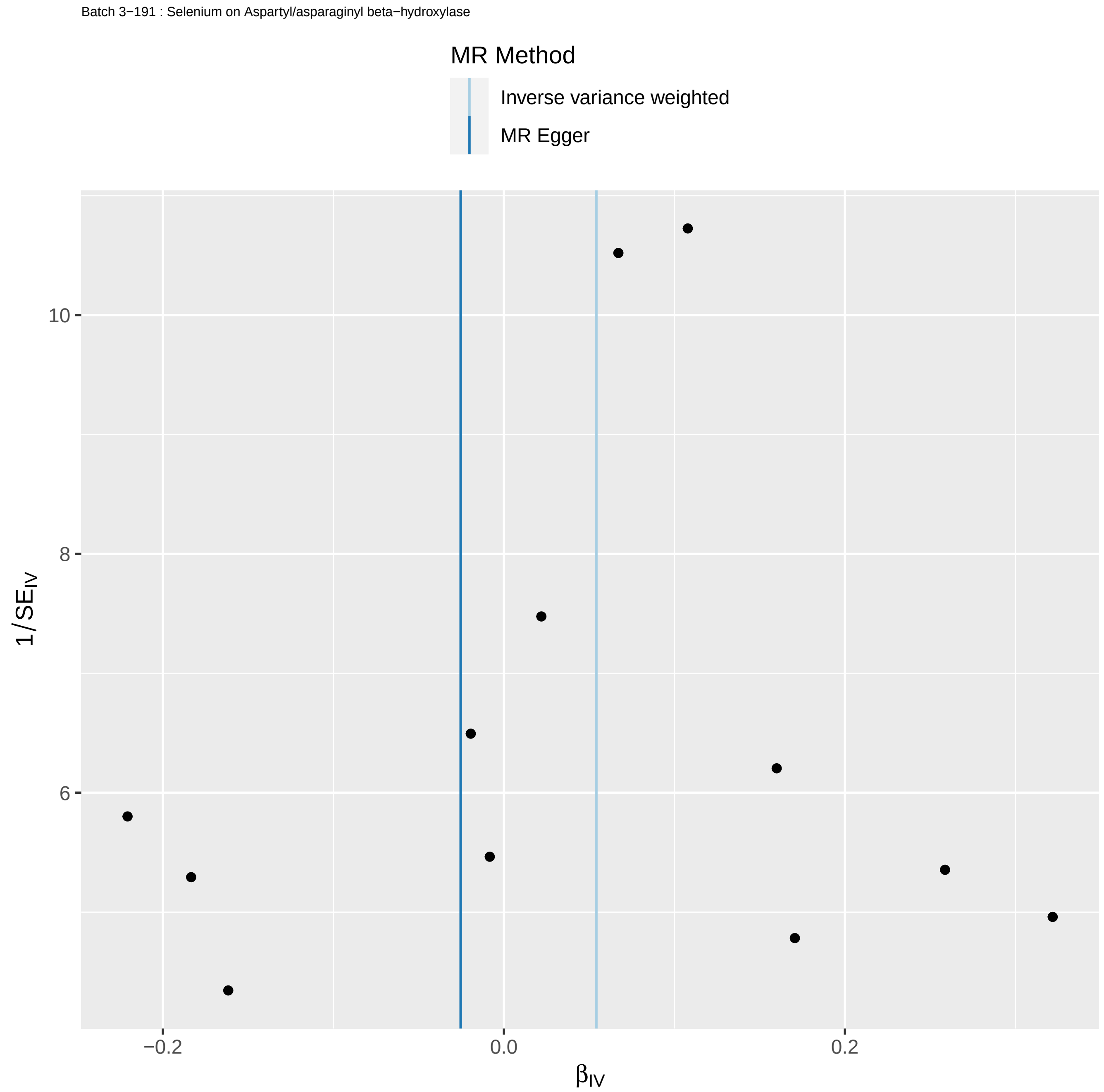
Batch 3−191 : Selenium on Aspartyl/asparaginyl beta−hydroxylase
MR Method
Inverse variance weighted
MR Egger
10
8
6
1/SE_IV
−0.2
0.0
0.2
β_IV

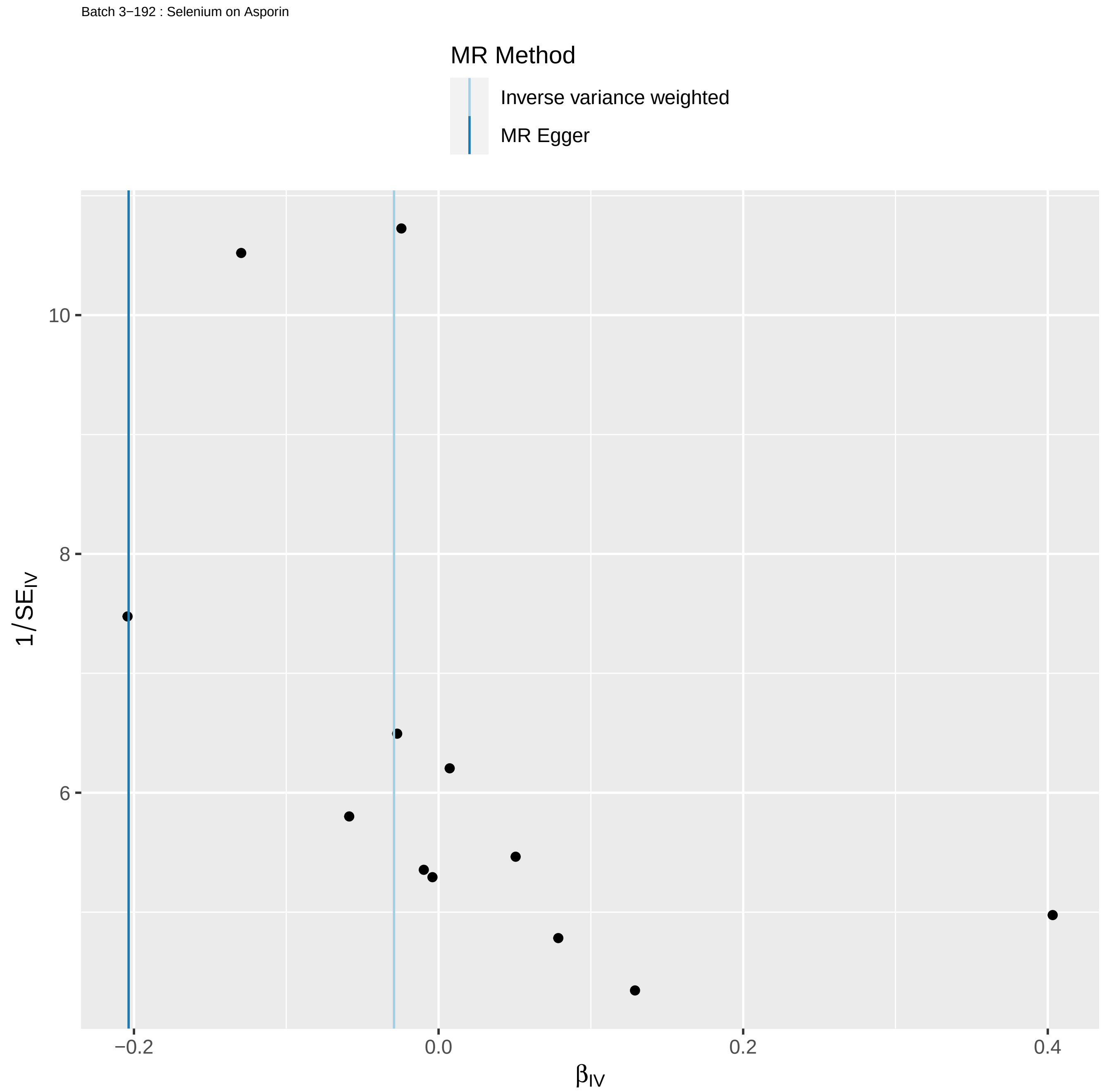
Batch 3−192 : Selenium on Asporin
MR Method
Inverse variance weighted
MR Egger
10
8
6
1/SE$_{IV}$
−0.2
0.0
0.2
0.4
β$_{IV}$

Batch 3−193 : Selenium on Retroviral−like aspartic protease 1

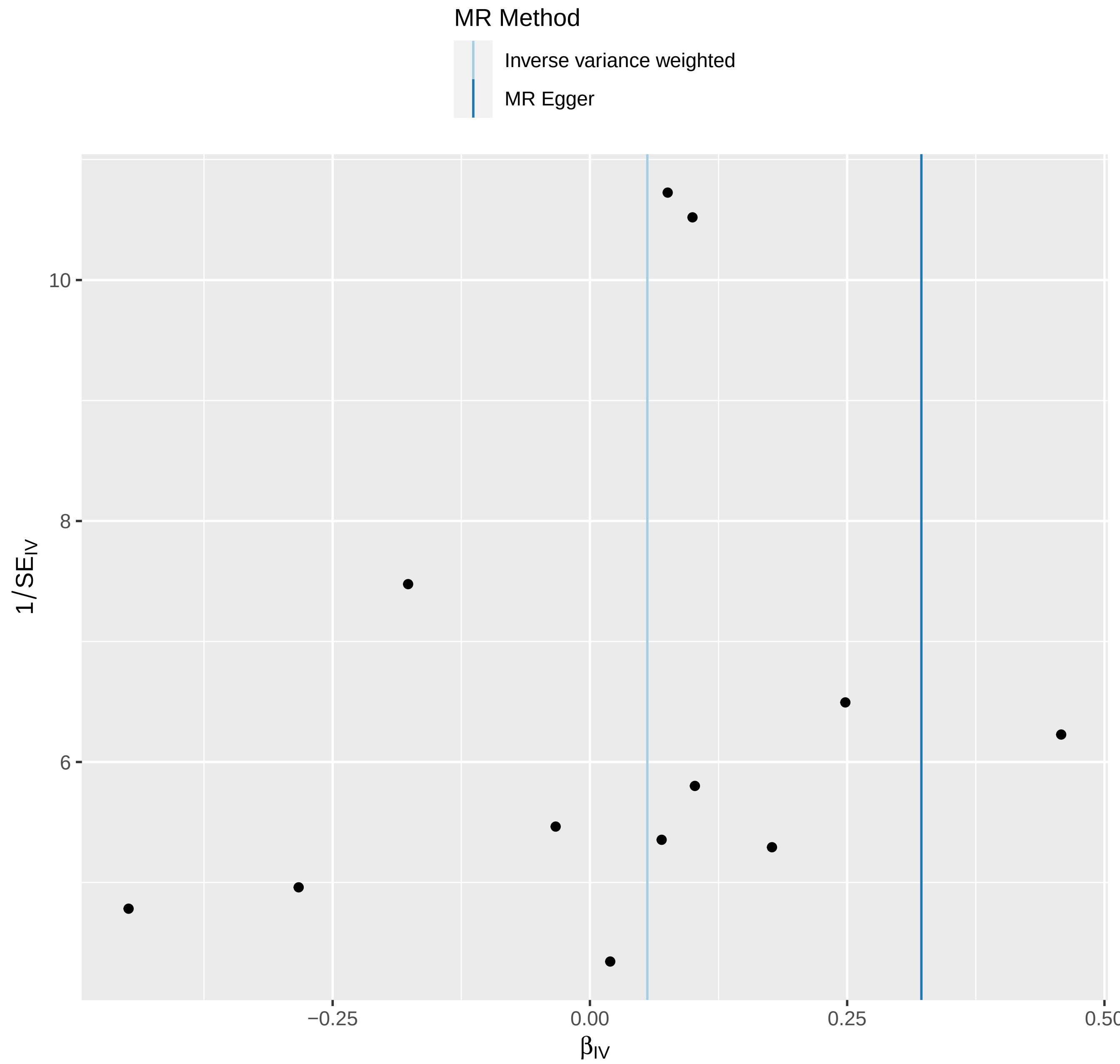

Batch 3−194 : Selenium on Astacin−like metalloendopeptidase

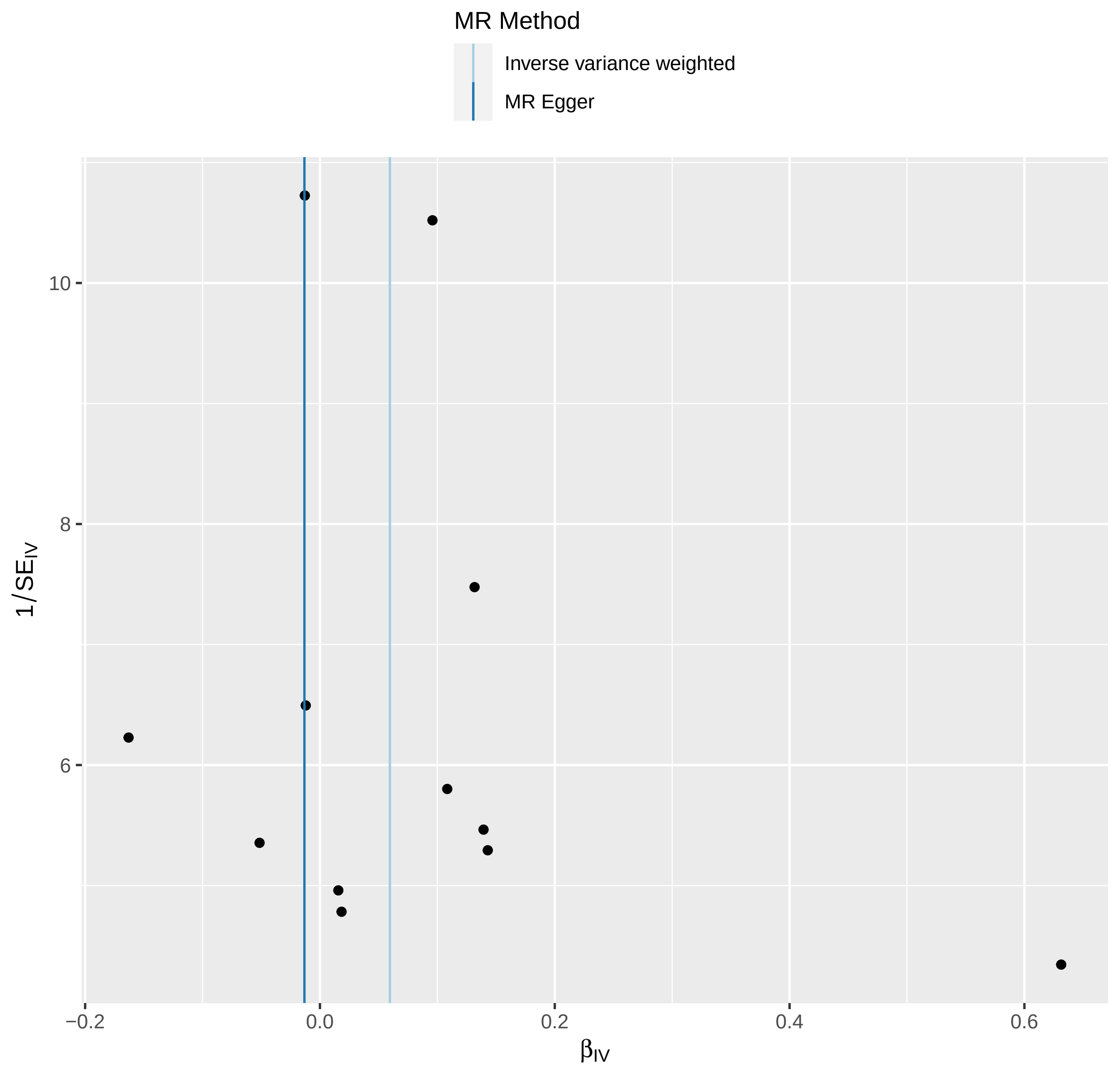

Batch 3−195 : Selenium on ATPase family AAA domain−containing protein 1

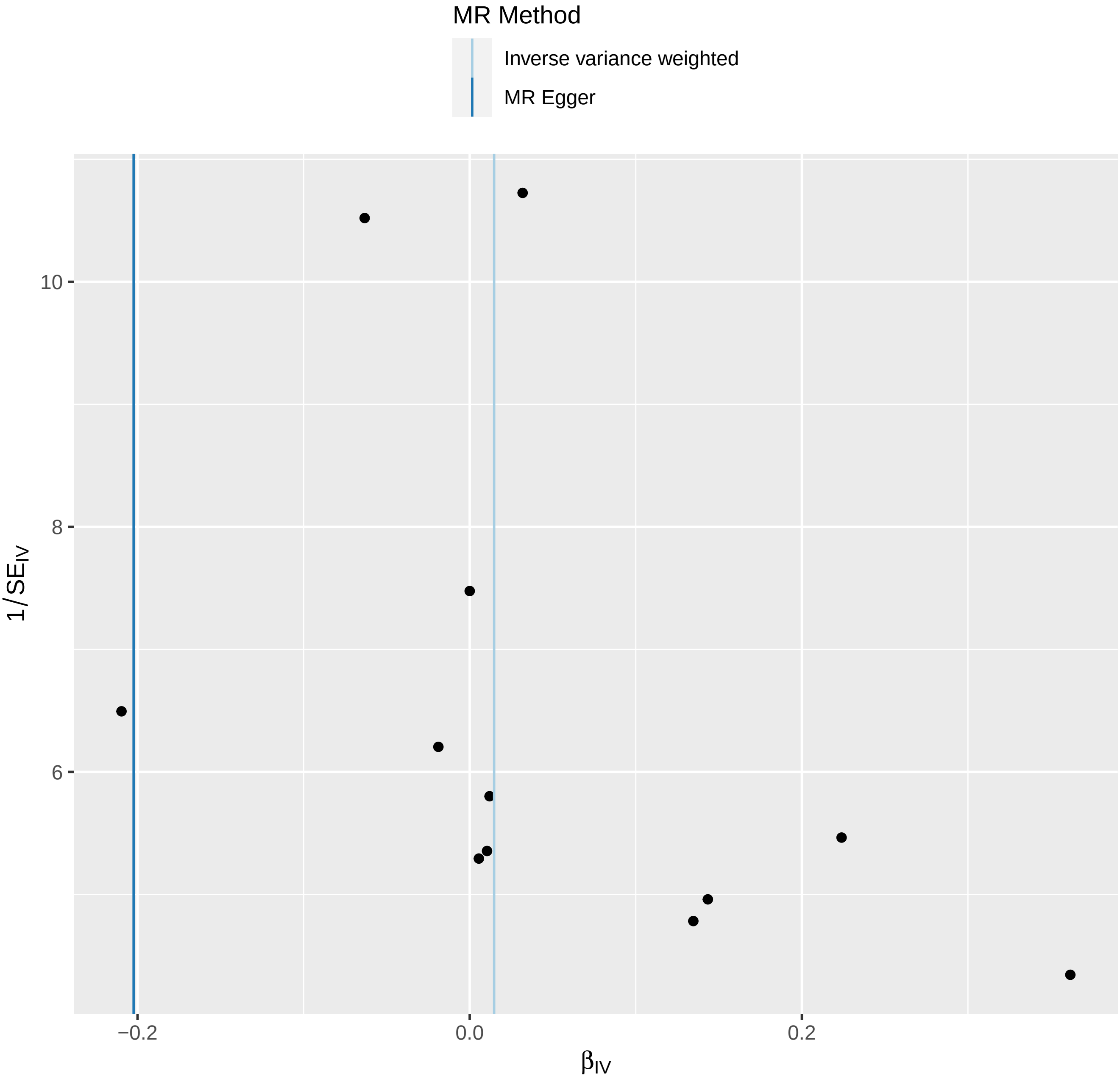

Batch 3−196 : Selenium on ATPase family AAA domain−containing protein 2

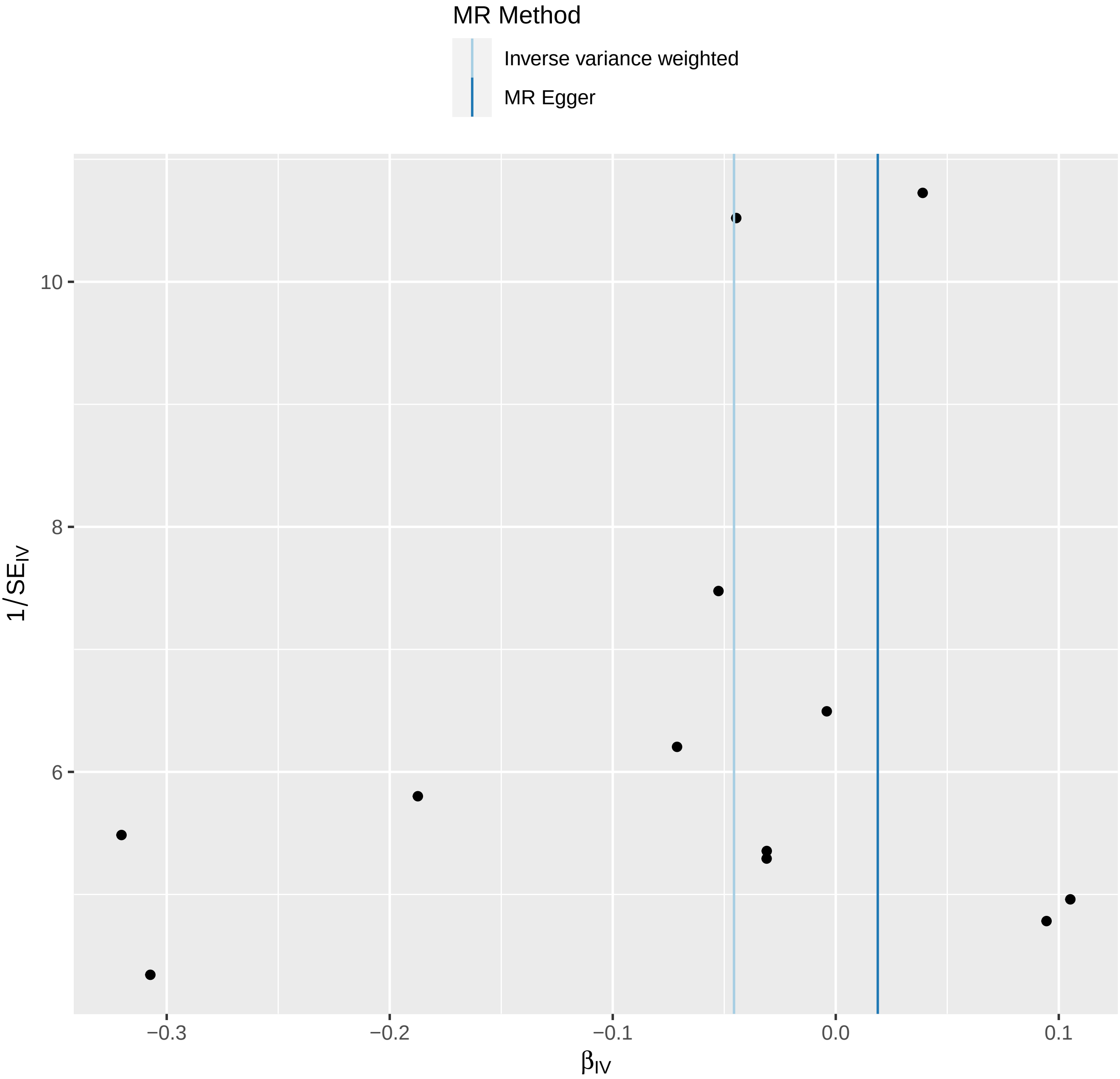

Batch 3−197 : Selenium on Cyclic AMP−dependent transcription factor ATF−6 alpha

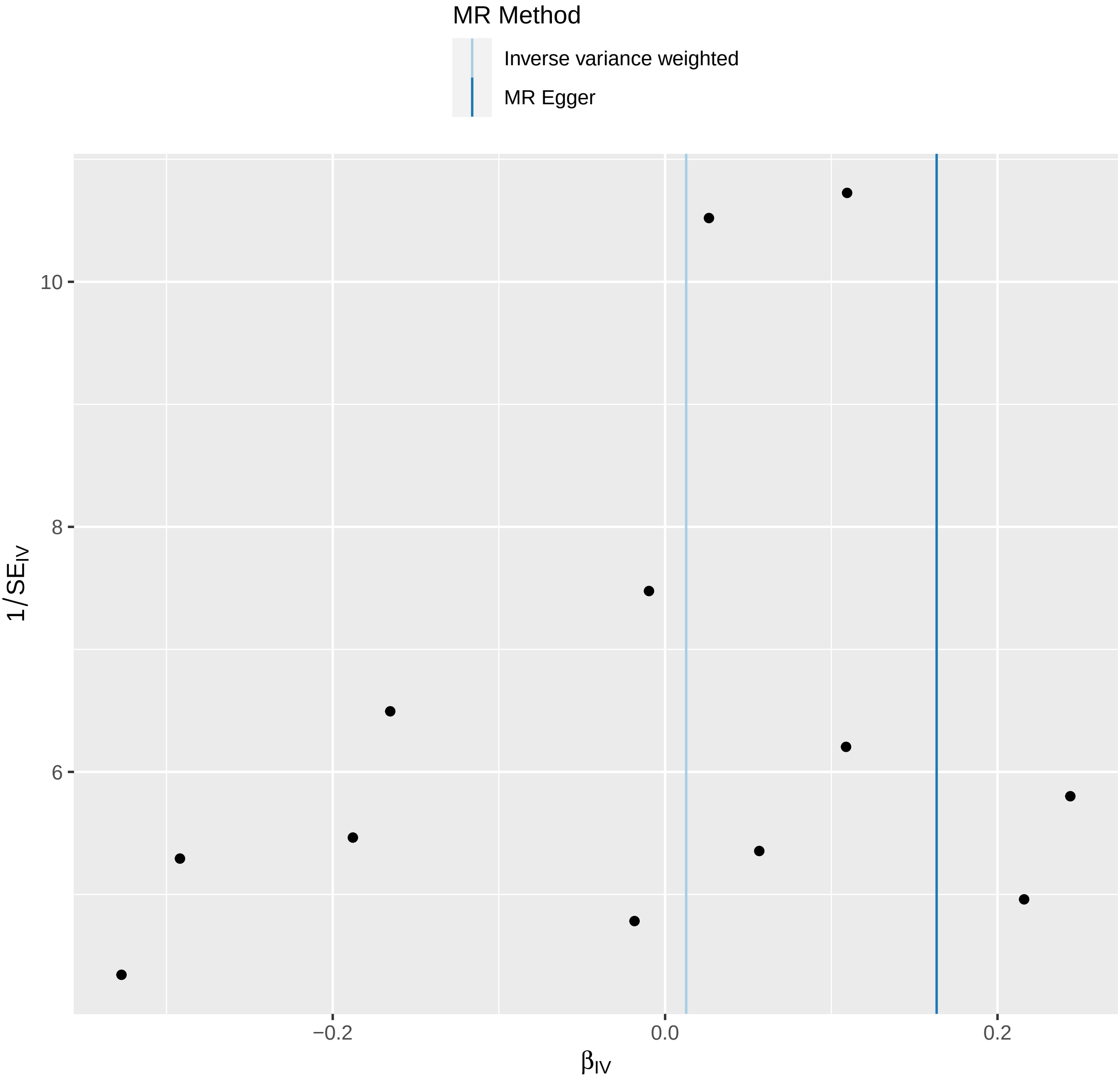

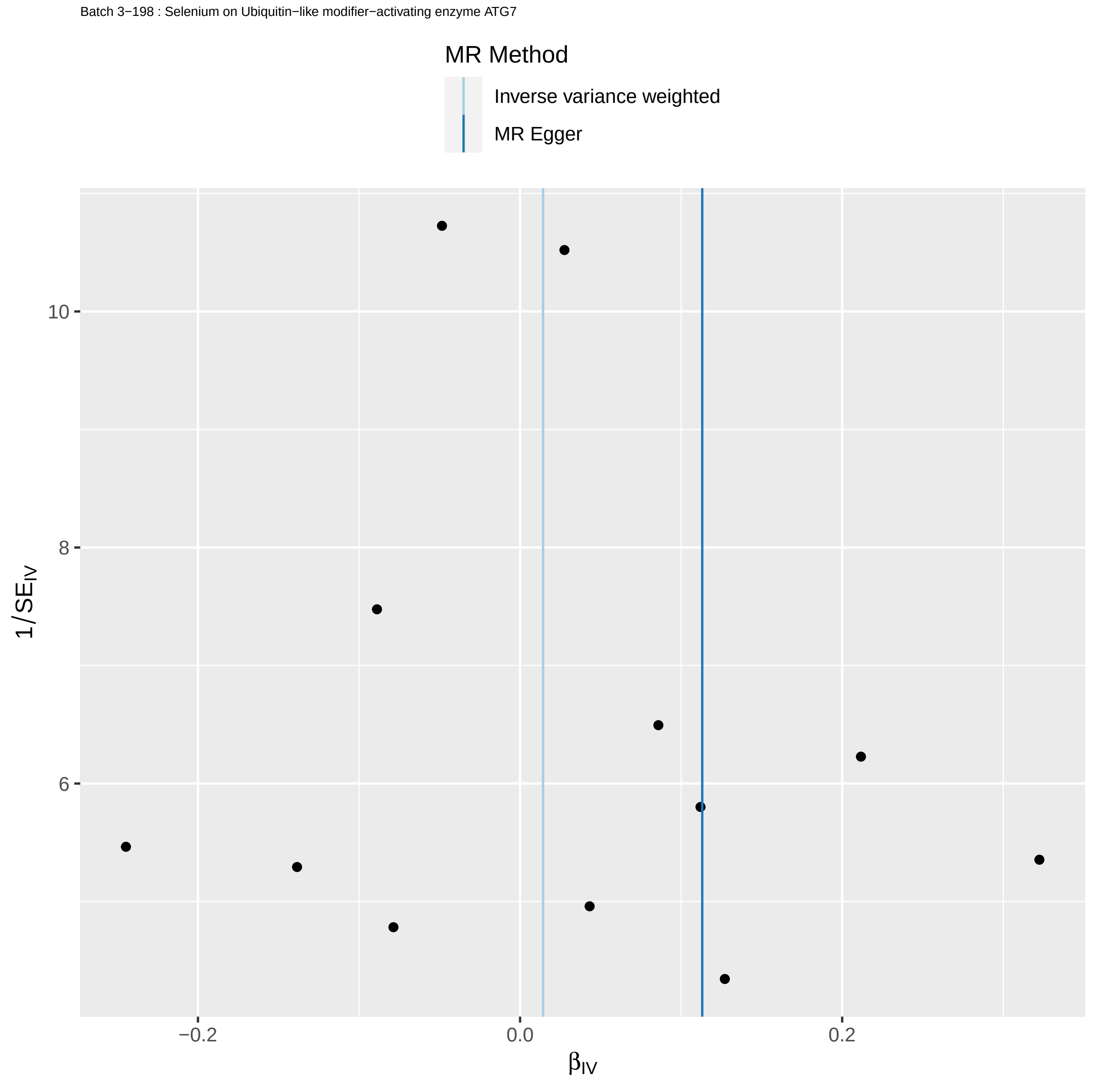

Batch 3−198 : Selenium on Ubiquitin−like modifier−activating enzyme ATG7
MR Method
Inverse variance weighted
MR Egger
10
8
6
1/SE_IV
−0.2
0.0
0.2
β_IV

Batch 3−199 : Selenium on Sodium/potassium−transporting ATPase subunit alpha−1

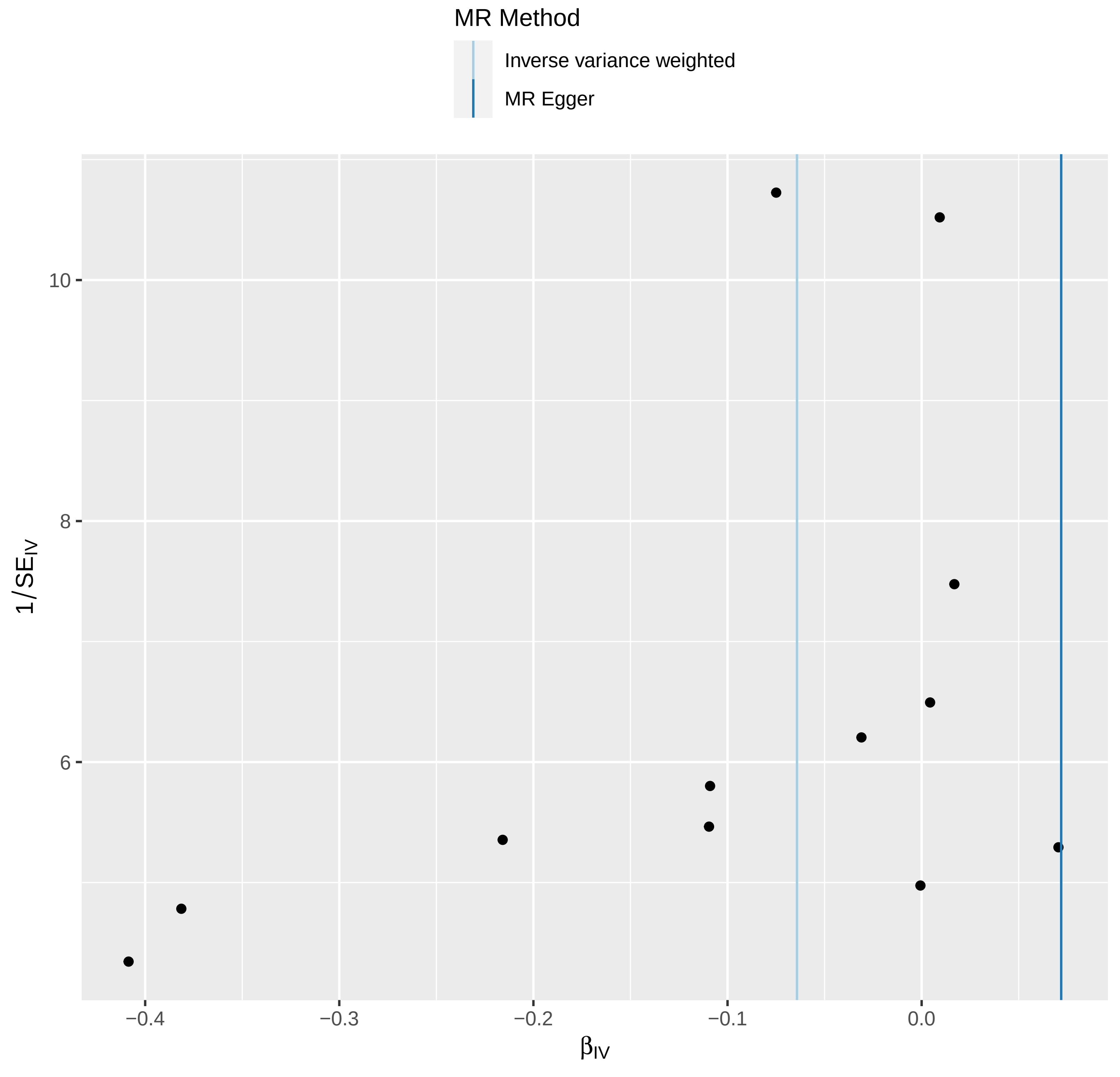

Batch 3−200 : Selenium on Sodium/potassium−transporting ATPase subunit beta−2

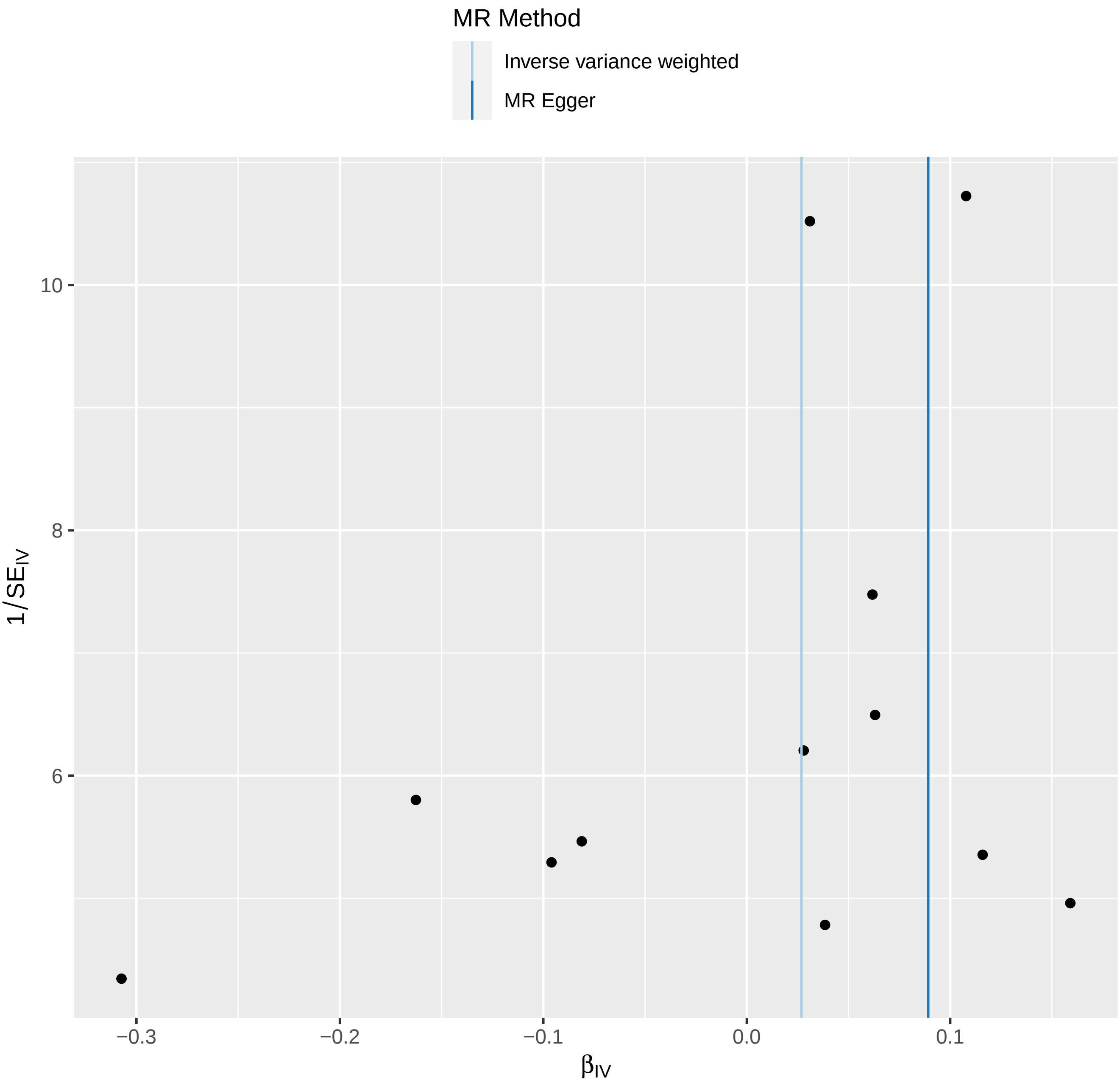

Batch 3−201 : Selenium on Sarcoplasmic/endoplasmic reticulum calcium ATPase 3

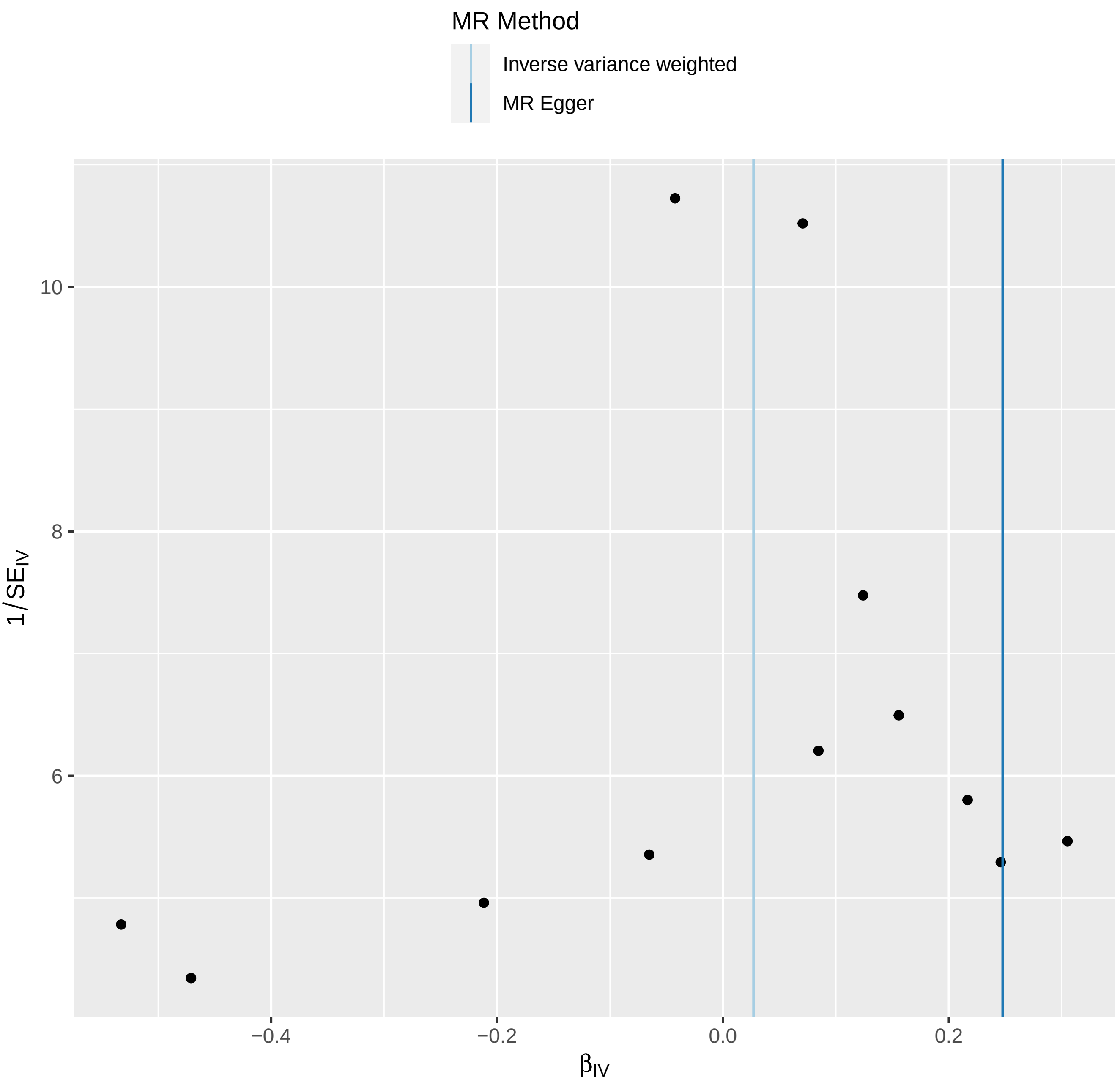

Batch 3−202 : Selenium on Potassium−transporting ATPase subunit beta

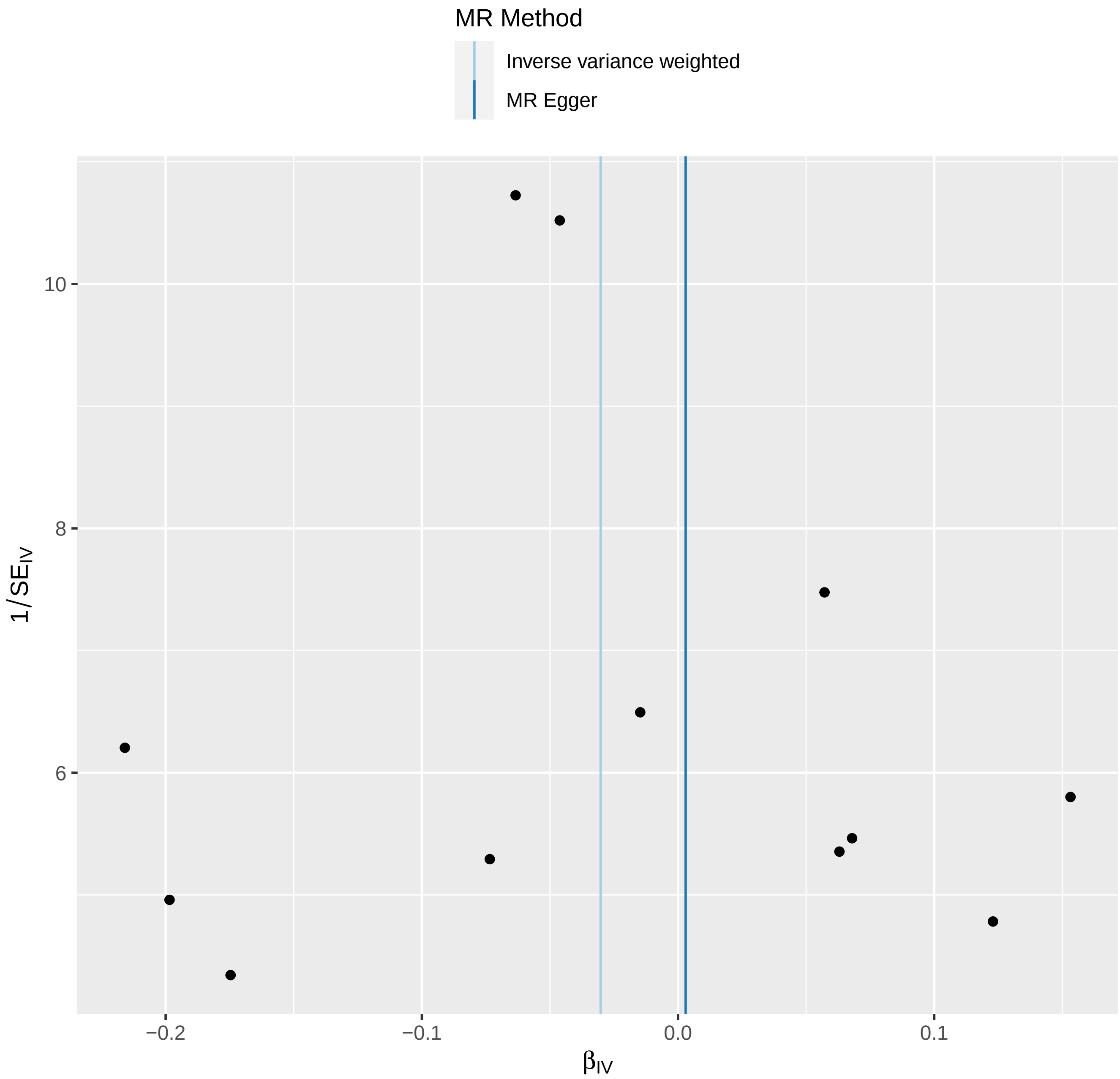

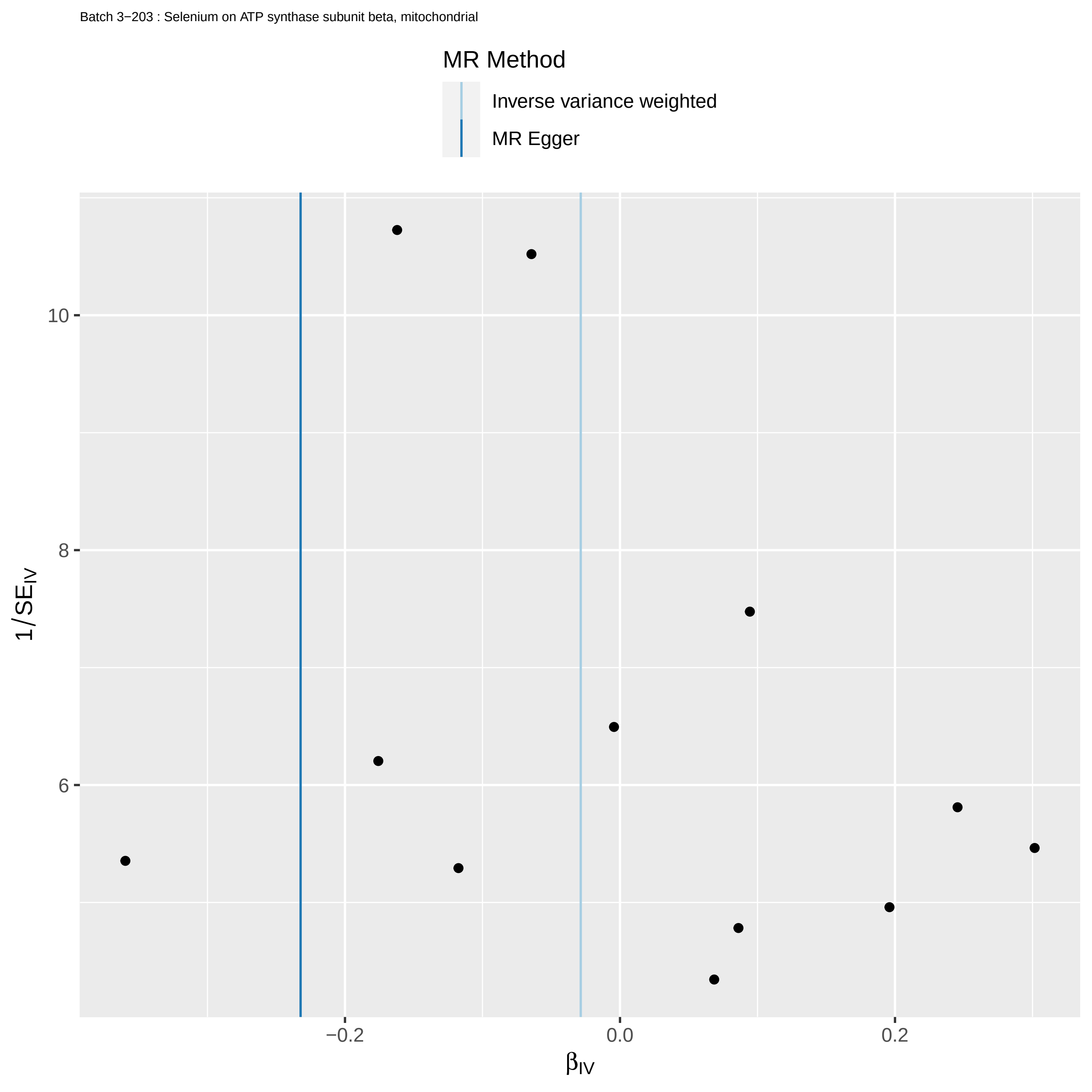

Batch 3−203 : Selenium on ATP synthase subunit beta, mitochondrial
MR Method
Inverse variance weighted
MR Egger
10
8
6
1/SE$_{IV}$
−0.2
0.0
0.2
β_{IV}

Batch 3−204 : Selenium on Aurora kinase A

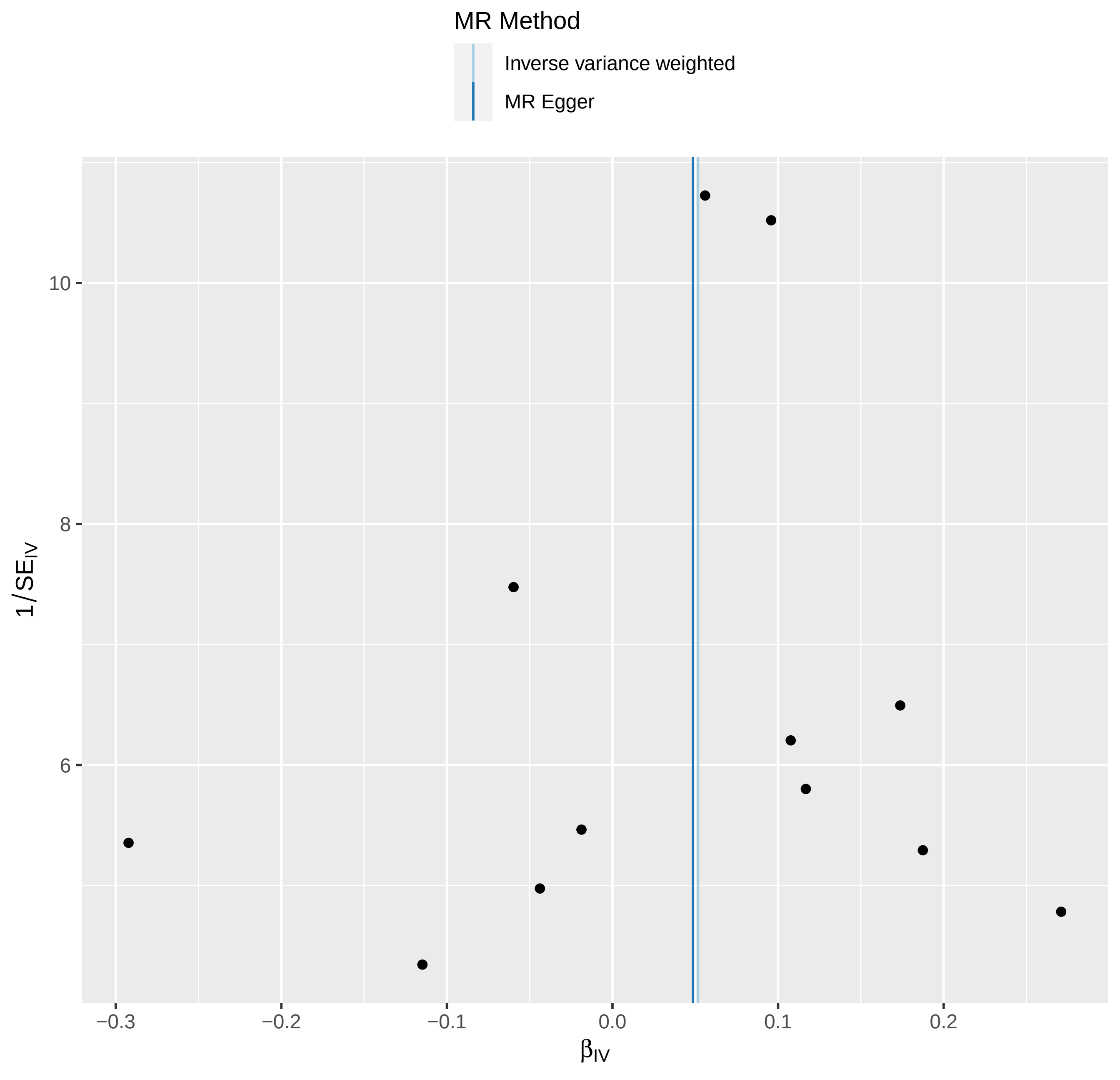

Batch 3−205 : Selenium on Aurora kinase B

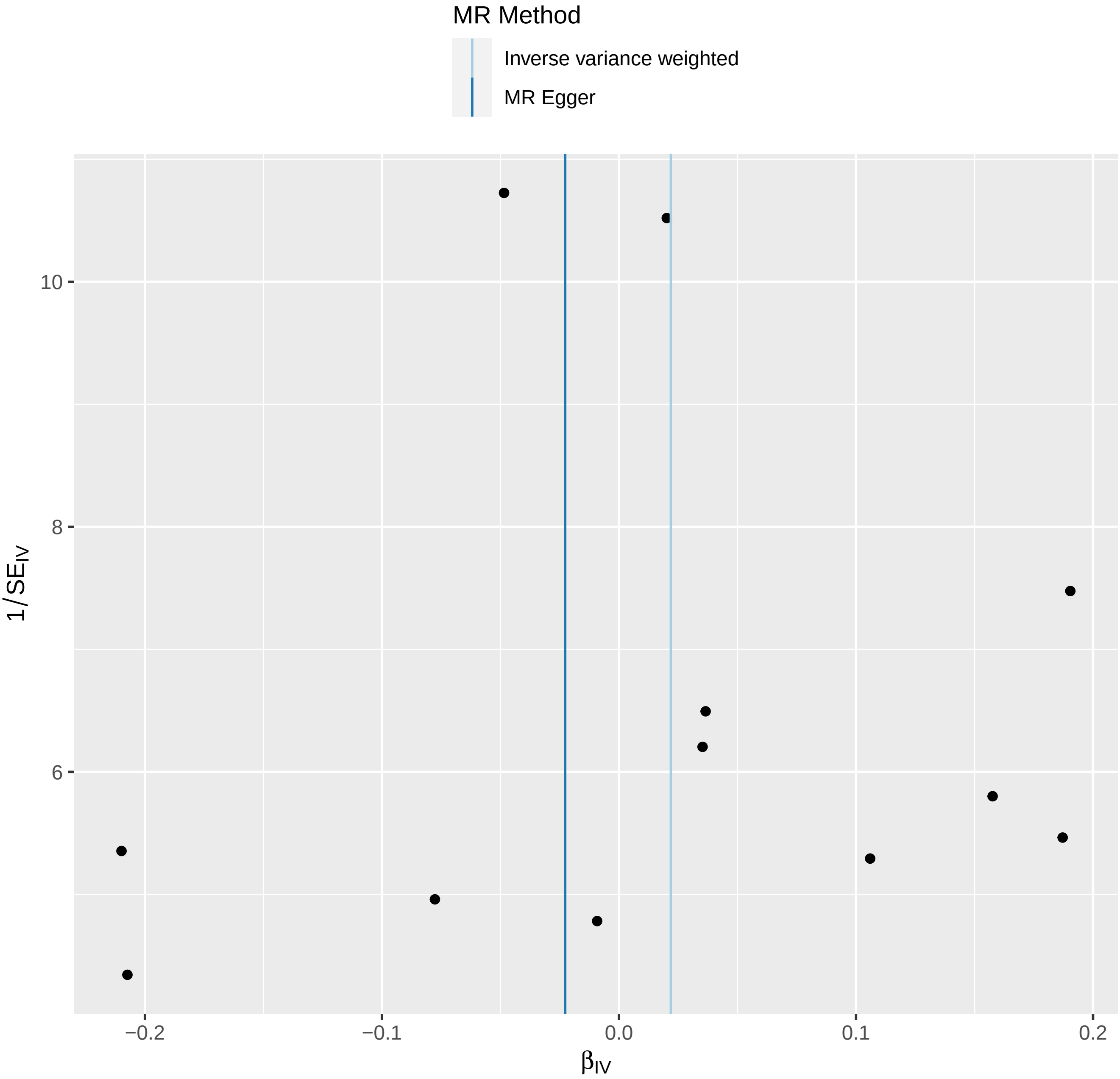

Batch 3−206 : Selenium on Axin−2

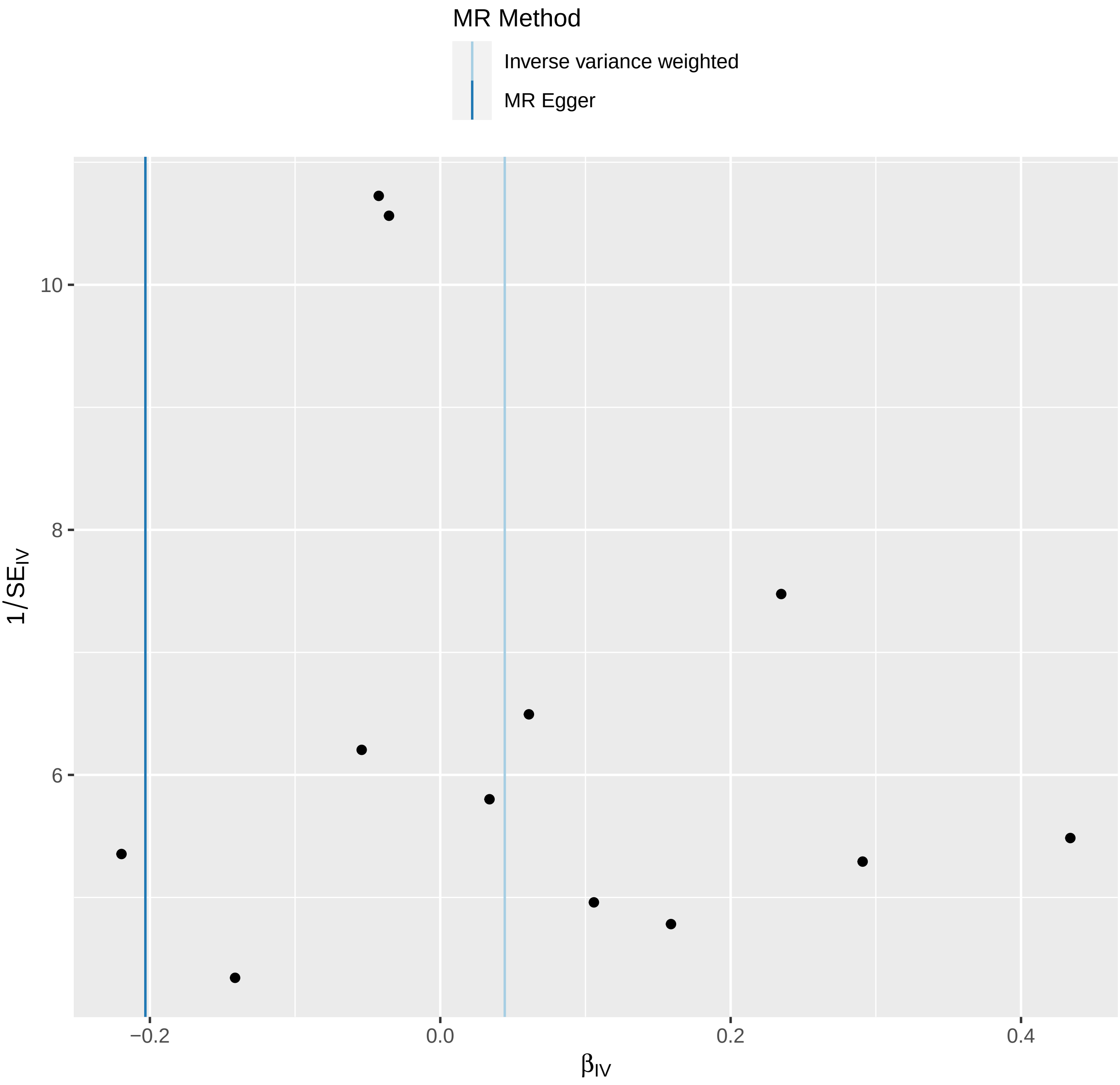

Batch 3−207 : Selenium on Axin−2

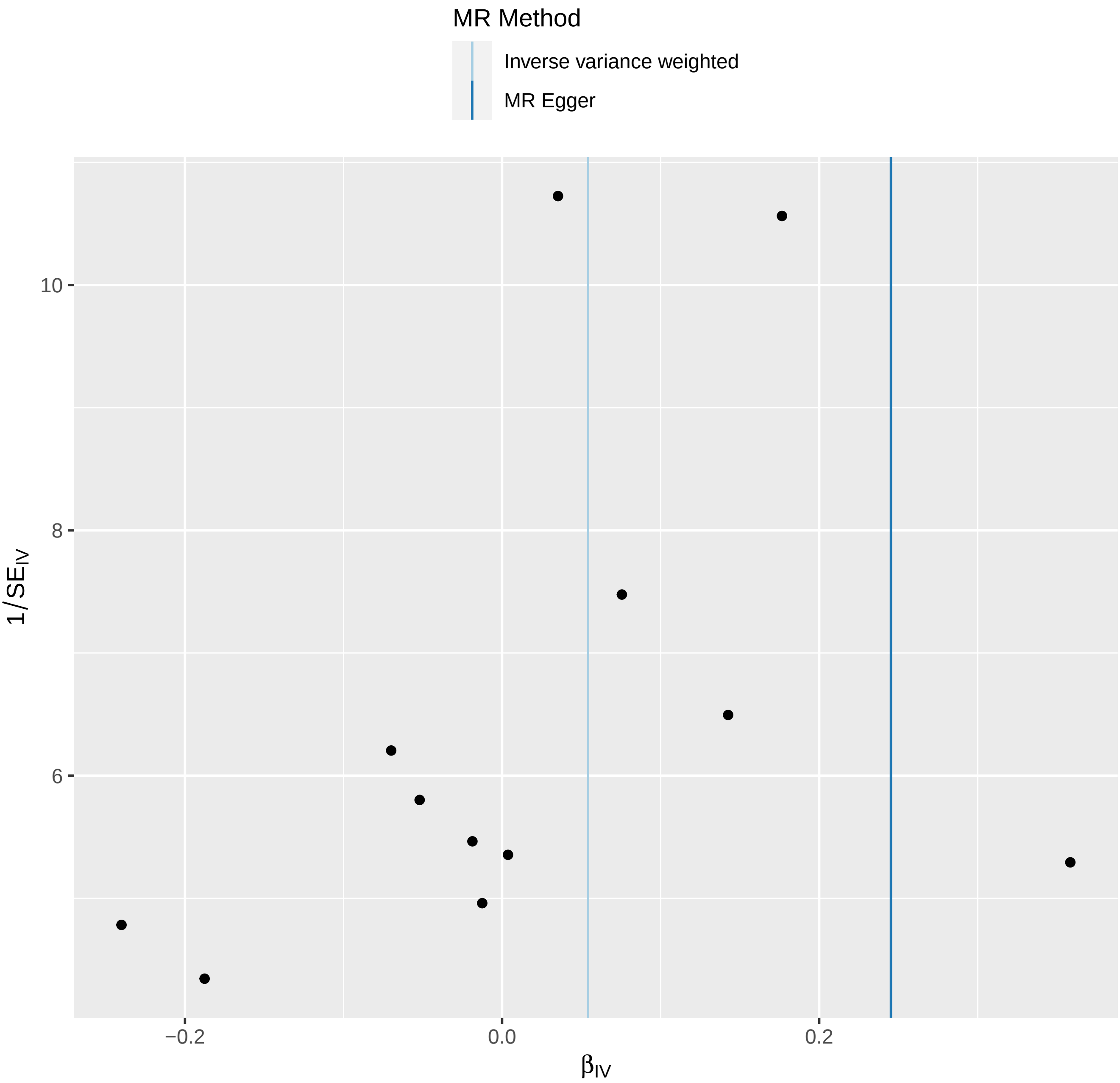

Batch 3−208 : Selenium on Zinc−alpha−2−glycoprotein

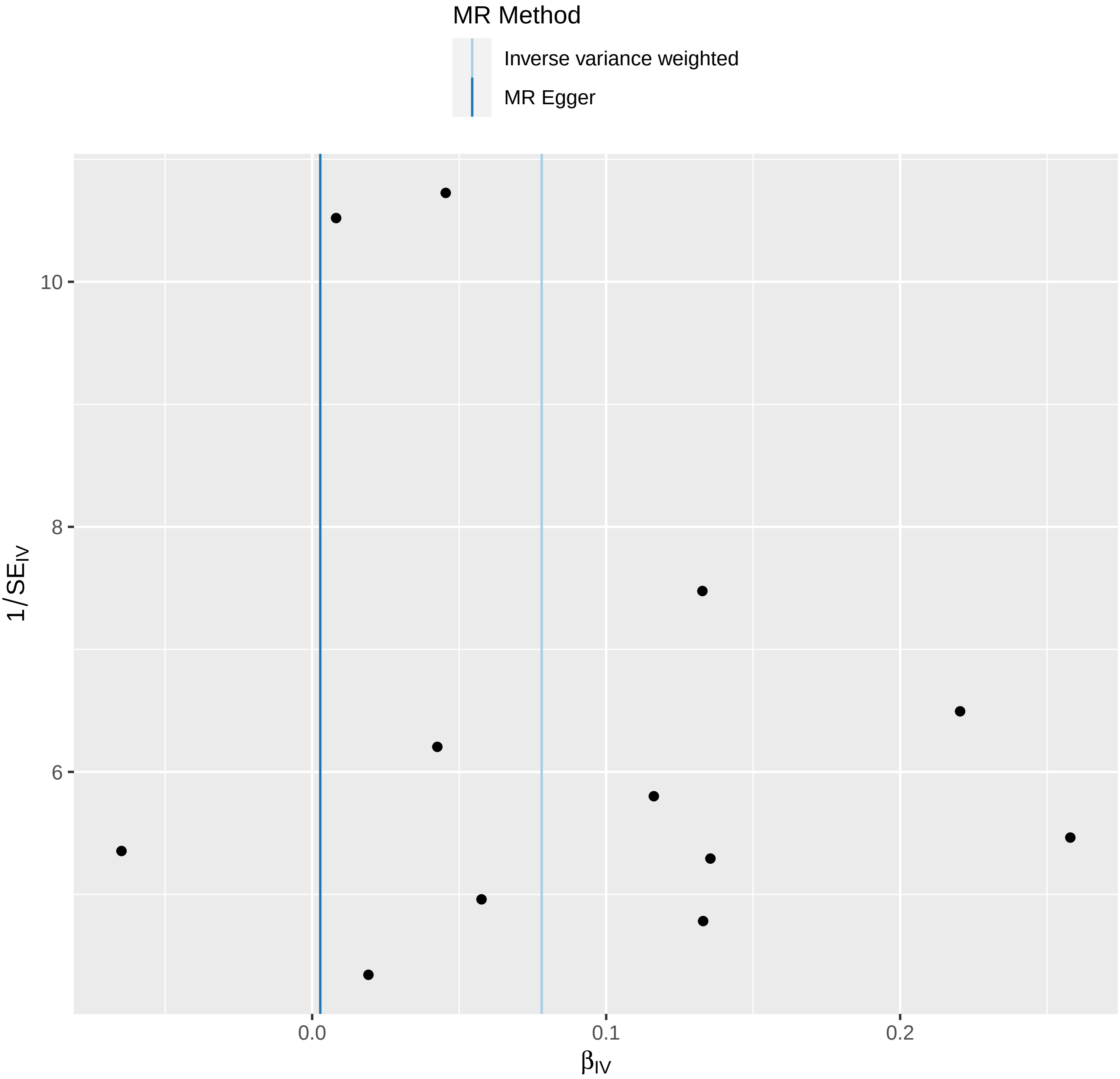

Batch 3−209 : Selenium on Beta−1,3−galactosyltransferase 6

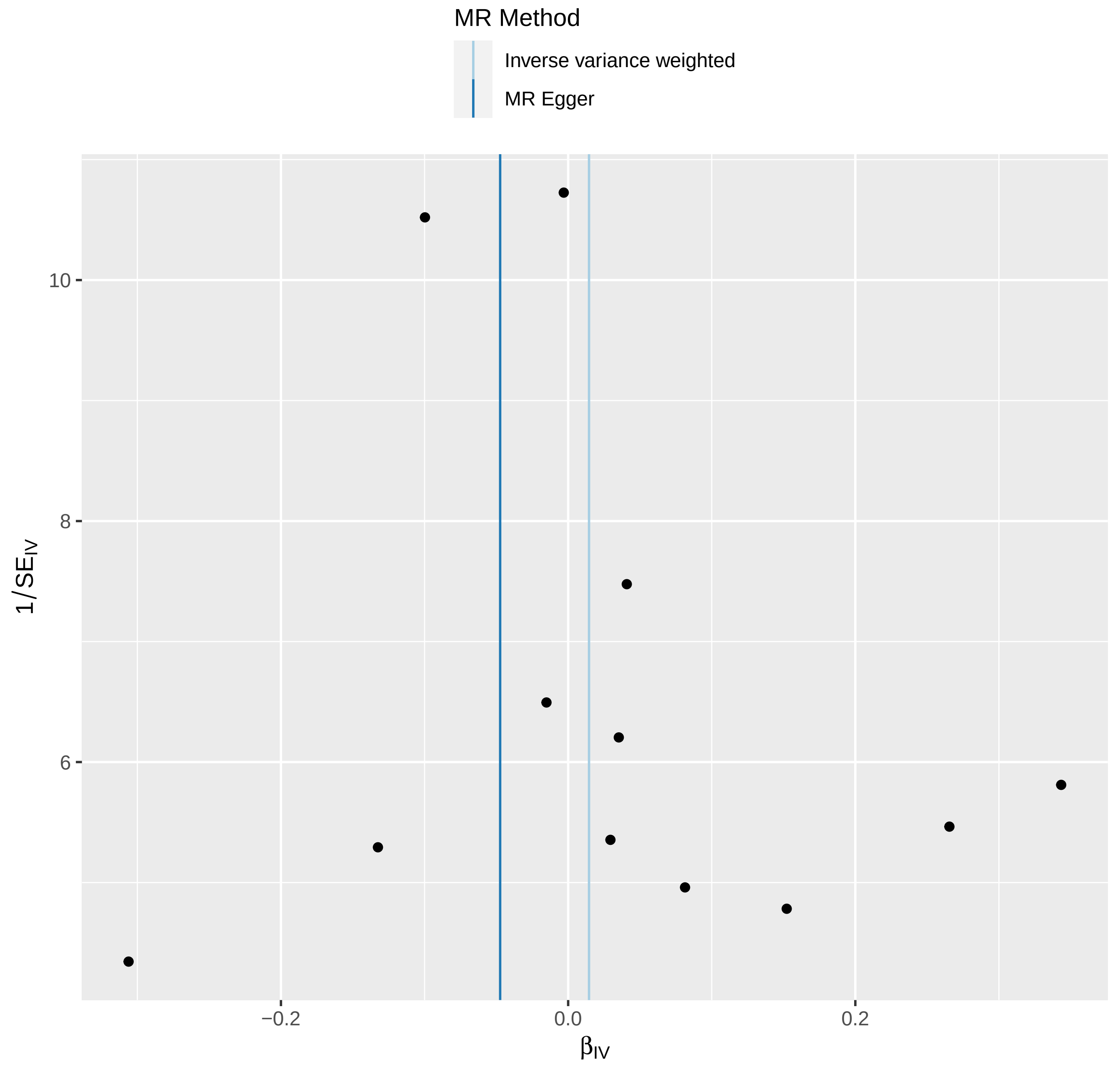

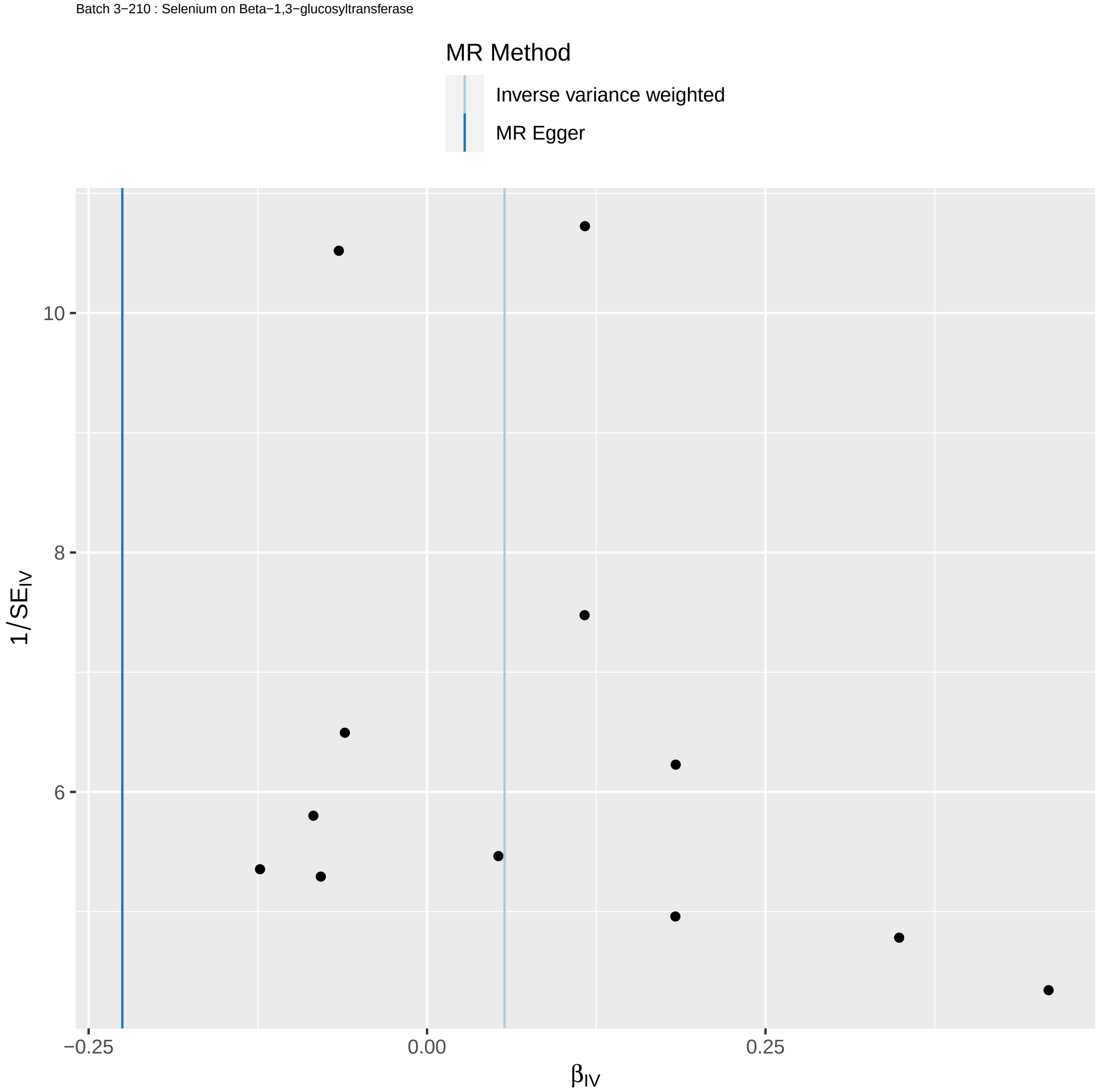
Batch 3−210 : Selenium on Beta−1,3−glucosyltransferase
MR Method
Inverse variance weighted
MR Egger
10
8
6
1/SE$_{IV}$
−0.25
0.00
0.25
β$_{IV}$

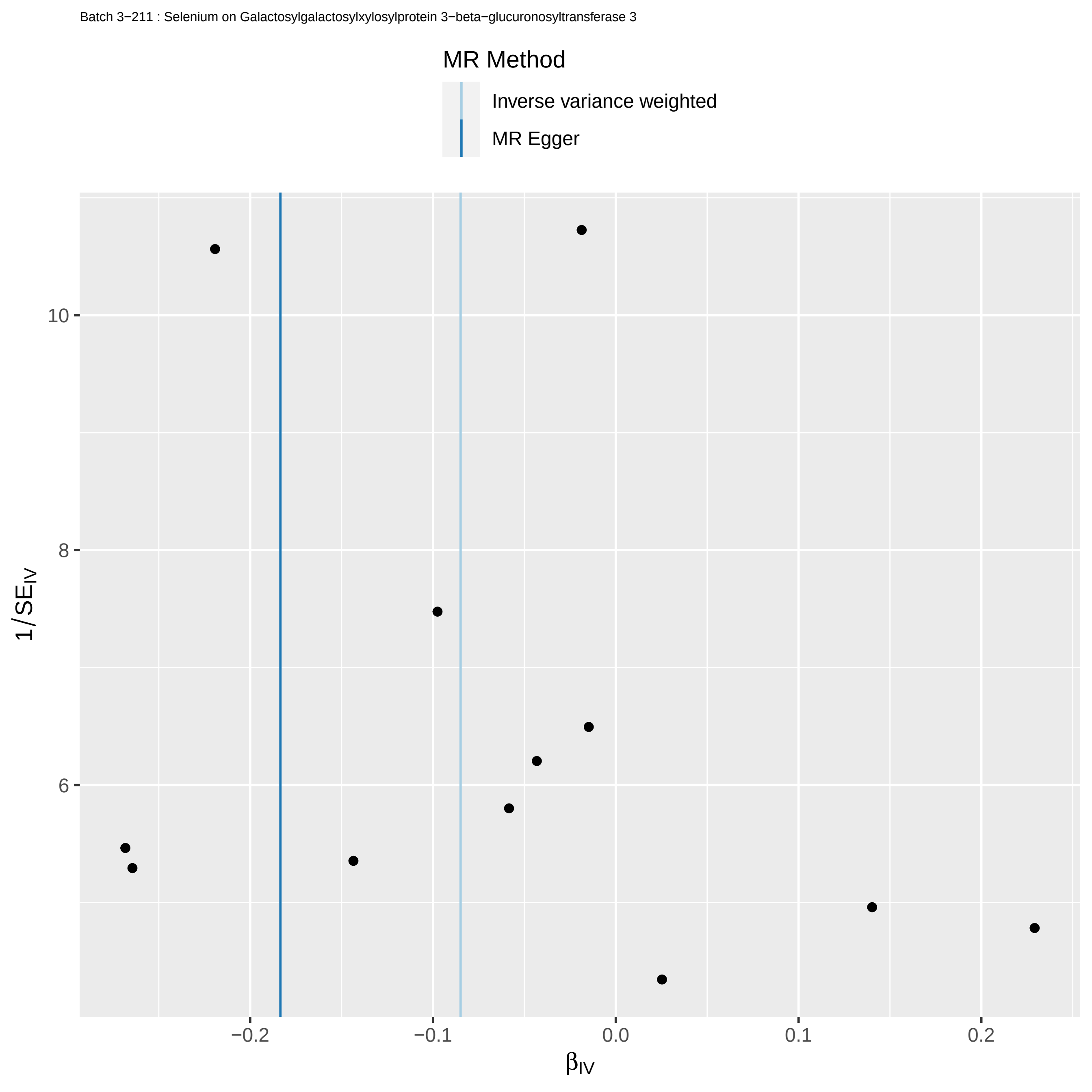

Batch 3−211 : Selenium on Galactosylgalactosylxylosylprotein 3−beta−glucuronosyltransferase 3
MR Method
Inverse variance weighted
MR Egger
10
8
6
1/SE_IV
−0.2
−0.1
0.0
0.1
0.2
β_IV

Batch 3−212 : Selenium on Beta−1,4−glucuronyltransferase 1

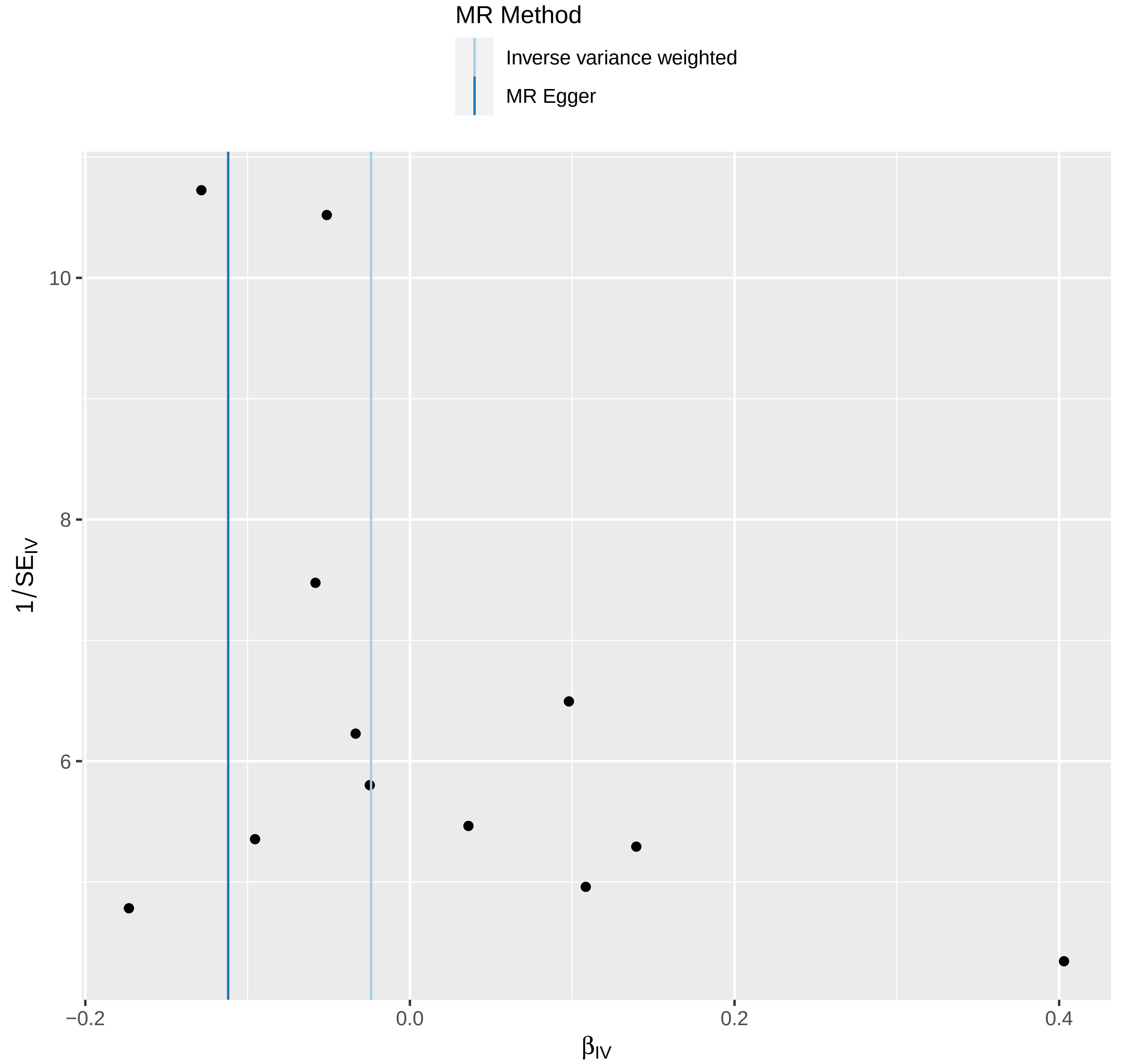

Batch 3−213 : Selenium on N−acetyllactosaminide beta−1,3−N−acetylglucosaminyltransferase 2

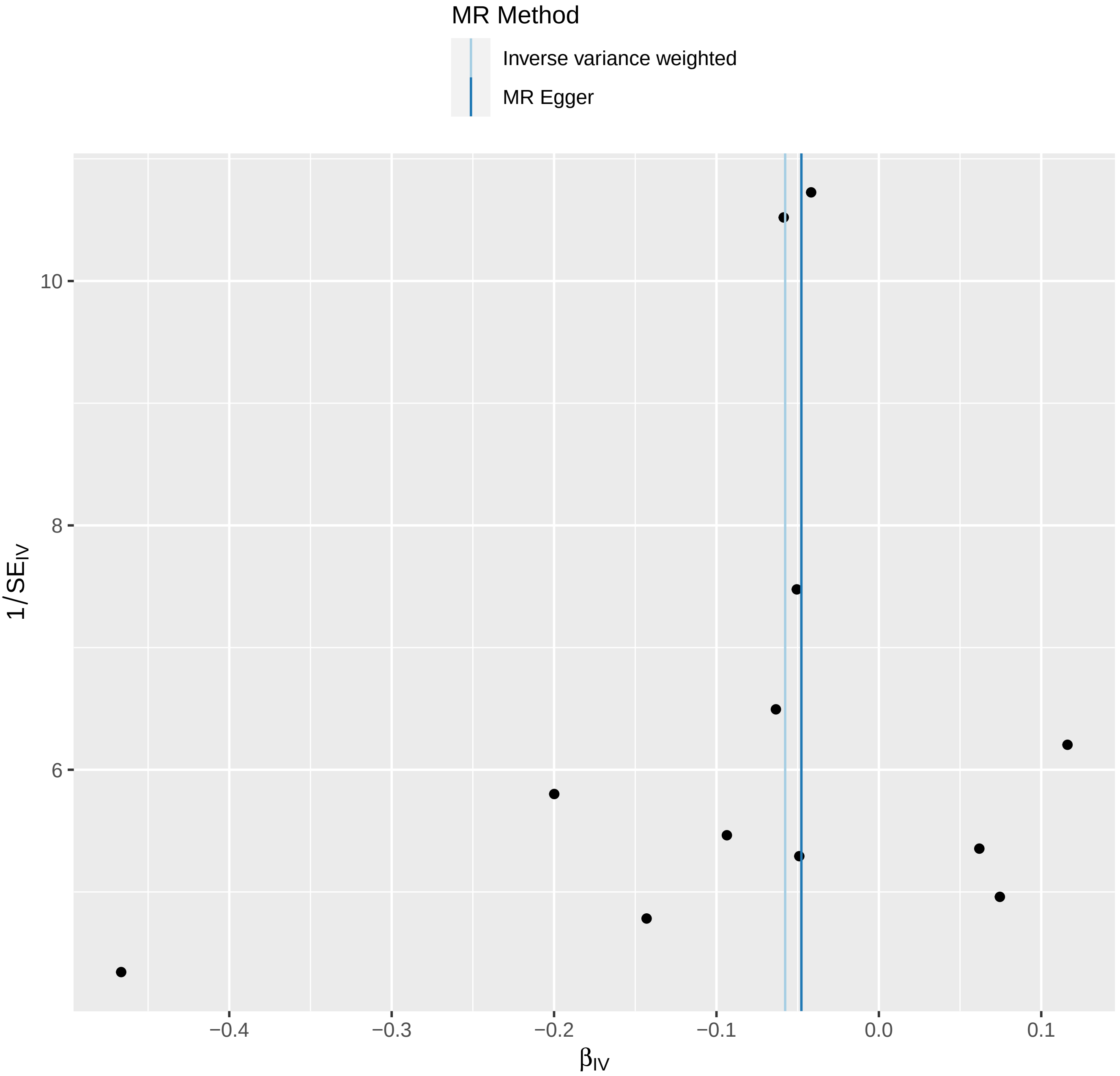

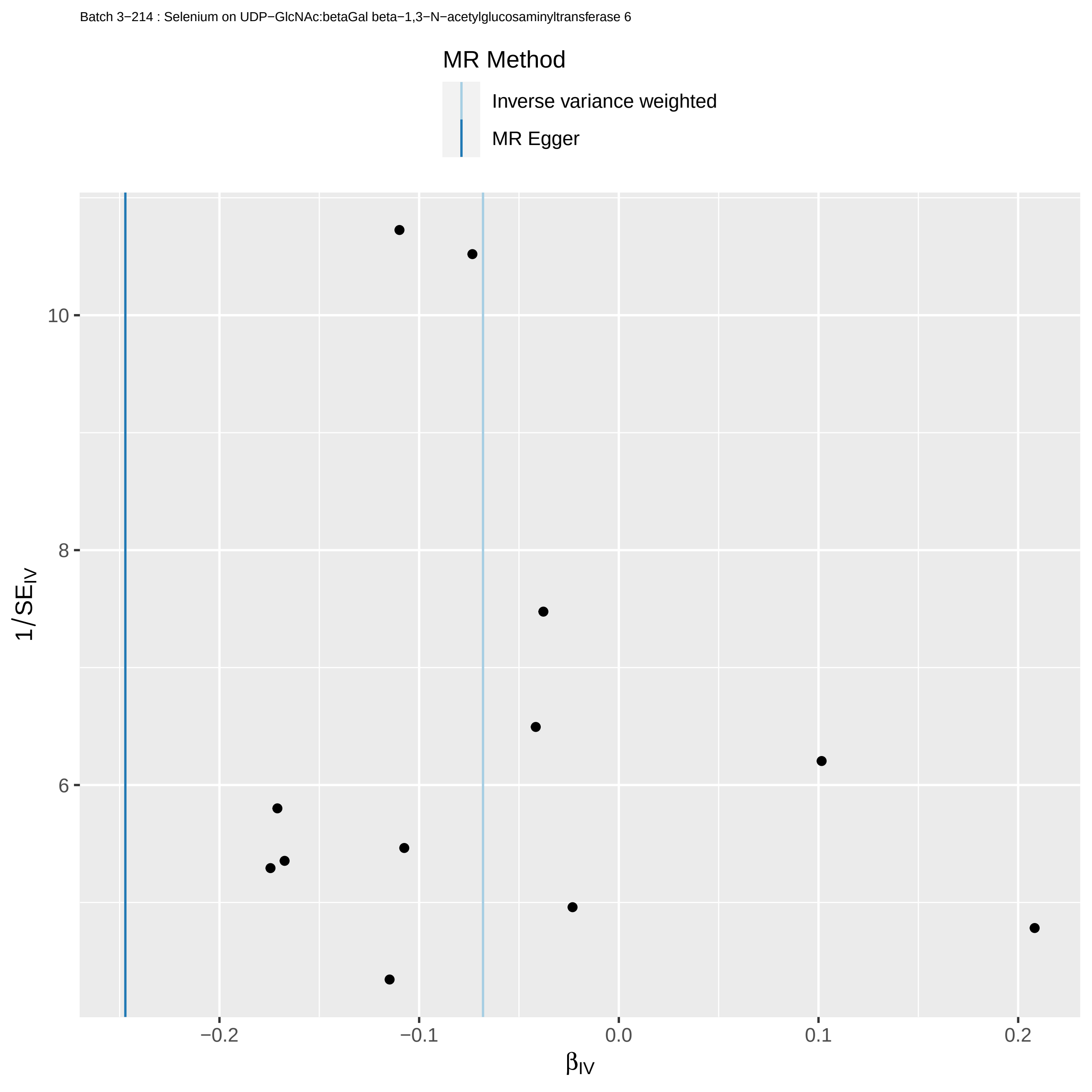

Batch 3−214 : Selenium on UDP−GlcNAc:betaGal beta−1,3−N−acetylglucosaminyltransferase 6
MR Method
Inverse variance weighted
MR Egger
10
8
6
1/SE_IV
−0.2
−0.1
0.0
0.1
0.2
β_IV

Batch 3−215 : Selenium on UDP−GlcNAc:betaGal beta−1,3−N−acetylglucosaminyltransferase 8

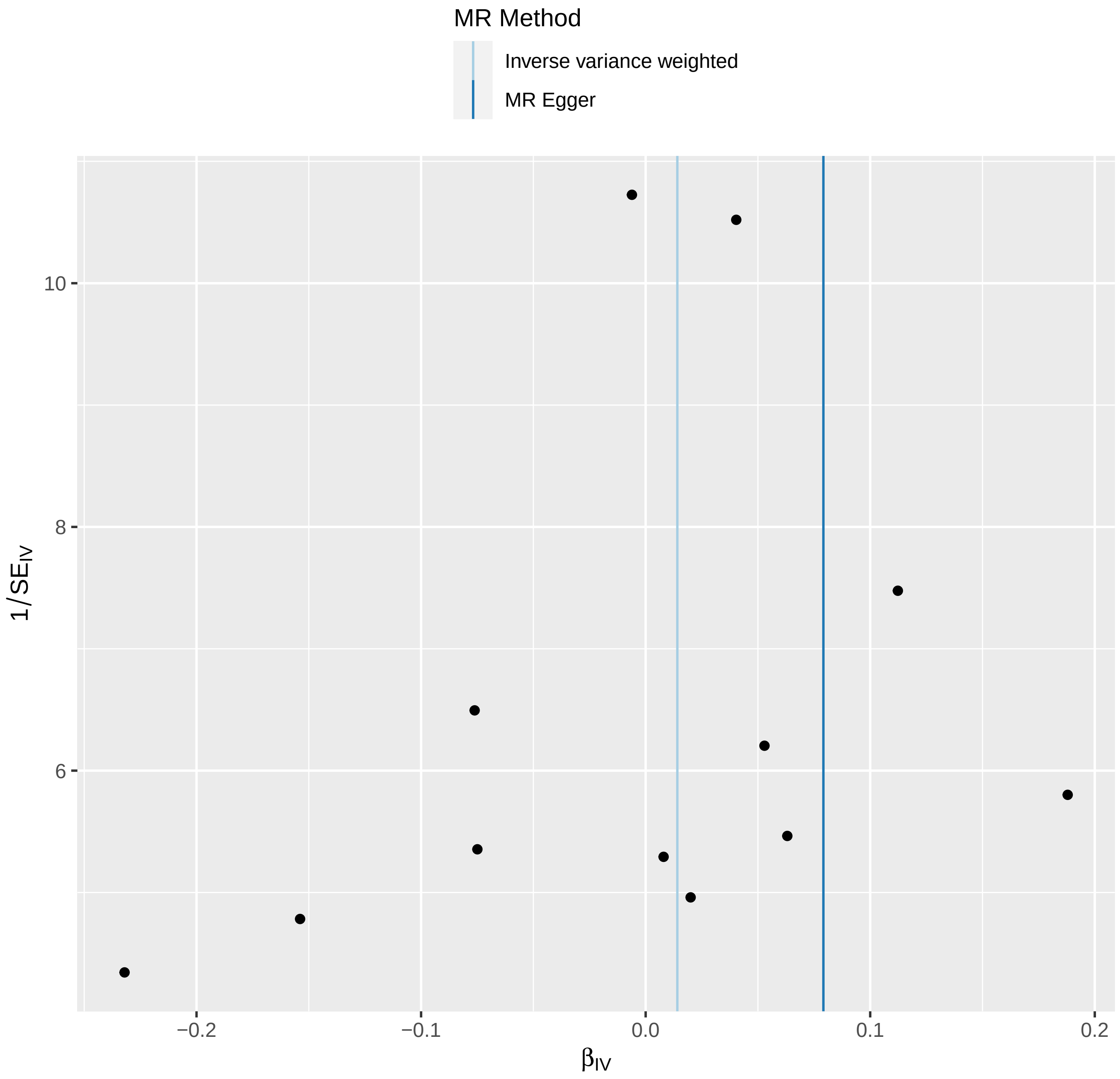

Batch 3−216 : Selenium on Beta−1,4−galactosyltransferase 1

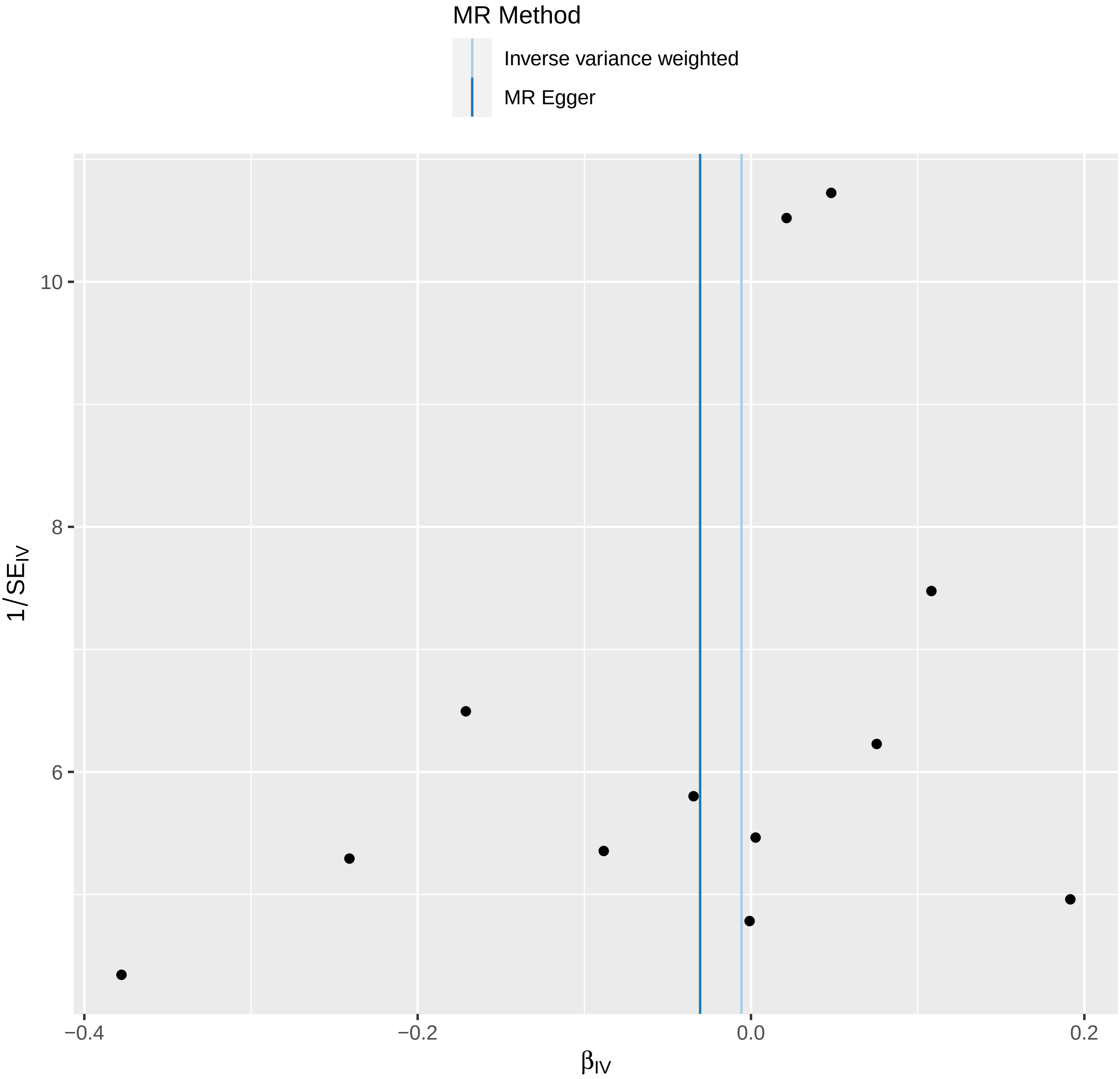

Batch 3−217 : Selenium on Beta−1,4−galactosyltransferase 2

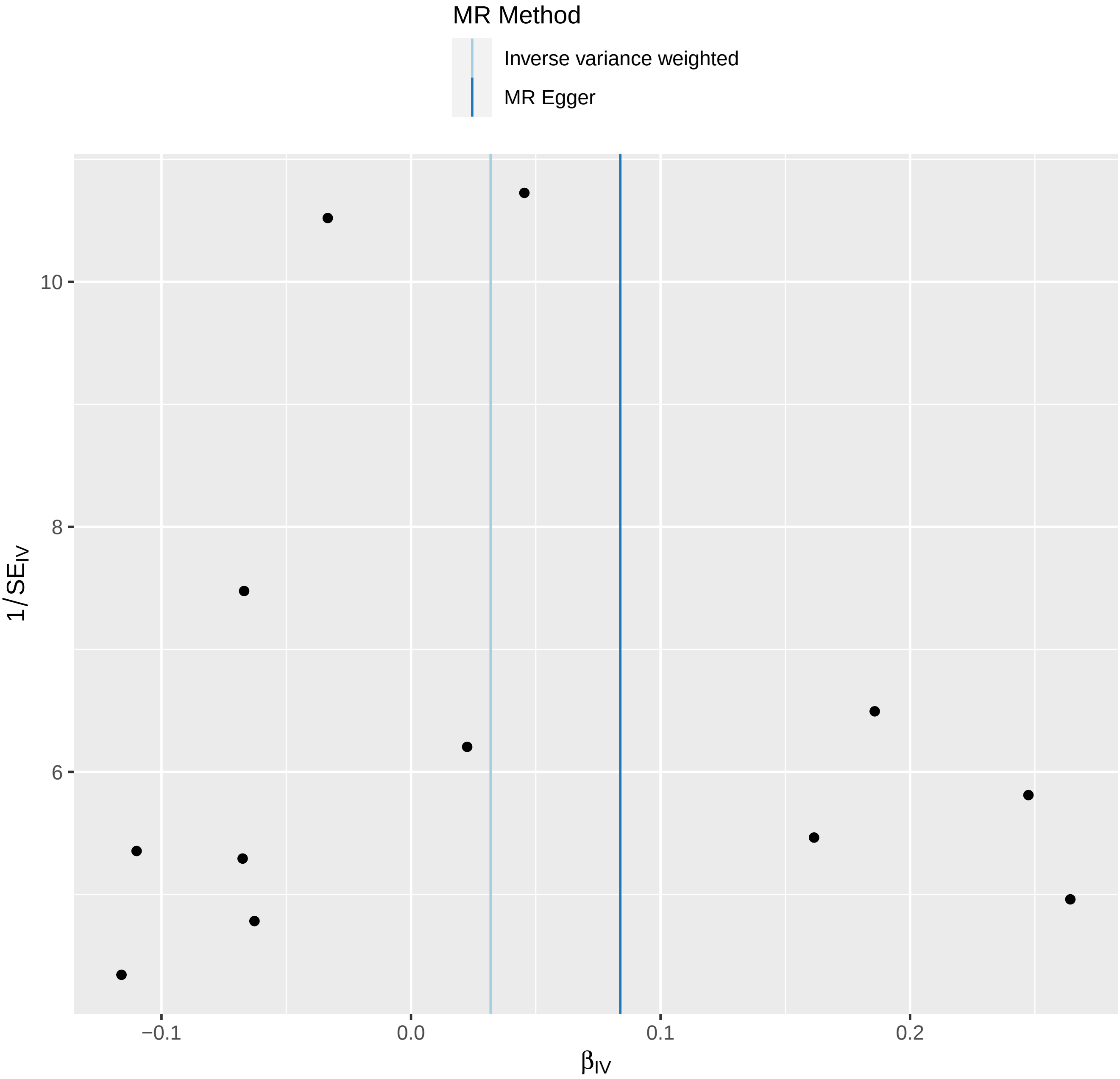

Batch 3−218 : Selenium on Beta−1,4−galactosyltransferase 3

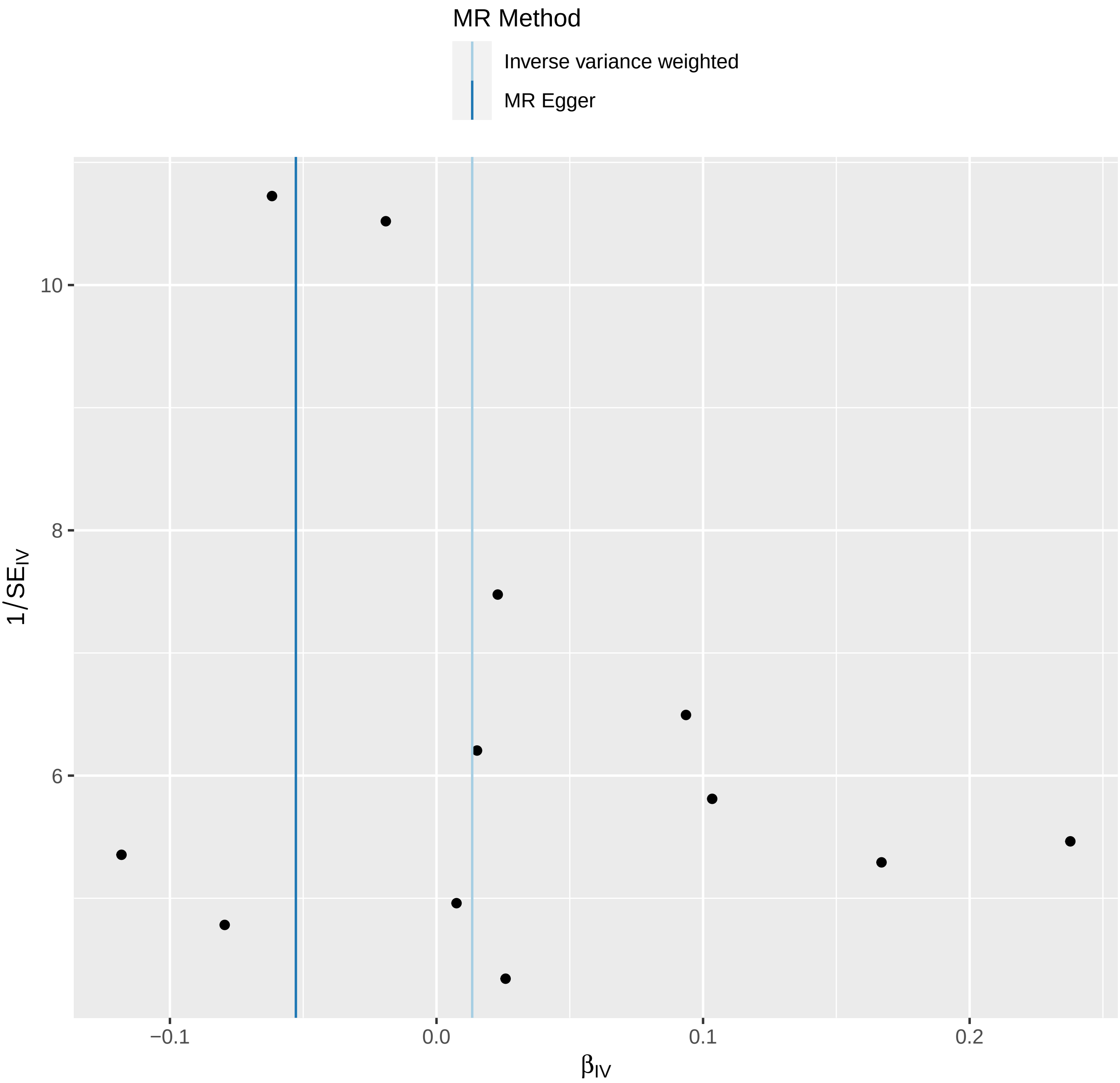

Batch 3−219 : Selenium on Beta−1,4−galactosyltransferase 5

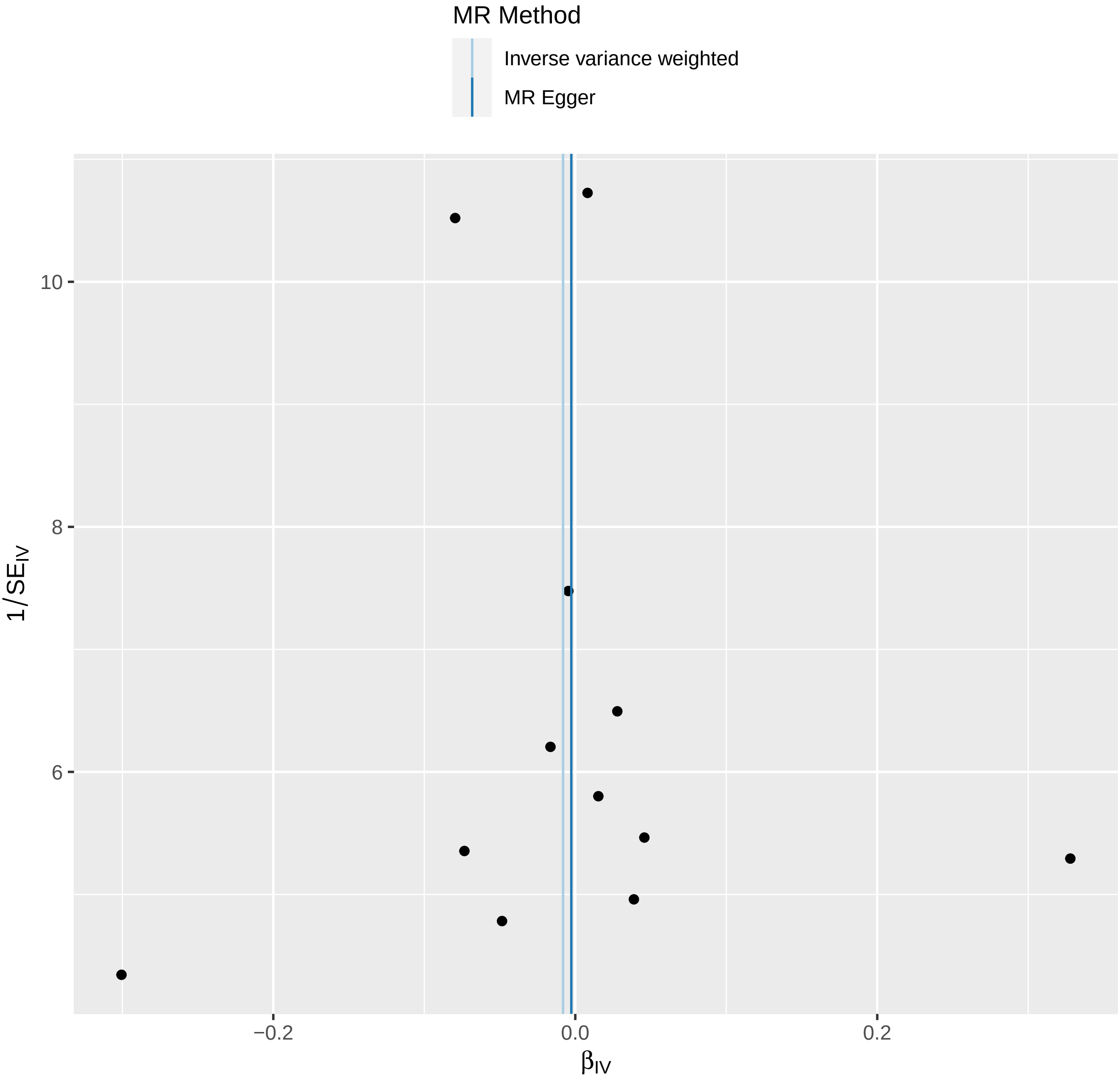

Batch 3−220 : Selenium on Beta−1,4−galactosyltransferase 6

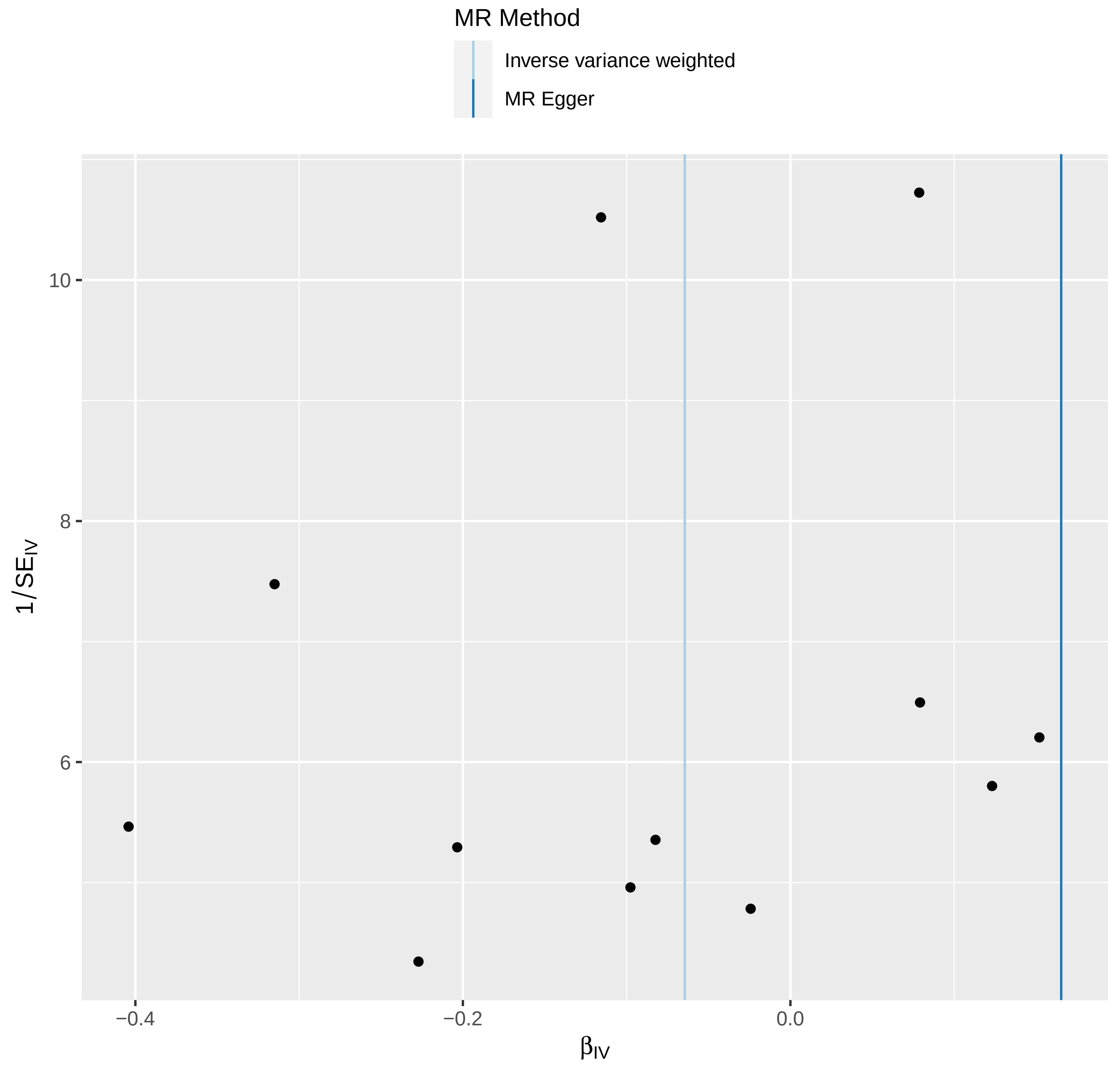

Batch 3−221 : Selenium on Beta−1,4−galactosyltransferase 7

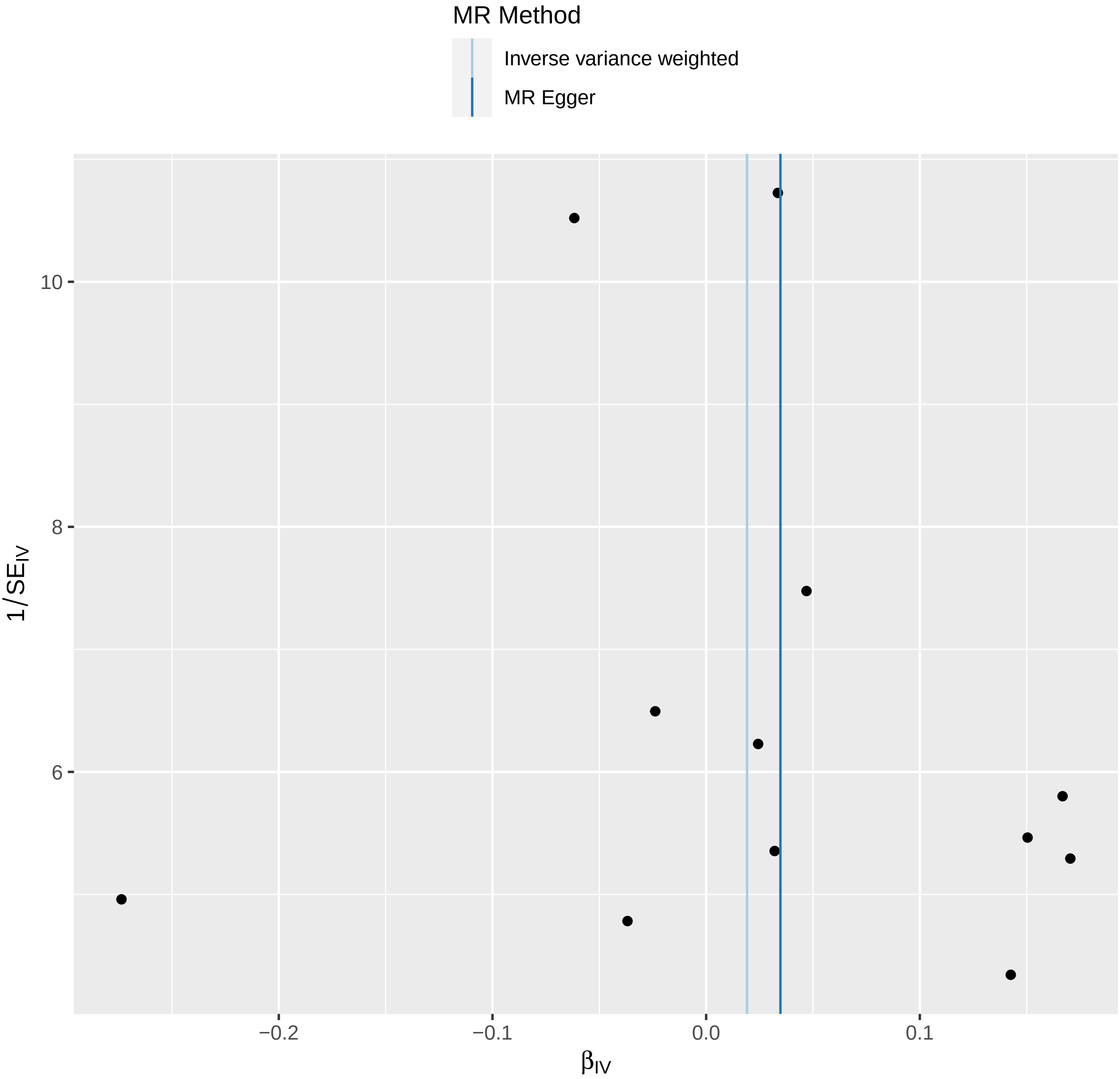

Batch 3−222 : Selenium on Transcription regulator protein BACH1

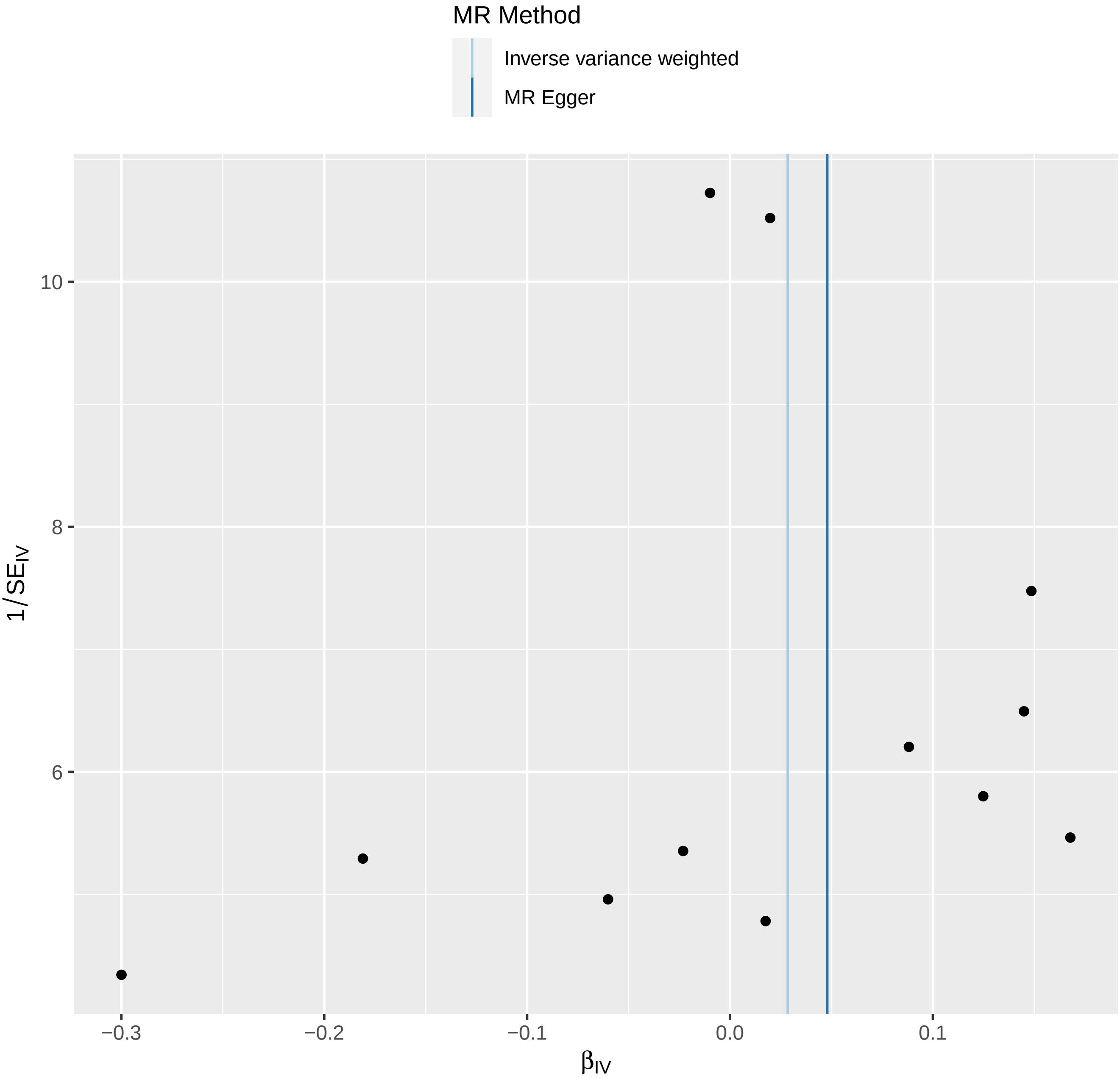

Batch 3−223 : Selenium on Transcription regulator protein BACH2

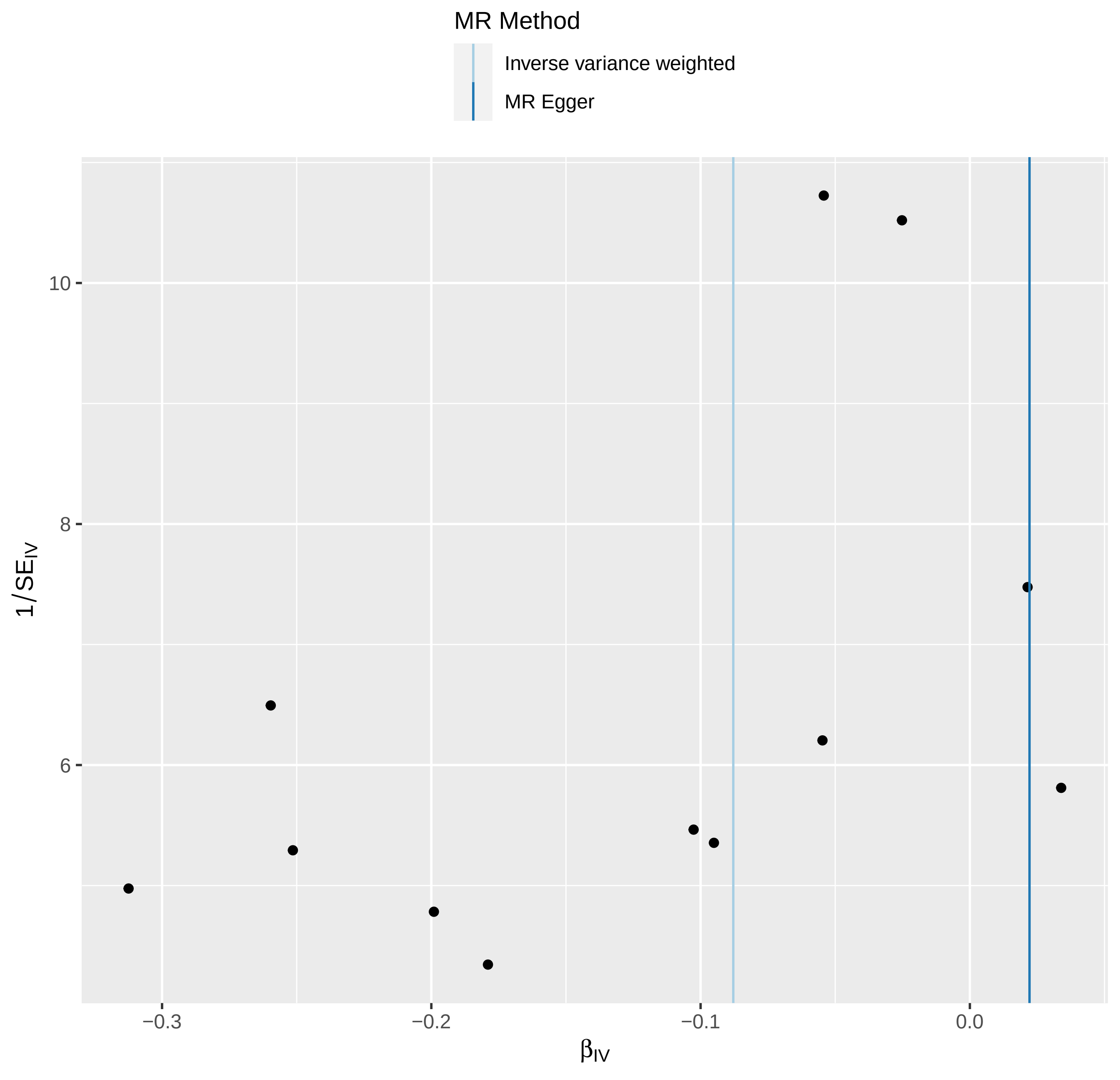

Batch 3−224 : Selenium on Bcl2−associated agonist of cell death

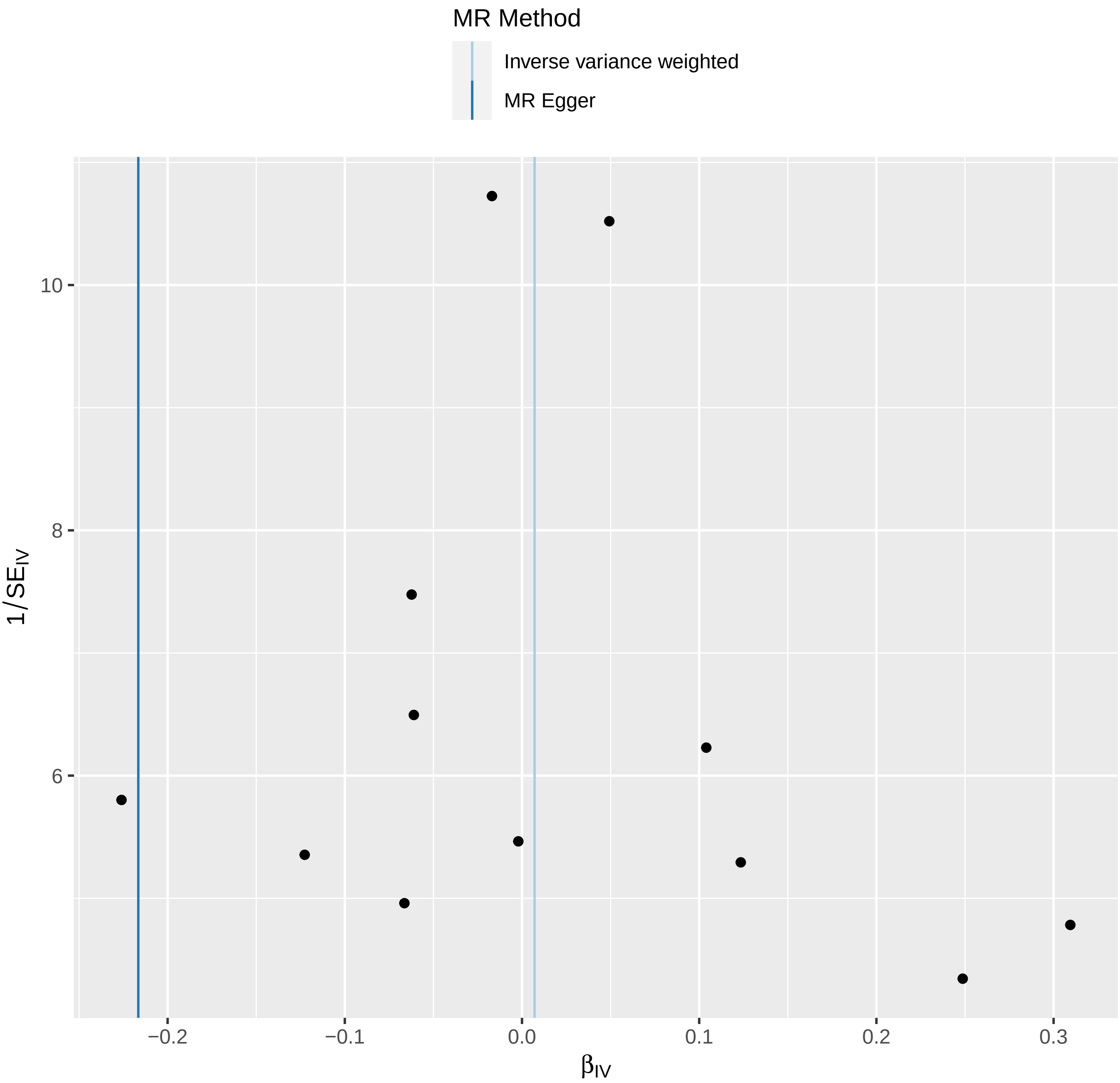

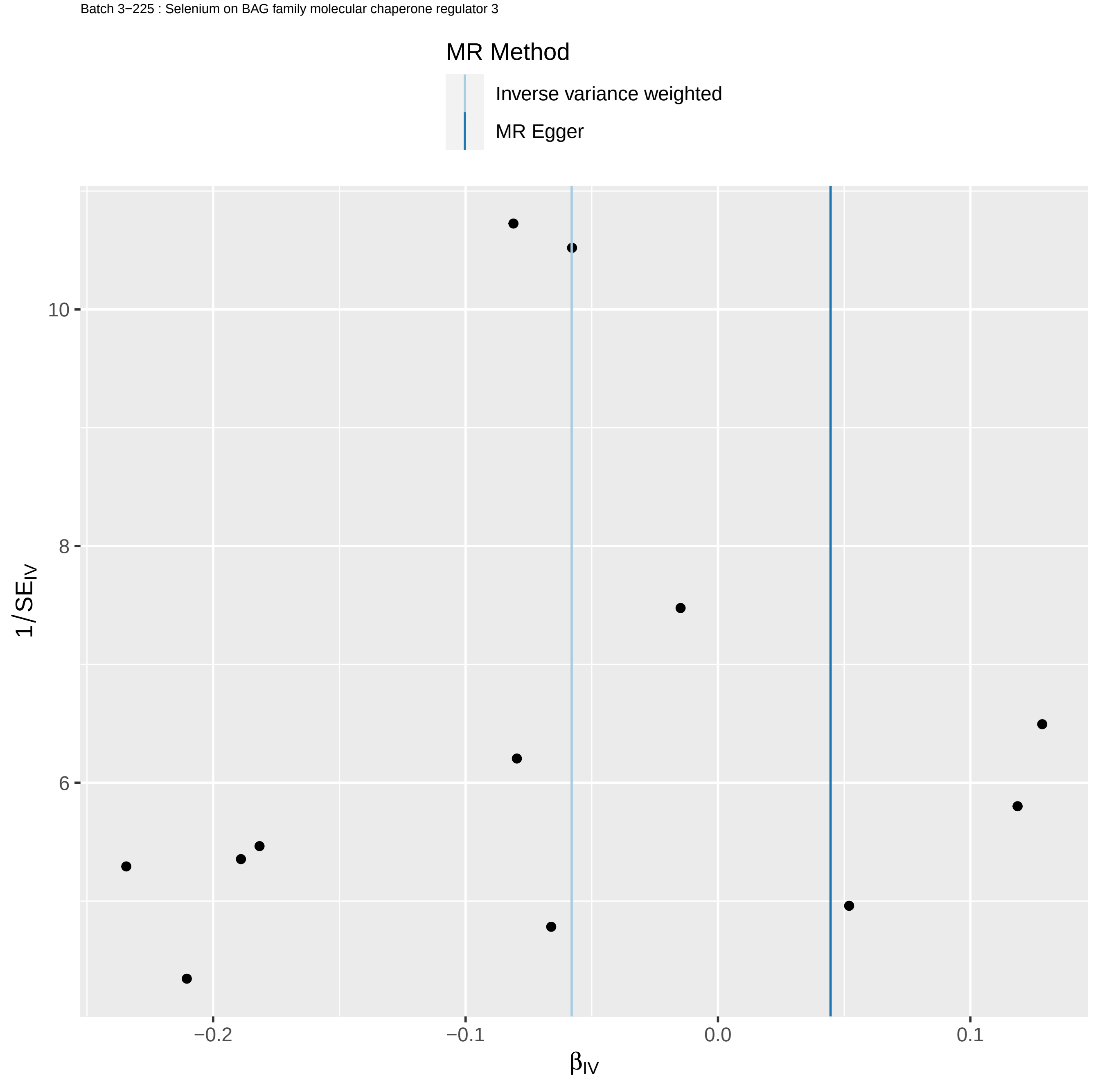
Batch 3−225 : Selenium on BAG family molecular chaperone regulator 3
MR Method
Inverse variance weighted
MR Egger
10
8
6
1/SE_IV
−0.2
−0.1
0.0
0.1
β_IV

Batch 3−226 : Selenium on BAG family molecular chaperone regulator 4

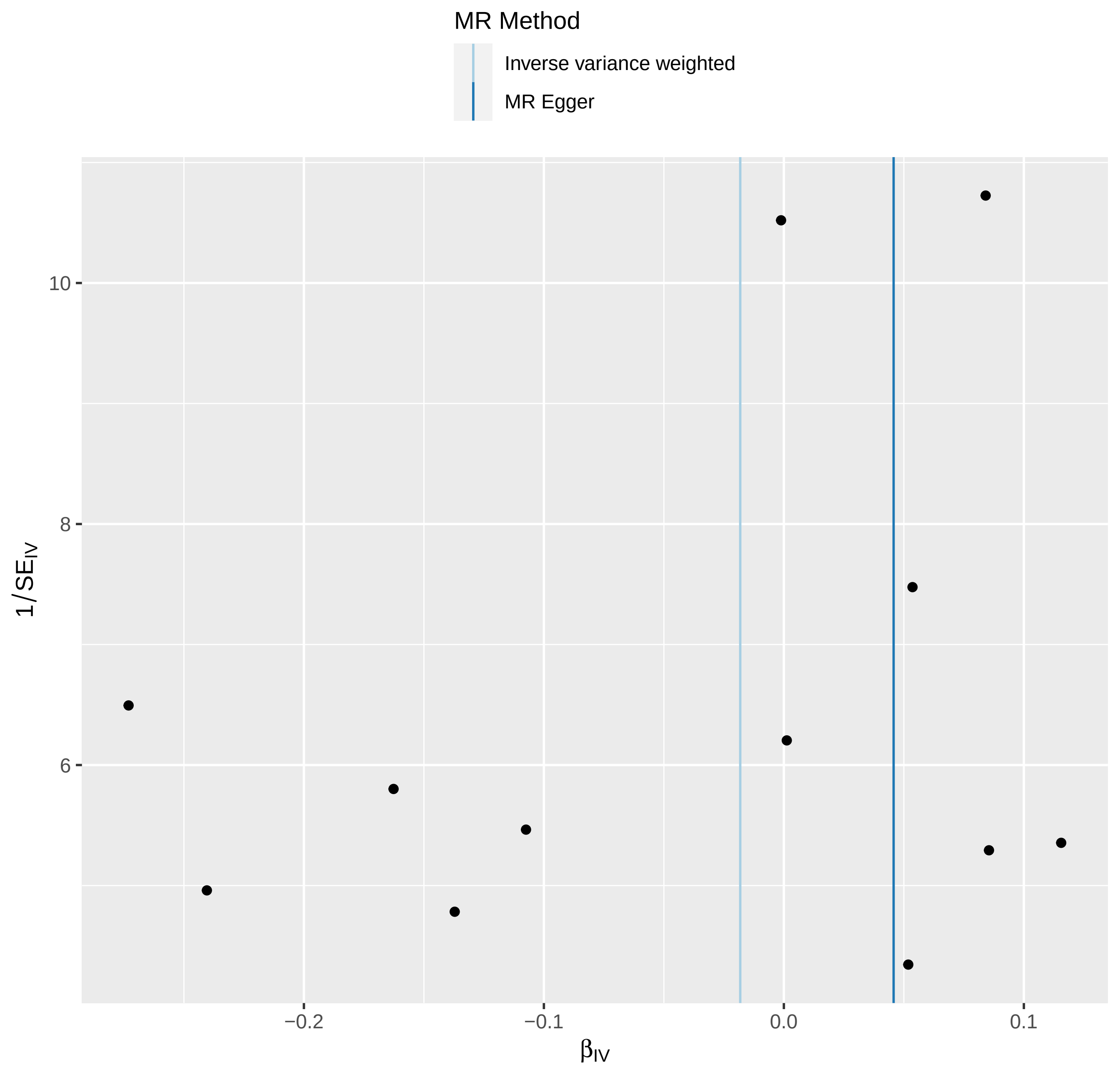

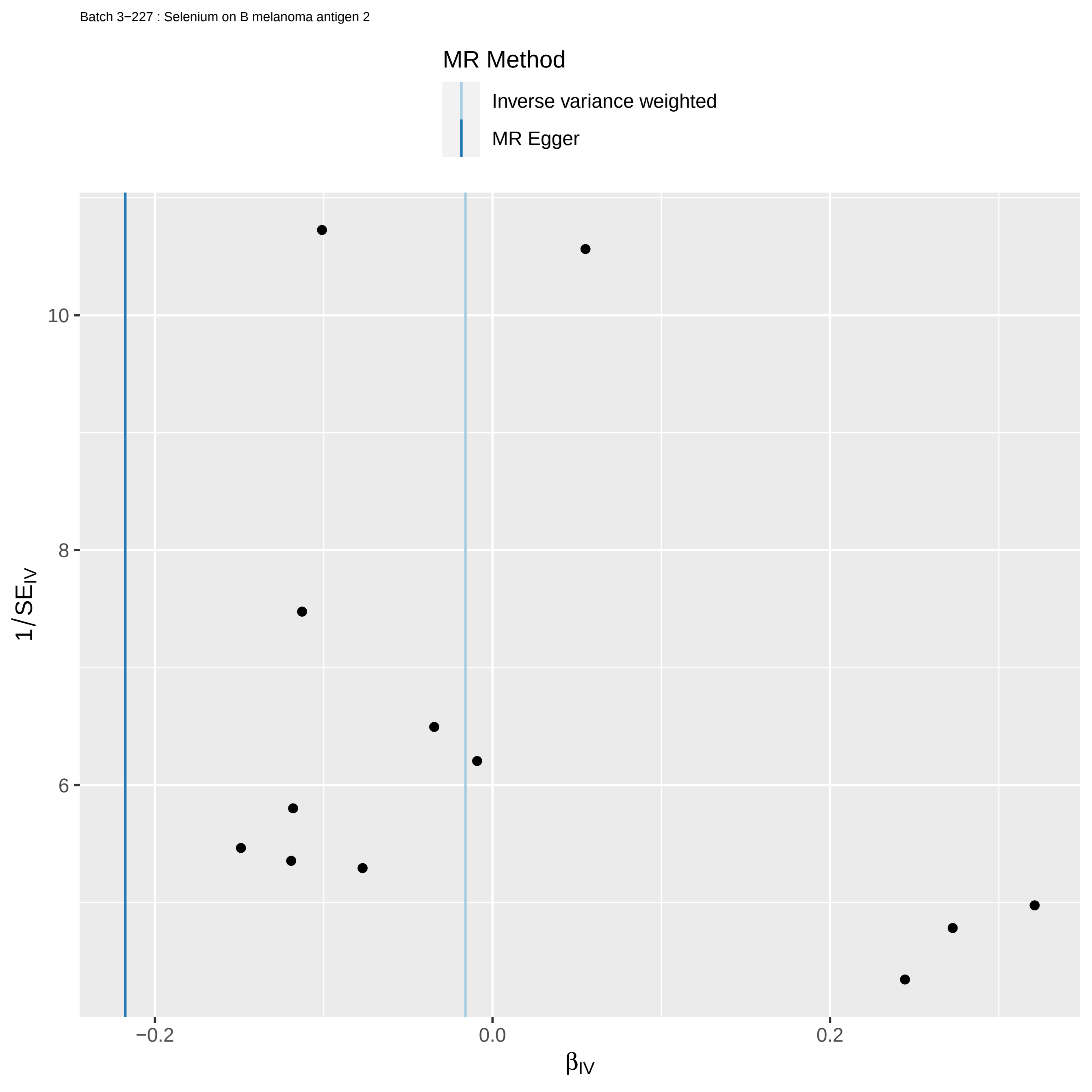

Batch 3−227 : Selenium on B melanoma antigen 2
MR Method
Inverse variance weighted
MR Egger
10
8
6
1/SE$_{IV}$
−0.2
0.0
0.2
β$_{IV}$

Batch 3−228 : Selenium on B melanoma antigen 3

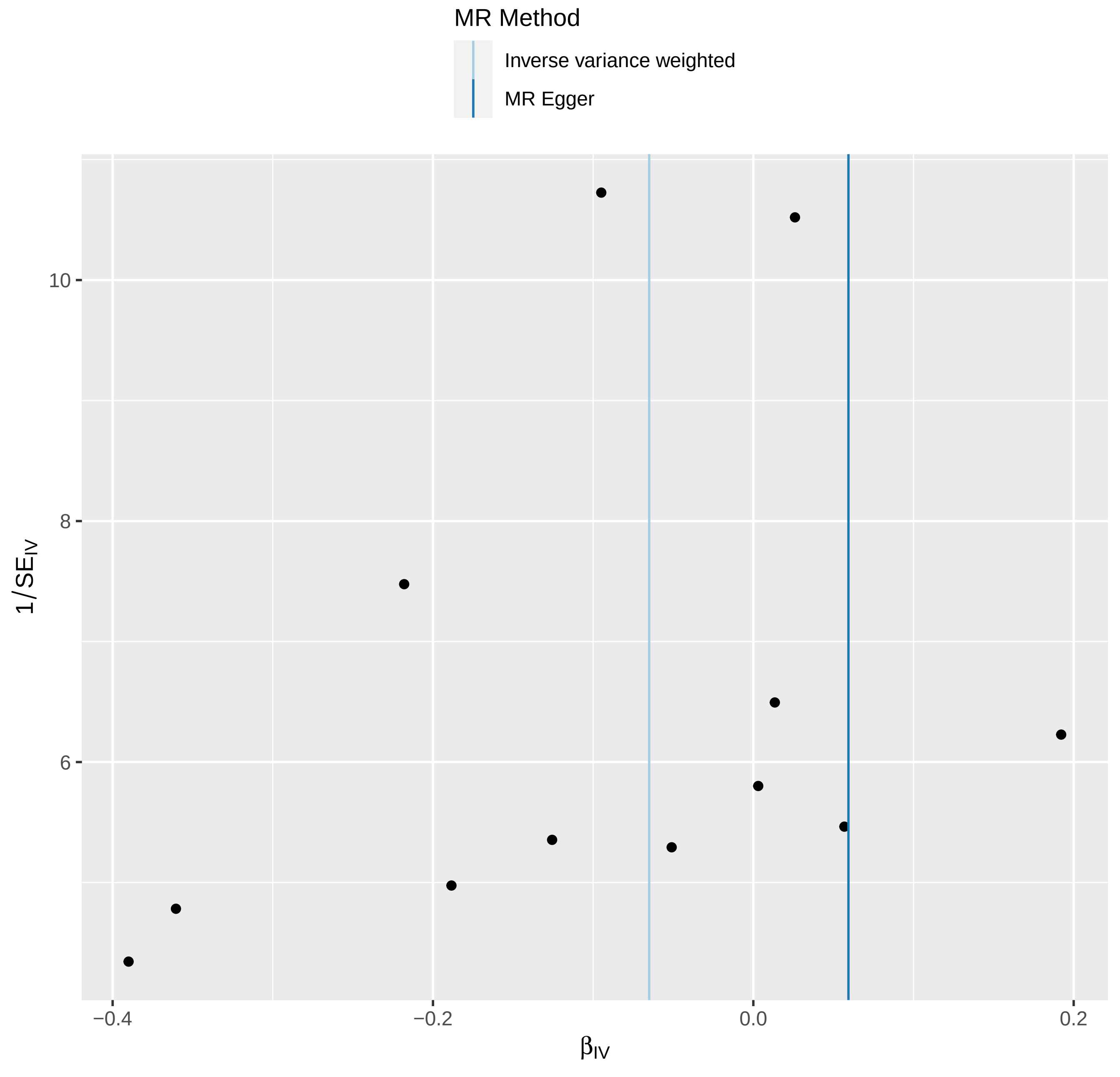

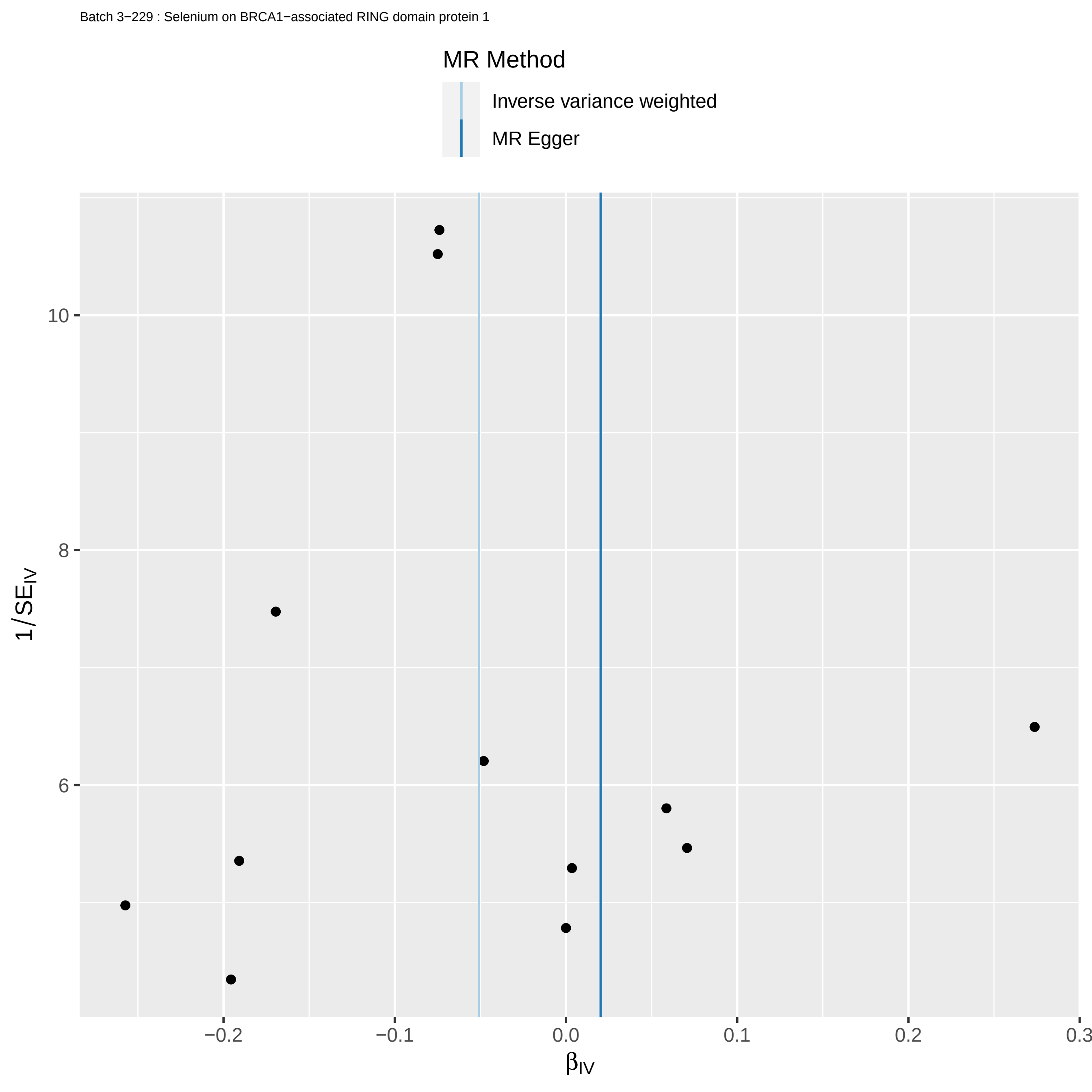

Batch 3−229 : Selenium on BRCA1−associated RING domain protein 1
MR Method
Inverse variance weighted
MR Egger
10
8
6
1/SE_IV
−0.2
−0.1
0.0
0.1
0.2
0.3
β_IV

Batch 3−230 : Selenium on Basic leucine zipper transcriptional factor ATF−like 3

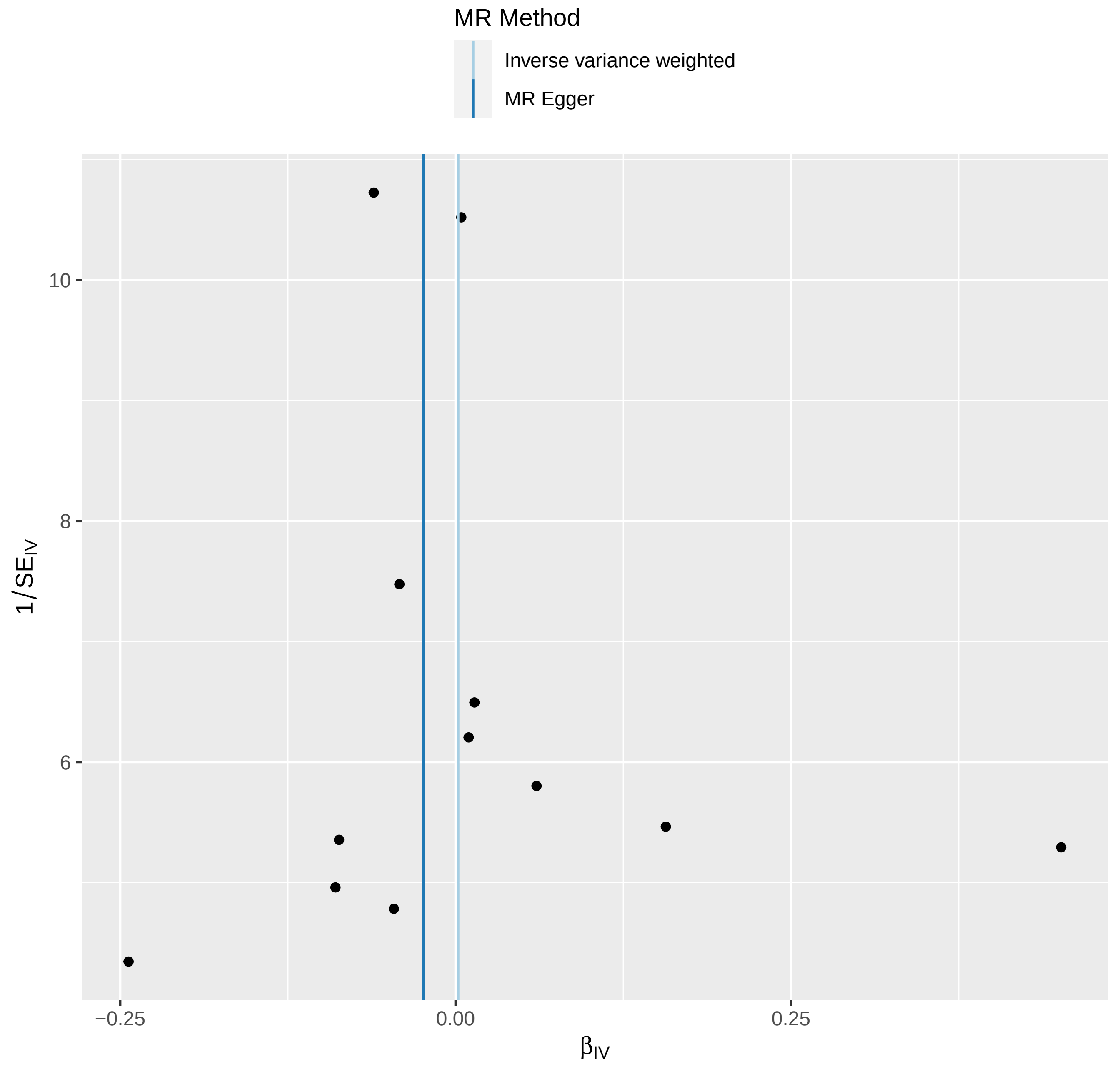

Batch 3−231 : Selenium on Basal Cell Adhesion Molecule

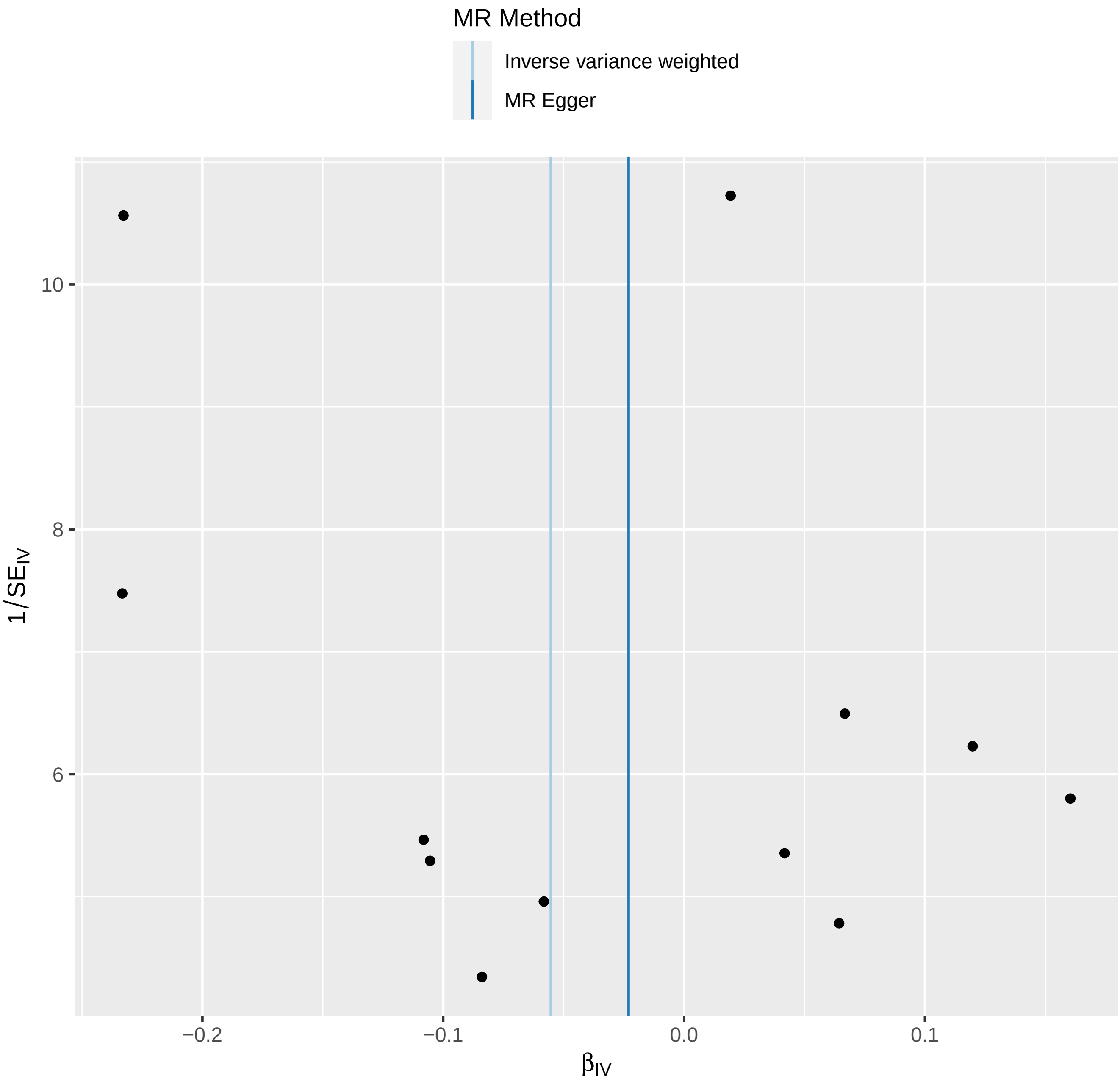

Batch 3−232 : Selenium on Brevican core protein

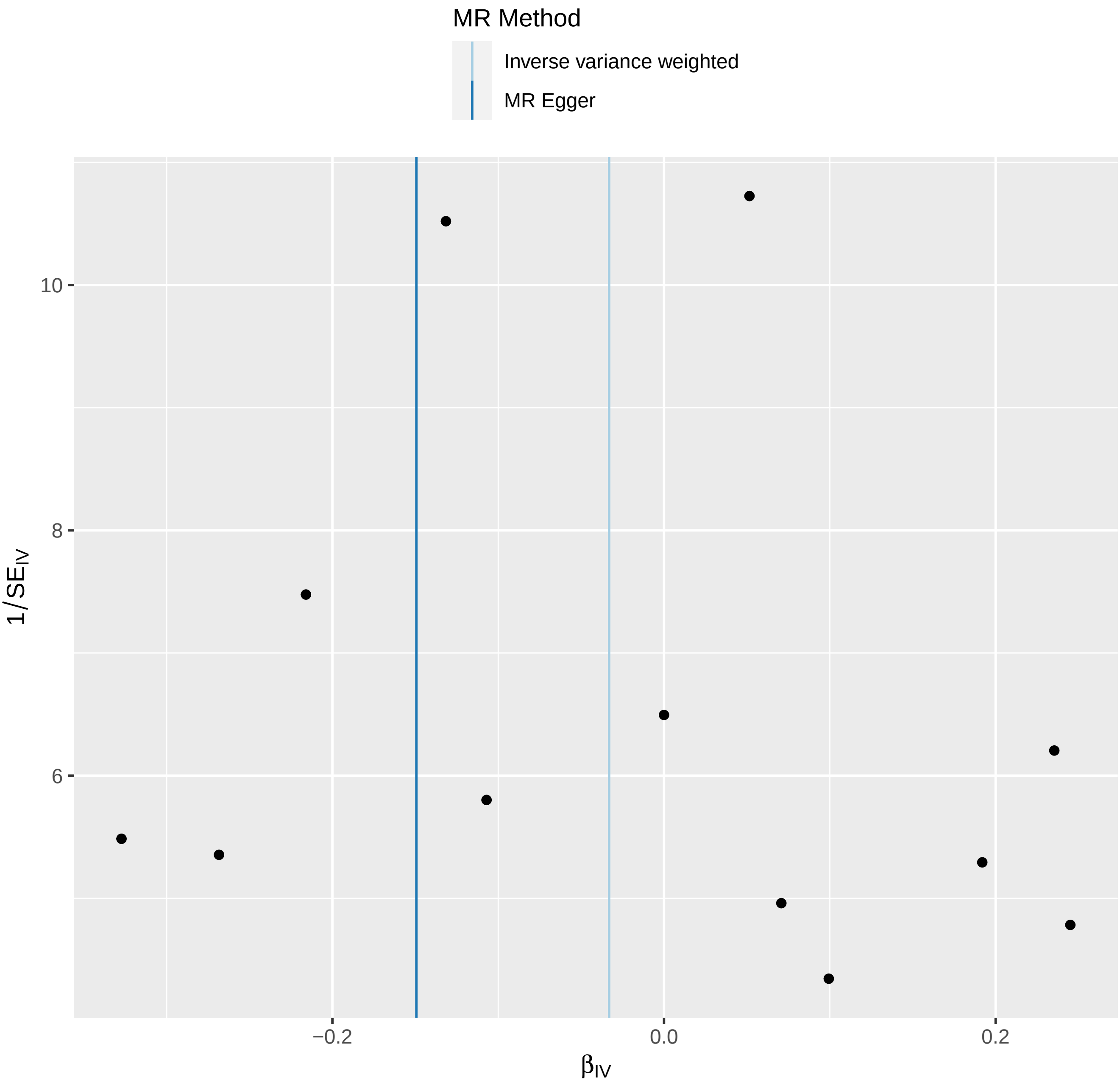

Batch 3−233 : Selenium on B−cell receptor−associated protein 29

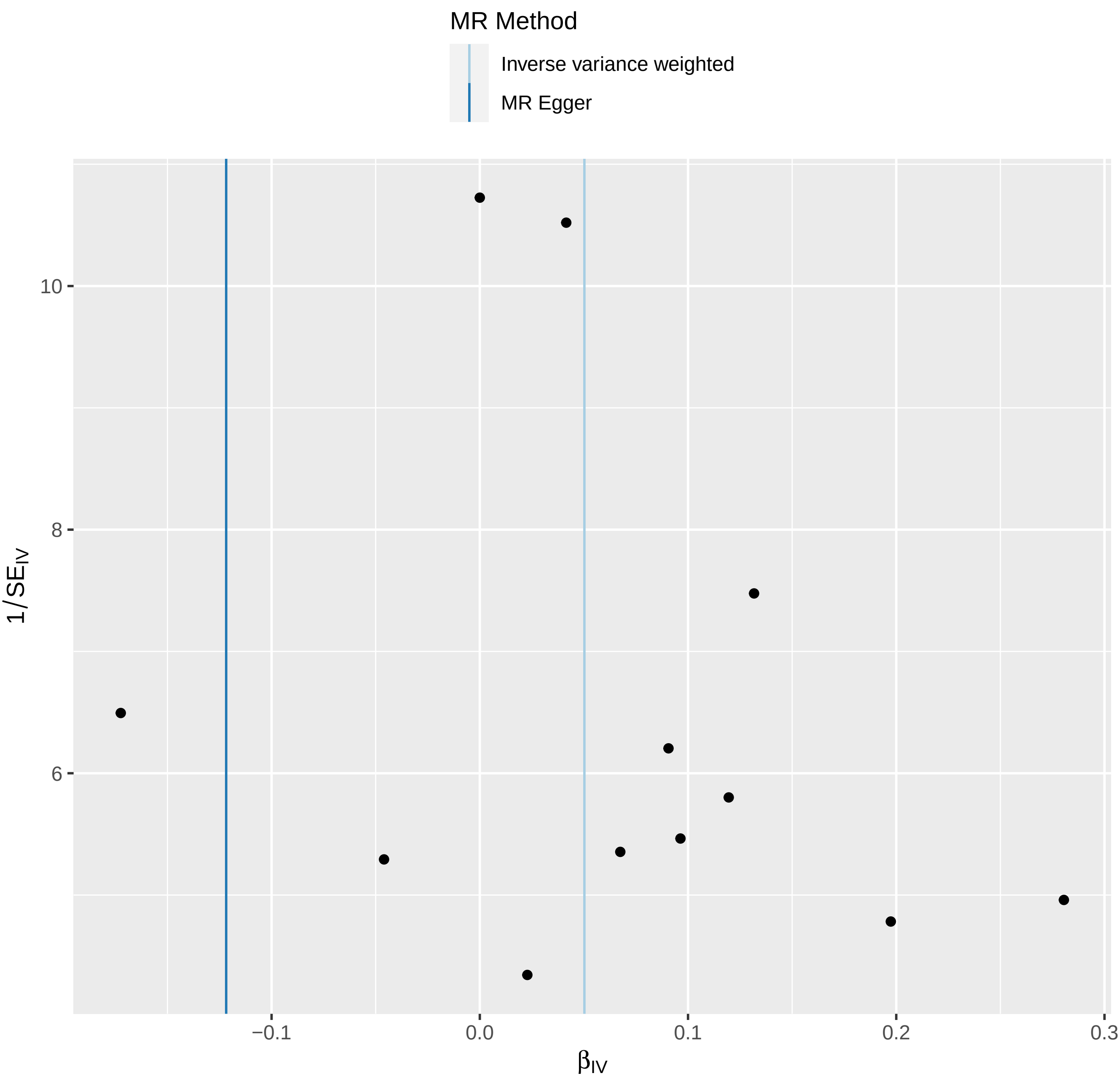

Batch 3−234 : Selenium on Breast cancer anti−estrogen resistance protein 3

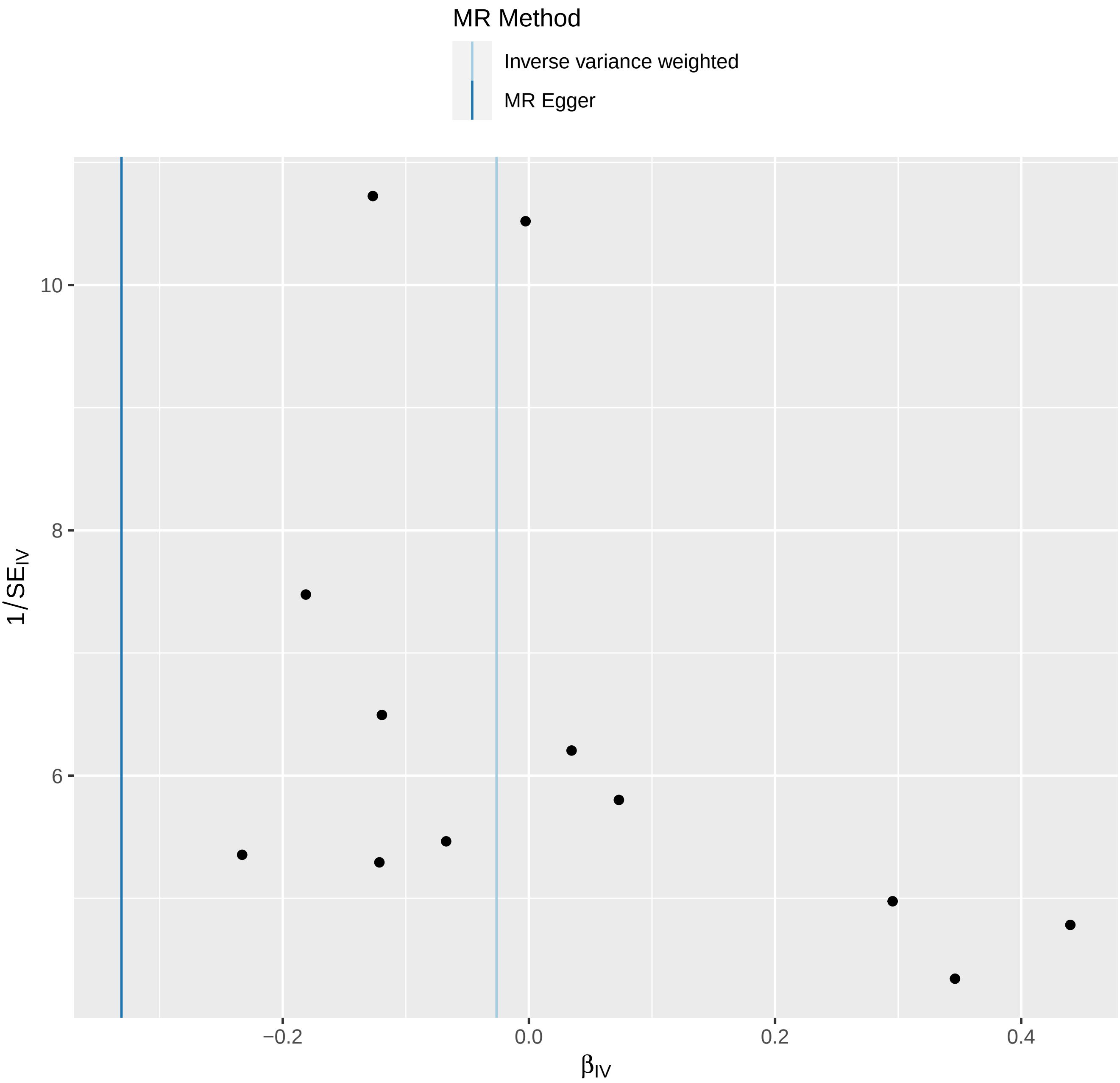

Batch 3−235 : Selenium on B−cell lymphoma/leukemia 10

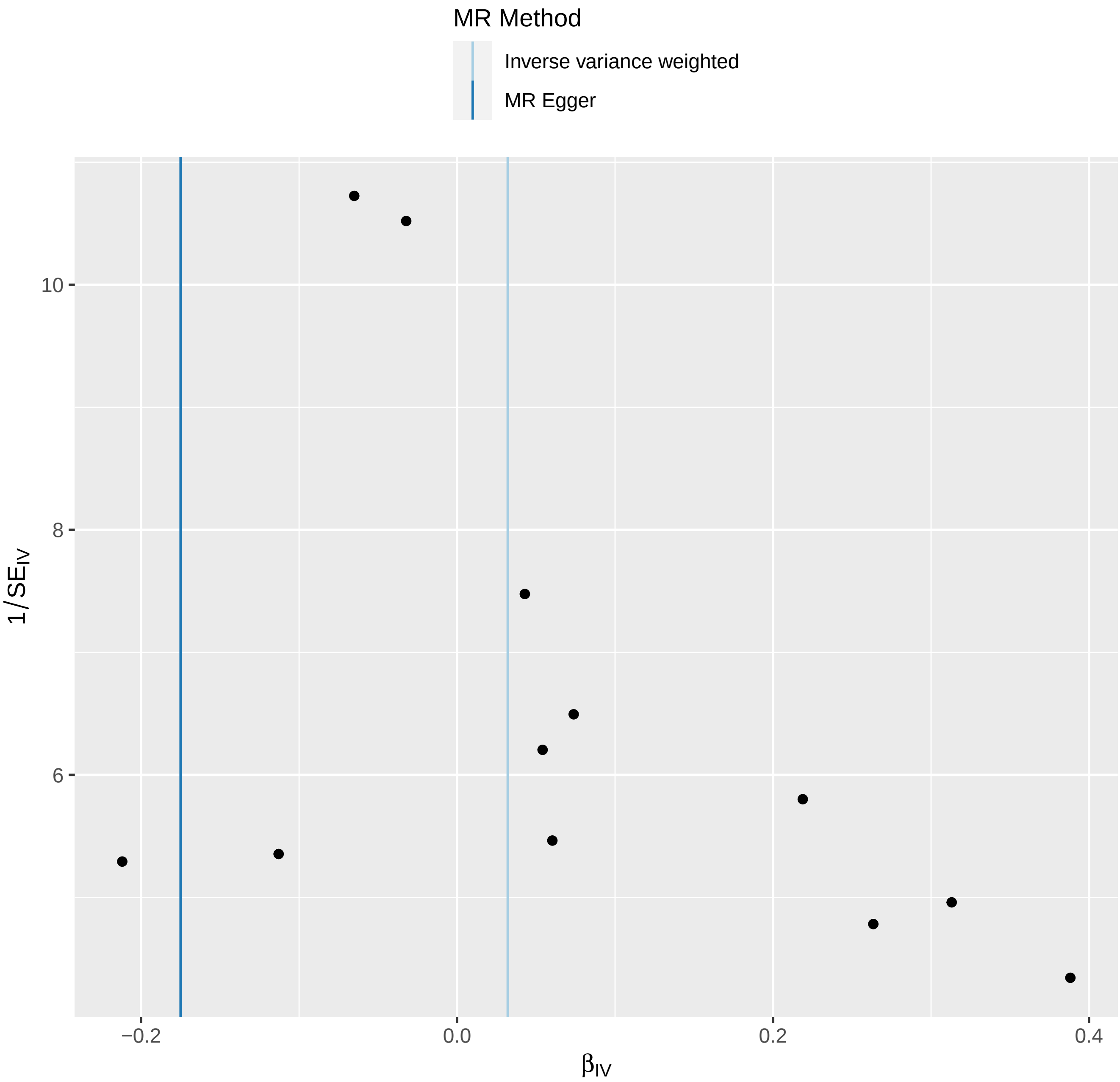

Batch 3−236 : Selenium on Apoptosis regulator Bcl−2

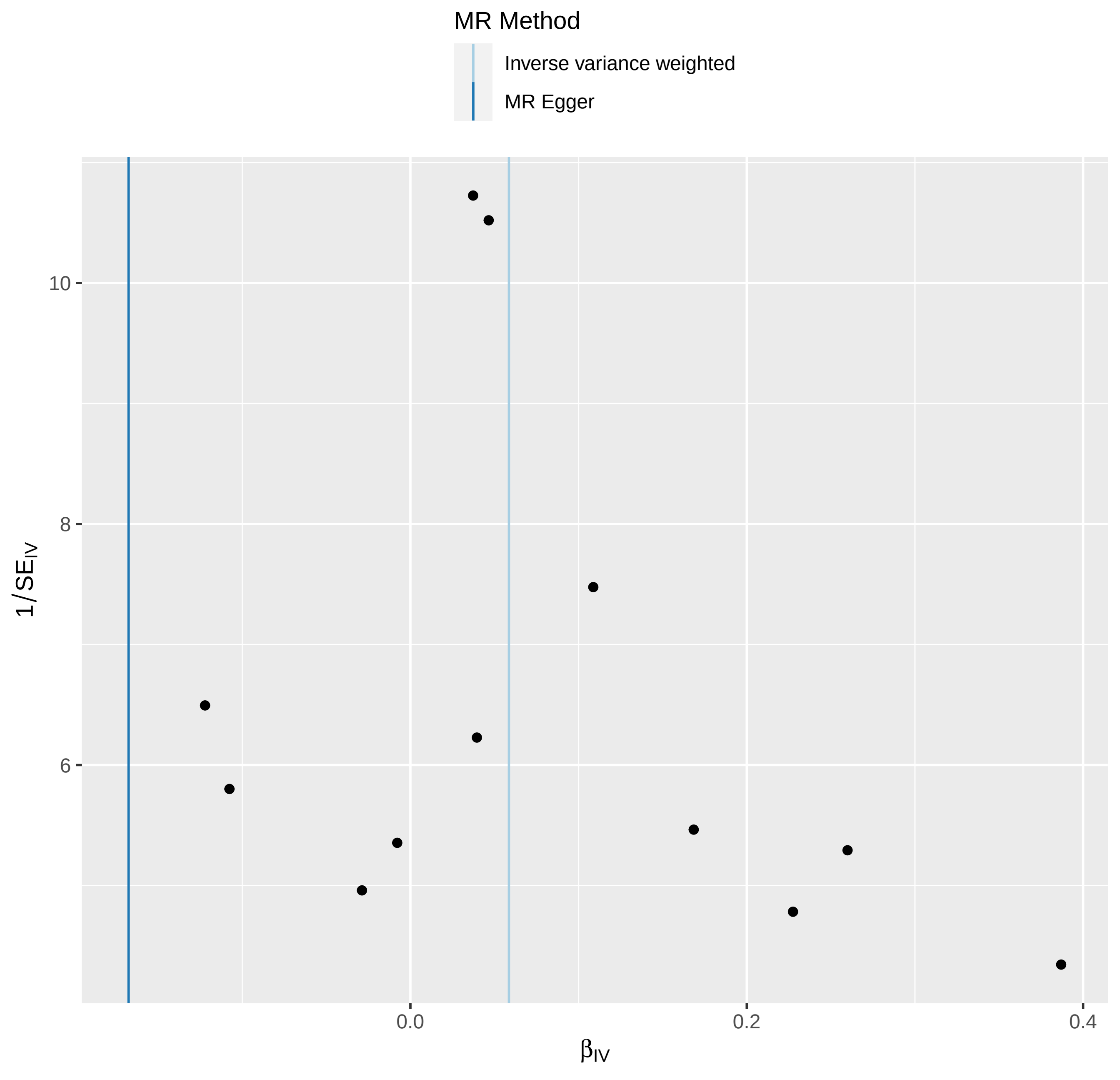

Batch 3−237 : Selenium on Bcl−2−related protein A1

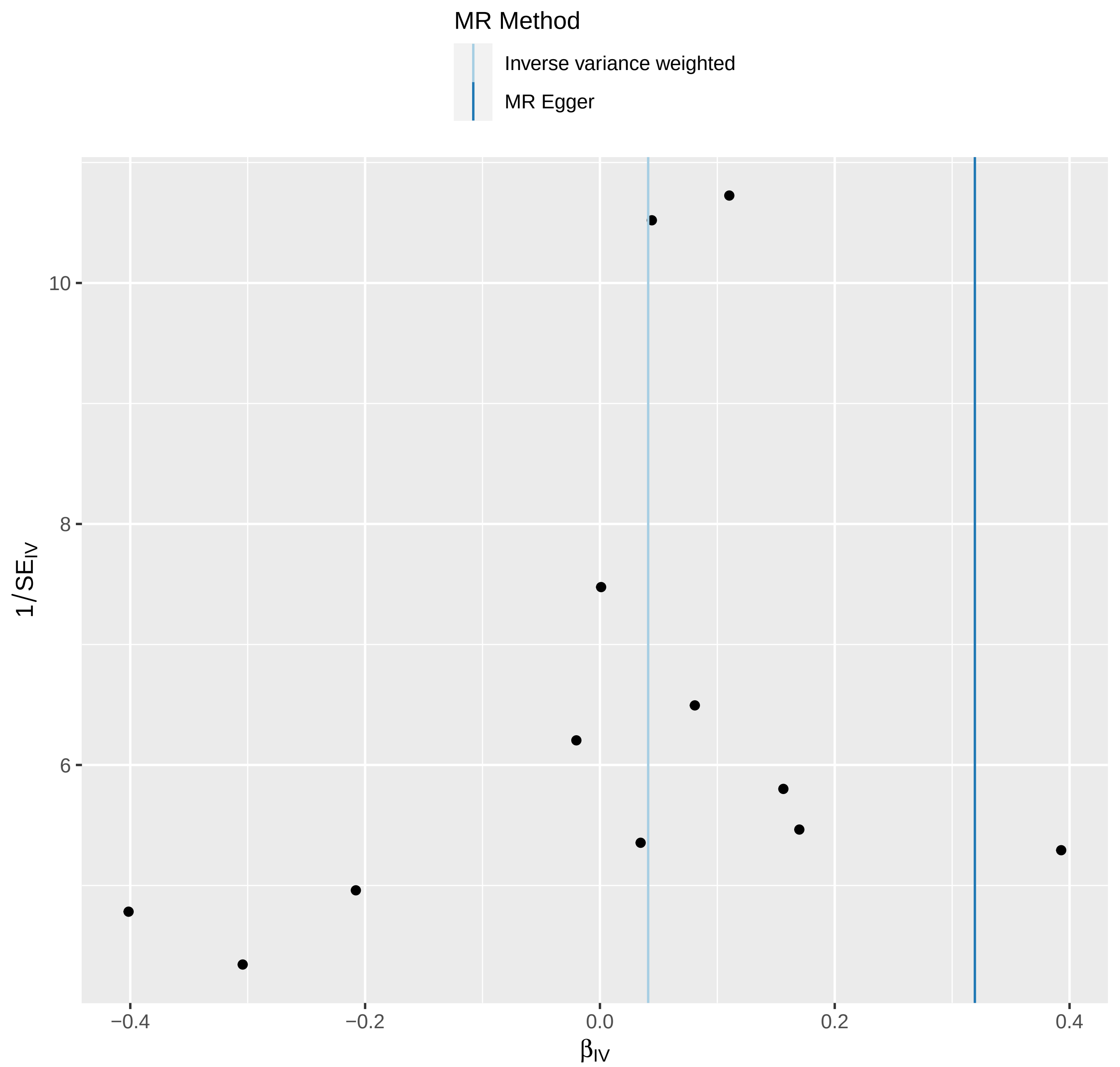

Batch 3−238 : Selenium on Bcl−2−like protein 1

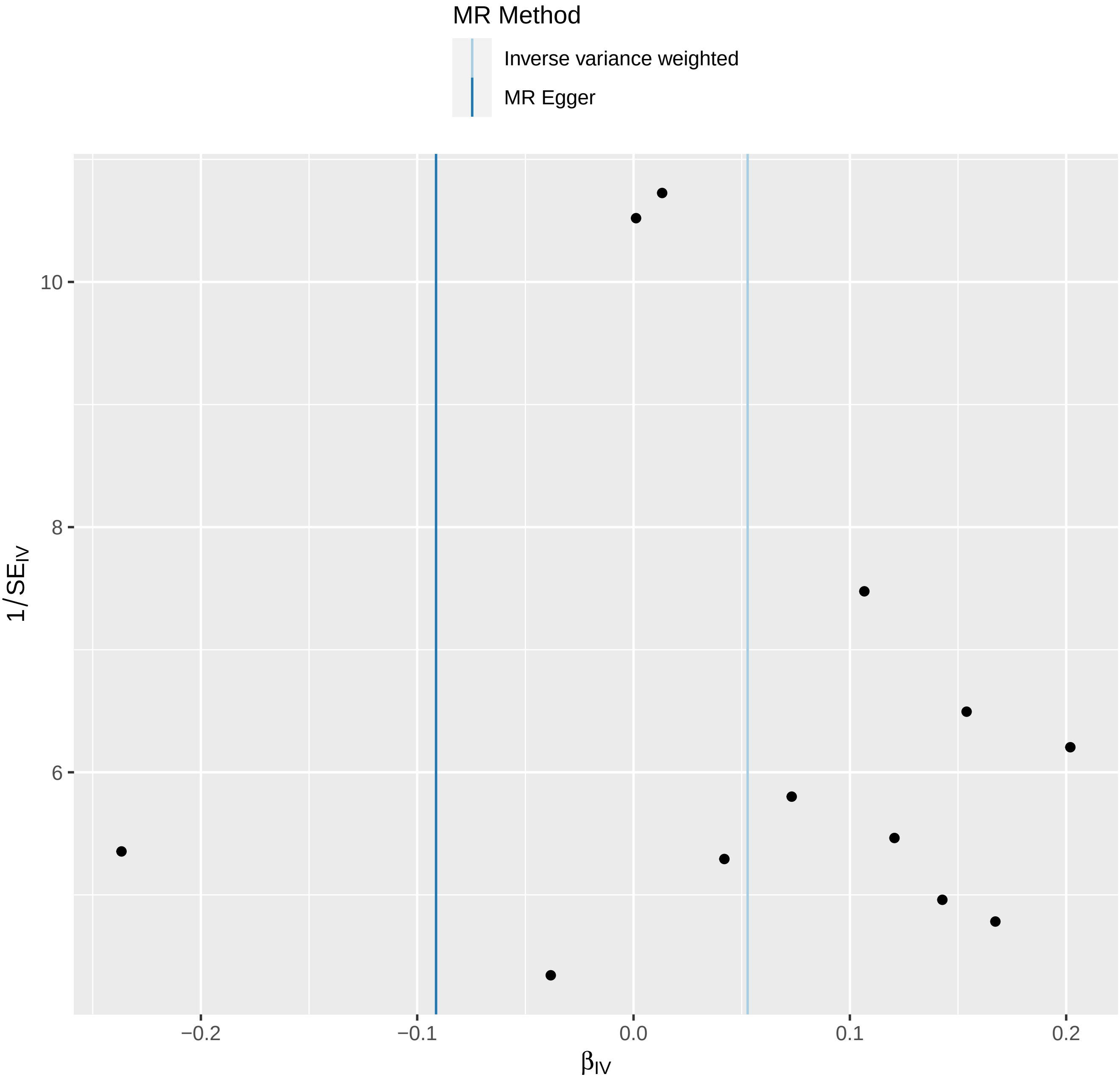

Batch 3−239 : Selenium on Bcl−2−like protein 2

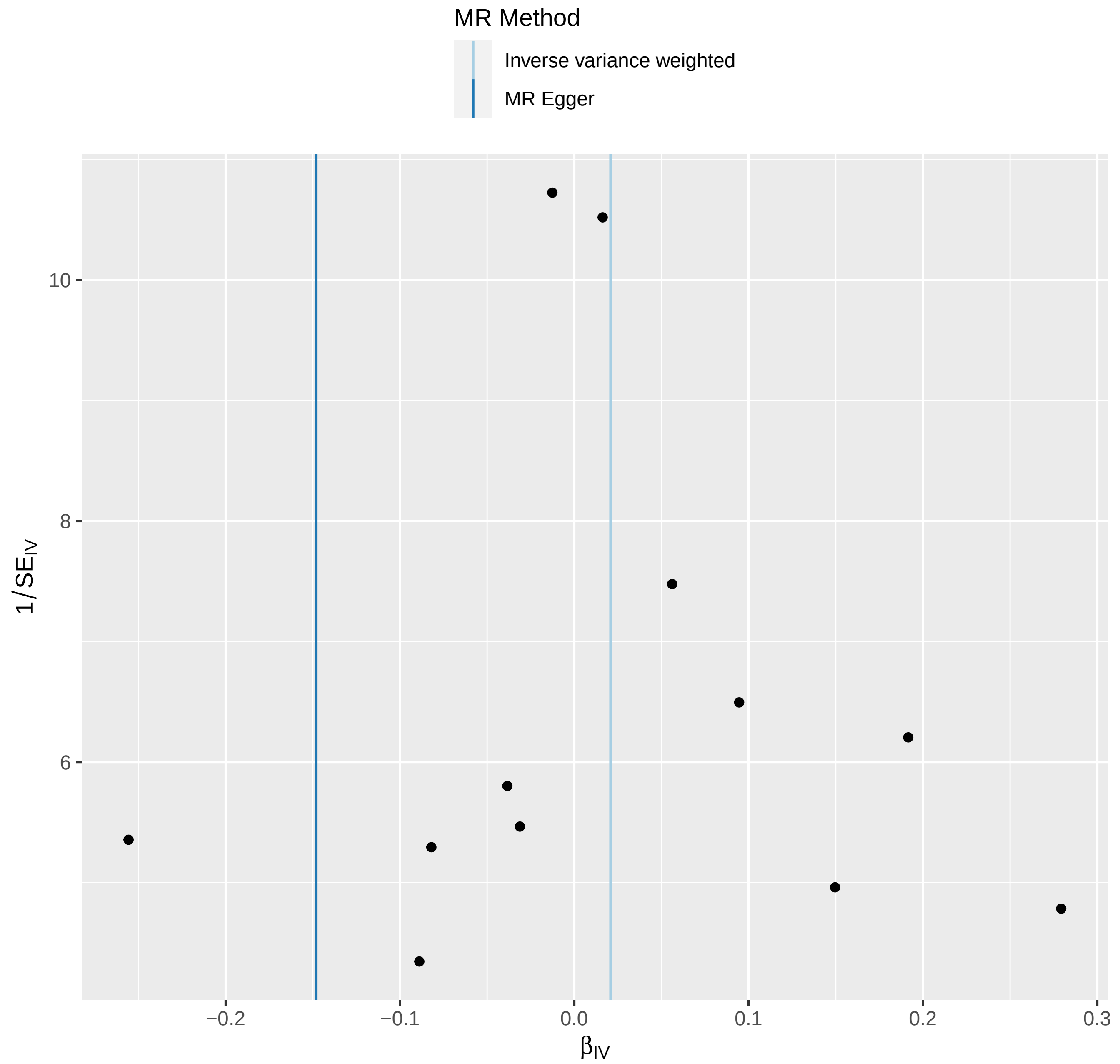

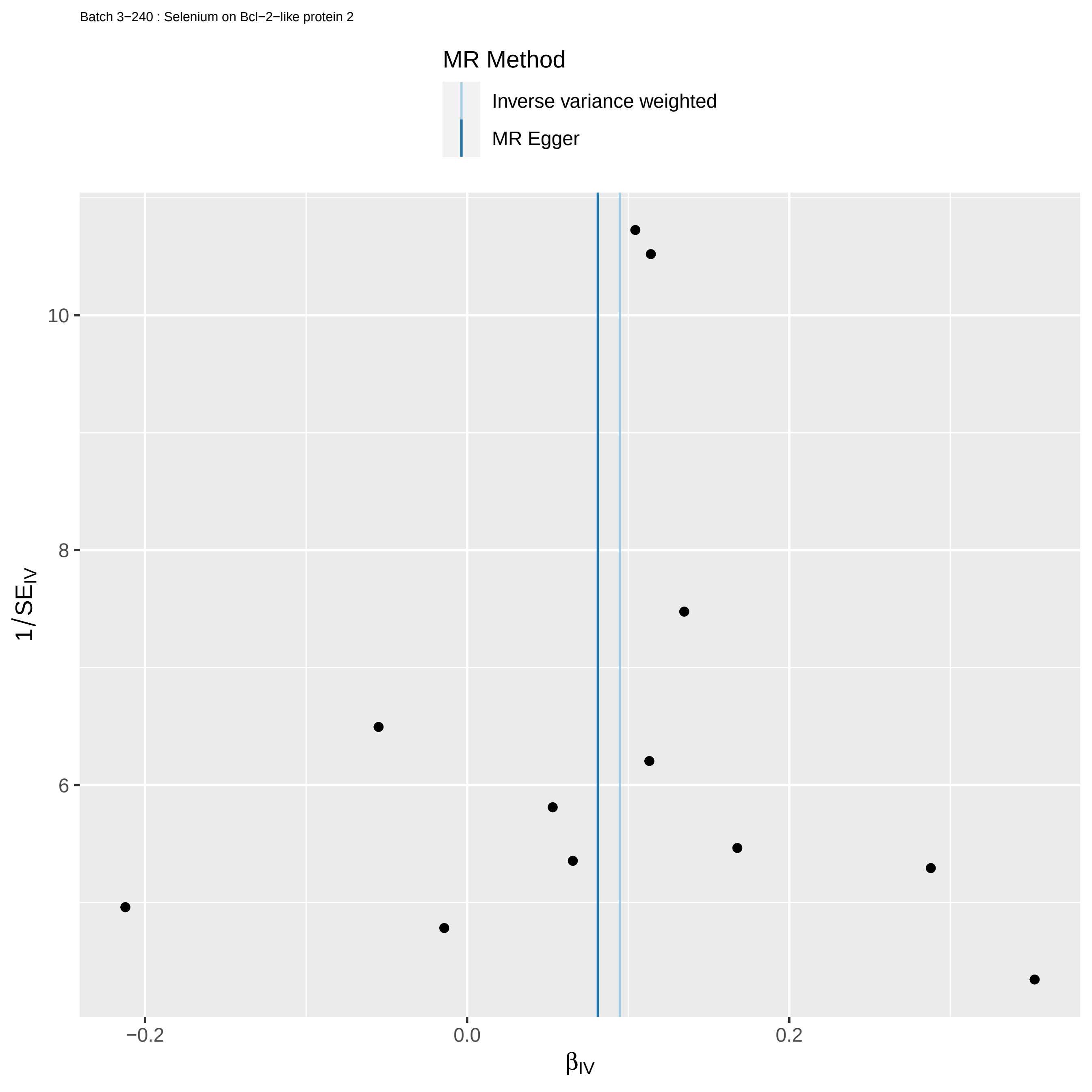
Batch 3−240 : Selenium on Bcl−2−like protein 2
MR Method
Inverse variance weighted
MR Egger
10
8
6
1/SE_IV
−0.2
0.0
0.2
β_IV

Batch 3−241 : Selenium on B−cell lymphoma 6 protein

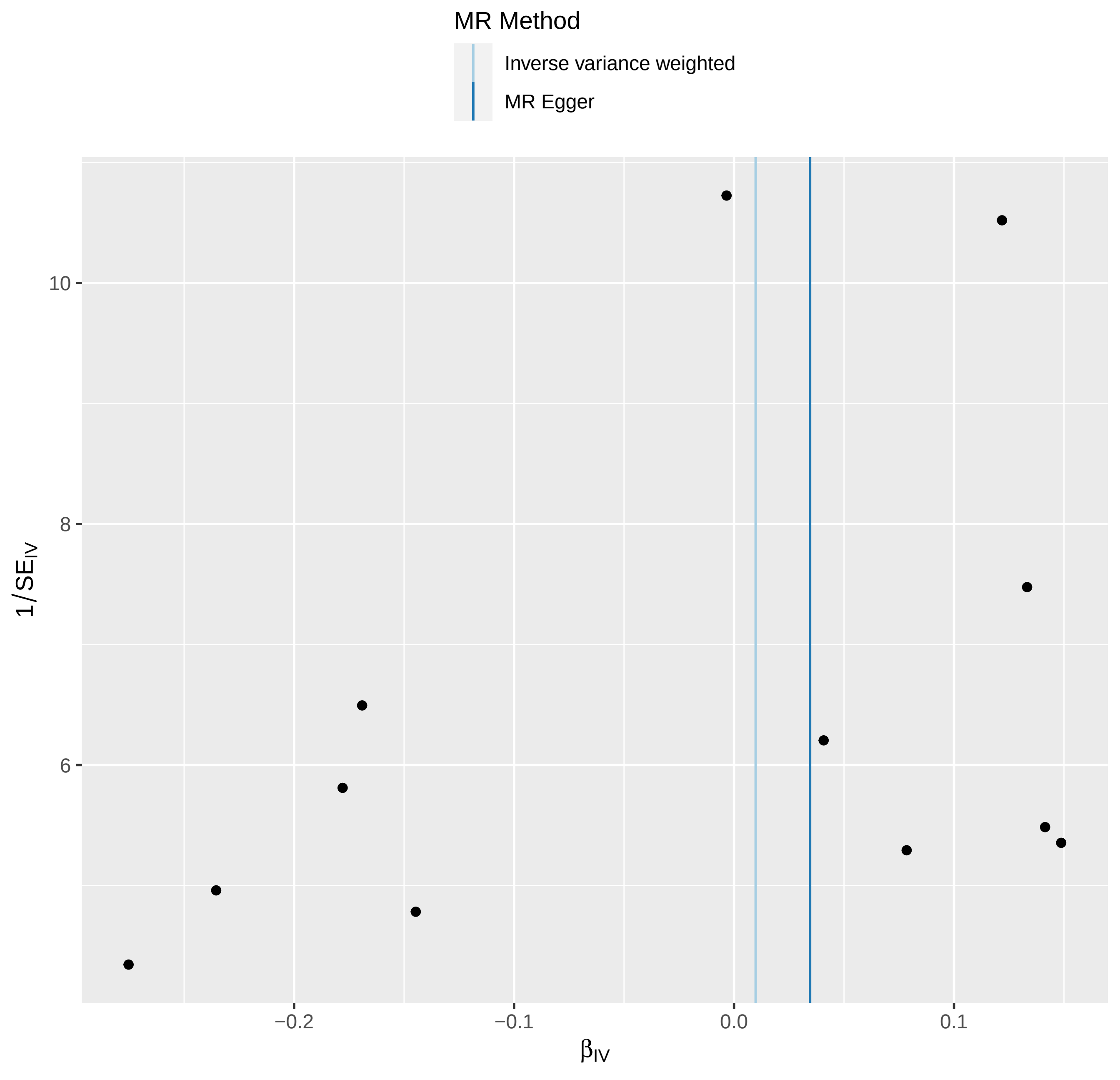

Batch 3−242 : Selenium on Brain−derived neurotrophic factor

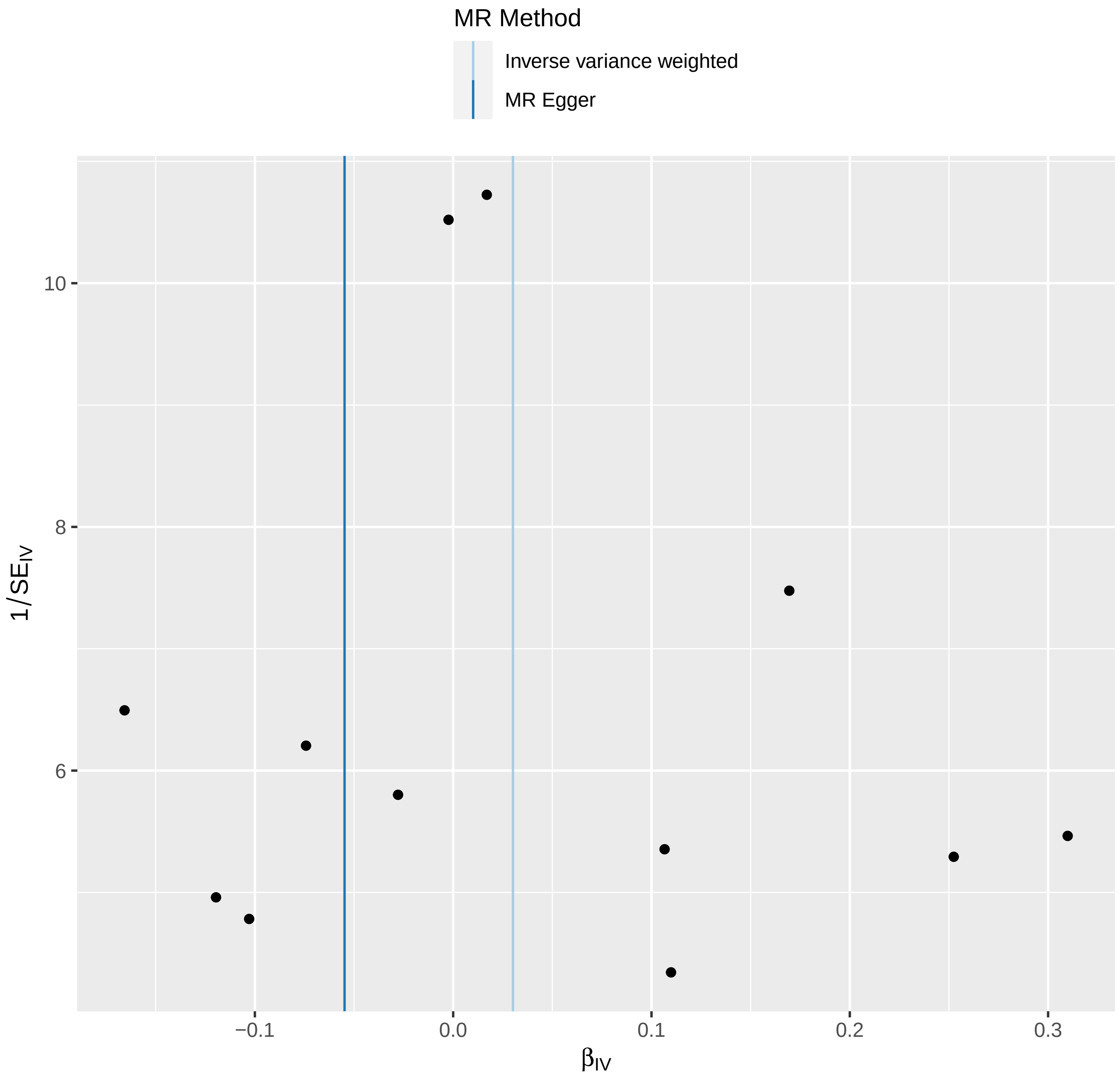

Batch 3−243 : Selenium on Transcription factor TFIIIB component B" homolog

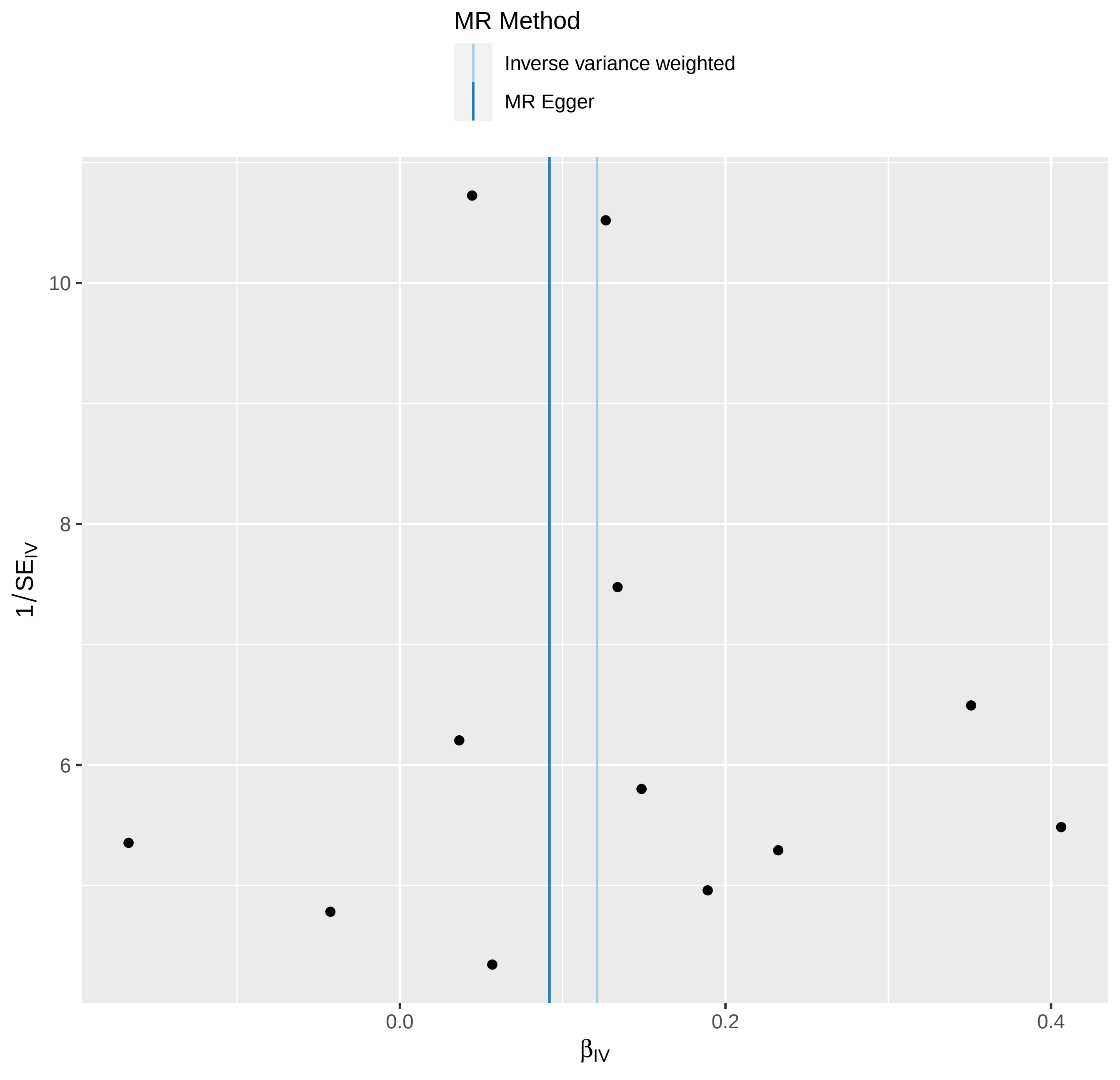

Batch 3−244 : Selenium on Beclin−1

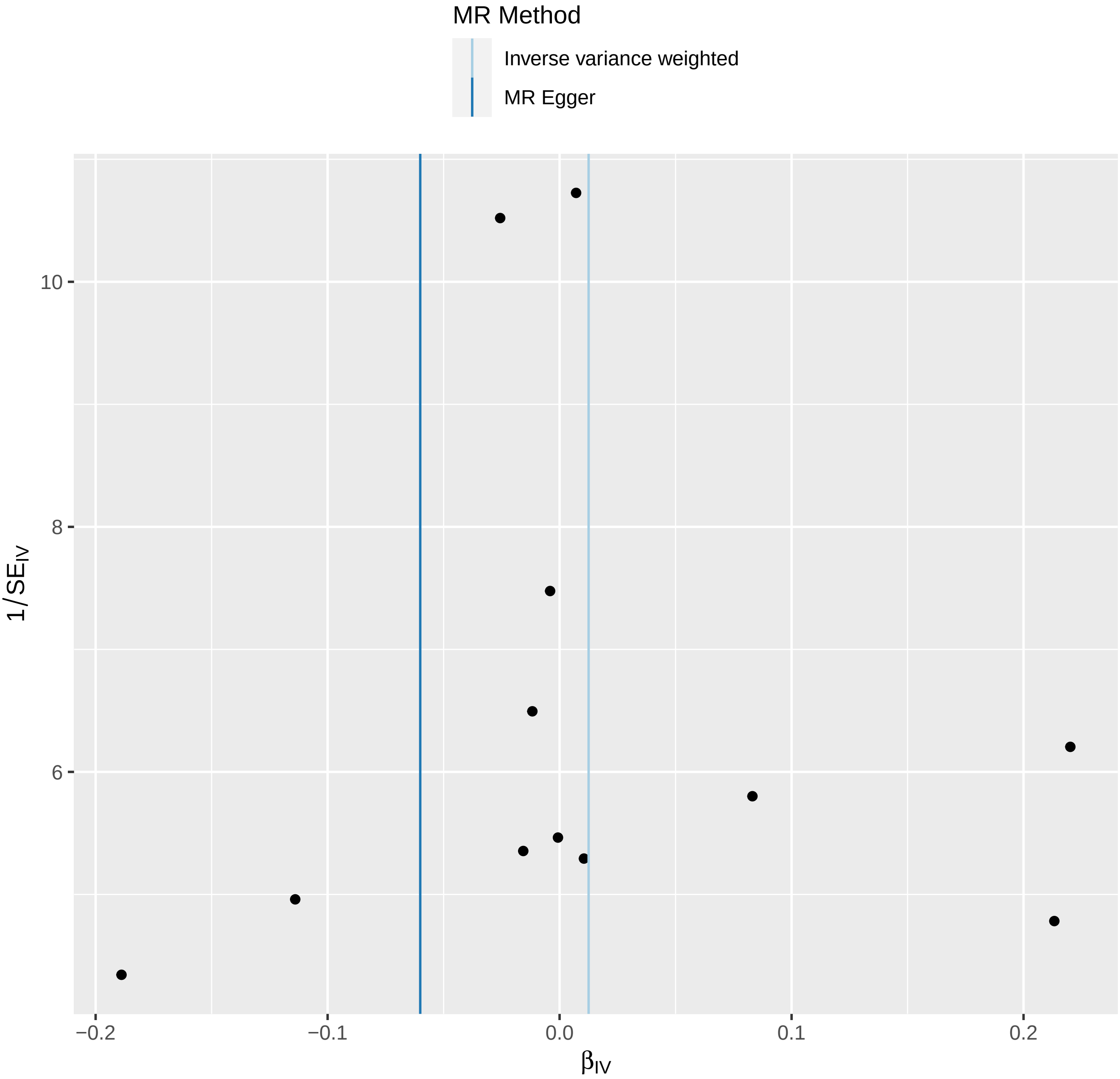

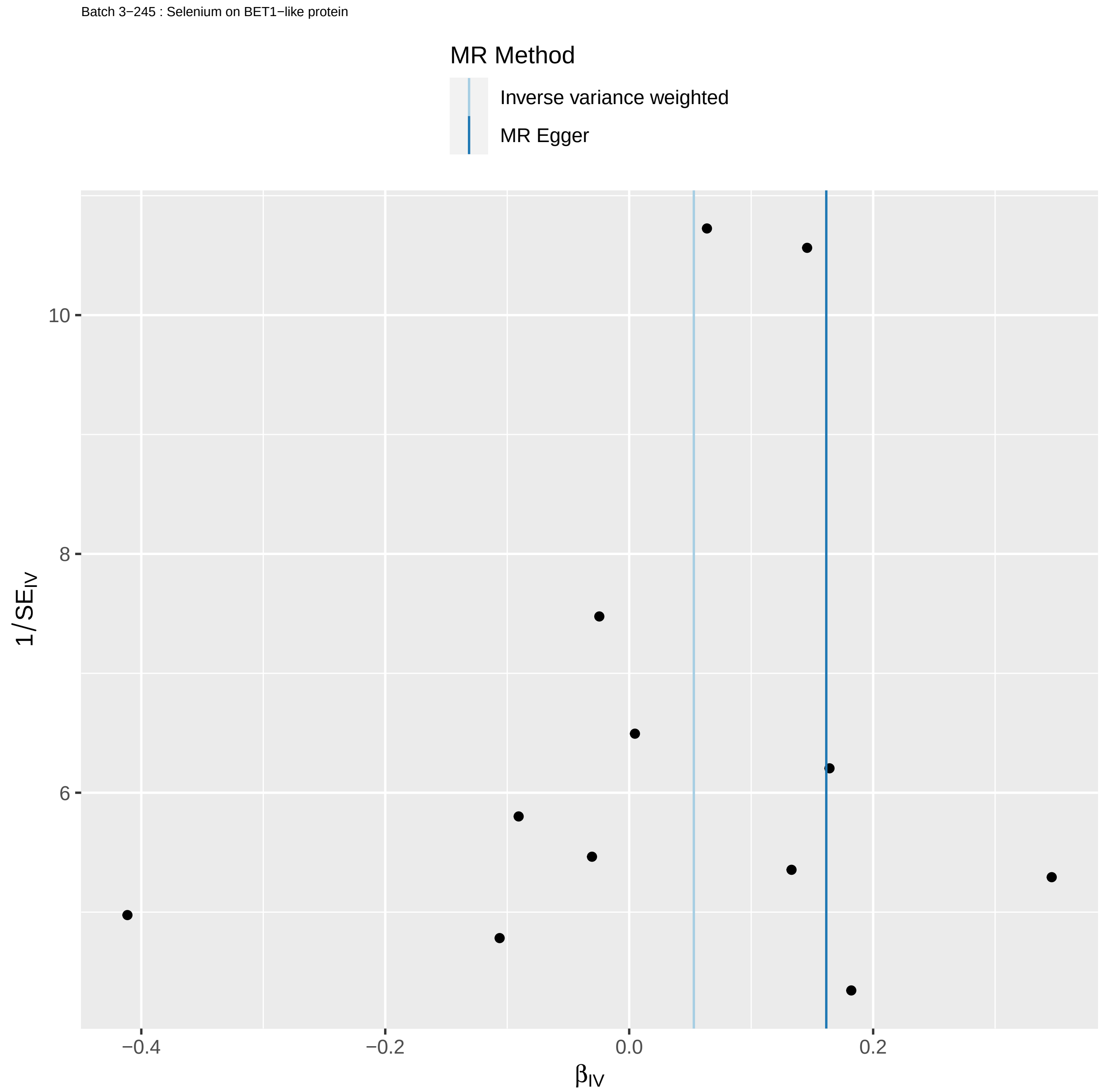
Batch 3−245 : Selenium on BET1−like protein
MR Method
Inverse variance weighted
MR Egger
10
8
6
1/SE_IV
−0.4
−0.2
0.0
0.2
β_IV

Batch 3−246 : Selenium on Osteocalcin

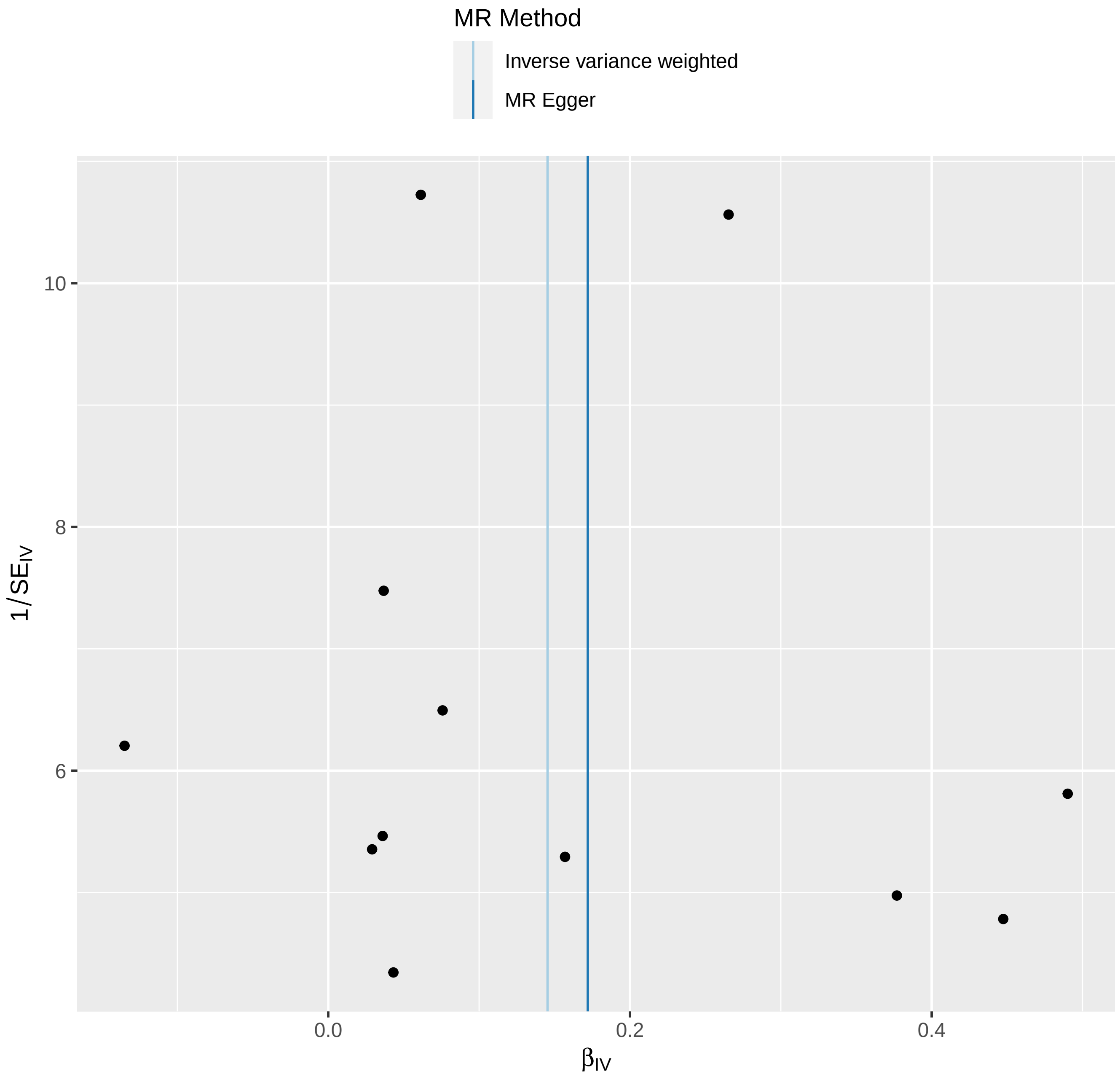

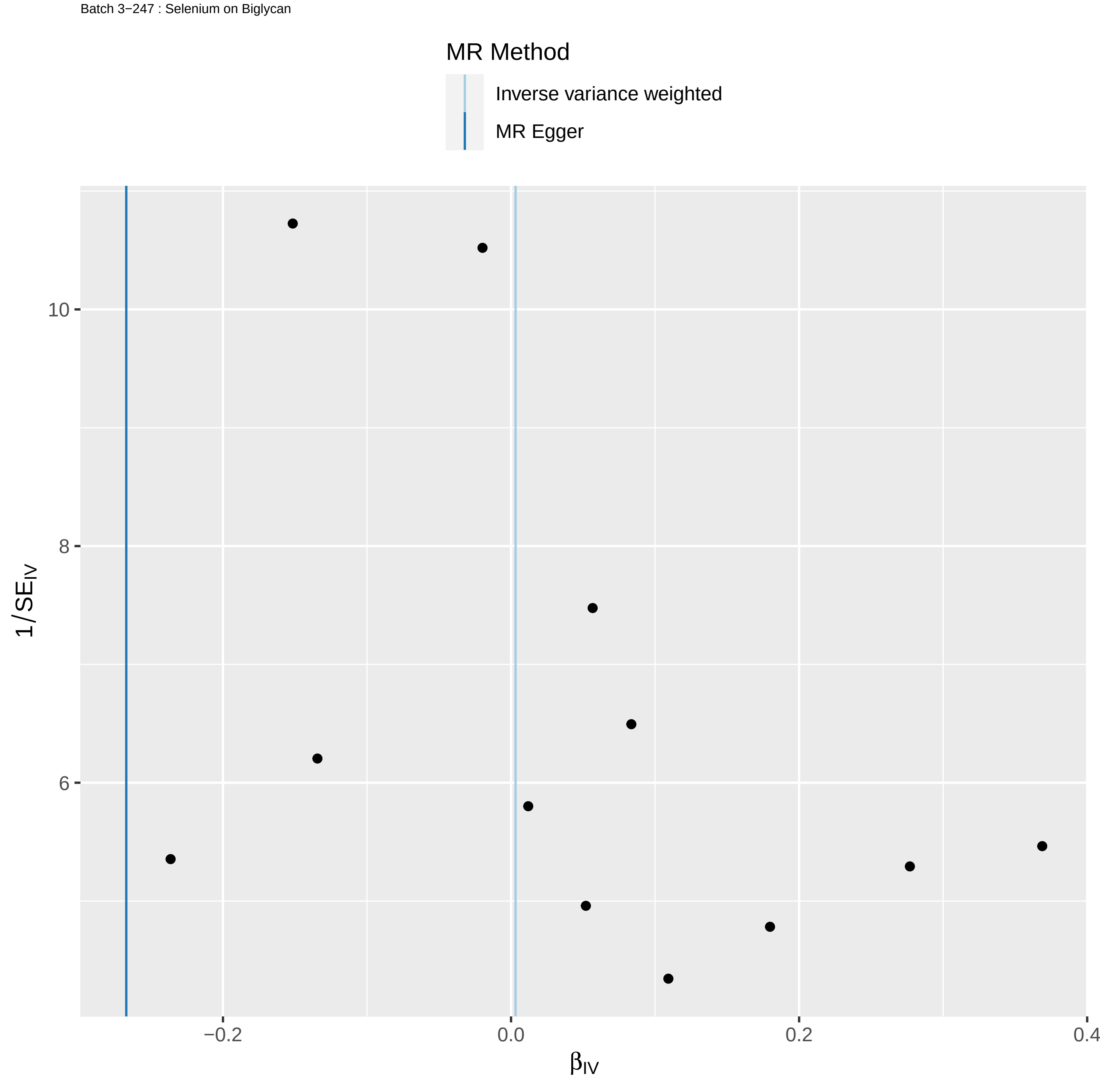

Batch 3−247 : Selenium on Biglycan
MR Method
Inverse variance weighted
MR Egger
10
8
6
1/SE_IV
−0.2
0.0
0.2
0.4
β_IV

Batch 3−248 : Selenium on Biglycan

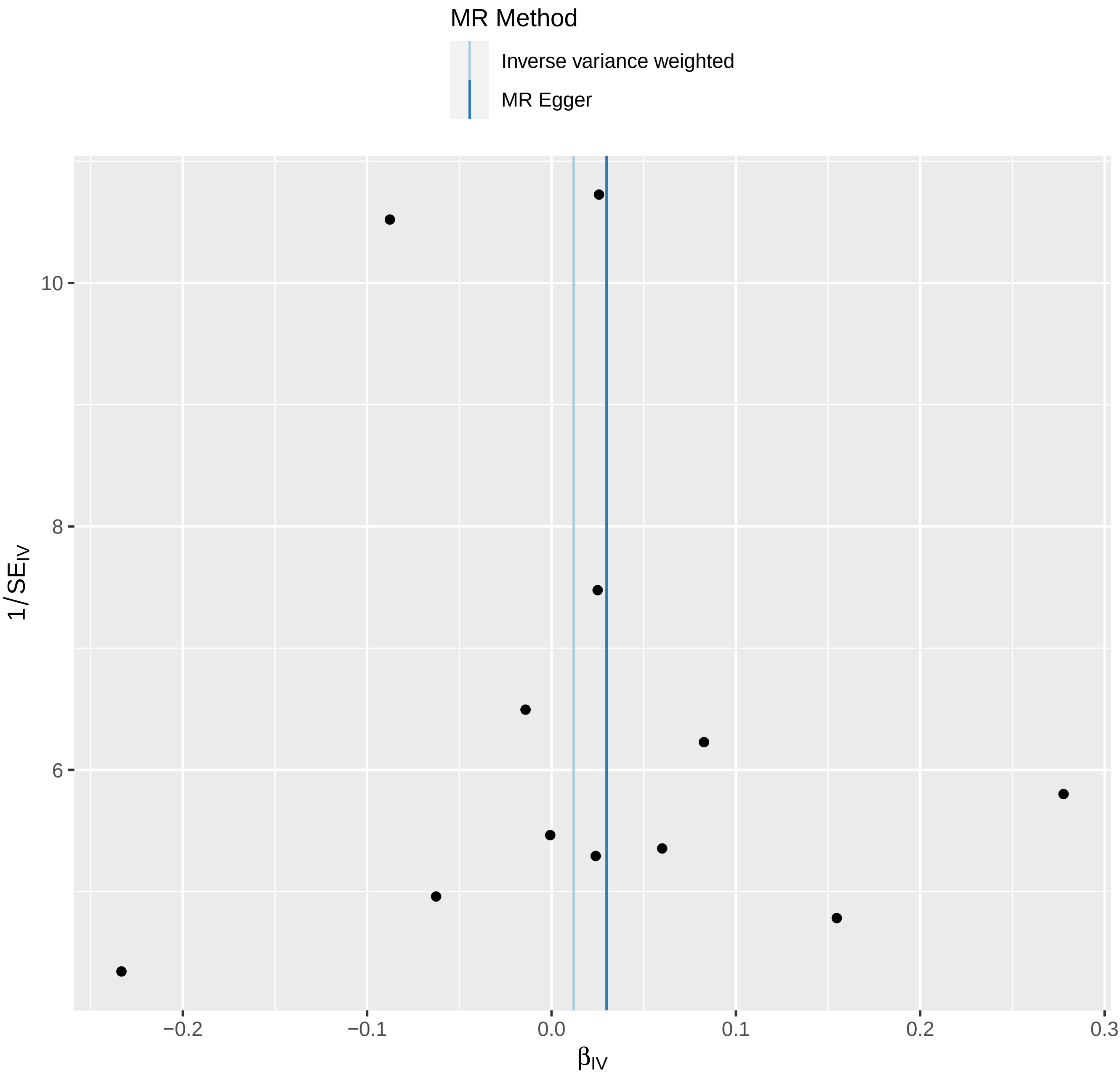

Batch 3−249 : Selenium on BH3−interacting domain death agonist

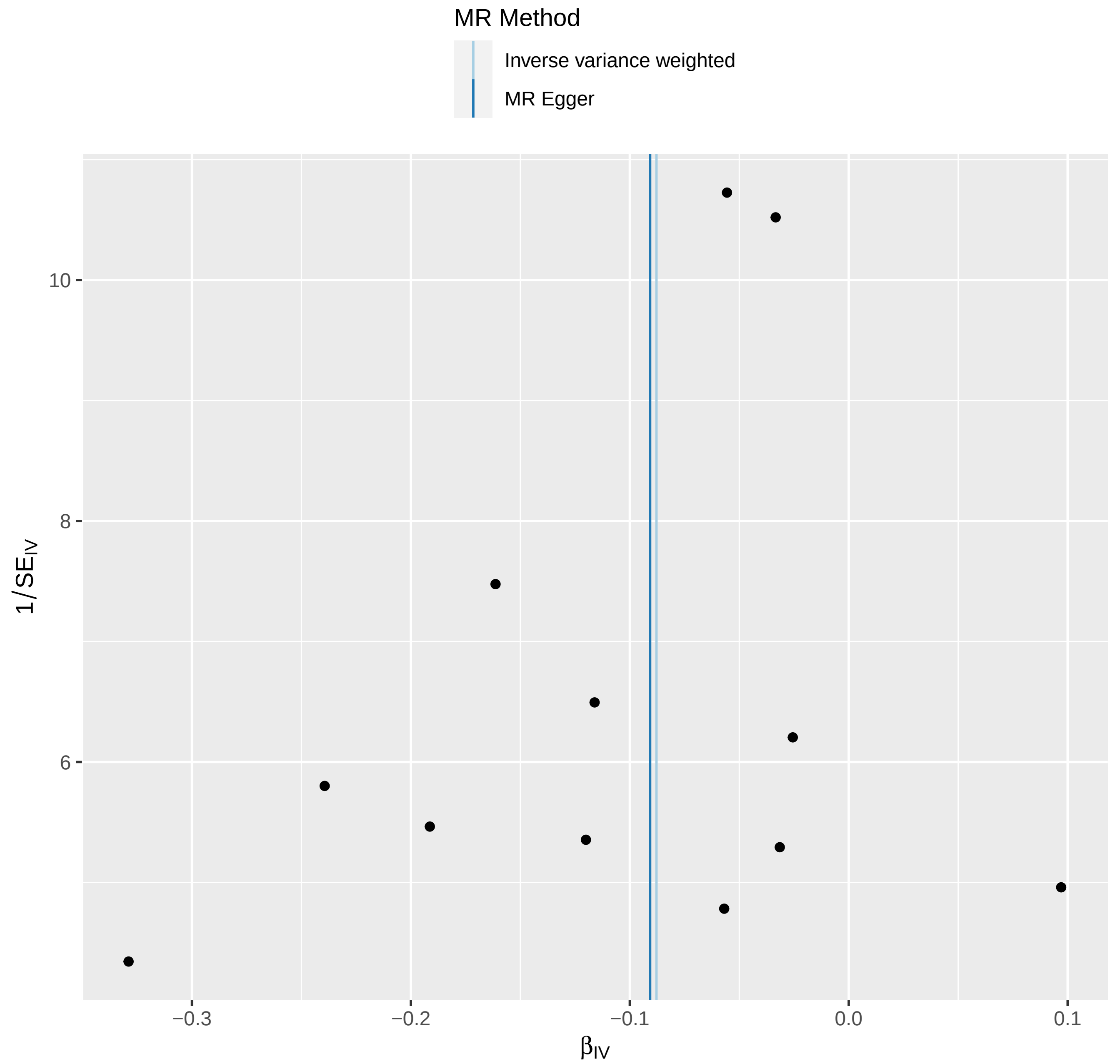

Batch 3−250 : Selenium on Myc box−dependent−interacting protein 1

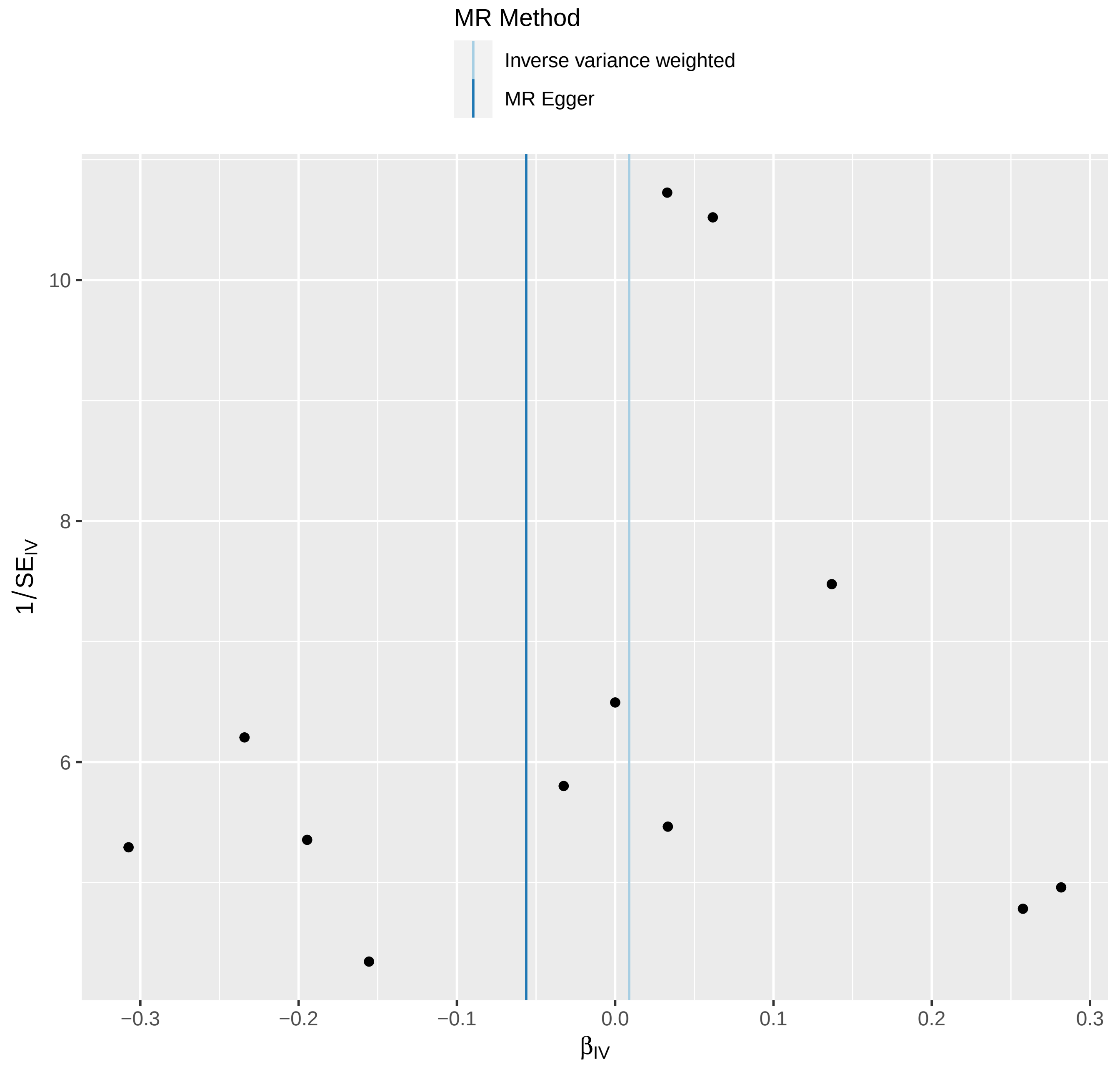

Batch 3−251 : Selenium on Baculoviral IAP repeat−containing protein 3

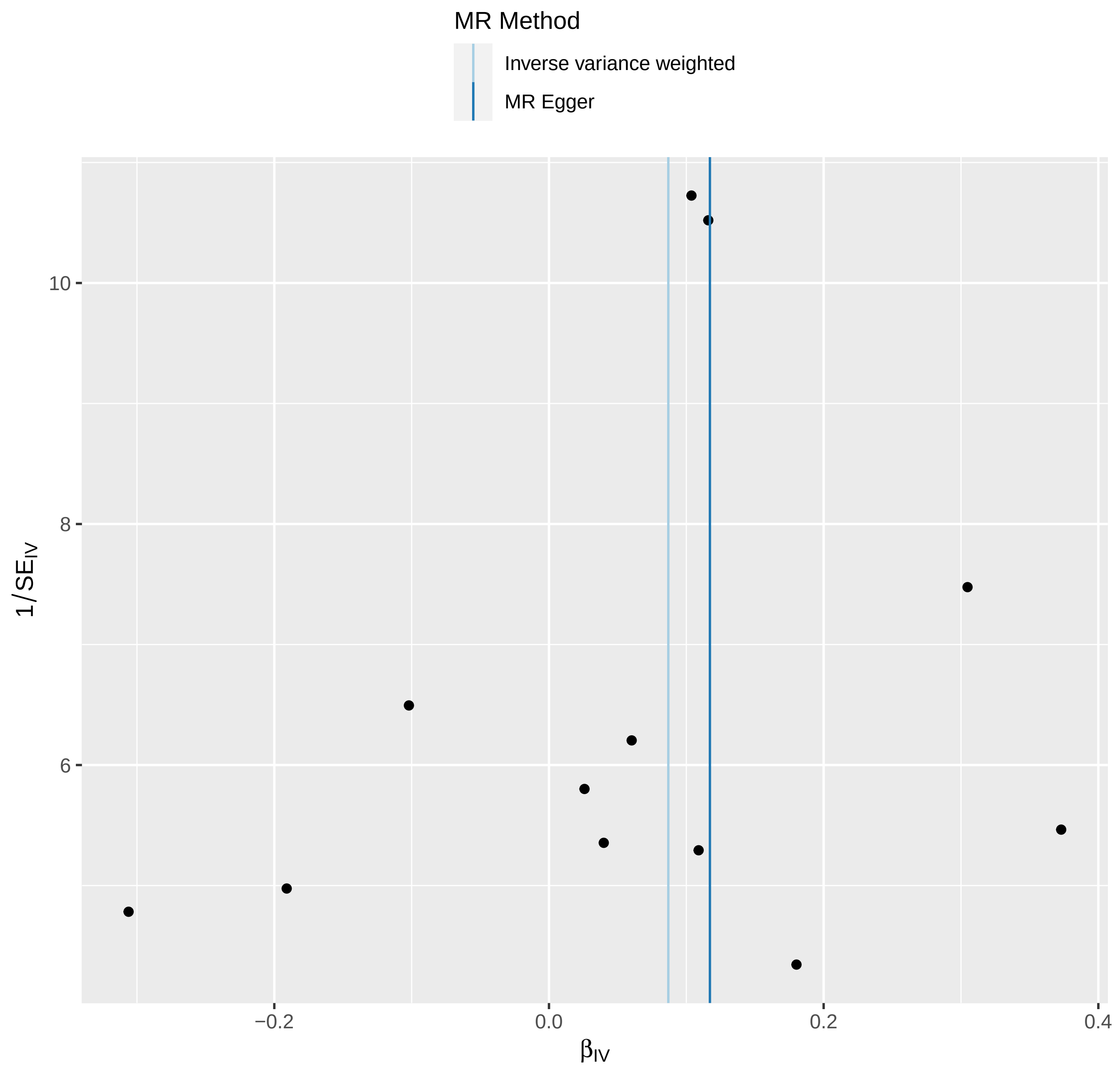

Batch 3−252 : Selenium on Baculoviral IAP repeat−containing protein 5

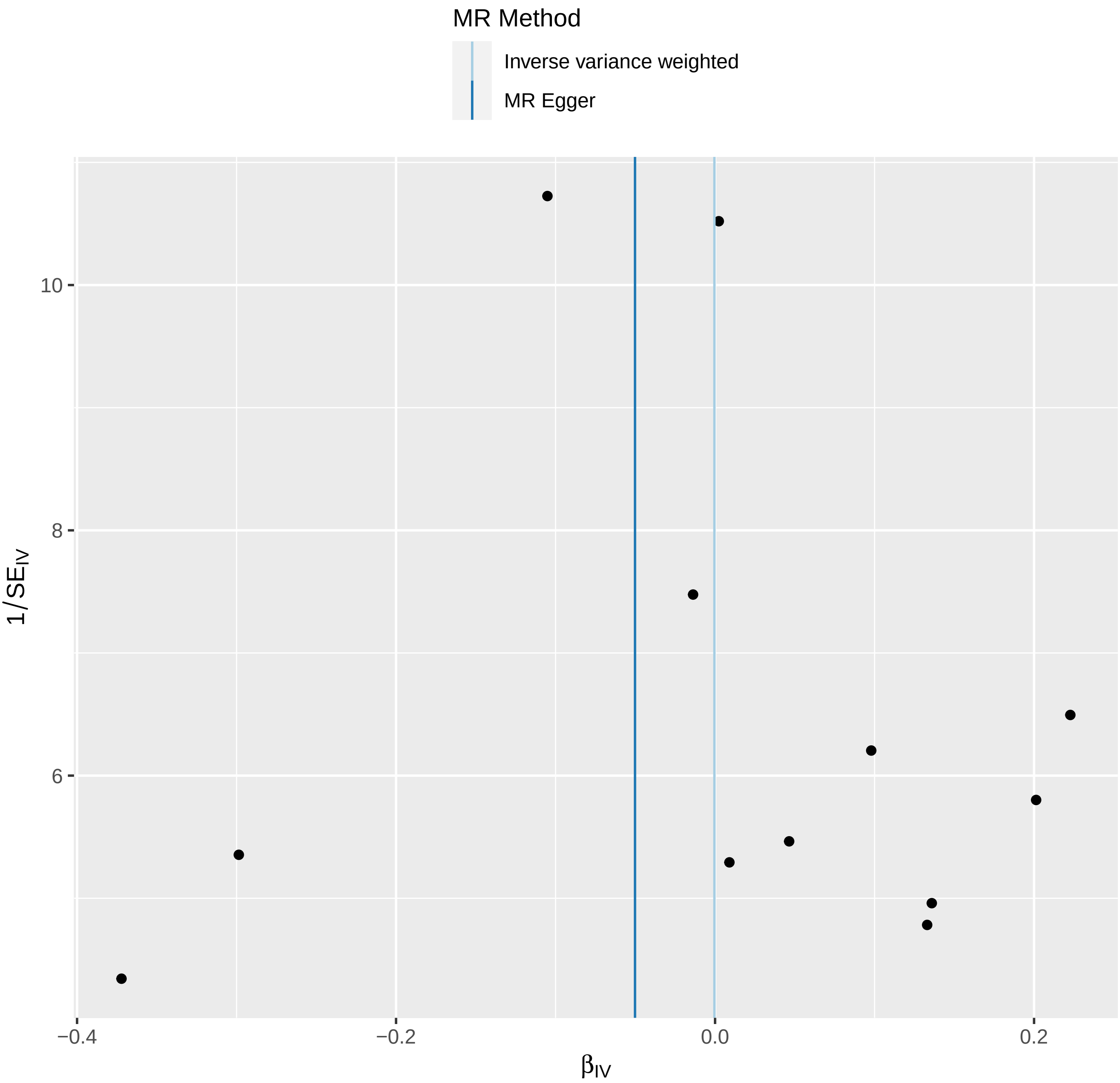

Batch 3−253 : Selenium on Biliverdin reductase A

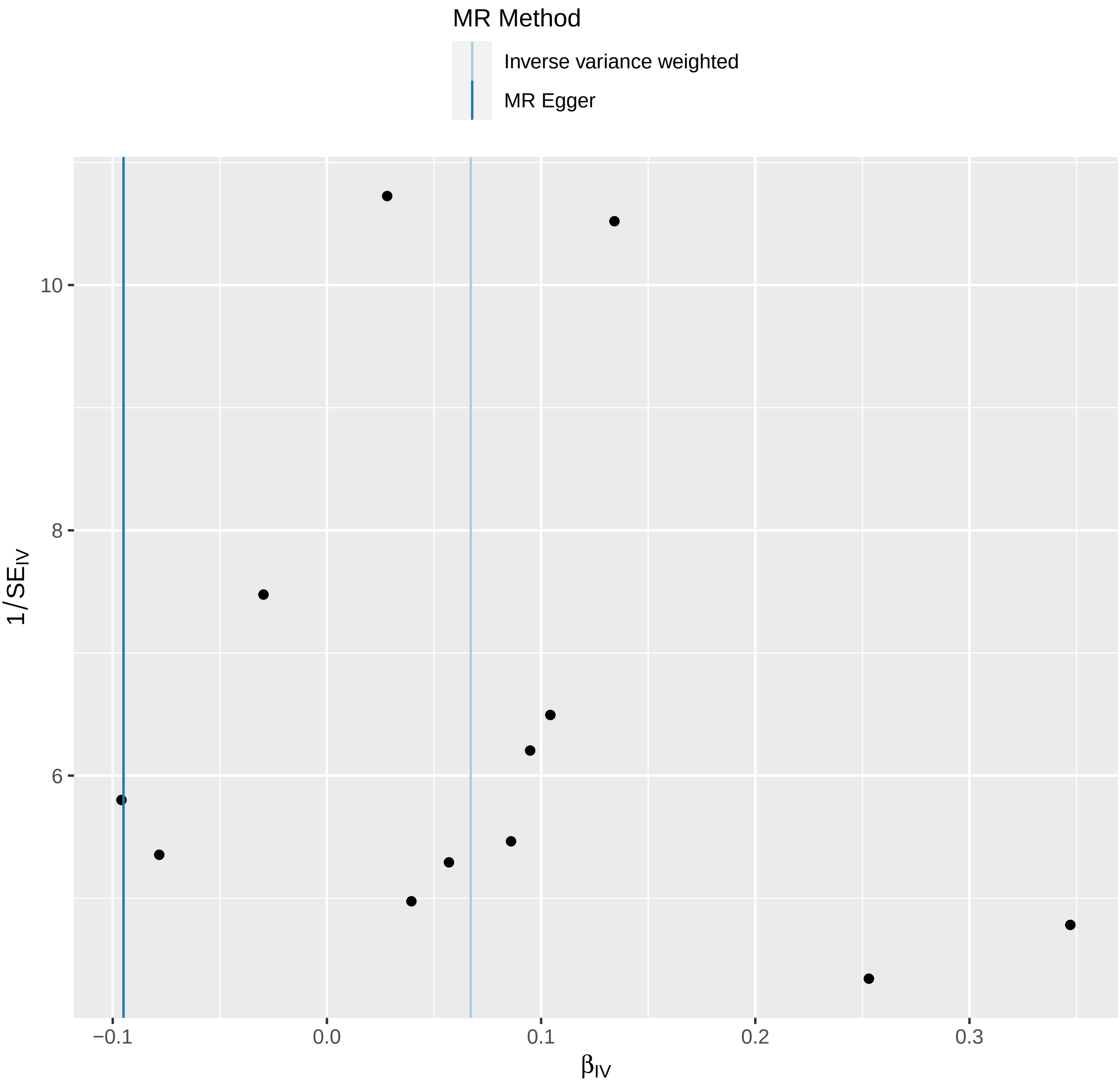

Batch 3−254 : Selenium on Bone morphogenetic protein 10

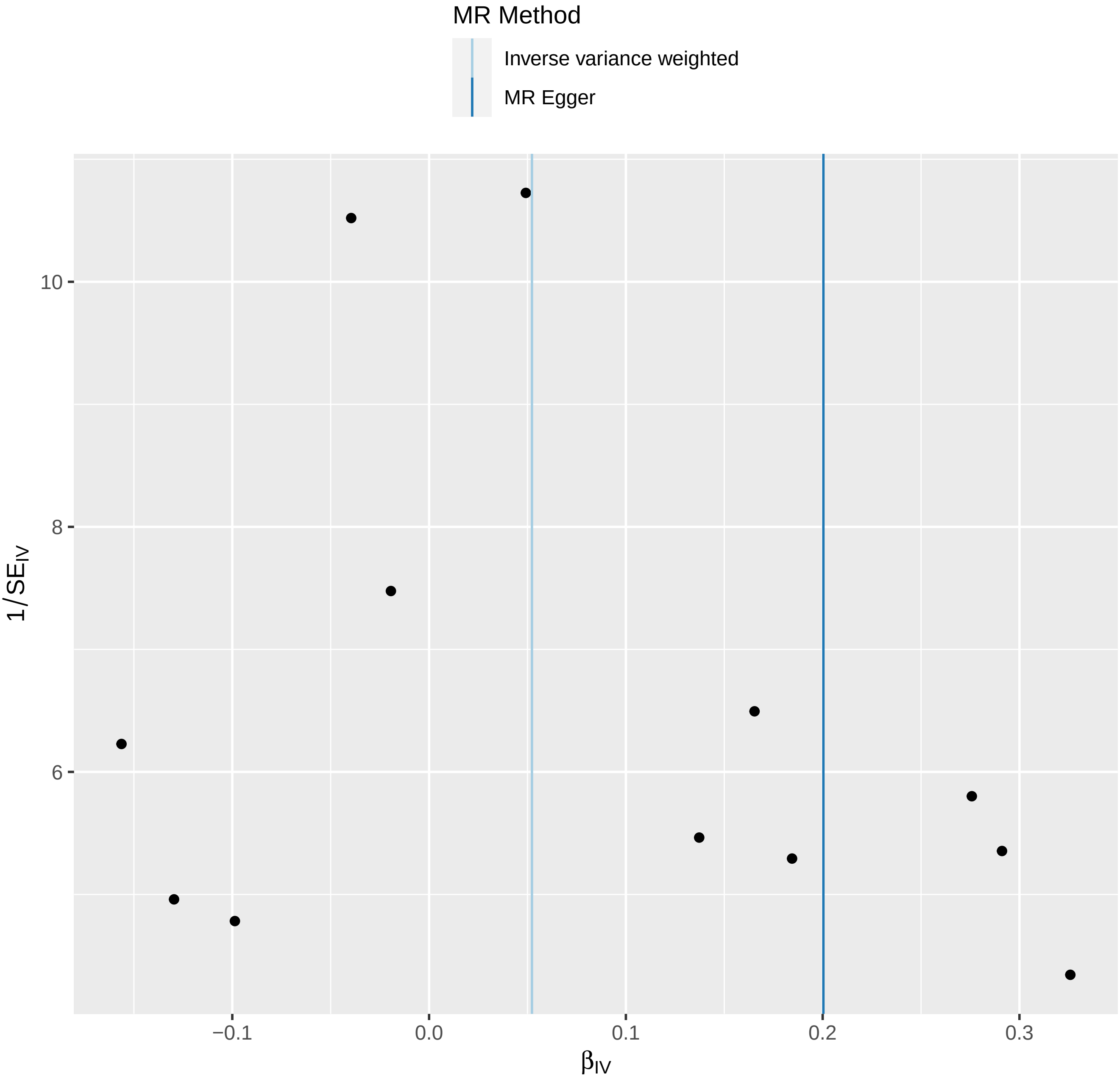

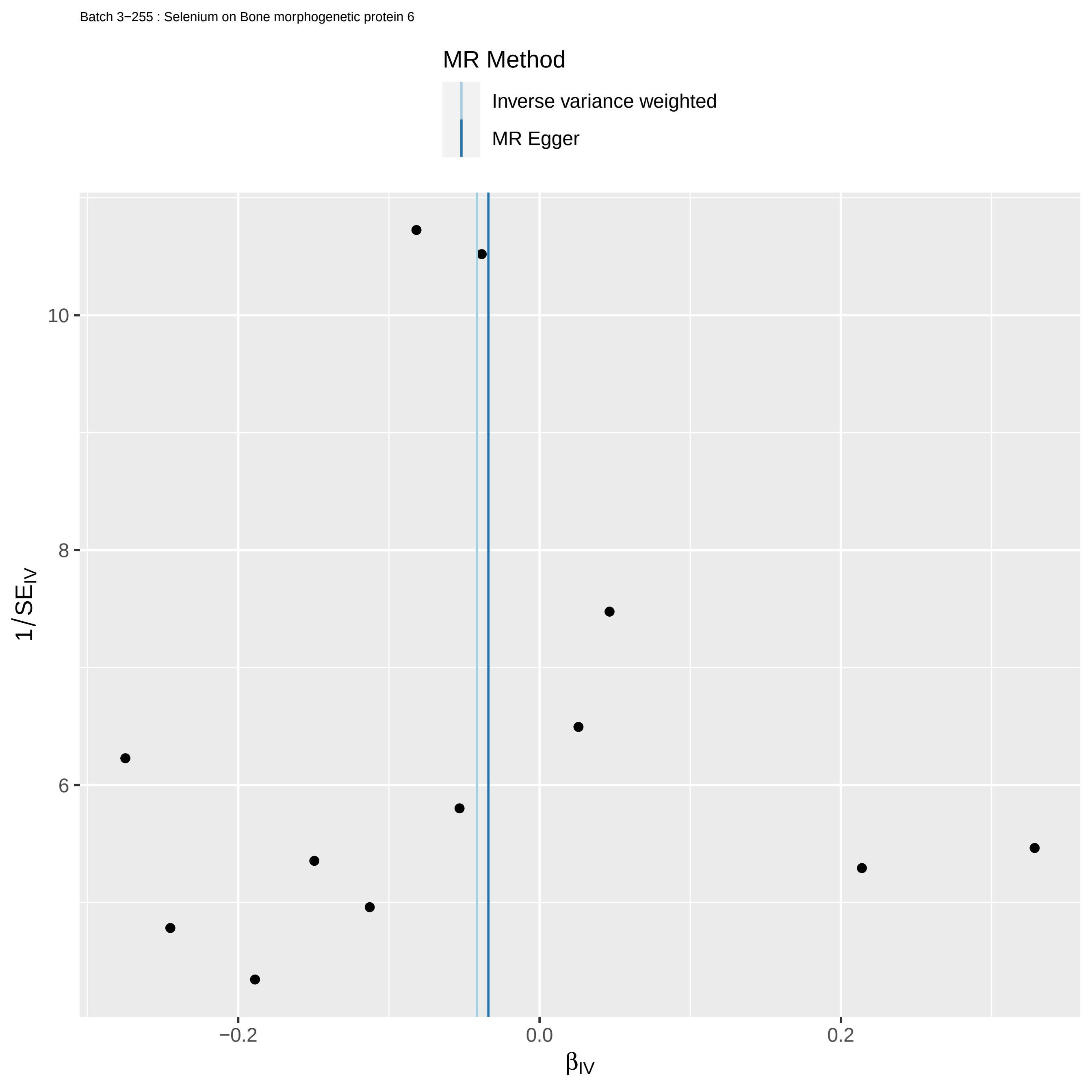

Batch 3−255 : Selenium on Bone morphogenetic protein 6
MR Method
Inverse variance weighted
MR Egger
10
8
6
1/SE$_{IV}$
−0.2
0.0
0.2
β$_{IV}$

Batch 3−256 : Selenium on Bone morphogenetic protein 6

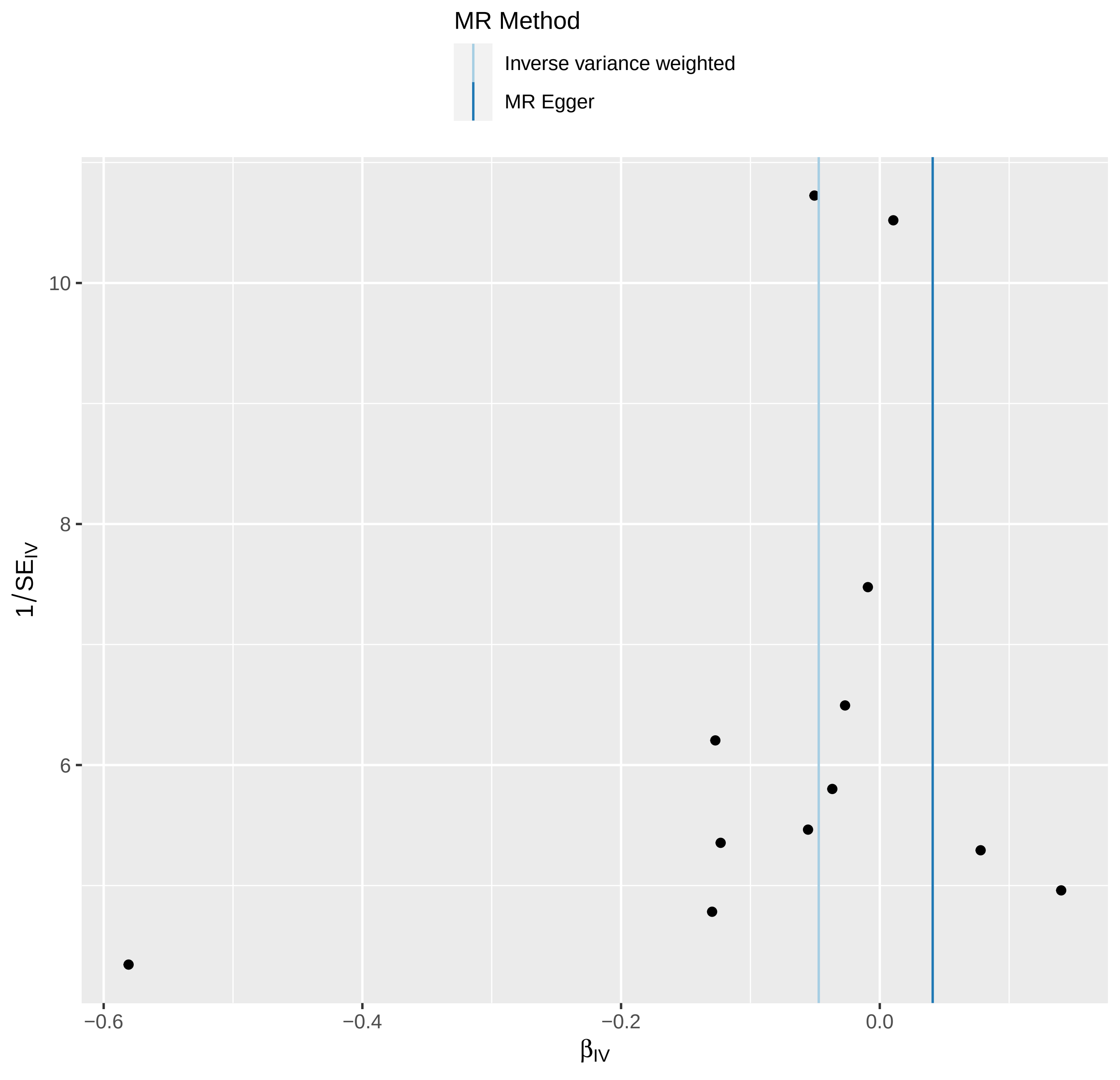

Batch 3−257 : Selenium on Bone morphogenetic protein 7

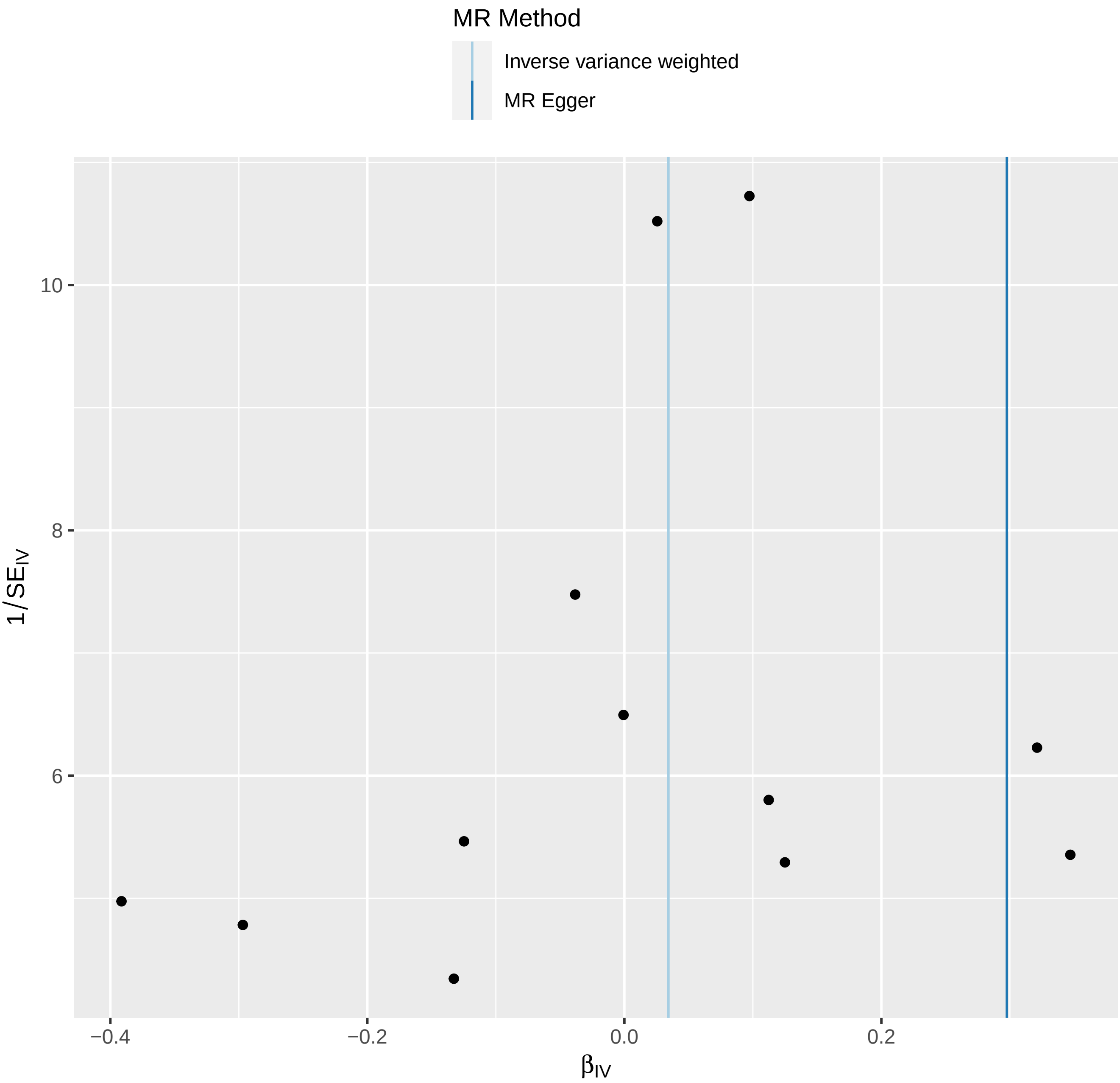

Batch 3−258 : Selenium on BMP−binding endothelial regulator protein

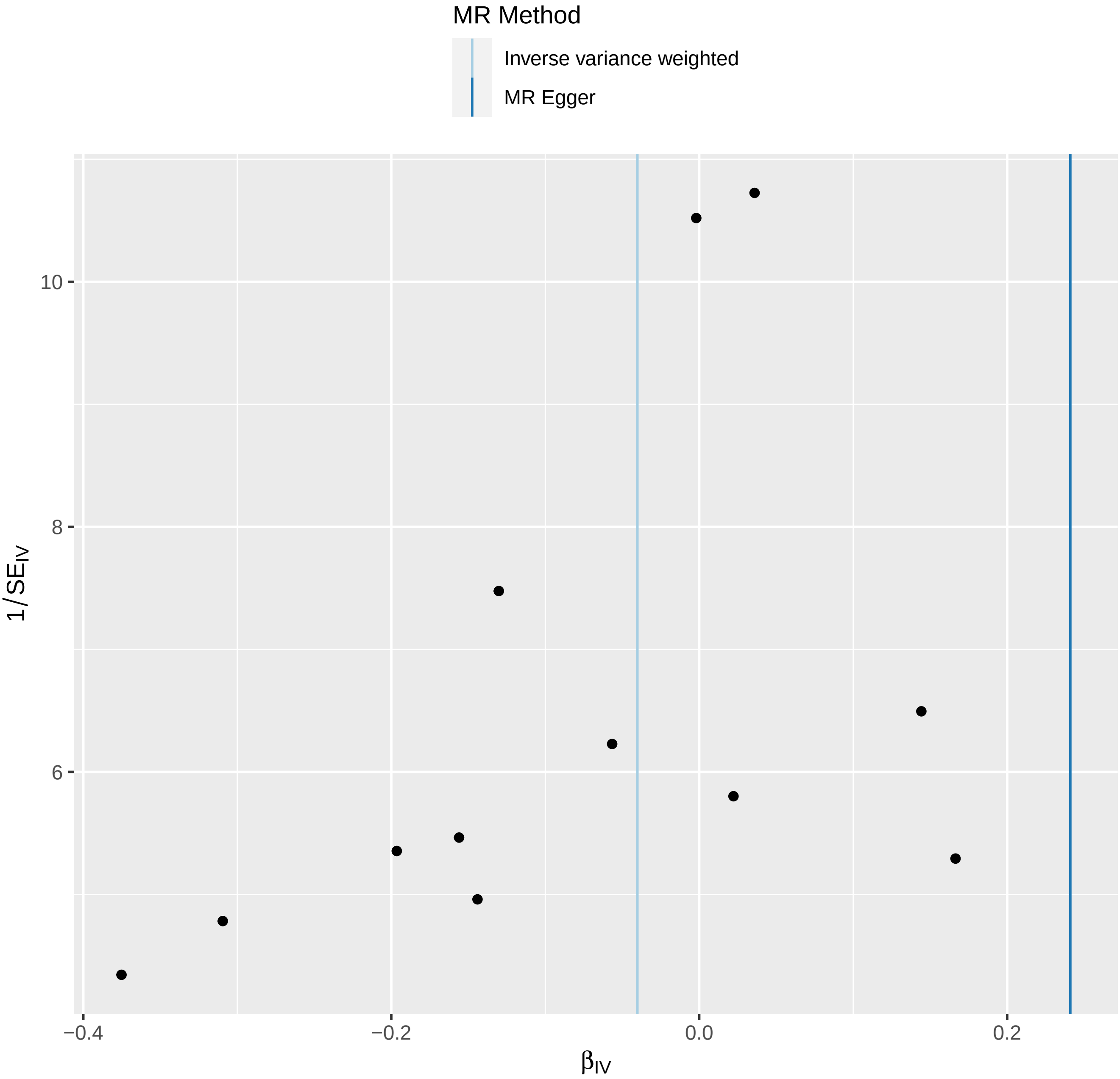

Batch 3−259 : Selenium on Bone morphogenetic protein receptor type−1A

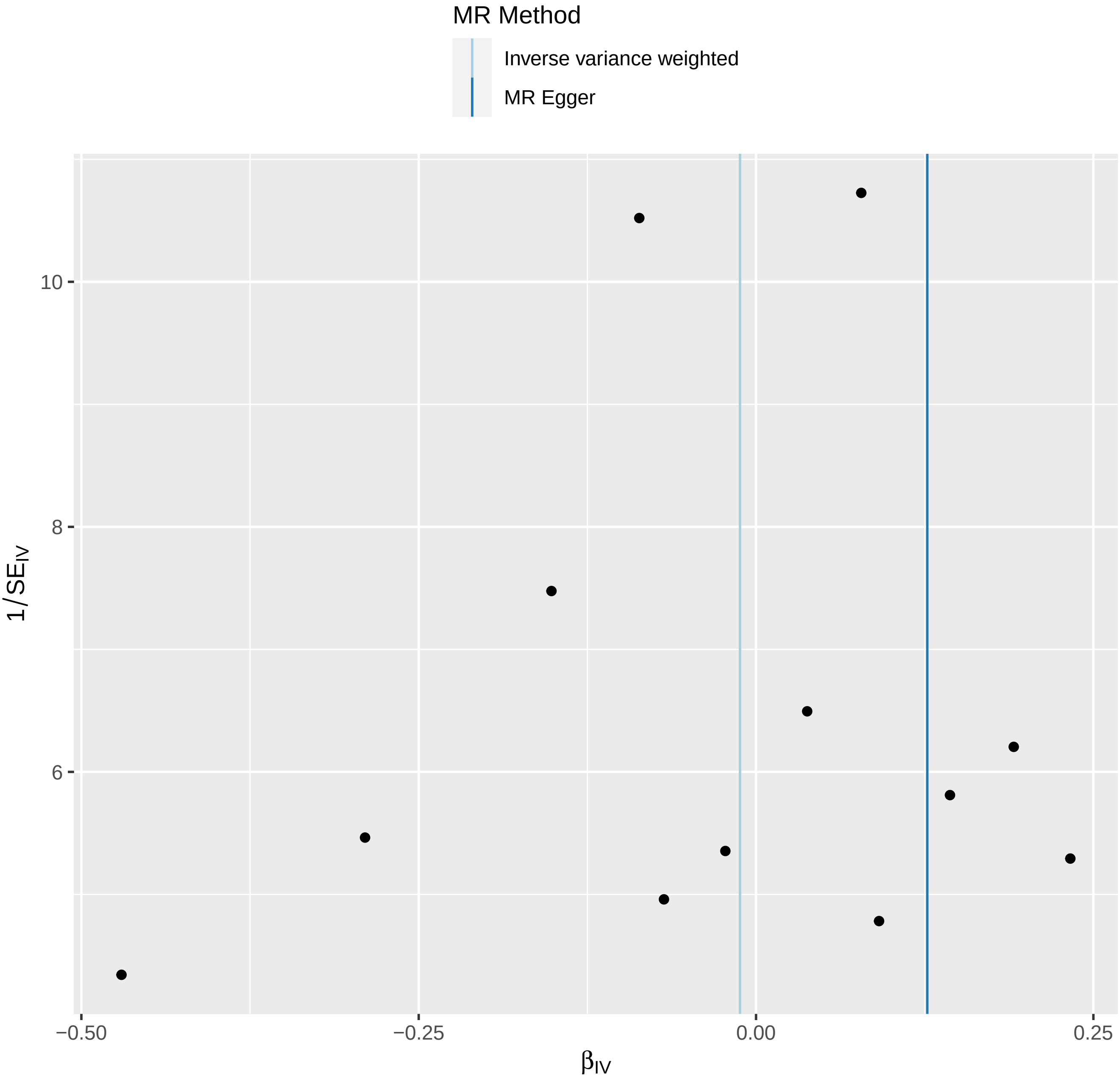

Batch 3−260 : Selenium on Cytoplasmic tyrosine−protein kinase BMX

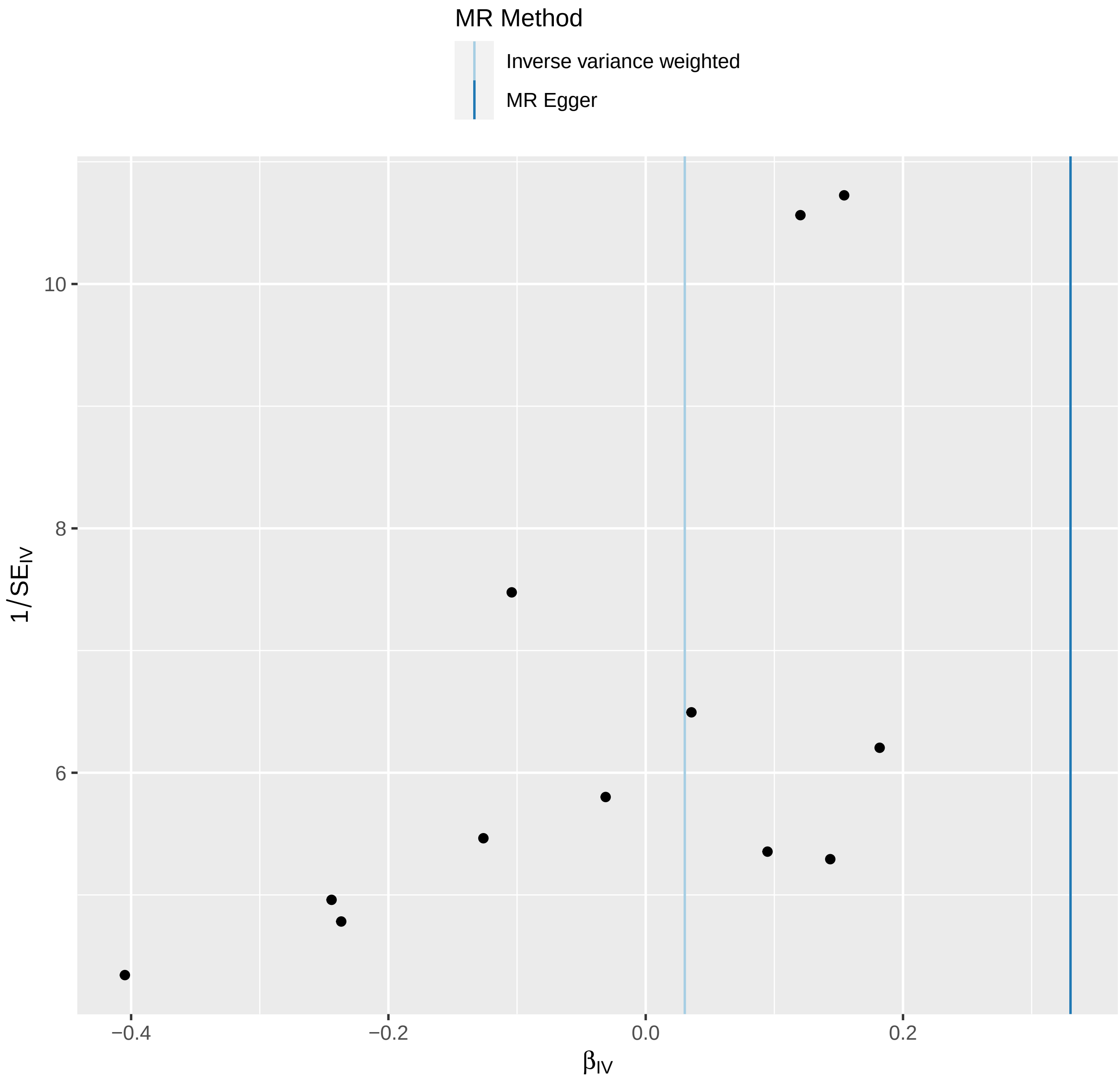

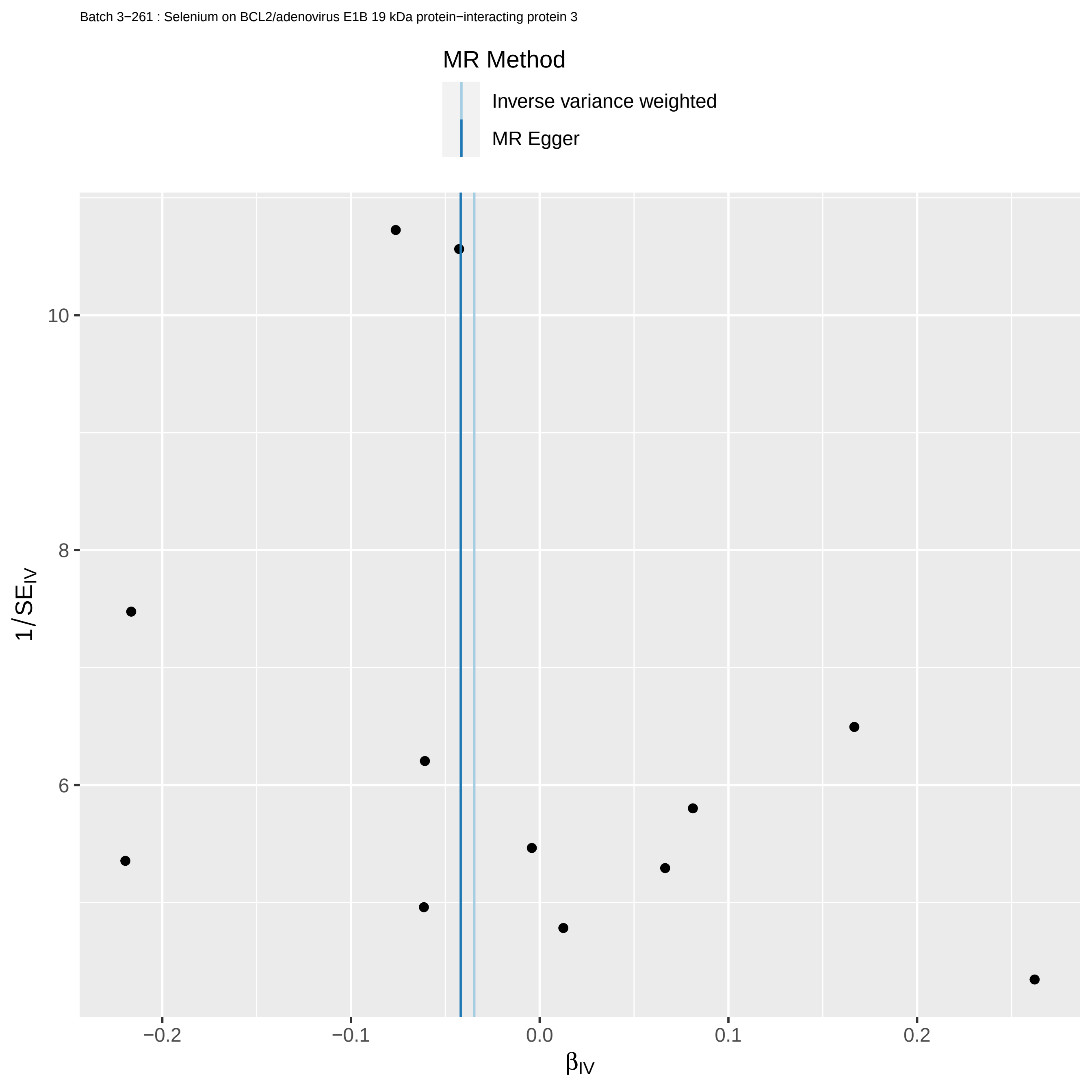

Batch 3−261 : Selenium on BCL2/adenovirus E1B 19 kDa protein−interacting protein 3
MR Method
Inverse variance weighted
MR Egger
10
8
6
1/SE$_{IV}$
−0.2
−0.1
0.0
0.1
0.2
β_{IV}

Batch 3−262 : Selenium on BCL2/adenovirus E1B 19 kDa protein−interacting protein 3−like

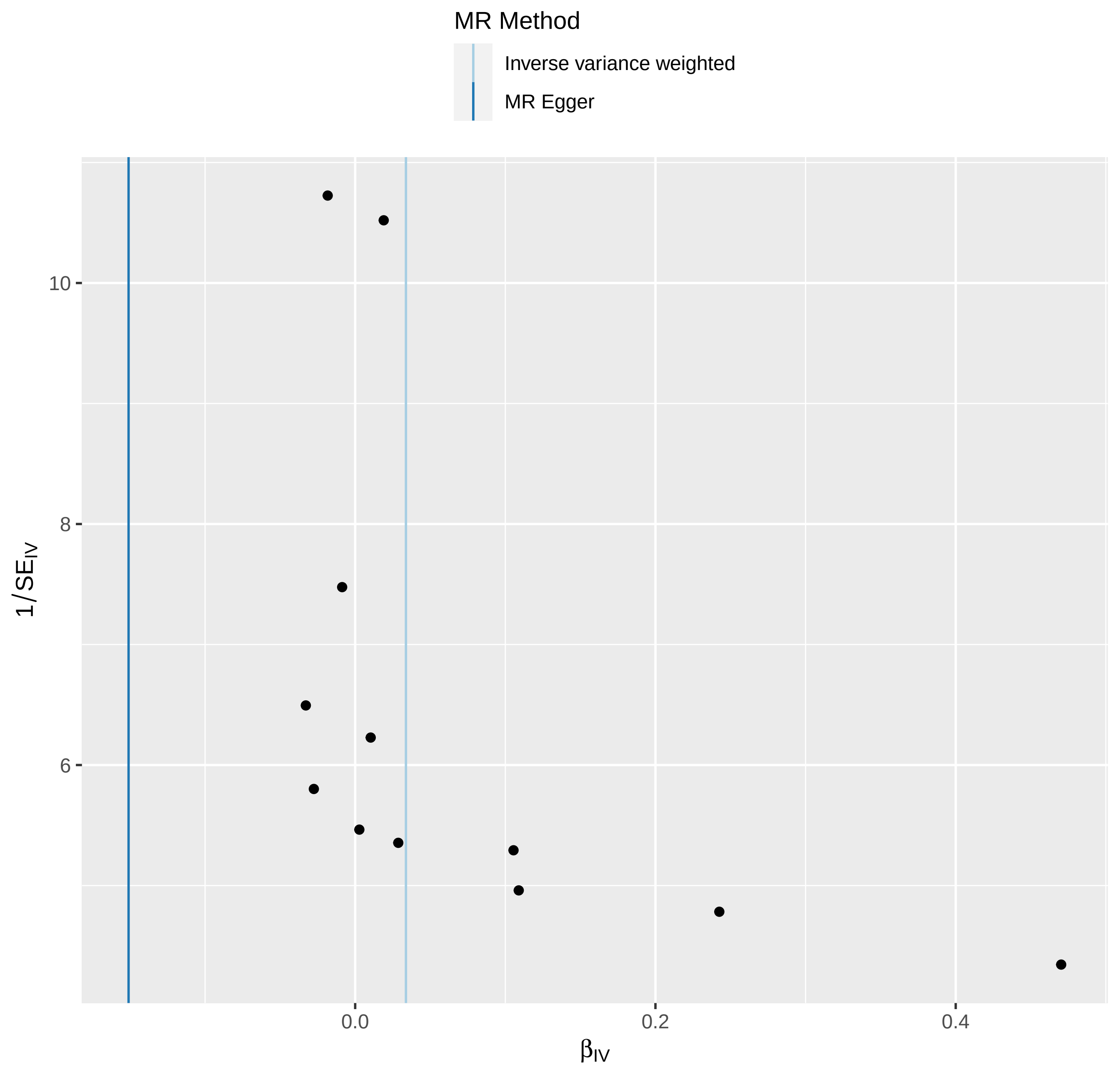

Batch 3−263 : Selenium on Brother of CDO

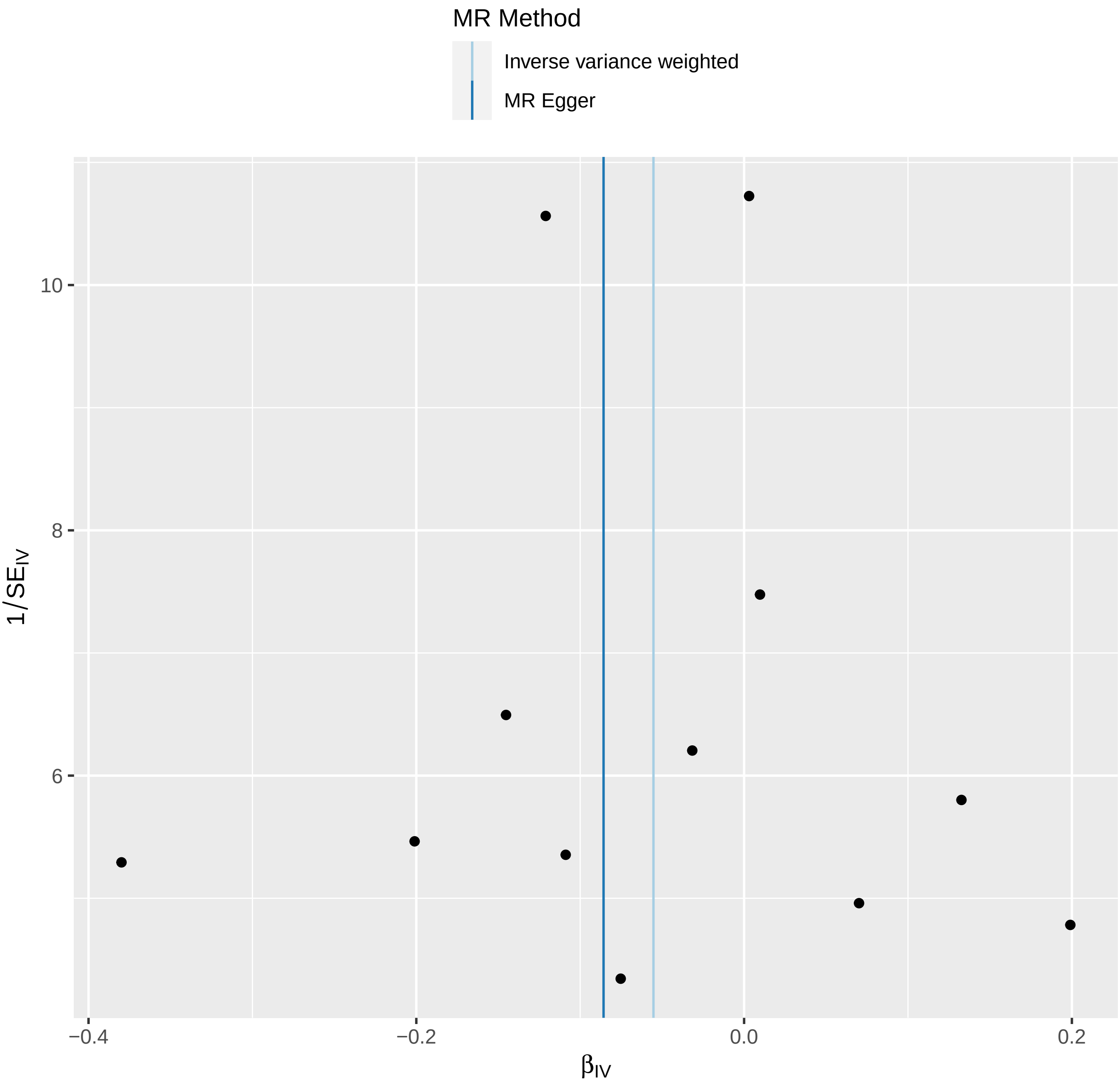

Batch 3−264 : Selenium on BolA−like protein 2

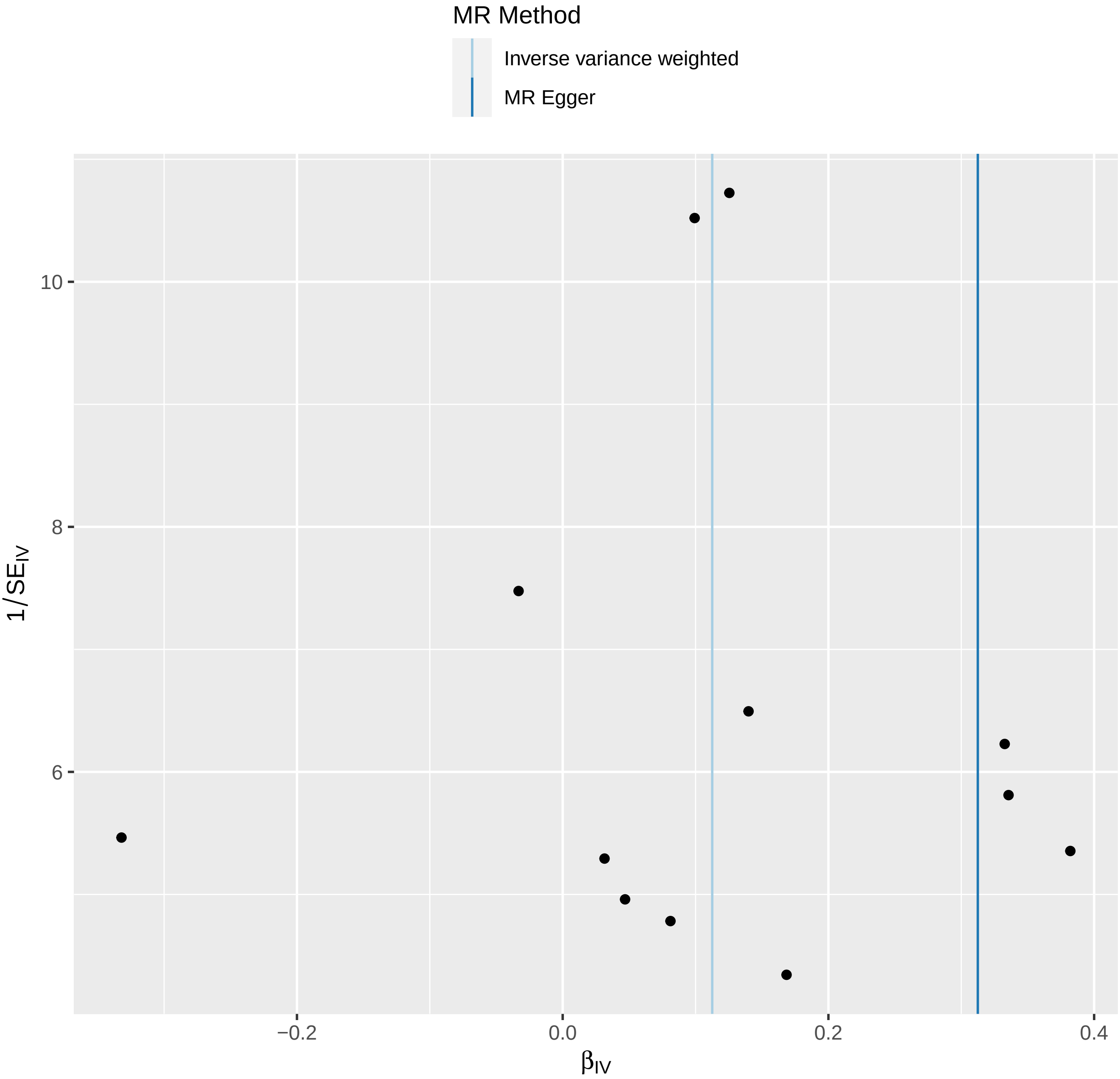

Batch 3−265 : Selenium on Bisphosphoglycerate mutase

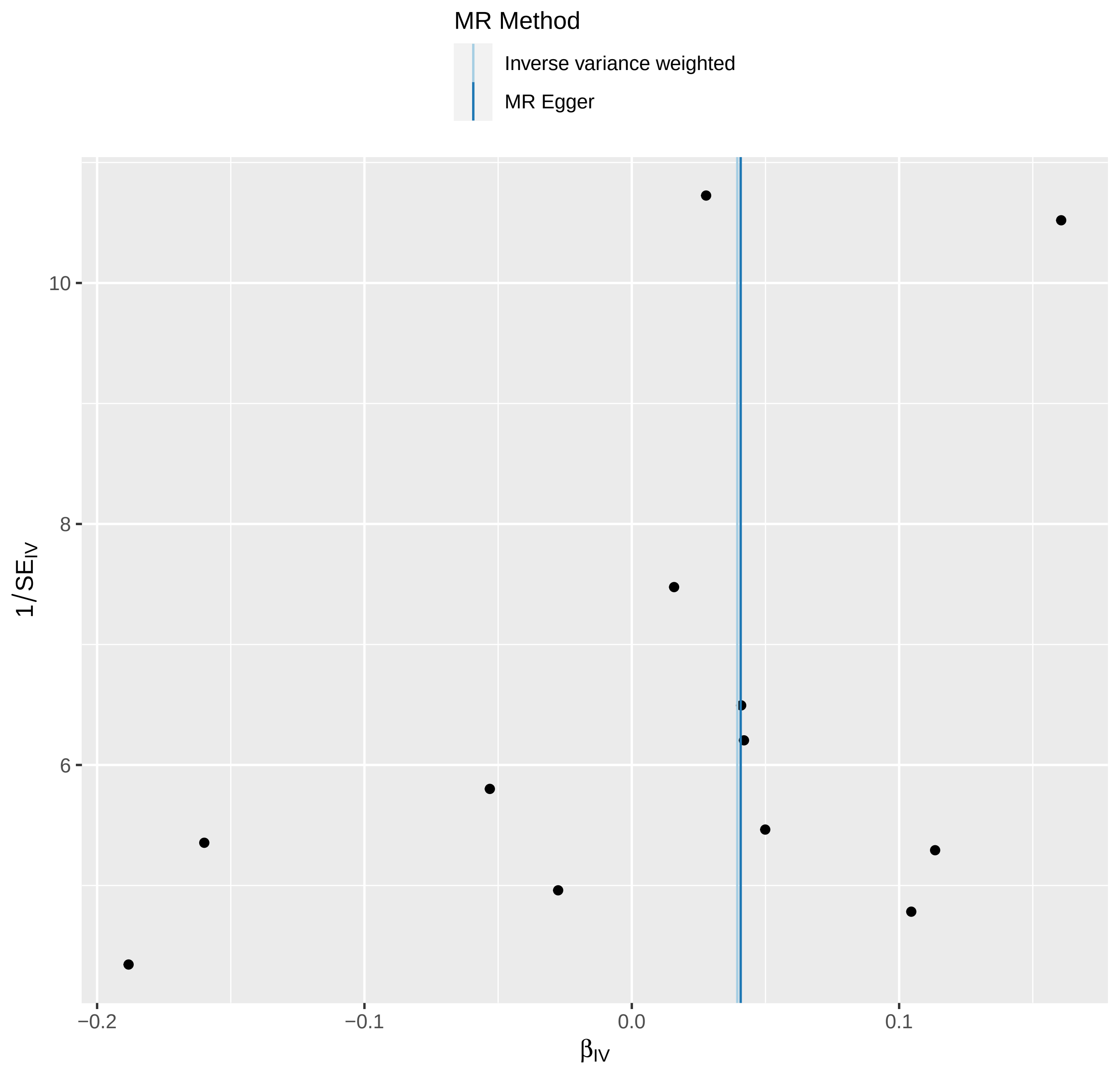

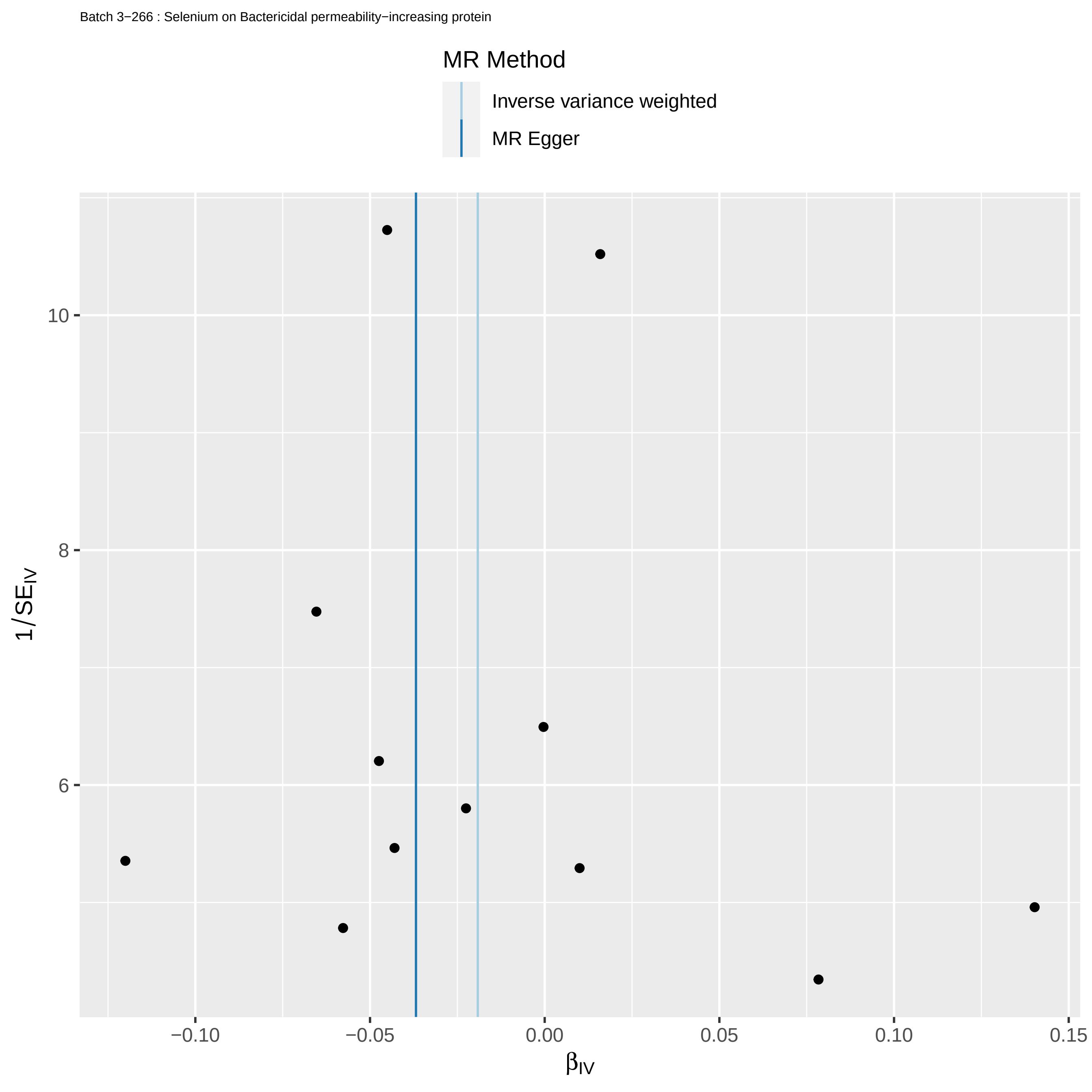

Batch 3−266 : Selenium on Bactericidal permeability−increasing protein
MR Method
Inverse variance weighted
MR Egger
10
8
6
1/SE_IV
−0.10
−0.05
0.00
0.05
0.10
0.15
β_IV

Batch 3−267 : Selenium on BPI fold−containing family A member 1

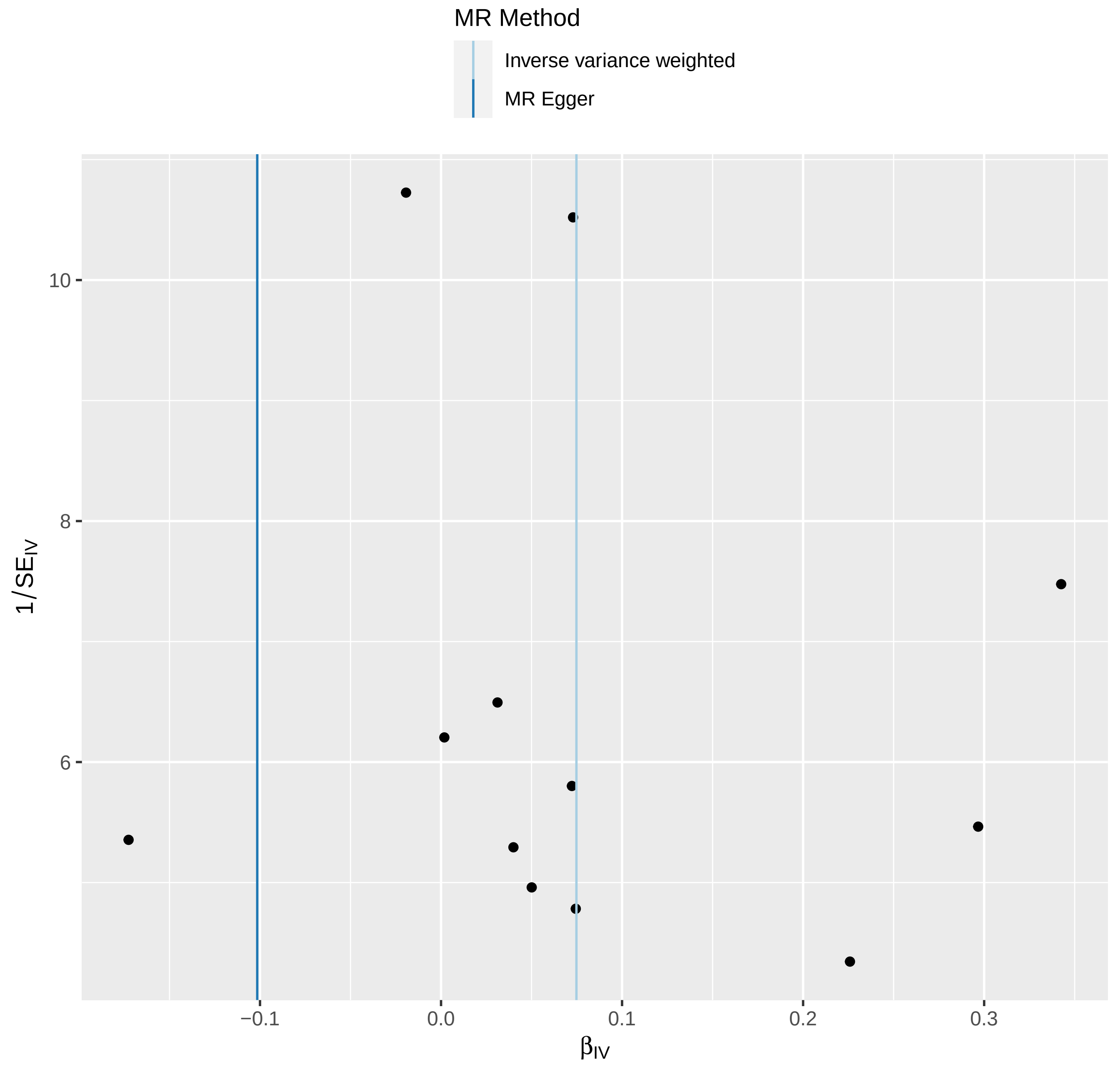

Batch 3−268 : Selenium on BPI fold−containing family B member 1

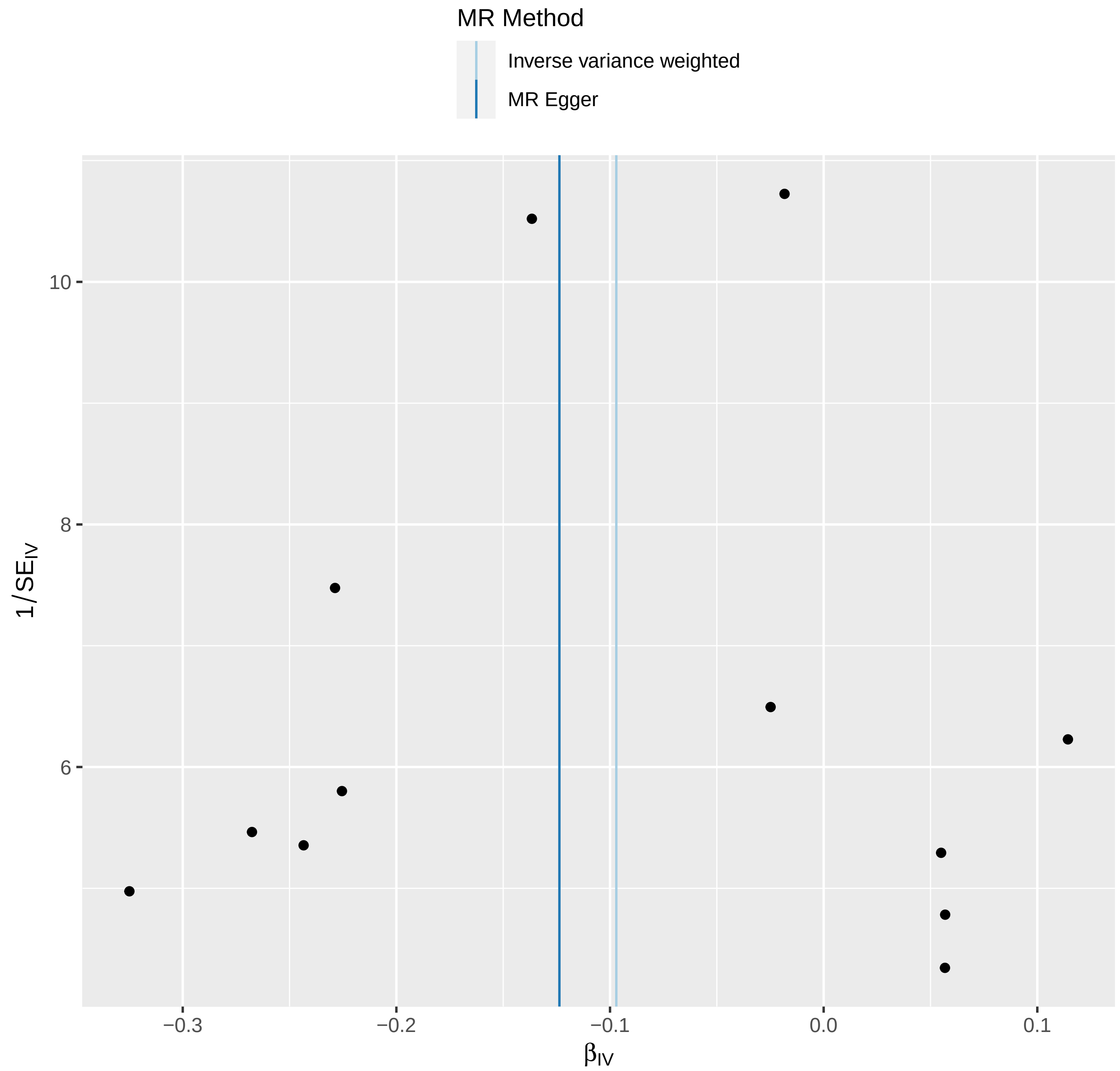

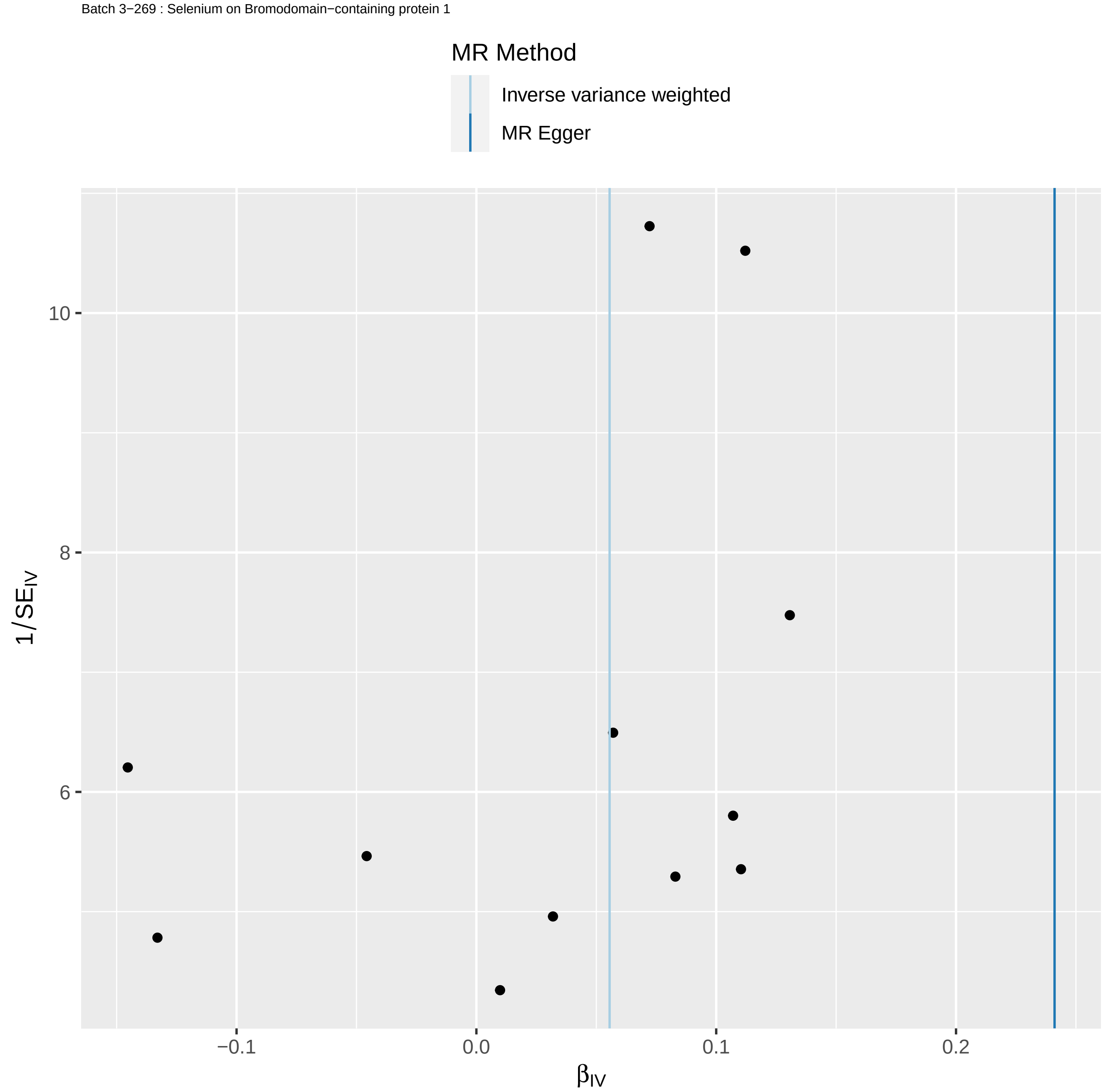
Batch 3−269 : Selenium on Bromodomain−containing protein 1
MR Method
Inverse variance weighted
MR Egger
10
8
6
1/SE_IV
−0.1
0.0
0.1
0.2
β_IV

Batch 3−270 : Selenium on Bromodomain testis−specific protein

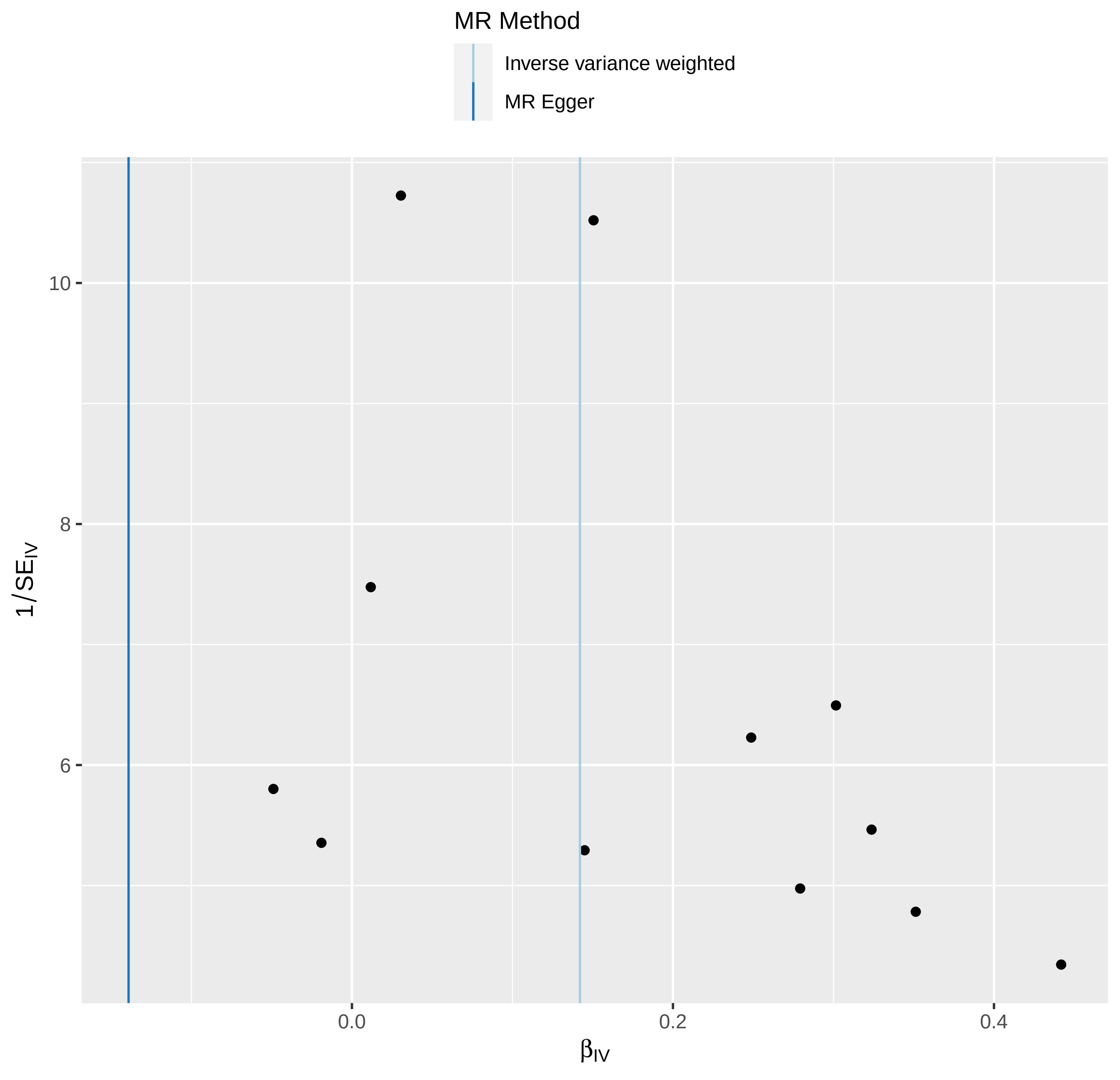

Batch 3−271 : Selenium on Transcription factor IIIB 90 kDa subunit

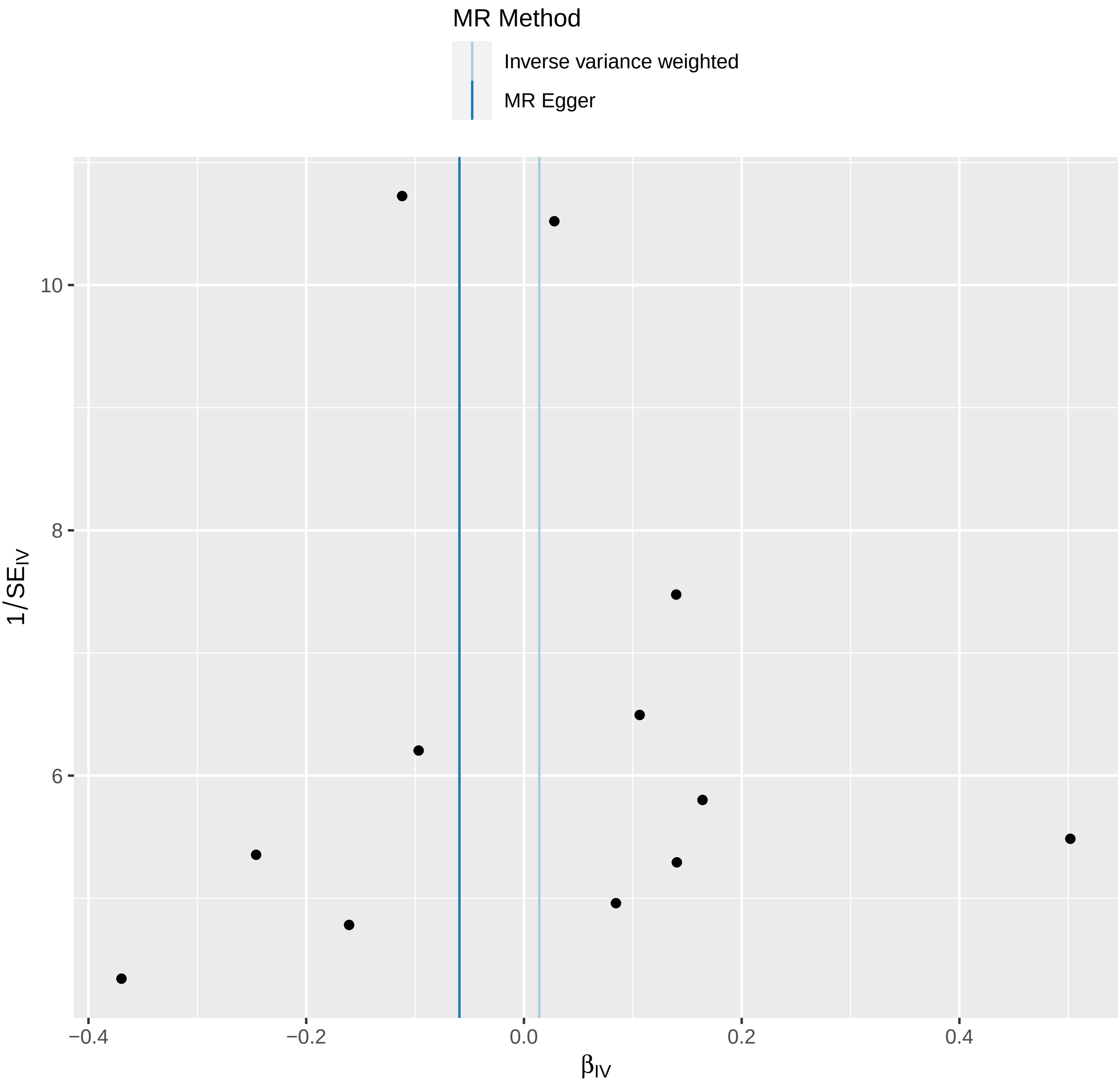

Batch 3−272 : Selenium on BRICHOS domain−containing protein 5

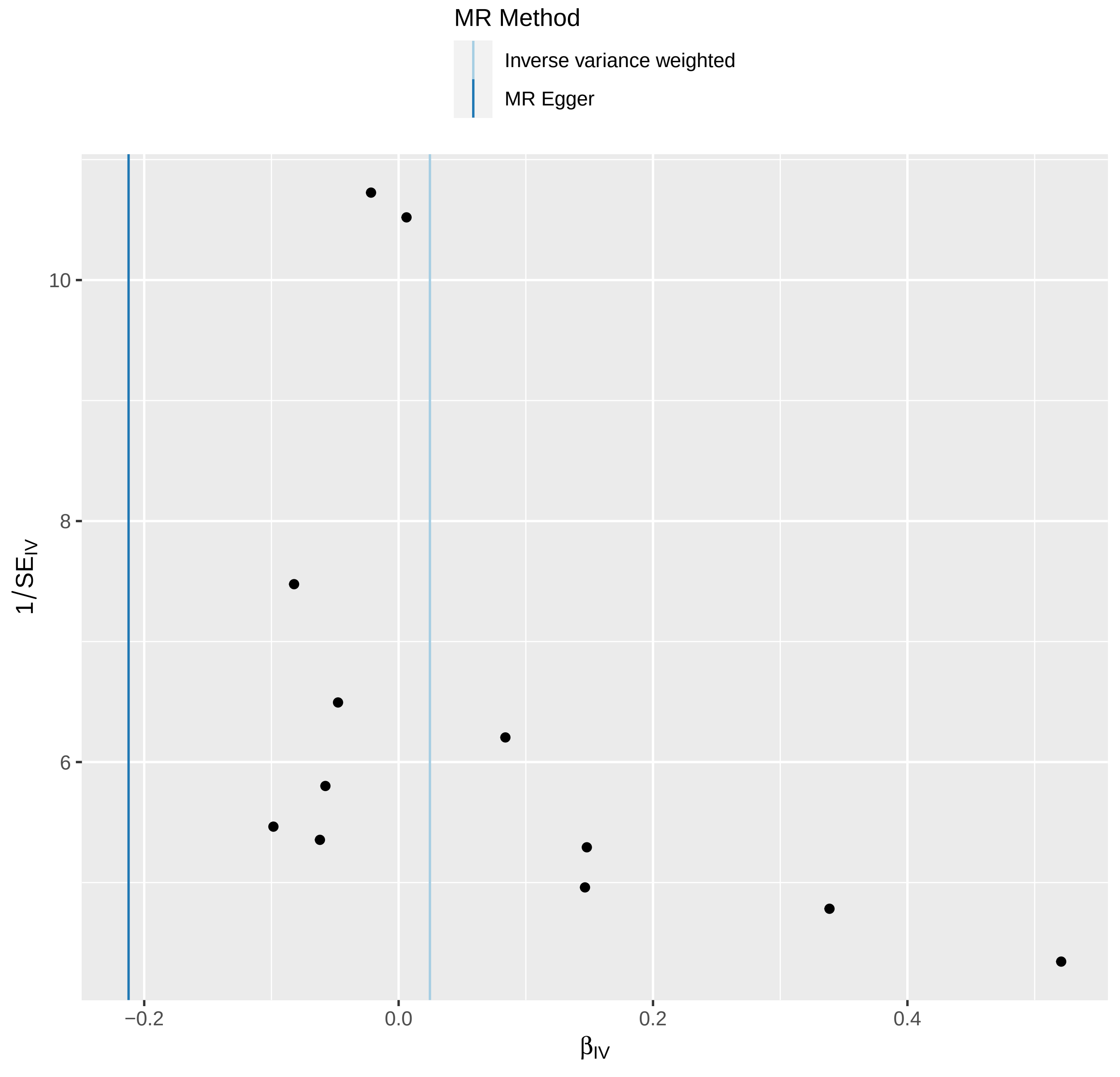

Batch 3−273 : Selenium on Peregrin

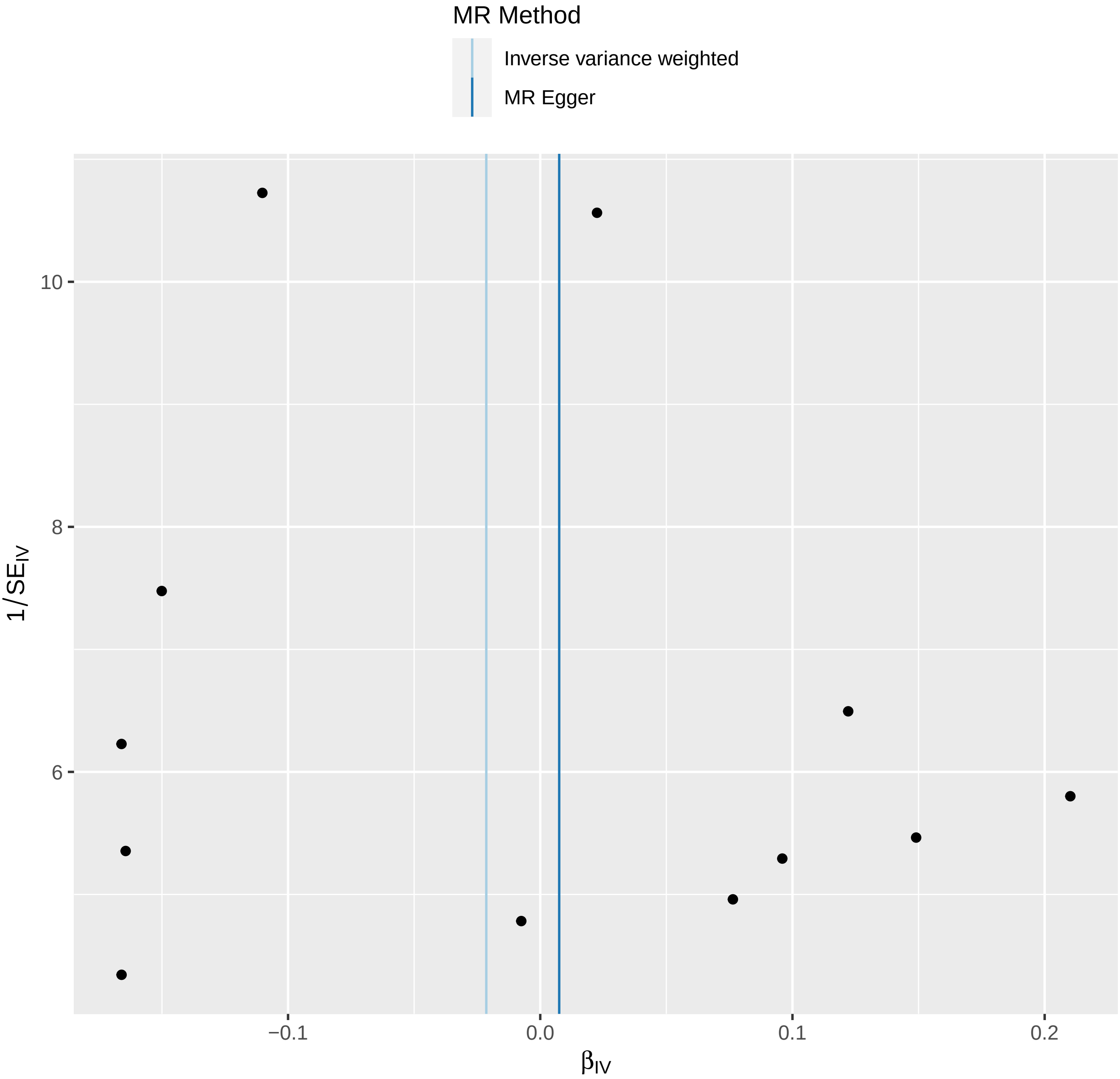

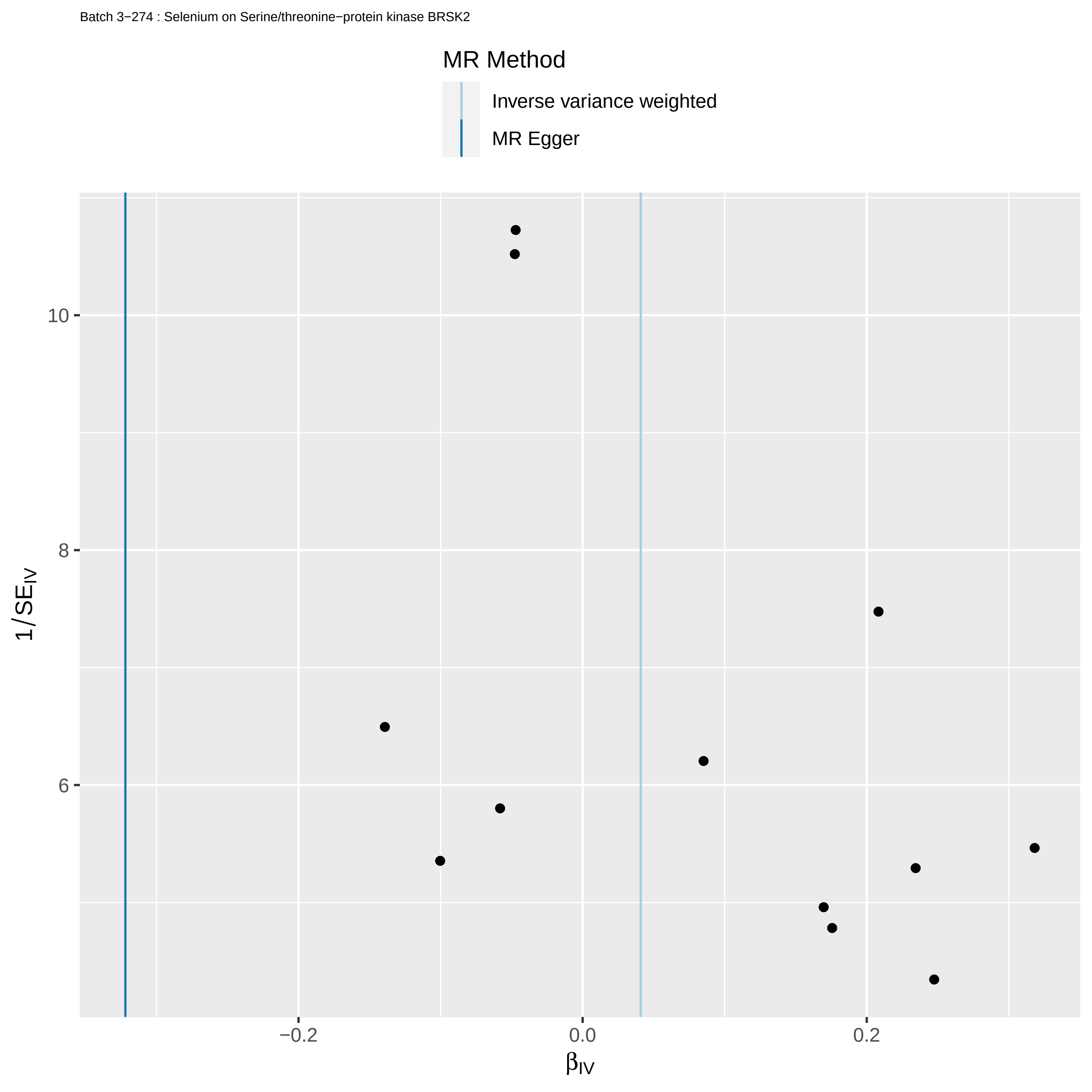
Batch 3−274 : Selenium on Serine/threonine−protein kinase BRSK2
MR Method
Inverse variance weighted
MR Egger
10
8
6
1/SE_IV
−0.2
0.0
0.2
β_IV

Batch 3−275 : Selenium on Basigin

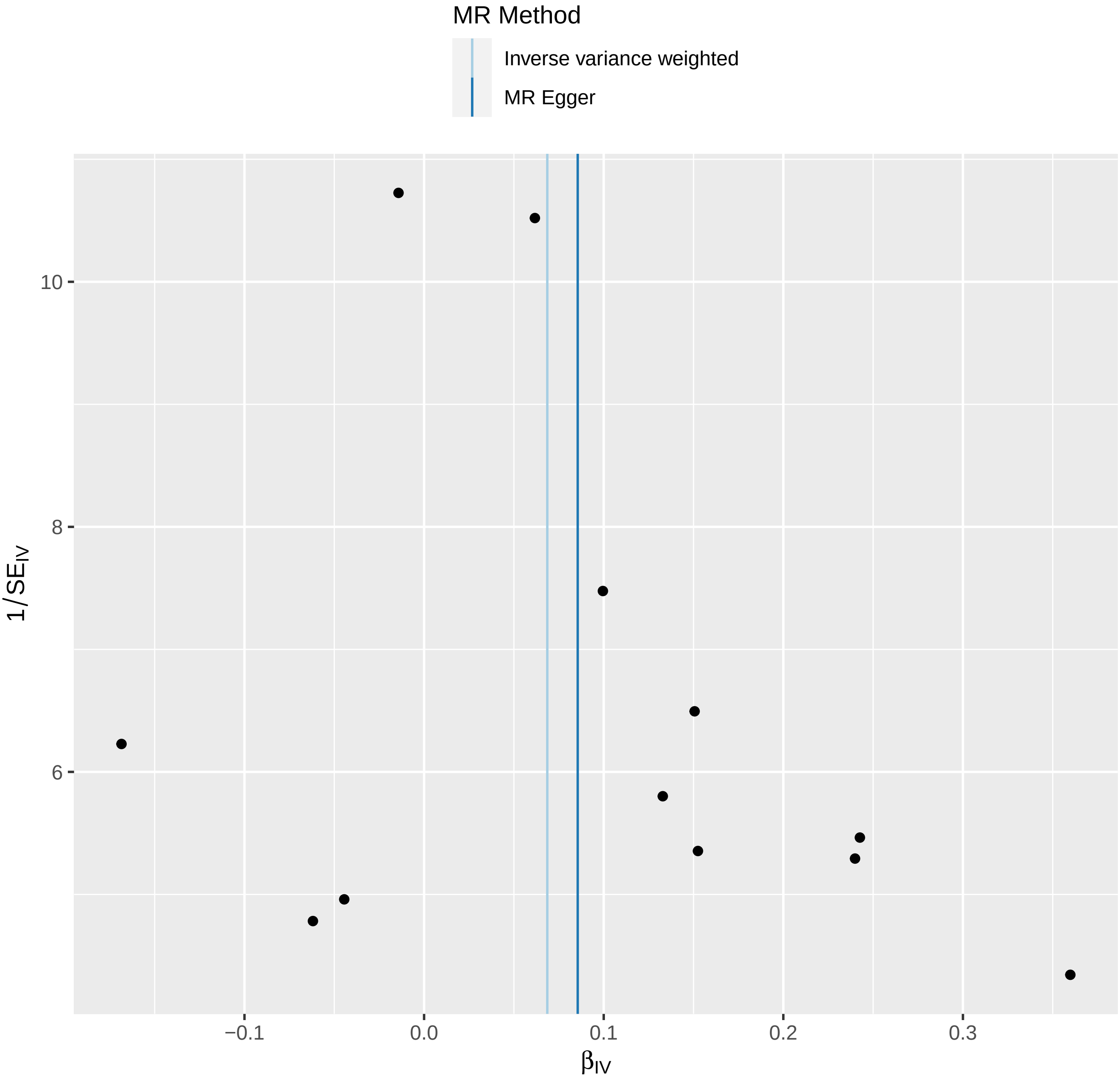

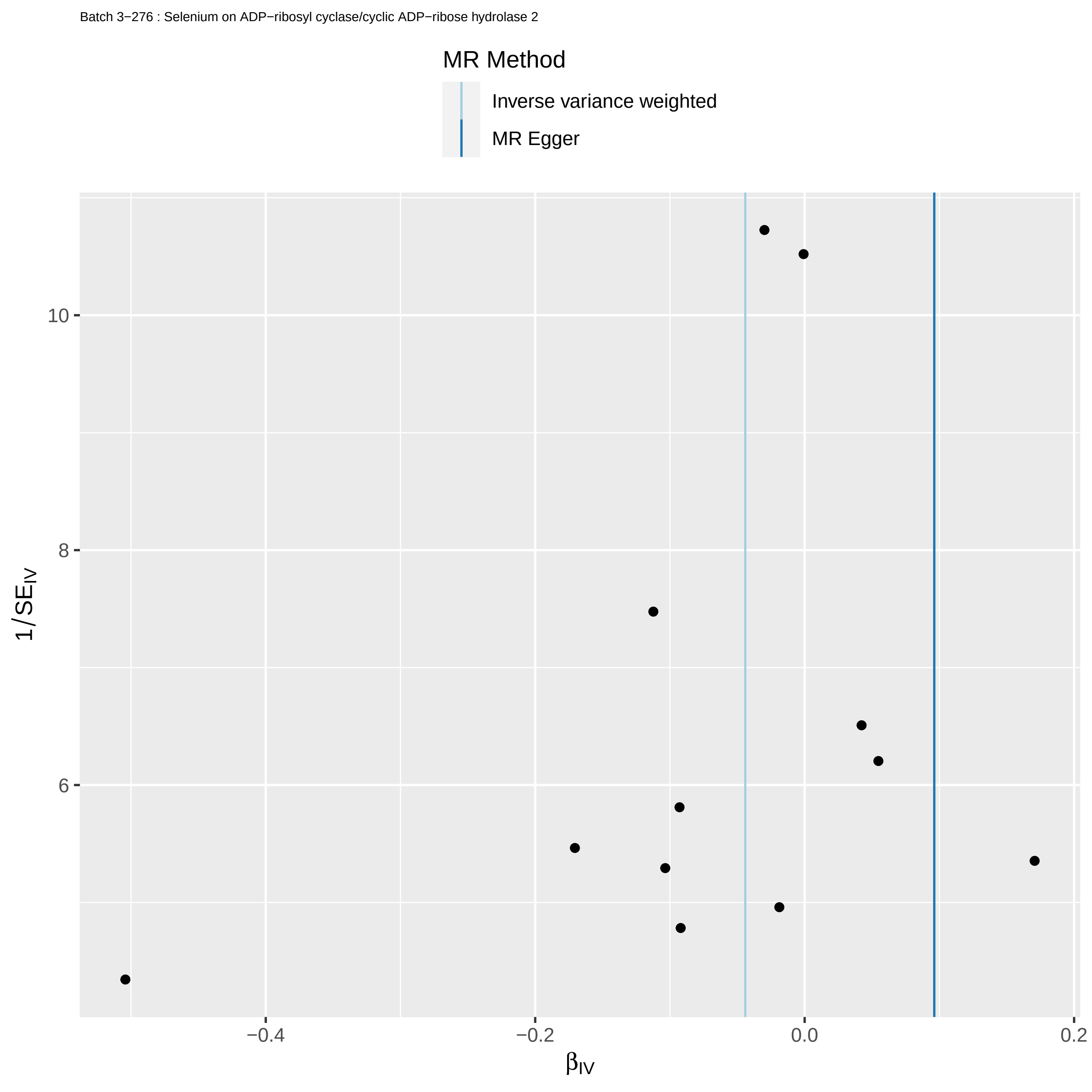
Batch 3−276 : Selenium on ADP−ribosyl cyclase/cyclic ADP−ribose hydrolase 2
MR Method
Inverse variance weighted
MR Egger
10
8
6
1/SE_IV
−0.4
−0.2
0.0
0.2
β_IV

Batch 3−277 : Selenium on Tyrosine−protein kinase BTK

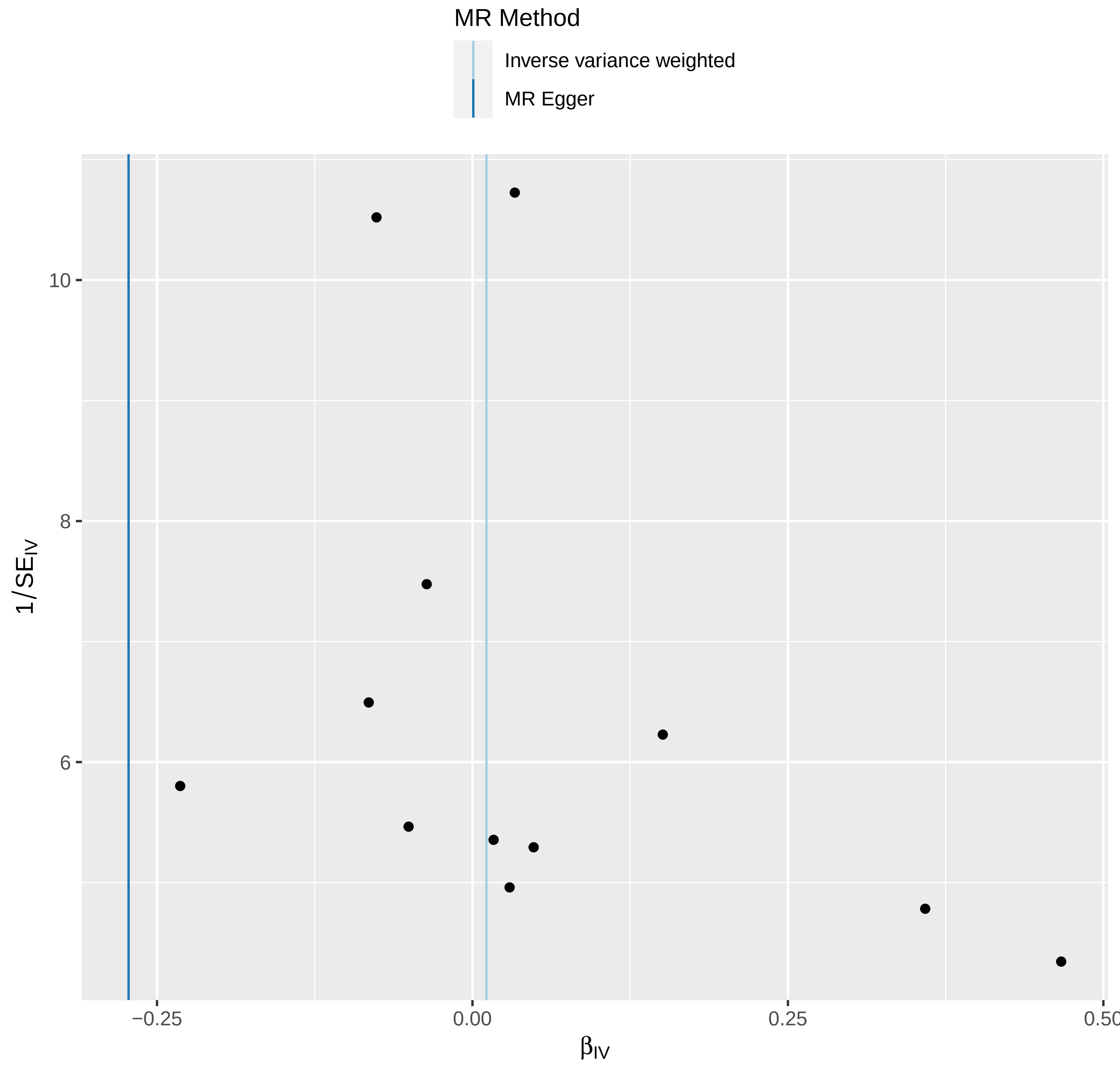

Batch 3−278 : Selenium on Butyrophilin−like protein 9

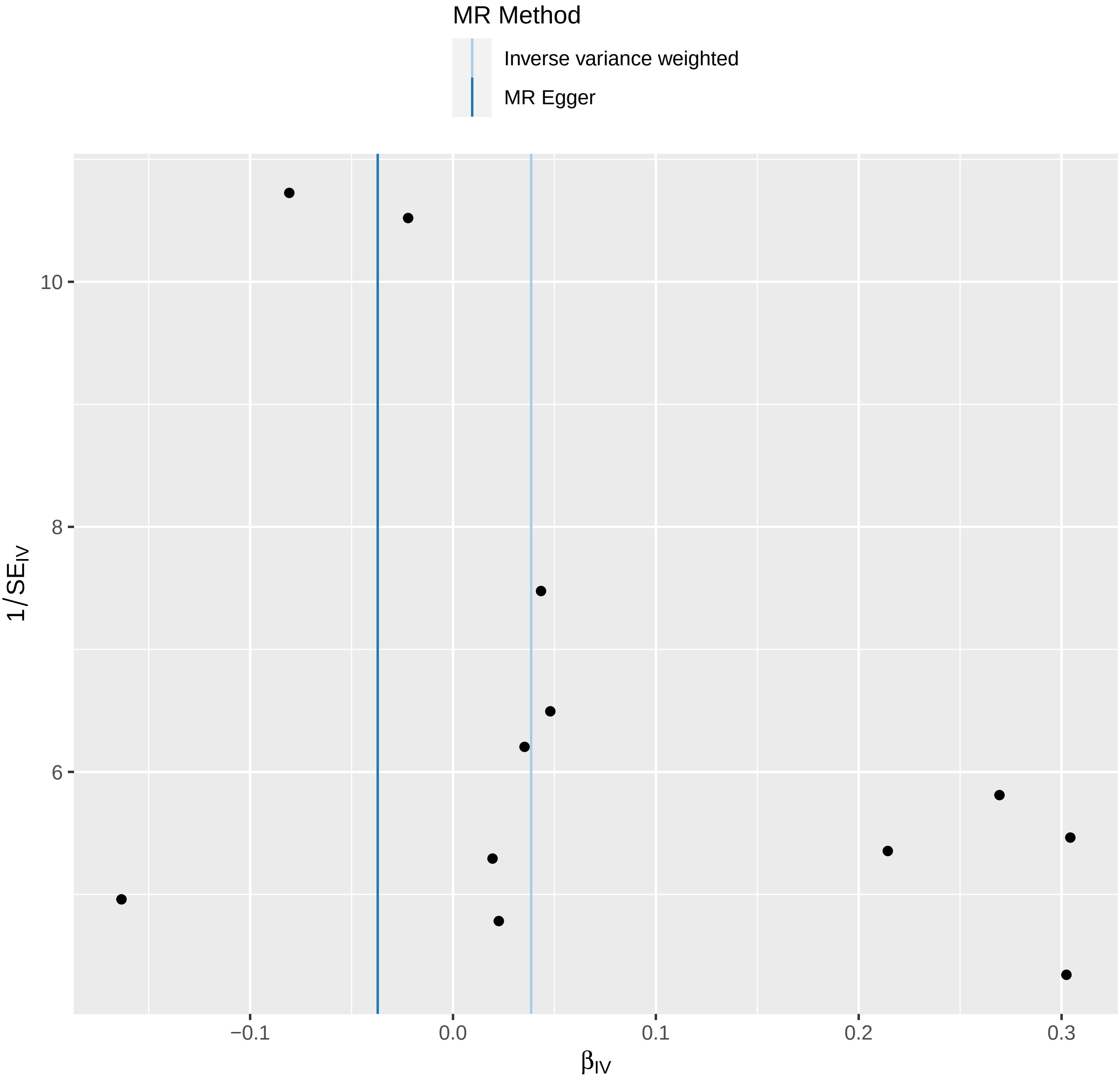

Batch 3−279 : Selenium on Mitotic checkpoint serine/threonine−protein kinase BUB1

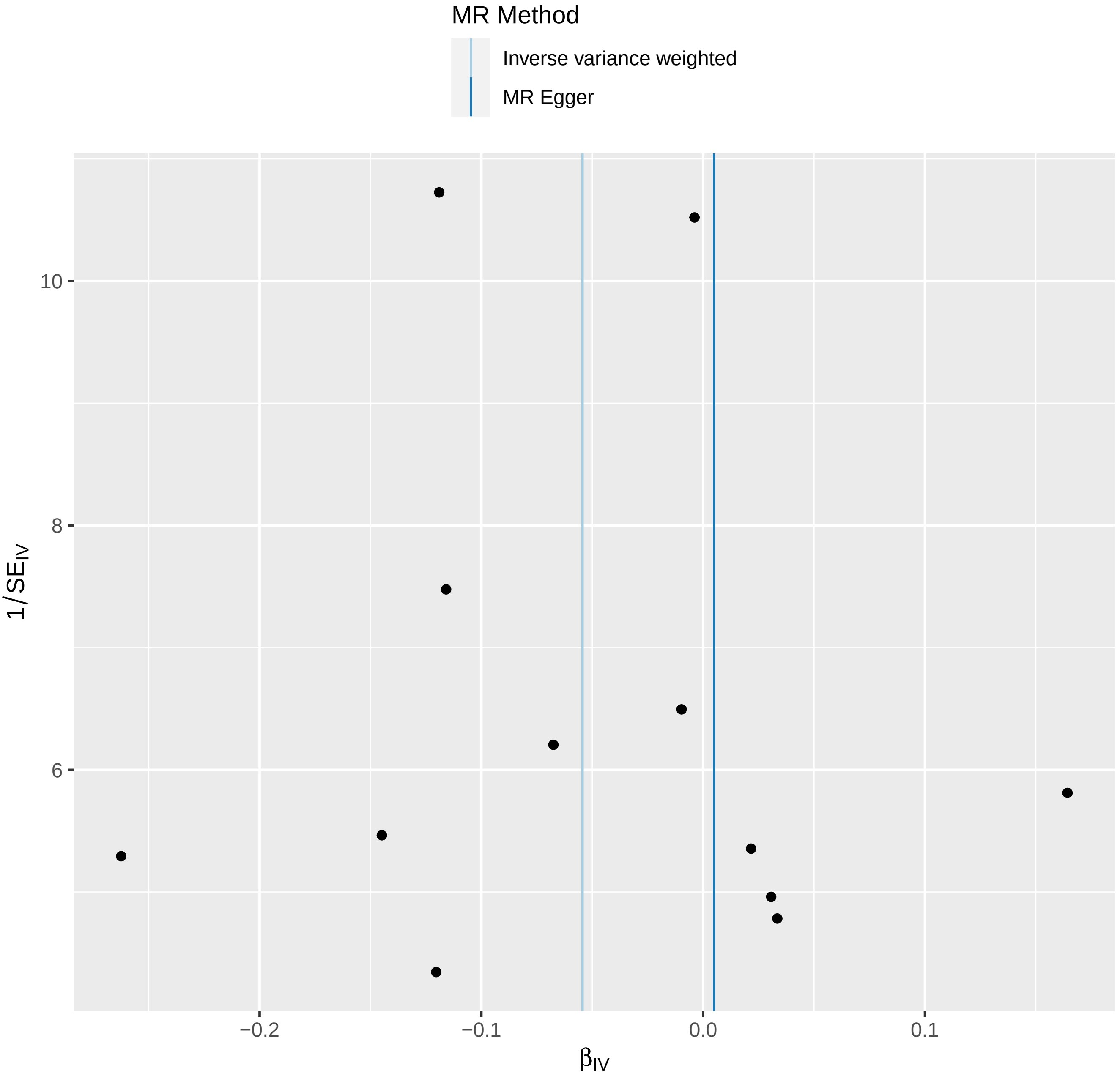

Batch 3−280 : Selenium on Uncharacterized protein C10orf35

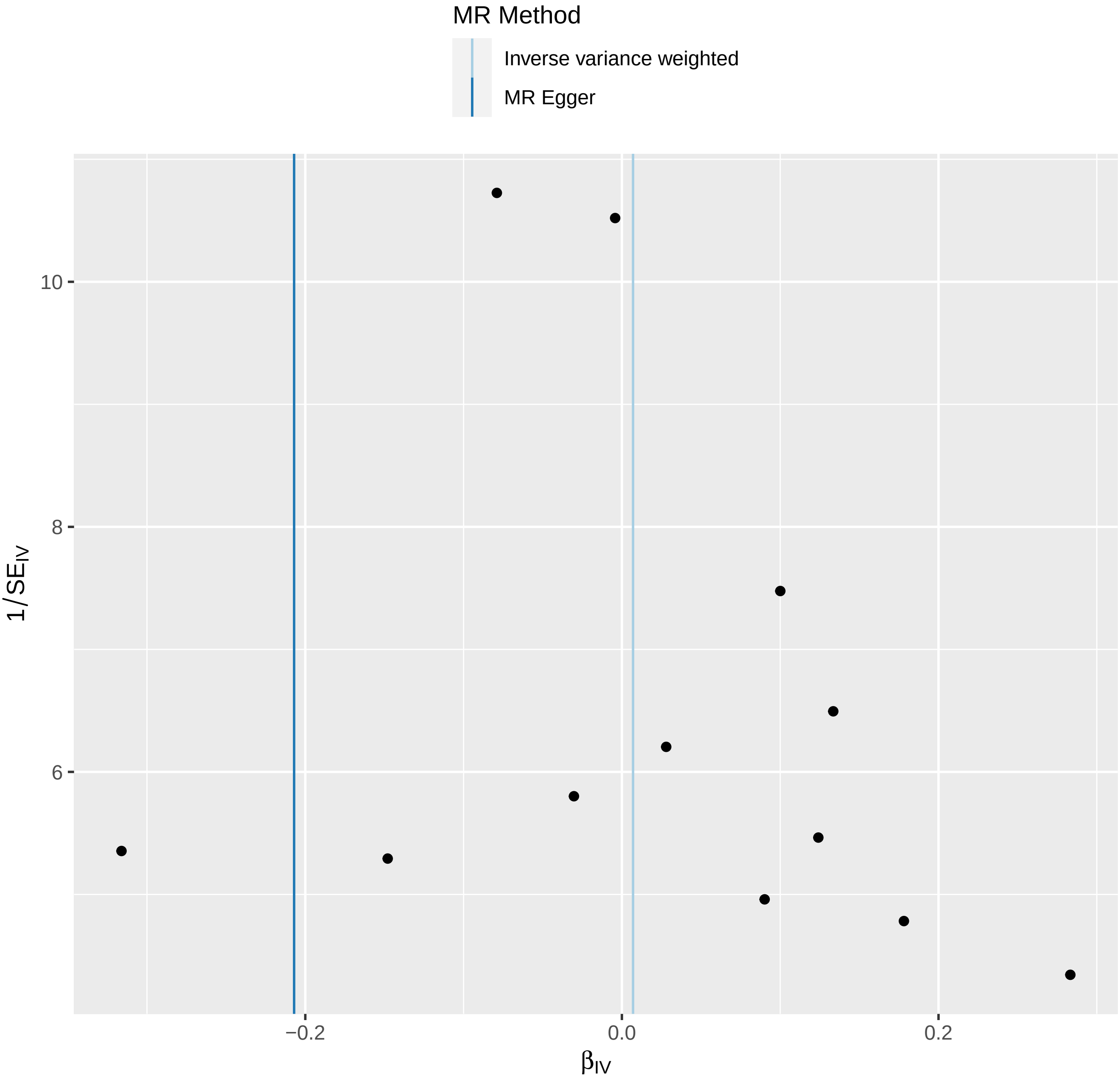

Batch 3−281 : Selenium on Platelet receptor Gi24

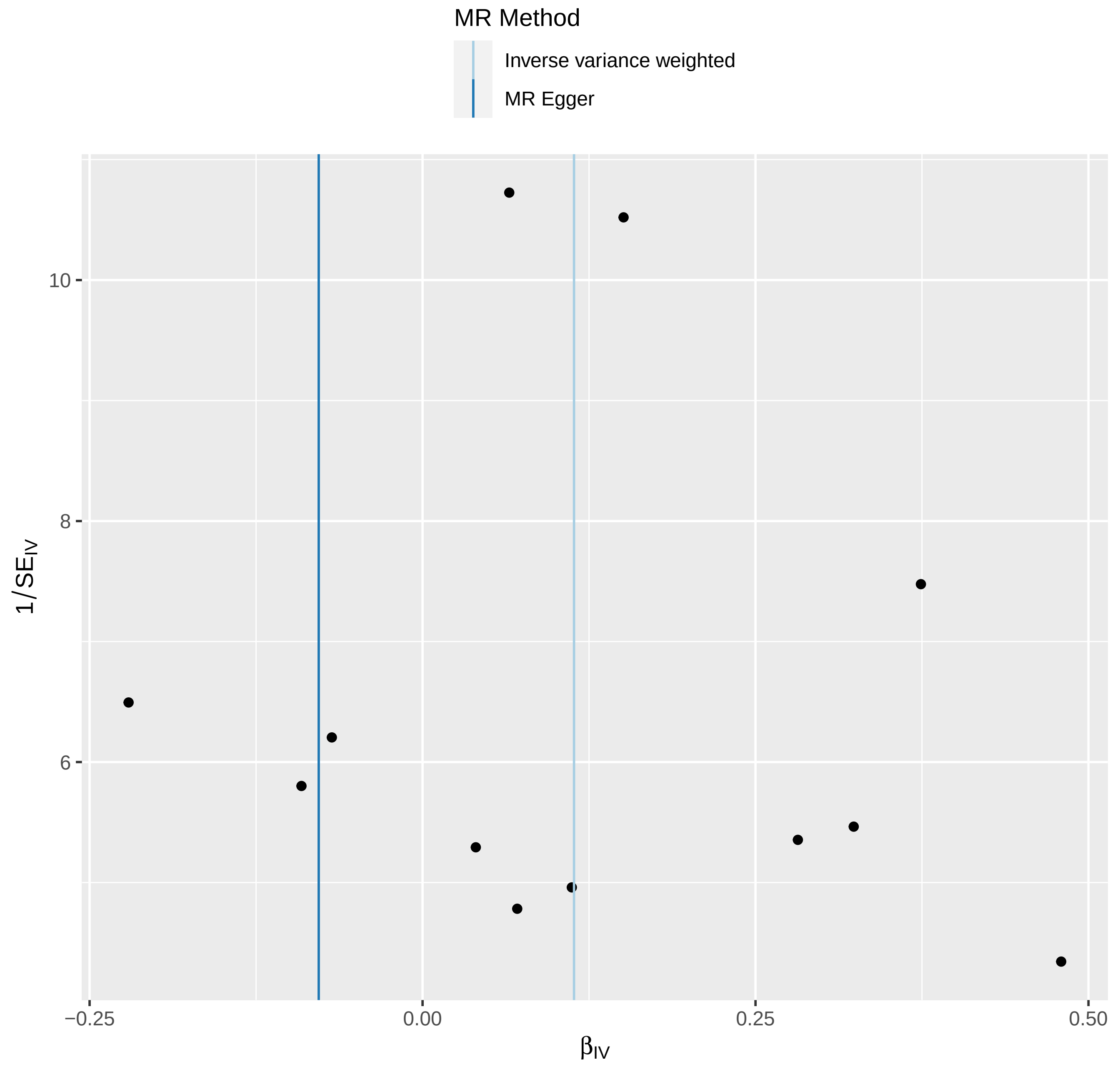

Batch 3−282 : Selenium on UPF0696 protein C11orf68

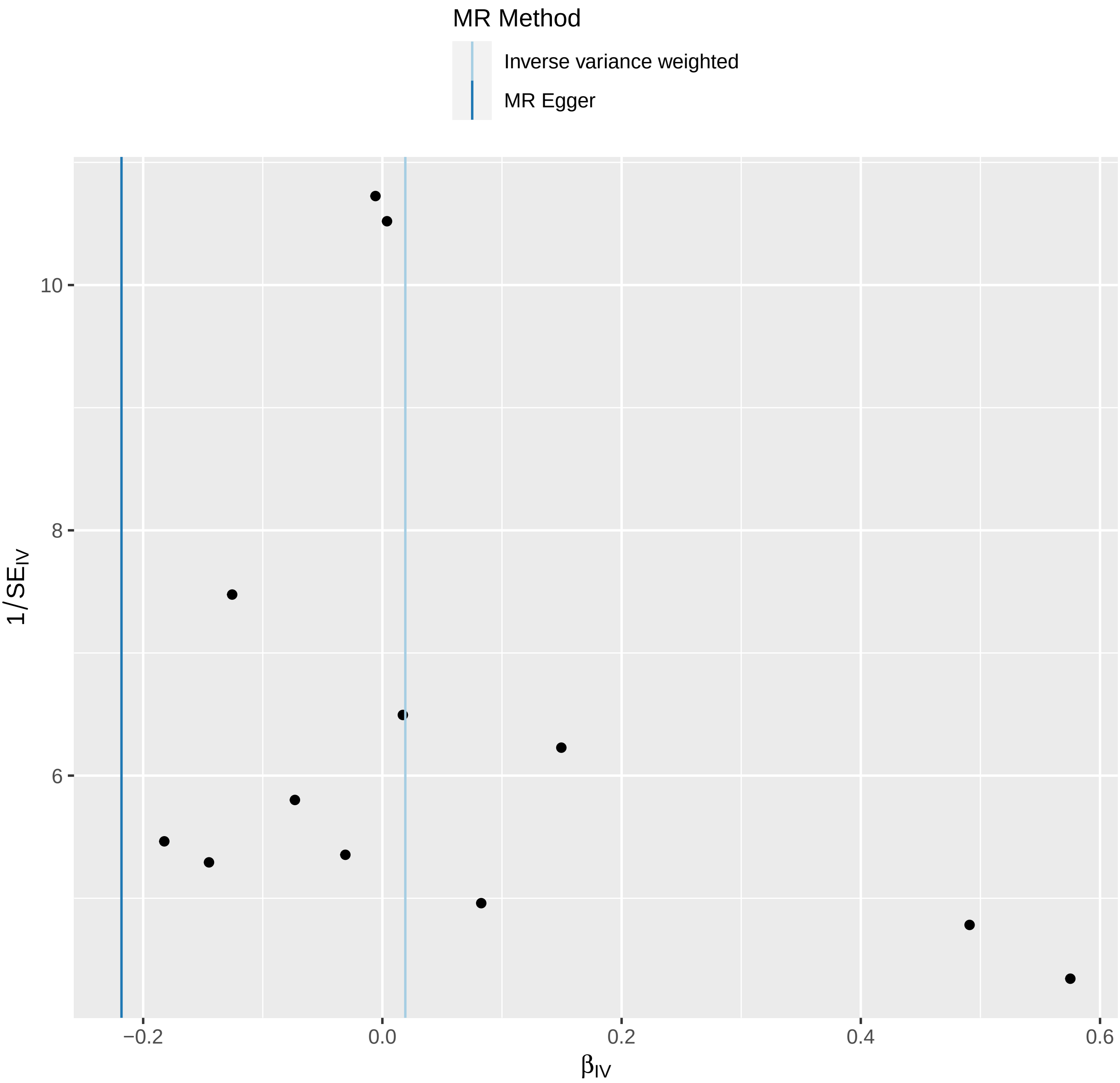

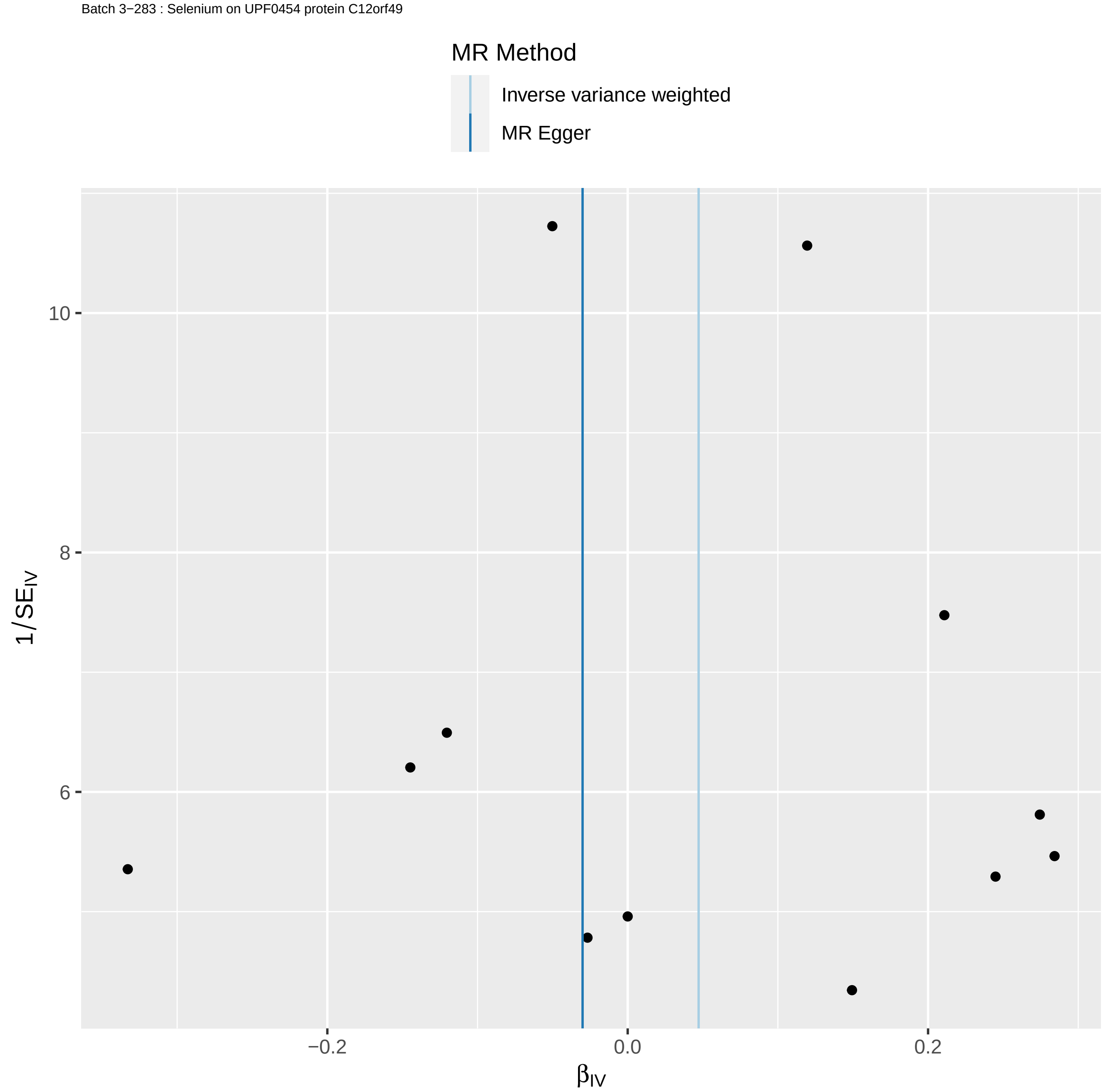

Batch 3−283 : Selenium on UPF0454 protein C12orf49
MR Method
Inverse variance weighted
MR Egger
10
8
6
1/SE_IV
−0.2
0.0
0.2
β_IV

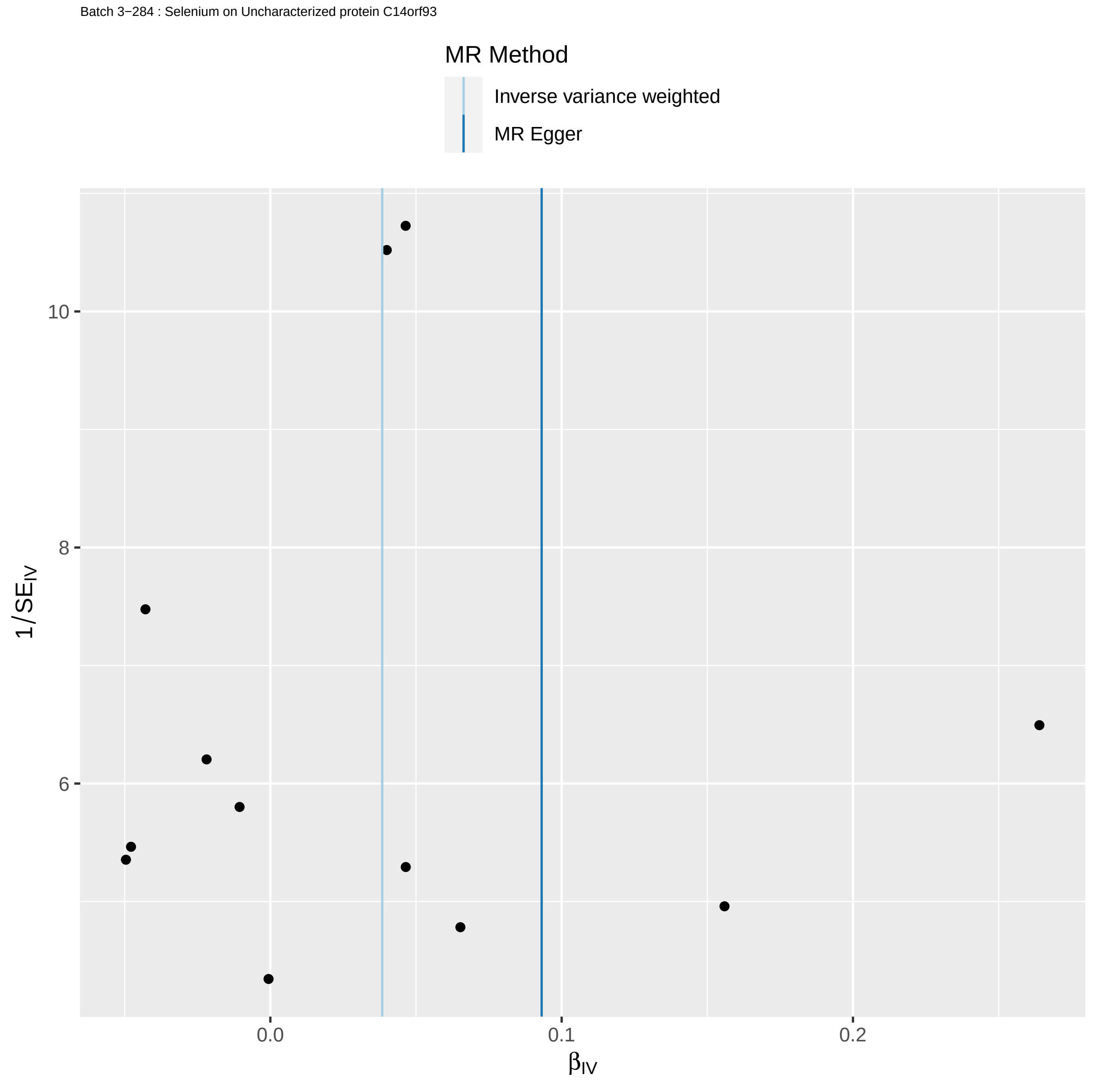

Batch 3−284 : Selenium on Uncharacterized protein C14orf93
MR Method
Inverse variance weighted
MR Egger
10
8
6
1/SE_IV
0.0
0.1
0.2
β_IV

Batch 3−285 : Selenium on Transmembrane protein C16orf54

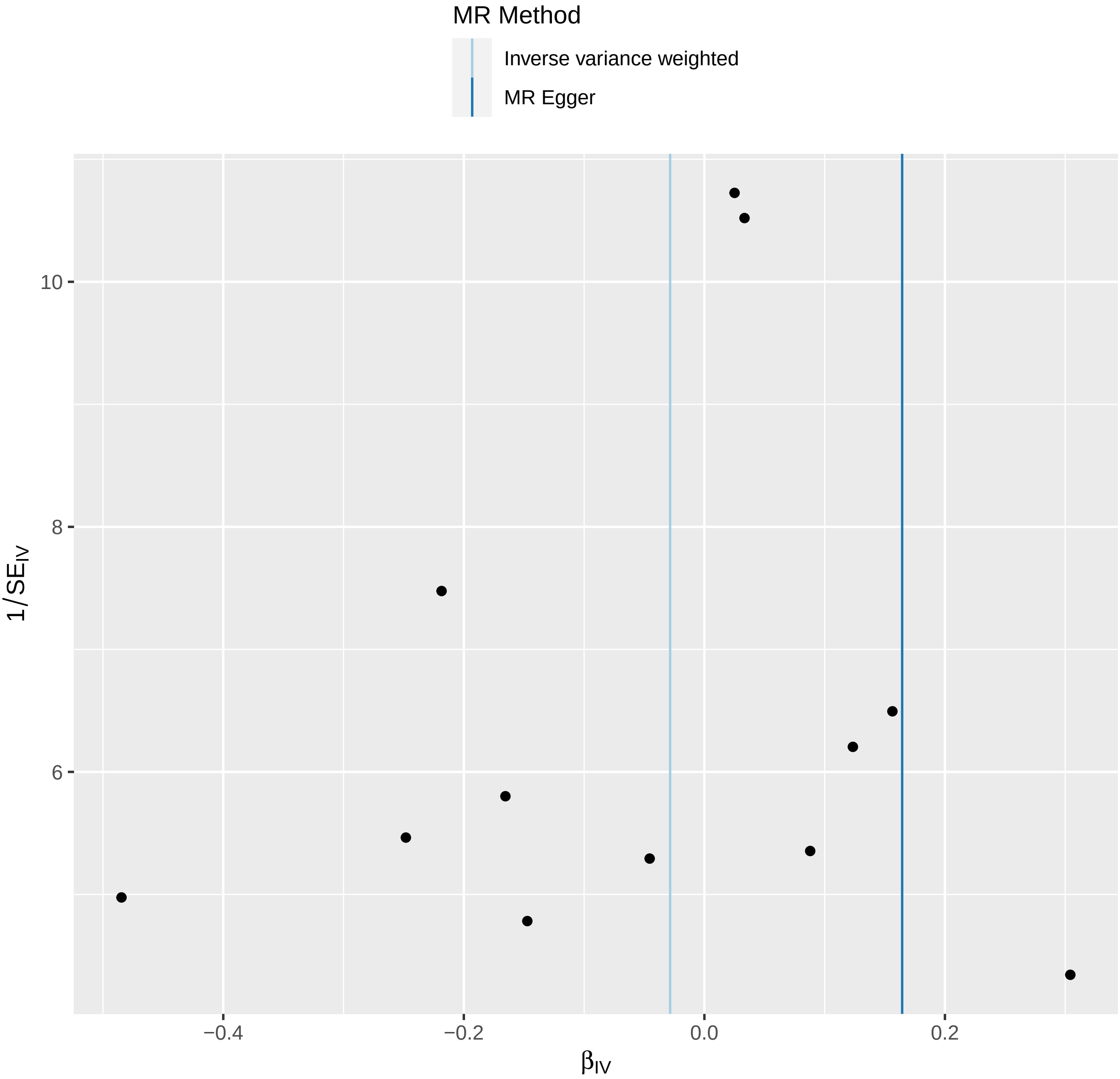

Batch 3−286 : Selenium on Uncharacterized protein C17orf67

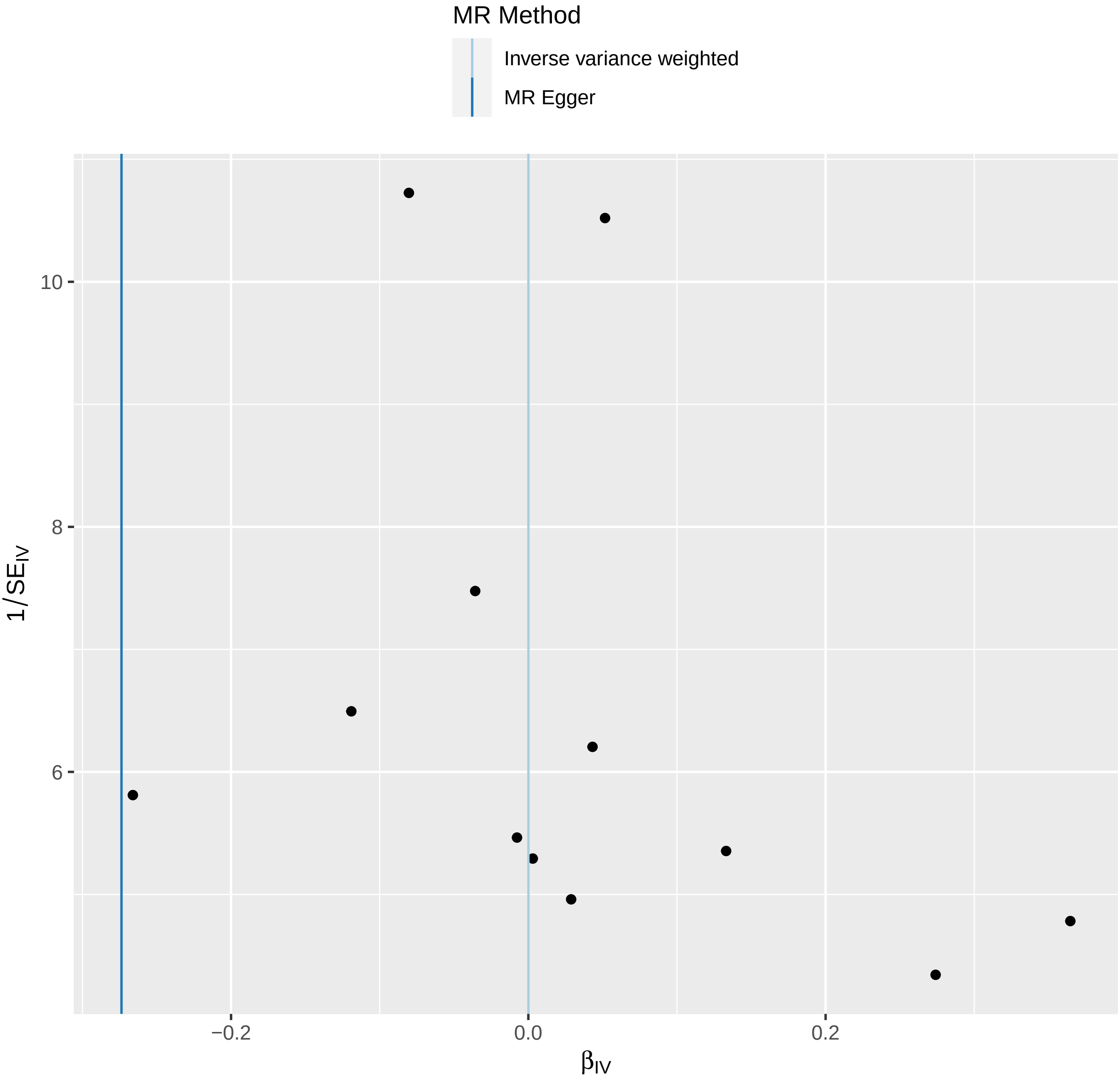

Batch 3−287 : Selenium on Uncharacterized protein C17orf78

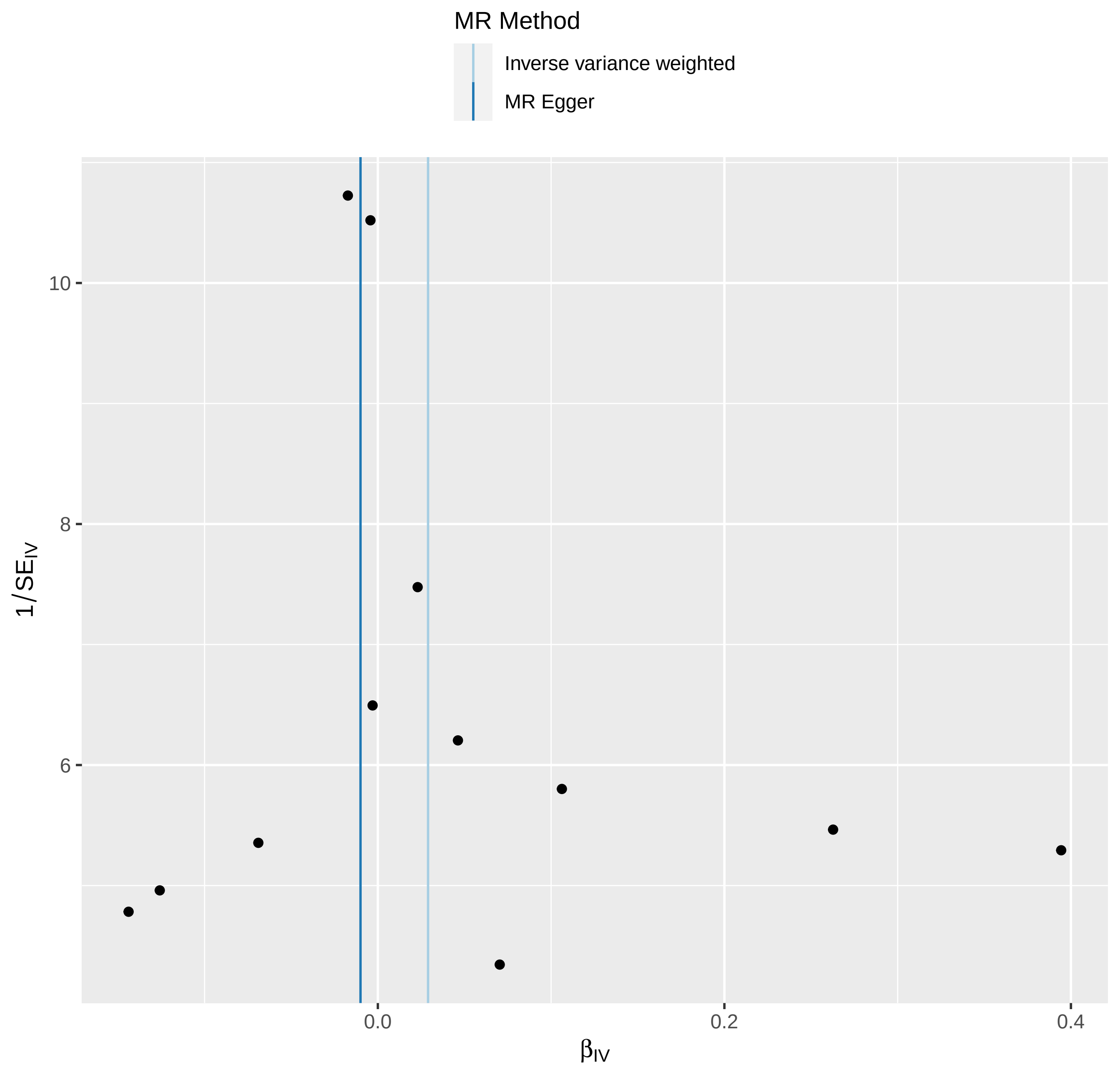

Batch 3−288 : Selenium on Uncharacterized protein C17orf78

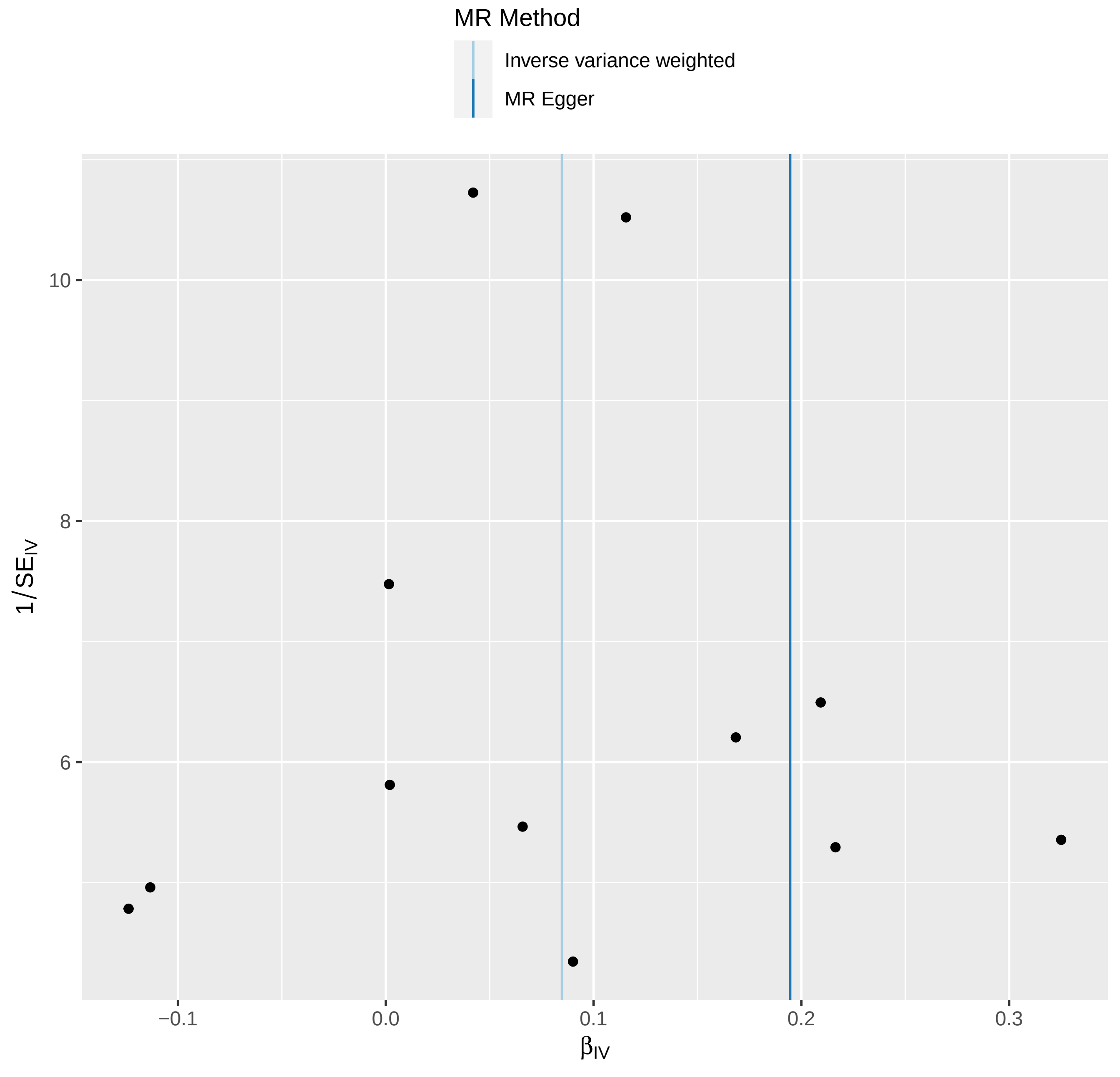

Batch 3−289 : Selenium on Uncharacterized protein C17orf89

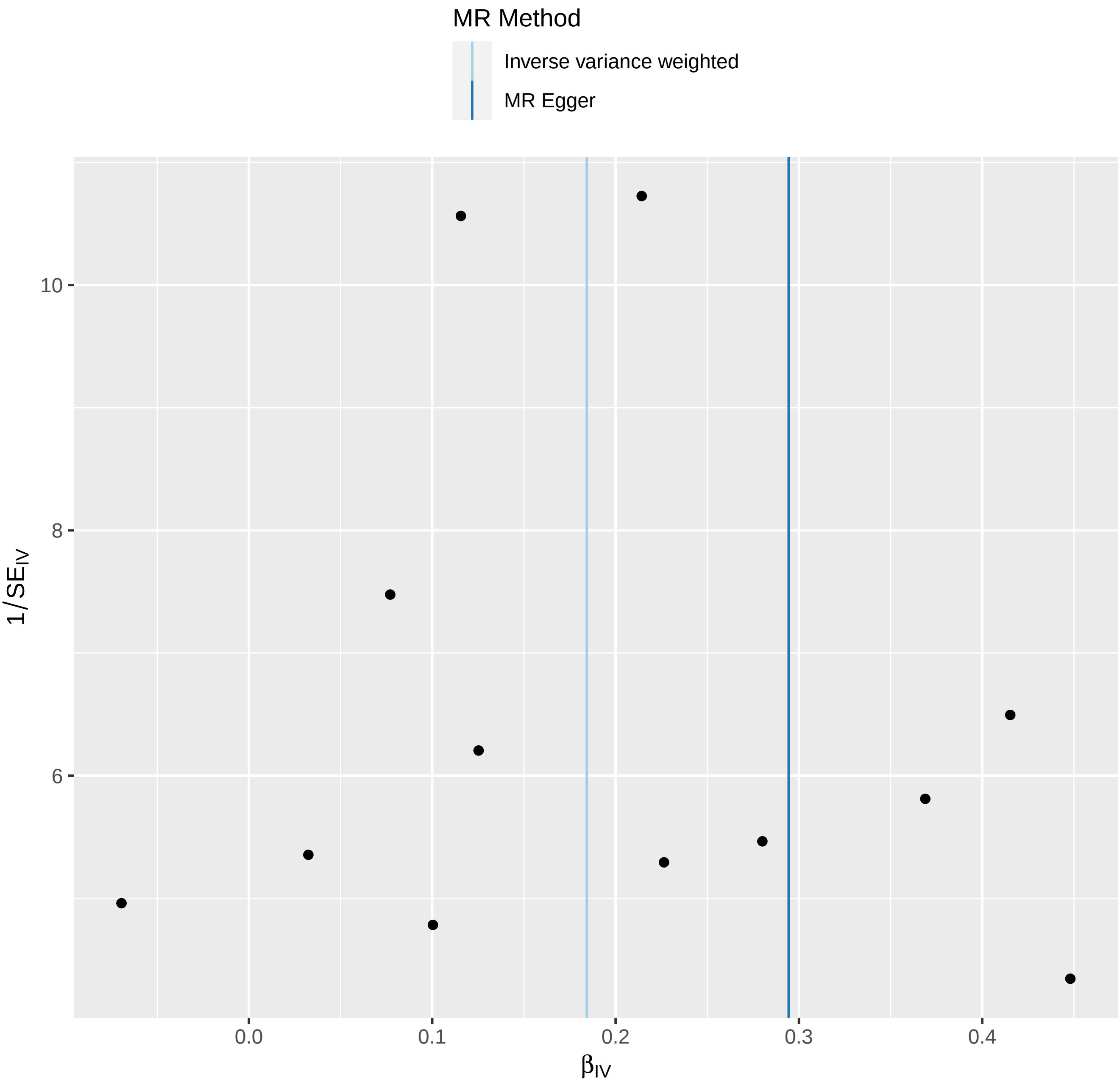

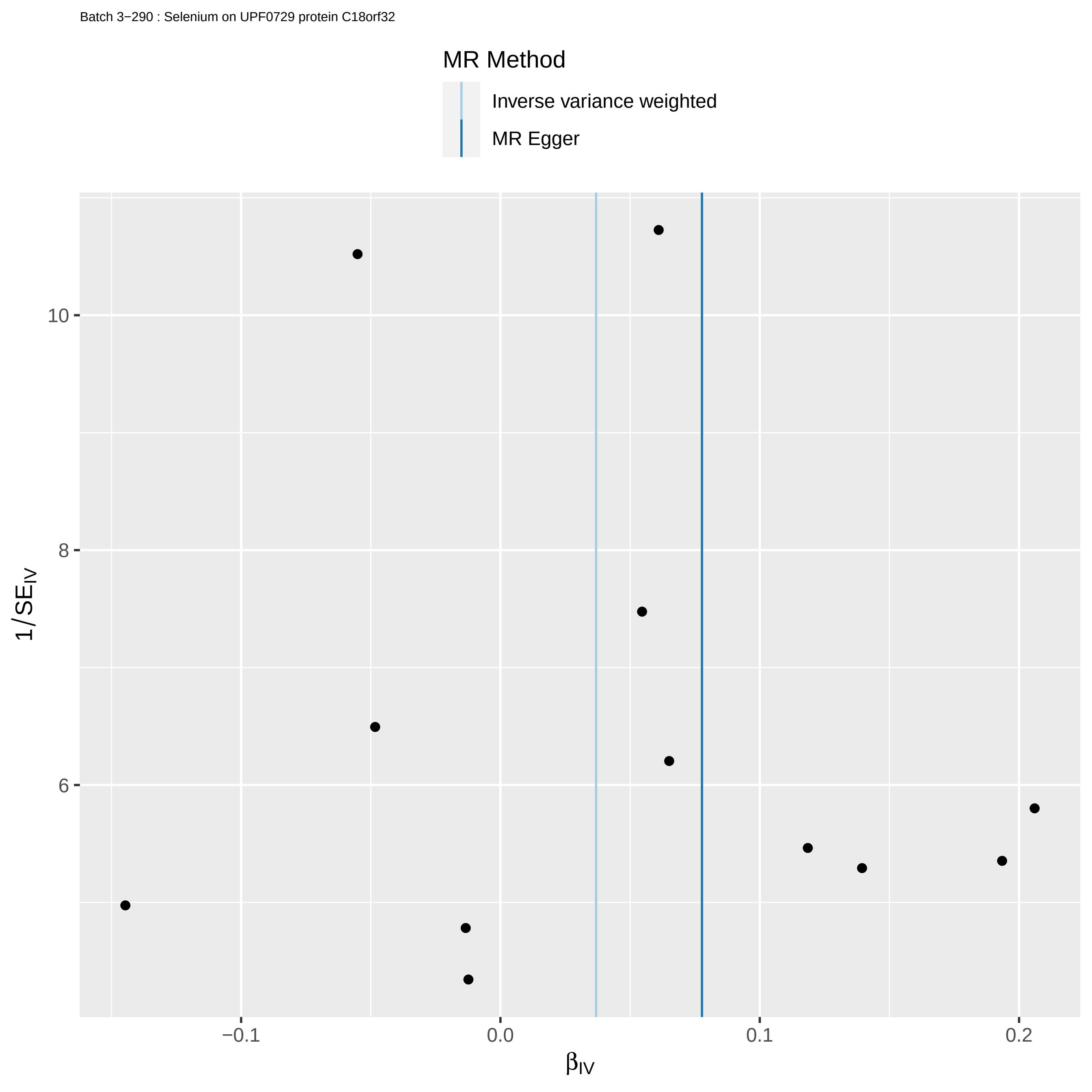

Batch 3−290 : Selenium on UPF0729 protein C18orf32
MR Method
Inverse variance weighted
MR Egger
10
8
6
1/SE_IV
−0.1
0.0
0.1
0.2
β_IV

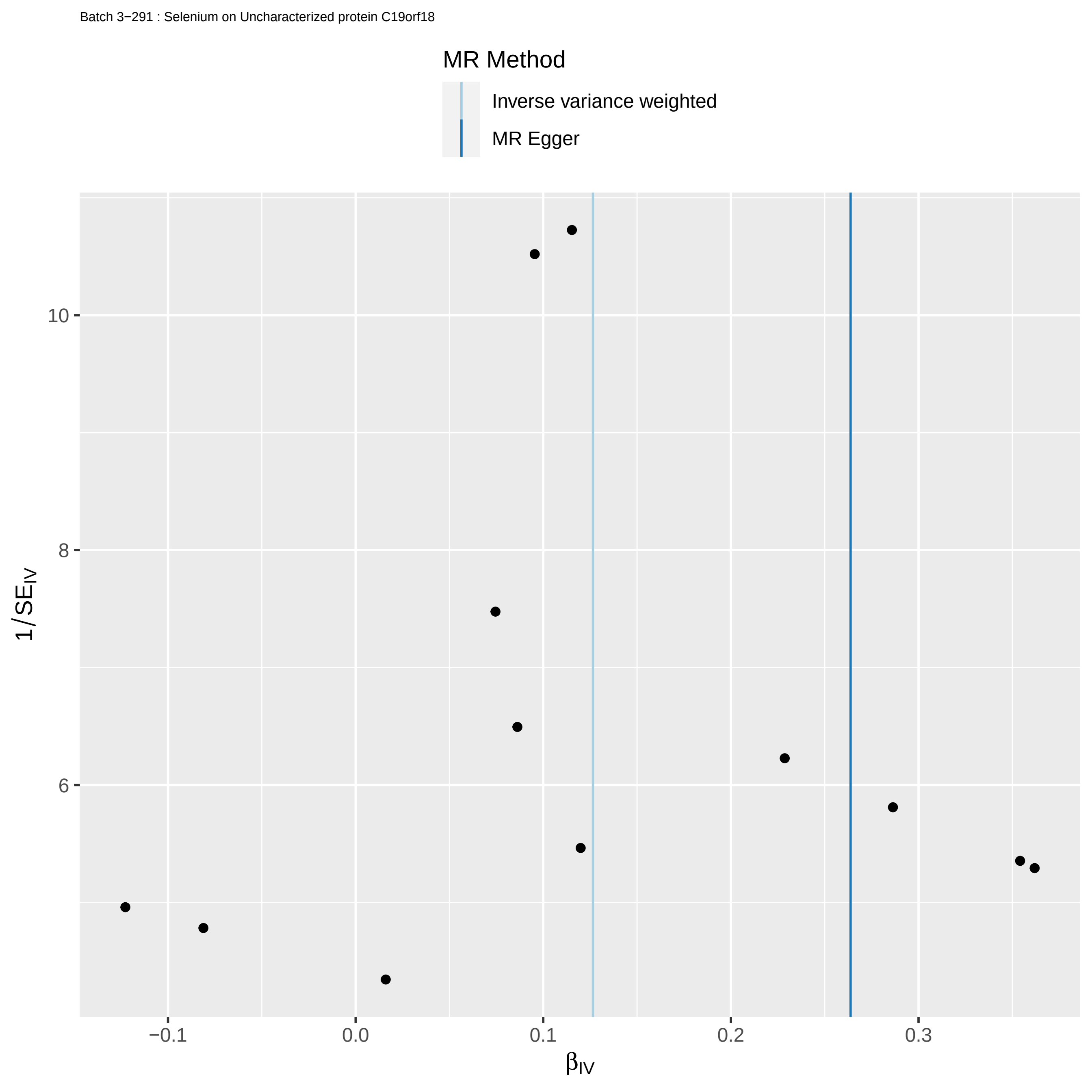
Batch 3−291 : Selenium on Uncharacterized protein C19orf18
MR Method
Inverse variance weighted
MR Egger
10
8
6
1/SE_IV
−0.1
0.0
0.1
0.2
0.3
β_IV

Batch 3−292 : Selenium on Angiopoietin−like protein 8

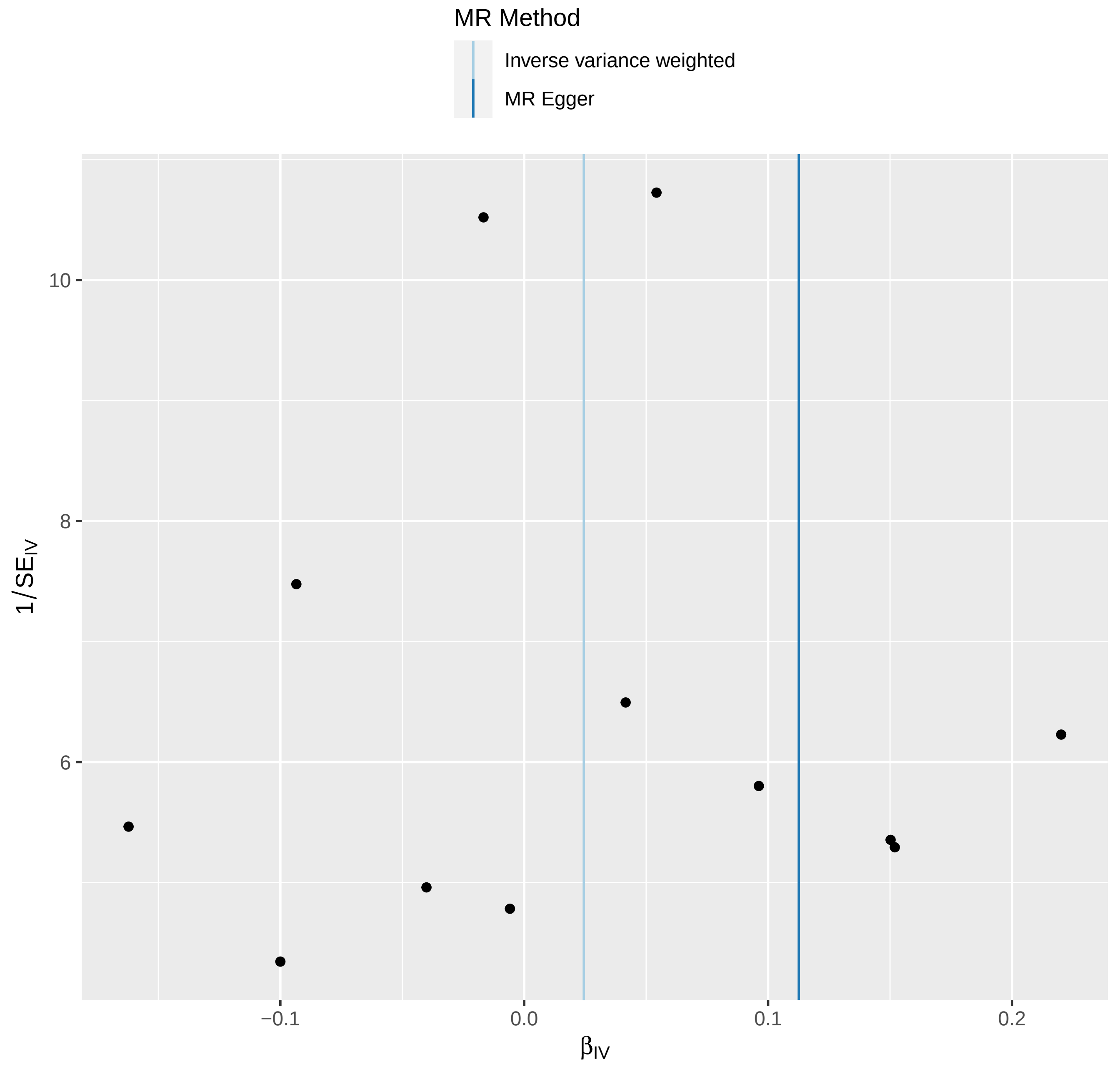

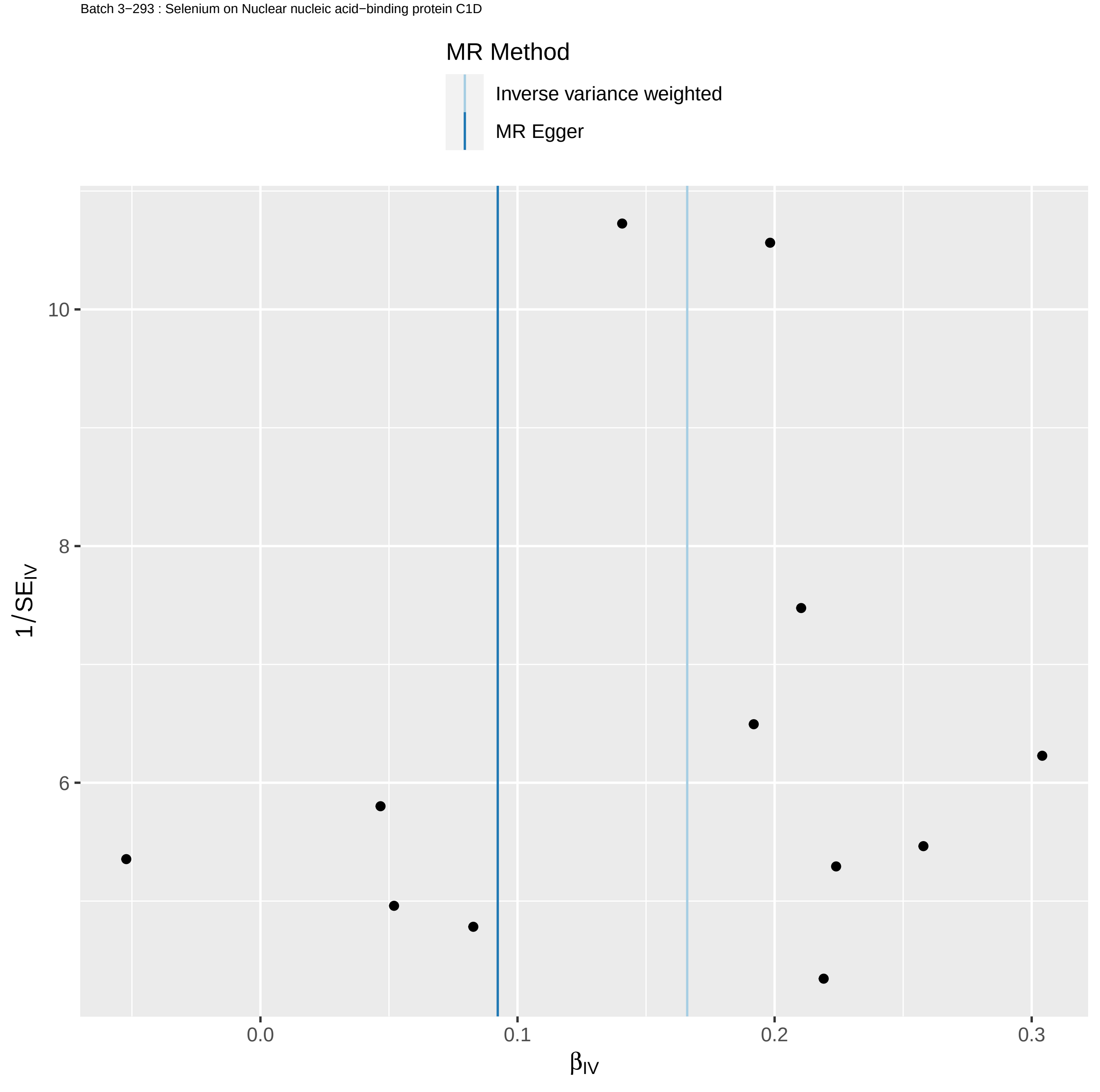
Batch 3−293 : Selenium on Nuclear nucleic acid−binding protein C1D
MR Method
Inverse variance weighted
MR Egger
10
8
6
1/SE_IV
0.0
0.1
0.2
0.3
β_IV

Batch 3−294 : Selenium on C1GALT1−specific chaperone 1

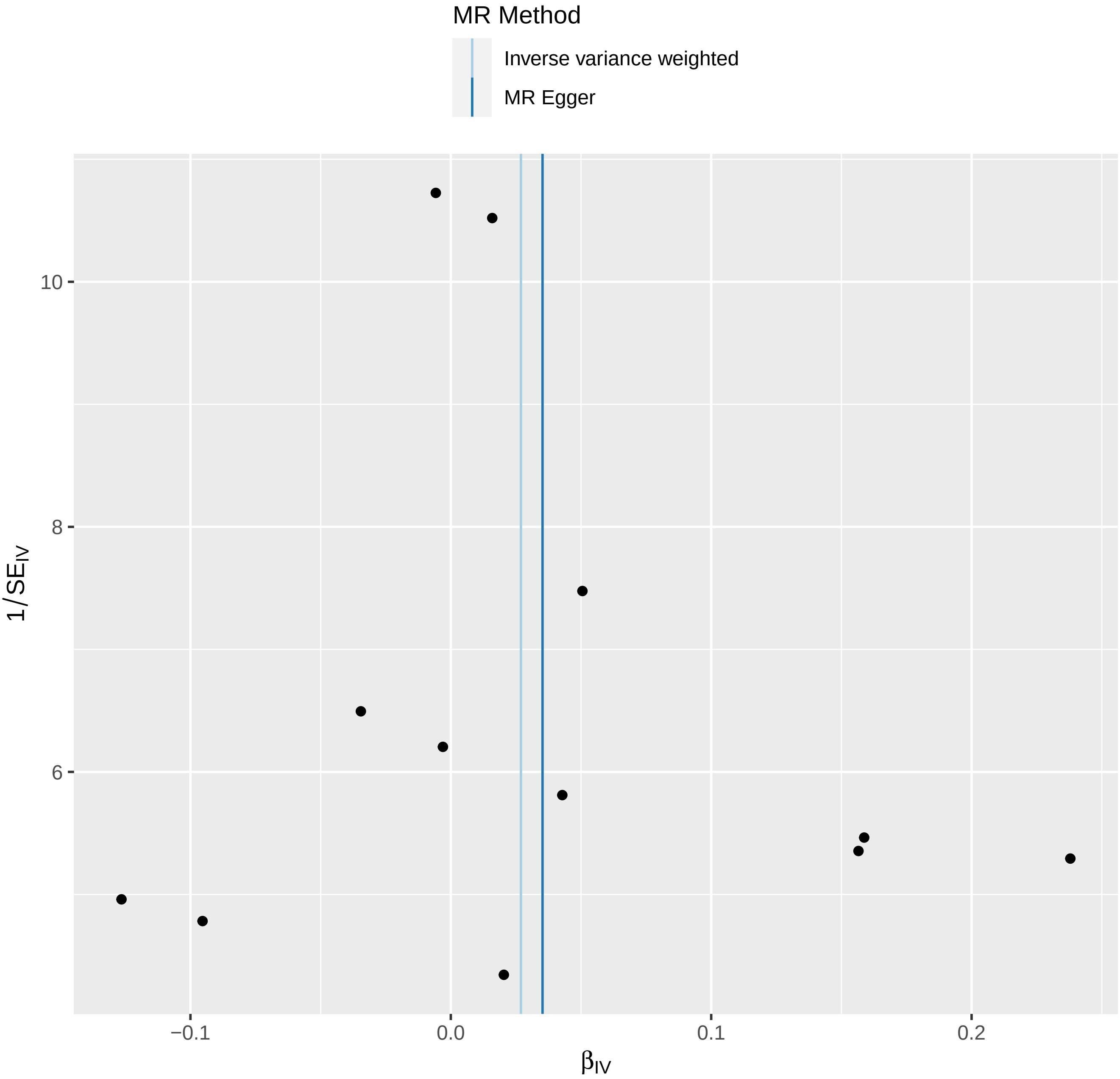

Batch 3−295 : Selenium on Uncharacterized protein C1orf115

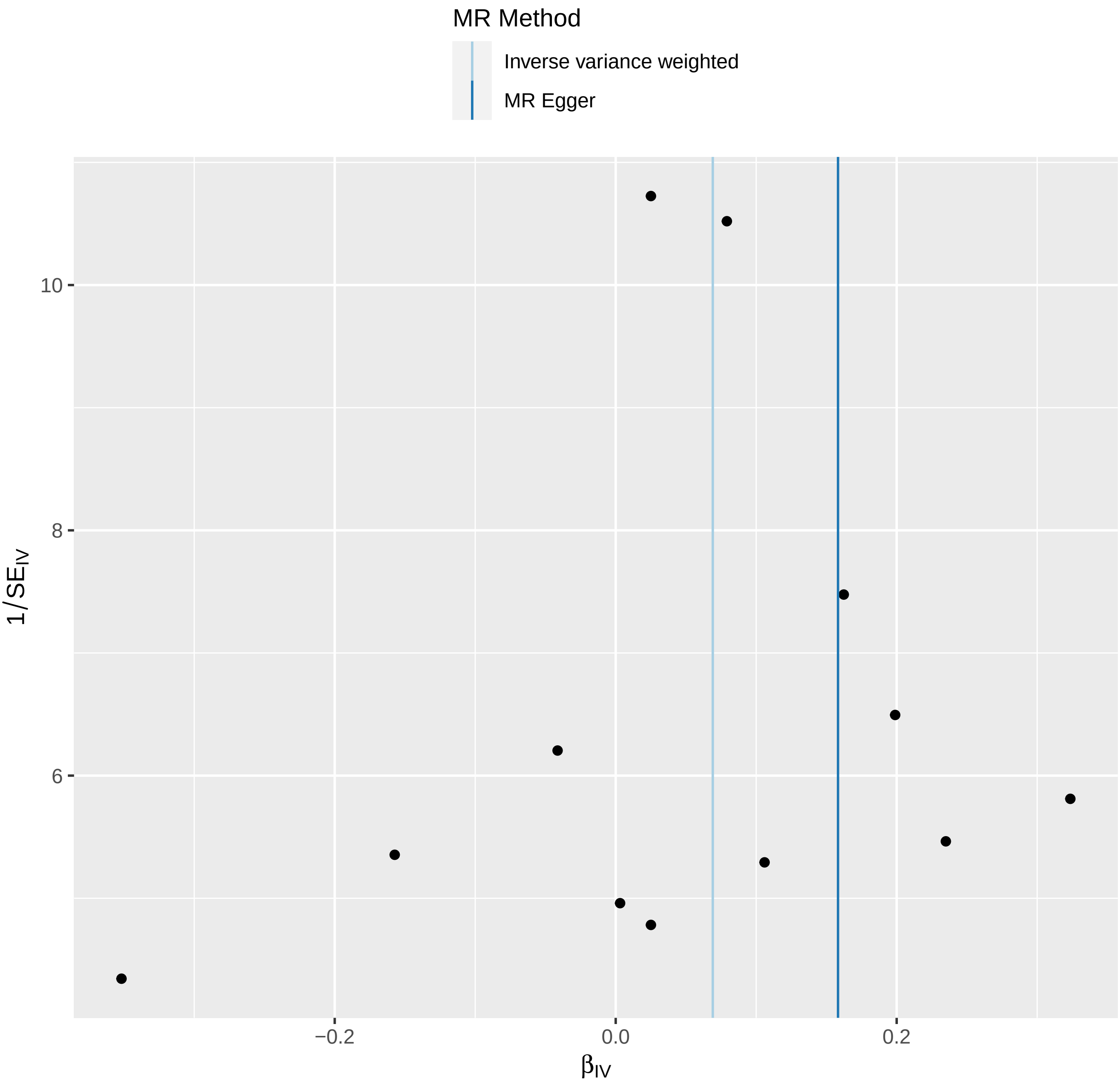

Batch 3−296 : Selenium on Uncharacterized protein C1orf185

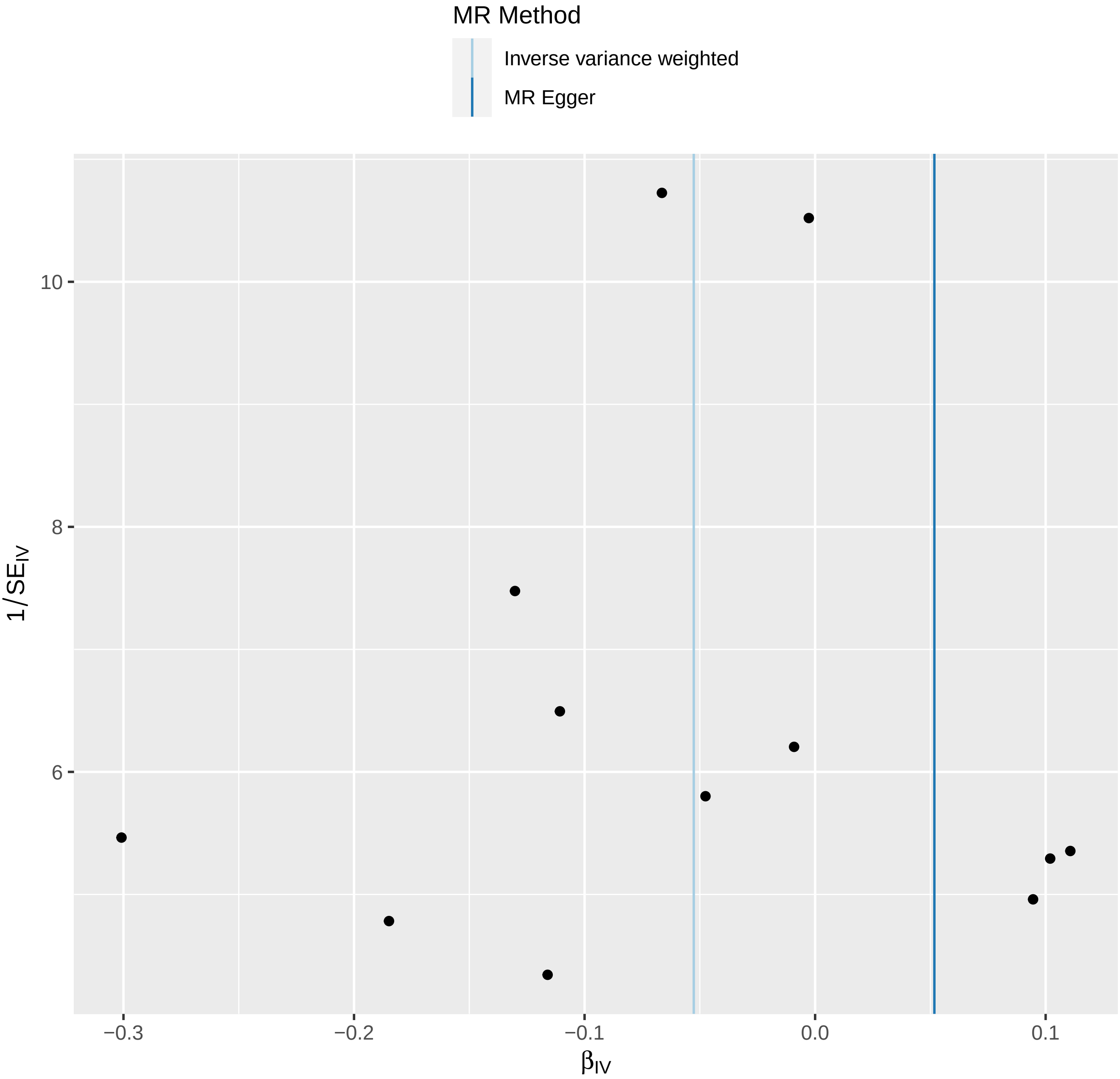

Batch 3−297 : Selenium on Uncharacterized protein C1orf198

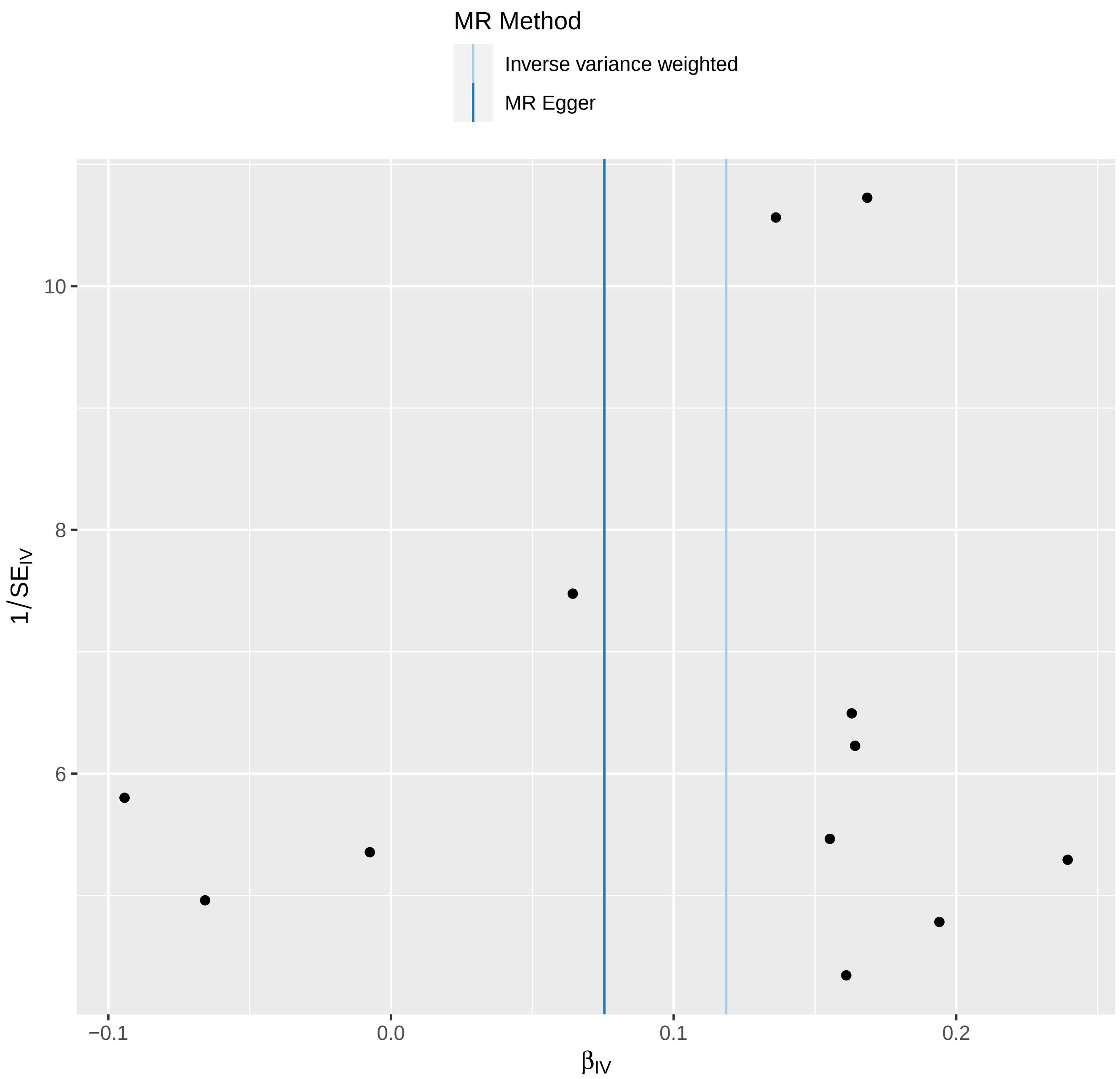

Batch 3−298 : Selenium on Type III endosome membrane protein TEMP

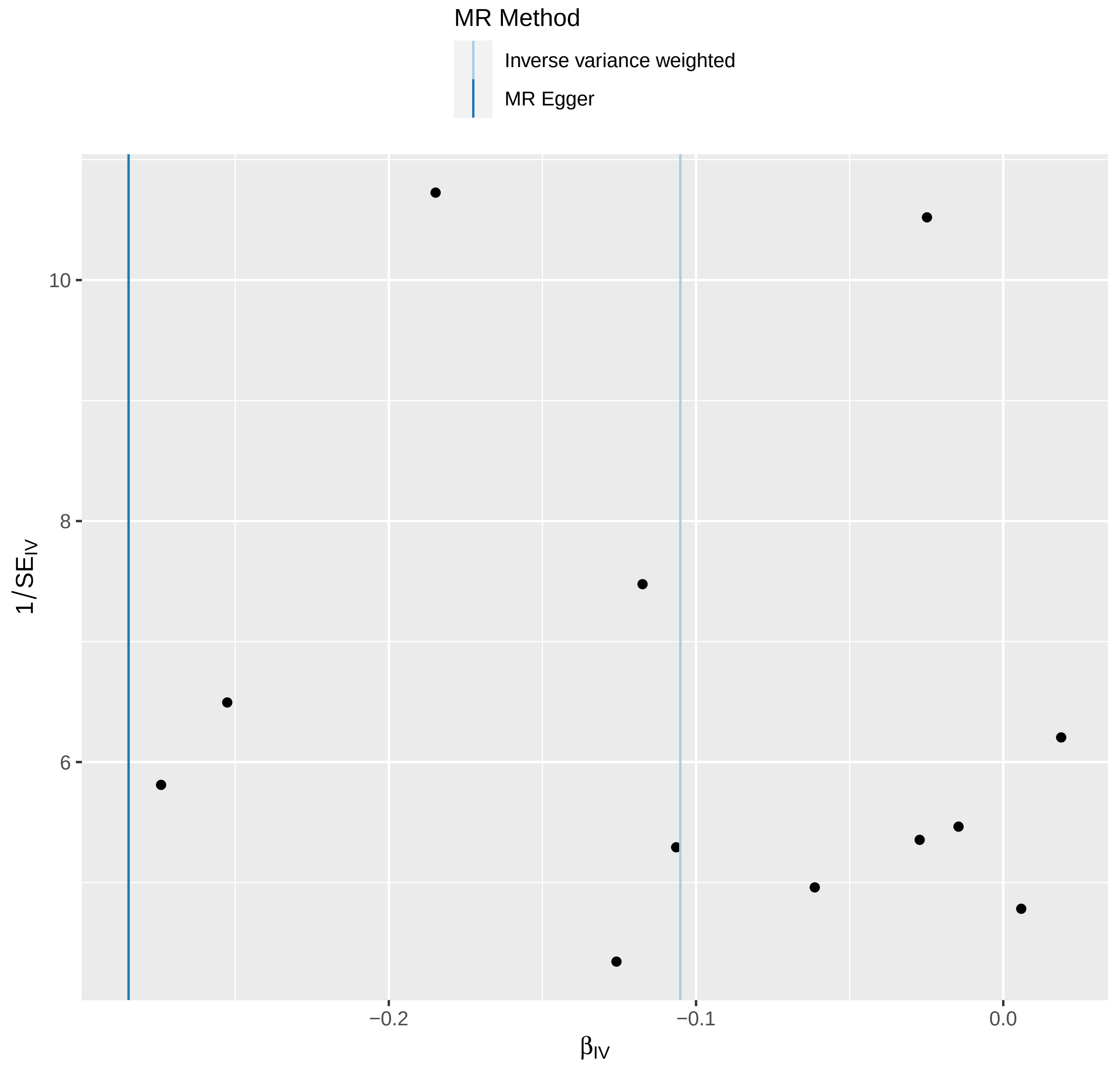

Batch 3−299 : Selenium on Uncharacterized protein C1orf226

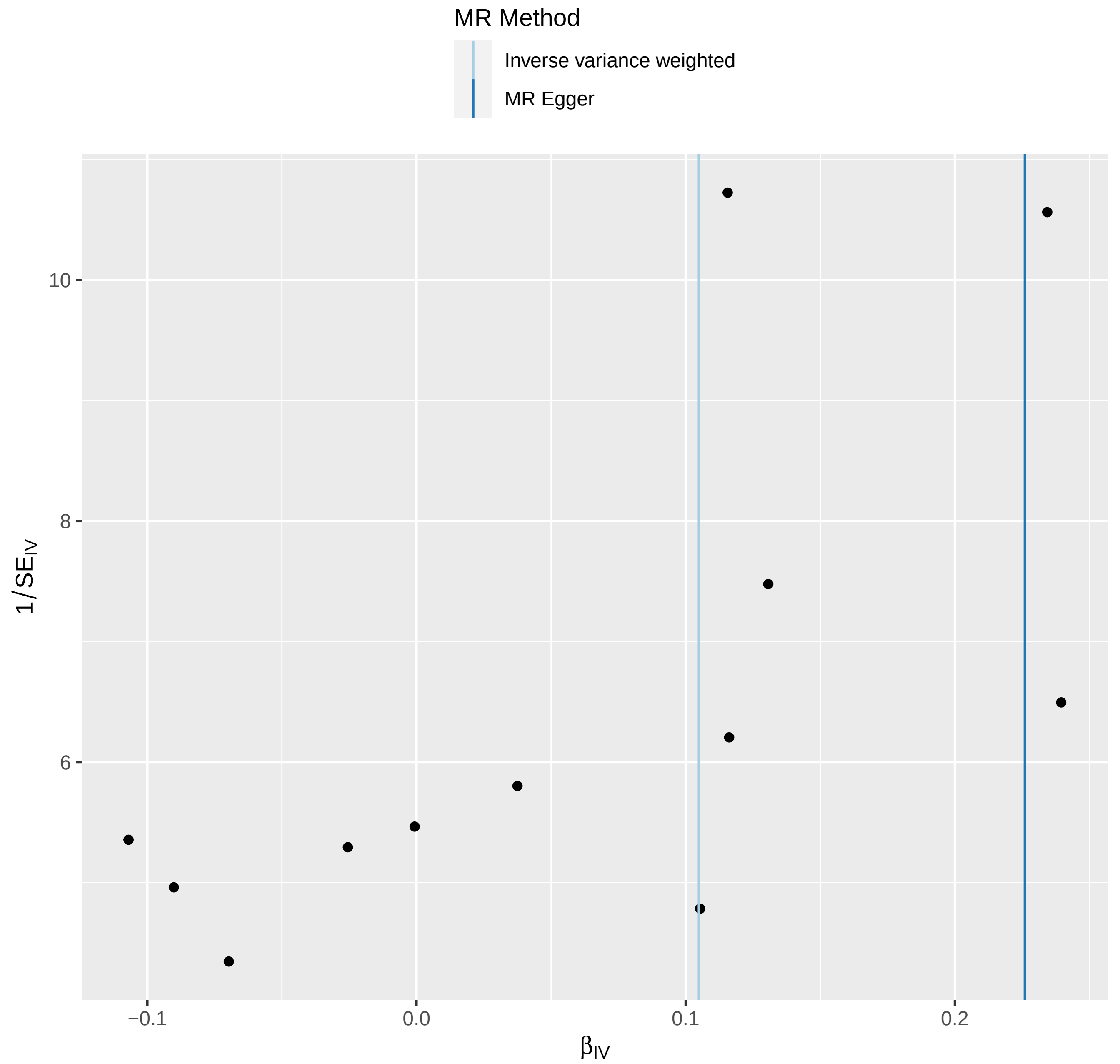

Batch 3−300 : Selenium on Complement component 1 Q subcomponent−binding protein, mitochondrial

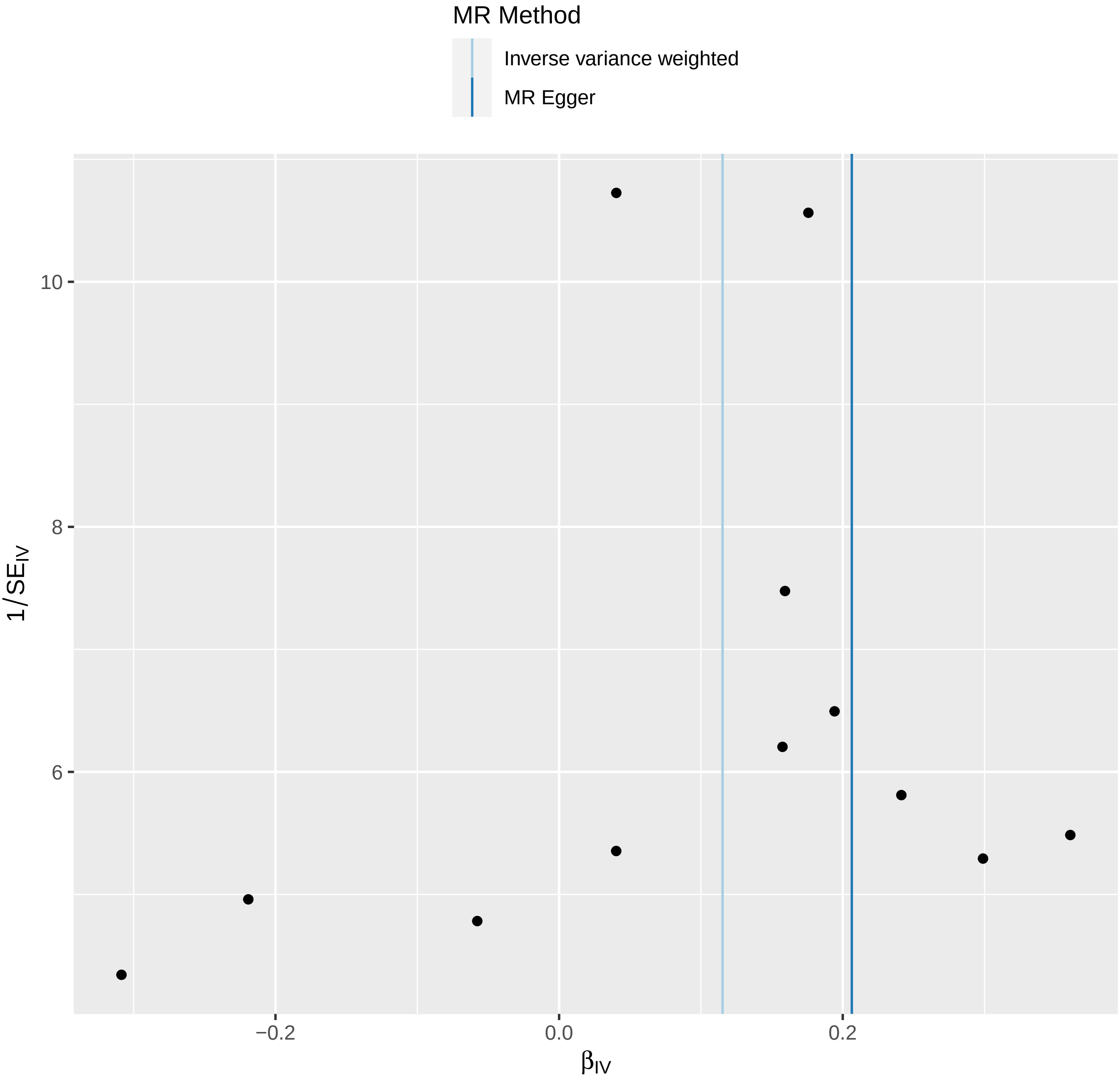

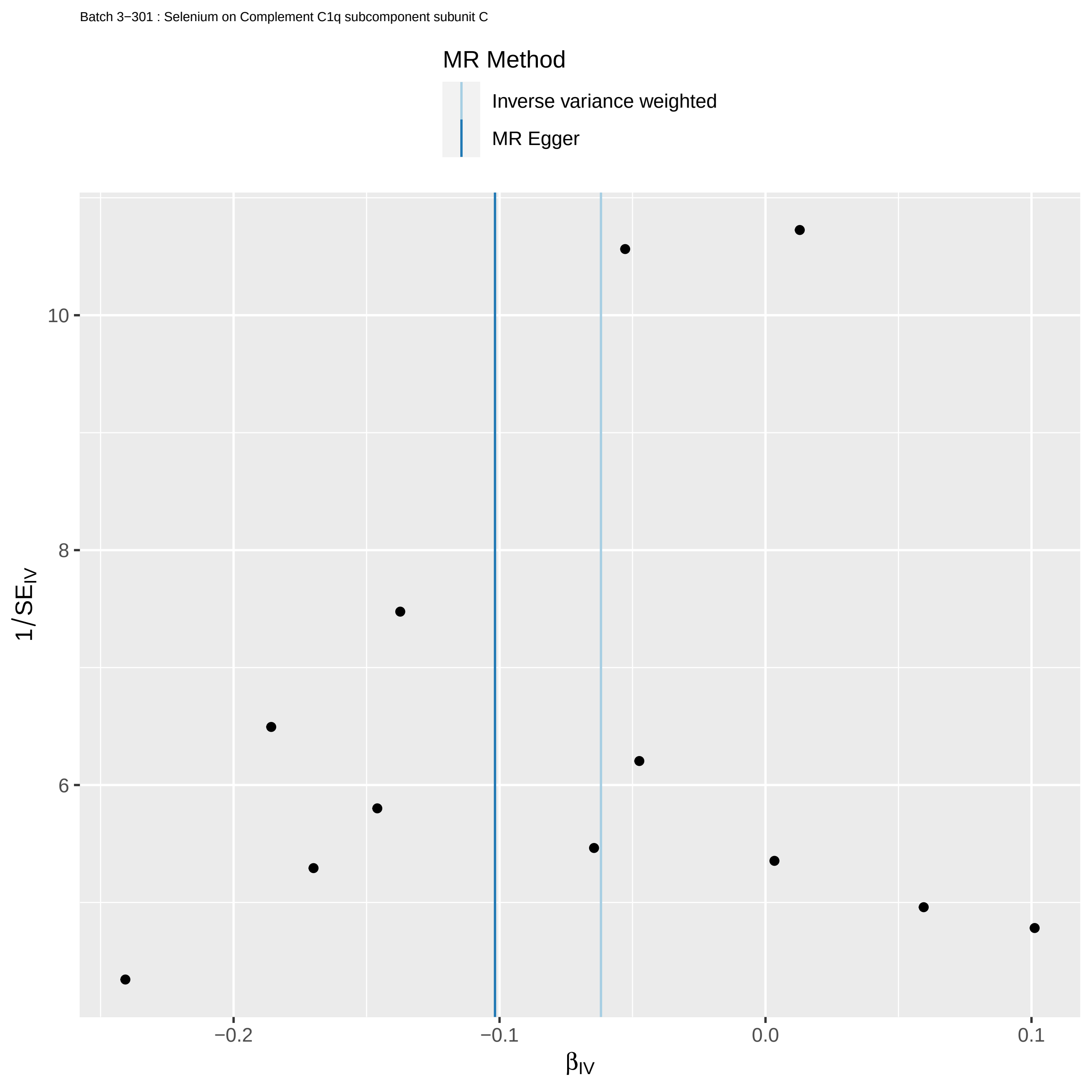

Batch 3−301 : Selenium on Complement C1q subcomponent subunit C
MR Method
Inverse variance weighted
MR Egger
10
8
6
1/SE_IV
−0.2
−0.1
0.0
0.1
β_IV

Batch 3−302 : Selenium on Complement C1q−like protein 4

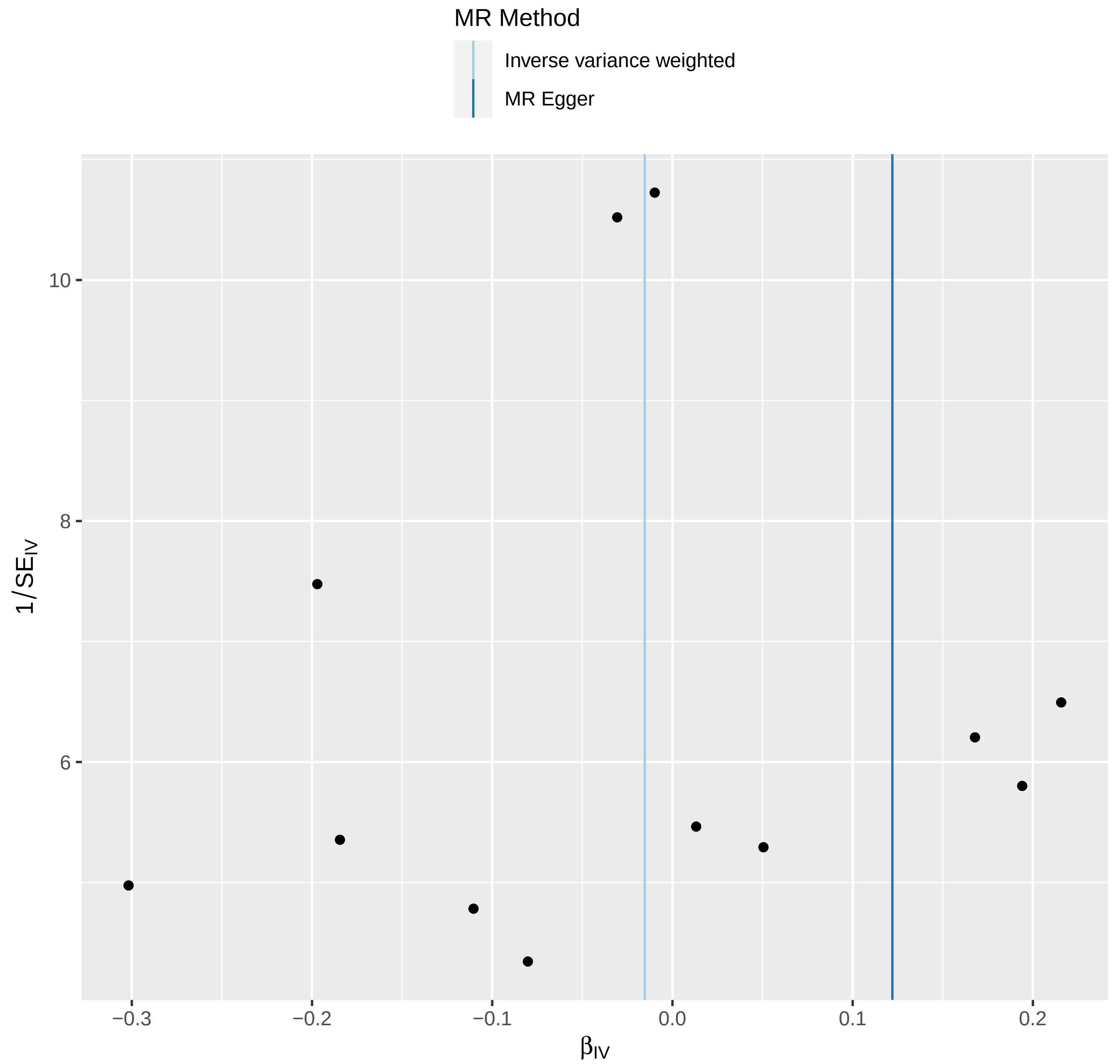

Batch 3−303 : Selenium on Complement C1q tumor necrosis factor−related protein 1

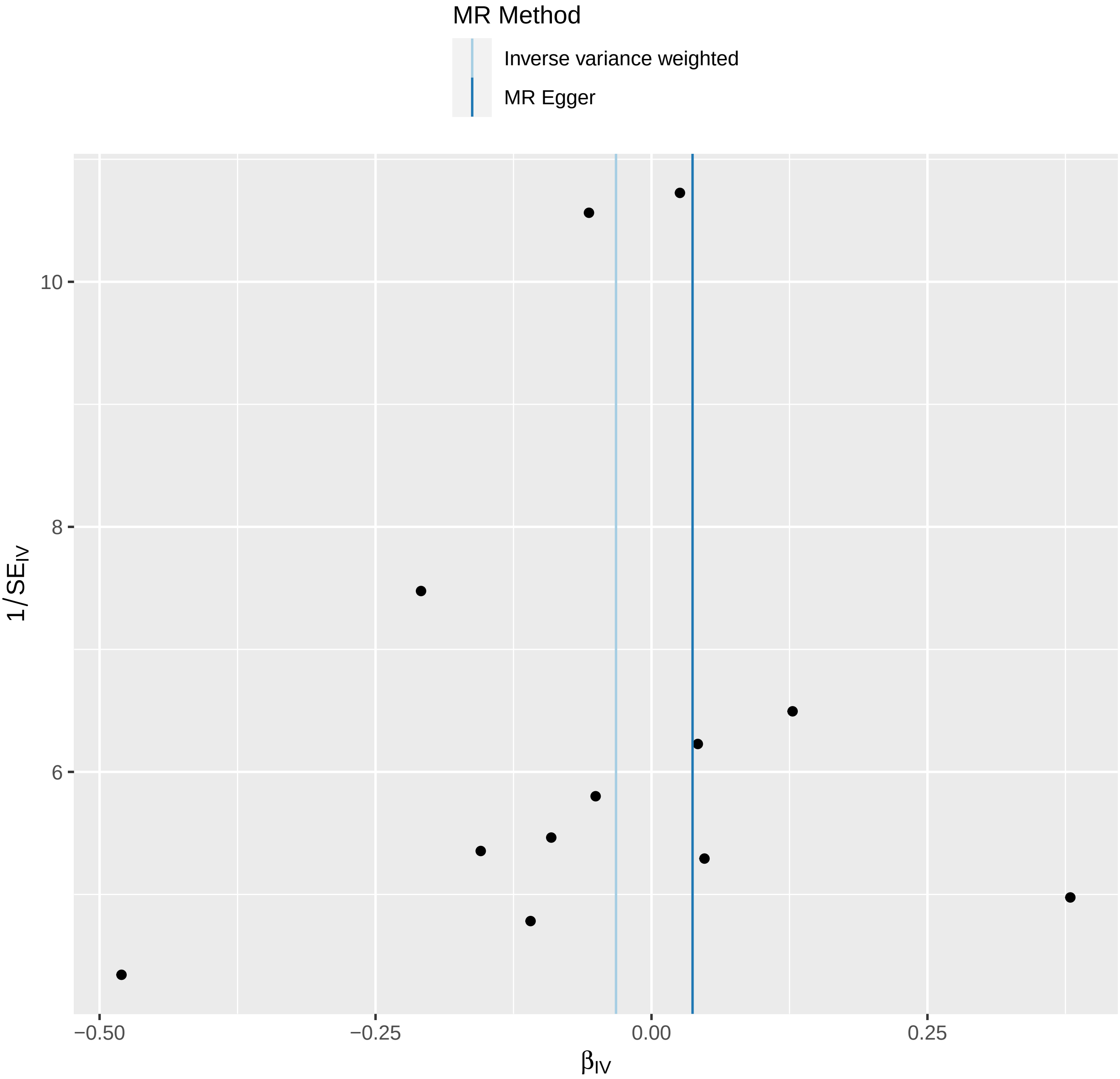

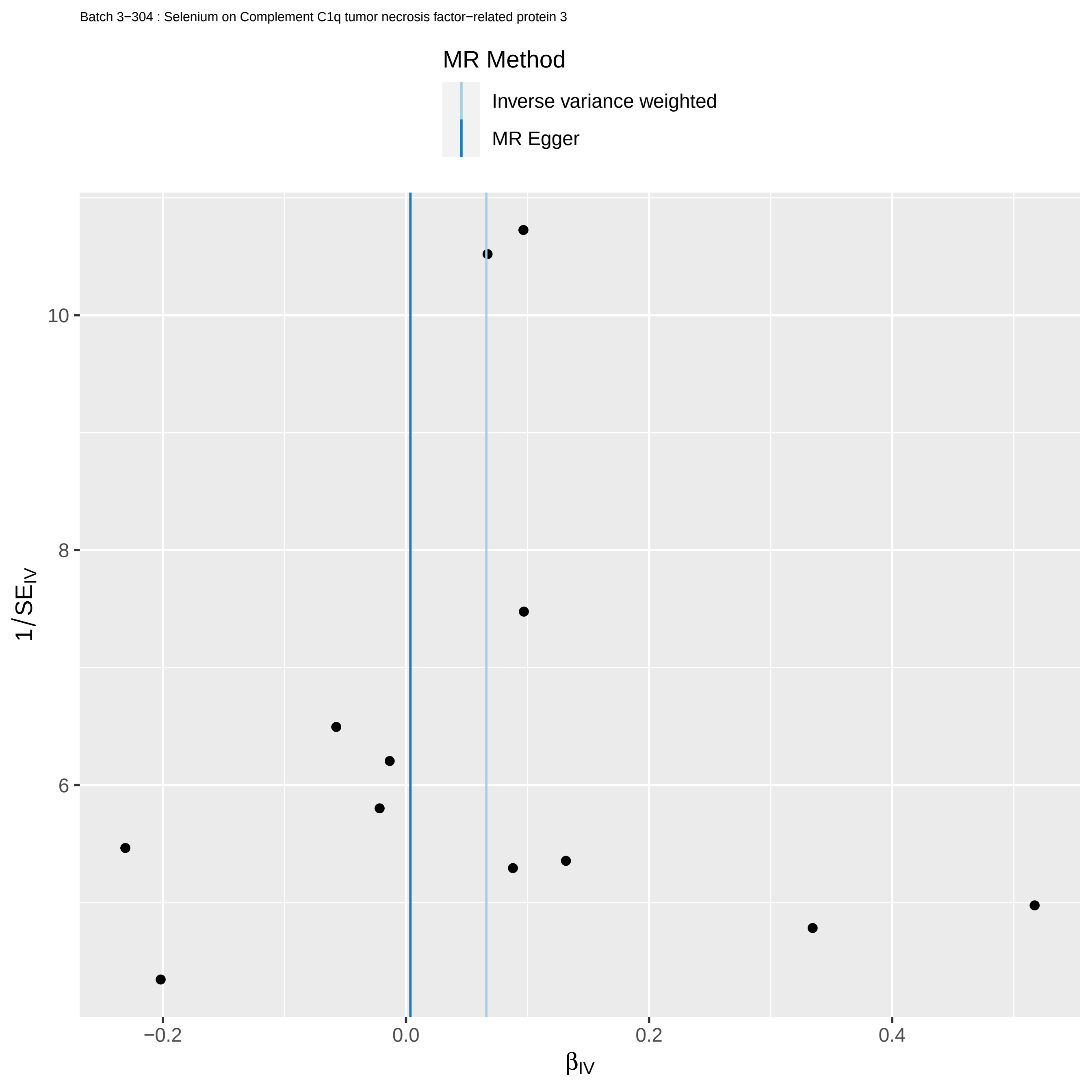

Batch 3−304 : Selenium on Complement C1q tumor necrosis factor−related protein 3
MR Method
Inverse variance weighted
MR Egger
10
8
6
1/SE$_{IV}$
−0.2
0.0
0.2
0.4
β$_{IV}$

Batch 3−305 : Selenium on Complement C1q tumor necrosis factor−related protein 5

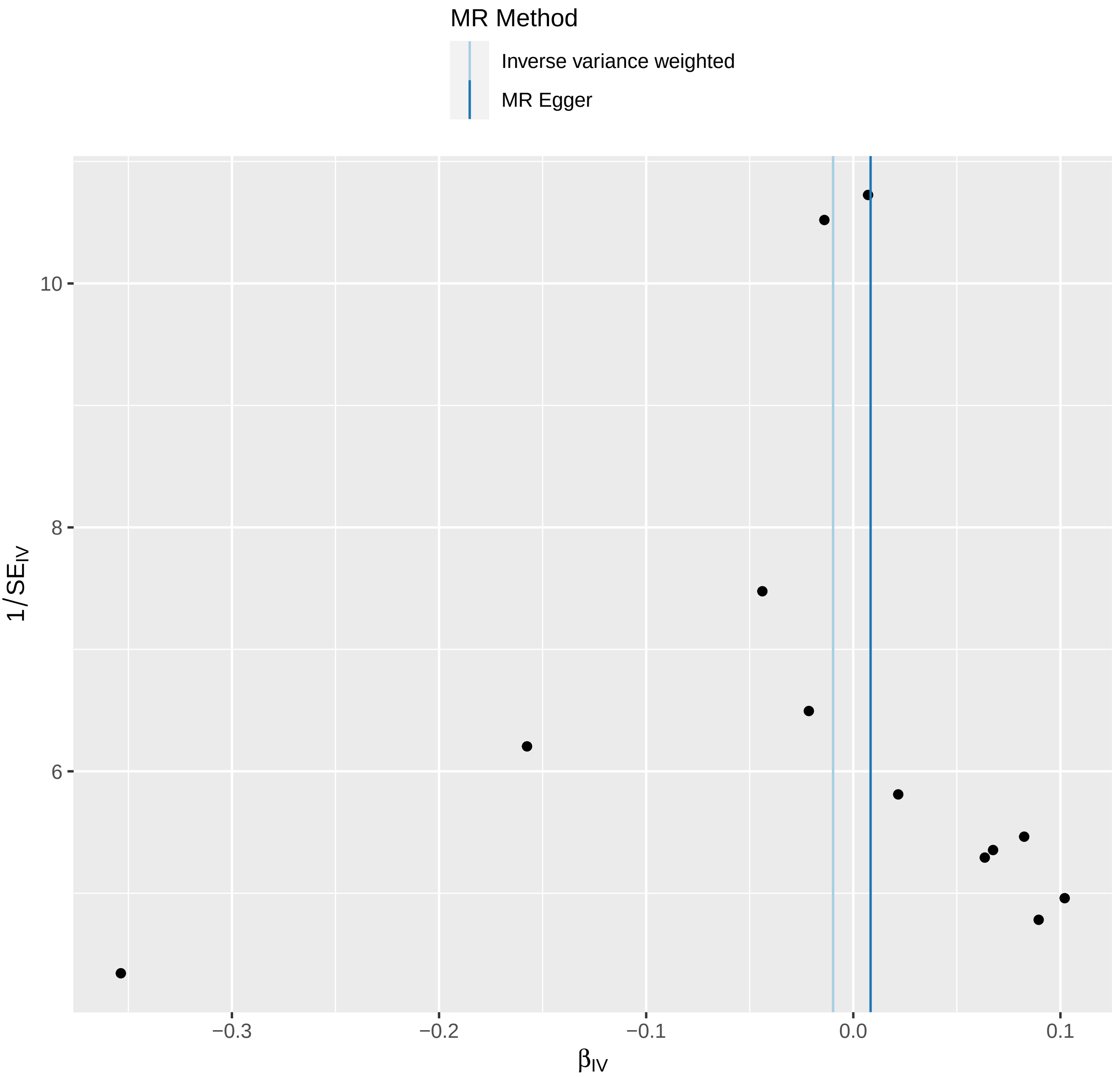

Batch 3−306 : Selenium on Complement C1q and tumor necrosis factor−related protein 9A

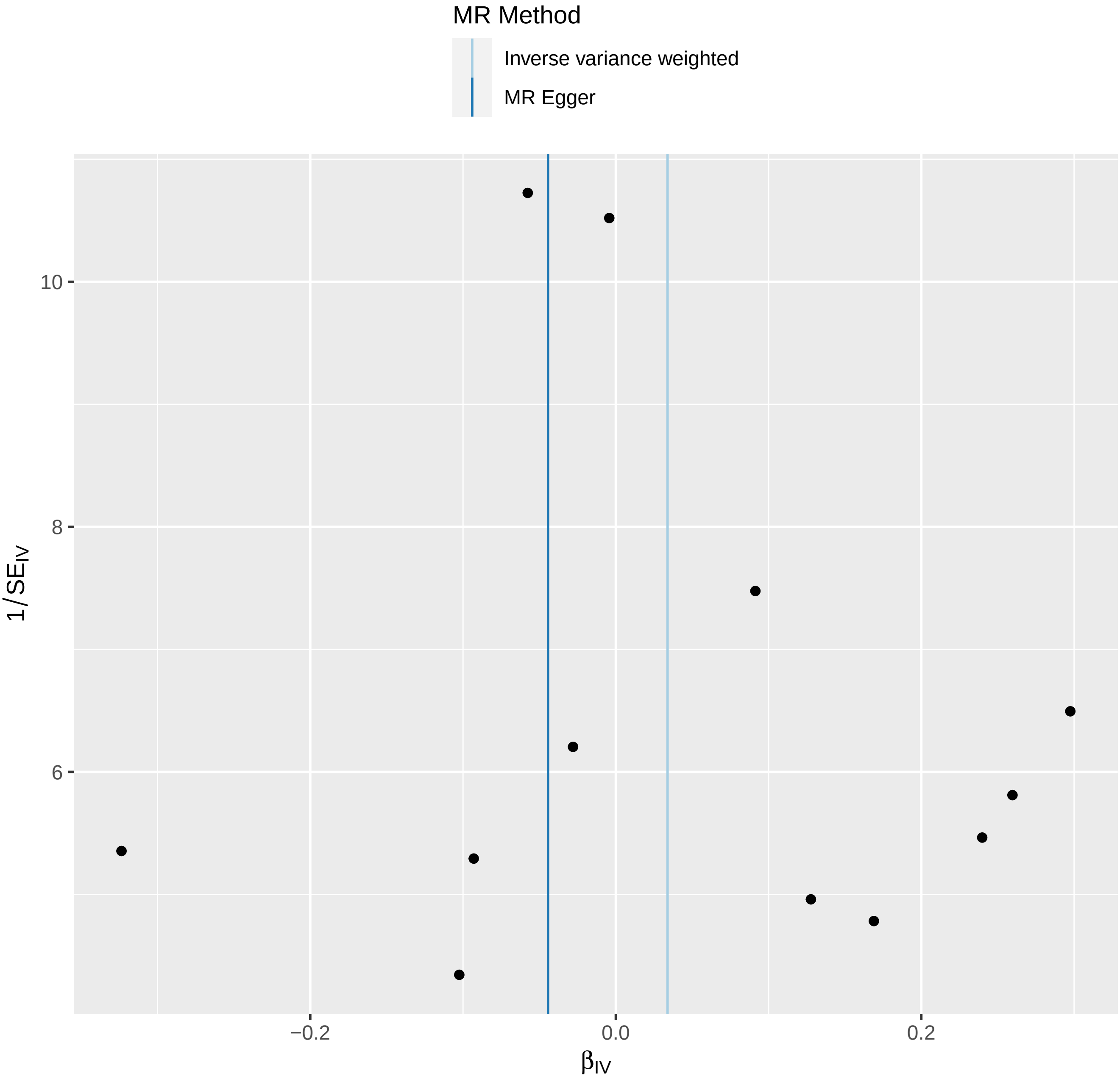

Batch 3−307 : Selenium on Complement C1r subcomponent−like protein

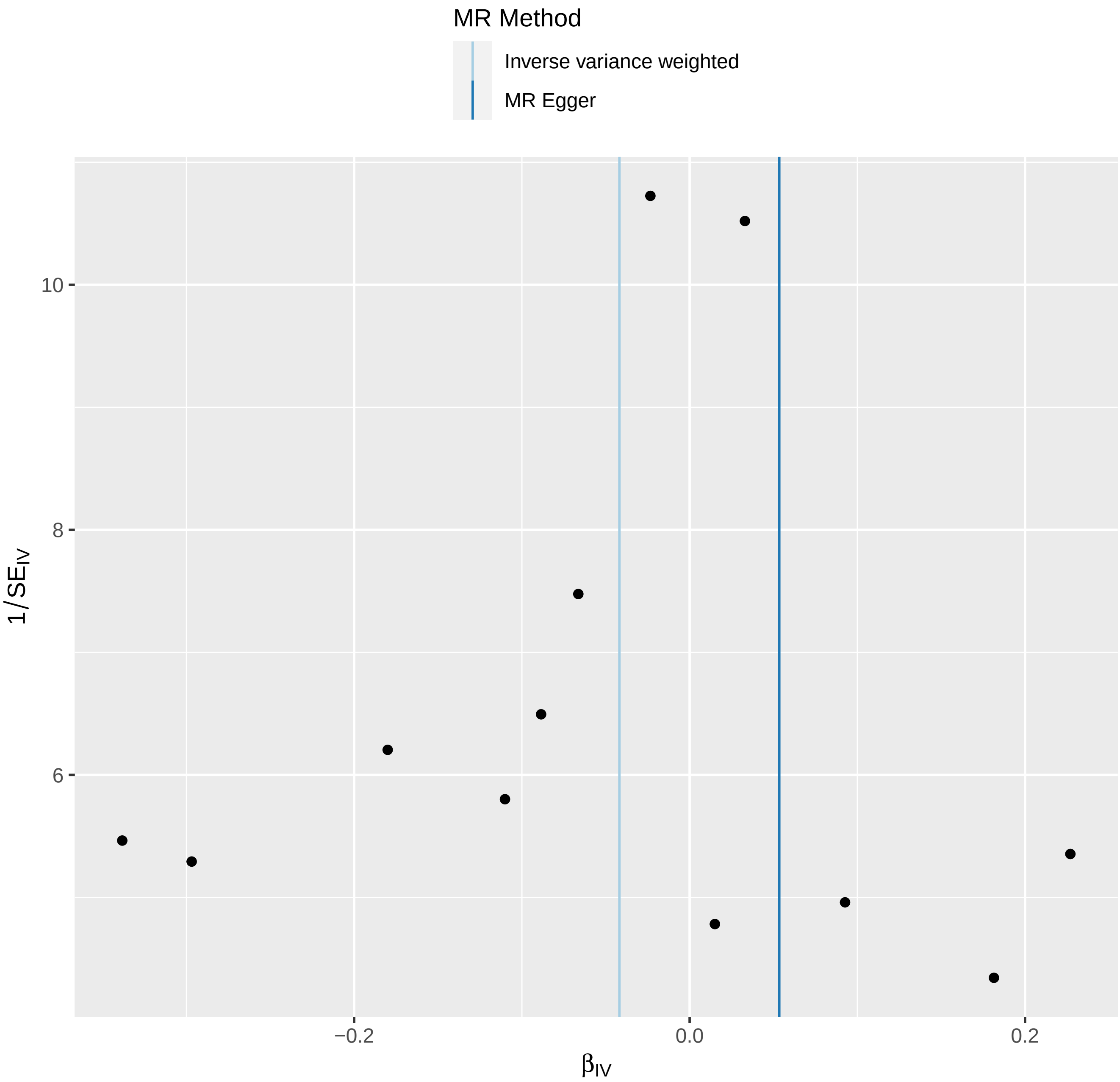

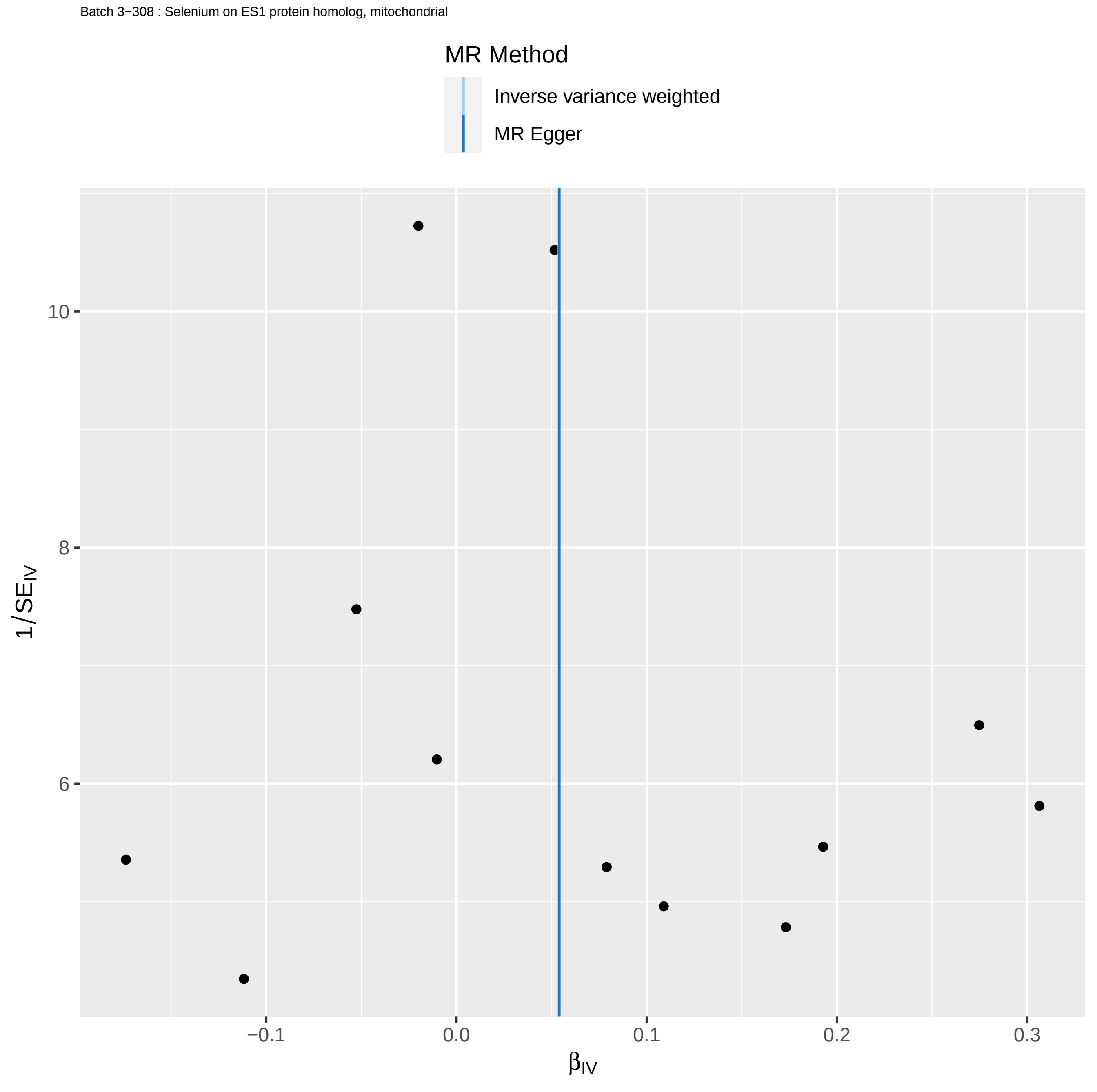
Batch 3−308 : Selenium on ES1 protein homolog, mitochondrial
MR Method
Inverse variance weighted
MR Egger
10
8
6
1/SE$_{IV}$
−0.1
0.0
0.1
0.2
0.3
β$_{IV}$

Batch 3−309 : Selenium on Uncharacterized protein C22orf15

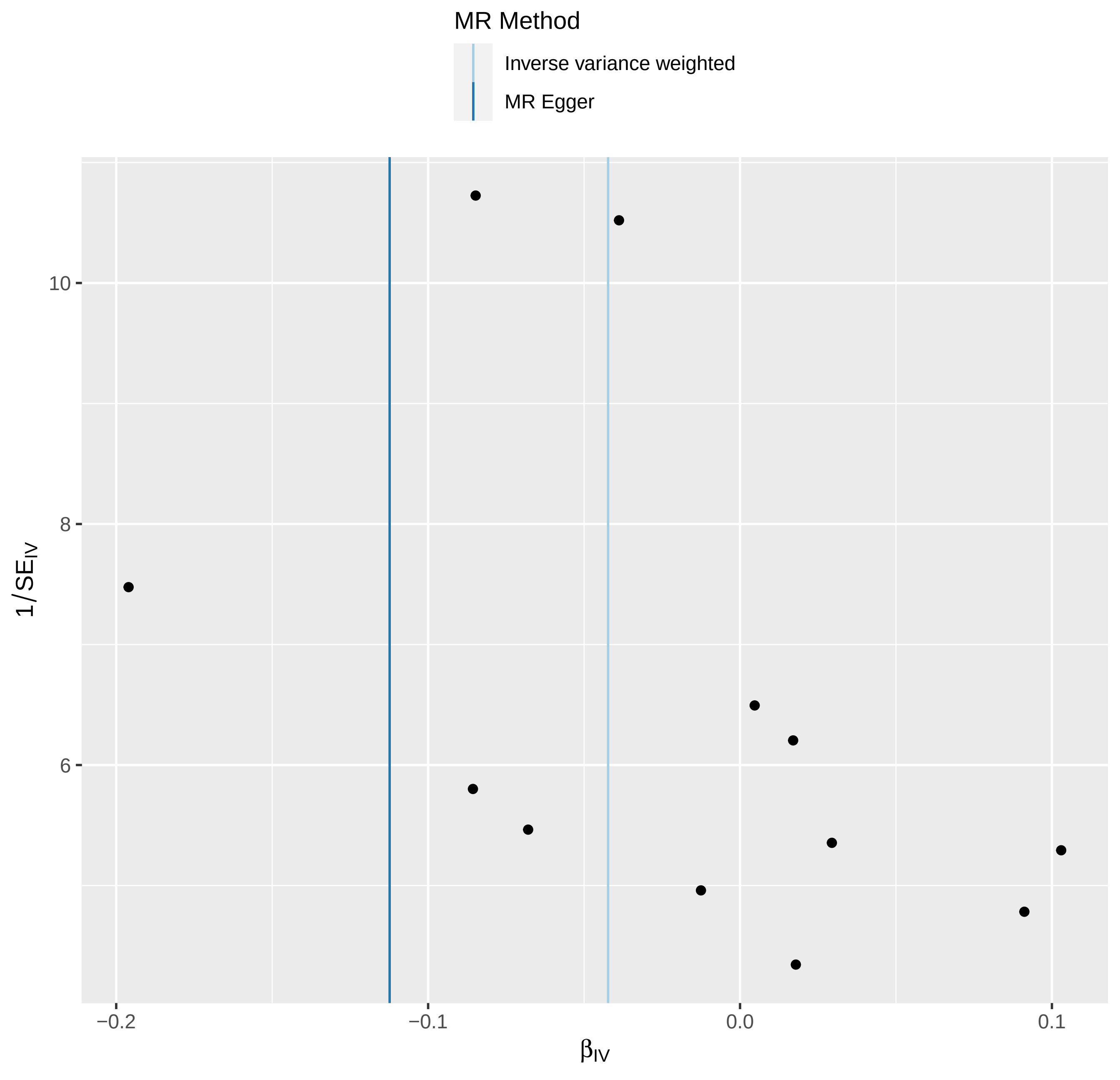

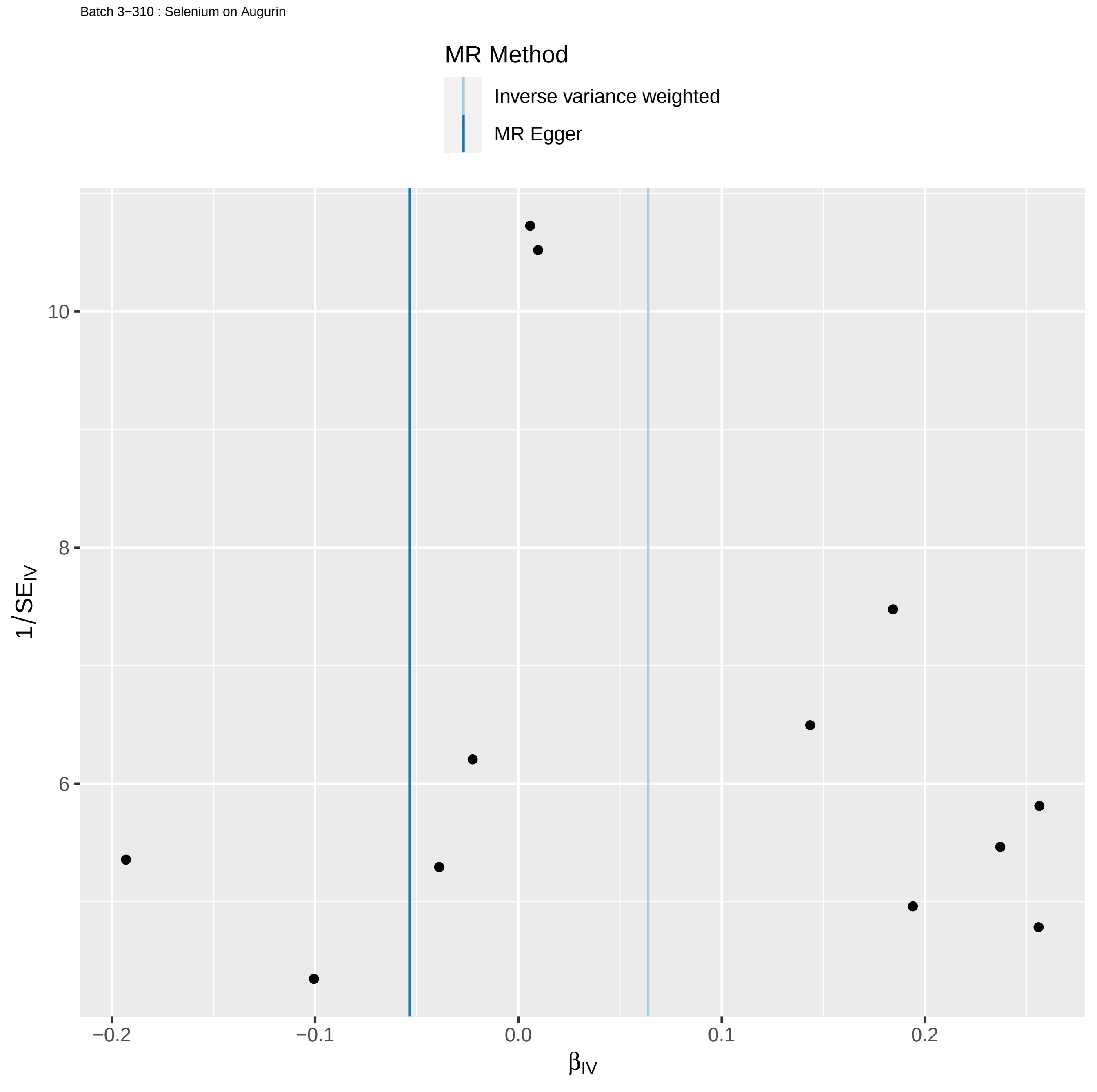

Batch 3−310 : Selenium on Augurin
MR Method
Inverse variance weighted
MR Egger
10
8
6
1/SE_IV
−0.2
−0.1
0.0
0.1
0.2
β_IV

Batch 3−311 : Selenium on Uncharacterized protein C2orf66

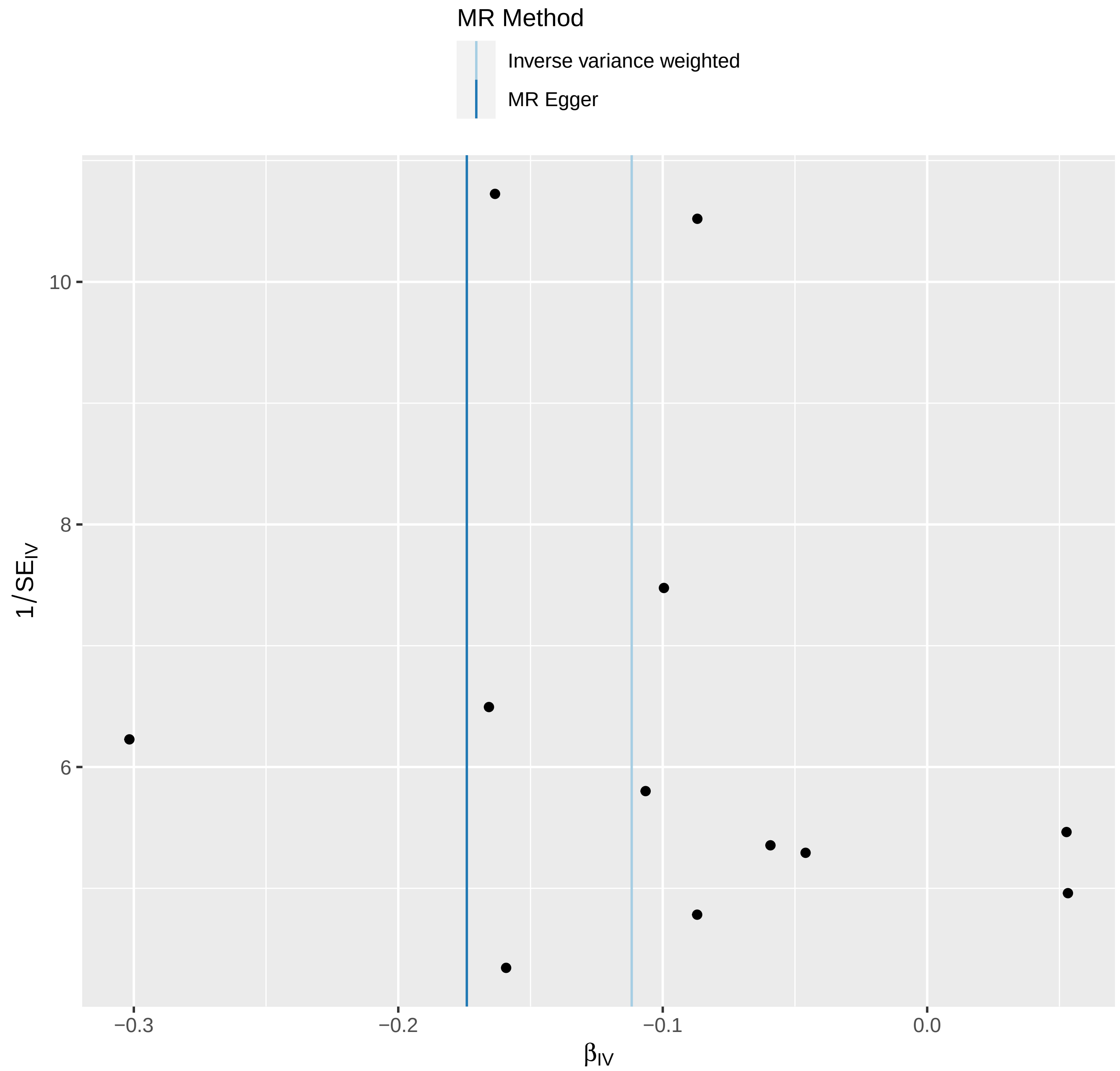

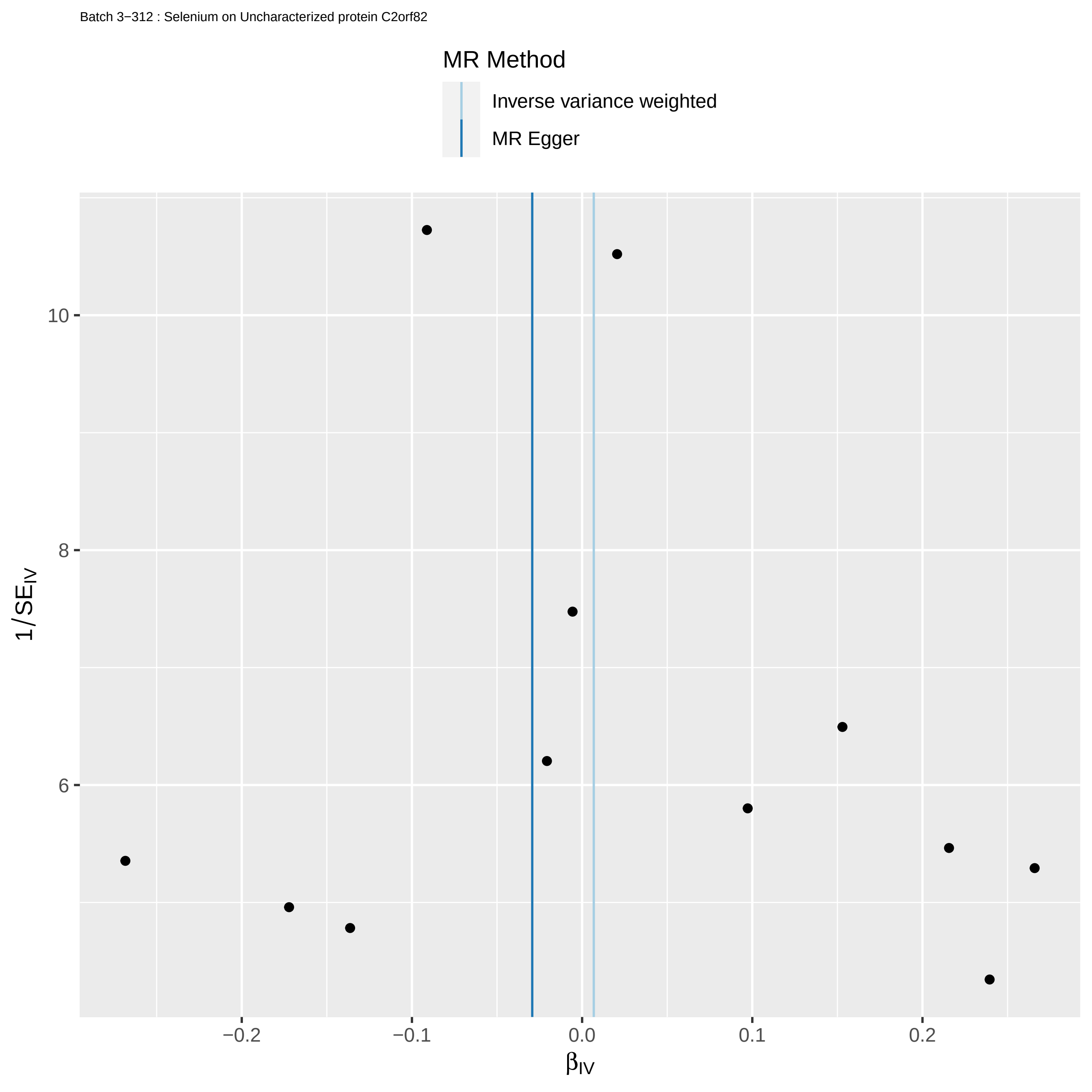

Batch 3−312 : Selenium on Uncharacterized protein C2orf82
MR Method
Inverse variance weighted
MR Egger
10
8
6
1/SE_IV
−0.2
−0.1
0.0
0.1
0.2
β_IV

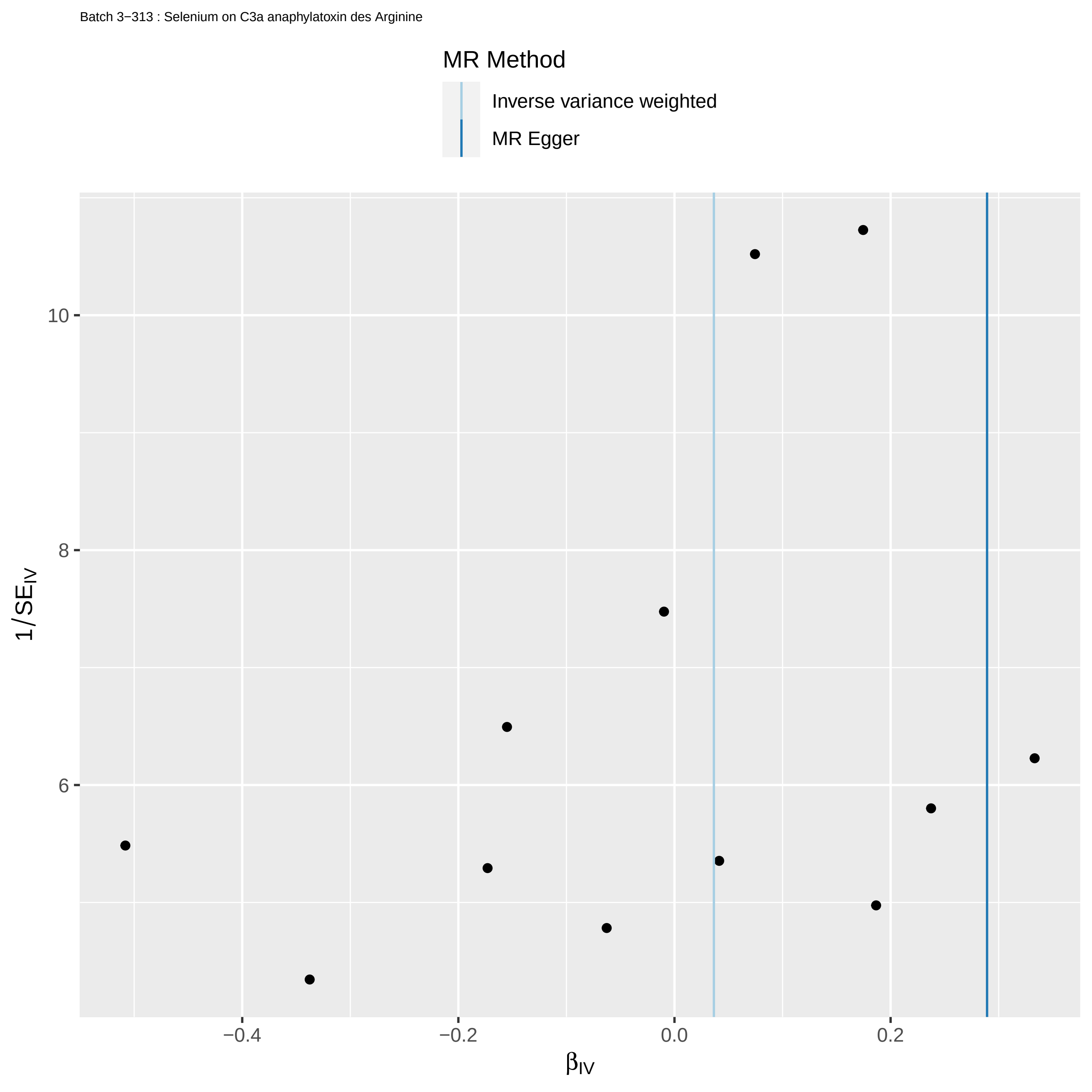
Batch 3−313 : Selenium on C3a anaphylatoxin des Arginine
MR Method
Inverse variance weighted
MR Egger
10
8
6
1/SE_IV
−0.4
−0.2
0.0
0.2
β_IV

Batch 3−314 : Selenium on Uncharacterized protein C3orf18

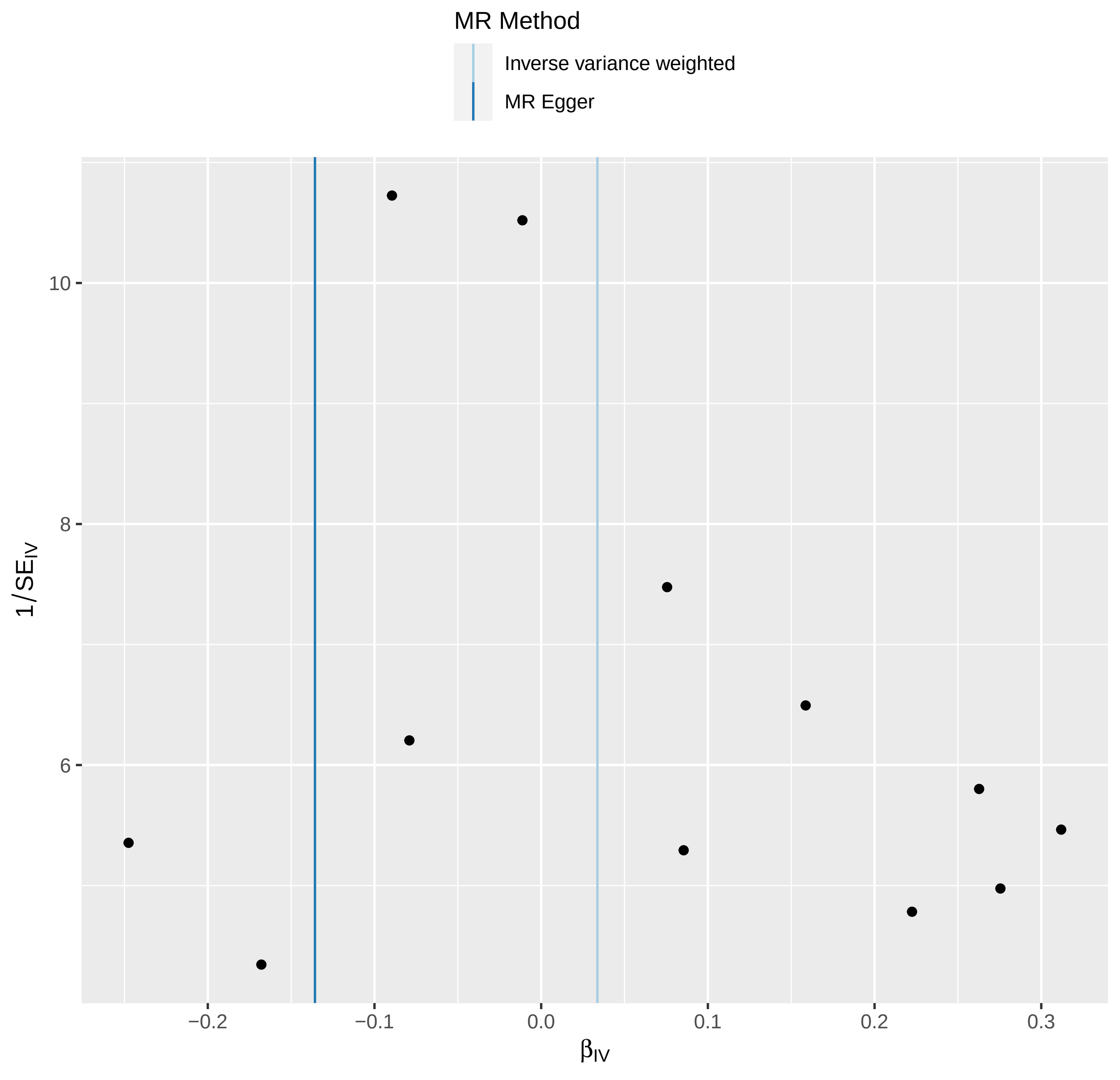

Batch 3−315 : Selenium on Complement C4

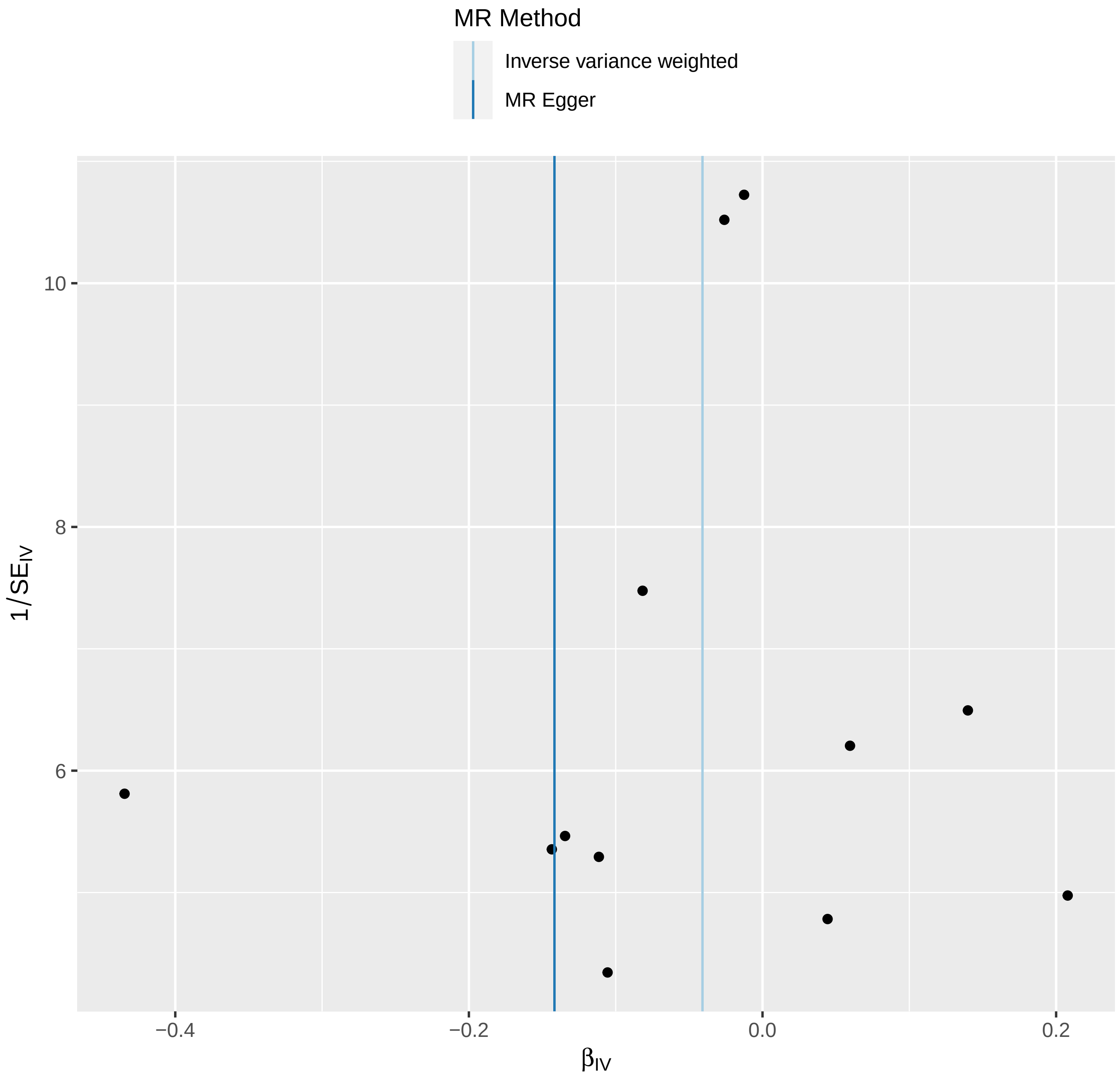

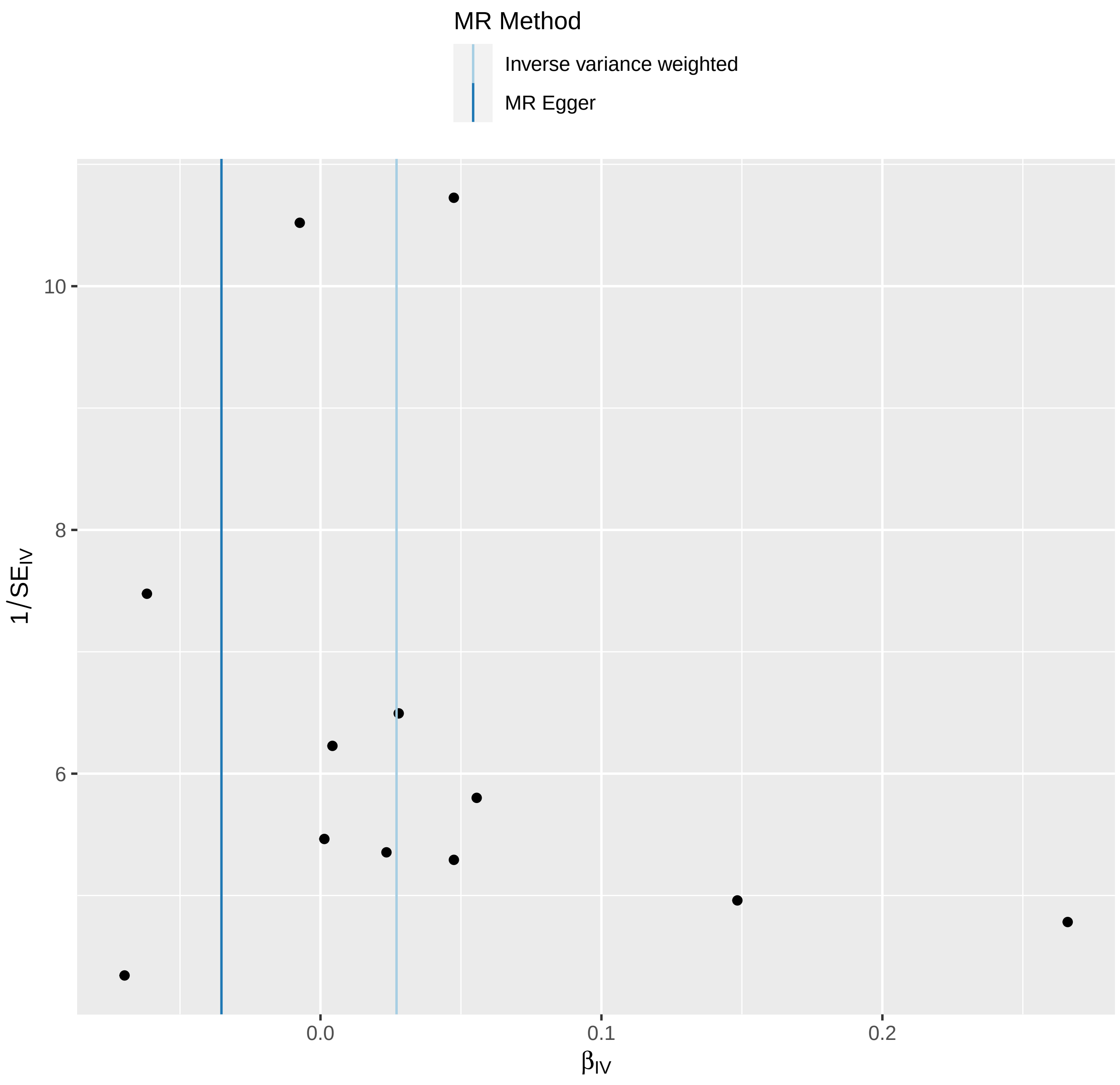
MR Method
Inverse variance weighted
MR Egger
10
8
6
1/SE_IV
0.0
0.1
0.2
β_IV

Batch 3−317 : Selenium on Glutaredoxin−like protein C5orf63

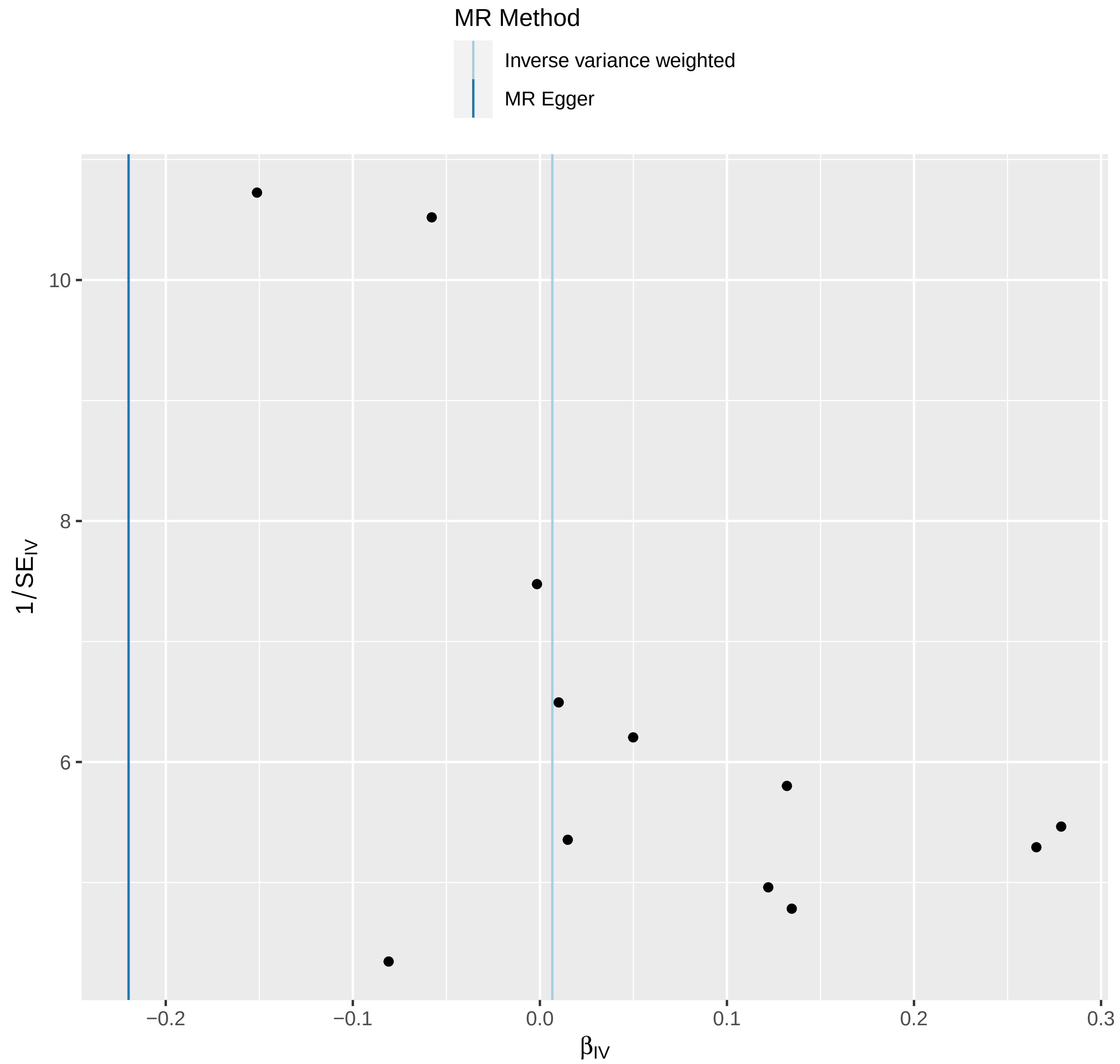

Batch 3−318 : Selenium on Complement component C6

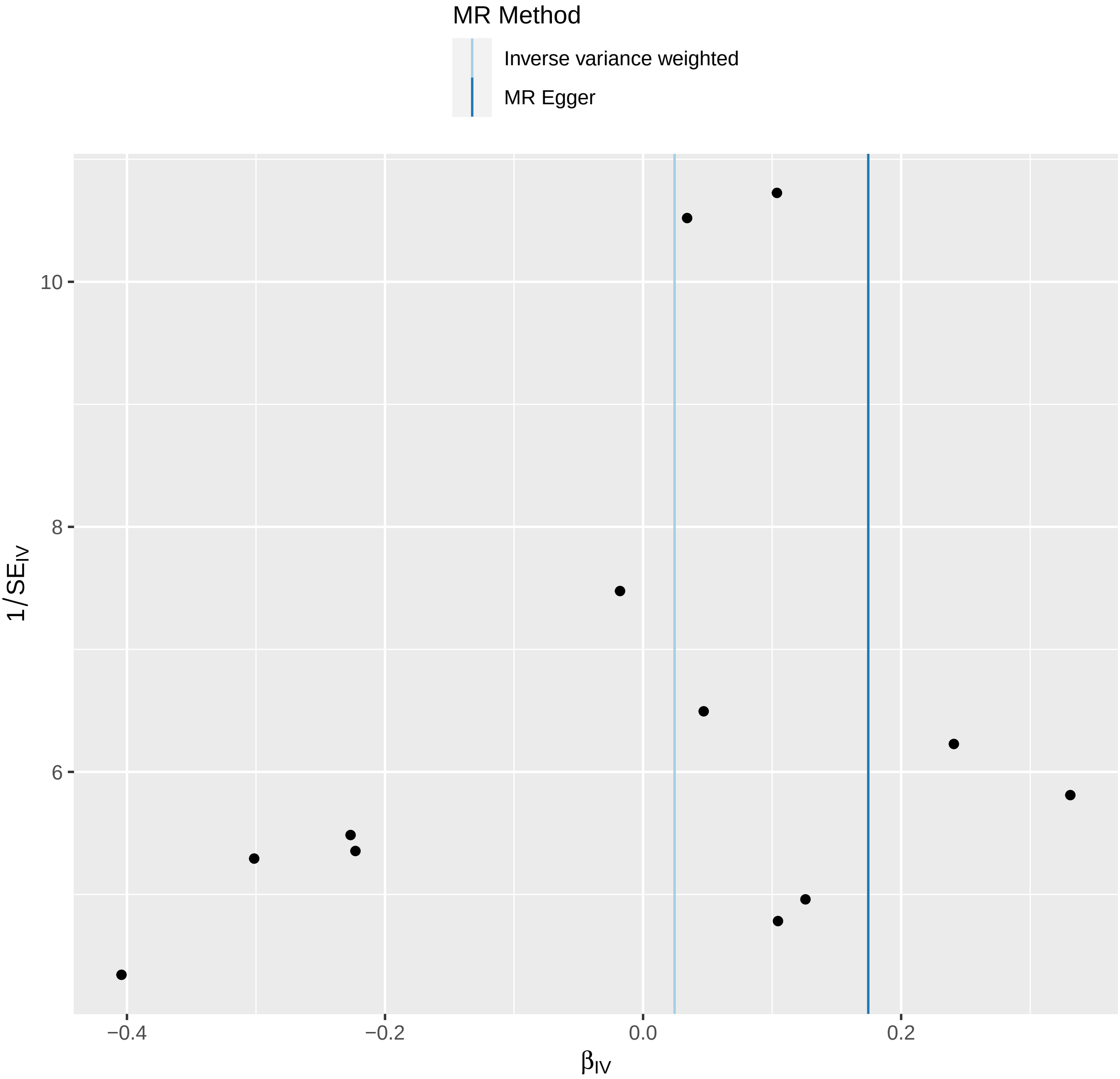

Batch 3−319 : Selenium on Uncharacterized protein C6orf226

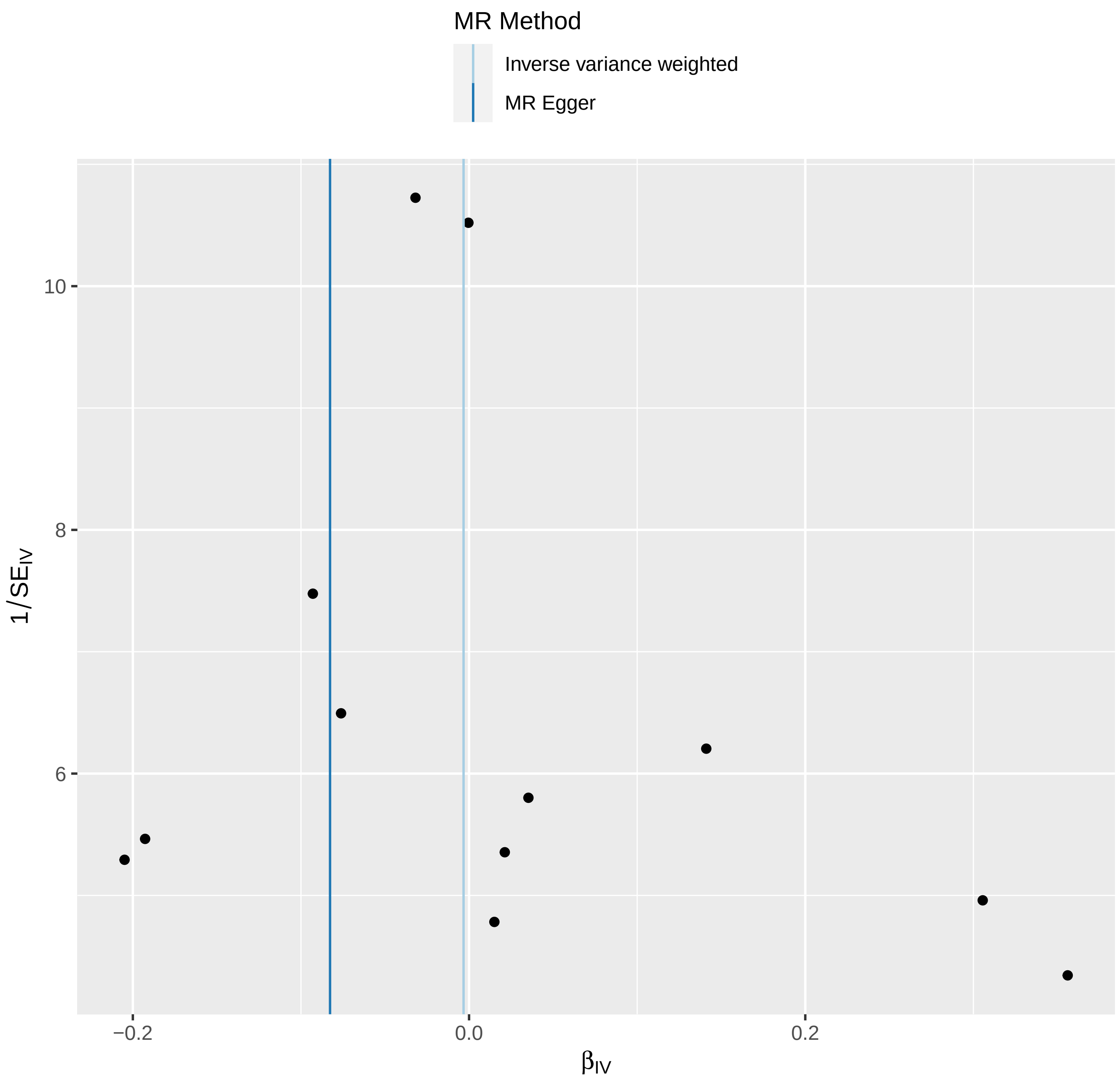

Batch 3−320 : Selenium on Bombesin receptor−activated protein C6orf89

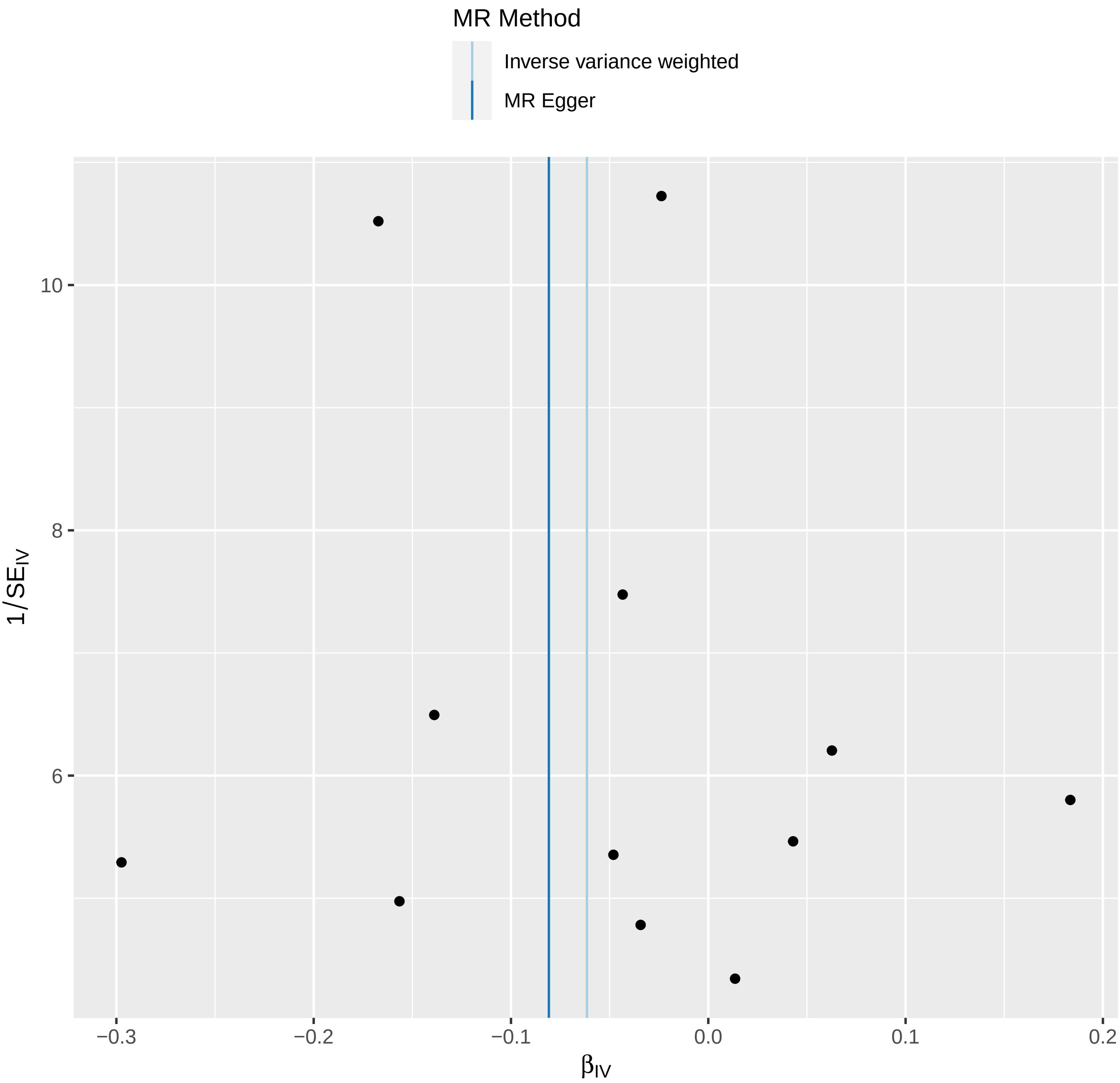

Batch 3−321 : Selenium on Uncharacterized protein C7orf69

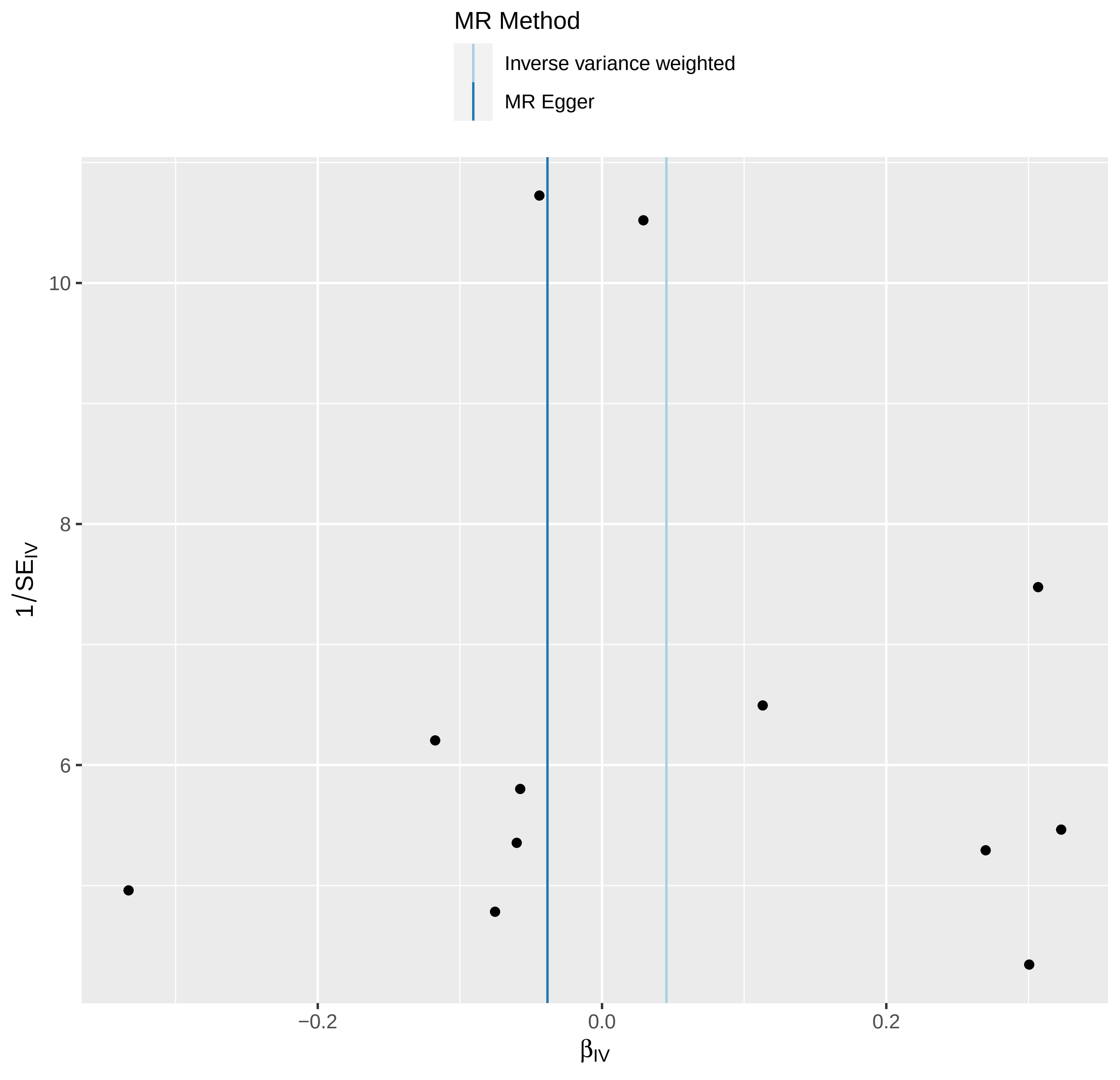

Batch 3−322 : Selenium on Complement component C8

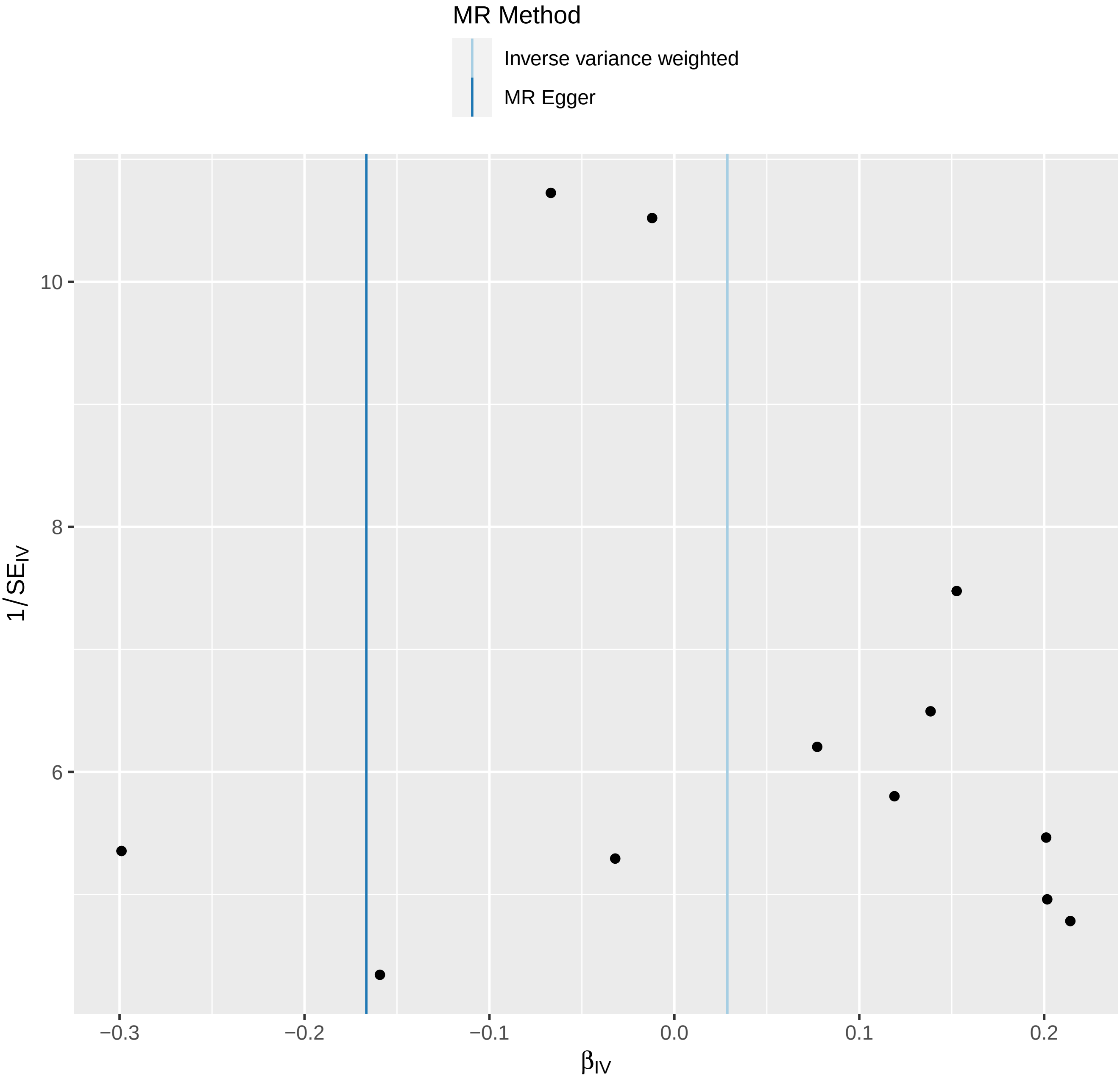

Batch 3−323 : Selenium on UPF0488 protein C8orf33

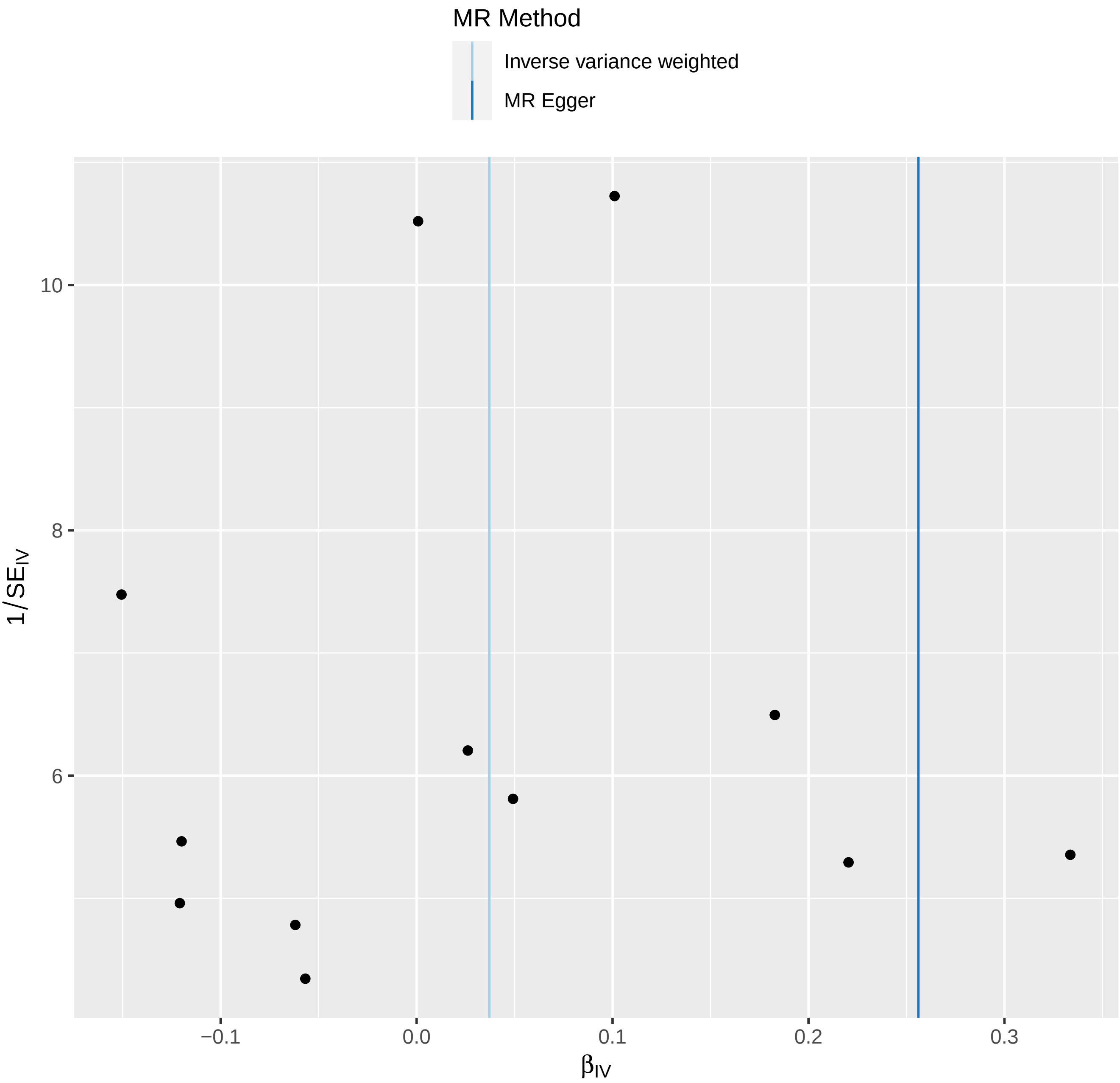

Batch 3−324 : Selenium on Complement component C9

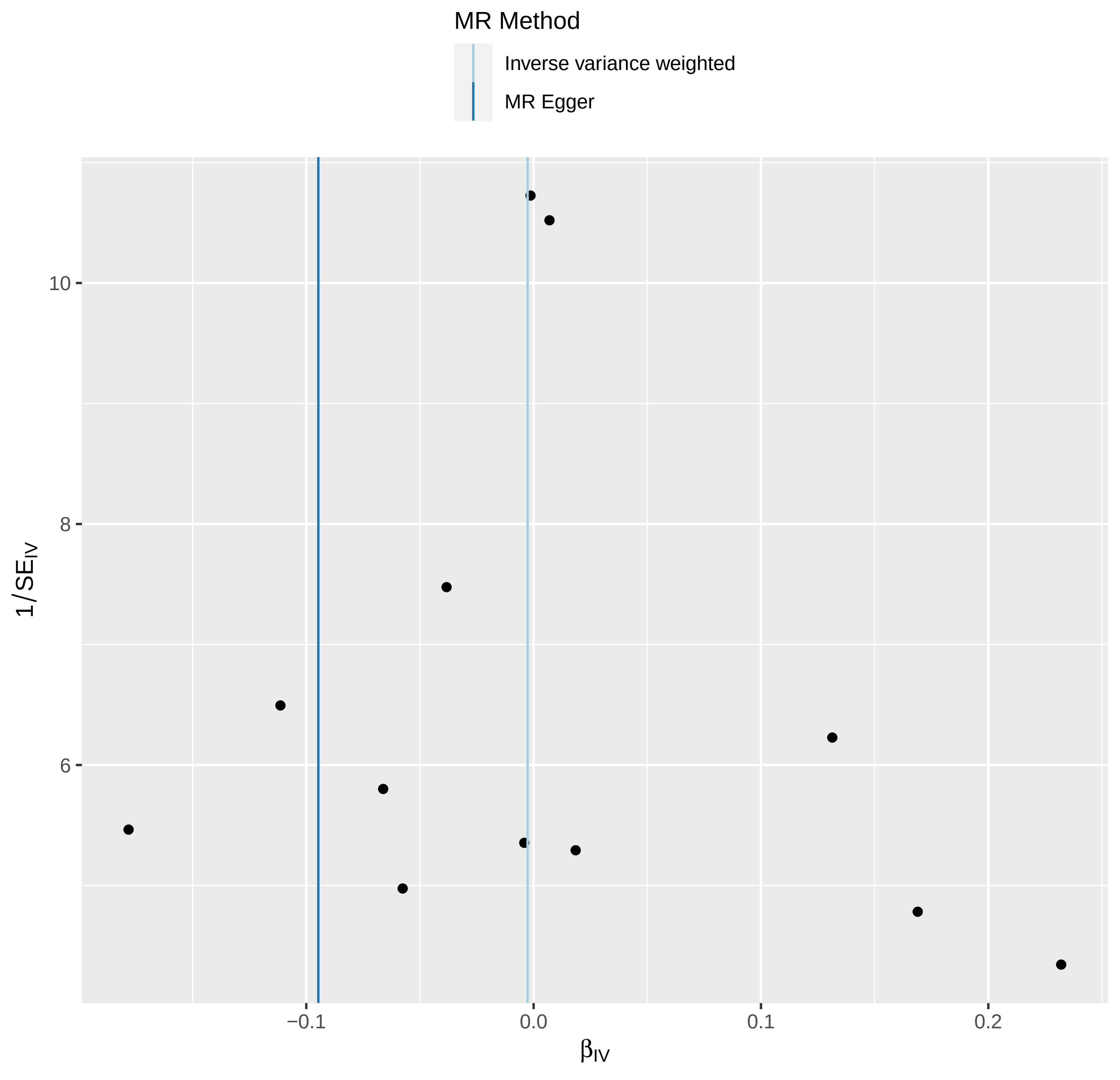

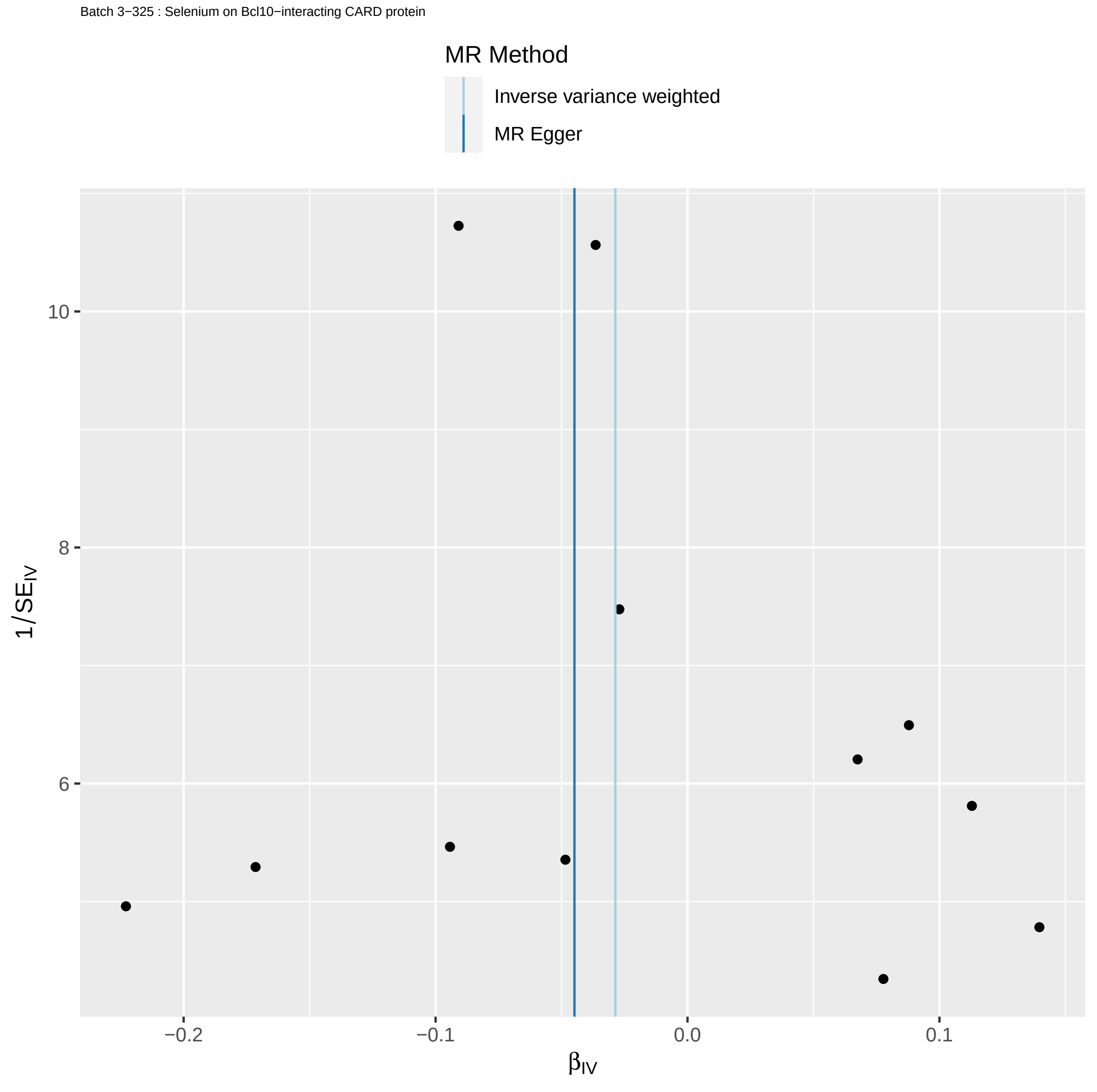

Batch 3−325 : Selenium on Bcl10−interacting CARD protein
MR Method
Inverse variance weighted
MR Egger
10
8
6
1/SE_IV
−0.2
−0.1
0.0
0.1
β_IV

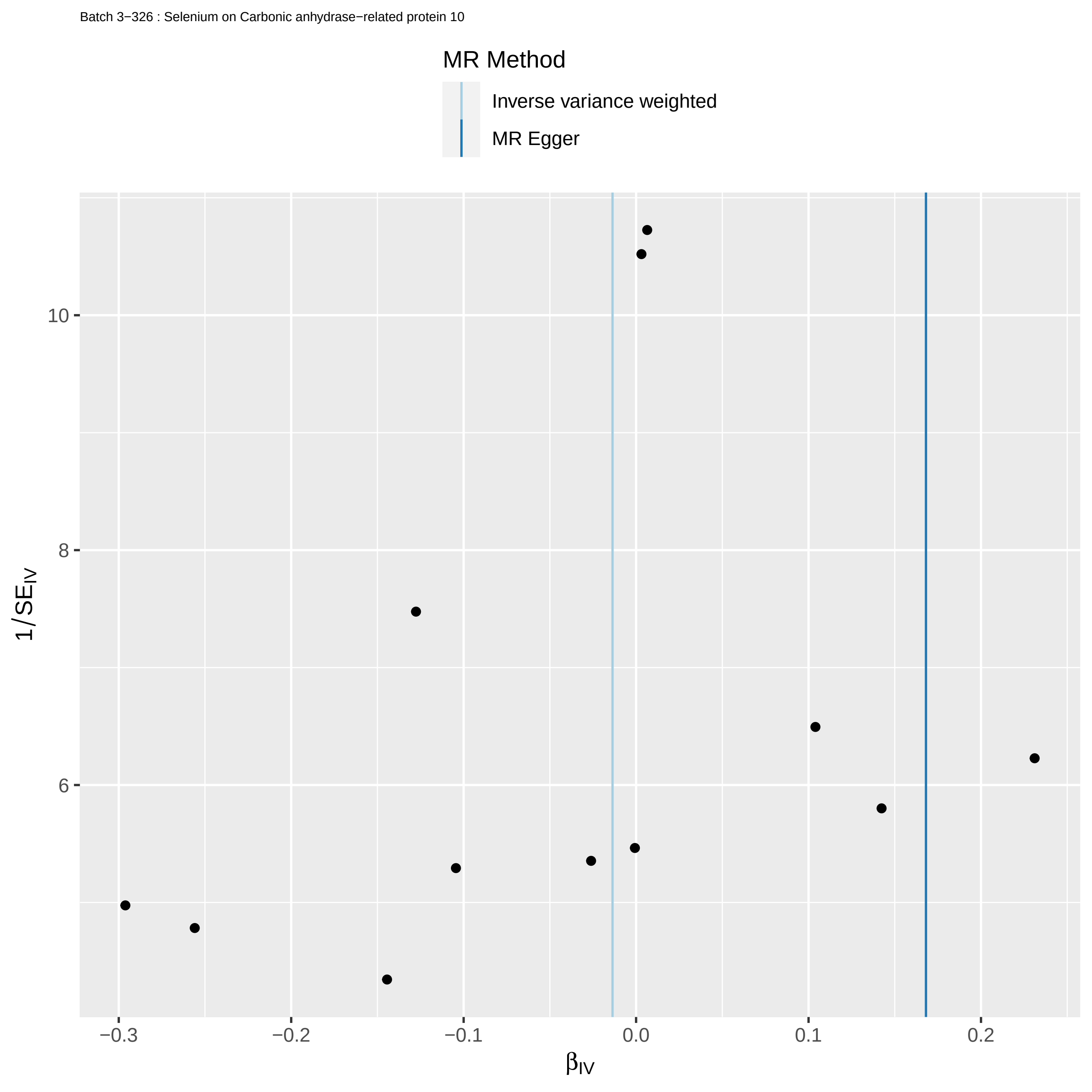

Batch 3−326 : Selenium on Carbonic anhydrase−related protein 10
MR Method
Inverse variance weighted
MR Egger
10
8
6
1/SE$_{IV}$
−0.3
−0.2
−0.1
0.0
0.1
0.2
β$_{IV}$

Batch 3−327 : Selenium on Carbonic anhydrase−related protein 10

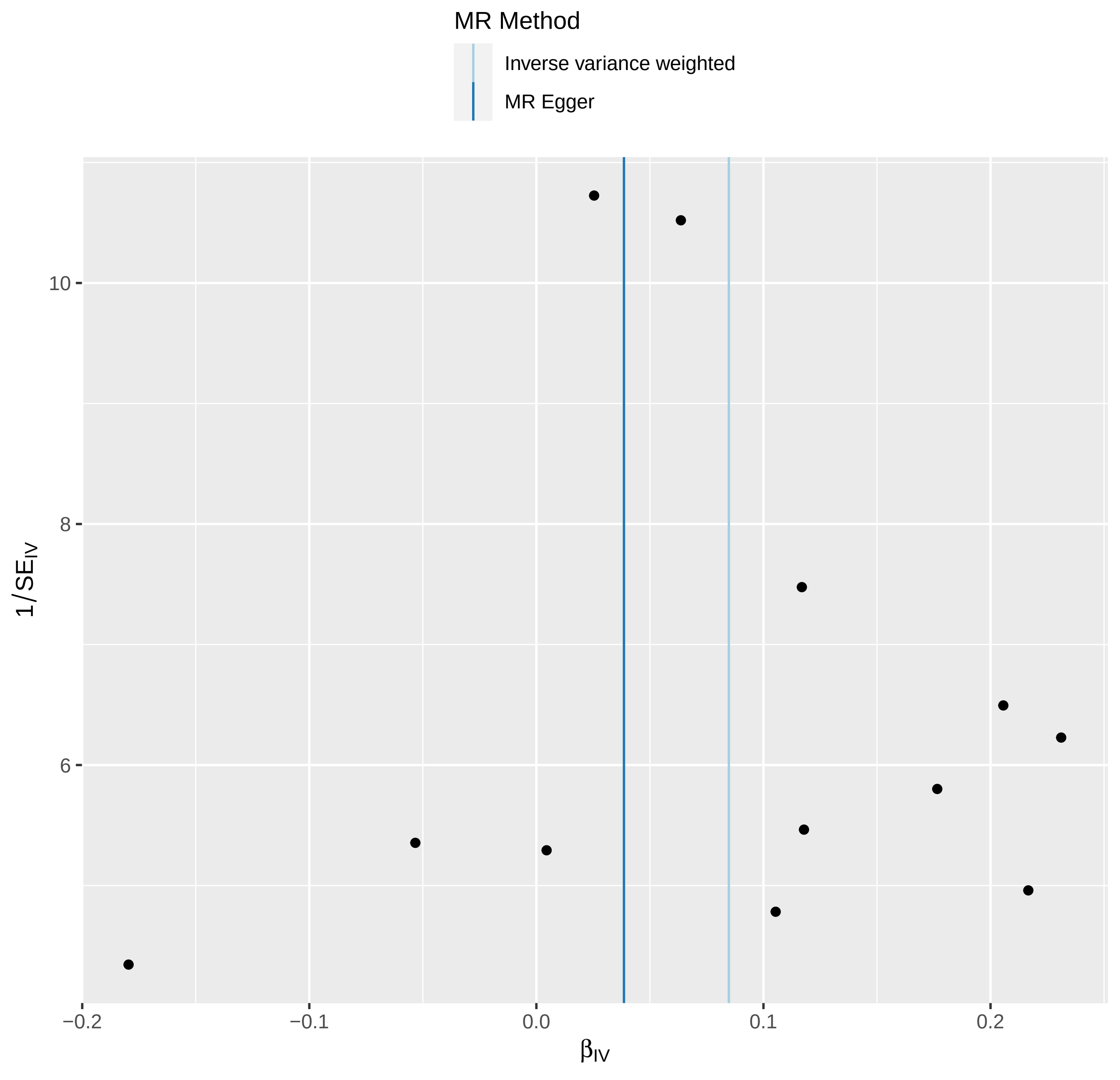

Batch 3−328 : Selenium on Carbonic anhydrase 1

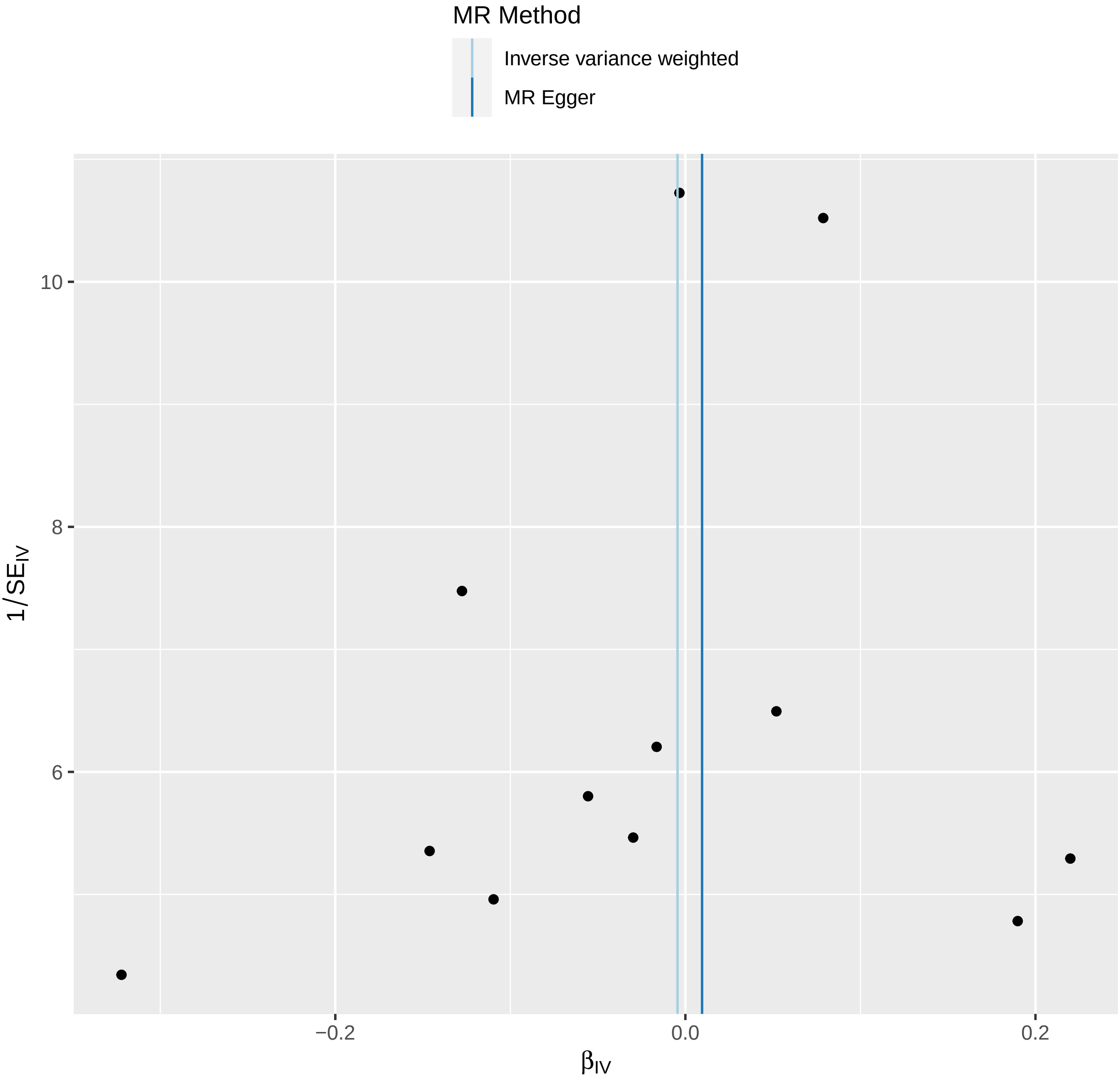

Batch 3−329 : Selenium on Carbonic anhydrase 2

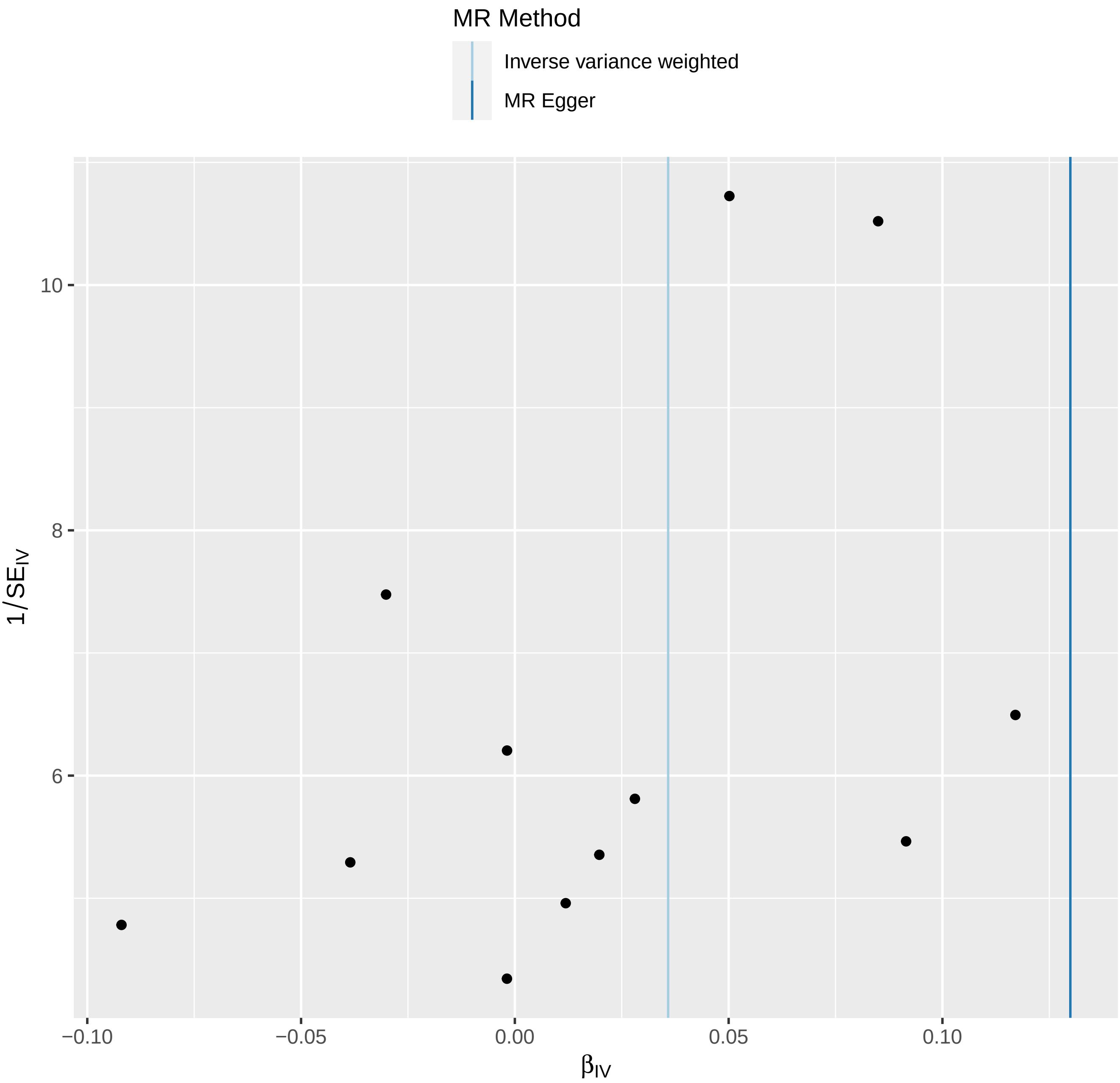

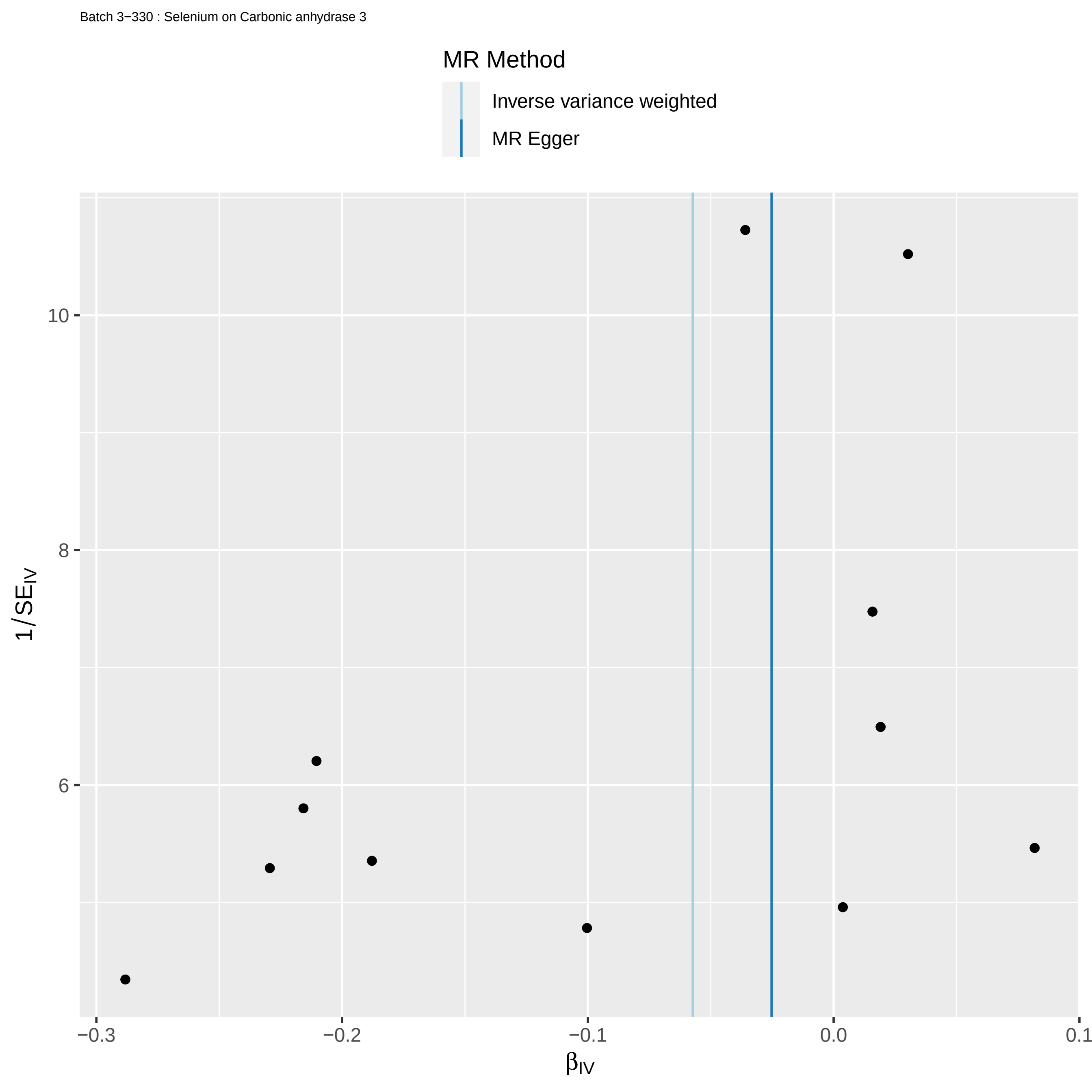
Batch 3−330 : Selenium on Carbonic anhydrase 3
MR Method
Inverse variance weighted
MR Egger
10
8
6
1/SE$_{IV}$
−0.3
−0.2
−0.1
0.0
0.1
β$_{IV}$

Batch 3−331 : Selenium on Carbonic anhydrase 4

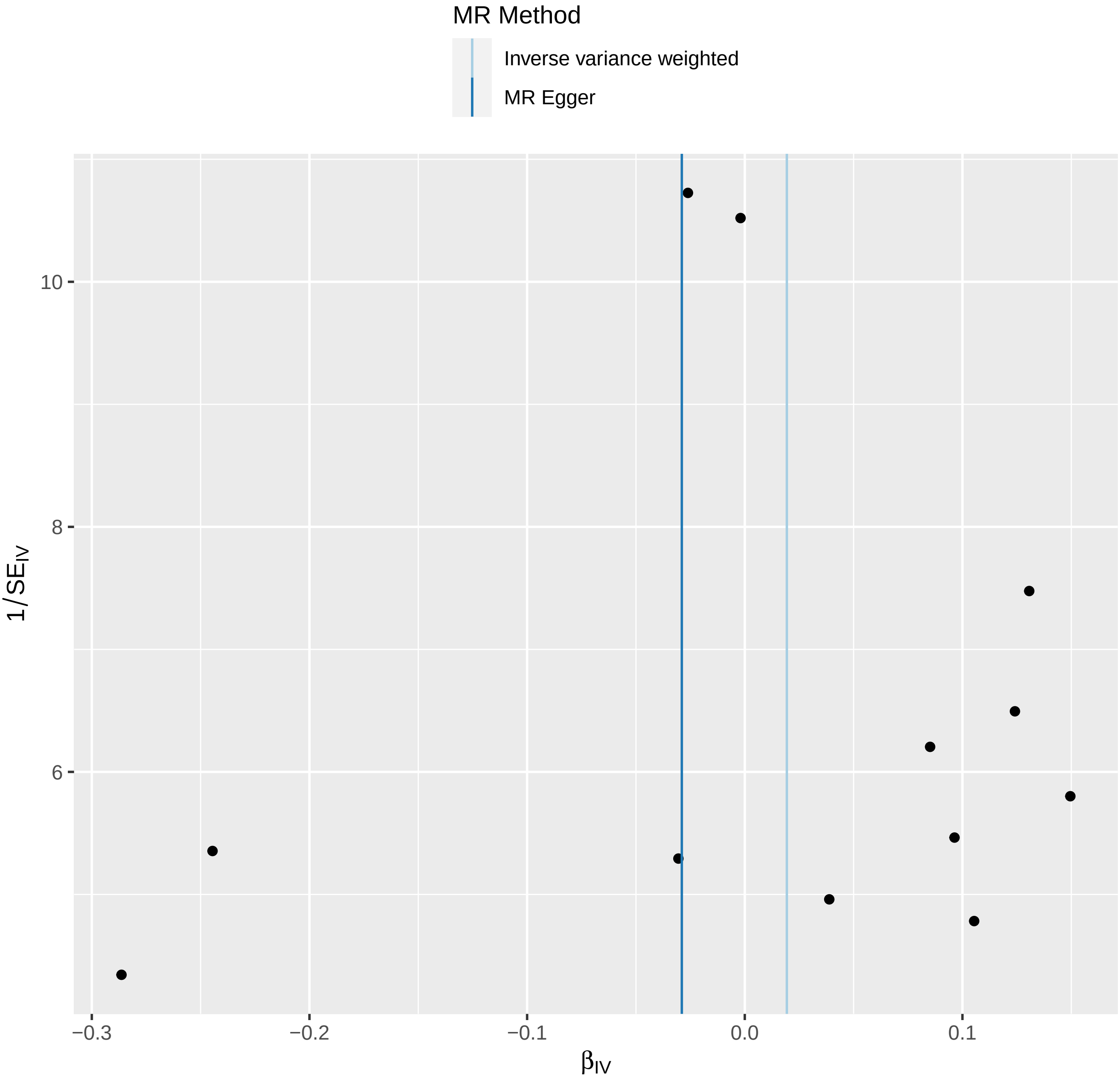

Batch 3−332 : Selenium on Carbonic anhydrase 5A, mitochondrial

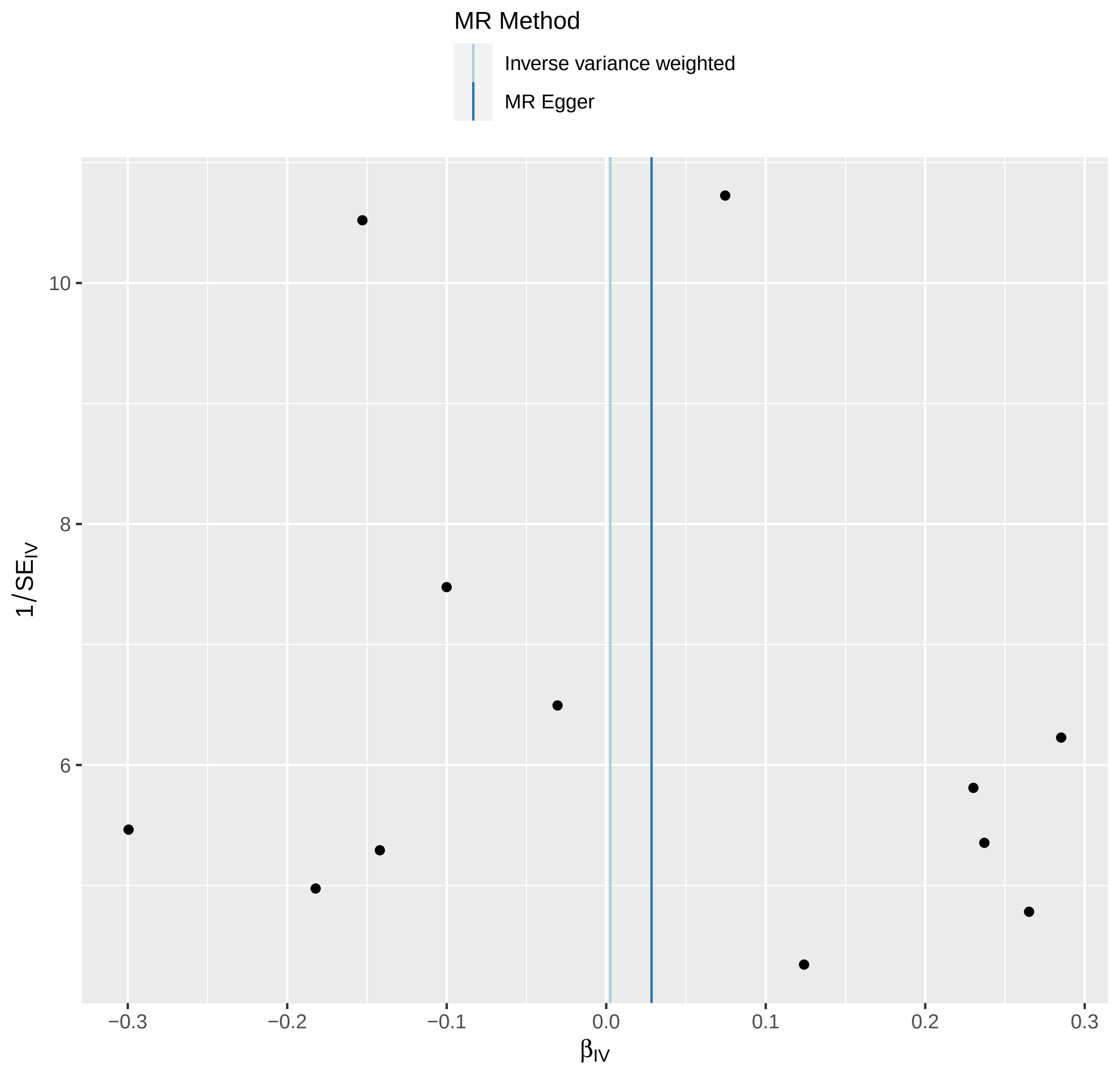

Batch 3−333 : Selenium on Carbonic anhydrase 6

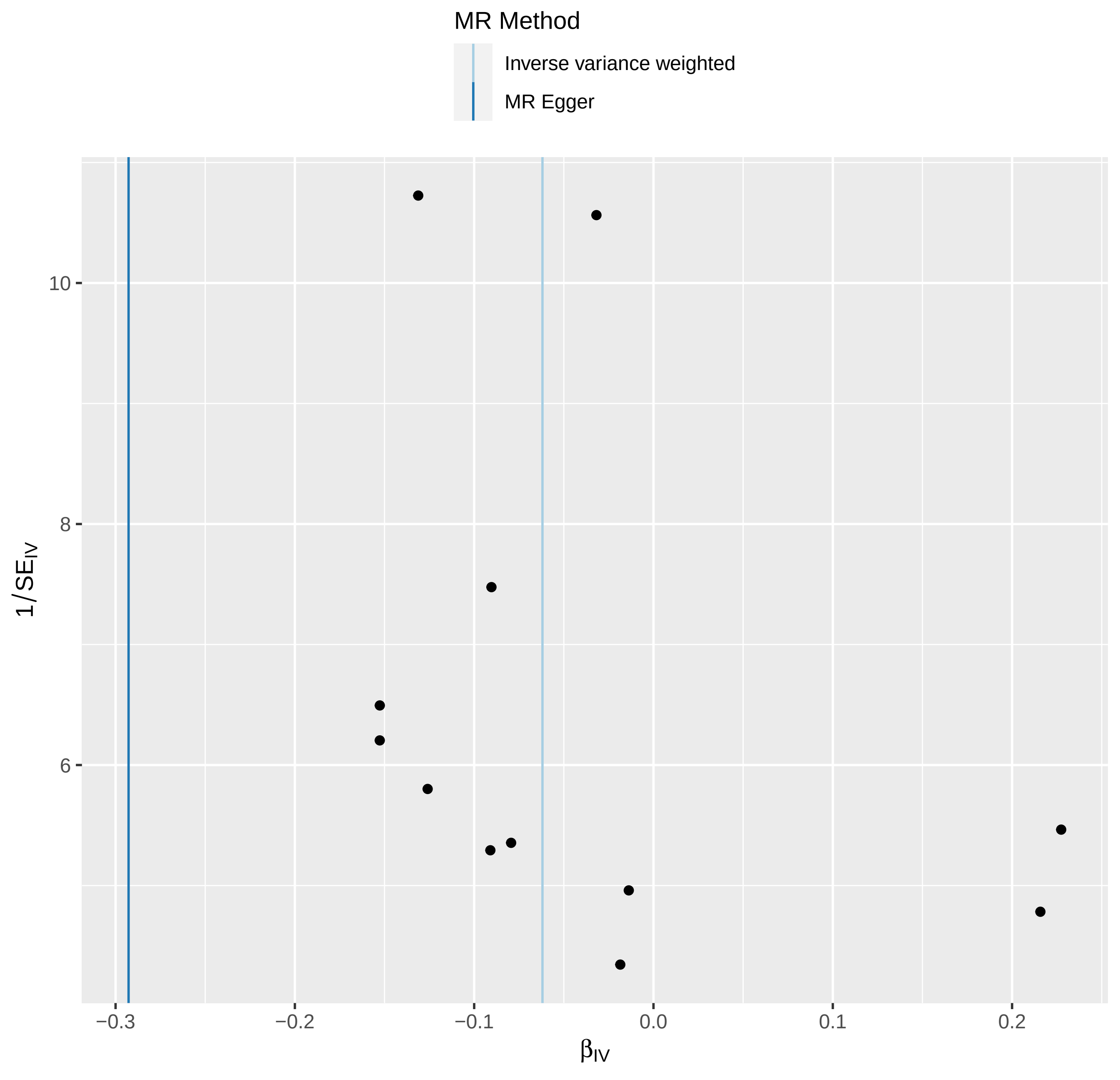

Batch 3−334 : Selenium on Carbonic anhydrase 9

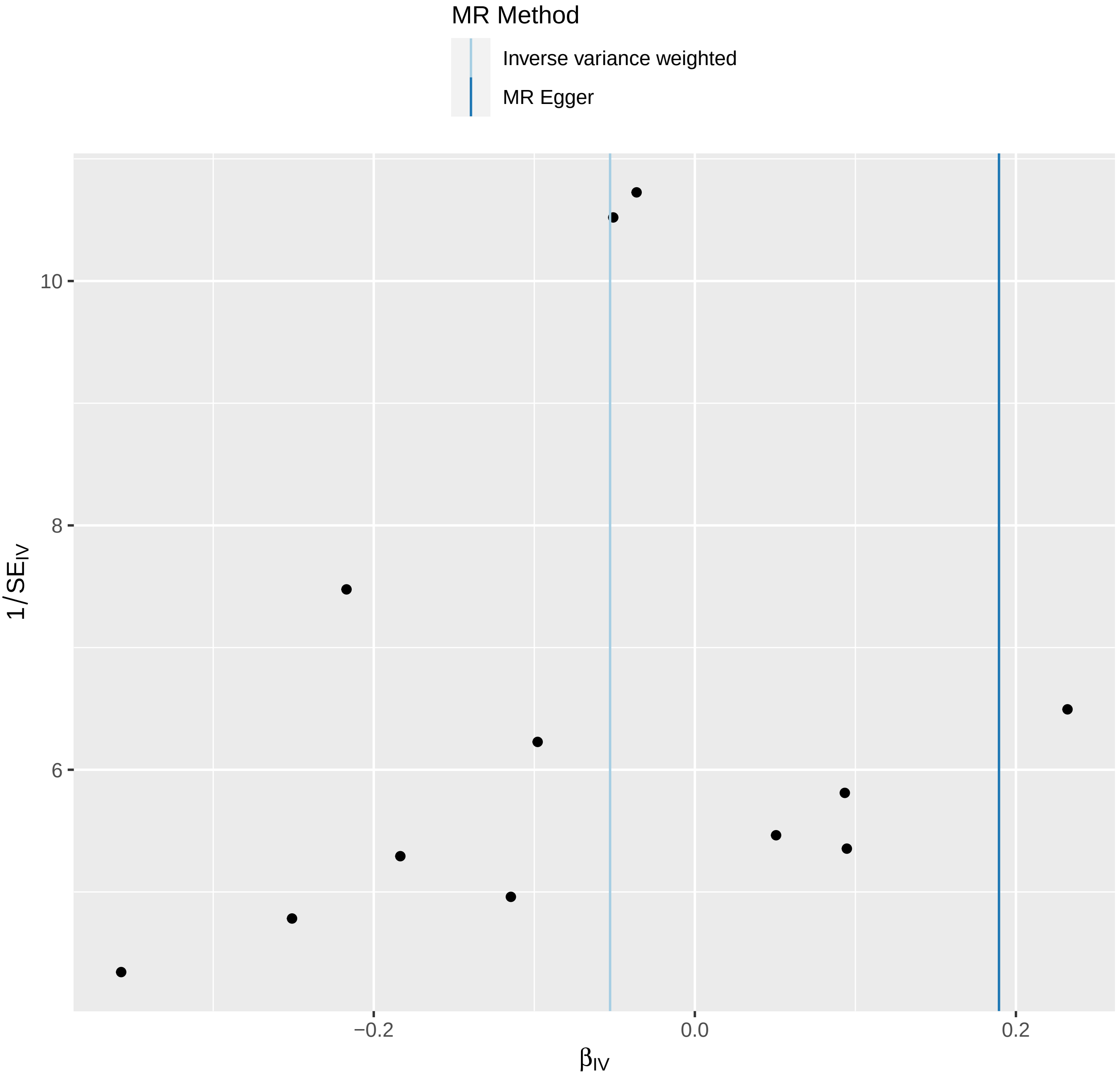

Batch 3−335 : Selenium on Voltage−dependent calcium channel subunit alpha−2/delta−3

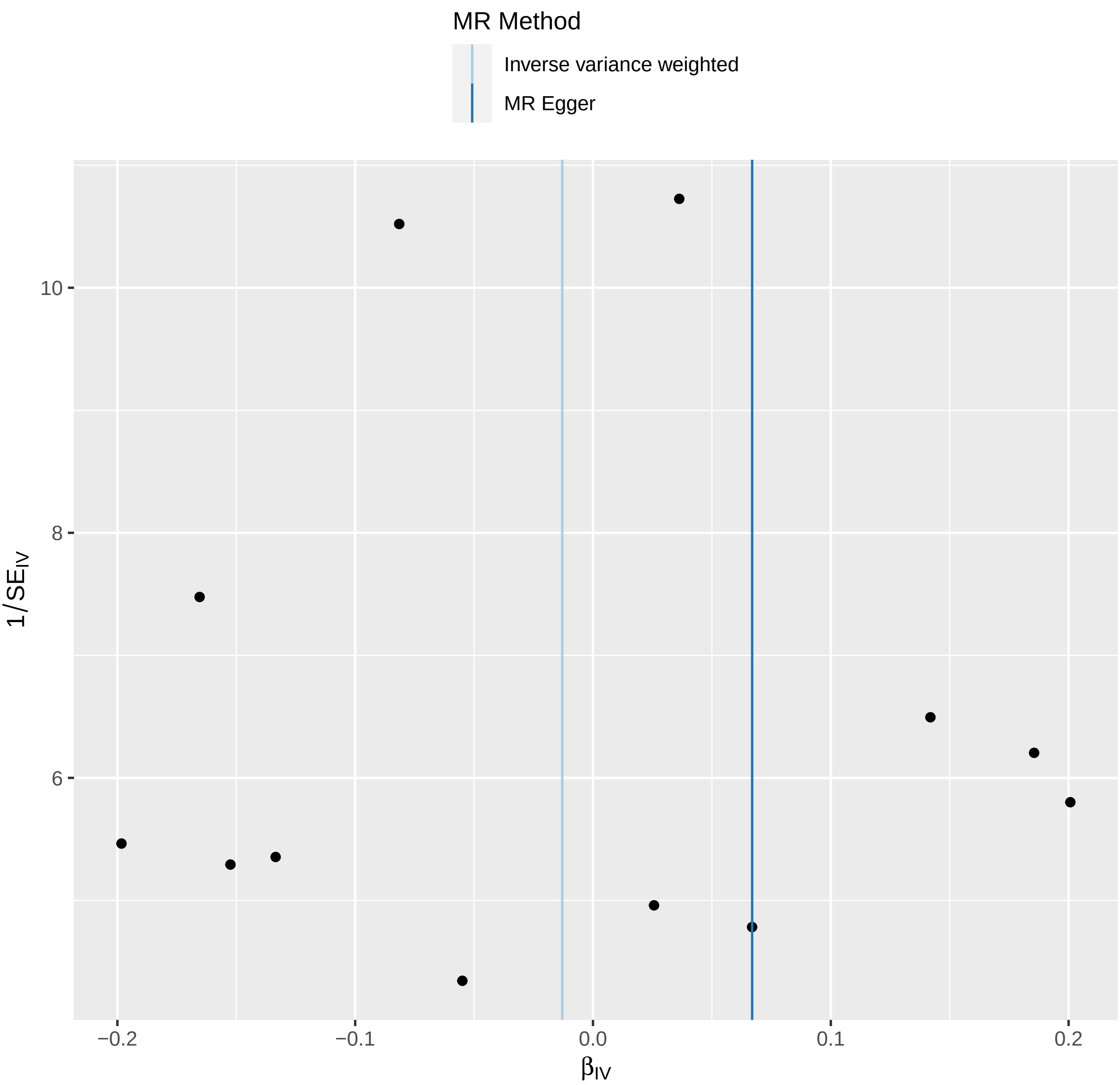

Batch 3−336 : Selenium on Calcyclin−binding protein

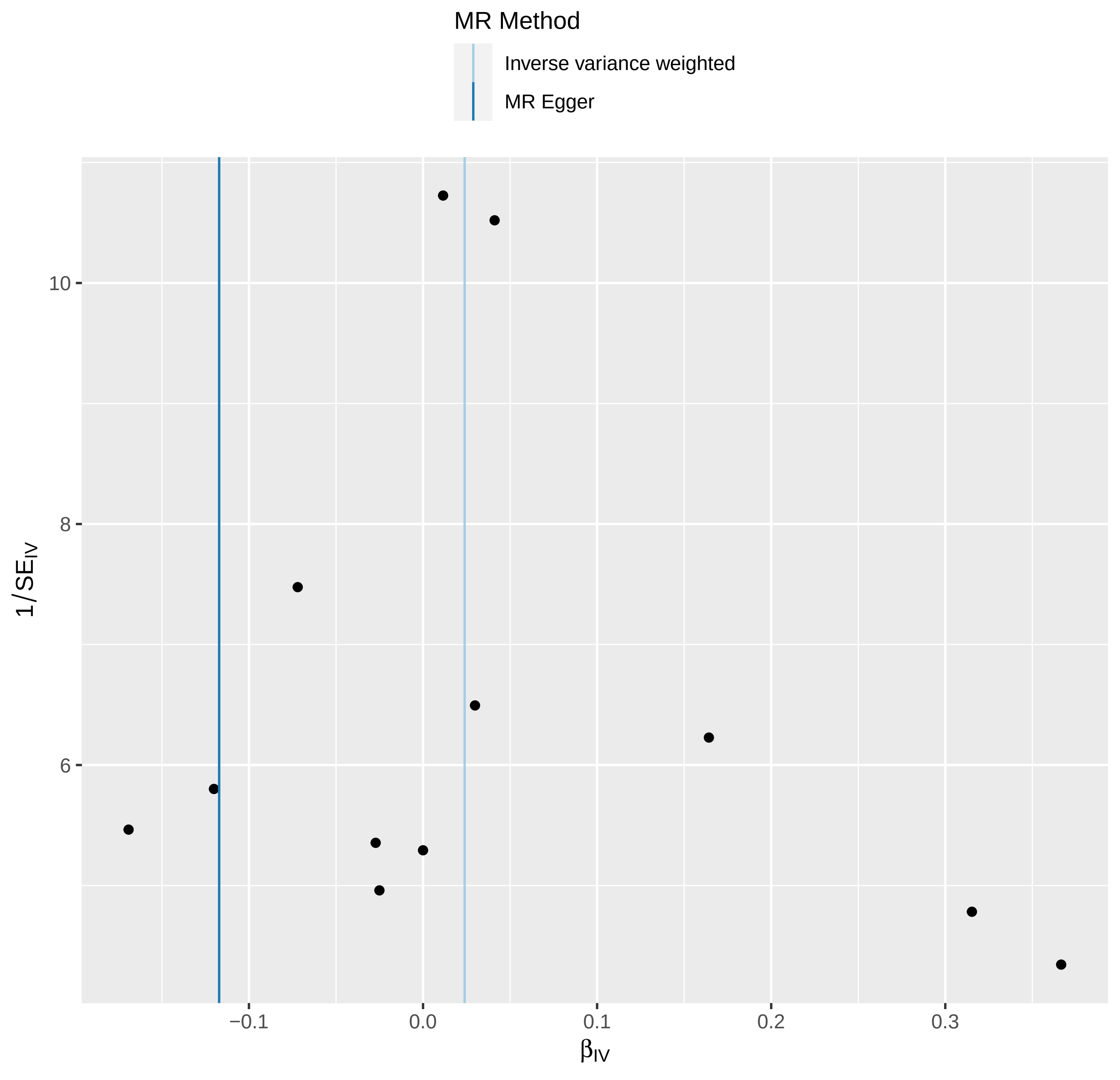

Batch 3−337 : Selenium on Cell adhesion molecule 1

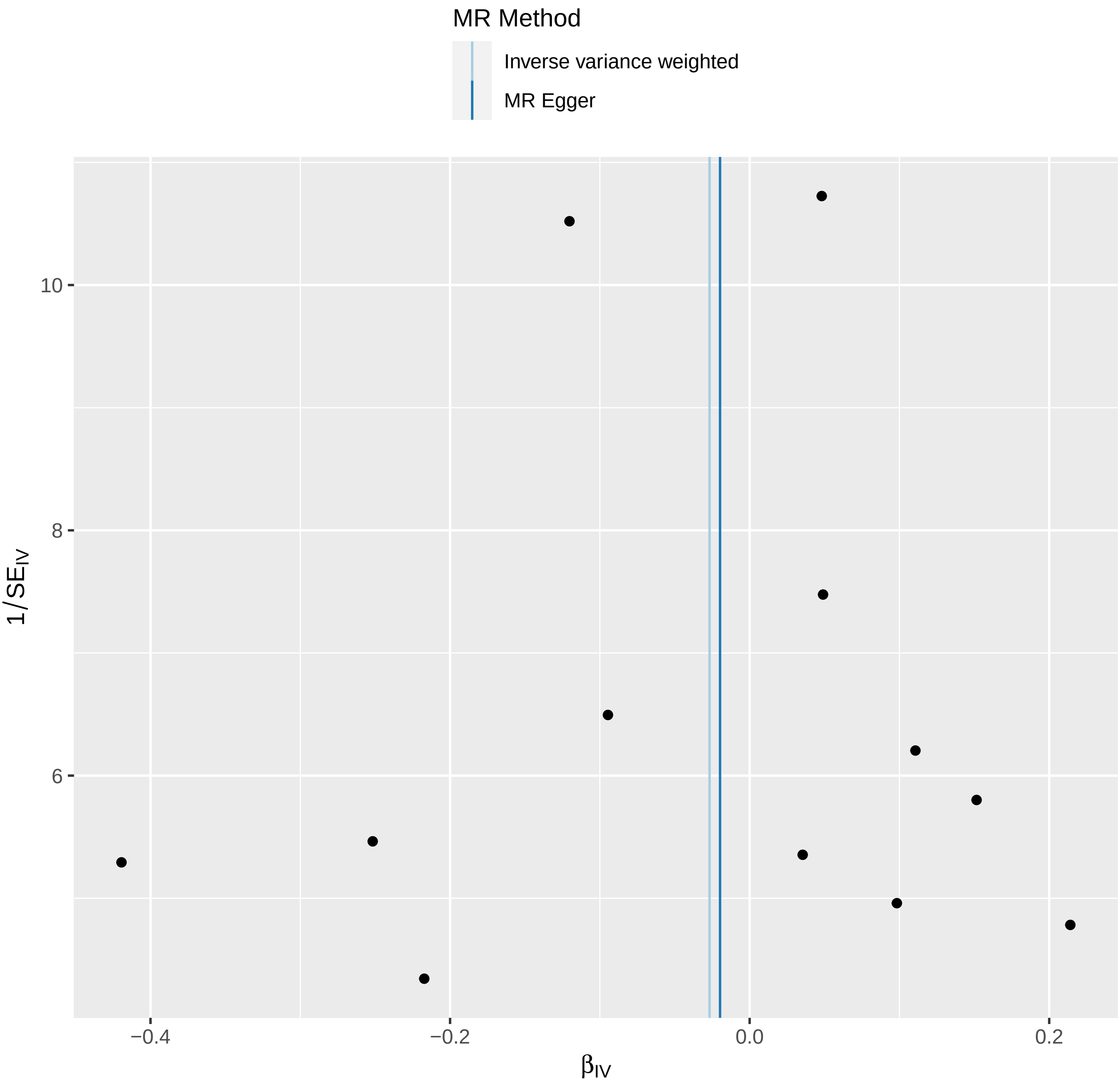

Batch 3−338 : Selenium on Cell adhesion molecule 3

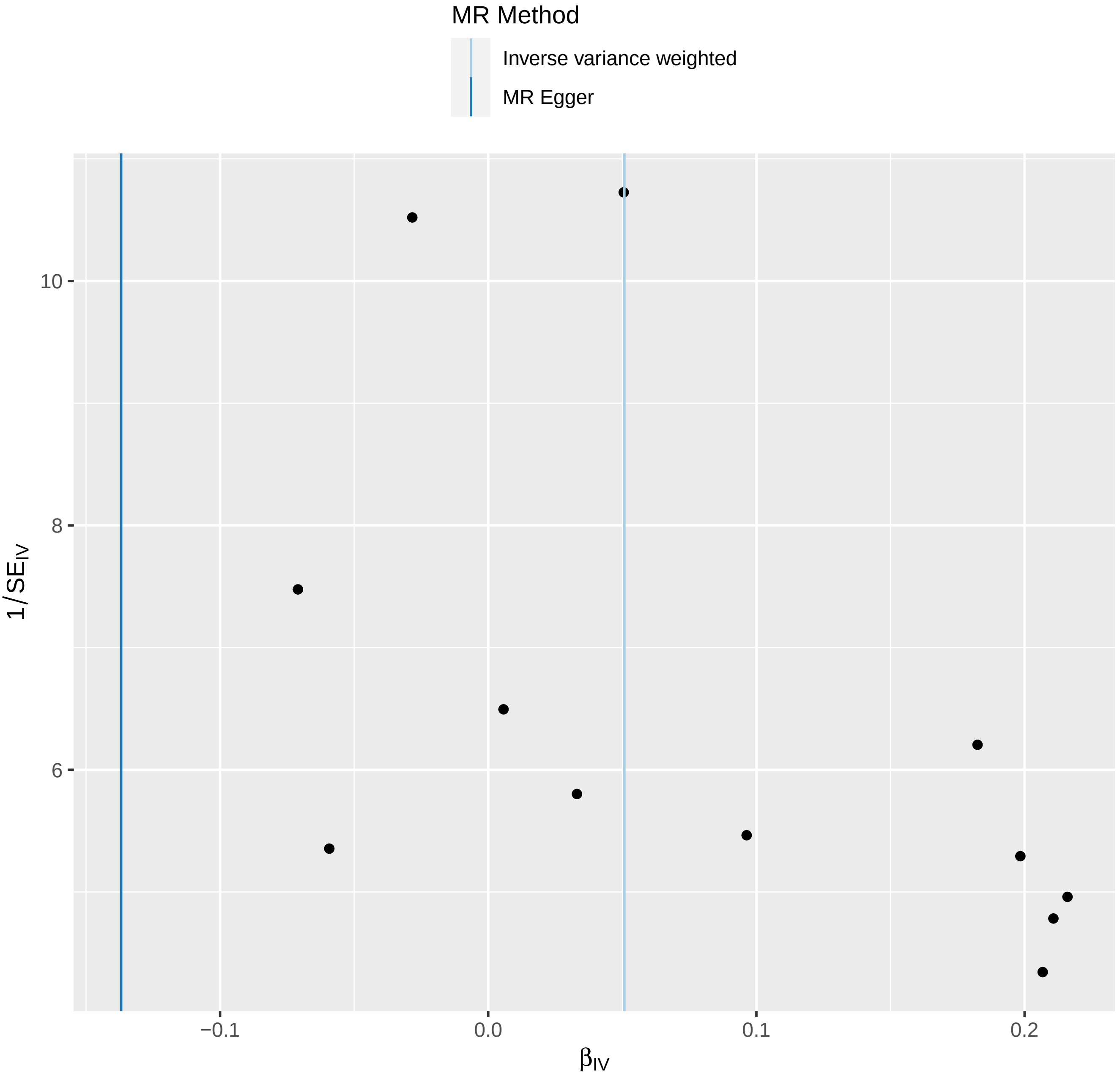

Batch 3−339 : Selenium on Cell adhesion molecule 4

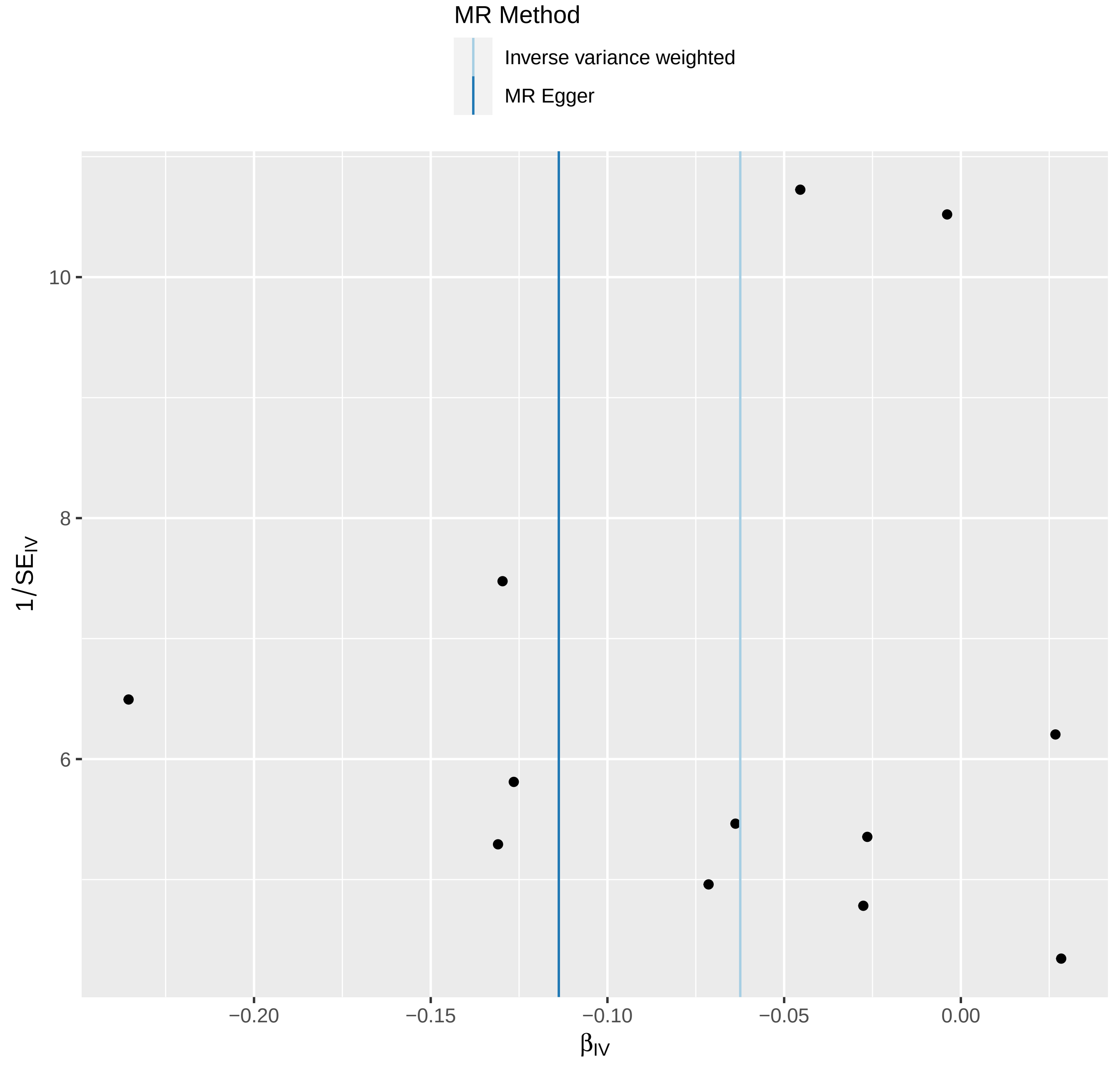

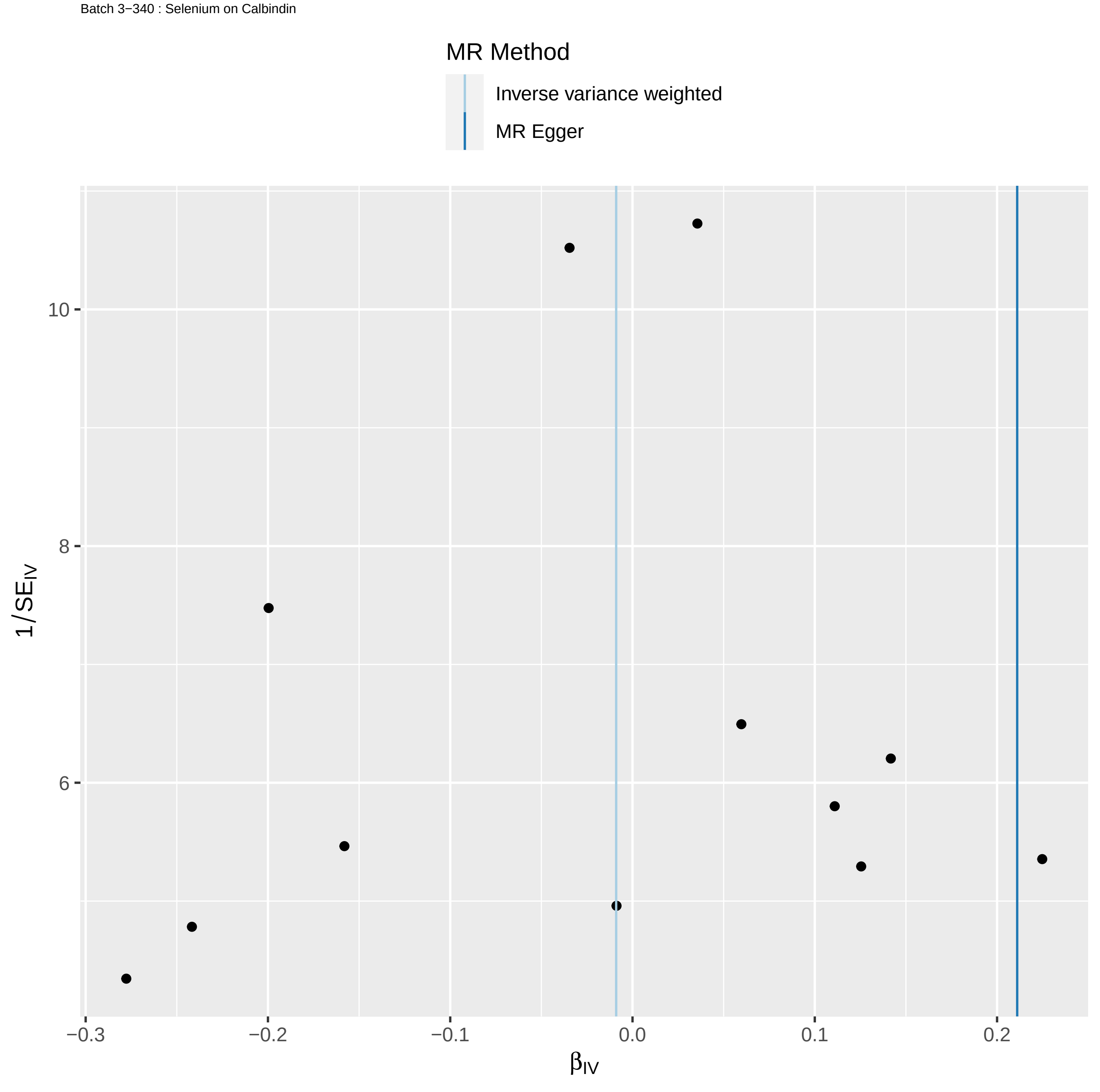
Batch 3−340 : Selenium on Calbindin
MR Method
Inverse variance weighted
MR Egger
10
8
6
1/SE_IV
−0.3
−0.2
−0.1
0.0
0.1
0.2
β_IV

Batch 3−341 : Selenium on Procalcitonin

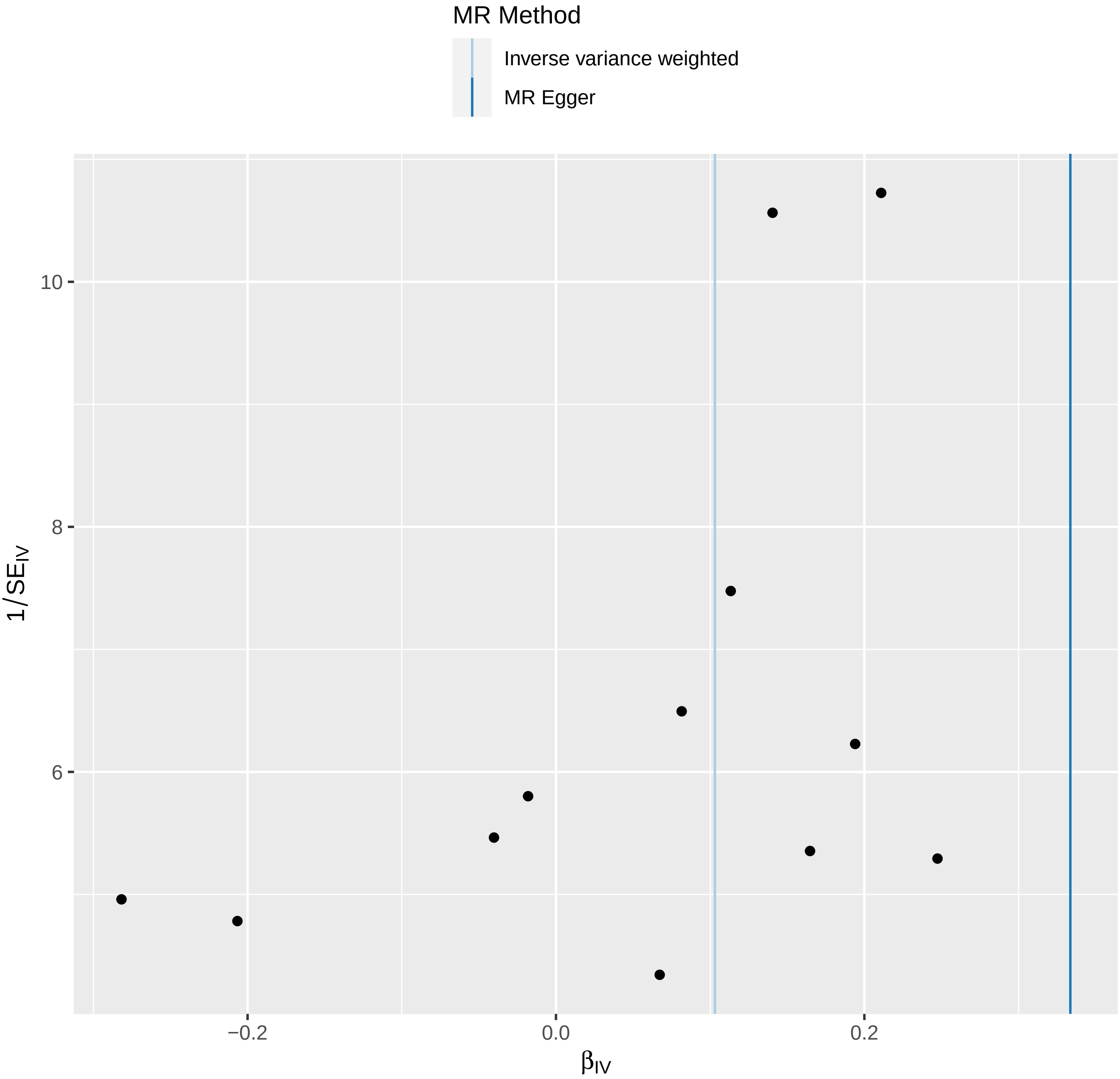

Batch 3−342 : Selenium on Calcium−binding and coiled−coil domain−containing protein 2

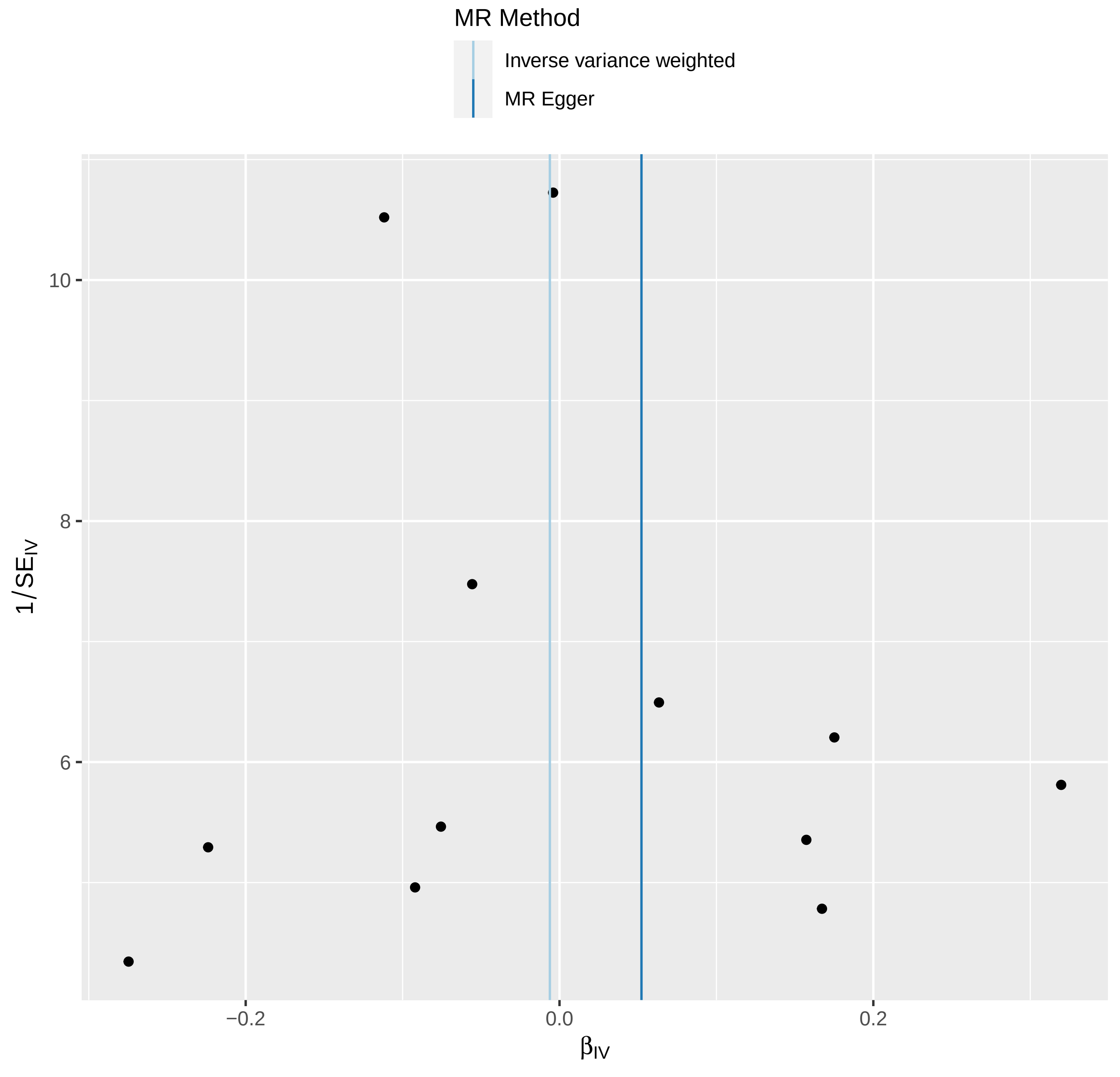

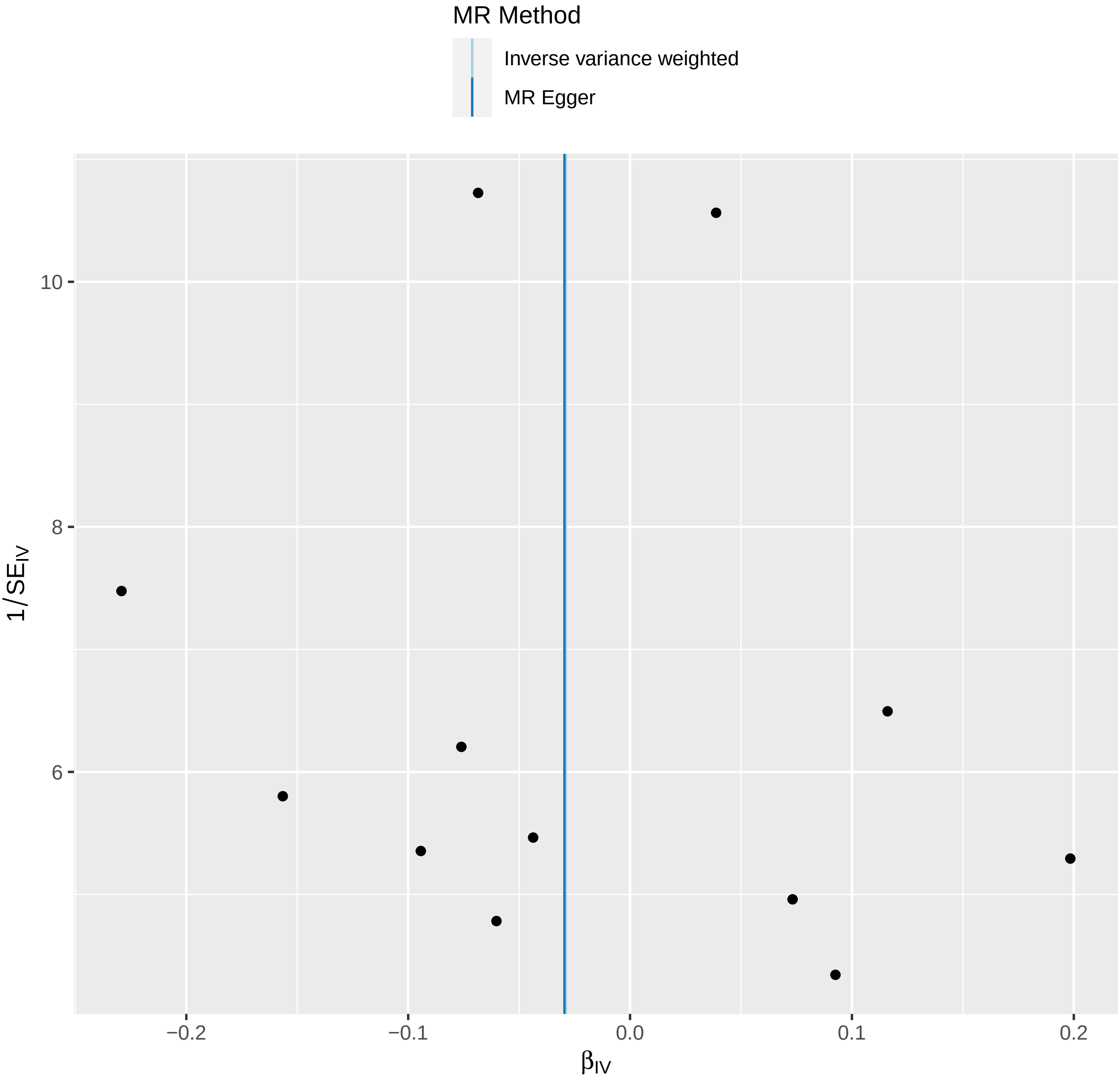
MR Method
Inverse variance weighted
MR Egger
10
8
6
1/SE_IV
−0.2
−0.1
0.0
0.1
0.2
β_IV

Batch 3−344 : Selenium on Calreticulin

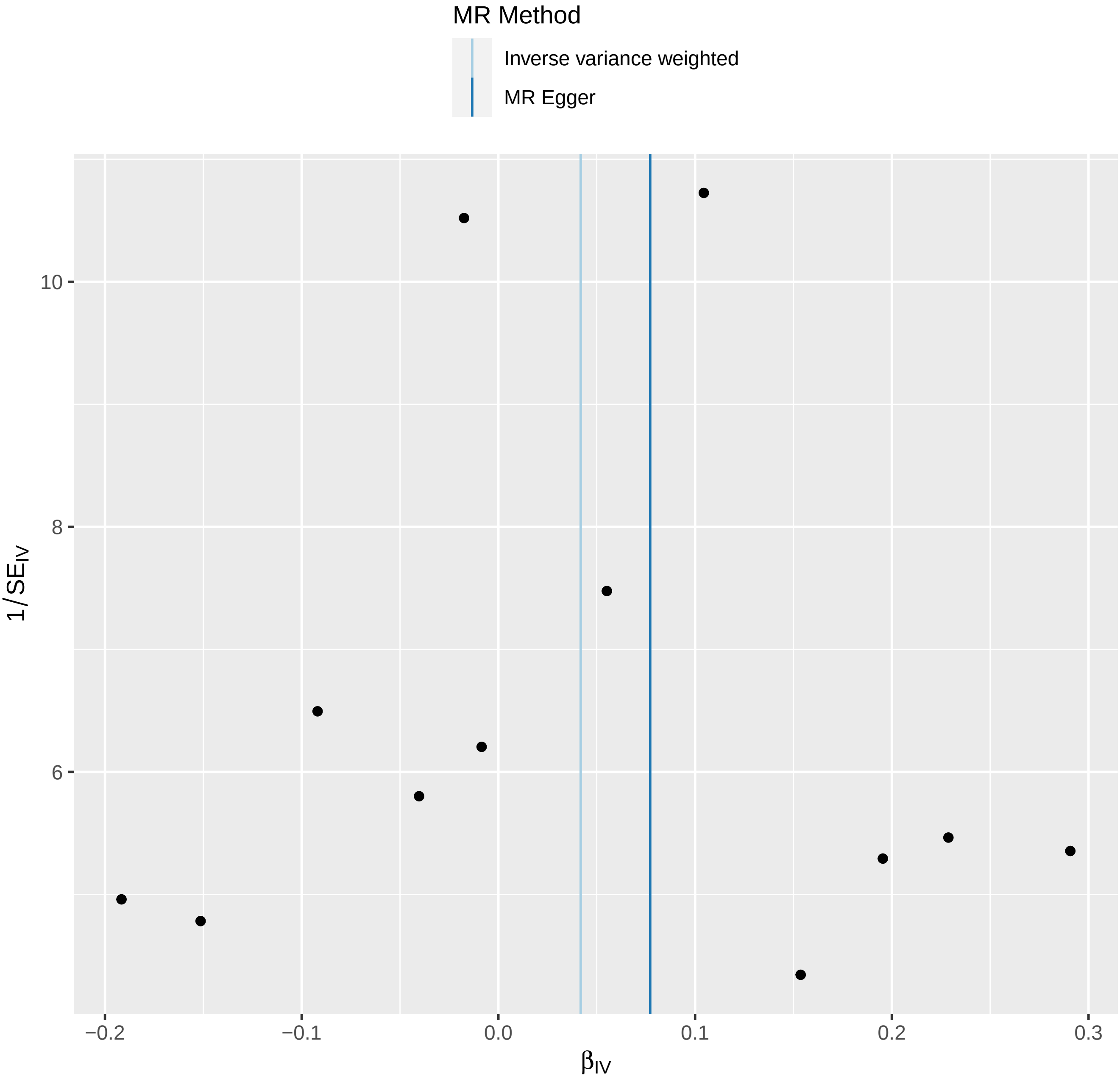

Batch 3−345 : Selenium on Neuron−specific vesicular protein calcyon

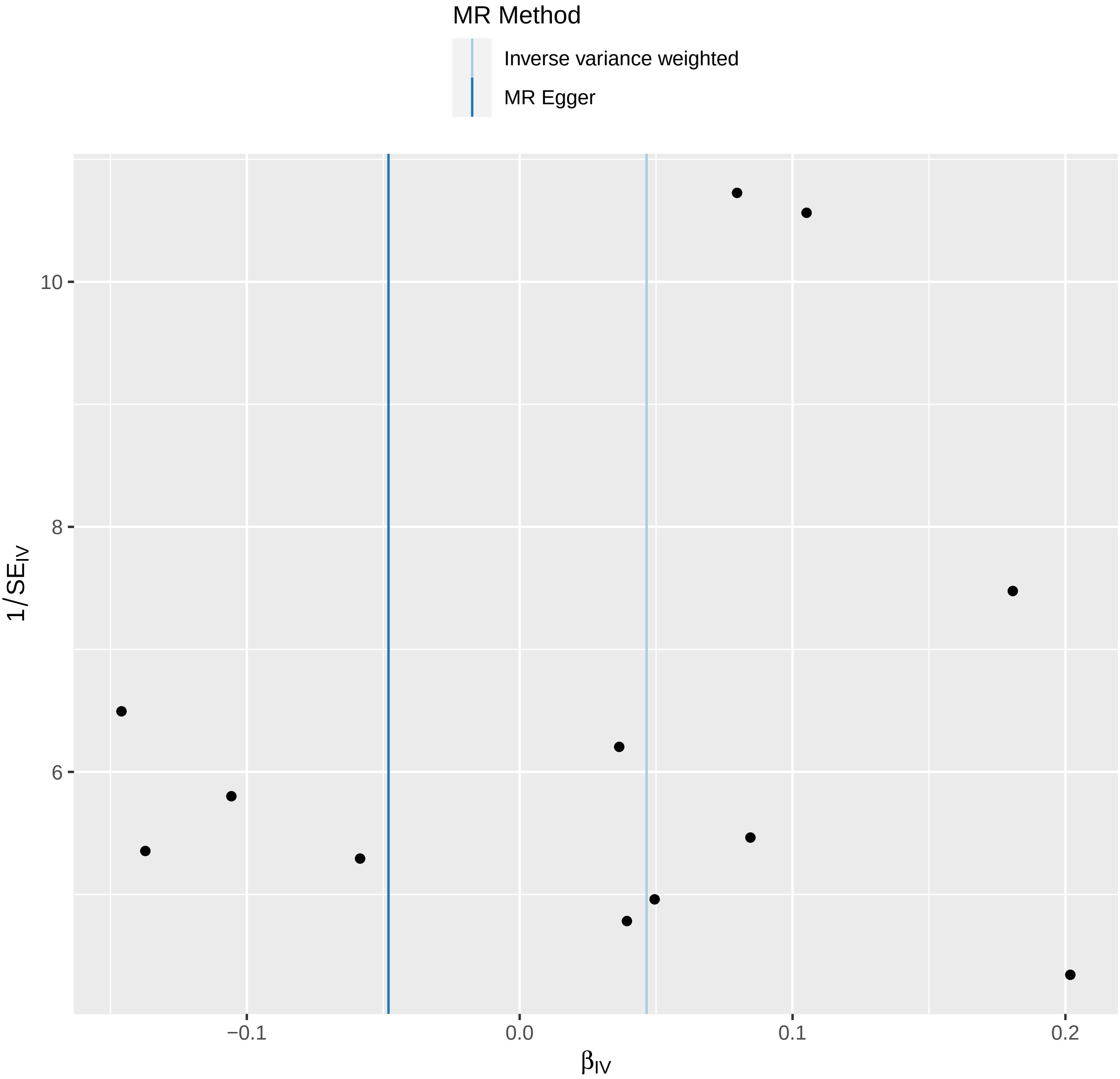

Batch 3−346 : Selenium on Calcium/calmodulin−dependent protein kinase type 1

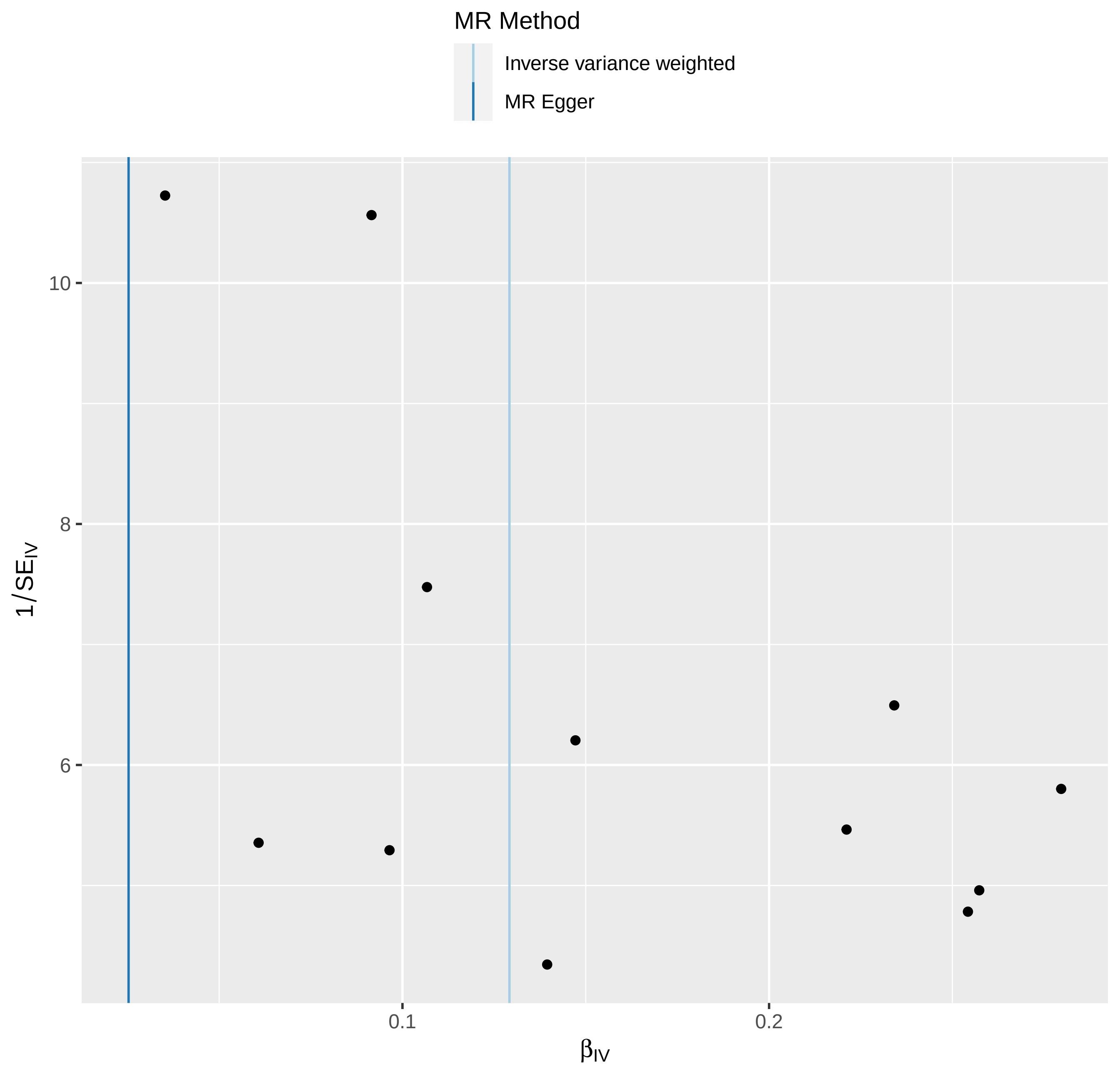

Batch 3−347 : Selenium on Calcium/calmodulin−dependent protein kinase type 1D

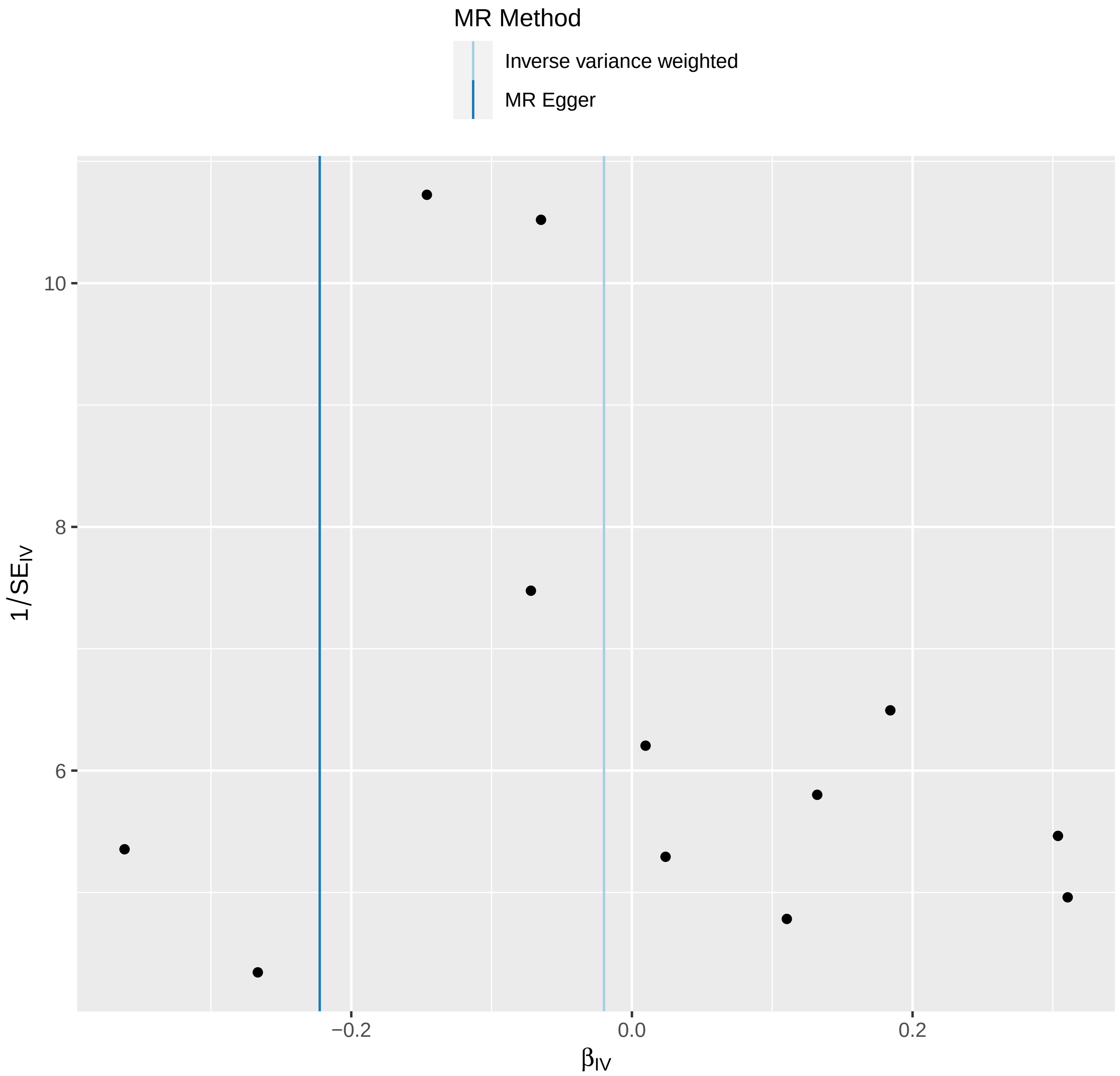

Batch 3−348 : Selenium on Calcium/calmodulin−dependent protein kinase type II subunit alpha

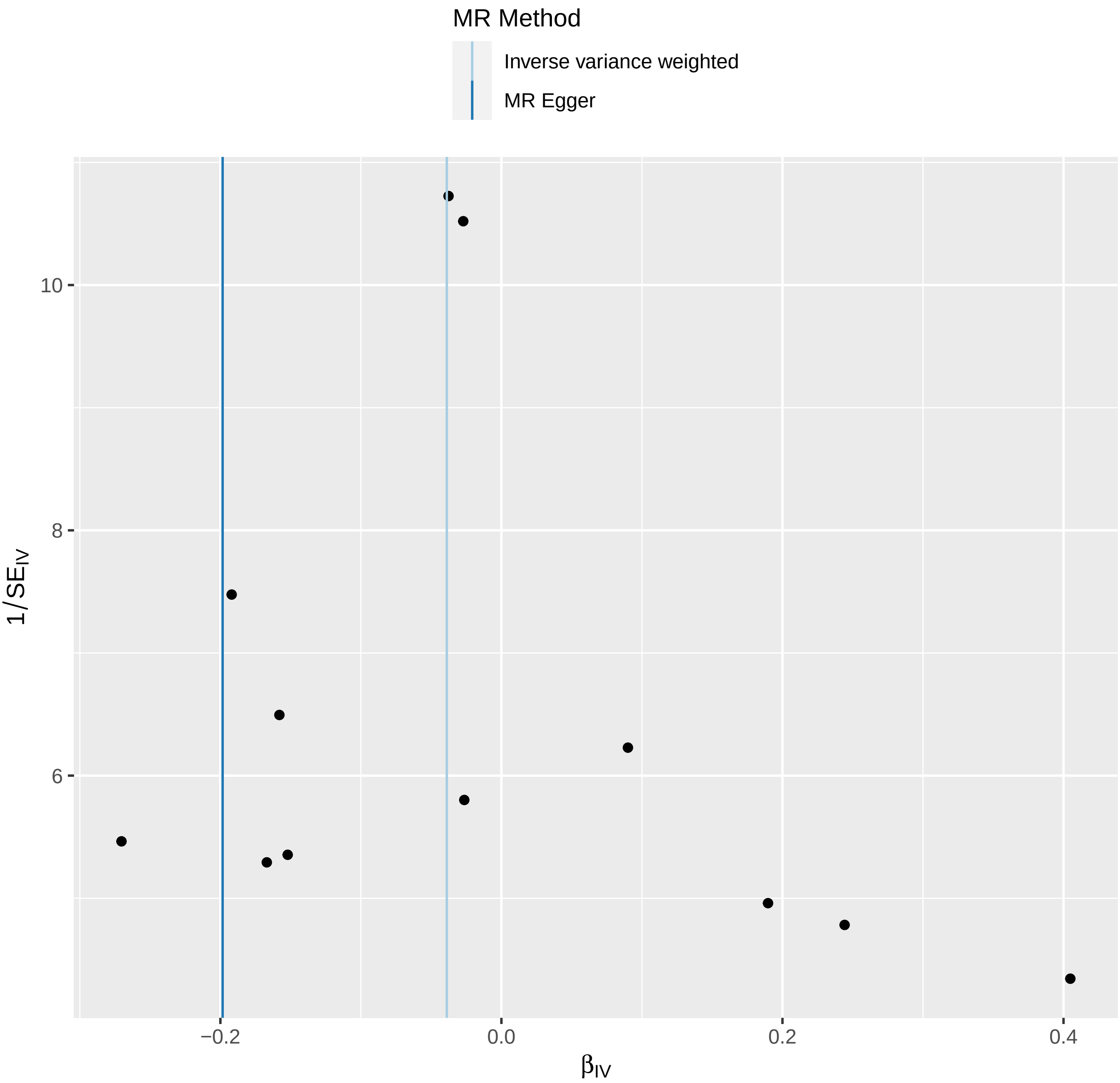

Batch 3−349 : Selenium on Calcium/calmodulin−dependent protein kinase type II subunit beta

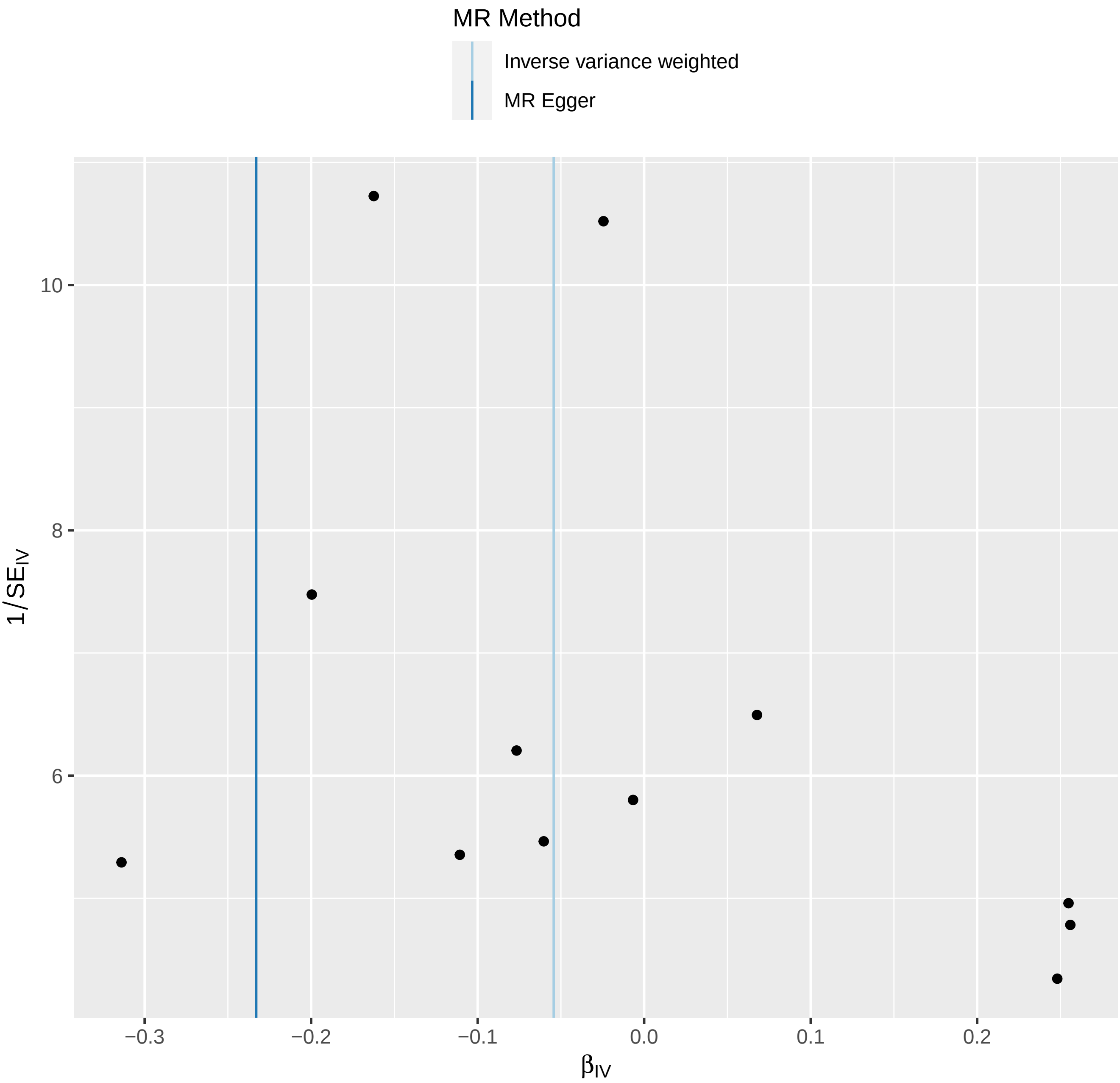

Batch 3−350 : Selenium on Calcium/calmodulin−dependent protein kinase kinase 1

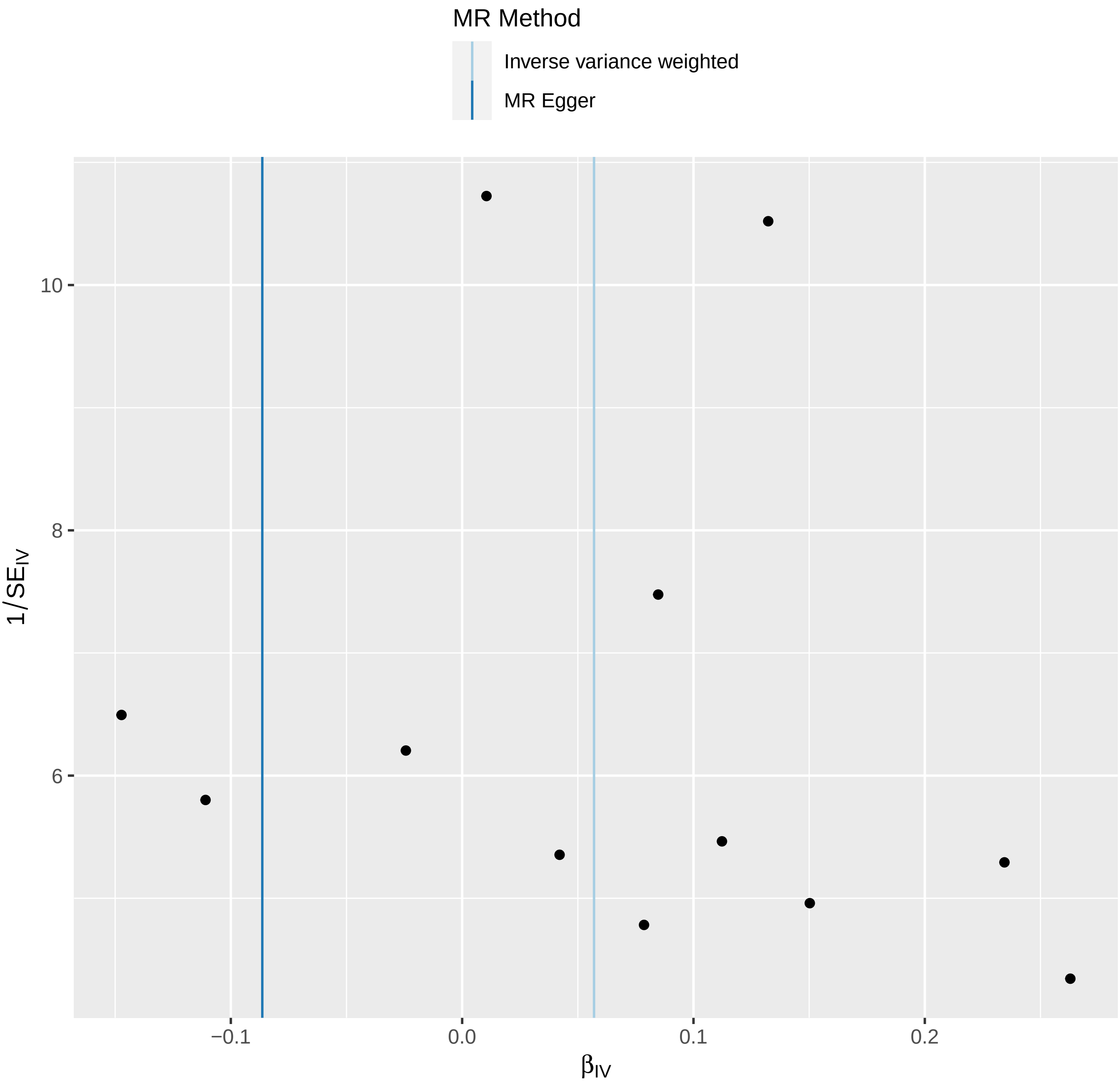

Batch 3−351 : Selenium on Cullin−associated NEDD8−dissociated protein 1

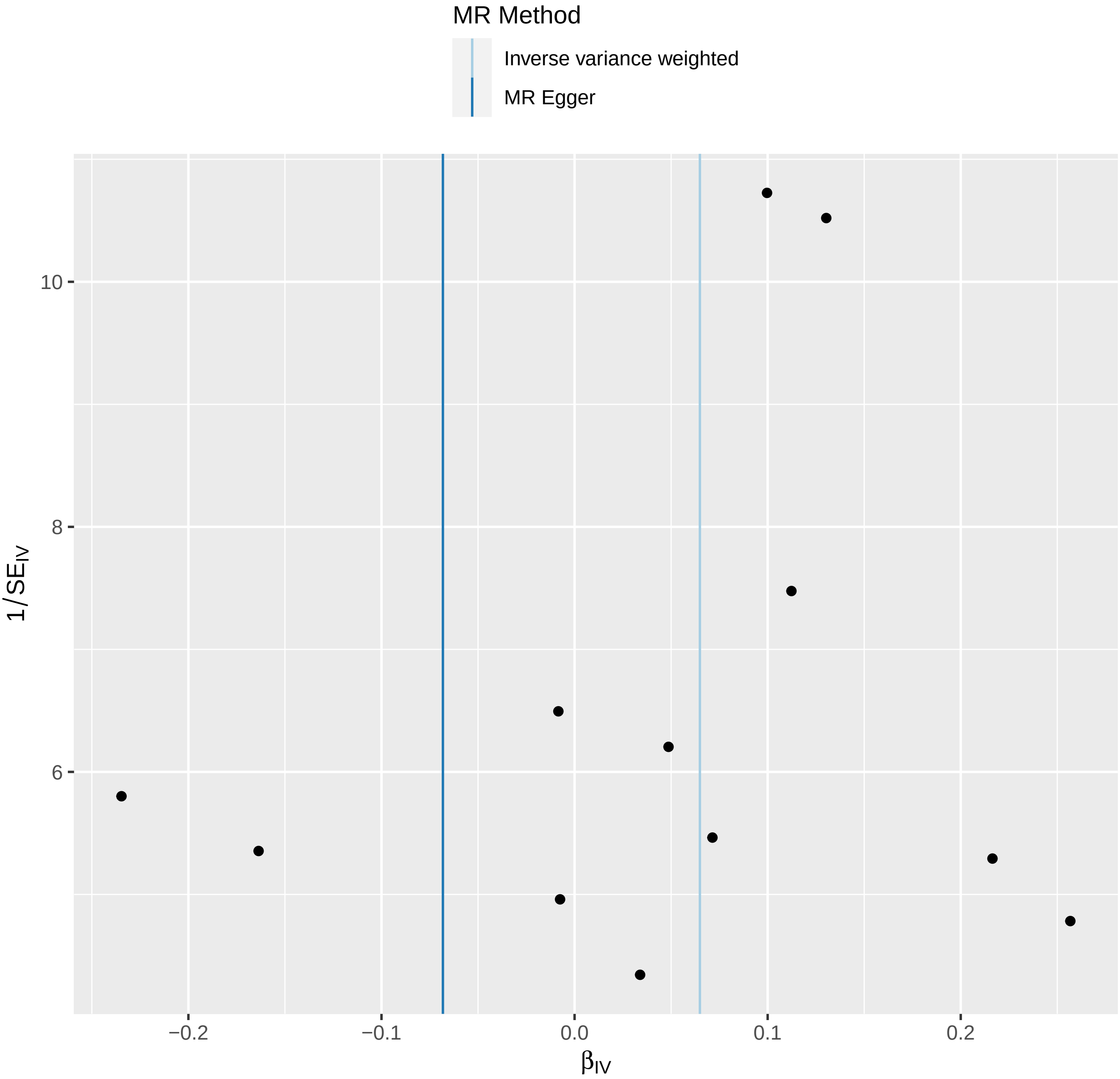

Batch 3−352 : Selenium on Soluble calcium−activated nucleotidase 1

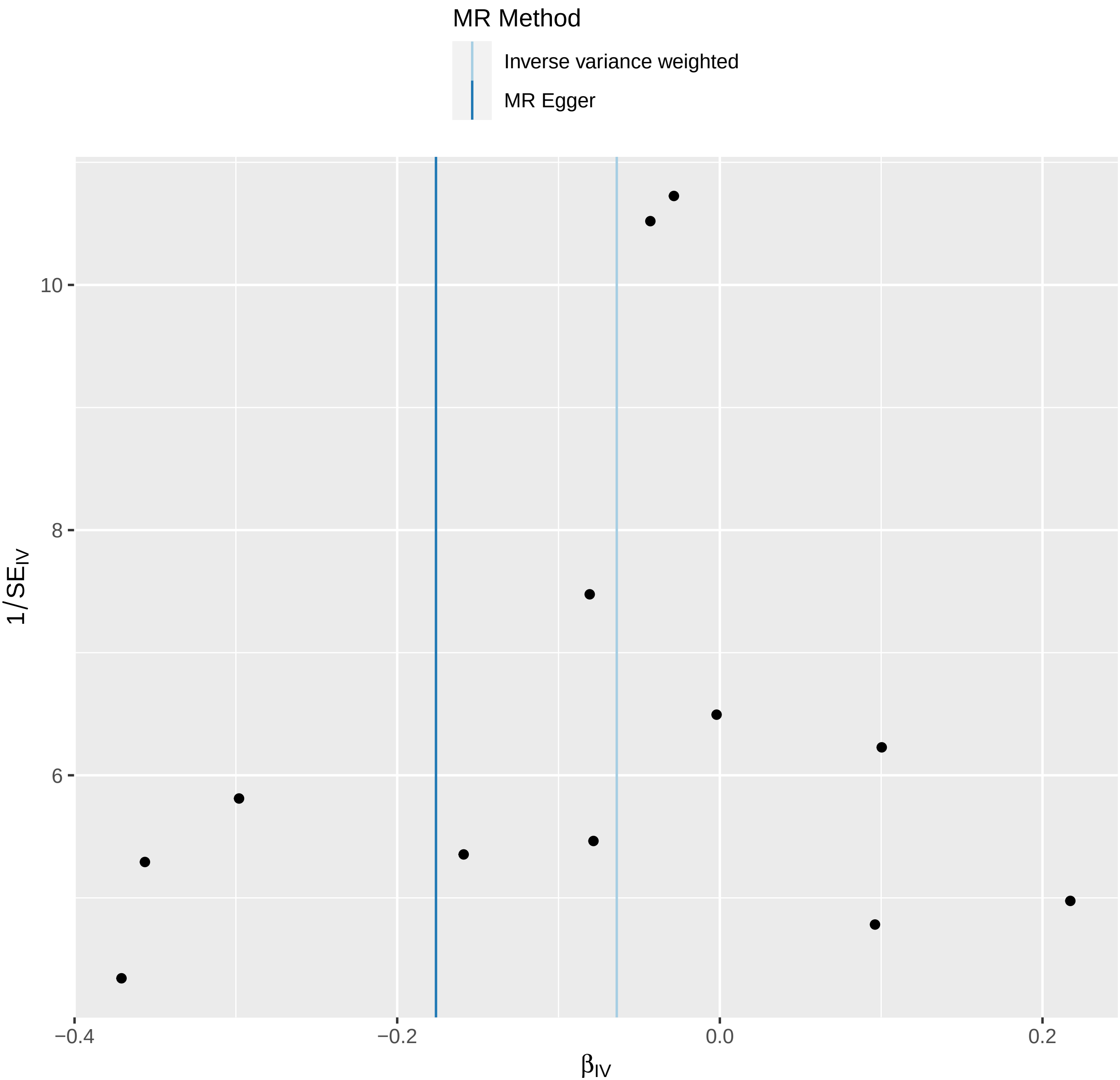

Batch 3−353 : Selenium on Calnexin

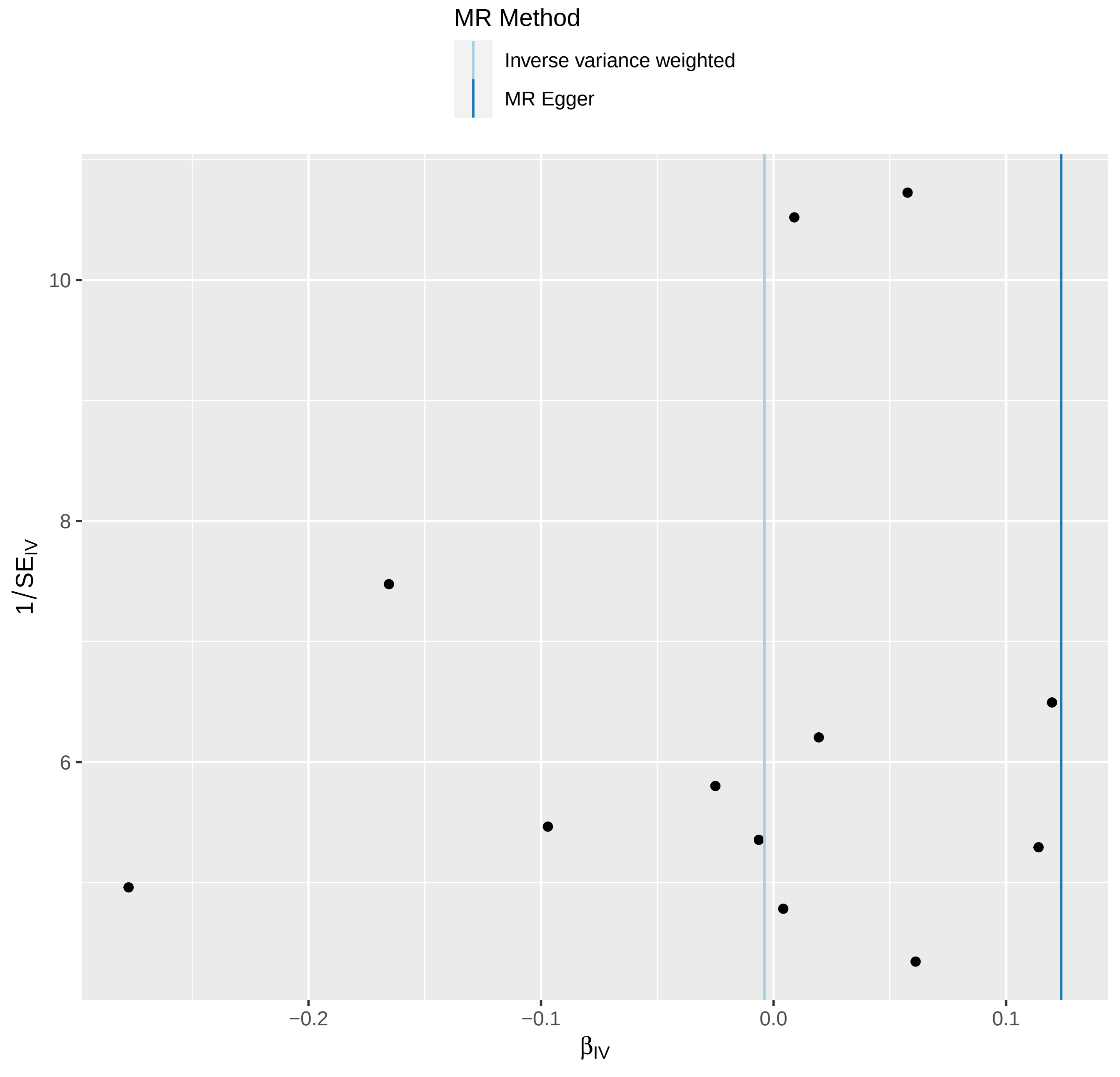

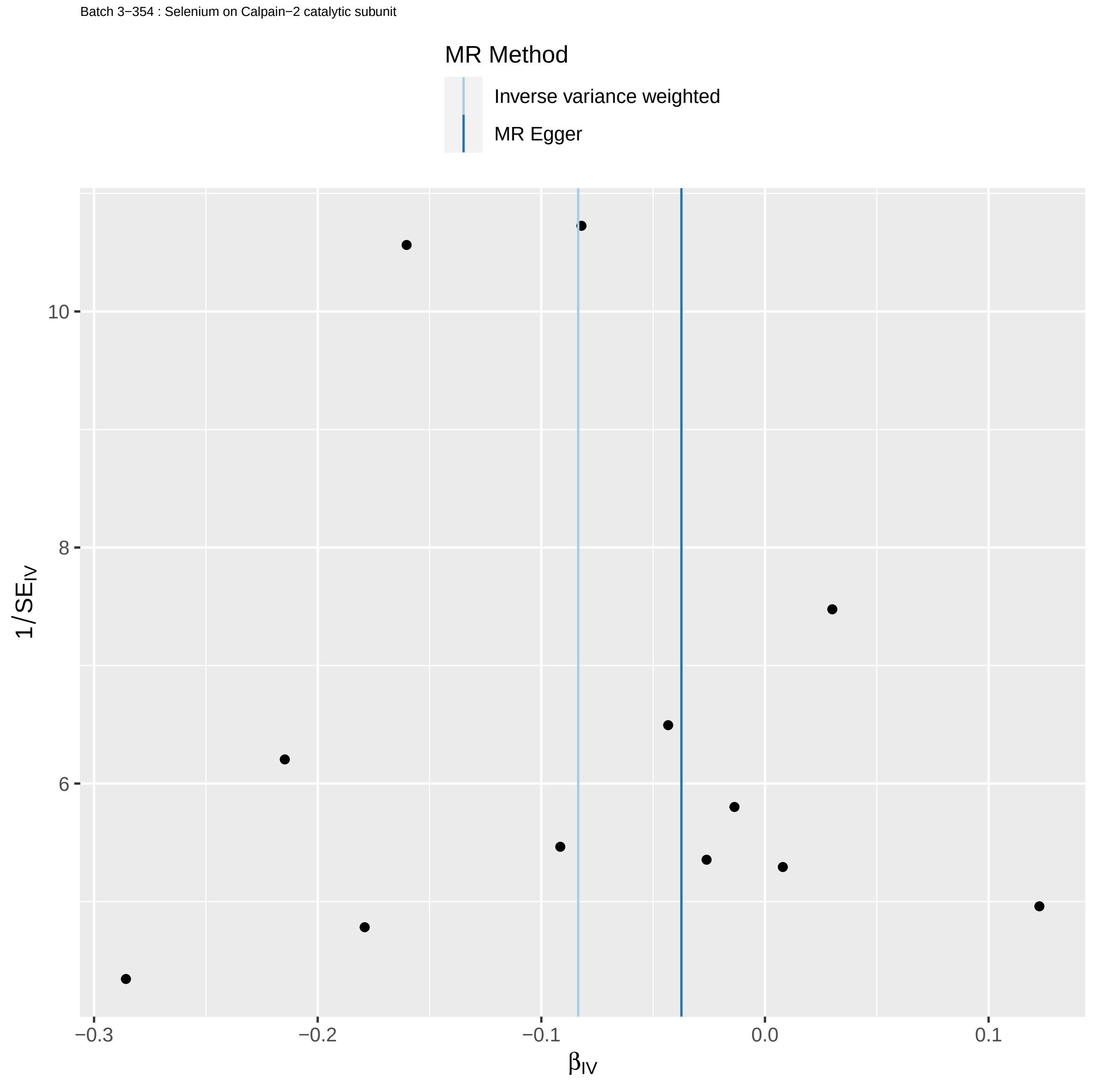
Batch 3−354 : Selenium on Calpain−2 catalytic subunit
MR Method
Inverse variance weighted
MR Egger
10
8
6
1/SE_IV
−0.3
−0.2
−0.1
0.0
0.1
β_IV

Batch 3−355 : Selenium on Calpain−3

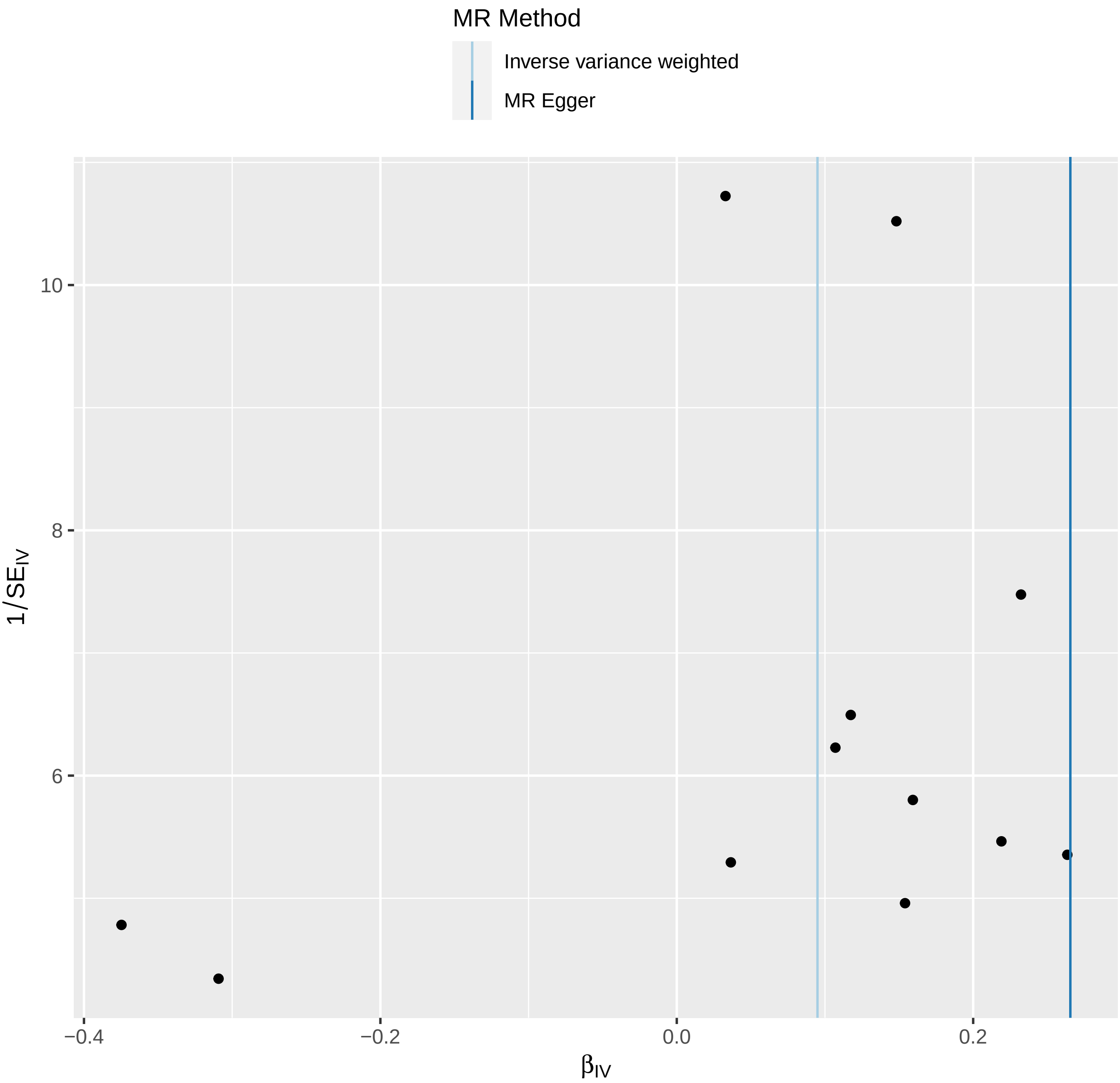

Batch 3−356 : Selenium on Calcyphosin−like protein

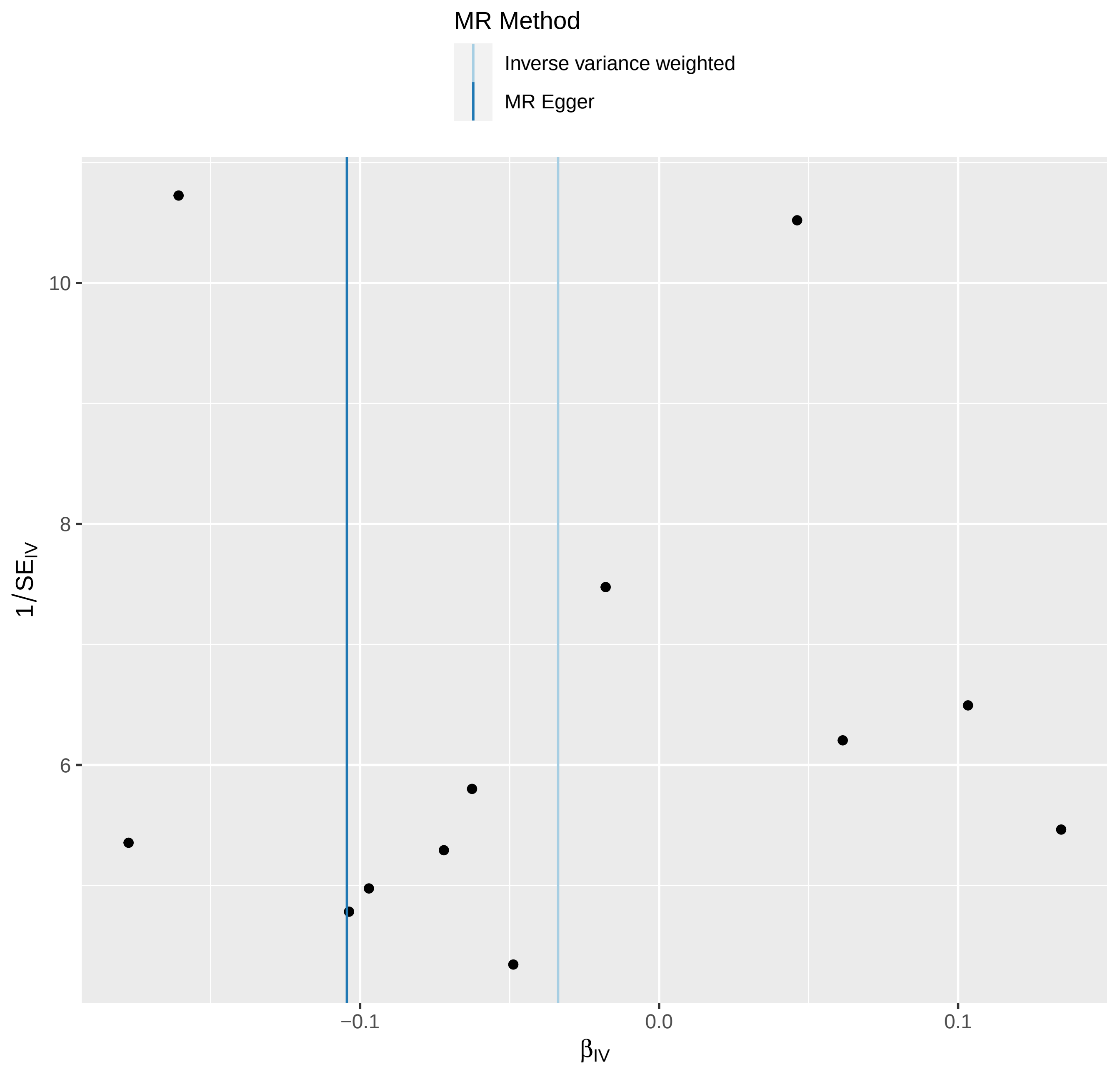

Batch 3−357 : Selenium on Calcium−regulated heat stable protein 1

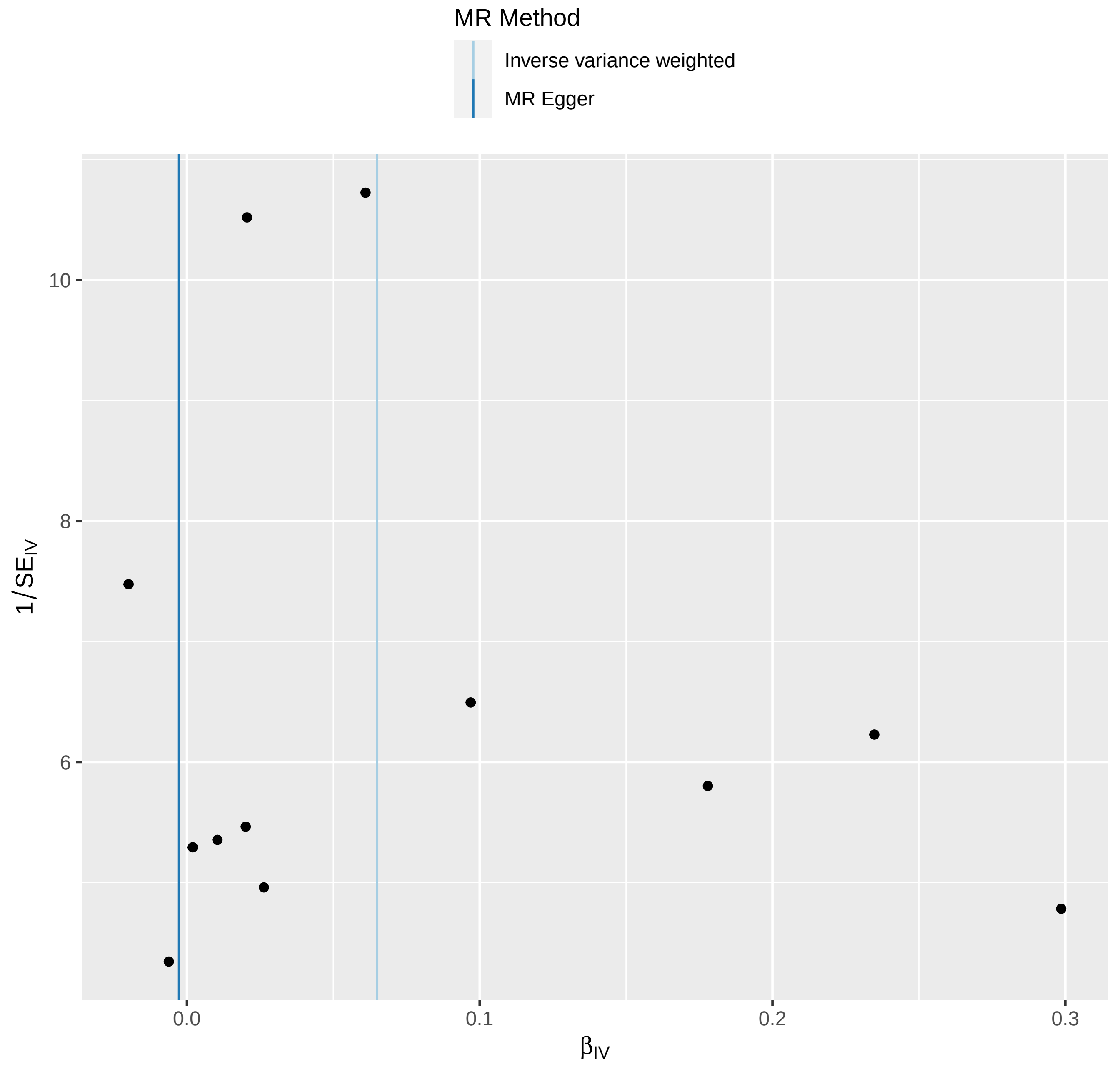

Batch 3−358 : Selenium on Cysteine−−tRNA ligase, cytoplasmic

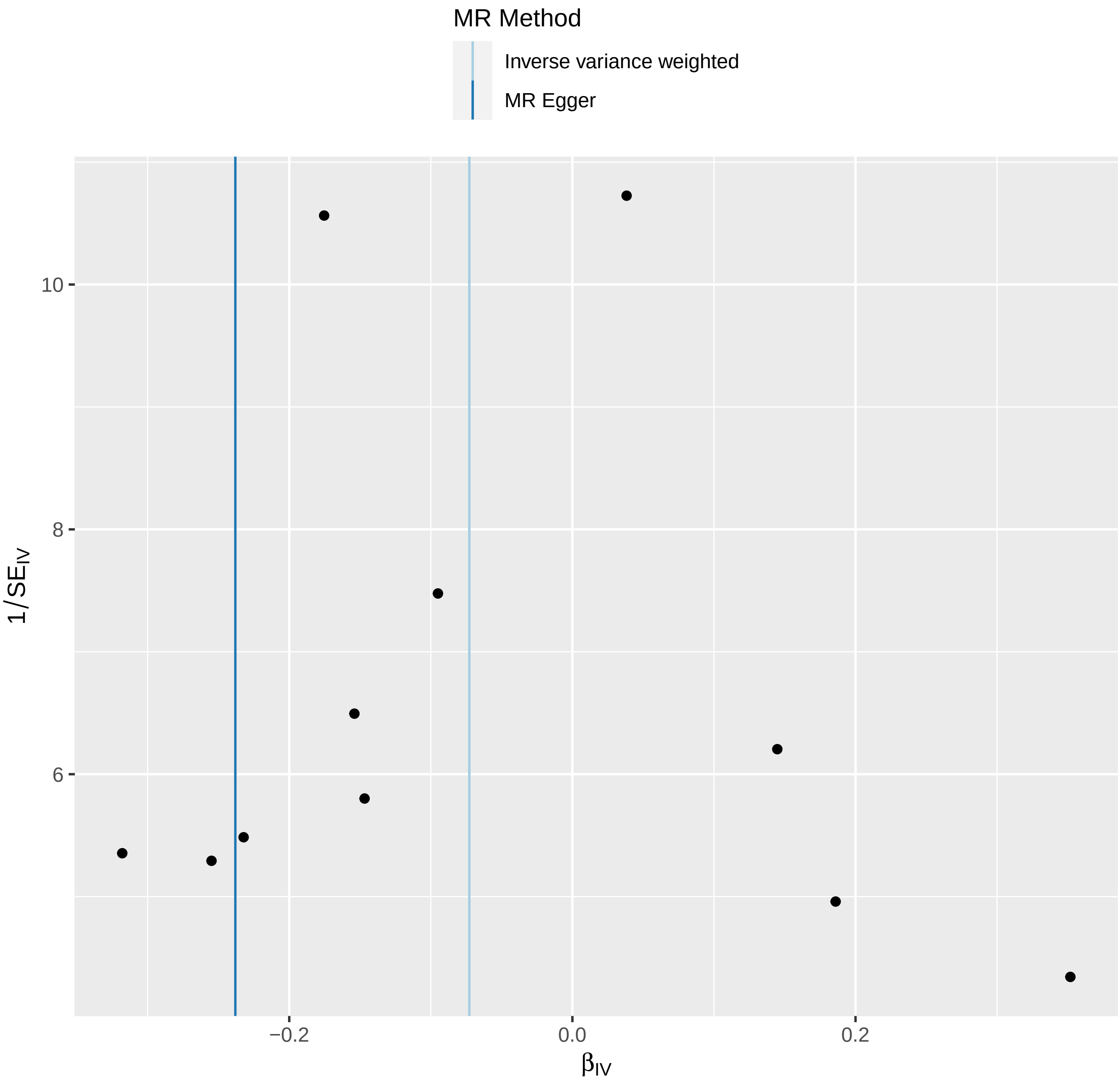

Batch 3−359 : Selenium on Cocaine− and amphetamine−regulated transcript protein

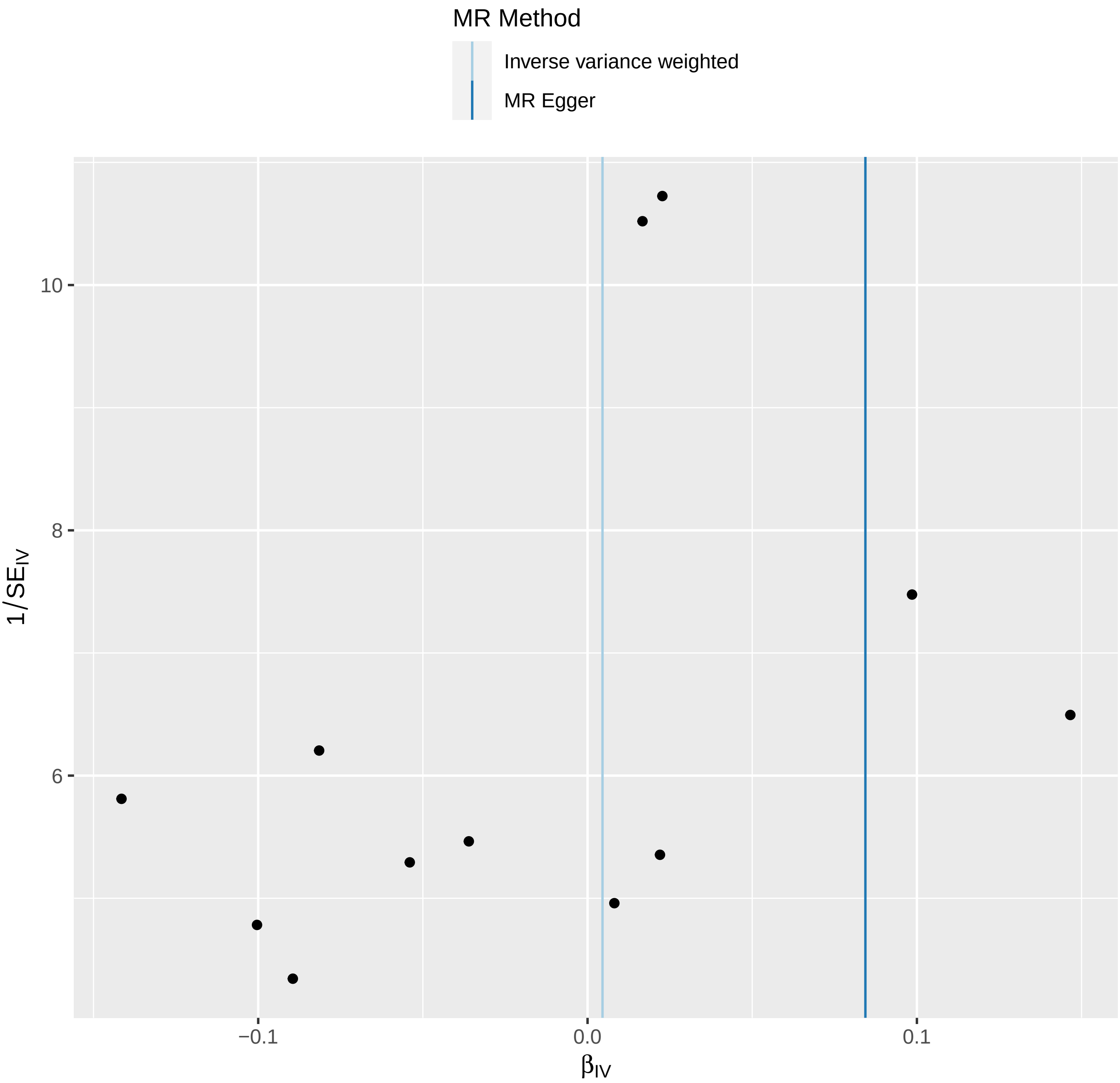

Batch 3−360 : Selenium on Protein CASC4

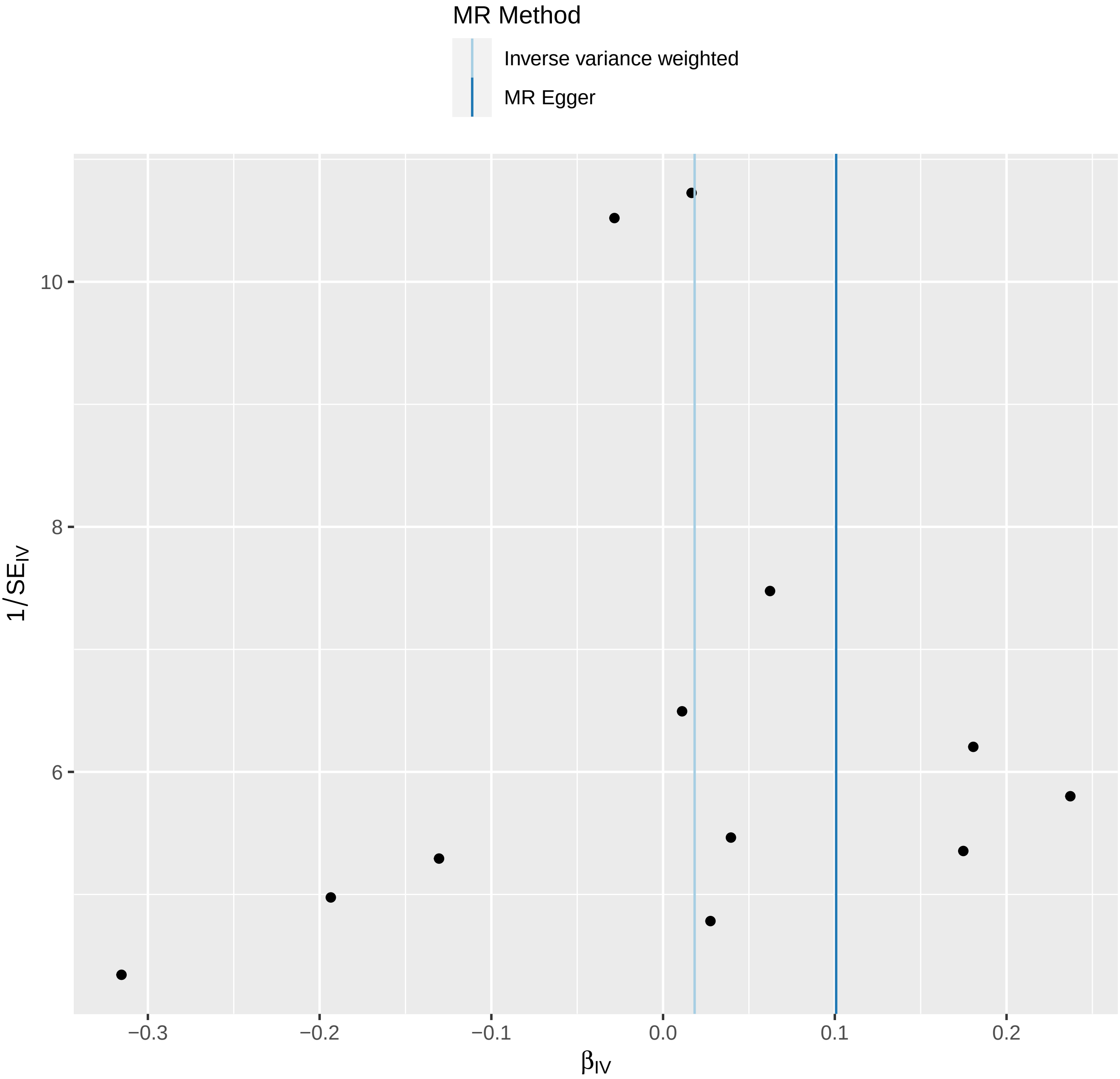

Batch 3−361 : Selenium on Protein CASC4

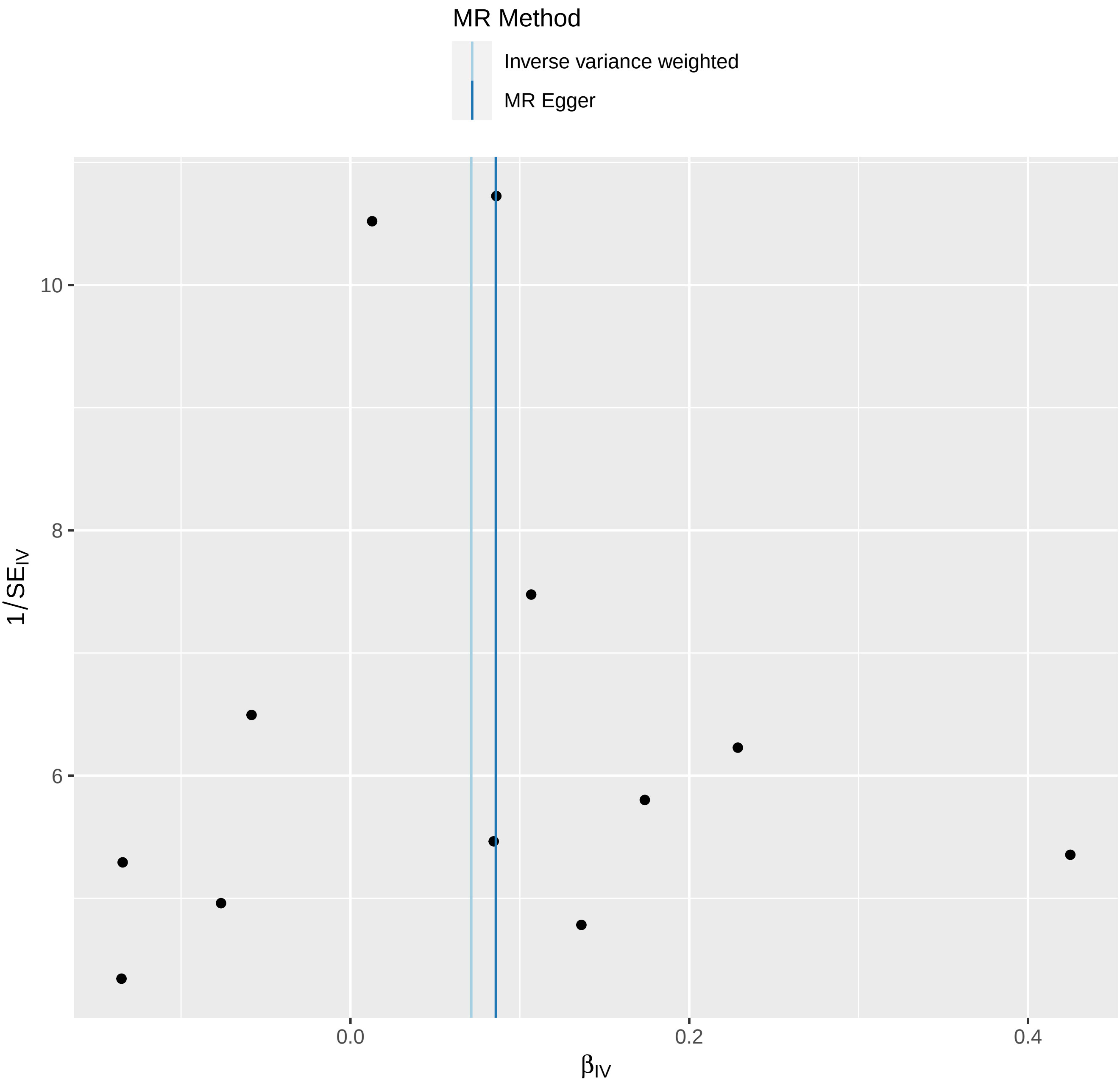

Batch 3−362 : Selenium on Caspase−10

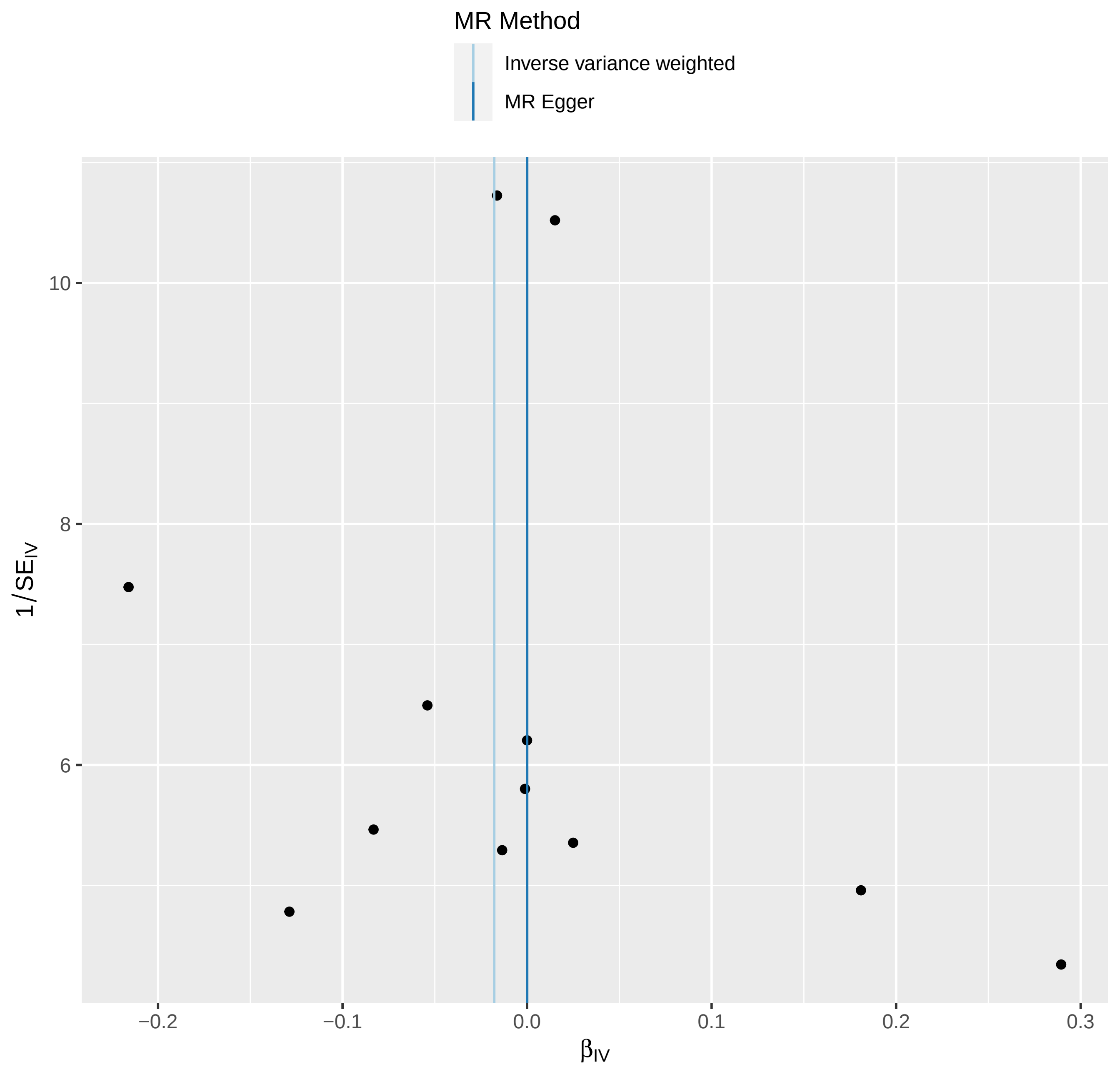

Batch 3−363 : Selenium on Caspase−2

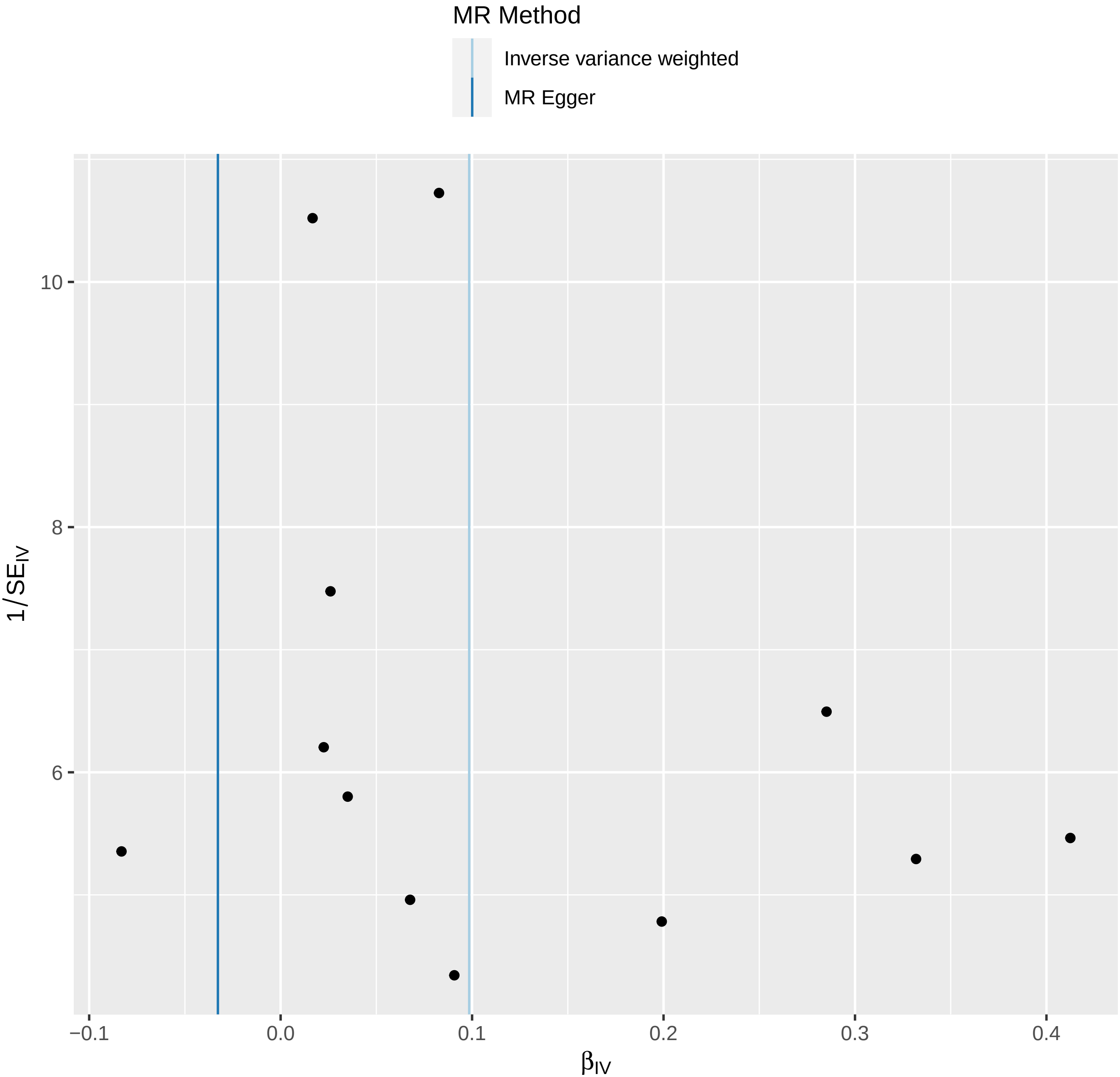

Batch 3−364 : Selenium on Caspase−3

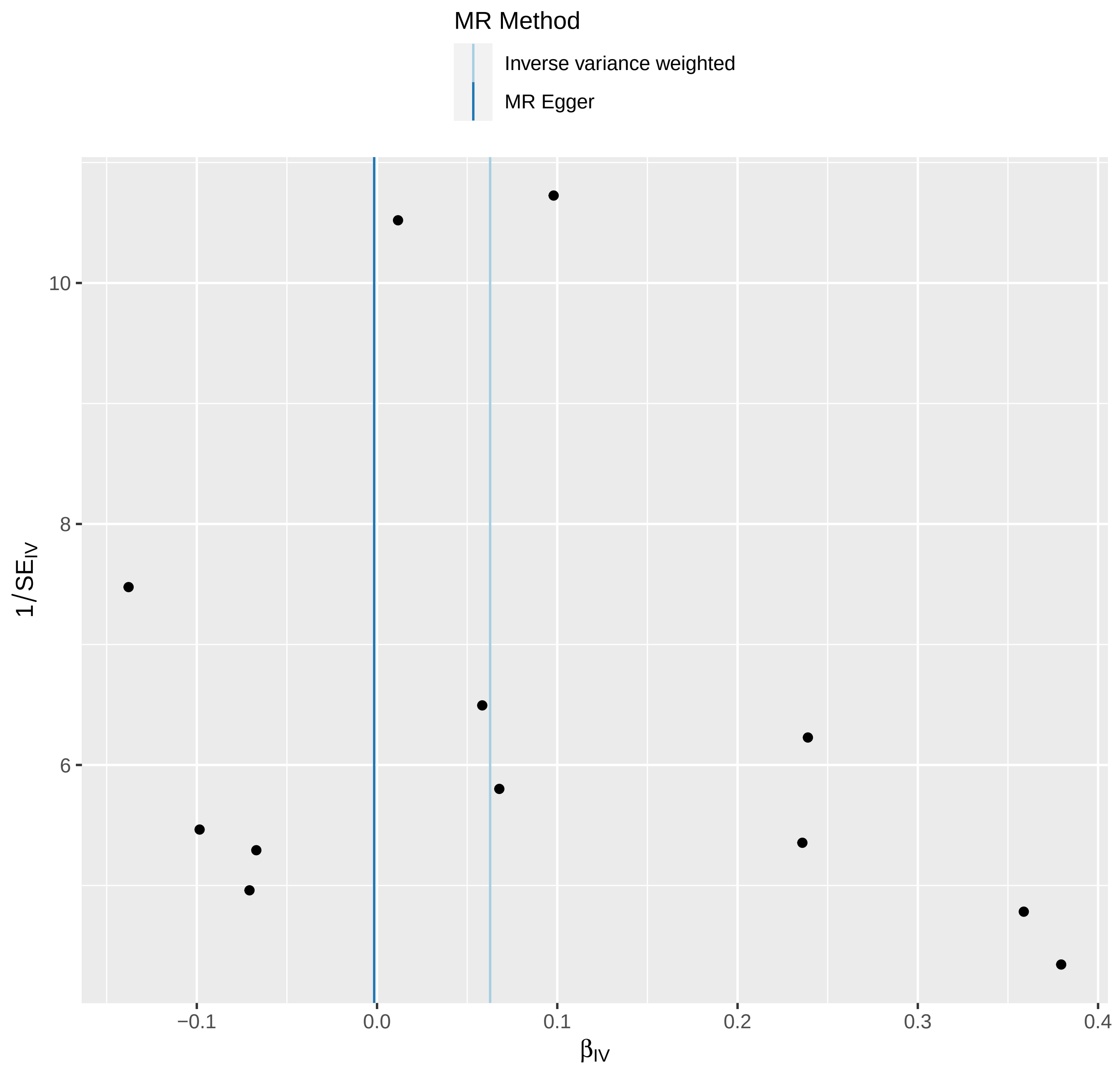

Batch 3−365 : Selenium on Cas scaffolding protein family member 4

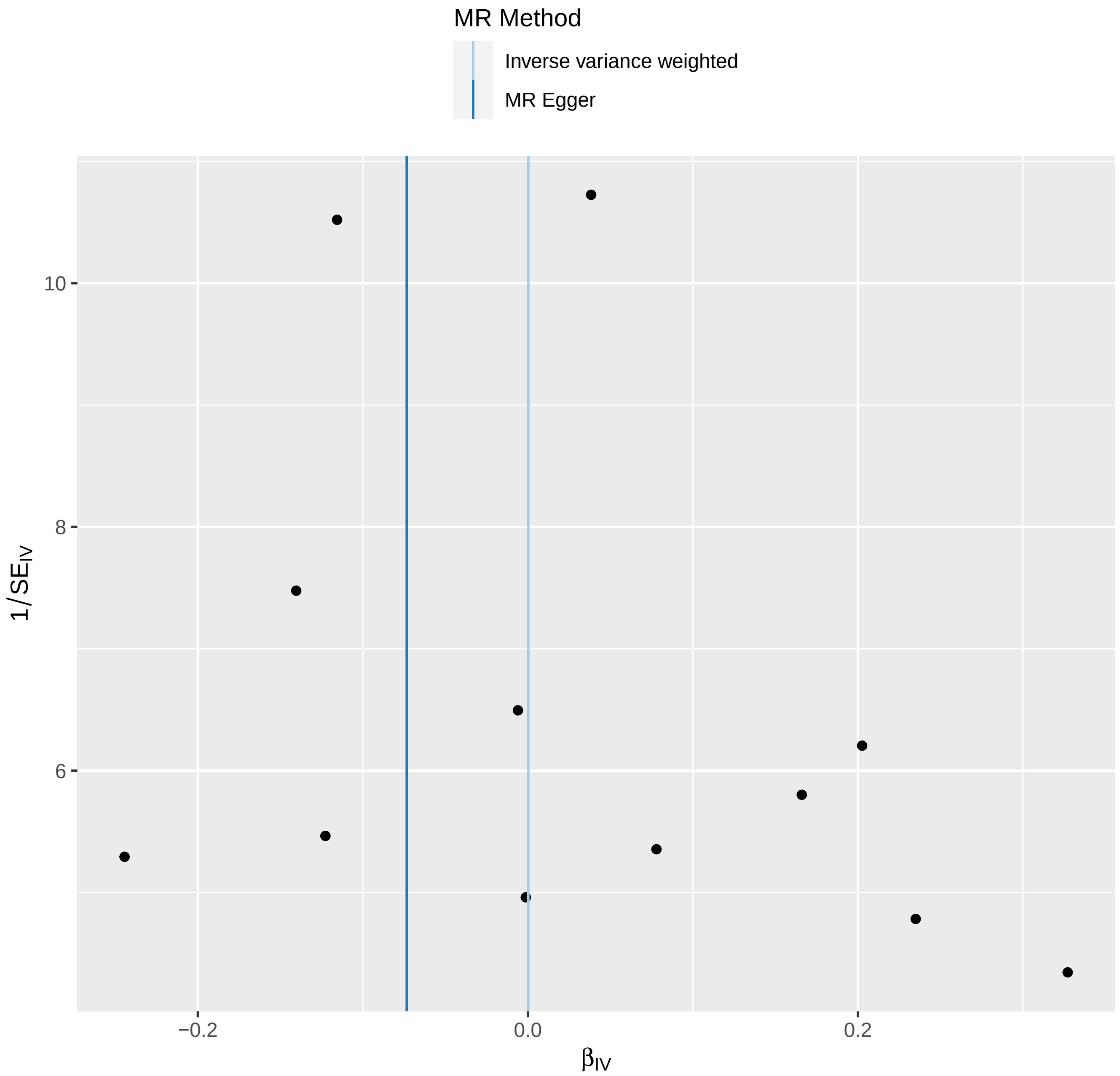

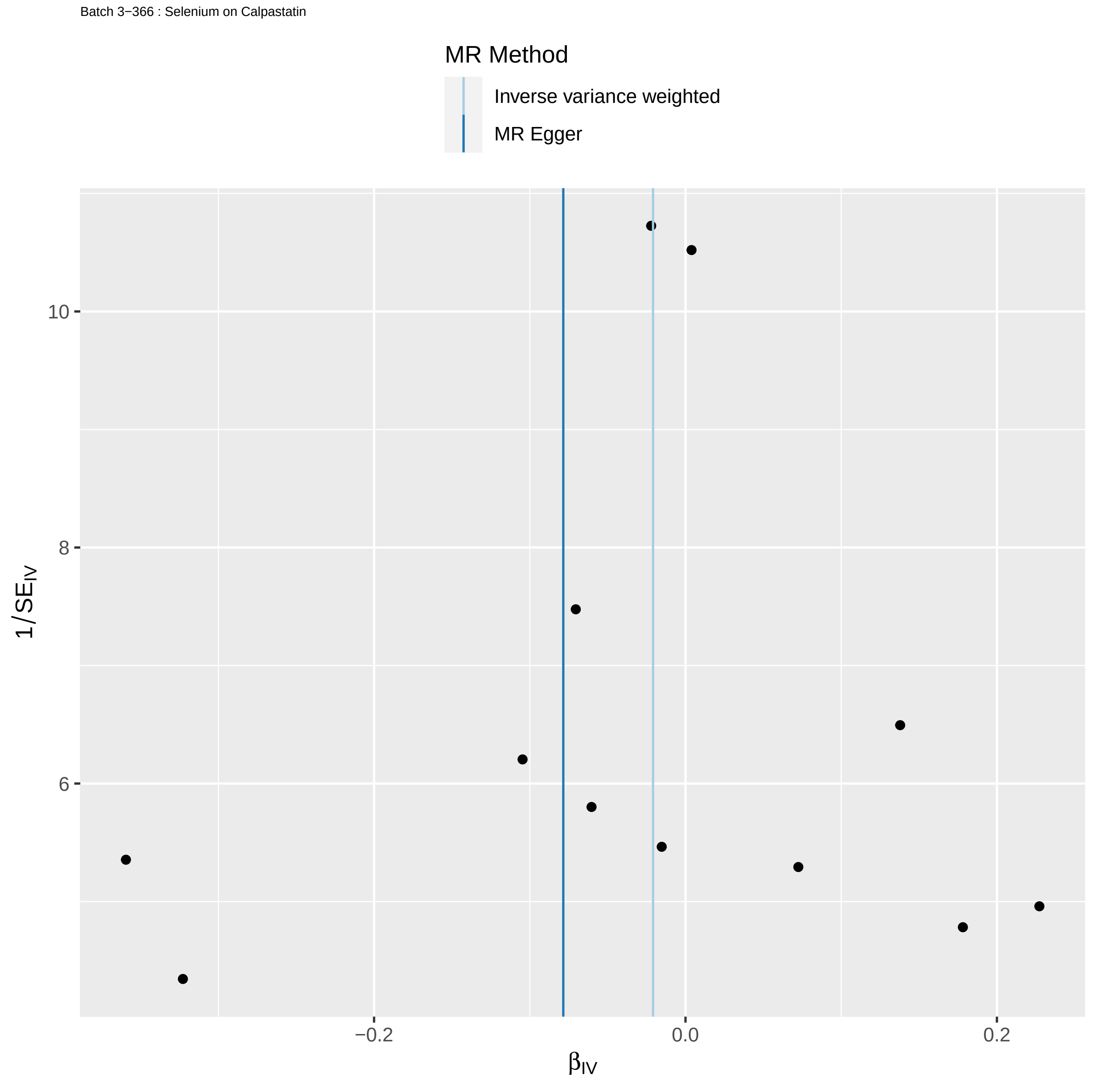

Batch 3−366 : Selenium on Calpastatin
MR Method
Inverse variance weighted
MR Egger
10
8
6
$1/SE_{IV}$
−0.2
0.0
0.2
β_{IV}

Batch 3−367 : Selenium on Catalase

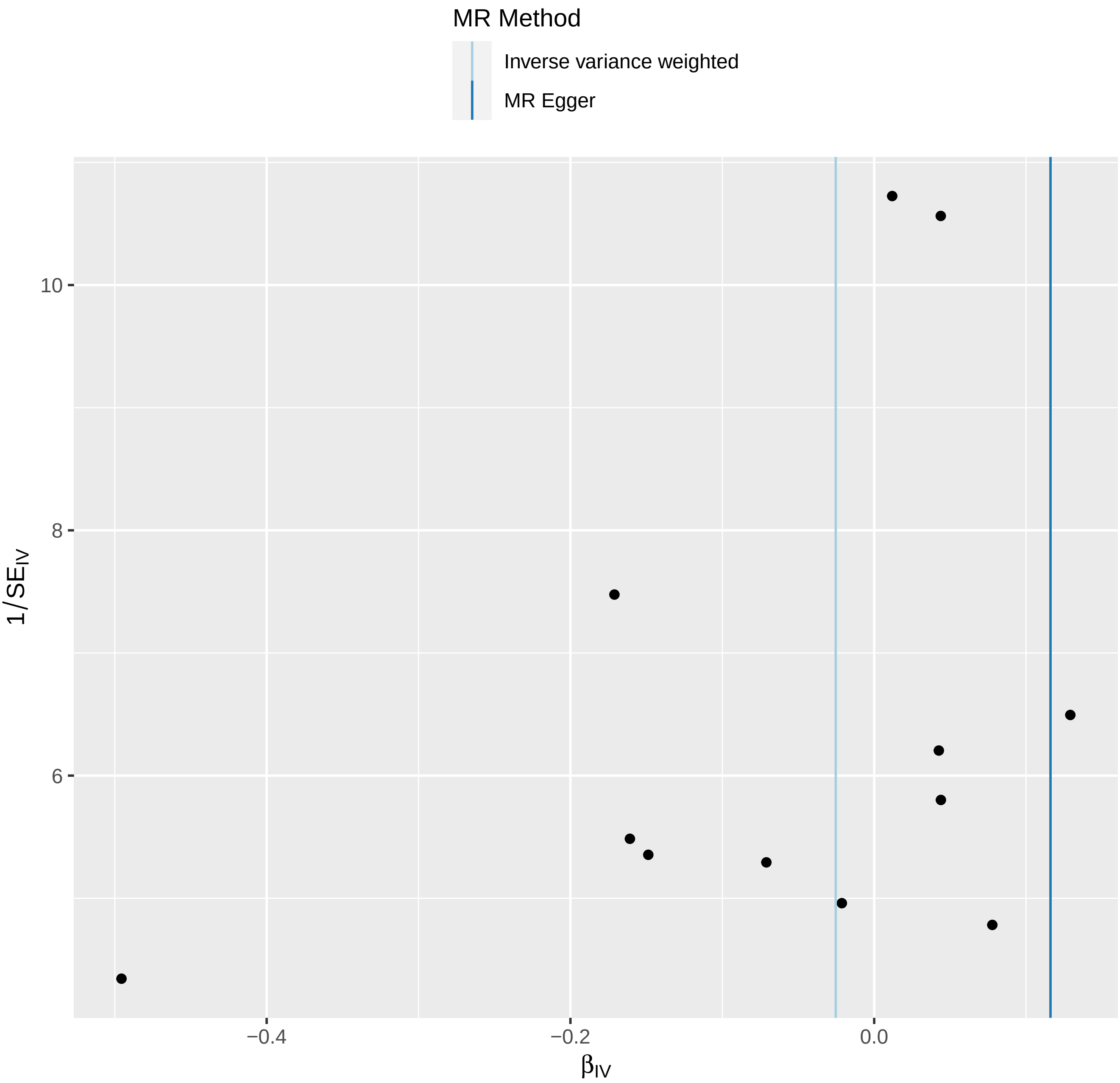

Batch 3−368 : Selenium on Caveolin−2

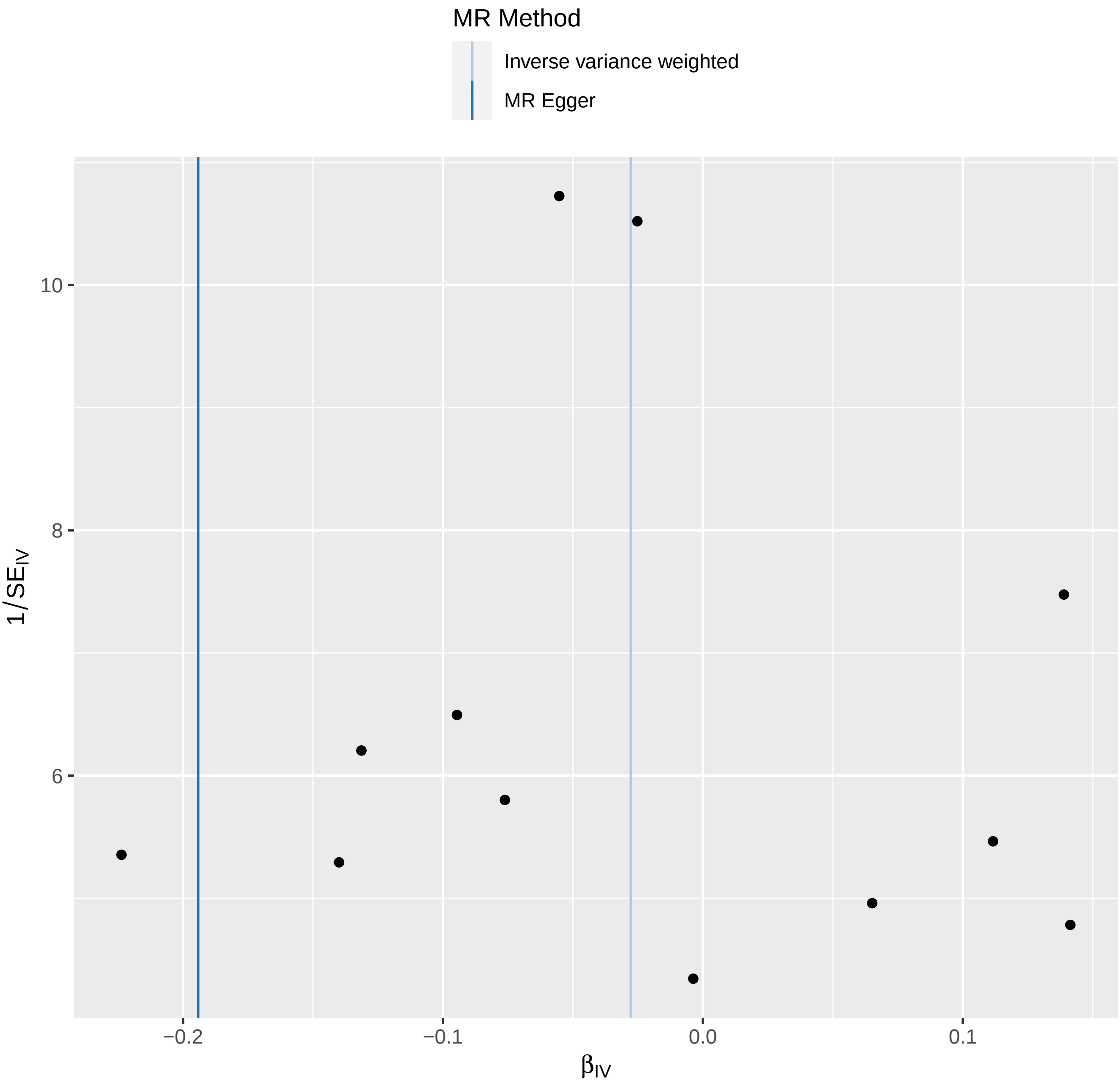

Batch 3−369 : Selenium on Core−binding factor subunit beta

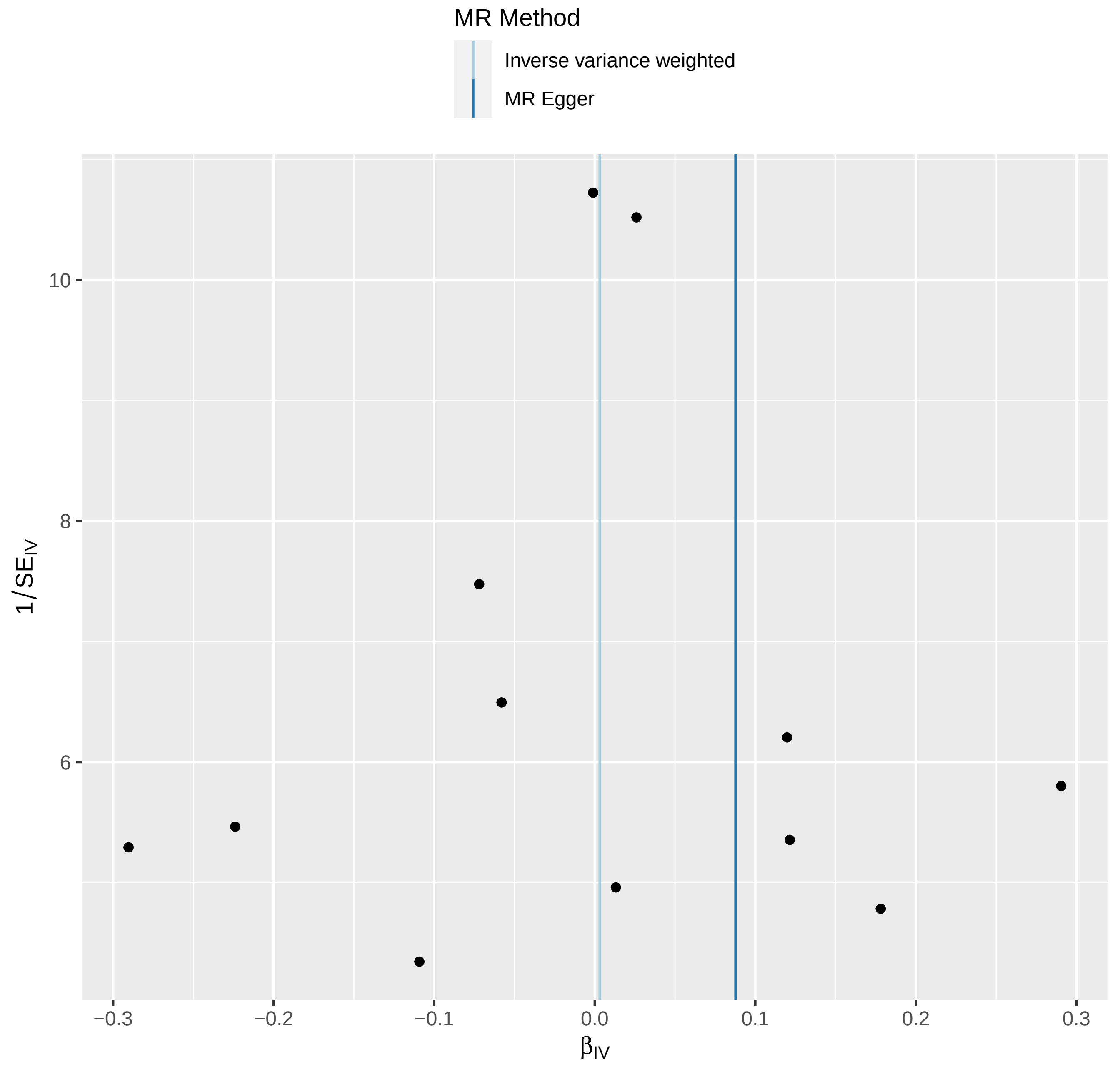

Batch 3−370 : Selenium on E3 ubiquitin−protein ligase CBL

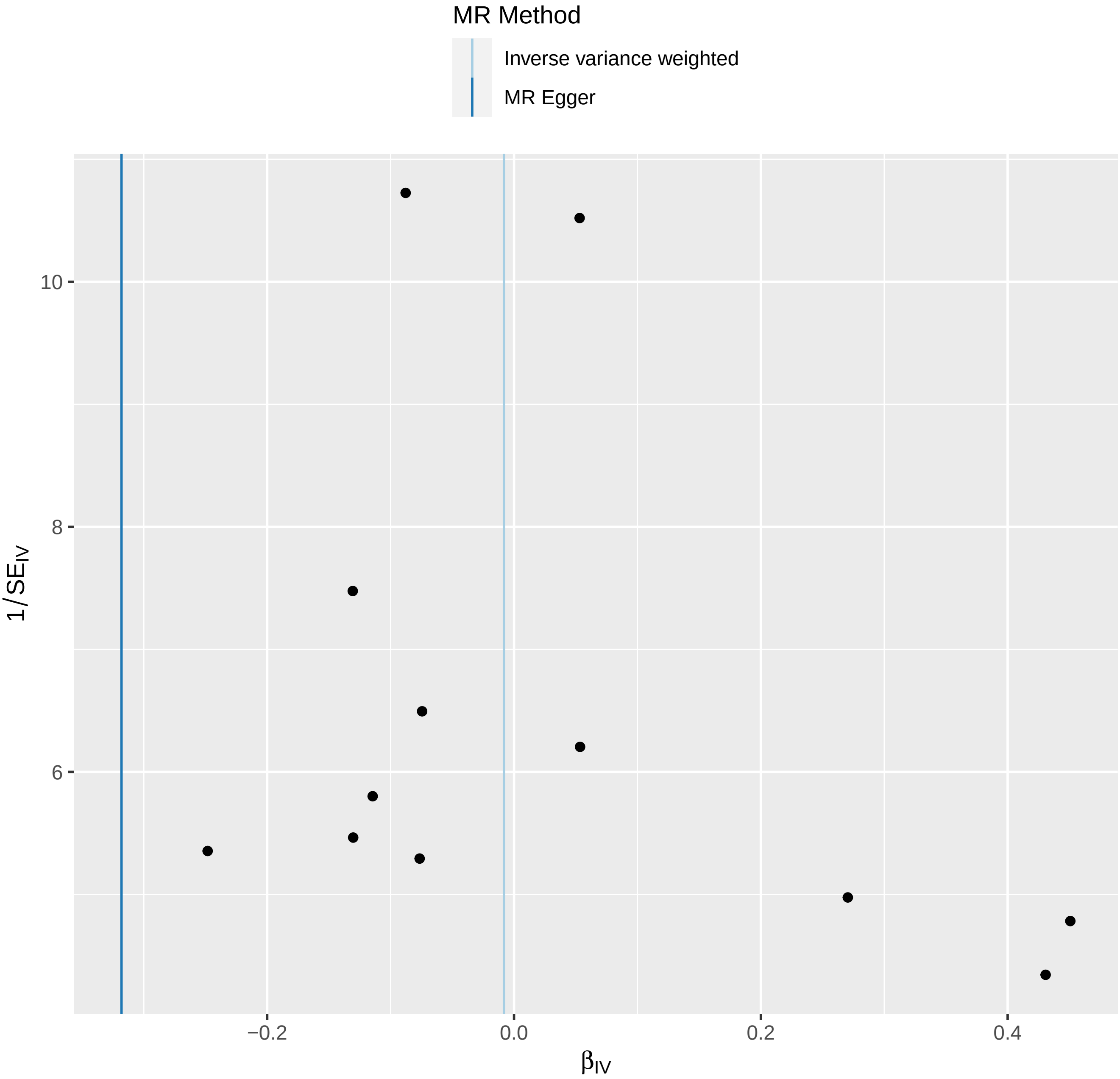

Batch 3−371 : Selenium on Cerebellin−1

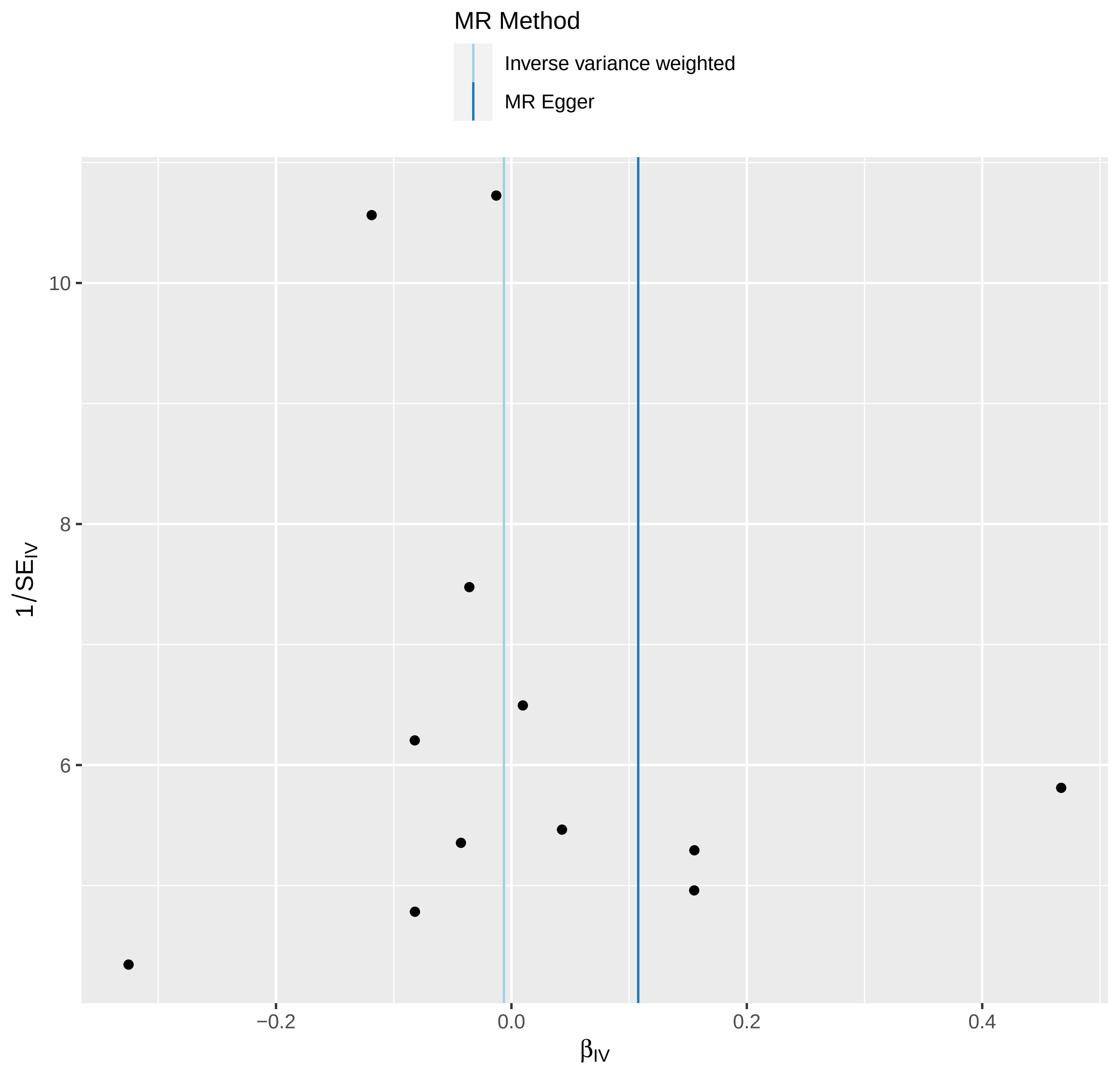

Batch 3−372 : Selenium on Cerebellin−4

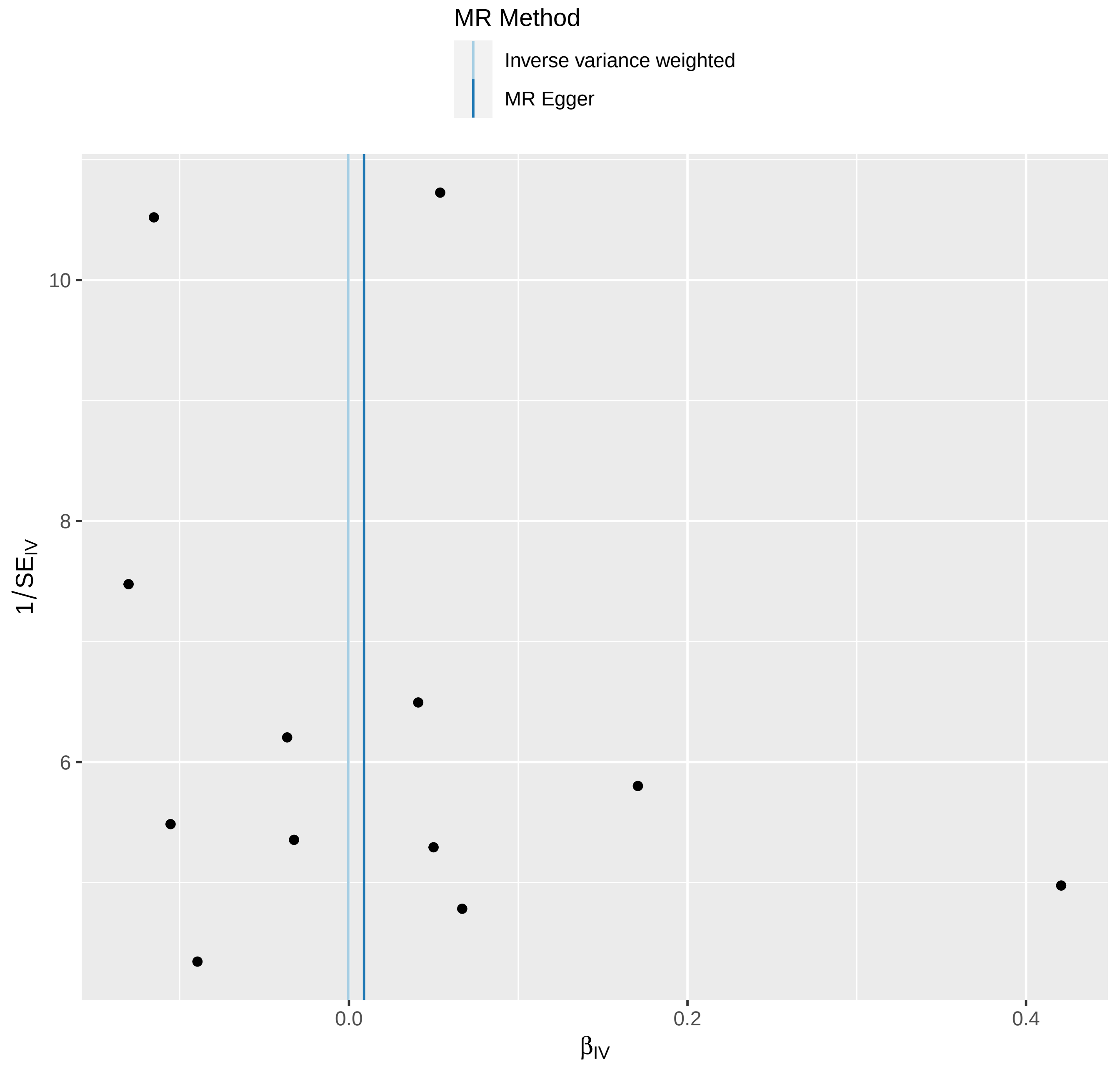

Batch 3−373 : Selenium on Carbonyl reductase [NADPH] 1

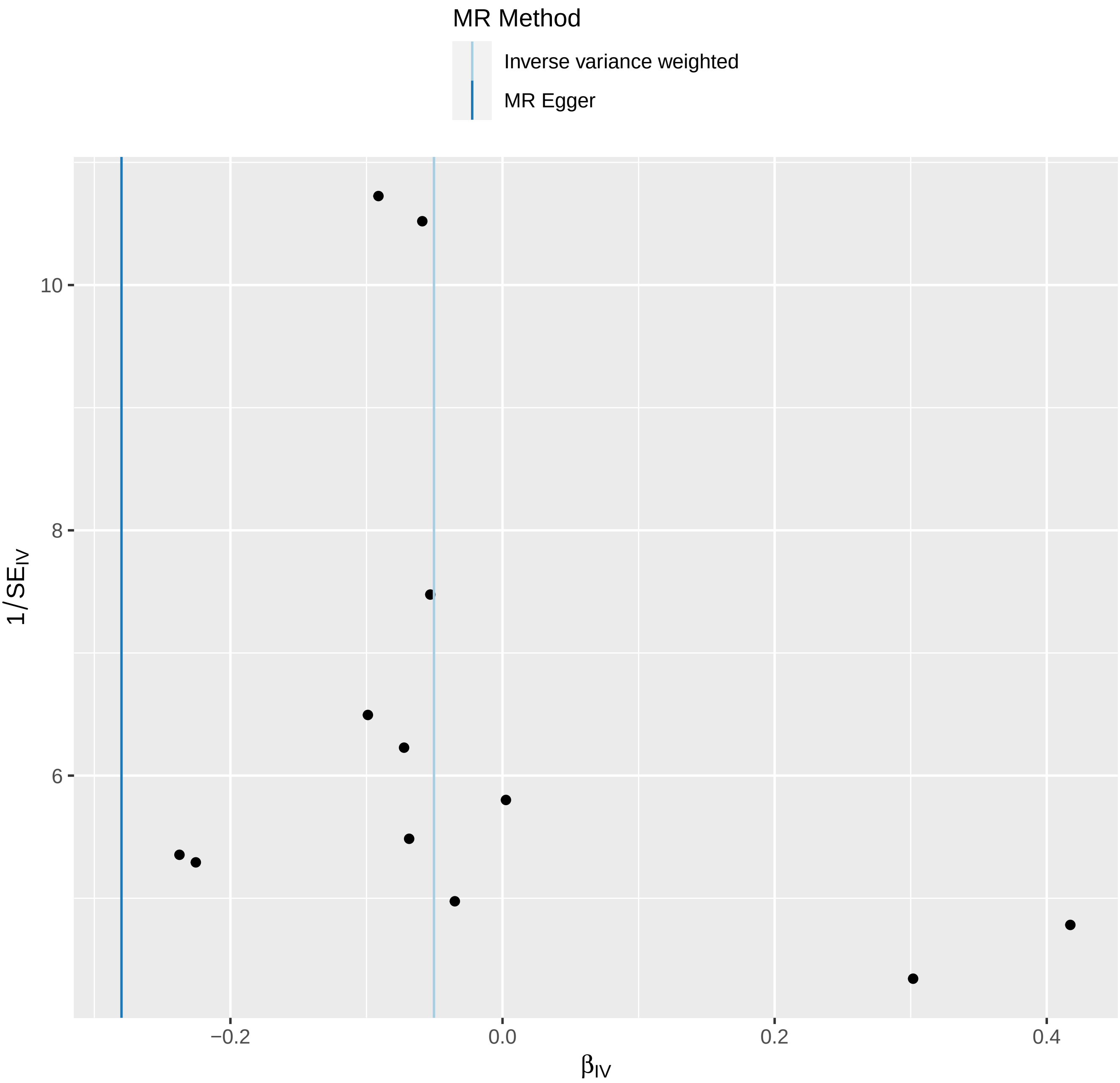

Batch 3−374 : Selenium on Carbonyl reductase [NADPH] 3

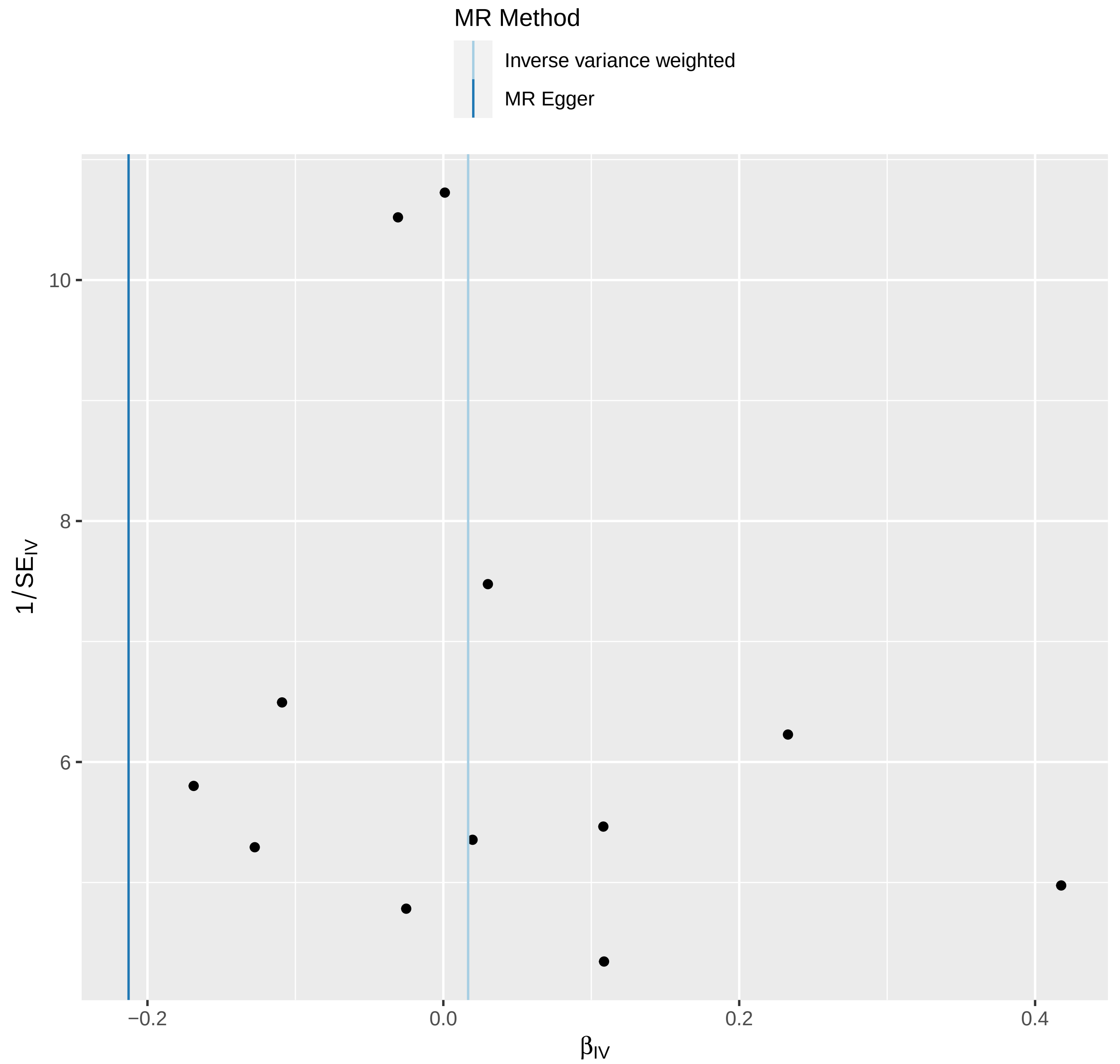

Batch 3−375 : Selenium on Cystathionine beta−synthase

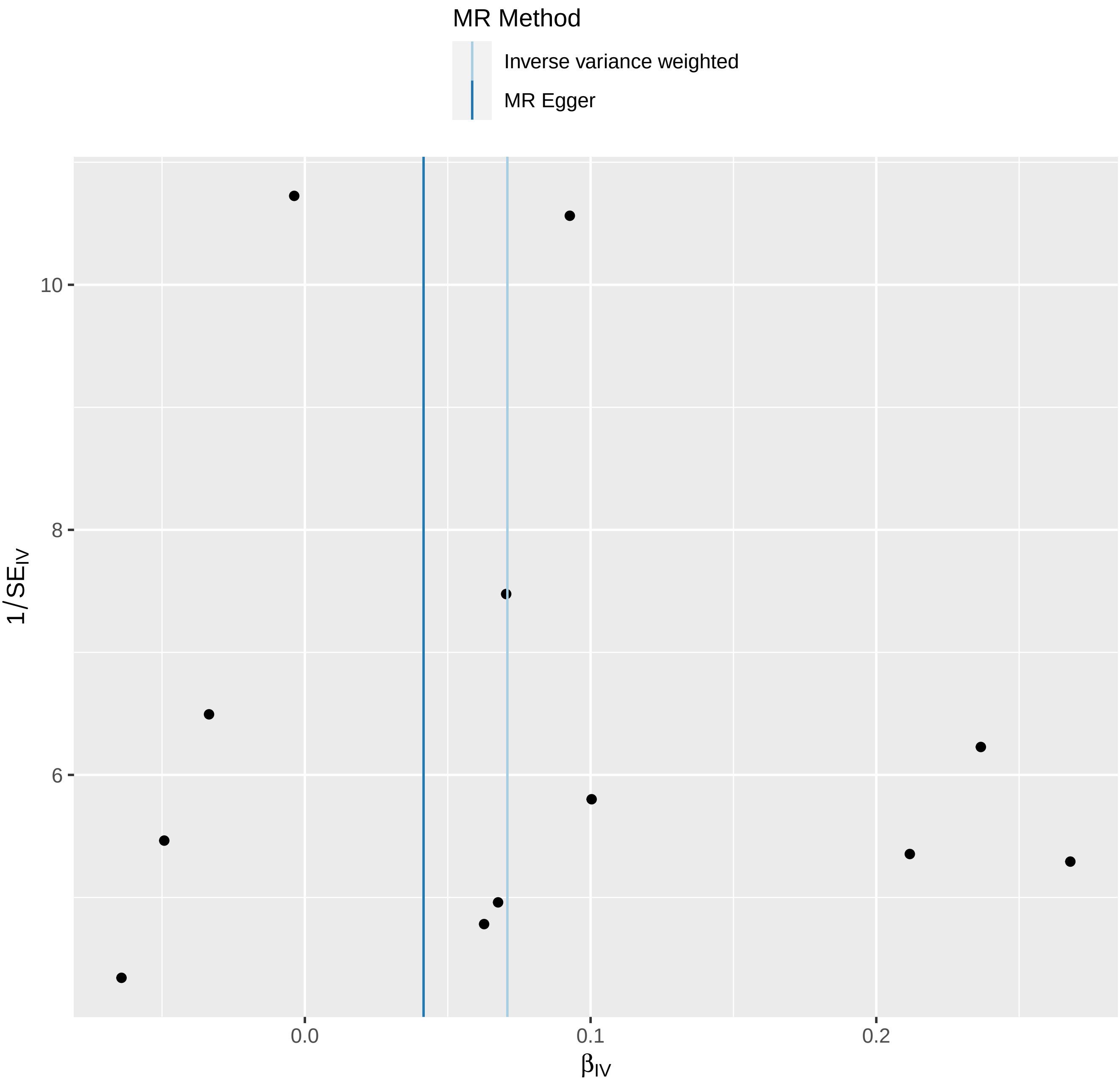

Batch 3−376 : Selenium on Chromobox protein homolog 5

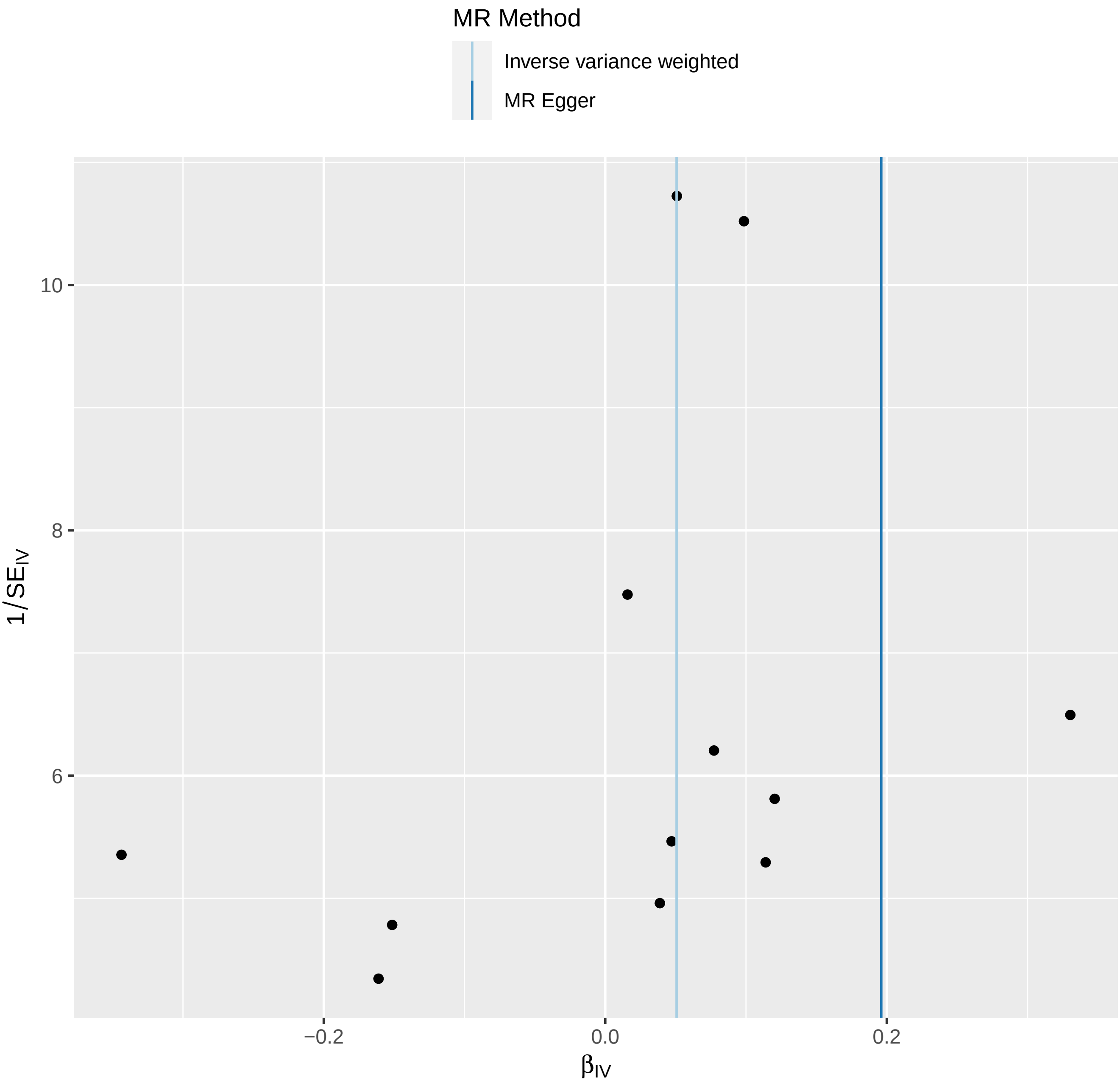

Batch 3−377 : Selenium on Chromobox protein homolog 7

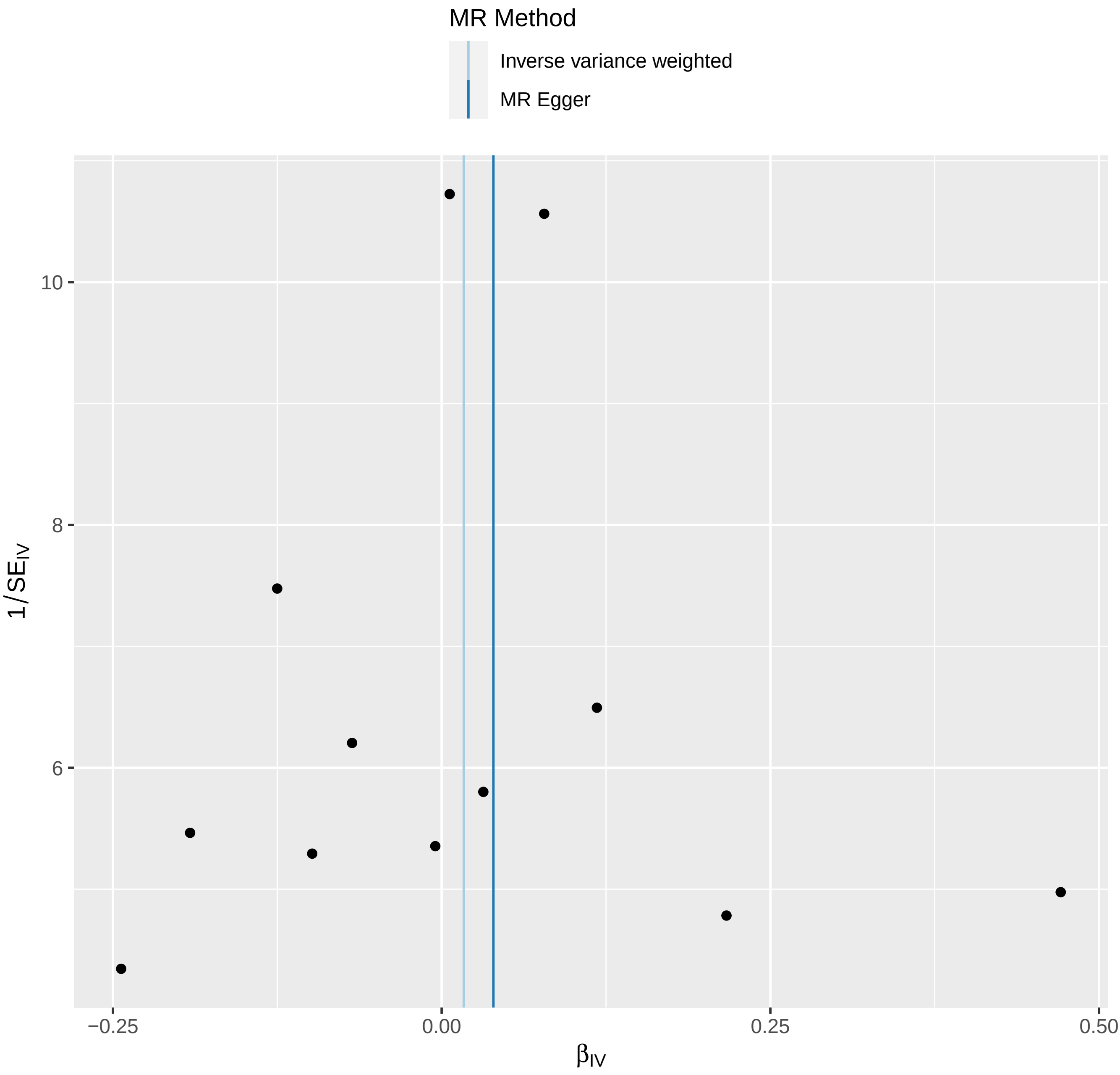

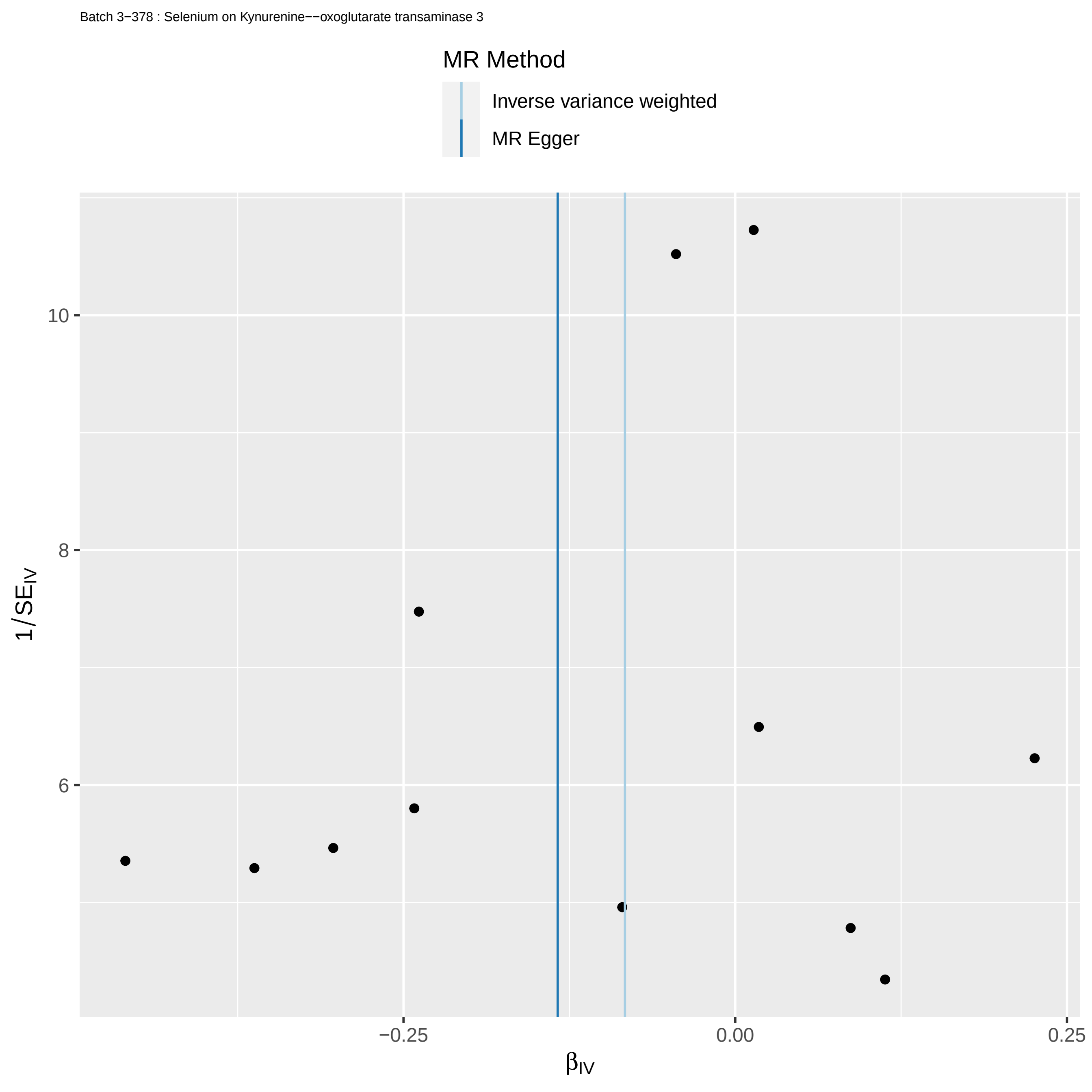
Batch 3−378 : Selenium on Kynurenine−−oxoglutarate transaminase 3
MR Method
Inverse variance weighted
MR Egger
10
8
6
1/SE$_{IV}$
−0.25
0.00
0.25
β$_{IV}$

Batch 3−379 : Selenium on SAGA−associated factor 29 homolog

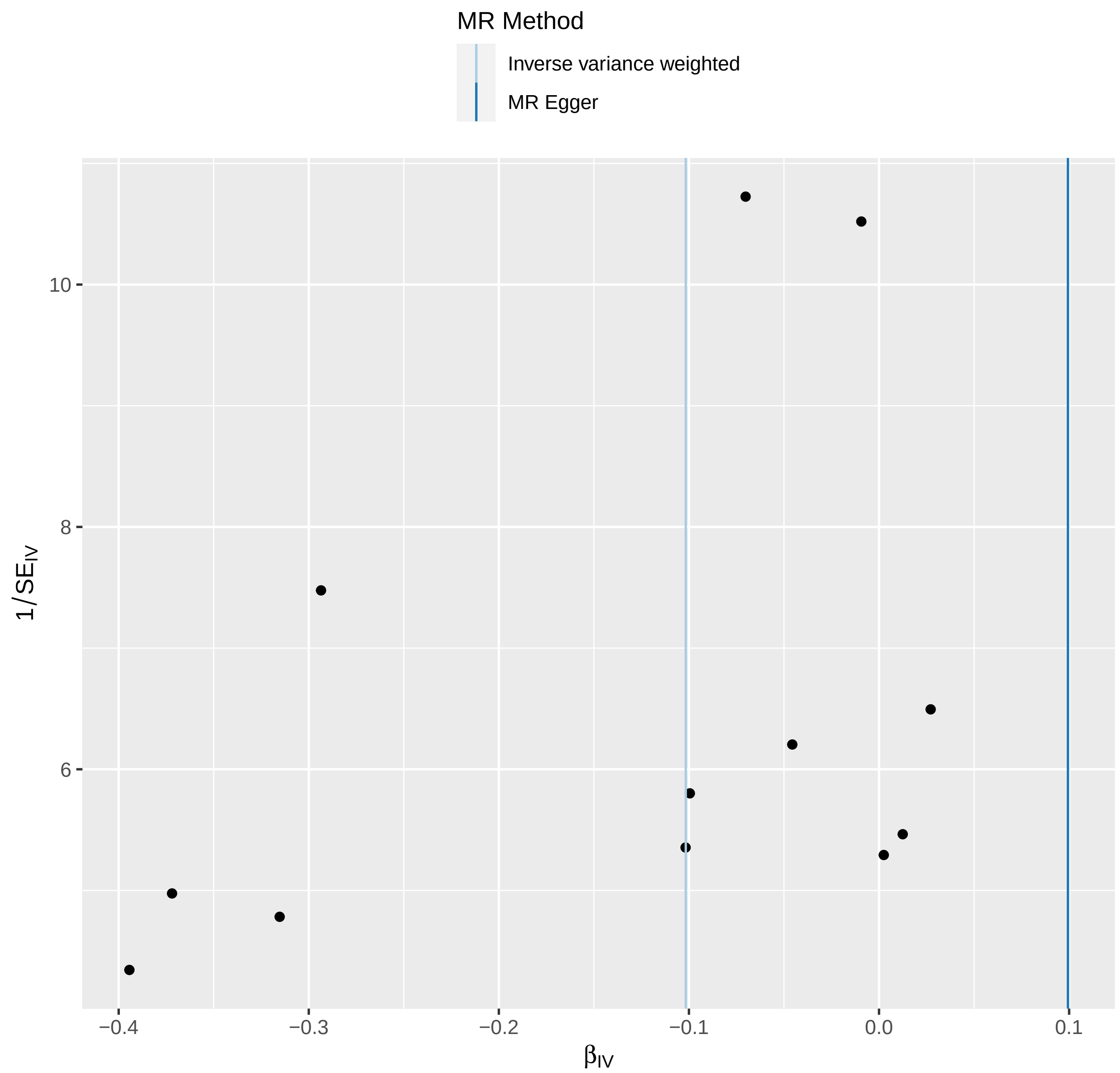

Batch 3−380 : Selenium on Coiled−coil domain−containing protein 126

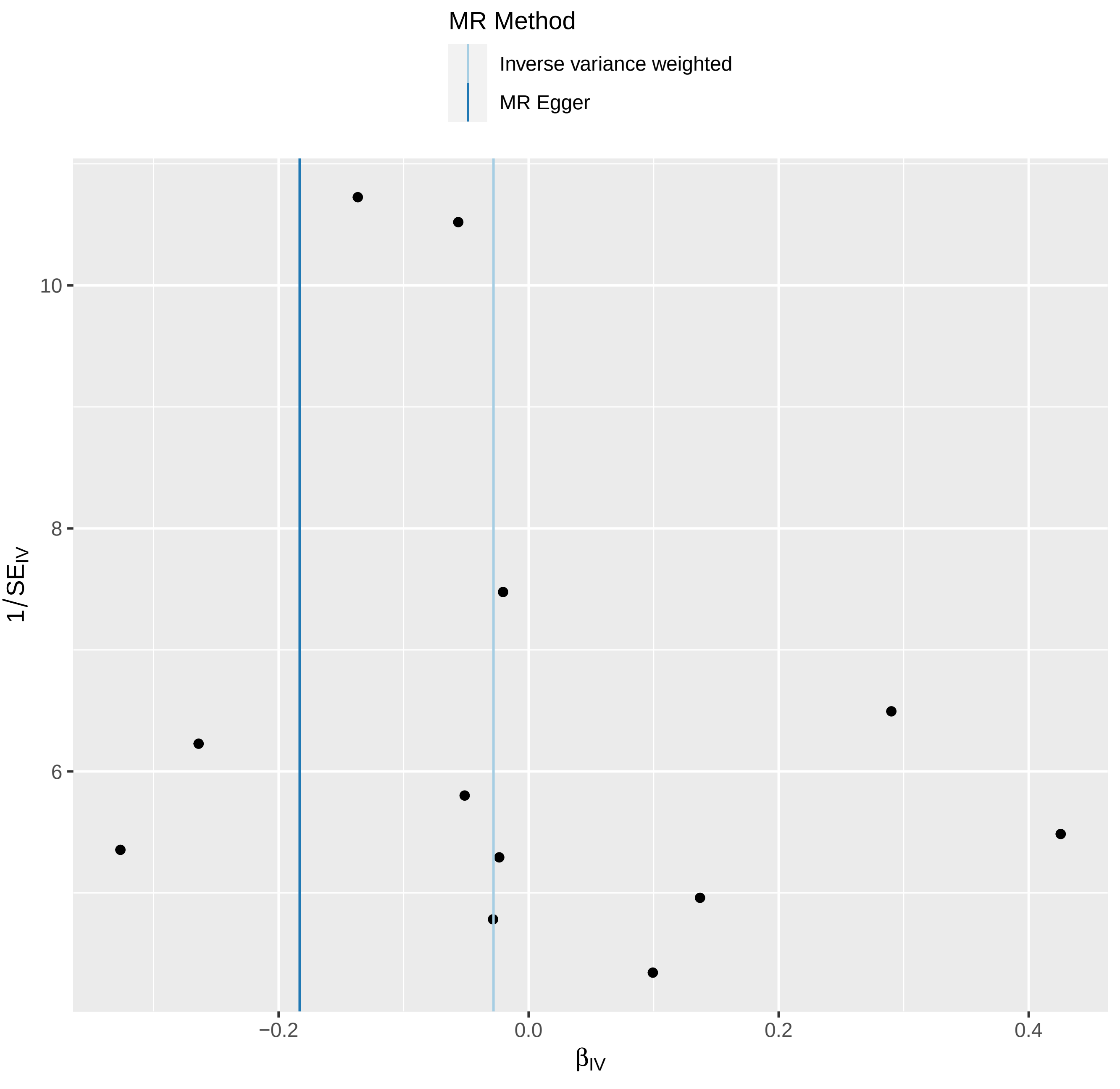

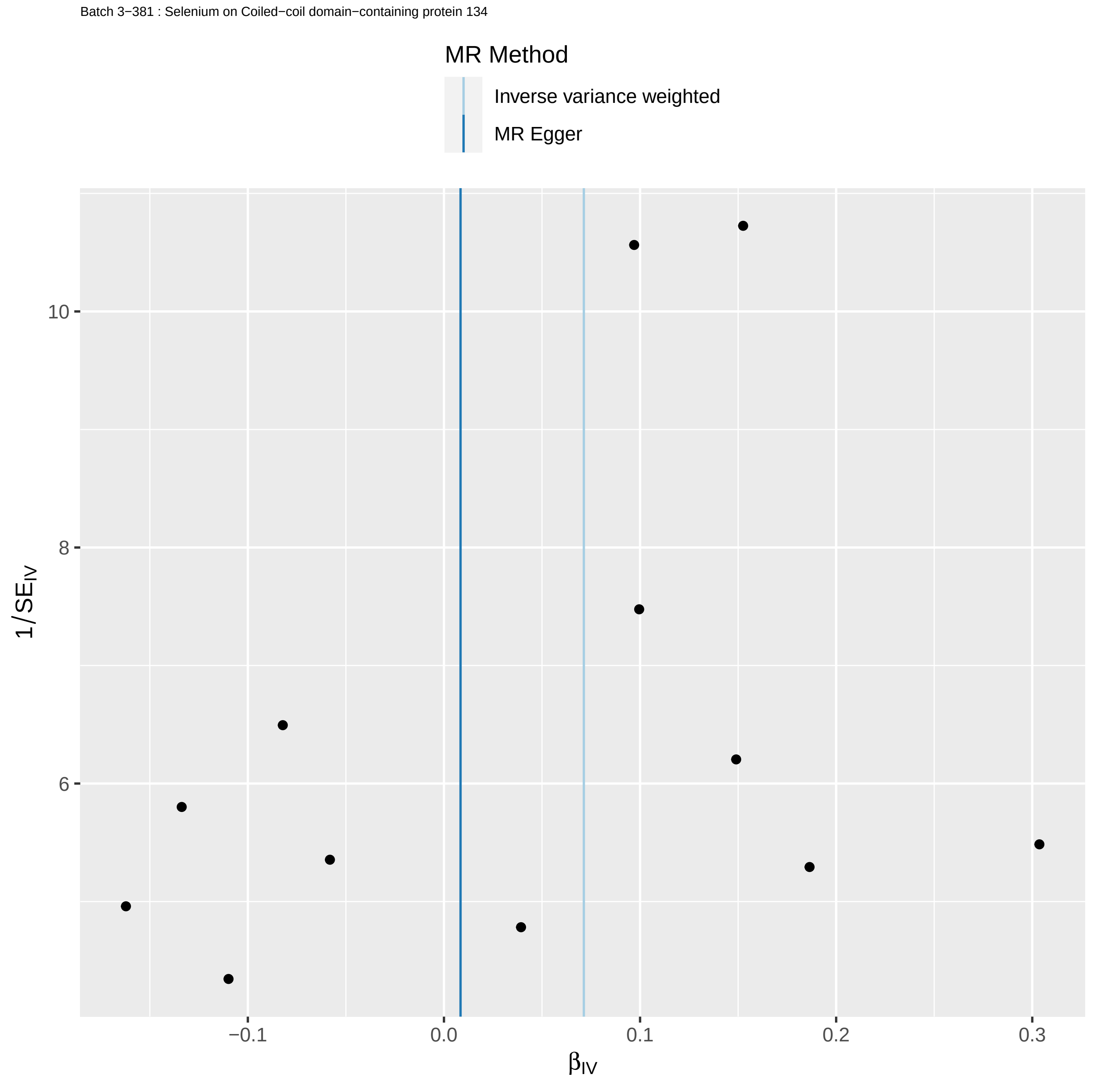
Batch 3−381 : Selenium on Coiled−coil domain−containing protein 134
MR Method
Inverse variance weighted
MR Egger
10
8
6
1/SE_IV
−0.1
0.0
0.1
0.2
0.3
β_IV

Batch 3−382 : Selenium on Coiled−coil domain−containing protein 167

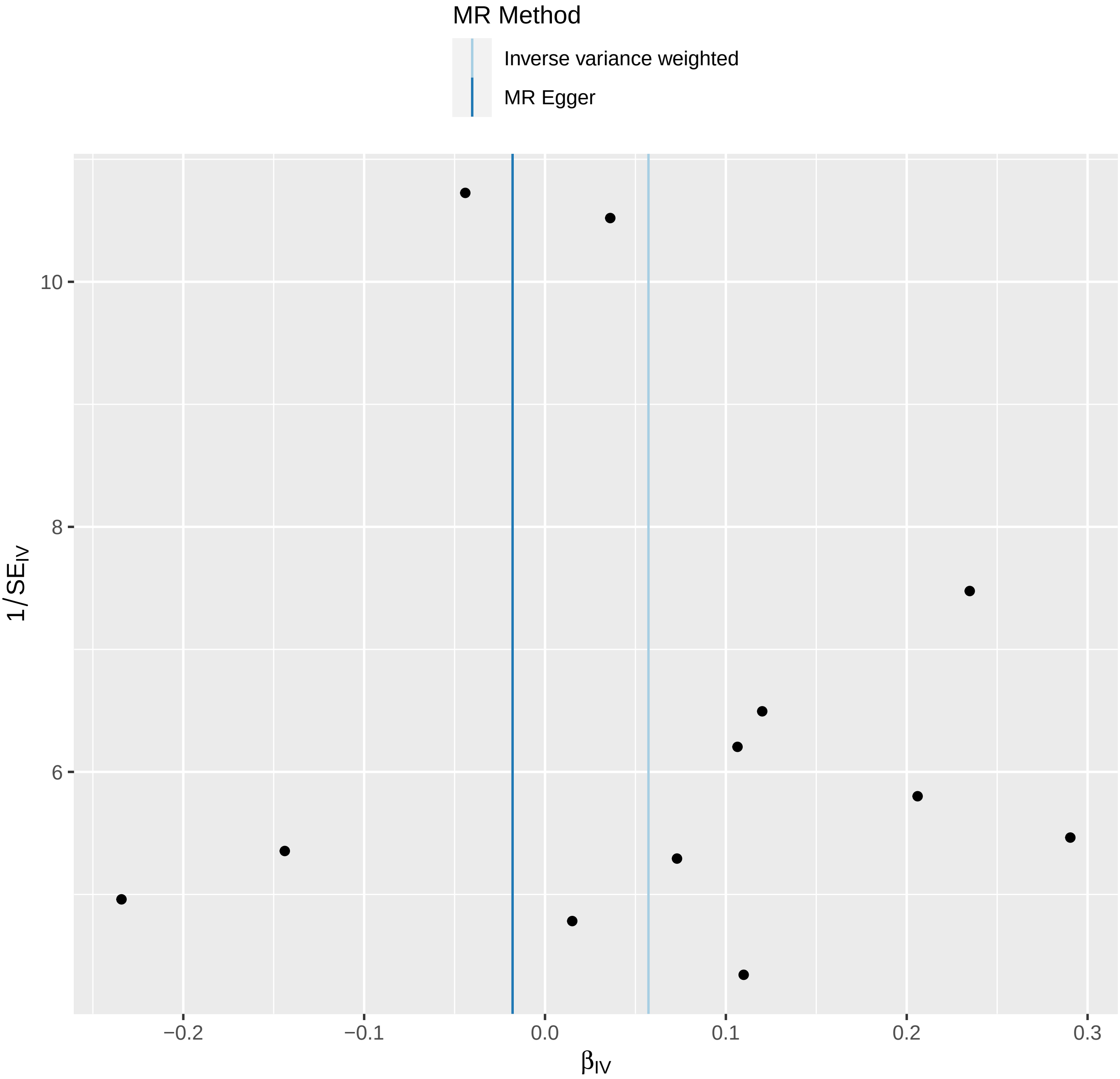

Batch 3−383 : Selenium on Coiled−coil domain−containing protein 51

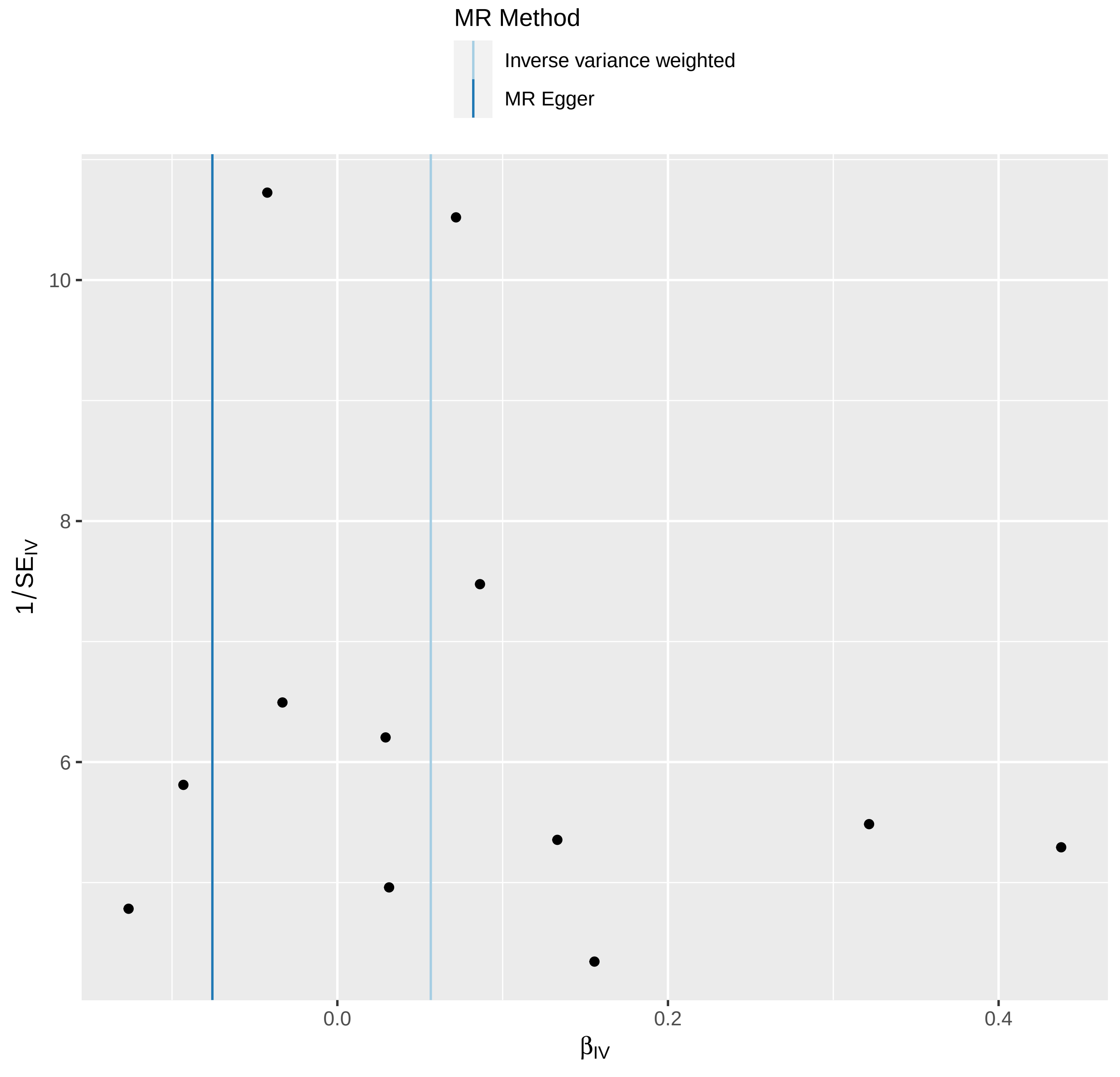

Batch 3−384 : Selenium on Coiled−coil domain−containing protein 80

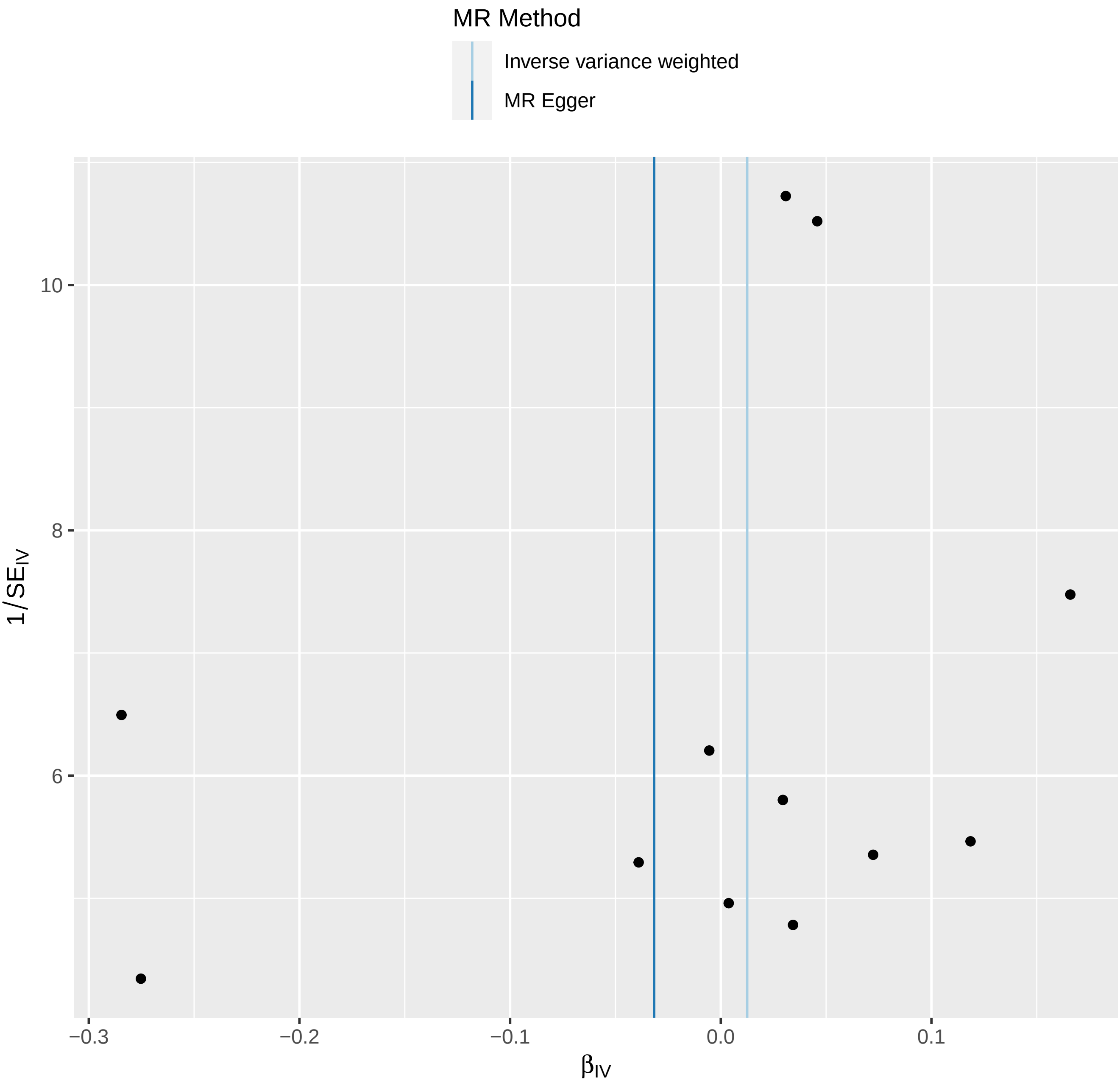

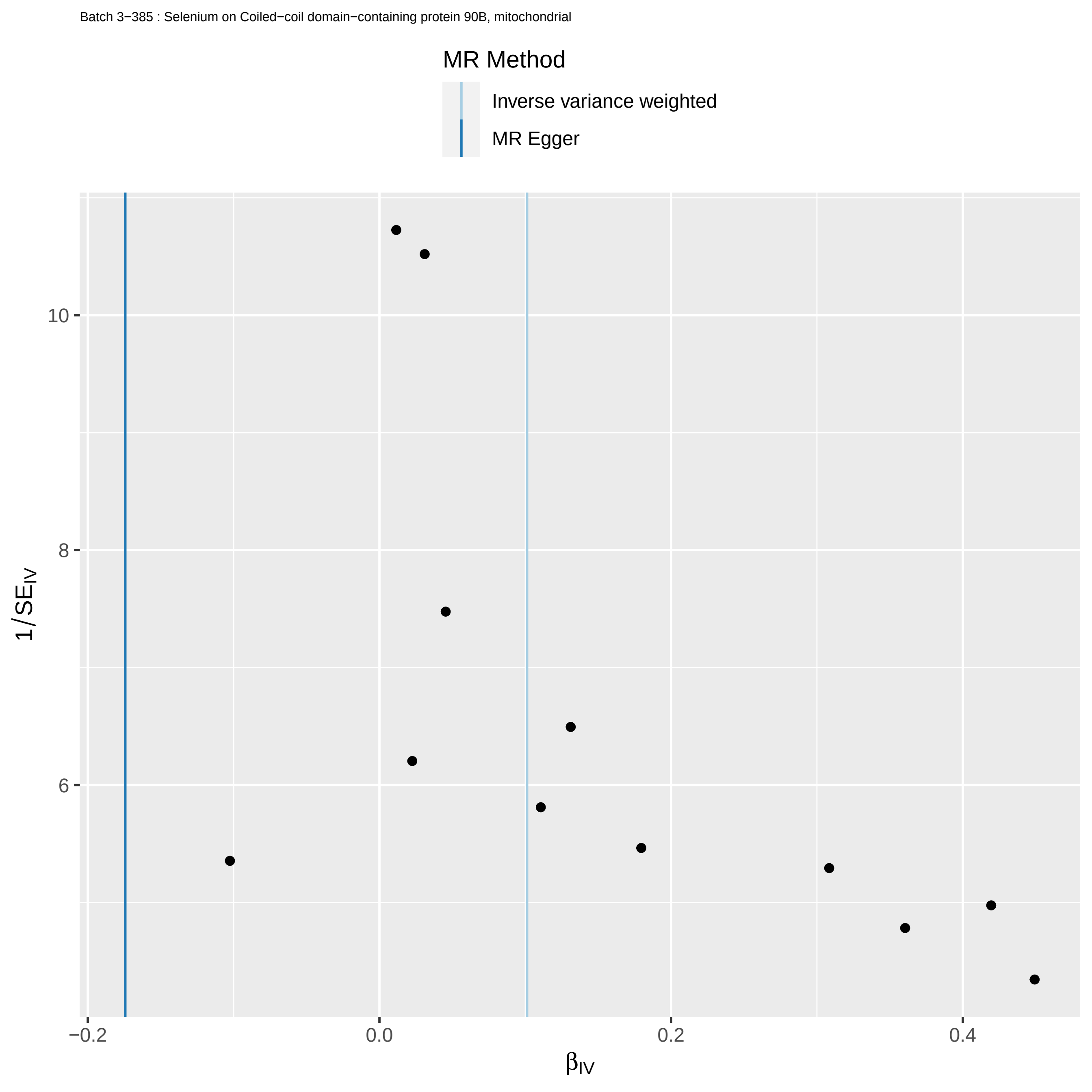

Batch 3−385 : Selenium on Coiled−coil domain−containing protein 90B, mitochondrial
MR Method
Inverse variance weighted
MR Egger
10
8
6
1/SE_IV
−0.2
0.0
0.2
0.4
β_IV

Batch 3−386 : Selenium on C−C motif chemokine 1

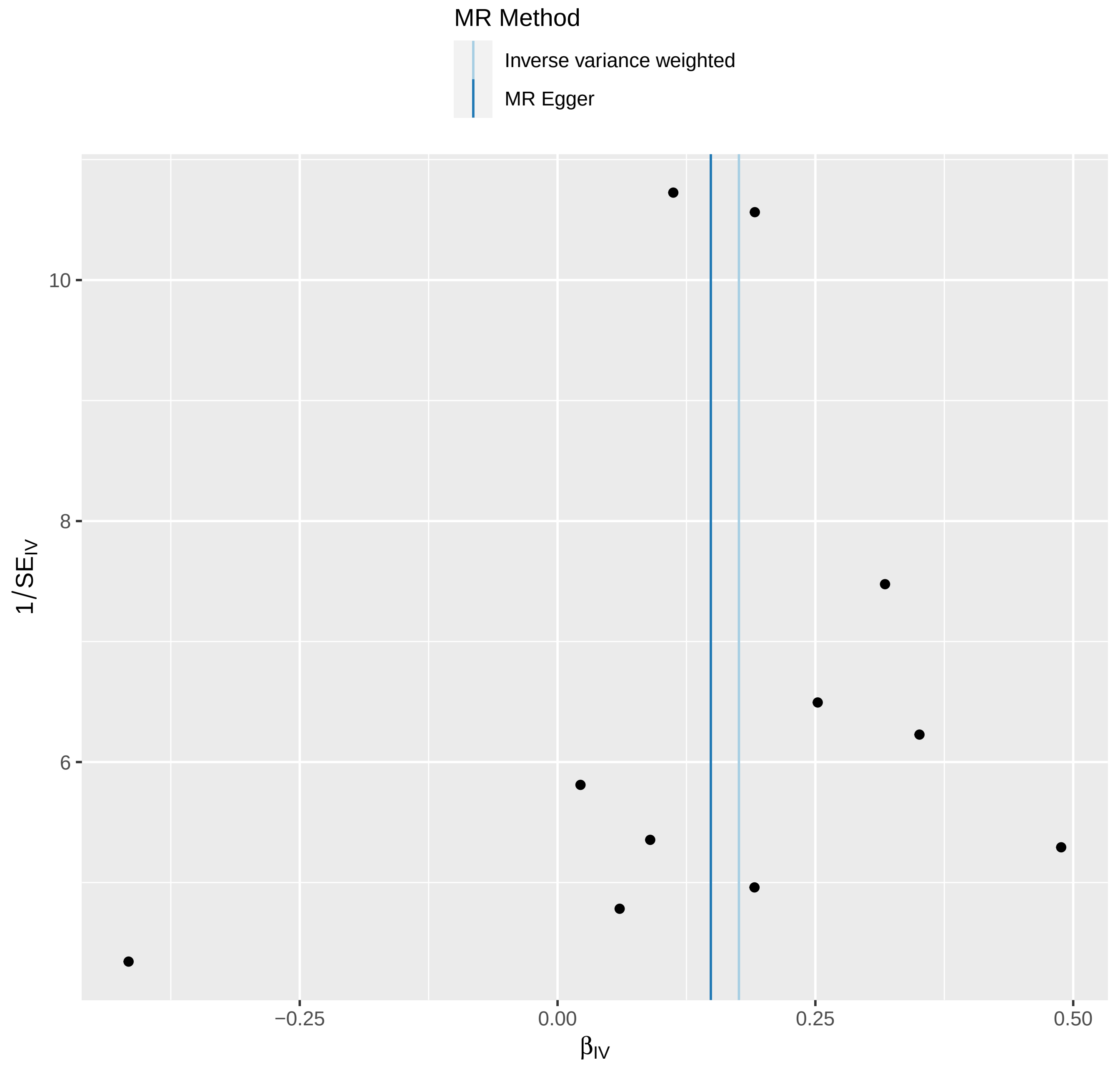

Batch 3−387 : Selenium on Eotaxin

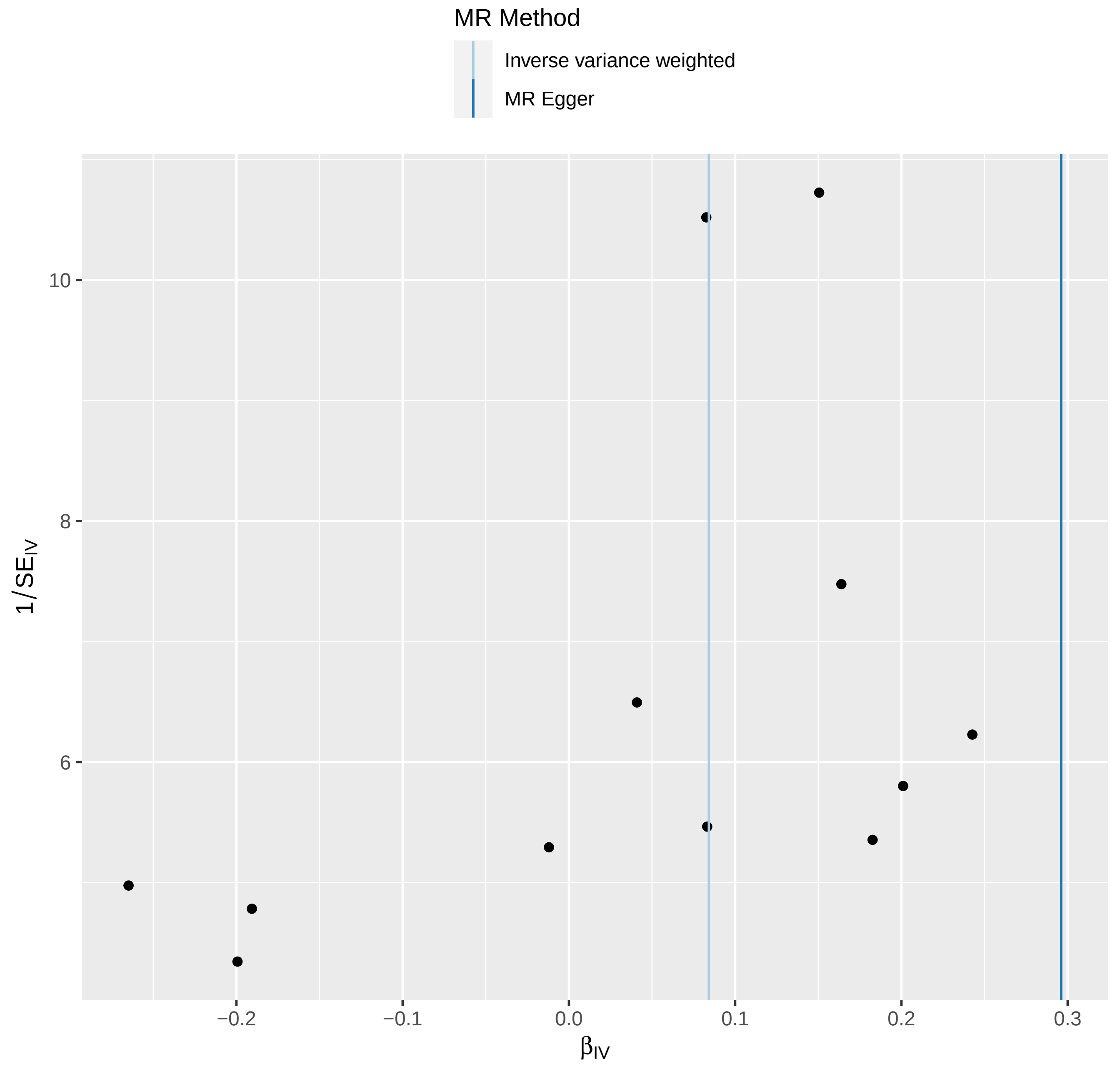

Batch 3−388 : Selenium on C−C motif chemokine 1

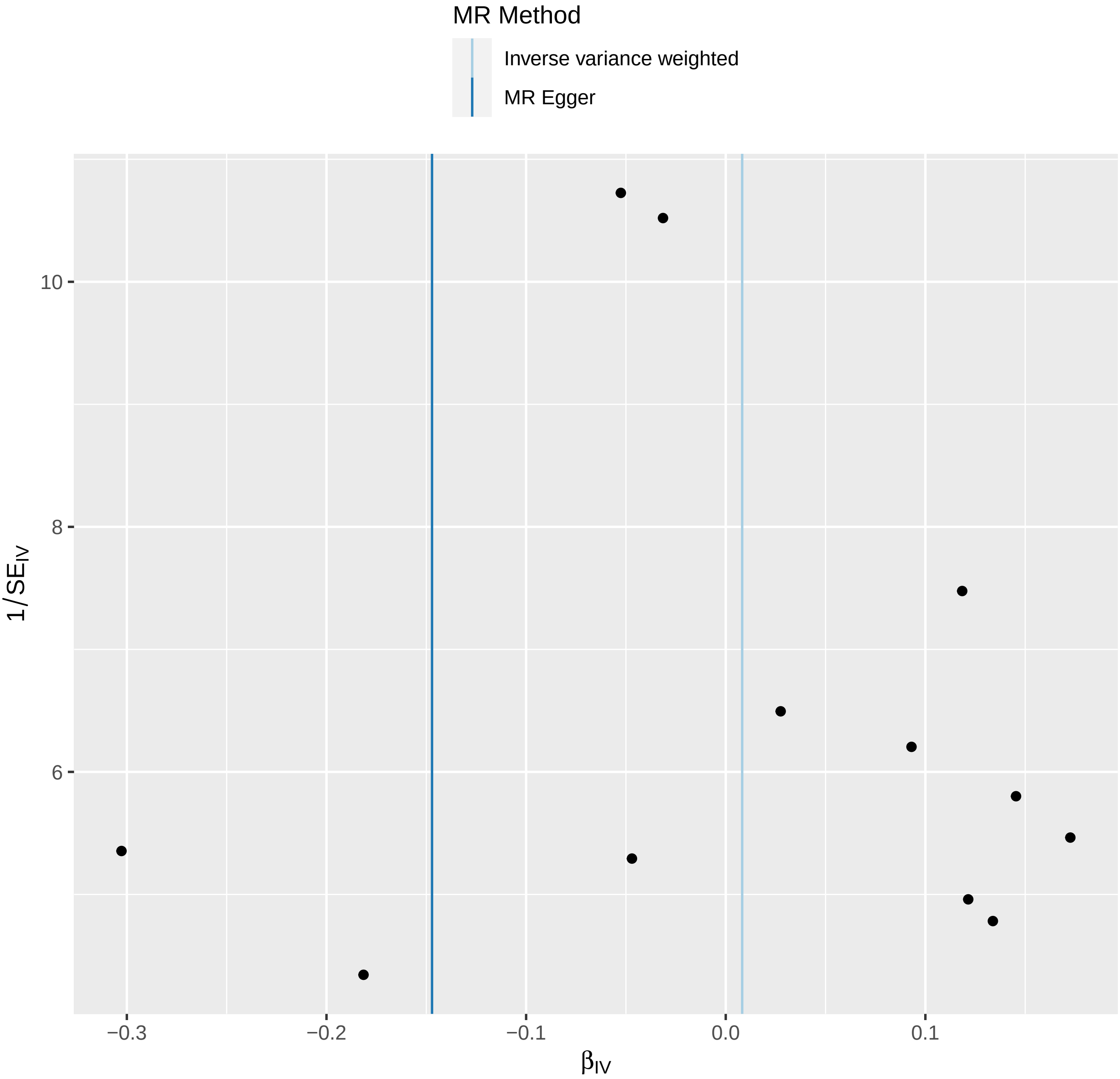

Batch 3−389 : Selenium on C−C motif chemokine 13

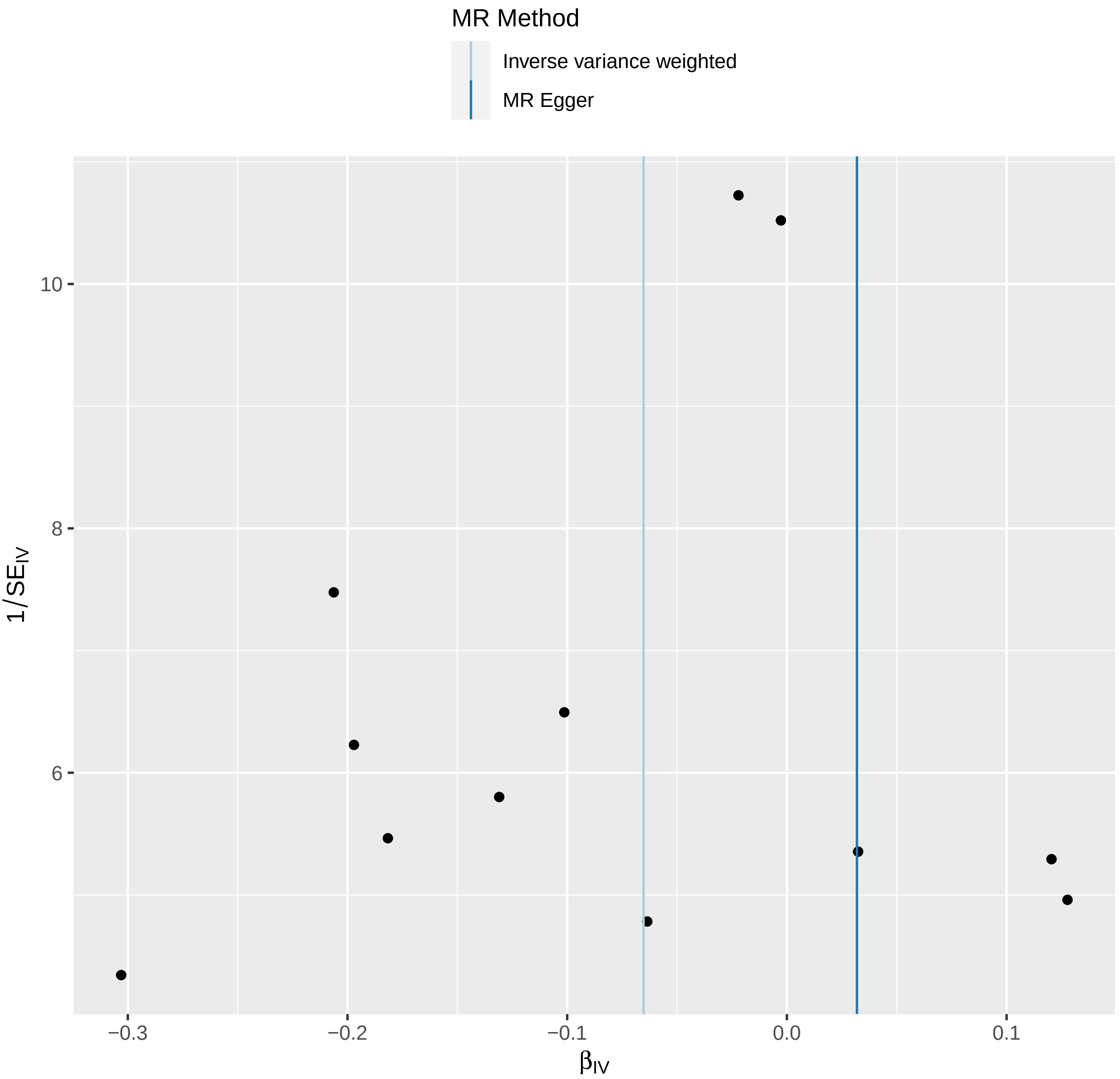

Batch 3−390 : Selenium on C−C motif chemokine 14

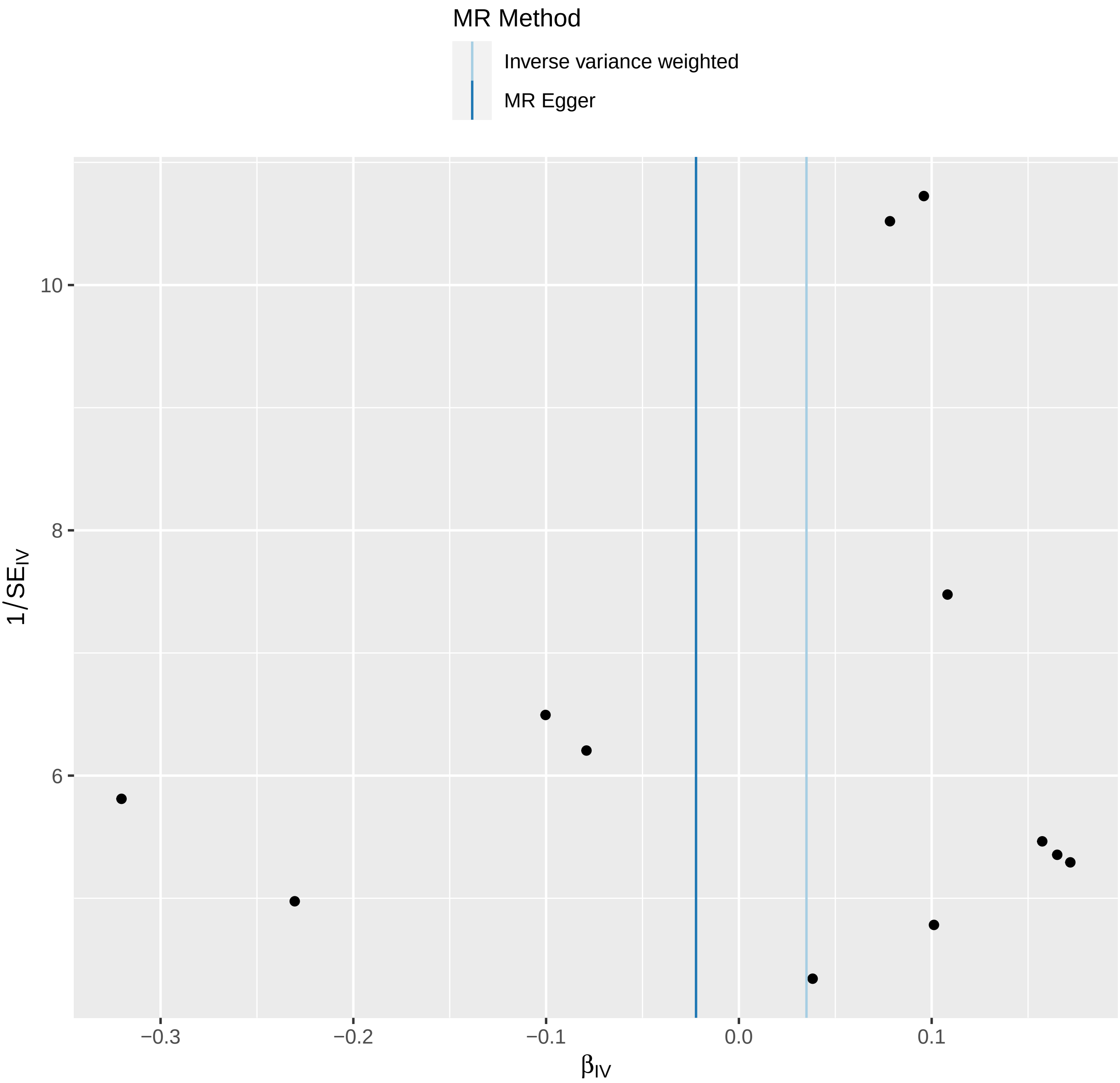

Batch 3−391 : Selenium on C−C motif chemokine 15

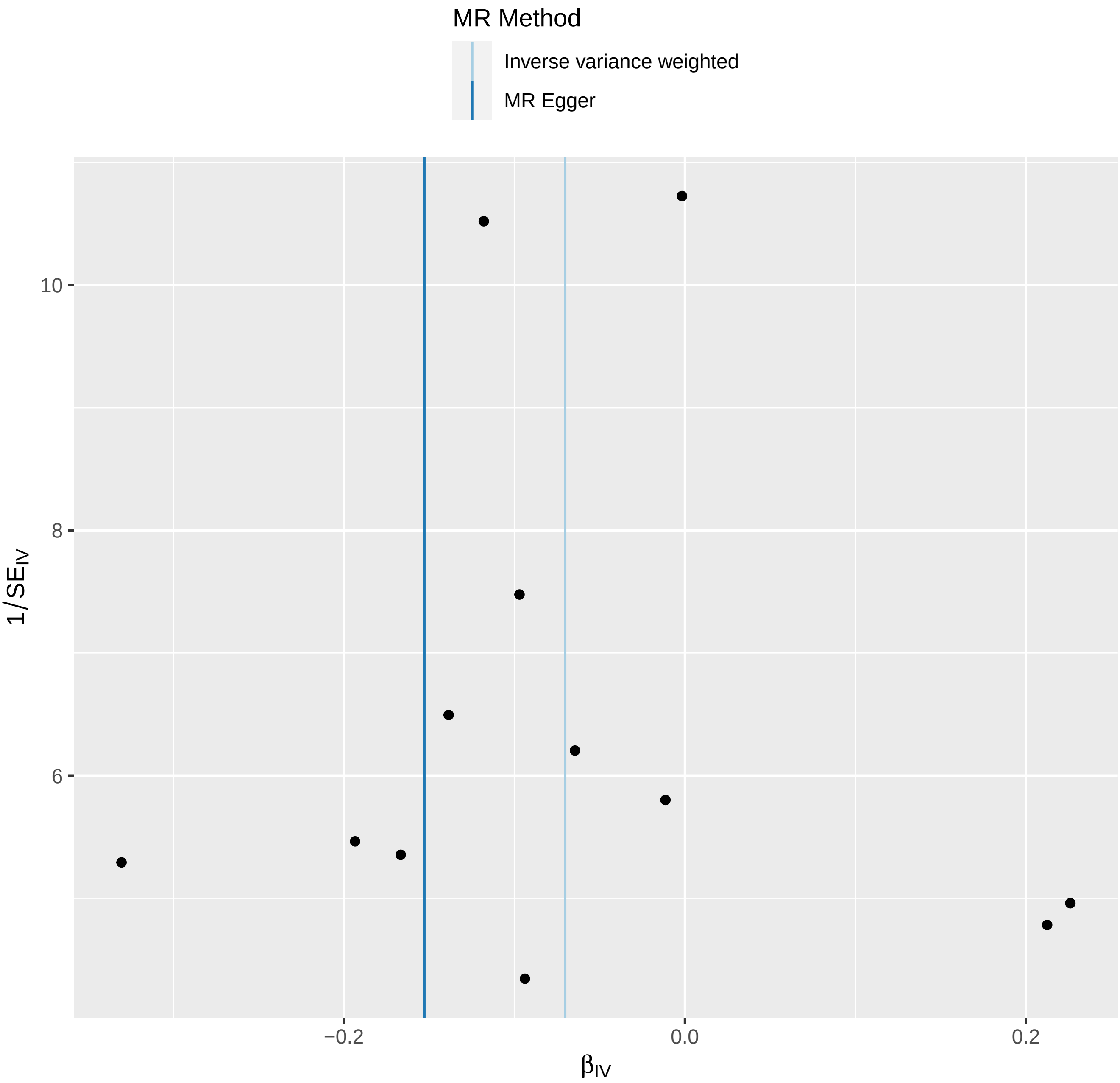

Batch 3−392 : Selenium on C−C motif chemokine 15

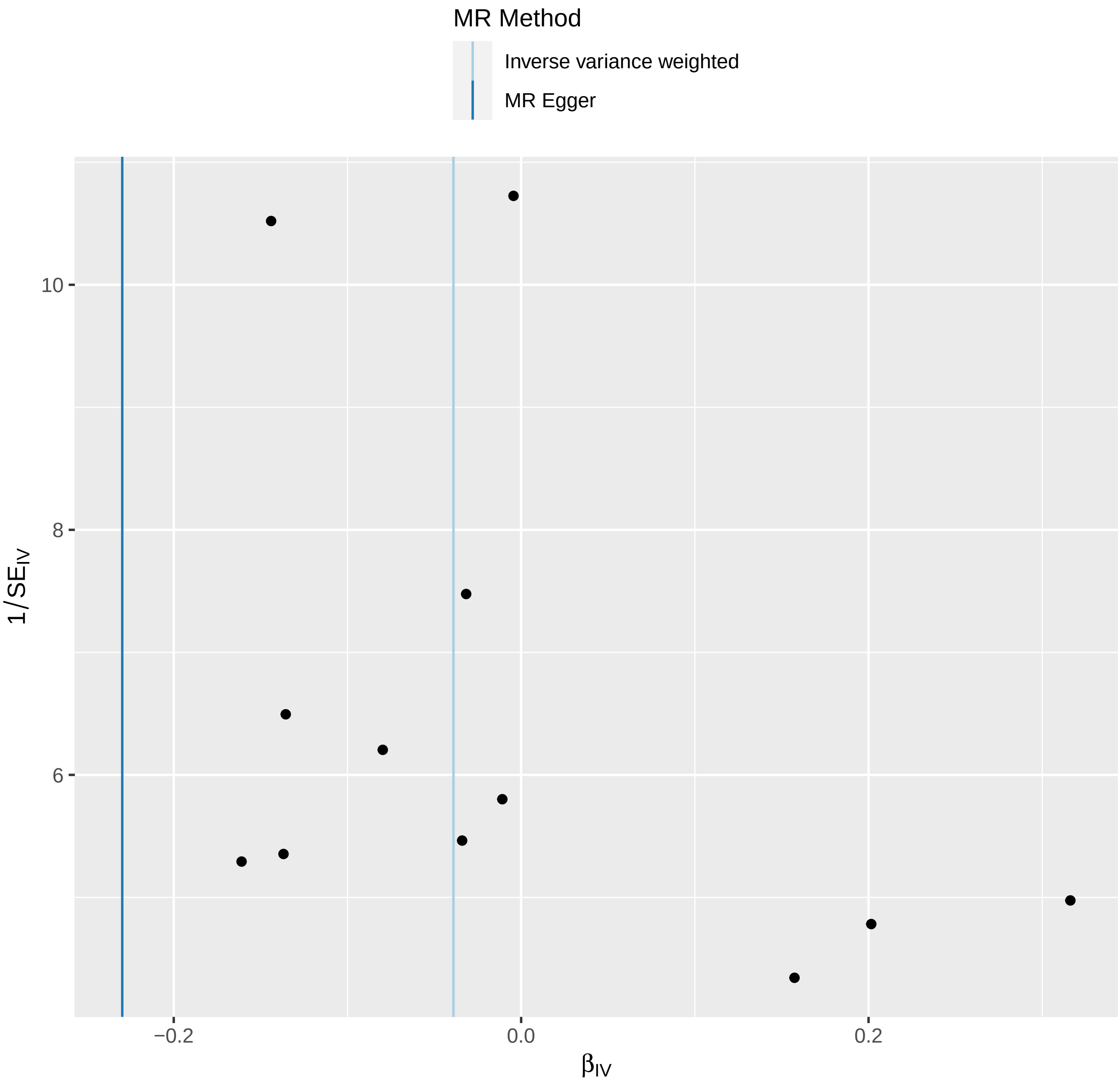

Batch 3−393 : Selenium on C−C motif chemokine 16

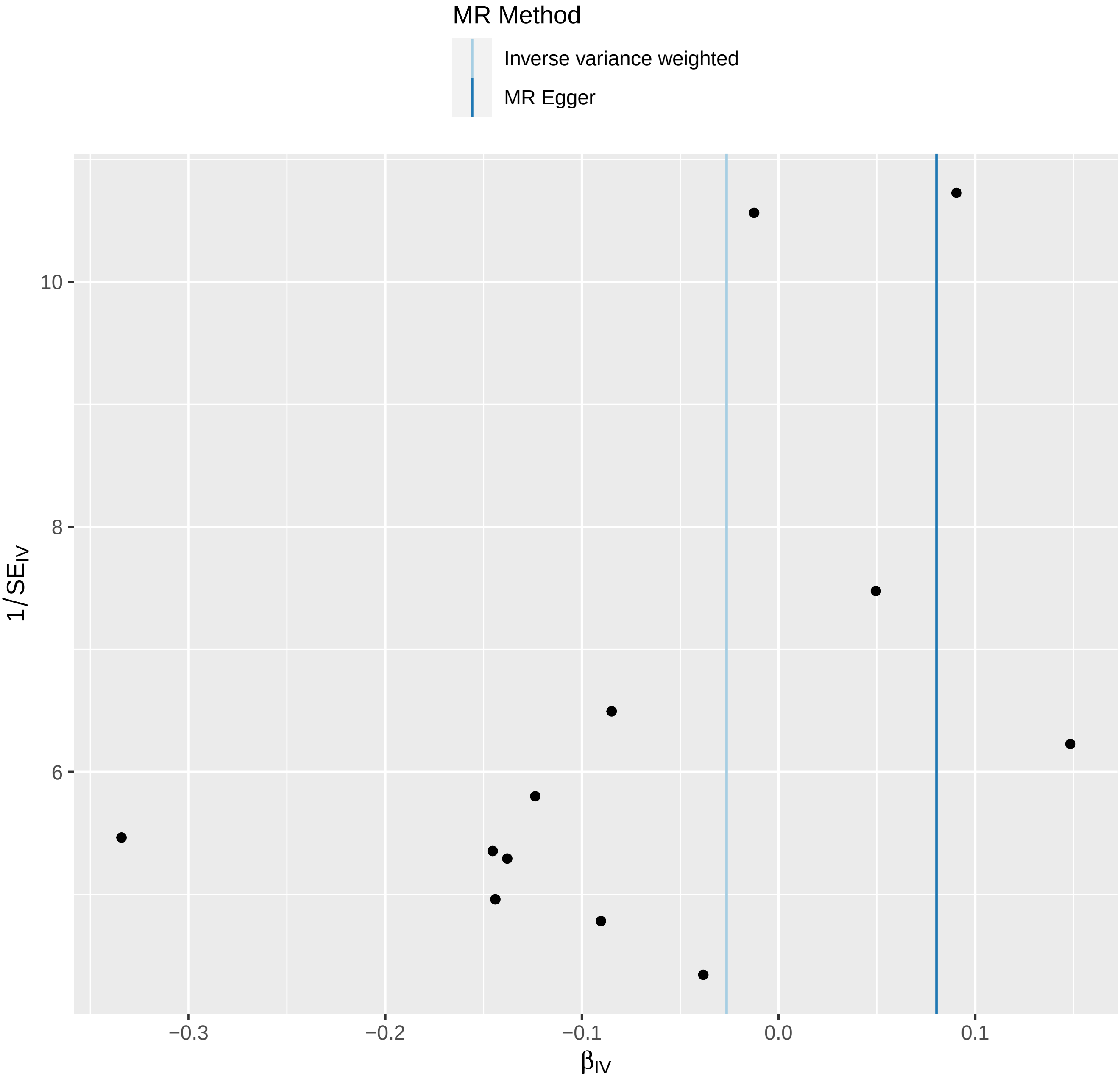

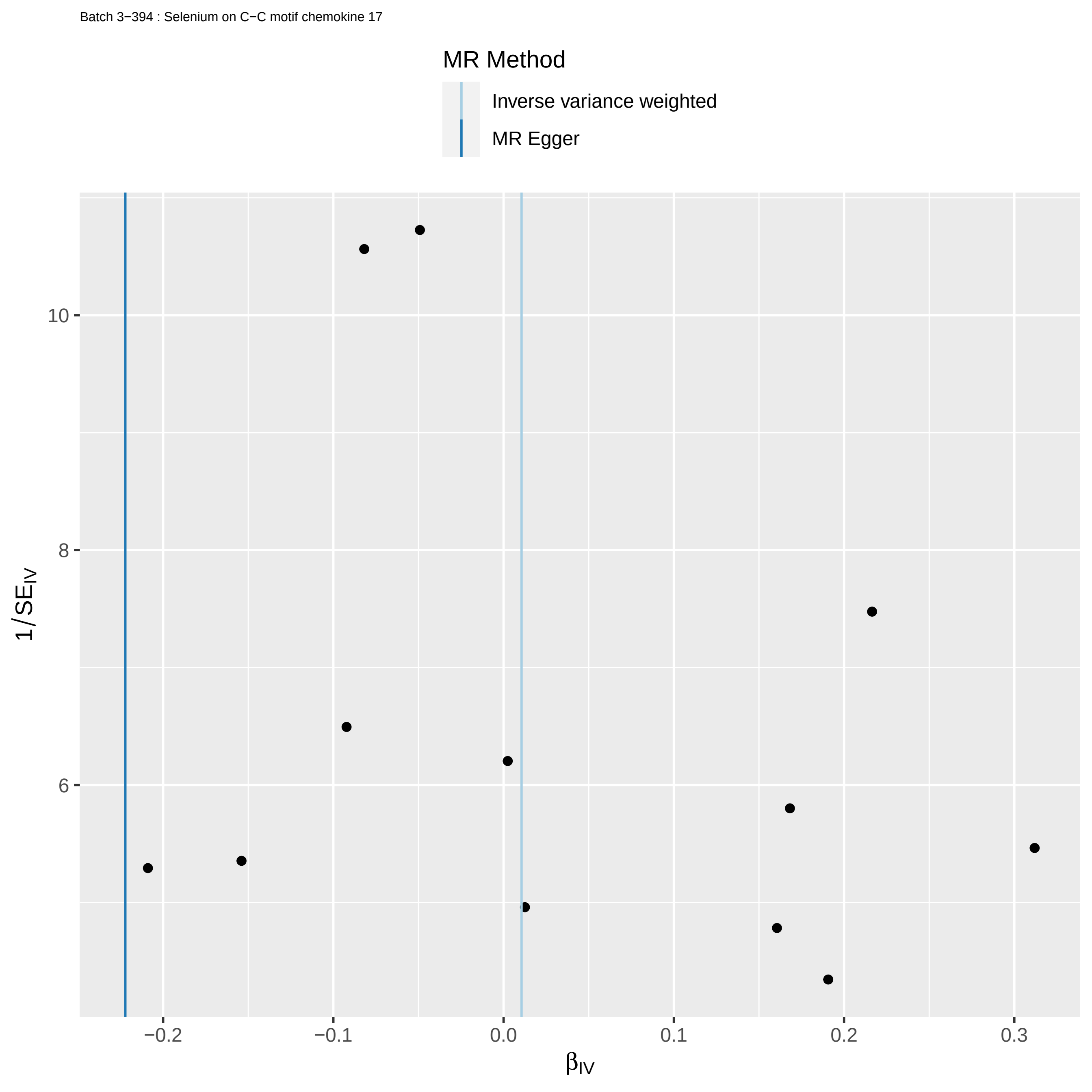

Batch 3−394 : Selenium on C−C motif chemokine 17
MR Method
Inverse variance weighted
MR Egger
10
8
6
1/SE_IV
−0.2
−0.1
0.0
0.1
0.2
0.3
β_IV

Batch 3−395 : Selenium on C−C motif chemokine 19

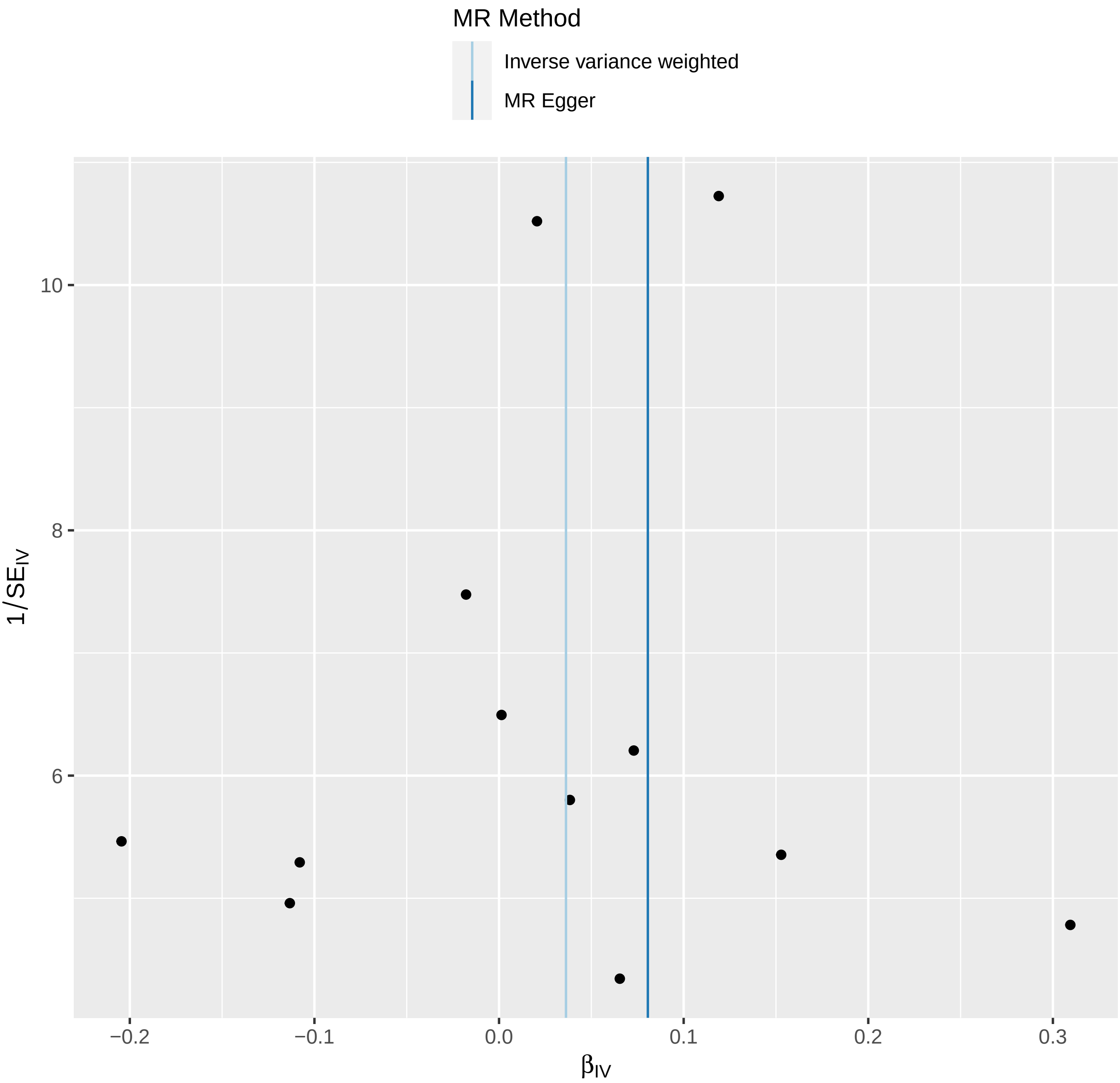

Batch 3−396 : Selenium on C−C motif chemokine 20

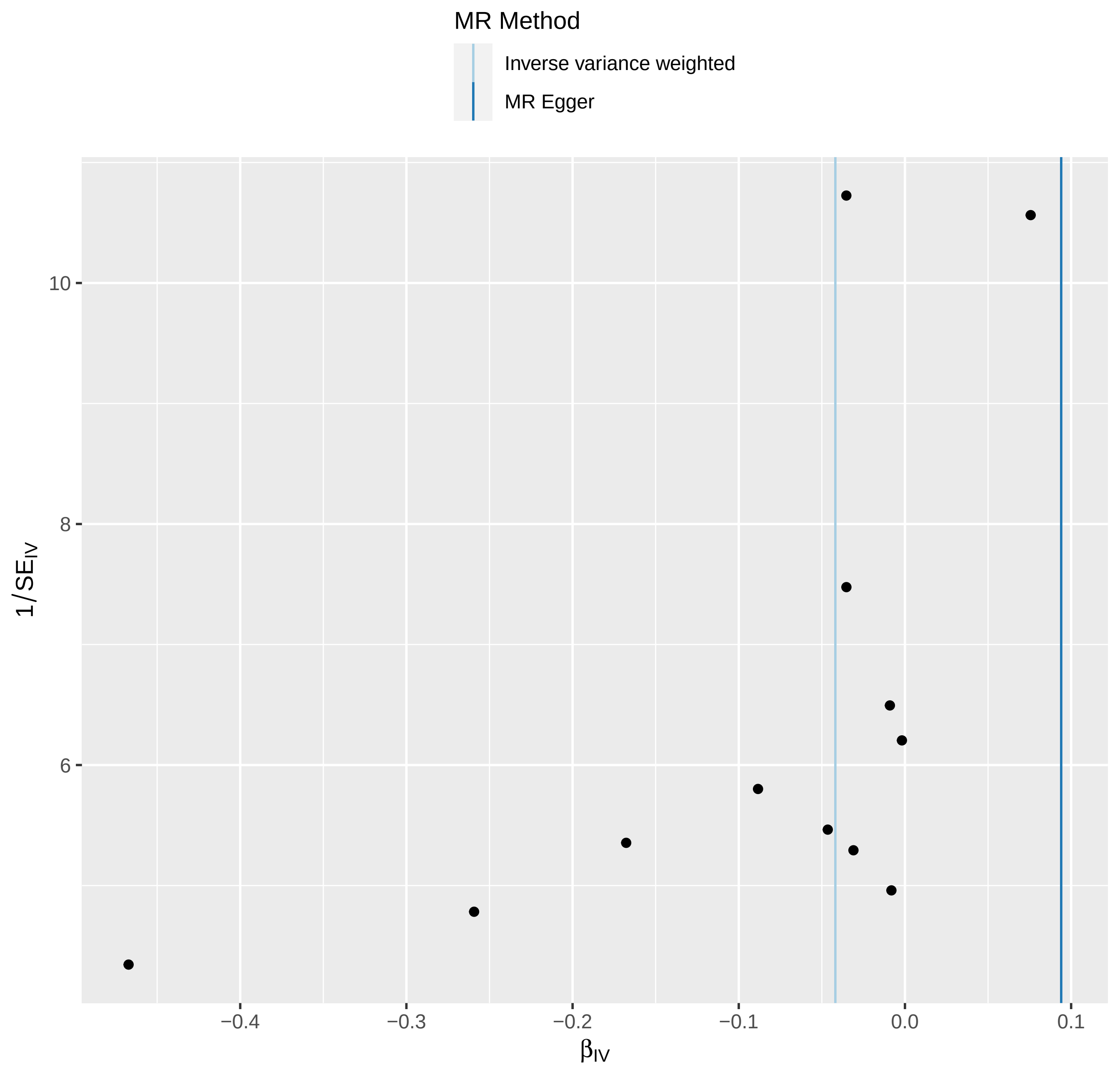

Batch 3−397 : Selenium on C−C motif chemokine 21

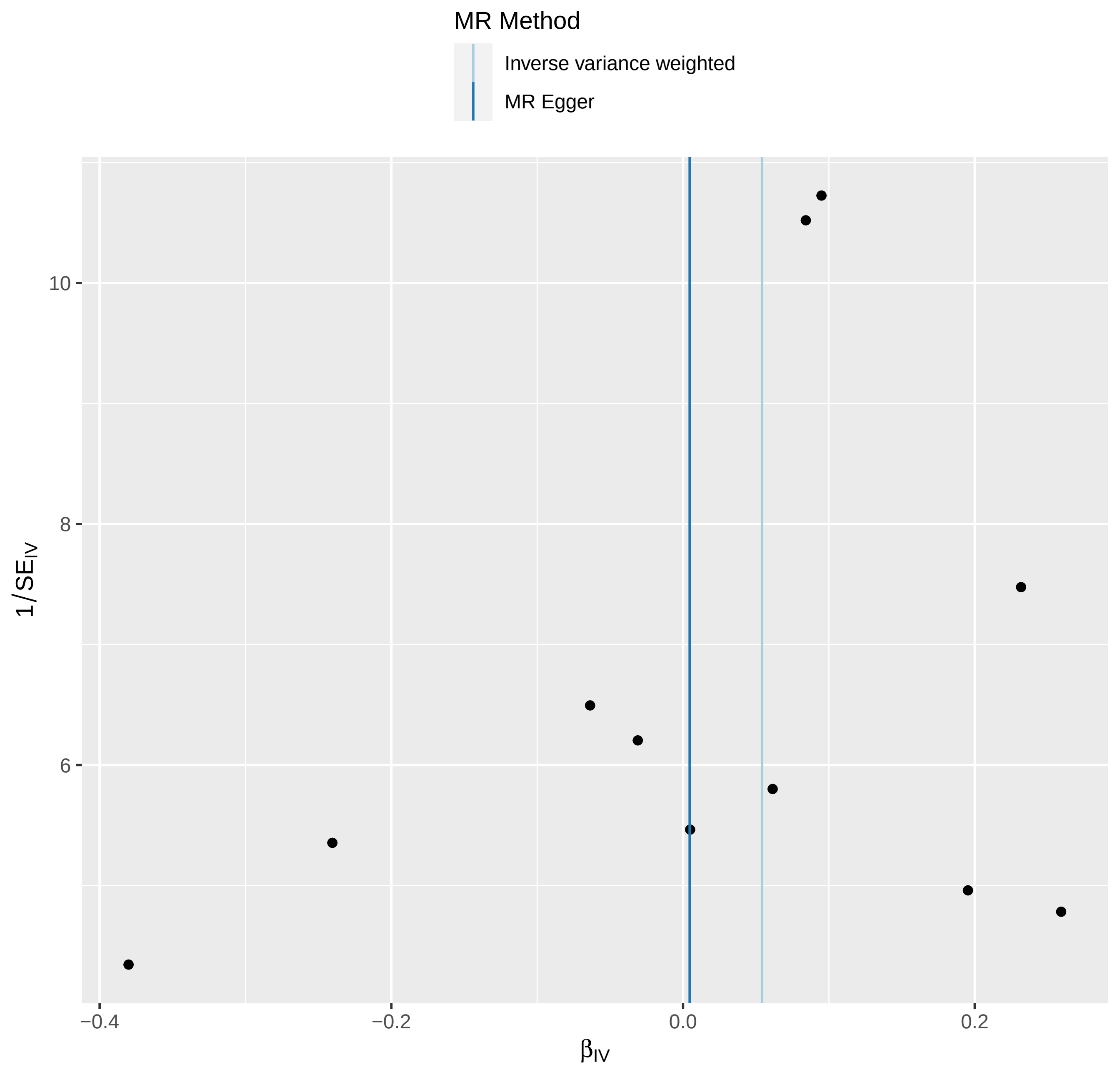

Batch 3−398 : Selenium on C−C motif chemokine 22

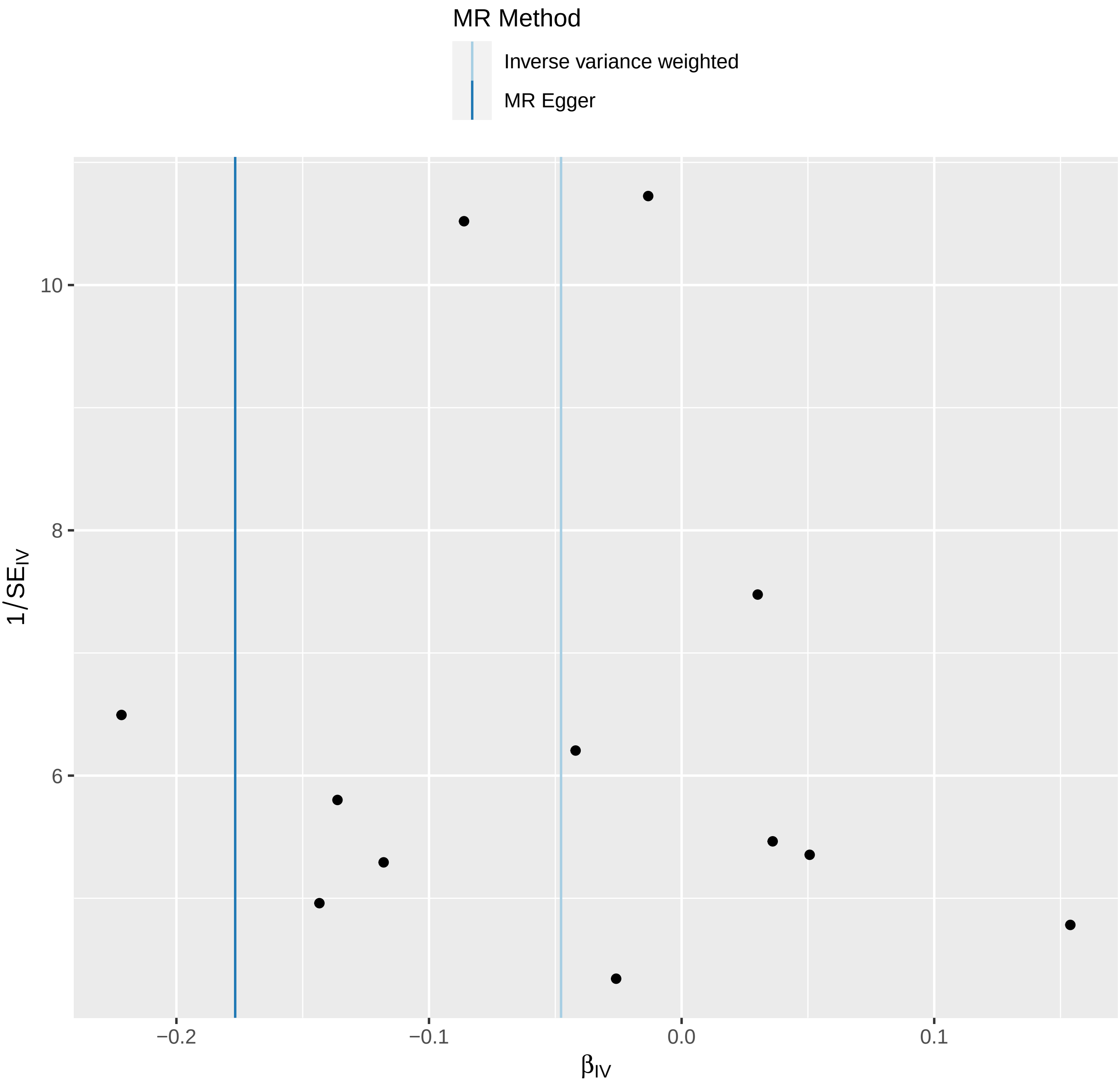

Batch 3−399 : Selenium on C−C motif chemokine 23

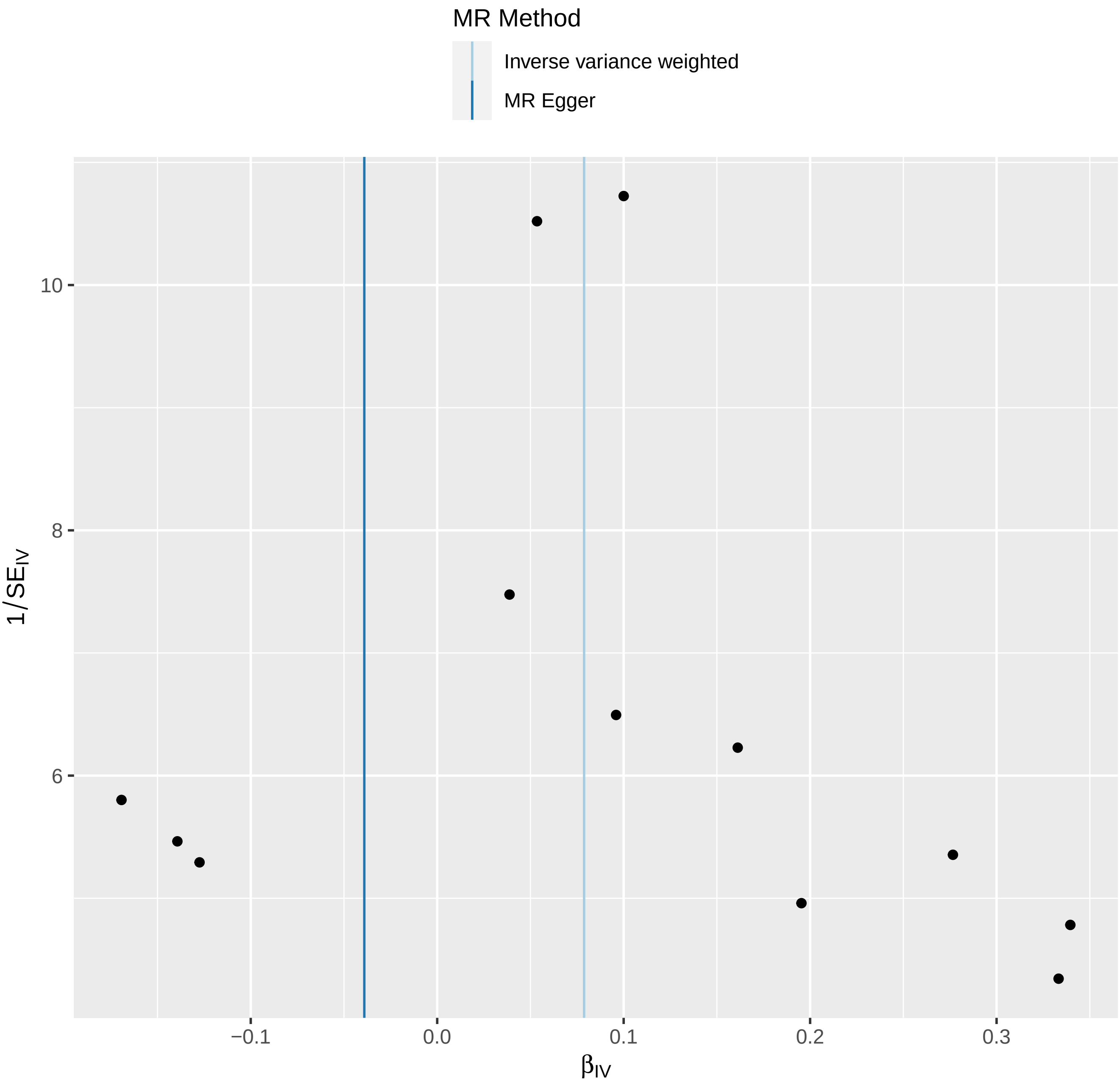

Batch 3−400 : Selenium on Ck−beta−8−1

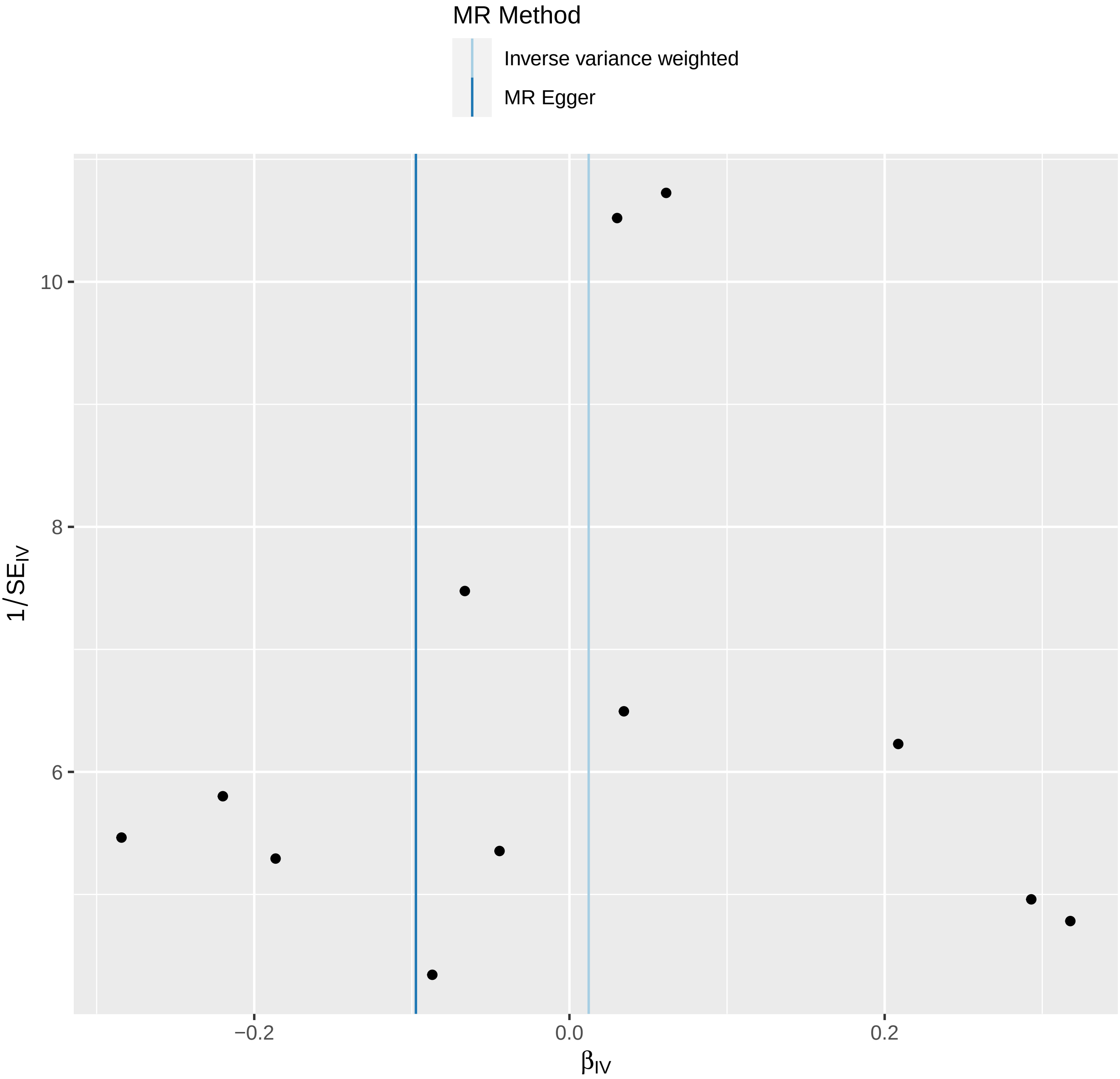

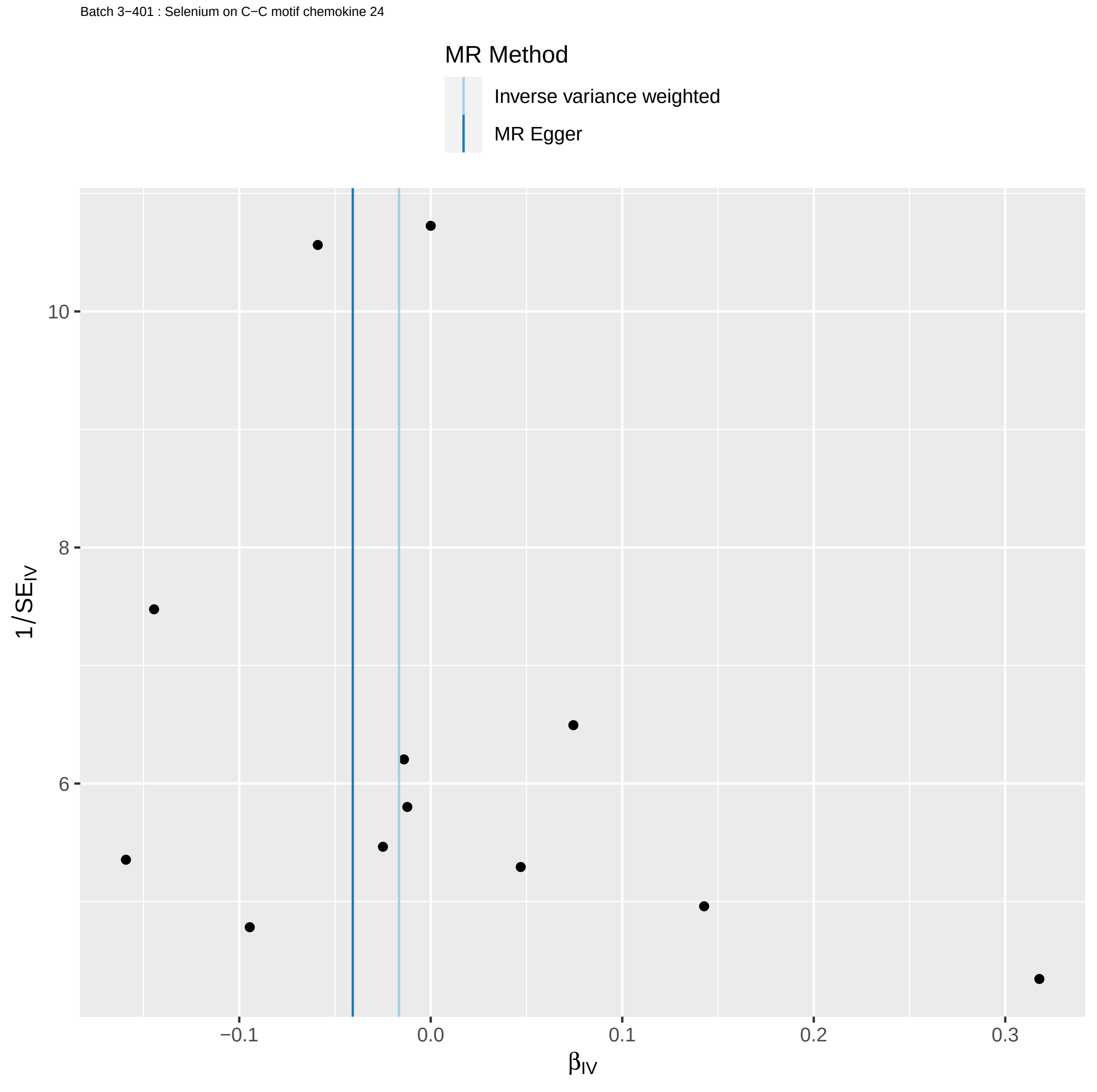

Batch 3−401 : Selenium on C−C motif chemokine 24
MR Method
Inverse variance weighted
MR Egger
10
8
6
1/SE_IV
−0.1
0.0
0.1
0.2
0.3
β_IV

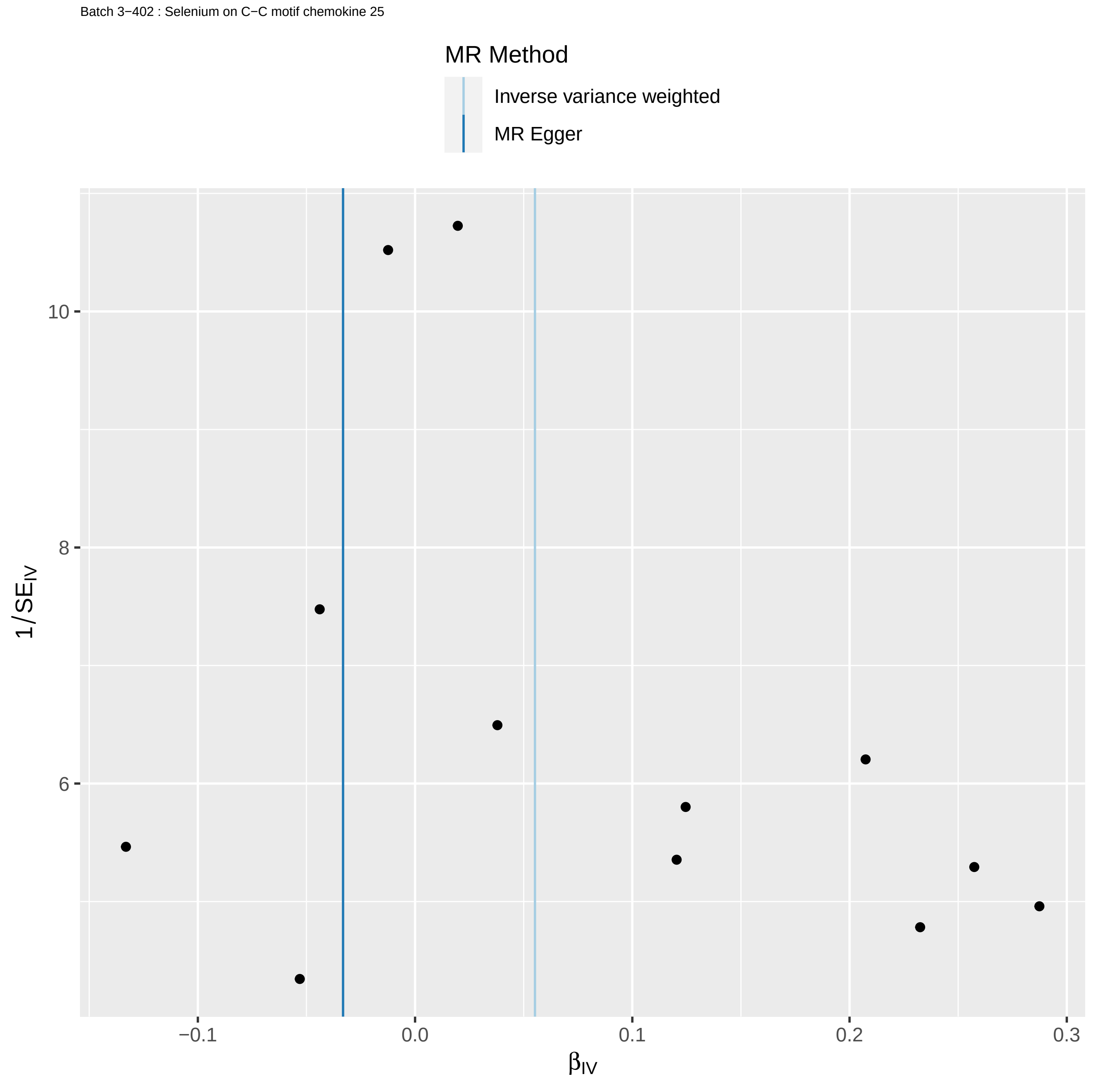

Batch 3−402 : Selenium on C−C motif chemokine 25
MR Method
Inverse variance weighted
MR Egger
10
8
6
$1/SE_{IV}$
−0.1
0.0
0.1
0.2
0.3
β_{IV}

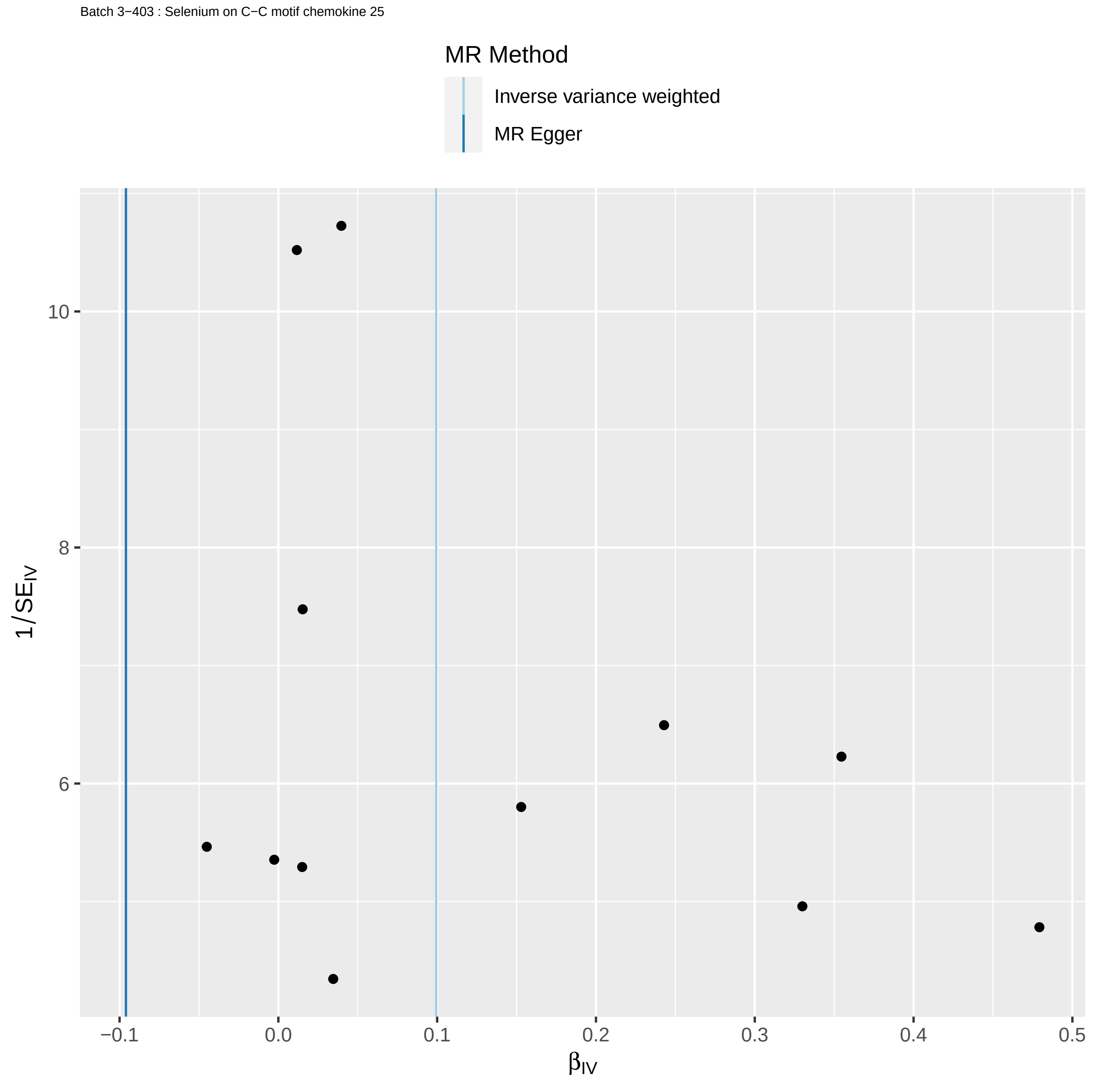

Batch 3−403 : Selenium on C−C motif chemokine 25
MR Method
Inverse variance weighted
MR Egger
10
8
6
1/SE_IV
−0.1
0.0
0.1
0.2
0.3
0.4
0.5
β_IV

Batch 3−404 : Selenium on C−C motif chemokine 26

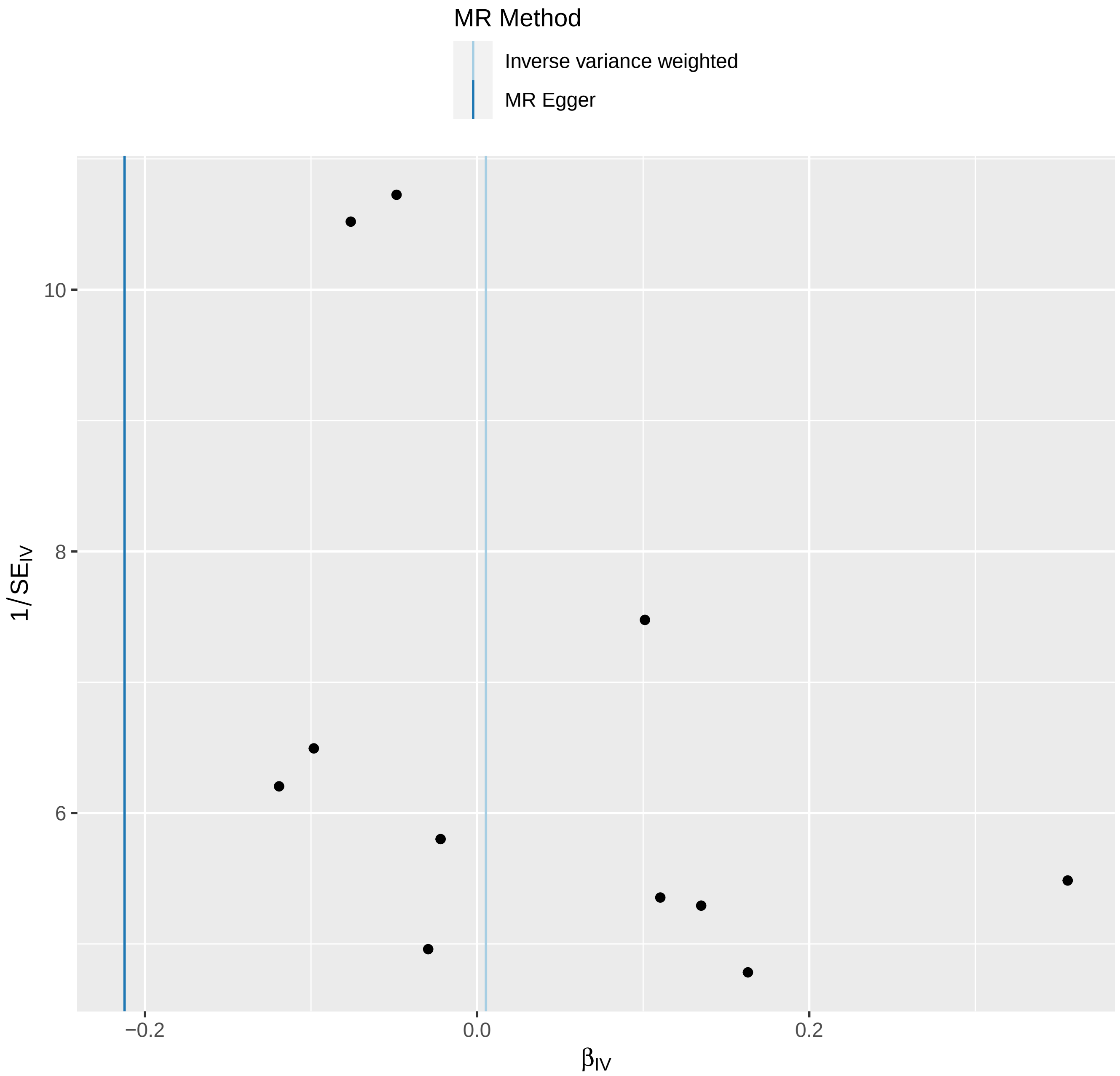

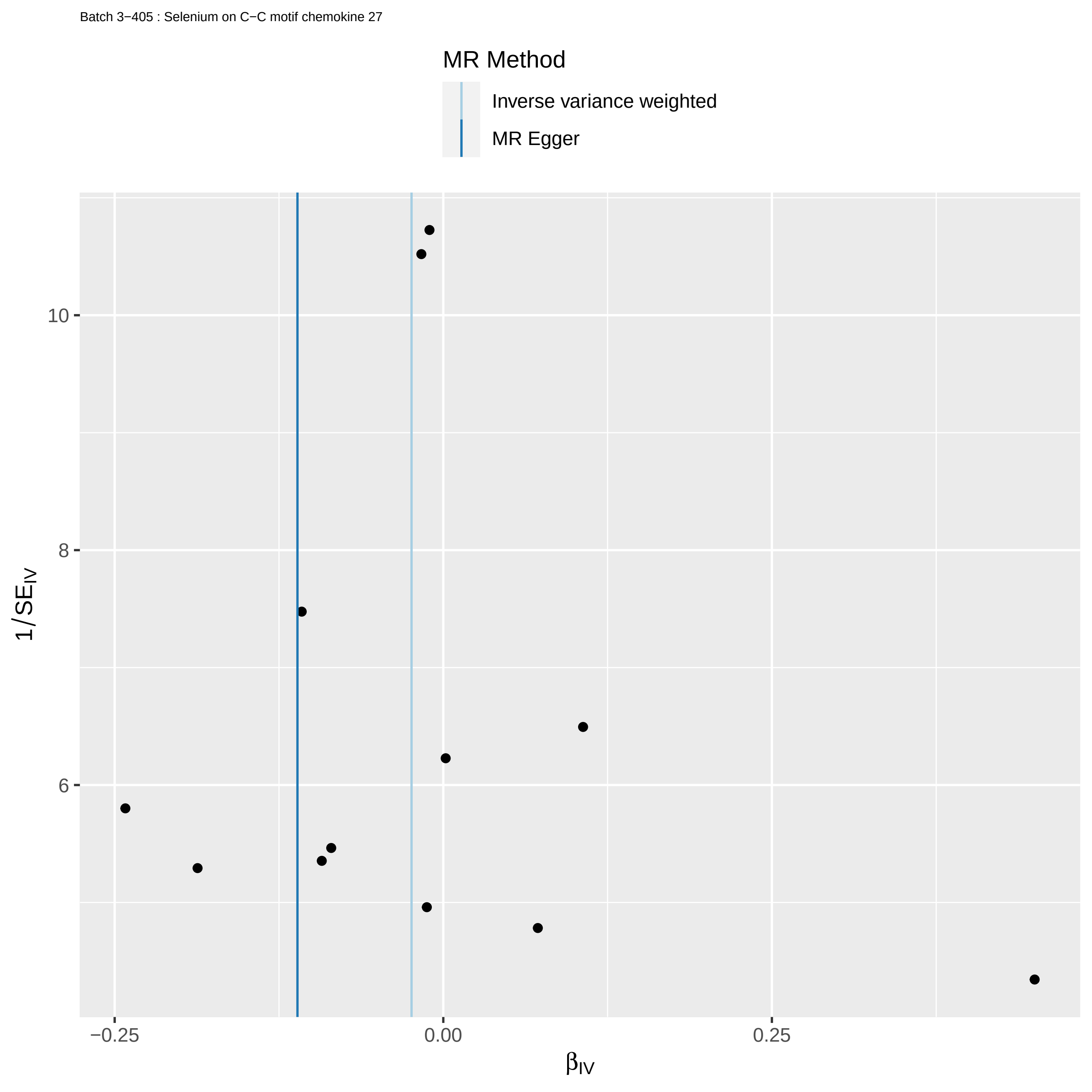

Batch 3−405 : Selenium on C−C motif chemokine 27
MR Method
Inverse variance weighted
MR Egger
10
8
6
1/SE_IV
−0.25
0.00
0.25
β_IV

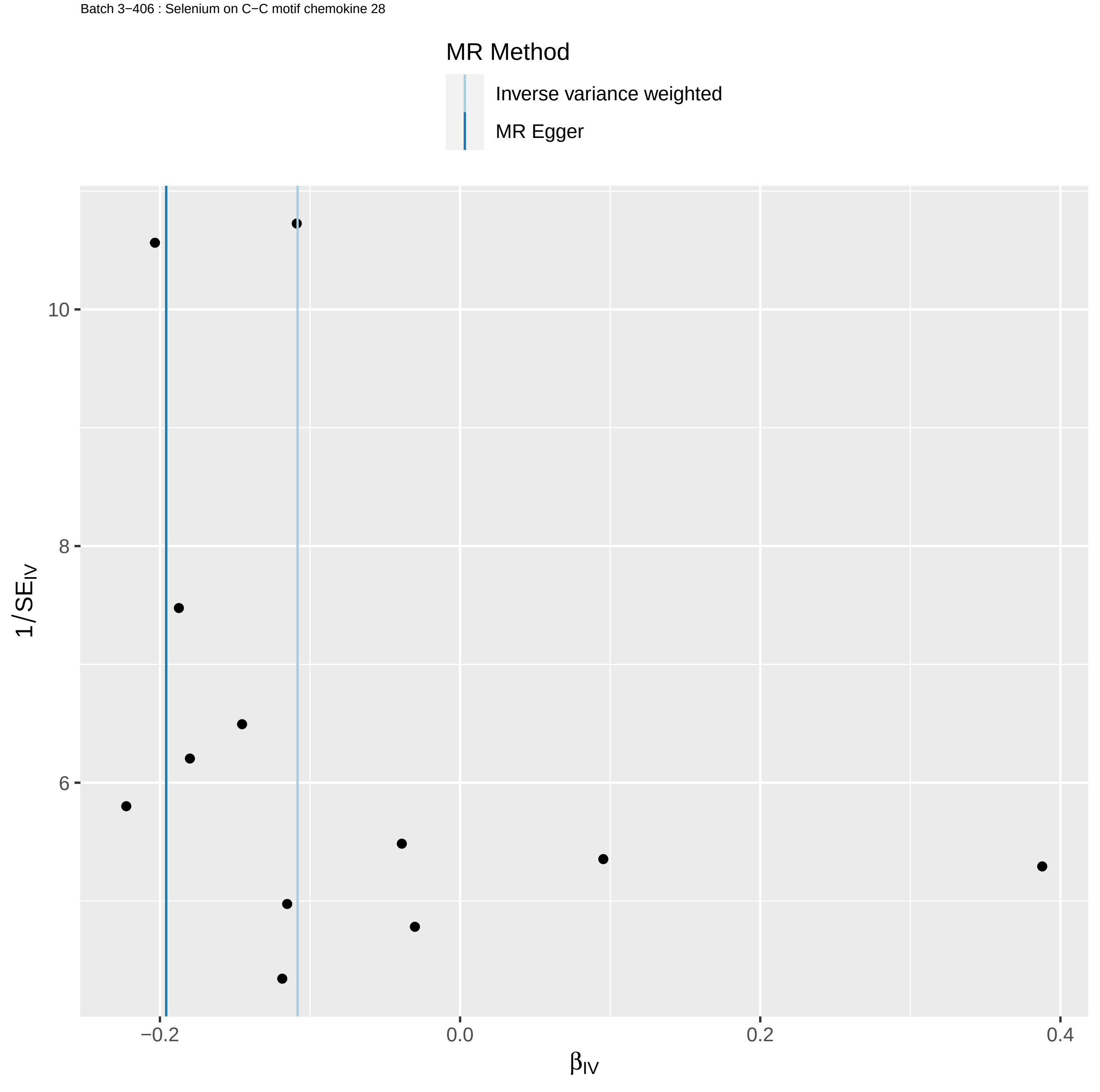
Batch 3−406 : Selenium on C−C motif chemokine 28
MR Method
Inverse variance weighted
MR Egger
10
8
6
1/SE_IV
−0.2
0.0
0.2
0.4
β_IV

Batch 3−407 : Selenium on C−C motif chemokine 3

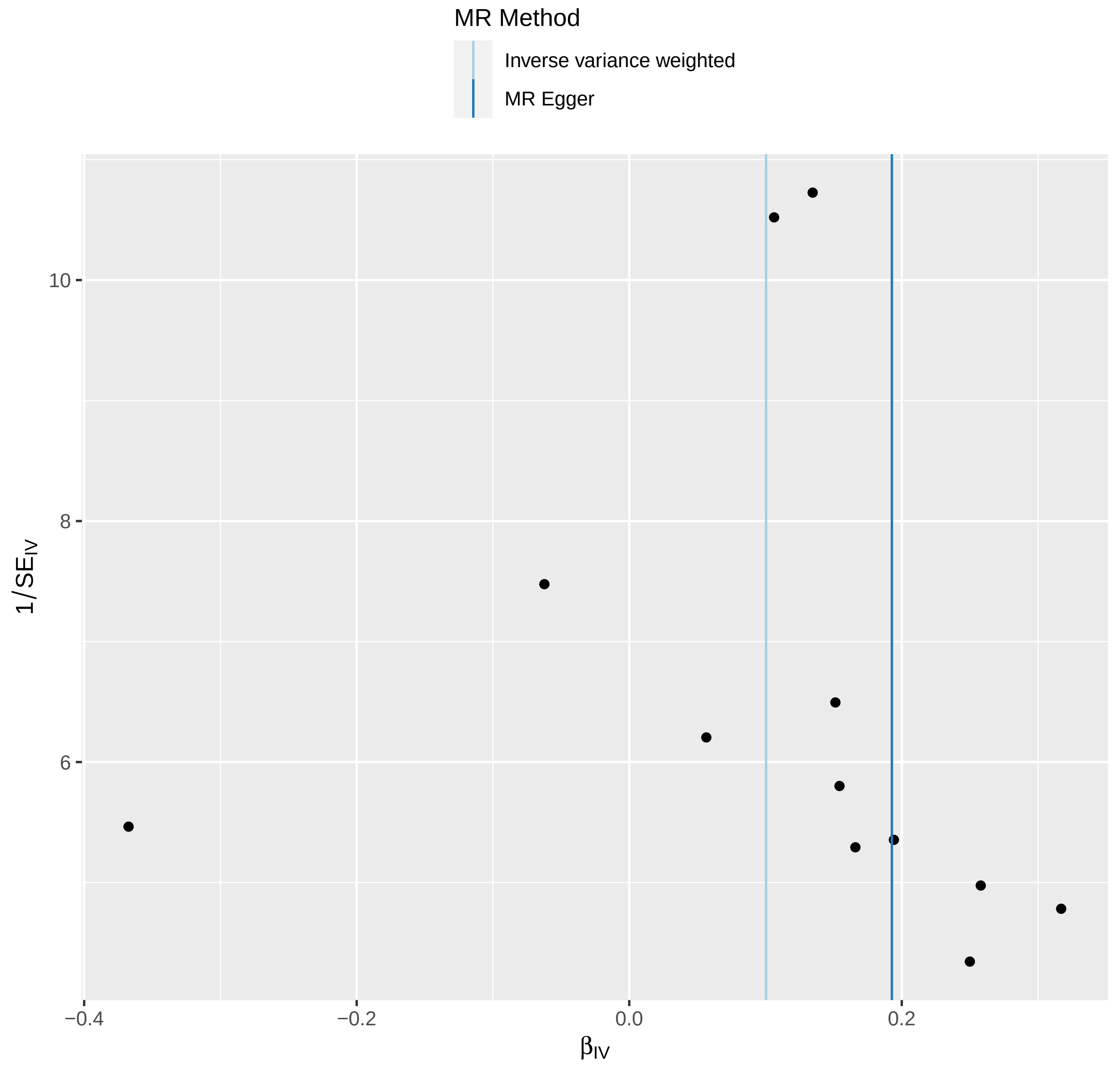

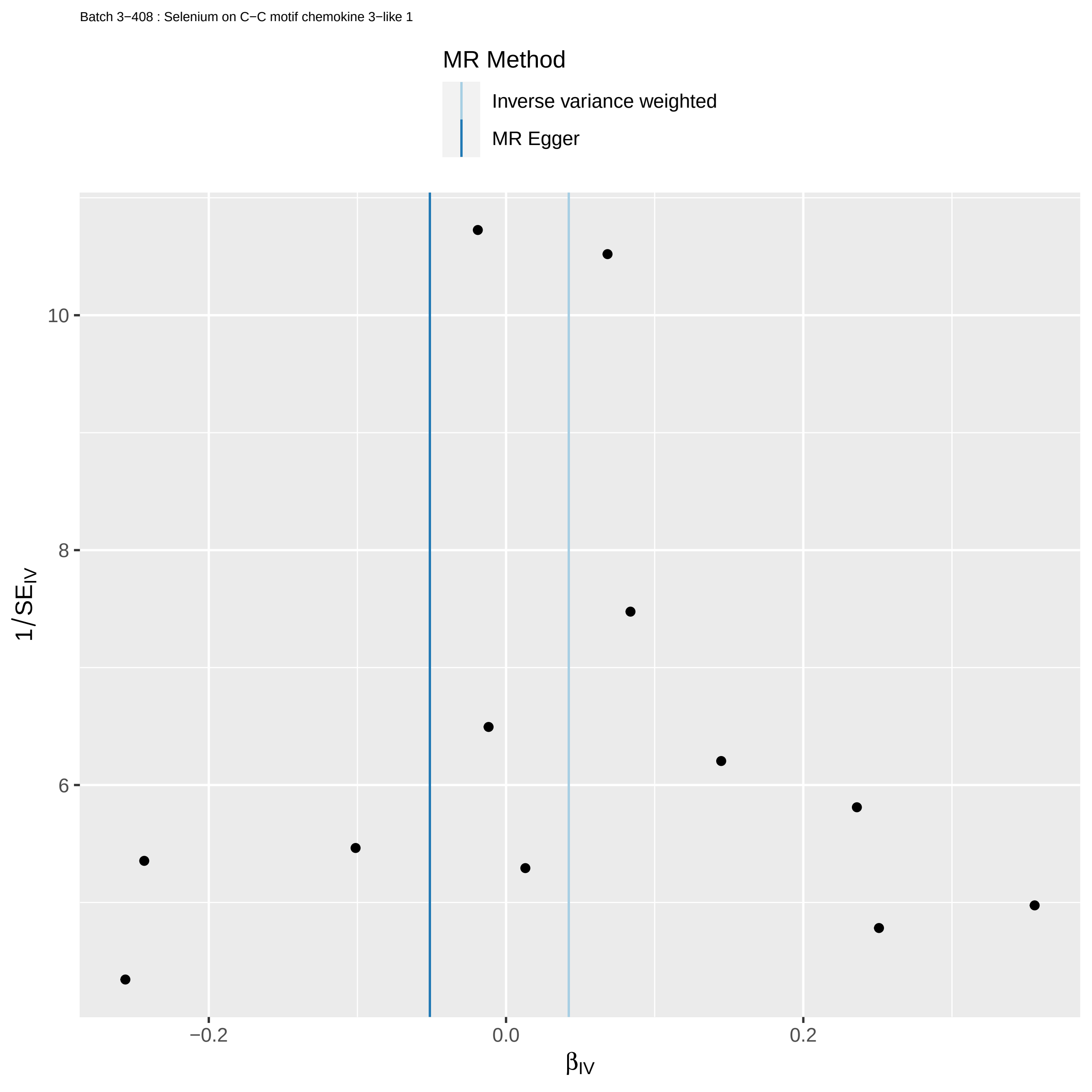
Batch 3−408 : Selenium on C−C motif chemokine 3−like 1
MR Method
Inverse variance weighted
MR Egger
10
8
6
1/SE_IV
−0.2
0.0
0.2
β_IV

Batch 3−409 : Selenium on C−C motif chemokine 5

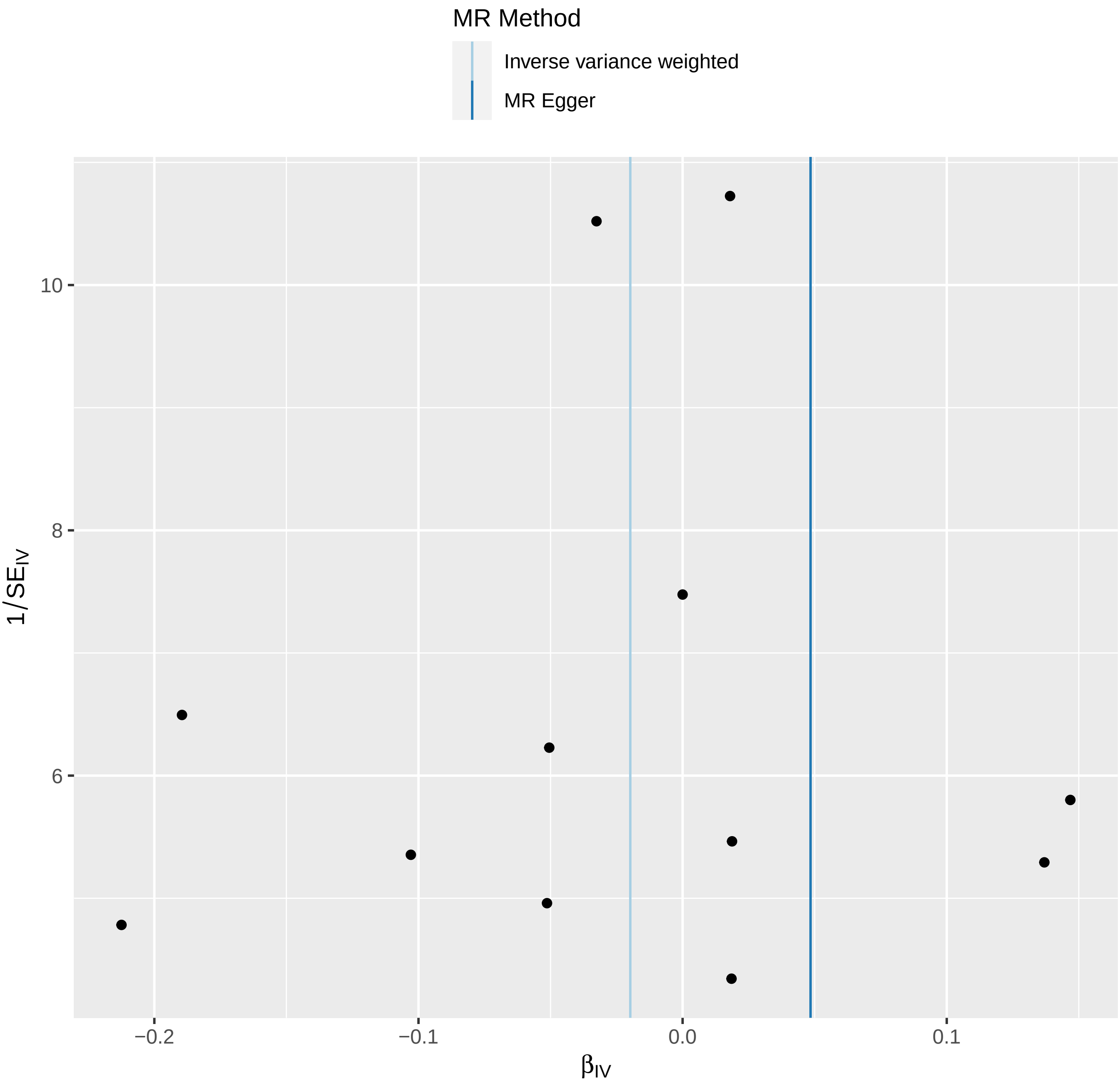

Batch 3−410 : Selenium on C−C motif chemokine 7

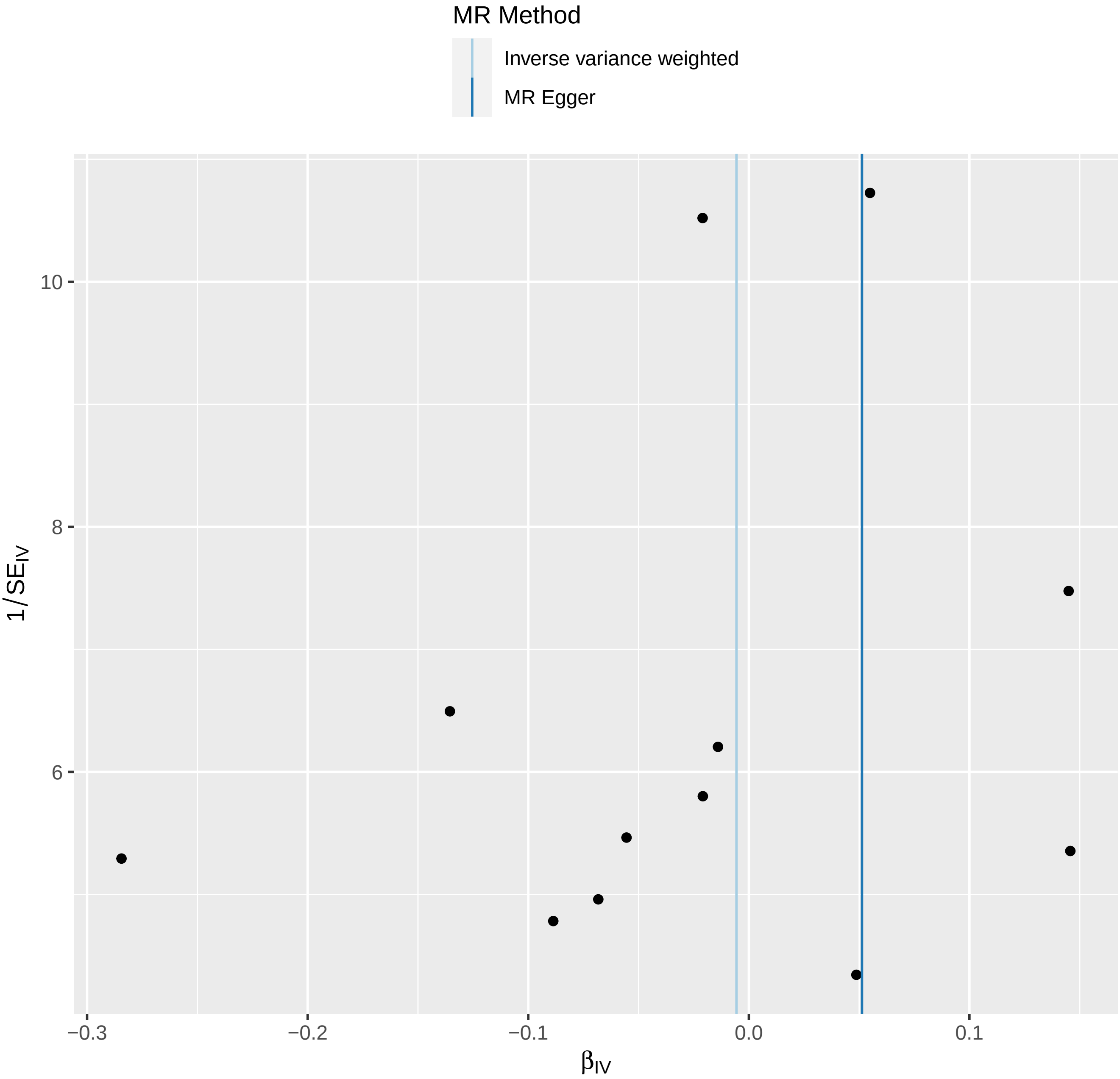

Batch 3−411 : Selenium on C−C motif chemokine 8

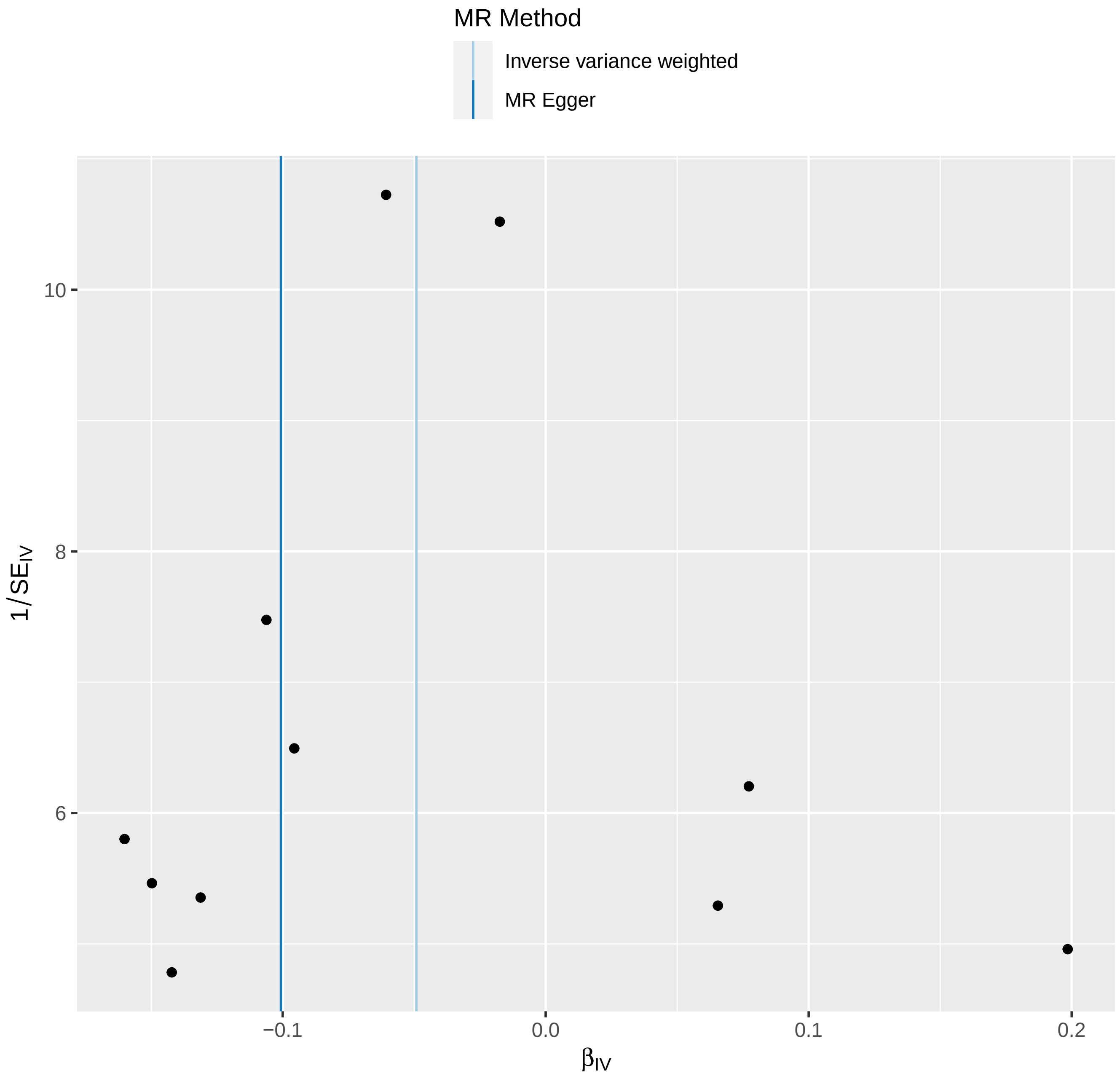

Batch 3−412 : Selenium on Cerebral cavernous malformations 2 protein

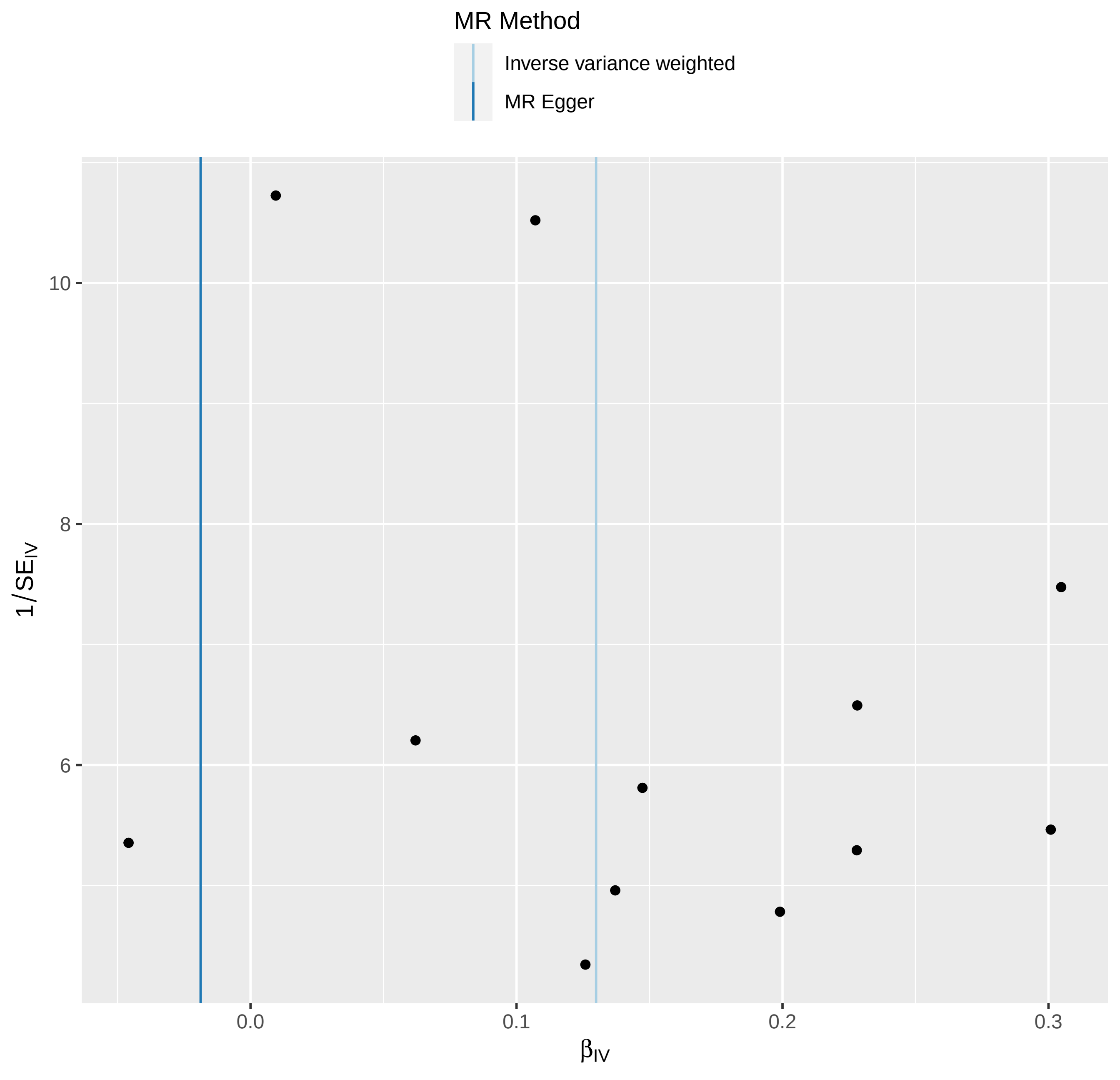

Batch 3−413 : Selenium on G2/mitotic−specific cyclin−B1

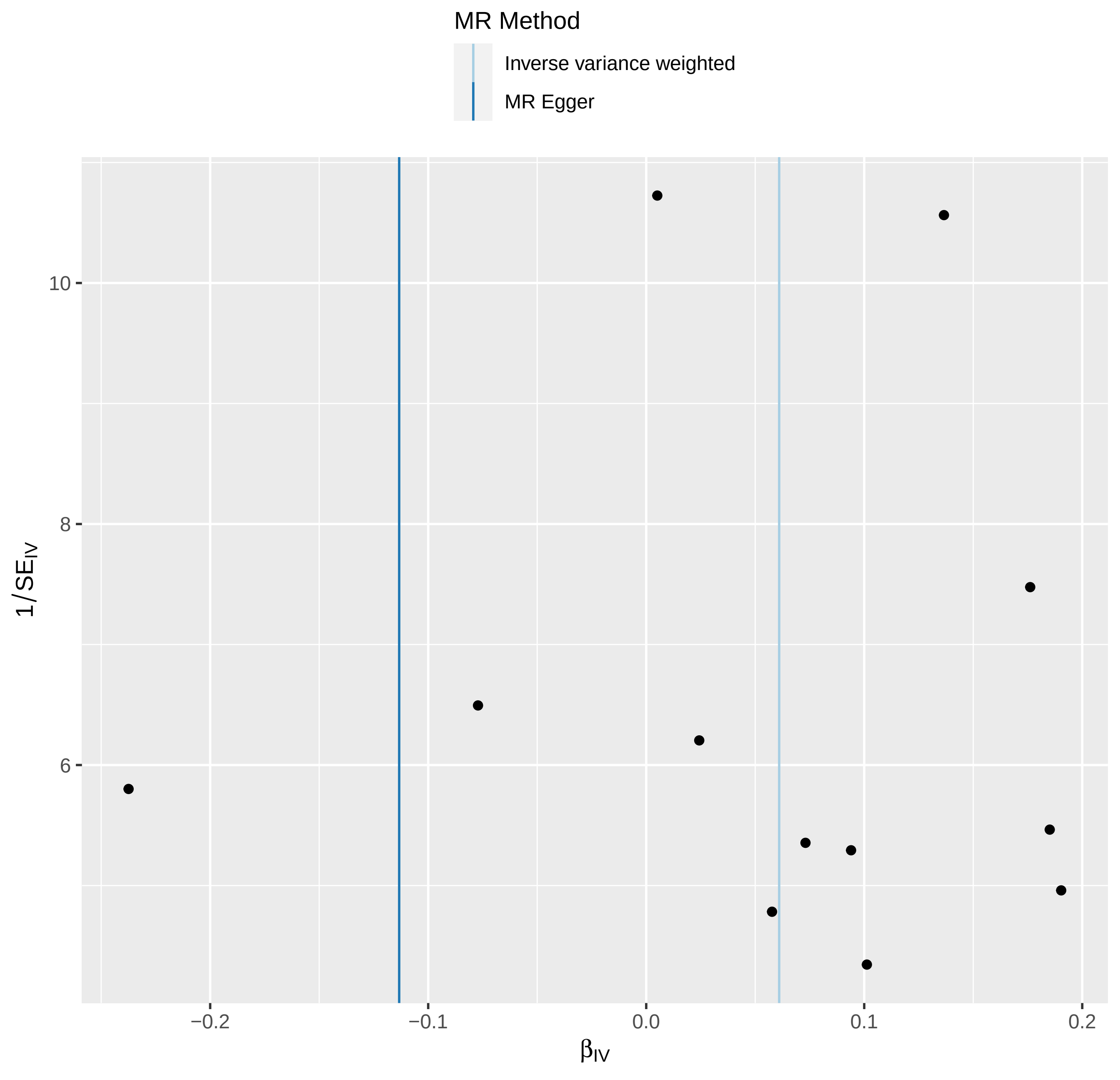

Batch 3−414 : Selenium on E3 ubiquitin−protein ligase CCNB1IP1

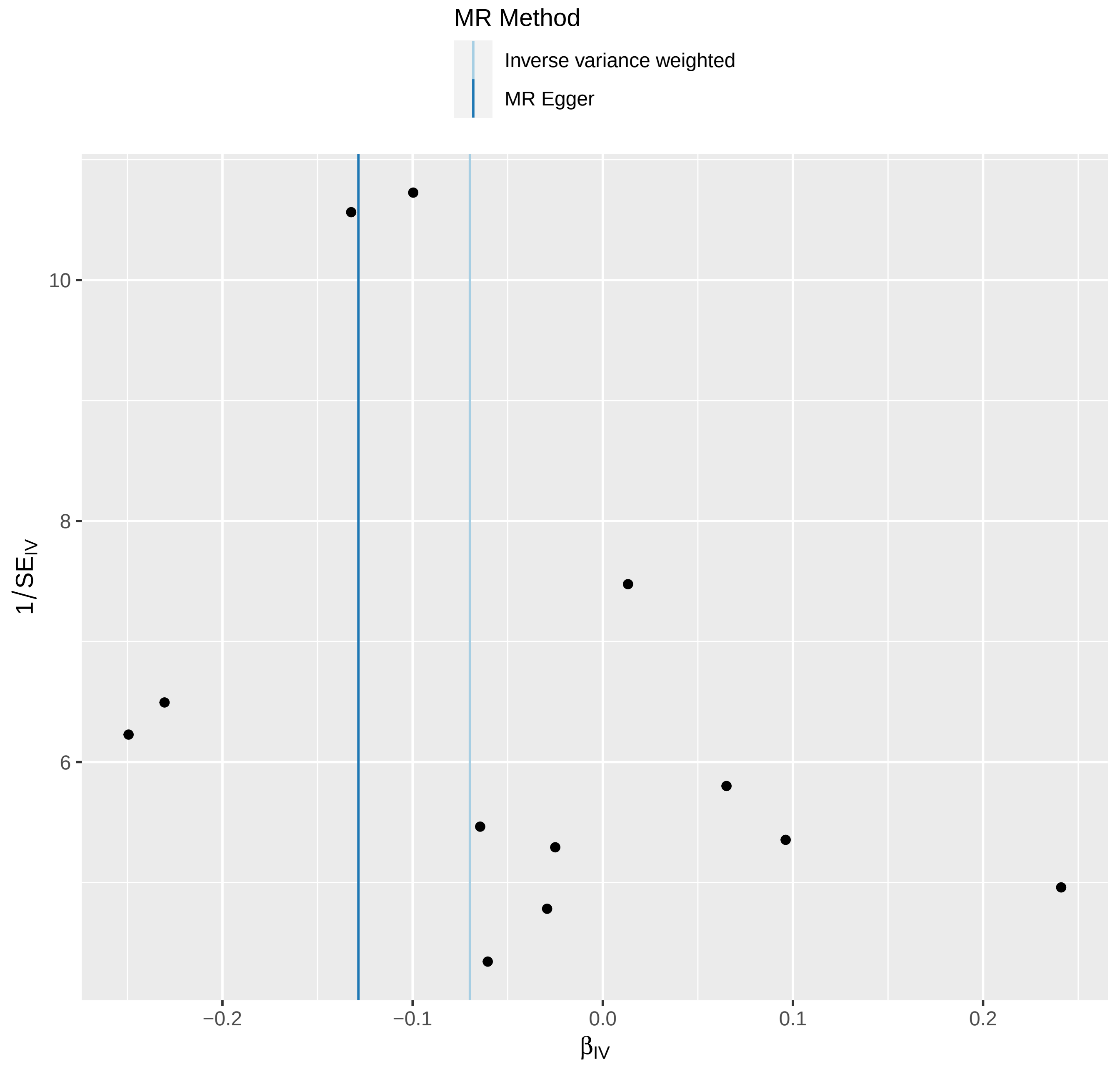

Batch 3−415 : Selenium on Cyclin−C

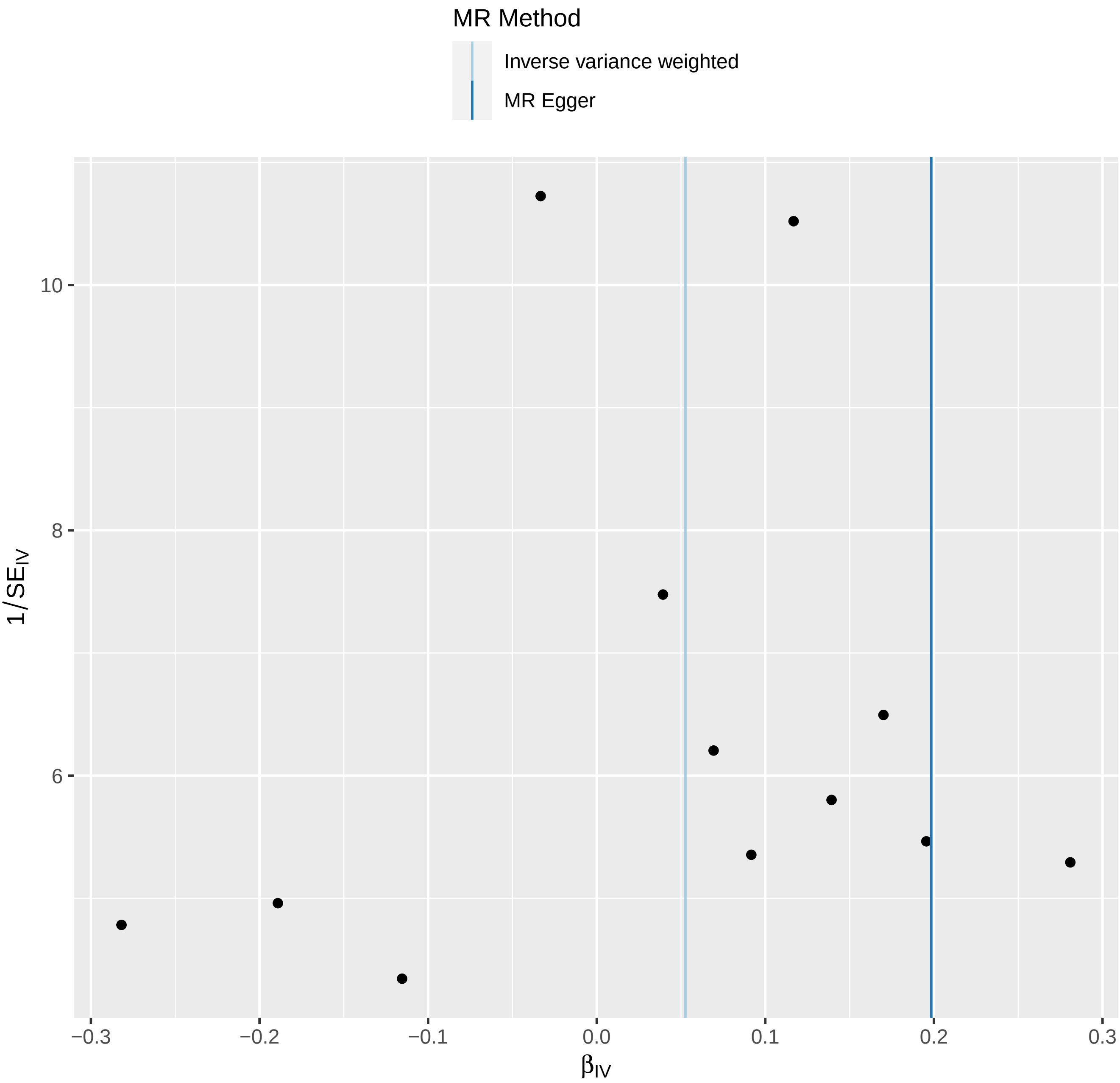

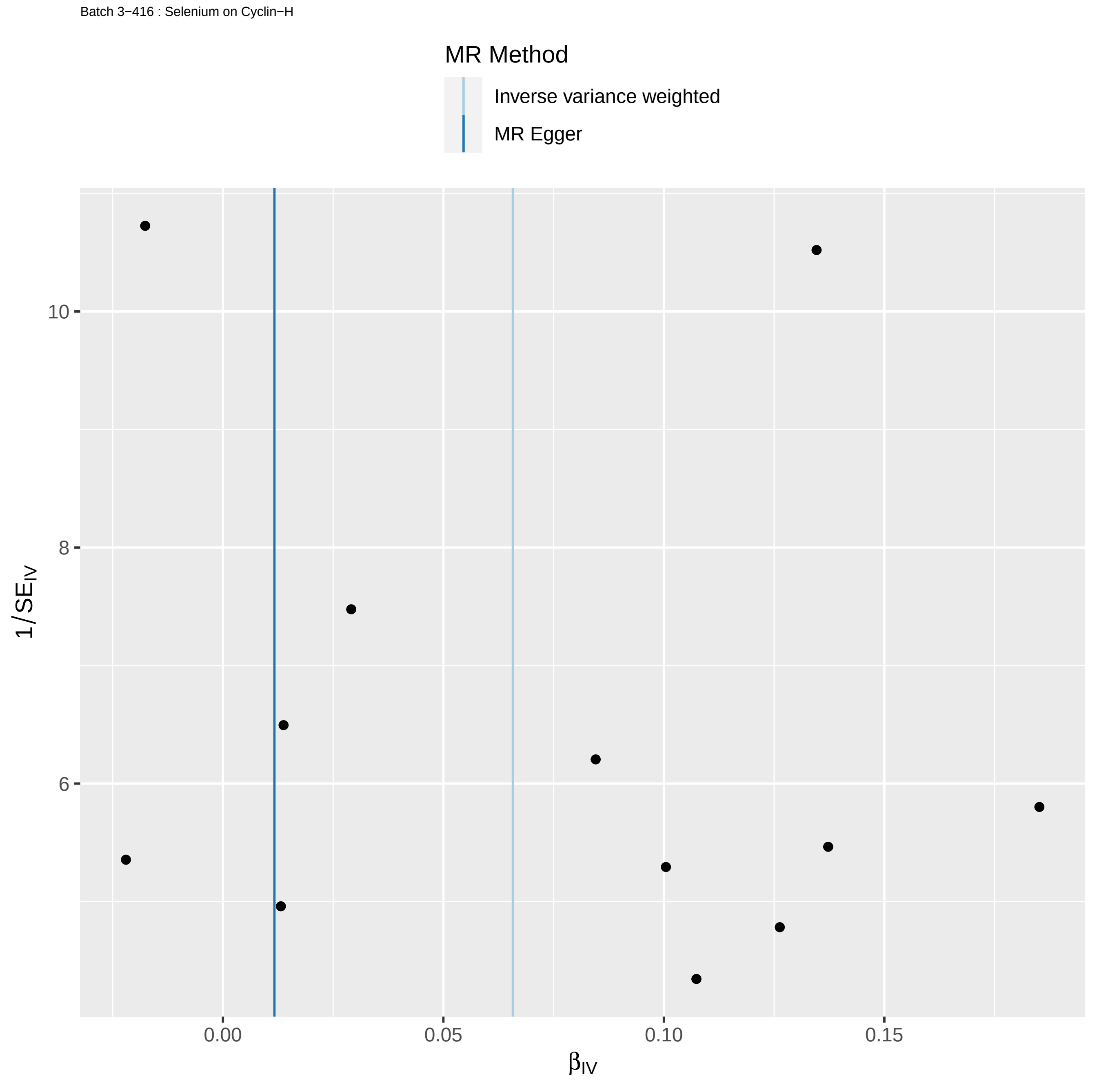

Batch 3−416 : Selenium on Cyclin−H
MR Method
Inverse variance weighted
MR Egger
10
8
6
1/SE_IV
0.00
0.05
0.10
0.15
β_IV

Batch 3−417 : Selenium on Cell cycle progression protein 1

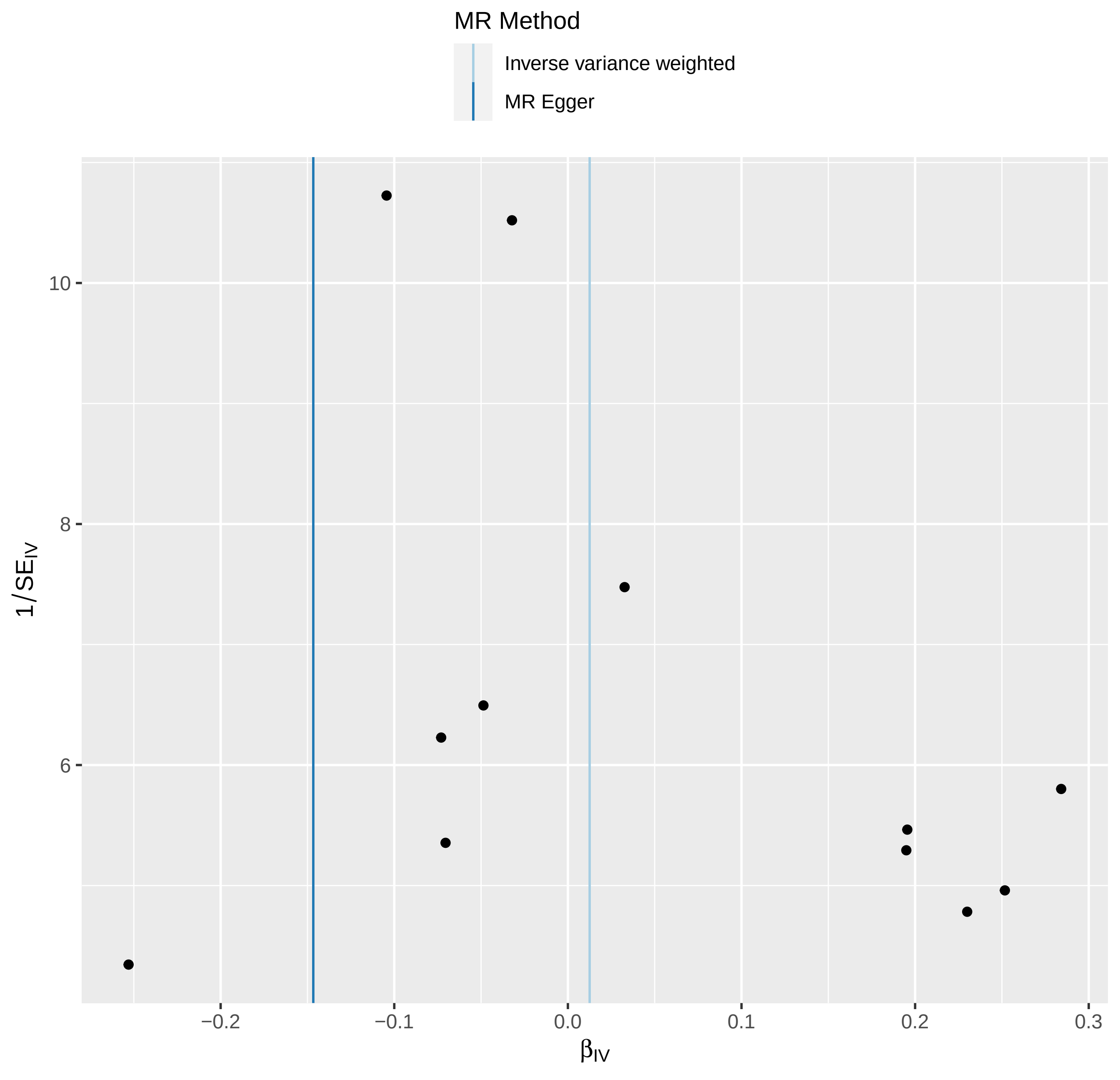

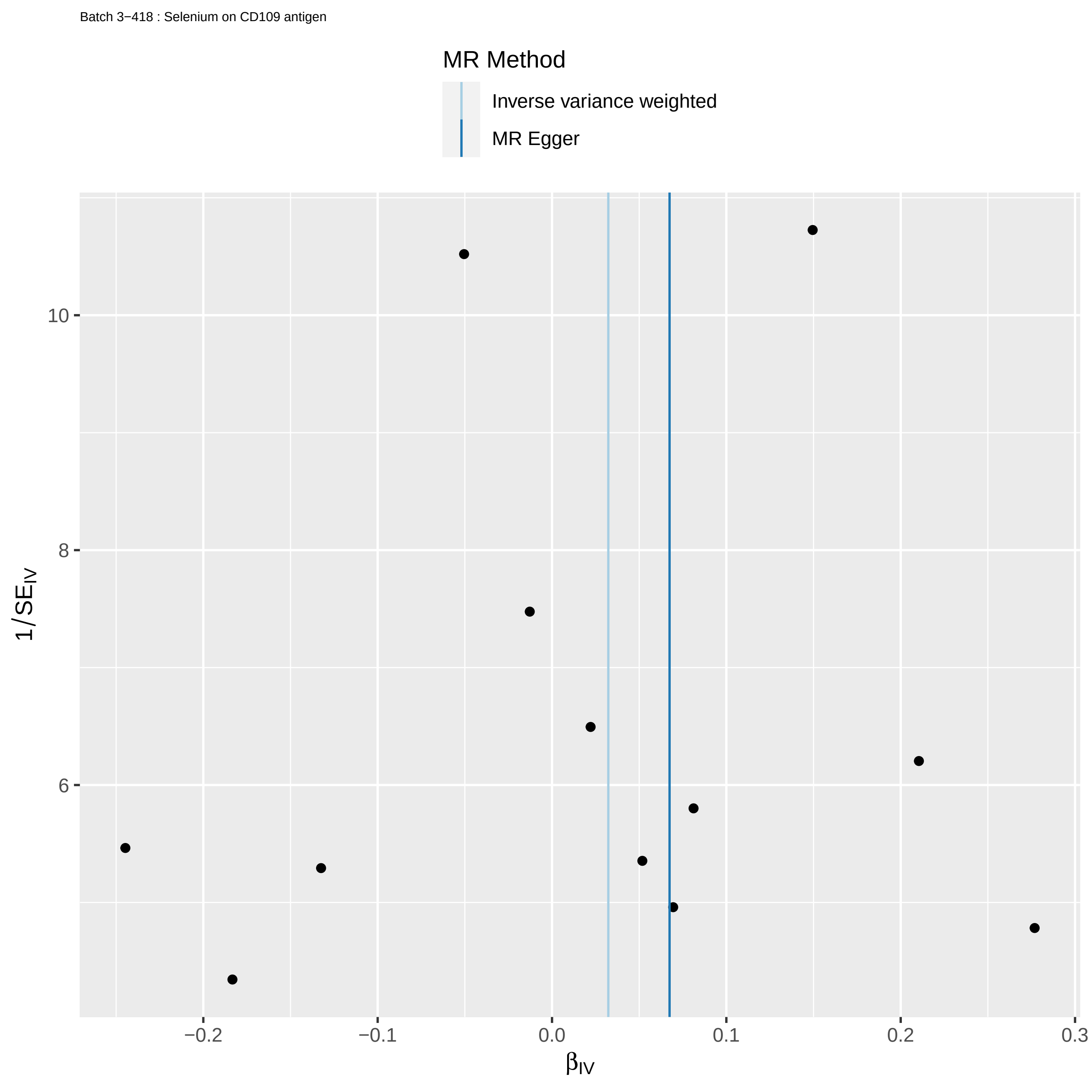

Batch 3−418 : Selenium on CD109 antigen
MR Method
Inverse variance weighted
MR Egger
10
8
6
1/SE_IV
−0.2
−0.1
0.0
0.1
0.2
0.3
β_IV

Batch 3−419 : Selenium on Scavenger receptor cysteine−rich type 1 protein M130

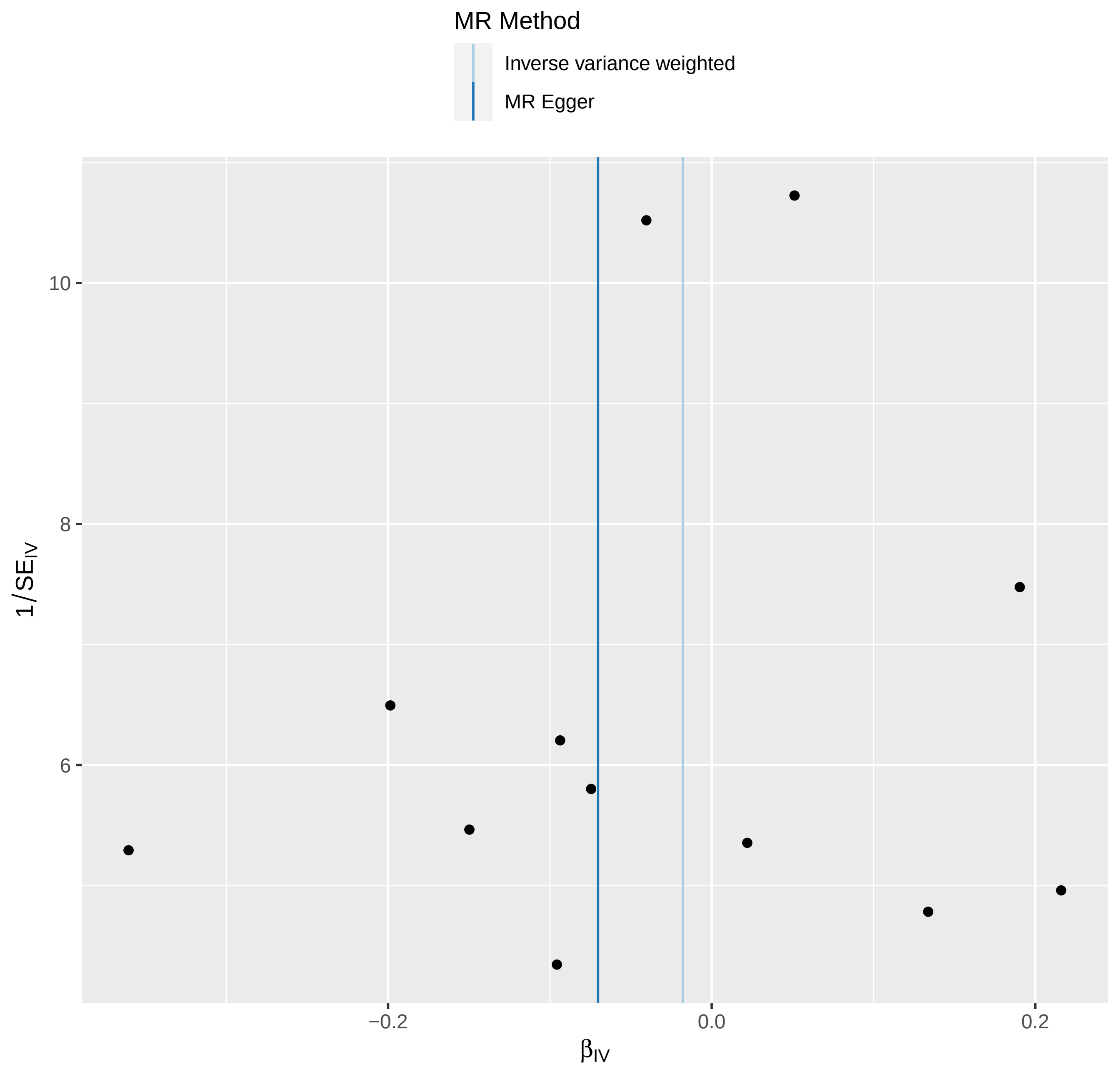

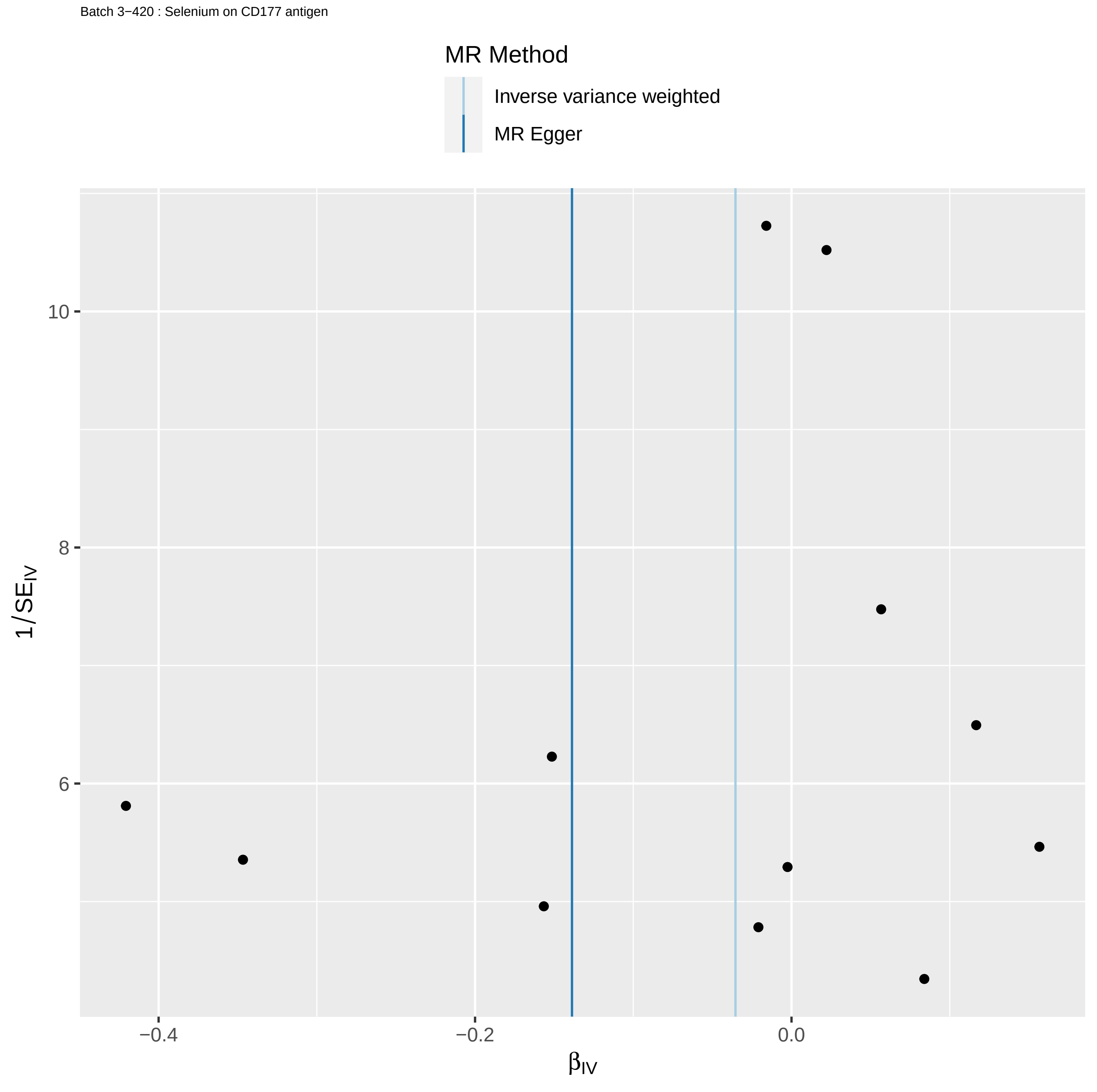

Batch 3−420 : Selenium on CD177 antigen
MR Method
Inverse variance weighted
MR Egger
10
8
6
1/SE_IV
−0.4
−0.2
0.0
β_IV

Batch 3−421 : Selenium on Antigen−presenting glycoprotein CD1d

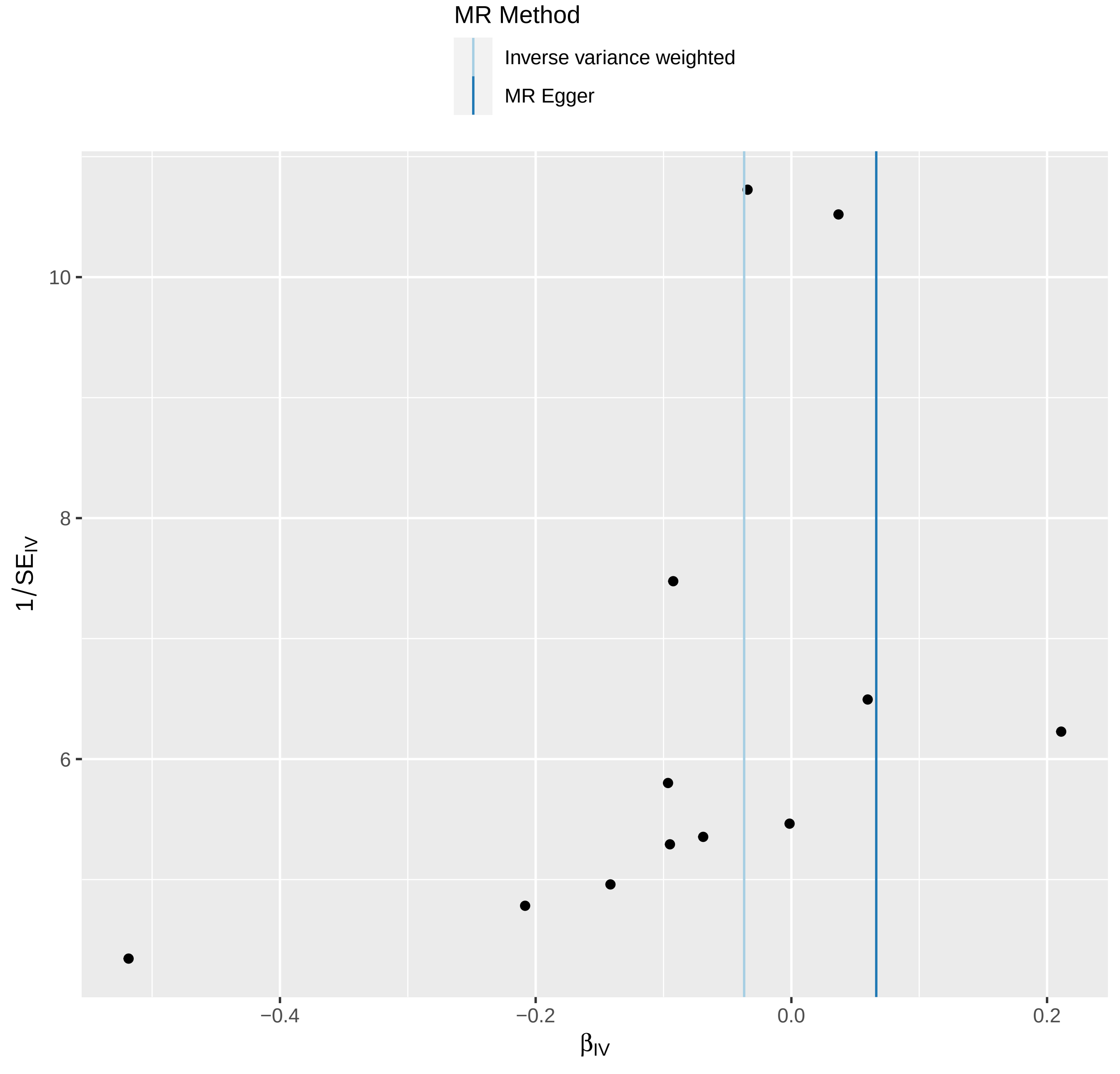

Batch 3−422 : Selenium on OX−2 membrane glycoprotein

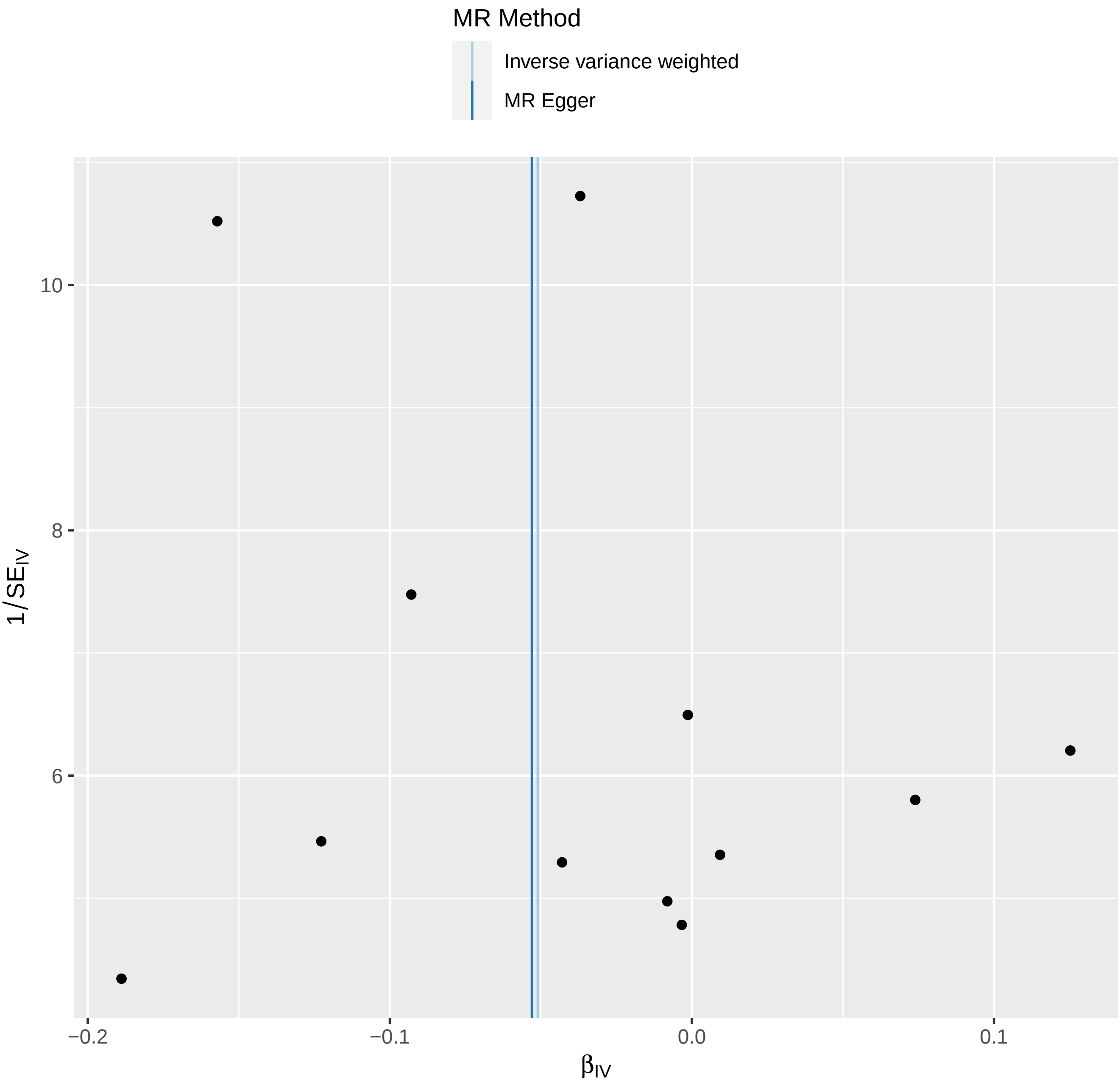

Batch 3−423 : Selenium on Cell surface glycoprotein CD200 receptor 1

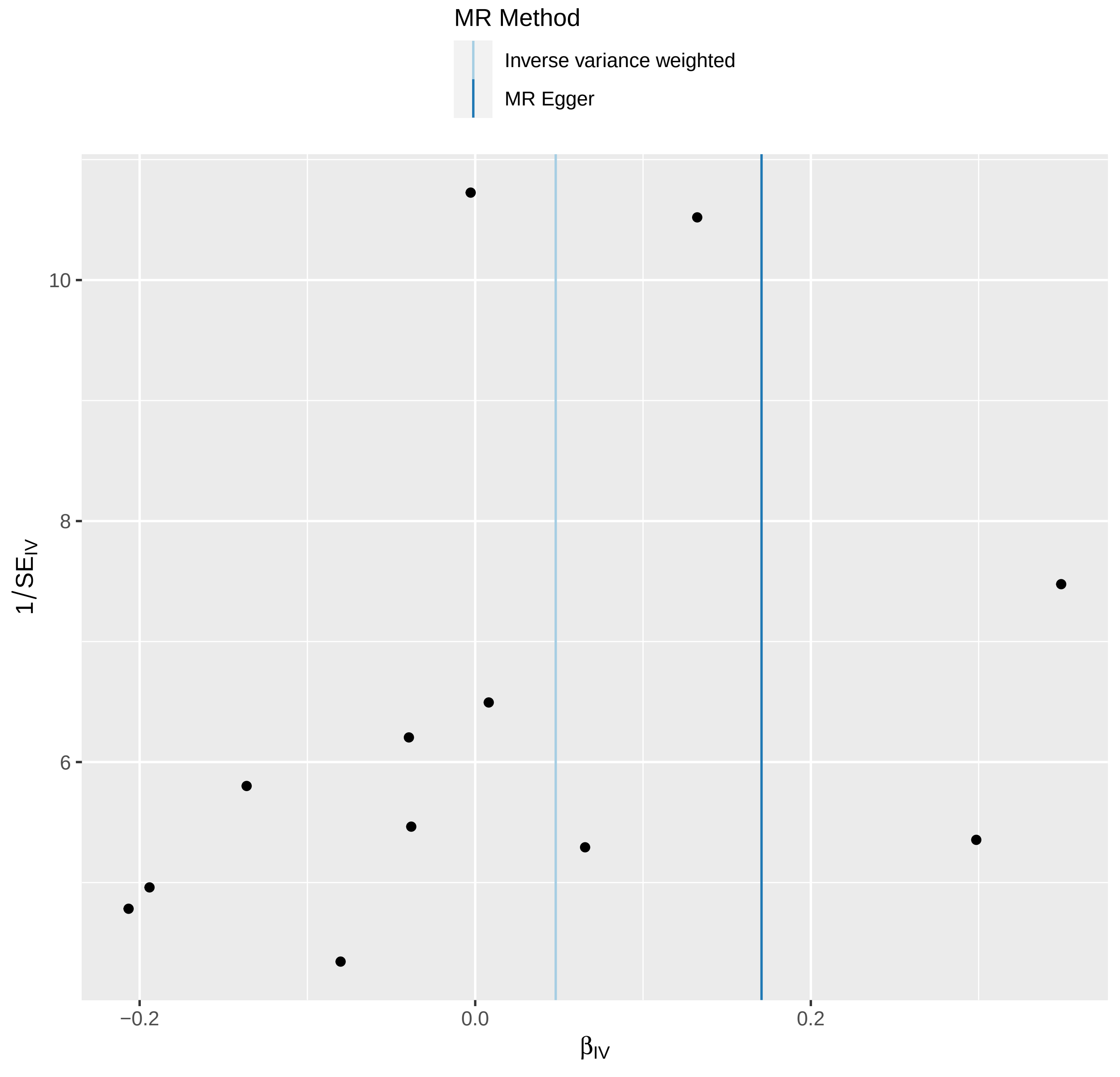

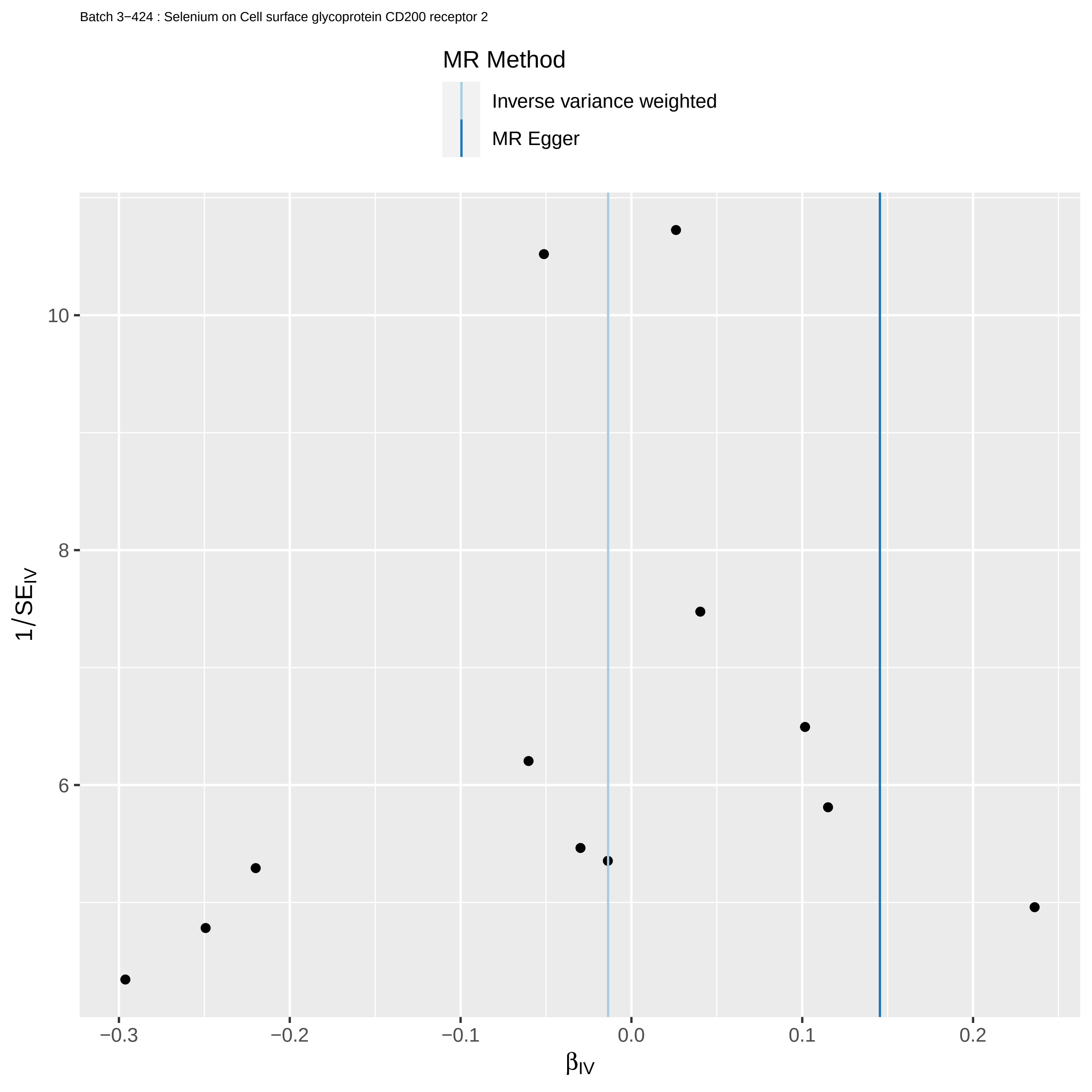
Batch 3−424 : Selenium on Cell surface glycoprotein CD200 receptor 2
MR Method
Inverse variance weighted
MR Egger
10
8
6
1/SE_IV
−0.3
−0.2
−0.1
0.0
0.1
0.2
β_IV

Batch 3−425 : Selenium on C−type lectin domain family 4 member K

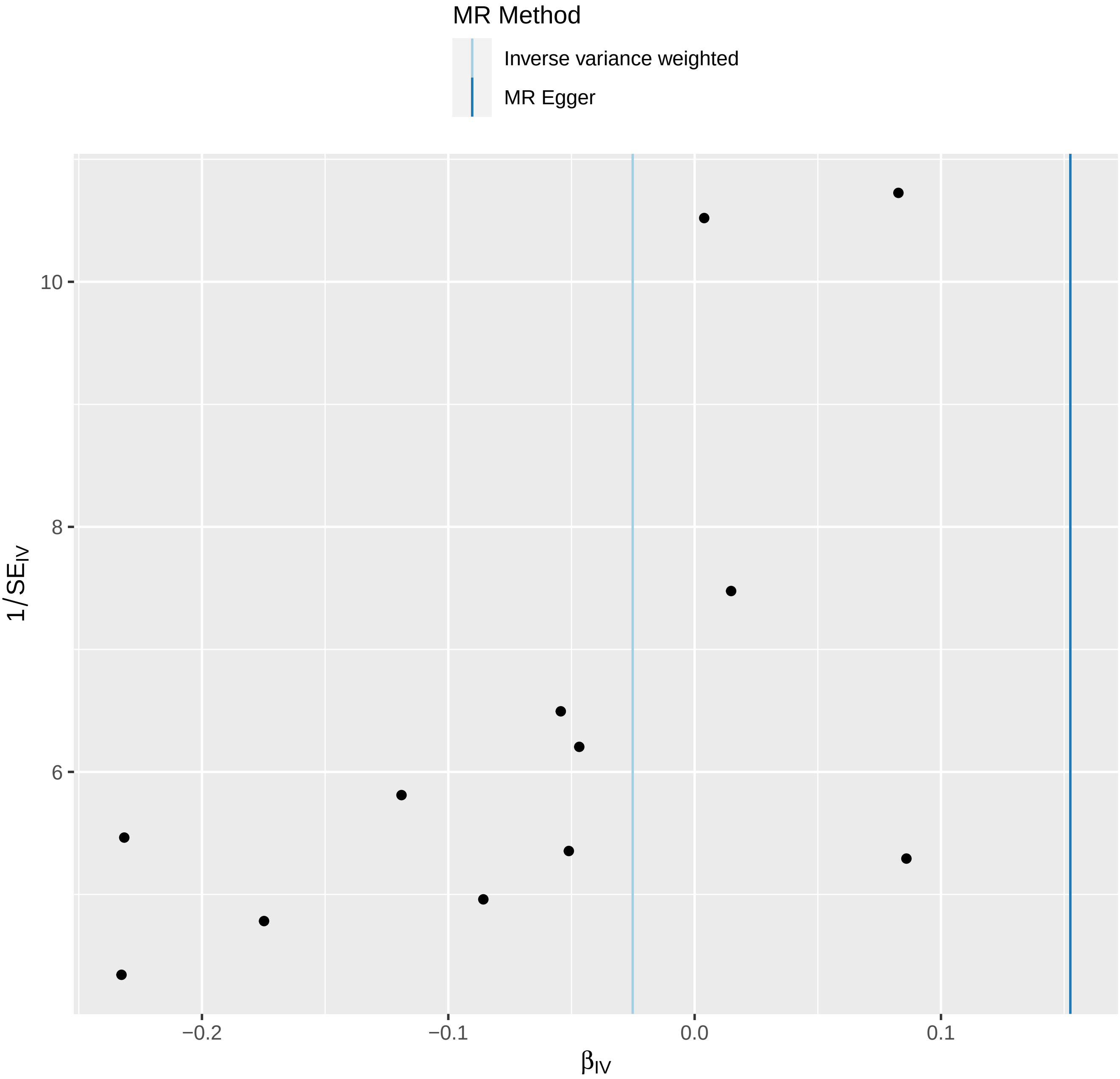

Batch 3−426 : Selenium on CD209 antigen

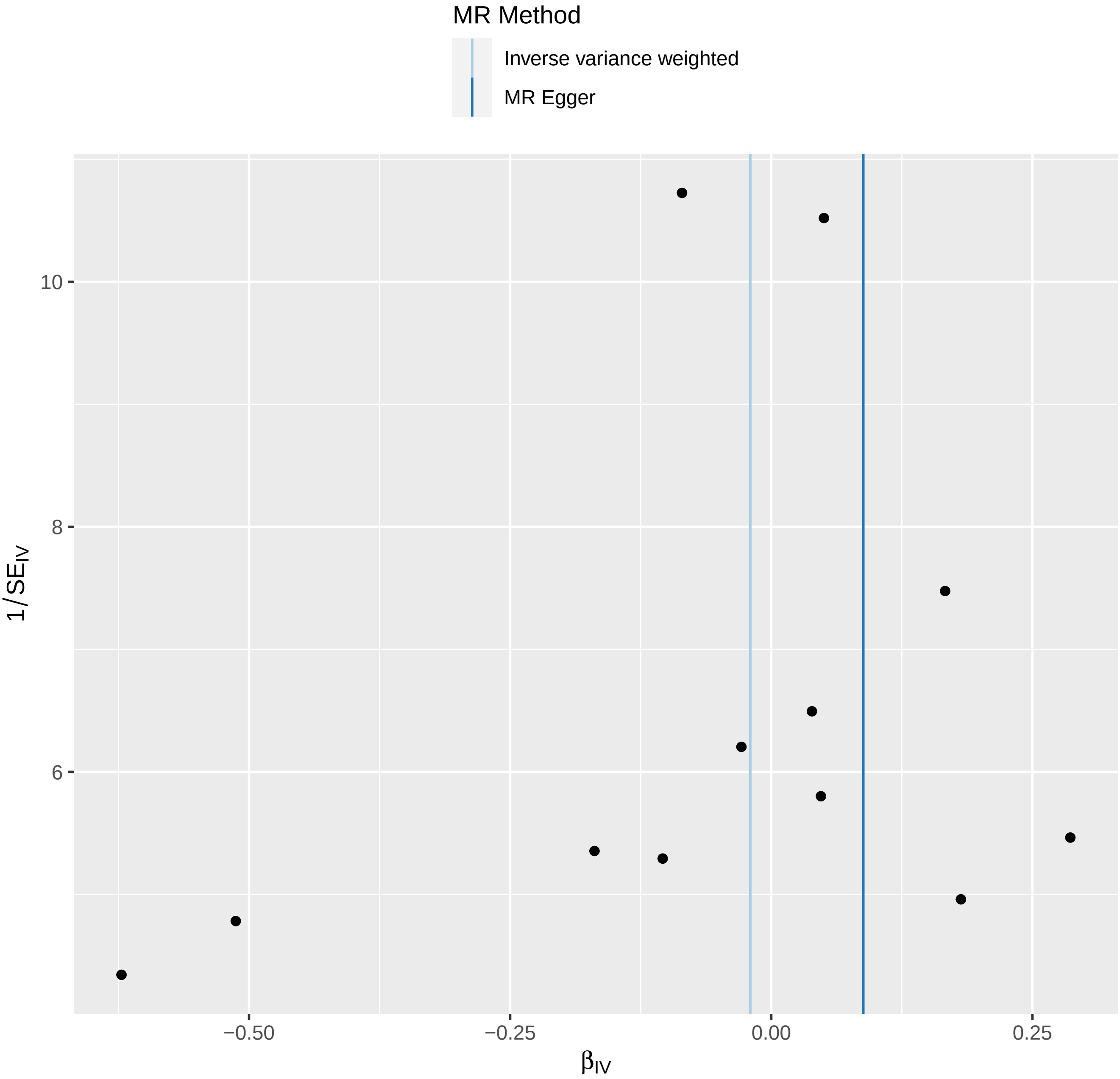

Batch 3−427 : Selenium on B−cell receptor CD22

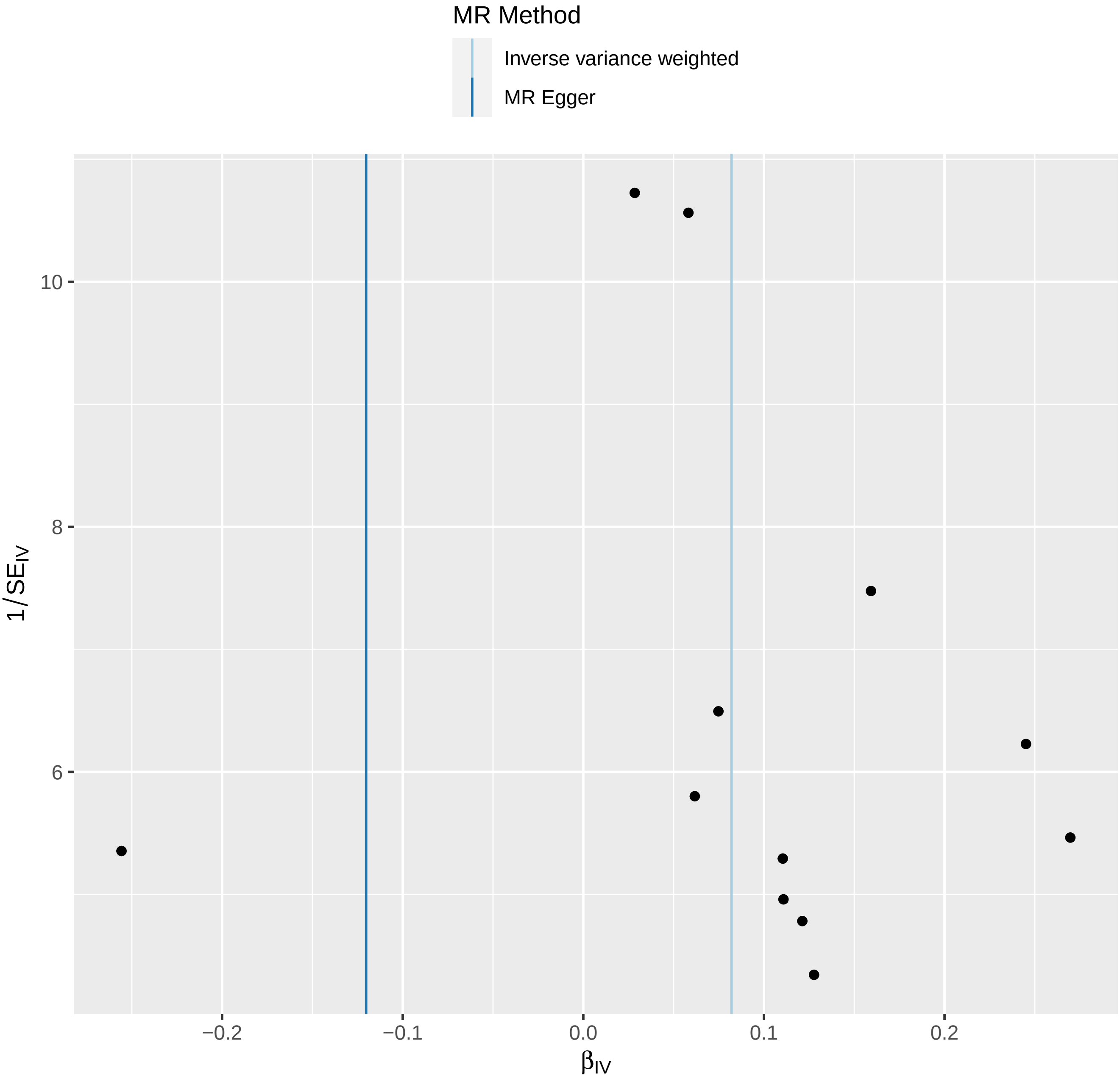

Batch 3−428 : Selenium on CD226 antigen

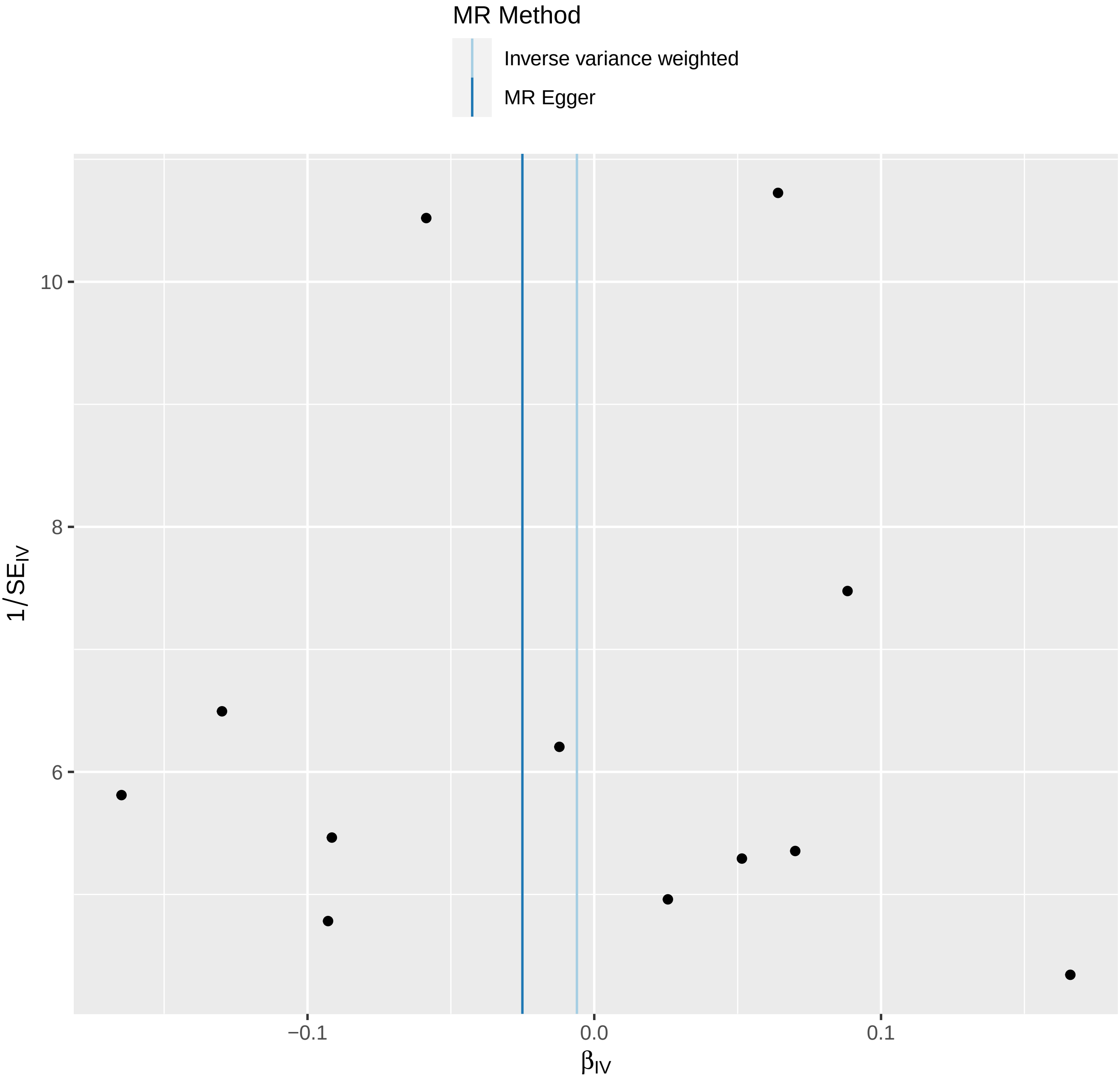

Batch 3−429 : Selenium on CD226 antigen

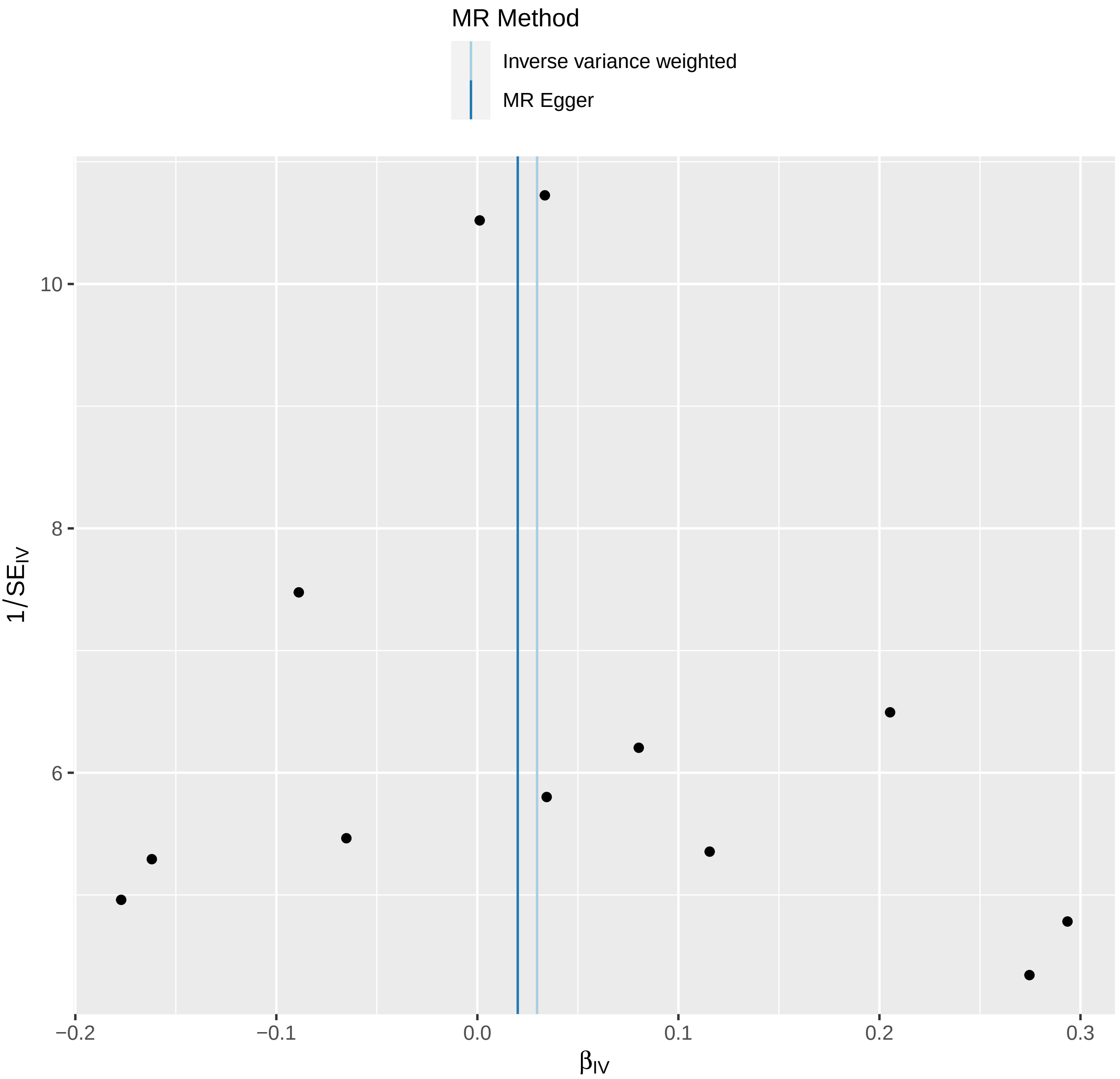

Batch 3−430 : Selenium on T−cell surface antigen CD2

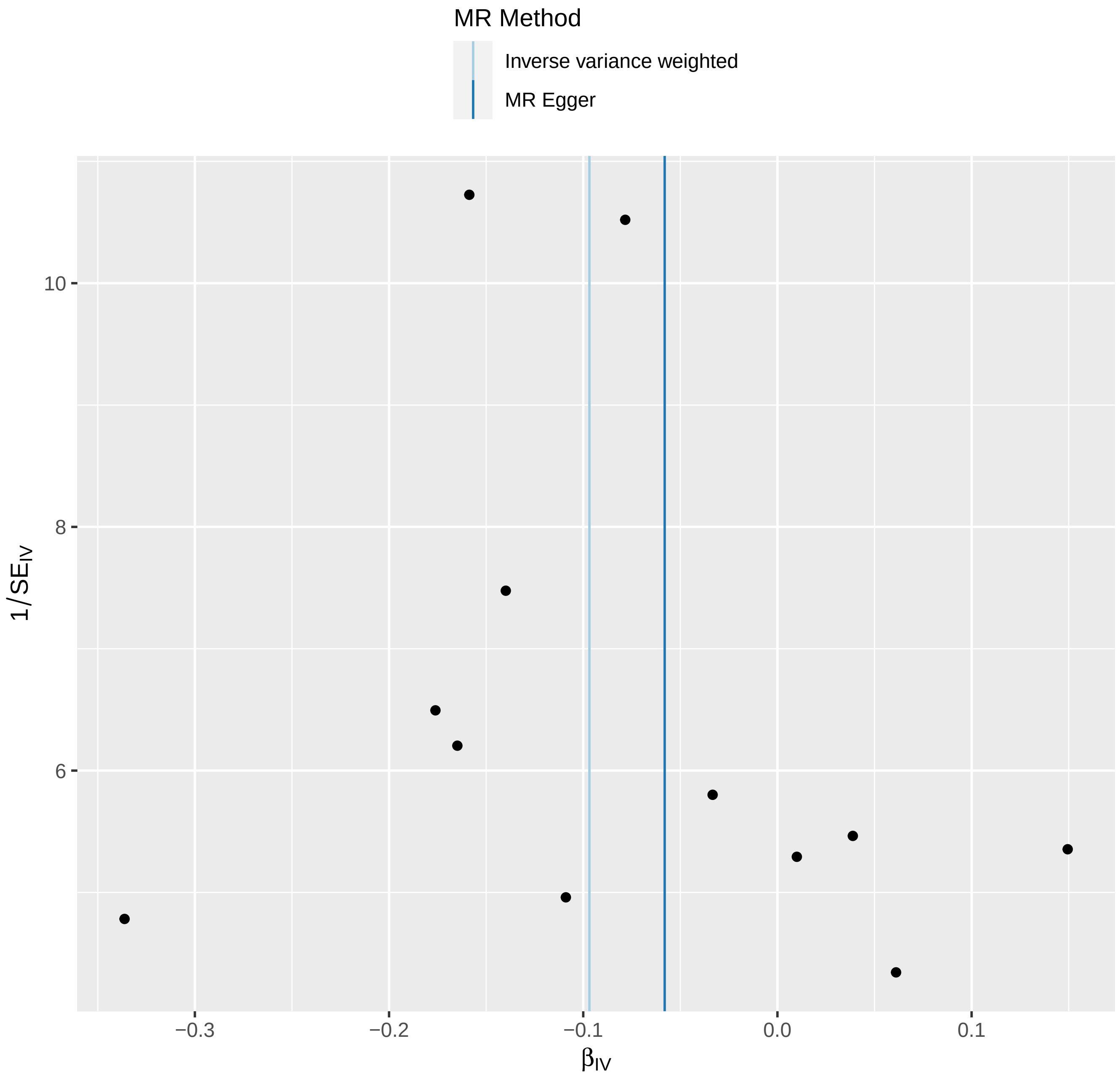

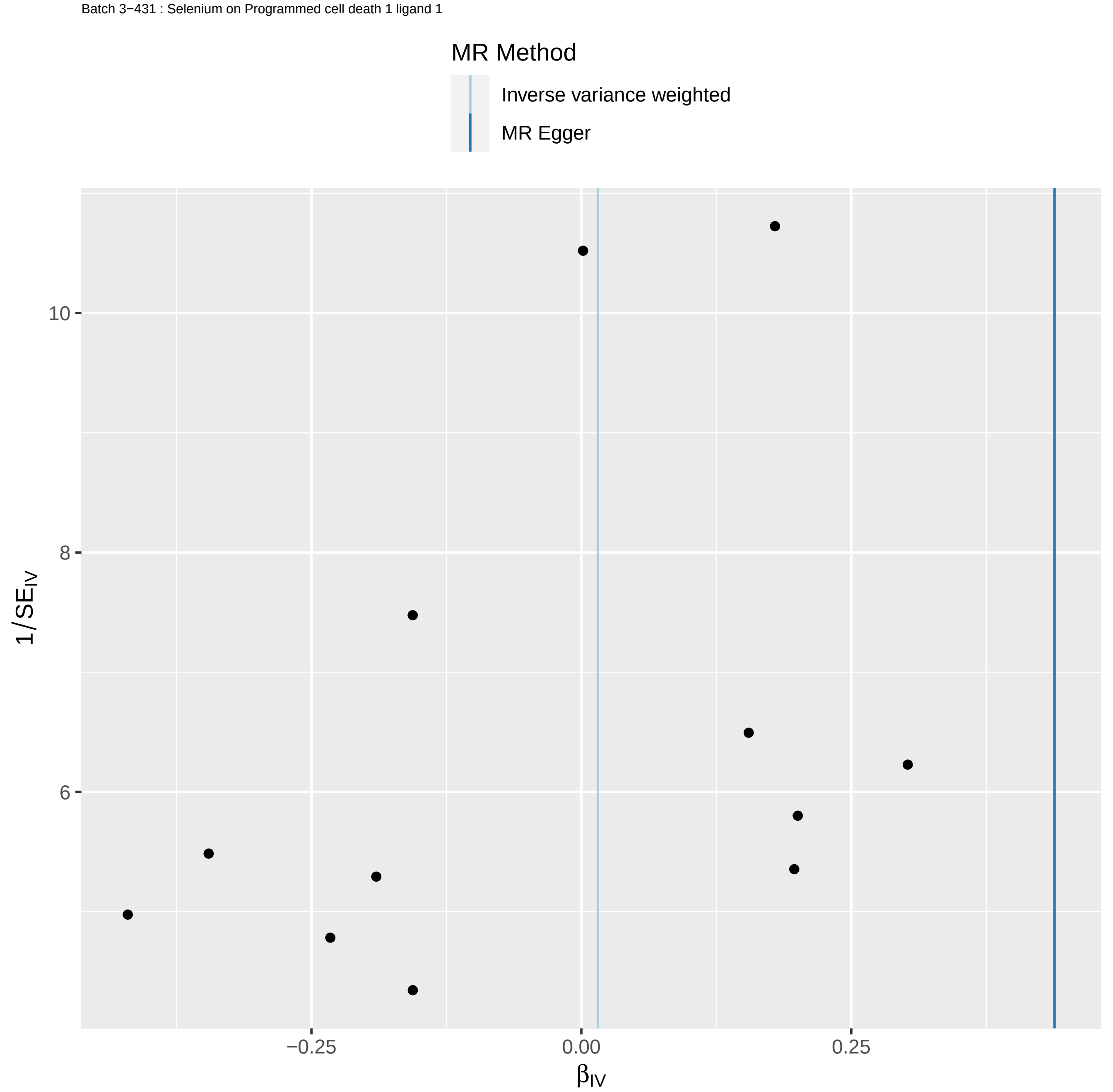
Batch 3−431 : Selenium on Programmed cell death 1 ligand 1
MR Method
Inverse variance weighted
MR Egger
10
8
6
1/SE_IV
−0.25
0.00
0.25
β_IV

Batch 3−432 : Selenium on CMRF35−like molecule 8

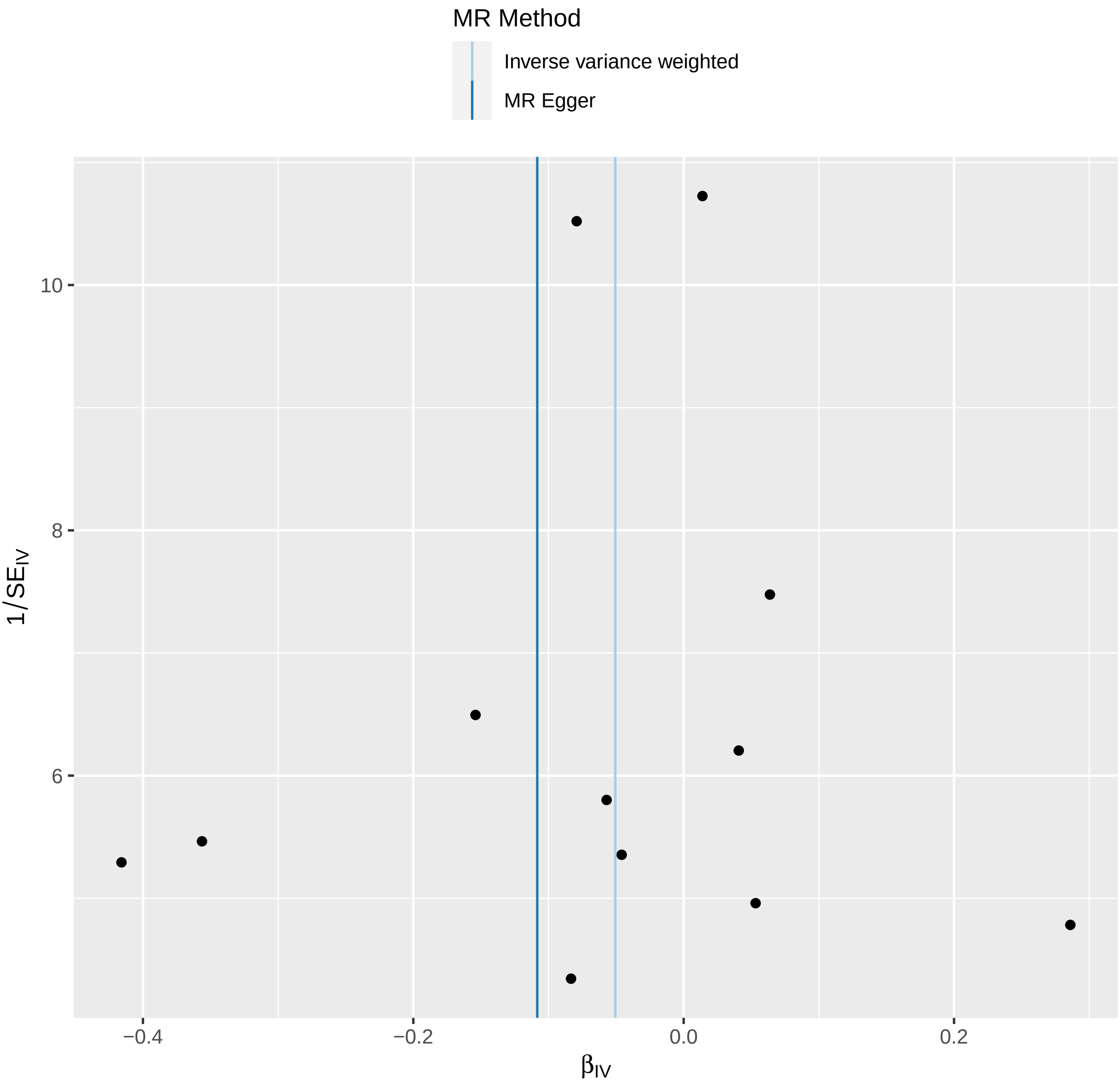

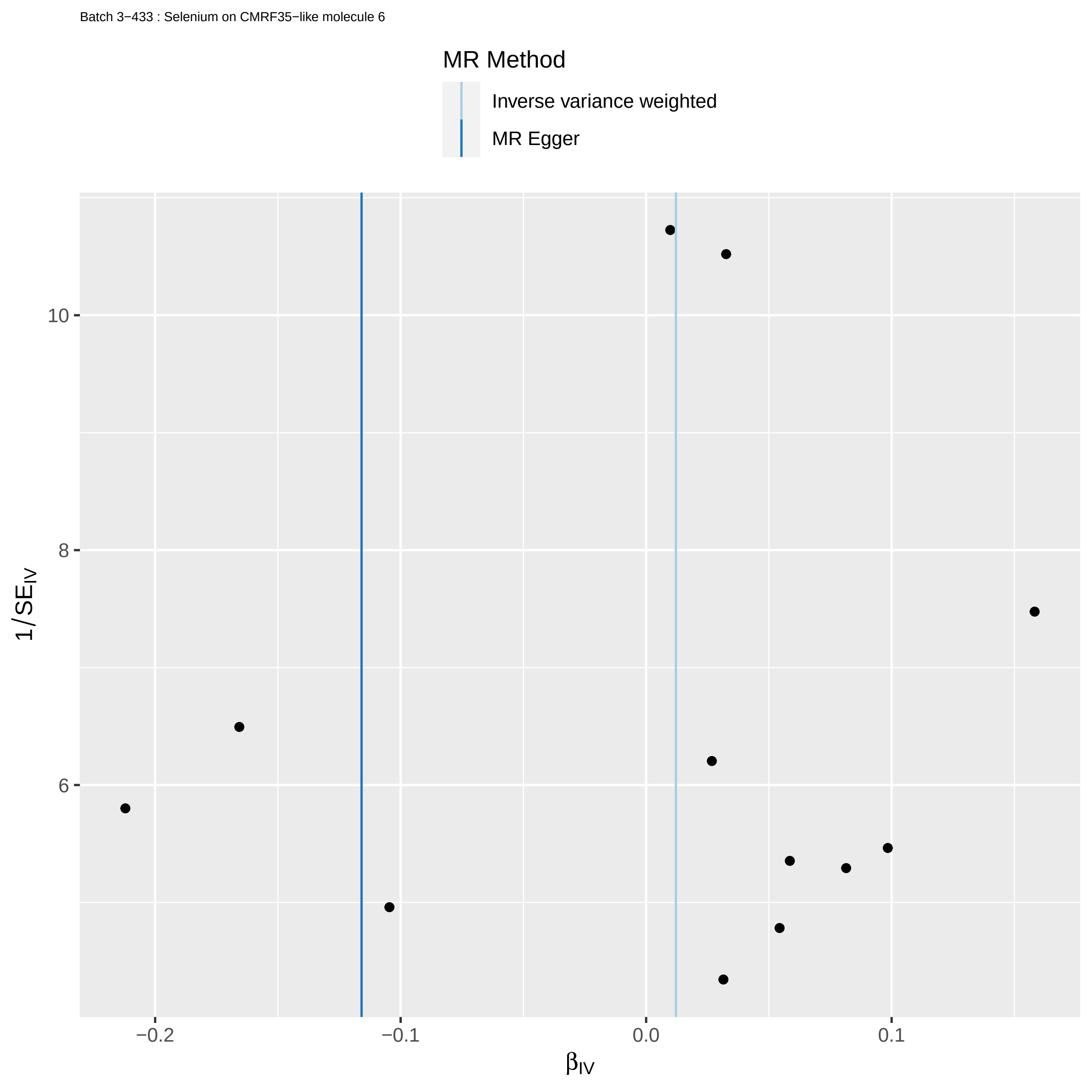
Batch 3−433 : Selenium on CMRF35−like molecule 6
MR Method
Inverse variance weighted
MR Egger
10
8
6
1/SE_IV
−0.2
−0.1
0.0
0.1
β_IV

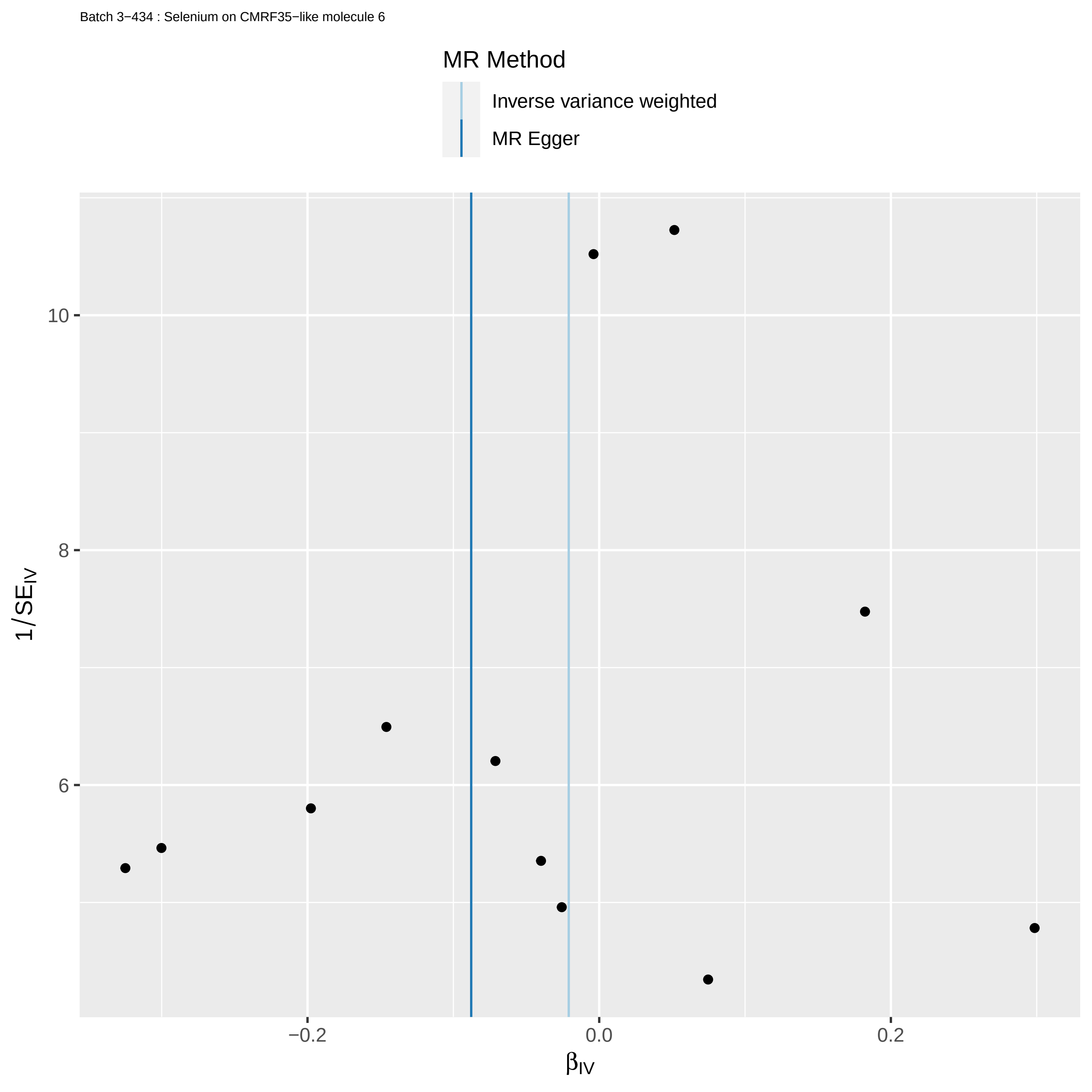
Batch 3−434 : Selenium on CMRF35−like molecule 6
MR Method
Inverse variance weighted
MR Egger
10
8
6
1/SE$_{IV}$
−0.2
0.0
0.2
β_{IV}

Batch 3−435 : Selenium on CMRF35−like molecule 2

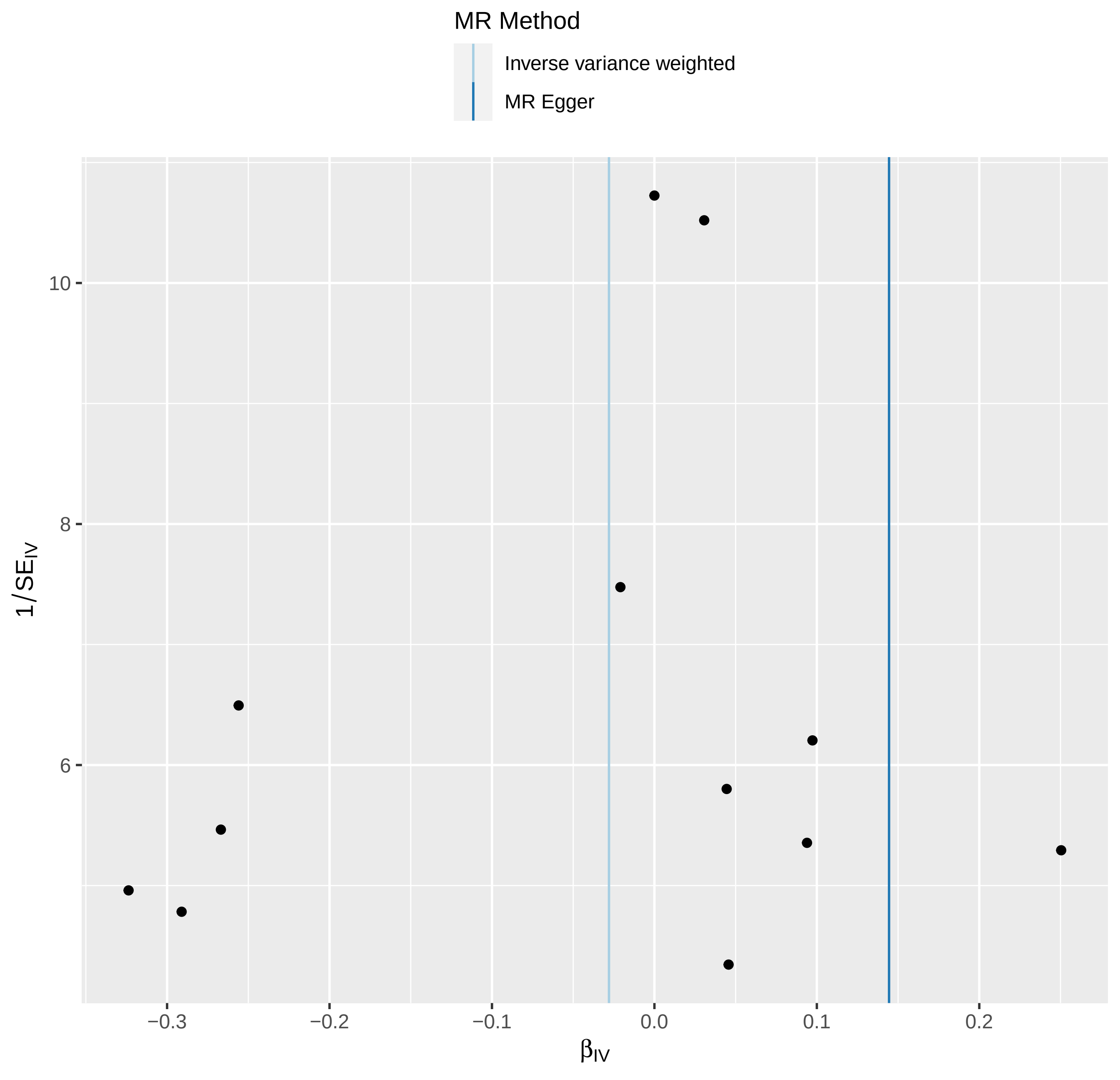

Batch 3−436 : Selenium on CMRF35−like molecule 7

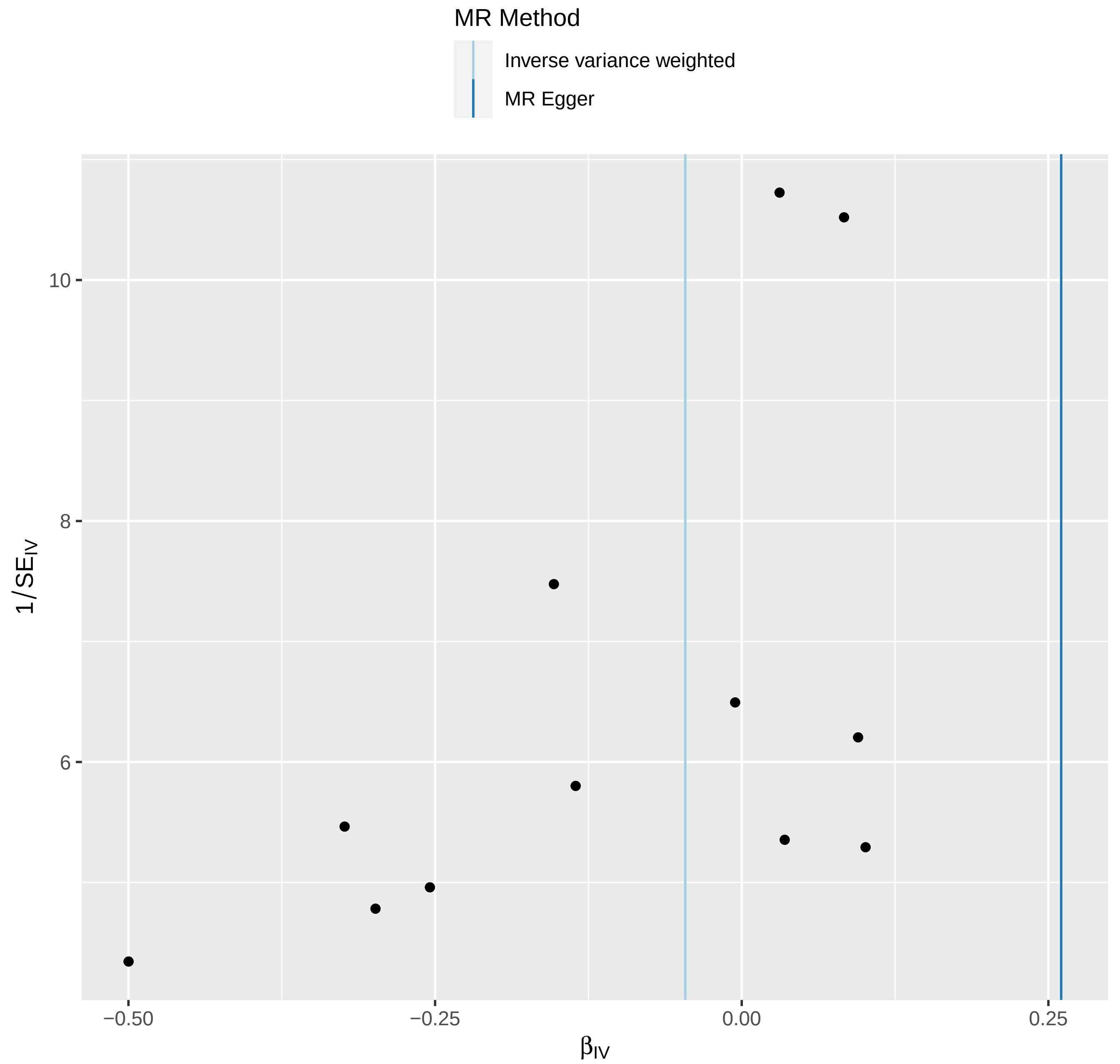

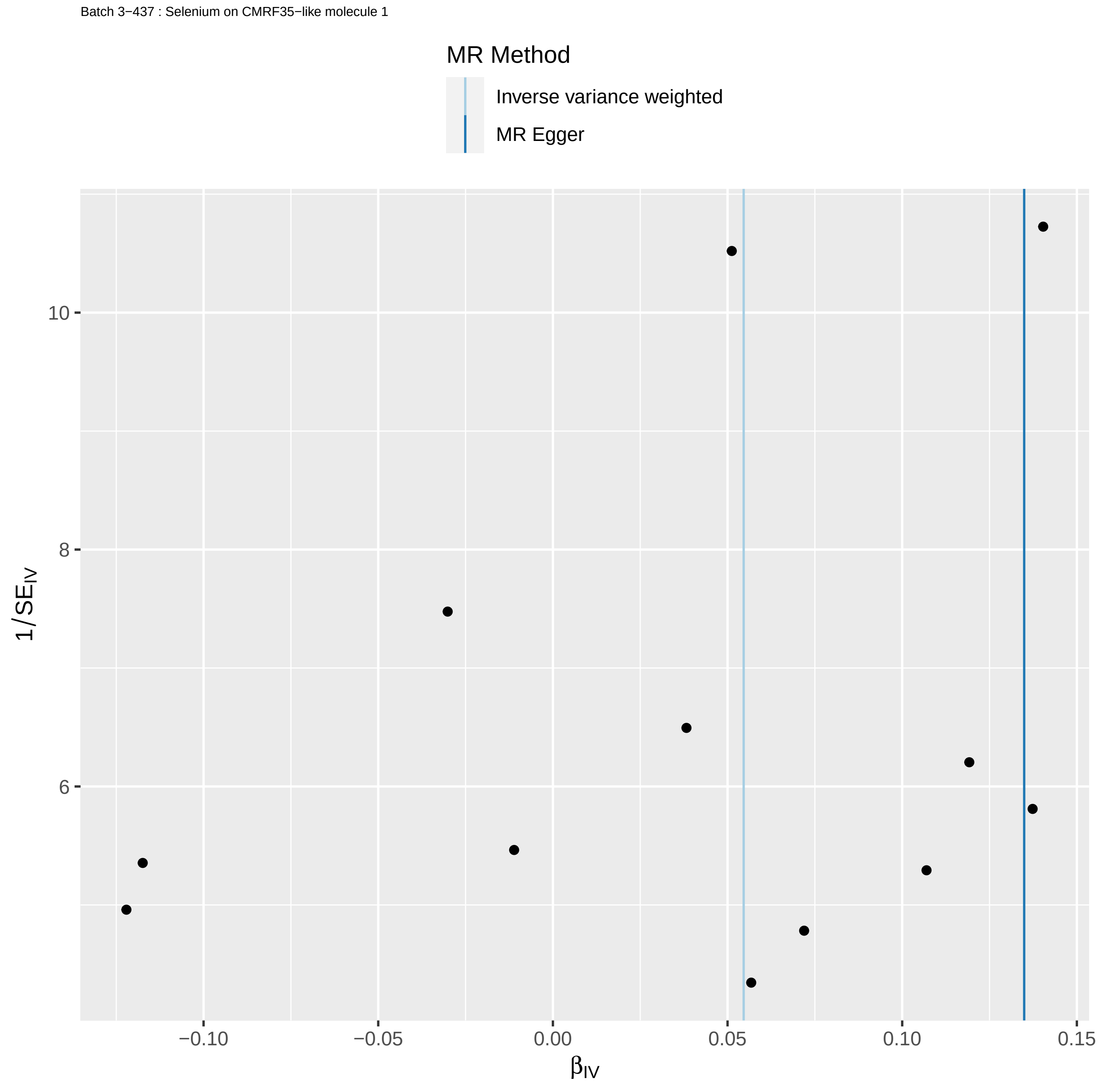
Batch 3−437 : Selenium on CMRF35−like molecule 1
MR Method
Inverse variance weighted
MR Egger
10
8
6
−0.10
−0.05
0.00
0.05
0.10
0.15
1/SE_IV
β_IV

Batch 3−438 : Selenium on CMRF35−like molecule 9

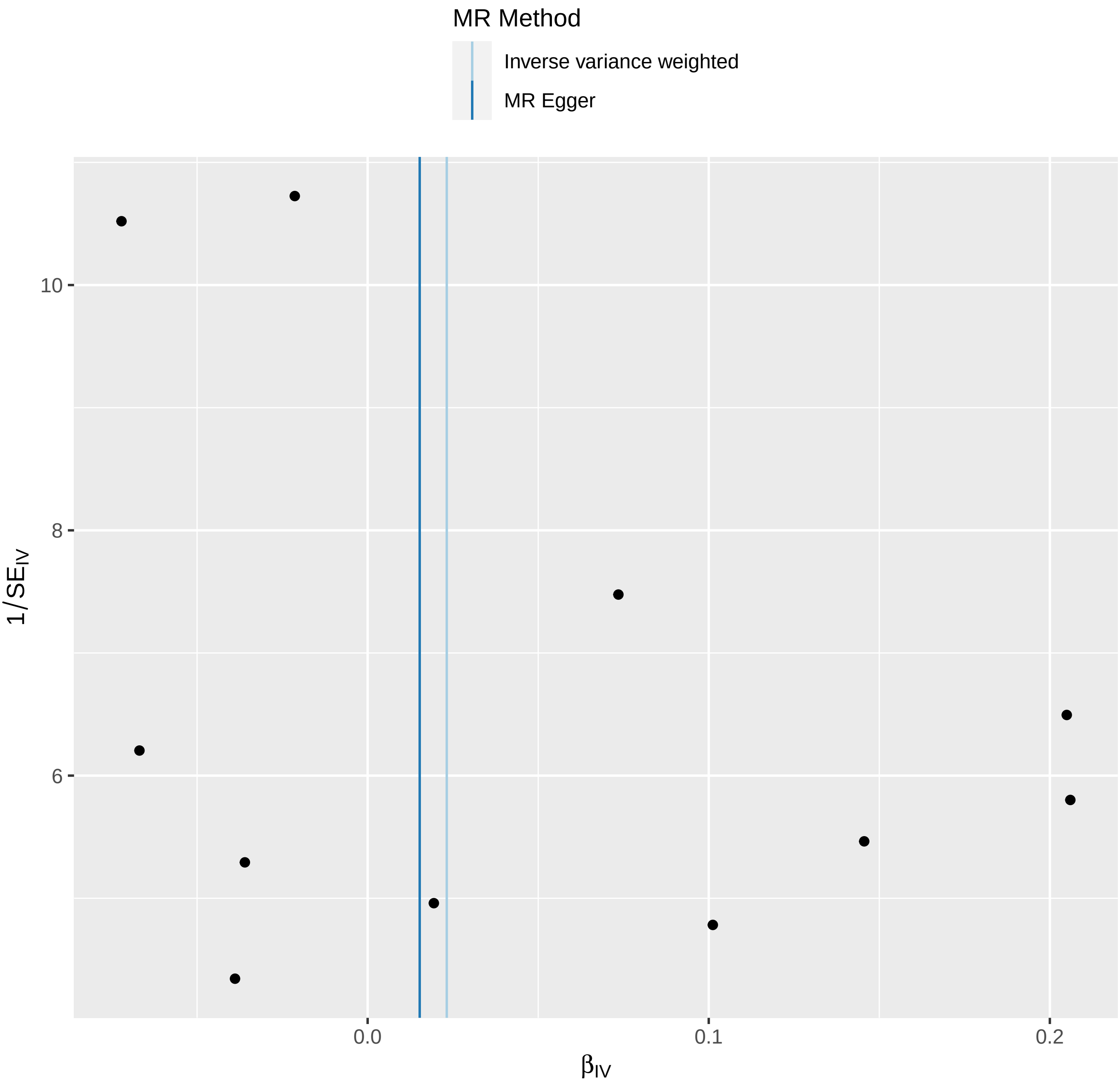

Batch 3−439 : Selenium on Myeloid cell surface antigen CD33

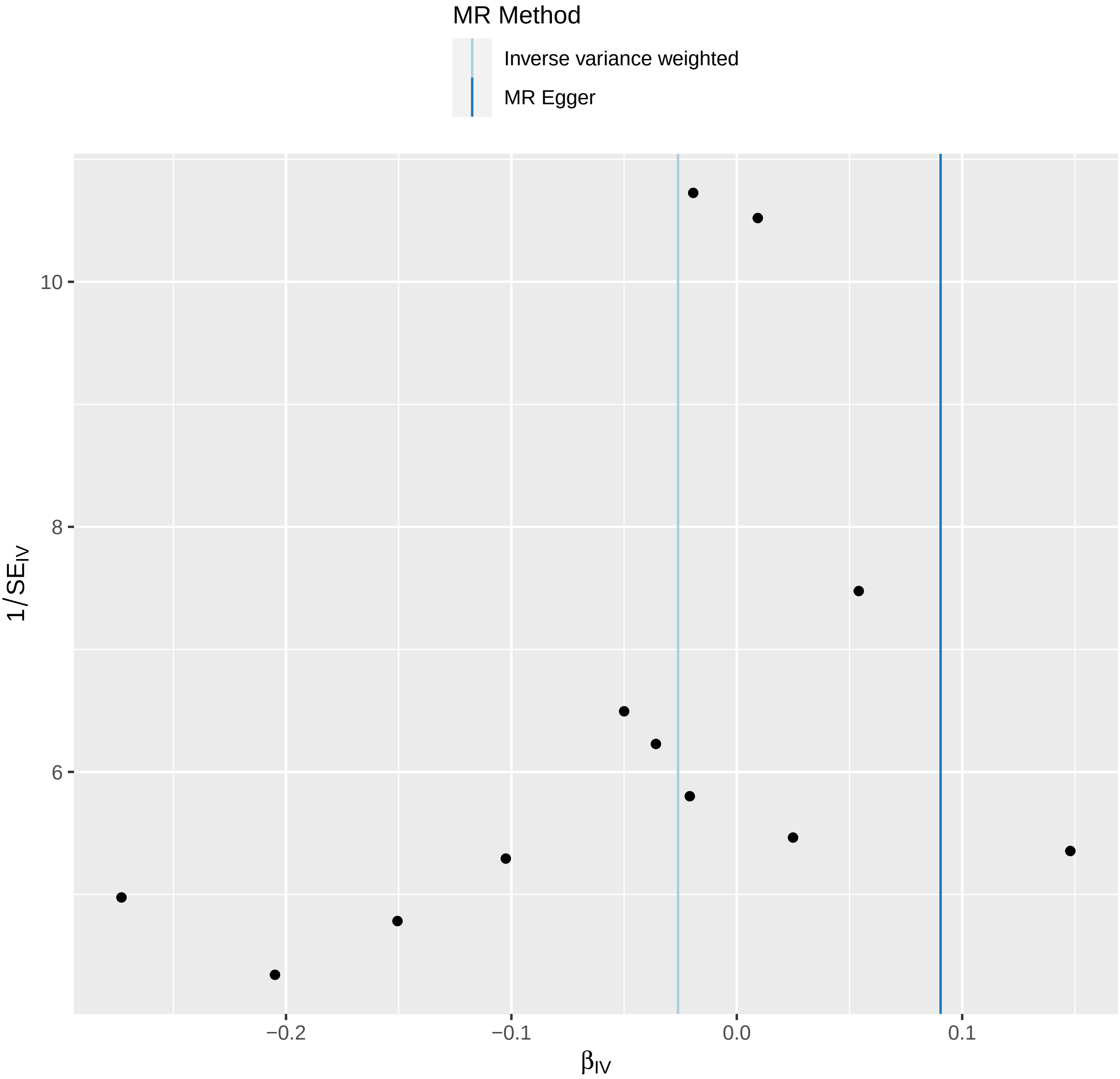

Batch 3−440 : Selenium on Hematopoietic progenitor cell antigen CD34

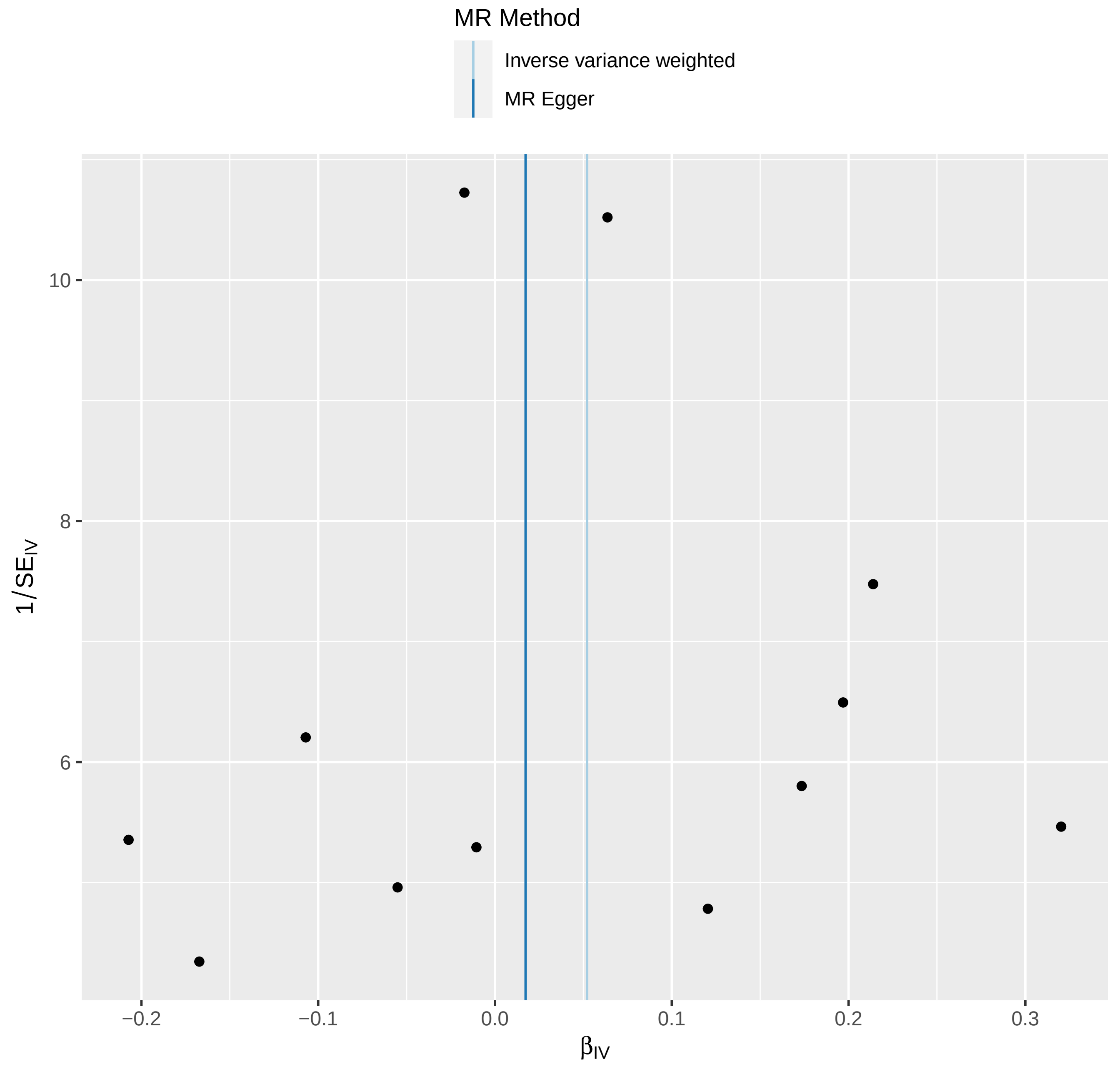

Batch 3−441 : Selenium on Hematopoietic progenitor cell antigen CD34

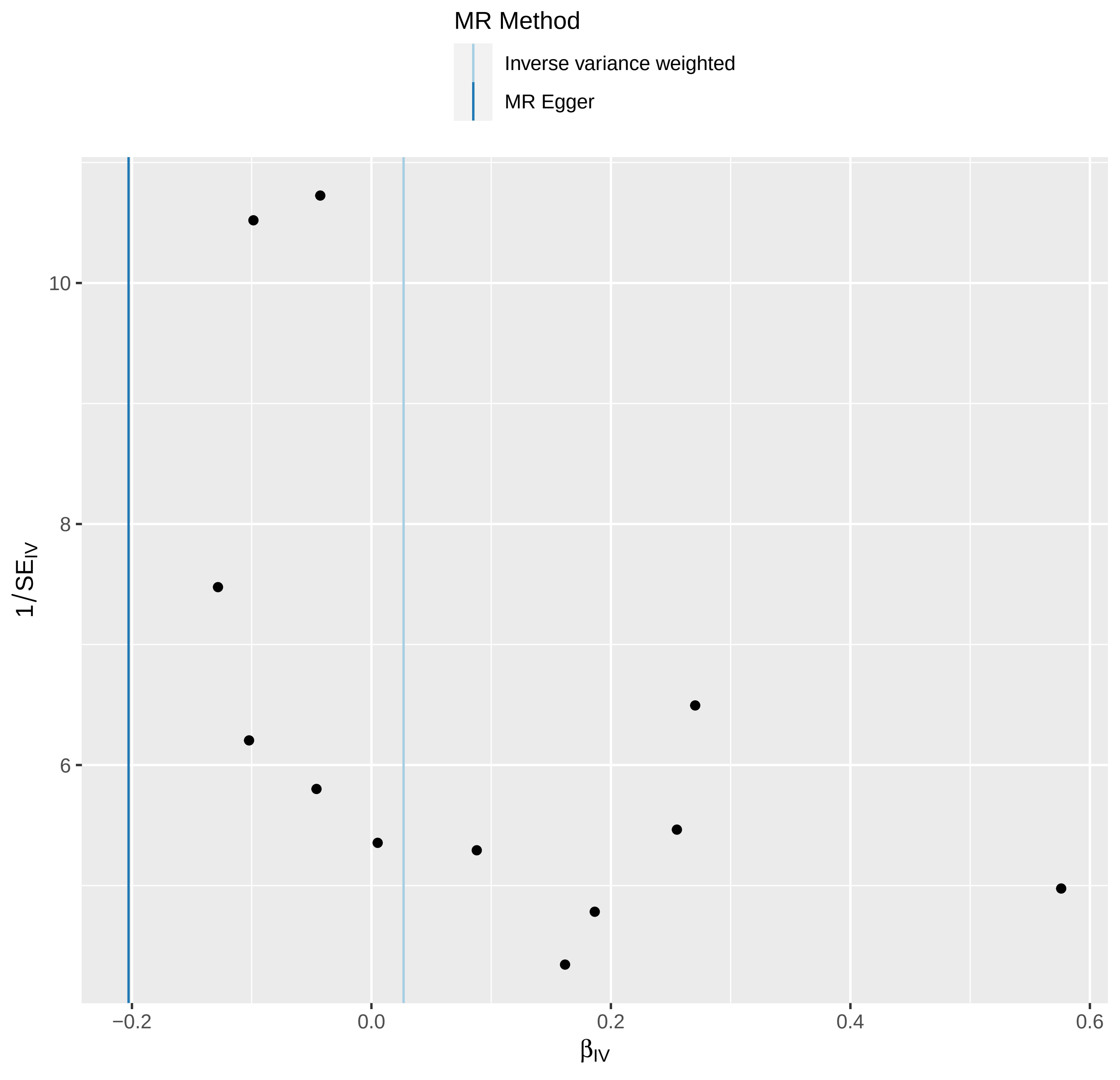

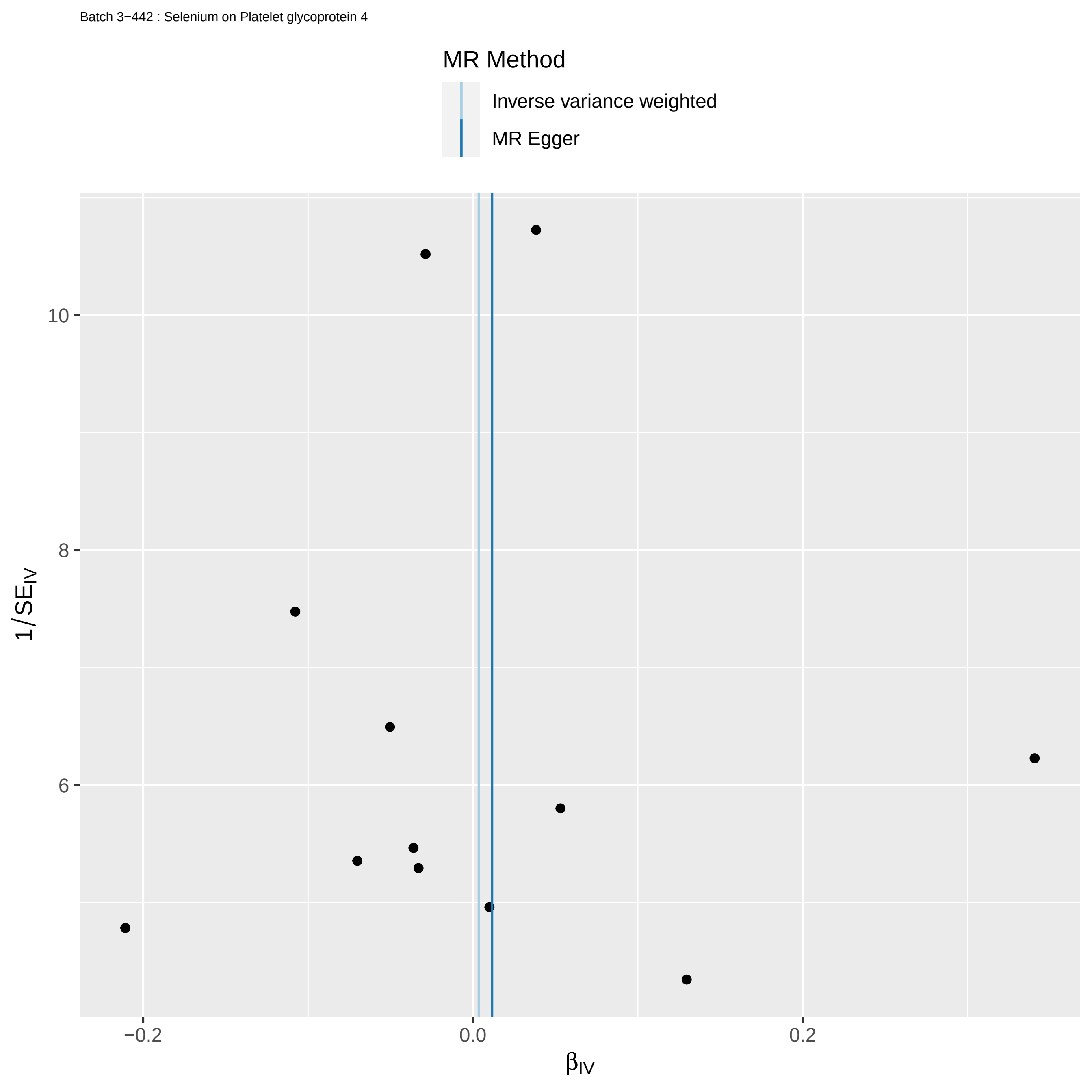

Batch 3−442 : Selenium on Platelet glycoprotein 4
MR Method
Inverse variance weighted
MR Egger
10
8
6
1/SE_IV
−0.2
0.0
0.2
β_IV

Batch 3−443 : Selenium on ADP−ribosyl cyclase/cyclic ADP−ribose hydrolase 1

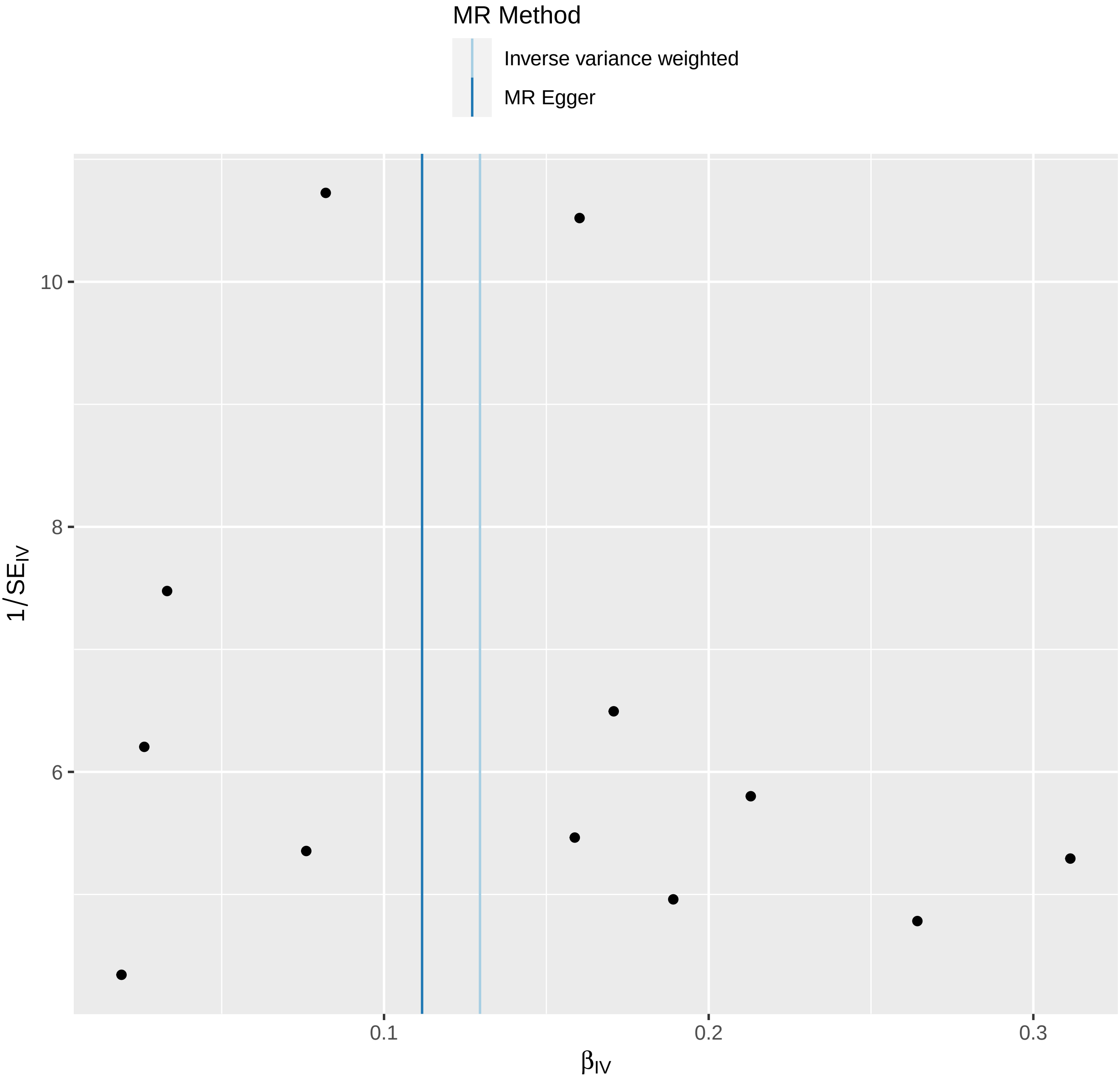

Batch 3−444 : Selenium on ADP−ribosyl cyclase/cyclic ADP−ribose hydrolase 1

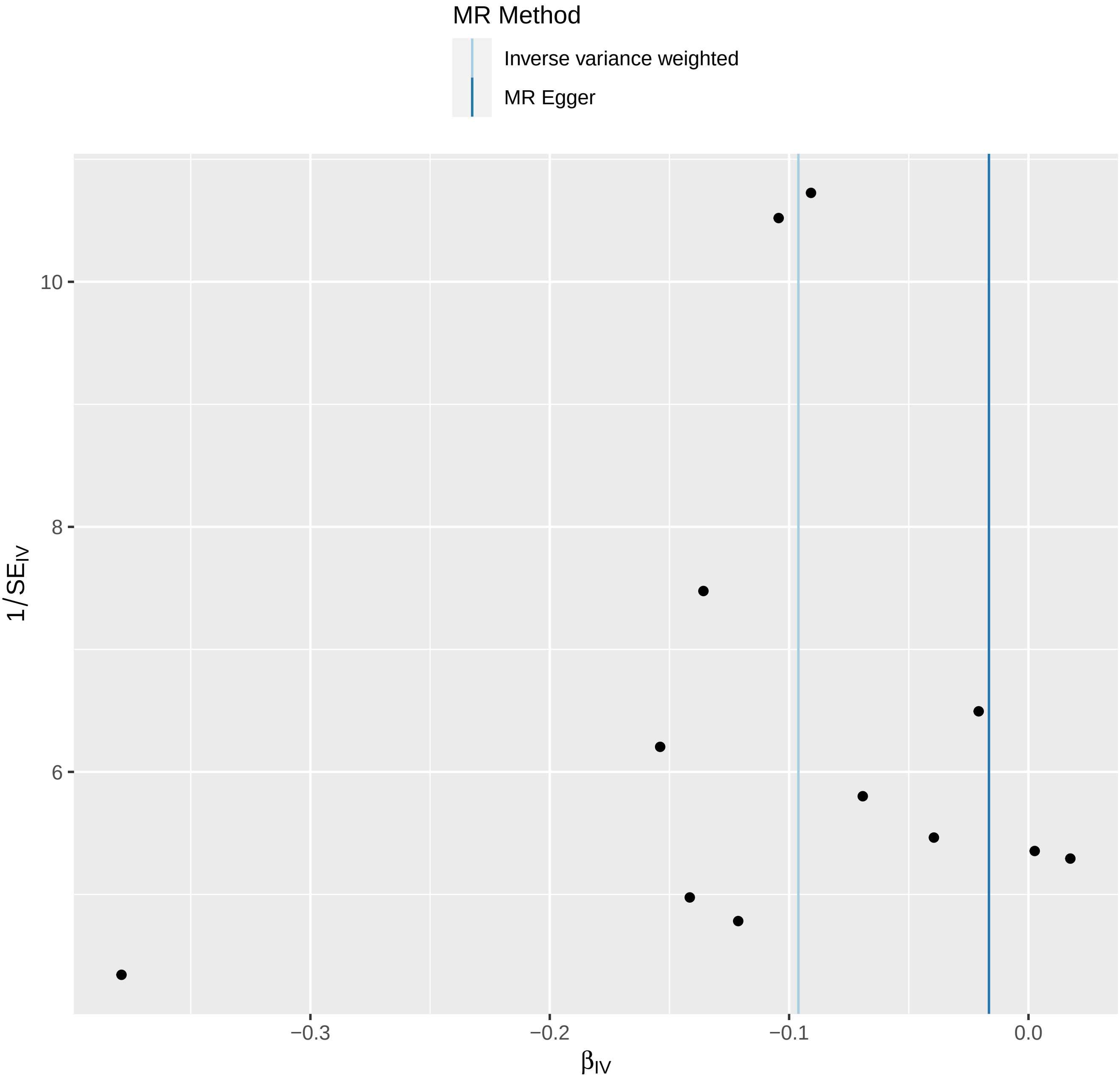

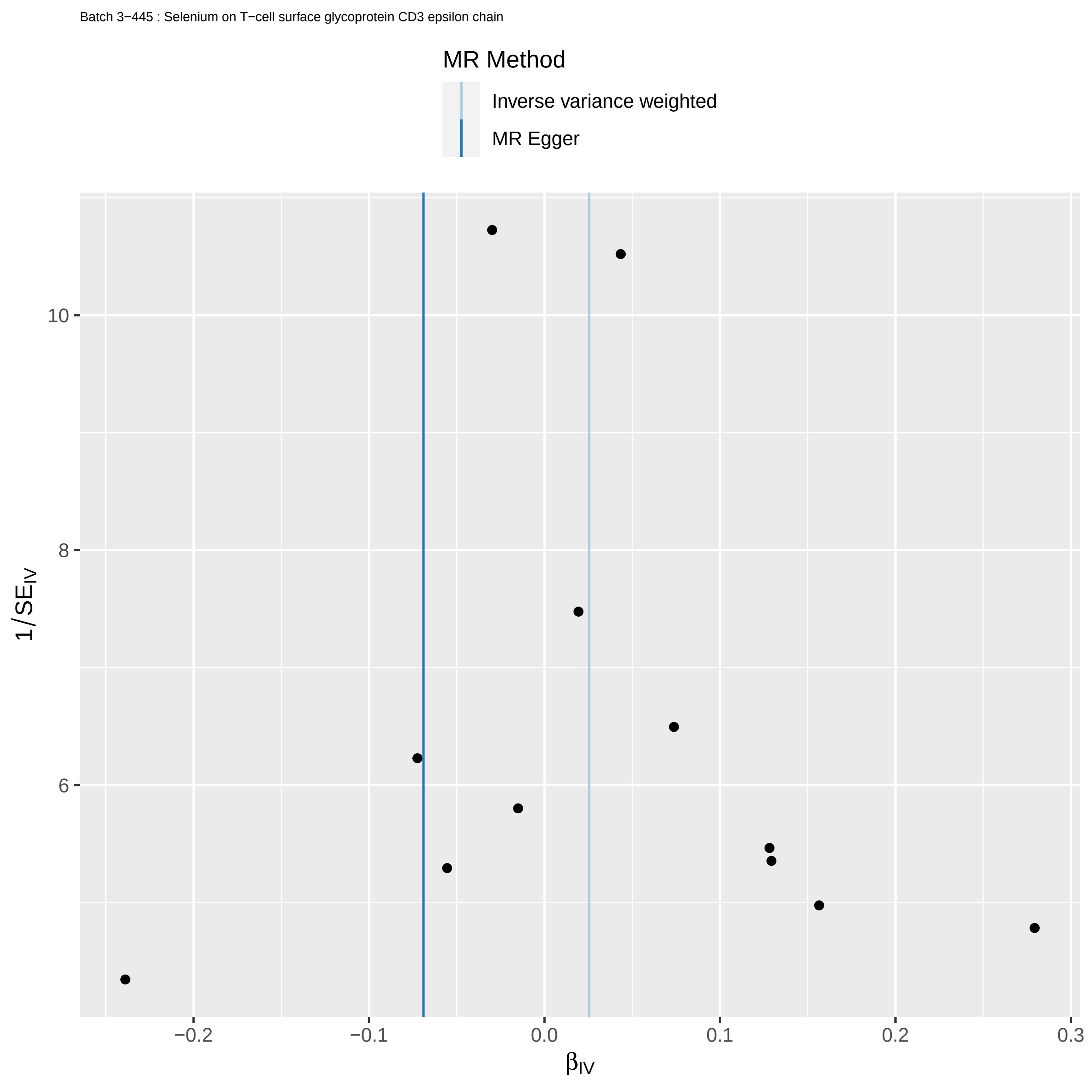

Batch 3−445 : Selenium on T−cell surface glycoprotein CD3 epsilon chain
MR Method
Inverse variance weighted
MR Egger
10
8
6
1/SE_IV
−0.2
−0.1
0.0
0.1
0.2
0.3
β_IV

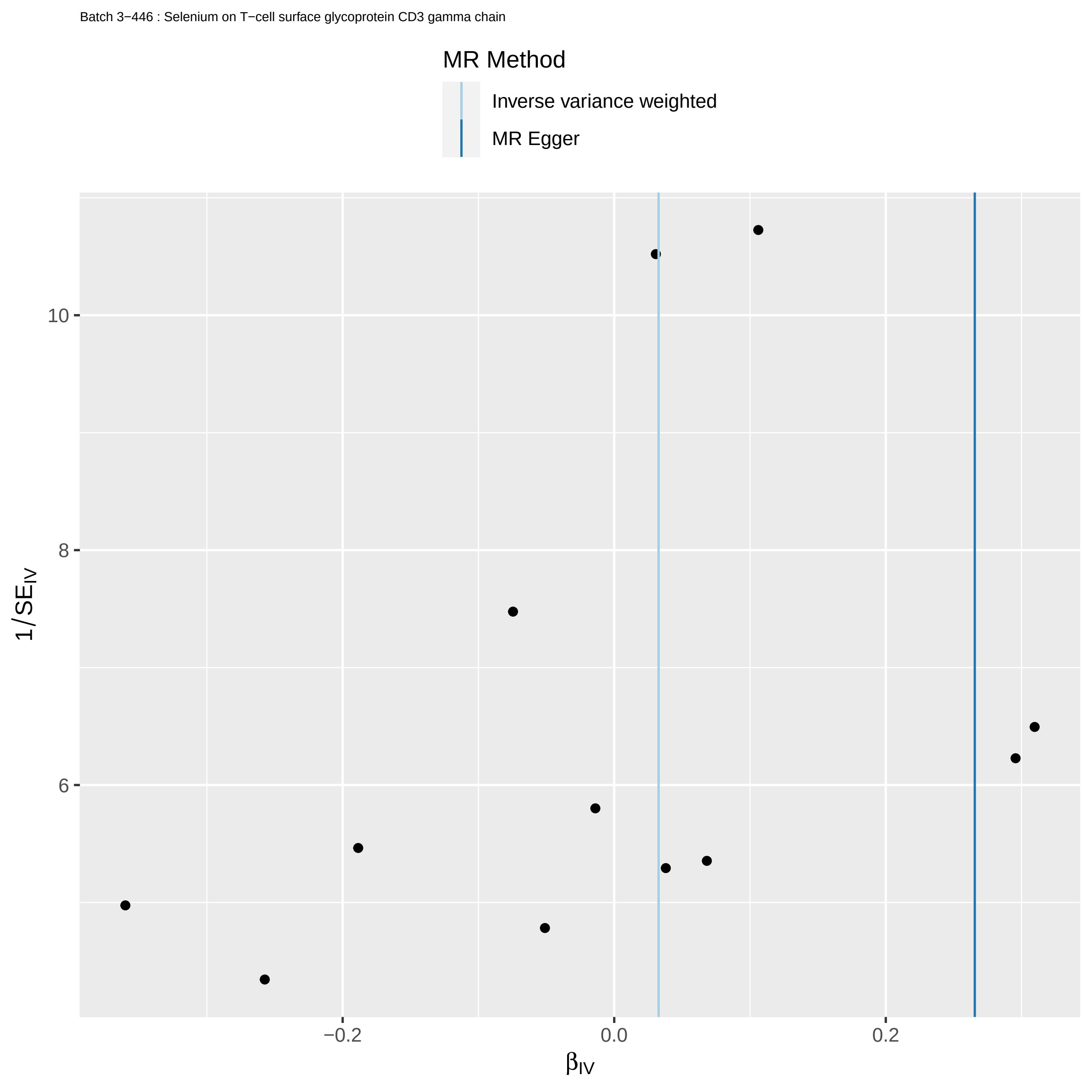
Batch 3−446 : Selenium on T−cell surface glycoprotein CD3 gamma chain
MR Method
Inverse variance weighted
MR Egger
10
8
6
1/SE_IV
−0.2
0.0
0.2
β_IV

Batch 3−447 : Selenium on CD40 ligand

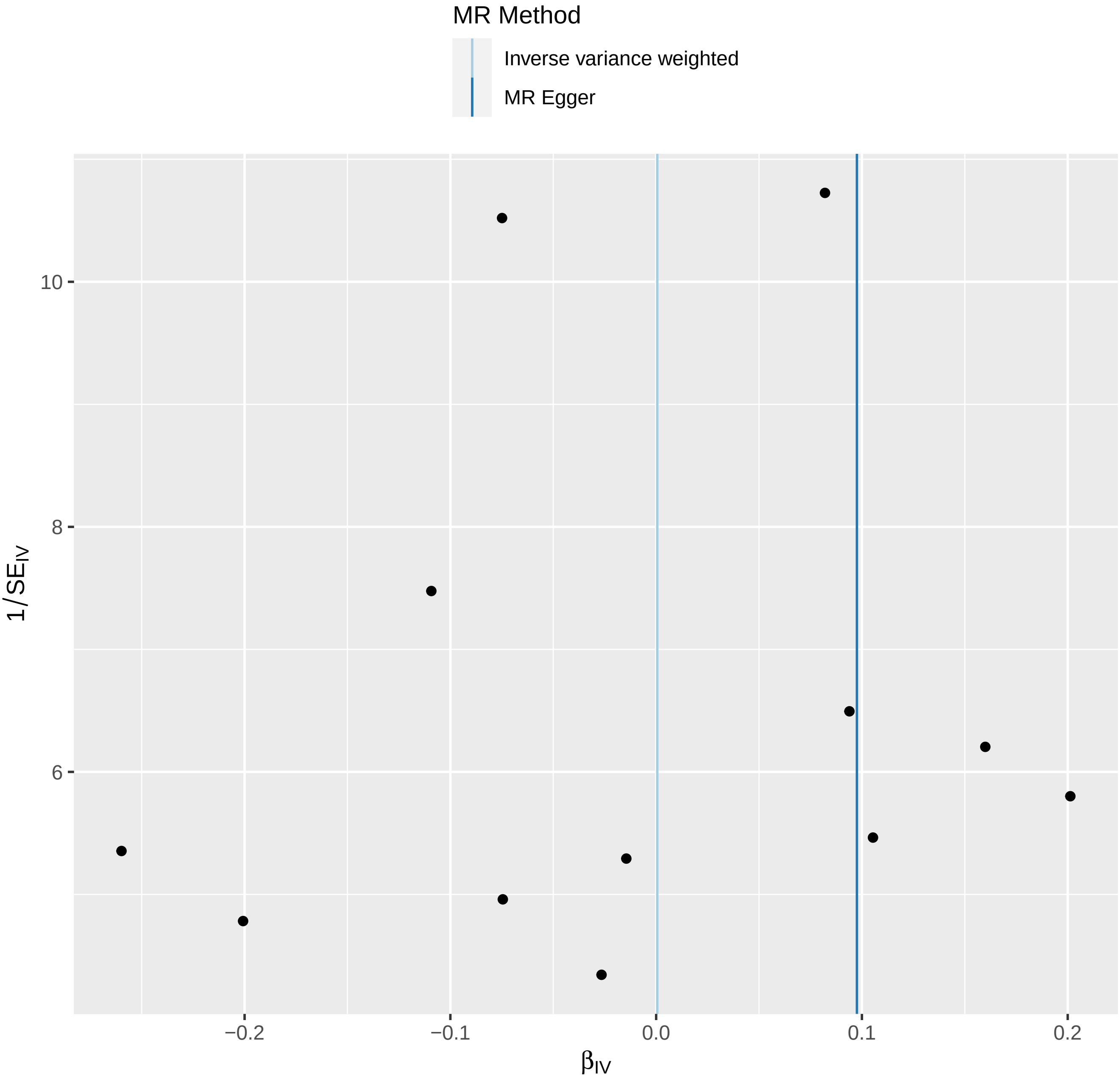

Batch 3−448 : Selenium on T−cell surface glycoprotein CD4

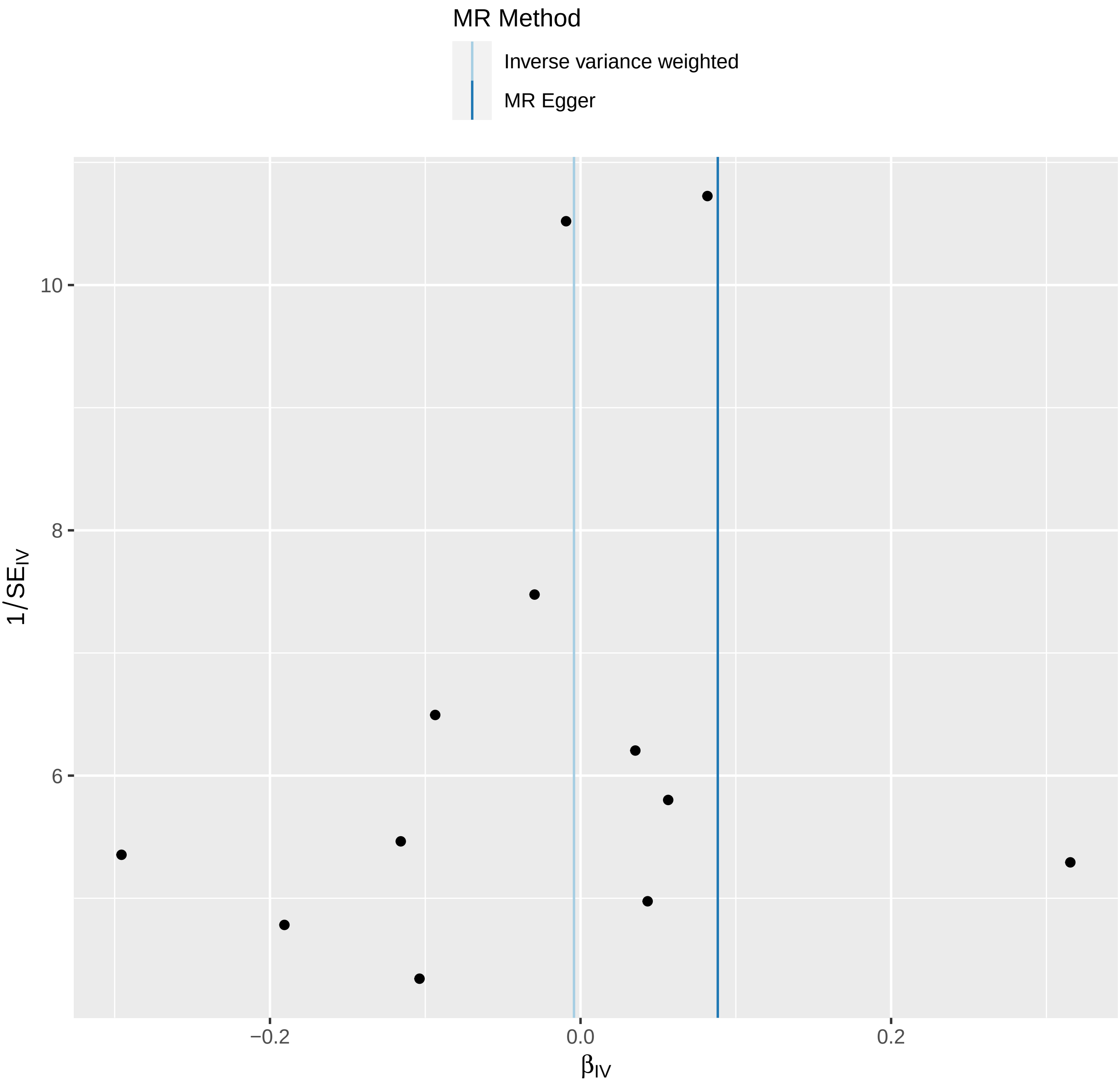

Batch 3−449 : Selenium on CD44 antigen

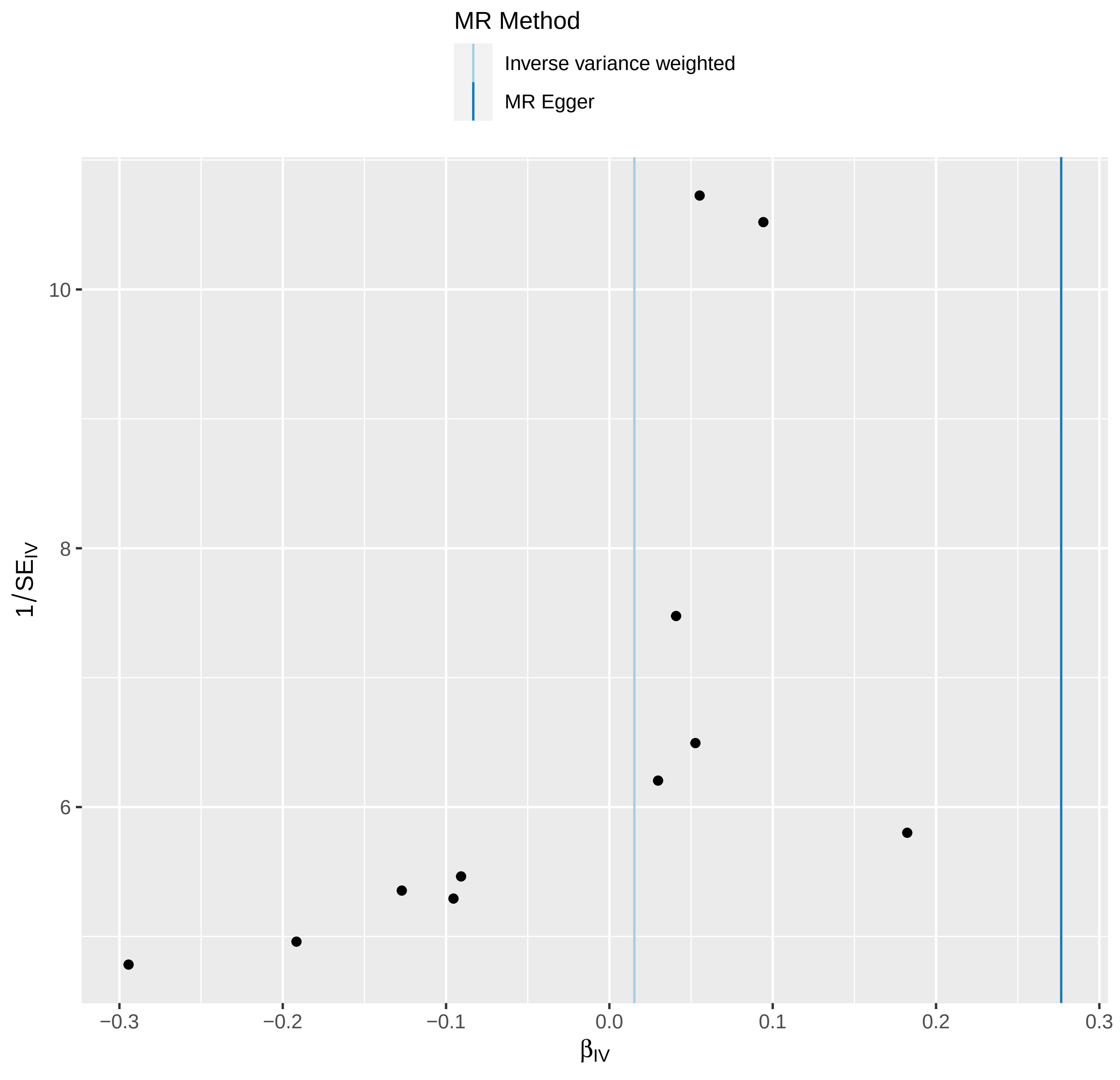

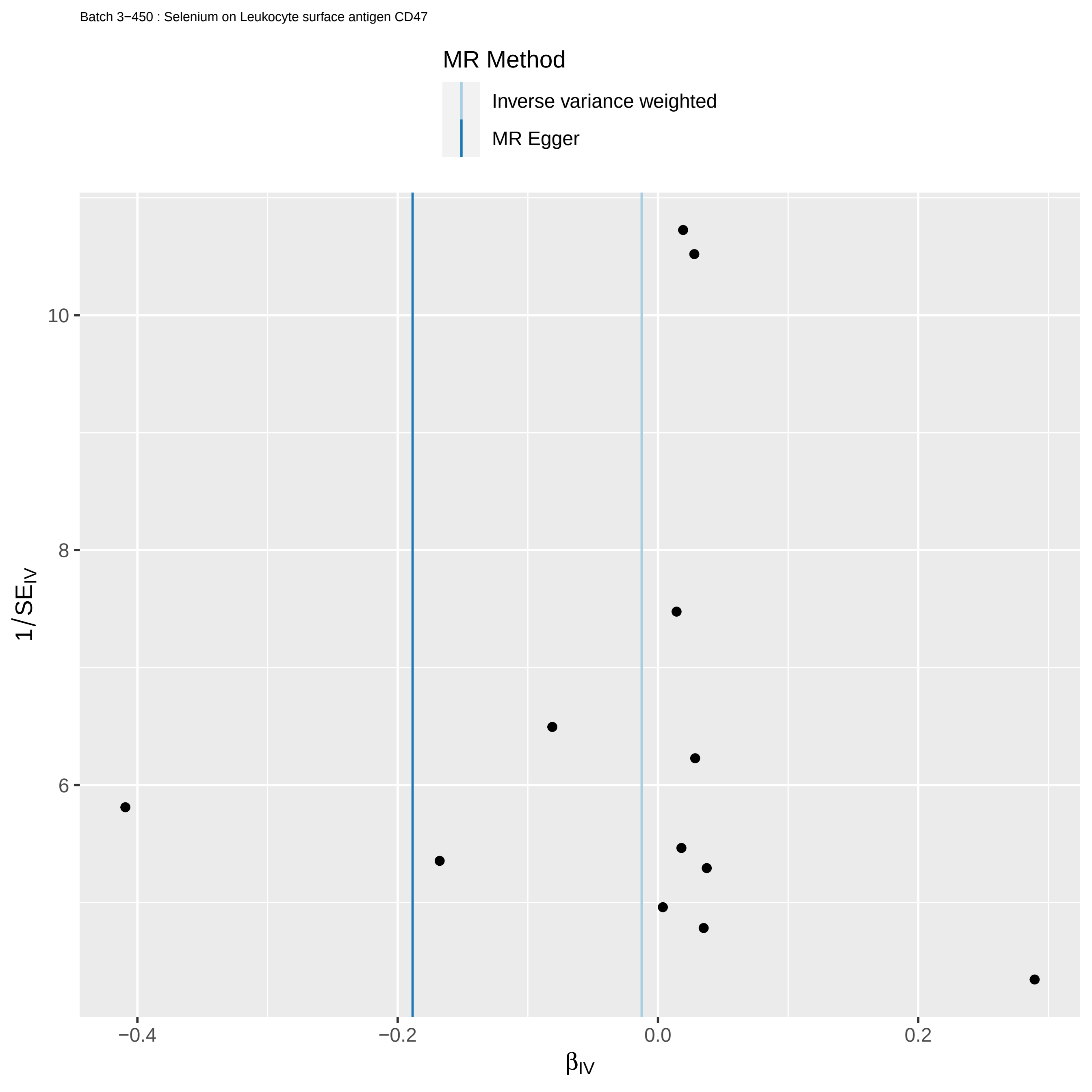

Batch 3−450 : Selenium on Leukocyte surface antigen CD47
MR Method
Inverse variance weighted
MR Egger
10
8
6
−0.4
−0.2
0.0
0.2
$1/SE_{IV}$
β_{IV}

Batch 3−451 : Selenium on CD48 antigen

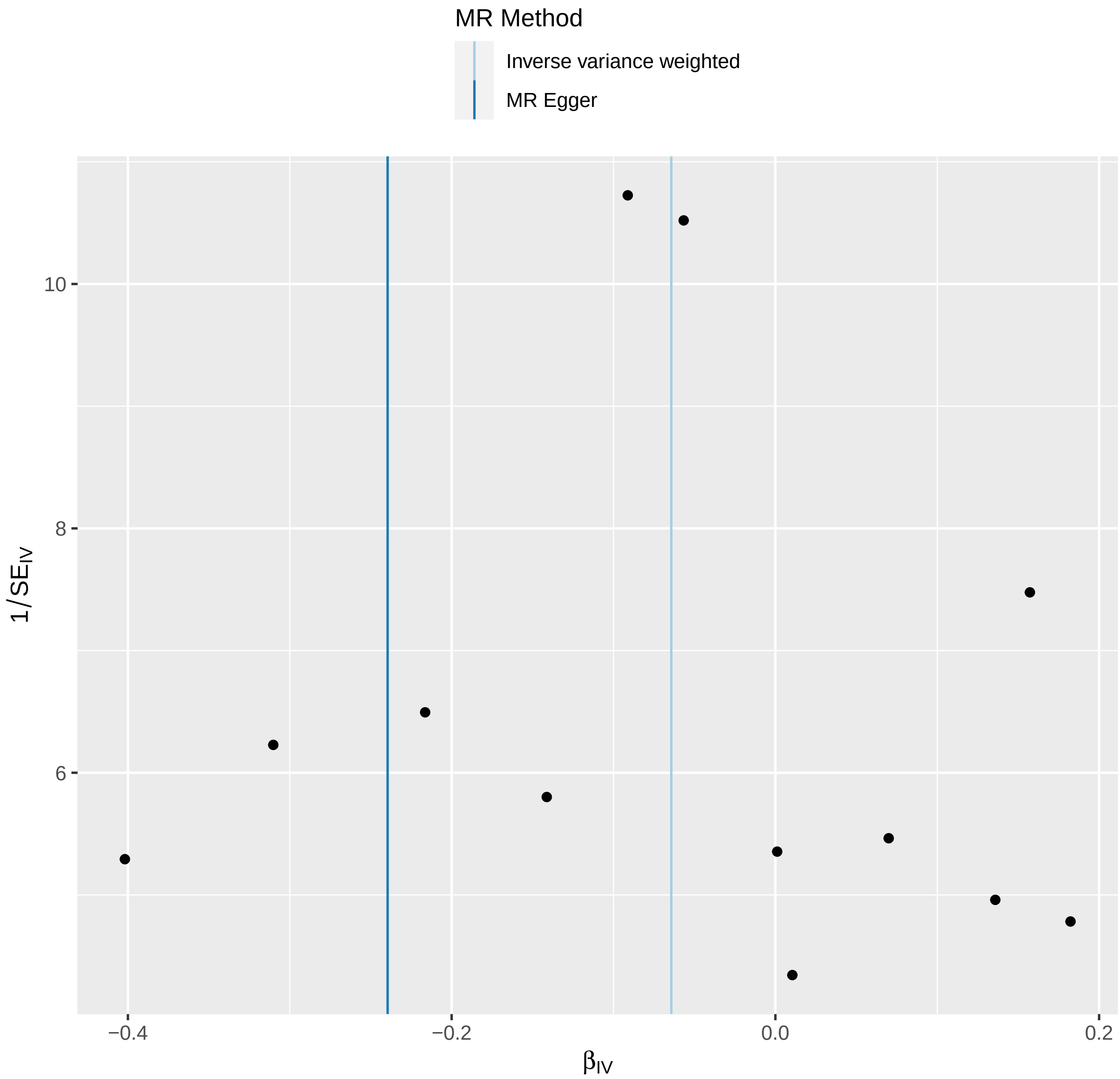

Batch 3−452 : Selenium on T−cell surface glycoprotein CD5

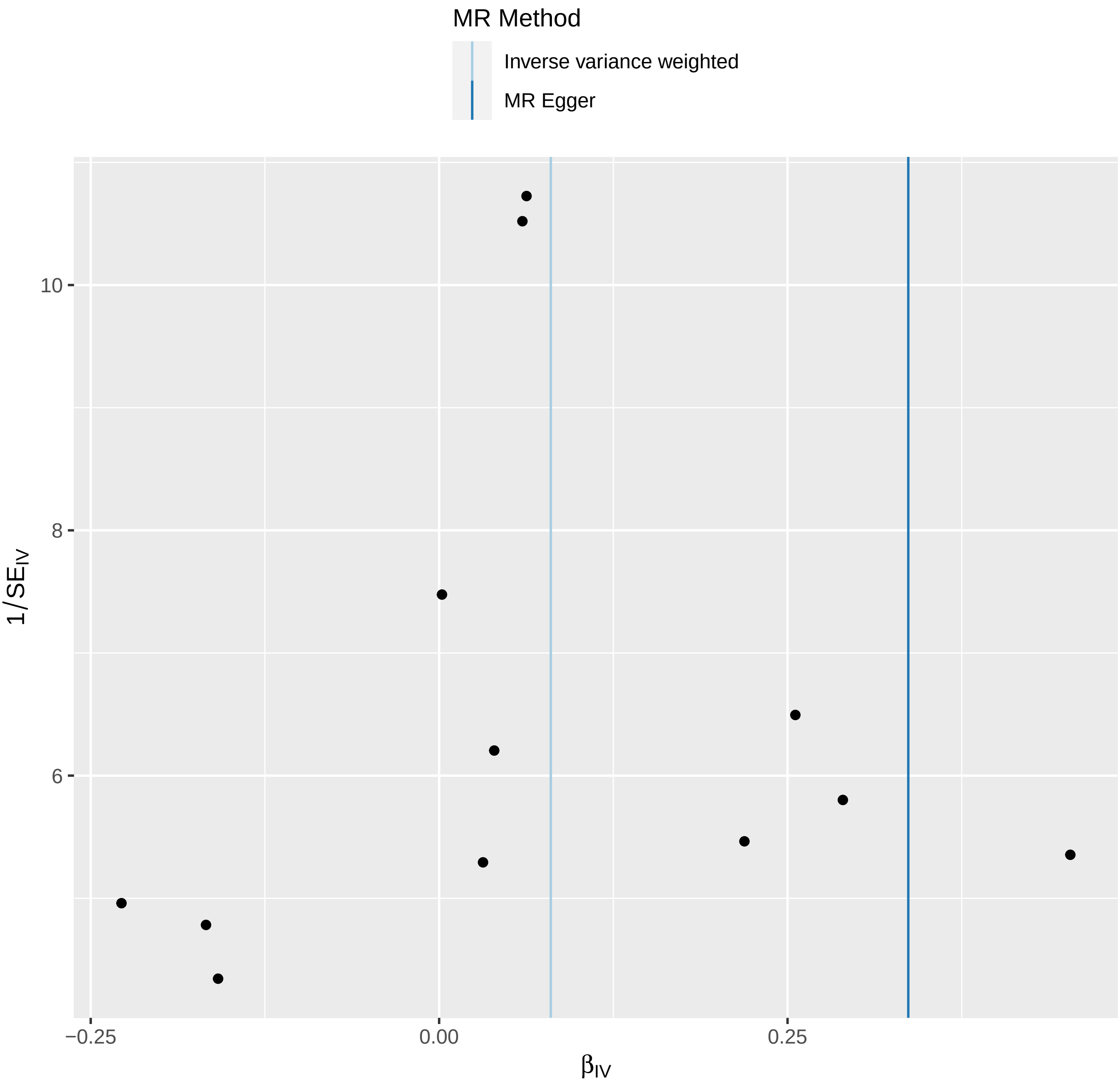

Batch 3−453 : Selenium on Complement decay−accelerating factor

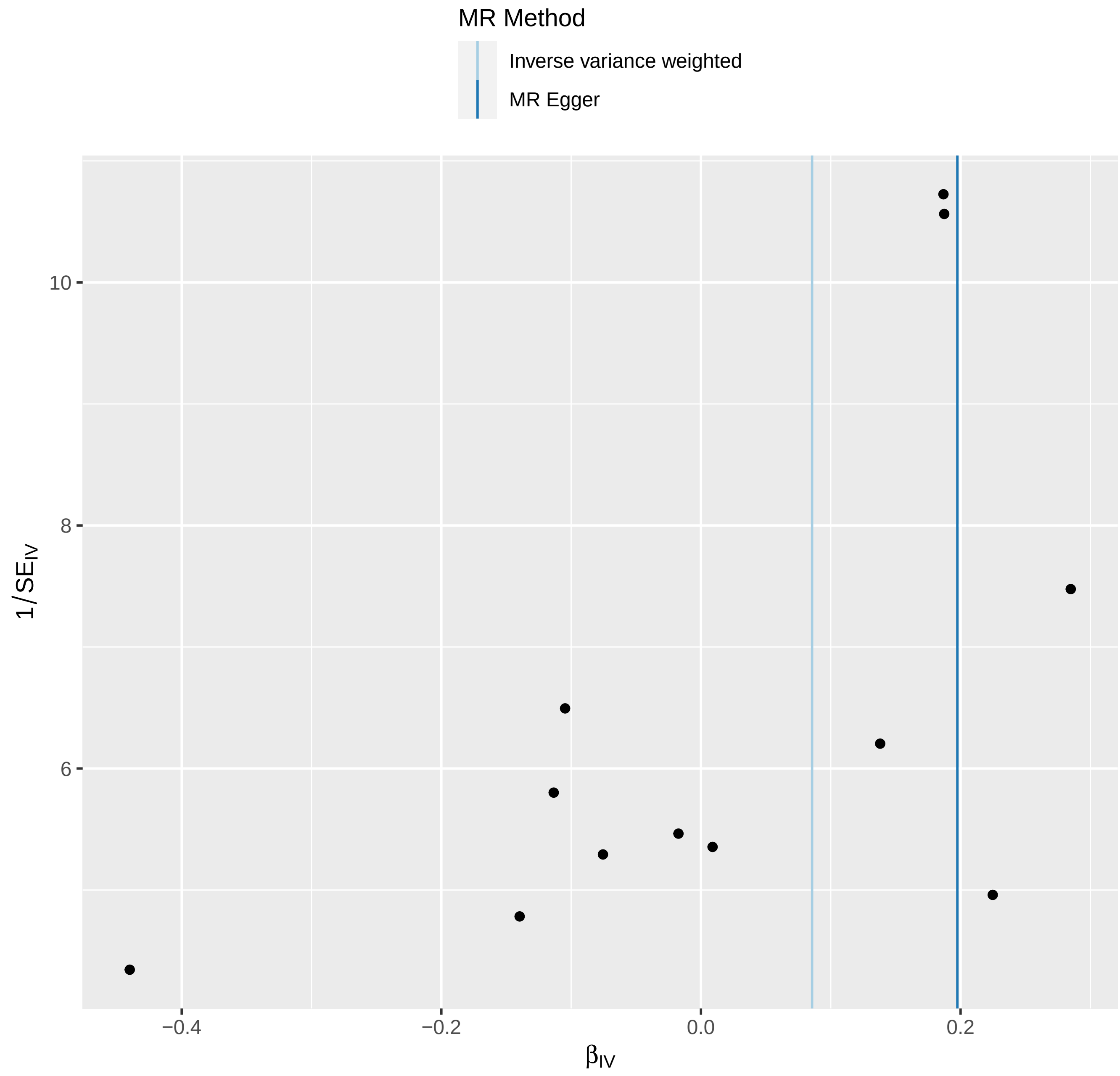

Batch 3−454 : Selenium on Lymphocyte function−associated antigen 3

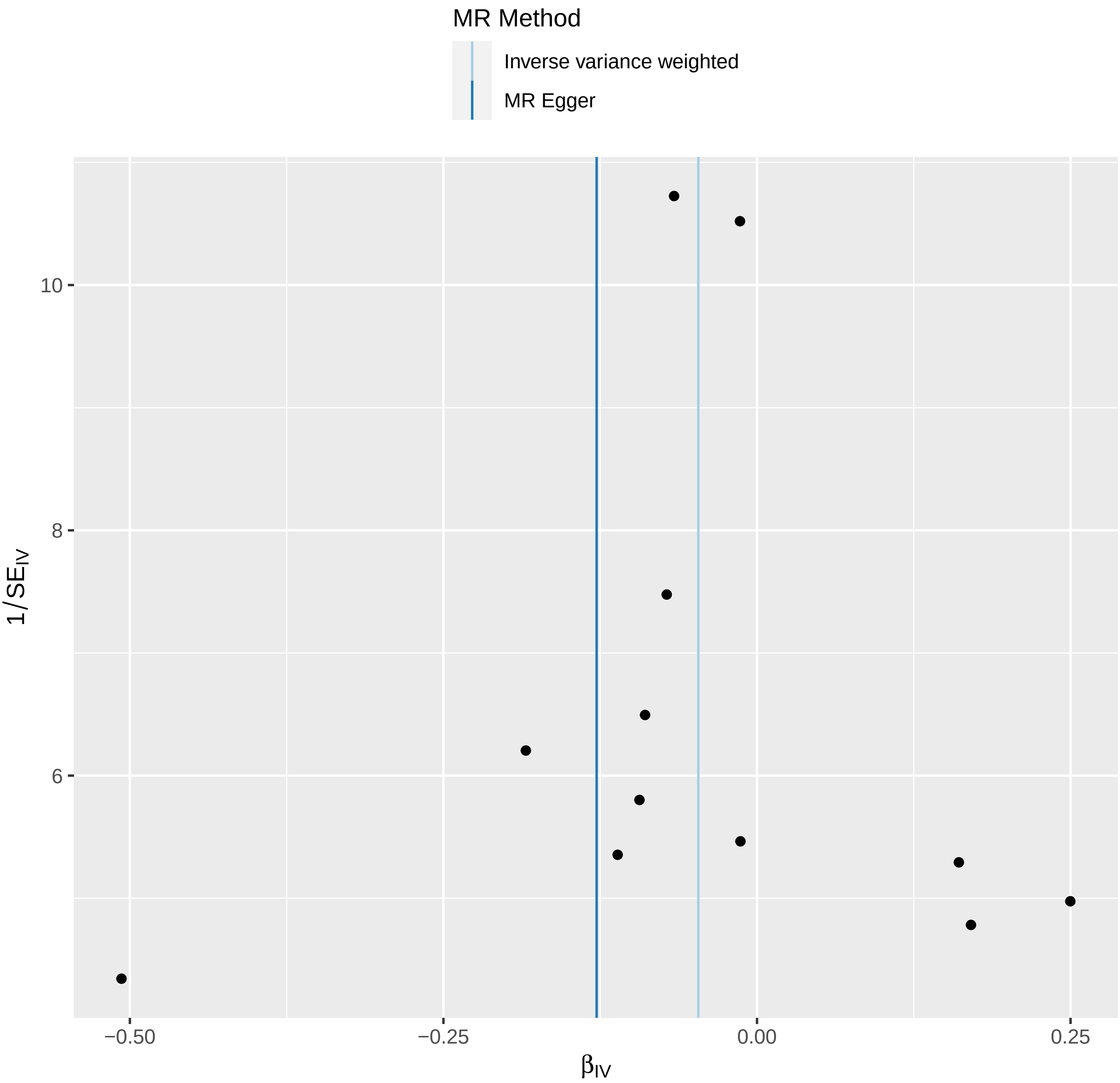

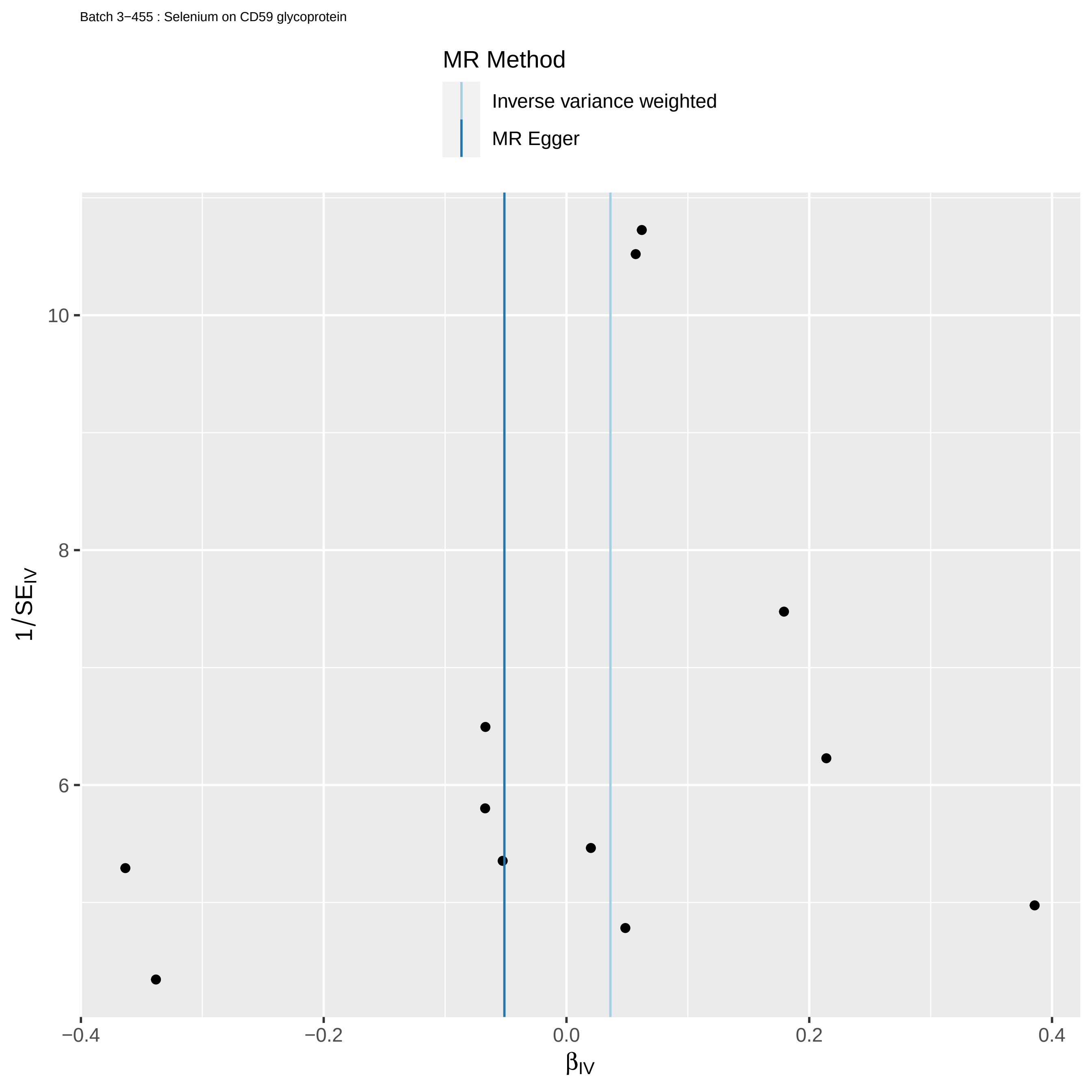
Batch 3−455 : Selenium on CD59 glycoprotein
MR Method
Inverse variance weighted
MR Egger
10
8
6
1/SE_IV
−0.4
−0.2
0.0
0.2
0.4
β_IV

Batch 3−456 : Selenium on CD63 antigen

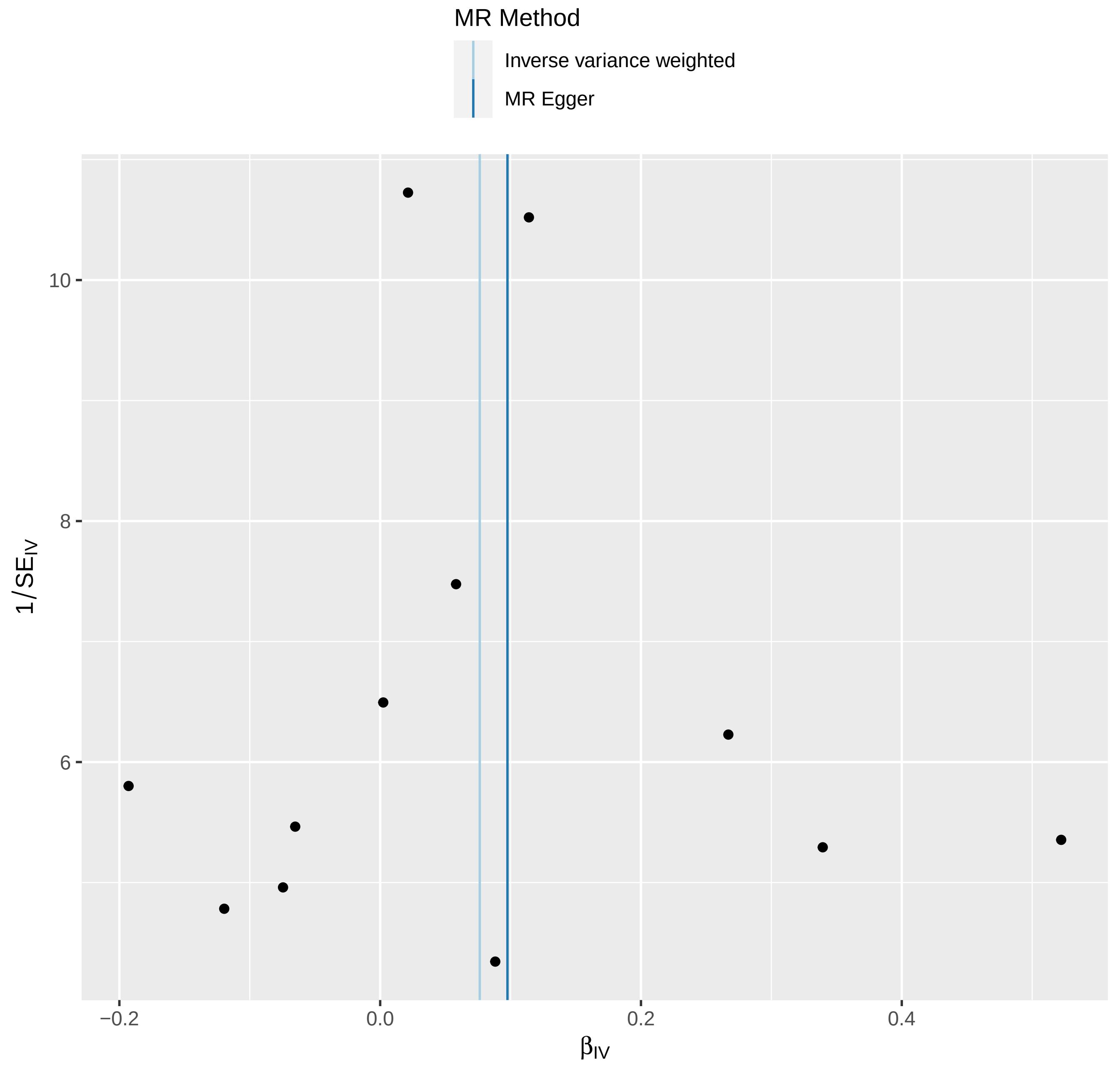

Batch 3−457 : Selenium on CD70 antigen

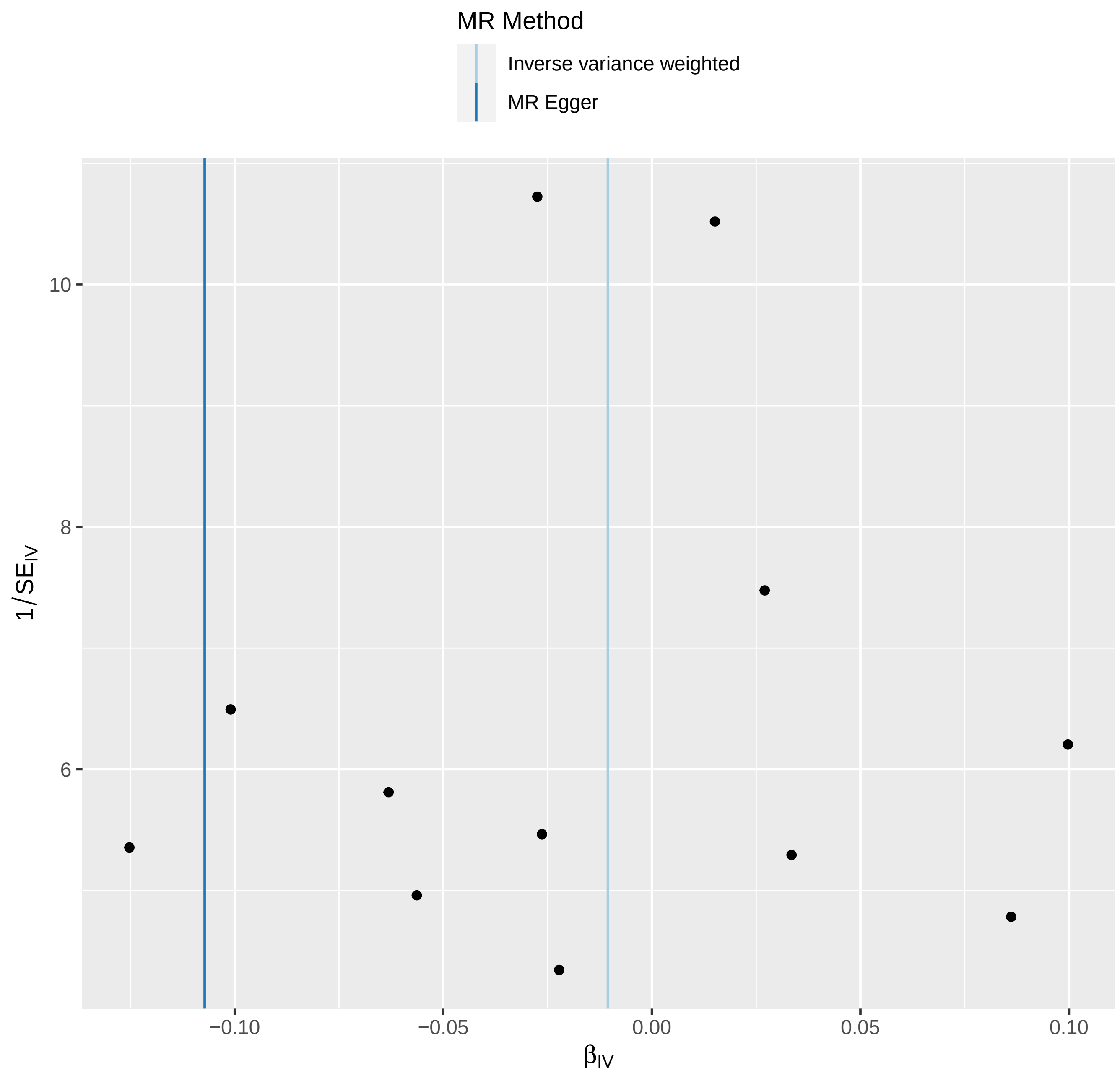

Batch 3−458 : Selenium on CD70 antigen

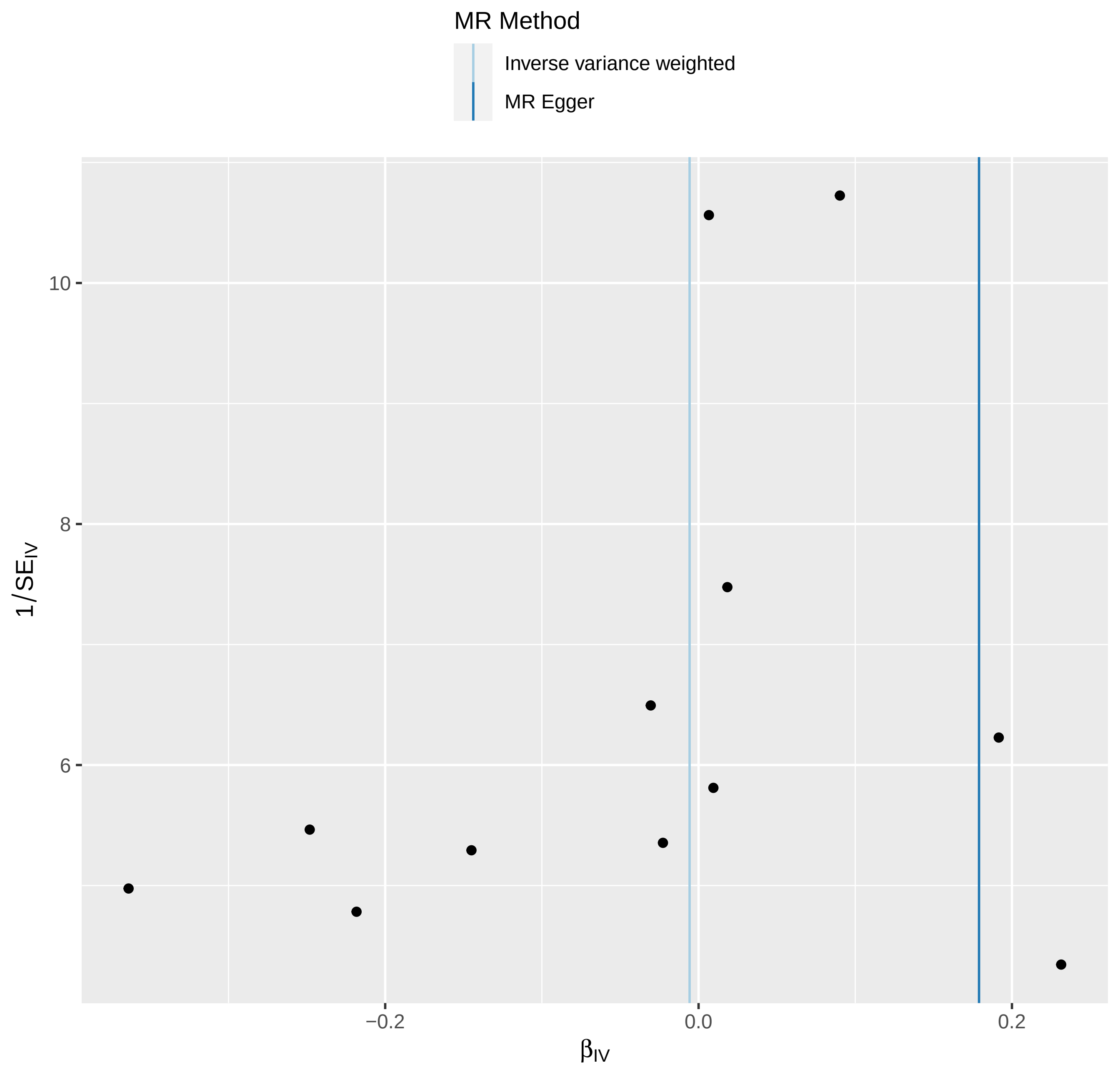

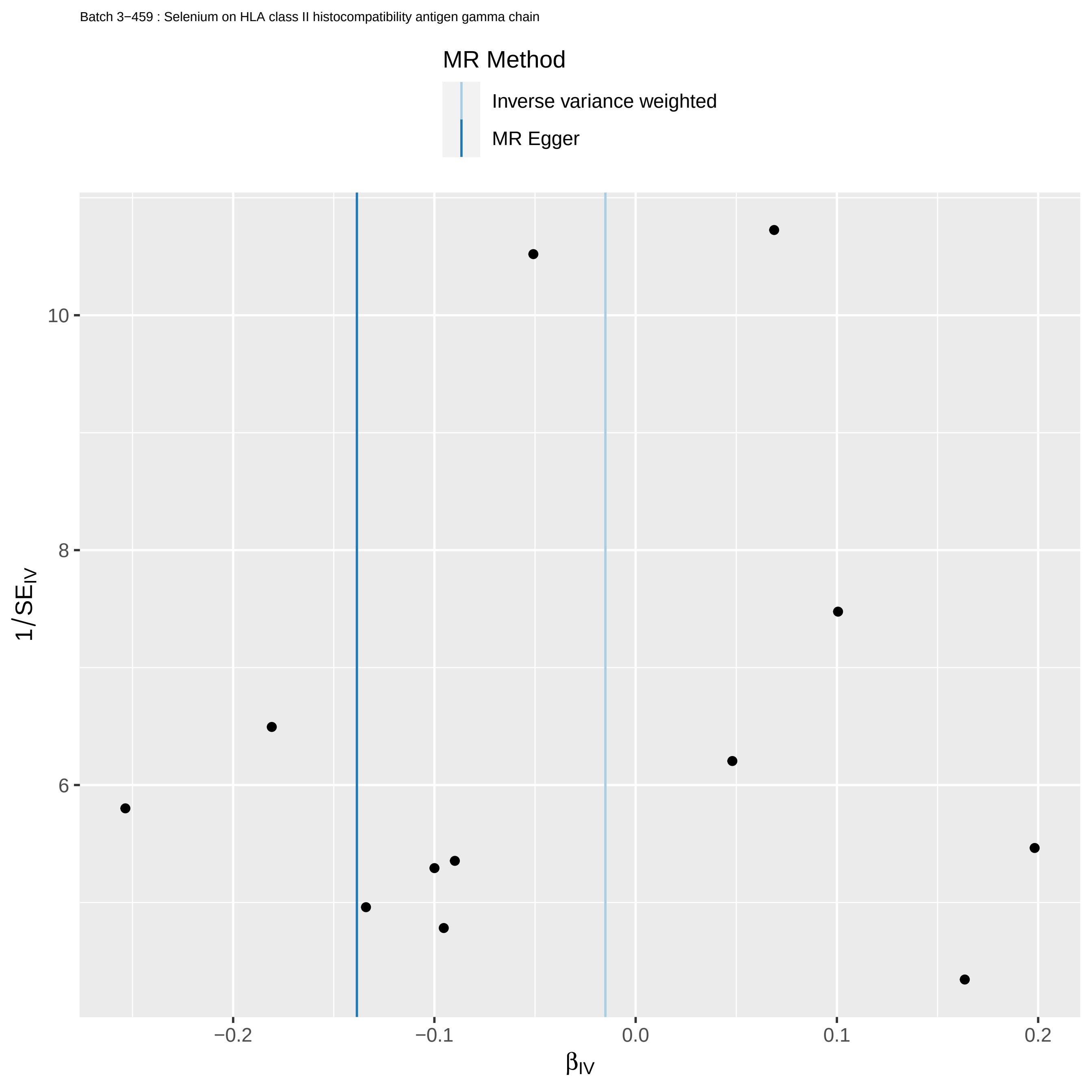

Batch 3−459 : Selenium on HLA class II histocompatibility antigen gamma chain
MR Method
Inverse variance weighted
MR Egger
10
8
6
1/SE_IV
−0.2
−0.1
0.0
0.1
0.2
β_IV

Batch 3−460 : Selenium on HLA class II histocompatibility antigen gamma chain

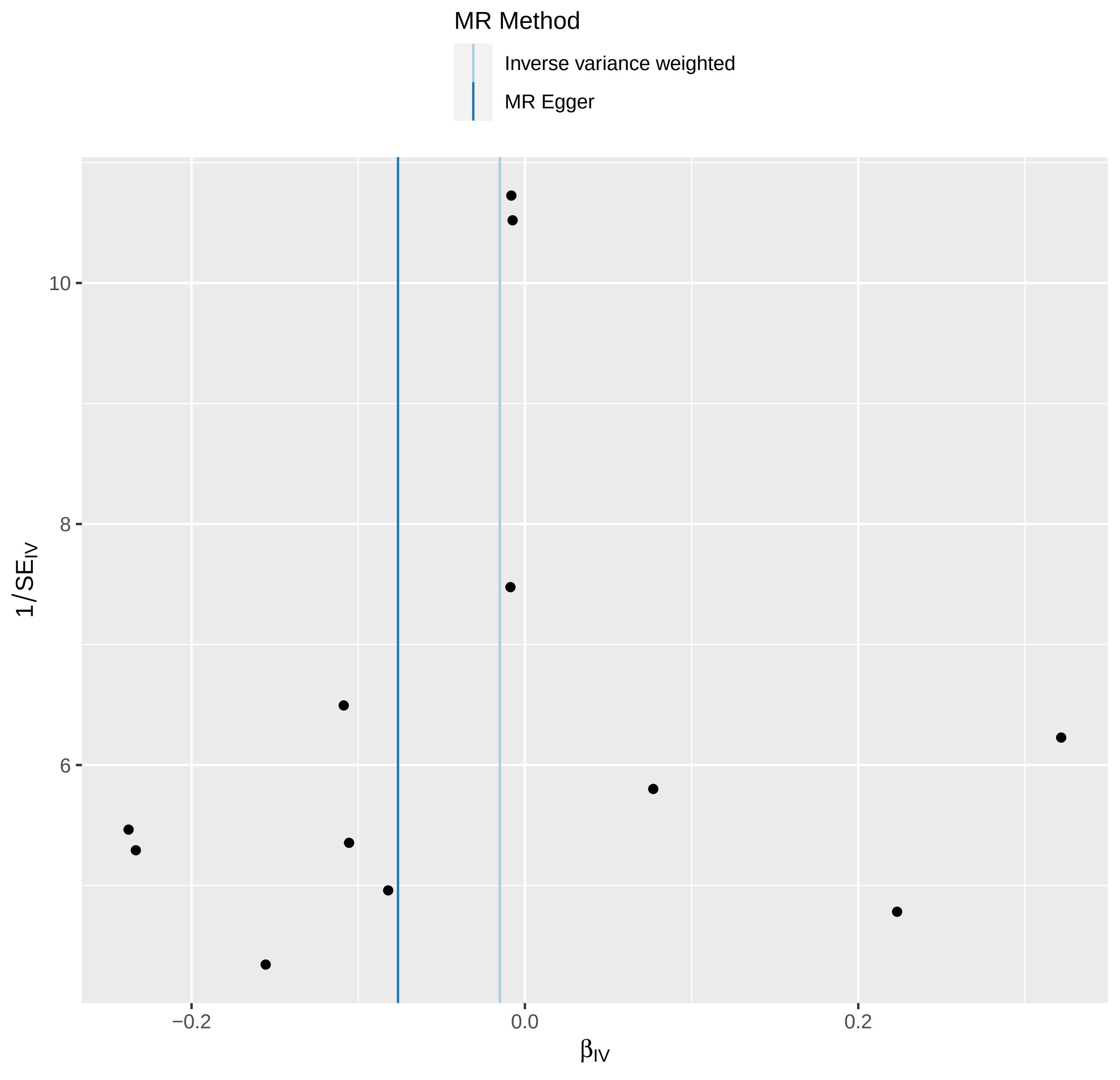

Batch 3−461 : Selenium on B−cell antigen receptor complex−associated protein alpha chain

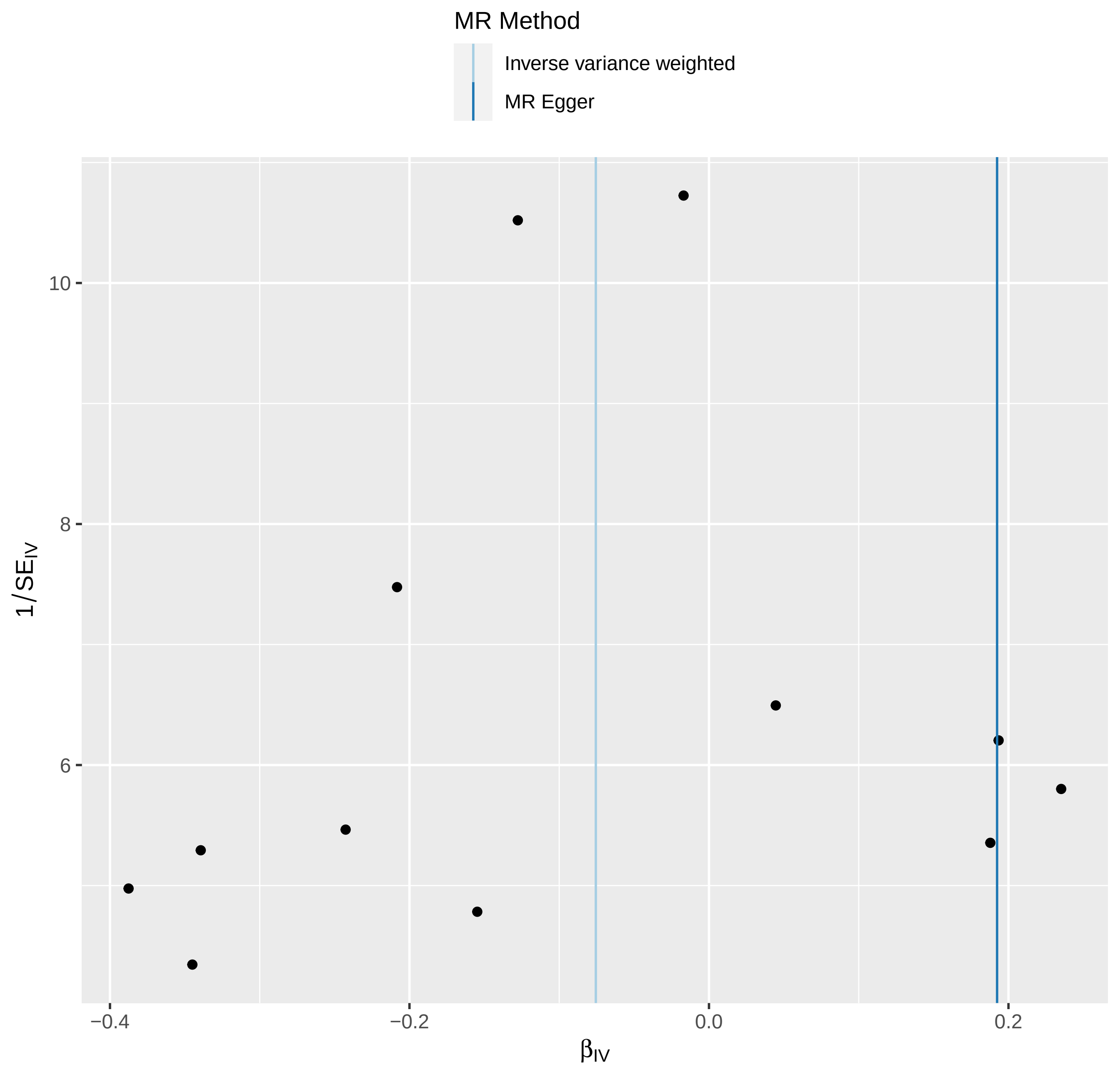

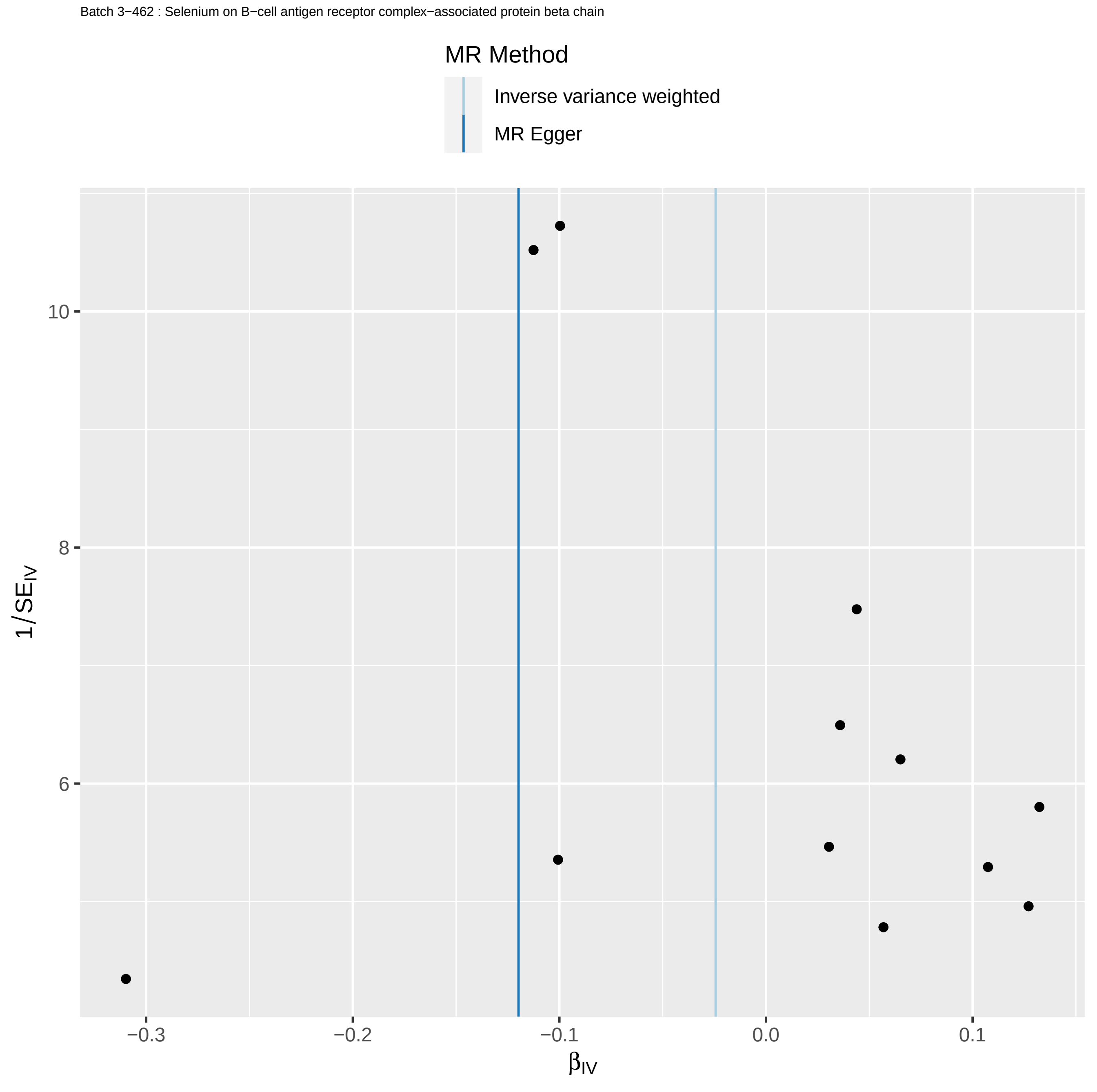

Batch 3−462 : Selenium on B−cell antigen receptor complex−associated protein beta chain
MR Method
Inverse variance weighted
MR Egger
10
8
6
1/SE_IV
−0.3
−0.2
−0.1
0.0
0.1
β_IV

Batch 3−463 : Selenium on T−lymphocyte activation antigen CD80

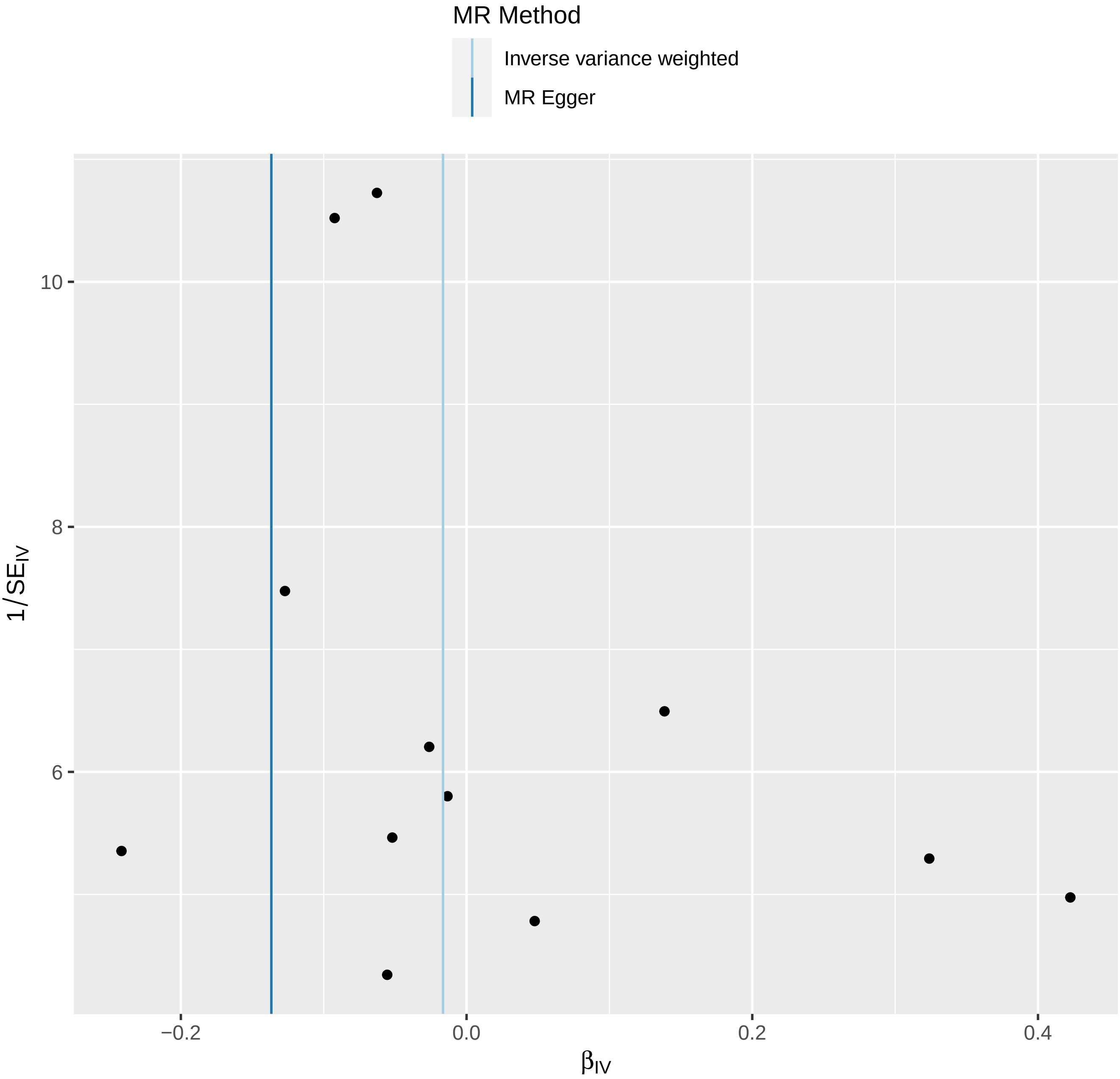

Batch 3−464 : Selenium on SLAM family member 5

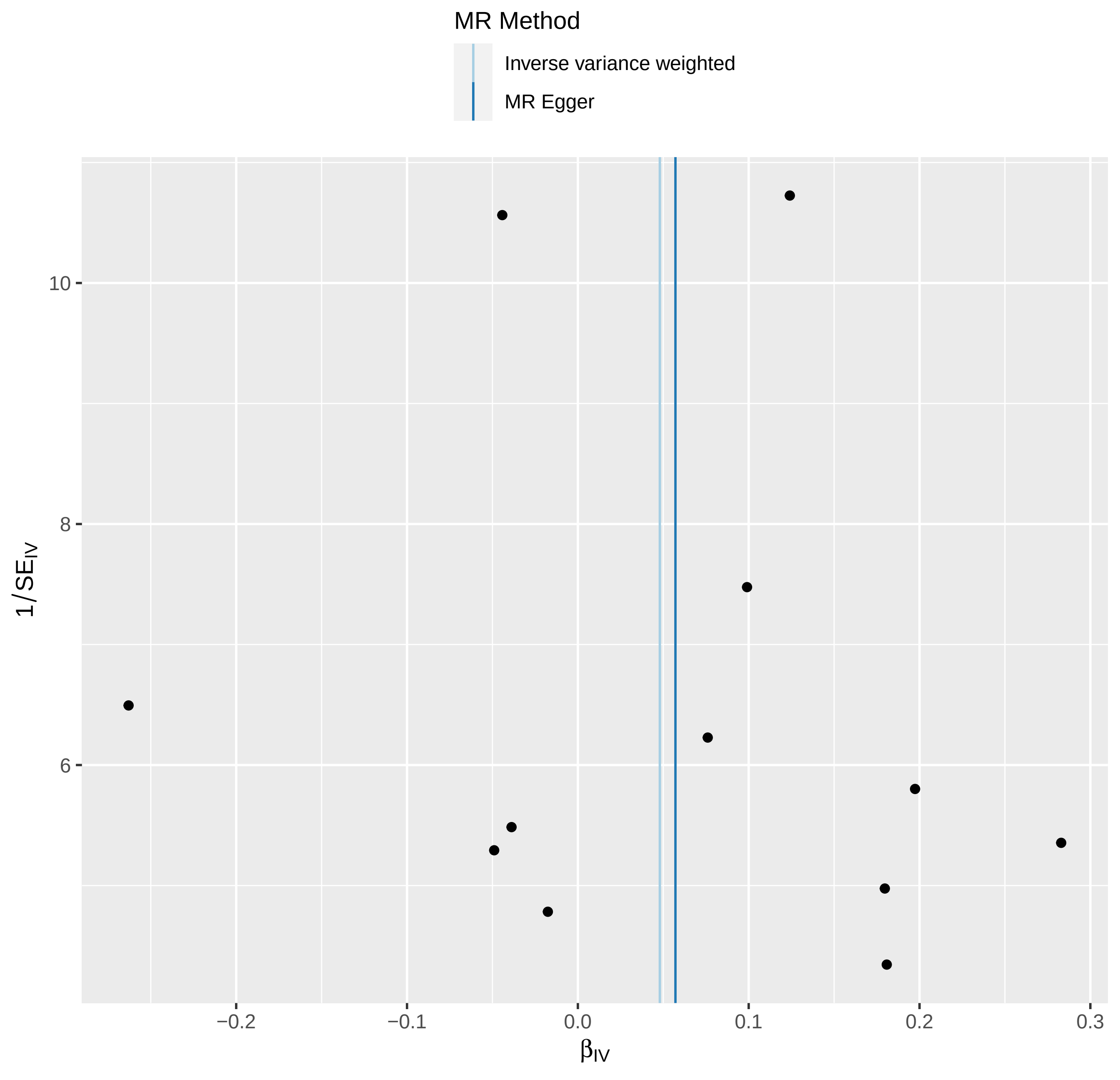

Batch 3−465 : Selenium on SLAM family member 5

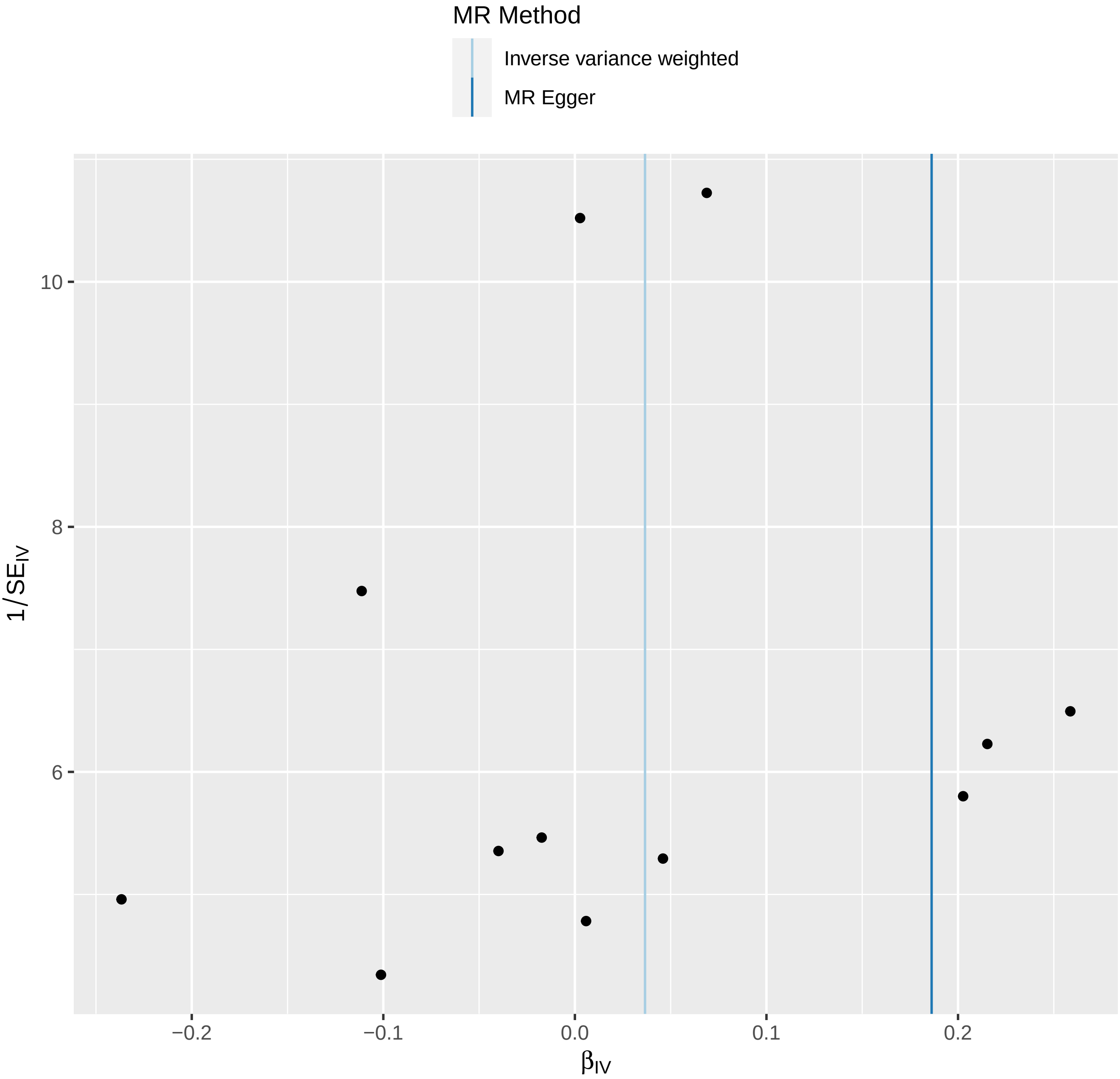

Batch 3−466 : Selenium on T−lymphocyte activation antigen CD86

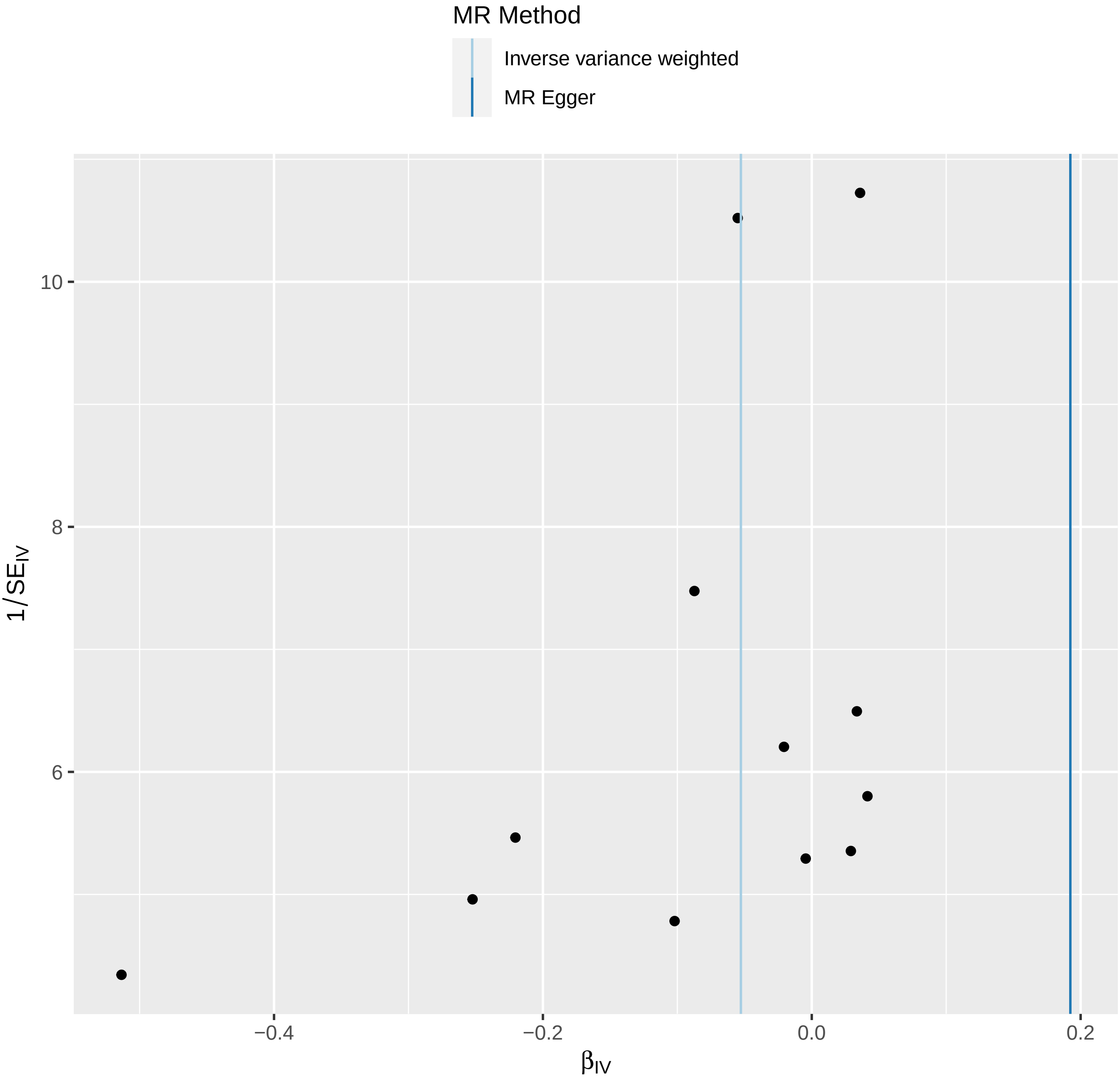

Batch 3−467 : Selenium on T−cell surface glycoprotein CD8 beta chain

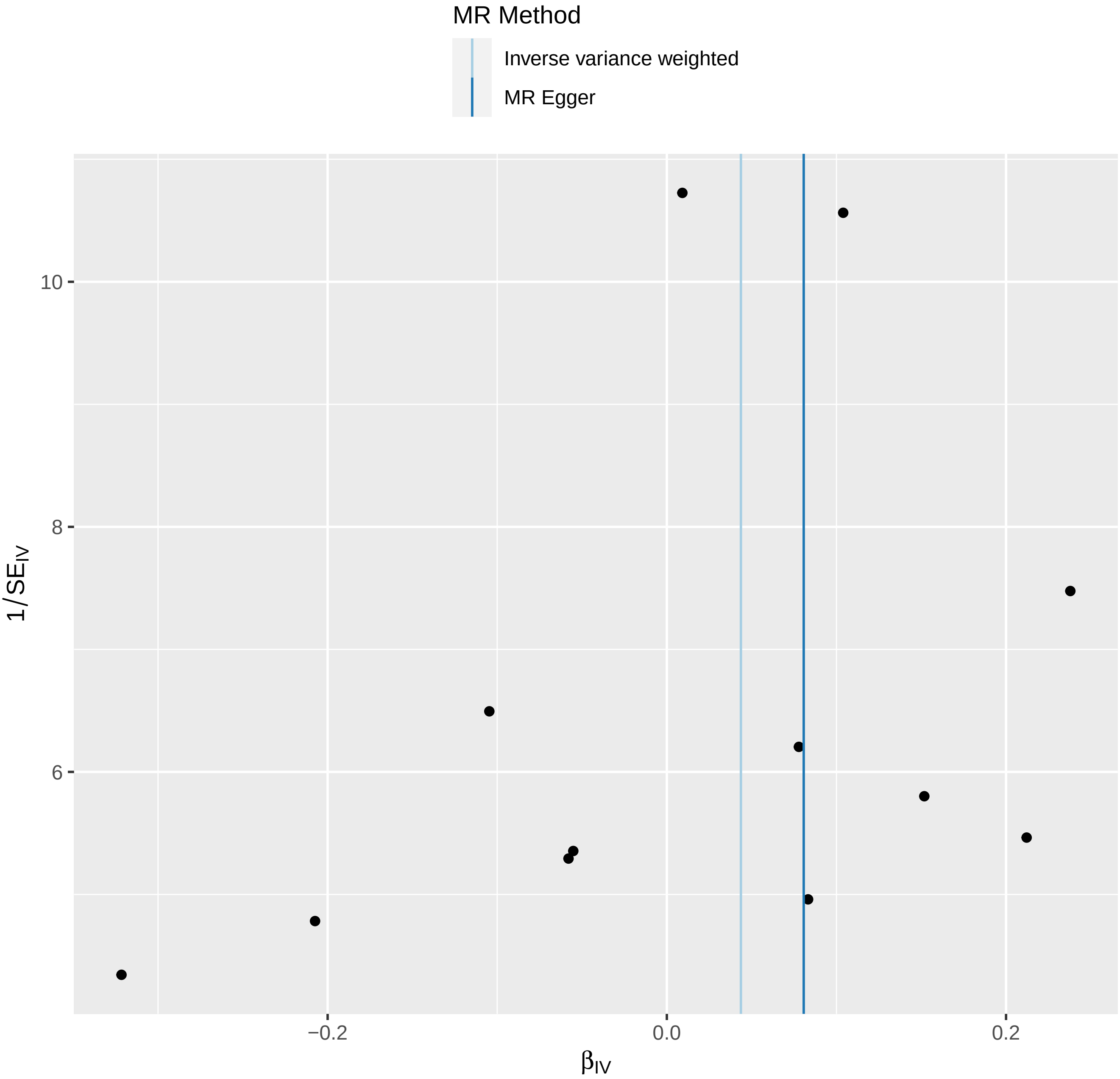

Batch 3−468 : Selenium on Complement component C1q receptor

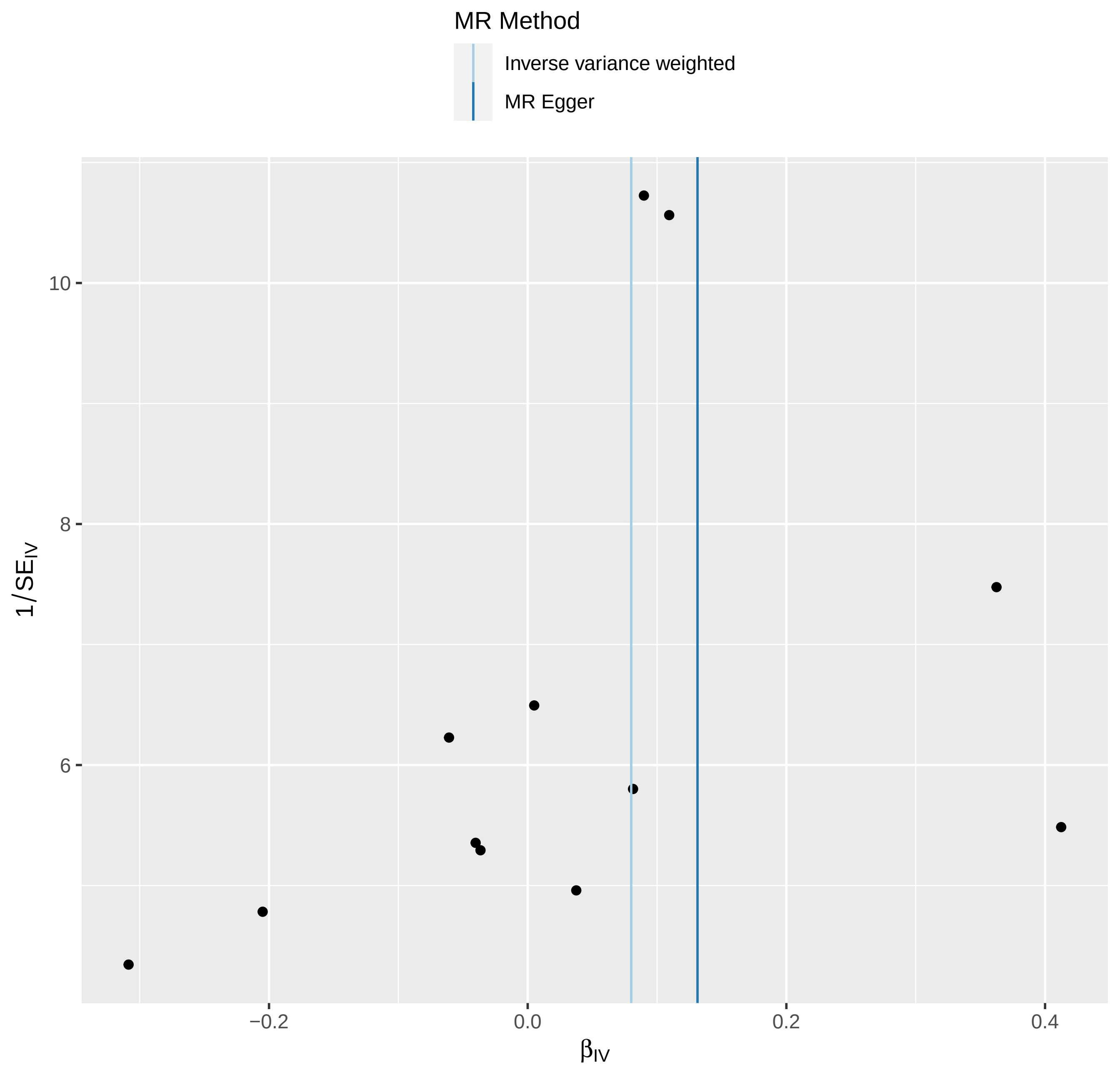

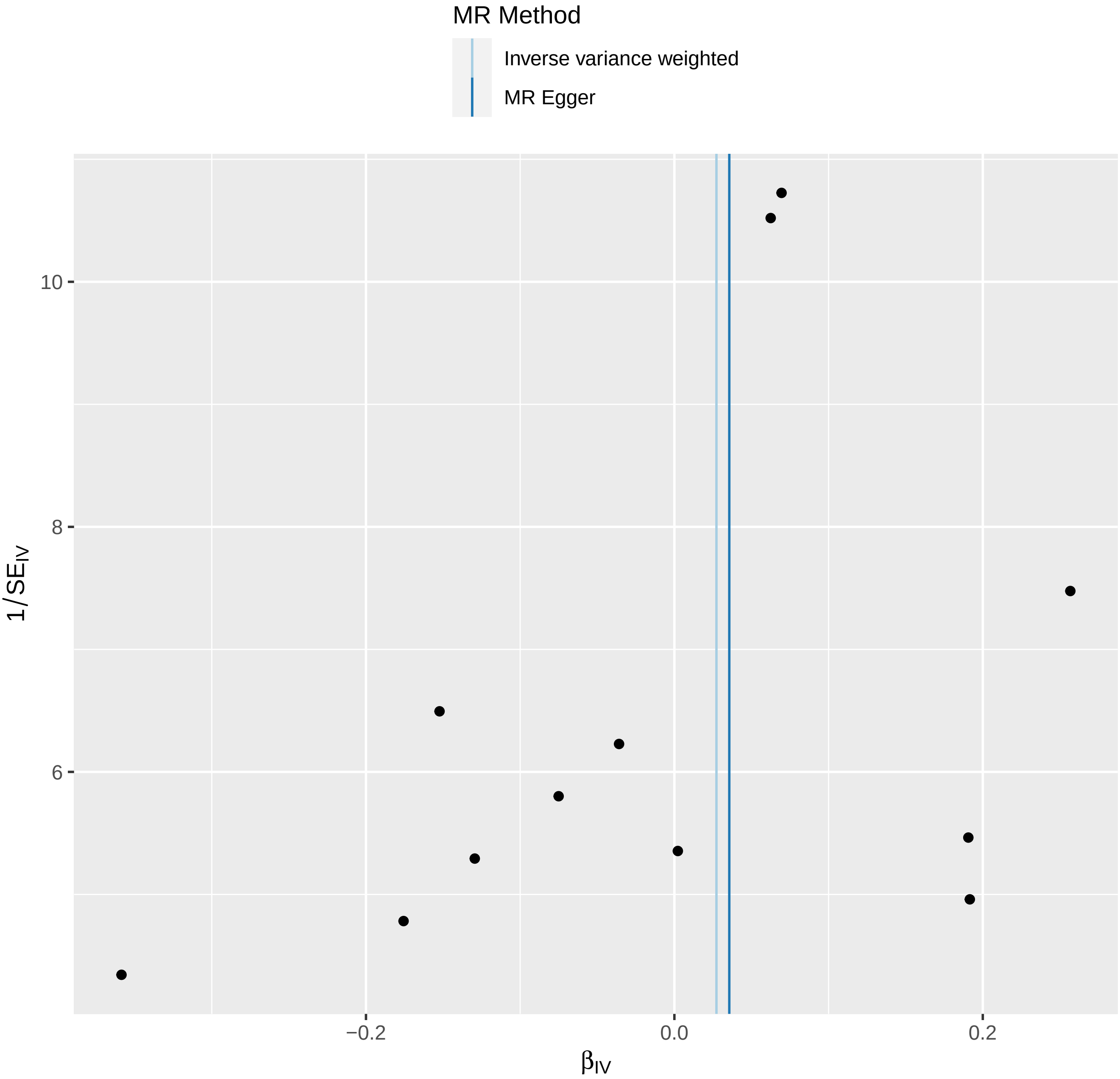
MR Method
Inverse variance weighted
MR Egger
10
8
6
1/SE$_{IV}$
−0.2
0.0
0.2
β$_{IV}$

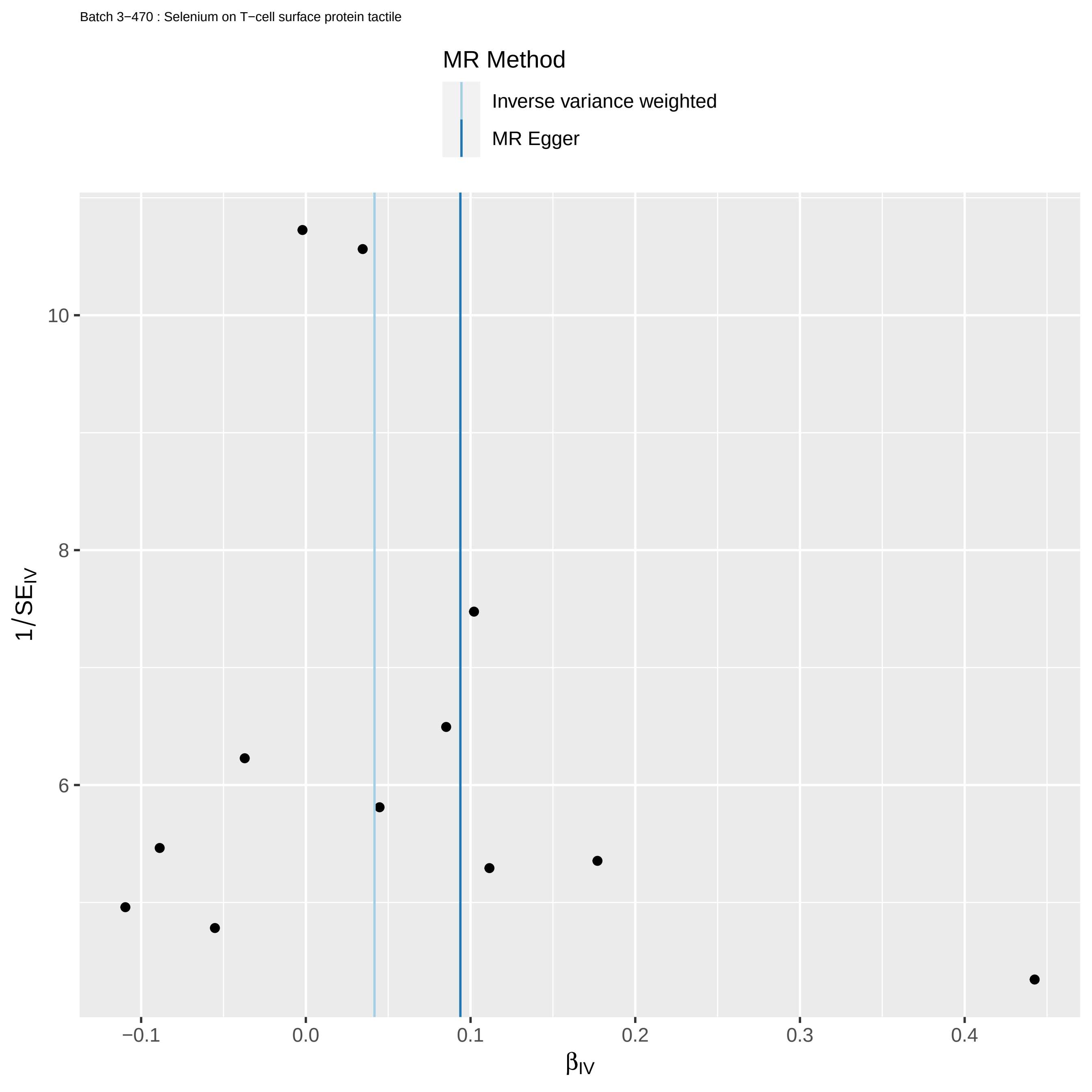
Batch 3−470 : Selenium on T−cell surface protein tactile
MR Method
Inverse variance weighted
MR Egger
1/SE$_{IV}$
10
8
6
−0.1
0.0
0.1
0.2
0.3
0.4
β$_{IV}$

Batch 3−471 : Selenium on CD97 antigen

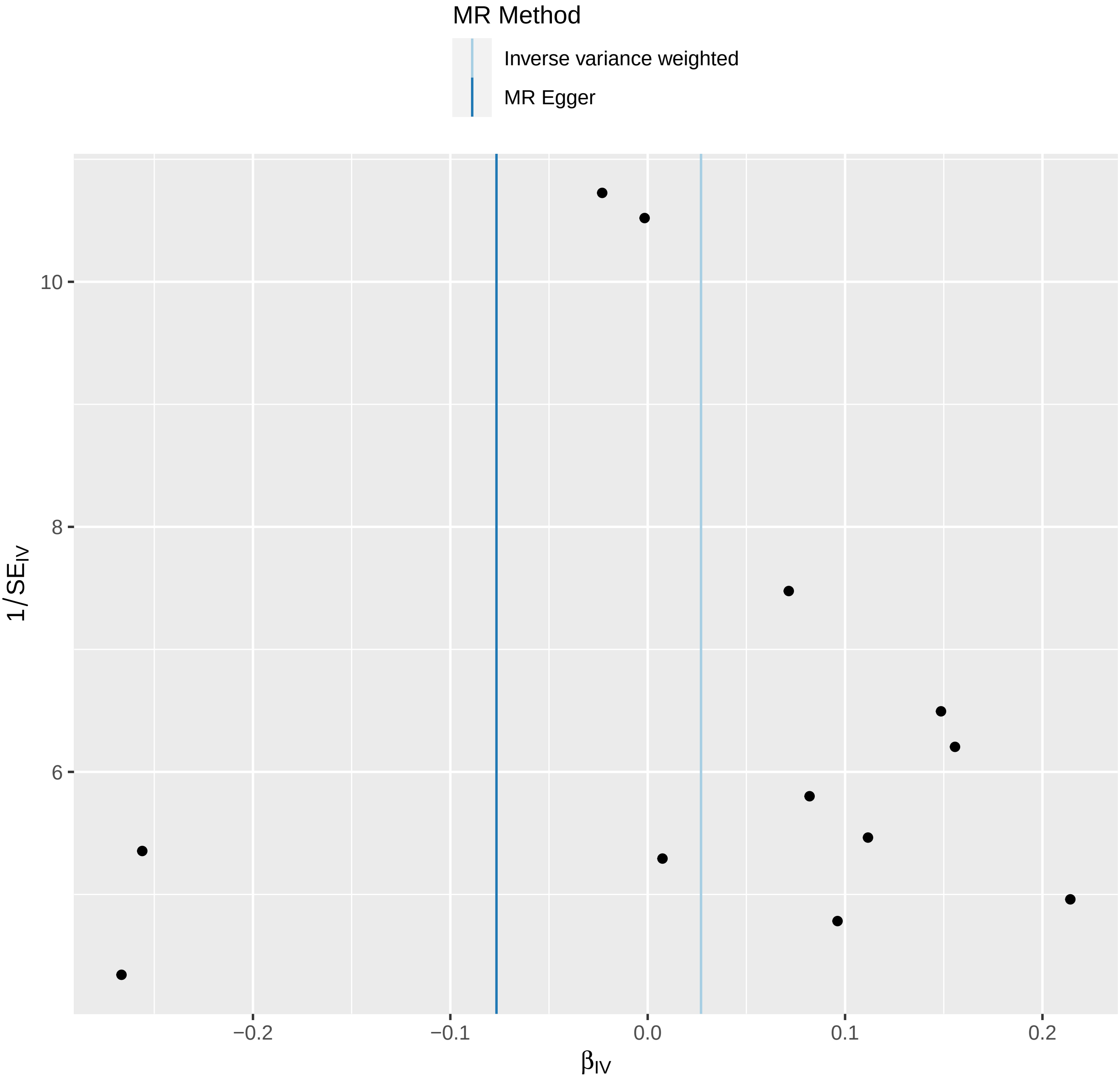

Batch 3−472 : Selenium on M−phase inducer phosphatase 2

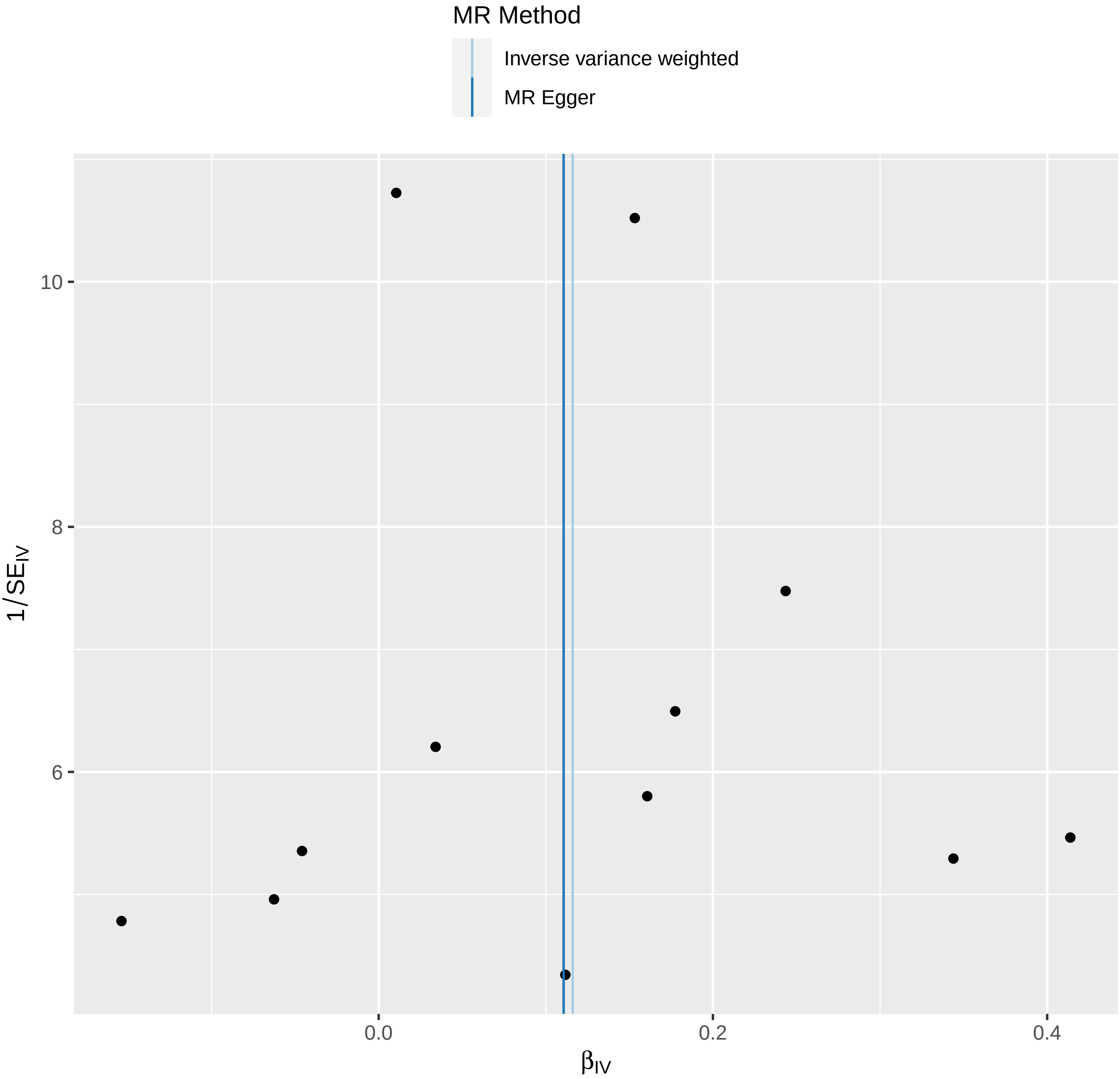

Batch 3−473 : Selenium on Cyclin−dependent kinase 1:G2/mitotic−specific cyclin−B1 complex

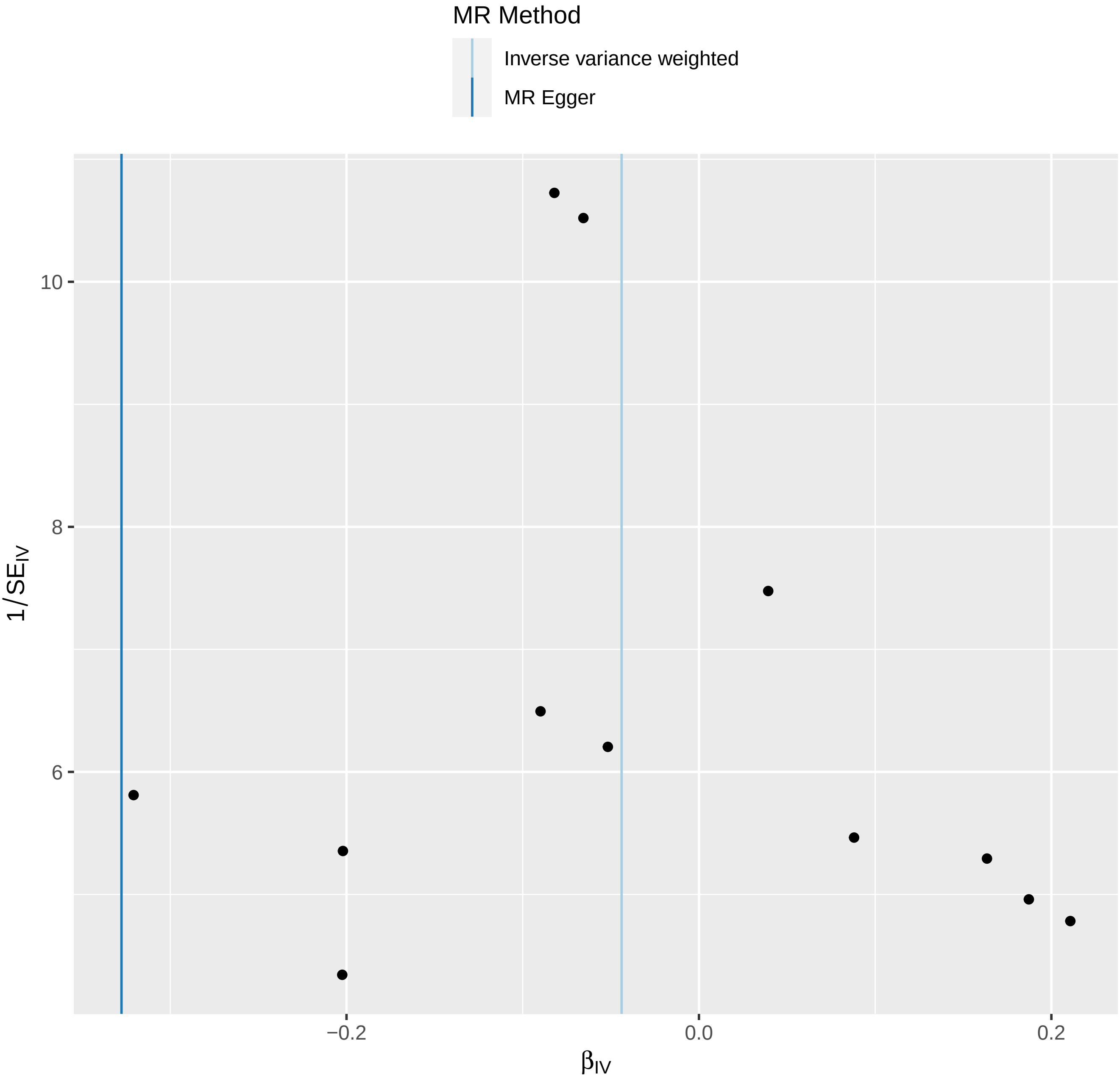

Batch 3−474 : Selenium on Cell division control protein 42 homolog

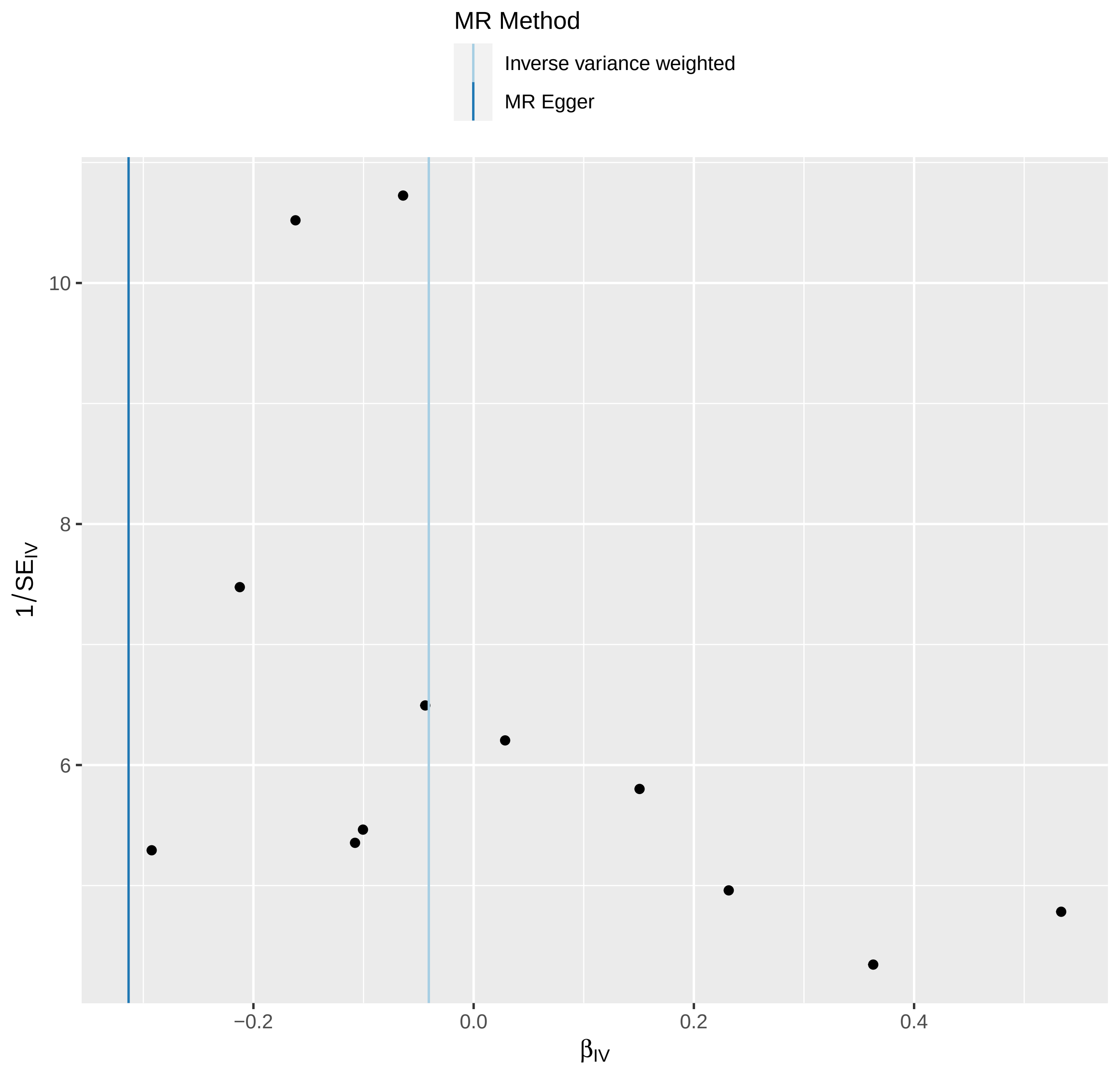

Batch 3−475 : Selenium on Serine/threonine−protein kinase MRCK alpha

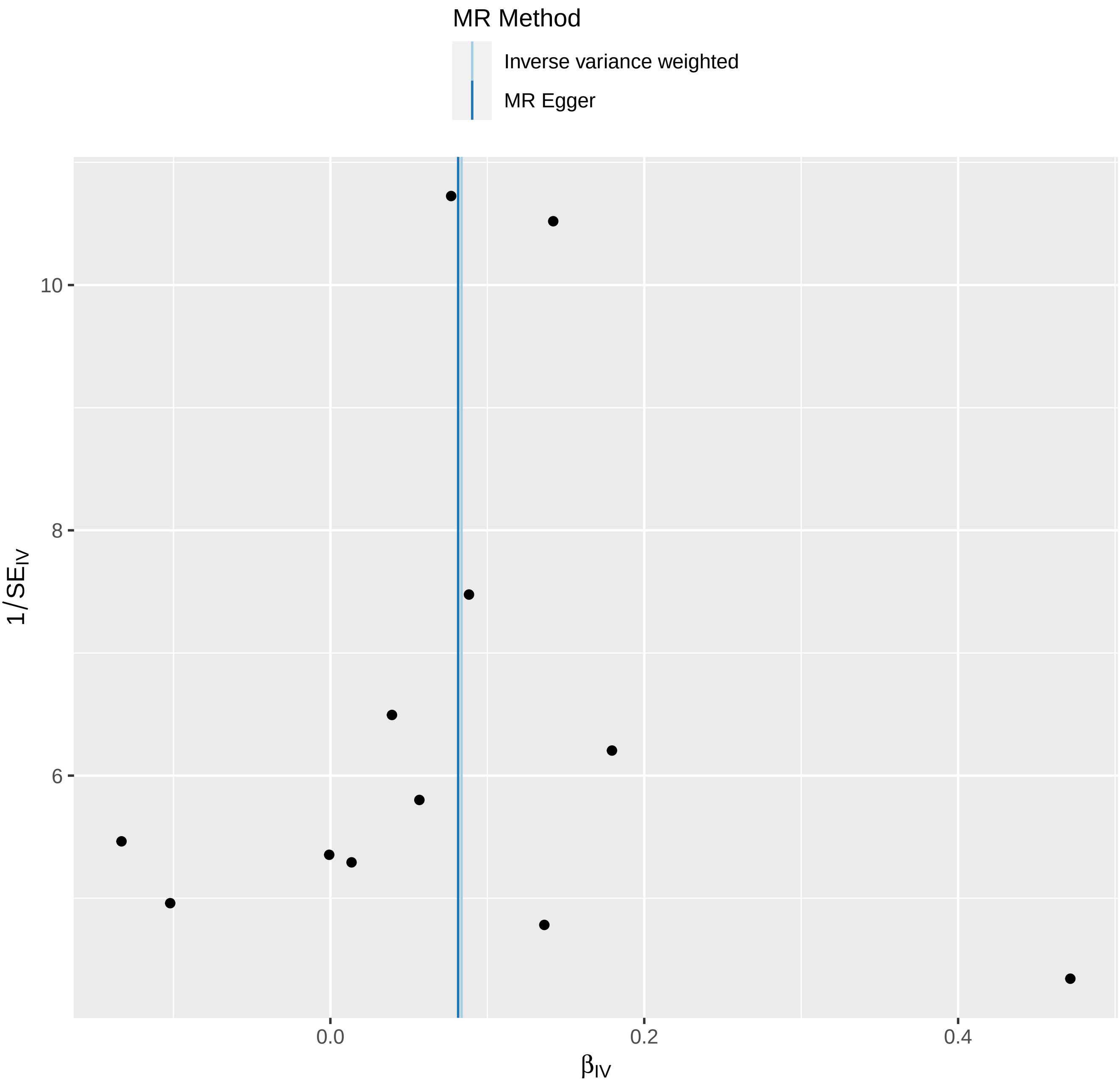

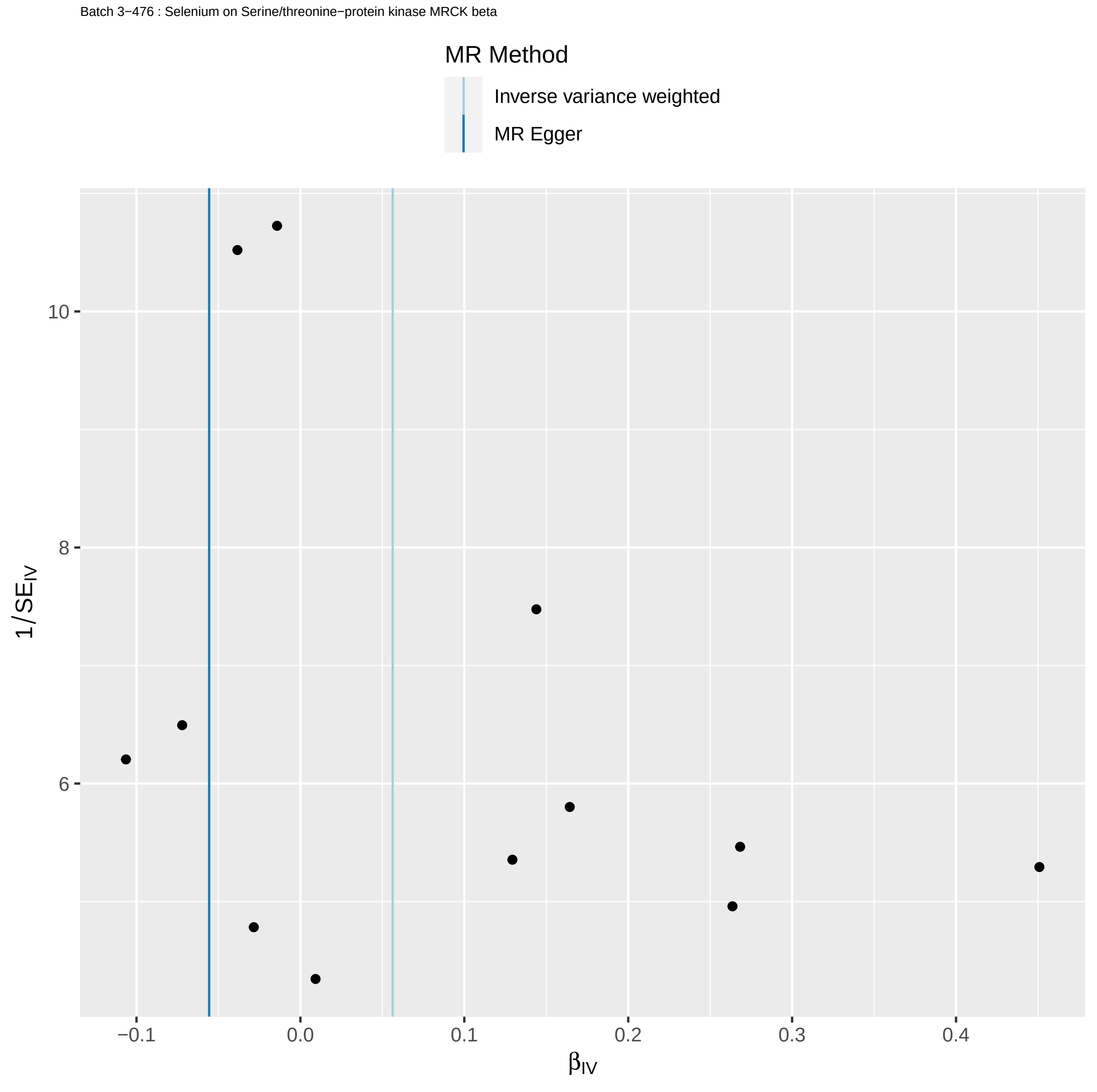
Batch 3−476 : Selenium on Serine/threonine−protein kinase MRCK beta
MR Method
Inverse variance weighted
MR Egger
1/SE_IV
10
8
6
−0.1
0.0
0.1
0.2
0.3
0.4
β_IV

Batch 3−477 : Selenium on CUB domain−containing protein 1

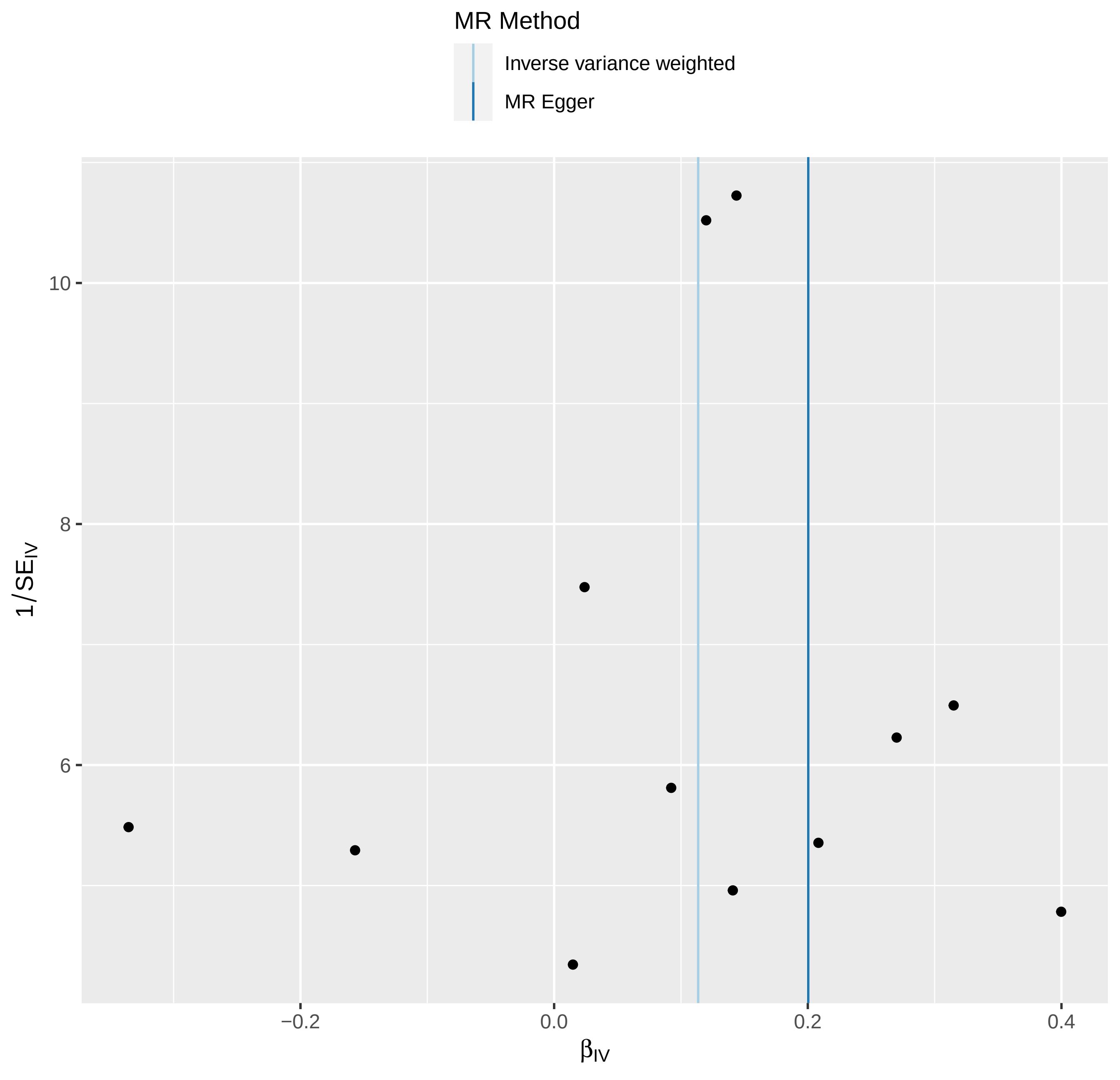

Batch 3−478 : Selenium on Cadherin−12

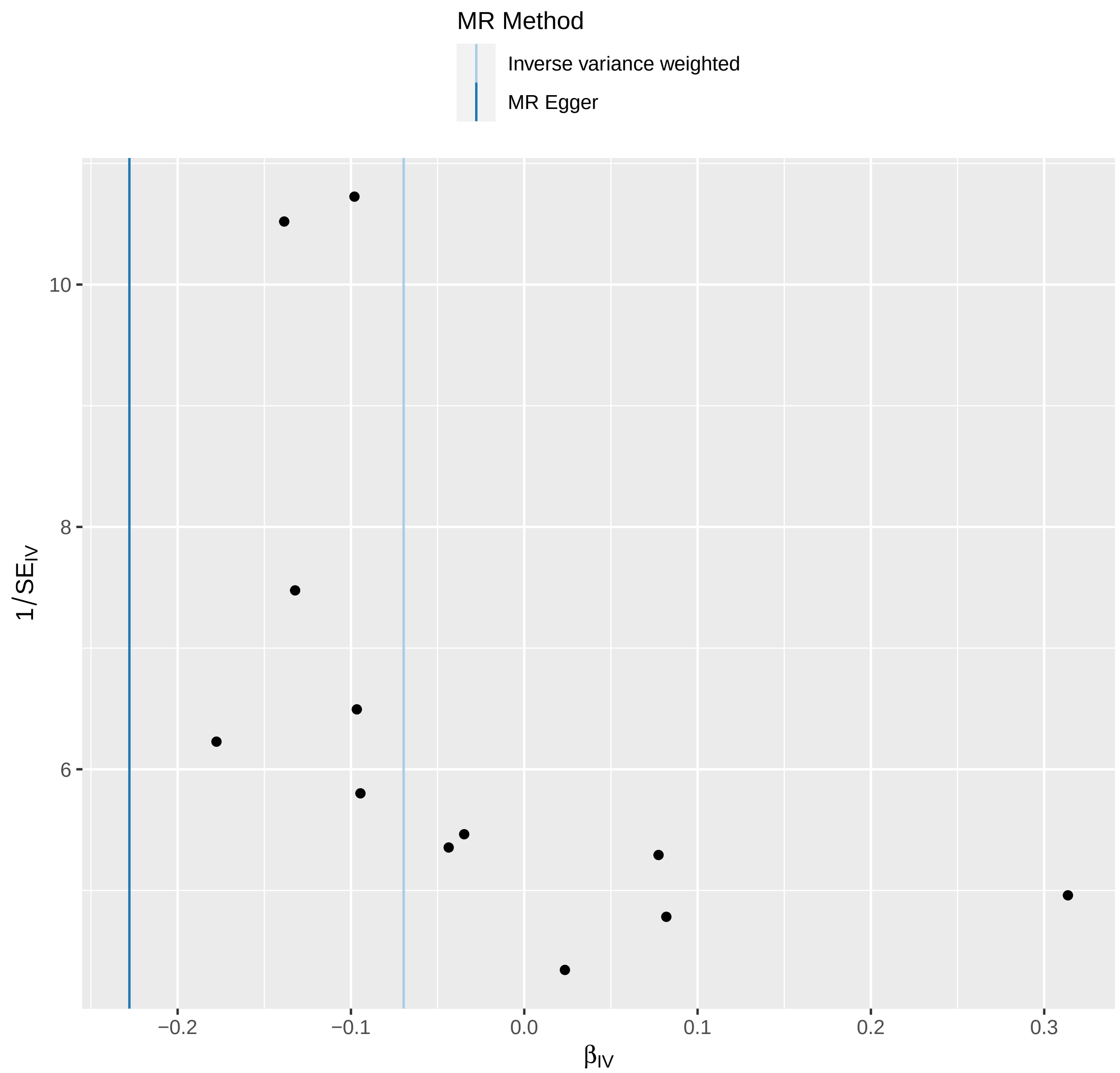

Batch 3−479 : Selenium on Cadherin−12

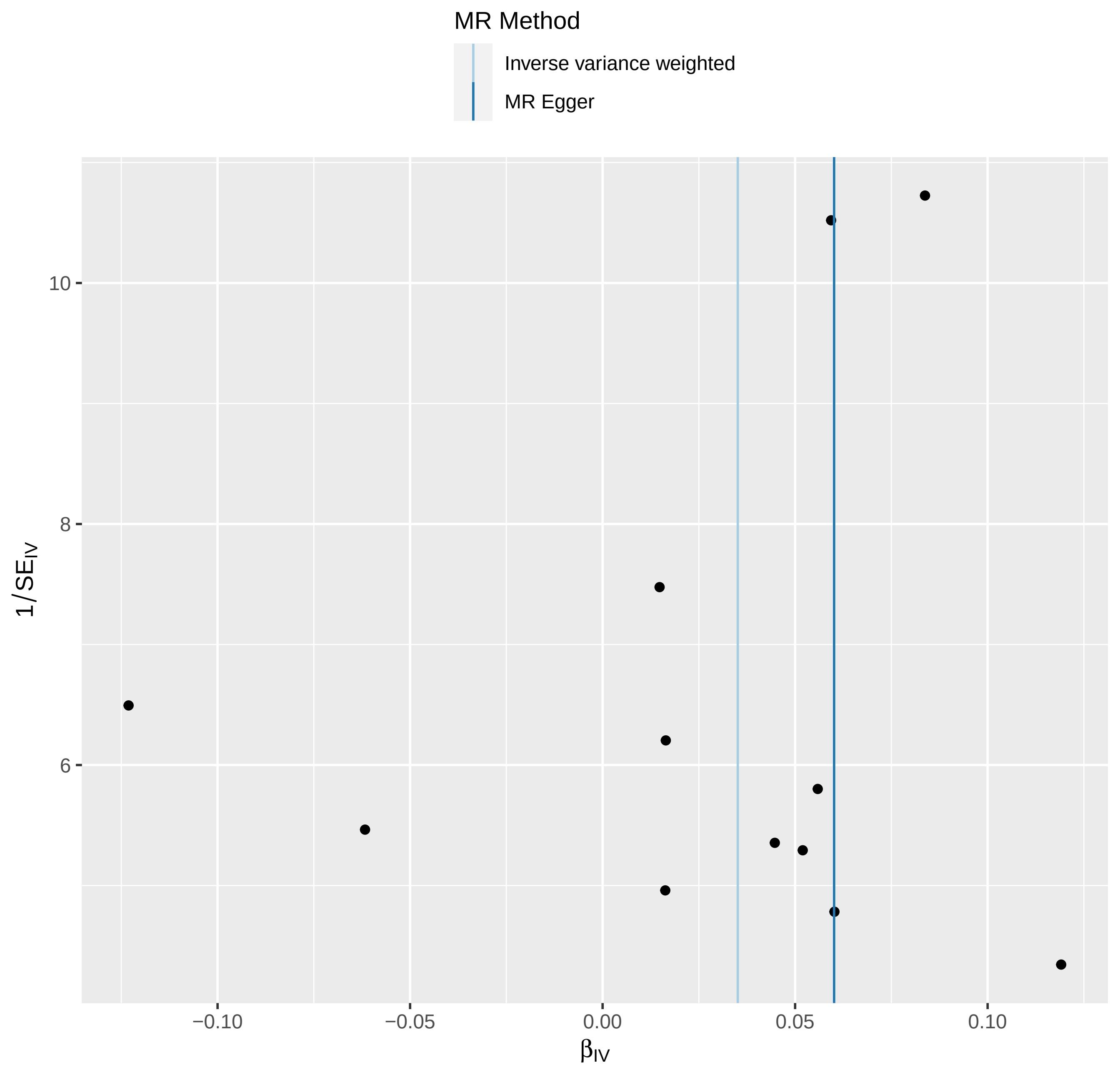

Batch 3−480 : Selenium on Cadherin−1

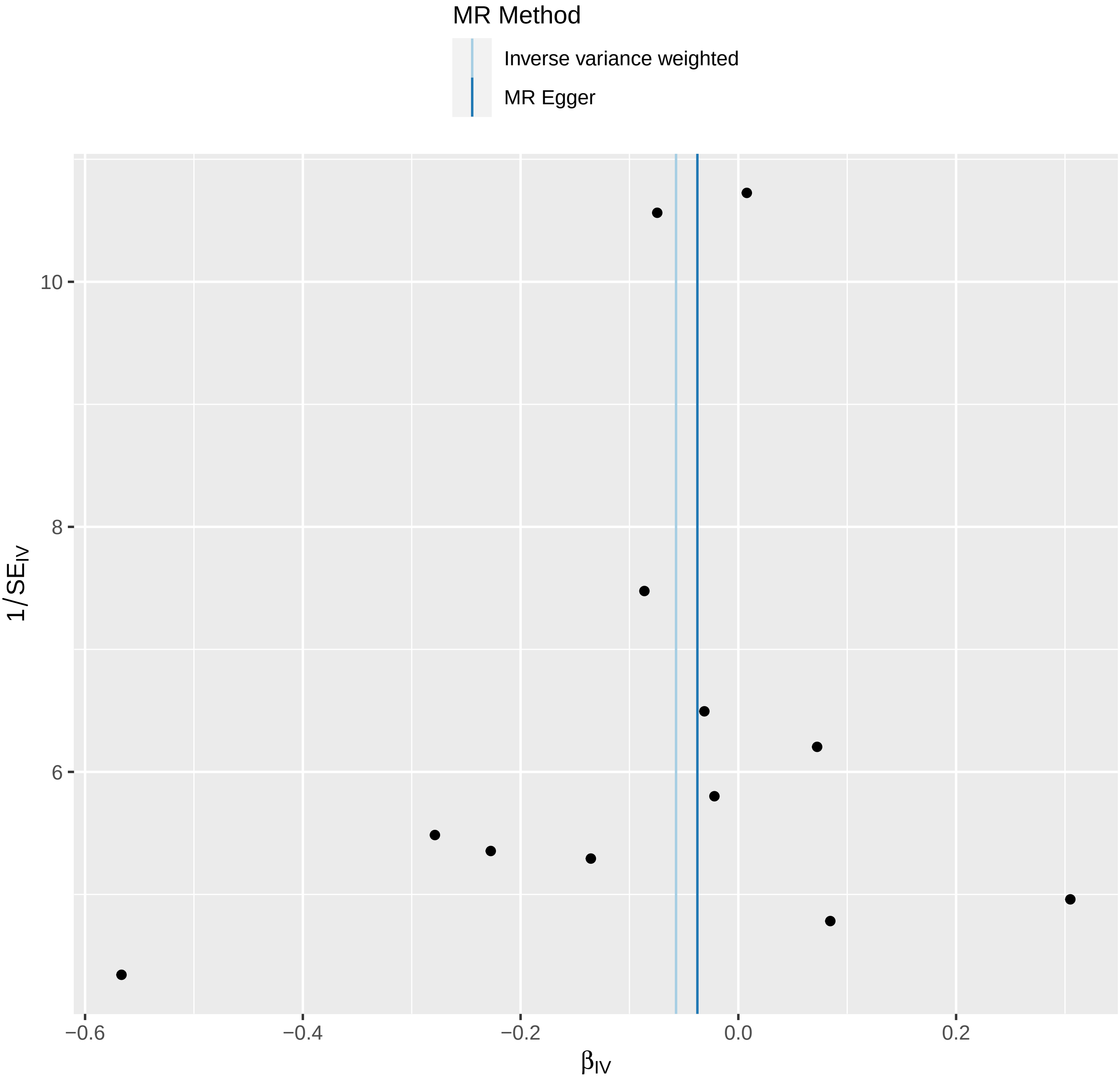

Batch 3−481 : Selenium on Cadherin−15

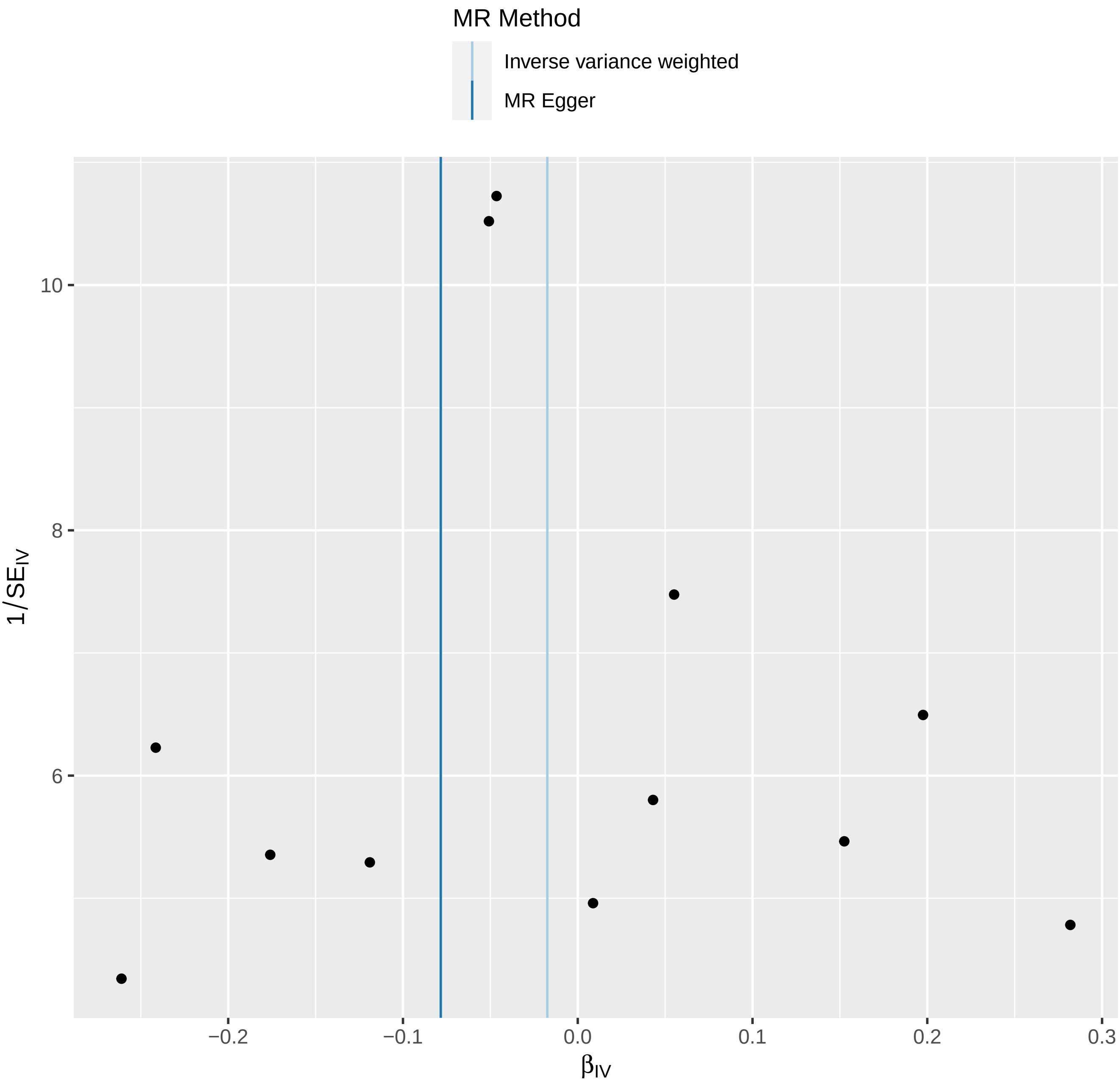

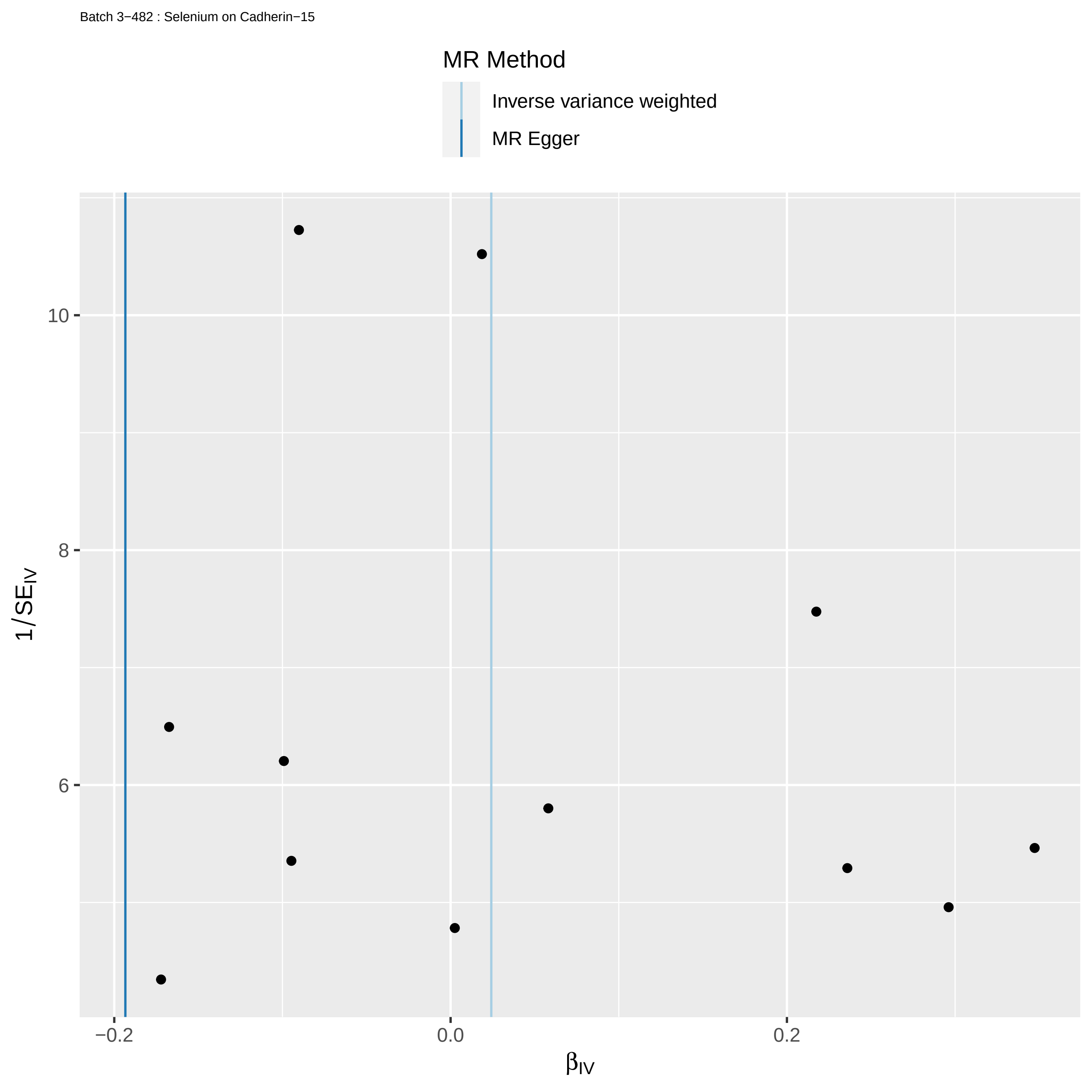
Batch 3−482 : Selenium on Cadherin−15
MR Method
Inverse variance weighted
MR Egger
10
8
6
1/SE_IV
−0.2
0.0
0.2
β_IV

Batch 3−483 : Selenium on Cadherin−2

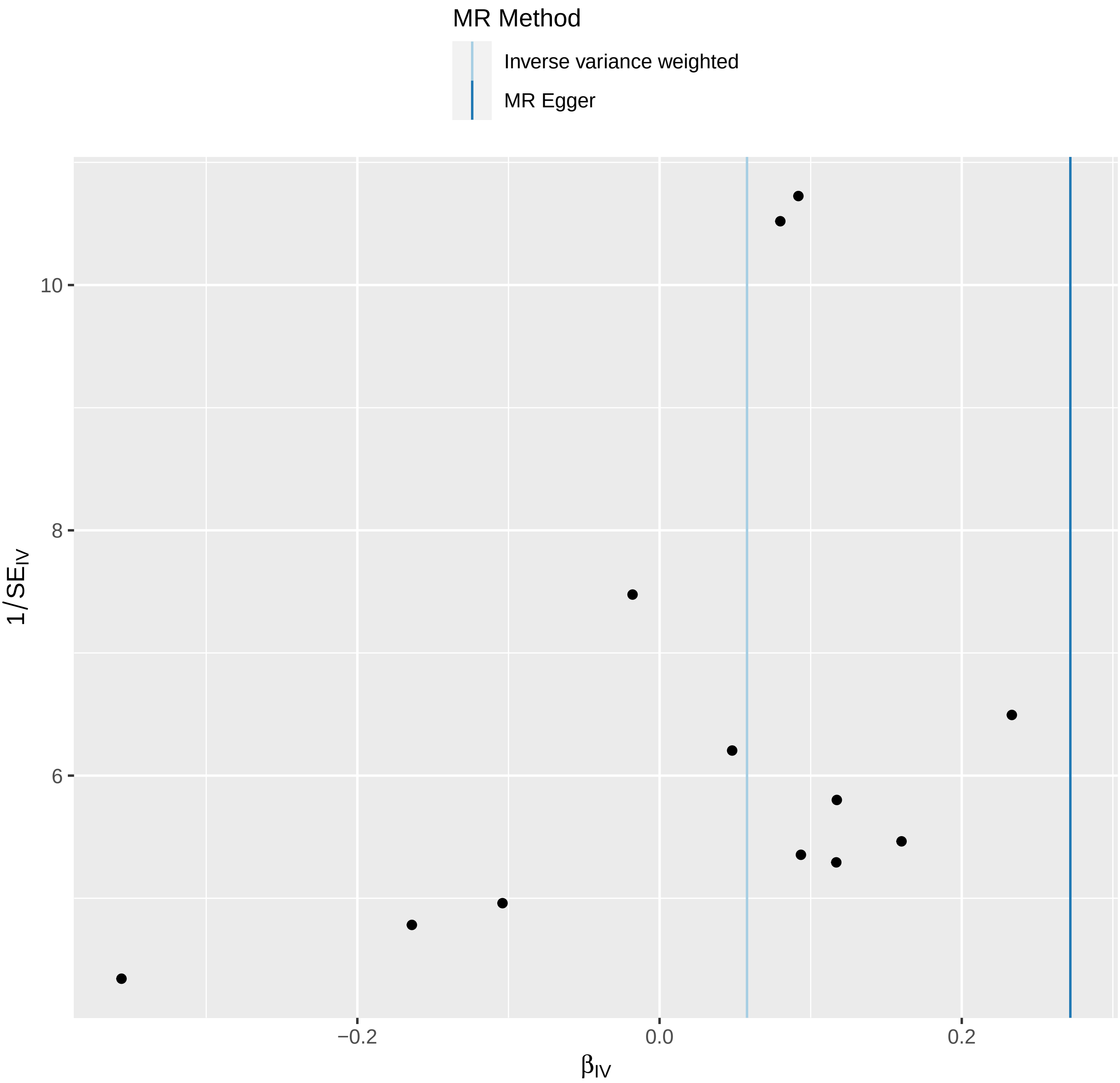

Batch 3−484 : Selenium on Cadherin−3

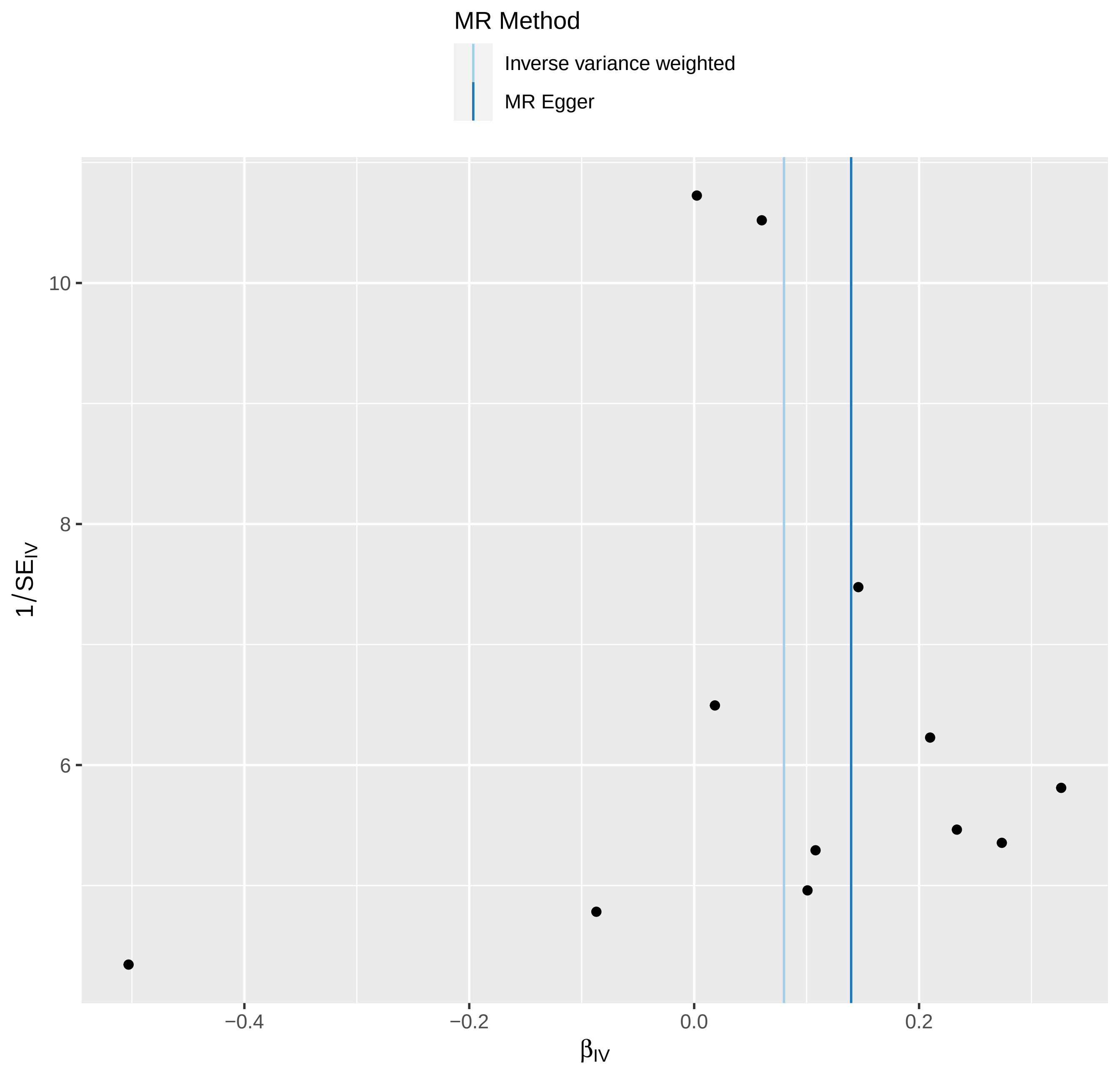

Batch 3−485 : Selenium on Cadherin−5

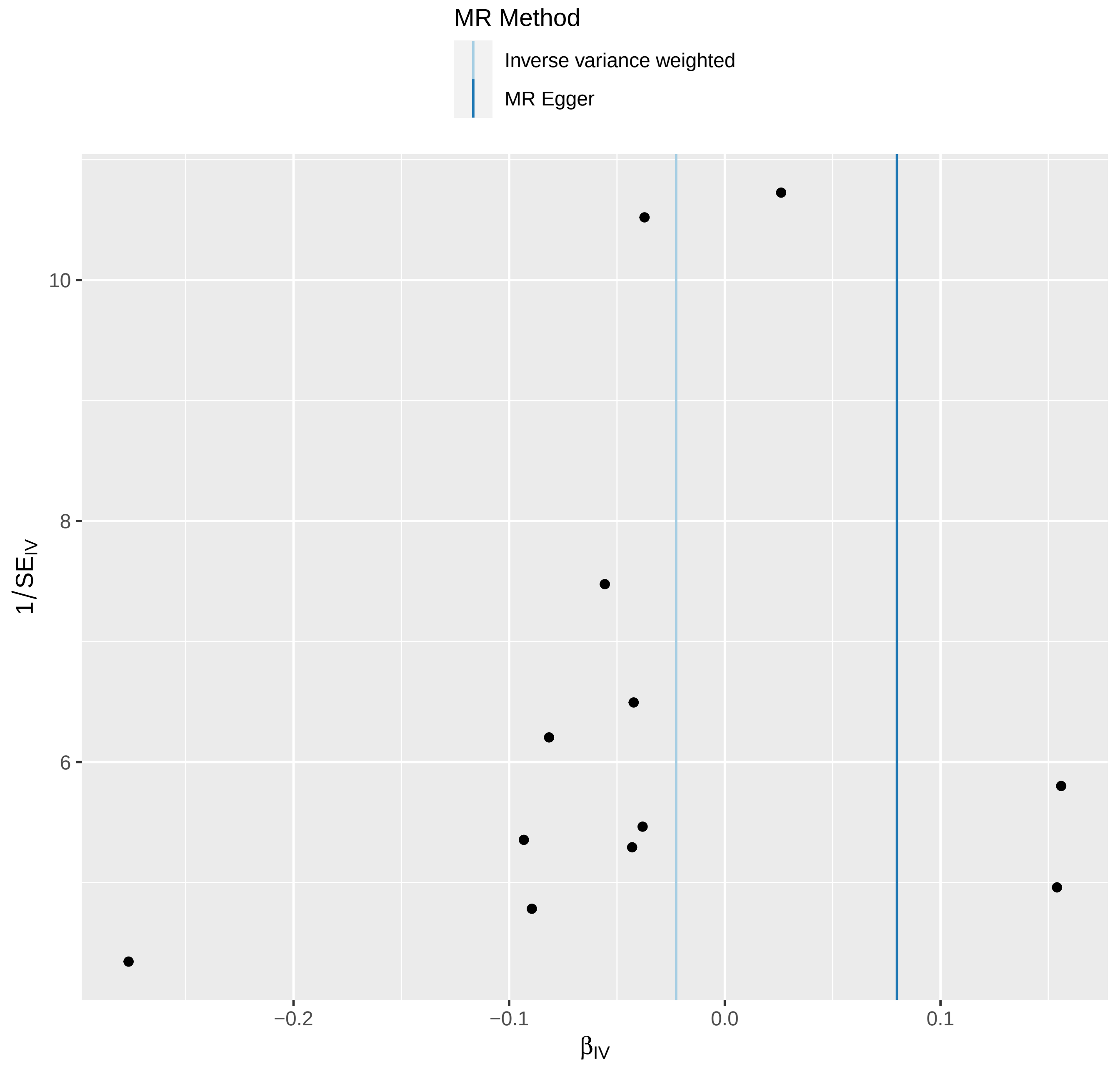

Batch 3−486 : Selenium on Cadherin−7

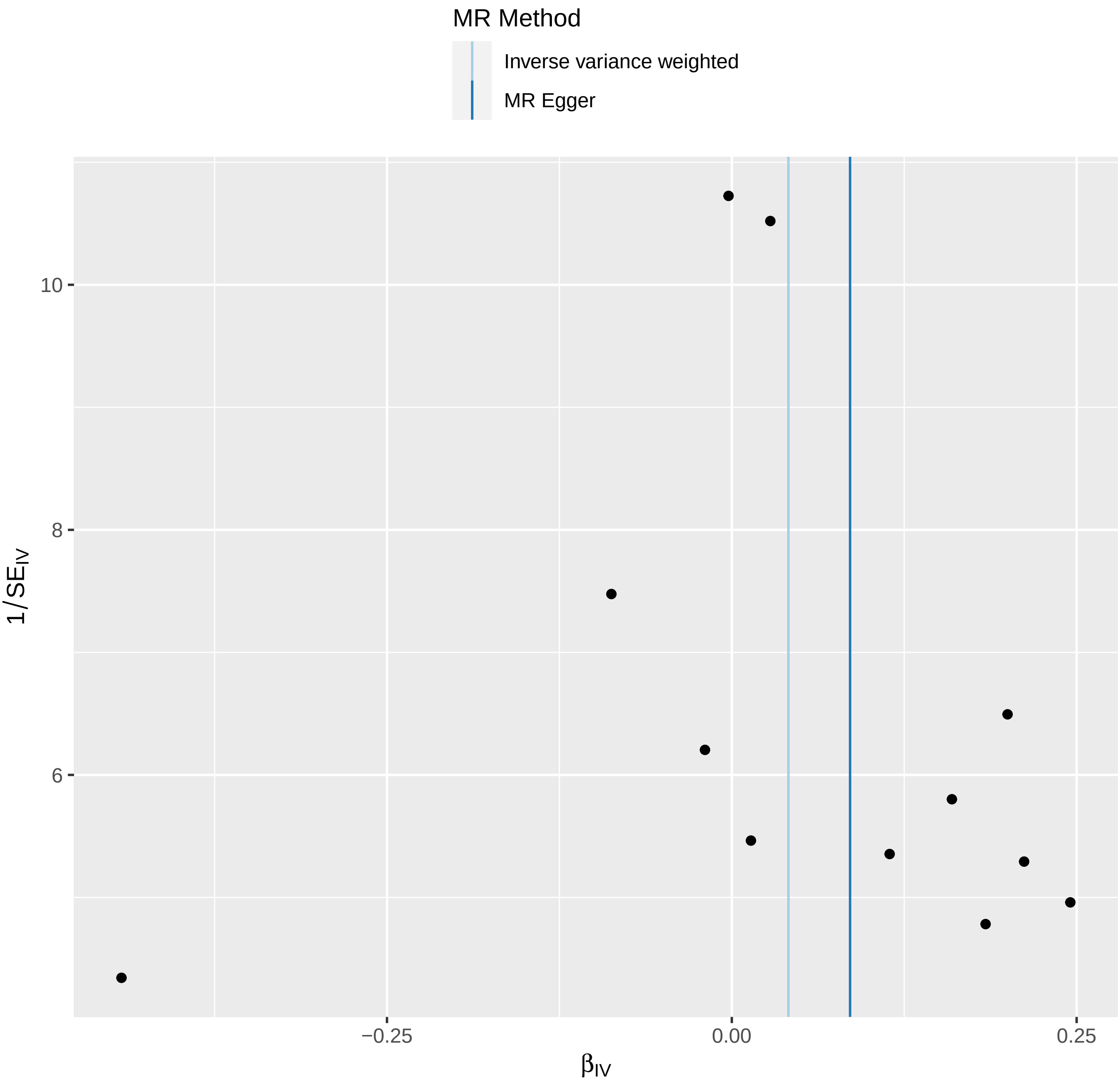

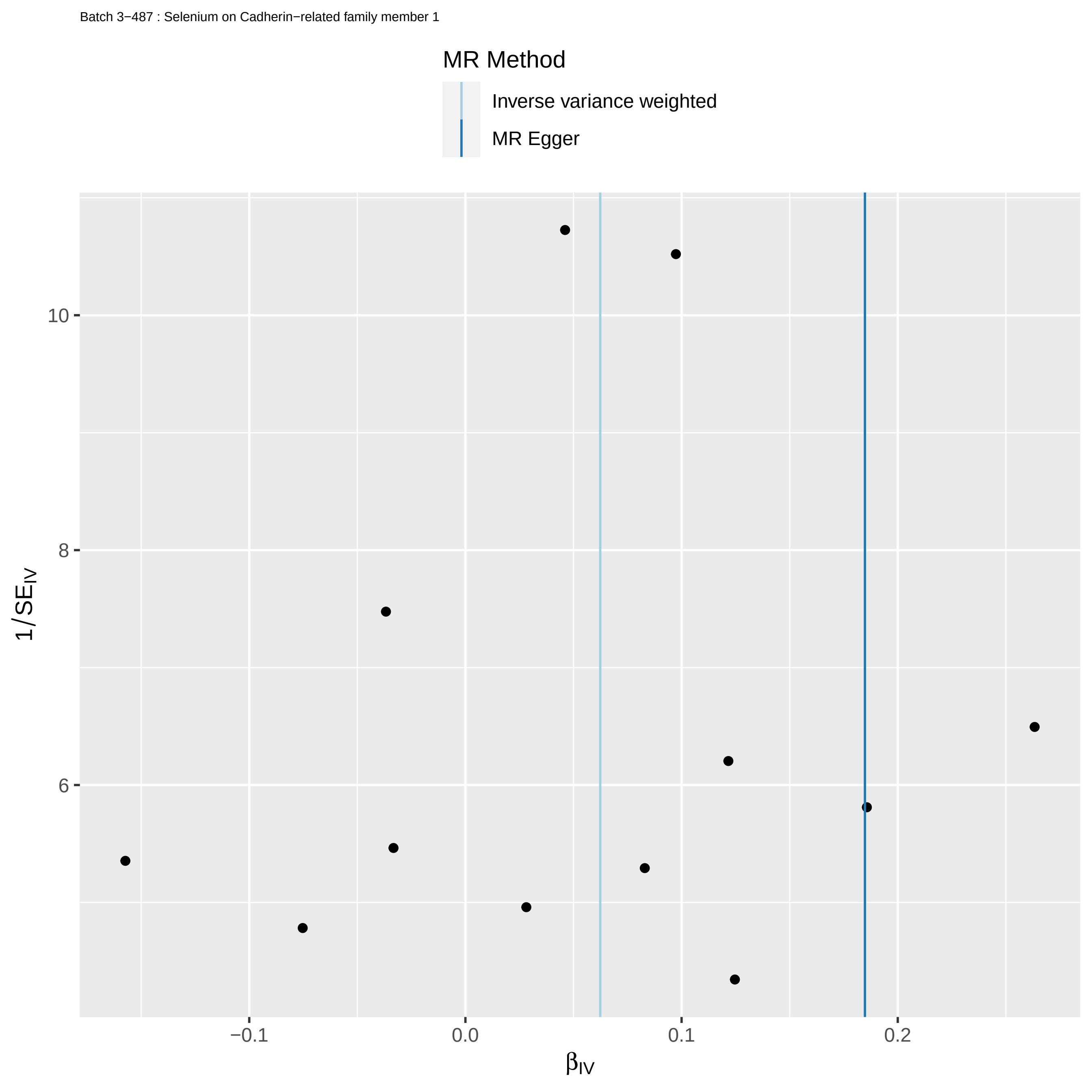

Batch 3−487 : Selenium on Cadherin−related family member 1
MR Method
Inverse variance weighted
MR Egger
10
8
6
1/SE_IV
−0.1
0.0
0.1
0.2
β_IV

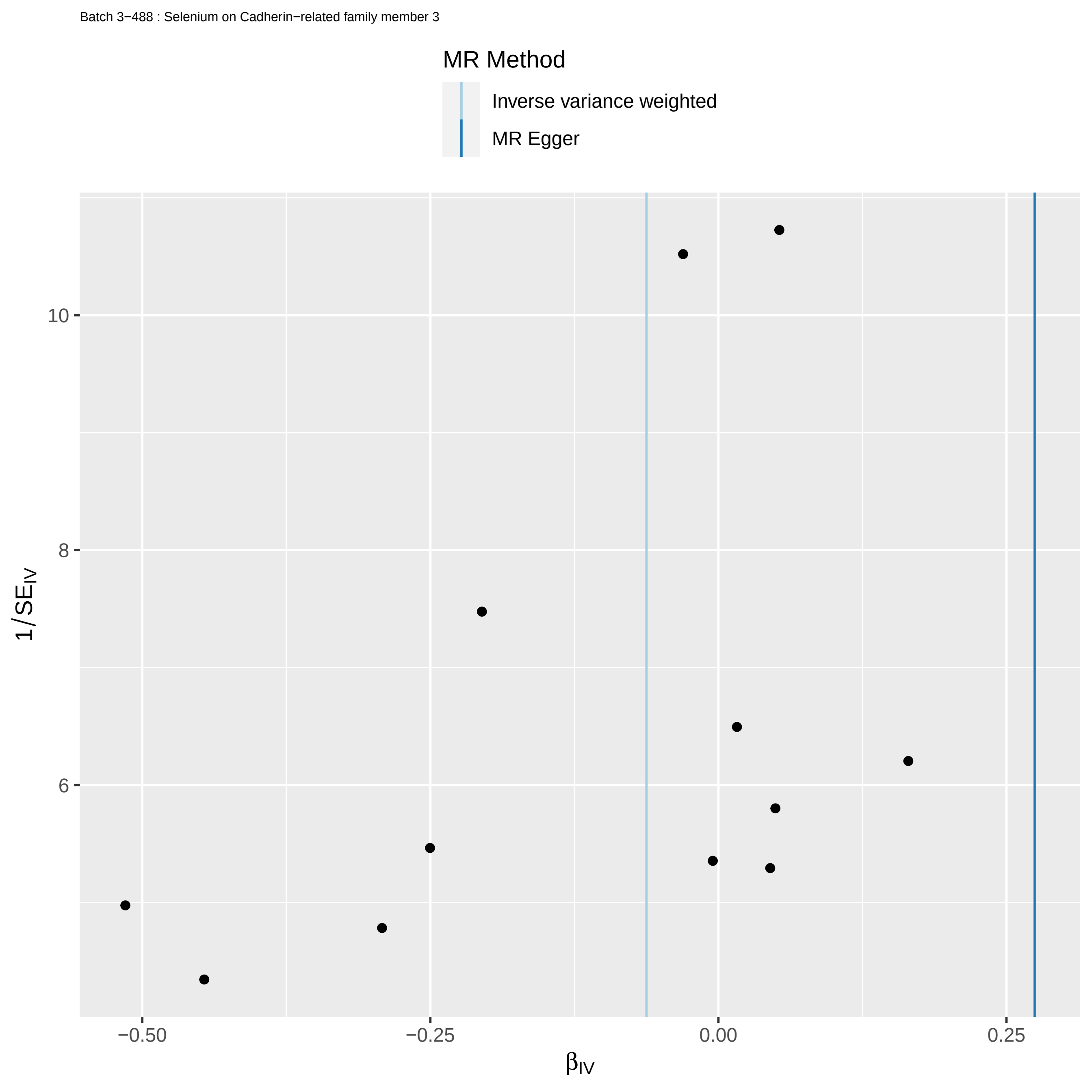
Batch 3−488 : Selenium on Cadherin−related family member 3
MR Method
Inverse variance weighted
MR Egger
10
8
6
1/SE_IV
−0.50
−0.25
0.00
0.25
β_IV

Batch 3−489 : Selenium on Cadherin−related family member 3

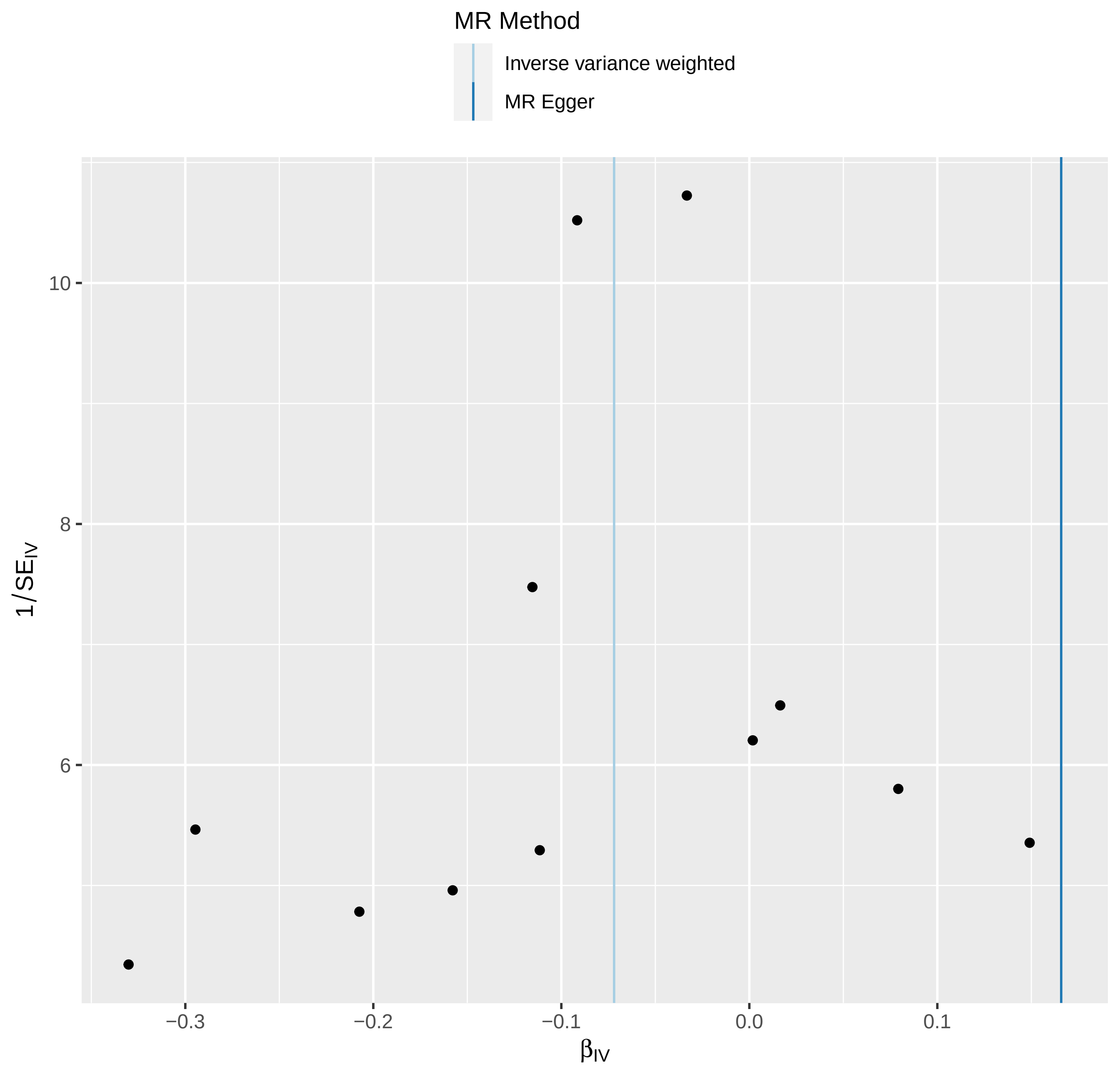

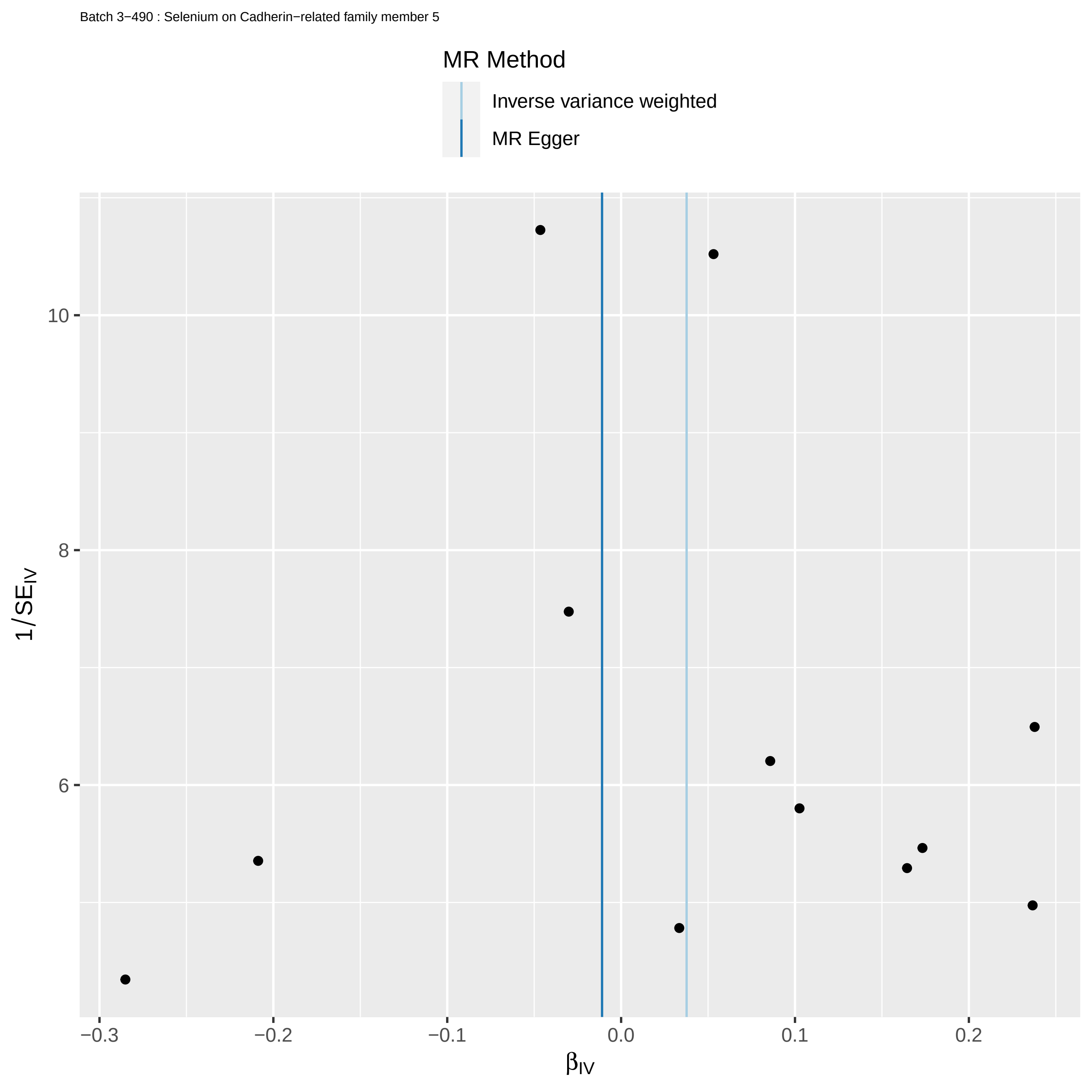

Batch 3−490 : Selenium on Cadherin−related family member 5
MR Method
Inverse variance weighted
MR Egger
10
8
6
1/SE$_{IV}$
−0.3
−0.2
−0.1
0.0
0.1
0.2
β$_{IV}$

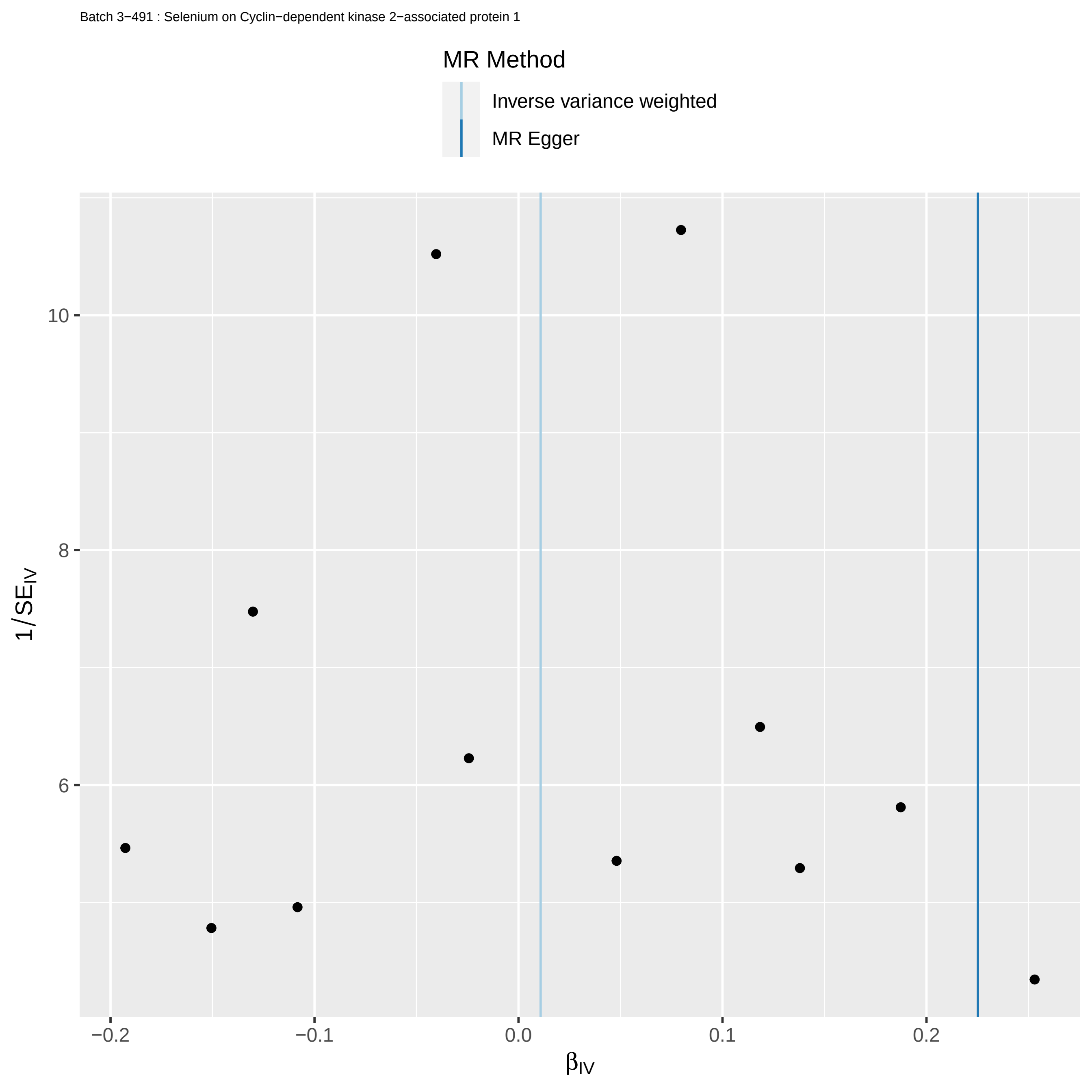

Batch 3−491 : Selenium on Cyclin−dependent kinase 2−associated protein 1
MR Method
Inverse variance weighted
MR Egger
10
8
6
$1/SE_{IV}$
−0.2
−0.1
0.0
0.1
0.2
β_{IV}

Batch 3−492 : Selenium on Cyclin−dependent kinase 2:Cyclin−A2 complex

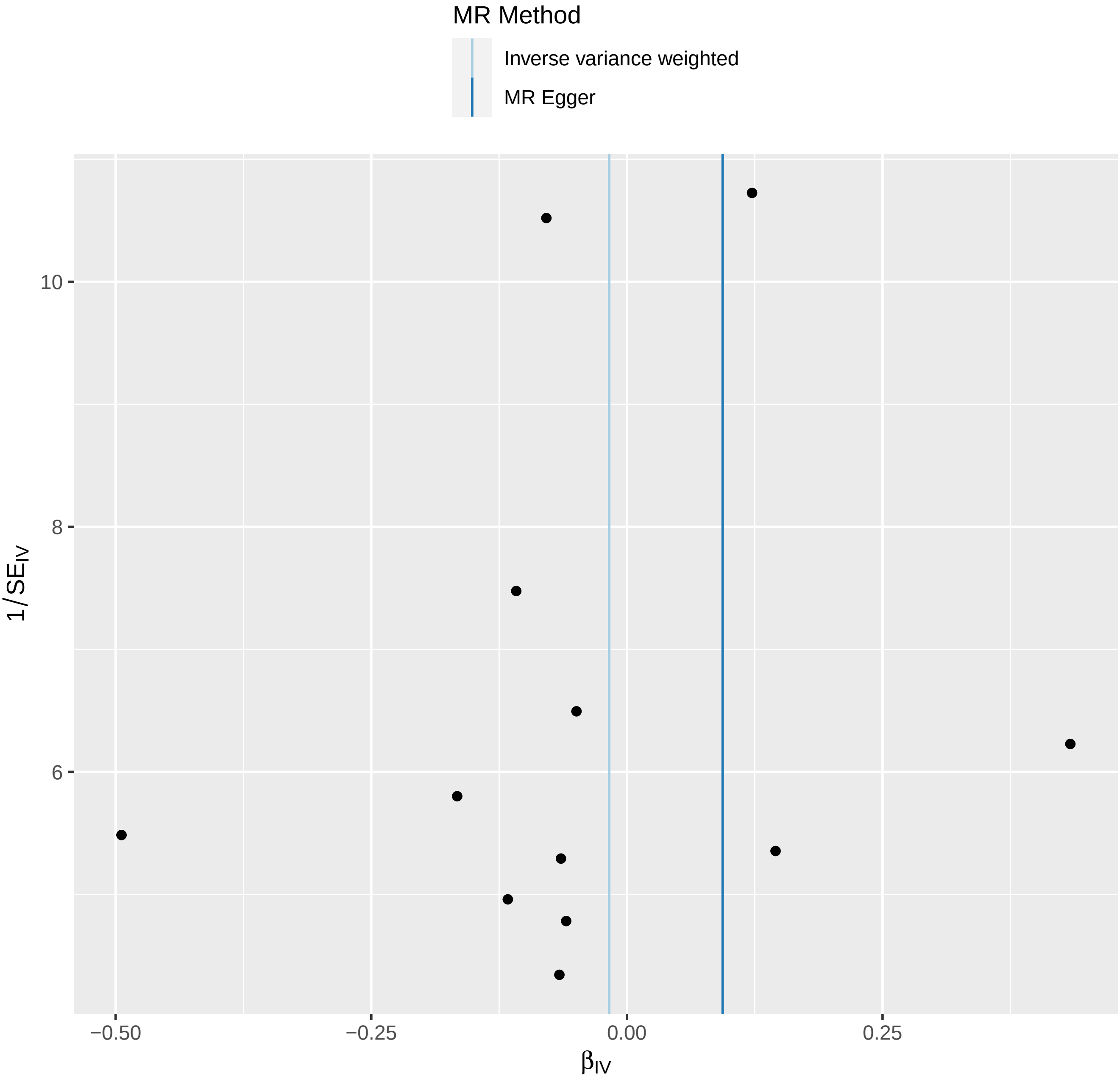

Batch 3−493 : Selenium on Cyclin−dependent kinase 5:Cyclin−dependent kinase 5 activator 1 complex

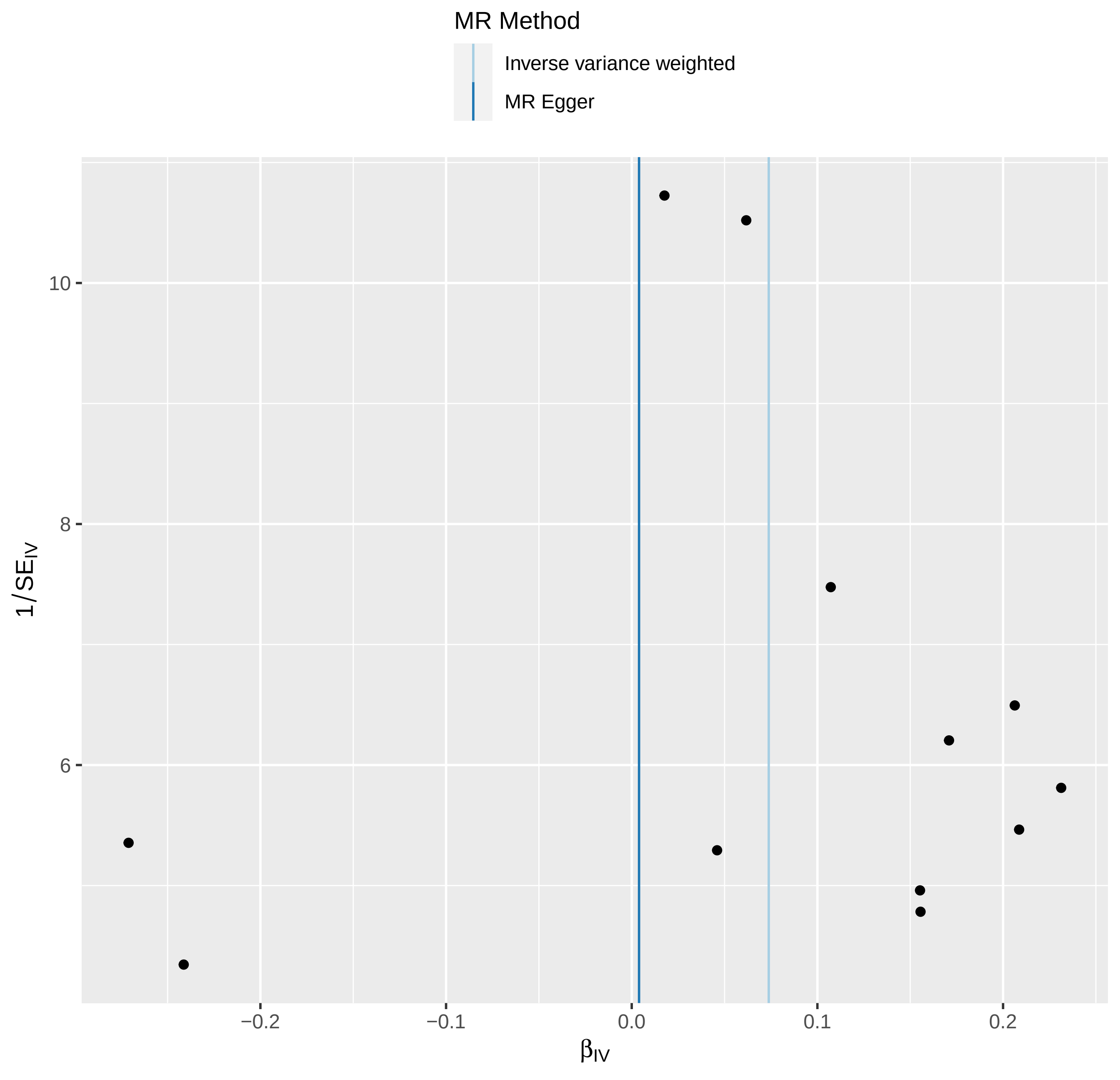

Batch 3−494 : Selenium on Cyclin−dependent kinase 8:Cyclin−C complex

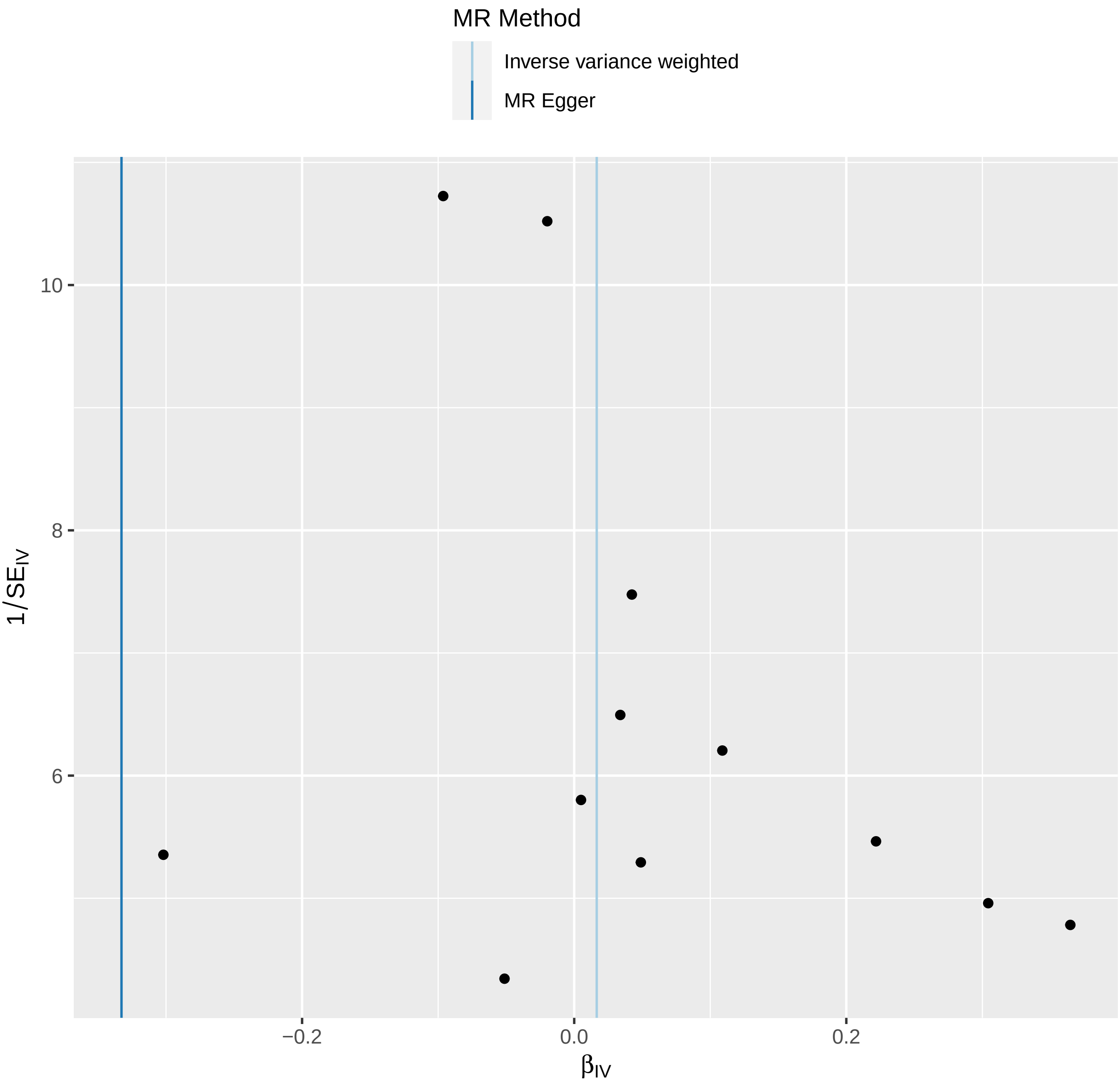

Batch 3−495 : Selenium on Cyclin−dependent kinase inhibitor 1B

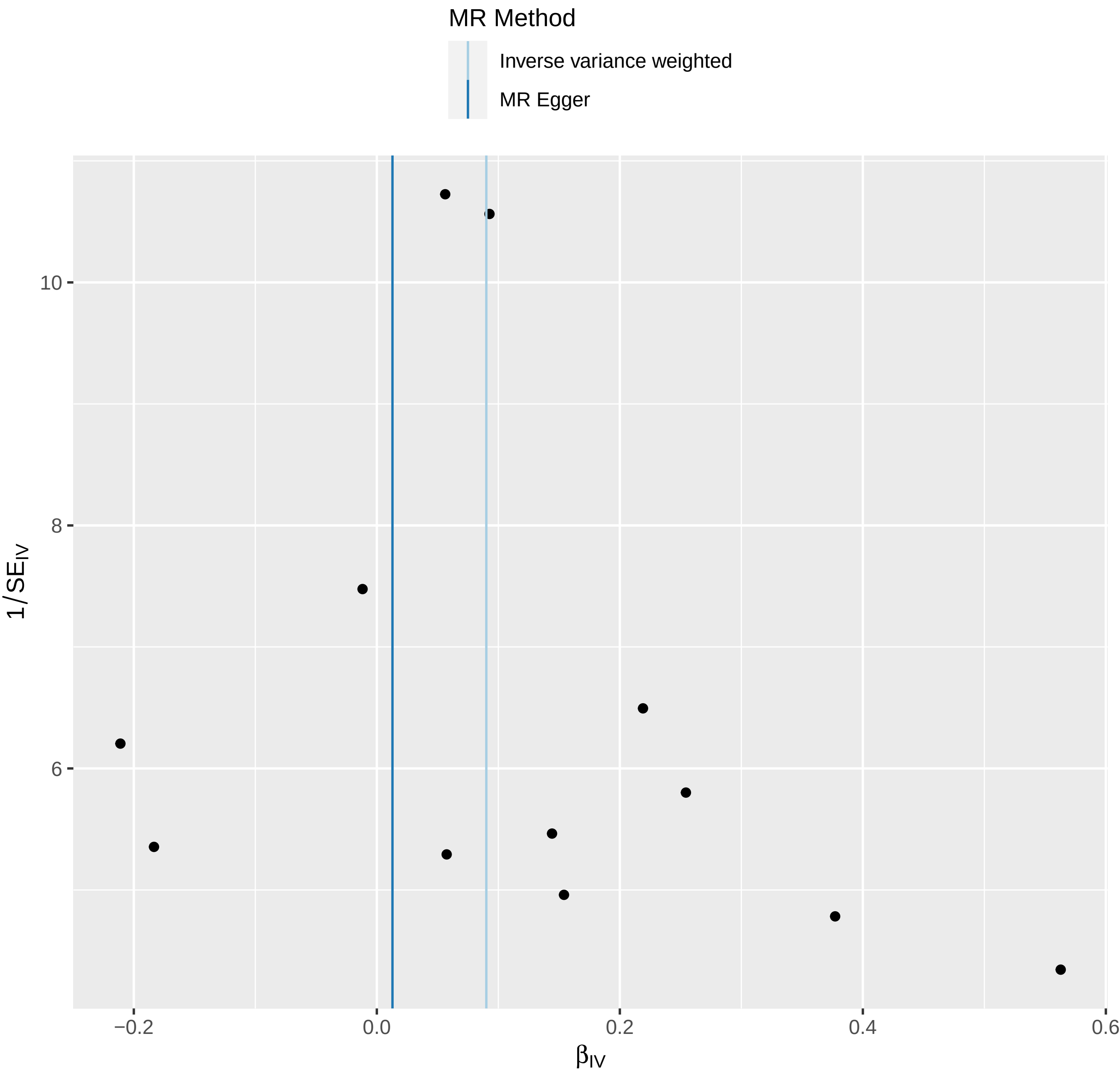

Batch 3−496 : Selenium on Cyclin−dependent kinase 4 inhibitor B

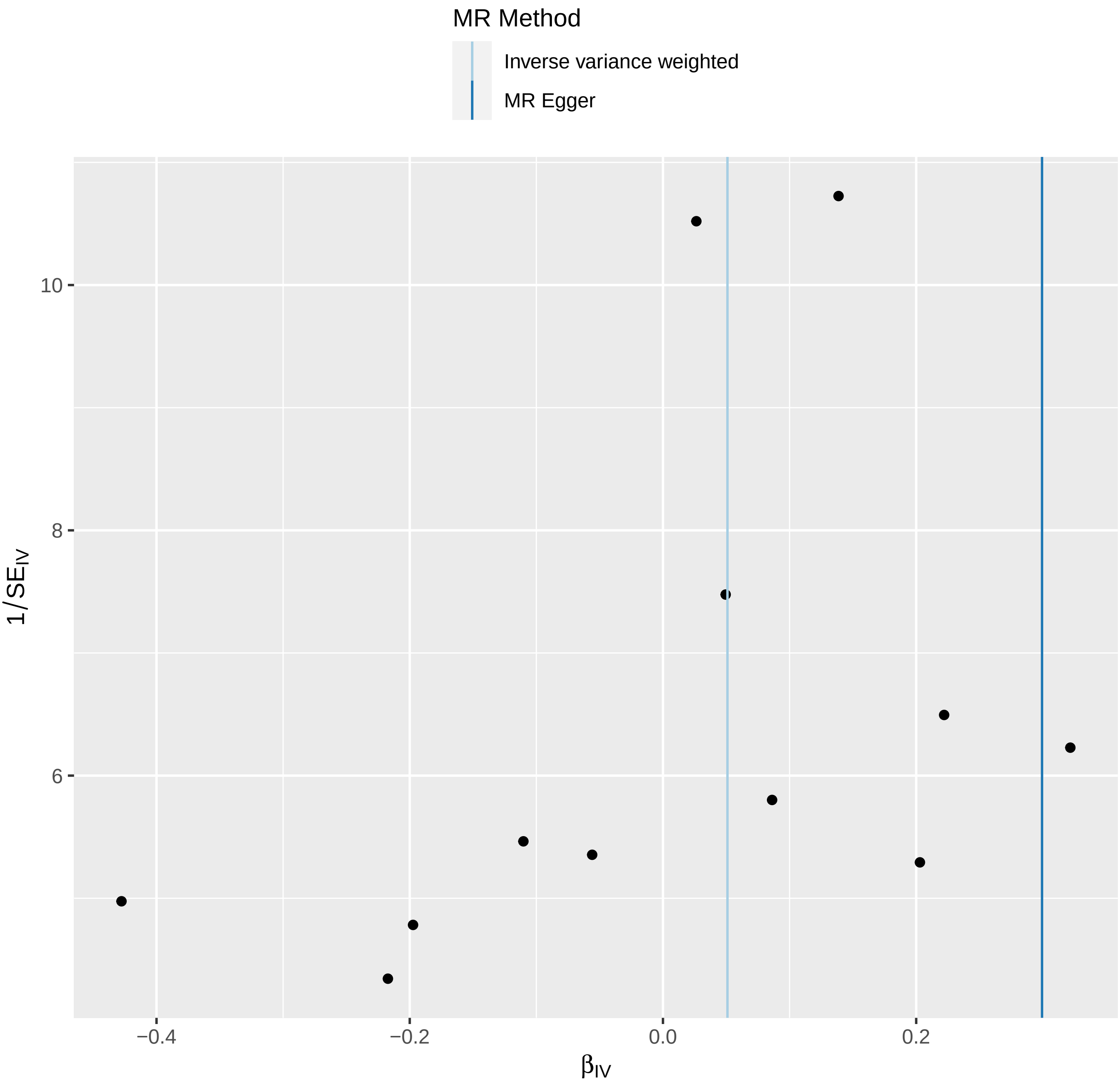

Batch 3−497 : Selenium on Cyclin−dependent kinase inhibitor 3

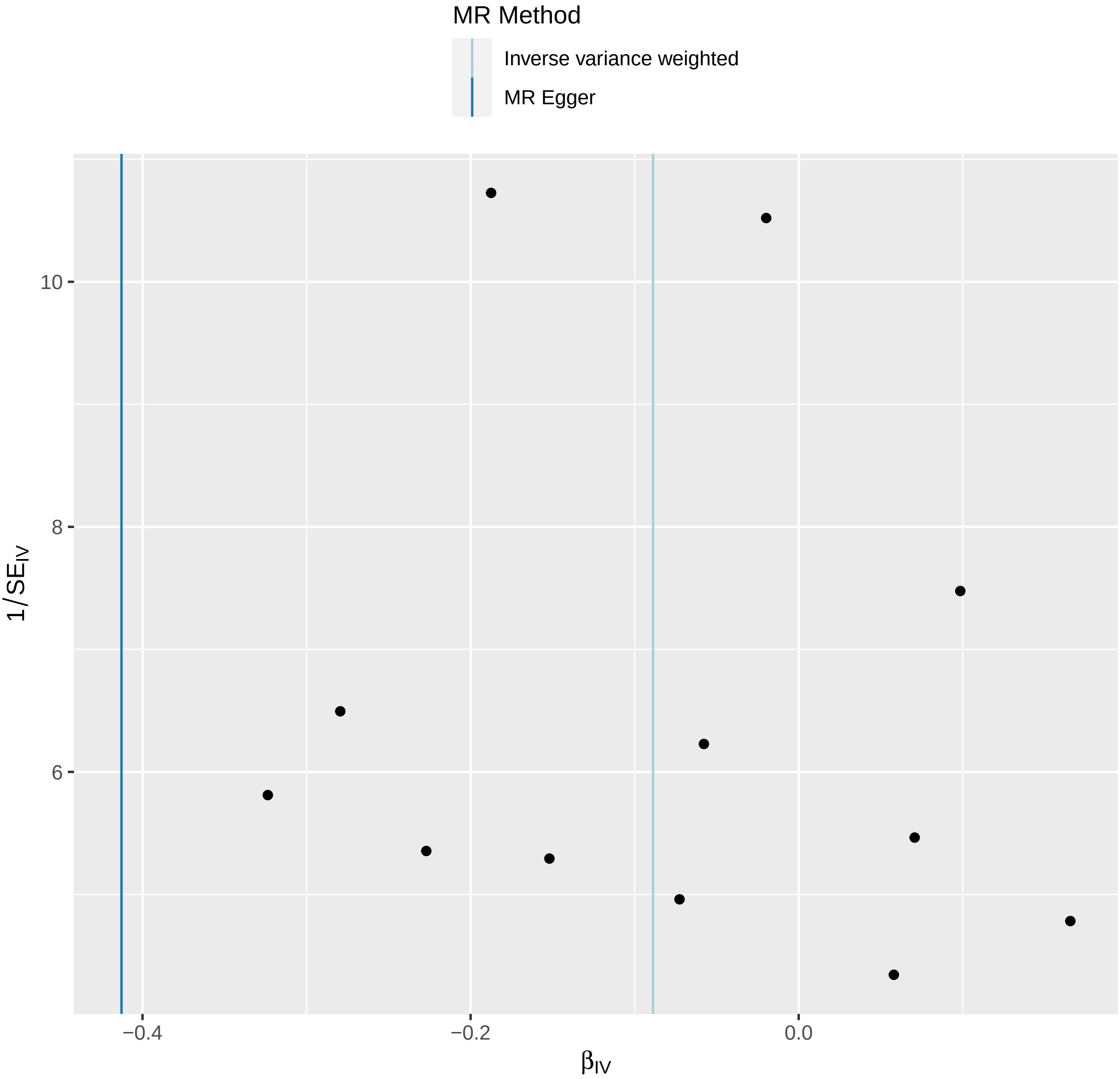

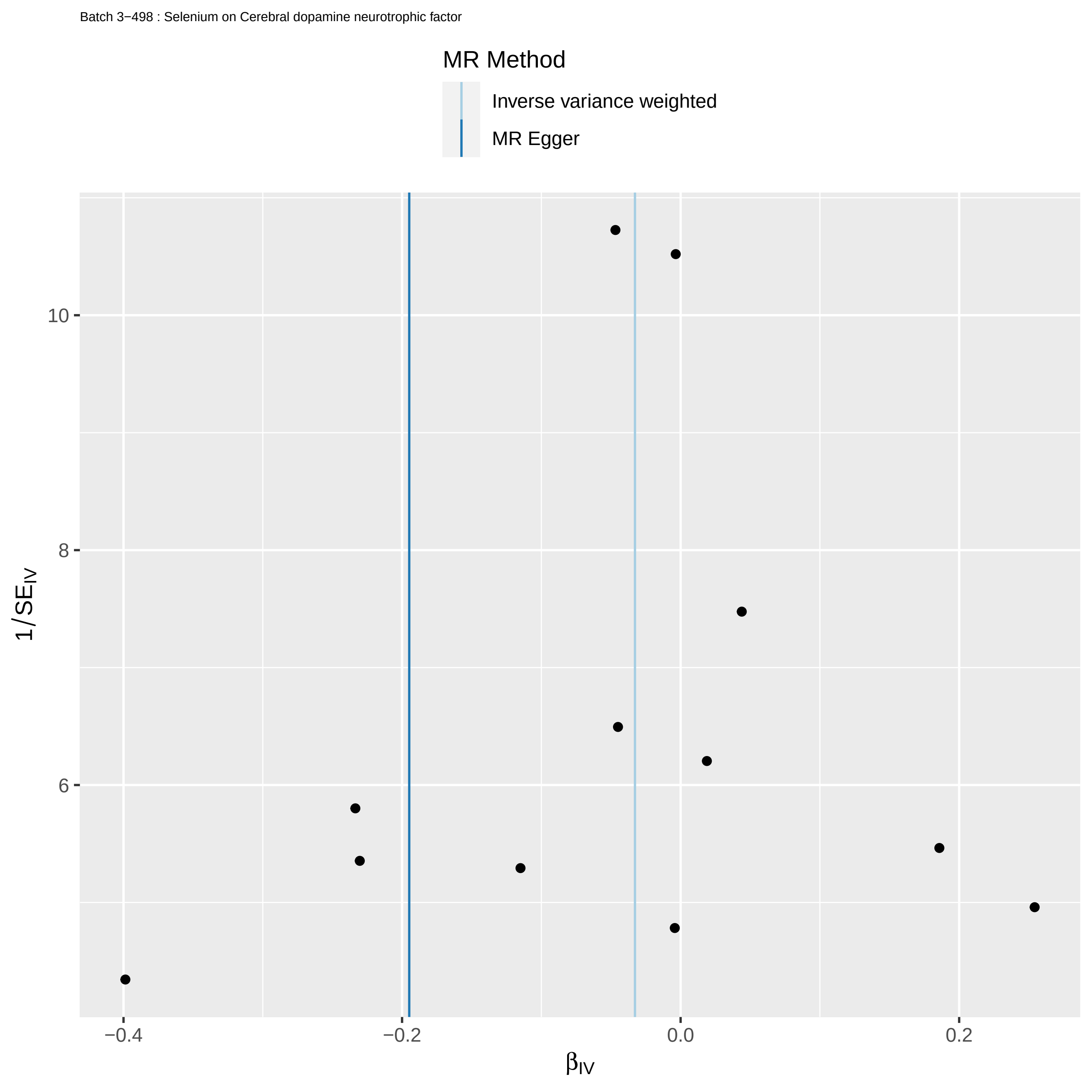

Batch 3−498 : Selenium on Cerebral dopamine neurotrophic factor
MR Method
Inverse variance weighted
MR Egger
10
8
6
1/SE$_{IV}$
−0.4
−0.2
0.0
0.2
β$_{IV}$

Batch 3−499 : Selenium on Cell adhesion molecule−related/down−regulated by oncogenes

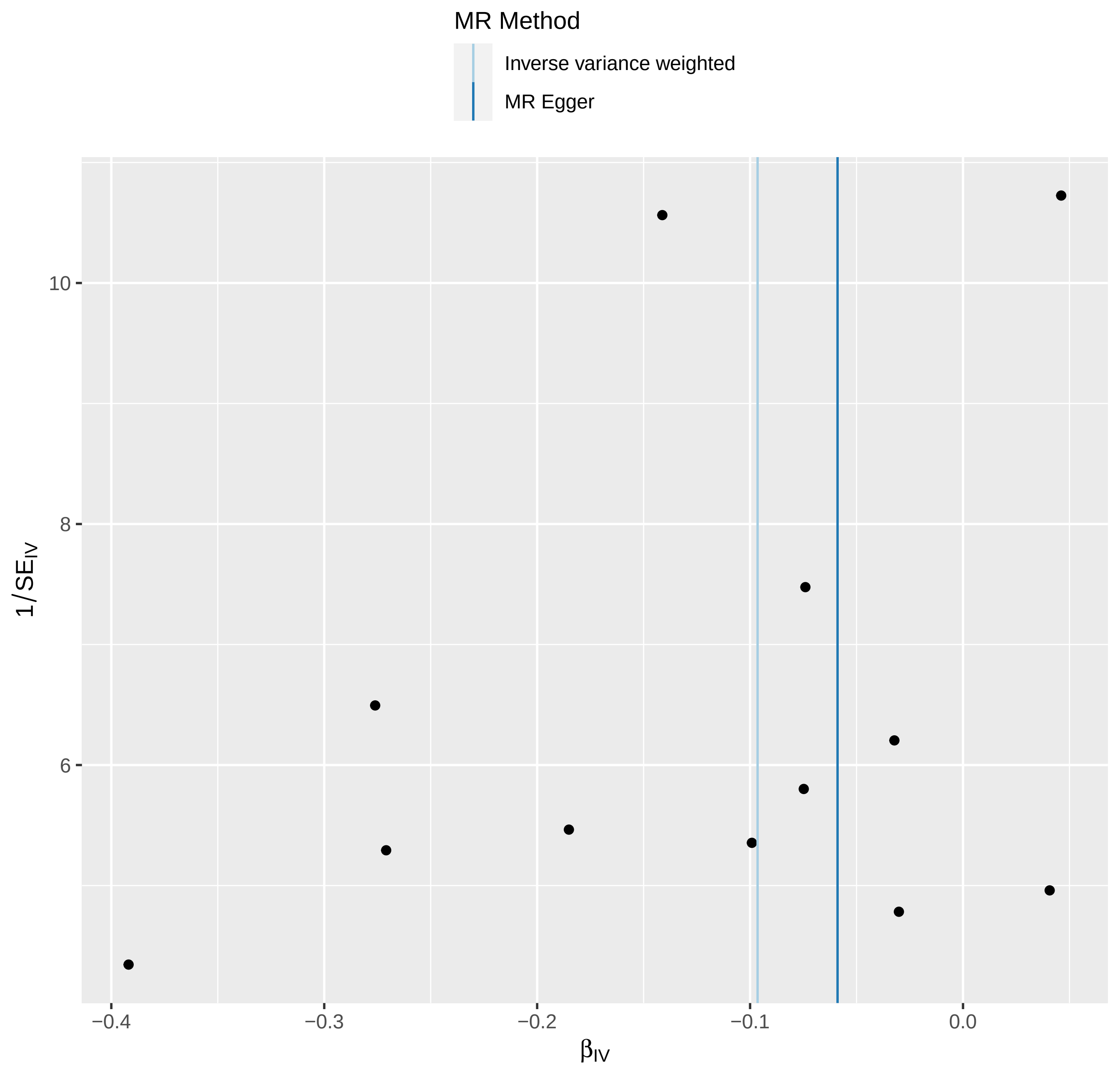

Batch 3−500 : Selenium on Corneodesmosin

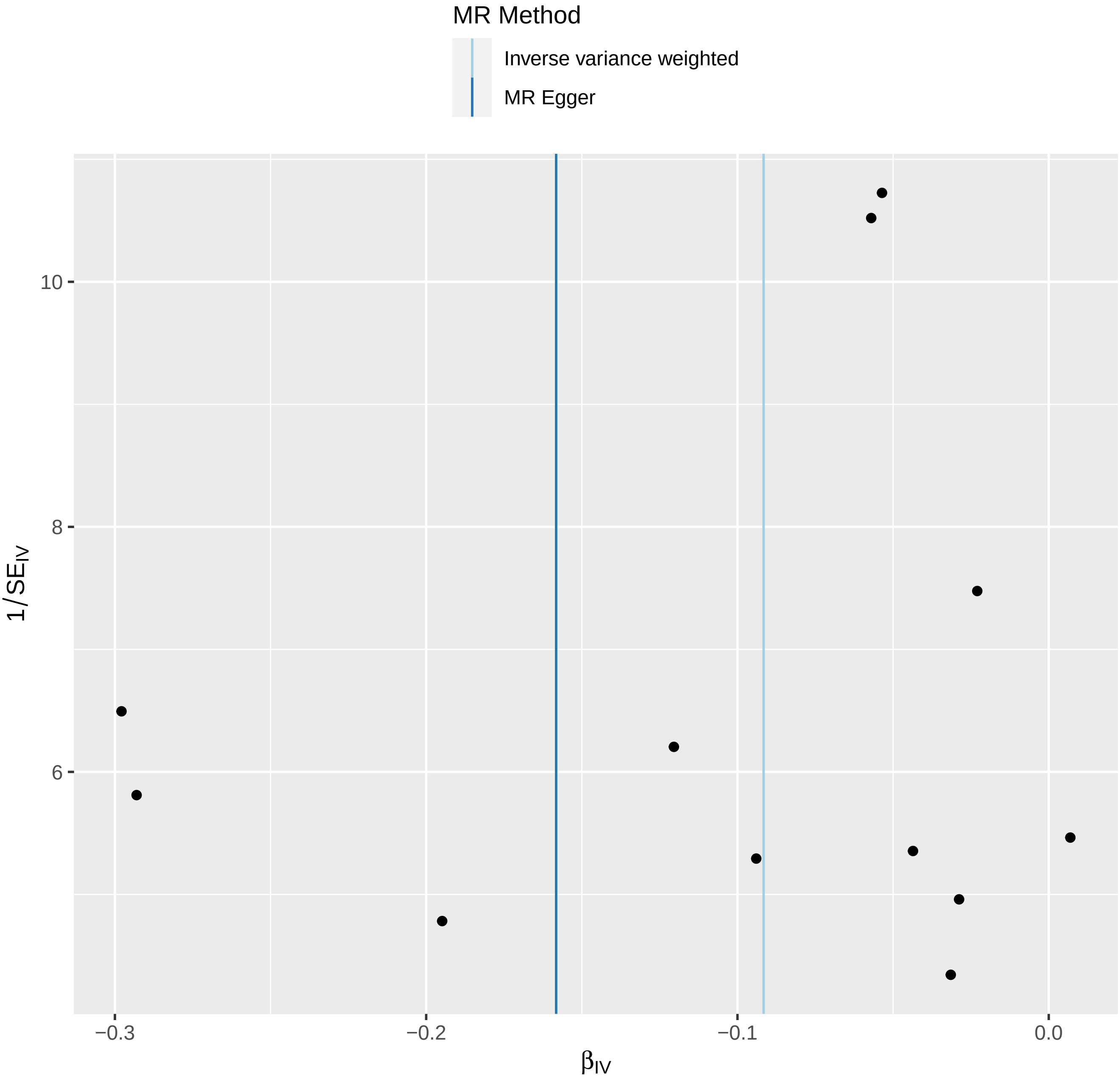

Batch 3−501 : Selenium on Testis−specific chromodomain protein Y 1

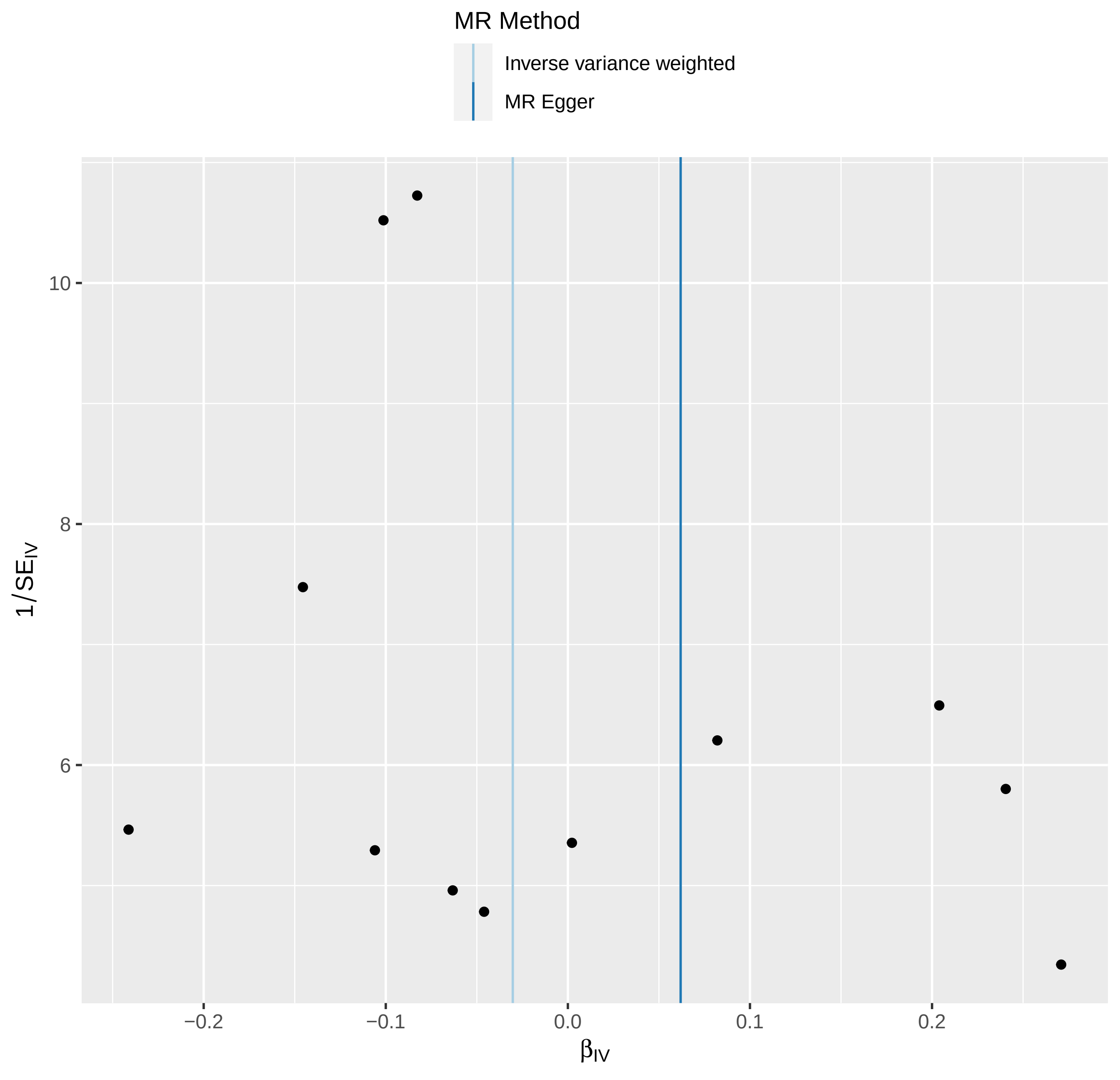

Batch 3−502 : Selenium on Carcinoembryonic antigen−related cell adhesion molecule 1

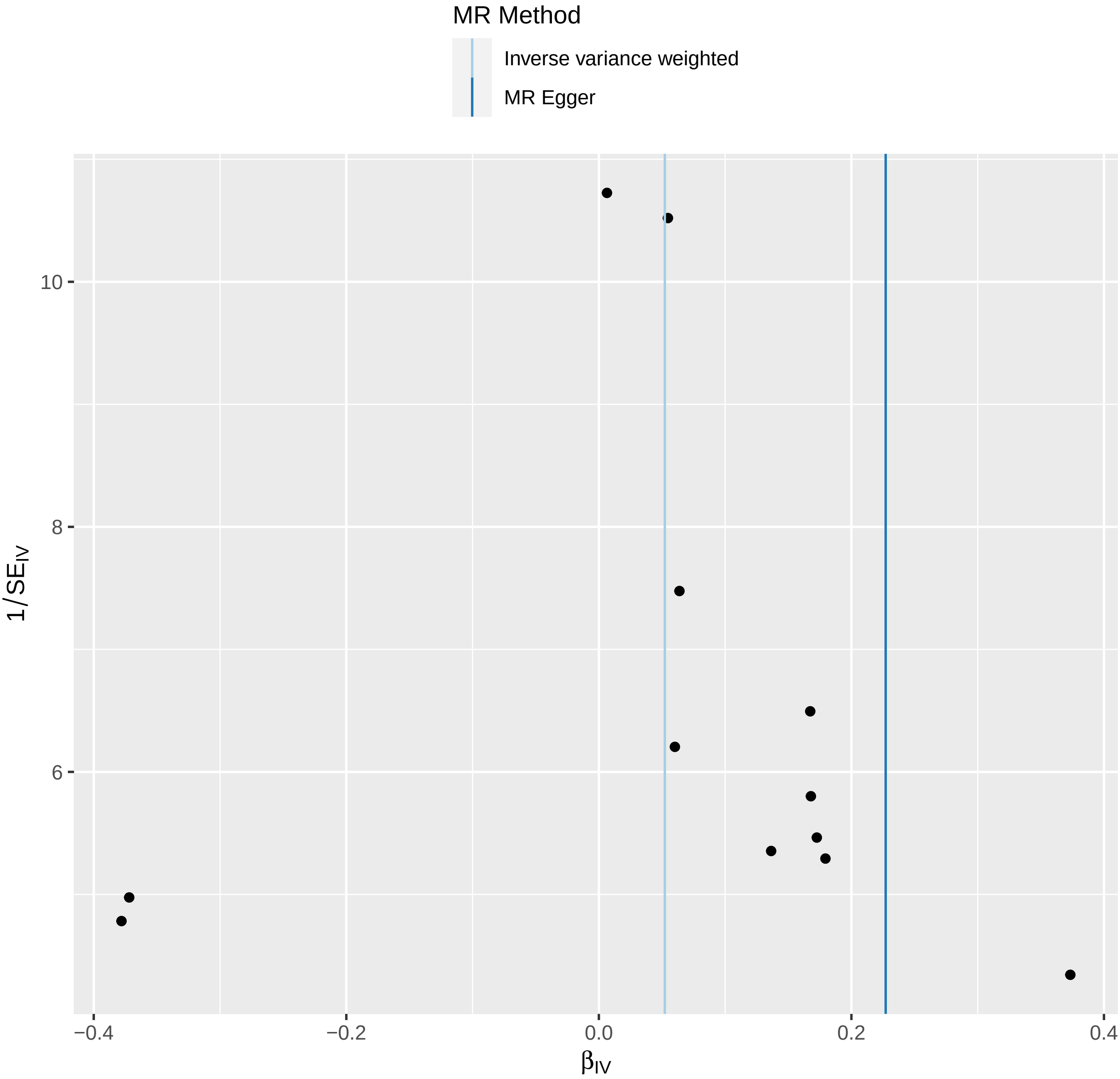

Batch 3−503 : Selenium on Carcinoembryonic antigen−related cell adhesion molecule 21

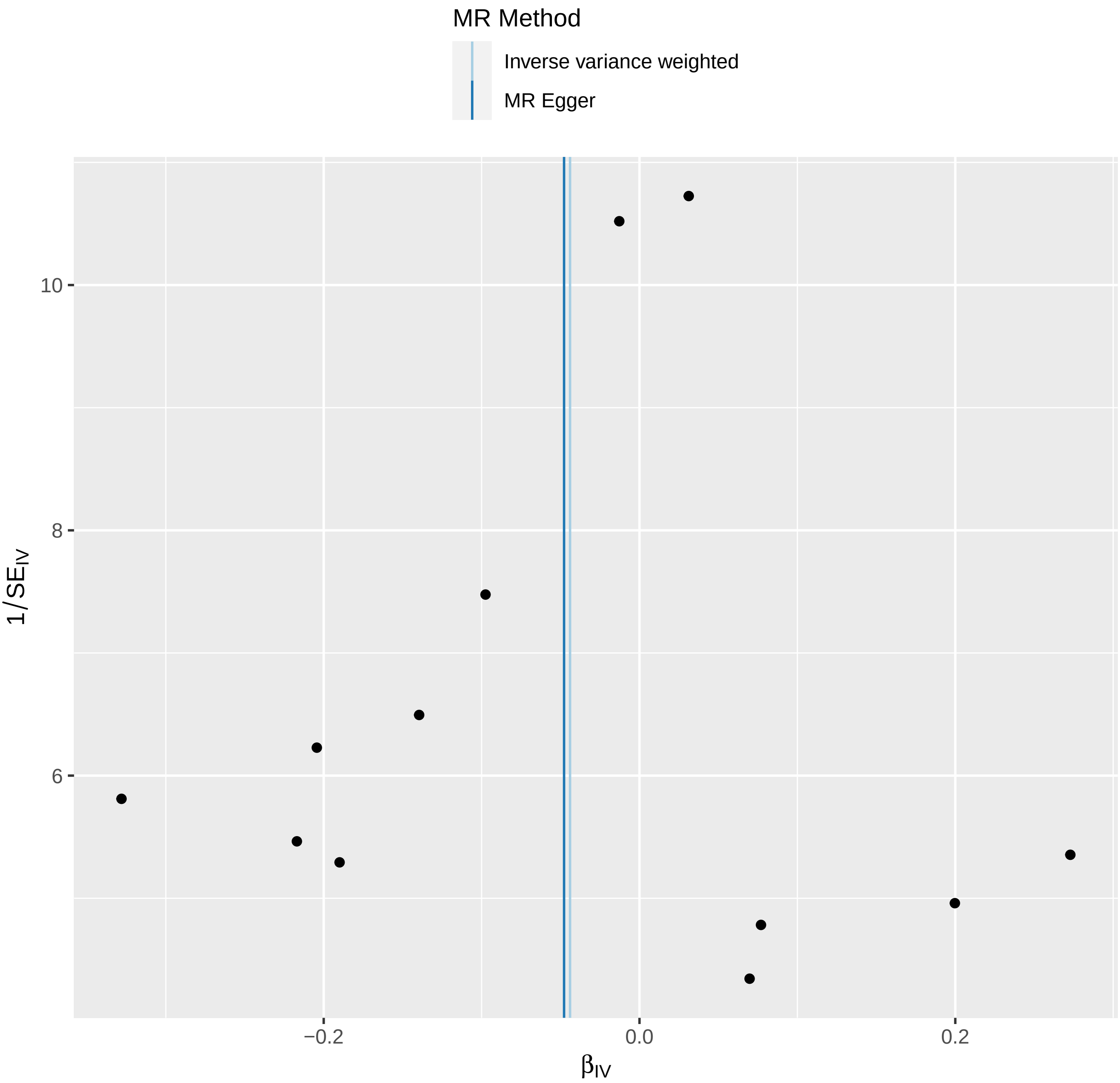

Batch 3−504 : Selenium on Carcinoembryonic antigen−related cell adhesion molecule 4

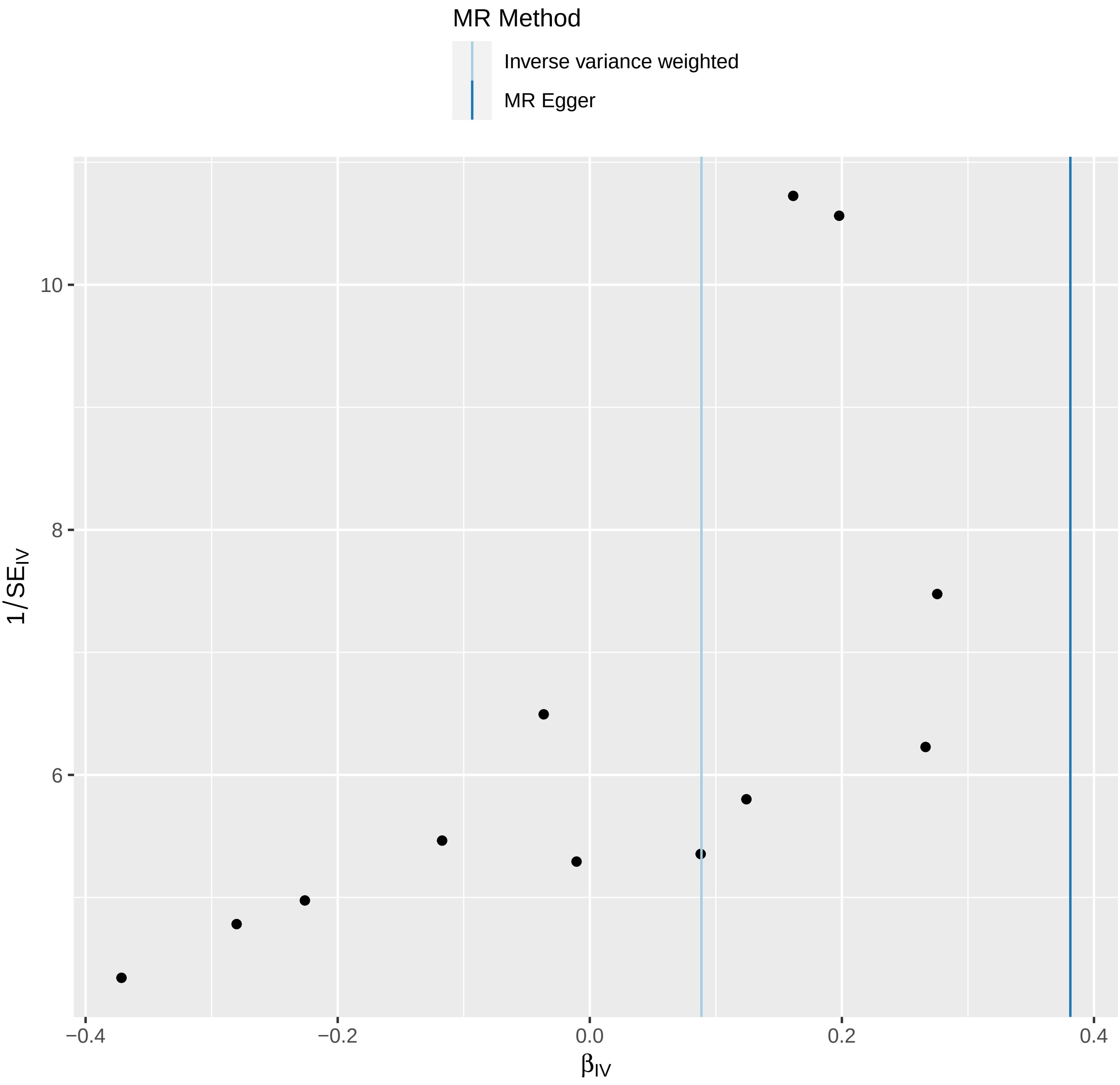

Batch 3−505 : Selenium on CCAAT/enhancer−binding protein beta

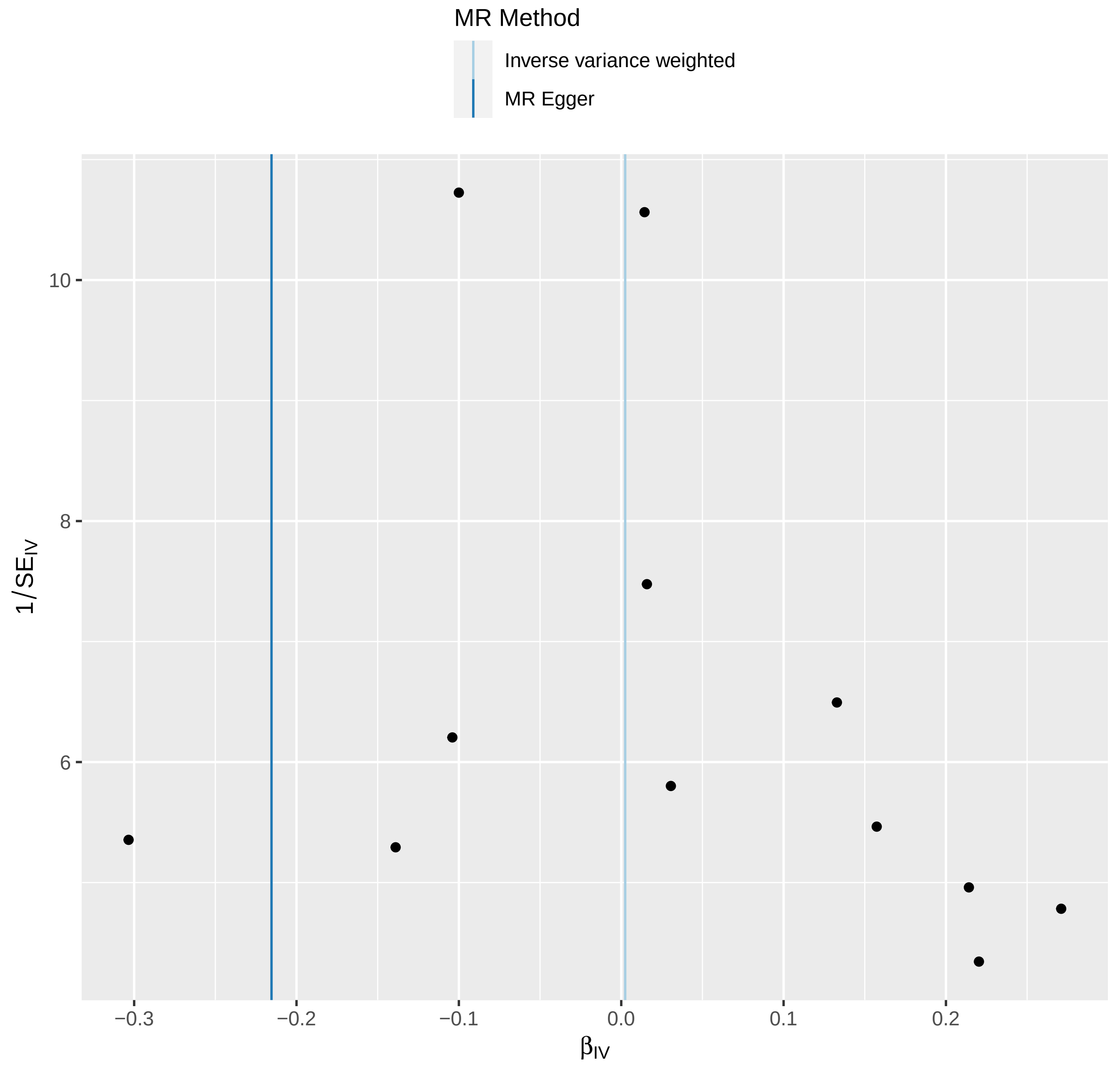

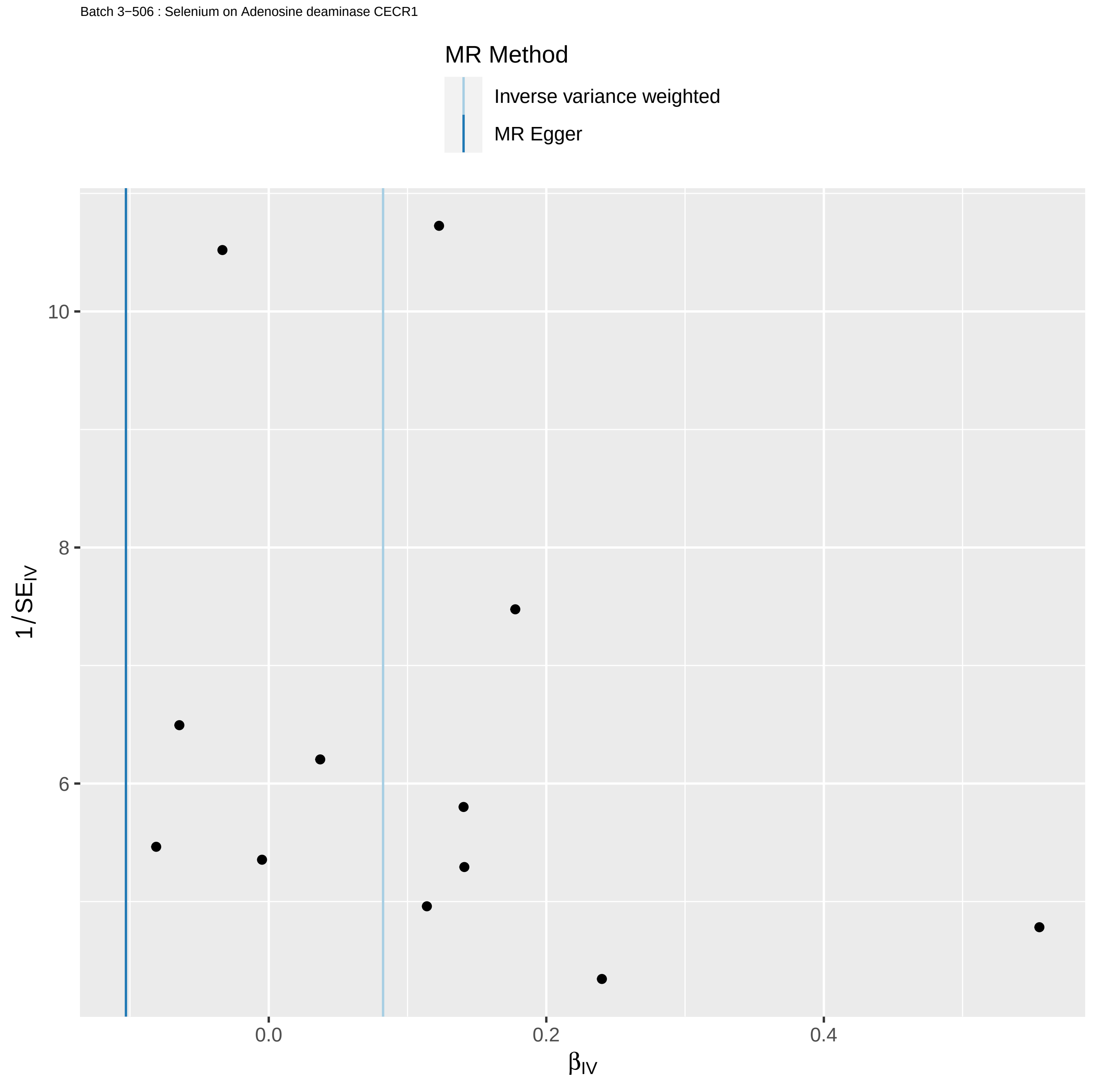
Batch 3−506 : Selenium on Adenosine deaminase CECR1
MR Method
Inverse variance weighted
MR Egger
10
8
6
1/SE_IV
0.0
0.2
0.4
β_IV

Batch 3−507 : Selenium on Bile salt−activated lipase

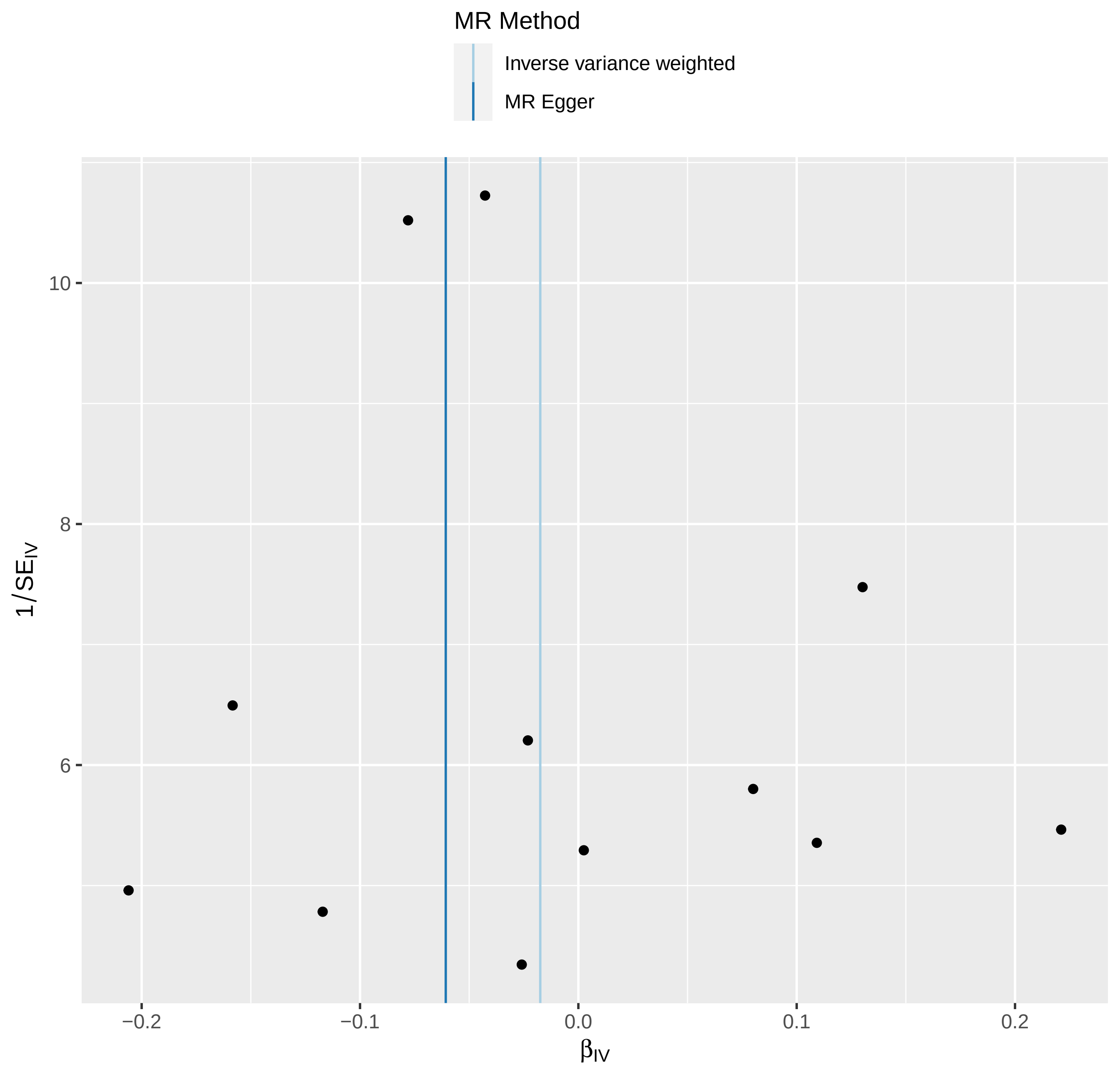

Batch 3−508 : Selenium on Chymotrypsin−like elastase family member 2A

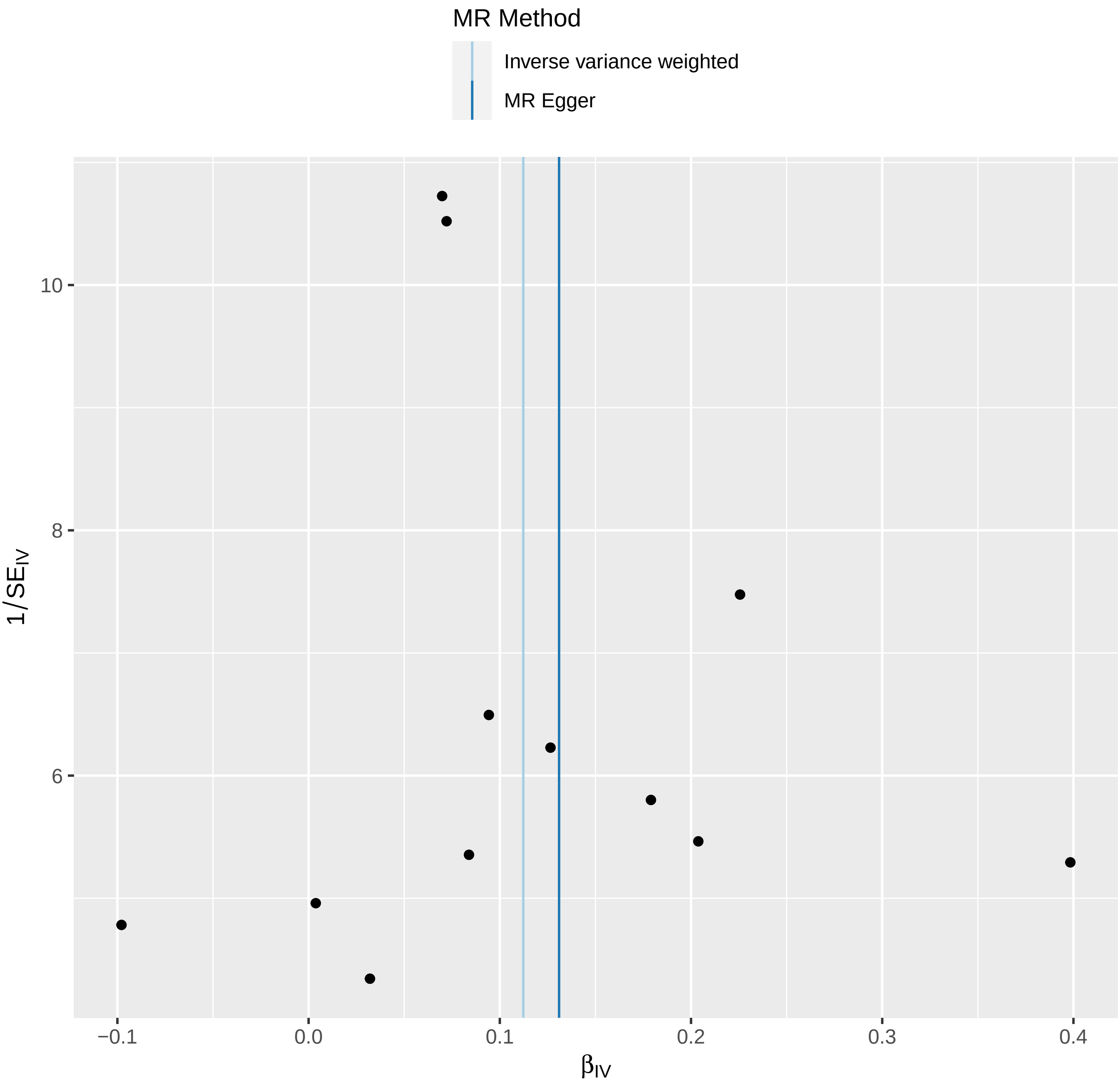

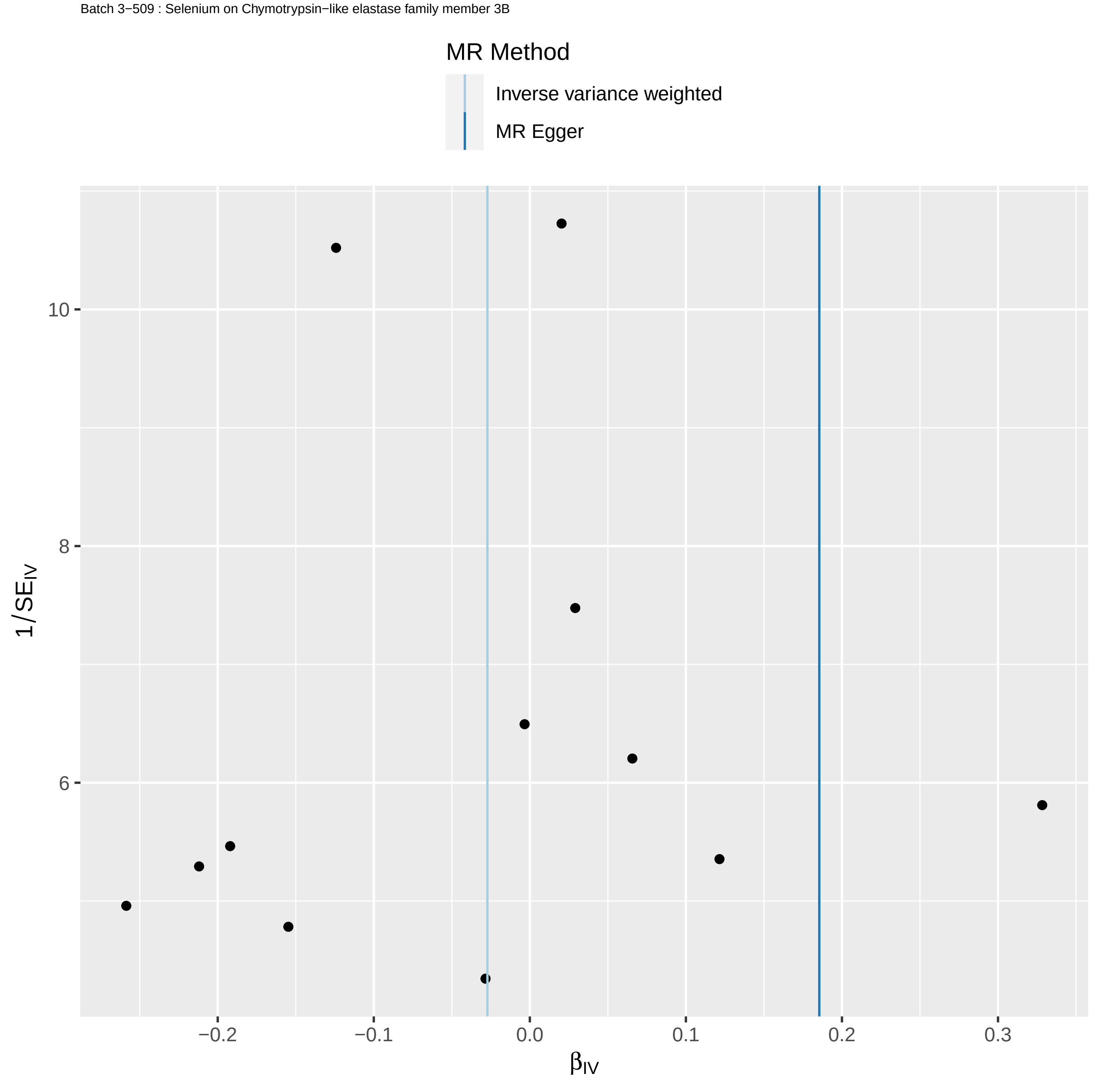

Batch 3−509 : Selenium on Chymotrypsin−like elastase family member 3B
MR Method
Inverse variance weighted
MR Egger
10
8
6
1/SE_IV
−0.2
−0.1
0.0
0.1
0.2
0.3
β_IV

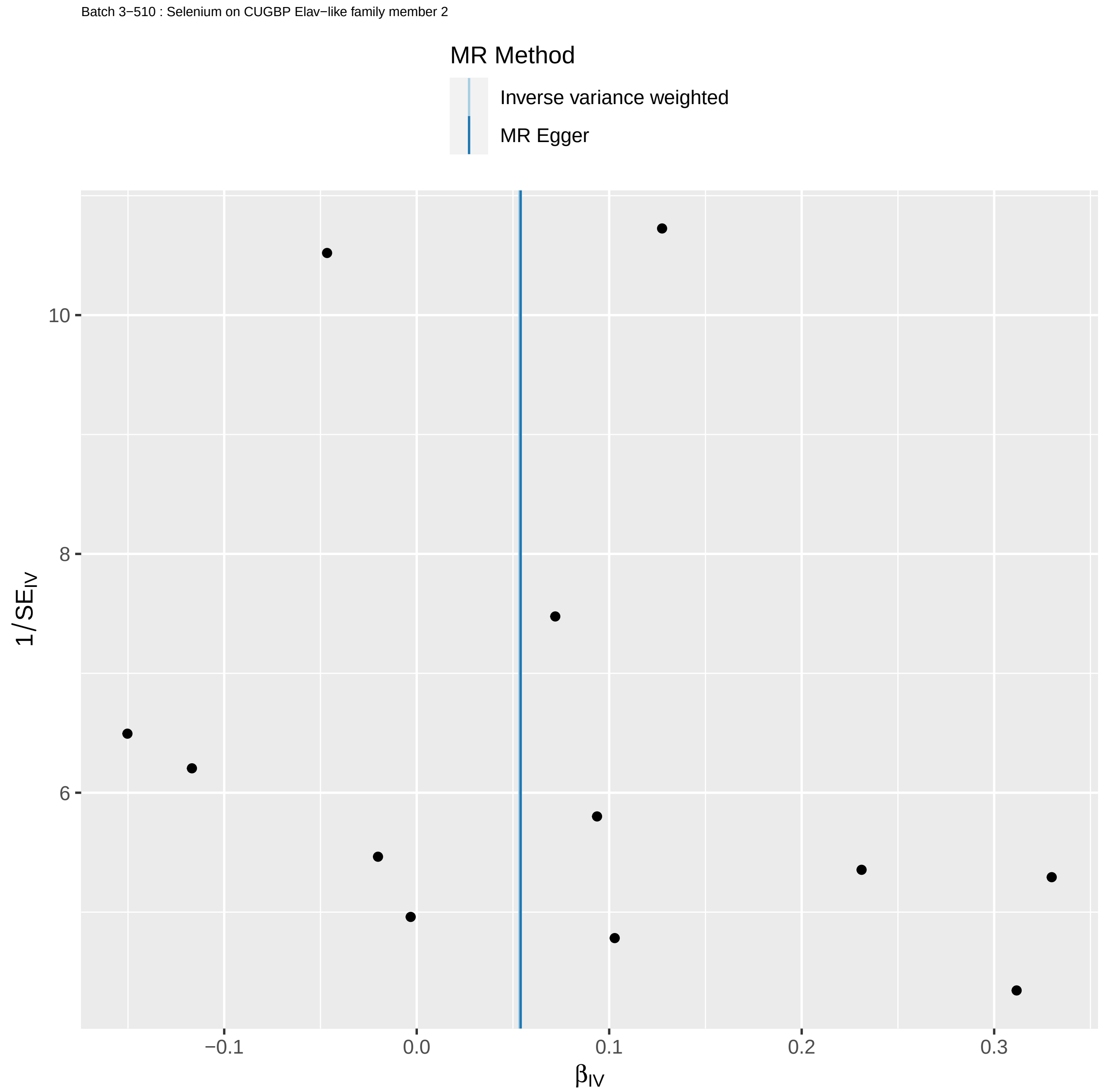
Batch 3−510 : Selenium on CUGBP Elav−like family member 2
MR Method
Inverse variance weighted
MR Egger
10
8
6
1/SE_IV
−0.1
0.0
0.1
0.2
0.3
β_IV

Batch 3−511 : Selenium on Centromere protein W

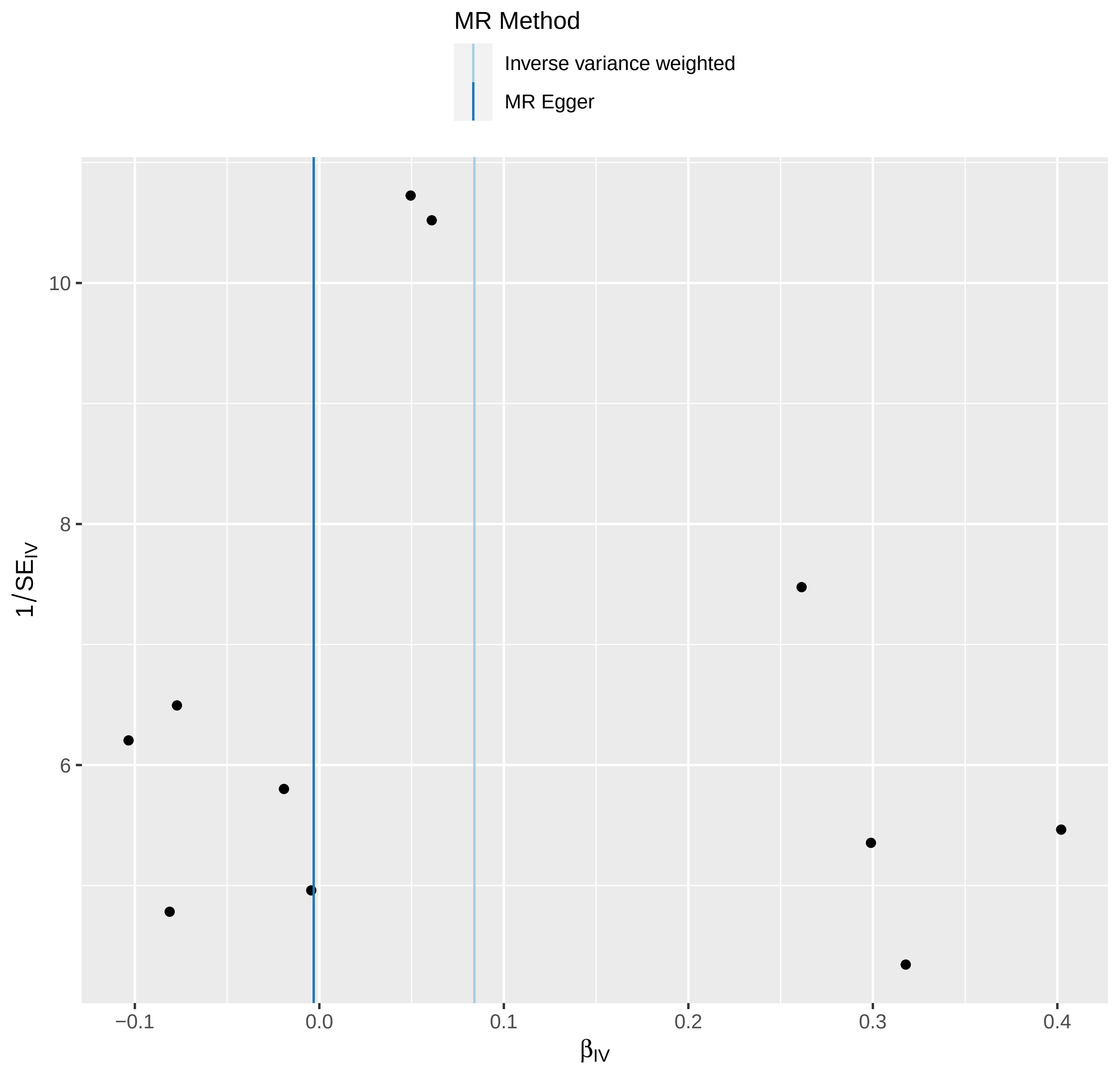

Batch 3−512 : Selenium on Centrosomal protein of 57 kDa

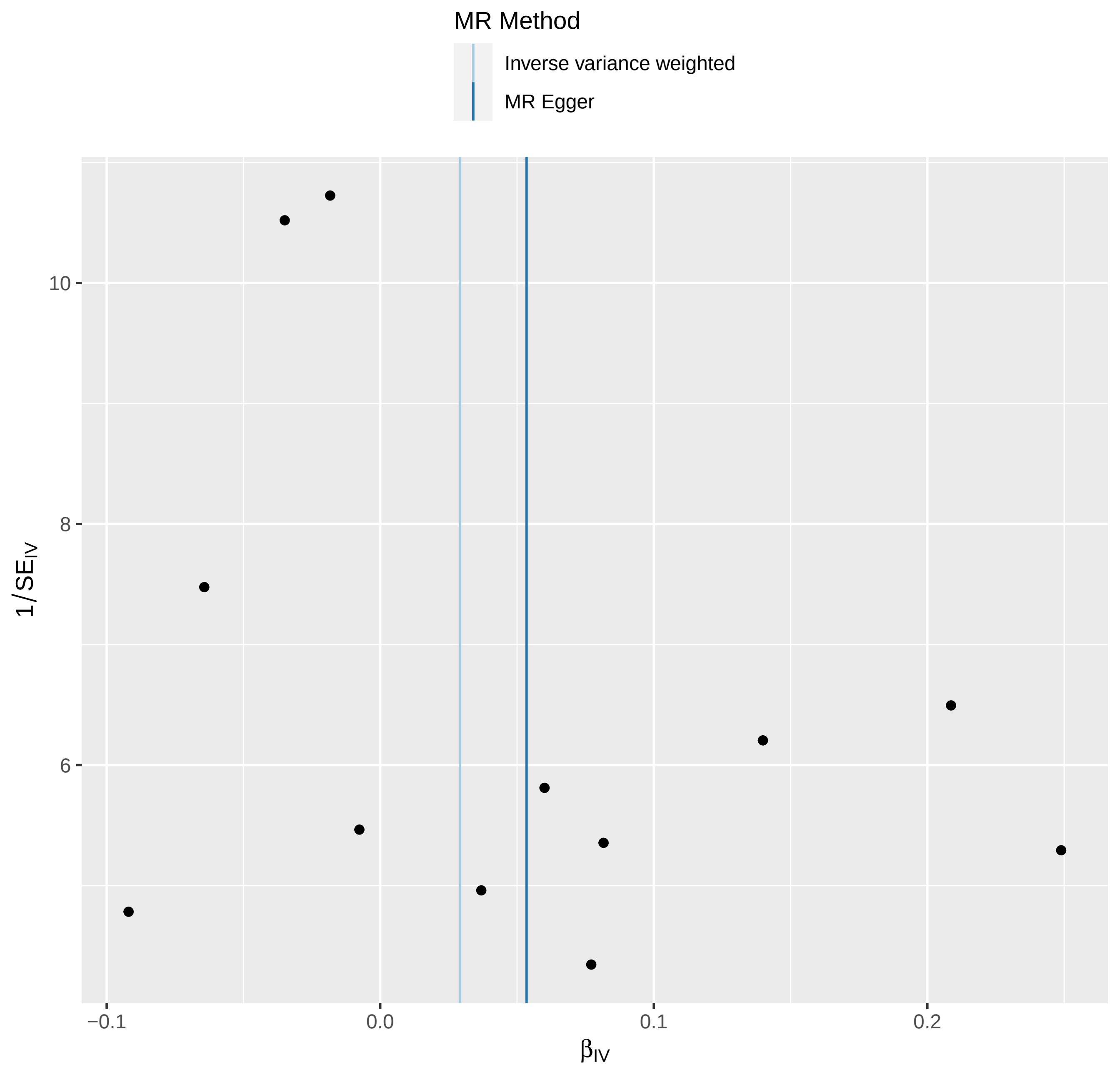

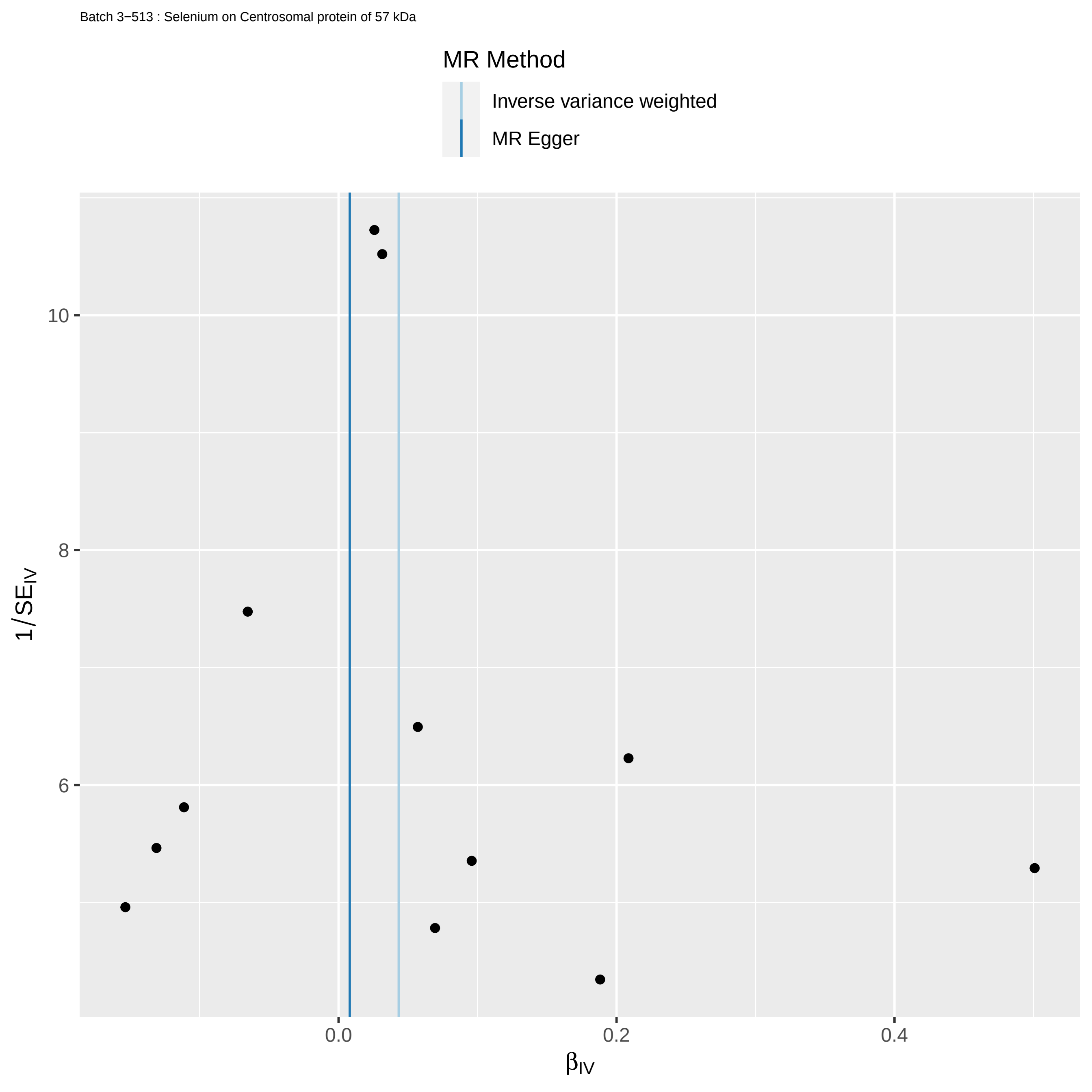

Batch 3−513 : Selenium on Centrosomal protein of 57 kDa
MR Method
Inverse variance weighted
MR Egger
10
8
6
0.0
0.2
0.4
$1/SE_{IV}$
β_{IV}

Batch 3−514 : Selenium on Centrosomal protein of 57 kDa

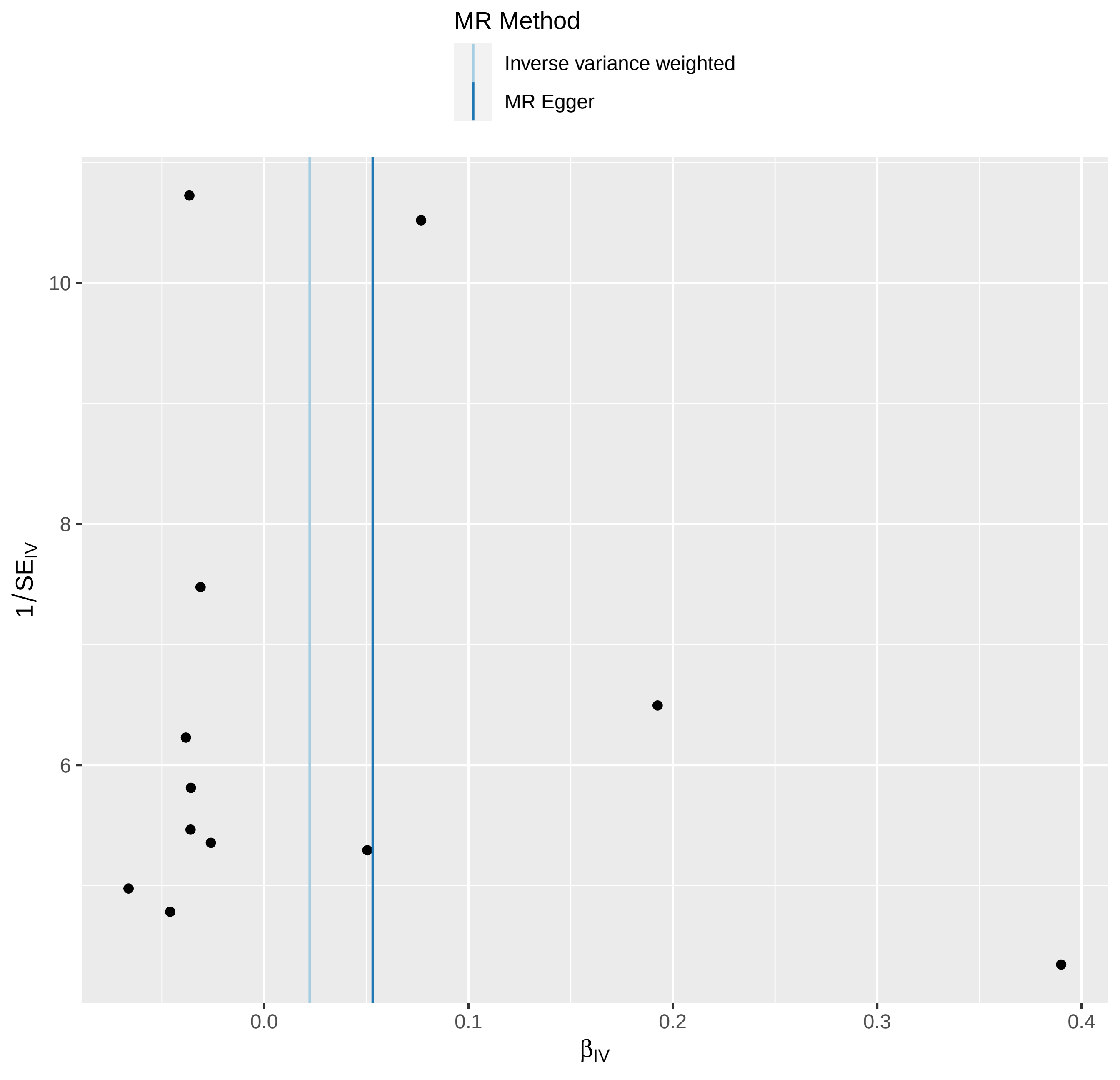

Batch 3−515 : Selenium on Ceramide synthase 5

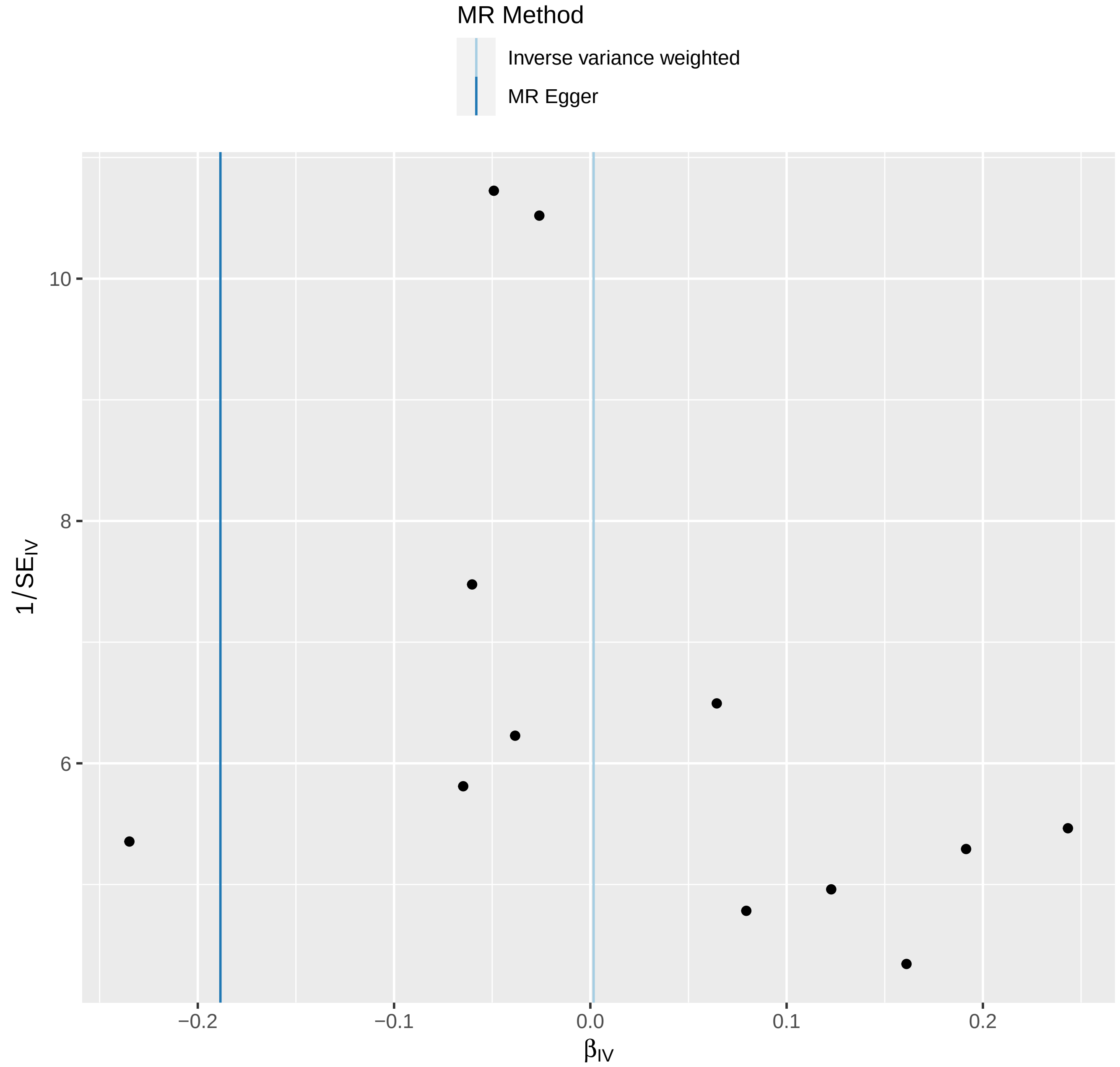

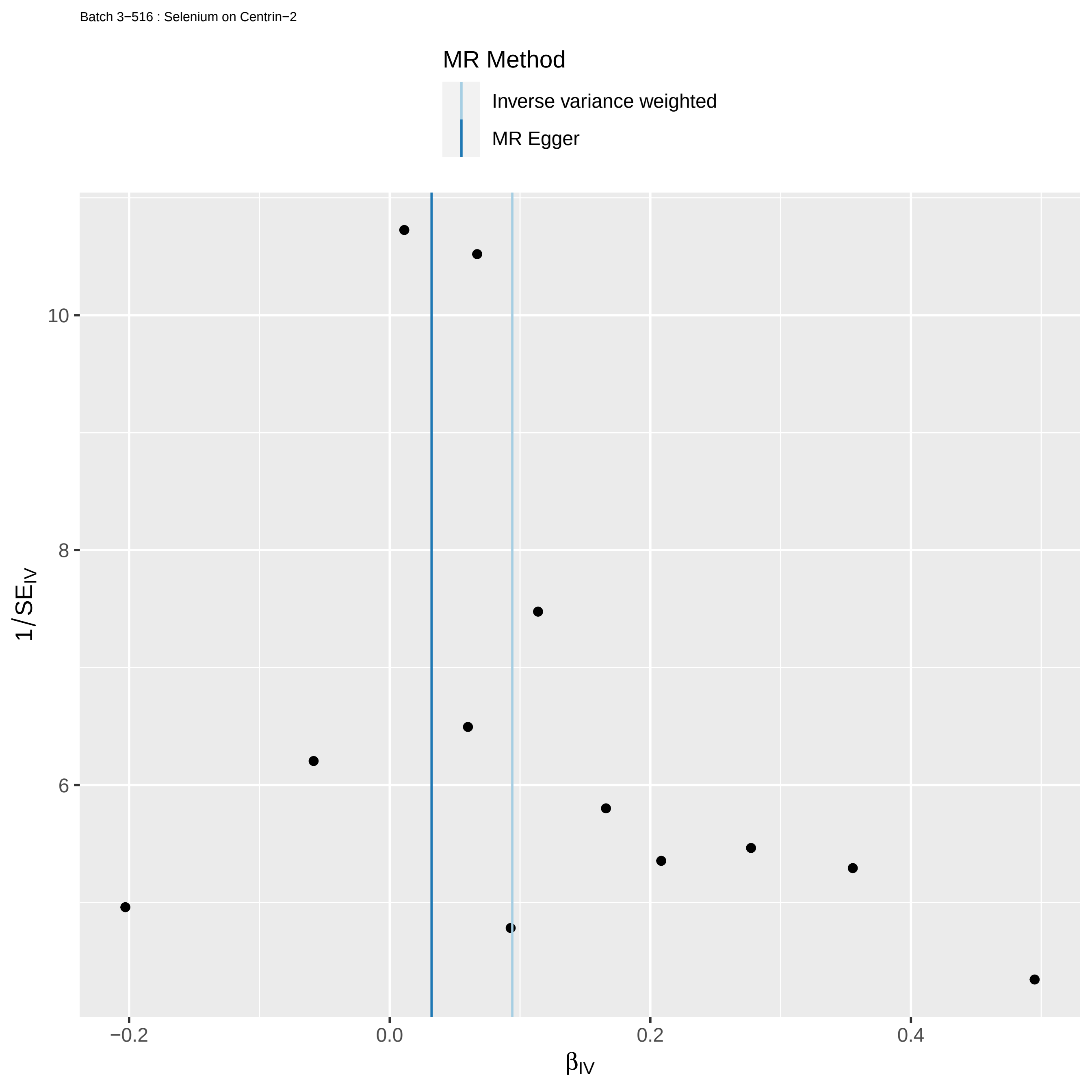
Batch 3−516 : Selenium on Centrin−2
MR Method
Inverse variance weighted
MR Egger
10
8
6
1/SE_IV
−0.2
0.0
0.2
0.4
β_IV

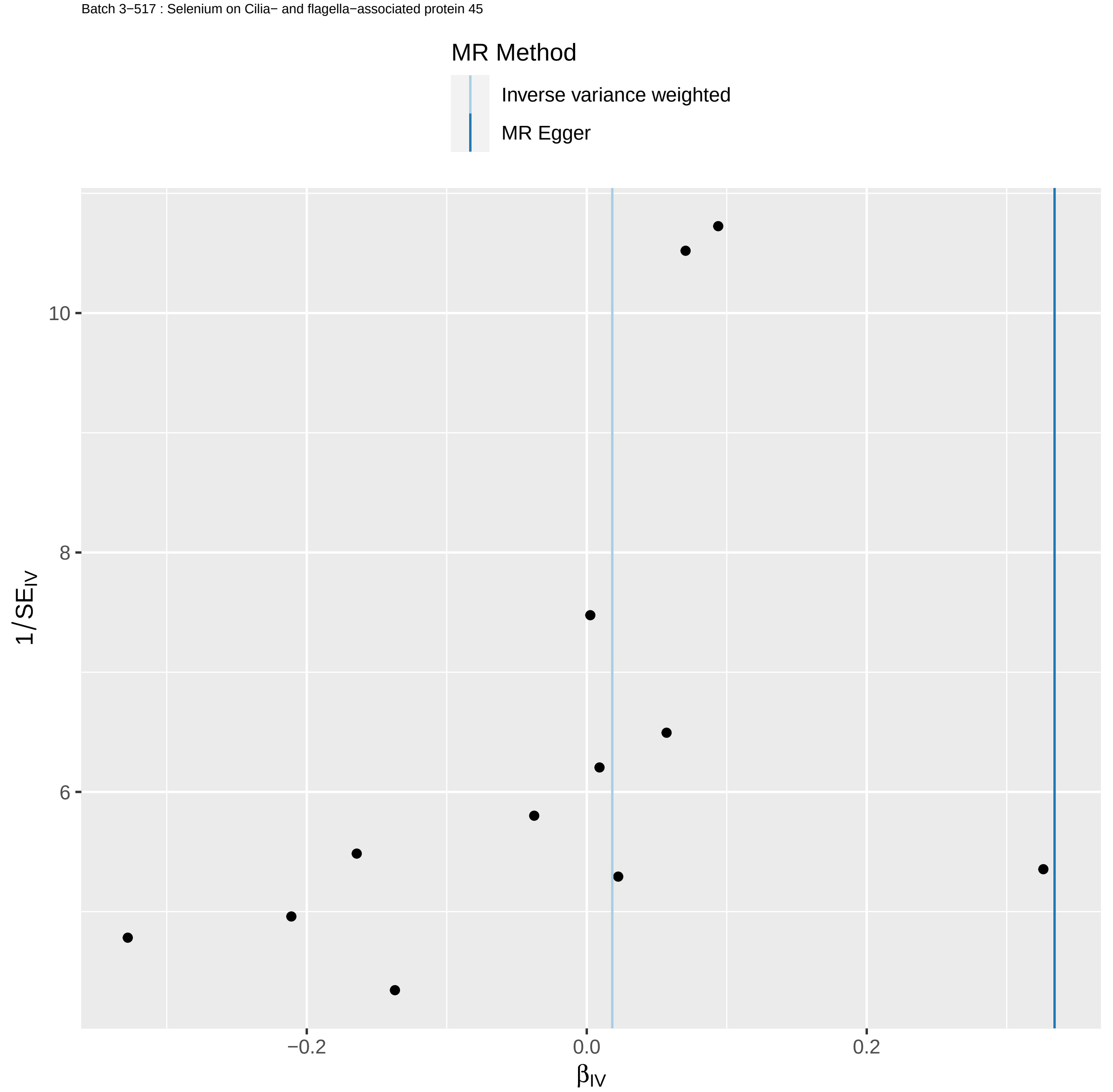
Batch 3−517 : Selenium on Cilia− and flagella−associated protein 45
MR Method
Inverse variance weighted
MR Egger
10
8
6
−0.2
0.0
0.2
$1/SE_{IV}$
β_{IV}

Batch 3−518 : Selenium on Complement factor B

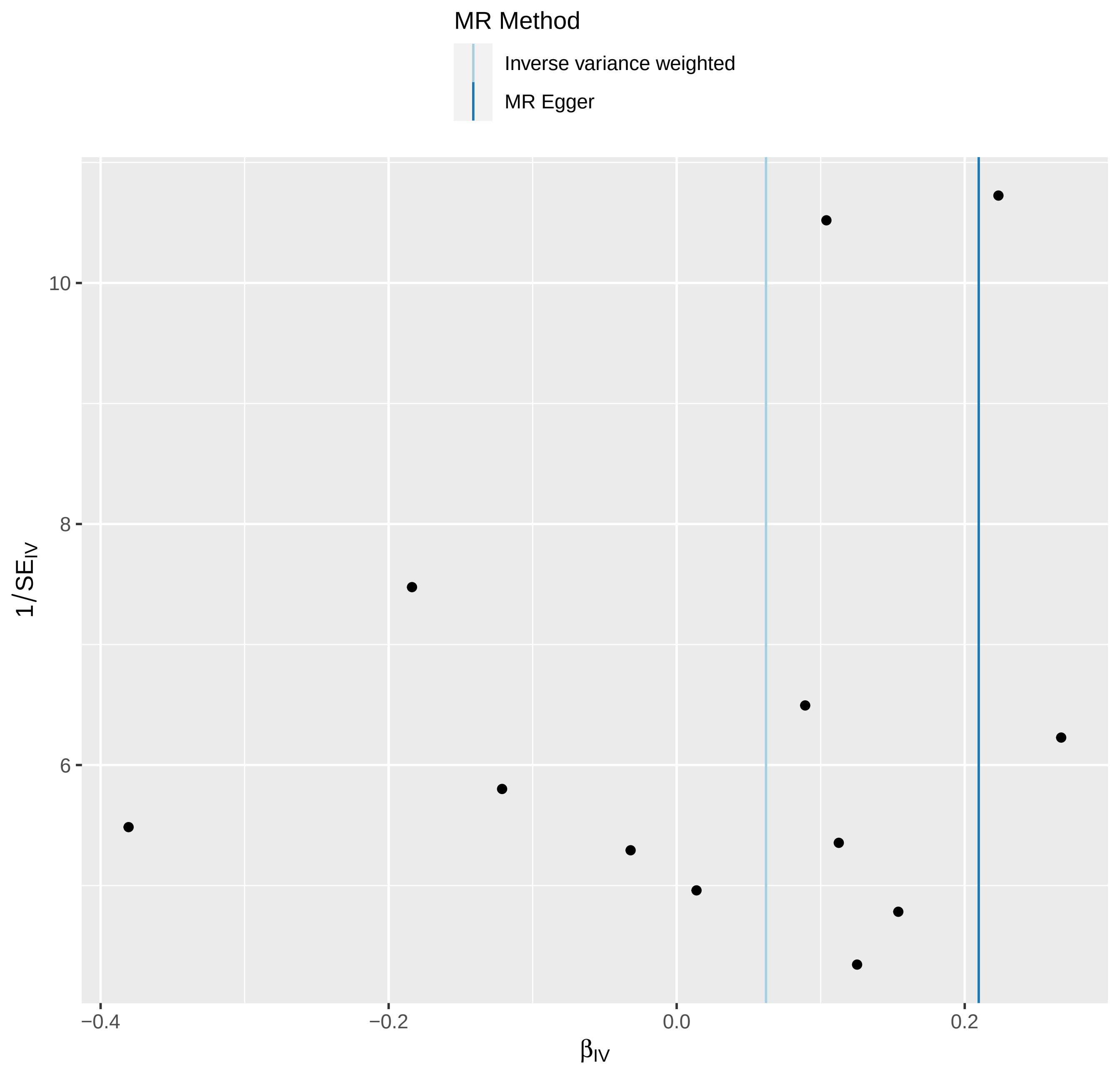

Batch 3−519 : Selenium on Cryptic protein

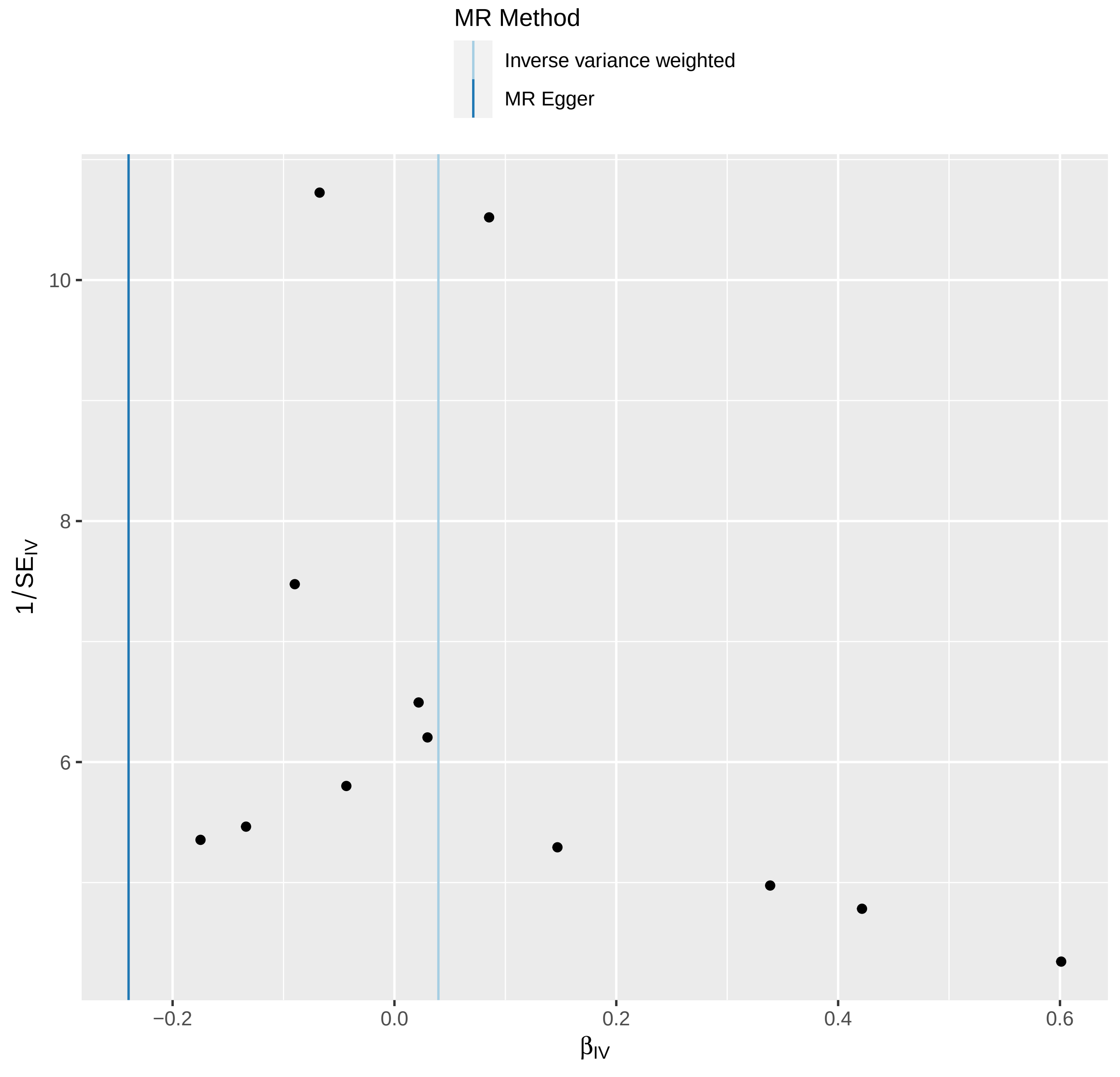

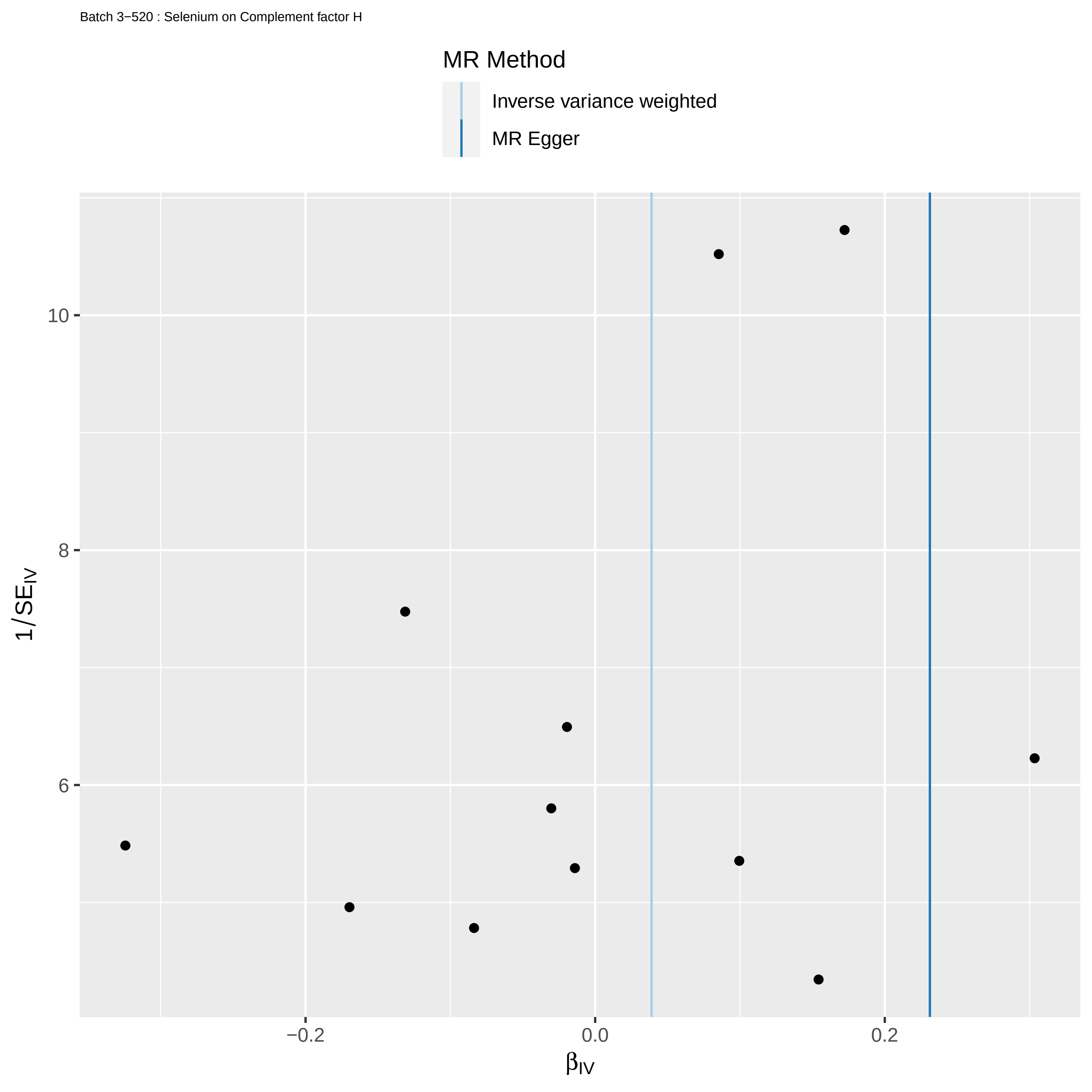

Batch 3−520 : Selenium on Complement factor H
MR Method
Inverse variance weighted
MR Egger
10
8
6
1/SE$_{IV}$
−0.2
0.0
0.2
β$_{IV}$

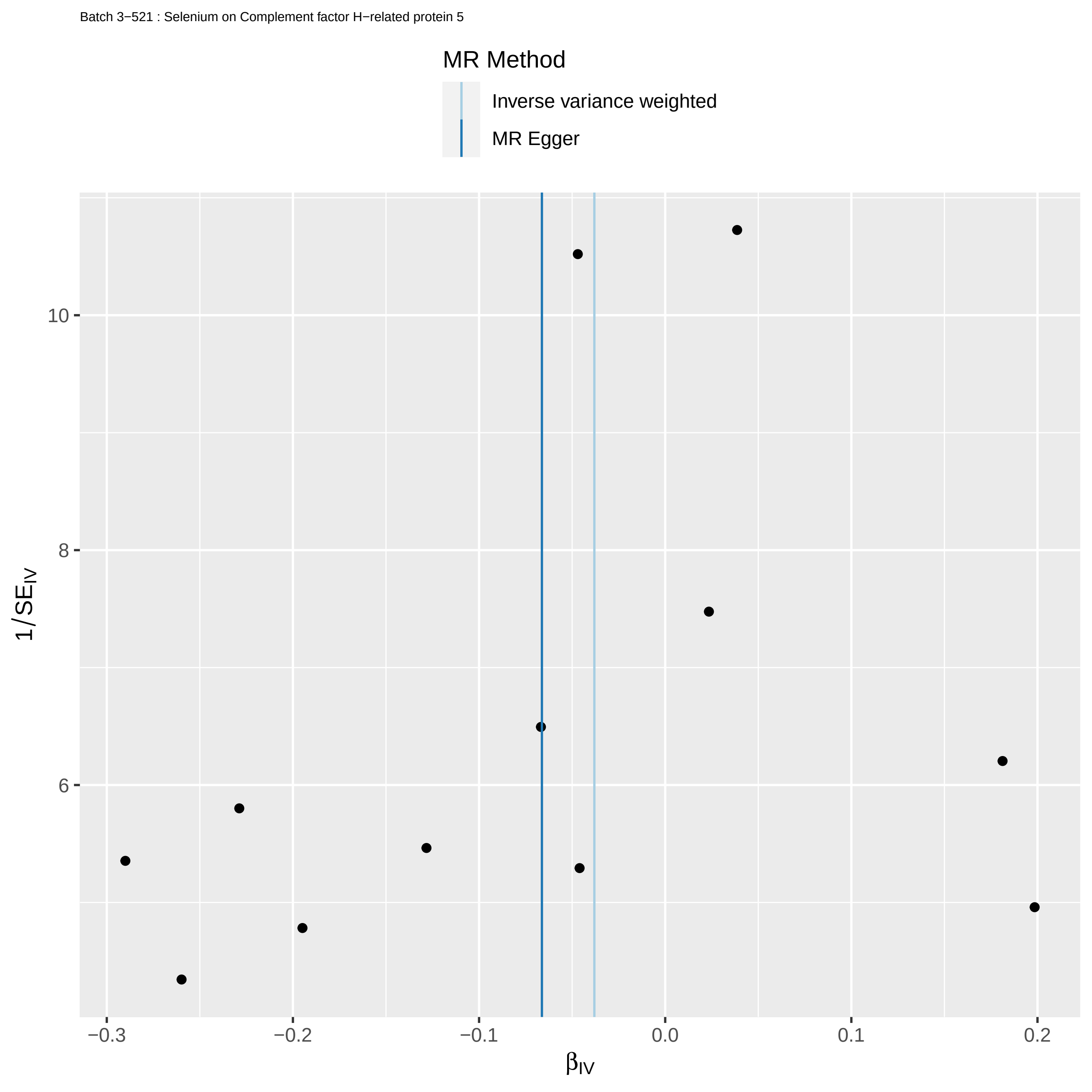

Batch 3−521 : Selenium on Complement factor H−related protein 5
MR Method
Inverse variance weighted
MR Egger
10
8
6
1/SE_IV
−0.3
−0.2
−0.1
0.0
0.1
0.2
β_IV

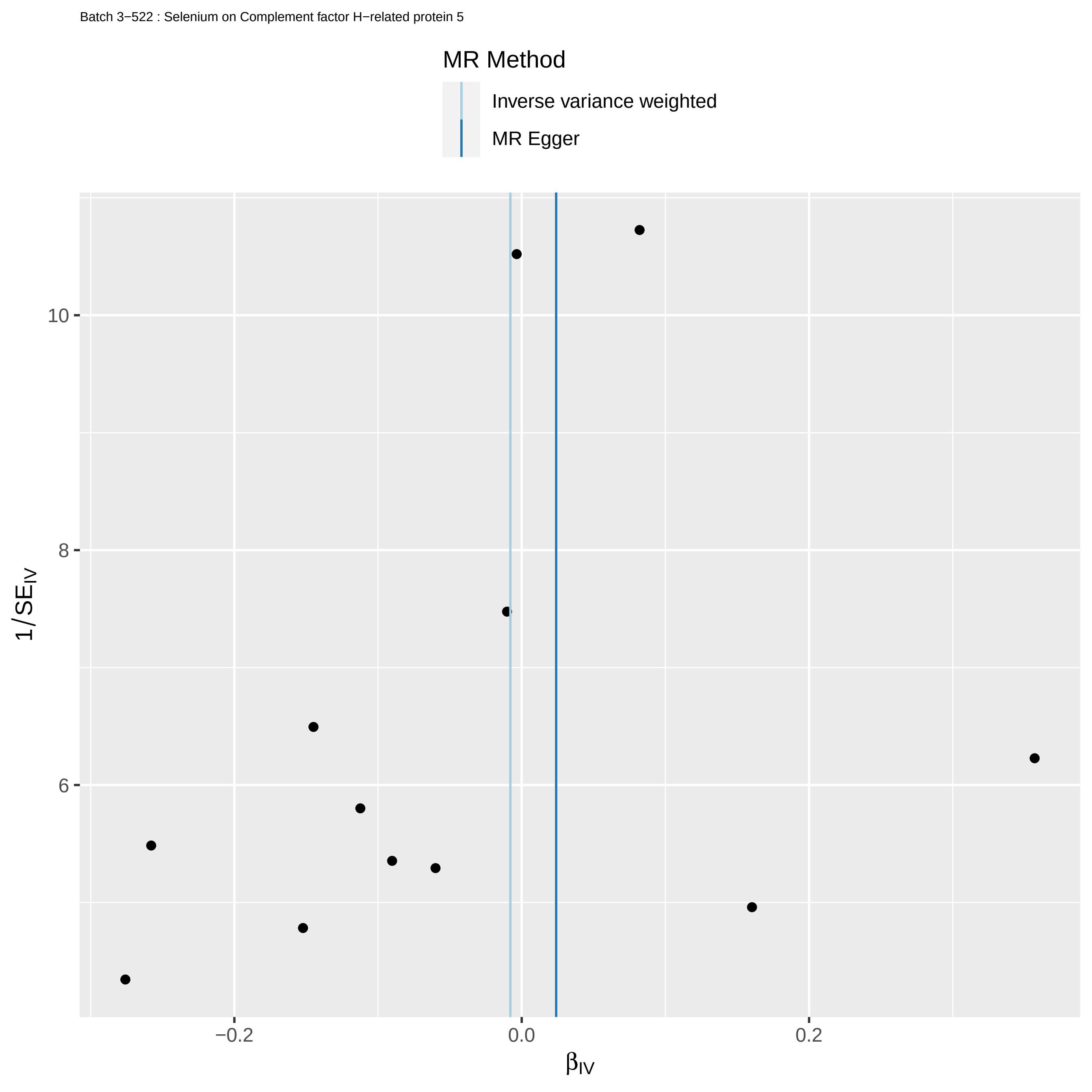

Batch 3−522 : Selenium on Complement factor H−related protein 5
MR Method
Inverse variance weighted
MR Egger
10
8
6
1/SE_IV
−0.2
0.0
0.2
β_IV

Batch 3−523 : Selenium on Complement factor I

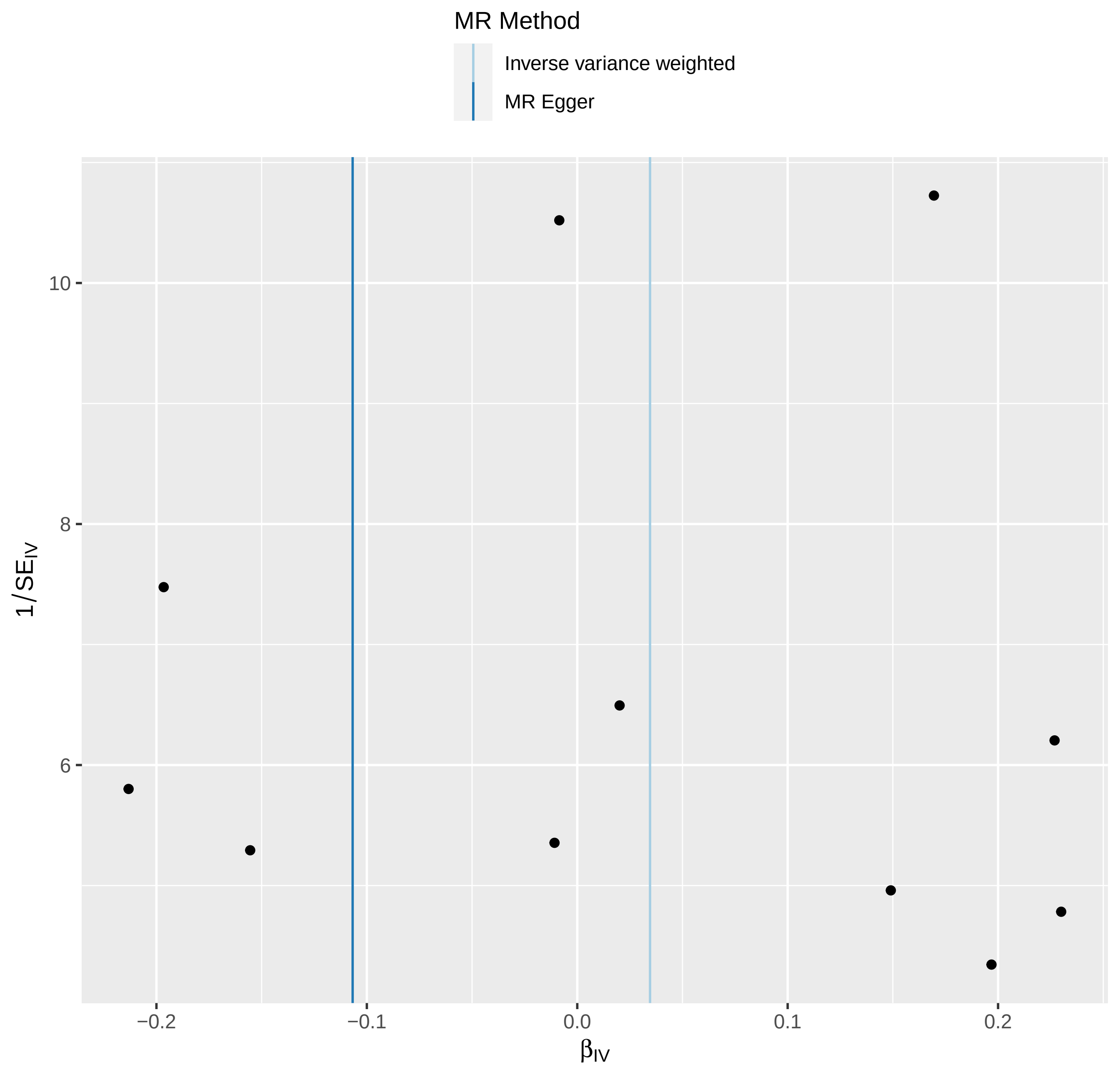

Batch 3−524 : Selenium on Cofilin−1

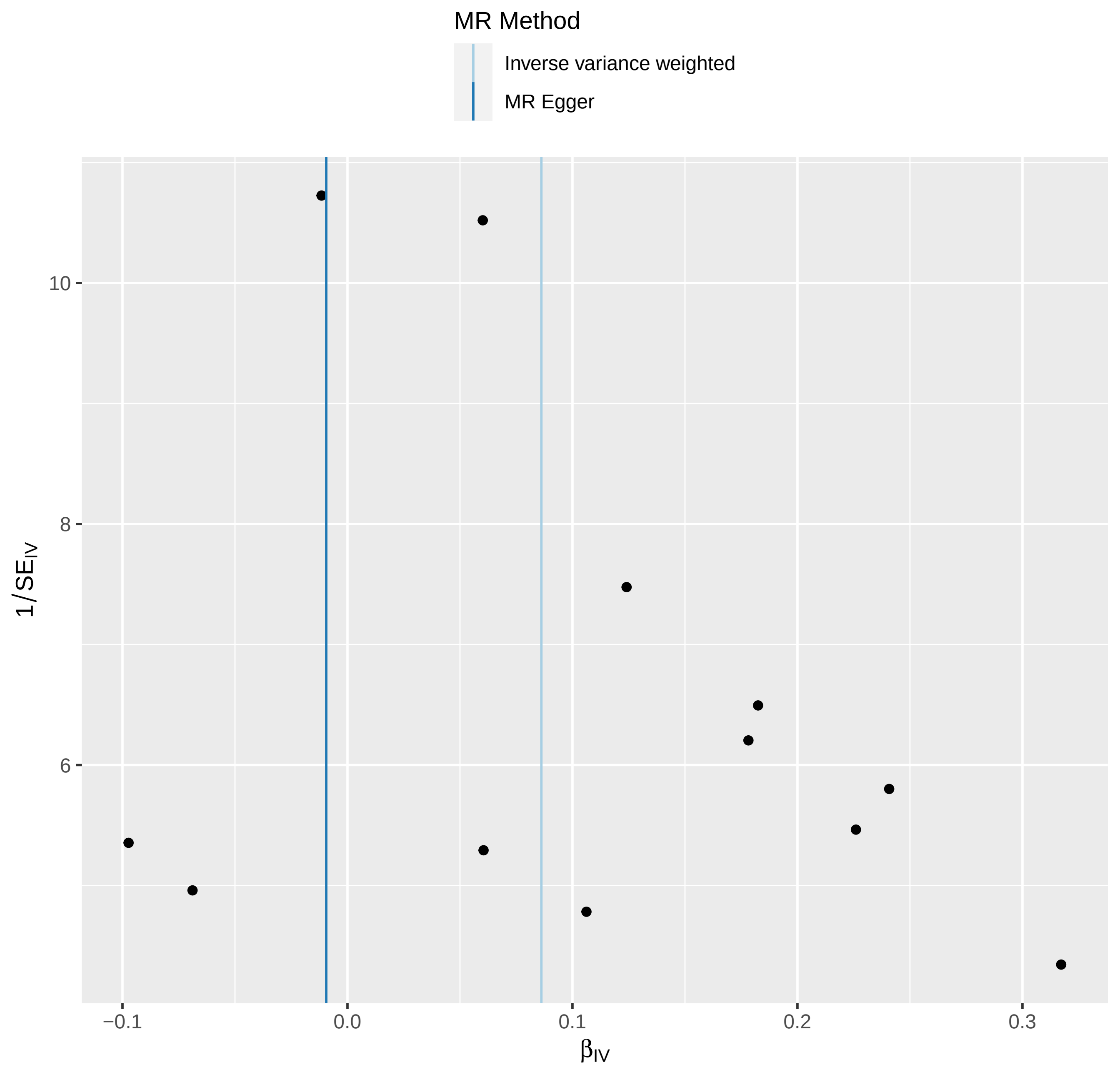

Batch 3−525 : Selenium on Properdin

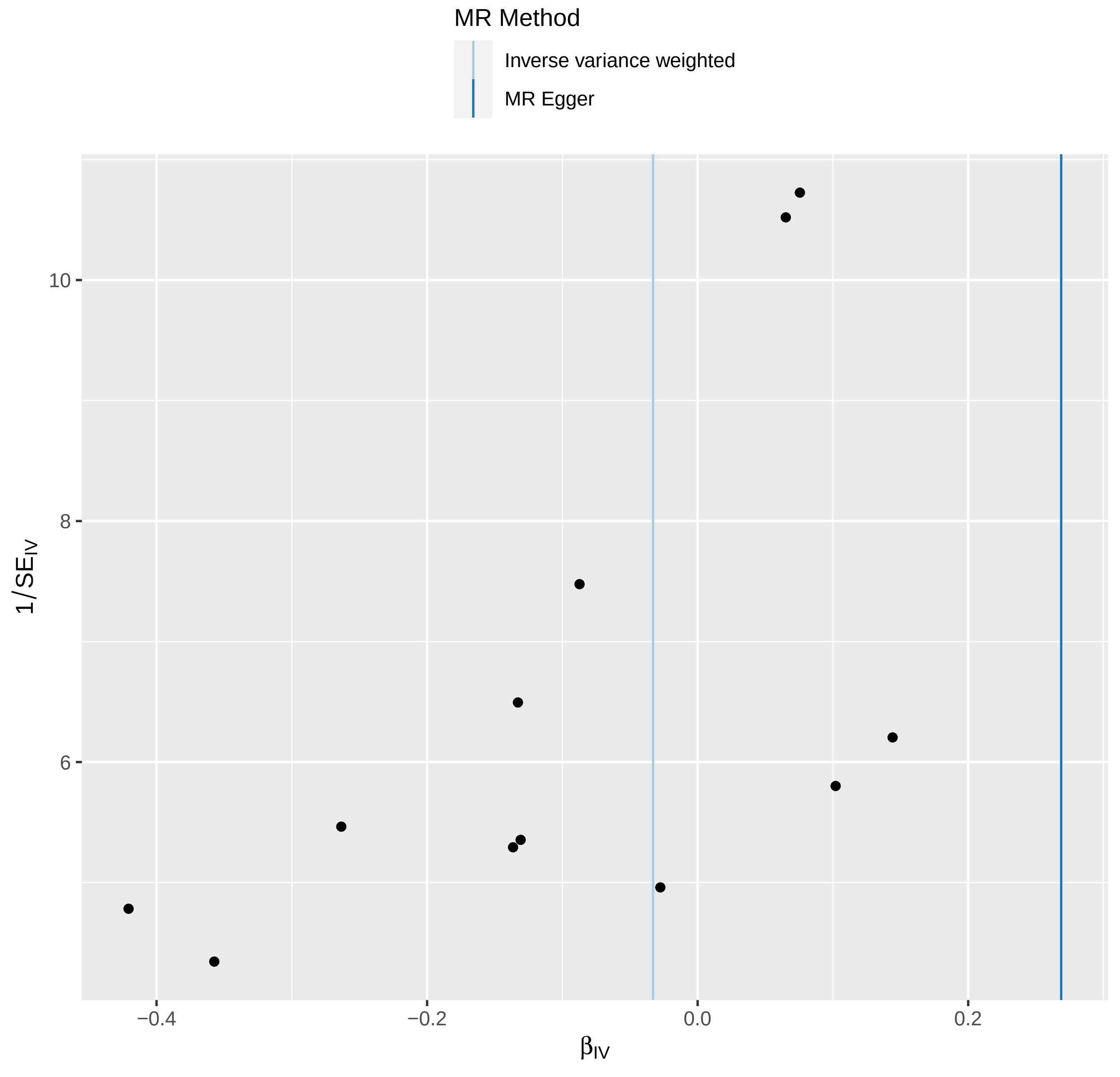

Batch 3−526 : Selenium on Glycoprotein hormones alpha chain

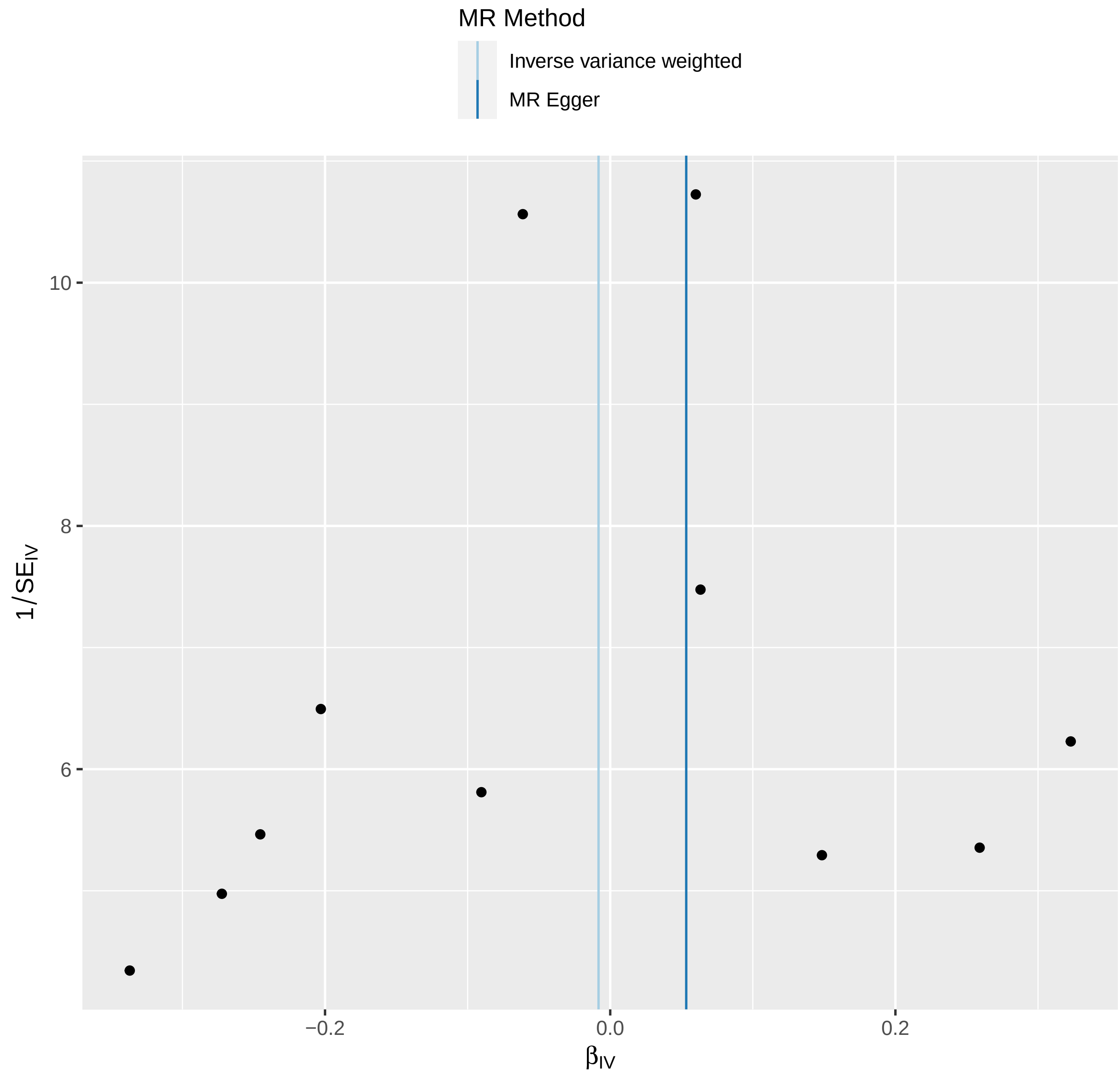

Batch 3−527 : Selenium on Human Chorionic Gonadotropin

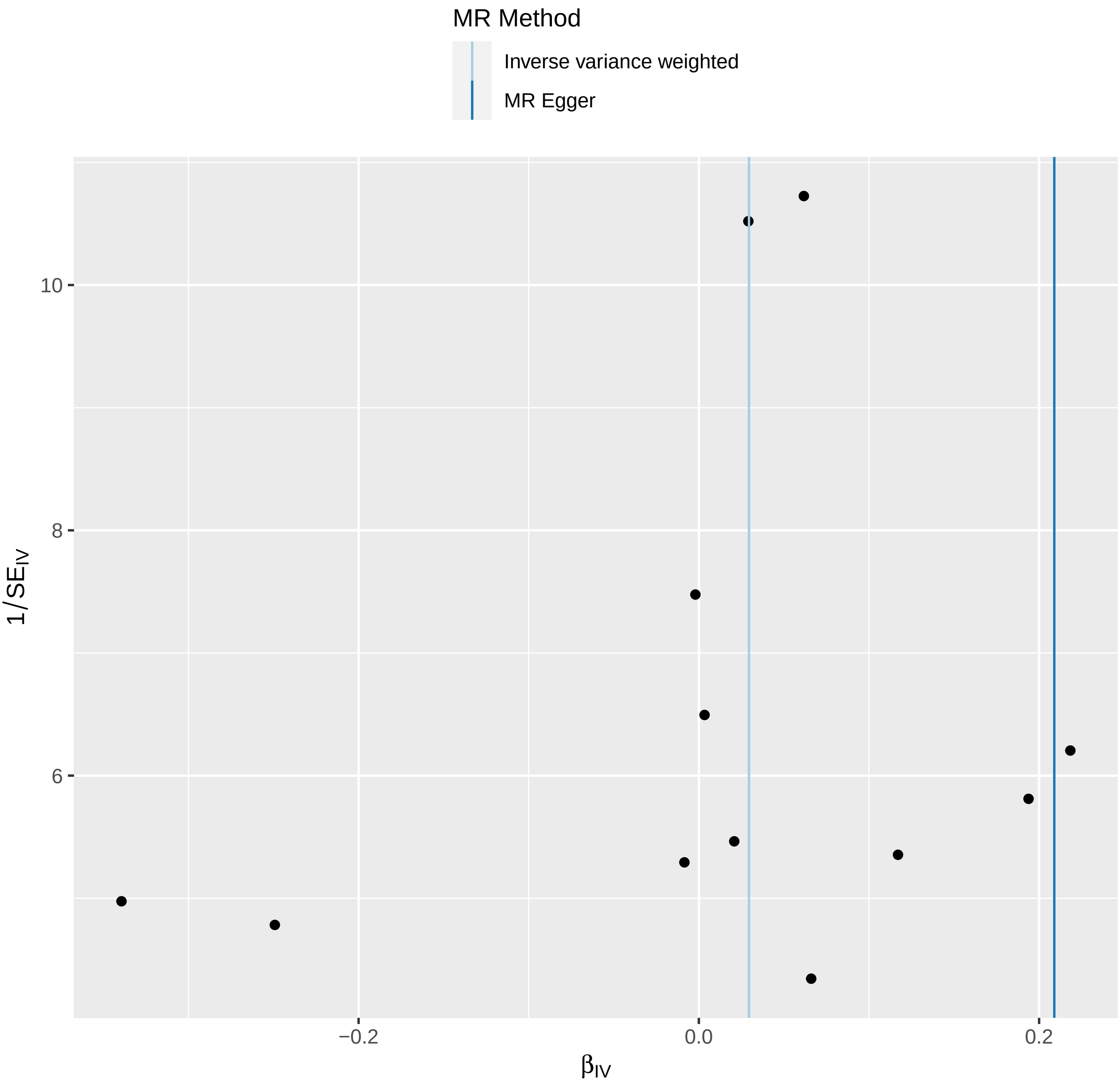

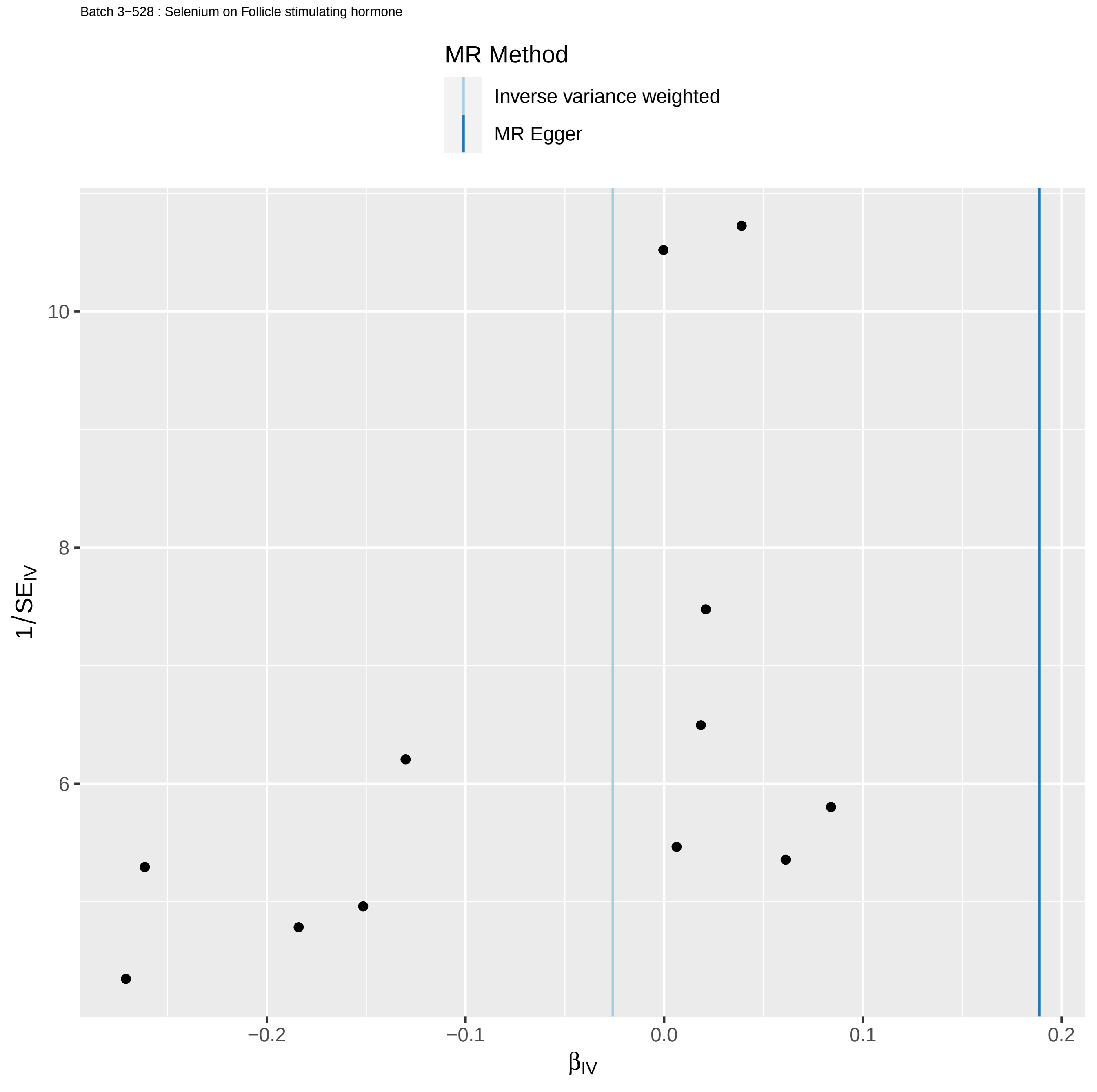
Batch 3−528 : Selenium on Follicle stimulating hormone
MR Method
Inverse variance weighted
MR Egger
10
8
6
1/SE$_{IV}$
−0.2
−0.1
0.0
0.1
0.2
β$_{IV}$

Batch 3−529 : Selenium on Luteinizing hormone

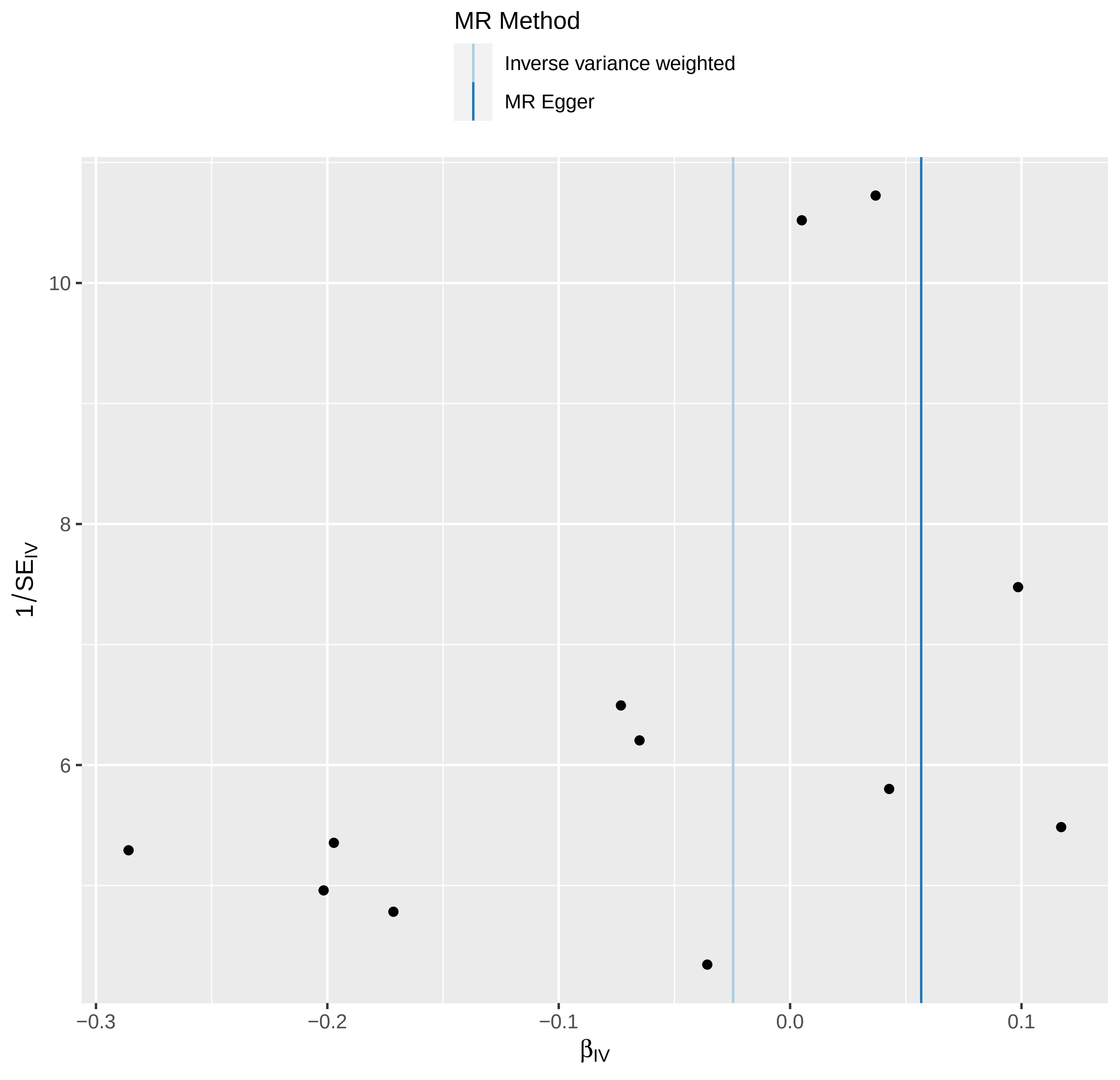

Batch 3−530 : Selenium on Thyroid Stimulating Hormone

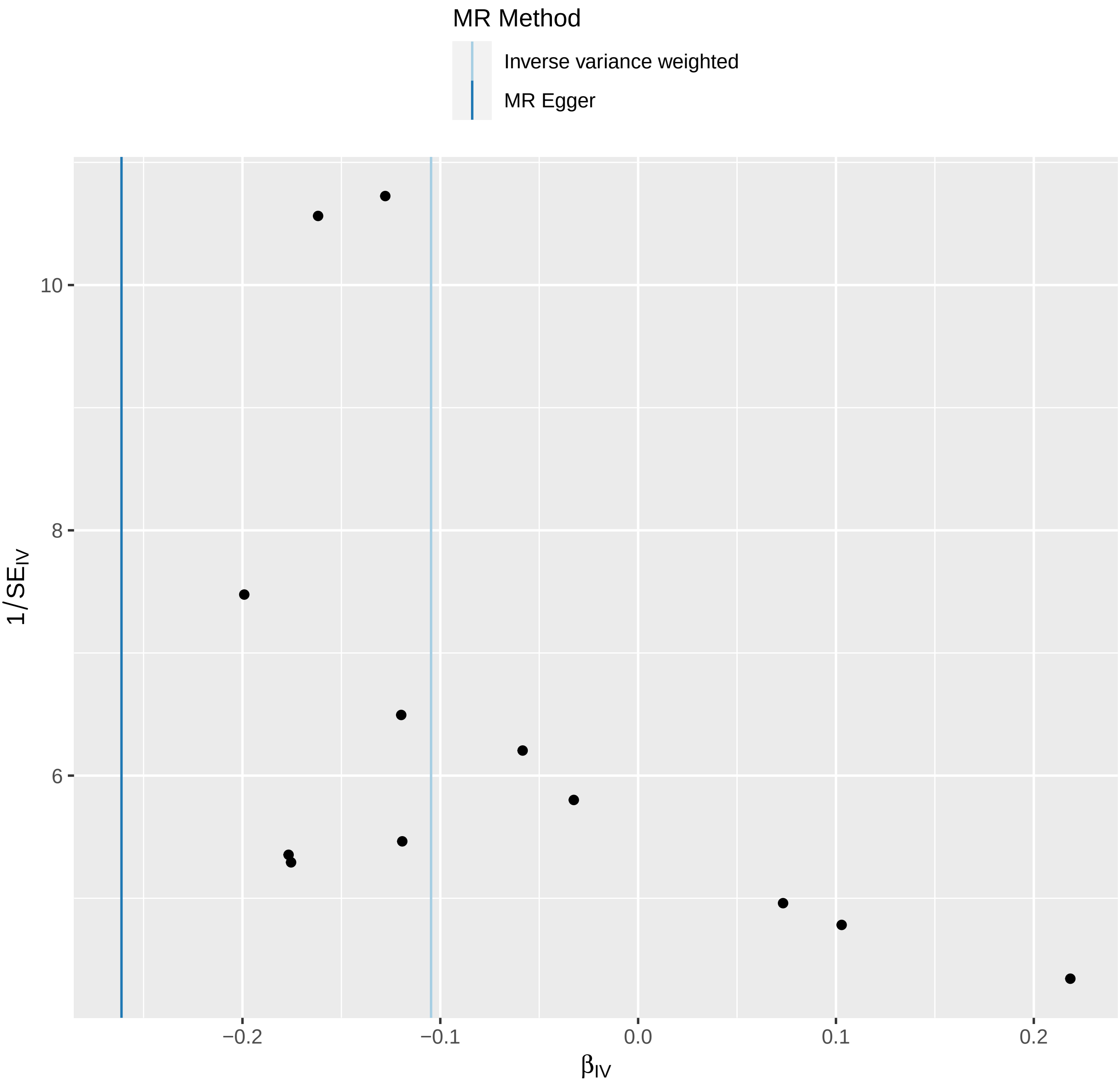

Batch 3−531 : Selenium on Choriogonadotropin subunit beta variant 2

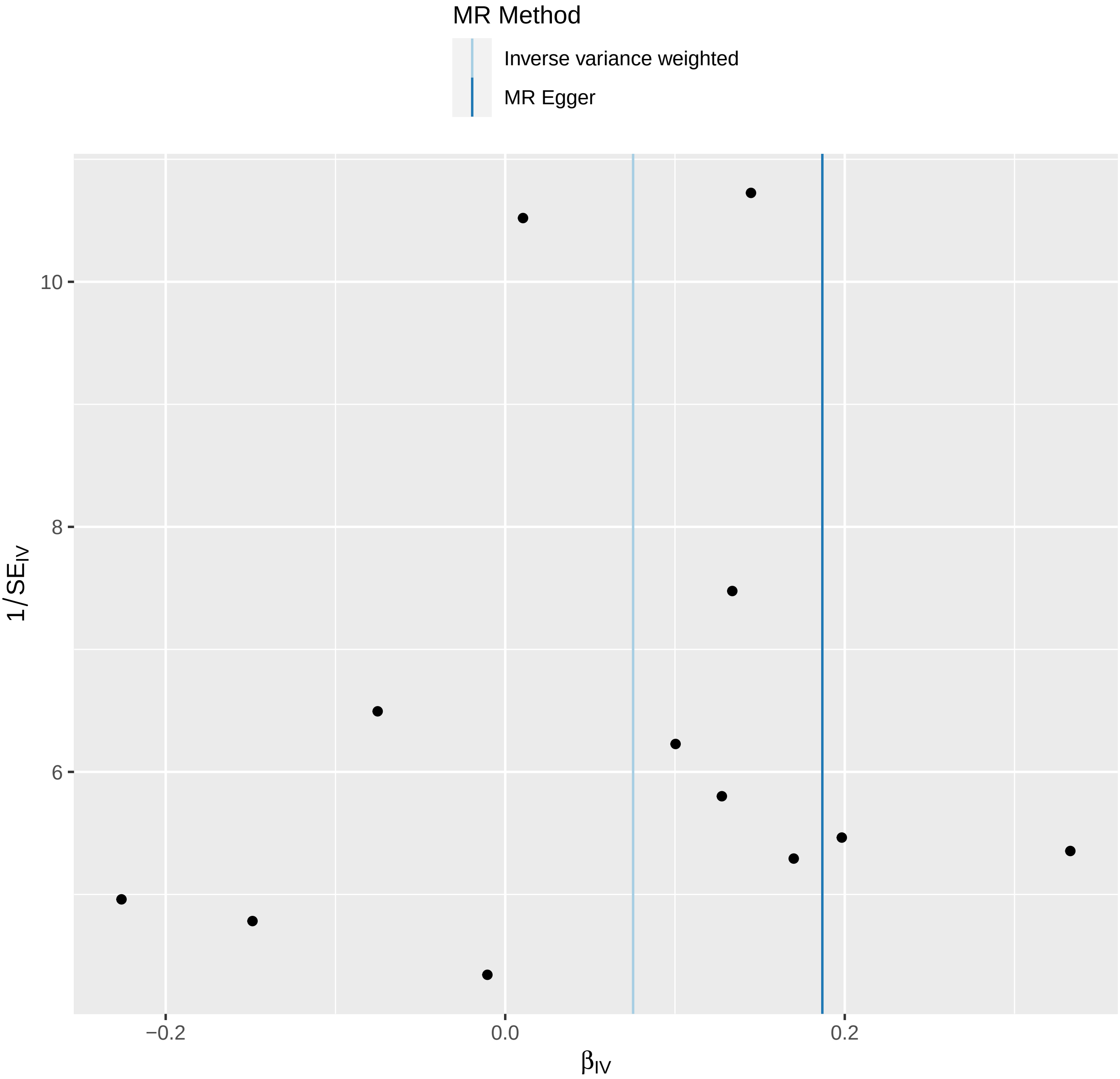

Batch 3−532 : Selenium on Cell growth regulator with EF hand domain protein 1

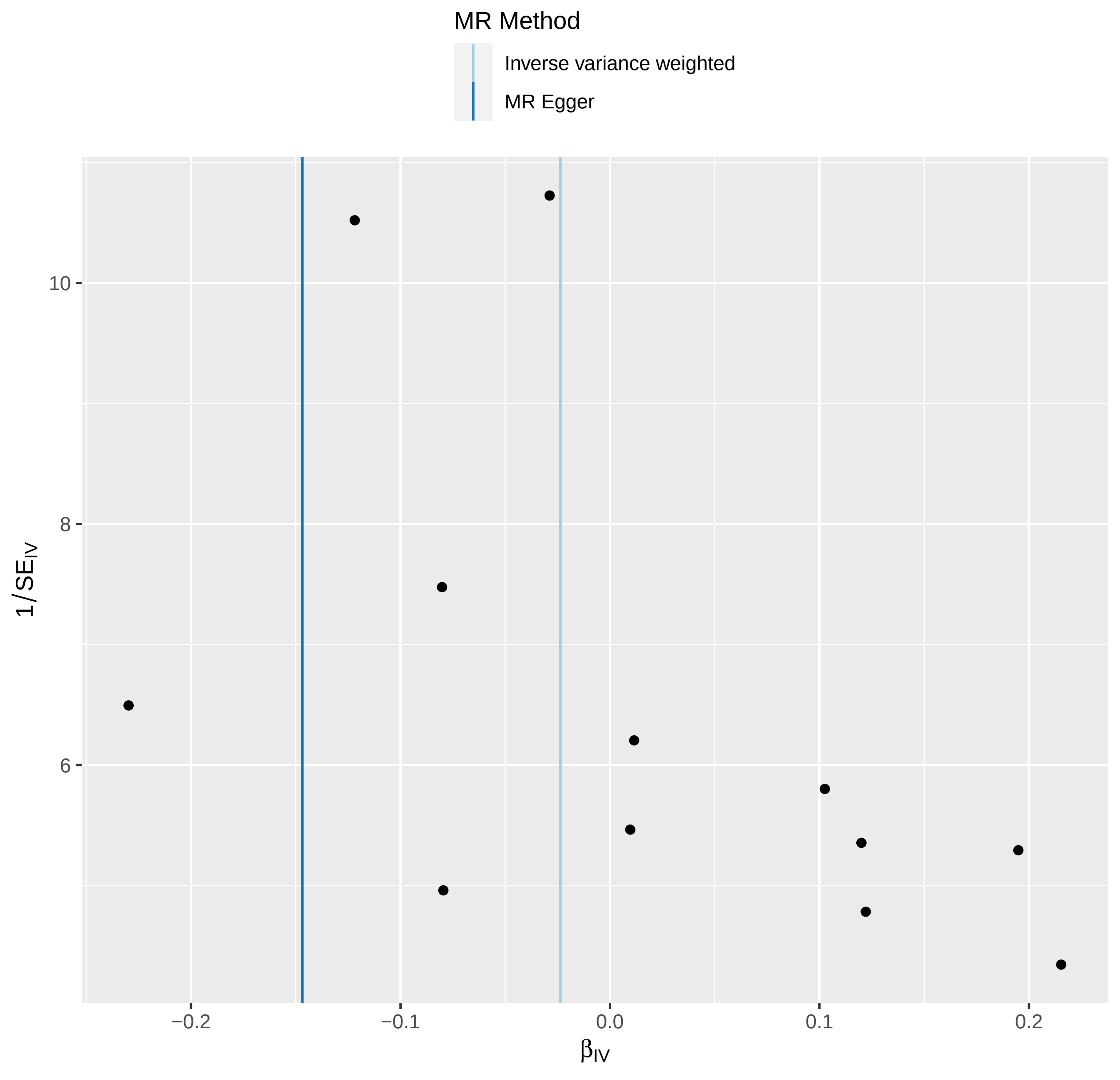

Batch 3−533 : Selenium on Chondroadherin

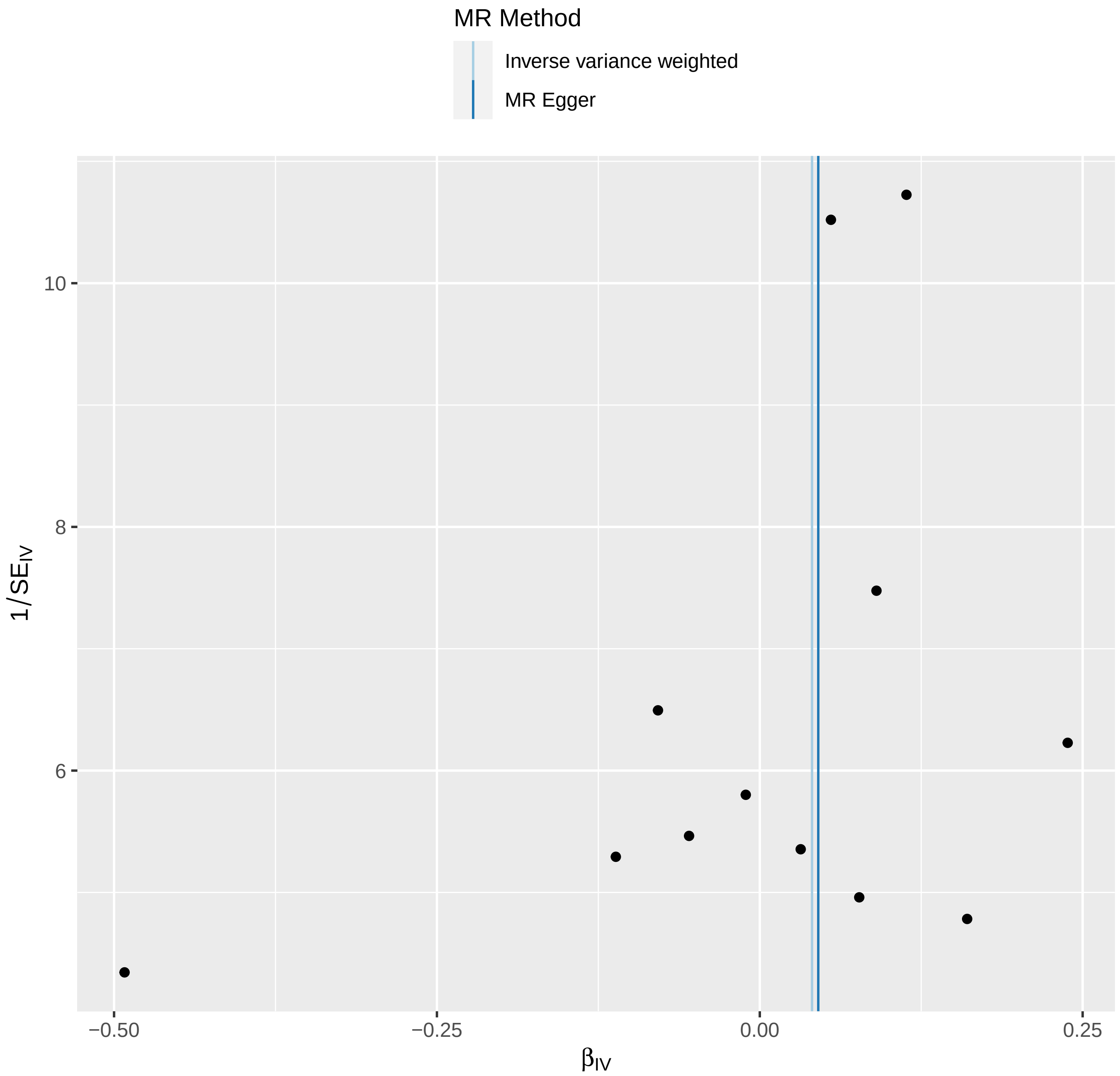

Batch 3−534 : Selenium on Coiled−coil−helix−coiled−coil−helix domain−containing protein 10, mitochondrial

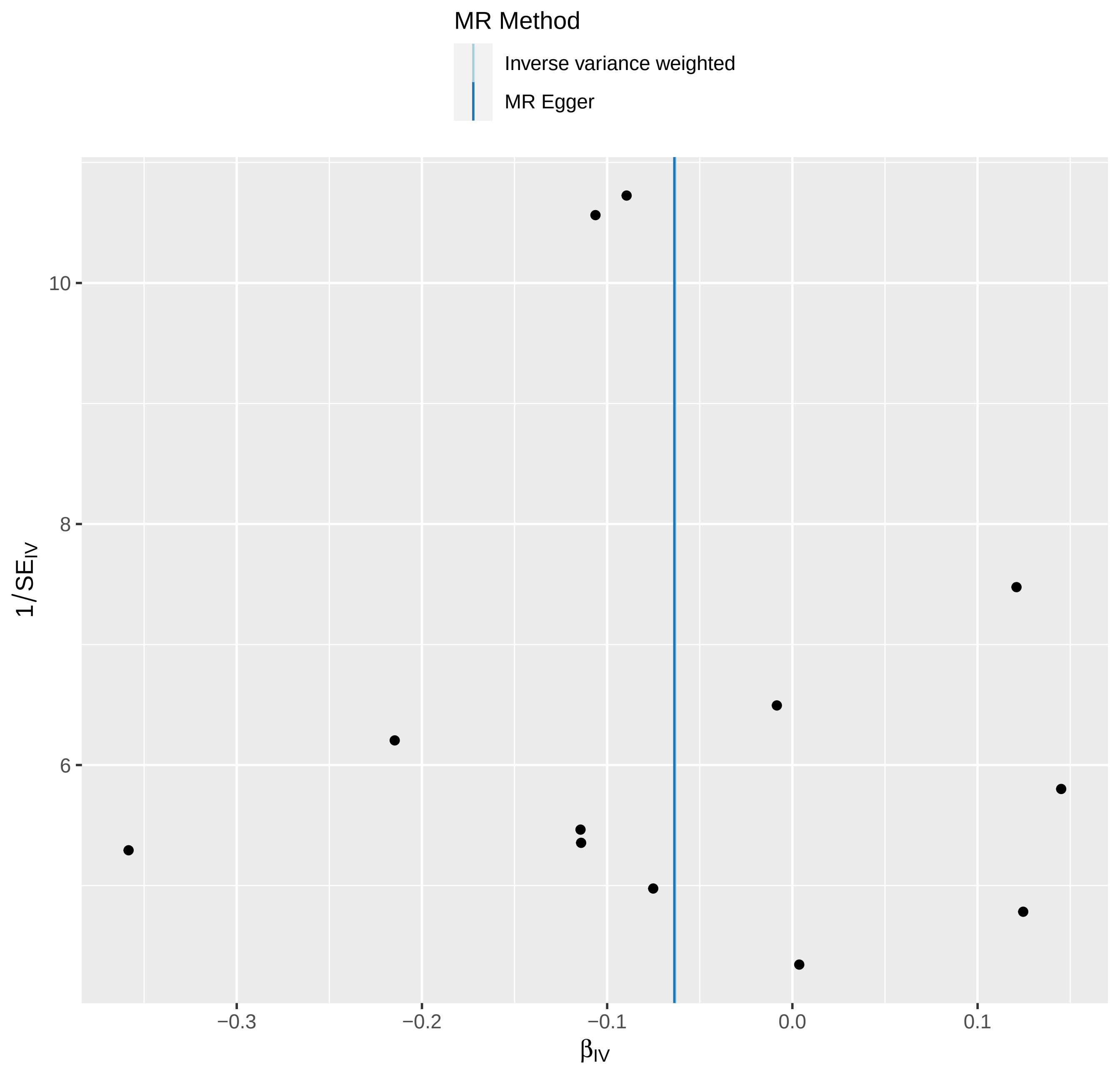

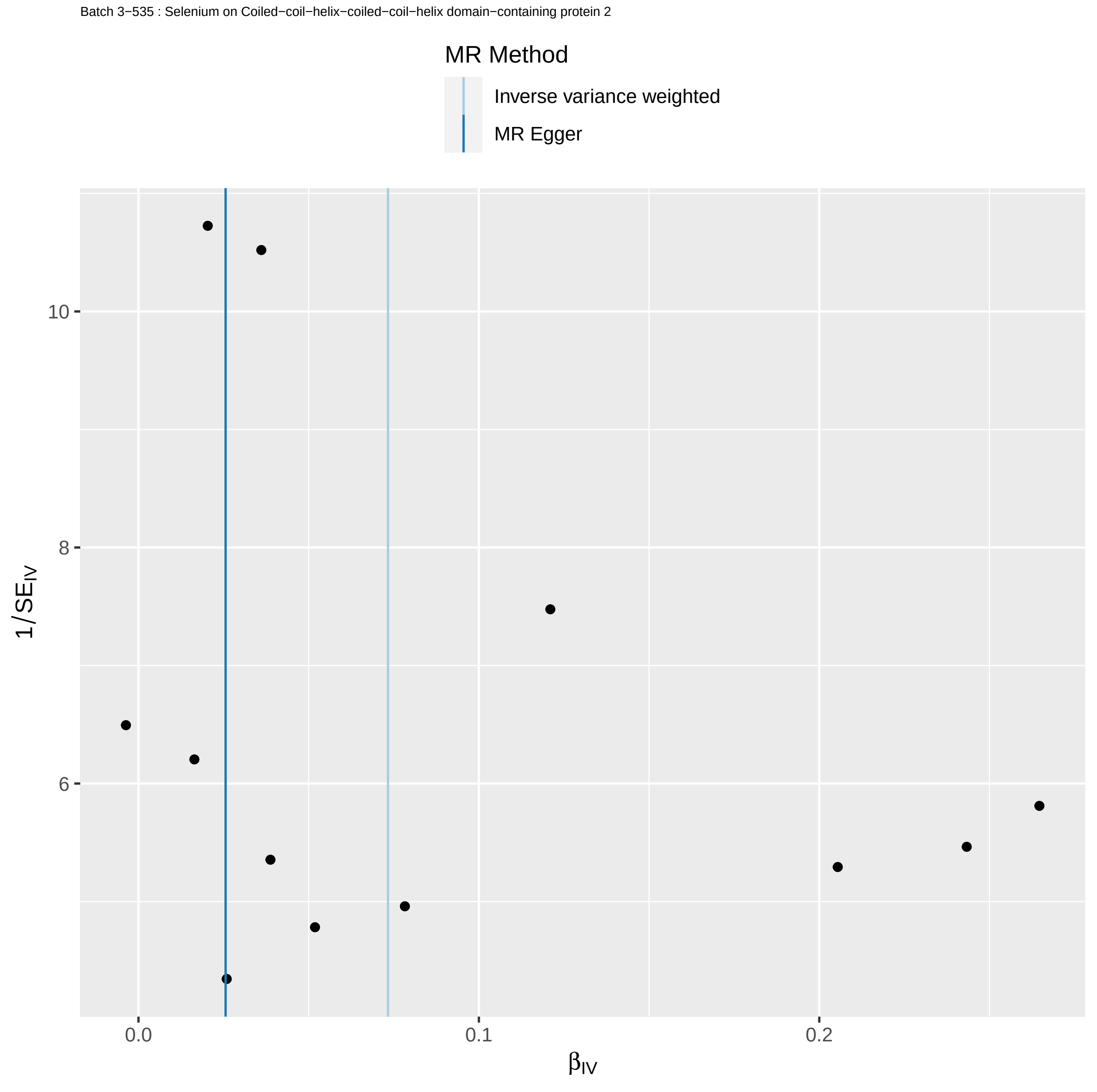

Batch 3−535 : Selenium on Coiled−coil−helix−coiled−coil−helix domain−containing protein 2
MR Method
Inverse variance weighted
MR Egger
10
8
6
1/SE_IV
0.0
0.1
0.2
β_IV

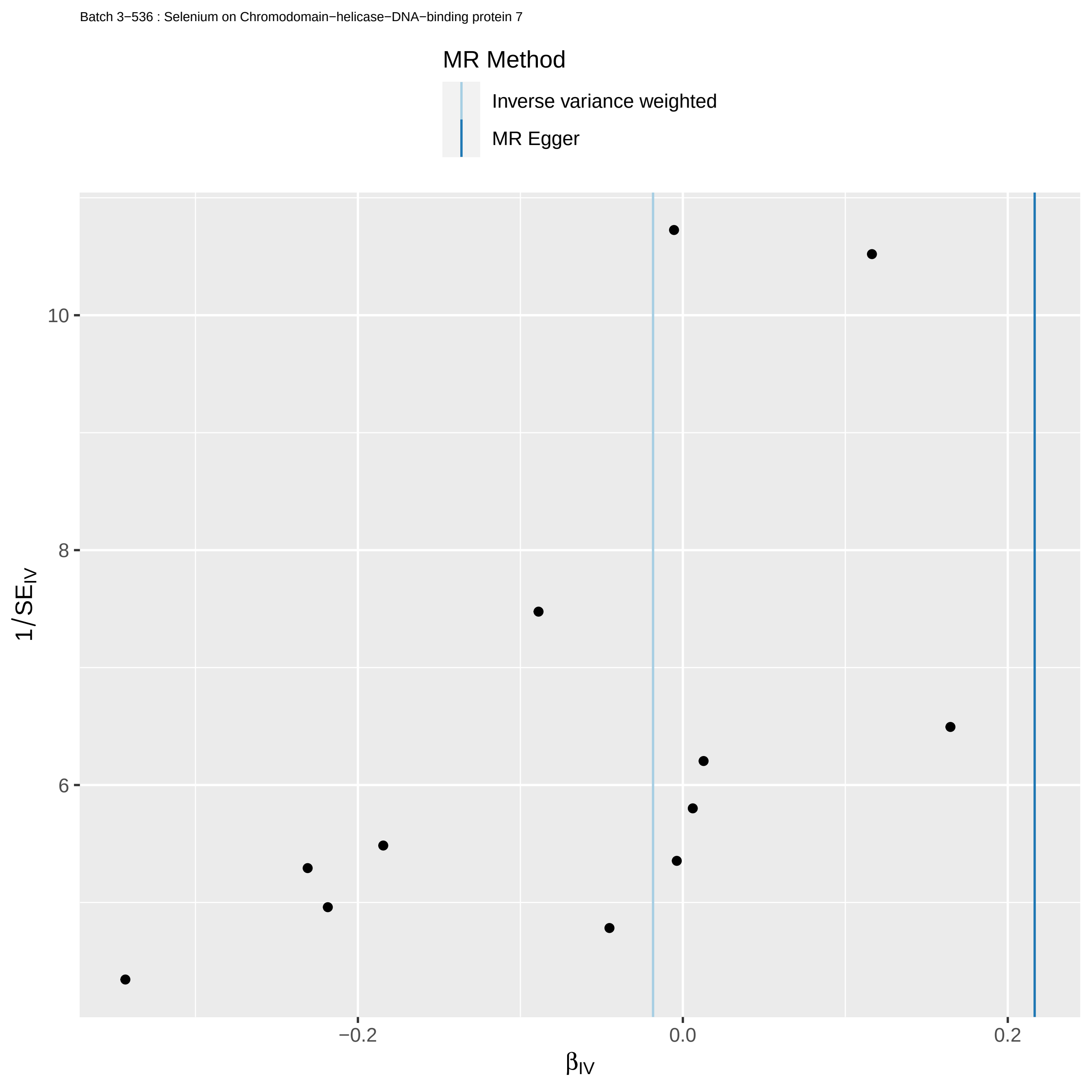

Batch 3−536 : Selenium on Chromodomain−helicase−DNA−binding protein 7
MR Method
Inverse variance weighted
MR Egger
10
8
6
1/SE_IV
−0.2
0.0
0.2
β_IV

Batch 3−537 : Selenium on Serine/threonine−protein kinase Chk1

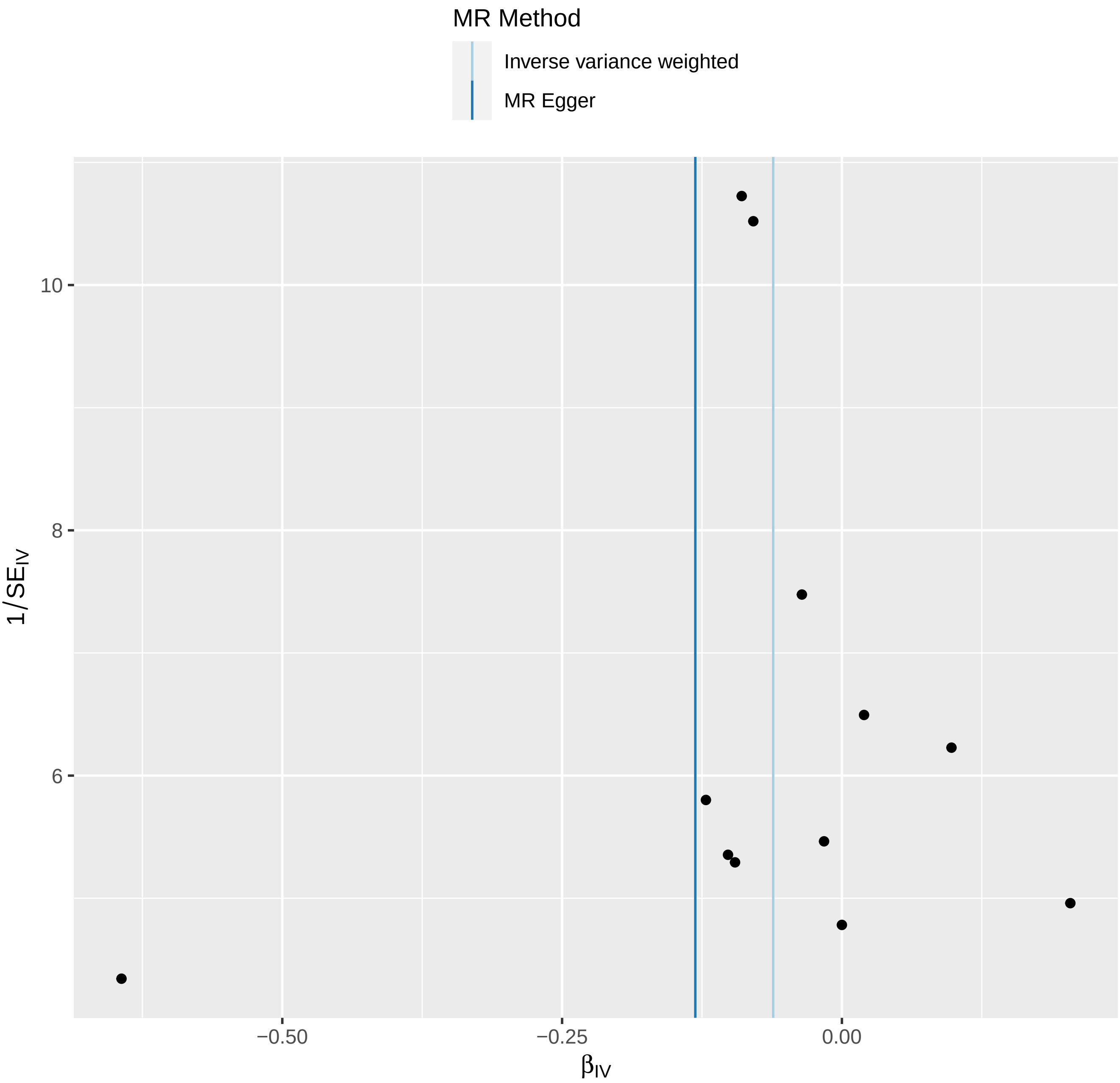

Batch 3−538 : Selenium on Chromogranin−A

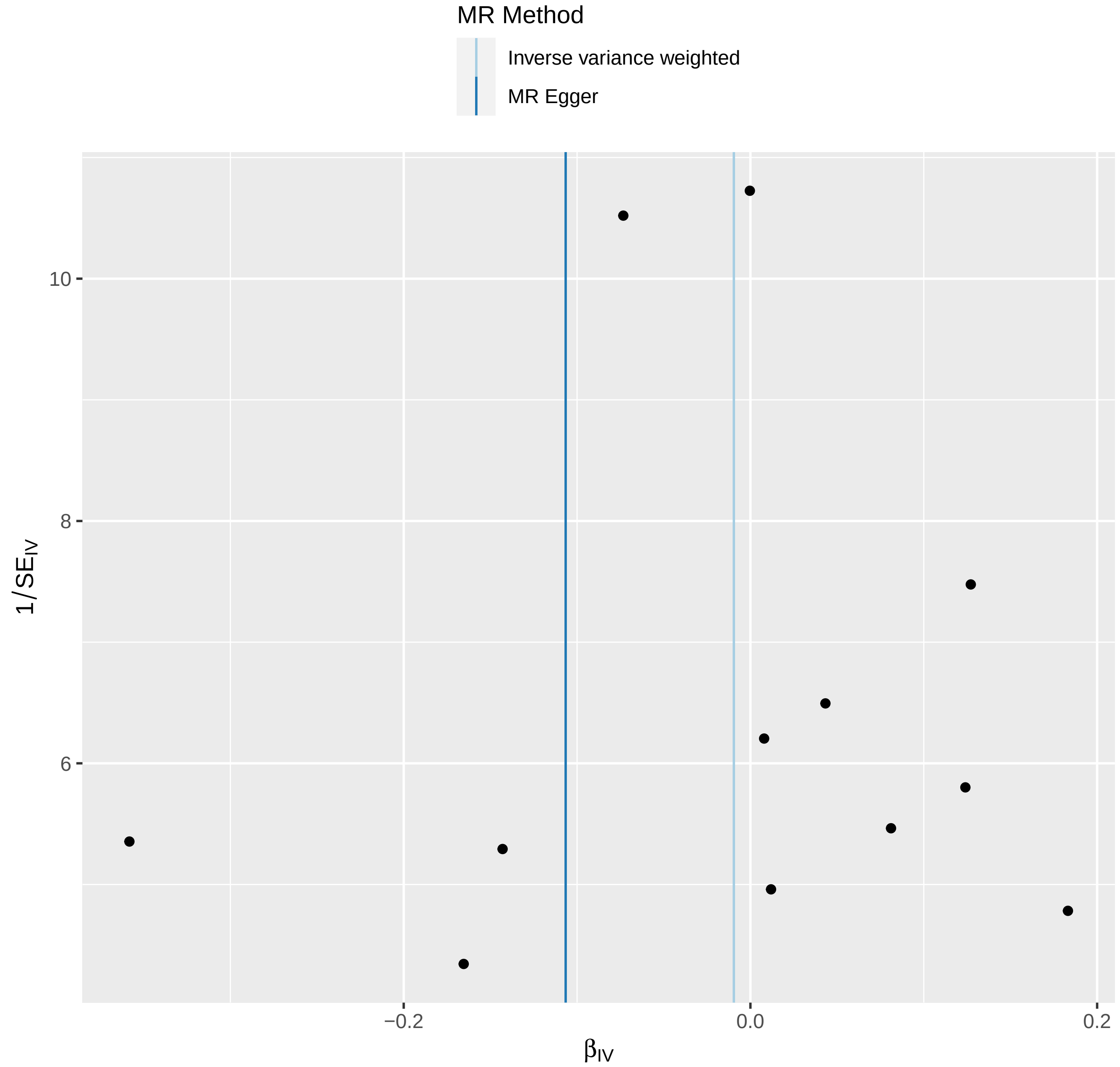

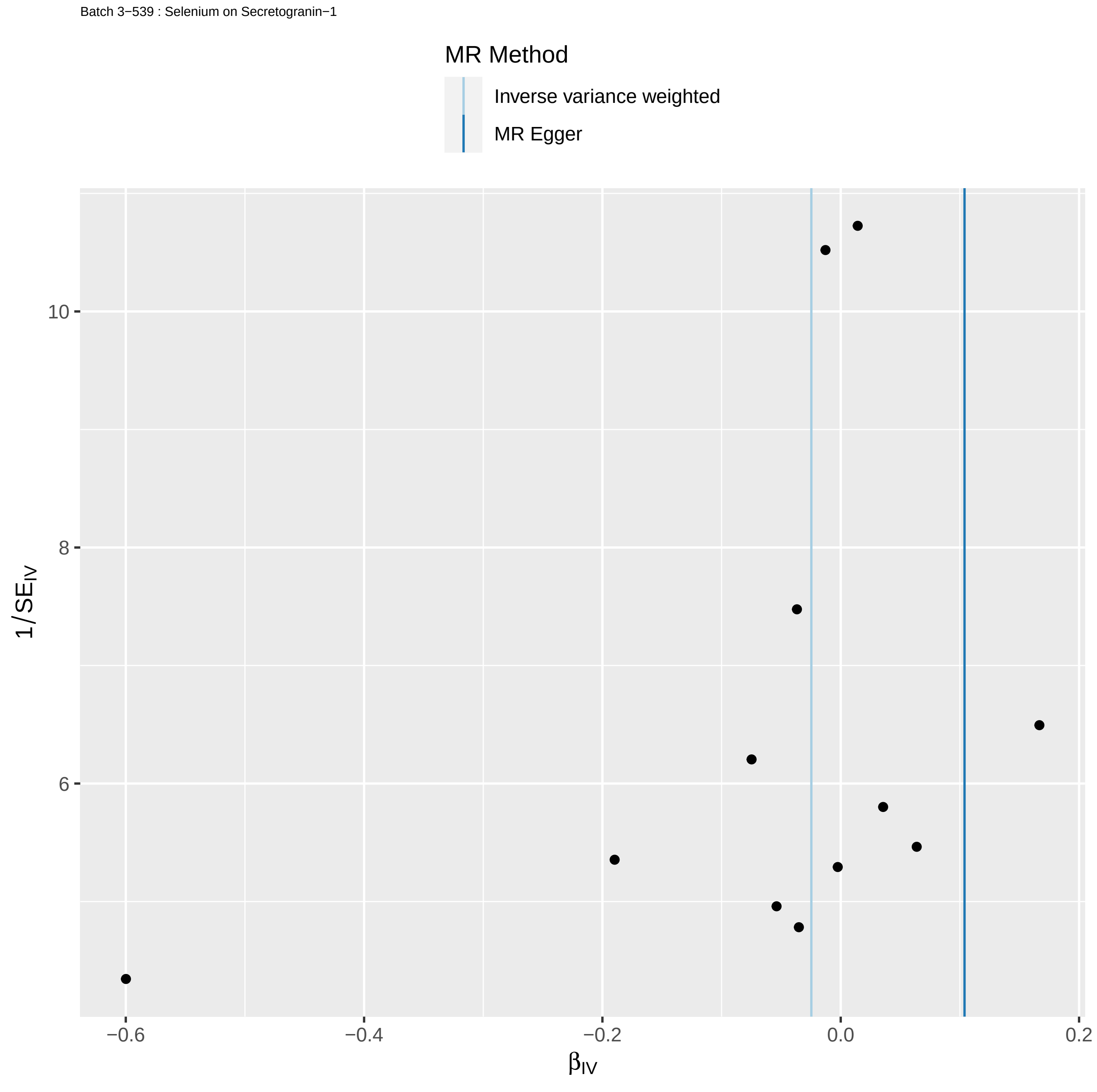

Batch 3−539 : Selenium on Secretogranin−1
MR Method
Inverse variance weighted
MR Egger
10
8
6
1/SE_IV
−0.6
−0.4
−0.2
0.0
0.2
β_IV

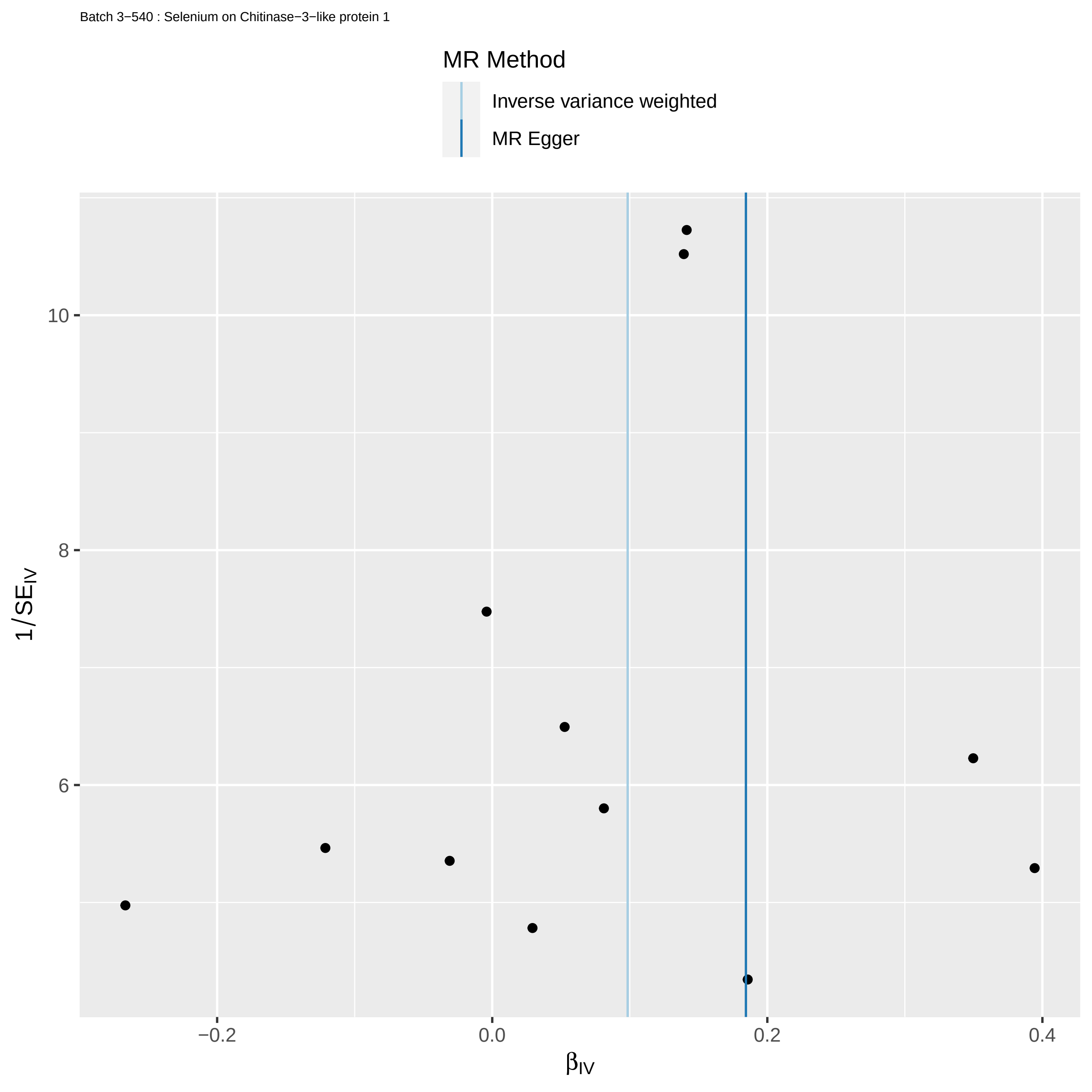

Batch 3−540 : Selenium on Chitinase−3−like protein 1
MR Method
Inverse variance weighted
MR Egger
10
8
6
$1/SE_{IV}$
−0.2
0.0
0.2
0.4
β_{IV}

Batch 3−541 : Selenium on Chitinase−3−like protein 2

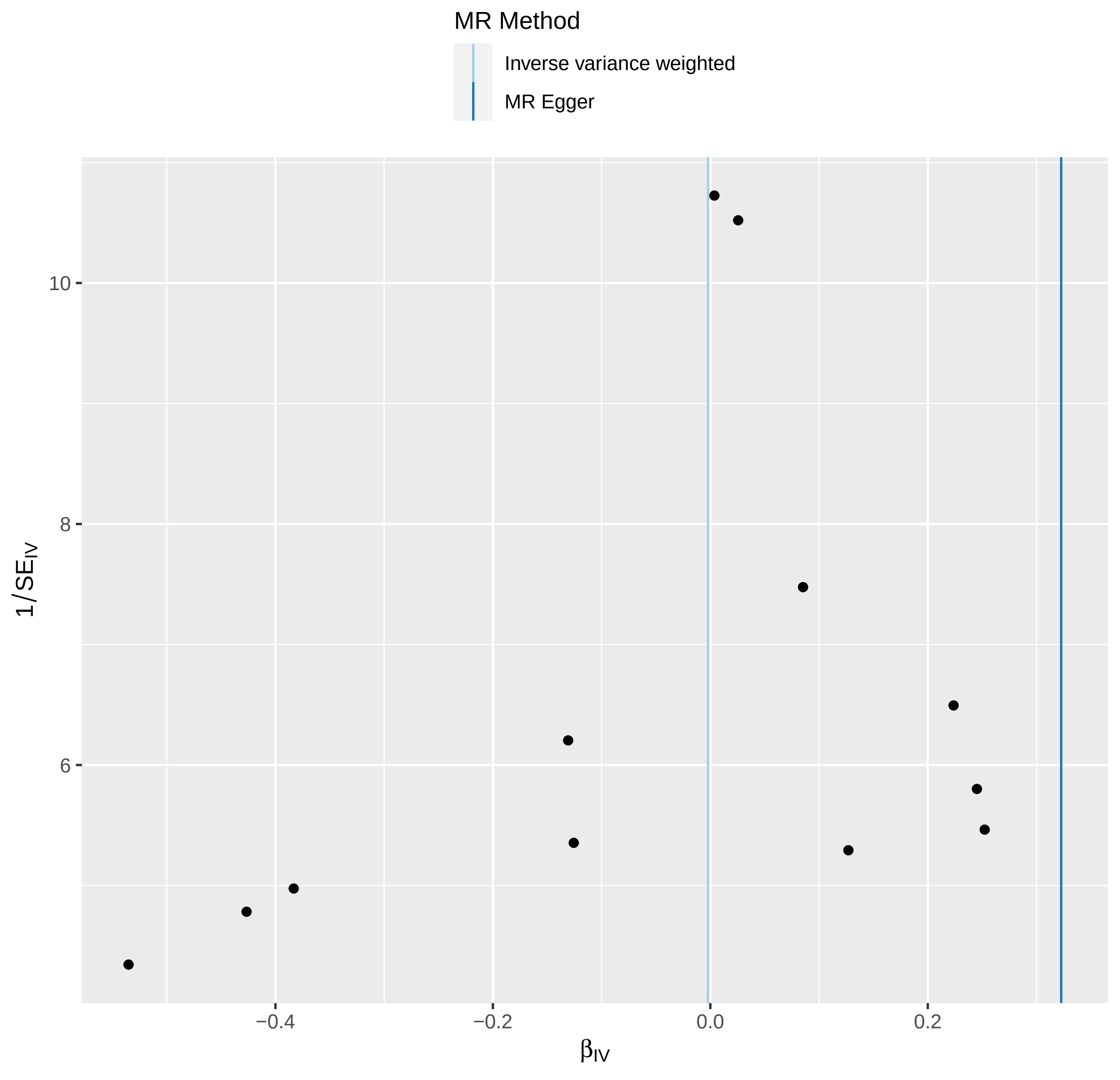

Batch 3−542 : Selenium on Cysteine−rich hydrophobic domain−containing protein 2

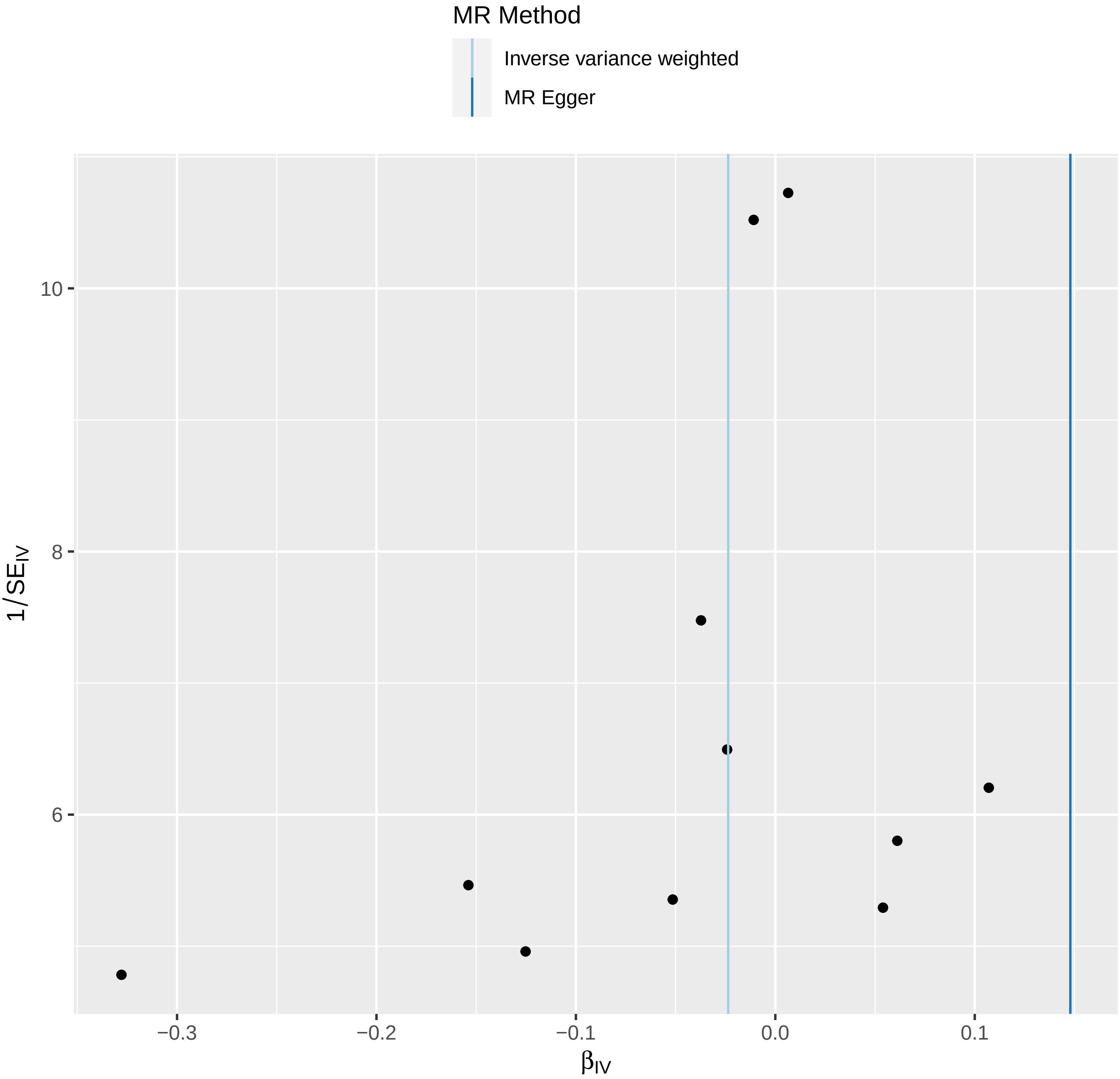

Batch 3−543 : Selenium on Chitotriosidase−1

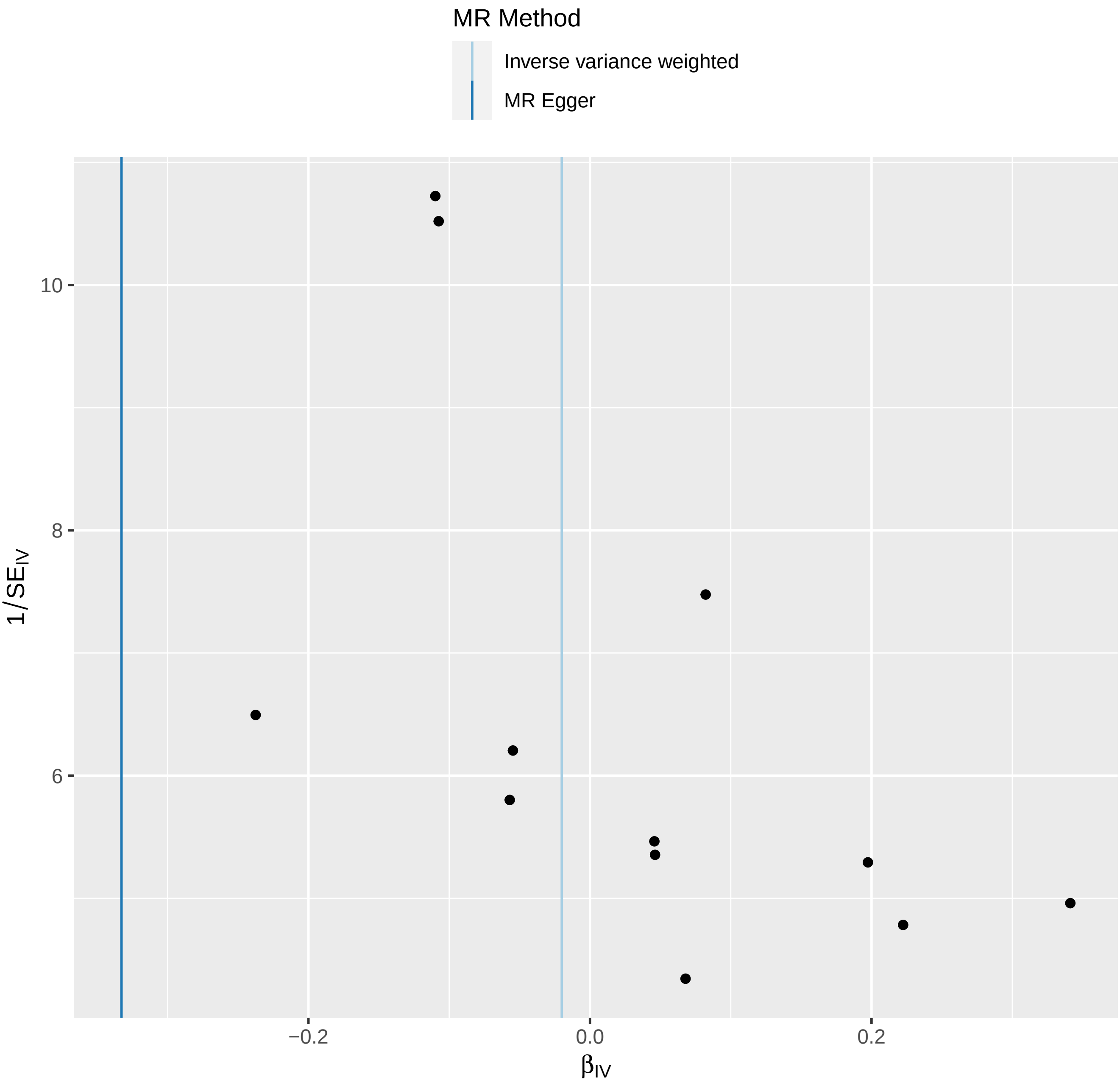

Batch 3−544 : Selenium on Choline/ethanolamine kinase

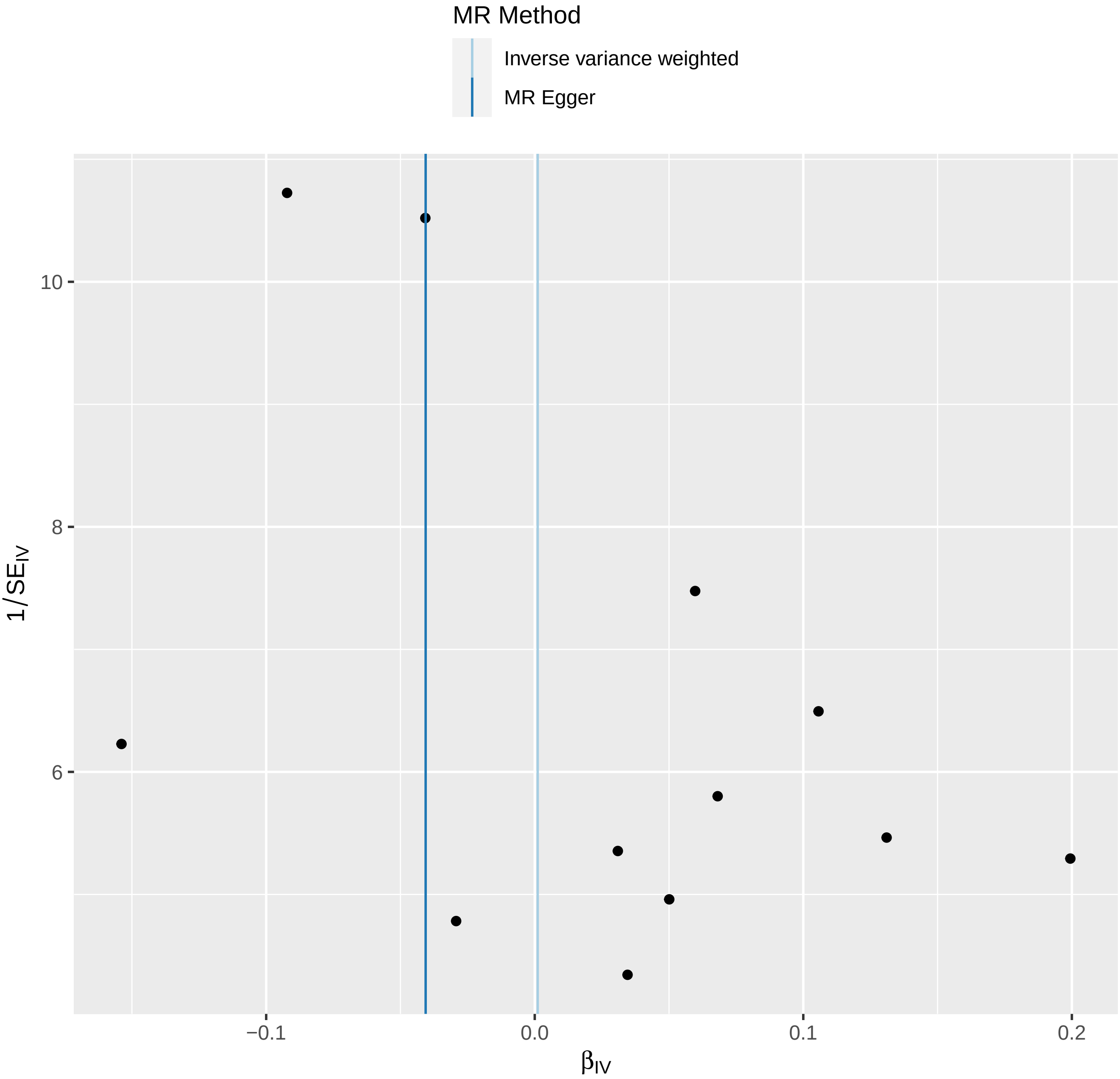

Batch 3−545 : Selenium on Neural cell adhesion molecule L1−like protein

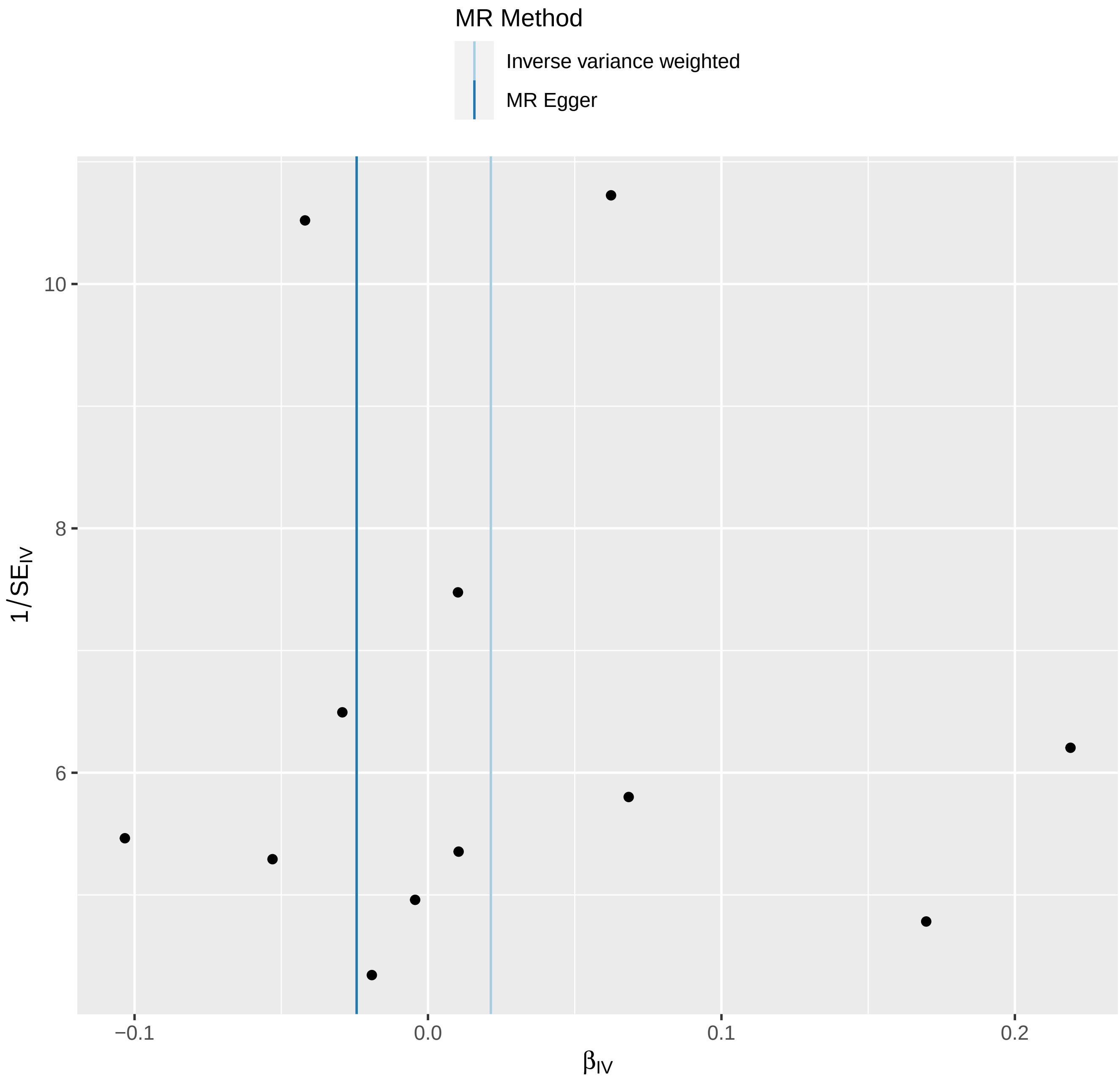

Batch 3−546 : Selenium on Calcineurin B homologous protein 1

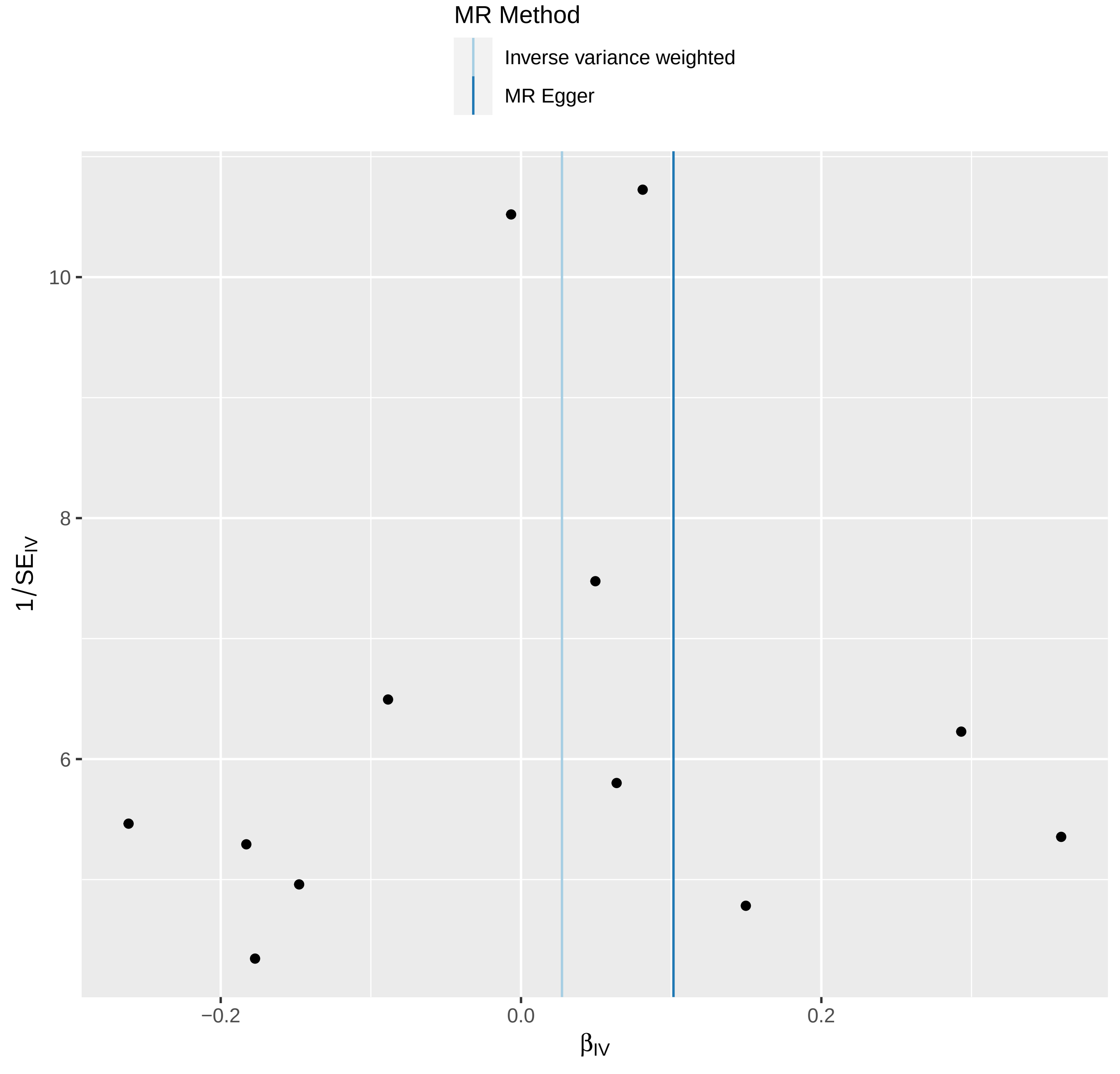

Batch 3−547 : Selenium on Chordin

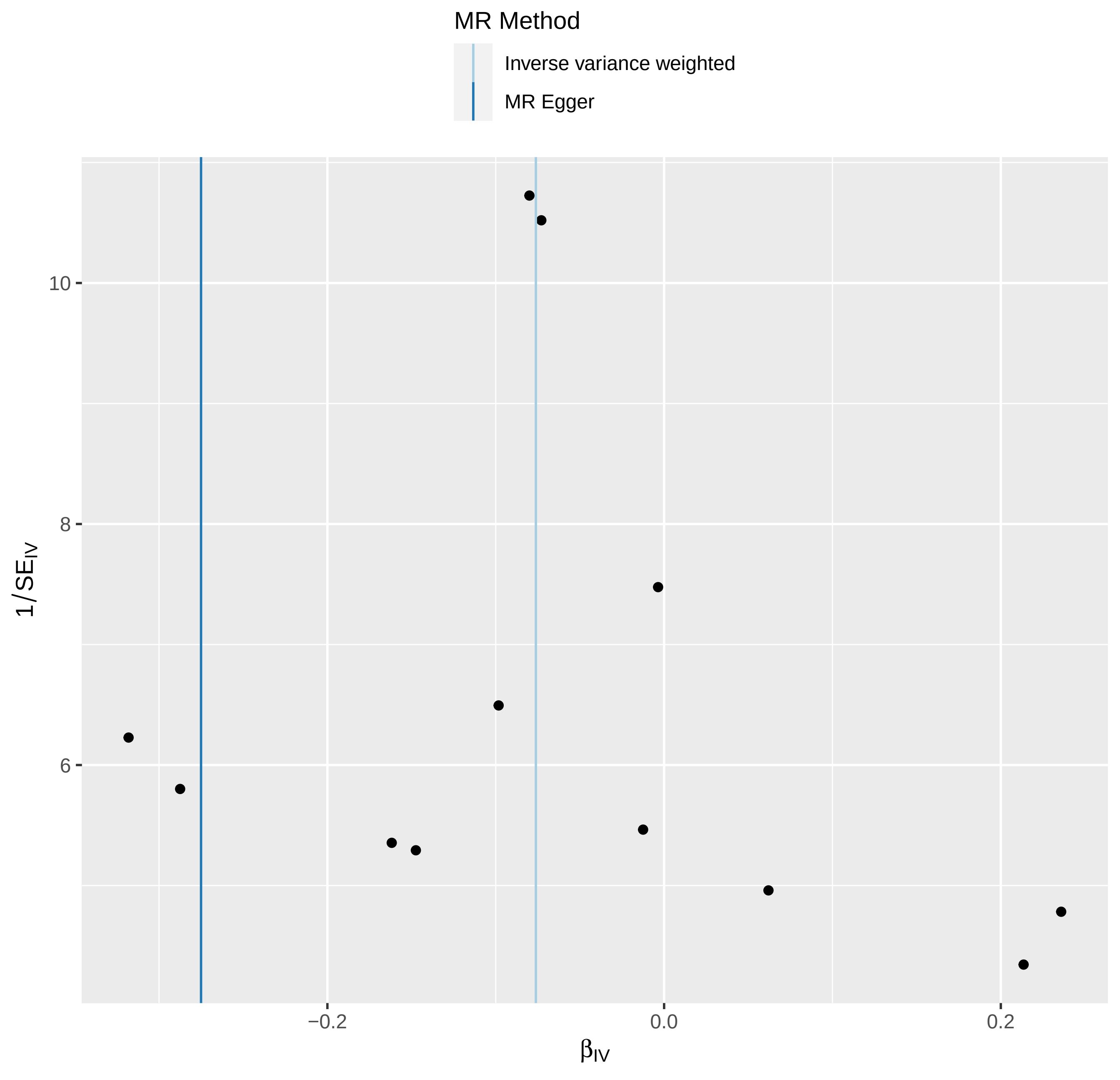

Batch 3−548 : Selenium on Chordin−like protein 1

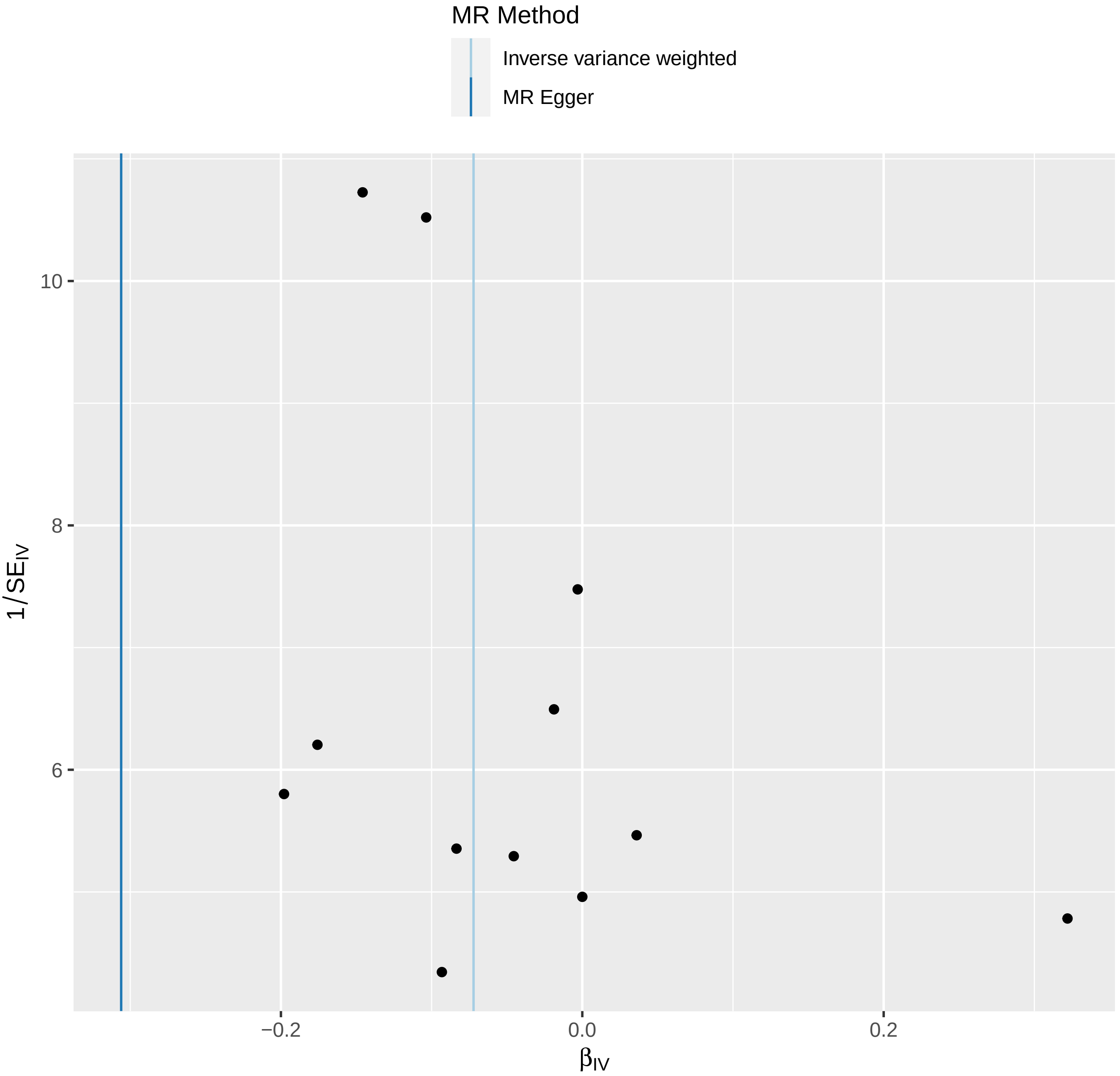

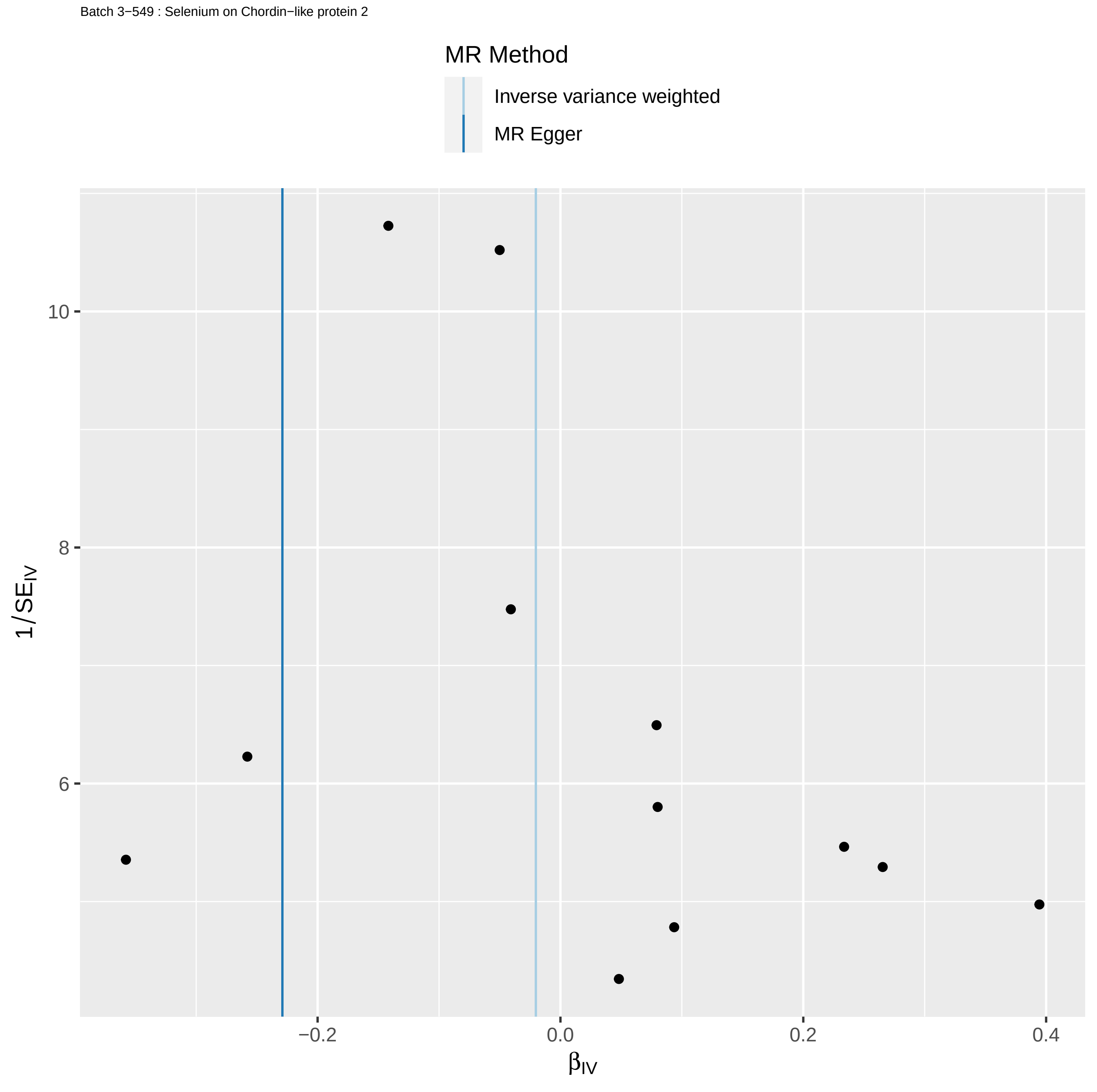
Batch 3−549 : Selenium on Chordin−like protein 2
MR Method
Inverse variance weighted
MR Egger
10
8
6
1/SE$_{IV}$
−0.2
0.0
0.2
0.4
β$_{IV}$

Batch 3−550 : Selenium on Carbohydrate sulfotransferase 11

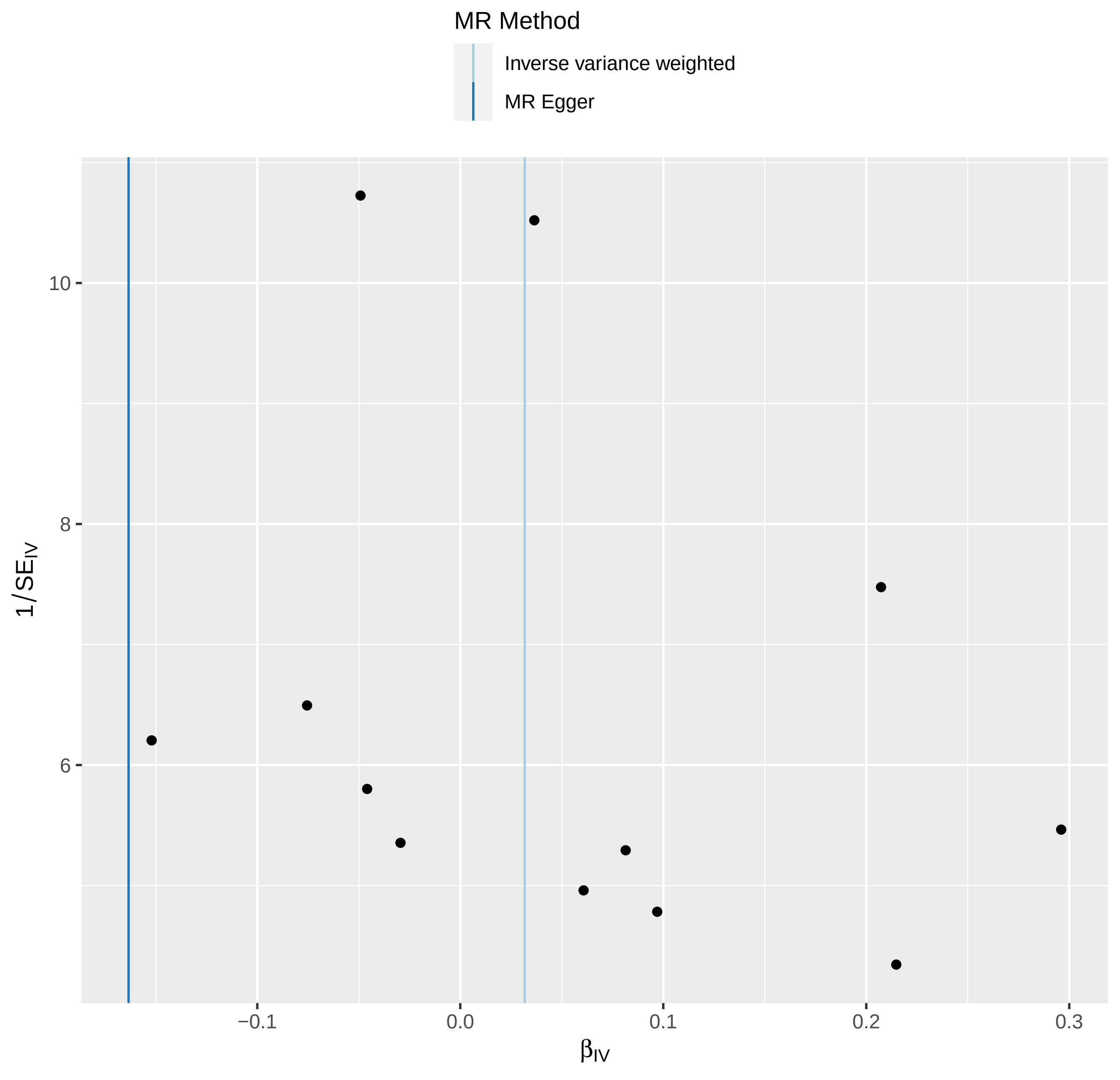

Batch 3−551 : Selenium on Carbohydrate sulfotransferase 14

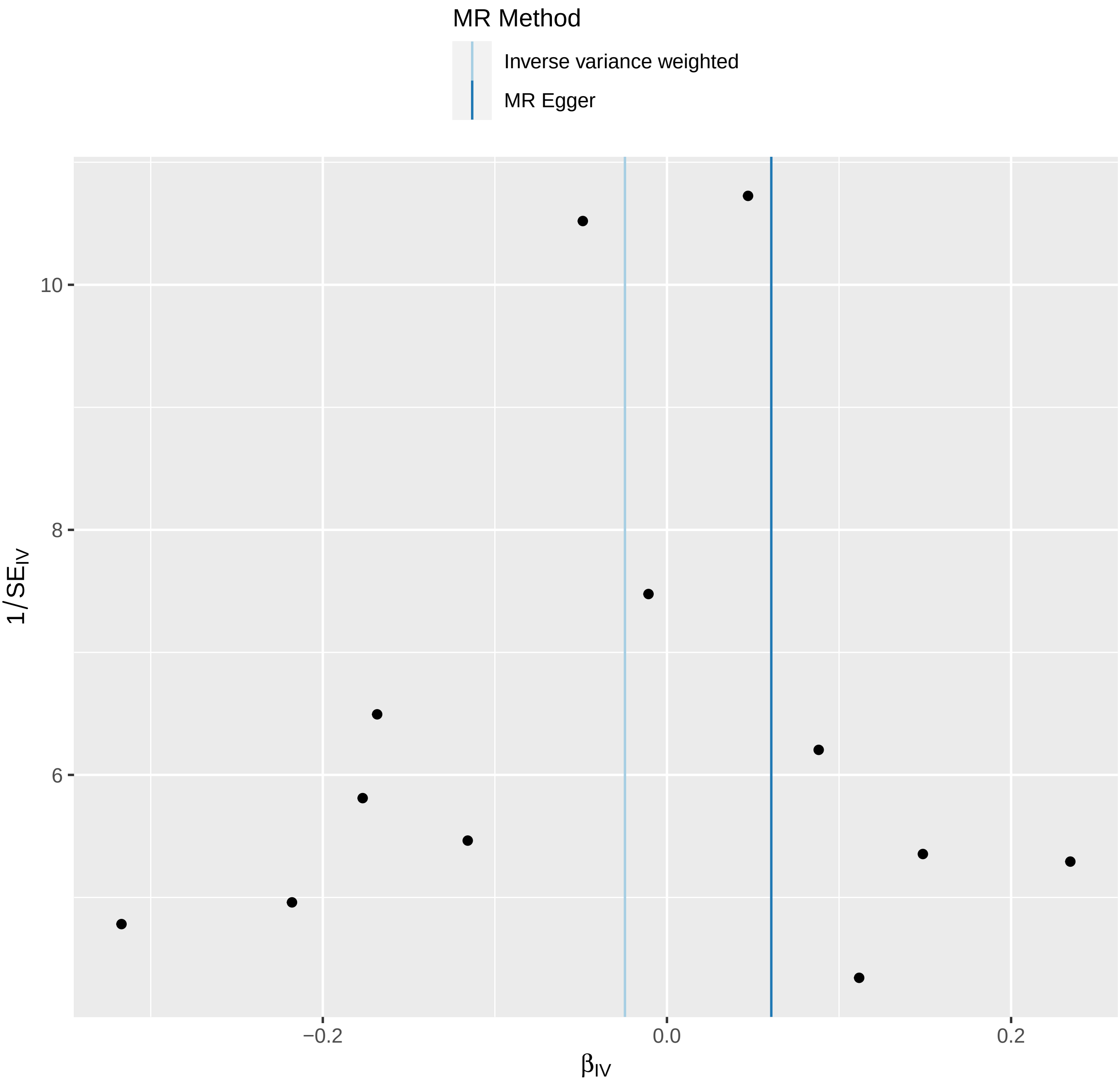

Batch 3−552 : Selenium on Carbohydrate sulfotransferase 15

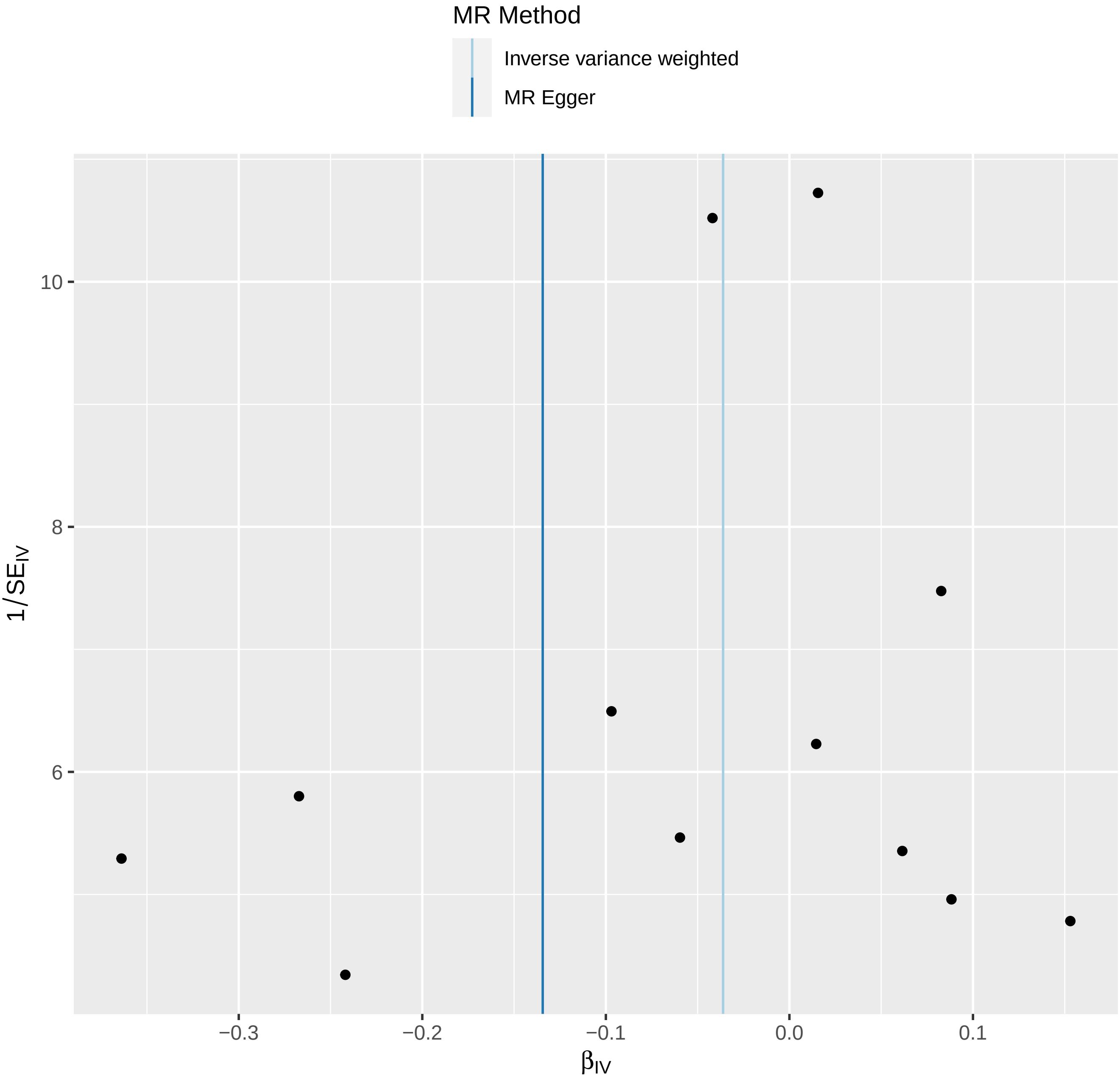

Batch 3−553 : Selenium on Carbohydrate sulfotransferase 15

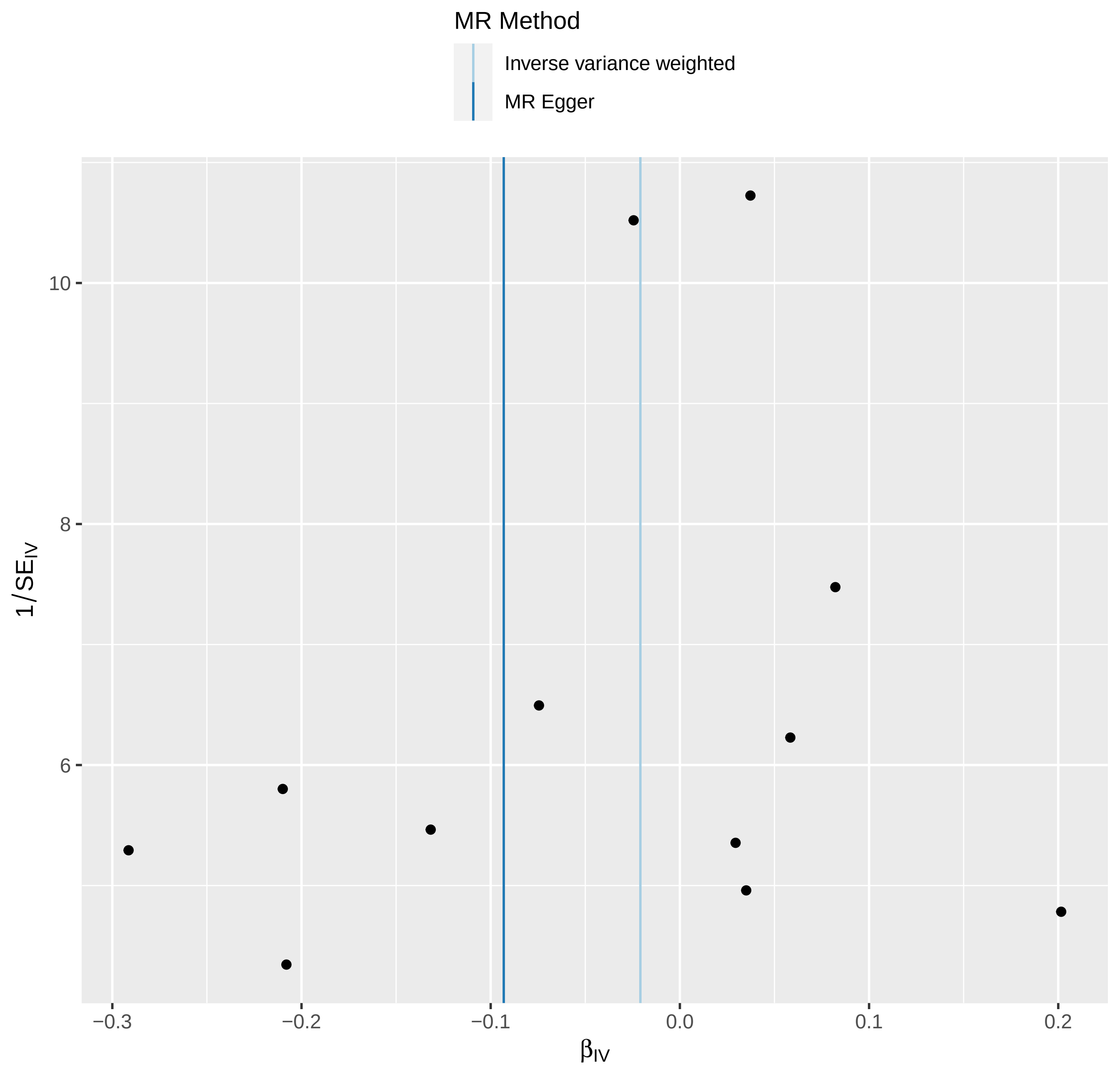

Batch 3−554 : Selenium on Carbohydrate sulfotransferase 1

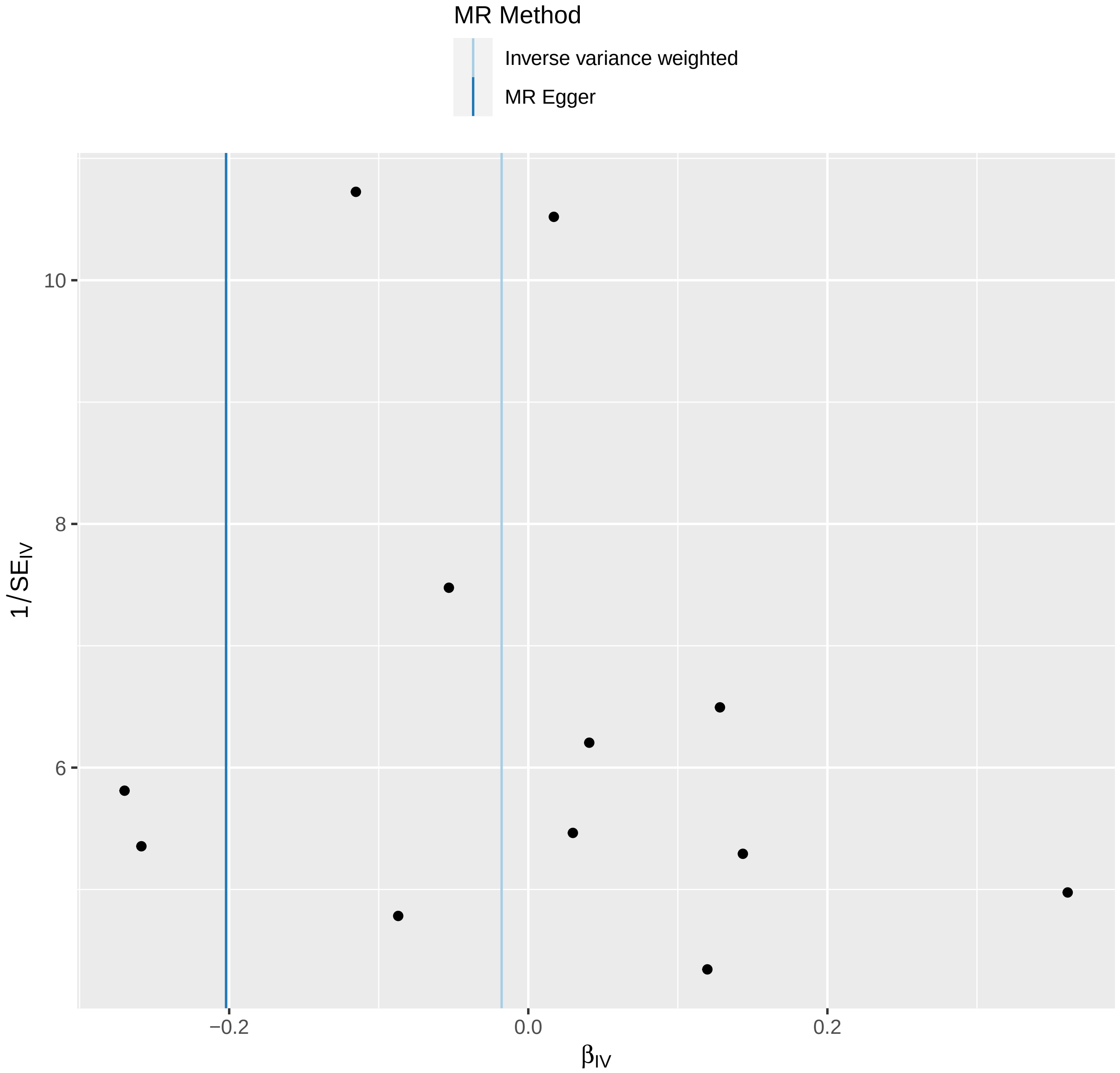

Batch 3−555 : Selenium on Carbohydrate sulfotransferase 2

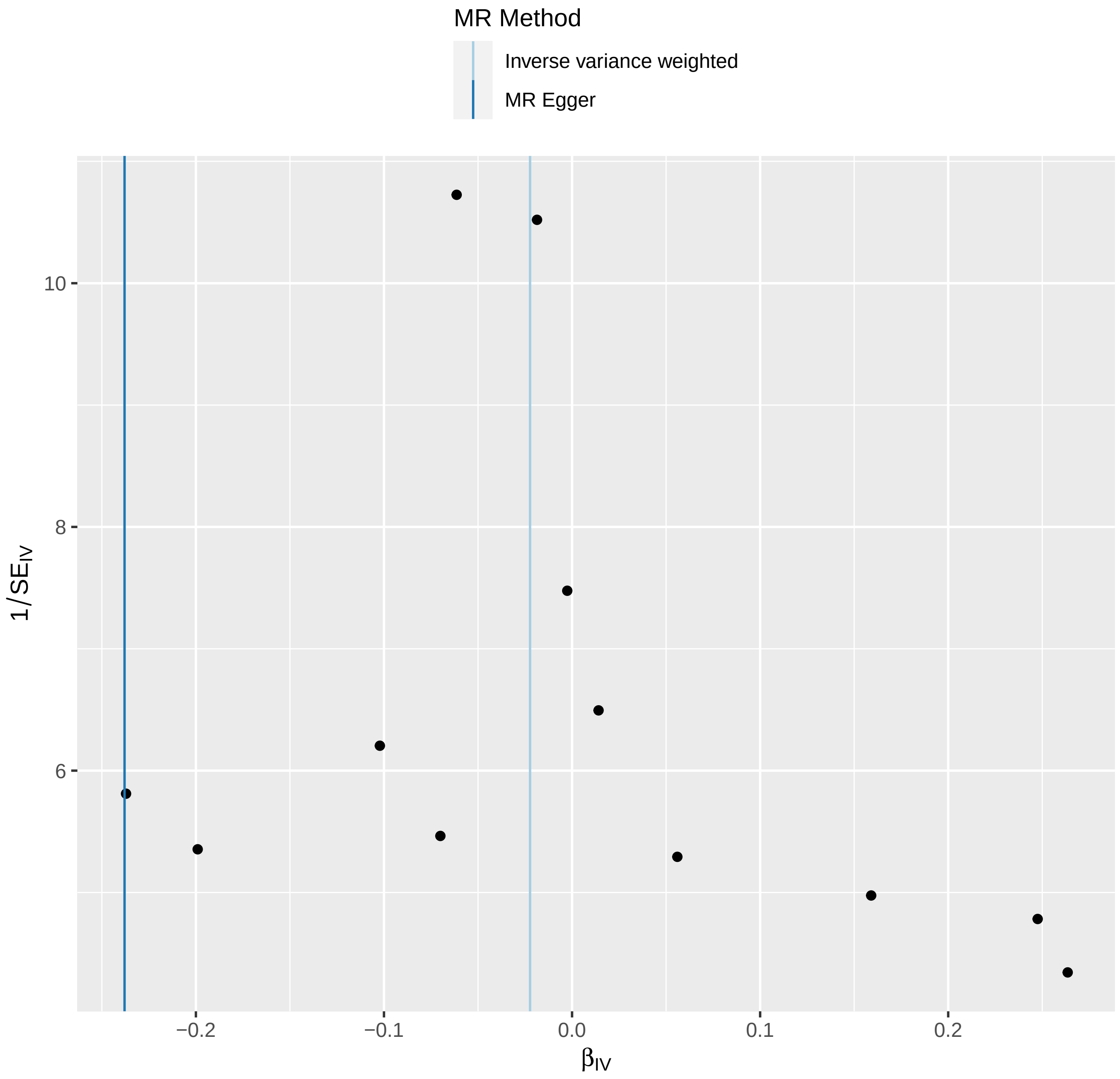

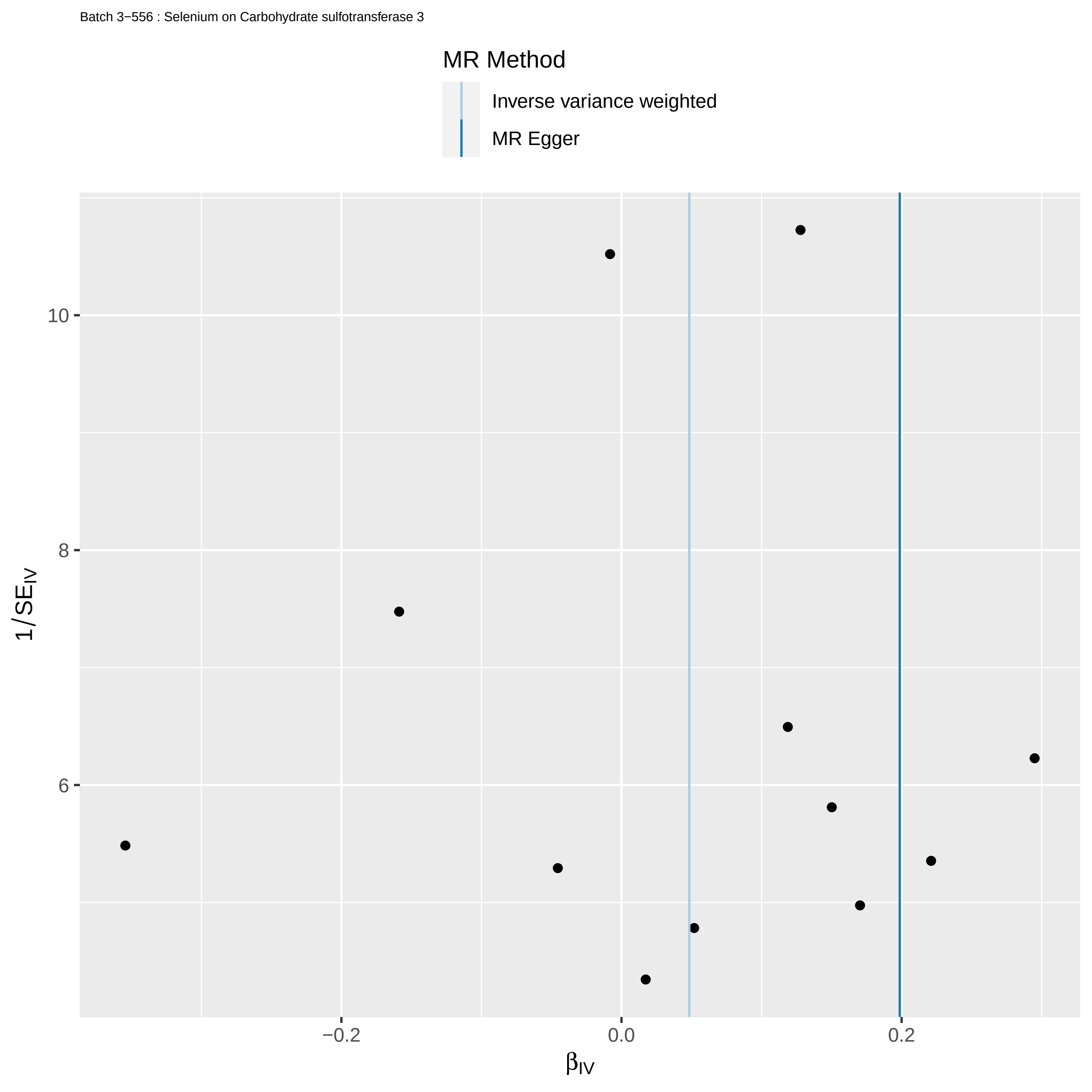
Batch 3−556 : Selenium on Carbohydrate sulfotransferase 3
MR Method
Inverse variance weighted
MR Egger
10
8
6
1/SE_IV
−0.2
0.0
0.2
β_IV

Batch 3−557 : Selenium on Carbohydrate sulfotransferase 5

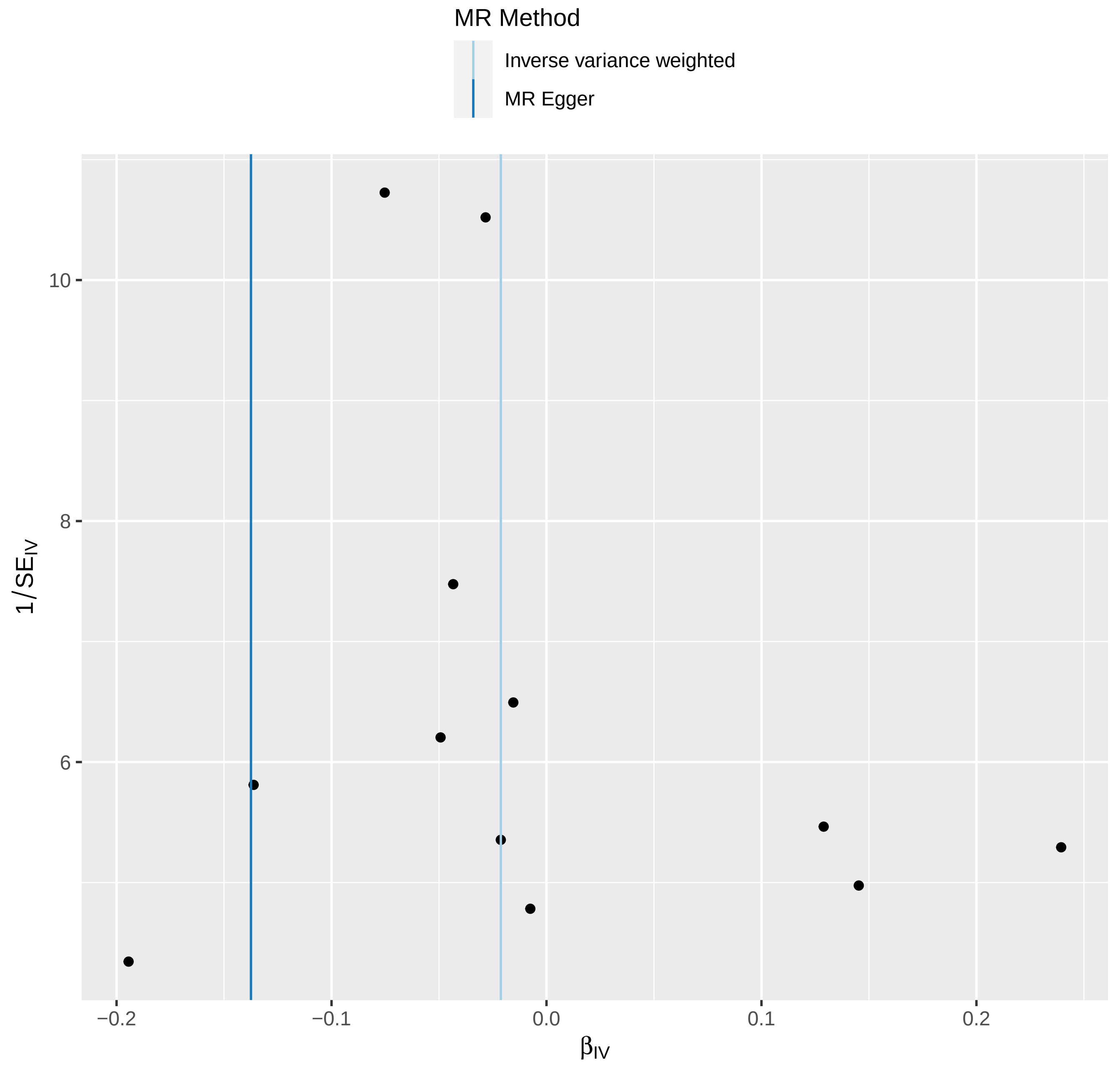

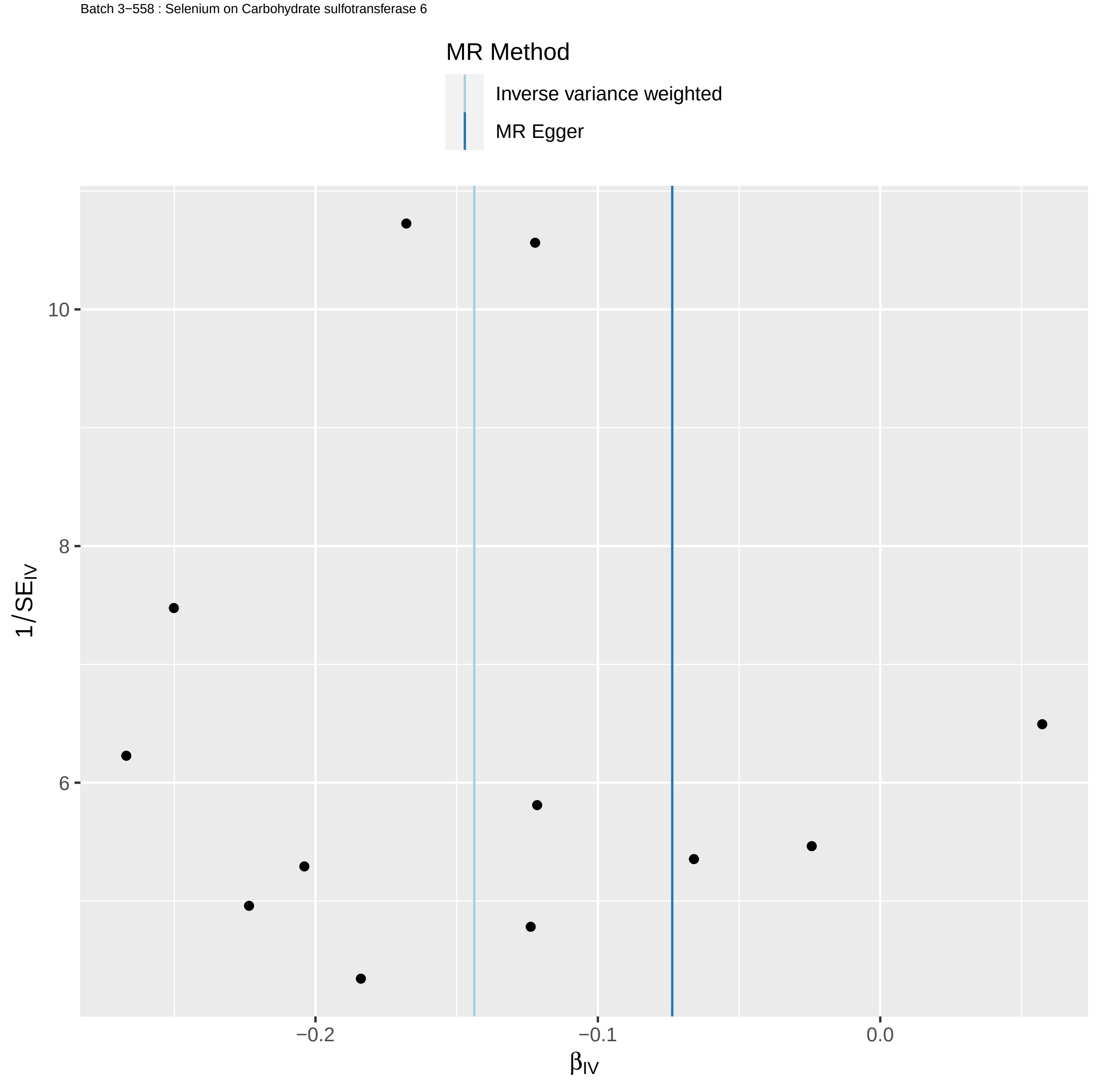
Batch 3−558 : Selenium on Carbohydrate sulfotransferase 6
MR Method
Inverse variance weighted
MR Egger
10
8
6
1/SE$_{IV}$
−0.2
−0.1
0.0
β$_{IV}$

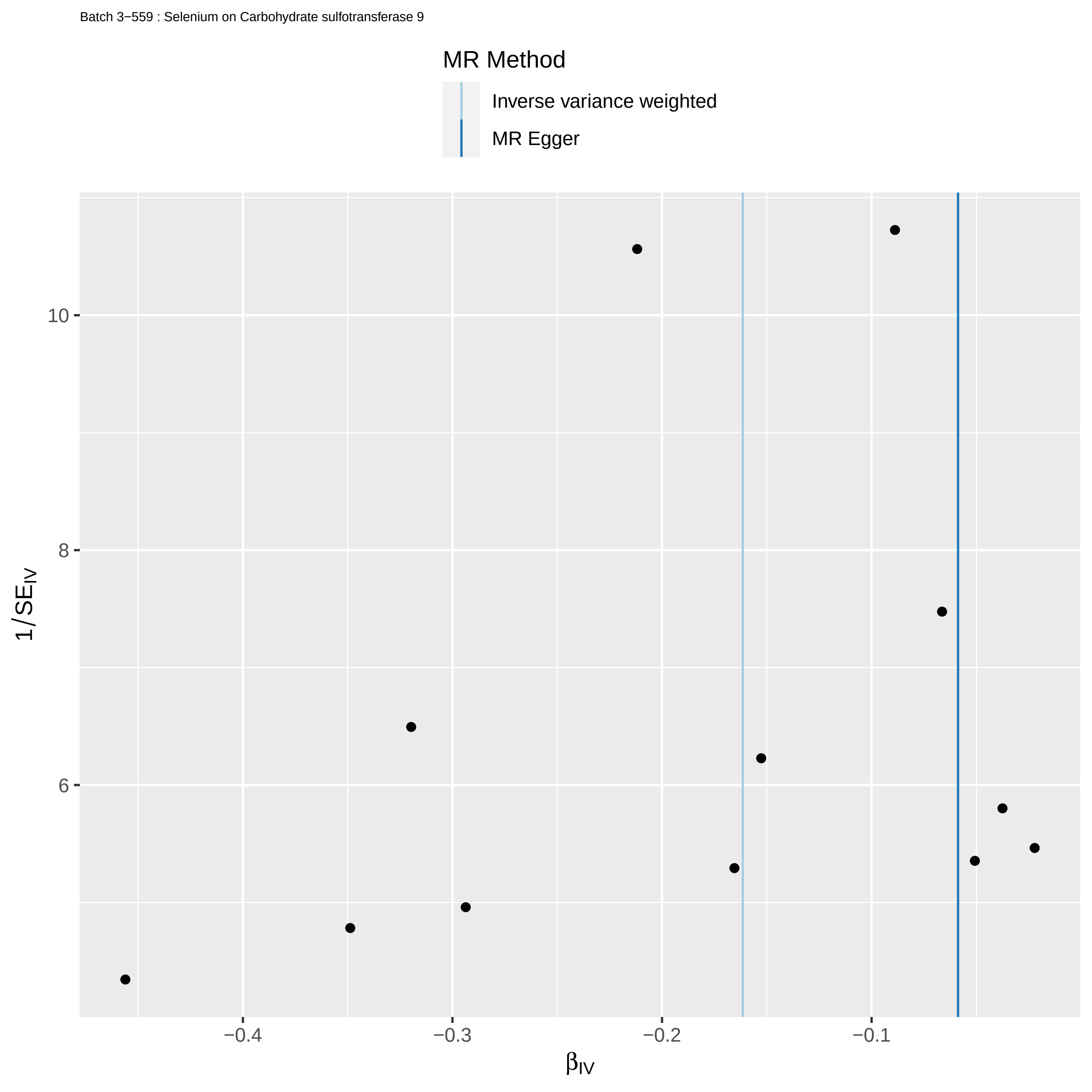

Batch 3−559 : Selenium on Carbohydrate sulfotransferase 9
MR Method
Inverse variance weighted
MR Egger
10
8
6
1/SE_IV
−0.4
−0.3
−0.2
−0.1
β_IV

Batch 3−560 : Selenium on Cartilage intermediate layer protein 1

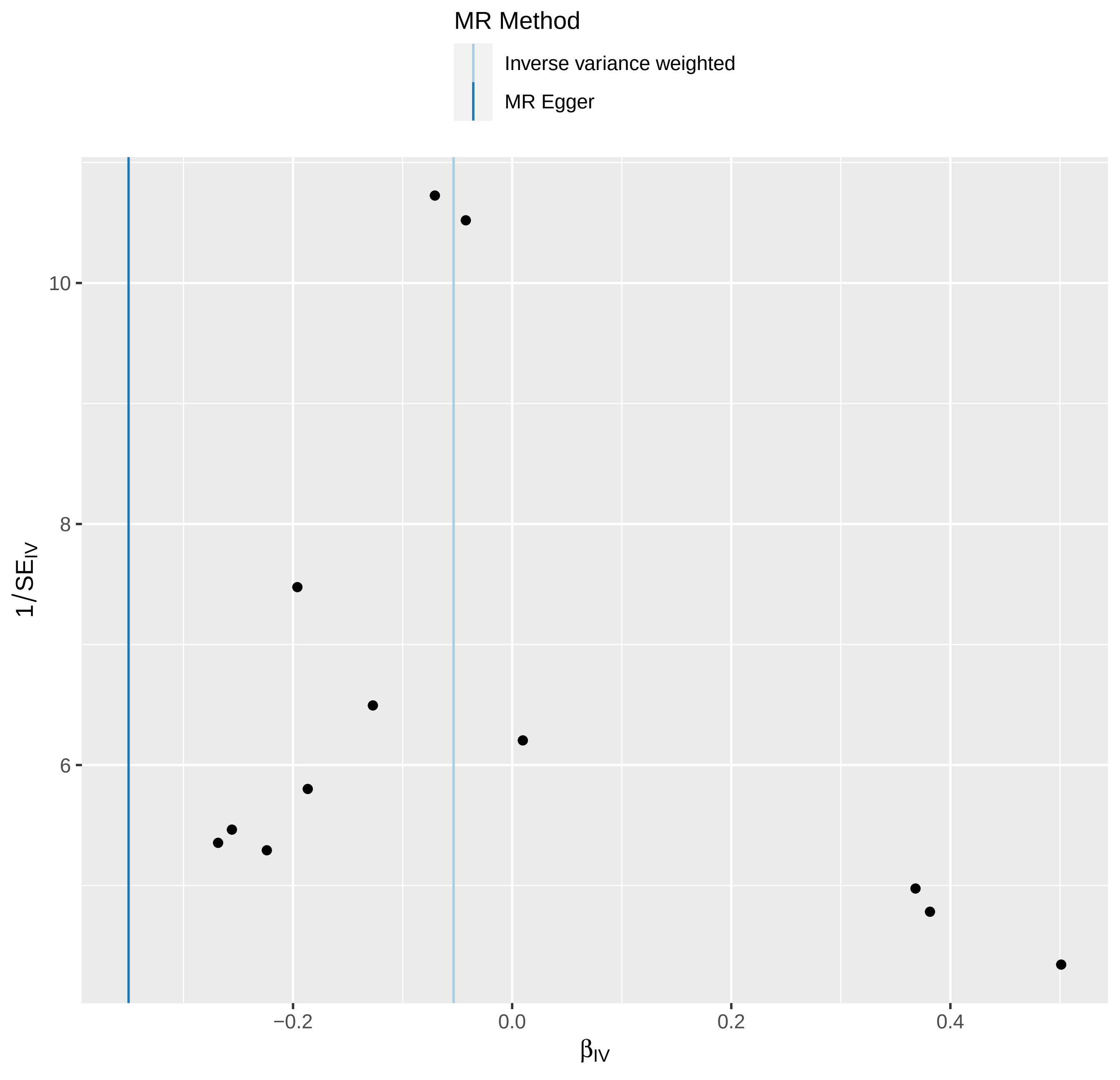

Batch 3−561 : Selenium on Cold−inducible RNA−binding protein

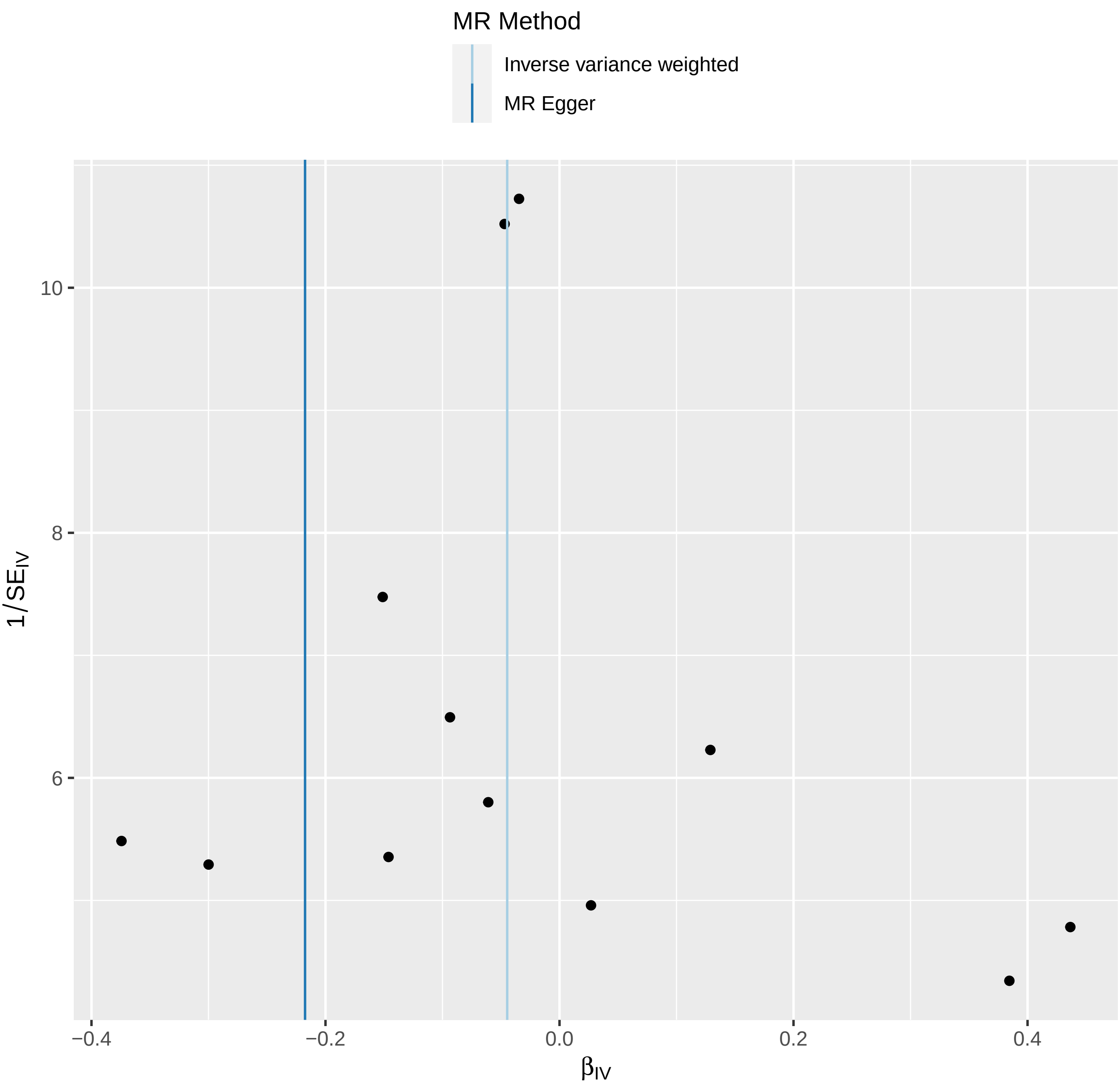

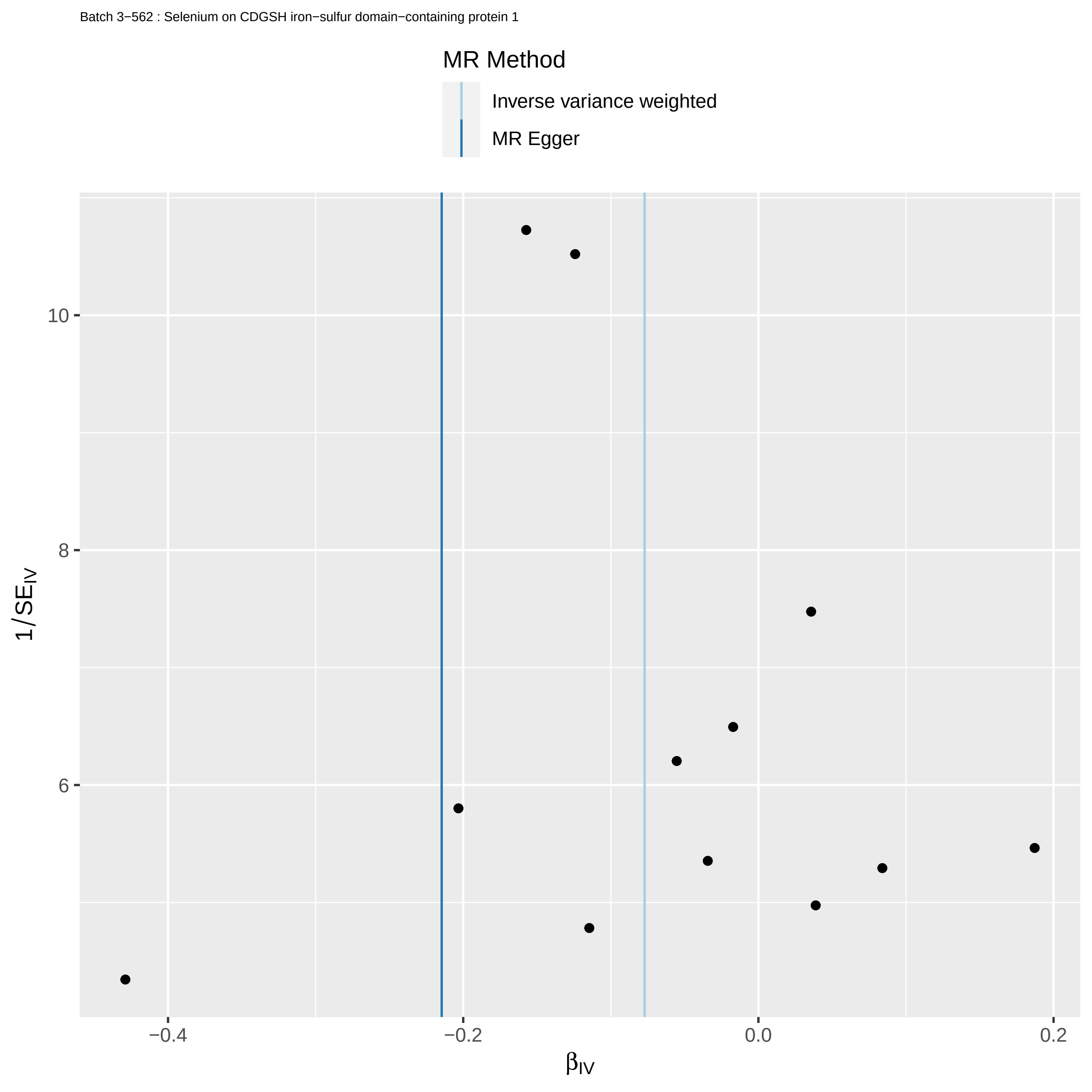
Batch 3−562 : Selenium on CDGSH iron−sulfur domain−containing protein 1
MR Method
Inverse variance weighted
MR Egger
10
8
6
1/SE_IV
−0.4
−0.2
0.0
0.2
β_IV

Batch 3−563 : Selenium on CDGSH iron−sulfur domain−containing protein 2

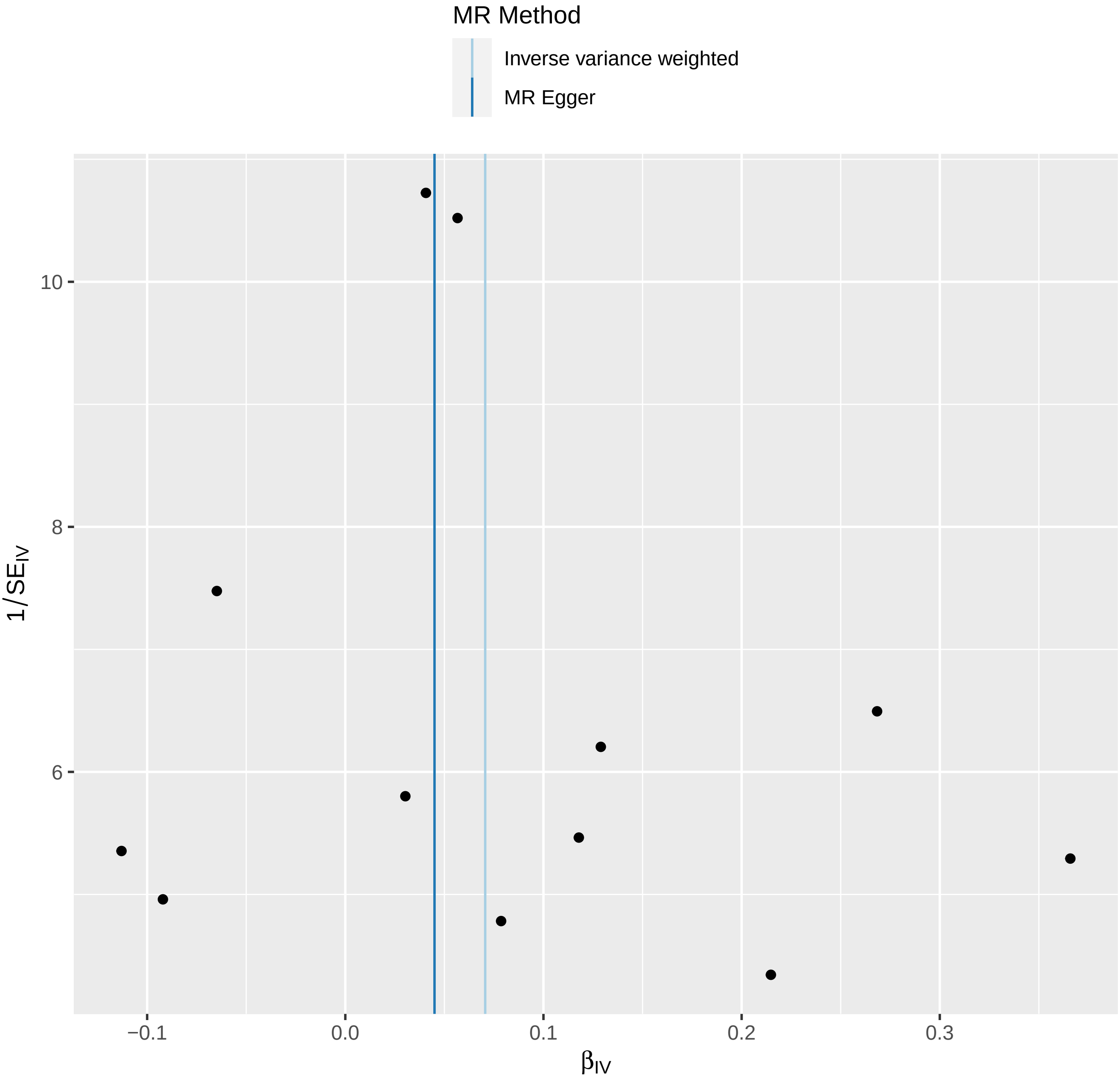

Batch 3−564 : Selenium on Cytoskeleton−associated protein 2

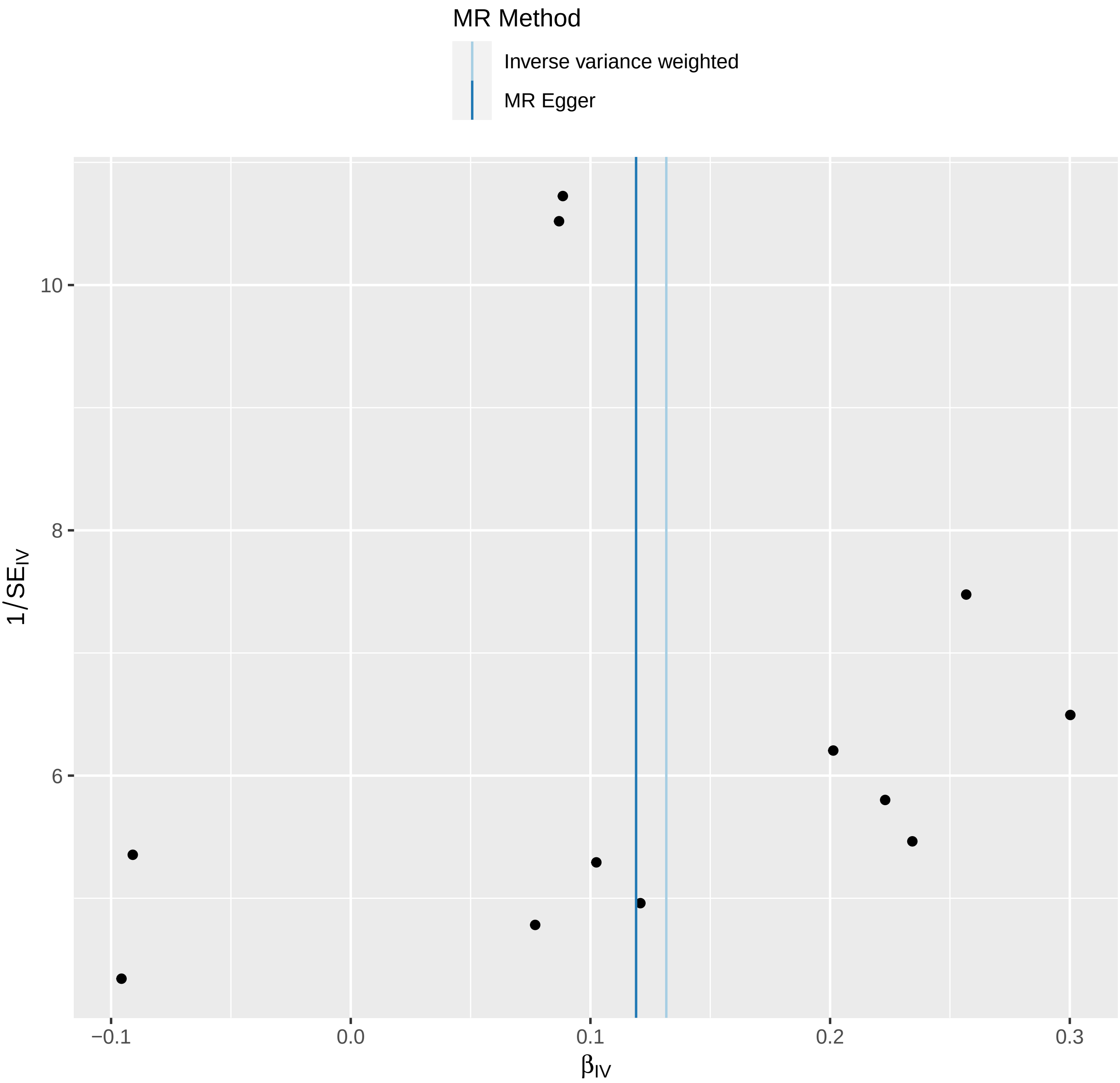

Batch 3−565 : Selenium on Creatine kinase B−type

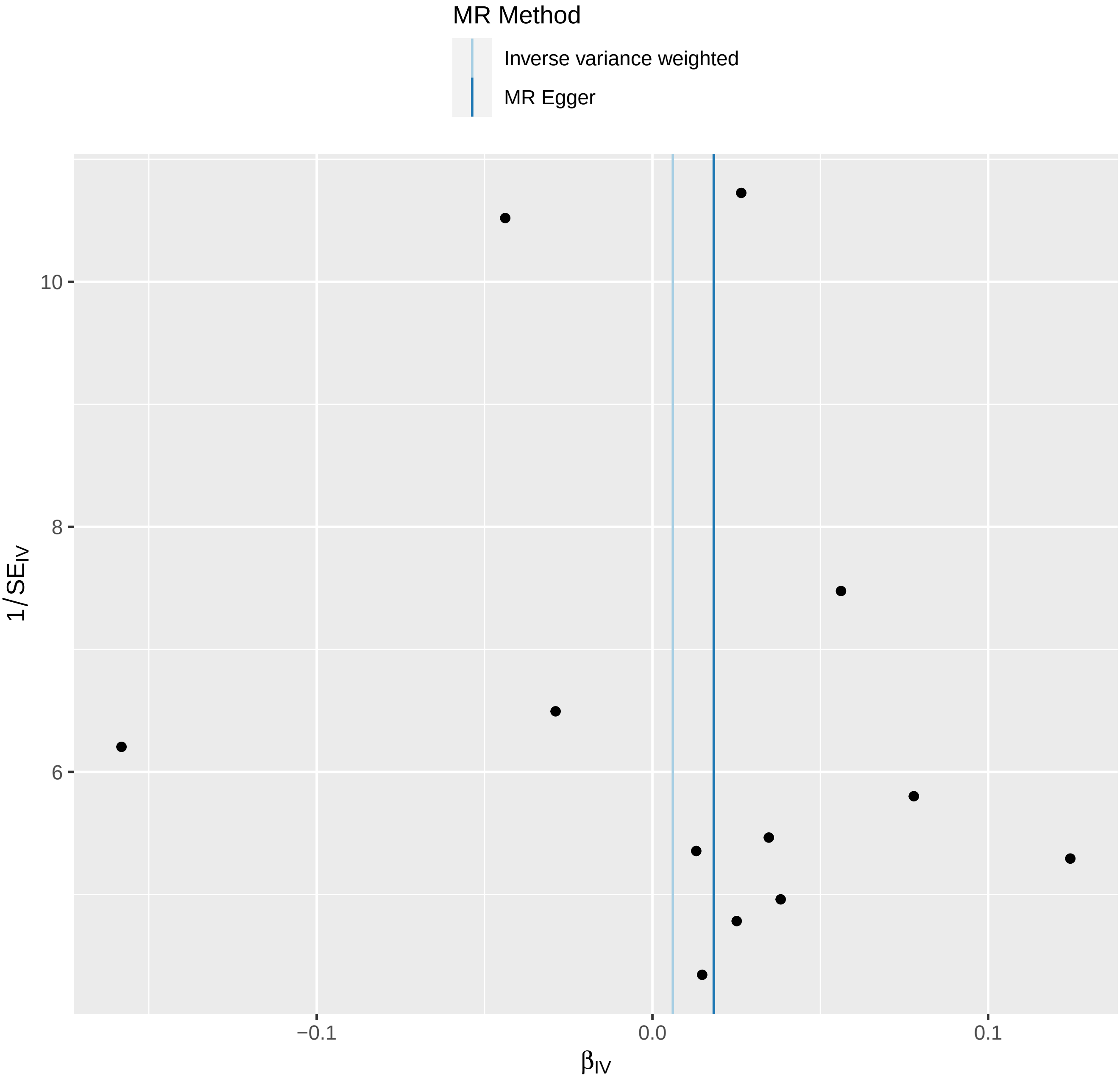

Batch 3−566 : Selenium on Creatine kinase M−type

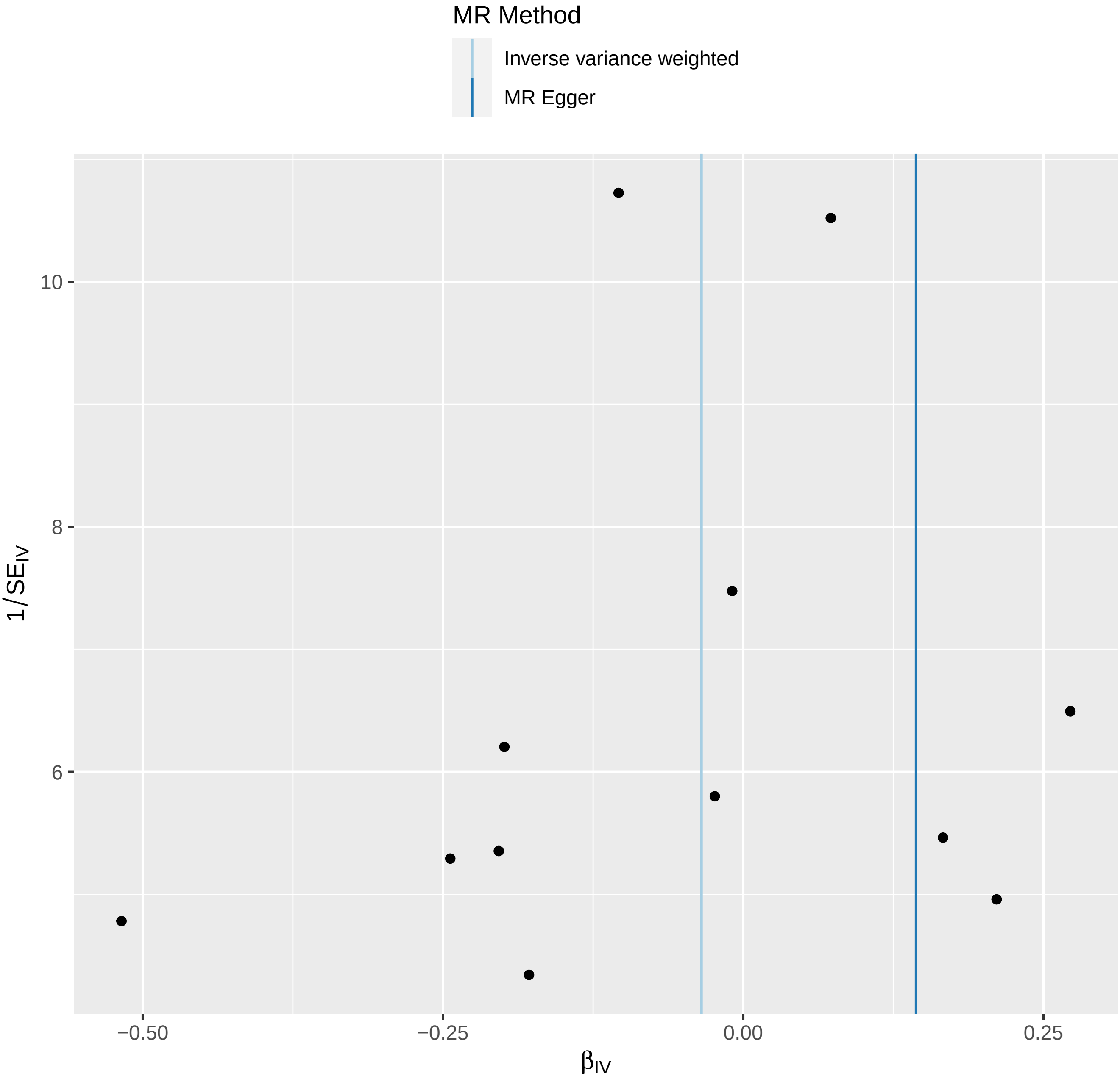

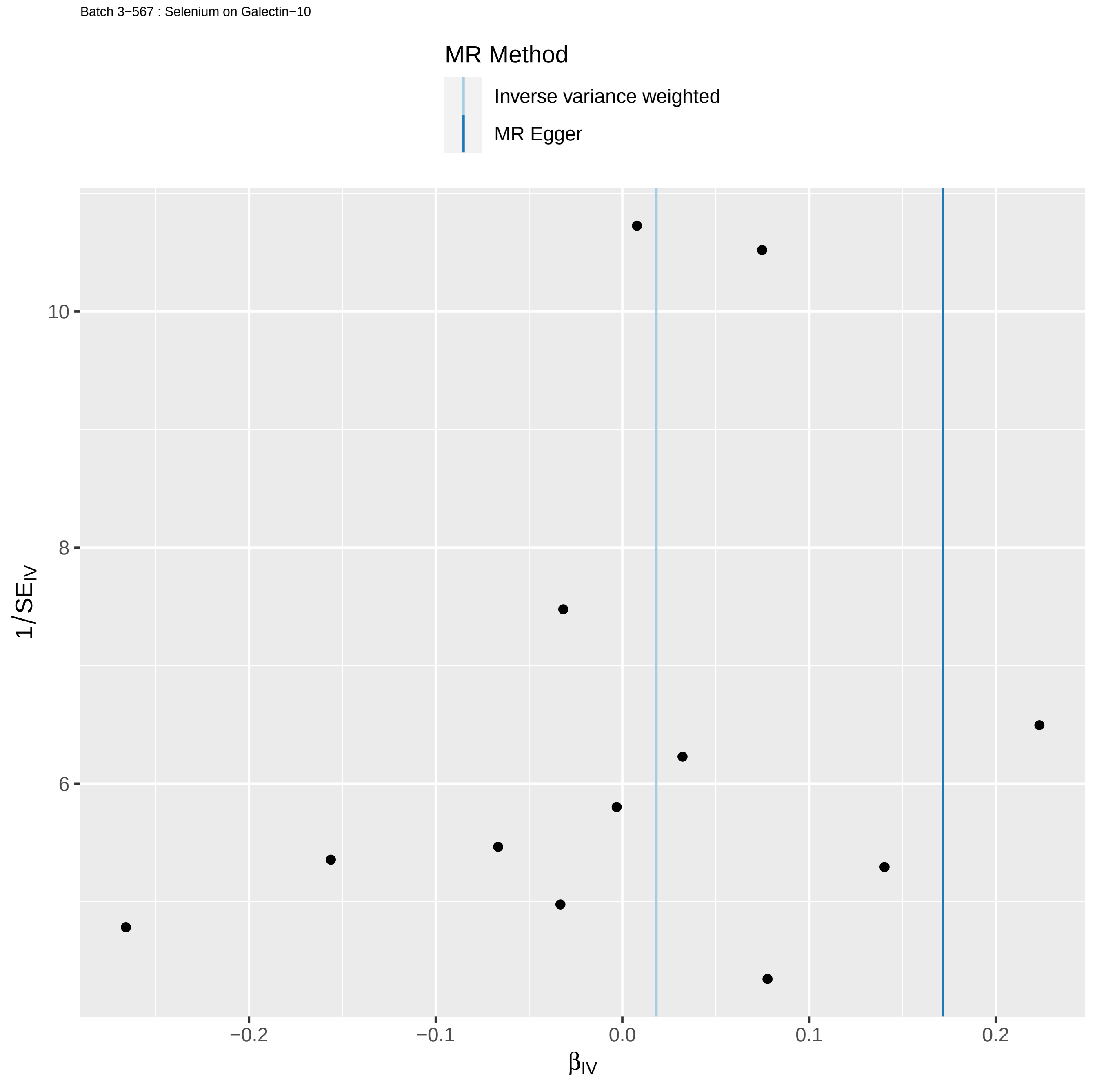
Batch 3−567 : Selenium on Galectin−10
MR Method
Inverse variance weighted
MR Egger
10
8
6
1/SE$_{IV}$
−0.2
−0.1
0.0
0.1
0.2
β$_{IV}$

Batch 3−568 : Selenium on C−type lectin domain family 10 member A

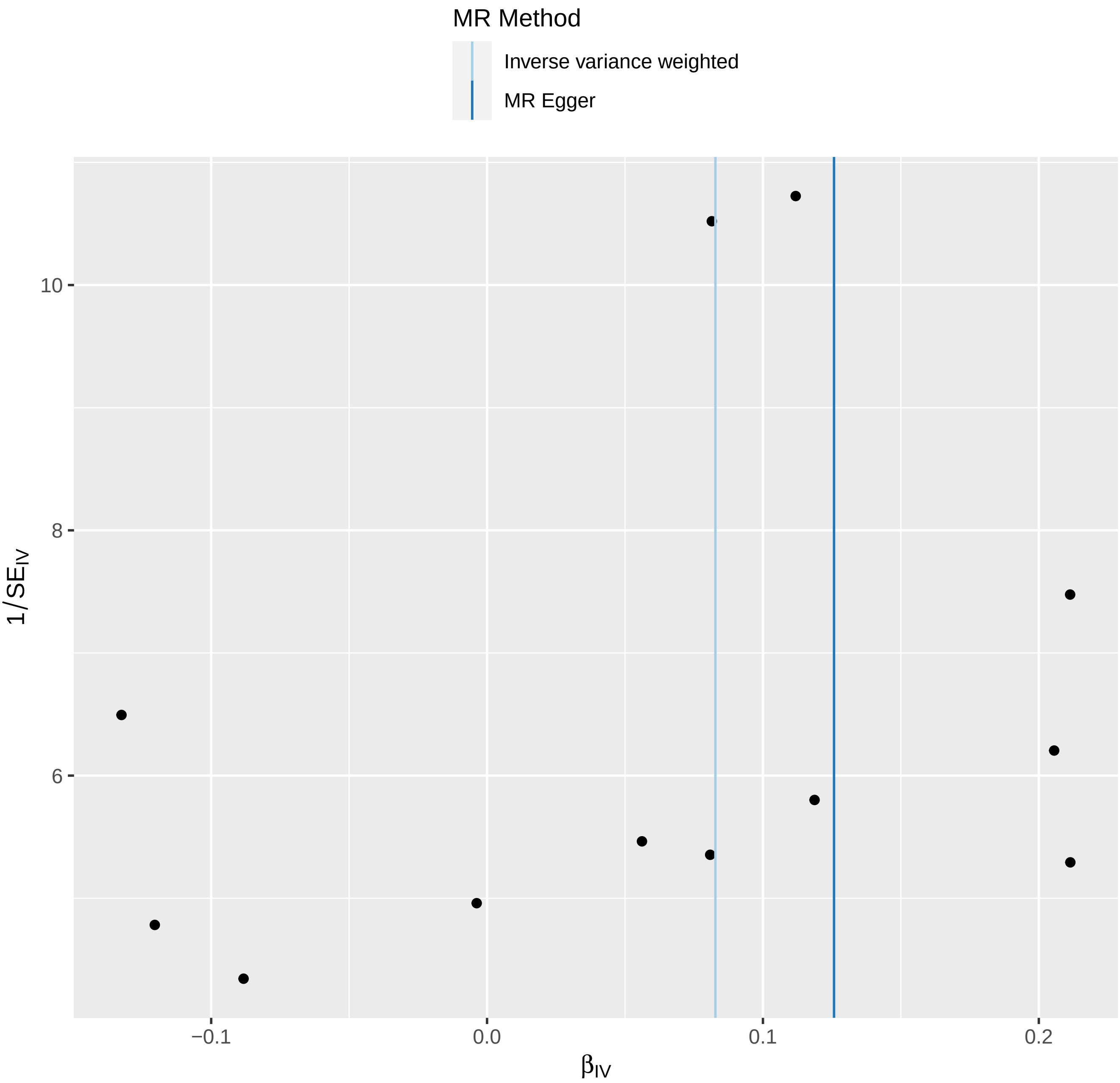

Batch 3−569 : Selenium on Stem Cell Growth Factor−beta

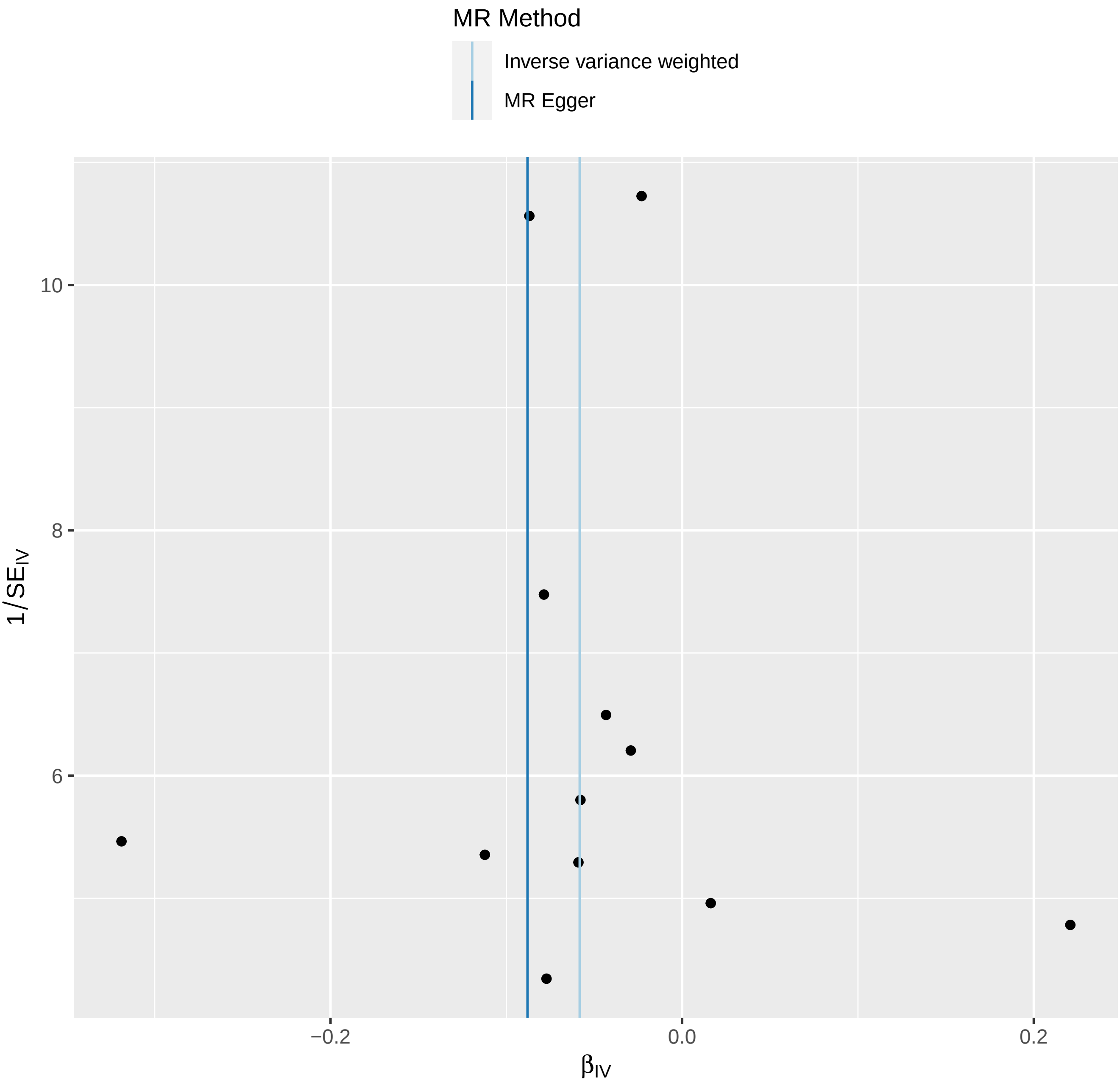

Batch 3−570 : Selenium on C−type lectin domain family 12 member A

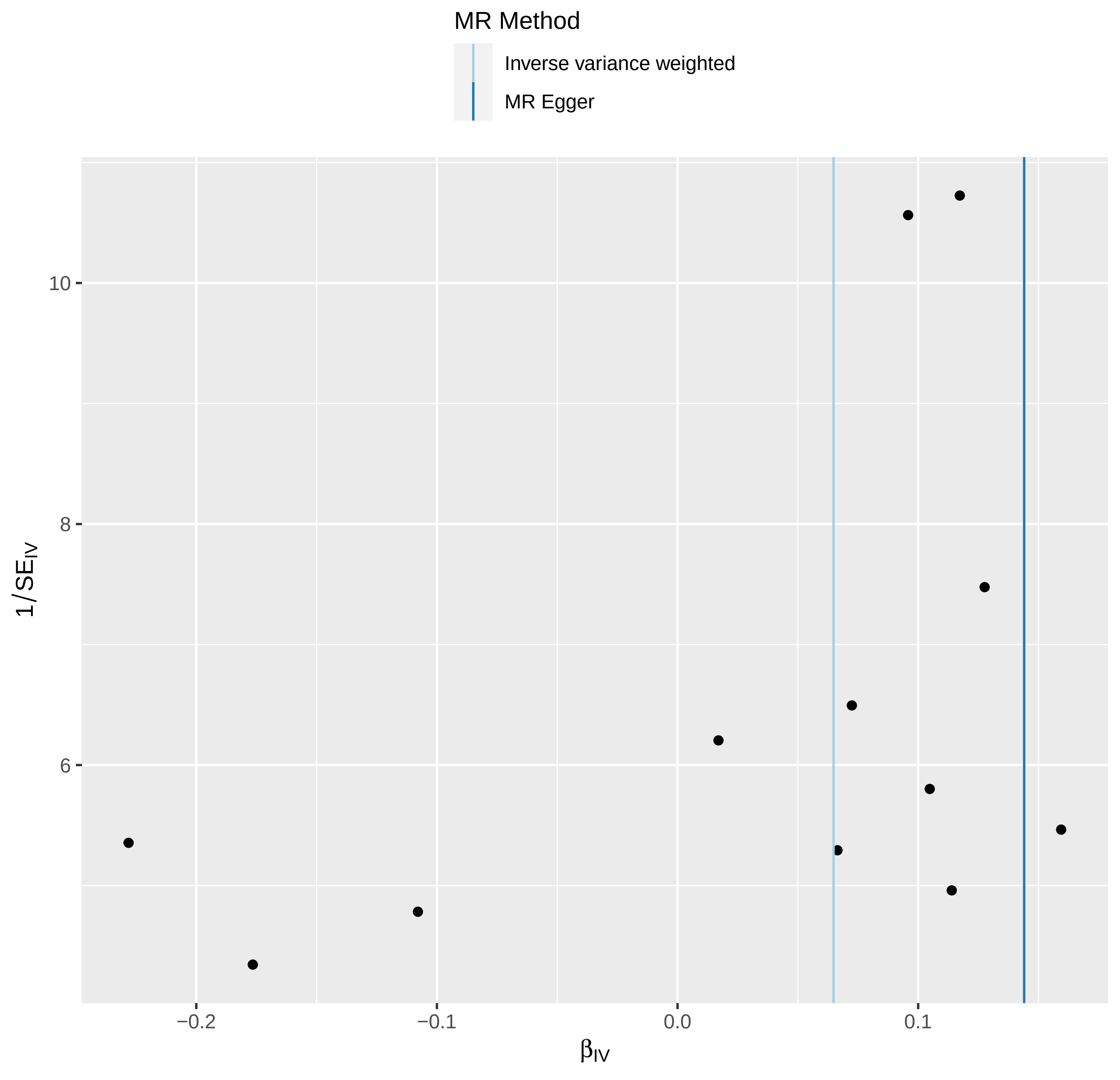

Batch 3−571 : Selenium on C−type lectin domain family 14 member A

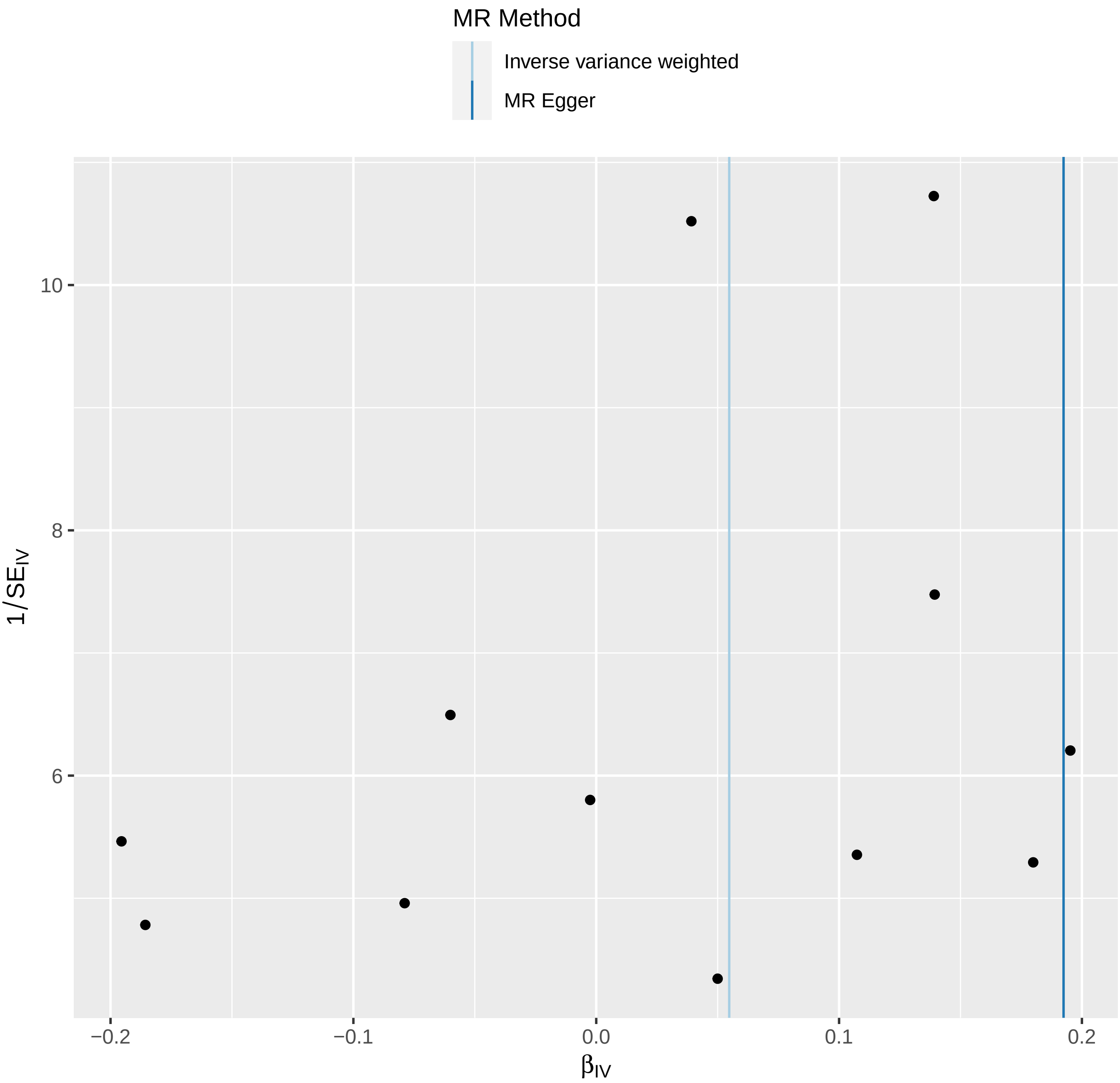

Batch 3−572 : Selenium on C−type lectin domain family 2 member A

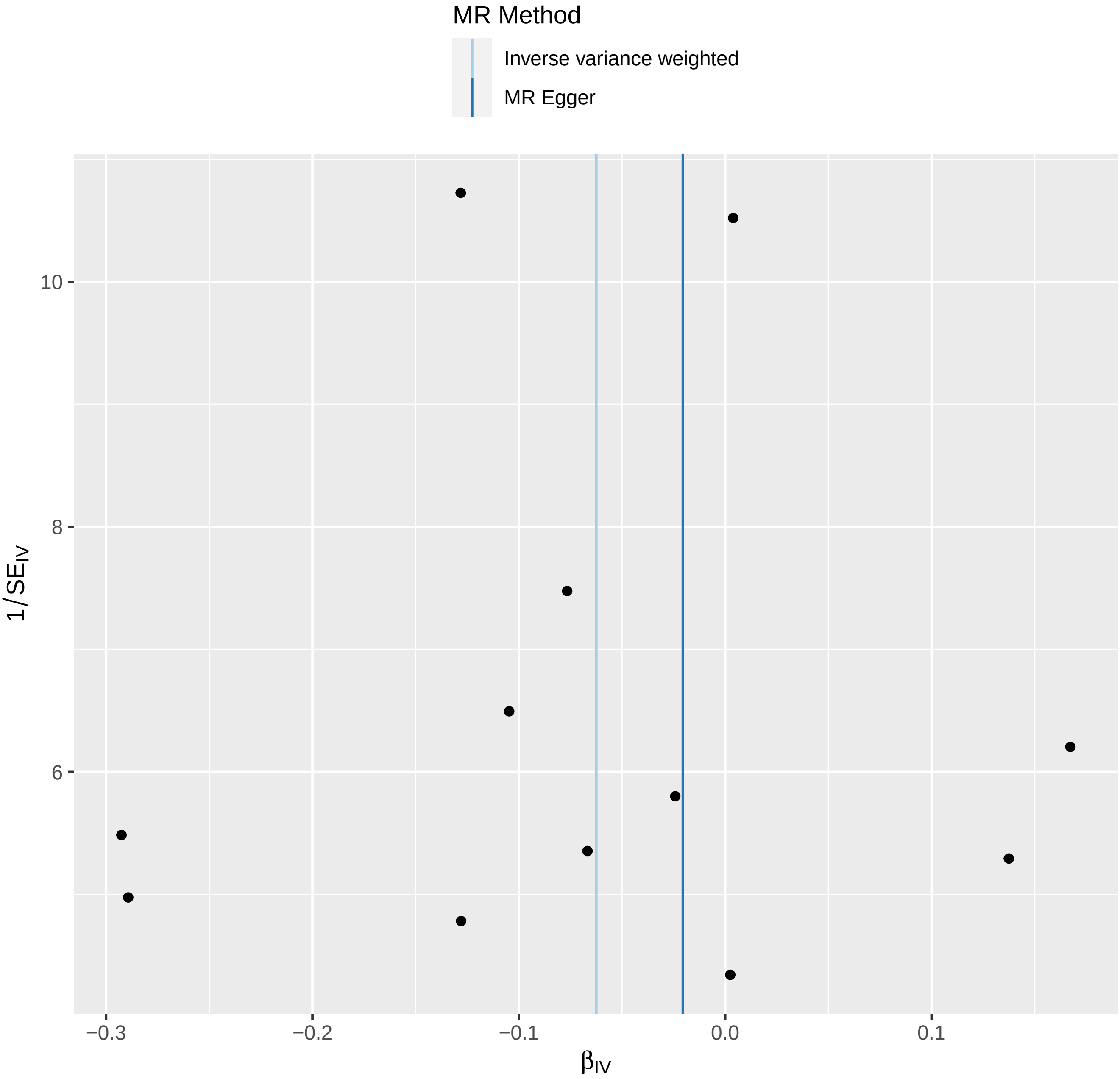

Batch 3−573 : Selenium on C−type lectin domain family 2 member B

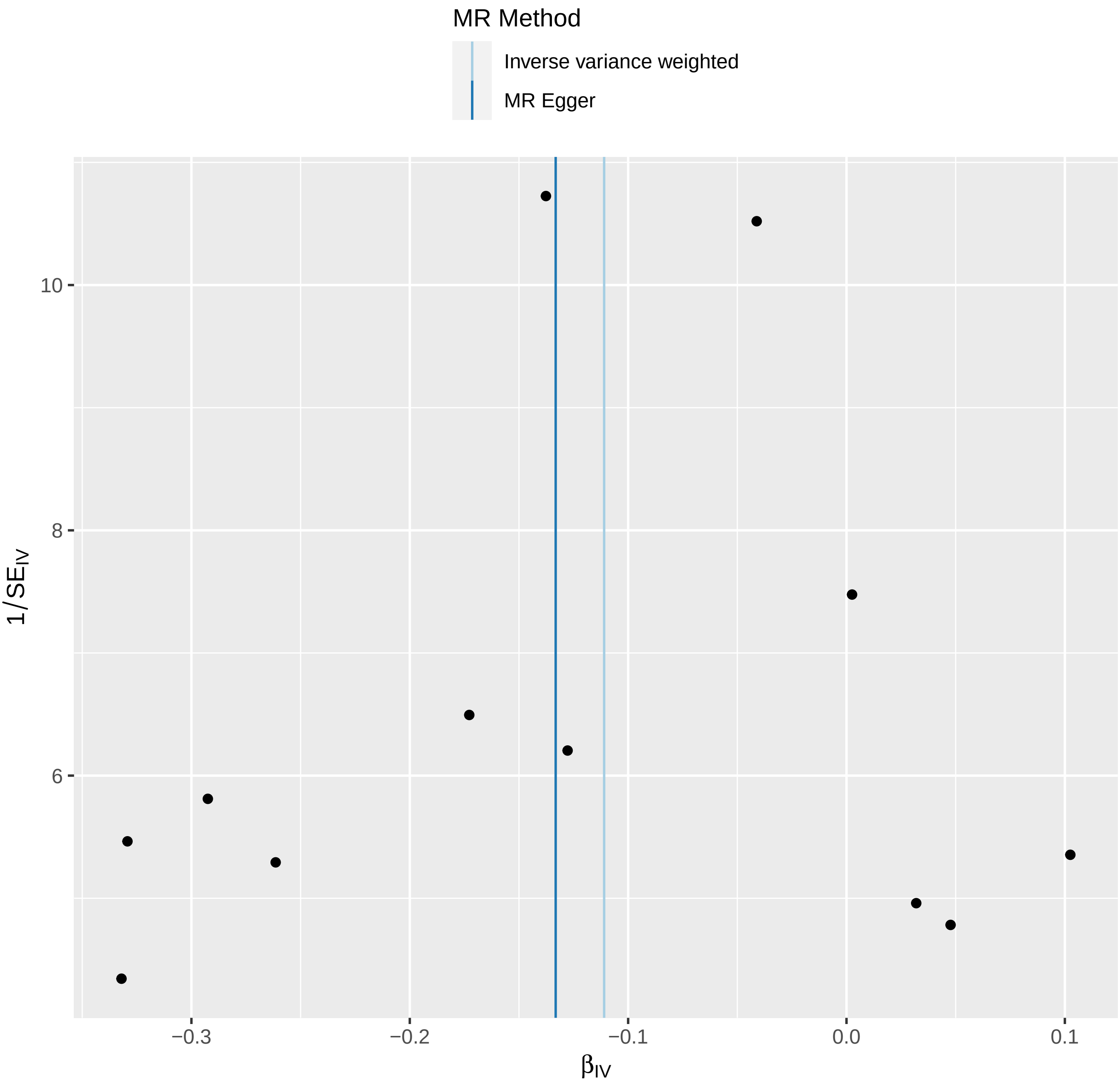

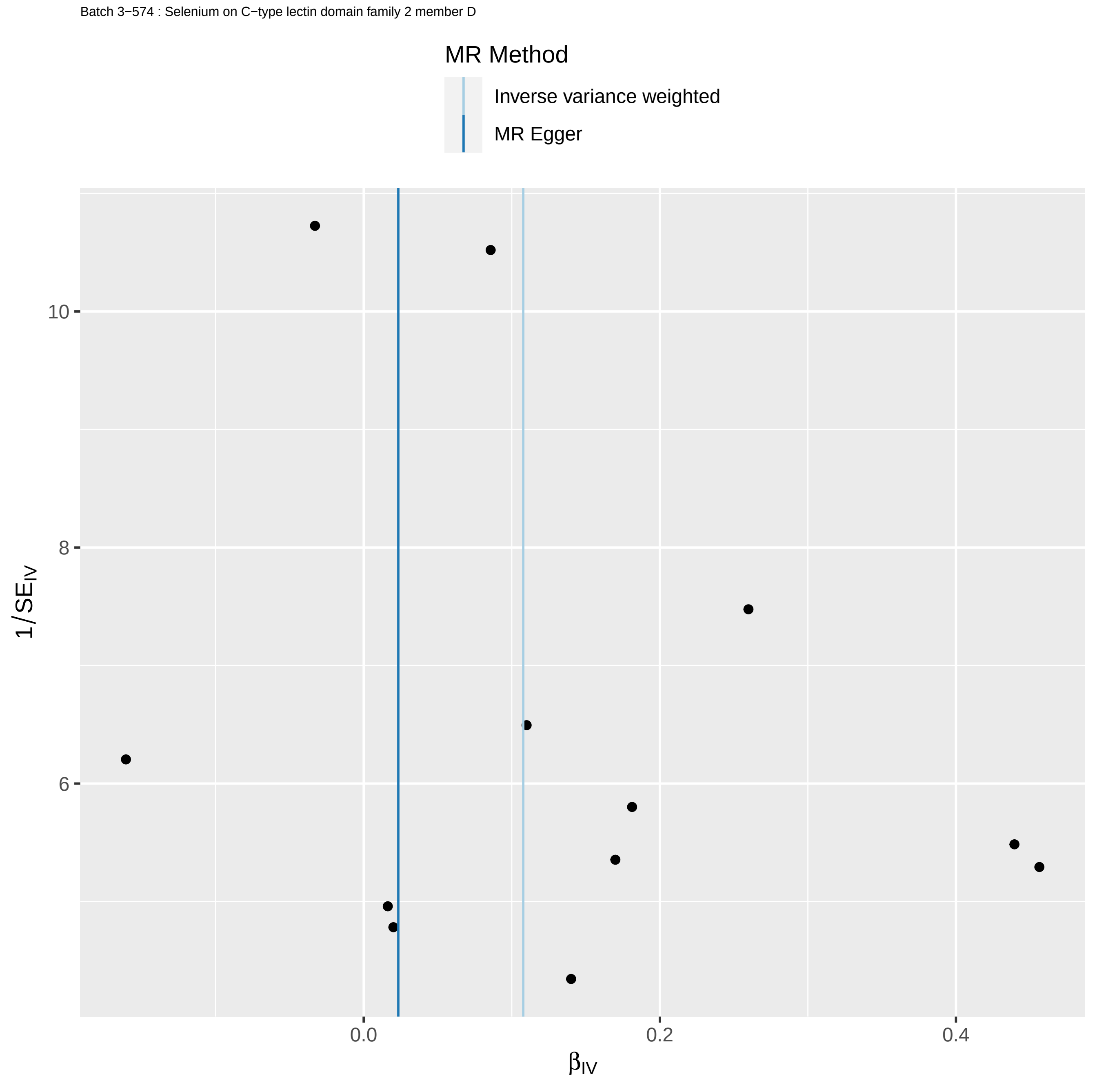

Batch 3−574 : Selenium on C−type lectin domain family 2 member D
MR Method
Inverse variance weighted
MR Egger
10
8
6
1/SE$_{IV}$
0.0
0.2
0.4
β$_{IV}$

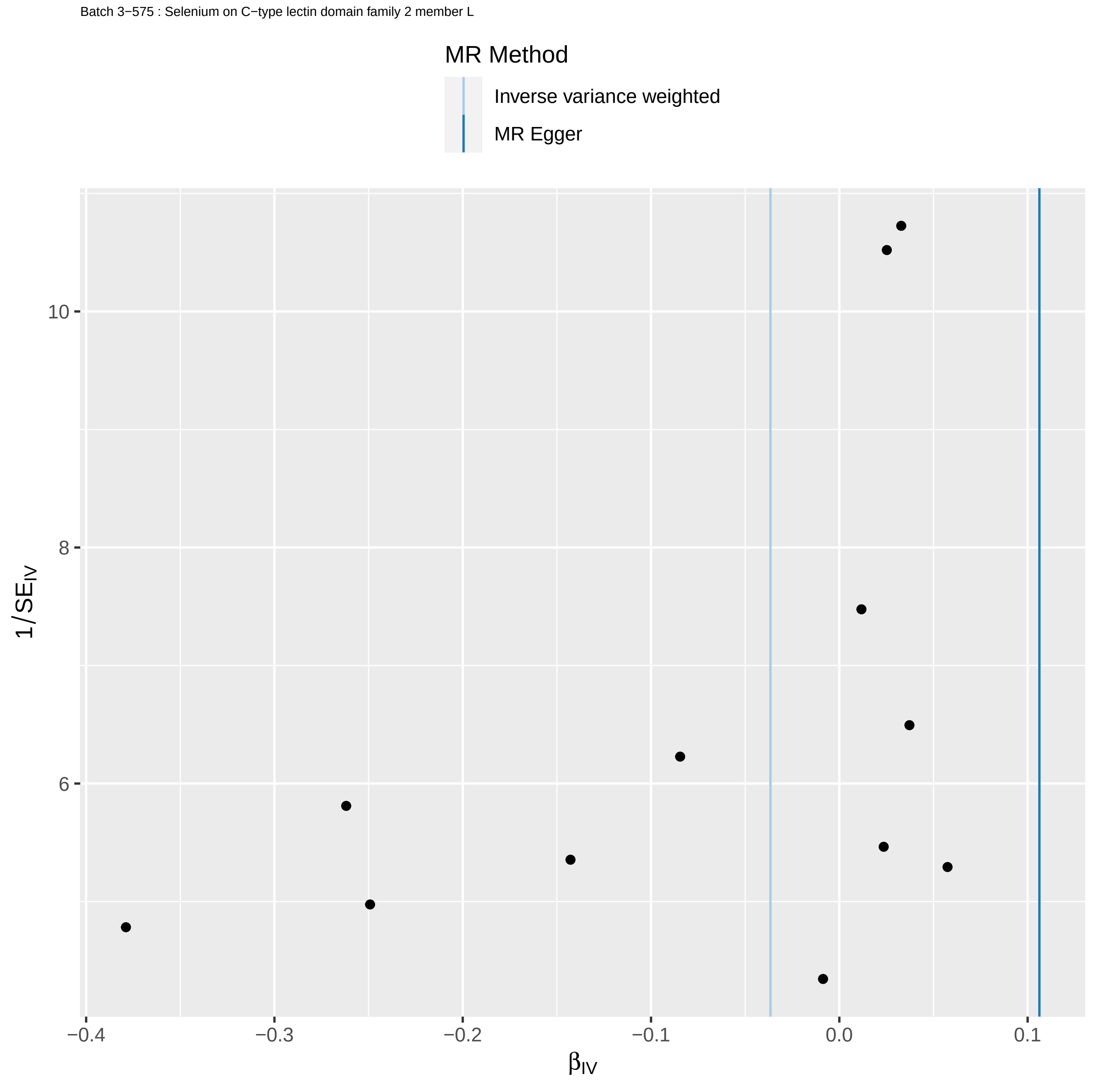

Batch 3−575 : Selenium on C−type lectin domain family 2 member L
MR Method
Inverse variance weighted
MR Egger
10
8
6
1/SE_IV
−0.4
−0.3
−0.2
−0.1
0.0
0.1
β_IV

Batch 3−576 : Selenium on C−type lectin domain family 4 member D

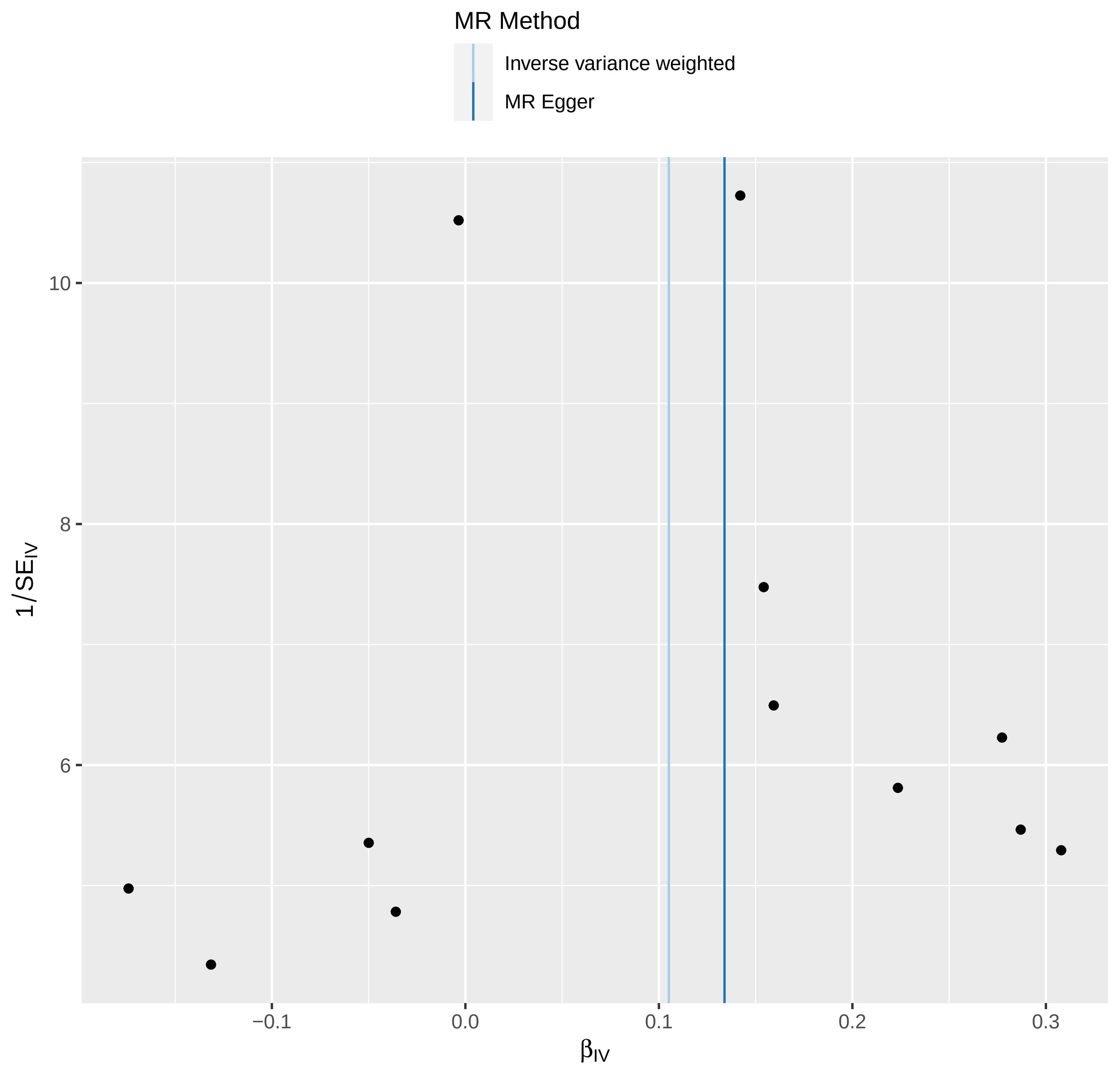

Batch 3−577 : Selenium on C−type lectin domain family 4 member E

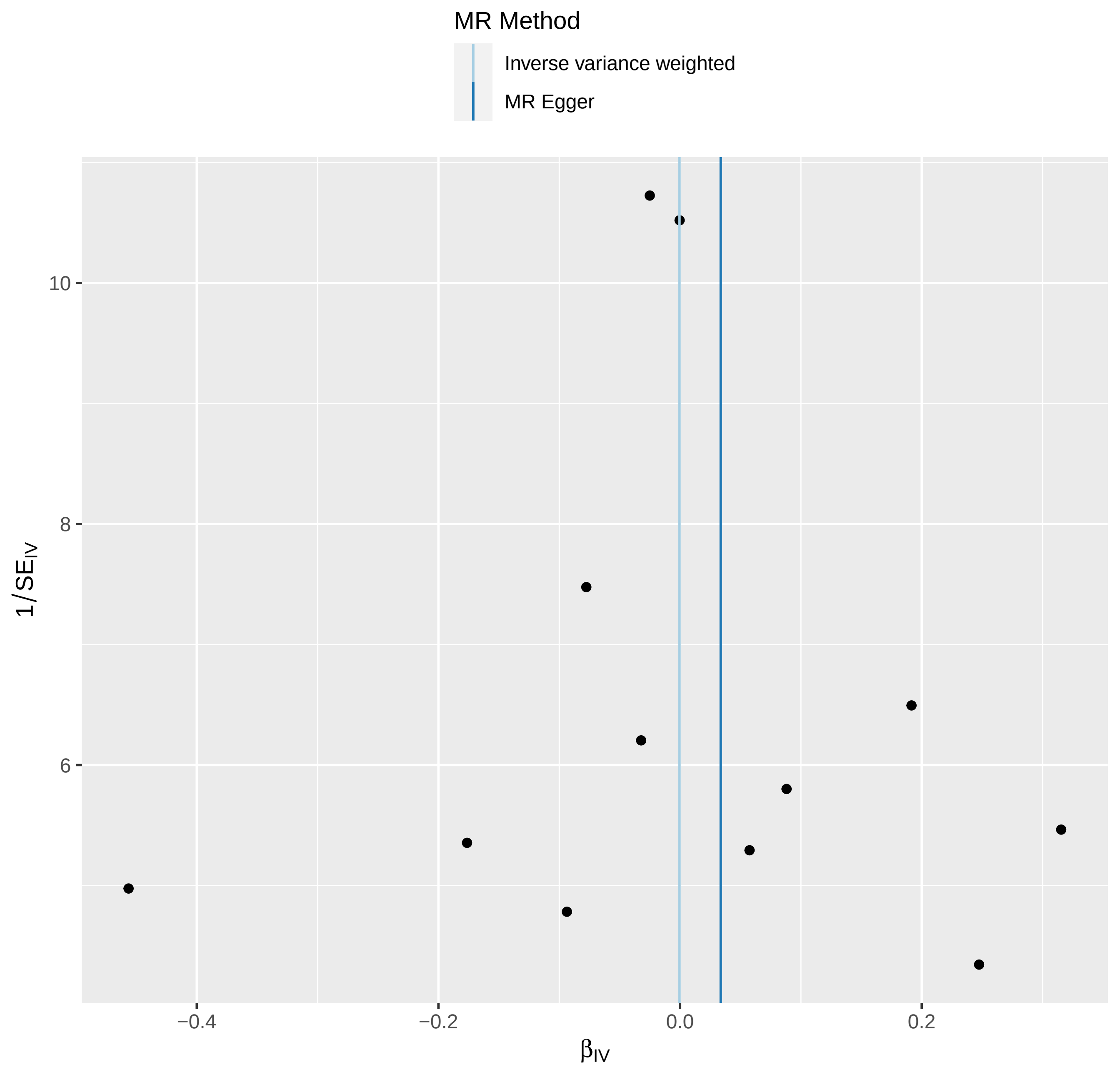

Batch 3−578 : Selenium on C−type lectin domain family 4 member M

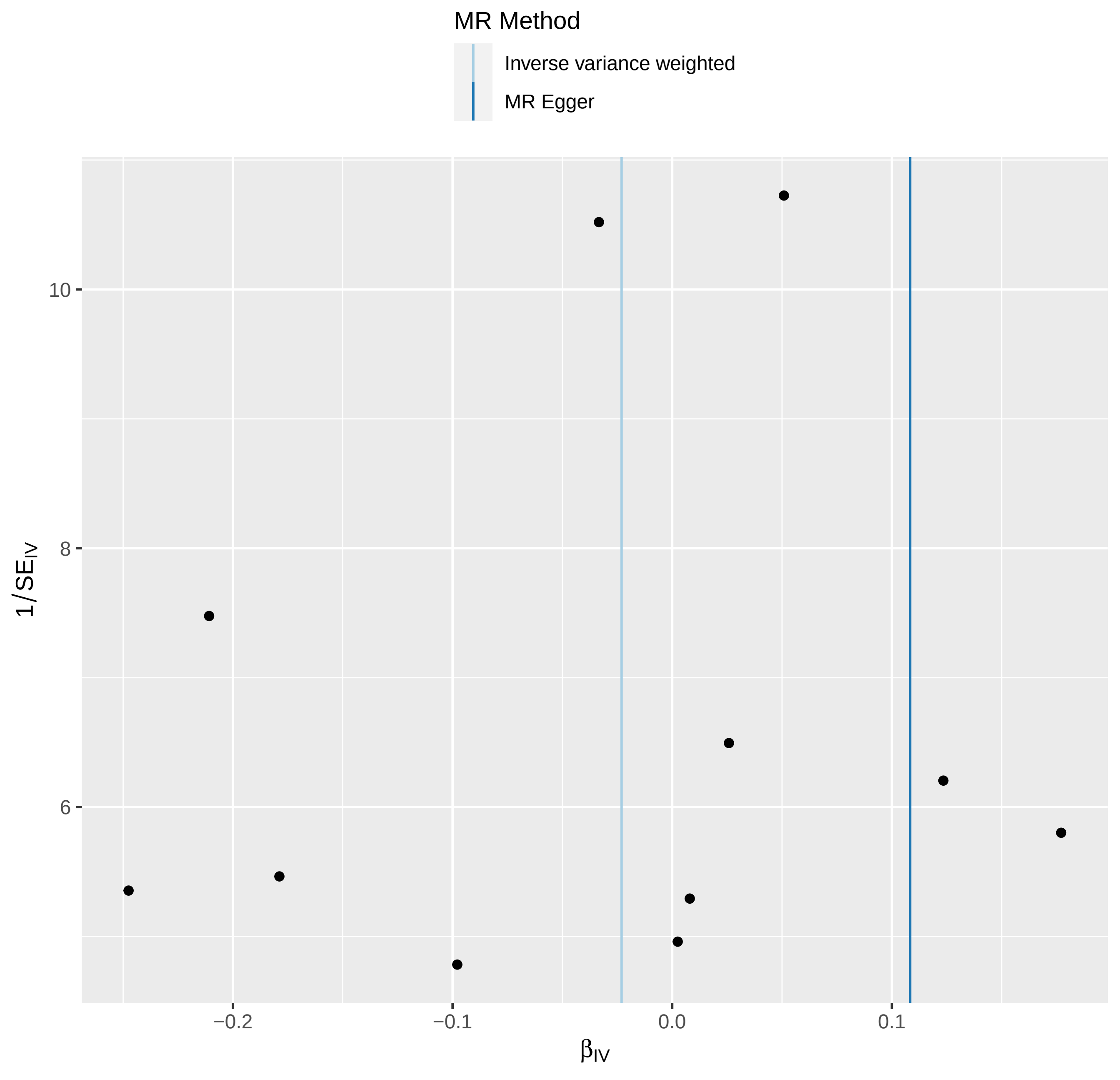

Batch 3−579 : Selenium on C−type lectin domain family 5 member A

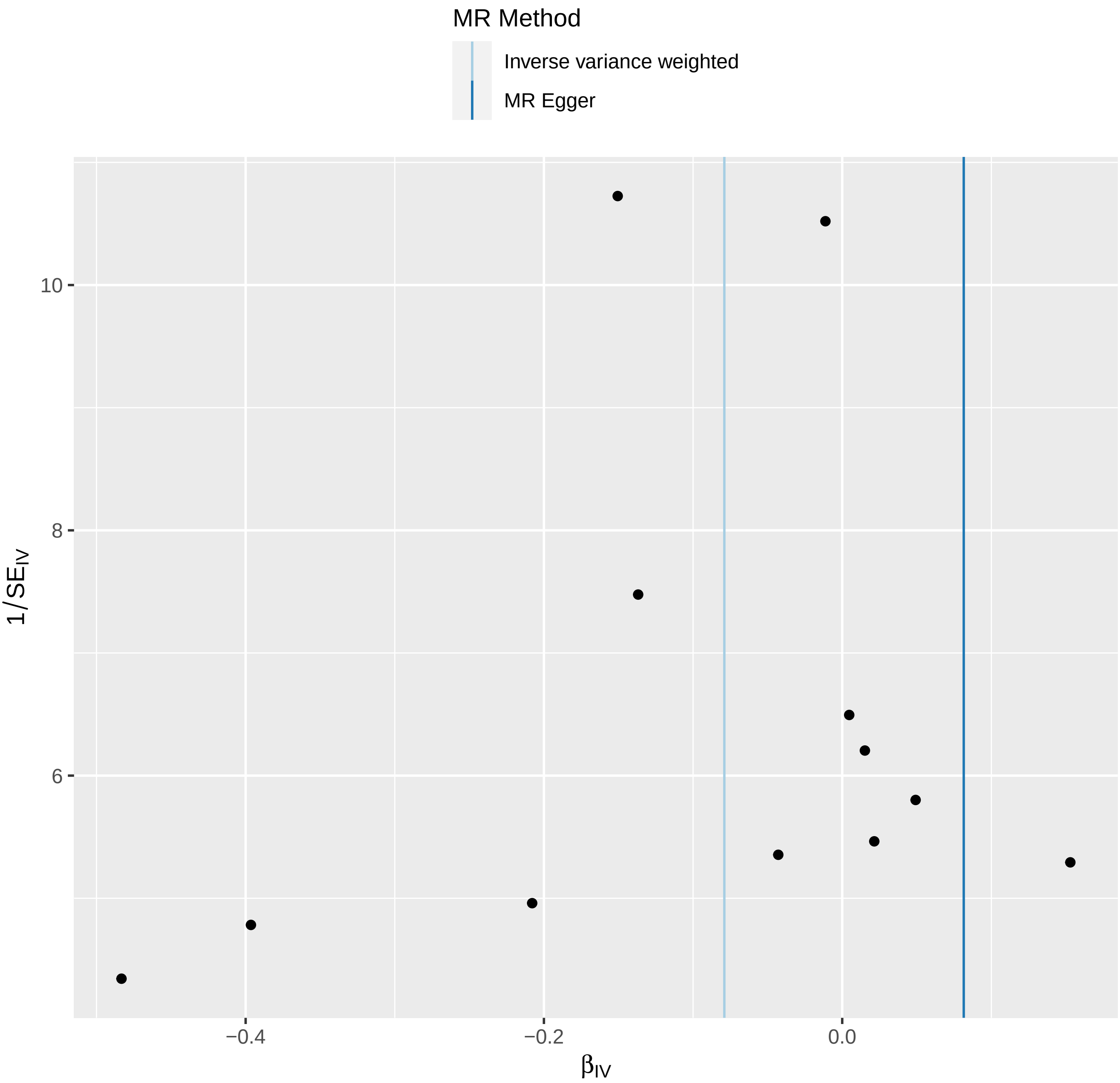

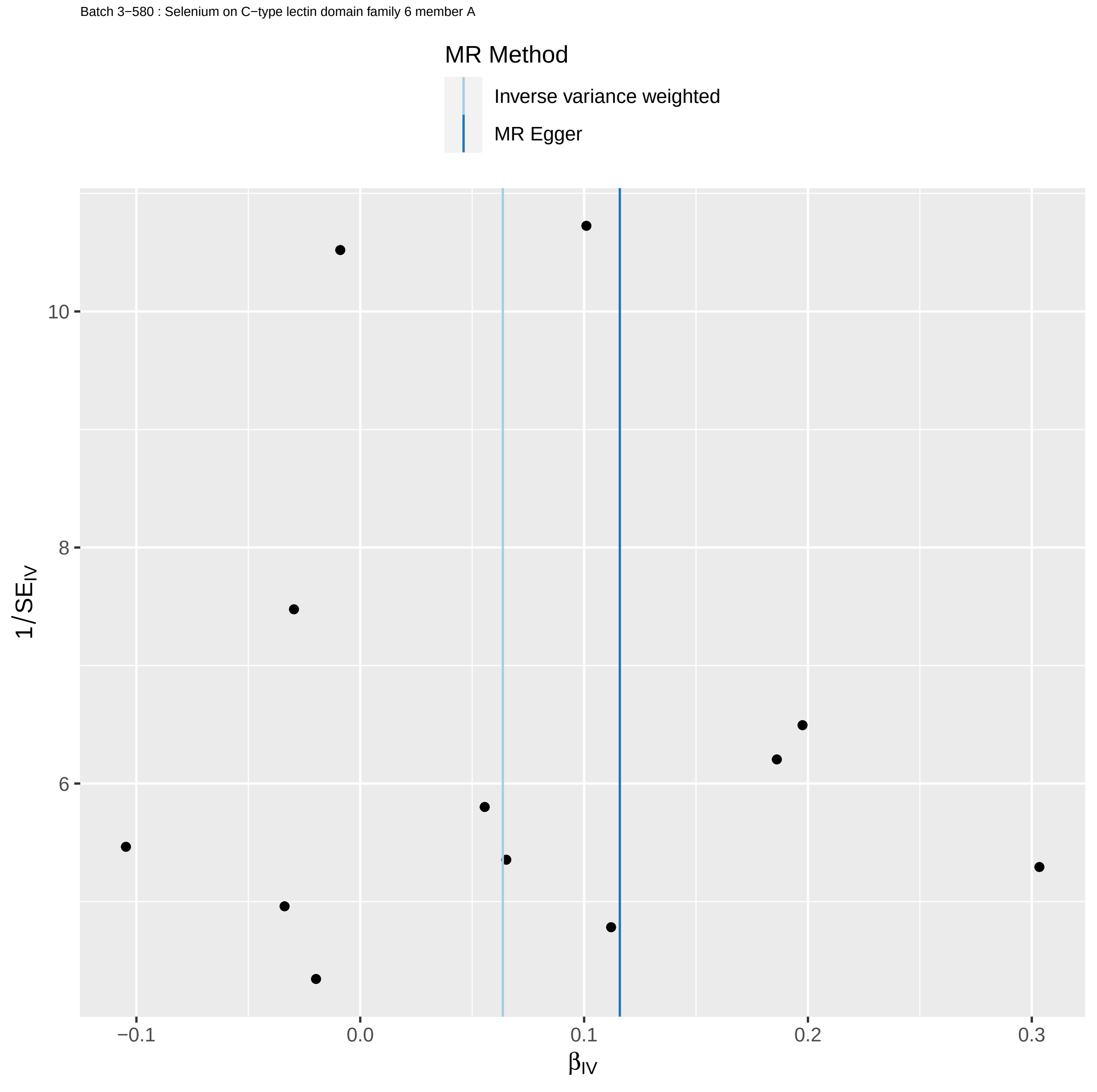
Batch 3−580 : Selenium on C−type lectin domain family 6 member A
MR Method
Inverse variance weighted
MR Egger
10
8
6
1/SE$_{IV}$
−0.1
0.0
0.1
0.2
0.3
β_{IV}

Batch 3−581 : Selenium on C−type lectin domain family 7 member A

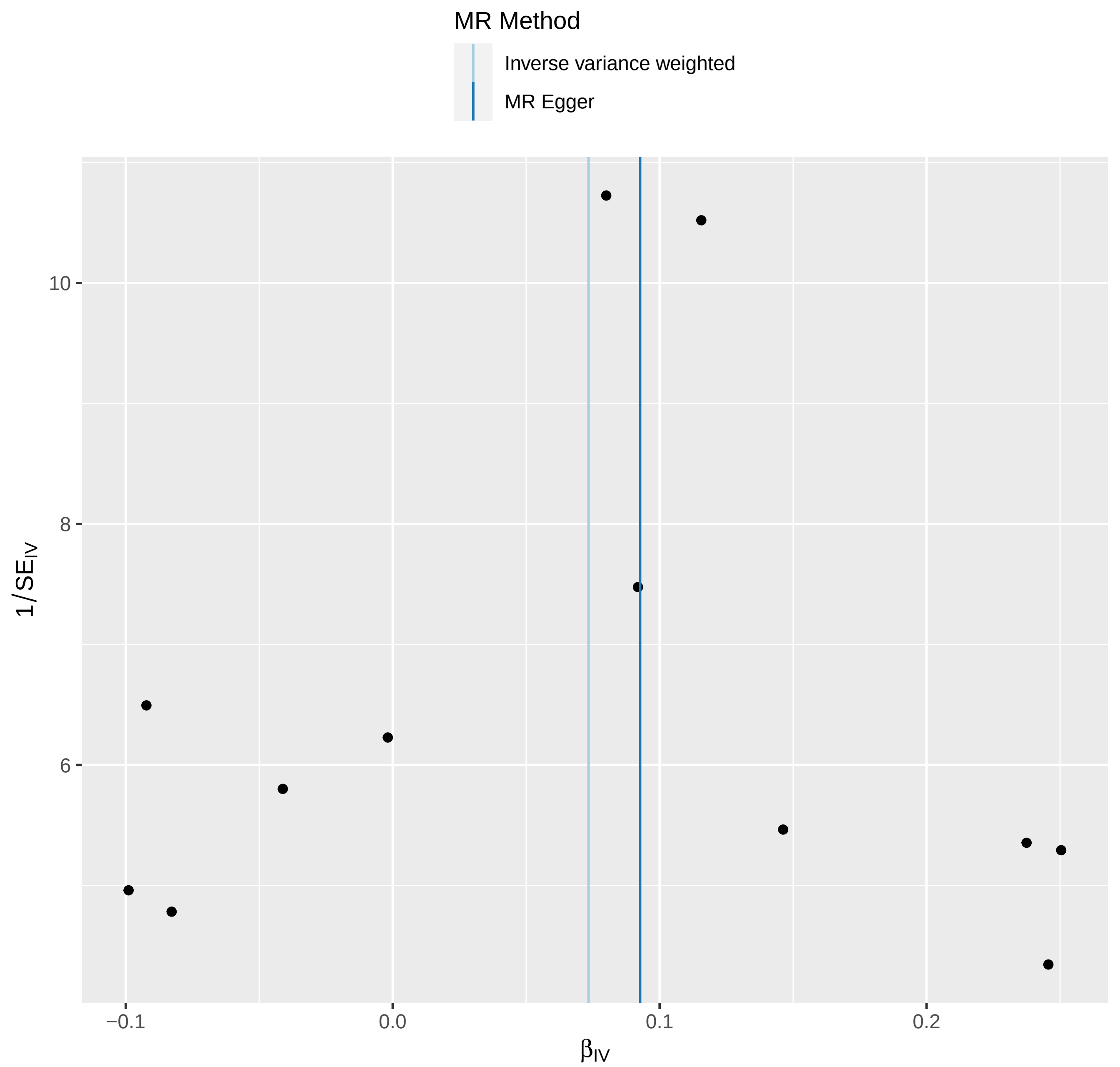

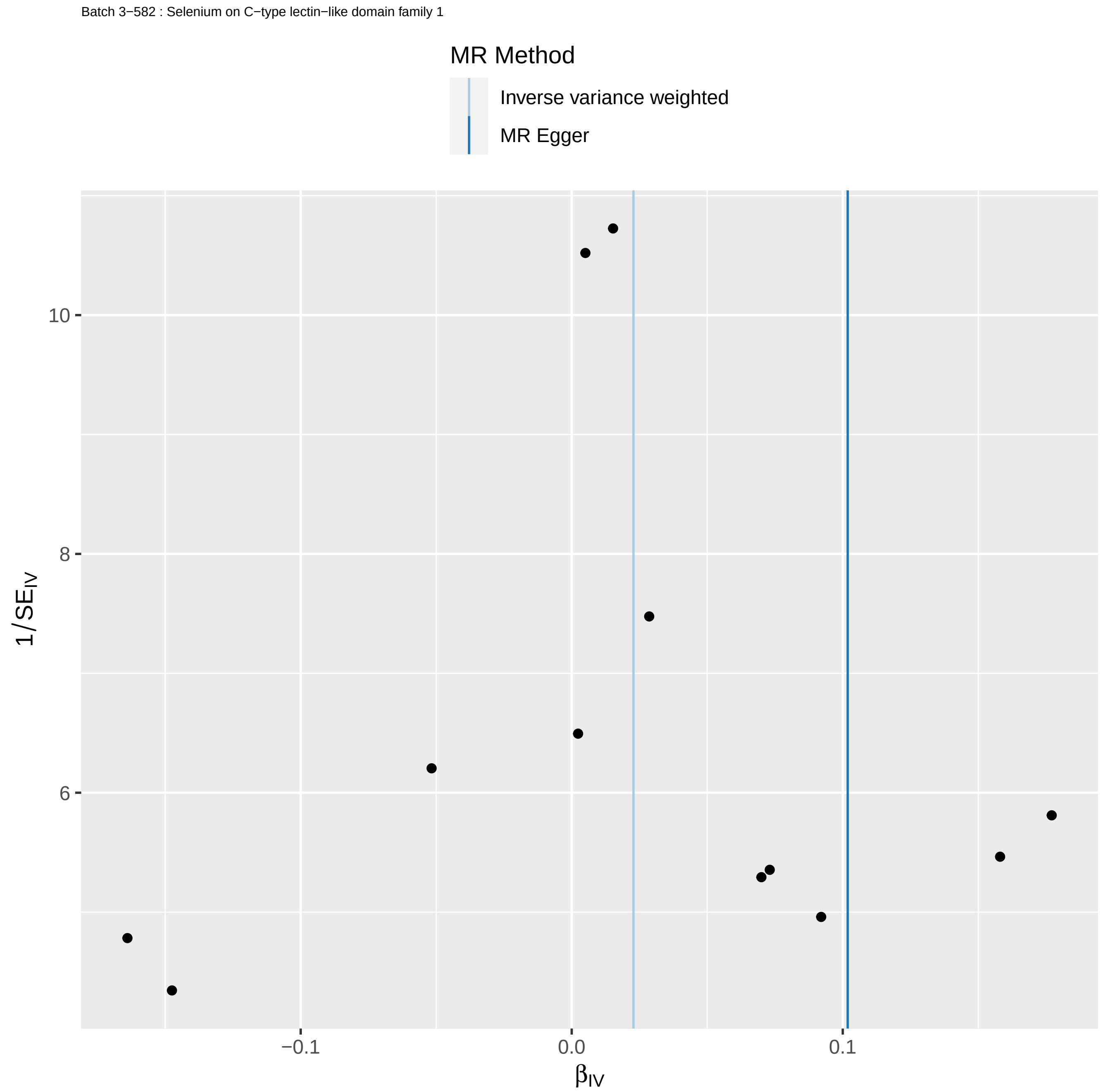

Batch 3−582 : Selenium on C−type lectin−like domain family 1
MR Method
Inverse variance weighted
MR Egger
10
8
6
1/SE_IV
−0.1
0.0
0.1
β_IV

Batch 3−583 : Selenium on Chloride intracellular channel protein 4

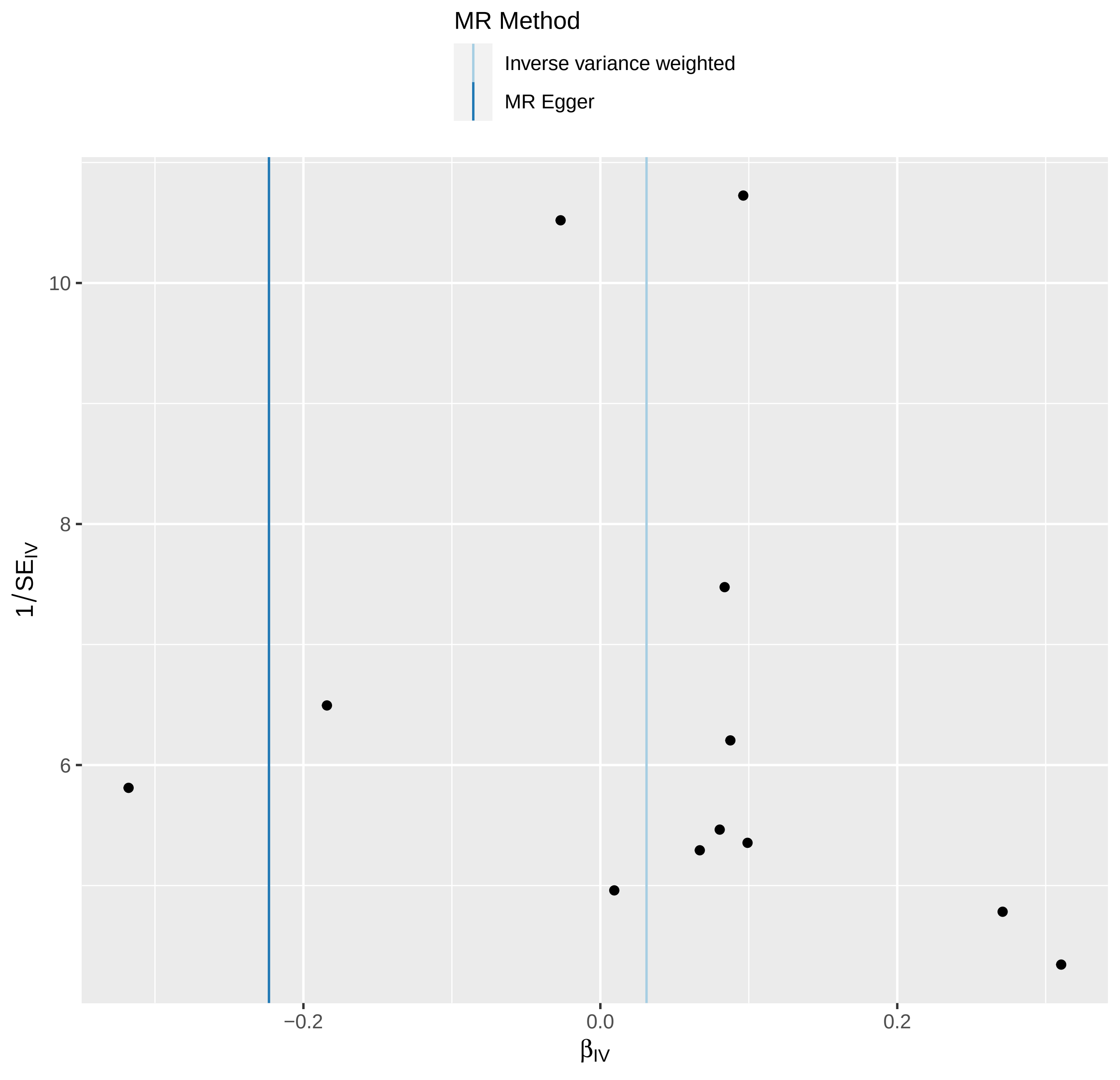

Batch 3−584 : Selenium on Chloride intracellular channel protein 5

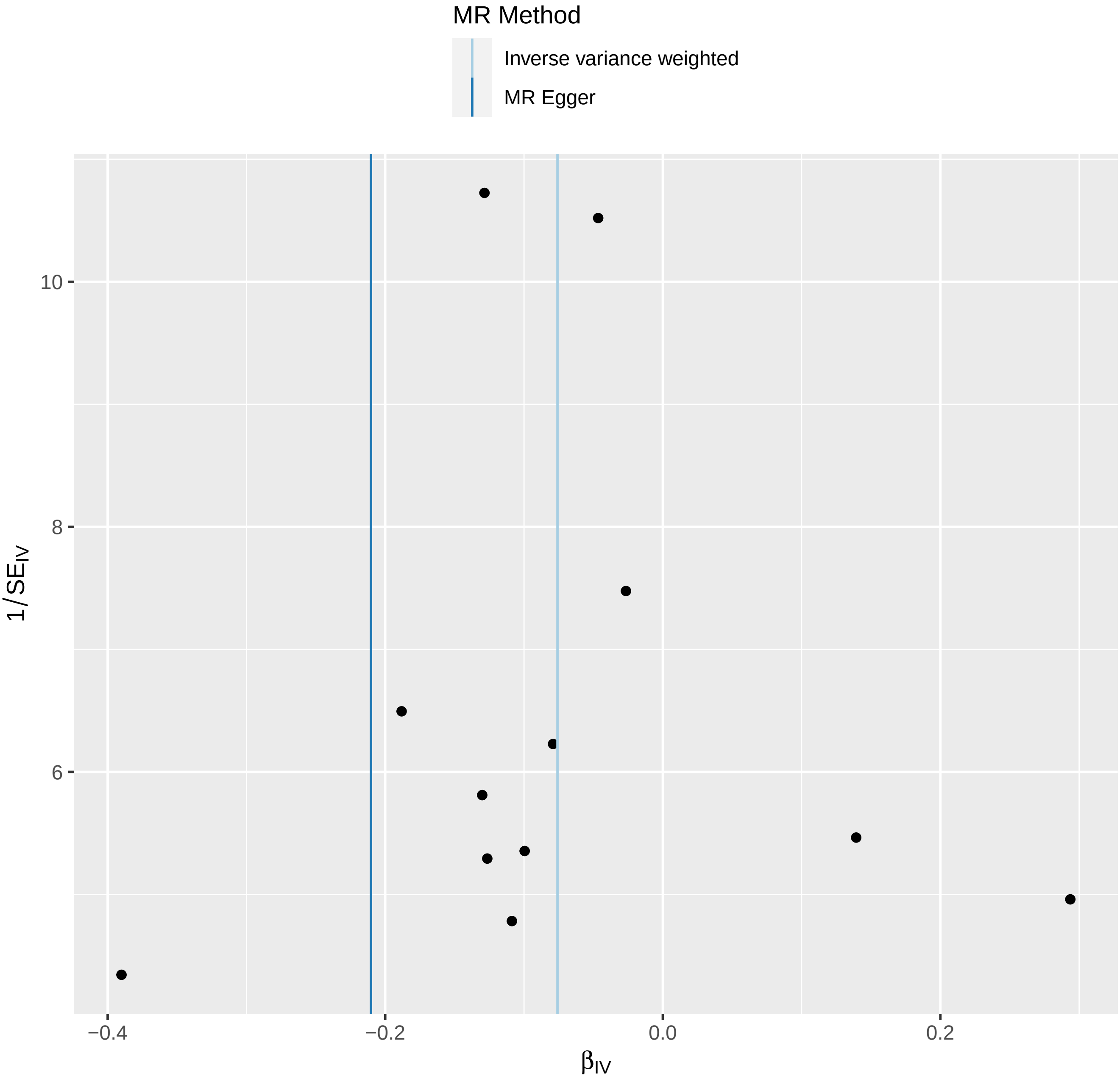

Batch 3−585 : Selenium on Clathrin interactor 1

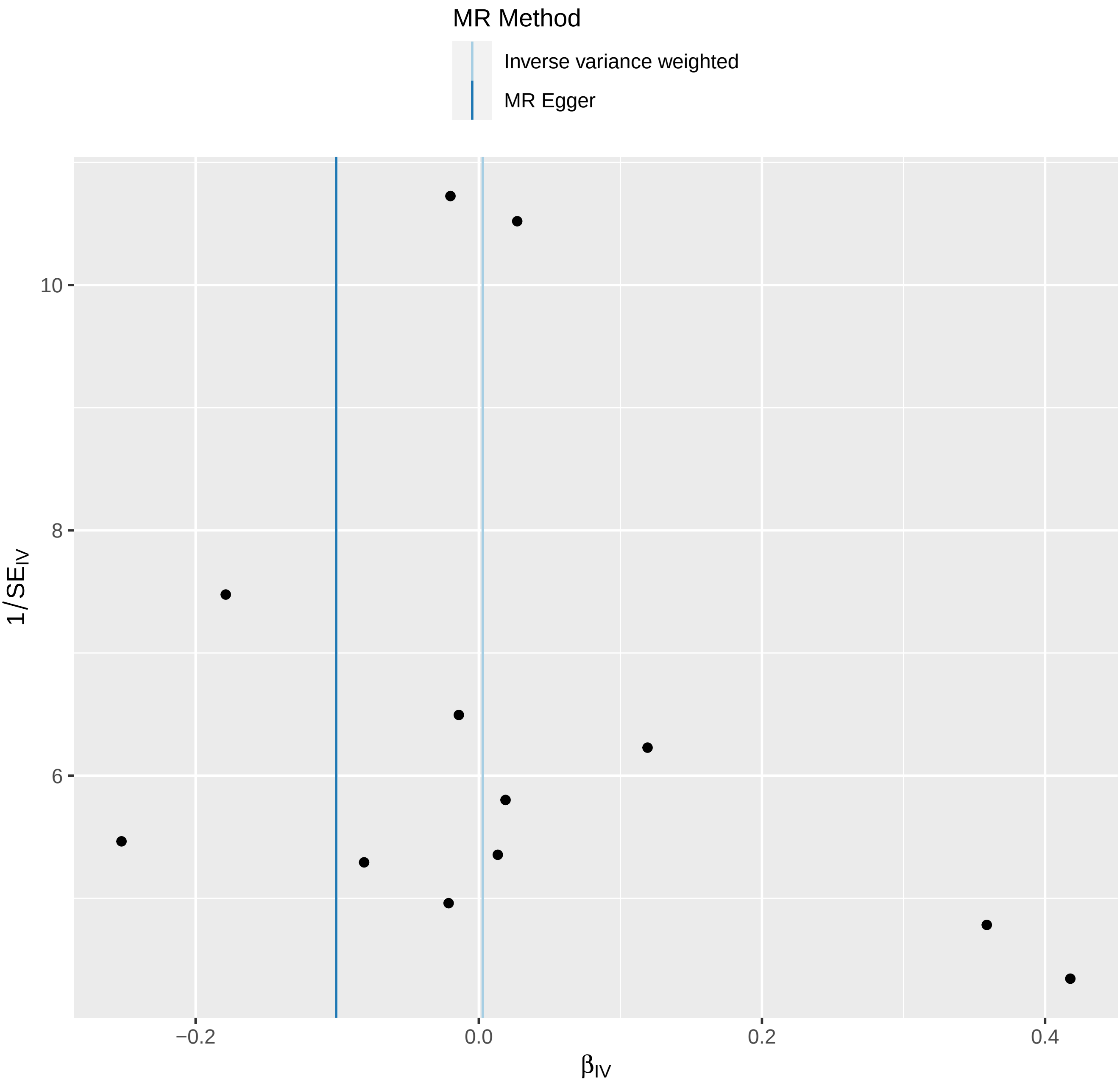

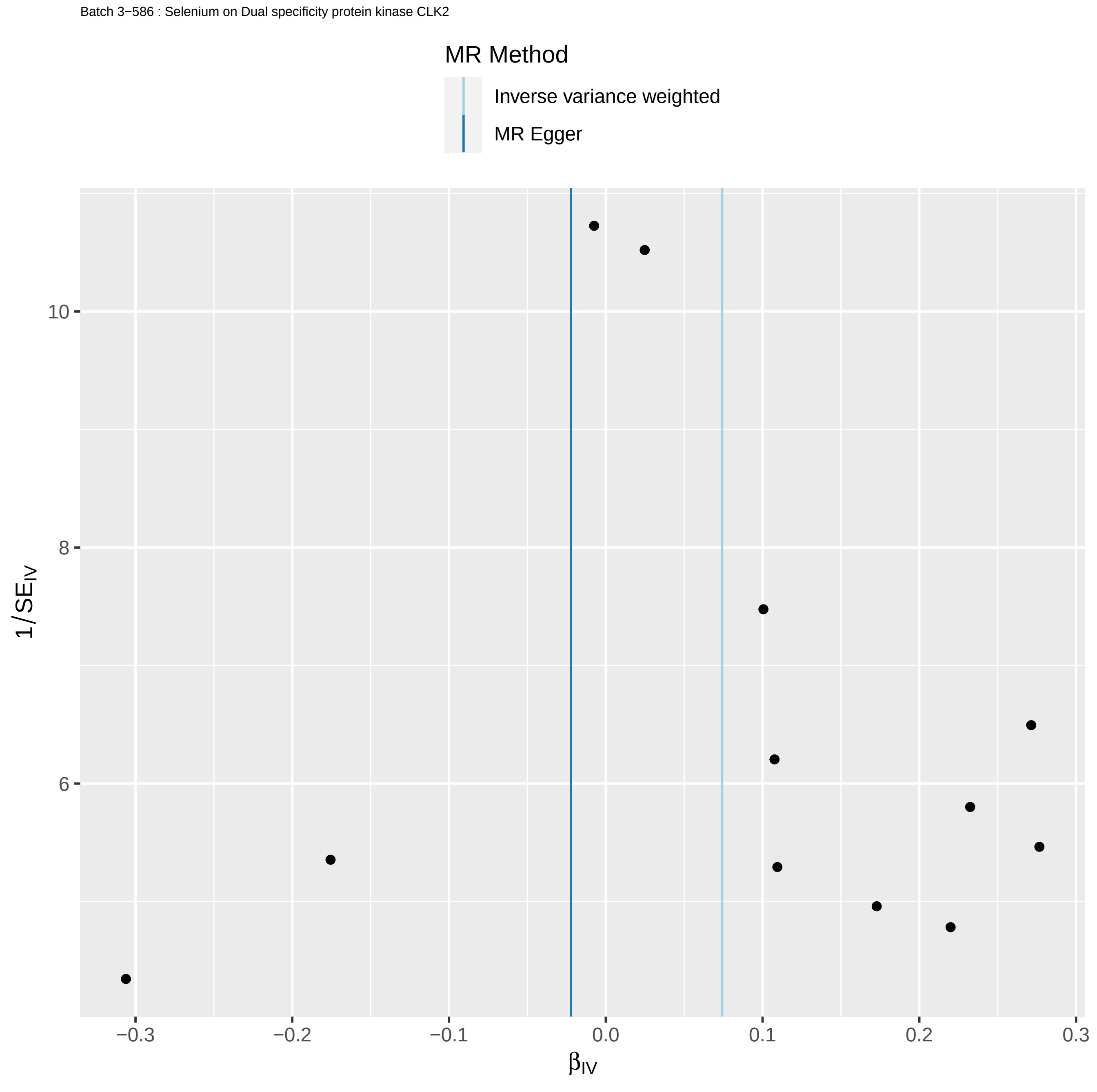
Batch 3−586 : Selenium on Dual specificity protein kinase CLK2
MR Method
Inverse variance weighted
MR Egger
1/SE$_{IV}$
10
8
6
−0.3
−0.2
−0.1
0.0
0.1
0.2
0.3
β$_{IV}$

Batch 3−587 : Selenium on CXADR−like membrane protein

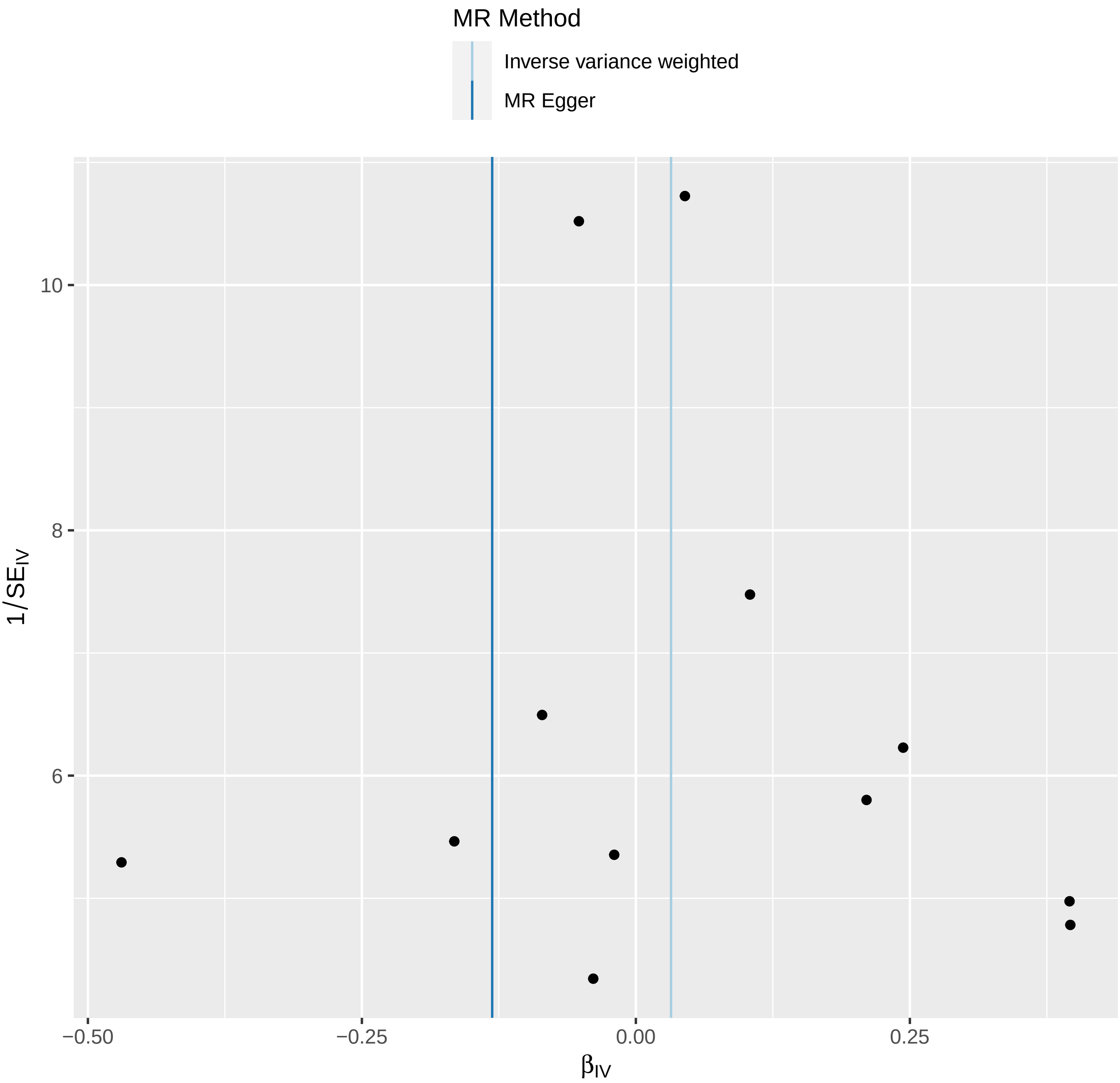

Batch 3−588 : Selenium on CXADR−like membrane protein

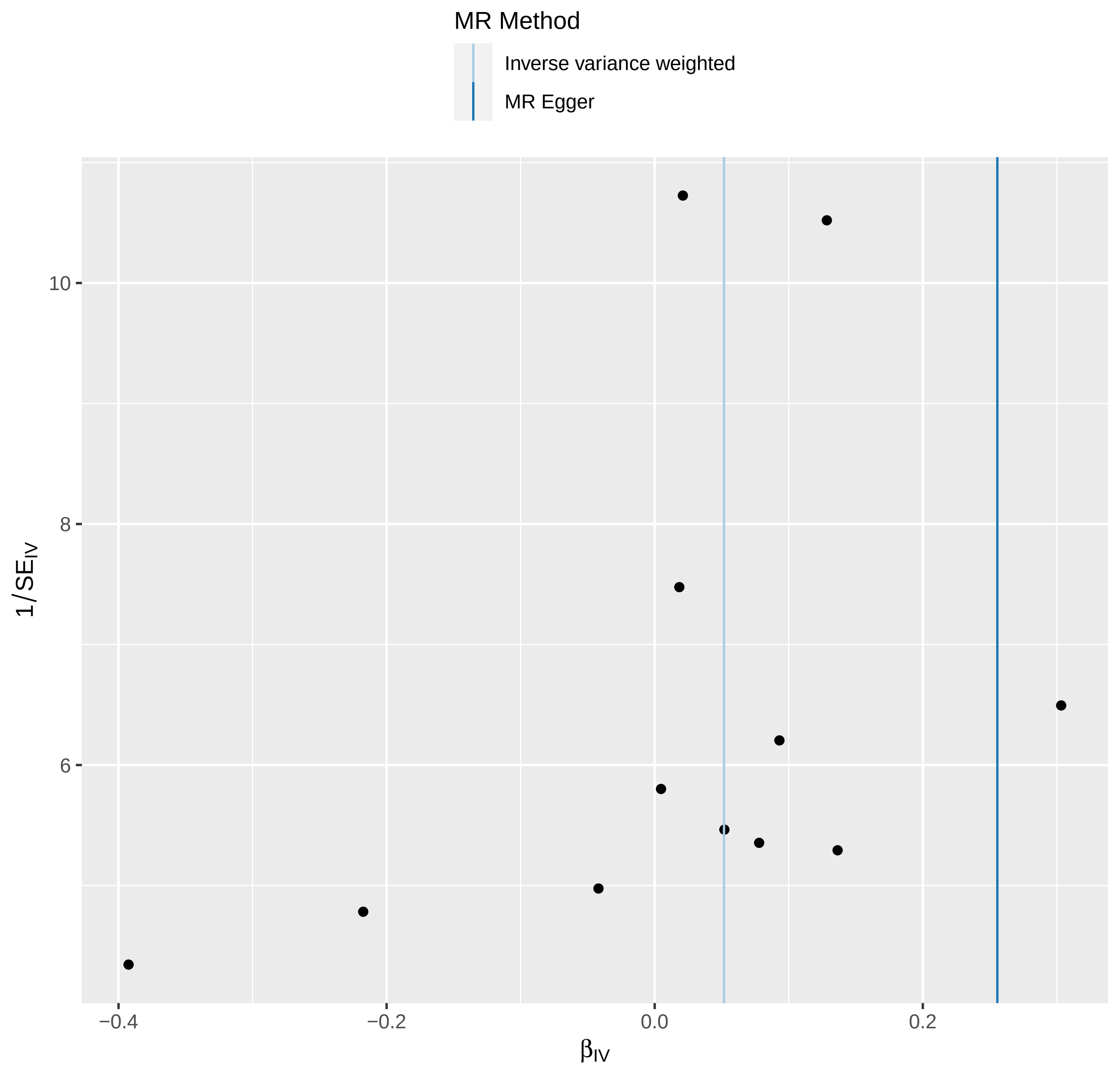

Batch 3−589 : Selenium on Ceroid−lipofuscinosis neuronal protein 5

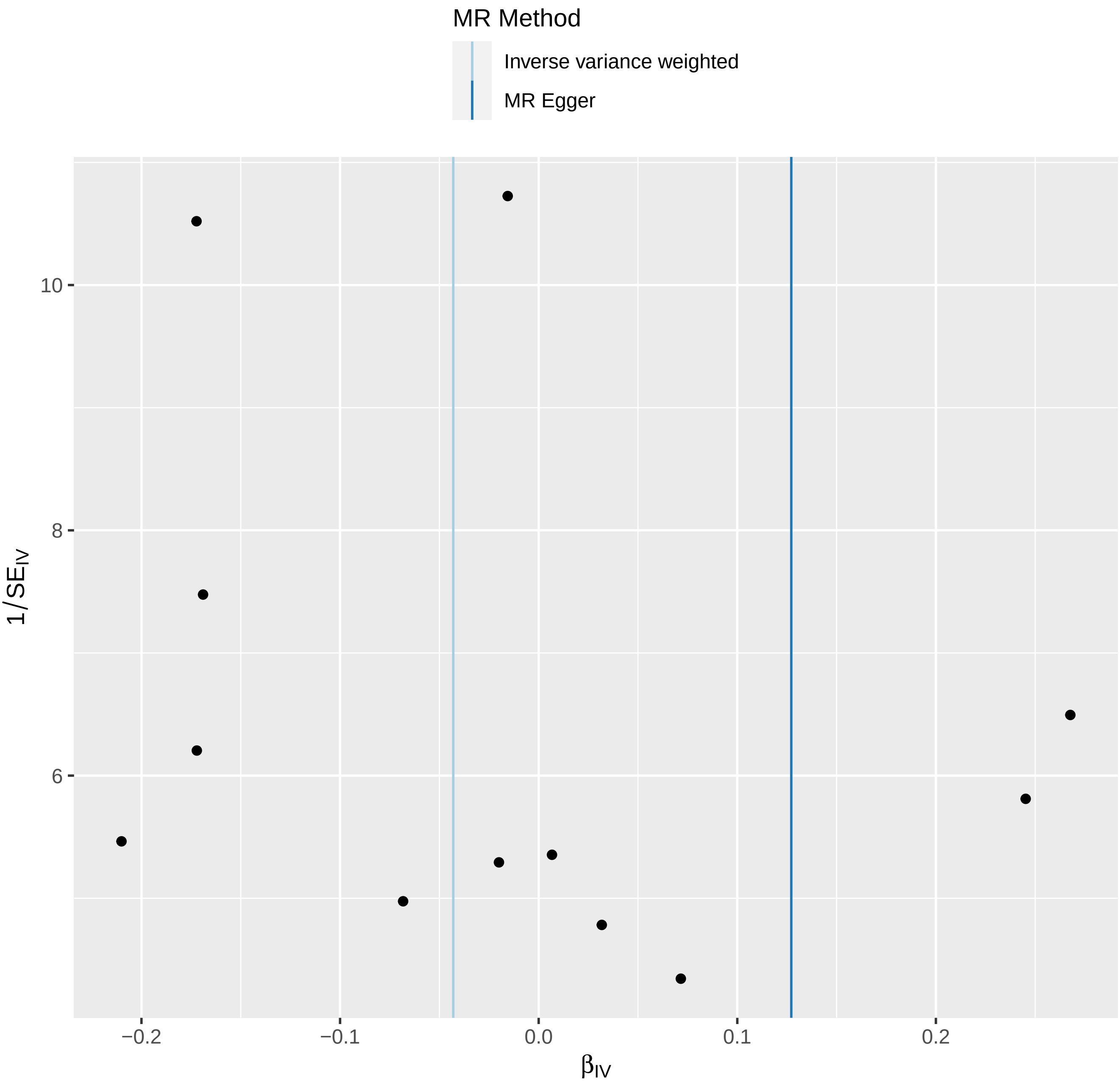

Batch 3−590 : Selenium on Ceroid−lipofuscinosis neuronal protein 5

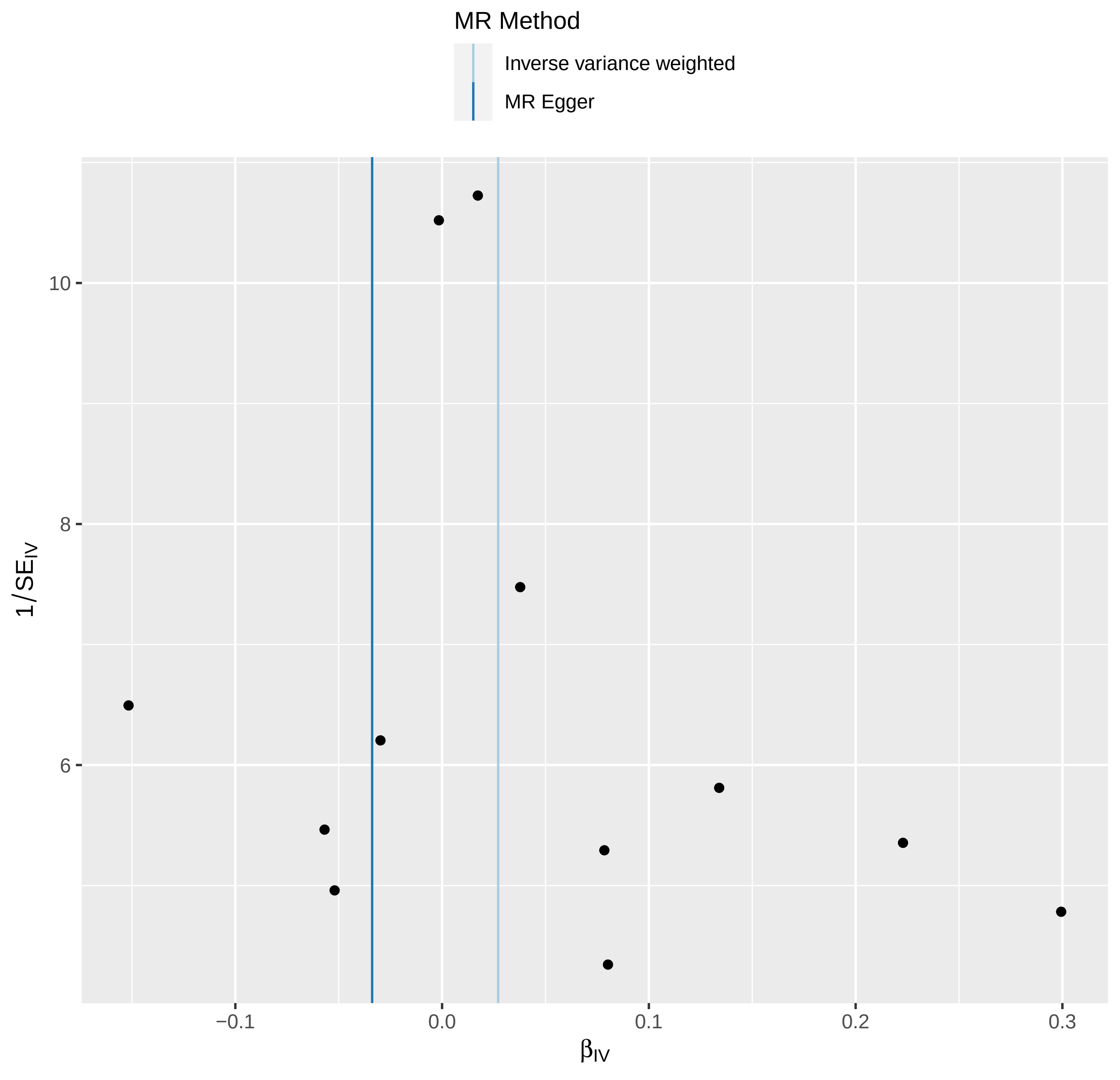

Batch 3−591 : Selenium on Caseinolytic peptidase B protein homolog

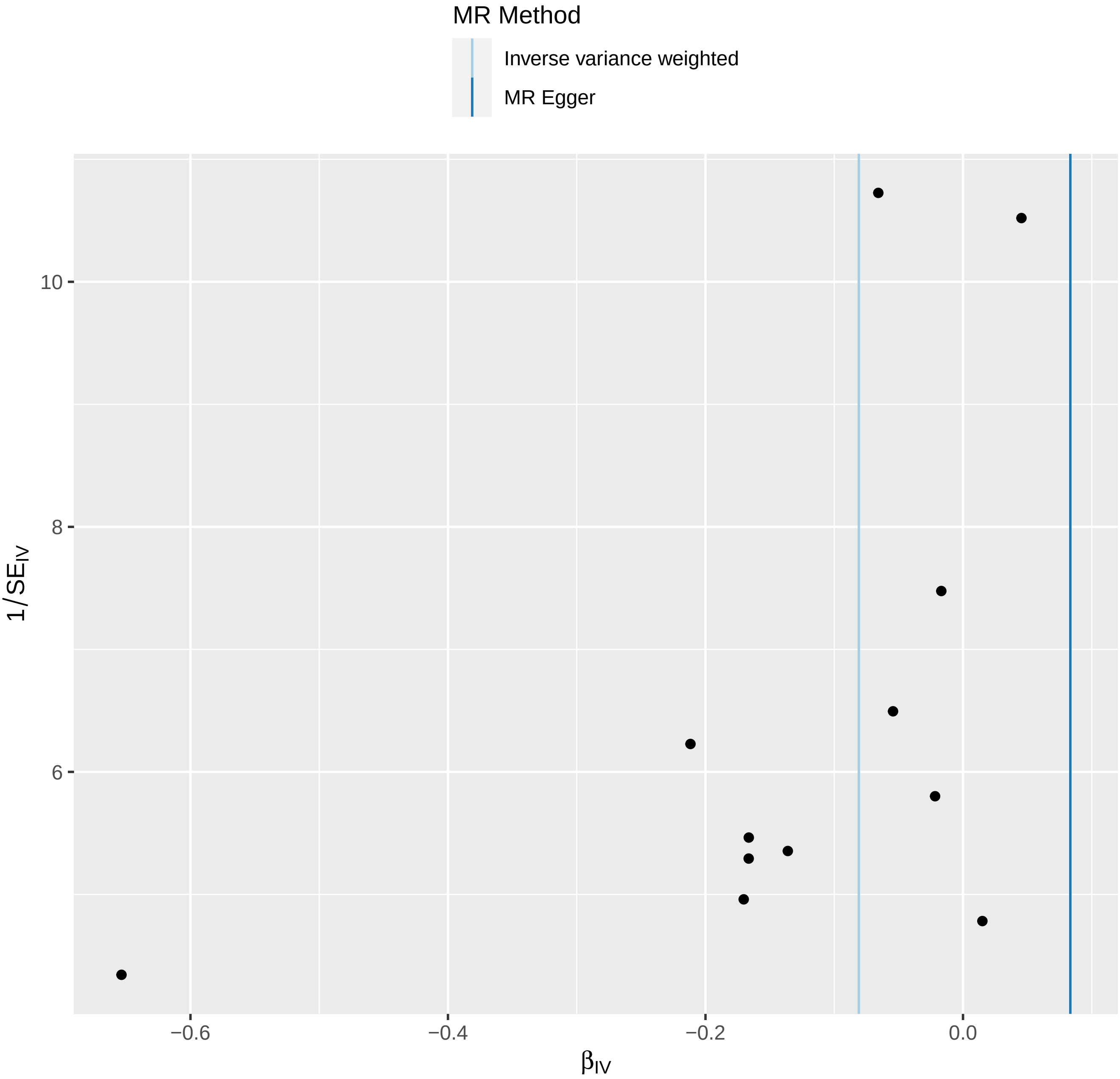

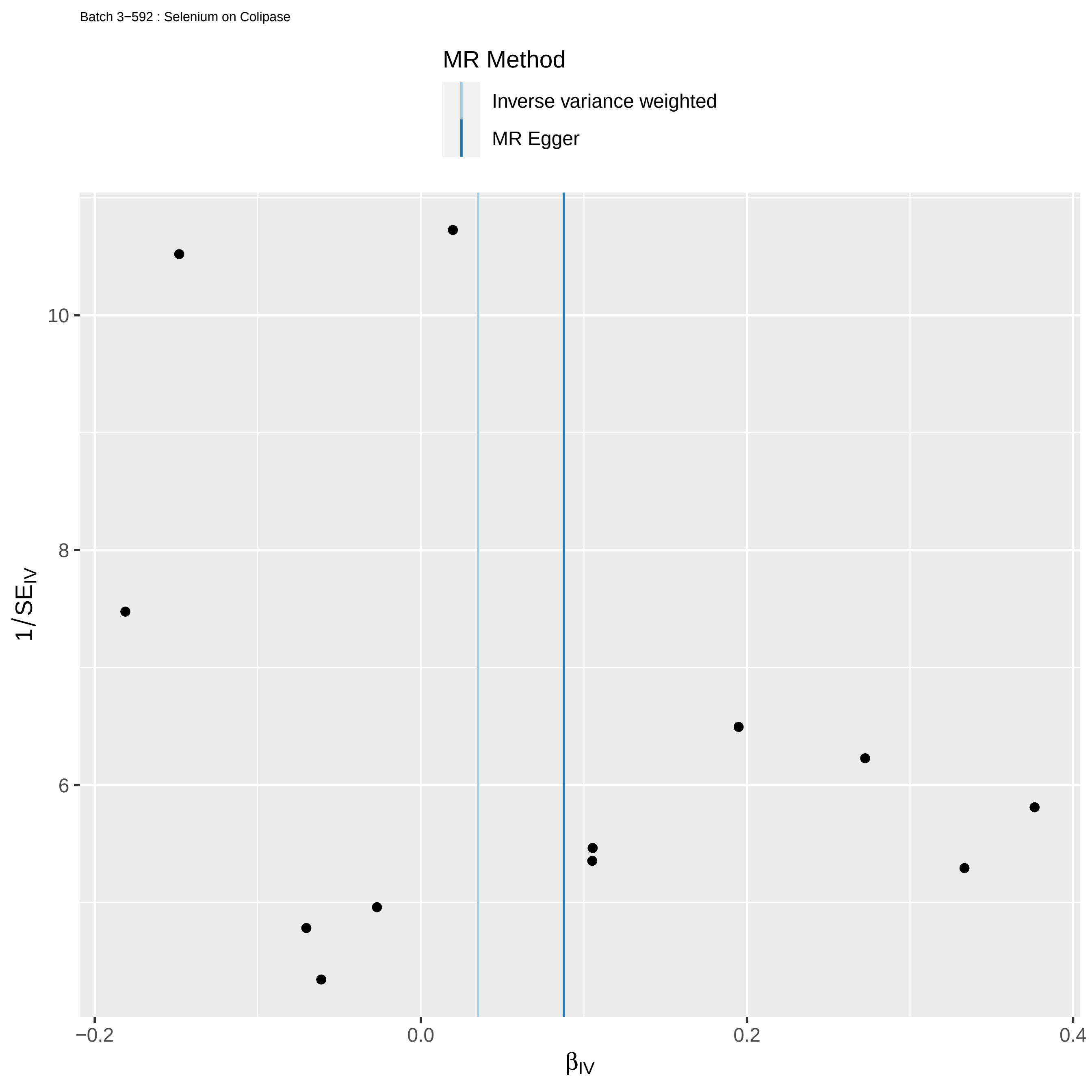

Batch 3−592 : Selenium on Colipase
MR Method
Inverse variance weighted
MR Egger
10
8
6
1/SE$_{IV}$
−0.2
0.0
0.2
0.4
β_{IV}

Batch 3−593 : Selenium on Colipase−like protein 2

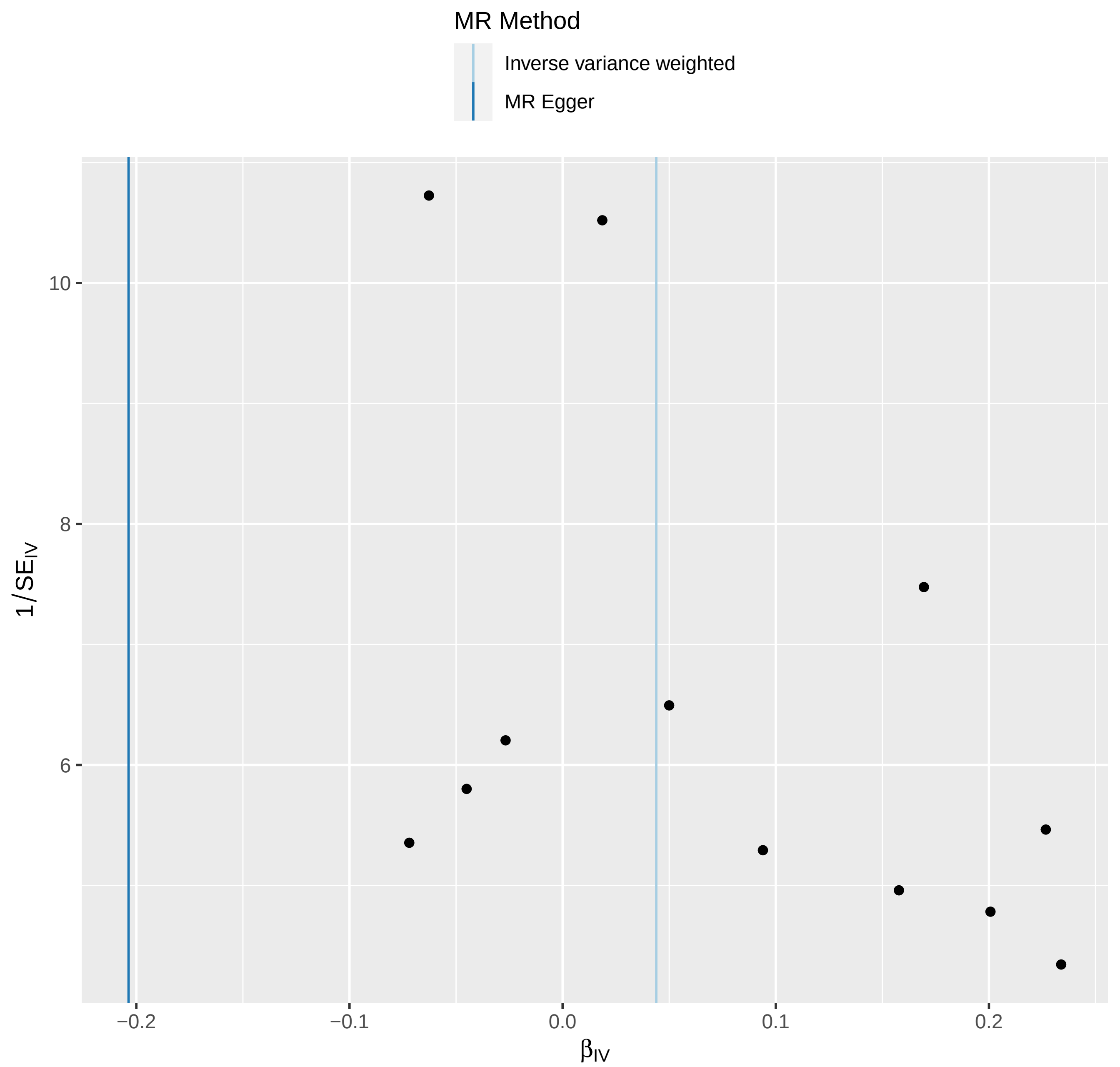

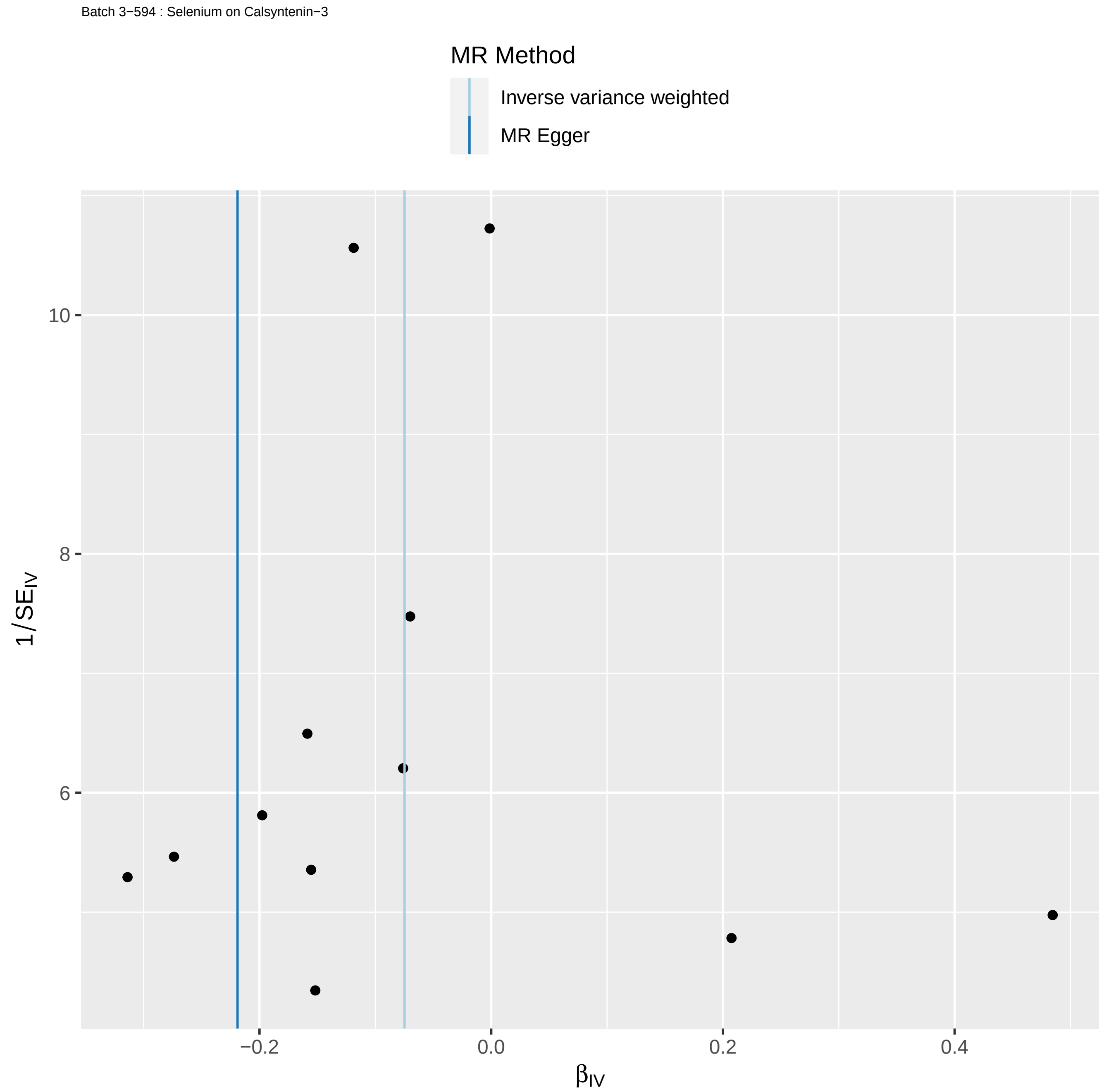
Batch 3−594 : Selenium on Calsyntenin−3
MR Method
Inverse variance weighted
MR Egger
10
8
6
1/SE$_{IV}$
−0.2
0.0
0.2
0.4
β$_{IV}$

Batch 3−595 : Selenium on Clathrin heavy chain 1

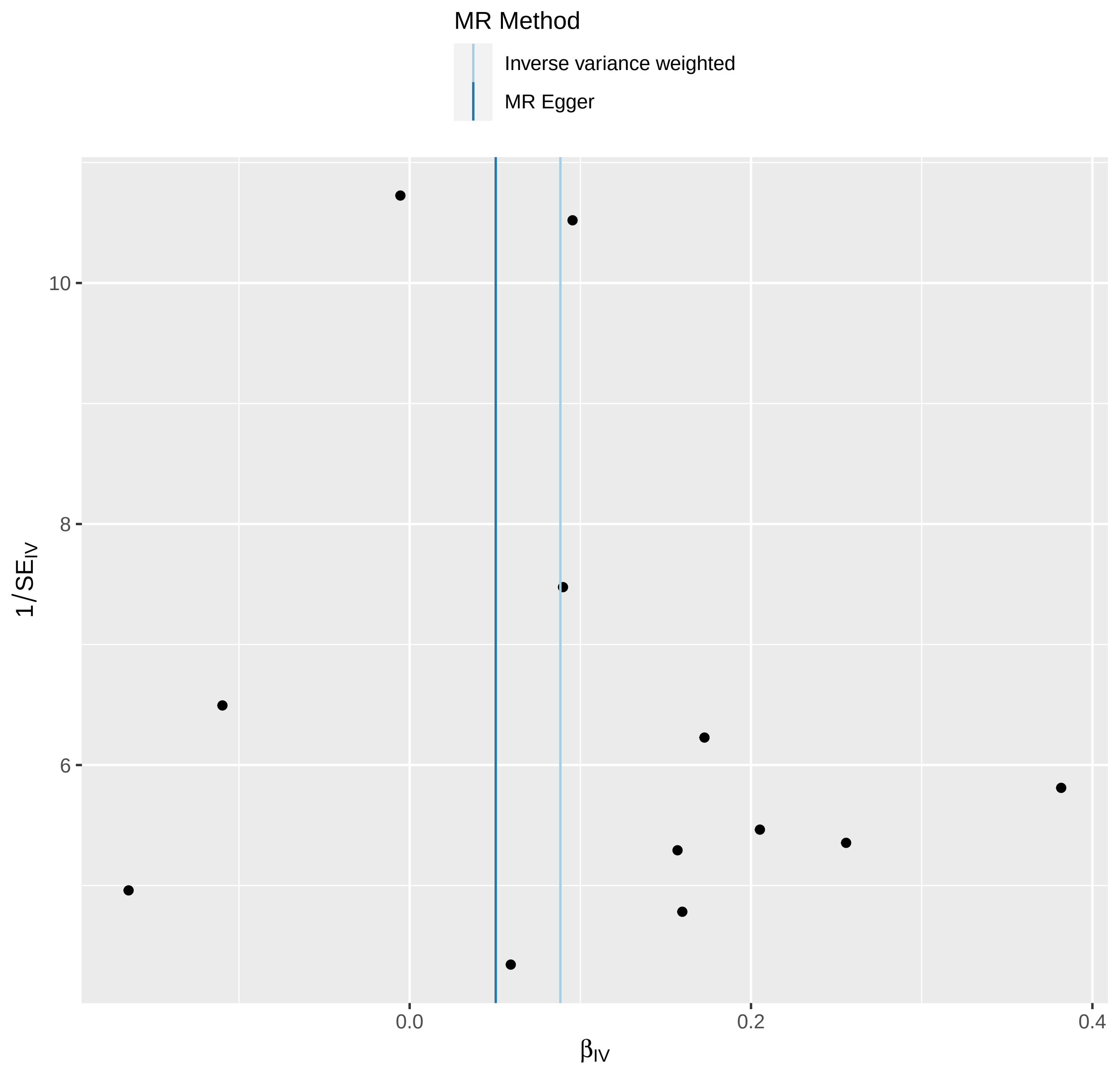

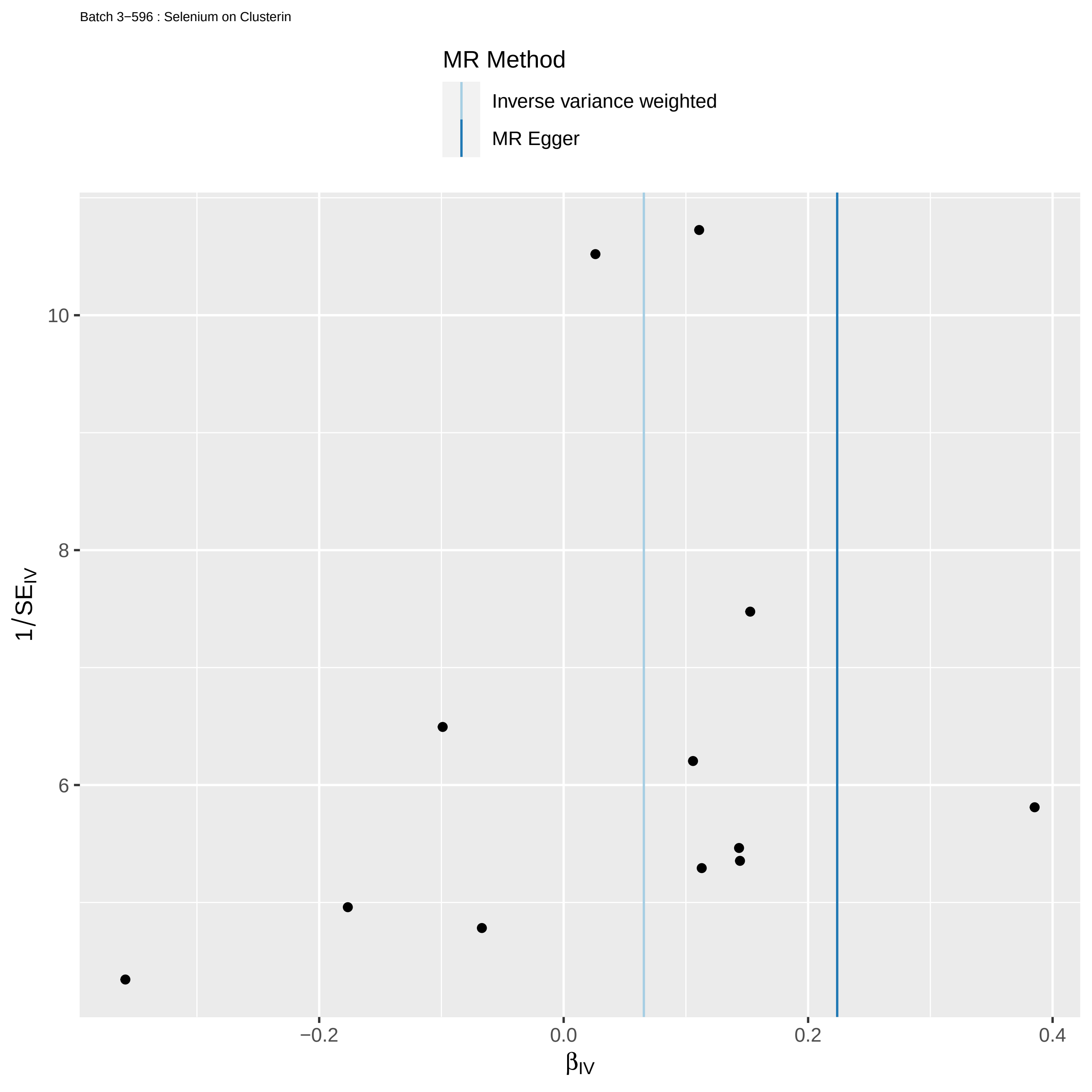

Batch 3−596 : Selenium on Clusterin
MR Method
Inverse variance weighted
MR Egger
10
8
6
1/SE_IV
−0.2
0.0
0.2
0.4
β_IV

Batch 3−597 : Selenium on Chymase

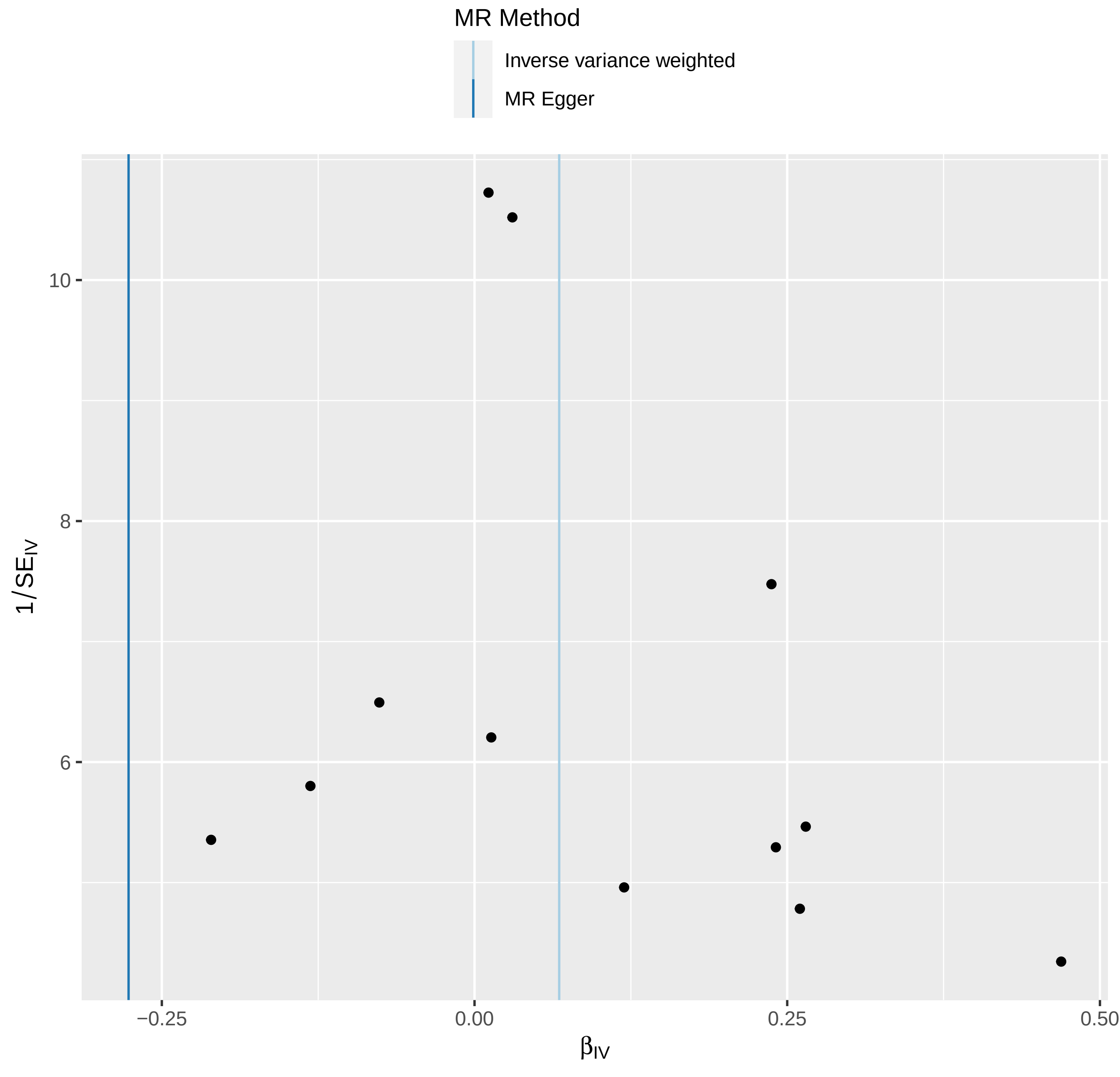

Batch 3−598 : Selenium on UMP−CMP kinase

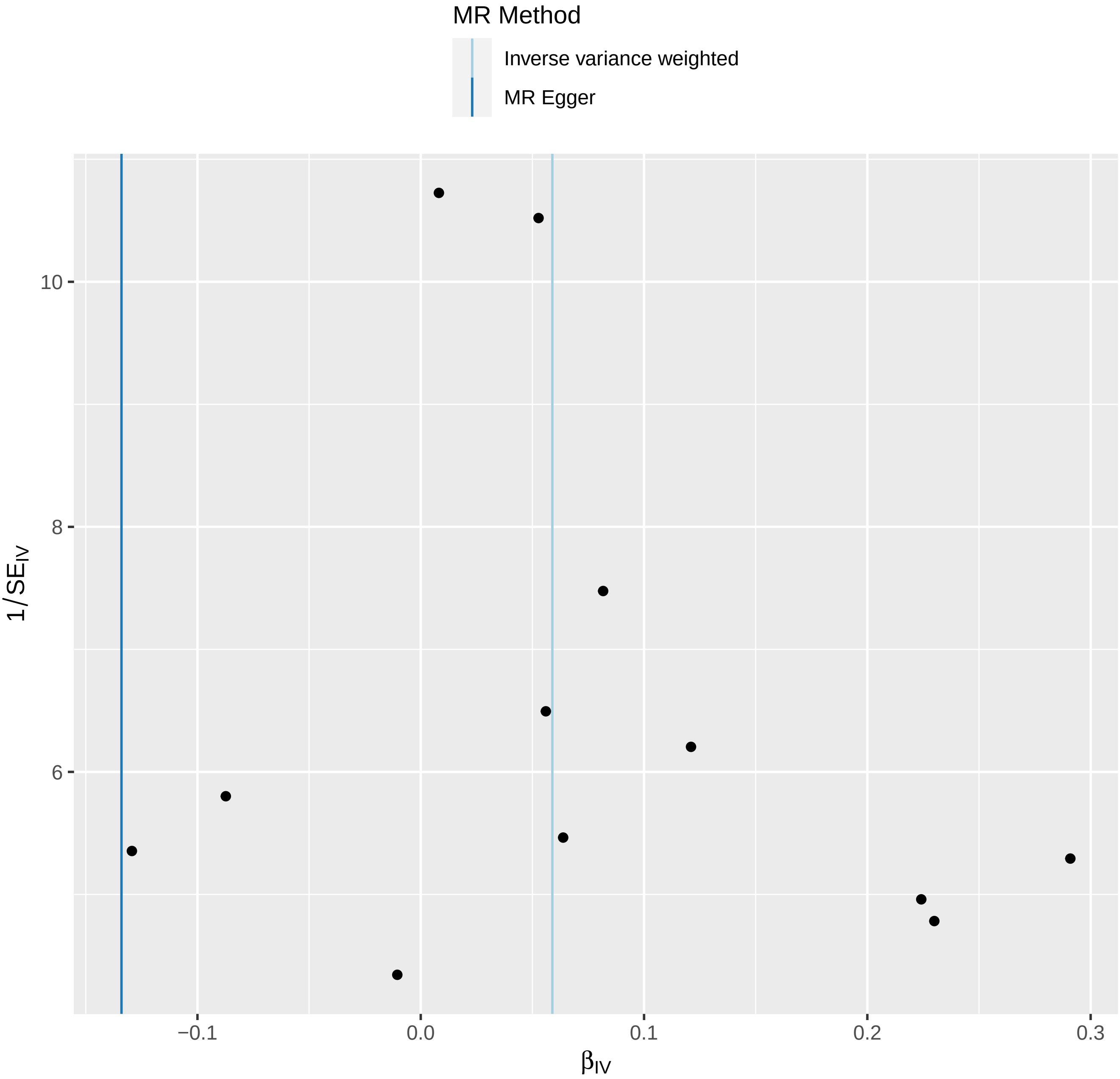

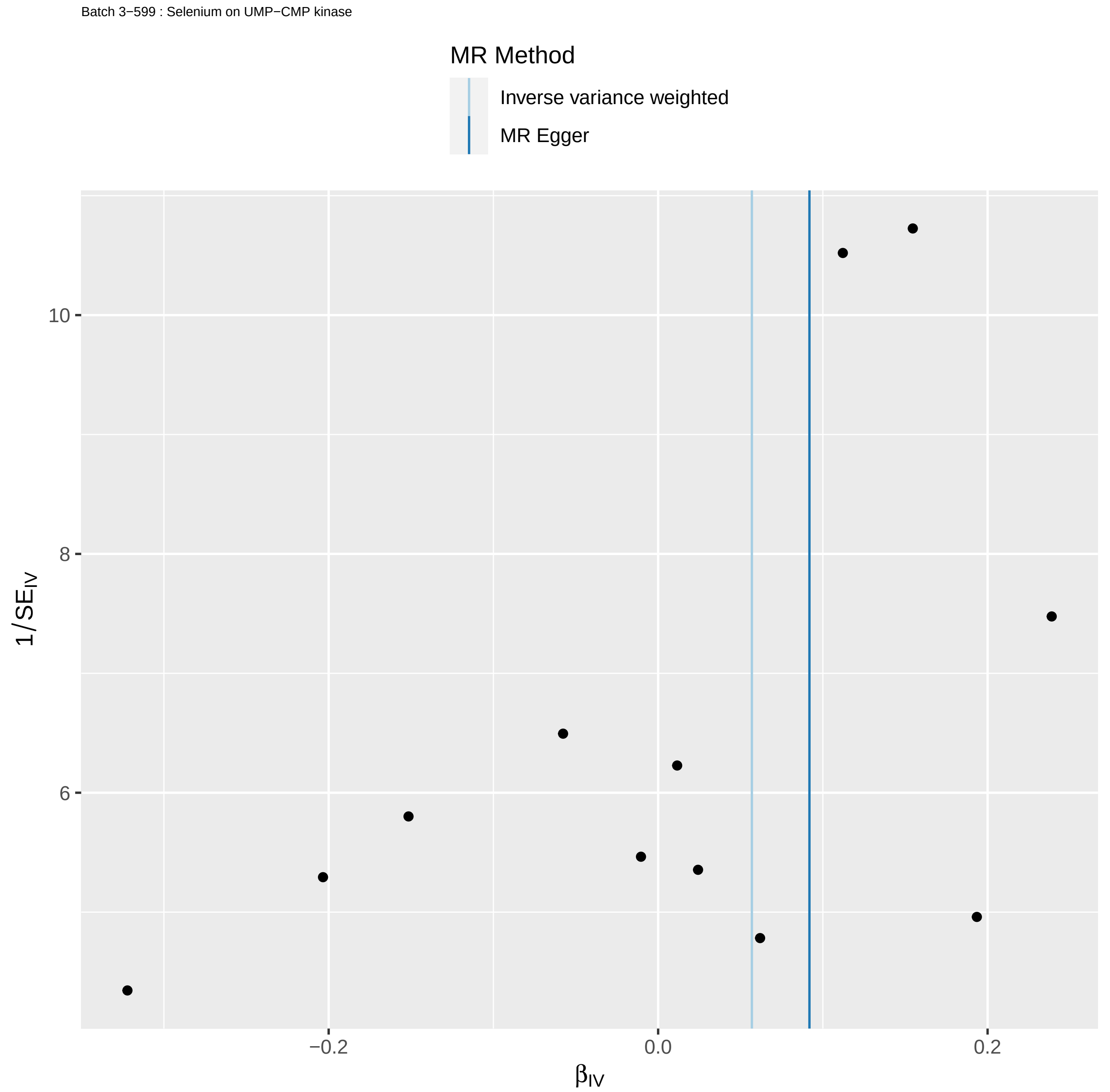

Batch 3−599 : Selenium on UMP−CMP kinase
MR Method
Inverse variance weighted
MR Egger
10
8
6
1/SE_IV
−0.2
0.0
0.2
β_IV

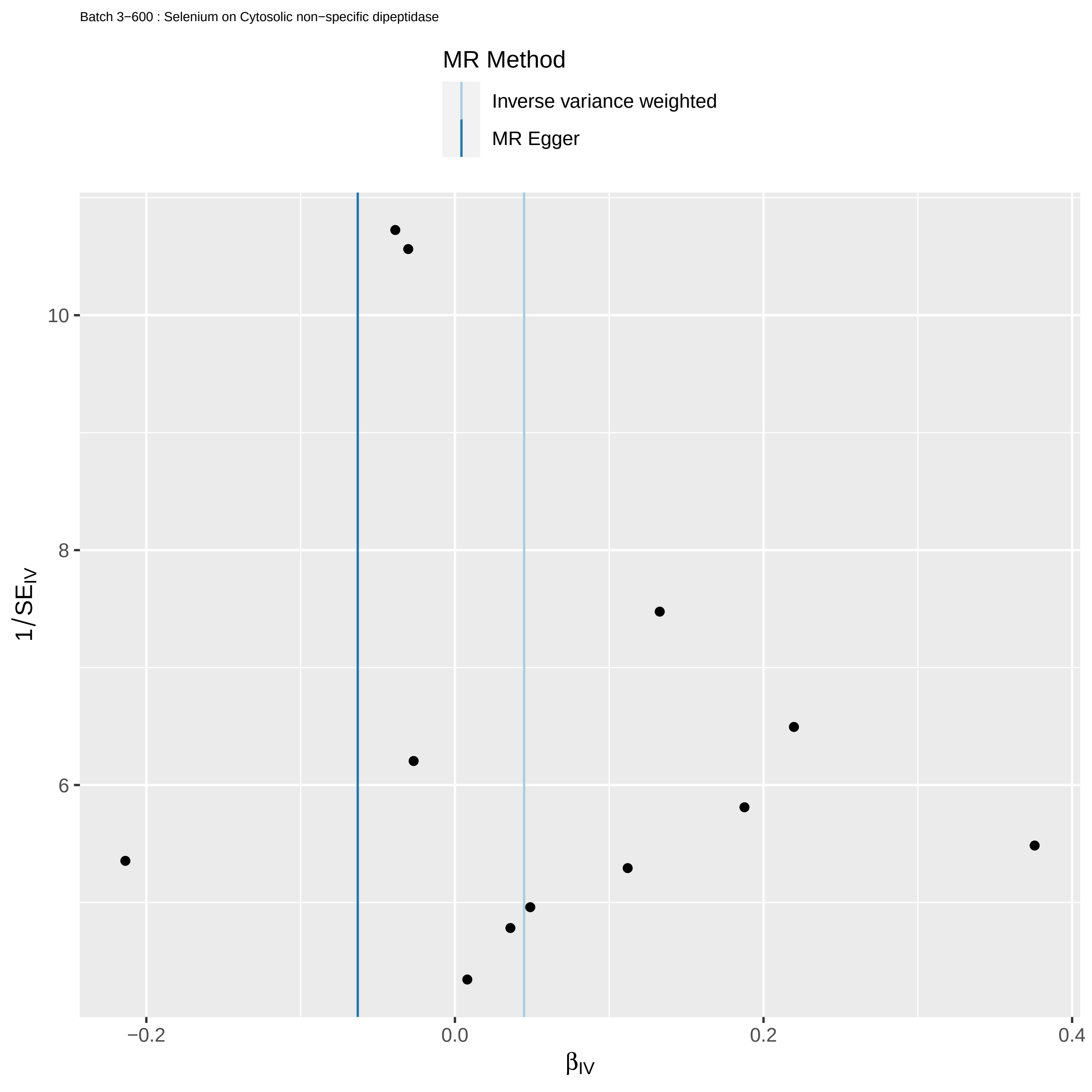
Batch 3−600 : Selenium on Cytosolic non−specific dipeptidase
MR Method
Inverse variance weighted
MR Egger
10
8
6
1/SE_IV
−0.2
0.0
0.2
0.4
β_IV

Batch 3−601 : Selenium on Nuclear envelope phosphatase−regulatory subunit 1

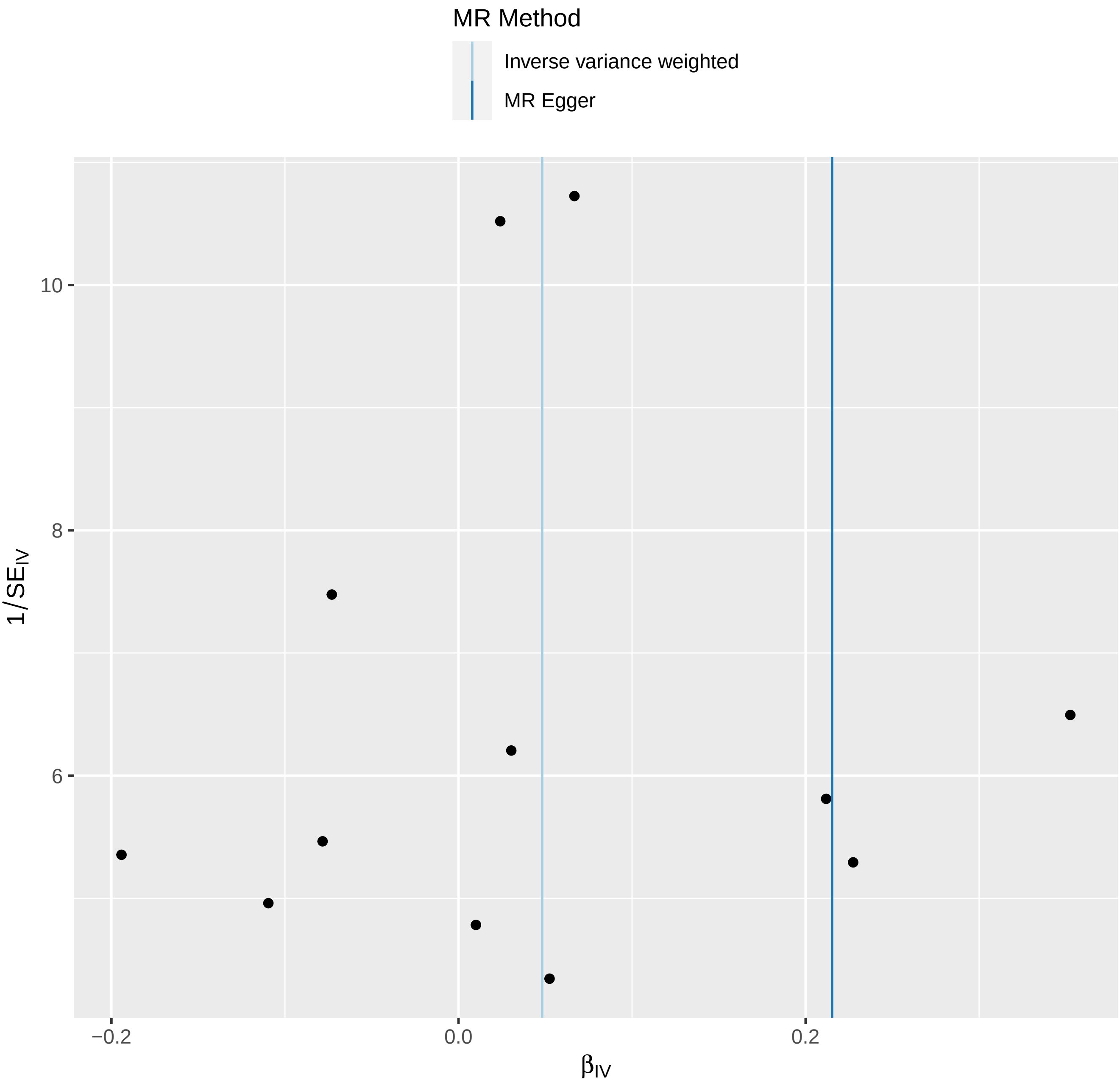

Batch 3−602 : Selenium on CCR4−NOT transcription complex subunit 1

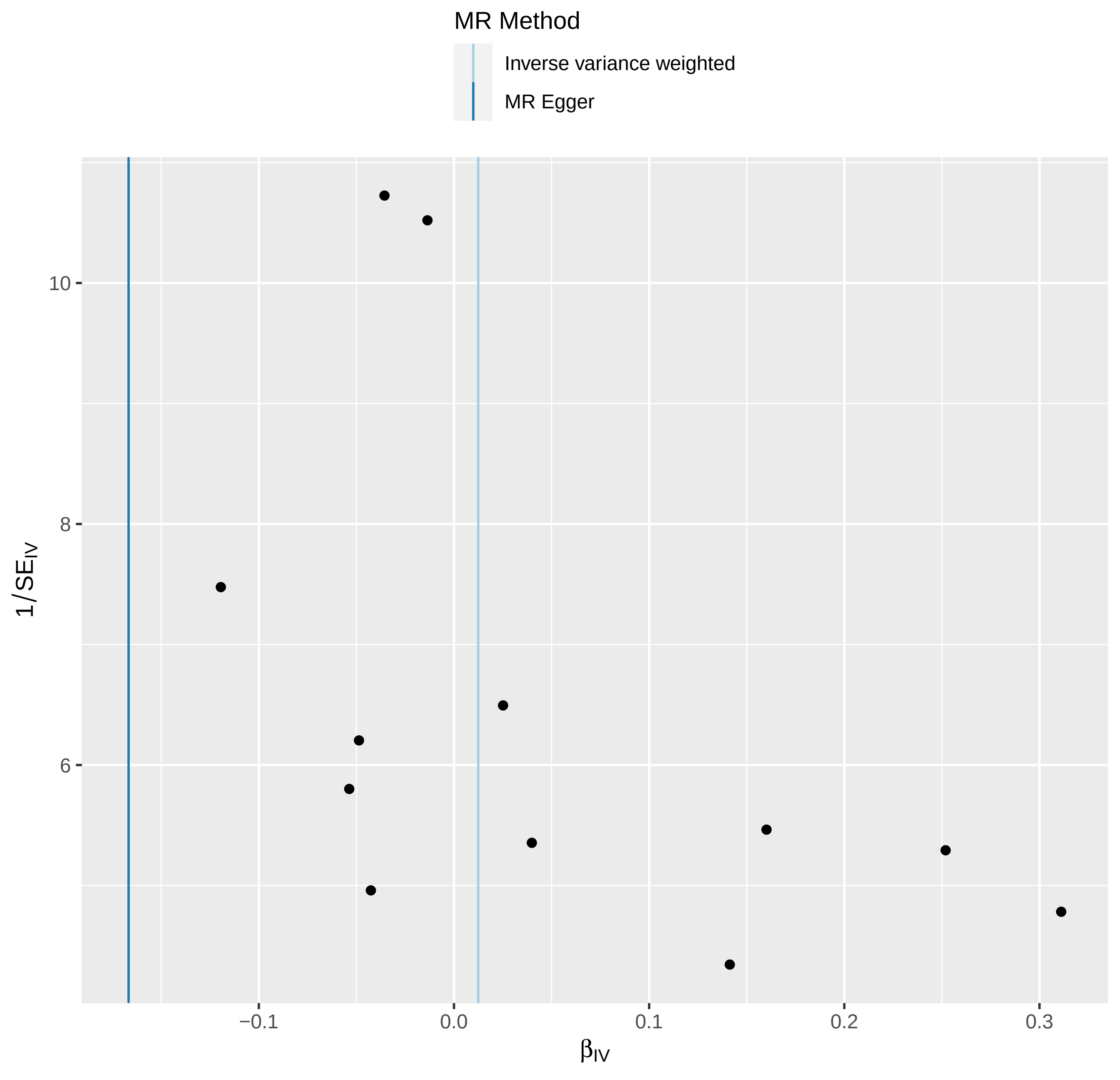

Batch 3−603 : Selenium on 2',3'−cyclic−nucleotide 3'−phosphodiesterase

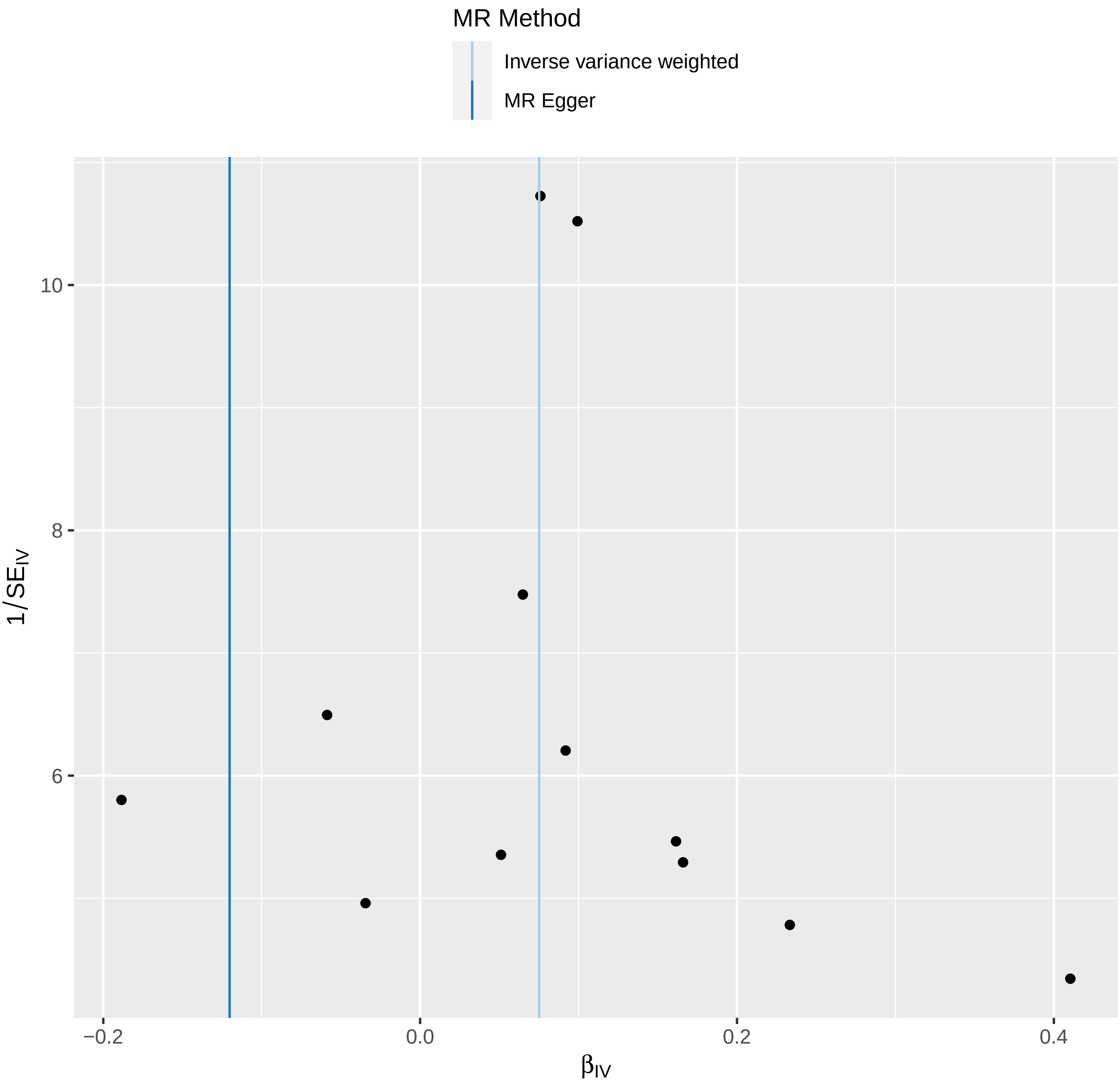

Batch 3−604 : Selenium on Ciliary neurotrophic factor

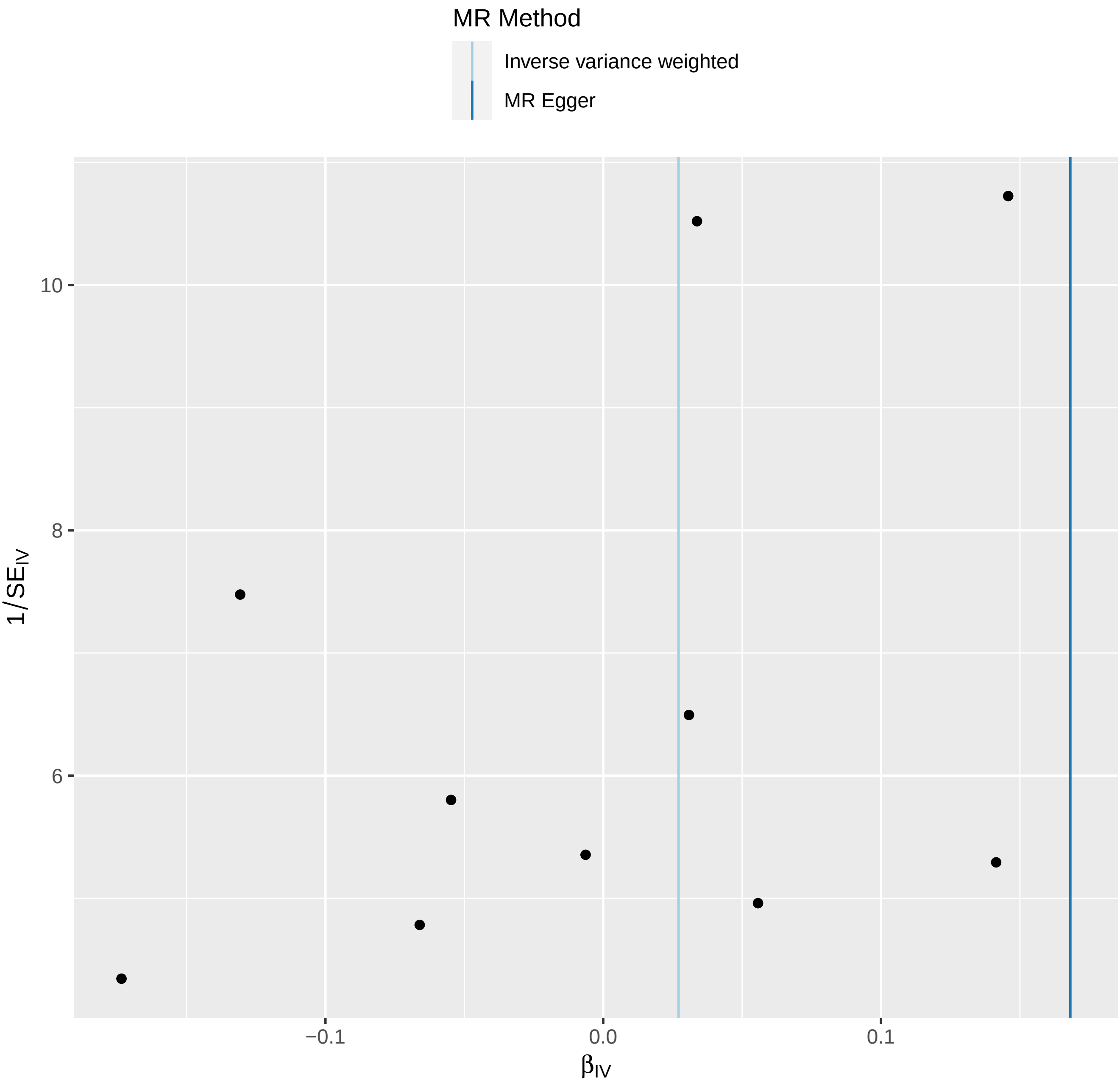

Batch 3−605 : Selenium on Ciliary neurotrophic factor receptor subunit alpha

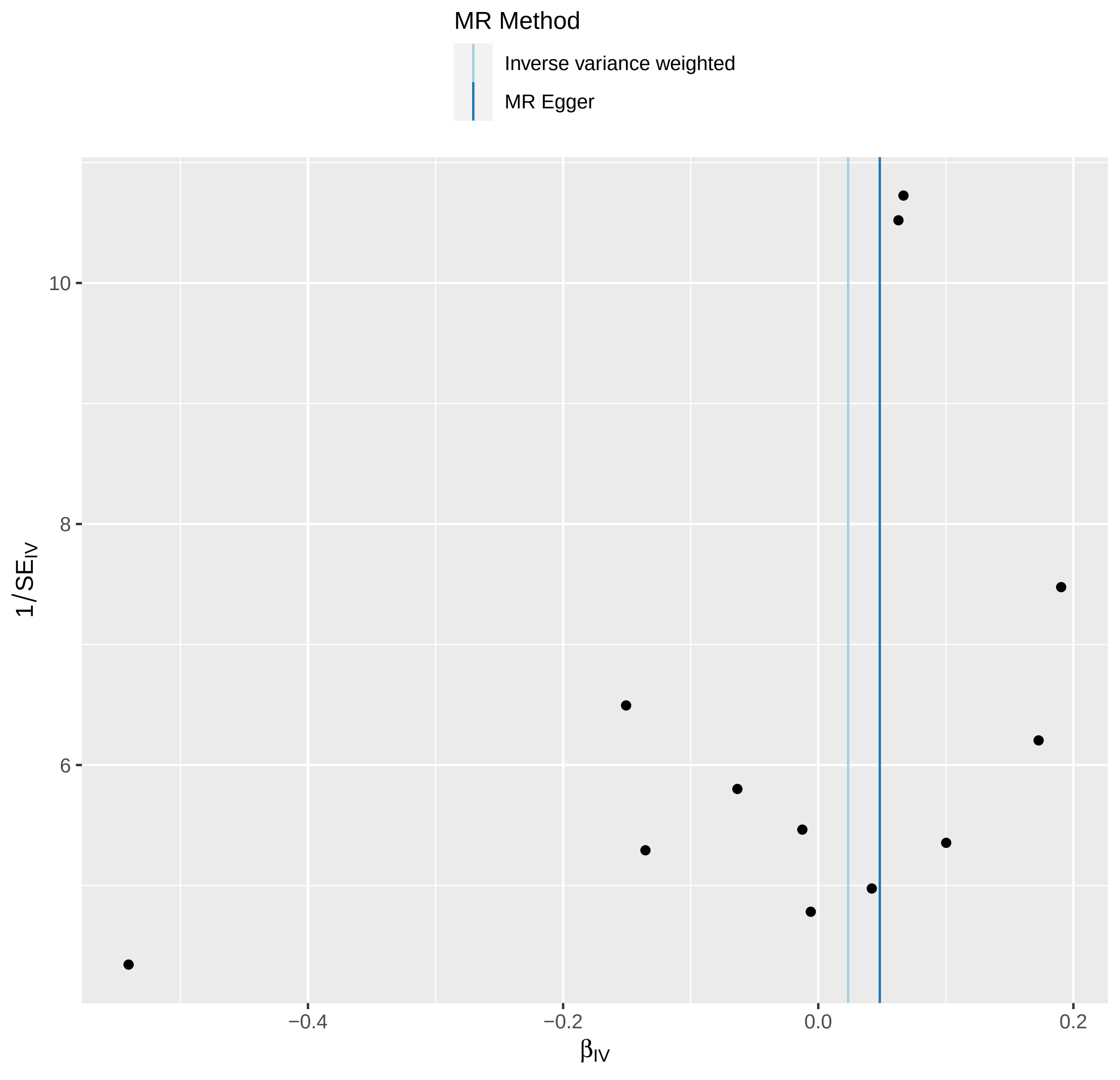

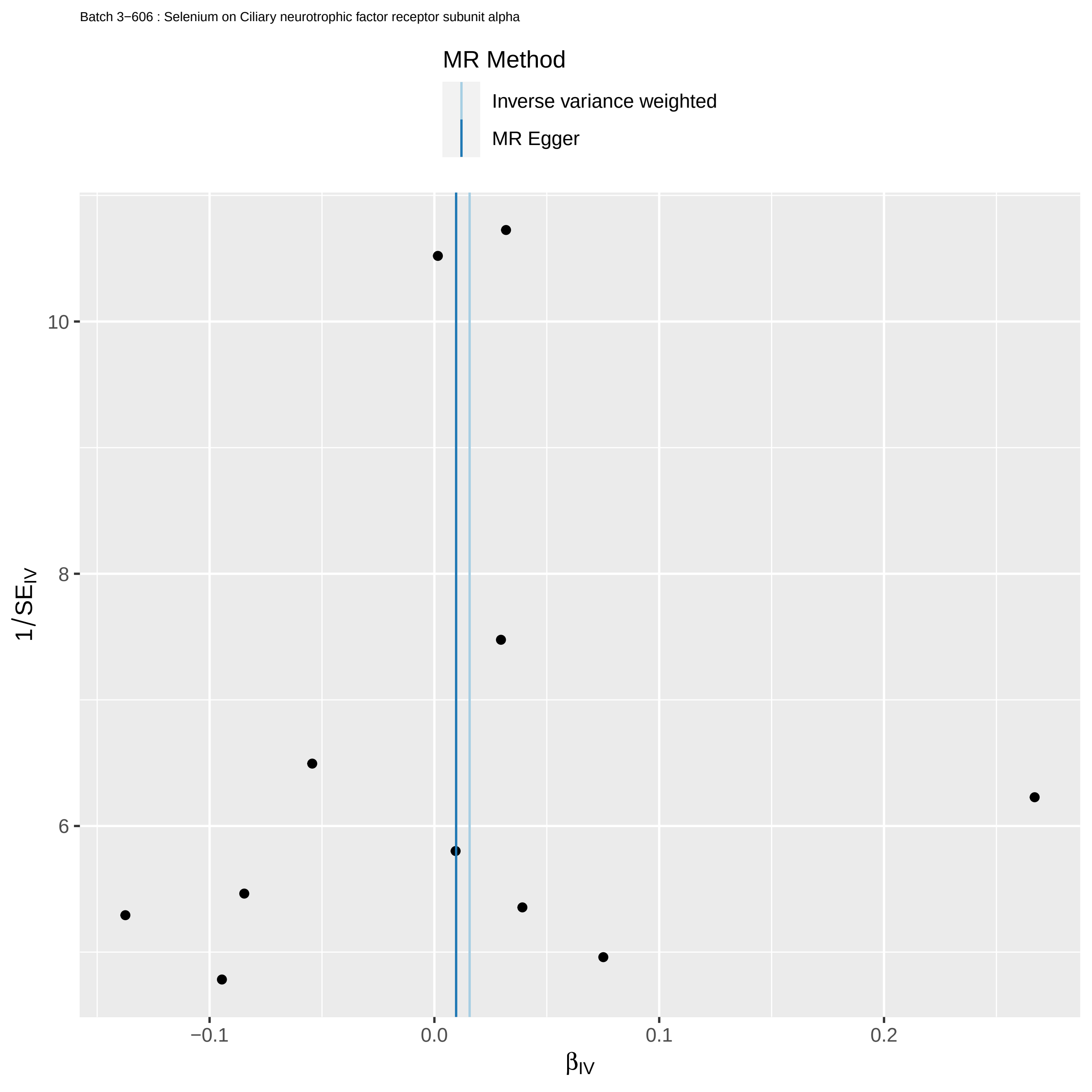
Batch 3−606 : Selenium on Ciliary neurotrophic factor receptor subunit alpha
MR Method
Inverse variance weighted
MR Egger
10
8
6
1/SE_IV
−0.1
0.0
0.1
0.2
β_IV

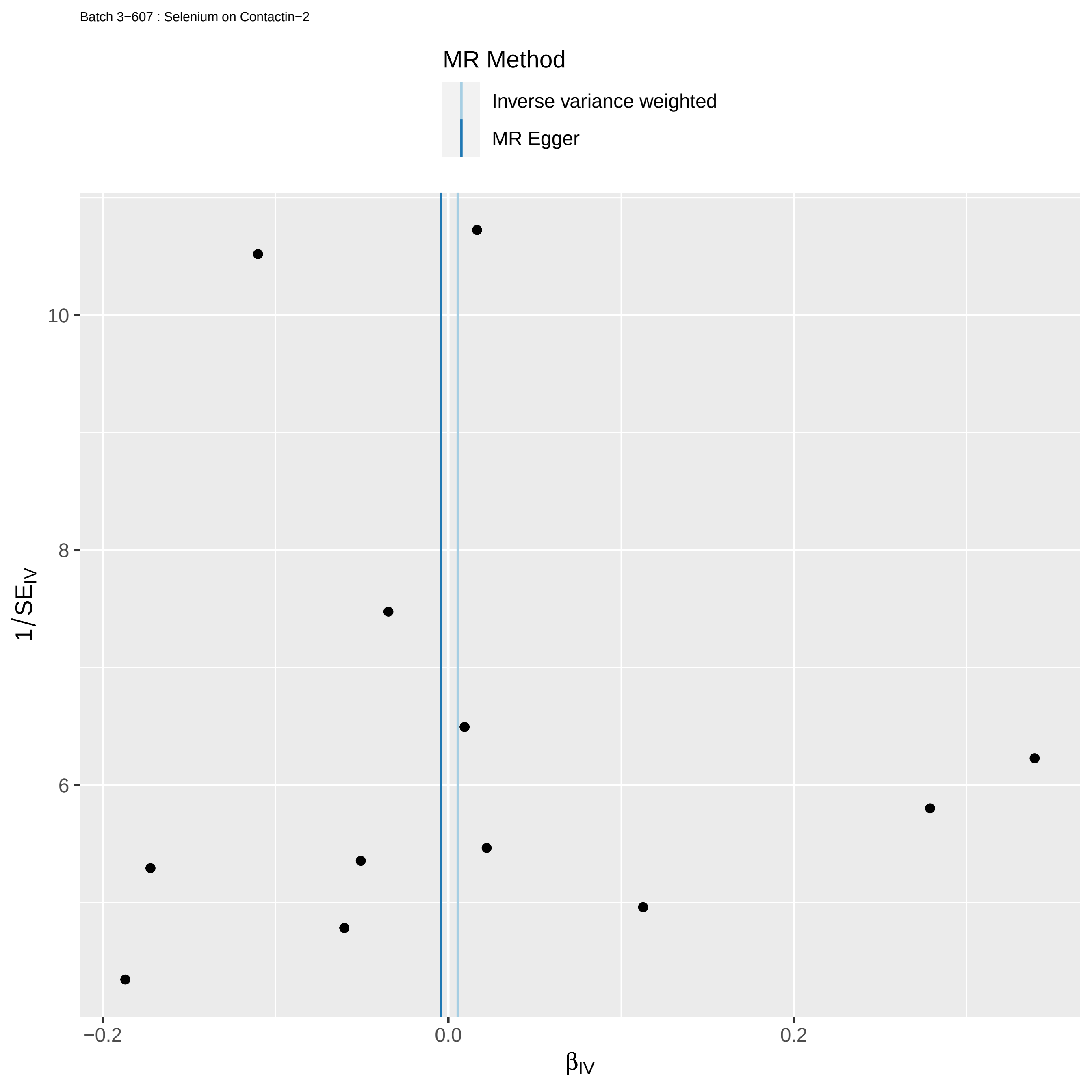
Batch 3−607 : Selenium on Contactin−2
MR Method
Inverse variance weighted
MR Egger
10
8
6
1/SE_IV
−0.2
0.0
0.2
β_IV

Batch 3−608 : Selenium on Contactin−4

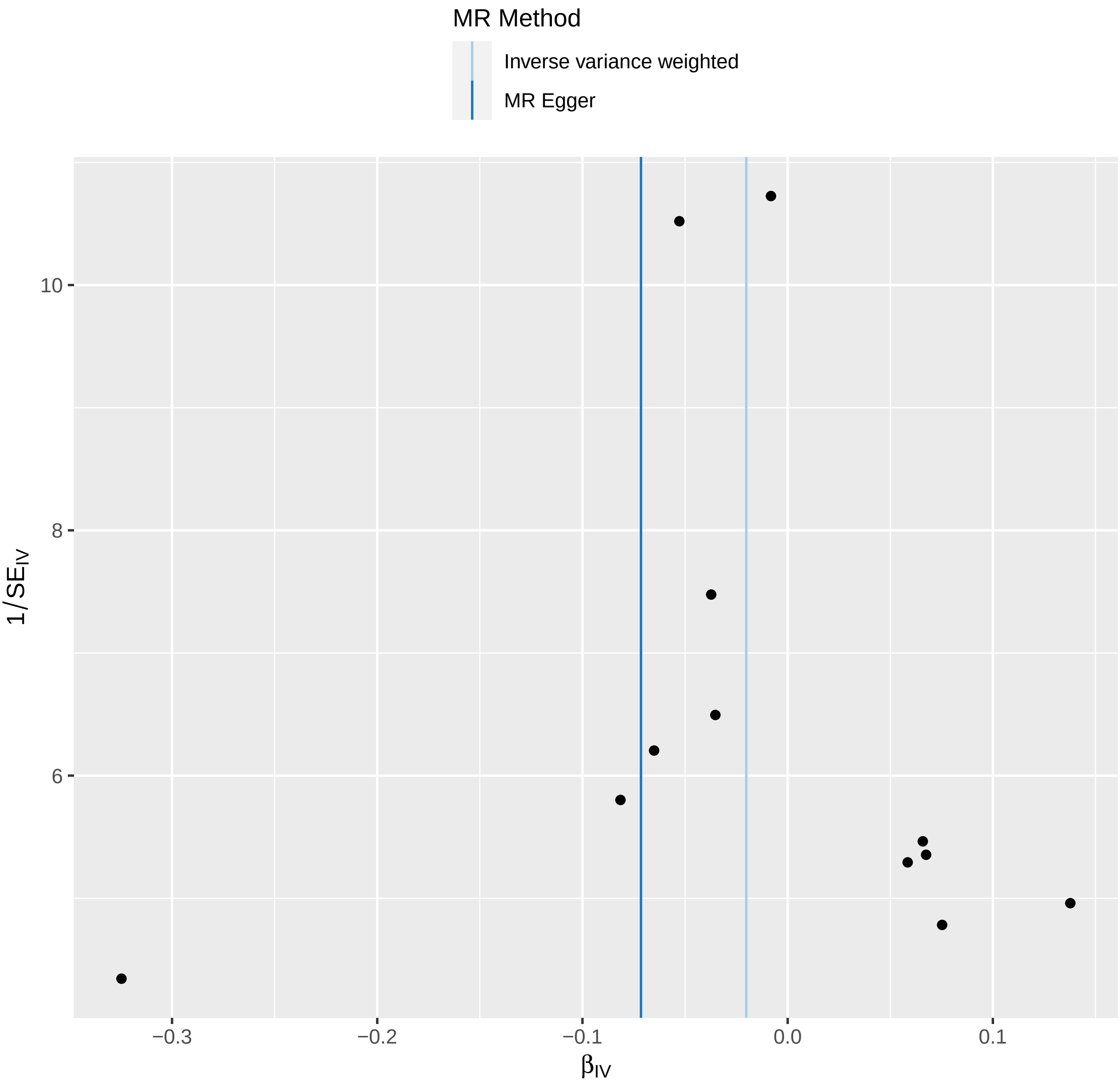

Batch 3−609 : Selenium on Contactin−5

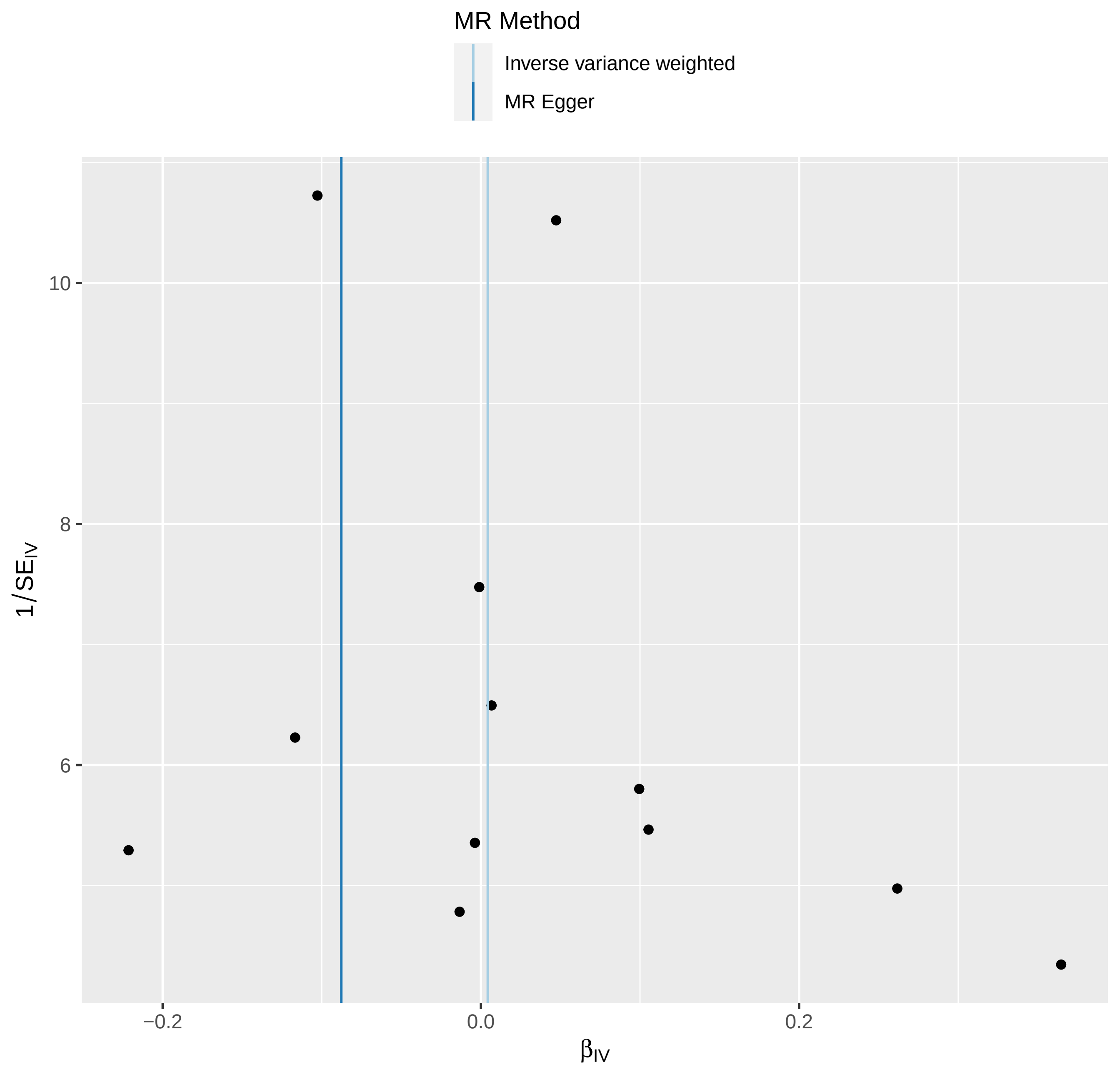

Batch 3−610 : Selenium on Contactin−associated protein 1

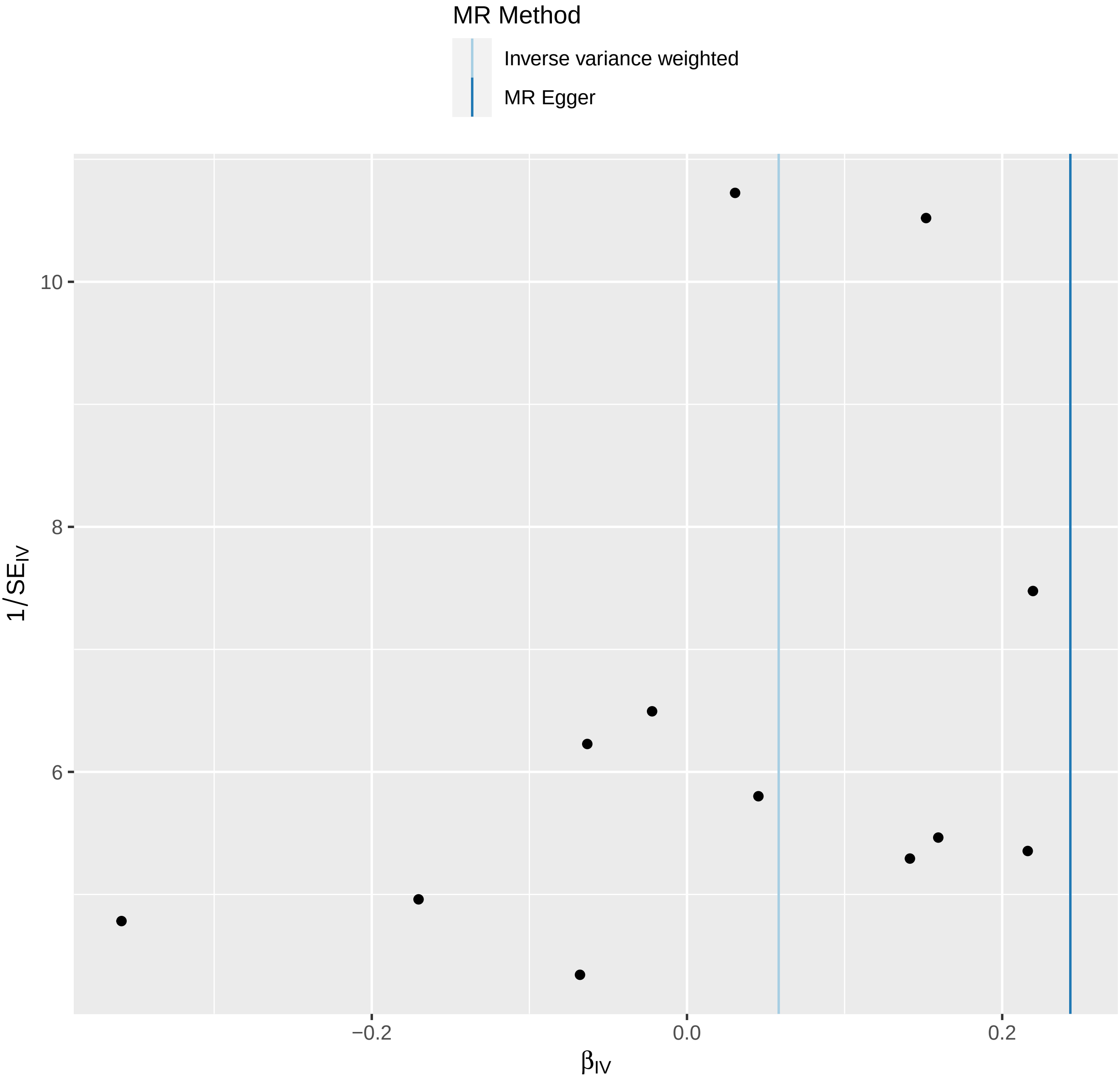

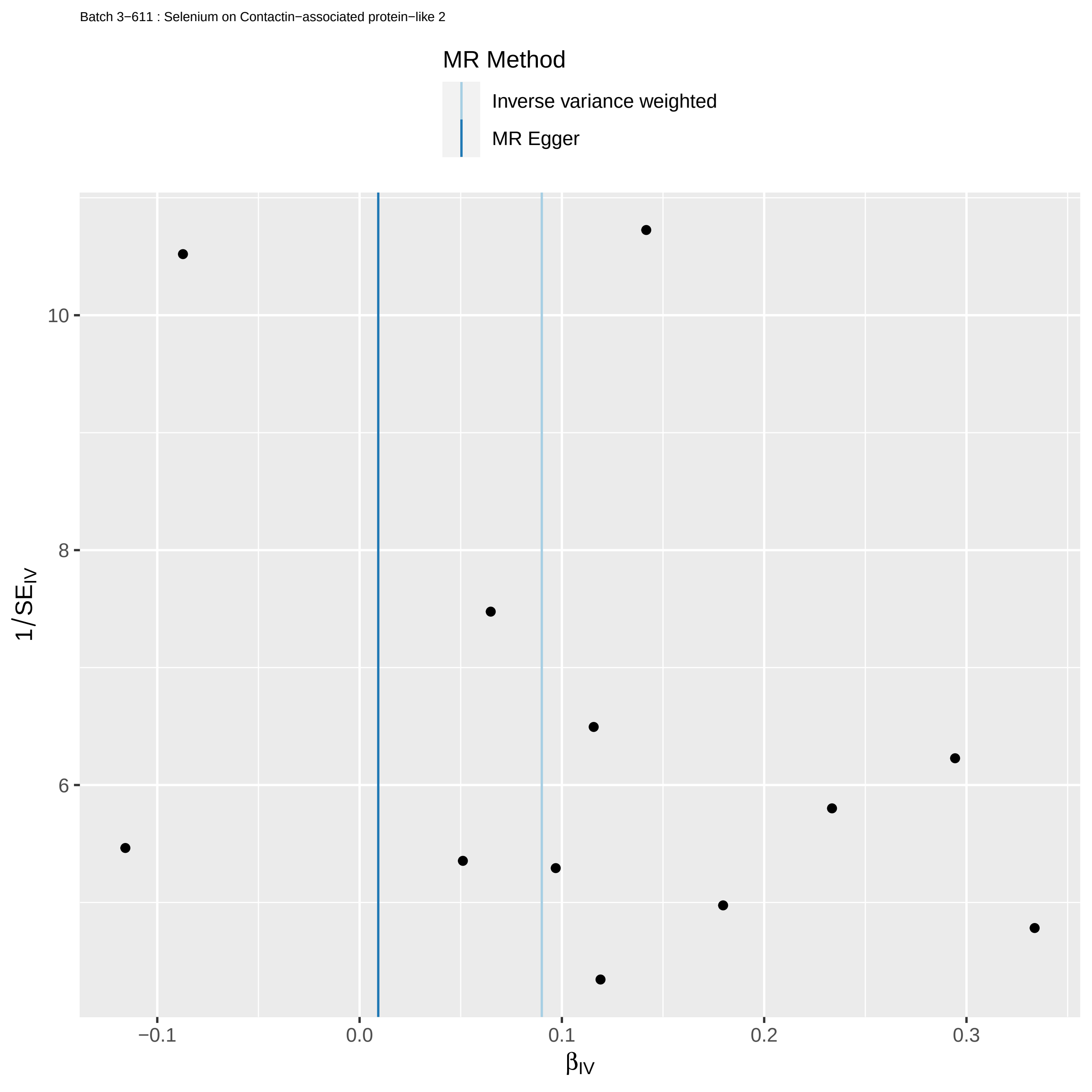

Batch 3−611 : Selenium on Contactin−associated protein−like 2
MR Method
Inverse variance weighted
MR Egger
10
8
6
$1/SE_{IV}$
−0.1
0.0
0.1
0.2
0.3
β_{IV}

Batch 3−612 : Selenium on Cytochrome c oxidase assembly factor 3 homolog, mitochondrial

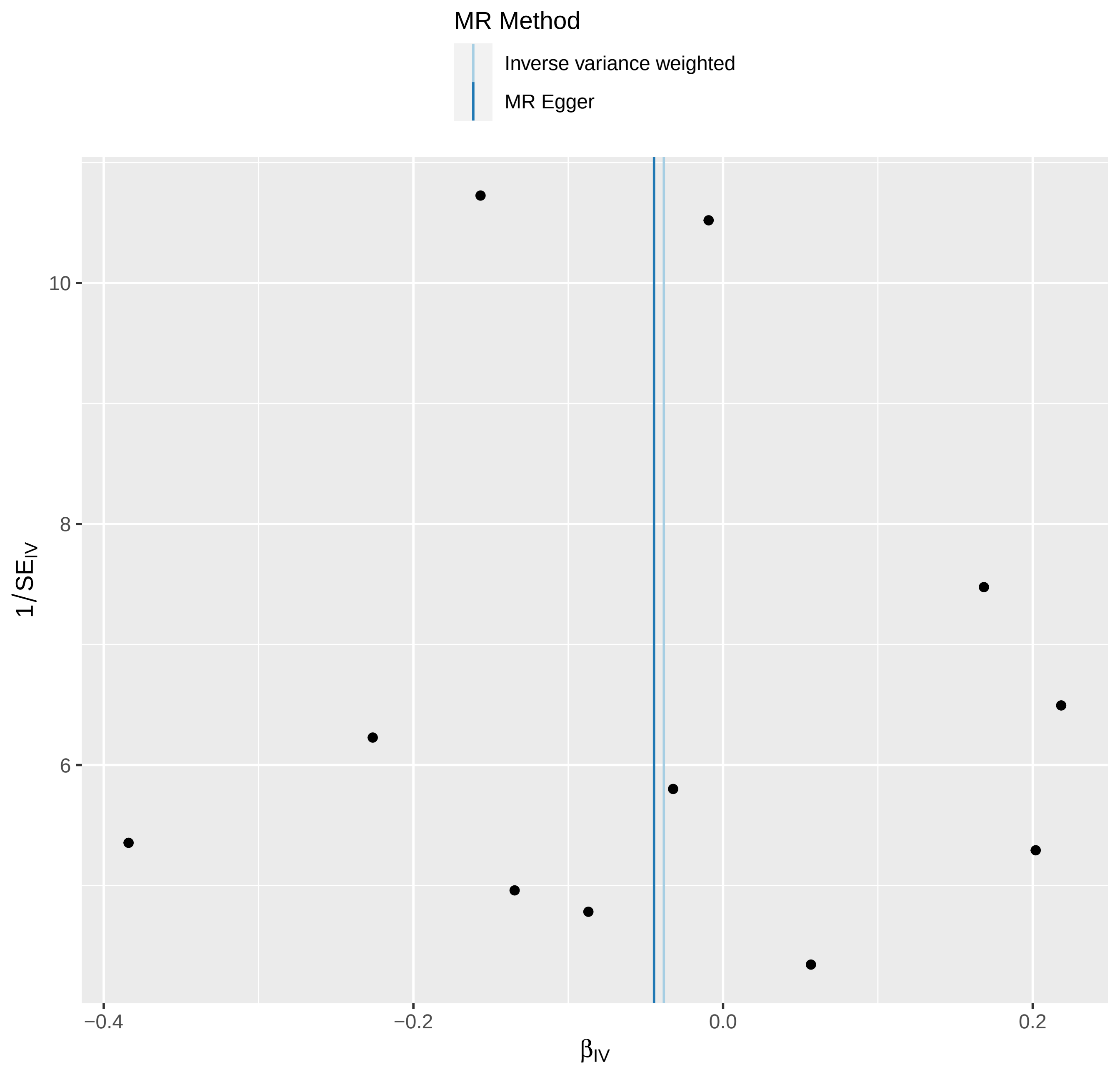

Batch 3−613 : Selenium on Cochlin

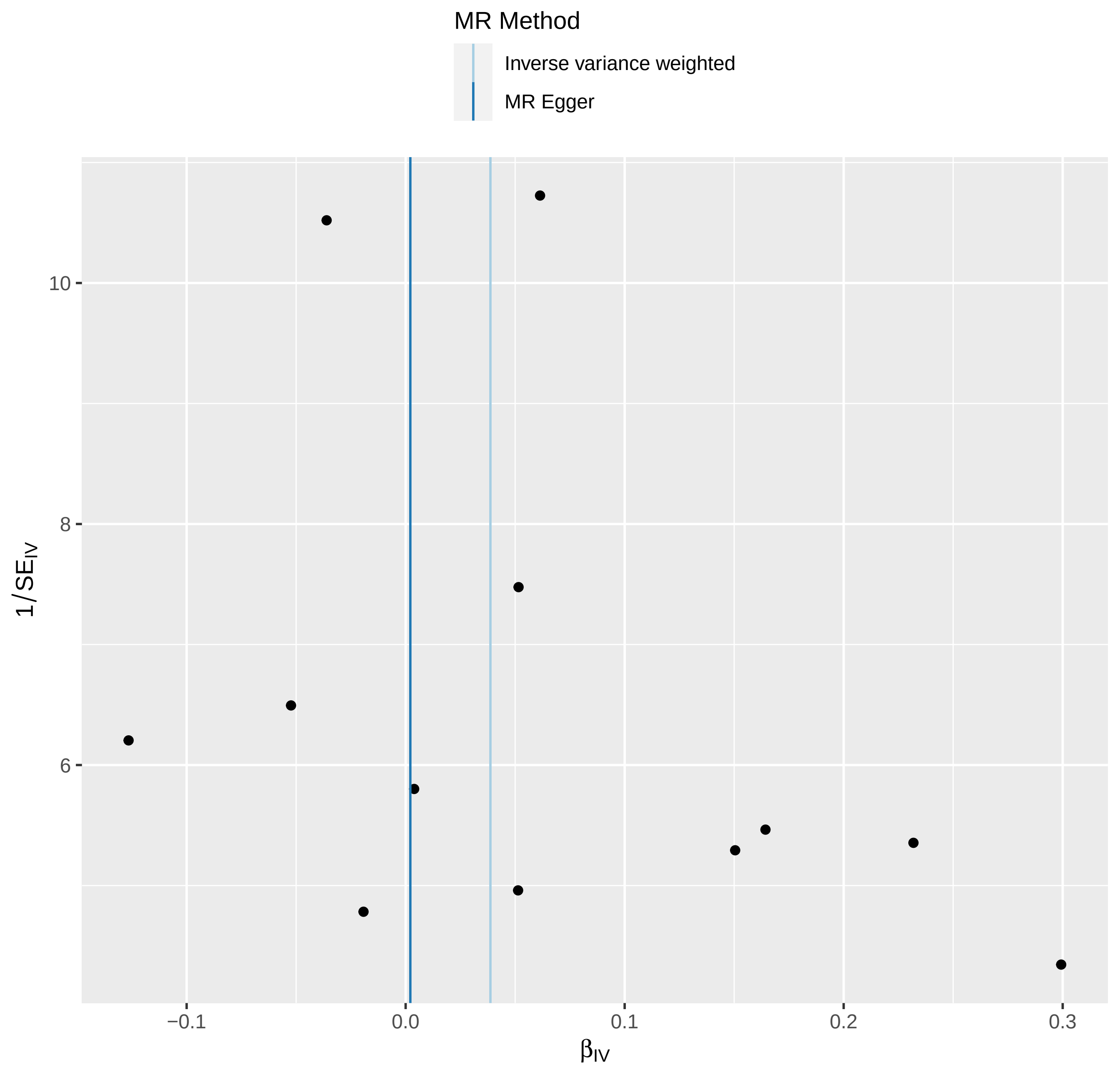

Batch 3−614 : Selenium on Conserved oligomeric Golgi complex subunit 8

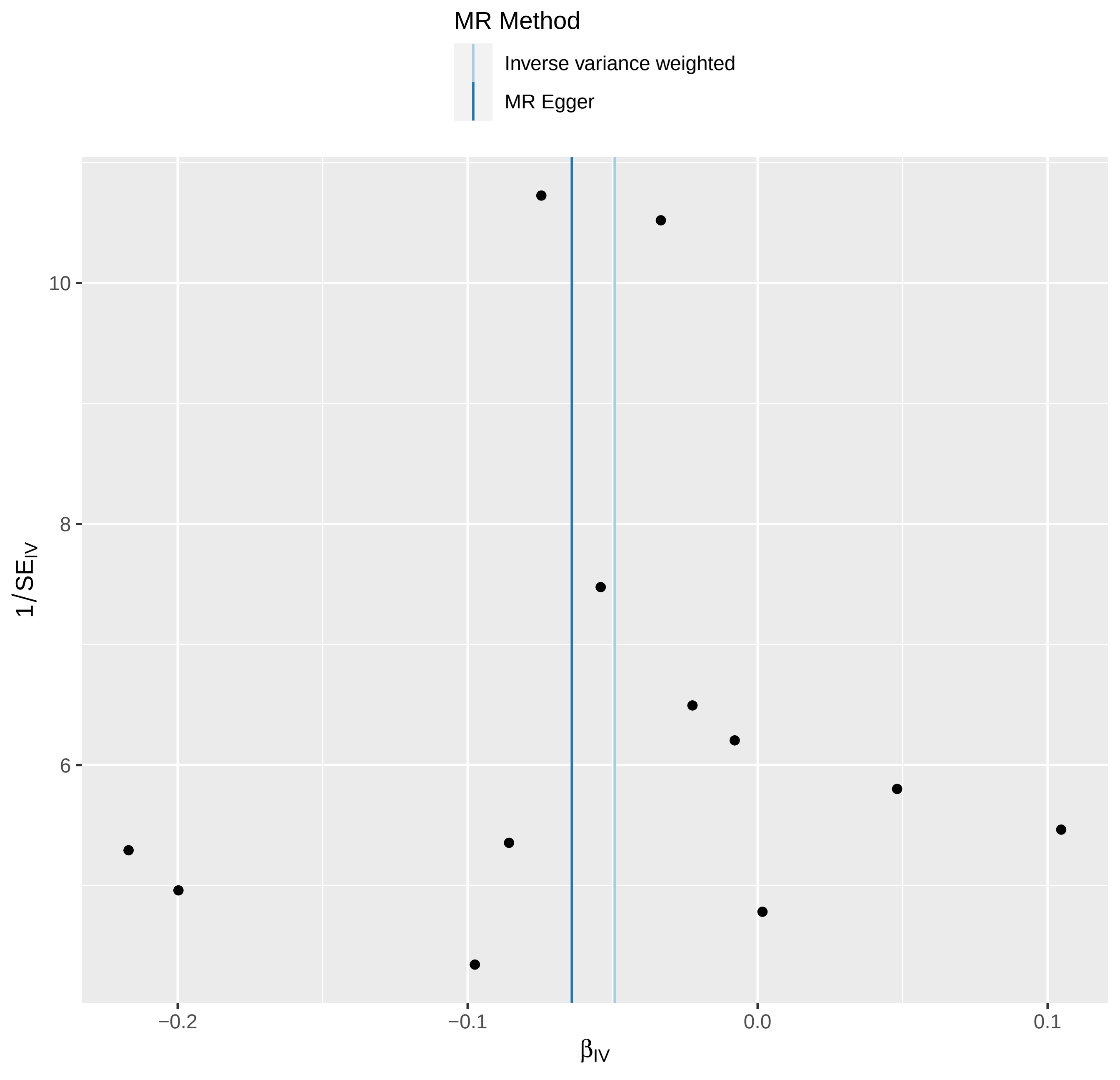

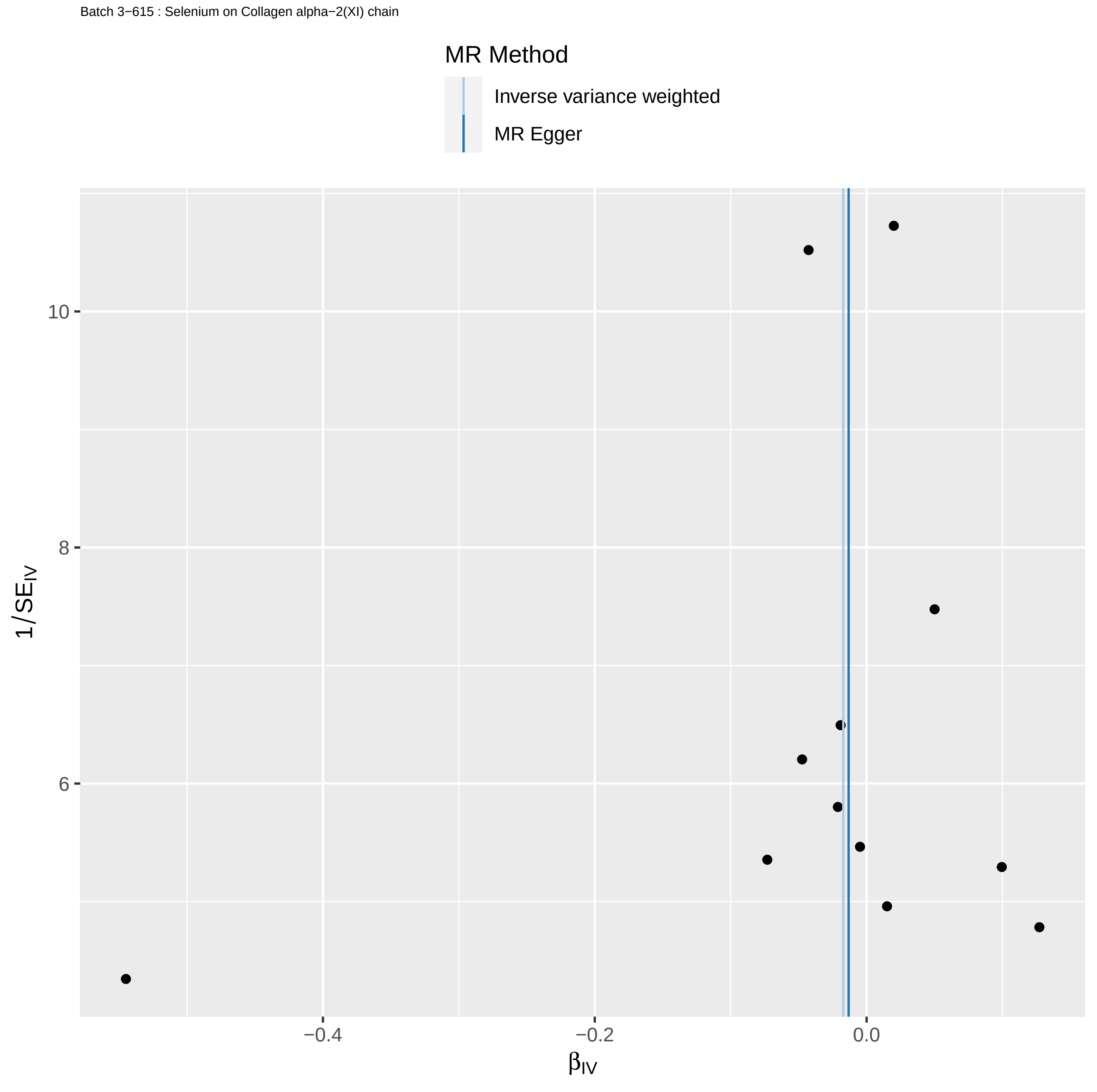
Batch 3−615 : Selenium on Collagen alpha−2(XI) chain
MR Method
Inverse variance weighted
MR Egger
10
8
6
1/SE$_{IV}$
−0.4
−0.2
0.0
β$_{IV}$

Batch 3−616 : Selenium on Collagen alpha−1(XV) chain

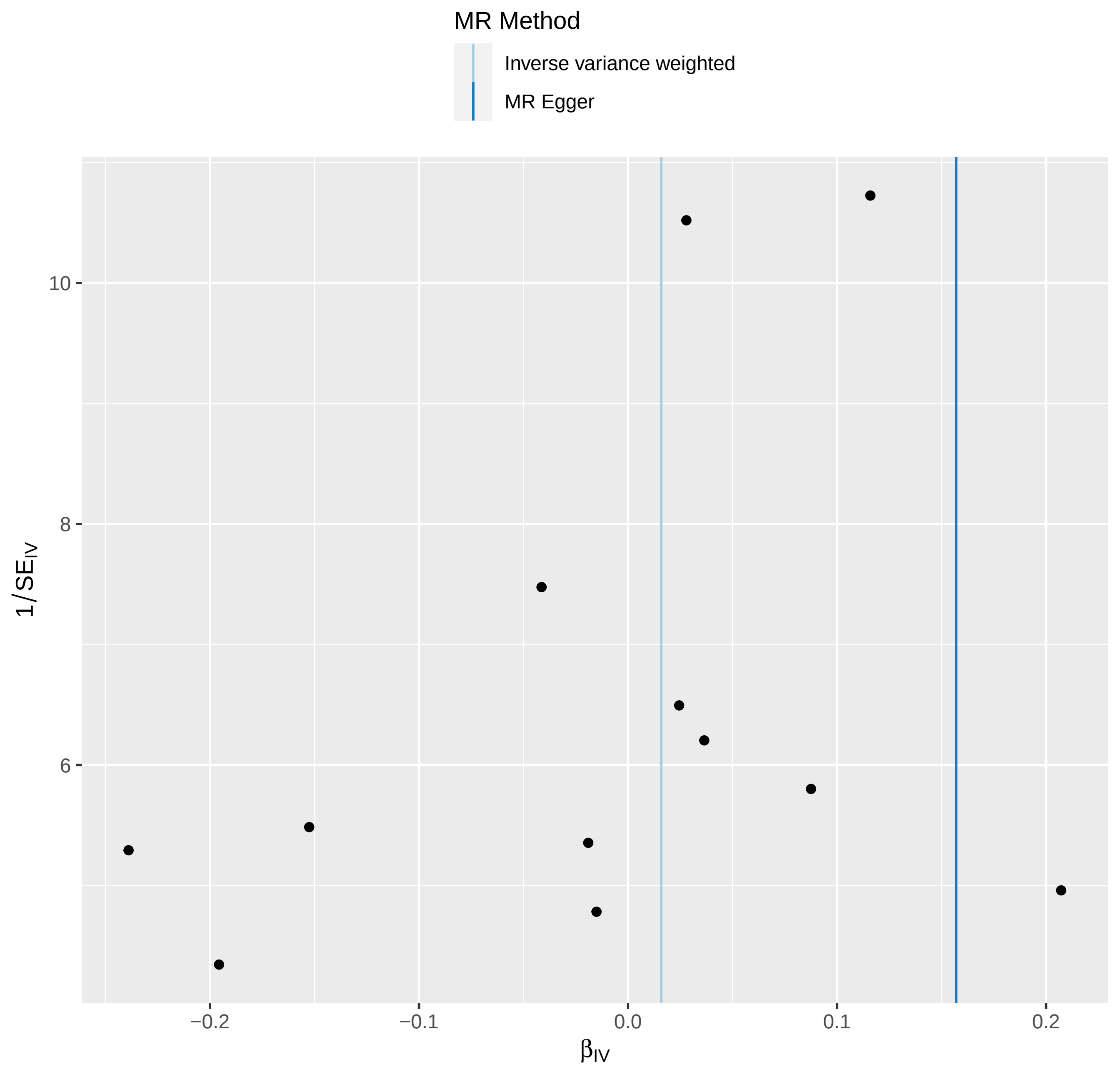

Batch 3−617 : Selenium on Endostatin

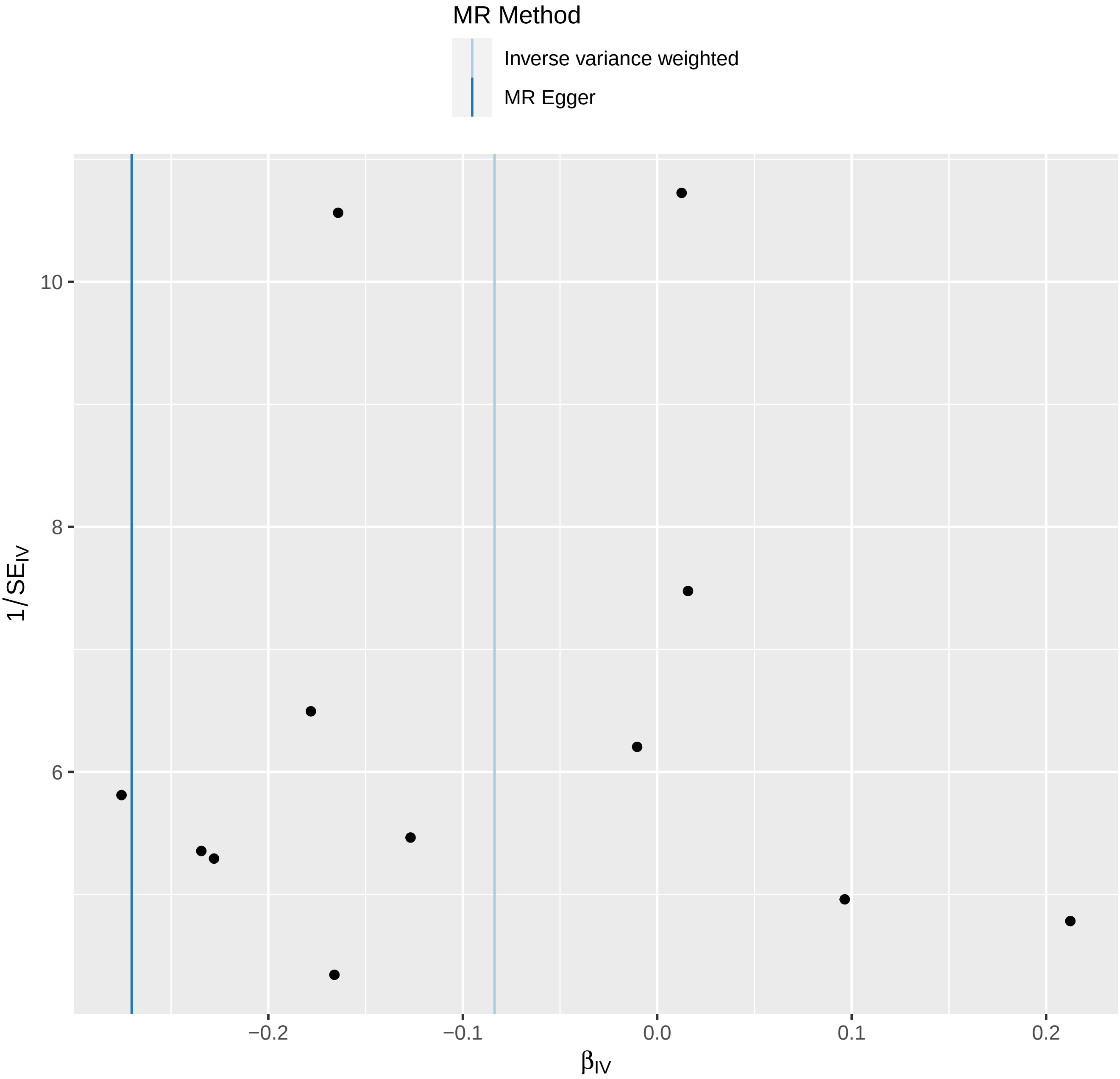

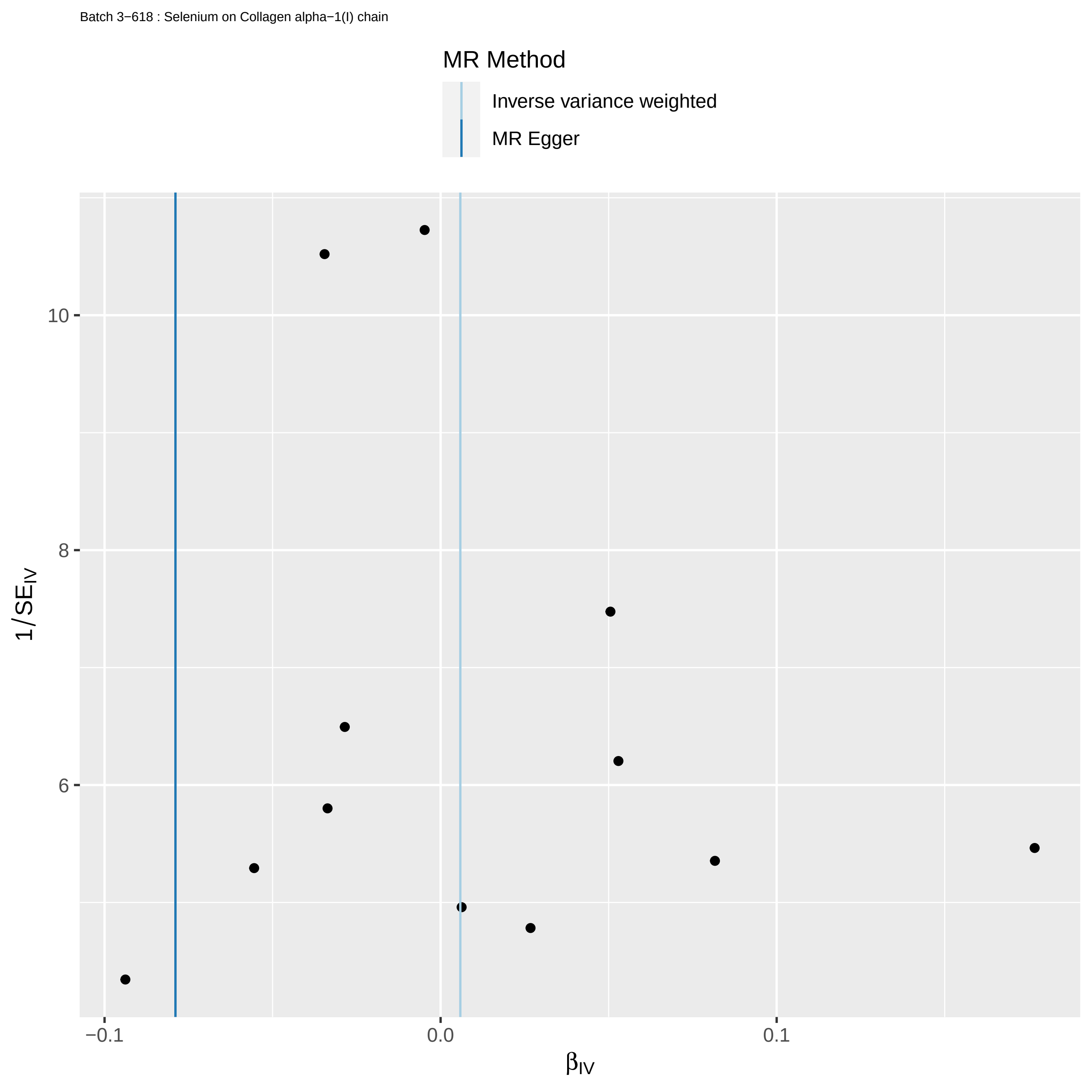

Batch 3−618 : Selenium on Collagen alpha−1(I) chain
MR Method
Inverse variance weighted
MR Egger
10
8
6
1/SE_IV
−0.1
0.0
0.1
β_IV

Batch 3−619 : Selenium on Collagen alpha−1(XX) chain

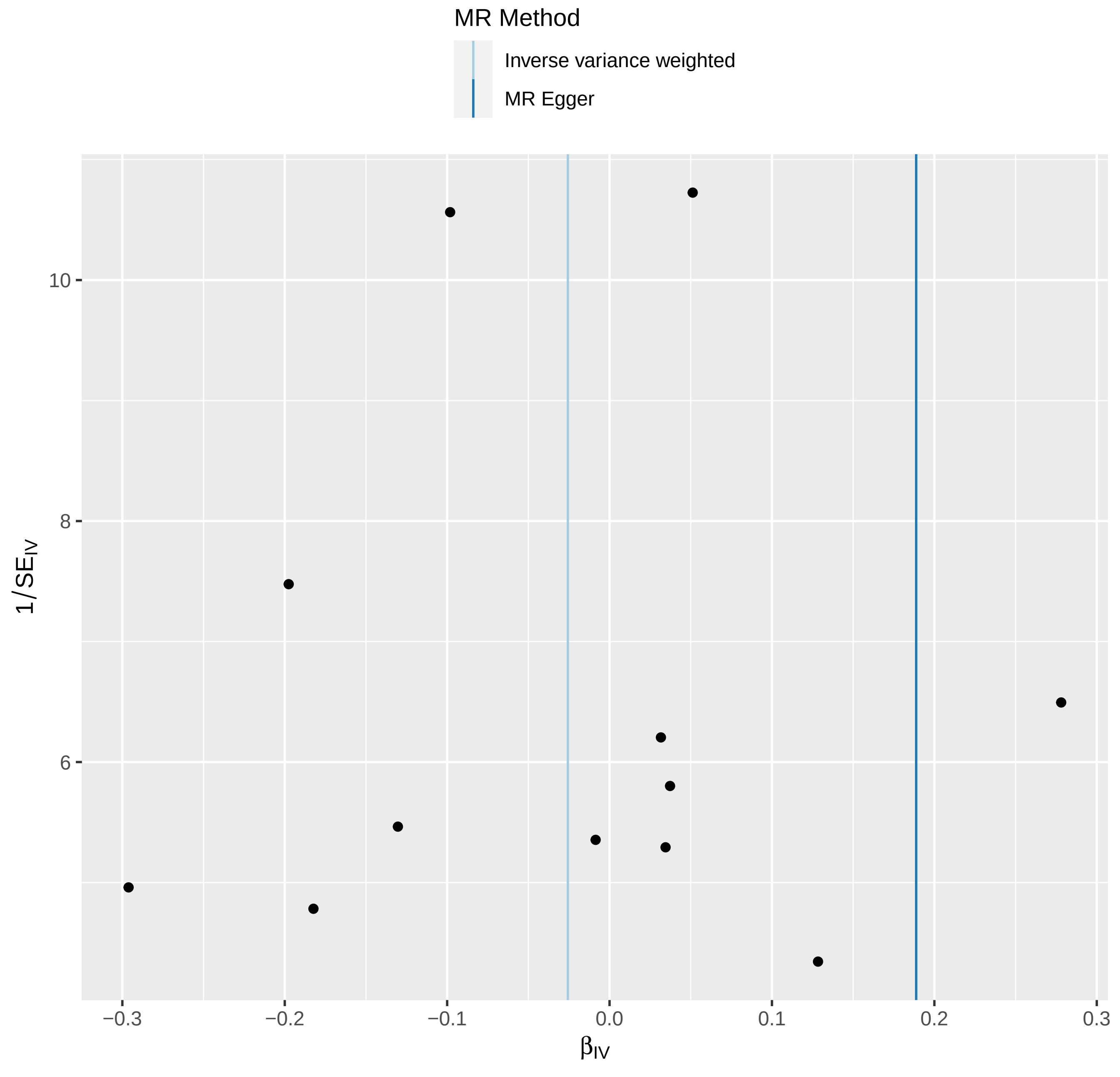

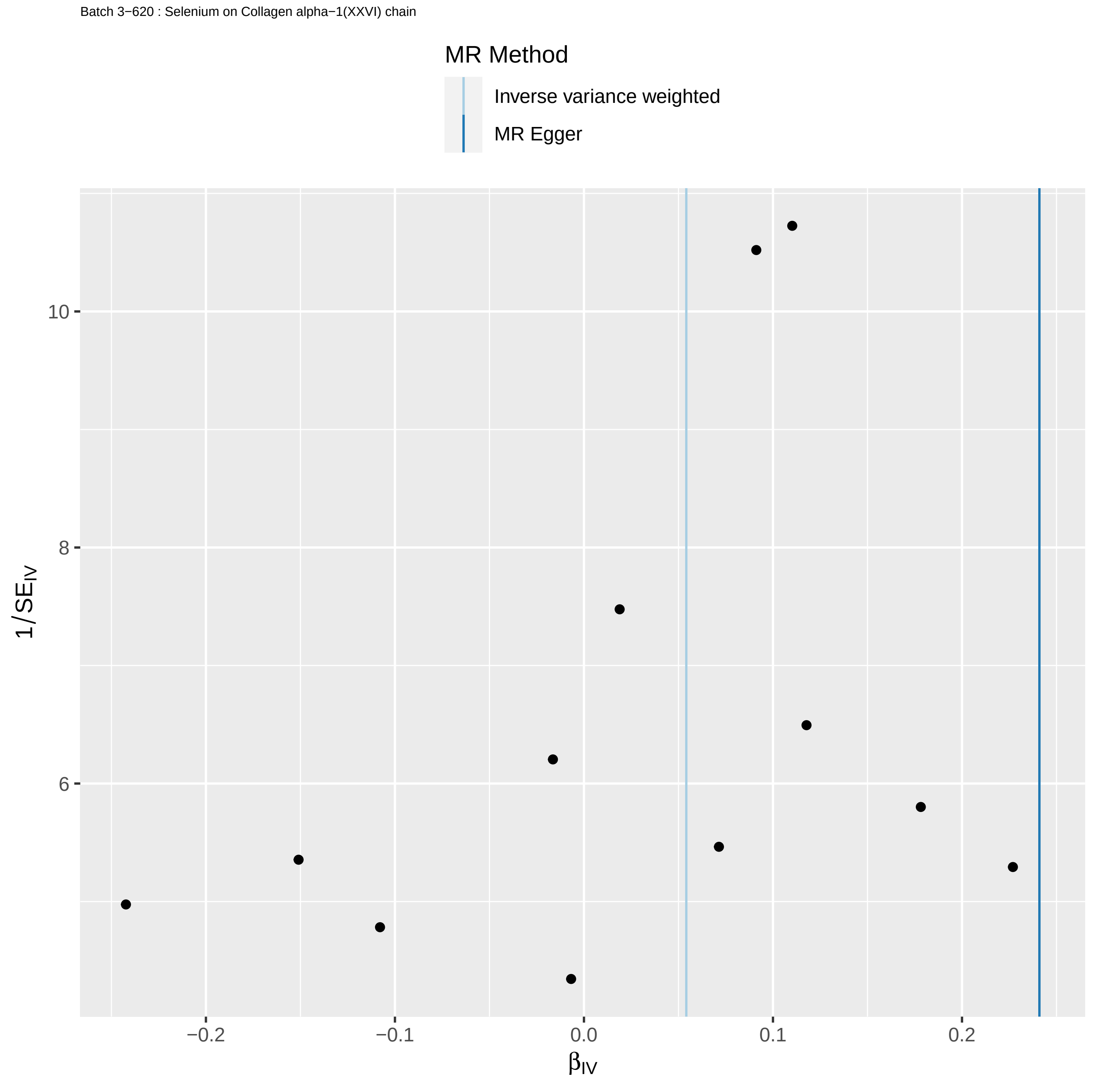

Batch 3−620 : Selenium on Collagen alpha−1(XXVI) chain
MR Method
Inverse variance weighted
MR Egger
10
8
6
1/SE$_{IV}$
−0.2
−0.1
0.0
0.1
0.2
β$_{IV}$

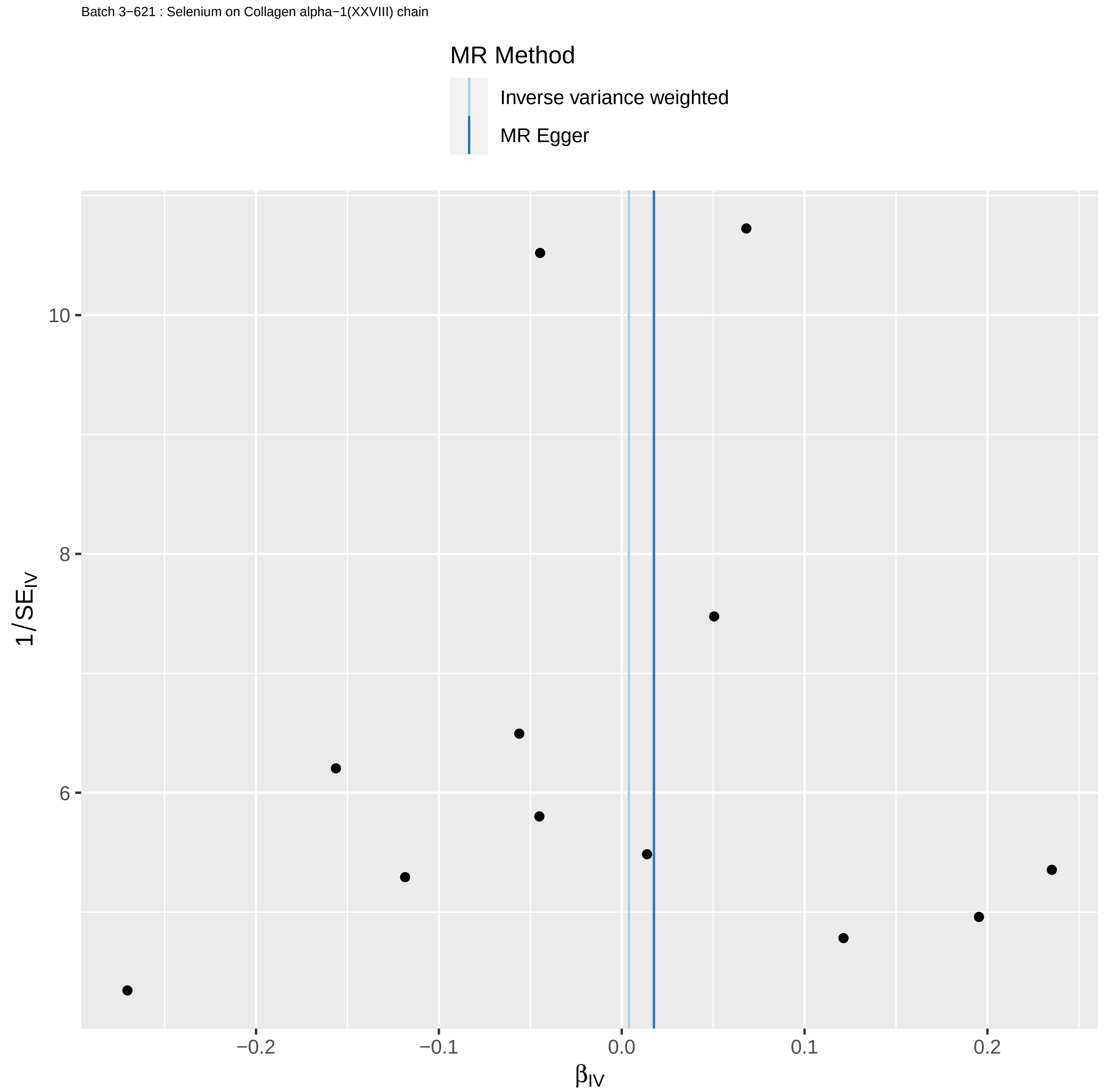

Batch 3−621 : Selenium on Collagen alpha−1(XXVIII) chain
MR Method
Inverse variance weighted
MR Egger
10
8
6
1/SE$_{IV}$
−0.2
−0.1
0.0
0.1
0.2
β_{IV}

Batch 3−622 : Selenium on Collagen alpha−1(VI) chain

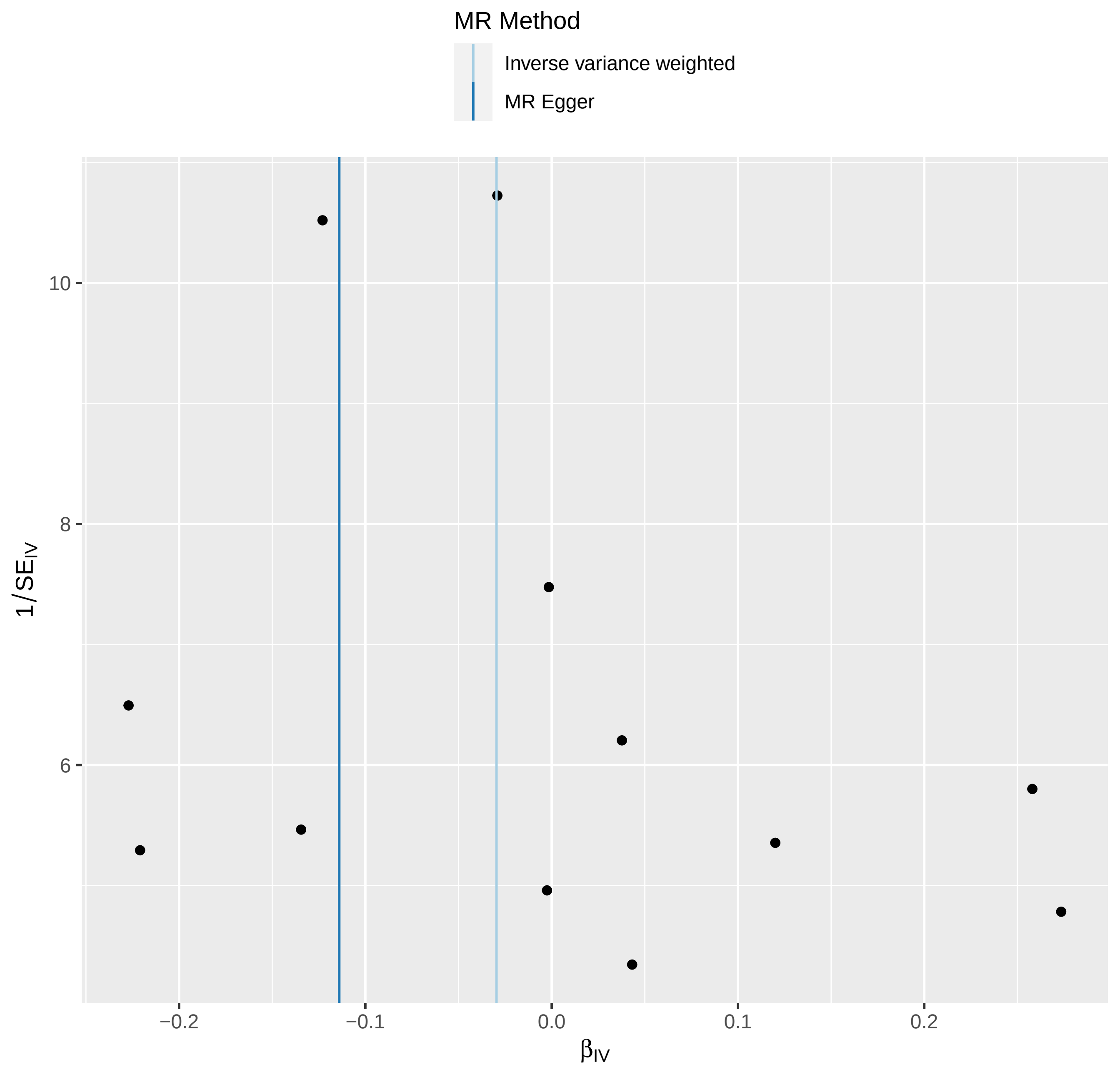

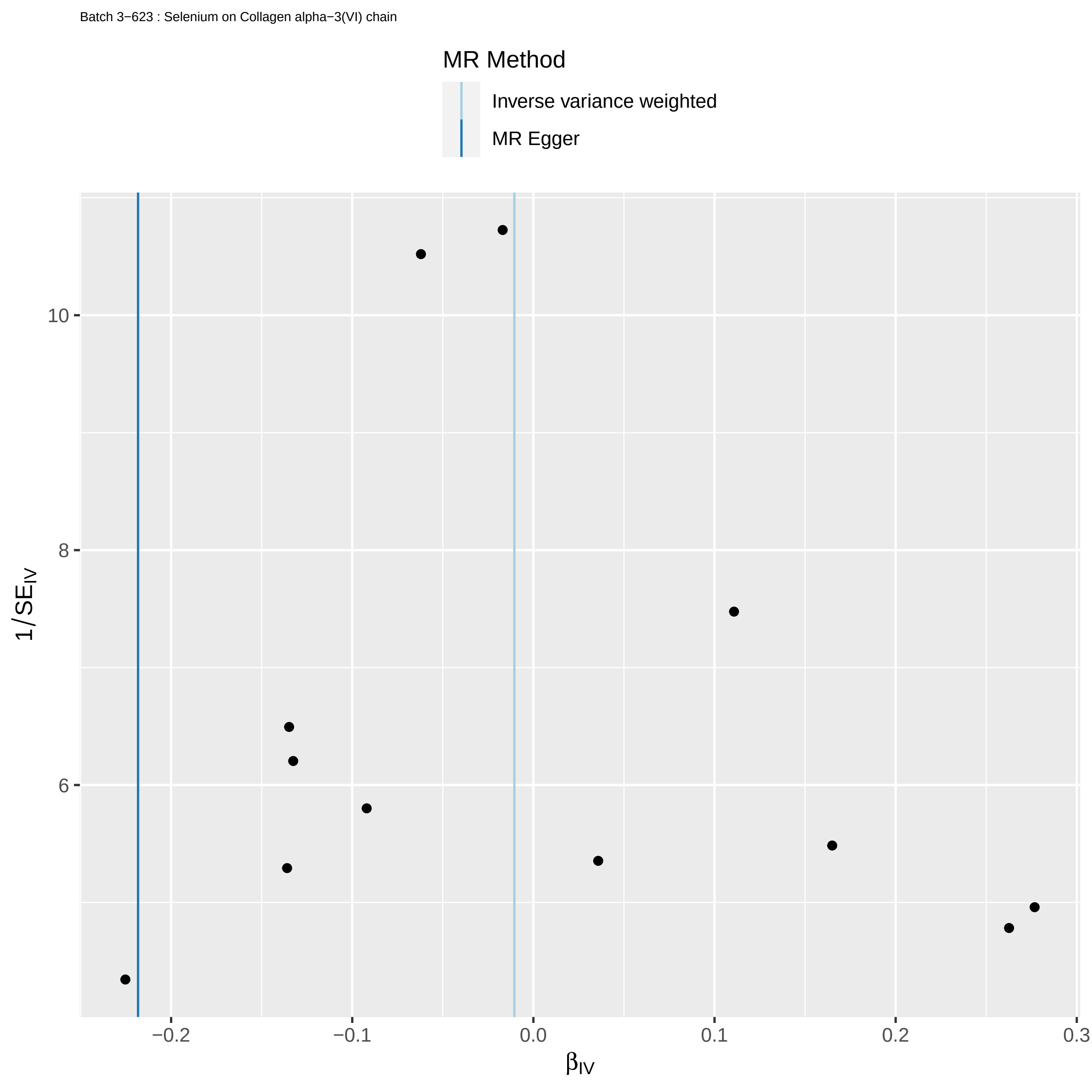

Batch 3−623 : Selenium on Collagen alpha−3(VI) chain
MR Method
Inverse variance weighted
MR Egger
10
8
6
1/SE_IV
−0.2
−0.1
0.0
0.1
0.2
0.3
β_IV

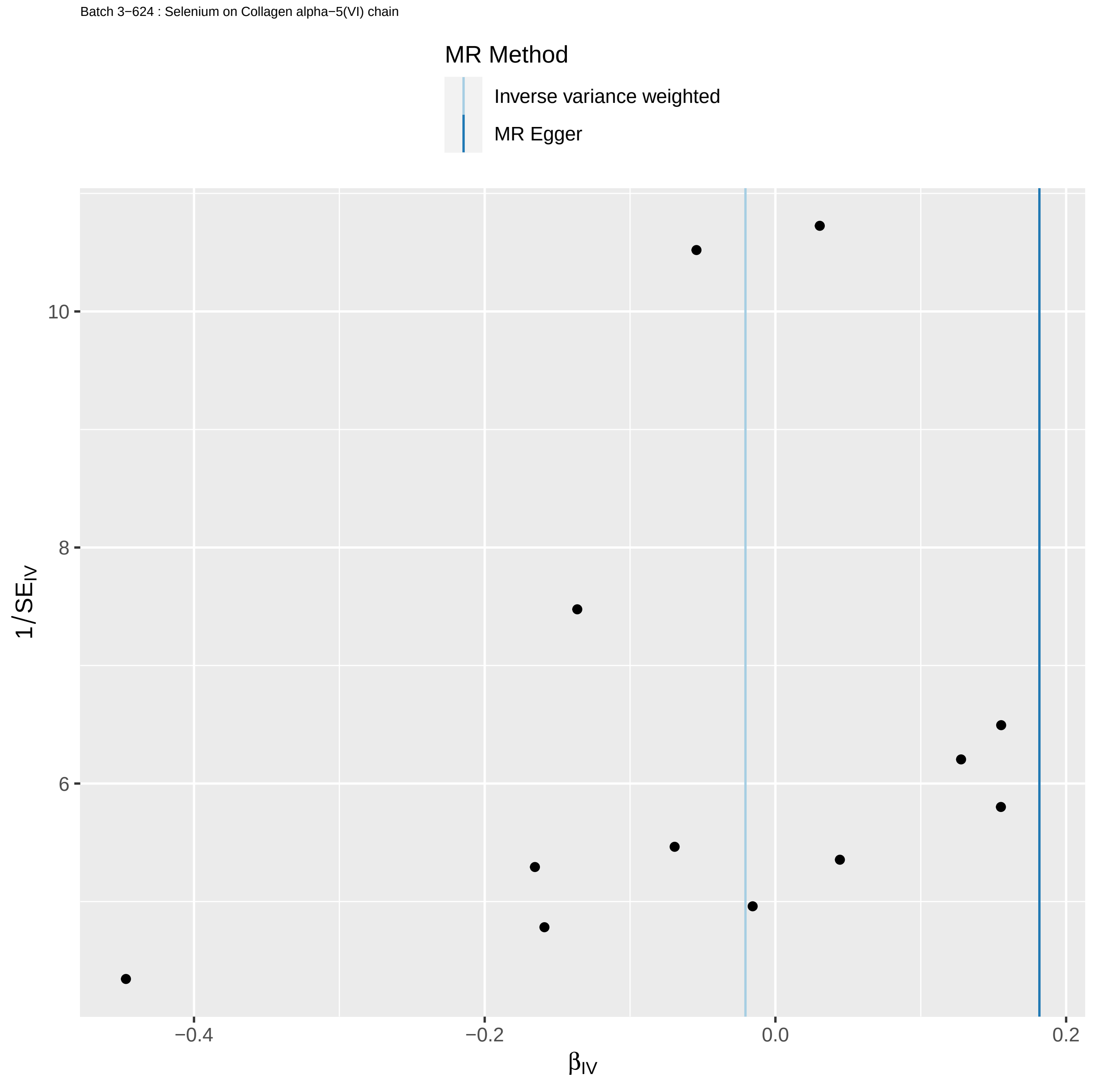

Batch 3−624 : Selenium on Collagen alpha−5(VI) chain
MR Method
Inverse variance weighted
MR Egger
10
8
6
1/SE_IV
−0.4
−0.2
0.0
0.2
β_IV

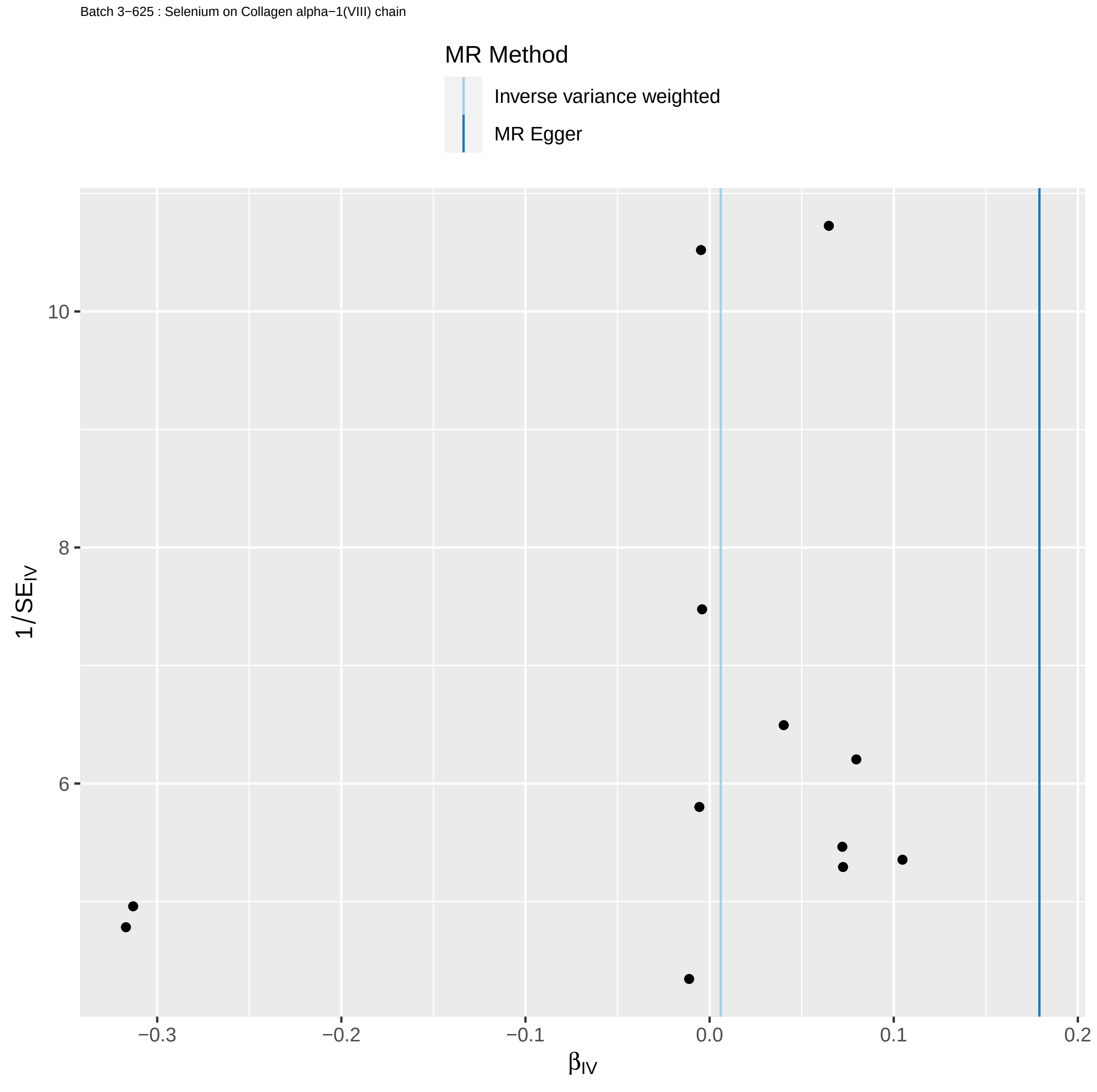

Batch 3−625 : Selenium on Collagen alpha−1(VIII) chain
MR Method
Inverse variance weighted
MR Egger
10
8
6
1/SE_IV
−0.3
−0.2
−0.1
0.0
0.1
0.2
β_IV

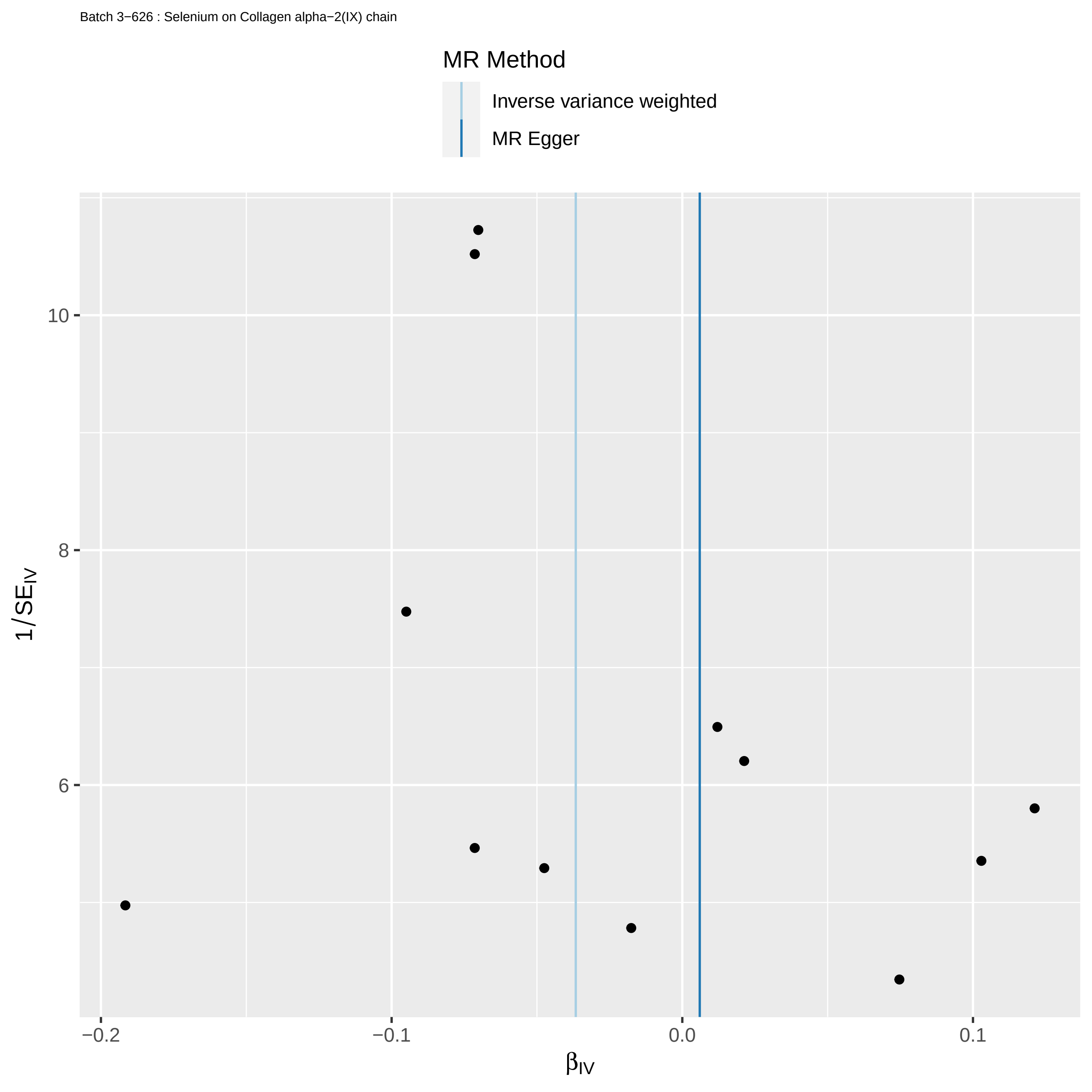
Batch 3−626 : Selenium on Collagen alpha−2(IX) chain
MR Method
Inverse variance weighted
MR Egger
10
8
6
1/SE_IV
−0.2
−0.1
0.0
0.1
β_IV

Batch 3−627 : Selenium on Collectin−10

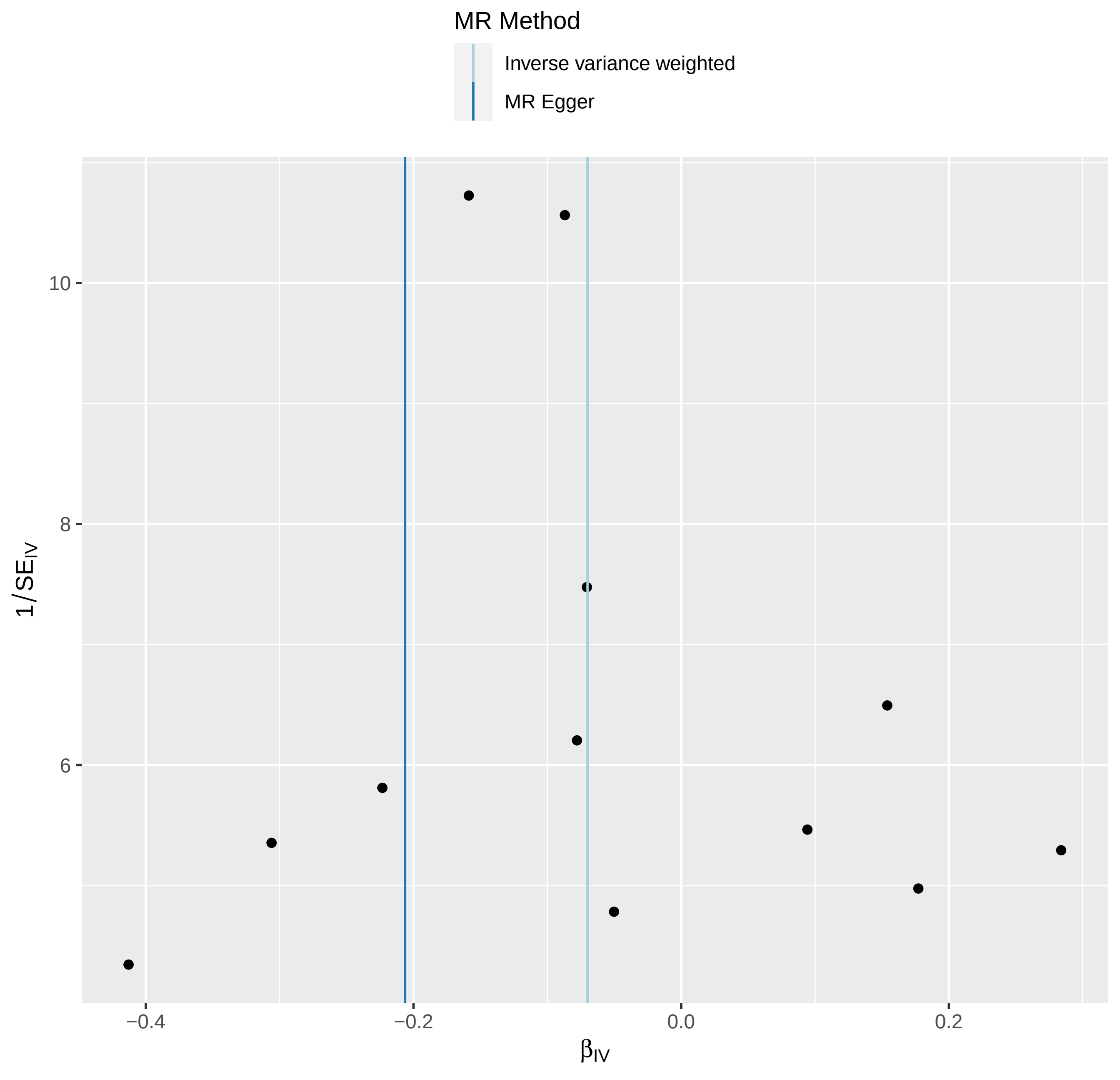

Batch 3−628 : Selenium on Collectin−11

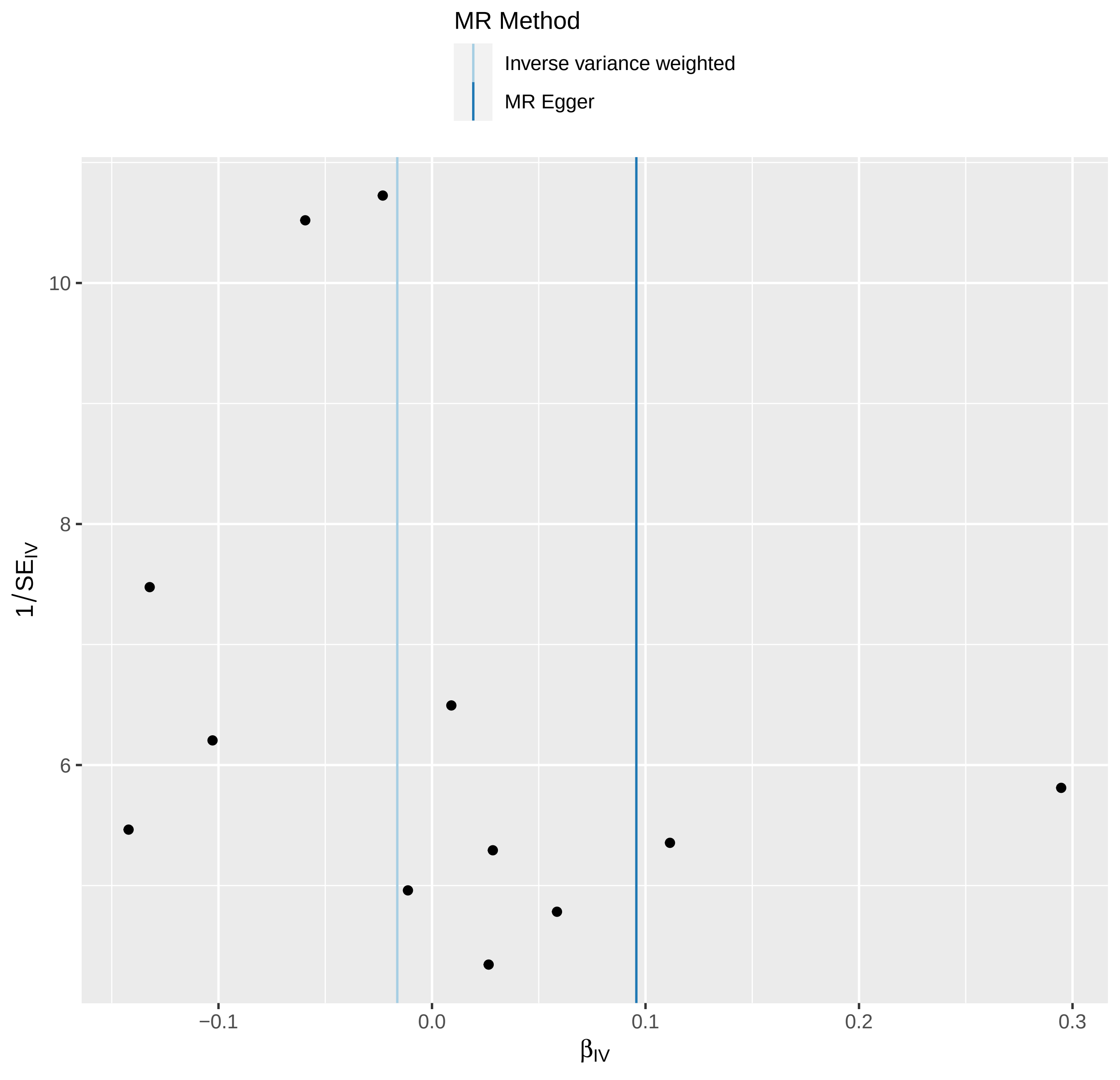

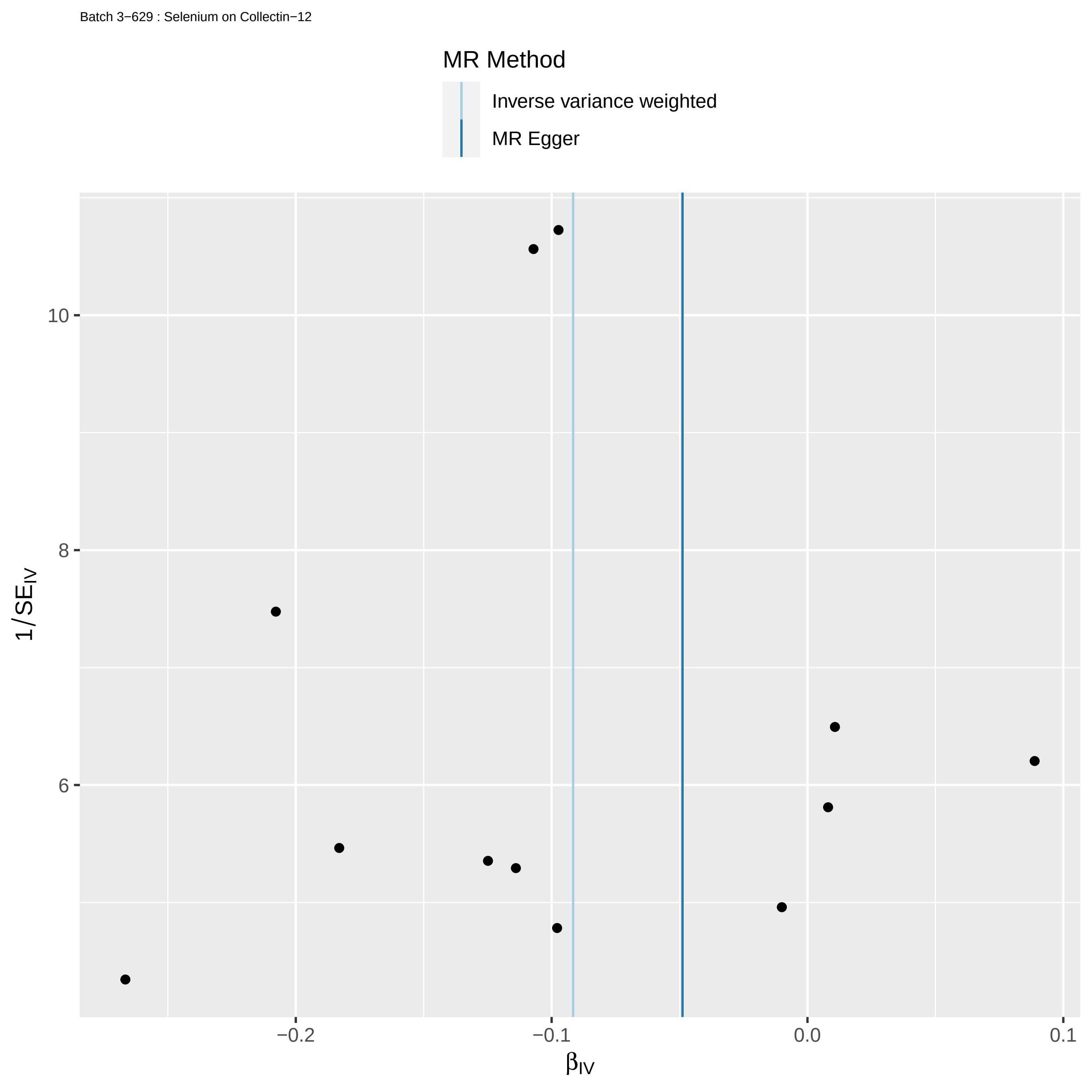
Batch 3−629 : Selenium on Collectin−12
MR Method
Inverse variance weighted
MR Egger
10
8
6
1/SE_IV
−0.2
−0.1
0.0
0.1
β_IV

Batch 3−630 : Selenium on Collectin−12

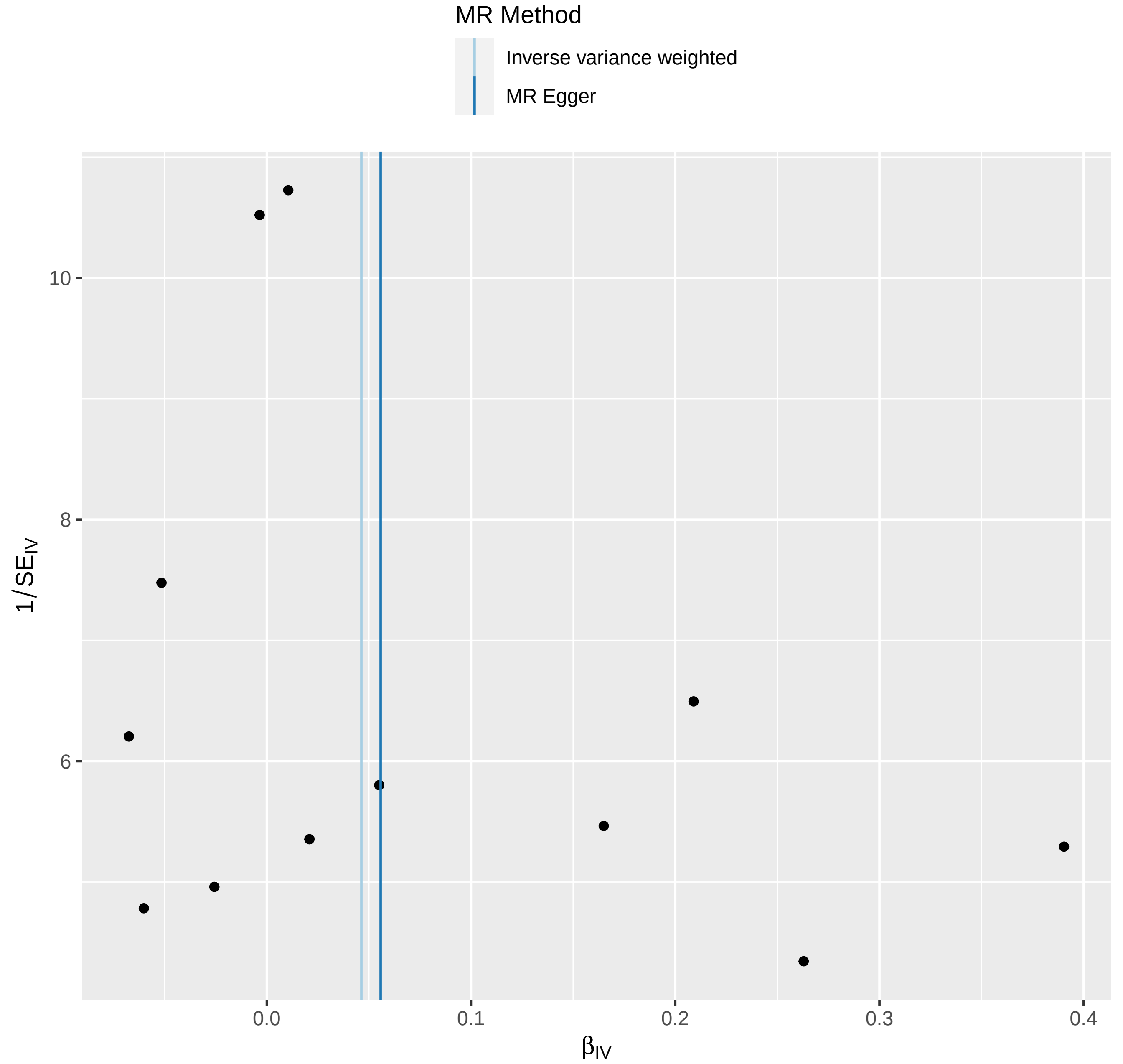

Batch 3−631 : Selenium on Procollagen galactosyltransferase 1

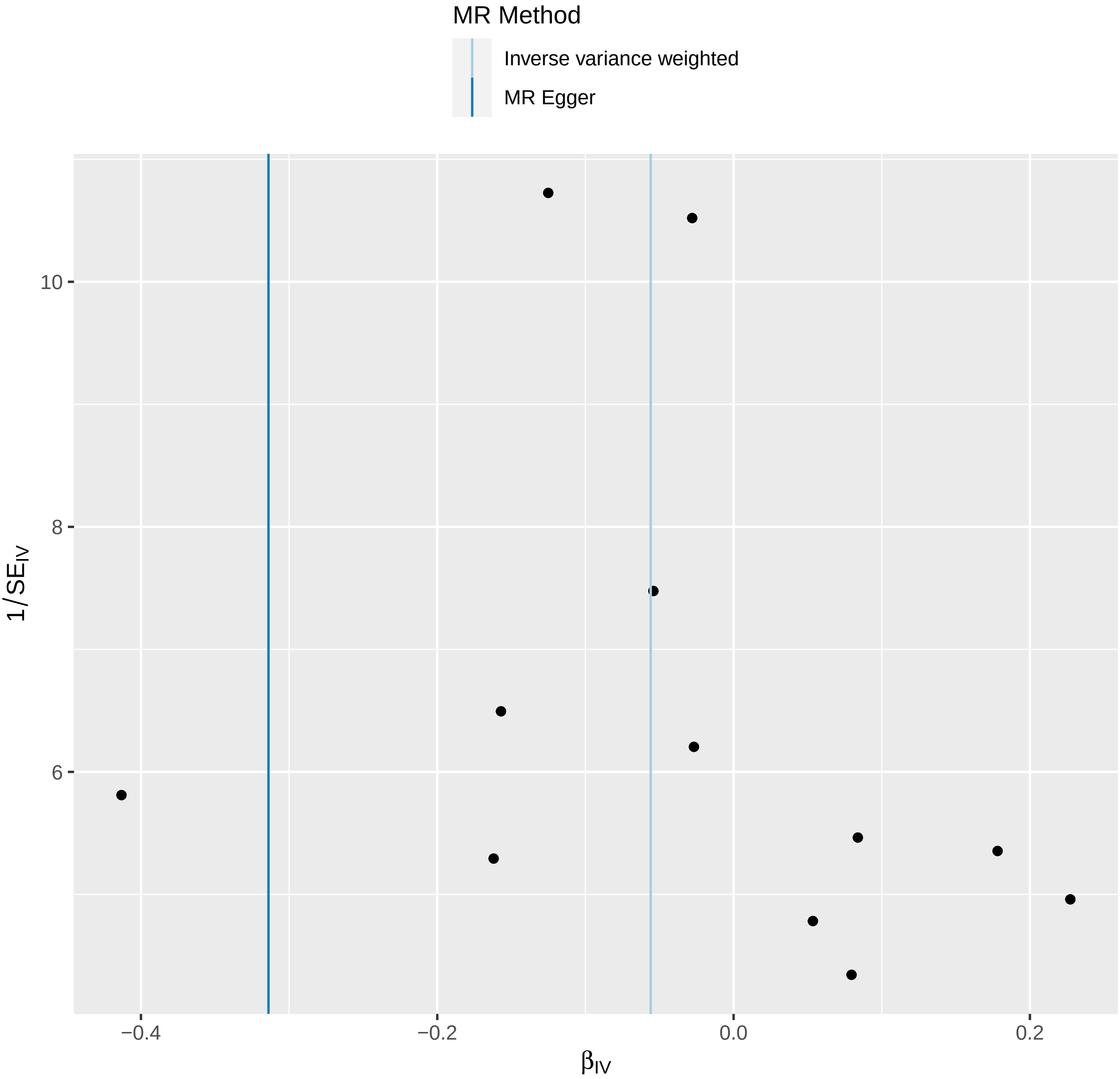

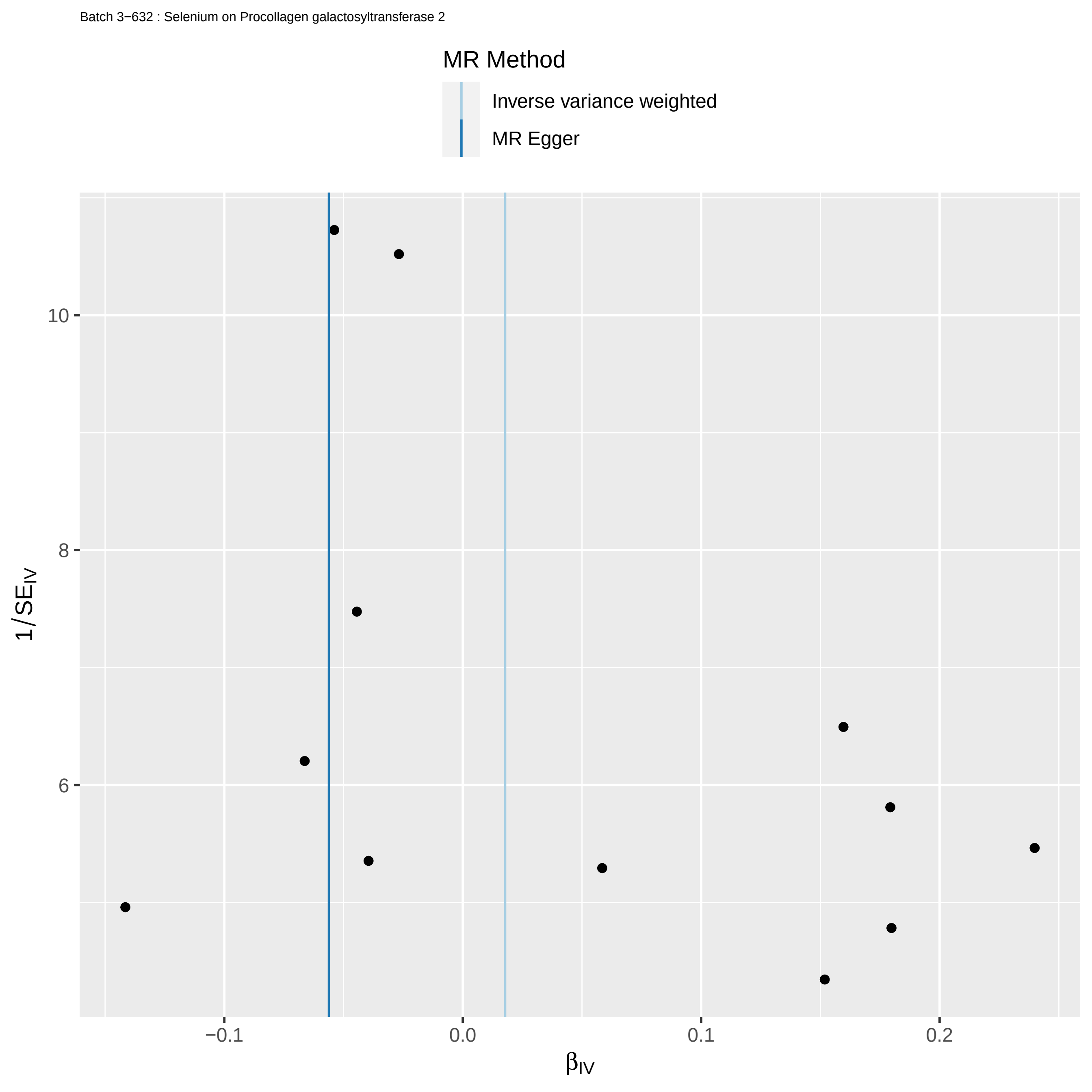

Batch 3−632 : Selenium on Procollagen galactosyltransferase 2
MR Method
Inverse variance weighted
MR Egger
10
8
6
1/SE_IV
−0.1
0.0
0.1
0.2
β_IV

Batch 3−633 : Selenium on COMM domain−containing protein 1

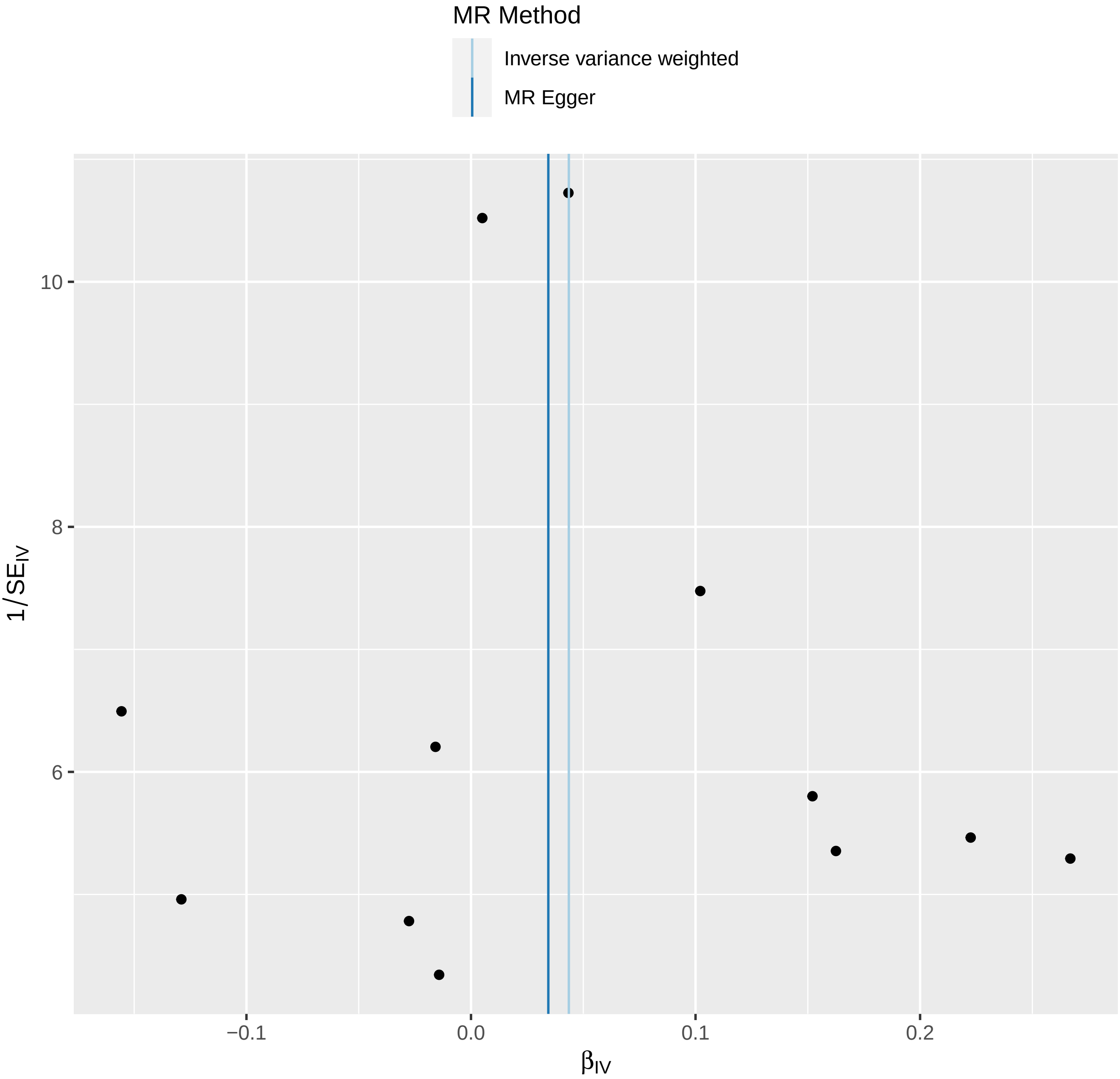

Batch 3−634 : Selenium on COMM domain−containing protein 7

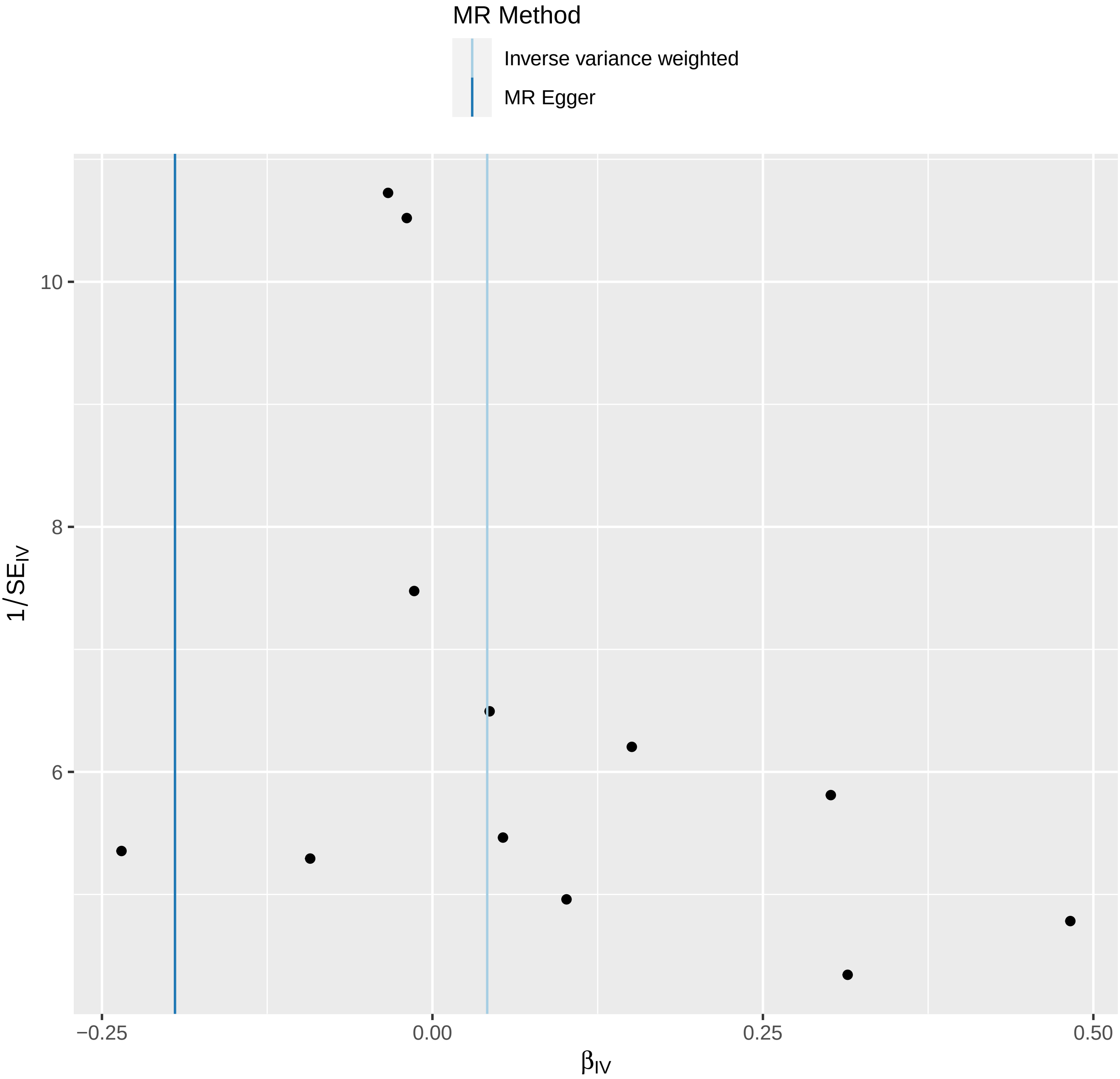

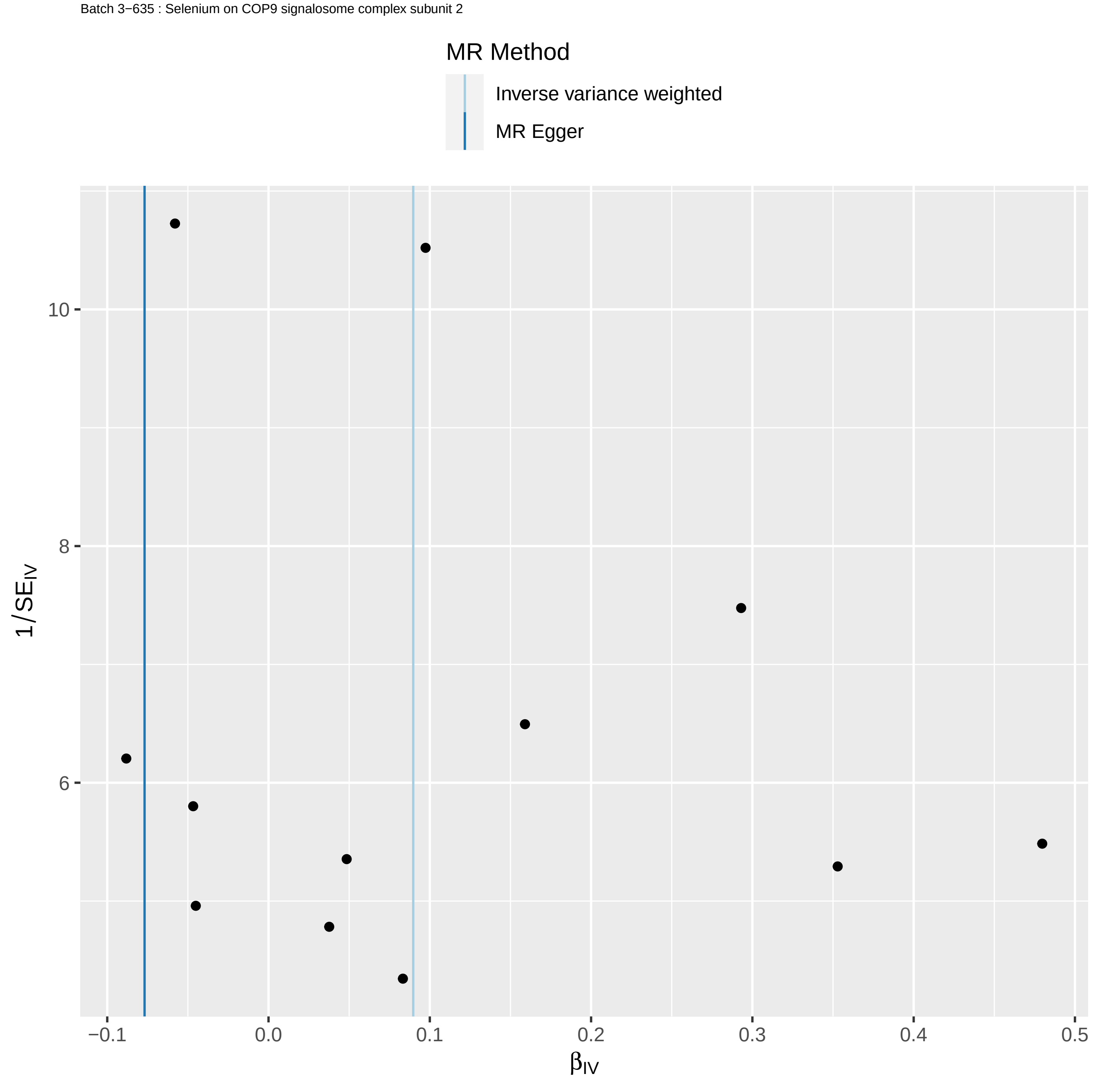
Batch 3−635 : Selenium on COP9 signalosome complex subunit 2
MR Method
Inverse variance weighted
MR Egger
10
8
6
1/SE_IV
−0.1
0.0
0.1
0.2
0.3
0.4
0.5
β_IV

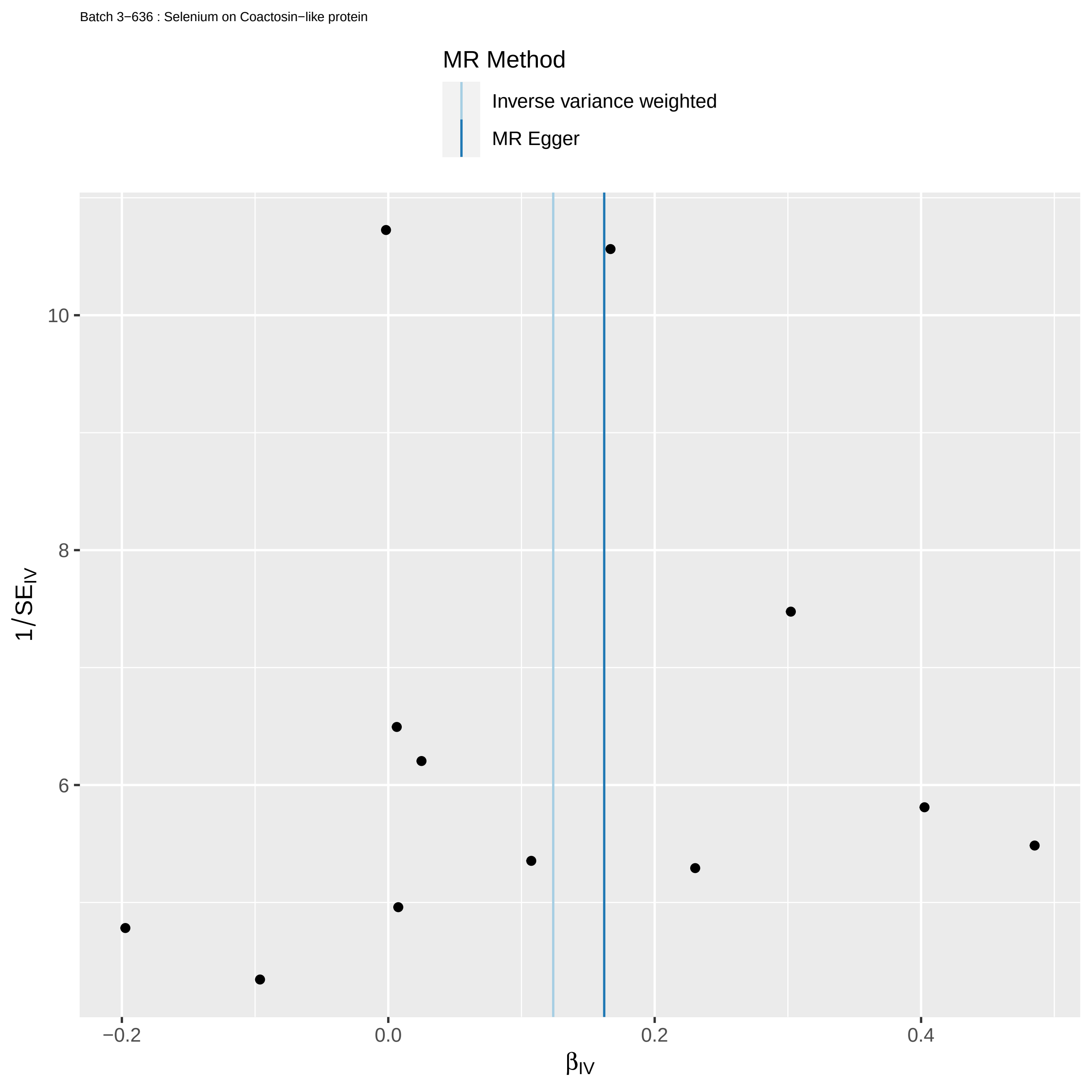

Batch 3−636 : Selenium on Coactosin−like protein
MR Method
Inverse variance weighted
MR Egger
10
8
6
1/SE$_{IV}$
−0.2
0.0
0.2
0.4
β$_{IV}$

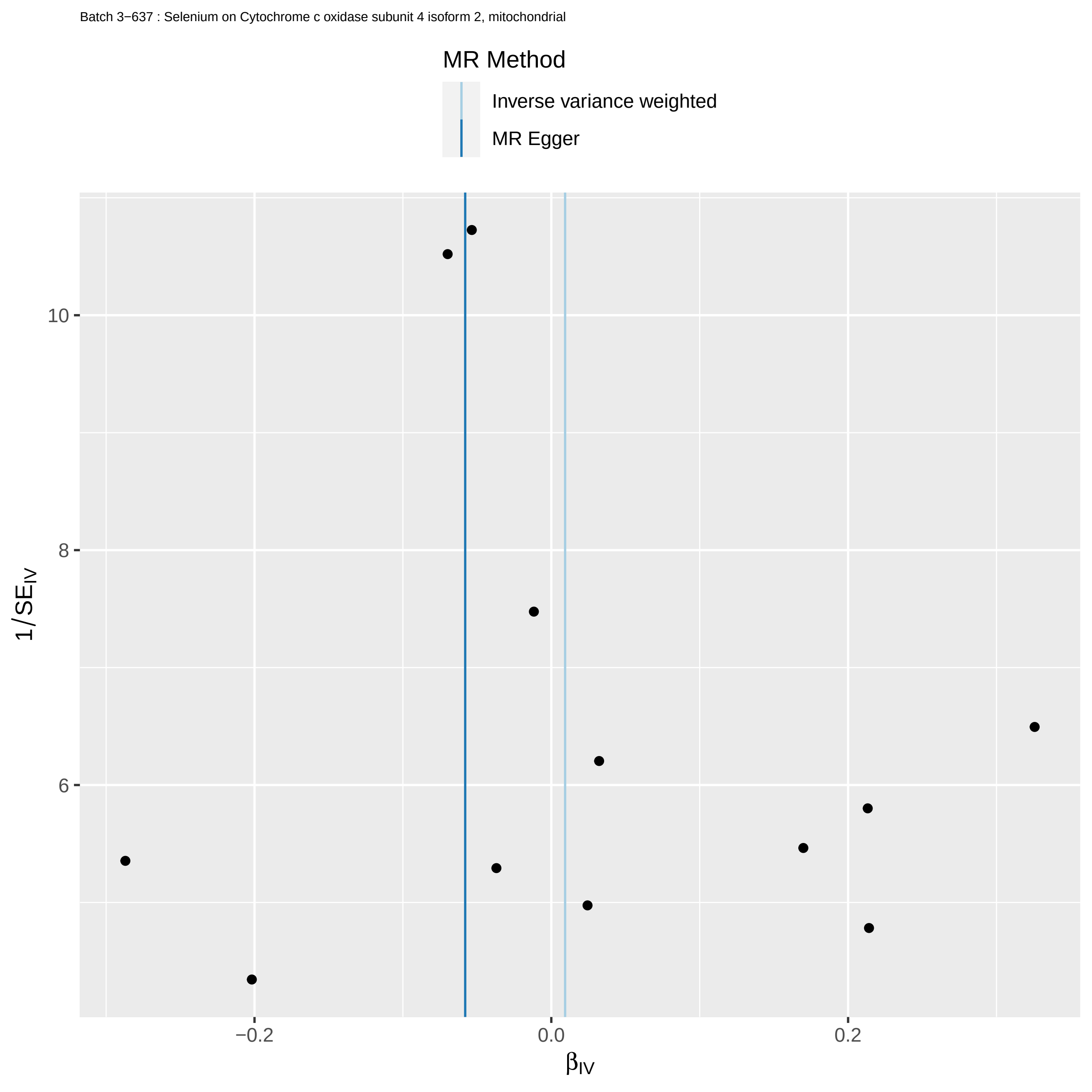

Batch 3−637 : Selenium on Cytochrome c oxidase subunit 4 isoform 2, mitochondrial
MR Method
Inverse variance weighted
MR Egger
10
8
6
1/SE$_{IV}$
−0.2
0.0
0.2
β$_{IV}$

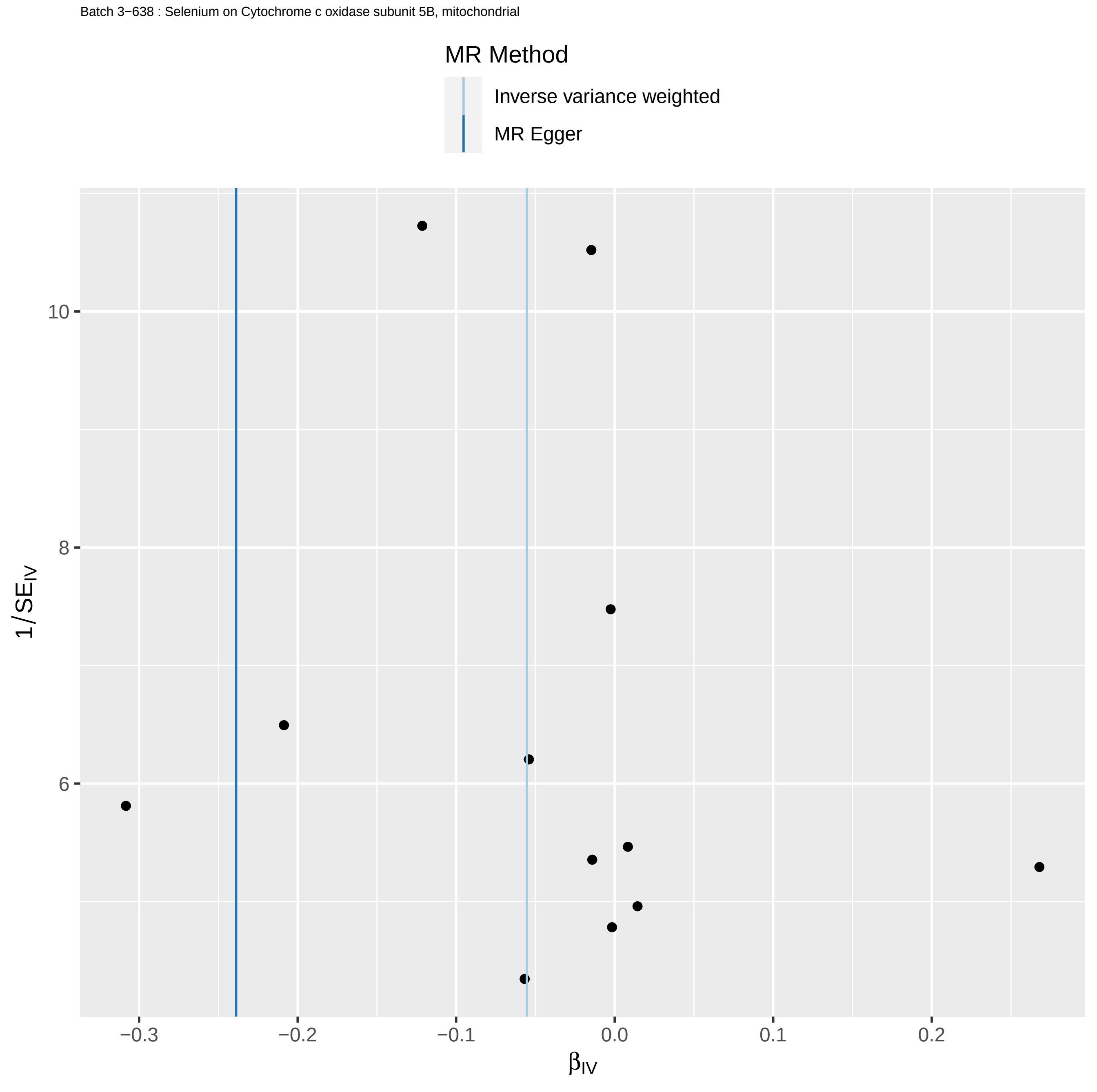

Batch 3−638 : Selenium on Cytochrome c oxidase subunit 5B, mitochondrial
MR Method
Inverse variance weighted
MR Egger
10
8
6
1/SE_IV
−0.3
−0.2
−0.1
0.0
0.1
0.2
β_IV

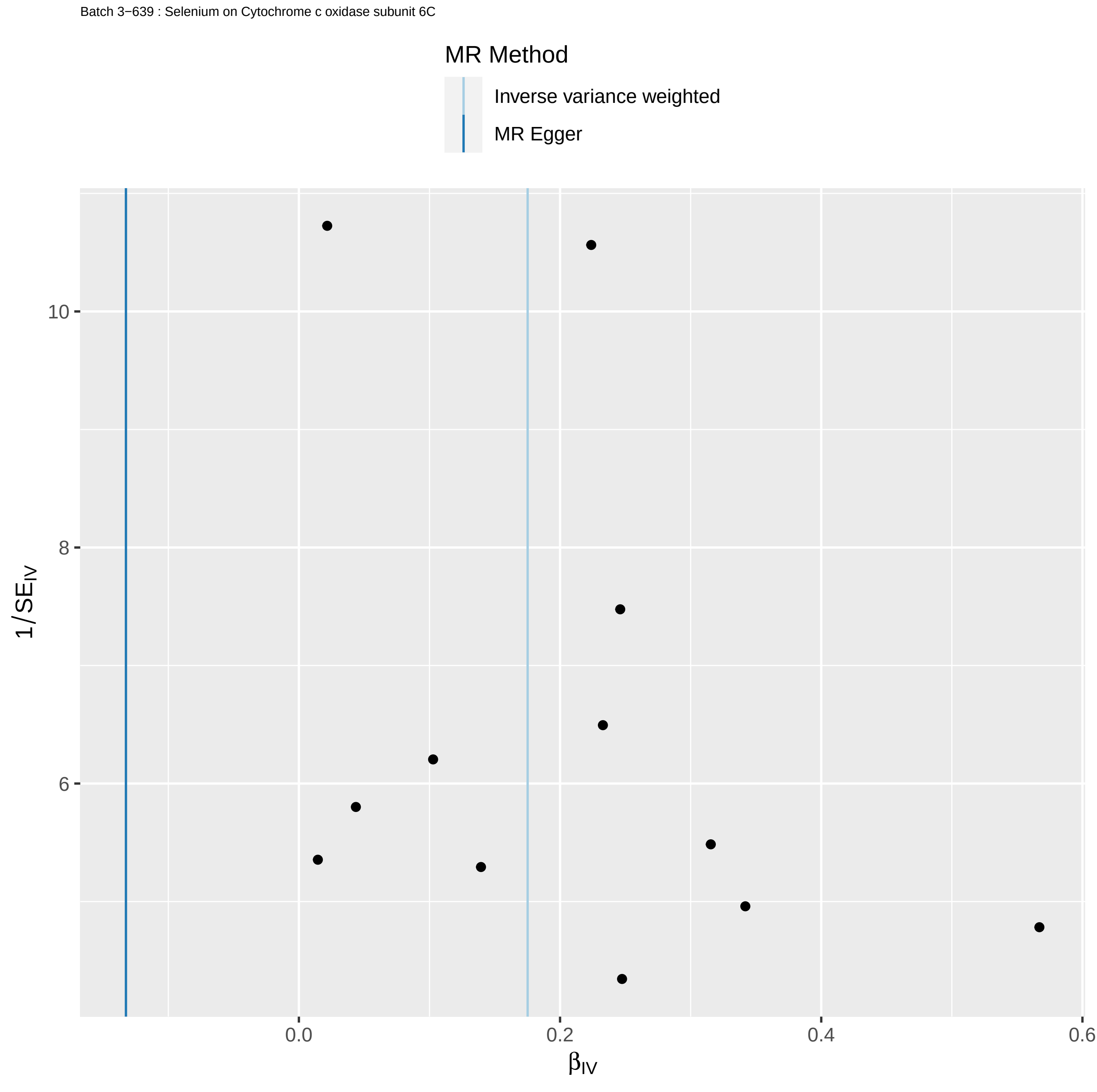

Batch 3−639 : Selenium on Cytochrome c oxidase subunit 6C
MR Method
Inverse variance weighted
MR Egger
10
8
6
$1/SE_{IV}$
0.0
0.2
0.4
0.6
β_{IV}

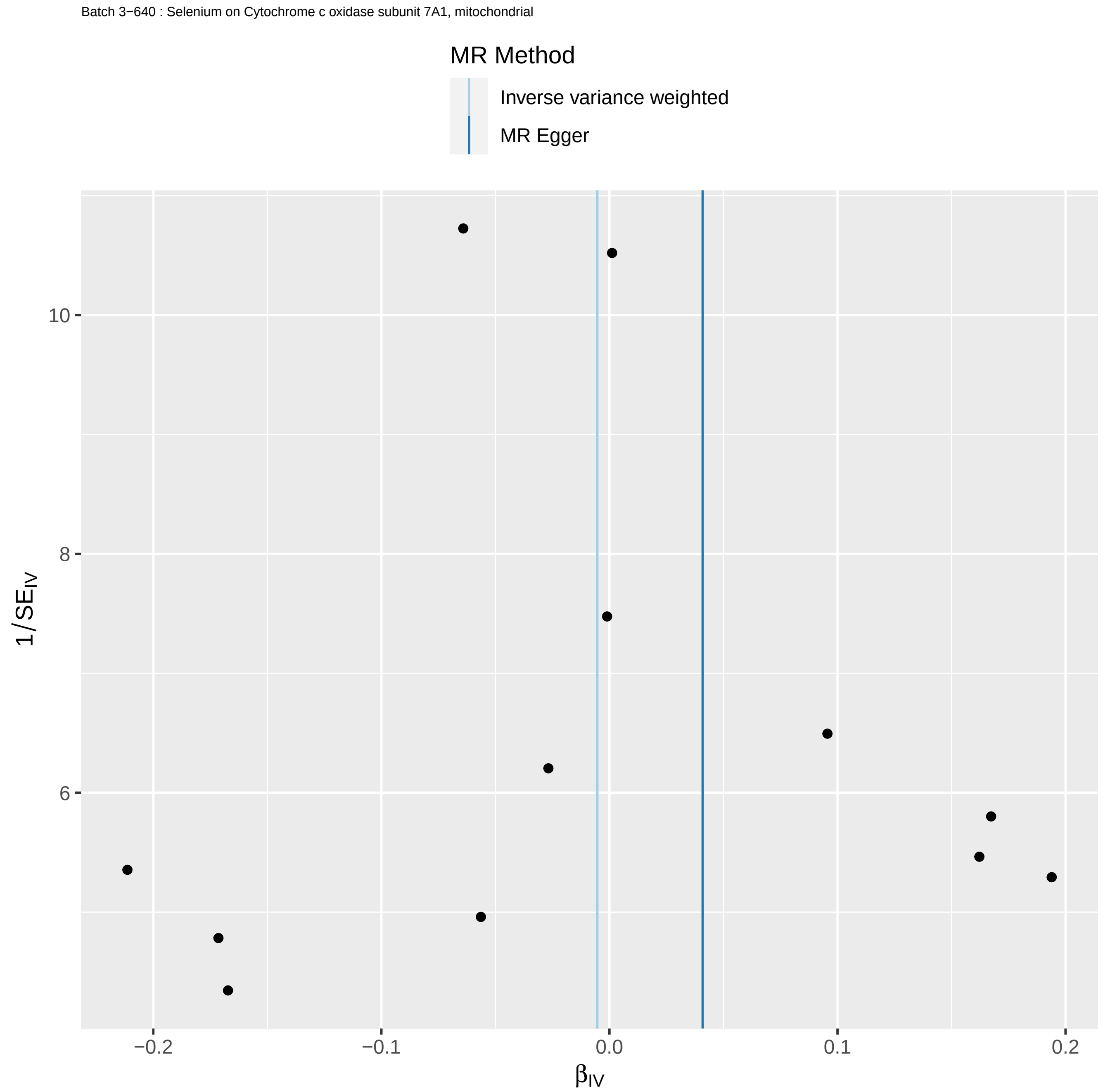

Batch 3−640 : Selenium on Cytochrome c oxidase subunit 7A1, mitochondrial
MR Method
Inverse variance weighted
MR Egger
10
8
6
1/SE$_{IV}$
−0.2
−0.1
0.0
0.1
0.2
β$_{IV}$

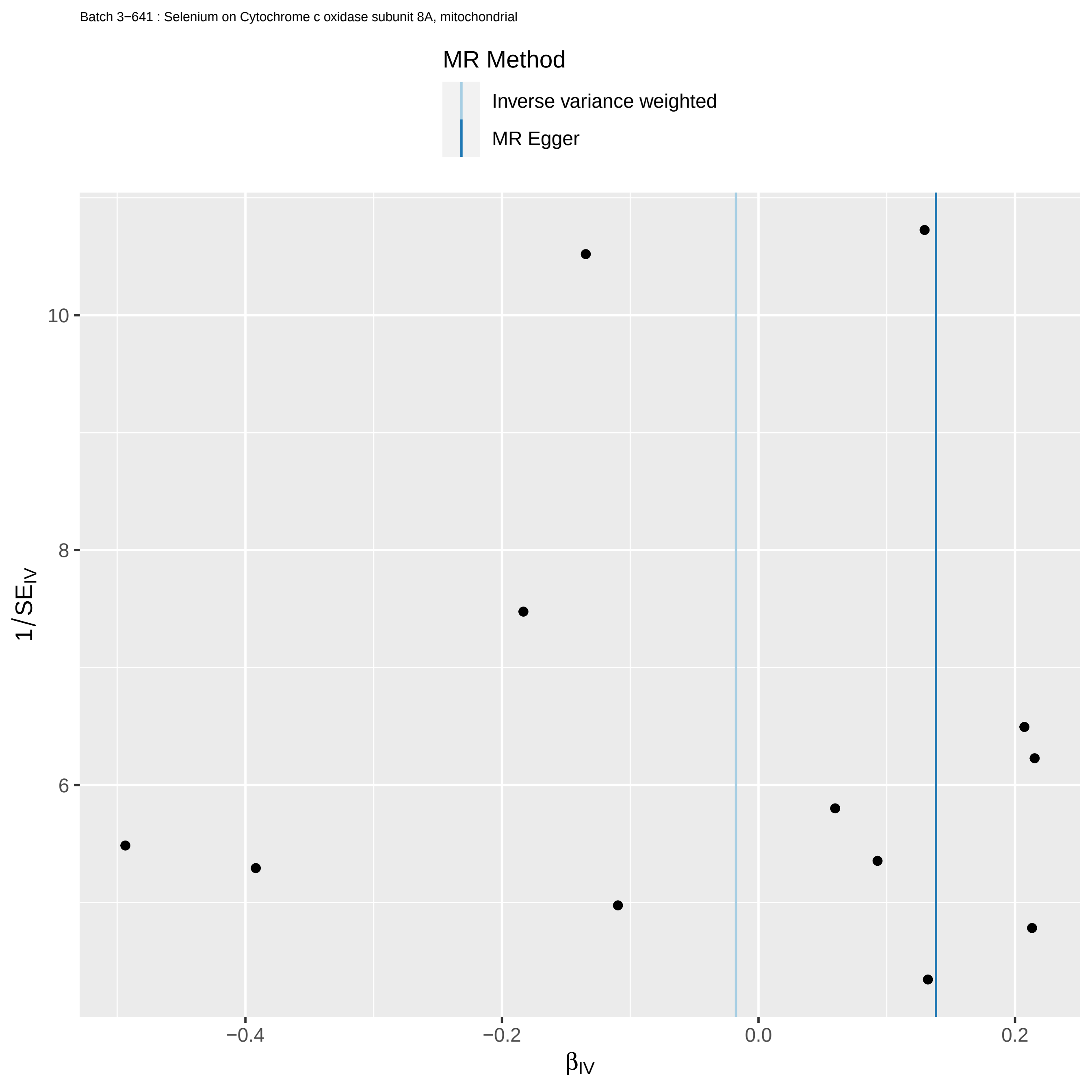

Batch 3−641 : Selenium on Cytochrome c oxidase subunit 8A, mitochondrial
MR Method
Inverse variance weighted
MR Egger
10
8
6
$1/SE_{IV}$
−0.4
−0.2
0.0
0.2
β_{IV}

Batch 3−642 : Selenium on Carboxypeptidase A2

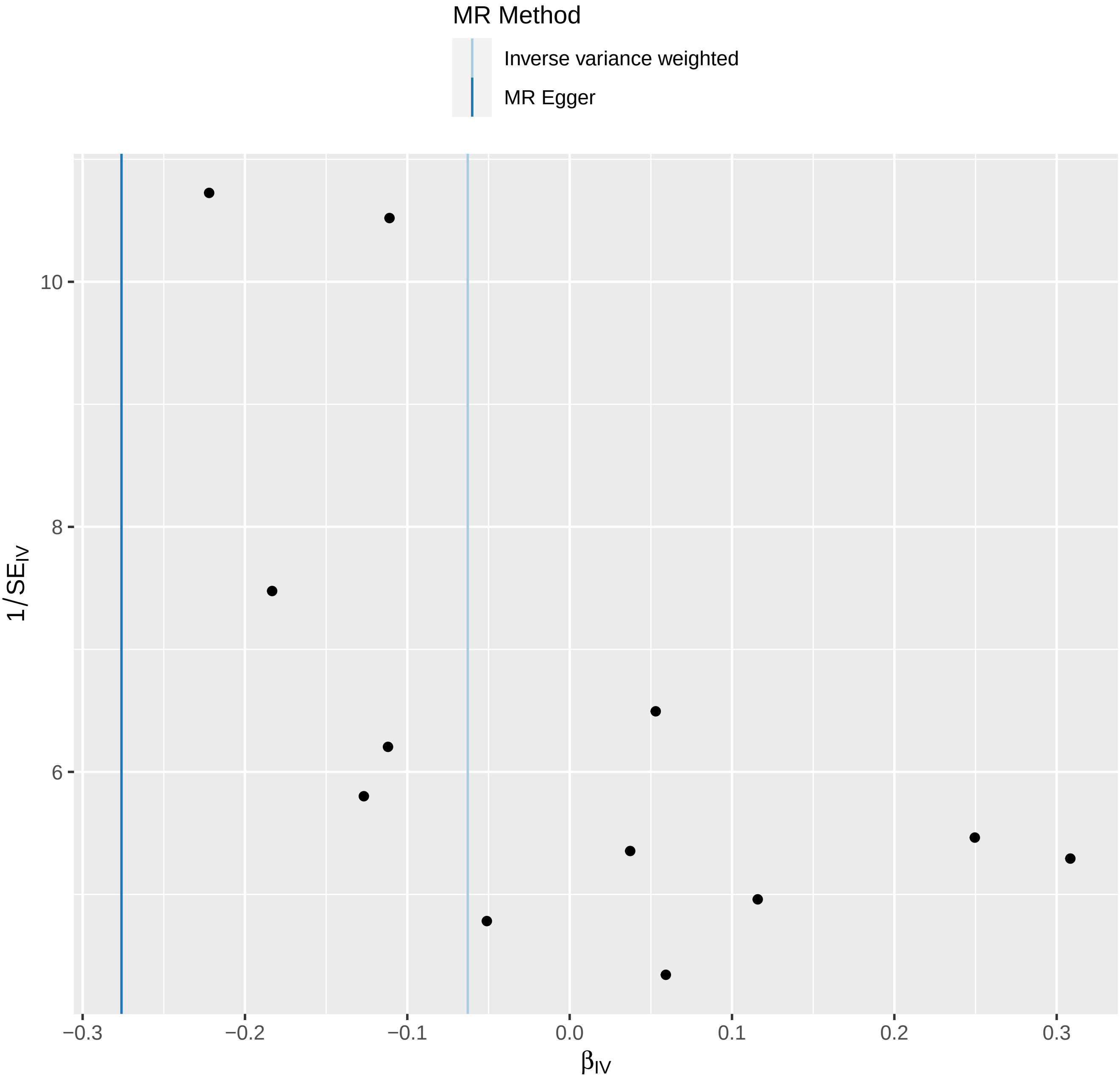

Batch 3−643 : Selenium on Carboxypeptidase A4

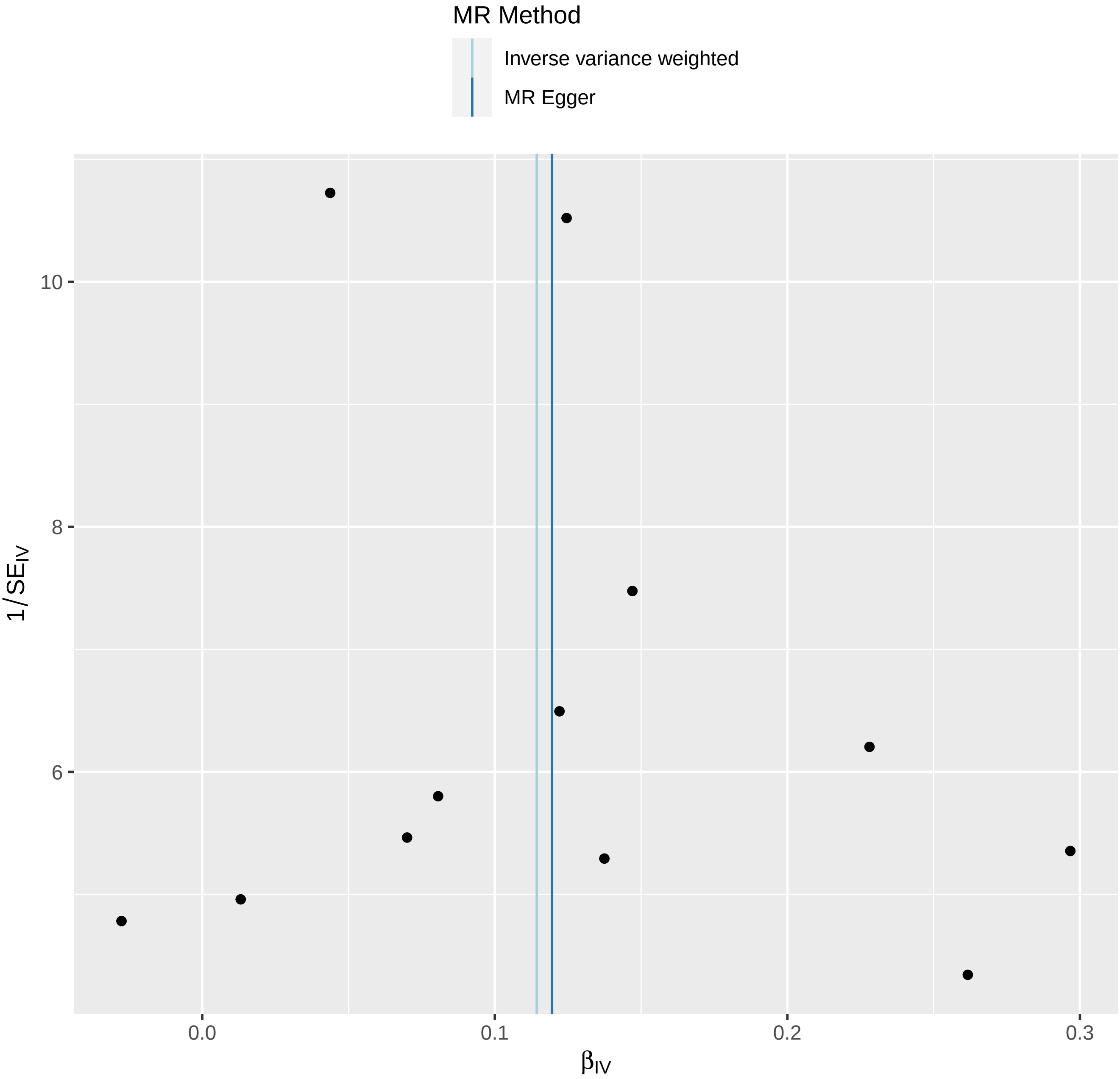

Batch 3−644 : Selenium on Carboxypeptidase B

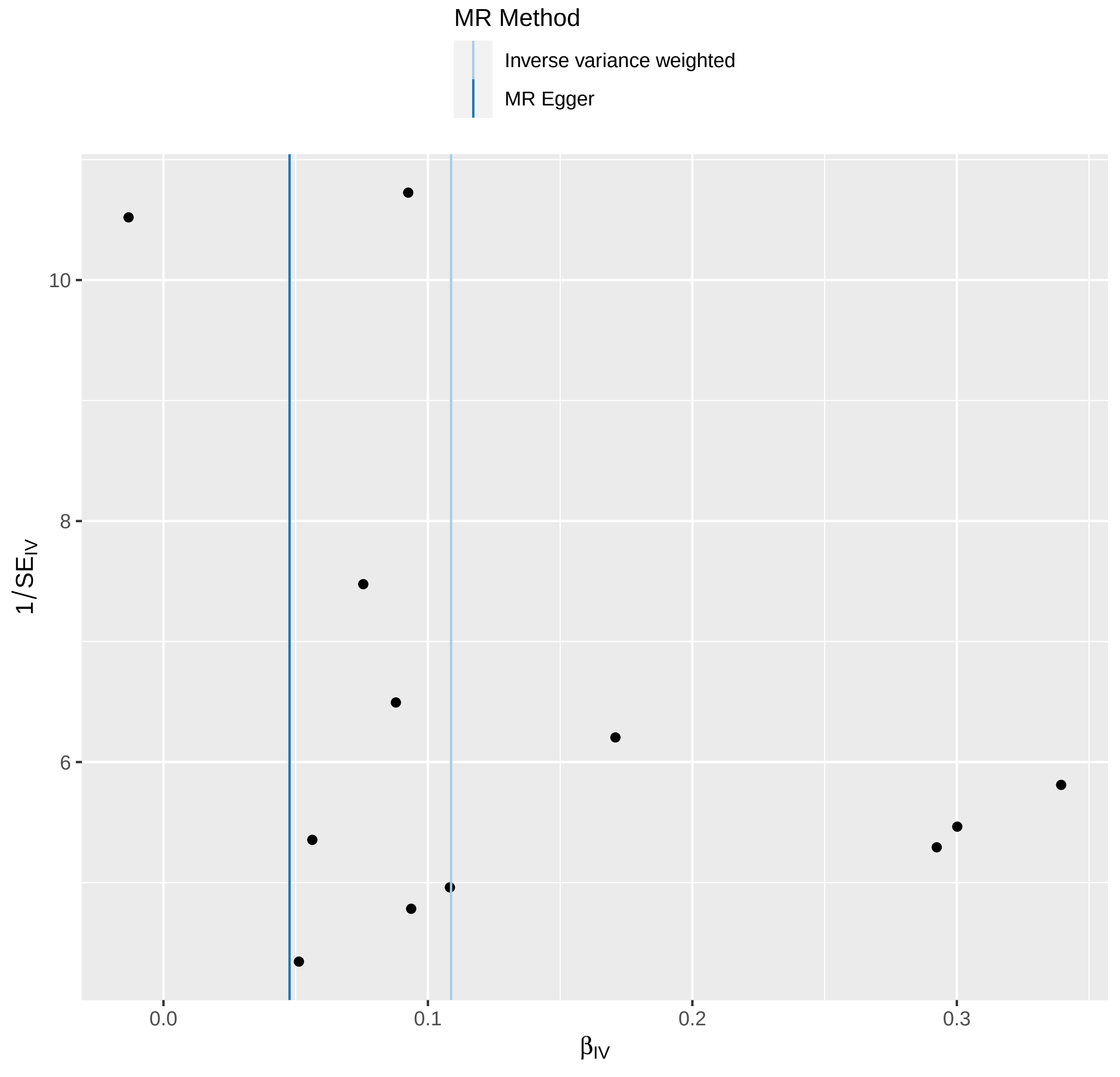

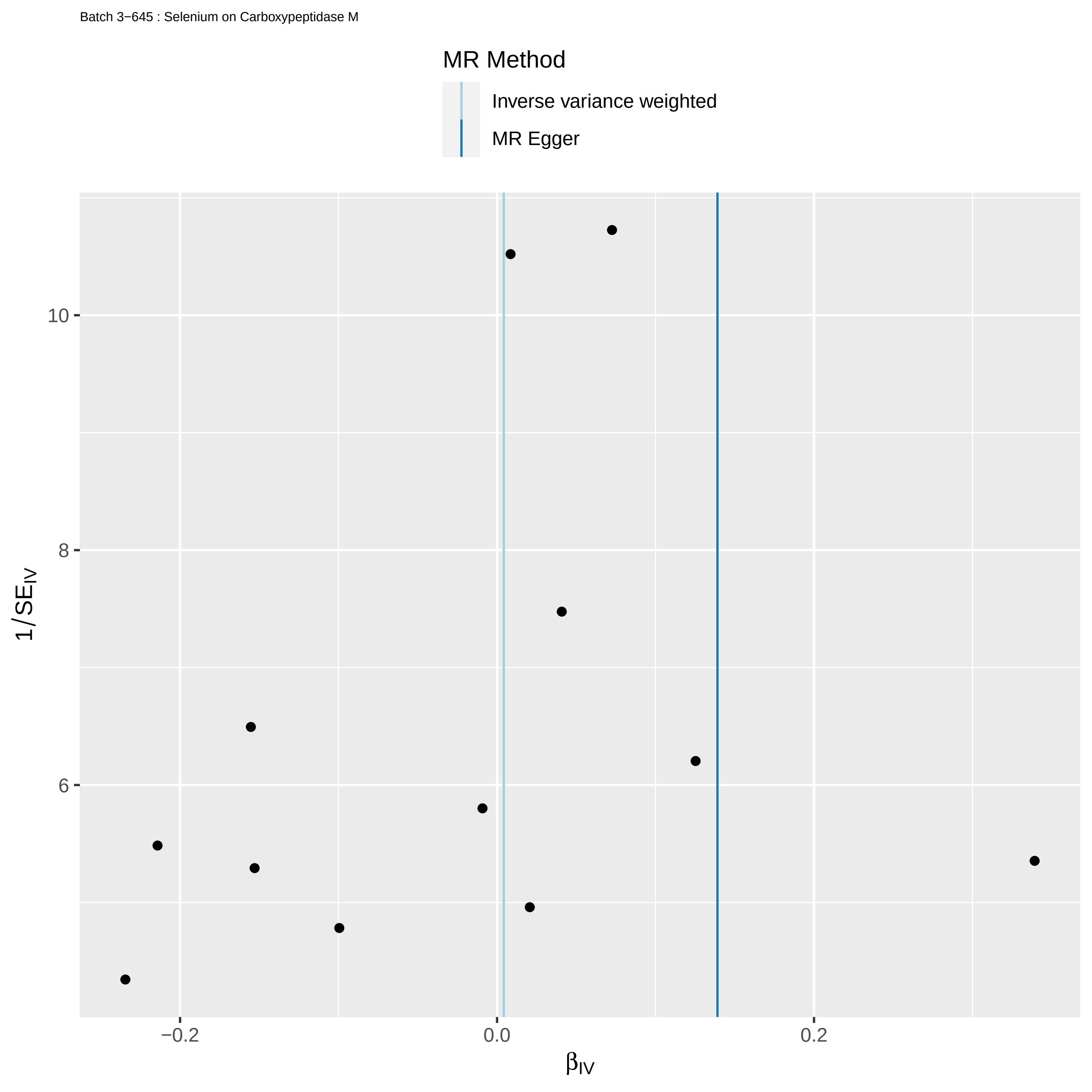

Batch 3−645 : Selenium on Carboxypeptidase M
MR Method
Inverse variance weighted
MR Egger
10
8
6
1/SE$_{IV}$
−0.2
0.0
0.2
β$_{IV}$

Batch 3−646 : Selenium on Copine−1

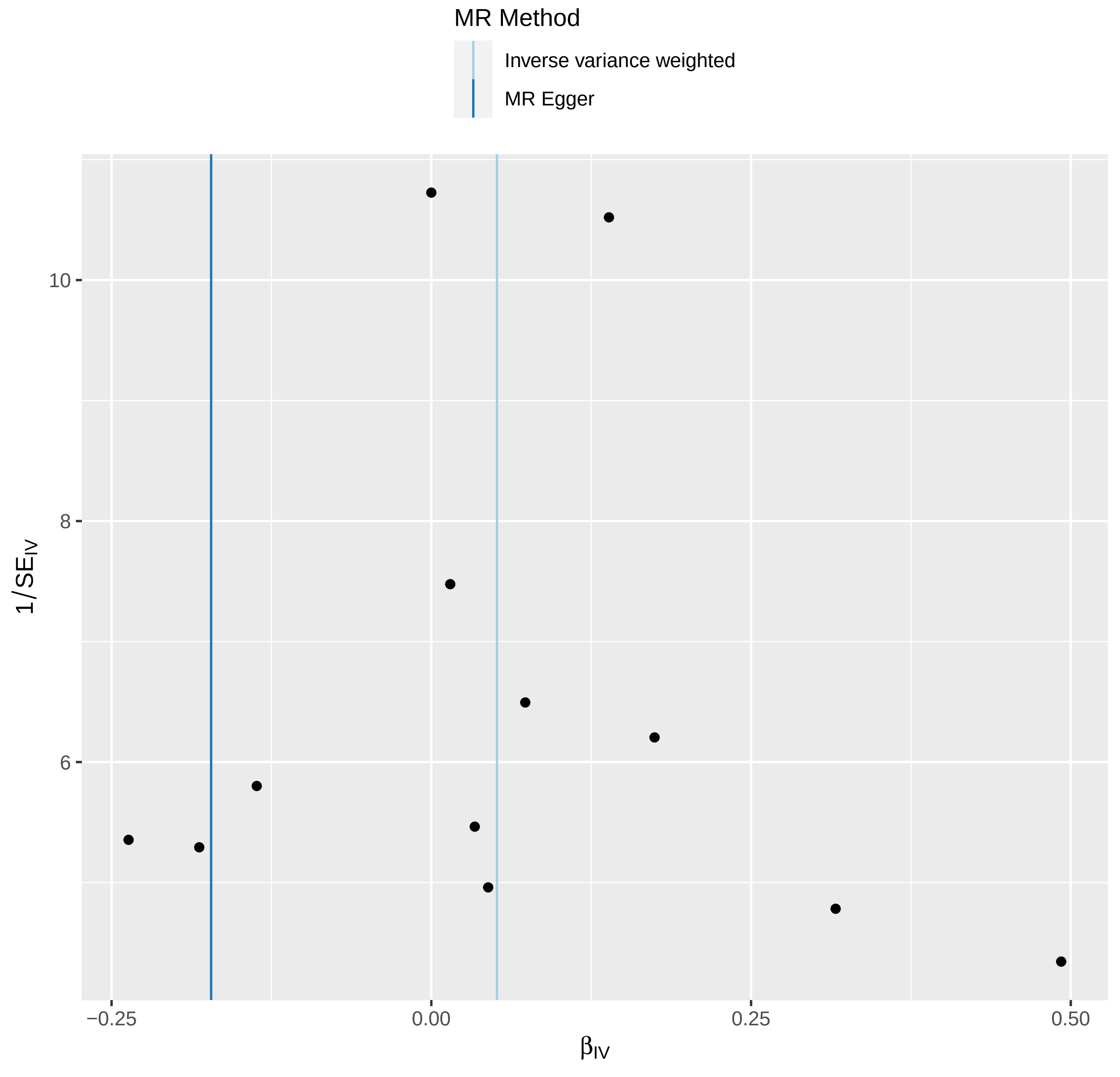

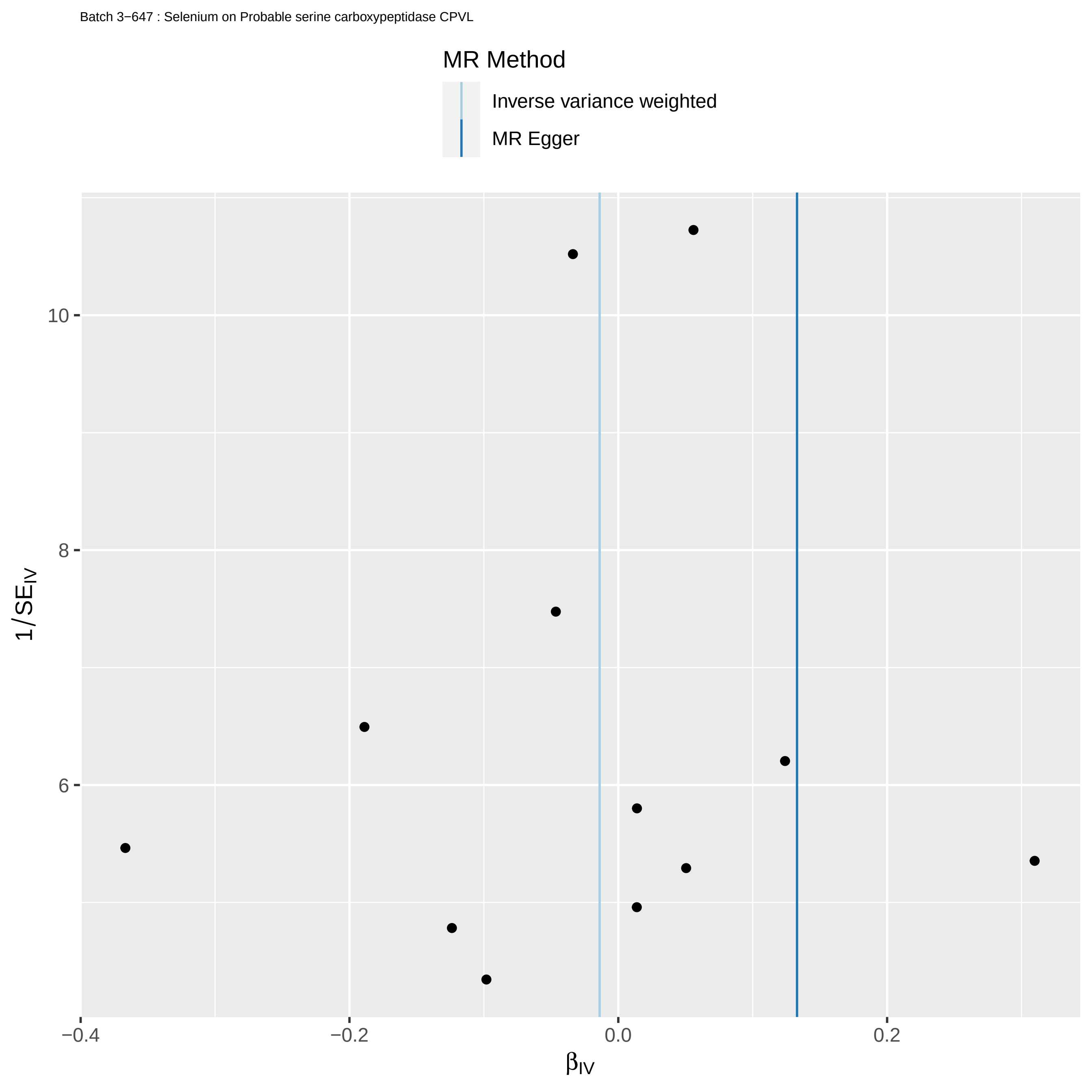
Batch 3−647 : Selenium on Probable serine carboxypeptidase CPVL
MR Method
Inverse variance weighted
MR Egger
10
8
6
1/SE_IV
−0.4
−0.2
0.0
0.2
β_IV

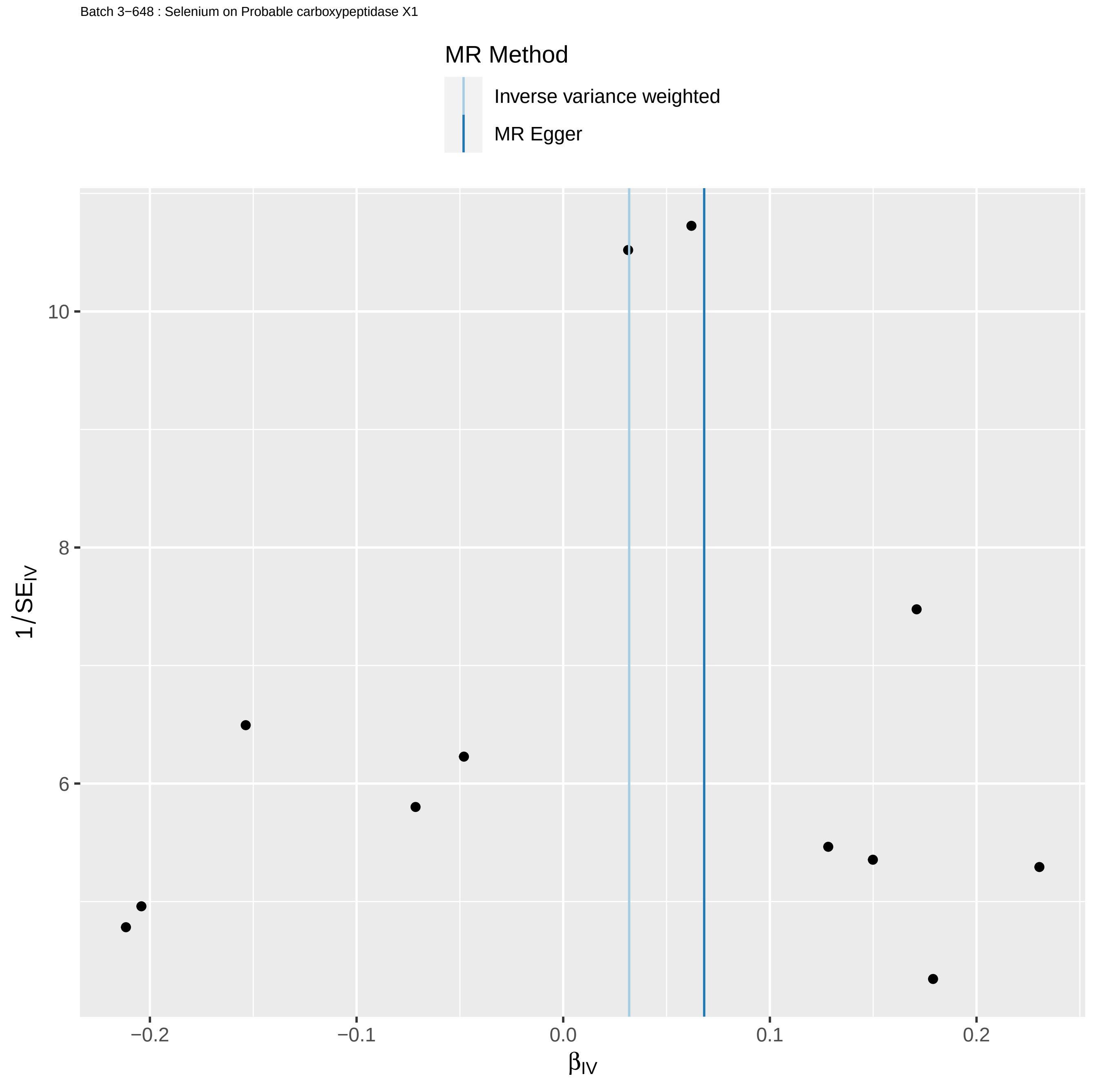
Batch 3−648 : Selenium on Probable carboxypeptidase X1
MR Method
Inverse variance weighted
MR Egger
10
8
6
$1/SE_{IV}$
−0.2
−0.1
0.0
0.1
0.2
β_{IV}

Batch 3−649 : Selenium on Carboxypeptidase Z

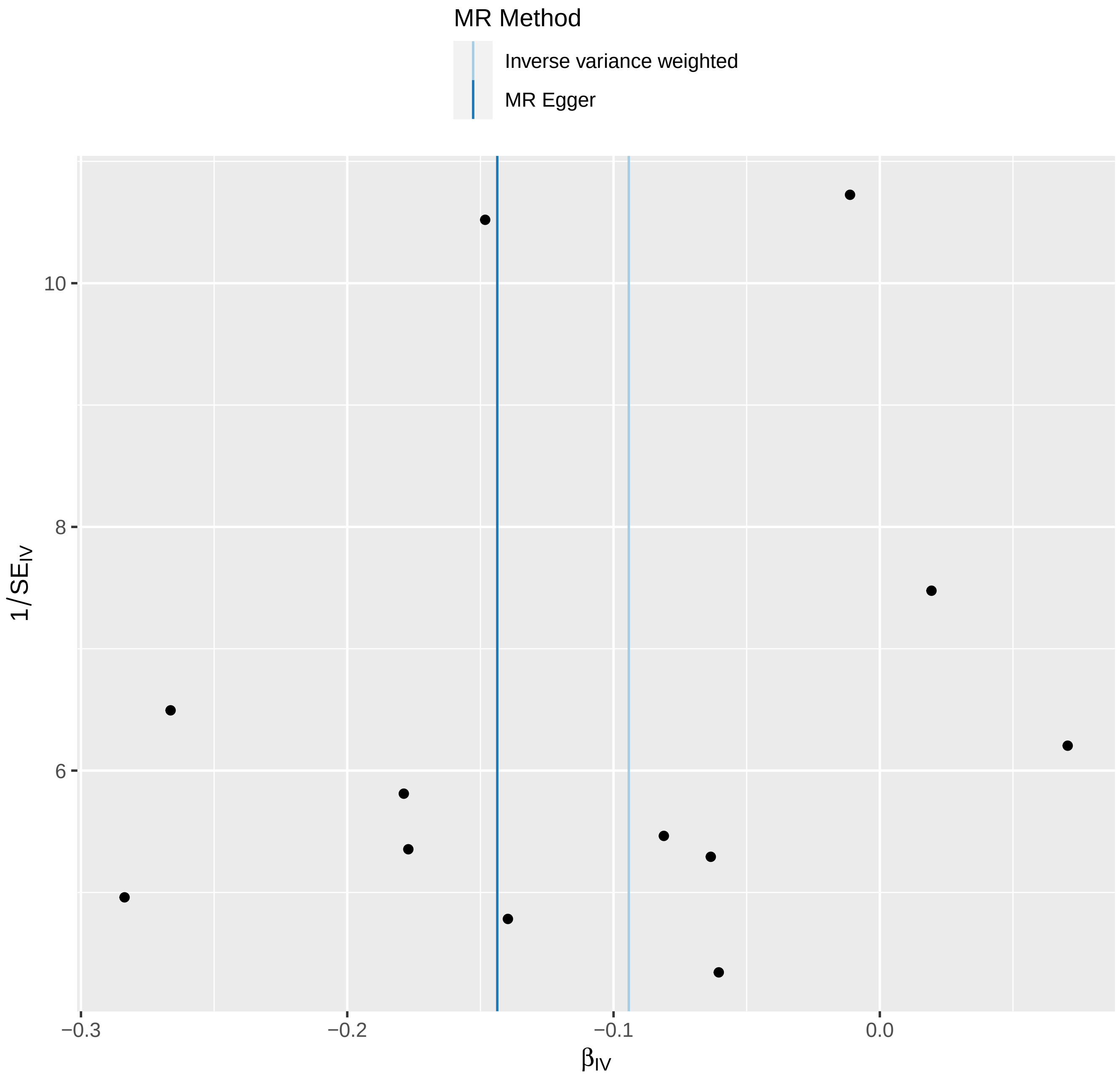

Batch 3−650 : Selenium on Cellular retinoic acid−binding protein 1

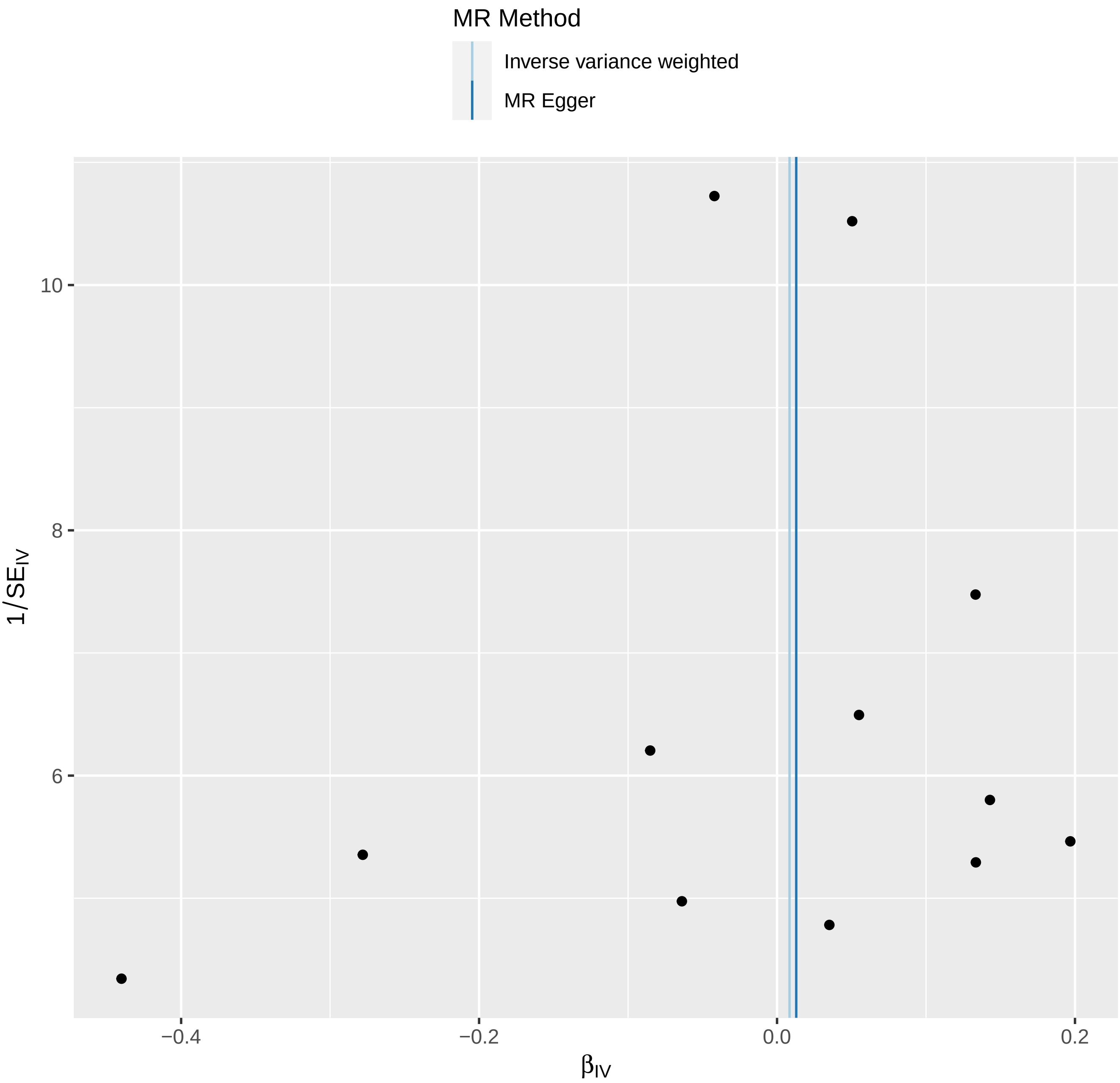

Batch 3−651 : Selenium on Cellular retinoic acid−binding protein 2

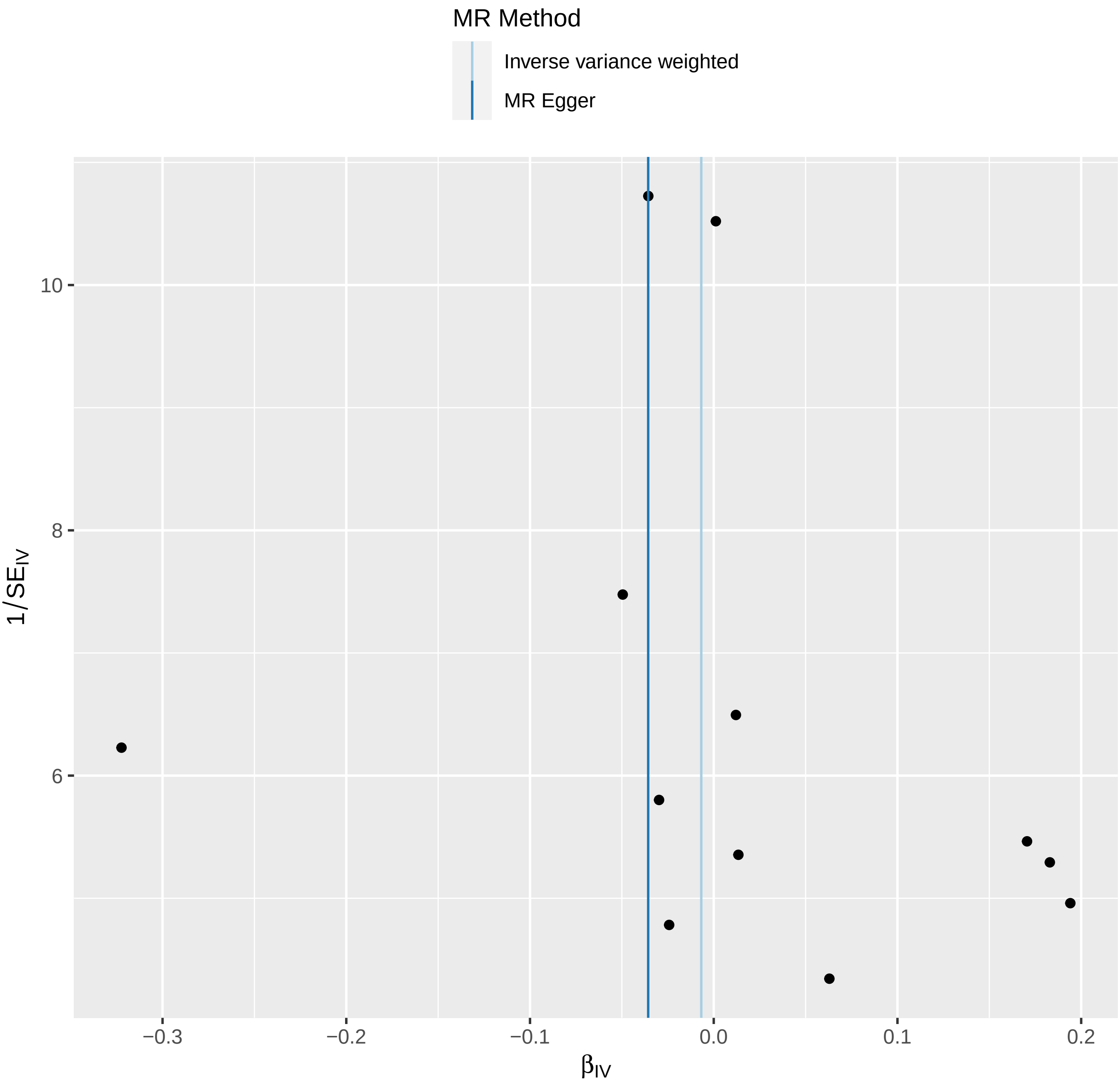

Batch 3−652 : Selenium on Protein crumbs homolog 1

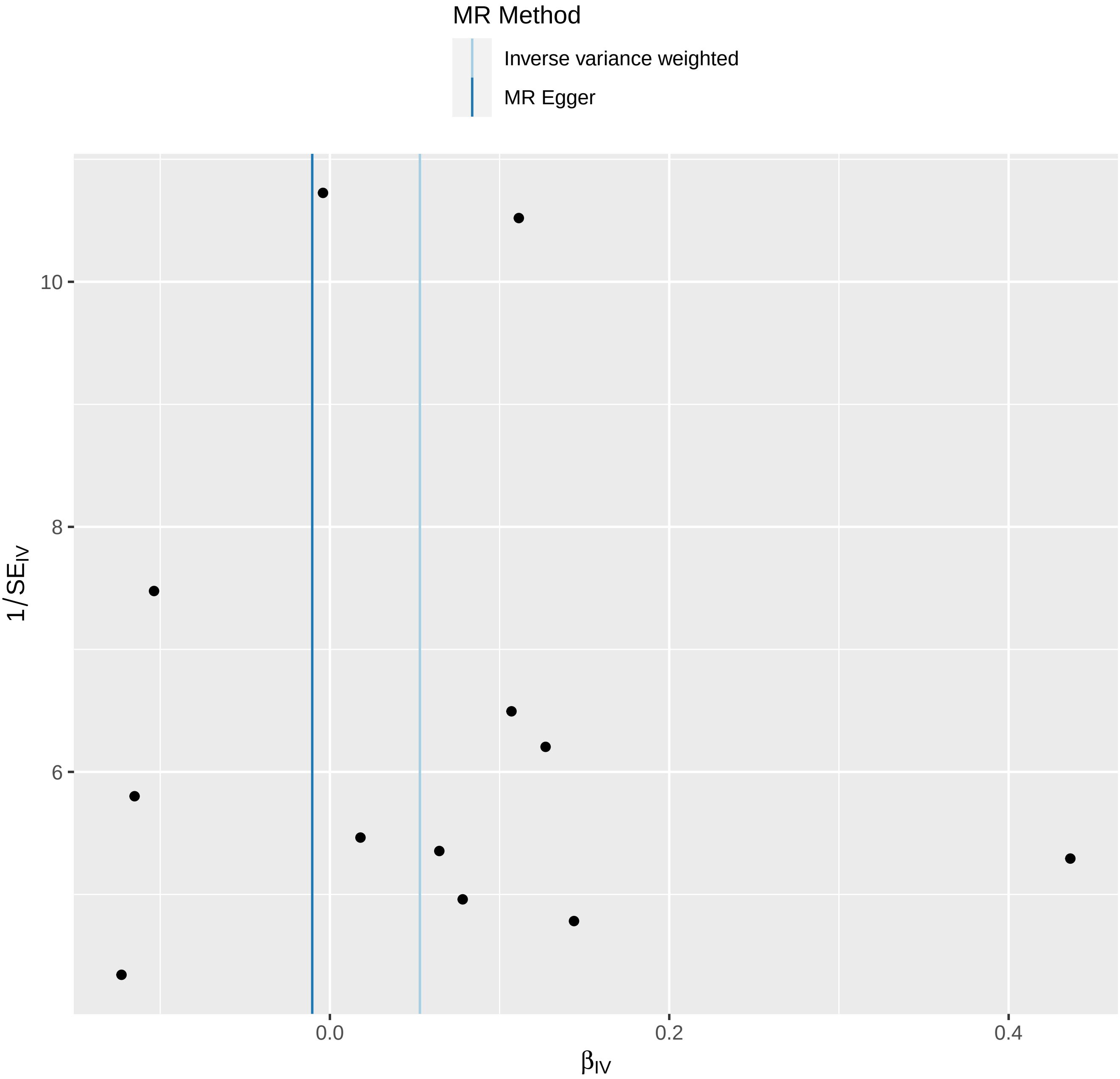

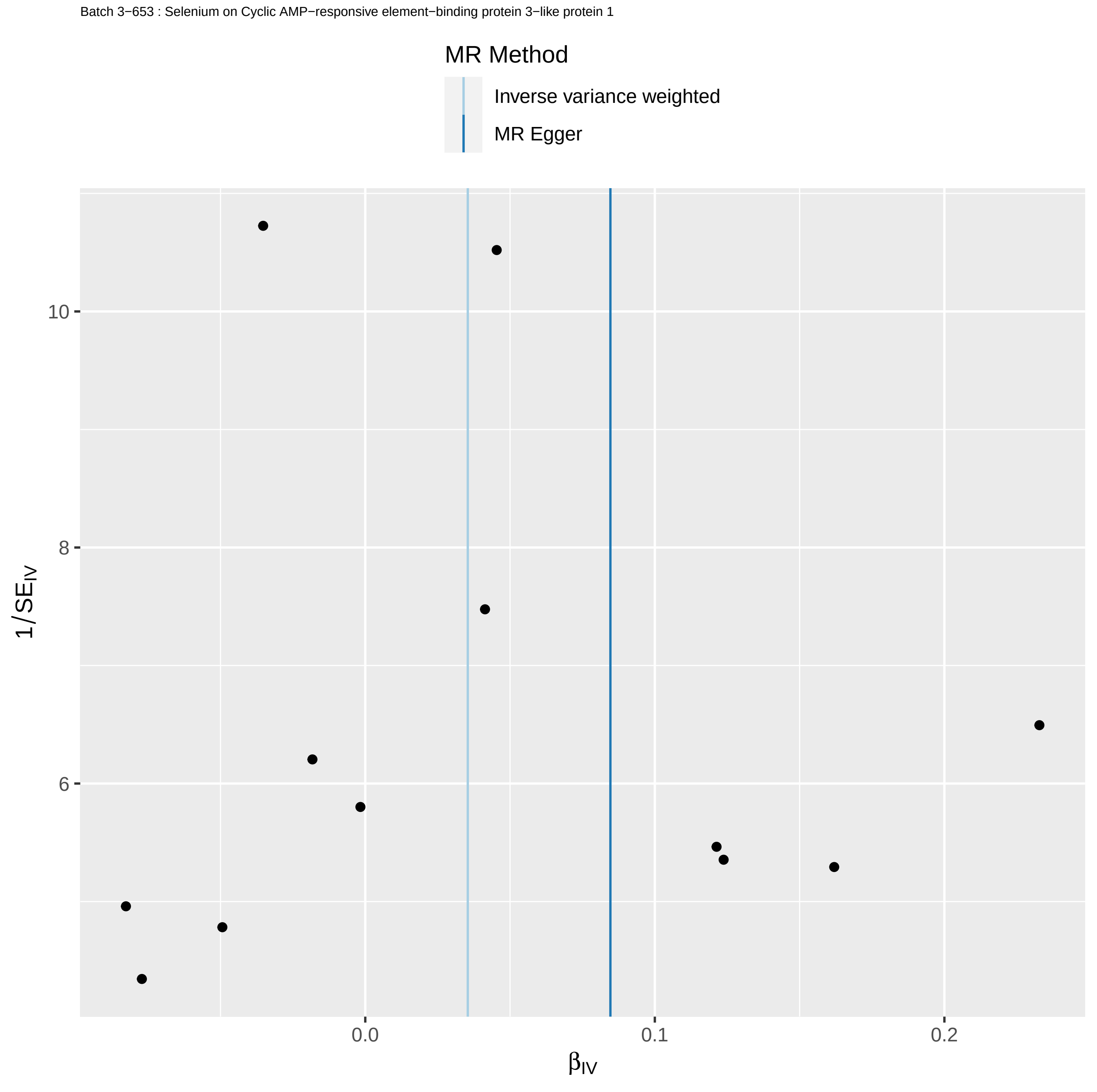

Batch 3−653 : Selenium on Cyclic AMP−responsive element−binding protein 3−like protein 1
MR Method
Inverse variance weighted
MR Egger
10
8
6
1/SE_IV
0.0
0.1
0.2
β_IV

Batch 3−654 : Selenium on Cyclic AMP−responsive element−binding protein 3−like protein 4

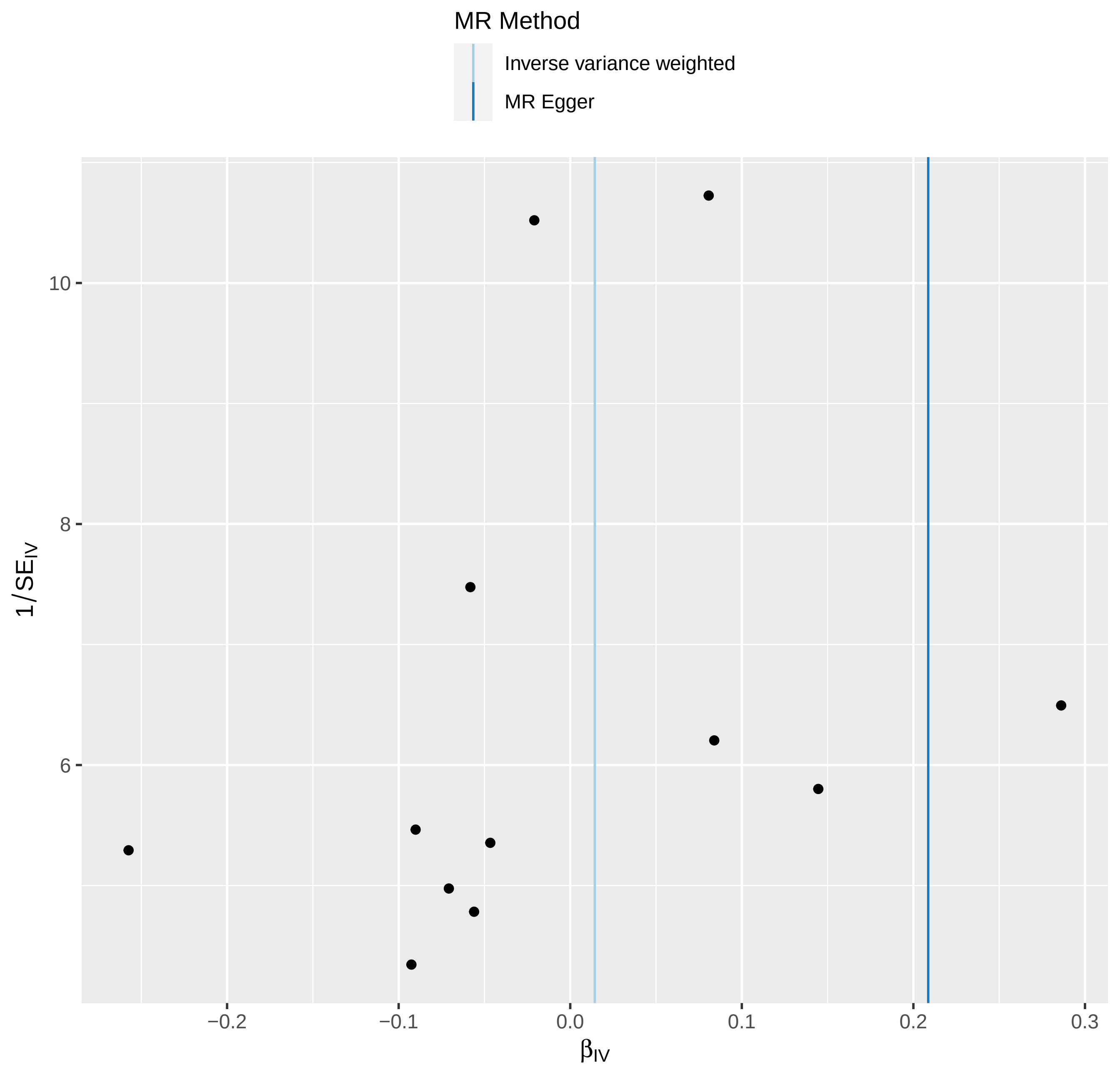

Batch 3−655 : Selenium on CREB−binding protein

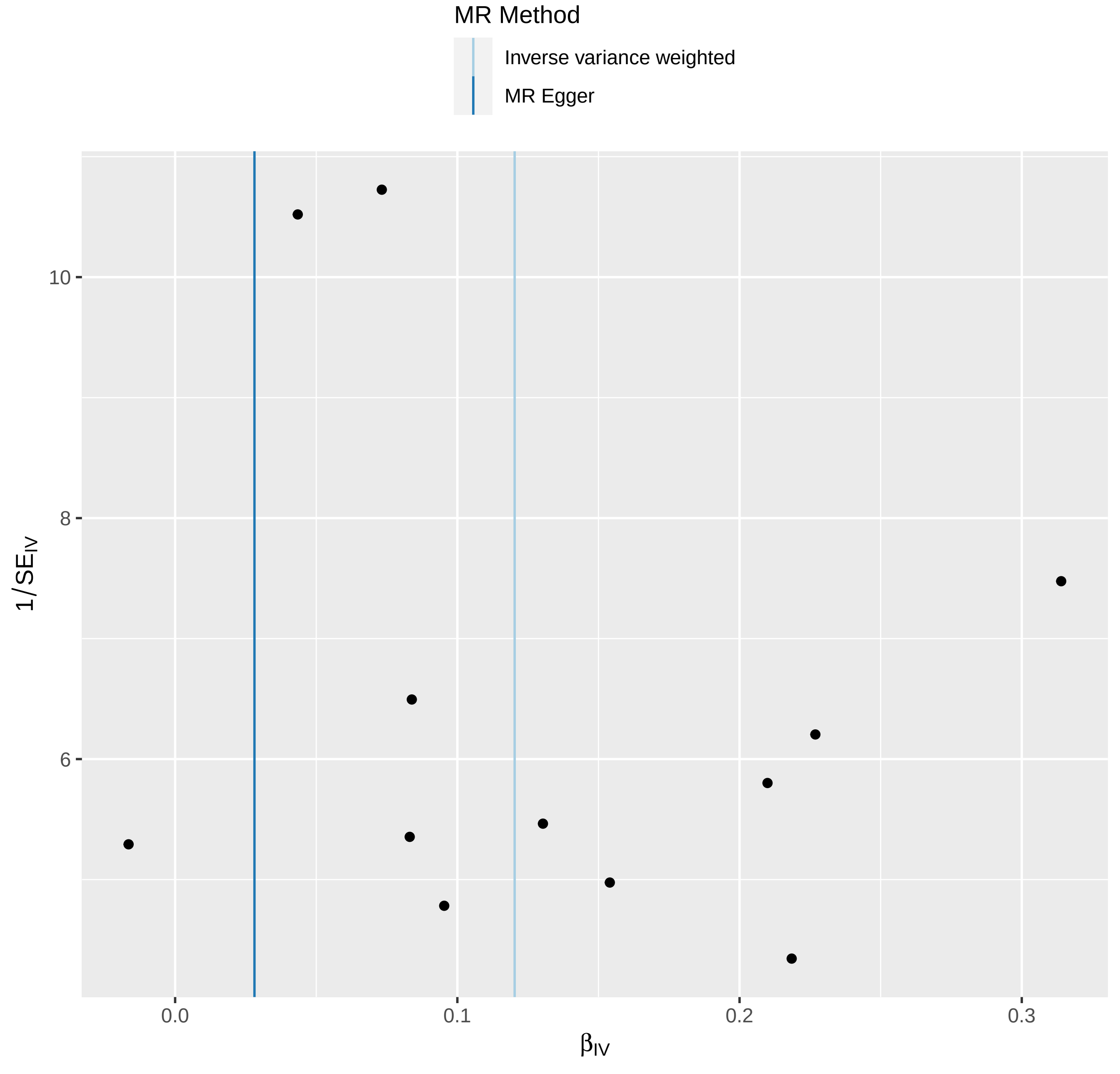

Batch 3−656 : Selenium on Protein CREG1

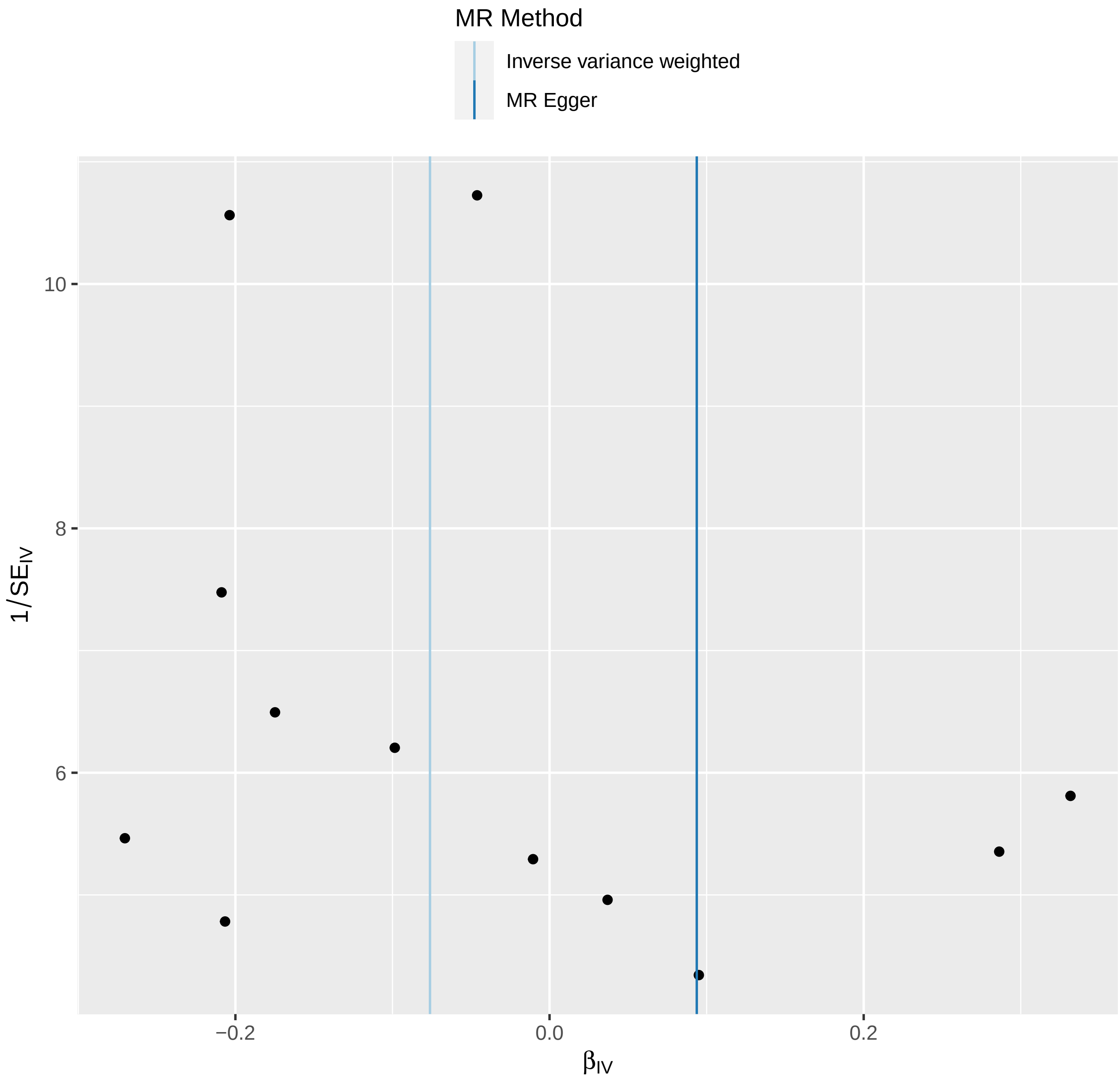

Batch 3−657 : Selenium on Cysteine−rich with EGF−like domain protein 1

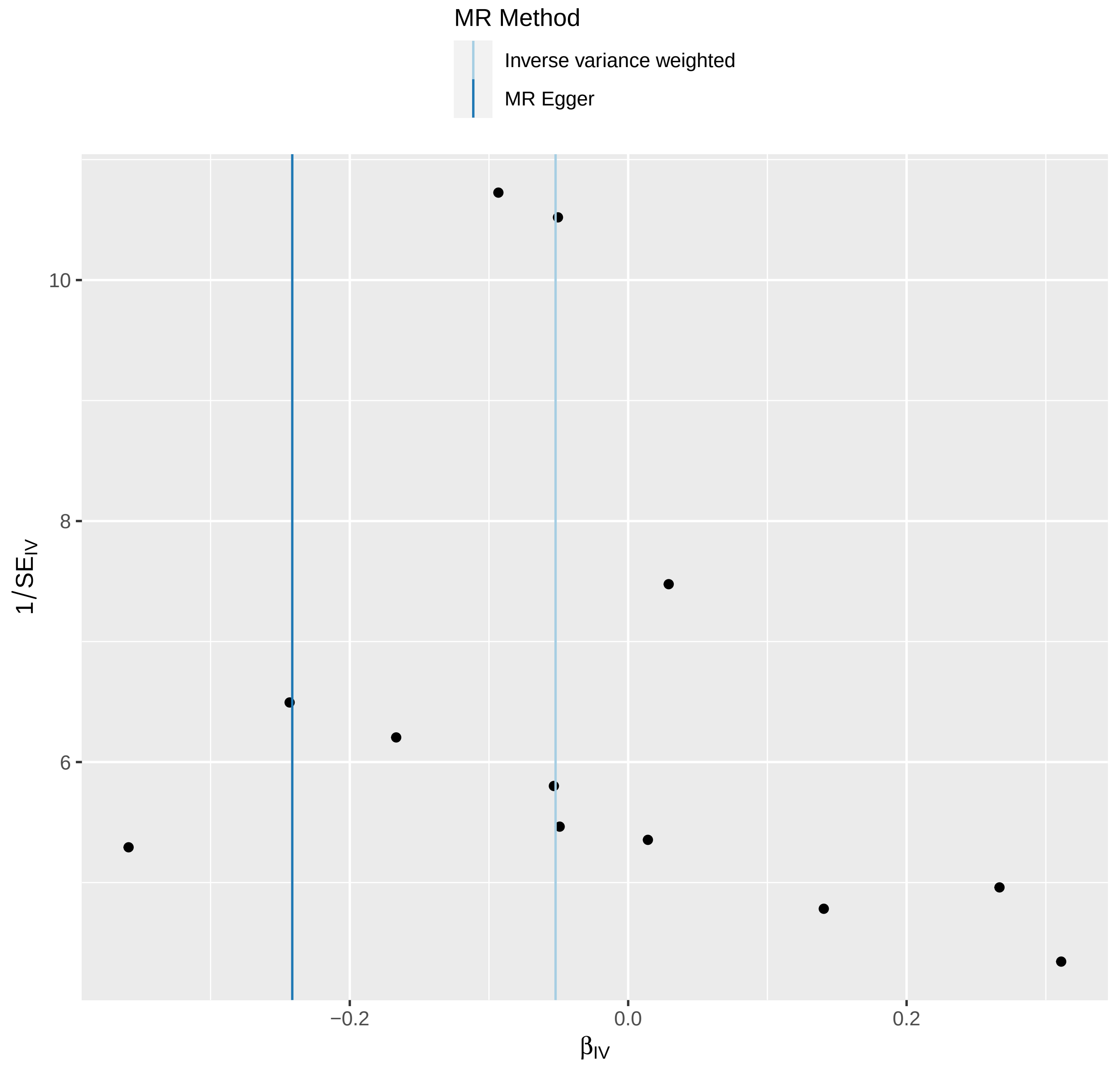

Batch 3−658 : Selenium on Corticoliberin

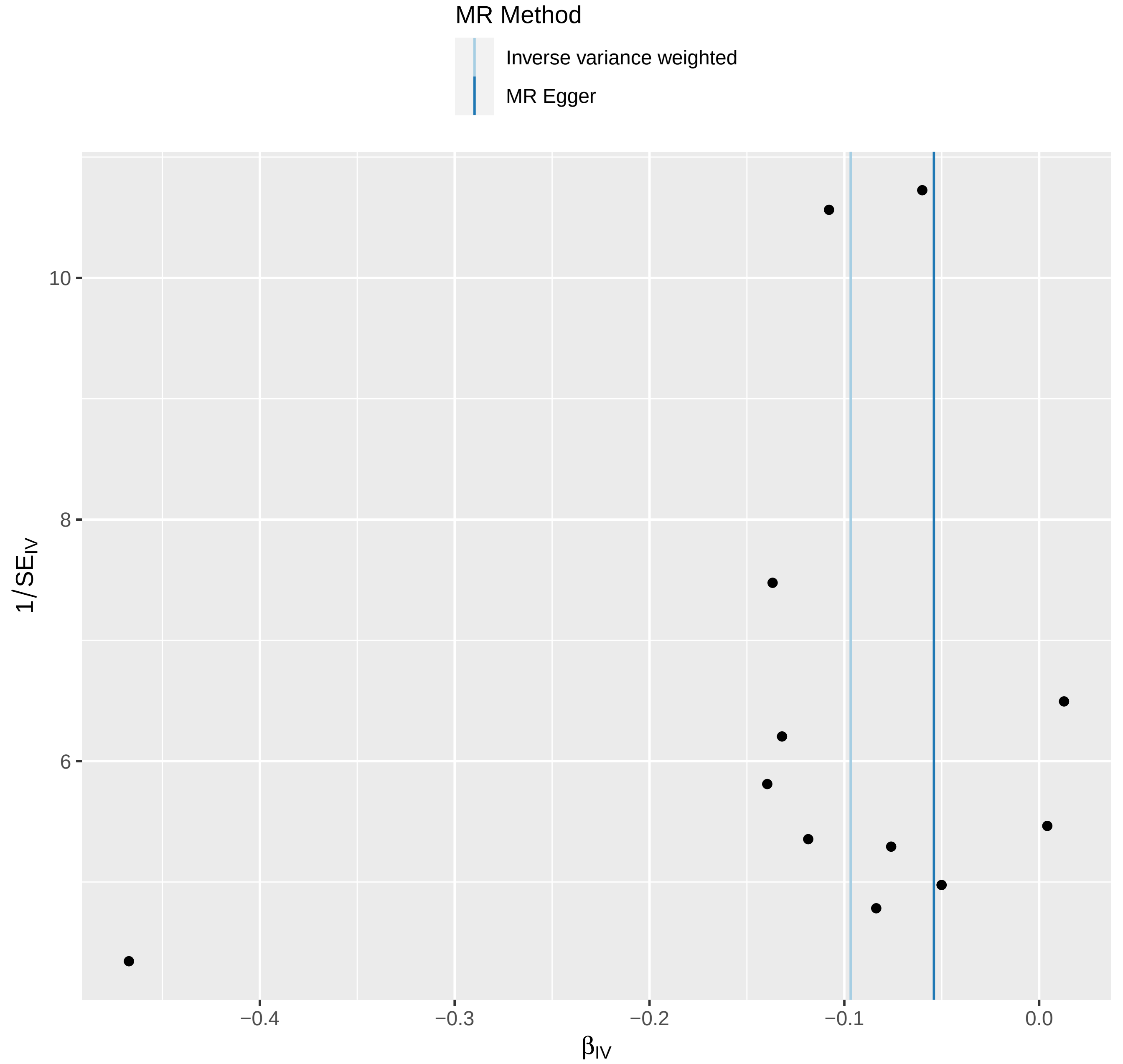

Batch 3−659 : Selenium on Corticotropin−releasing factor−binding protein

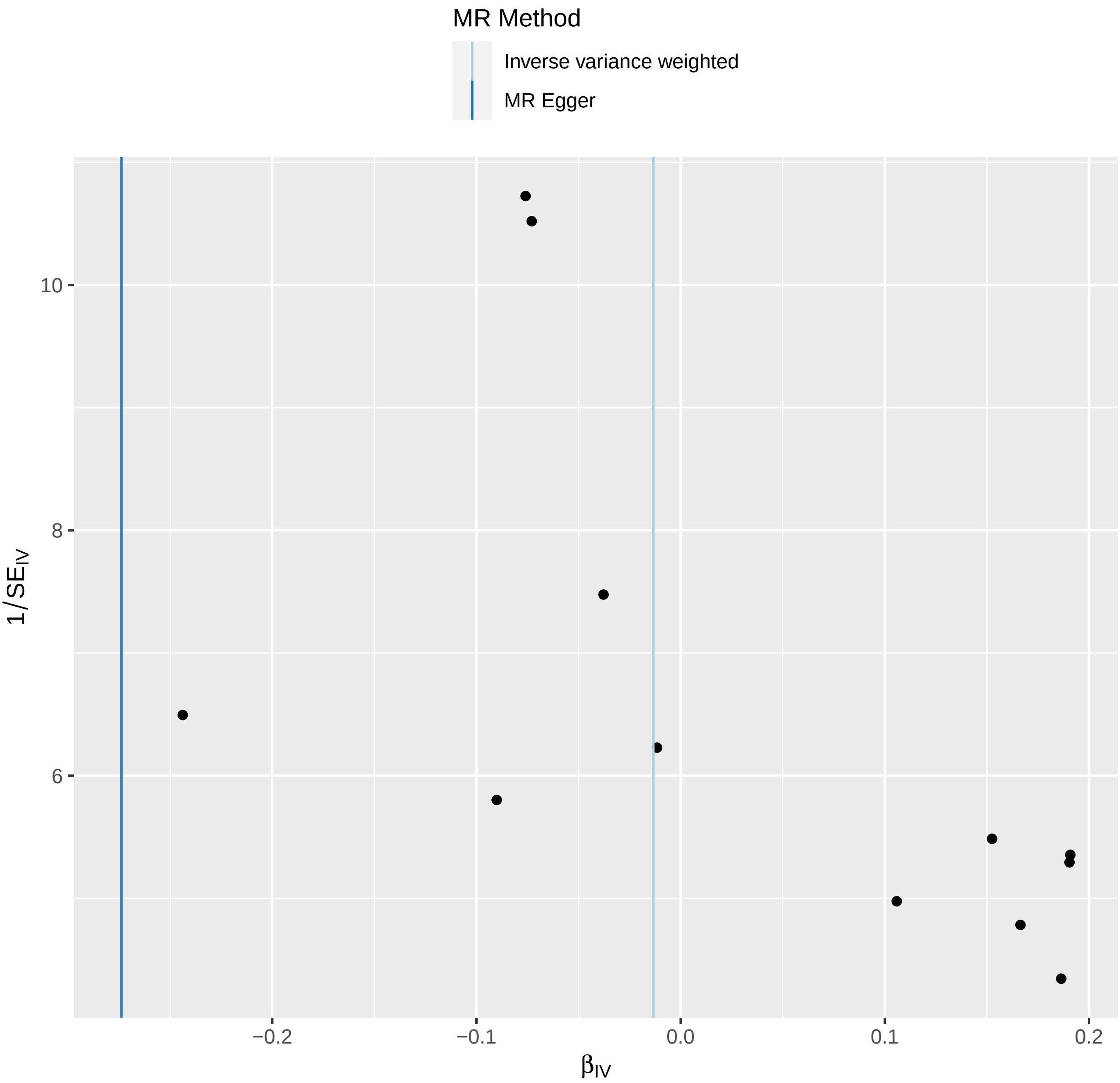

Batch 3−660 : Selenium on Cysteine−rich motor neuron 1 protein

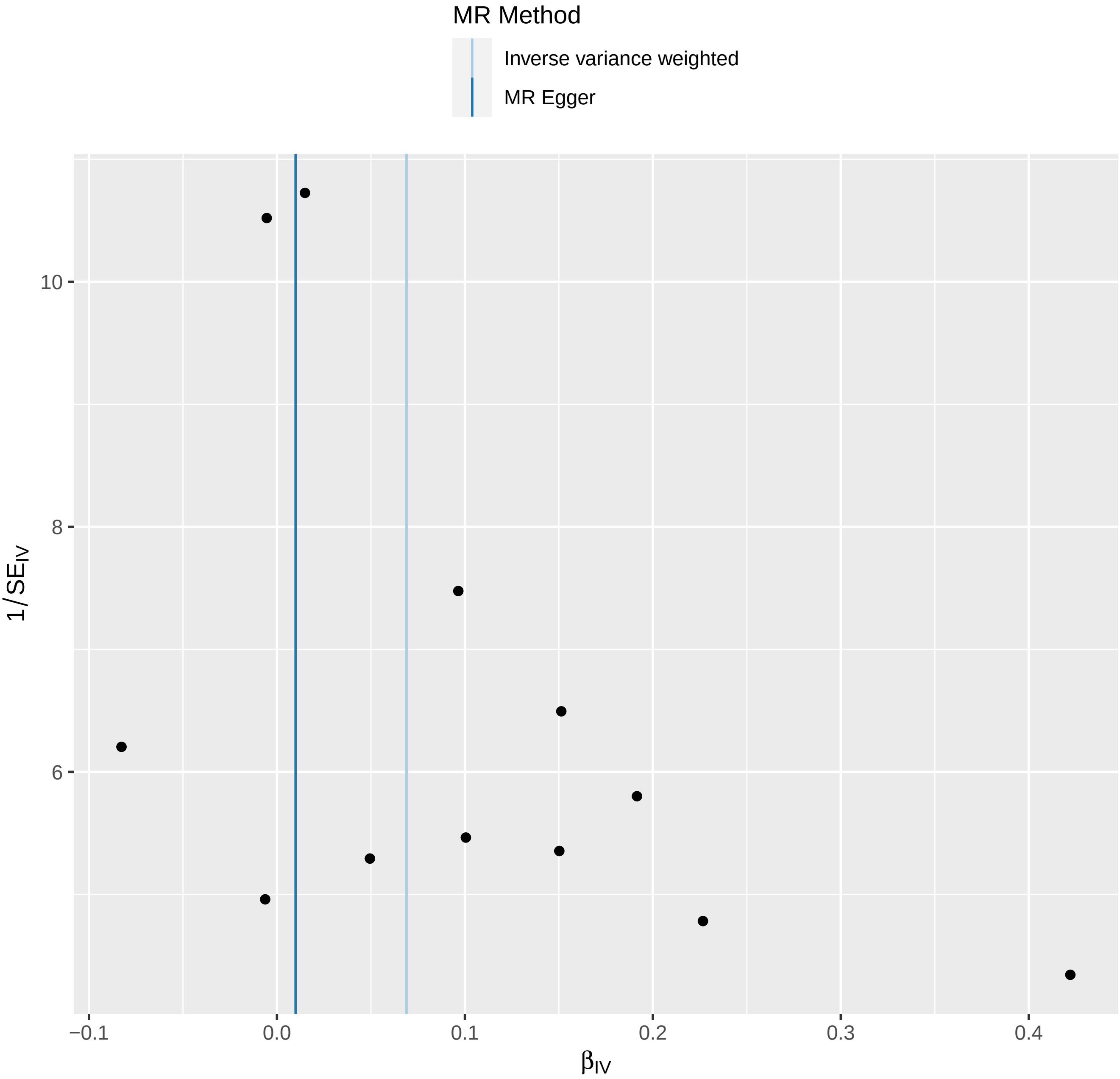

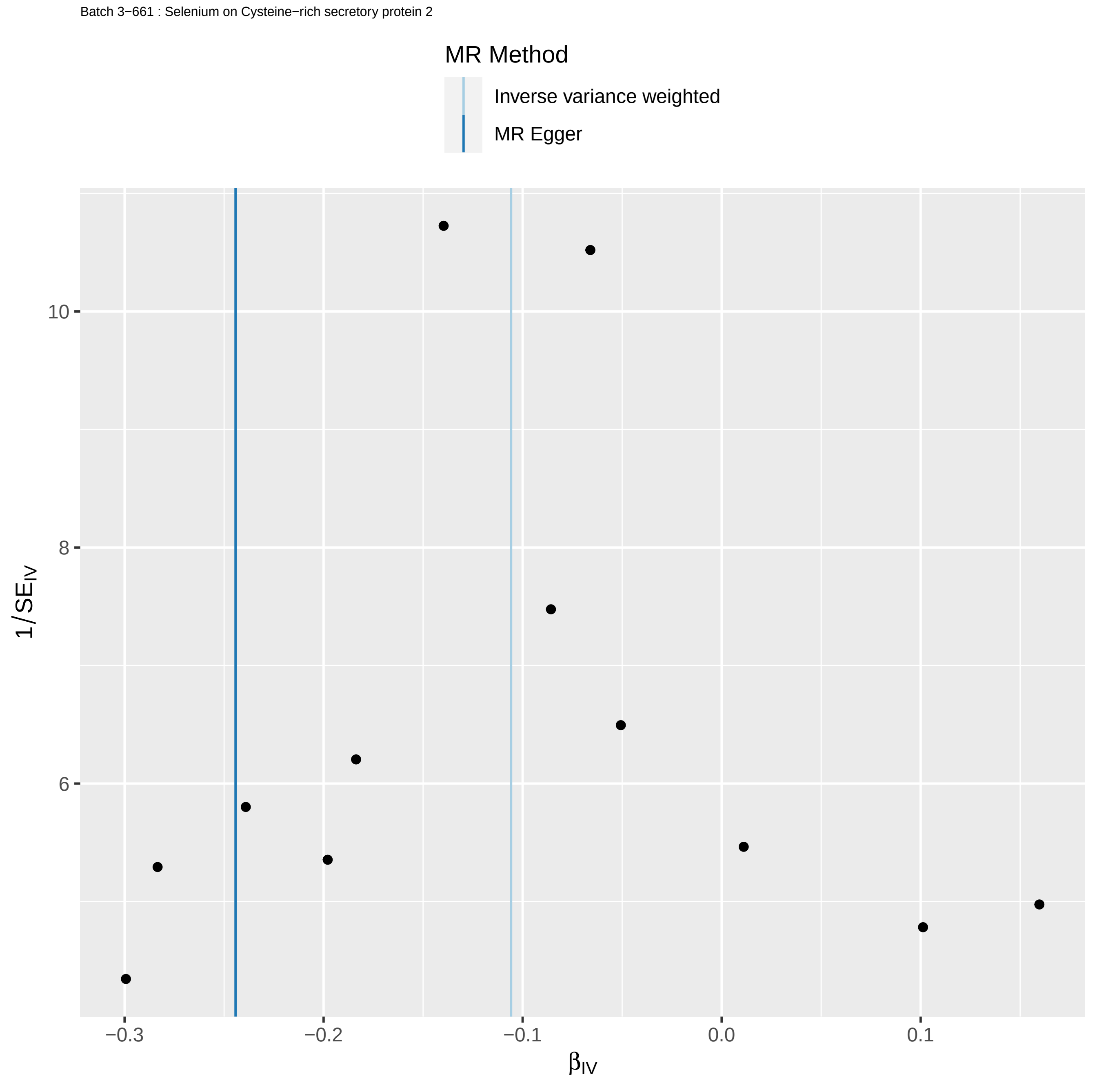
Batch 3−661 : Selenium on Cysteine−rich secretory protein 2
MR Method
Inverse variance weighted
MR Egger
10
8
6
$1/SE_{IV}$
−0.3
−0.2
−0.1
0.0
0.1
β_{IV}

Batch 3−662 : Selenium on Cysteine−rich secretory protein 3

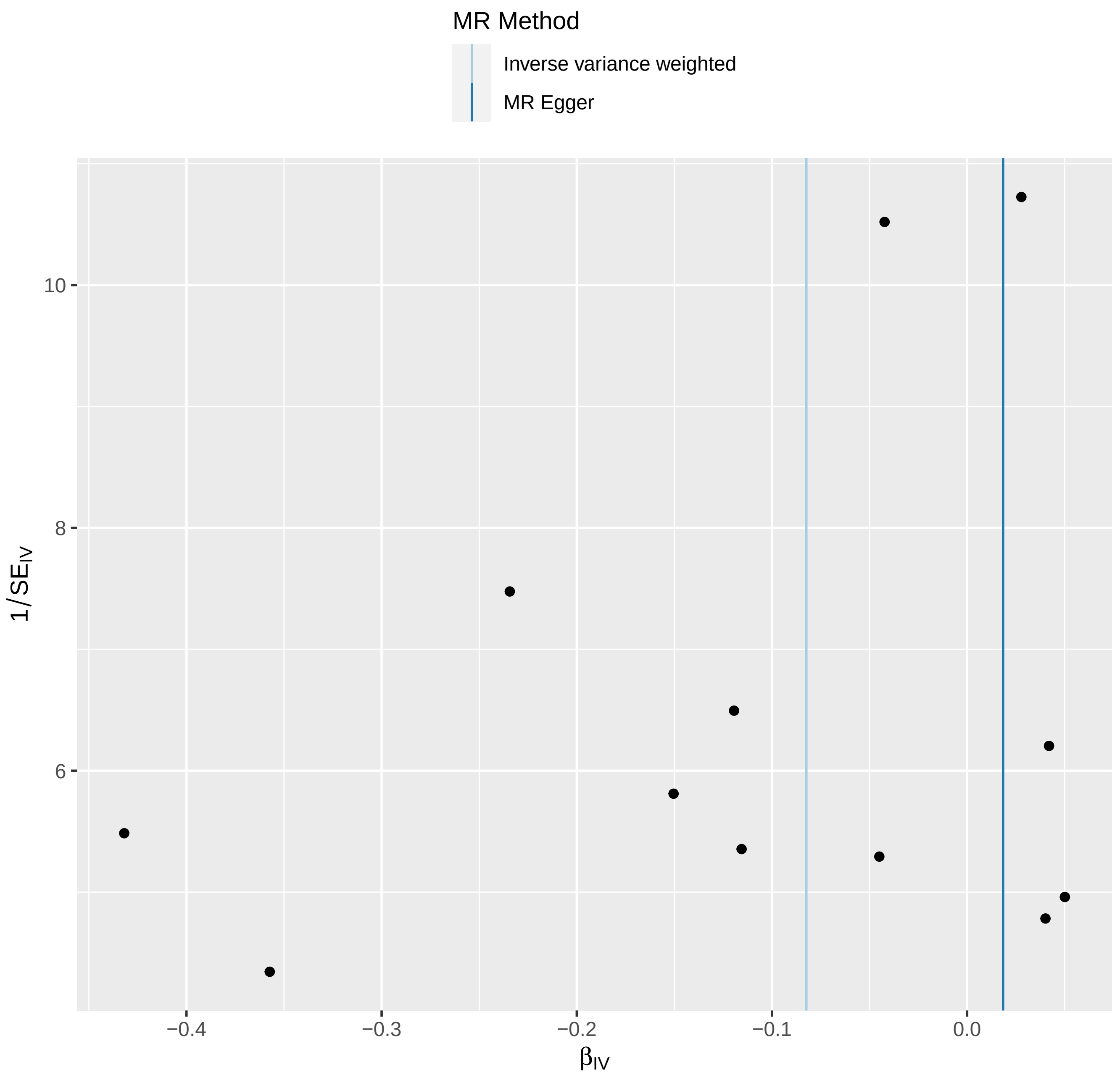

Batch 3−663 : Selenium on Cysteine−rich secretory protein LCCL domain−containing 2

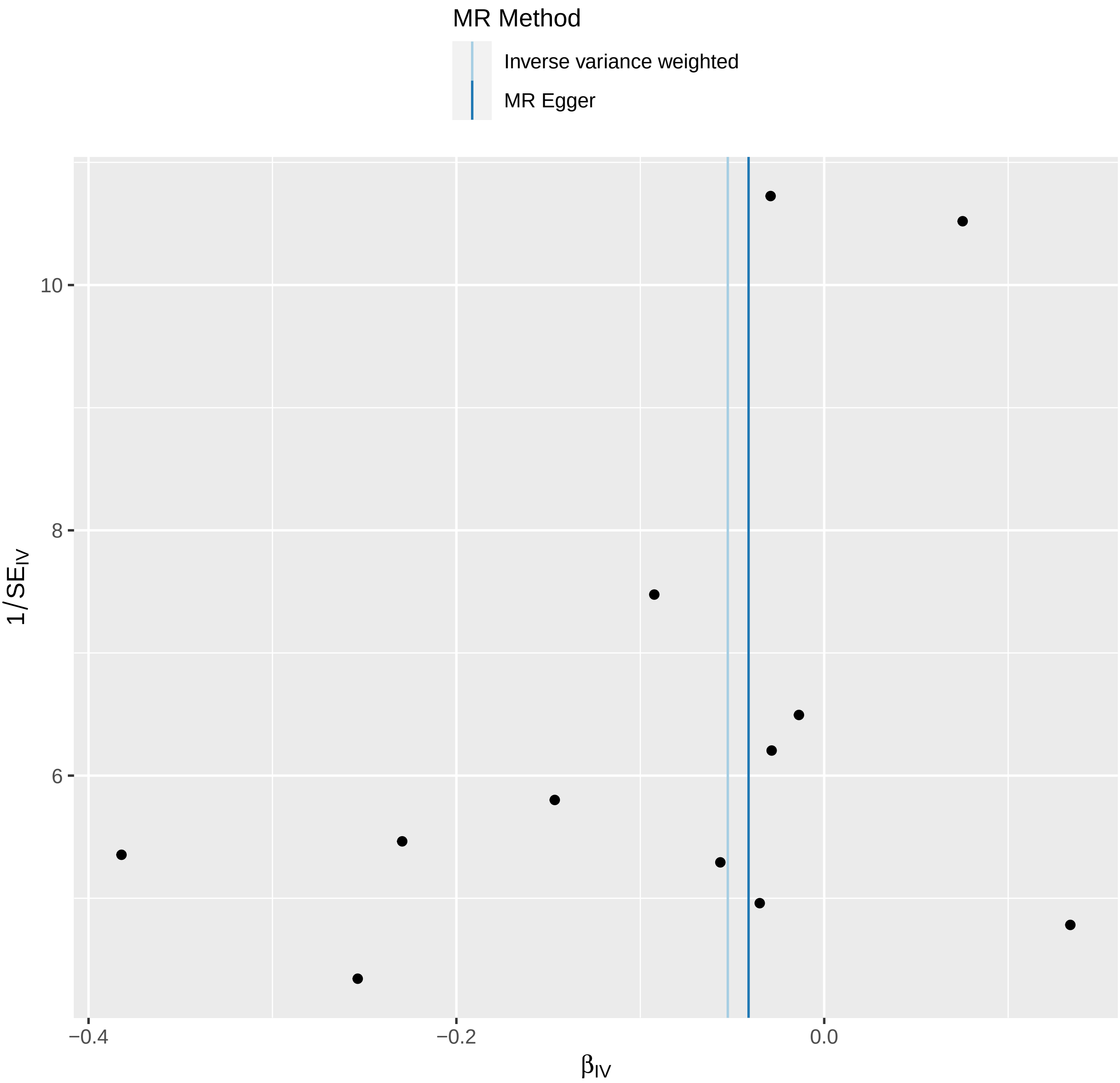

Batch 3−664 : Selenium on Adapter molecule crk

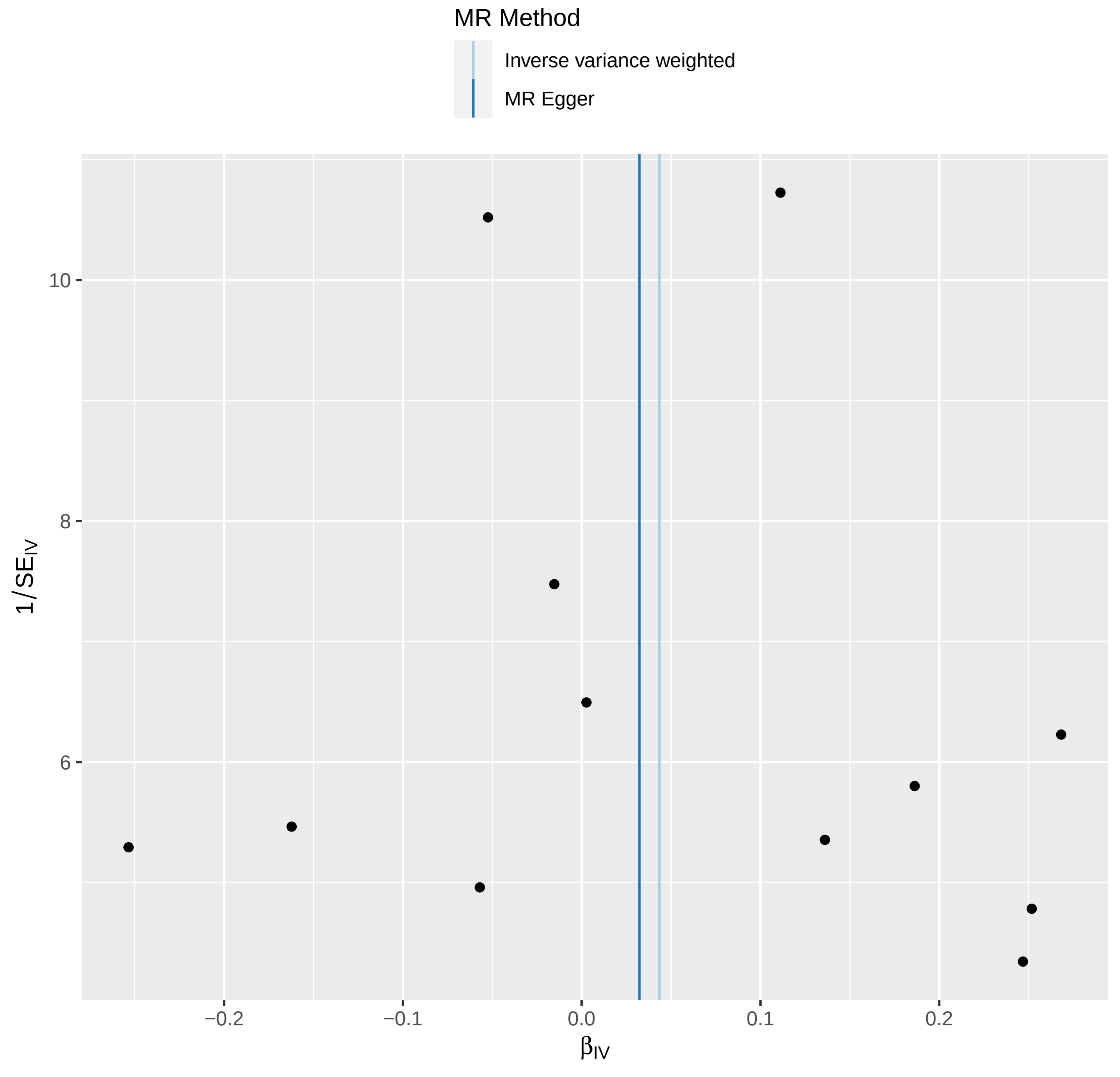

Batch 3−665 : Selenium on Crk−like protein

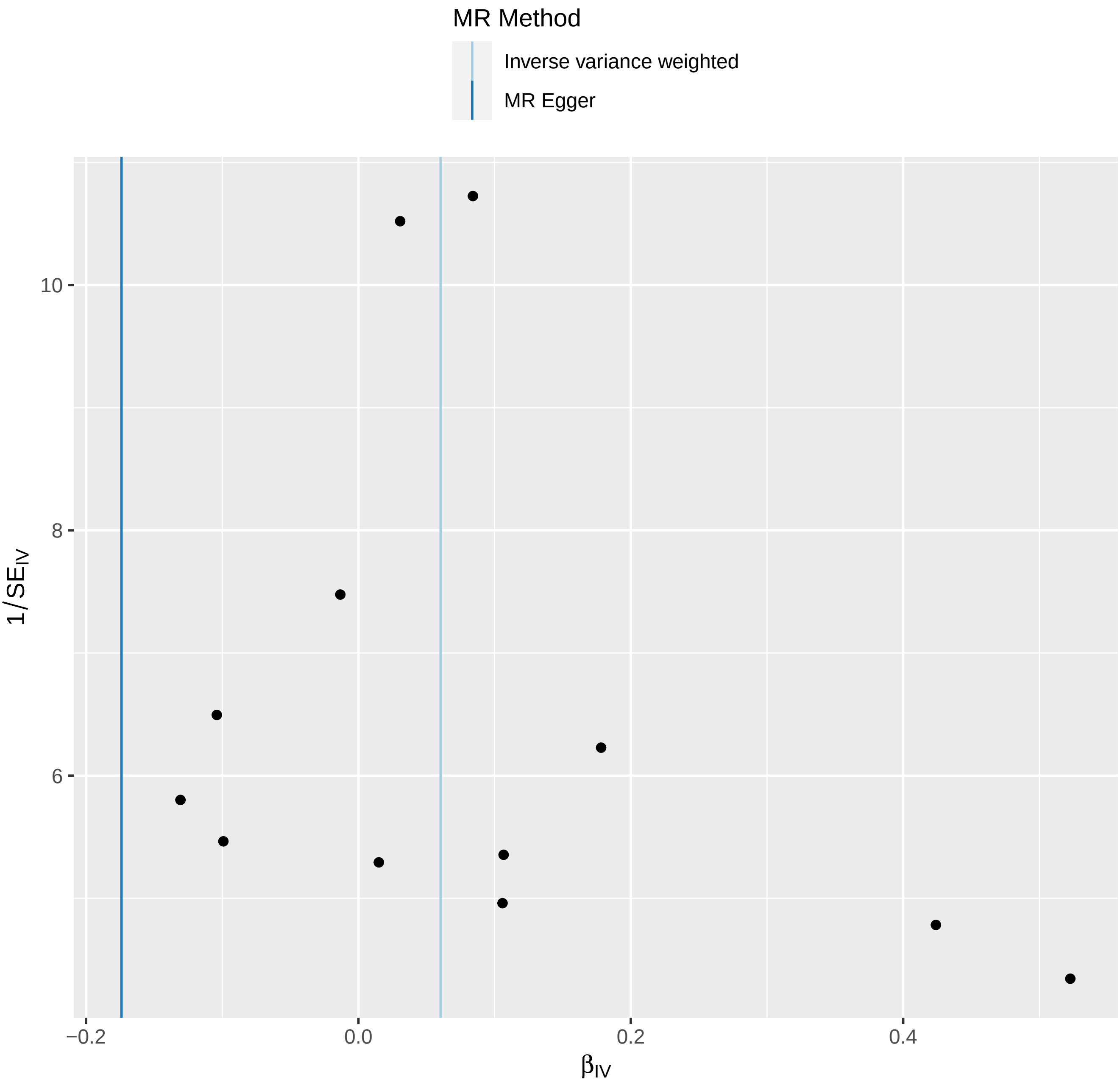

Batch 3−666 : Selenium on Cytokine receptor−like factor 1:Cardiotrophin−like cytokine factor 1 Complex

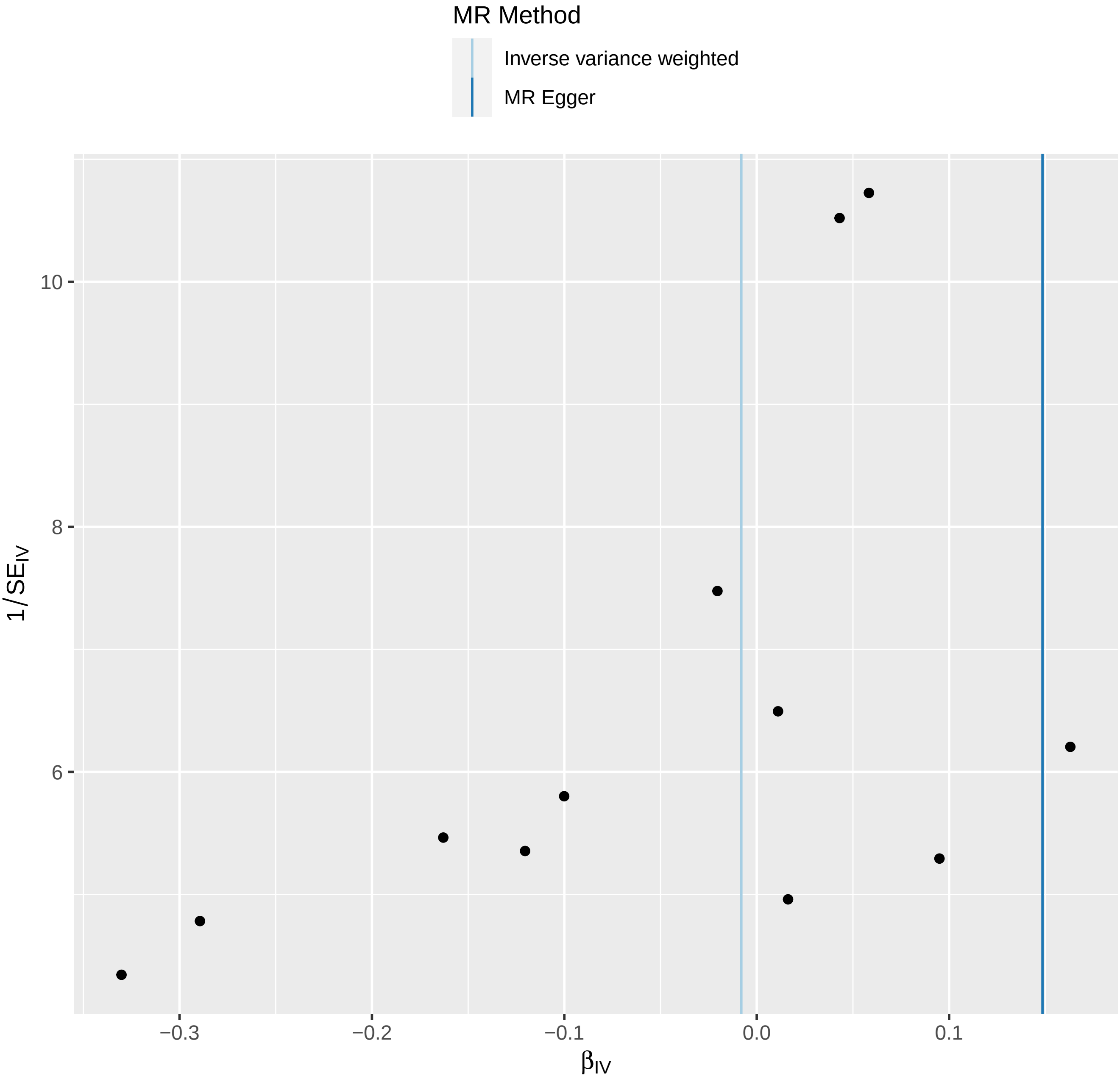

Batch 3−667 : Selenium on Cytokine receptor−like factor 2

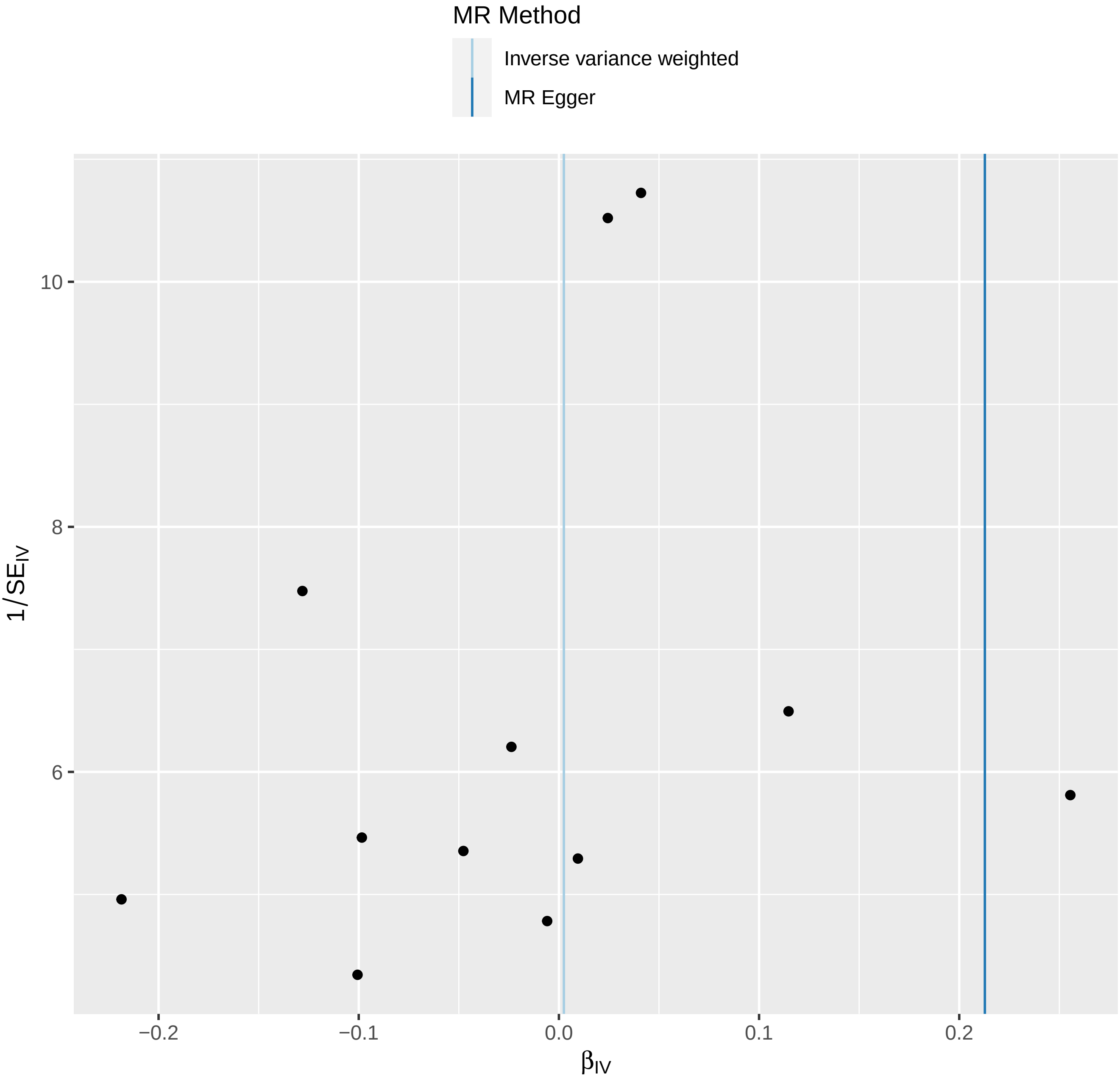

Batch 3−668 : Selenium on Cytokine receptor−like factor 2

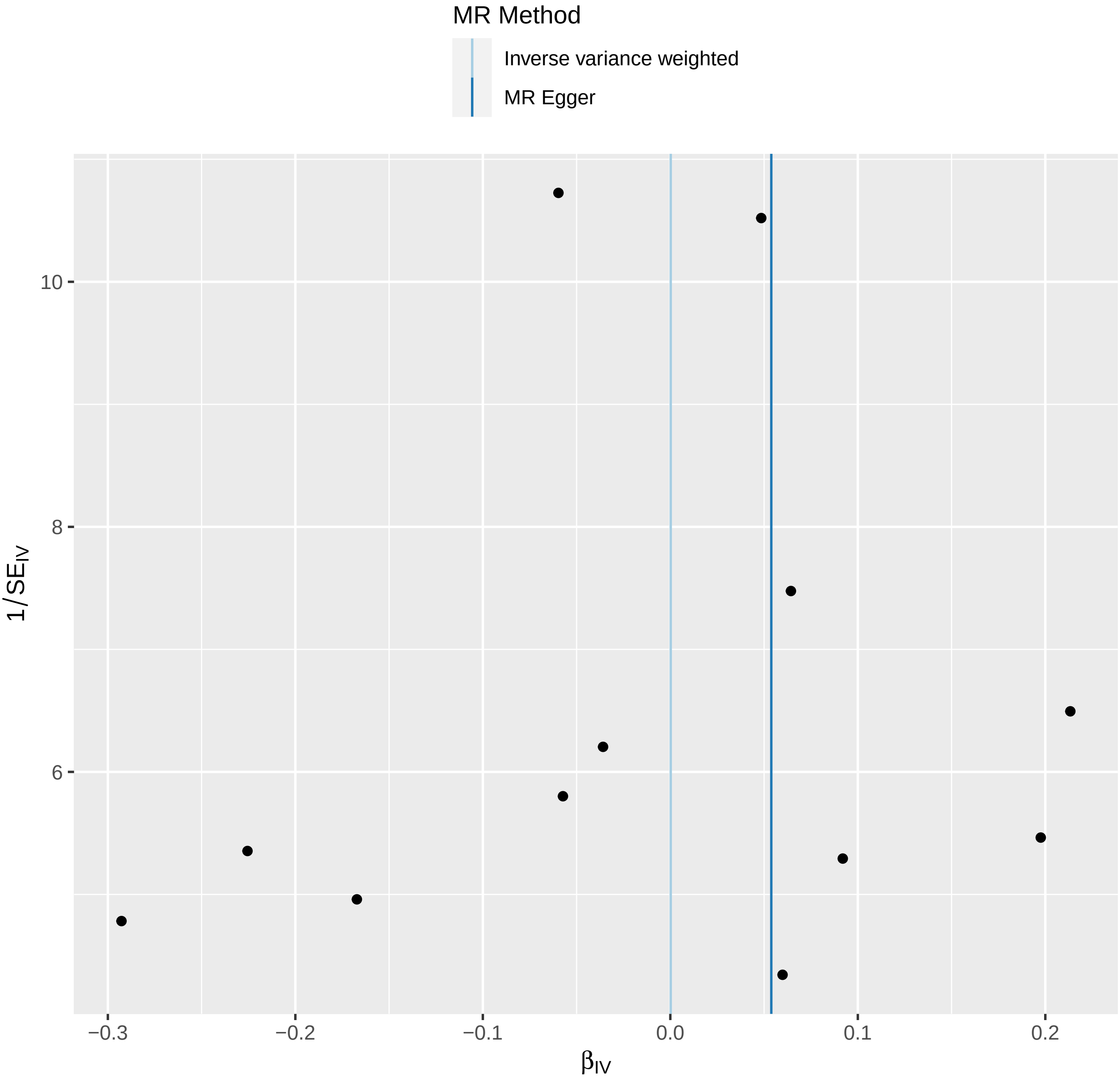

Batch 3−669 : Selenium on Peroxisomal carnitine O−octanoyltransferase

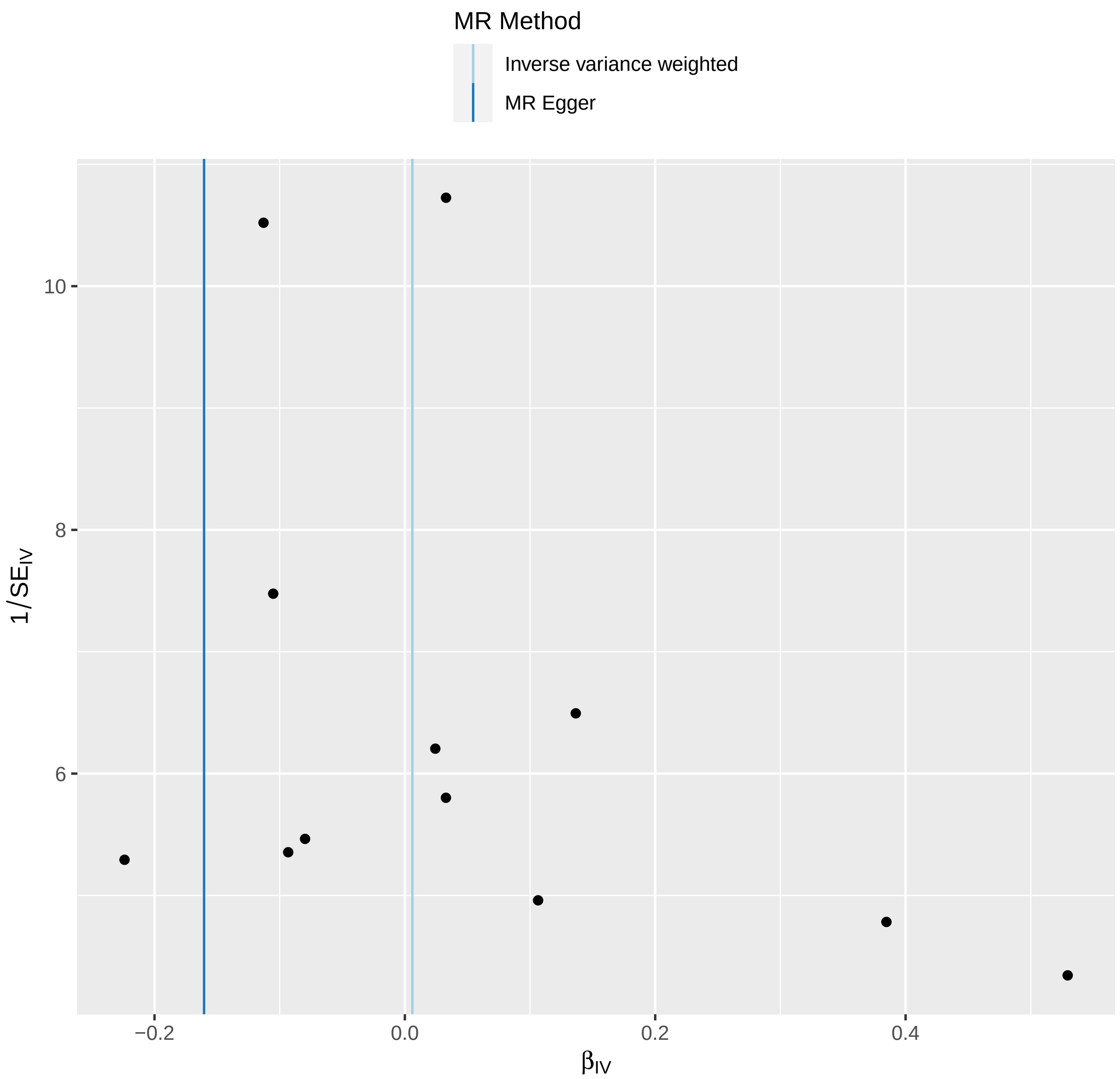

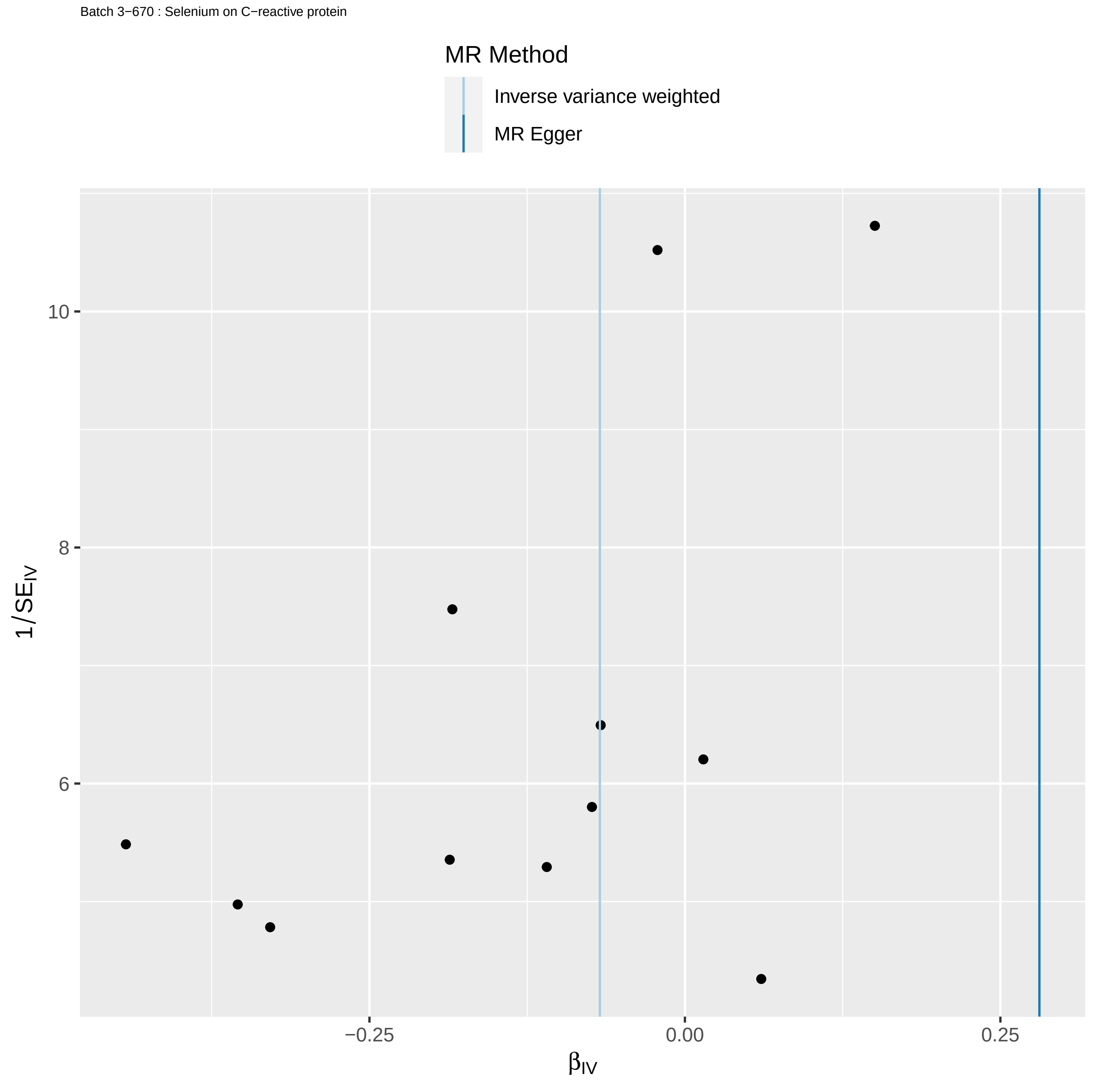
Batch 3−670 : Selenium on C−reactive protein
MR Method
Inverse variance weighted
MR Egger
10
8
6
1/SE_IV
−0.25
0.00
0.25
β_IV

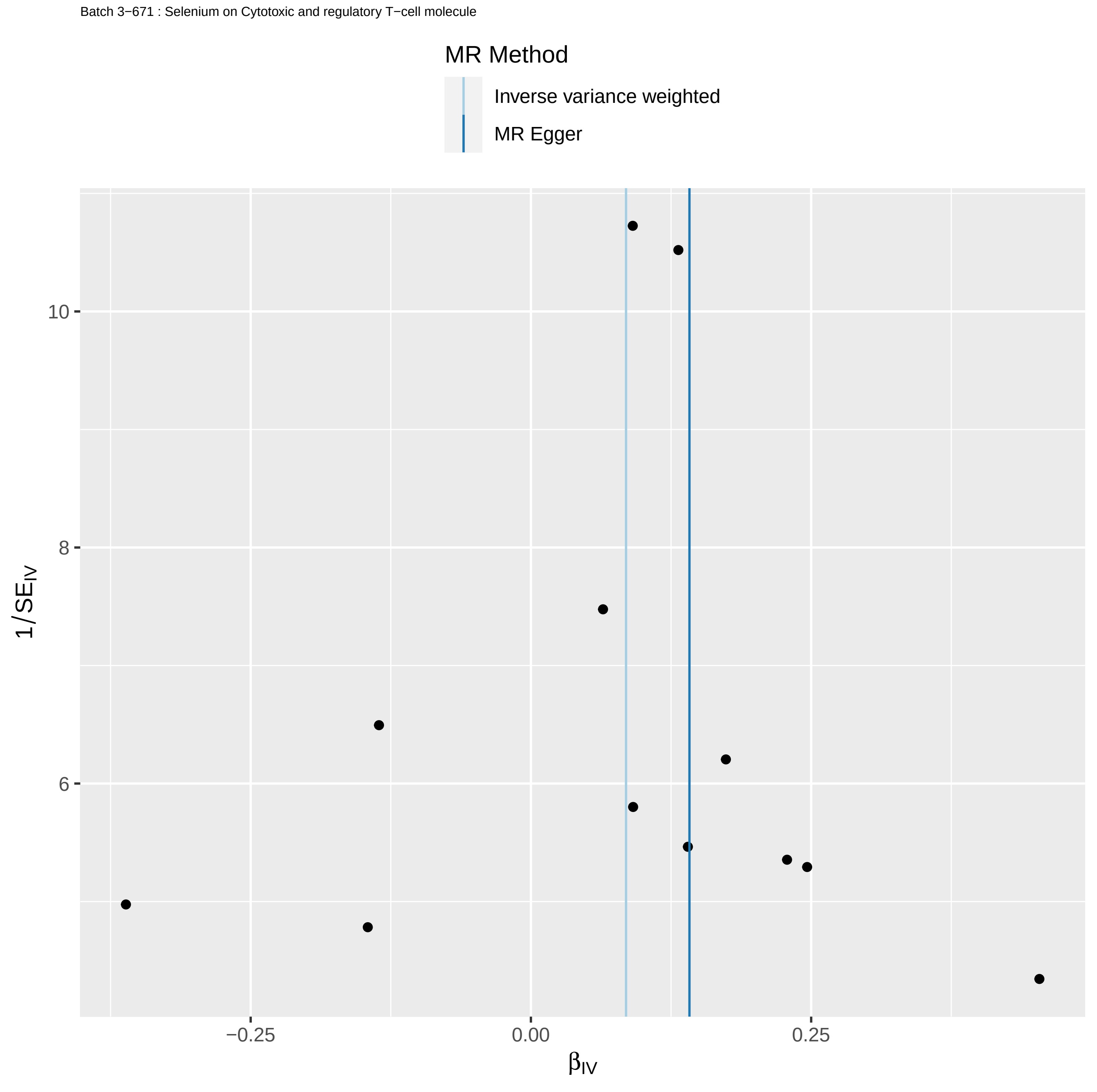
Batch 3−671 : Selenium on Cytotoxic and regulatory T−cell molecule
MR Method
Inverse variance weighted
MR Egger
10
8
6
1/SE_IV
−0.25
0.00
0.25
β_IV

Batch 3−672 : Selenium on Cytotoxic and regulatory T−cell molecule

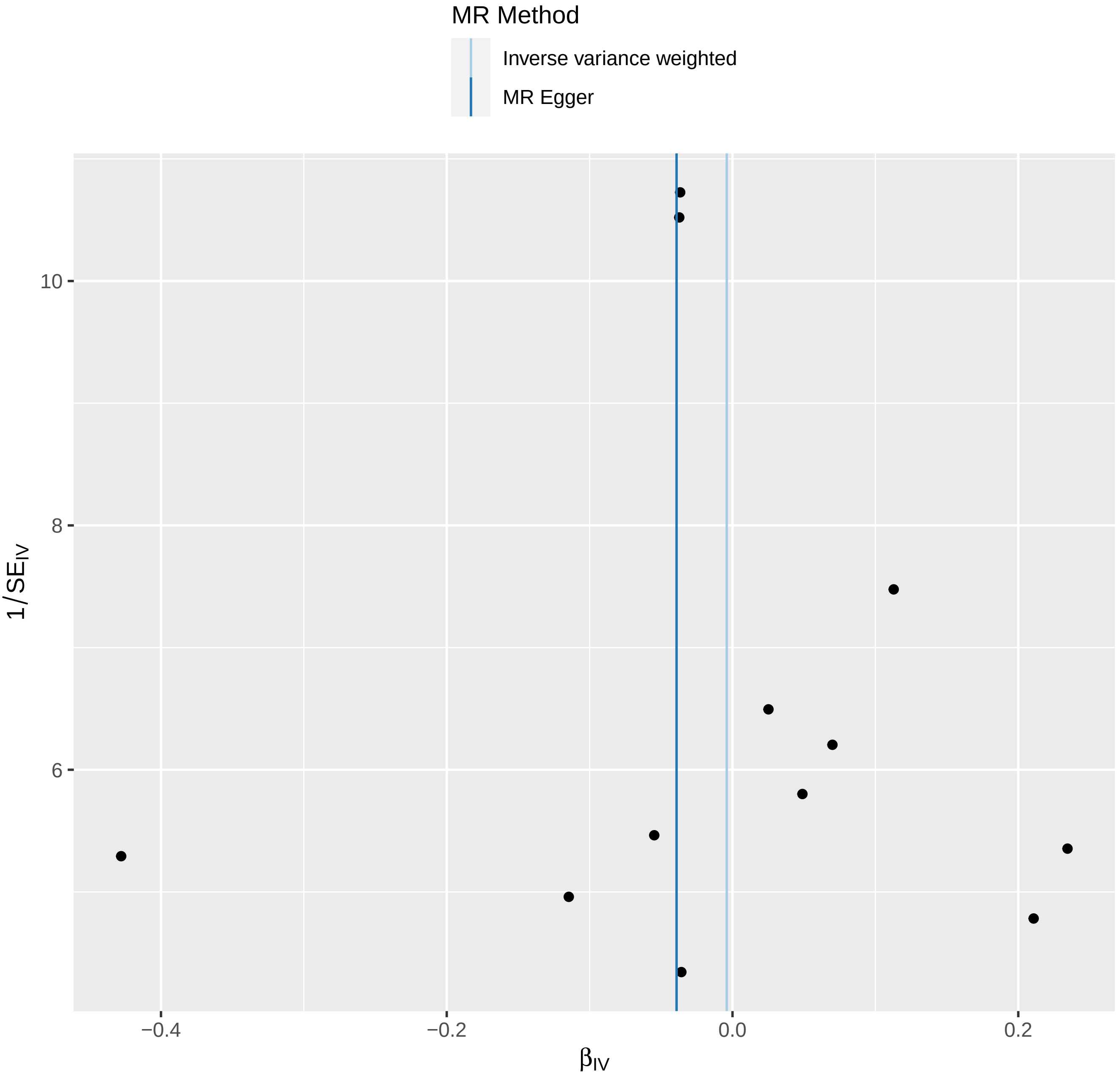

Batch 3−673 : Selenium on Alpha−crystallin A chain

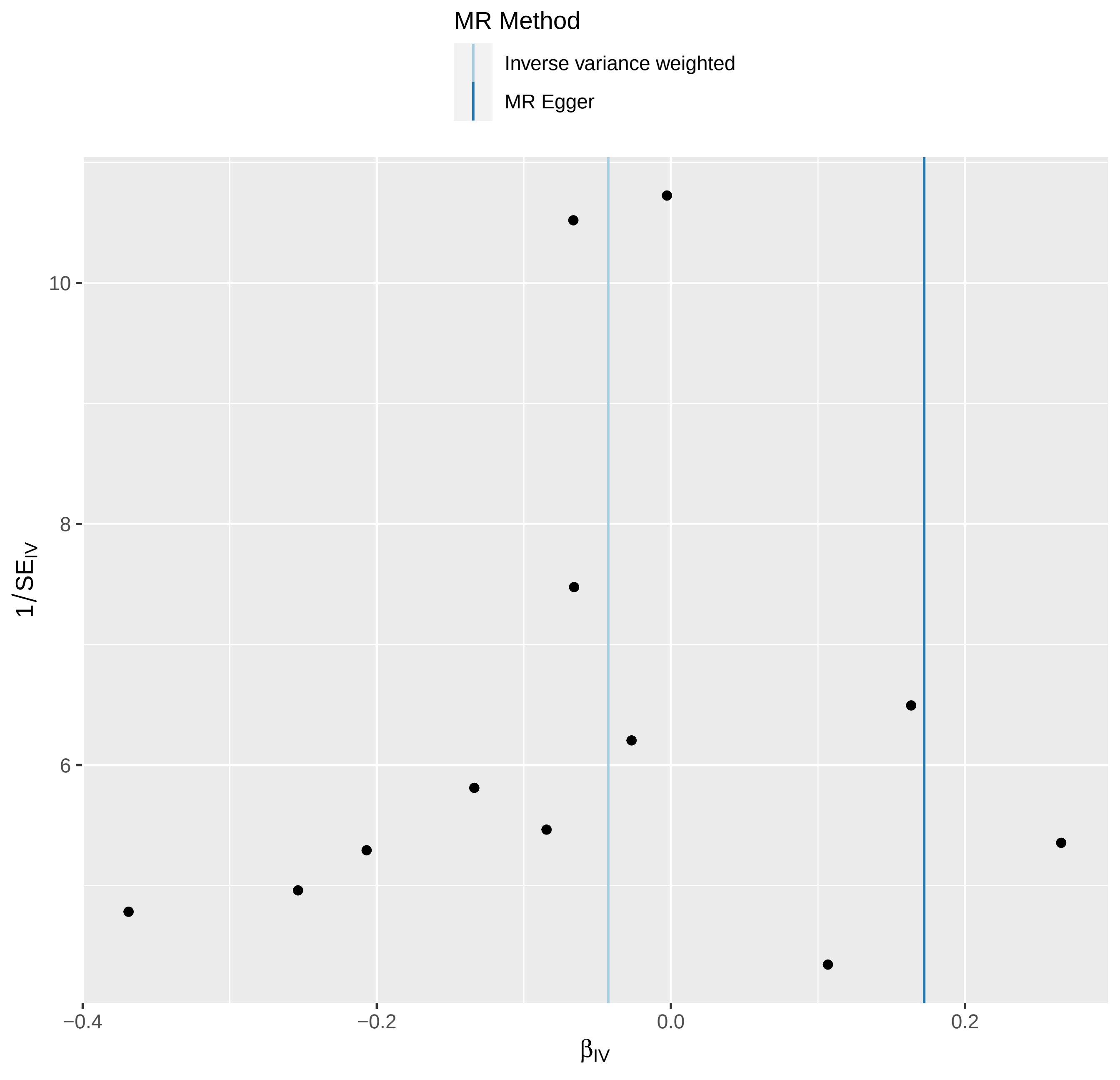

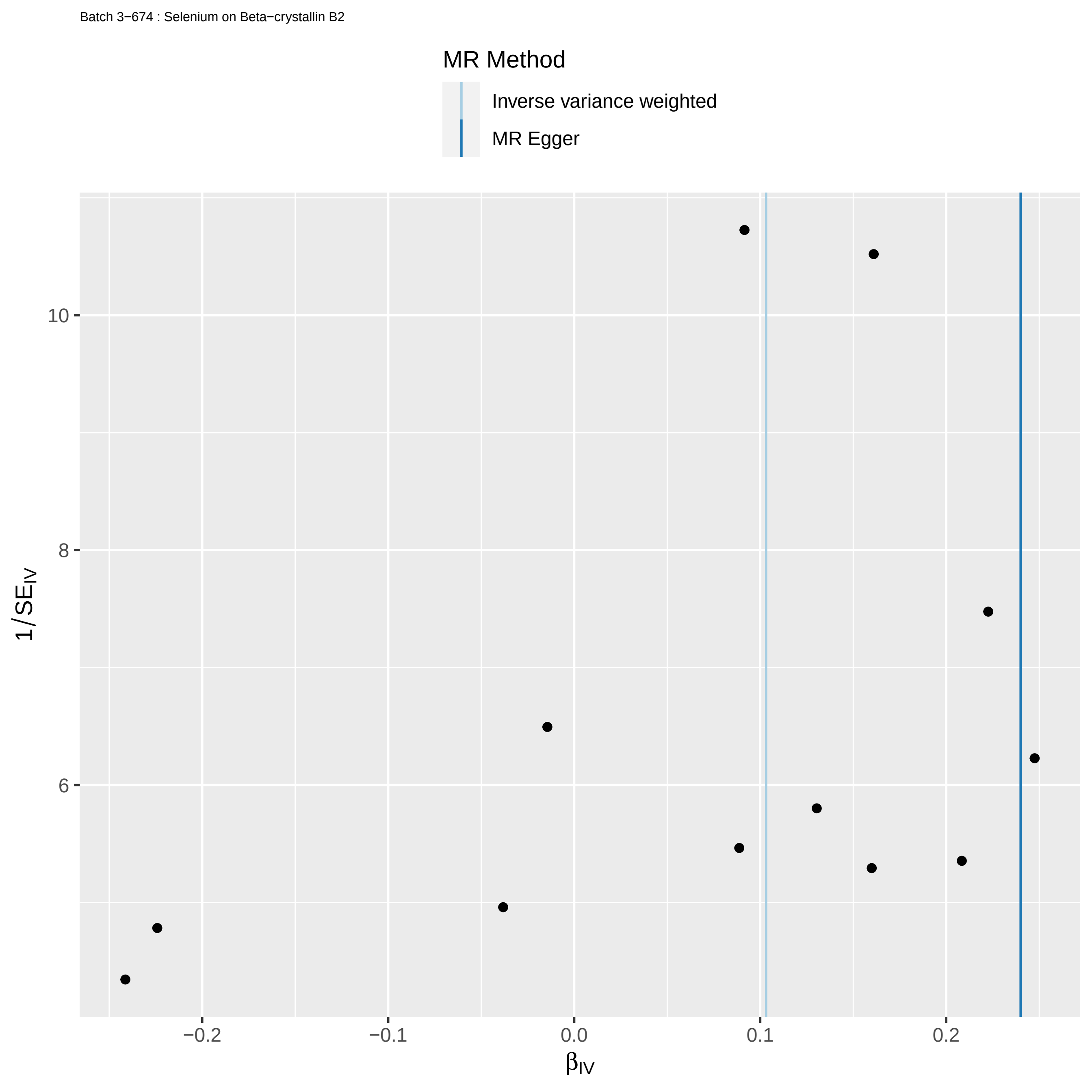

Batch 3−674 : Selenium on Beta−crystallin B2
MR Method
Inverse variance weighted
MR Egger
10
8
6
1/SE$_{IV}$
−0.2
−0.1
0.0
0.1
0.2
β$_{IV}$

Batch 3−675 : Selenium on Gamma−crystallin D

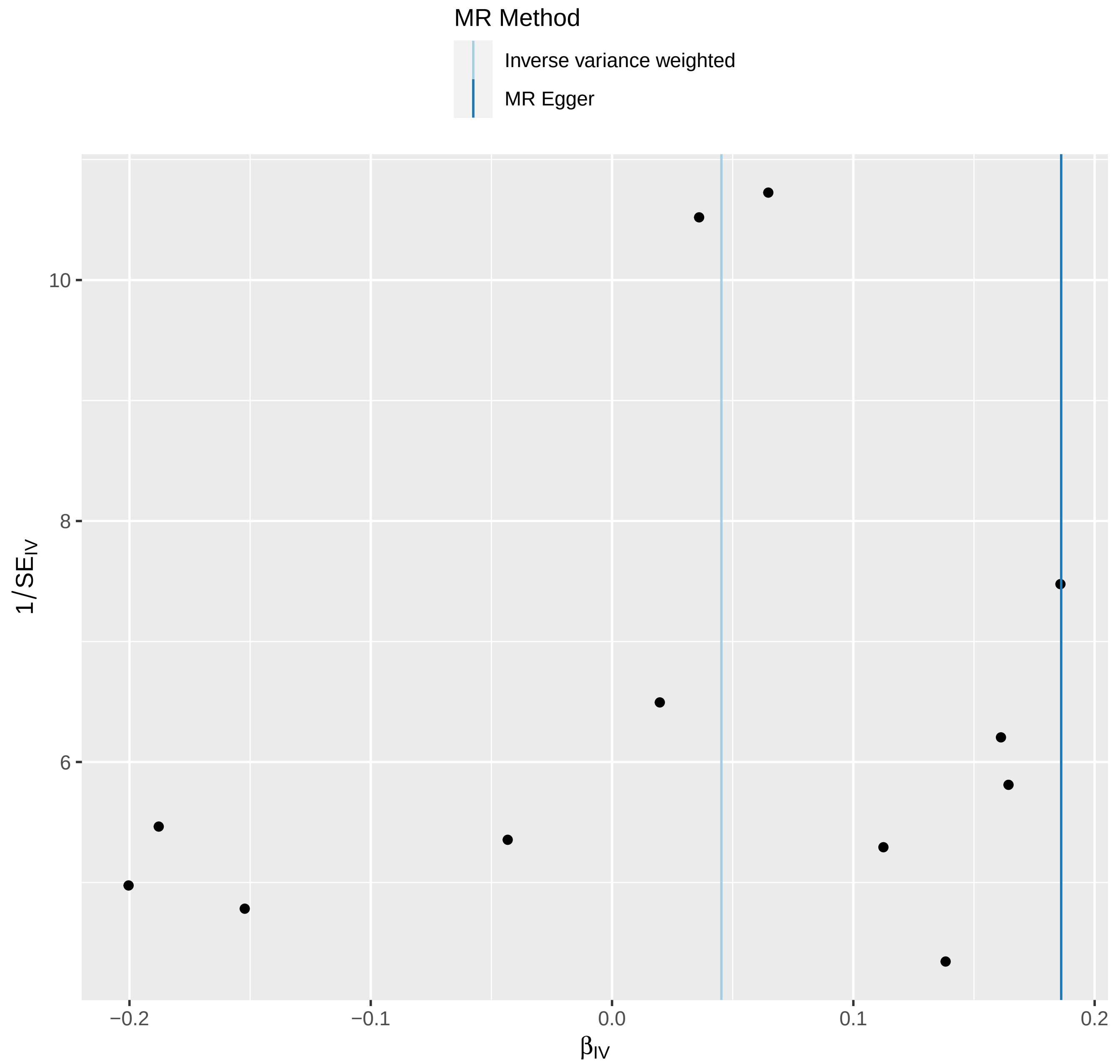

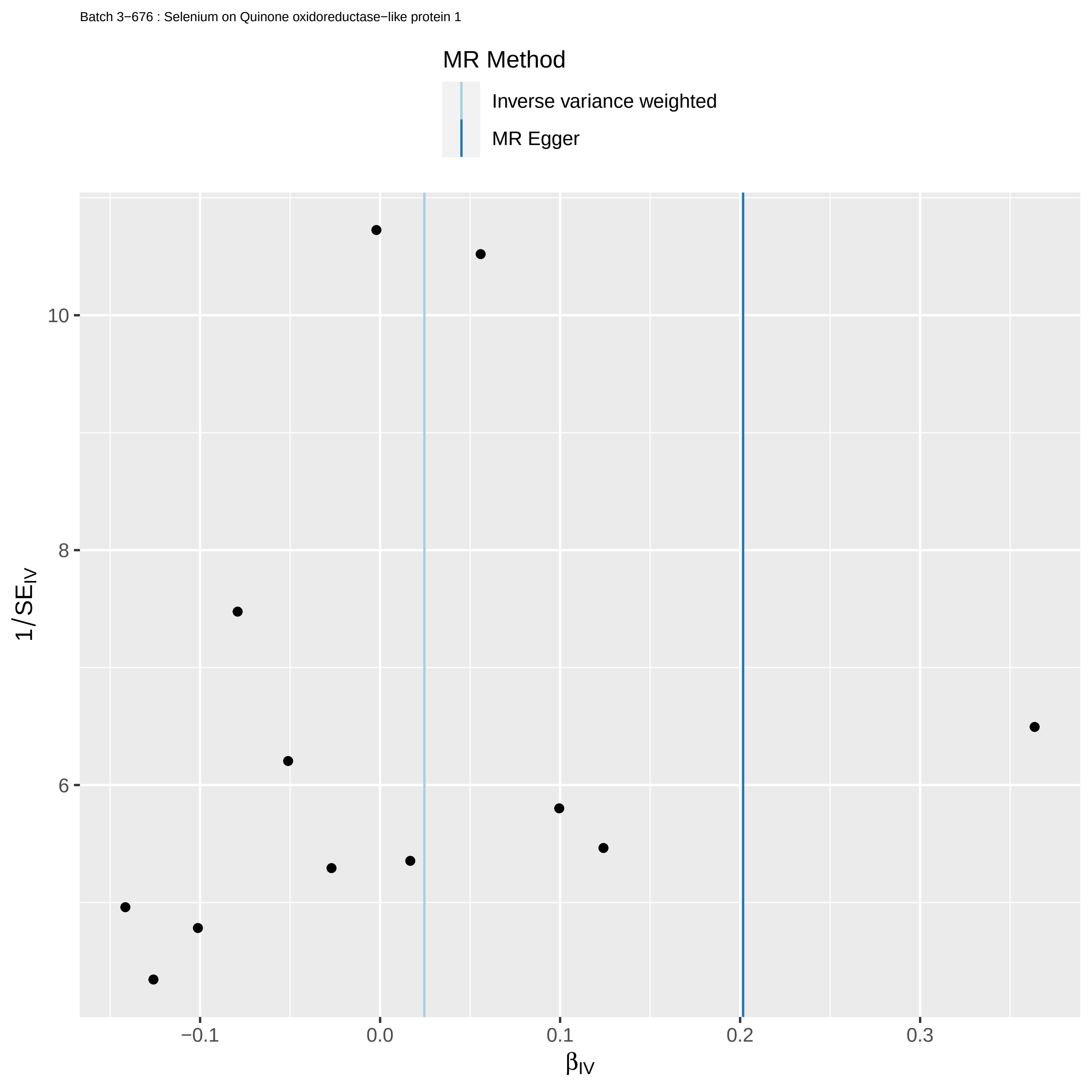

Batch 3−676 : Selenium on Quinone oxidoreductase−like protein 1
MR Method
Inverse variance weighted
MR Egger
10
8
6
1/SE_IV
−0.1
0.0
0.1
0.2
0.3
β_IV

Batch 3−677 : Selenium on Putative chondrosarcoma−associated gene 1 protein

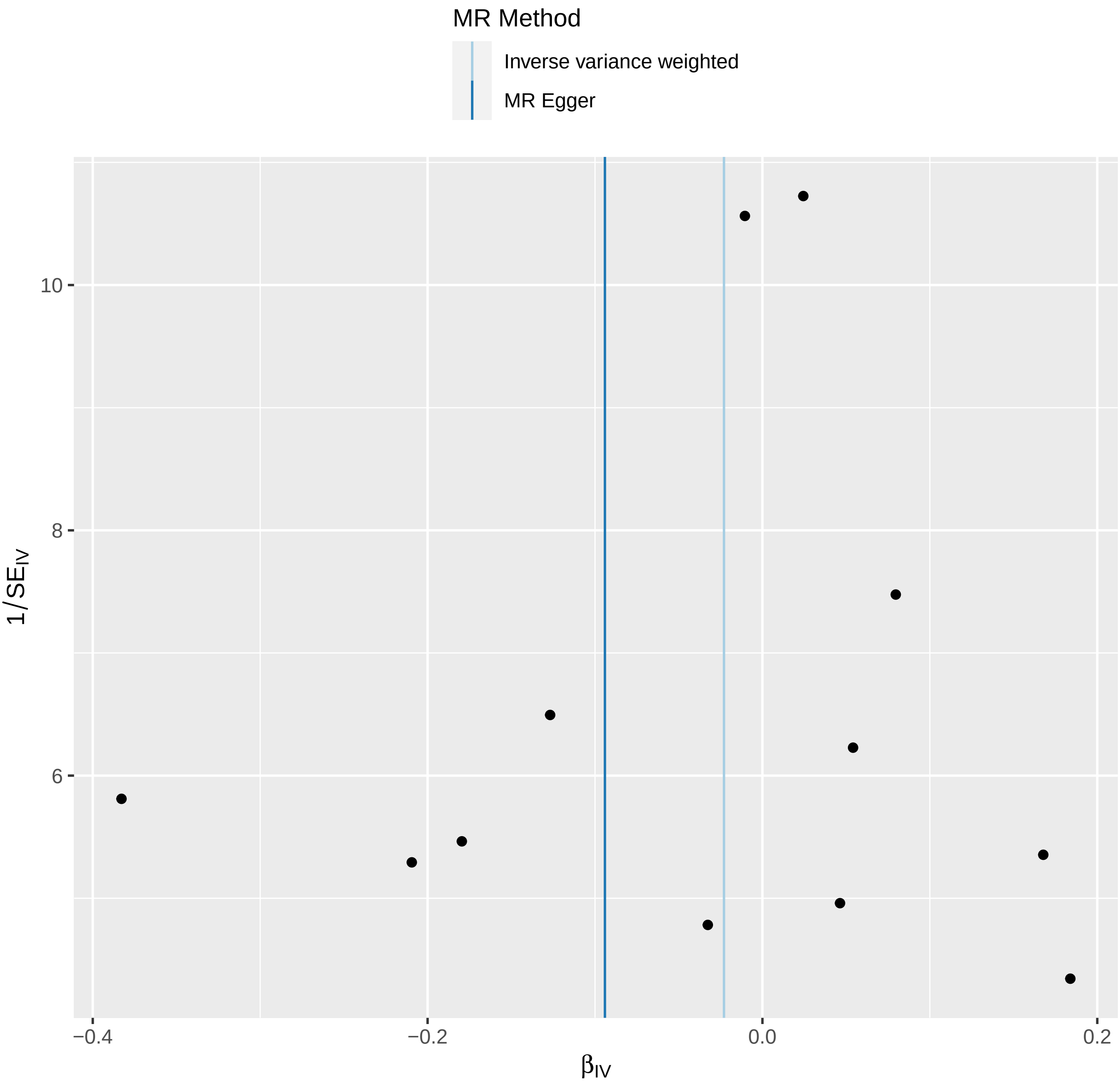

Batch 3−678 : Selenium on Cold shock domain−containing protein C2

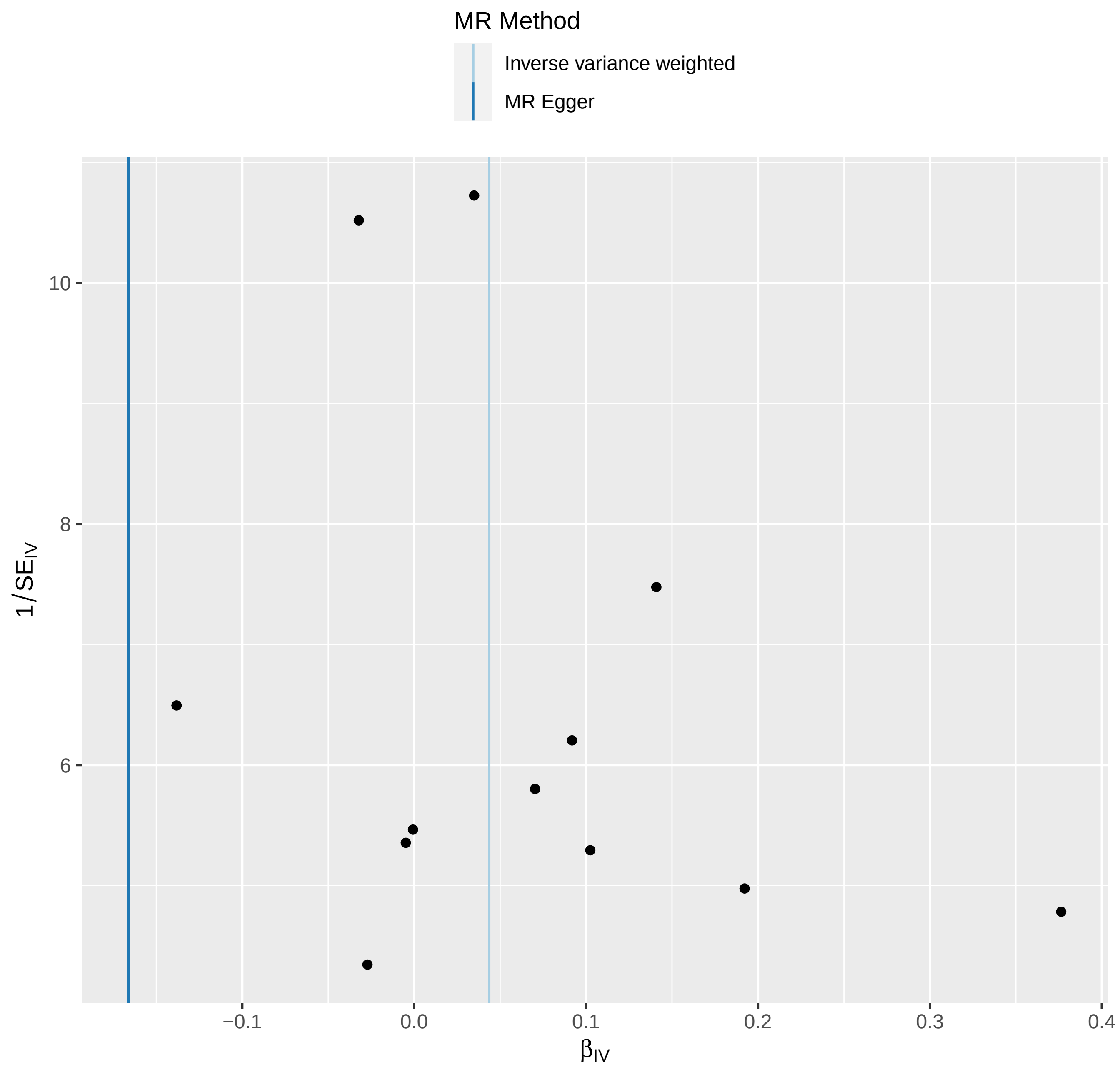

Batch 3−679 : Selenium on Cold shock domain−containing protein E1

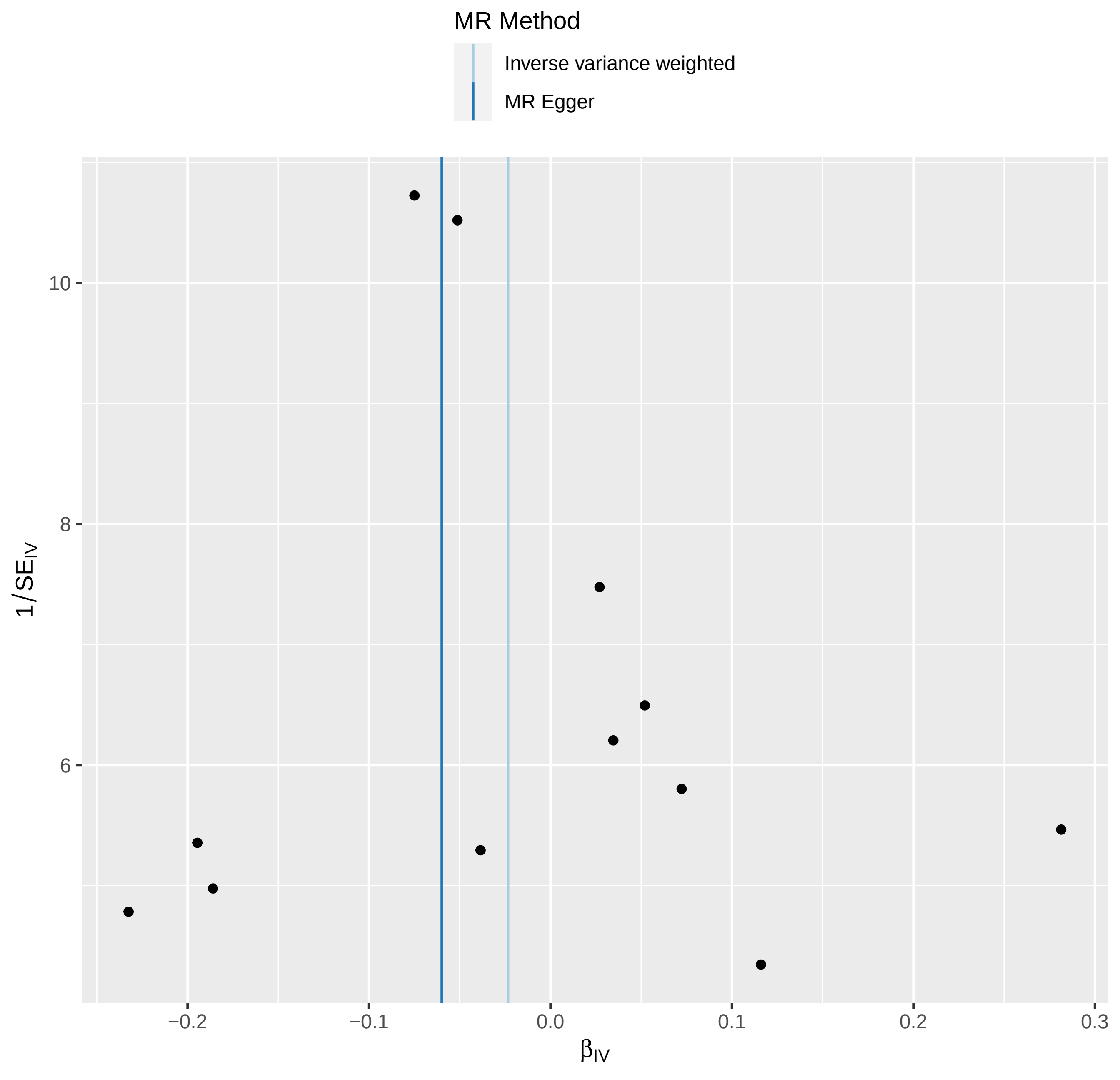

Batch 3−680 : Selenium on Macrophage colony−stimulating factor 1

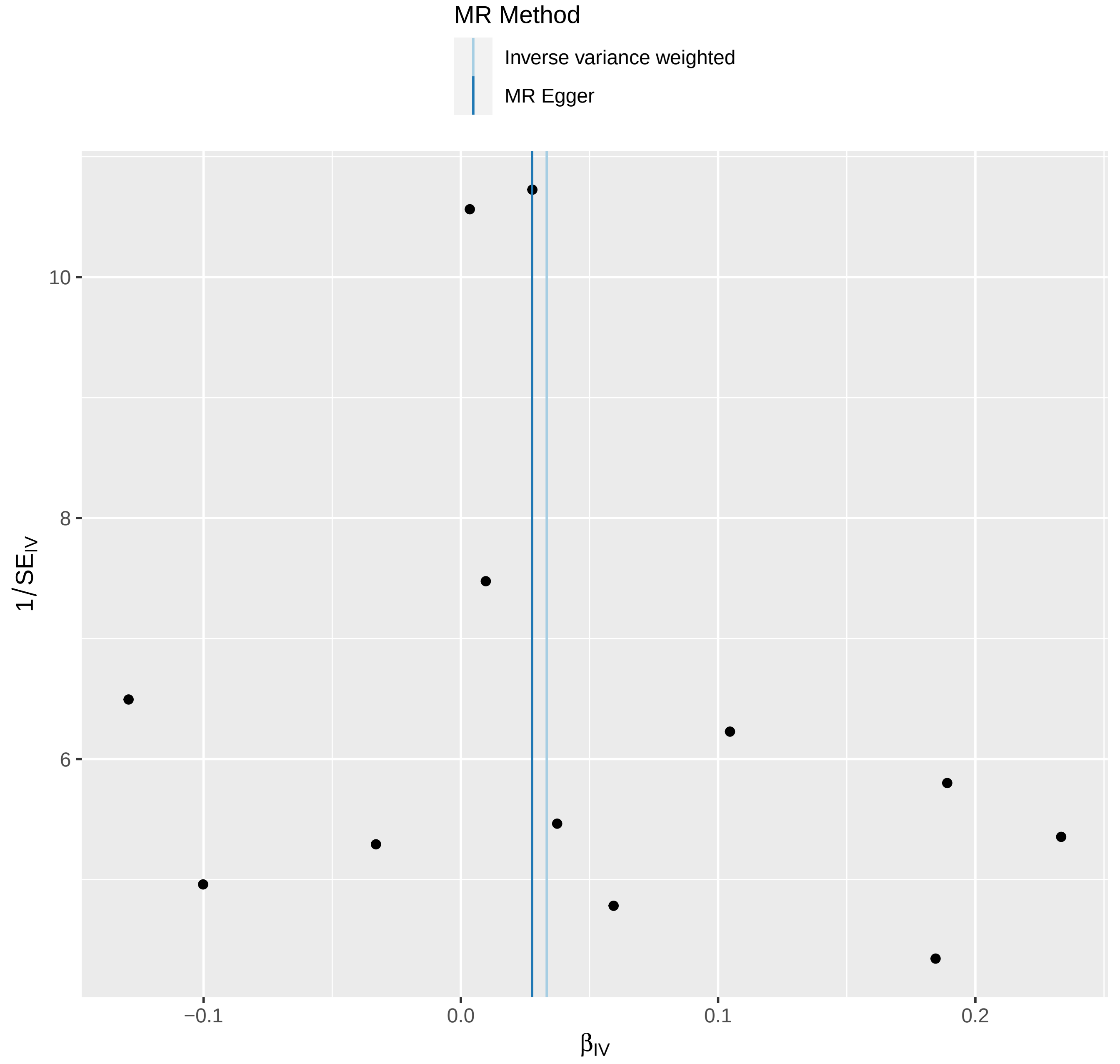

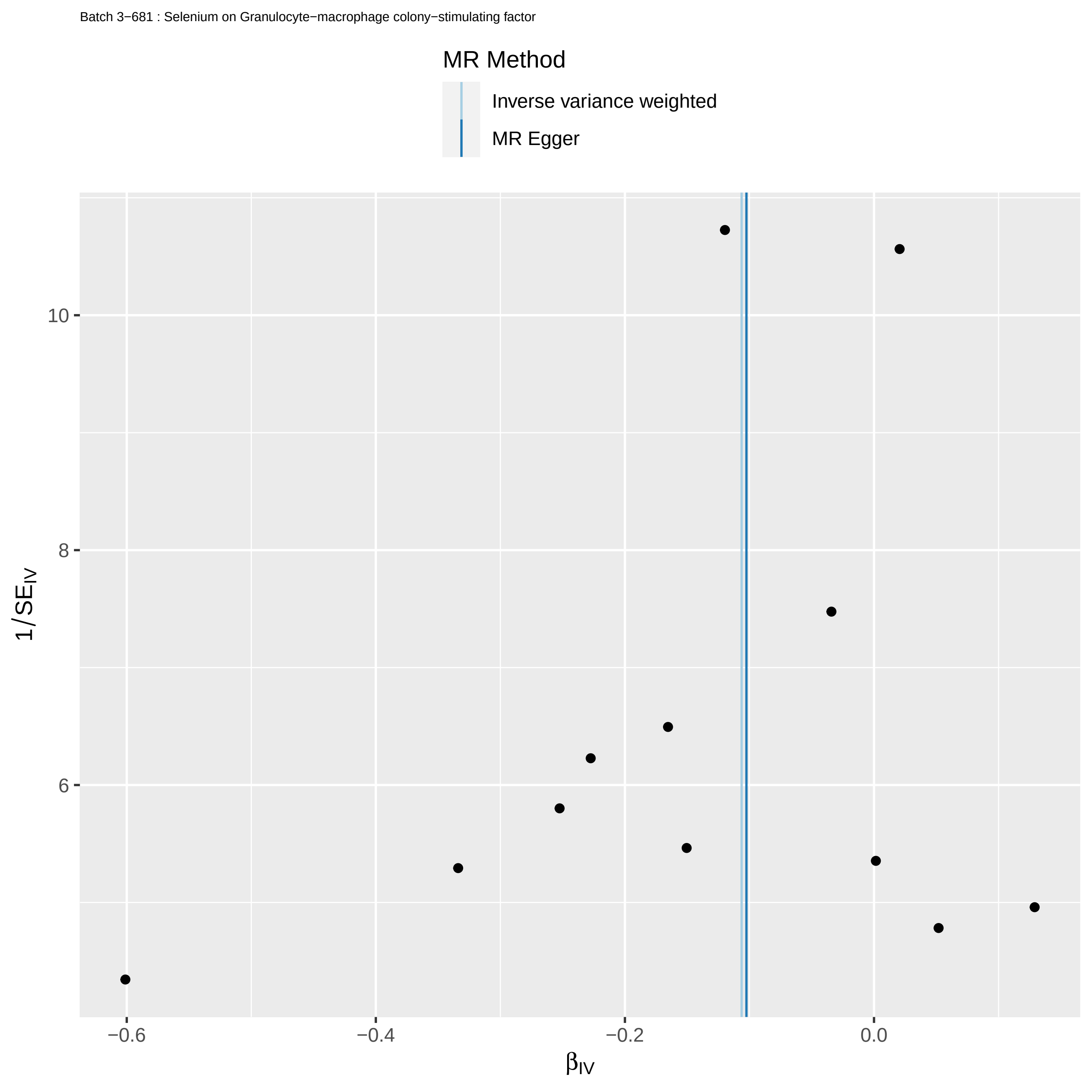
Batch 3−681 : Selenium on Granulocyte−macrophage colony−stimulating factor
MR Method
Inverse variance weighted
MR Egger
10
8
6
1/SE_IV
−0.6
−0.4
−0.2
0.0
β_IV

Batch 3−682 : Selenium on Granulocyte−macrophage colony−stimulating factor receptor subunit alpha

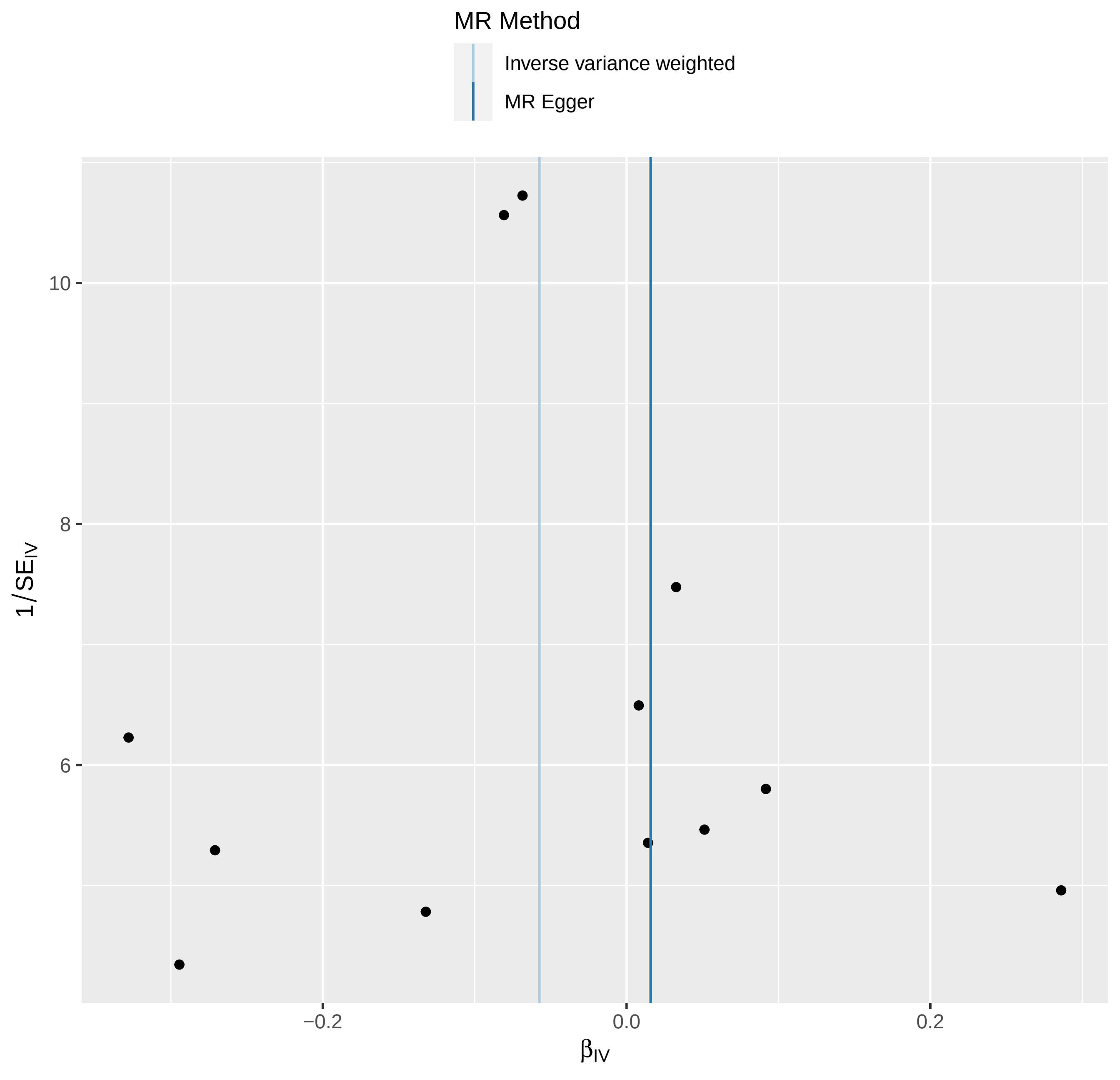

Batch 3−683 : Selenium on Cytokine receptor common subunit beta

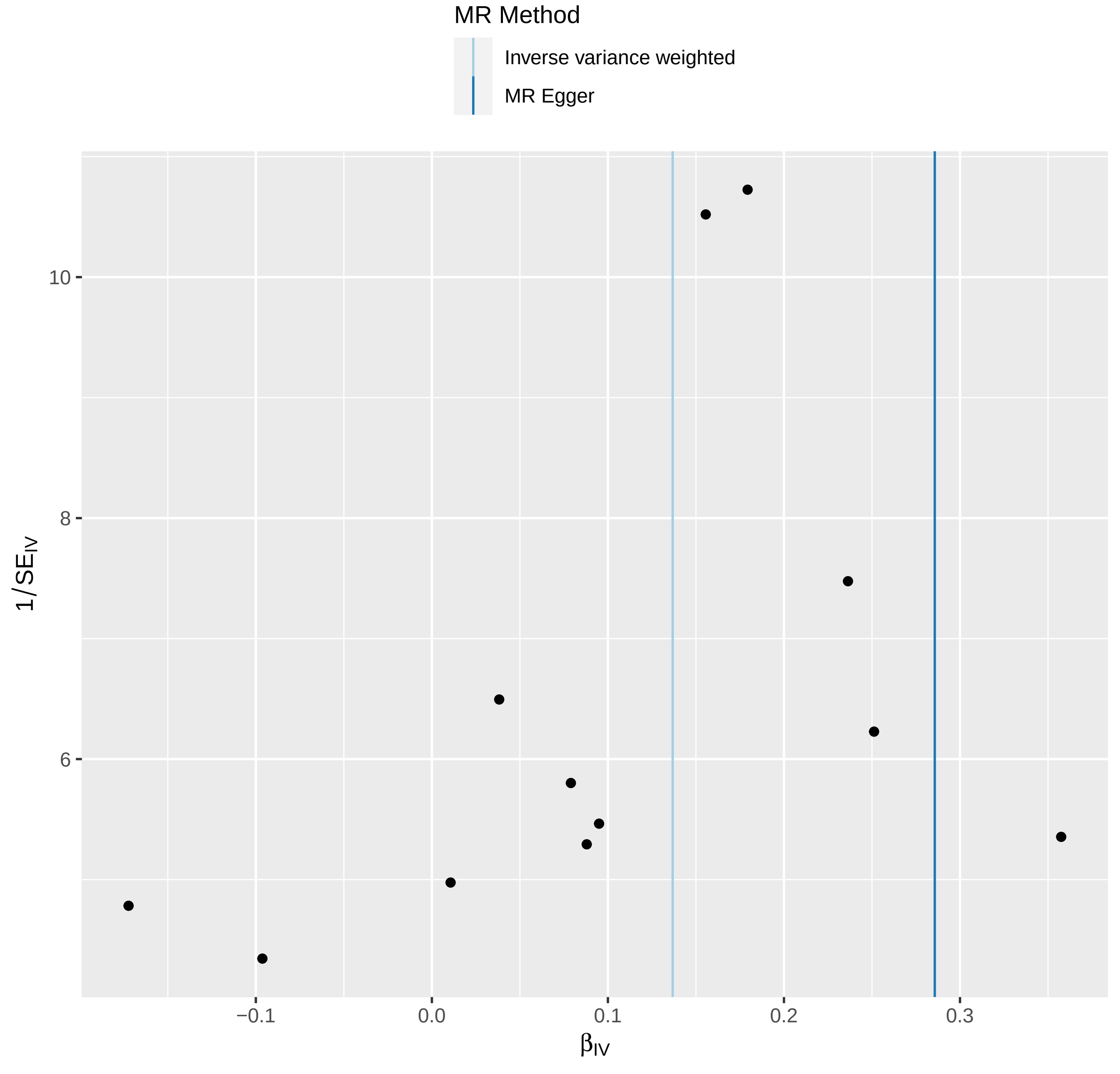

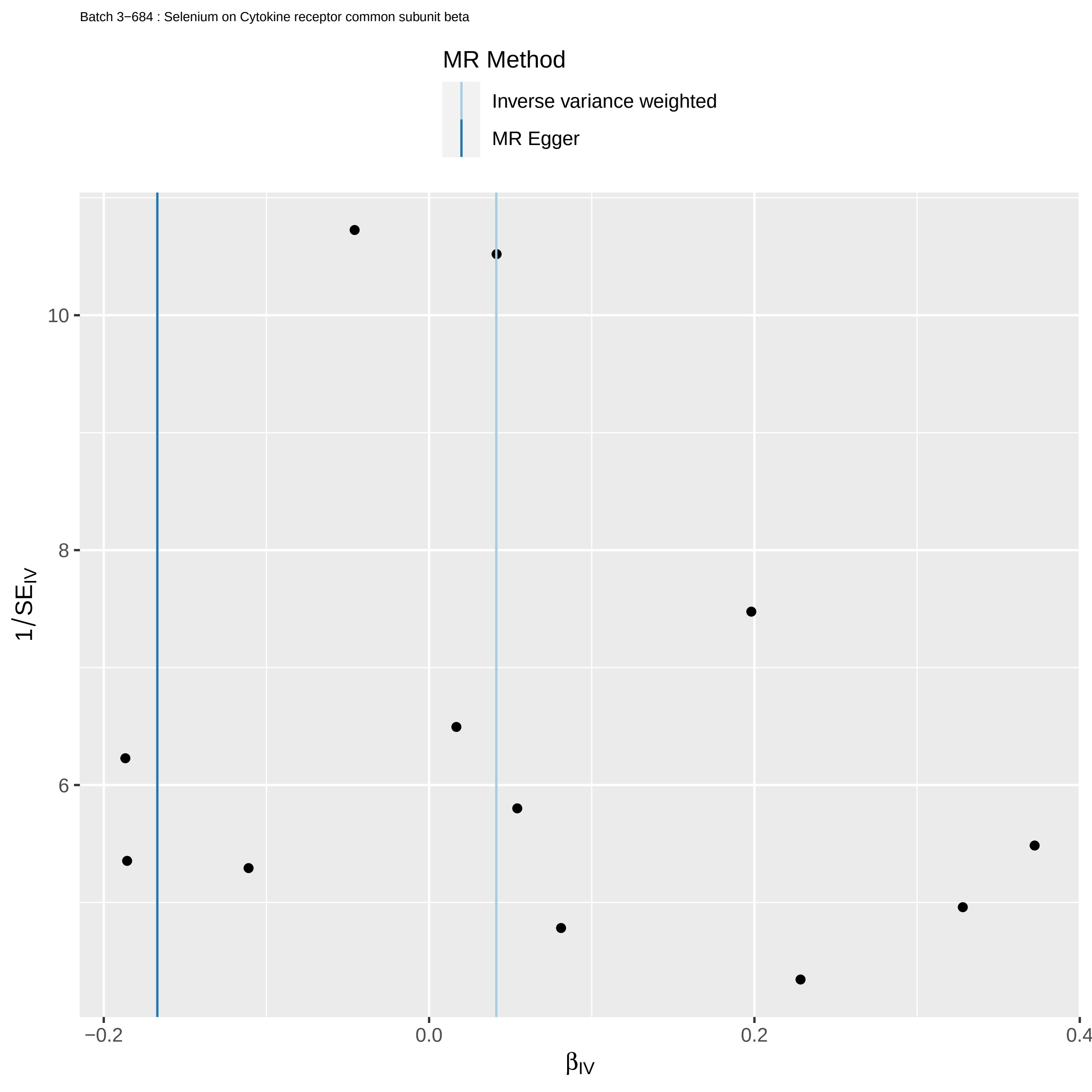
Batch 3−684 : Selenium on Cytokine receptor common subunit beta
MR Method
Inverse variance weighted
MR Egger
10
8
6
1/SE_IV
−0.2
0.0
0.2
0.4
β_IV

Batch 3−685 : Selenium on Granulocyte colony−stimulating factor

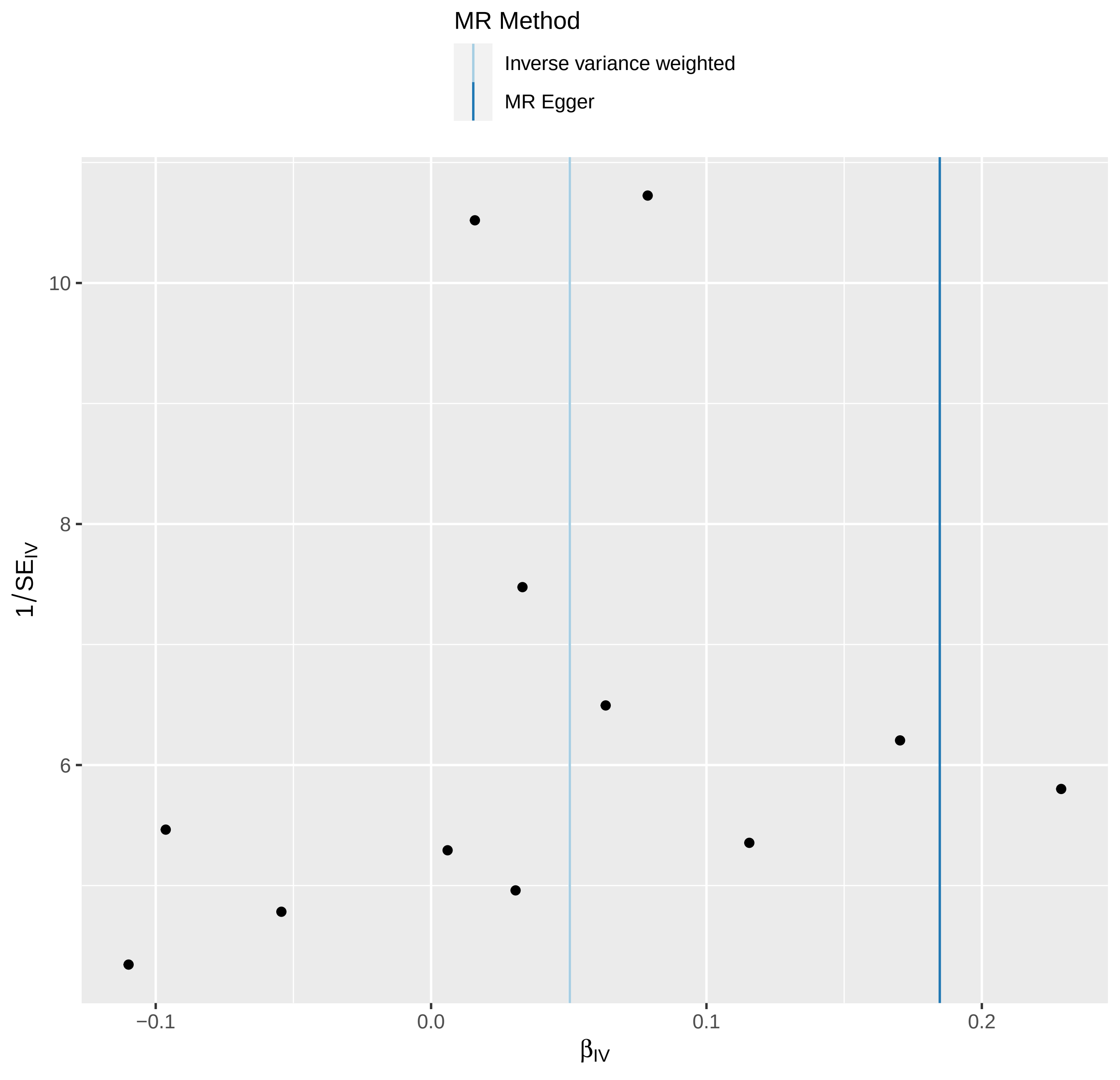

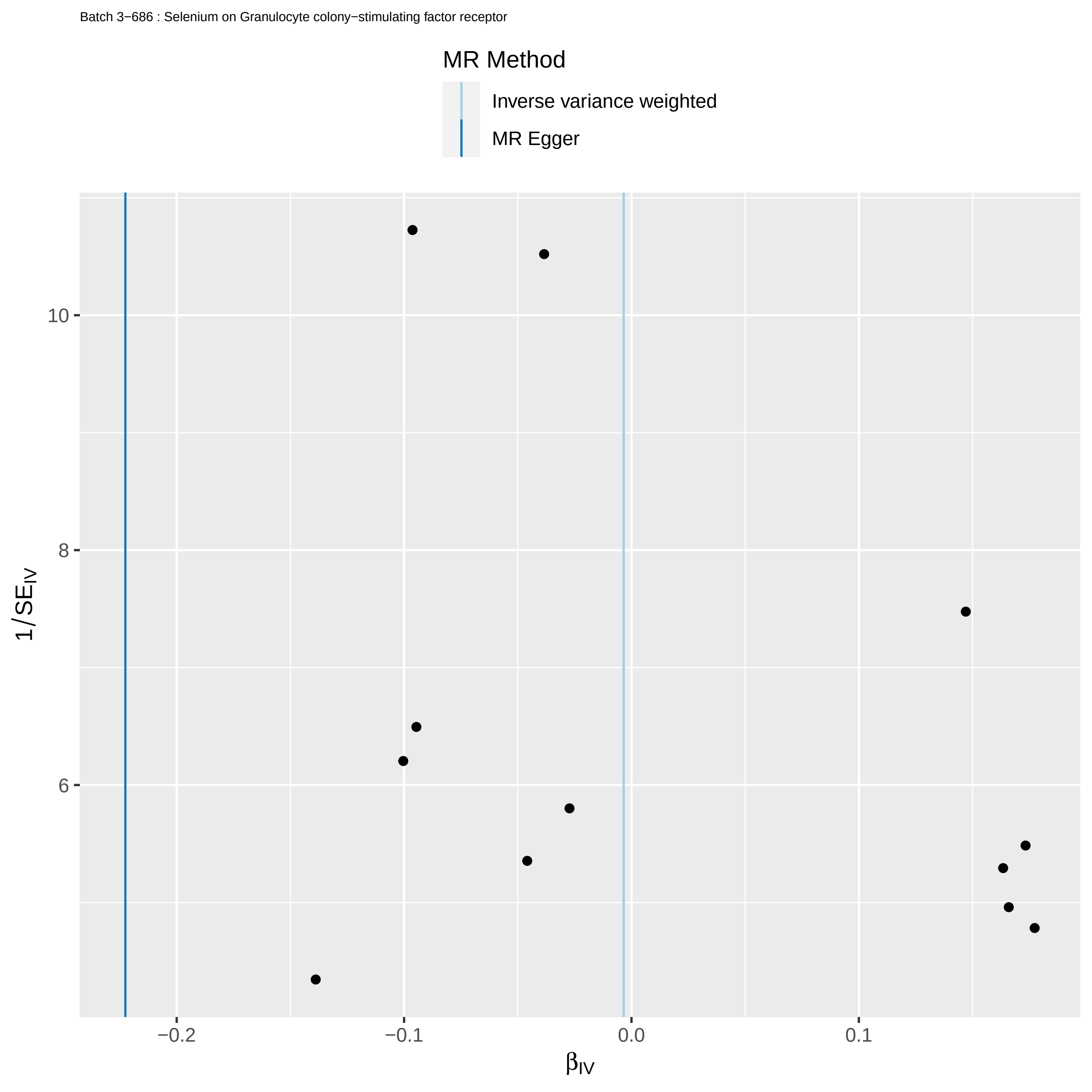
Batch 3−686 : Selenium on Granulocyte colony−stimulating factor receptor
MR Method
Inverse variance weighted
MR Egger
10
8
6
1/SE$_{IV}$
−0.2
−0.1
0.0
0.1
β$_{IV}$

Batch 3−687 : Selenium on Chondroitin sulfate N−acetylgalactosaminyltransferase 2

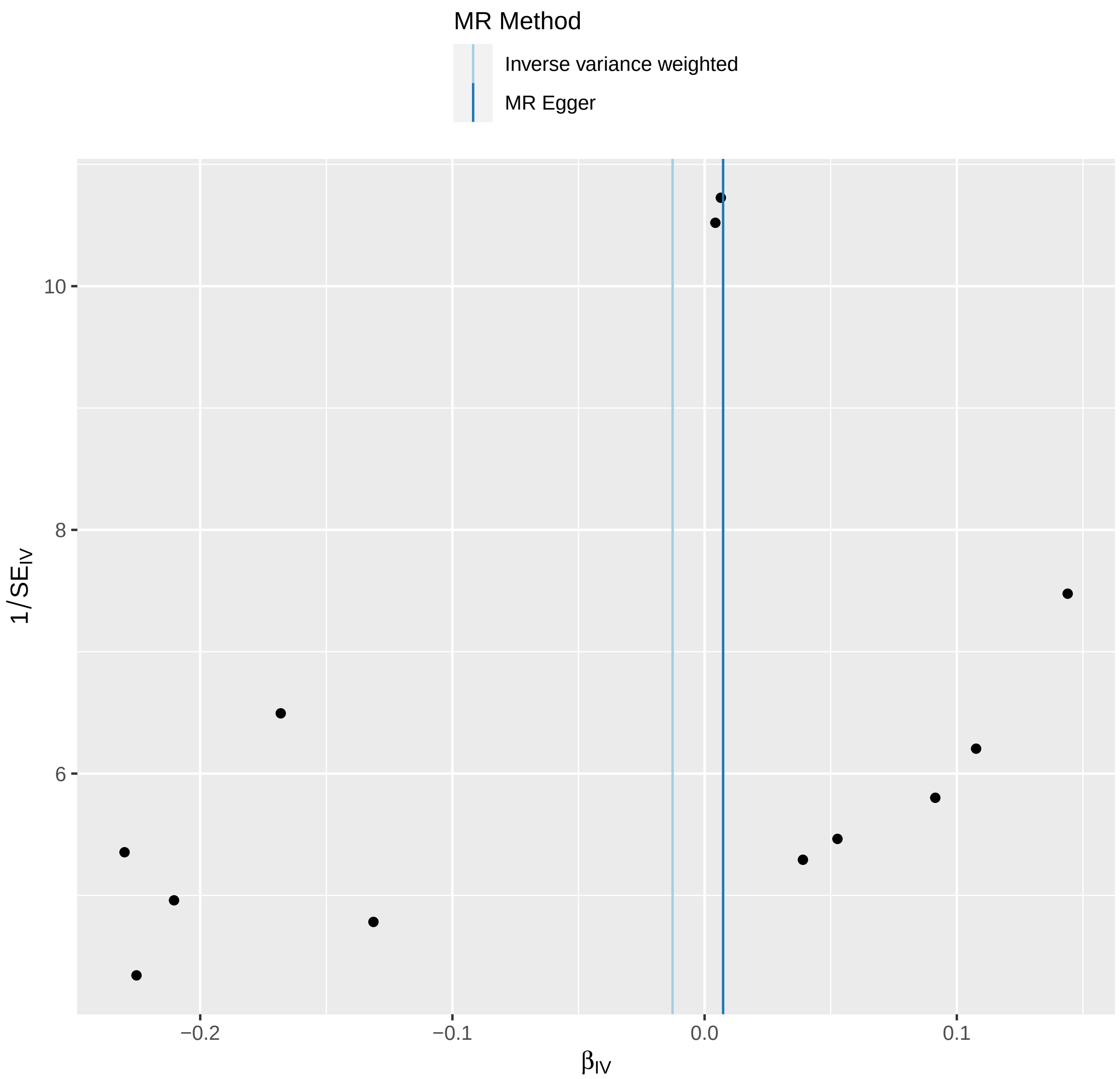

Batch 3−688 : Selenium on Chorionic somatomammotropin hormone

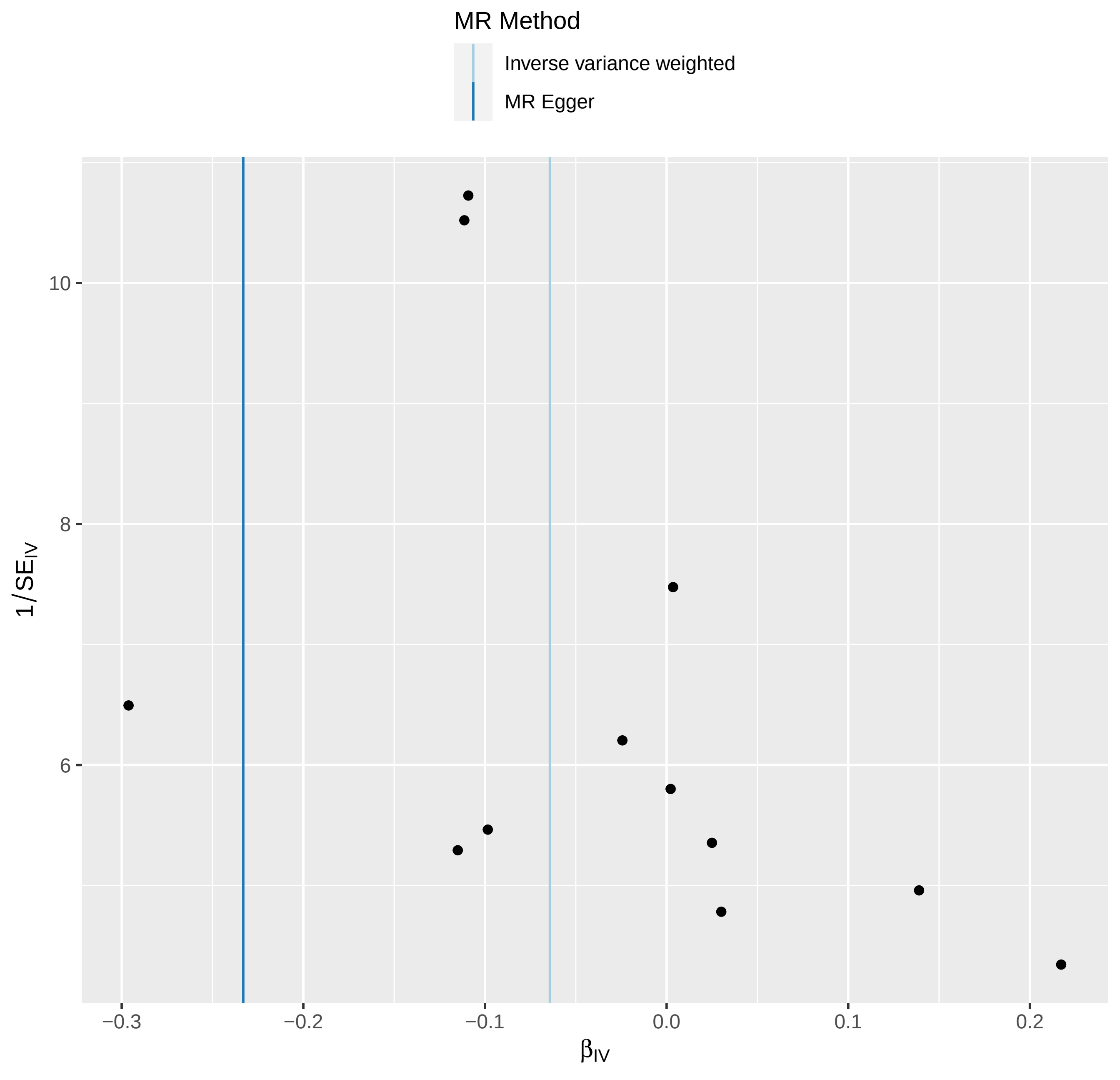

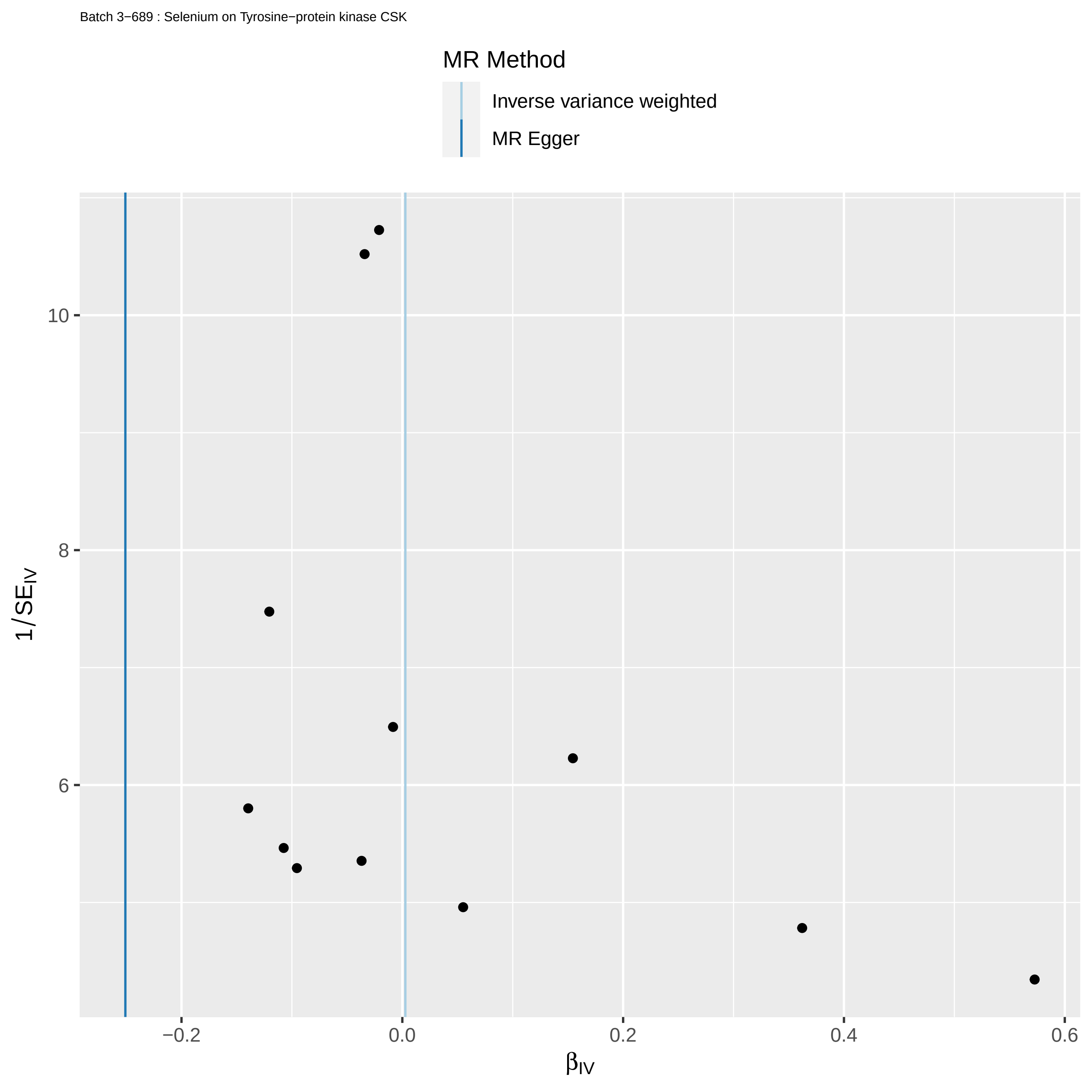
Batch 3−689 : Selenium on Tyrosine−protein kinase CSK
MR Method
Inverse variance weighted
MR Egger
10
8
6
1/SE_IV
−0.2
0.0
0.2
0.4
0.6
β_IV

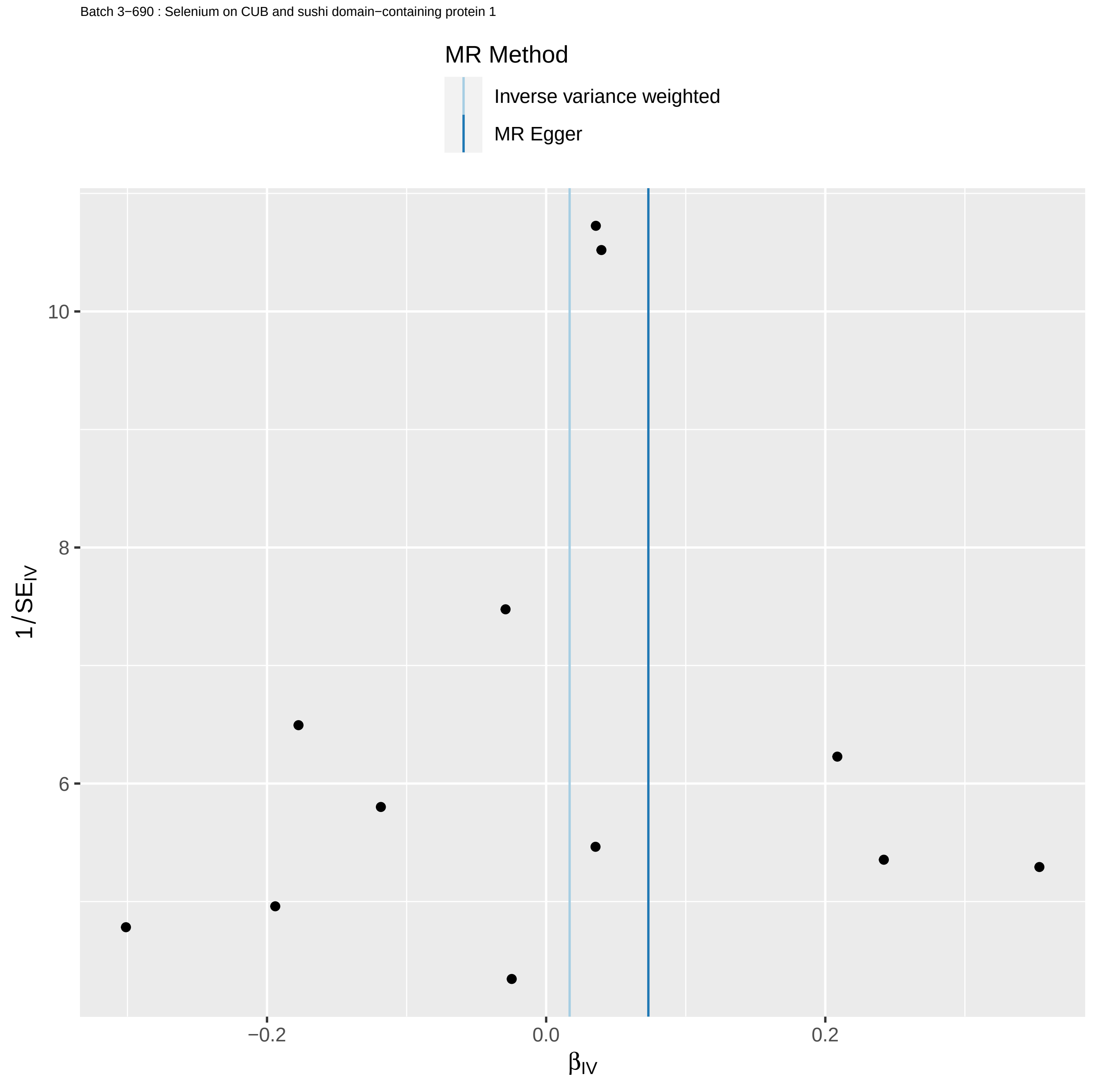

Batch 3−690 : Selenium on CUB and sushi domain−containing protein 1
MR Method
Inverse variance weighted
MR Egger
10
8
6
1/SE_IV
−0.2
0.0
0.2
β_IV

Batch 3−691 : Selenium on CUB and sushi domain−containing protein 2

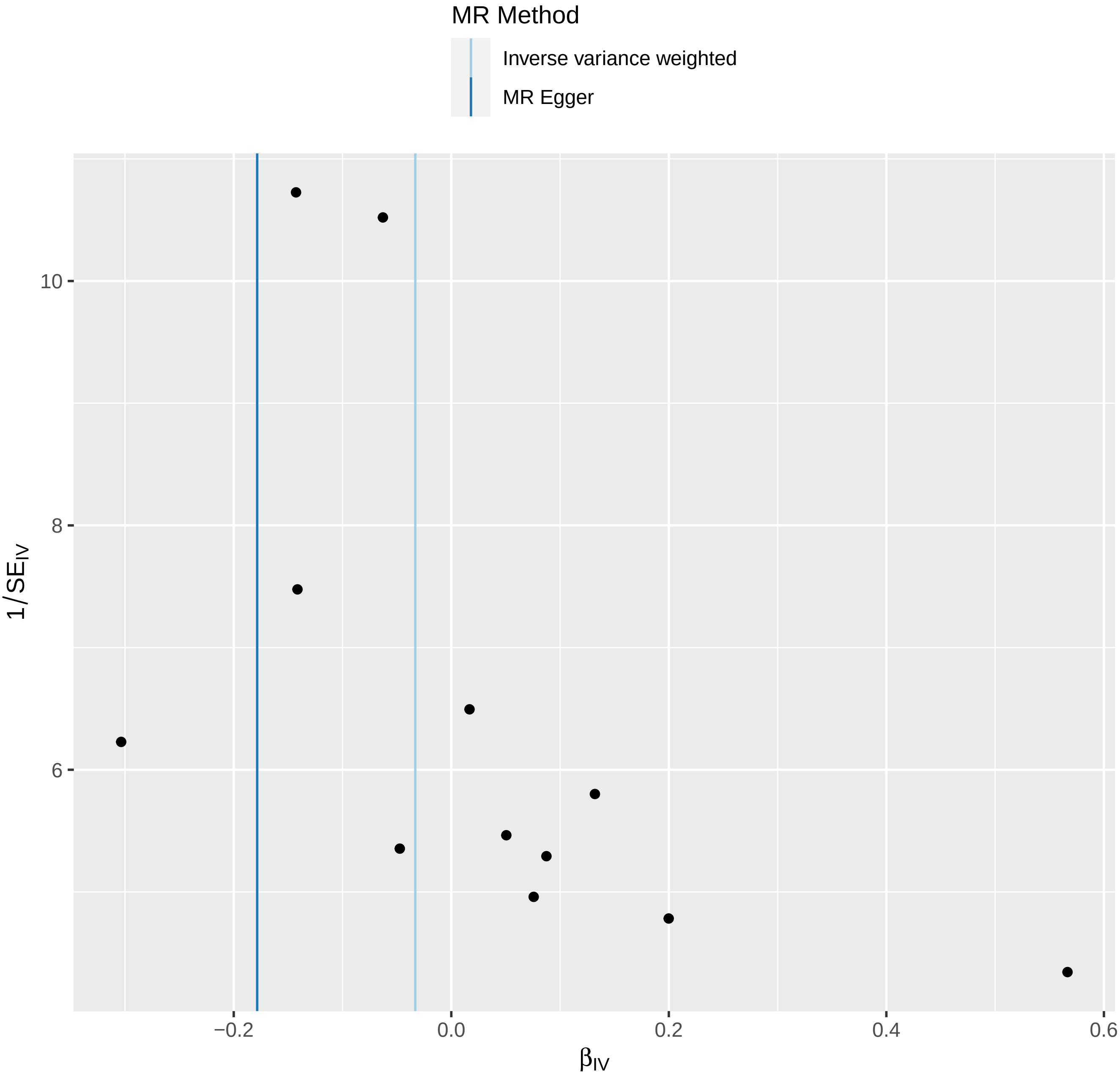

Batch 3−692 : Selenium on alpha−S1−casein

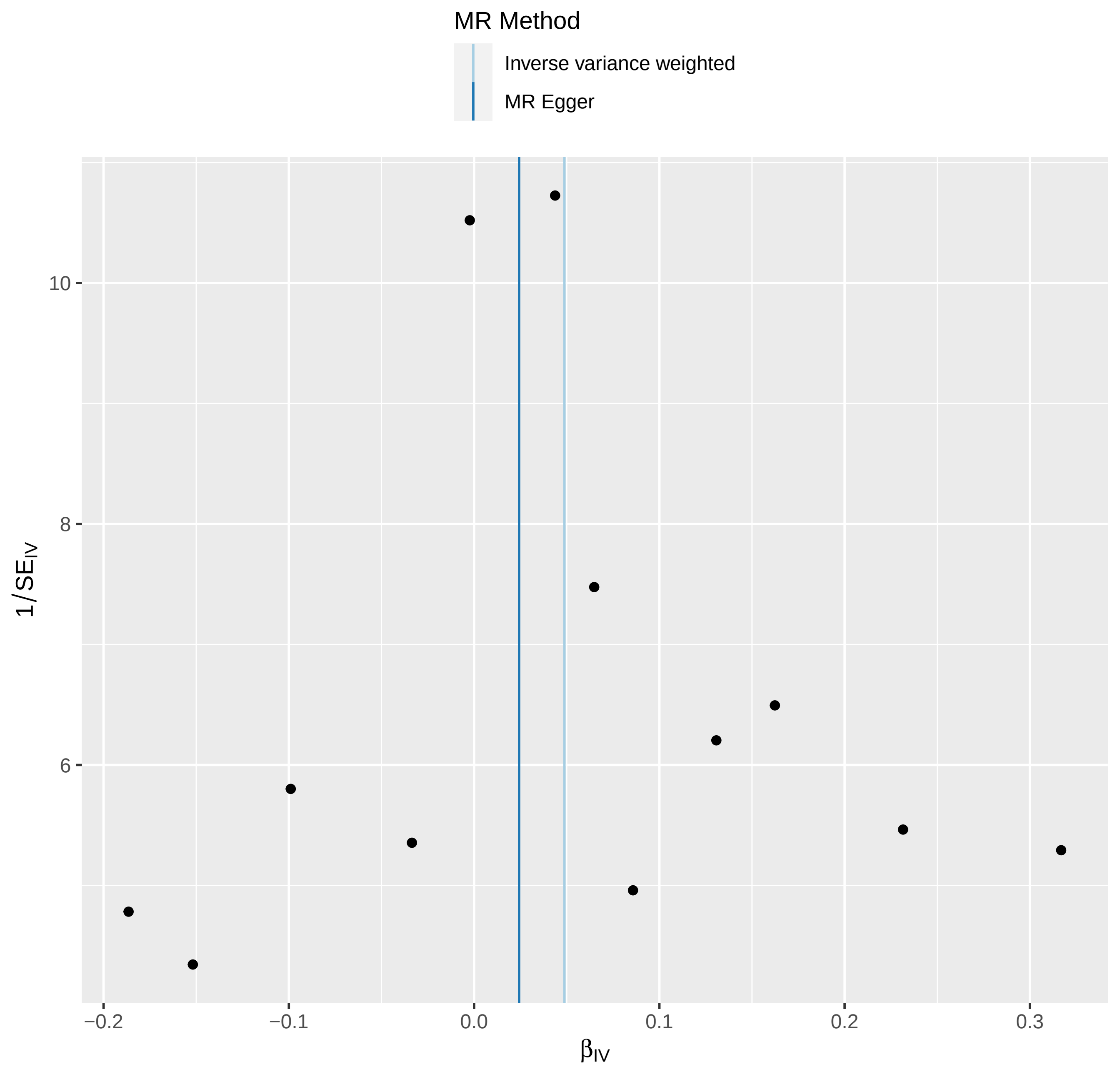

Batch 3−693 : Selenium on Casein kinase I isoform delta

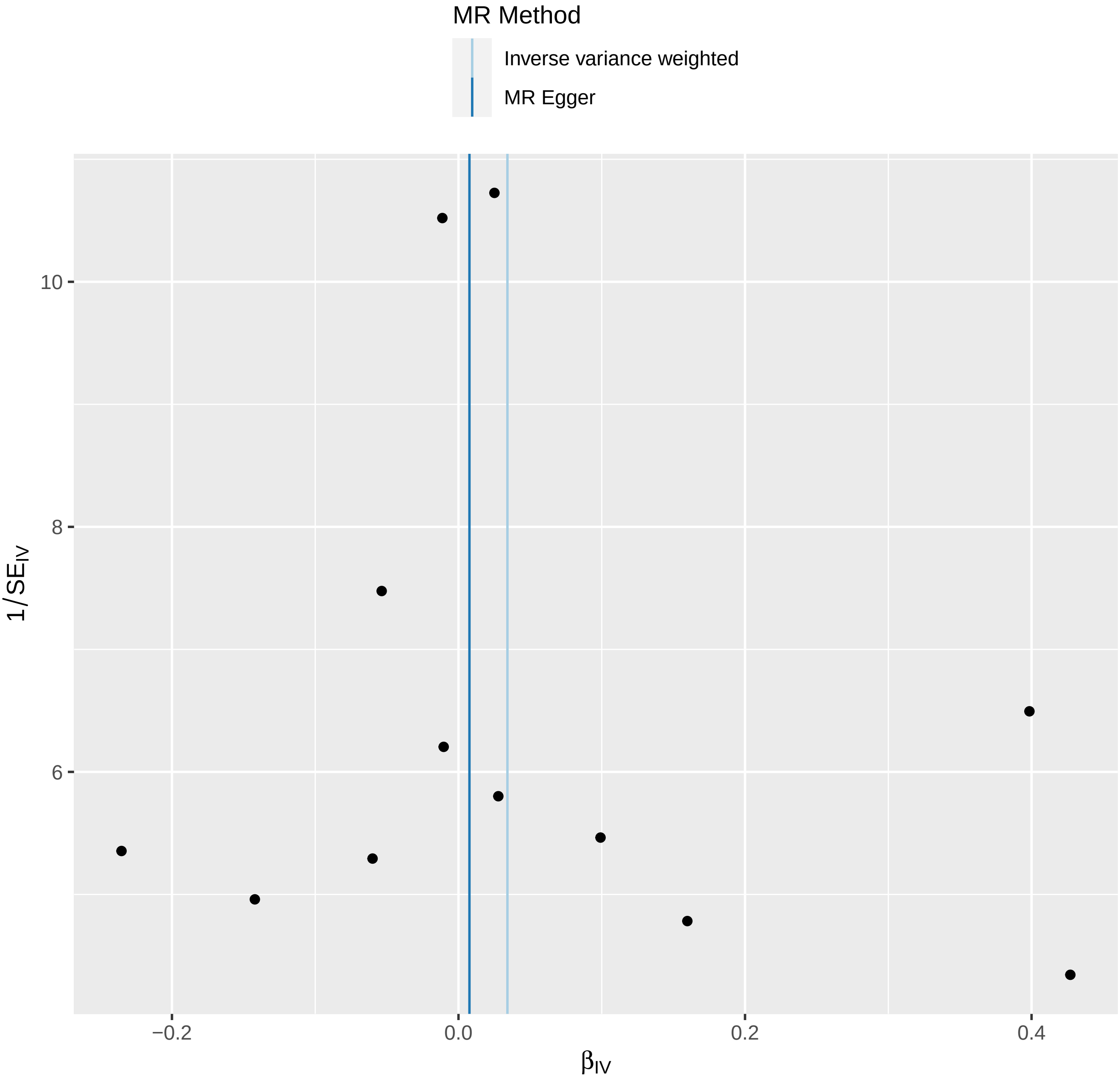

Batch 3−694 : Selenium on Casein kinase I isoform gamma−2

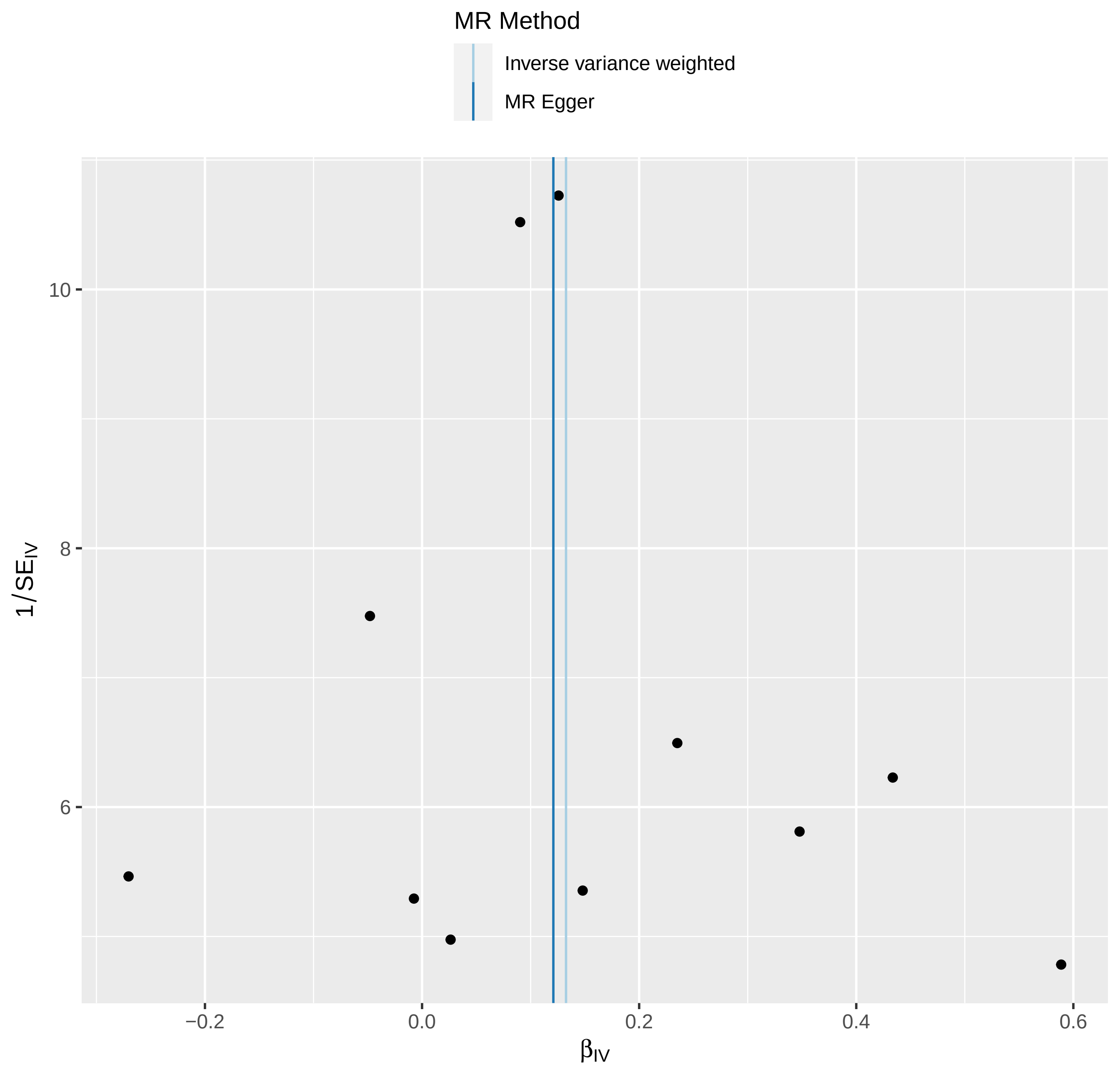

Batch 3−695 : Selenium on Casein kinase II subunit alpha

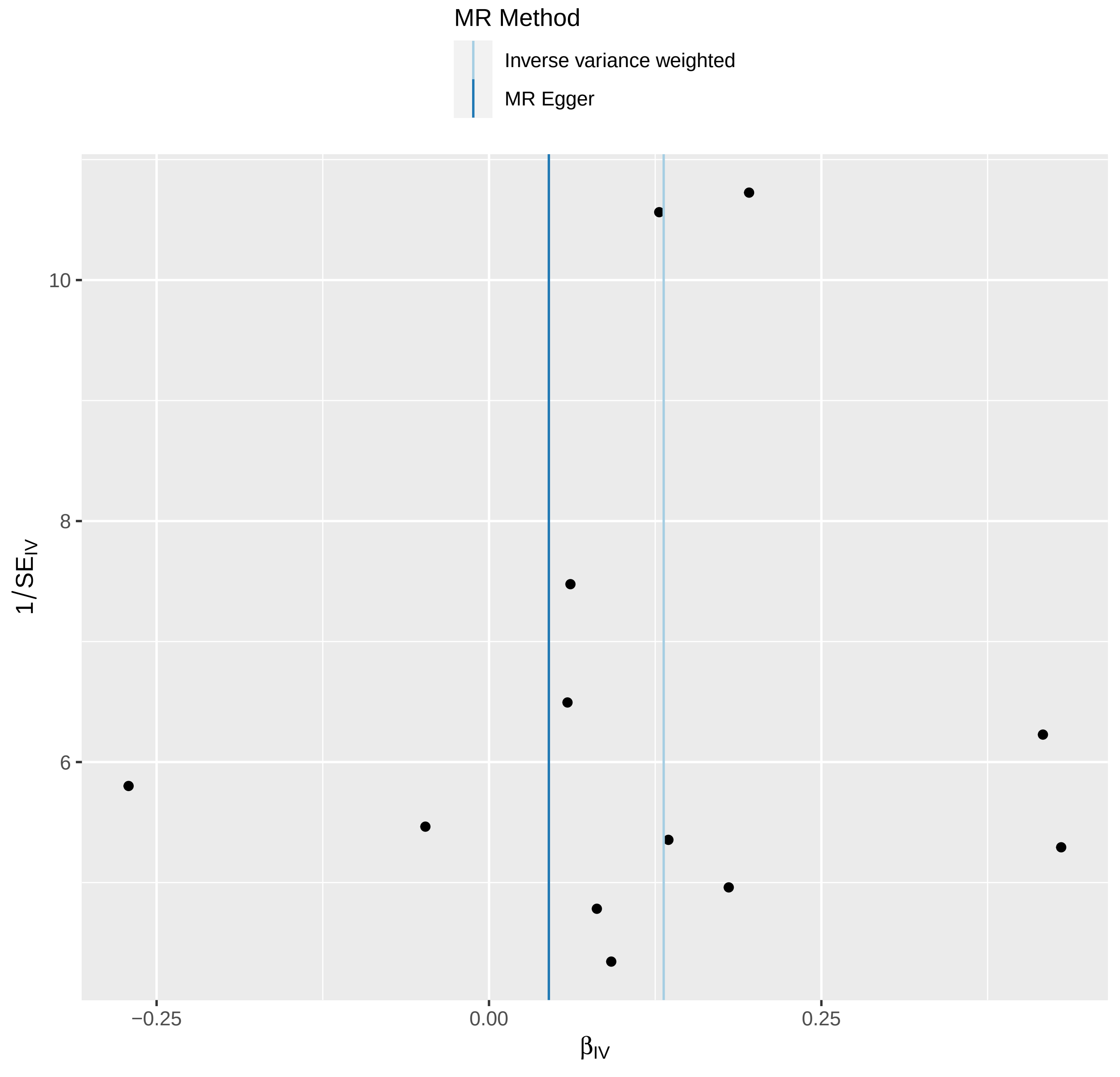

Batch 3−696 : Selenium on Casein kinase II subunit alpha

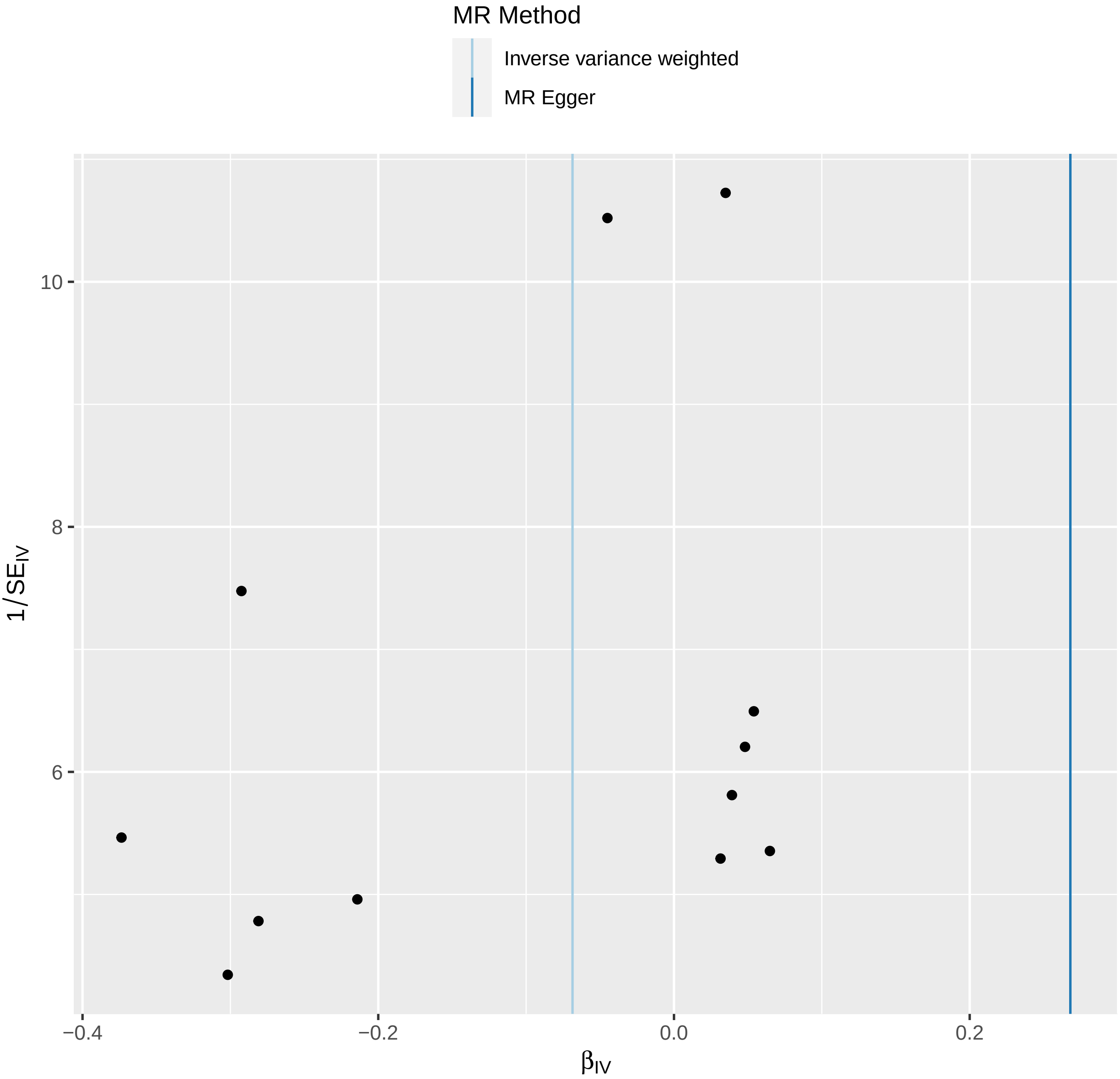

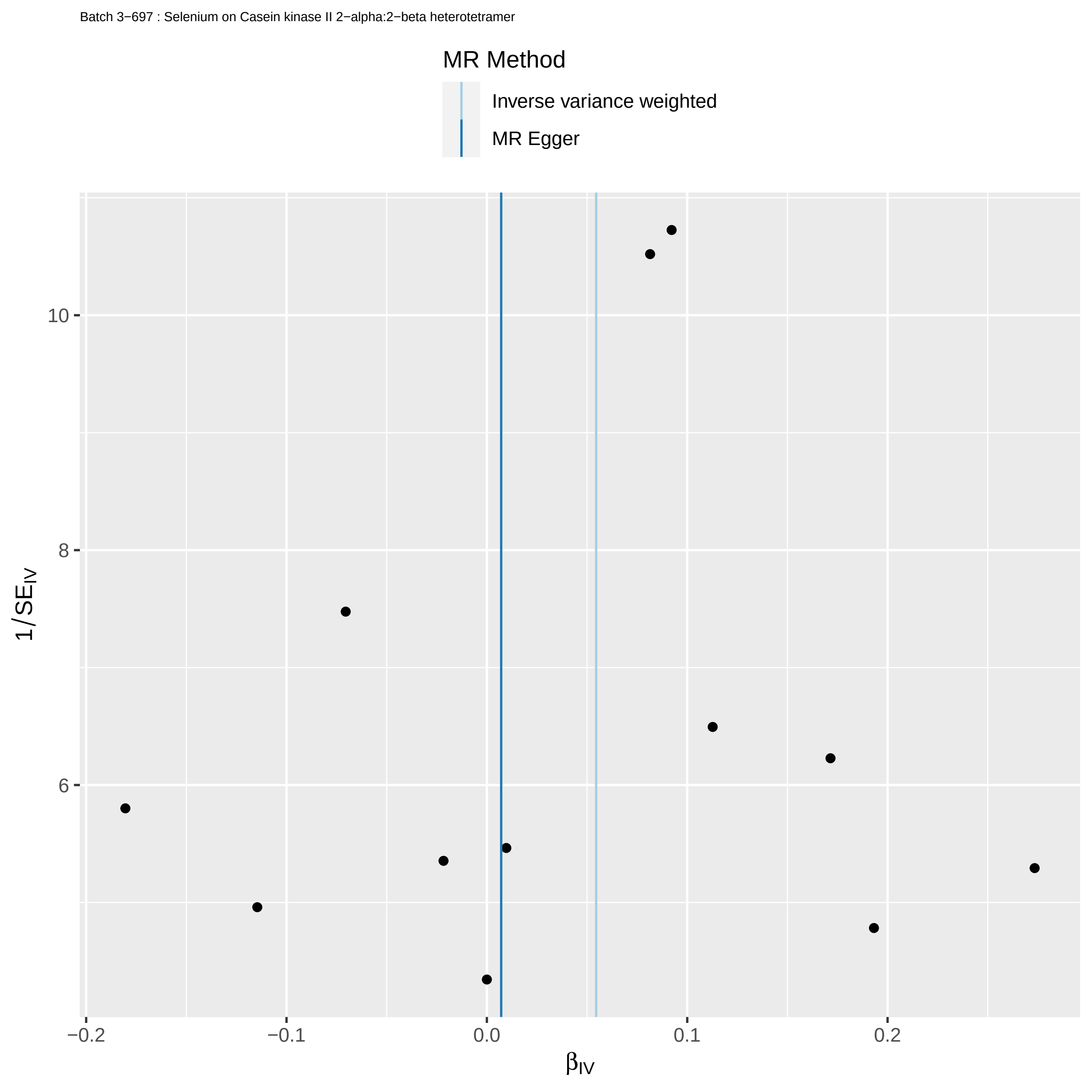
Batch 3−697 : Selenium on Casein kinase II 2−alpha:2−beta heterotetramer
MR Method
Inverse variance weighted
MR Egger
10
8
6
1/SE_IV
−0.2
−0.1
0.0
0.1
0.2
β_IV

Batch 3−698 : Selenium on Casein kinase II 2−alpha':2−beta heterotetramer

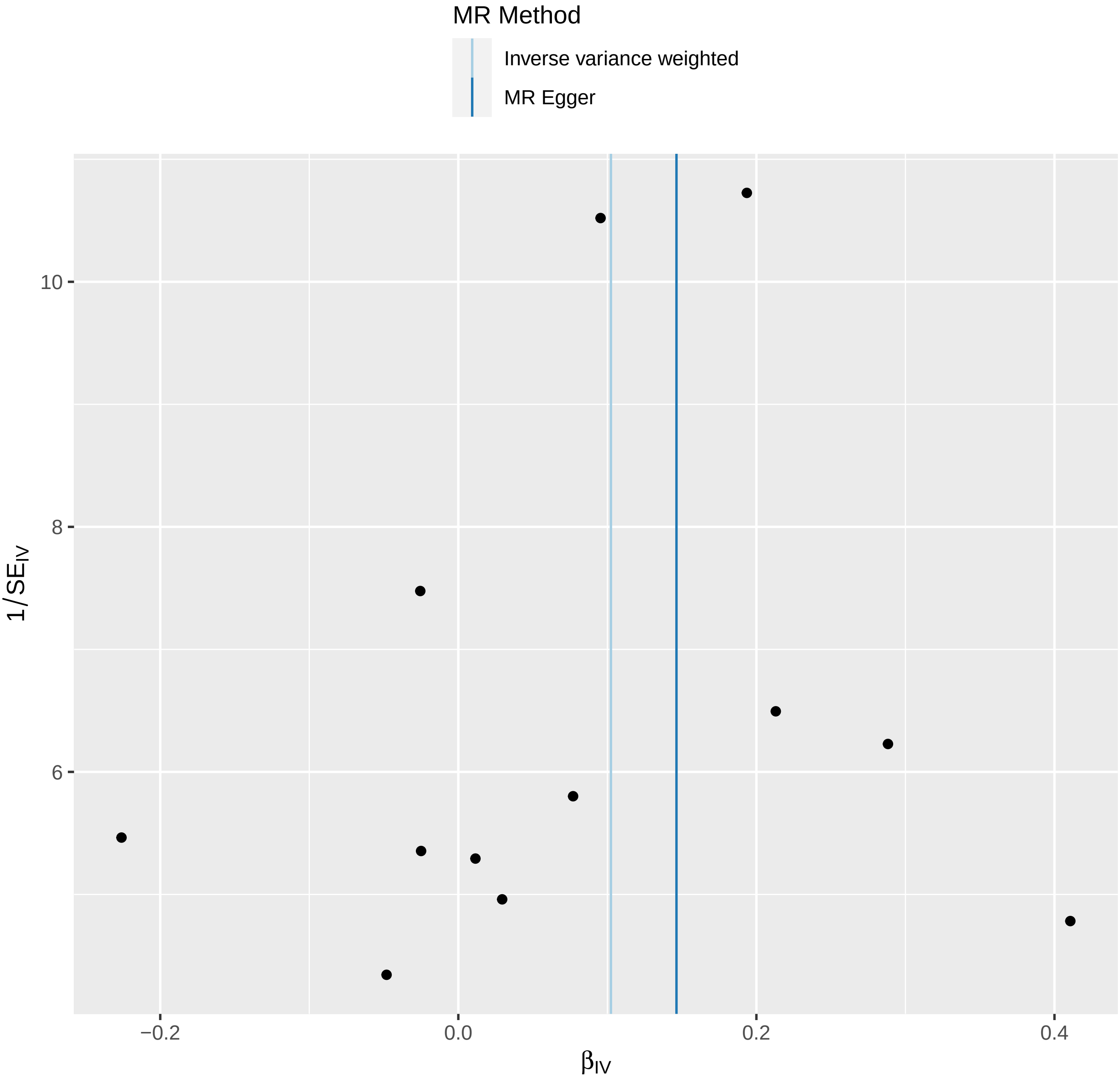

Batch 3−699 : Selenium on Chondroitin sulfate proteoglycan 4

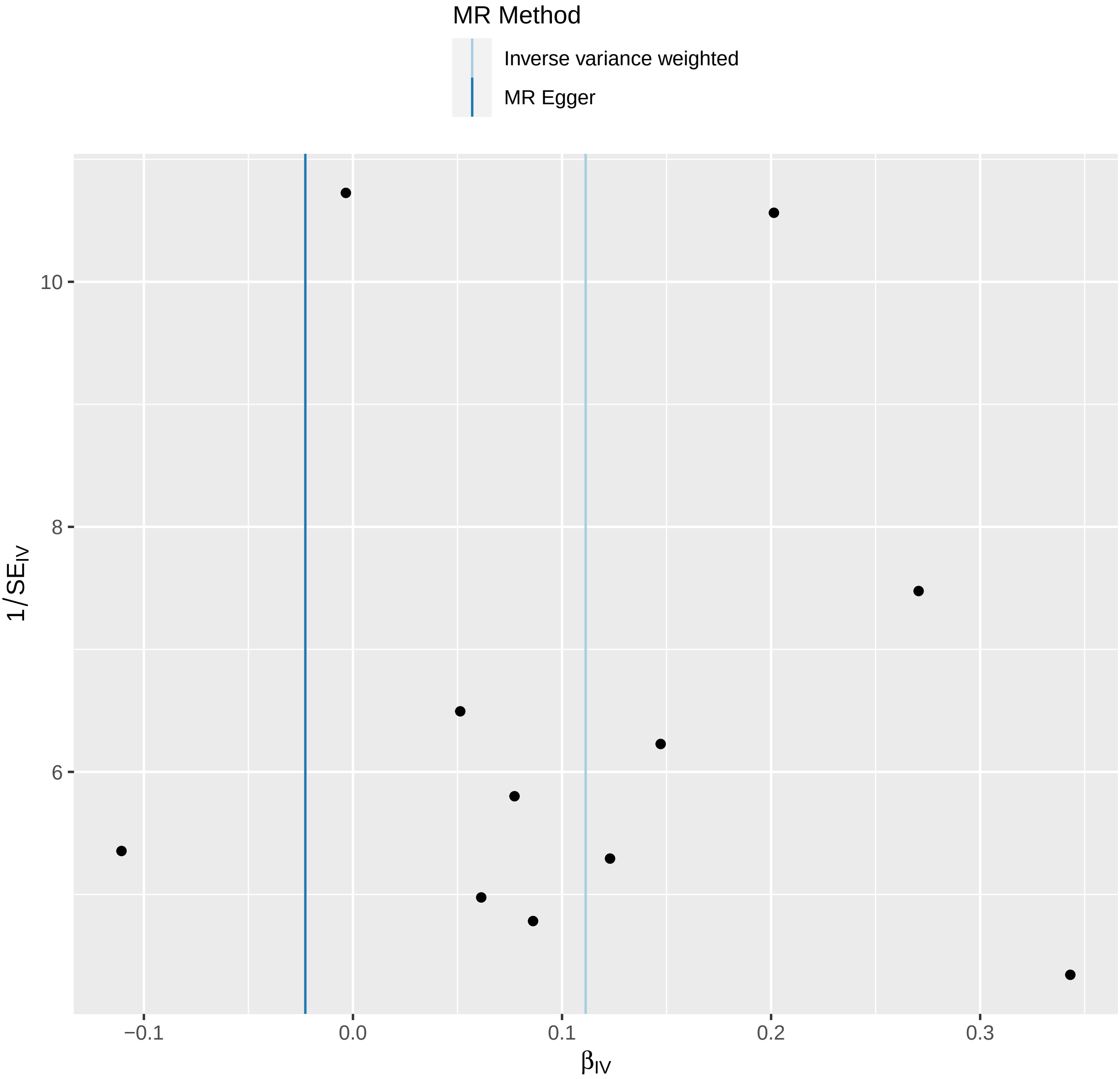

Batch 3−700 : Selenium on Cysteine and glycine−rich protein 2

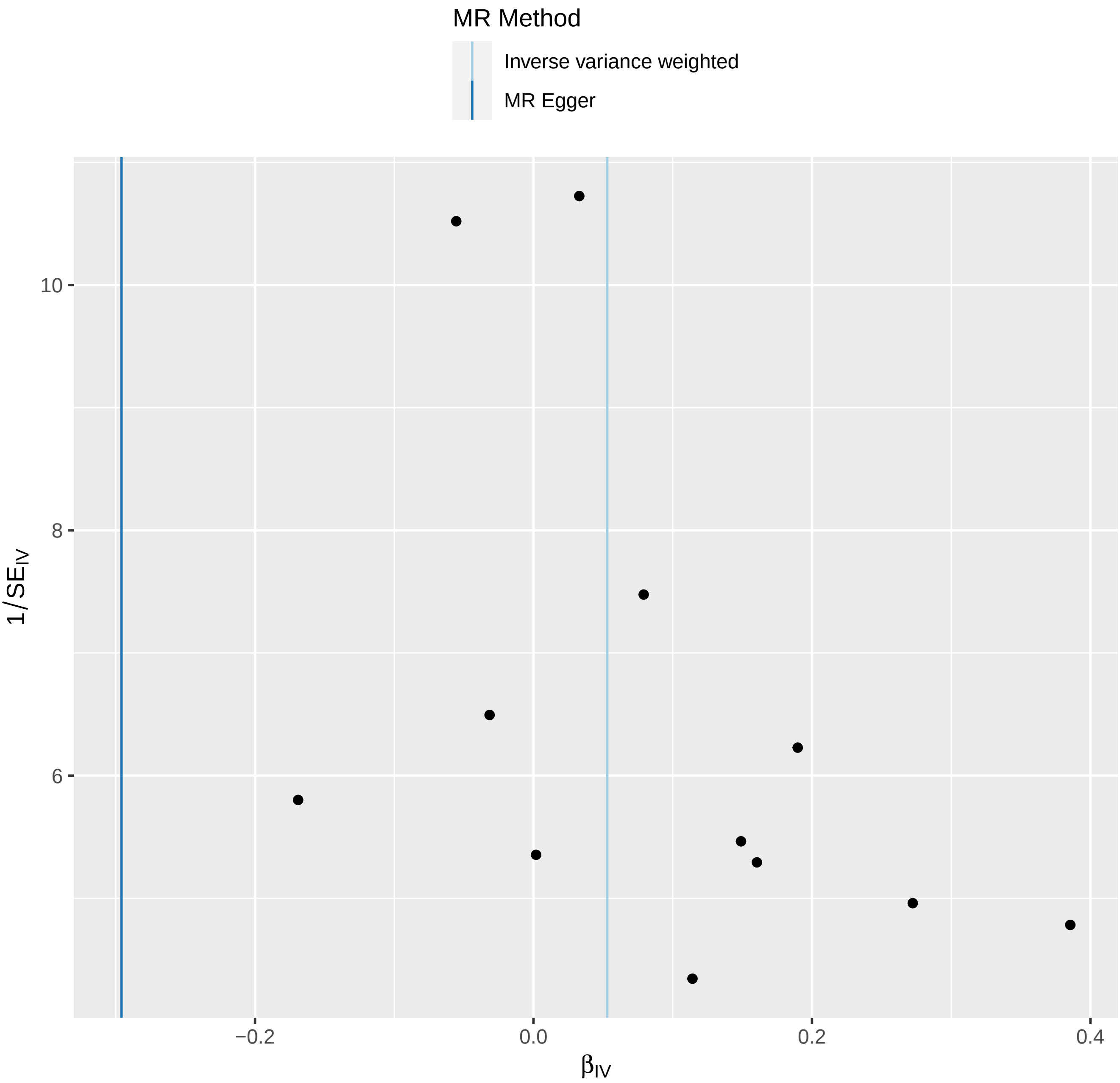

Batch 3−701 : Selenium on Cysteine and glycine−rich protein 3

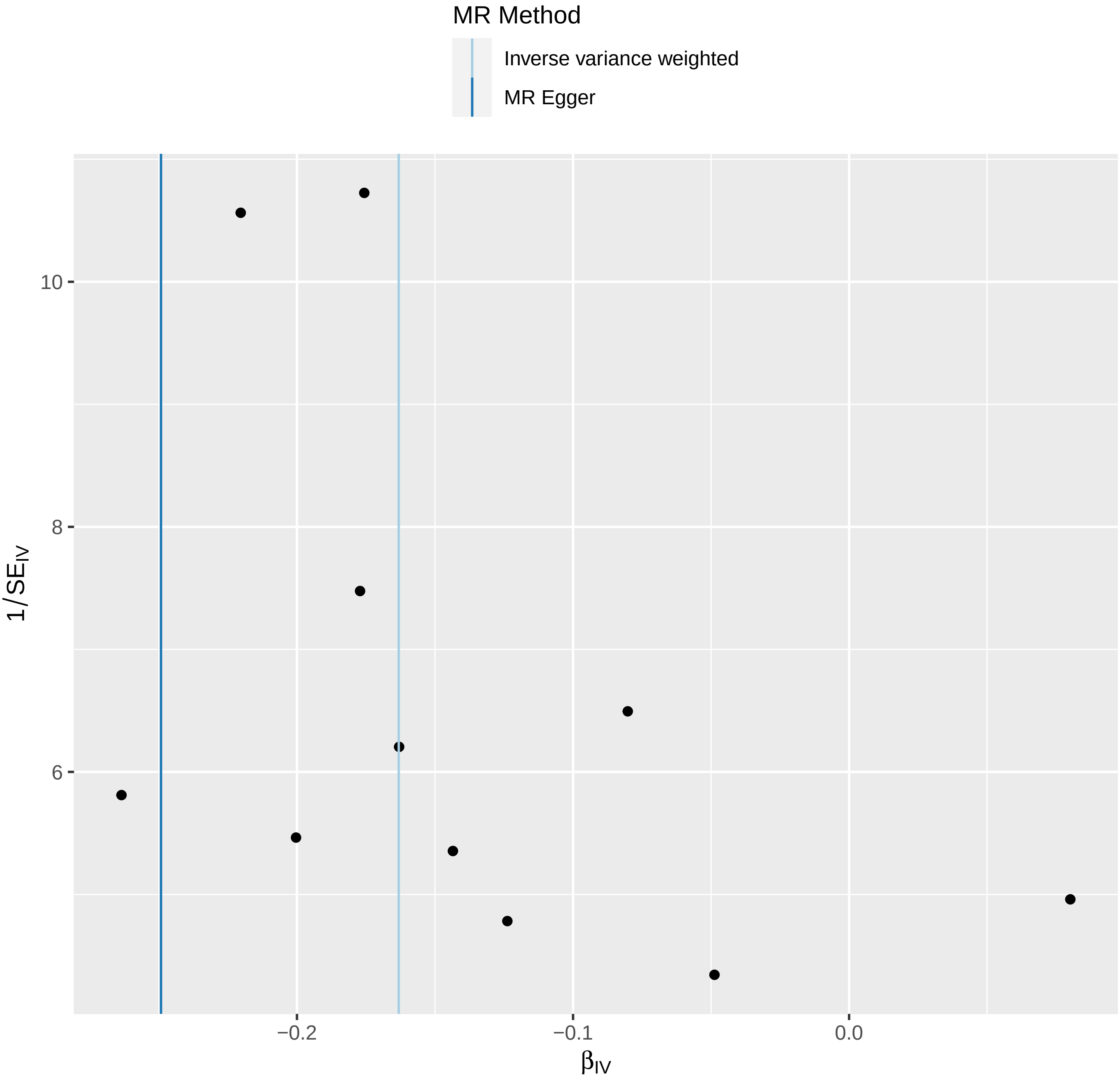

Batch 3−702 : Selenium on Cystatin−D

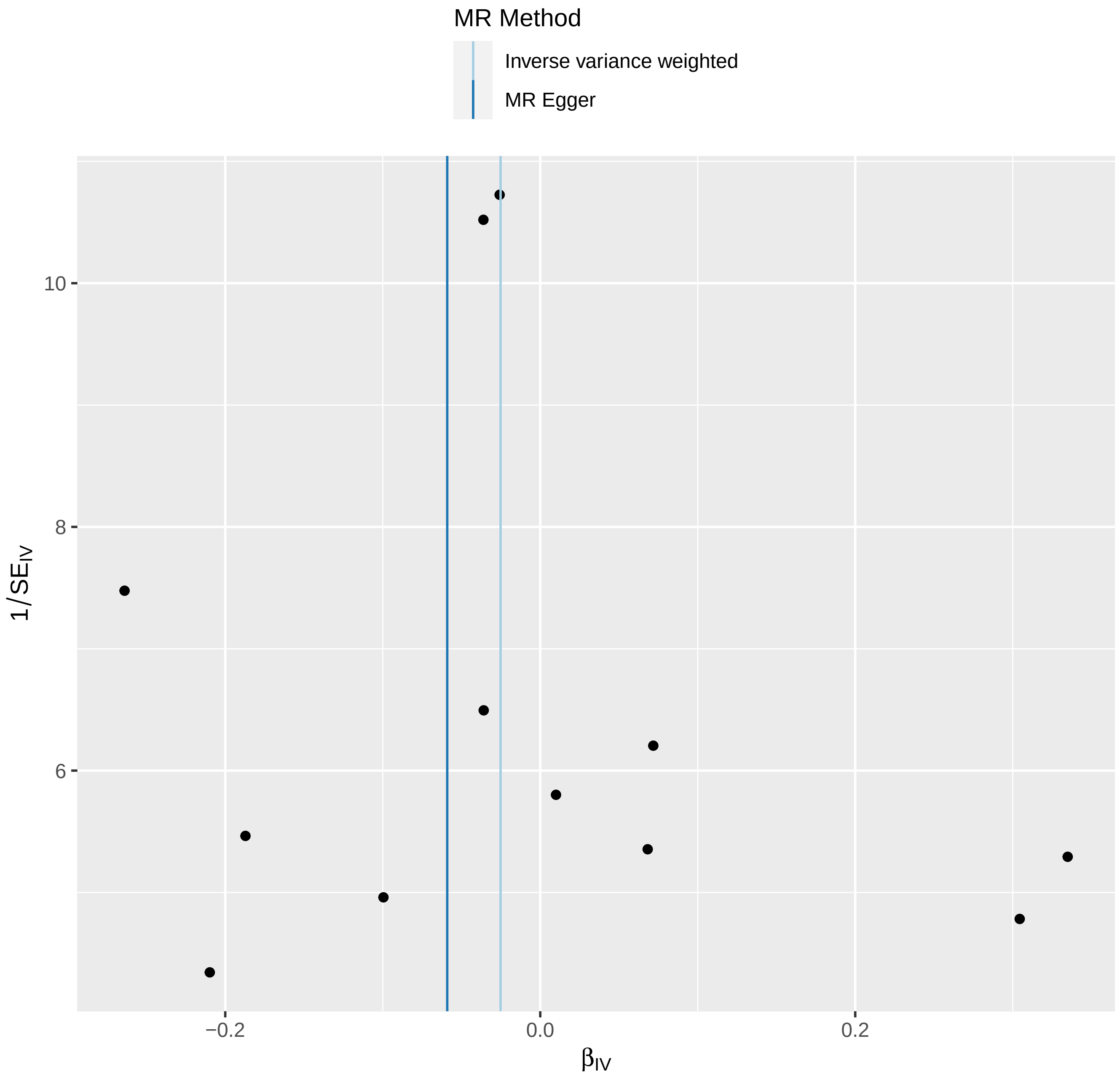

Batch 3−703 : Selenium on Cystatin−M

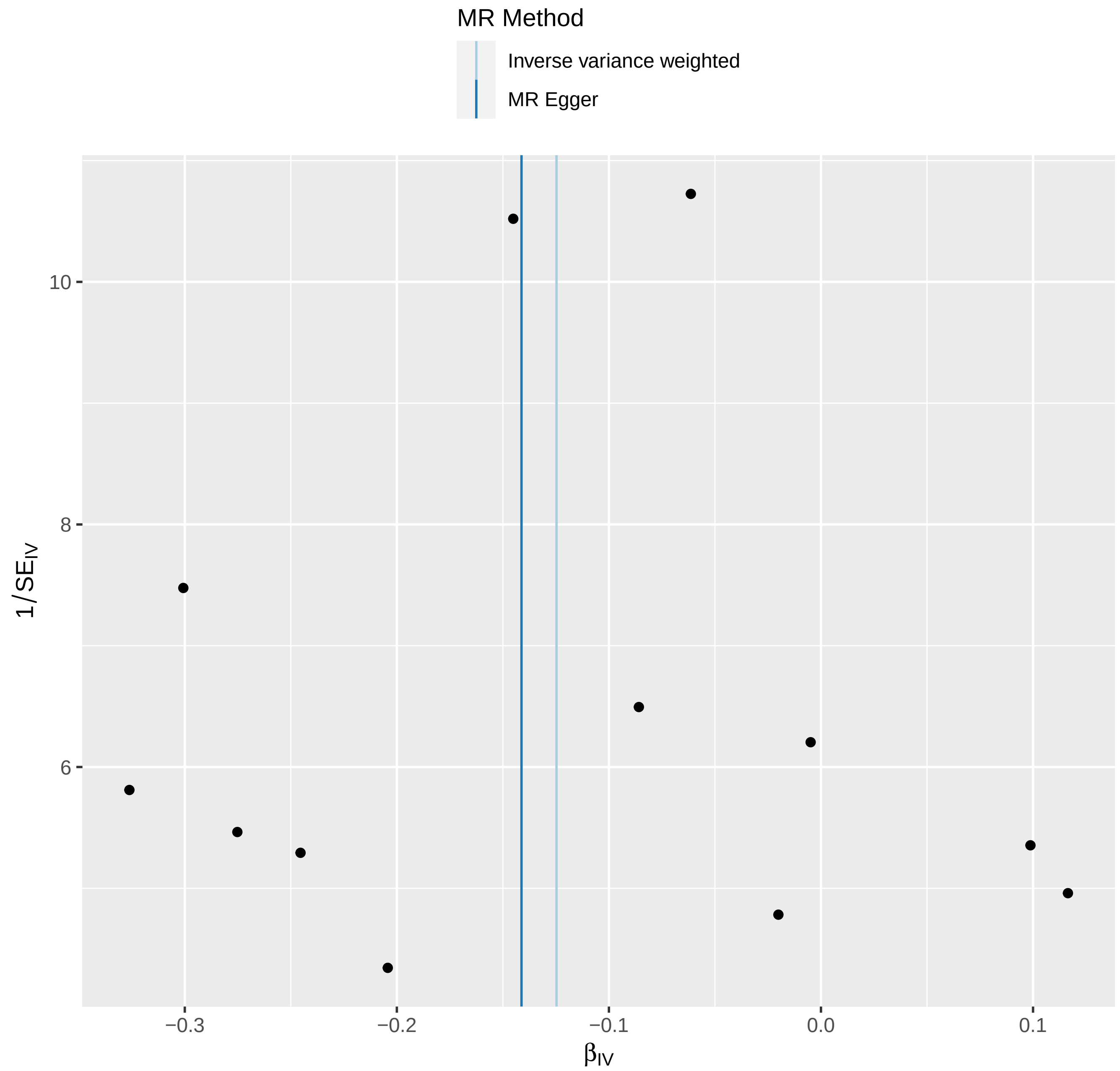

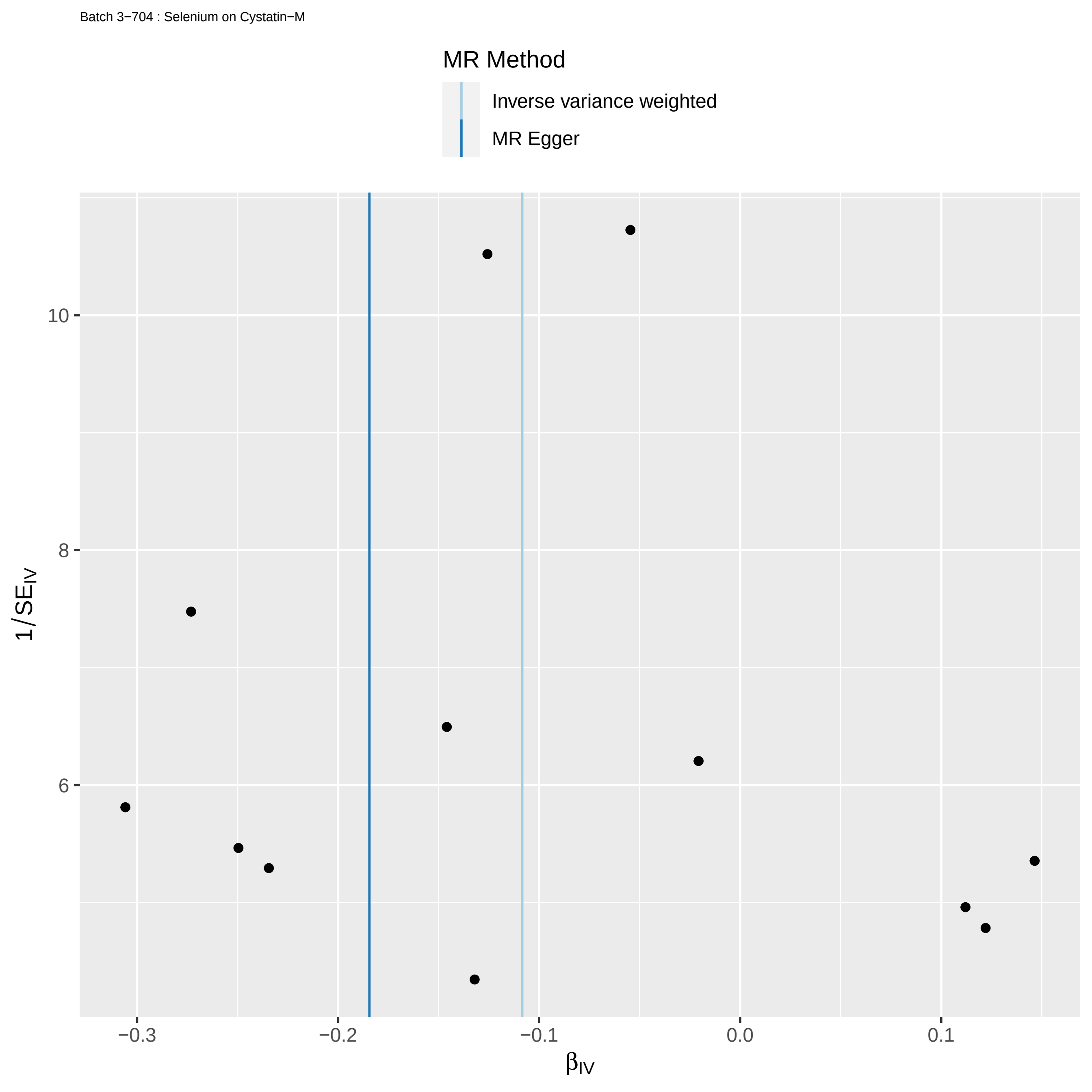

Batch 3−704 : Selenium on Cystatin−M
MR Method
Inverse variance weighted
MR Egger
10
8
6
1/SE_IV
−0.3
−0.2
−0.1
0.0
0.1
β_IV

Batch 3−705 : Selenium on Cystatin−F

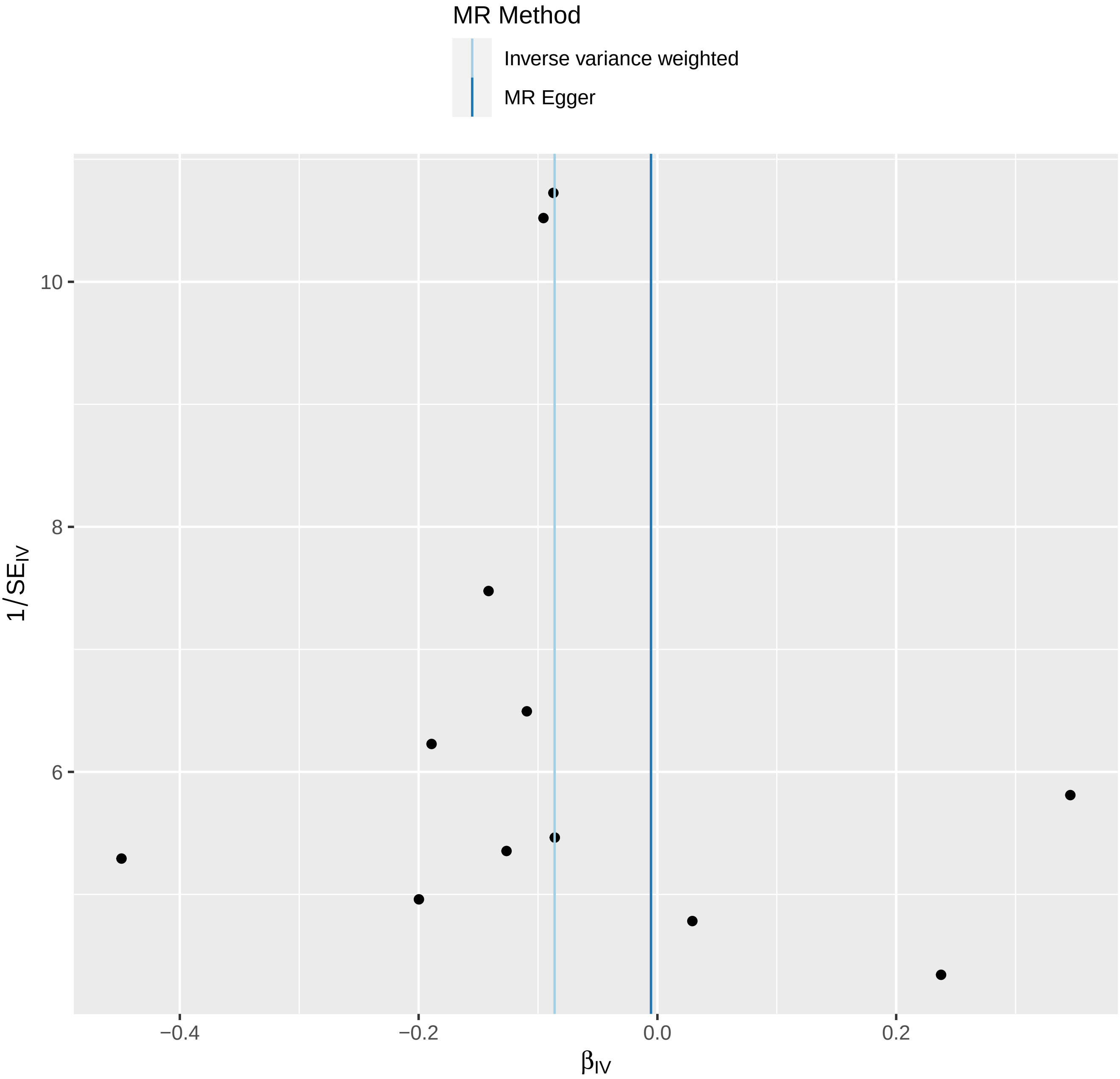

Batch 3−706 : Selenium on Cystatin−8

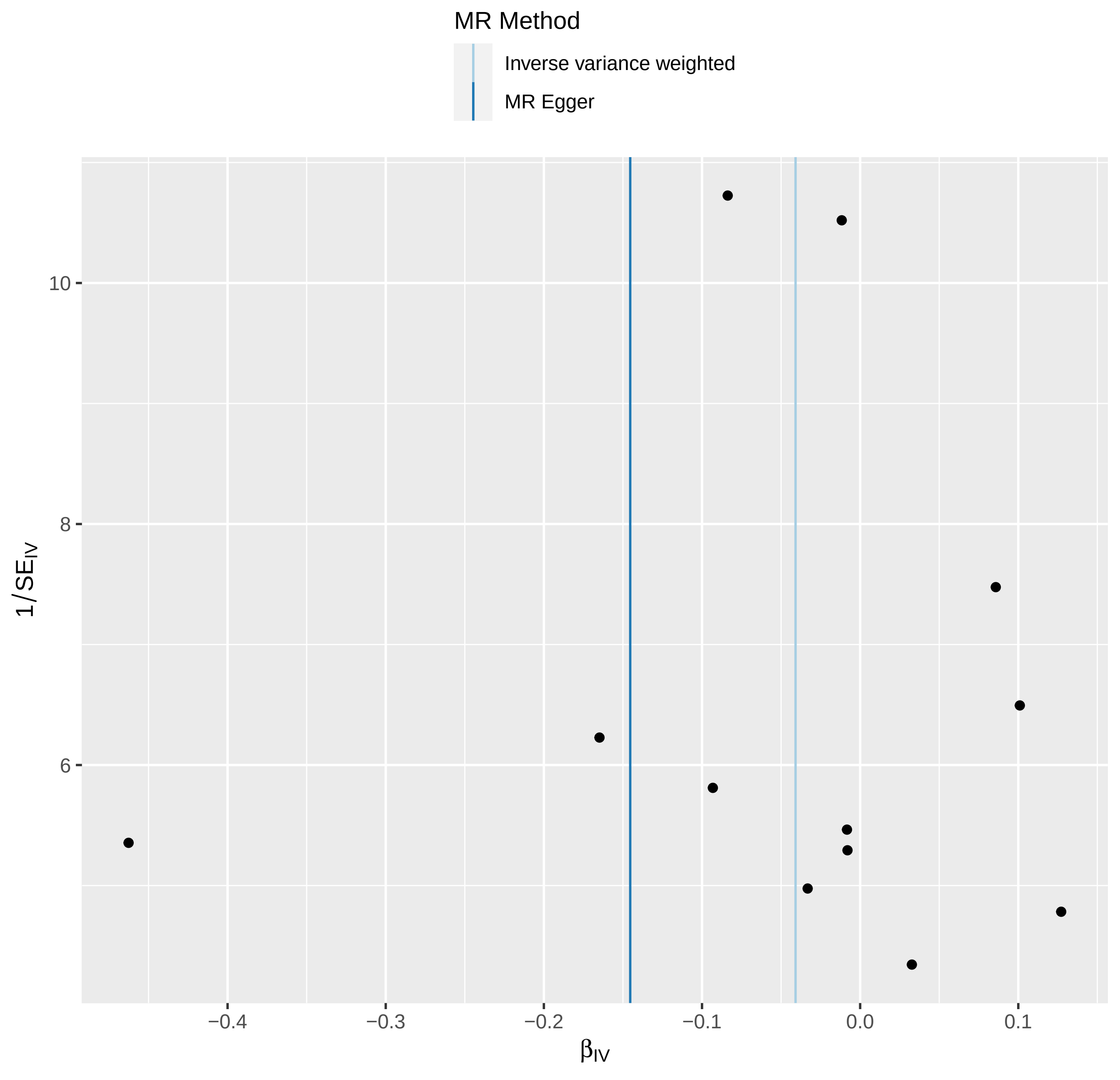

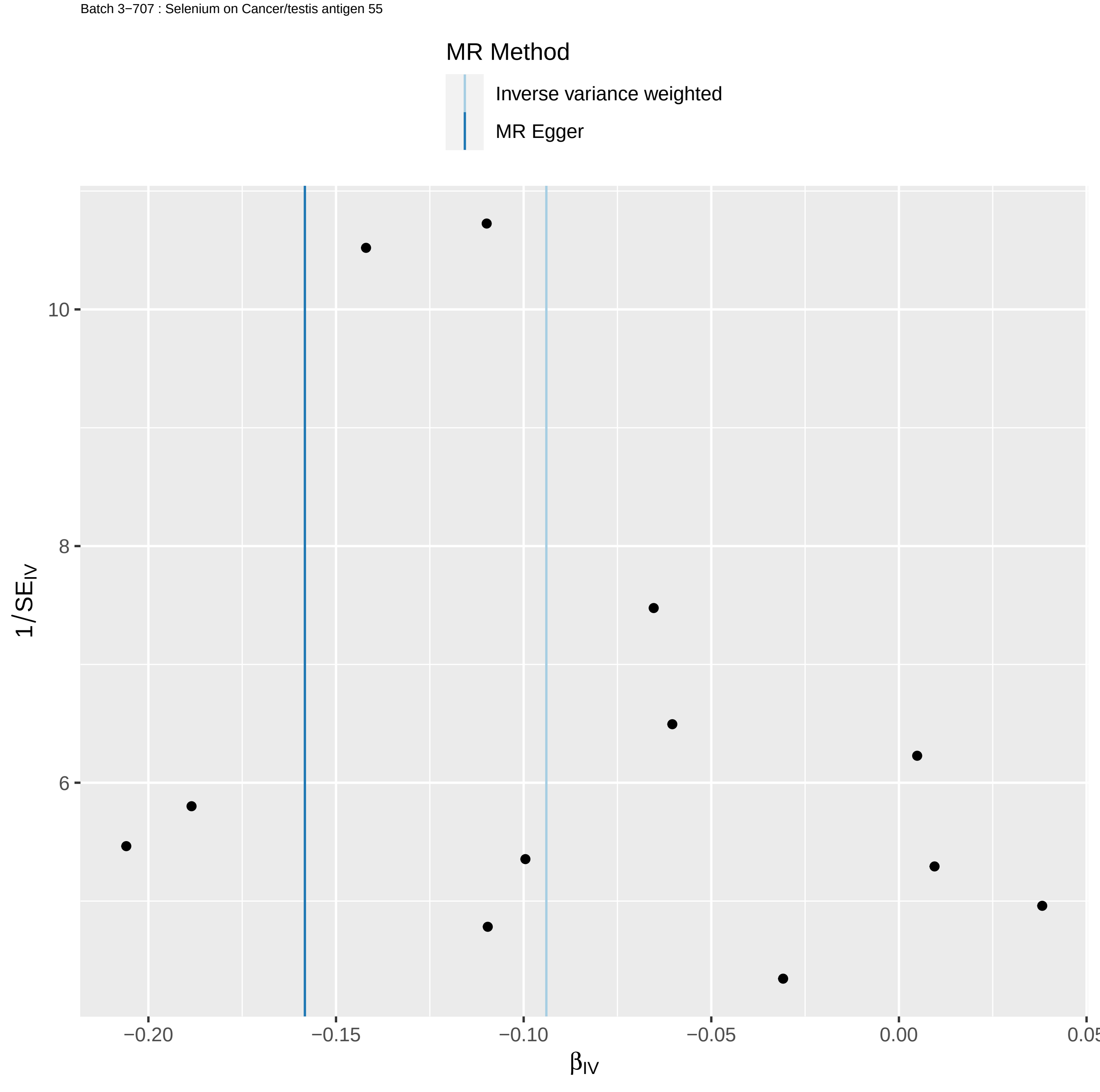
Batch 3−707 : Selenium on Cancer/testis antigen 55
MR Method
Inverse variance weighted
MR Egger
10
8
6
1/SE_IV
−0.20
−0.15
−0.10
−0.05
0.00
0.05
β_IV

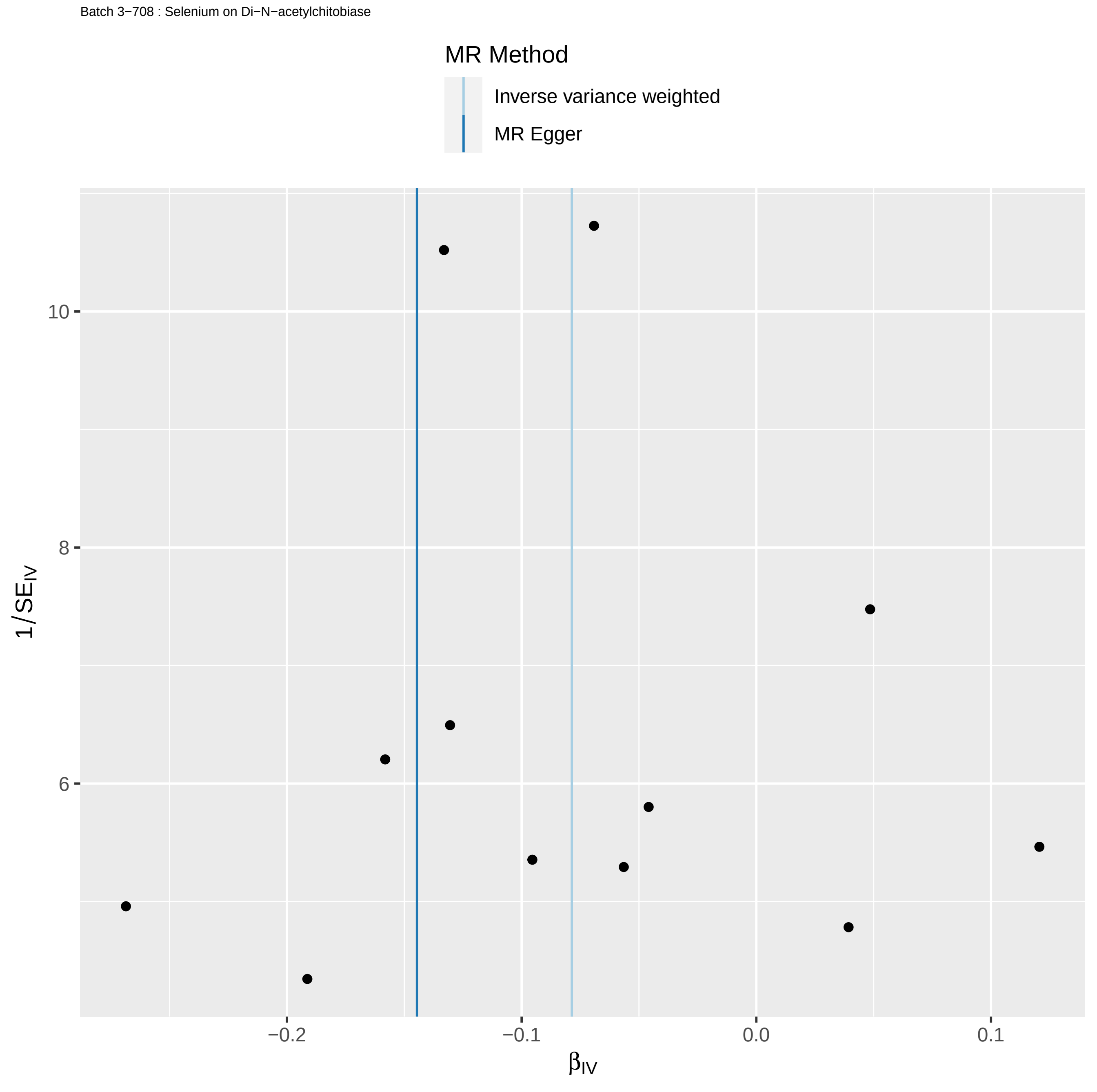
Batch 3−708 : Selenium on Di−N−acetylchitobiase
MR Method
Inverse variance weighted
MR Egger
10
8
6
1/SE_IV
−0.2
−0.1
0.0
0.1
β_IV

Batch 3−709 : Selenium on Transcriptional repressor CTCF

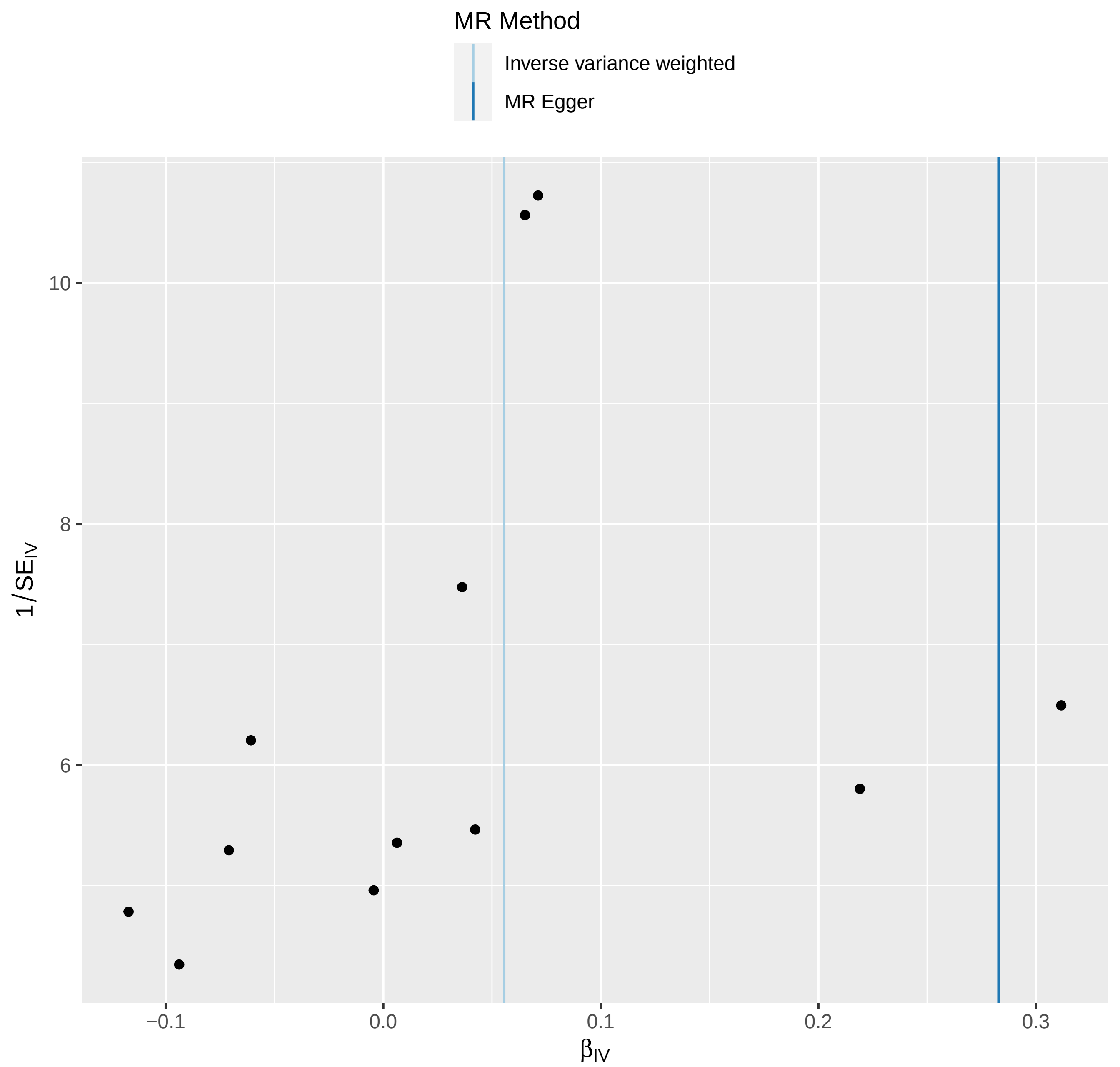

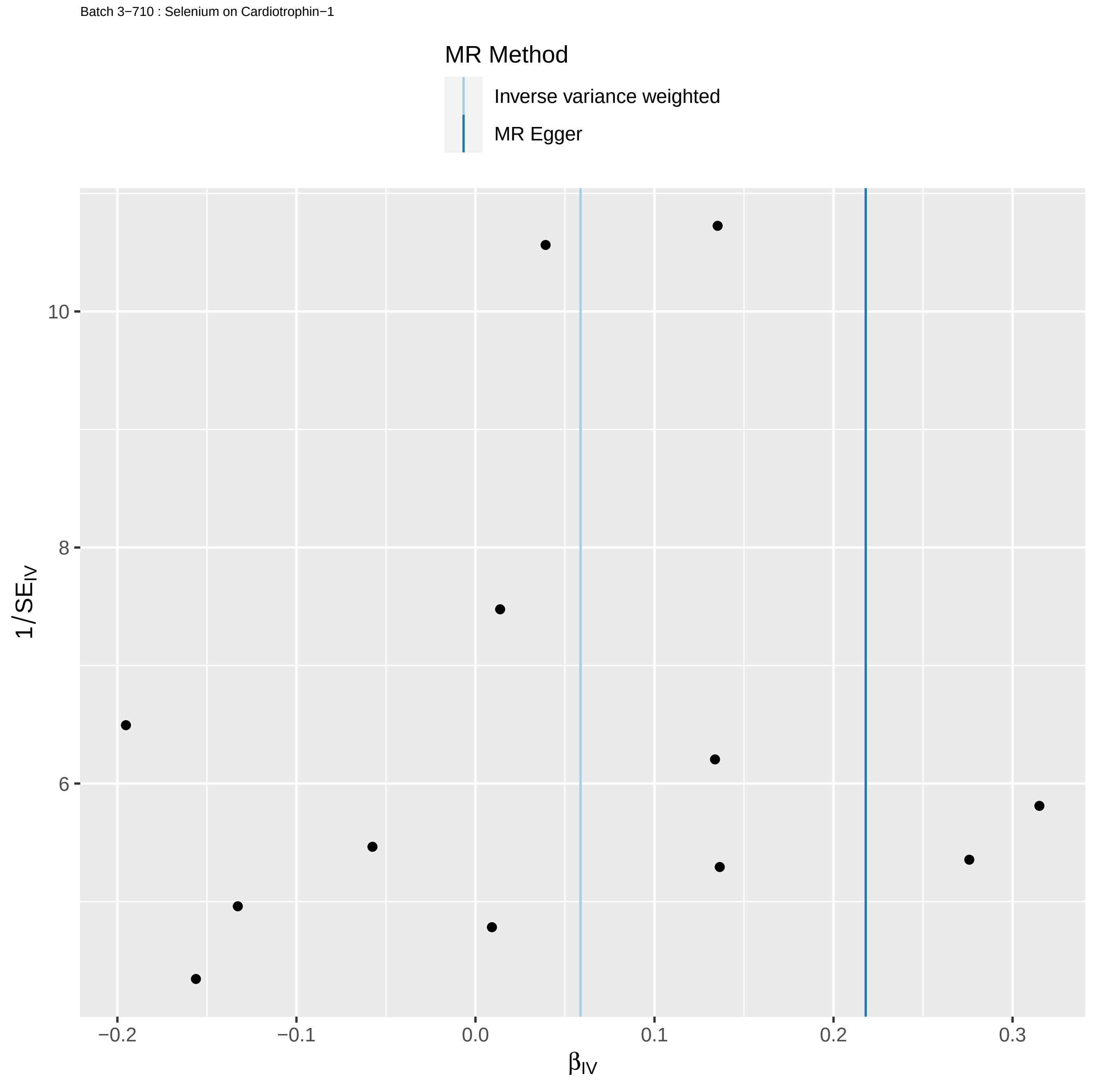
Batch 3−710 : Selenium on Cardiotrophin−1
MR Method
Inverse variance weighted
MR Egger
10
8
6
1/SE_IV
−0.2
−0.1
0.0
0.1
0.2
0.3
β_IV

Batch 3−711 : Selenium on Cardiotrophin−1

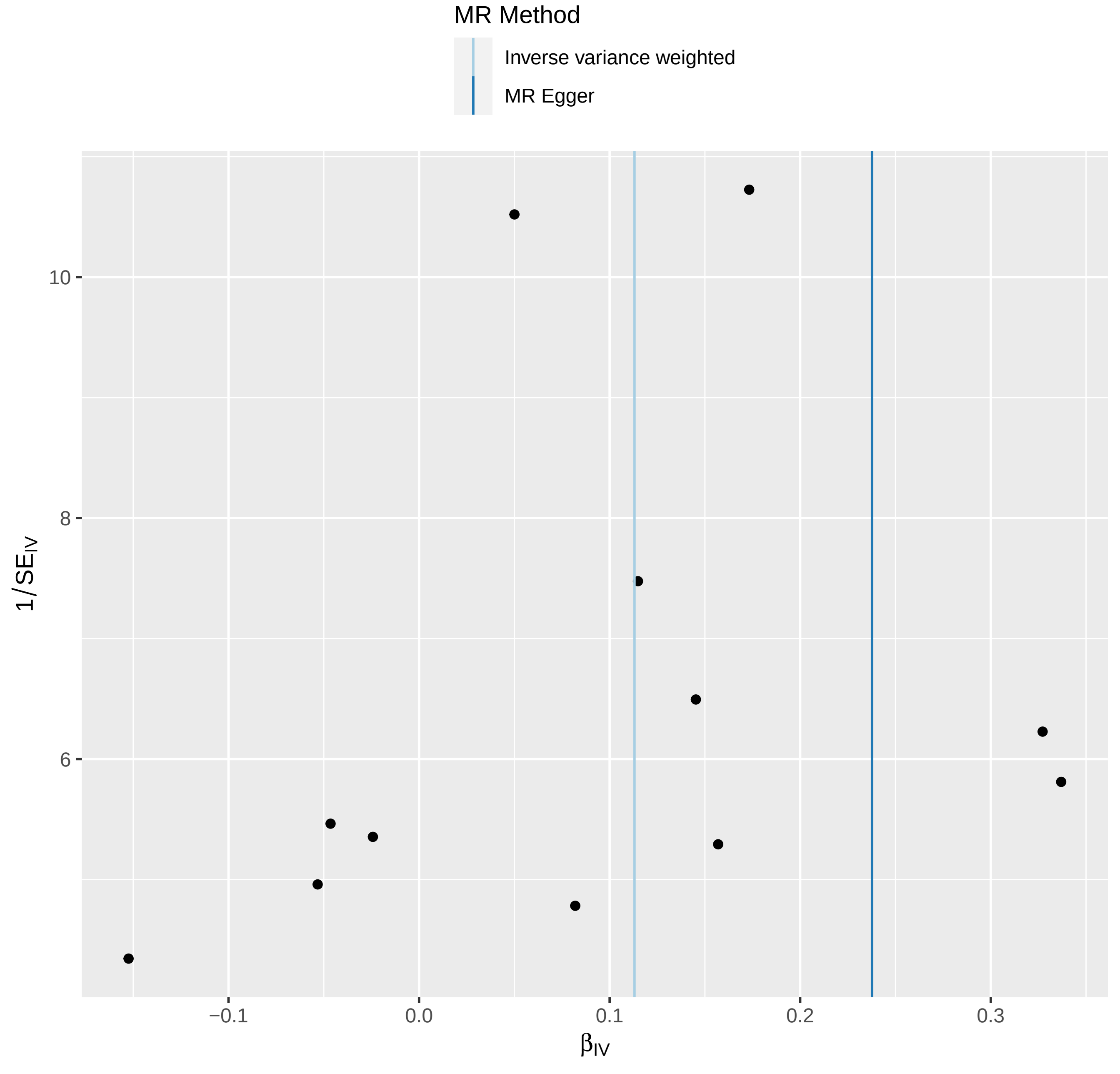

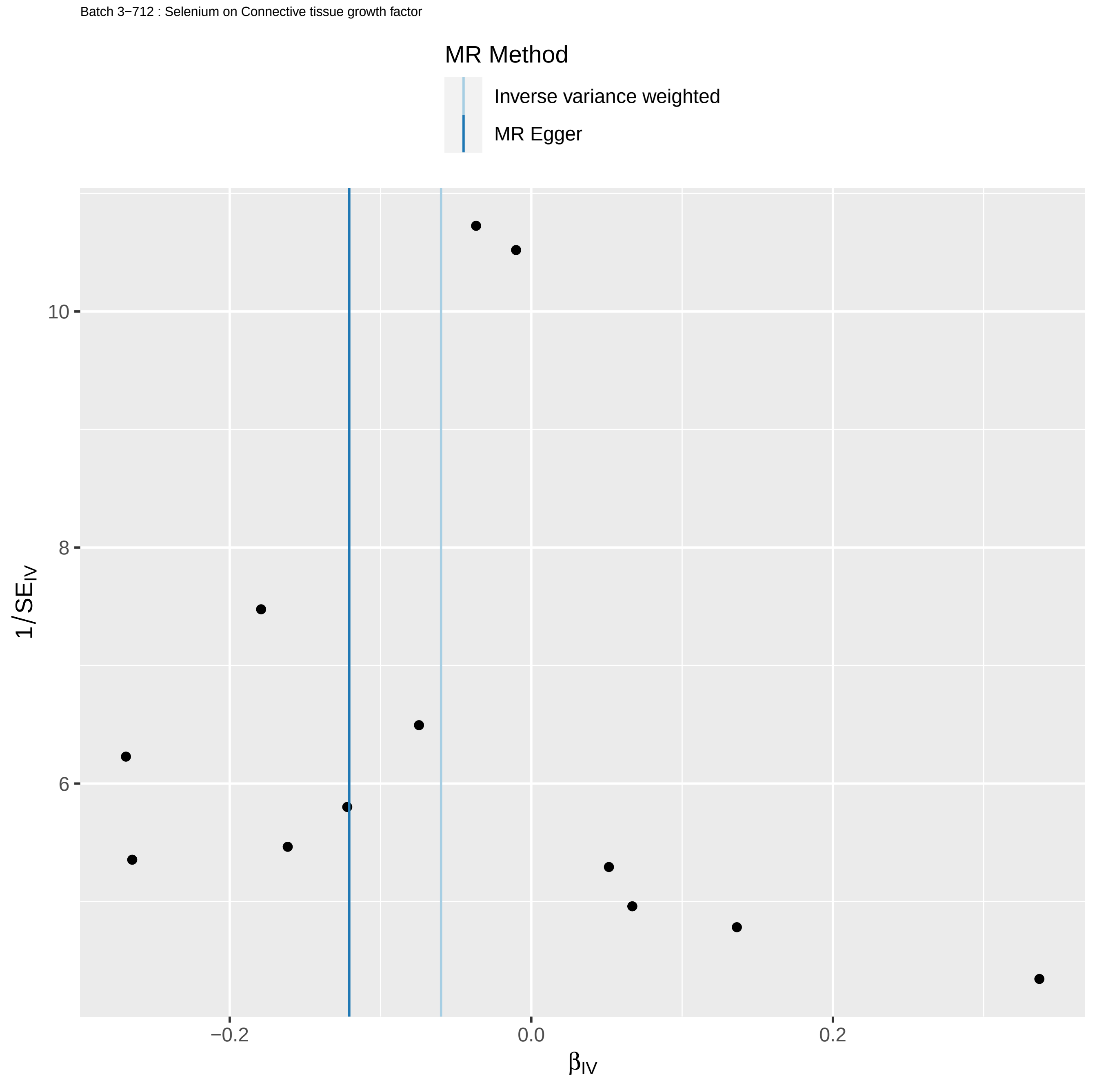
Batch 3−712 : Selenium on Connective tissue growth factor
MR Method
Inverse variance weighted
MR Egger
10
8
6
−0.2
0.0
0.2
$1/SE_{IV}$
β_{IV}

Batch 3−713 : Selenium on Catenin alpha−2

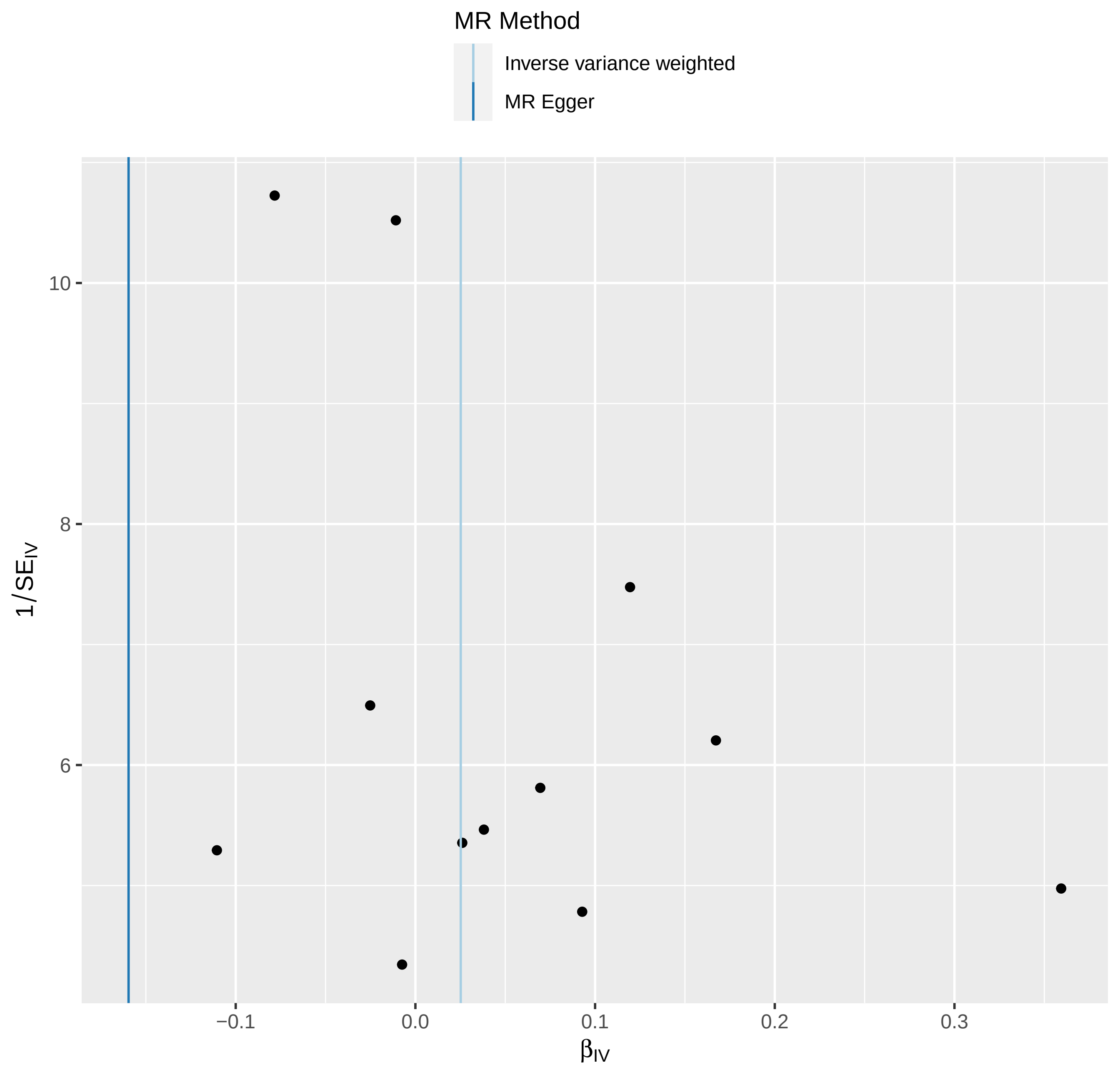

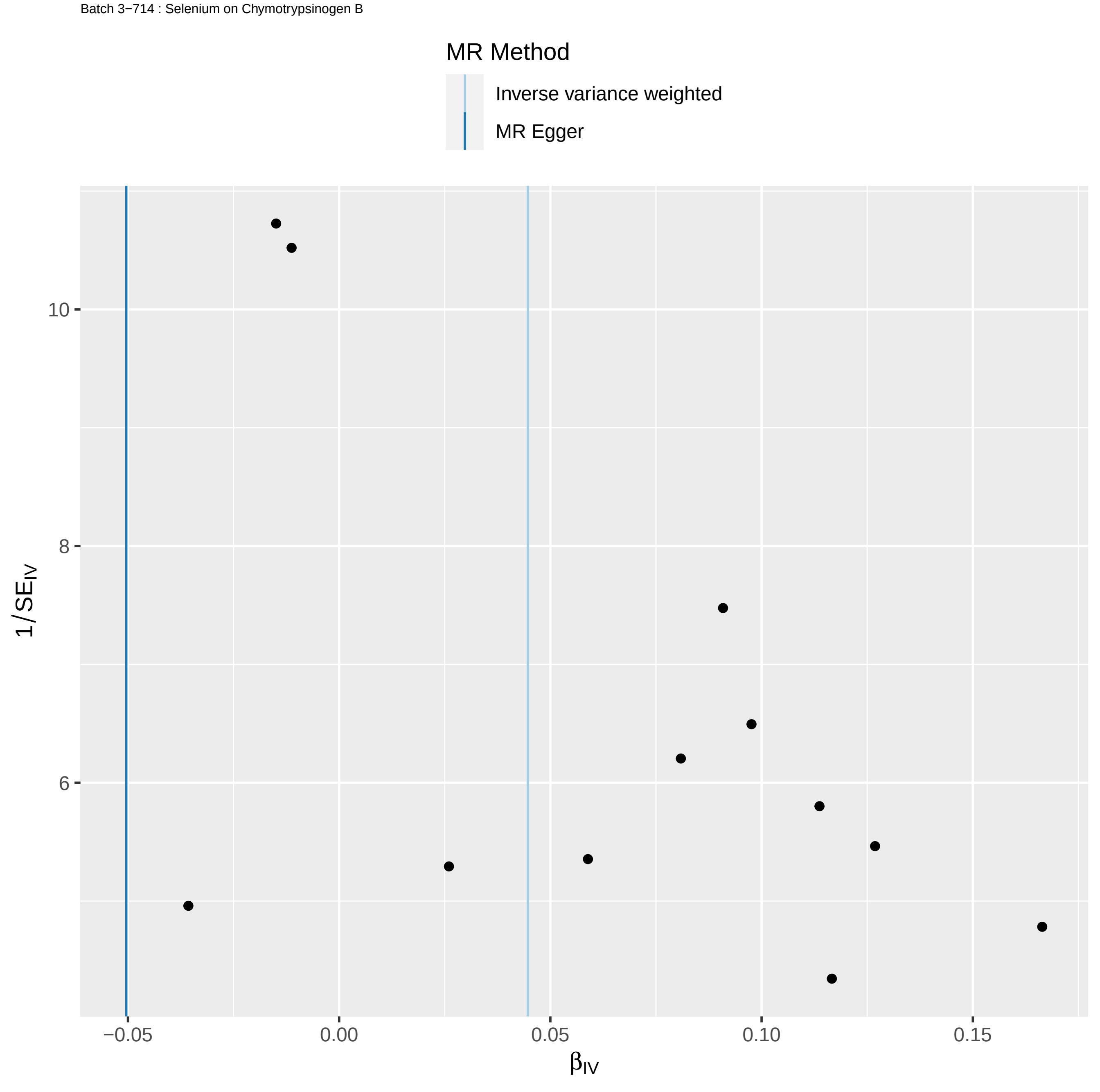

Batch 3−714 : Selenium on Chymotrypsinogen B
MR Method
Inverse variance weighted
MR Egger
10
8
6
1/SE_IV
−0.05
0.00
0.05
0.10
0.15
β_IV

Batch 3−715 : Selenium on Chymotrypsin−C

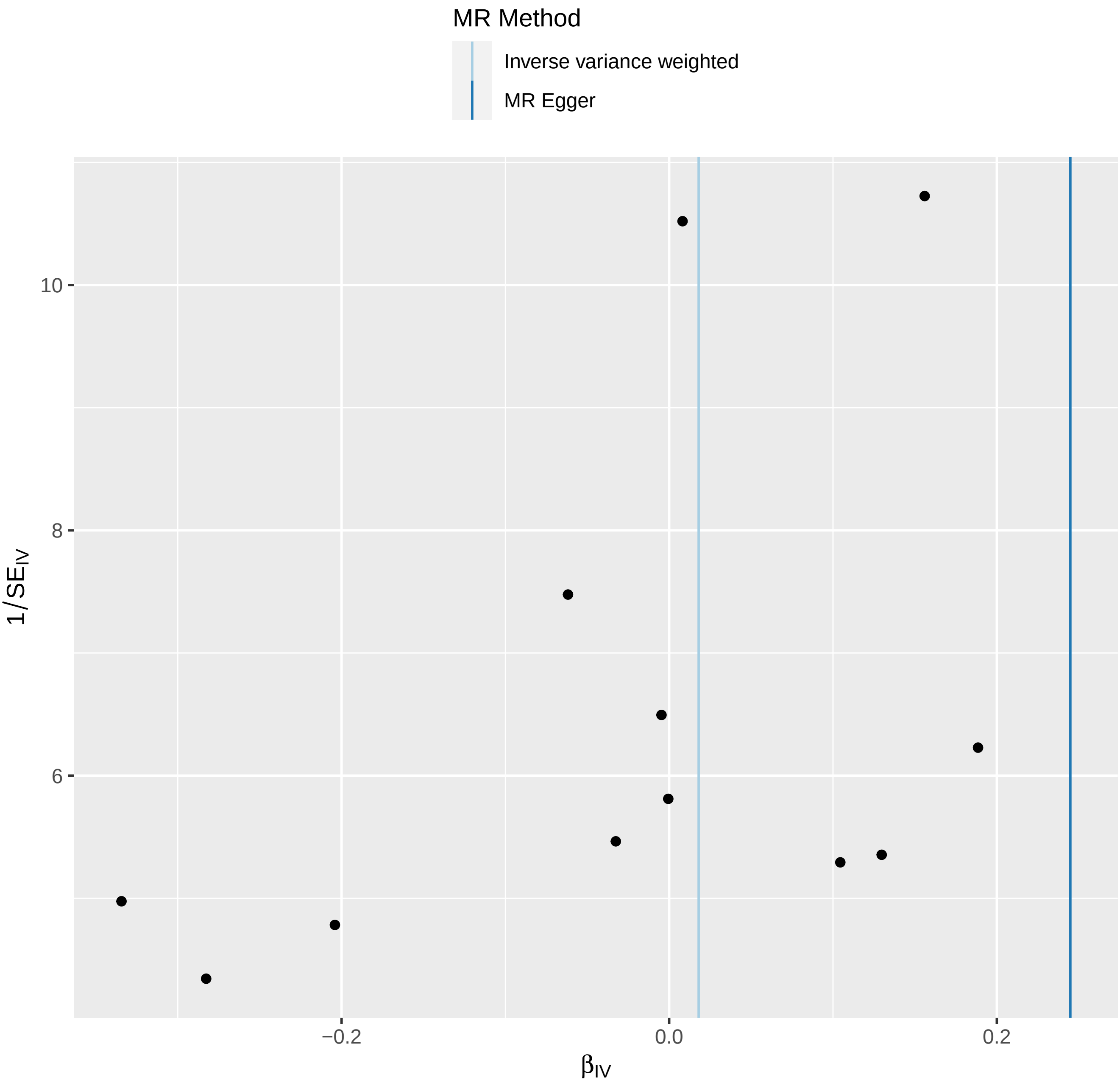

Batch 3−716 : Selenium on Chymotrypsin−like protease CTRL−1

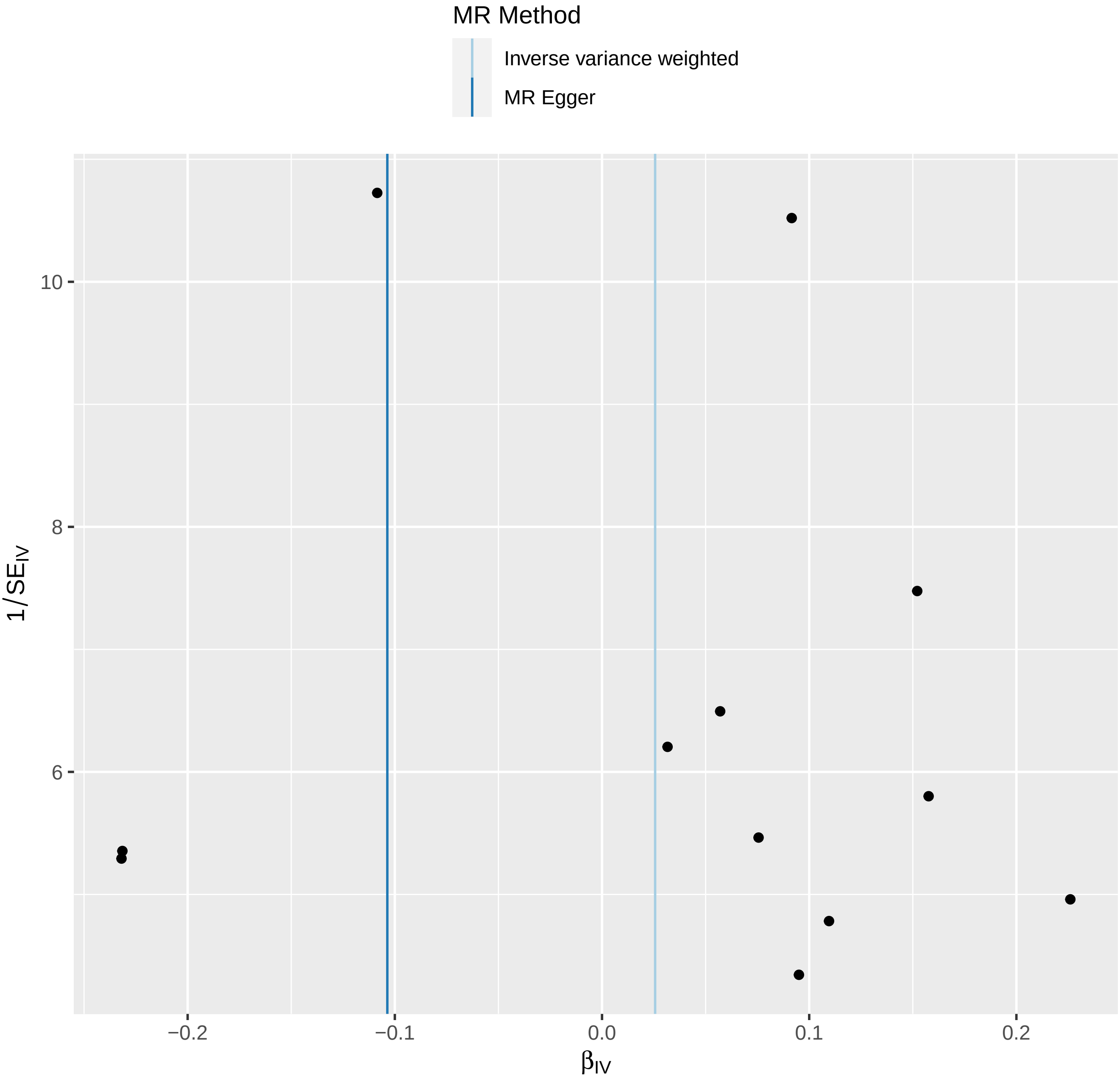

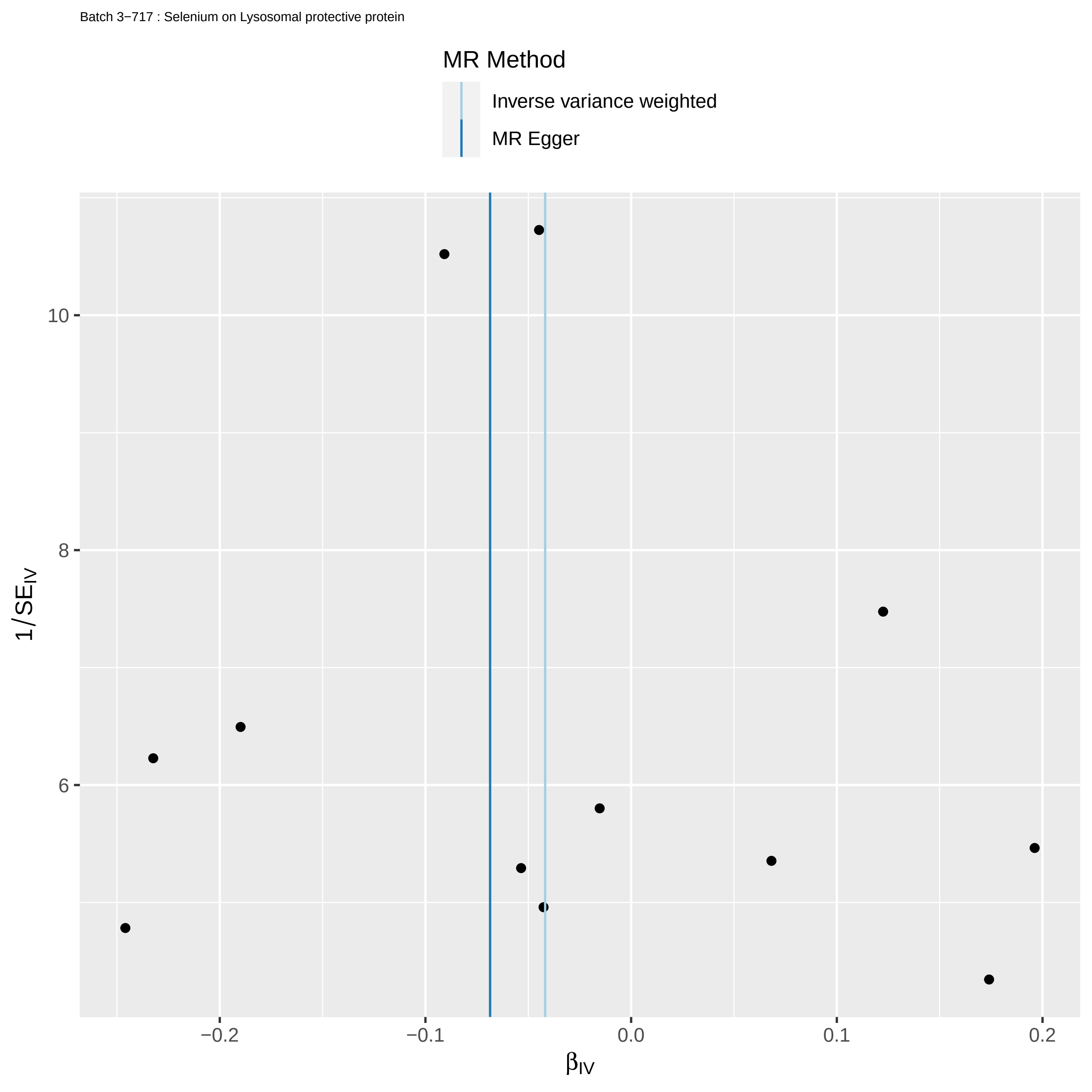

Batch 3−717 : Selenium on Lysosomal protective protein
MR Method
Inverse variance weighted
MR Egger
10
8
6
1/SE_IV
−0.2
−0.1
0.0
0.1
0.2
β_IV

Batch 3−718 : Selenium on Cathepsin B

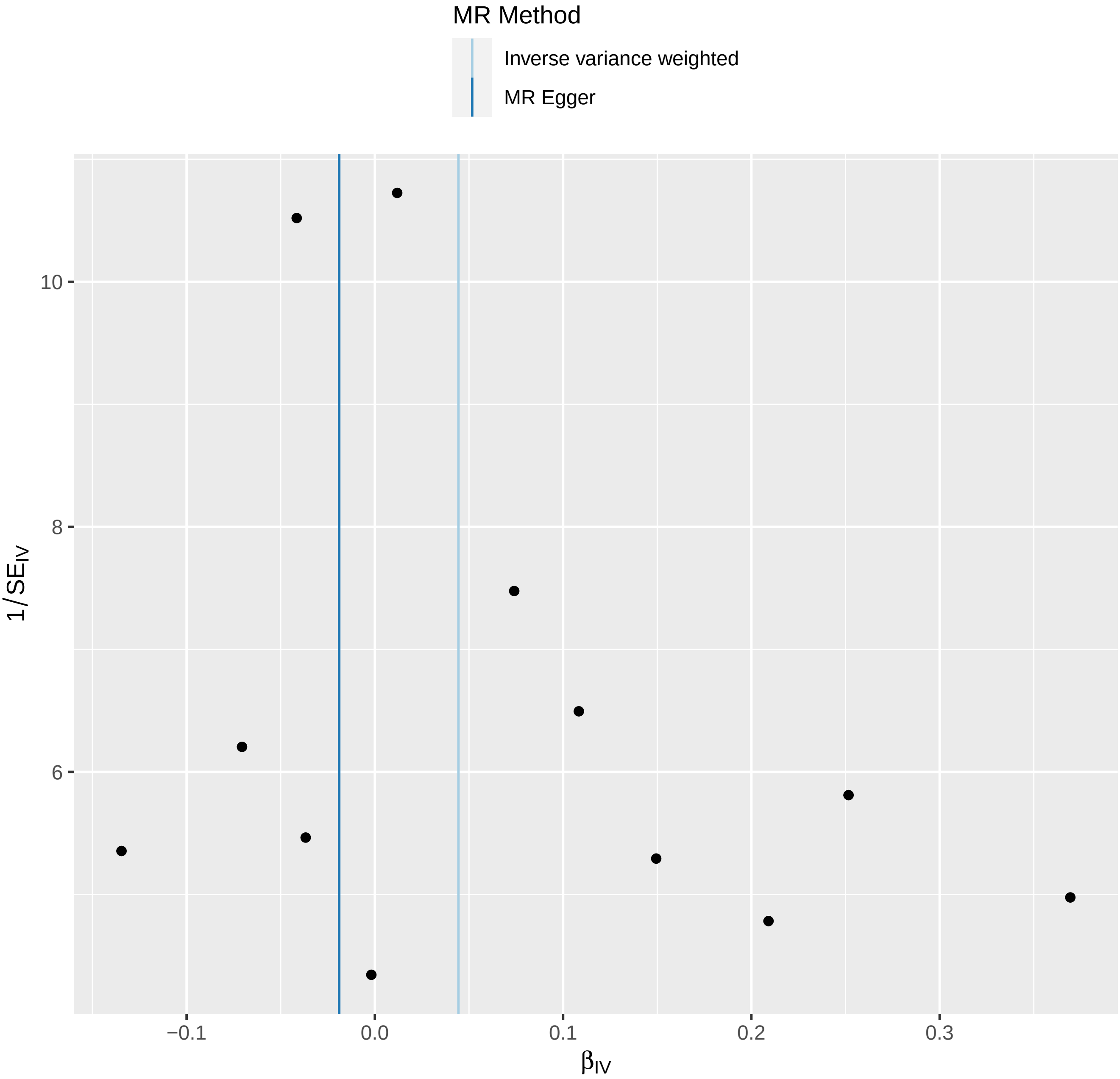

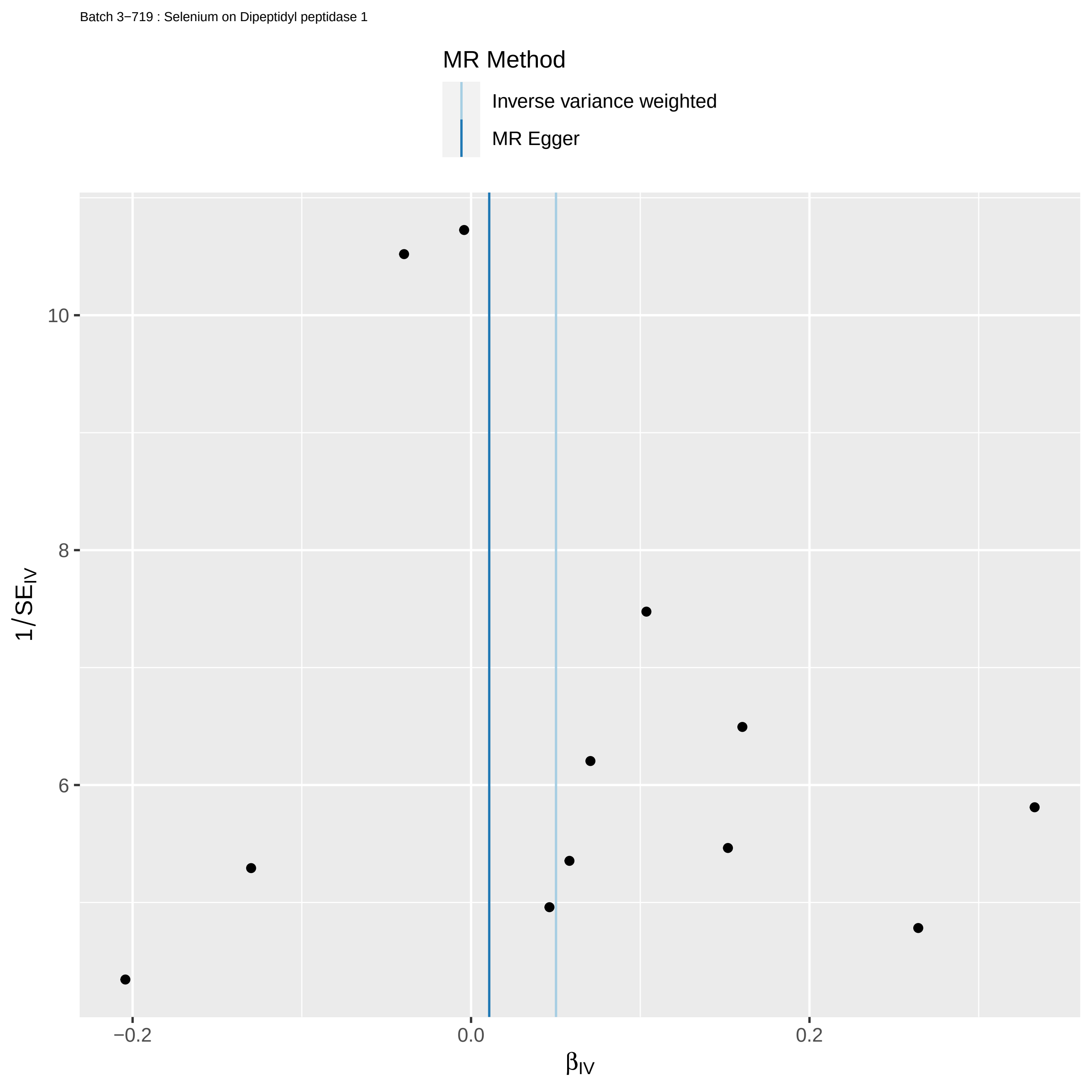
Batch 3−719 : Selenium on Dipeptidyl peptidase 1
MR Method
Inverse variance weighted
MR Egger
10
8
6
1/SE_IV
−0.2
0.0
0.2
β_IV

Batch 3−720 : Selenium on Cathepsin E

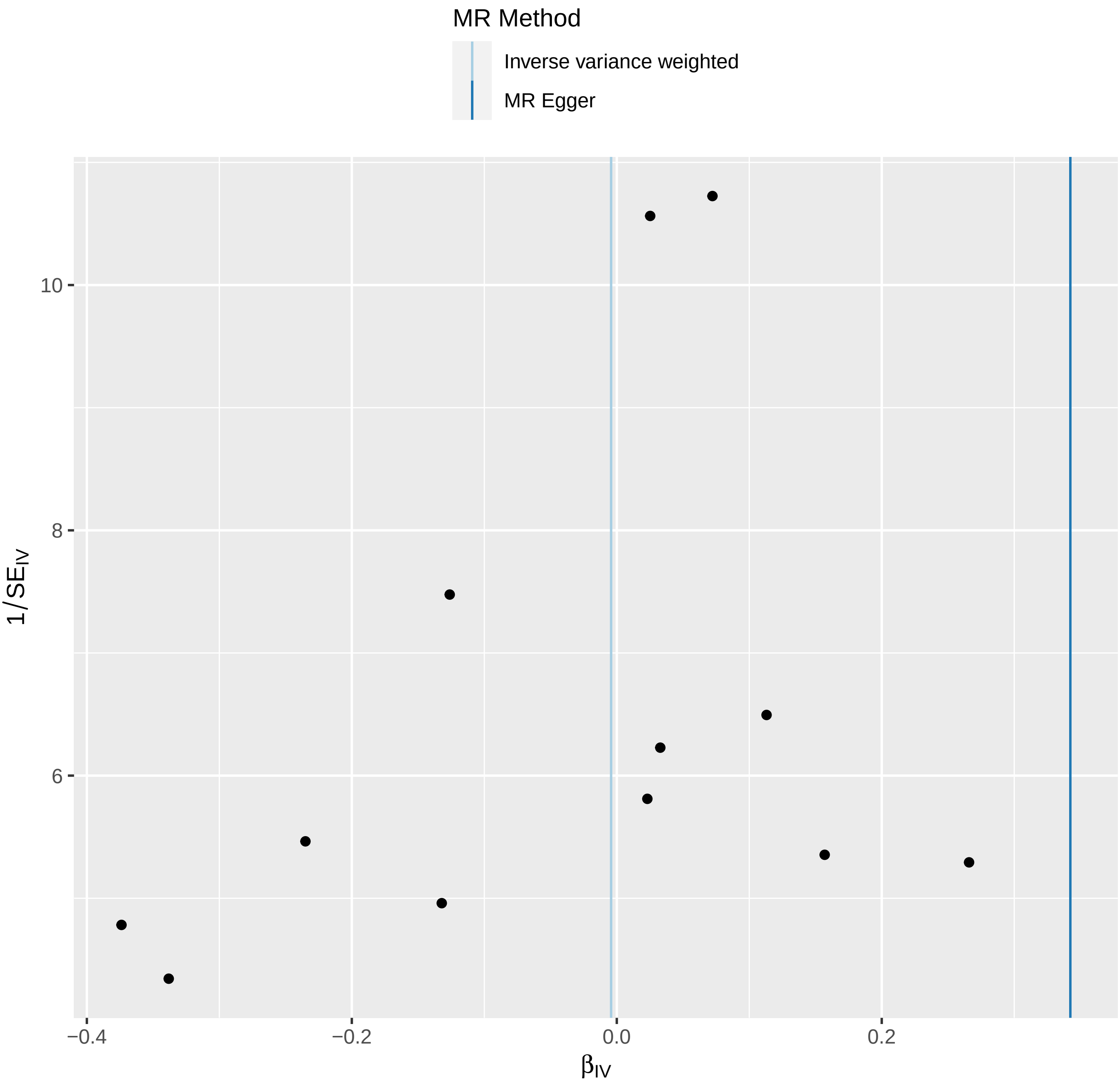

Batch 3−721 : Selenium on Cathepsin F

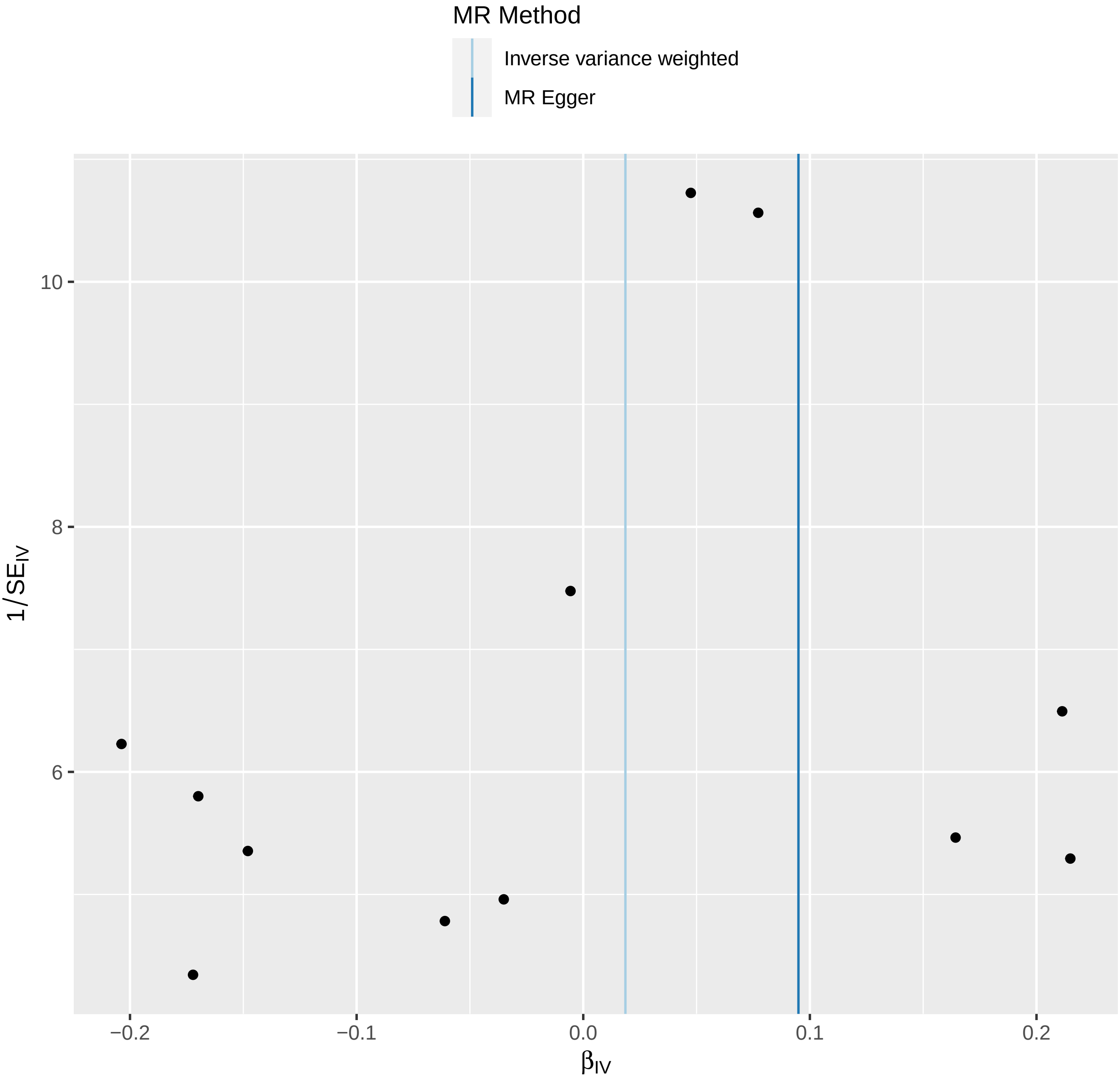

Batch 3−722 : Selenium on Cathepsin F

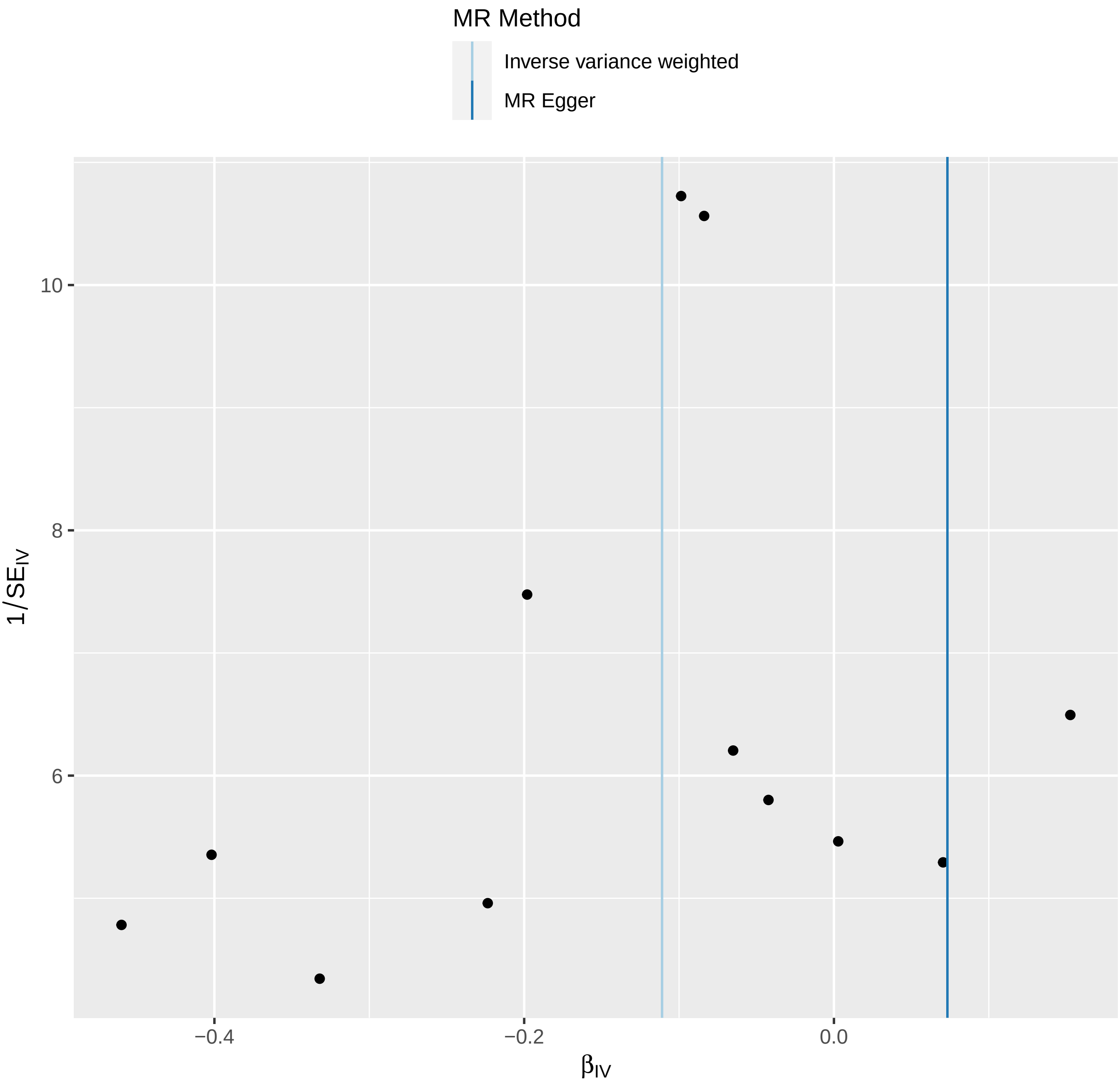

Batch 3−723 : Selenium on Cathepsin G

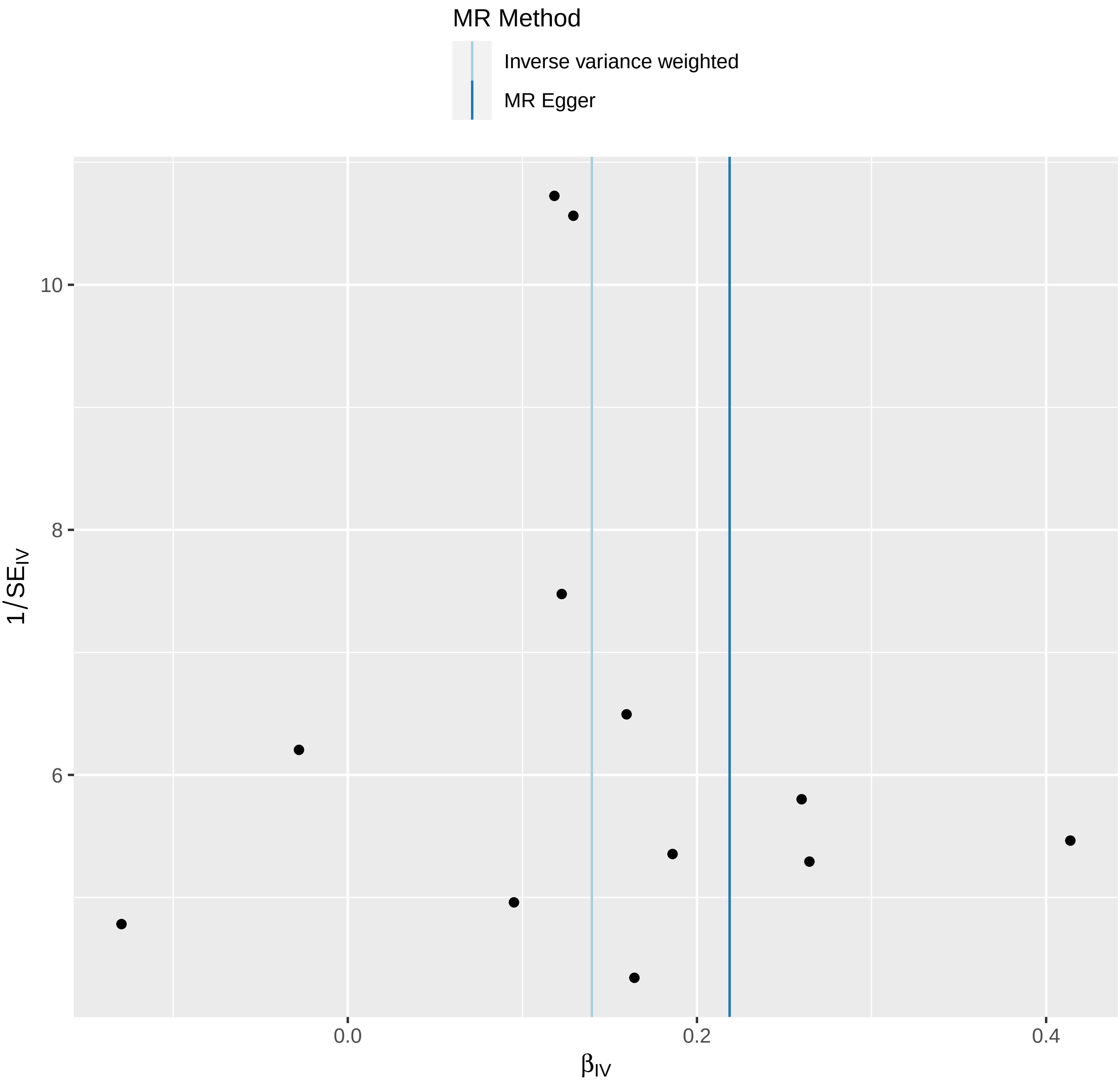

Batch 3−724 : Selenium on Cathepsin H

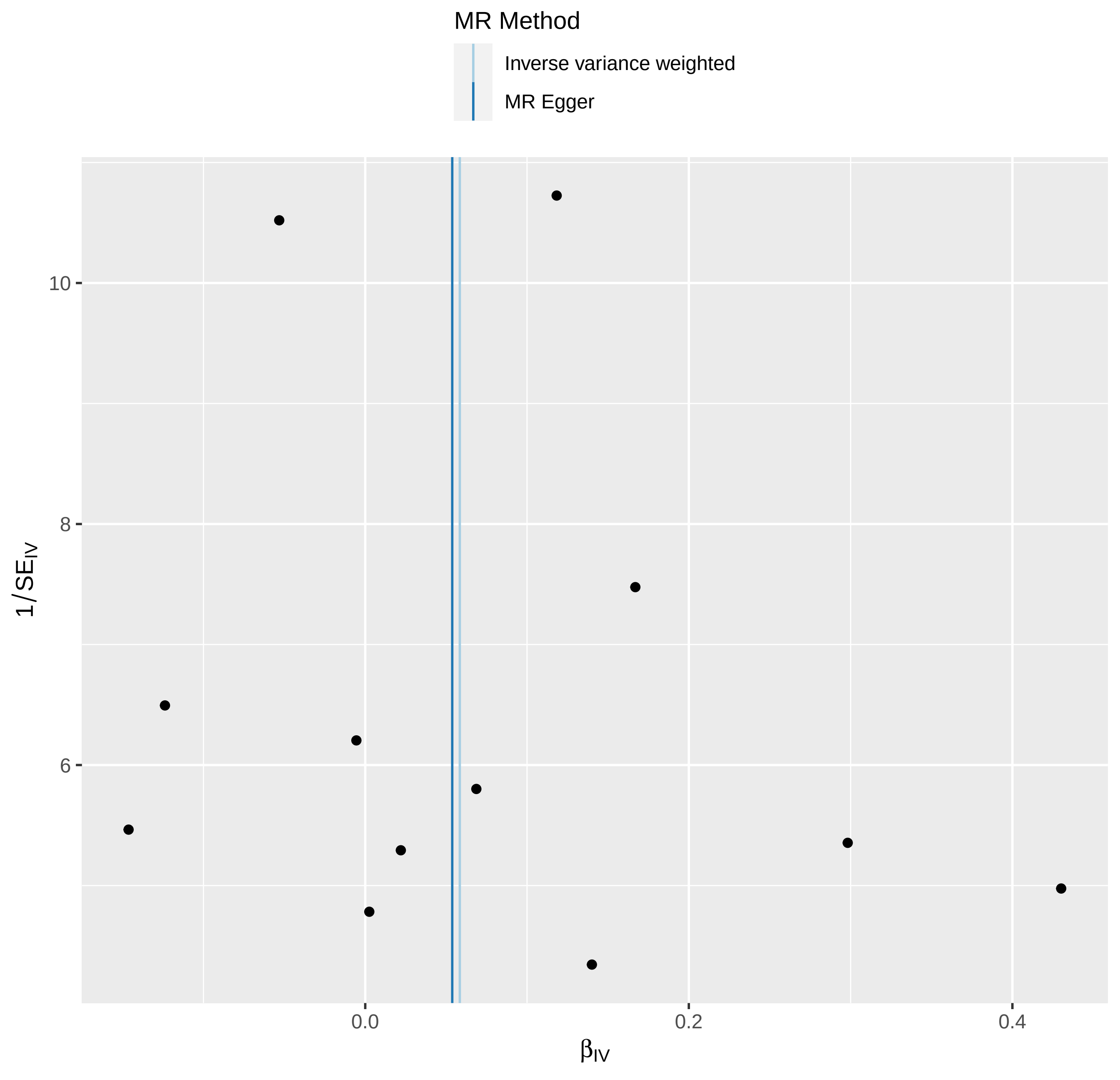

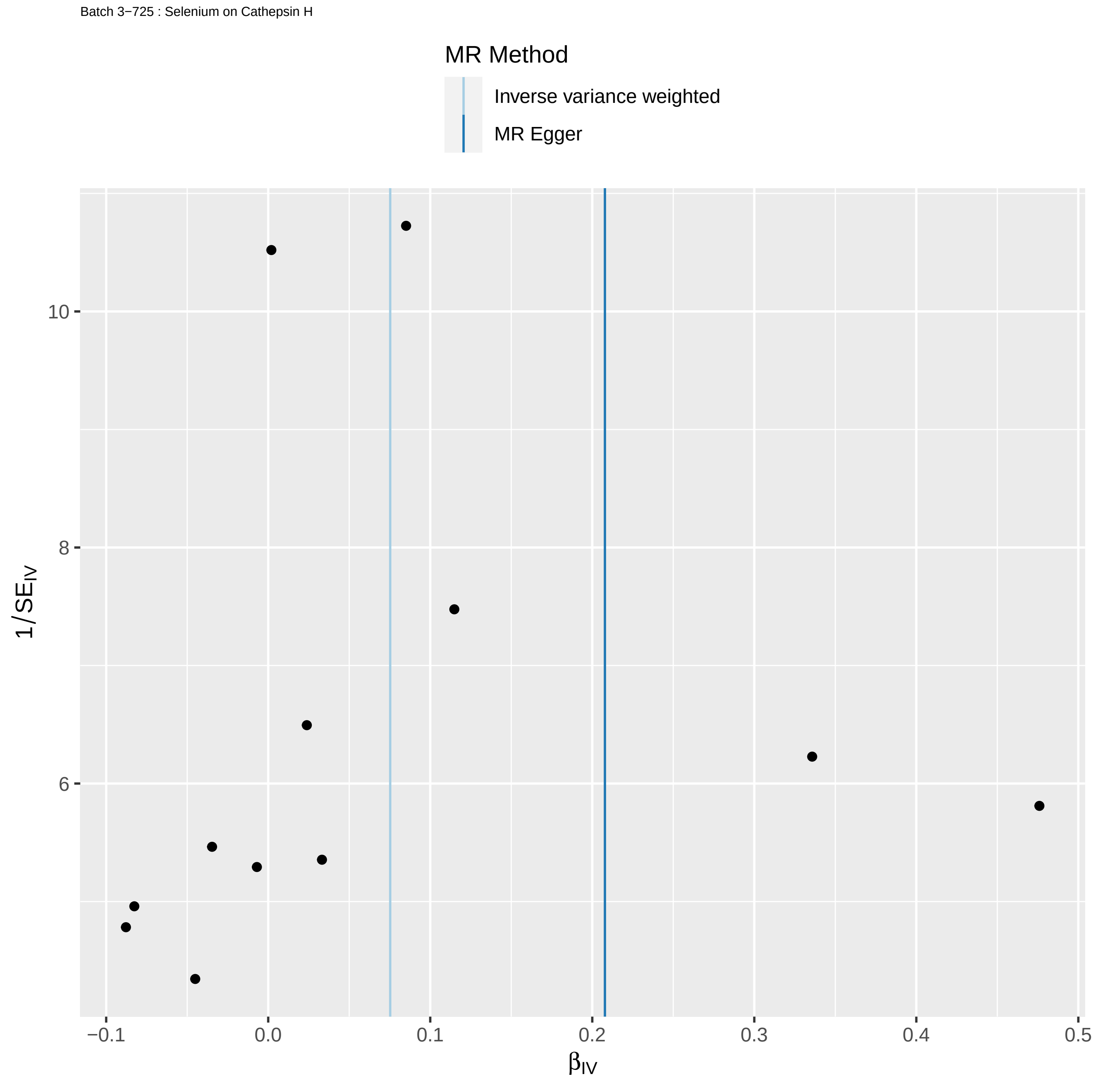
Batch 3−725 : Selenium on Cathepsin H
MR Method
Inverse variance weighted
MR Egger
10
8
6
1/SE$_{IV}$
−0.1
0.0
0.1
0.2
0.3
0.4
0.5
β$_{IV}$

Batch 3−726 : Selenium on Cathepsin O

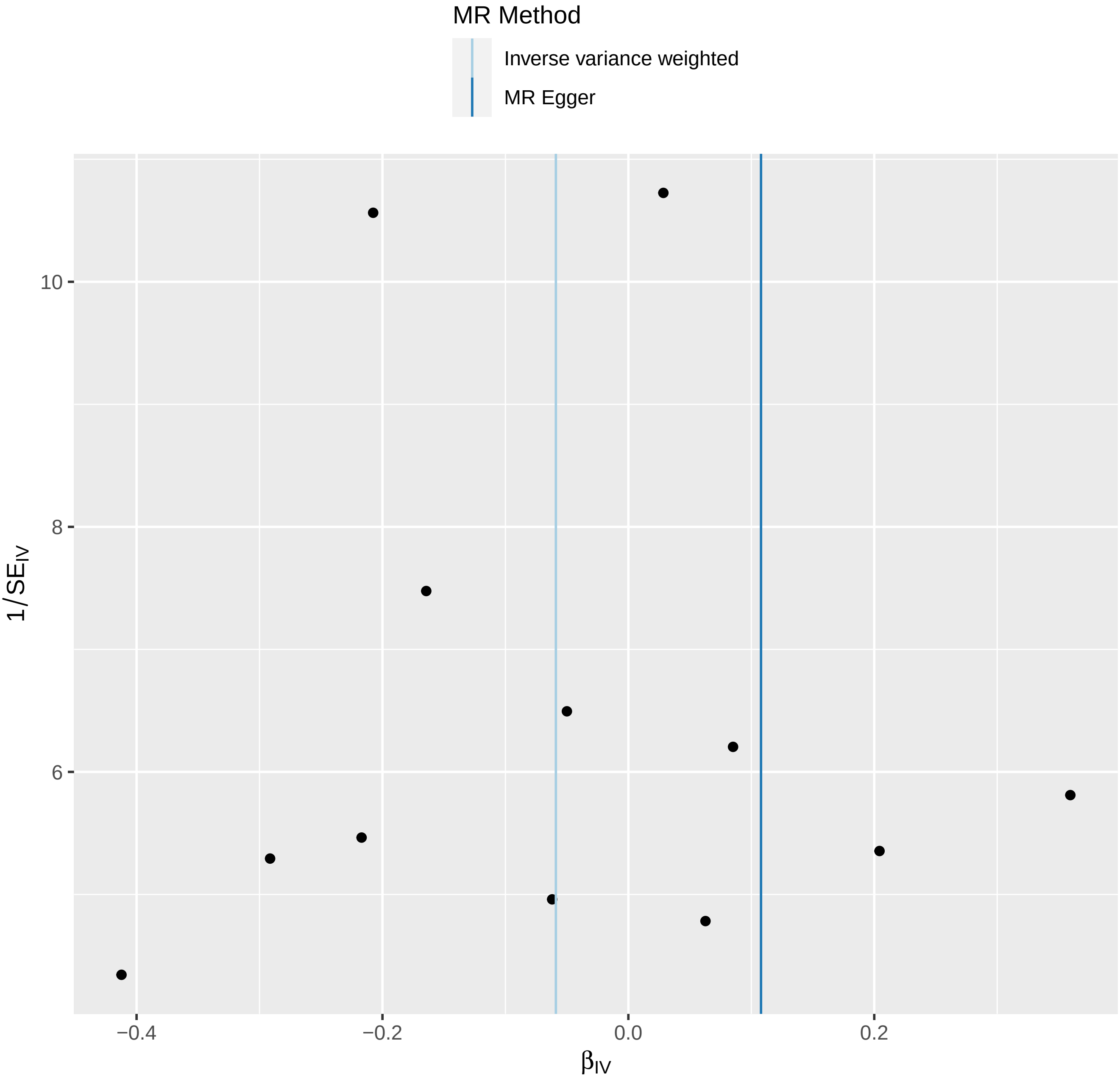

Batch 3−727 : Selenium on Cathepsin S

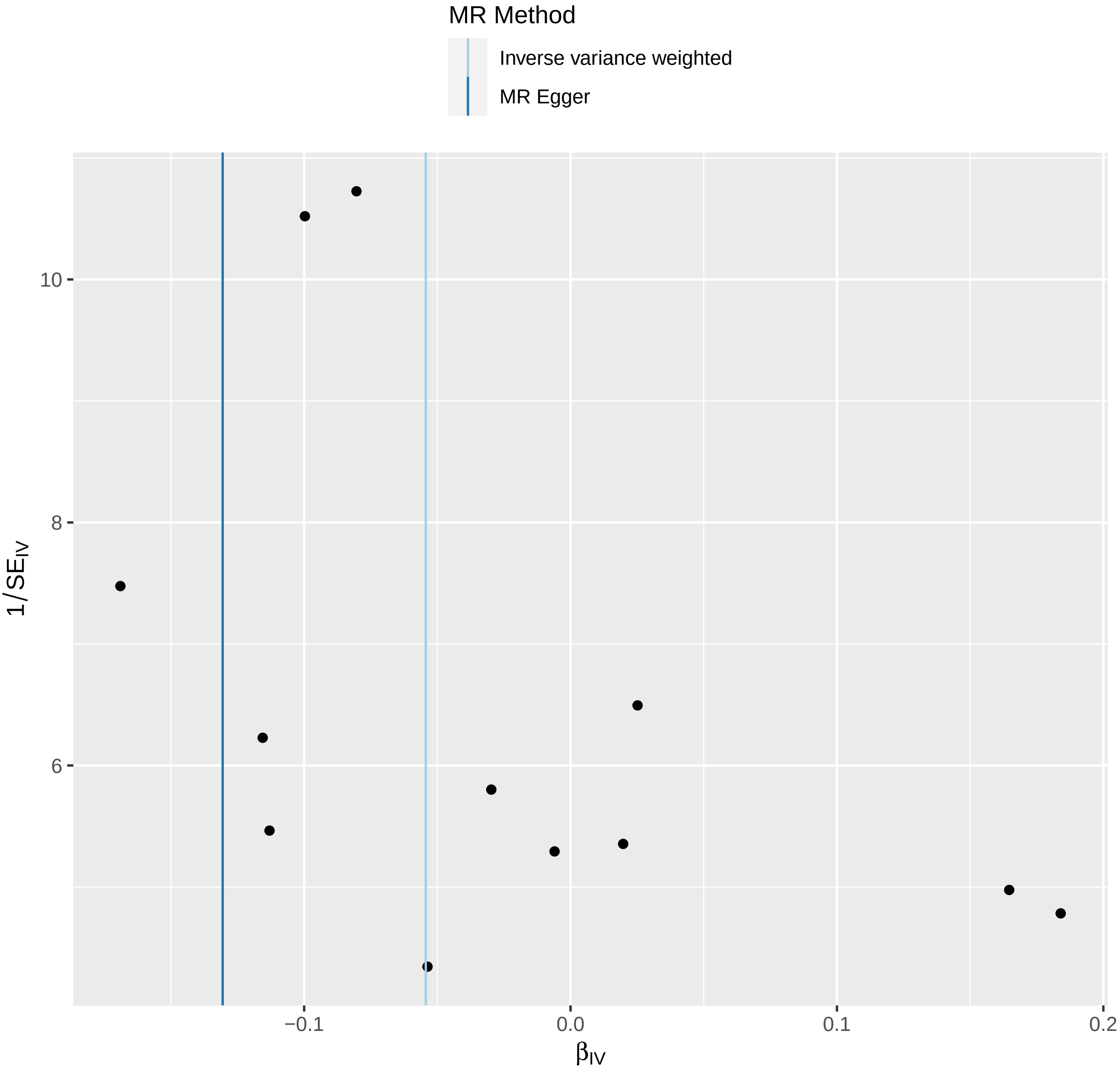

Batch 3−728 : Selenium on Cathepsin L2

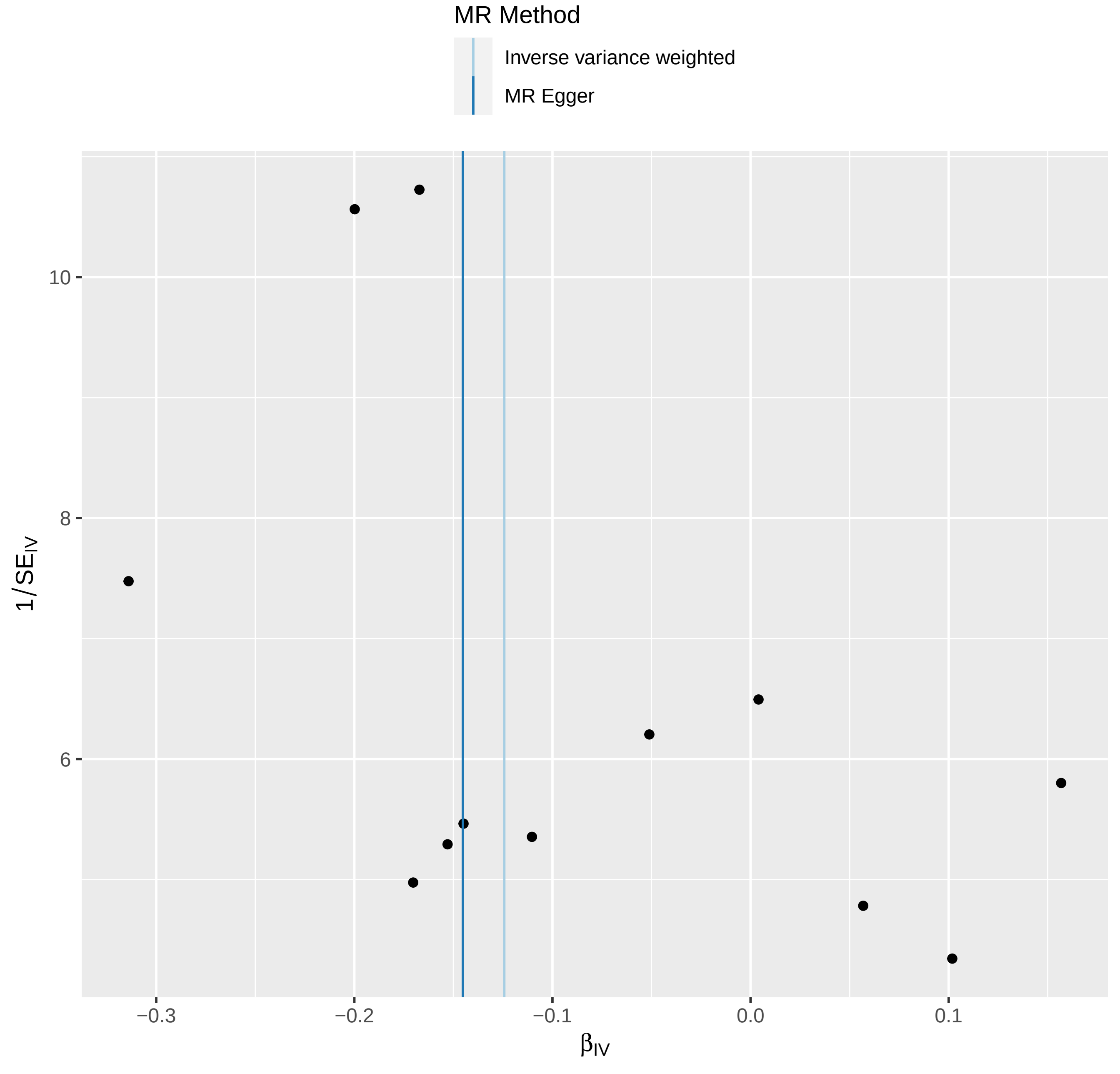

Batch 3−729 : Selenium on Cathepsin Z

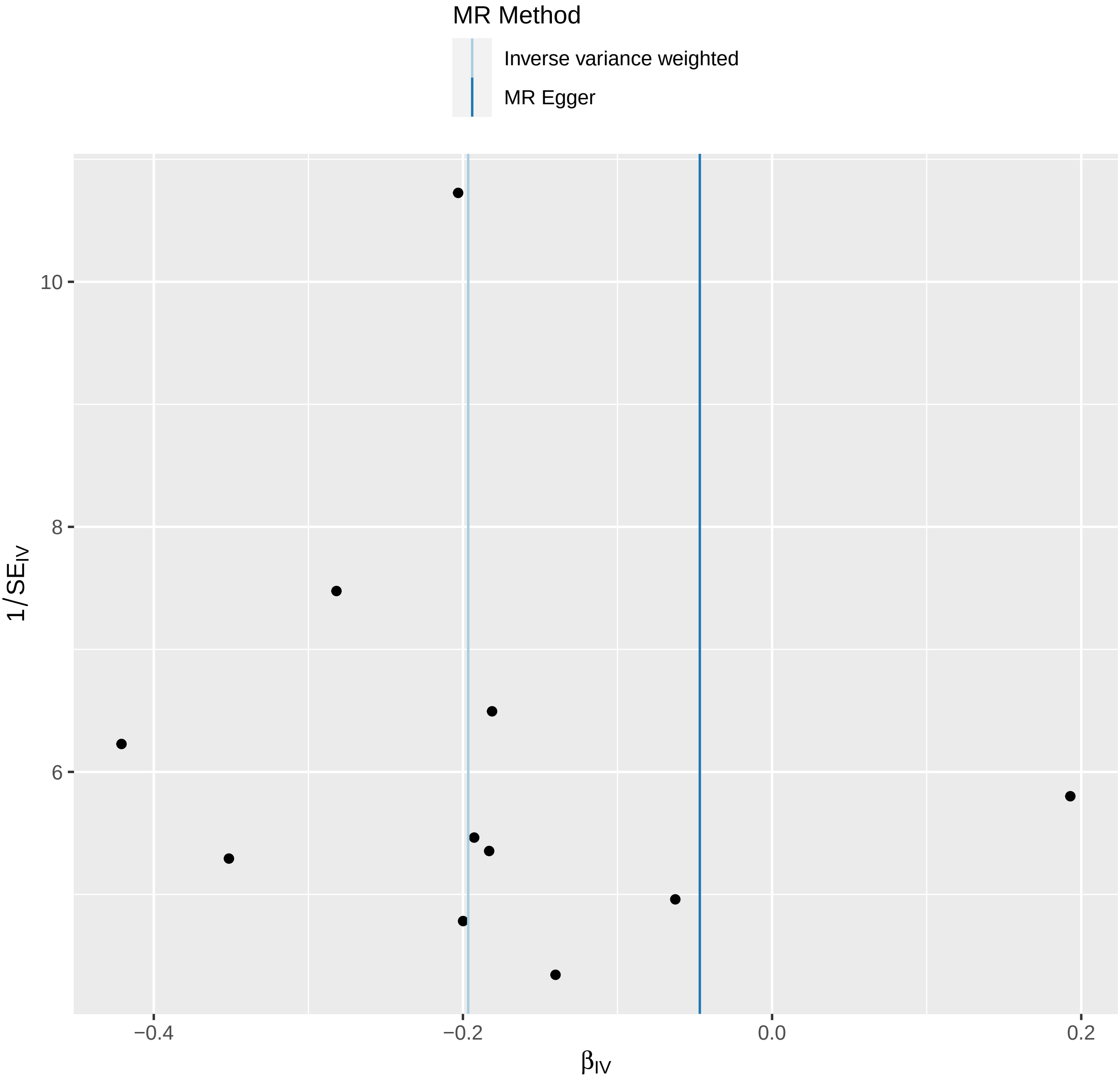

Batch 3−730 : Selenium on Cortexin−3

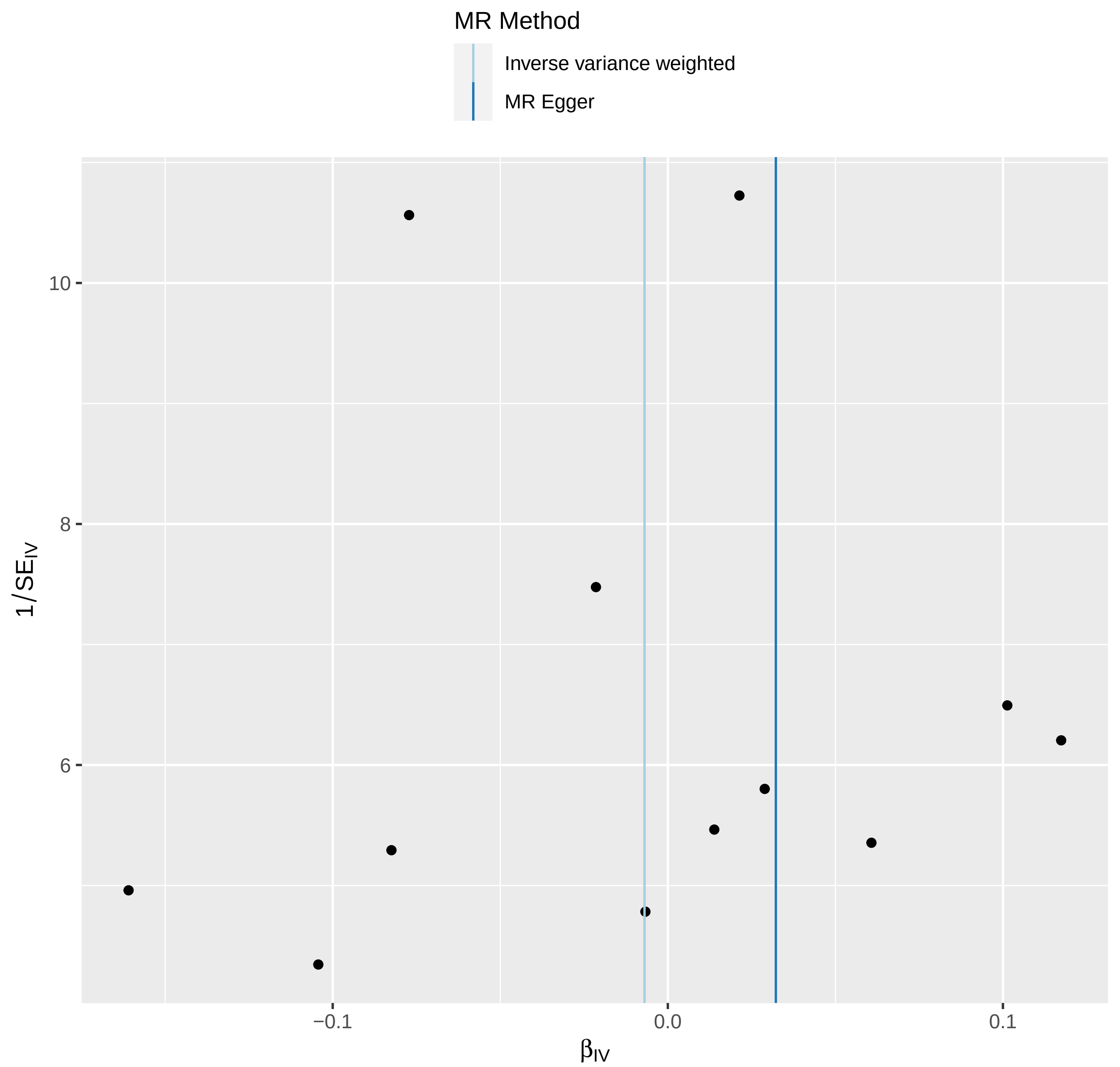

Batch 3−731 : Selenium on cubilin

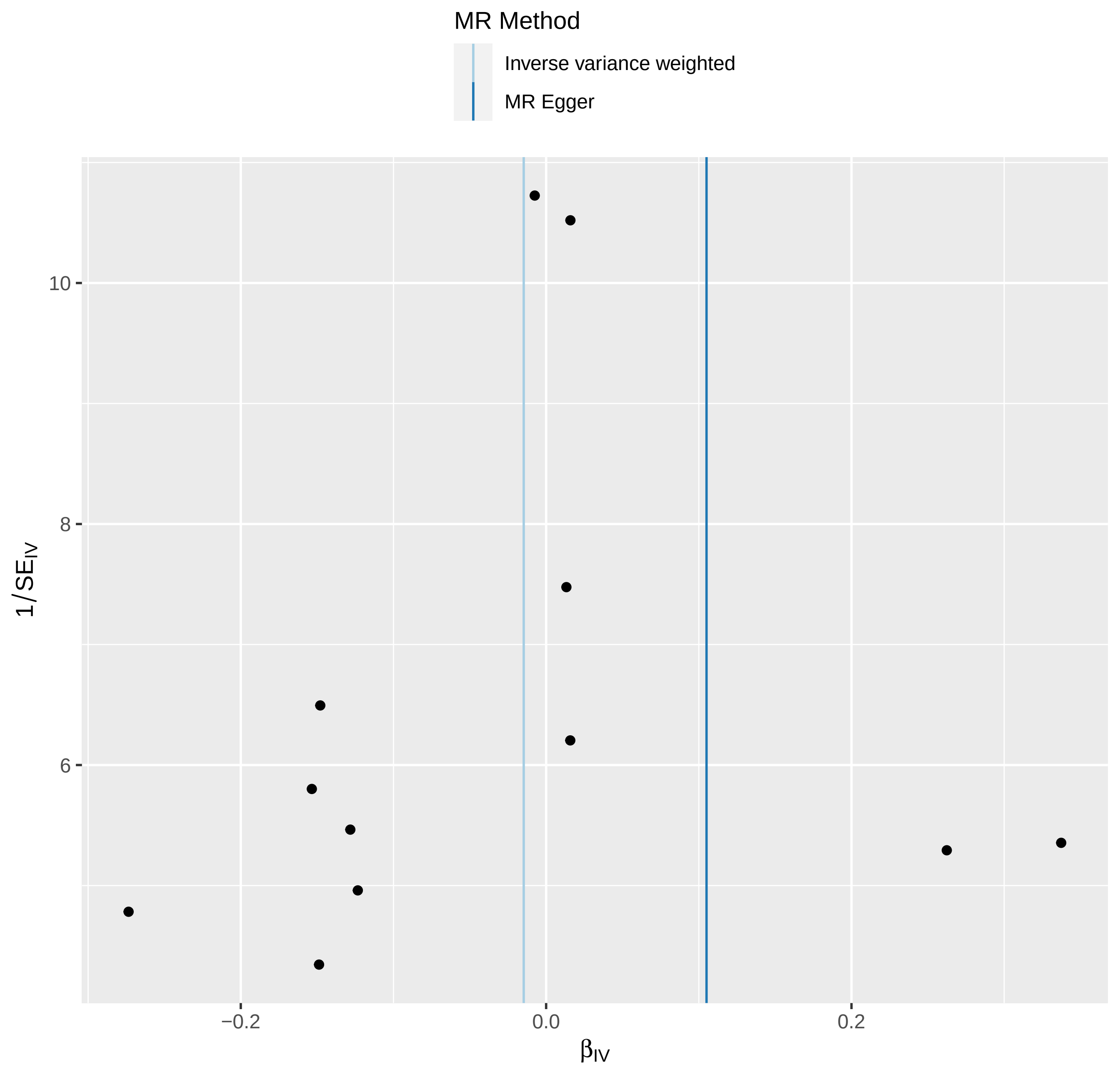

Batch 3−732 : Selenium on Cullin−3

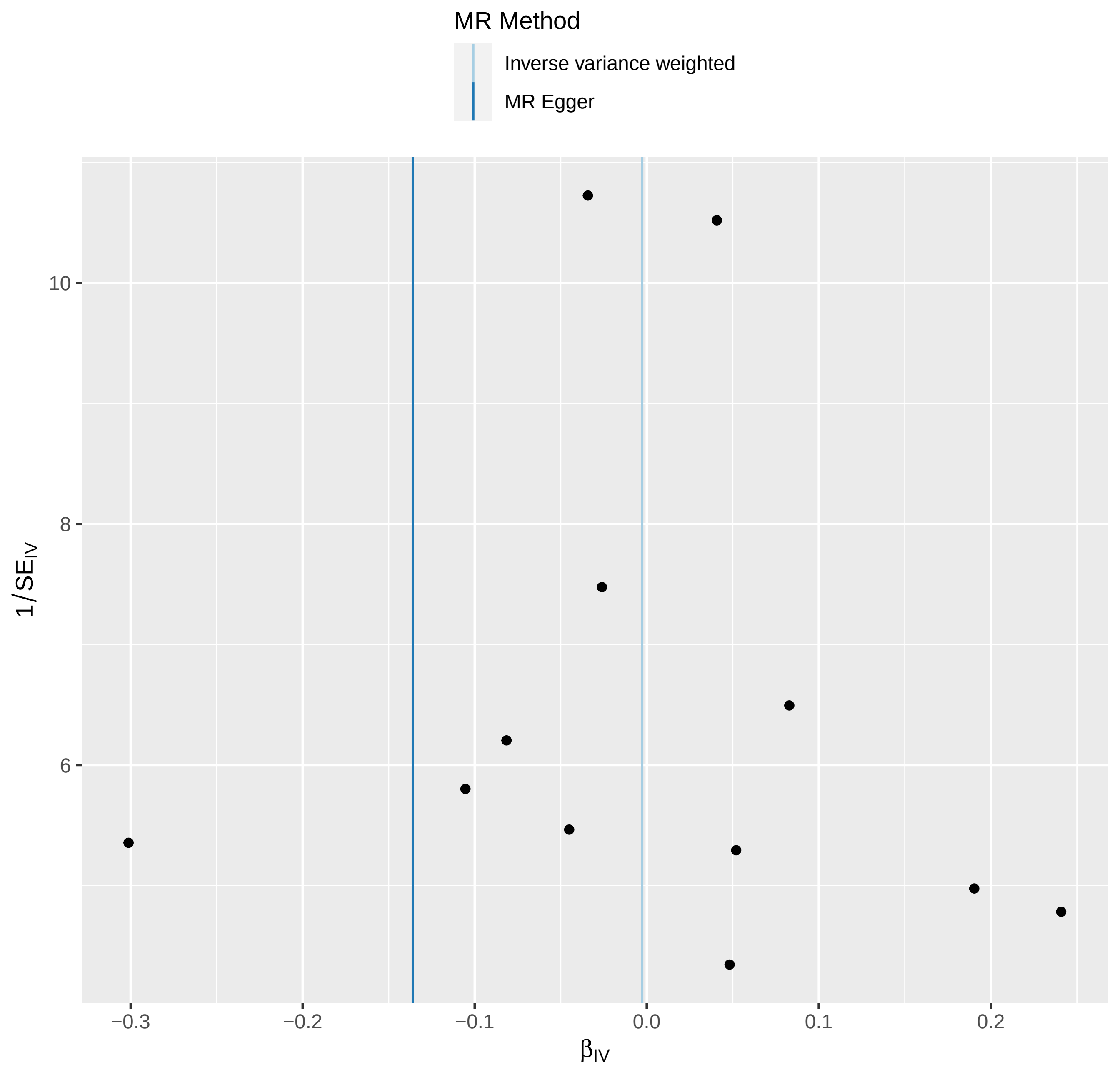

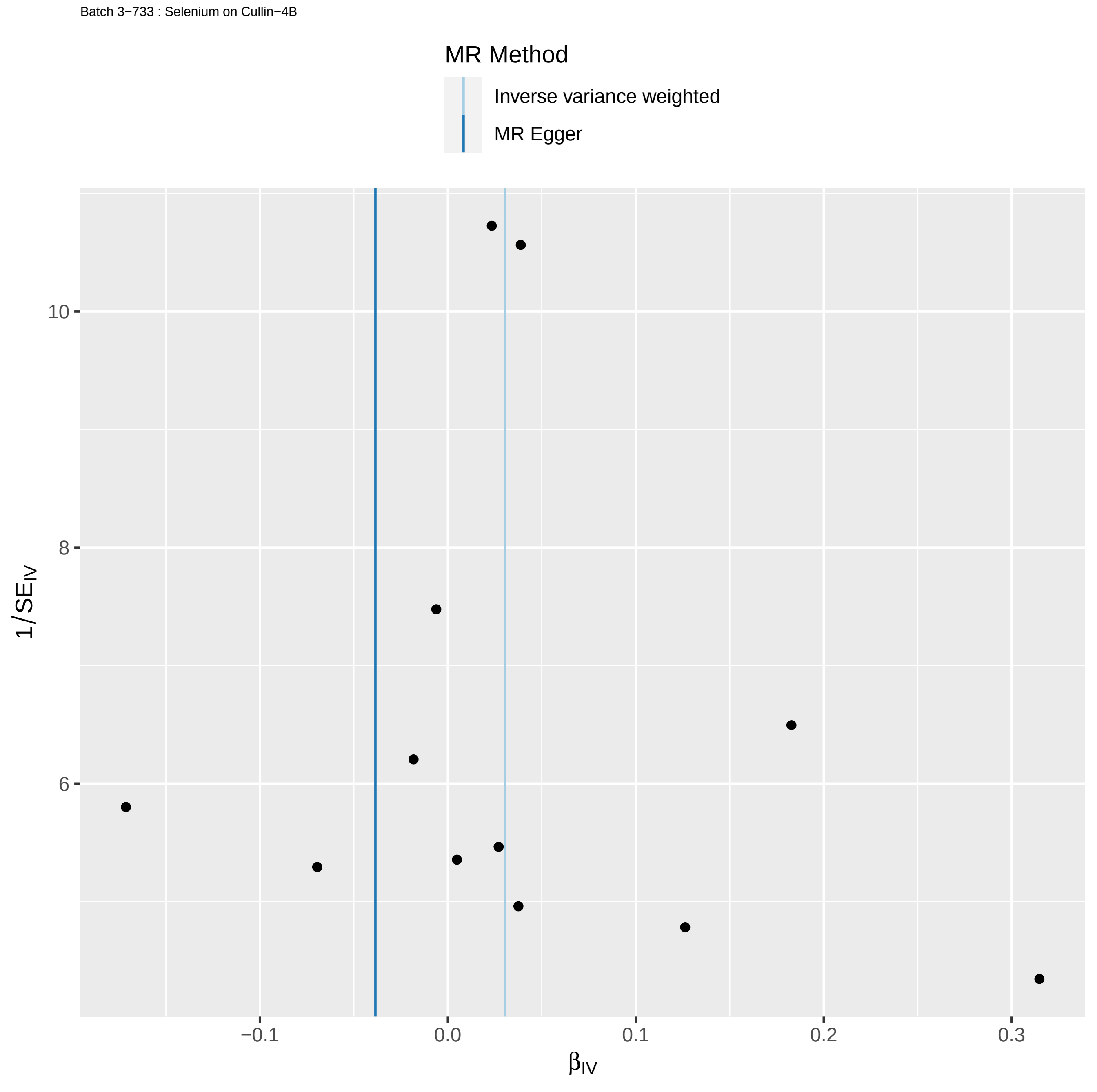

Batch 3−733 : Selenium on Cullin−4B
MR Method
Inverse variance weighted
MR Egger
10
8
6
1/SE_IV
−0.1
0.0
0.1
0.2
0.3
β_IV

Batch 3−734 : Selenium on Cullin−9

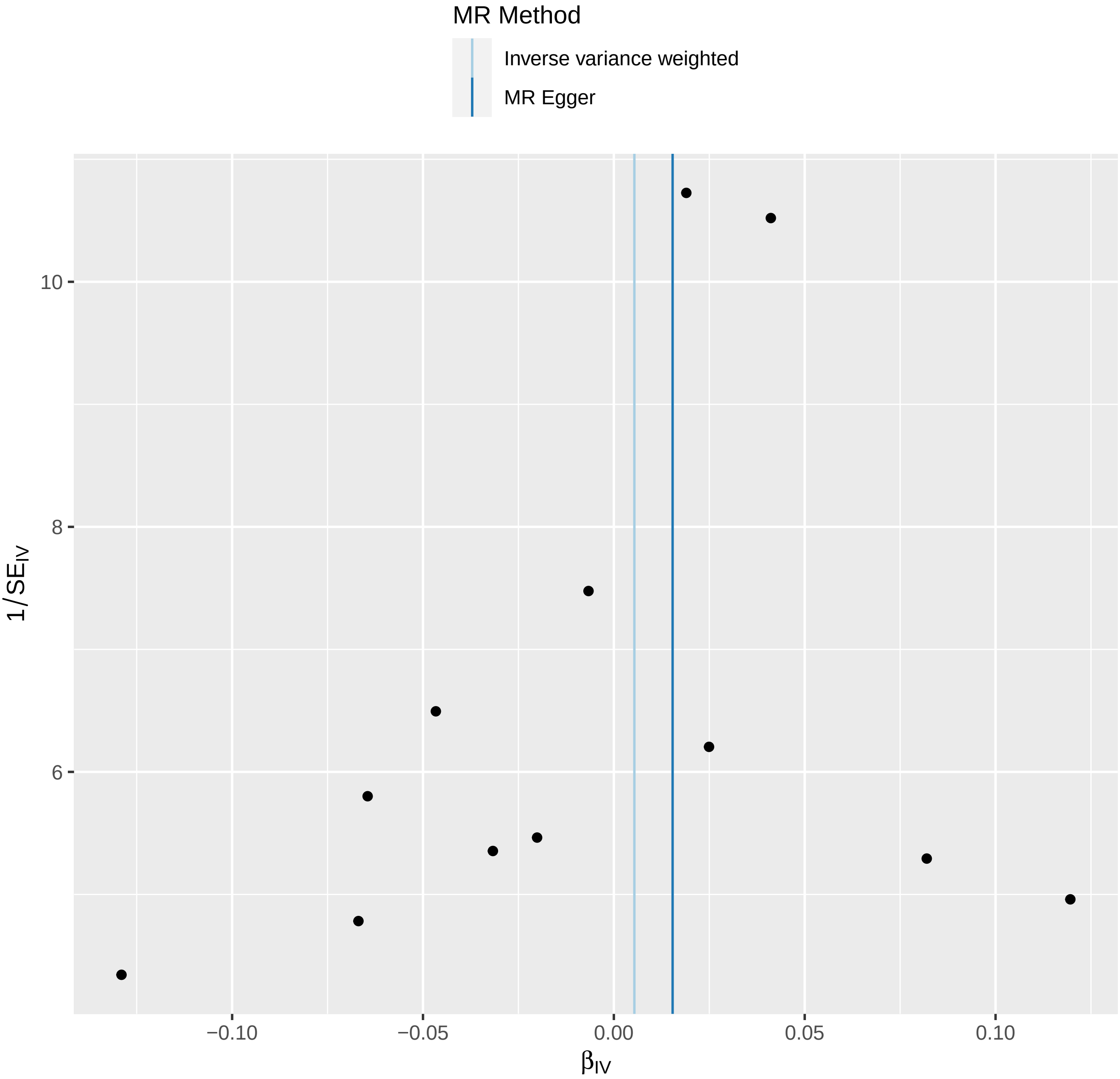

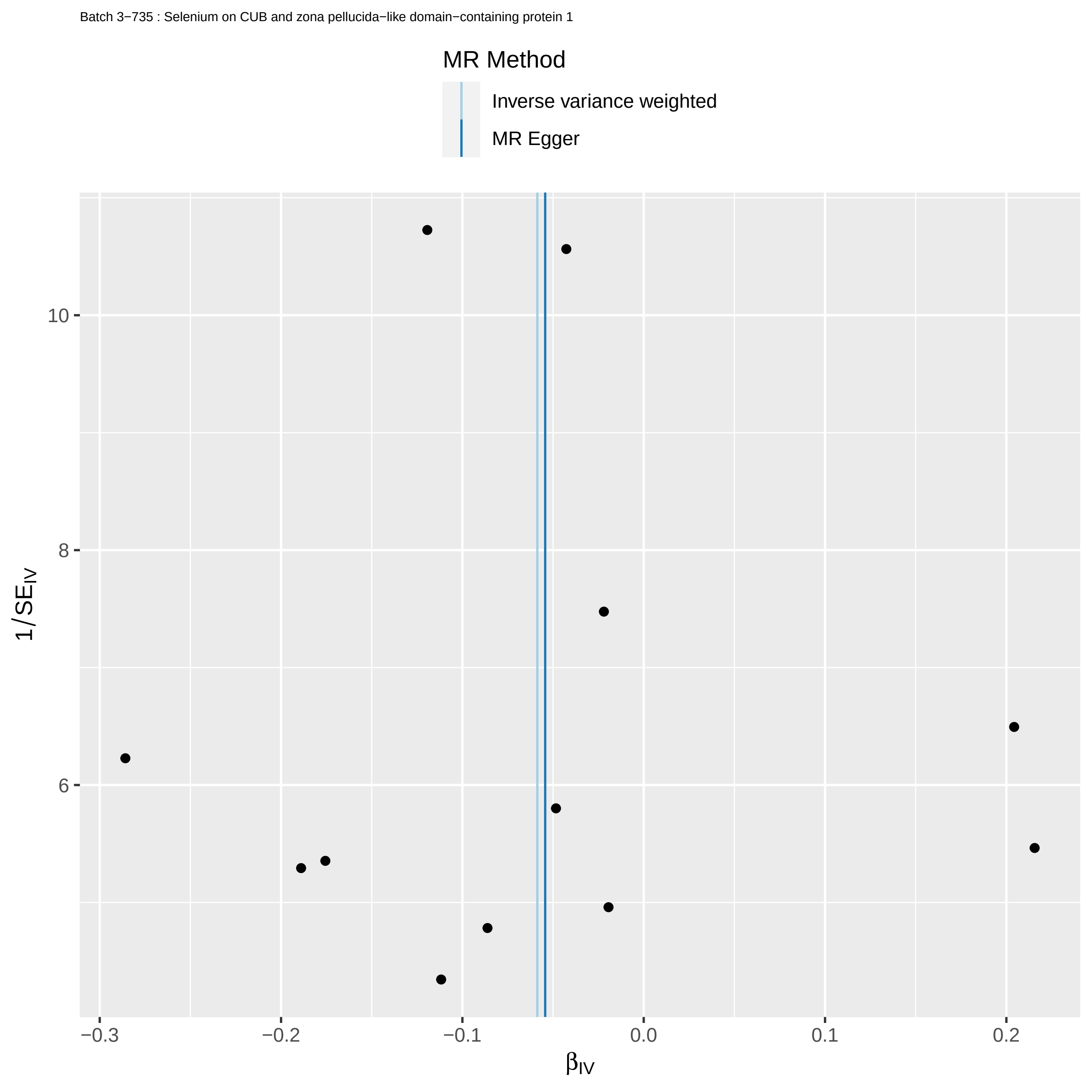
Batch 3−735 : Selenium on CUB and zona pellucida−like domain−containing protein 1
MR Method
Inverse variance weighted
MR Egger
10
8
6
1/SE$_{IV}$
−0.3
−0.2
−0.1
0.0
0.1
0.2
β$_{IV}$

Batch 3−736 : Selenium on Fractalkine

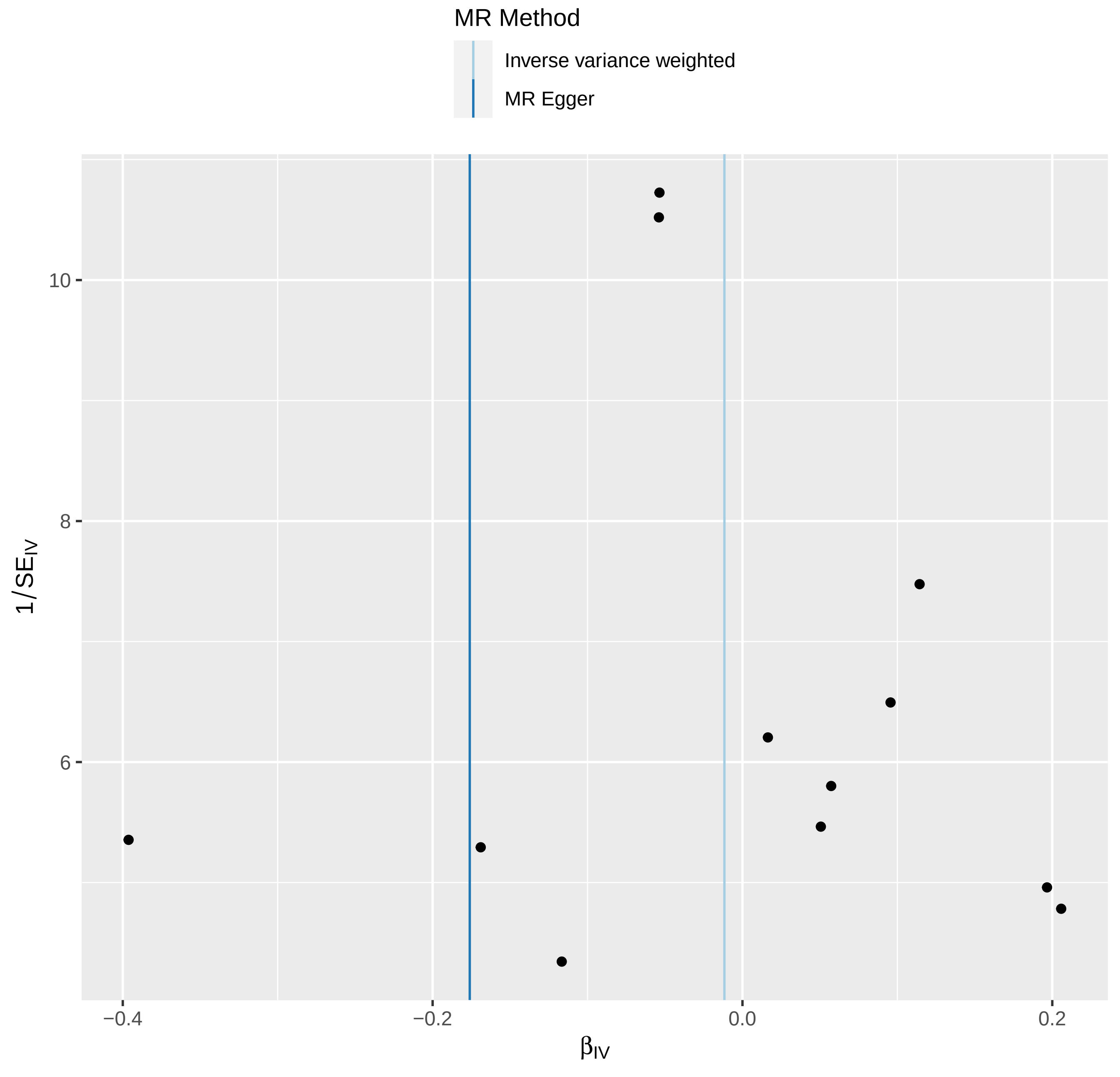

Batch 3−737 : Selenium on Coxsackievirus and adenovirus receptor

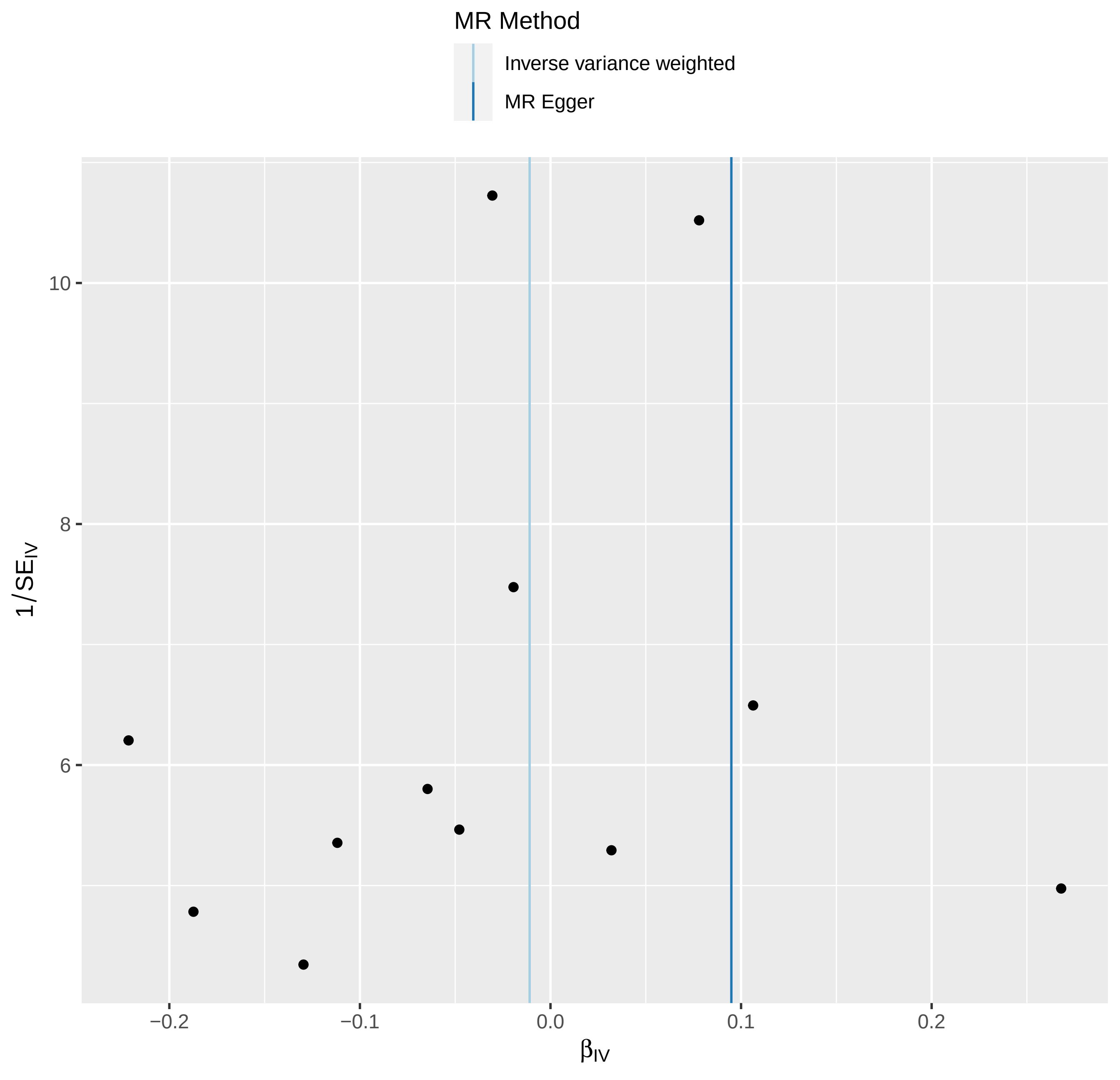

Batch 3−738 : Selenium on C−X−C motif chemokine 10

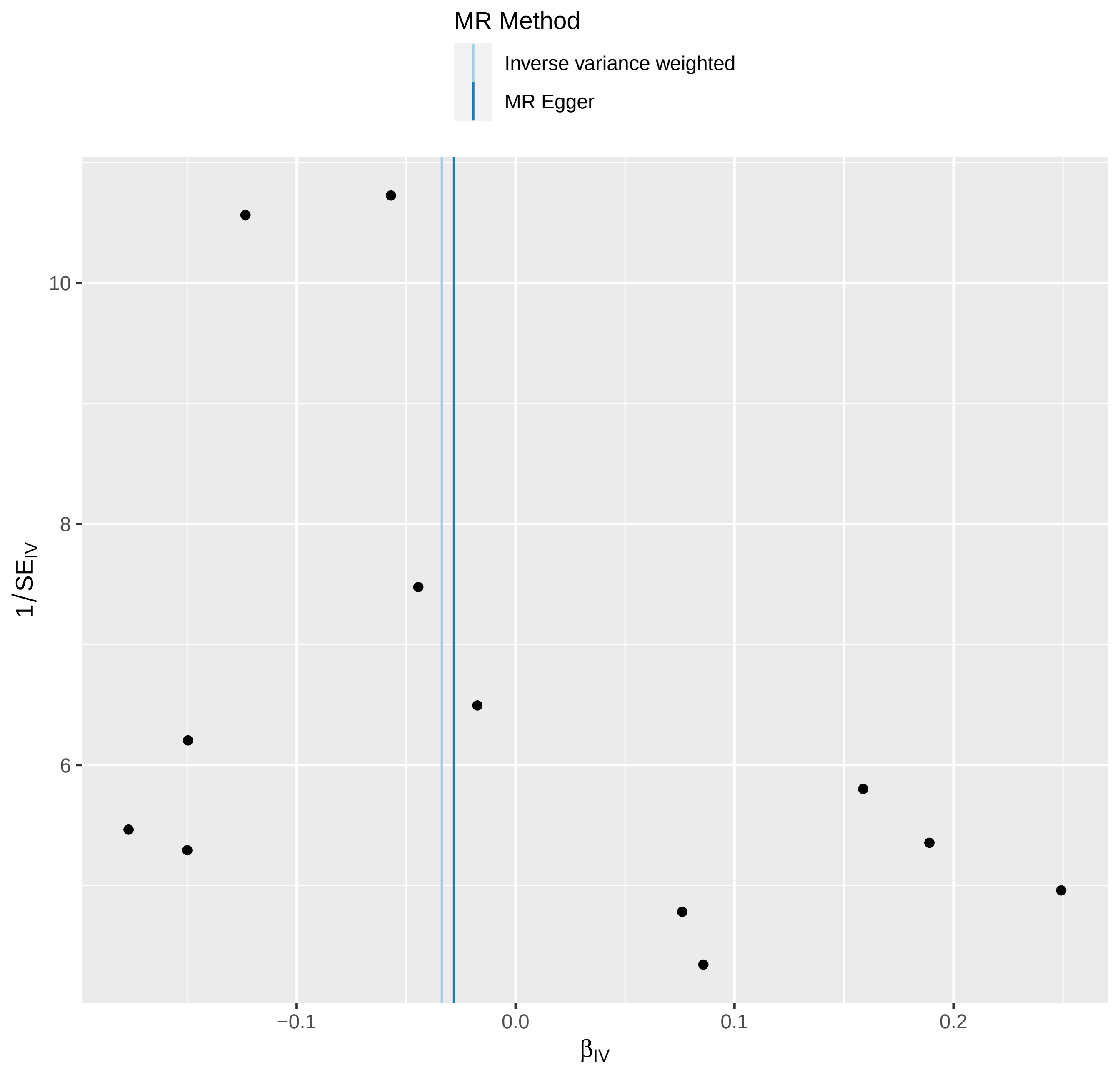

Batch 3−739 : Selenium on C−X−C motif chemokine 11

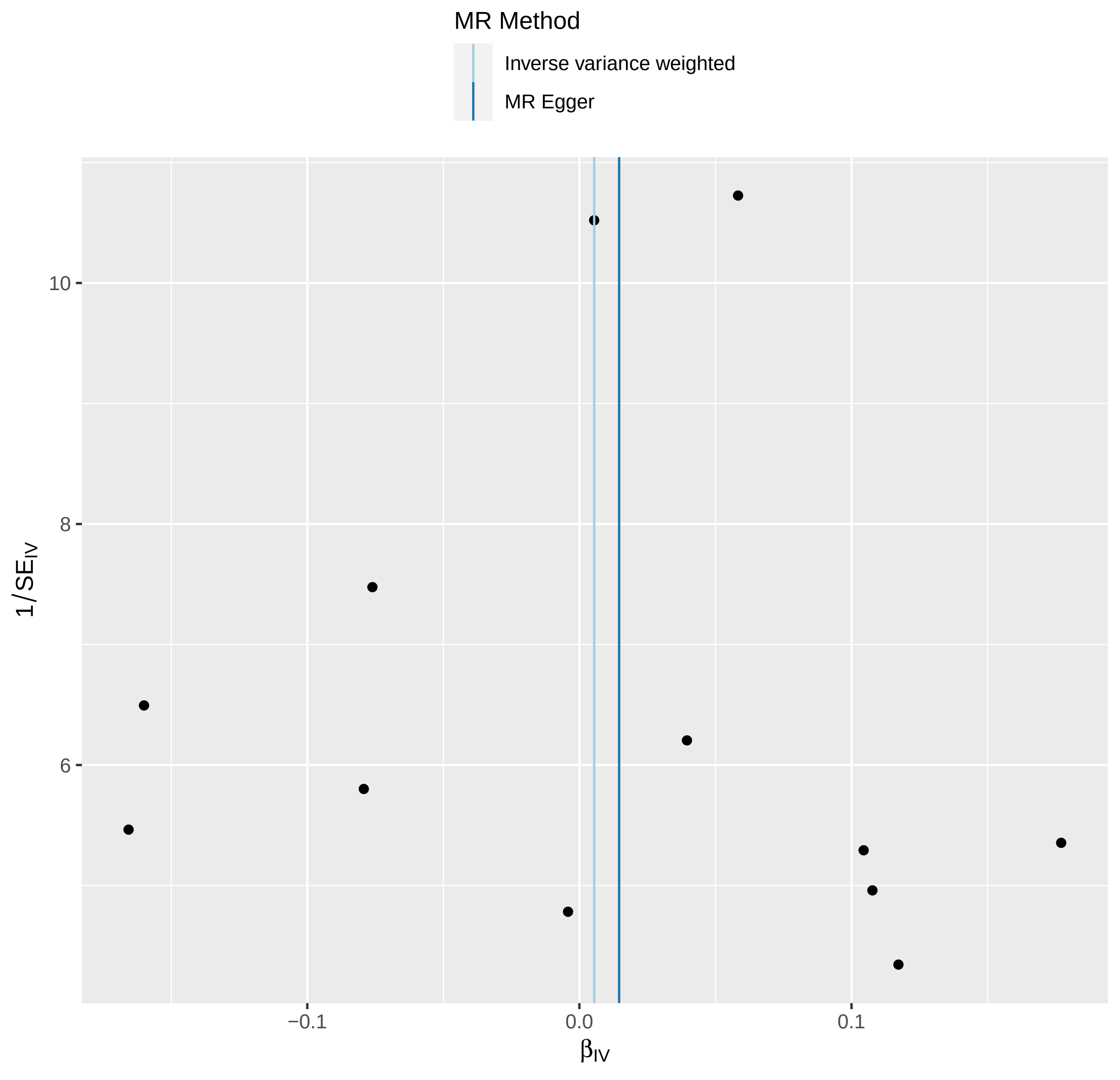

Batch 3−740 : Selenium on Stromal cell−derived factor 1

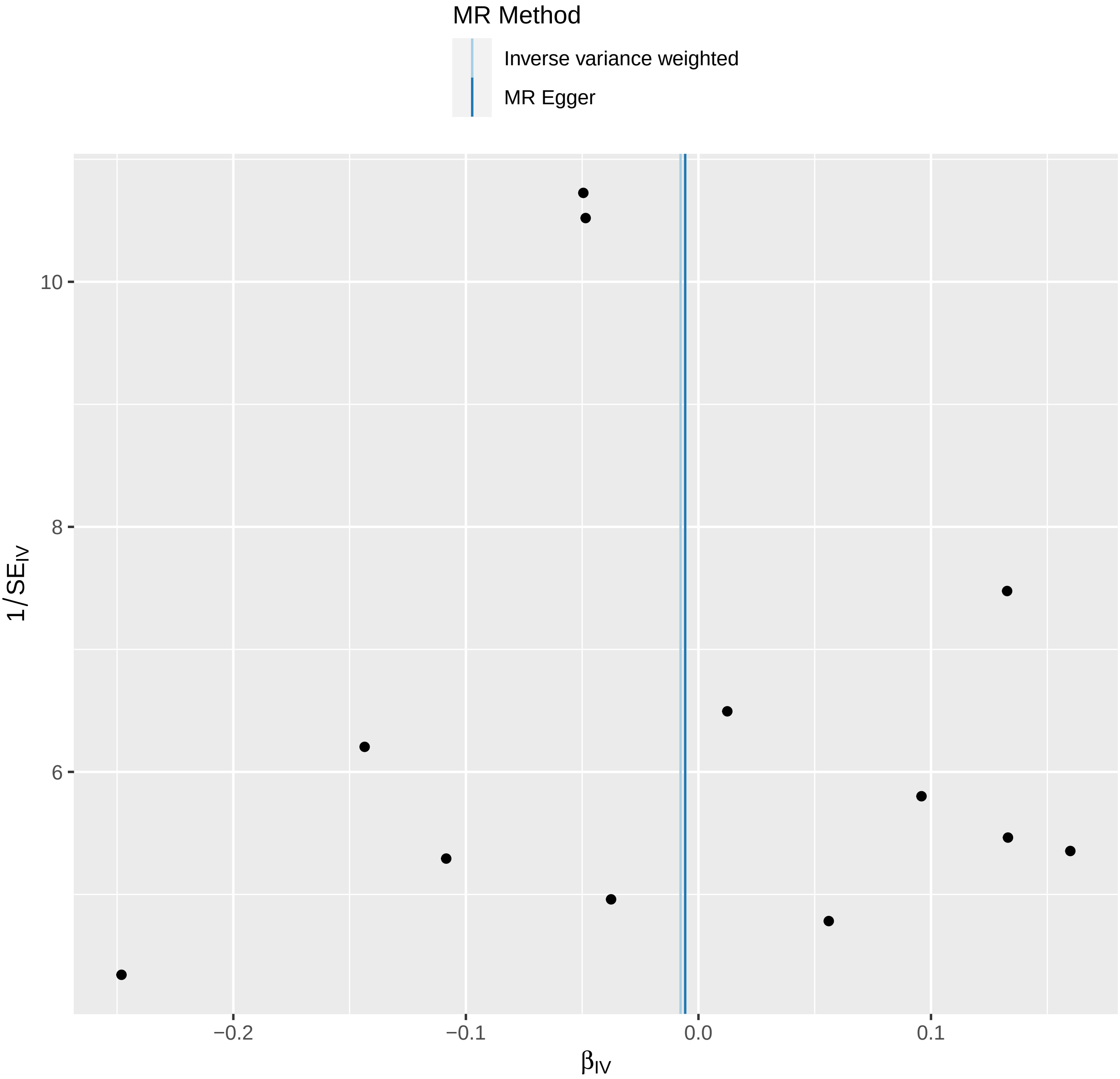

Batch 3−741 : Selenium on Stromal cell−derived factor 1

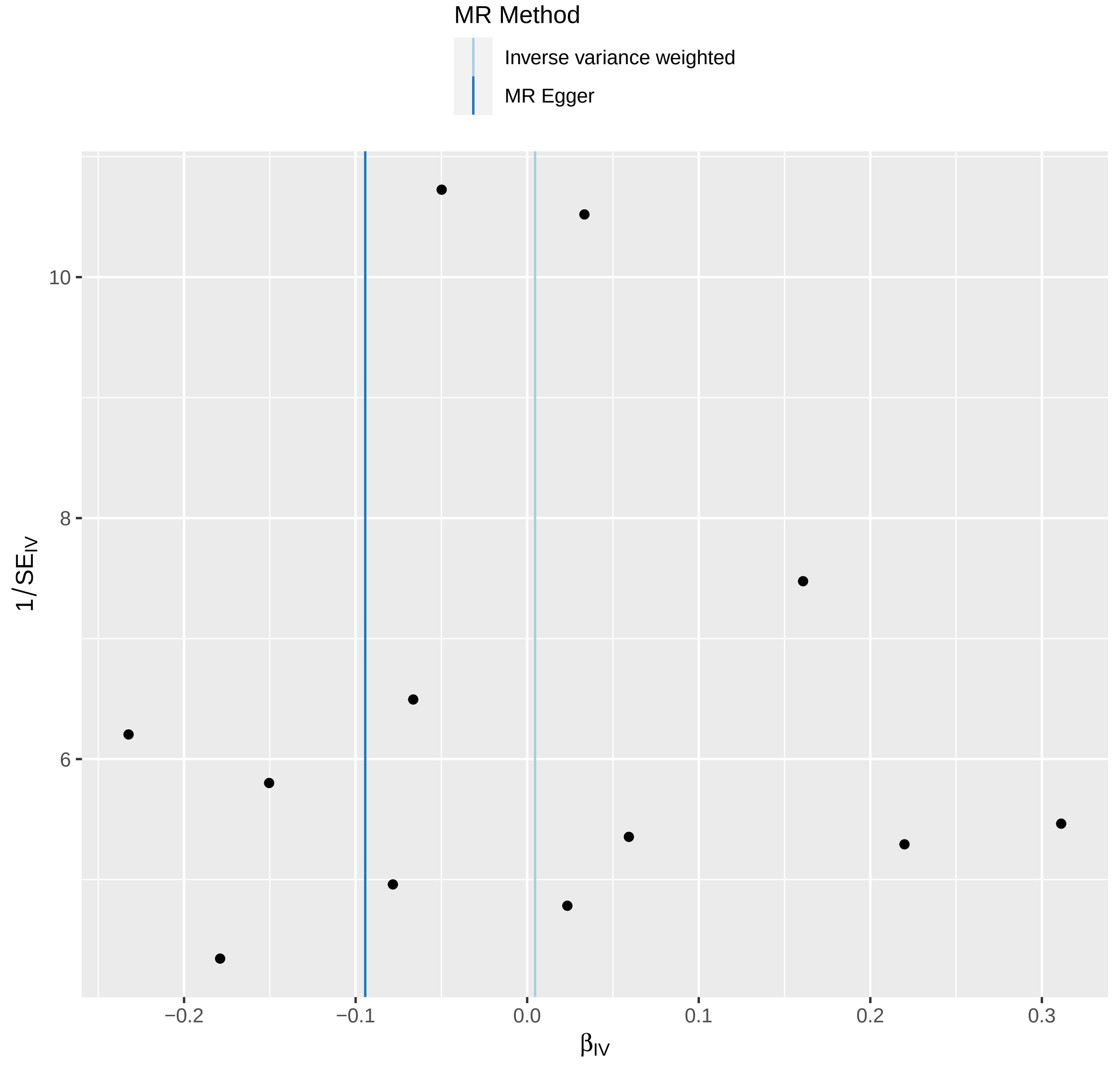

Batch 3−742 : Selenium on Growth−regulated alpha protein

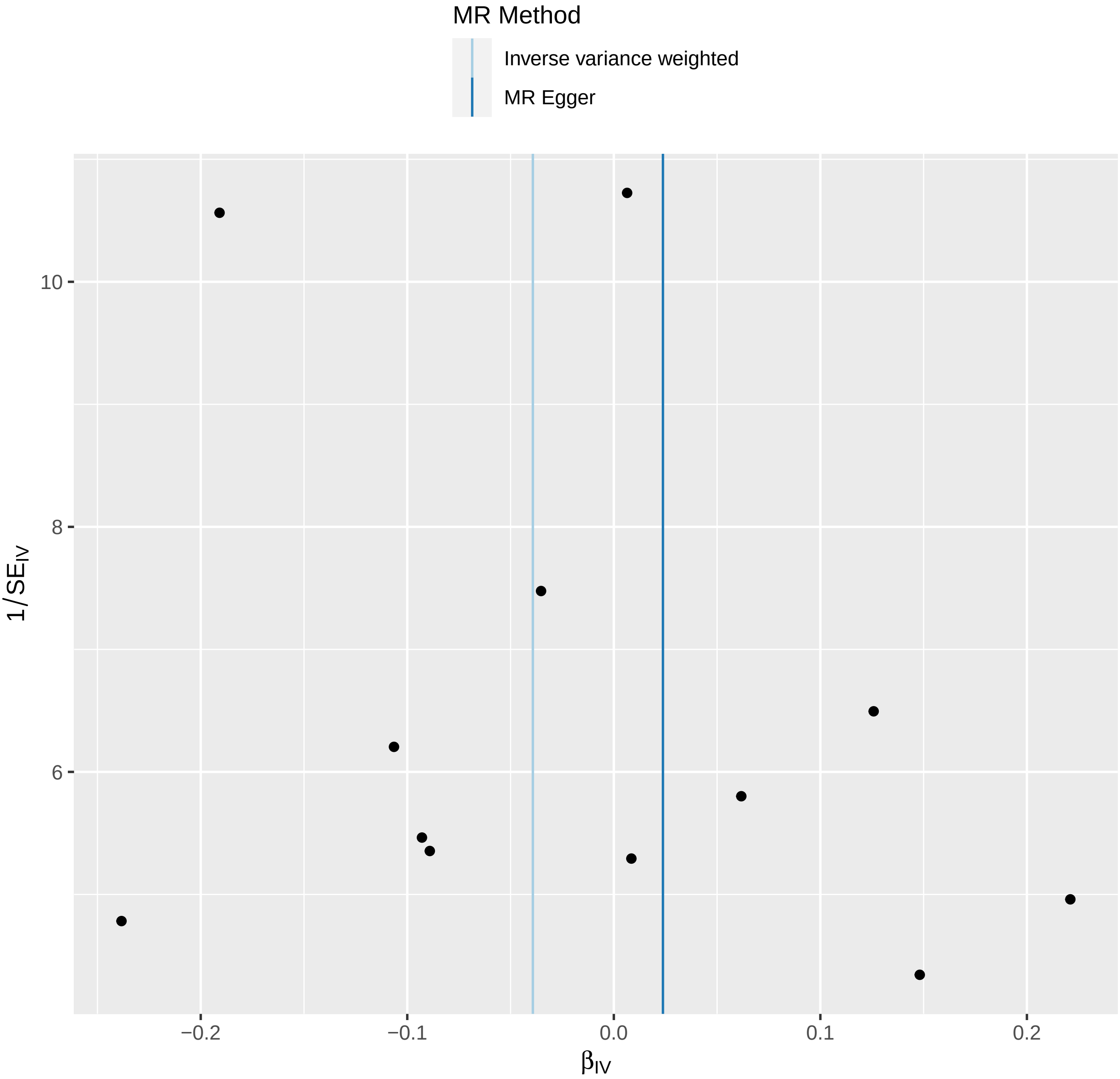

Batch 3−743 : Selenium on C−X−C motif chemokine 13

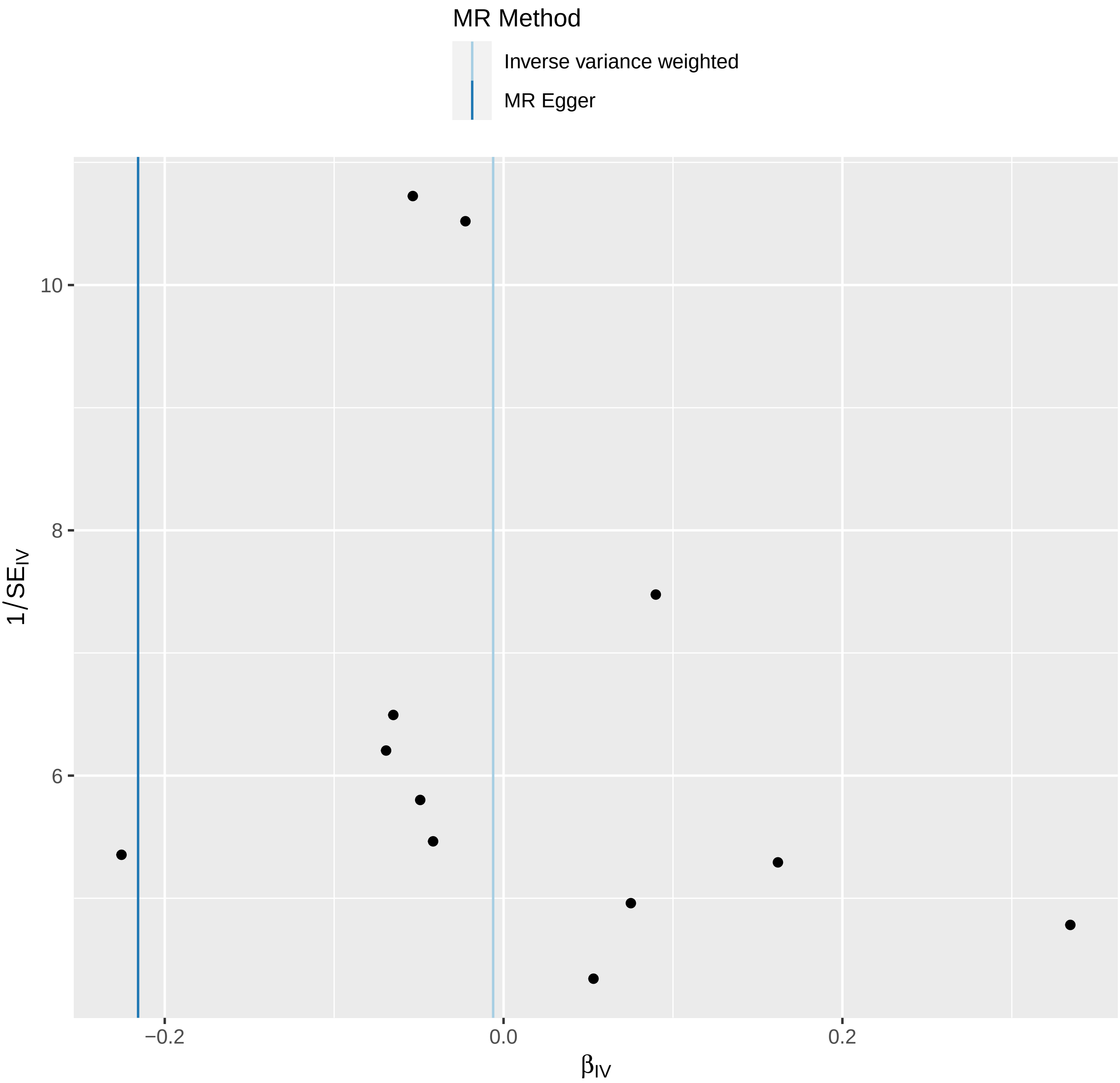

Batch 3−744 : Selenium on C−X−C motif chemokine 14

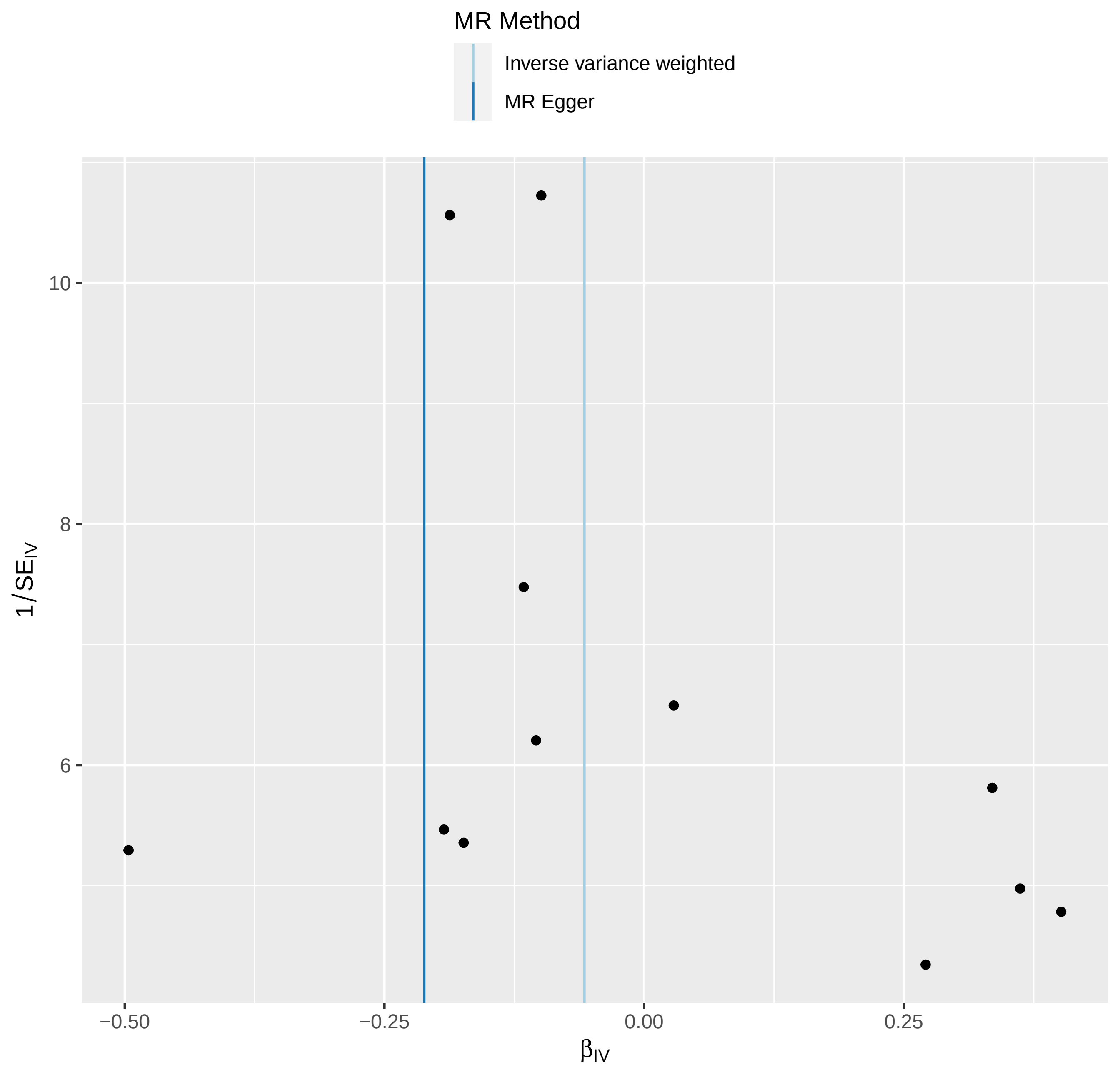

Batch 3−745 : Selenium on C−X−C motif chemokine 16

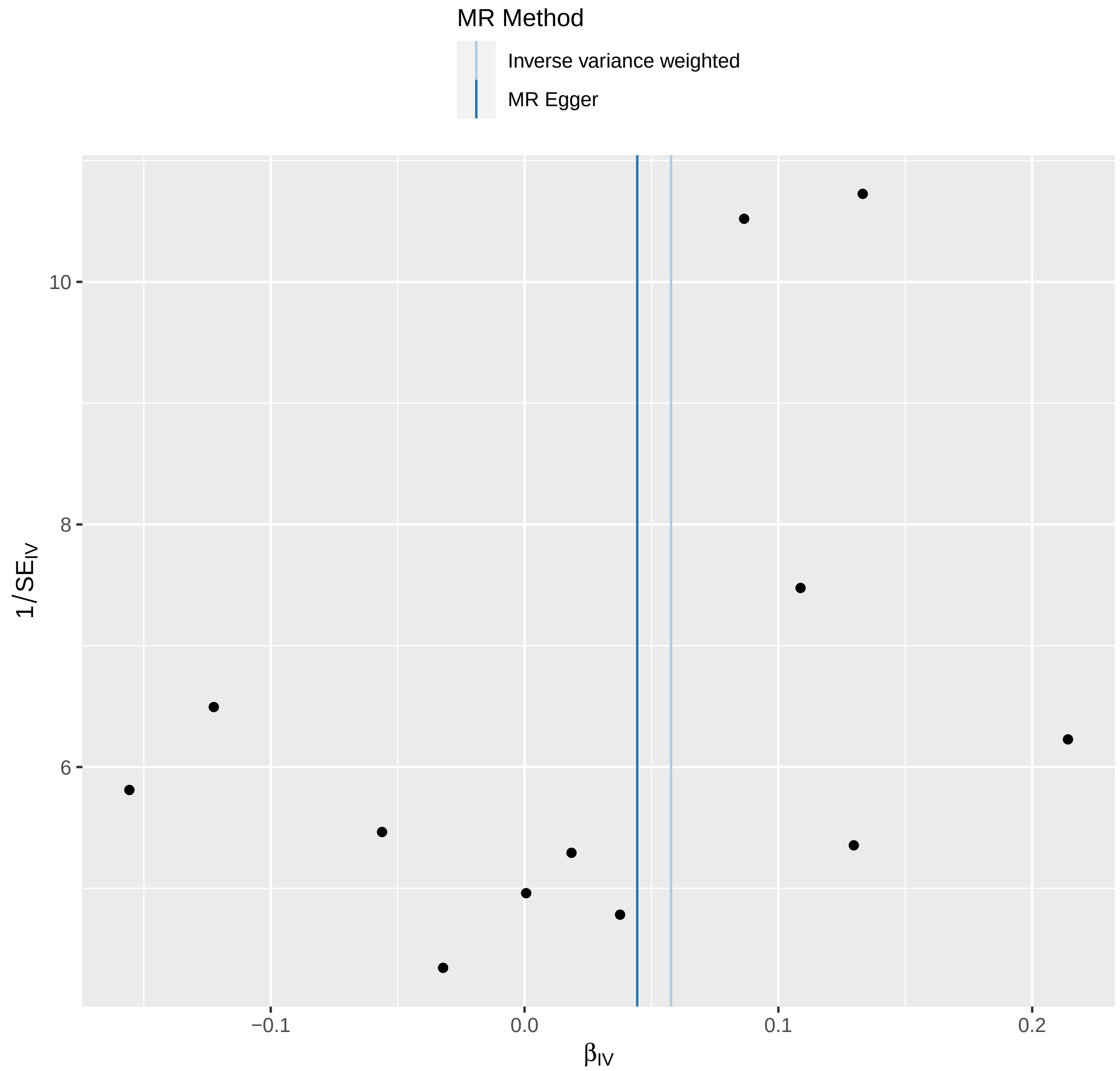

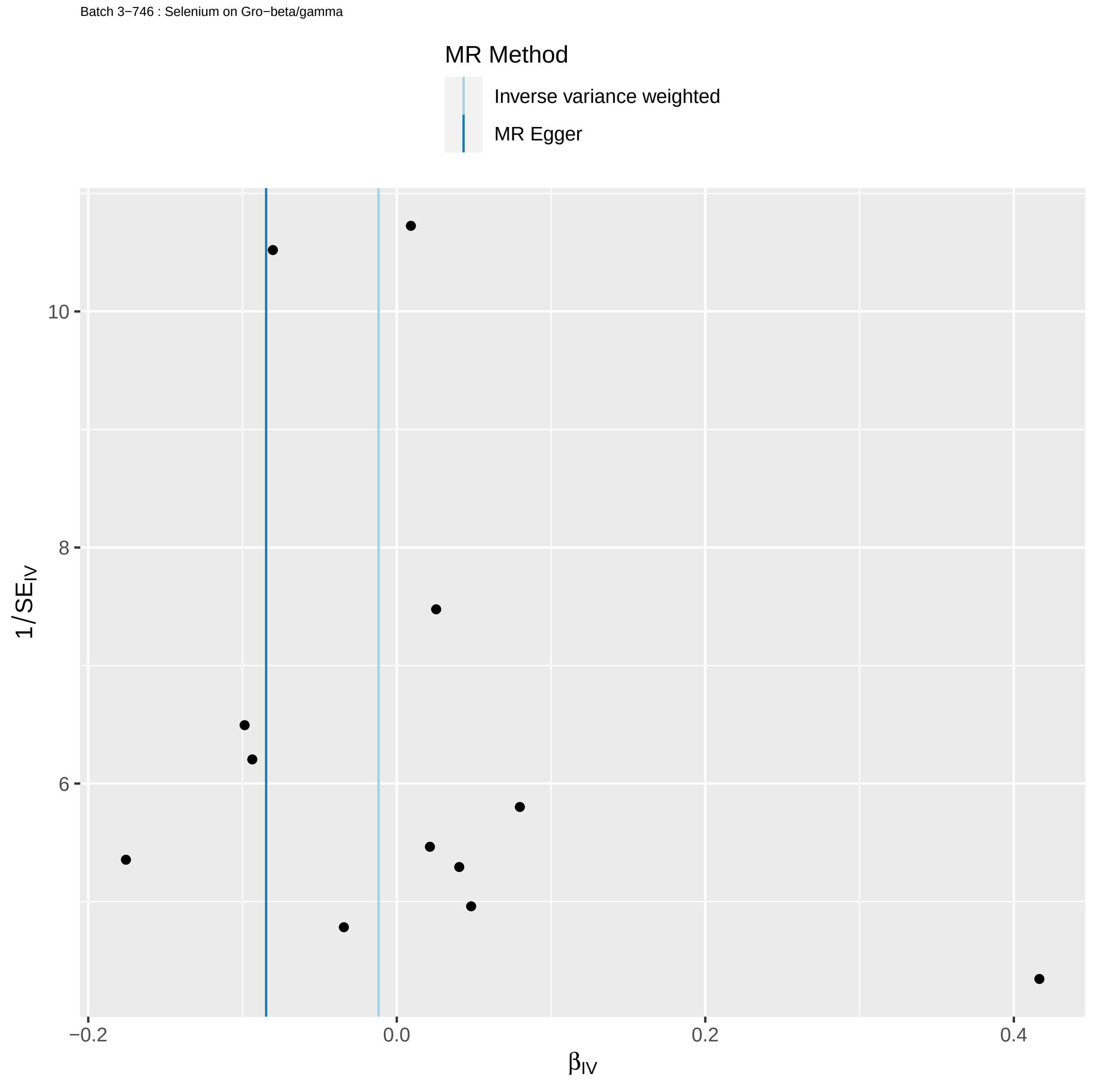
Batch 3−746 : Selenium on Gro−beta/gamma
MR Method
Inverse variance weighted
MR Egger
10
8
6
1/SE$_{IV}$
−0.2
0.0
0.2
0.4
β$_{IV}$

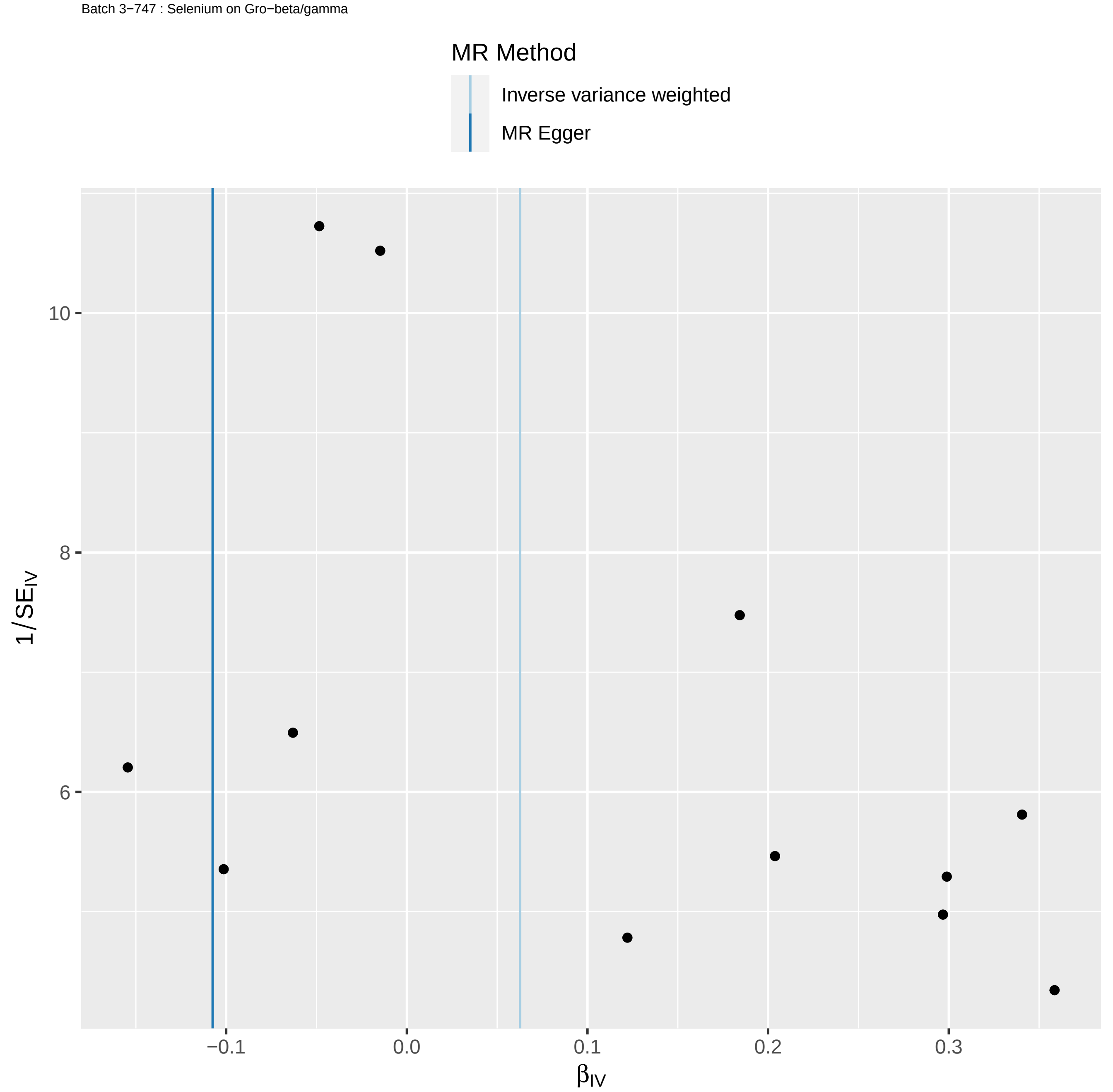
Batch 3−747 : Selenium on Gro−beta/gamma
MR Method
Inverse variance weighted
MR Egger
10
8
6
1/SE_IV
−0.1
0.0
0.1
0.2
0.3
β_IV

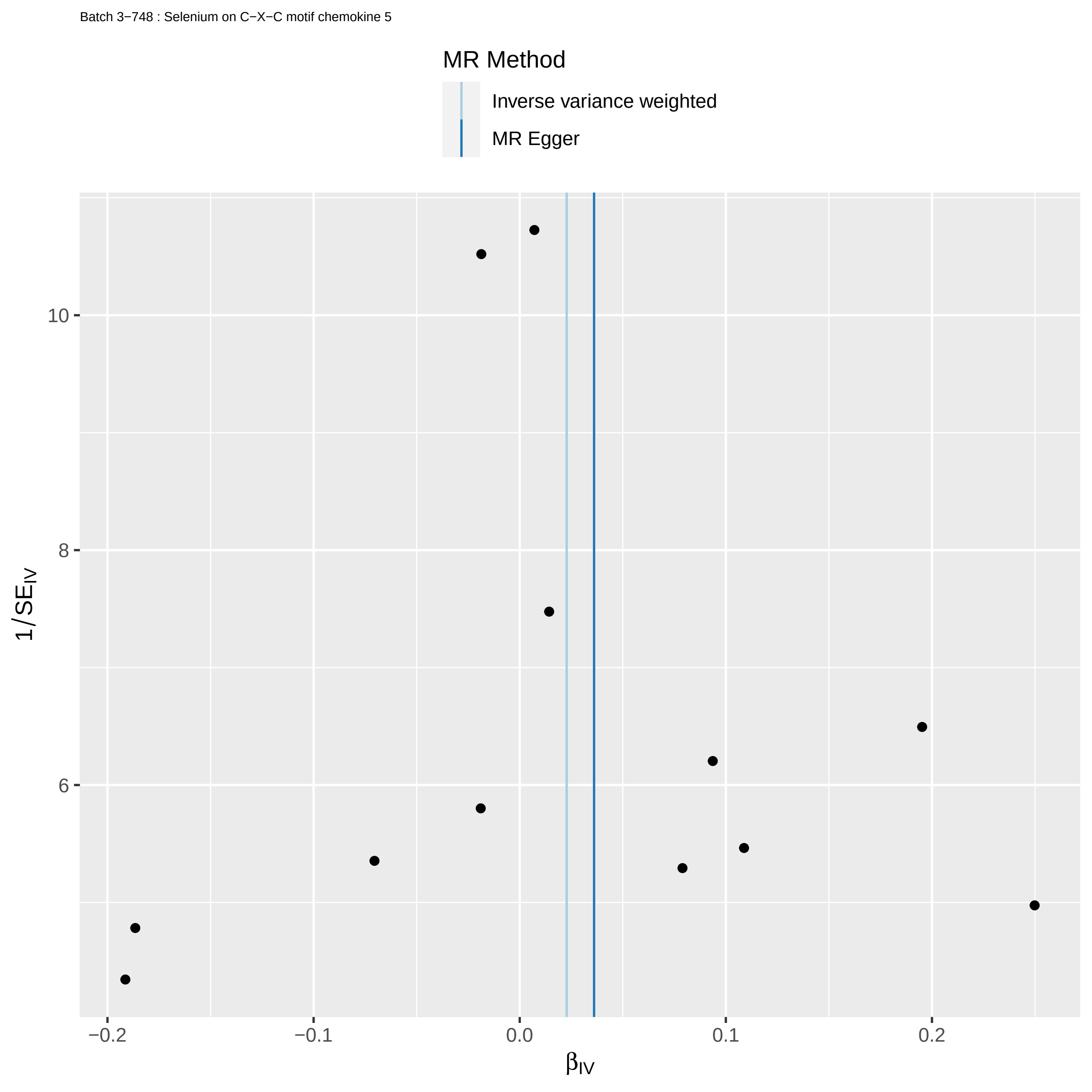
Batch 3−748 : Selenium on C−X−C motif chemokine 5
MR Method
Inverse variance weighted
MR Egger
10
8
6
$1/SE_{IV}$
−0.2
−0.1
0.0
0.1
0.2
β_{IV}

Batch 3−749 : Selenium on Interleukin−8

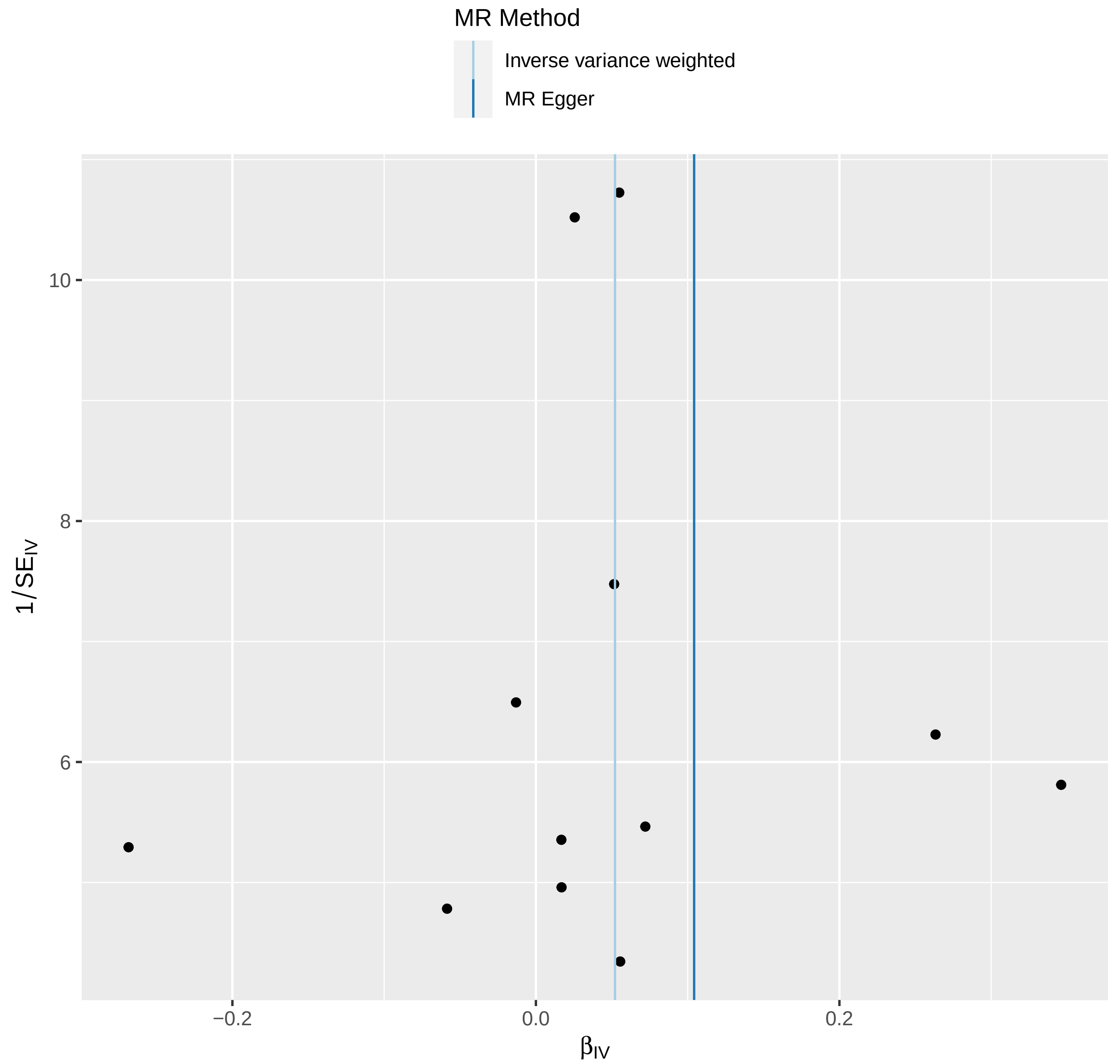

Batch 3−750 : Selenium on C−X−C motif chemokine 9

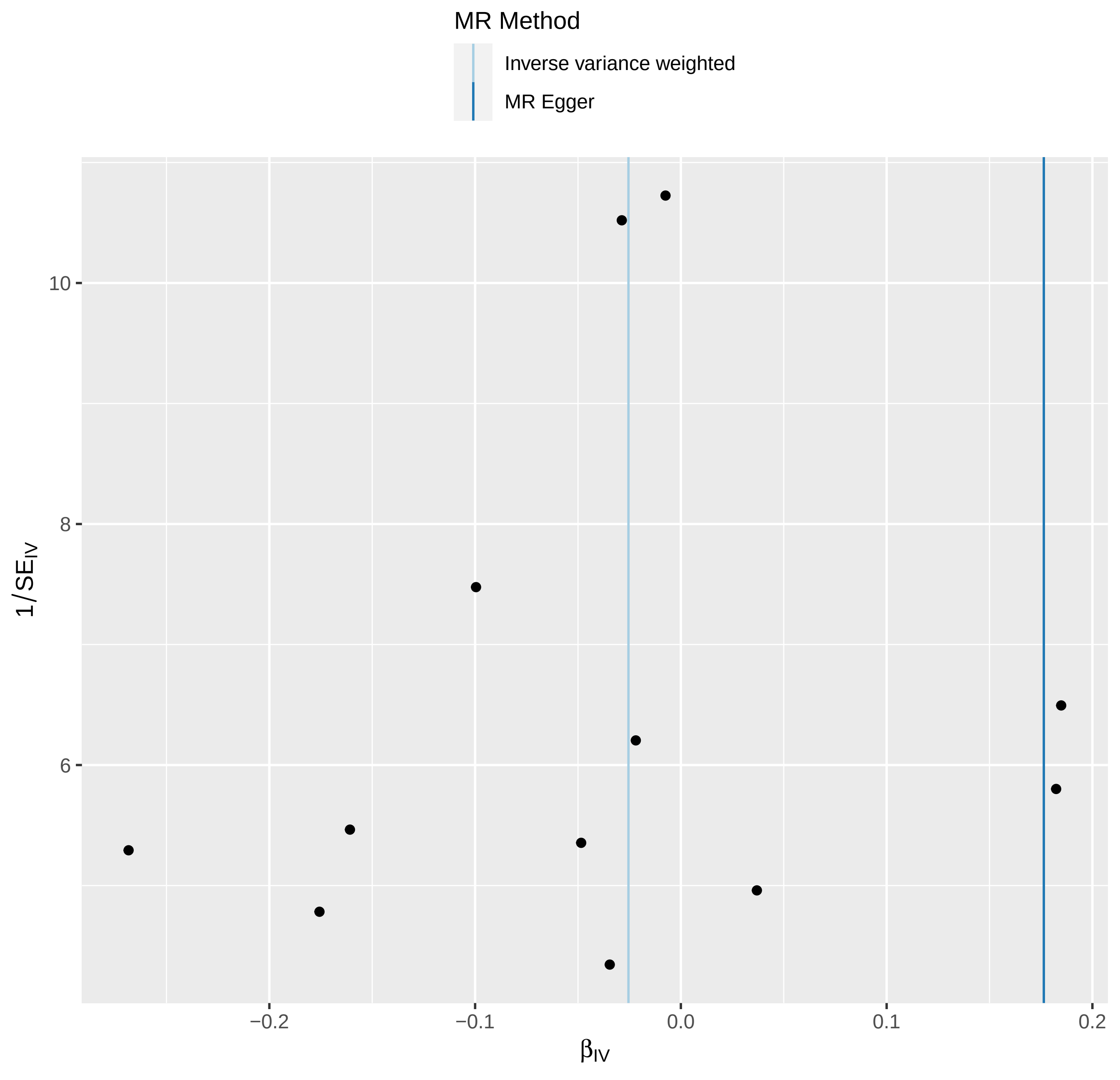

Batch 3−751 : Selenium on C−X−C motif chemokine 9

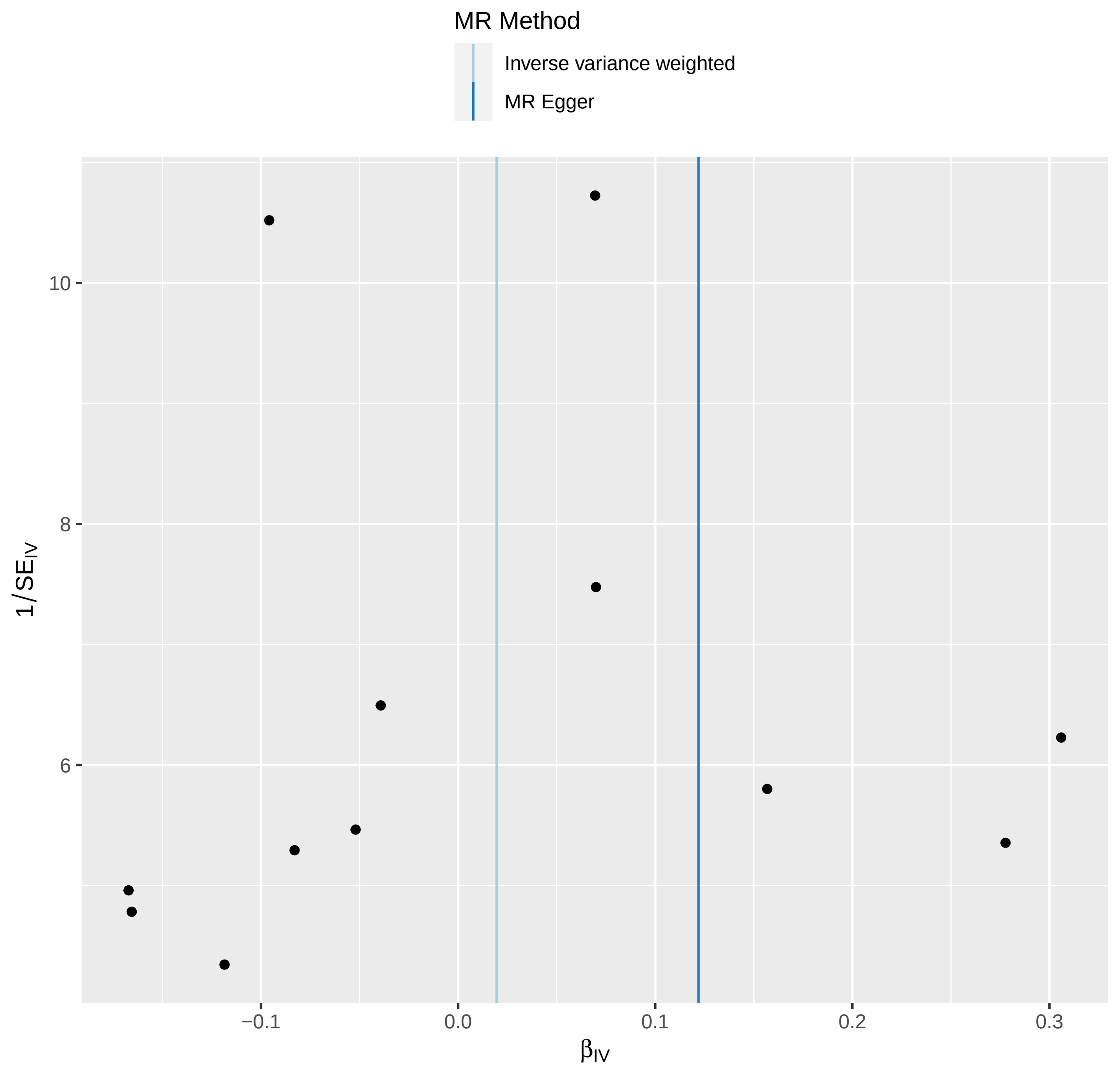

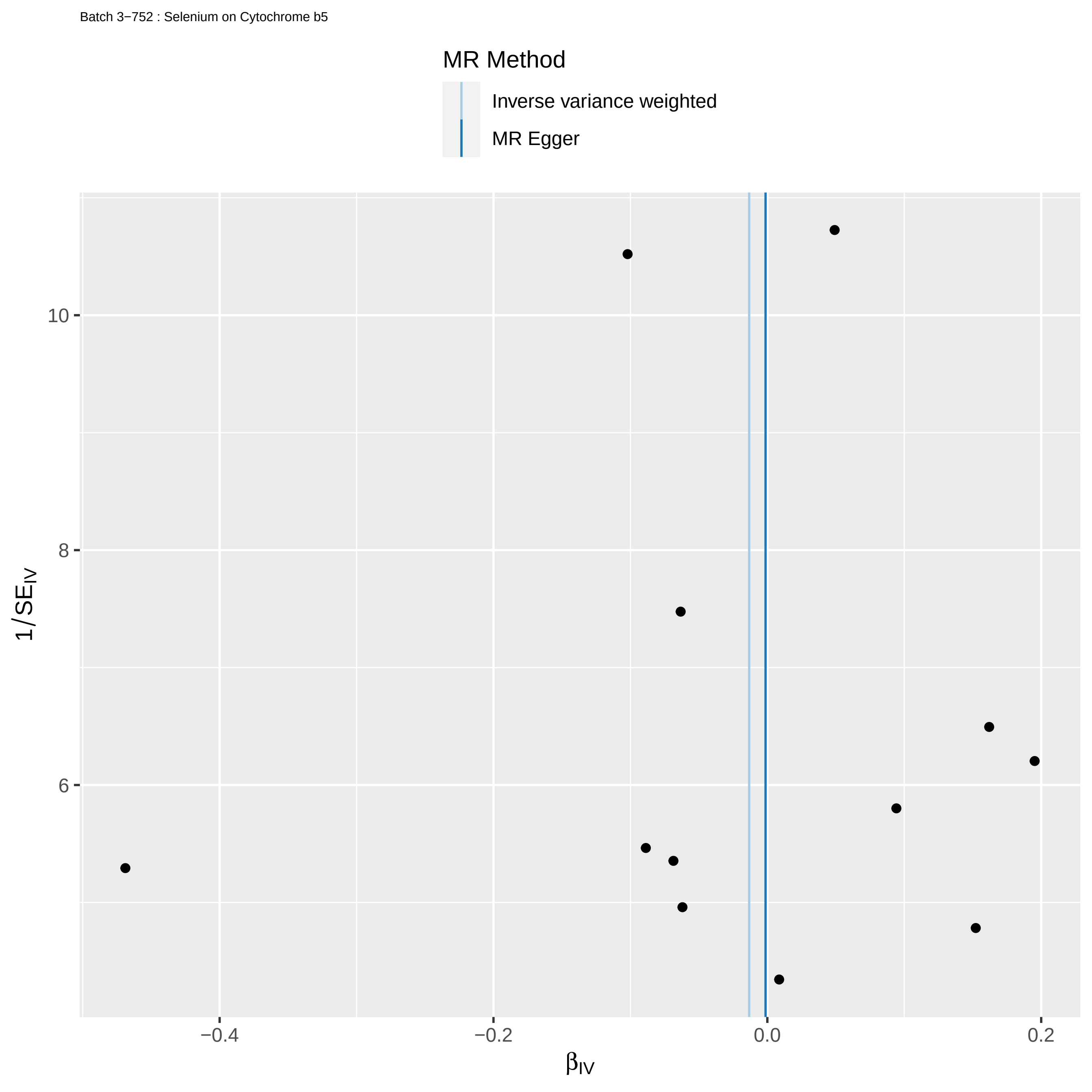

Batch 3−752 : Selenium on Cytochrome b5
MR Method
Inverse variance weighted
MR Egger
10
8
6
1/SE_IV
−0.4
−0.2
0.0
0.2
β_IV

Batch 3−753 : Selenium on NADH−cytochrome b5 reductase 3

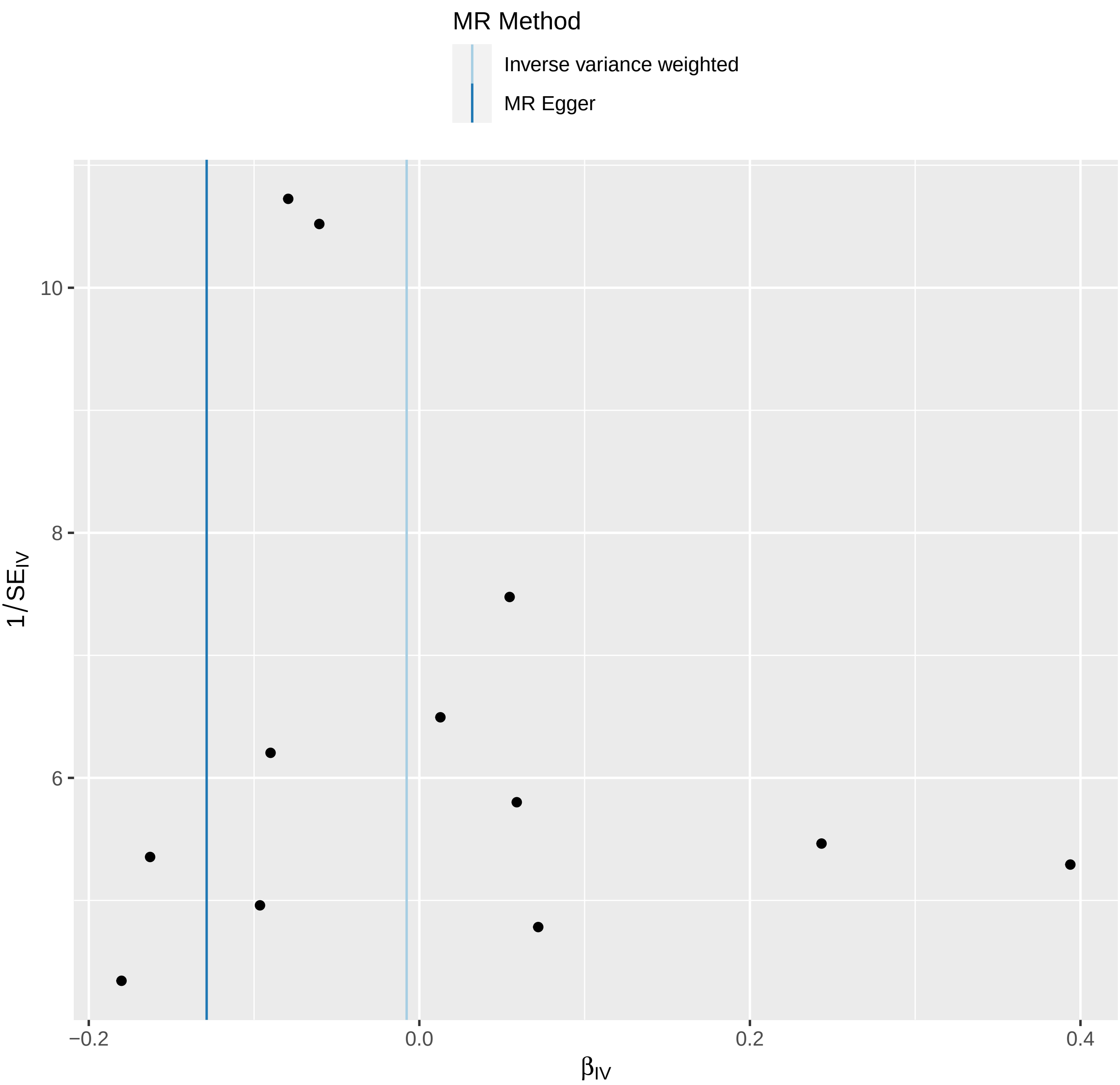

Batch 3−754 : Selenium on Cytochrome c

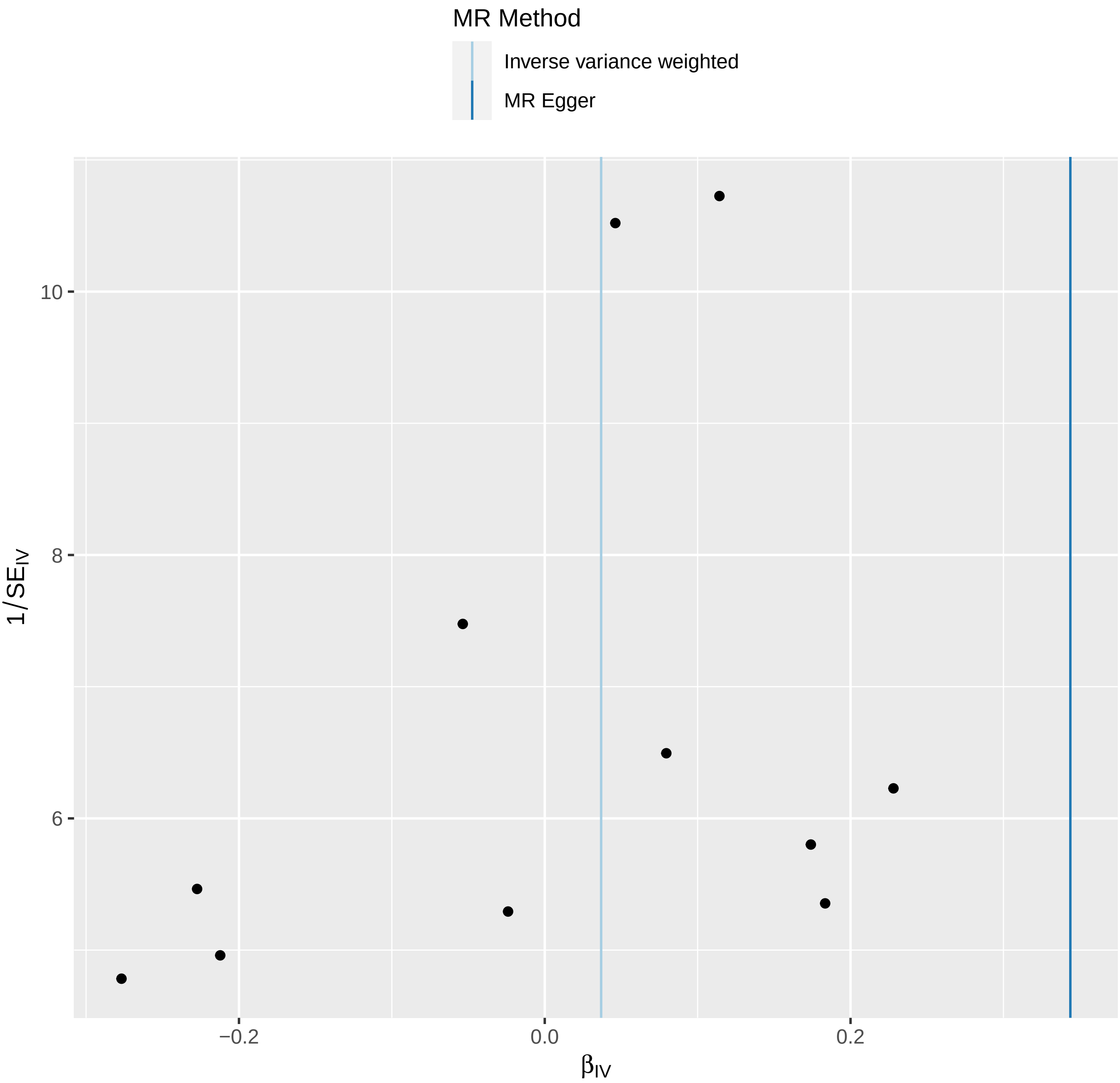

Batch 3−755 : Selenium on Cytoglobin

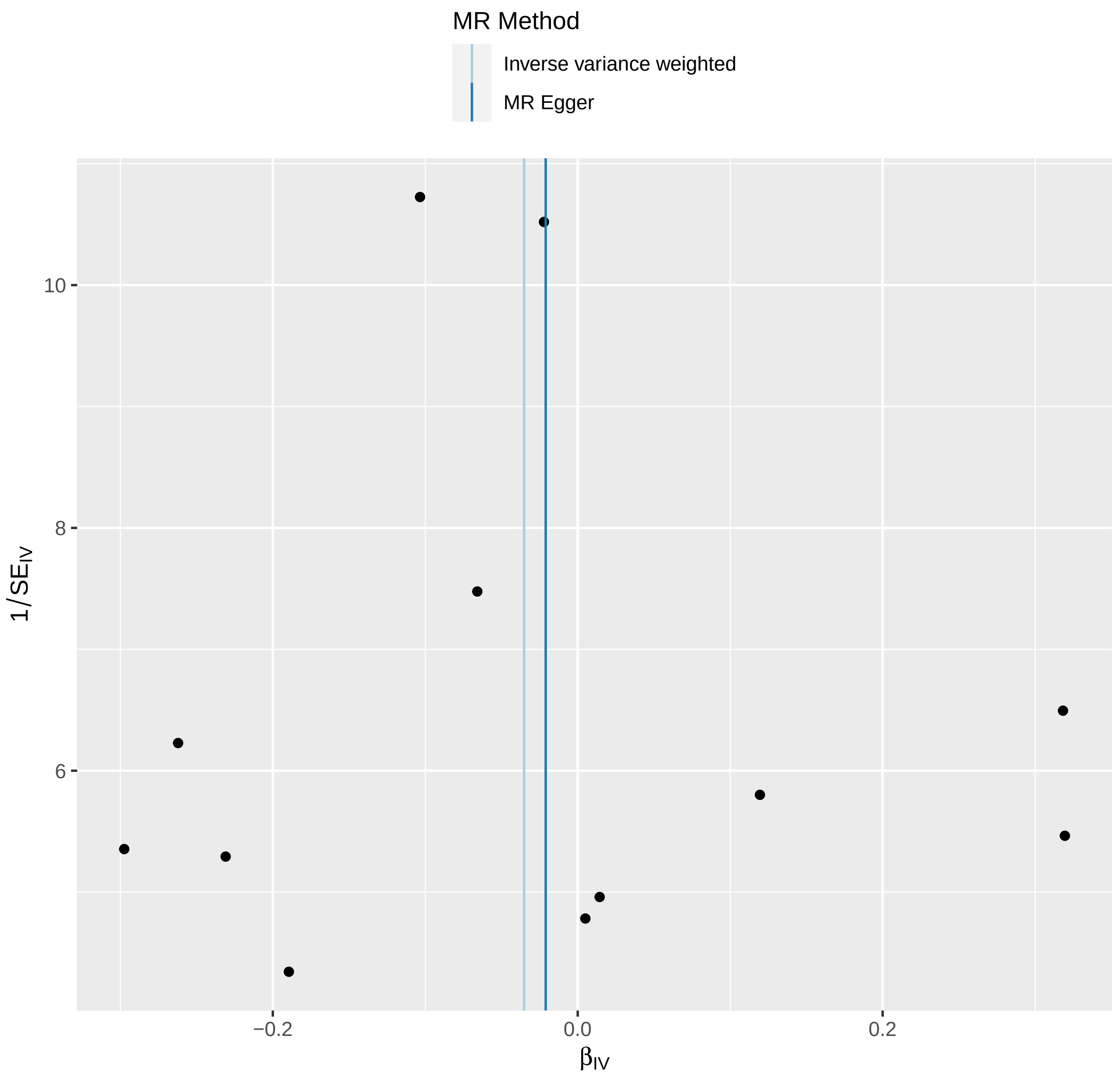

Batch 3−756 : Selenium on Cytochrome P450 3A4

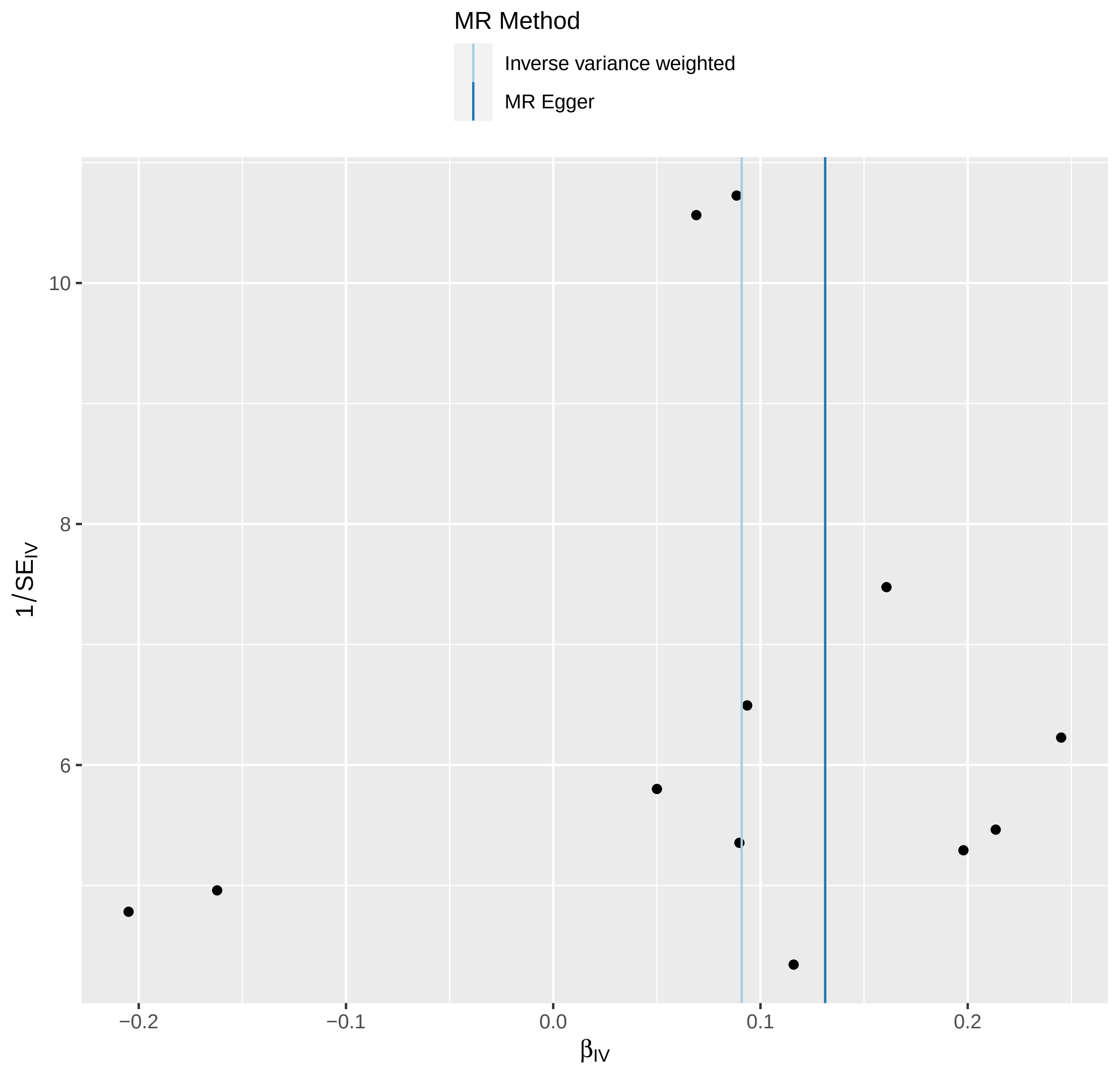

Batch 3−757 : Selenium on Cytochrome P450 3A4

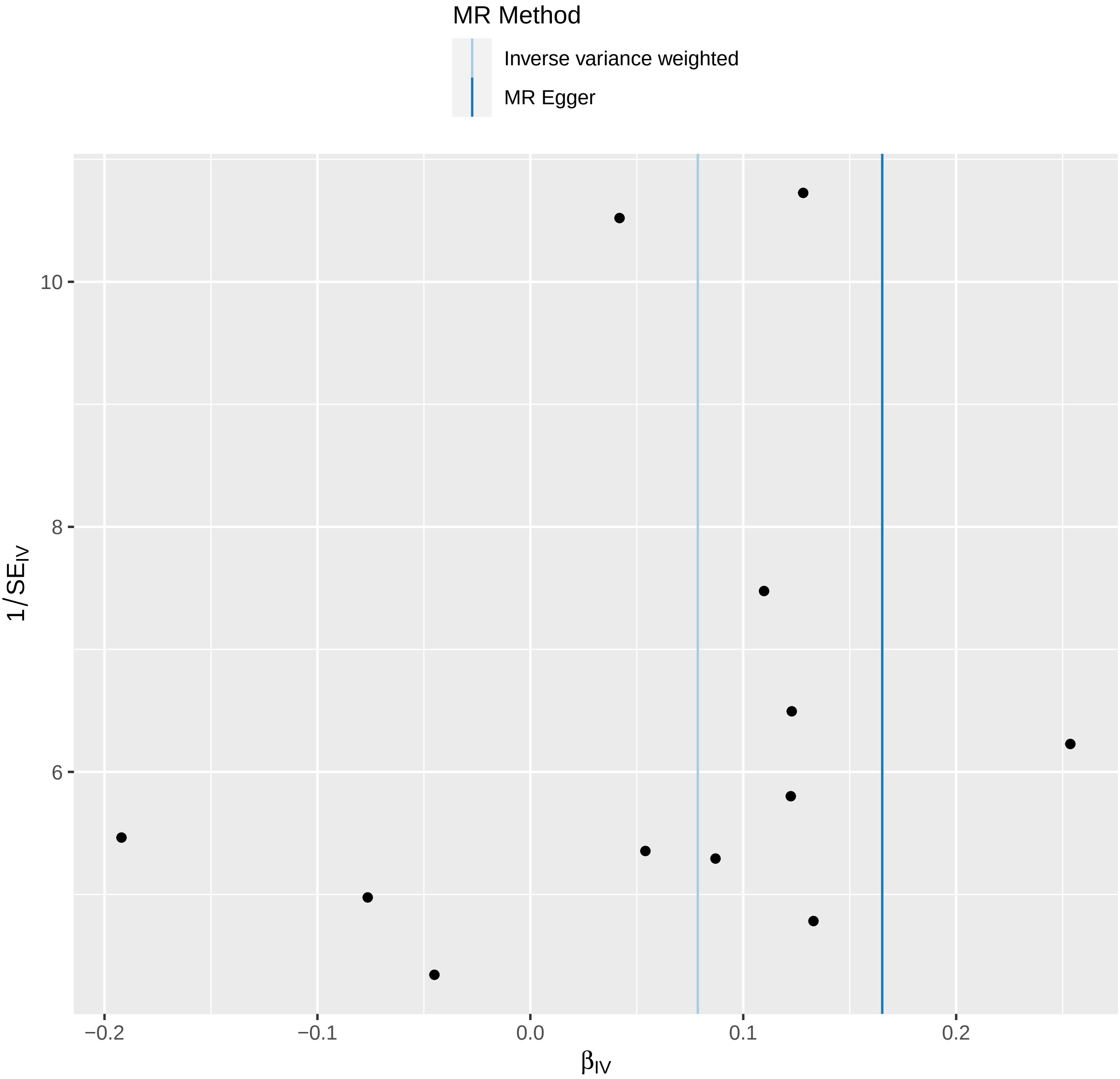

Batch 3−758 : Selenium on Protein CYR61

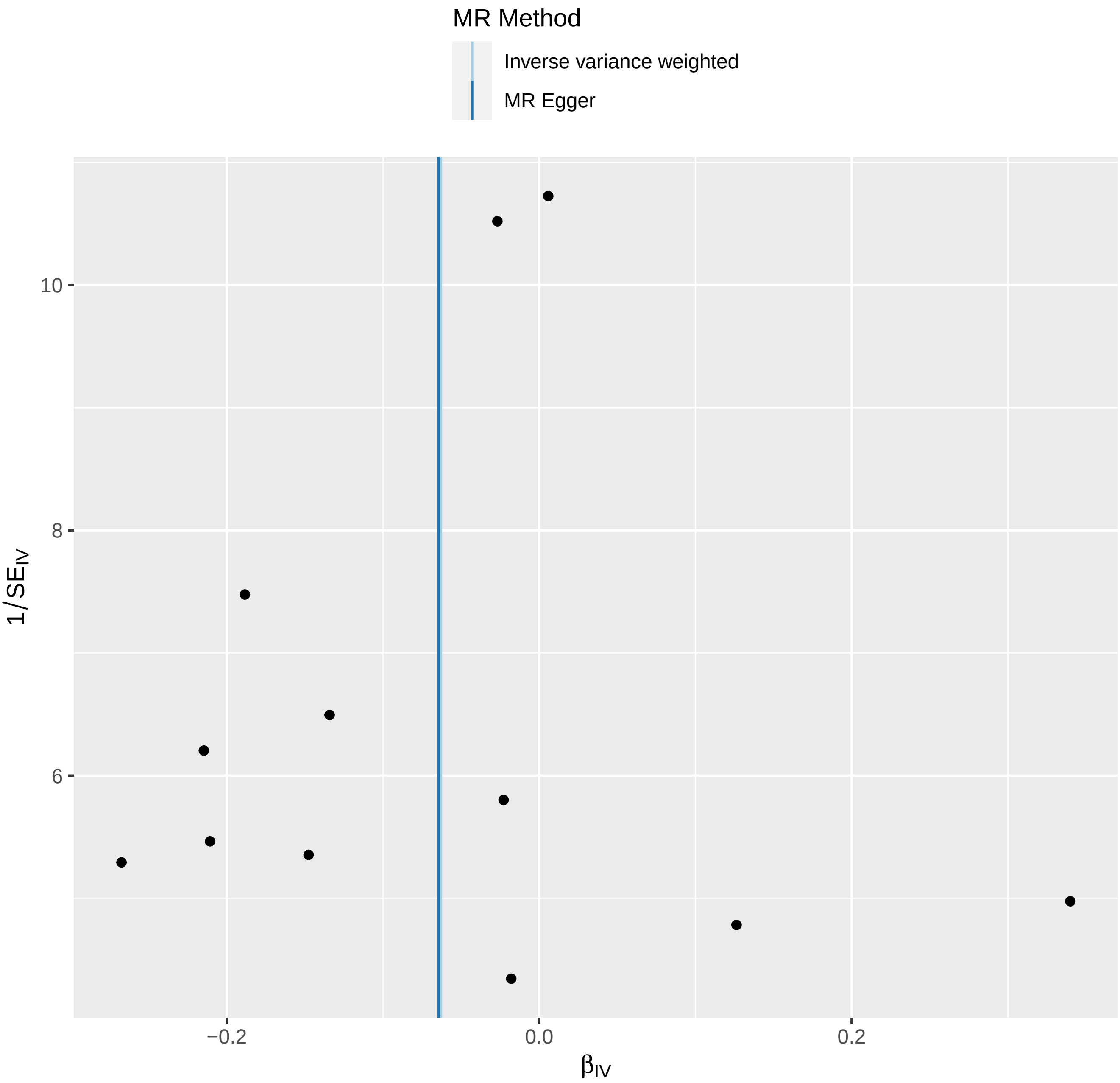

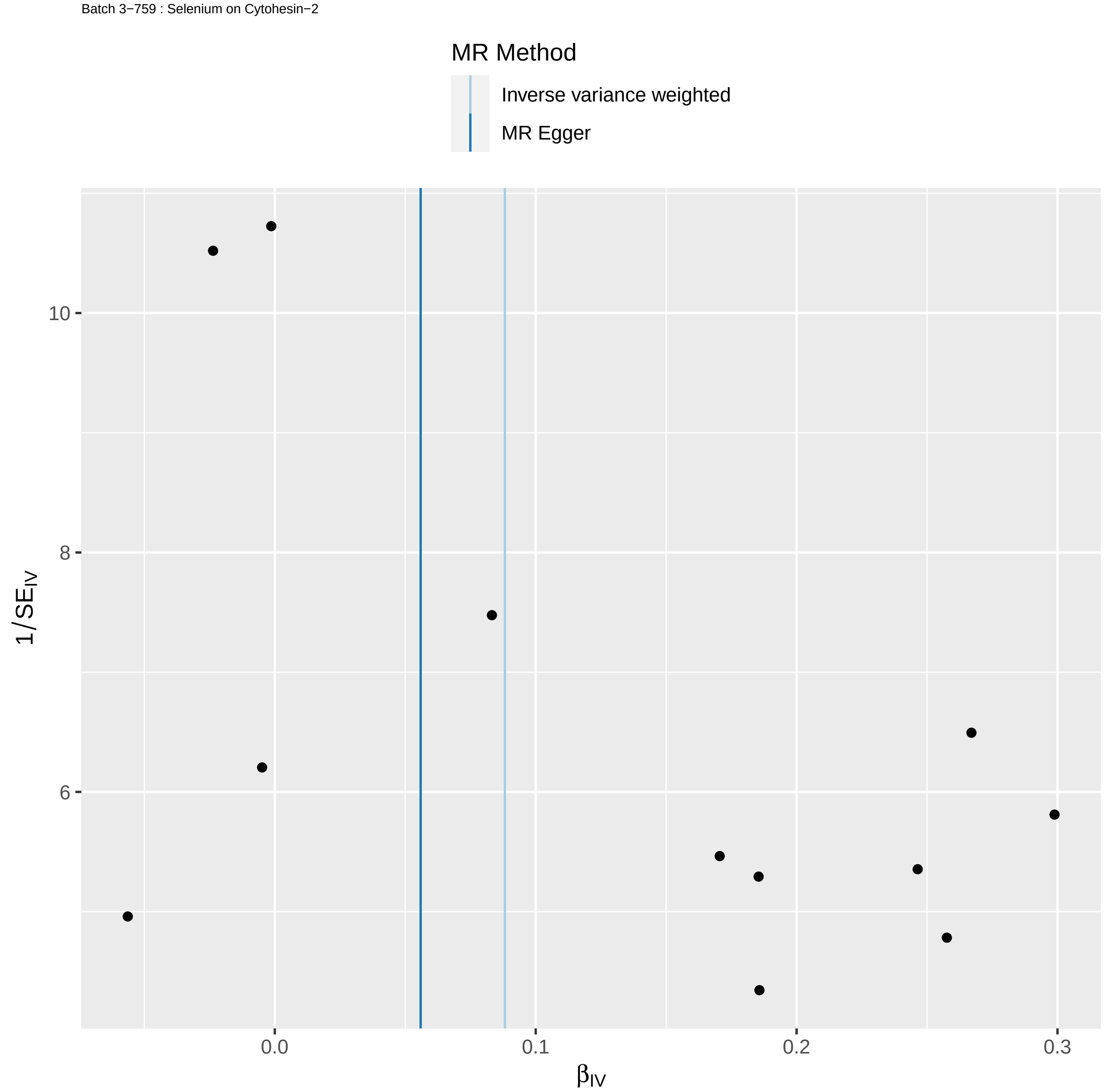

Batch 3−759 : Selenium on Cytohesin−2
MR Method
Inverse variance weighted
MR Egger
10
8
6
1/SE_IV
0.0
0.1
0.2
0.3
β_IV

Batch 3−760 : Selenium on Cytohesin−4

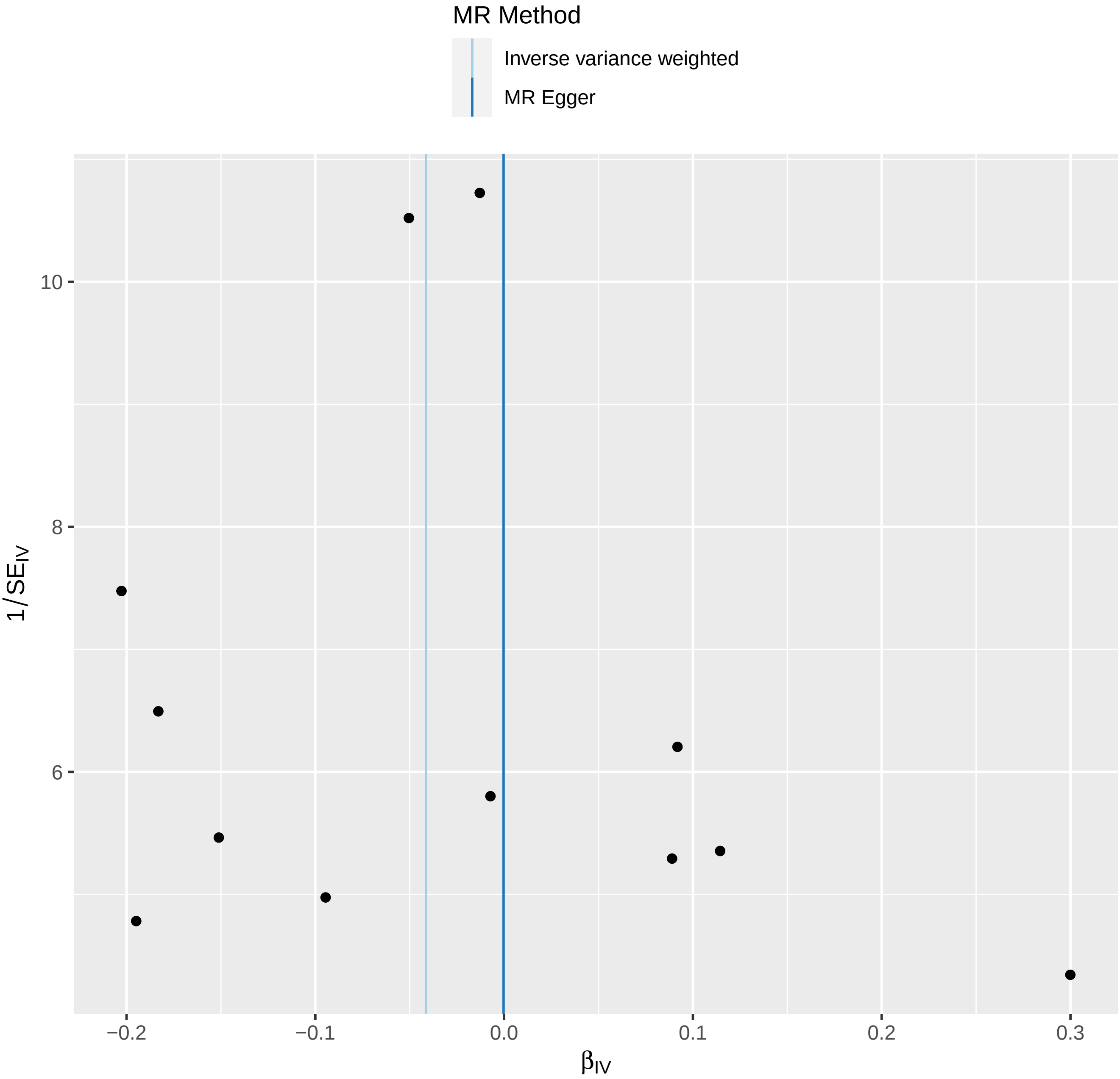

Batch 3−761 : Selenium on Cytohesin−interacting protein

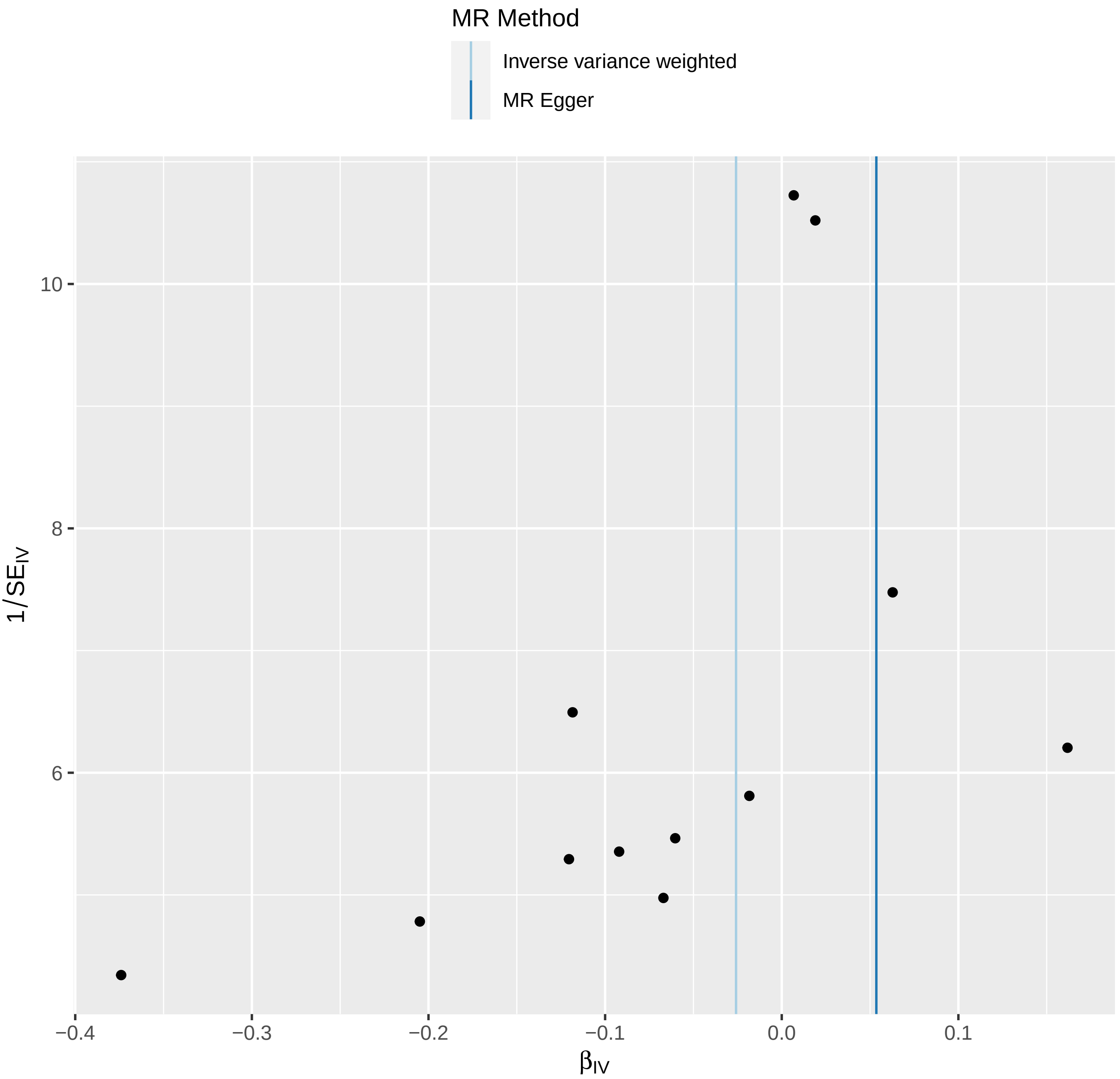

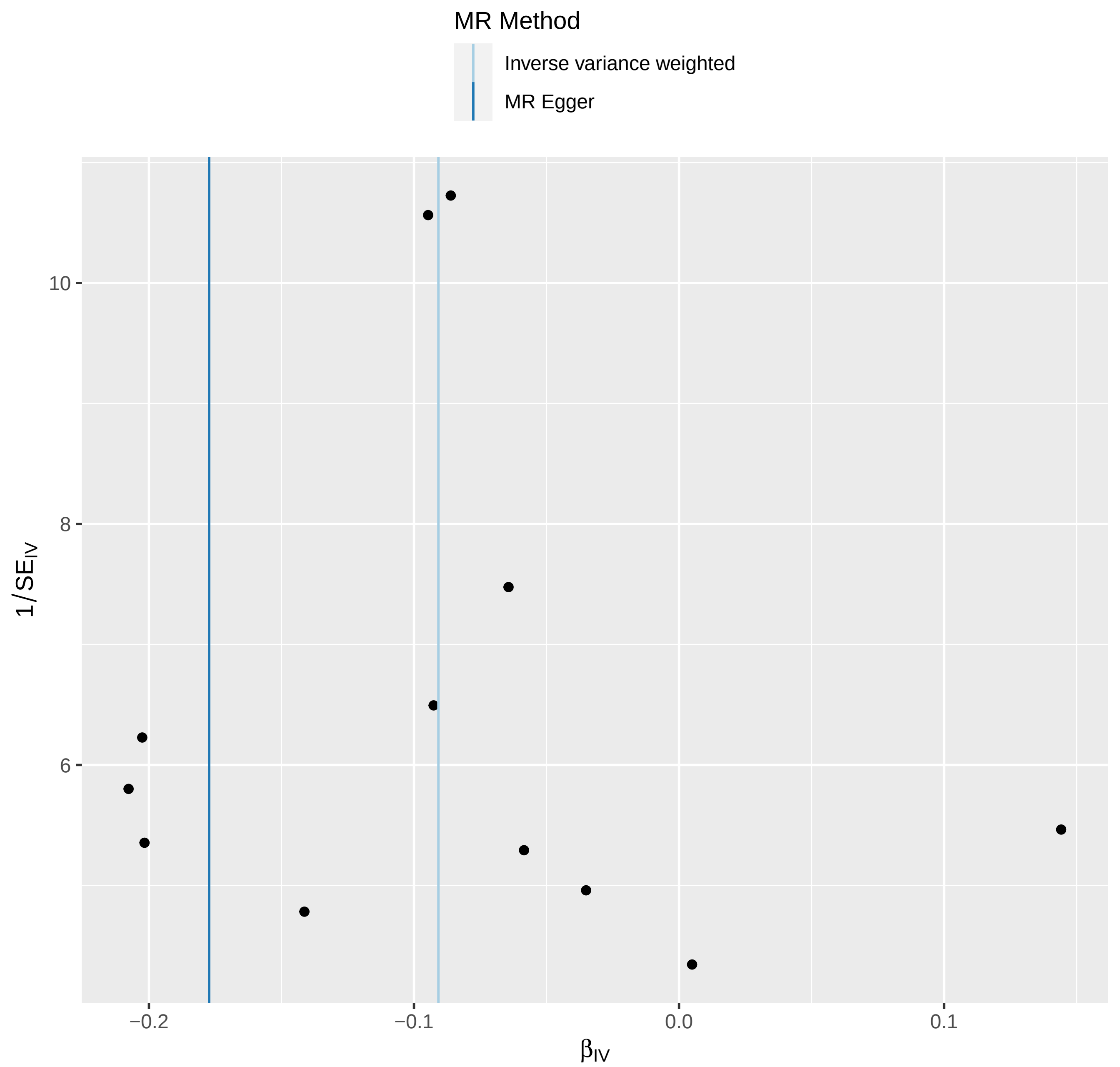
MR Method
Inverse variance weighted
MR Egger
10
8
6
1/SE$_{IV}$
−0.2
−0.1
0.0
0.1
β_{IV}

Batch 3−763 : Selenium on Death−associated protein kinase 2

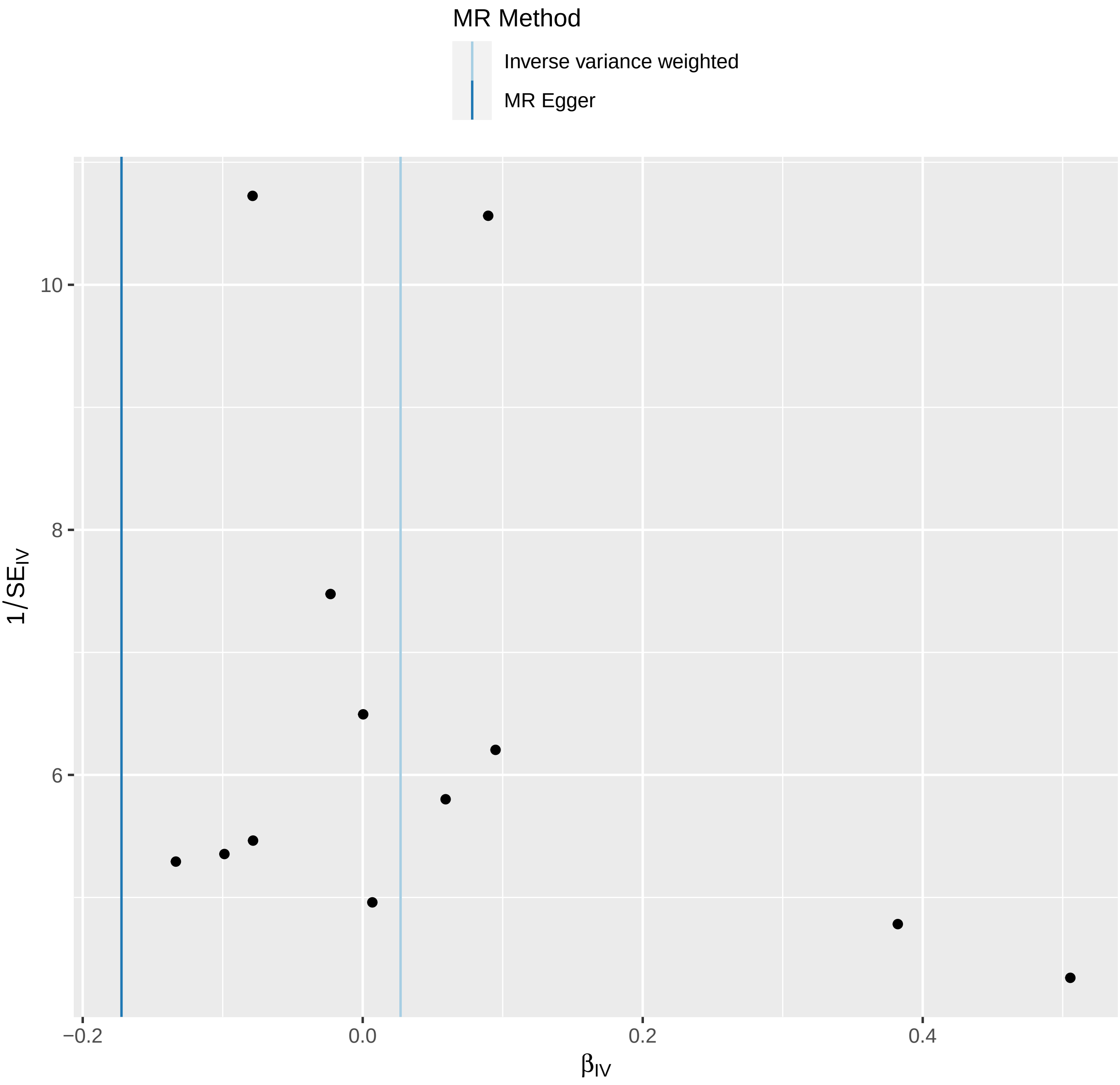

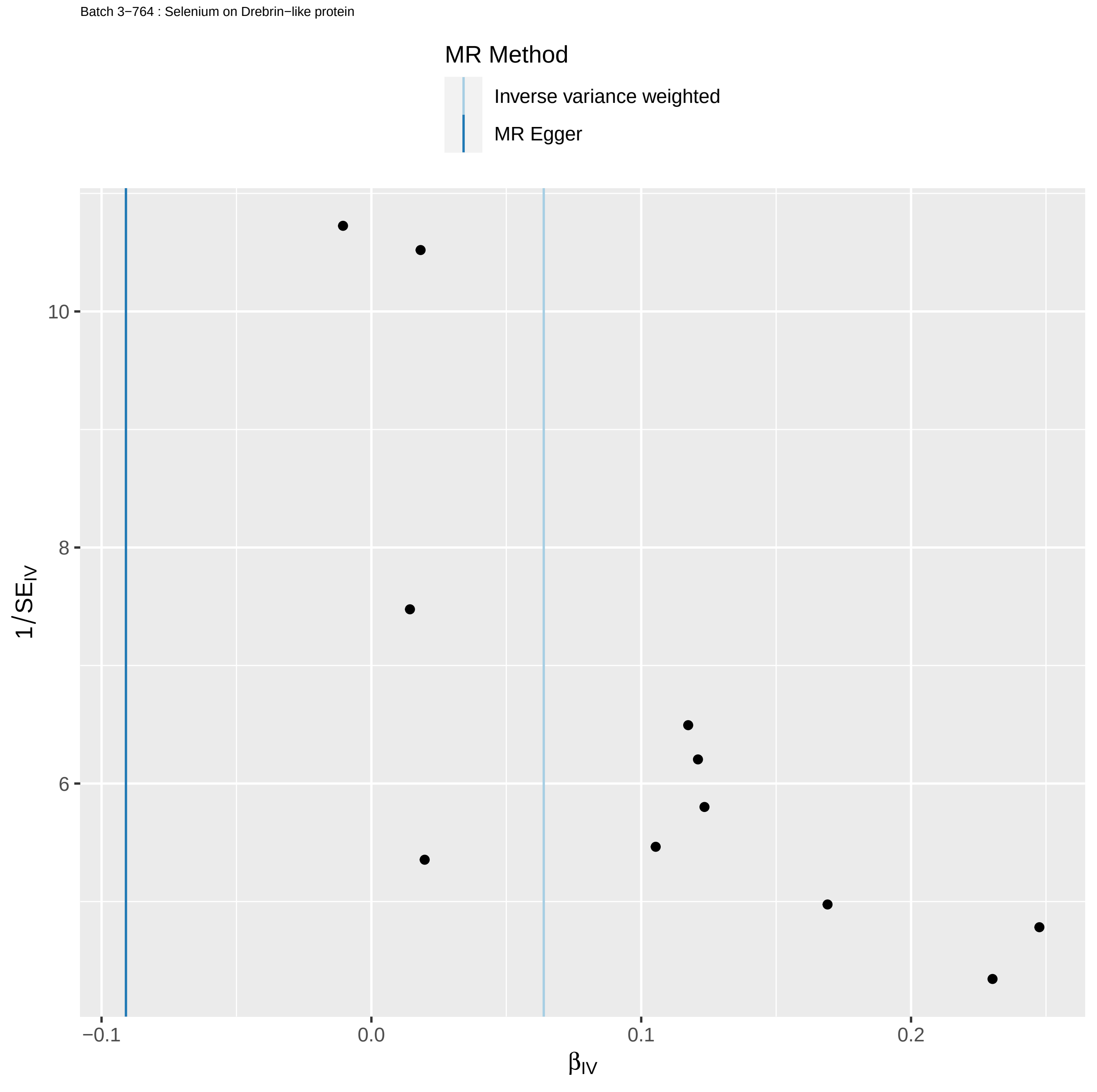
Batch 3−764 : Selenium on Drebrin−like protein
MR Method
Inverse variance weighted
MR Egger
10
8
6
1/SE_IV
−0.1
0.0
0.1
0.2
β_IV

Batch 3−765 : Selenium on DDB1− and CUL4−associated factor 5

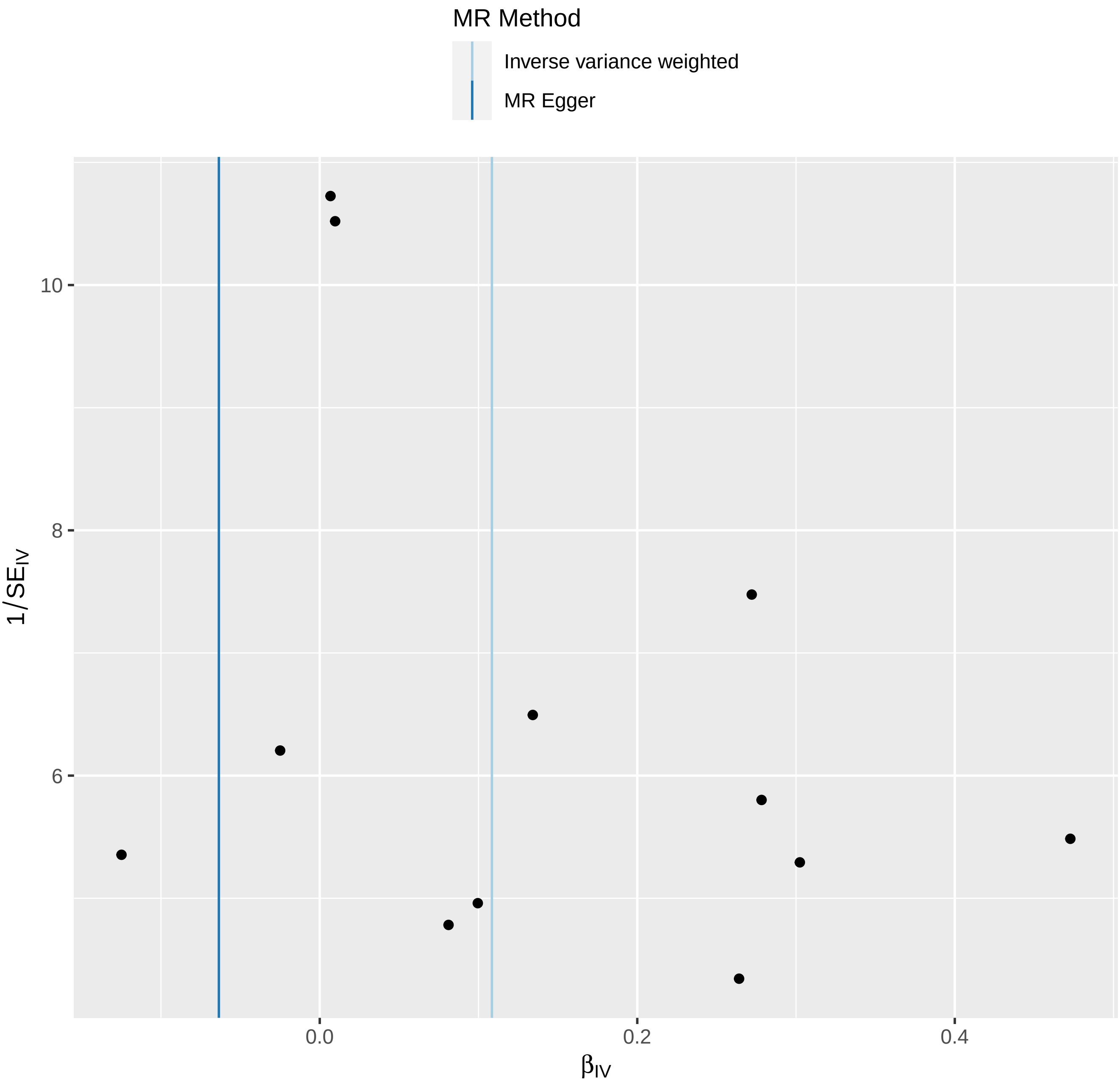

Batch 3−766 : Selenium on Discoidin, CUB and LCCL domain−containing protein 2

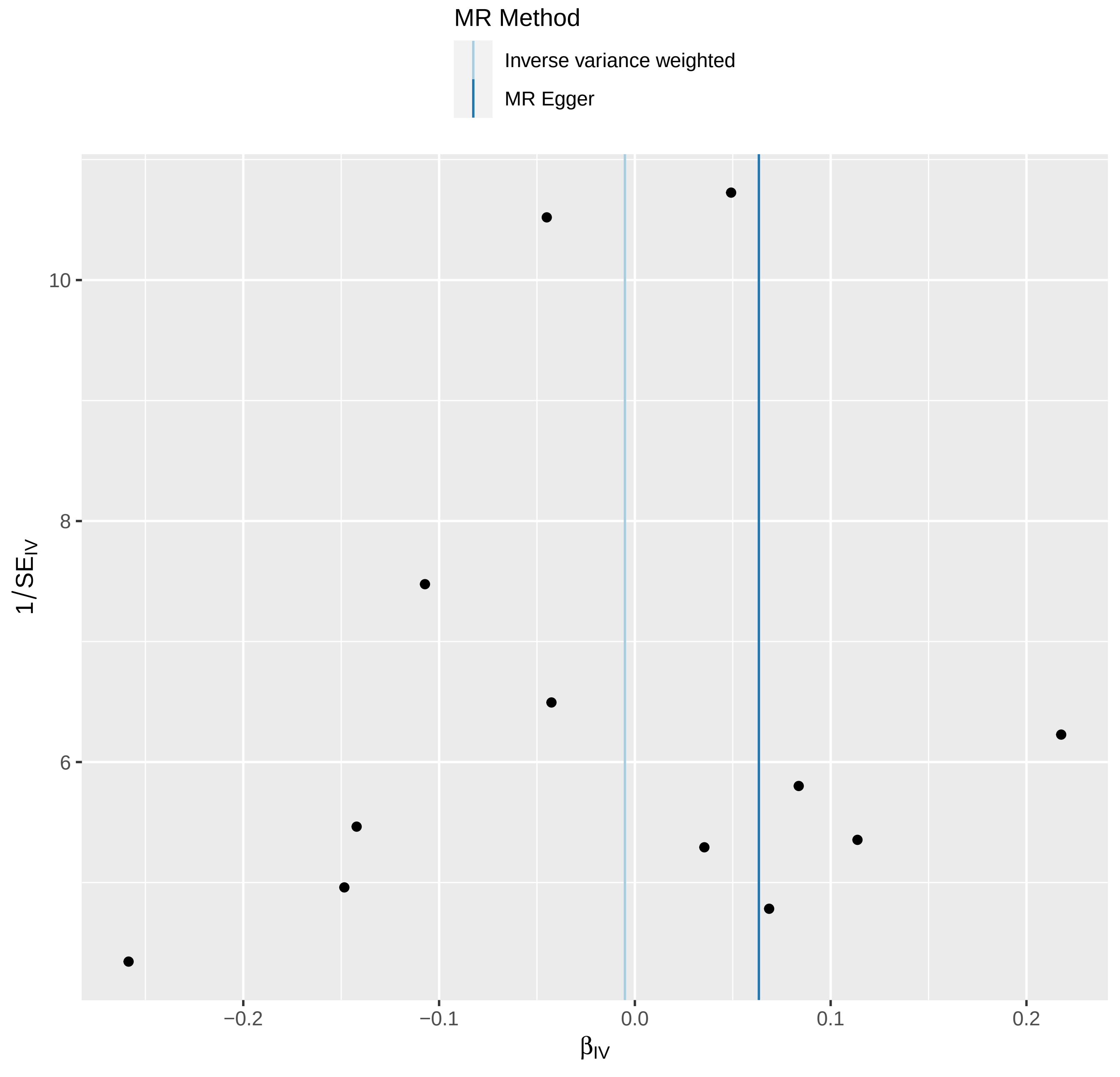

Batch 3−767 : Selenium on Deoxycytidine kinase

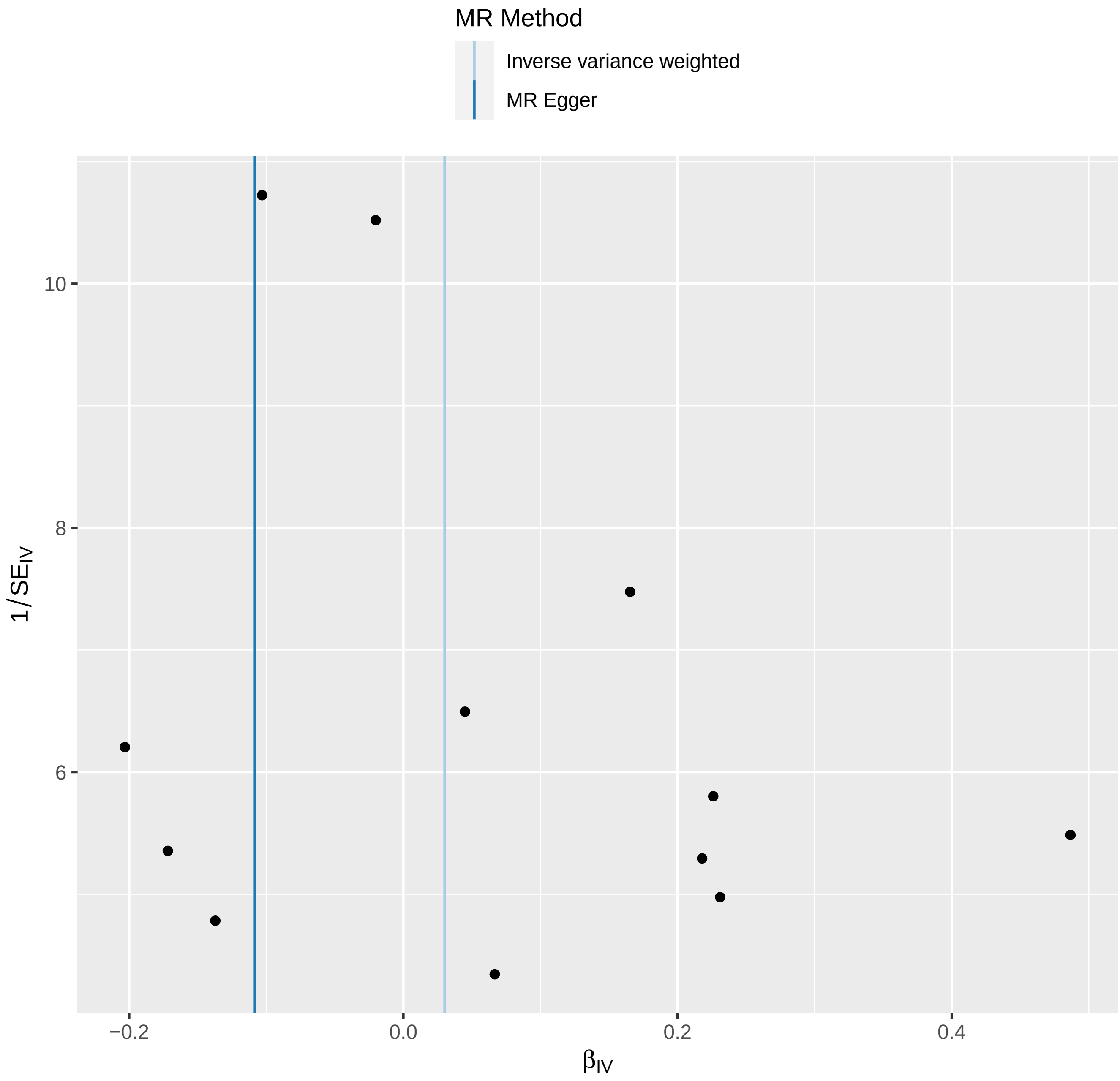

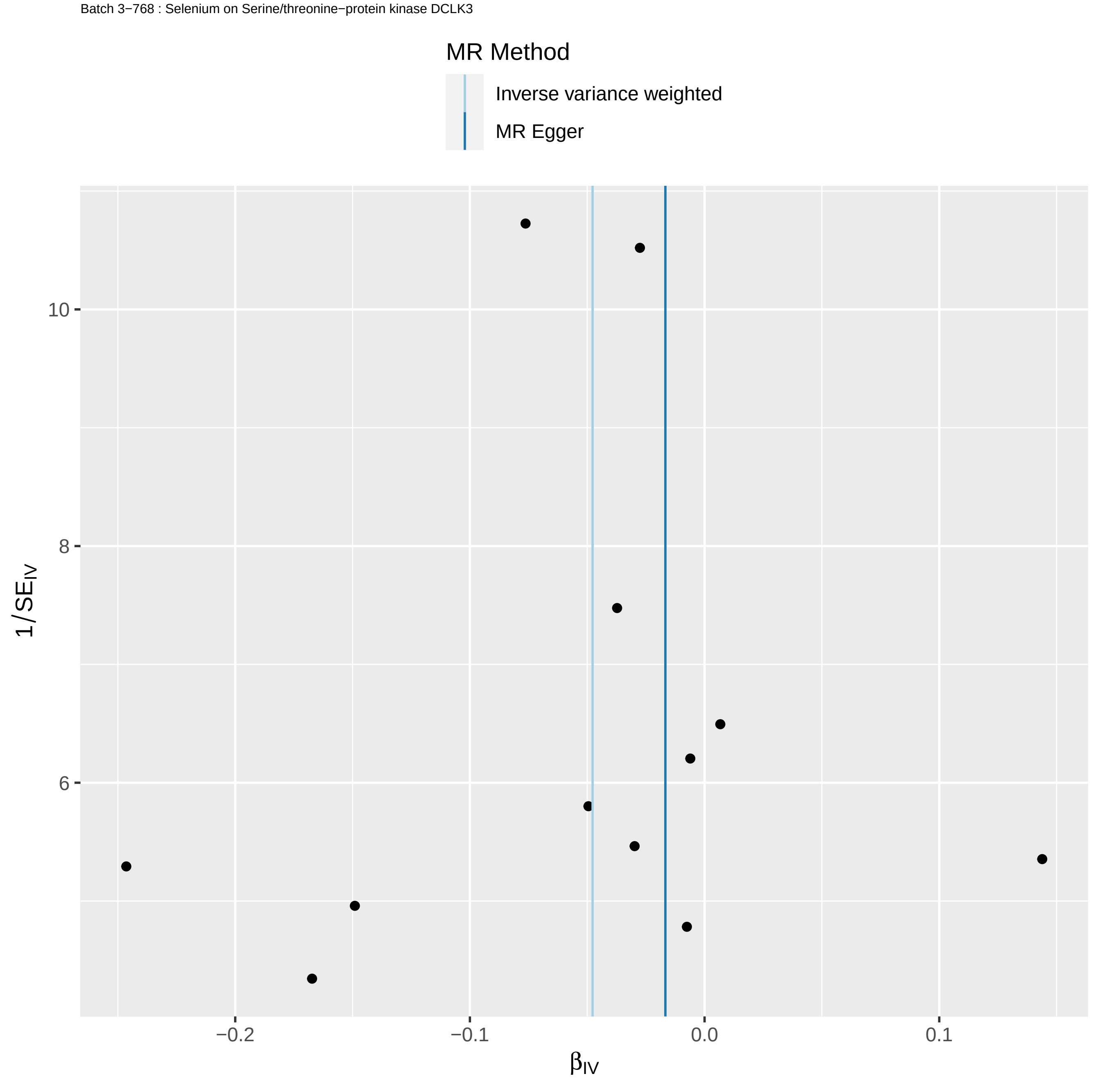

Batch 3−768 : Selenium on Serine/threonine−protein kinase DCLK3
MR Method
Inverse variance weighted
MR Egger
10
8
6
1/SE_IV
−0.2
−0.1
0.0
0.1
β_IV

Batch 3−769 : Selenium on Decorin

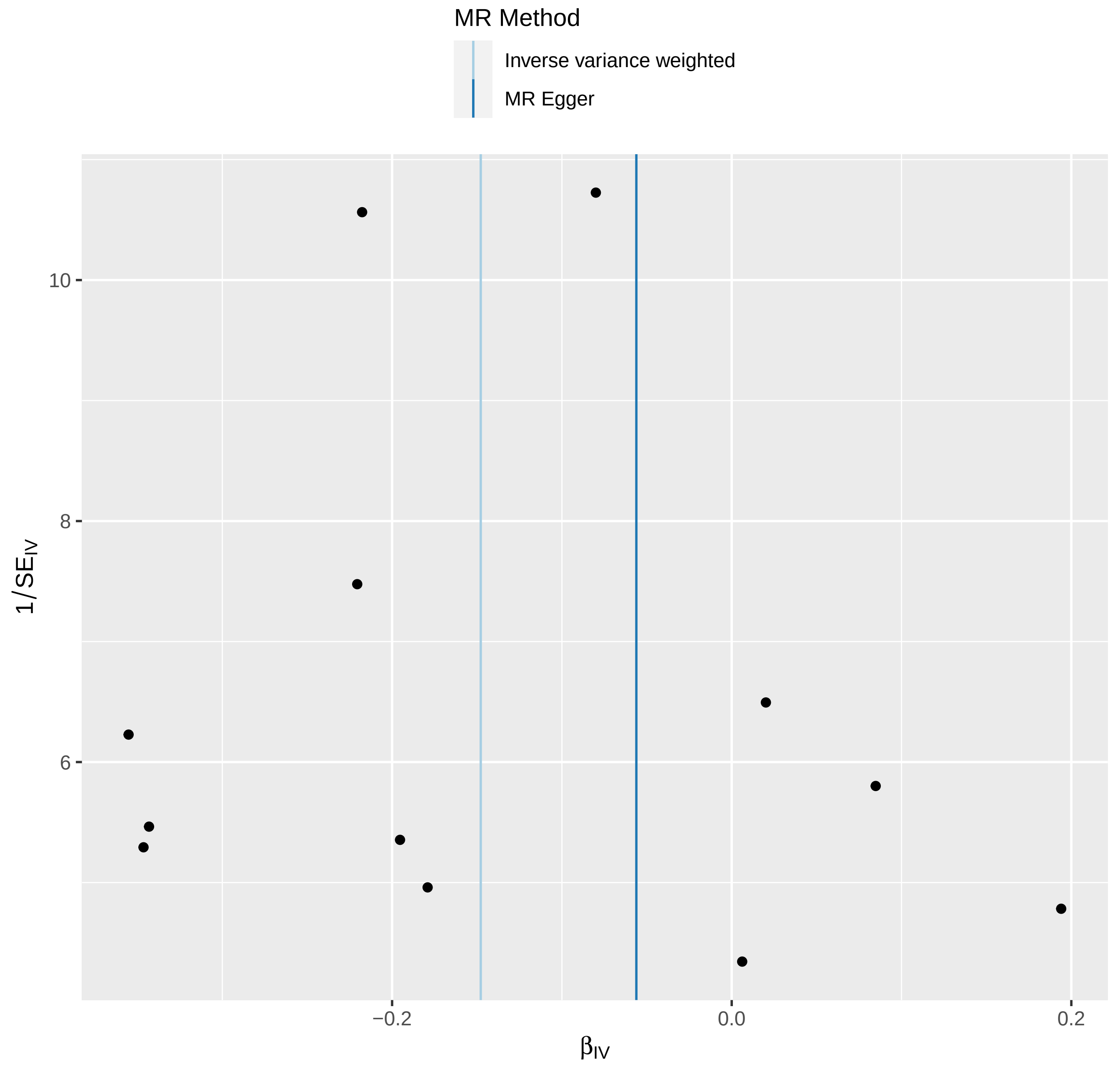

Batch 3−770 : Selenium on mRNA−decapping enzyme 1A

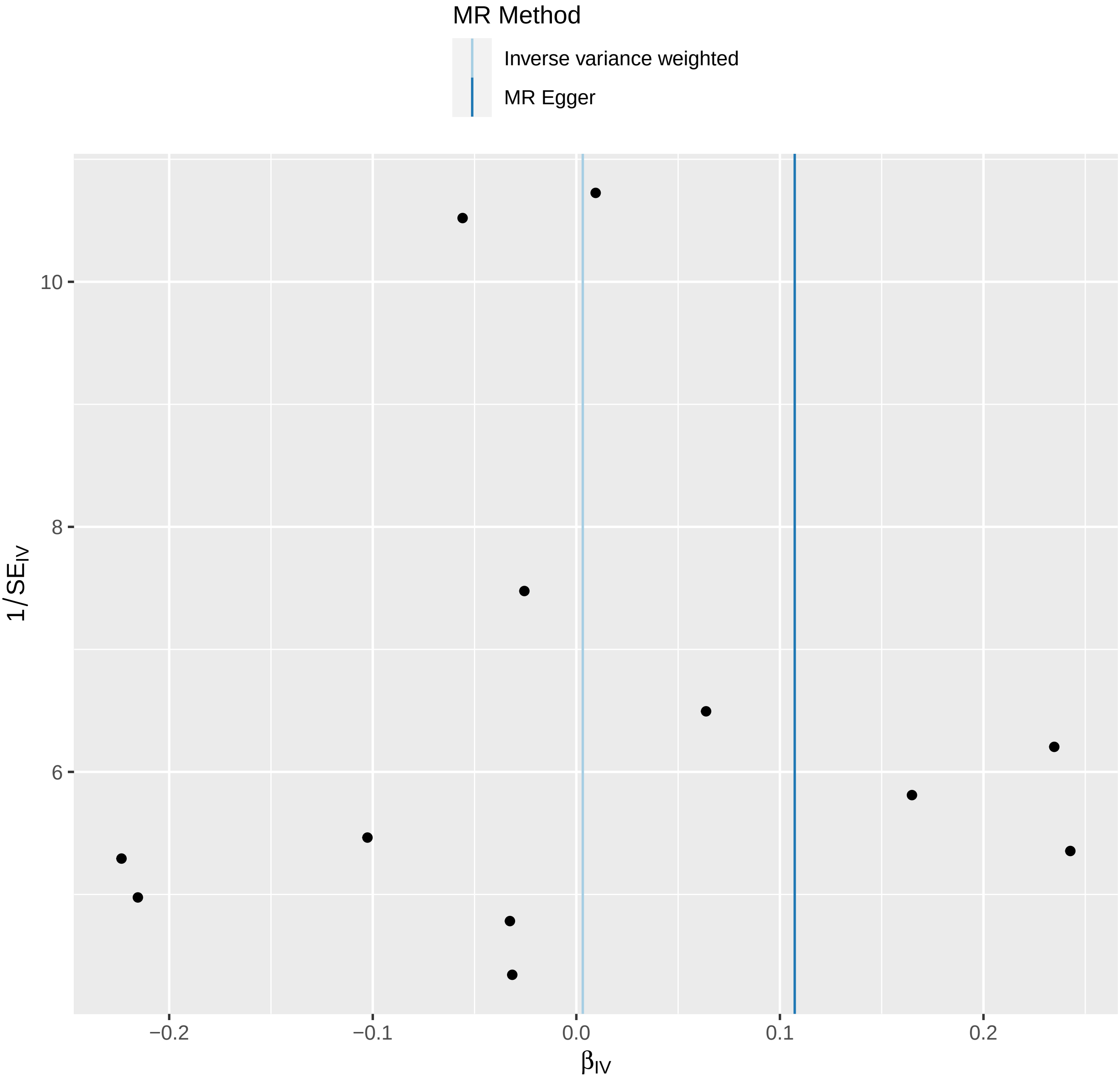

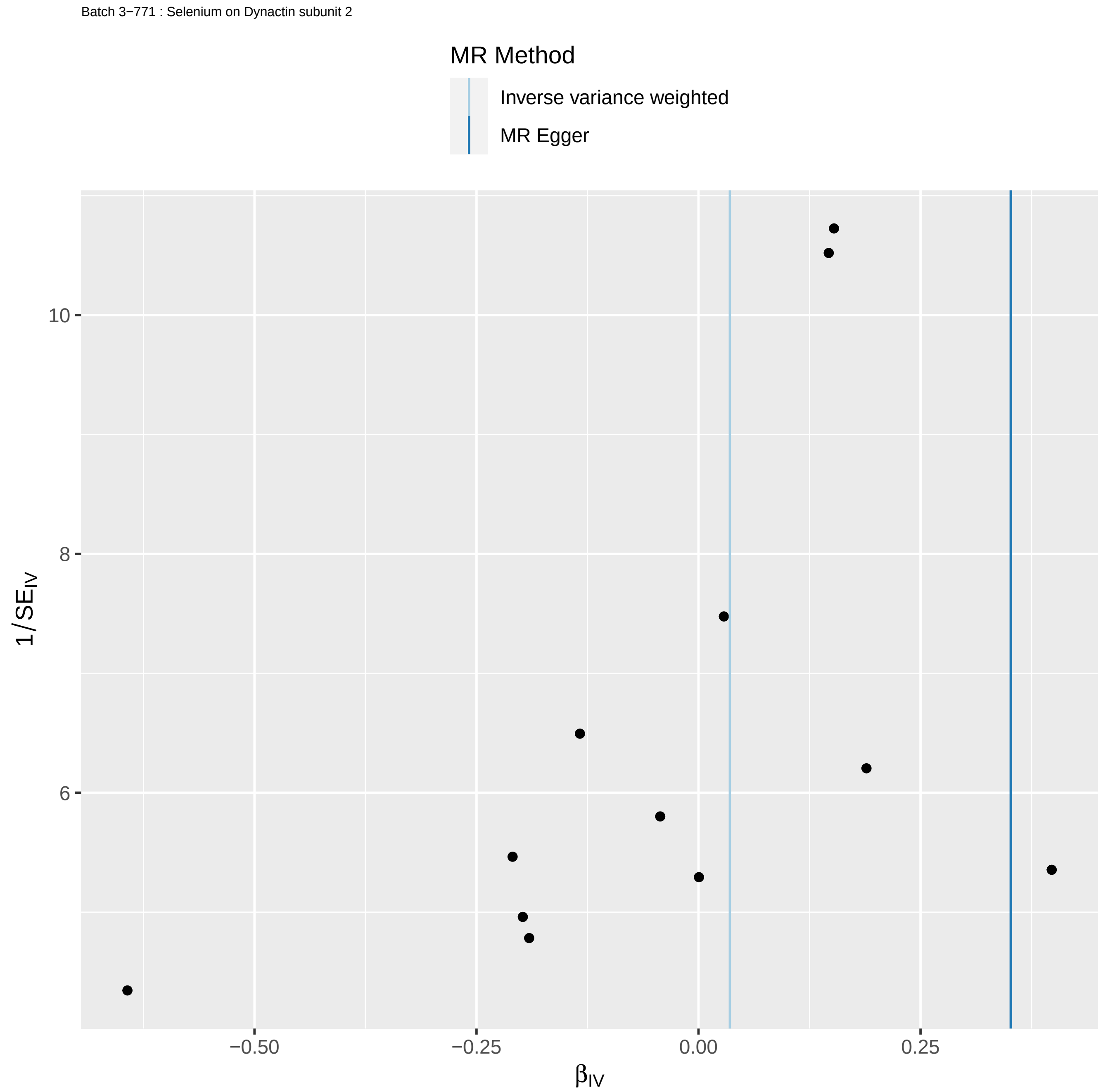
Batch 3−771 : Selenium on Dynactin subunit 2
MR Method
Inverse variance weighted
MR Egger
10
8
6
$1/SE_{IV}$
−0.50
−0.25
0.00
0.25
β_{IV}

Batch 3−772 : Selenium on dCTP pyrophosphatase 1

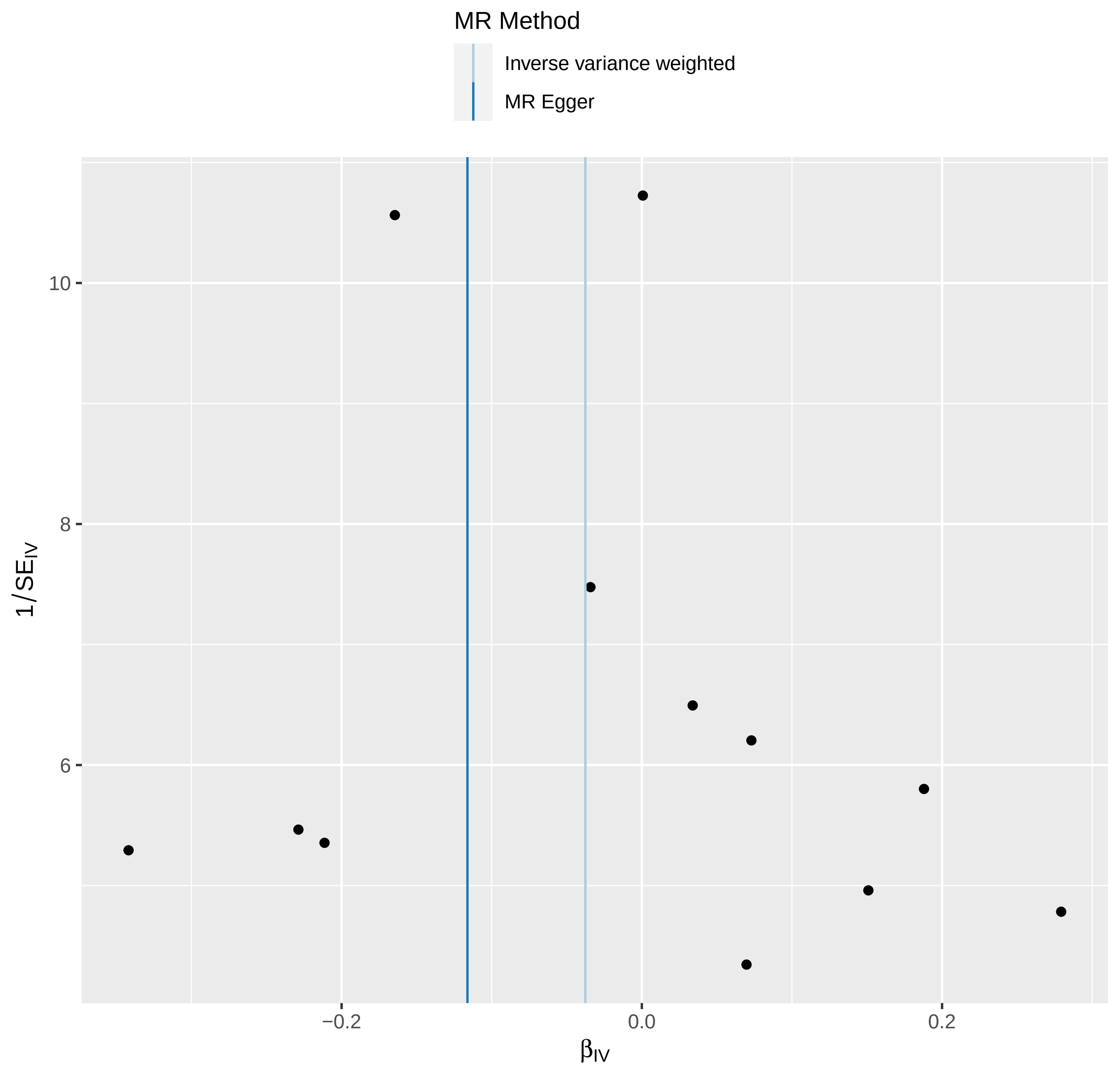

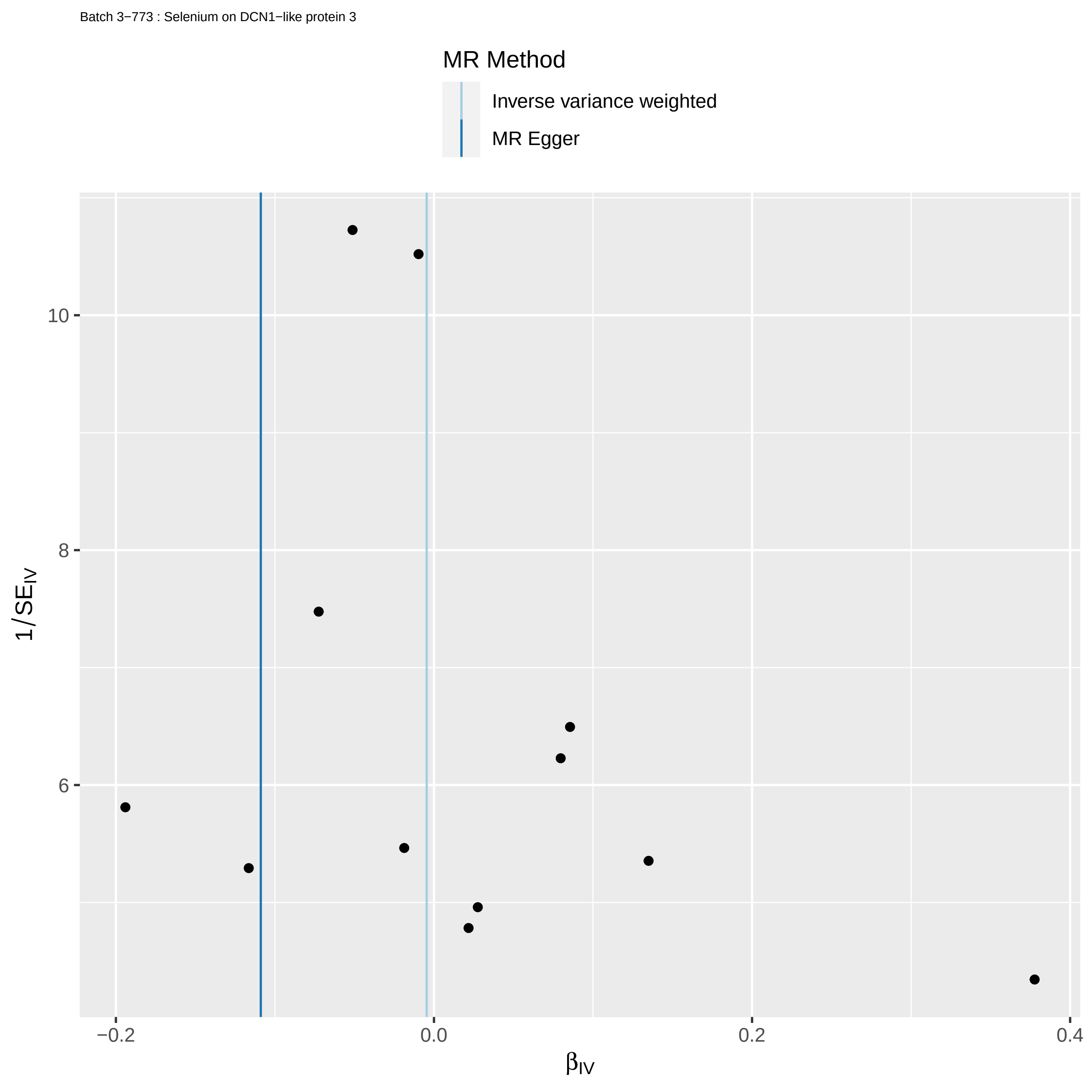

Batch 3−773 : Selenium on DCN1−like protein 3
MR Method
Inverse variance weighted
MR Egger
10
8
6
1/SE_IV
−0.2
0.0
0.2
0.4
β_IV

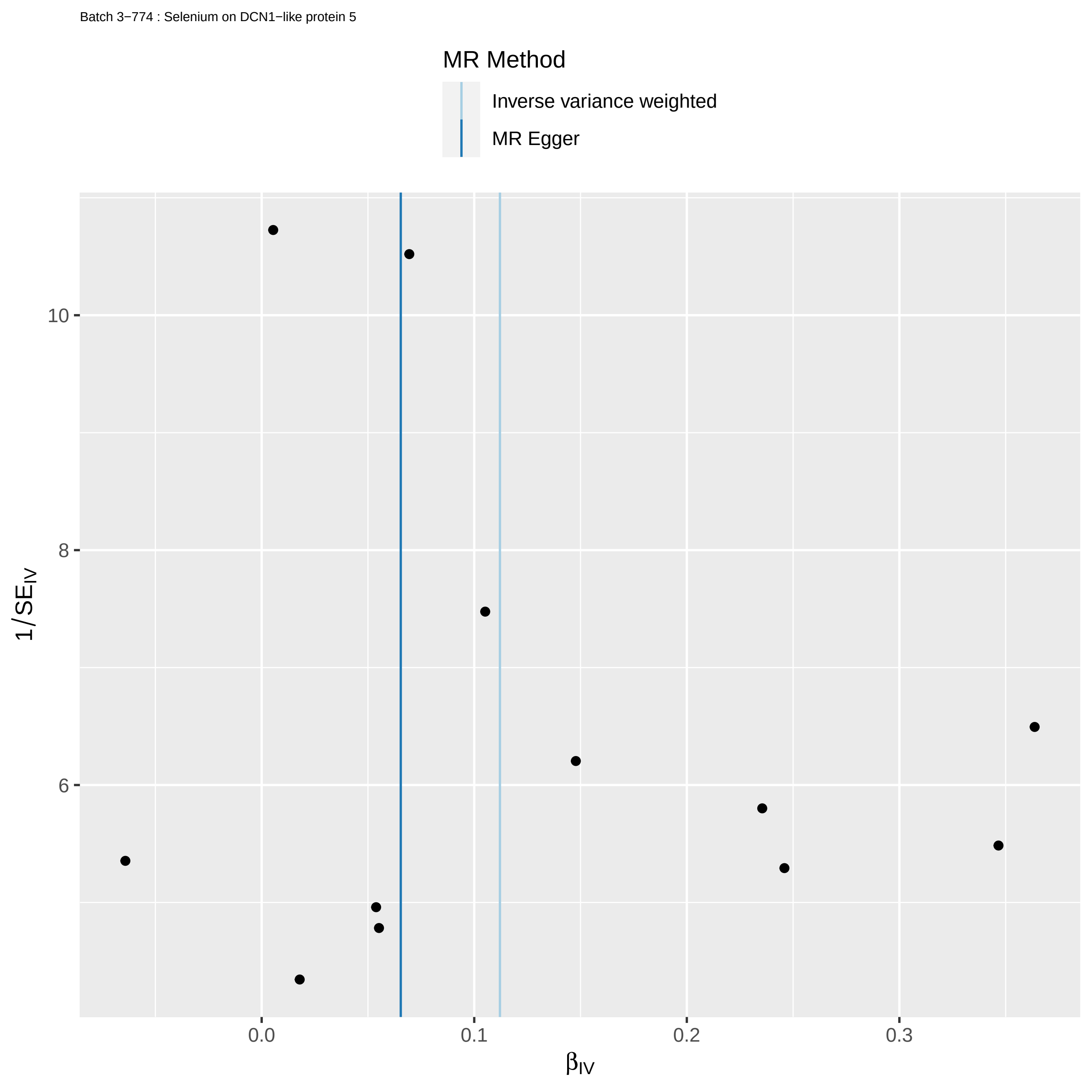

Batch 3−774 : Selenium on DCN1−like protein 5
MR Method
Inverse variance weighted
MR Egger
10
8
6
1/SE_IV
0.0
0.1
0.2
0.3
β_IV

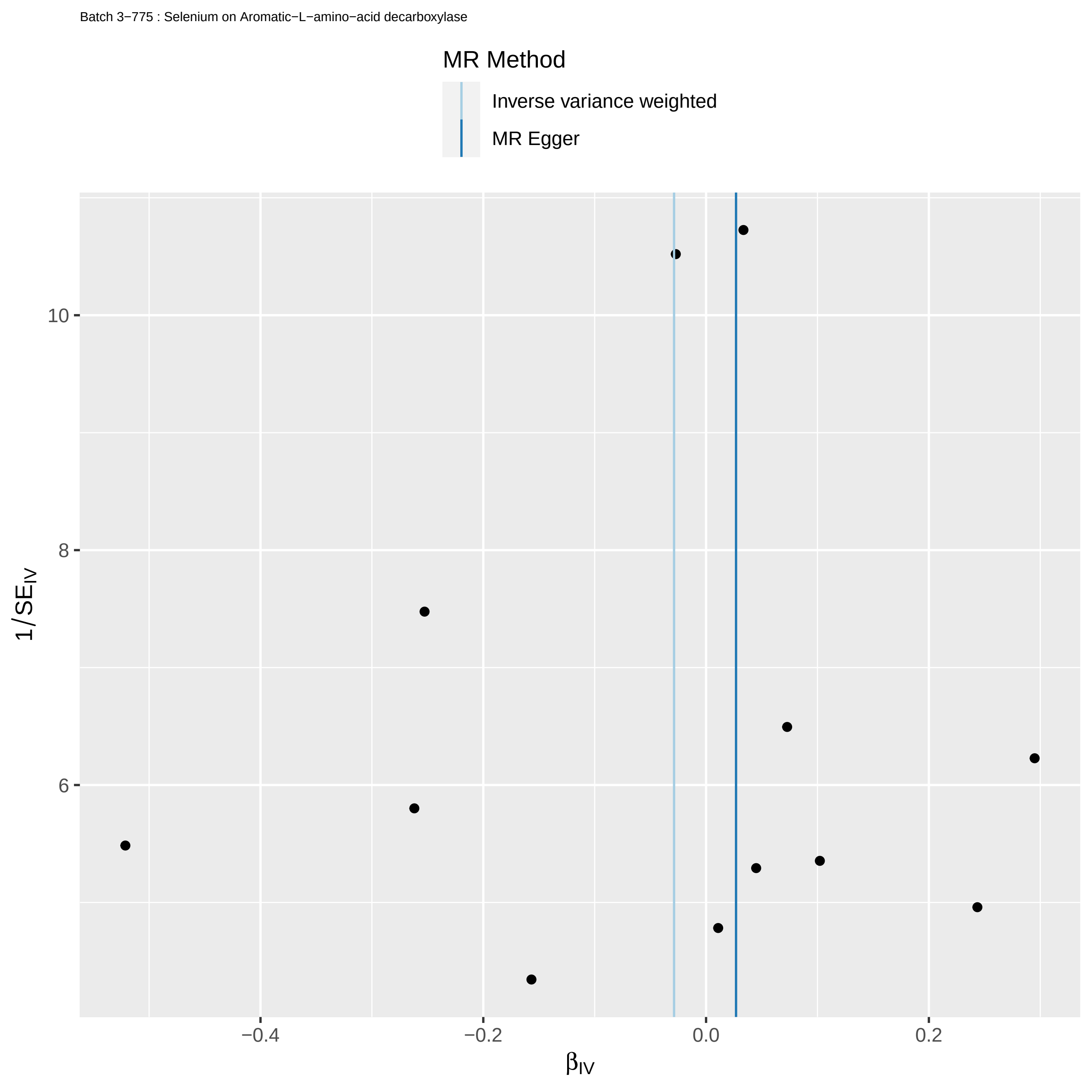

Batch 3−775 : Selenium on Aromatic−L−amino−acid decarboxylase
MR Method
Inverse variance weighted
MR Egger
10
8
6
1/SE_IV
−0.4
−0.2
0.0
0.2
β_IV

Batch 3−776 : Selenium on Discoidin domain−containing receptor 2

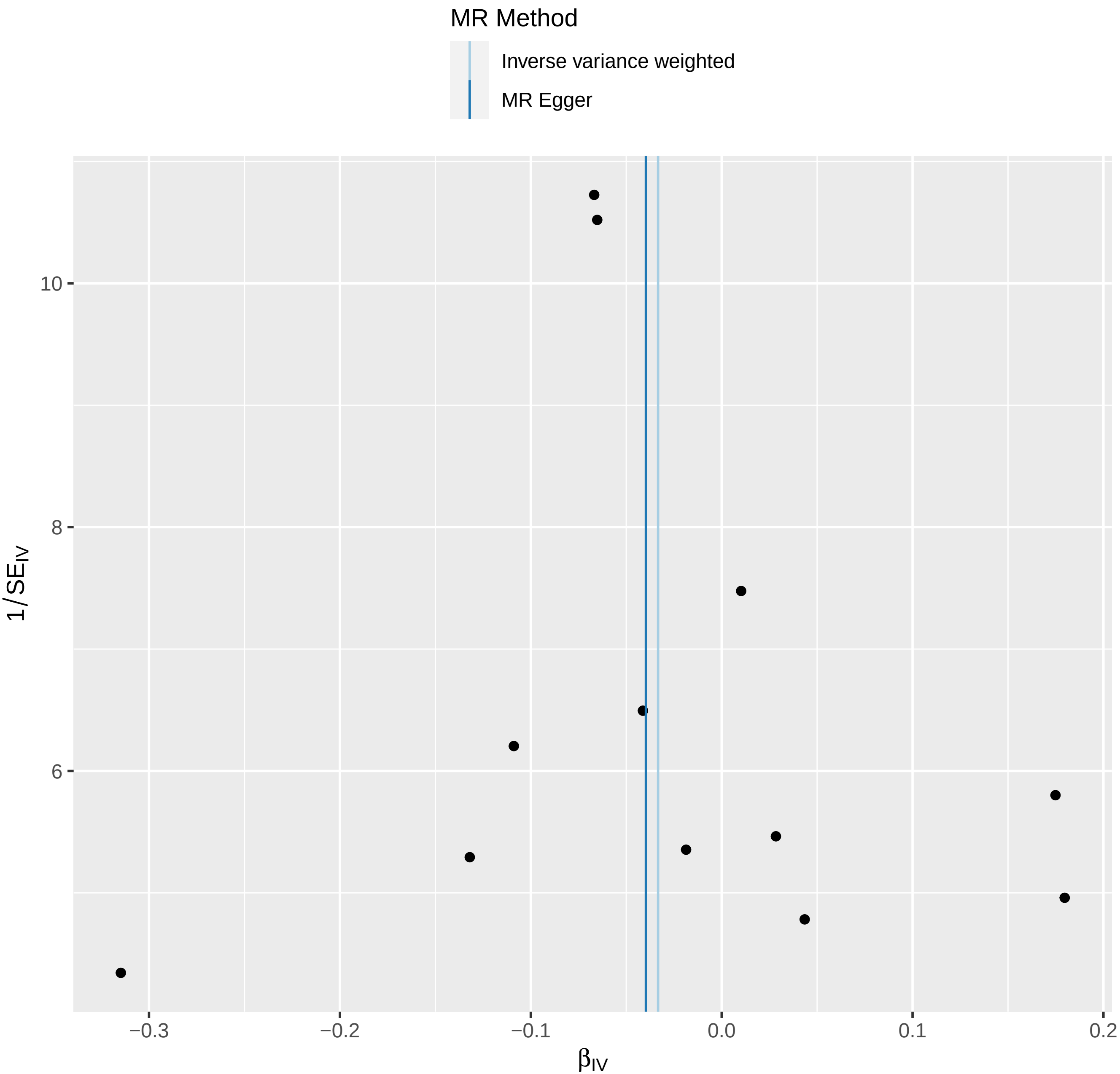

Batch 3−777 : Selenium on ATP−dependent RNA helicase DDX25

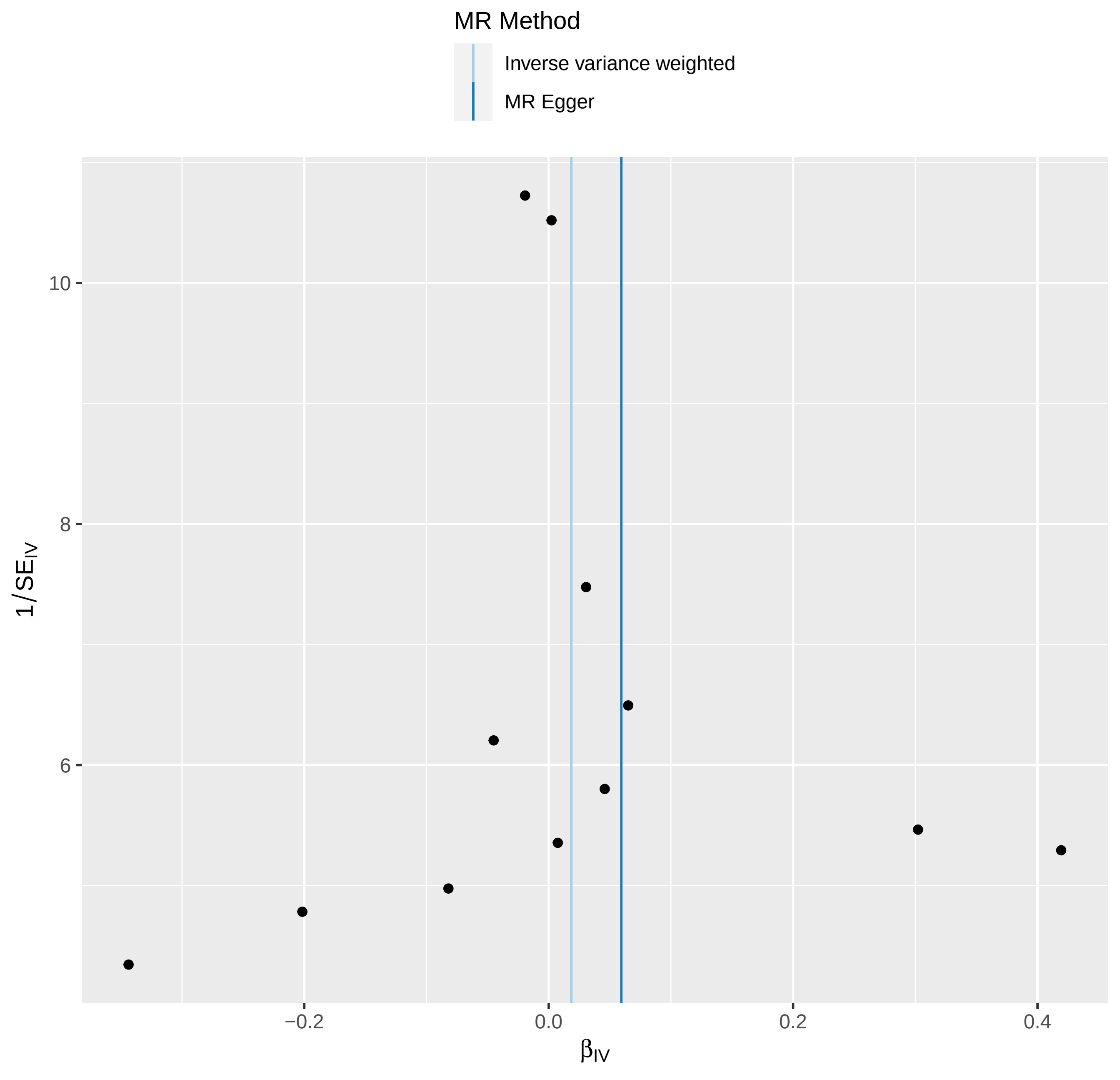

Batch 3−778 : Selenium on Spliceosome RNA helicase DDX39B

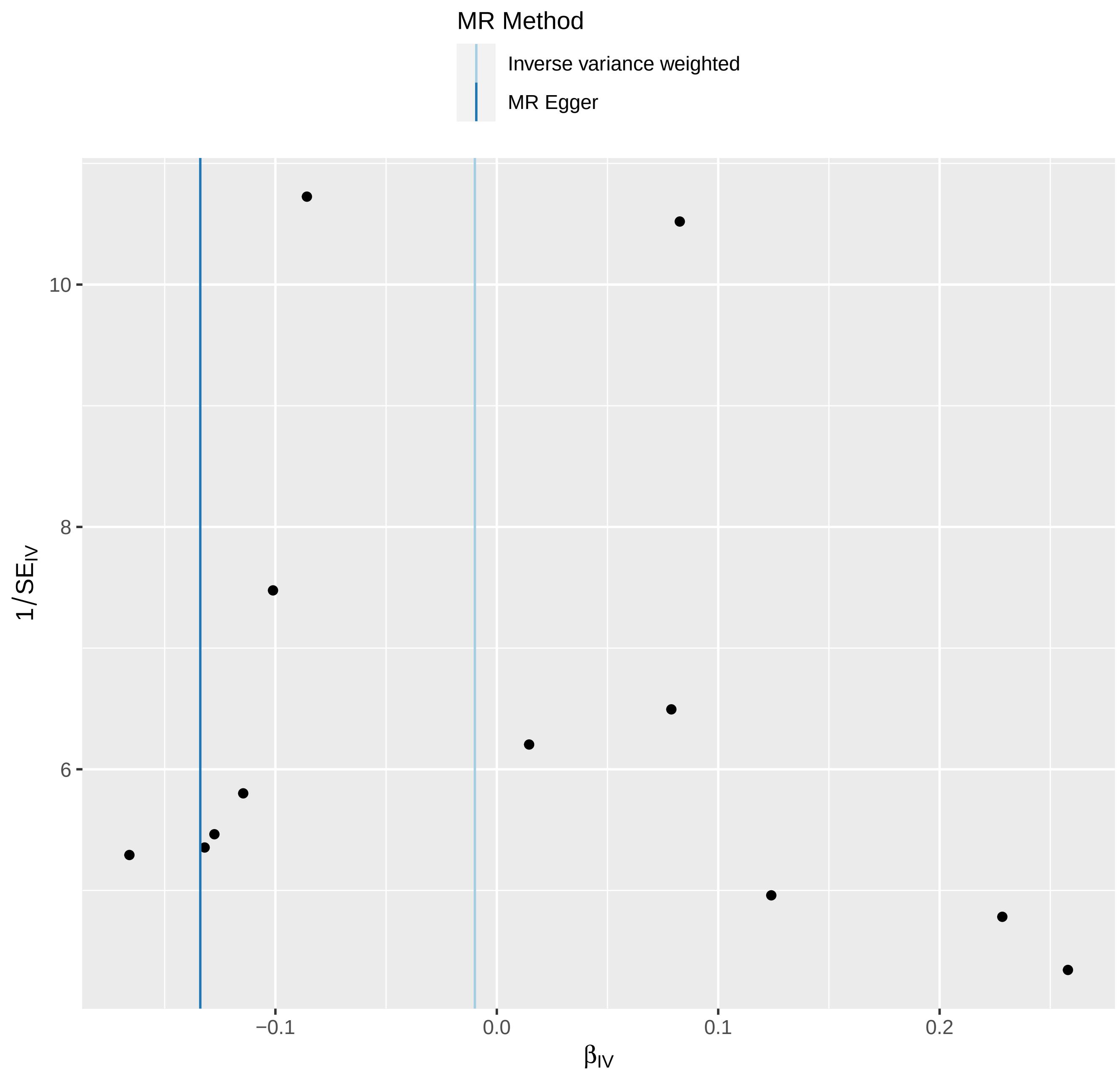

Batch 3−779 : Selenium on Probable ATP−dependent RNA helicase DDX58

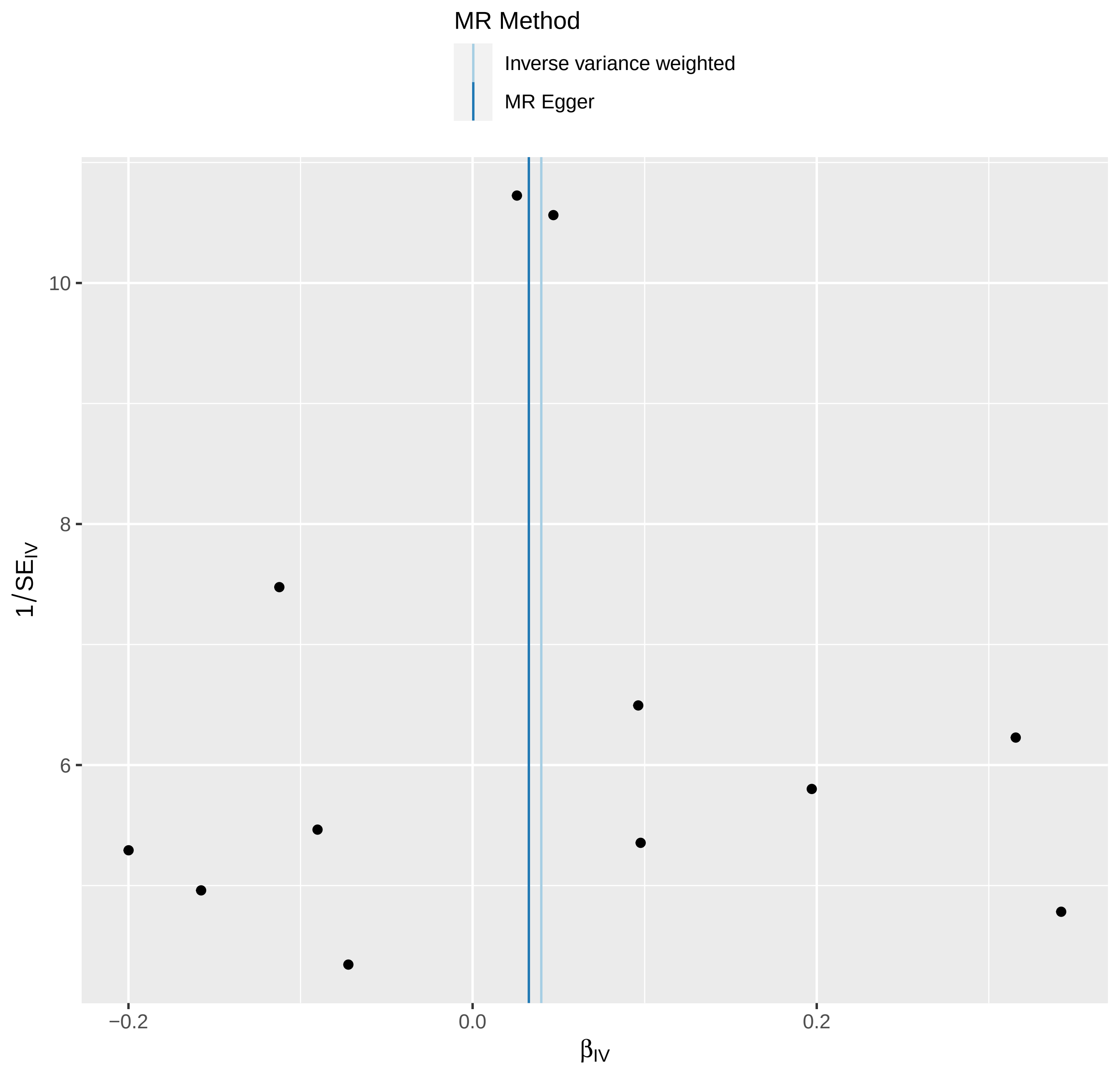

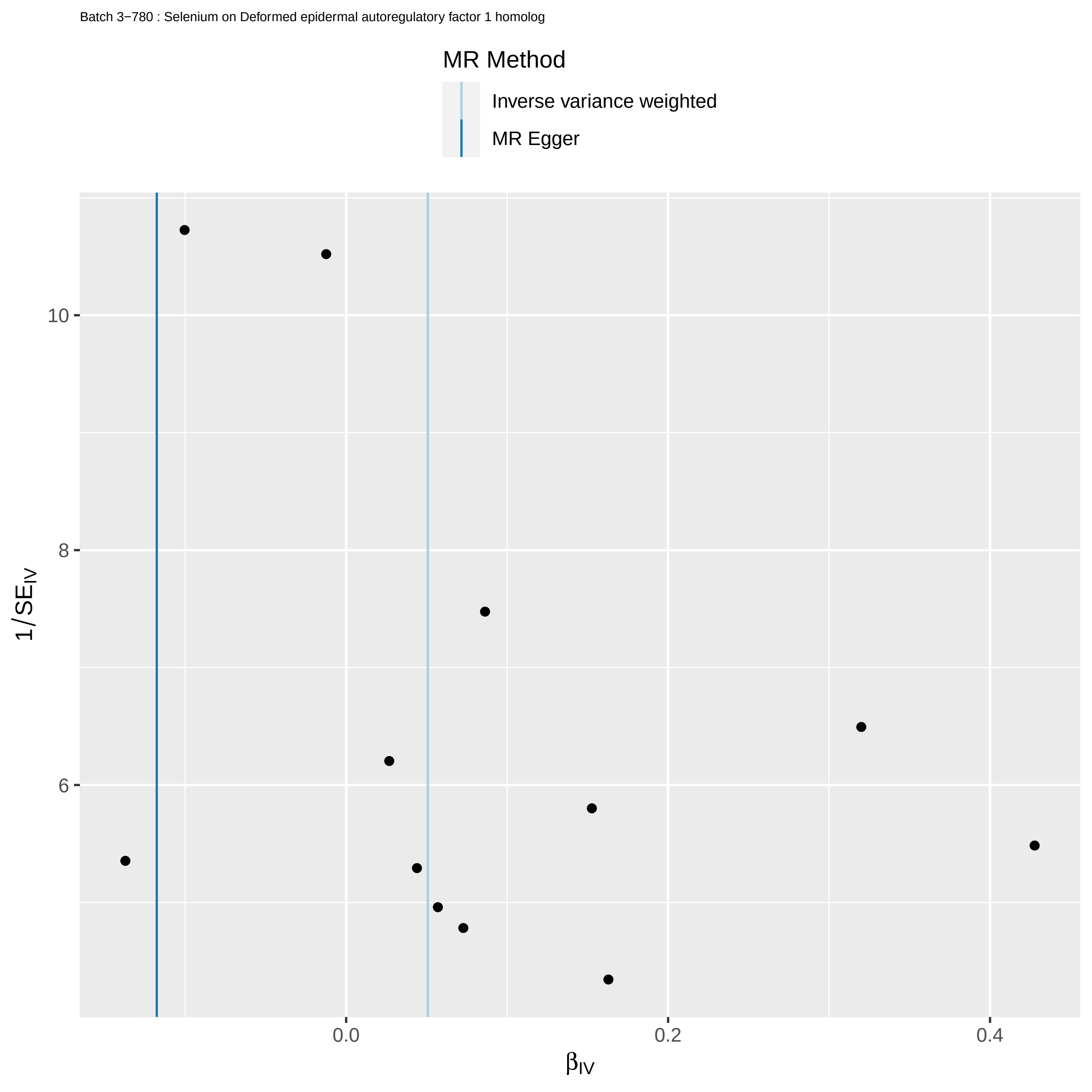
Batch 3−780 : Selenium on Deformed epidermal autoregulatory factor 1 homolog
MR Method
Inverse variance weighted
MR Egger
10
8
6
1/SE_IV
0.0
0.2
0.4
β_IV

Batch 3−781 : Selenium on Beta−defensin 103

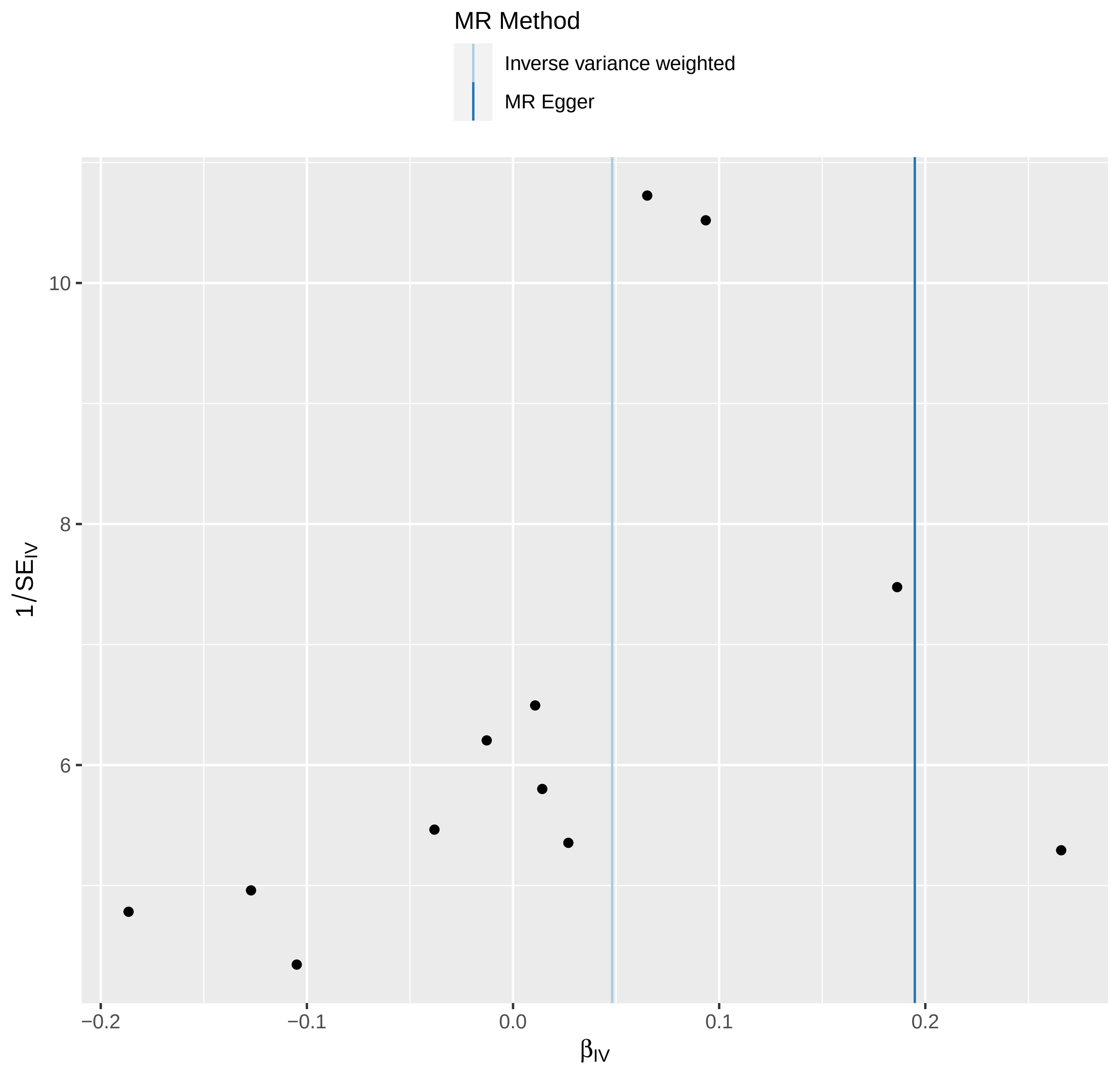

Batch 3−782 : Selenium on Beta−defensin 104

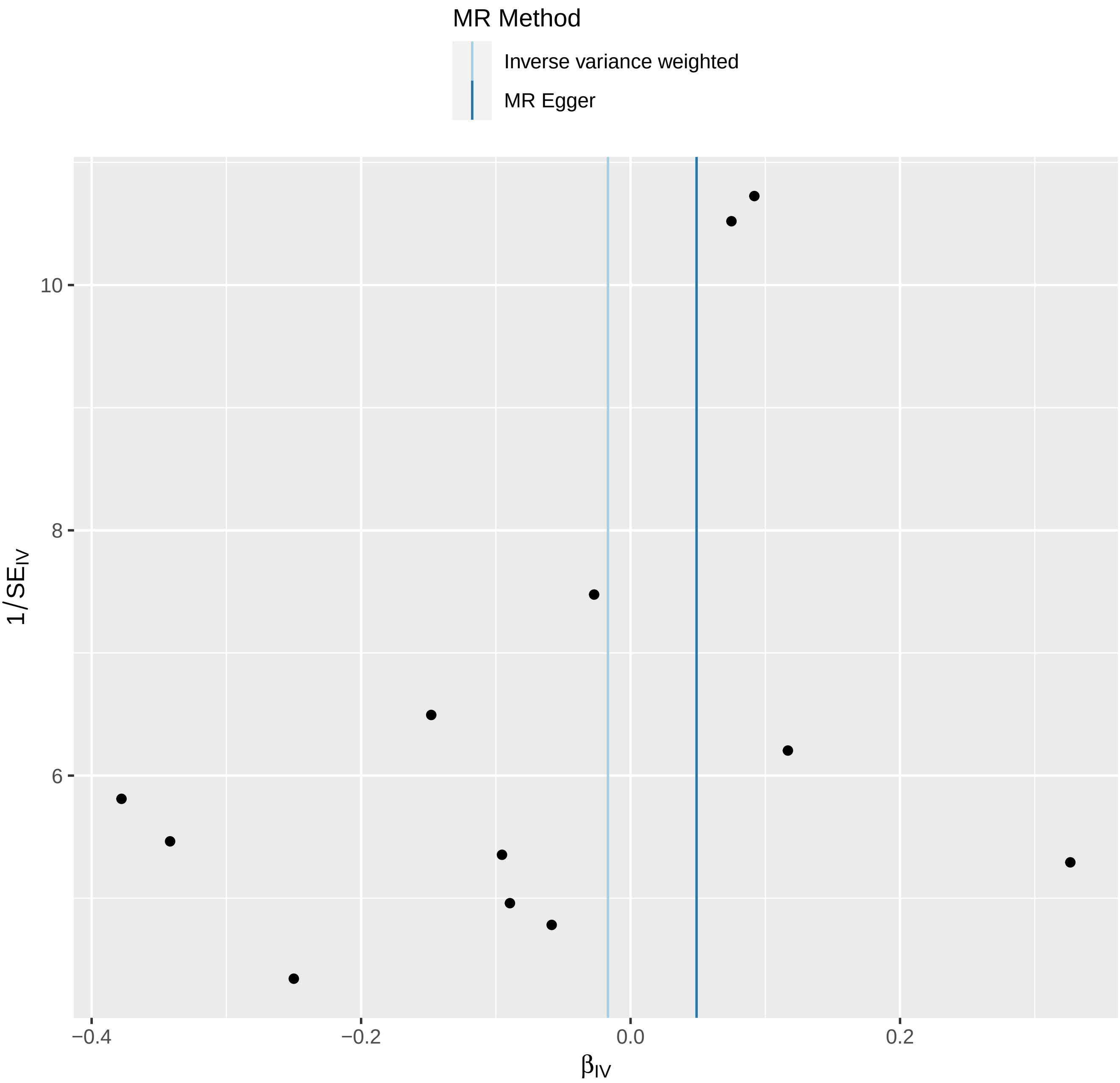

Batch 3−783 : Selenium on Beta−defensin 105

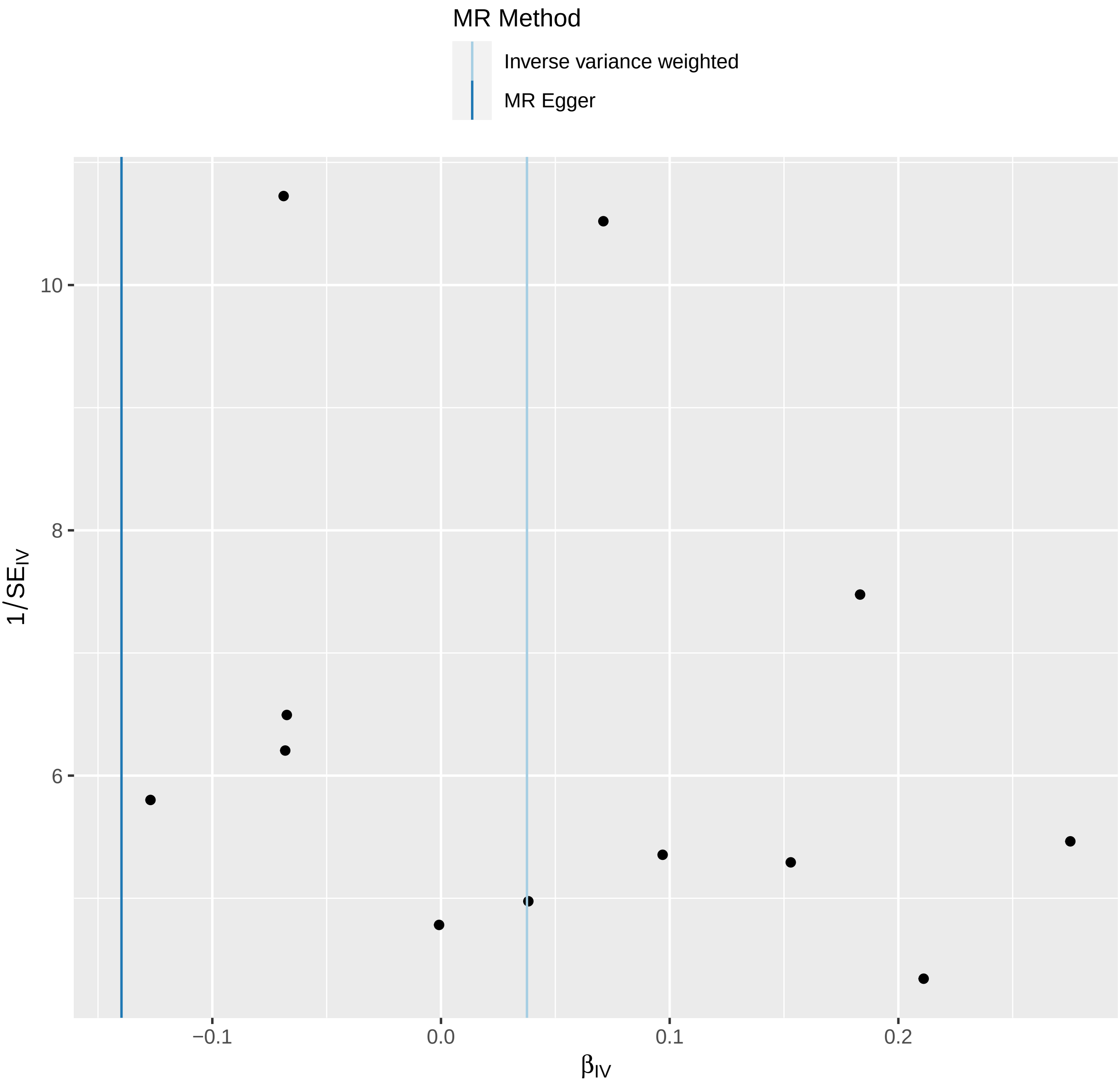

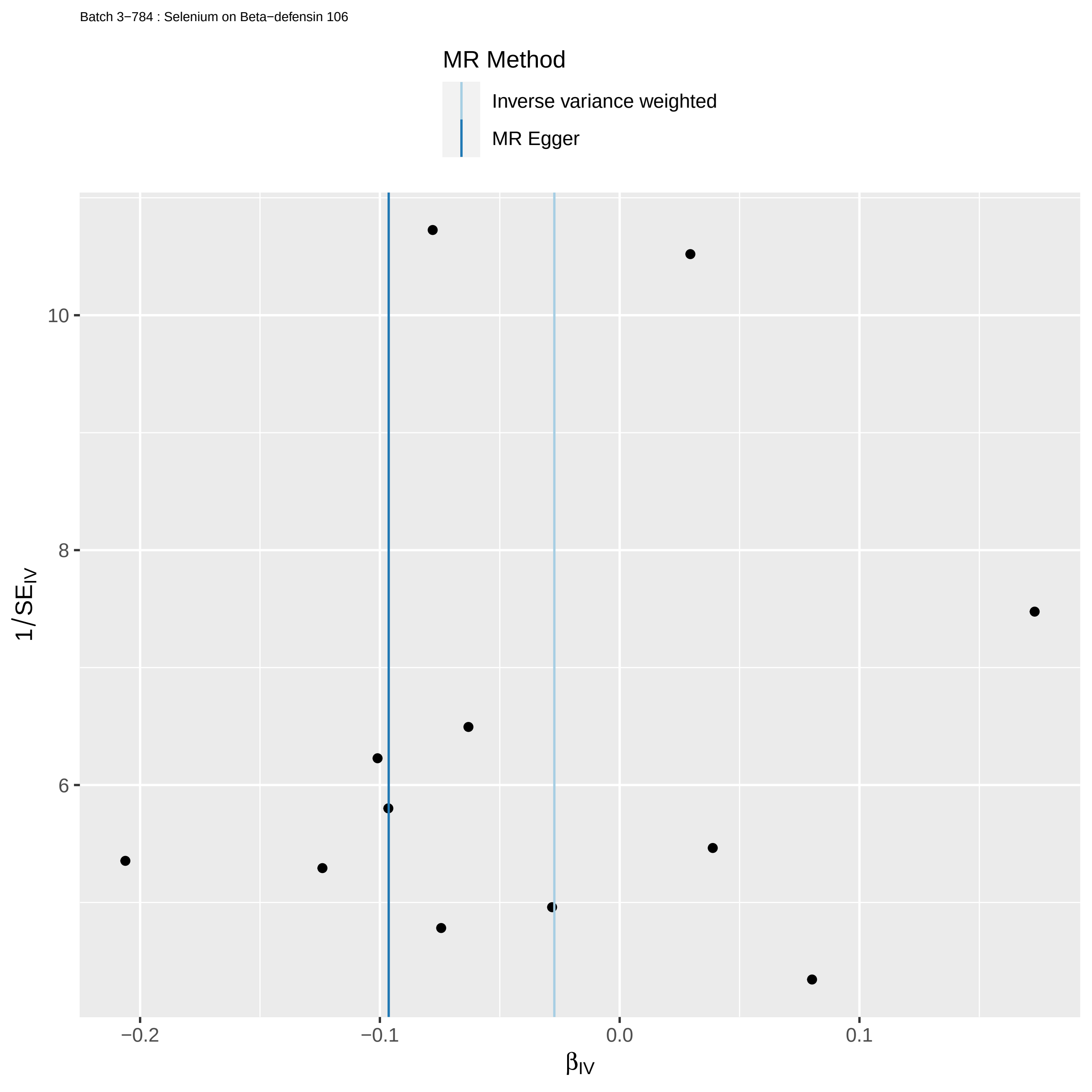

Batch 3−784 : Selenium on Beta−defensin 106
MR Method
Inverse variance weighted
MR Egger
10
8
6
1/SE_IV
−0.2
−0.1
0.0
0.1
β_IV

Batch 3−785 : Selenium on Beta−defensin 107

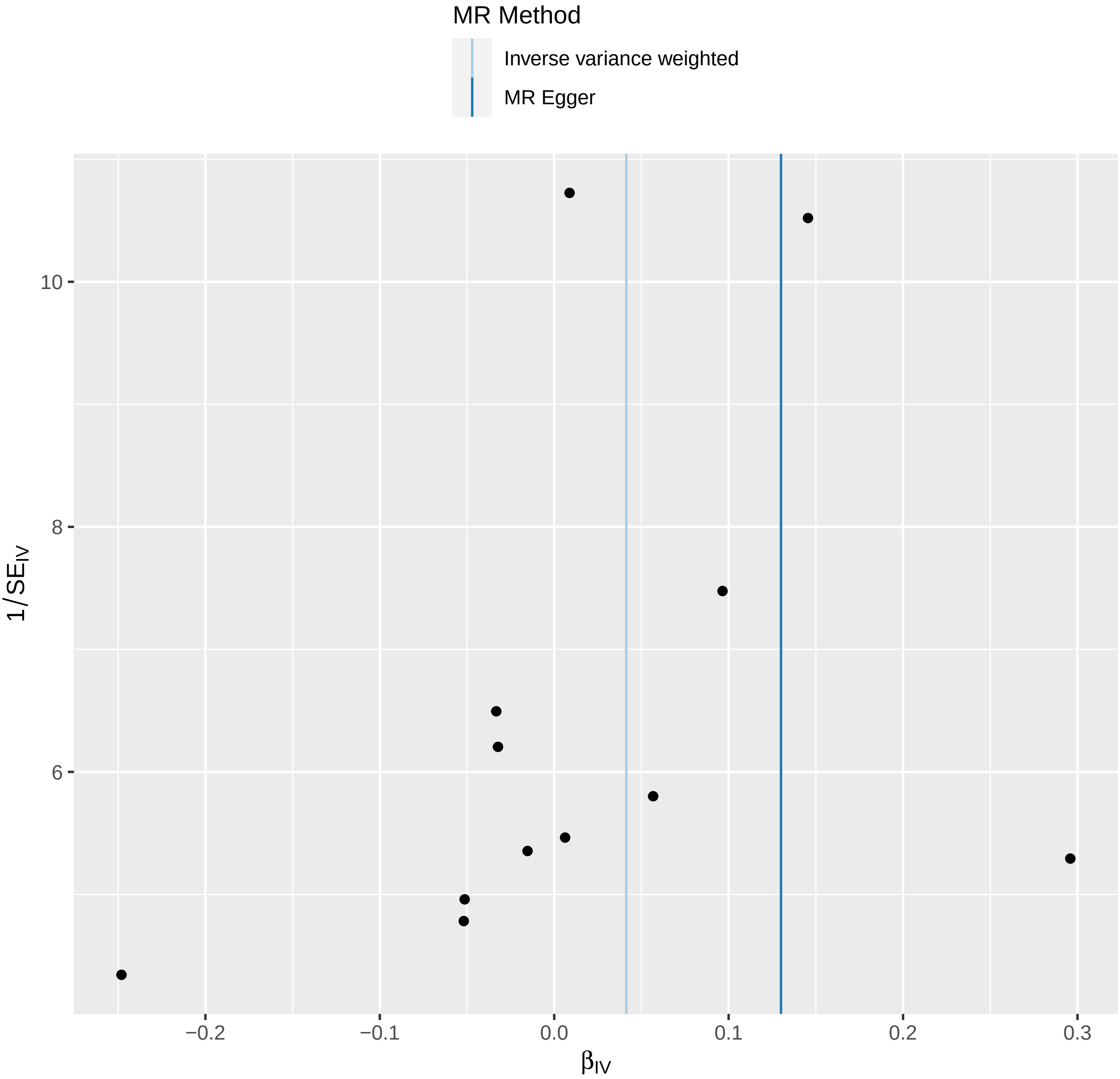

Batch 3−786 : Selenium on Beta−defensin 108B

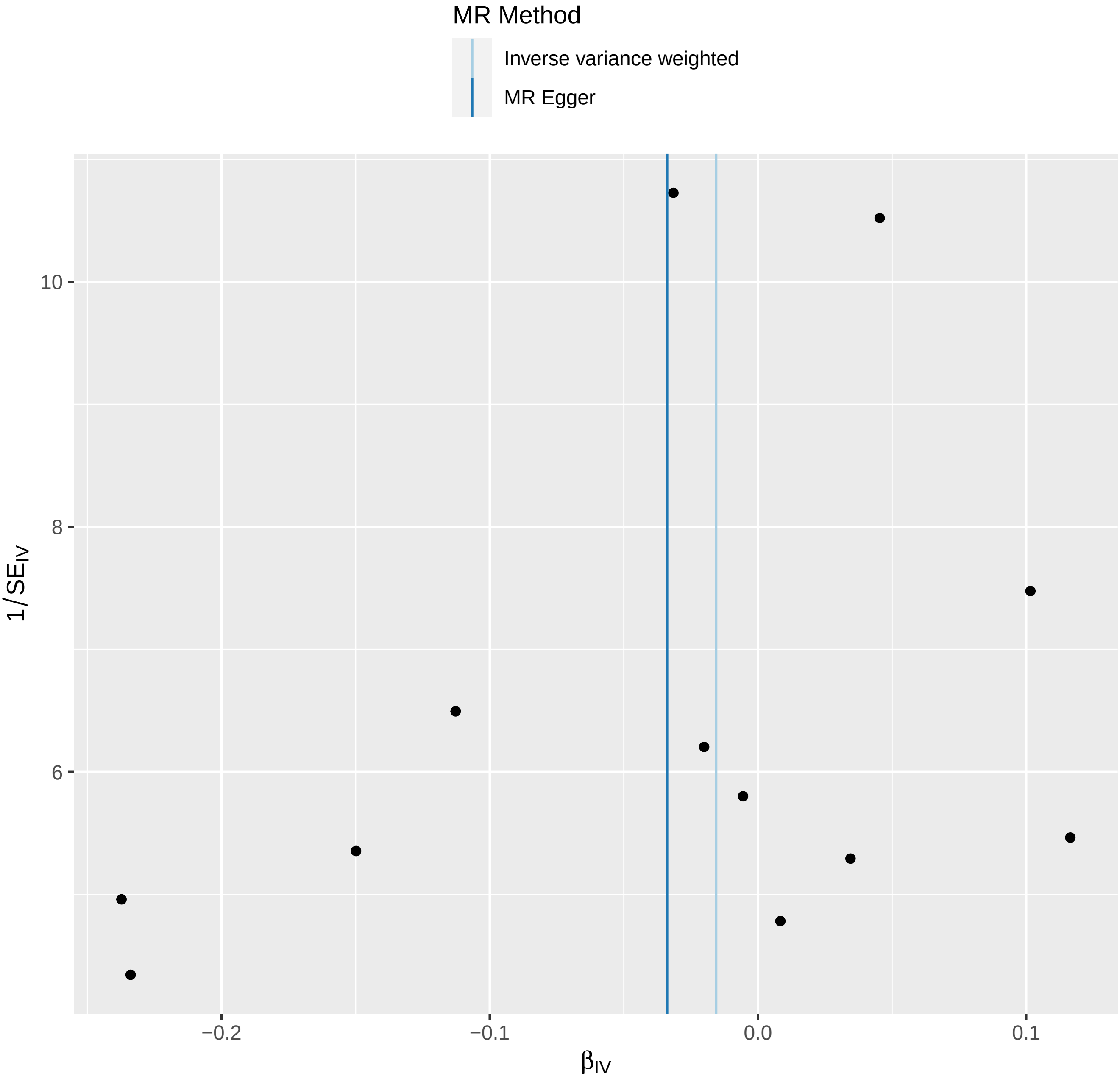

Batch 3−787 : Selenium on Beta−defensin 110

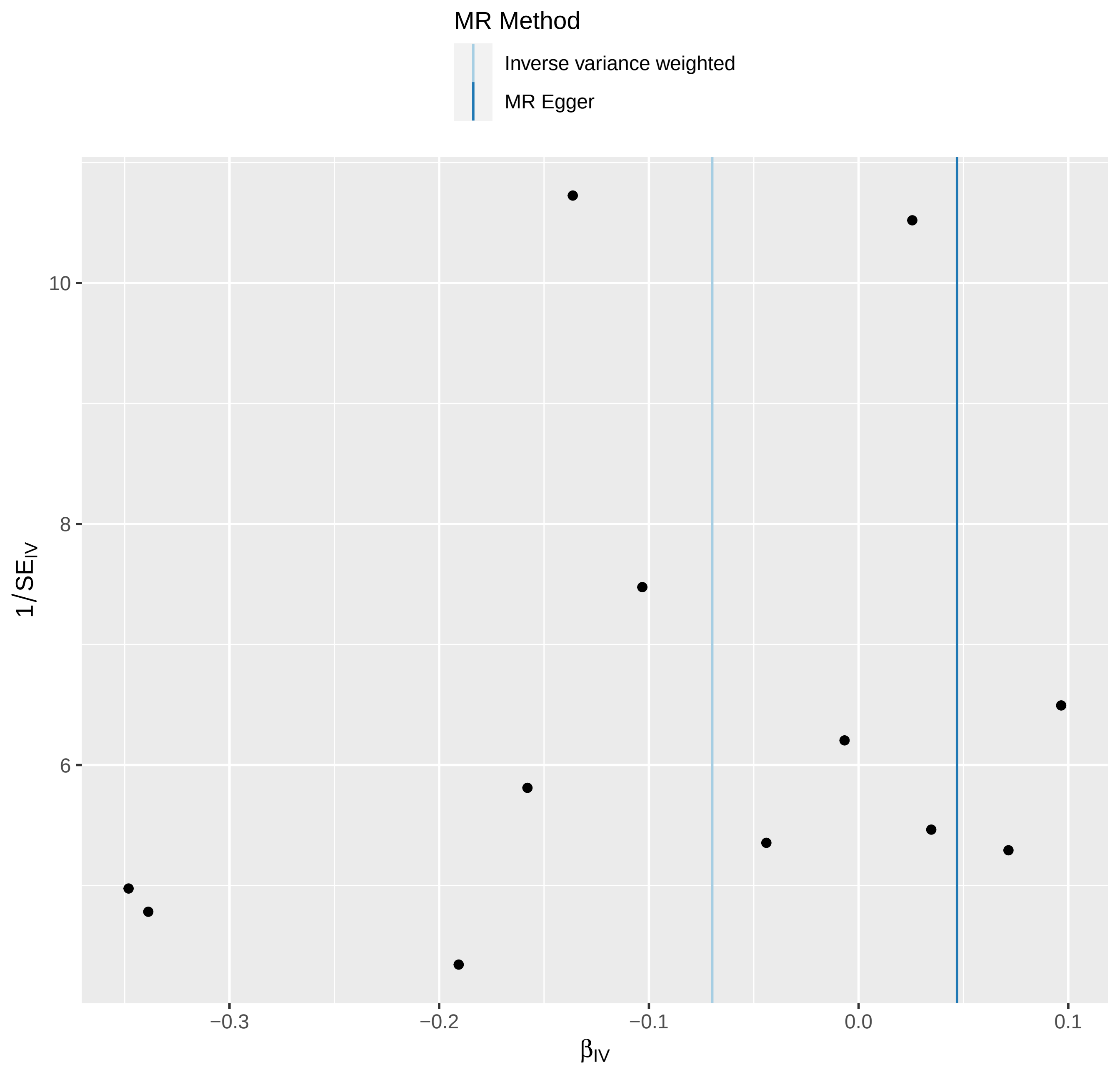

Batch 3−788 : Selenium on Beta−defensin 112

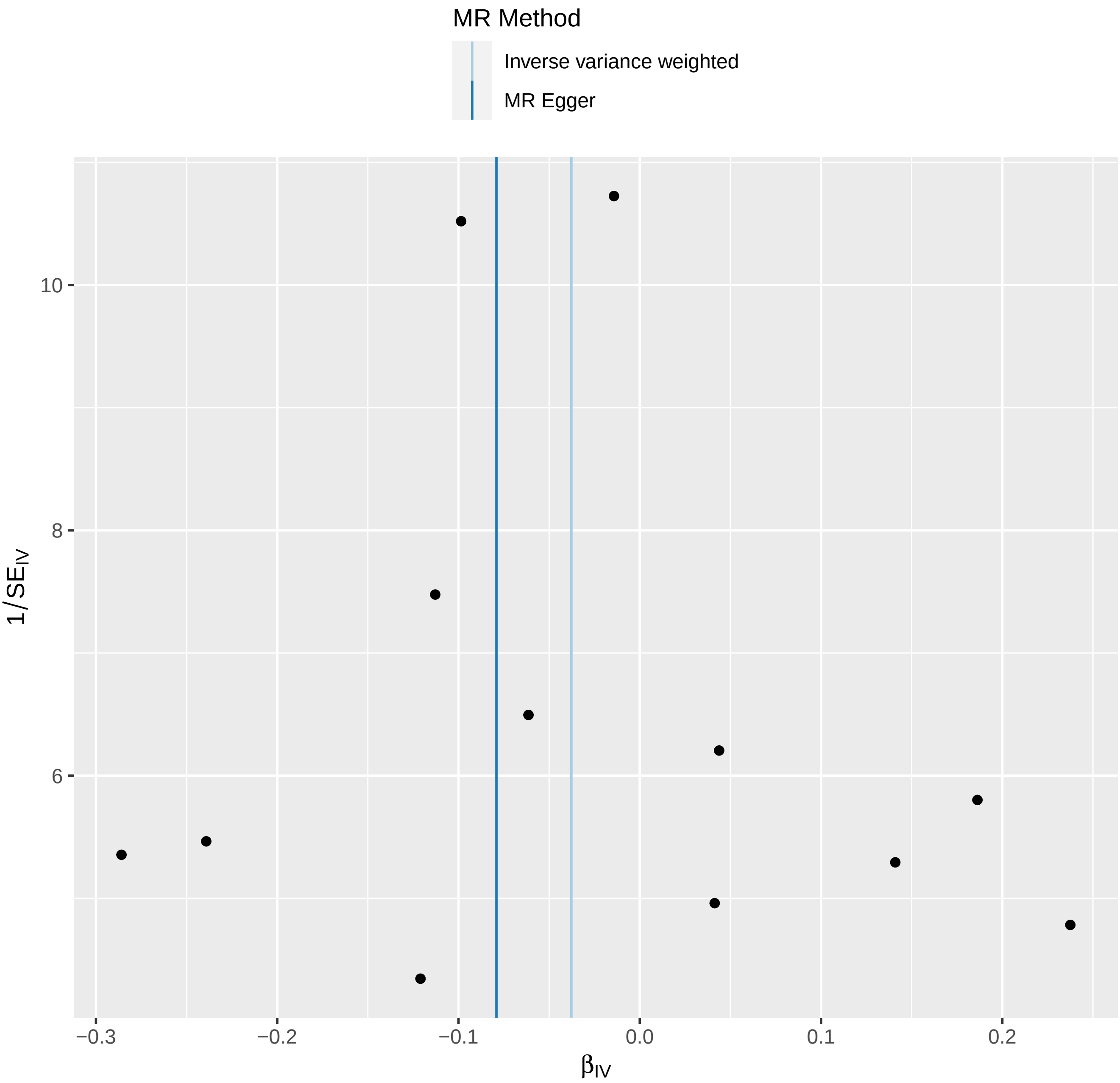

Batch 3−789 : Selenium on Beta−defensin 113

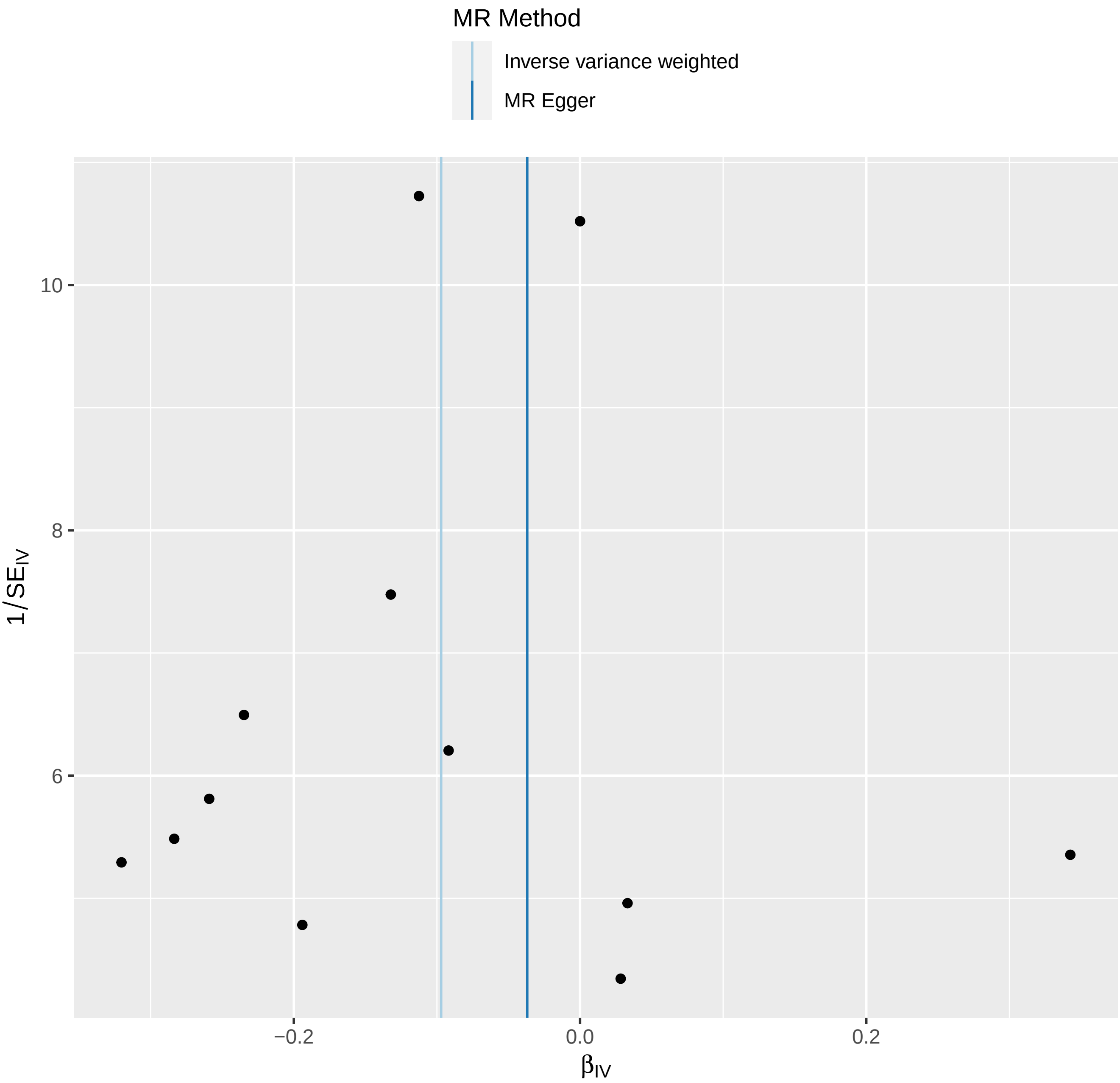

Batch 3−790 : Selenium on Beta−defensin 115

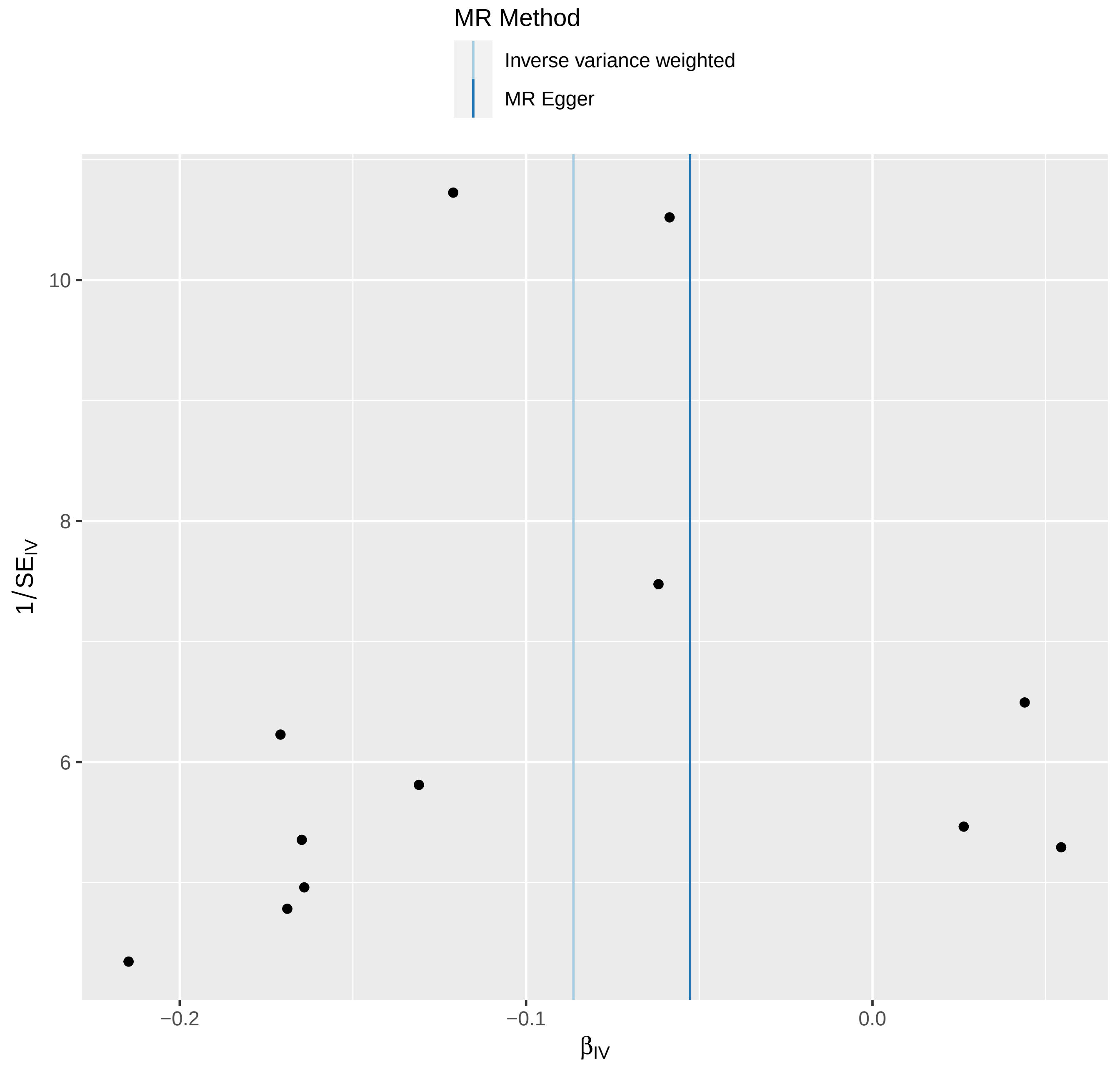

Batch 3−791 : Selenium on Beta−defensin 116

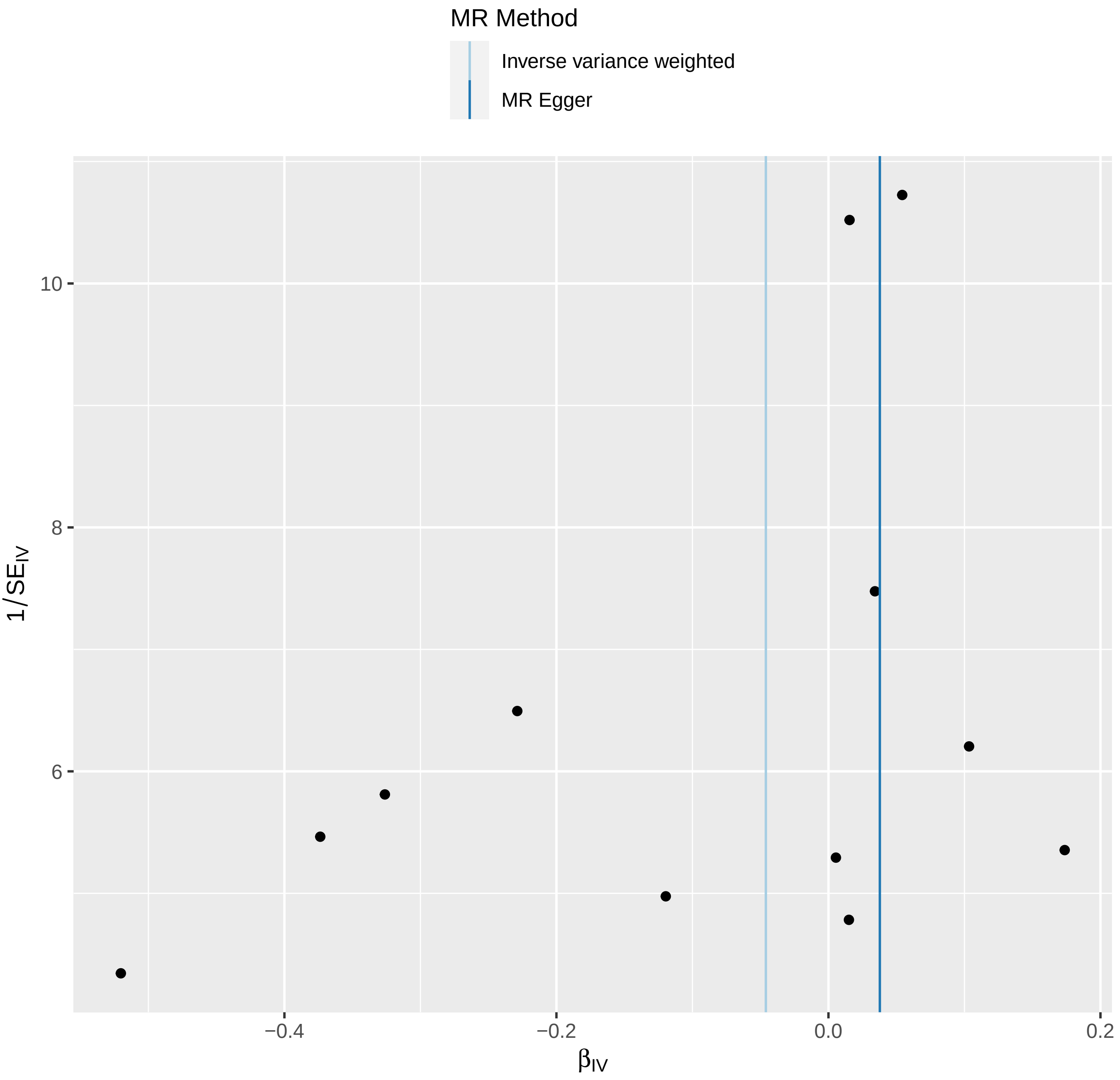

Batch 3−792 : Selenium on Beta−defensin 118

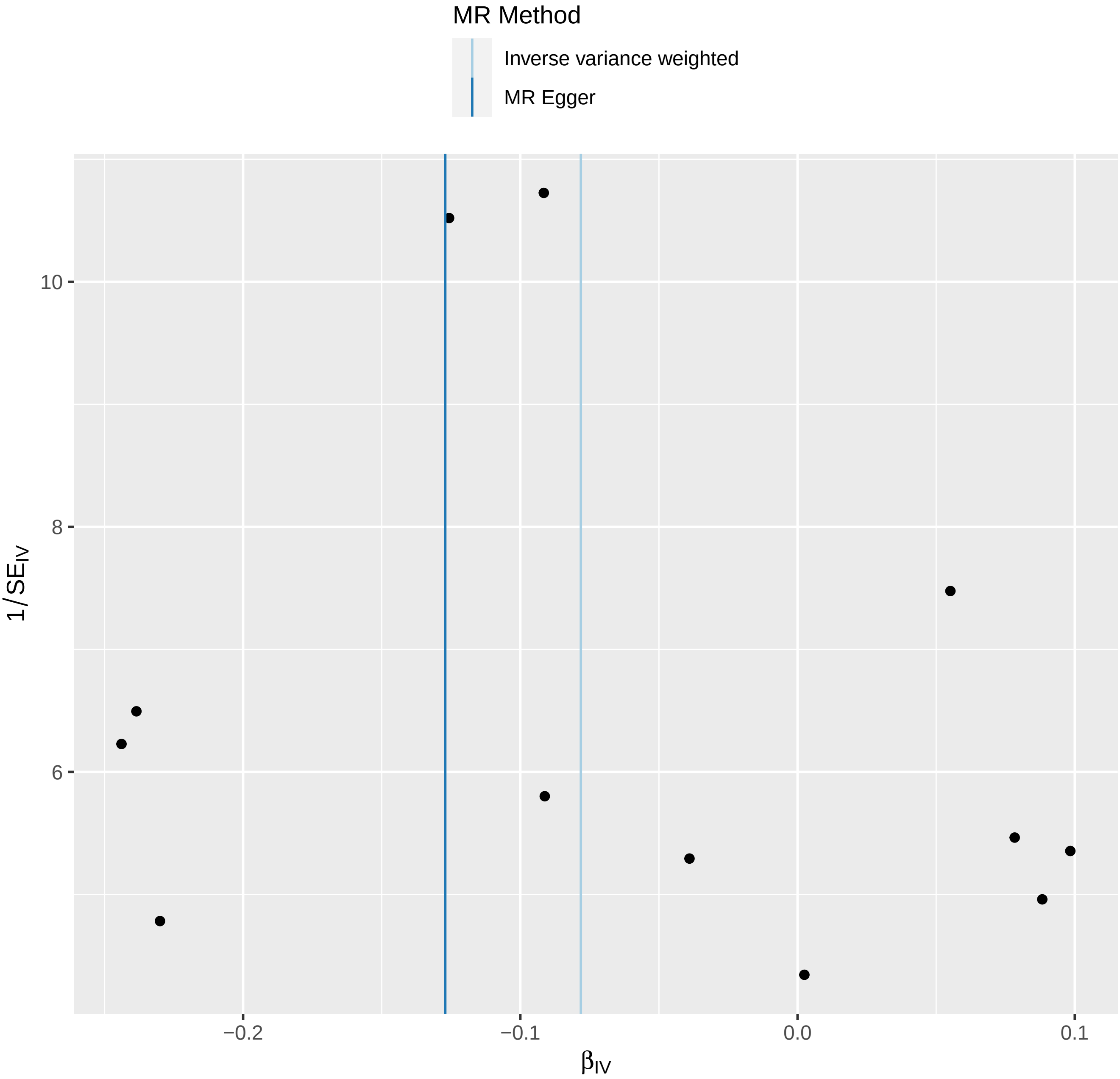

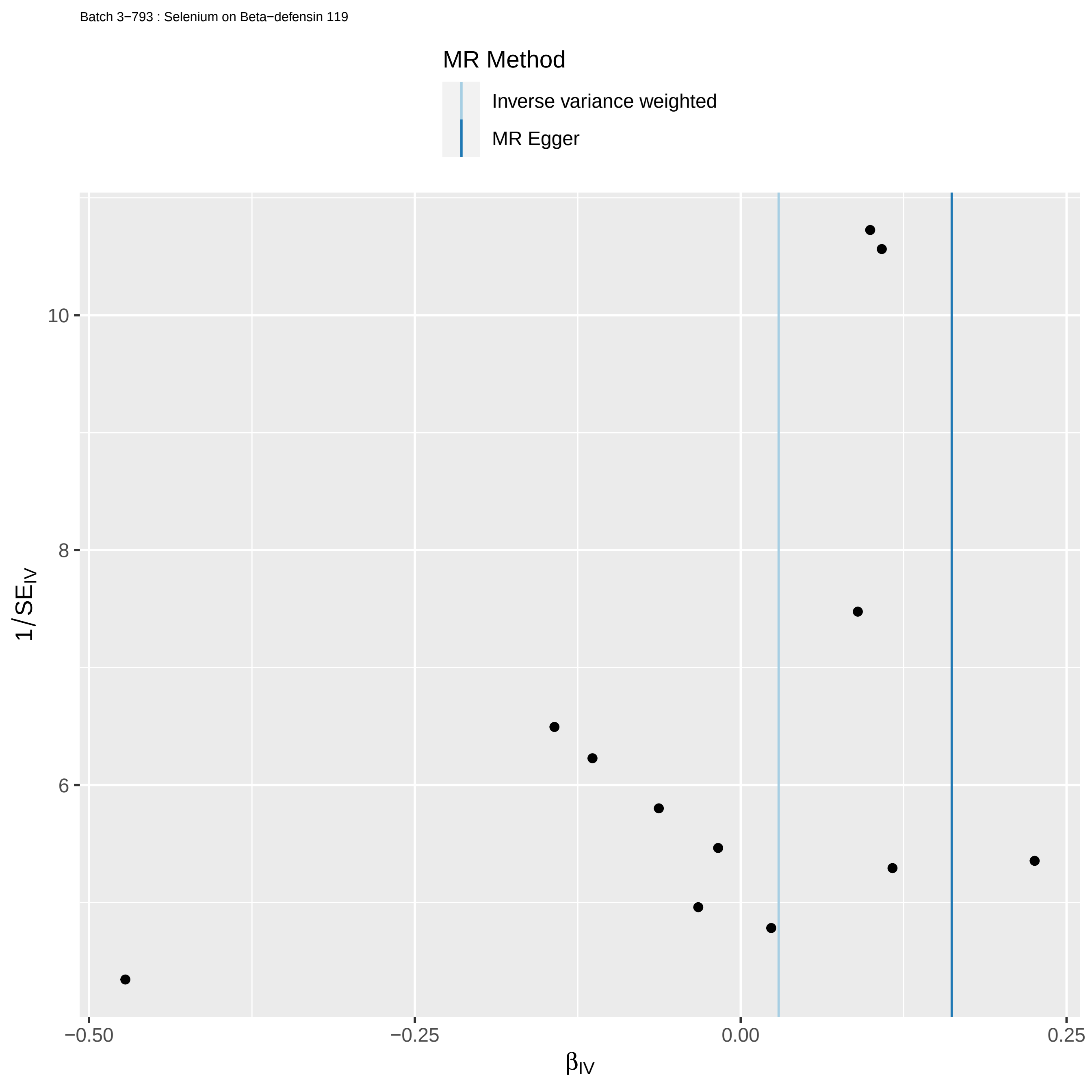

Batch 3−793 : Selenium on Beta−defensin 119
MR Method
Inverse variance weighted
MR Egger
10
8
6
1/SE_IV
−0.50
−0.25
0.00
0.25
β_IV

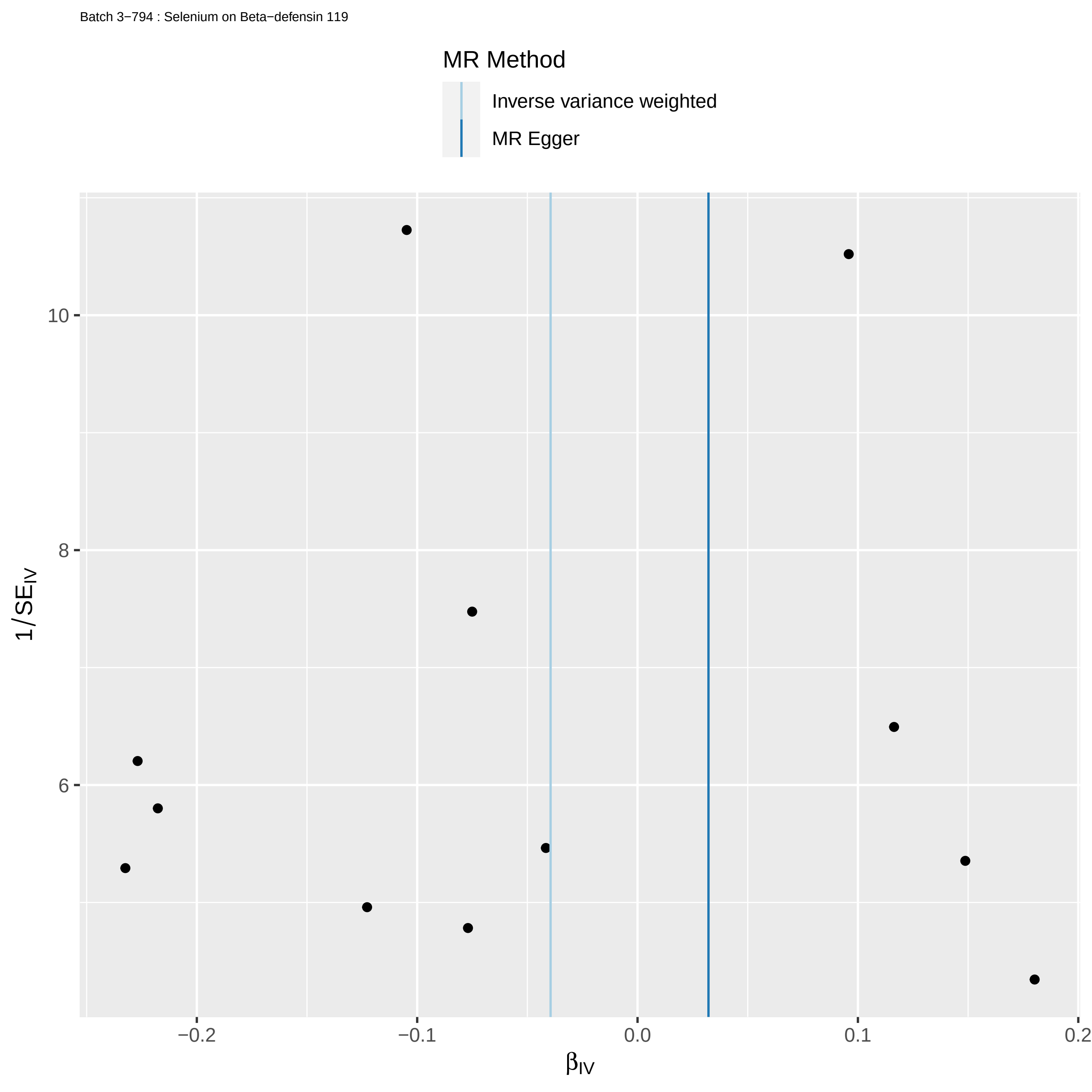

Batch 3−794 : Selenium on Beta−defensin 119
MR Method
Inverse variance weighted
MR Egger
10
8
6
1/SE_IV
−0.2
−0.1
0.0
0.1
0.2
β_IV

Batch 3−795 : Selenium on Beta−defensin 119

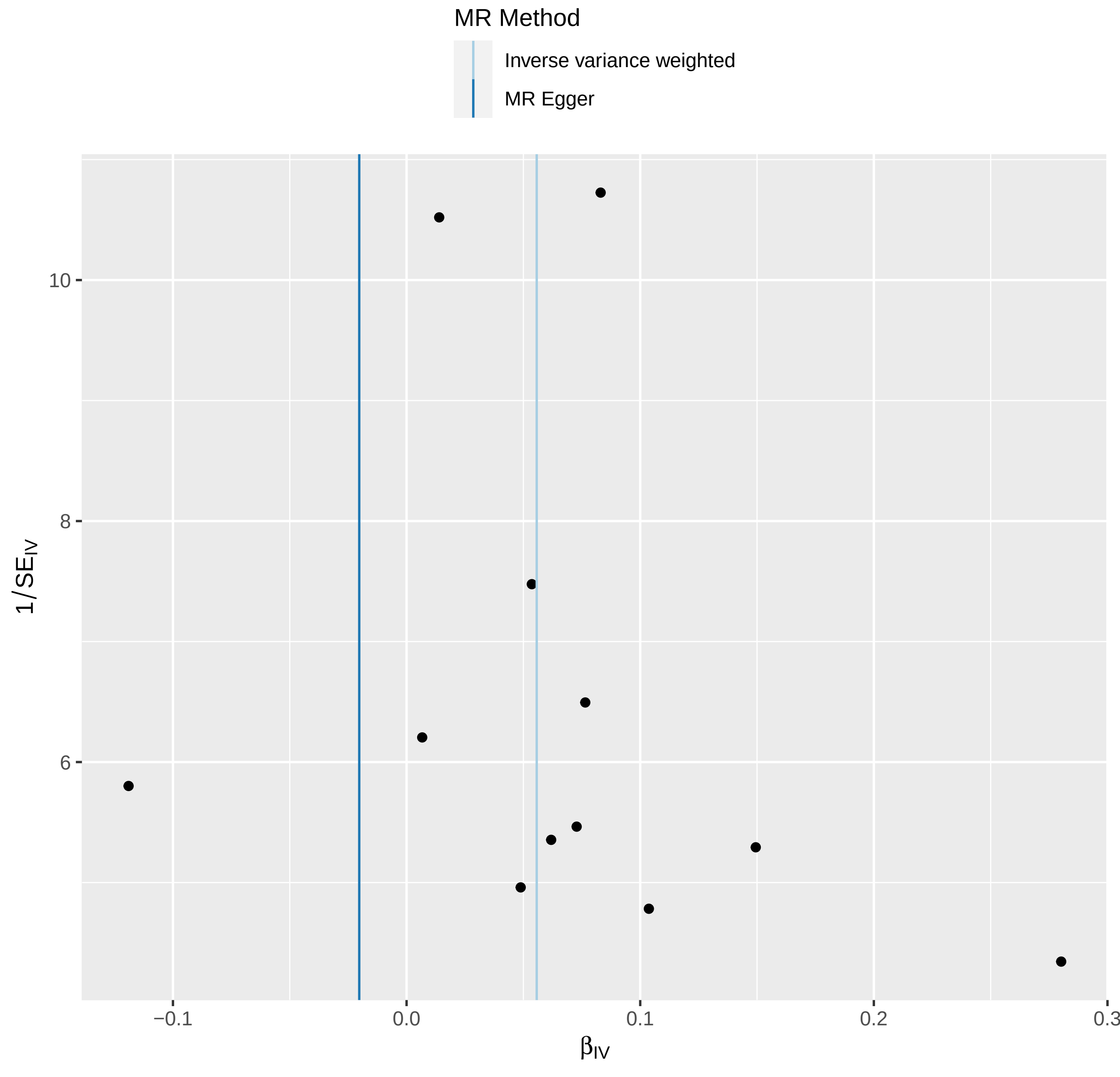

Batch 3−796 : Selenium on Beta−defensin 119

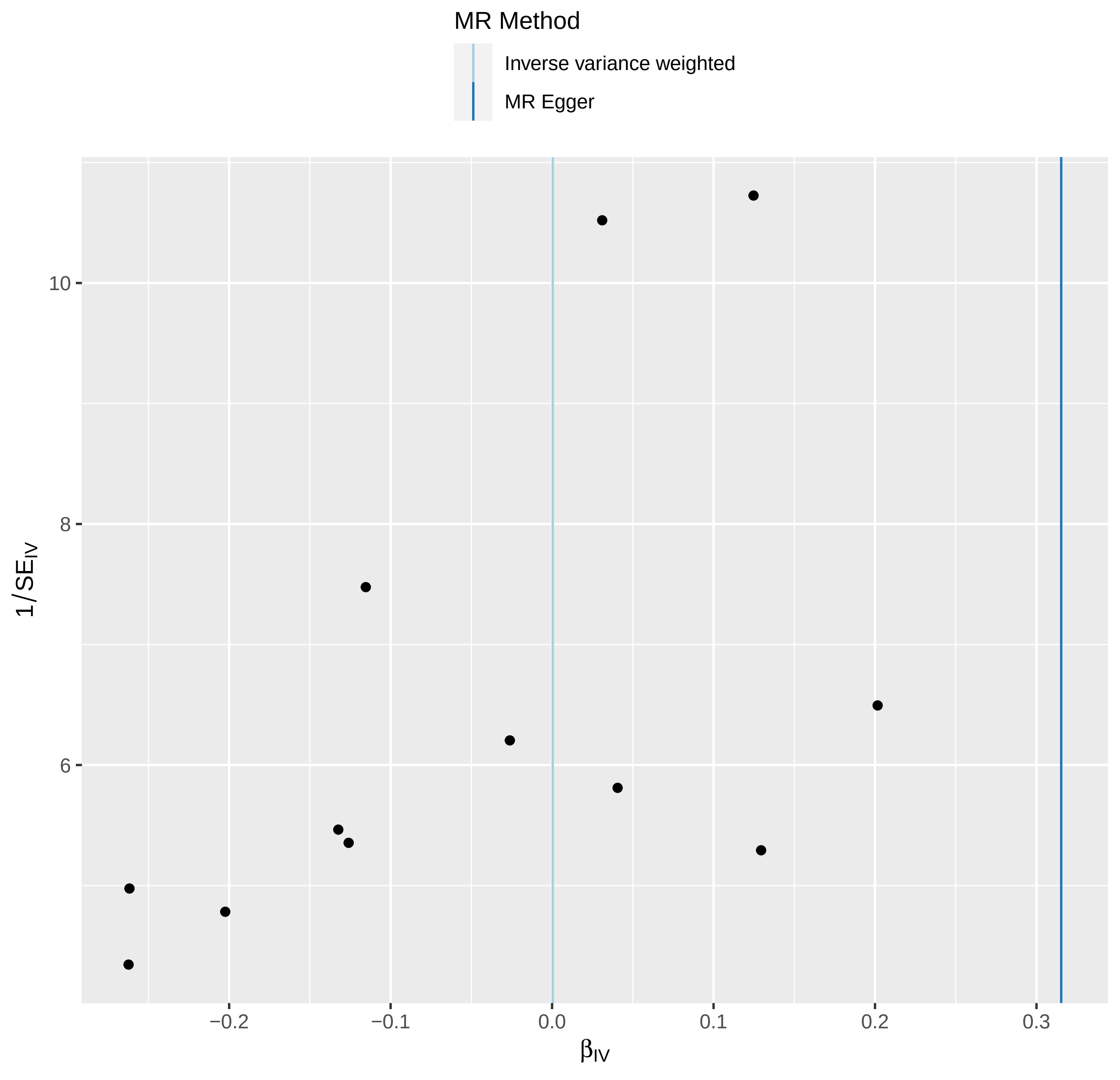

Batch 3−797 : Selenium on Beta−defensin 121

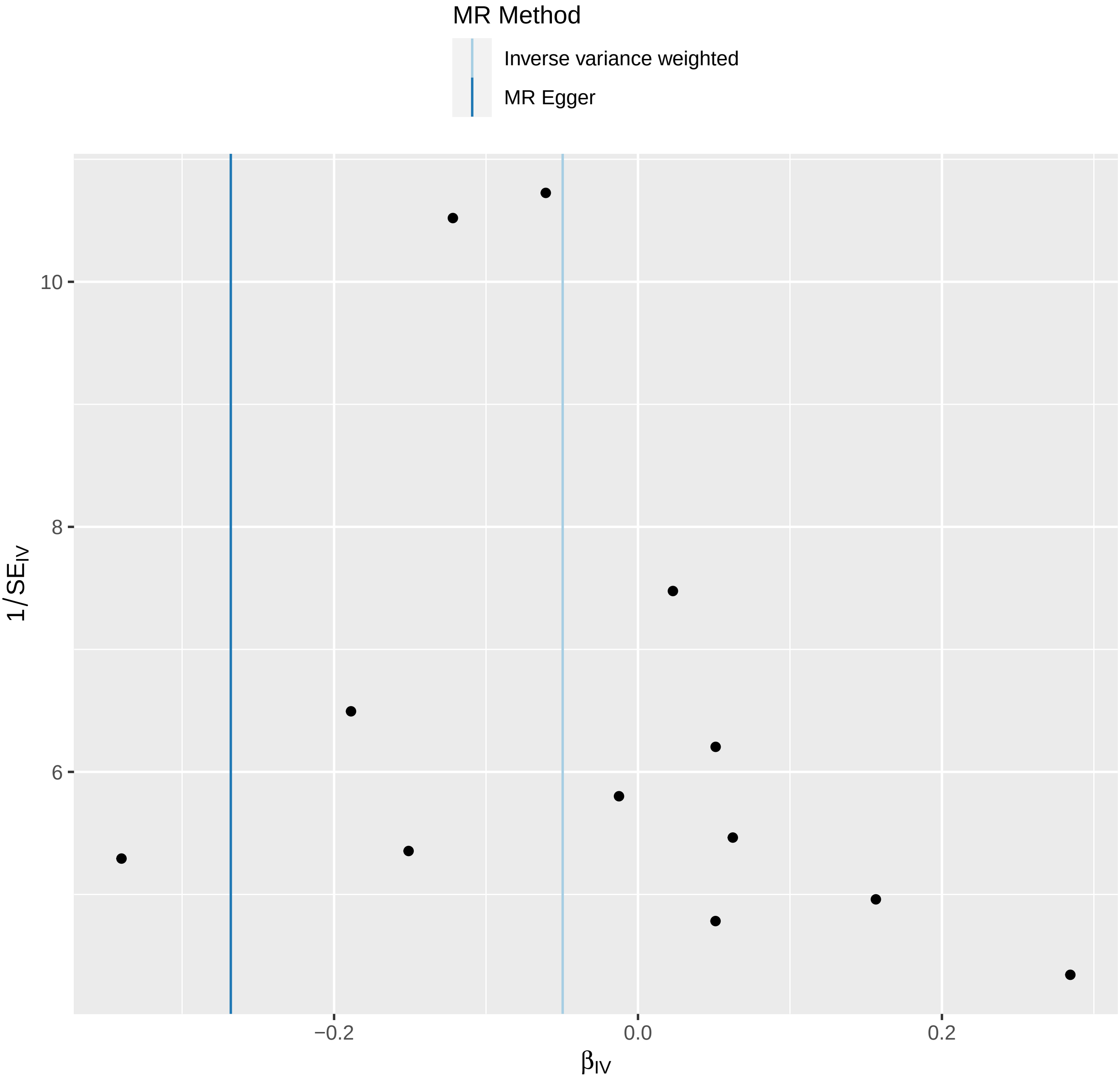

Batch 3−798 : Selenium on Beta−defensin 123

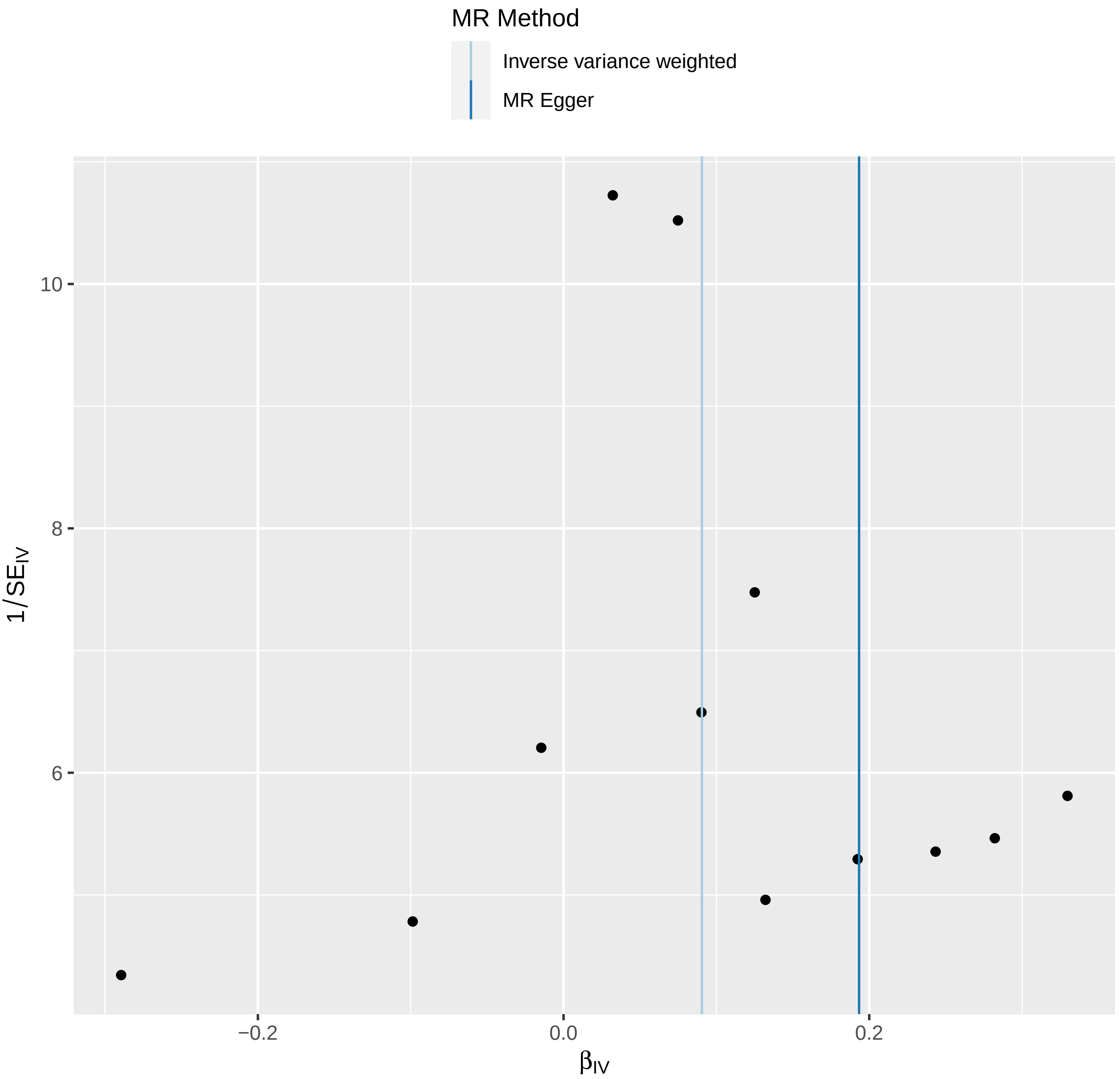

Batch 3−799 : Selenium on Beta−defensin 125

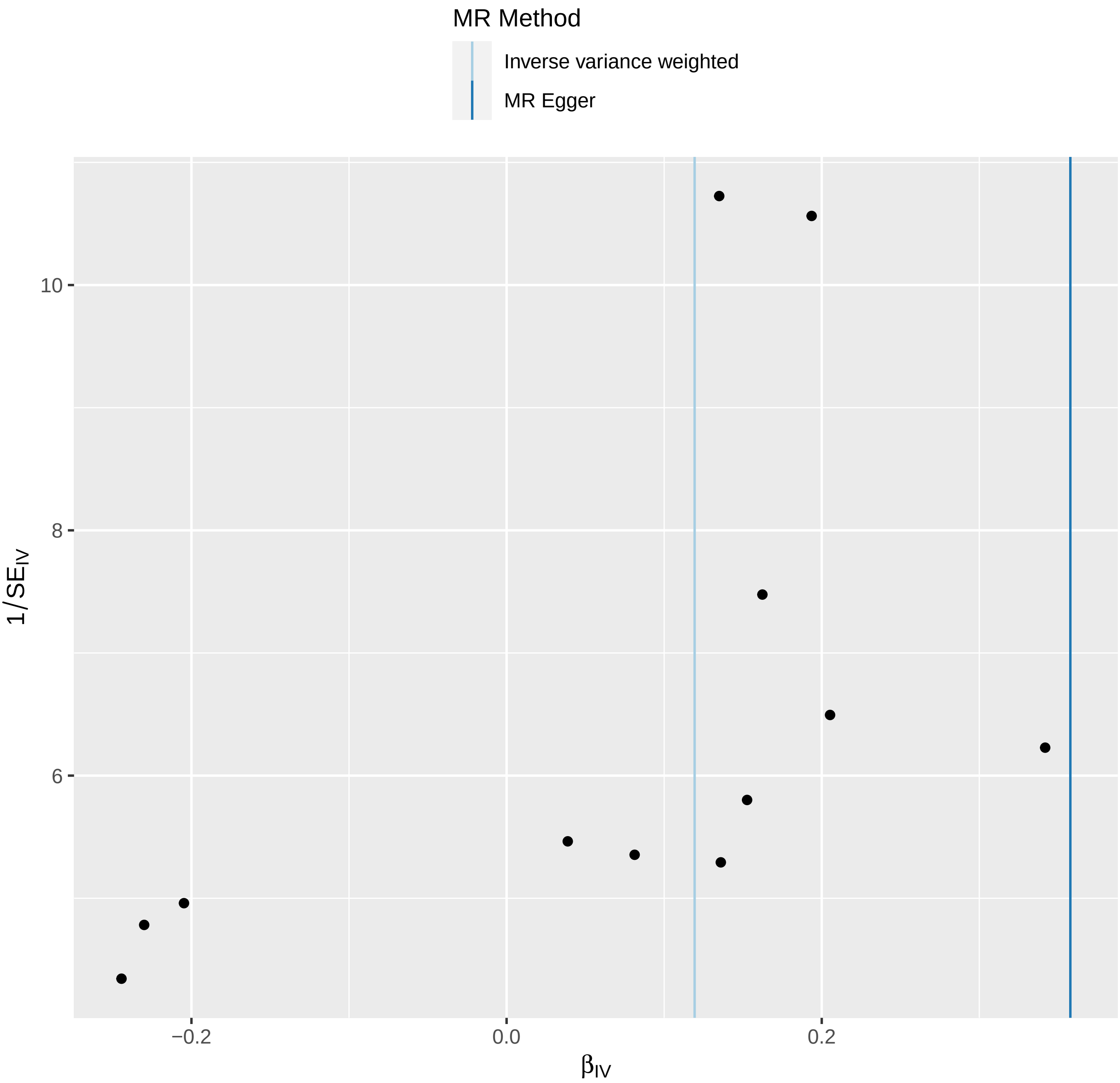

Batch 3−800 : Selenium on Beta−defensin 128

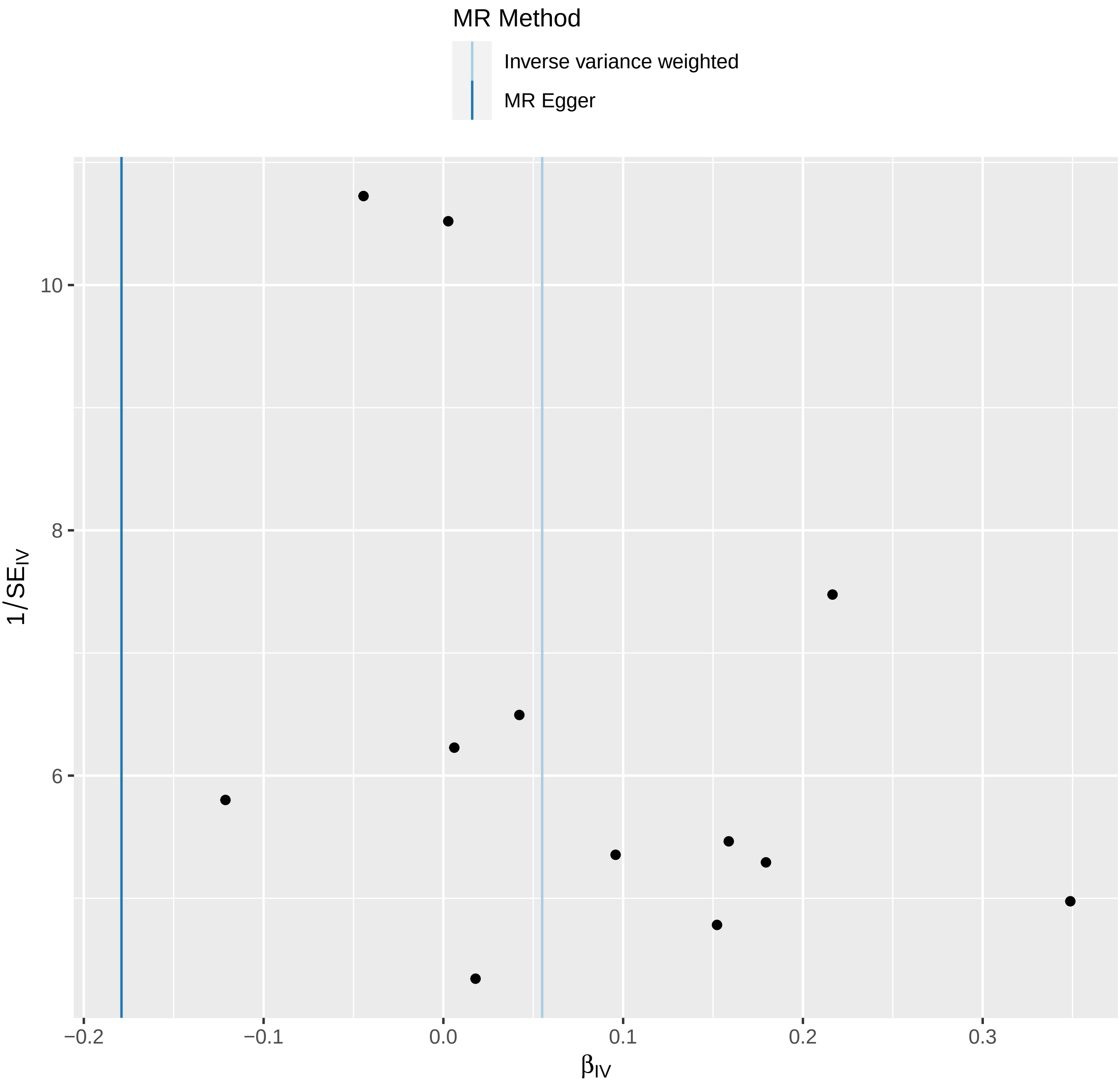

Batch 3−801 : Selenium on Beta−defensin 134

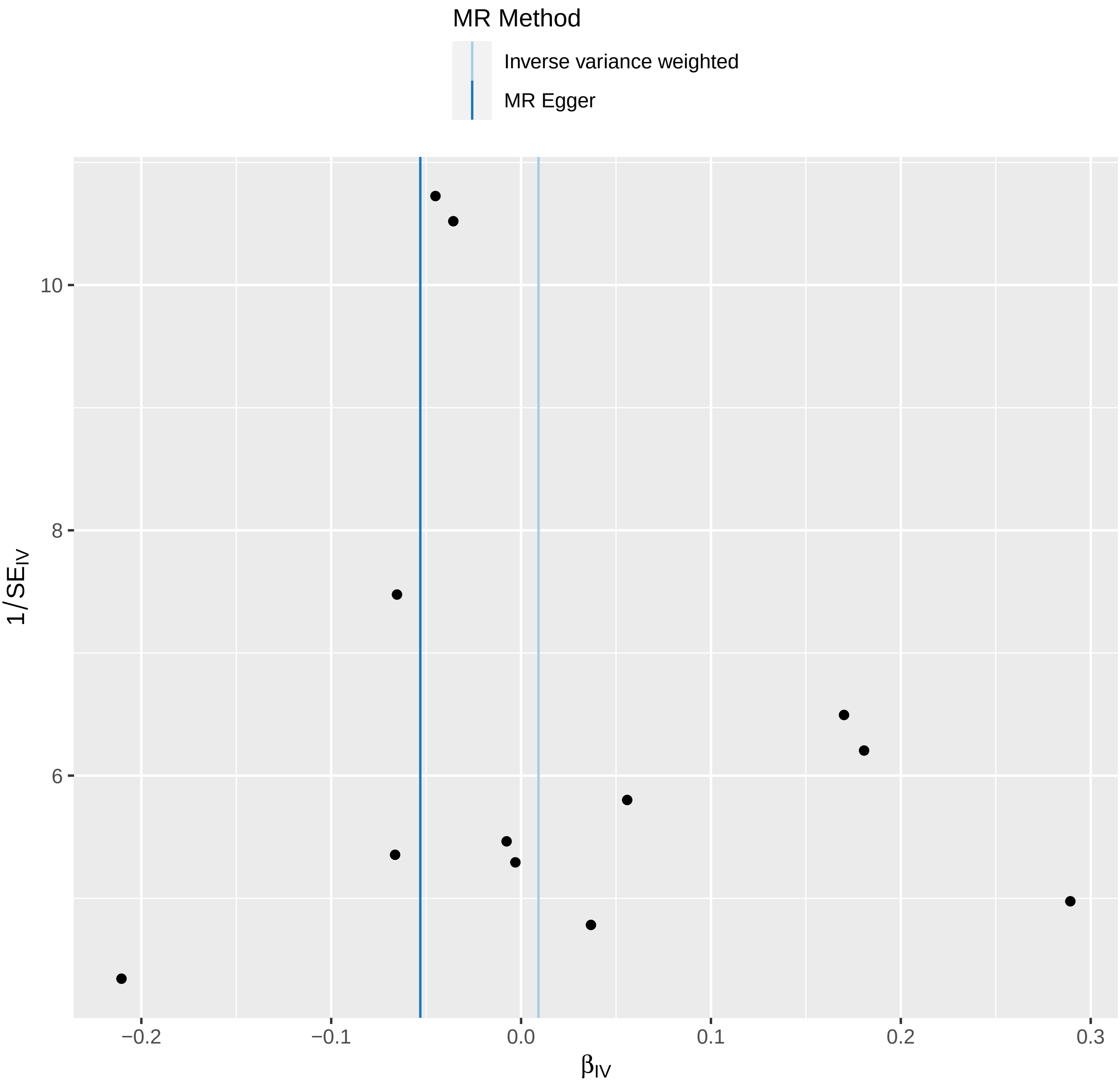

Batch 3−802 : Selenium on Beta−defensin 135

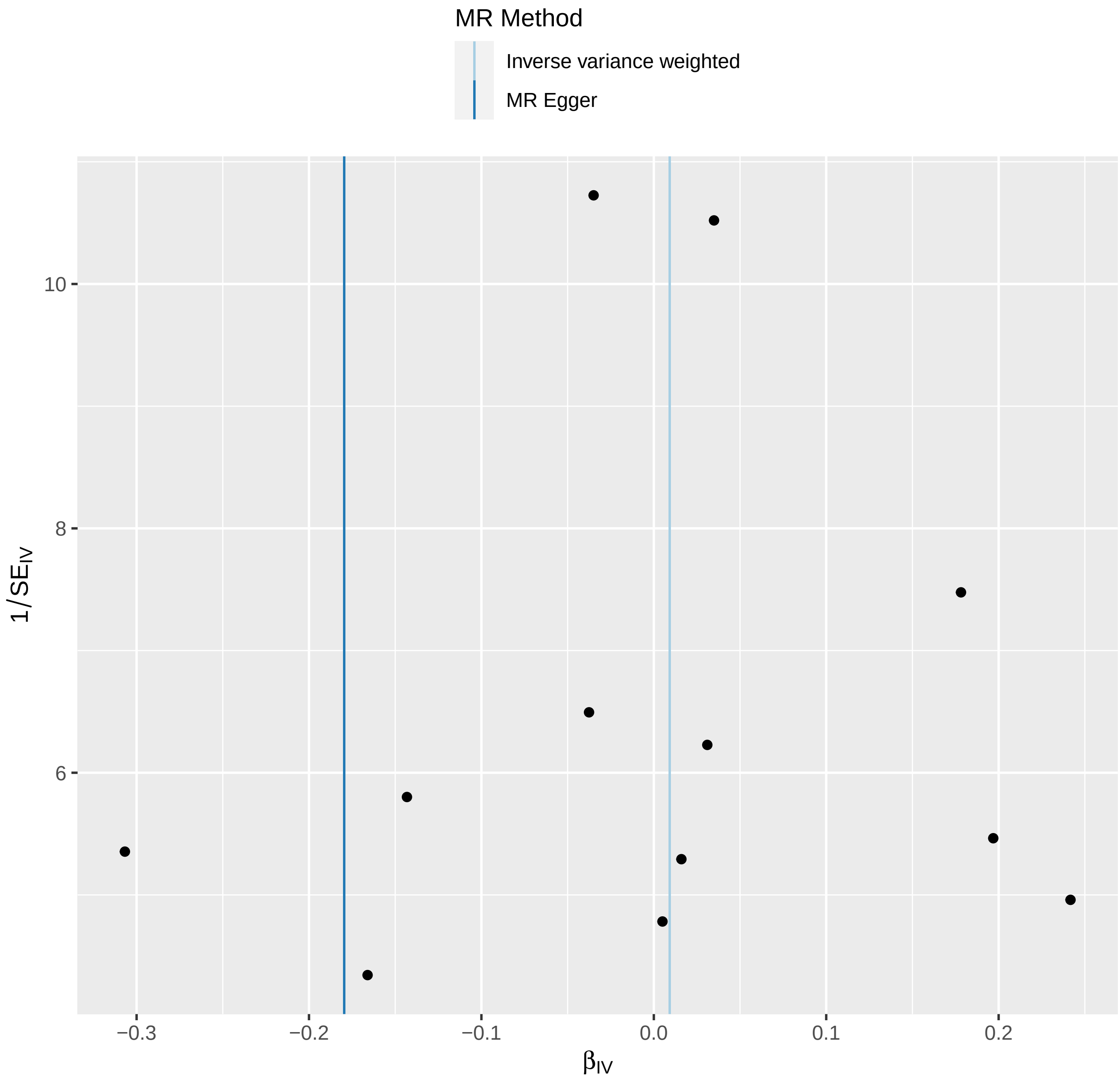

Batch 3−803 : Selenium on Beta−defensin 136

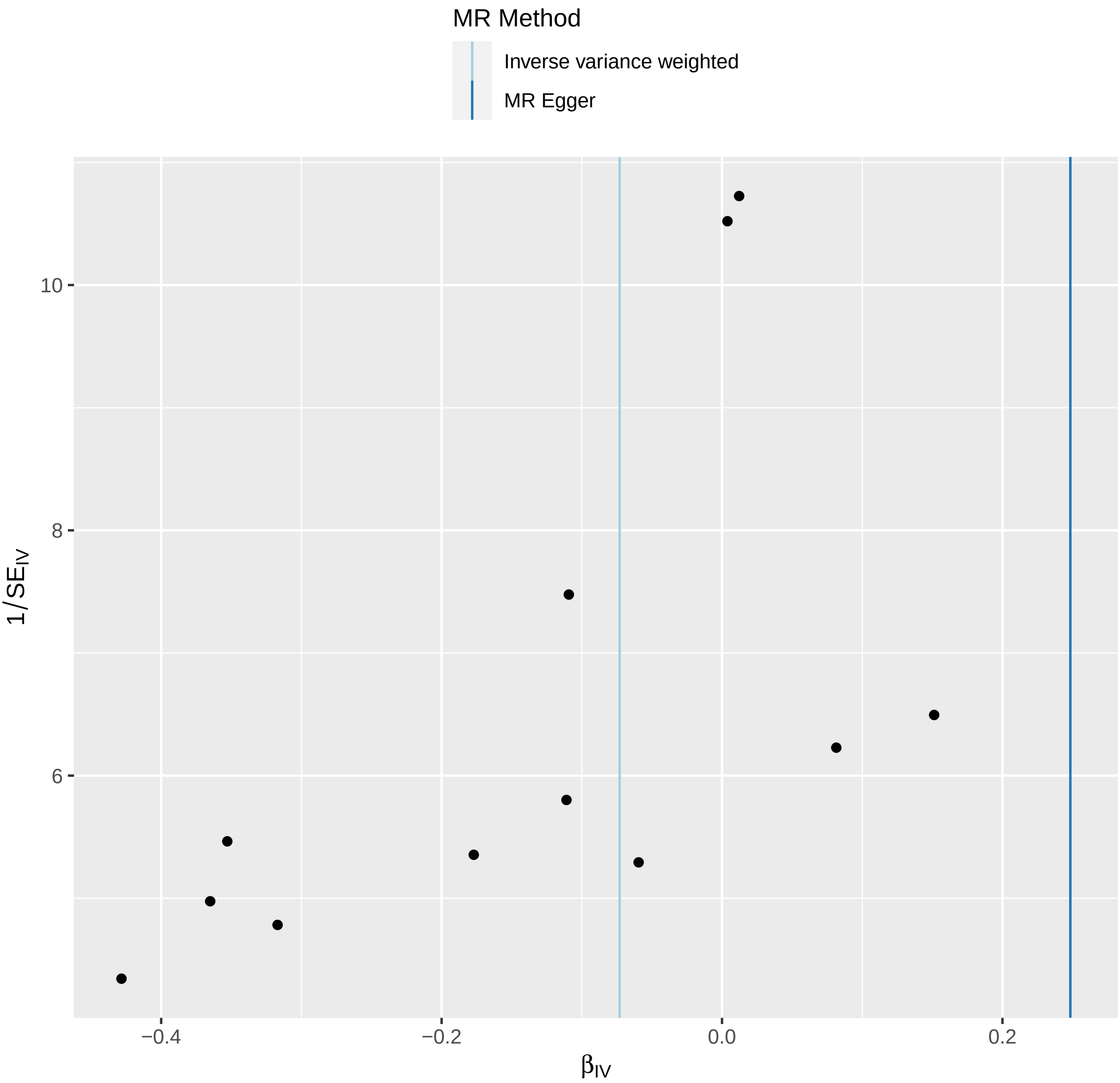

Batch 3−804 : Selenium on Beta−defensin 1

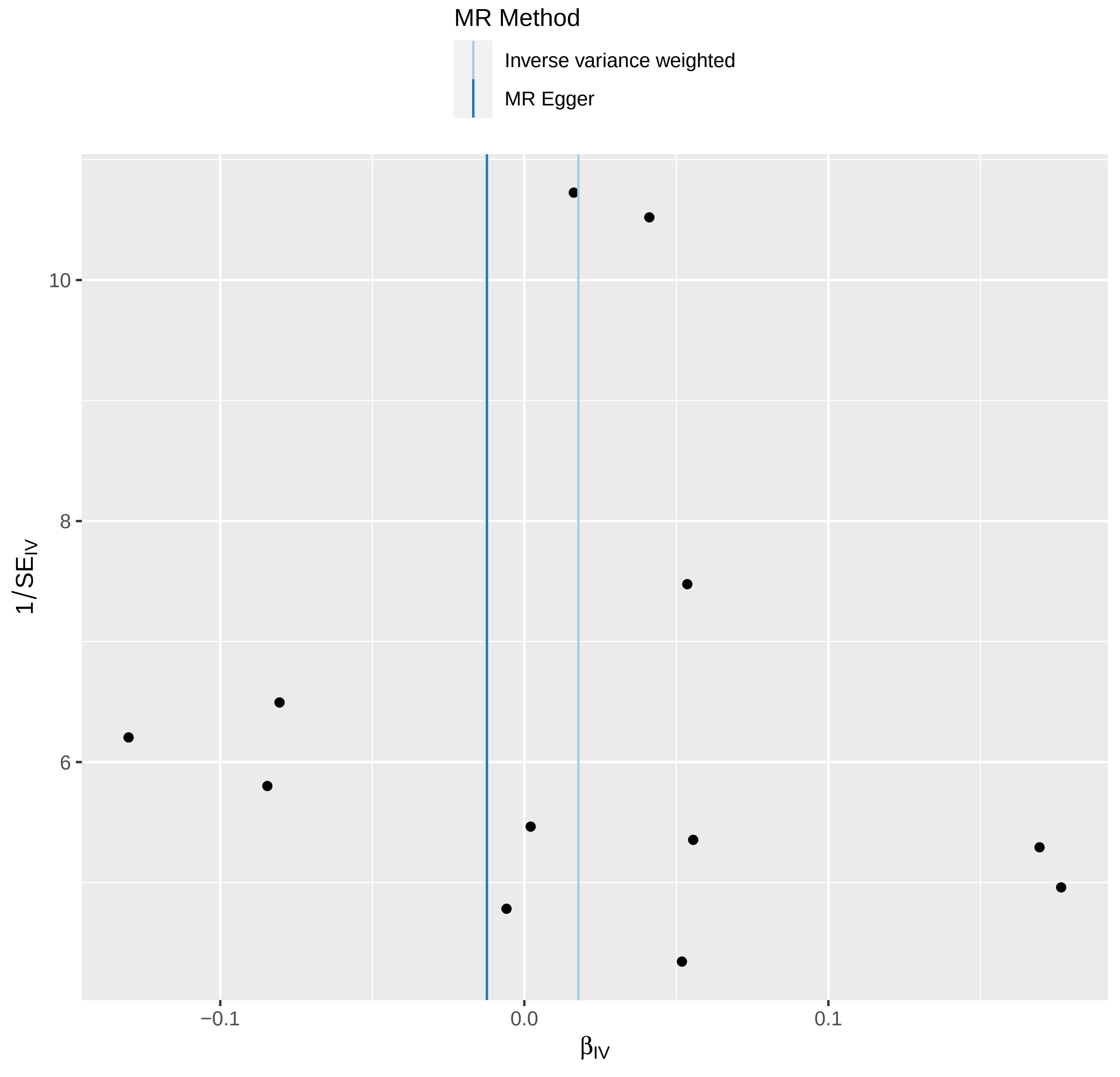

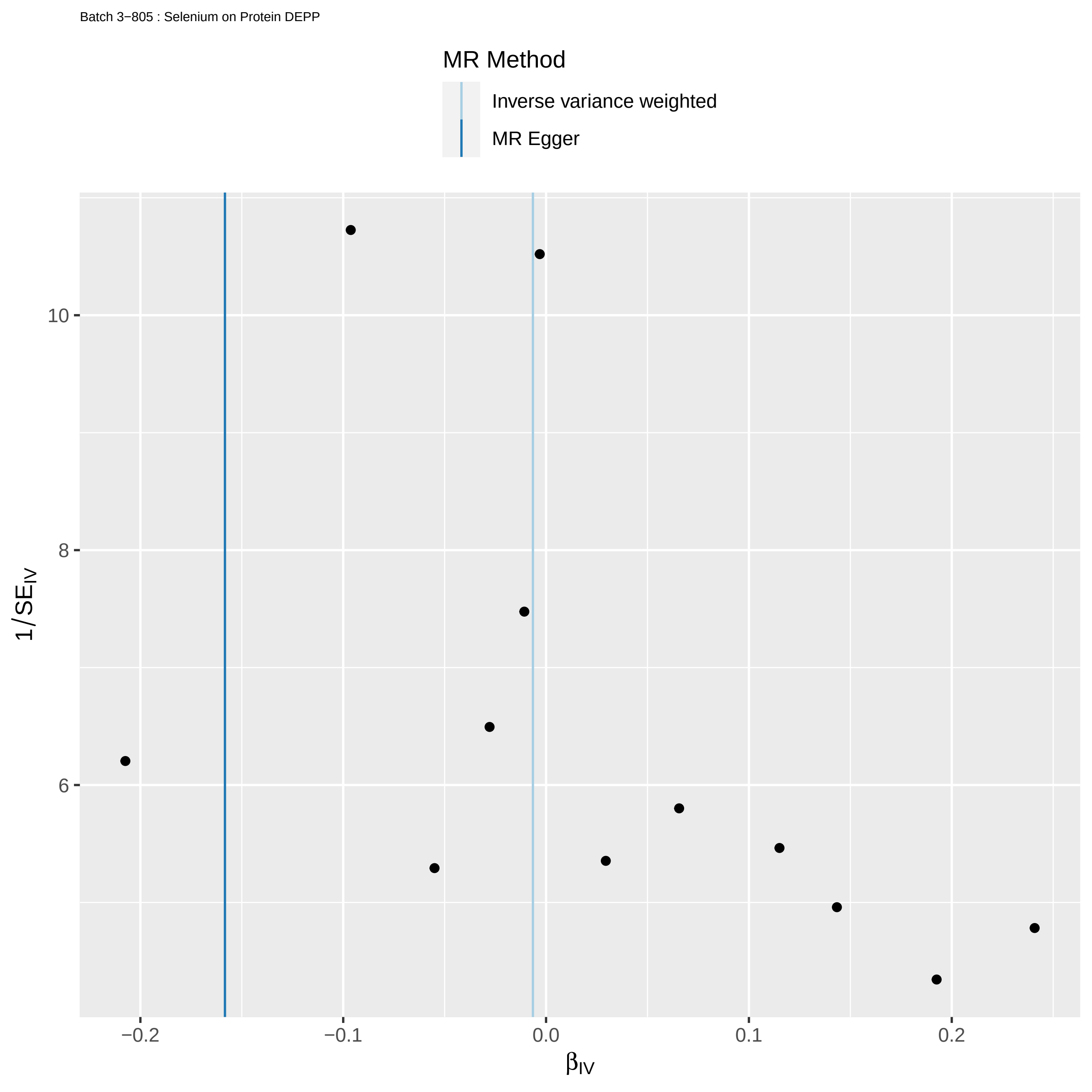
Batch 3−805 : Selenium on Protein DEPP
MR Method
Inverse variance weighted
MR Egger
10
8
6
1/SE_IV
−0.2
−0.1
0.0
0.1
0.2
β_IV

Batch 3−806 : Selenium on Derlin−1

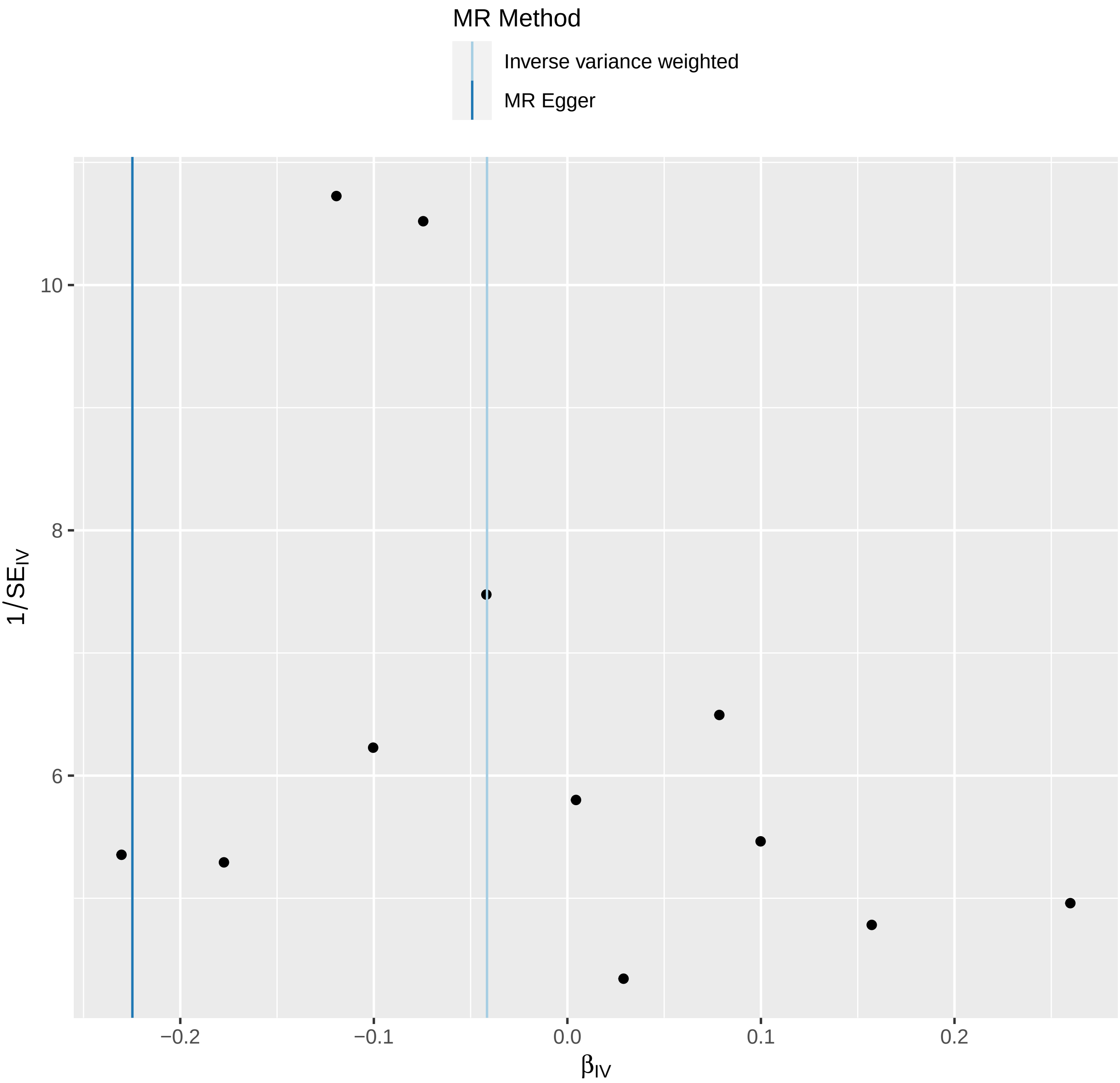

Batch 3−807 : Selenium on Desmin

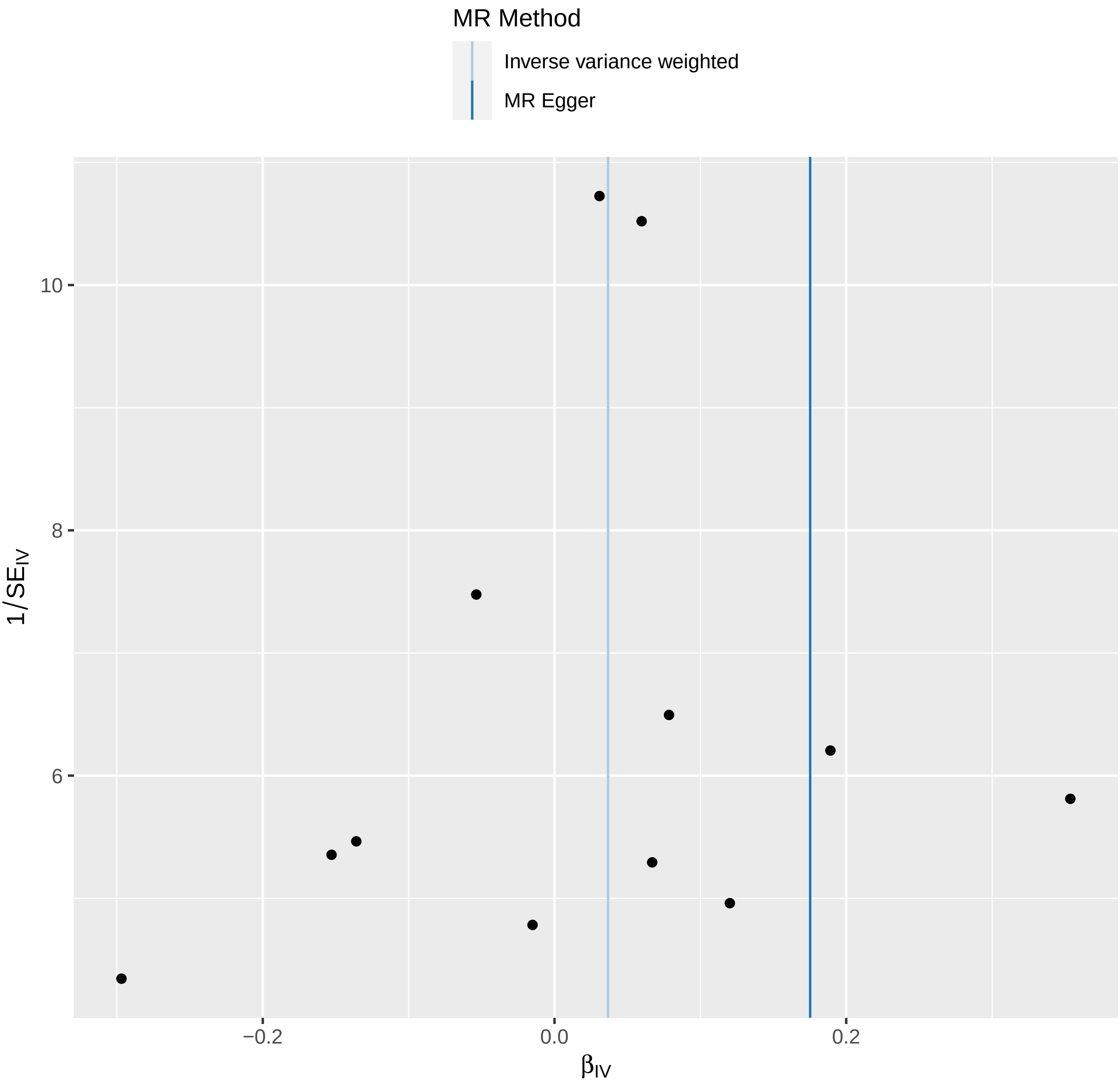

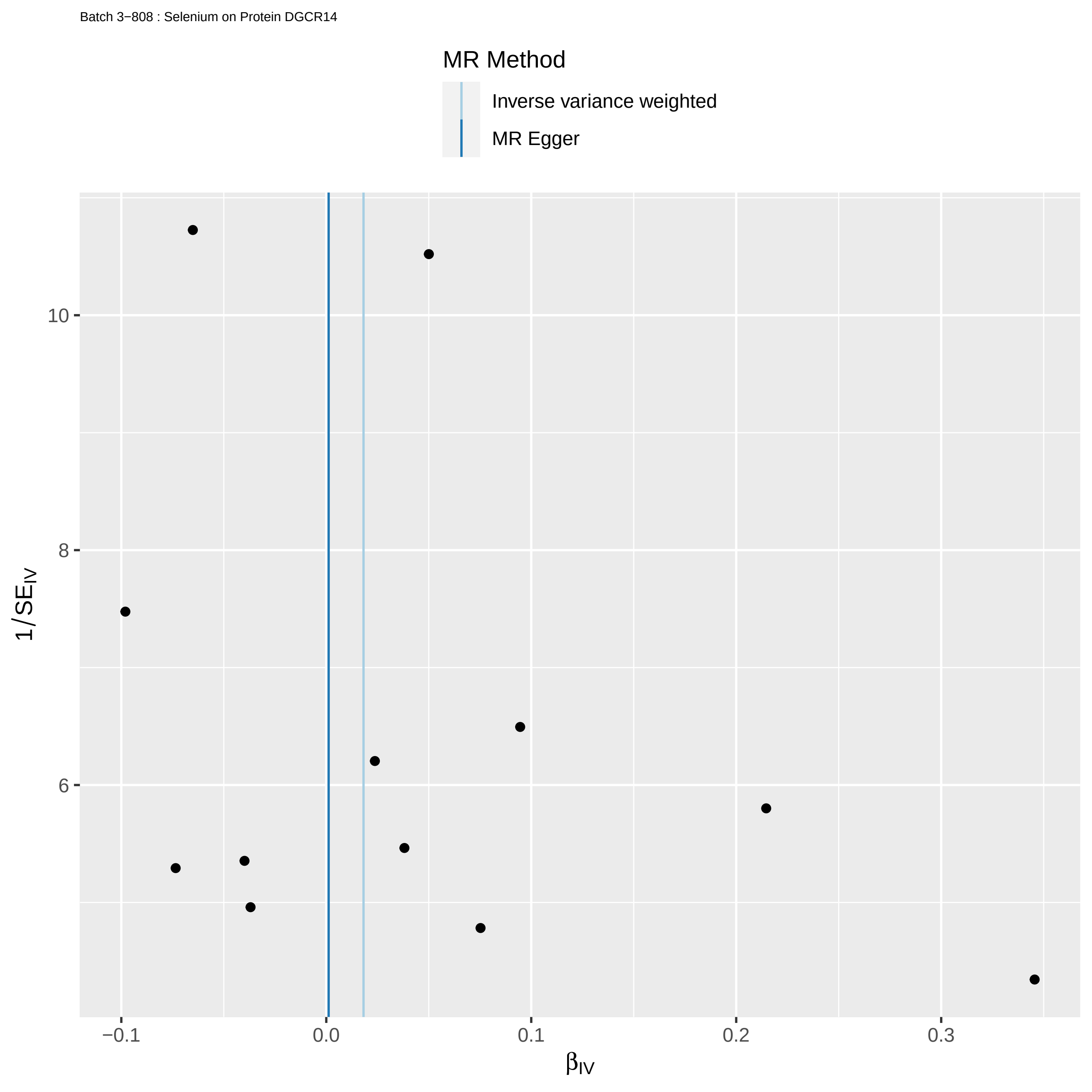

Batch 3−808 : Selenium on Protein DGCR14
MR Method
Inverse variance weighted
MR Egger
10
8
6
1/SE_IV
−0.1
0.0
0.1
0.2
0.3
β_IV

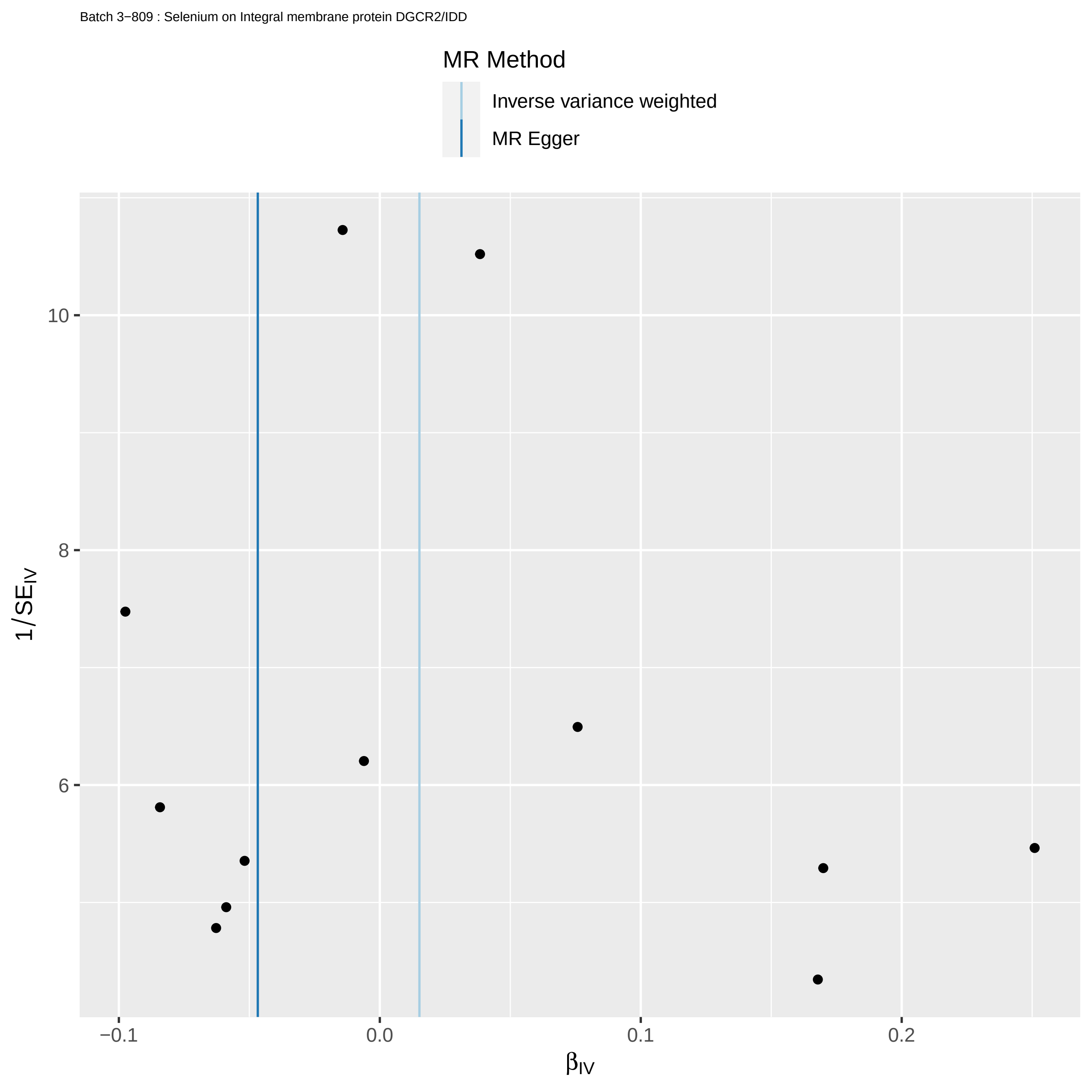
Batch 3−809 : Selenium on Integral membrane protein DGCR2/IDD
MR Method
Inverse variance weighted
MR Egger
10
8
6
1/SE_IV
−0.1
0.0
0.1
0.2
β_IV

Batch 3−810 : Selenium on Protein DGCR6

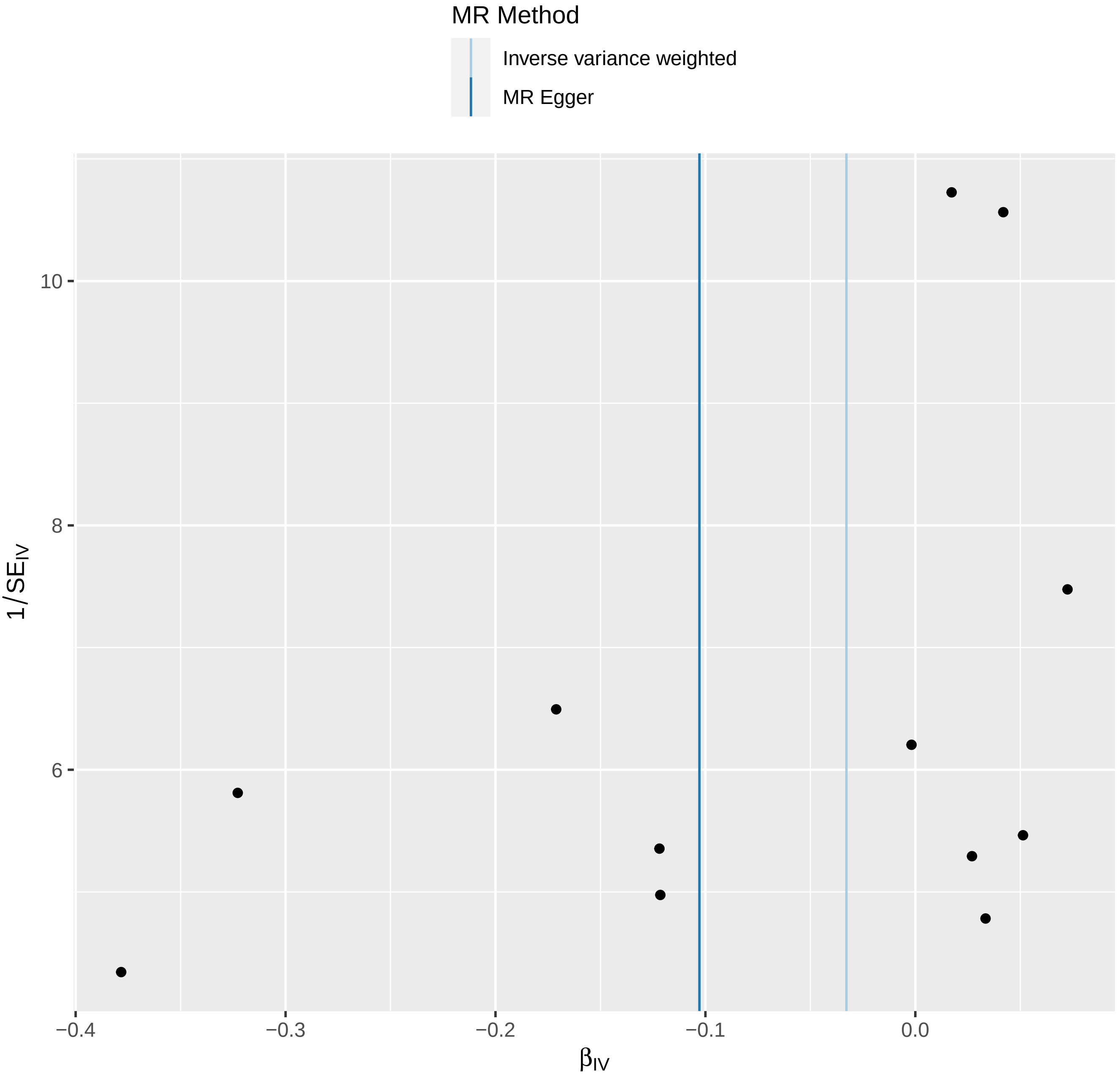

Batch 3−811 : Selenium on Diacylglycerol kinase beta

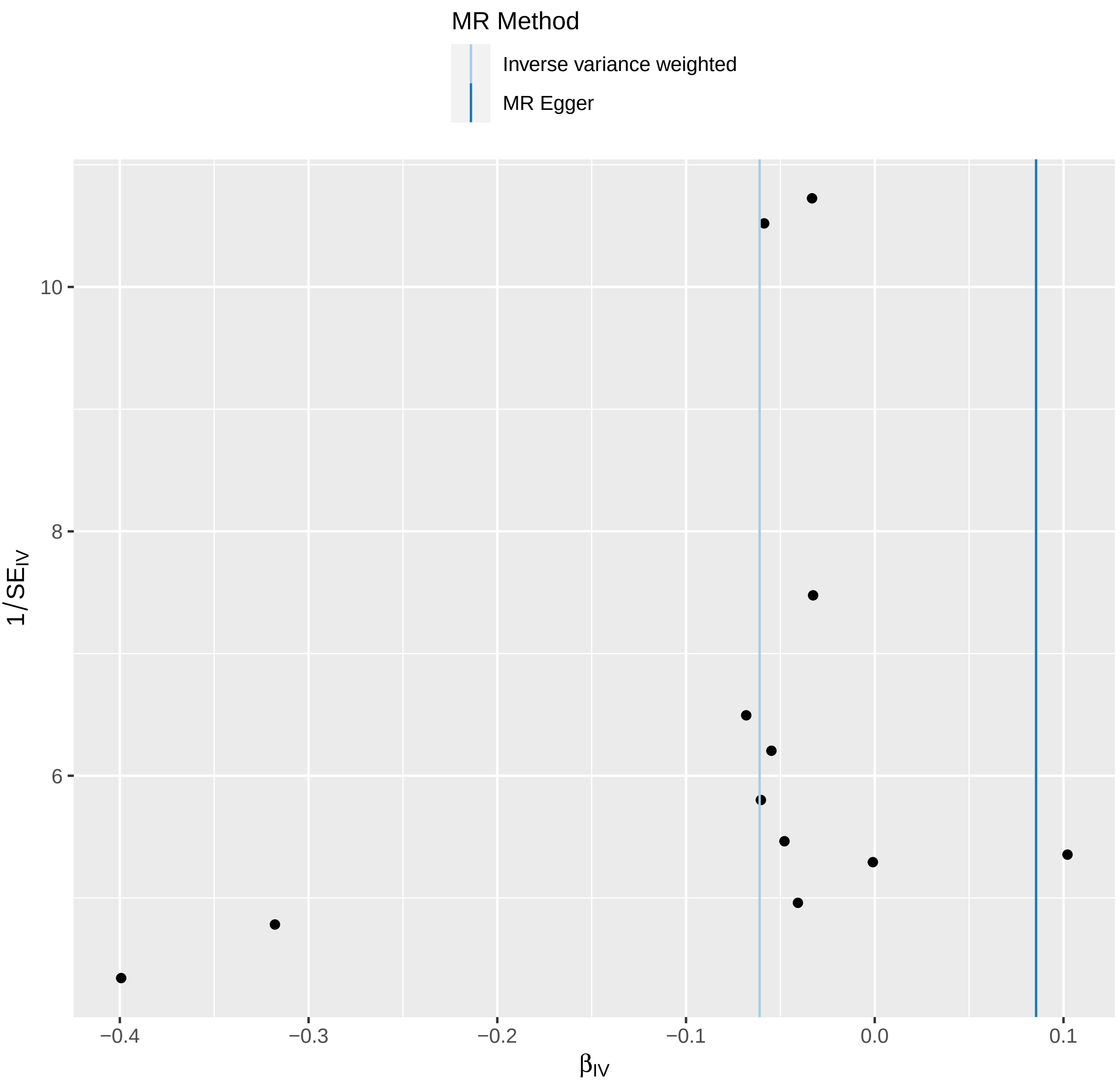

Batch 3−812 : Selenium on Dihydrofolate reductase

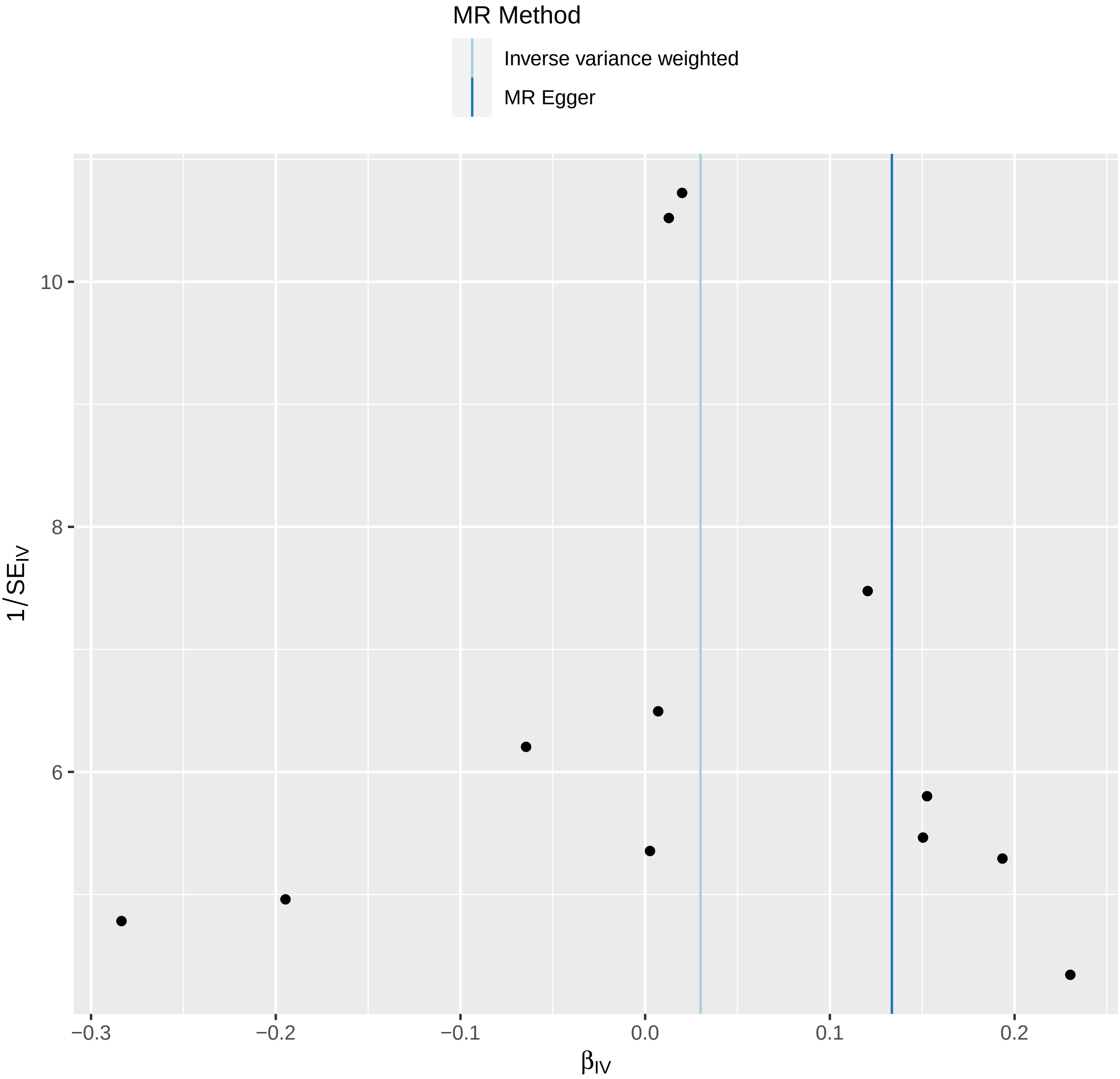

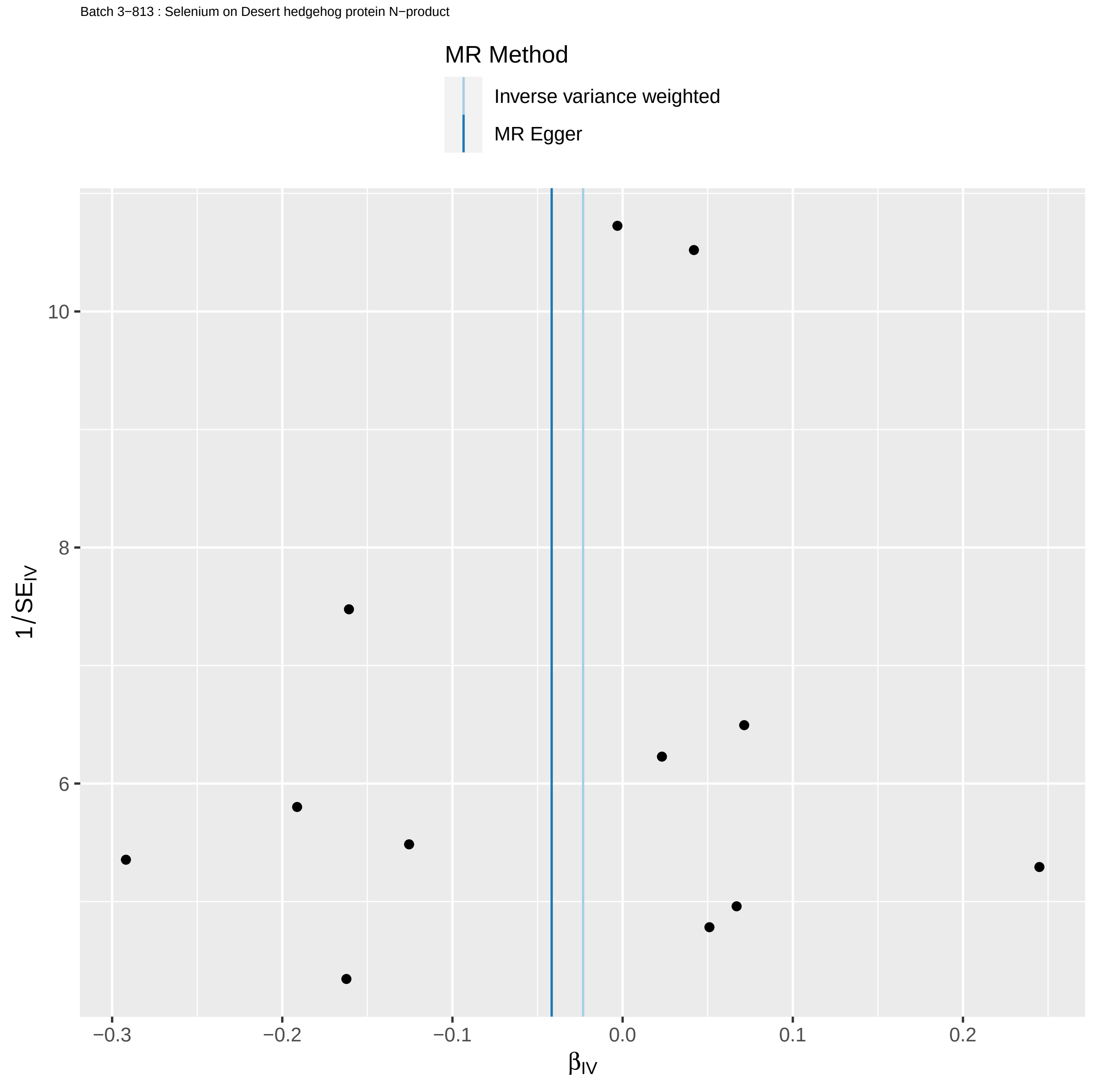

Batch 3−813 : Selenium on Desert hedgehog protein N−product
MR Method
Inverse variance weighted
MR Egger
10
8
6
$1/SE_{IV}$
−0.3
−0.2
−0.1
0.0
0.1
0.2
β_{IV}

Batch 3−814 : Selenium on Pre−mRNA−splicing factor ATP−dependent RNA helicase PRP16

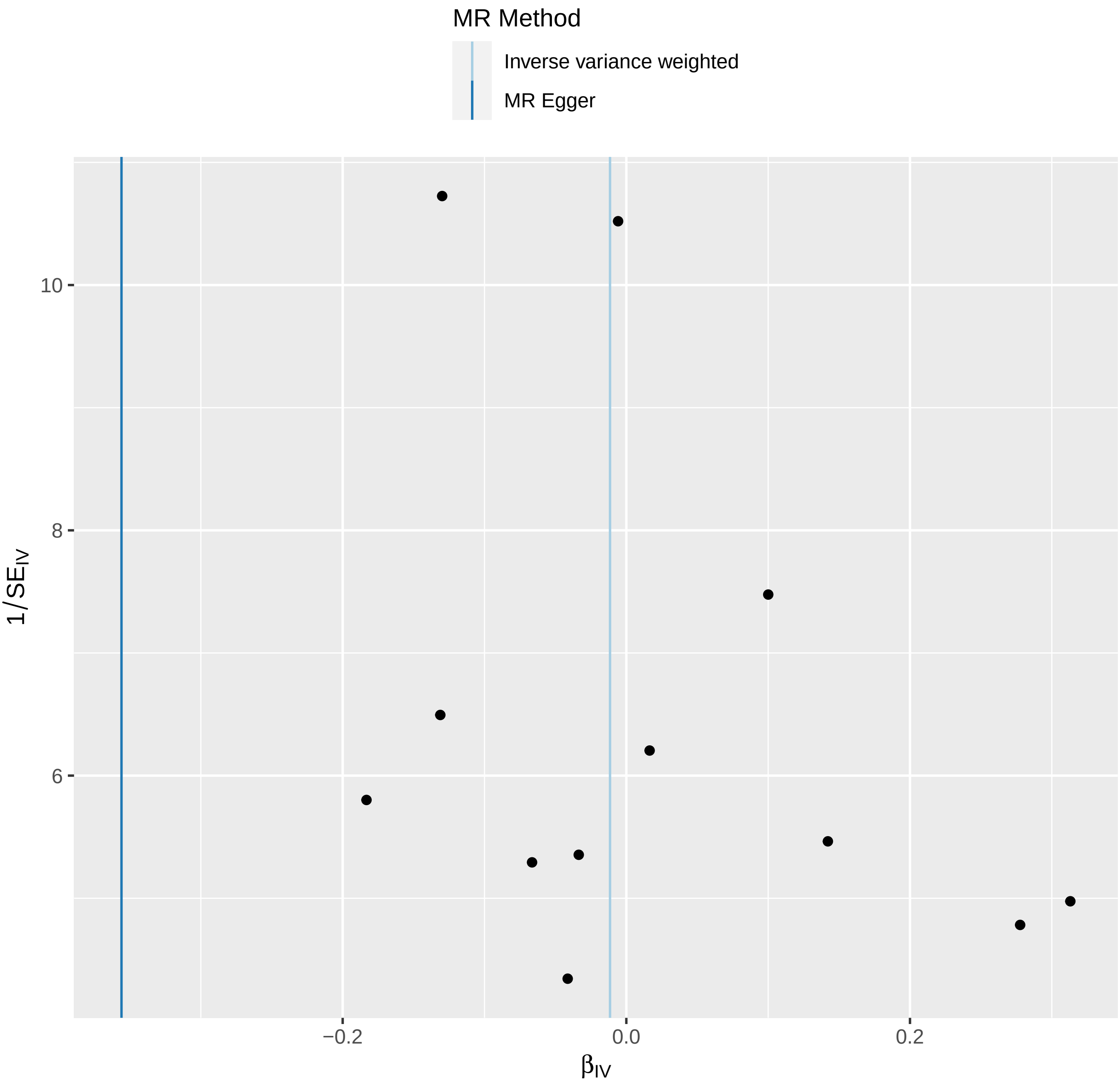

Batch 3−815 : Selenium on Probable ATP−dependent RNA helicase DHX58

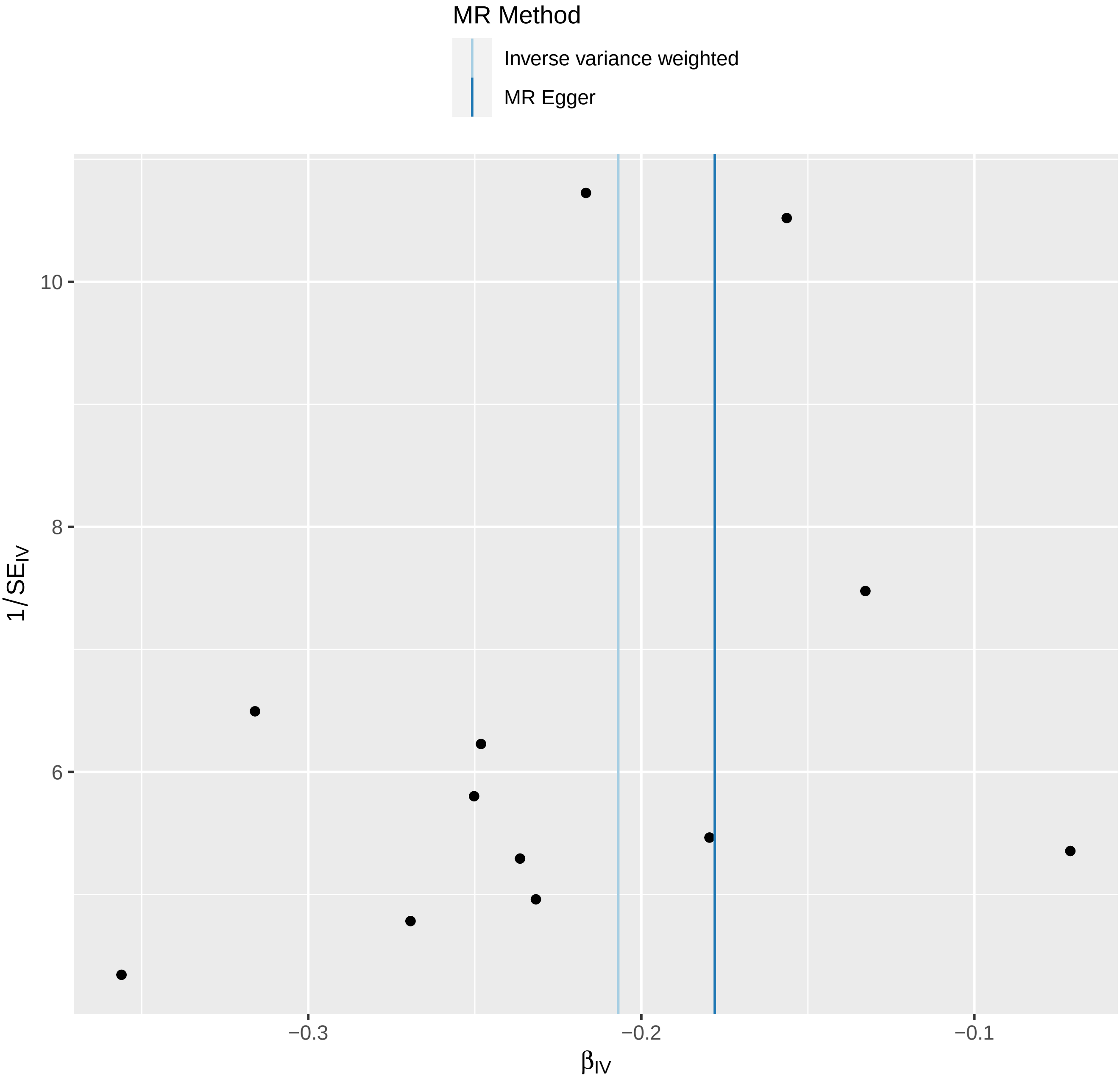

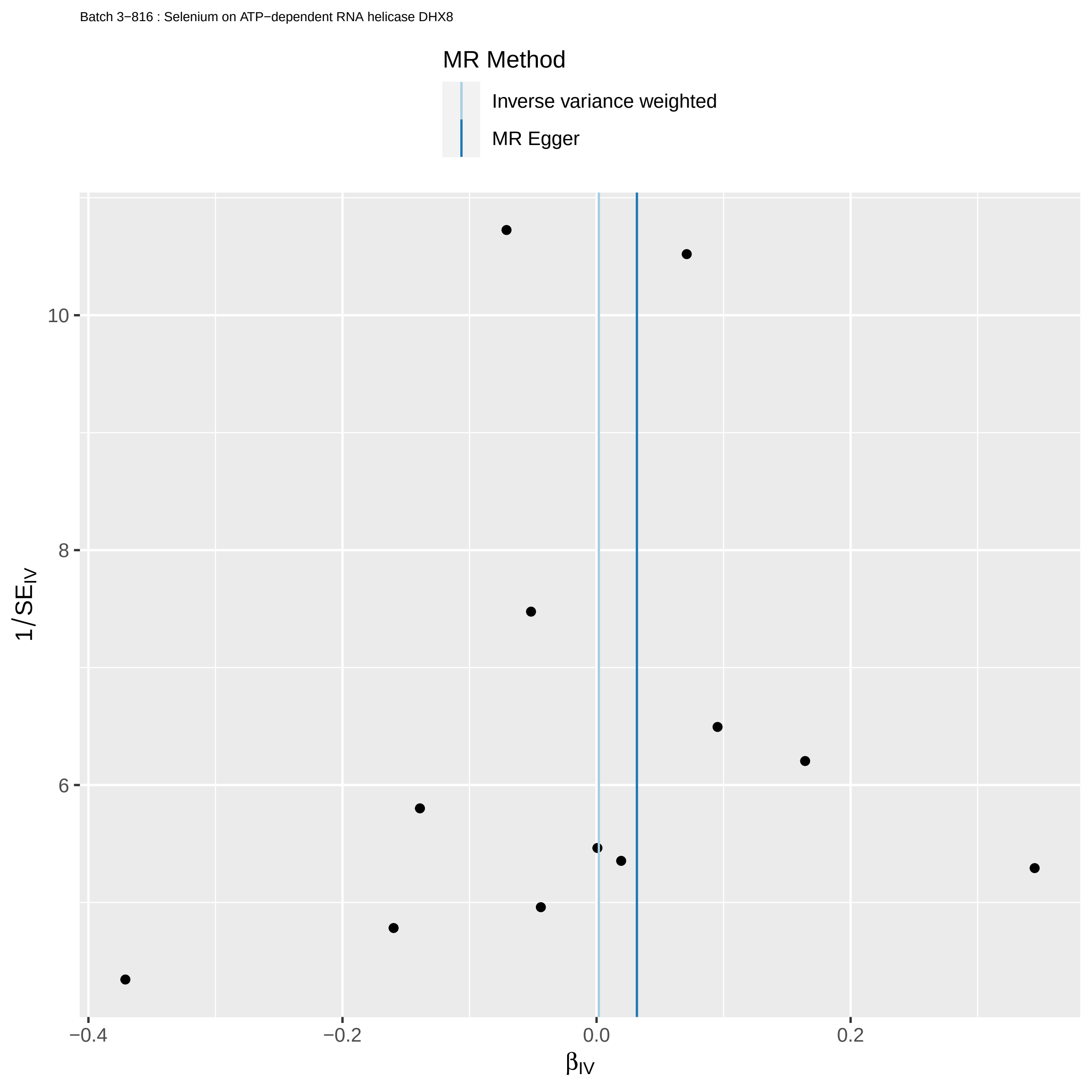

Batch 3−816 : Selenium on ATP−dependent RNA helicase DHX8
MR Method
Inverse variance weighted
MR Egger
10
8
6
$1/SE_{IV}$
−0.4
−0.2
0.0
0.2
β_{IV}

Batch 3−817 : Selenium on ATP−dependent RNA helicase A

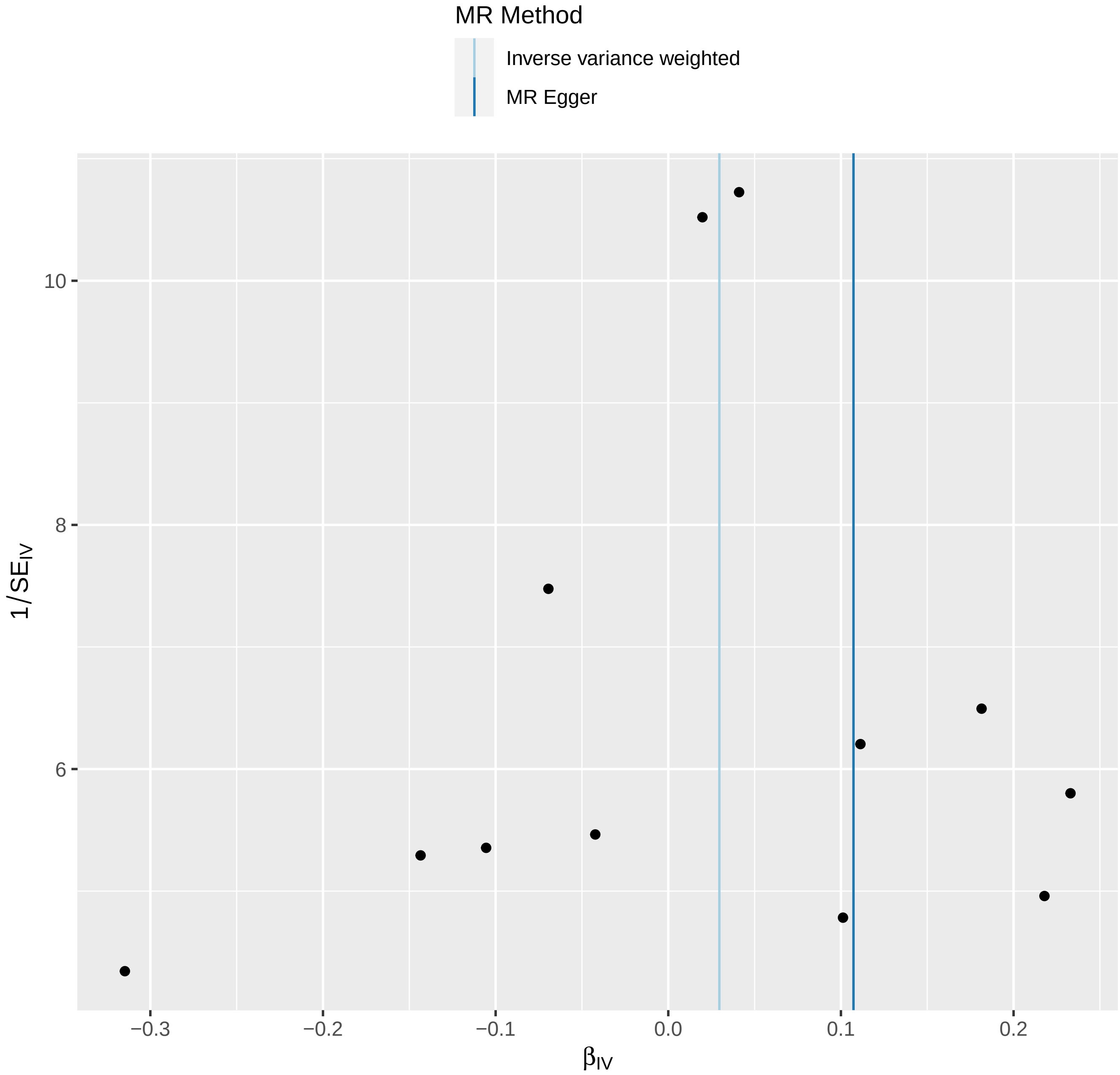

Batch 3−818 : Selenium on Diablo homolog, mitochondrial

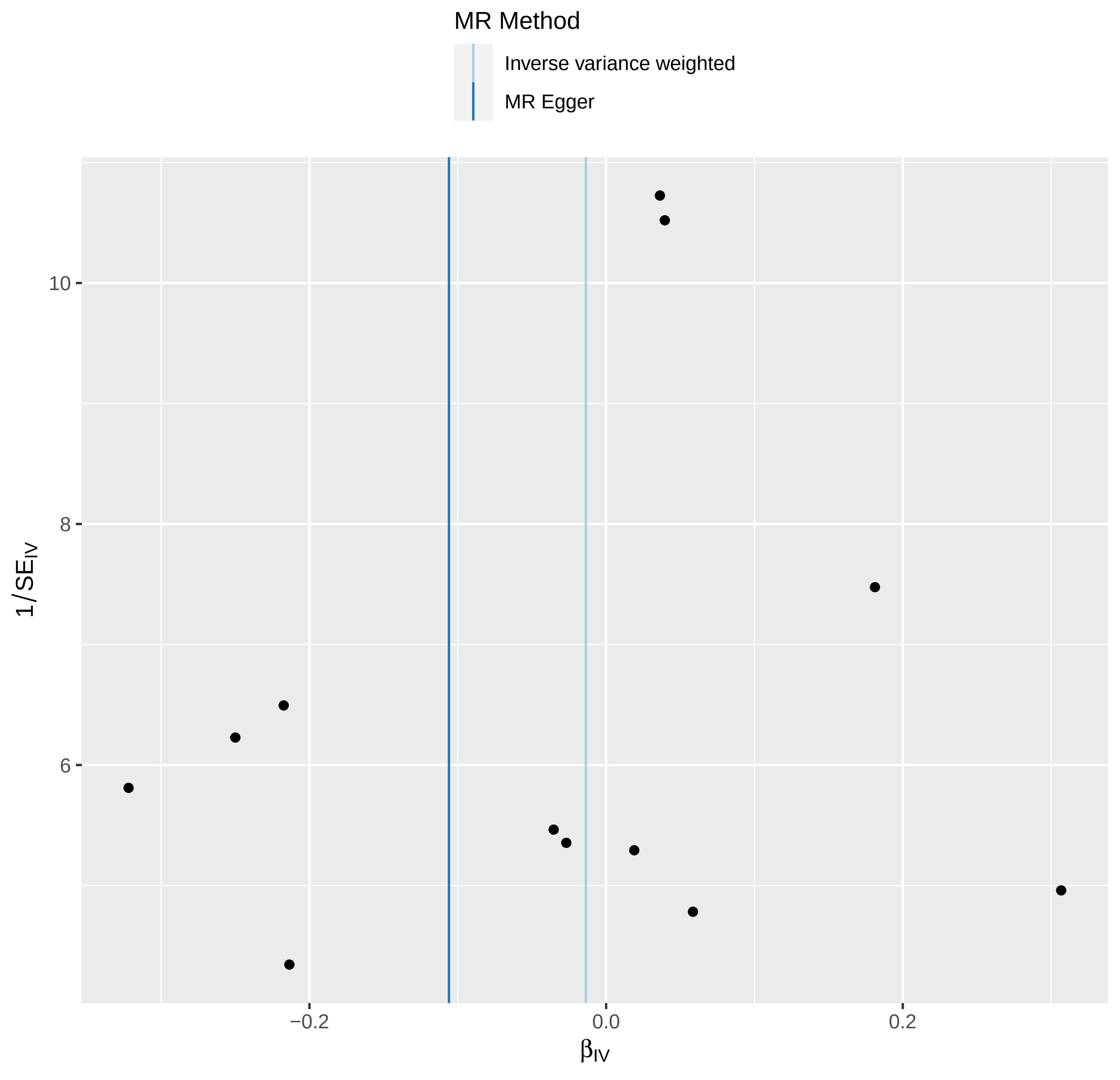

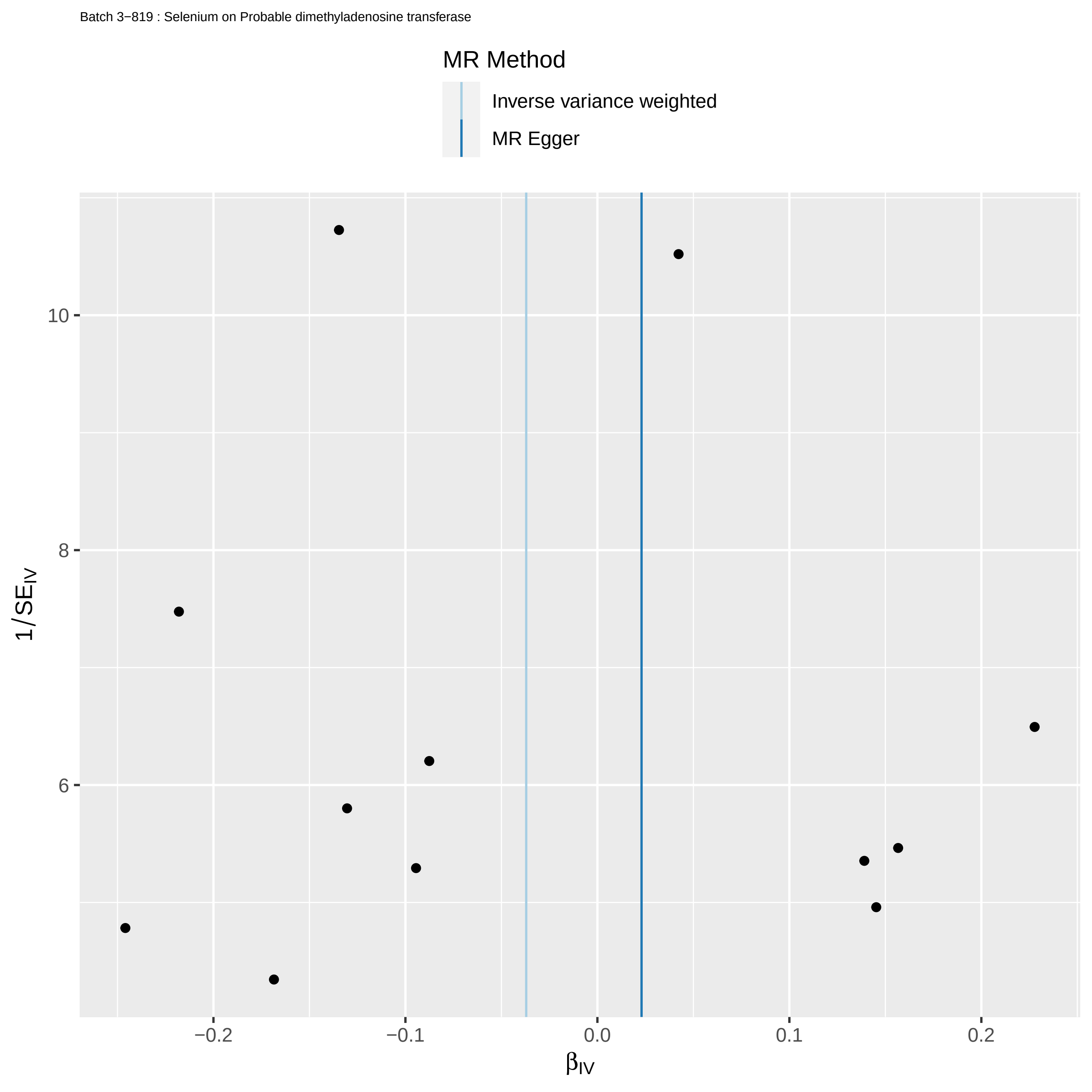
Batch 3−819 : Selenium on Probable dimethyladenosine transferase
MR Method
Inverse variance weighted
MR Egger
10
8
6
1/SE$_{IV}$
−0.2
−0.1
0.0
0.1
0.2
β$_{IV}$

Batch 3−820 : Selenium on GTP−binding protein Di−Ras3

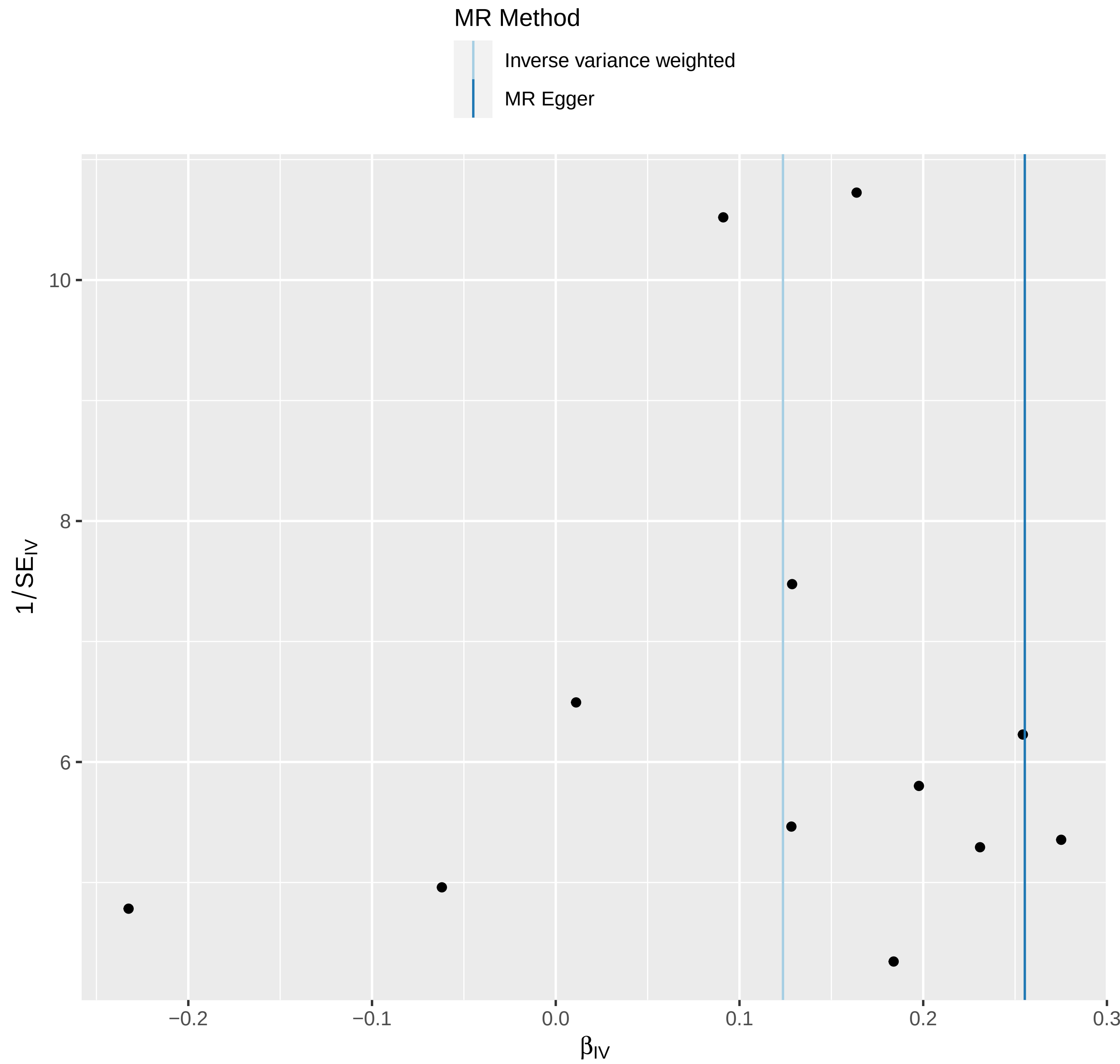

Batch 3−821 : Selenium on Dickkopf−related protein 1

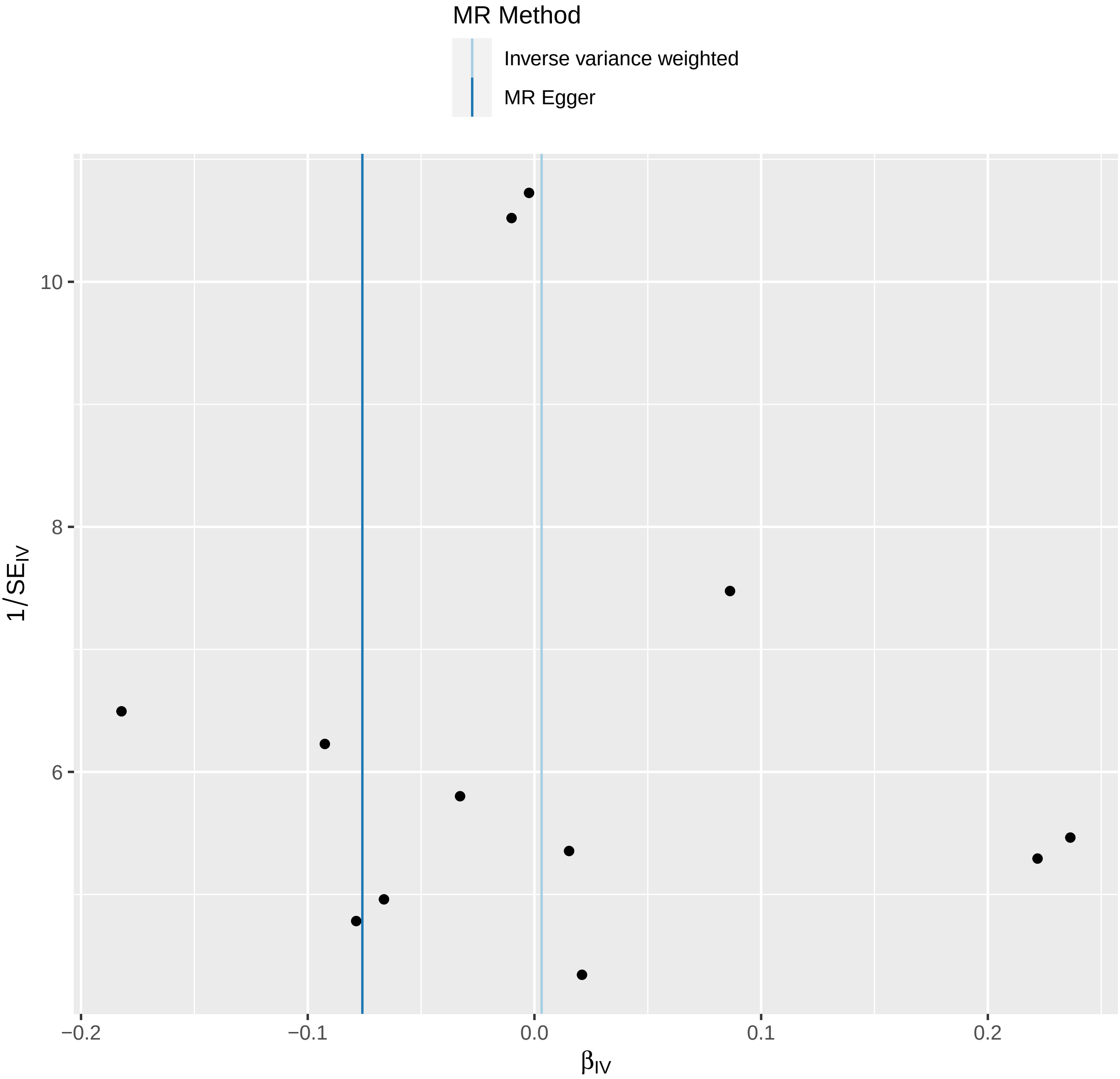

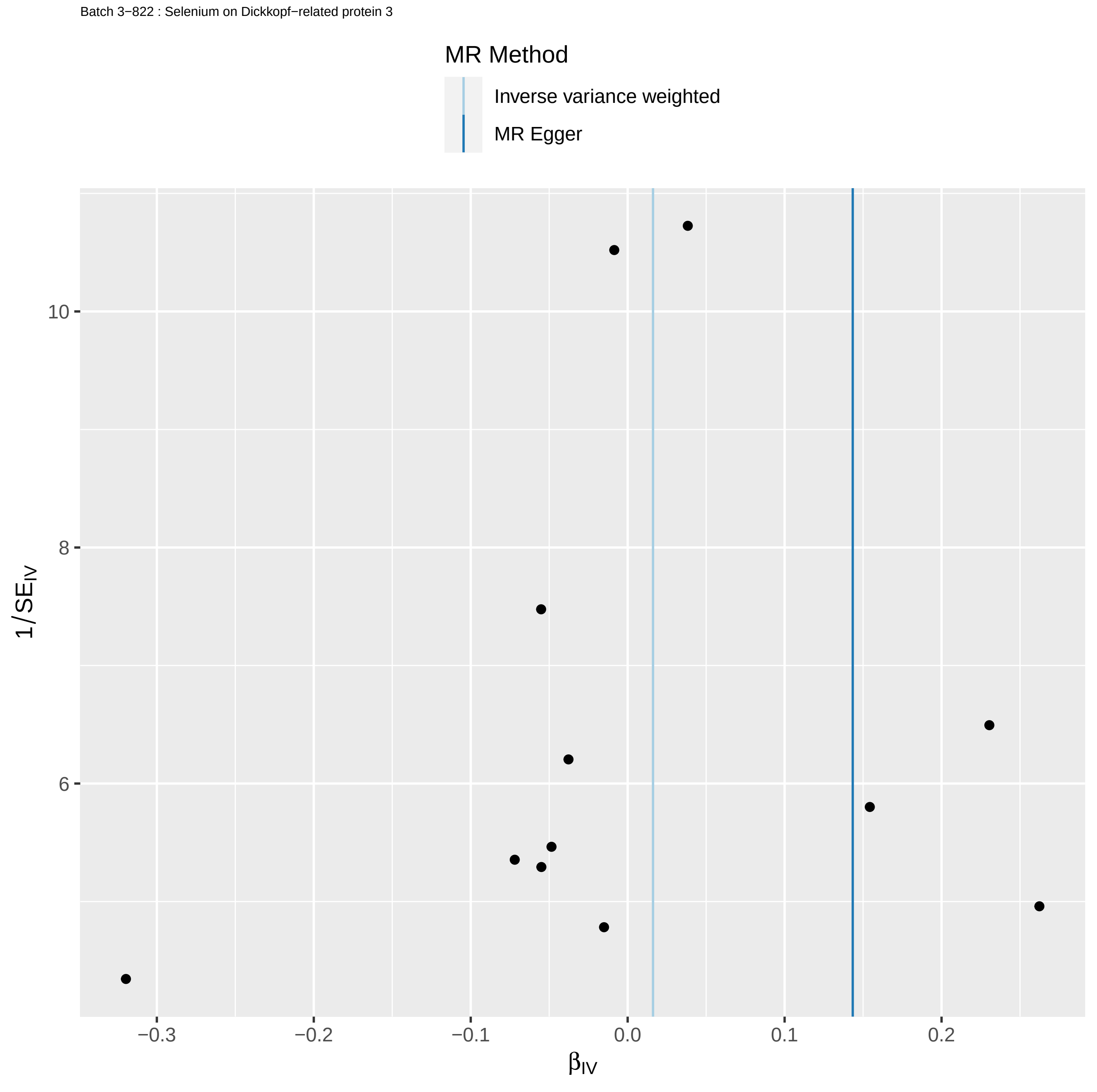
Batch 3−822 : Selenium on Dickkopf−related protein 3
MR Method
Inverse variance weighted
MR Egger
10
8
6
1/SE_IV
−0.3
−0.2
−0.1
0.0
0.1
0.2
β_IV

Batch 3−823 : Selenium on Dickkopf−related protein 4

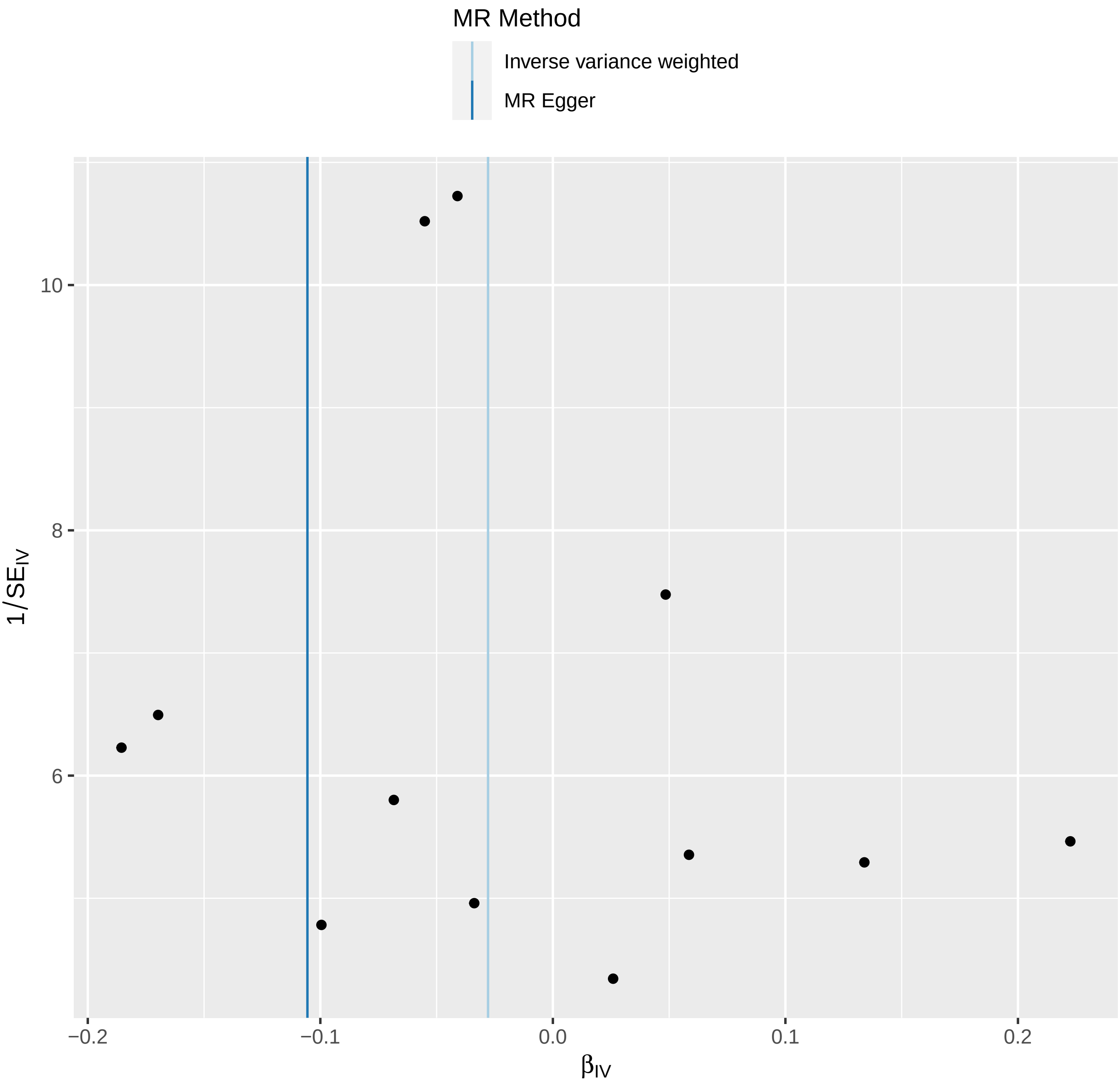

Batch 3−824 : Selenium on Dickkopf−like protein 1

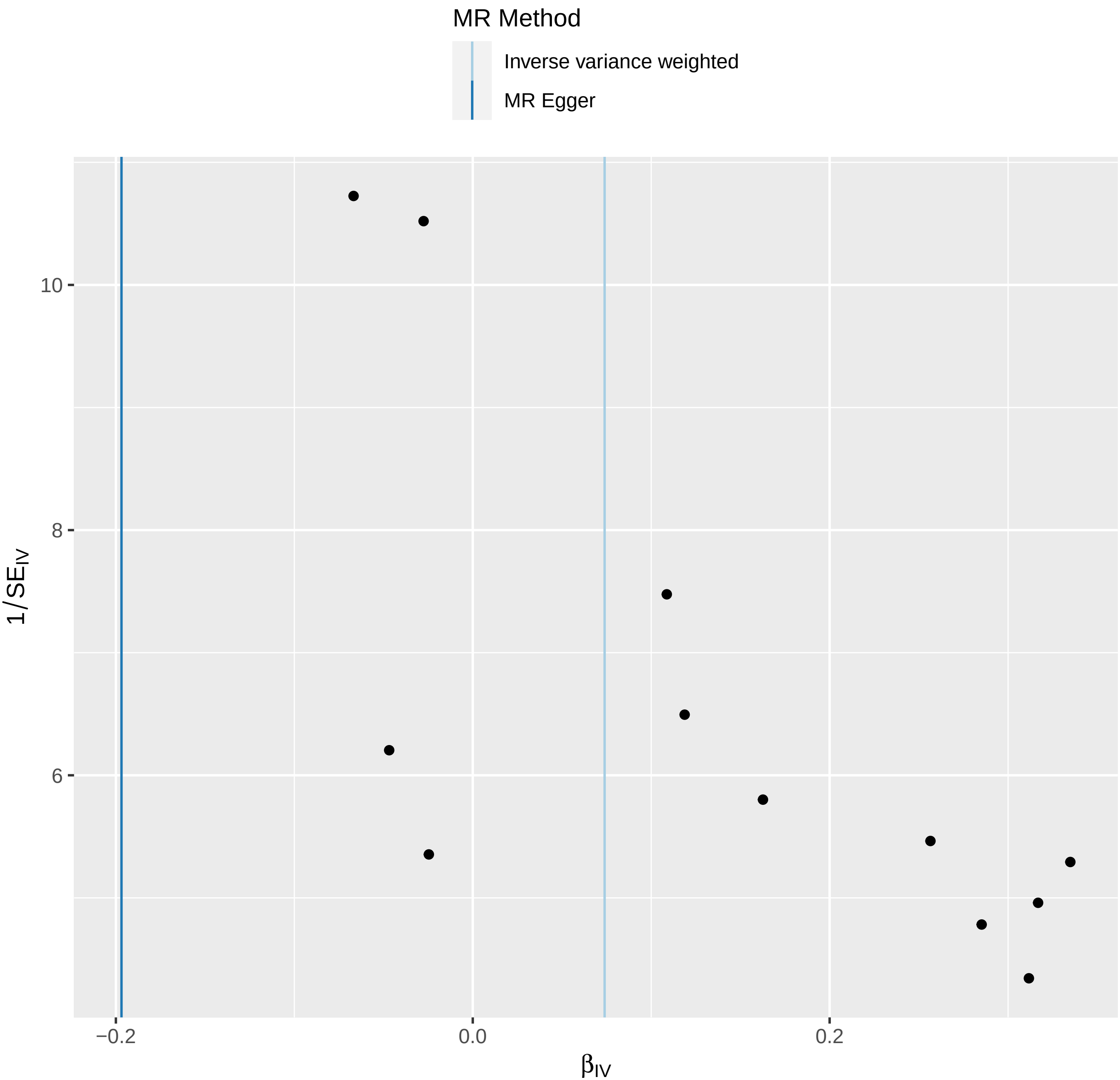

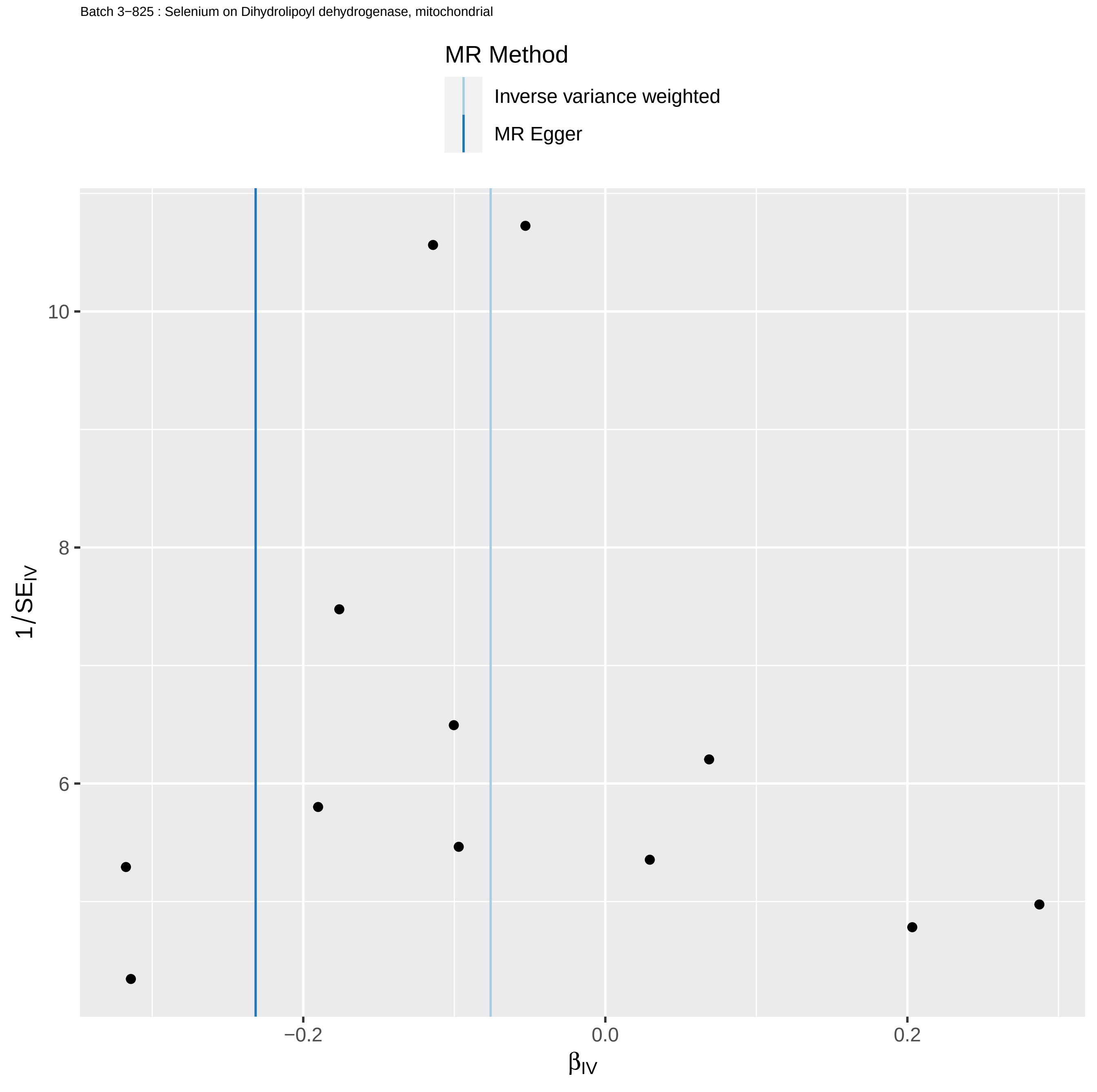
Batch 3−825 : Selenium on Dihydrolipoyl dehydrogenase, mitochondrial
MR Method
Inverse variance weighted
MR Egger
10
8
6
1/SE_IV
−0.2
0.0
0.2
β_IV

Batch 3−826 : Selenium on Disks large homolog 3

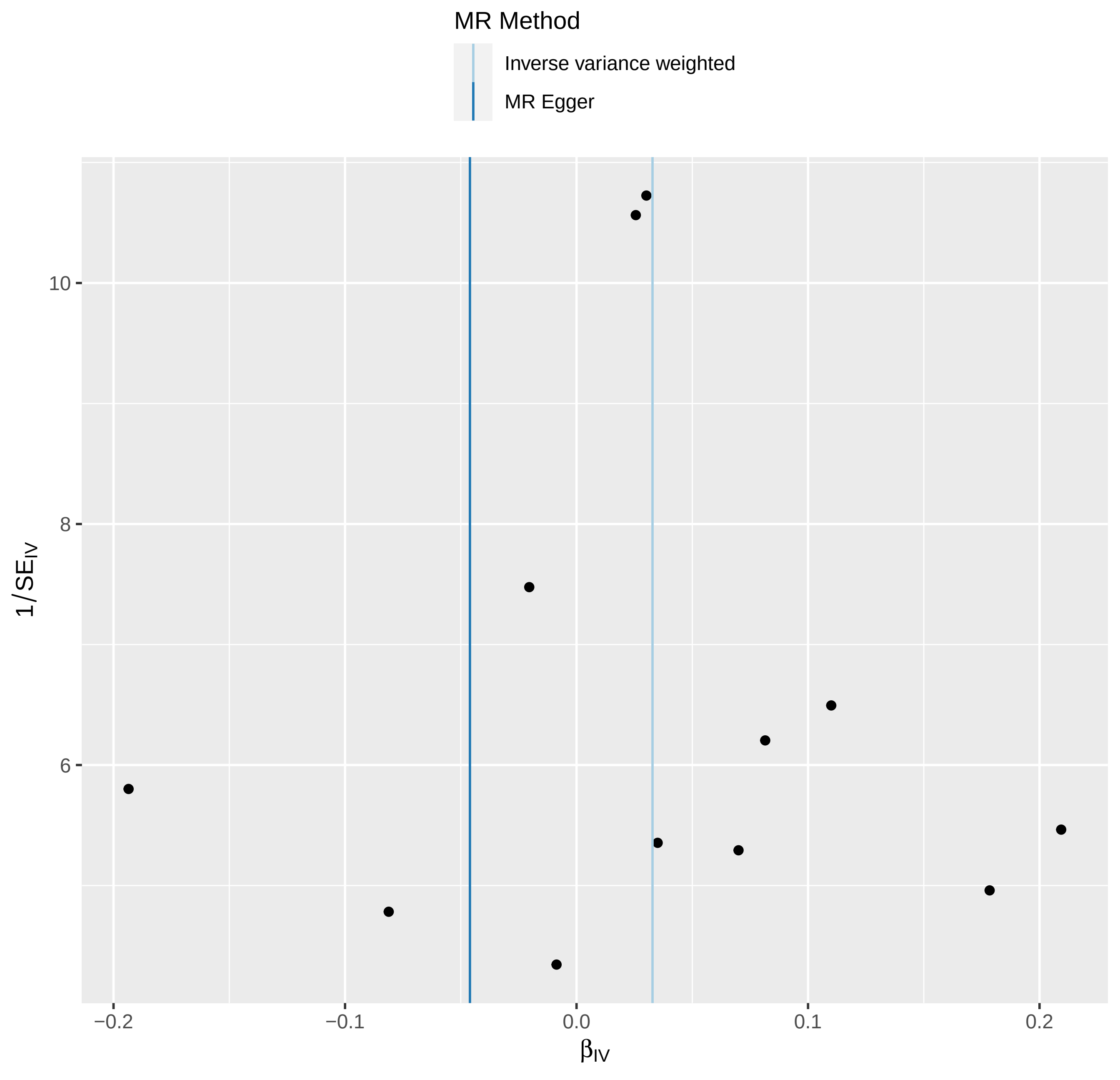

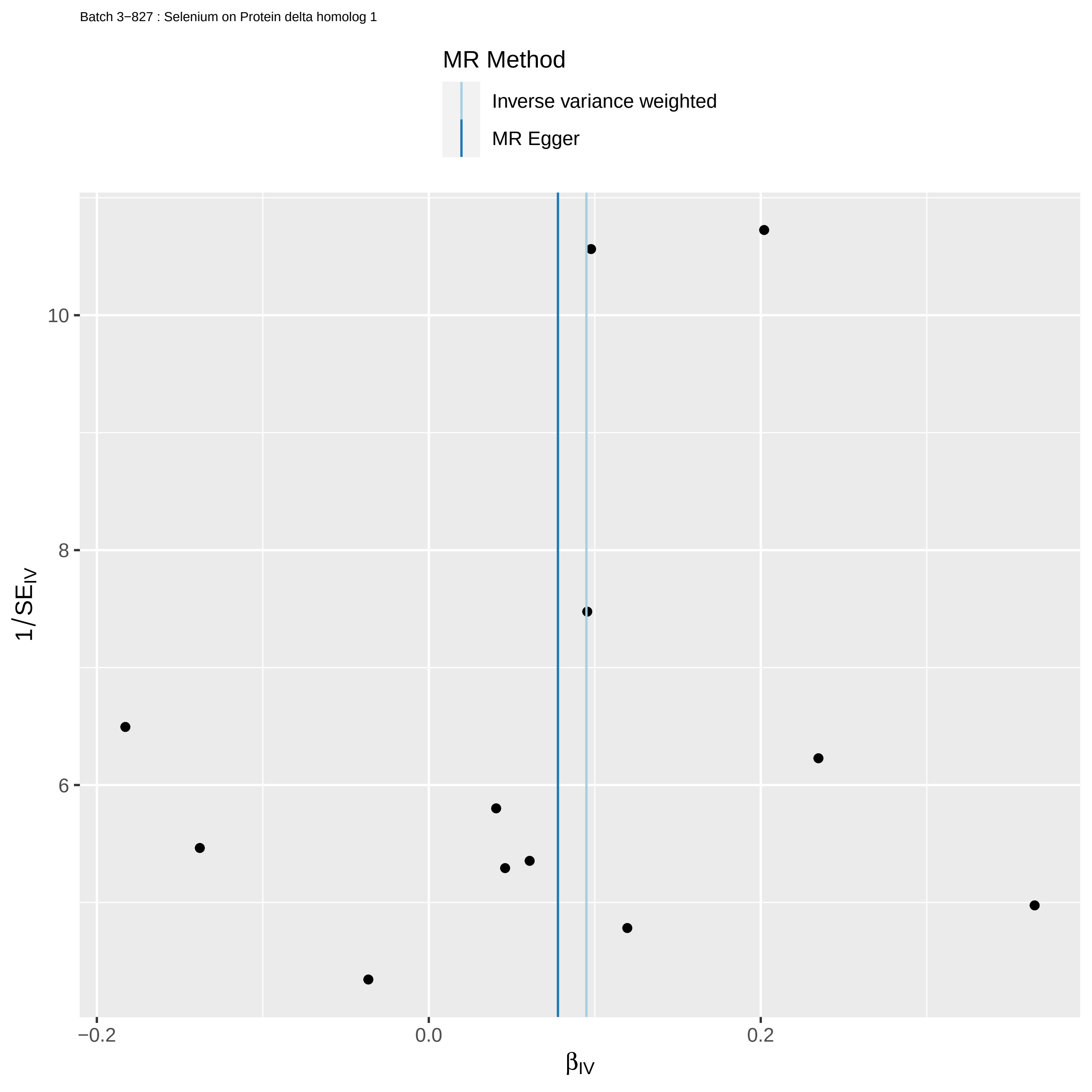
Batch 3−827 : Selenium on Protein delta homolog 1
MR Method
Inverse variance weighted
MR Egger
10
8
6
1/SE_IV
−0.2
0.0
0.2
β_IV

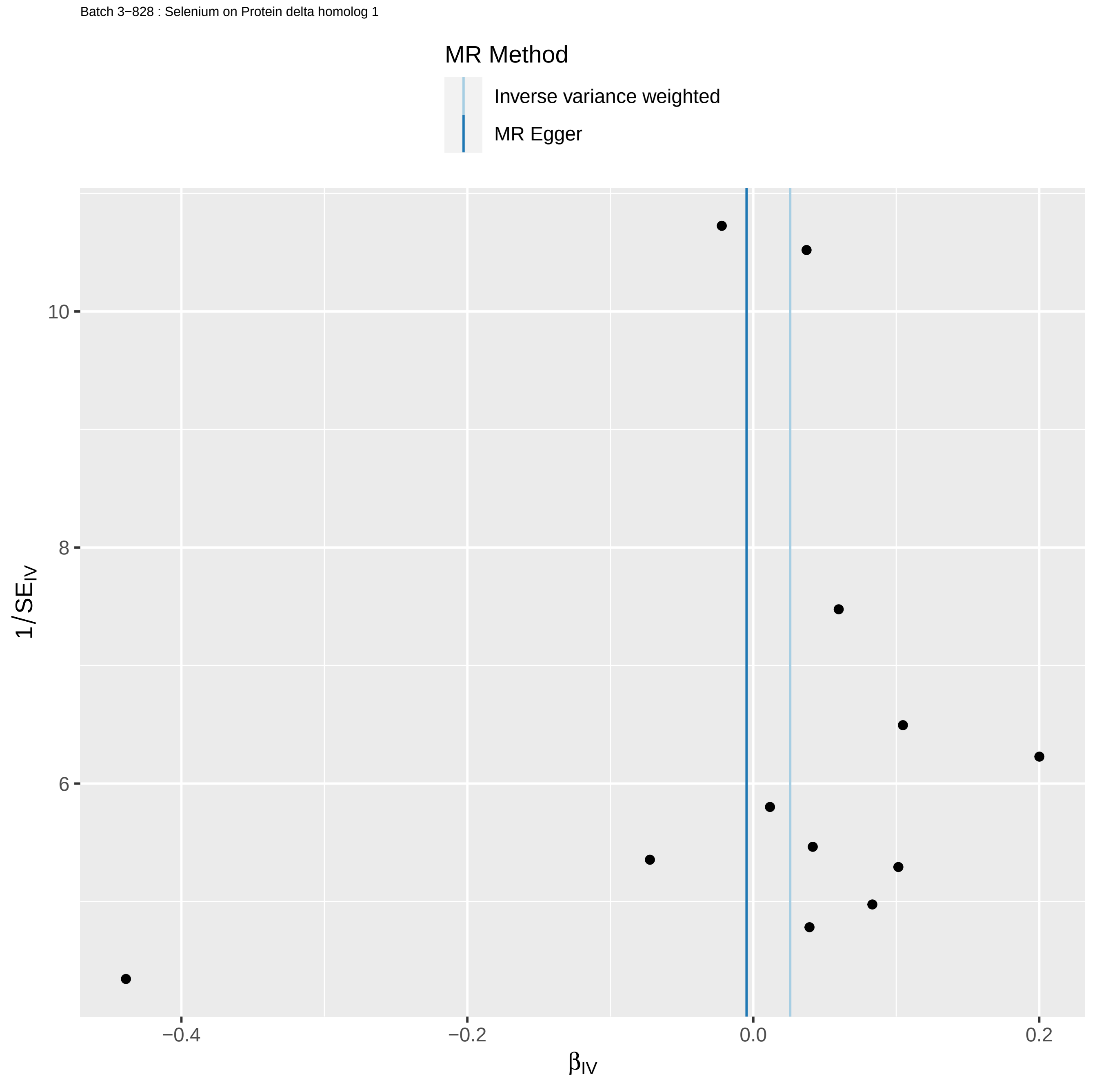
Batch 3−828 : Selenium on Protein delta homolog 1
MR Method
Inverse variance weighted
MR Egger
10
8
6
1/SE_IV
−0.4
−0.2
0.0
0.2
β_IV

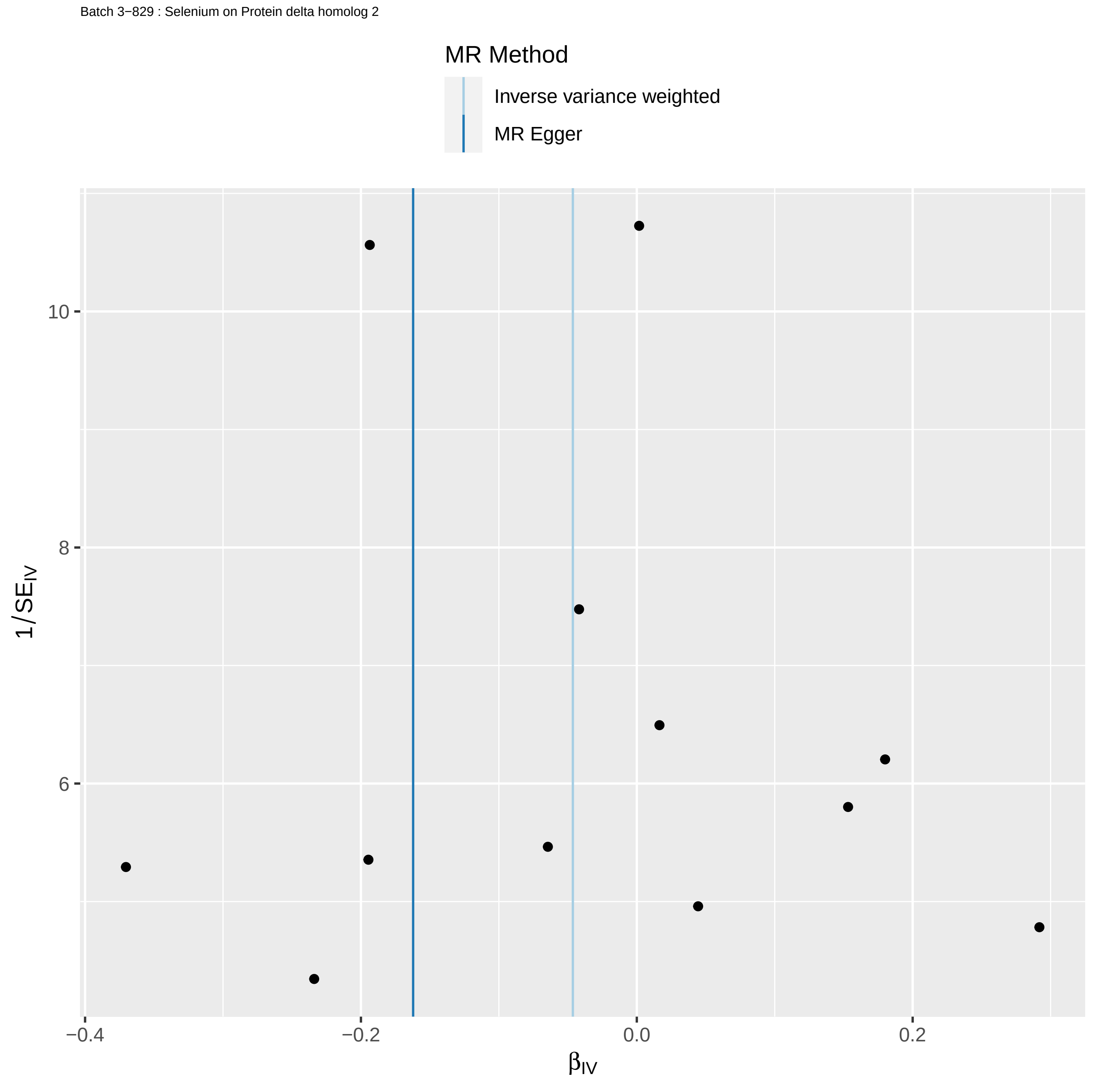
Batch 3−829 : Selenium on Protein delta homolog 2
MR Method
Inverse variance weighted
MR Egger
10
8
6
1/SE_IV
−0.4
−0.2
0.0
0.2
β_IV

Batch 3−830 : Selenium on Delta−like protein 1

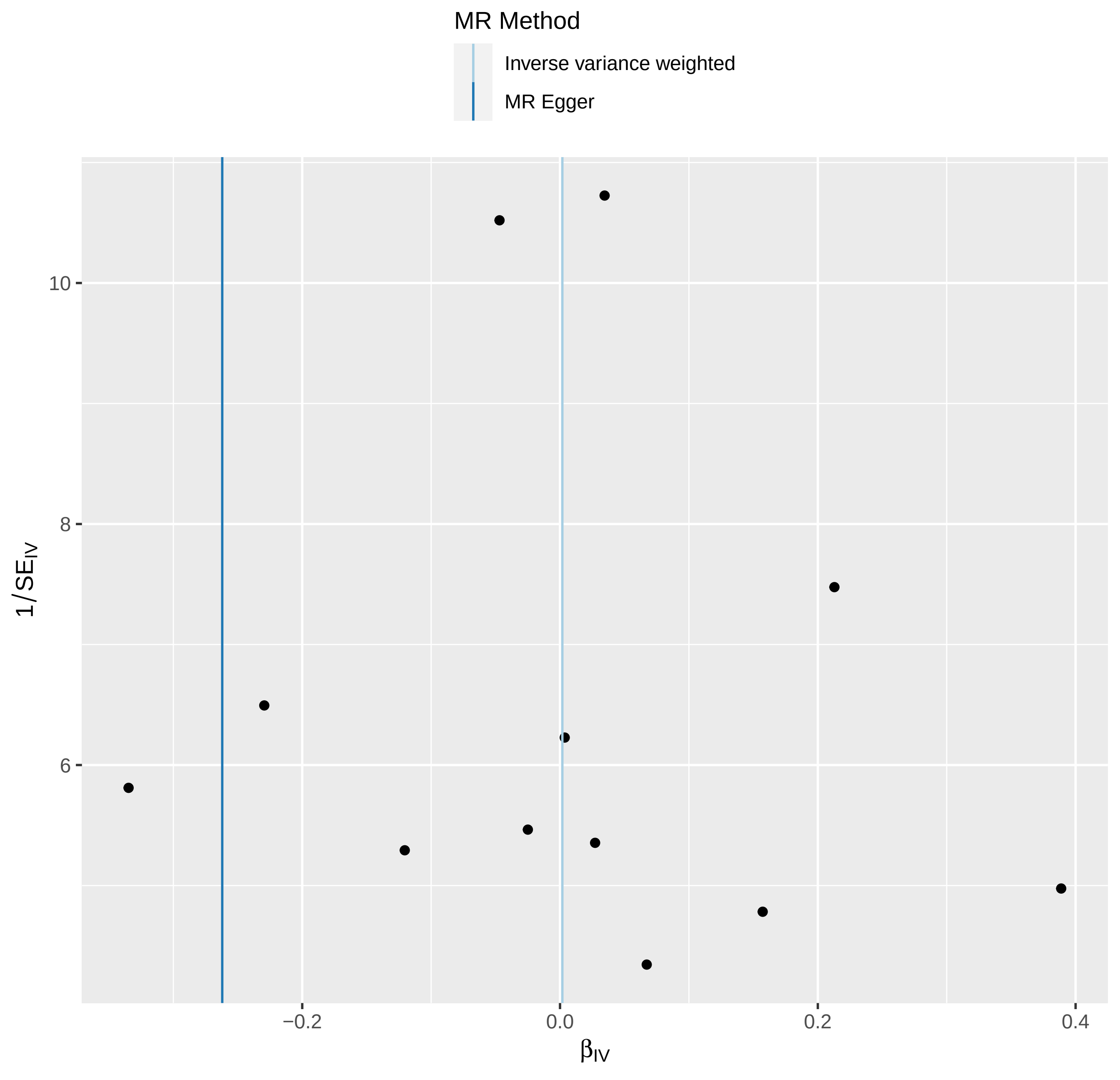

Batch 3−831 : Selenium on Delta−like protein 1

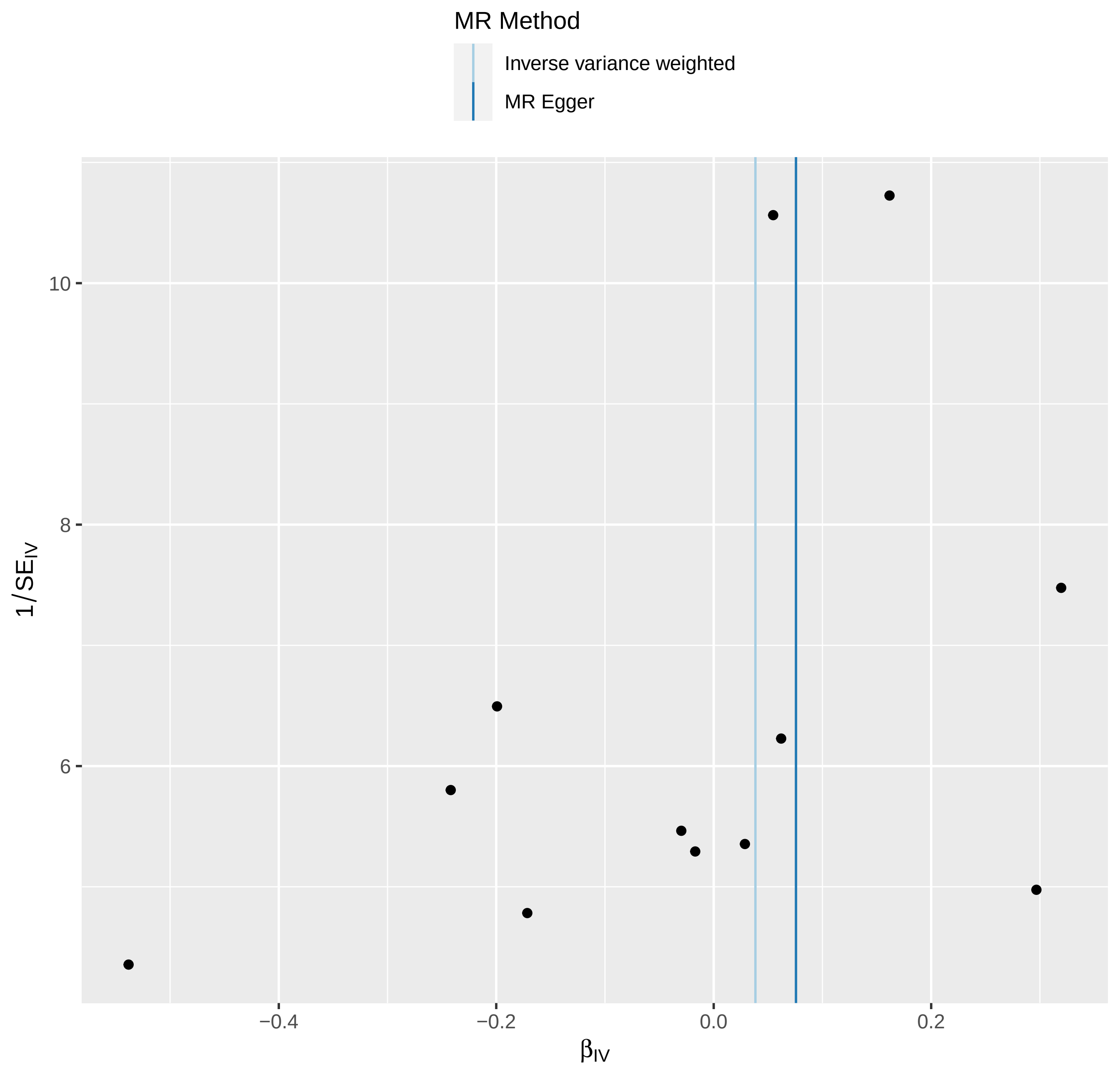

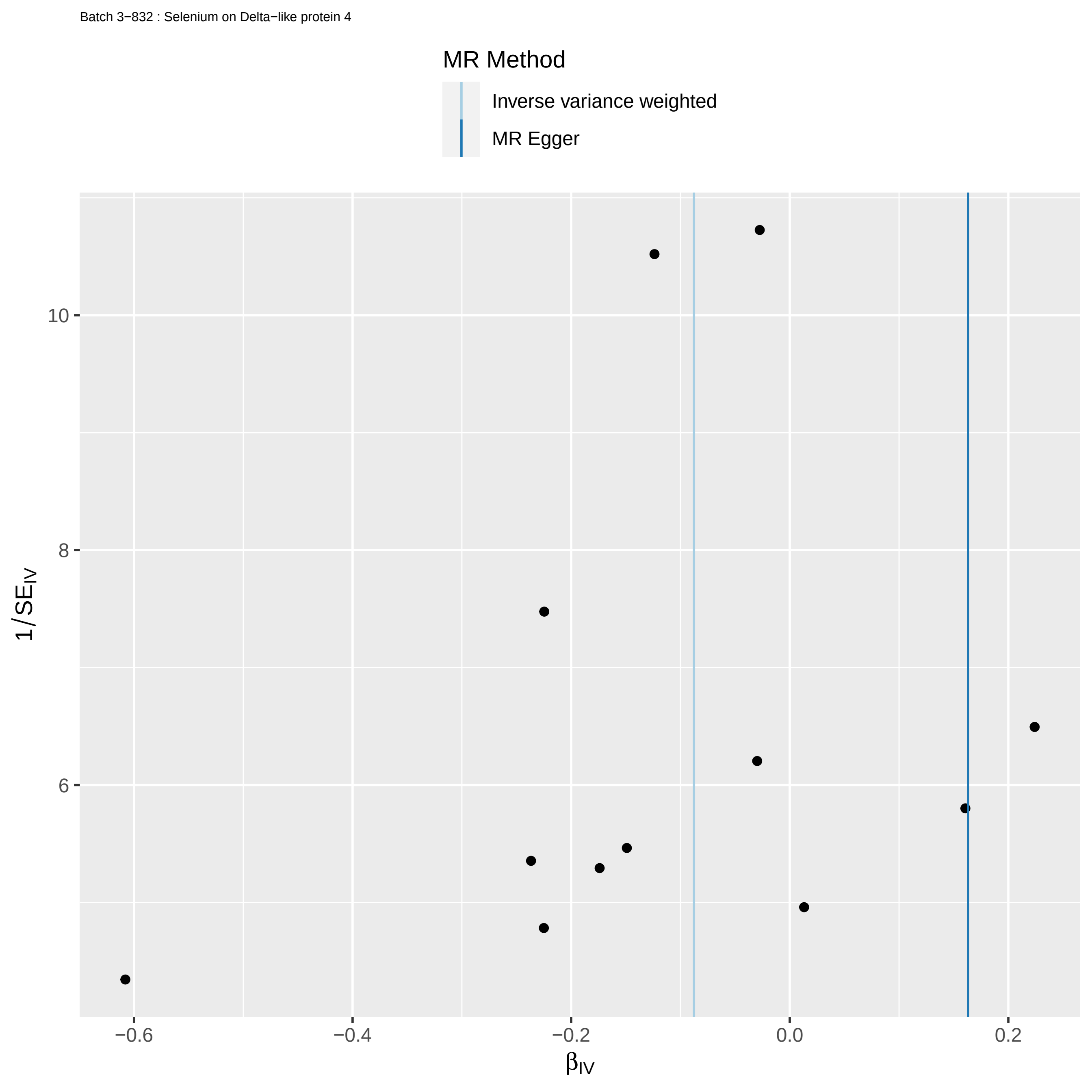

Batch 3−832 : Selenium on Delta−like protein 4
MR Method
Inverse variance weighted
MR Egger
10
8
6
1/SE_IV
−0.6
−0.4
−0.2
0.0
0.2
β_IV

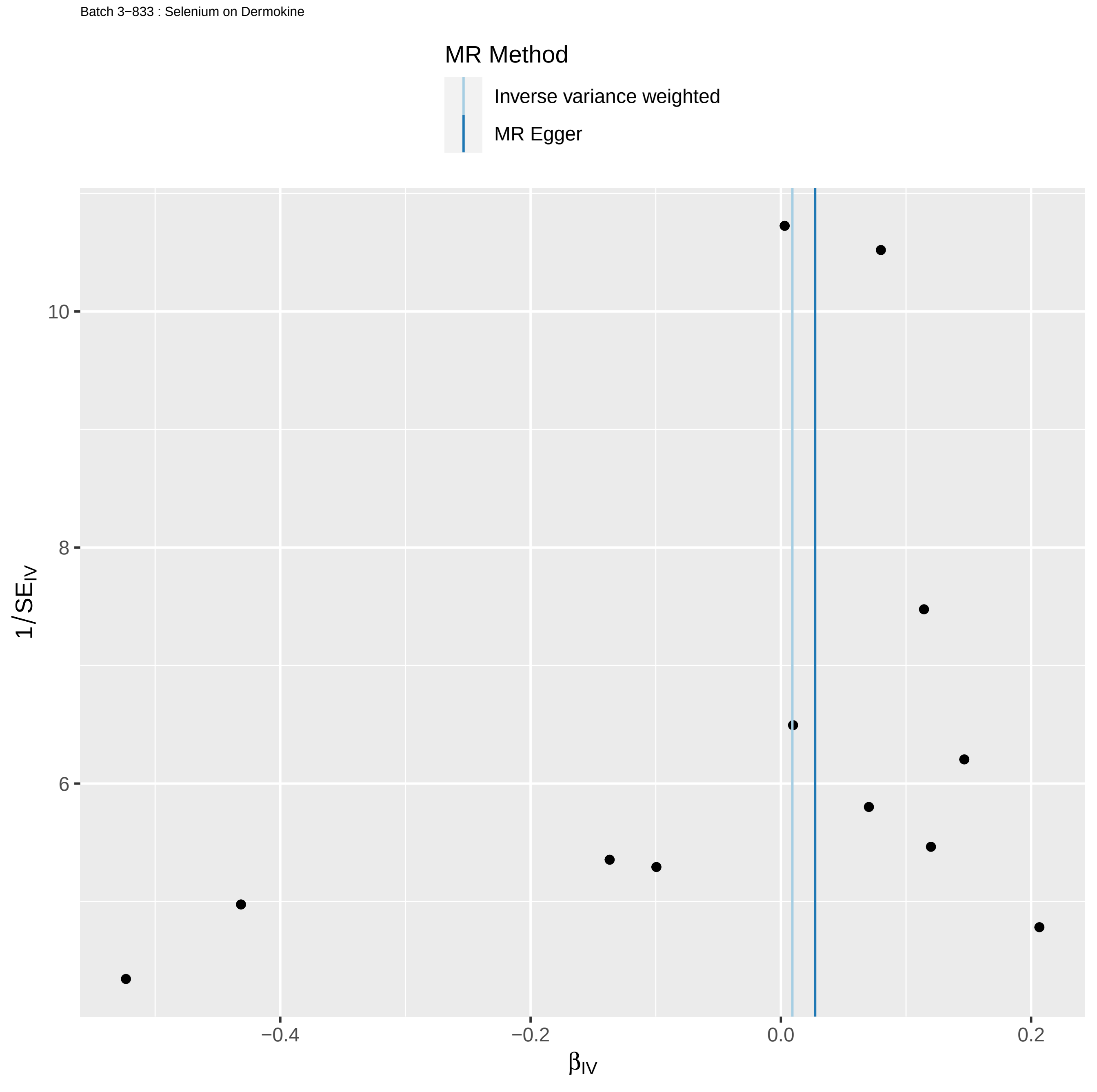

Batch 3−833 : Selenium on Dermokine
MR Method
Inverse variance weighted
MR Egger
10
8
6
1/SE_IV
−0.4
−0.2
0.0
0.2
β_IV

Batch 3−834 : Selenium on Myotonin−protein kinase

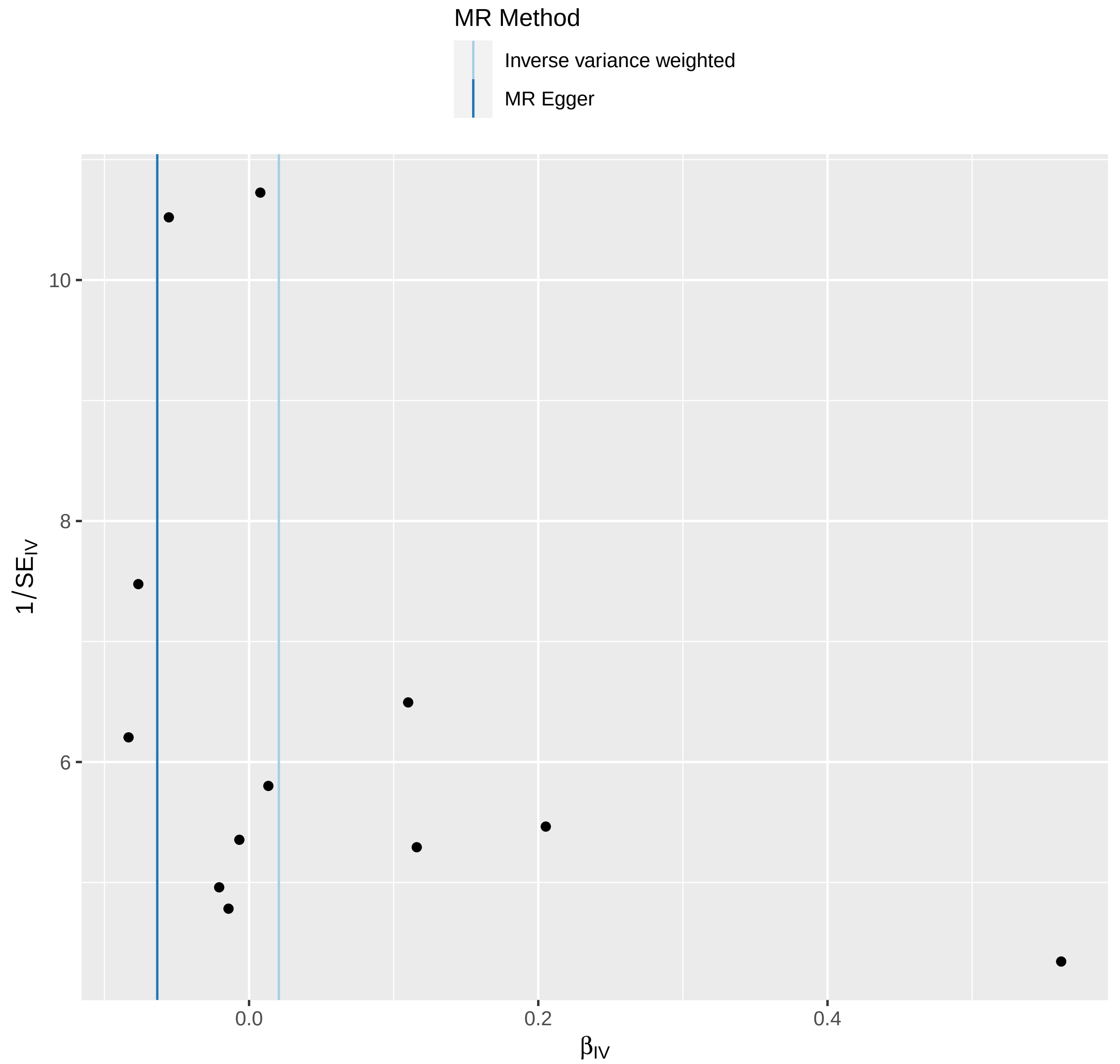

Batch 3−835 : Selenium on DnaJ homolog subfamily A member 4

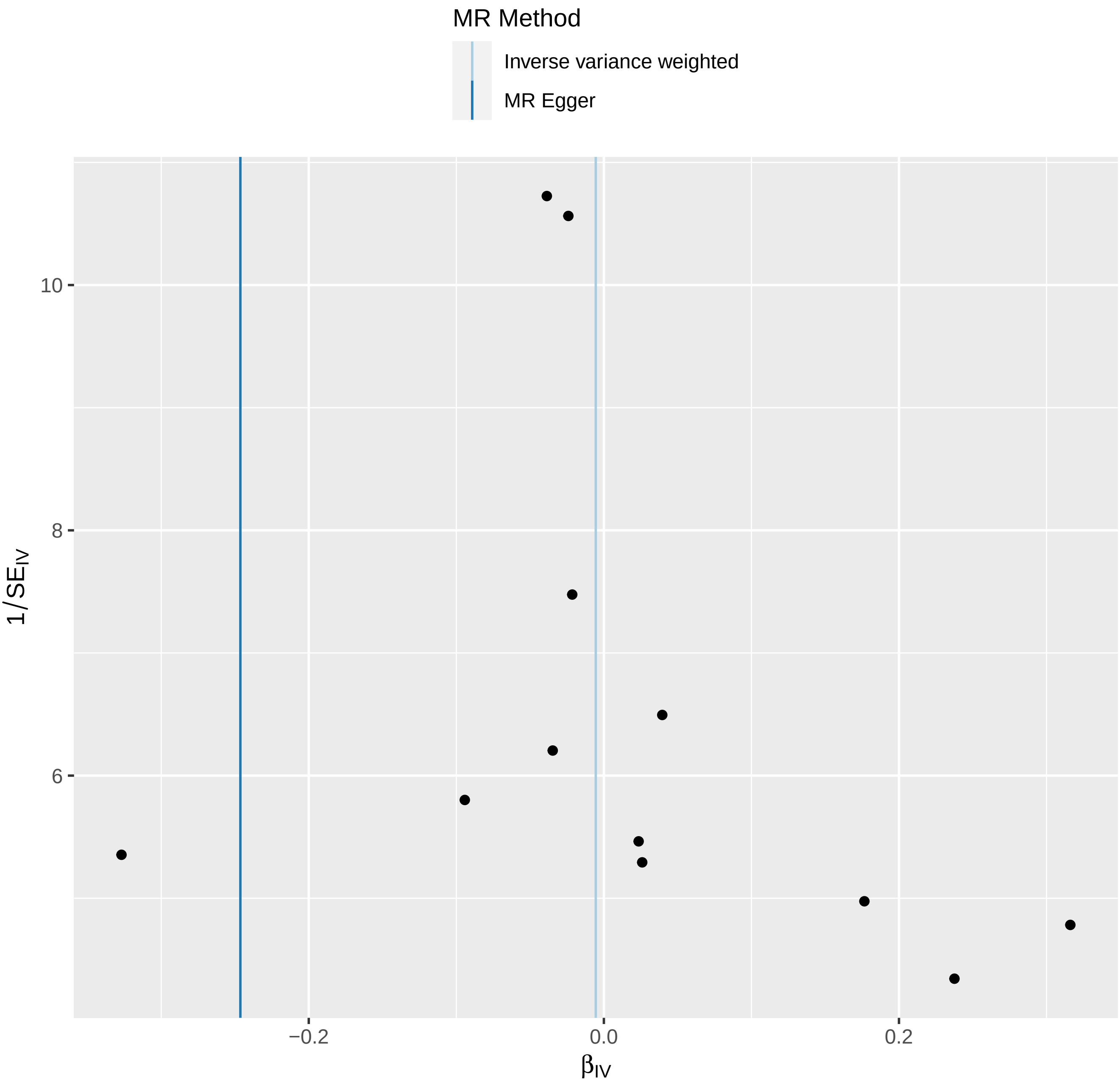

Batch 3−836 : Selenium on DnaJ homolog subfamily B member 11

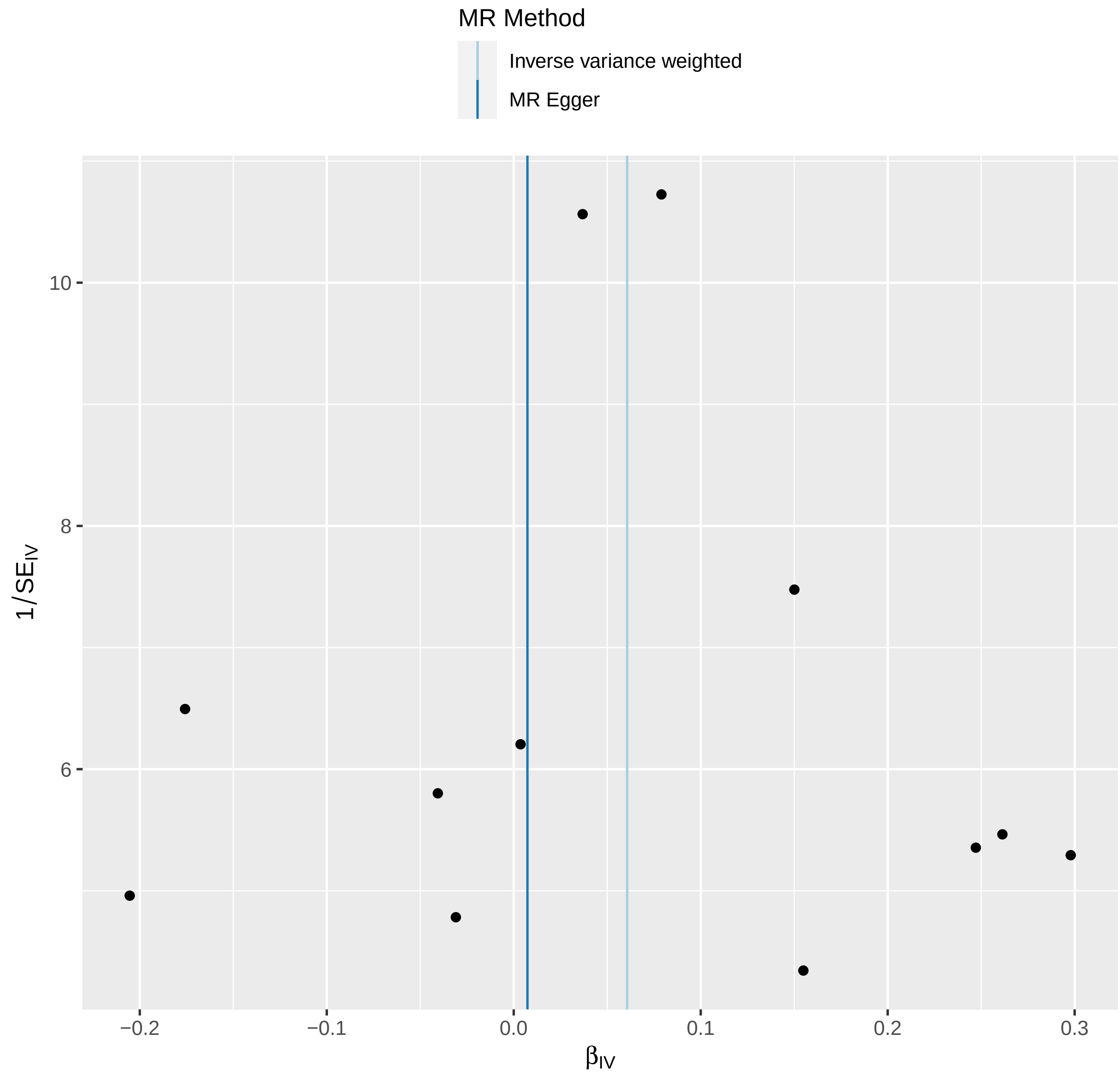

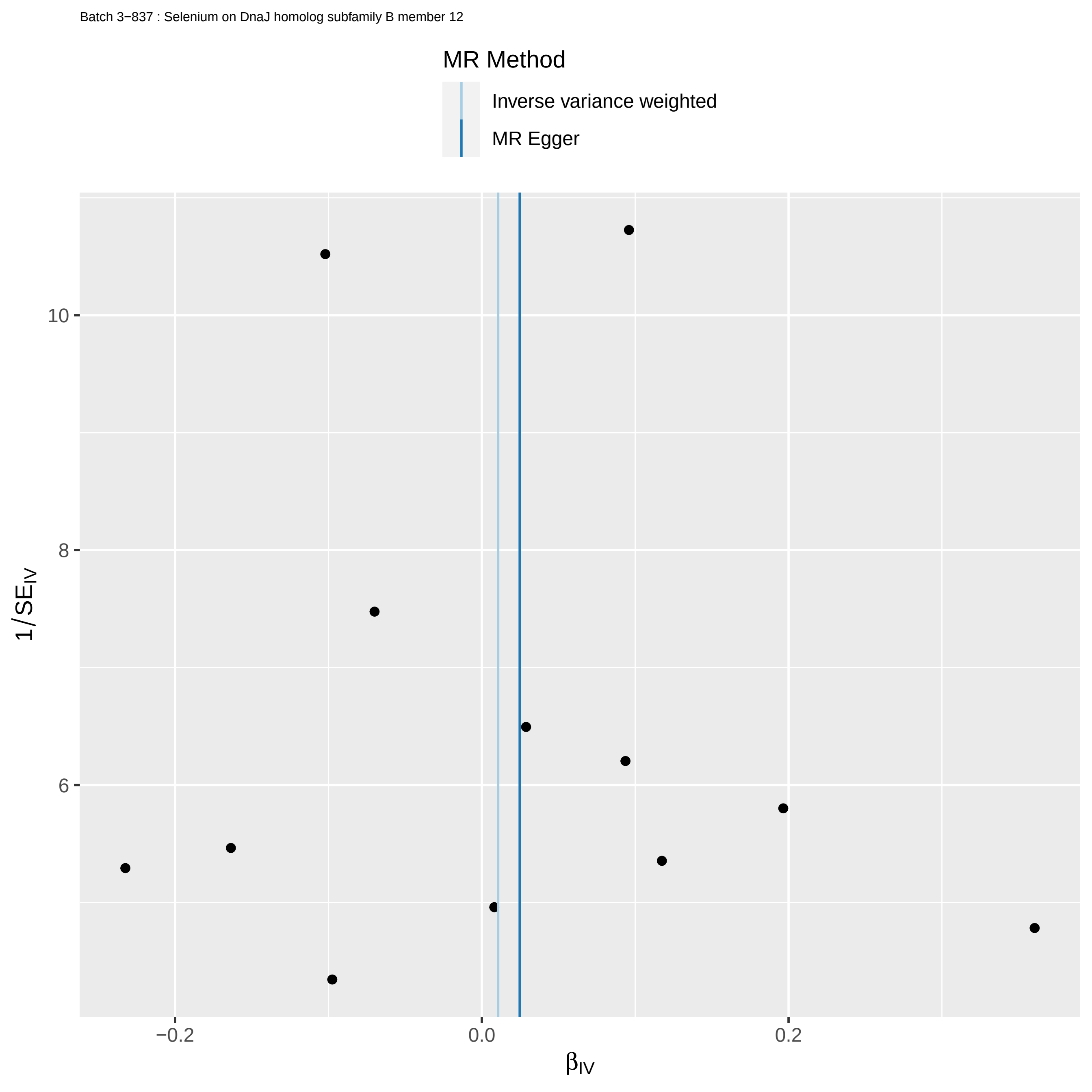
Batch 3−837 : Selenium on DnaJ homolog subfamily B member 12
MR Method
Inverse variance weighted
MR Egger
10
8
6
1/SE_IV
−0.2
0.0
0.2
β_IV

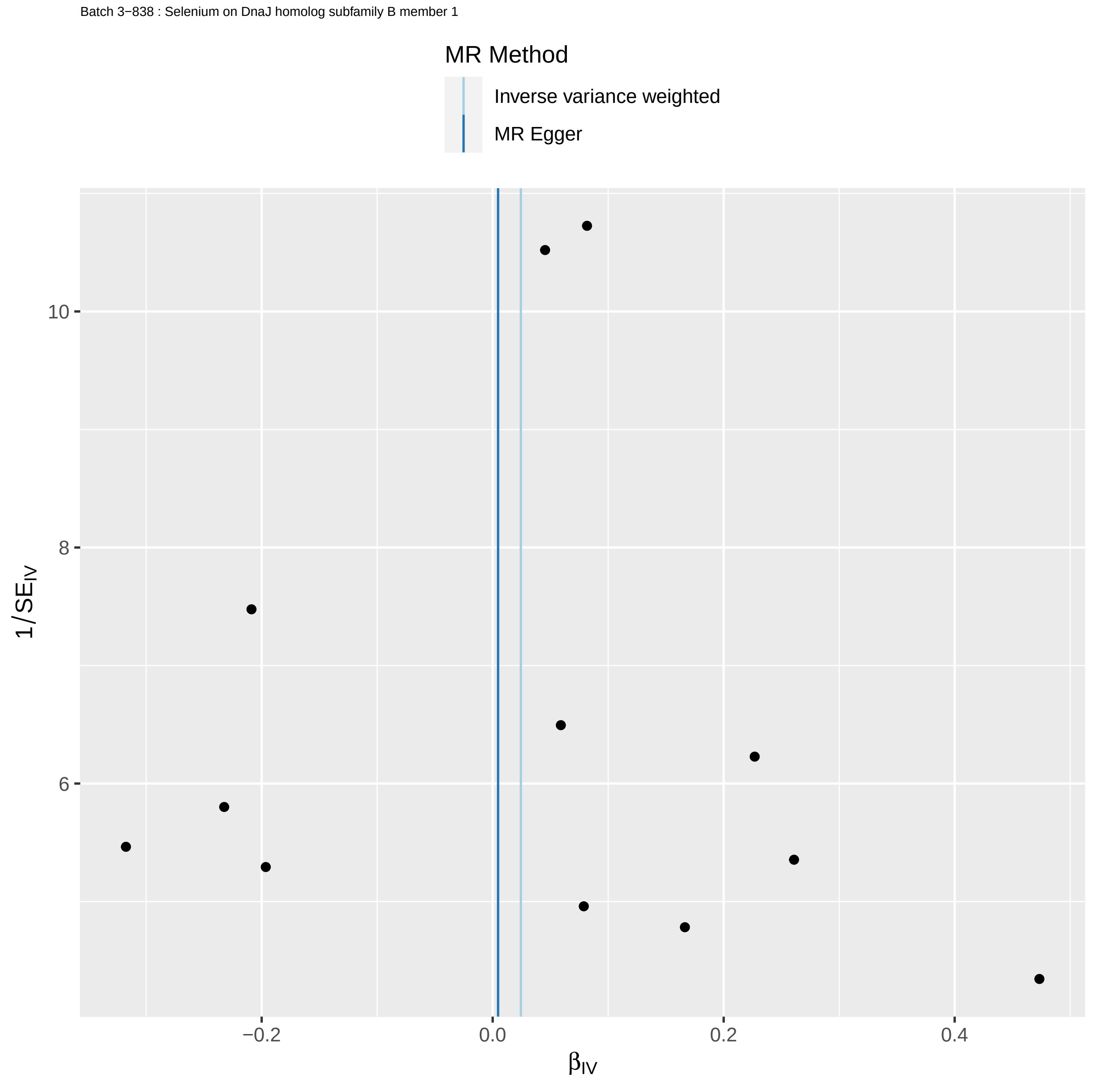
Batch 3−838 : Selenium on DnaJ homolog subfamily B member 1
MR Method
Inverse variance weighted
MR Egger
10
8
6
1/SE_IV
−0.2
0.0
0.2
0.4
β_IV

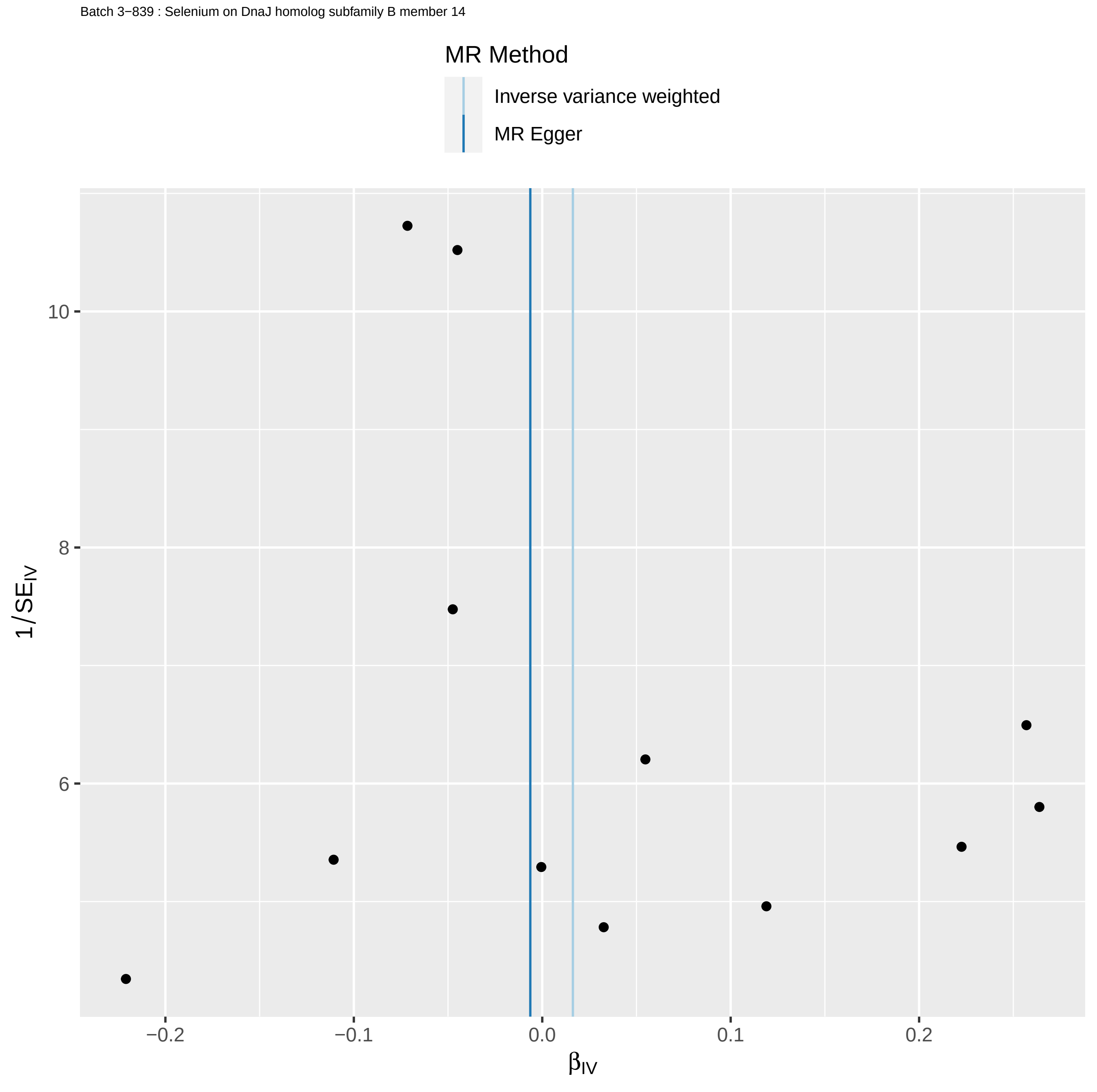

Batch 3−839 : Selenium on DnaJ homolog subfamily B member 14
MR Method
Inverse variance weighted
MR Egger
1/SE_IV
10
8
6
−0.2
−0.1
0.0
0.1
0.2
β_IV

Batch 3−840 : Selenium on DnaJ homolog subfamily B member 14

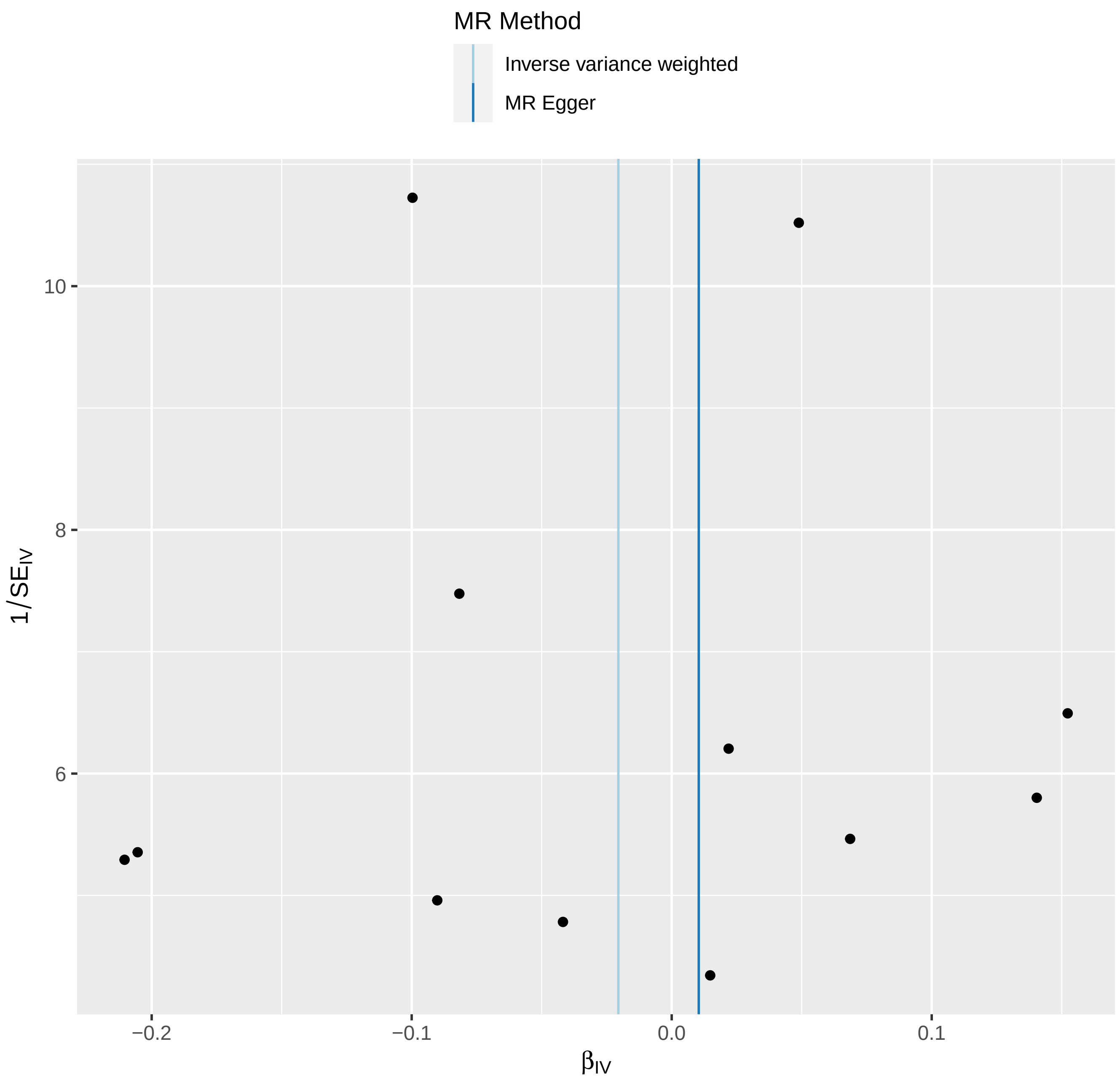

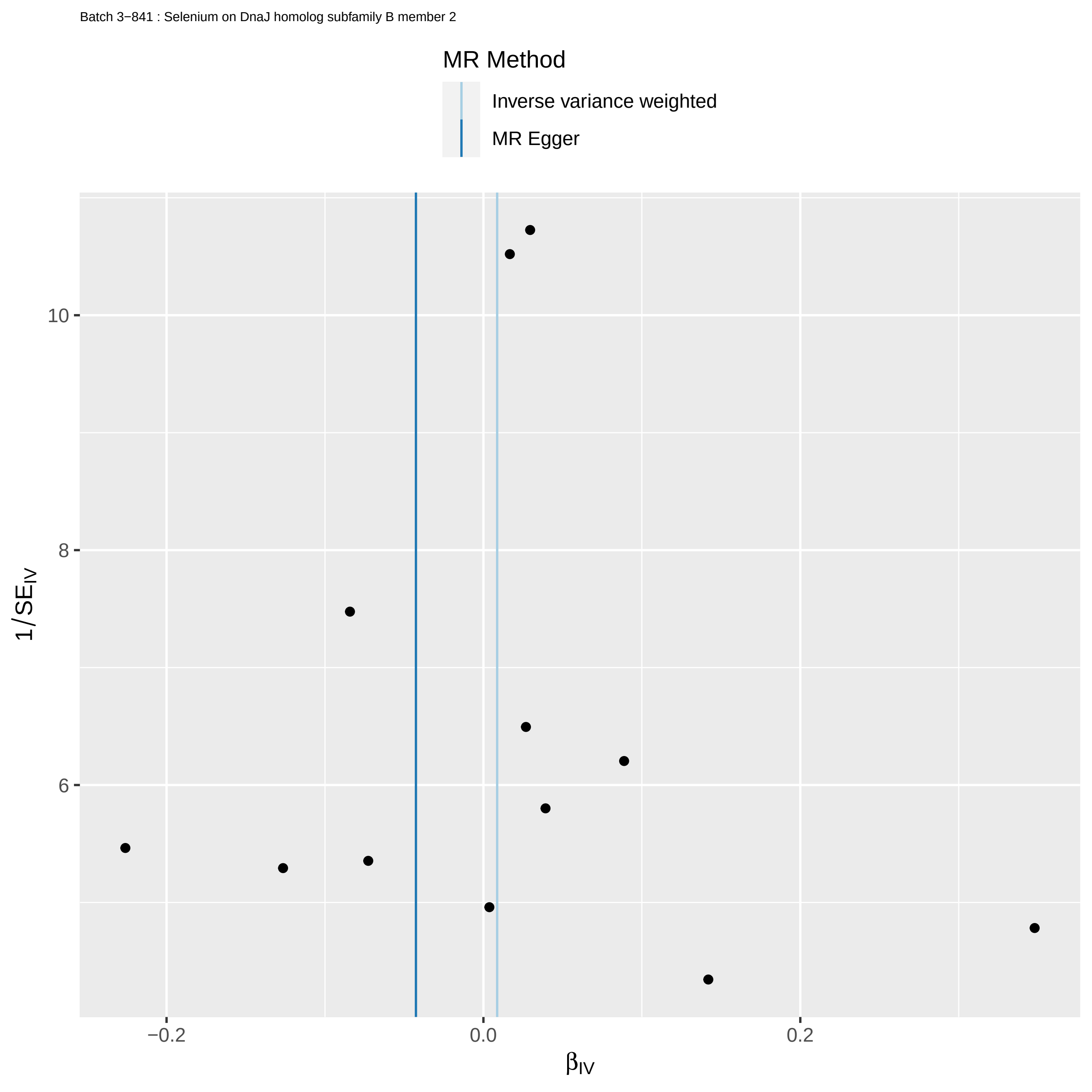

Batch 3−841 : Selenium on DnaJ homolog subfamily B member 2
MR Method
Inverse variance weighted
MR Egger
10
8
6
1/SE$_{IV}$
−0.2
0.0
0.2
β_{IV}

Batch 3−842 : Selenium on DnaJ homolog subfamily B member 9

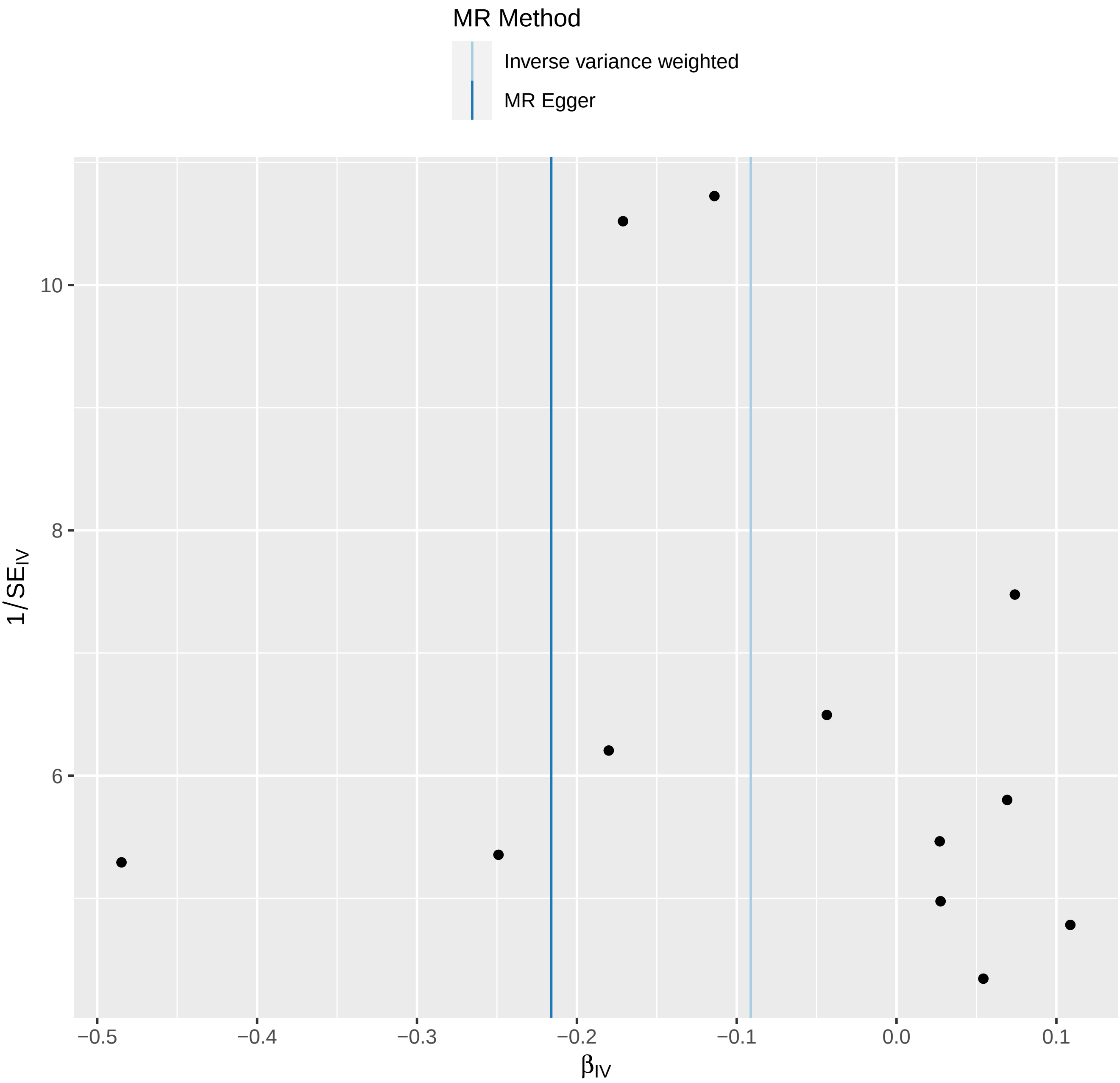

Batch 3−843 : Selenium on DnaJ homolog subfamily C member 10

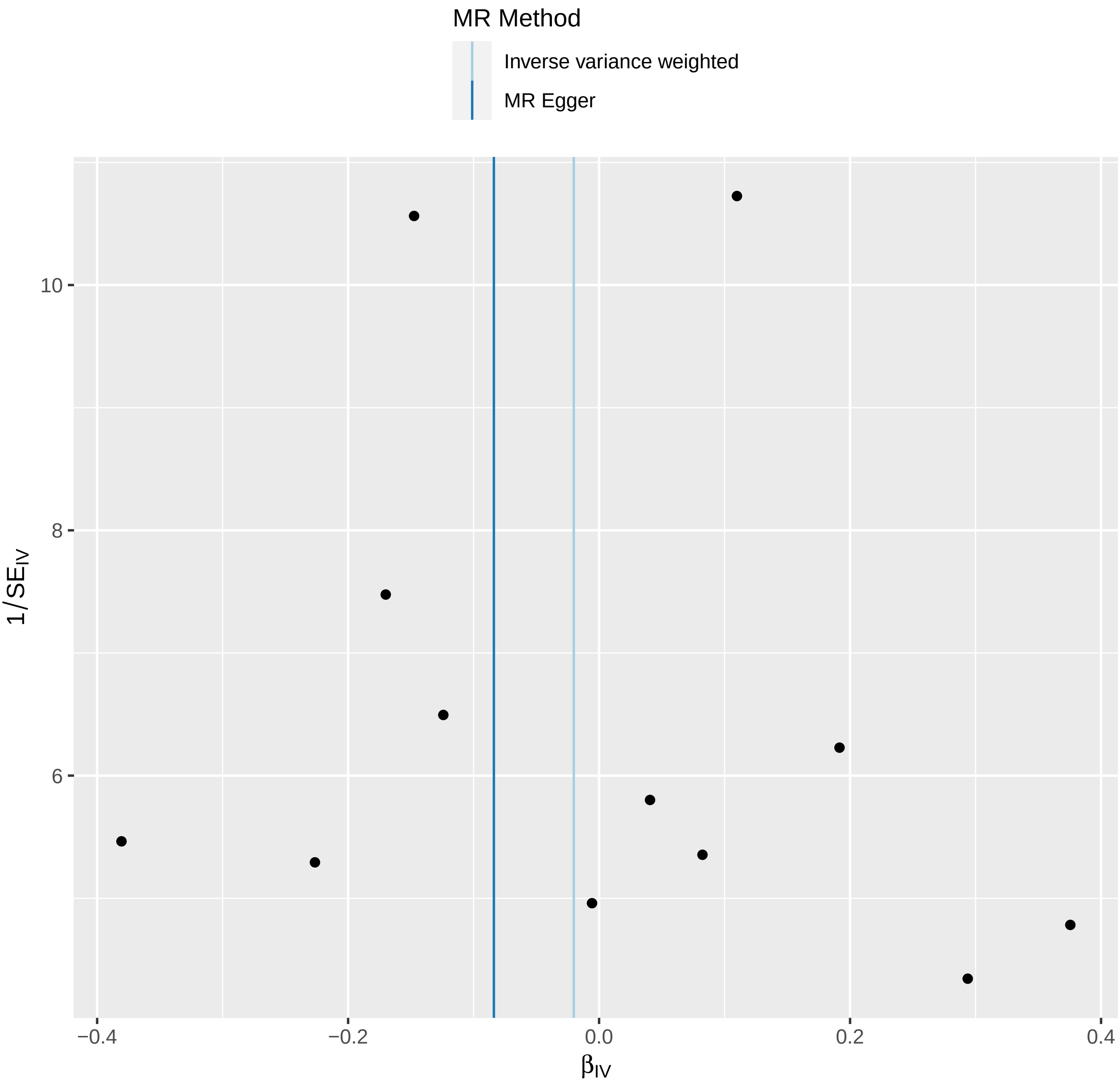

Batch 3−844 : Selenium on DnaJ homolog subfamily C member 15

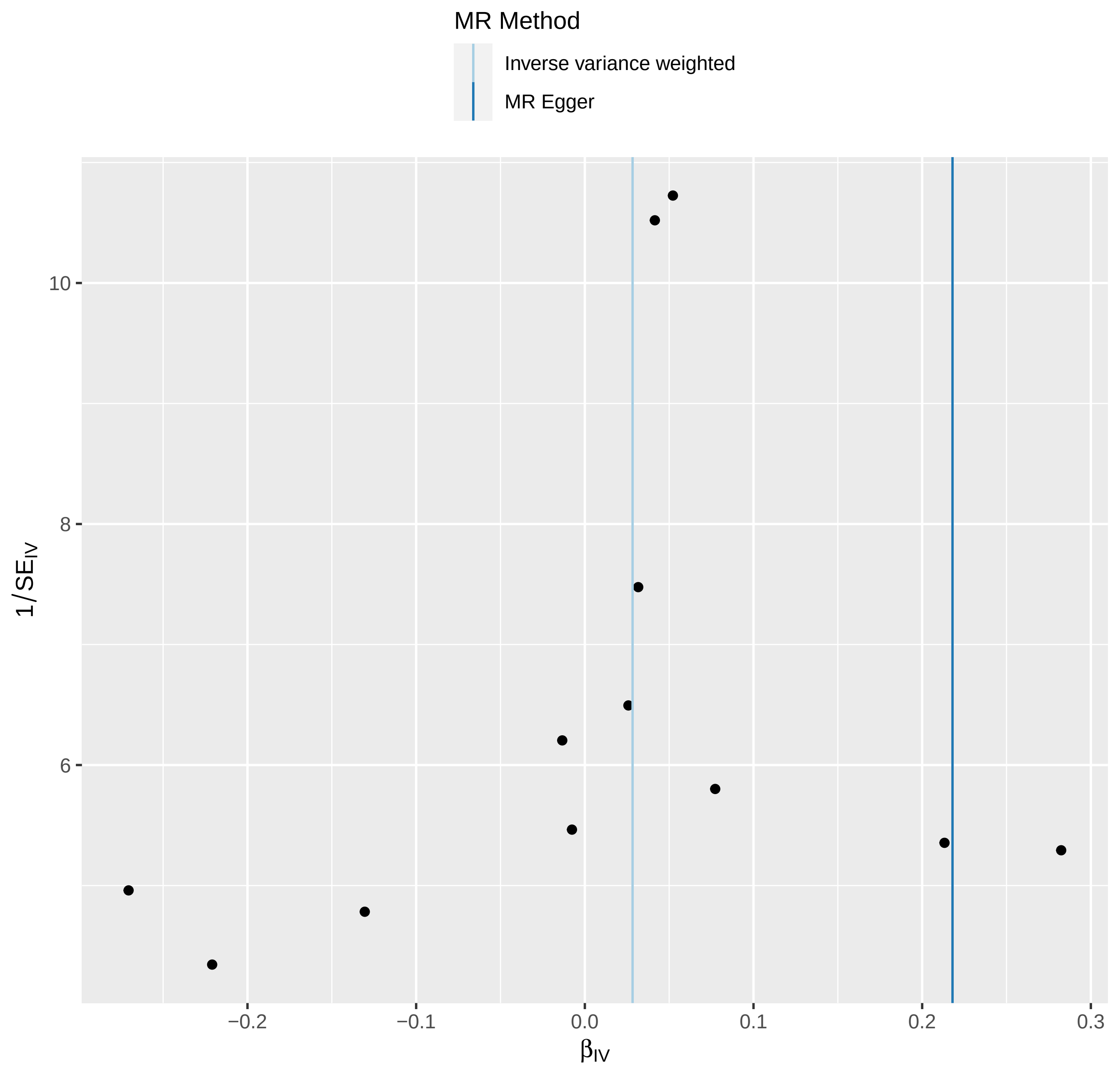

Batch 3−845 : Selenium on DnaJ homolog subfamily C member 17

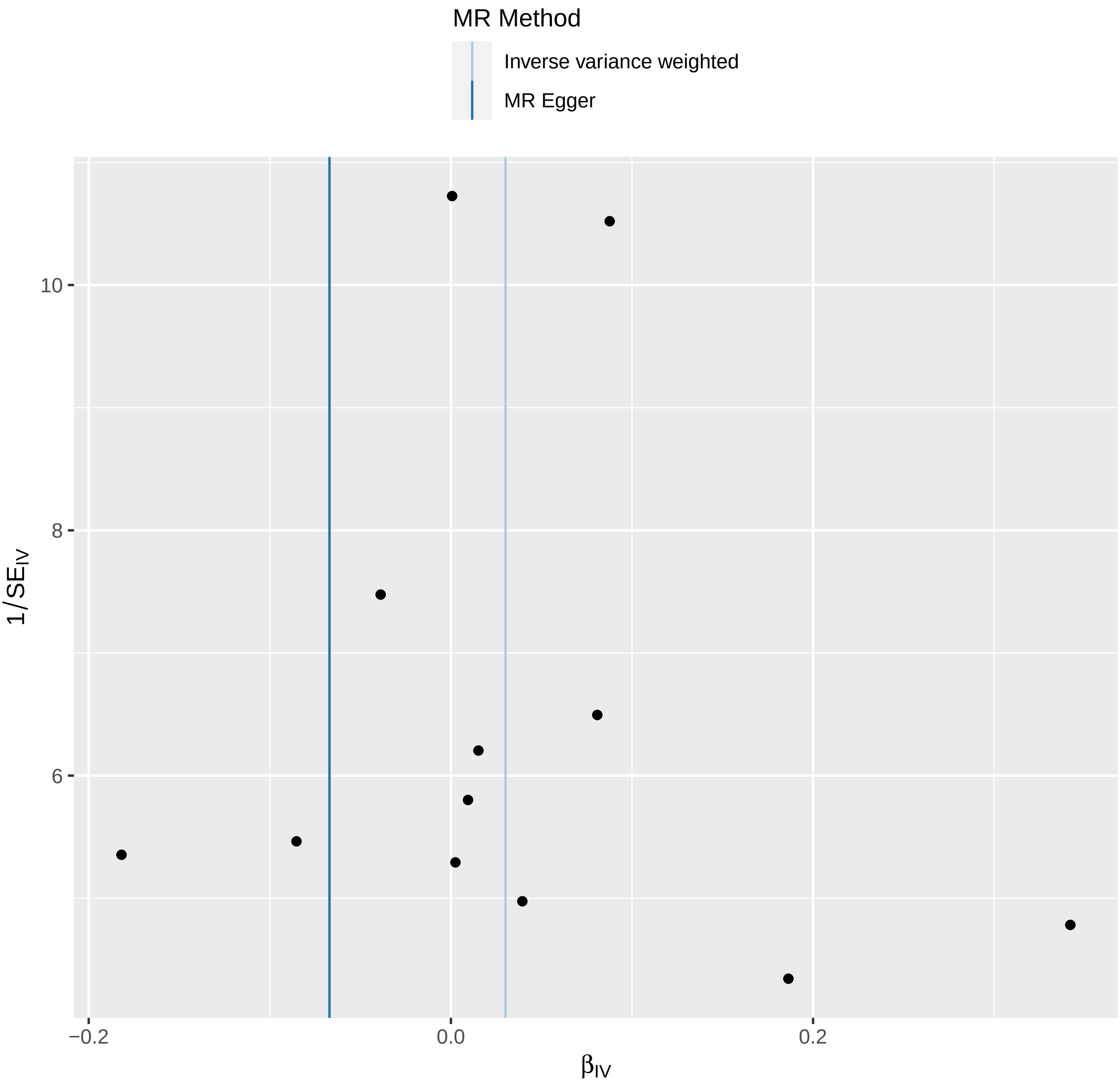

Batch 3−846 : Selenium on DnaJ homolog subfamily C member 18

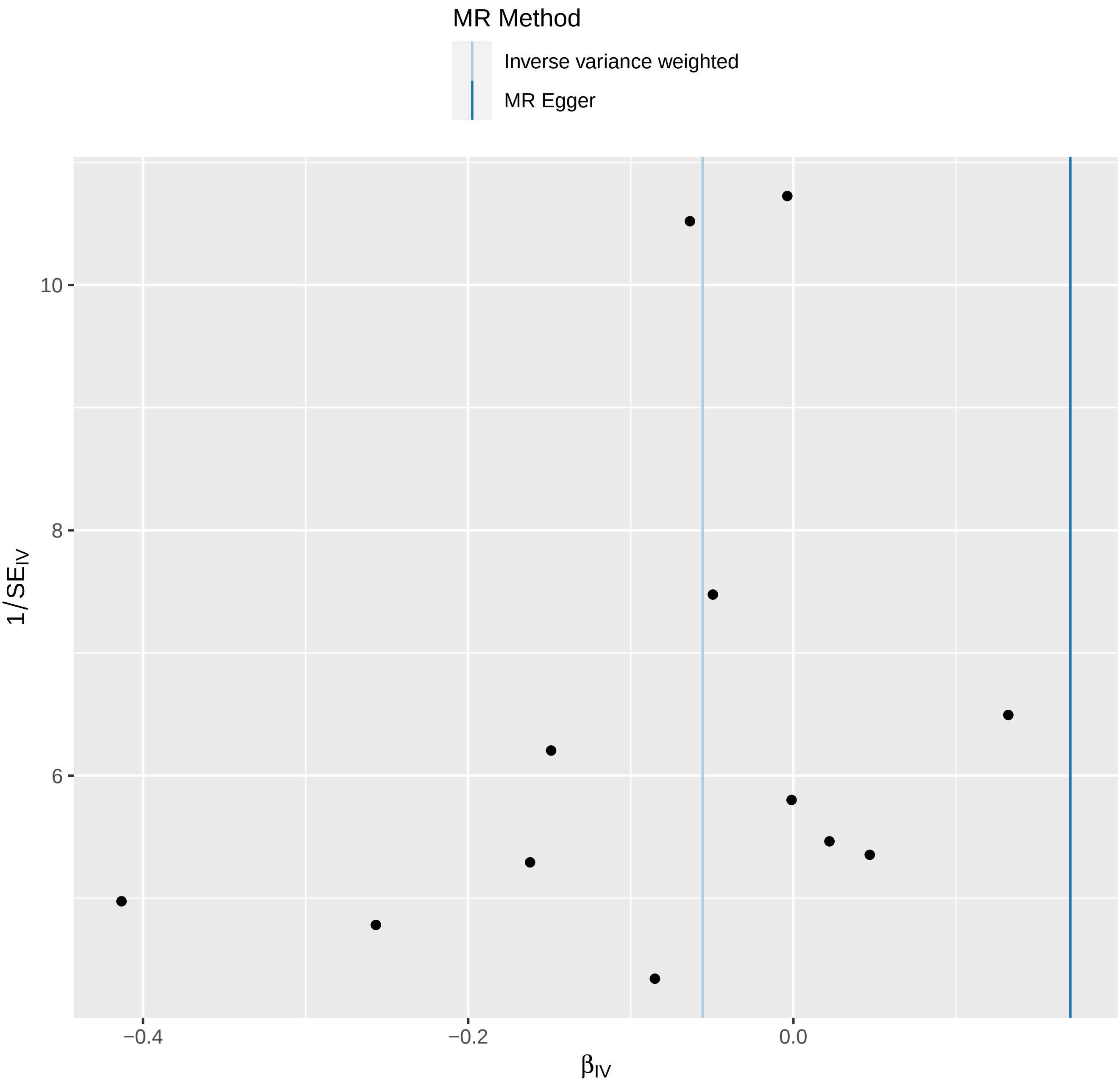

Batch 3−847 : Selenium on Mitochondrial import inner membrane translocase subunit TIM14

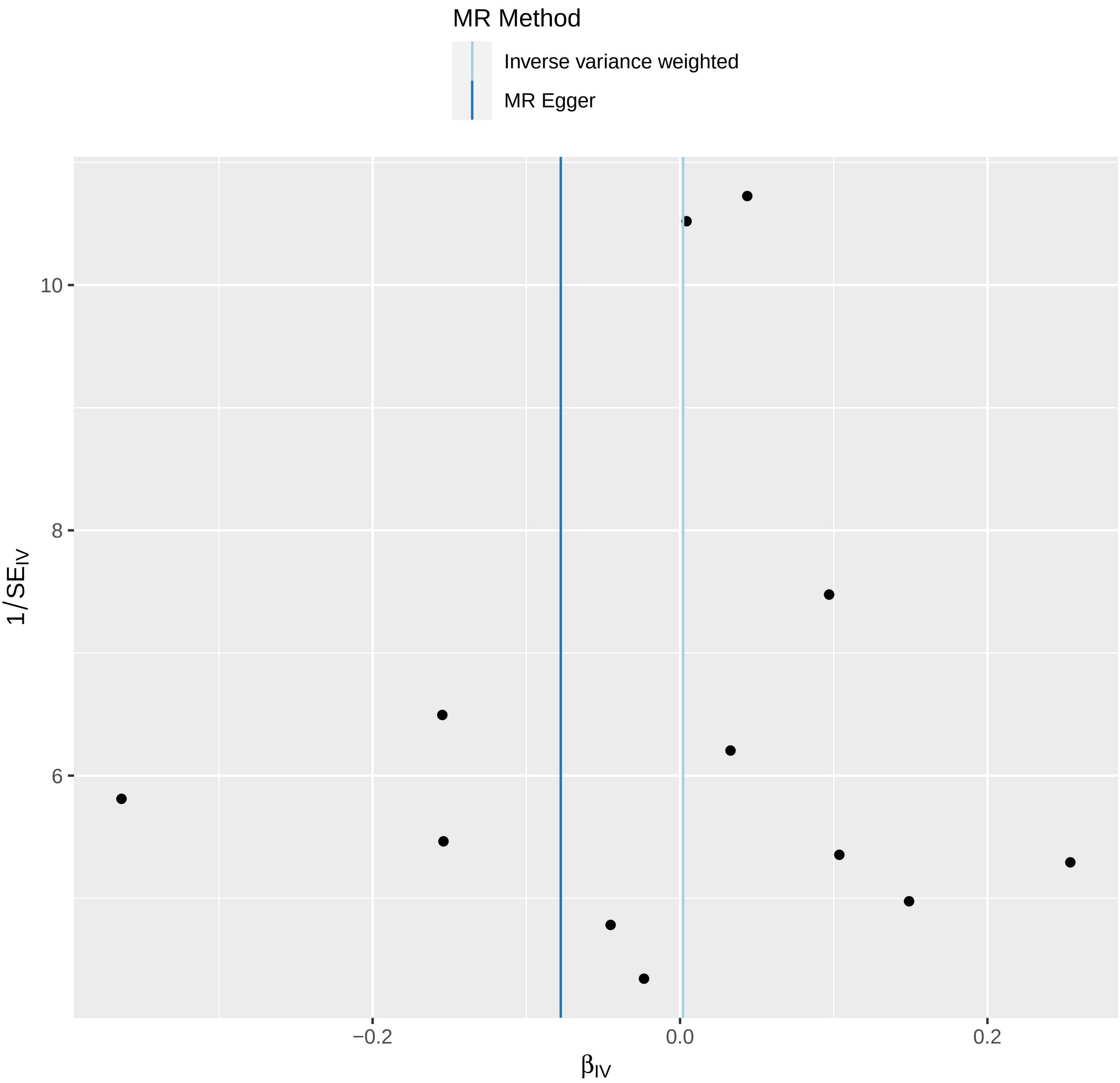

Batch 3−848 : Selenium on DnaJ homolog subfamily C member 27

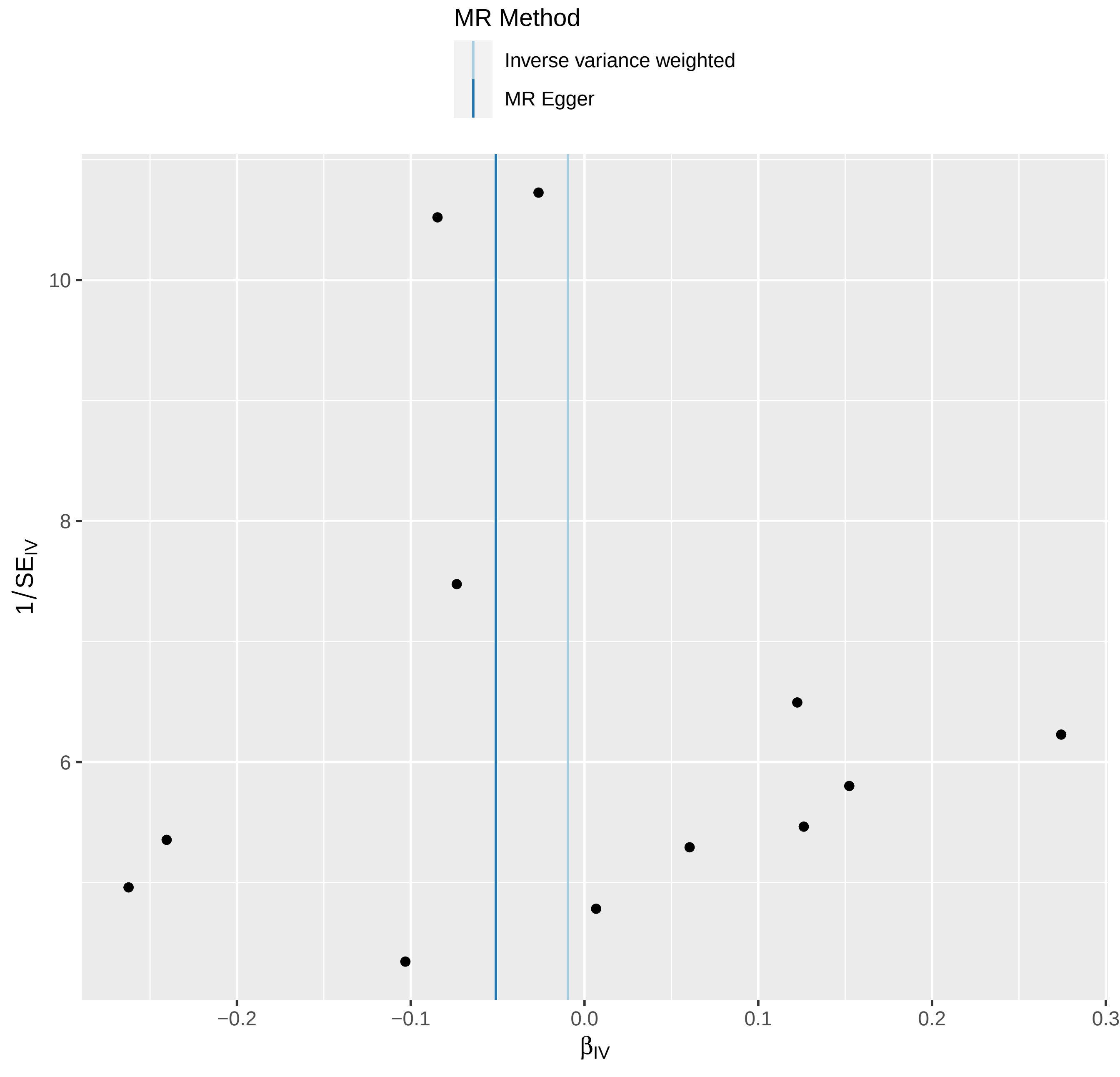

Batch 3−849 : Selenium on DnaJ homolog subfamily C member 30

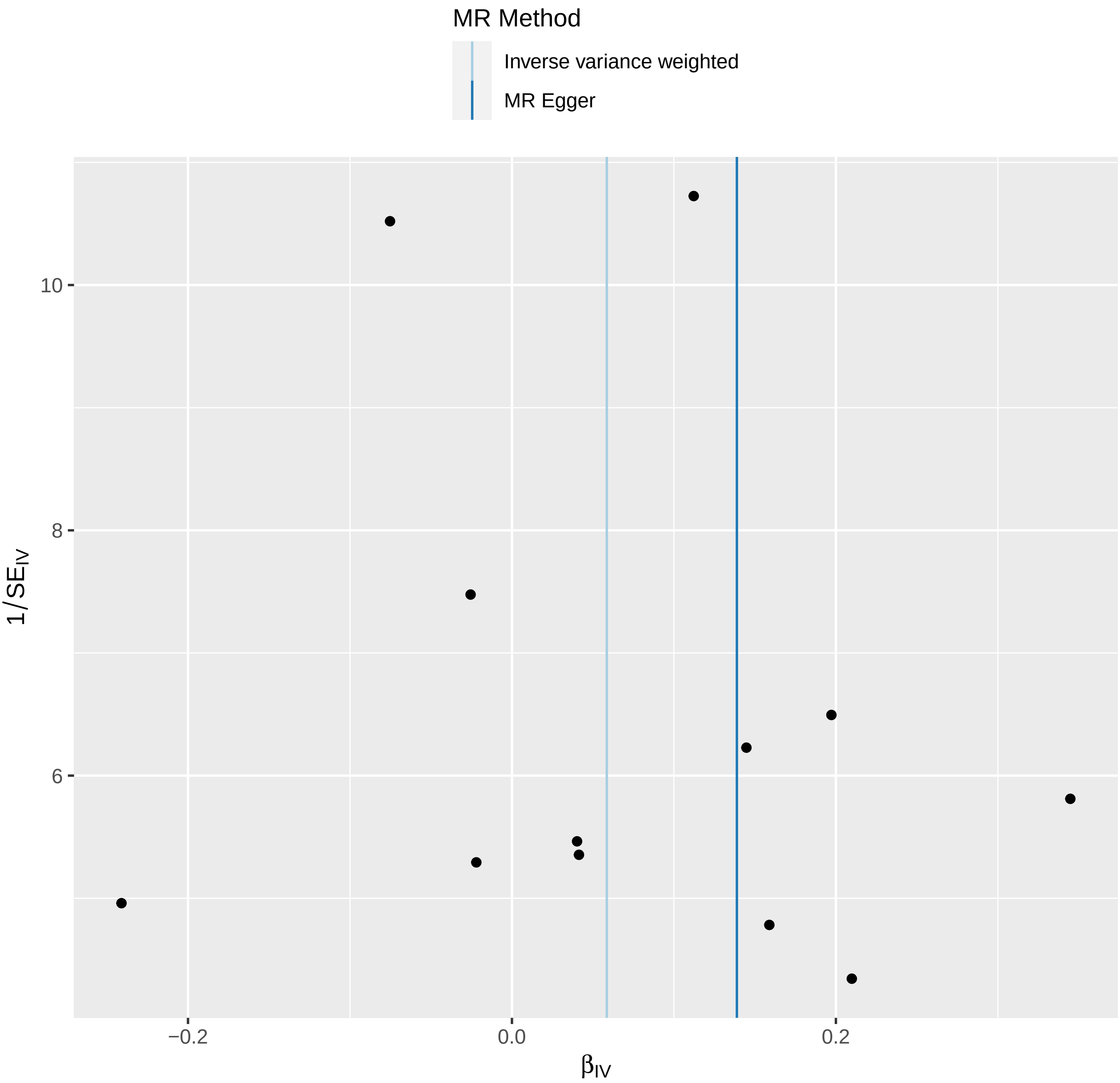

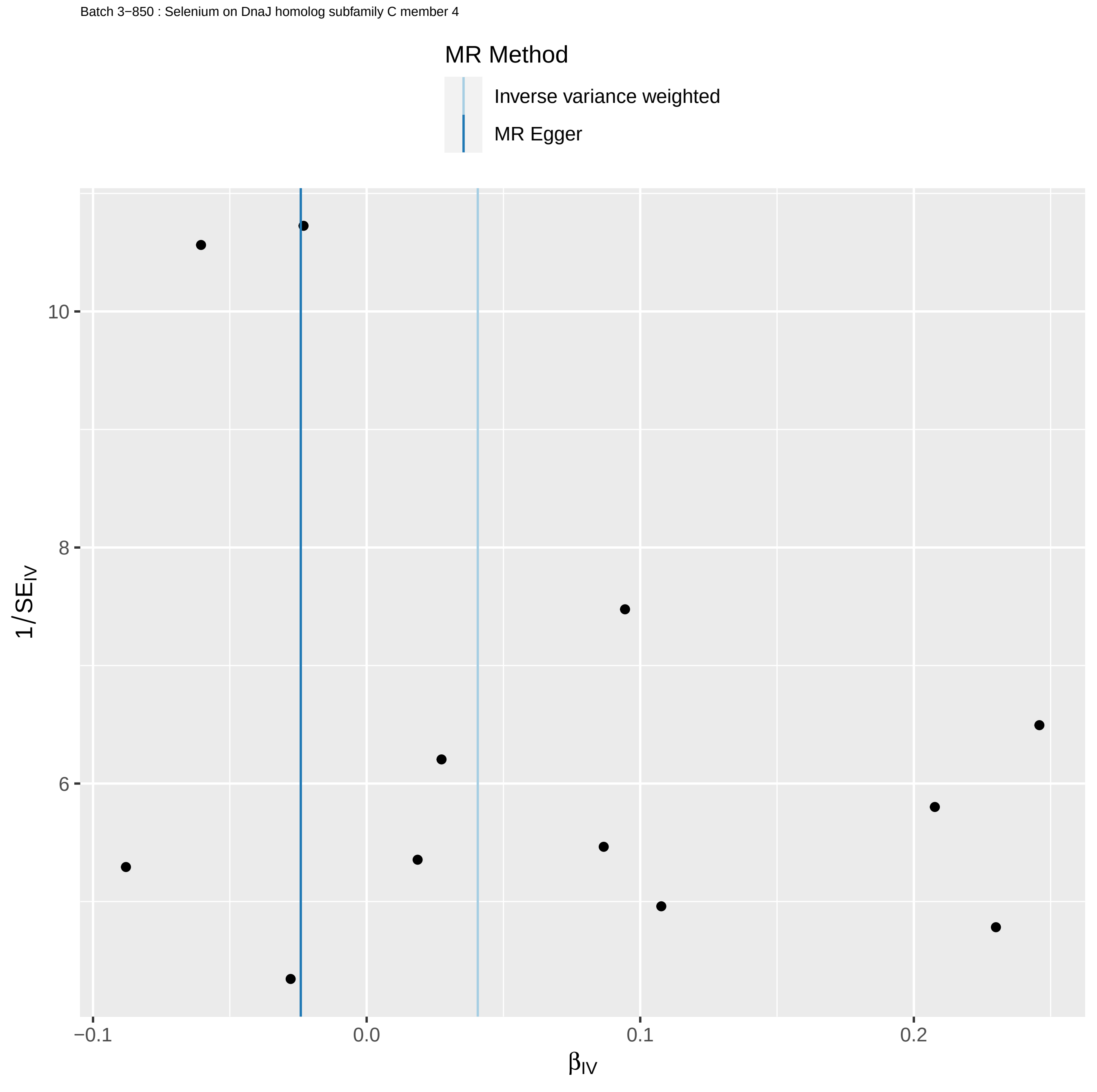

Batch 3−850 : Selenium on DnaJ homolog subfamily C member 4
MR Method
Inverse variance weighted
MR Egger
10
8
6
1/SE_IV
−0.1
0.0
0.1
0.2
β_IV

Batch 3−851 : Selenium on Deoxyribonuclease−2−beta

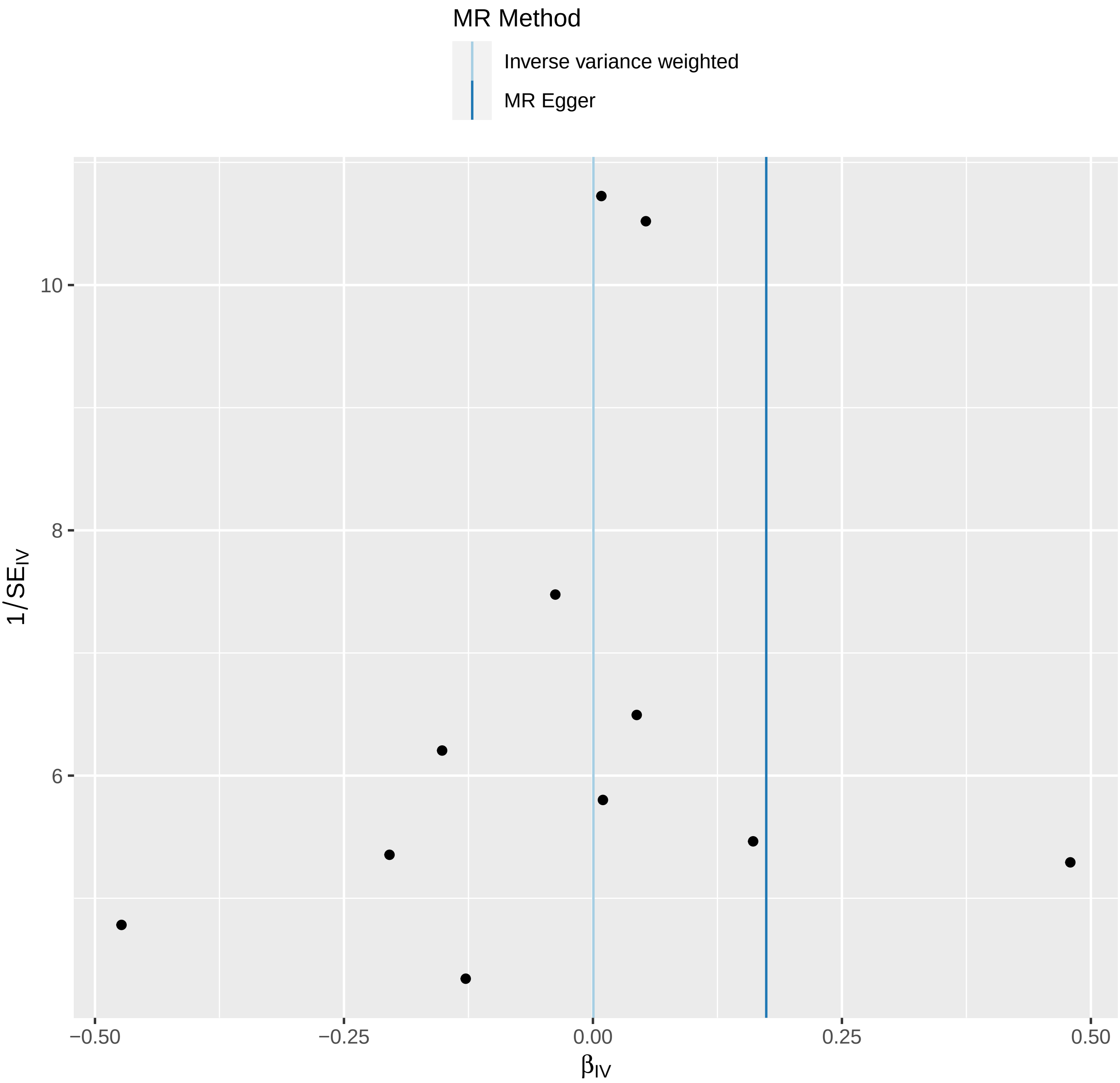

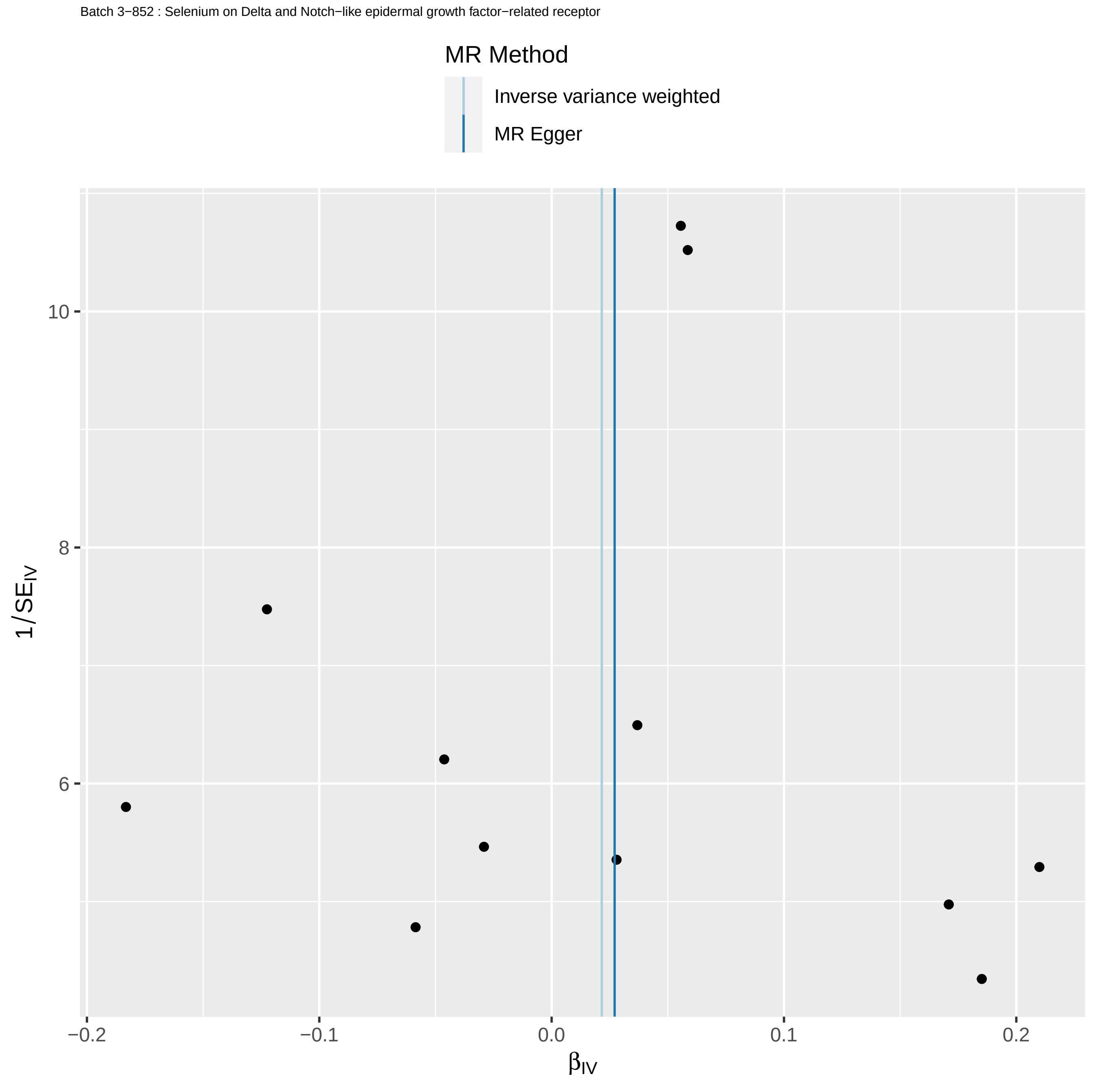

Batch 3−852 : Selenium on Delta and Notch−like epidermal growth factor−related receptor
MR Method
Inverse variance weighted
MR Egger
10
8
6
1/SE_IV
−0.2
−0.1
0.0
0.1
0.2
β_IV

Batch 3−853 : Selenium on Delta and Notch−like epidermal growth factor−related receptor

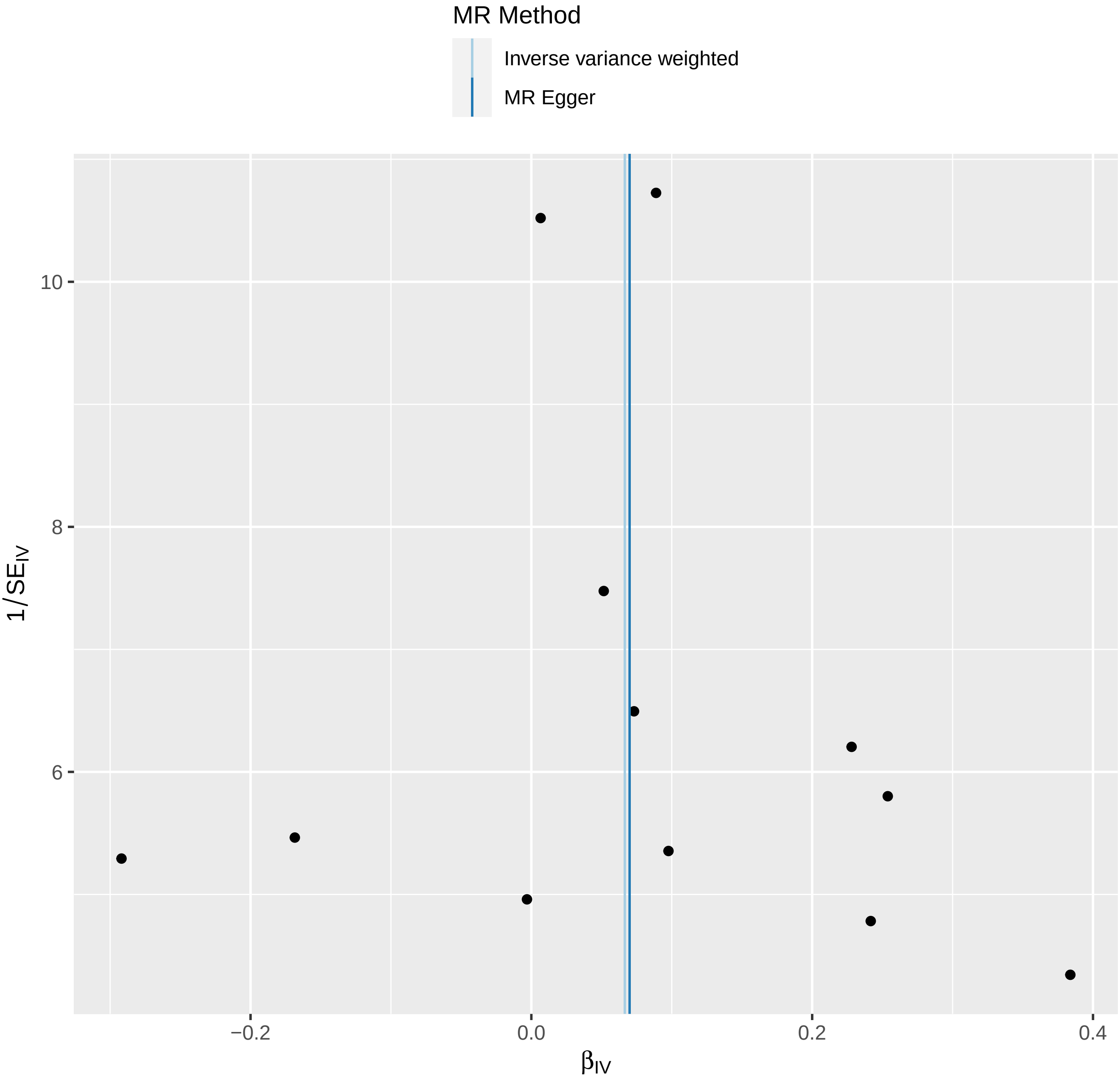

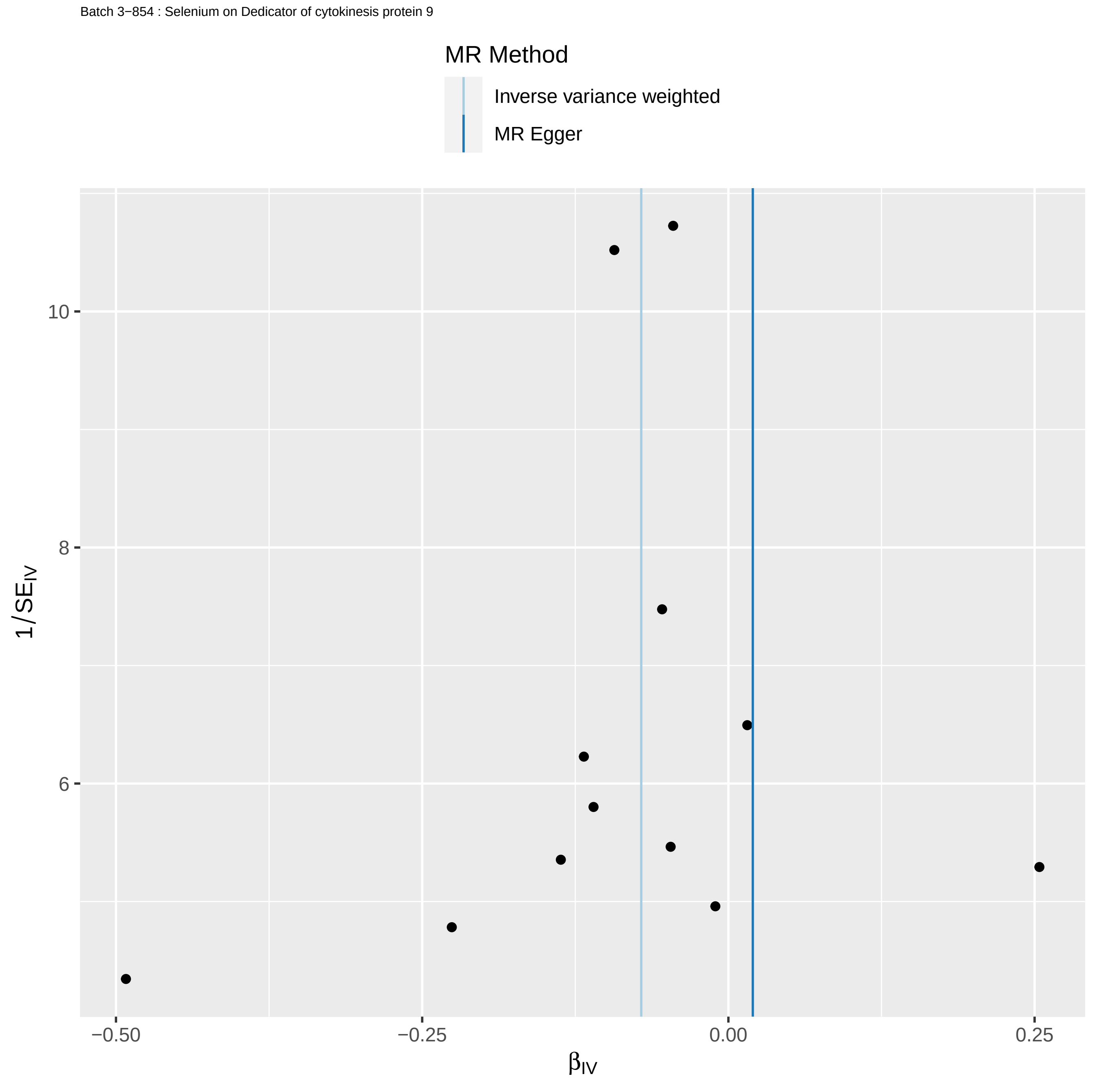

Batch 3−854 : Selenium on Dedicator of cytokinesis protein 9
MR Method
Inverse variance weighted
MR Egger
10
8
6
1/SE$_{IV}$
−0.50
−0.25
0.00
0.25
β$_{IV}$

Batch 3−855 : Selenium on Docking protein 2

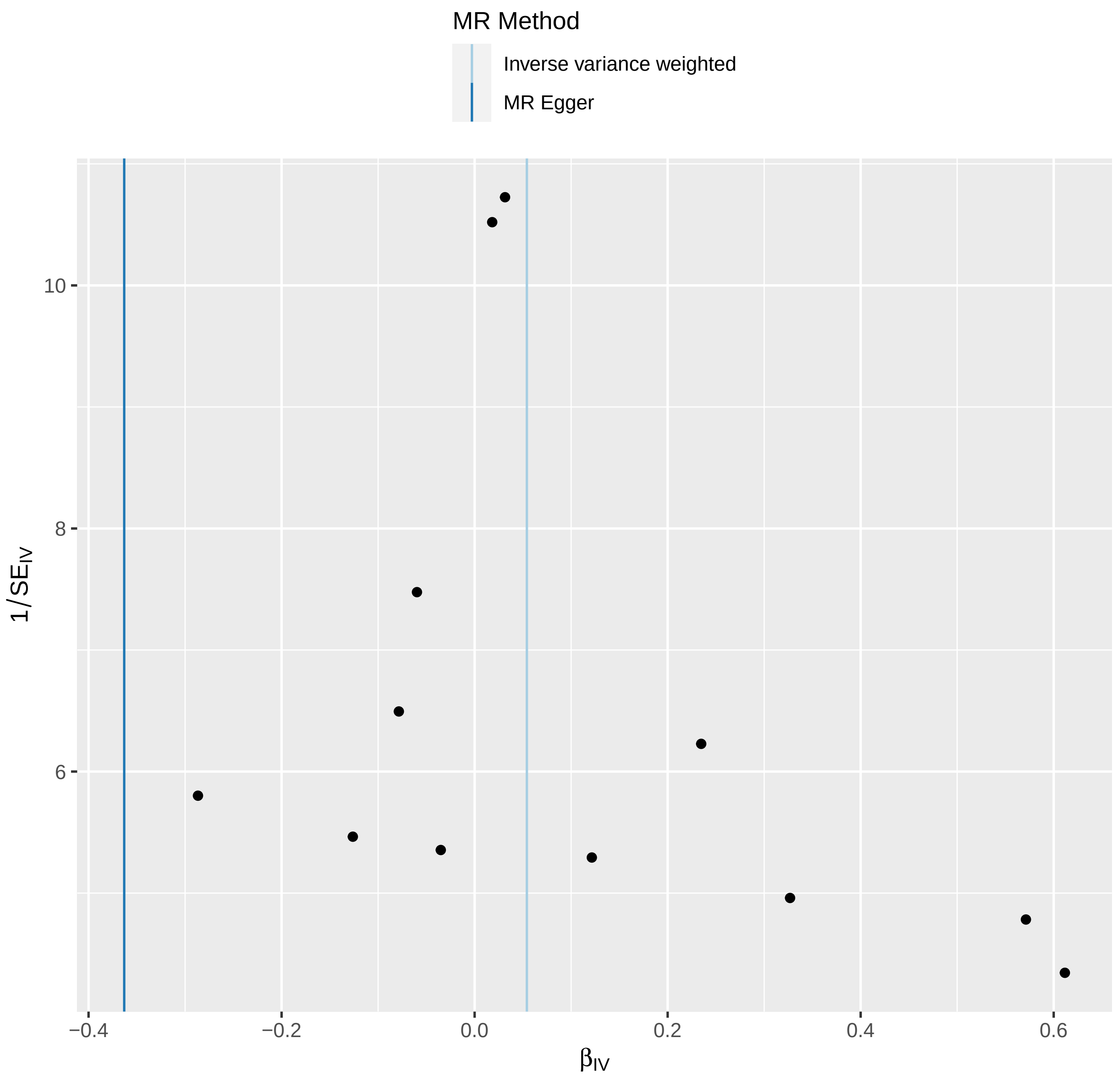

Batch 3−856 : Selenium on Dipeptidase 1

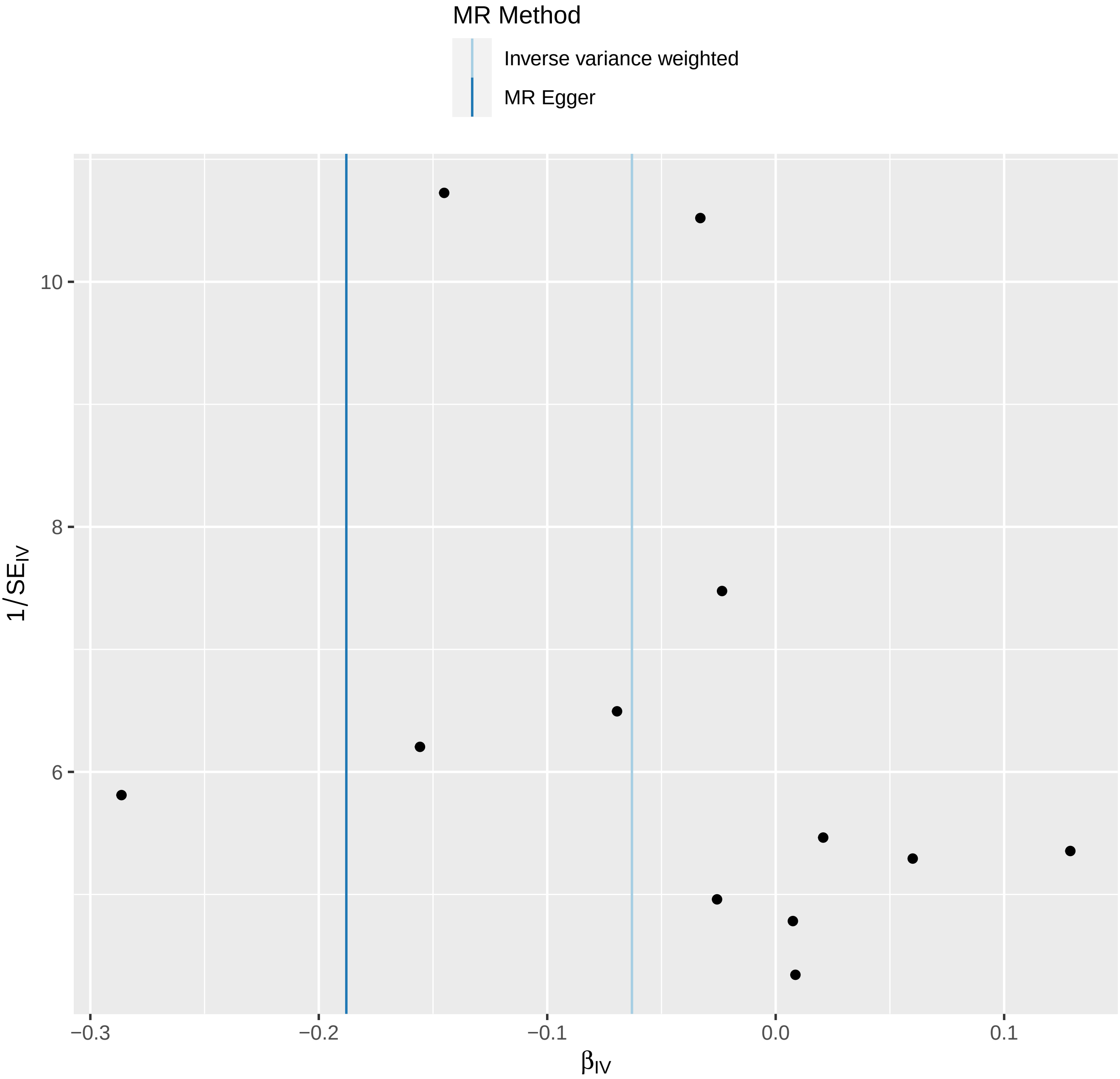

Batch 3−857 : Selenium on Dipeptidase 2

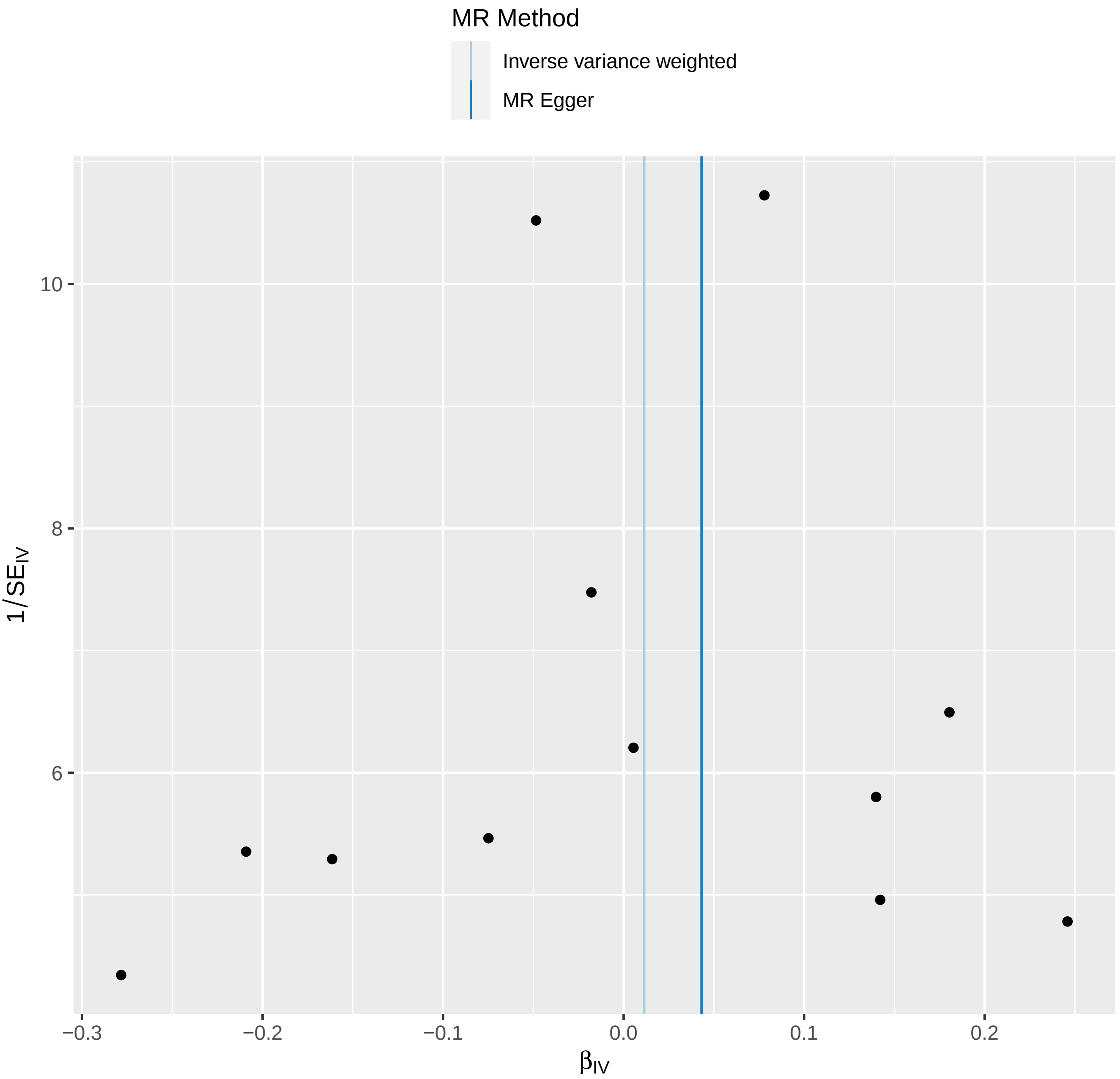

Batch 3−858 : Selenium on Inactive dipeptidyl peptidase 10

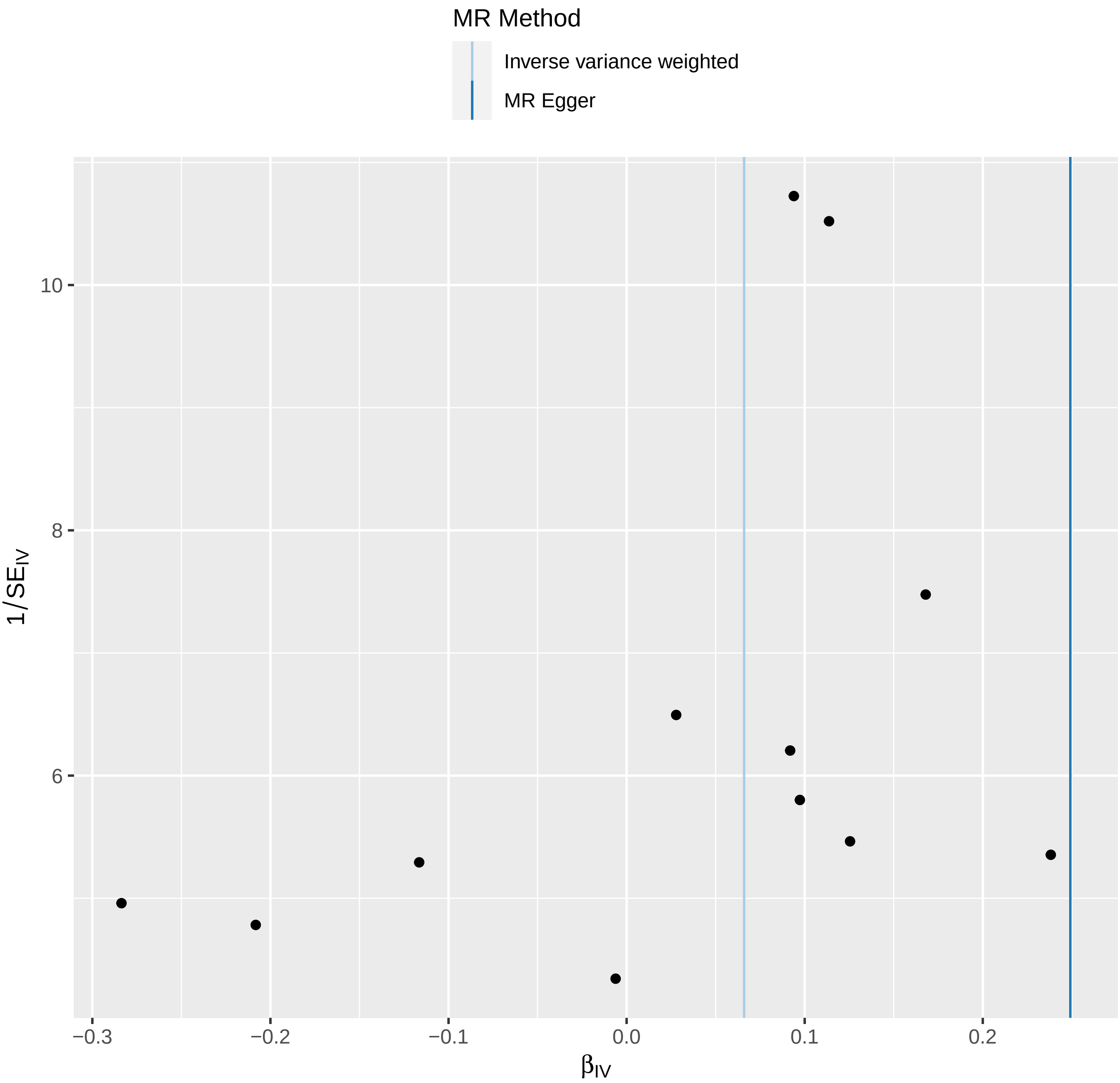

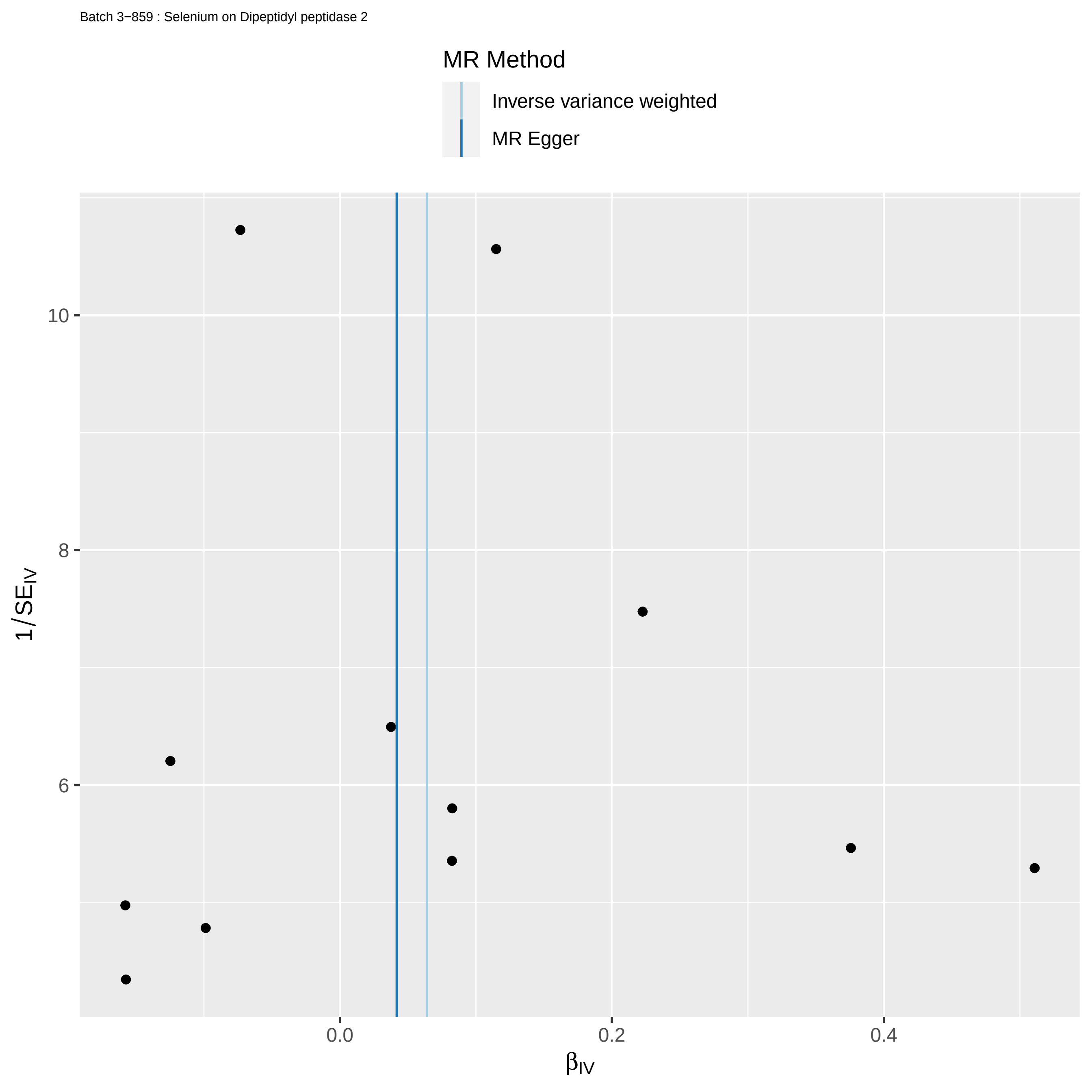

Batch 3−859 : Selenium on Dipeptidyl peptidase 2
MR Method
Inverse variance weighted
MR Egger
10
8
6
1/SE$_{IV}$
0.0
0.2
0.4
β_{IV}

Batch 3−860 : Selenium on Dipeptidyl peptidase 2

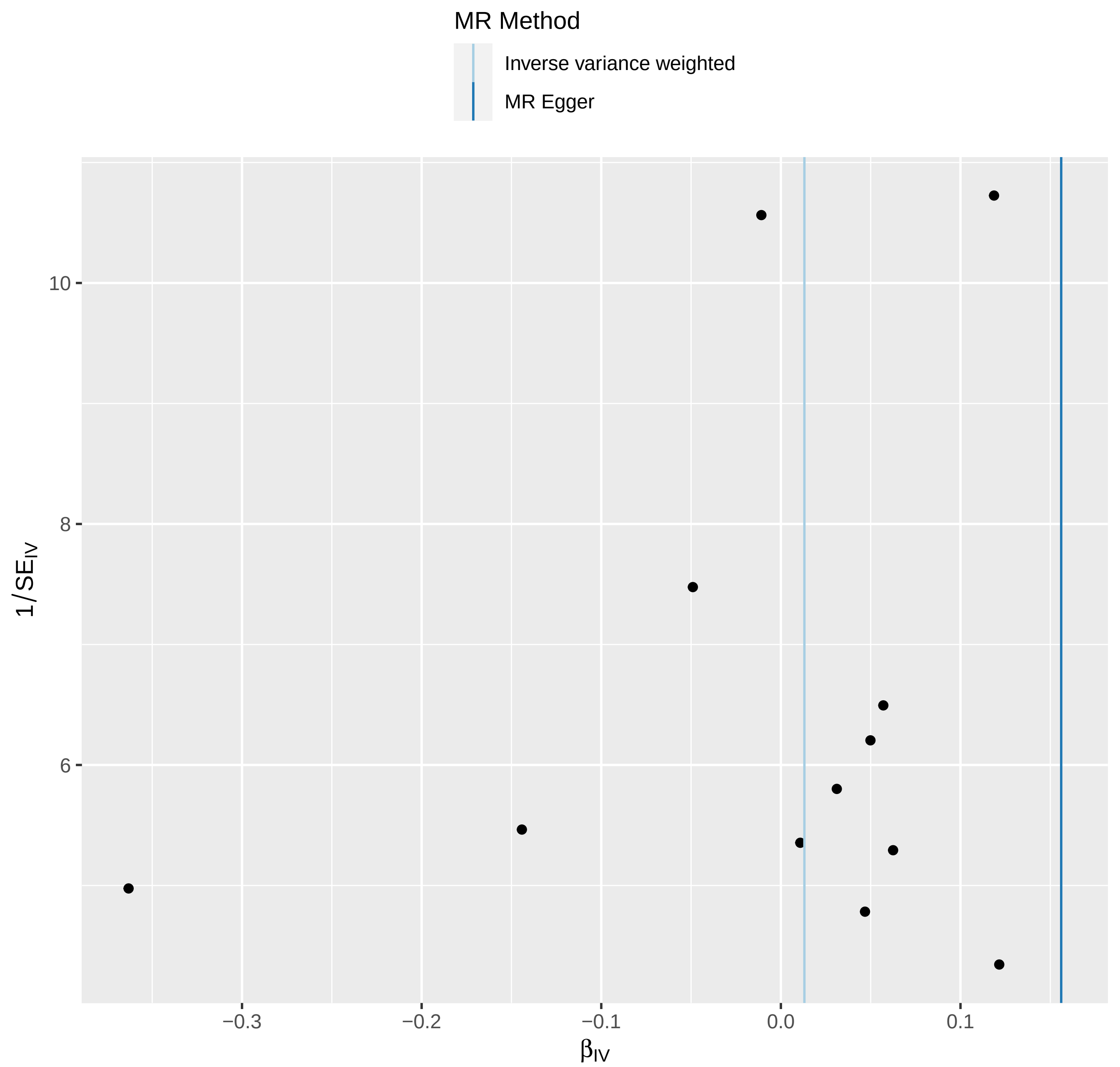

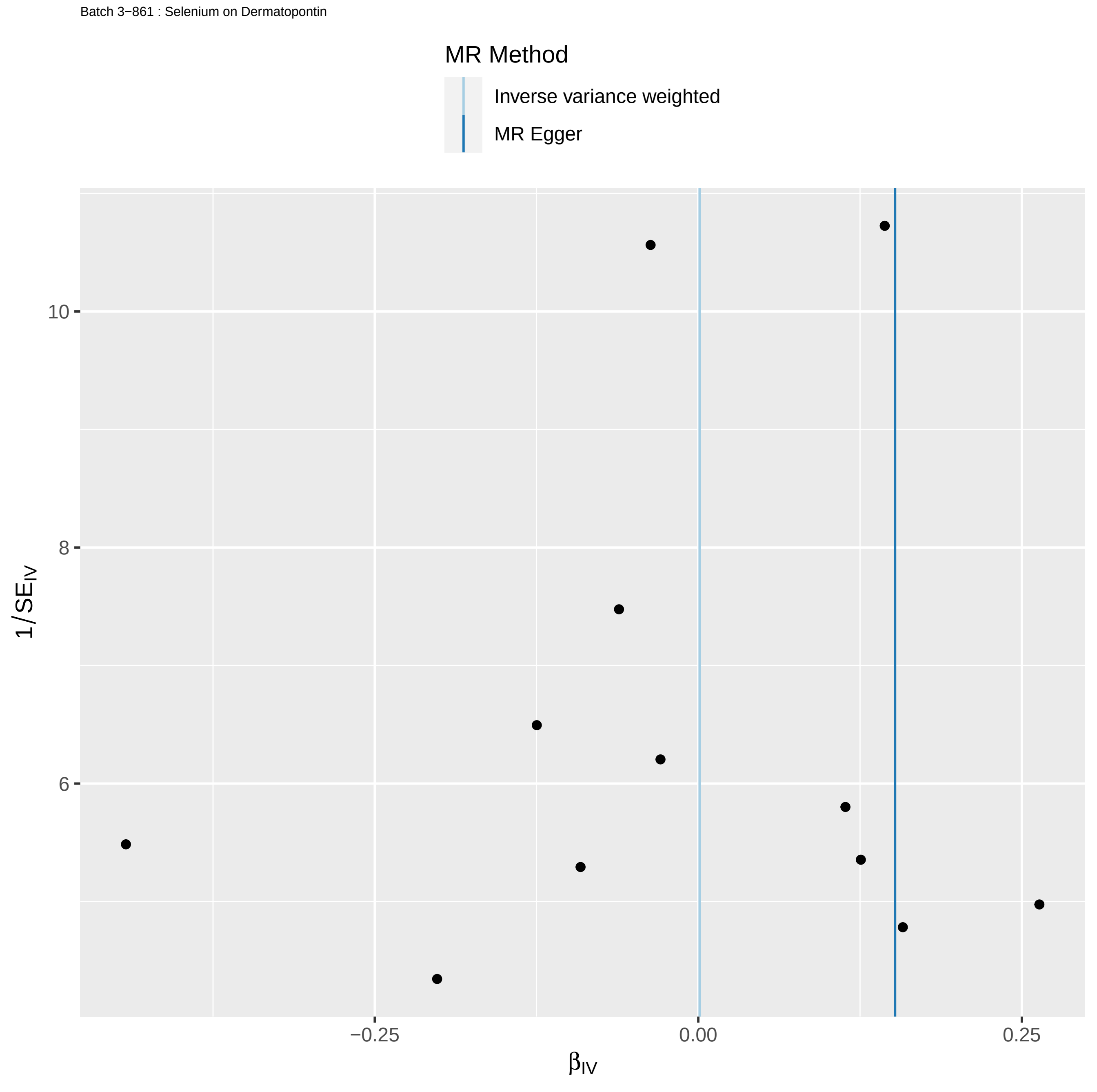
Batch 3−861 : Selenium on Dermatopontin
MR Method
Inverse variance weighted
MR Egger
10
8
6
1/SE_IV
−0.25
0.00
0.25
β_IV

Batch 3−862 : Selenium on Protein dpy−30 homolog

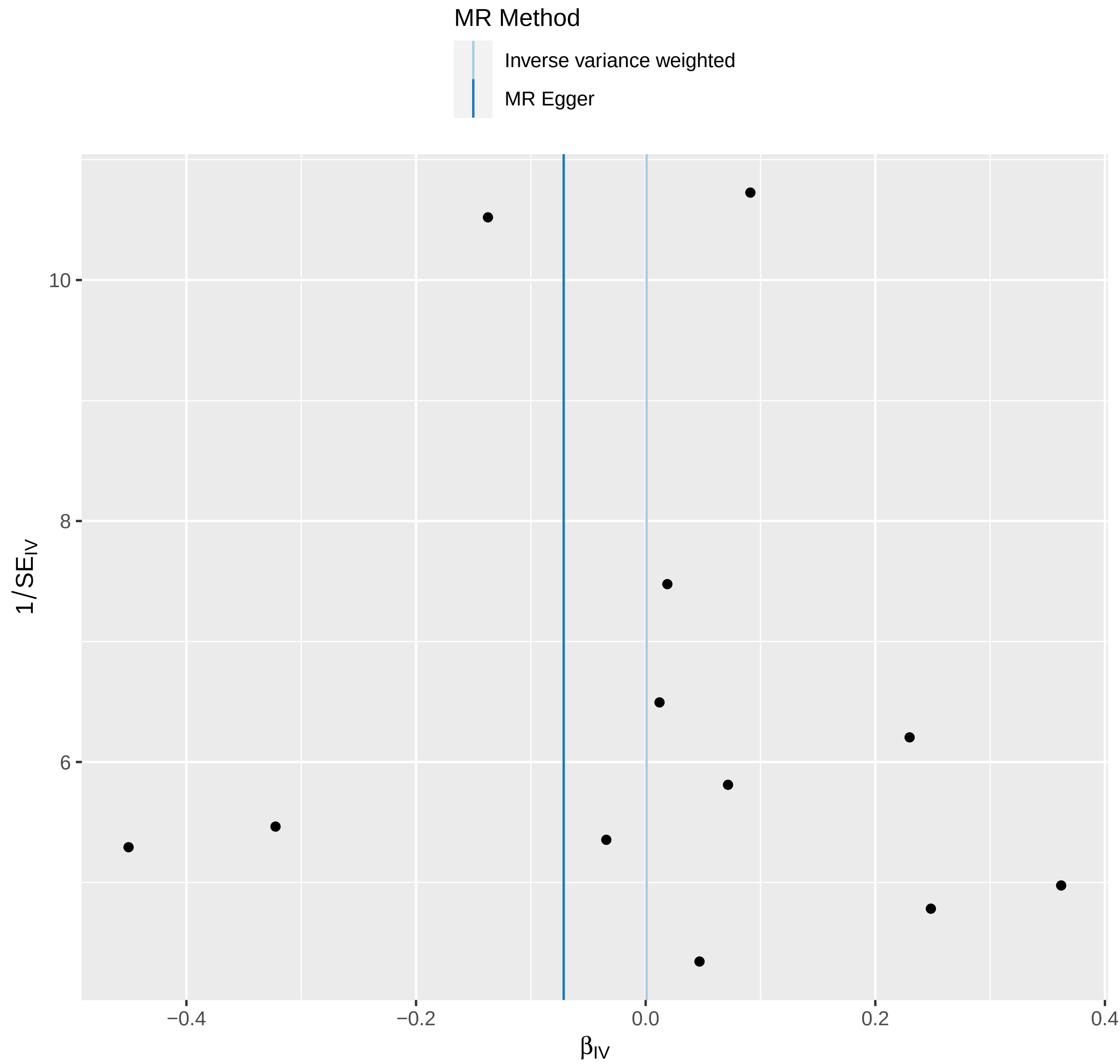

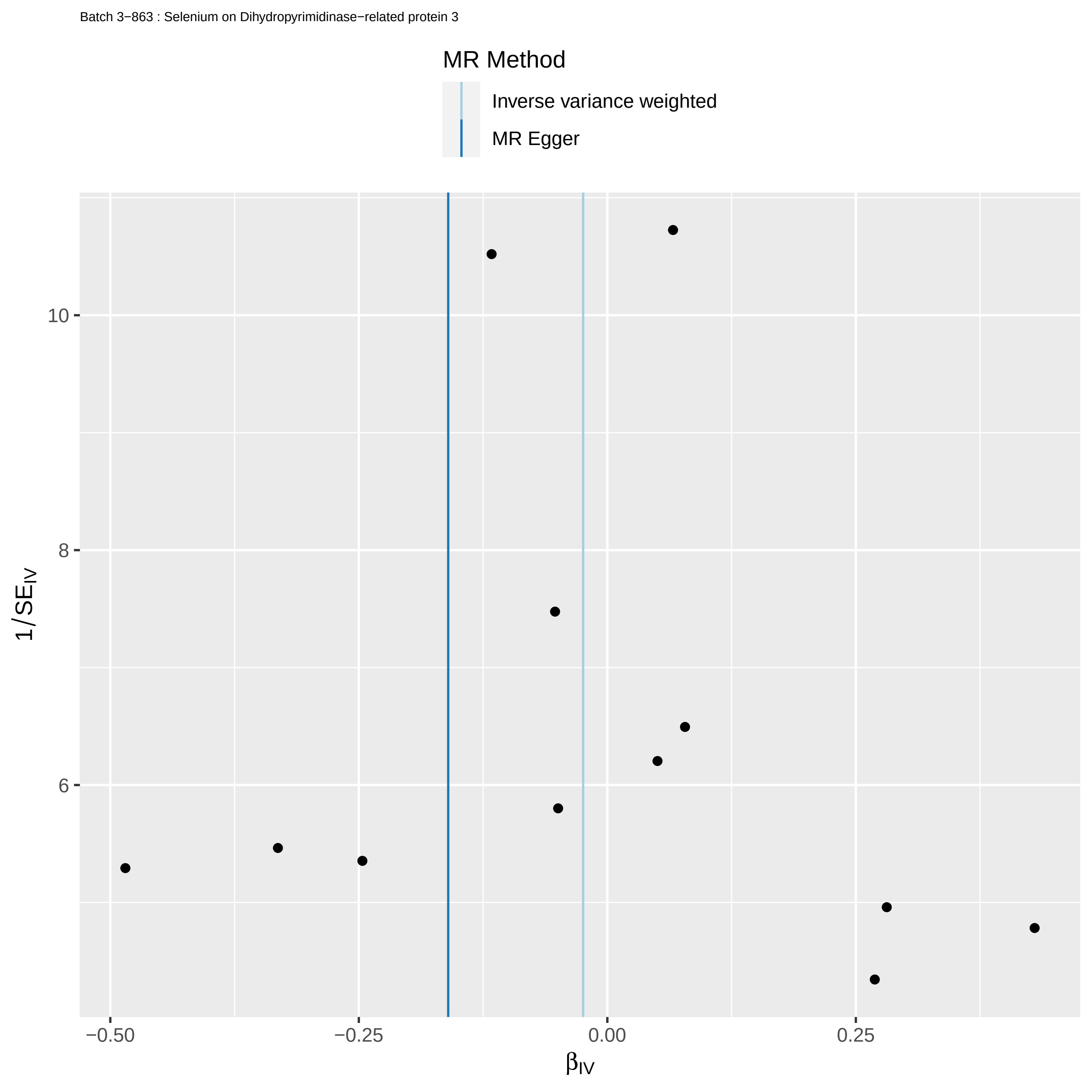
Batch 3−863 : Selenium on Dihydropyrimidinase−related protein 3
MR Method
Inverse variance weighted
MR Egger
10
8
6
1/SE_IV
−0.50
−0.25
0.00
0.25
β_IV

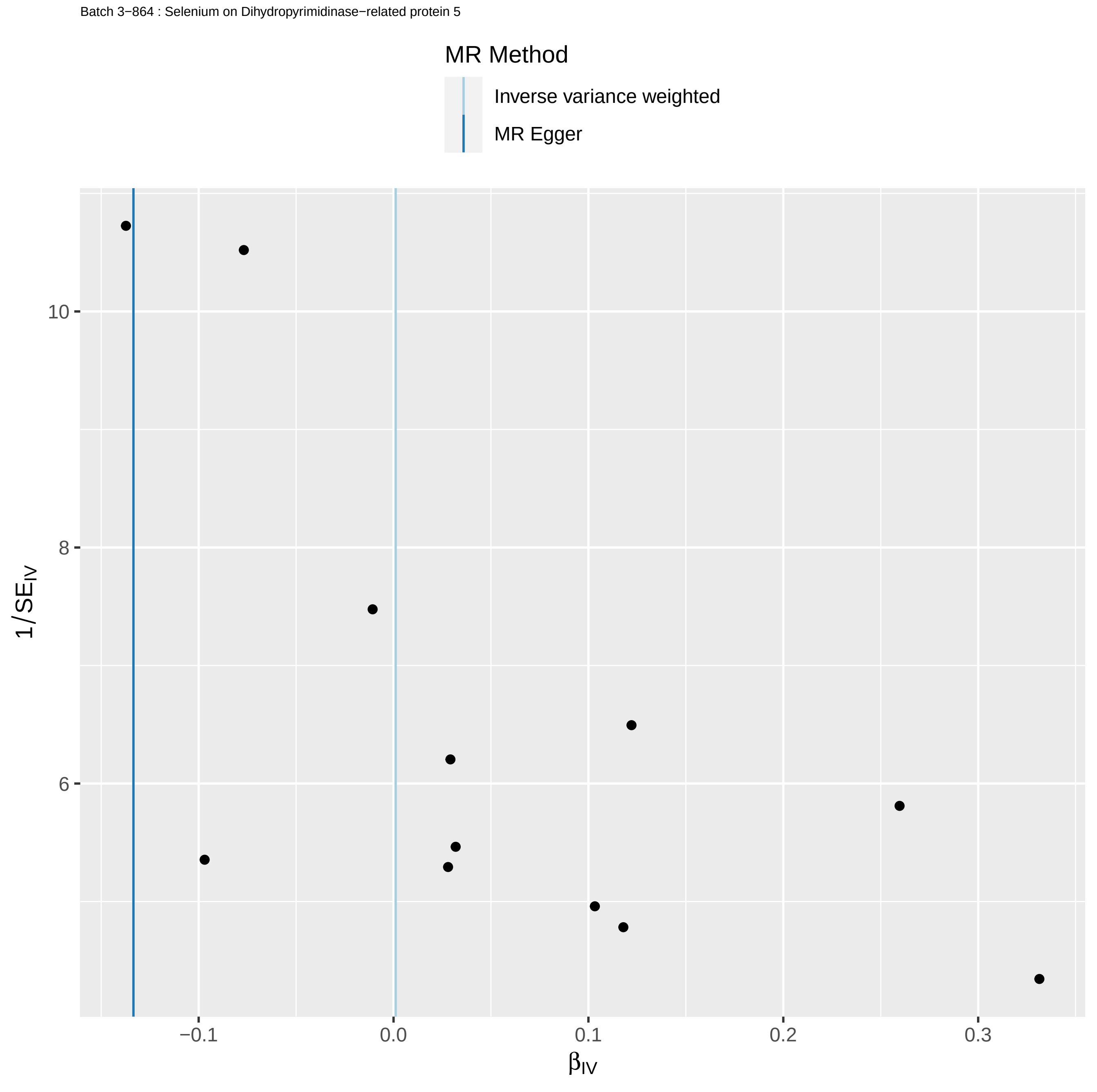
Batch 3−864 : Selenium on Dihydropyrimidinase−related protein 5
MR Method
Inverse variance weighted
MR Egger
10
8
6
1/SE_IV
−0.1
0.0
0.1
0.2
0.3
β_IV

Batch 3−865 : Selenium on Dorsal root ganglia homeobox protein

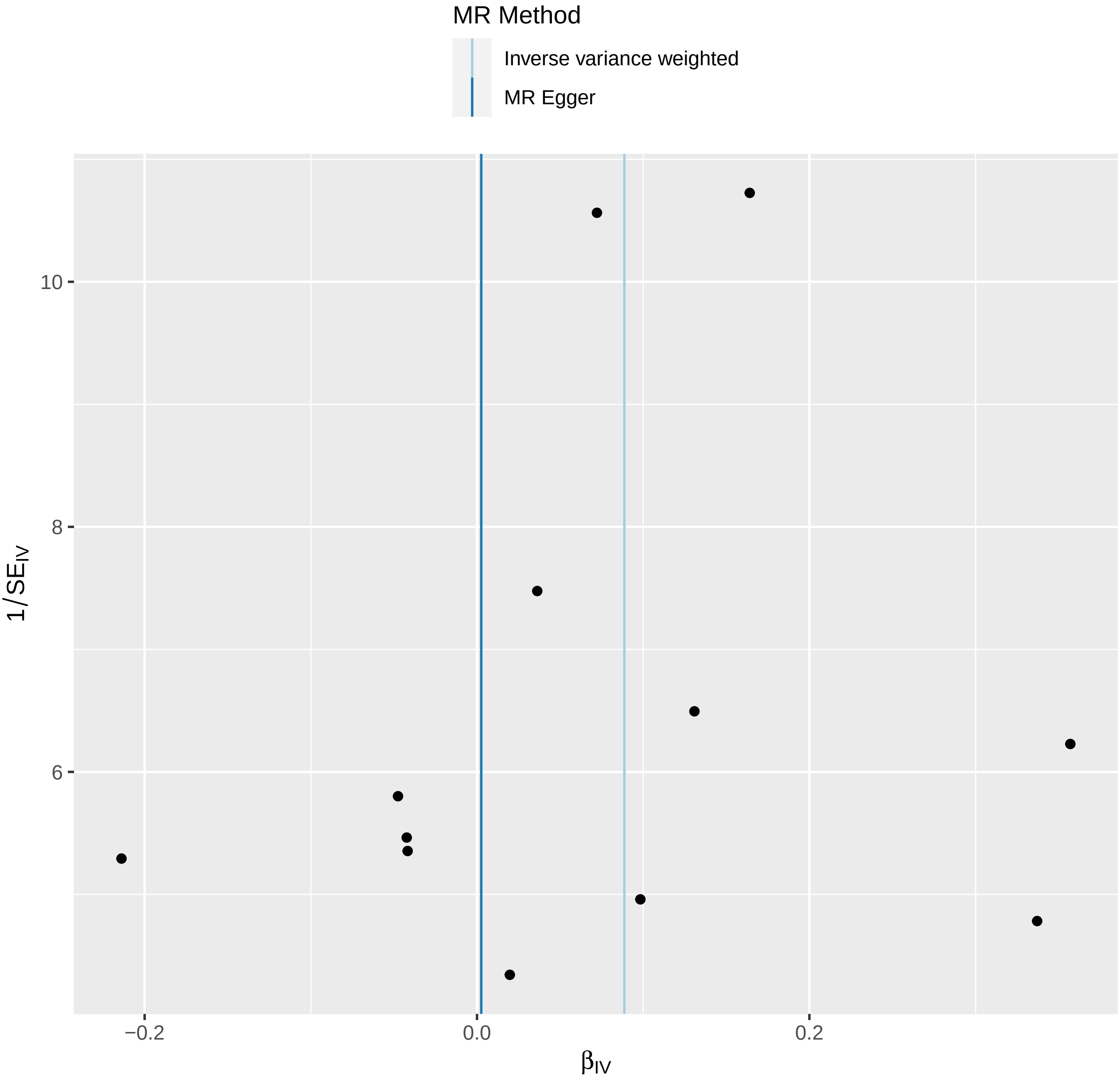

Batch 3−866 : Selenium on Desmocollin−2

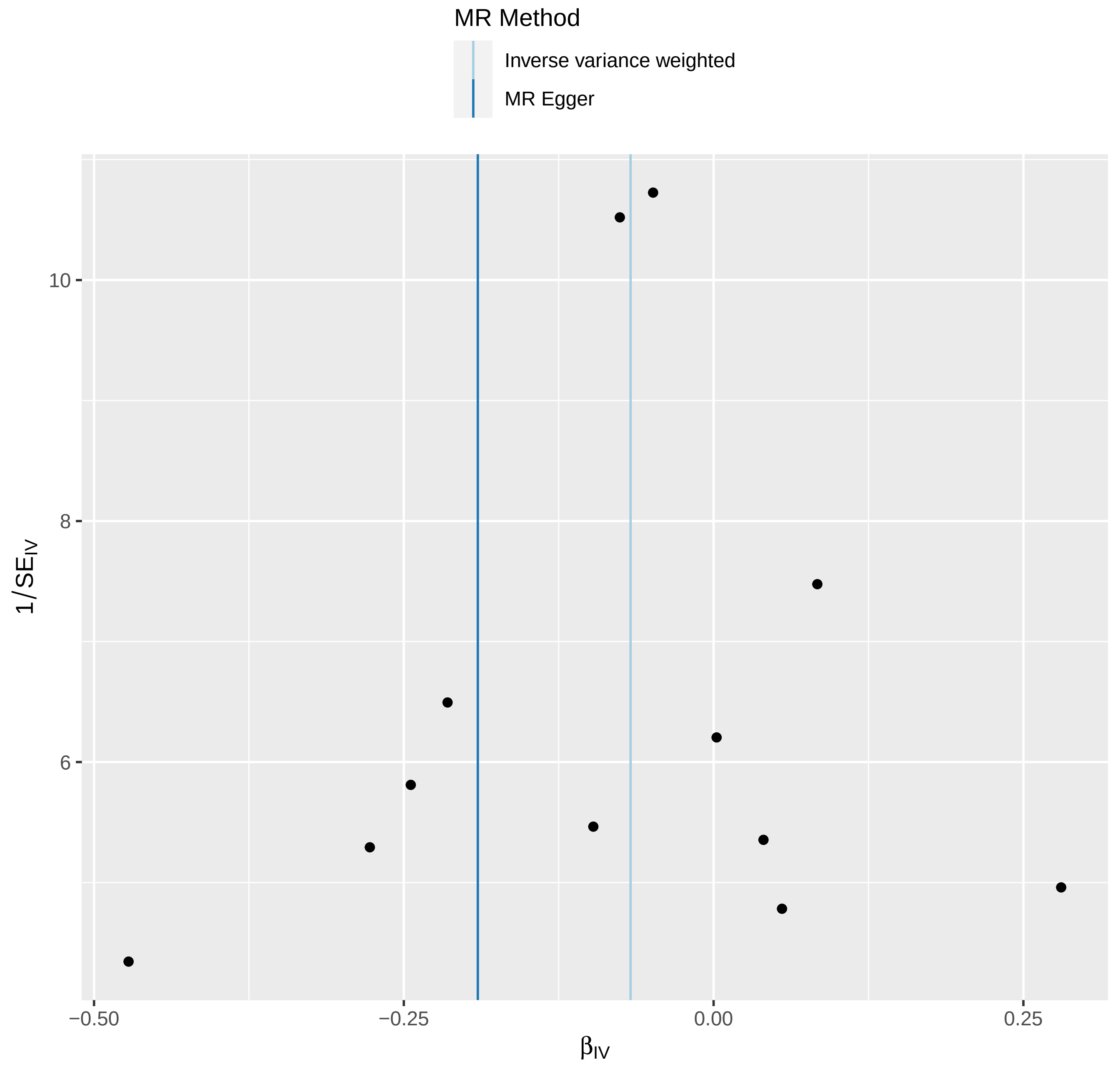

Batch 3−867 : Selenium on Desmocollin−3

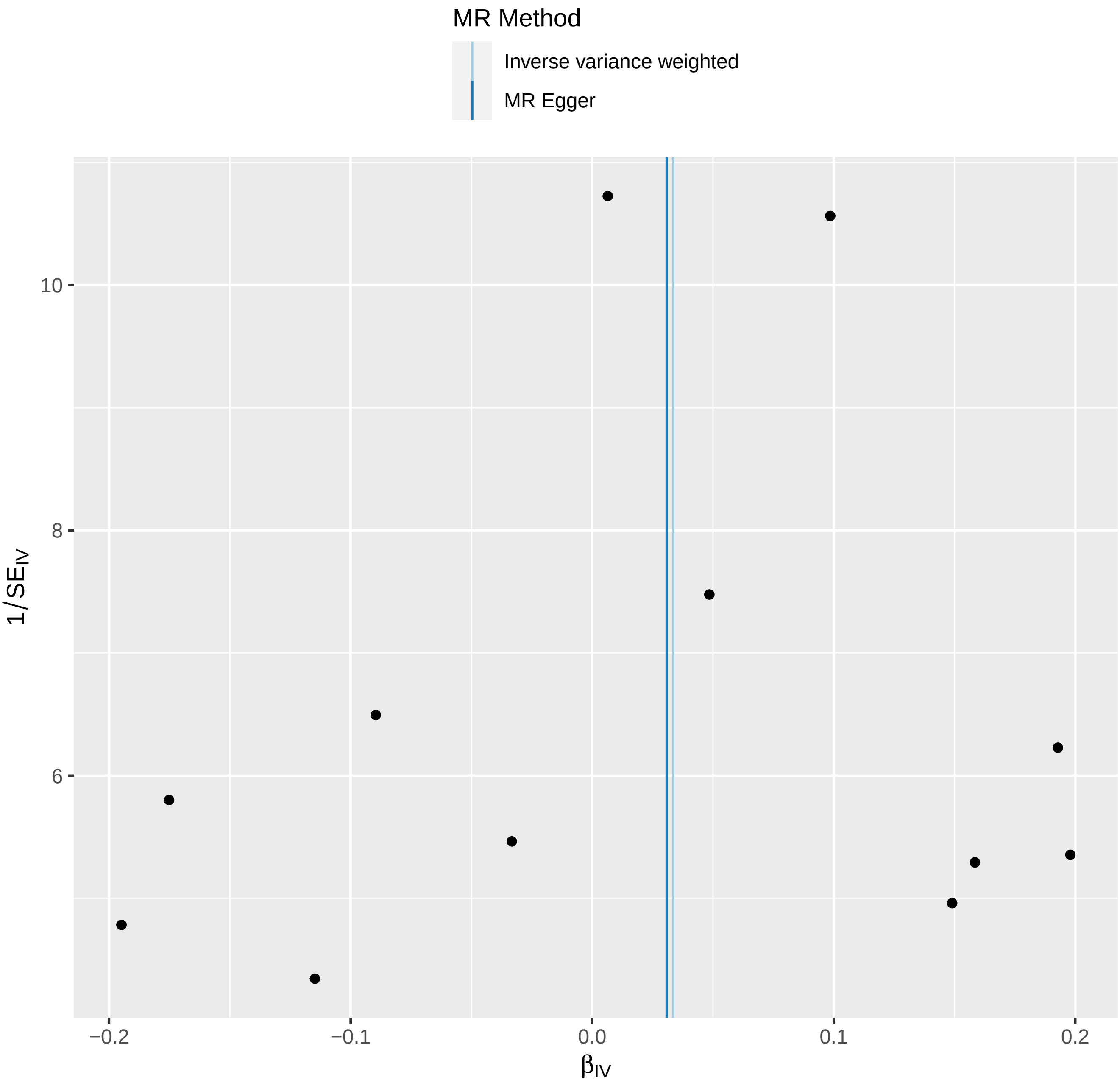

Batch 3−868 : Selenium on Down syndrome cell adhesion molecule

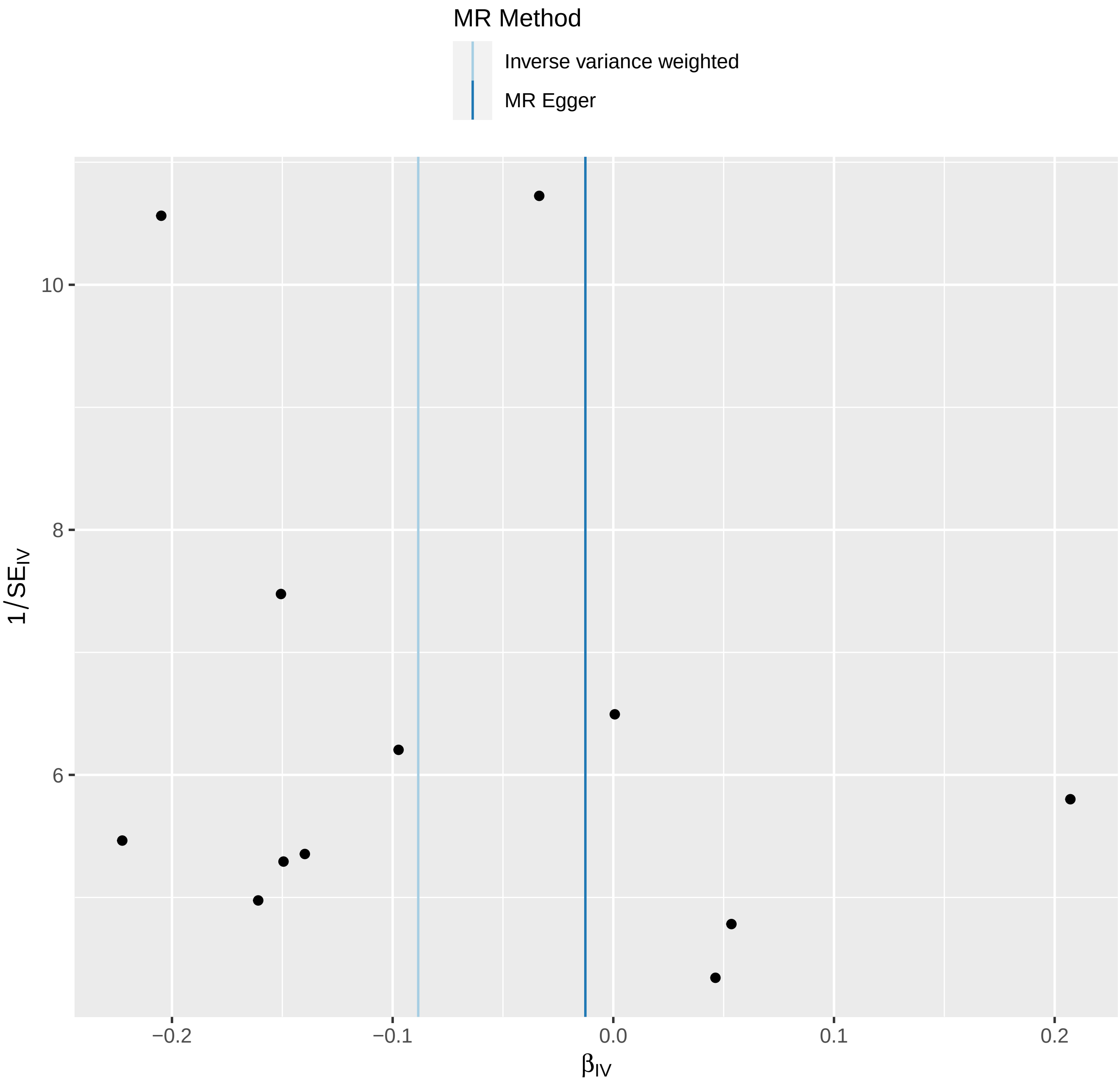

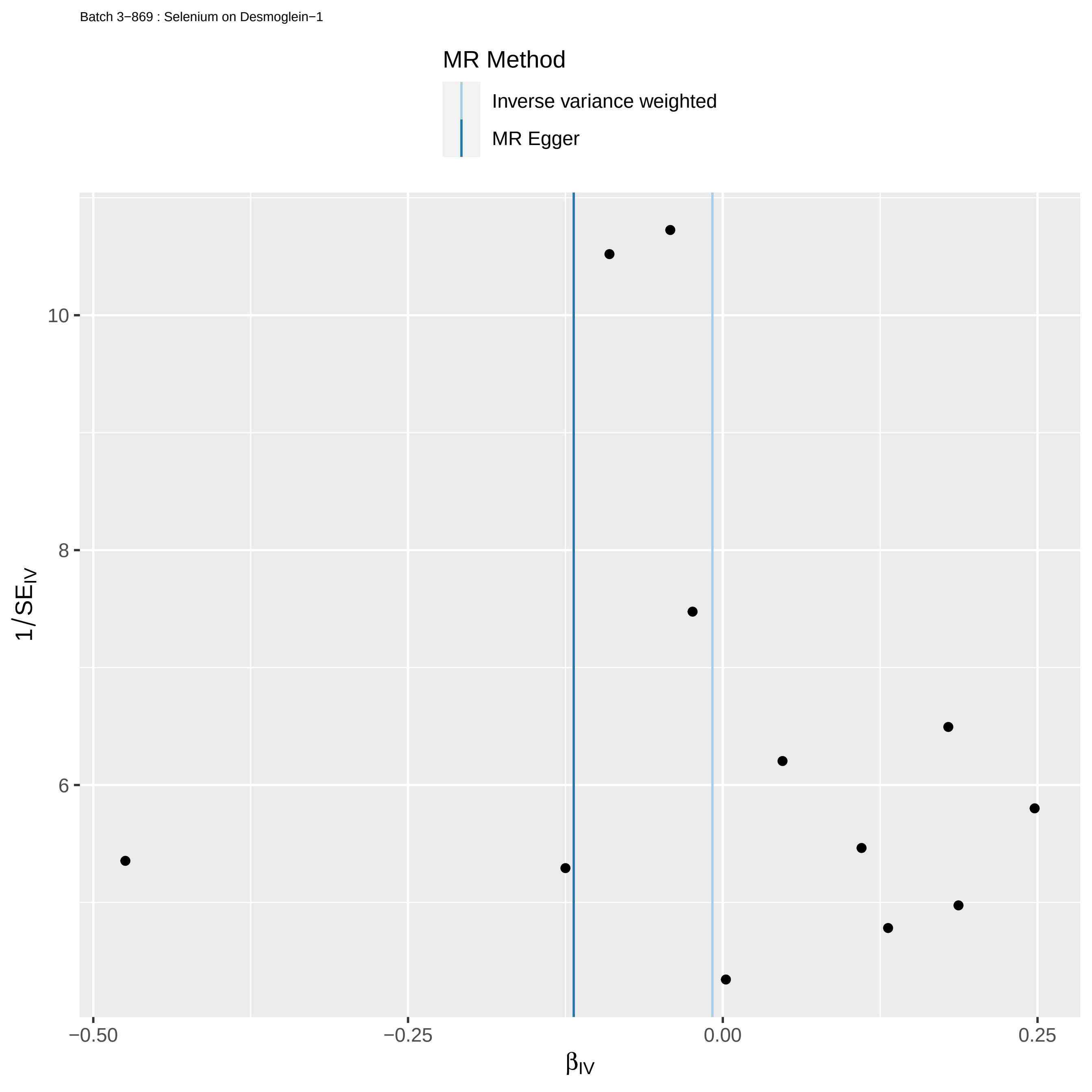
Batch 3−869 : Selenium on Desmoglein−1
MR Method
Inverse variance weighted
MR Egger
10
8
6
1/SE$_{IV}$
−0.50
−0.25
0.00
0.25
β$_{IV}$

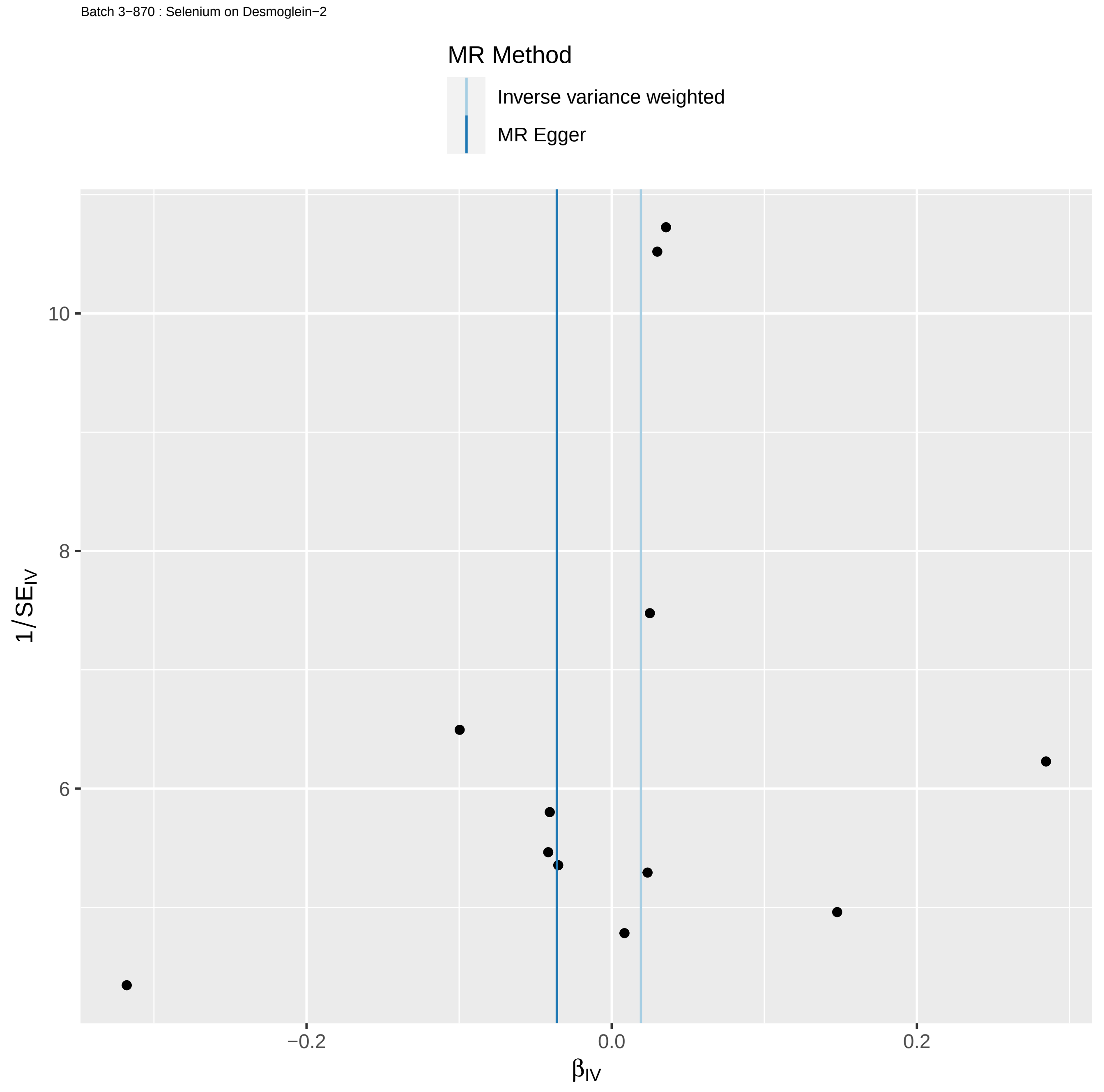
Batch 3−870 : Selenium on Desmoglein−2
MR Method
Inverse variance weighted
MR Egger
10
8
6
1/SE_IV
−0.2
0.0
0.2
β_IV

Batch 3−871 : Selenium on E3 ubiquitin−protein ligase DTX1

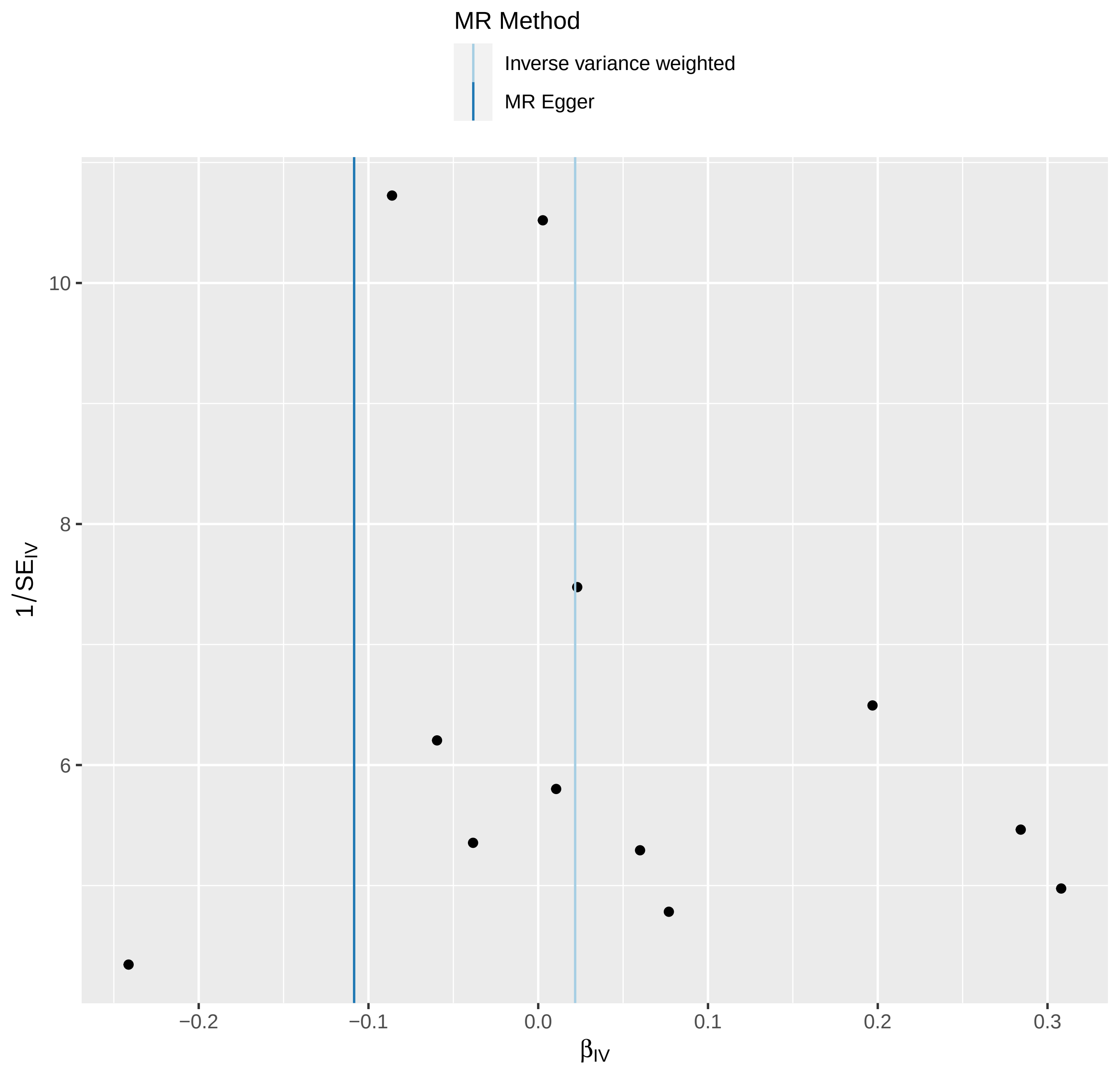

Batch 3−872 : Selenium on E3 ubiquitin−protein ligase DTX3L

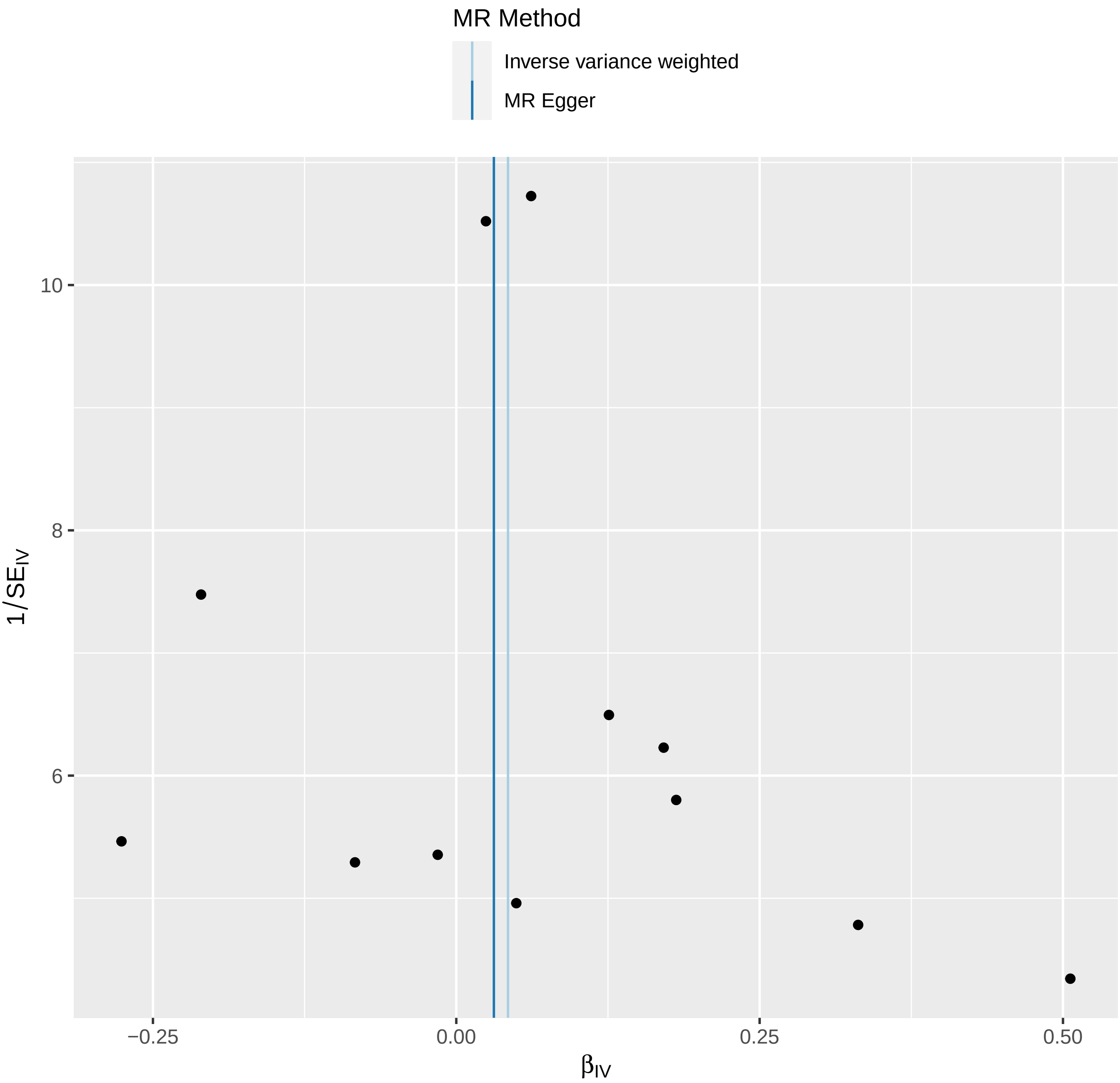

Batch 3−873 : Selenium on Dual specificity protein phosphatase 13 isoform A

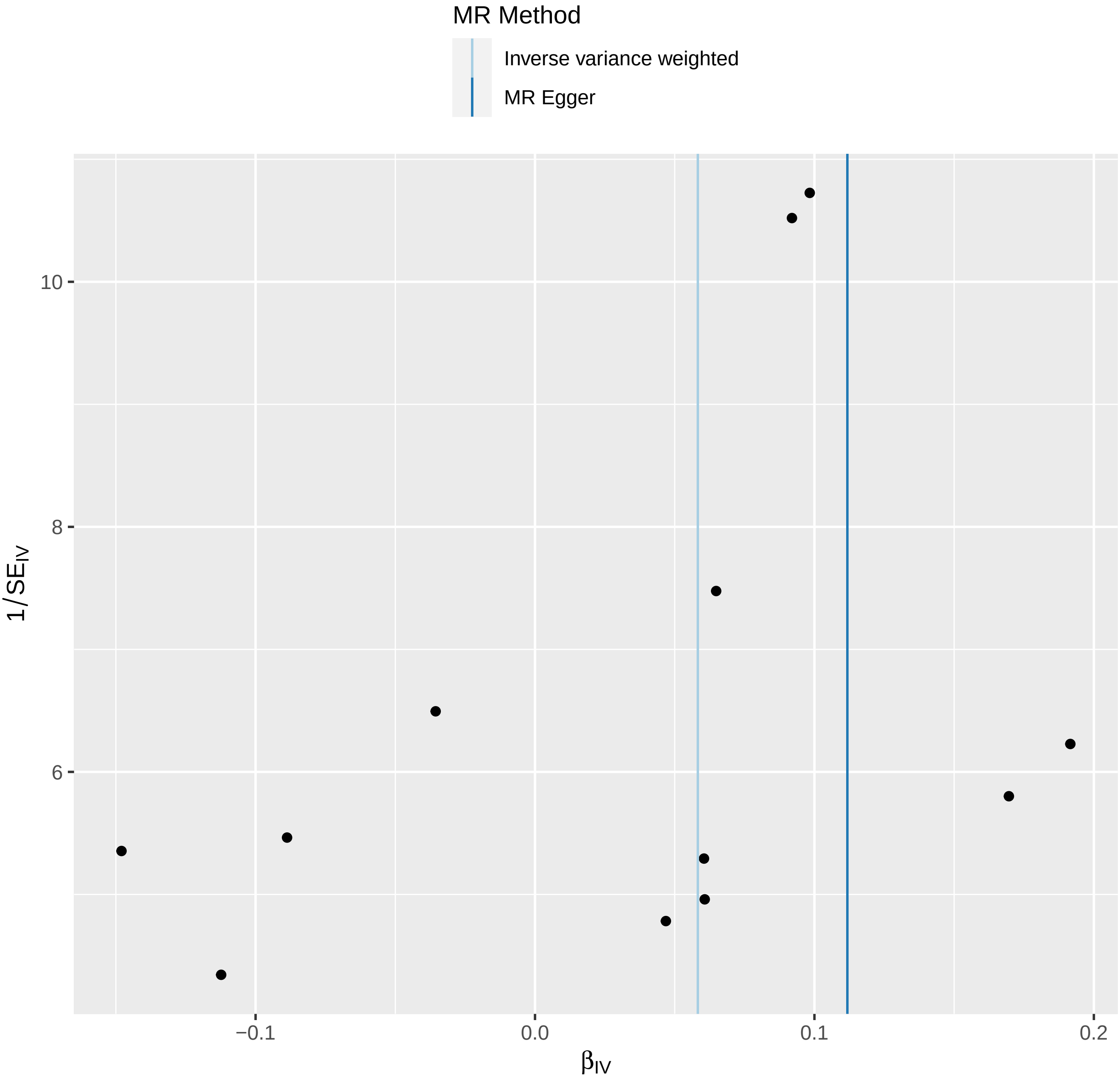

Batch 3−874 : Selenium on Dual specificity protein phosphatase 15

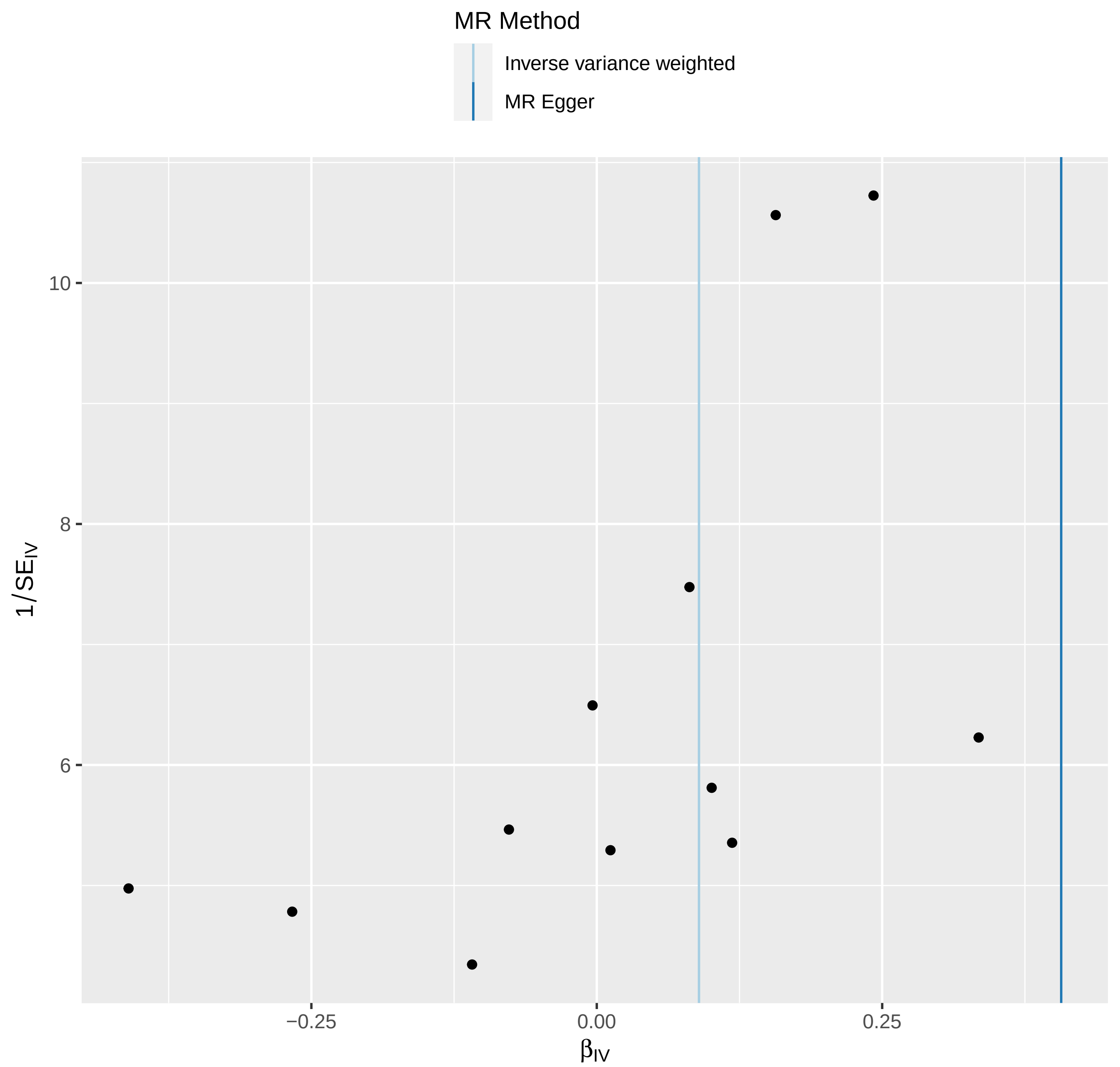

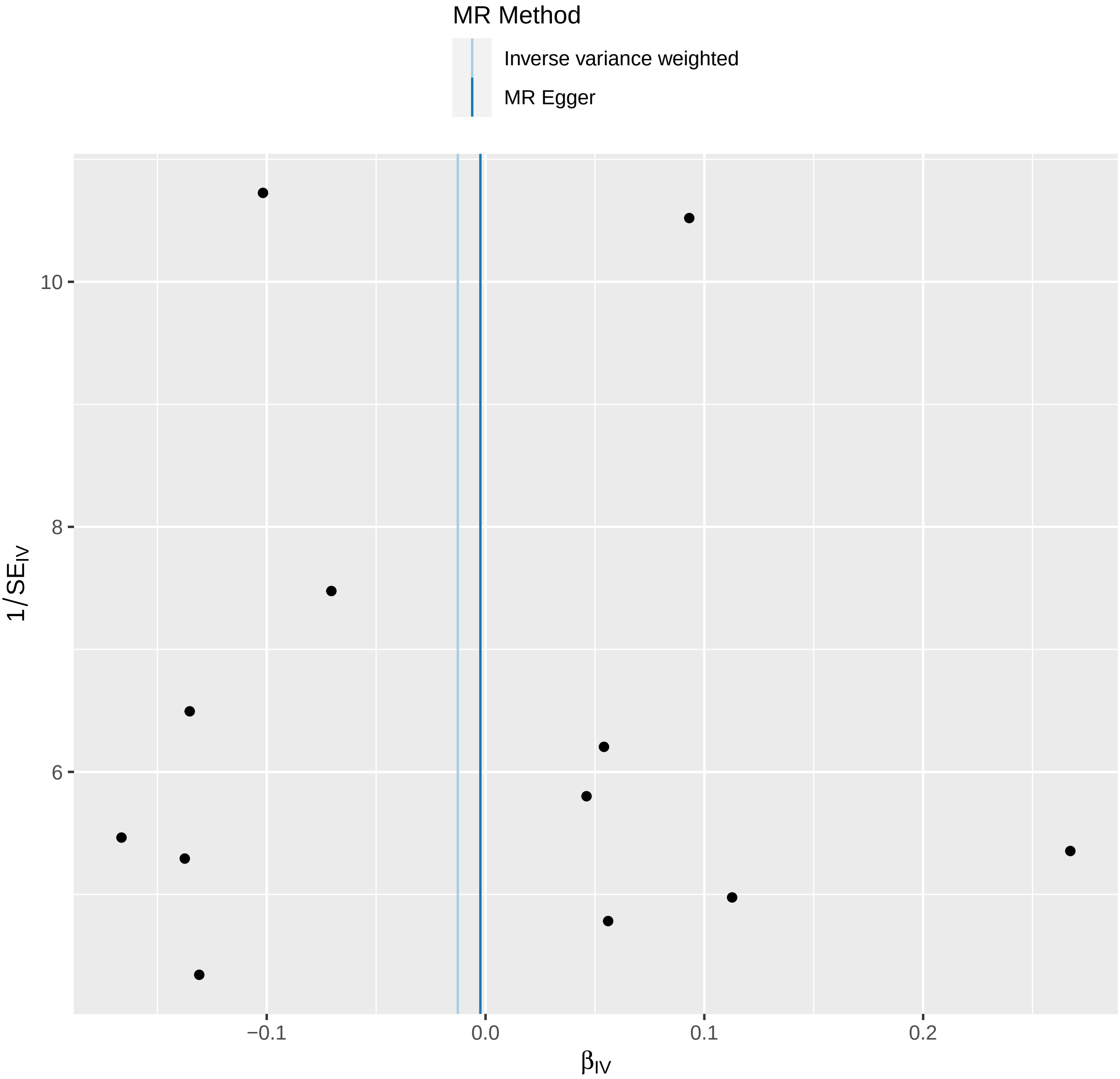

MR Method
Inverse variance weighted
MR Egger
10
8
6
1/SE_IV
−0.1
0.0
0.1
0.2
β_IV

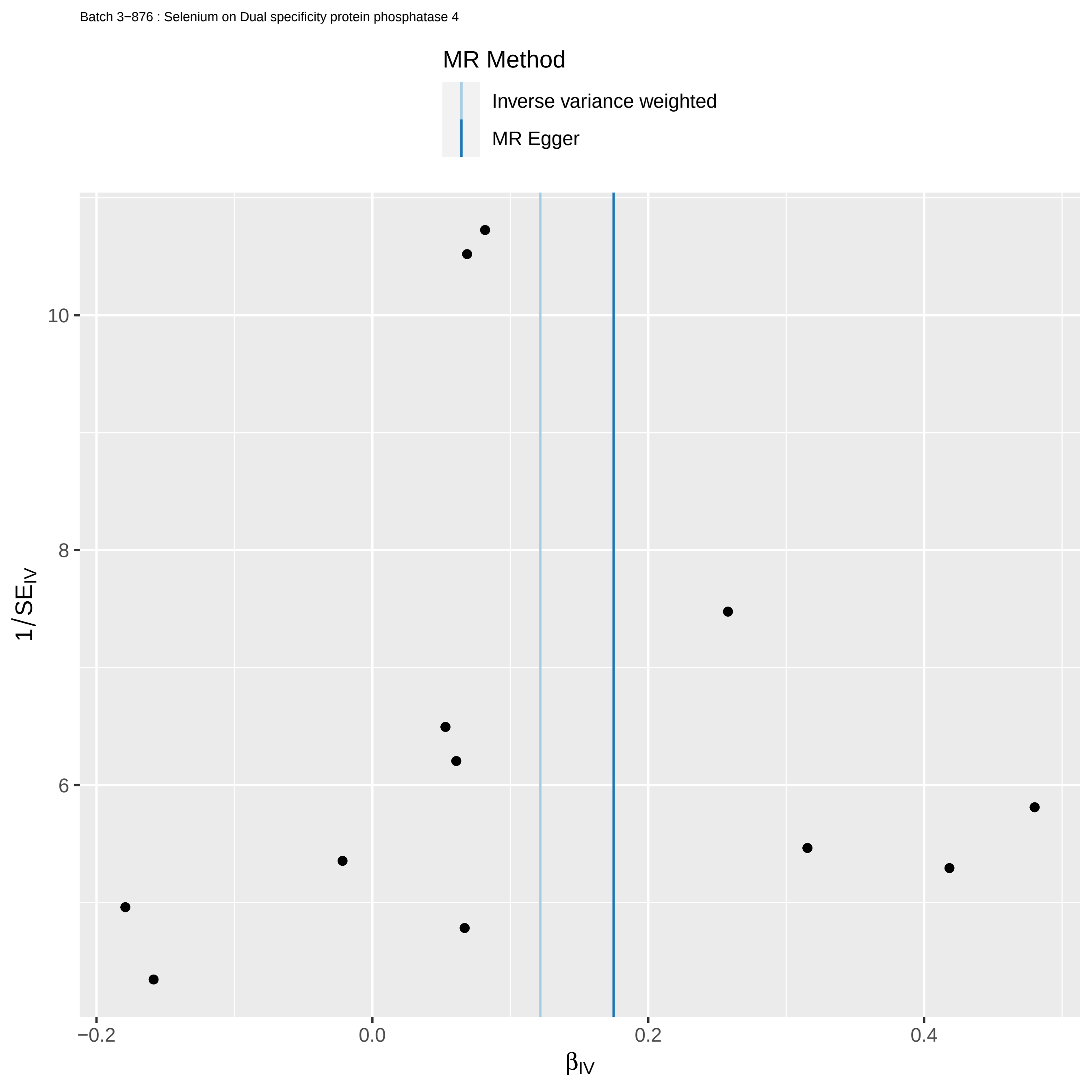
Batch 3−876 : Selenium on Dual specificity protein phosphatase 4
MR Method
Inverse variance weighted
MR Egger
10
8
6
1/SE_IV
−0.2
0.0
0.2
0.4
β_IV

Batch 3−877 : Selenium on Dual specificity protein phosphatase 6

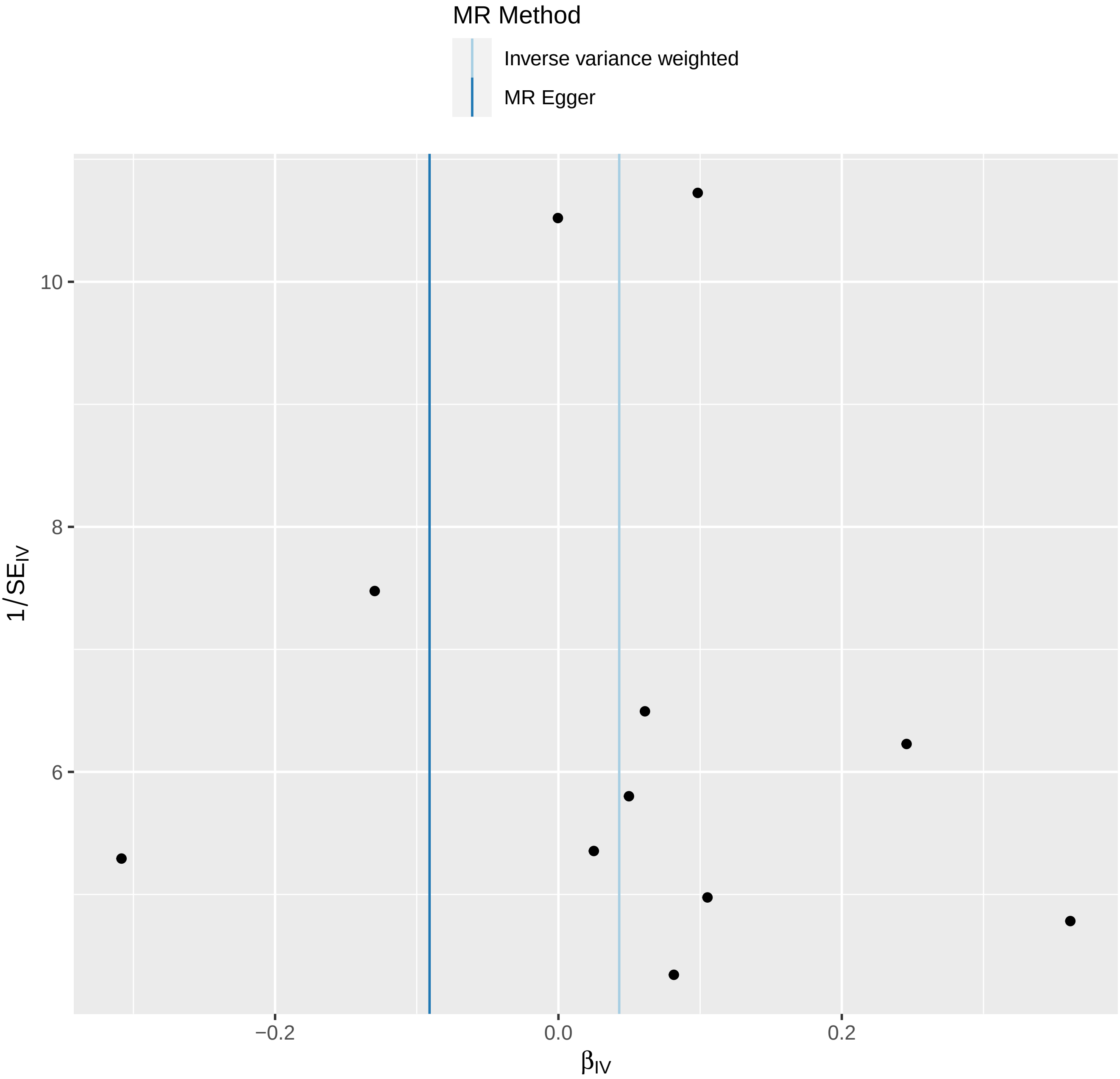

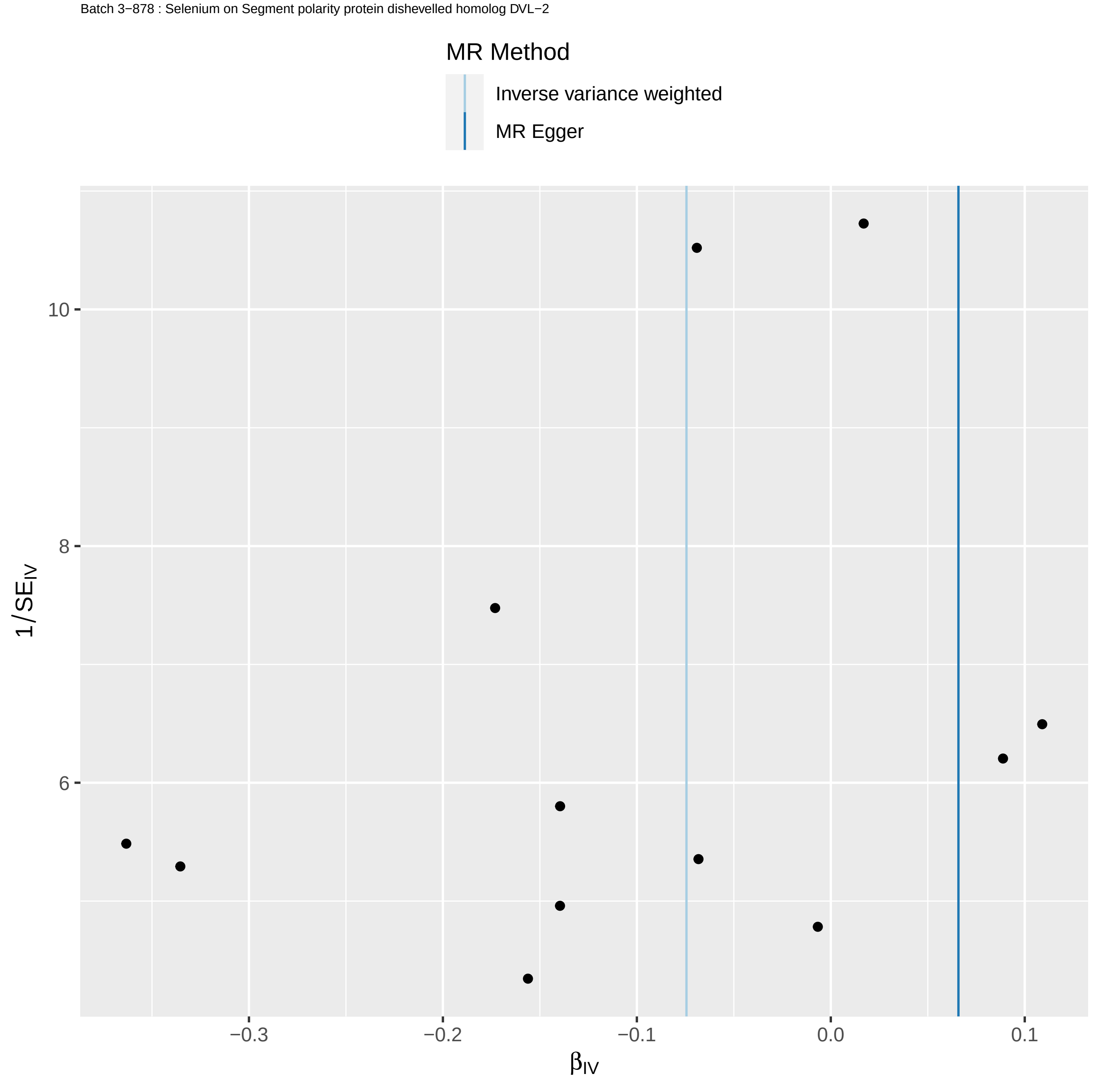
Batch 3−878 : Selenium on Segment polarity protein dishevelled homolog DVL−2
MR Method
Inverse variance weighted
MR Egger
10
8
6
1/SE$_{IV}$
−0.3
−0.2
−0.1
0.0
0.1
β$_{IV}$

Batch 3−879 : Selenium on Dynactin−associated protein

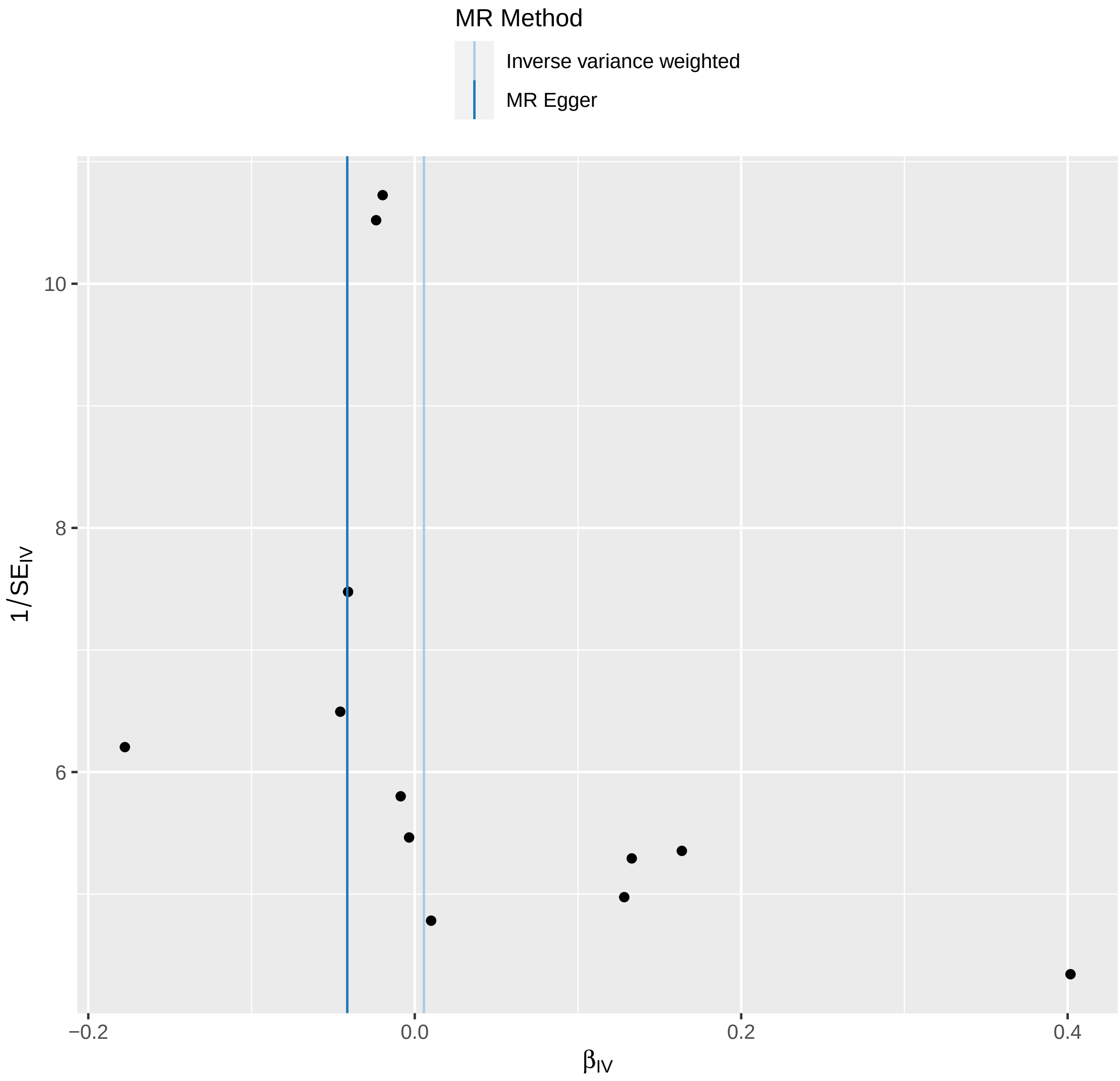

Batch 3−880 : Selenium on Dynein light chain 1, cytoplasmic

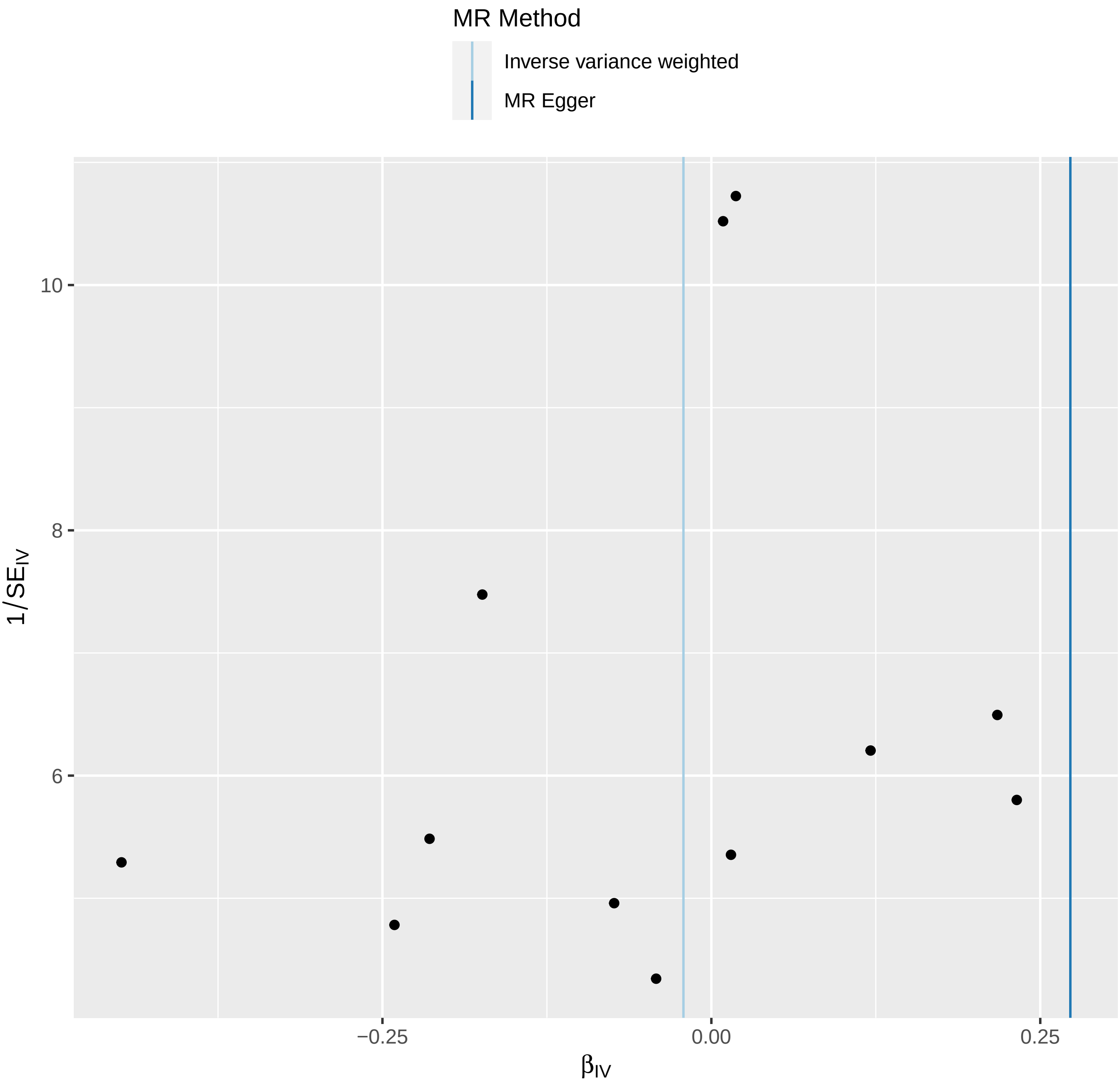

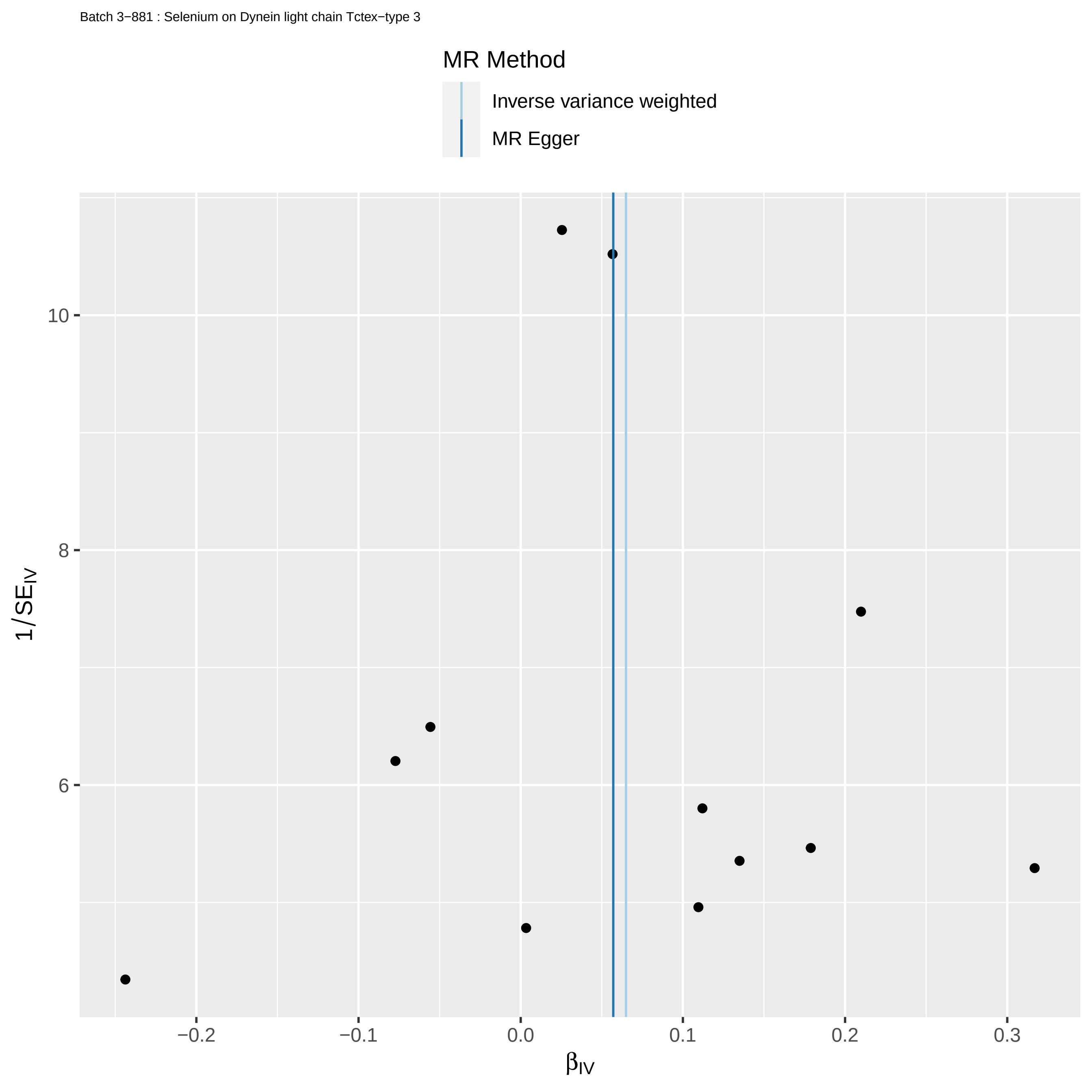

Batch 3−881 : Selenium on Dynein light chain Tctex−type 3
MR Method
Inverse variance weighted
MR Egger
10
8
6
1/SE_IV
−0.2
−0.1
0.0
0.1
0.2
0.3
β_IV

Batch 3−882 : Selenium on Dual specificity tyrosine−phosphorylation−regulated kinase 3

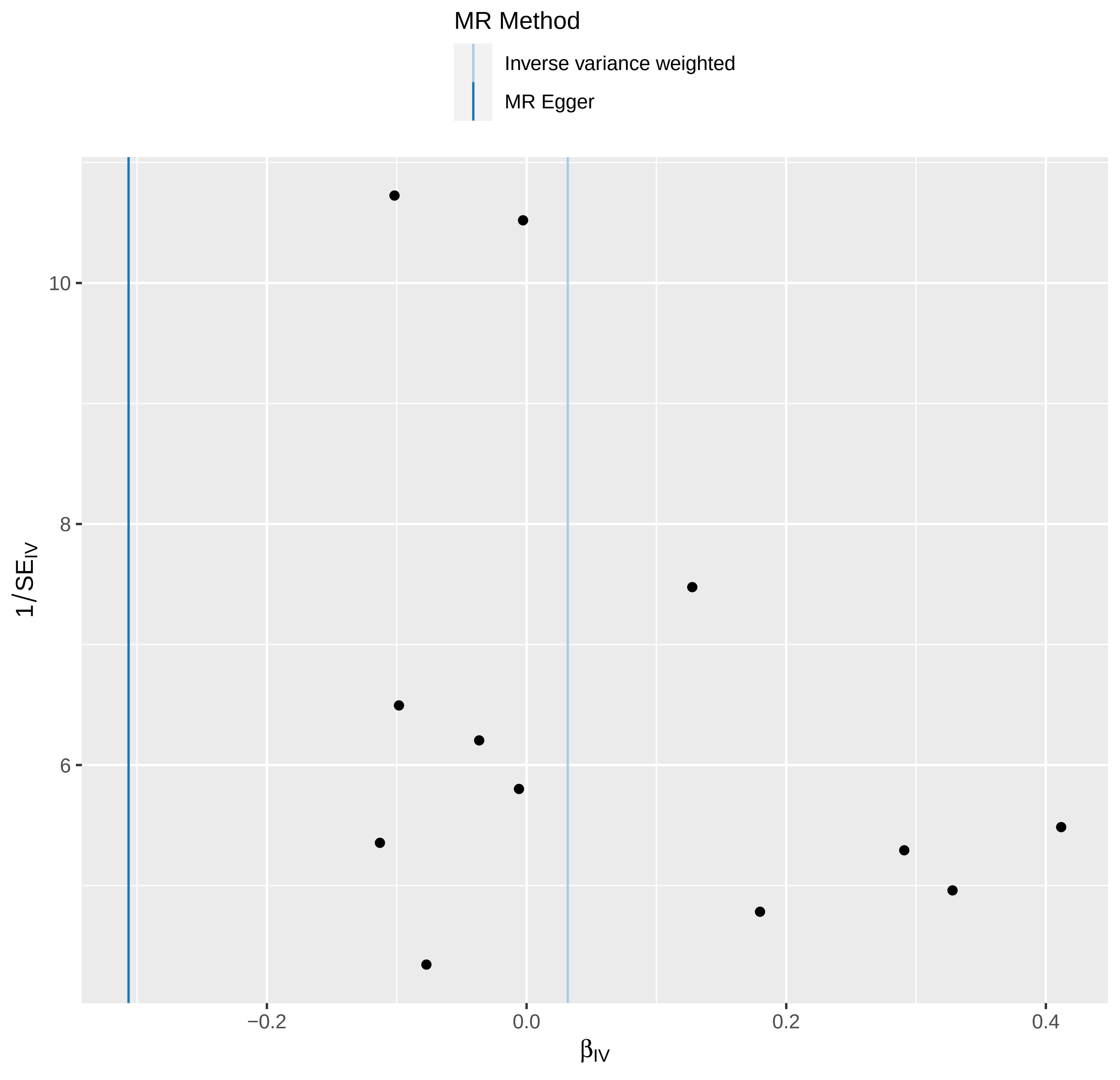

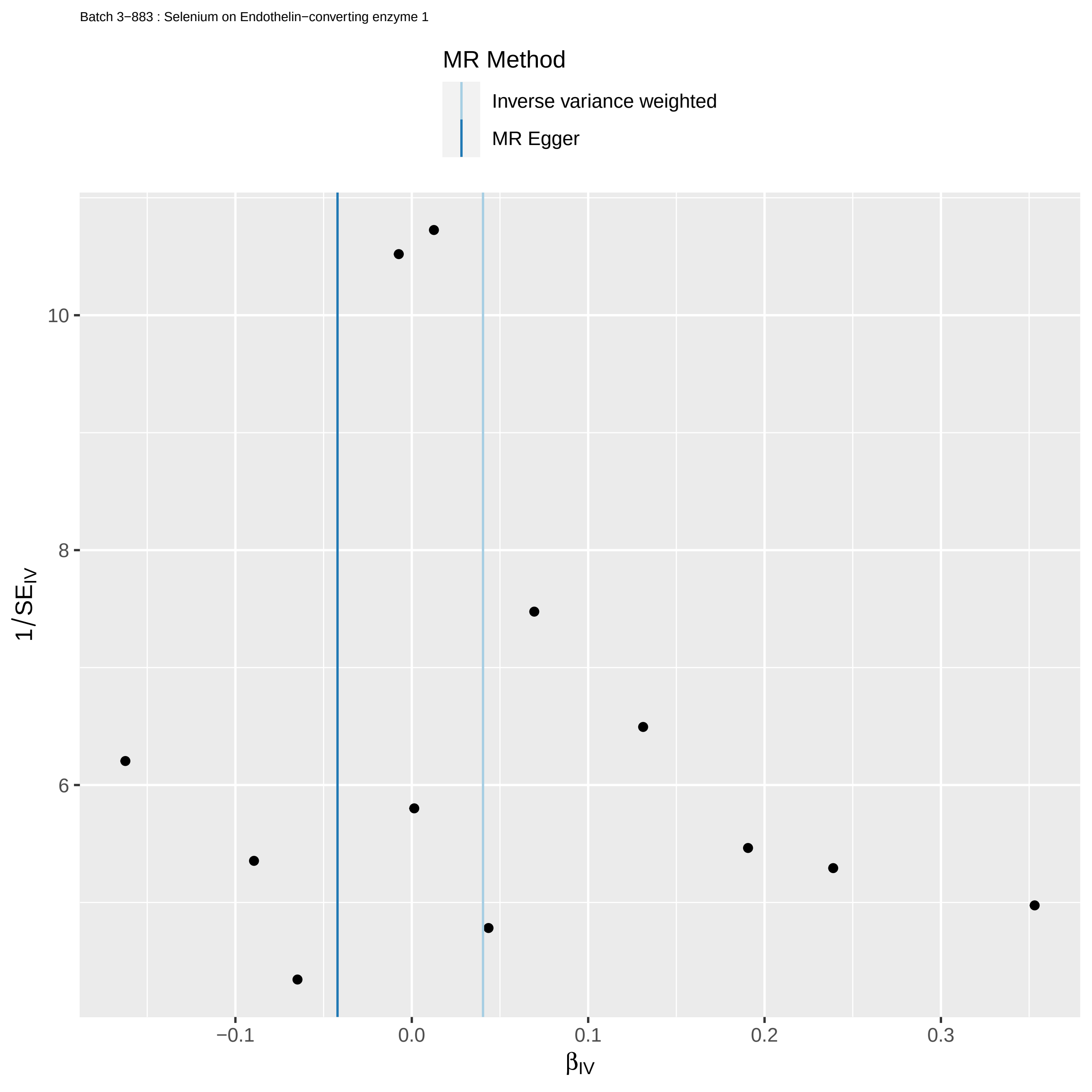
Batch 3−883 : Selenium on Endothelin−converting enzyme 1
MR Method
Inverse variance weighted
MR Egger
10
8
6
$1/SE_{IV}$
−0.1
0.0
0.1
0.2
0.3
β_{IV}

Batch 3−884 : Selenium on Endothelin−converting enzyme 1

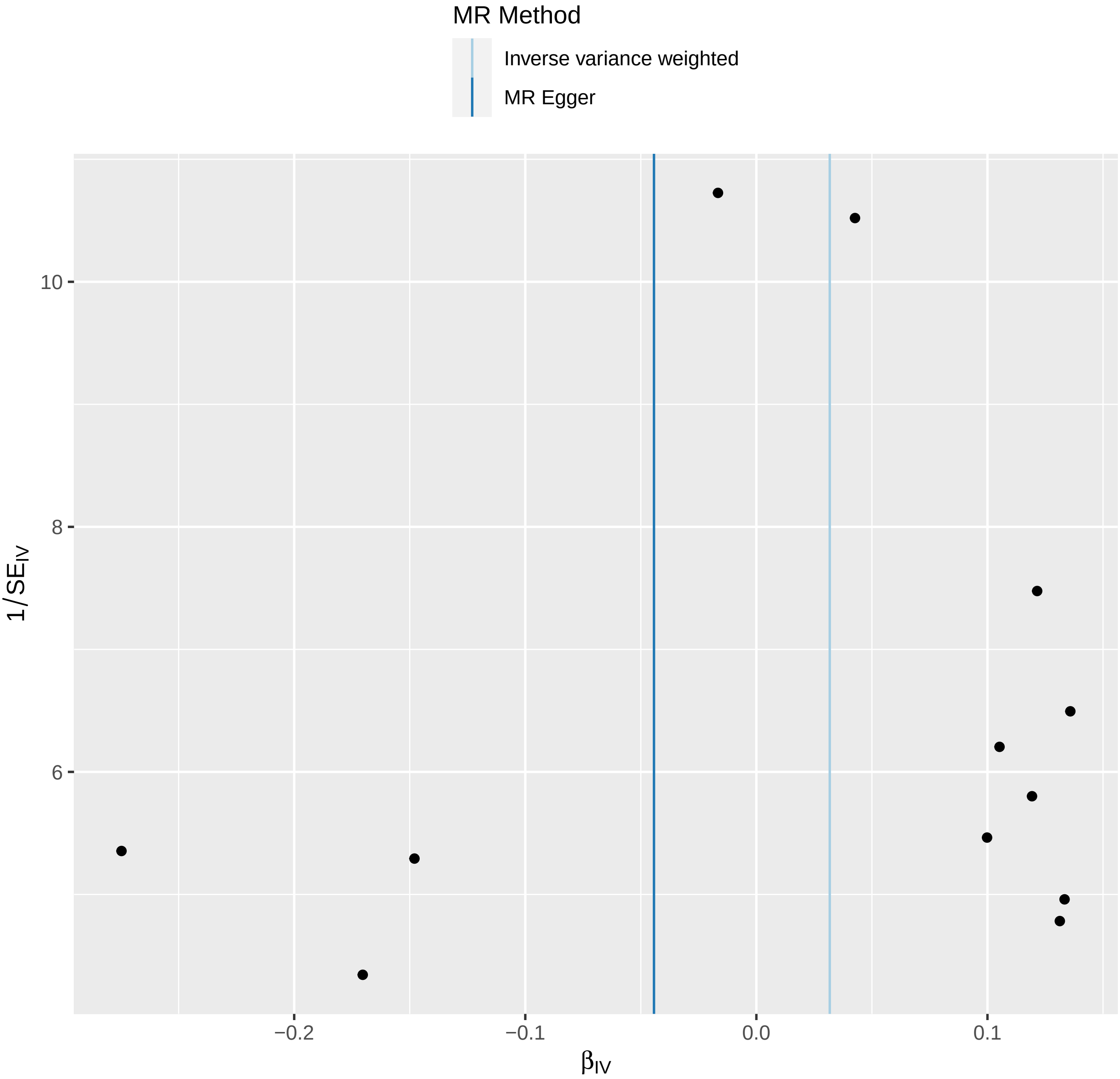

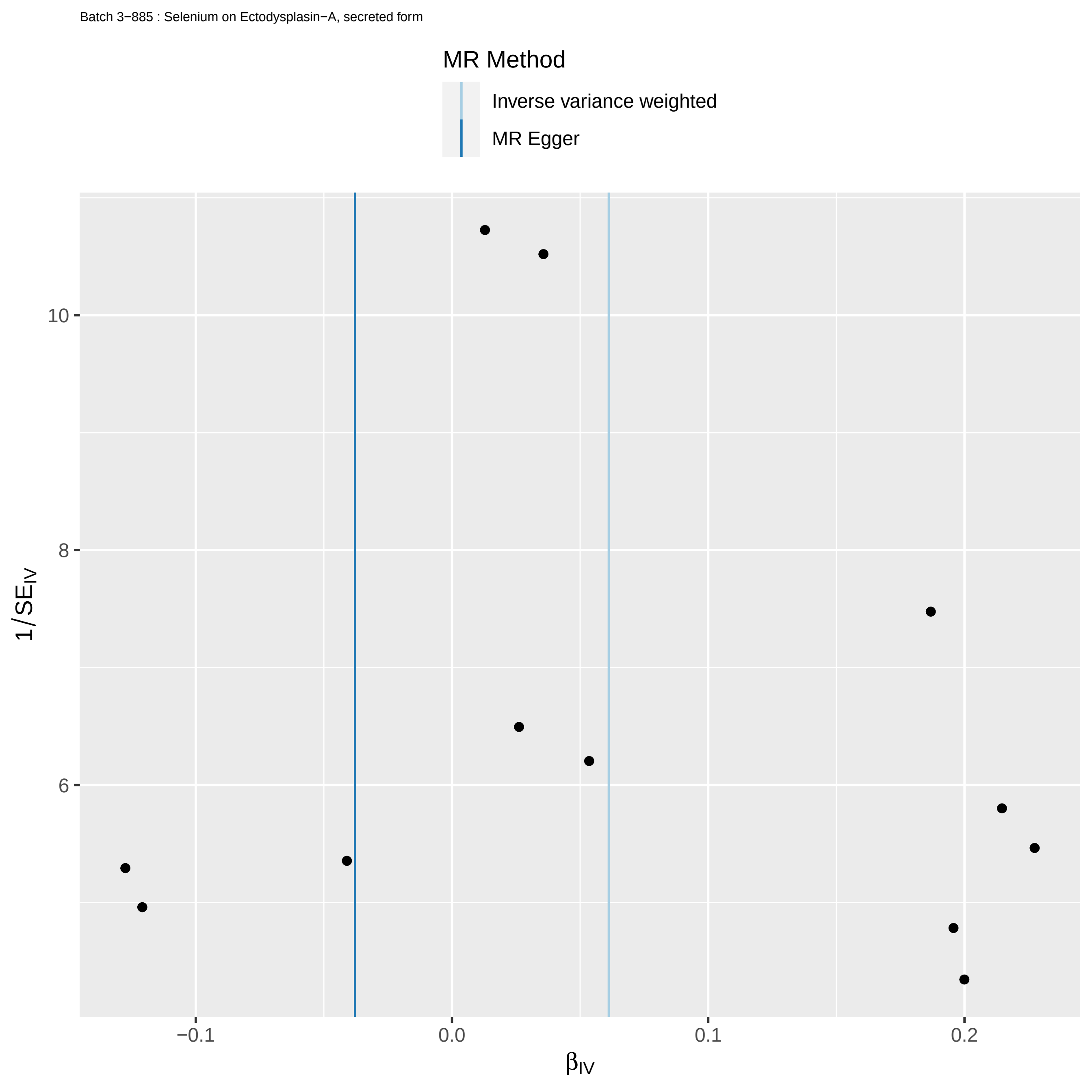
Batch 3−885 : Selenium on Ectodysplasin−A, secreted form
MR Method
Inverse variance weighted
MR Egger
10
8
6
1/SE$_{IV}$
−0.1
0.0
0.1
0.2
β_{IV}

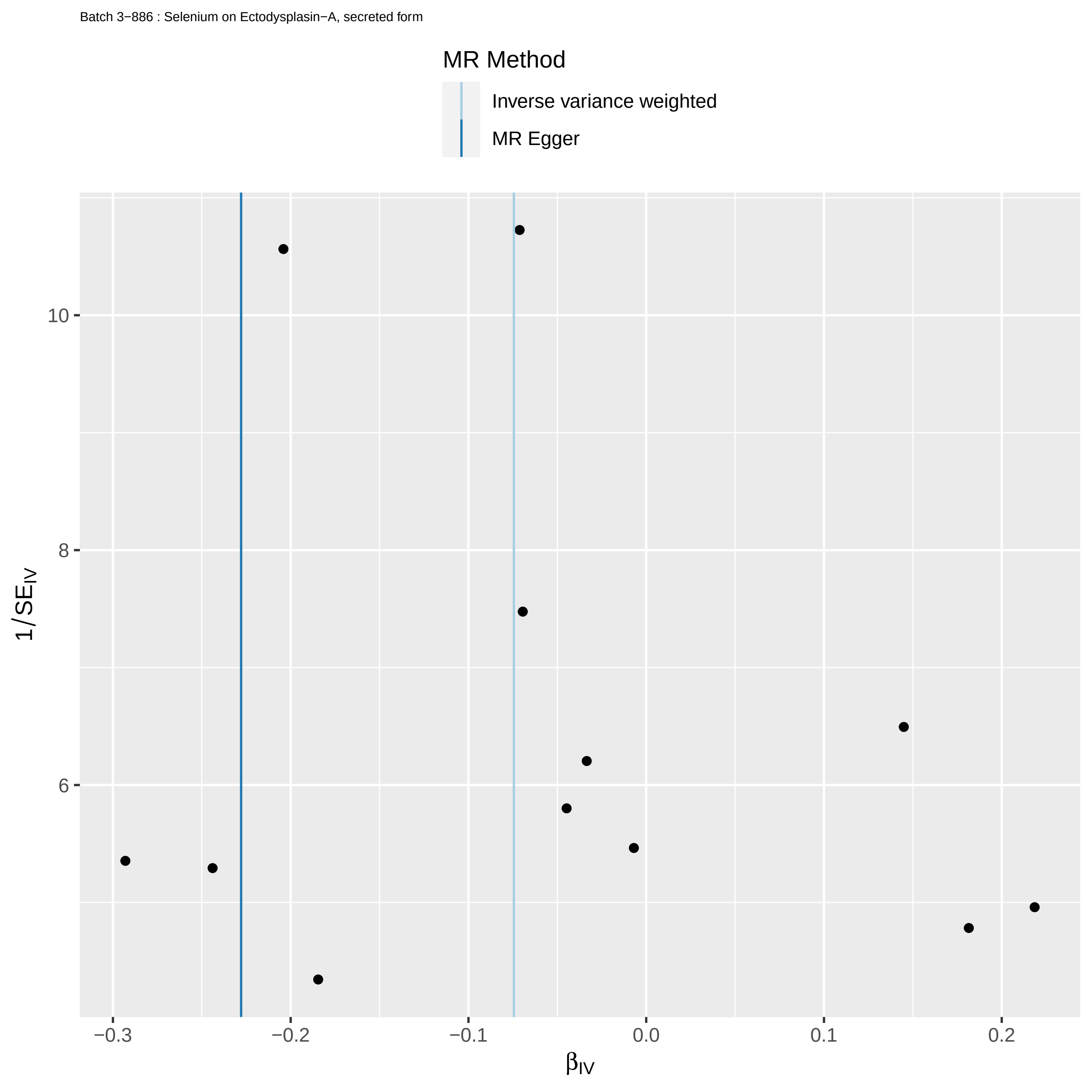
Batch 3−886 : Selenium on Ectodysplasin−A, secreted form
MR Method
Inverse variance weighted
MR Egger
10
8
6
1/SE$_{IV}$
−0.3
−0.2
−0.1
0.0
0.1
0.2
β_{IV}

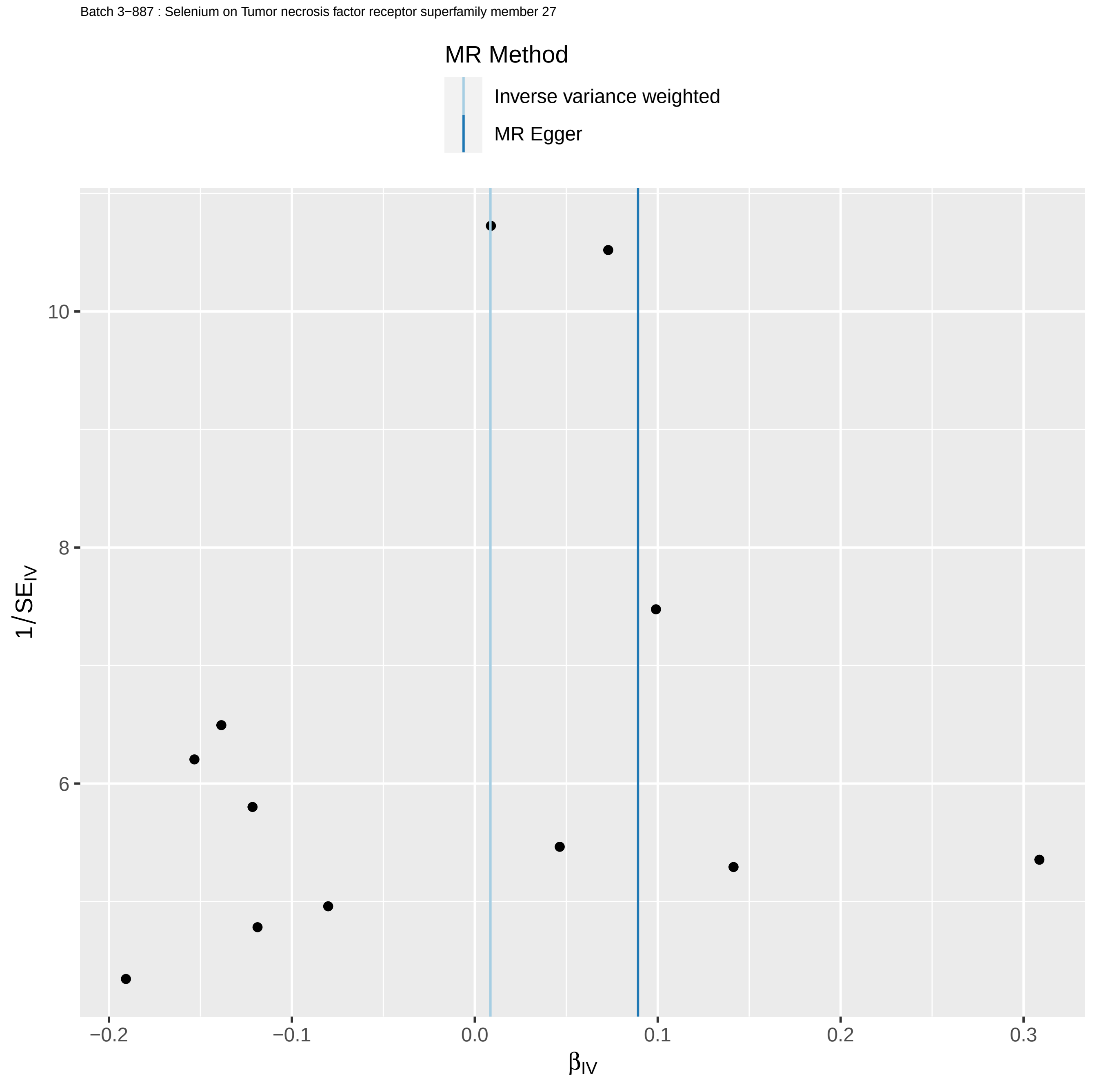
Batch 3−887 : Selenium on Tumor necrosis factor receptor superfamily member 27
MR Method
Inverse variance weighted
MR Egger
10
8
6
1/SE_IV
−0.2
−0.1
0.0
0.1
0.2
0.3
β_IV

Batch 3−888 : Selenium on Tumor necrosis factor receptor superfamily member EDAR

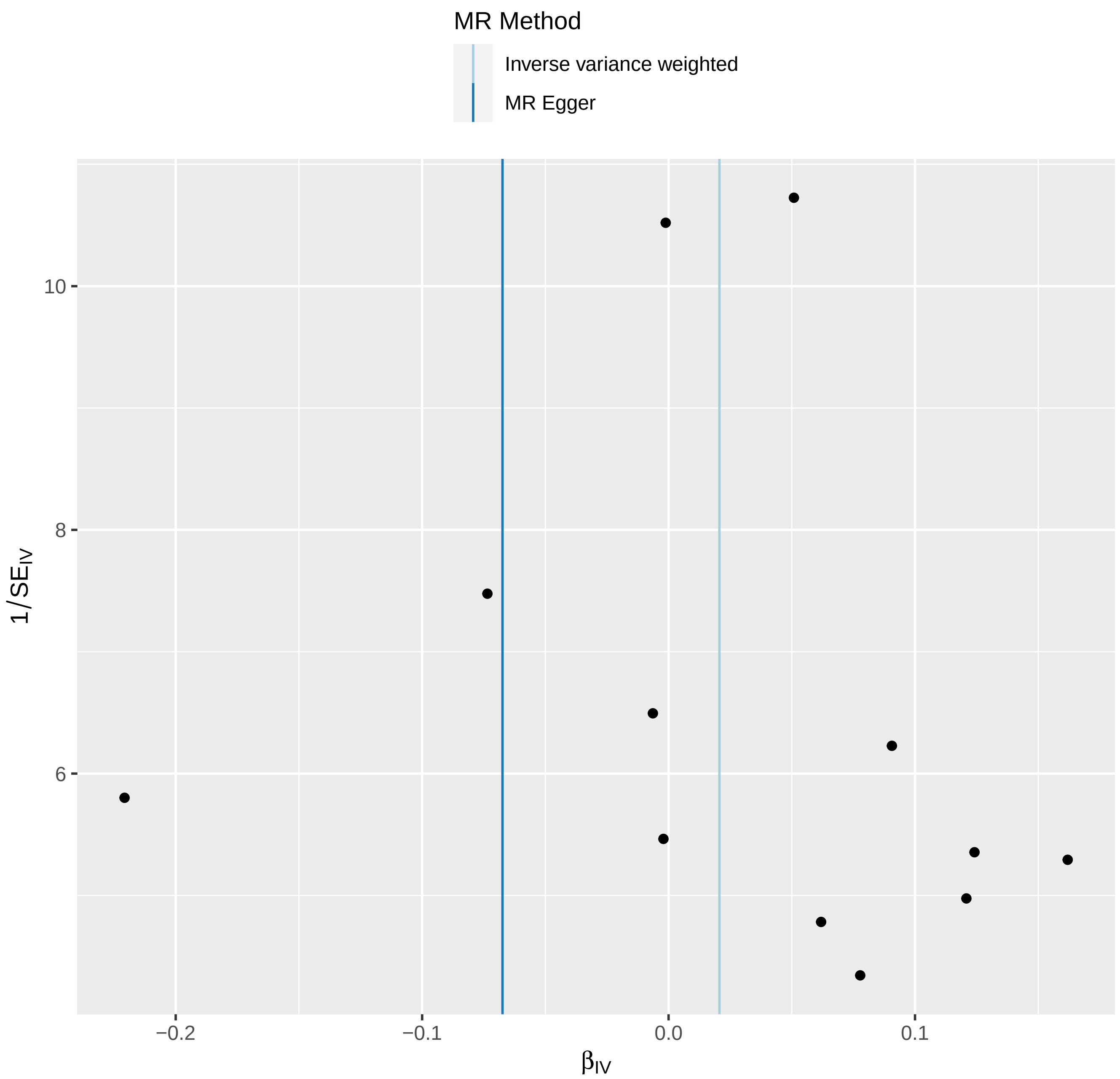

Batch 3−889 : Selenium on Enhancer of mRNA−decapping protein 4

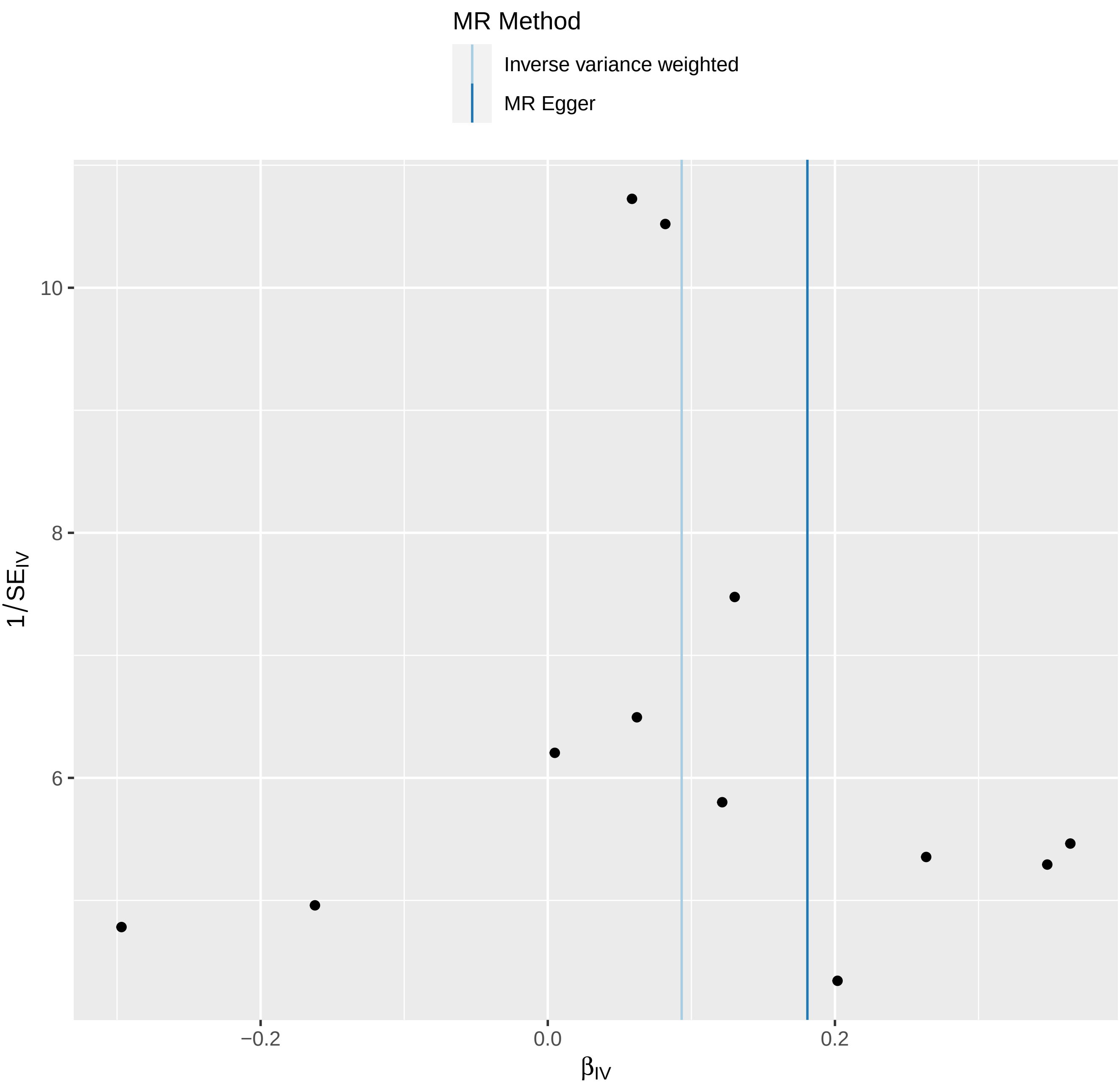

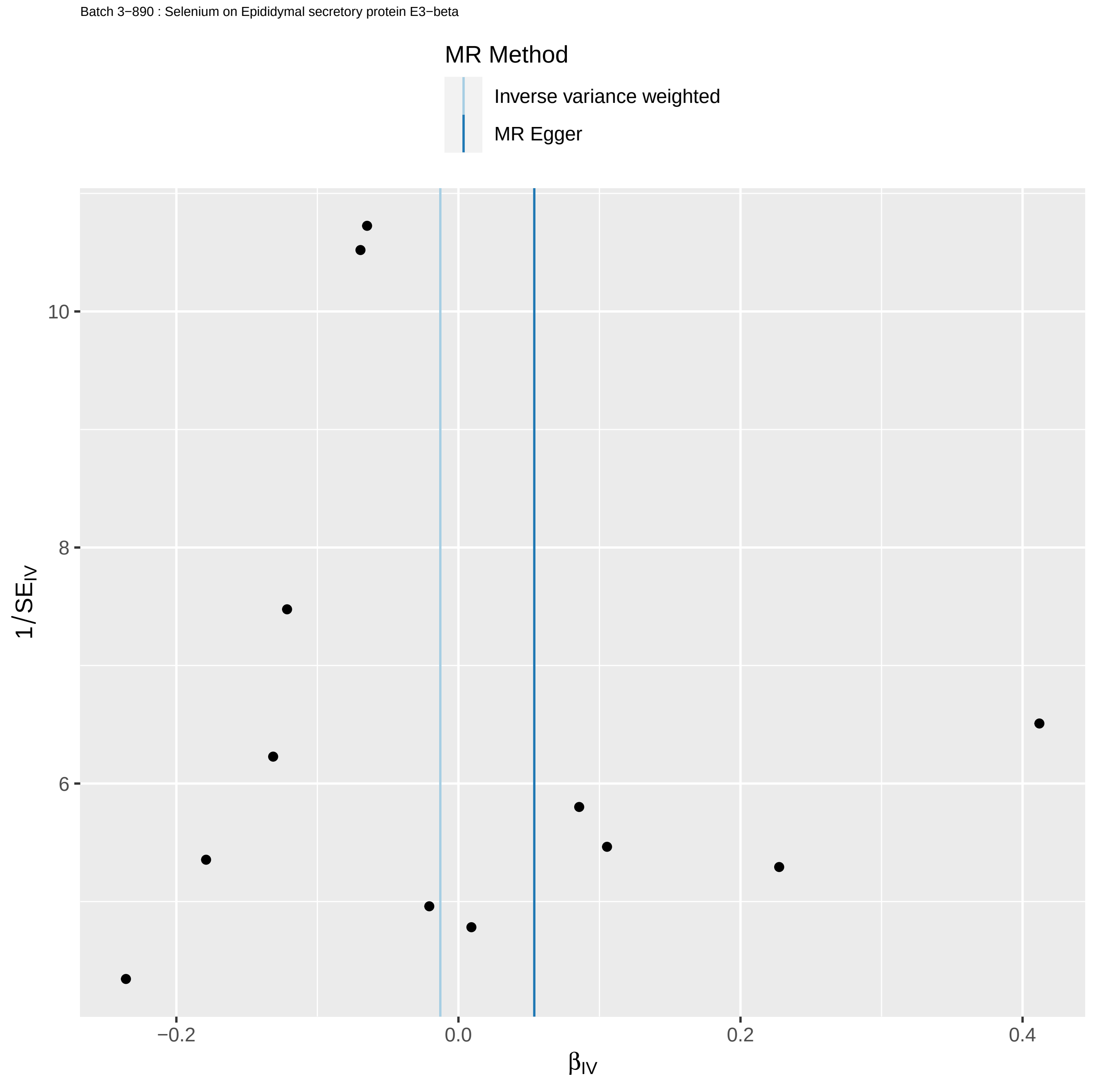
Batch 3−890 : Selenium on Epididymal secretory protein E3−beta
MR Method
Inverse variance weighted
MR Egger
10
8
6
1/SE_IV
−0.2
0.0
0.2
0.4
β_IV

Batch 3−891 : Selenium on Endothelial differentiation−related factor 1

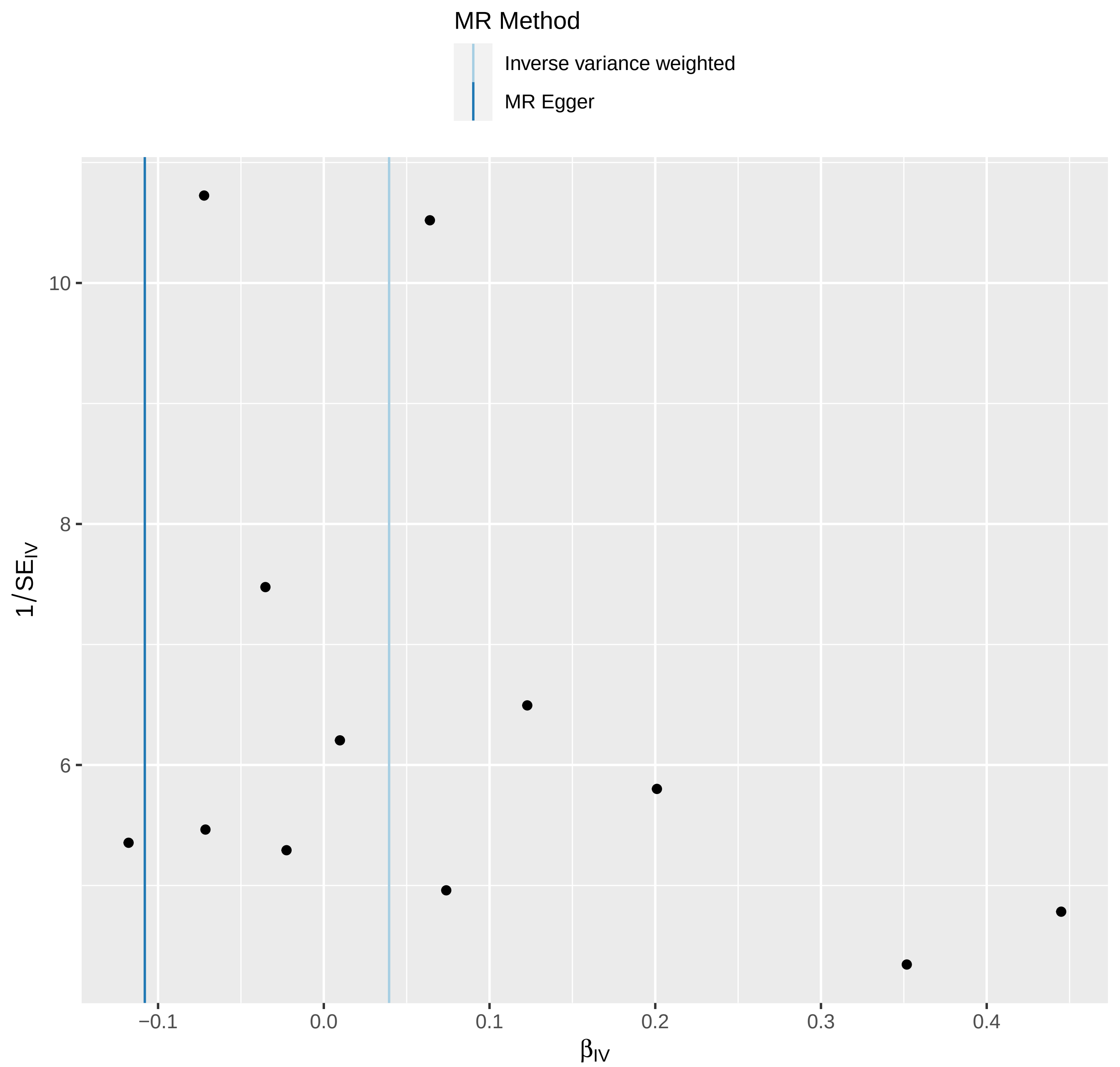

Batch 3−892 : Selenium on Endothelin−2

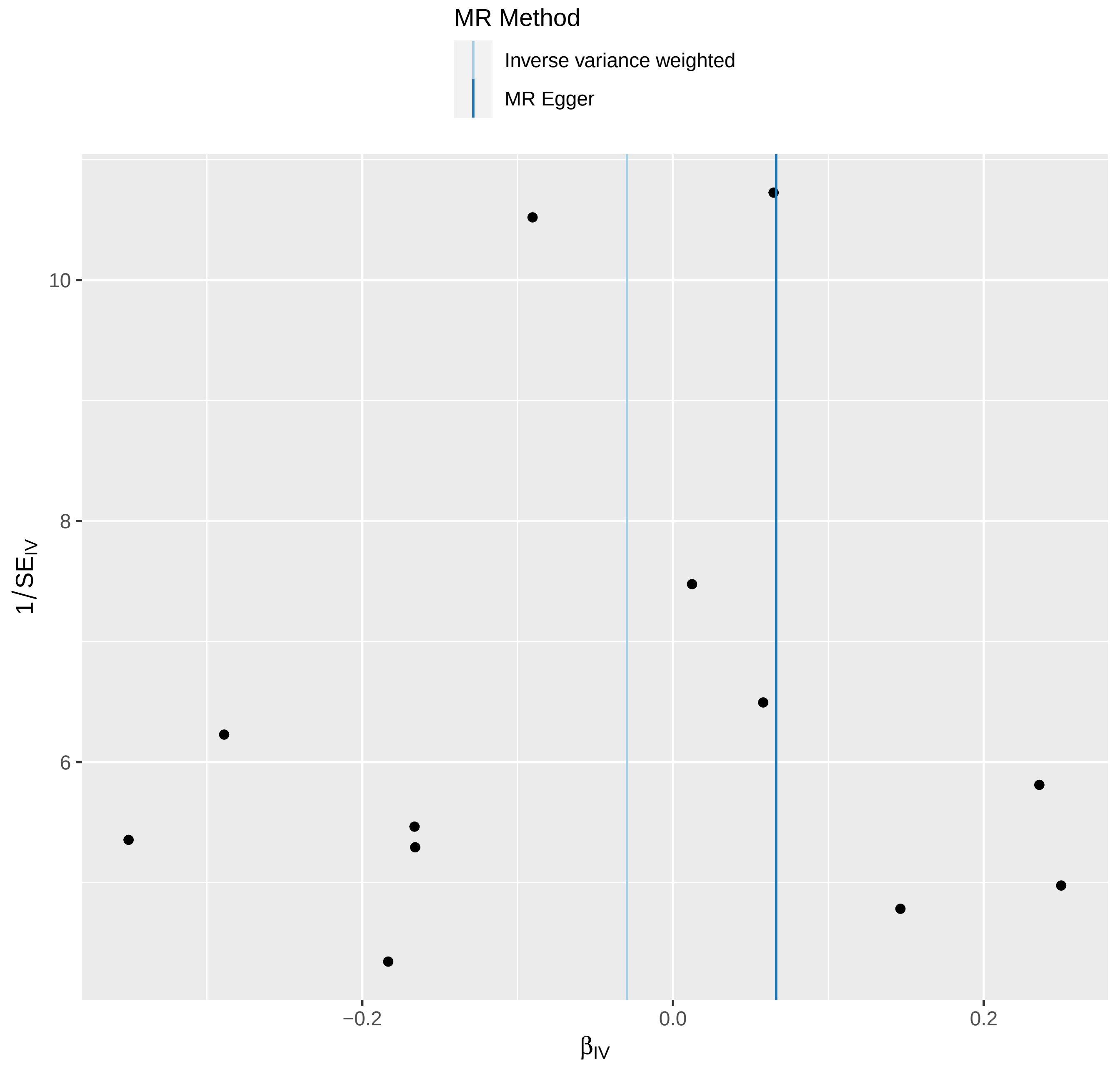

Batch 3−893 : Selenium on Early endosome antigen 1

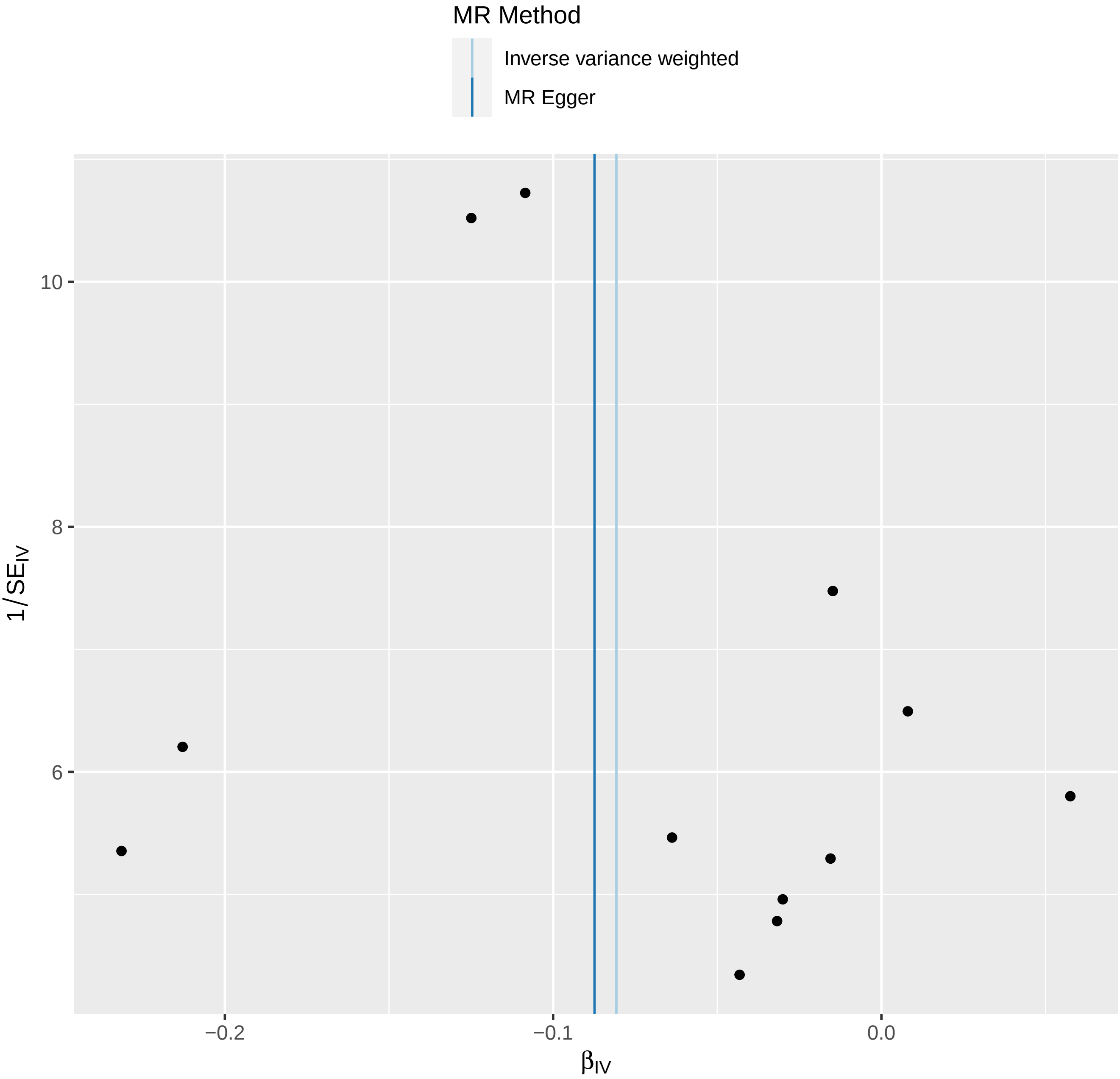

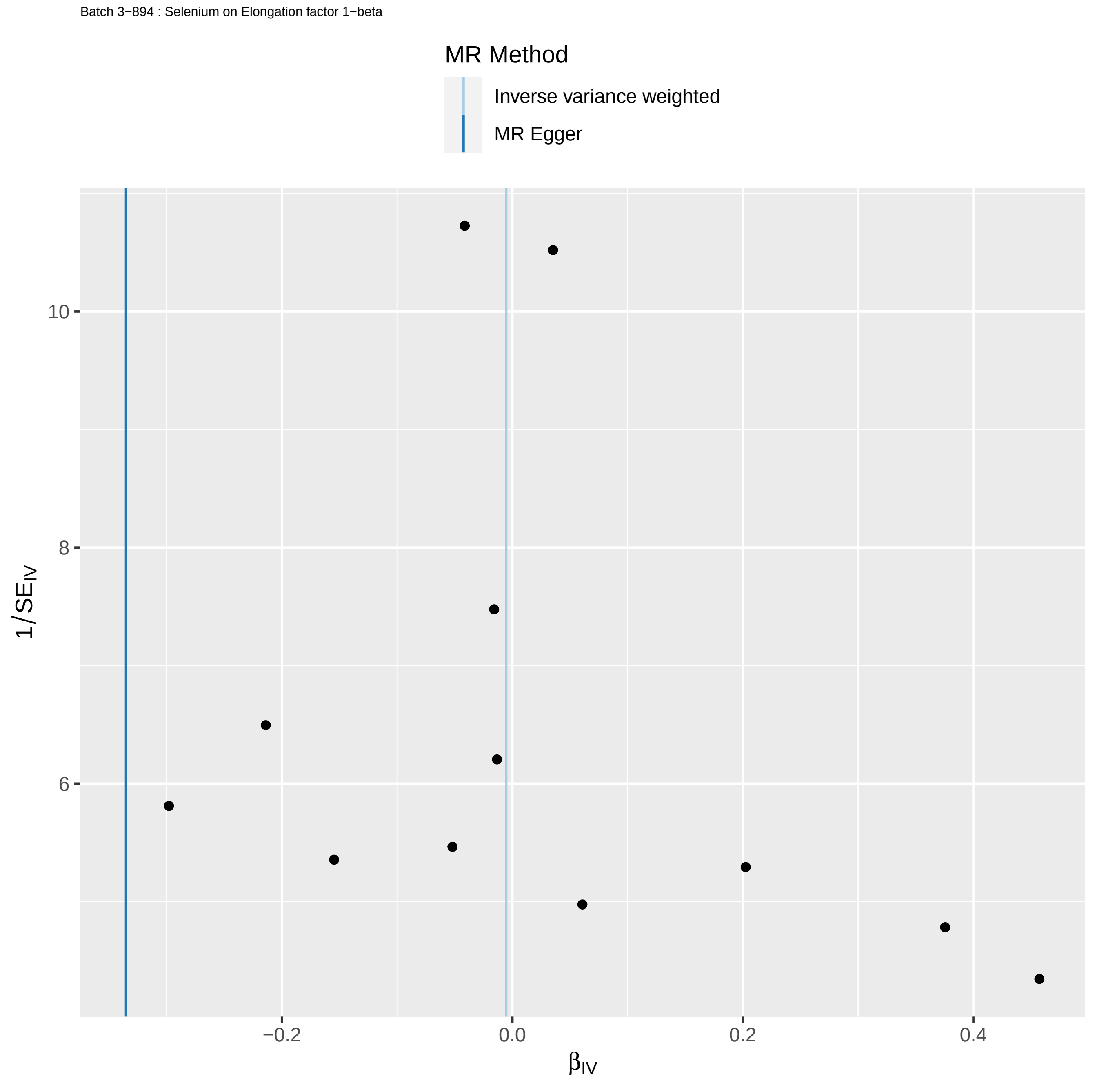
Batch 3−894 : Selenium on Elongation factor 1−beta
MR Method
Inverse variance weighted
MR Egger
10
8
6
1/SE_IV
−0.2
0.0
0.2
0.4
β_IV

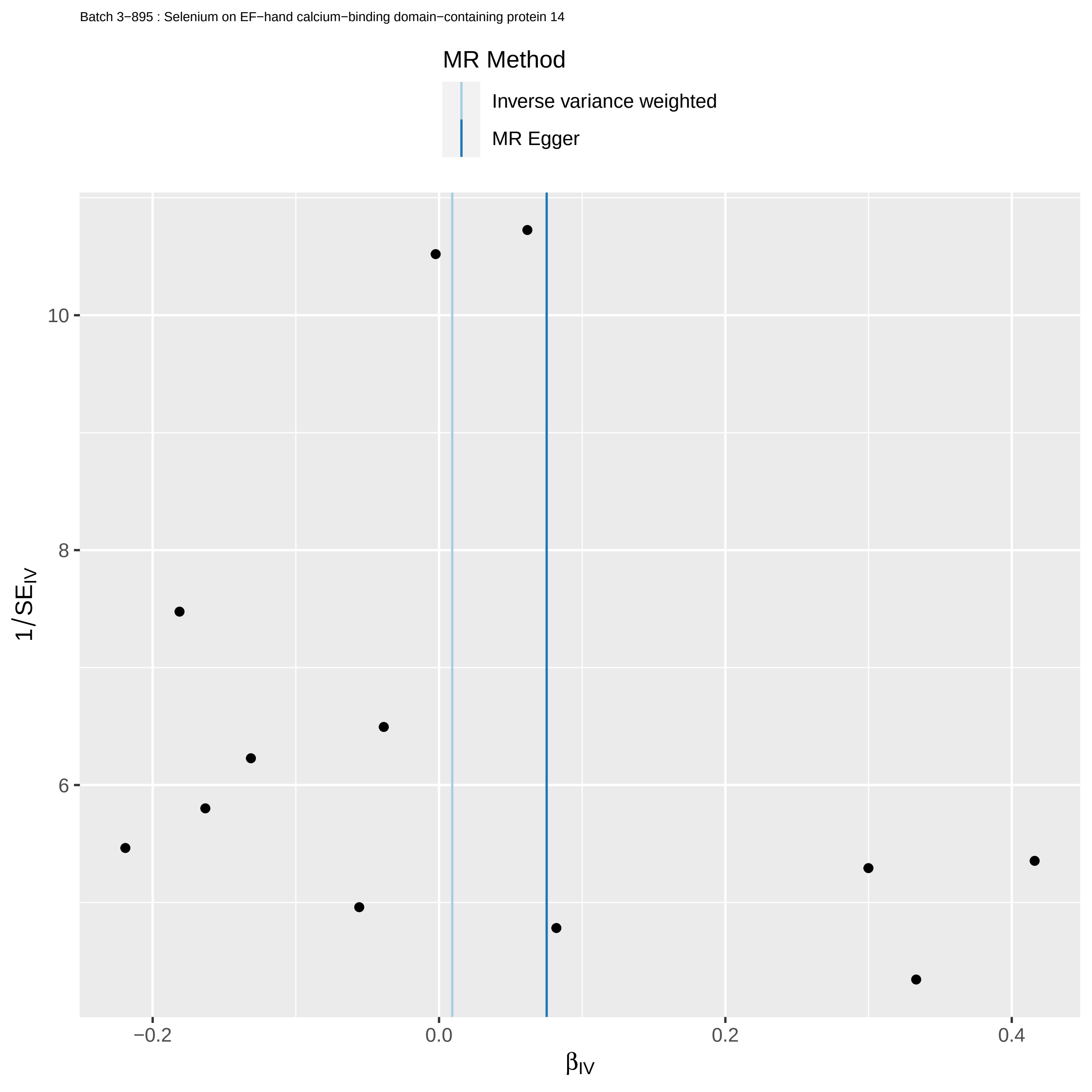
Batch 3−895 : Selenium on EF−hand calcium−binding domain−containing protein 14
MR Method
Inverse variance weighted
MR Egger
10
8
6
1/SE_IV
−0.2
0.0
0.2
0.4
β_IV

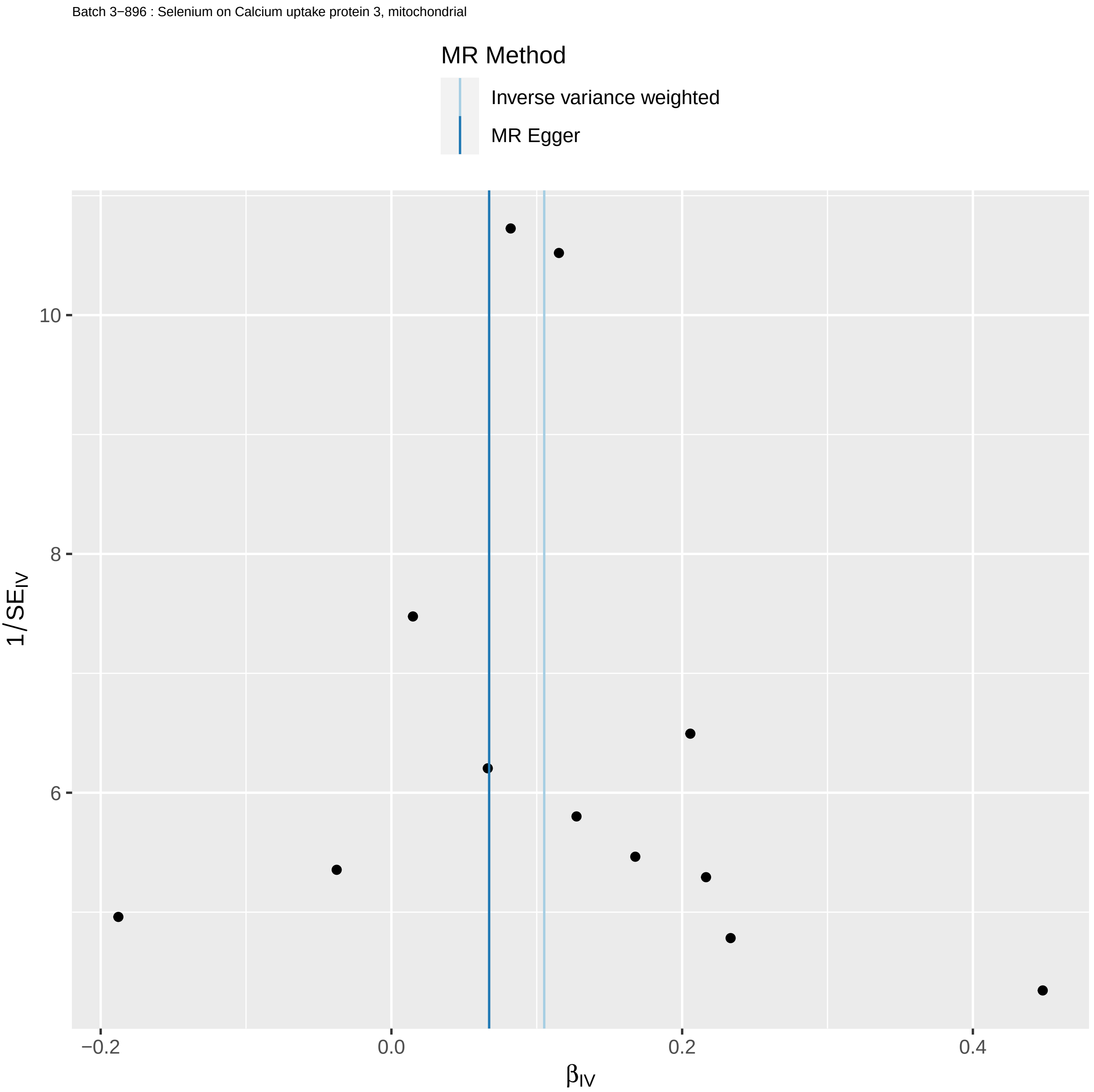
Batch 3−896 : Selenium on Calcium uptake protein 3, mitochondrial
MR Method
Inverse variance weighted
MR Egger
10
8
6
$1/SE_{IV}$
−0.2
0.0
0.2
0.4
β_{IV}

Batch 3−897 : Selenium on Ephrin−A2

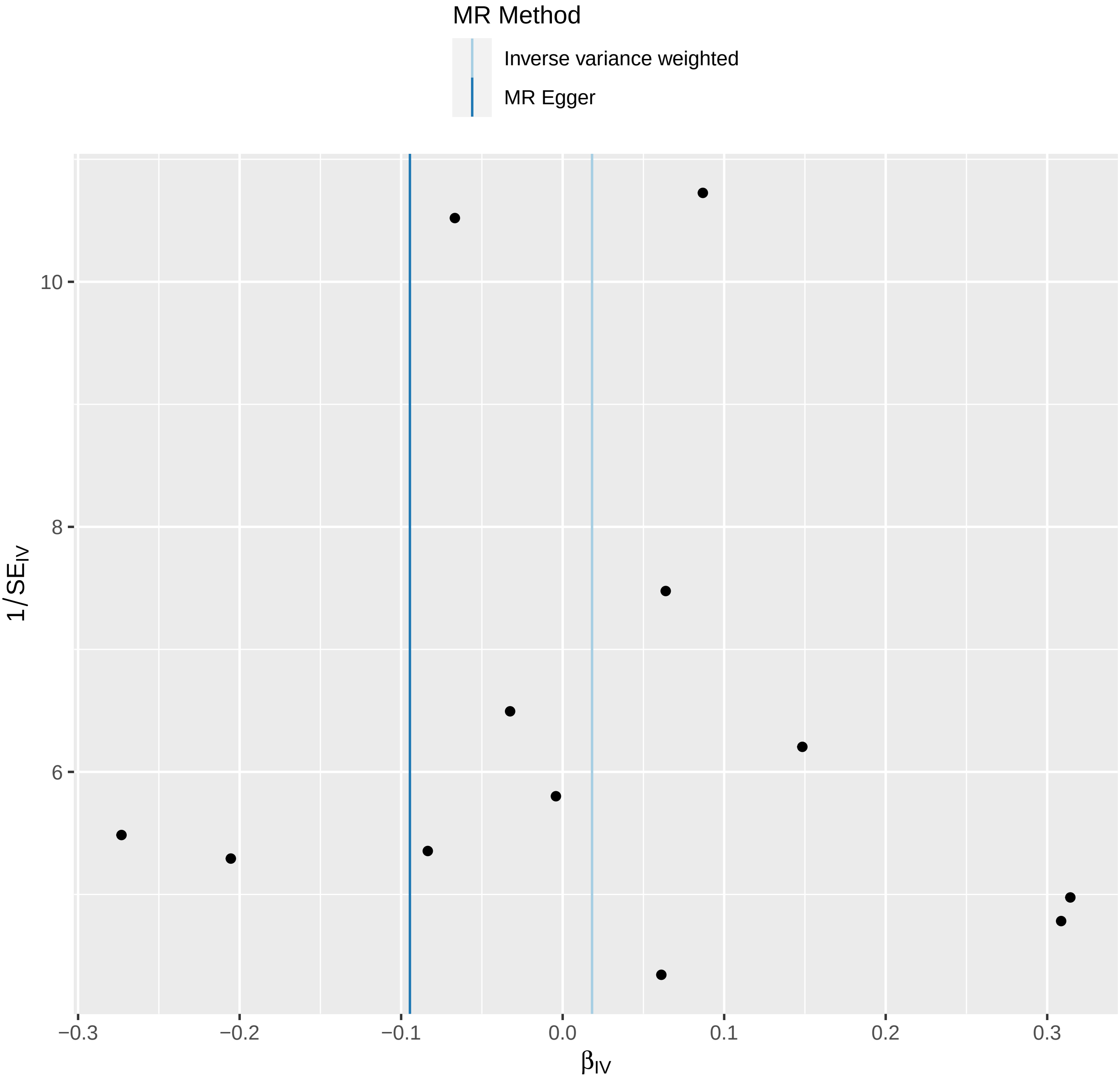

Batch 3−898 : Selenium on Ephrin−A3

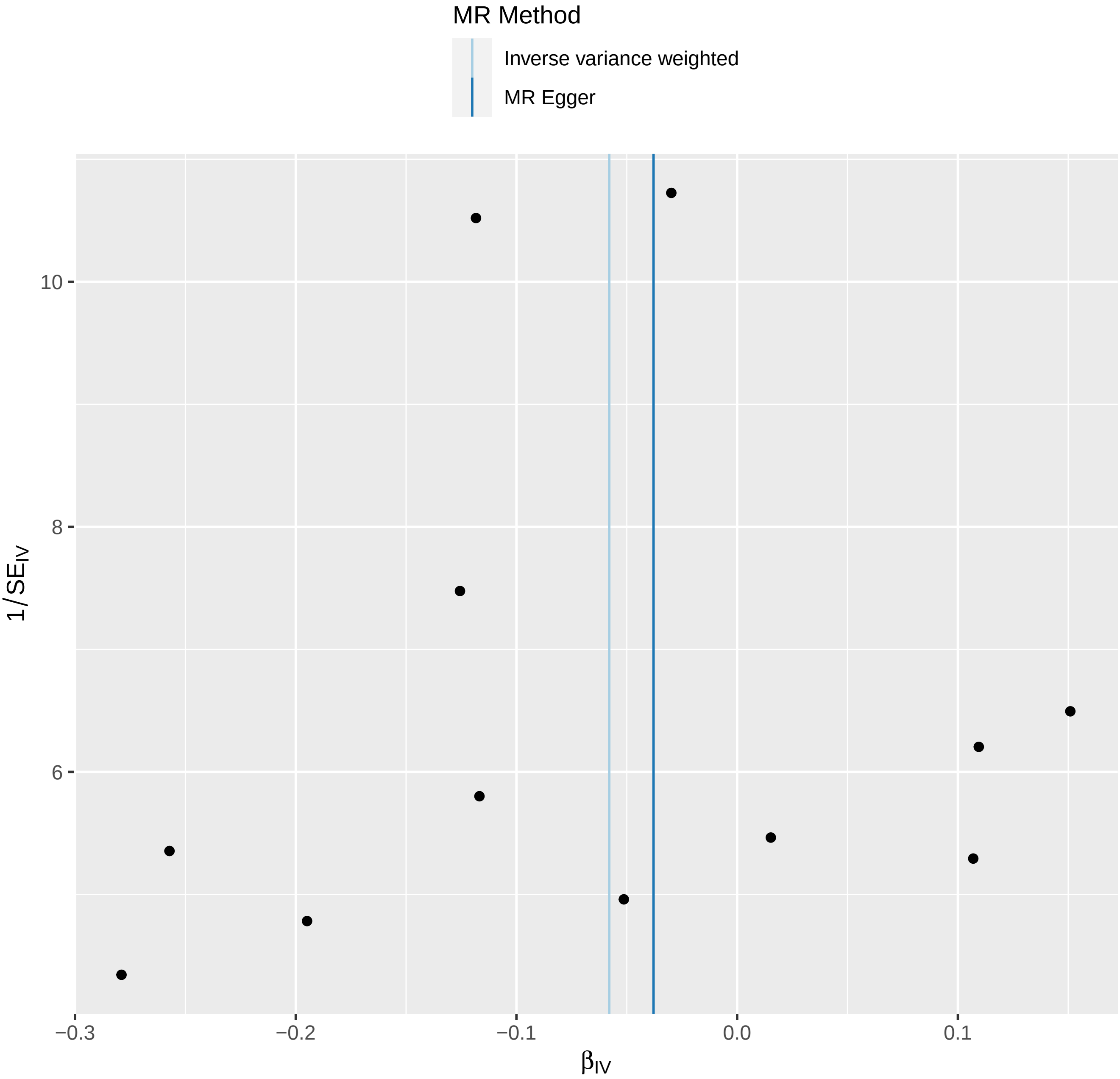

Batch 3−899 : Selenium on Ephrin−A3

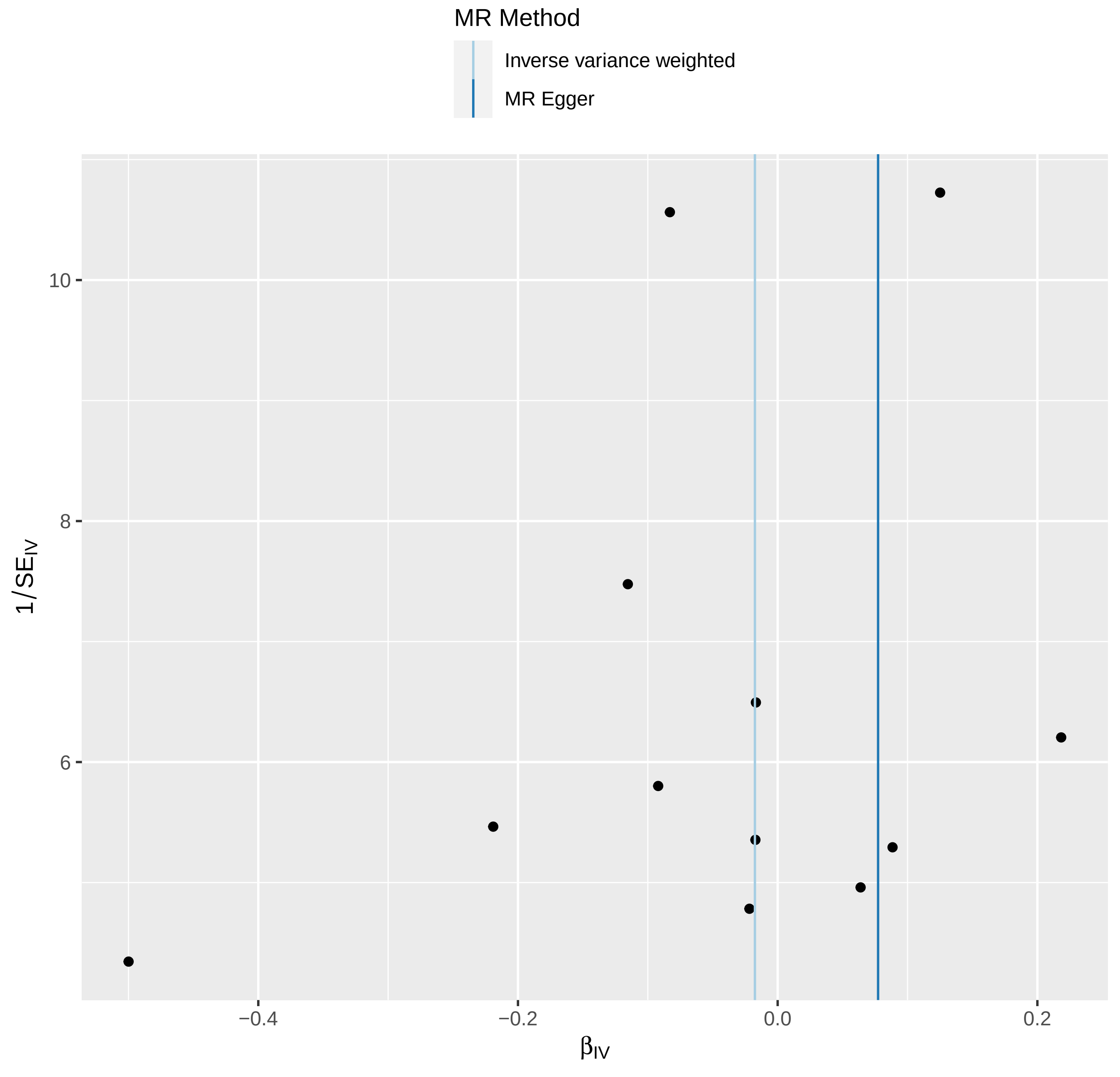

Batch 3−900 : Selenium on Ephrin−A4

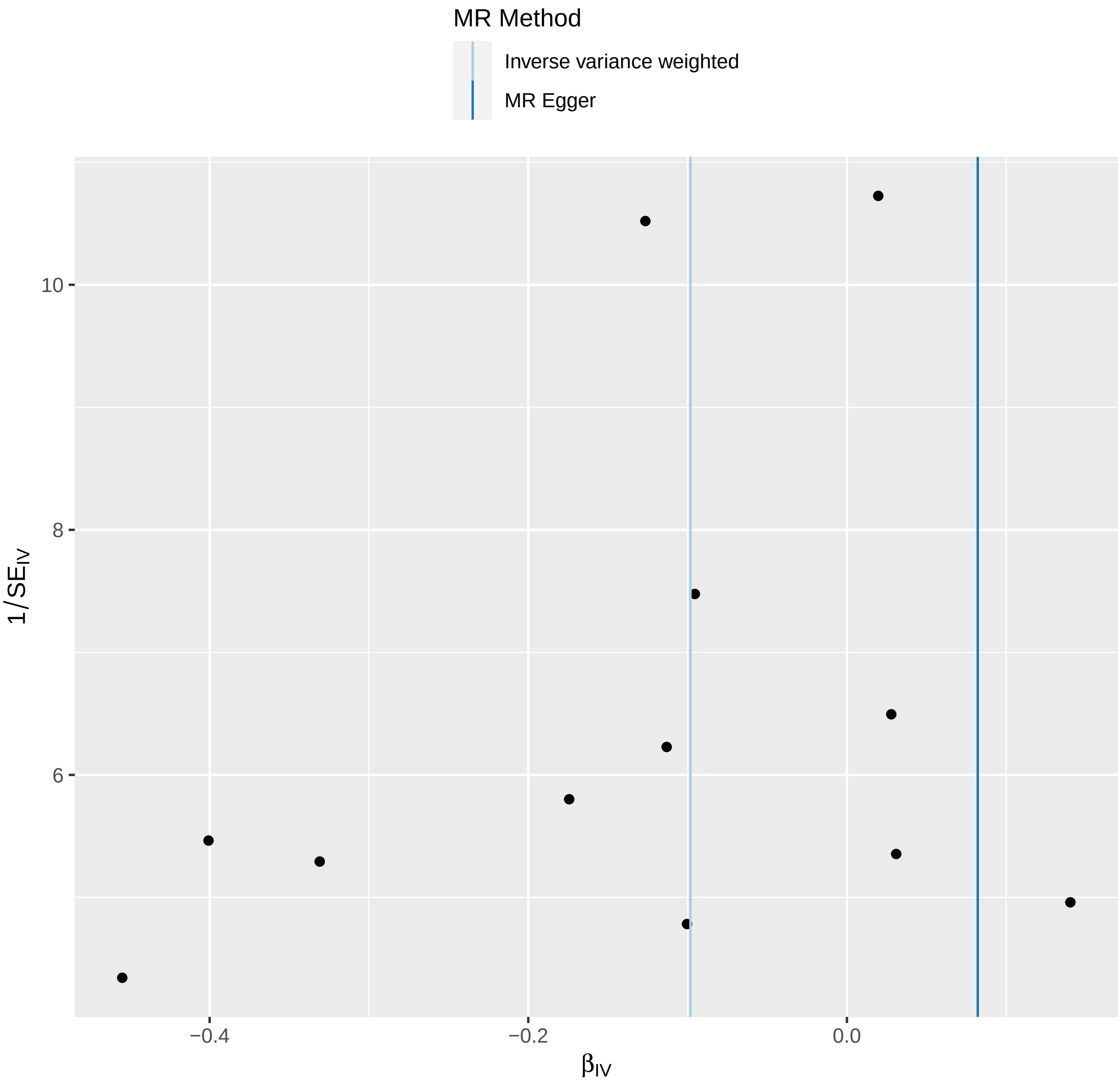

Batch 3−901 : Selenium on Ephrin−A5

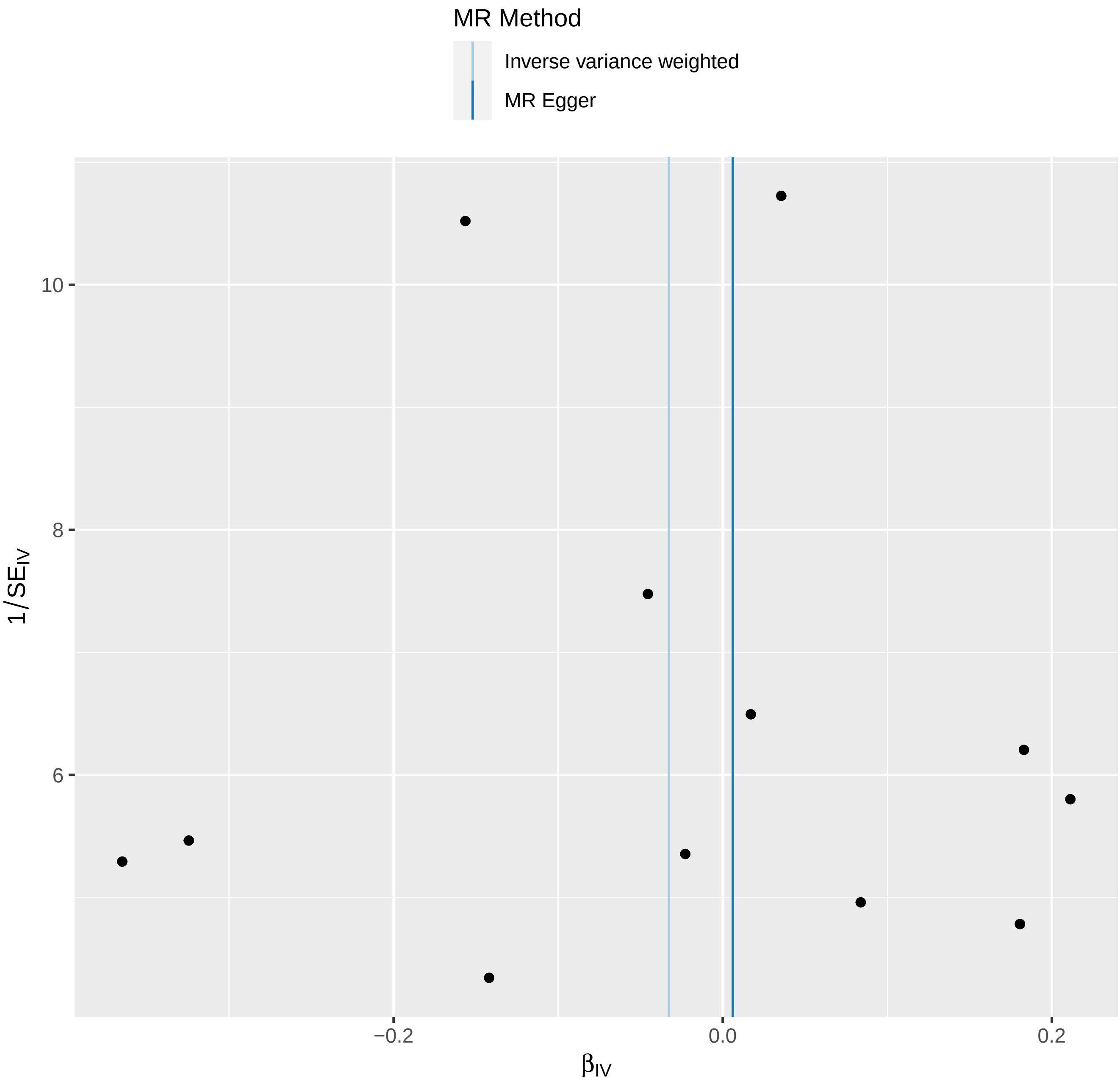

Batch 3−902 : Selenium on Ephrin−B1

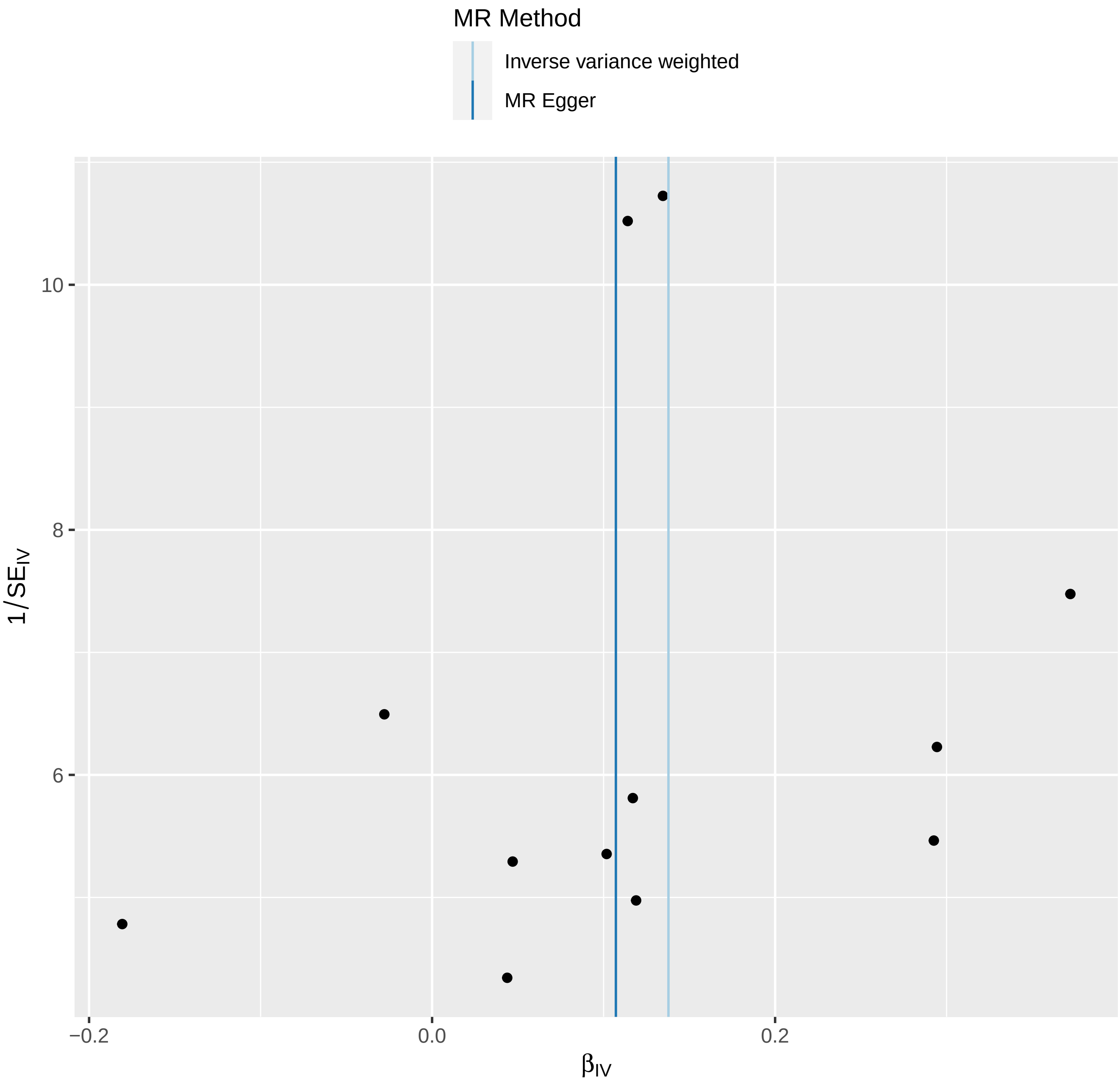

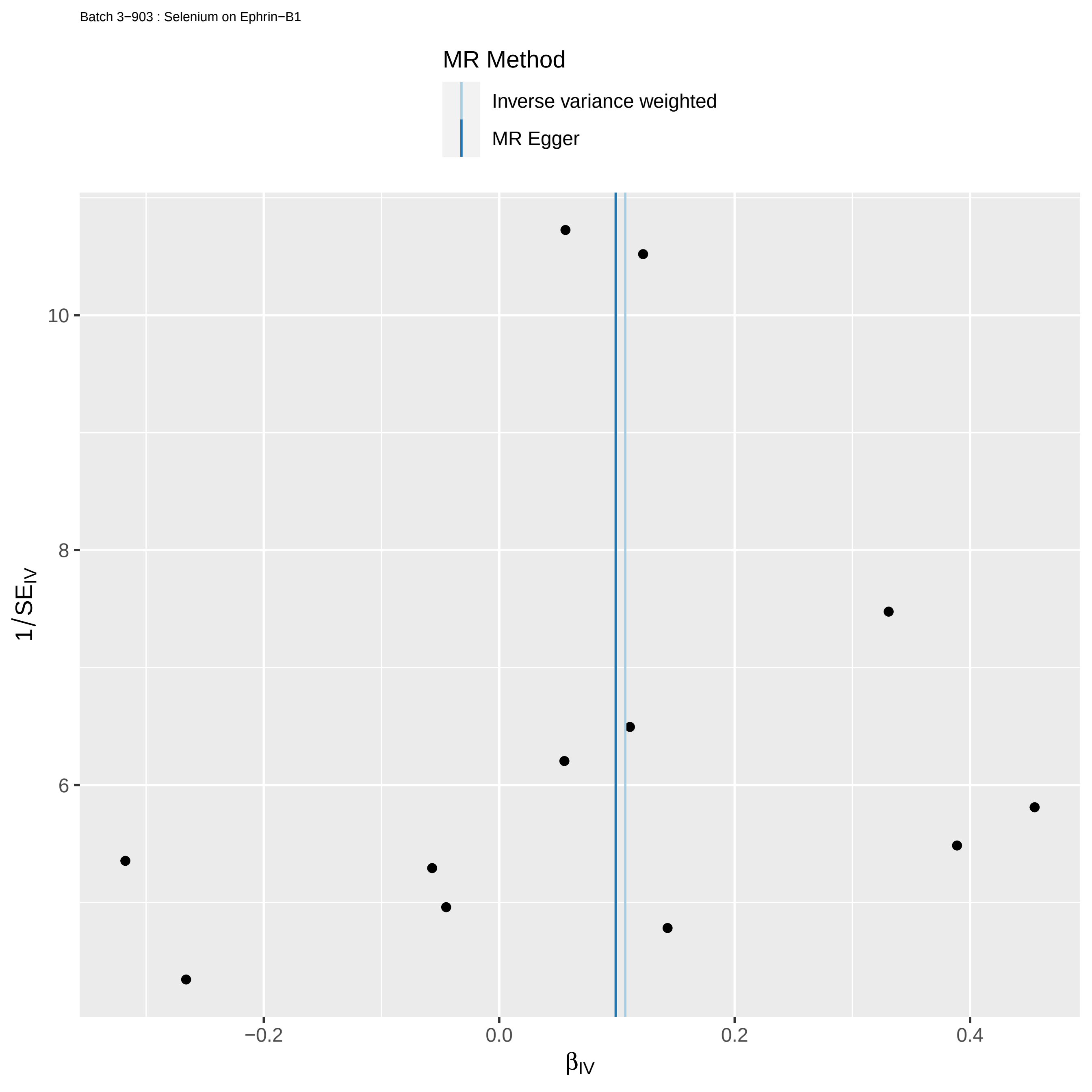

Batch 3−903 : Selenium on Ephrin−B1
MR Method
Inverse variance weighted
MR Egger
10
8
6
1/SE_IV
−0.2
0.0
0.2
0.4
β_IV

Batch 3−904 : Selenium on Ephrin−B2

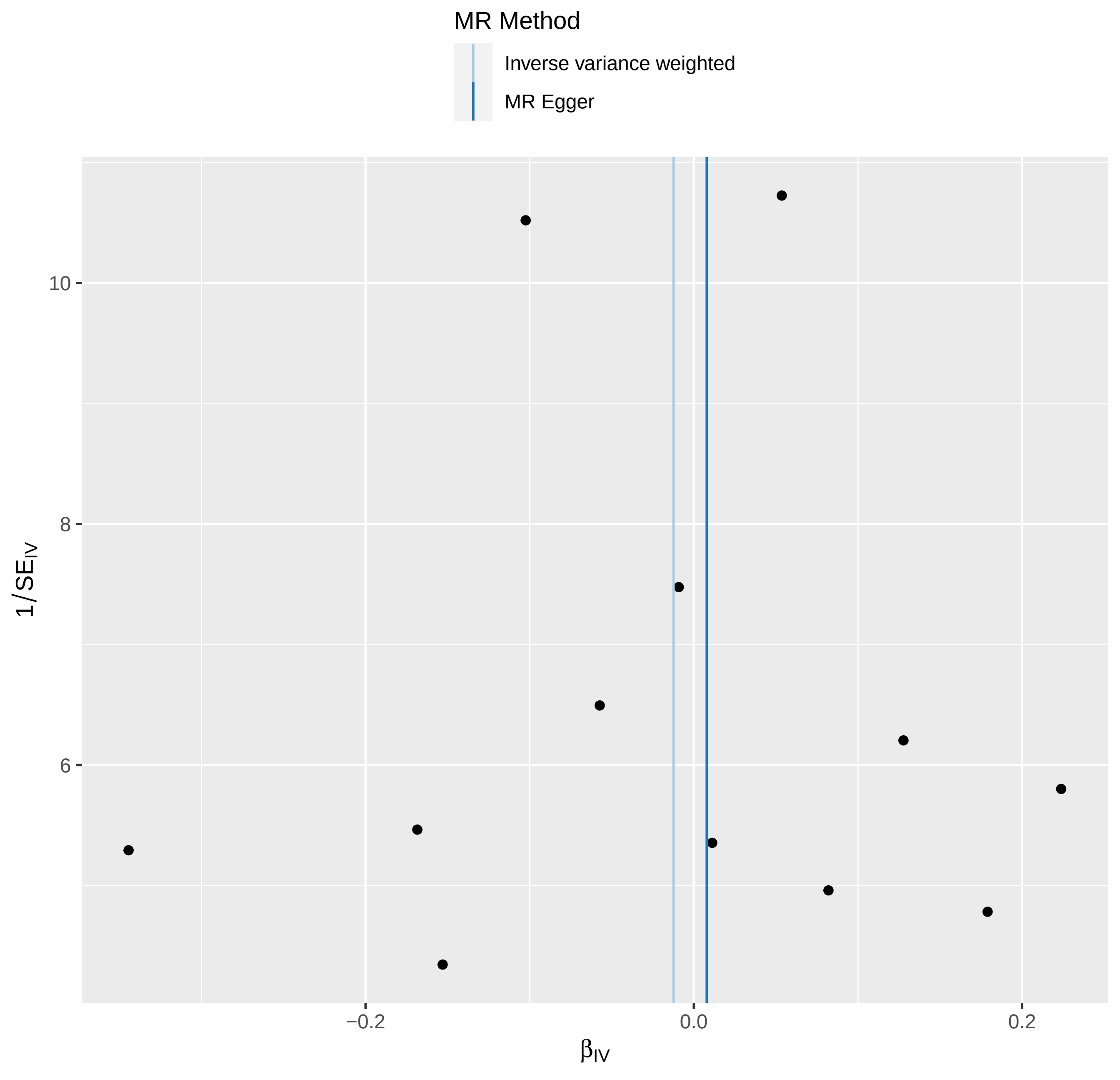

Batch 3−905 : Selenium on Ephrin−B2

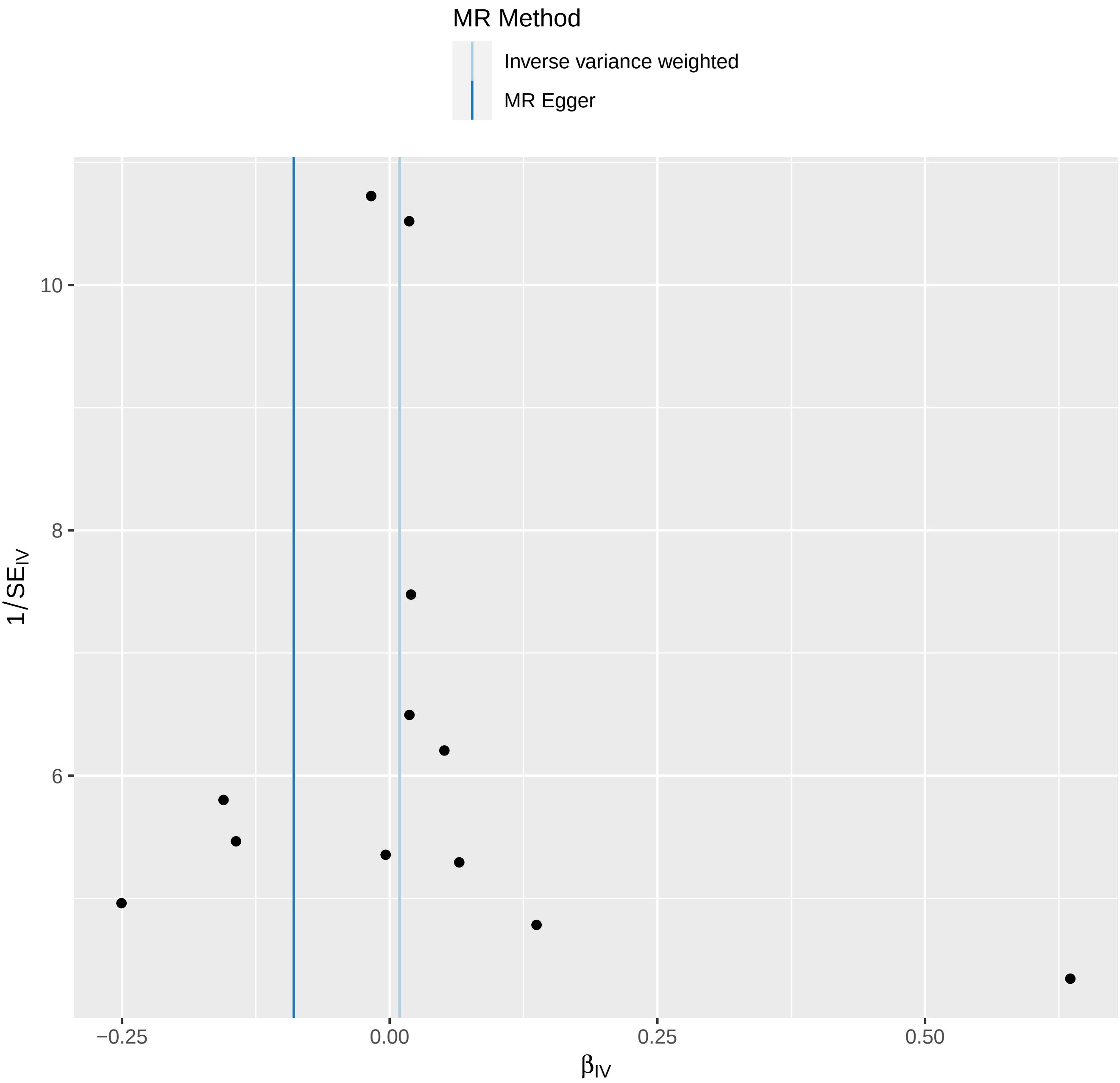

Batch 3−906 : Selenium on Ephrin−B3

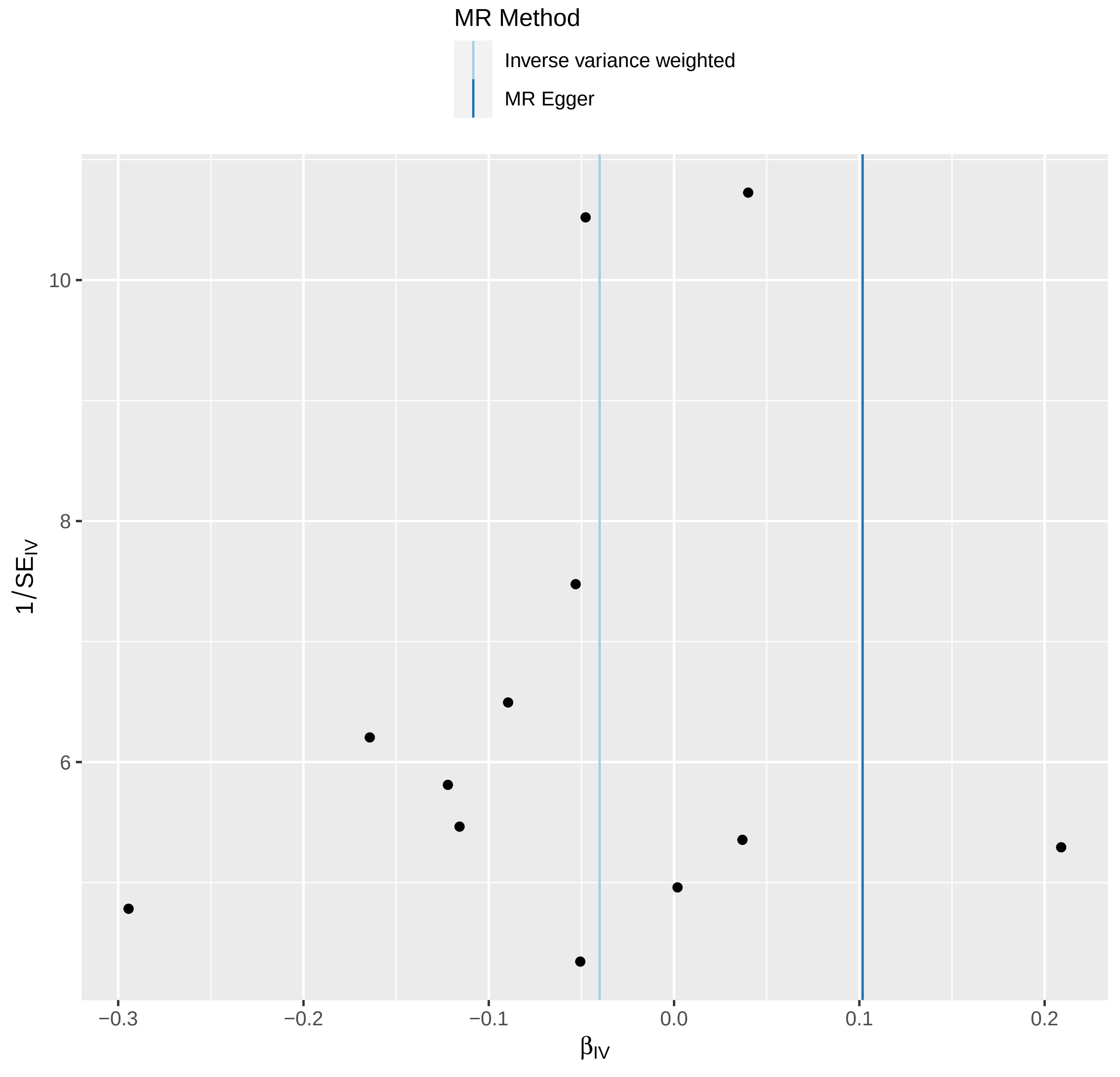

Batch 3−907 : Selenium on Ephrin−B3

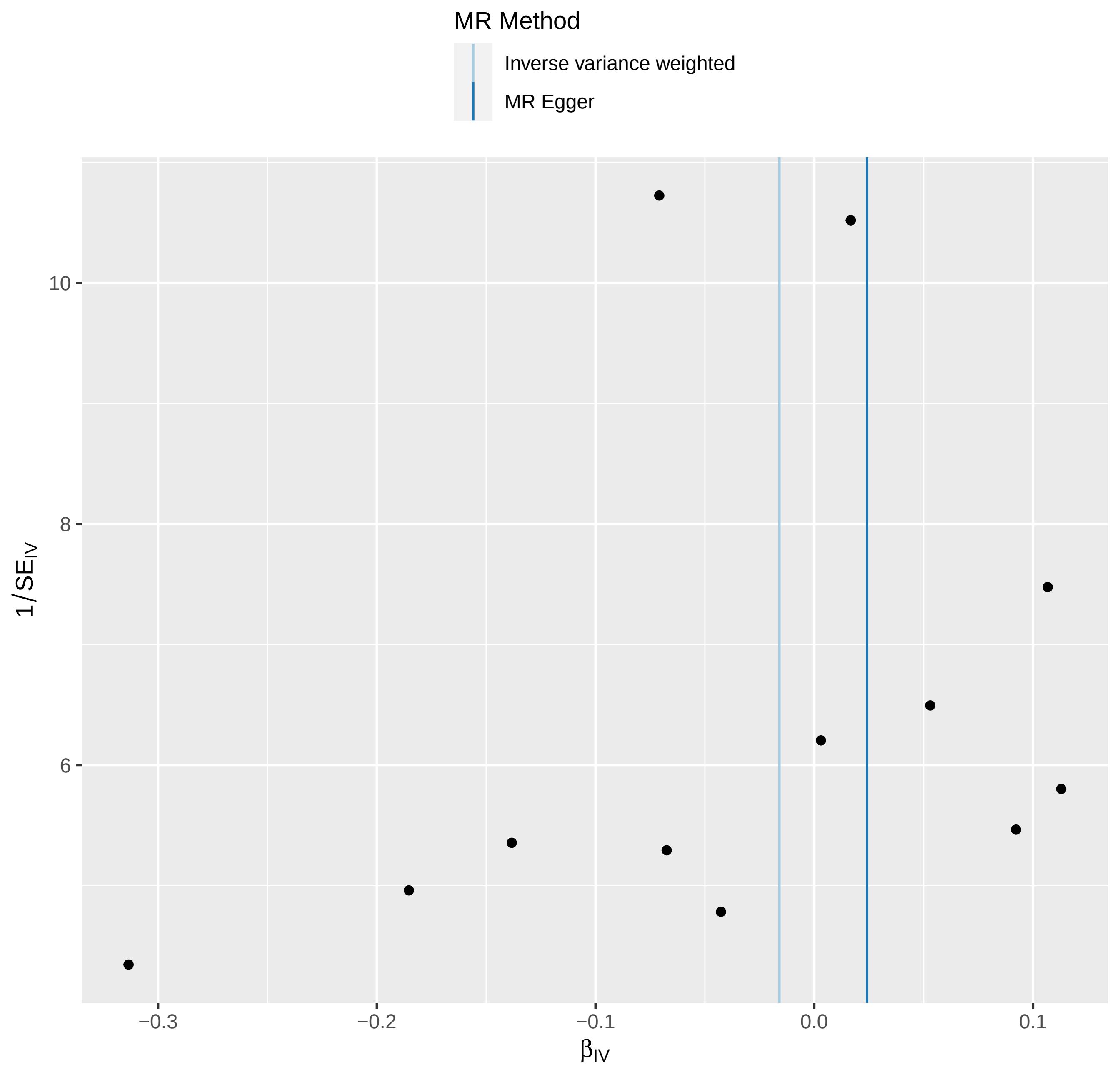

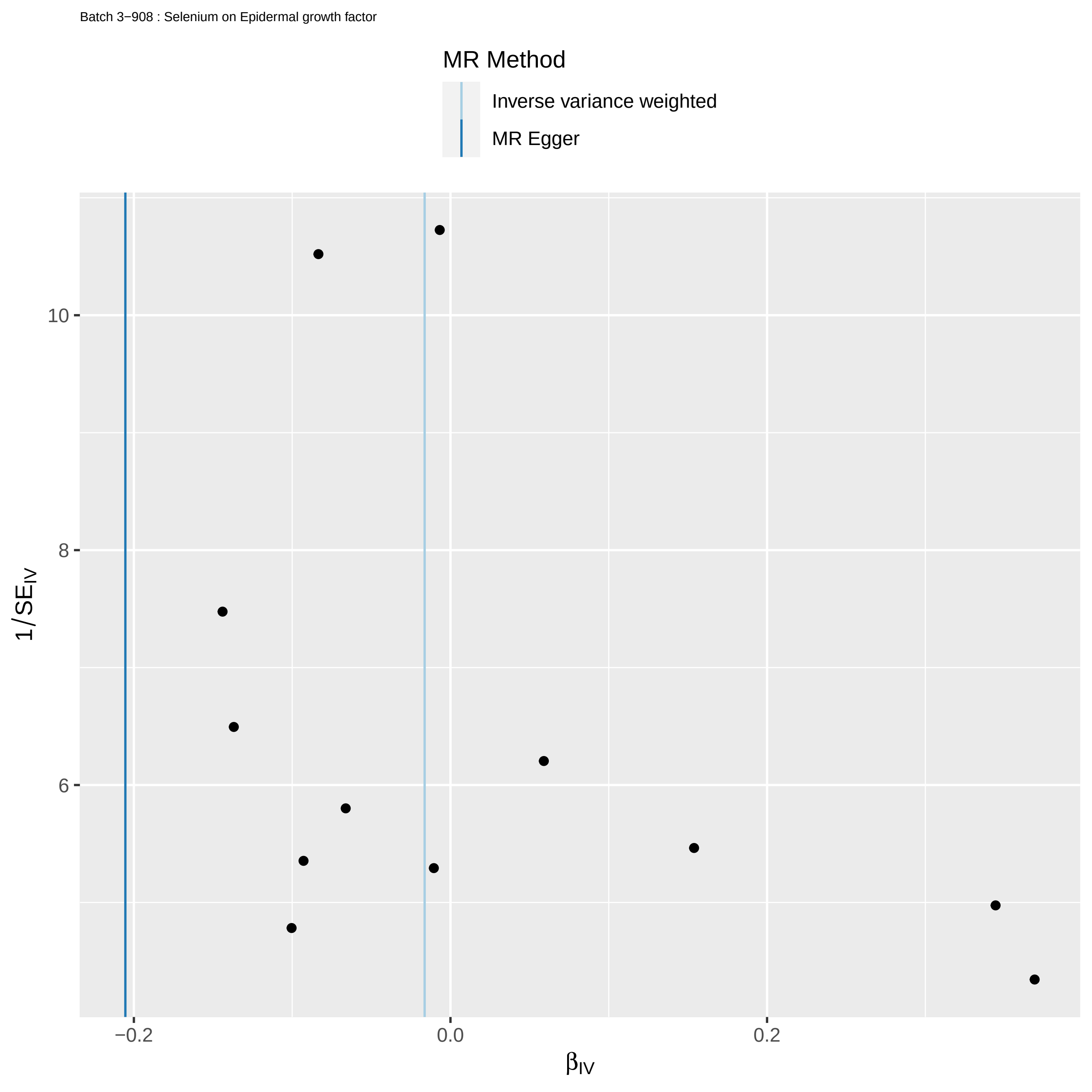

Batch 3−908 : Selenium on Epidermal growth factor
MR Method
Inverse variance weighted
MR Egger
10
8
6
1/SE_IV
−0.2
0.0
0.2
β_IV

Batch 3−909 : Selenium on Epidermal growth factor receptor

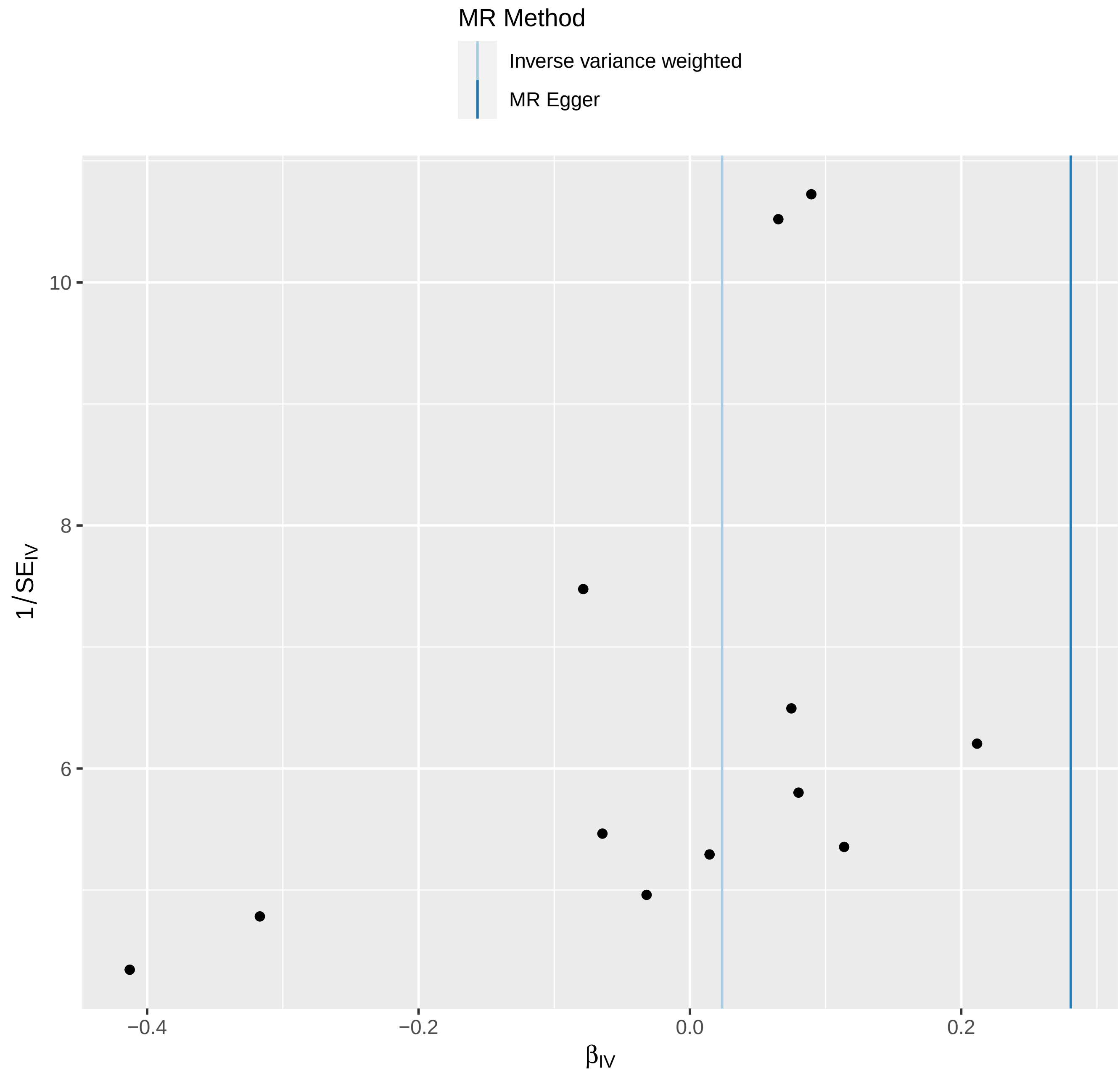

Batch 3−910 : Selenium on Epidermal growth factor receptor variant III

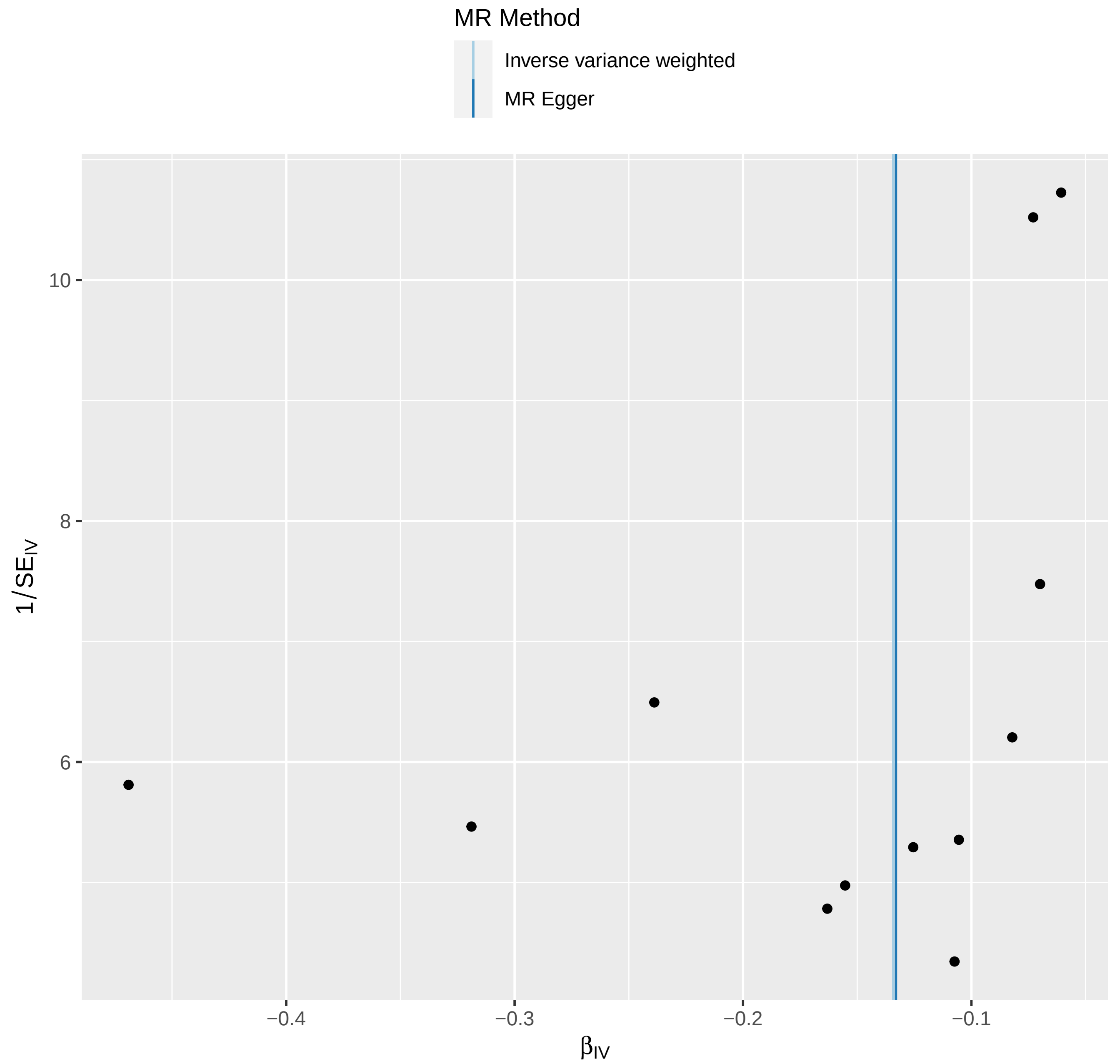

Batch 3−911 : Selenium on EH domain−binding protein 1

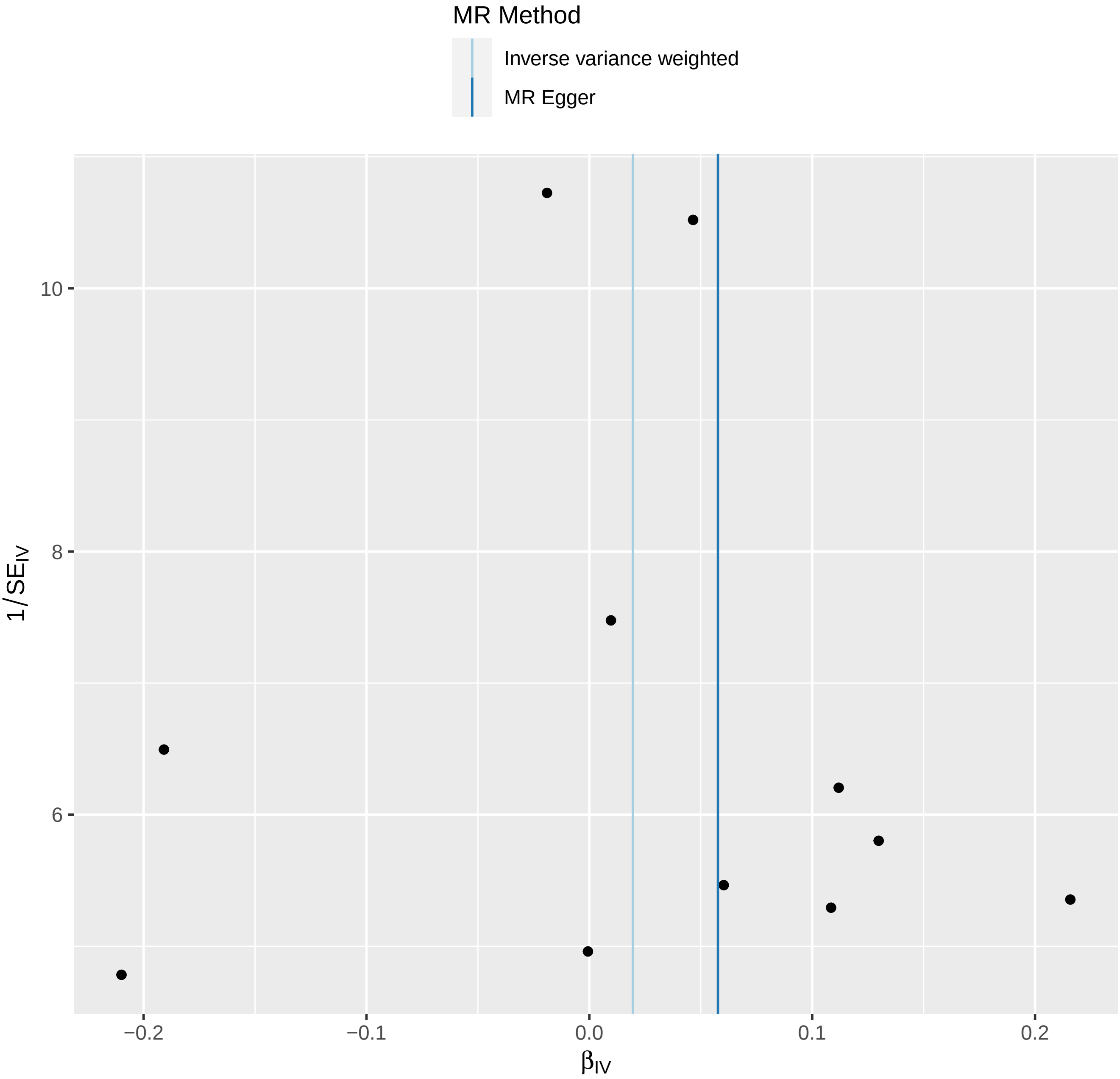

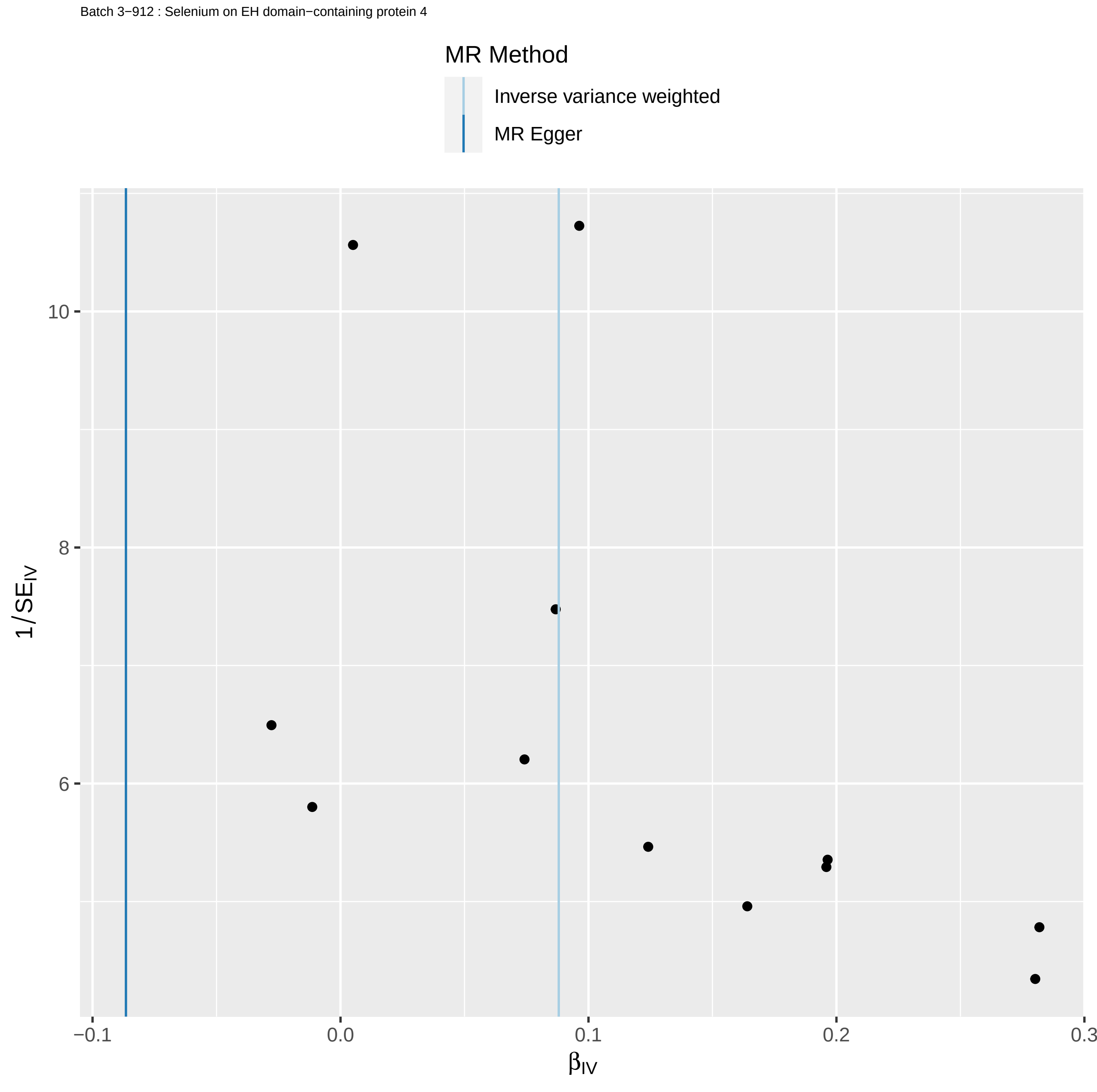

Batch 3−912 : Selenium on EH domain−containing protein 4
MR Method
Inverse variance weighted
MR Egger
10
8
6
1/SE_IV
−0.1
0.0
0.1
0.2
0.3
β_IV

Batch 3−913 : Selenium on ETS homologous factor

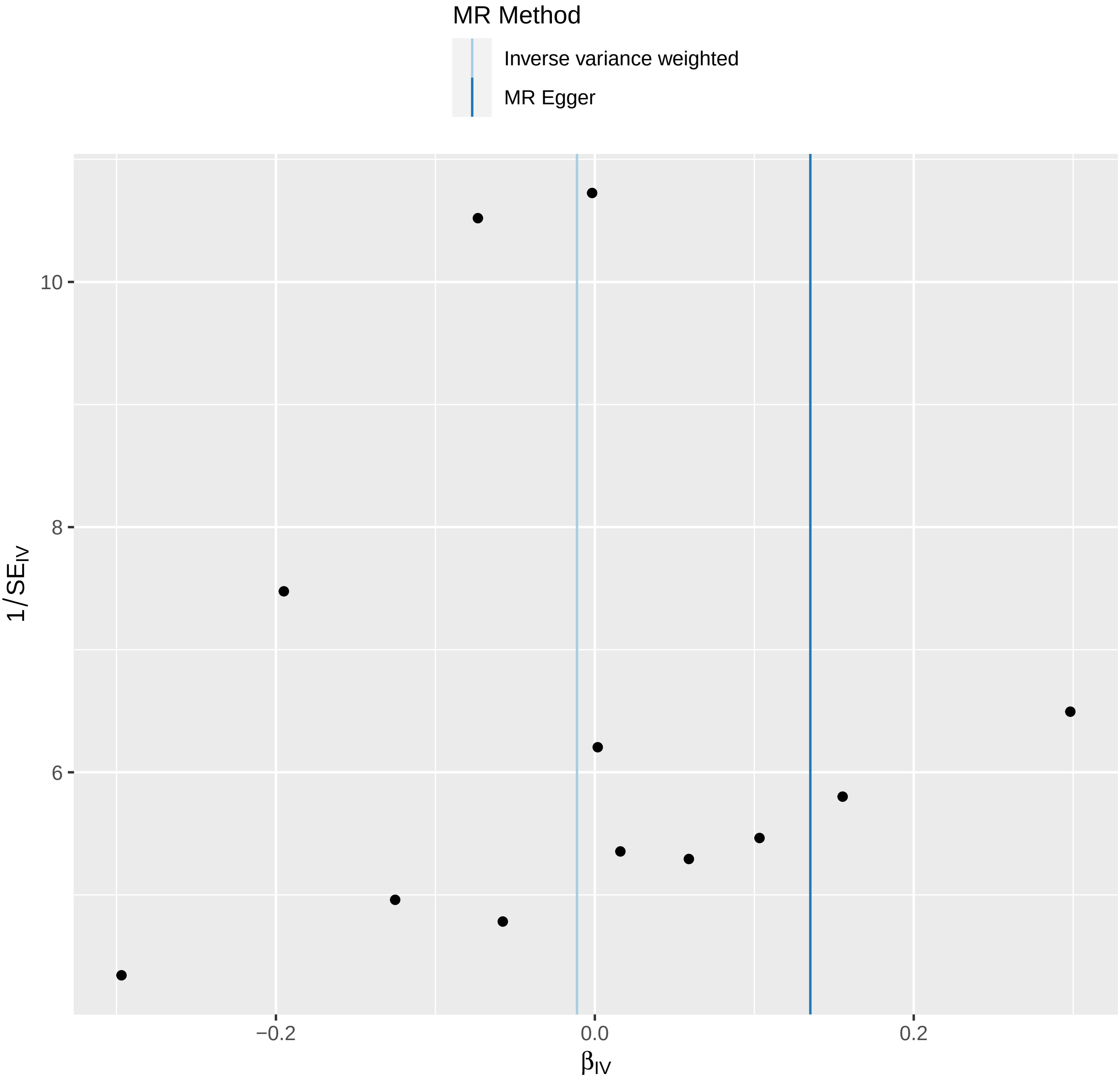

Batch 3−914 : Selenium on Histone−lysine N−methyltransferase EHMT2

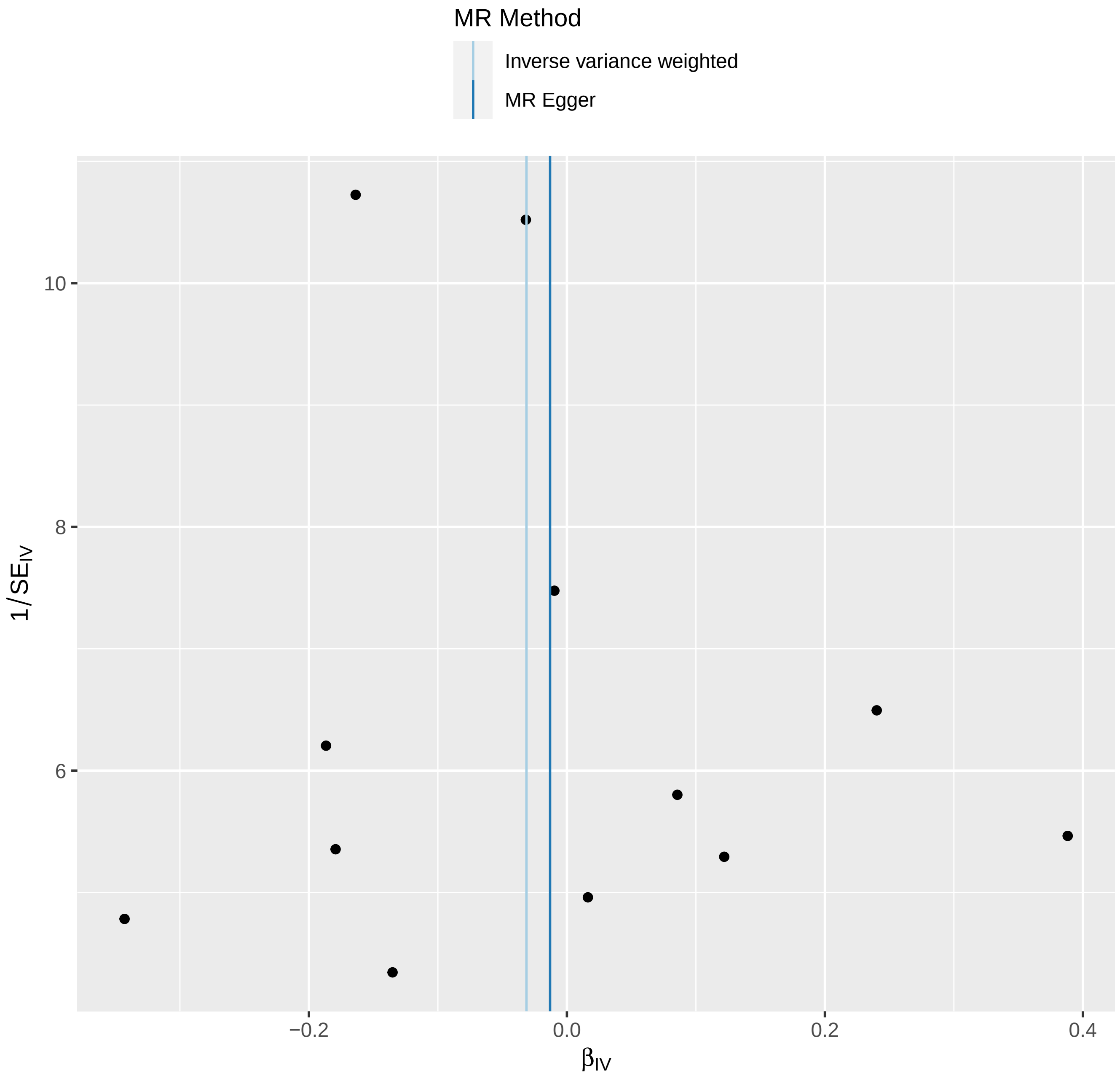

Batch 3−915 : Selenium on EP300−interacting inhibitor of differentiation 3

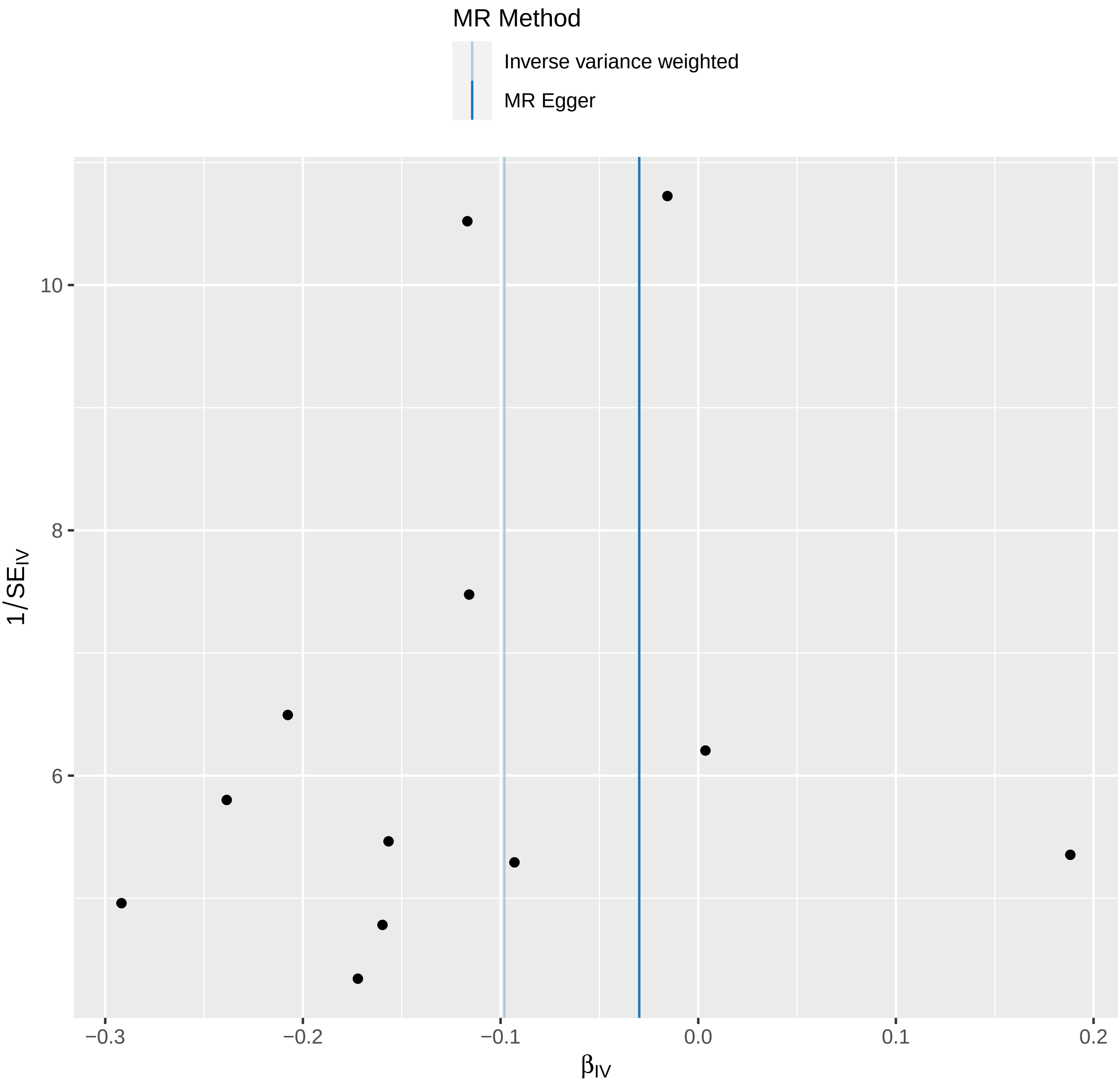

Batch 3−916 : Selenium on Probable RNA−binding protein EIF1AD

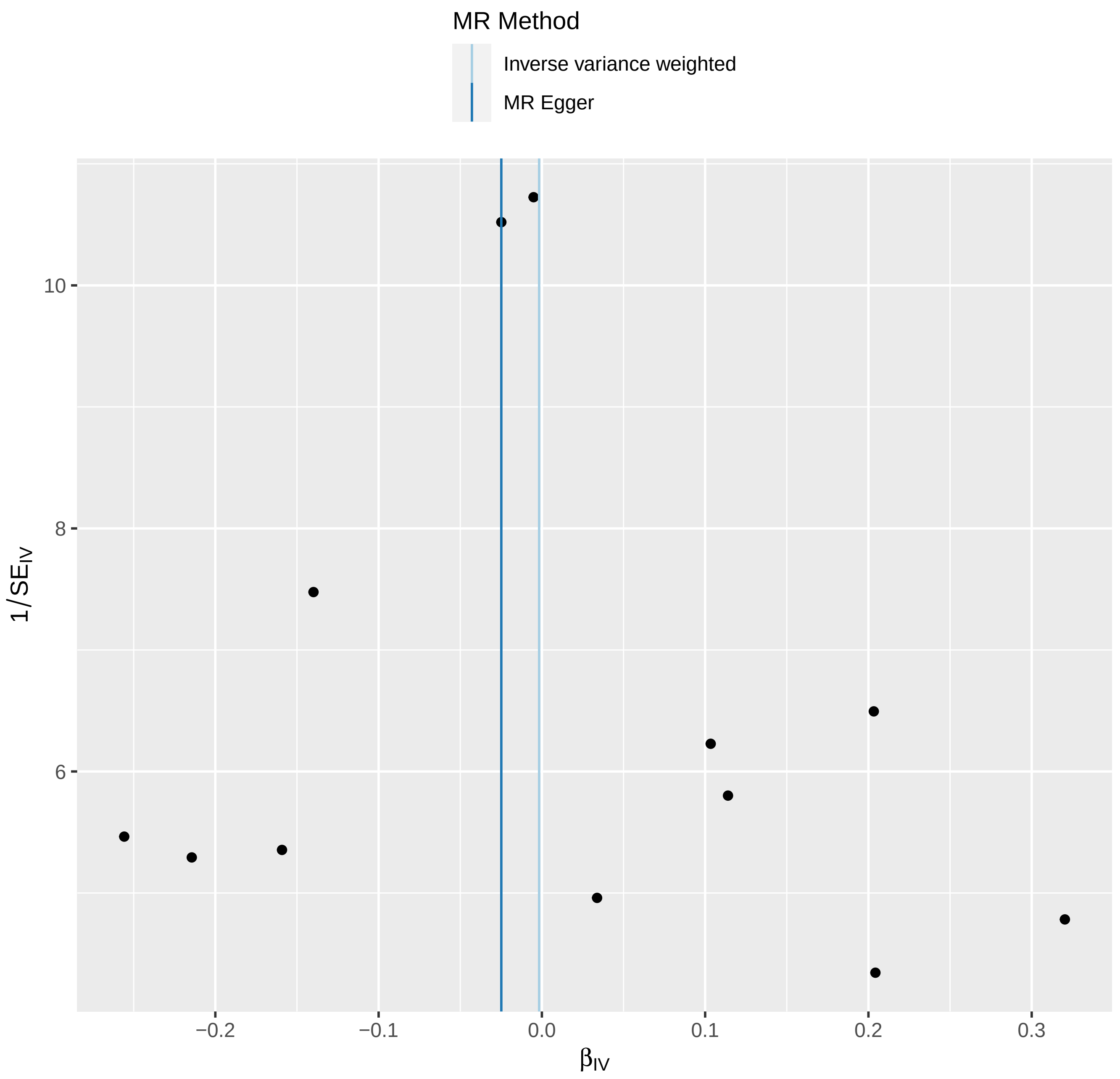

Batch 3−917 : Selenium on Eukaryotic translation initiation factor 1A, X−chromosomal

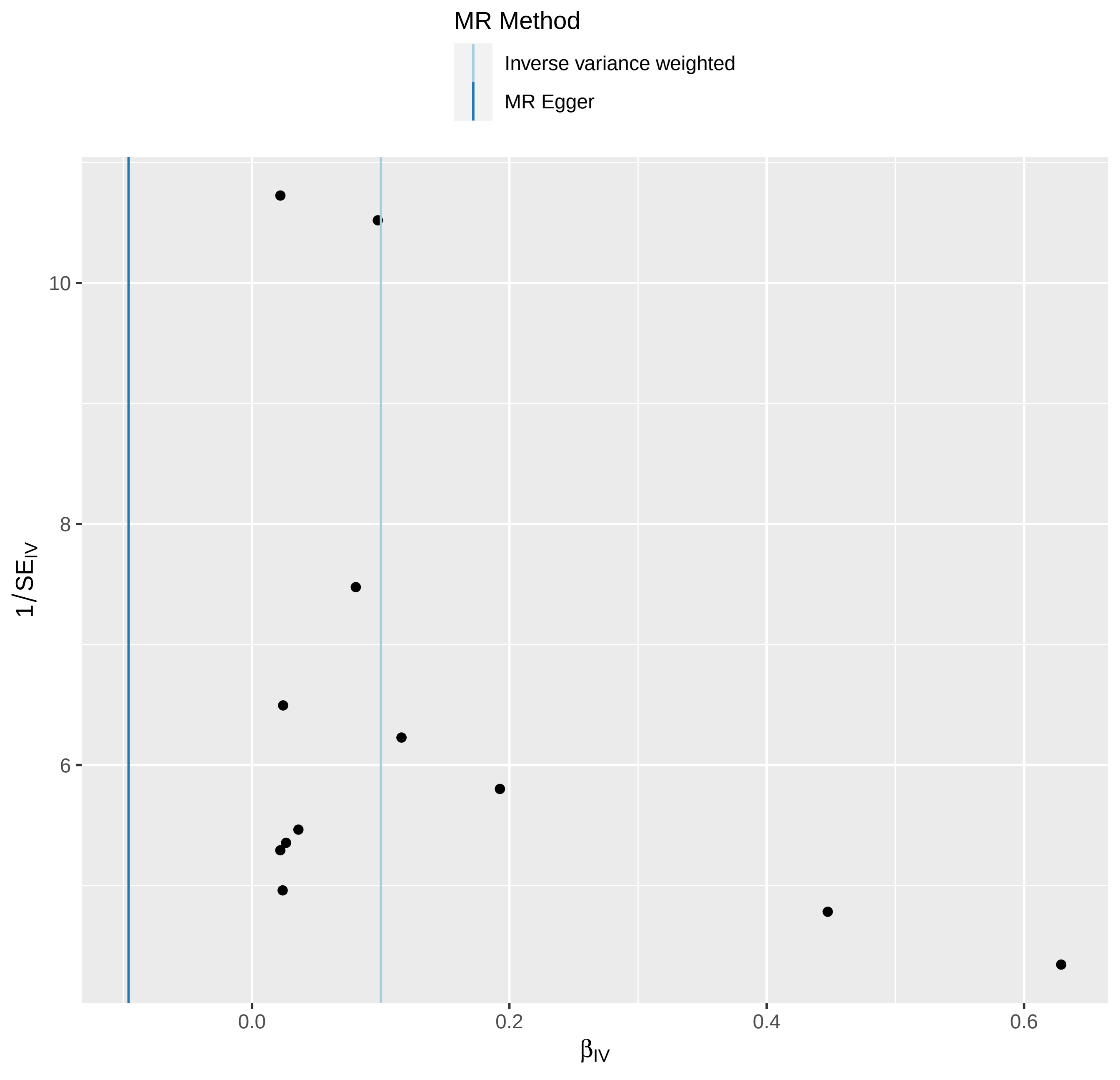

Batch 3−918 : Selenium on Translation initiation factor eIF−2B subunit alpha

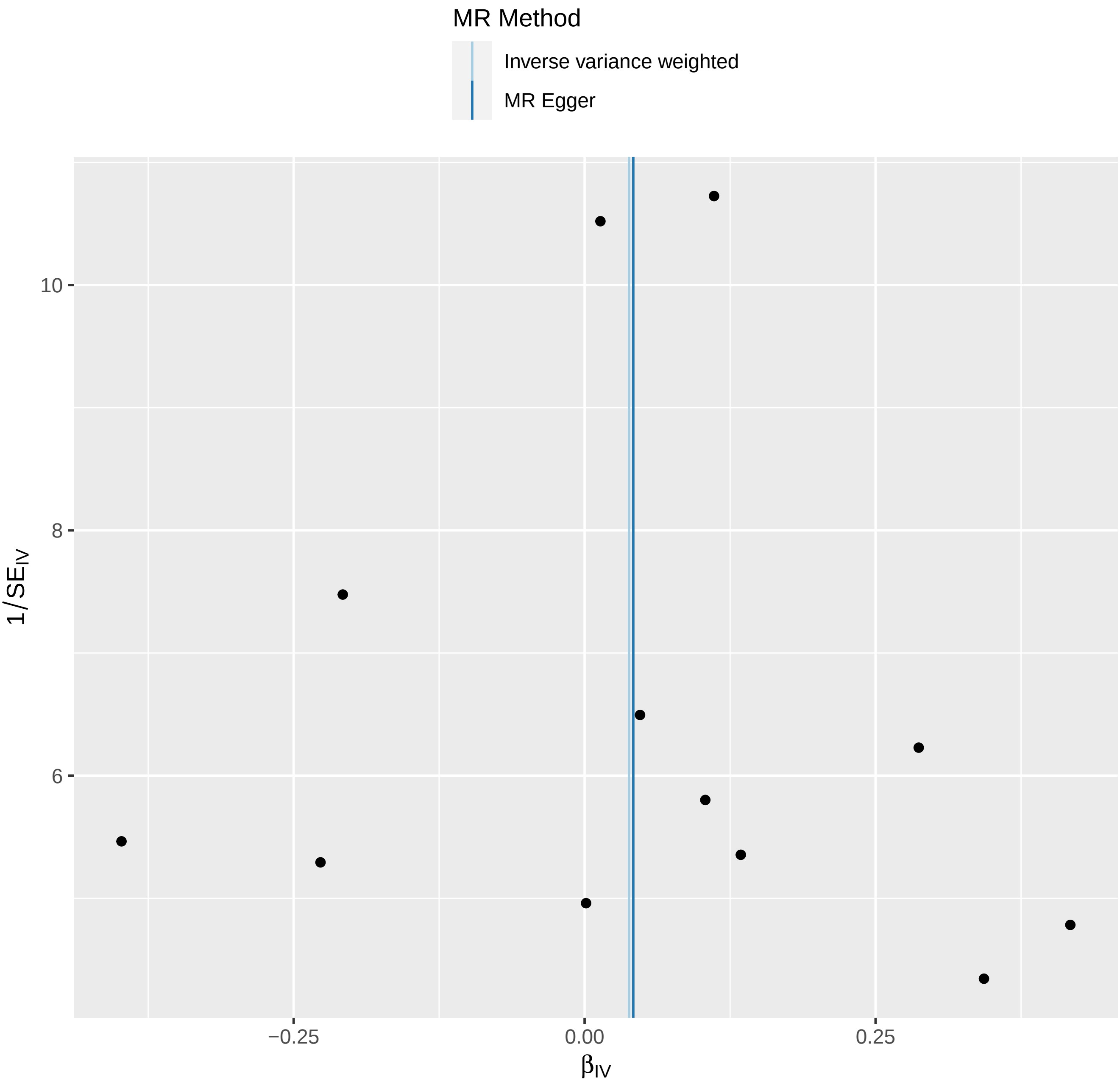

Batch 3−919 : Selenium on Eukaryotic translation initiation factor 3 subunit G

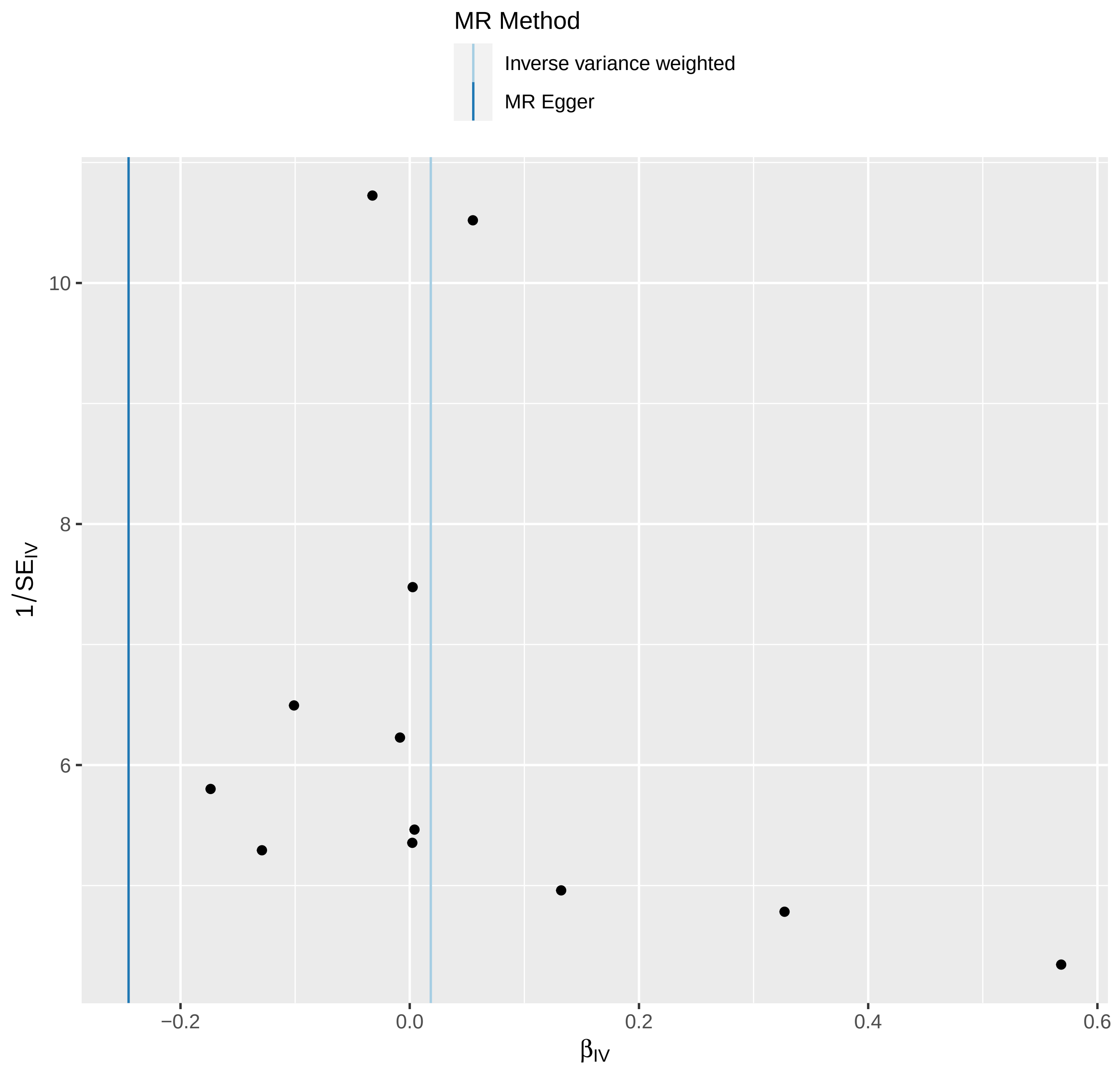

Batch 3−920 : Selenium on Eukaryotic translation initiation factor 3 subunit J

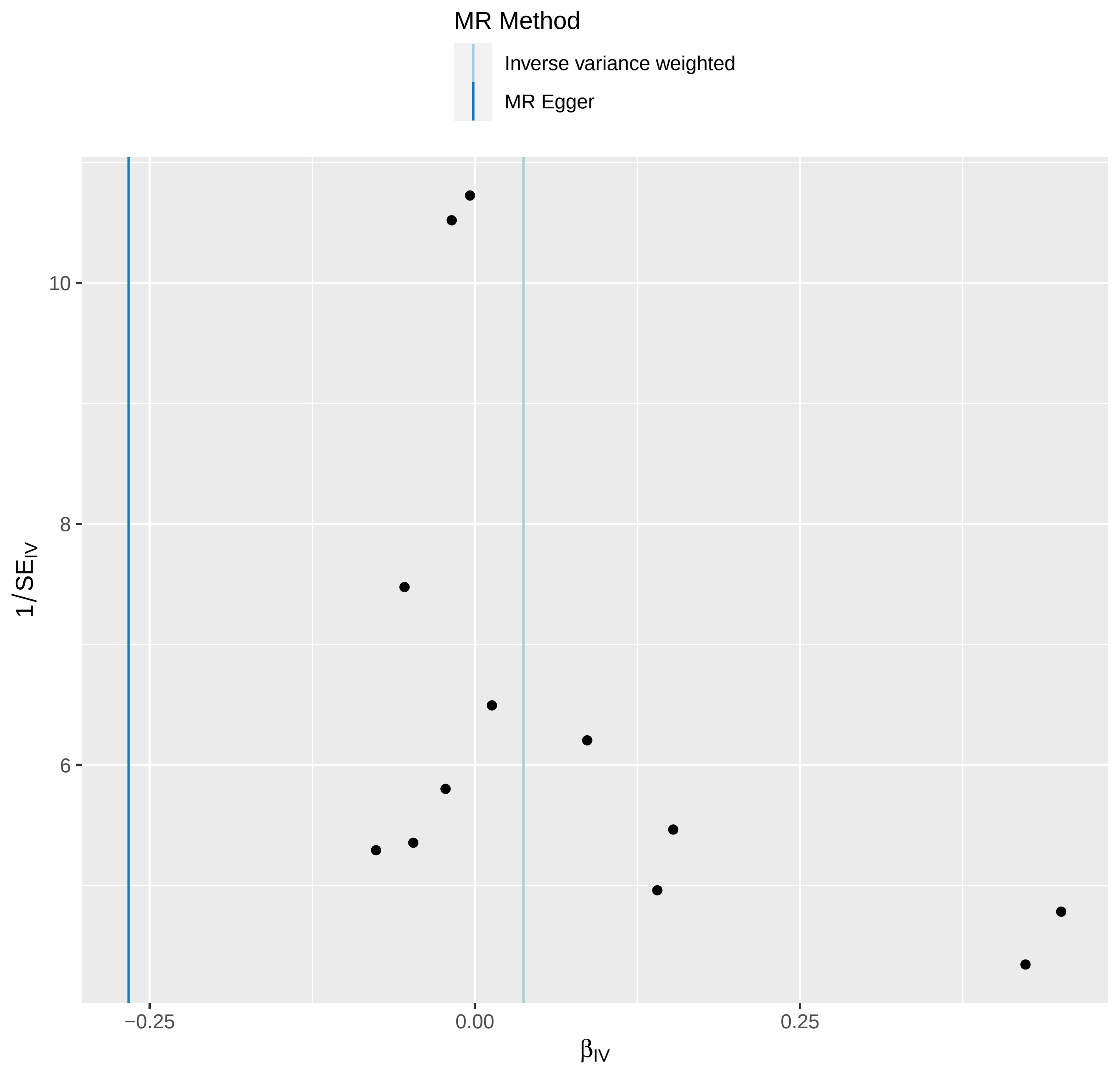

Batch 3−921 : Selenium on Eukaryotic initiation factor 4A−III

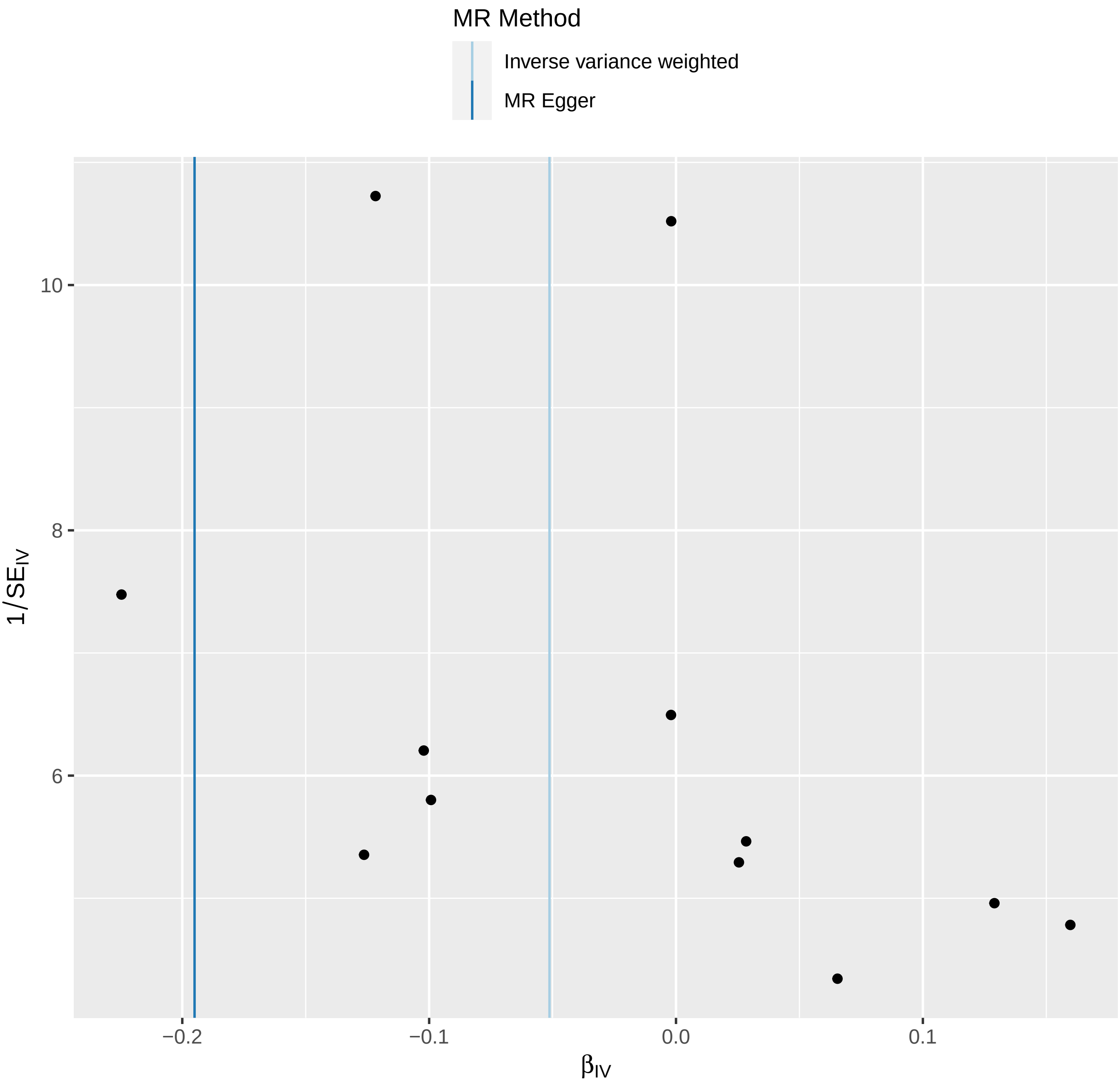

Batch 3−922 : Selenium on Eukaryotic translation initiation factor 4B

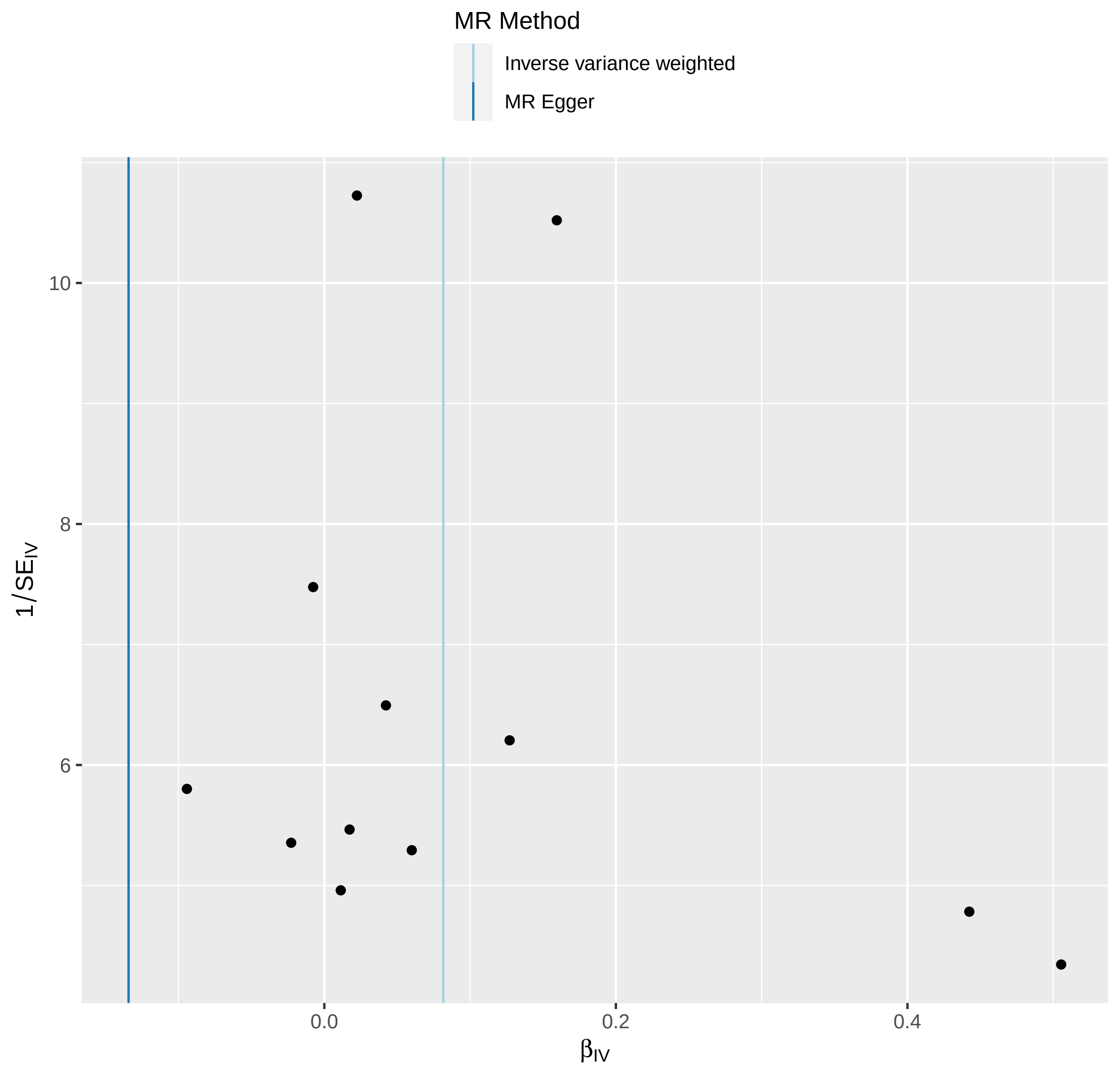

Batch 3−923 : Selenium on Eukaryotic translation initiation factor 4E type 2

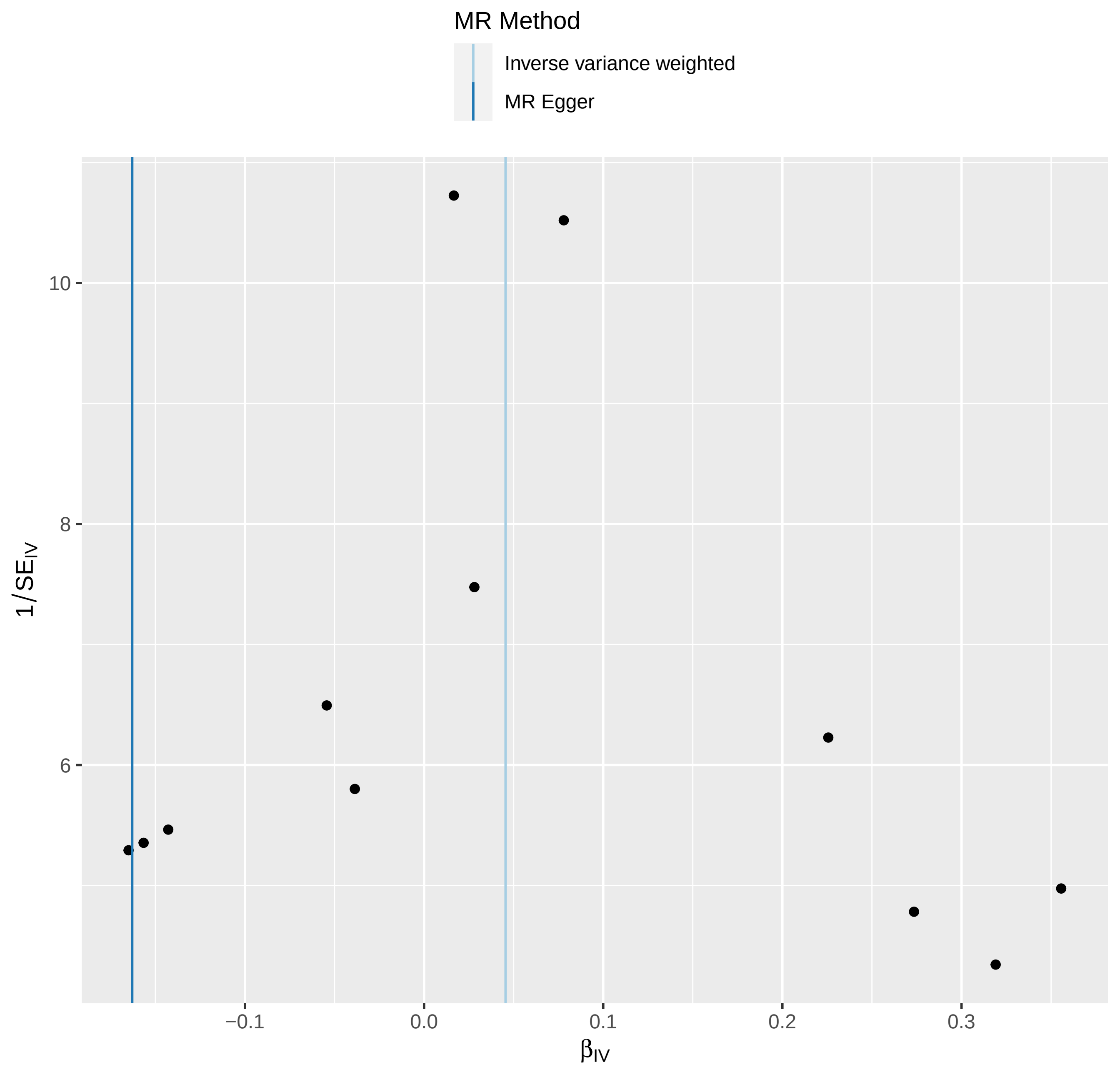

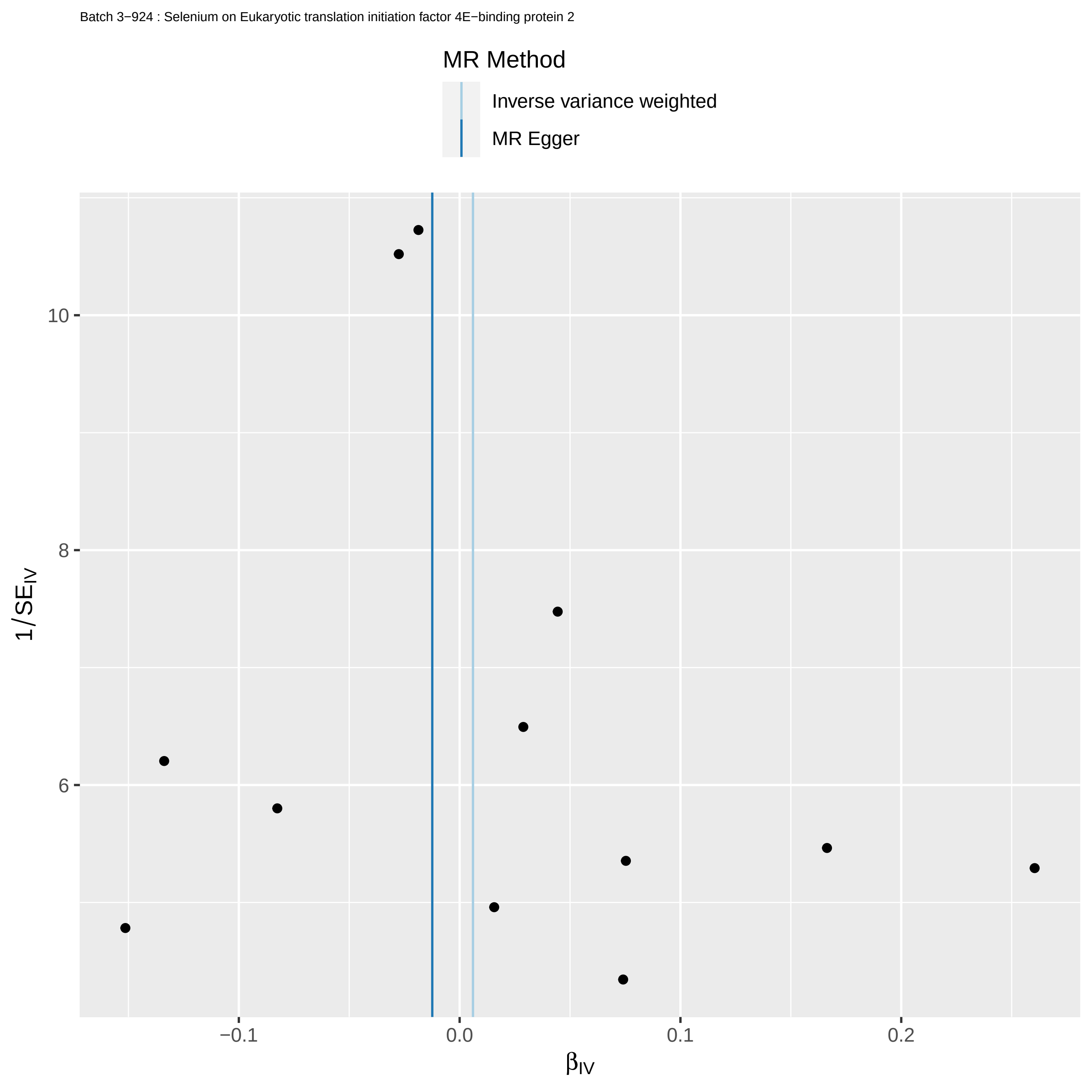

Batch 3−924 : Selenium on Eukaryotic translation initiation factor 4E−binding protein 2
MR Method
Inverse variance weighted
MR Egger
10
8
6
1/SE_IV
−0.1
0.0
0.1
0.2
β_IV

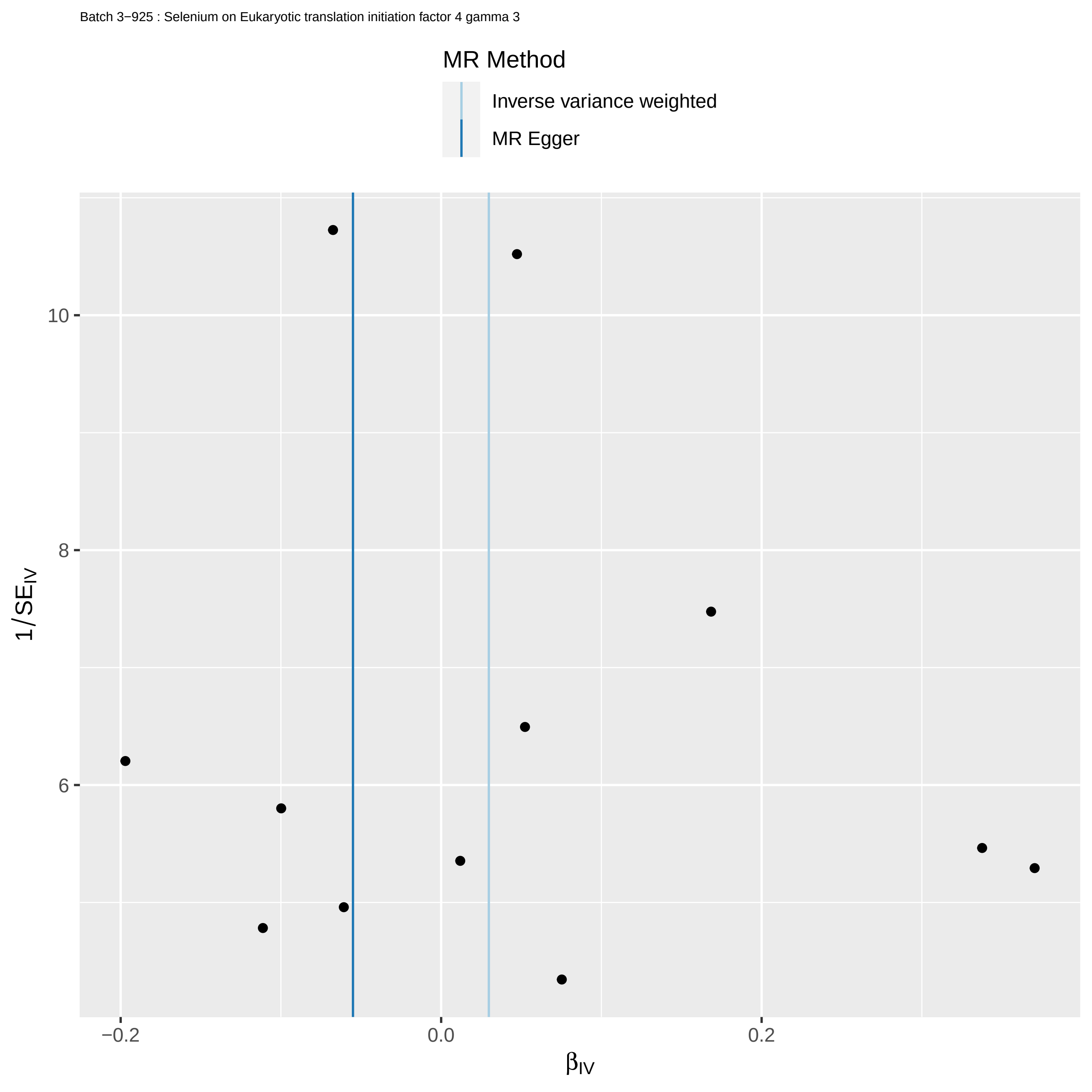
Batch 3−925 : Selenium on Eukaryotic translation initiation factor 4 gamma 3
MR Method
Inverse variance weighted
MR Egger
10
8
6
1/SE_IV
−0.2
0.0
0.2
β_IV

Batch 3−926 : Selenium on Eukaryotic translation initiation factor 5

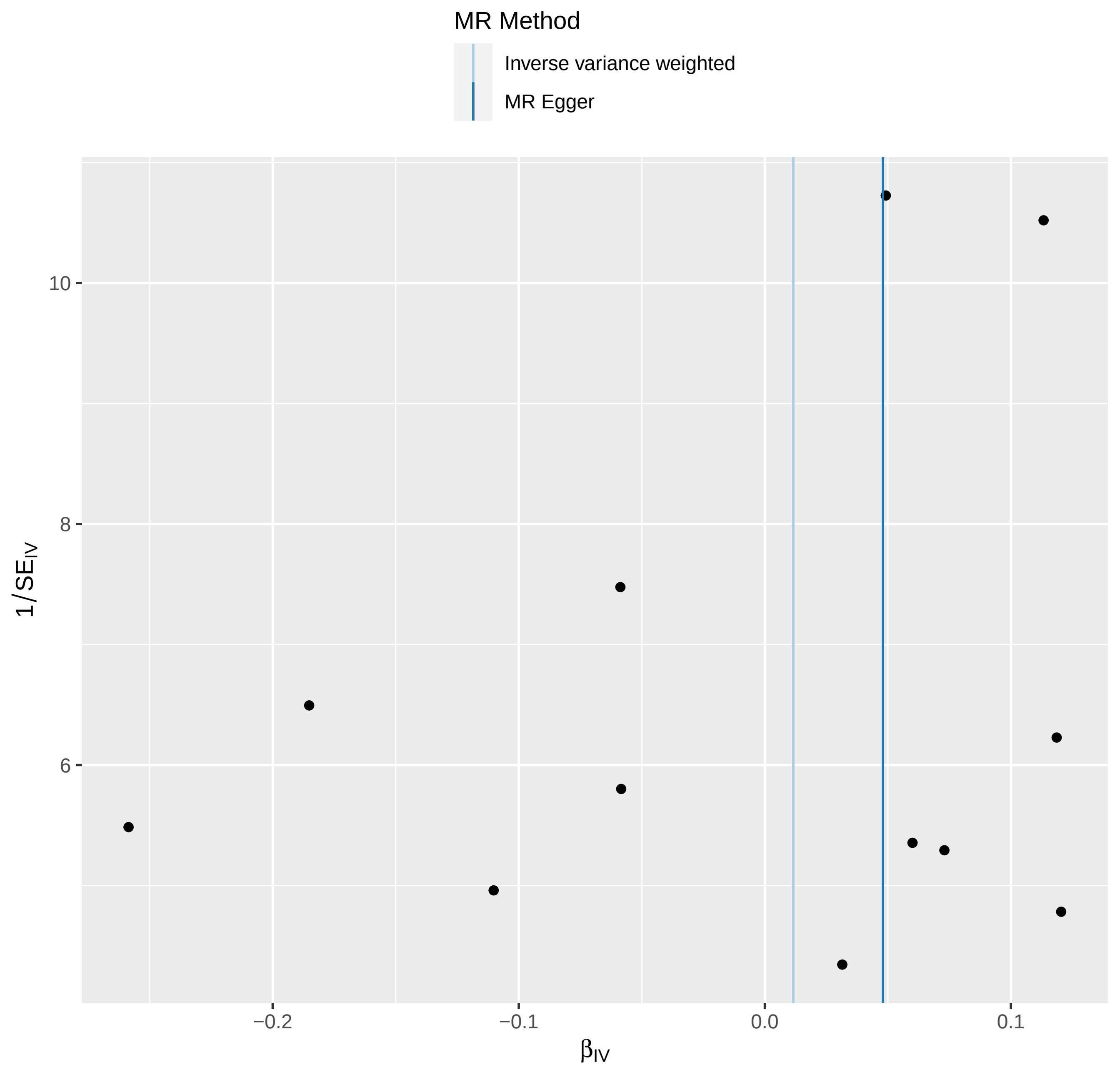

Batch 3−927 : Selenium on Eukaryotic translation initiation factor 5A−1

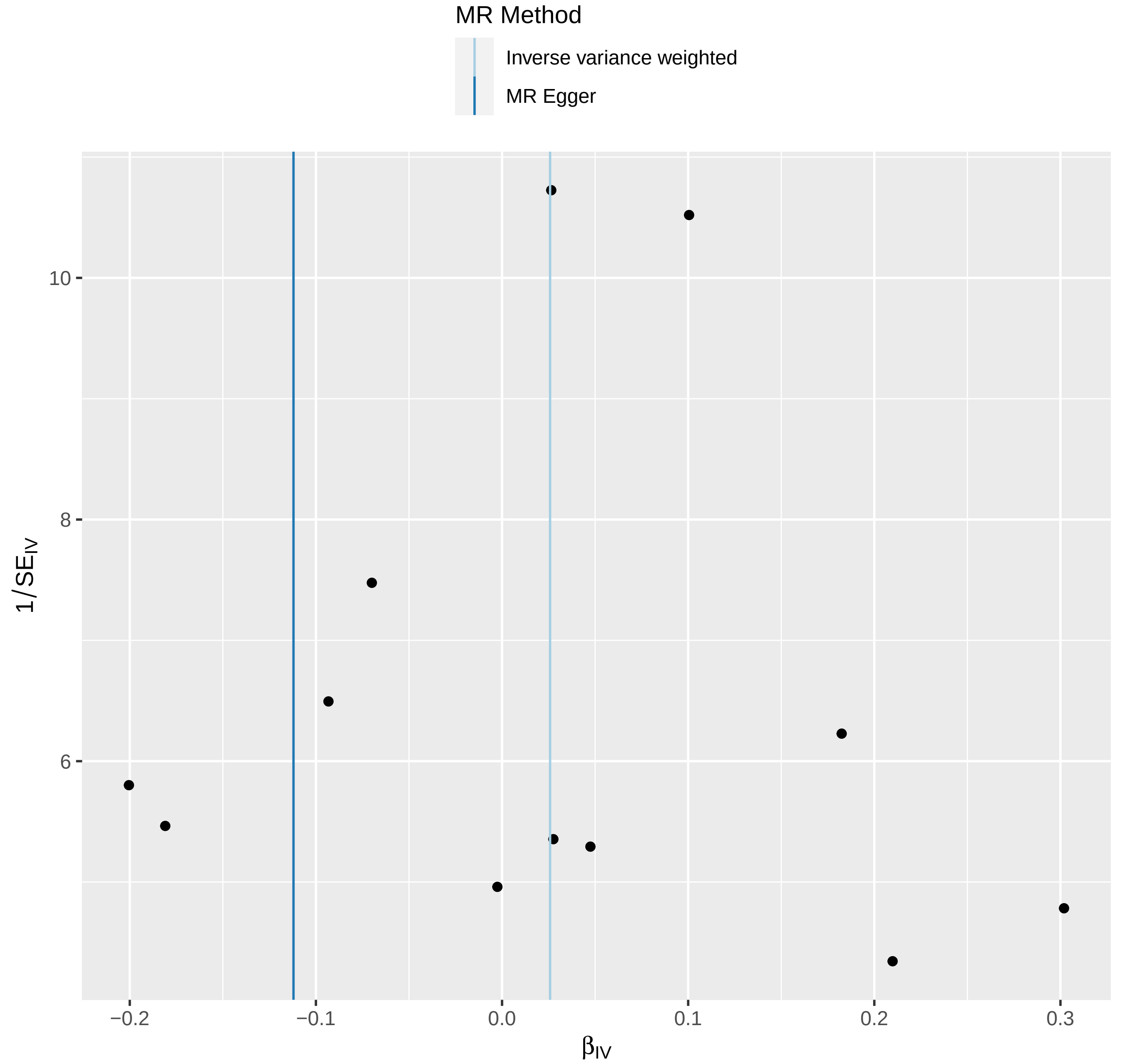

Batch 3−928 : Selenium on ELAV−like protein 1

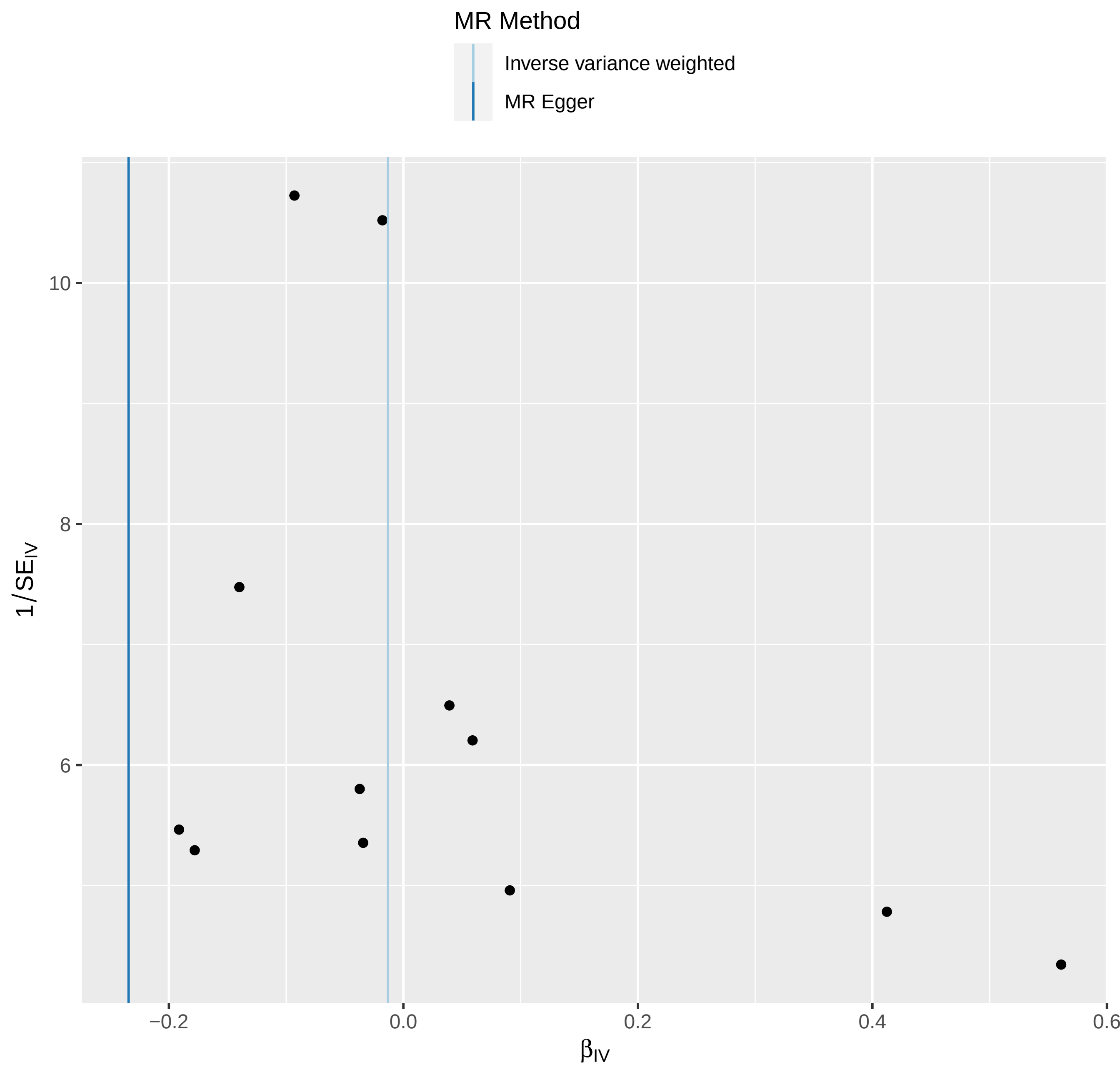

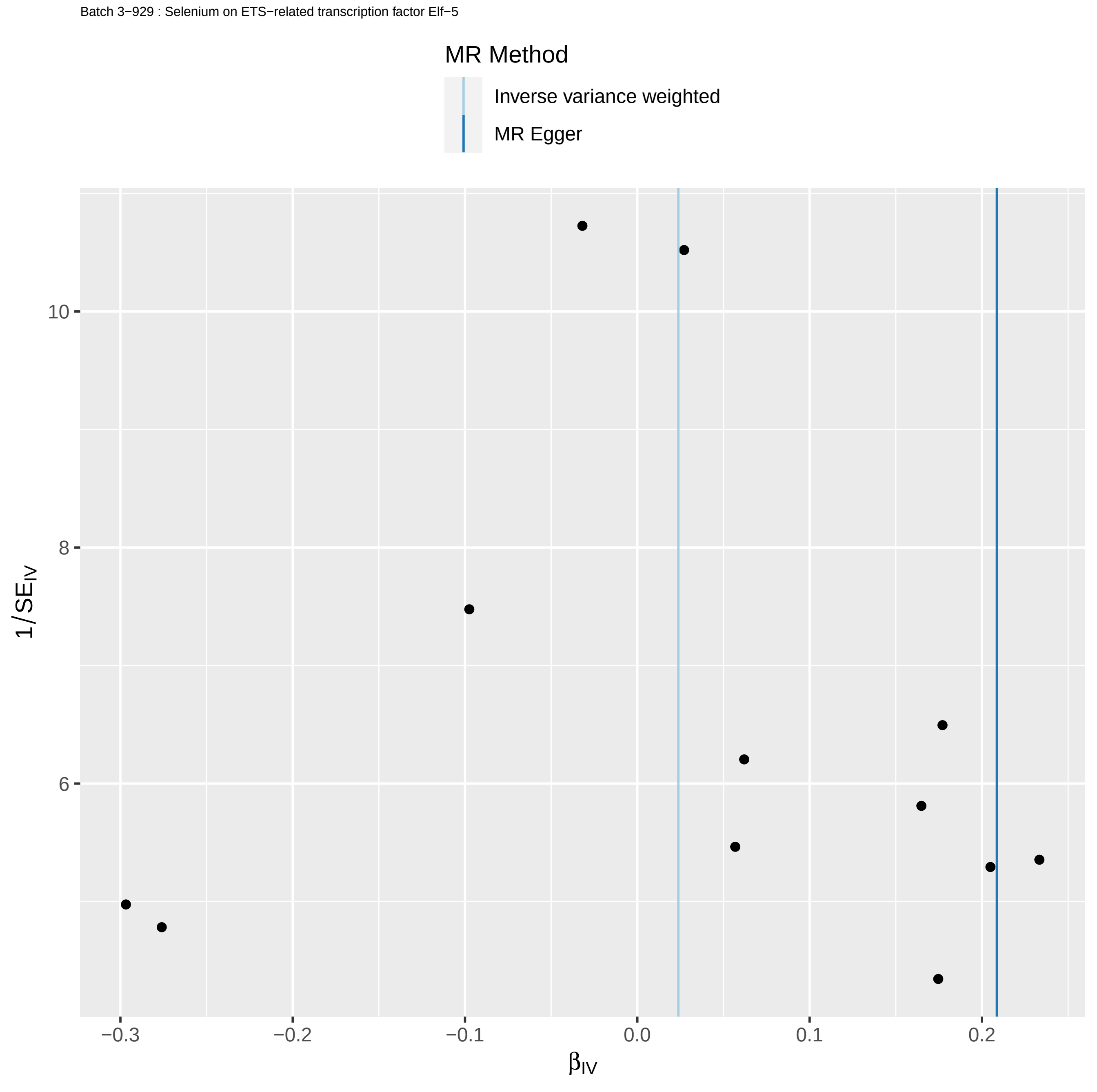

Batch 3−929 : Selenium on ETS−related transcription factor Elf−5
MR Method
Inverse variance weighted
MR Egger
10
8
6
−0.3
−0.2
−0.1
0.0
0.1
0.2
1/SE_IV
β_IV

Batch 3−930 : Selenium on ETS domain−containing protein Elk−1

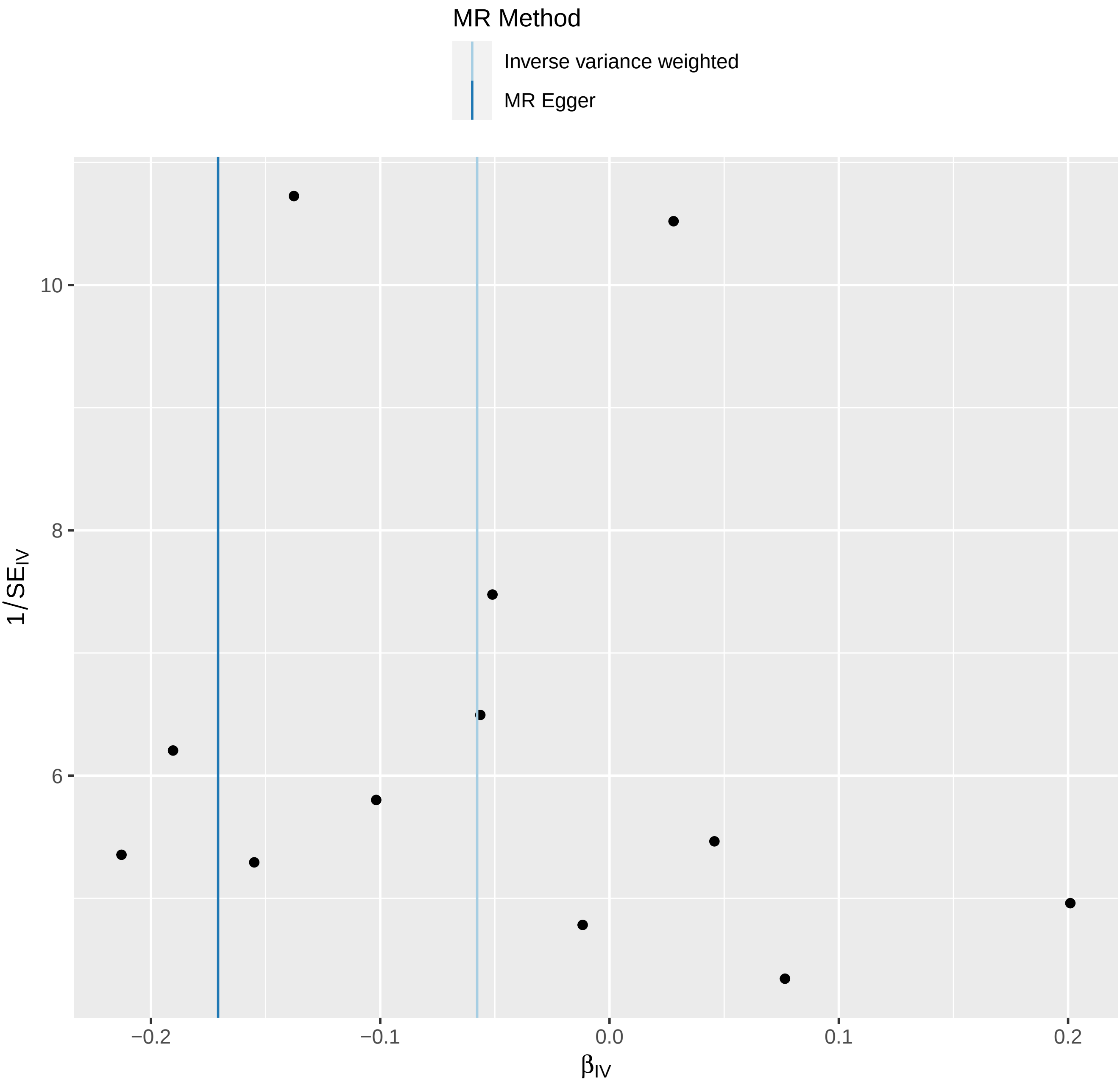

Batch 3−931 : Selenium on ETS domain−containing protein Elk−3

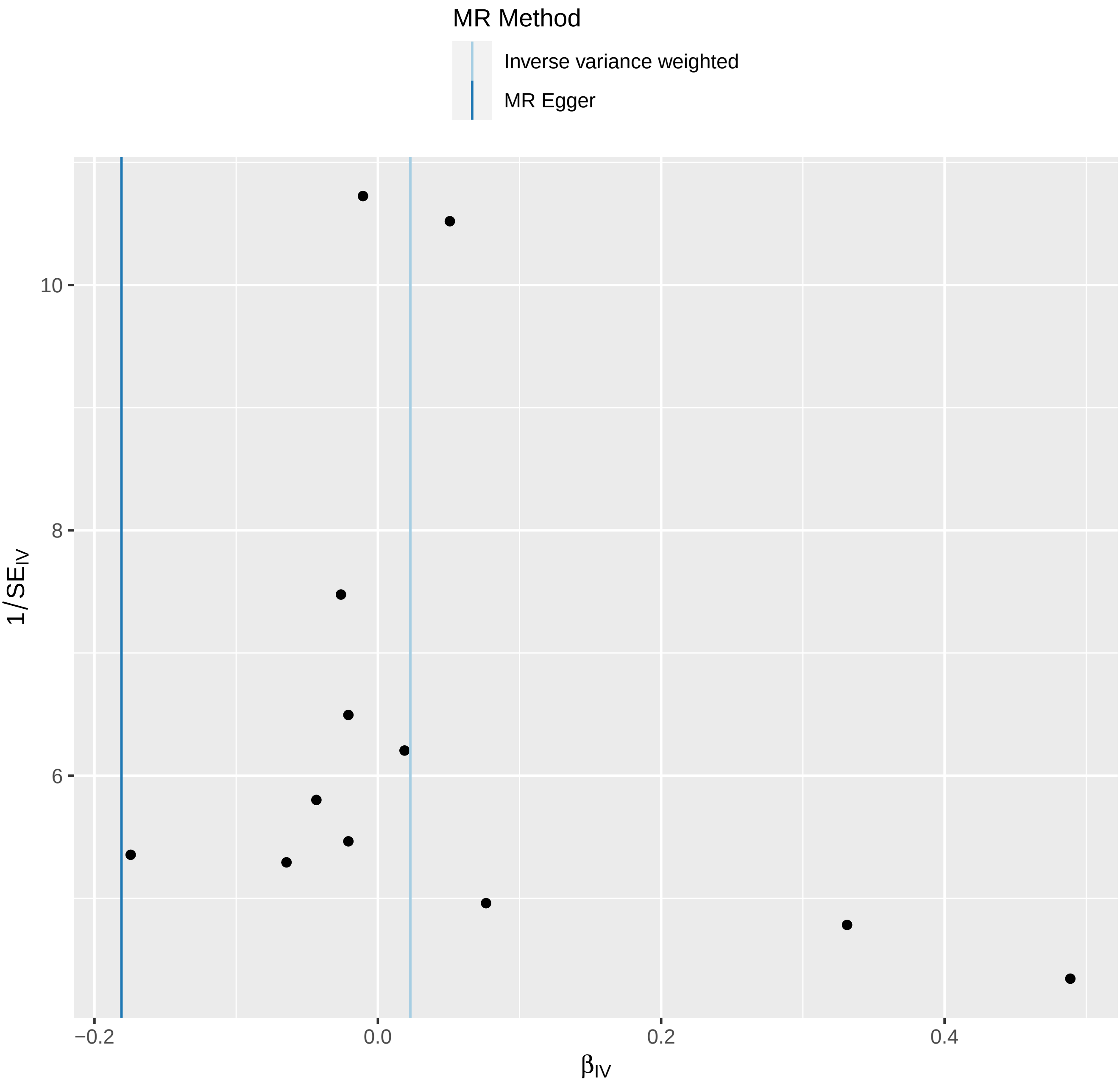

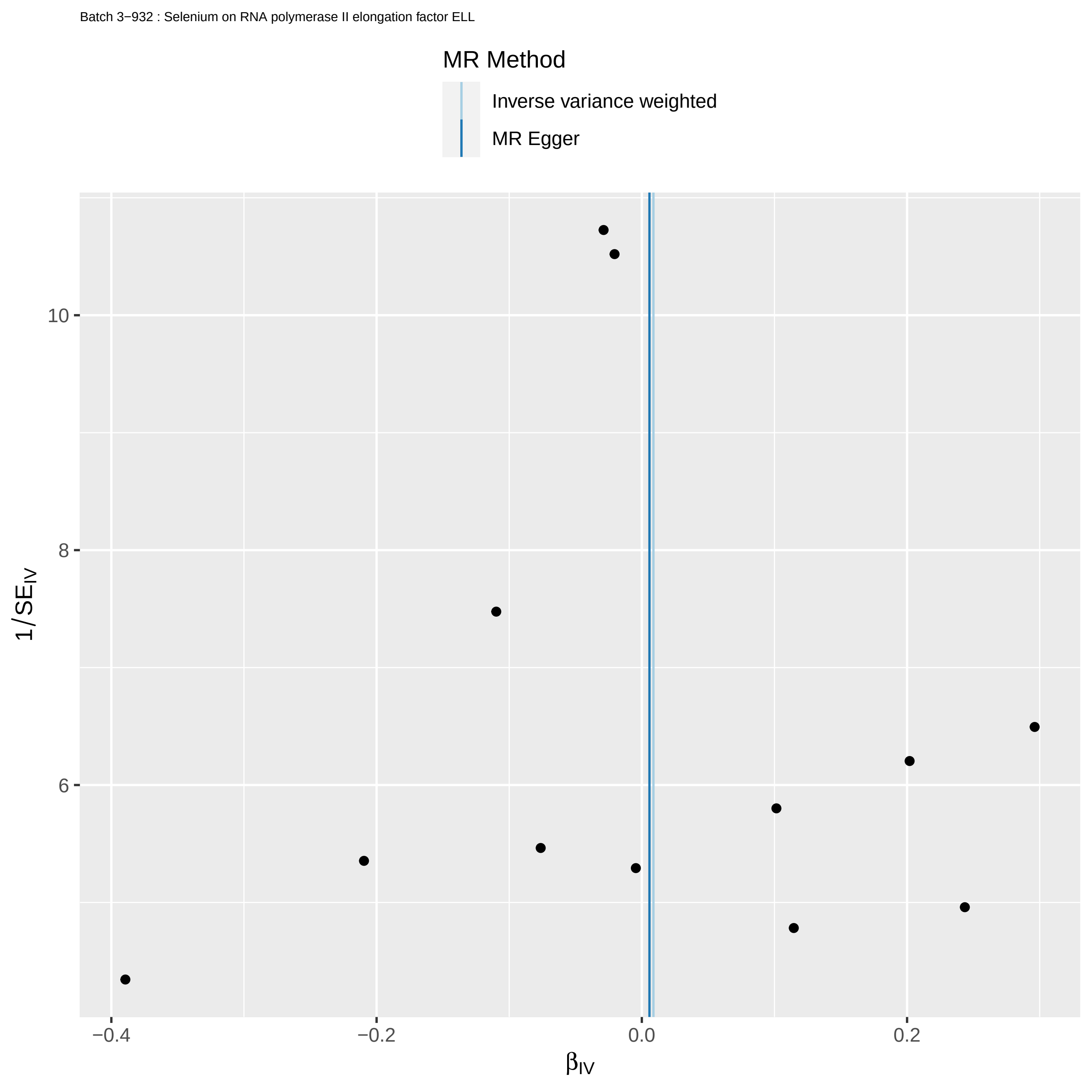
Batch 3−932 : Selenium on RNA polymerase II elongation factor ELL
MR Method
Inverse variance weighted
MR Egger
10
8
6
1/SE_IV
−0.4
−0.2
0.0
0.2
β_IV

Batch 3−933 : Selenium on RNA polymerase II elongation factor ELL2

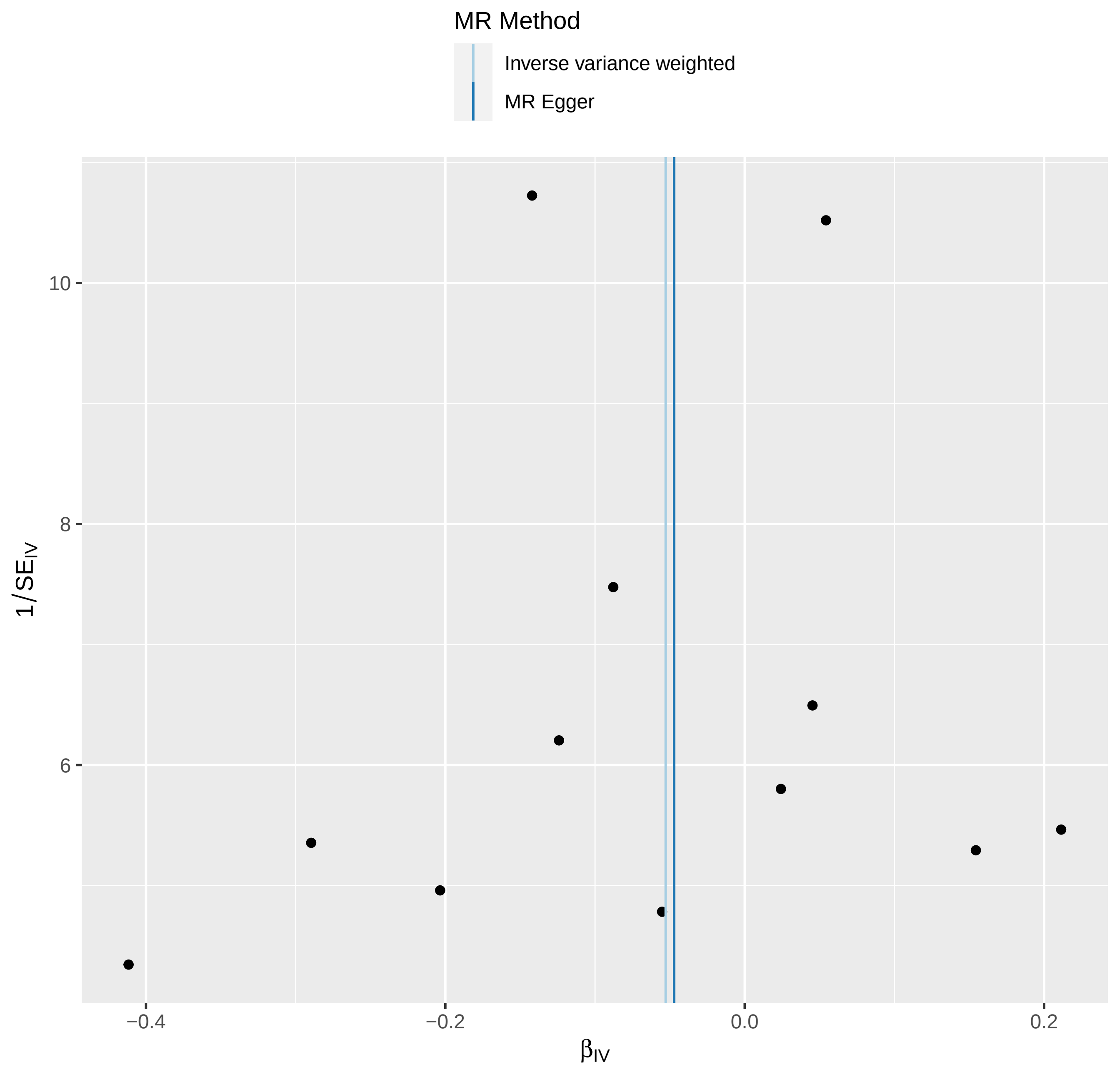

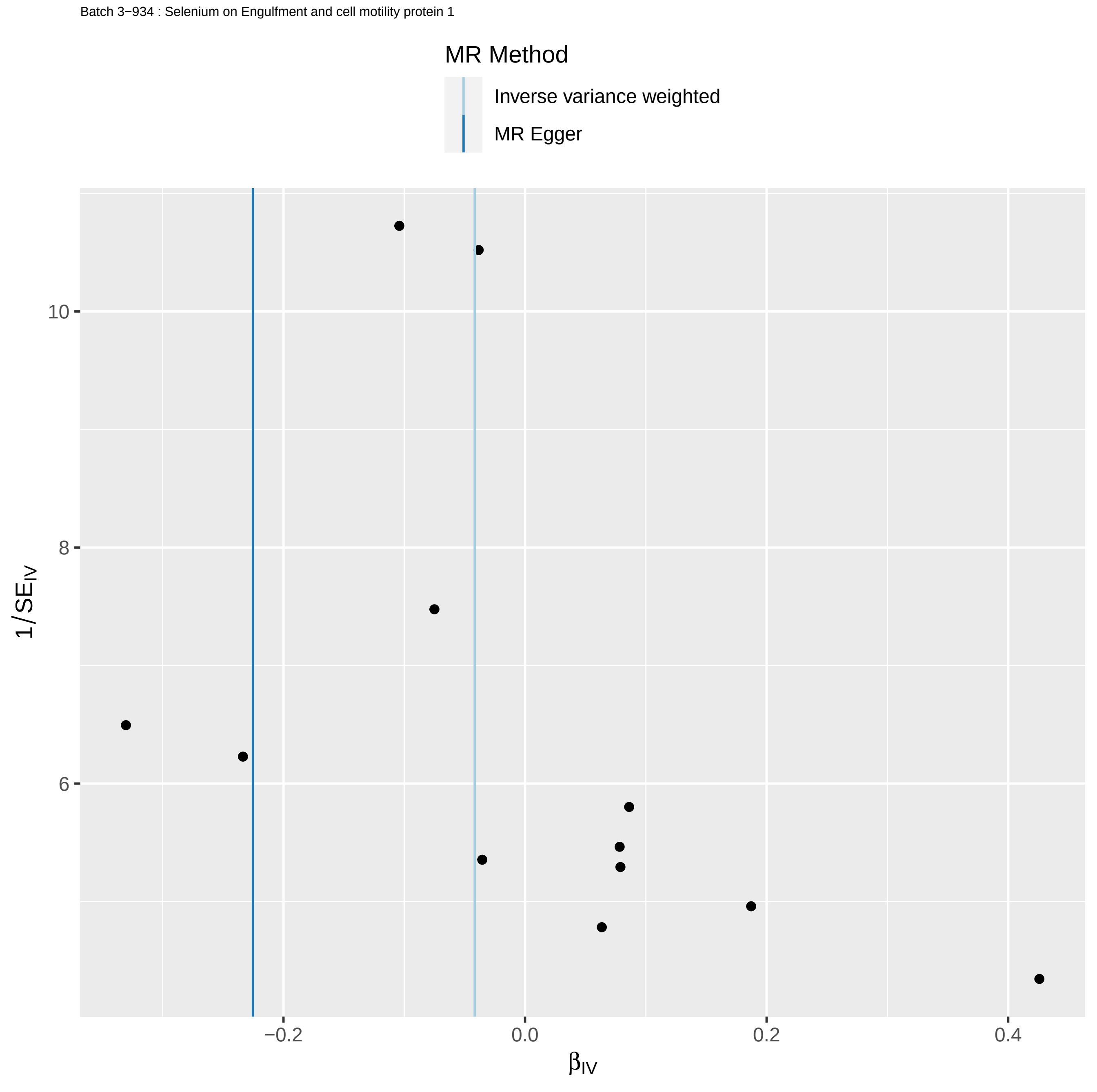
Batch 3−934 : Selenium on Engulfment and cell motility protein 1
MR Method
Inverse variance weighted
MR Egger
10
8
6
1/SE_IV
−0.2
0.0
0.2
0.4
β_IV

Batch 3−935 : Selenium on ER membrane protein complex subunit 1

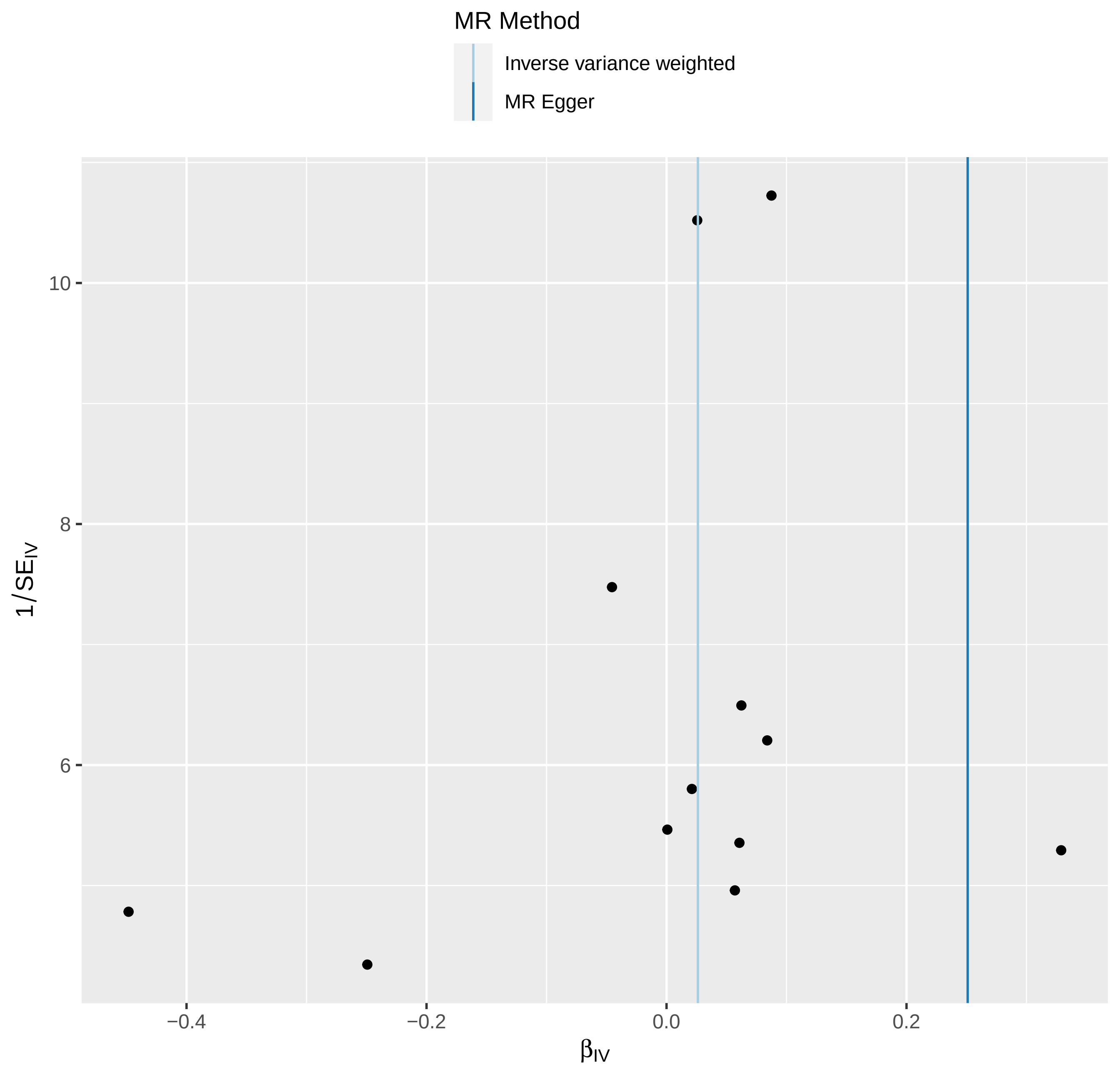

Batch 3−936 : Selenium on ER membrane protein complex subunit 4

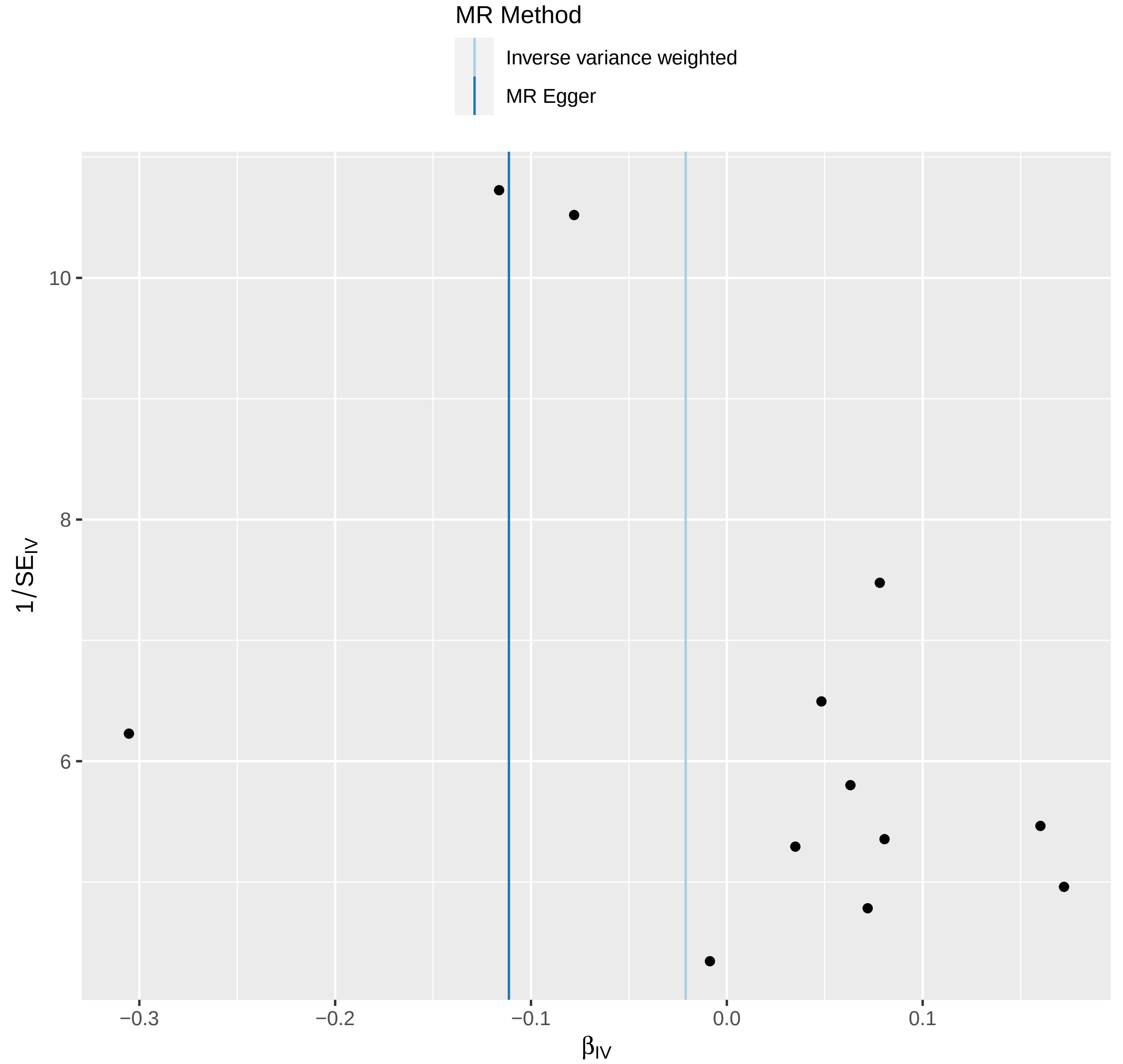

Batch 3−937 : Selenium on EMI domain−containing protein 1

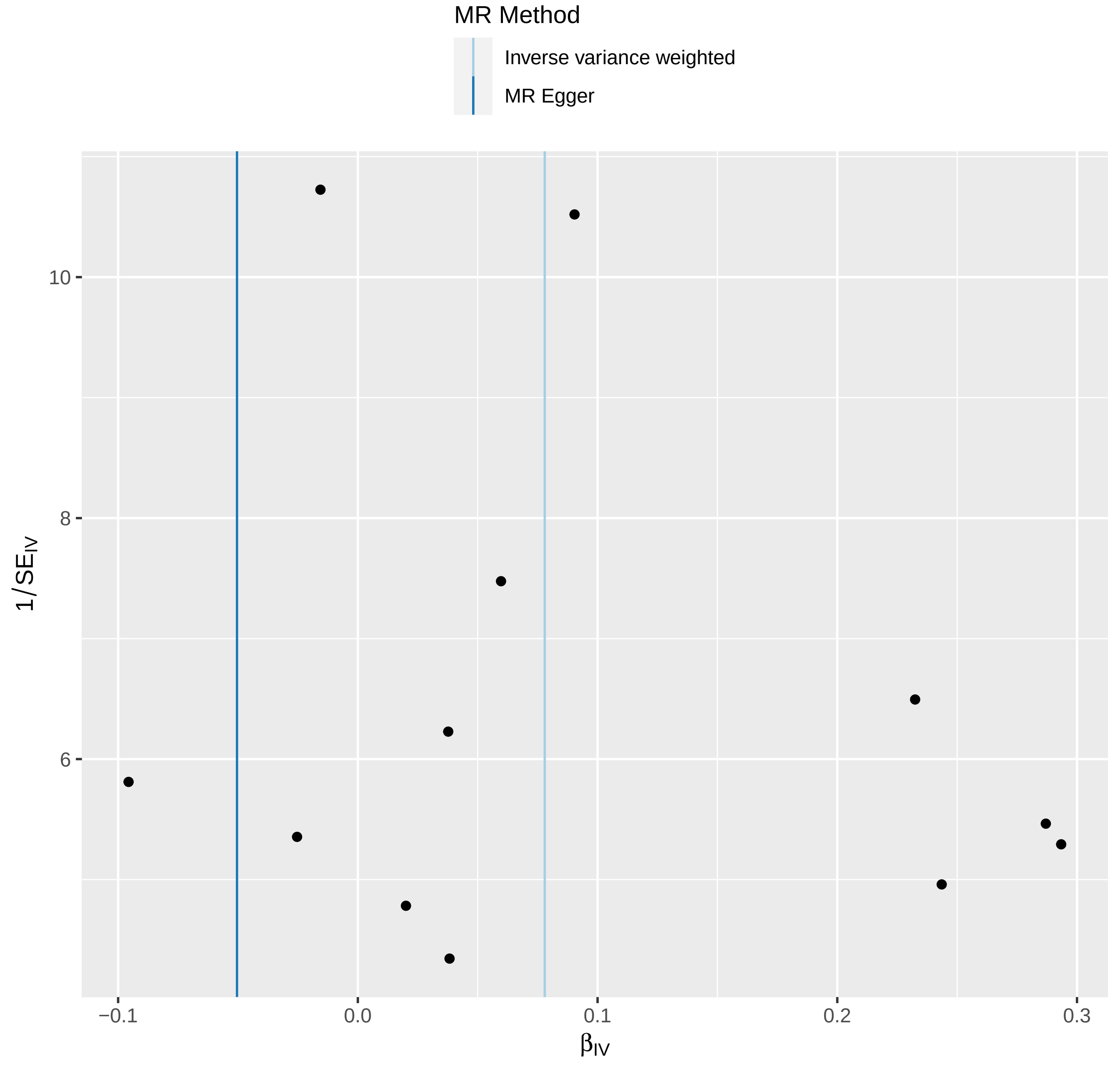

Batch 3−938 : Selenium on EMILIN−3

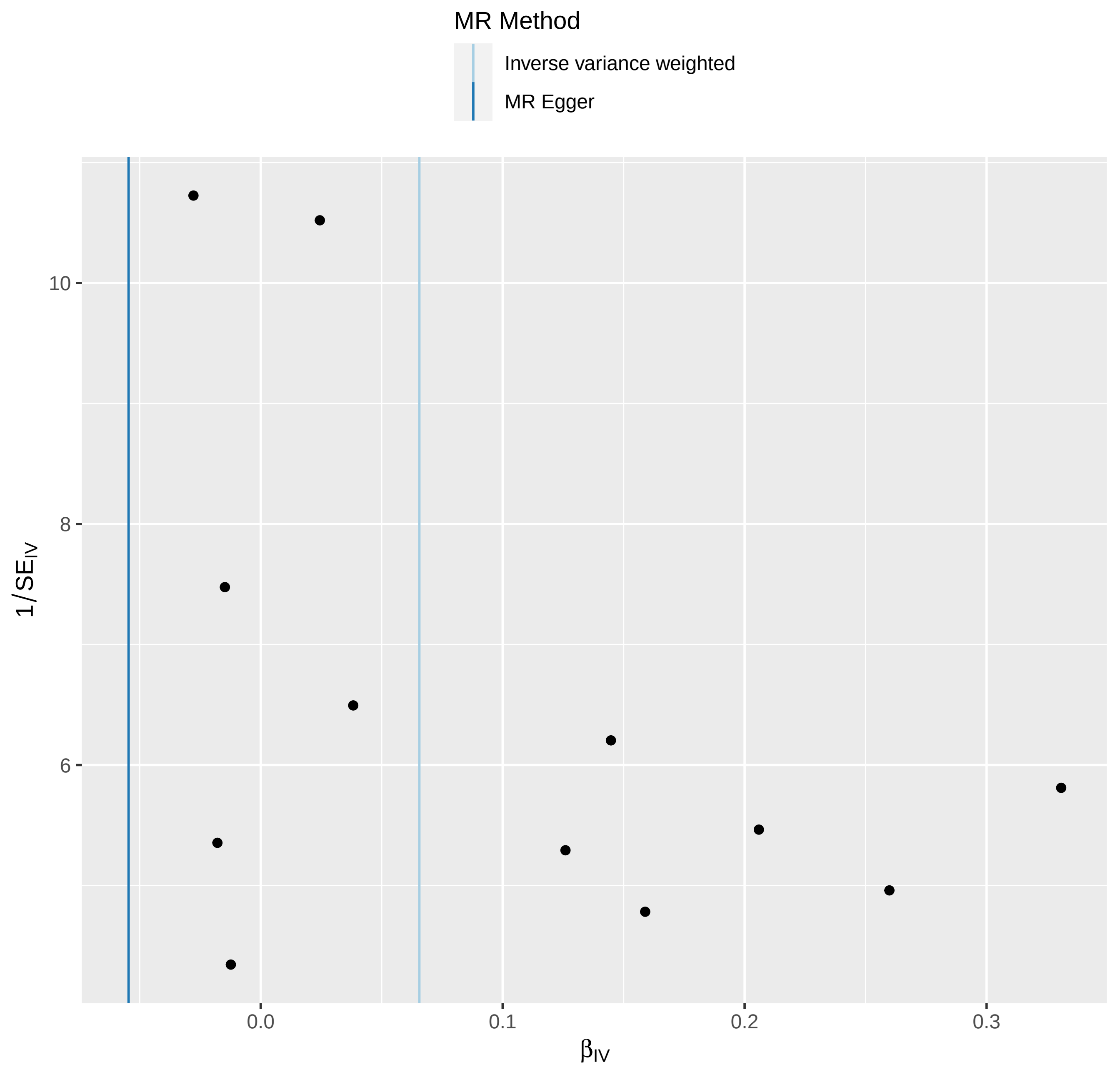

Batch 3−939 : Selenium on EMILIN−3

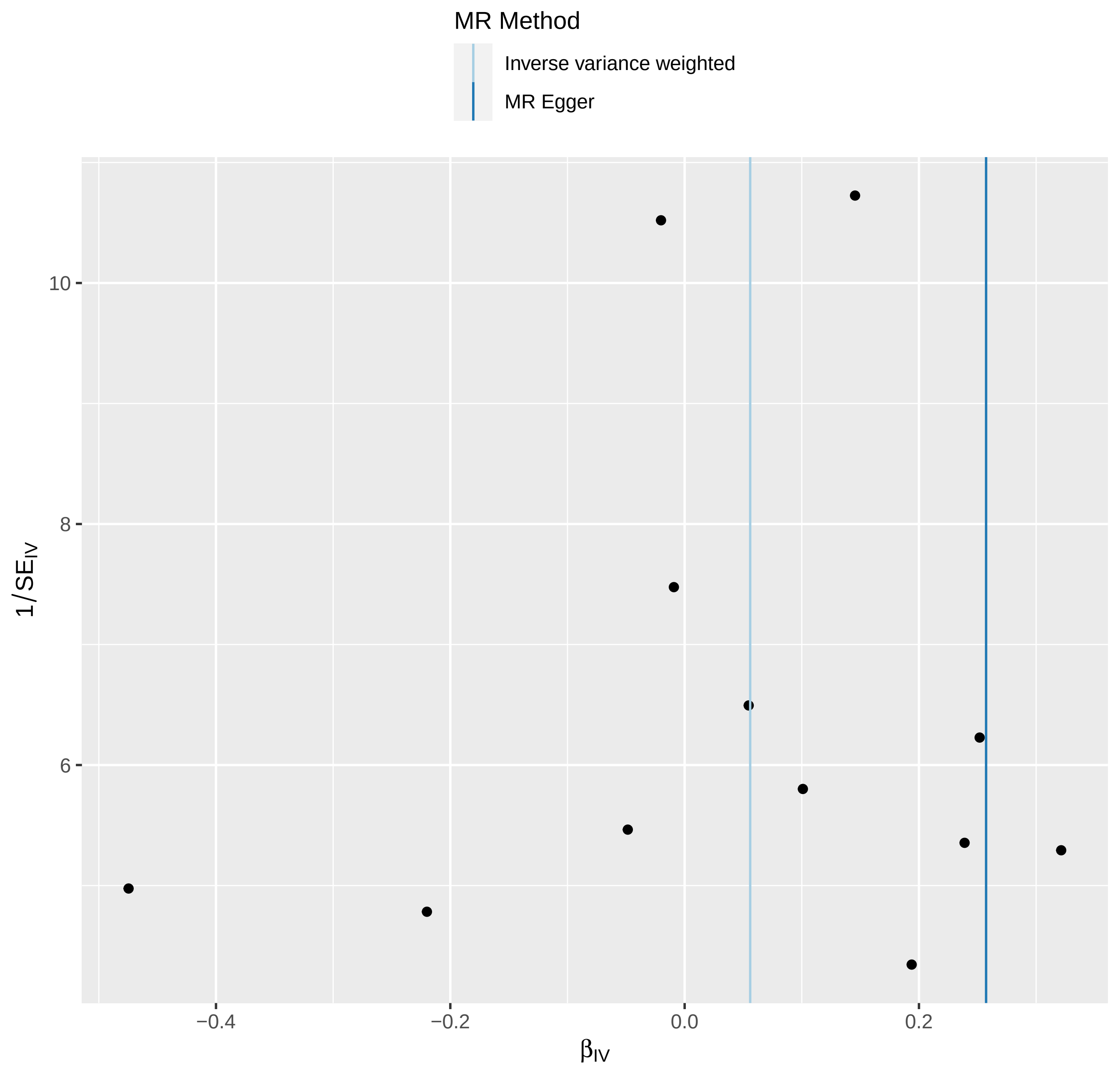

Batch 3−940 : Selenium on EMILIN−3

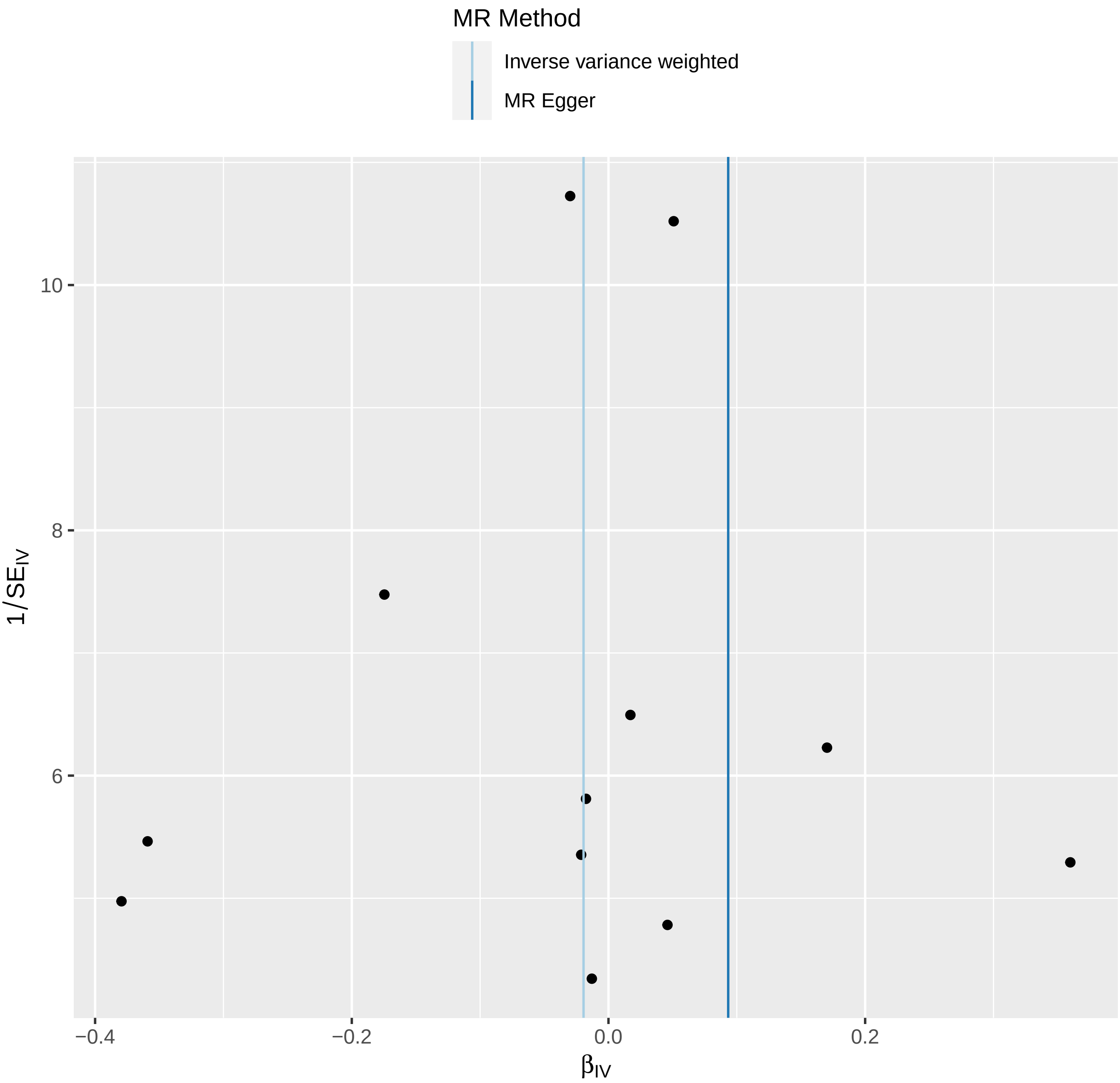

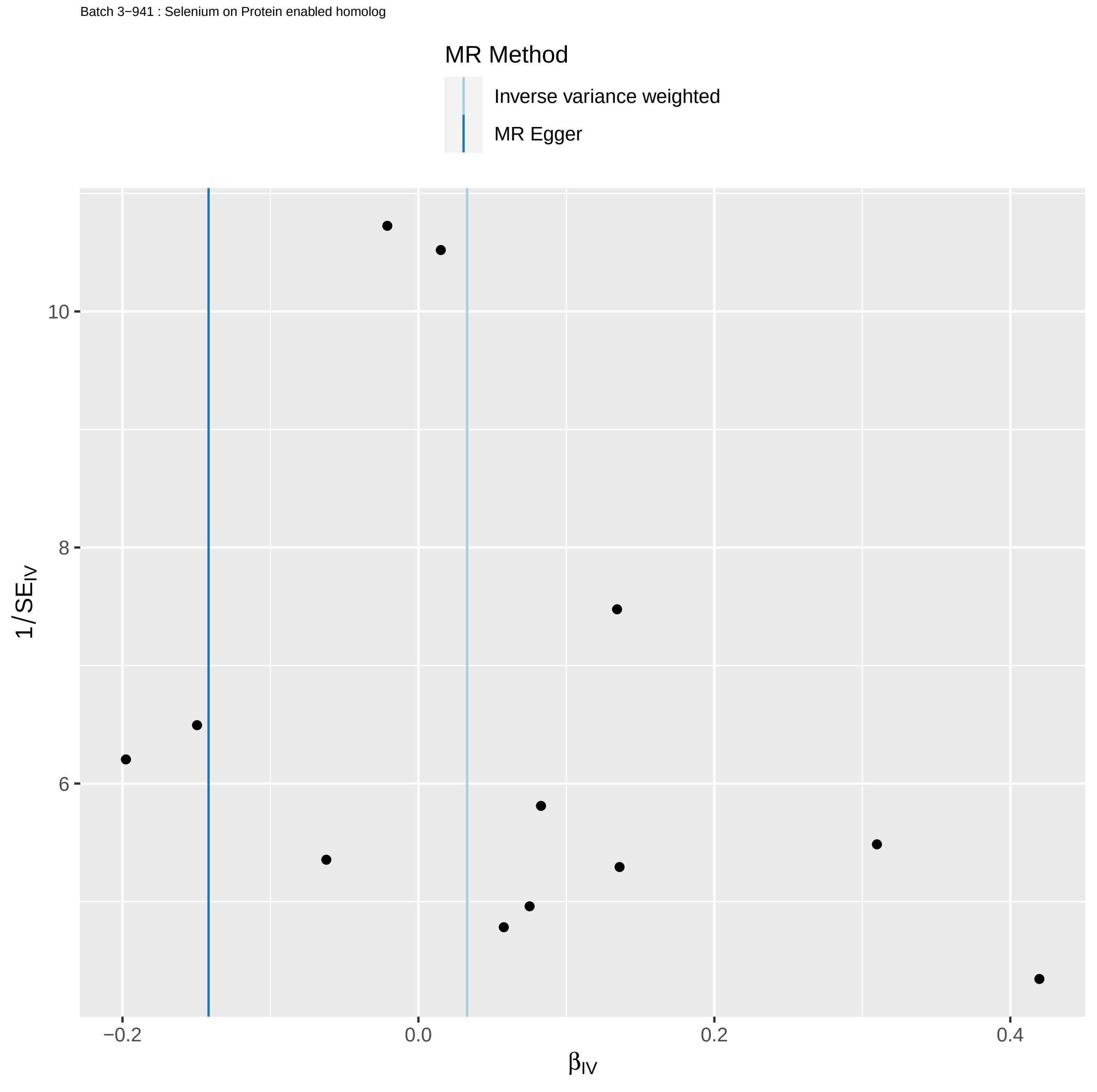
Batch 3−941 : Selenium on Protein enabled homolog
MR Method
Inverse variance weighted
MR Egger
10
8
6
1/SE_IV
−0.2
0.0
0.2
0.4
β_IV

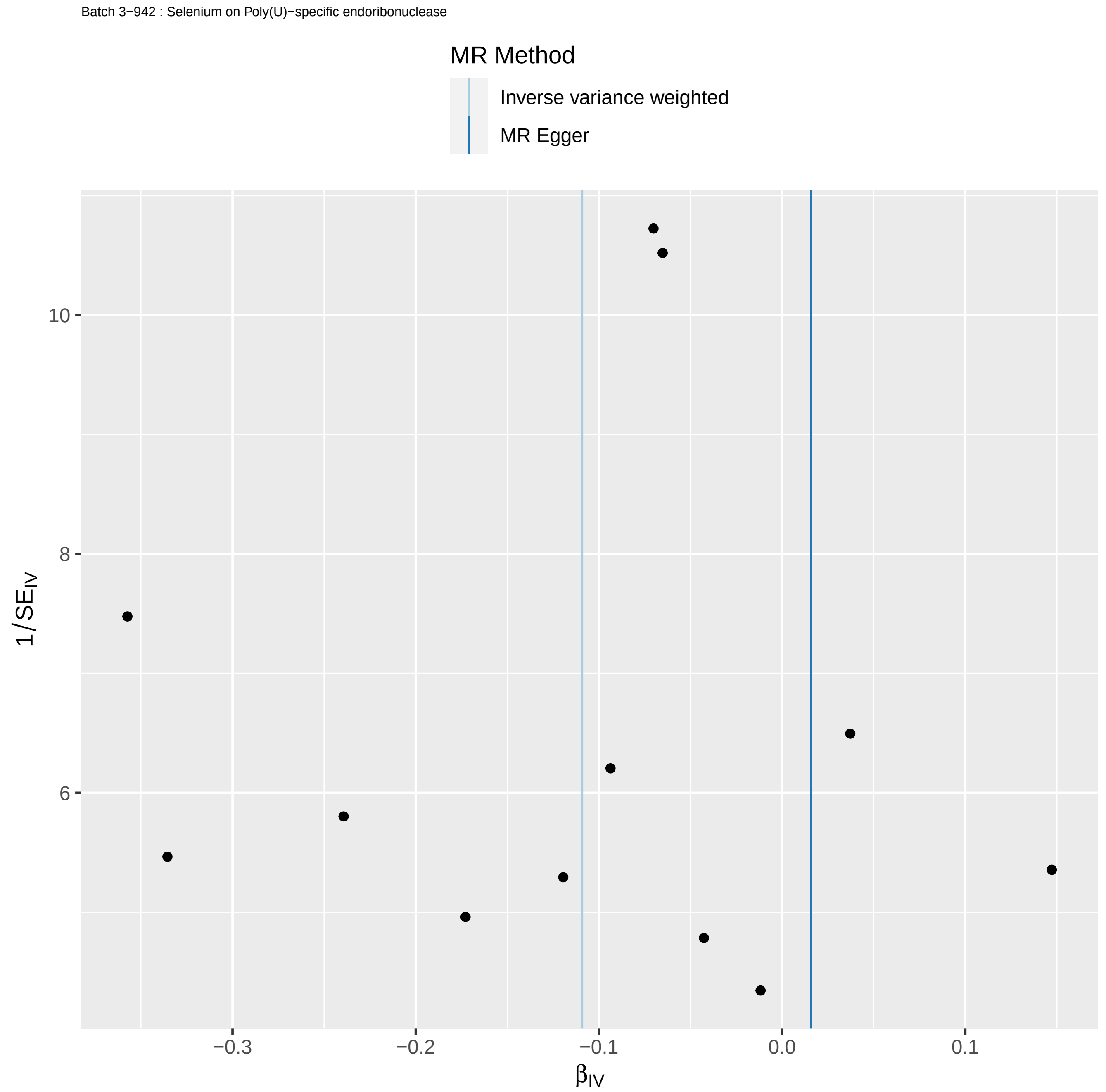

Batch 3−942 : Selenium on Poly(U)−specific endoribonuclease
MR Method
Inverse variance weighted
MR Egger
10
8
6
1/SE_IV
−0.3
−0.2
−0.1
0.0
0.1
β_IV

Batch 3−943 : Selenium on Endoglin

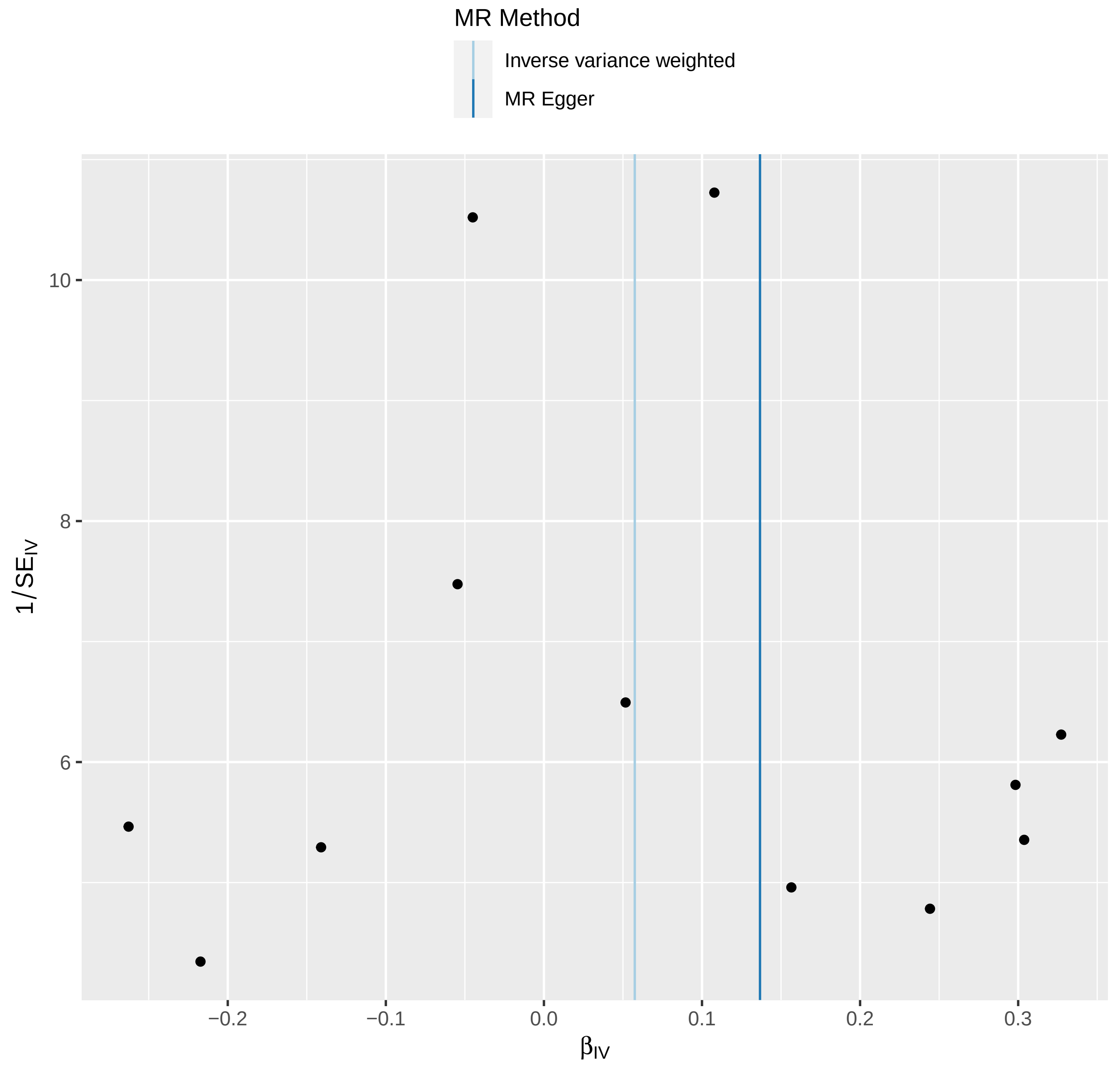

Batch 3−944 : Selenium on Gamma−enolase

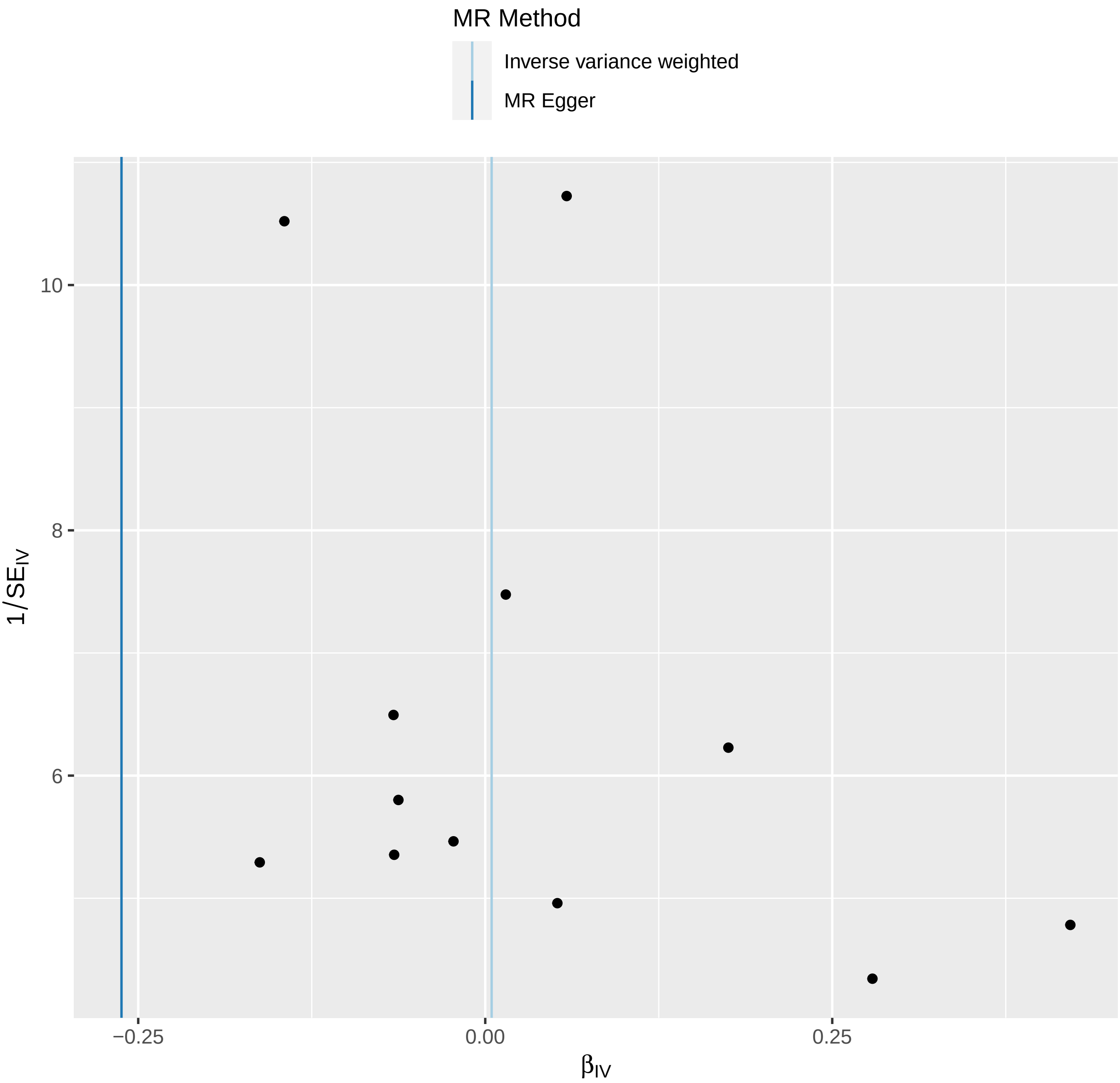

Batch 3−945 : Selenium on Gamma−enolase

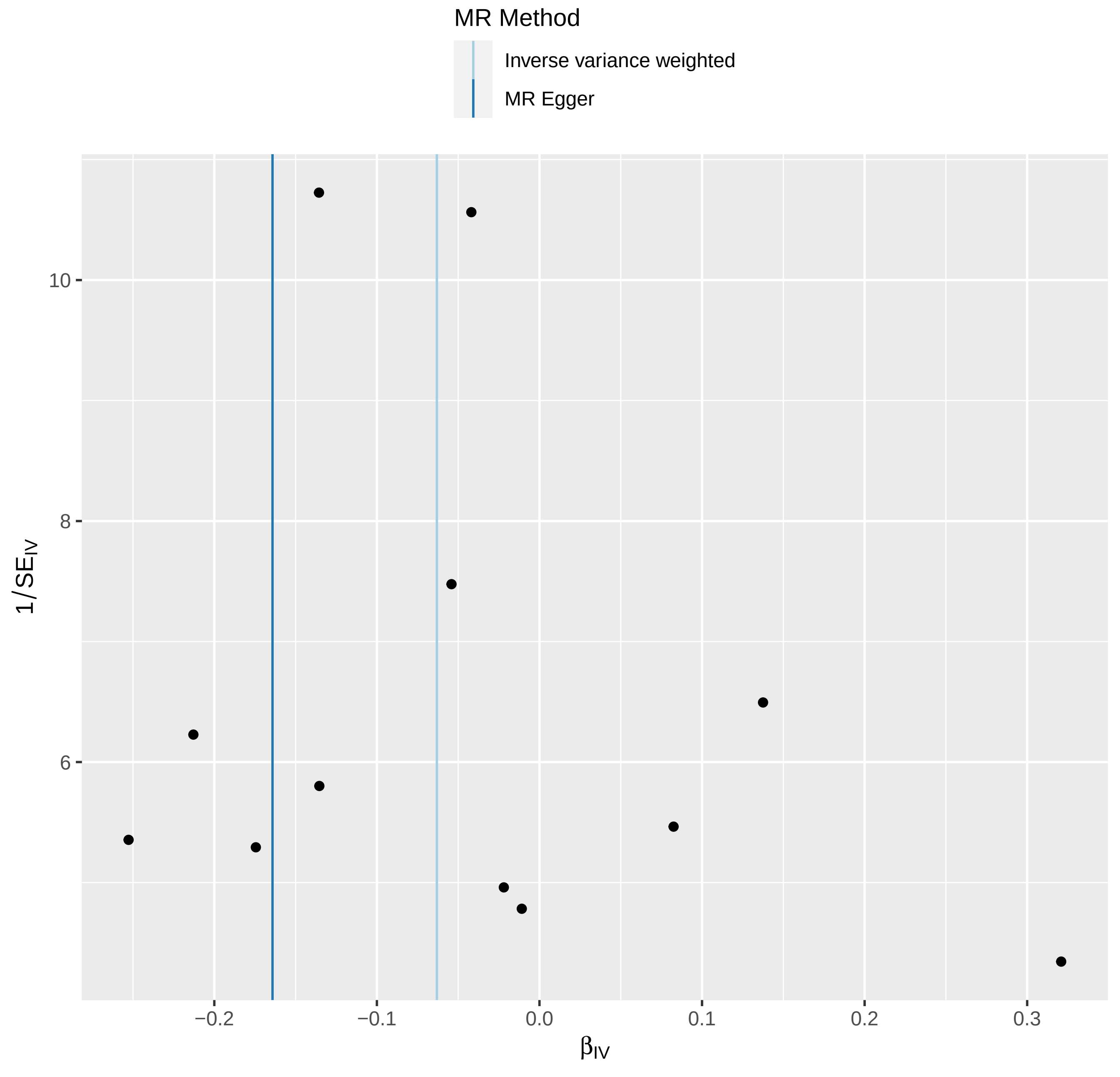

Batch 3−946 : Selenium on Ecto−NOX disulfide−thiol exchanger 2

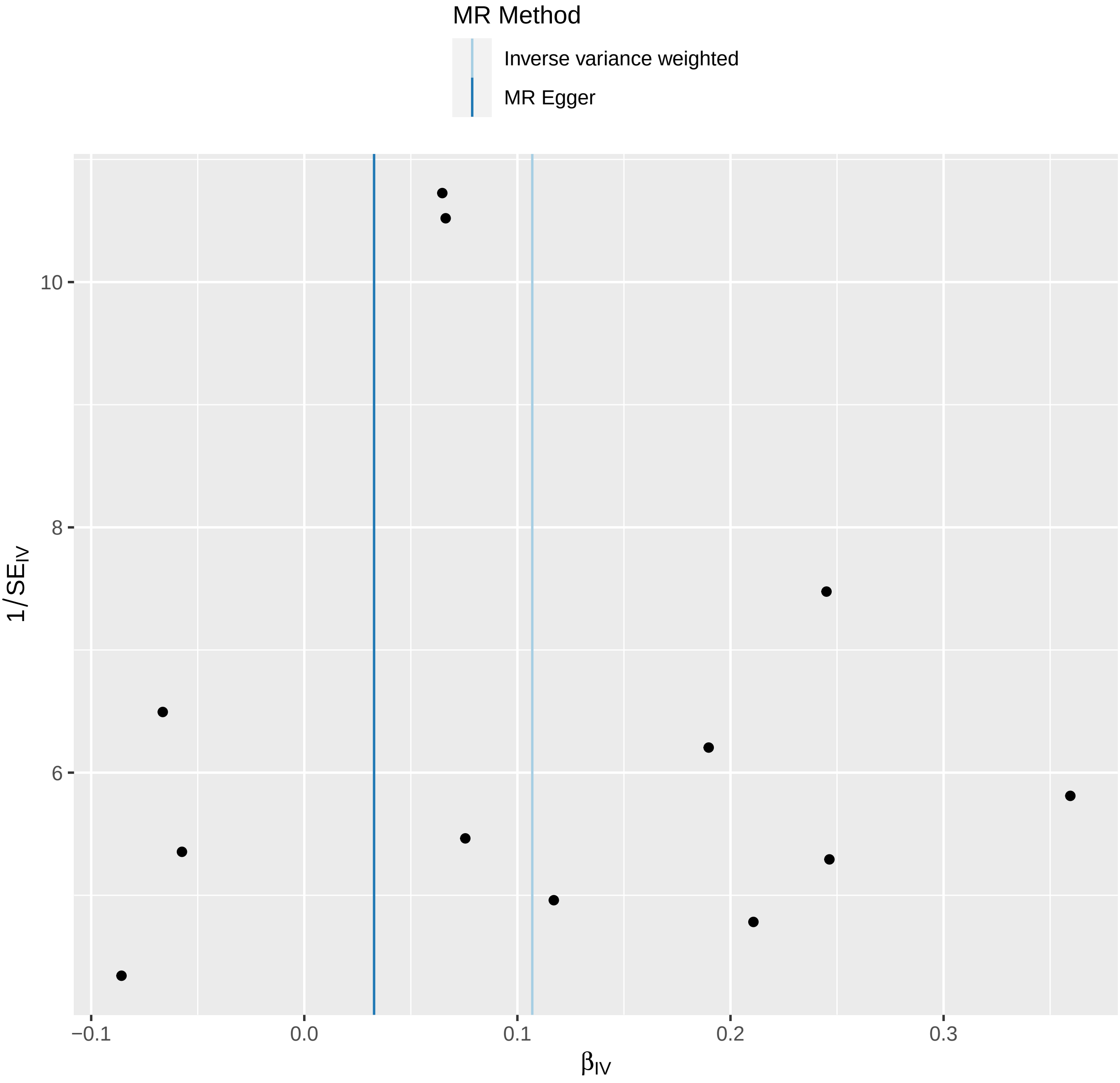

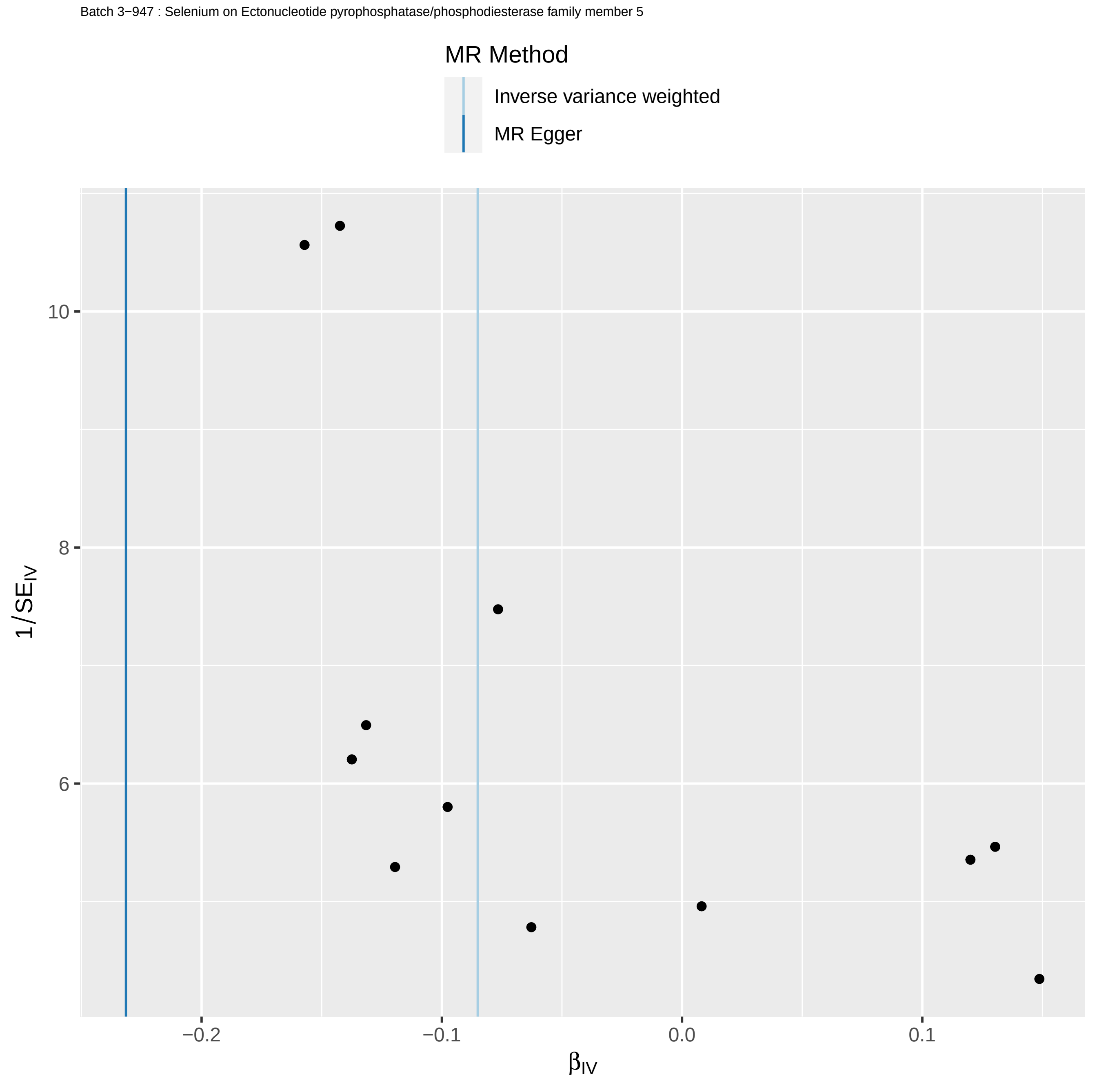

Batch 3−947 : Selenium on Ectonucleotide pyrophosphatase/phosphodiesterase family member 5
MR Method
Inverse variance weighted
MR Egger
10
8
6
1/SE_IV
−0.2
−0.1
0.0
0.1
β_IV

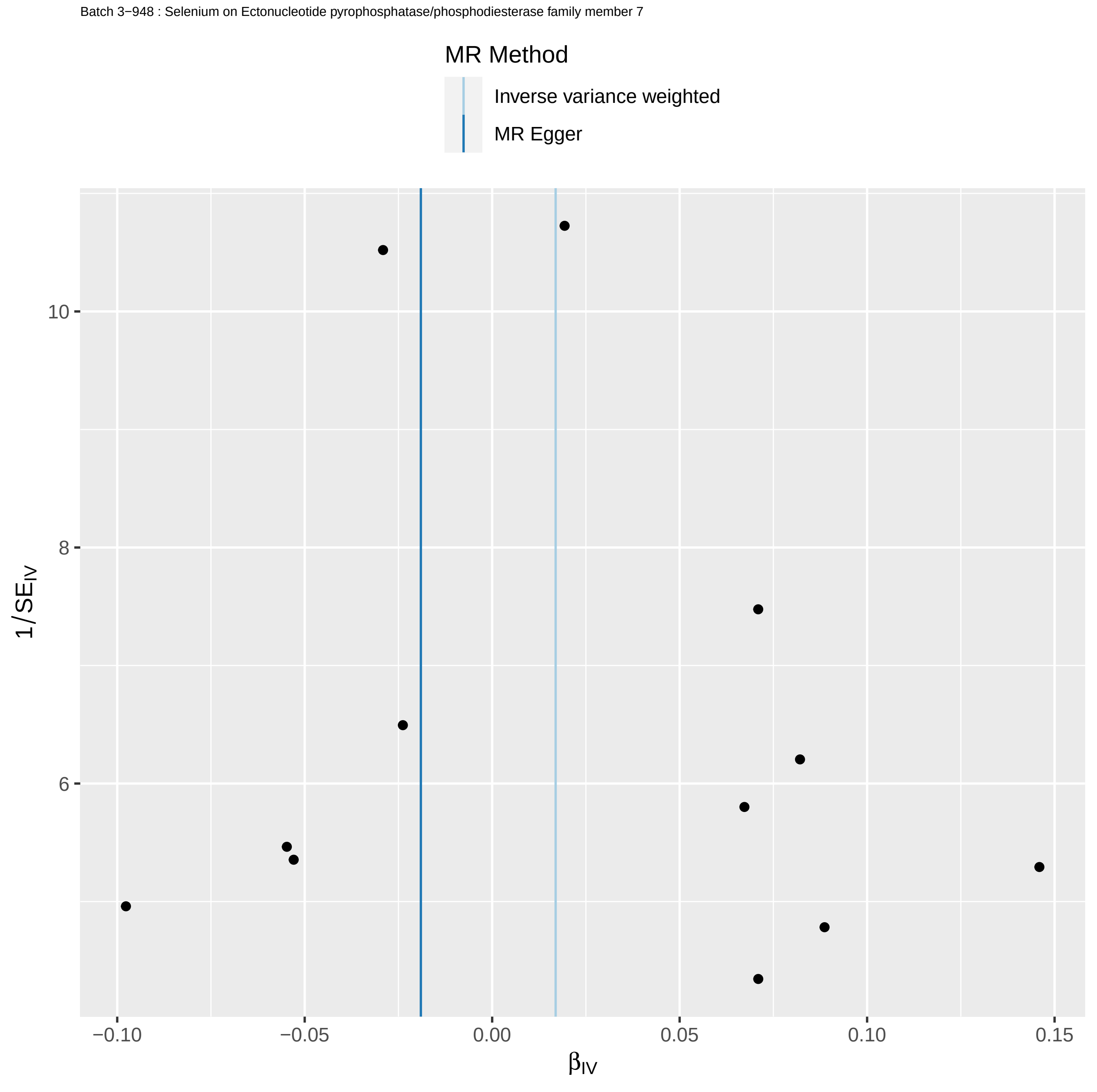

Batch 3−948 : Selenium on Ectonucleotide pyrophosphatase/phosphodiesterase family member 7
MR Method
Inverse variance weighted
MR Egger
10
8
6
1/SE_IV
−0.10
−0.05
0.00
0.05
0.10
0.15
β_IV

Batch 3−949 : Selenium on AP−4 complex accessory subunit tepsin

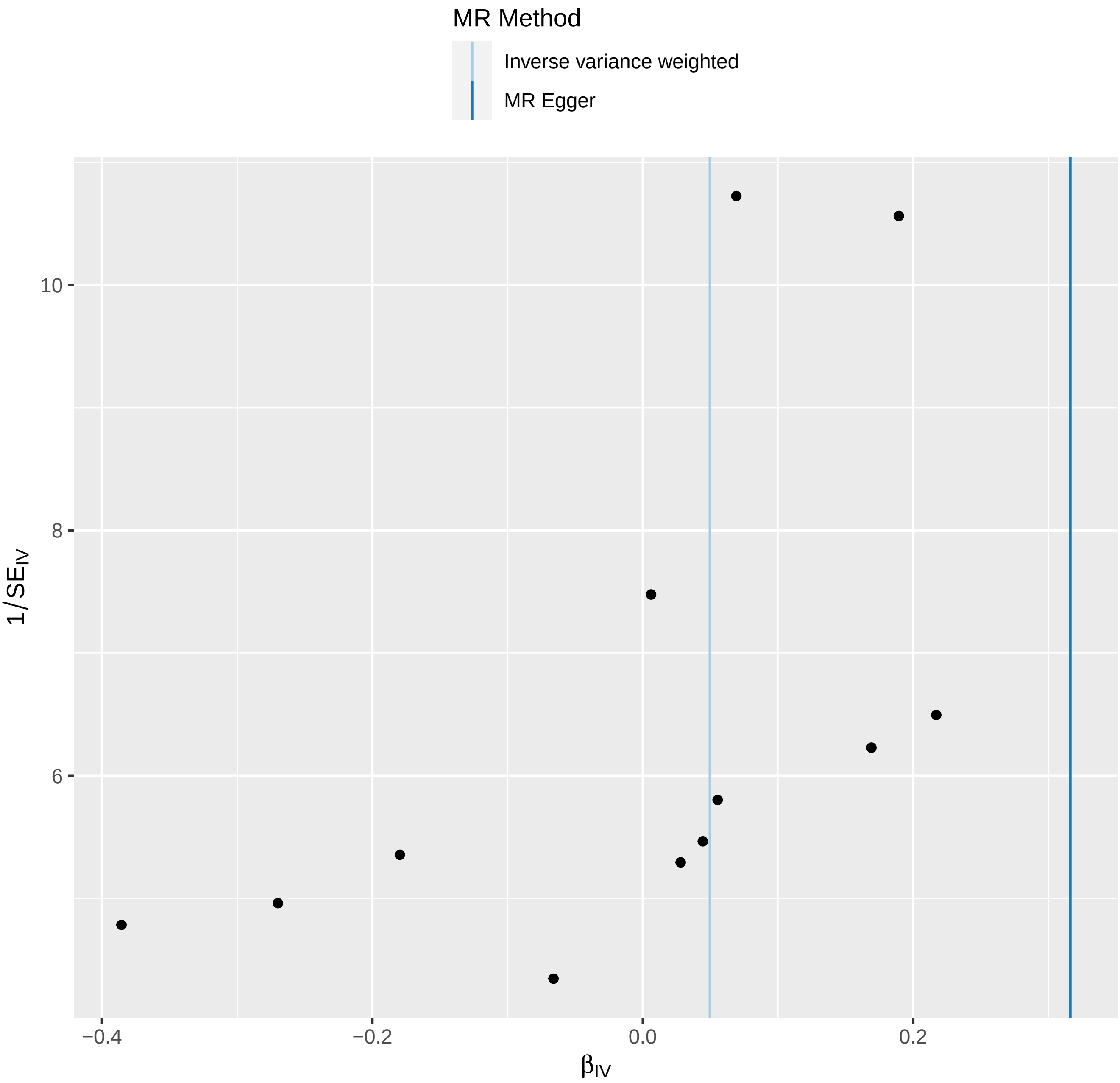

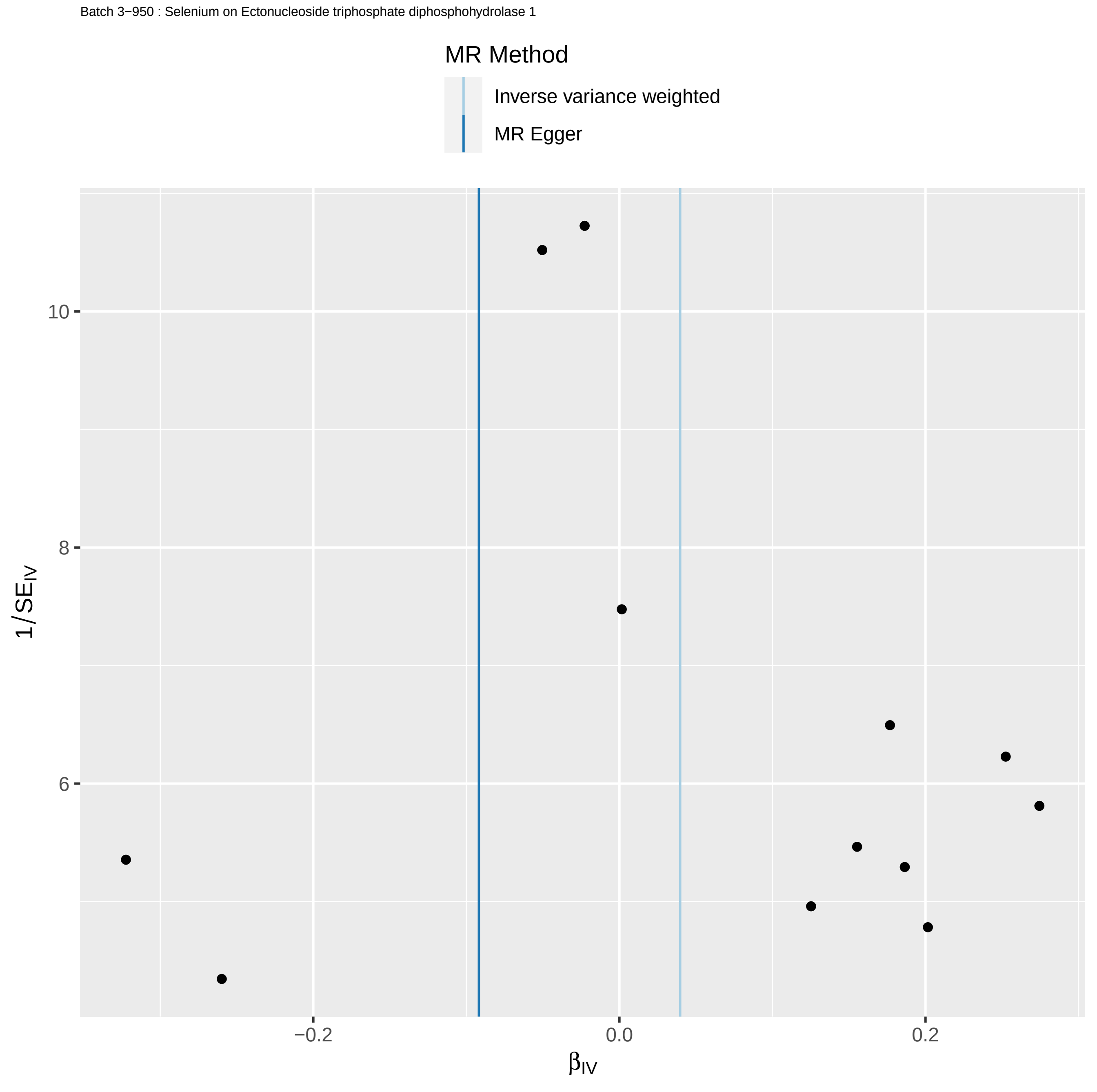
Batch 3−950 : Selenium on Ectonucleoside triphosphate diphosphohydrolase 1
MR Method
Inverse variance weighted
MR Egger
10
8
6
1/SE_IV
−0.2
0.0
0.2
β_IV

Batch 3−951 : Selenium on Ectonucleoside triphosphate diphosphohydrolase 1

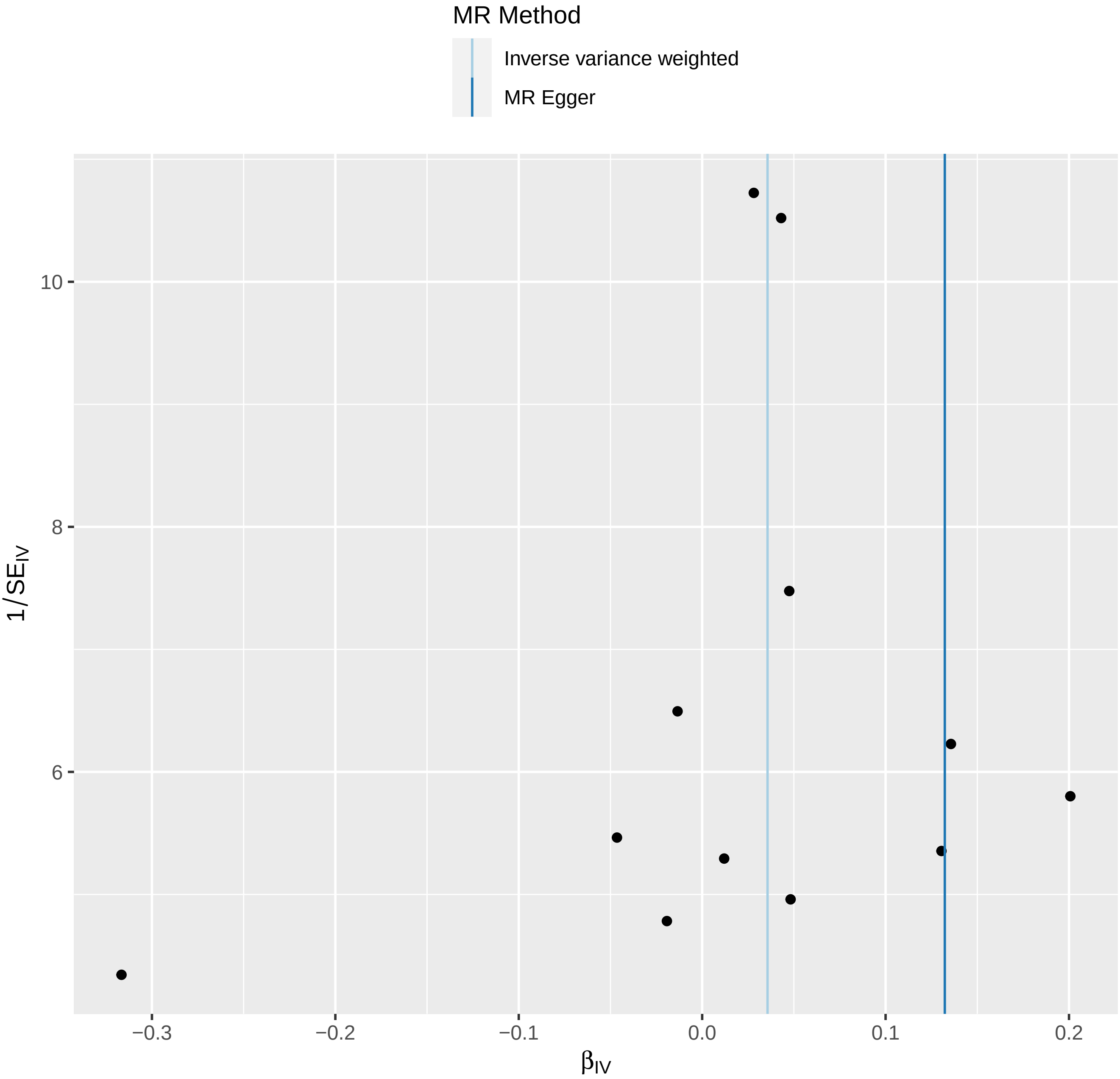

Batch 3−952 : Selenium on Ectonucleoside triphosphate diphosphohydrolase 3

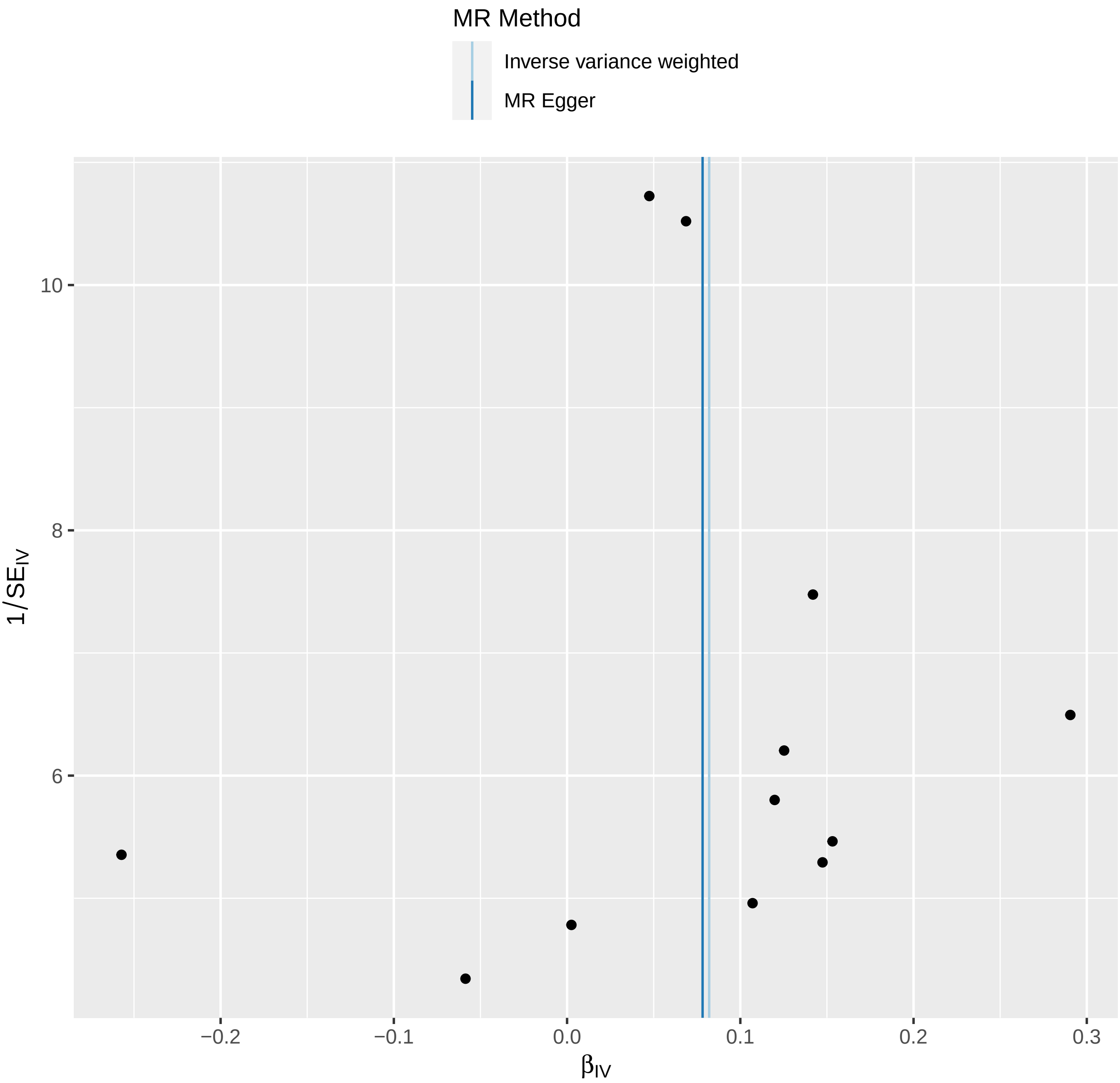

Batch 3−953 : Selenium on Ectonucleoside triphosphate diphosphohydrolase 5

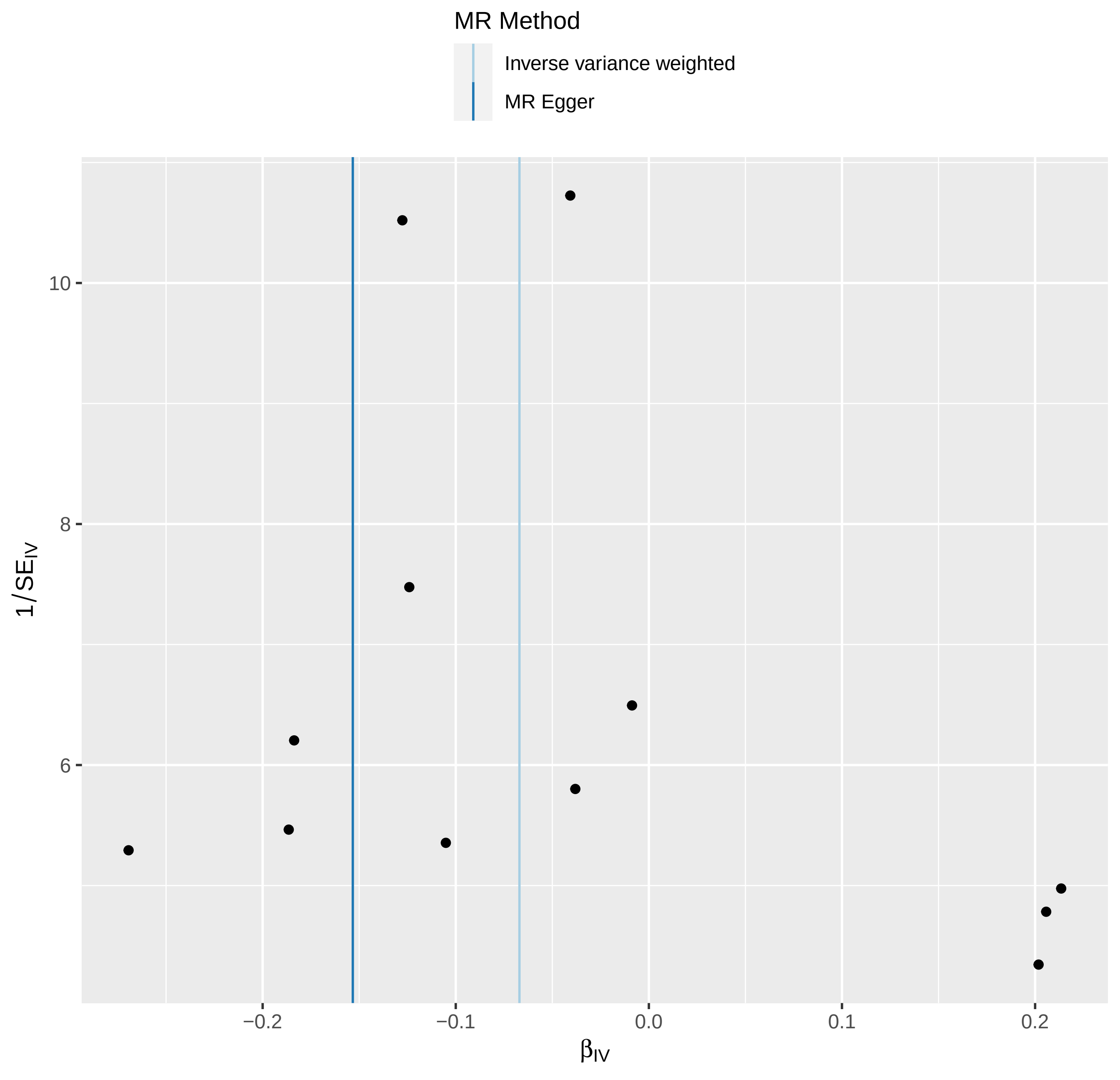

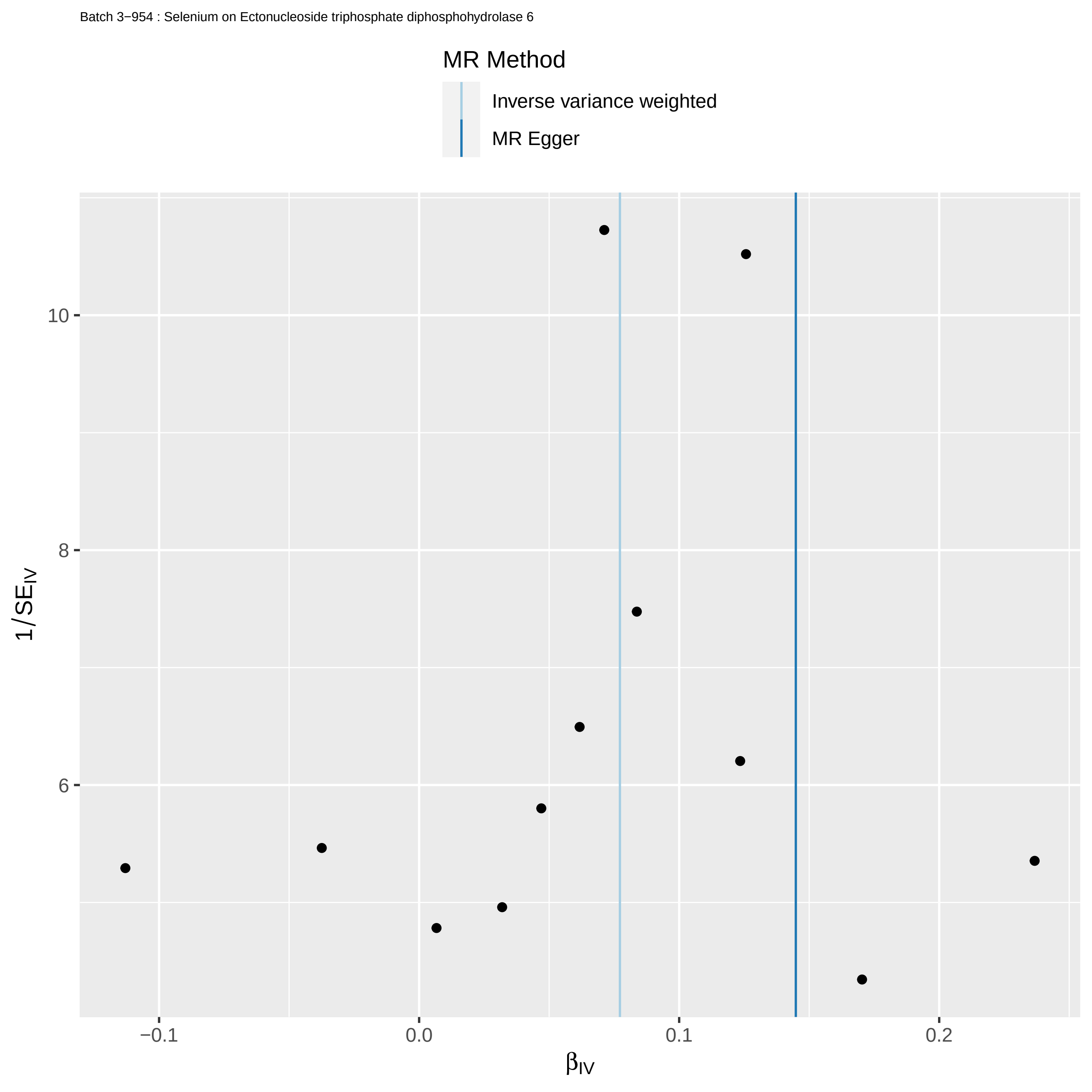
Batch 3−954 : Selenium on Ectonucleoside triphosphate diphosphohydrolase 6
MR Method
Inverse variance weighted
MR Egger
1/SE_IV
10
8
6
−0.1
0.0
0.1
0.2
β_IV

Batch 3−955 : Selenium on Ephrin type−A receptor 10

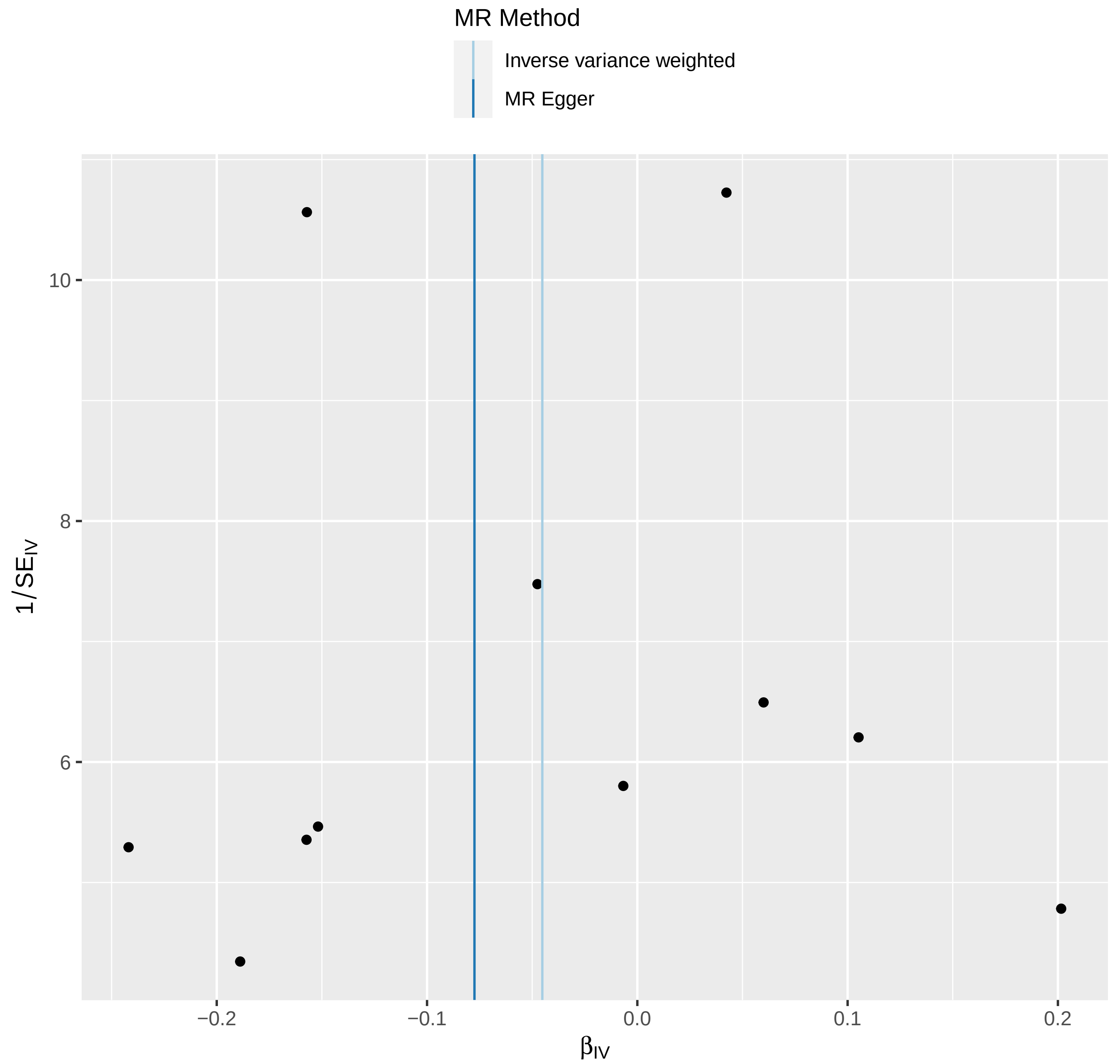

Batch 3−956 : Selenium on Ephrin type−A receptor 1

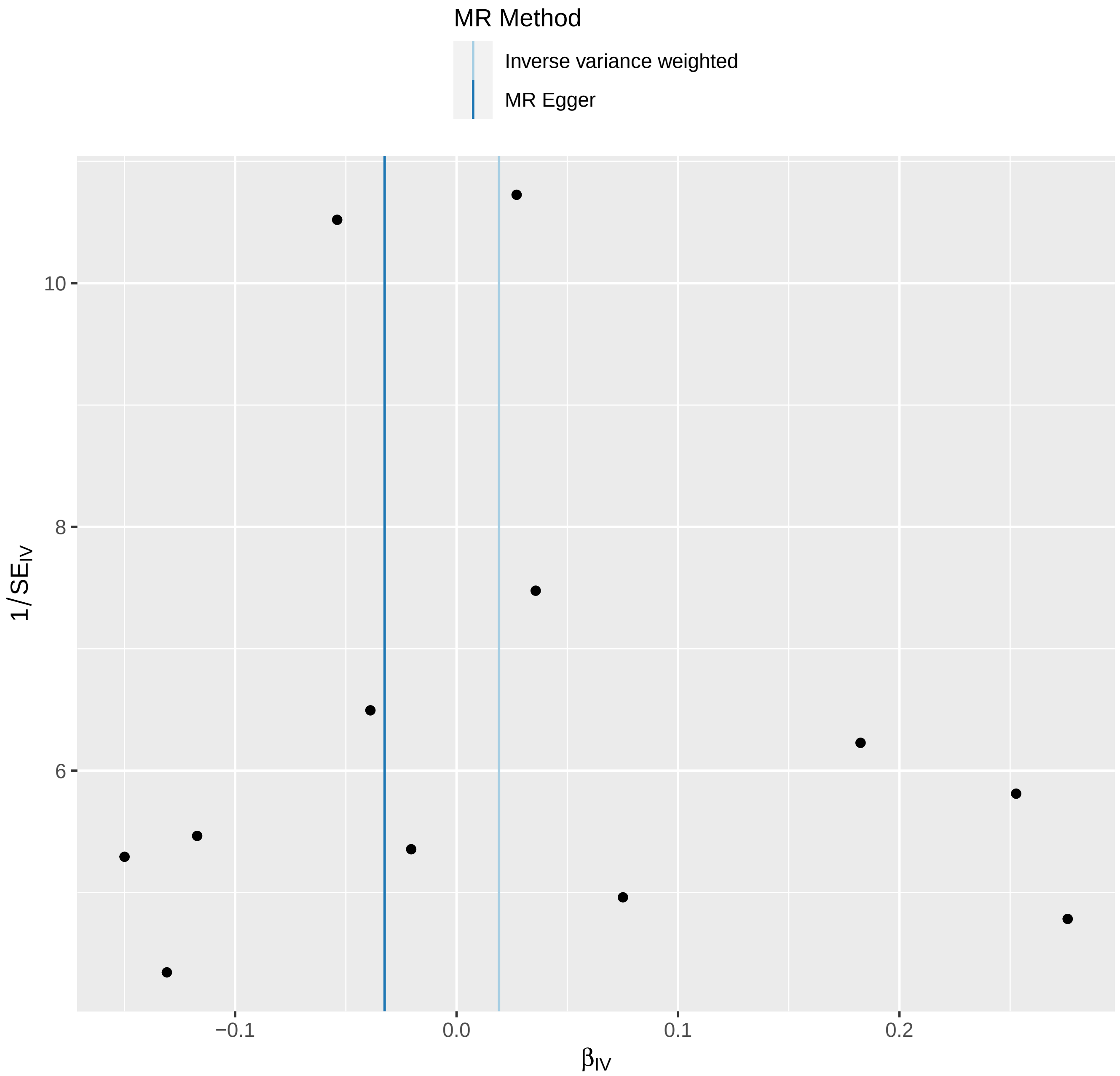

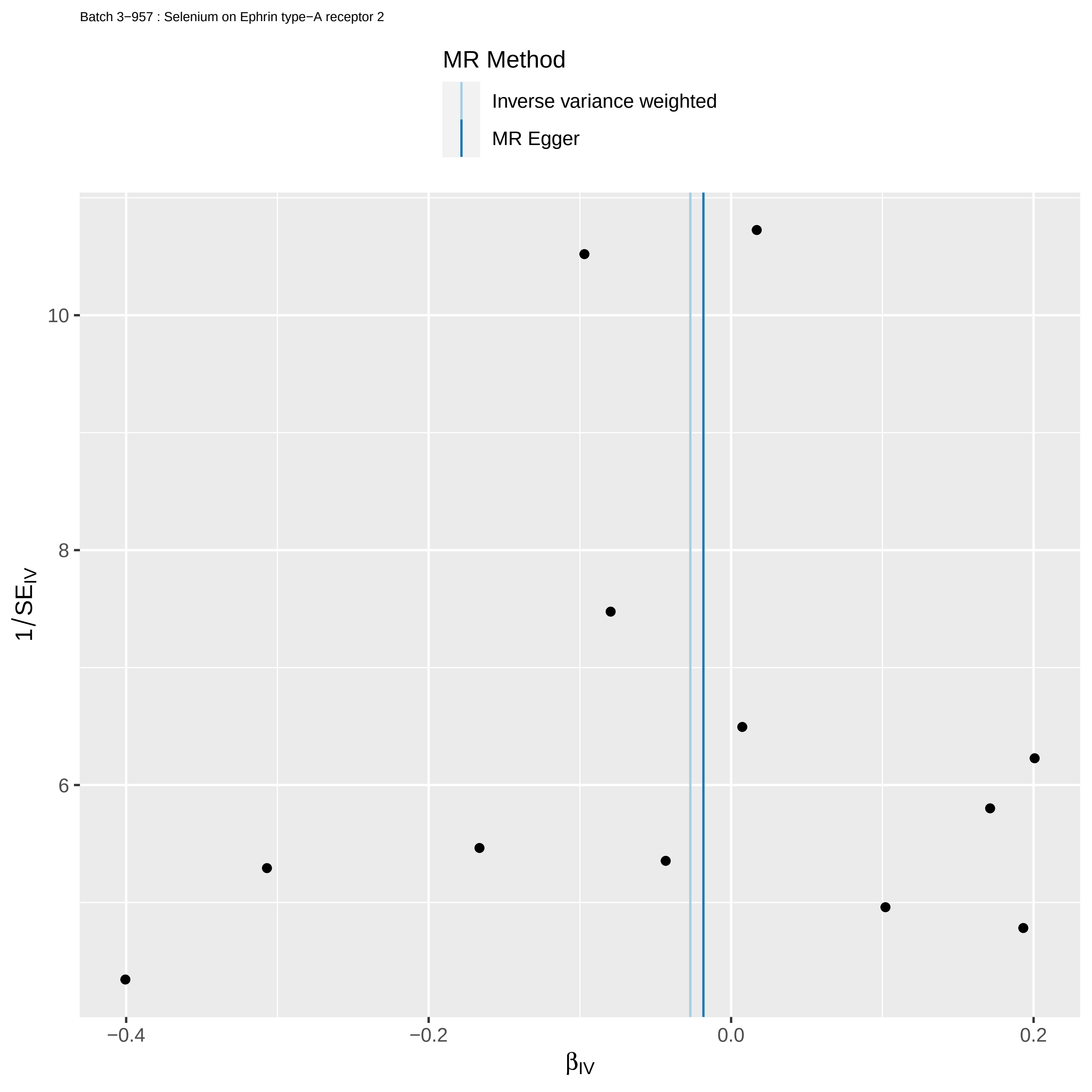
Batch 3−957 : Selenium on Ephrin type−A receptor 2
MR Method
Inverse variance weighted
MR Egger
10
8
6
1/SE$_{IV}$
−0.4
−0.2
0.0
0.2
β_{IV}

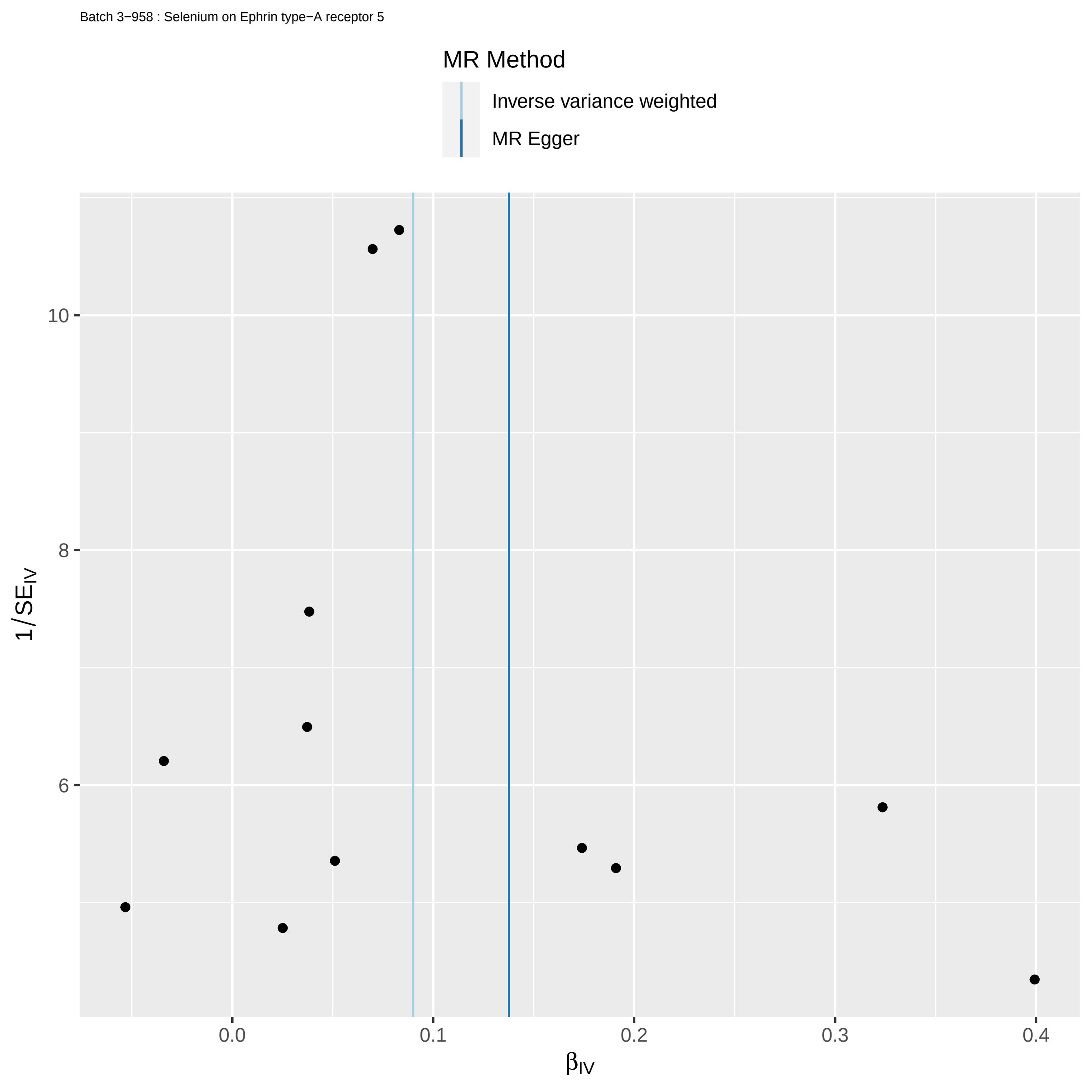

Batch 3−958 : Selenium on Ephrin type−A receptor 5
MR Method
Inverse variance weighted
MR Egger
10
8
6
1/SE_IV
0.0
0.1
0.2
0.3
0.4
β_IV

Batch 3−959 : Selenium on Ephrin type−A receptor 5

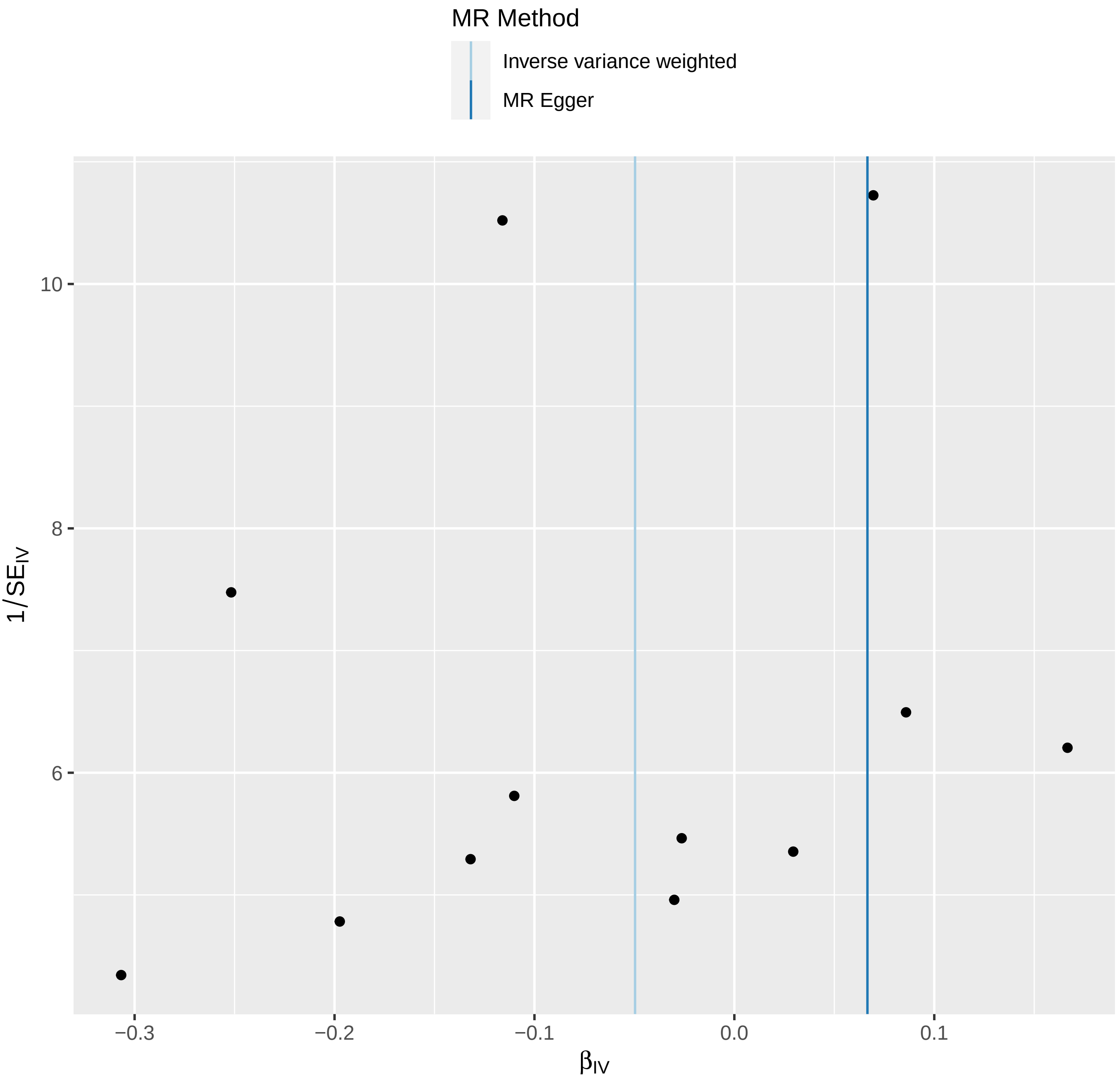

Batch 3−960 : Selenium on Ephrin type−B receptor 2

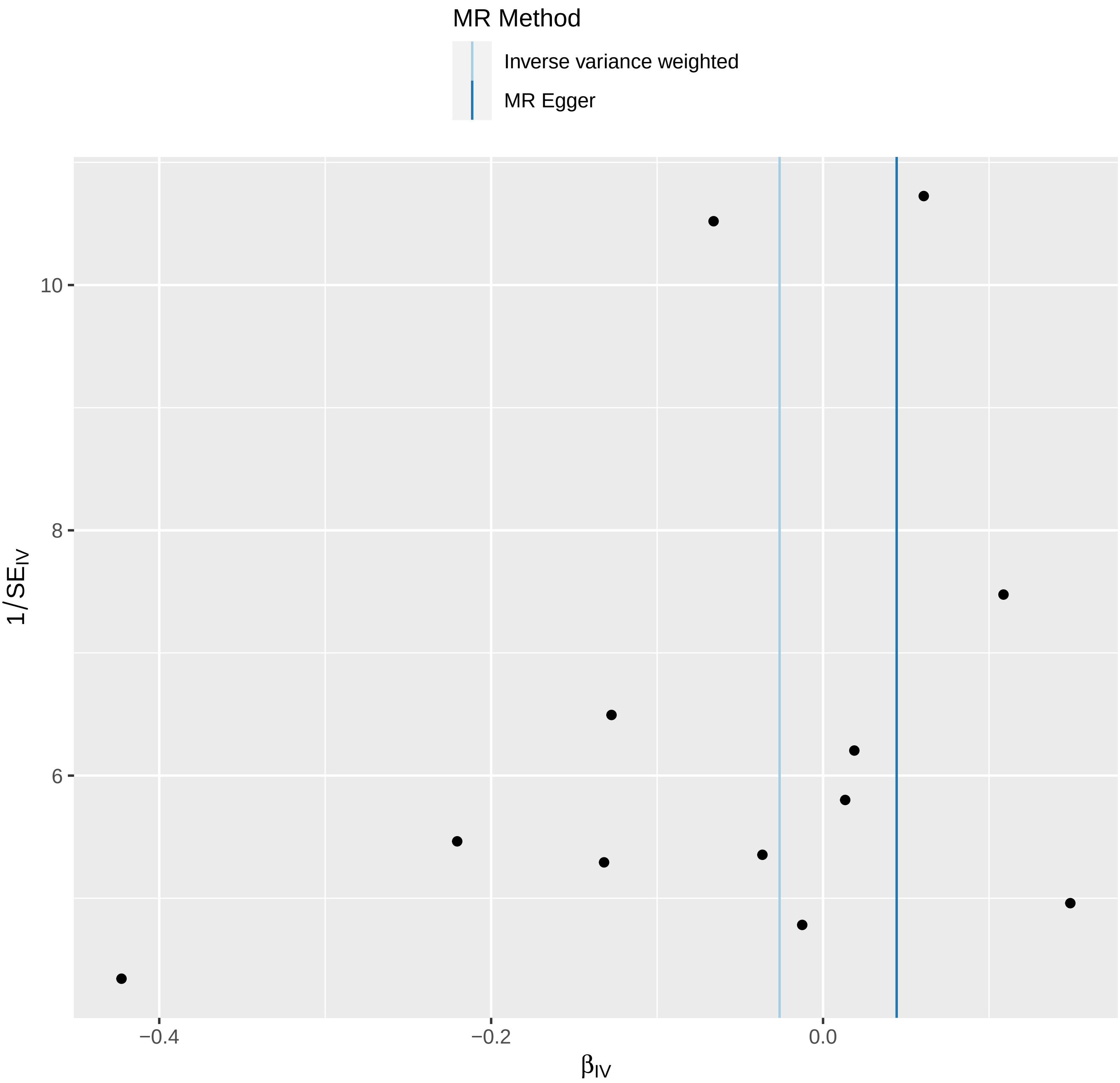

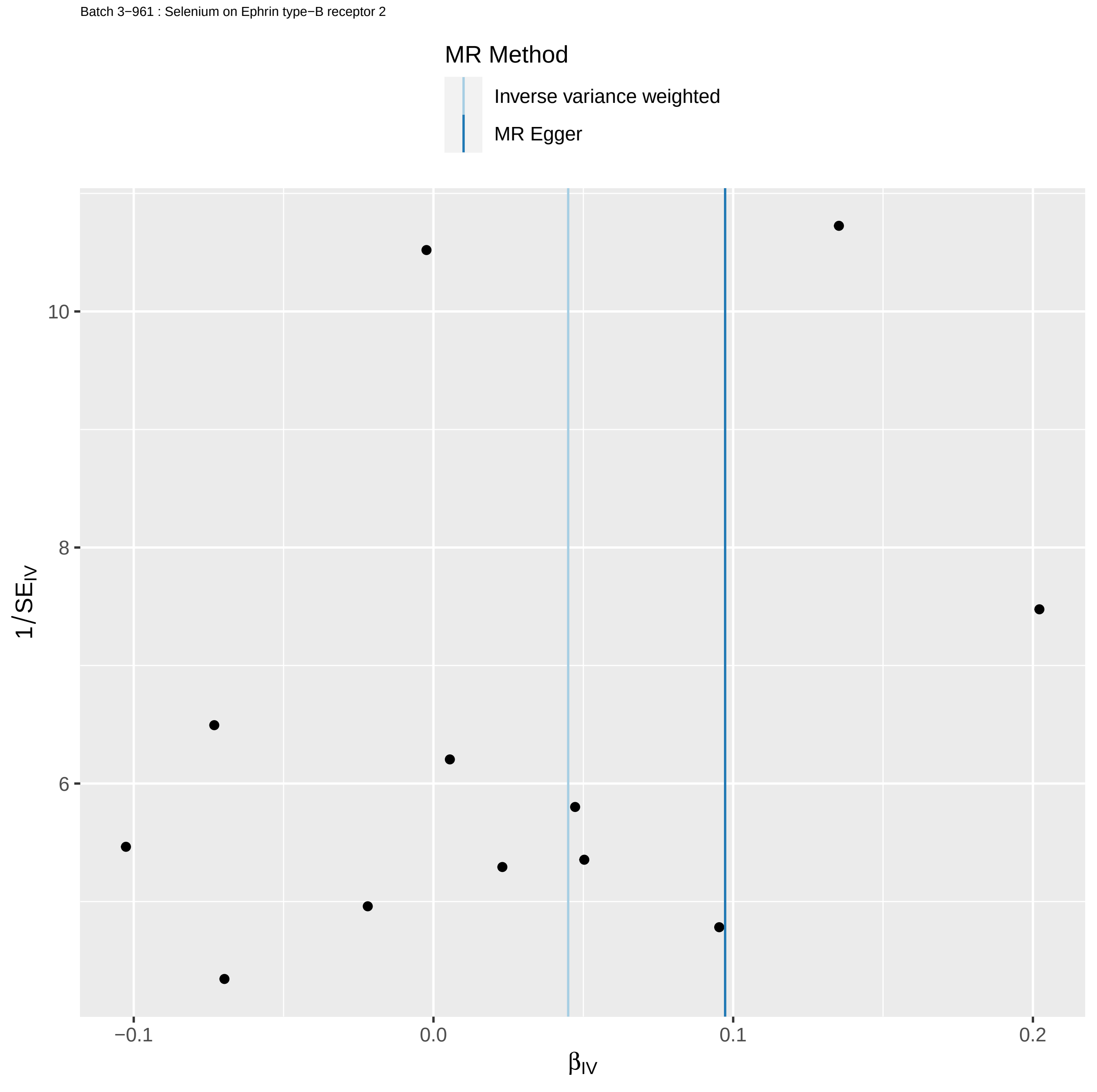

Batch 3−961 : Selenium on Ephrin type−B receptor 2
MR Method
Inverse variance weighted
MR Egger
10
8
6
1/SE$_{IV}$
−0.1
0.0
0.1
0.2
β_{IV}

Batch 3−962 : Selenium on Ephrin type−B receptor 2

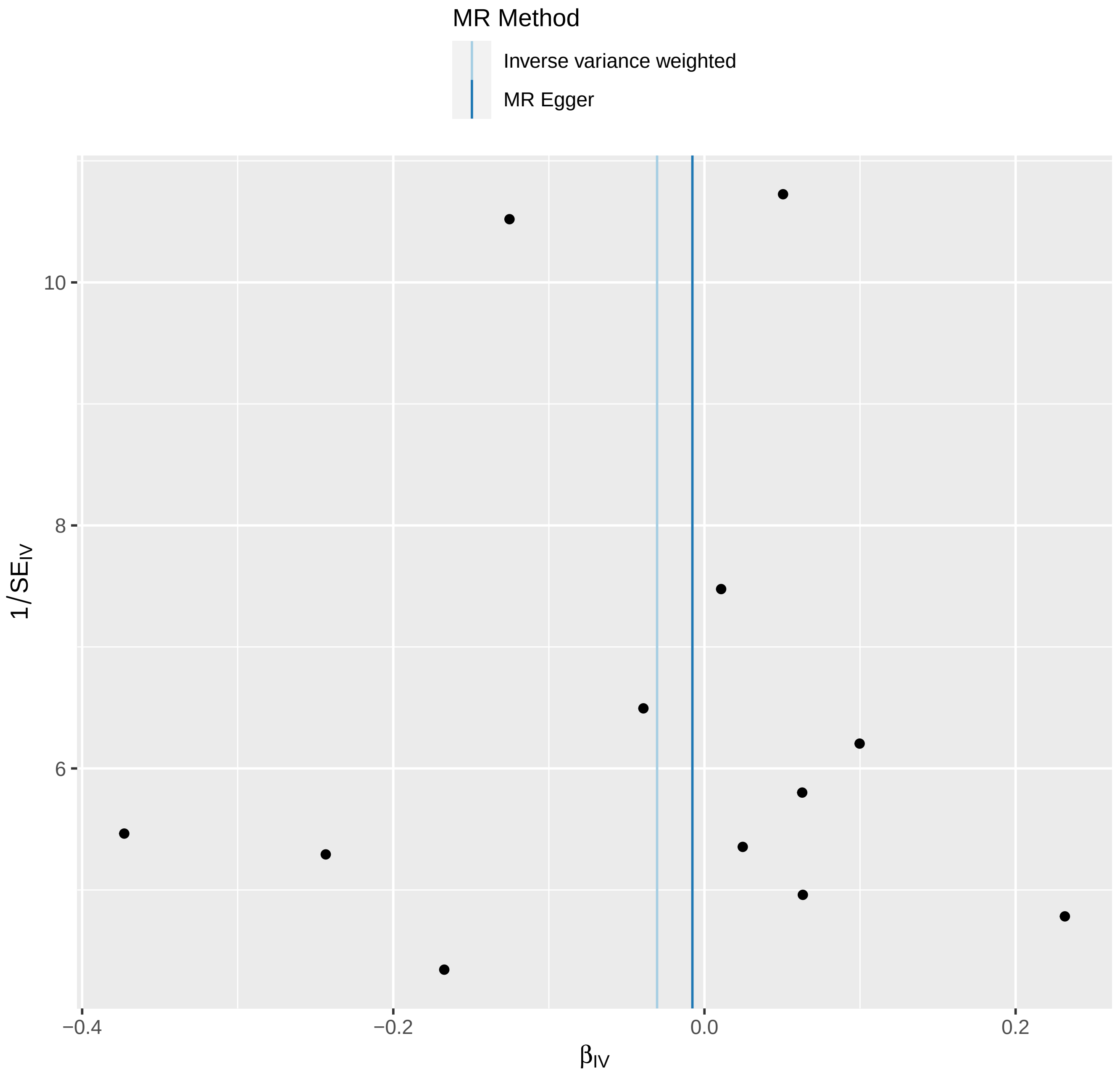

Batch 3−963 : Selenium on Ephrin type−B receptor 3

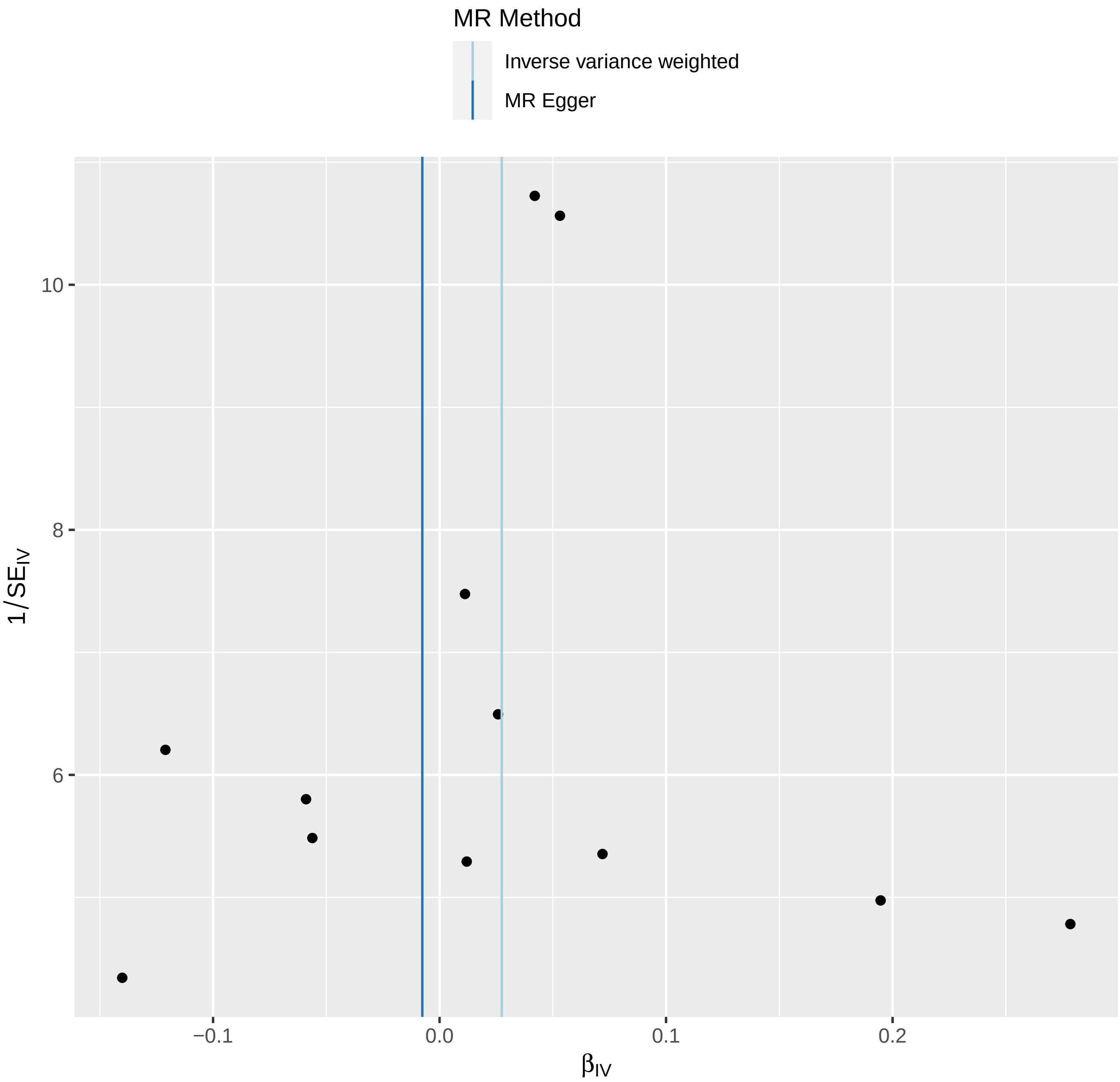

Batch 3−964 : Selenium on Ephrin type−B receptor 4

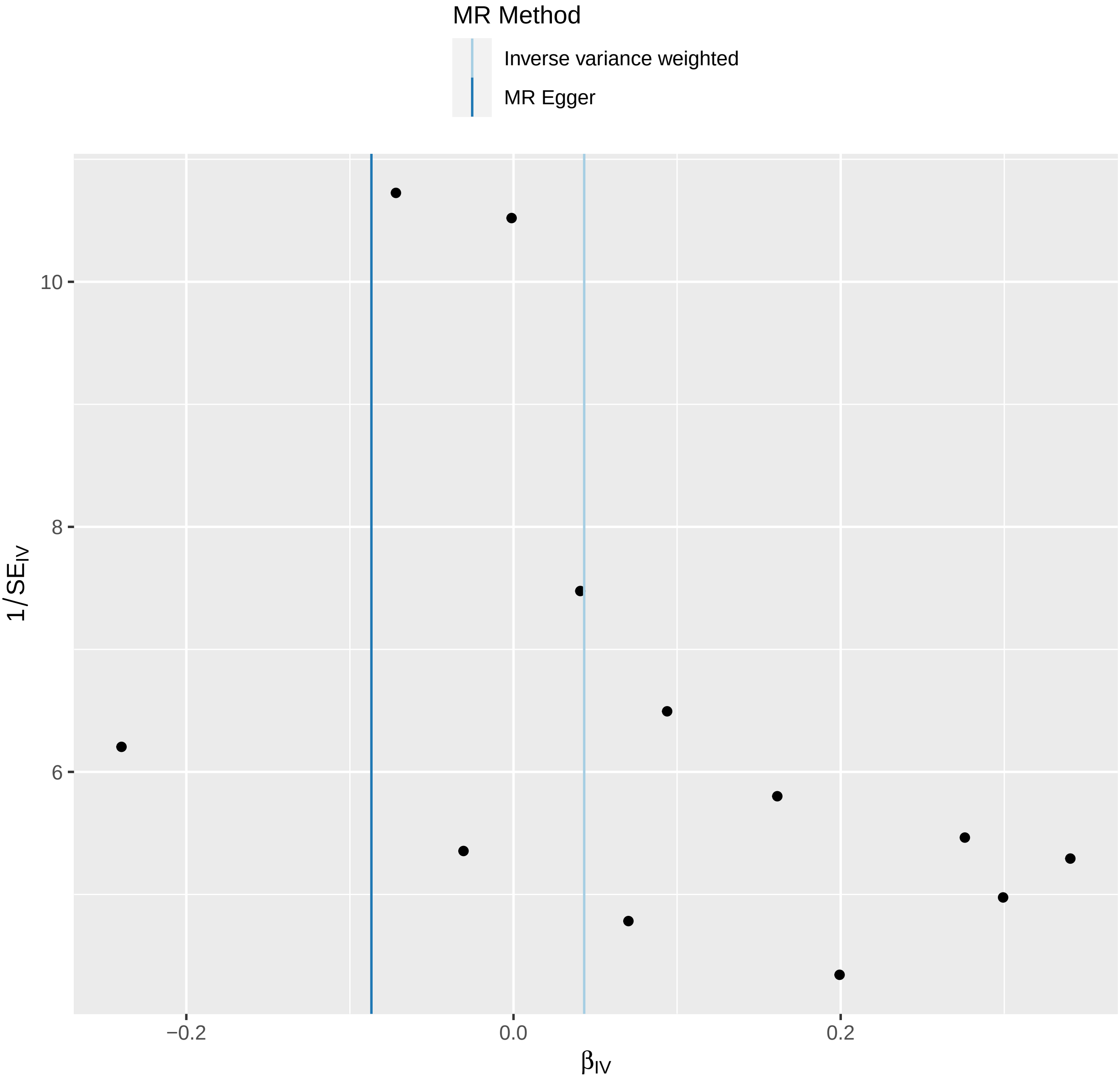

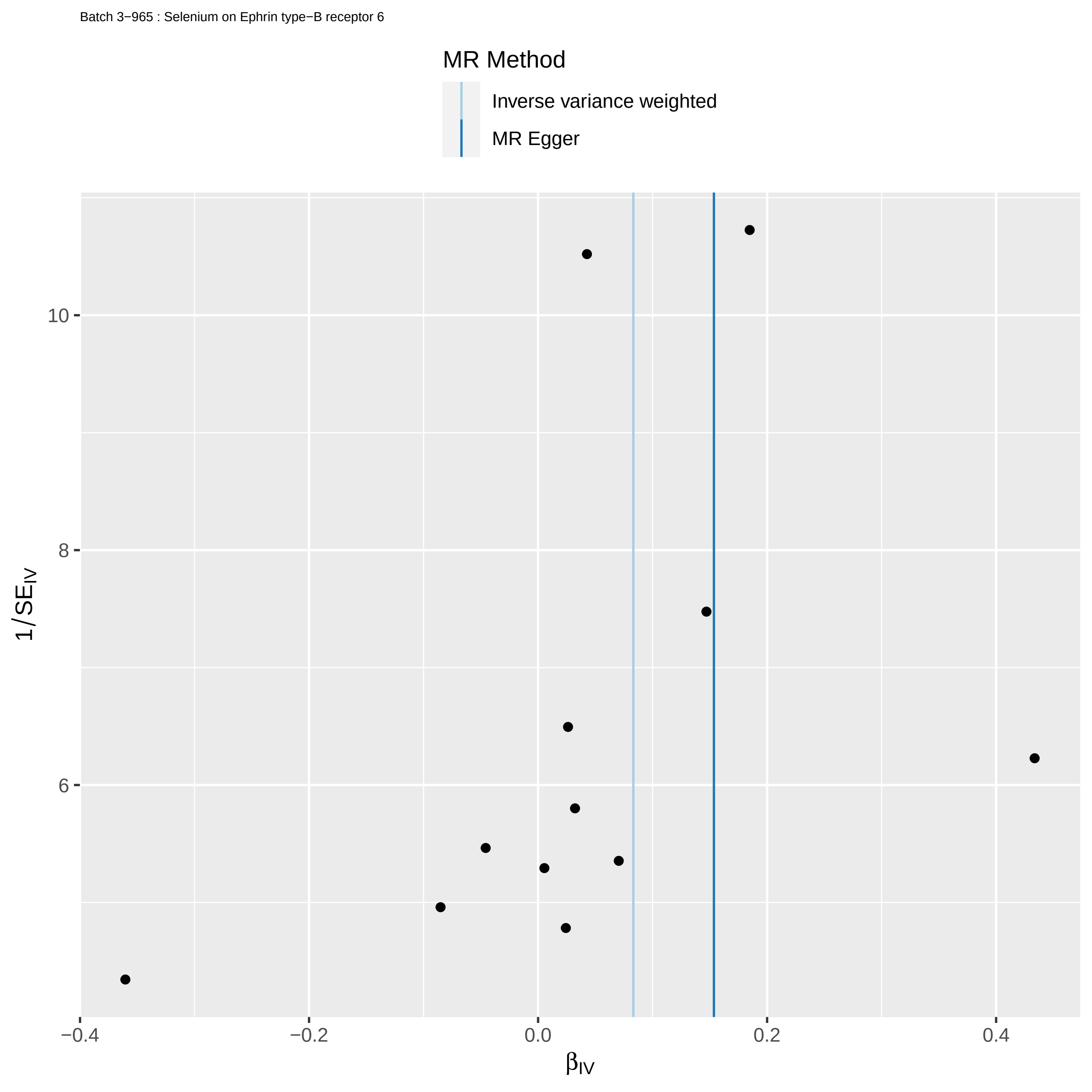
Batch 3−965 : Selenium on Ephrin type−B receptor 6
MR Method
Inverse variance weighted
MR Egger
10
8
6
1/SE_IV
−0.4
−0.2
0.0
0.2
0.4
β_IV

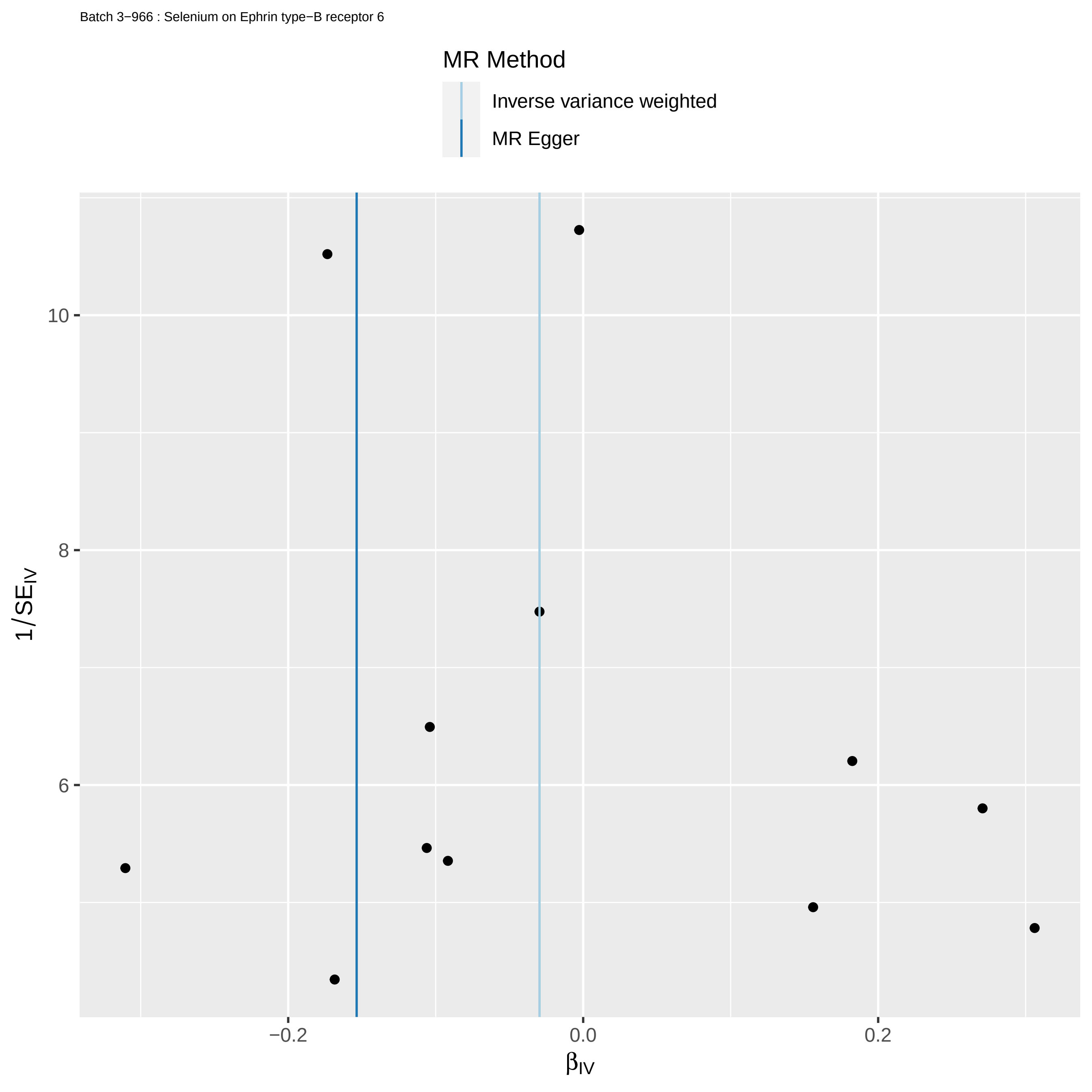
Batch 3−966 : Selenium on Ephrin type−B receptor 6
MR Method
Inverse variance weighted
MR Egger
10
8
6
1/SE_IV
−0.2
0.0
0.2
β_IV

Batch 3−967 : Selenium on Erythropoietin

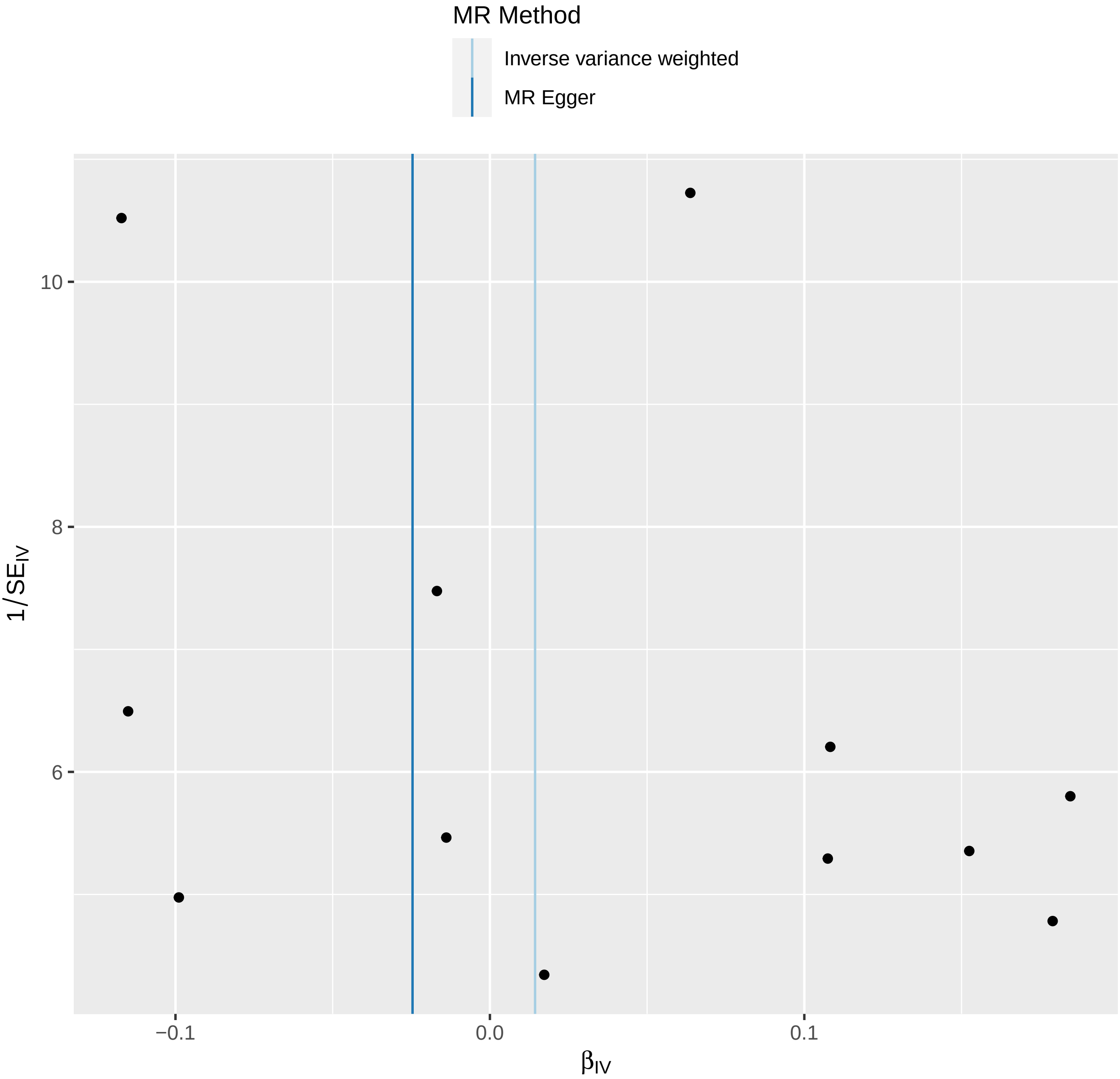

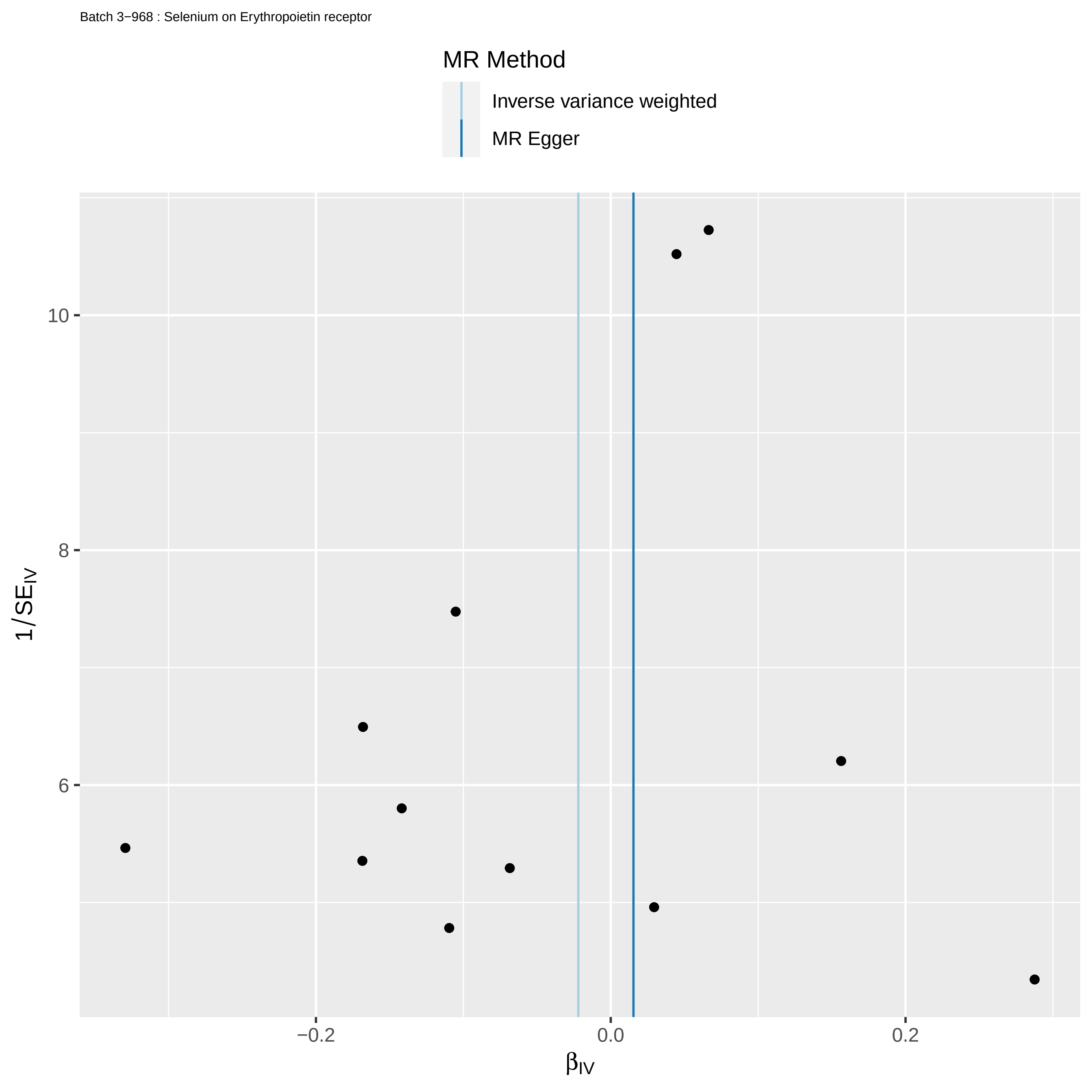
Batch 3−968 : Selenium on Erythropoietin receptor
MR Method
Inverse variance weighted
MR Egger
10
8
6
1/SE_IV
−0.2
0.0
0.2
β_IV

Batch 3−969 : Selenium on Epidermal growth factor receptor substrate 15−like 1

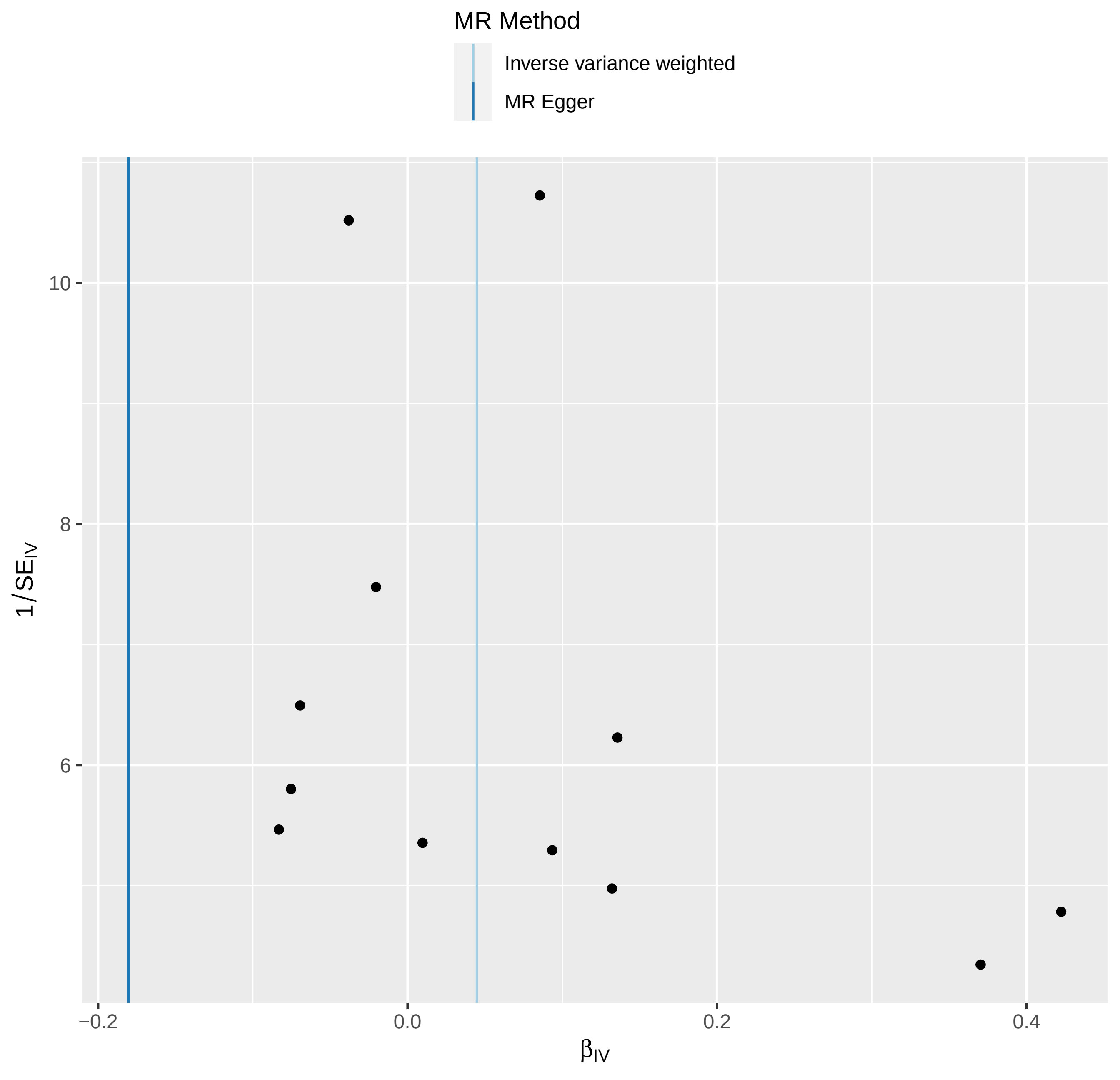

Batch 3−970 : Selenium on Epiphycan

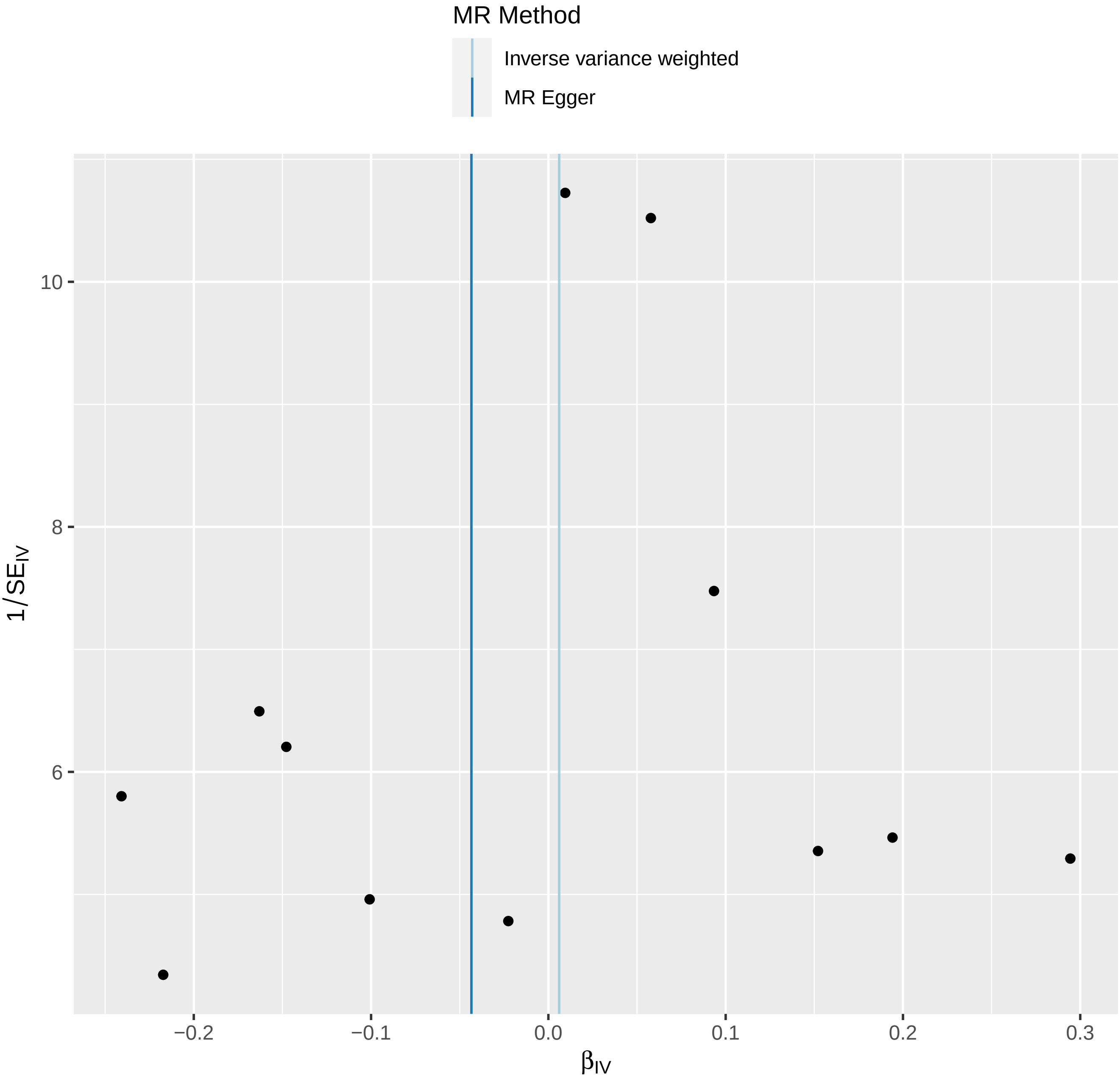

Batch 3−971 : Selenium on Endoplasmic reticulum aminopeptidase 1

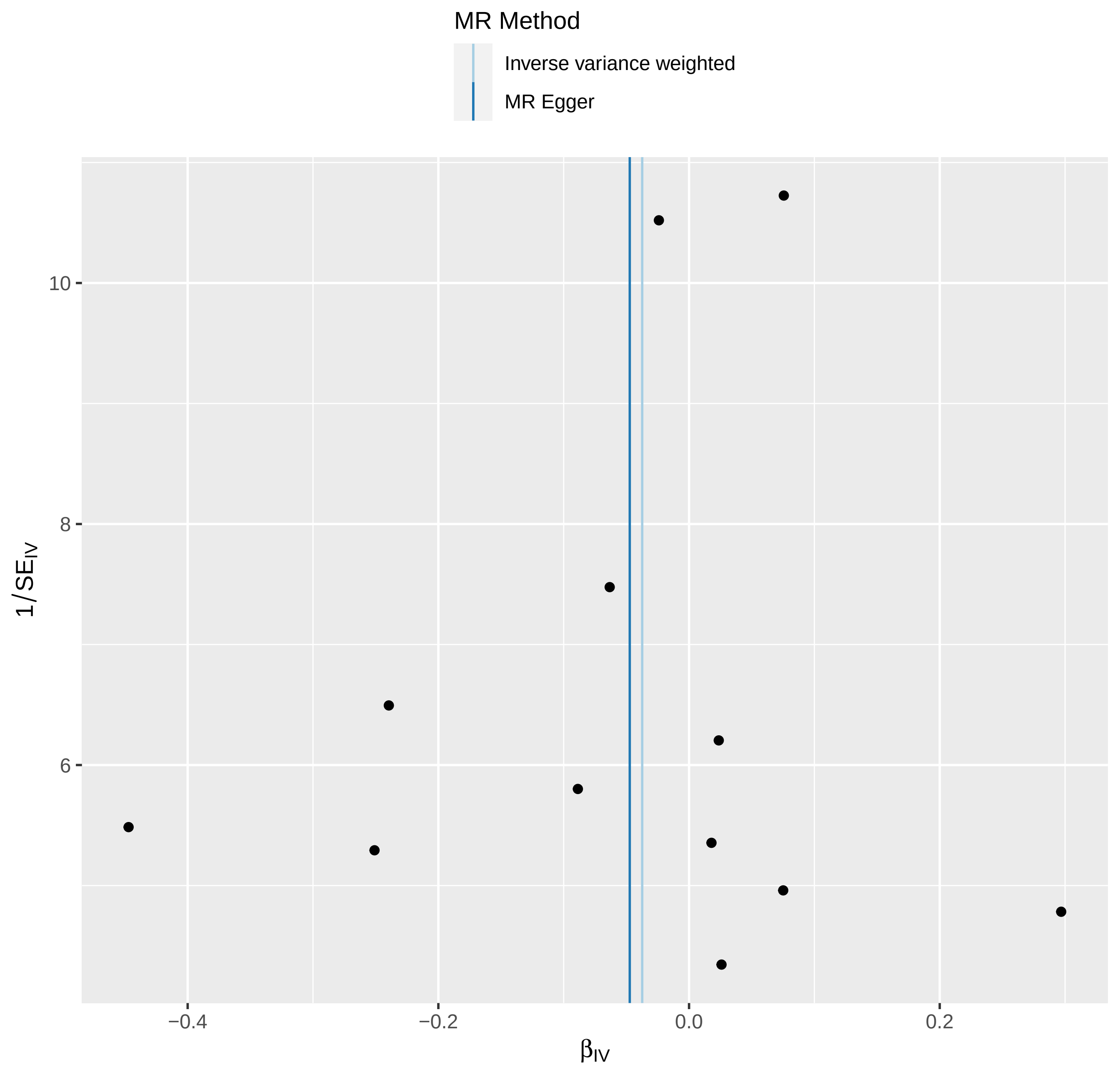

Batch 3−972 : Selenium on Endoplasmic reticulum aminopeptidase 2

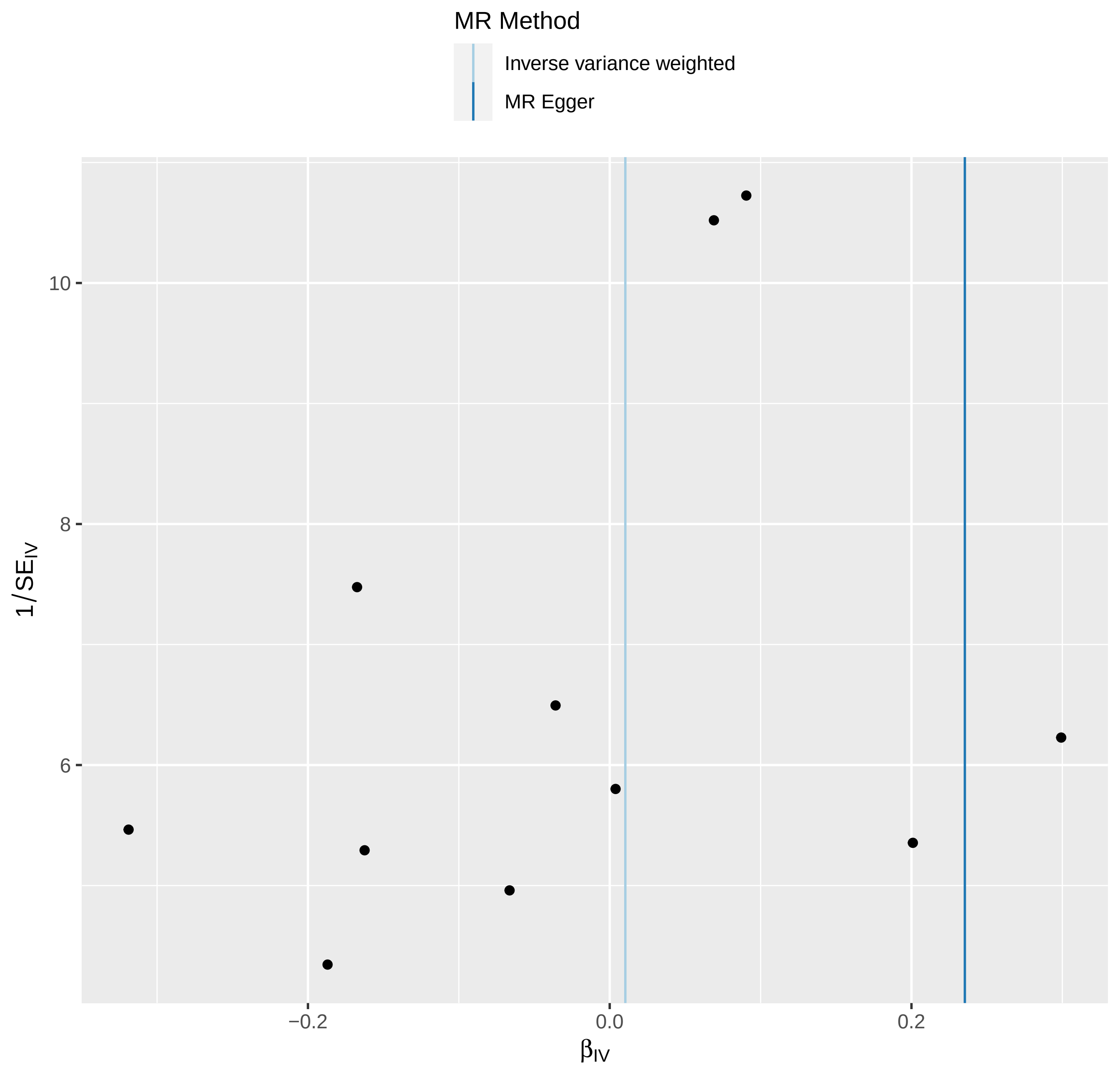

Batch 3−973 : Selenium on Receptor tyrosine−protein kinase erbB−2

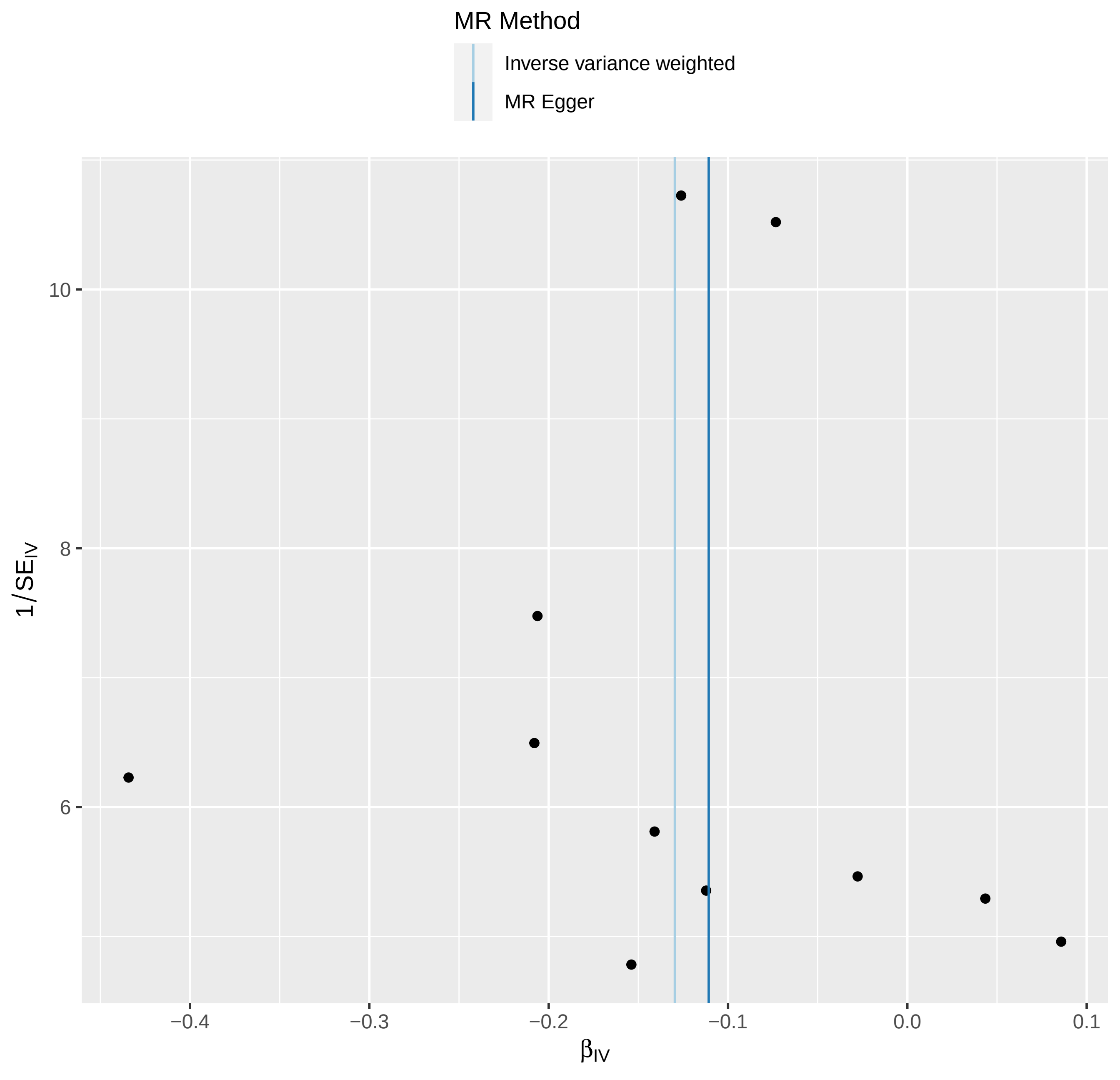

Batch 3−974 : Selenium on Receptor tyrosine−protein kinase erbB−3

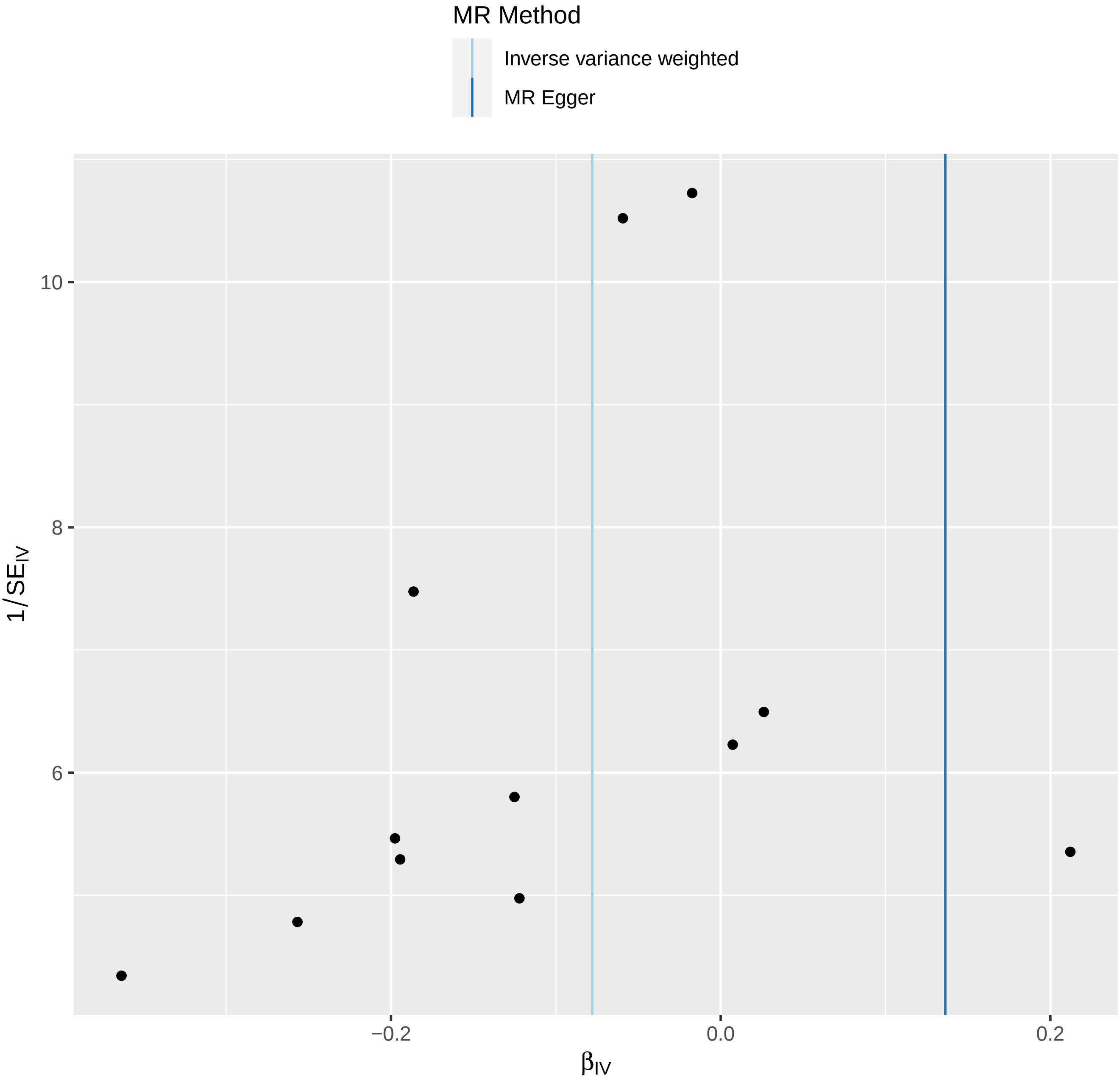

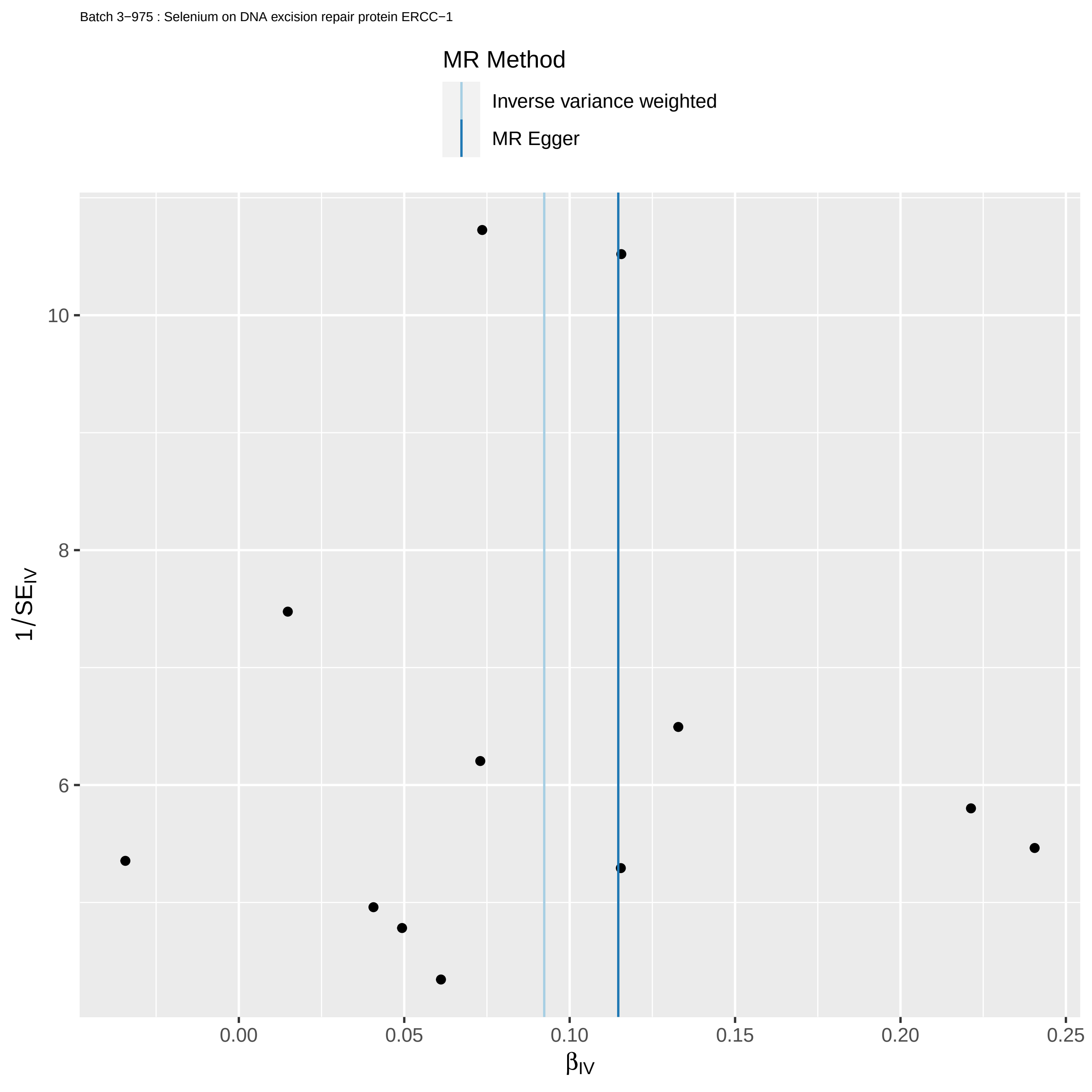

Batch 3−975 : Selenium on DNA excision repair protein ERCC−1
MR Method
Inverse variance weighted
MR Egger
10
8
6
1/SE_IV
0.00
0.05
0.10
0.15
0.20
0.25
β_IV

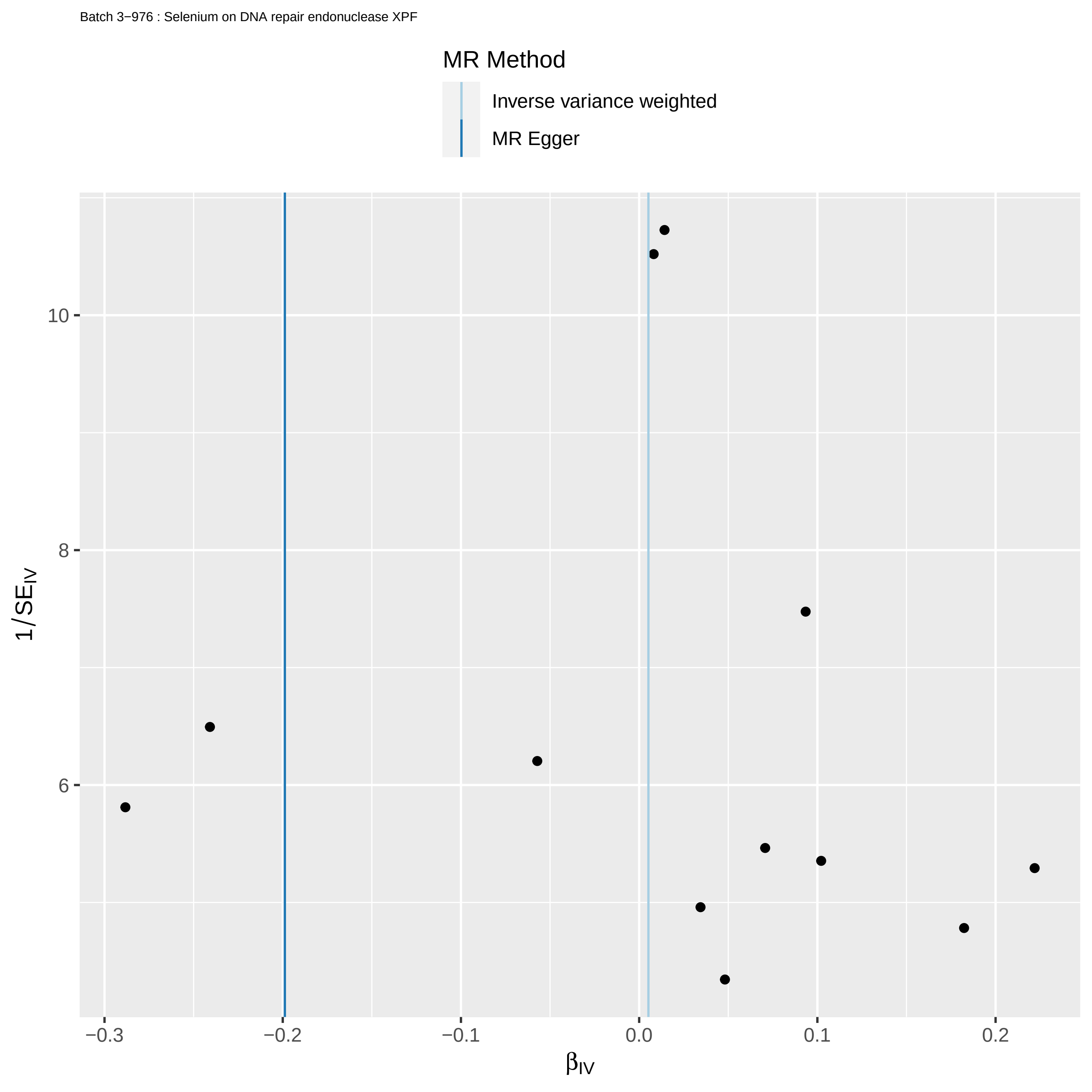

Batch 3−976 : Selenium on DNA repair endonuclease XPF
MR Method
Inverse variance weighted
MR Egger
10
8
6
1/SE_IV
−0.3
−0.2
−0.1
0.0
0.1
0.2
β_IV

Batch 3−977 : Selenium on Epiregulin

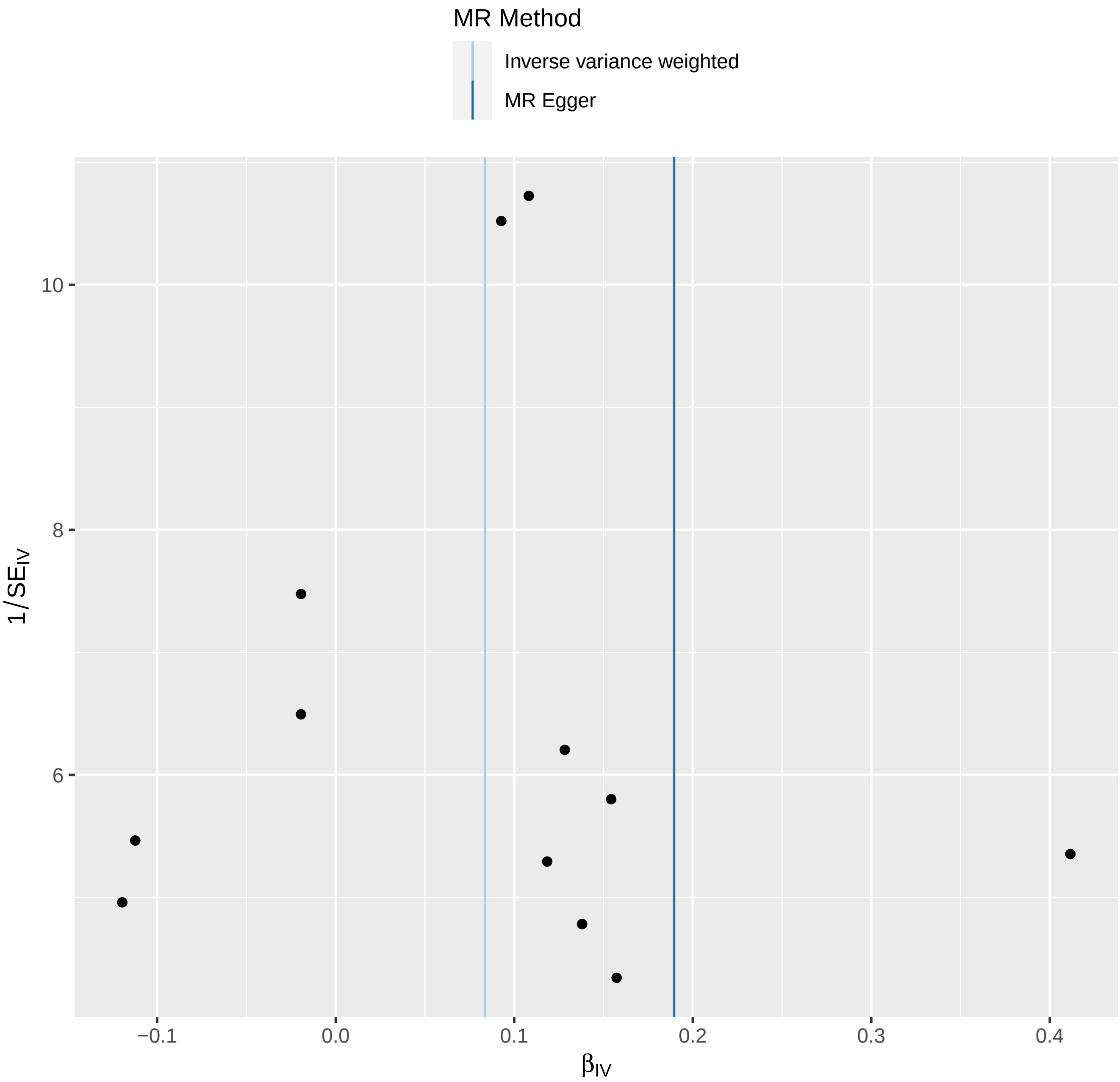

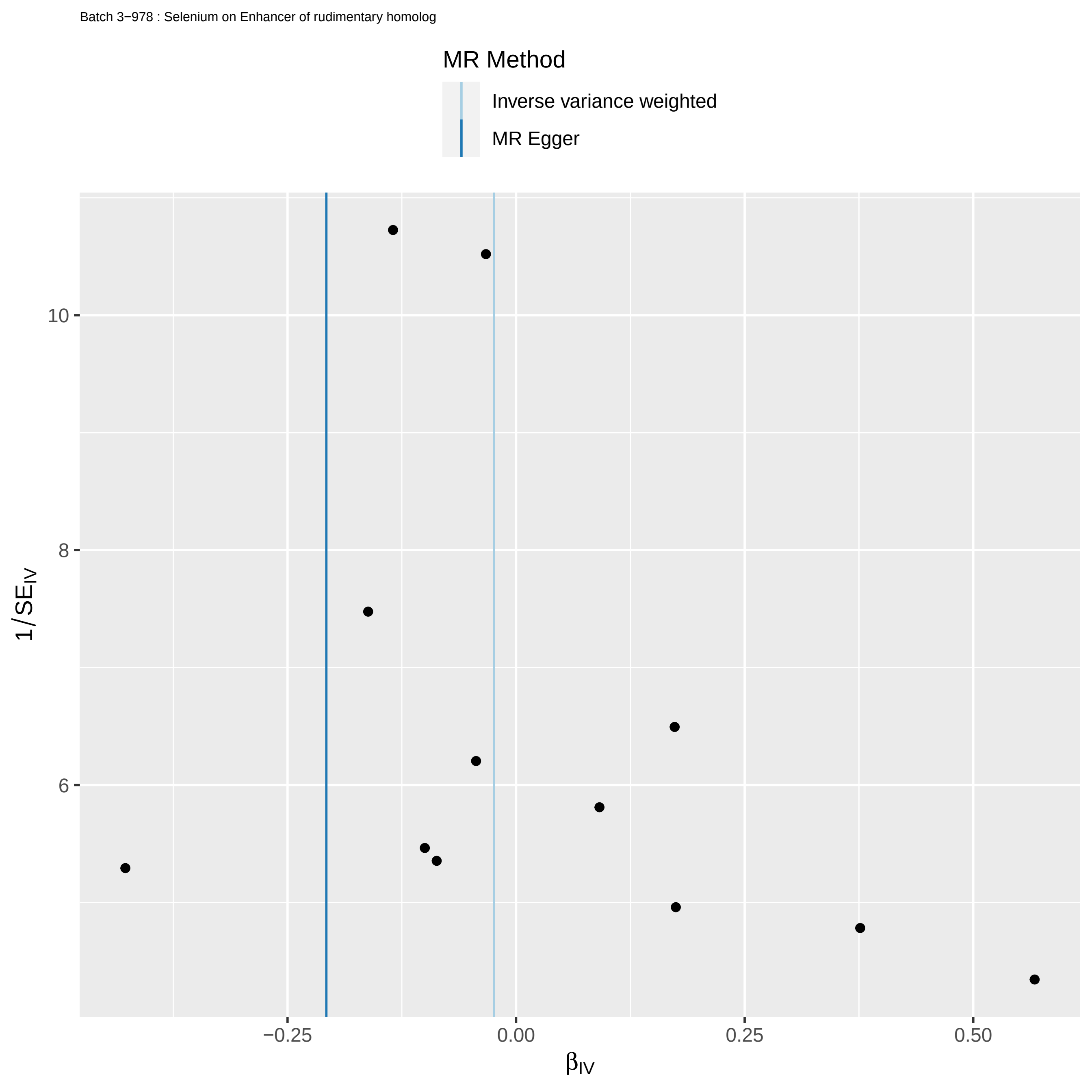
Batch 3−978 : Selenium on Enhancer of rudimentary homolog
MR Method
Inverse variance weighted
MR Egger
10
8
6
1/SE_IV
−0.25
0.00
0.25
0.50
β_IV

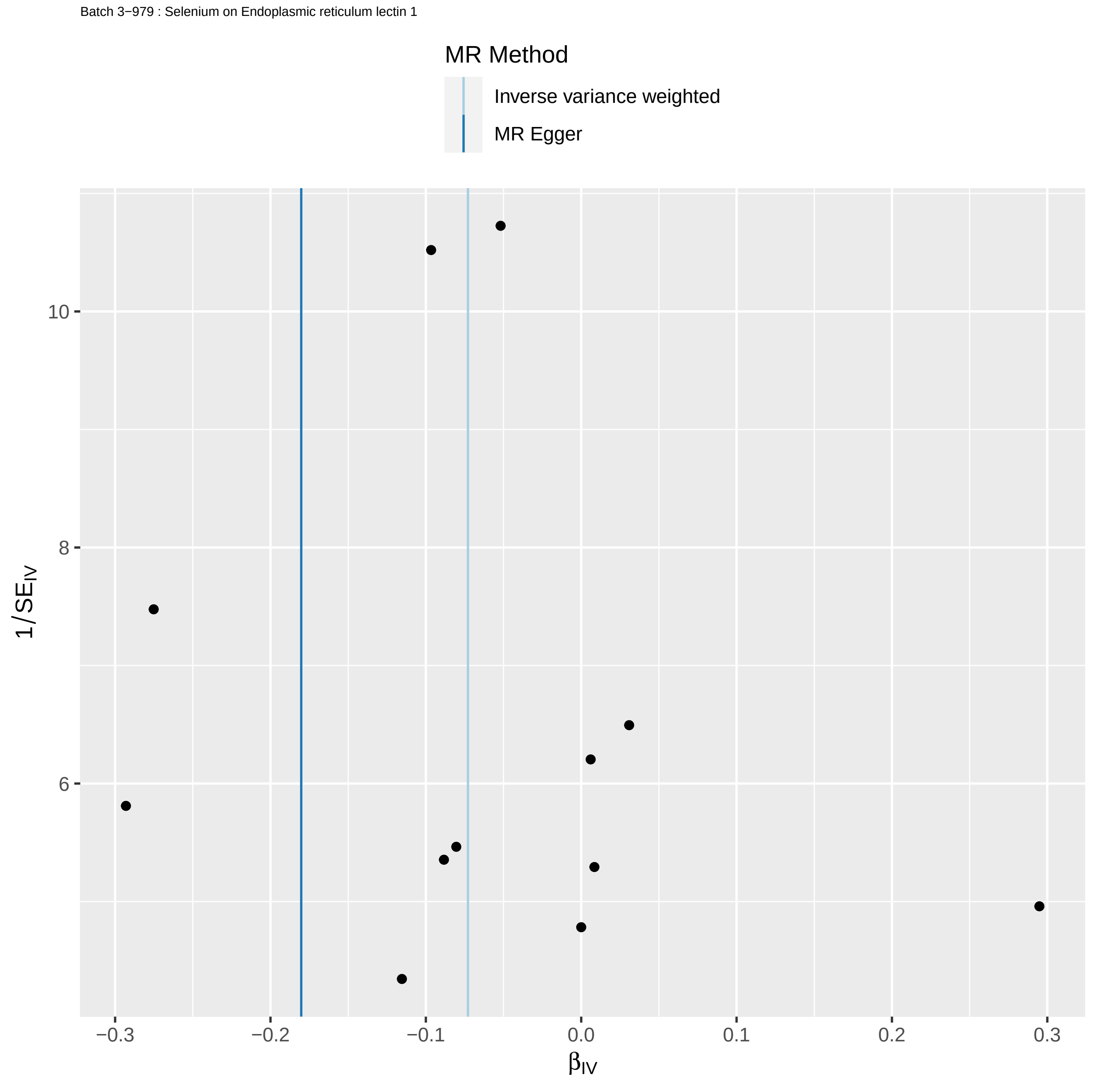

Batch 3−979 : Selenium on Endoplasmic reticulum lectin 1
MR Method
Inverse variance weighted
MR Egger
10
8
6
1/SE$_{IV}$
−0.3
−0.2
−0.1
0.0
0.1
0.2
0.3
β$_{IV}$

Batch 3−980 : Selenium on Erlin−1

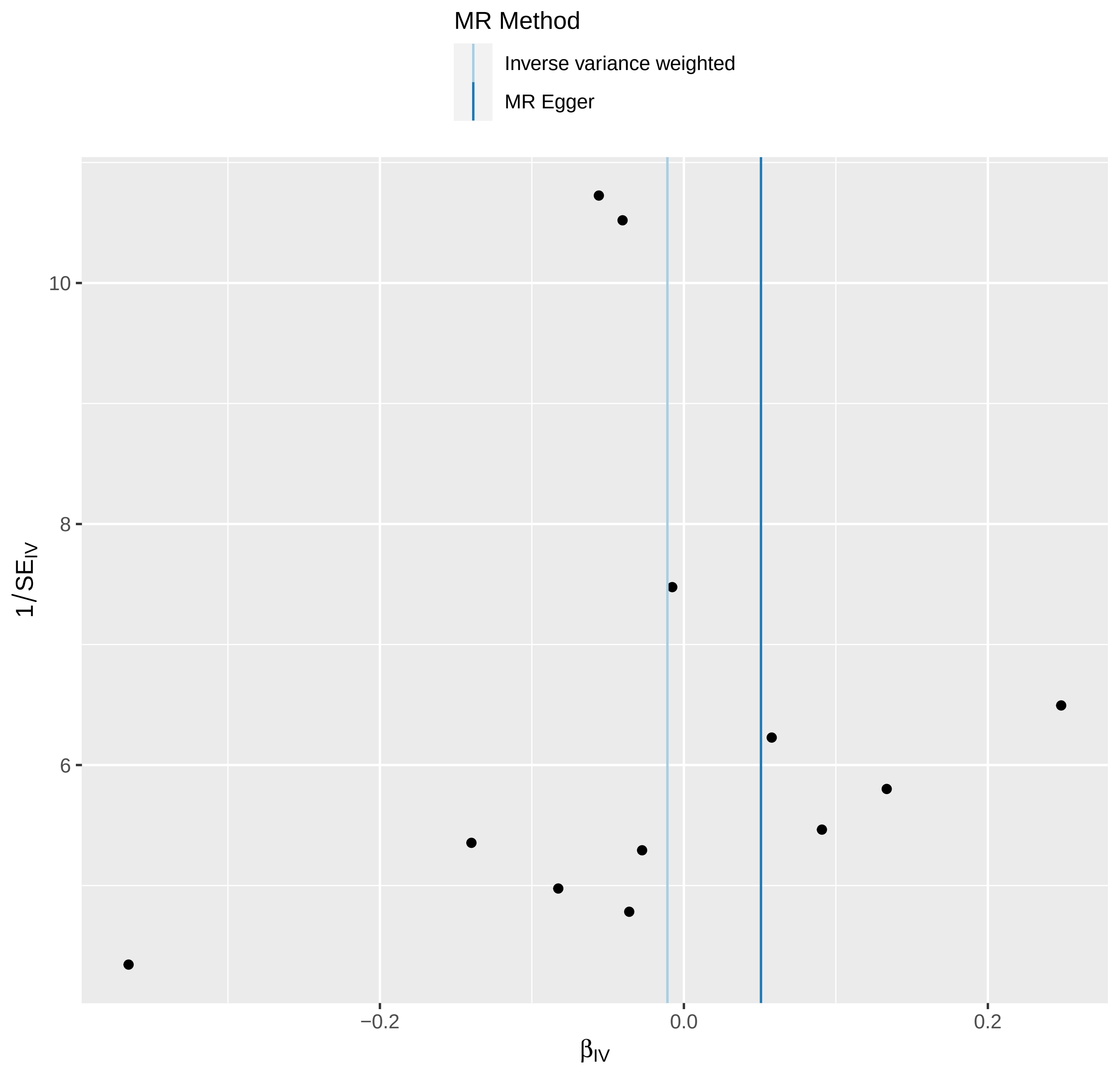

Batch 3−981 : Selenium on Erythroid membrane−associated protein

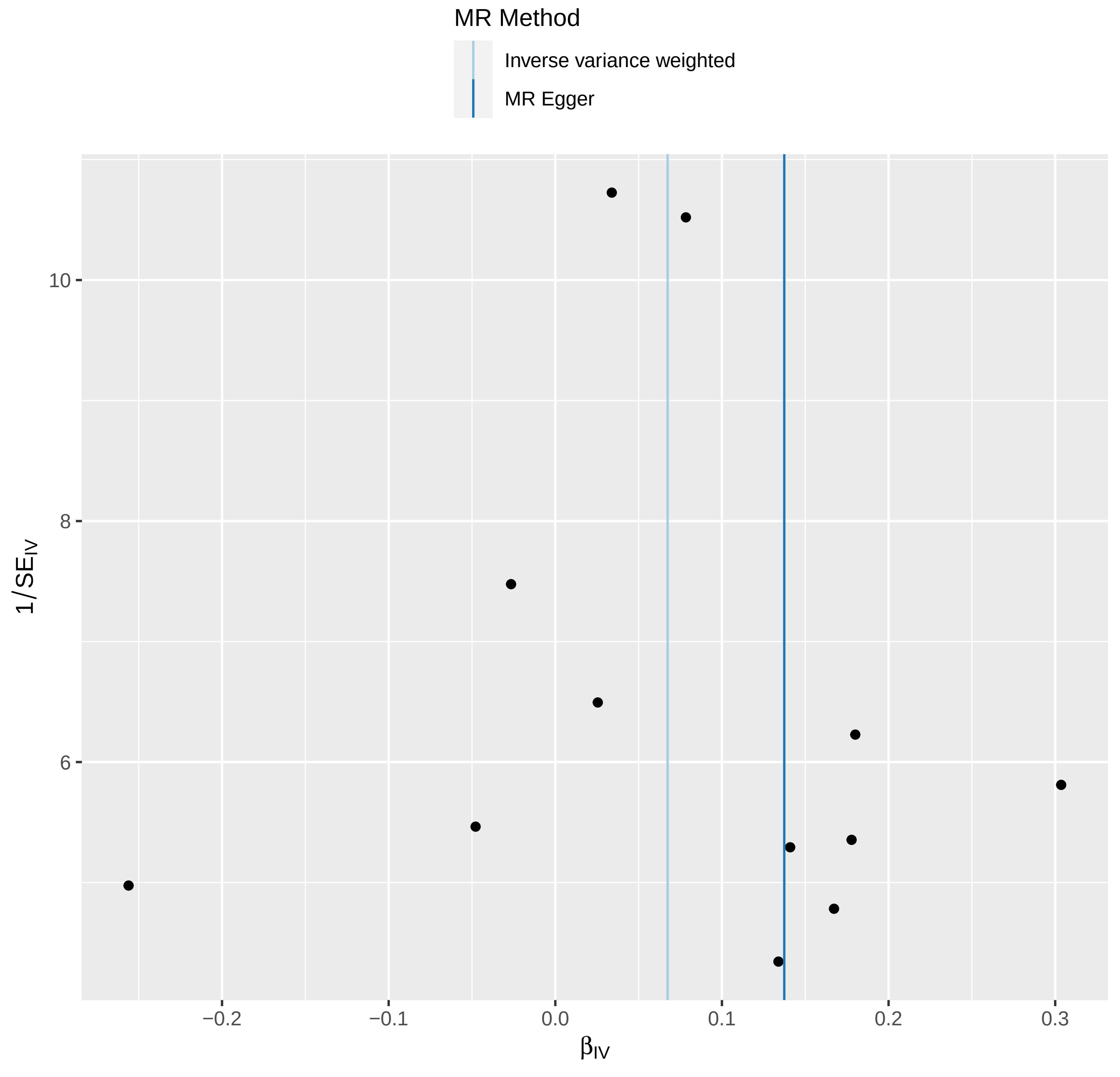

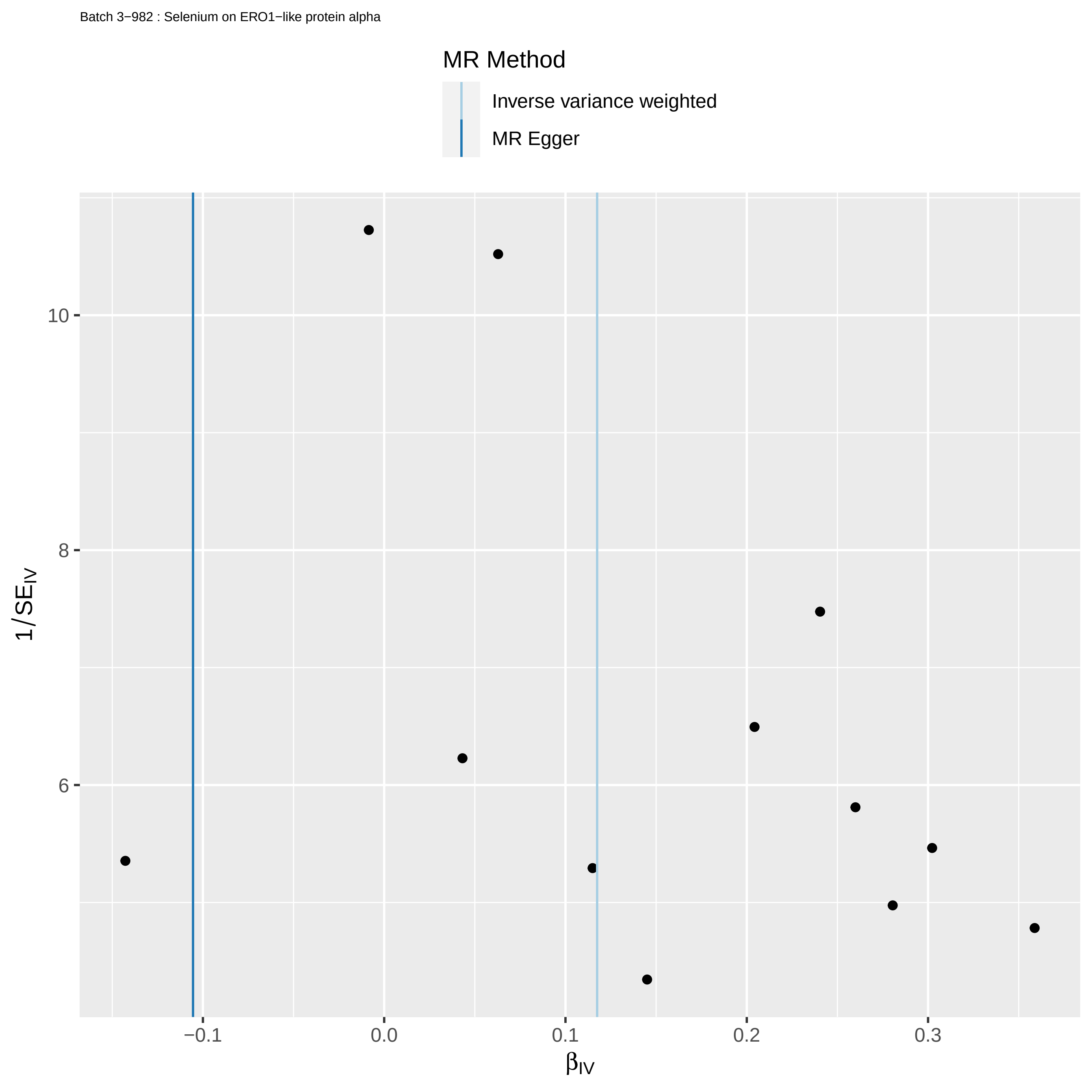

Batch 3−982 : Selenium on ERO1−like protein alpha
MR Method
Inverse variance weighted
MR Egger
10
8
6
1/SE_IV
−0.1
0.0
0.1
0.2
0.3
β_IV

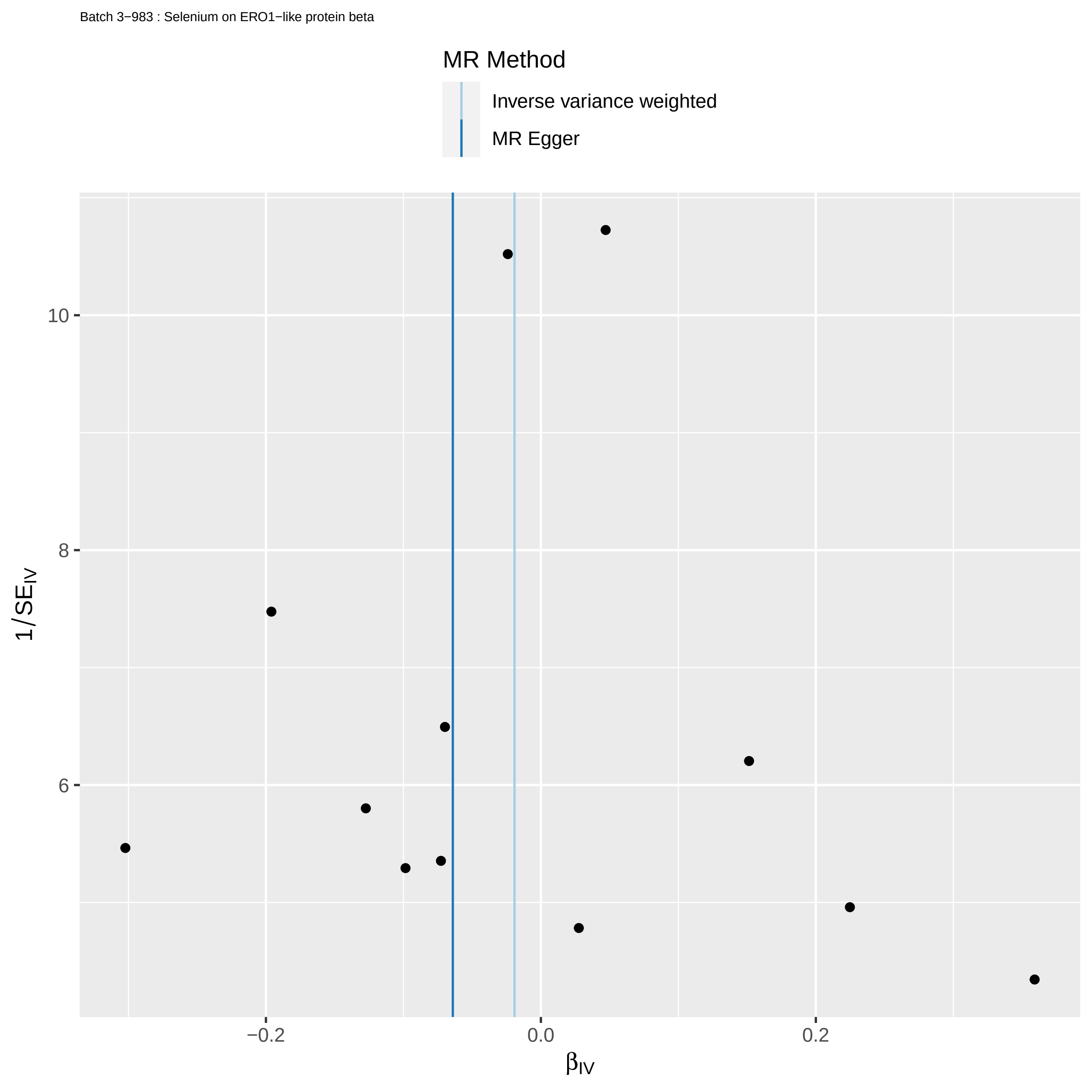

Batch 3−983 : Selenium on ERO1−like protein beta
MR Method
Inverse variance weighted
MR Egger
10
8
6
1/SE_IV
−0.2
0.0
0.2
β_IV

Batch 3−984 : Selenium on Endoplasmic reticulum resident protein 29

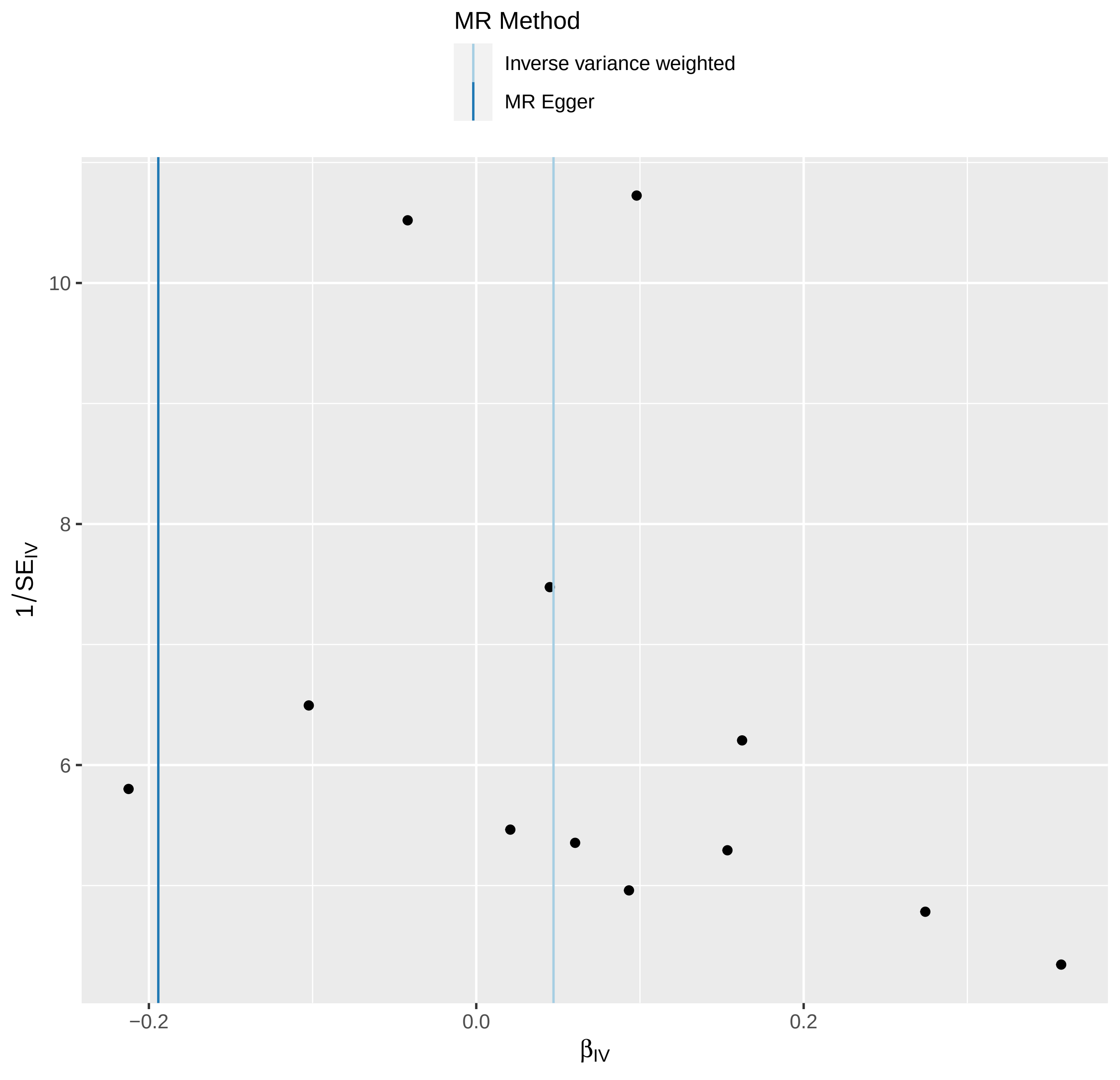

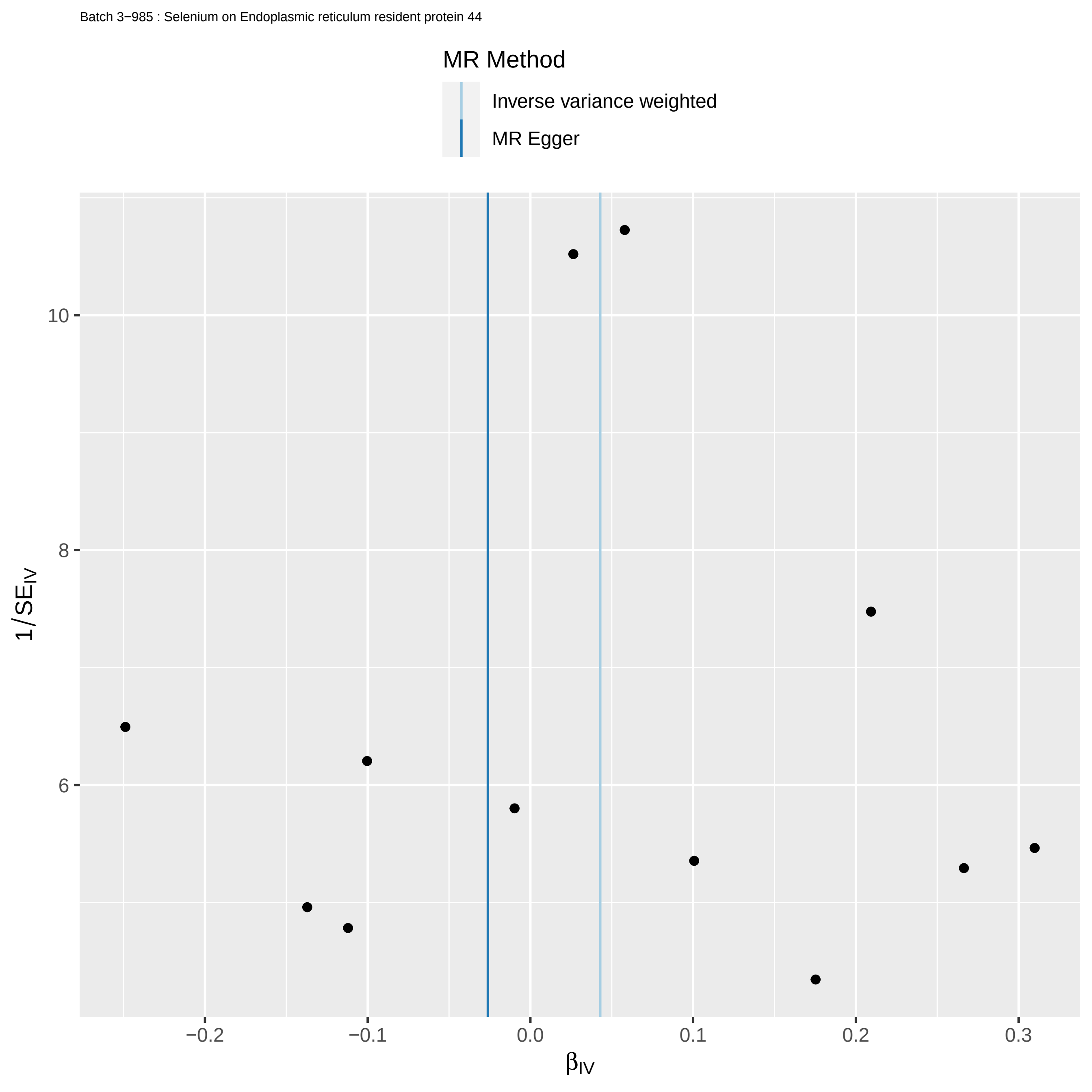

Batch 3−985 : Selenium on Endoplasmic reticulum resident protein 44
MR Method
Inverse variance weighted
MR Egger
10
8
6
−0.2
−0.1
0.0
0.1
0.2
0.3
1/SE_IV
β_IV

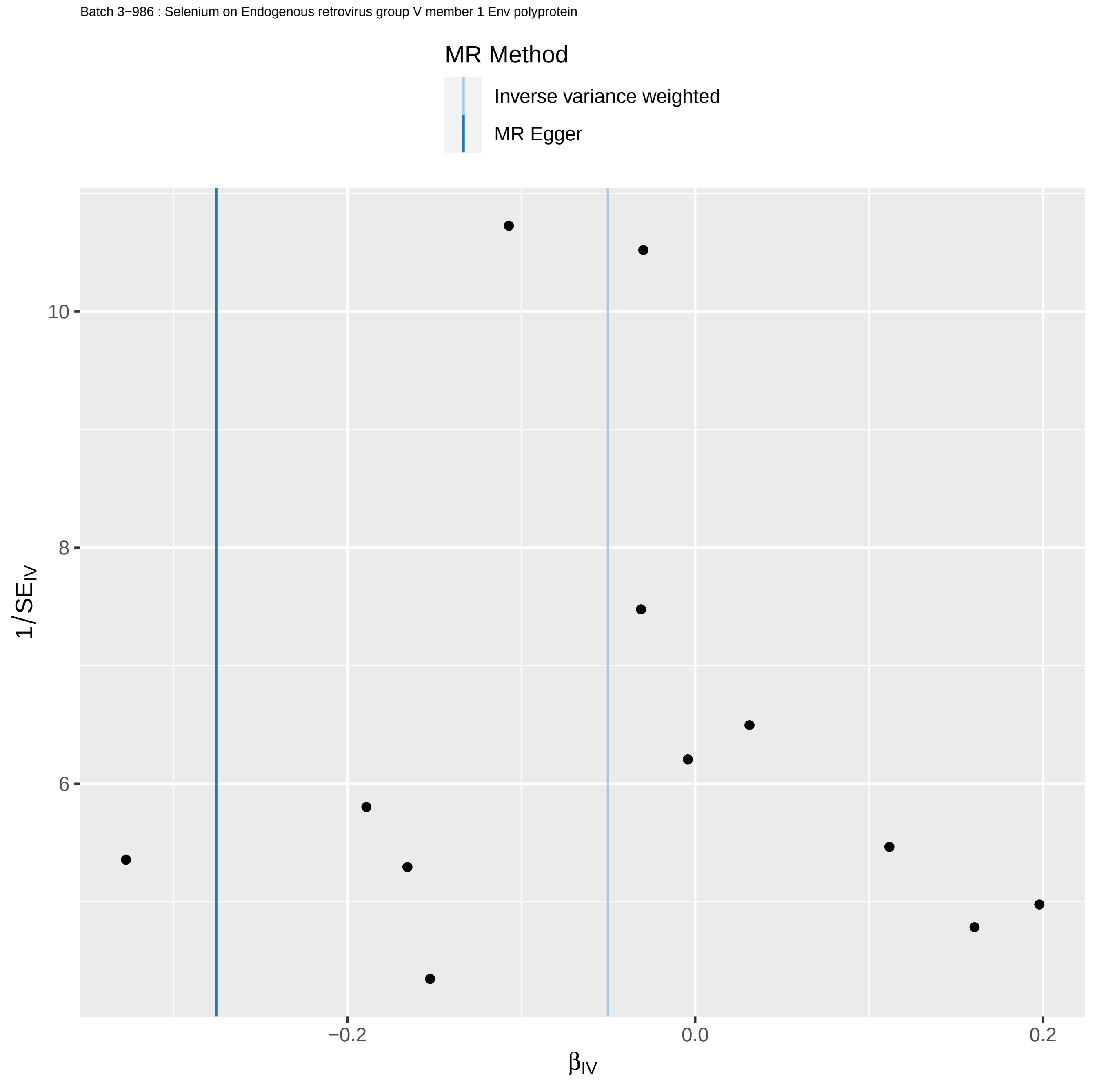

Batch 3−986 : Selenium on Endogenous retrovirus group V member 1 Env polyprotein
MR Method
Inverse variance weighted
MR Egger
10
8
6
1/SE_IV
−0.2
0.0
0.2
β_IV

Batch 3−987 : Selenium on Endothelial cell−selective adhesion molecule

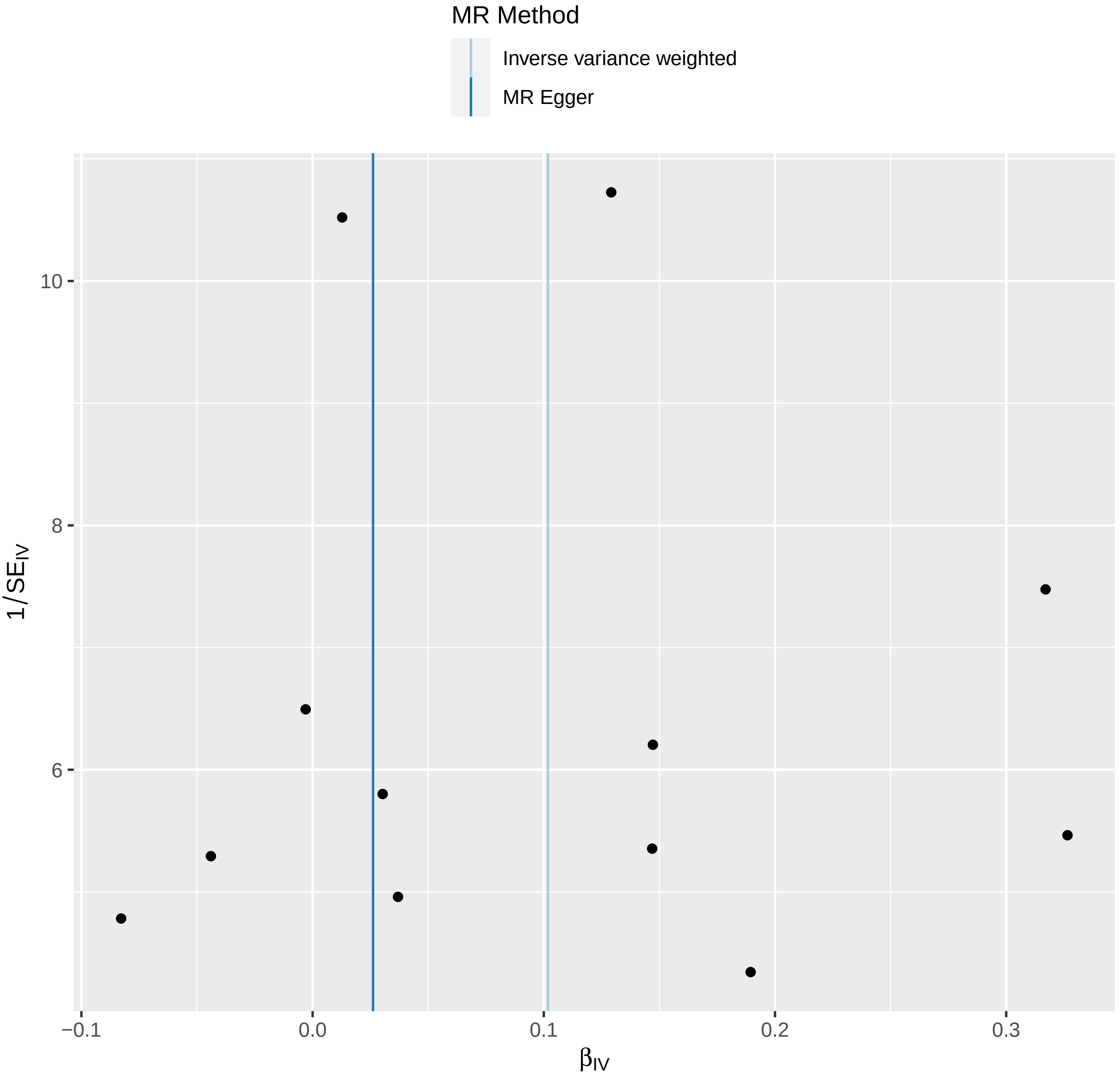

Batch 3−988 : Selenium on Endothelial cell−selective adhesion molecule

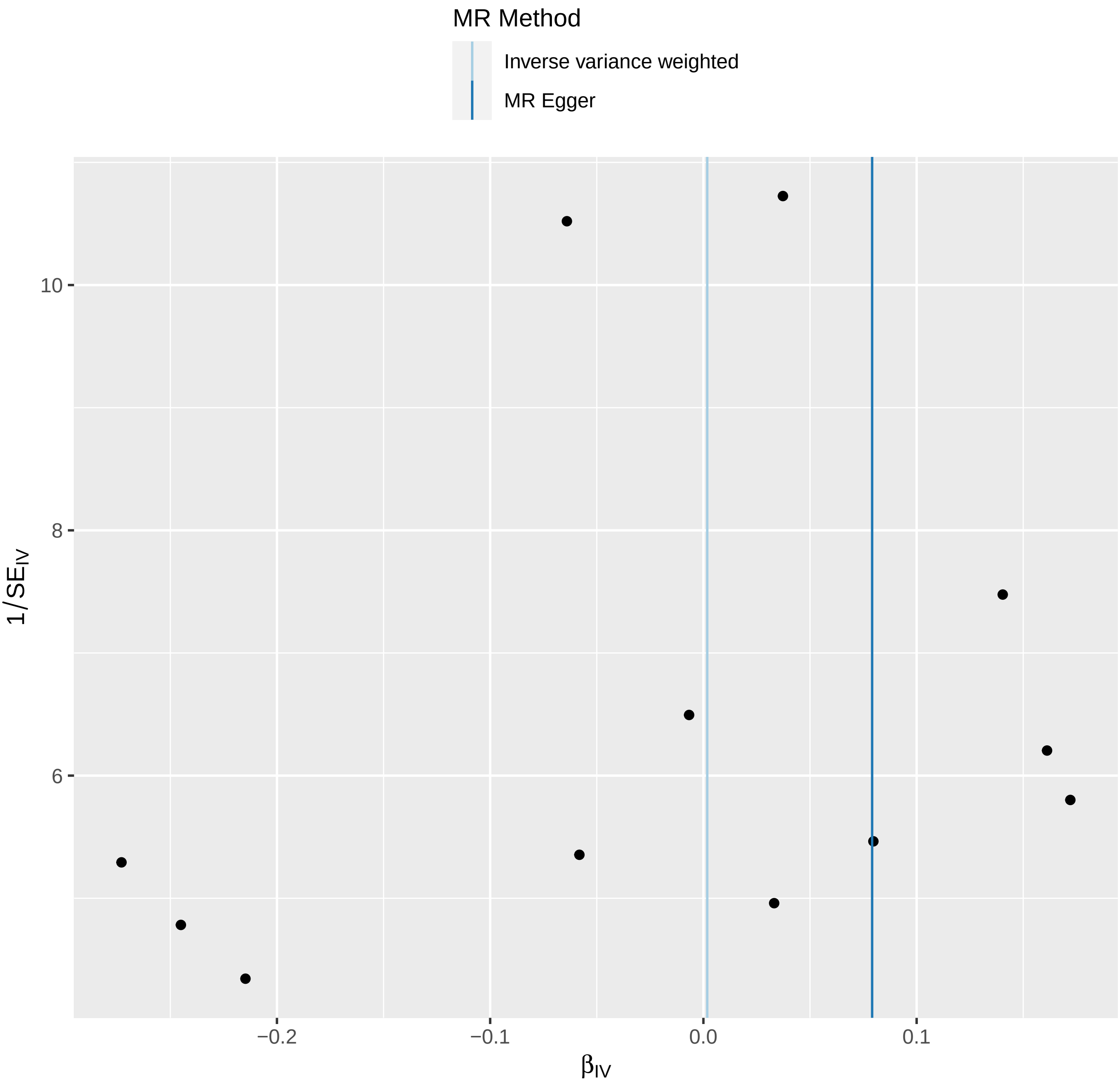

Batch 3−989 : Selenium on S−formylglutathione hydrolase

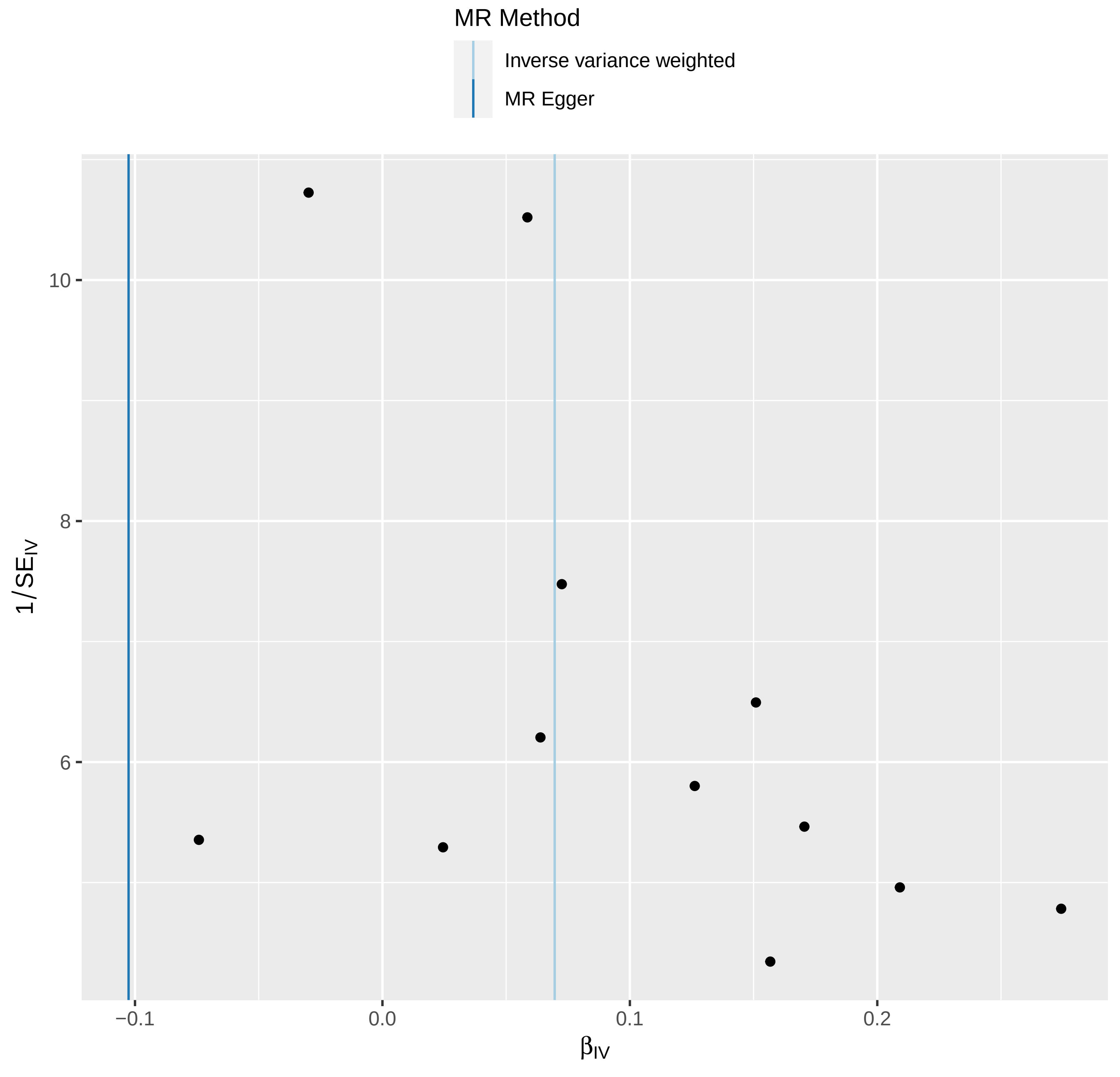

Batch 3−990 : Selenium on Endothelial cell−specific molecule 1

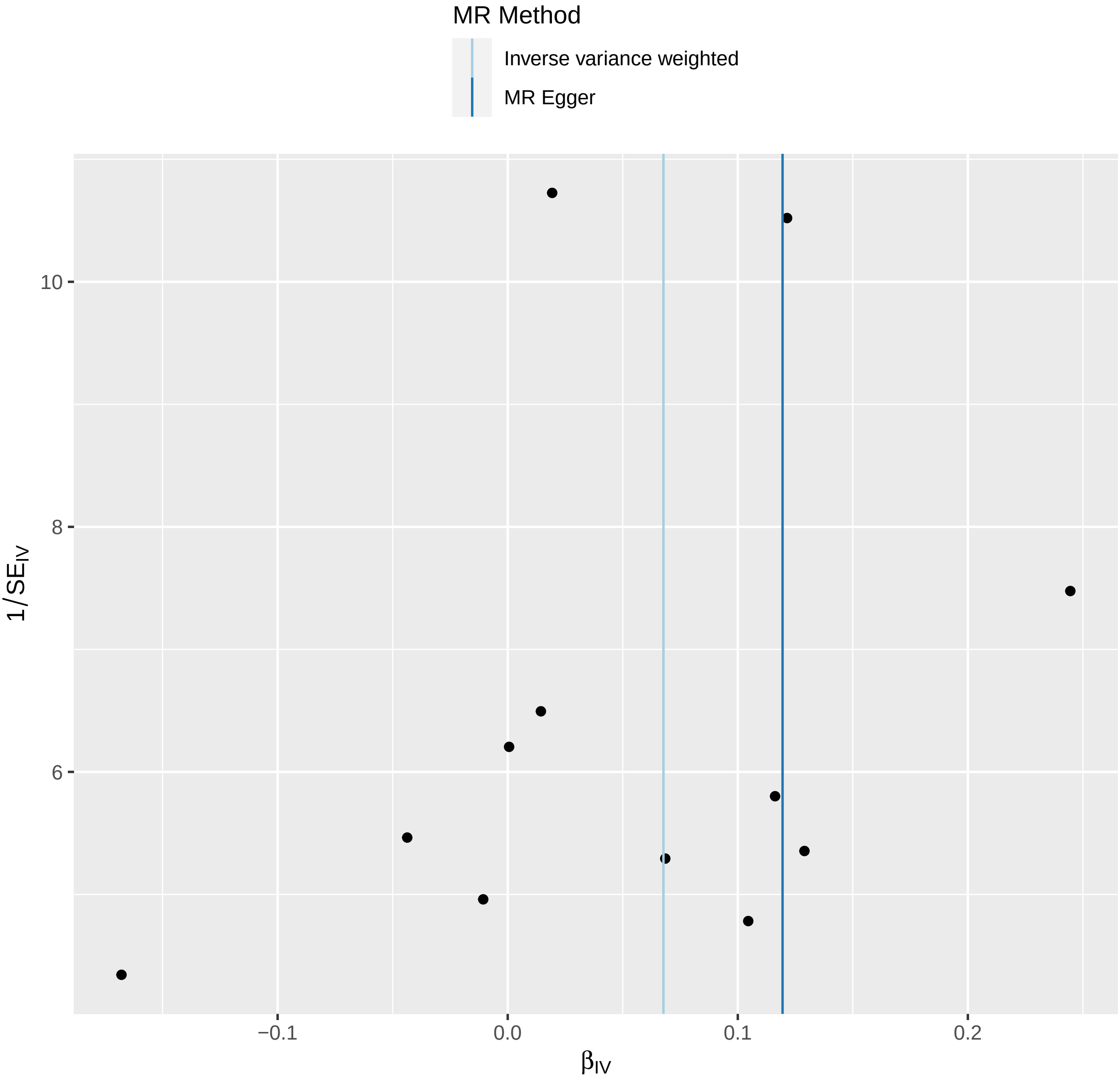

Batch 3−991 : Selenium on Estrogen receptor

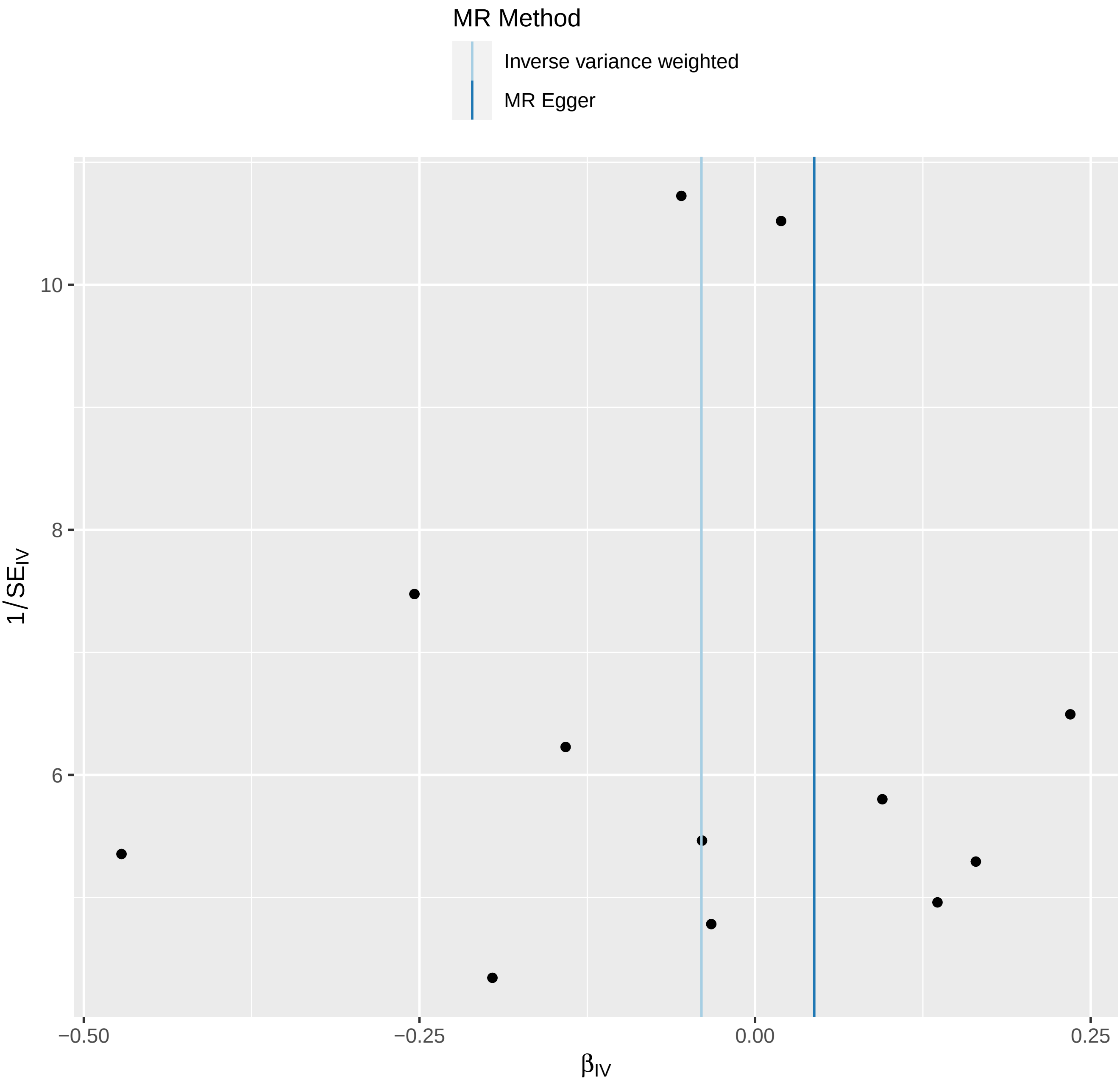

Batch 3−992 : Selenium on Persulfide dioxygenase ETHE1, mitochondrial

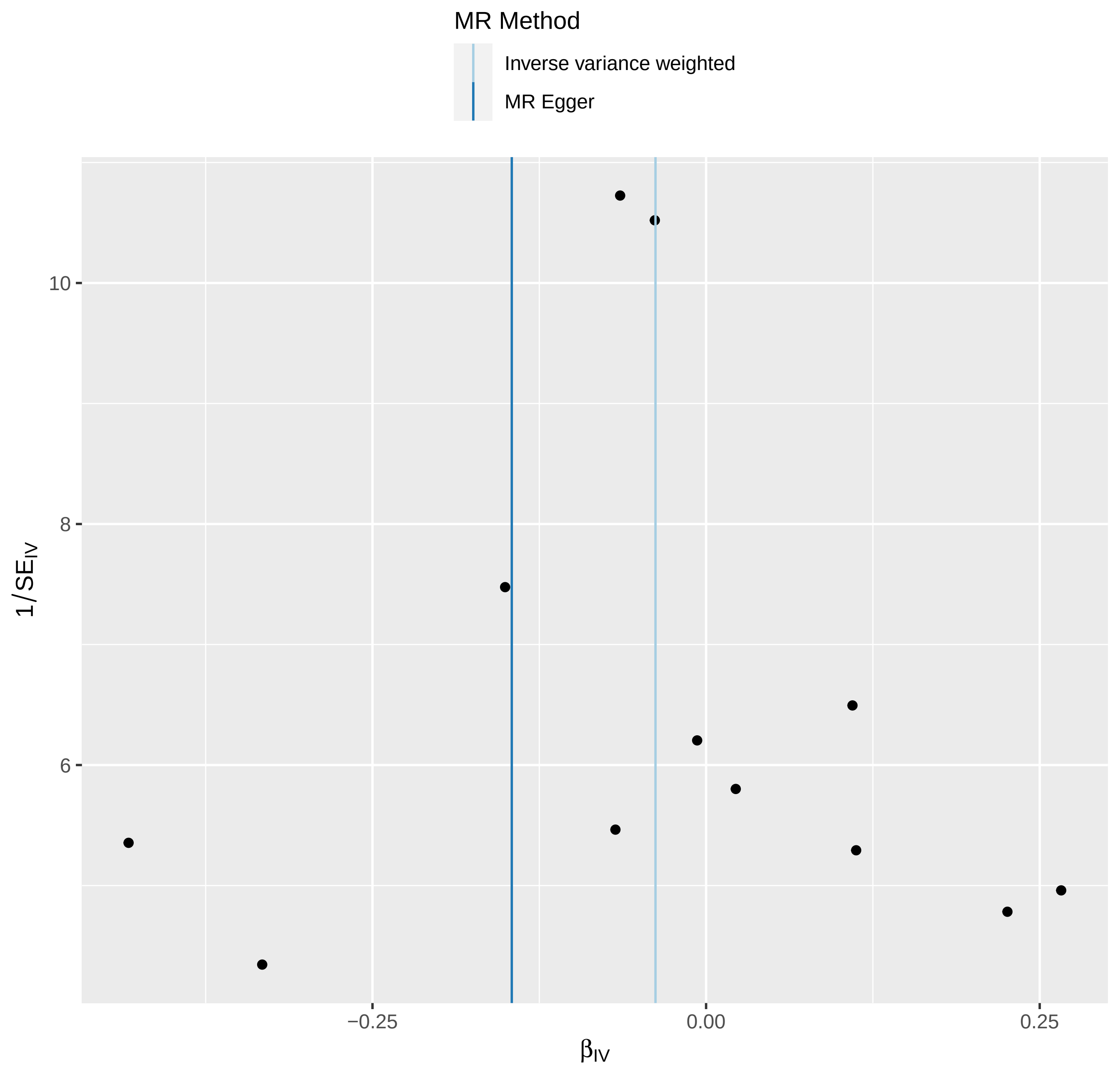

Batch 3−993 : Selenium on Ethanolamine kinase 1

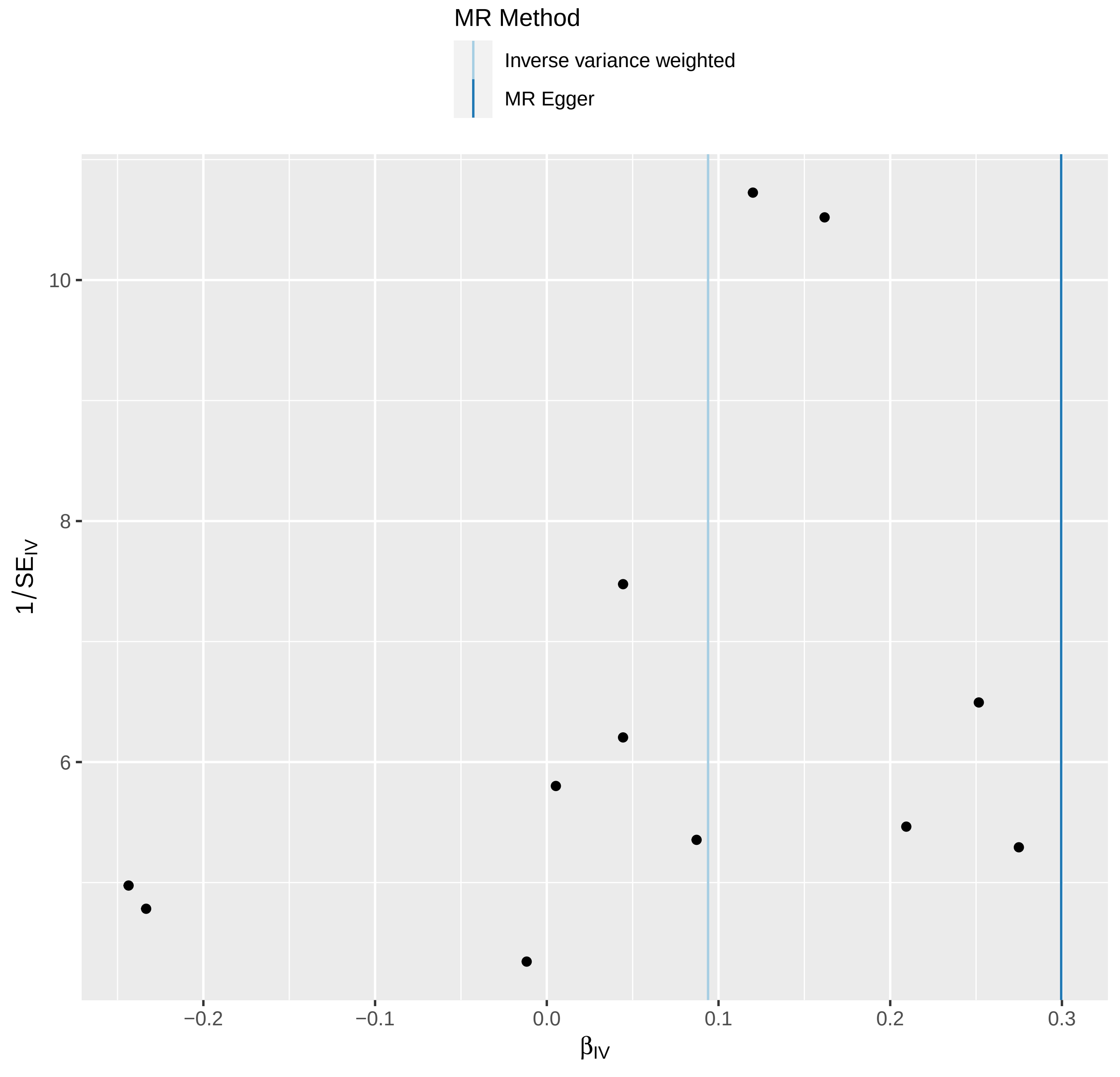

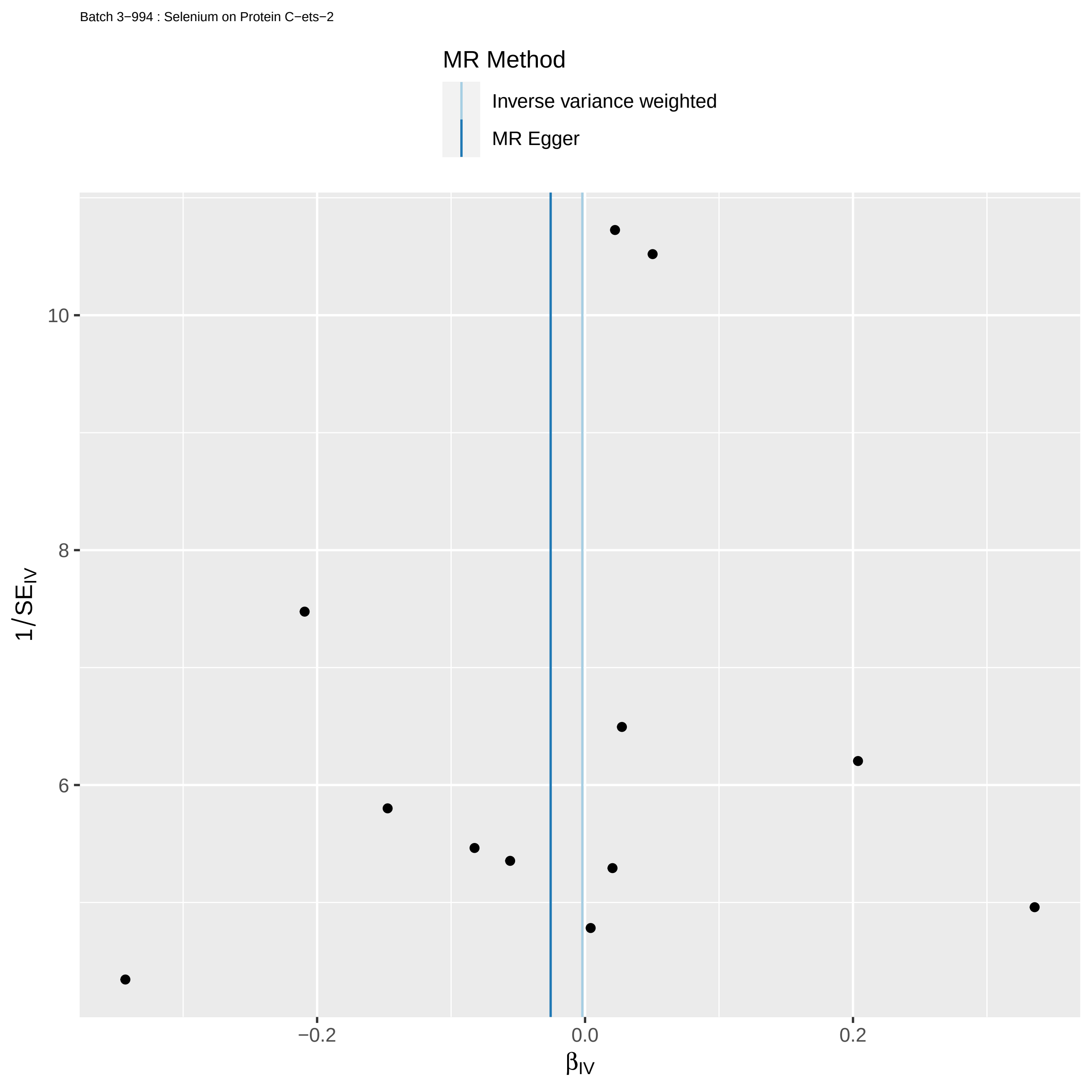

Batch 3−994 : Selenium on Protein C−ets−2
MR Method
Inverse variance weighted
MR Egger
10
8
6
1/SE$_{IV}$
−0.2
0.0
0.2
β$_{IV}$

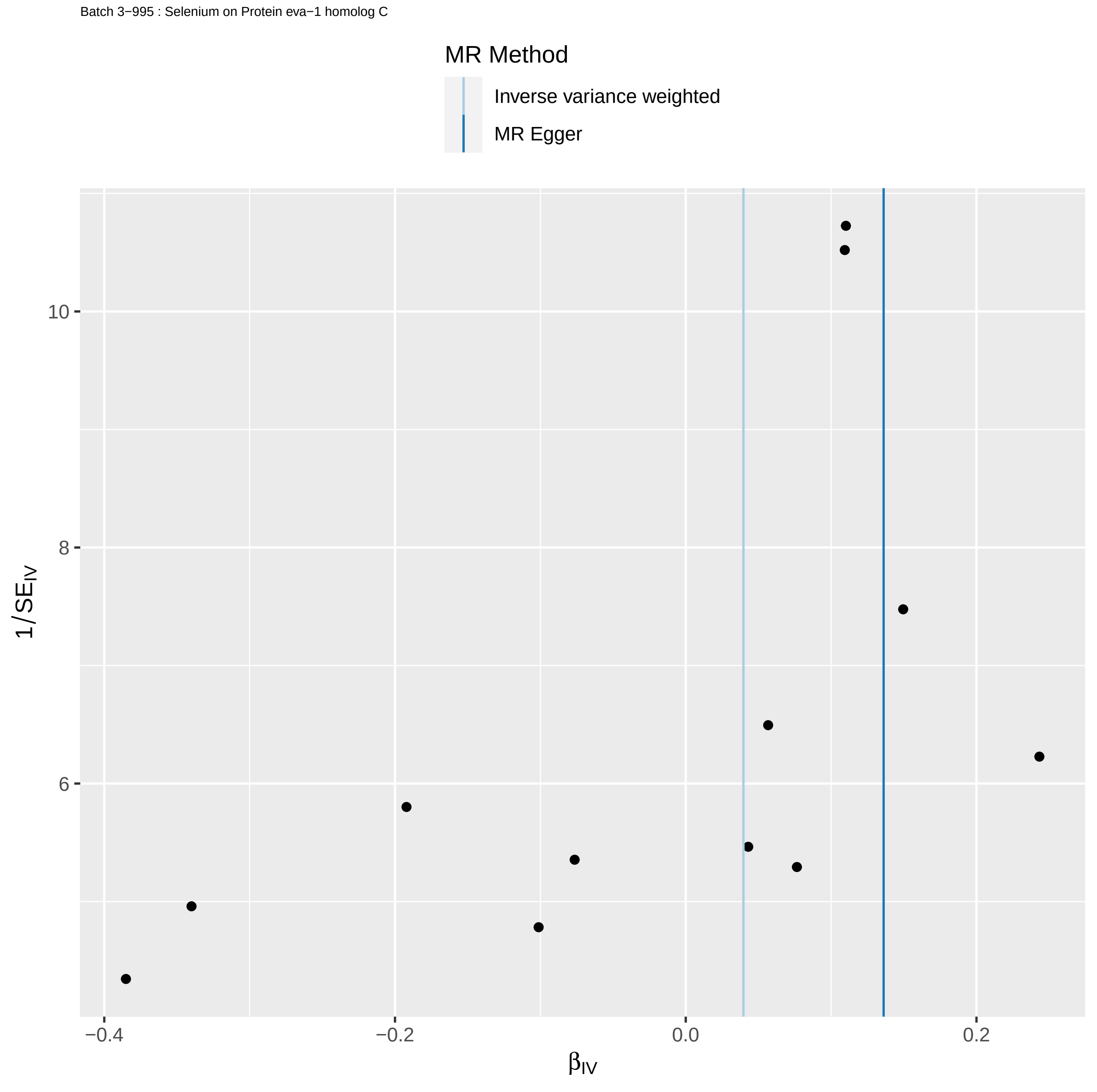

Batch 3−995 : Selenium on Protein eva−1 homolog C
MR Method
Inverse variance weighted
MR Egger
10
8
6
1/SE$_{IV}$
−0.4
−0.2
0.0
0.2
β$_{IV}$

Batch 3−996 : Selenium on Protein eva−1 homolog C

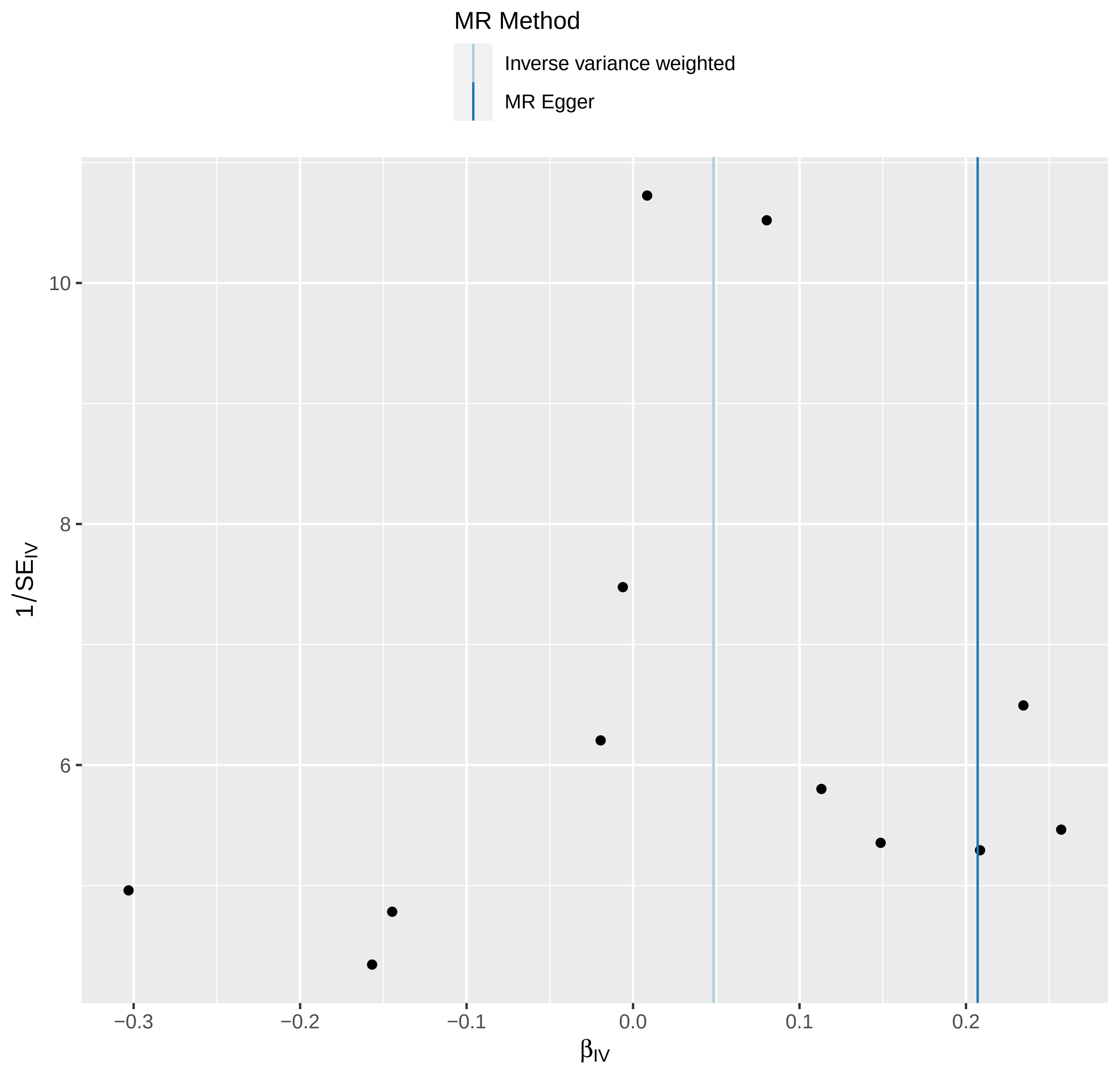

Batch 3−997 : Selenium on Protein EVI2B

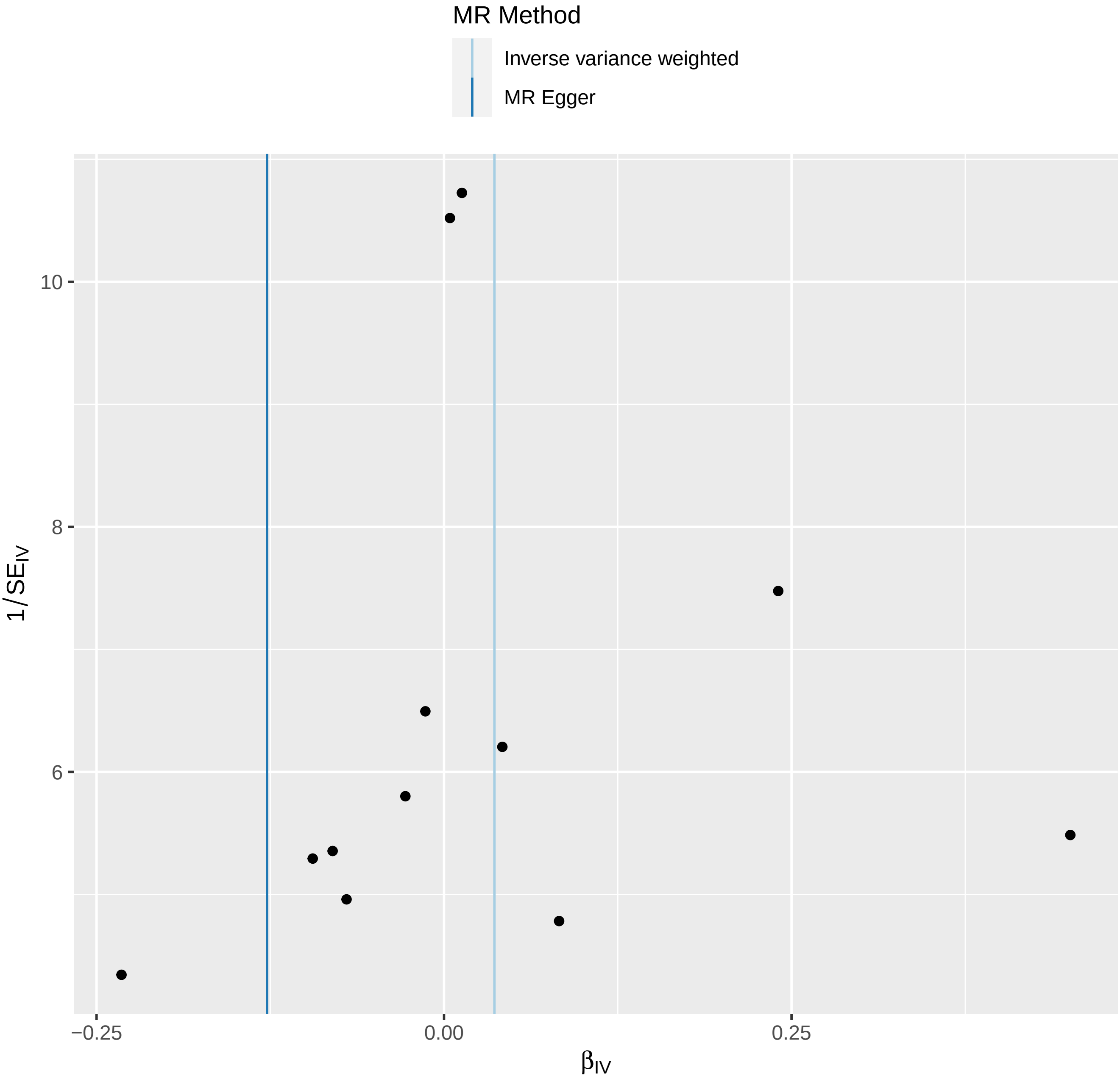

Batch 3−998 : Selenium on Ena/VASP−like protein

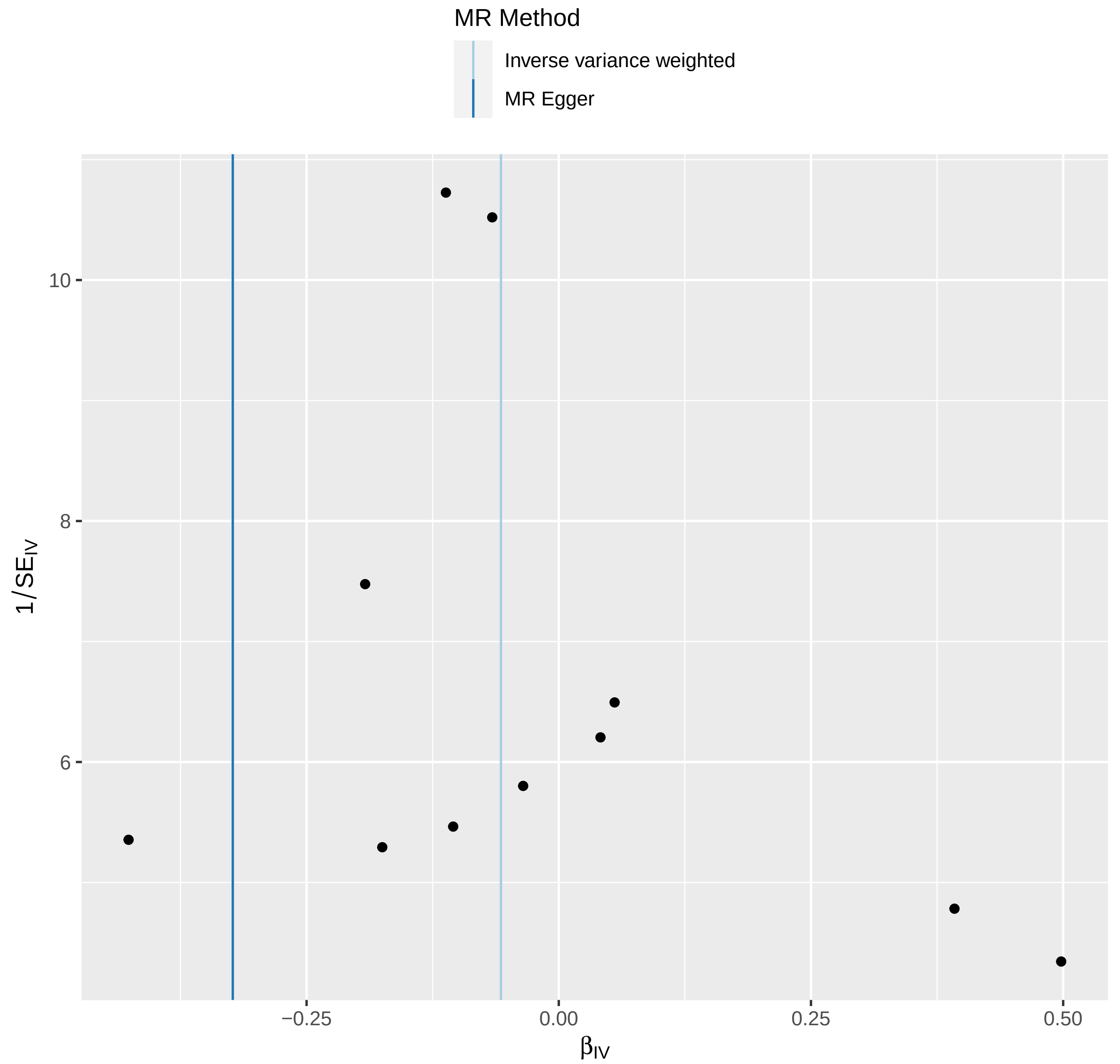

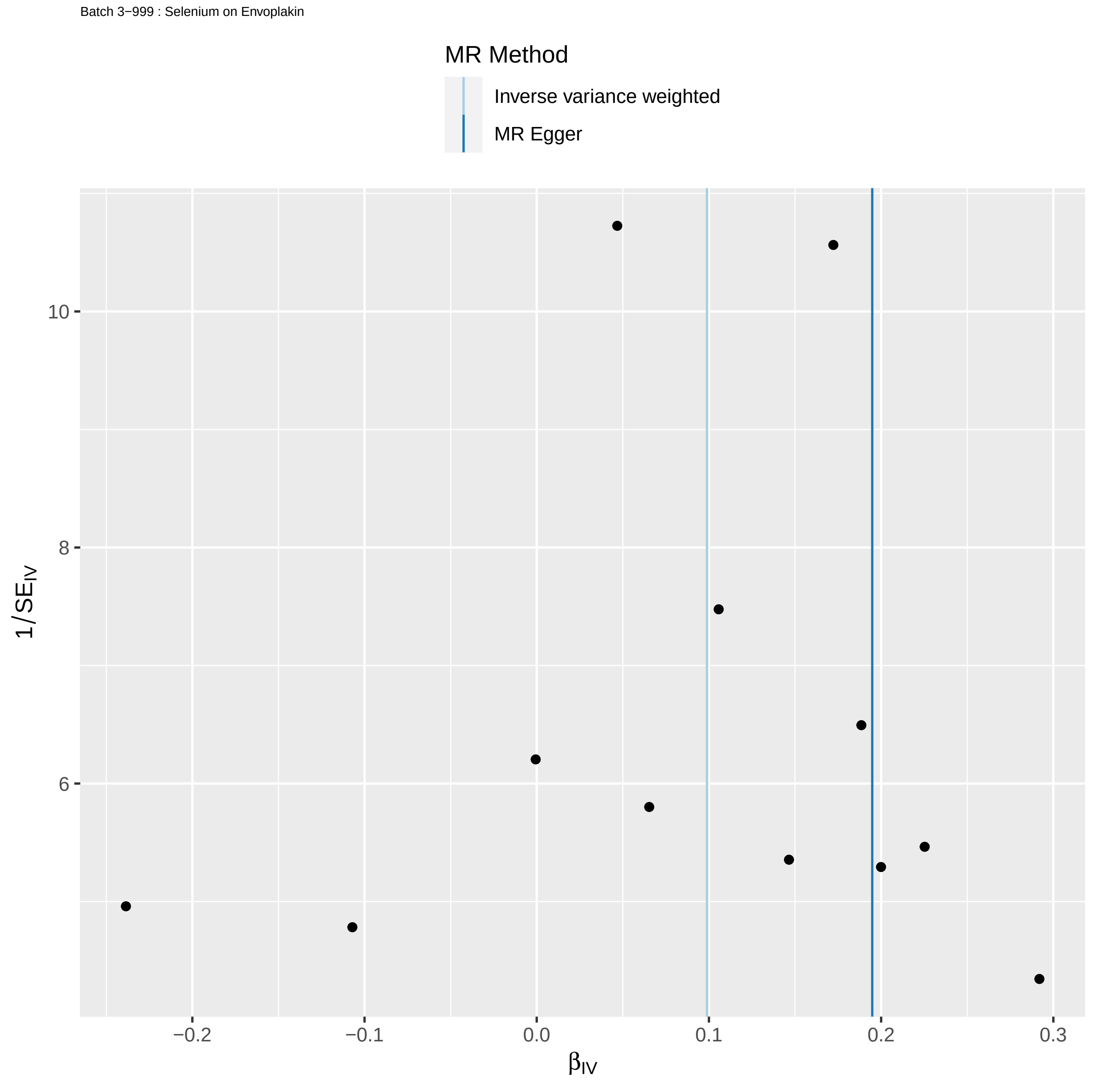

Batch 3−999 : Selenium on Envoplakin
MR Method
Inverse variance weighted
MR Egger
10
8
6
1/SE_IV
−0.2
−0.1
0.0
0.1
0.2
0.3
β_IV

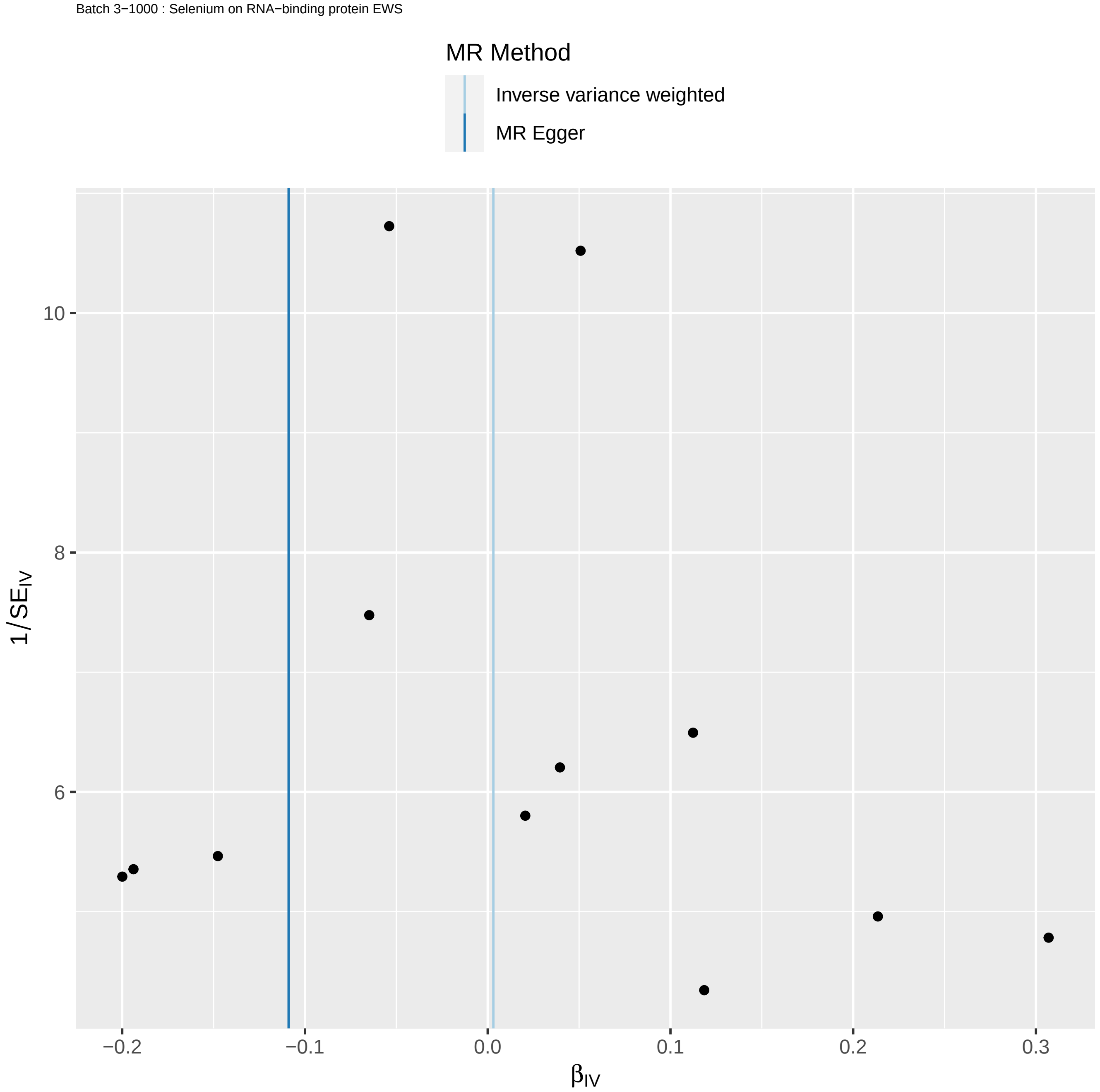
Batch 3−1000 : Selenium on RNA−binding protein EWS
MR Method
Inverse variance weighted
MR Egger
10
8
6
1/SE_IV
−0.2
−0.1
0.0
0.1
0.2
0.3
β_IV

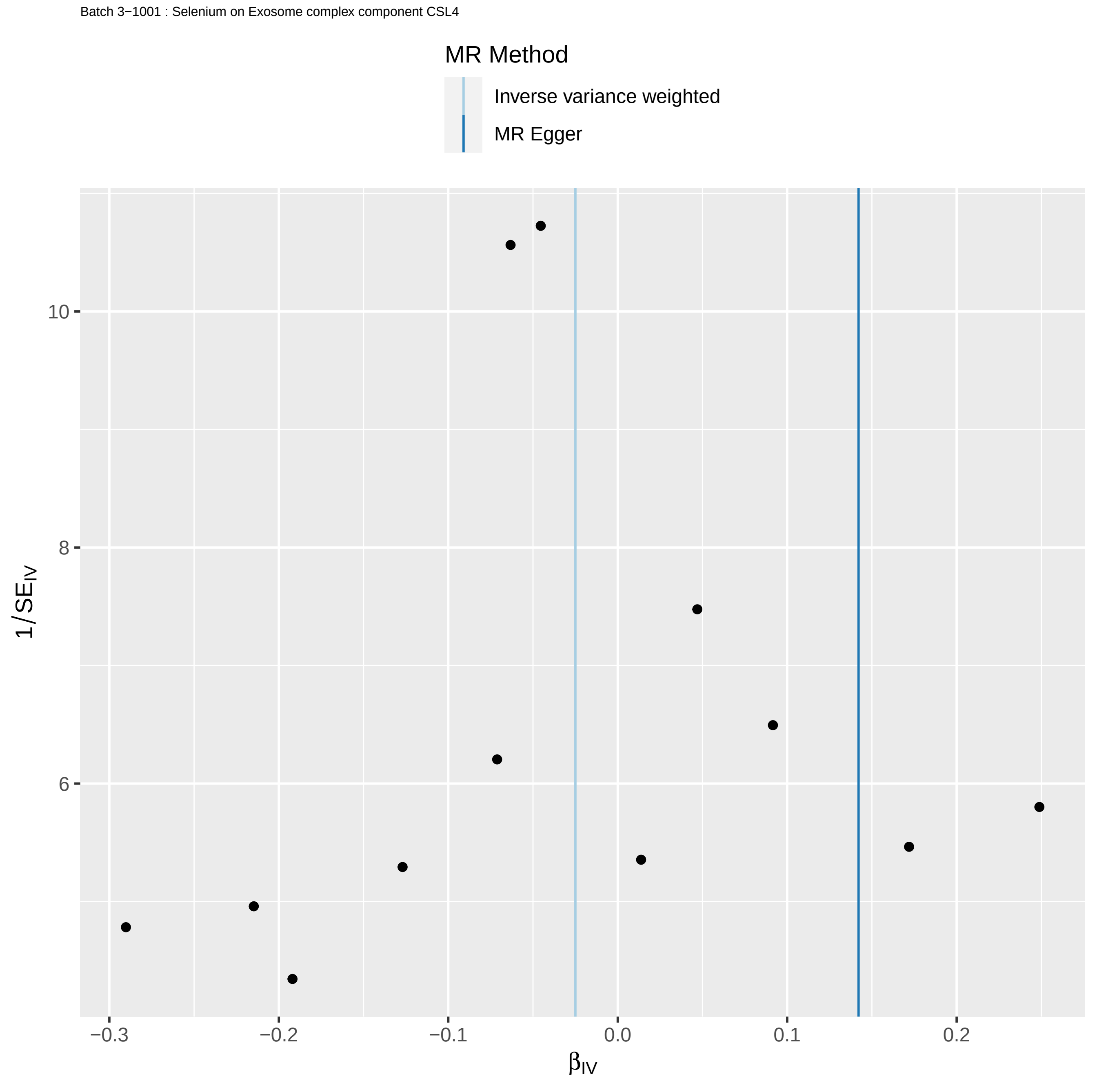

Batch 3−1001 : Selenium on Exosome complex component CSL4
MR Method
Inverse variance weighted
MR Egger
10
8
6
1/SE_IV
−0.3
−0.2
−0.1
0.0
0.1
0.2
β_IV

Batch 3−1002 : Selenium on Exosome complex component RRP40

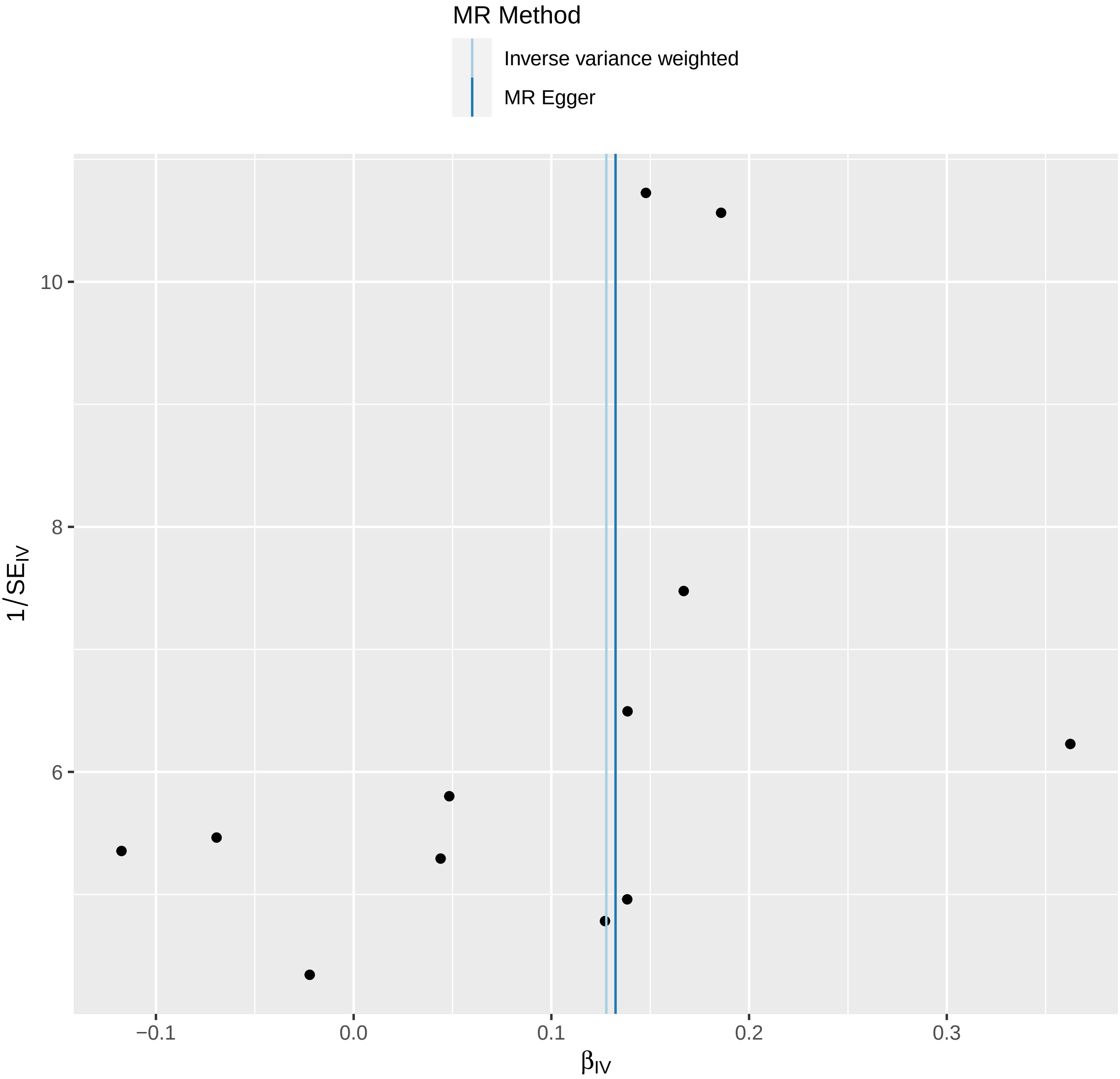

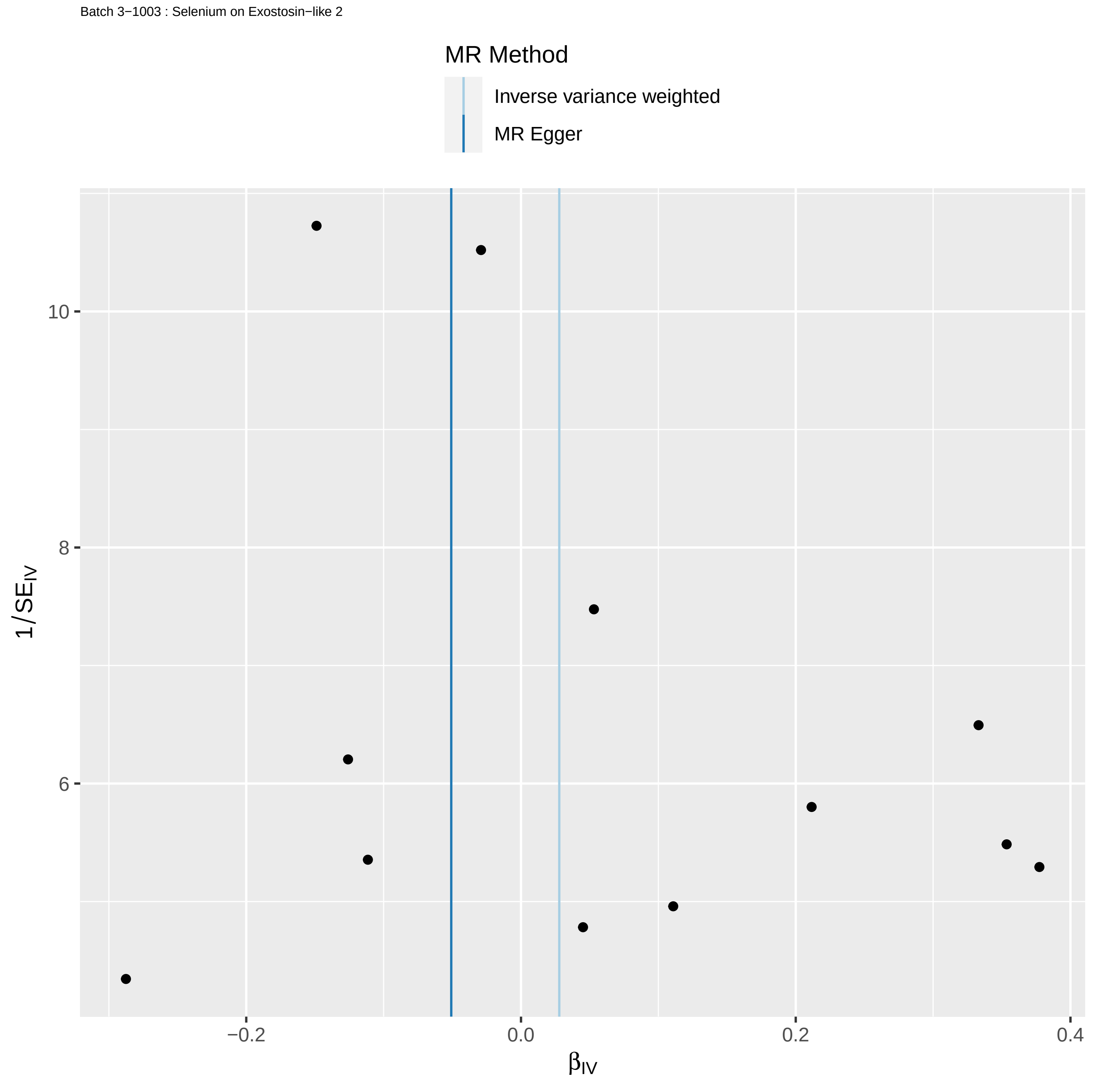

Batch 3−1003 : Selenium on Exostosin−like 2
MR Method
Inverse variance weighted
MR Egger
10
8
6
1/SE_IV
−0.2
0.0
0.2
0.4
β_IV

Batch 3−1004 : Selenium on Ezrin

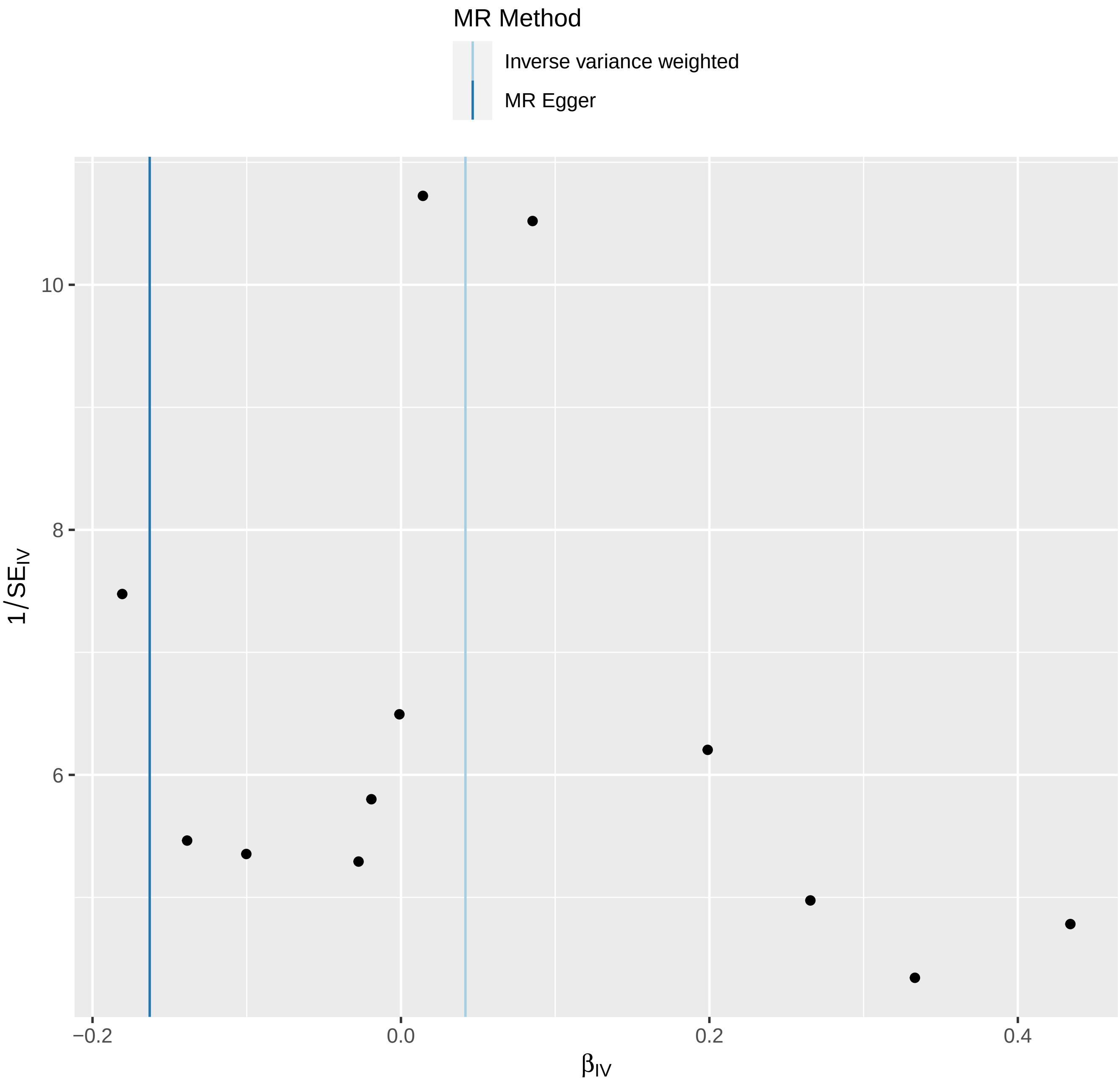

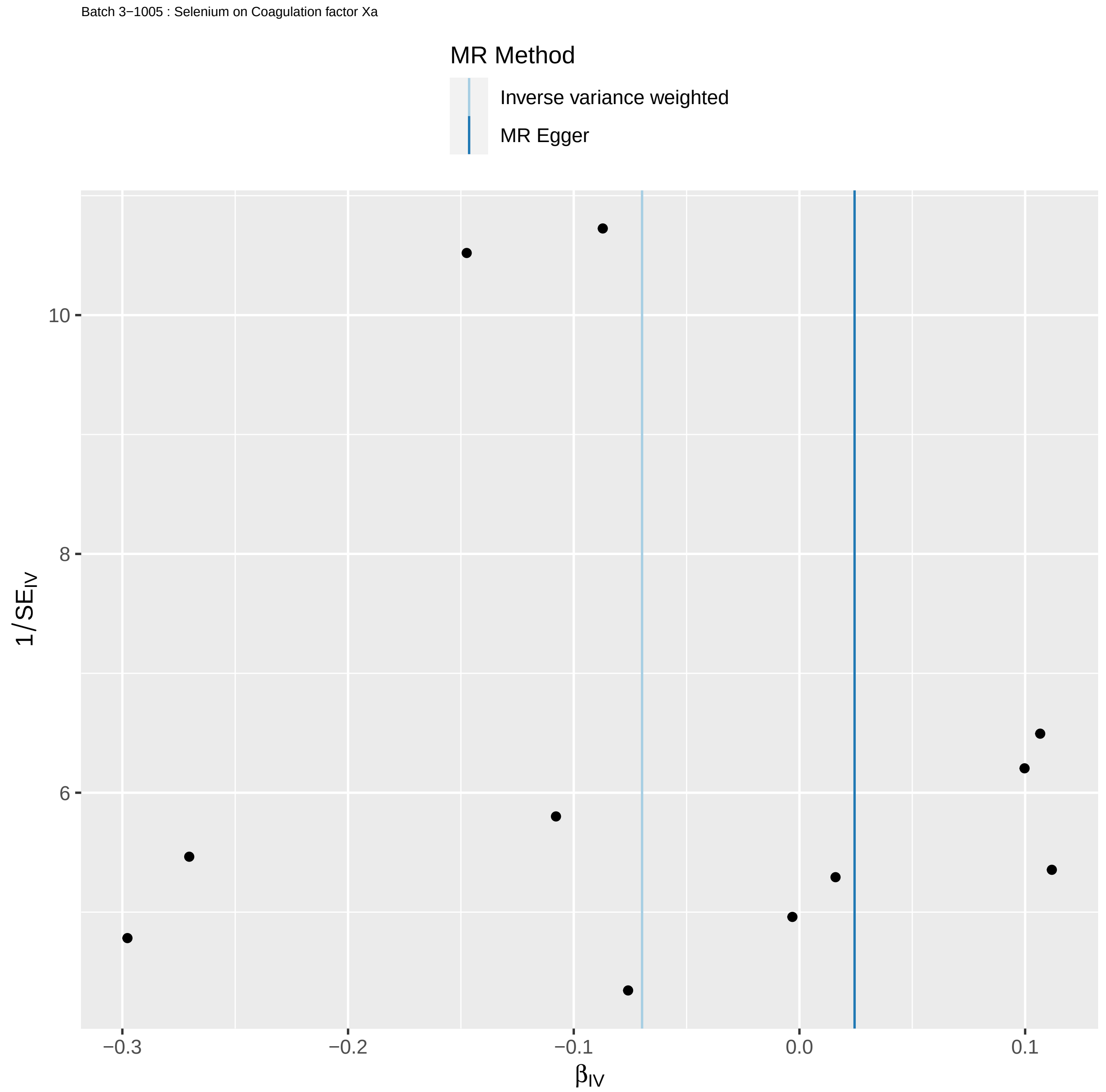
Batch 3−1005 : Selenium on Coagulation factor Xa
MR Method
Inverse variance weighted
MR Egger
10
8
6
1/SE_IV
−0.3
−0.2
−0.1
0.0
0.1
β_IV

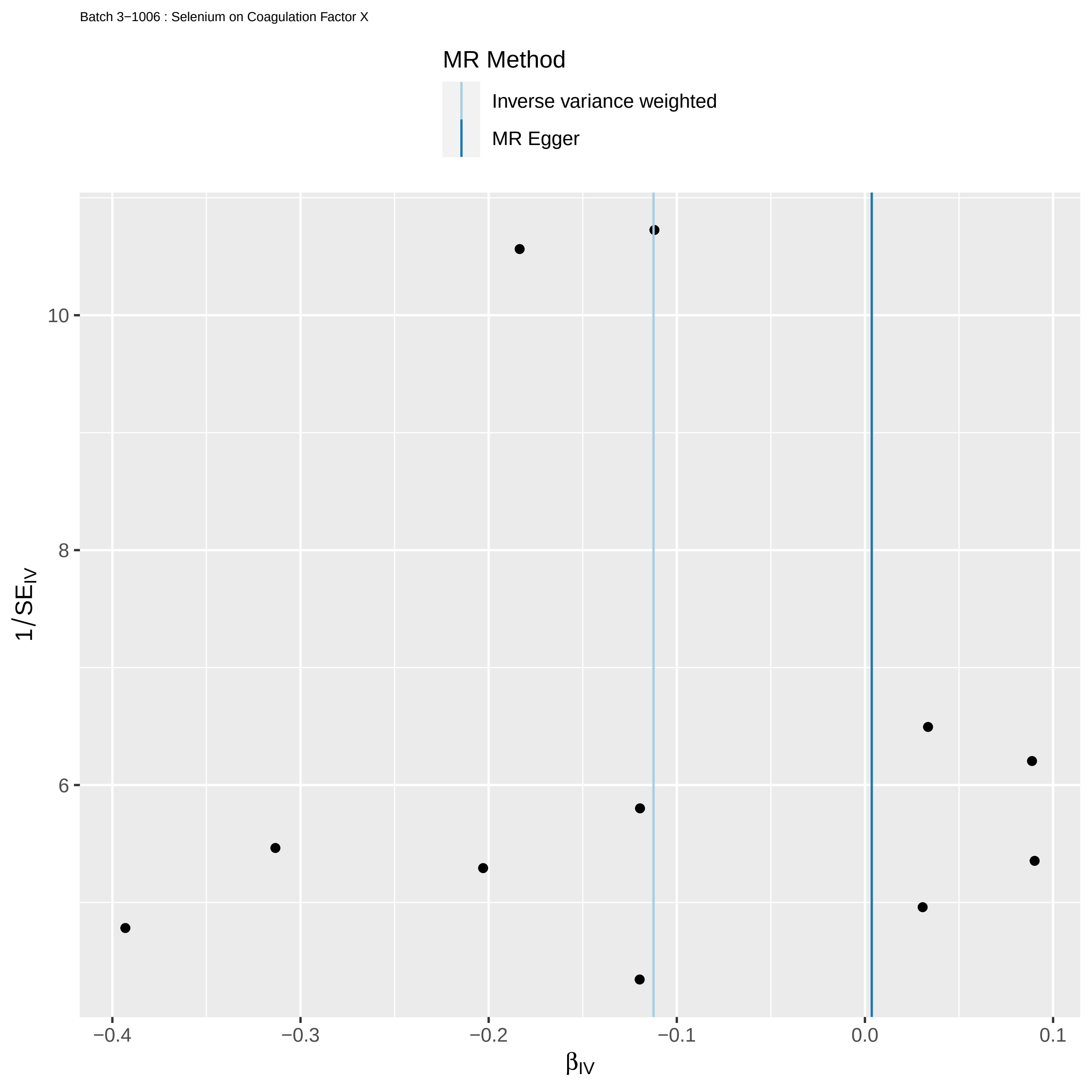

Batch 3−1006 : Selenium on Coagulation Factor X
MR Method
Inverse variance weighted
MR Egger
10
8
6
1/SE_IV
−0.4
−0.3
−0.2
−0.1
0.0
0.1
β_IV

Batch 3−1007 : Selenium on Prothrombin

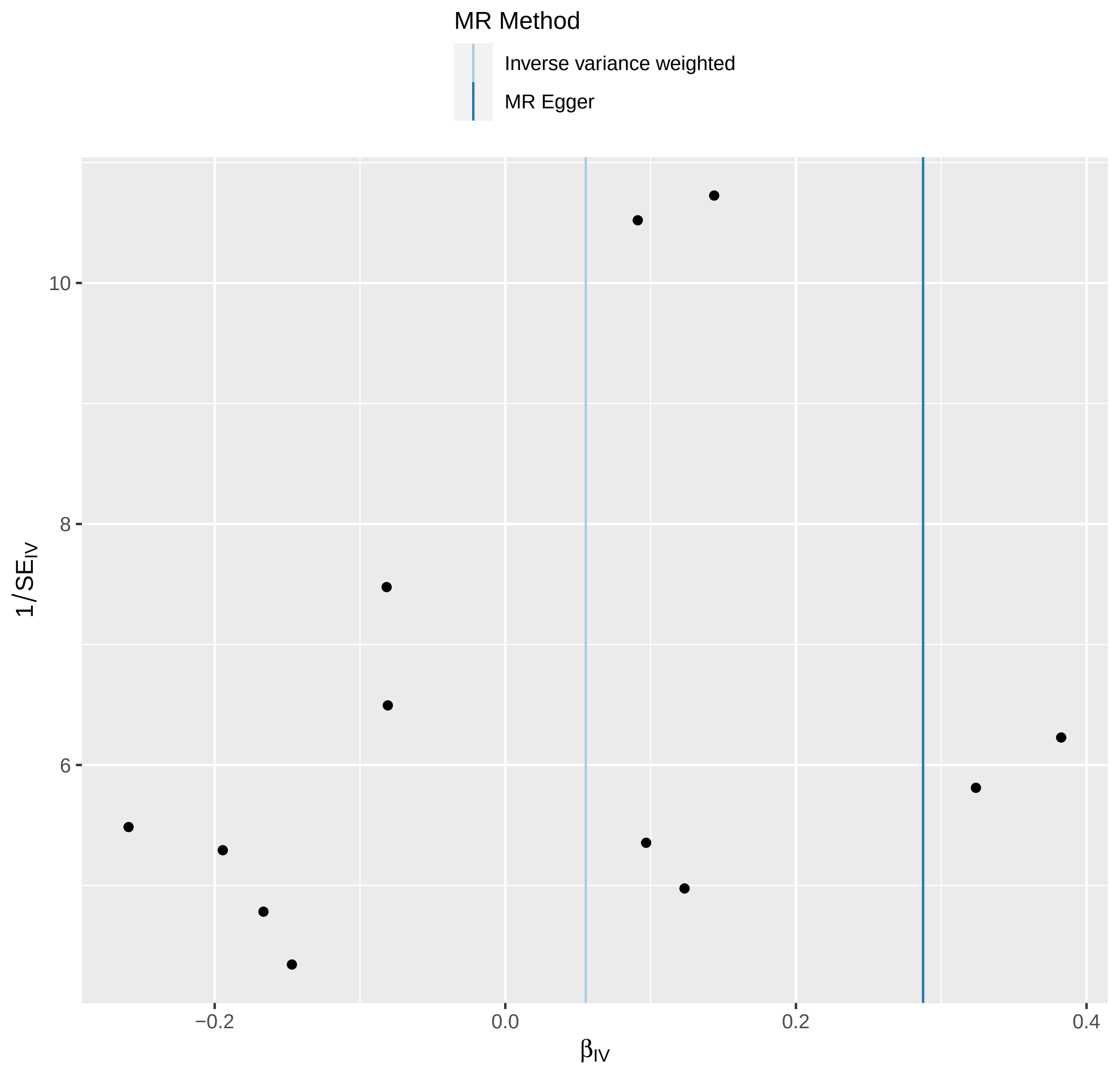

Batch 3−1008 : Selenium on Tissue Factor

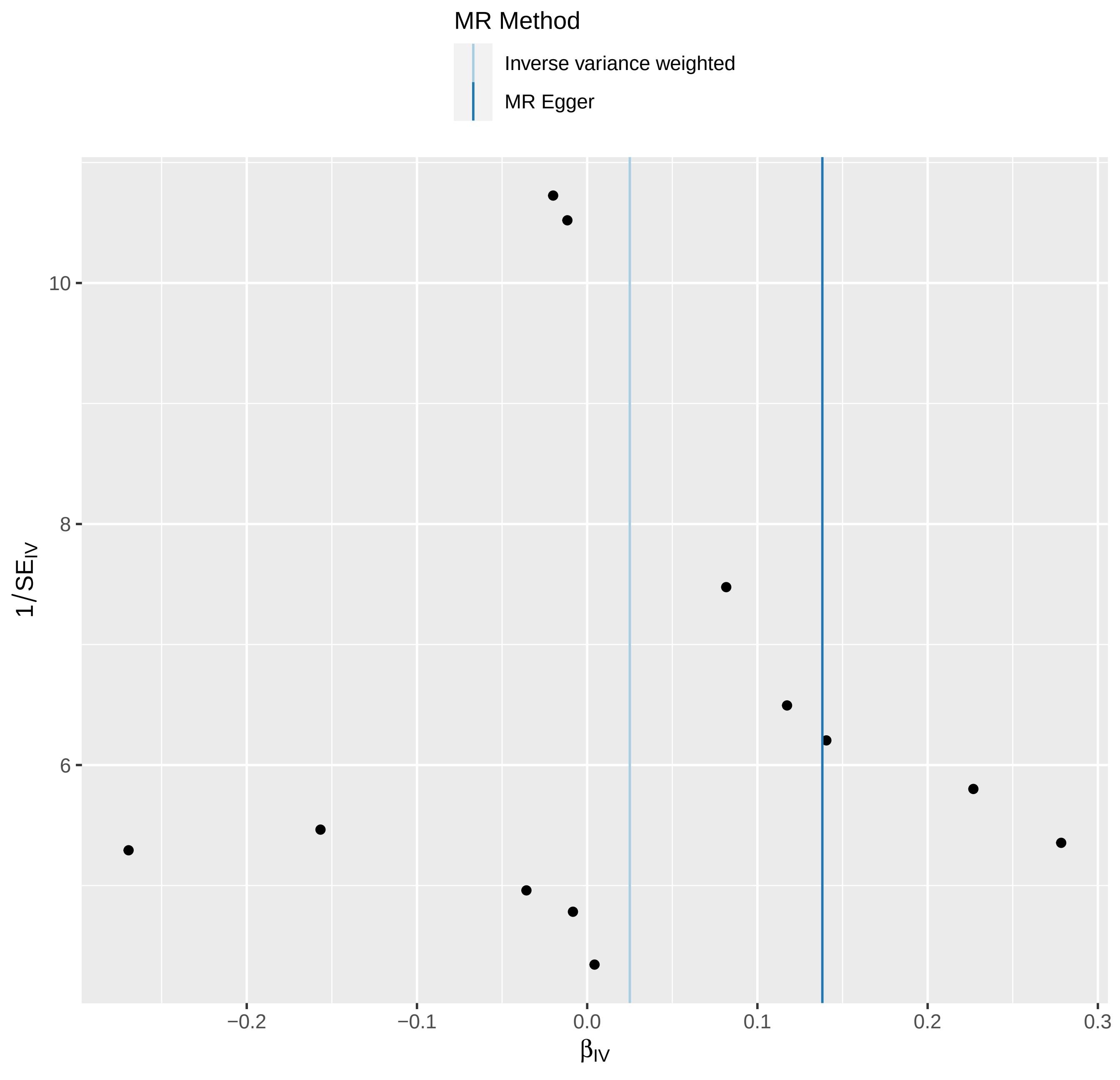

Batch 3−1009 : Selenium on Coagulation Factor VIII

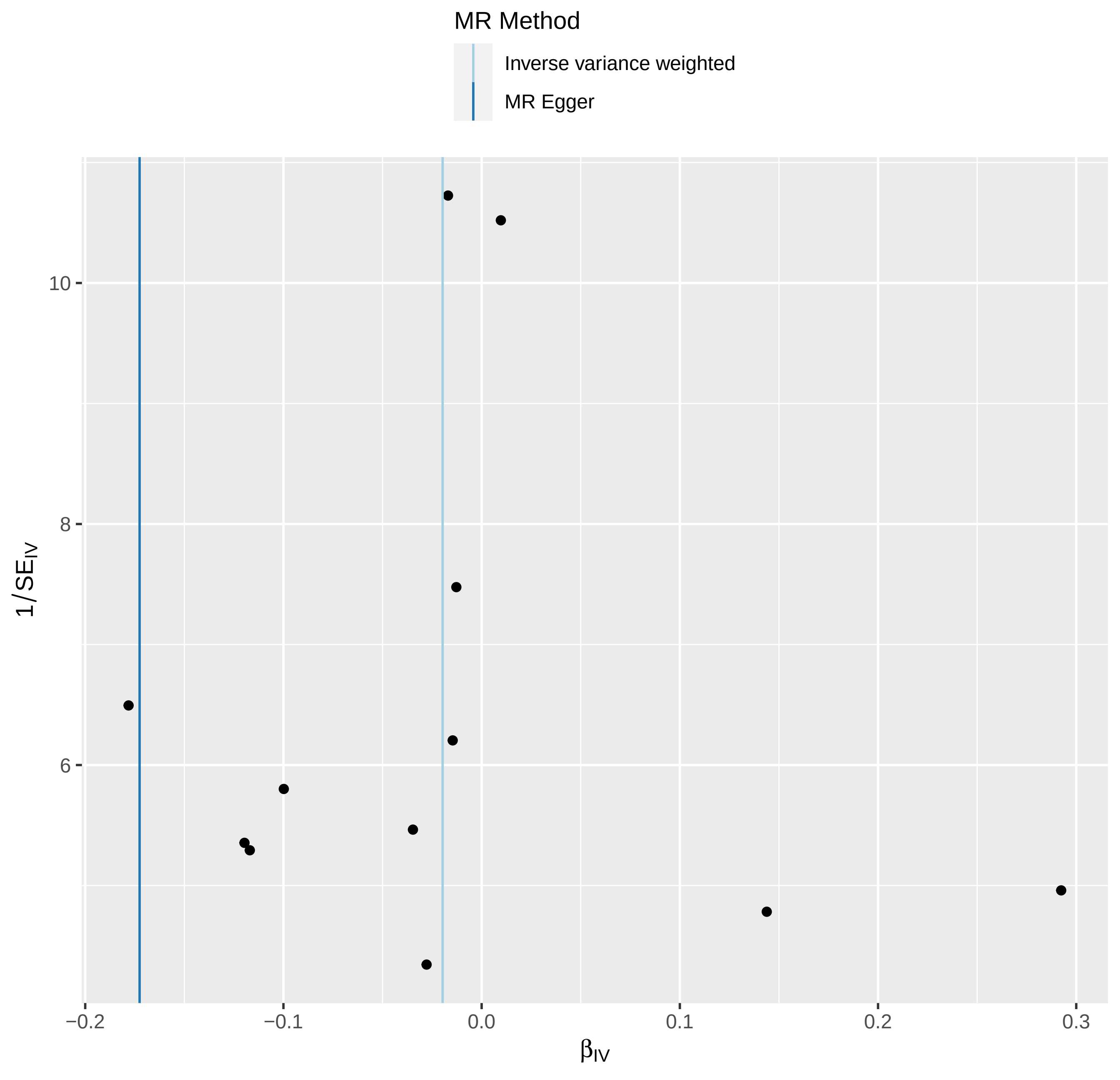

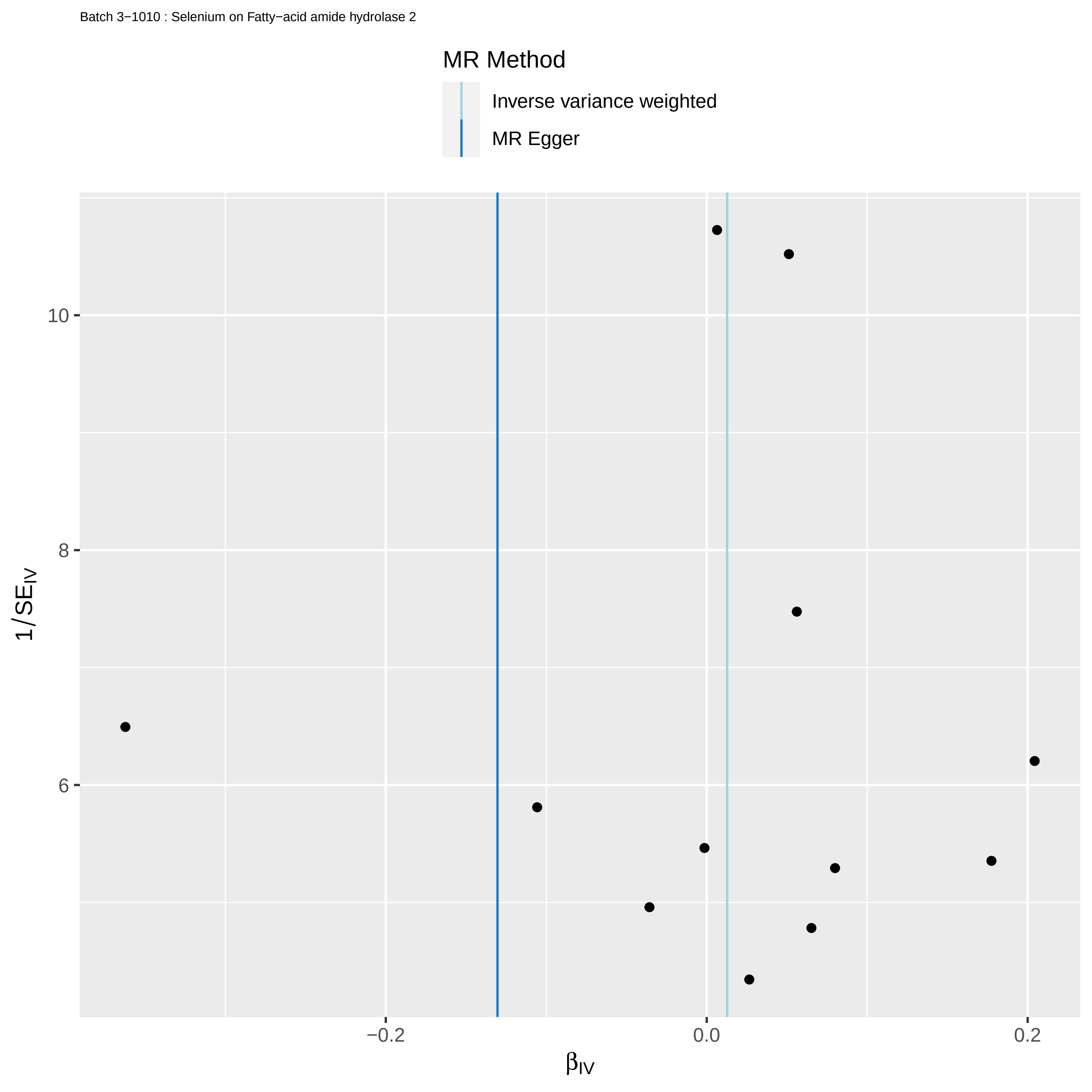

Batch 3−1010 : Selenium on Fatty−acid amide hydrolase 2
MR Method
Inverse variance weighted
MR Egger
10
8
6
1/SE_IV
−0.2
0.0
0.2
β_IV

Batch 3−1011 : Selenium on Fatty acid−binding protein, liver

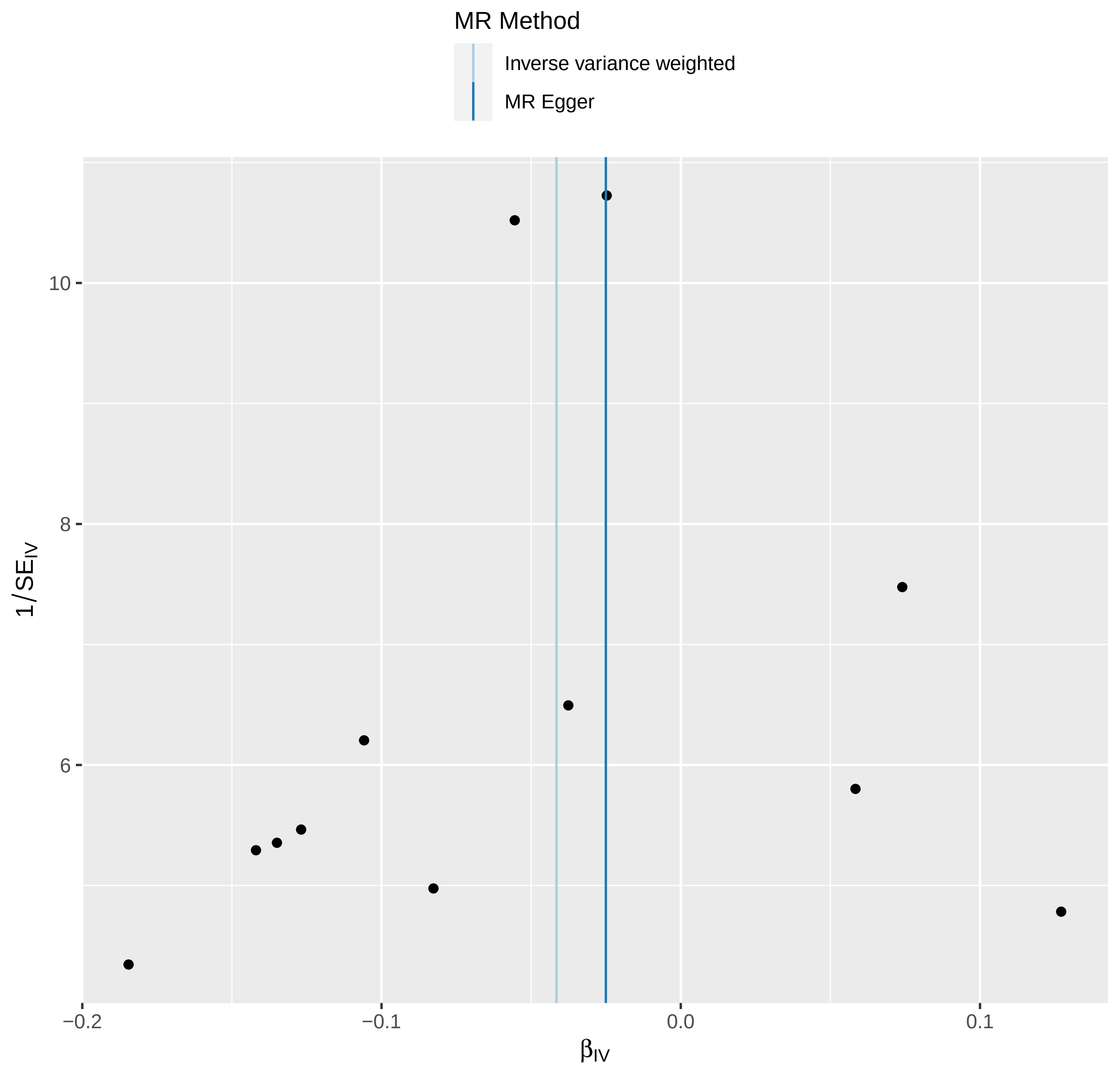

Batch 3−1012 : Selenium on Fatty acid−binding protein, heart

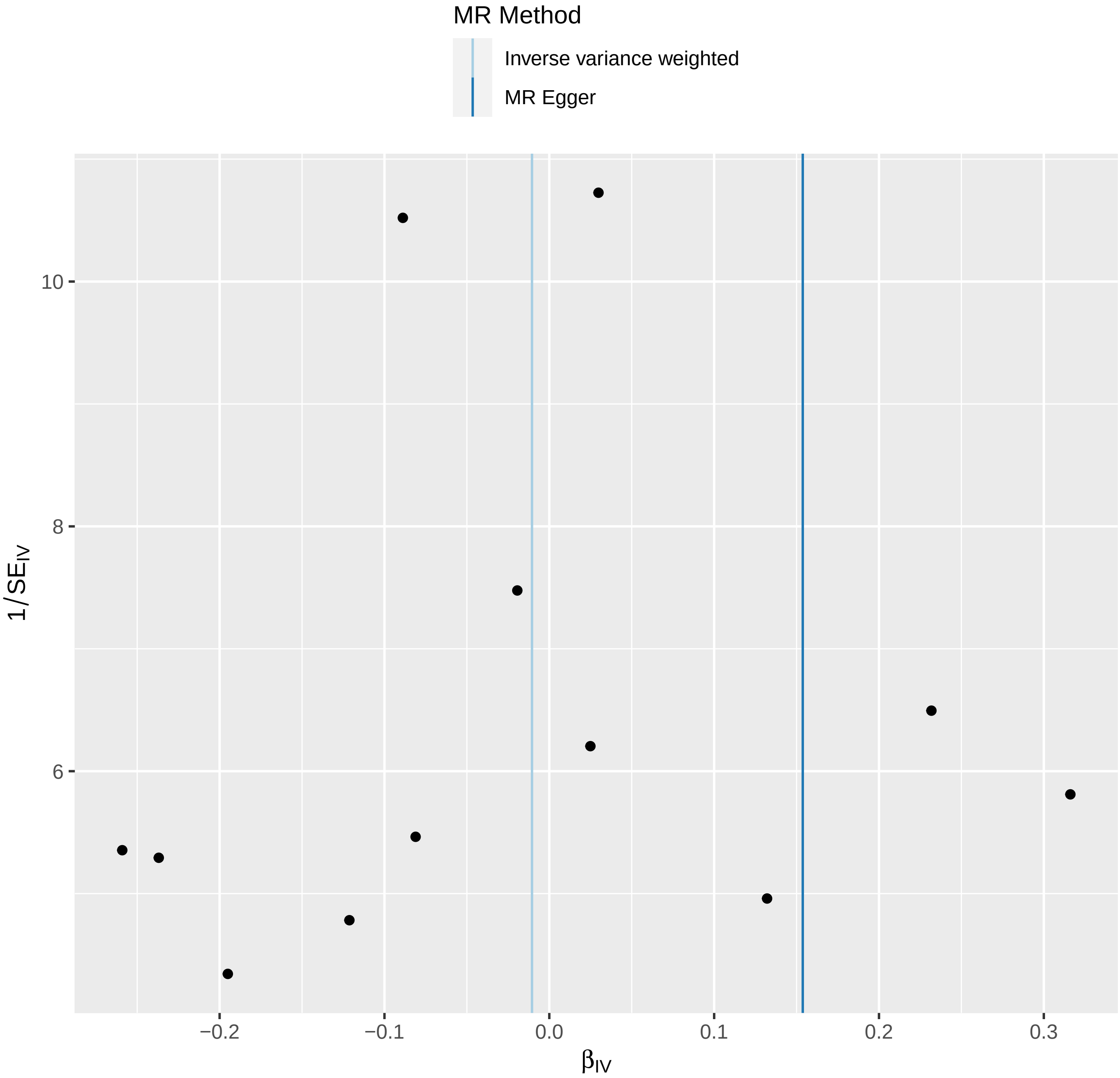

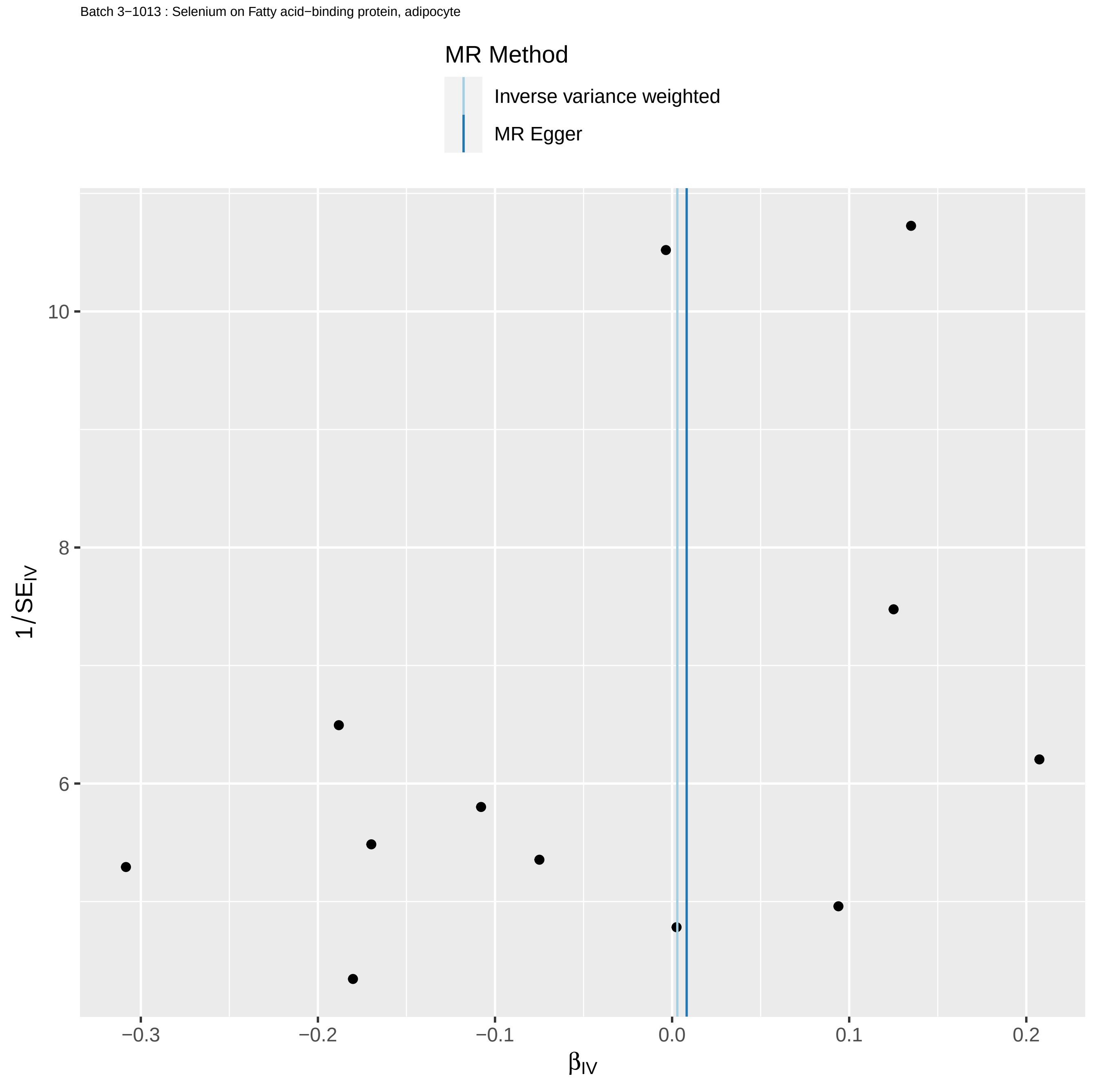

Batch 3−1013 : Selenium on Fatty acid−binding protein, adipocyte
MR Method
Inverse variance weighted
MR Egger
10
8
6
1/SE_IV
−0.3
−0.2
−0.1
0.0
0.1
0.2
β_IV

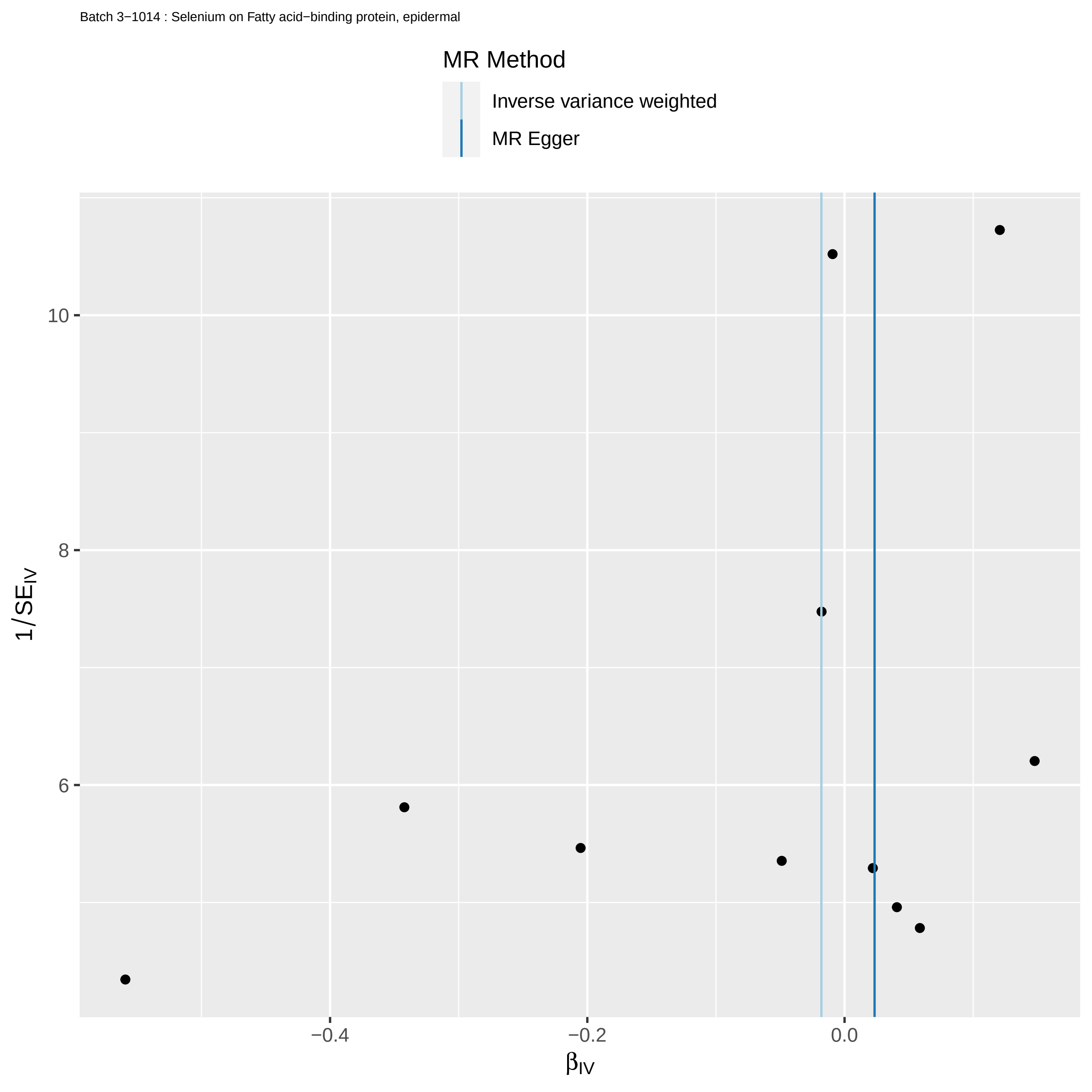
Batch 3−1014 : Selenium on Fatty acid−binding protein, epidermal
MR Method
Inverse variance weighted
MR Egger
10
8
6
1/SE_IV
−0.4
−0.2
0.0
β_IV

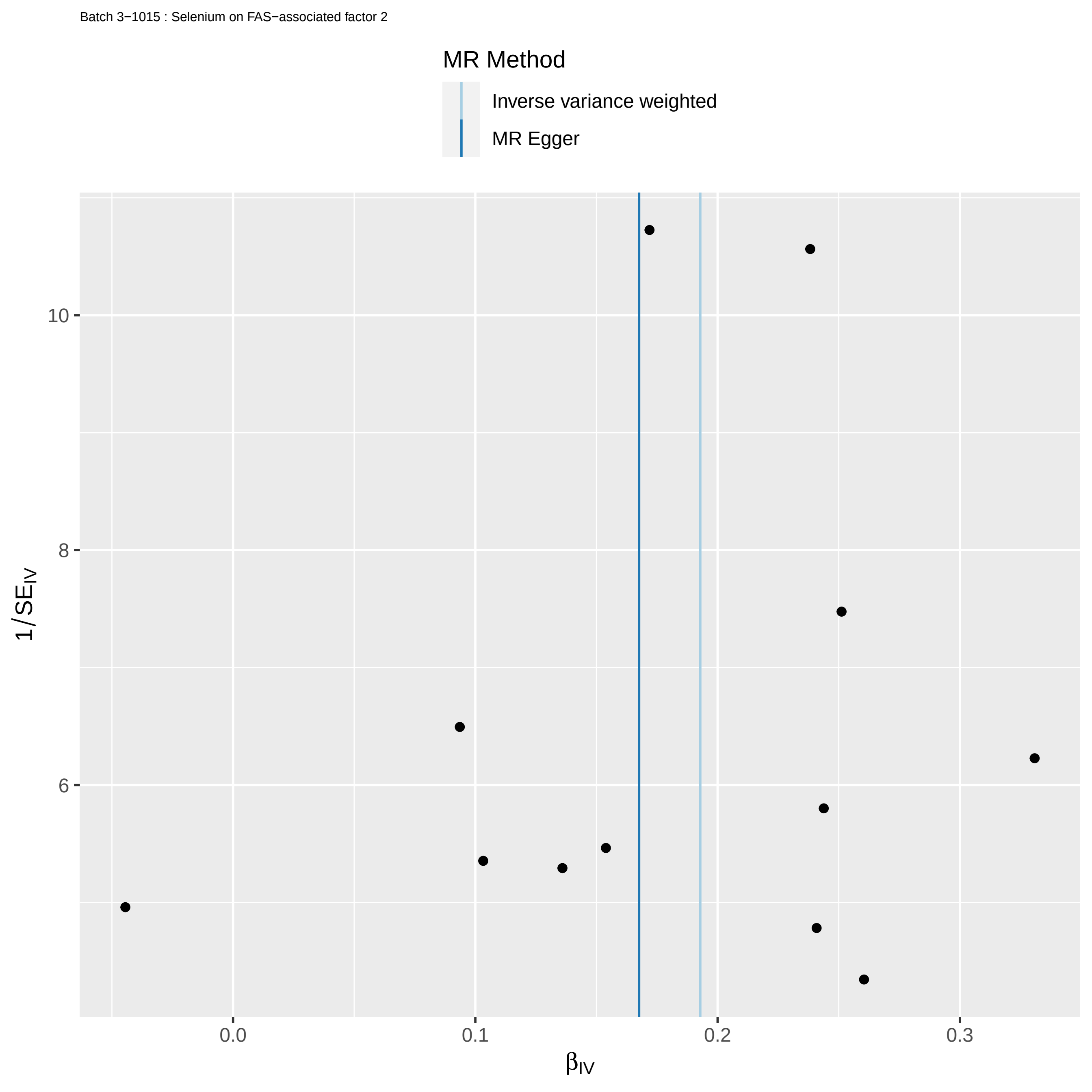
Batch 3−1015 : Selenium on FAS−associated factor 2
MR Method
Inverse variance weighted
MR Egger
10
8
6
1/SE_IV
0.0
0.1
0.2
0.3
β_IV

Batch 3−1016 : Selenium on FAS−associated factor 2

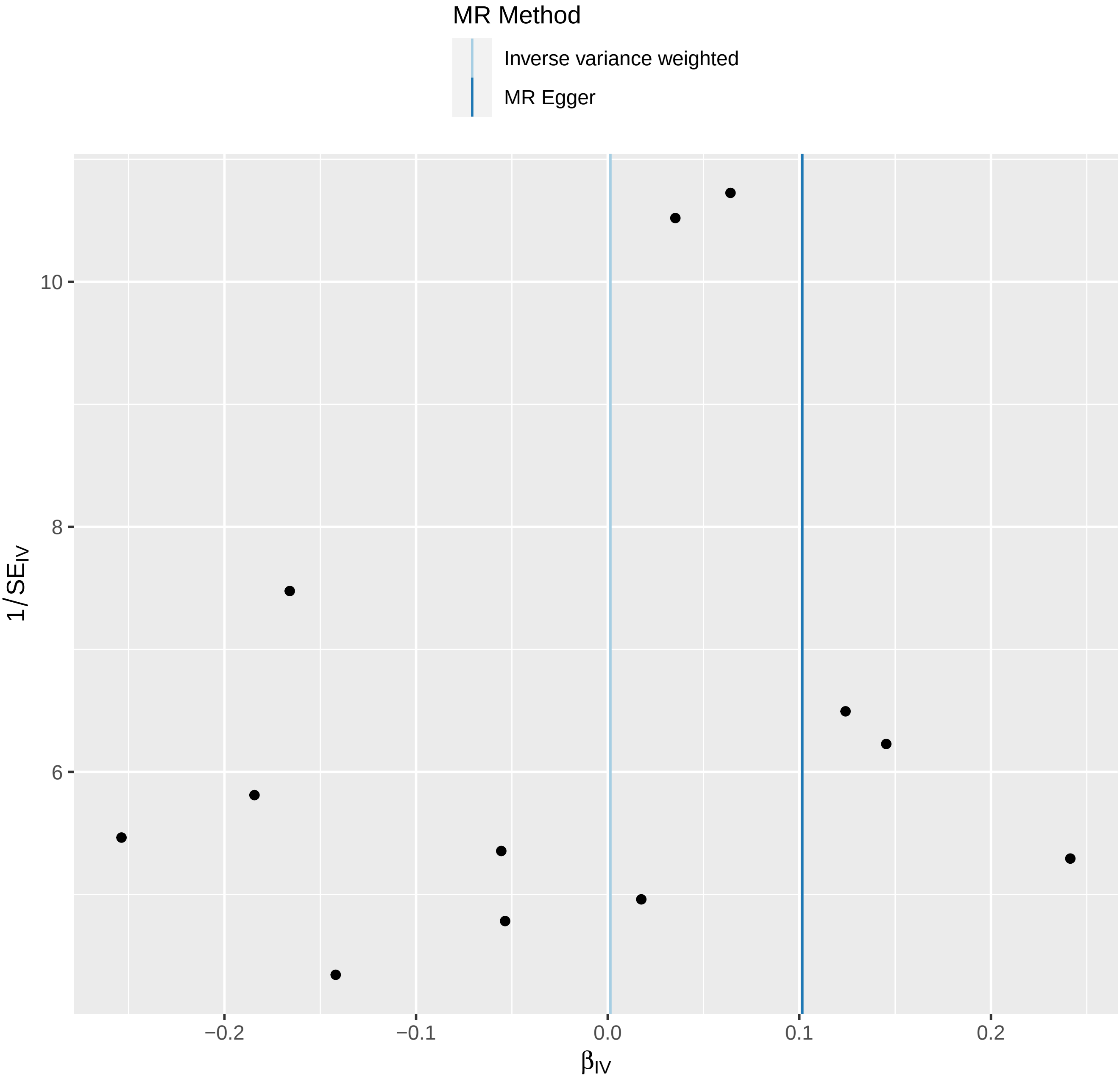

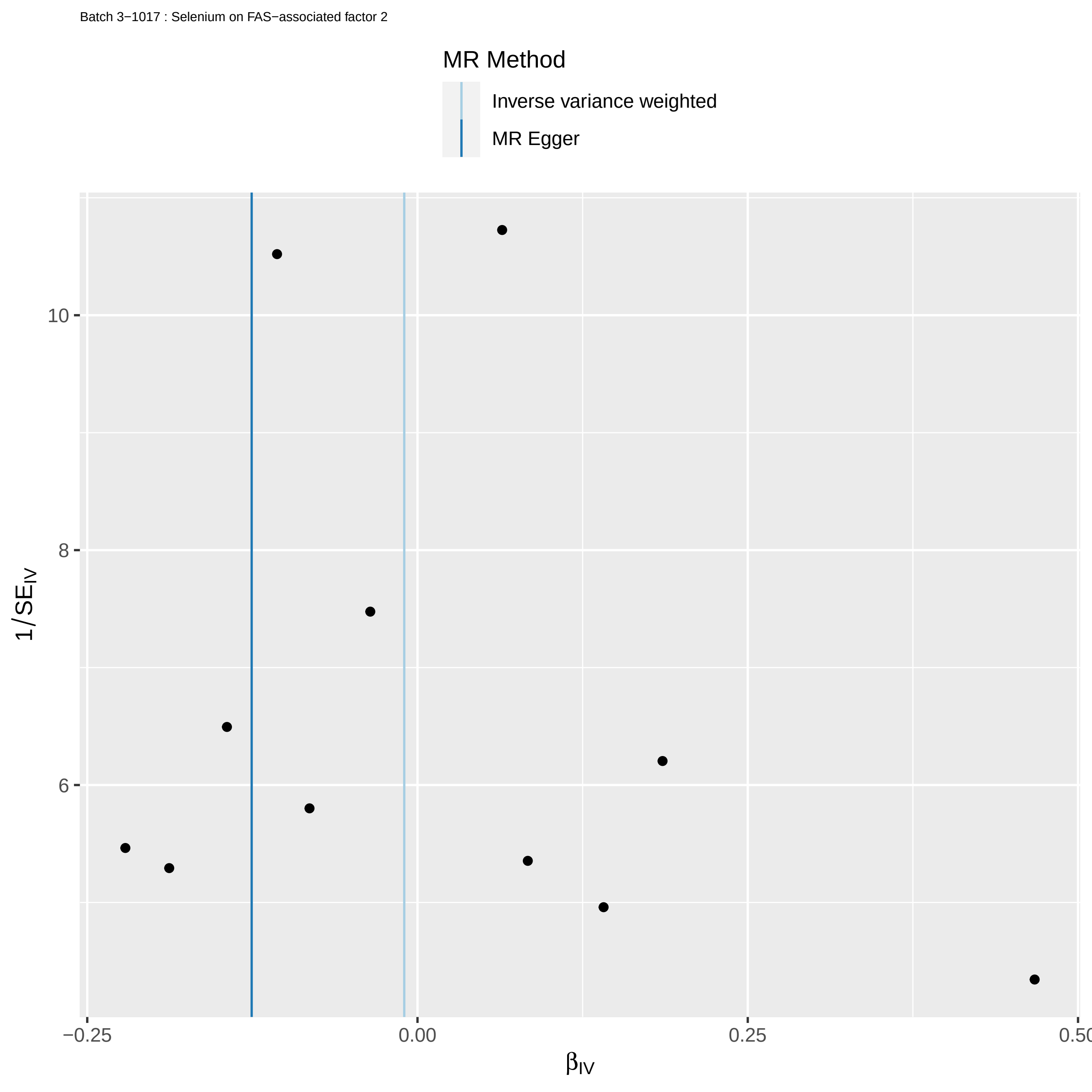
Batch 3−1017 : Selenium on FAS−associated factor 2
MR Method
Inverse variance weighted
MR Egger
10
8
6
1/SE_IV
−0.25
0.00
0.25
0.50
β_IV

Batch 3−1018 : Selenium on Fumarylacetoacetase

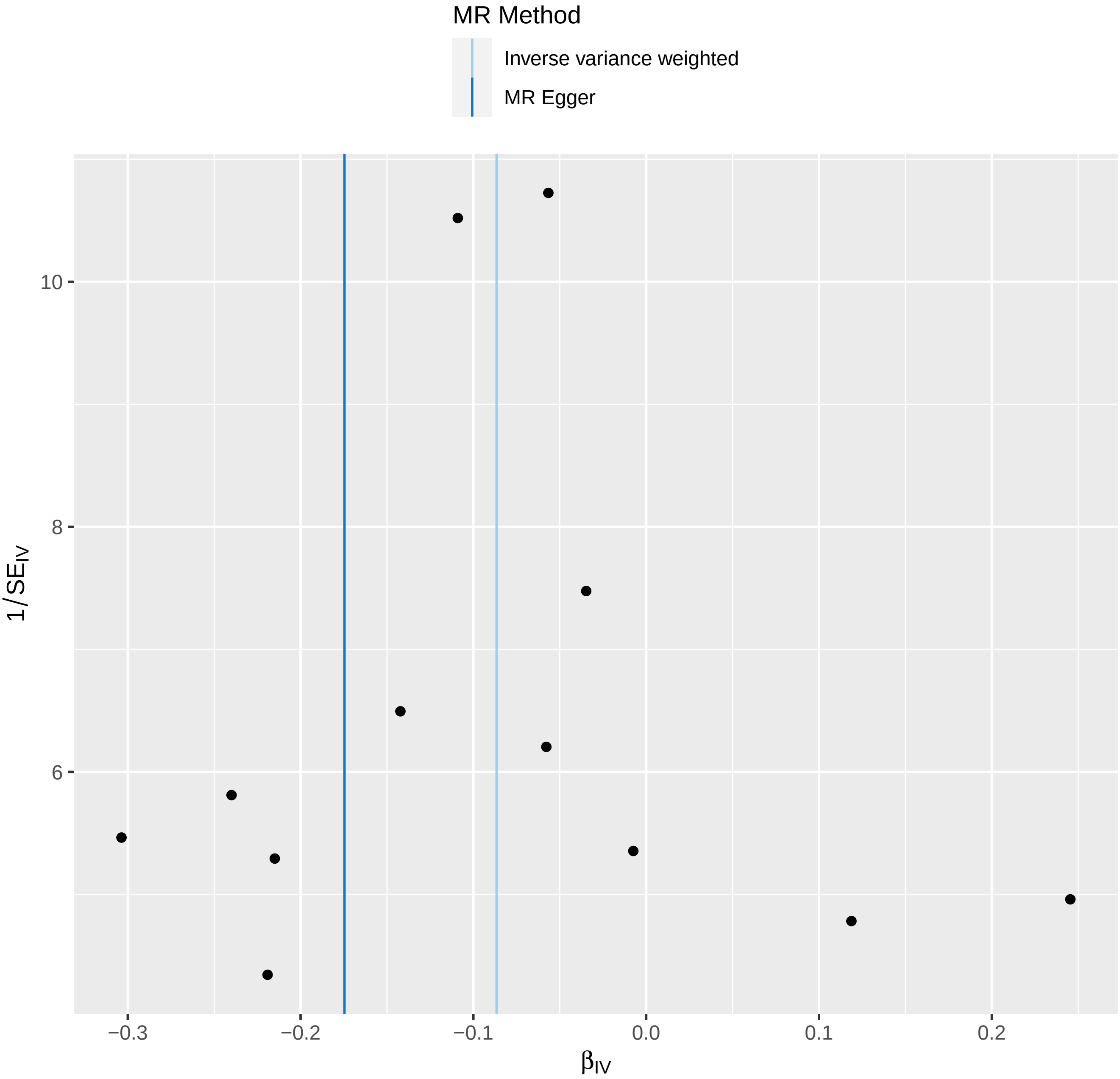

Batch 3−1019 : Selenium on Fas apoptotic inhibitory molecule 3

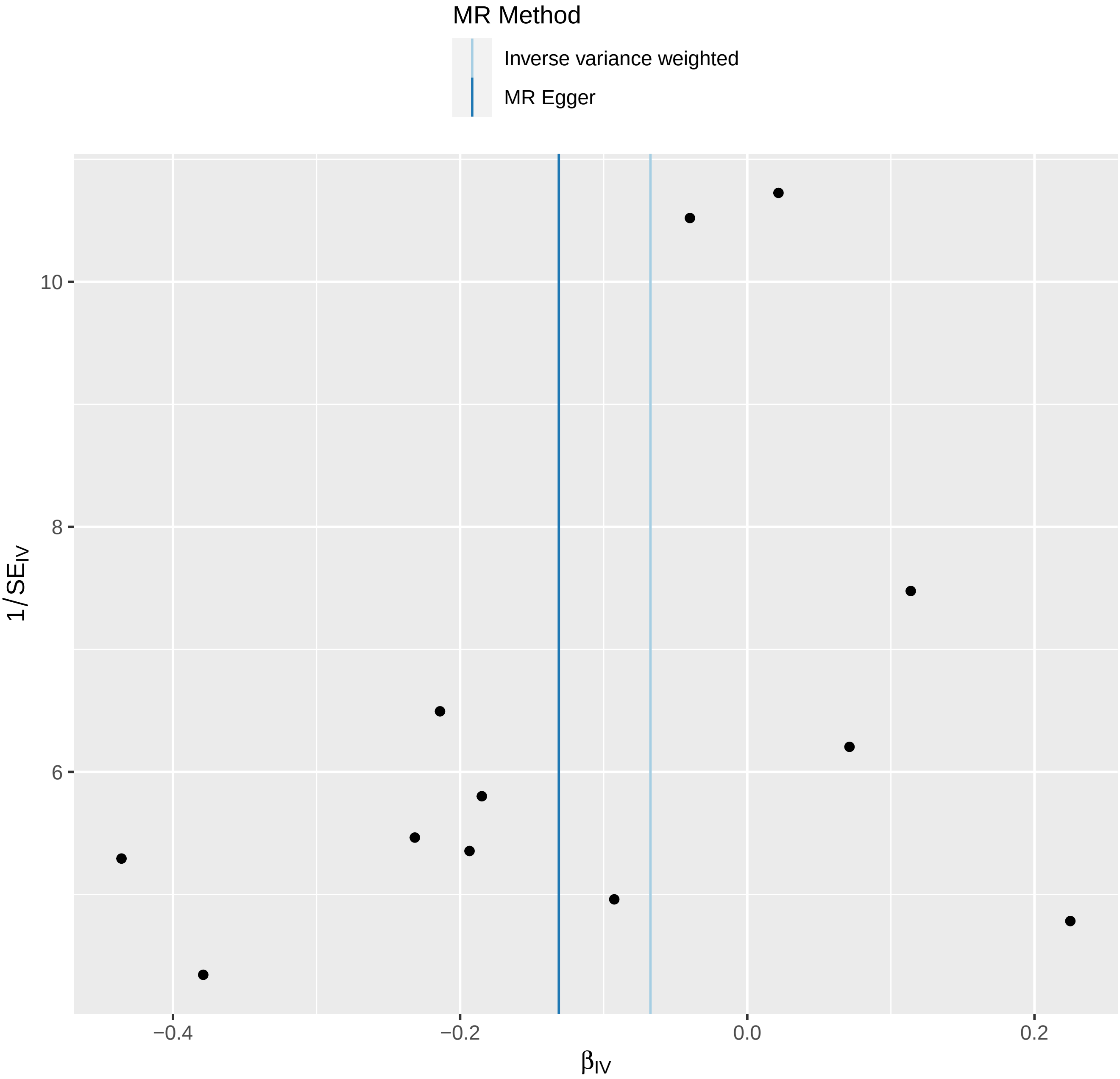

Batch 3−1020 : Selenium on Protein FAM107A

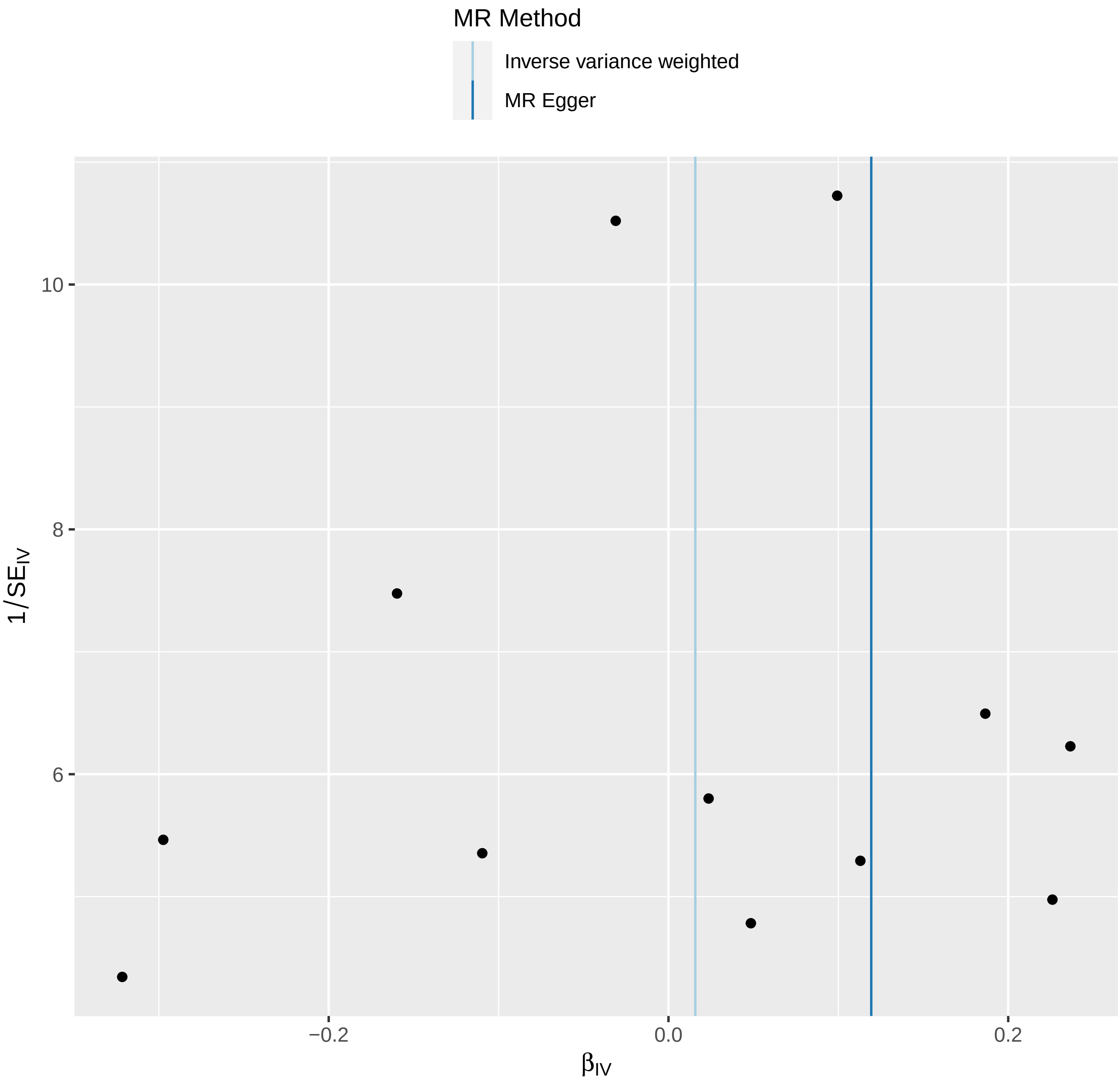

Batch 3−1021 : Selenium on Protein FAM107B

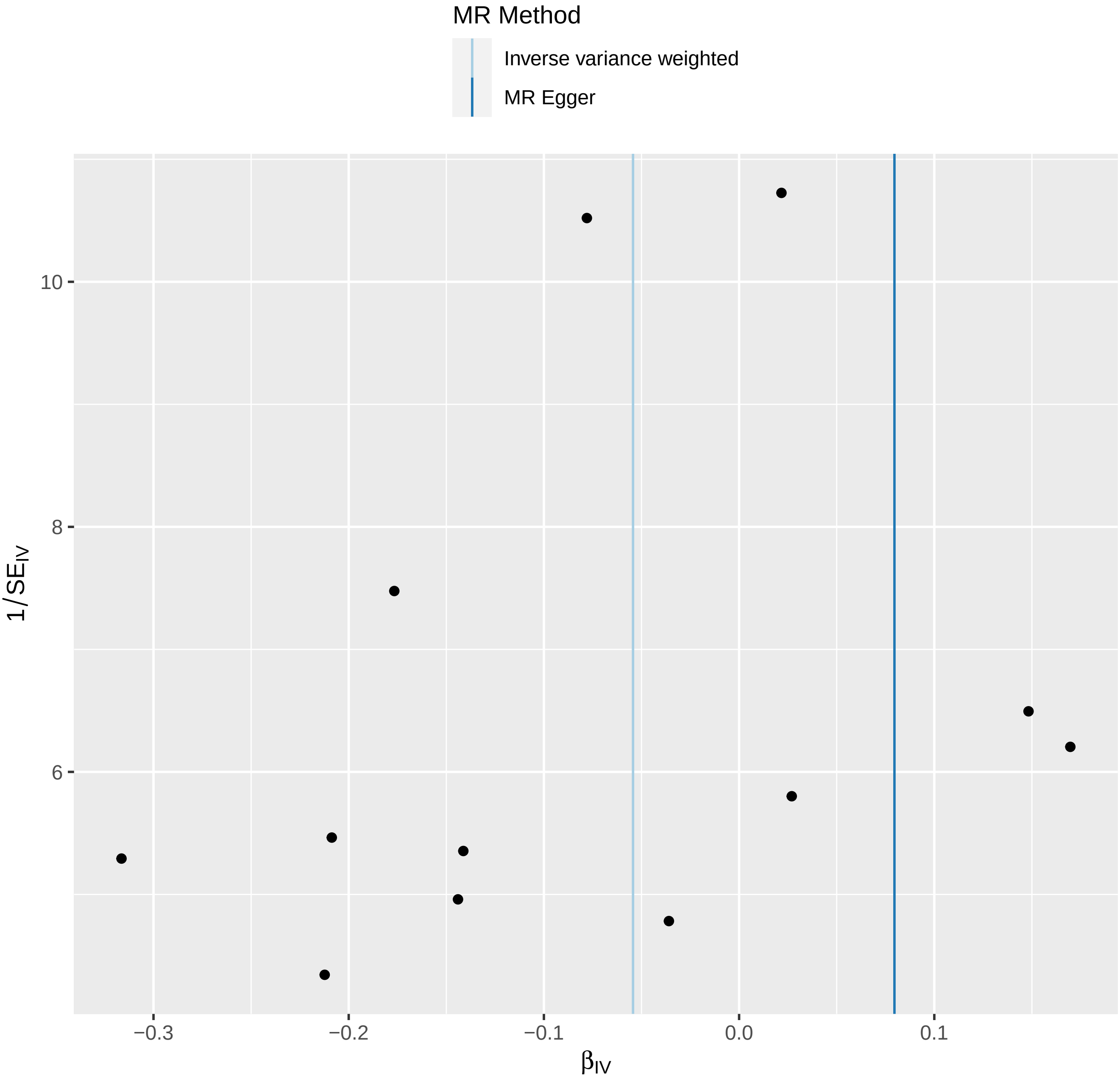

Batch 3−1022 : Selenium on Protein FAM107B

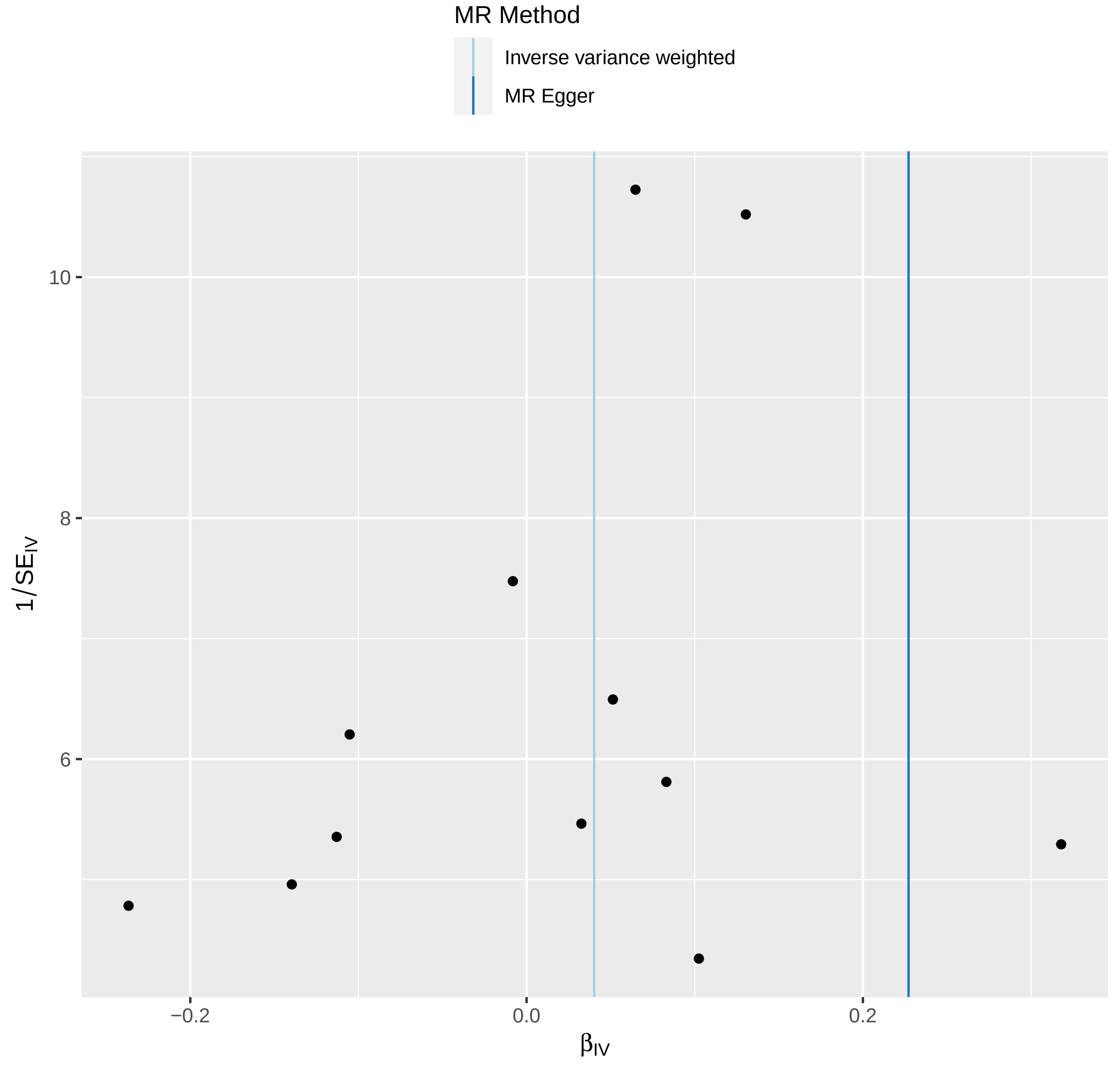

Batch 3−1023 : Selenium on Protein FAM134B

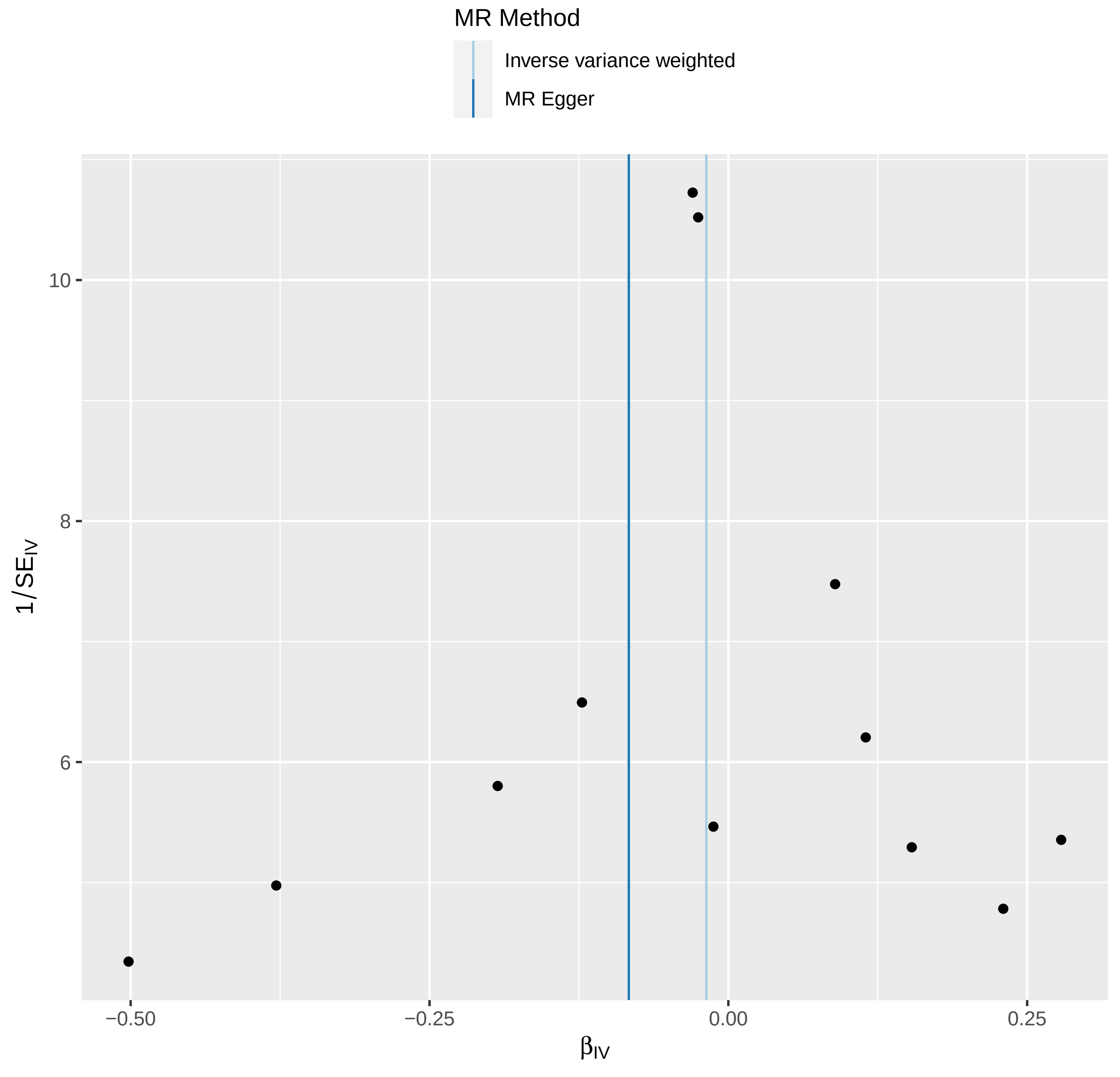

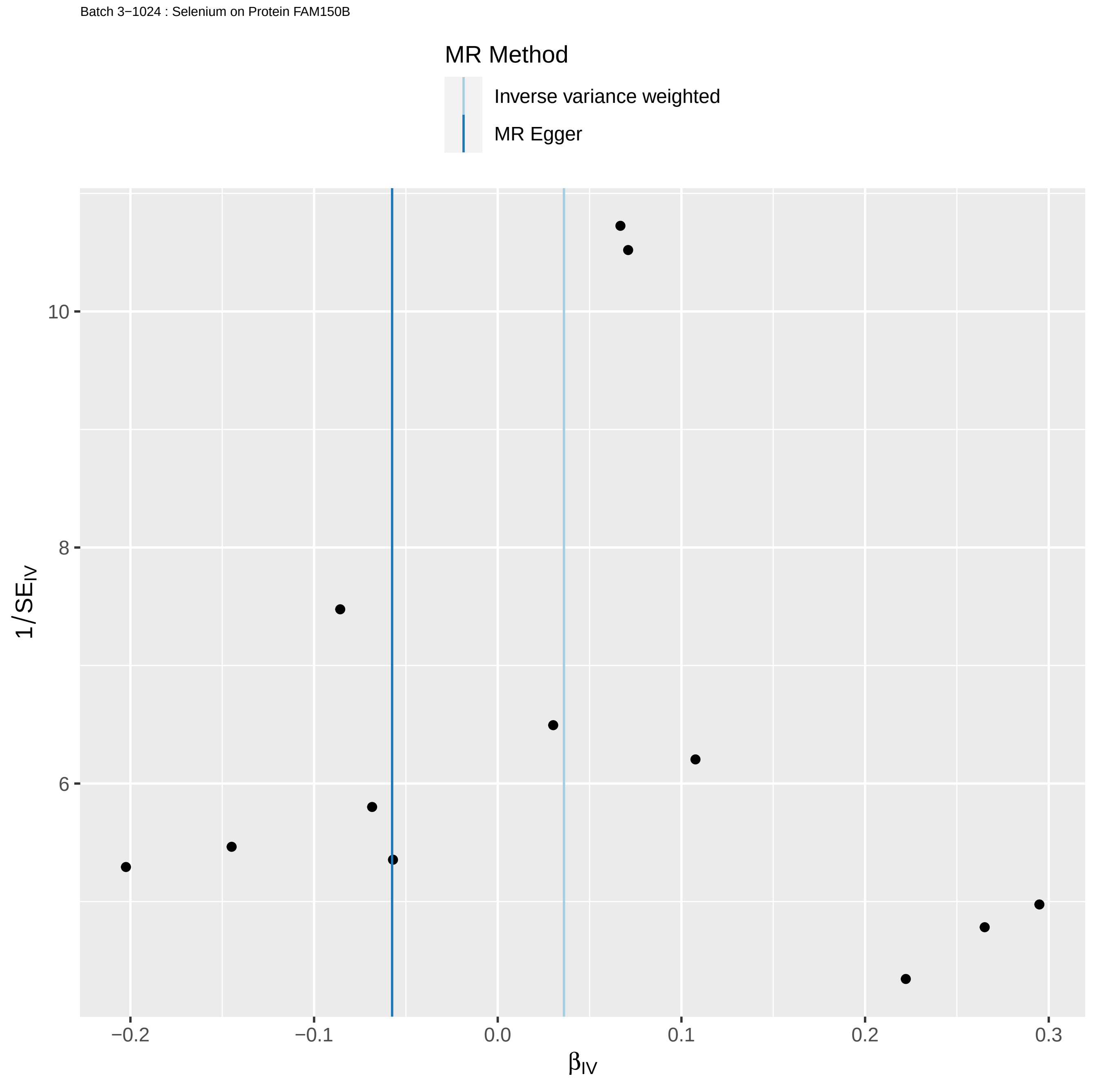

Batch 3−1024 : Selenium on Protein FAM150B
MR Method
Inverse variance weighted
MR Egger
10
8
6
1/SE_IV
−0.2
−0.1
0.0
0.1
0.2
0.3
β_IV

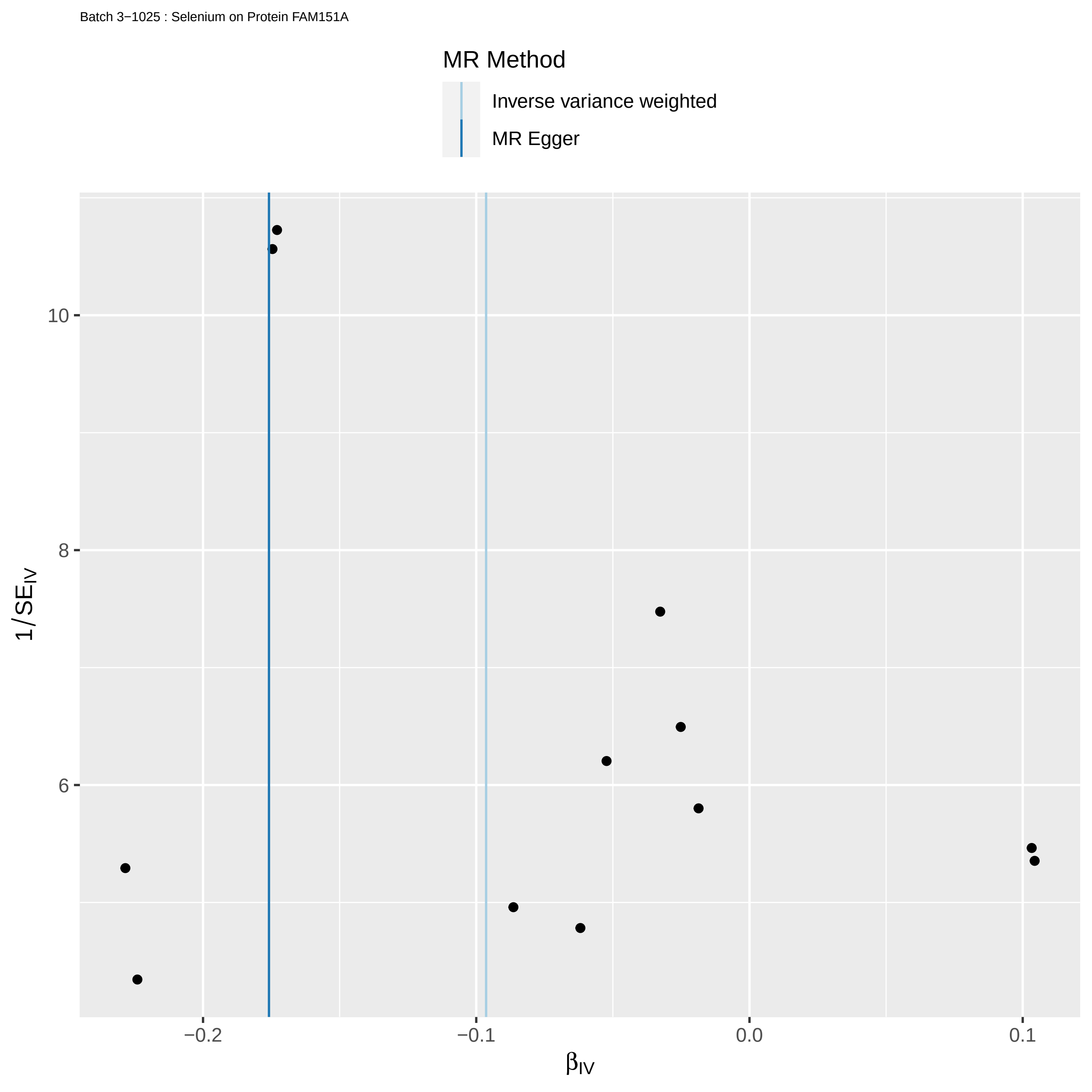

Batch 3−1025 : Selenium on Protein FAM151A
MR Method
Inverse variance weighted
MR Egger
10
8
6
1/SE_IV
−0.2
−0.1
0.0
0.1
β_IV

Batch 3−1026 : Selenium on Membrane protein FAM159B

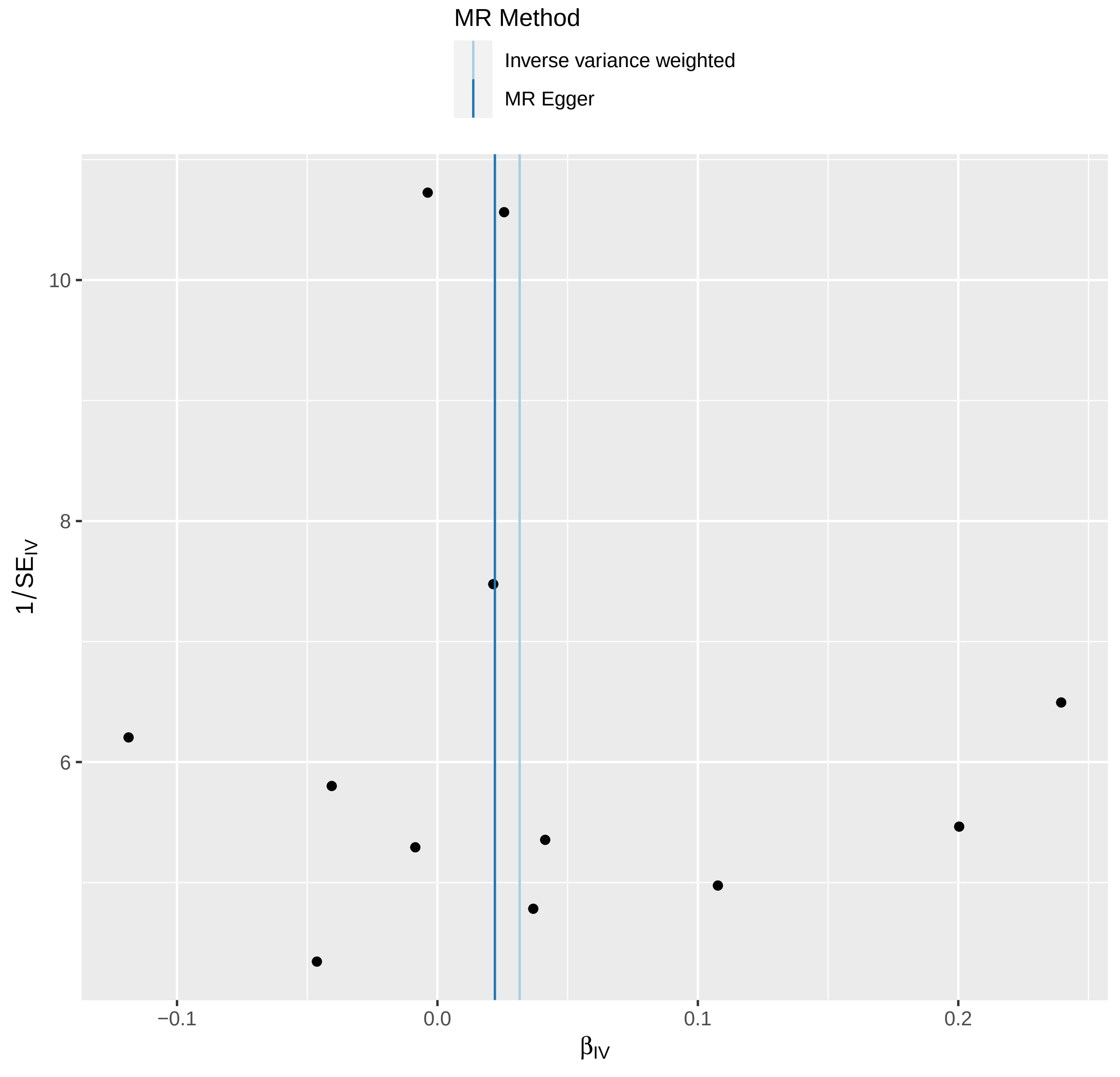

Batch 3−1027 : Selenium on Protein FAM163A

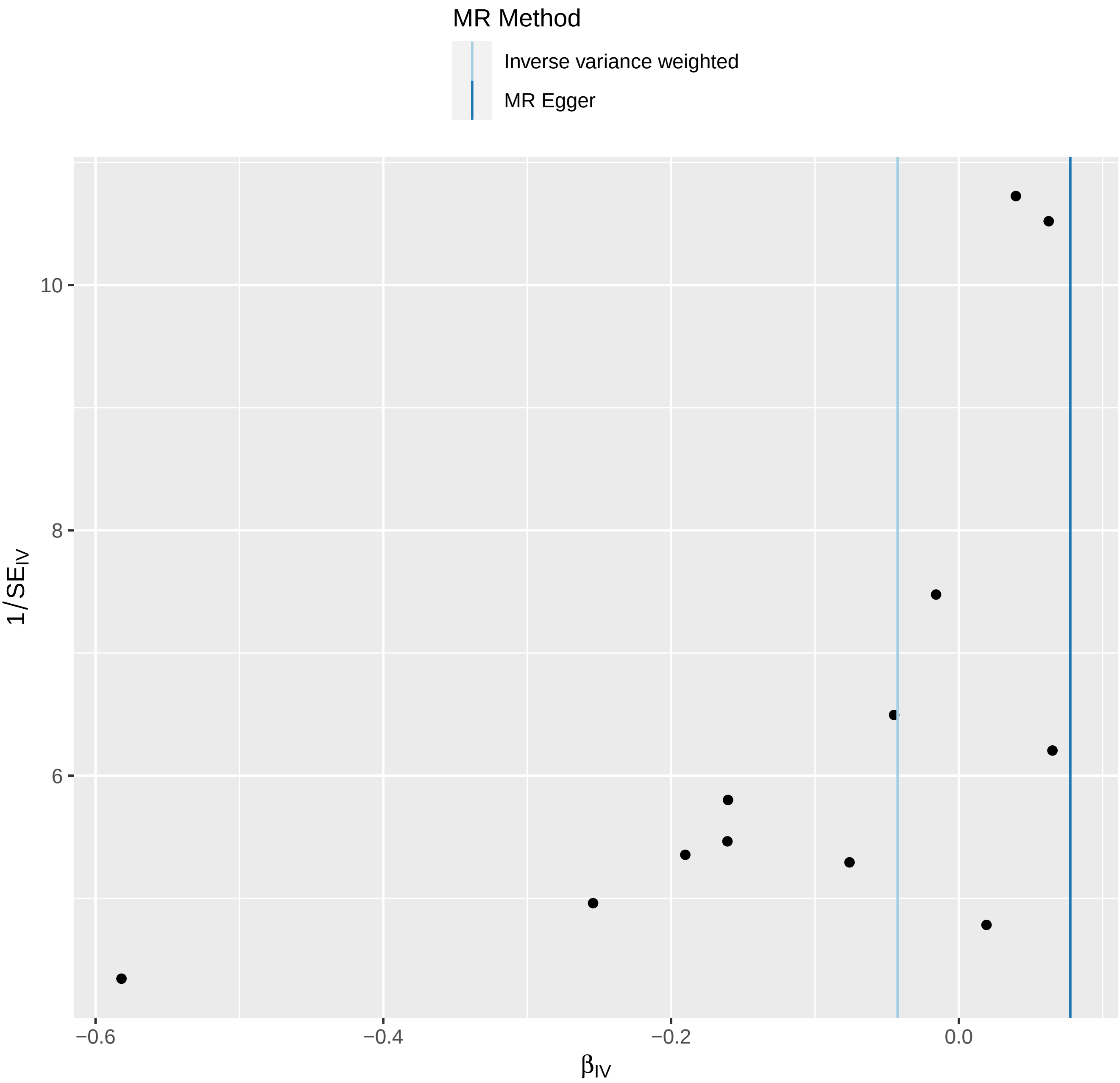

Batch 3−1028 : Selenium on Protein FAM163B

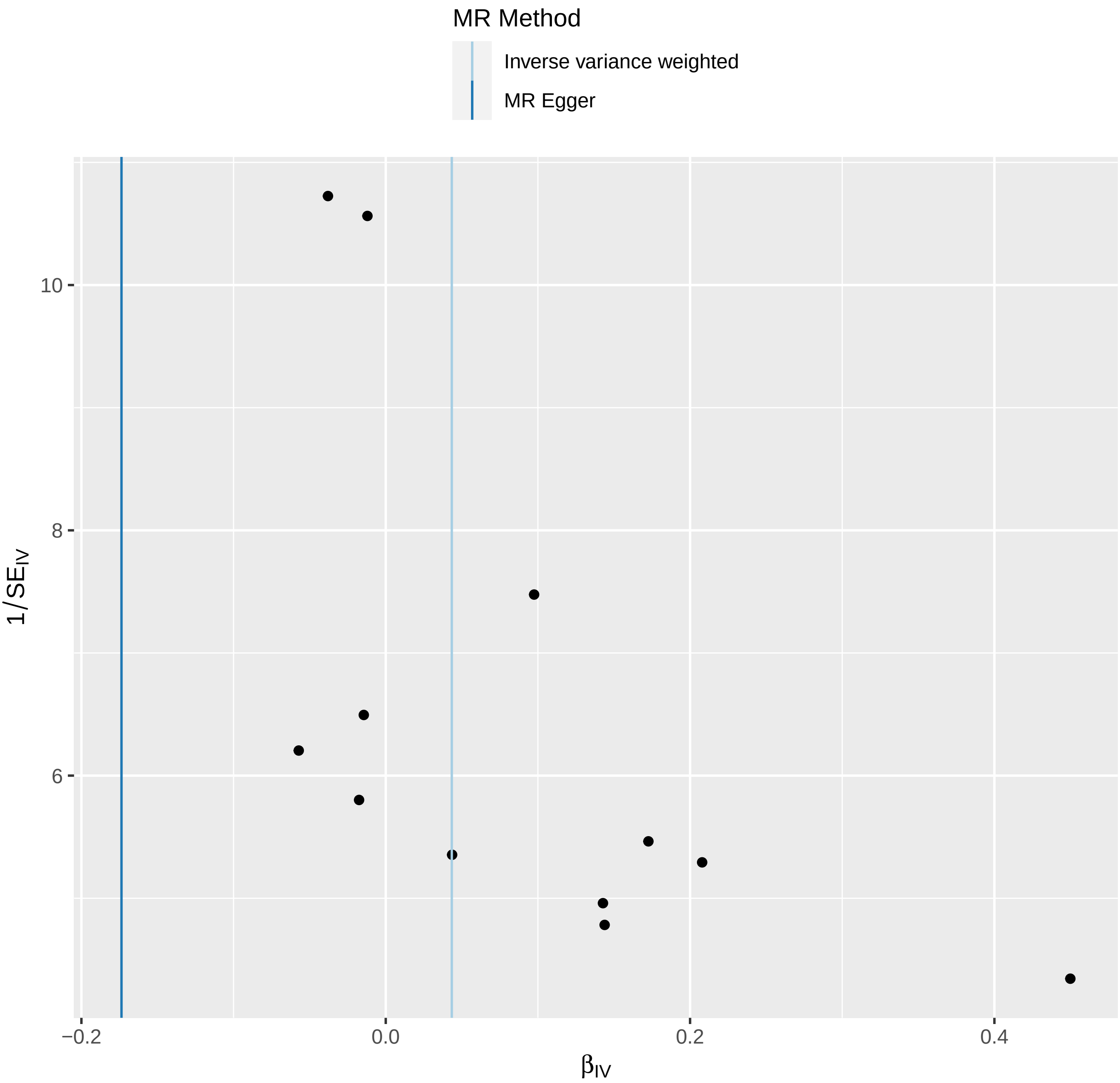

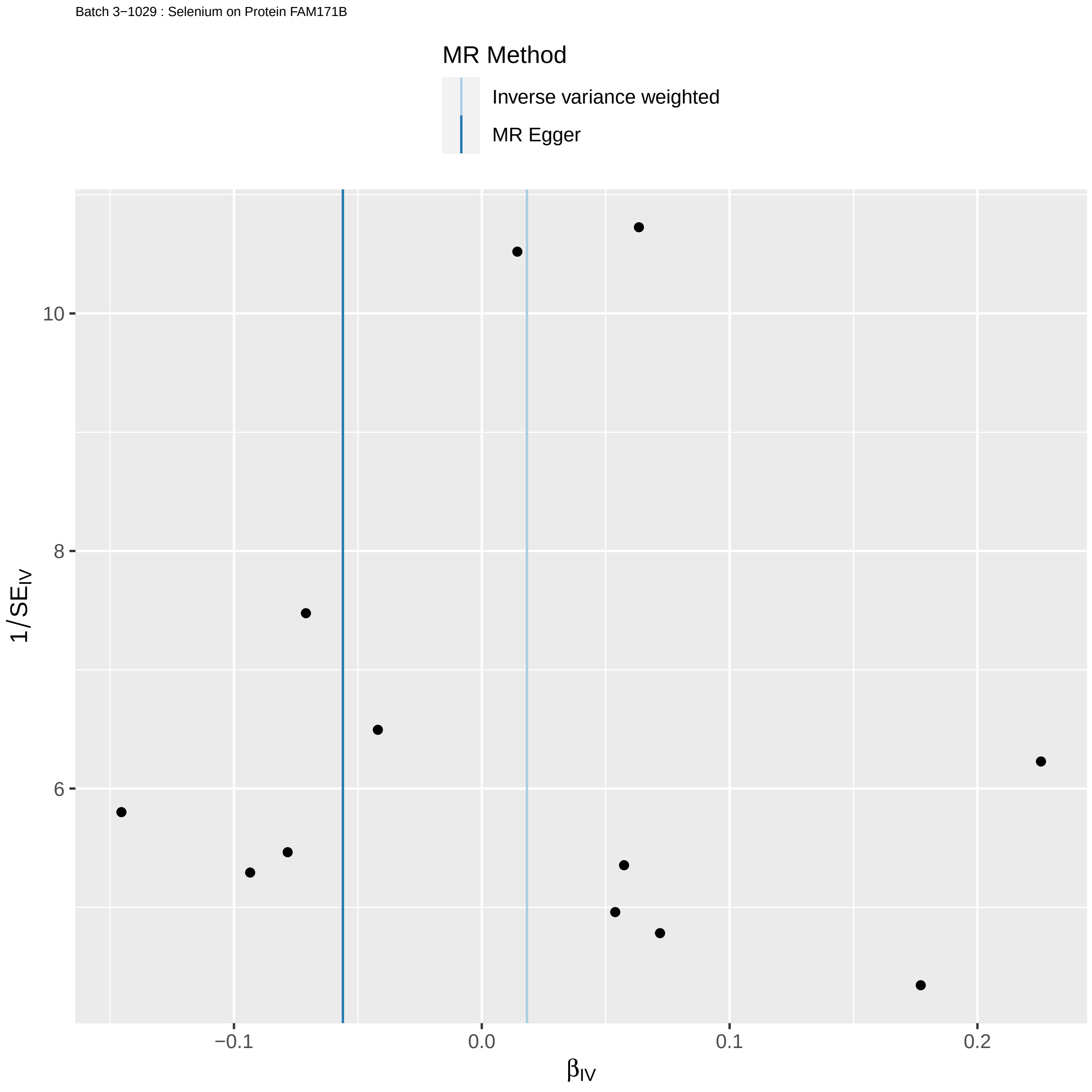
Batch 3−1029 : Selenium on Protein FAM171B
MR Method
Inverse variance weighted
MR Egger
10
8
6
1/SE_IV
−0.1
0.0
0.1
0.2
β_IV

Batch 3−1030 : Selenium on Protein FAM171B

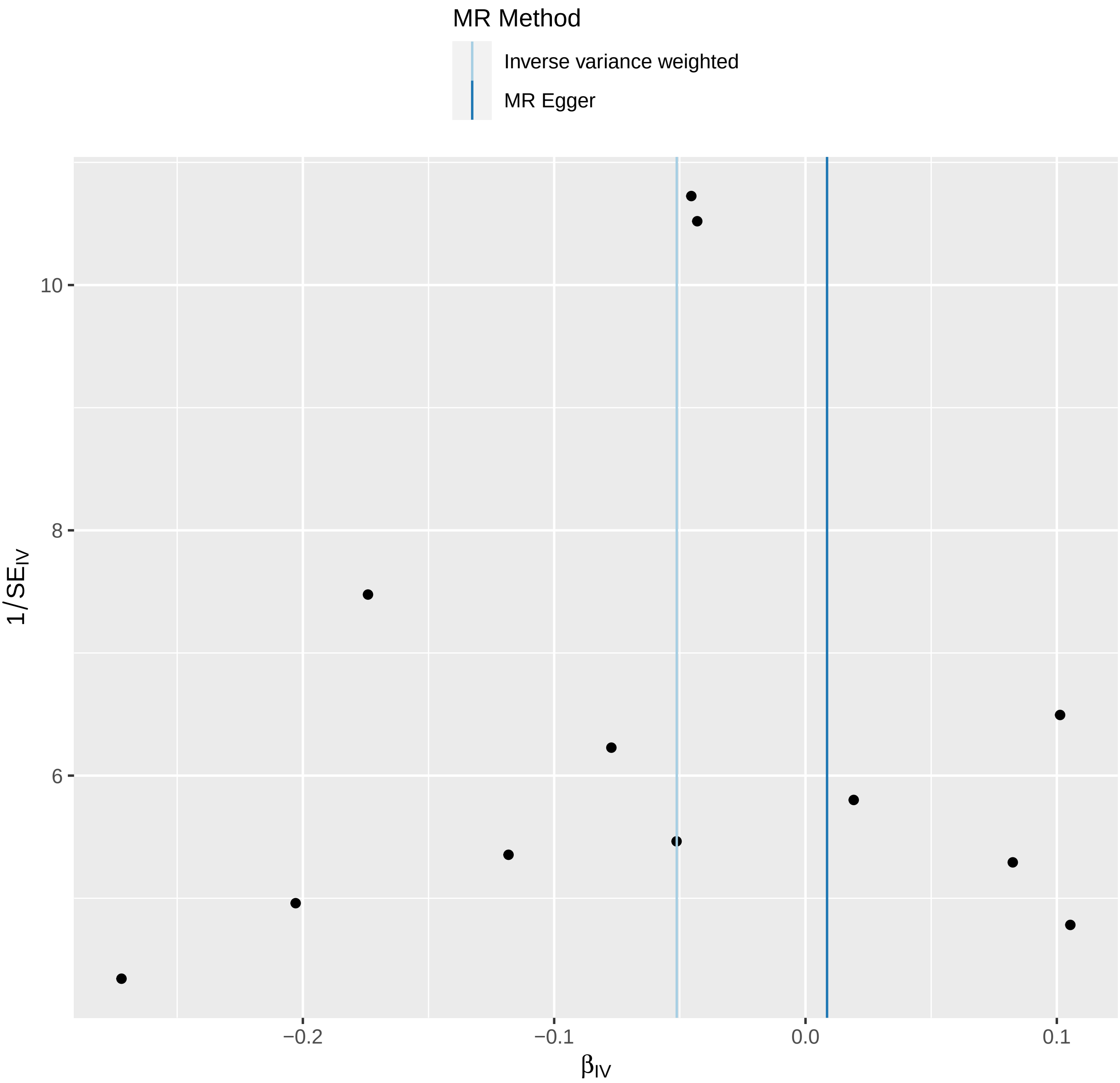

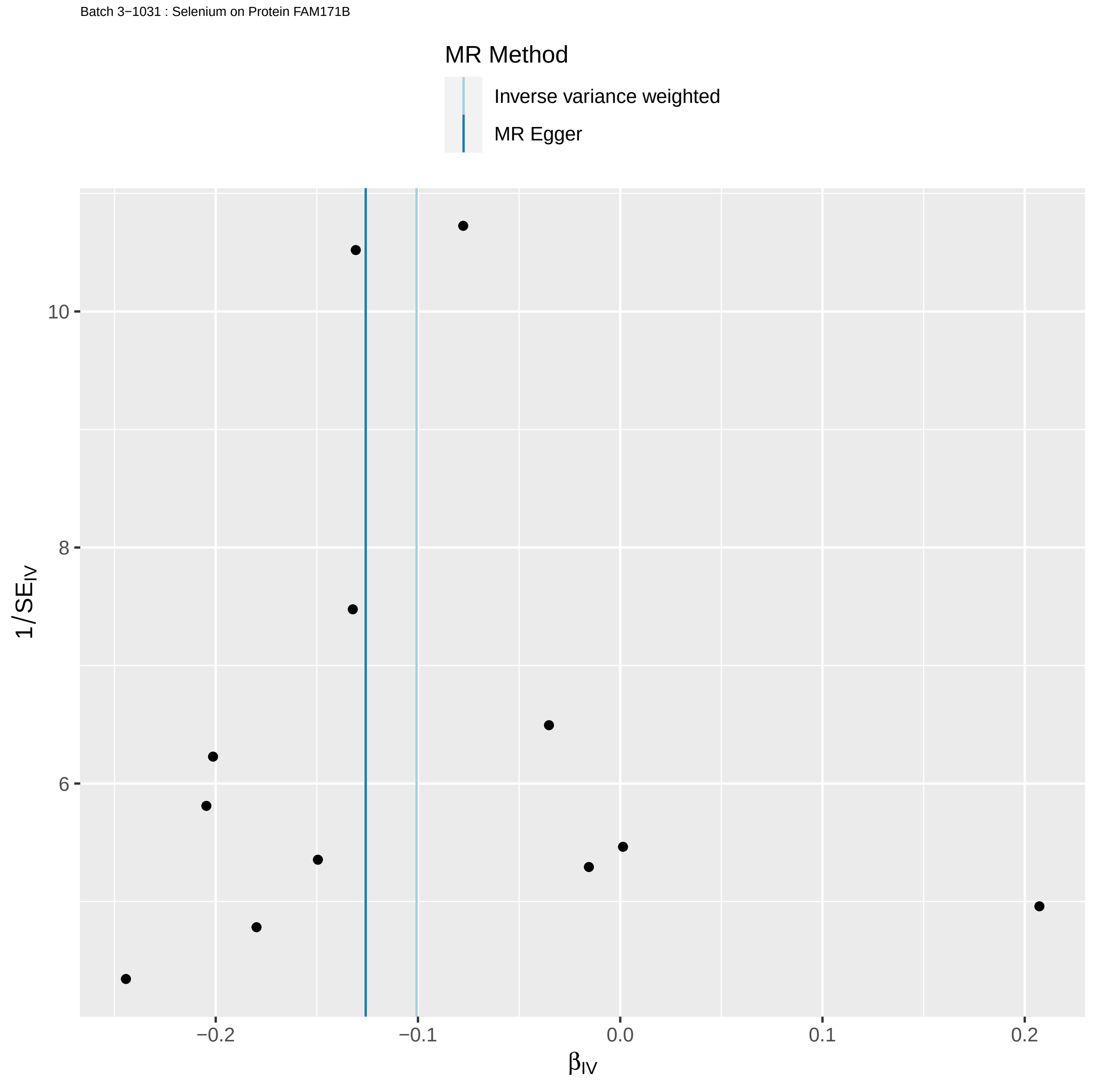

Batch 3−1031 : Selenium on Protein FAM171B
MR Method
Inverse variance weighted
MR Egger
10
8
6
1/SE_IV
−0.2
−0.1
0.0
0.1
0.2
β_IV

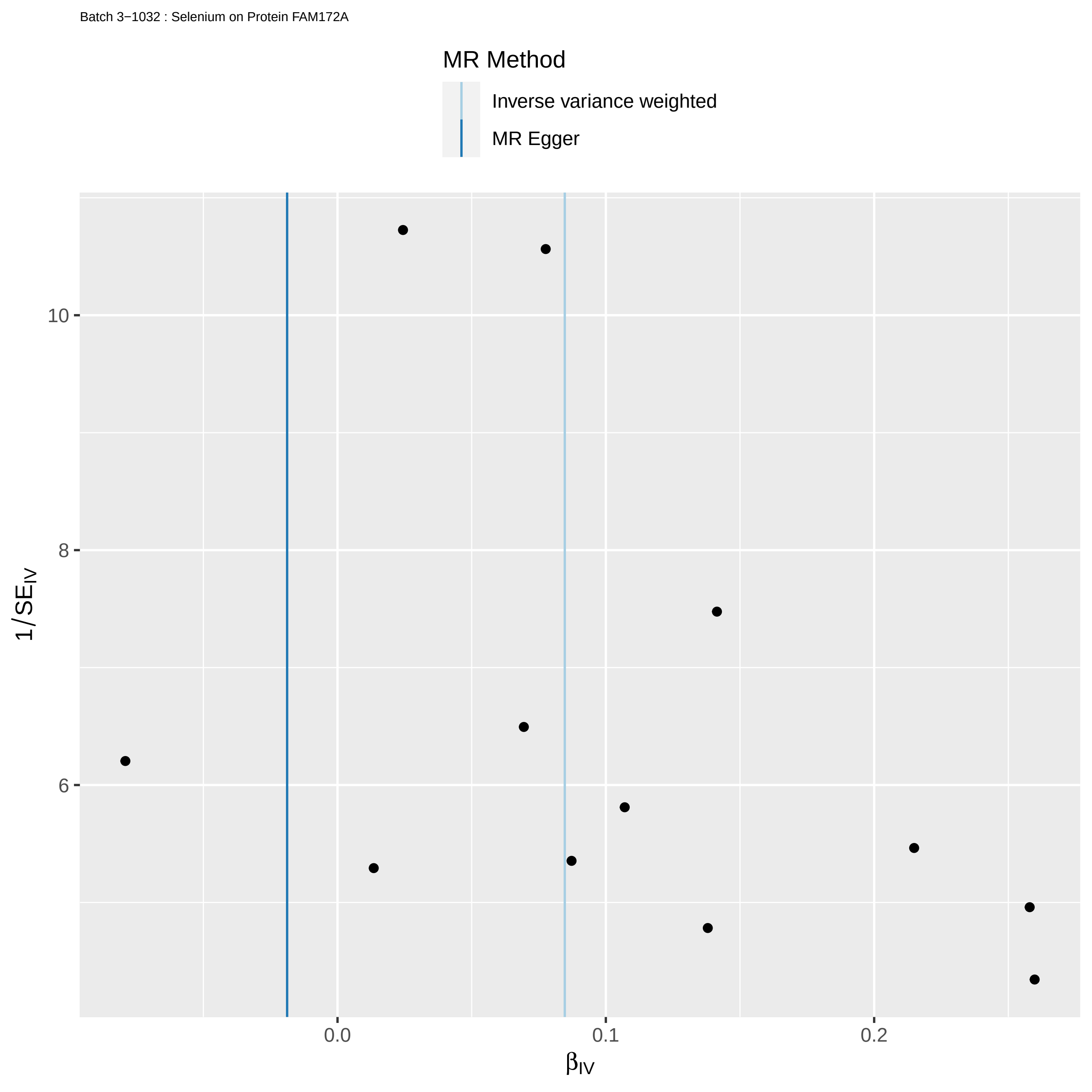

Batch 3−1032 : Selenium on Protein FAM172A
MR Method
Inverse variance weighted
MR Egger
10
8
6
1/SE_IV
0.0
0.1
0.2
β_IV

Batch 3−1033 : Selenium on Protein FAM173A

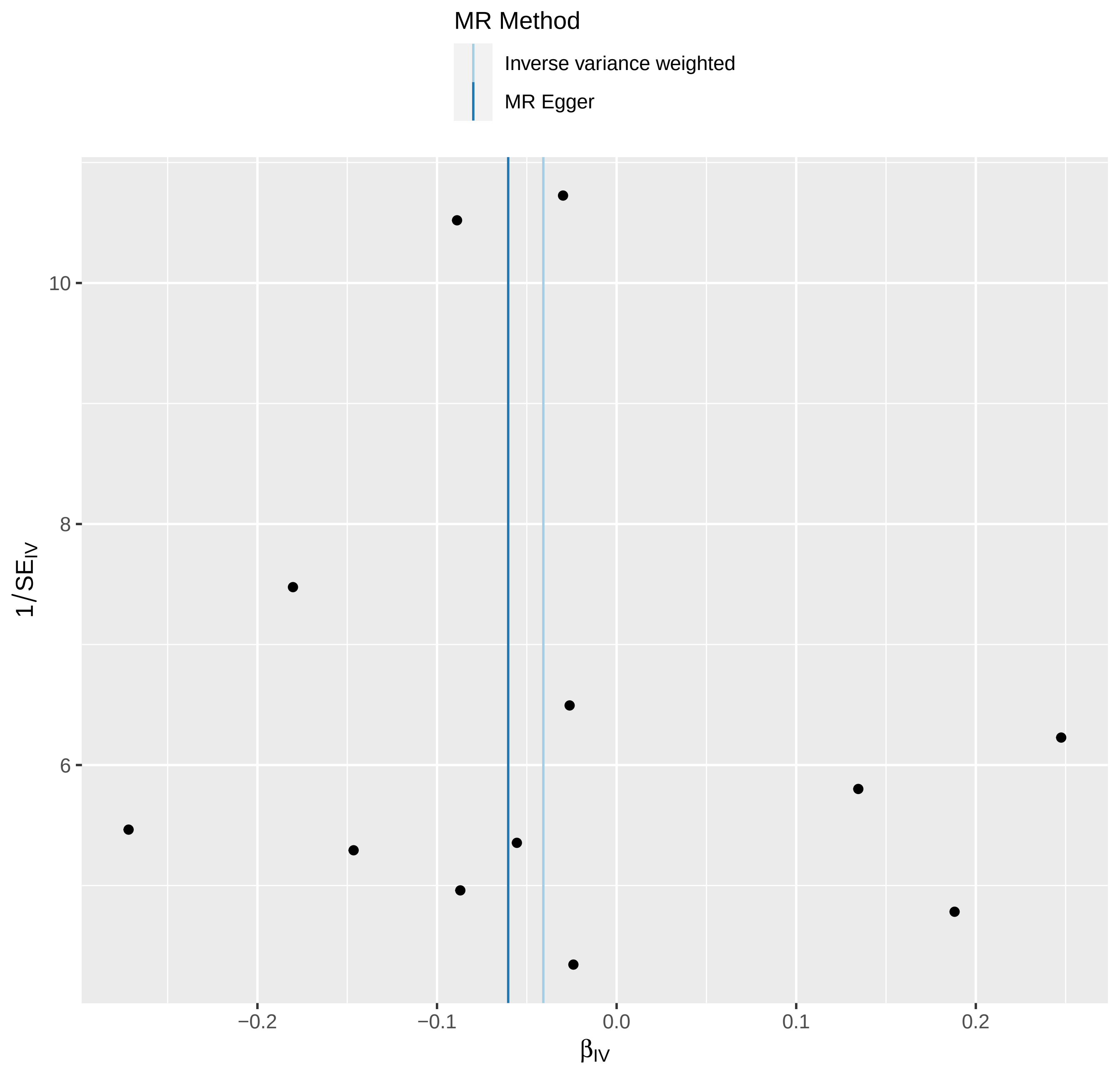

Batch 3−1034 : Selenium on Membrane protein FAM174A

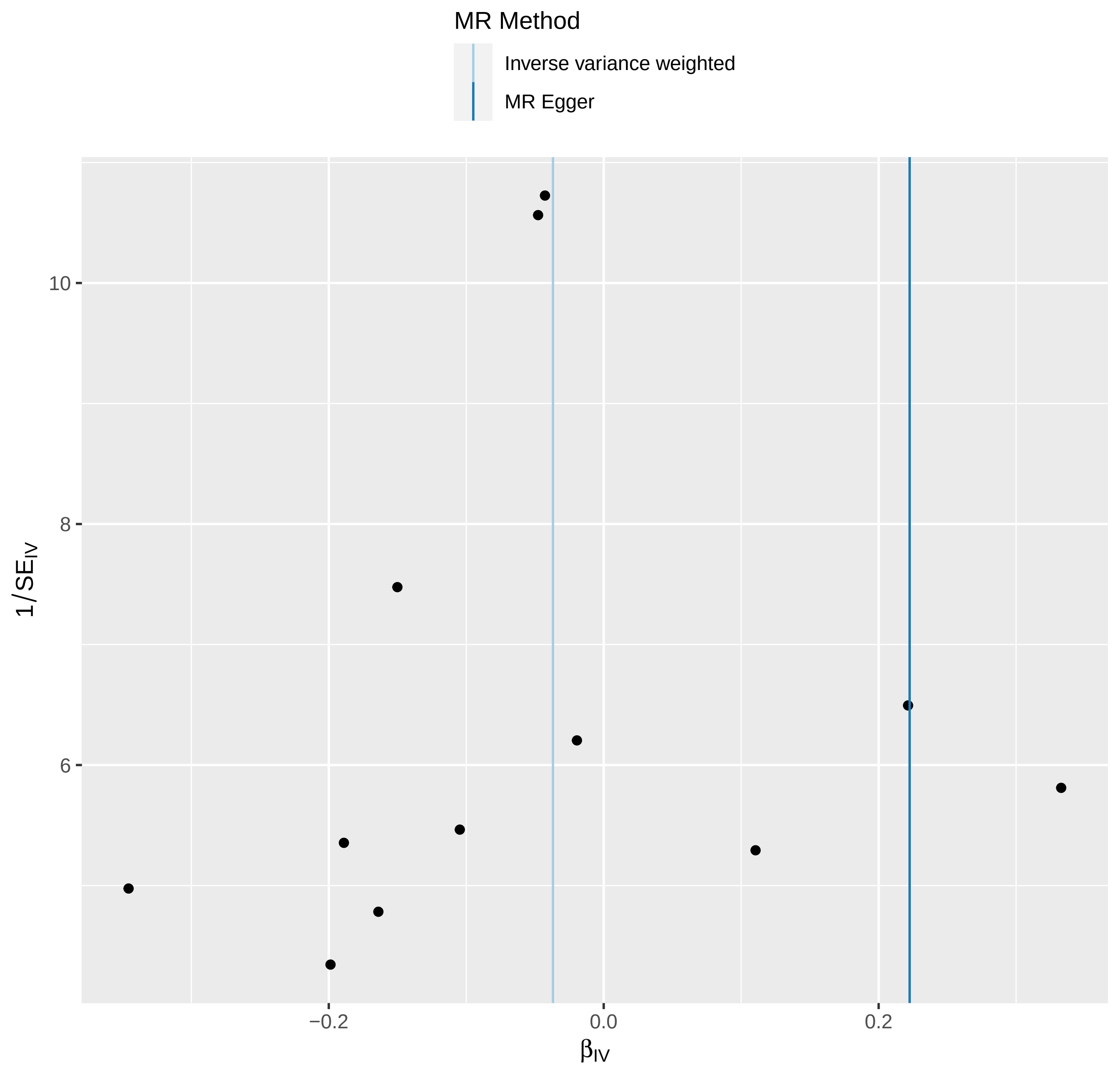

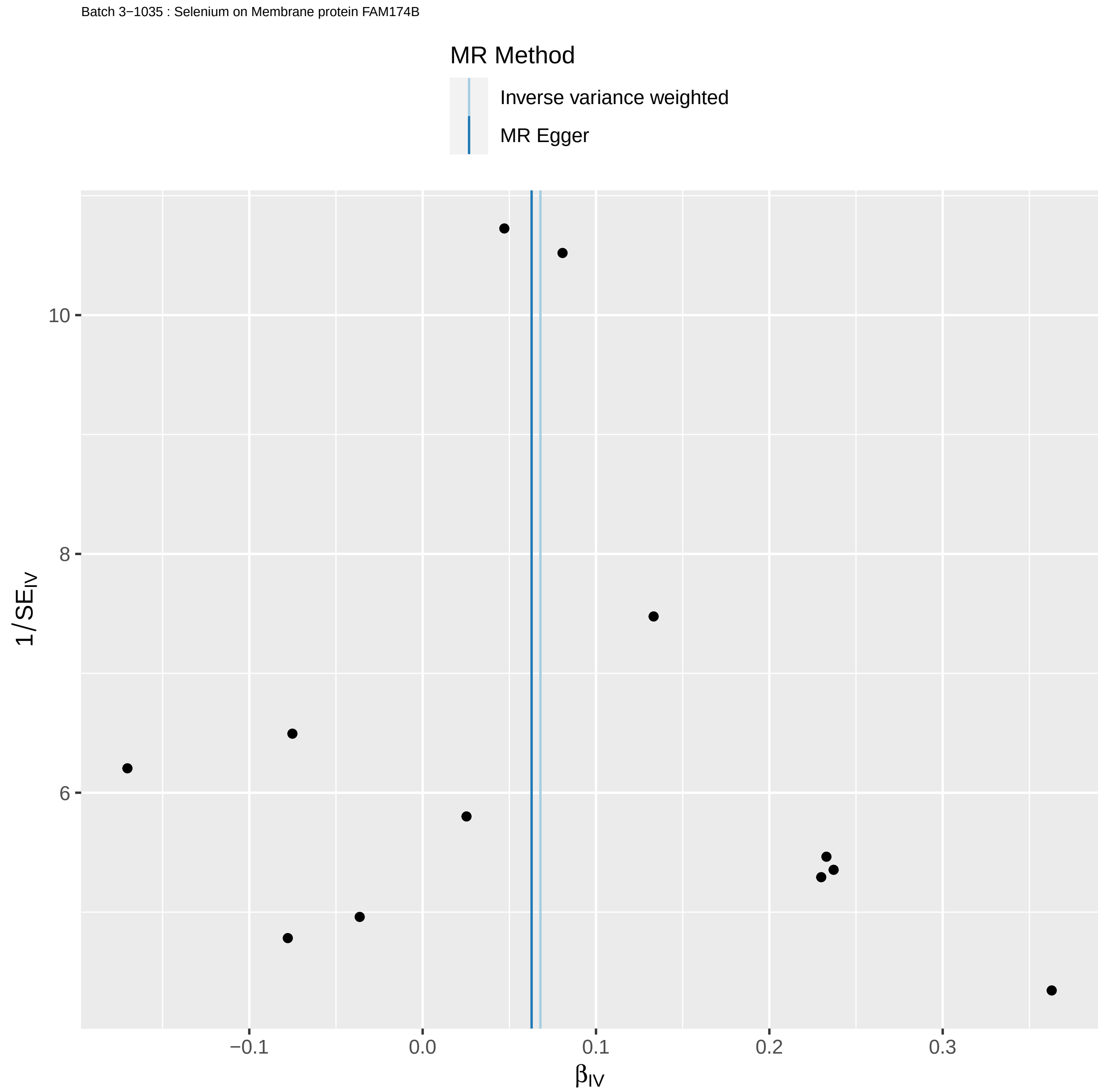

Batch 3−1035 : Selenium on Membrane protein FAM174B
MR Method
Inverse variance weighted
MR Egger
10
8
6
1/SE_IV
−0.1
0.0
0.1
0.2
0.3
β_IV

Batch 3−1036 : Selenium on BRISC complex subunit Abro1

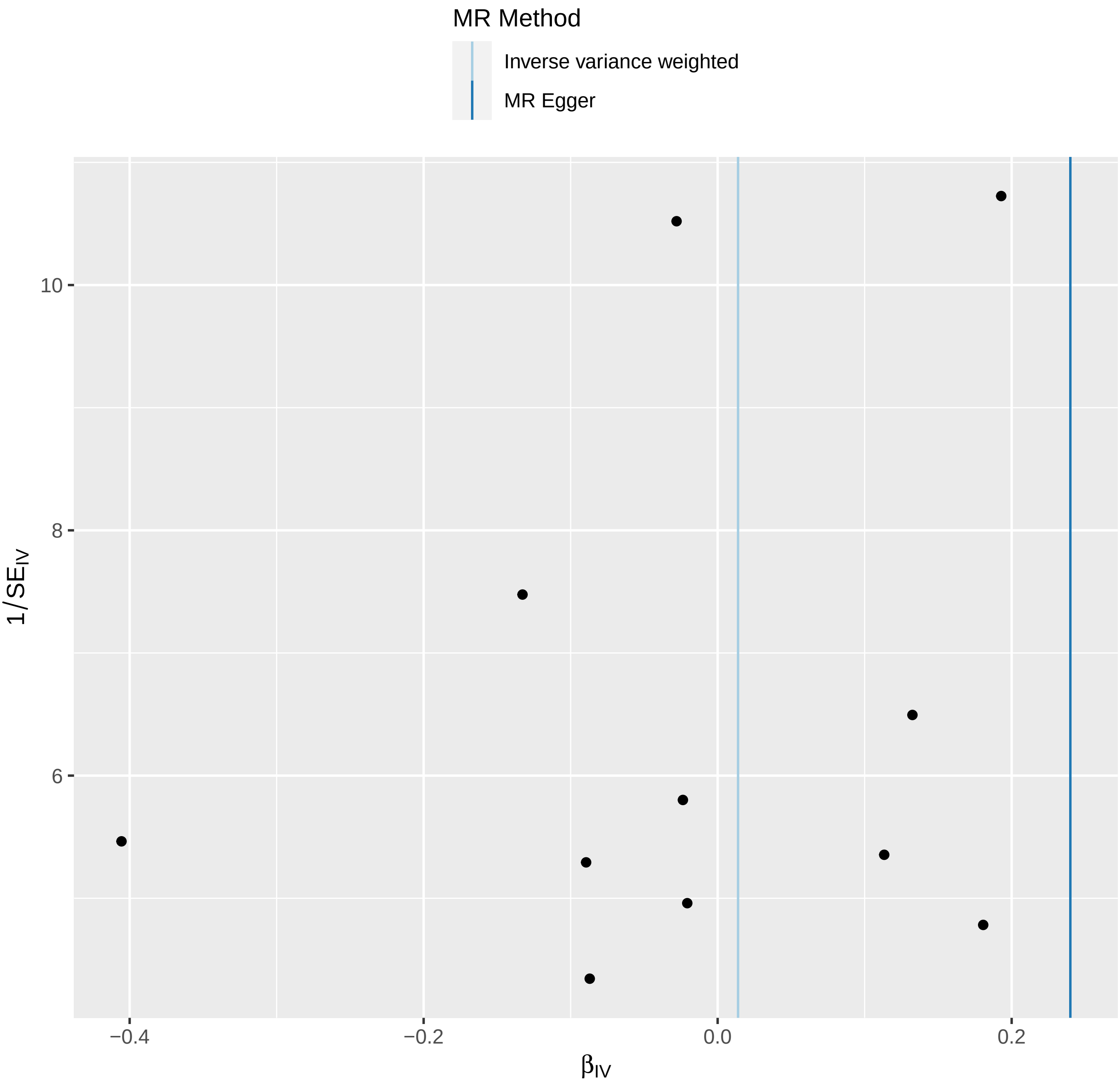

Batch 3−1037 : Selenium on Protein FAM177A1

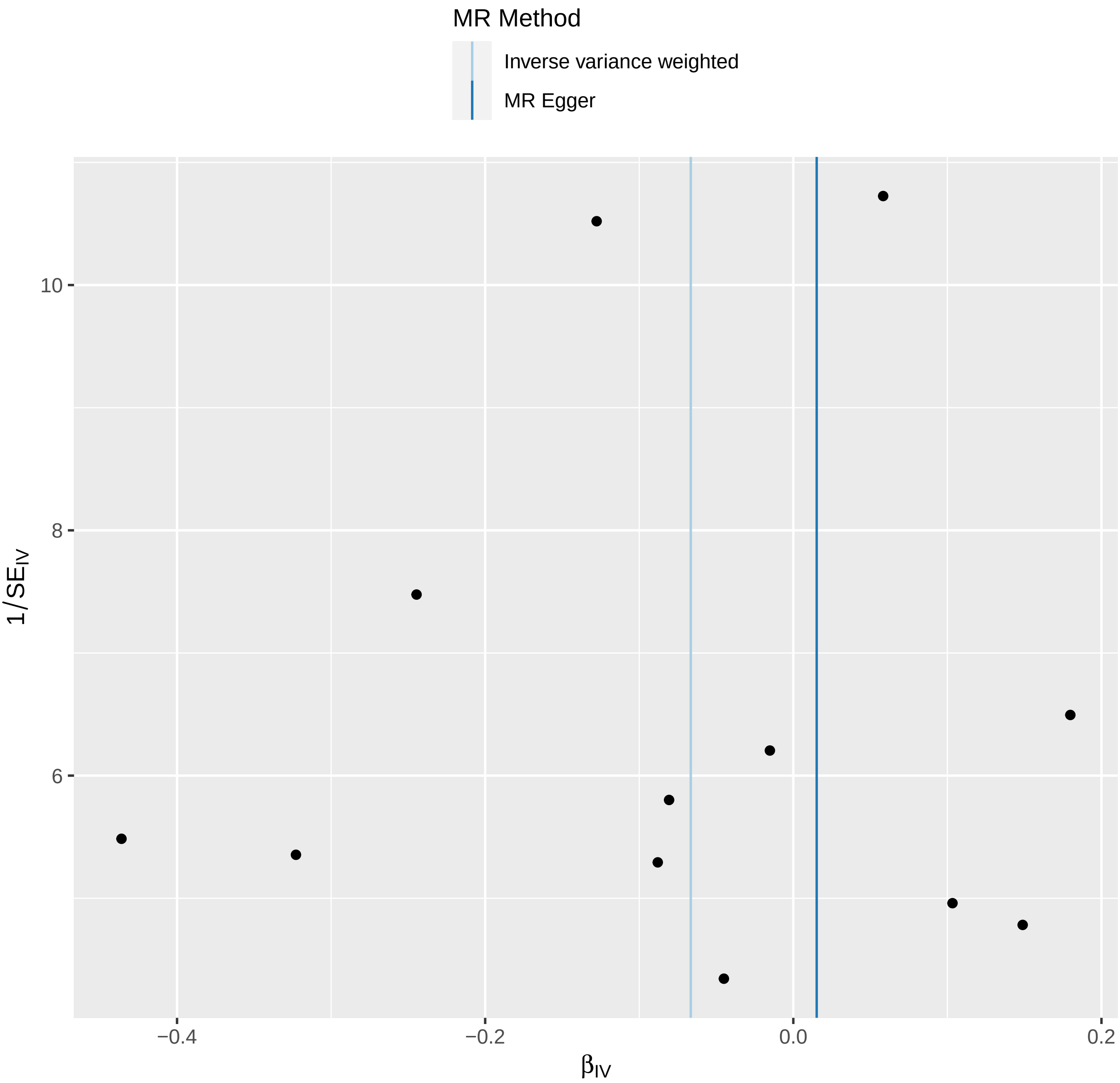

Batch 3−1038 : Selenium on Protein FAM189A2

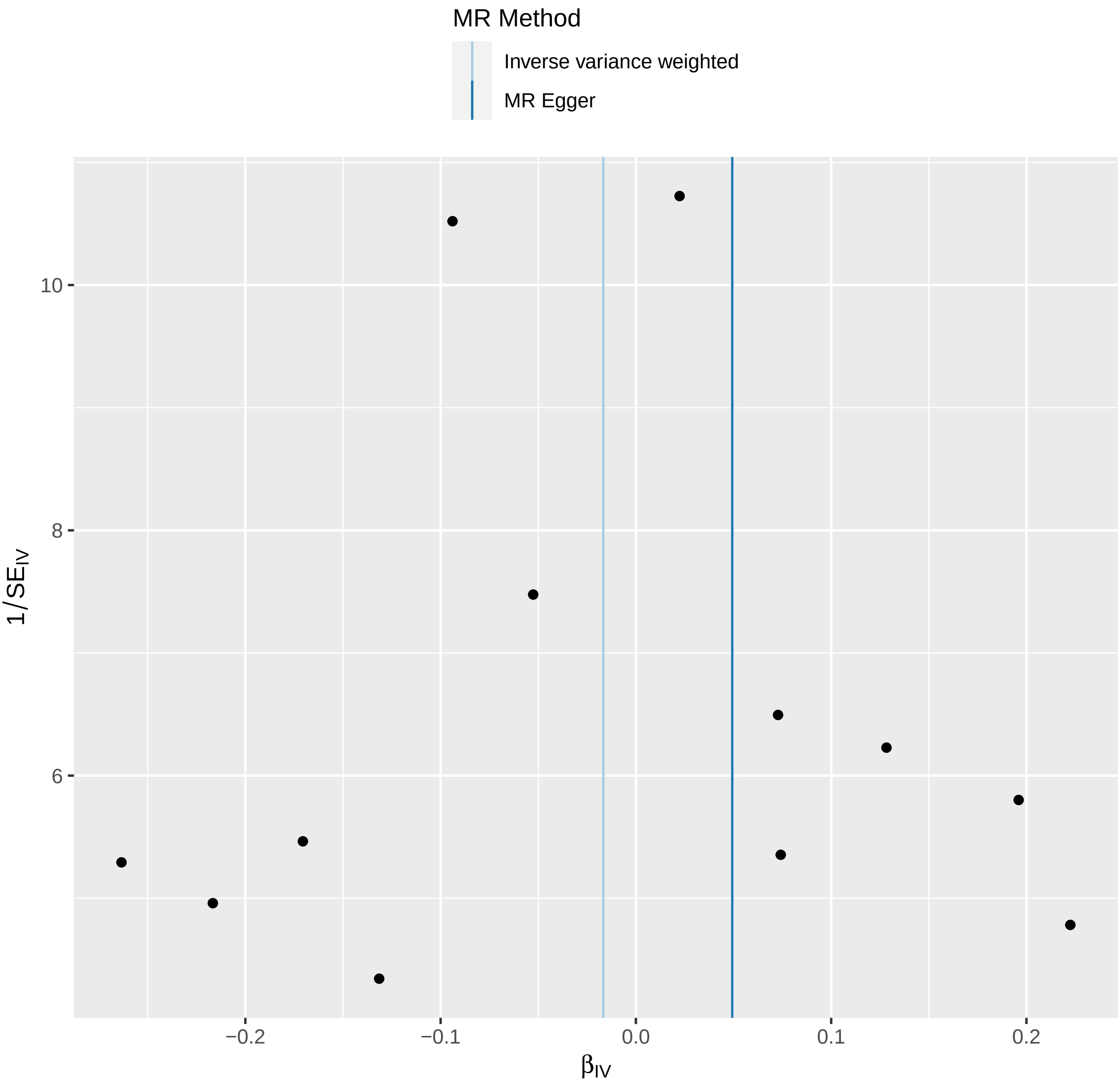

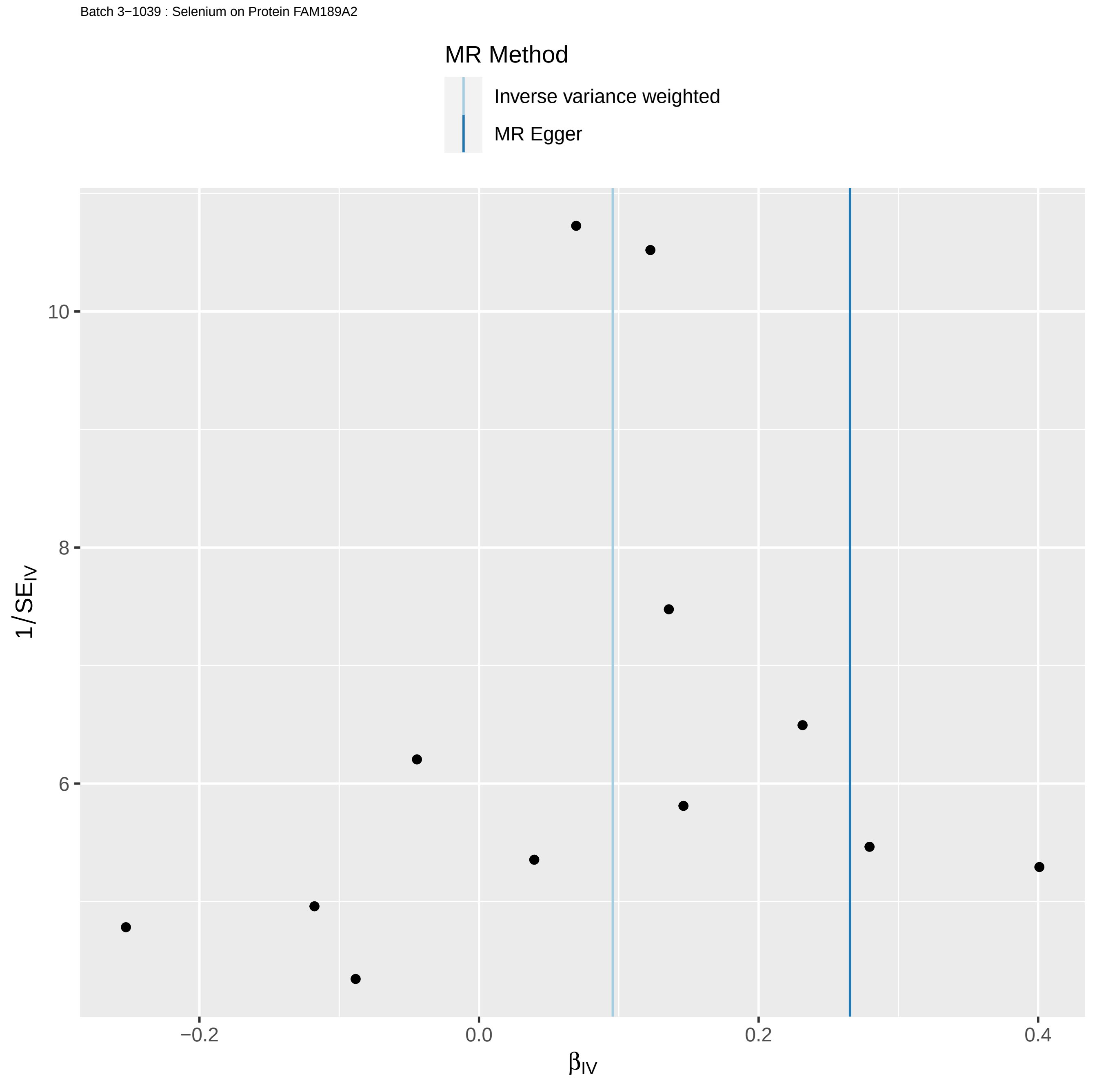
Batch 3−1039 : Selenium on Protein FAM189A2
MR Method
Inverse variance weighted
MR Egger
10
8
6
1/SE$_{IV}$
−0.2
0.0
0.2
0.4
β$_{IV}$

Batch 3−1040 : Selenium on Protein FAM19A2

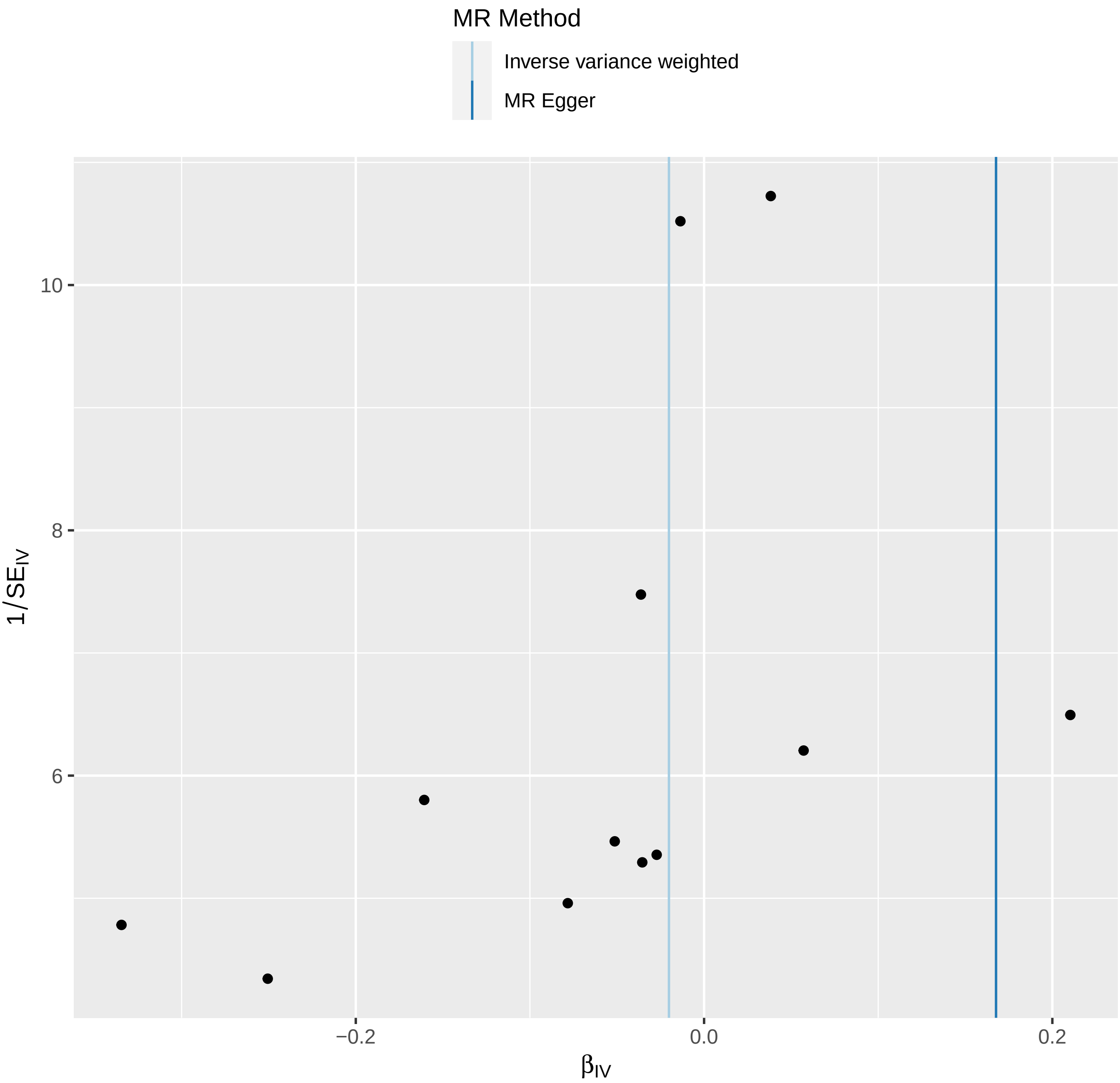

Batch 3−1041 : Selenium on Protein FAM19A3

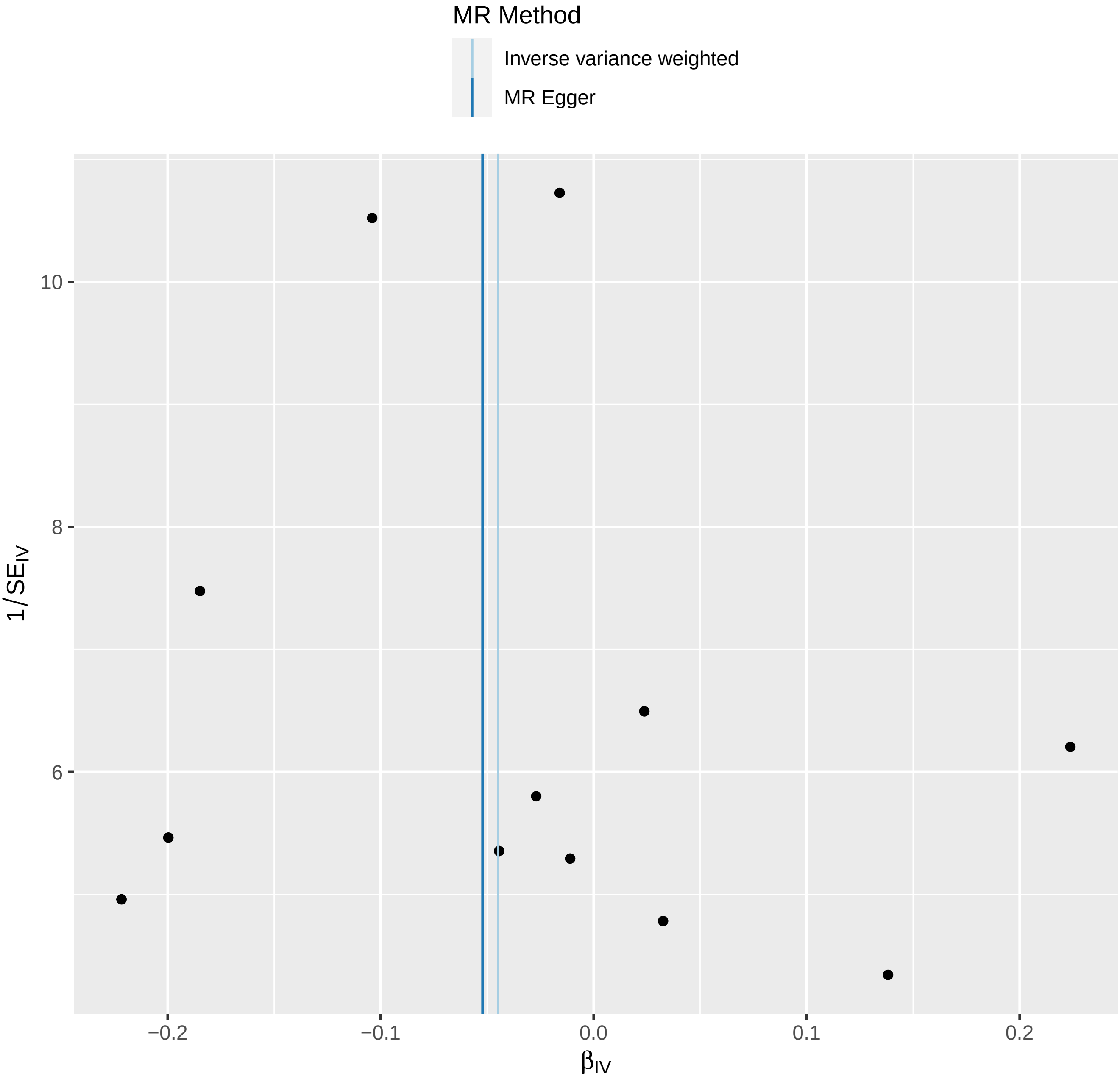

Batch 3−1042 : Selenium on Protein FAM19A4

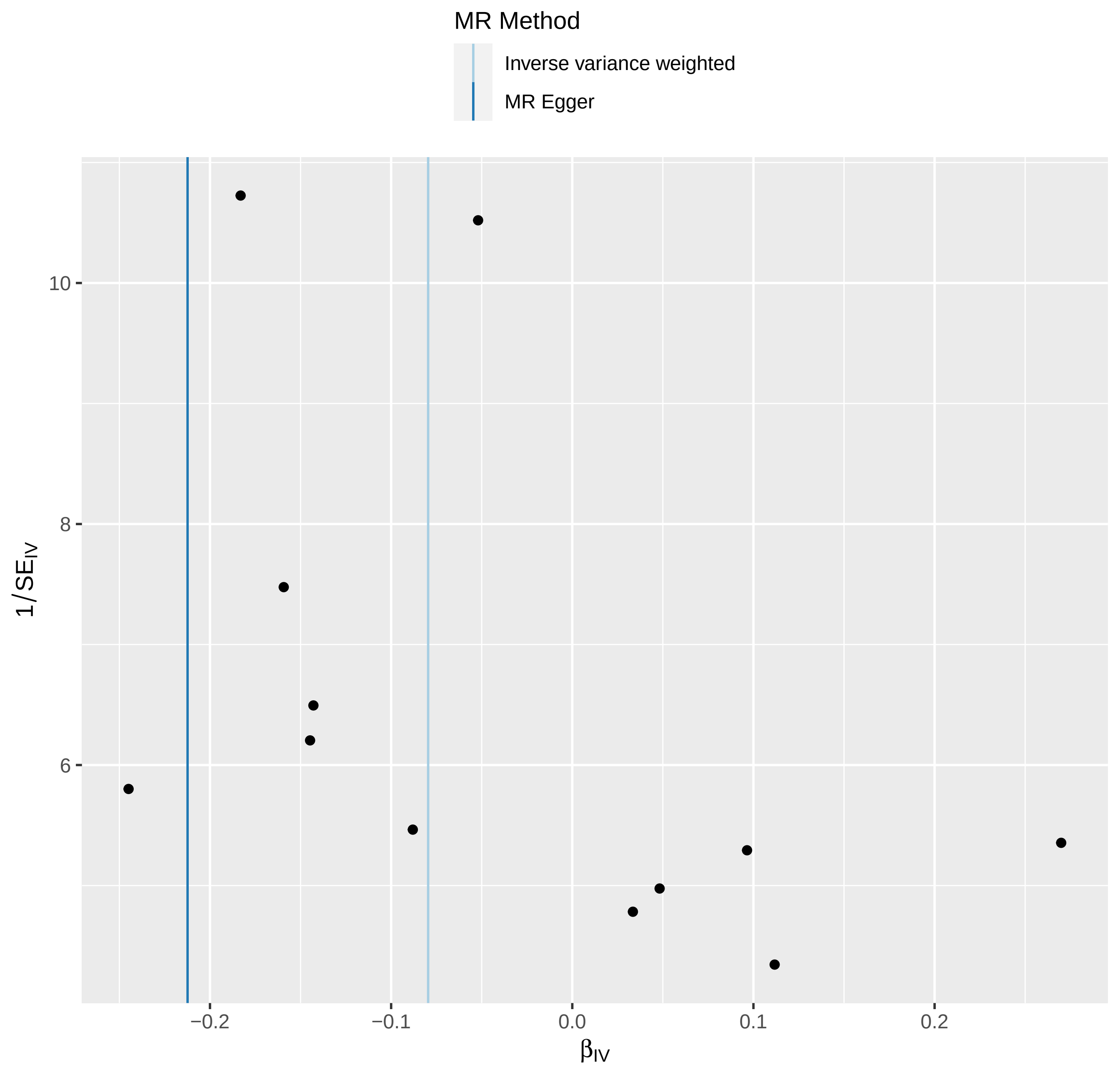

Batch 3−1043 : Selenium on Protein FAM19A5

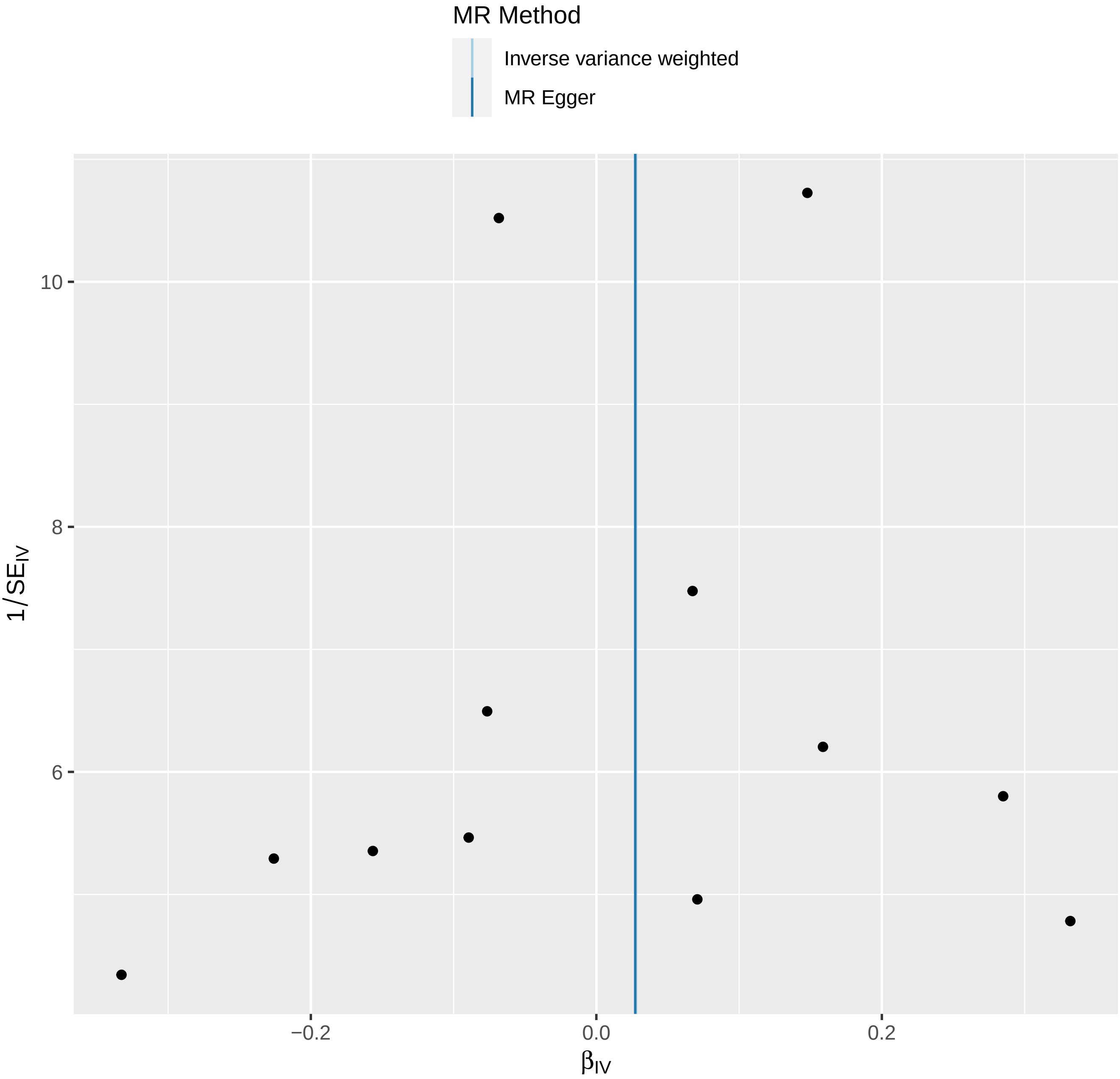

Batch 3−1044 : Selenium on Protein FAM209B

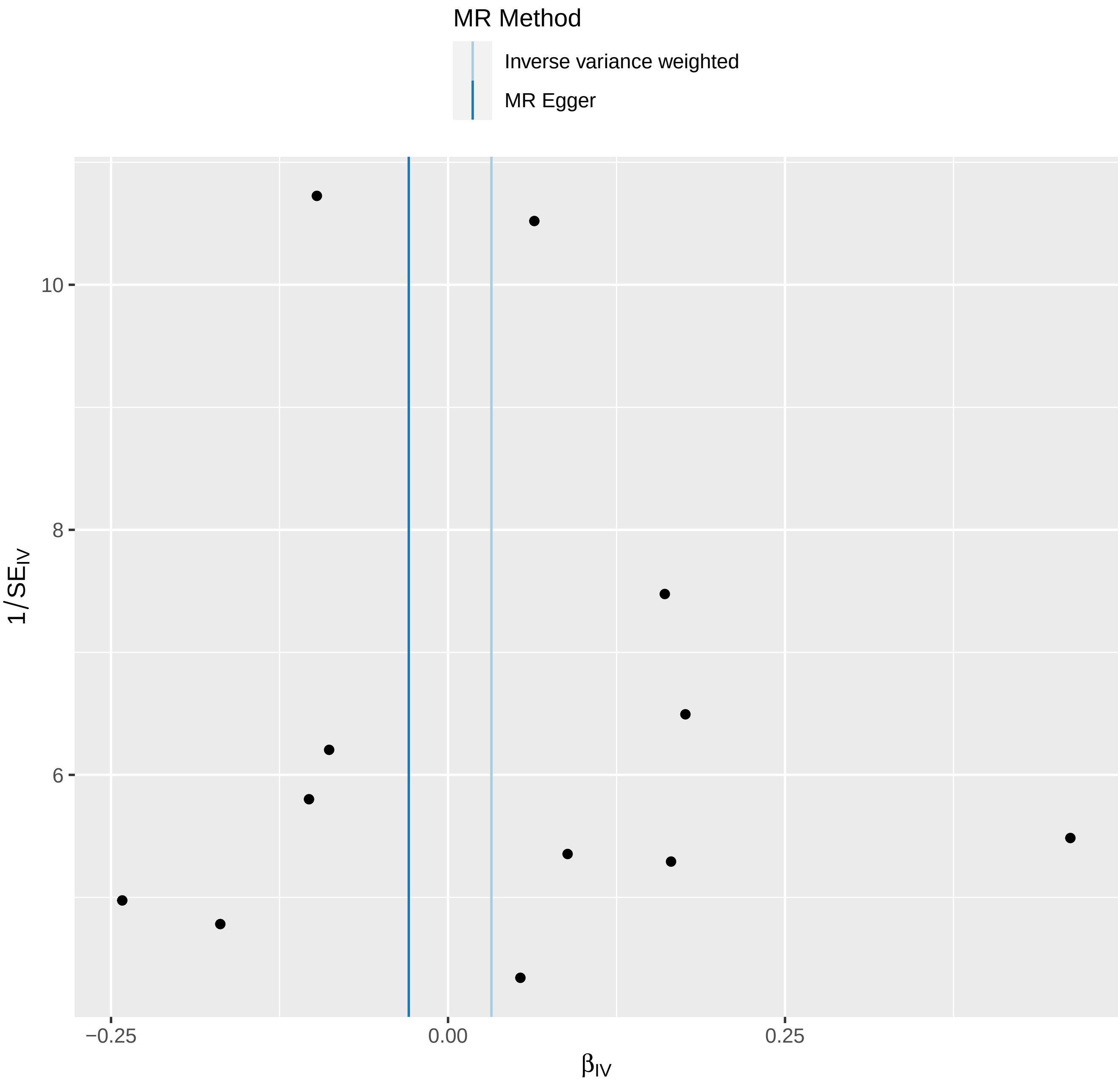

Batch 3−1045 : Selenium on Pseudokinase FAM20A

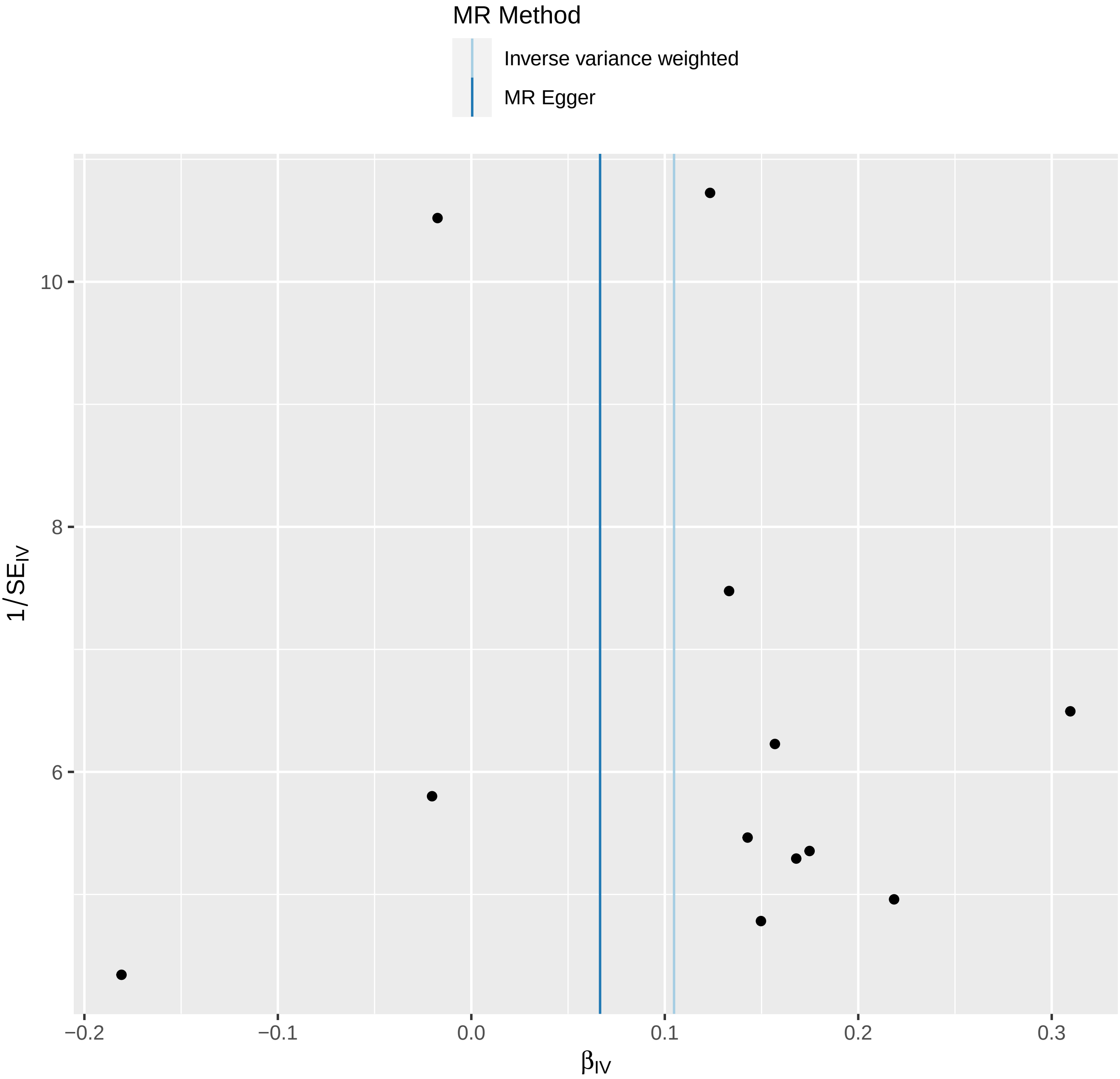

Batch 3−1046 : Selenium on Glycosaminoglycan xylosylkinase

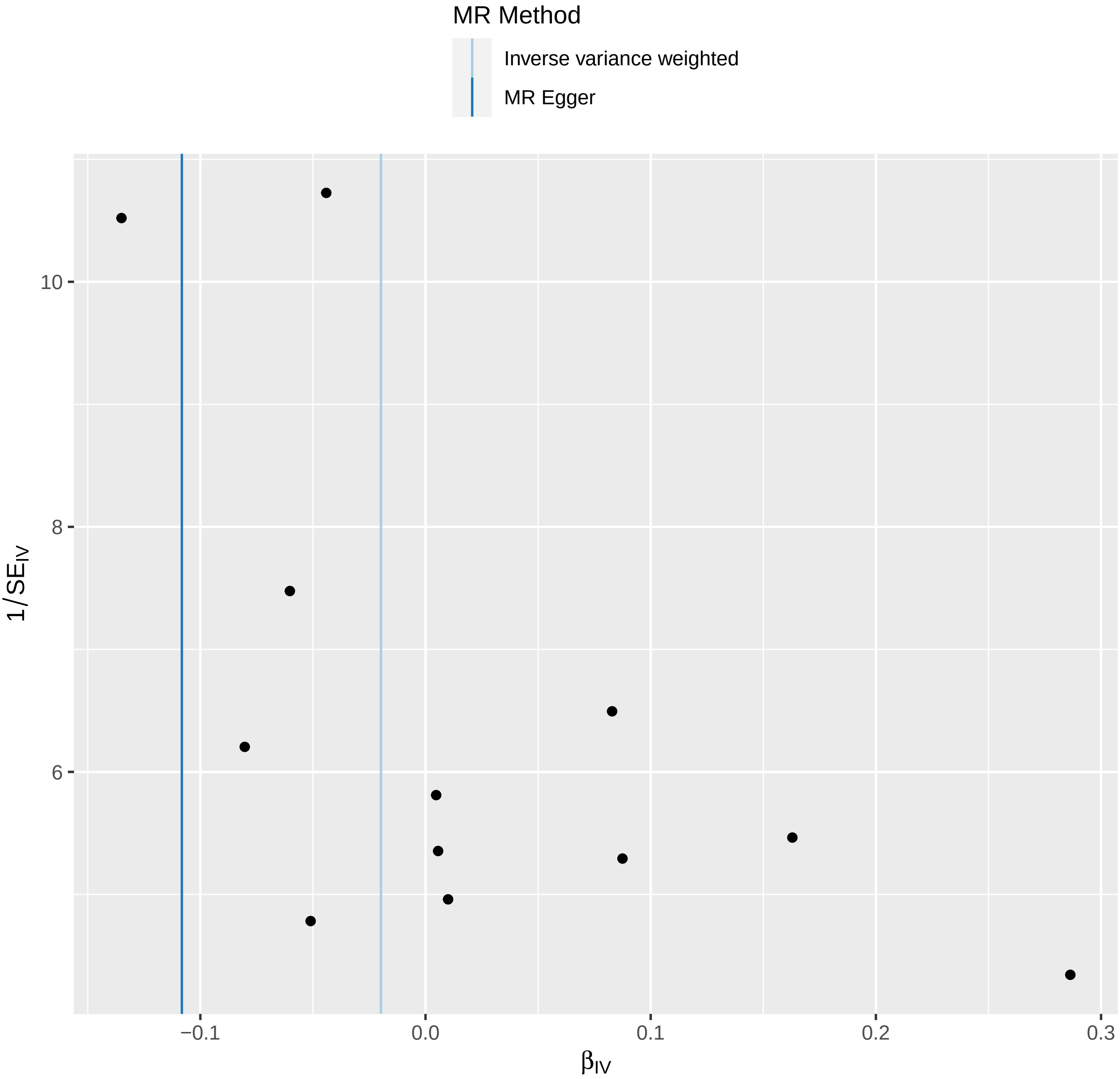

Batch 3−1047 : Selenium on Redox−regulatory protein FAM213A

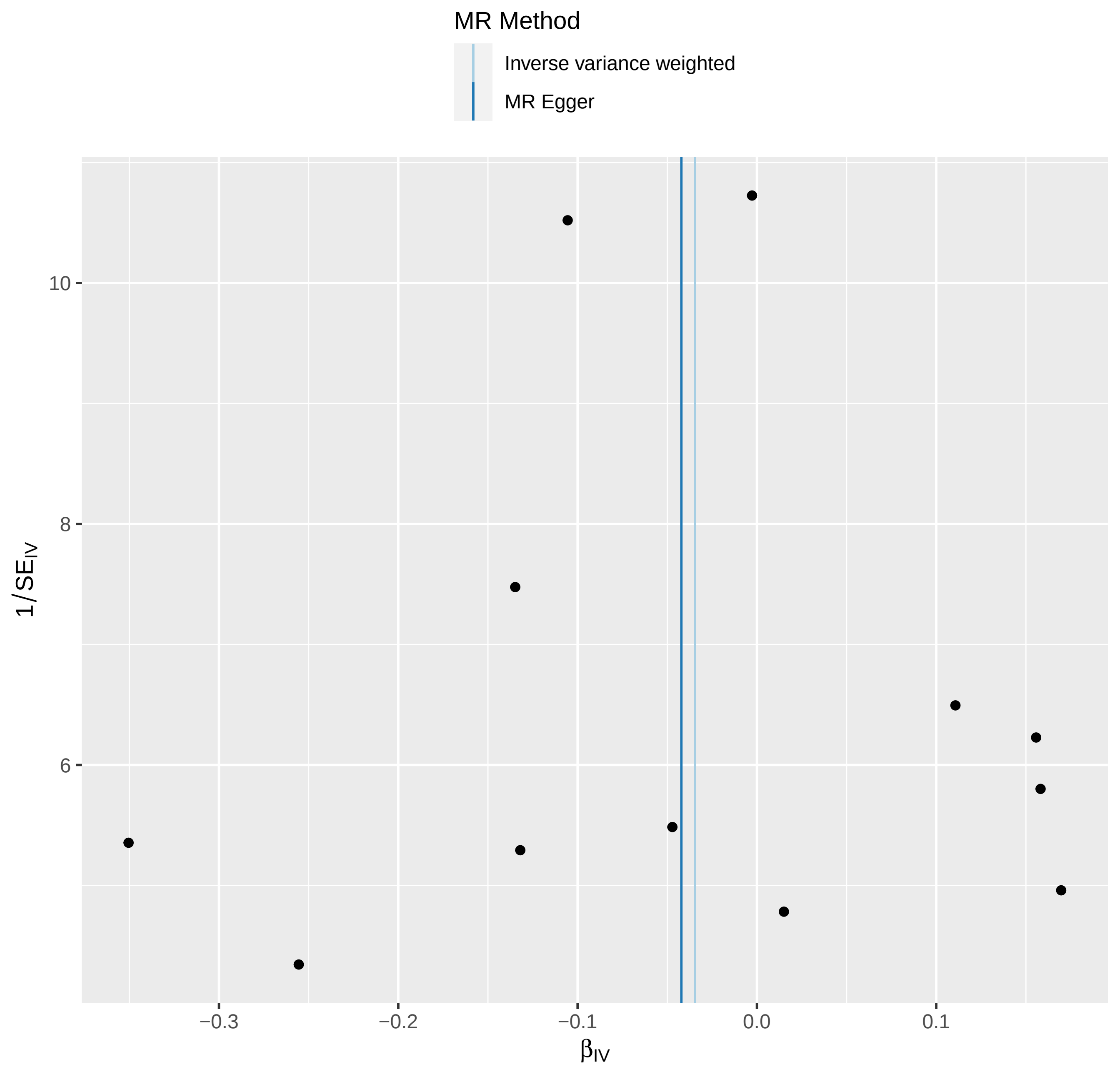

Batch 3−1048 : Selenium on Protein FAM24B

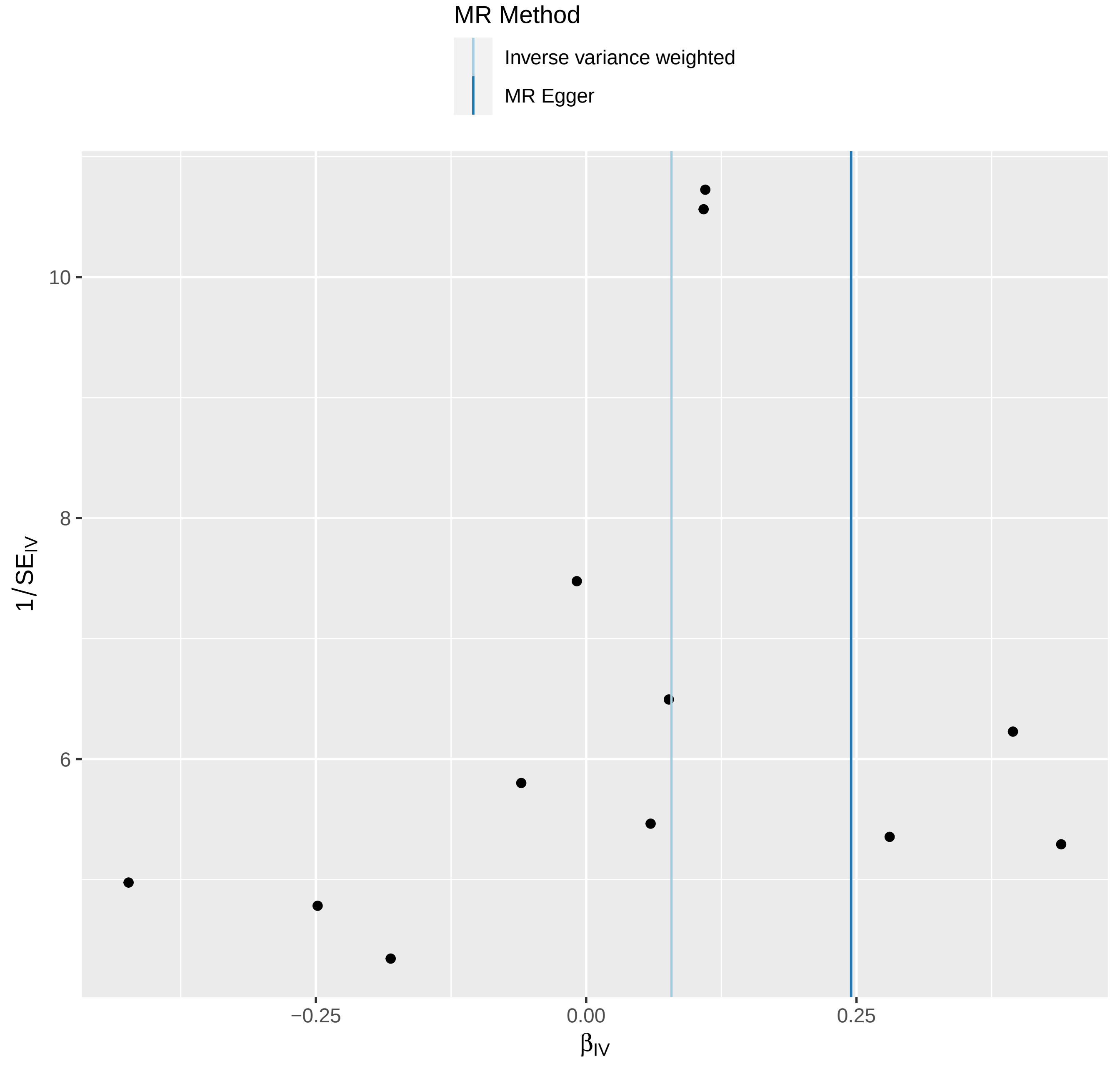

Batch 3−1049 : Selenium on Protein FAM3B

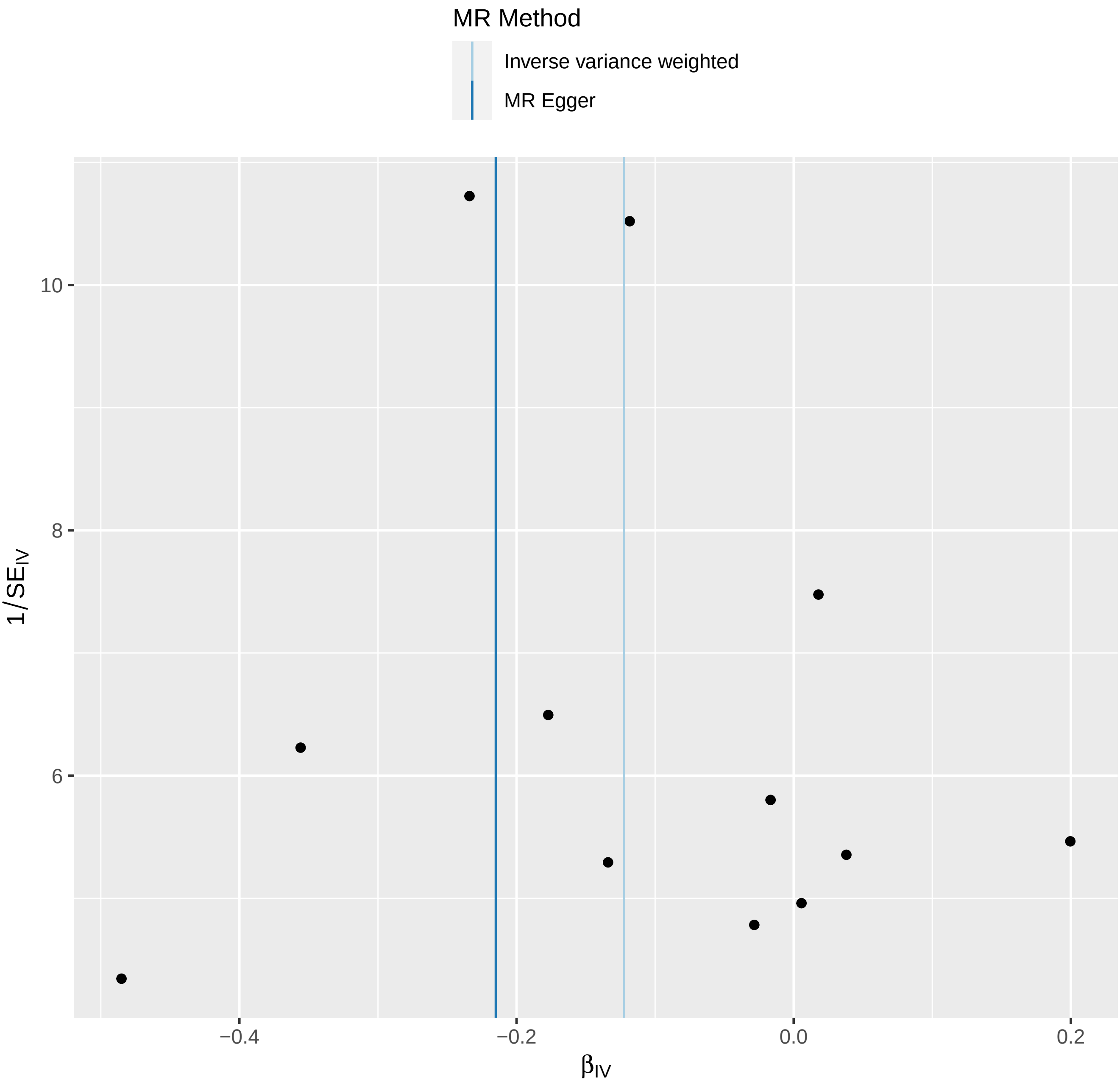

Batch 3−1050 : Selenium on Protein FAM3B

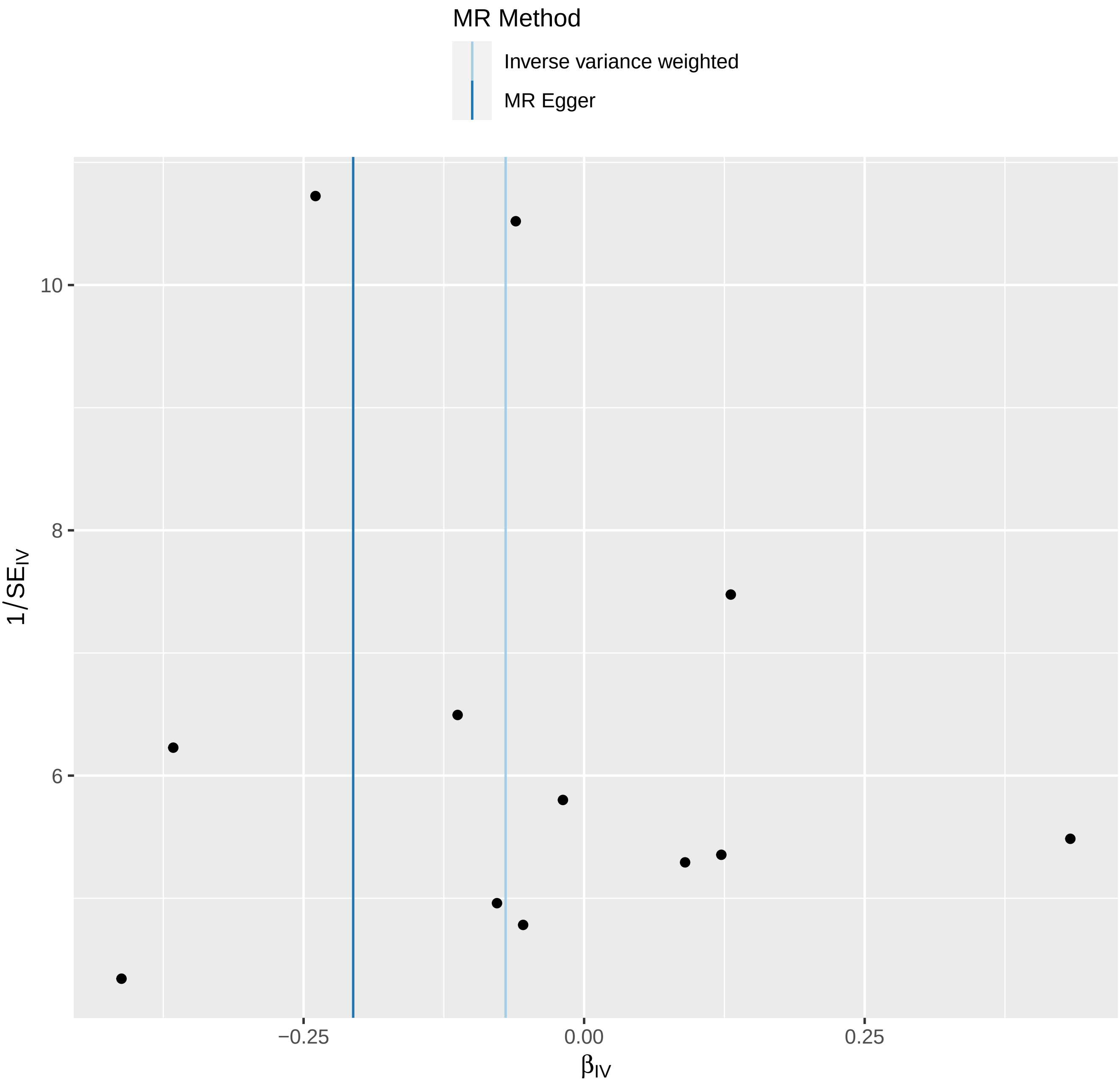

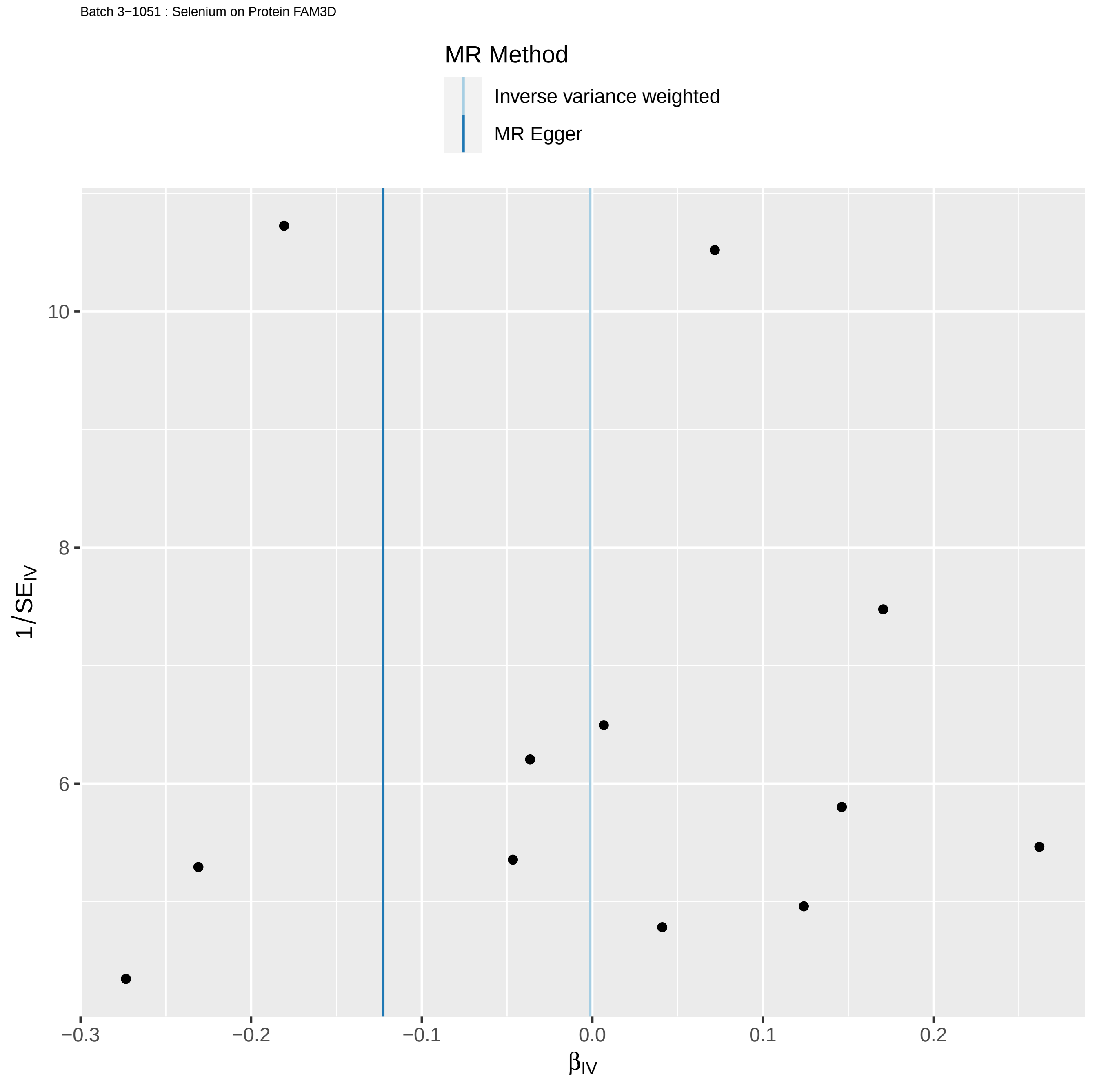

Batch 3−1051 : Selenium on Protein FAM3D
MR Method
Inverse variance weighted
MR Egger
10
8
6
1/SE_IV
−0.3
−0.2
−0.1
0.0
0.1
0.2
β_IV

Batch 3−1052 : Selenium on MIP18 family protein FAM96A

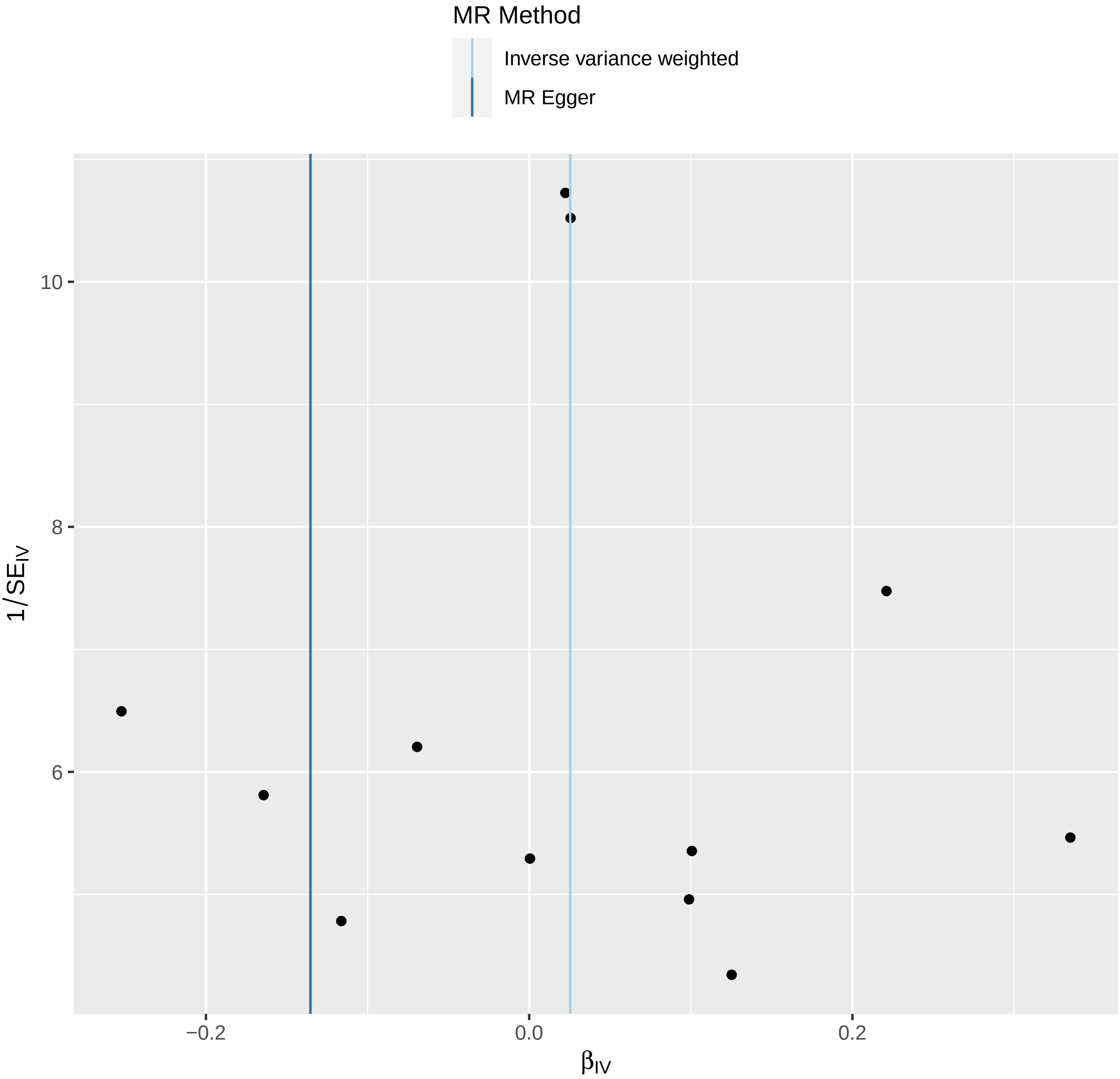

Batch 3−1053 : Selenium on E3 ubiquitin−protein ligase FANCL

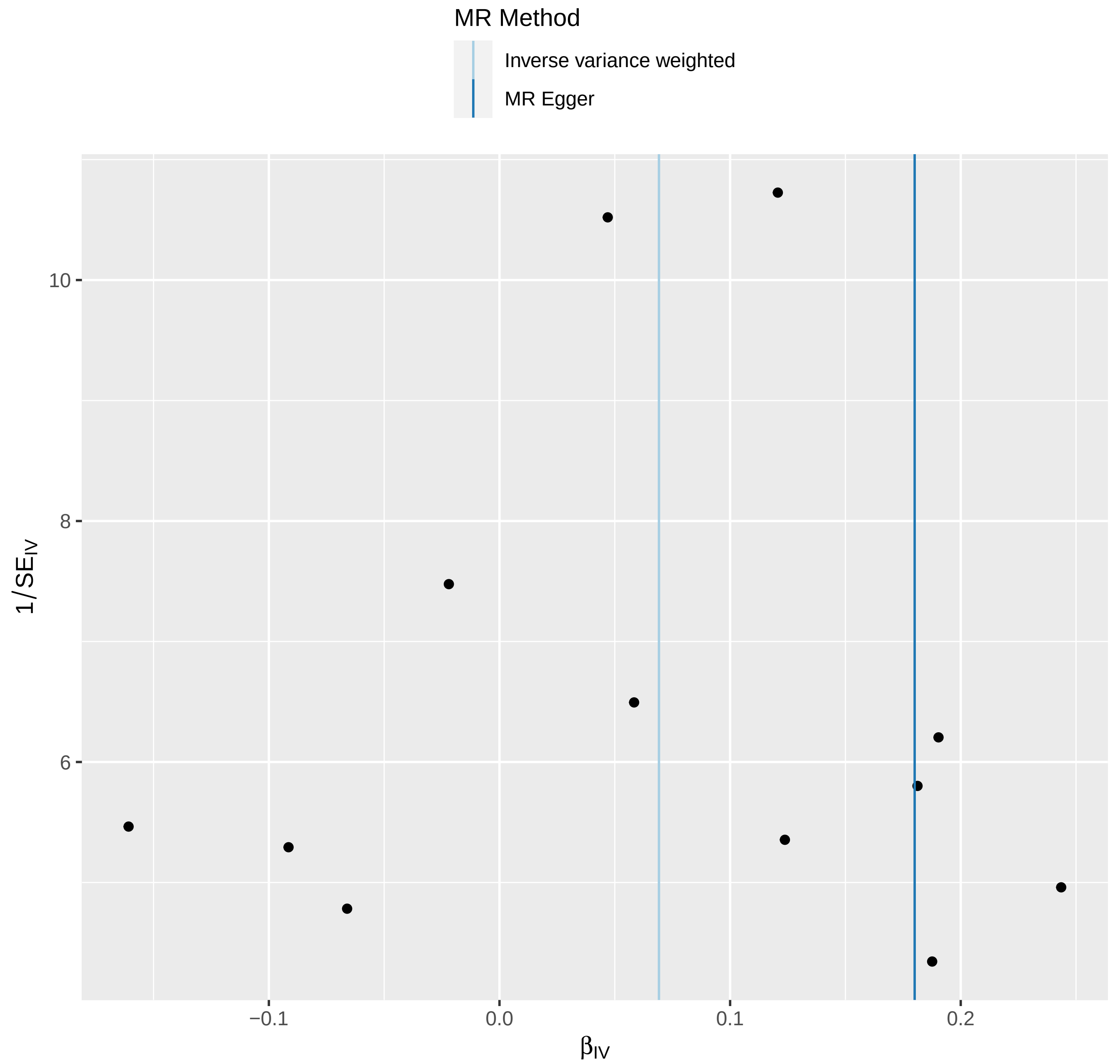

Batch 3−1054 : Selenium on Prolyl endopeptidase FAP

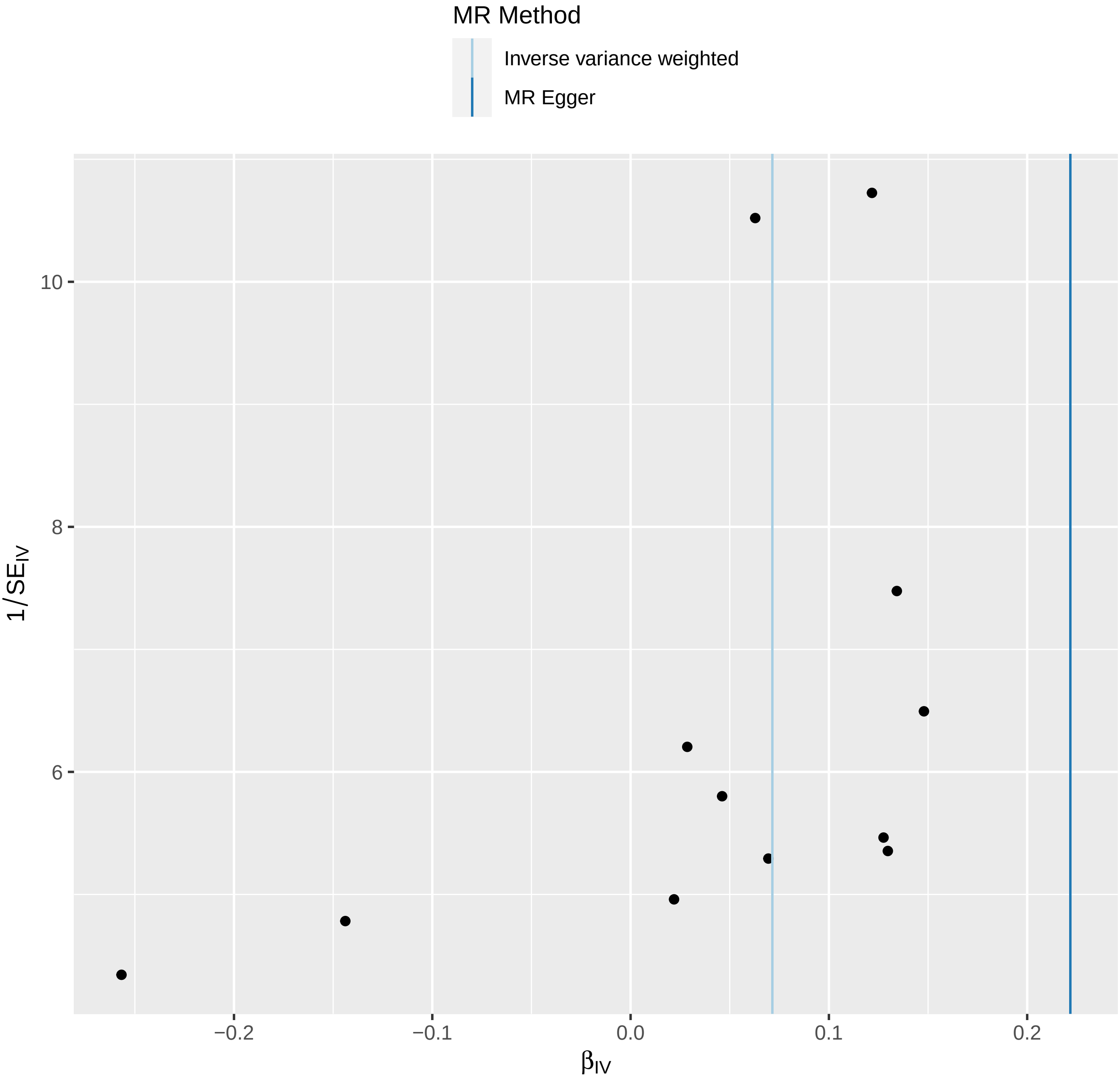

Batch 3−1055 : Selenium on Phenylalanine−−tRNA ligase, mitochondrial

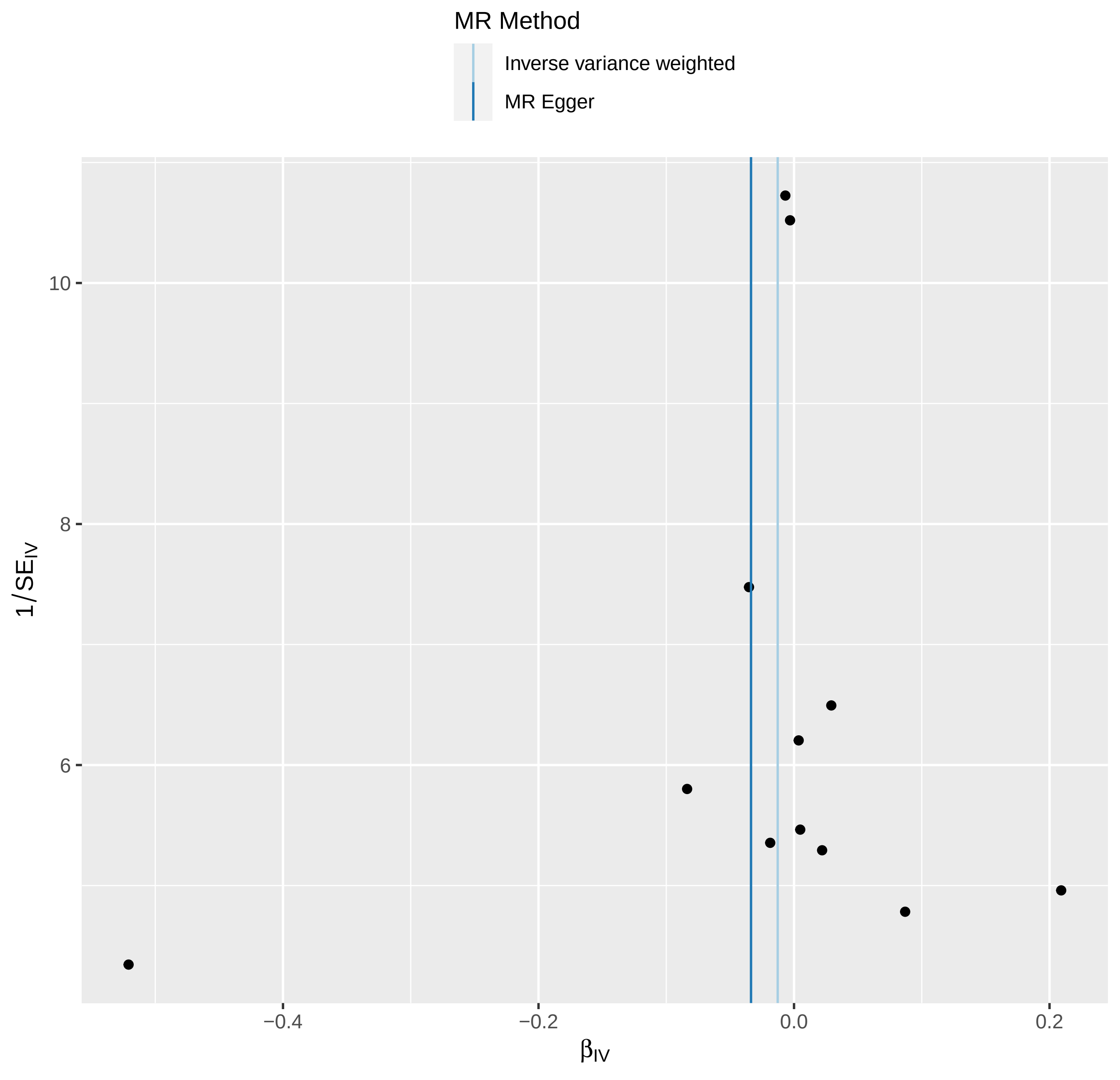

Batch 3−1056 : Selenium on Tumor necrosis factor receptor superfamily member 6

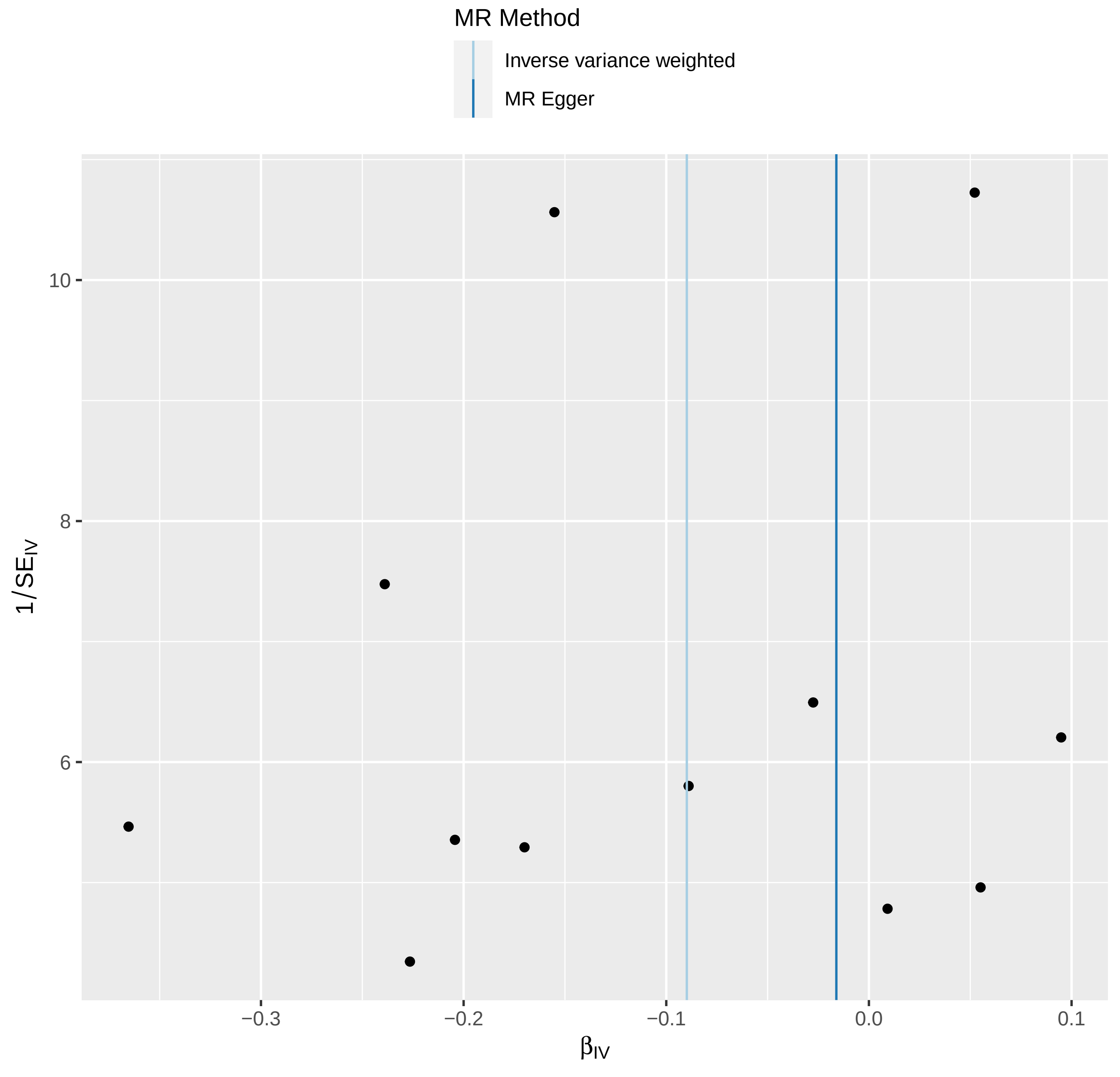

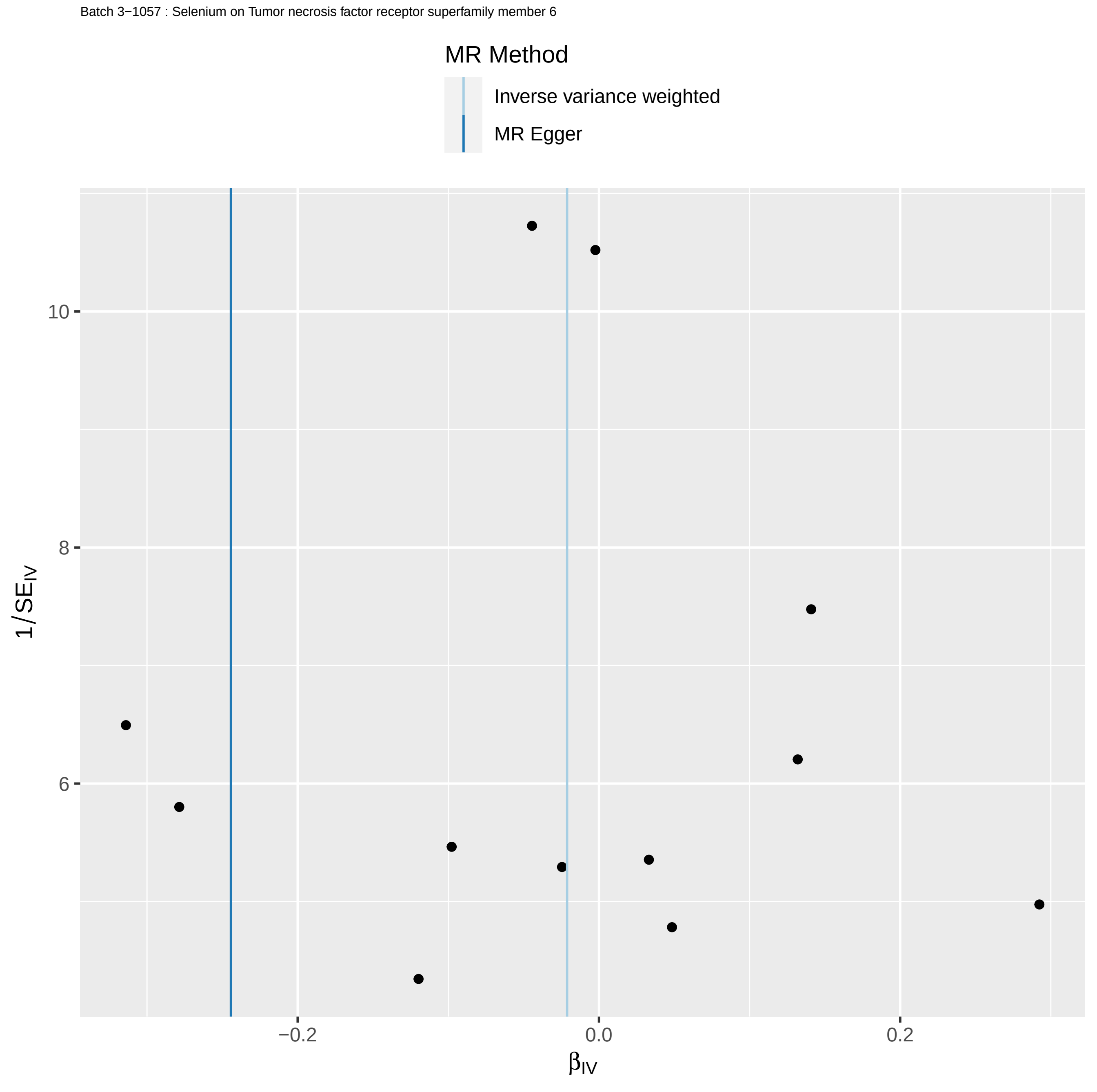
Batch 3−1057 : Selenium on Tumor necrosis factor receptor superfamily member 6
MR Method
Inverse variance weighted
MR Egger
10
8
6
1/SE_IV
−0.2
0.0
0.2
β_IV

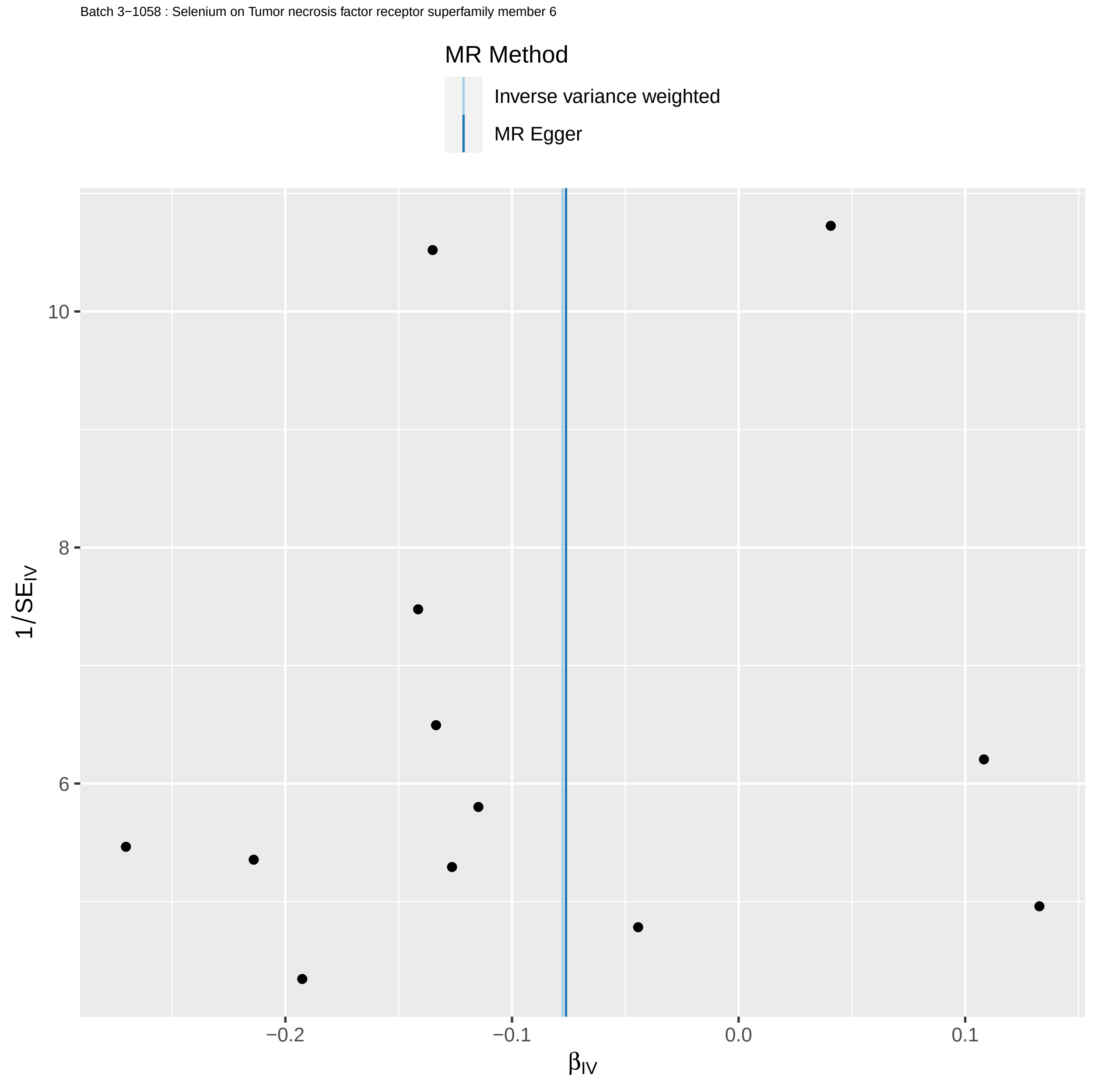

Batch 3−1058 : Selenium on Tumor necrosis factor receptor superfamily member 6
MR Method
Inverse variance weighted
MR Egger
10
8
6
1/SE_IV
−0.2
−0.1
0.0
0.1
β_IV

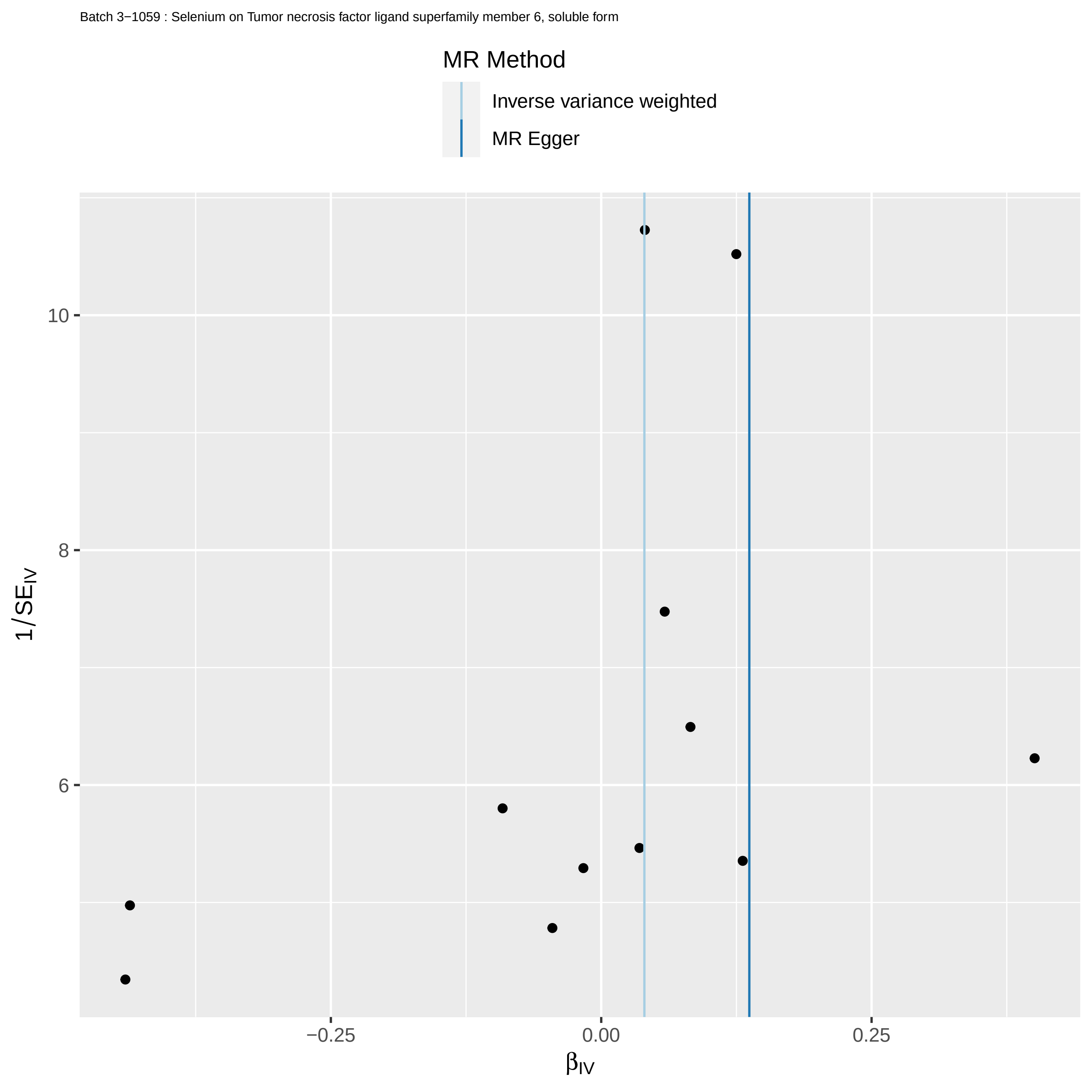

Batch 3−1059 : Selenium on Tumor necrosis factor ligand superfamily member 6, soluble form
MR Method
Inverse variance weighted
MR Egger
10
8
6
$1/SE_{IV}$
−0.25
0.00
0.25
β_{IV}

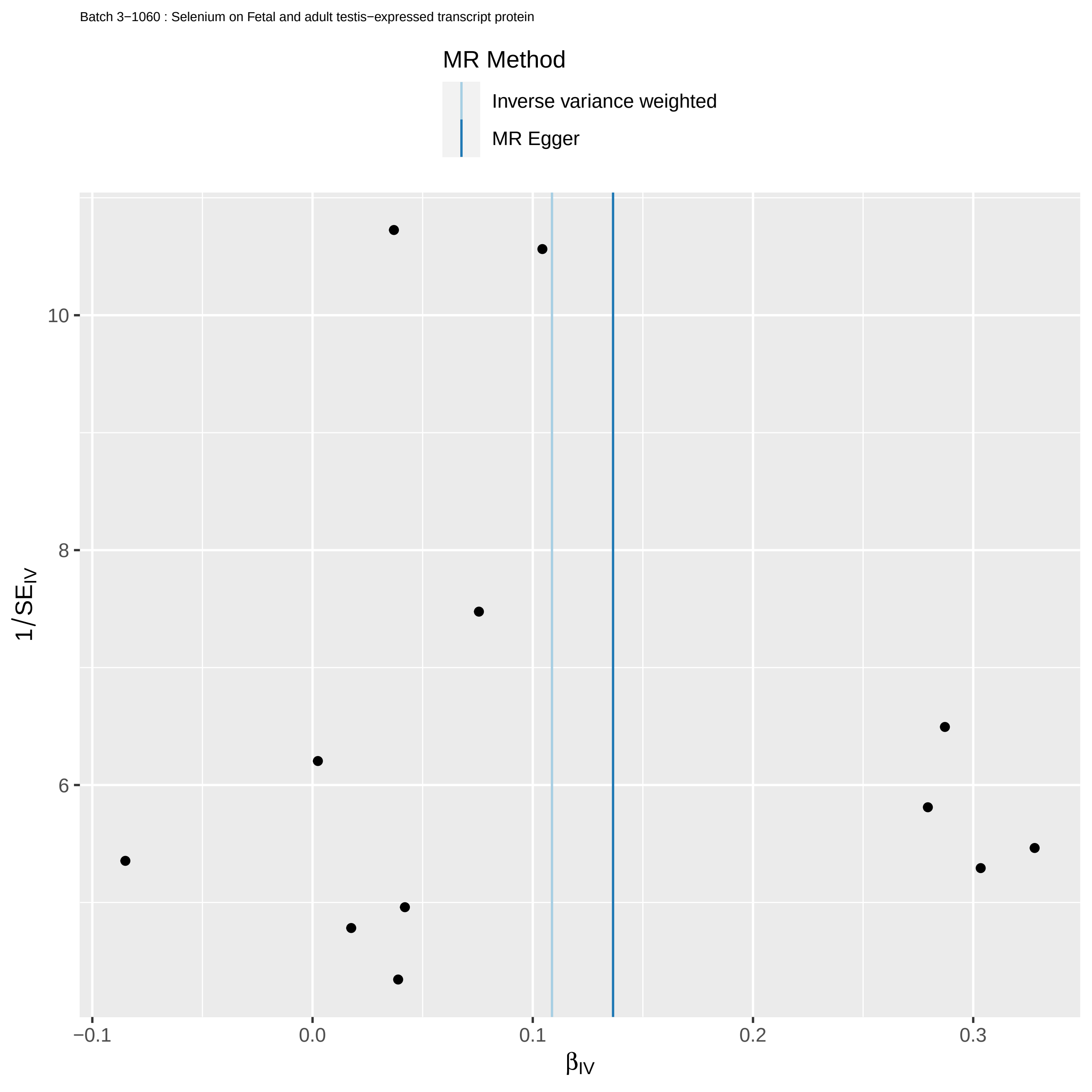

Batch 3−1060 : Selenium on Fetal and adult testis−expressed transcript protein
MR Method
Inverse variance weighted
MR Egger
10
8
6
−0.1
0.0
0.1
0.2
0.3
β_IV
1/SE_IV

Batch 3−1061 : Selenium on Fatty acid hydroxylase domain−containing protein 2

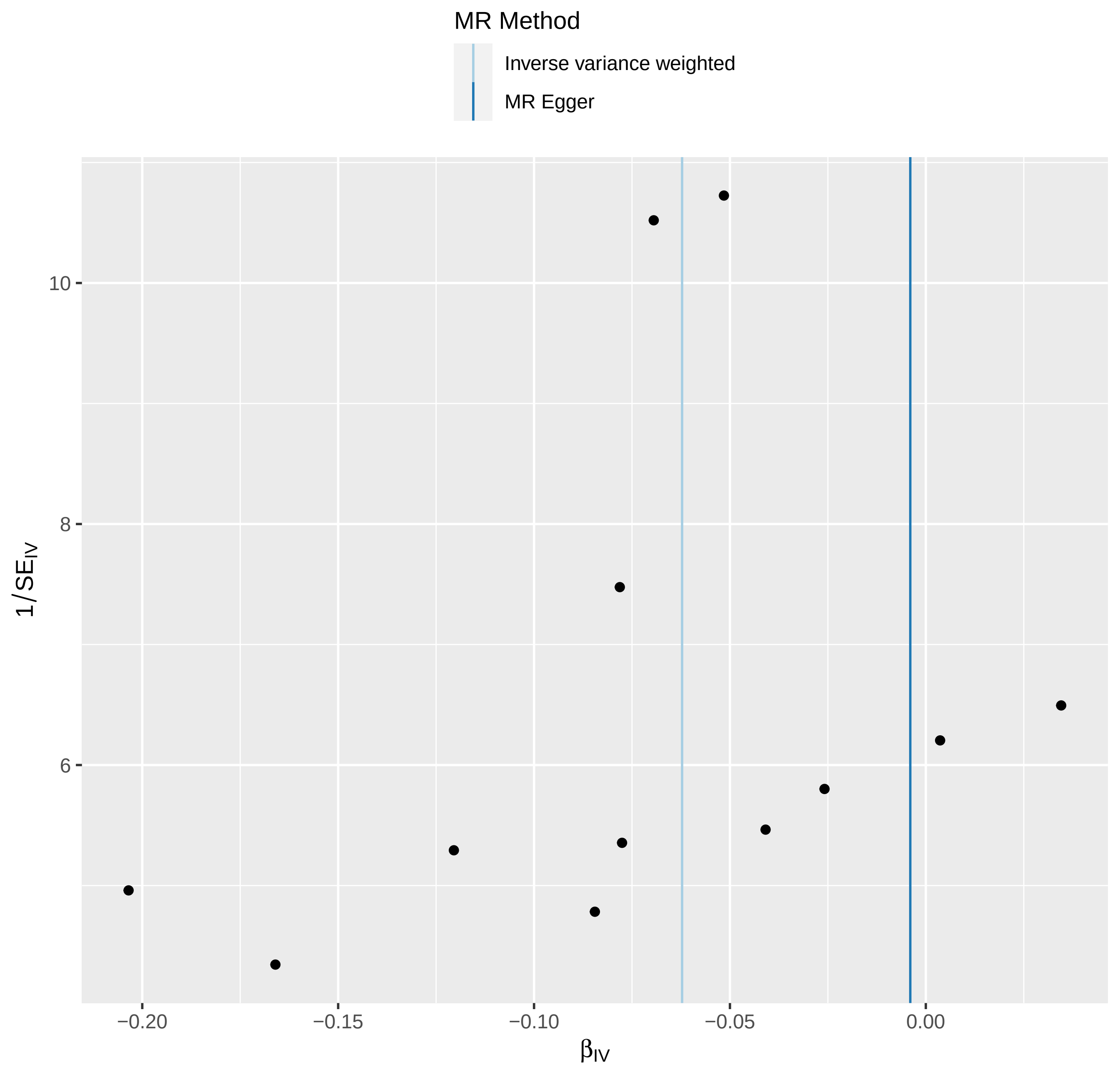

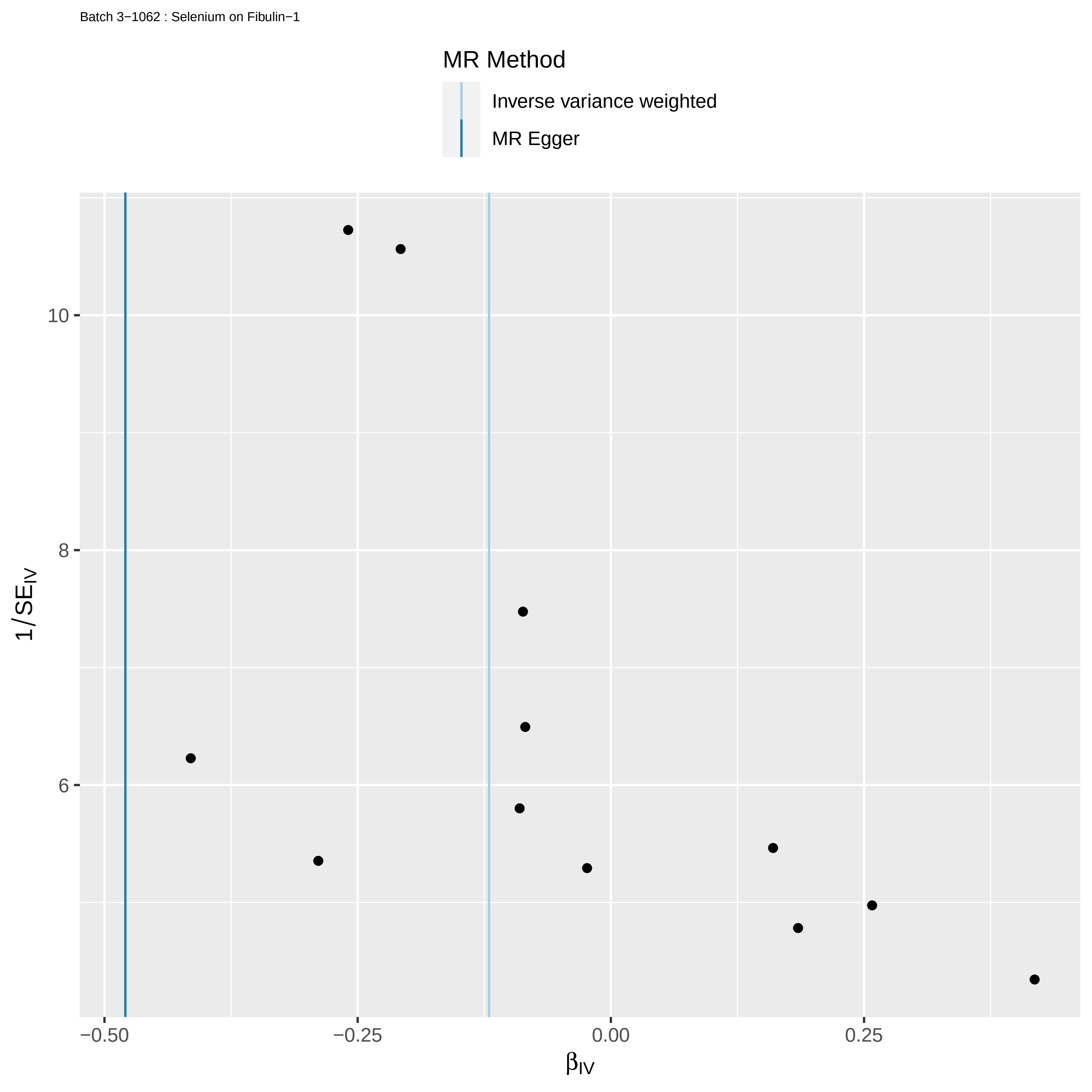

Batch 3−1062 : Selenium on Fibulin−1
MR Method
Inverse variance weighted
MR Egger
10
8
6
1/SE_IV
−0.50
−0.25
0.00
0.25
β_IV

Batch 3−1063 : Selenium on Fructose−1,6−bisphosphatase 1

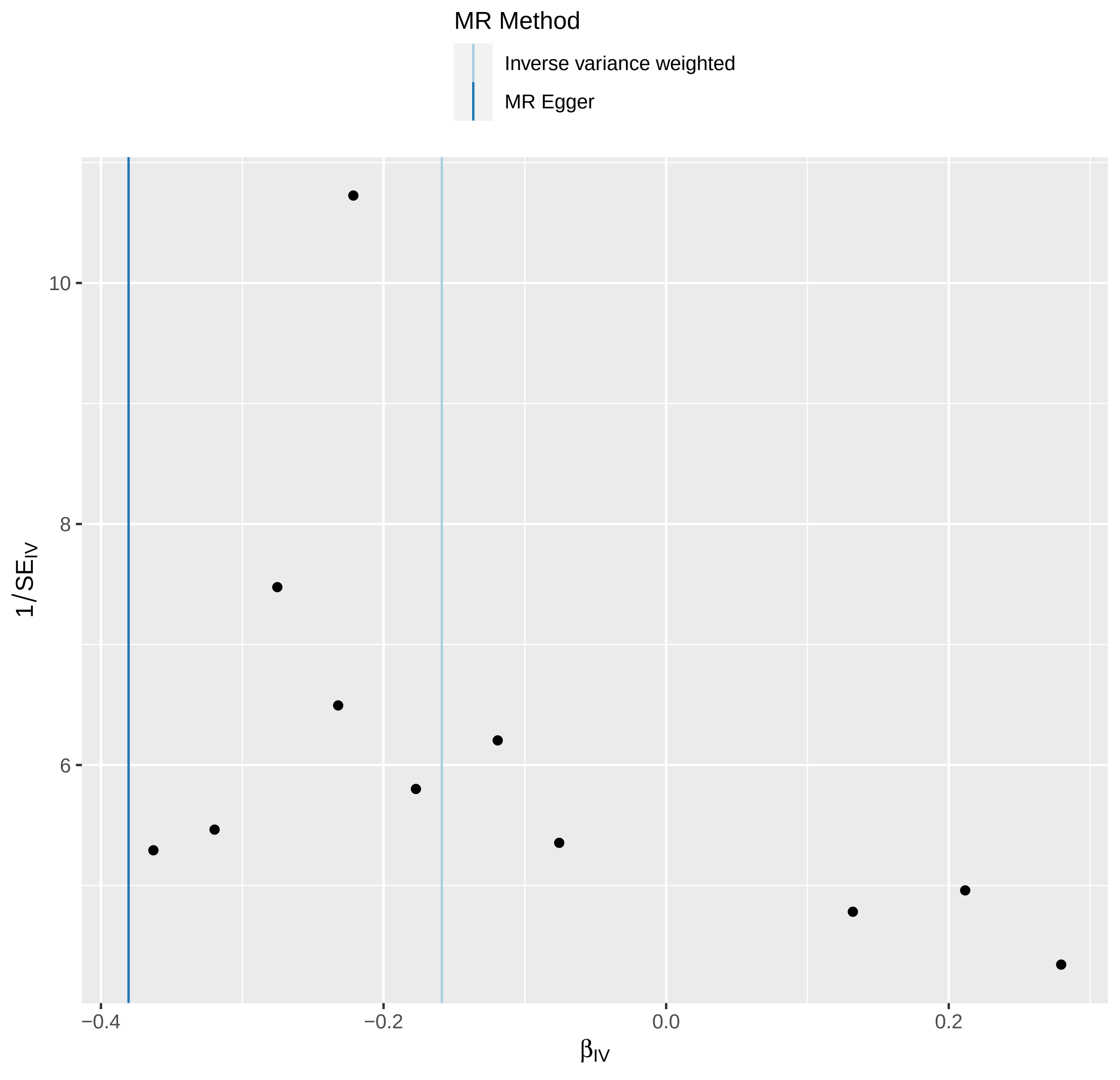

Batch 3−1064 : Selenium on Fructose−1,6−bisphosphatase isozyme 2

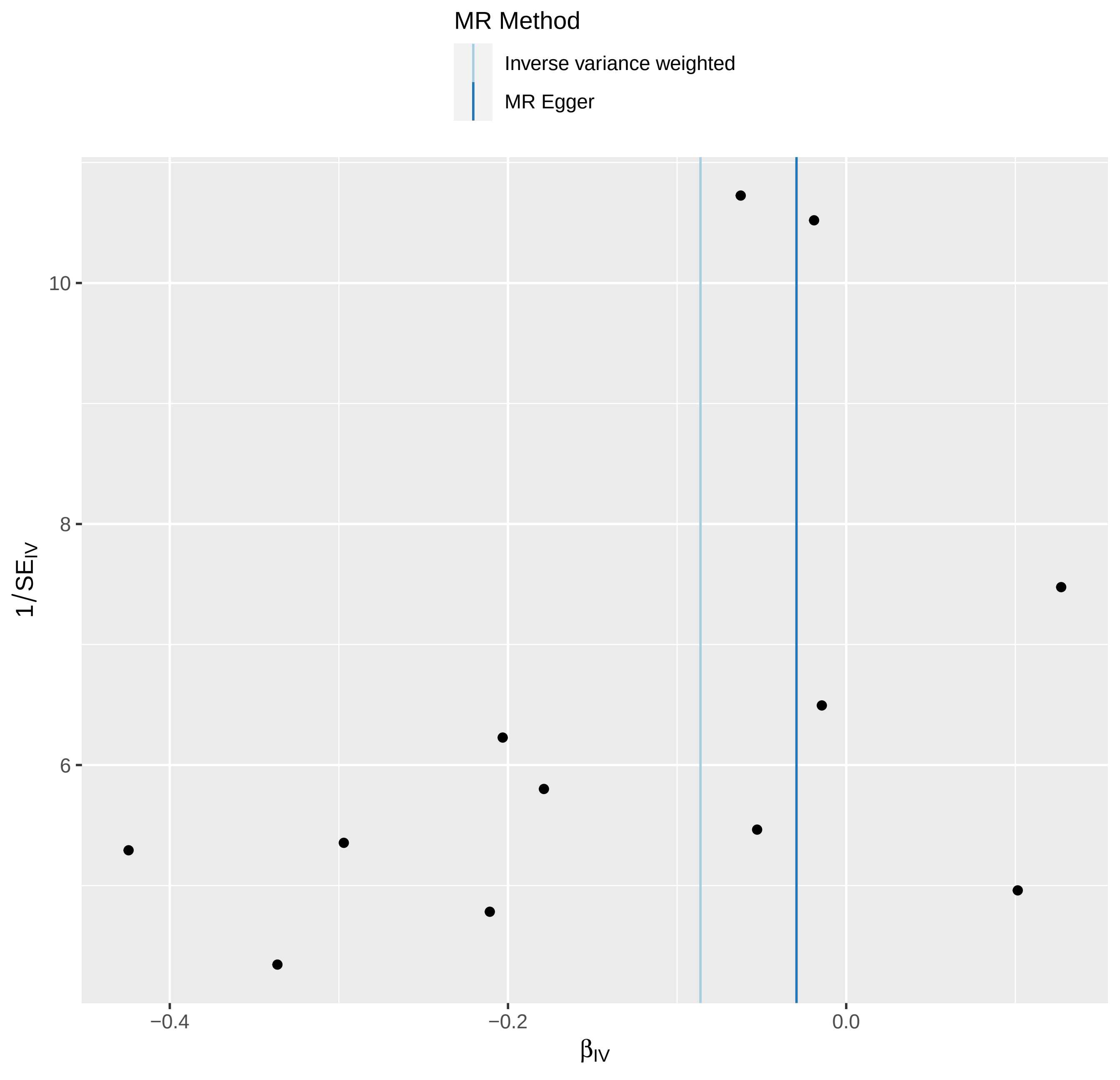

Batch 3−1065 : Selenium on F−box/LRR−repeat protein 4

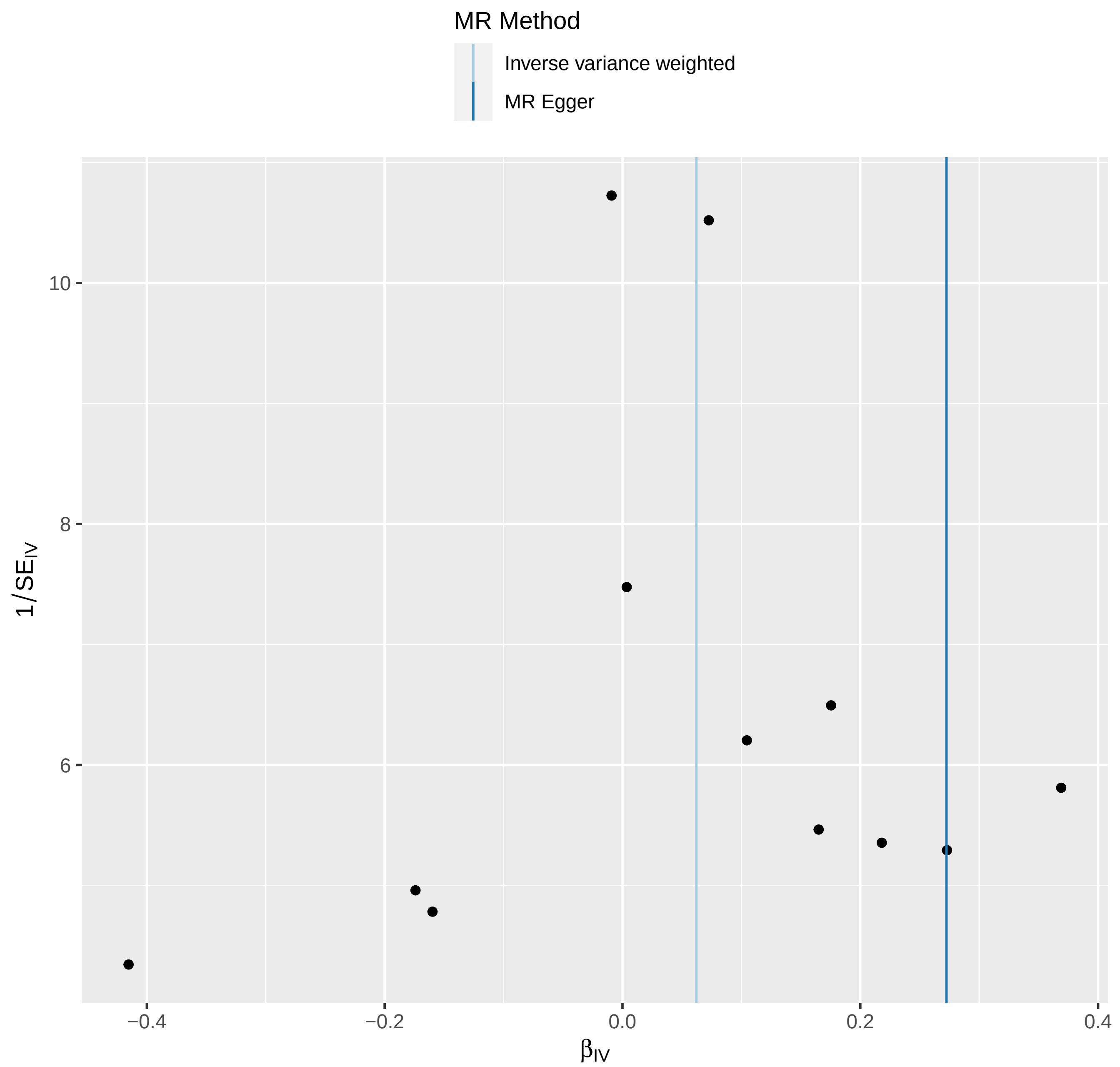

Batch 3−1066 : Selenium on F−box/LRR−repeat protein 4

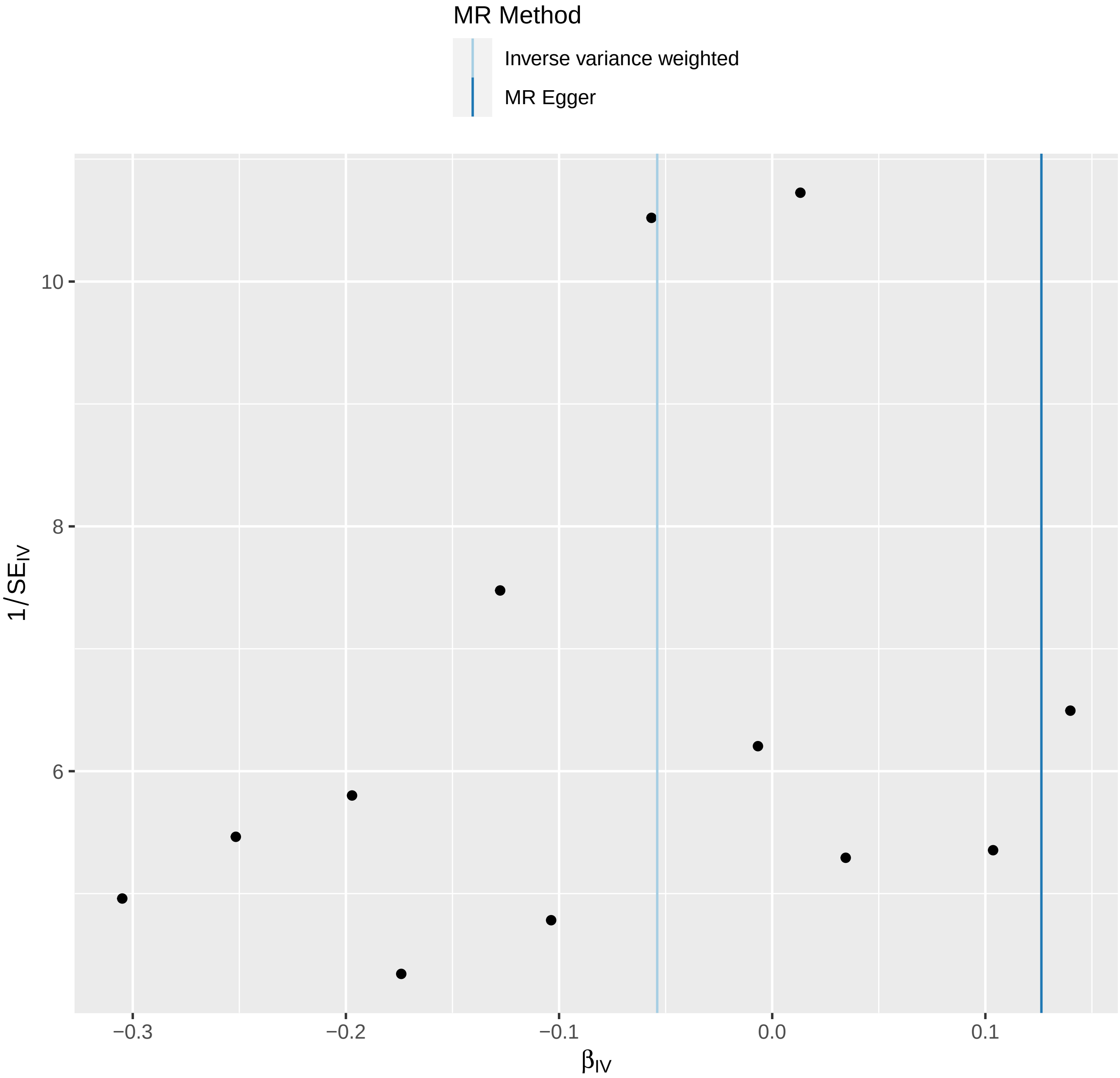

Batch 3−1067 : Selenium on F−box/LRR−repeat protein 4

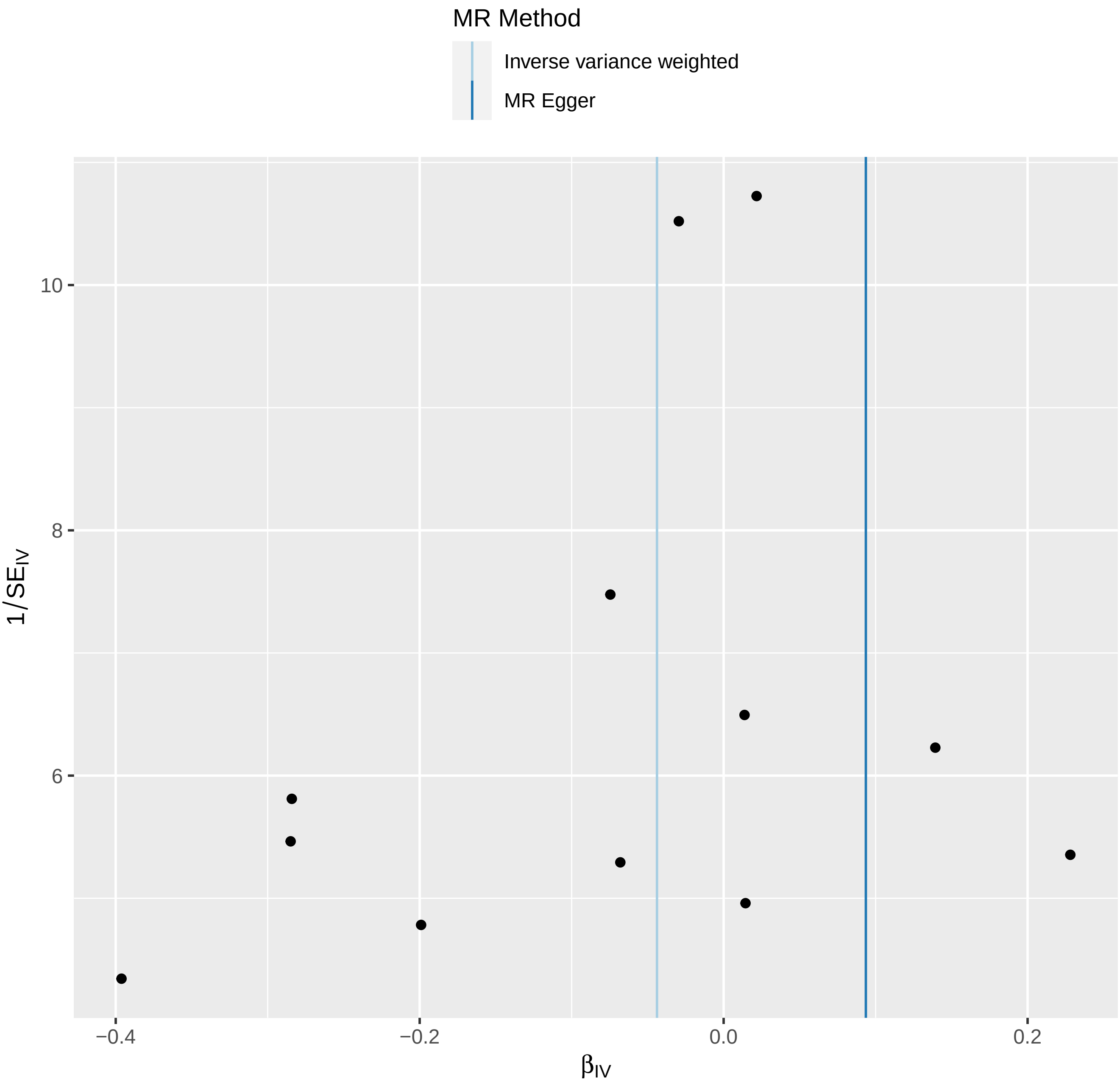

Batch 3−1068 : Selenium on F−box/LRR−repeat protein 5

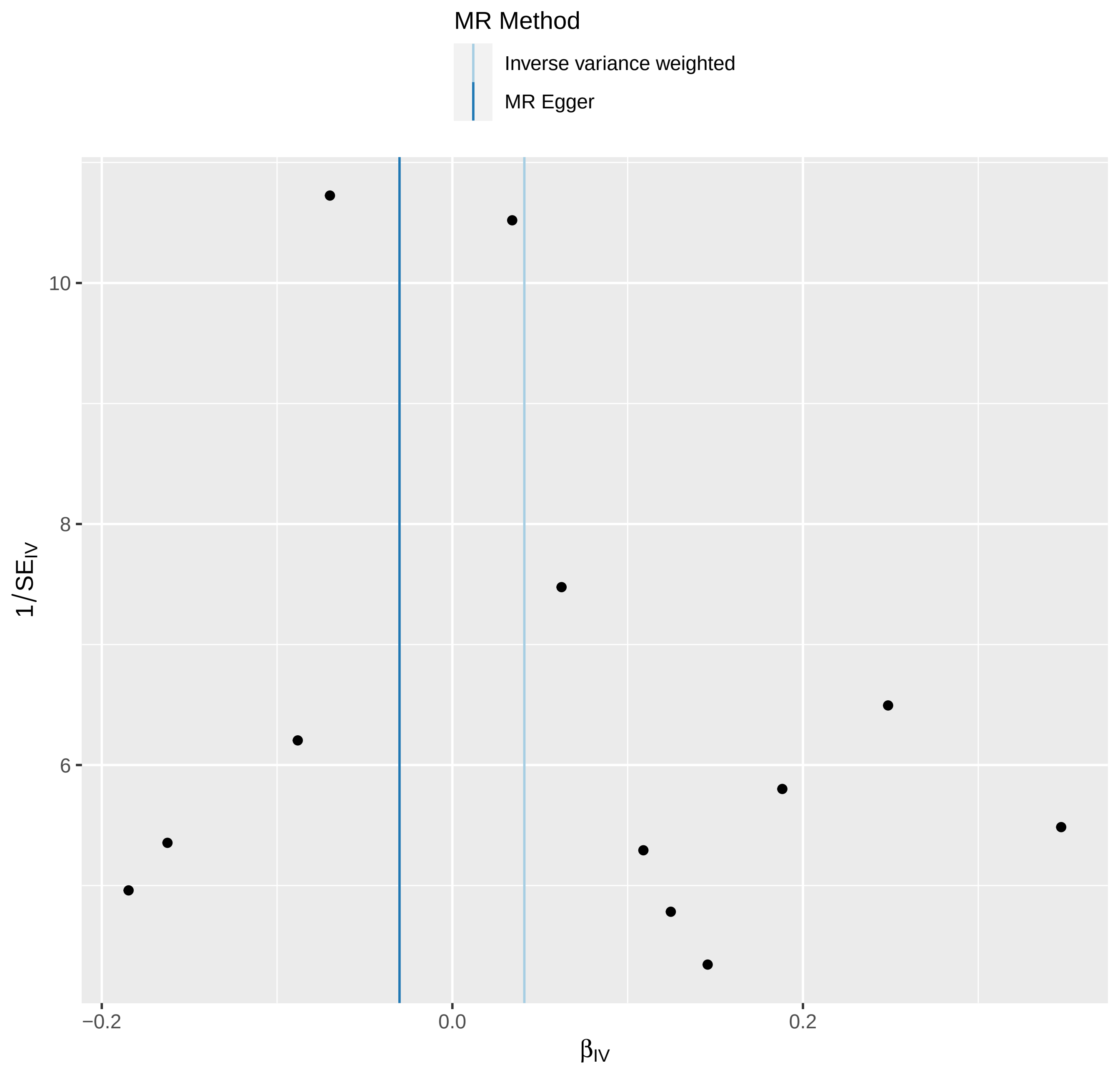

Batch 3−1069 : Selenium on F−box only protein 3

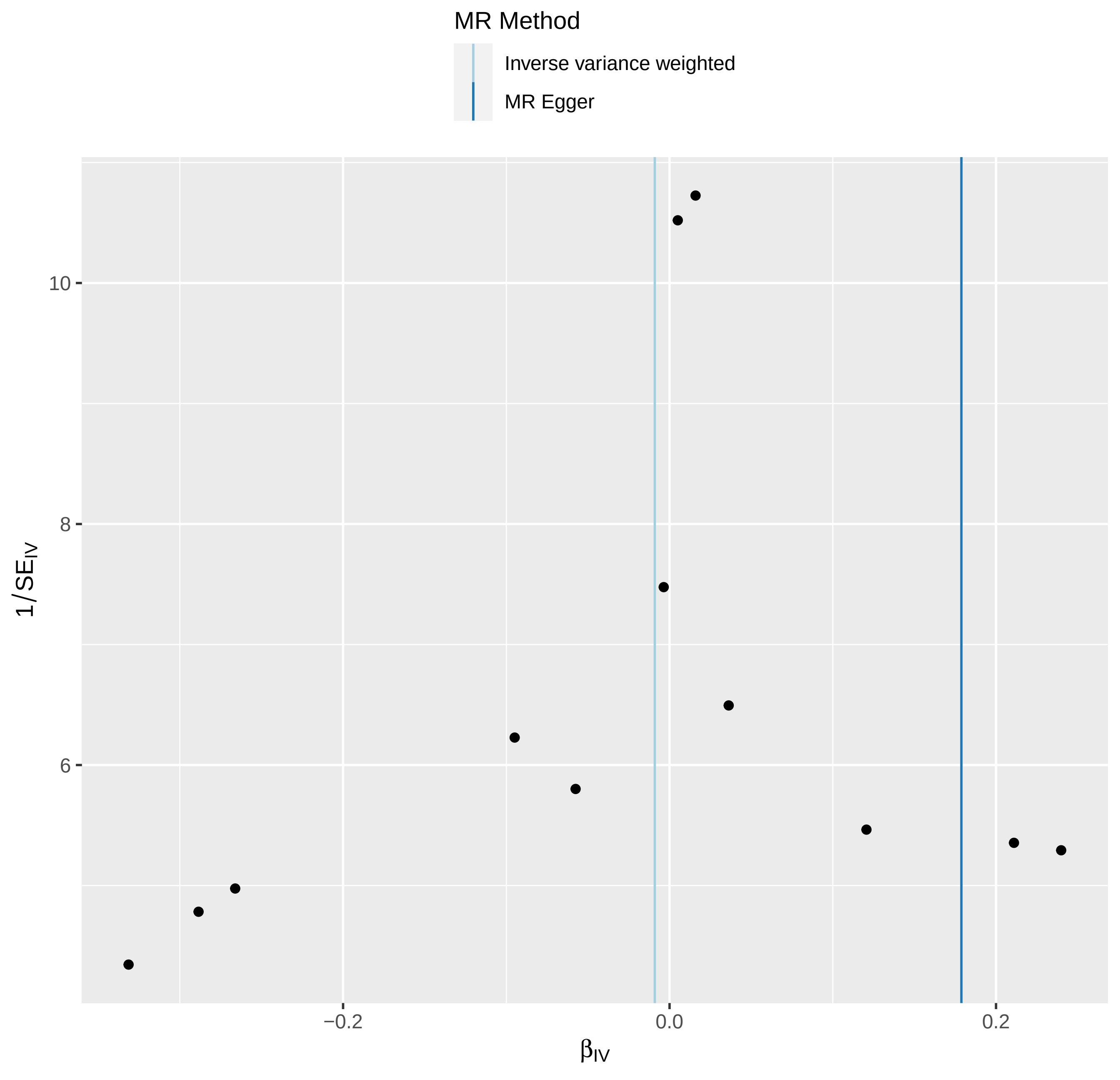

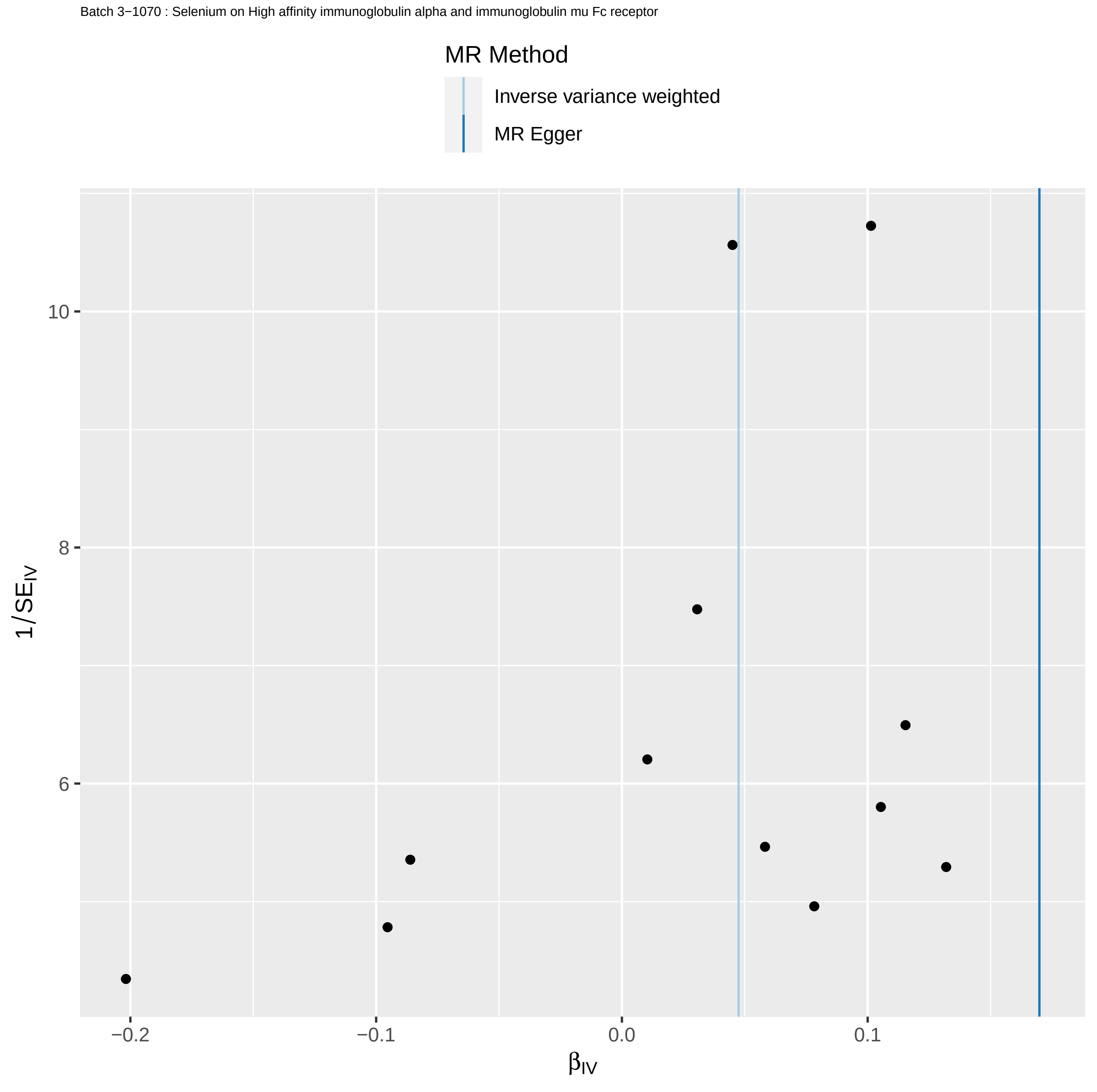

Batch 3−1070 : Selenium on High affinity immunoglobulin alpha and immunoglobulin mu Fc receptor
MR Method
Inverse variance weighted
MR Egger
10
8
6
1/SE_IV
−0.2
−0.1
0.0
0.1
β_IV

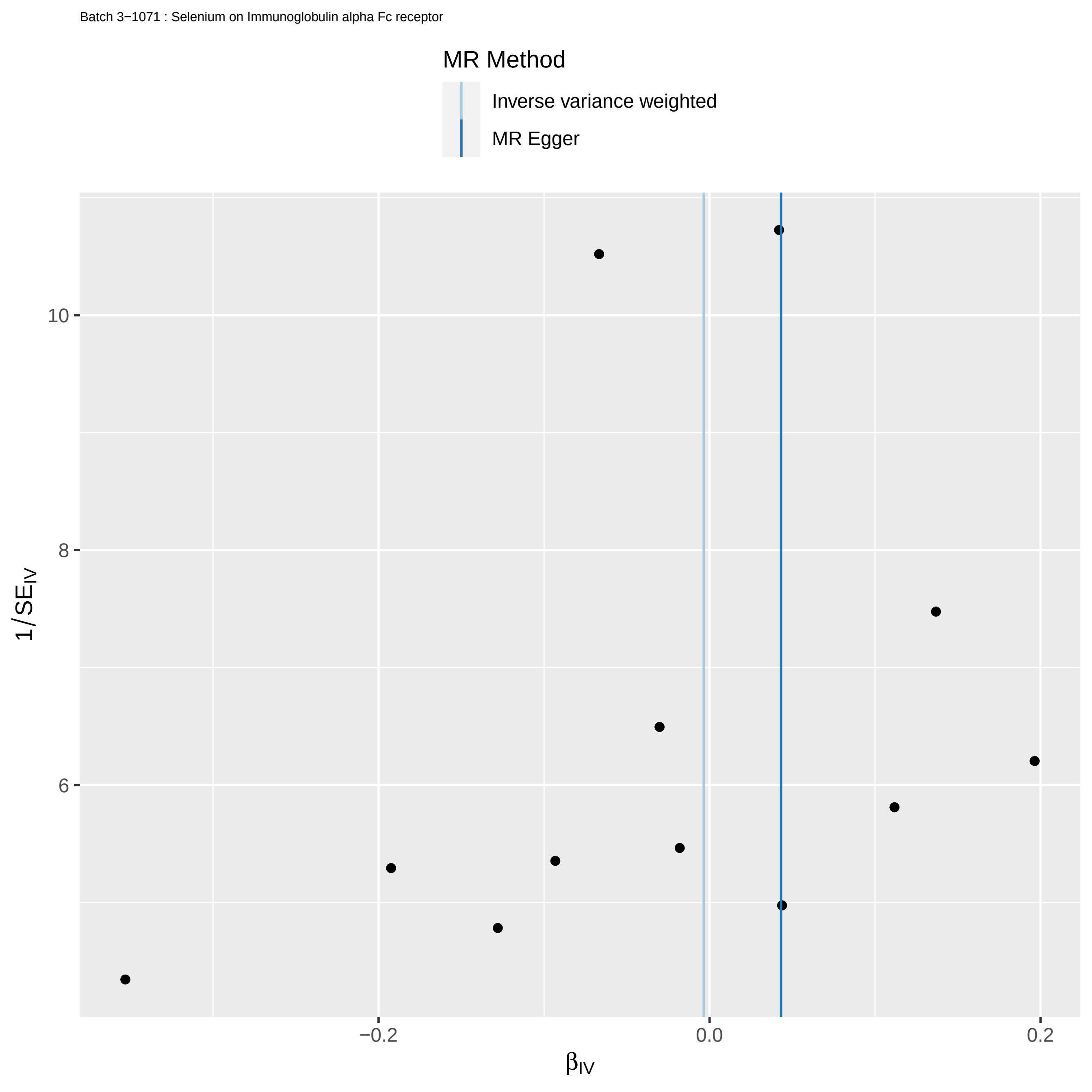

Batch 3−1071 : Selenium on Immunoglobulin alpha Fc receptor
MR Method
Inverse variance weighted
MR Egger
10
8
6
1/SE_IV
−0.2
0.0
0.2
β_IV

Batch 3−1072 : Selenium on Low affinity immunoglobulin epsilon Fc receptor

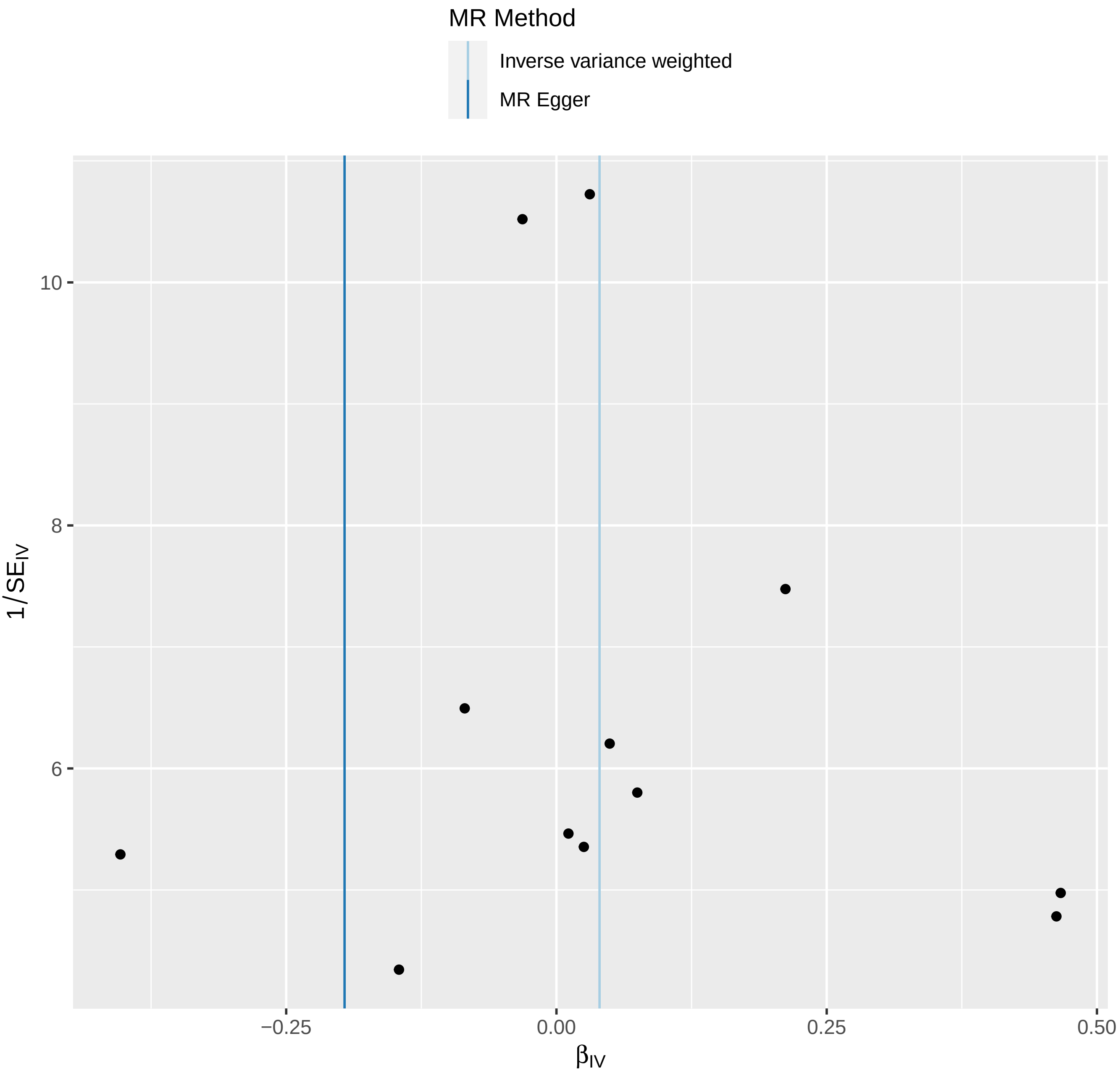

Batch 3−1073 : Selenium on High affinity immunoglobulin gamma Fc receptor I

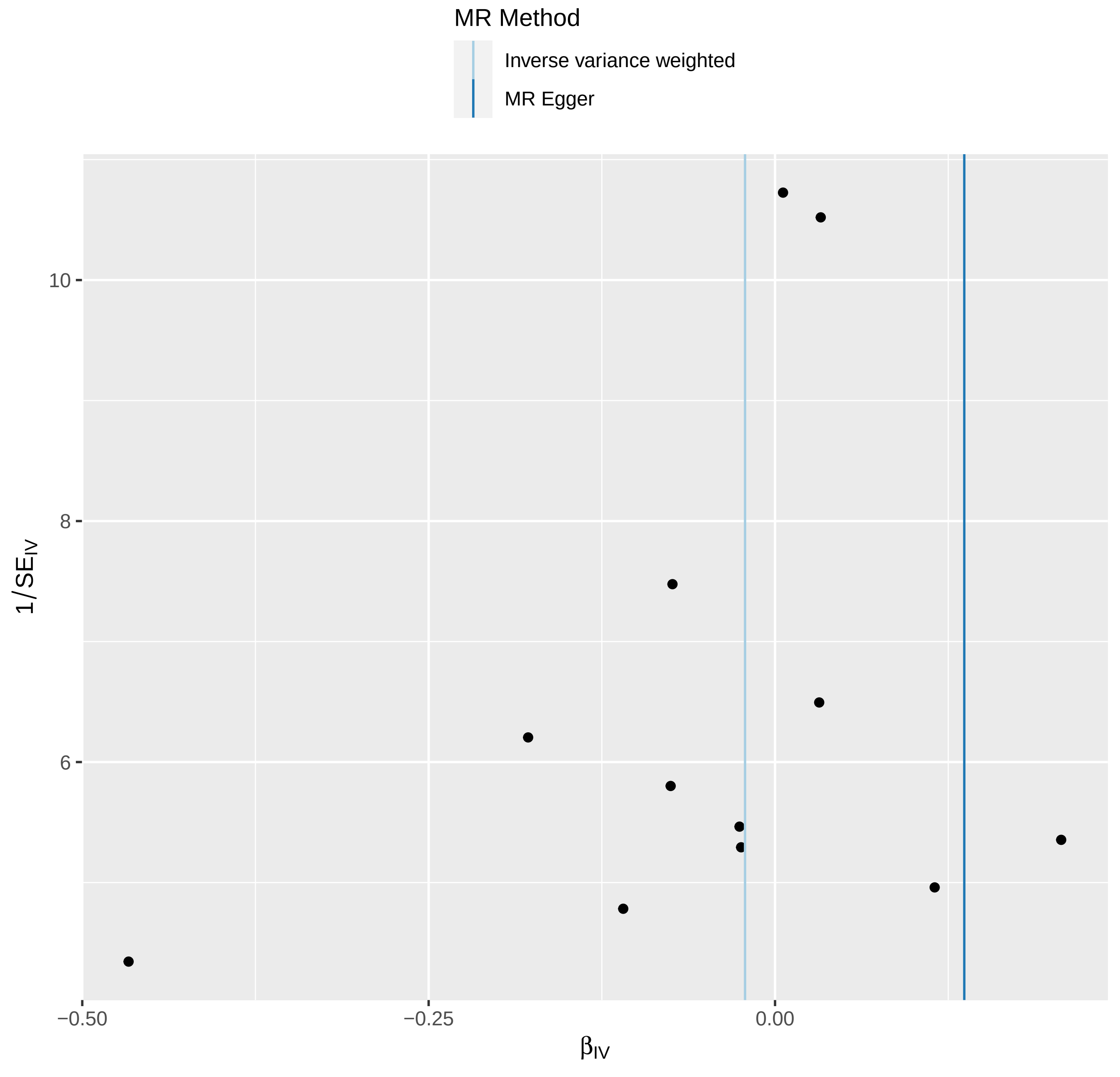

Batch 3−1074 : Selenium on Low affinity immunoglobulin gamma Fc region receptor II−a

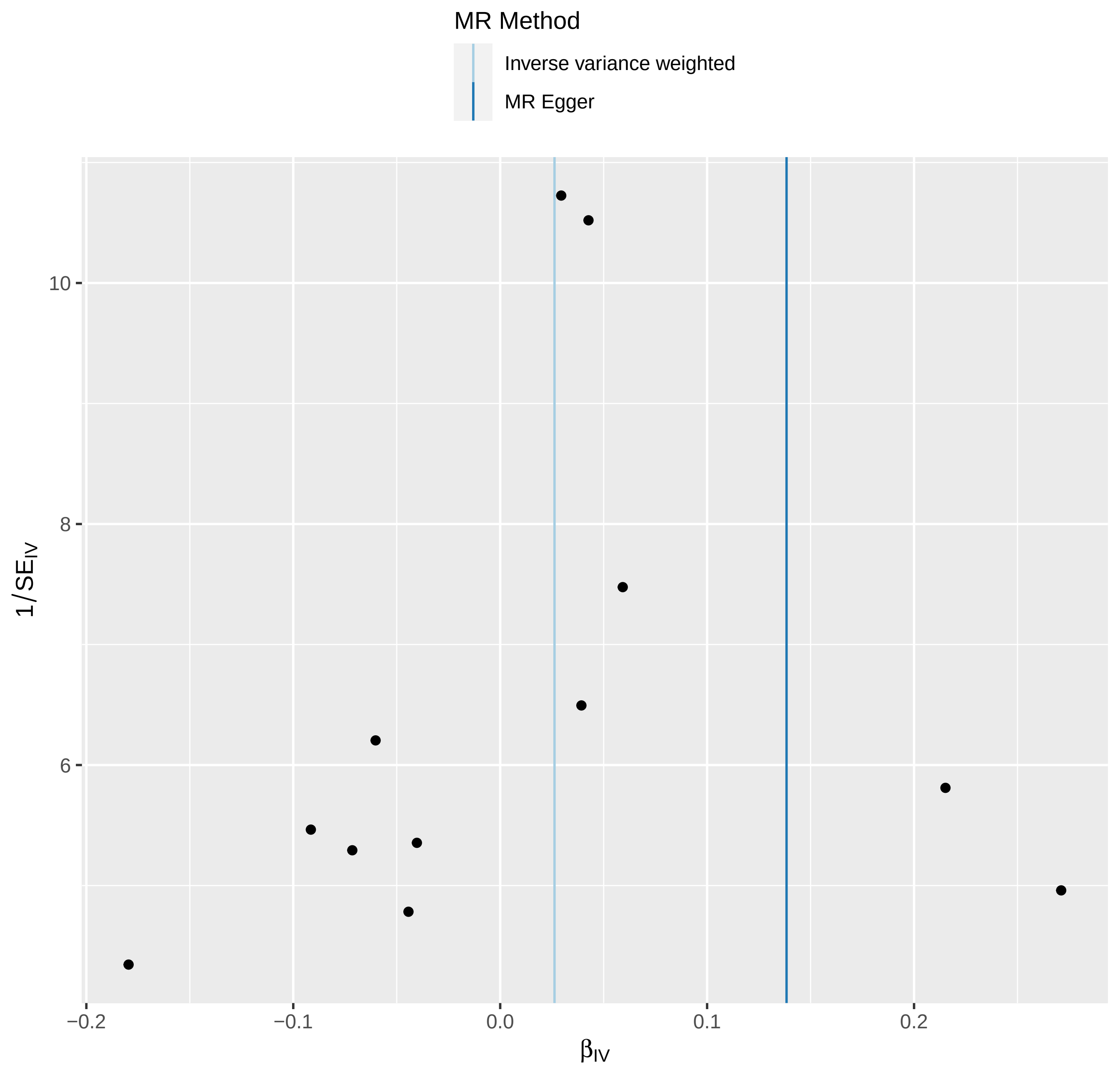

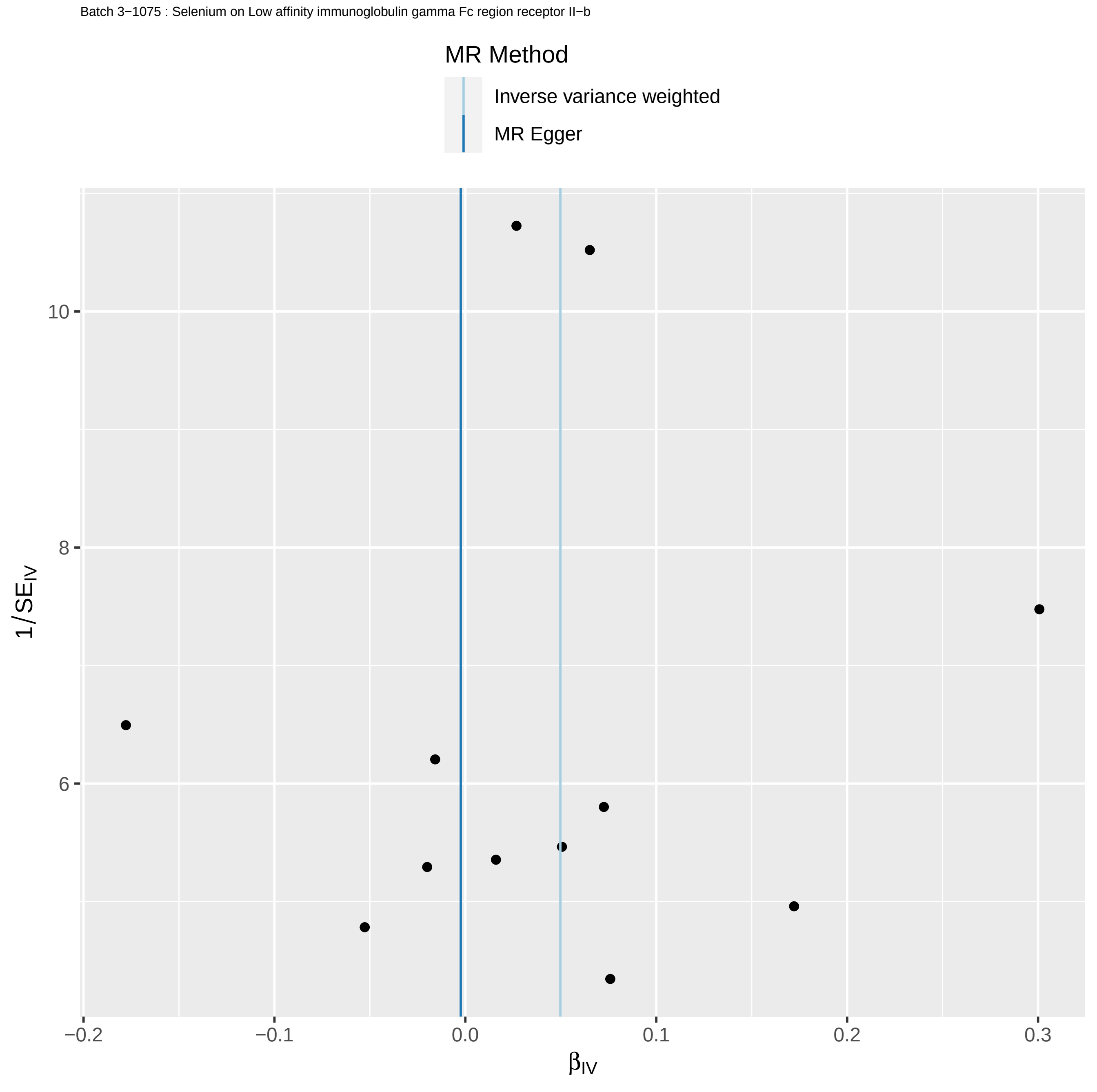

Batch 3−1075 : Selenium on Low affinity immunoglobulin gamma Fc region receptor II−b
MR Method
Inverse variance weighted
MR Egger
10
8
6
1/SE$_{IV}$
−0.2
−0.1
0.0
0.1
0.2
0.3
β$_{IV}$

Batch 3−1076 : Selenium on Low affinity immunoglobulin gamma Fc region receptor III−B

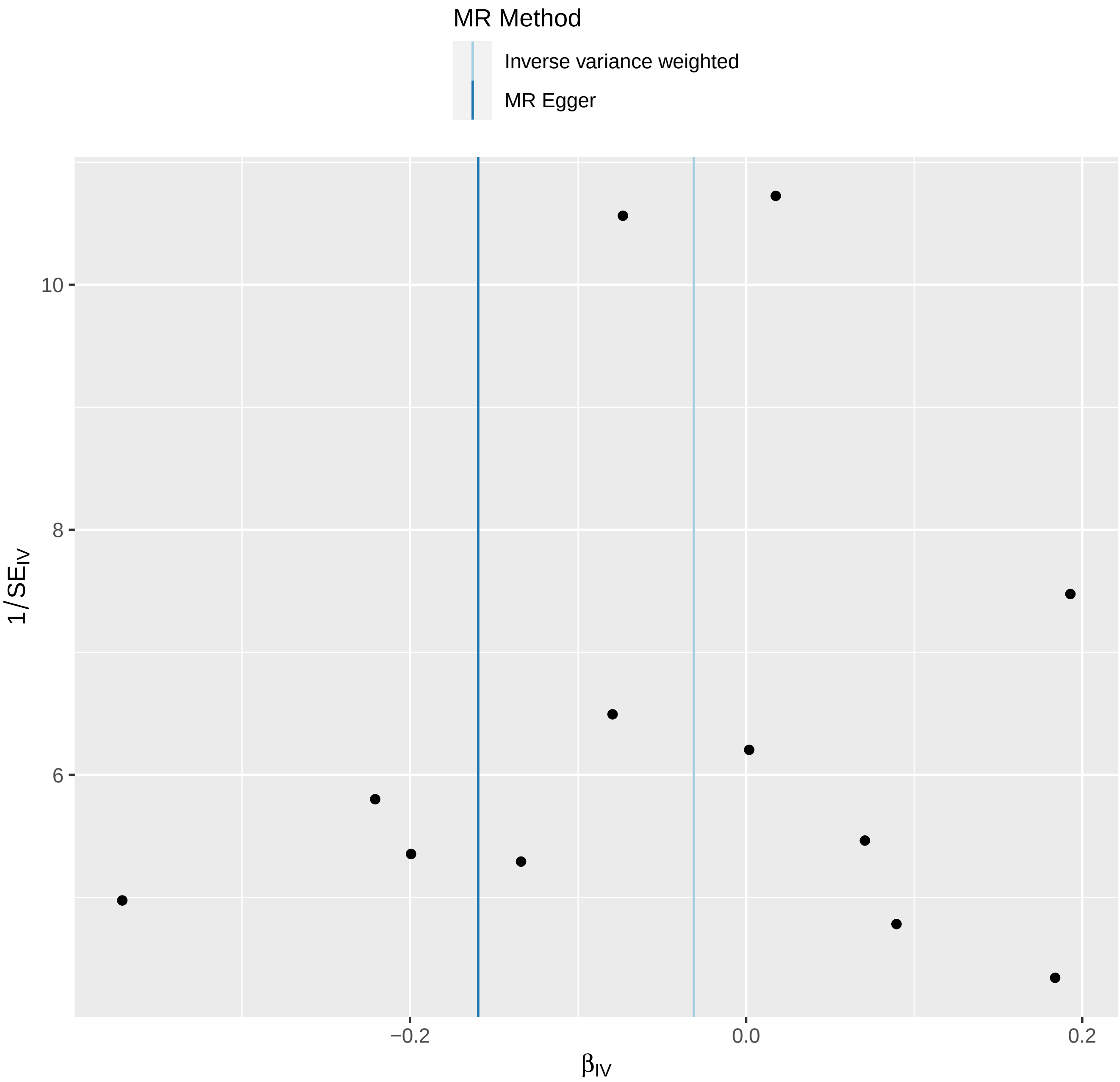

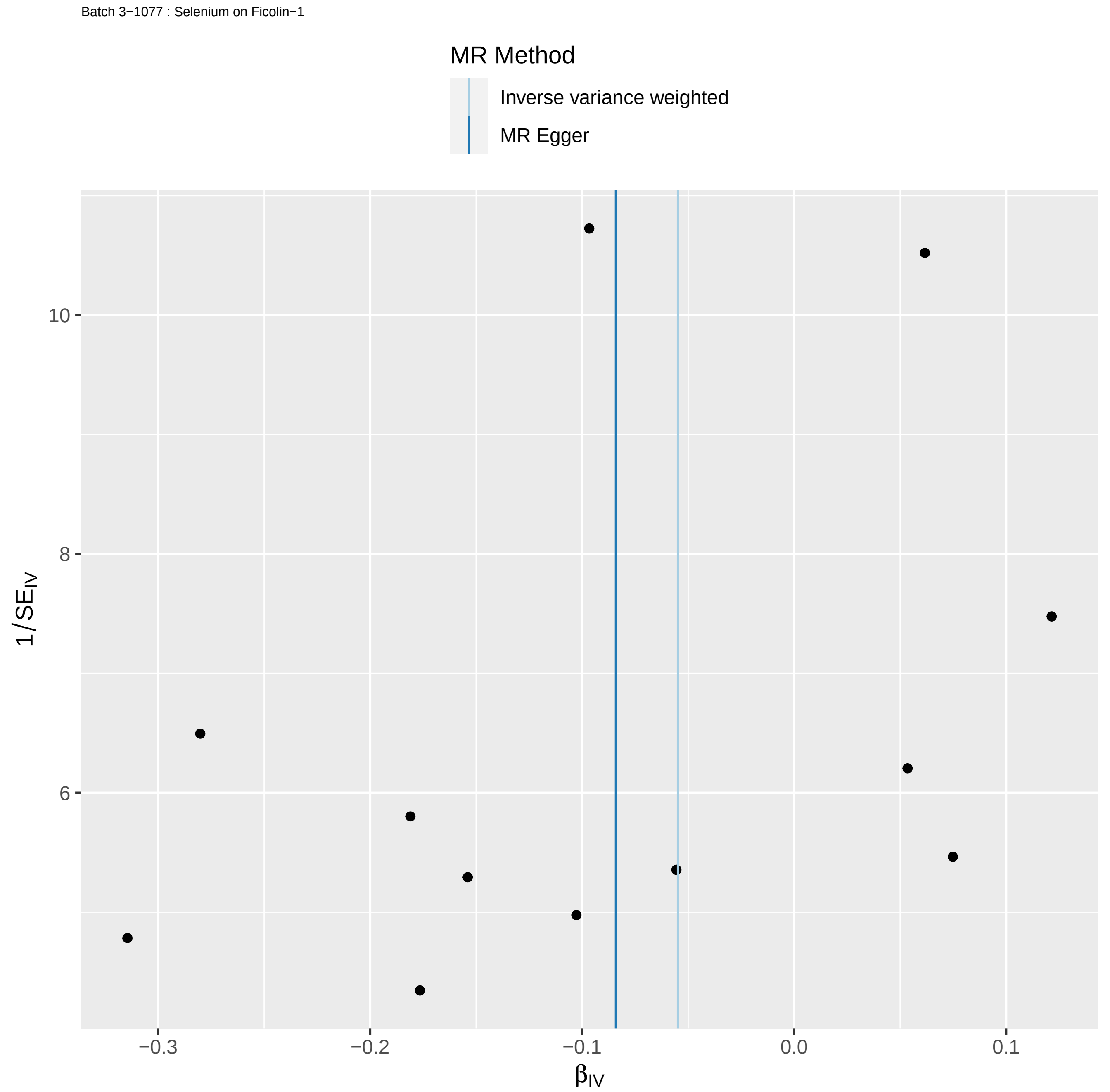

Batch 3−1077 : Selenium on Ficolin−1
MR Method
Inverse variance weighted
MR Egger
10
8
6
1/SE_IV
−0.3
−0.2
−0.1
0.0
0.1
β_IV

Batch 3−1078 : Selenium on Ficolin−2

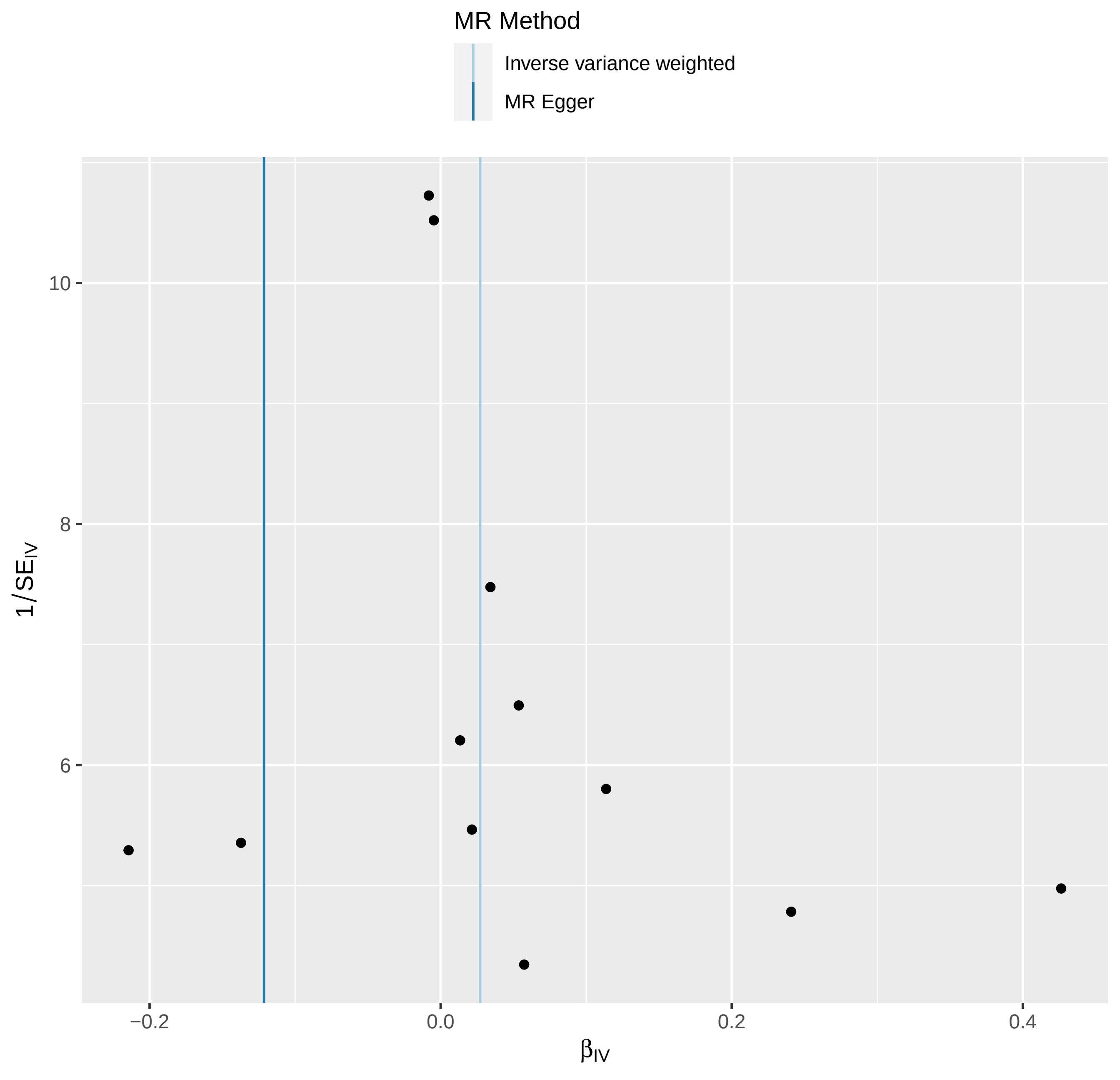

Batch 3−1079 : Selenium on Fc receptor−like protein 1

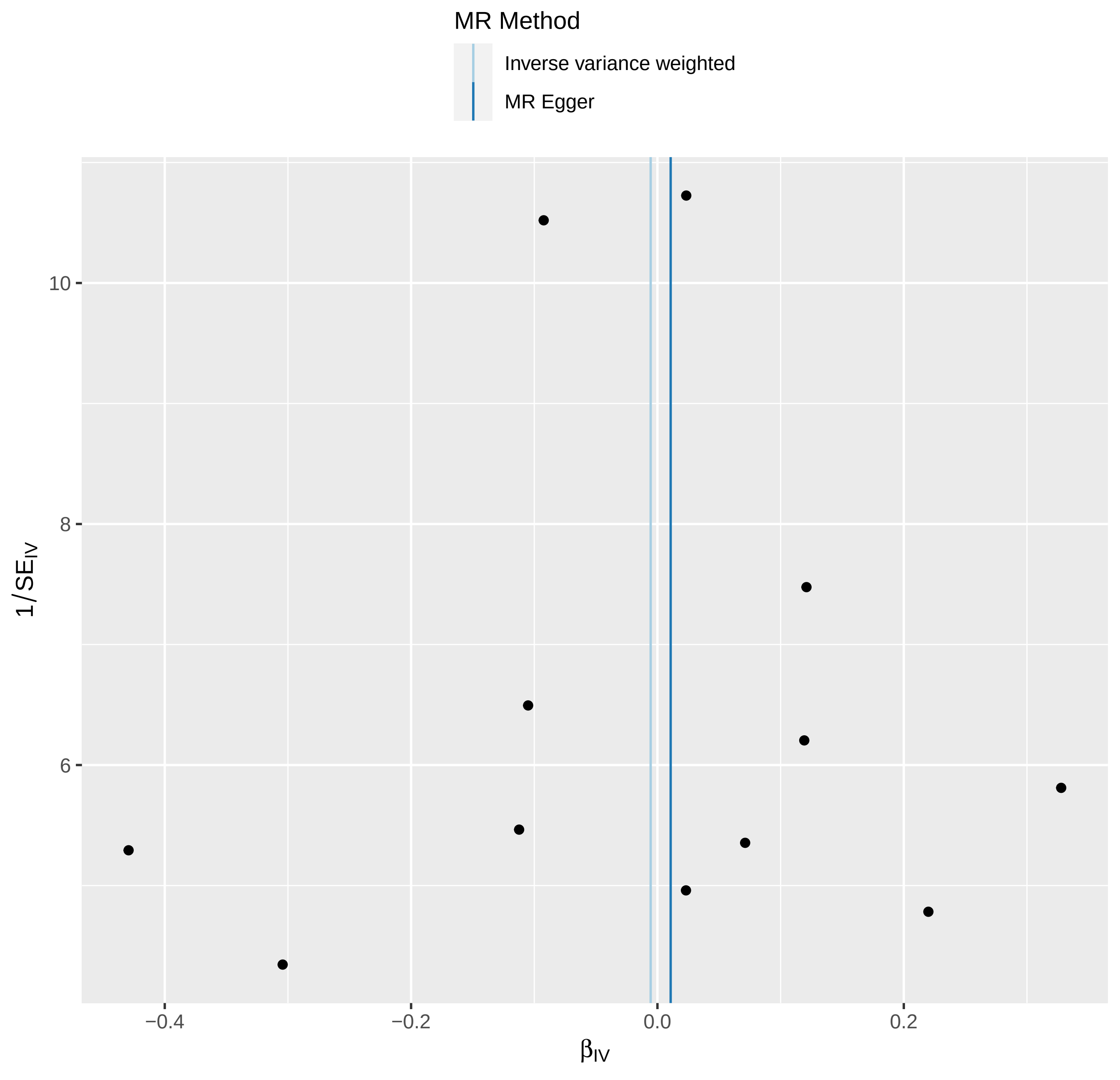

Batch 3−1080 : Selenium on Fc receptor−like protein 2

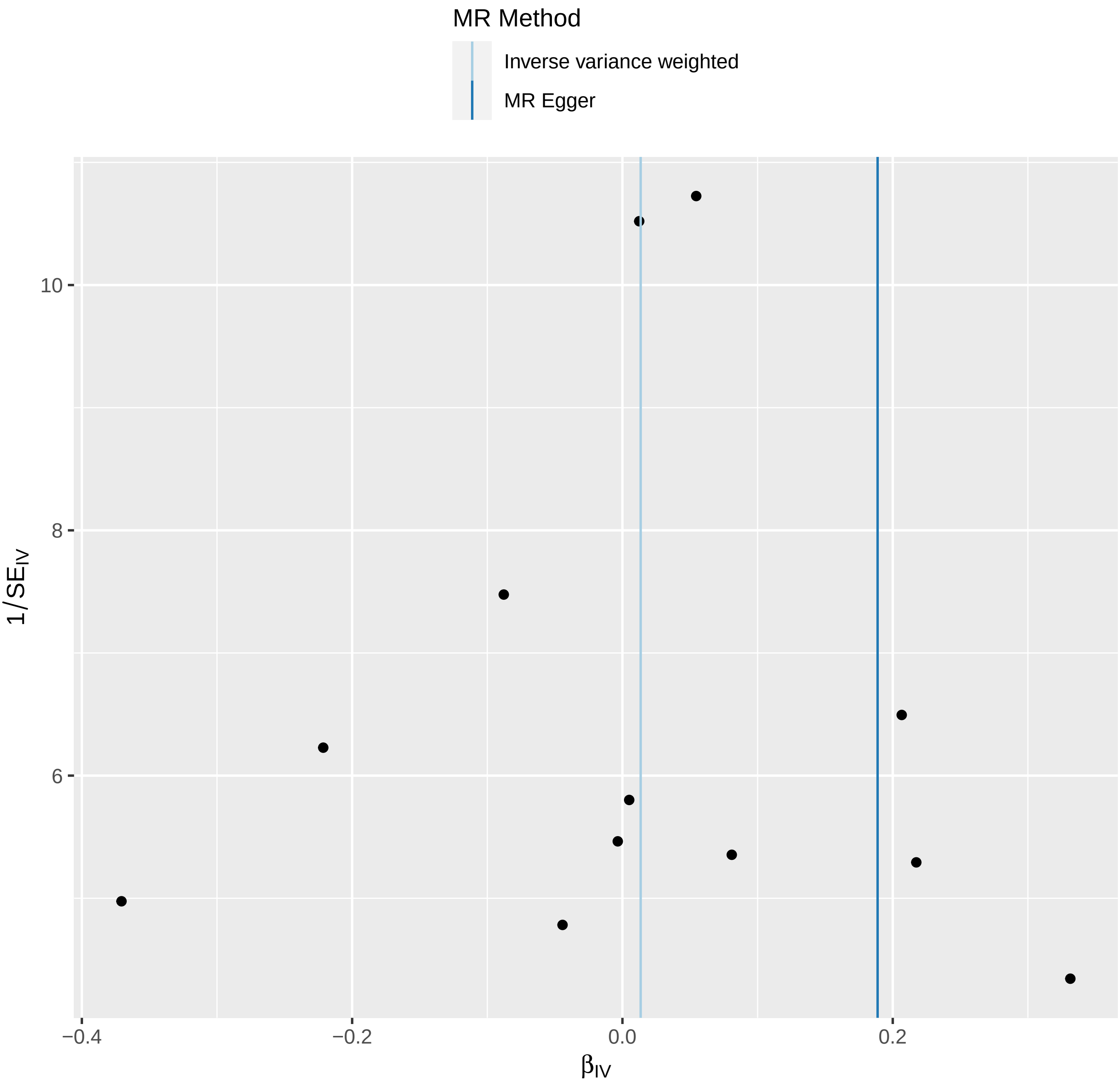

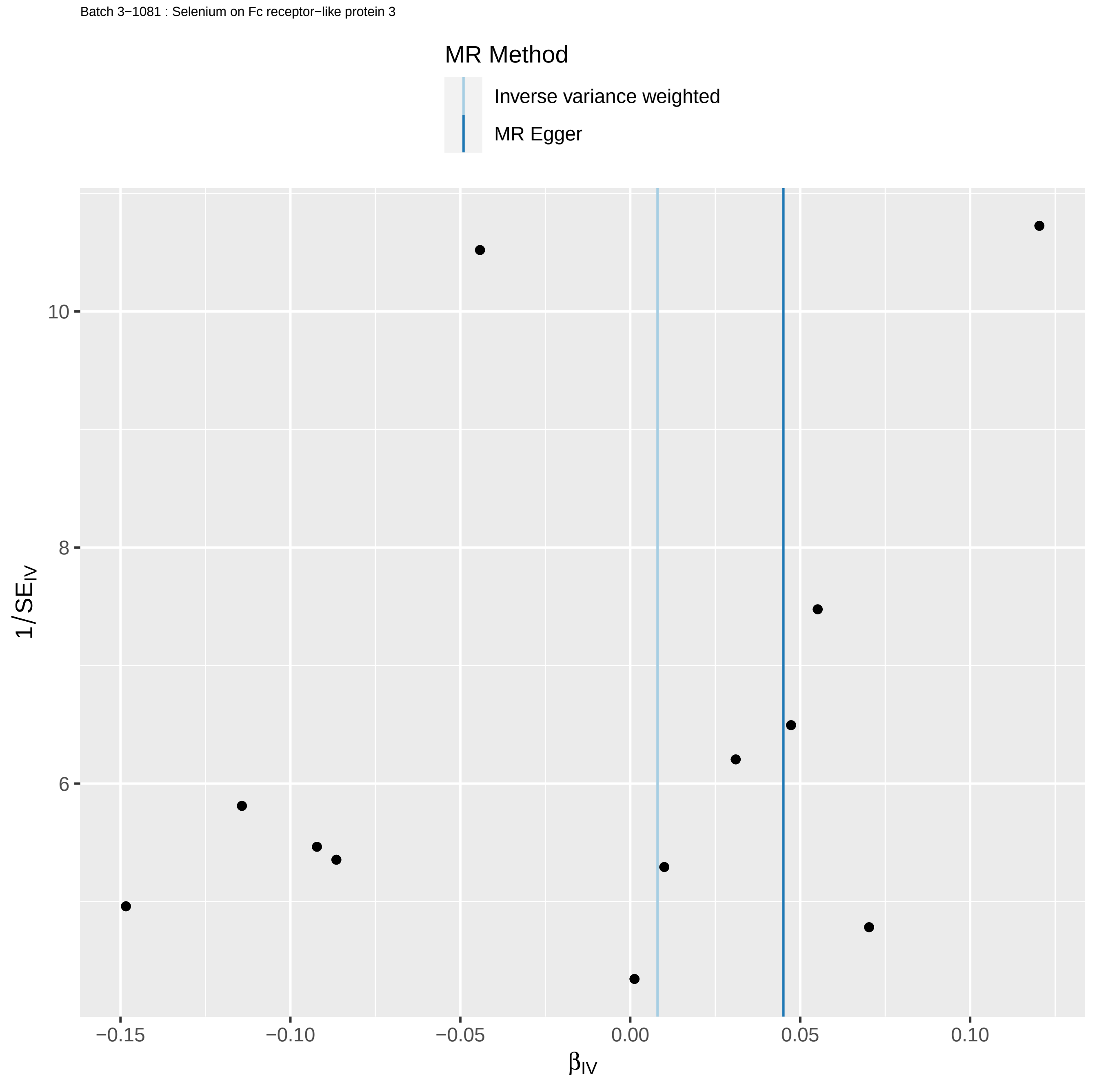

Batch 3−1081 : Selenium on Fc receptor−like protein 3
MR Method
Inverse variance weighted
MR Egger
10
8
6
1/SE_IV
−0.15
−0.10
−0.05
0.00
0.05
0.10
β_IV

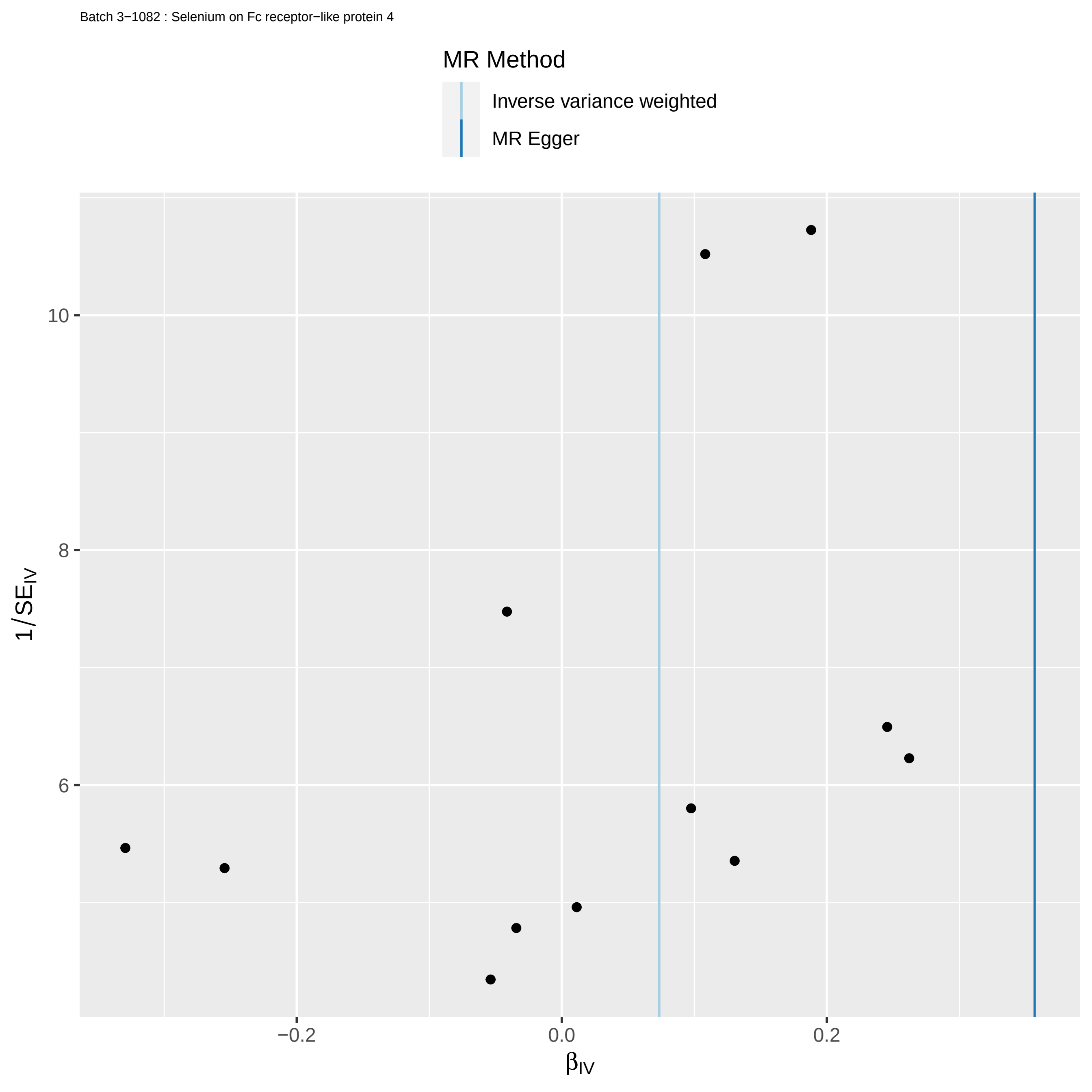
Batch 3−1082 : Selenium on Fc receptor−like protein 4
MR Method
Inverse variance weighted
MR Egger
10
8
6
1/SE_IV
−0.2
0.0
0.2
β_IV

Batch 3−1083 : Selenium on Fc receptor−like protein 5

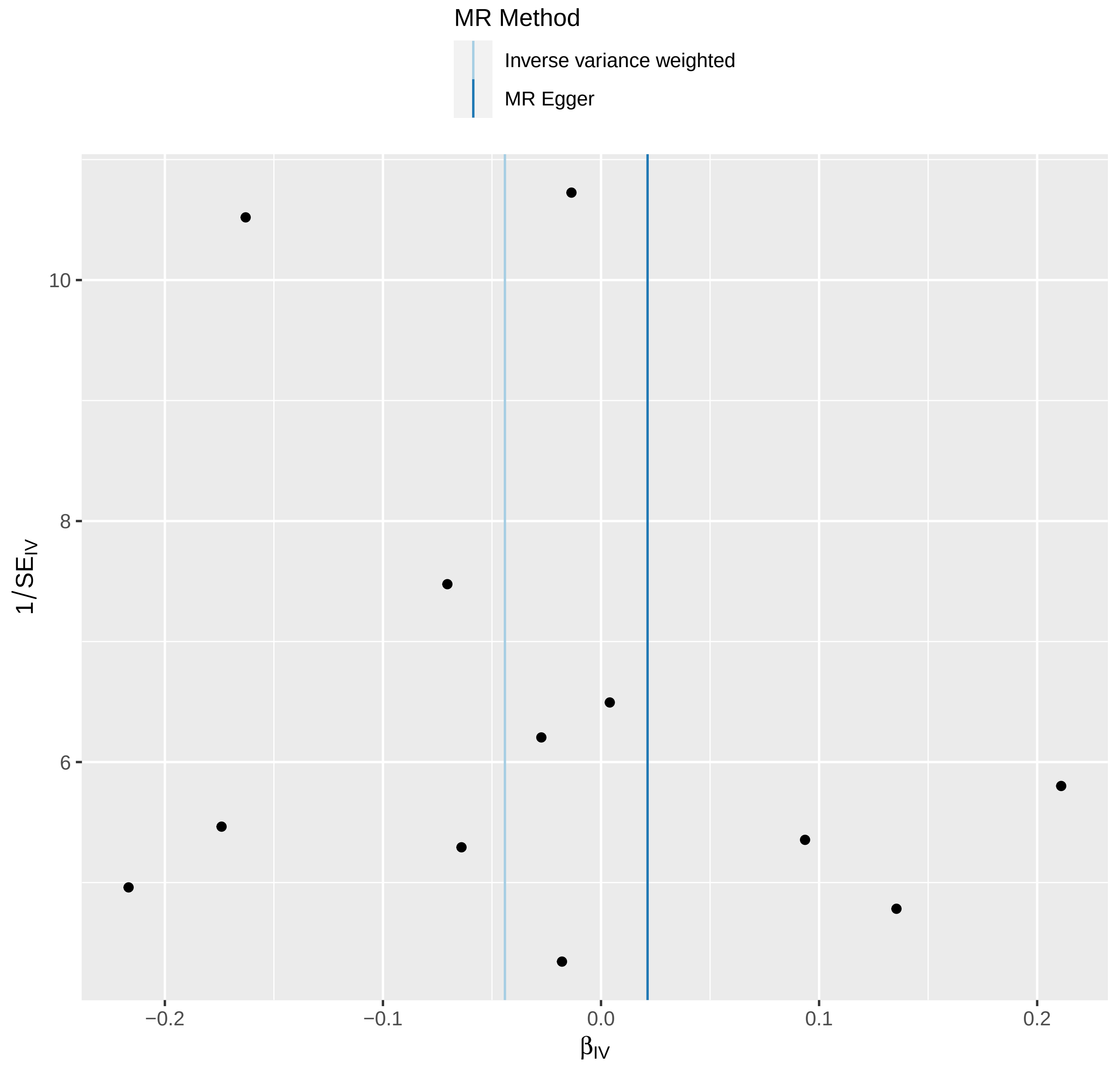

Batch 3−1084 : Selenium on Fc receptor−like protein 6

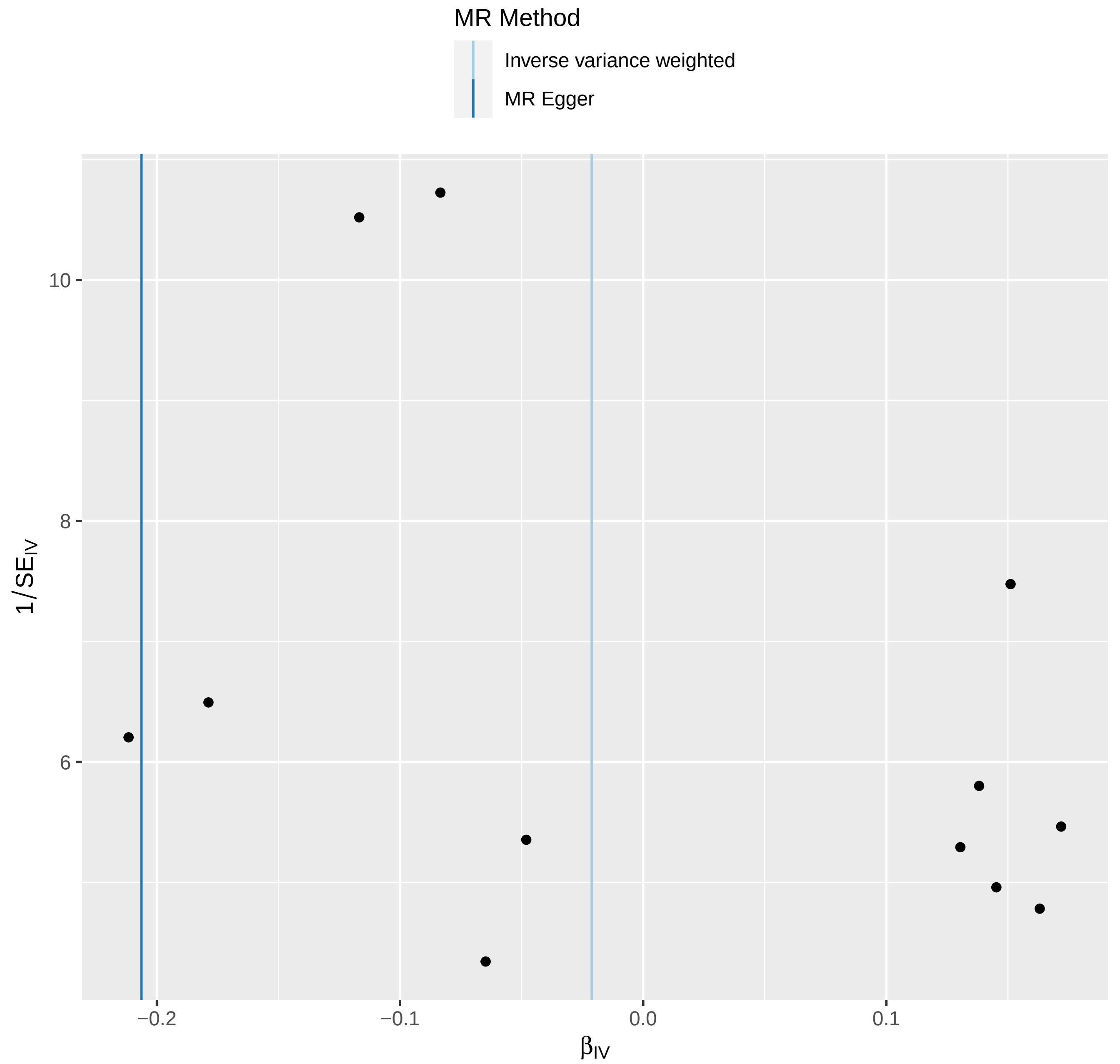

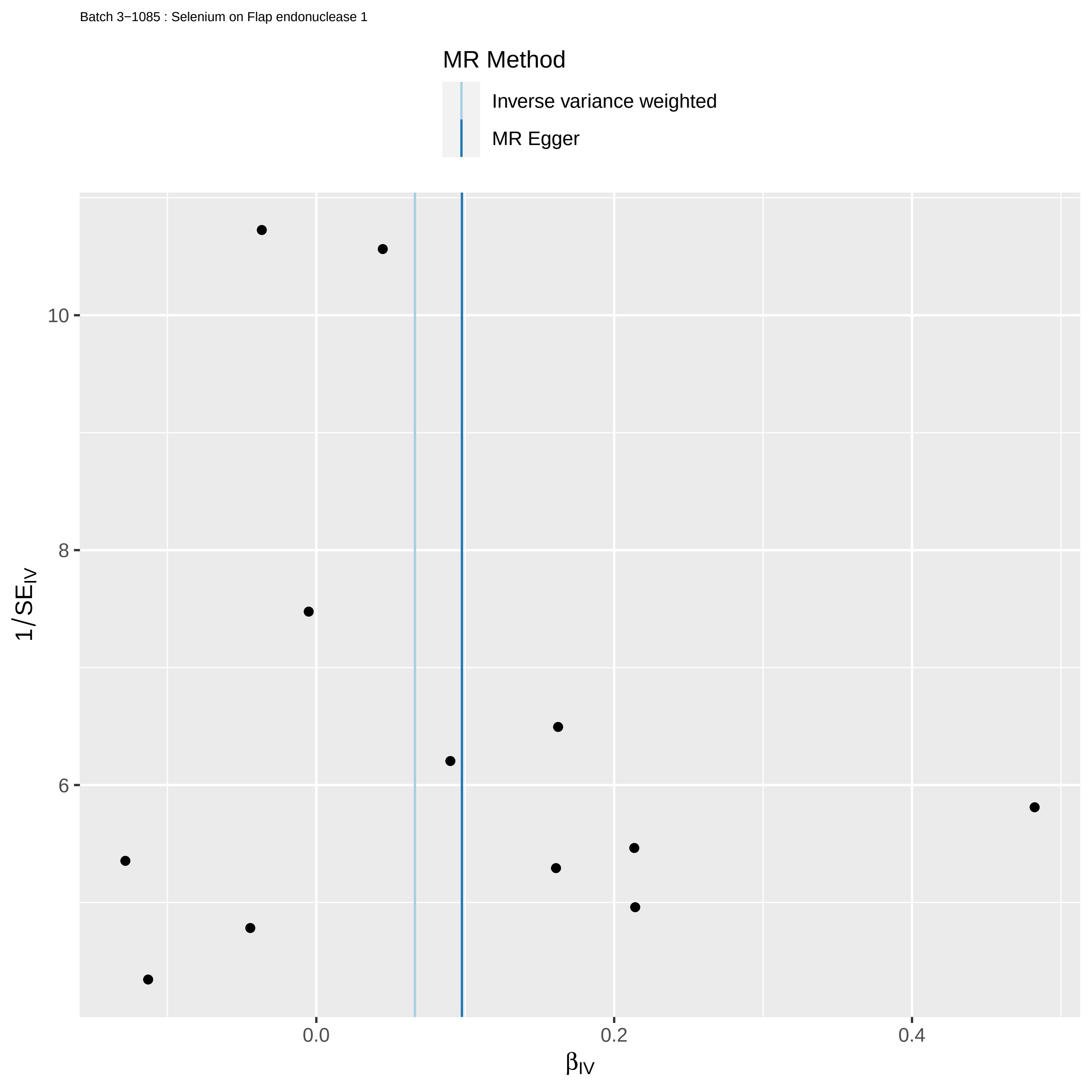

Batch 3−1085 : Selenium on Flap endonuclease 1
MR Method
Inverse variance weighted
MR Egger
10
8
6
$1/SE_{IV}$
0.0
0.2
0.4
β_{IV}

Batch 3−1086 : Selenium on D−dimer

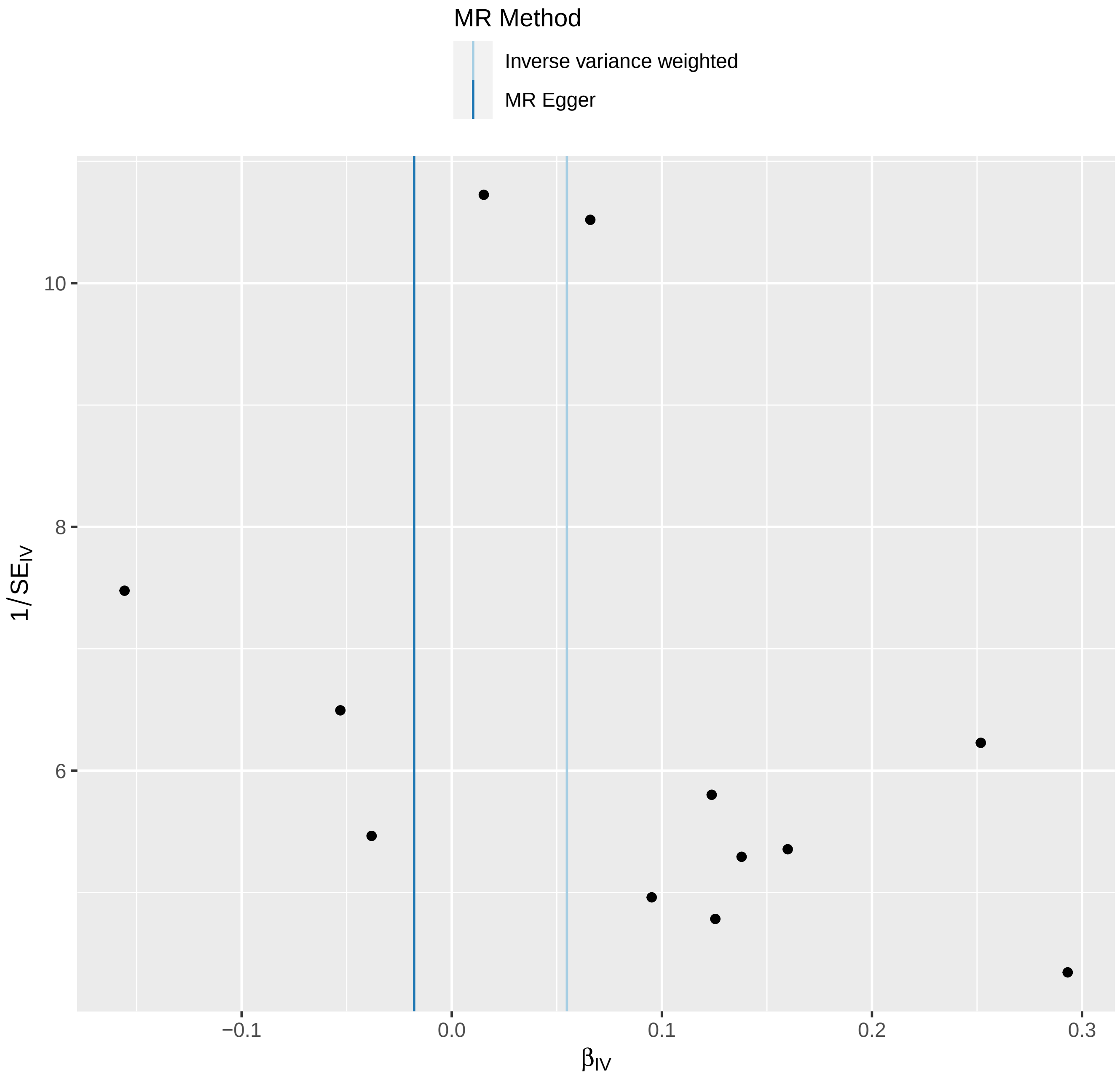

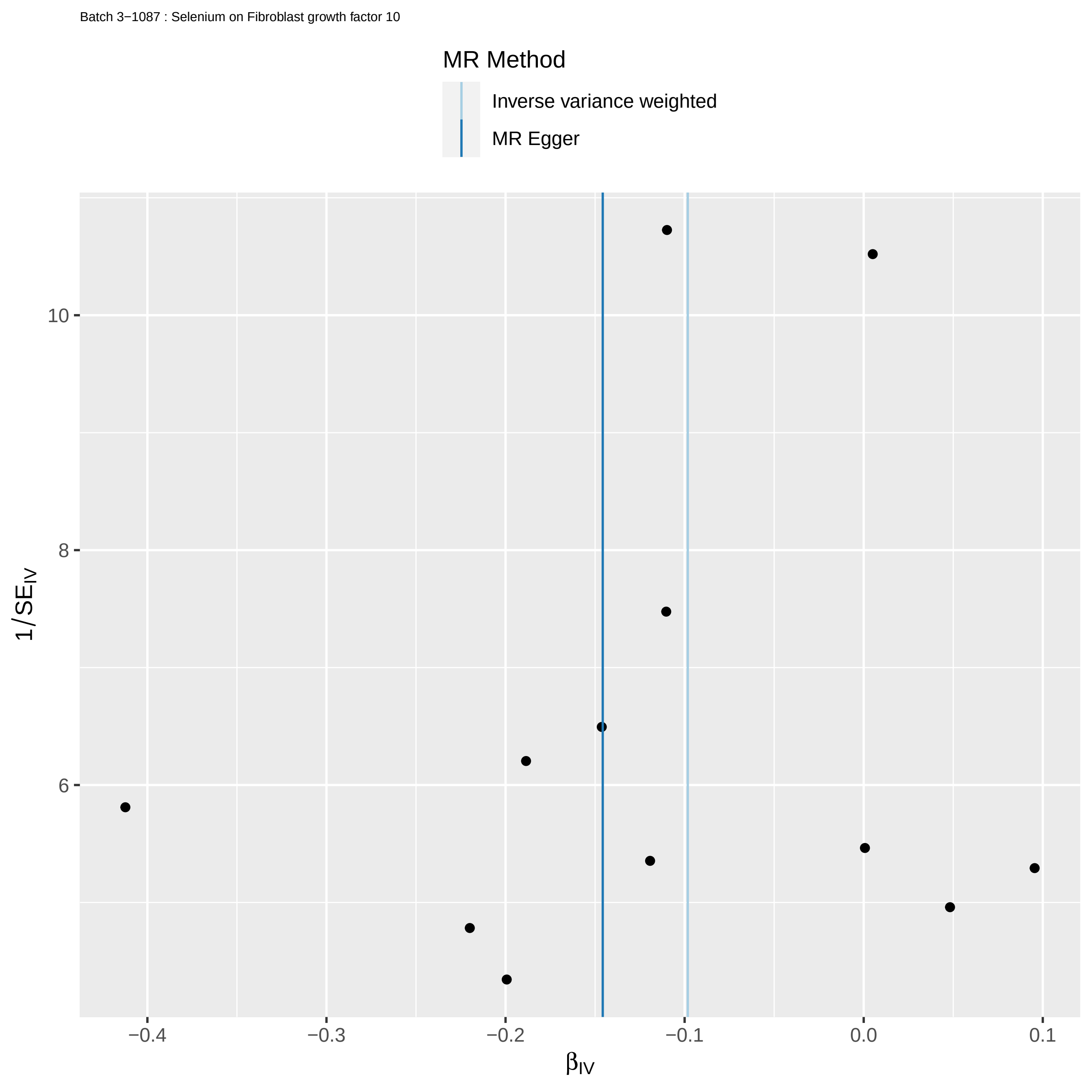
Batch 3−1087 : Selenium on Fibroblast growth factor 10
MR Method
Inverse variance weighted
MR Egger
10
8
6
1/SE_IV
−0.4
−0.3
−0.2
−0.1
0.0
0.1
β_IV

Batch 3−1088 : Selenium on Fibroblast growth factor 1

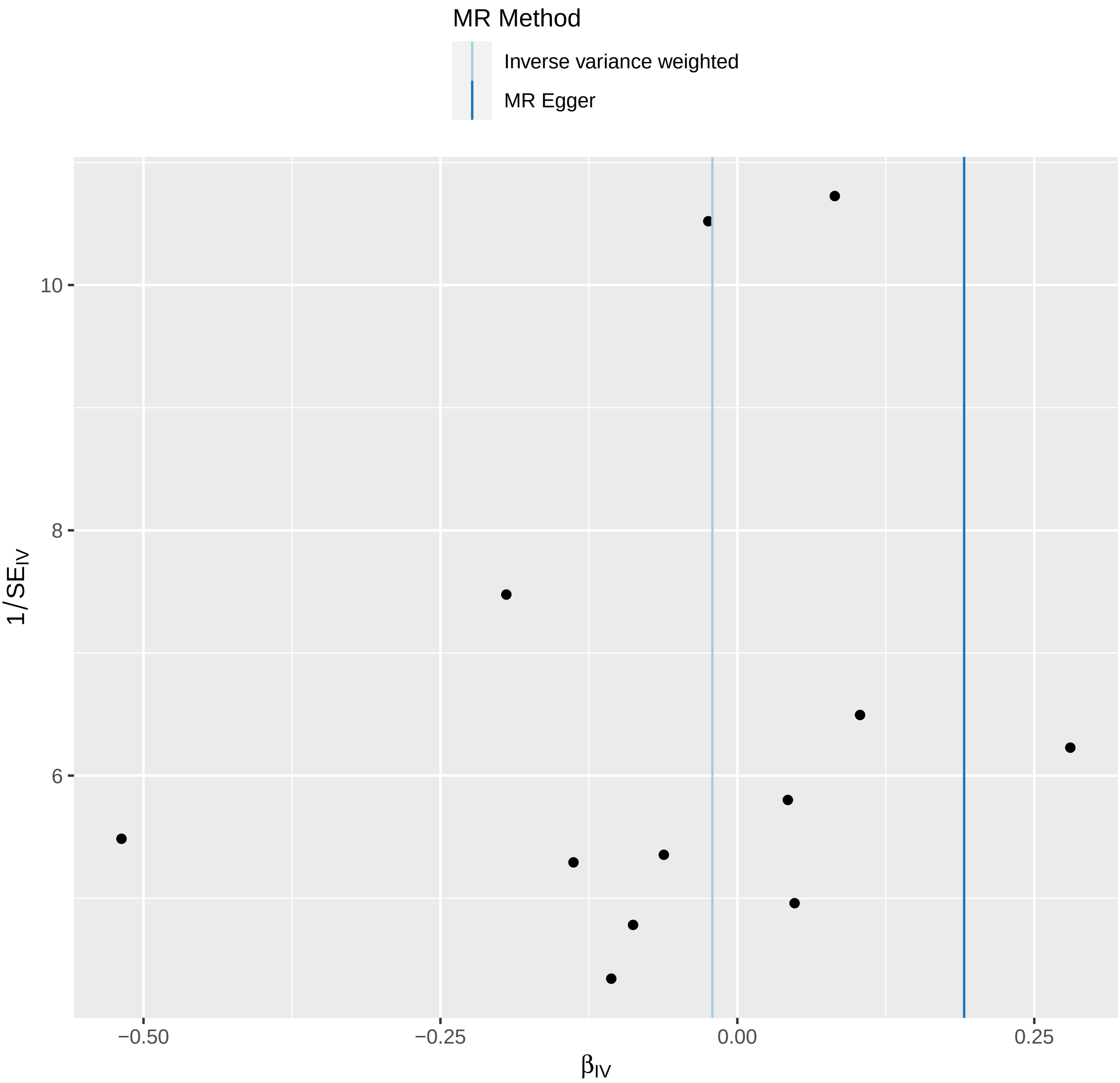

Batch 3−1089 : Selenium on Fibroblast growth factor 16

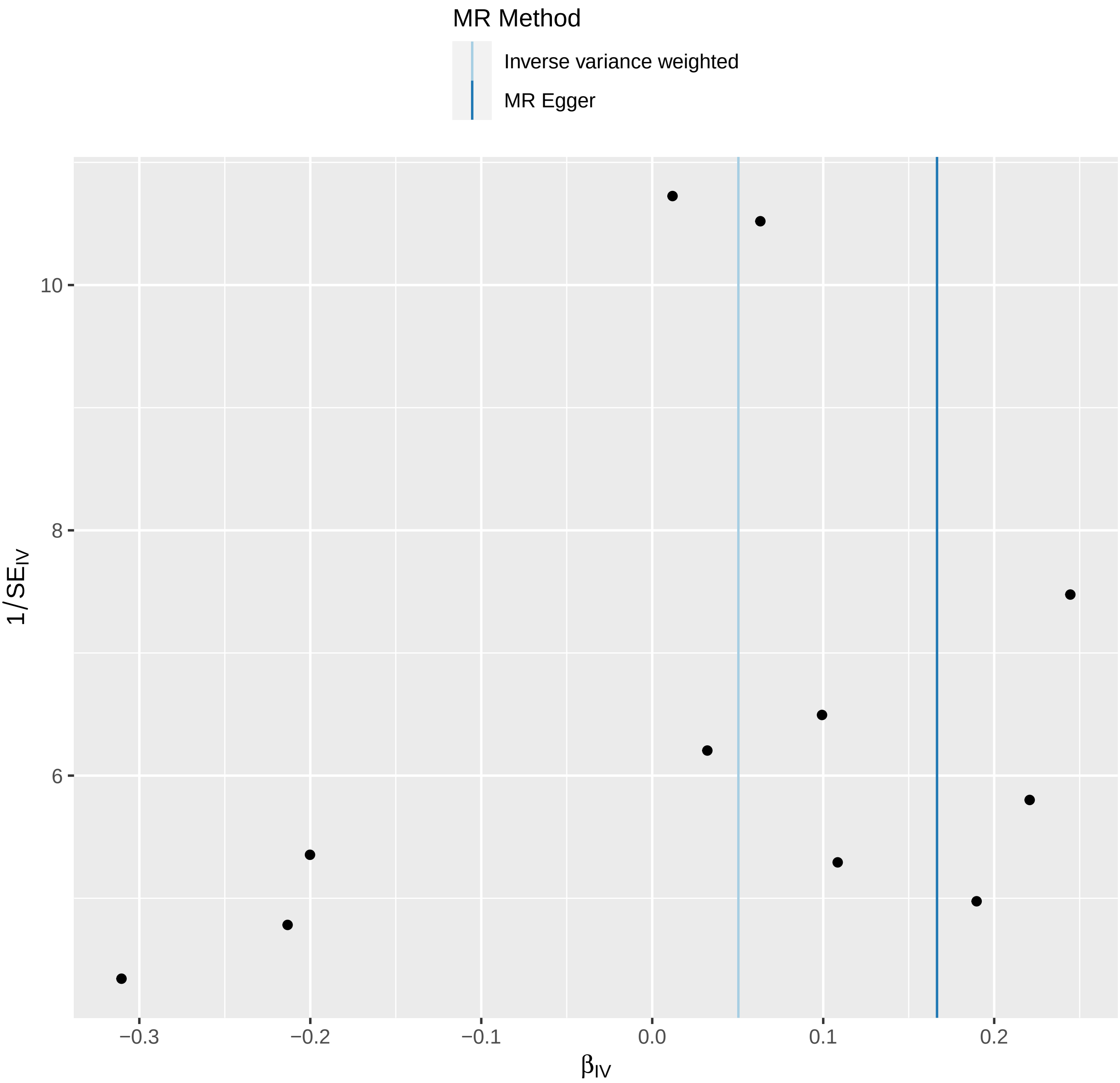

Batch 3−1090 : Selenium on Fibroblast growth factor 16

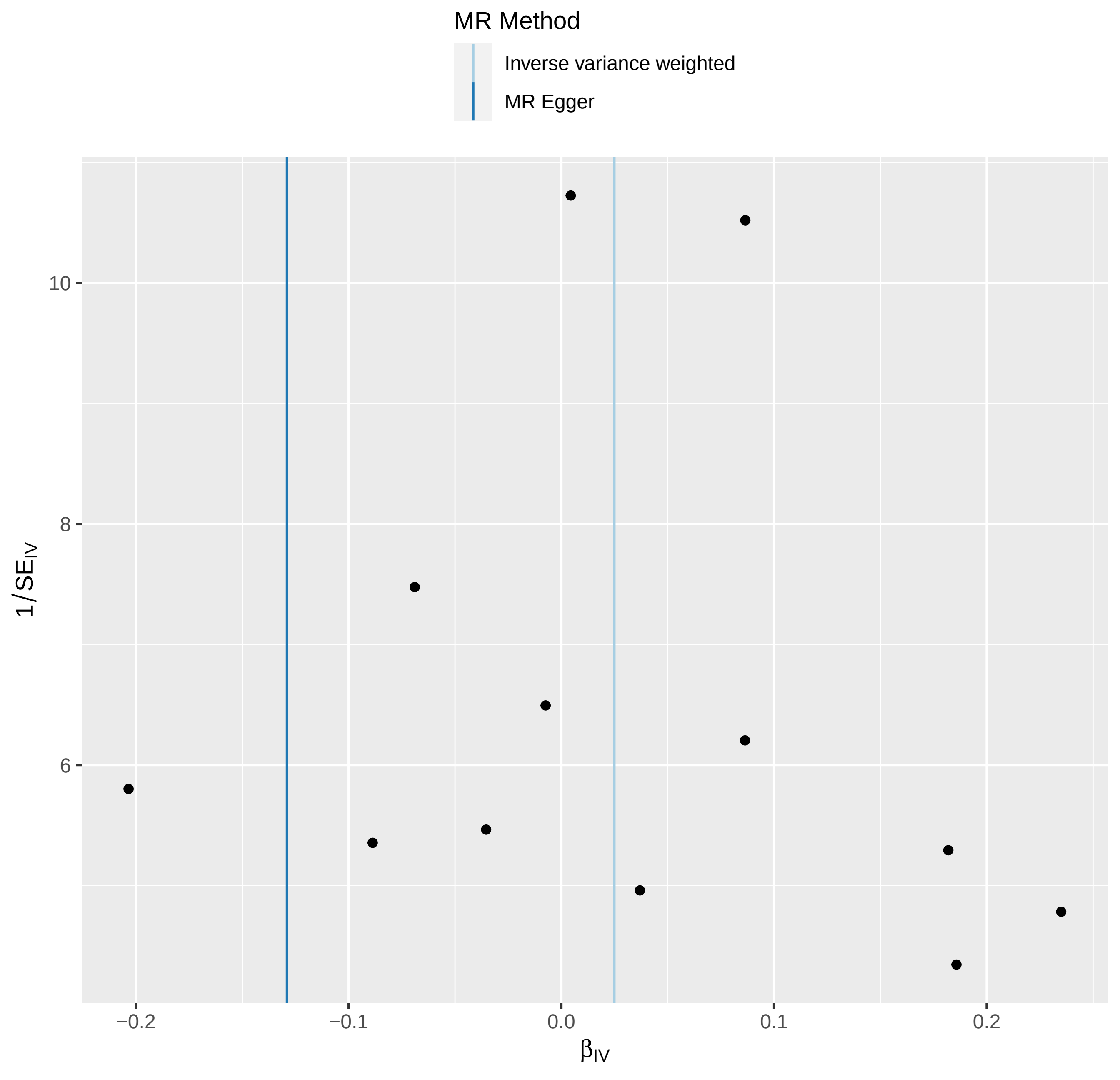

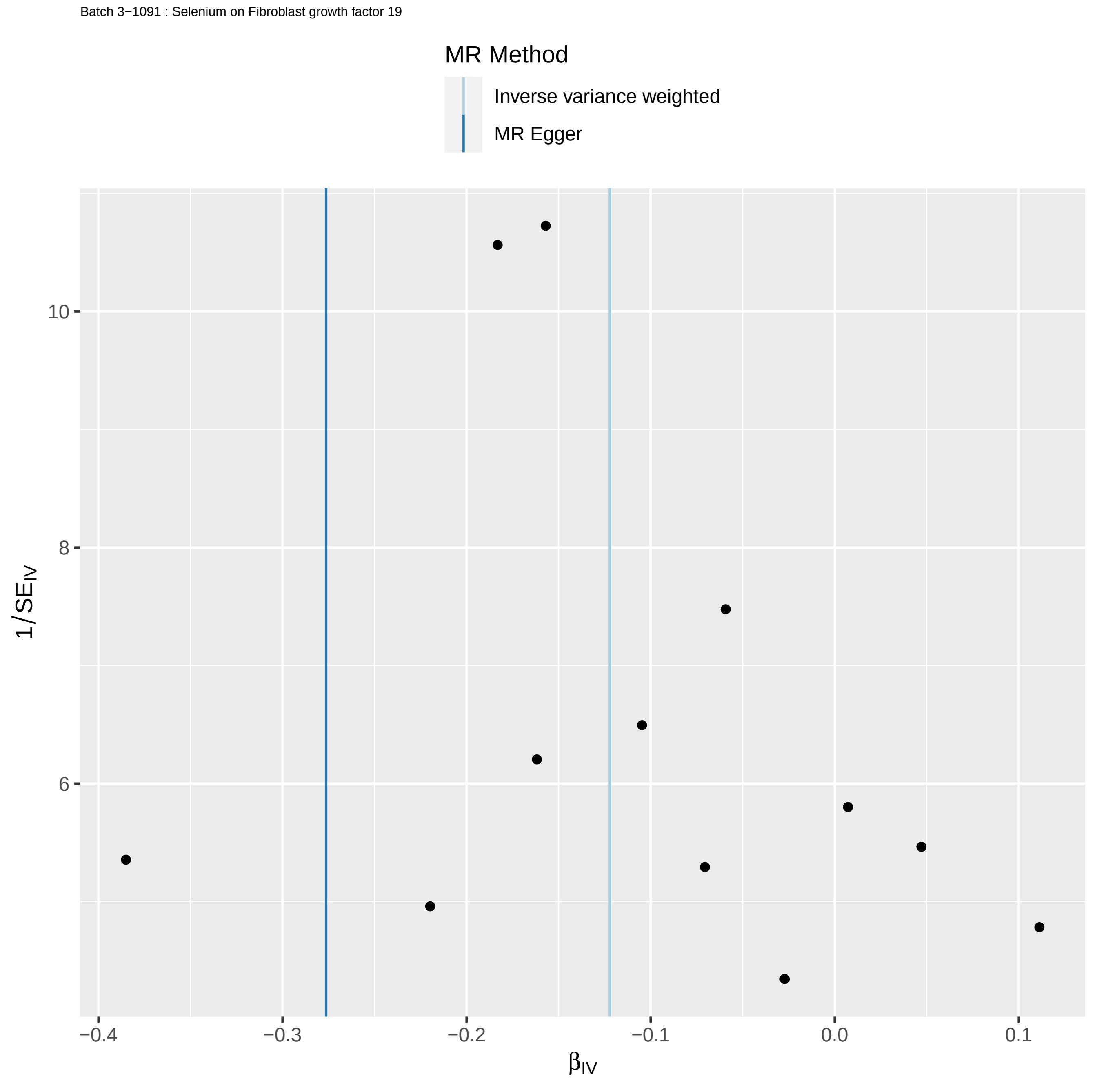
Batch 3−1091 : Selenium on Fibroblast growth factor 19
MR Method
Inverse variance weighted
MR Egger
10
8
6
1/SE$_{IV}$
−0.4
−0.3
−0.2
−0.1
0.0
0.1
β$_{IV}$

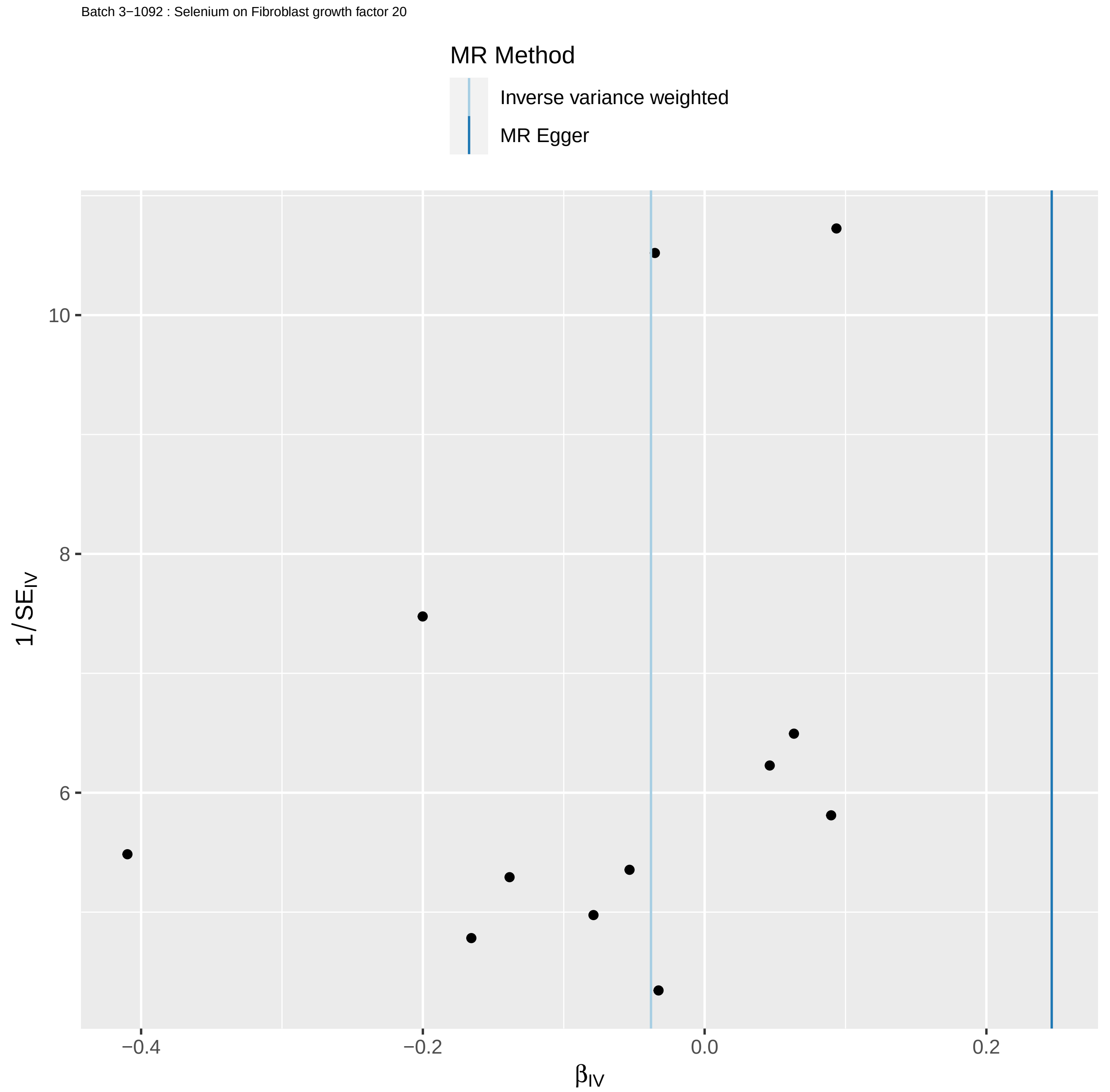

Batch 3−1092 : Selenium on Fibroblast growth factor 20
MR Method
Inverse variance weighted
MR Egger
10
8
6
1/SE_IV
−0.4
−0.2
0.0
0.2
β_IV

Batch 3−1093 : Selenium on Fibroblast growth factor 23

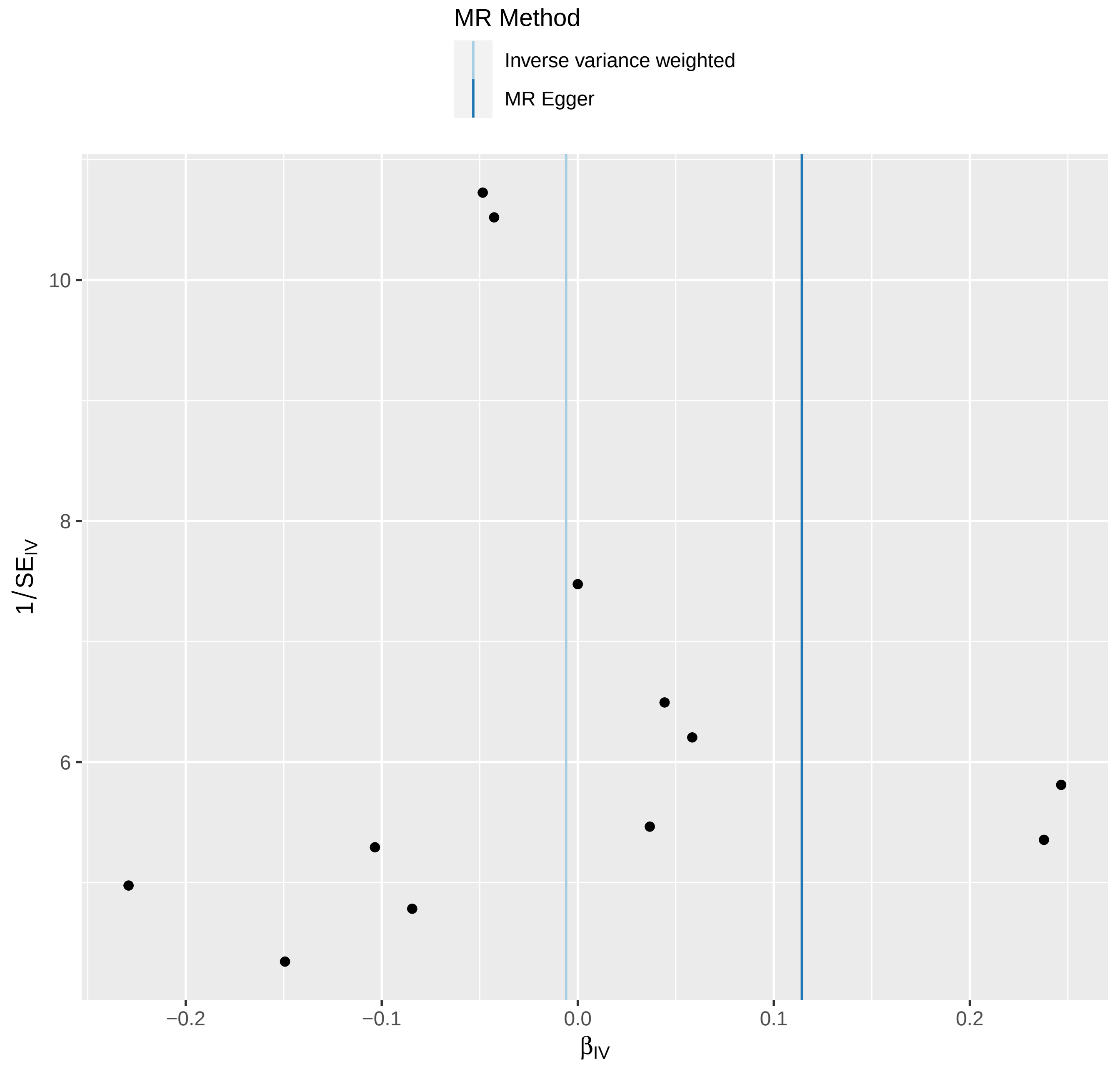

Batch 3−1094 : Selenium on Fibroblast growth factor 4

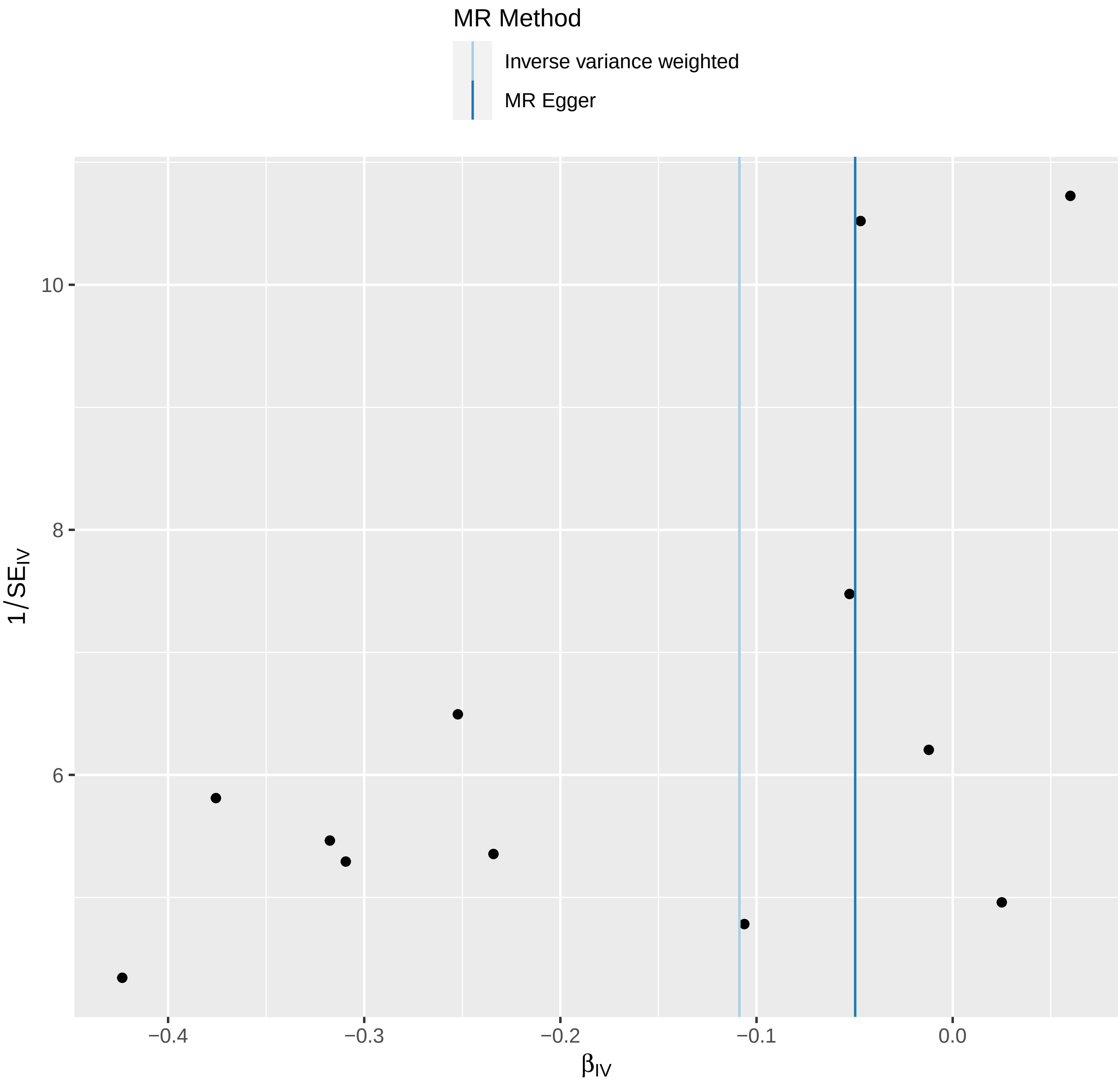

Batch 3−1095 : Selenium on Fibroblast growth factor 5

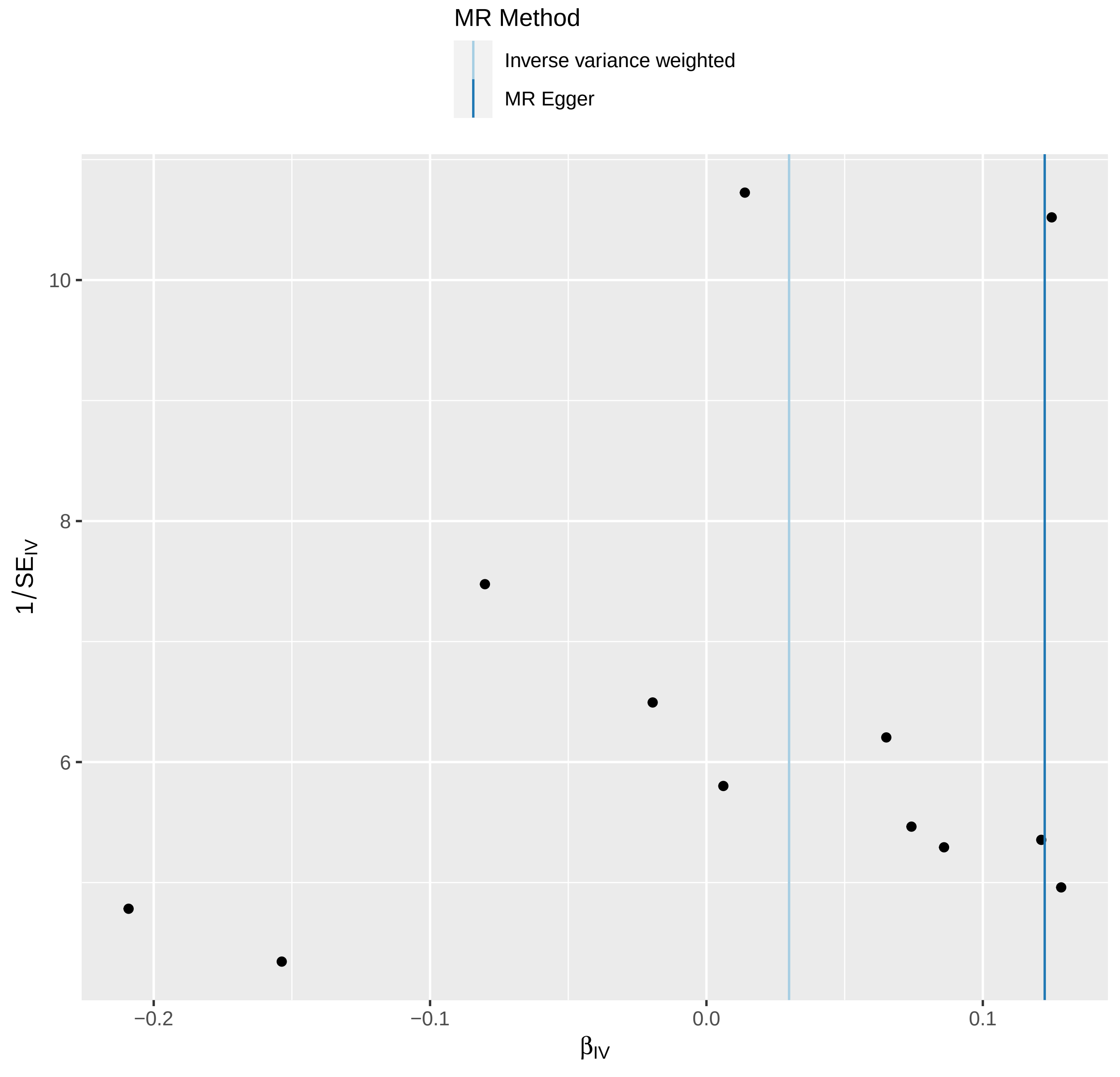

Batch 3−1096 : Selenium on Fibroblast growth factor 6

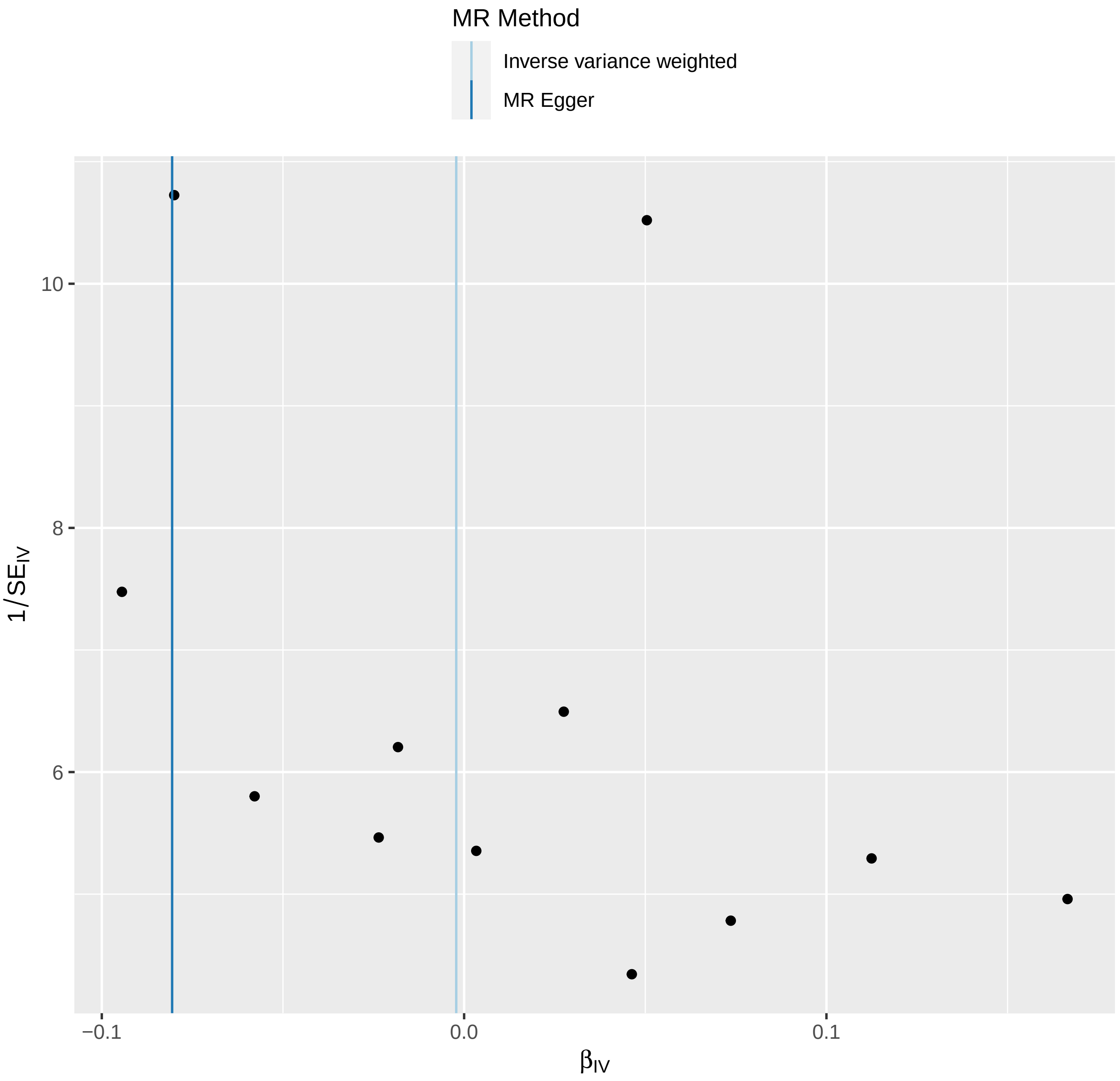

Batch 3−1097 : Selenium on Fibroblast growth factor 7

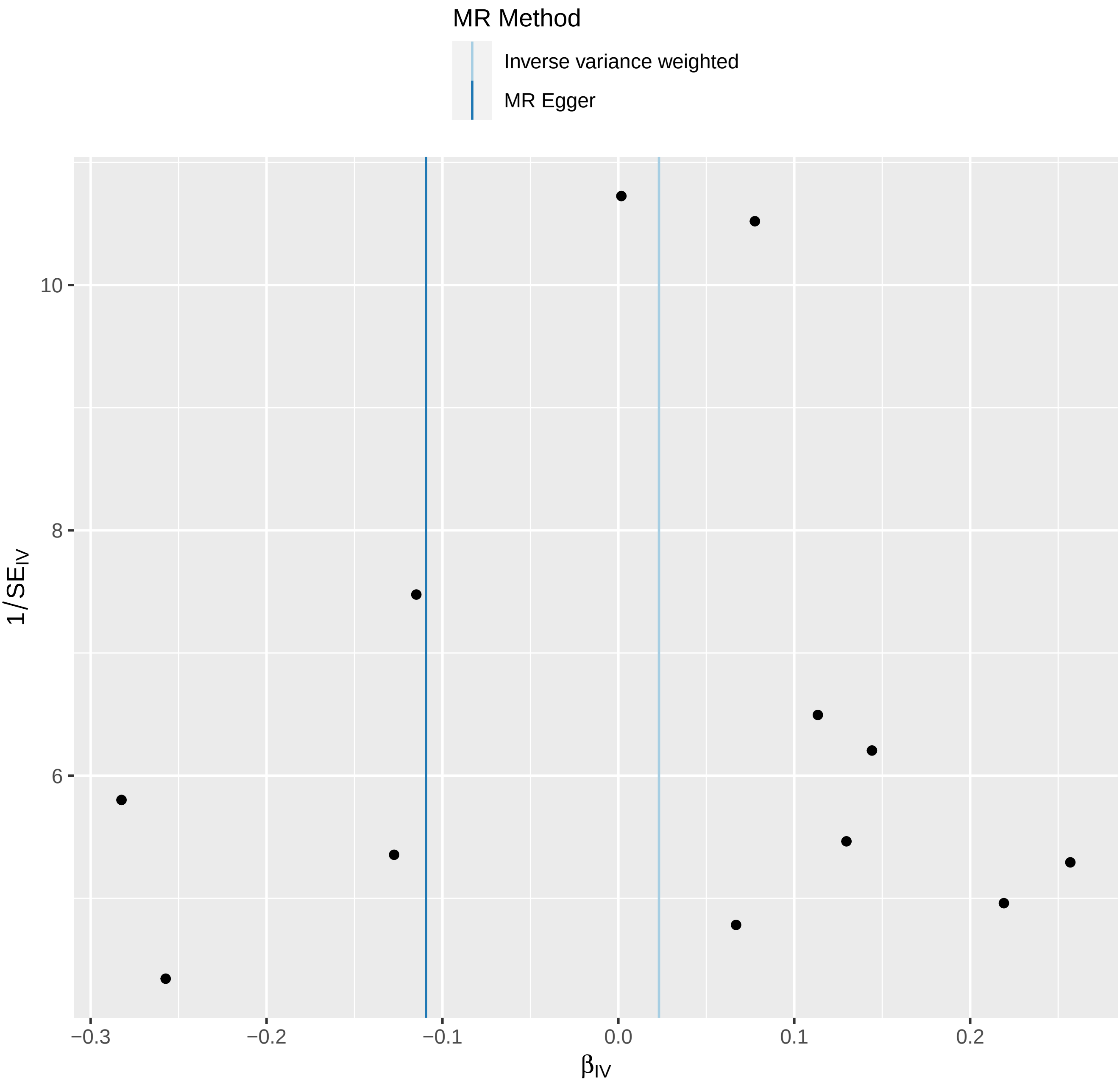

Batch 3−1098 : Selenium on Fibroblast growth factor 7

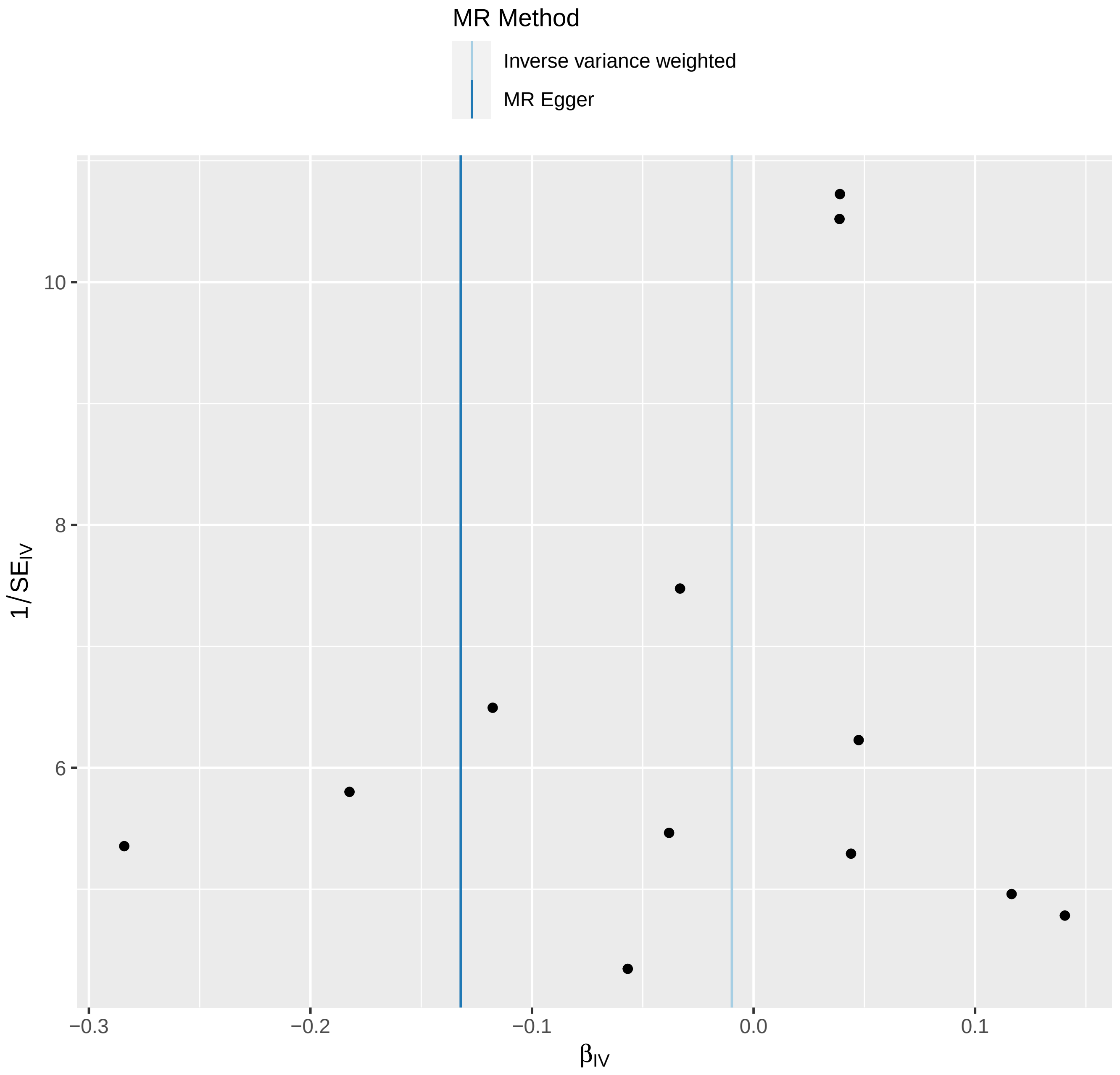

Batch 3−1099 : Selenium on Fibroblast growth factor 8 isoform B

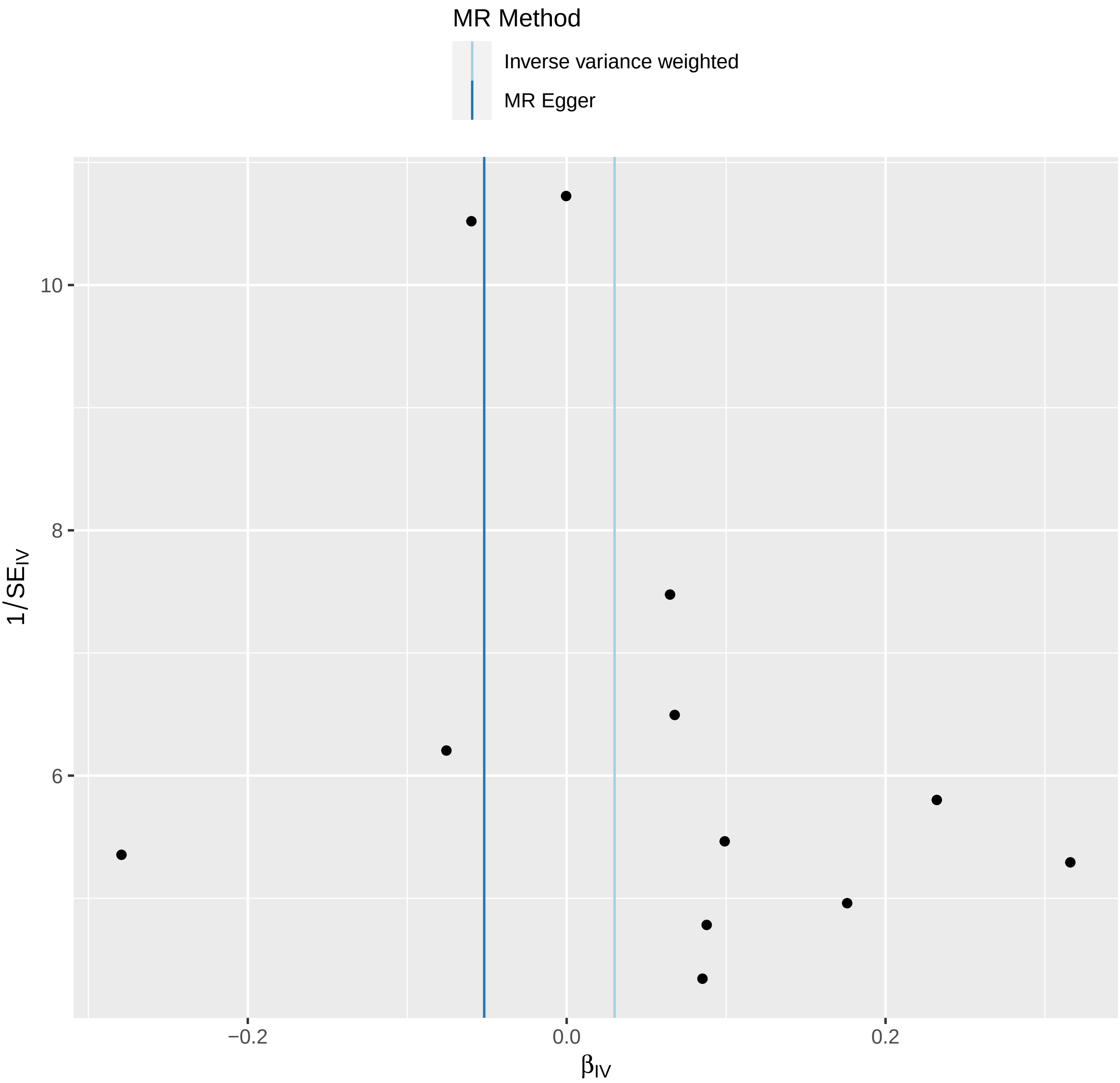

Batch 3−1100 : Selenium on Fibroblast growth factor 8 isoform A

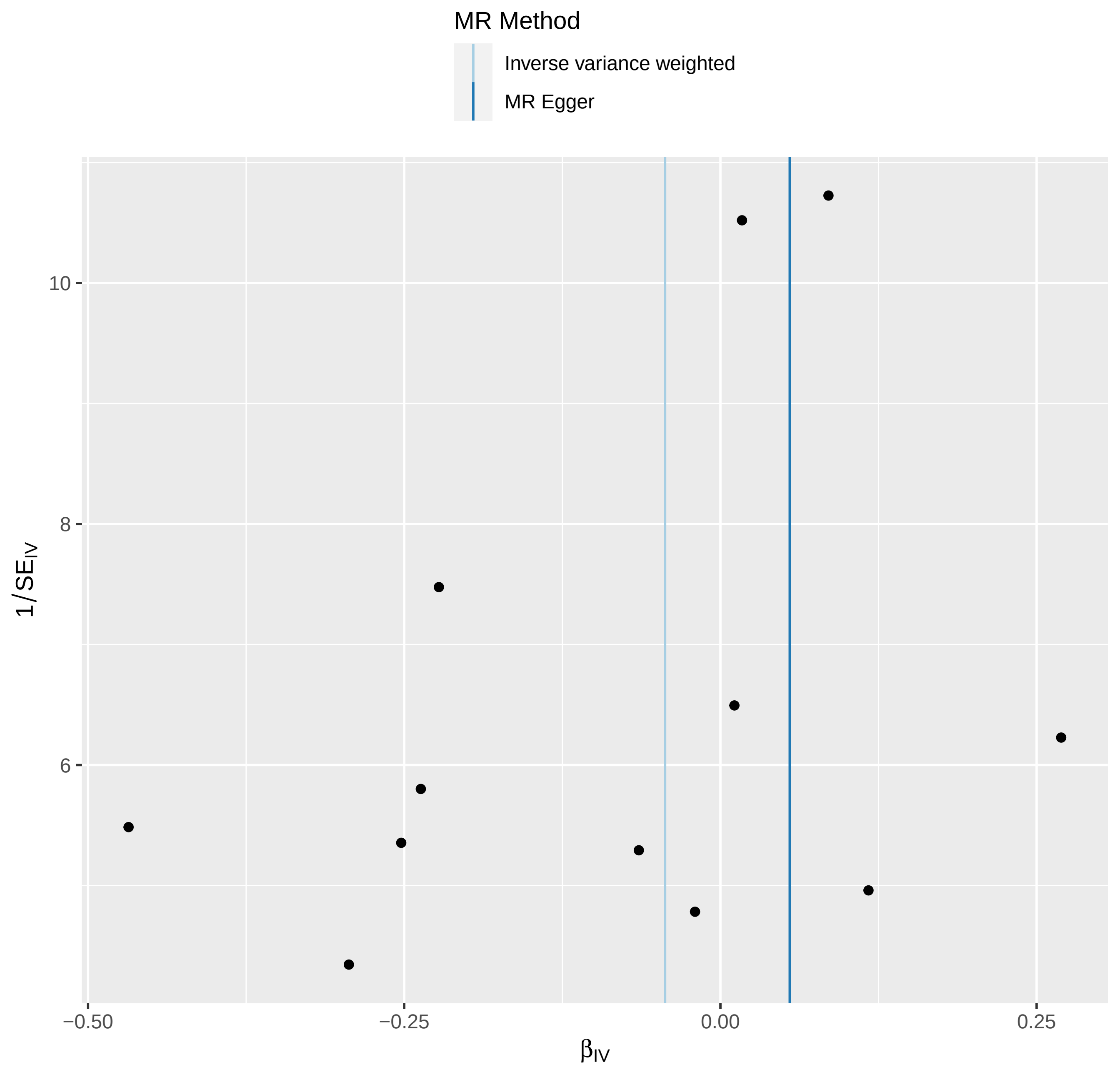

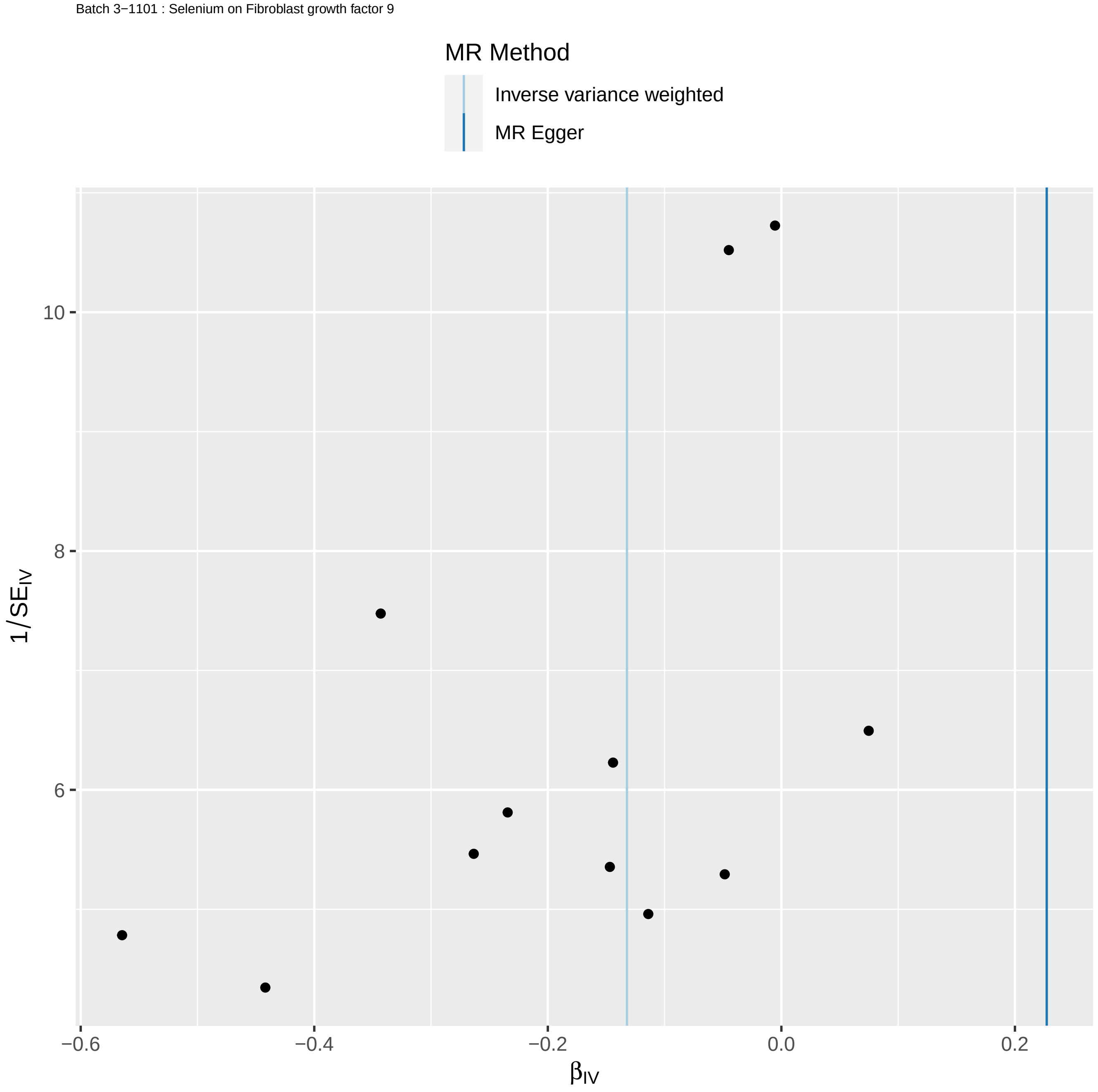

Batch 3−1101 : Selenium on Fibroblast growth factor 9
MR Method
Inverse variance weighted
MR Egger
10
8
6
1/SE_IV
−0.6
−0.4
−0.2
0.0
0.2
β_IV

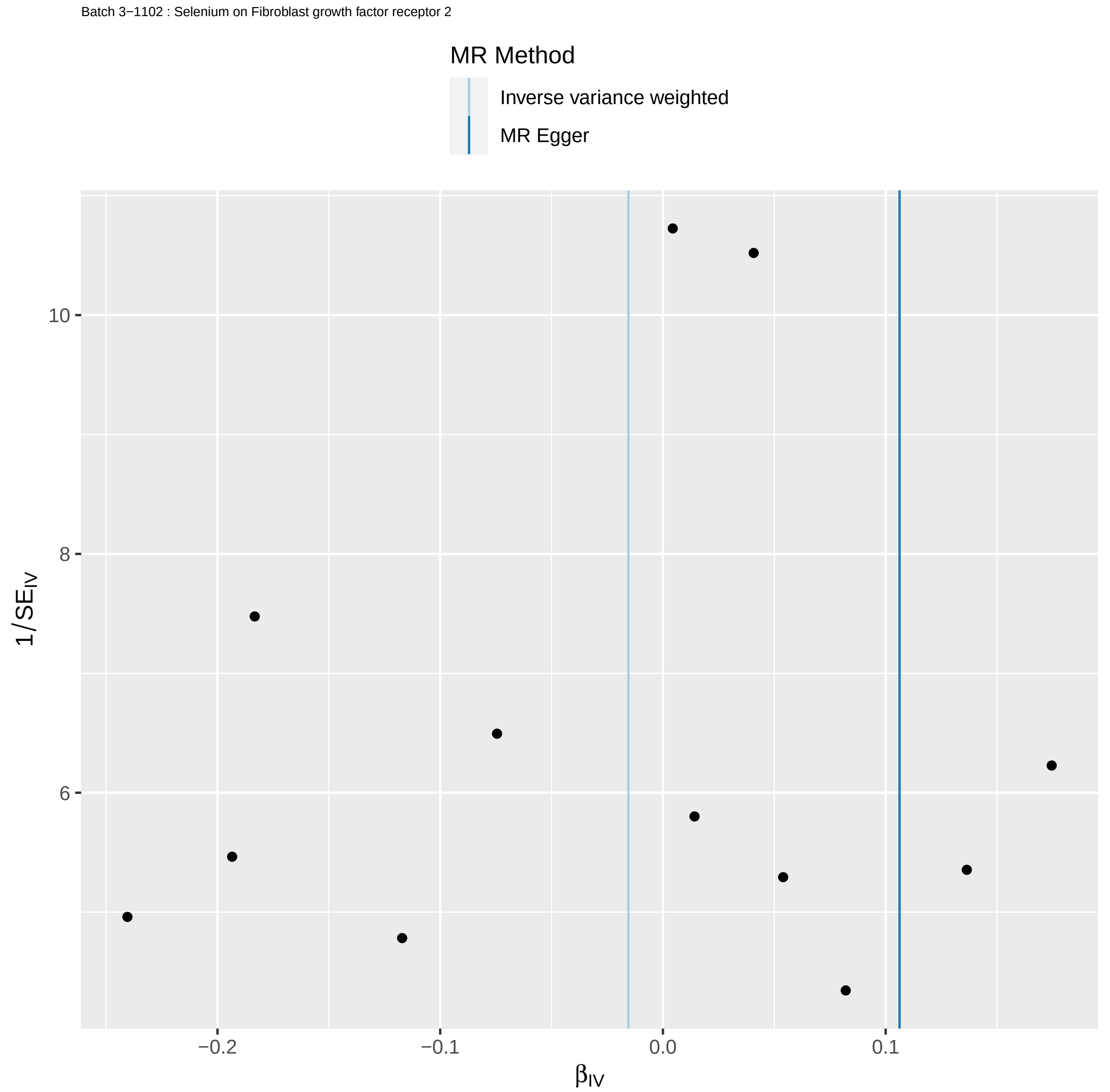

Batch 3−1102 : Selenium on Fibroblast growth factor receptor 2
MR Method
Inverse variance weighted
MR Egger
10
8
6
1/SE_IV
−0.2
−0.1
0.0
0.1
β_IV

Batch 3−1103 : Selenium on Fibroblast growth factor receptor 3

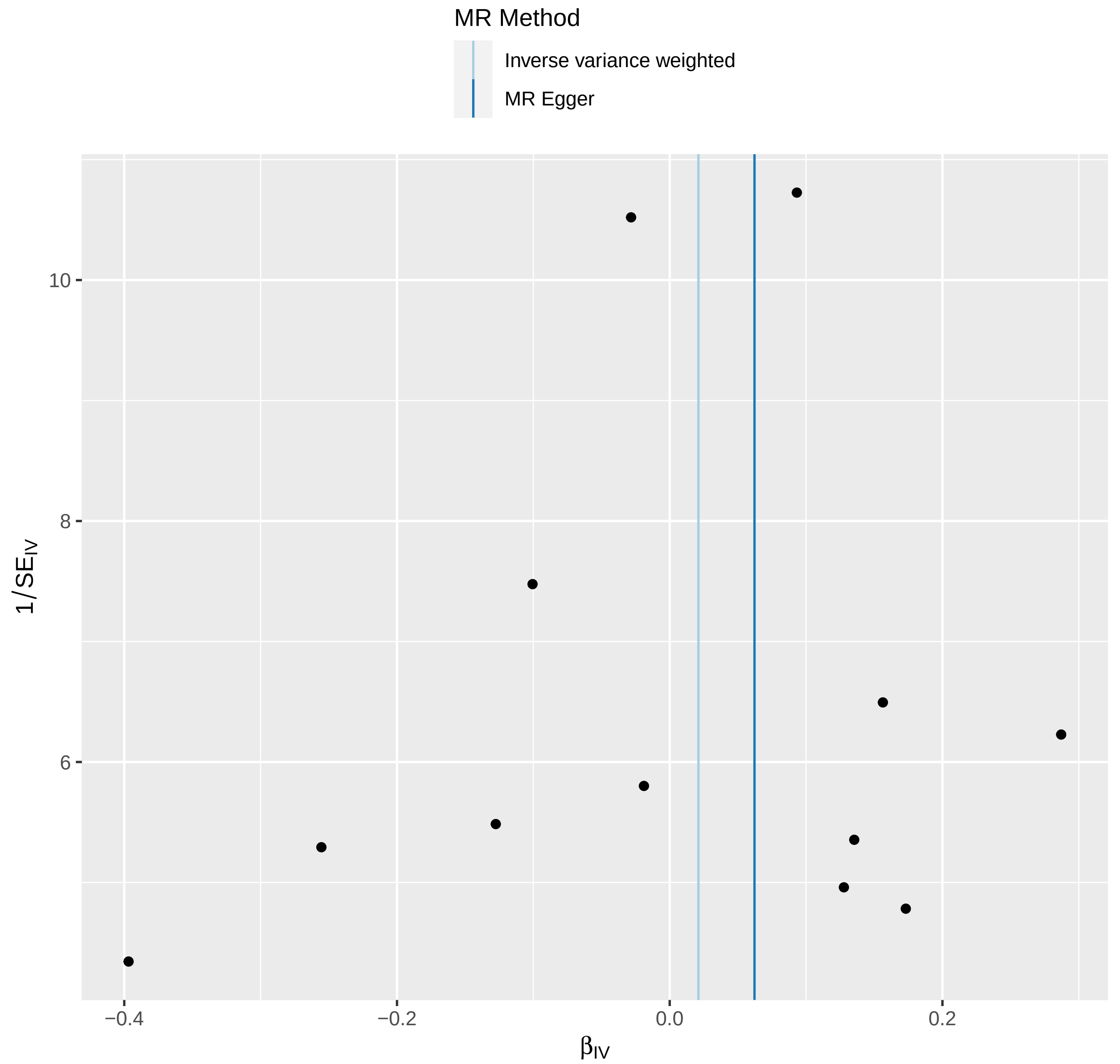

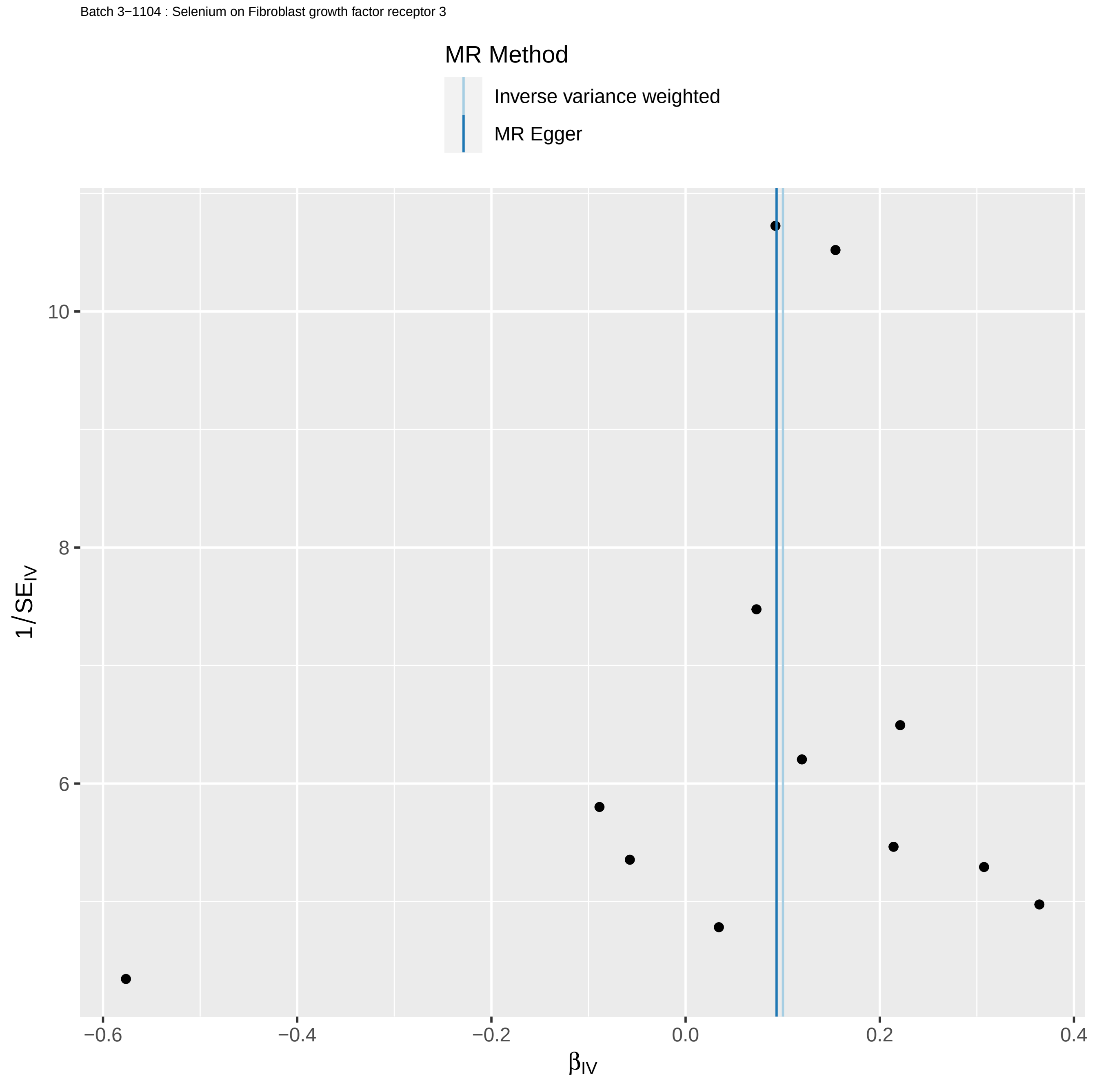
Batch 3−1104 : Selenium on Fibroblast growth factor receptor 3
MR Method
Inverse variance weighted
MR Egger
10
8
6
1/SE$_{IV}$
−0.6
−0.4
−0.2
0.0
0.2
0.4
β$_{IV}$

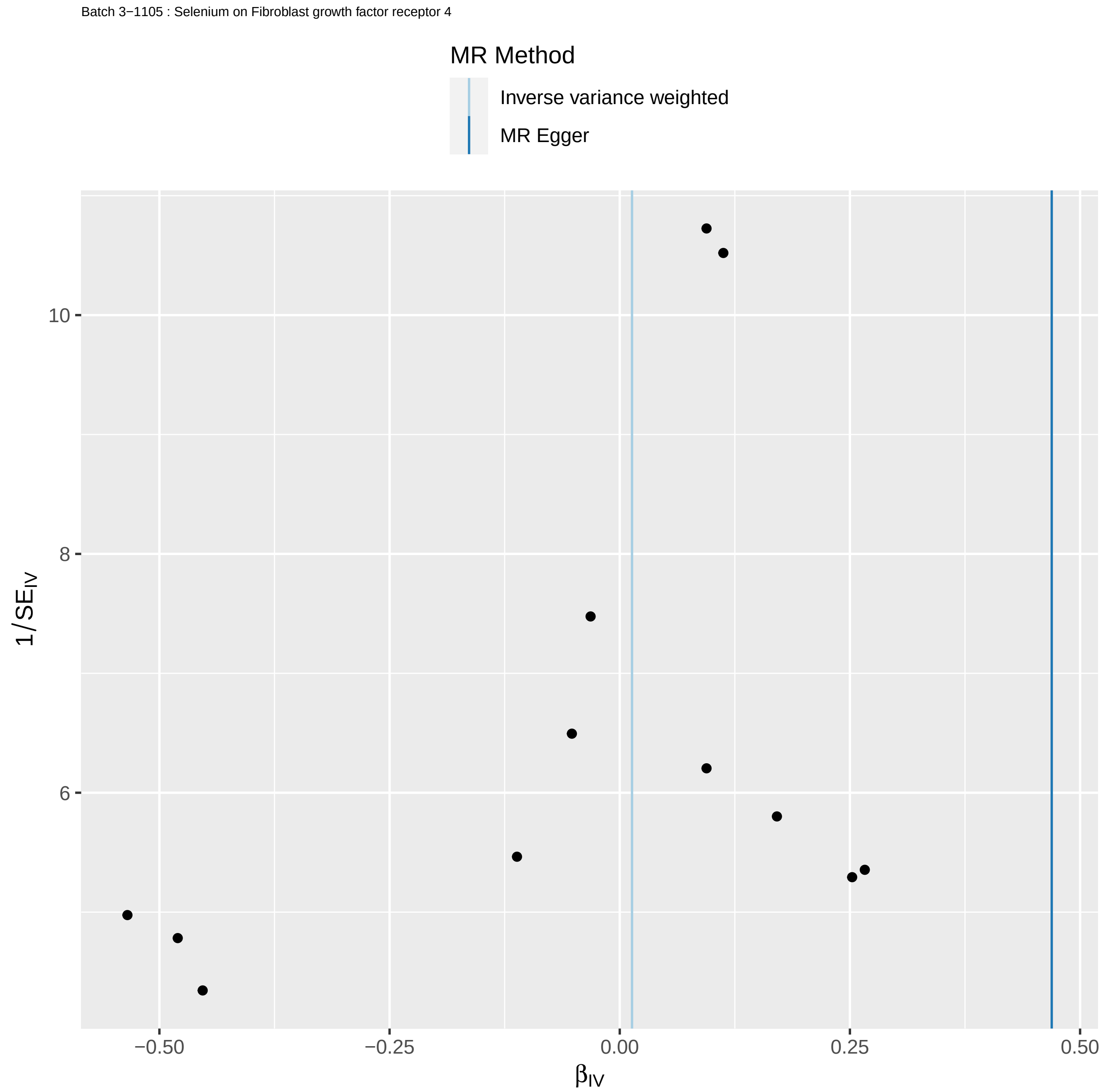

Batch 3−1105 : Selenium on Fibroblast growth factor receptor 4
MR Method
Inverse variance weighted
MR Egger
10
8
6
1/SE_IV
−0.50
−0.25
0.00
0.25
0.50
β_IV

Batch 3−1106 : Selenium on Tyrosine−protein kinase Fgr

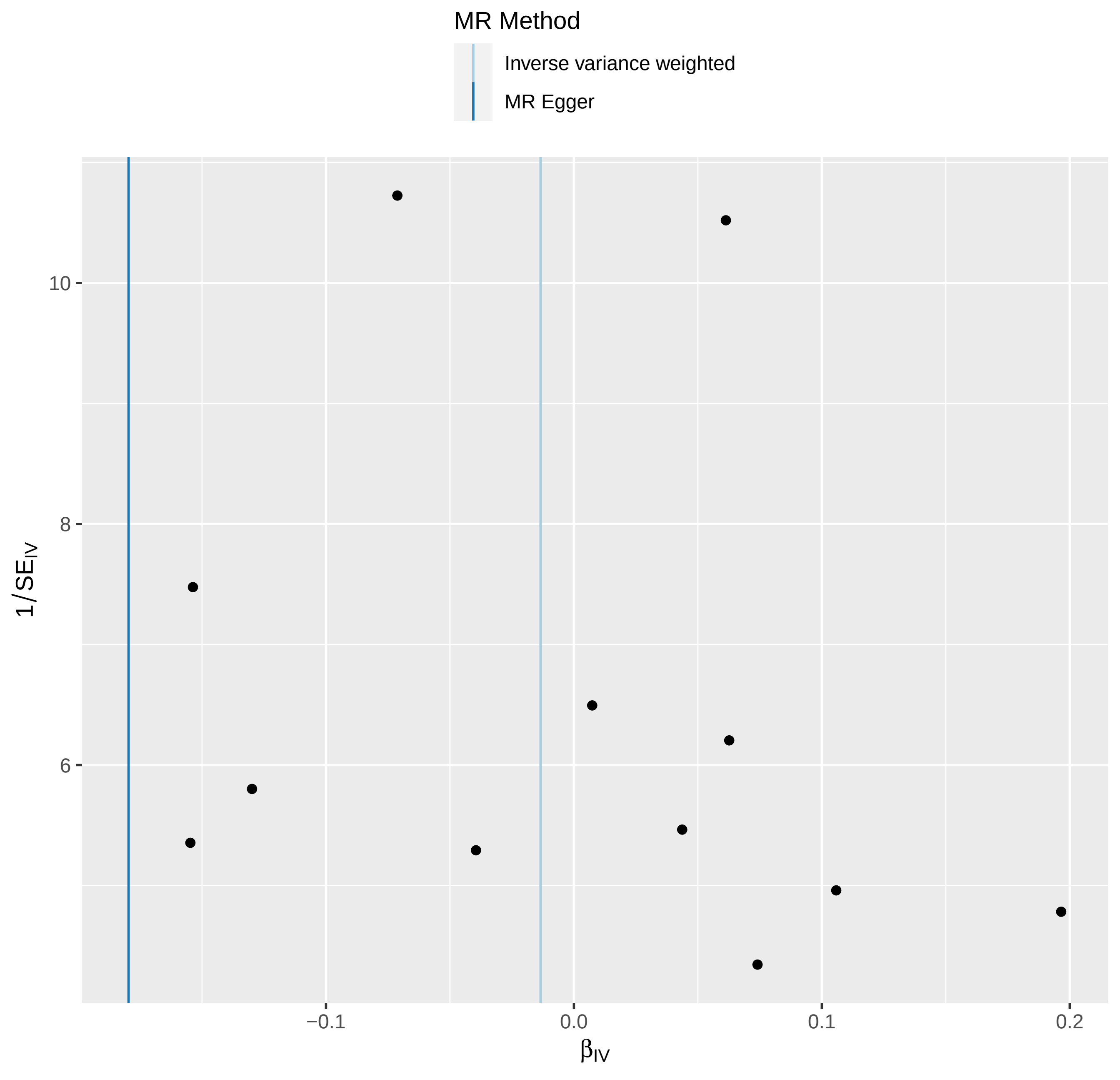

Batch 3−1107 : Selenium on Bis(5'−adenosyl)−triphosphatase

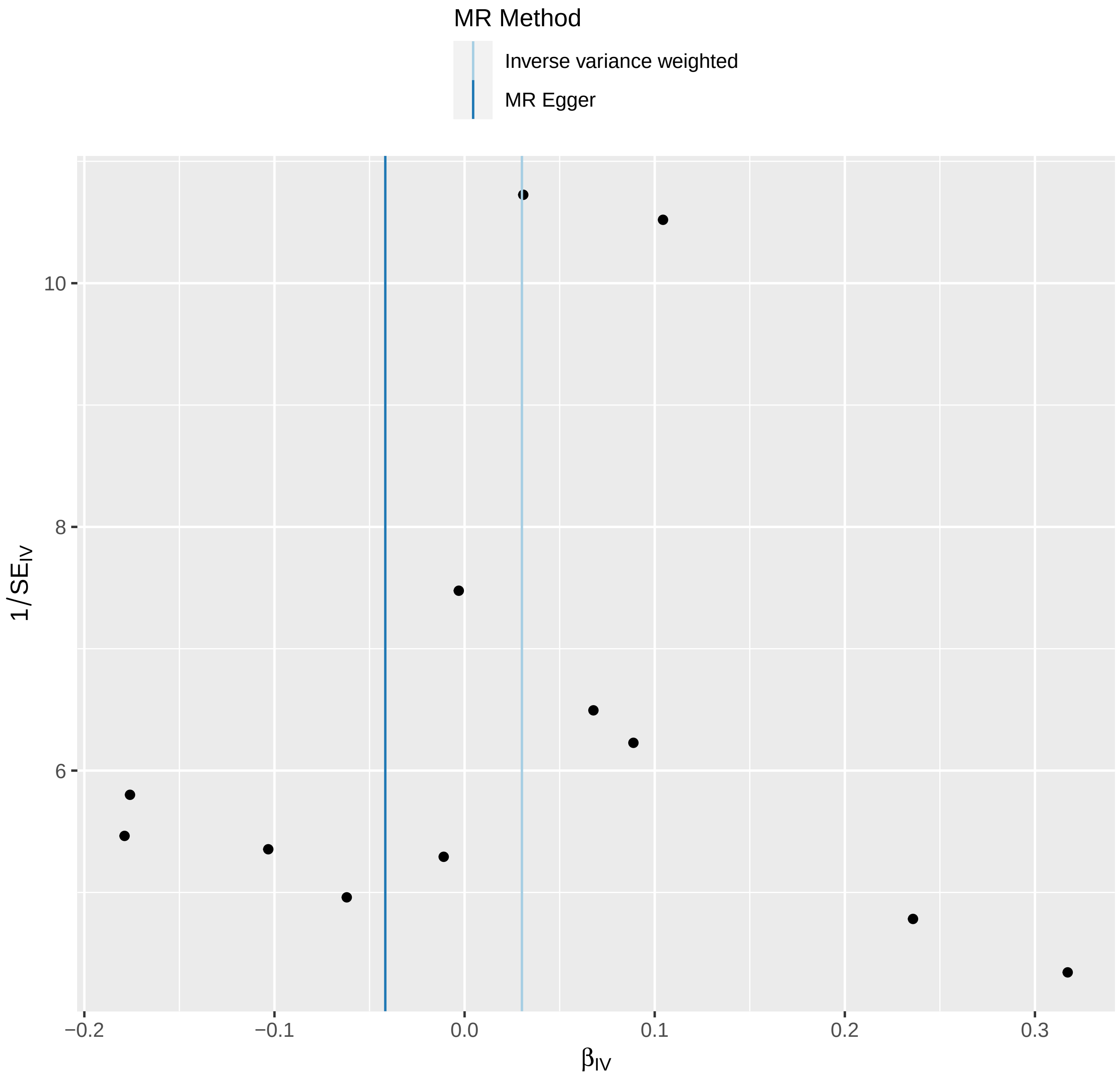

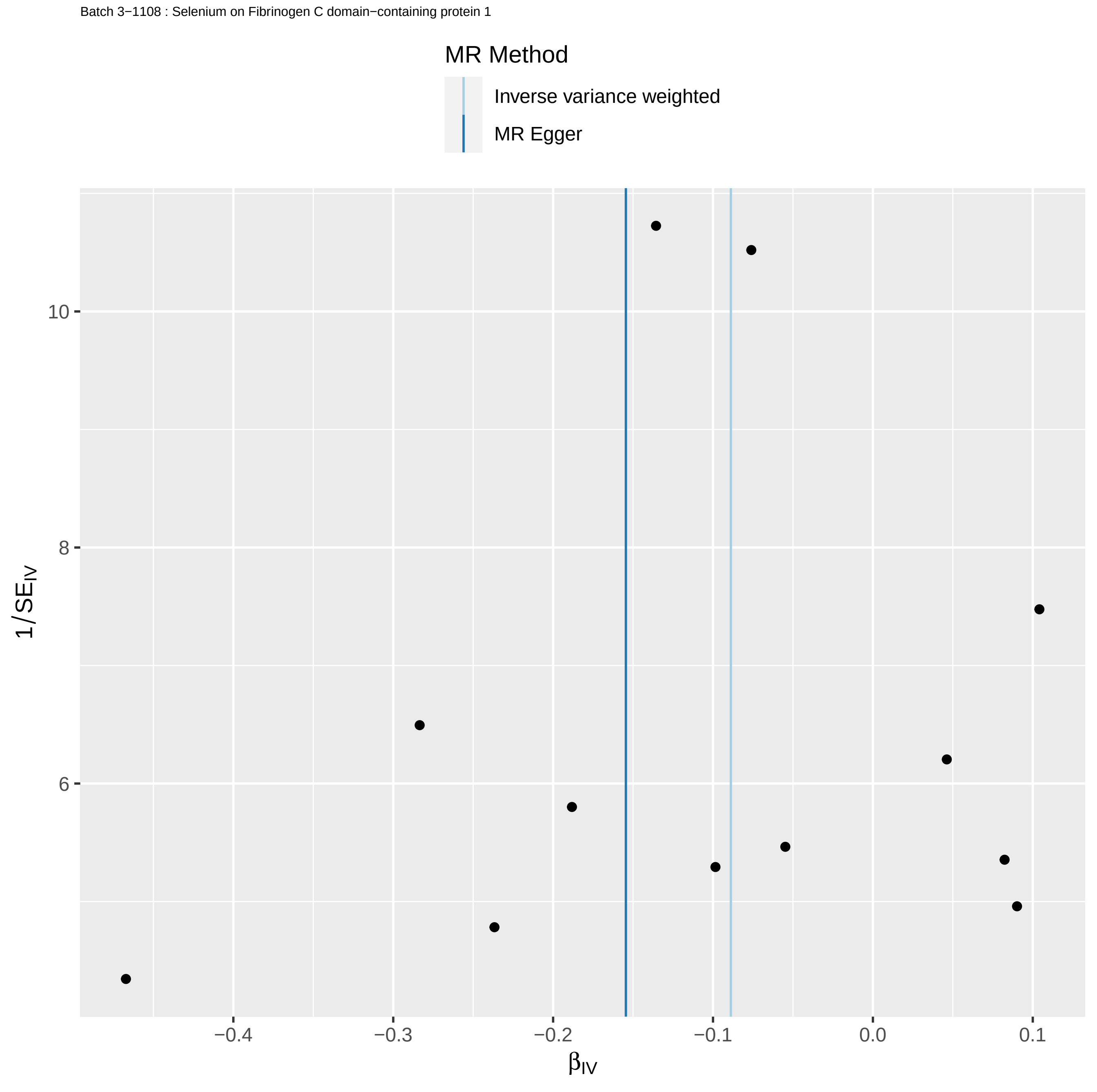

Batch 3−1108 : Selenium on Fibrinogen C domain−containing protein 1
MR Method
Inverse variance weighted
MR Egger
10
8
6
1/SE_IV
−0.4
−0.3
−0.2
−0.1
0.0
0.1
β_IV

Batch 3−1109 : Selenium on Polyphosphoinositide phosphatase

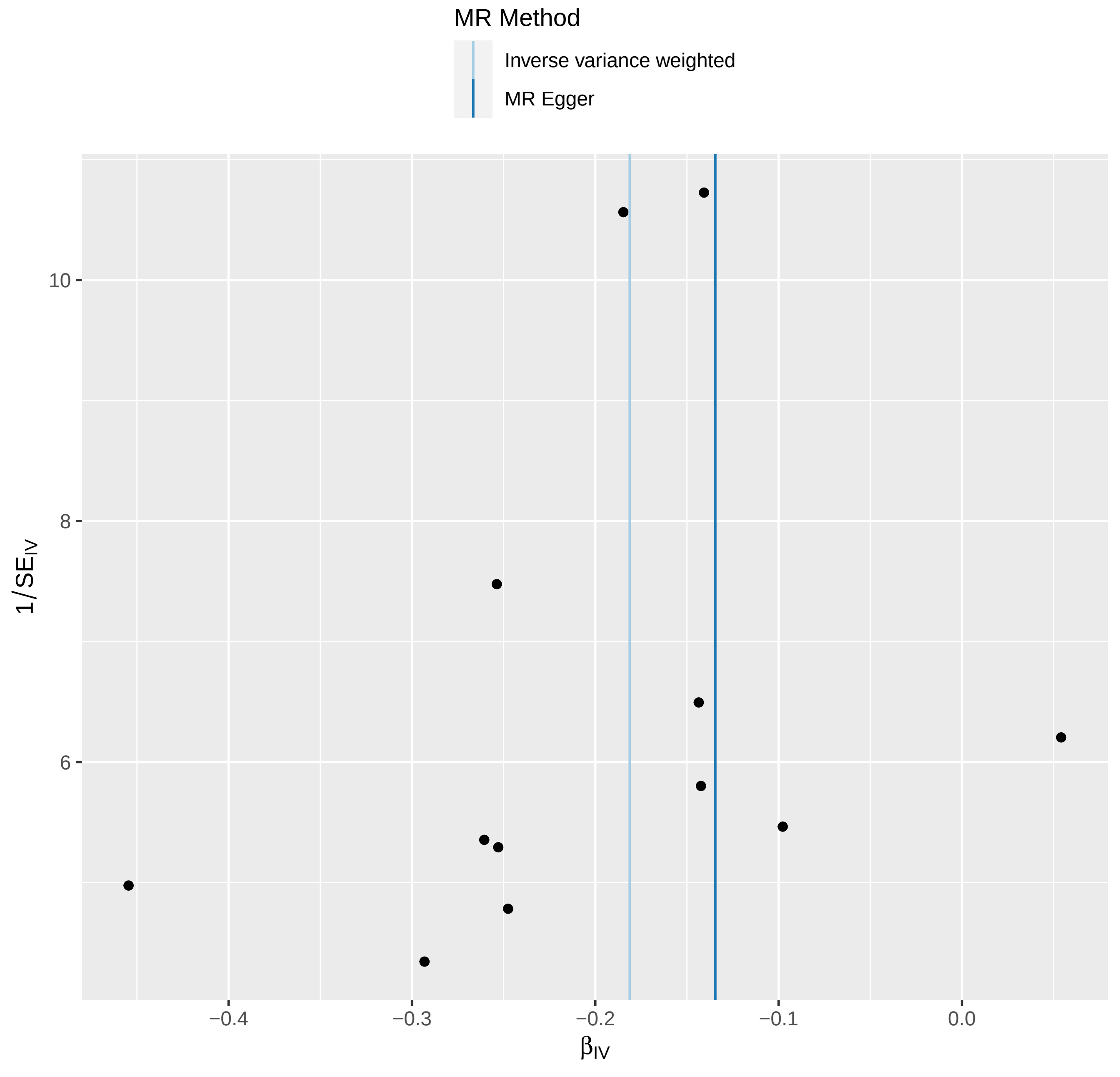

Batch 3−1110 : Selenium on Vascular endothelial growth factor D

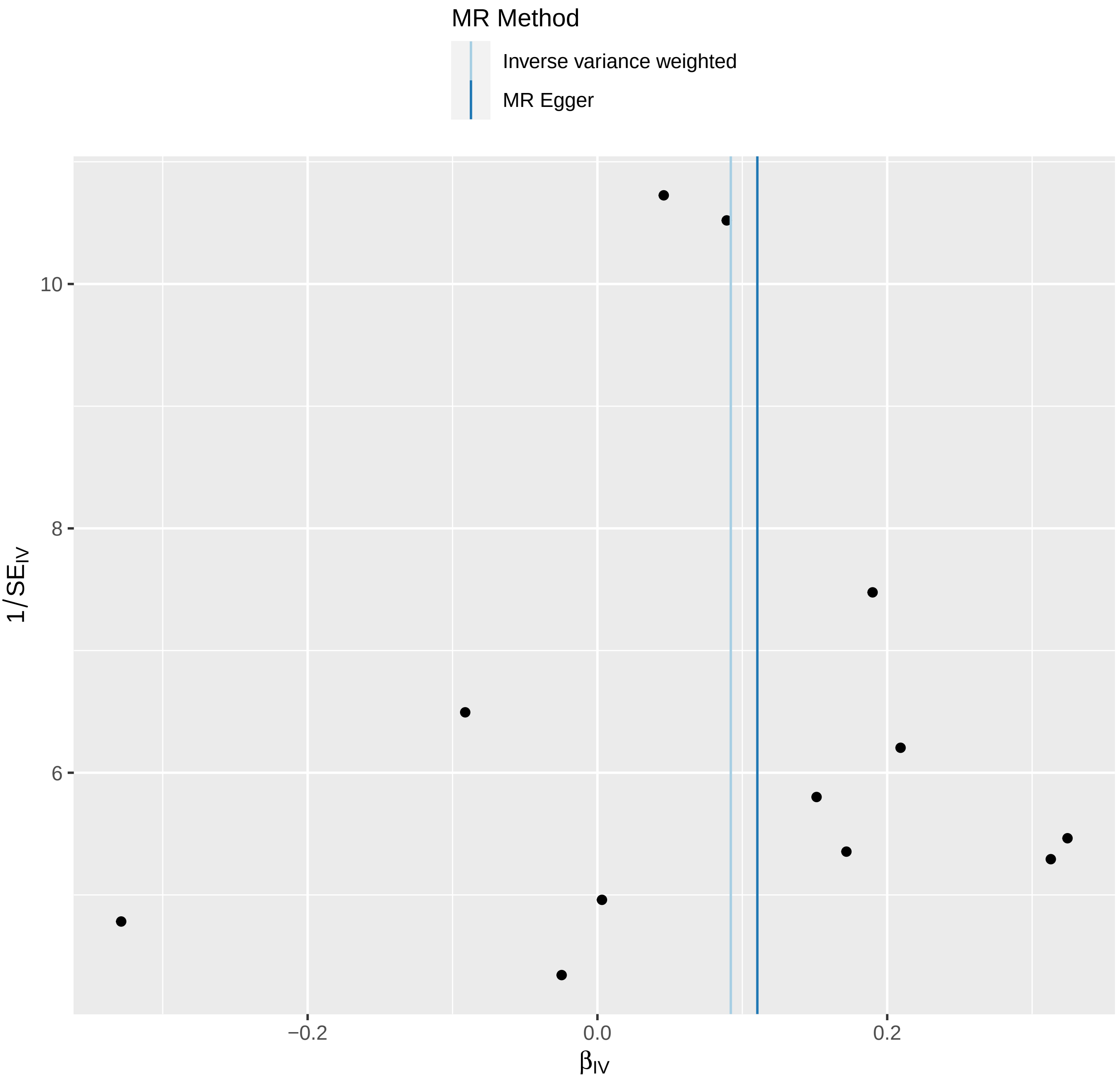

Batch 3−1111 : Selenium on Vascular endothelial growth factor D

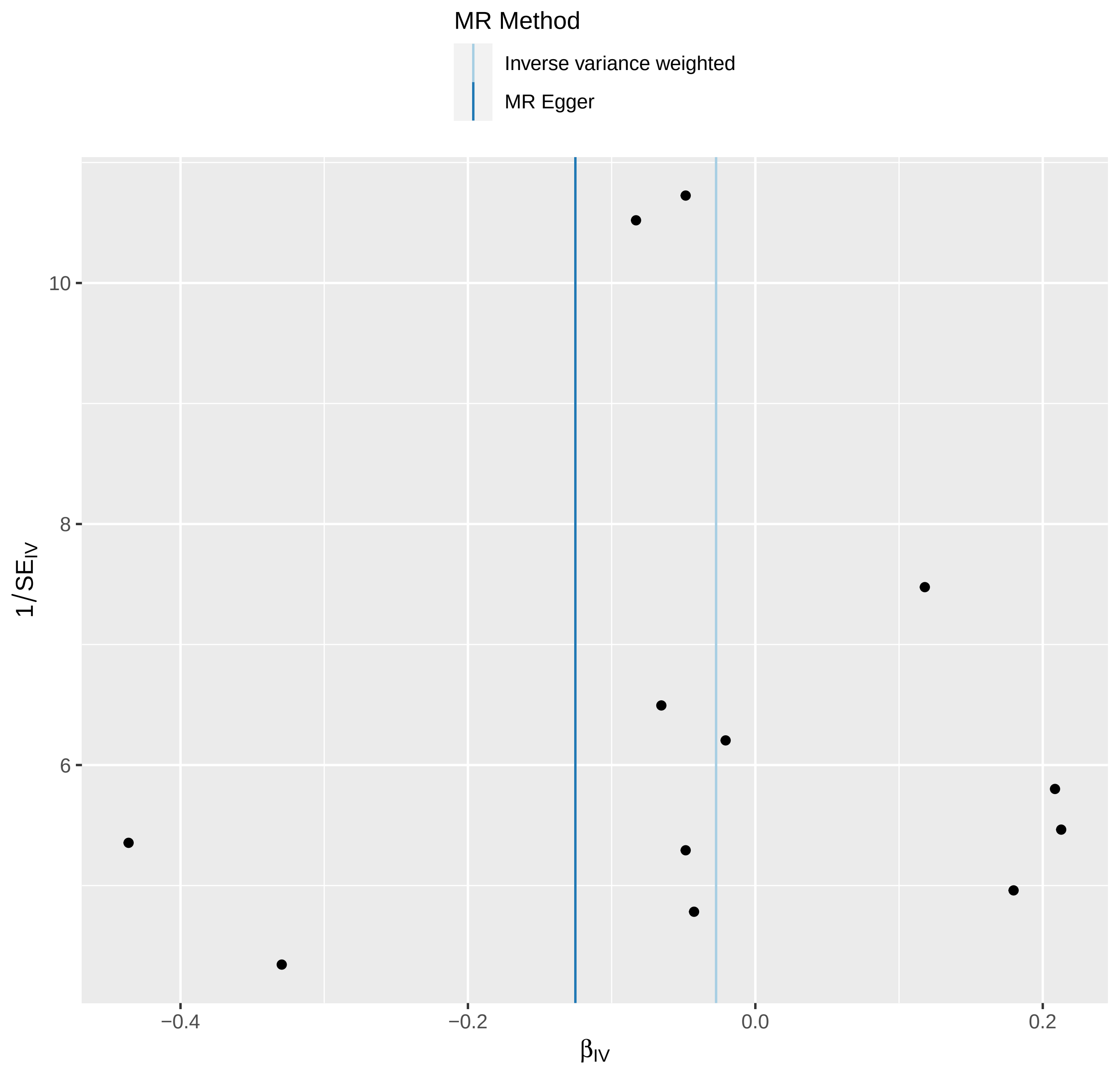

Batch 3−1112 : Selenium on Four−jointed box protein 1

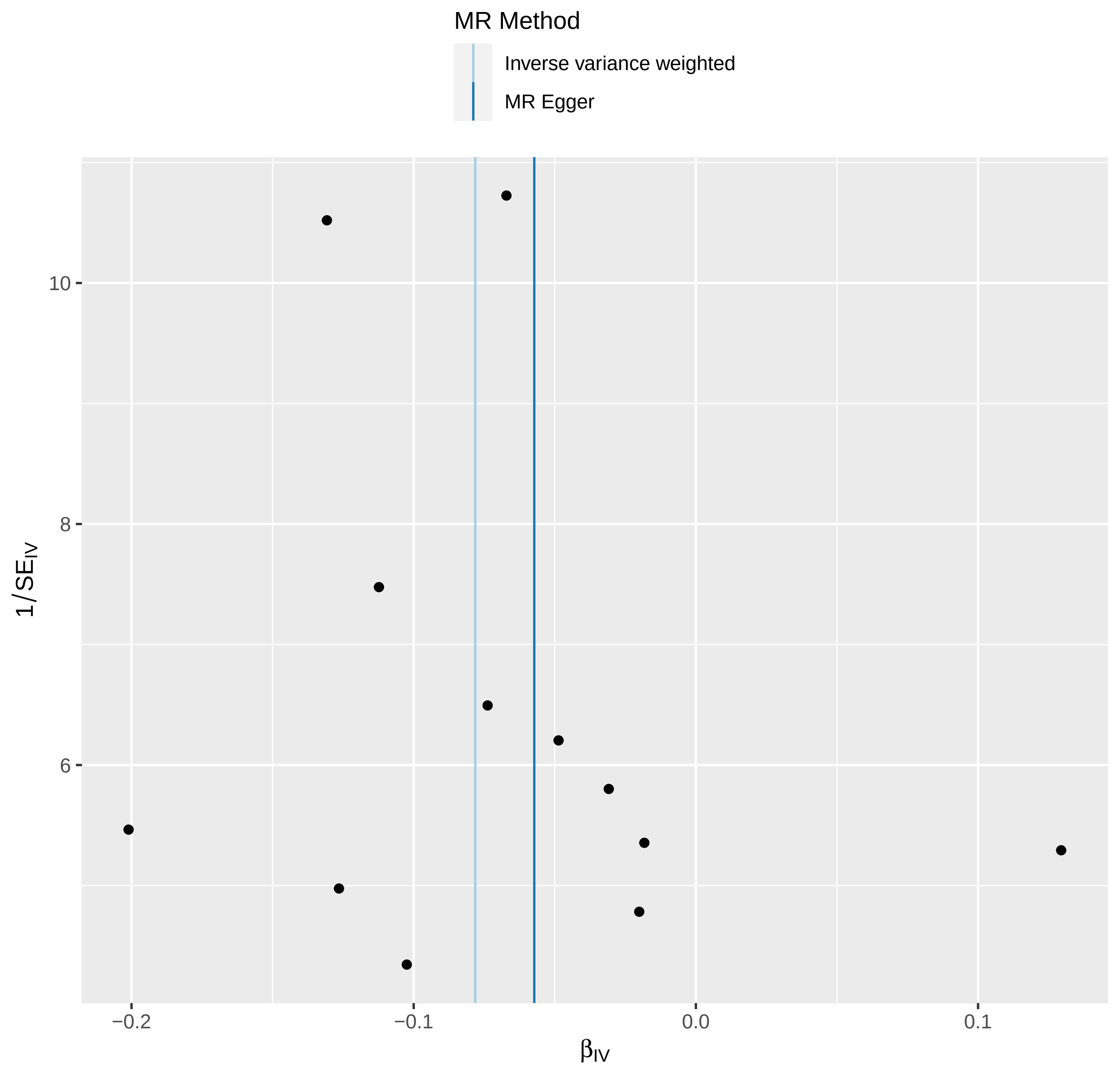

Batch 3−1113 : Selenium on Peptidyl−prolyl cis−trans isomerase FKBP14

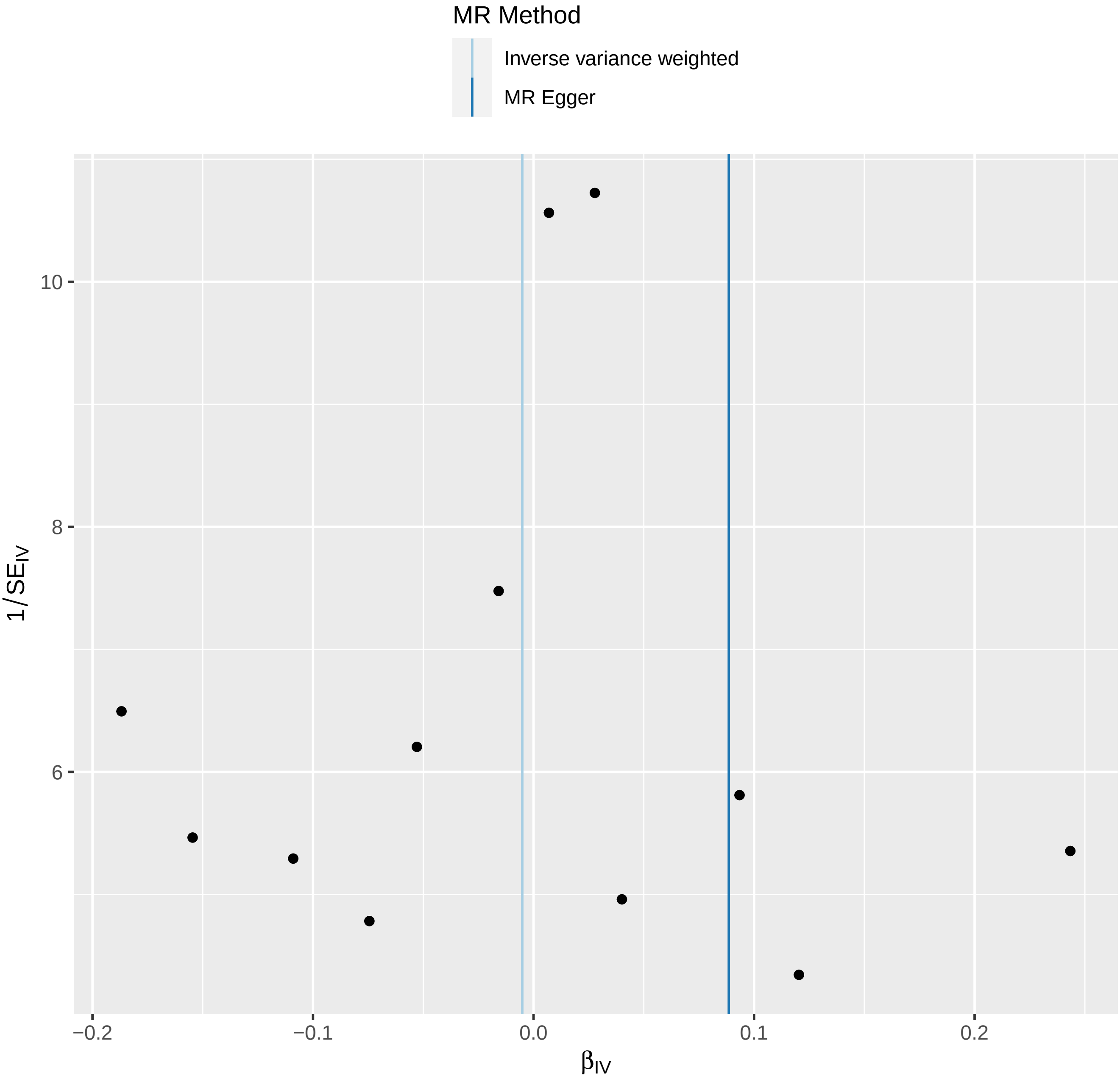

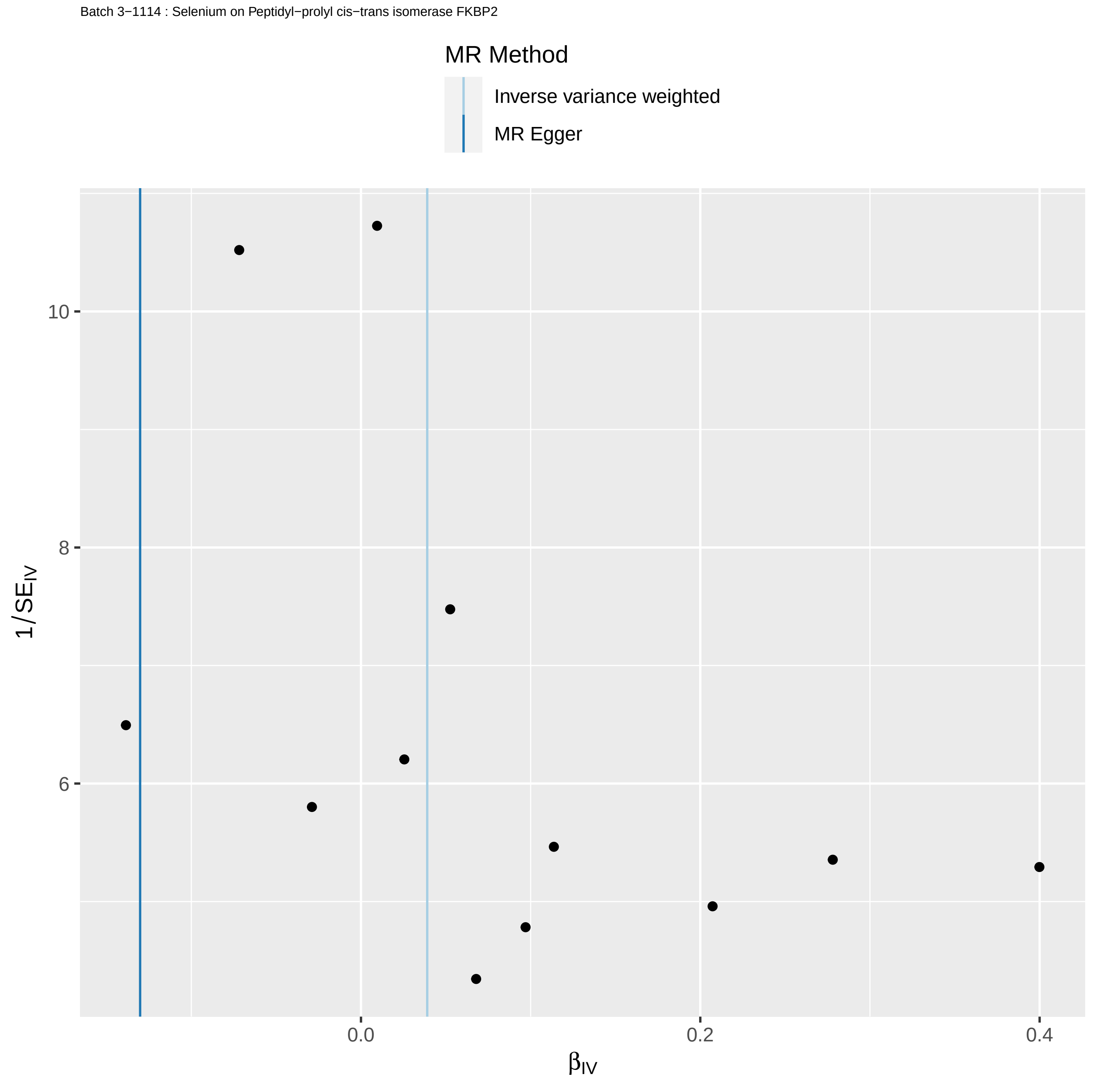

Batch 3−1114 : Selenium on Peptidyl−prolyl cis−trans isomerase FKBP2
MR Method
Inverse variance weighted
MR Egger
10
8
6
1/SE_IV
0.0
0.2
0.4
β_IV

Batch 3−1115 : Selenium on Inactive peptidyl−prolyl cis−trans isomerase FKBP6

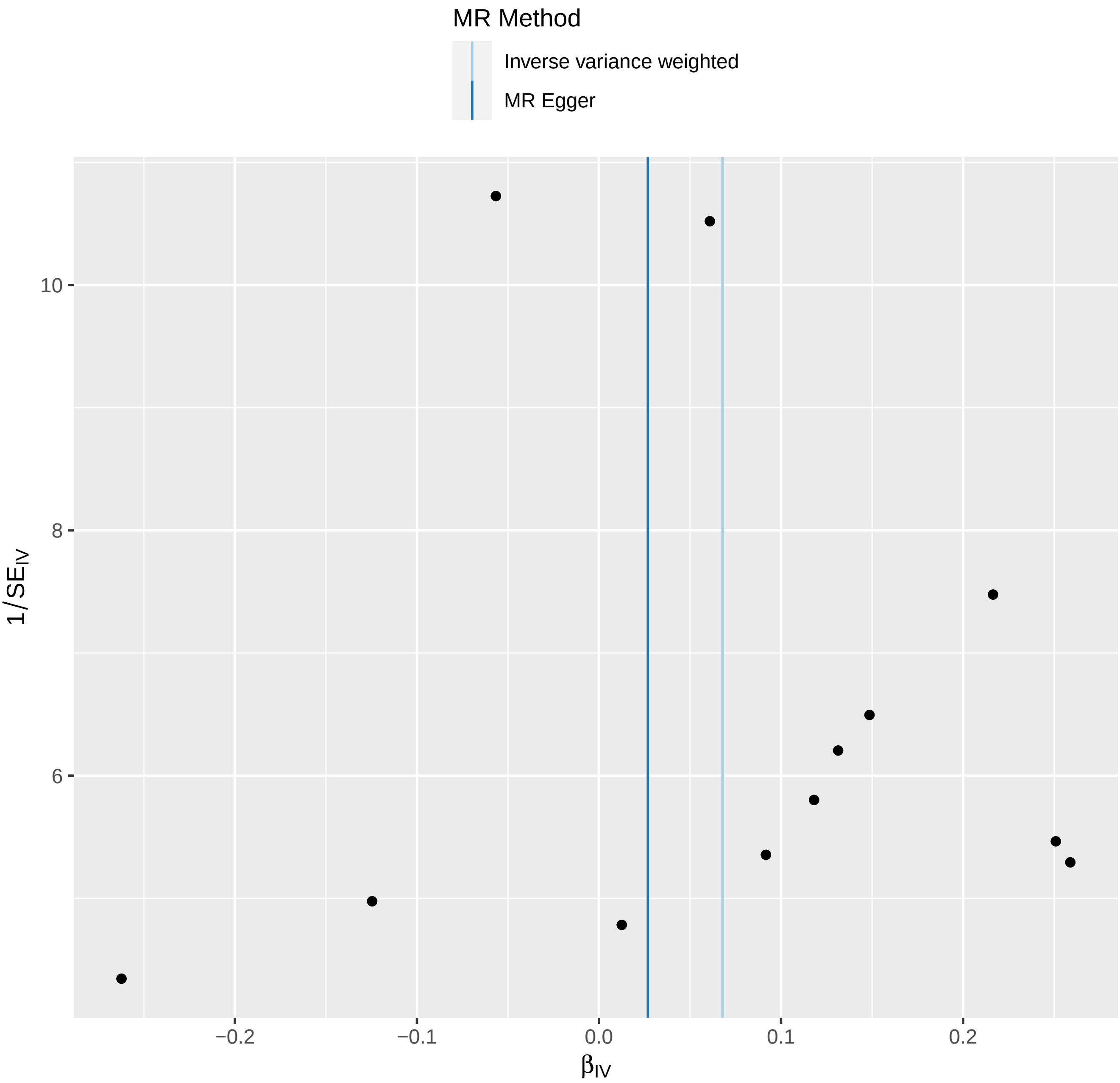

Batch 3−1116 : Selenium on Peptidyl−prolyl cis−trans isomerase FKBP7

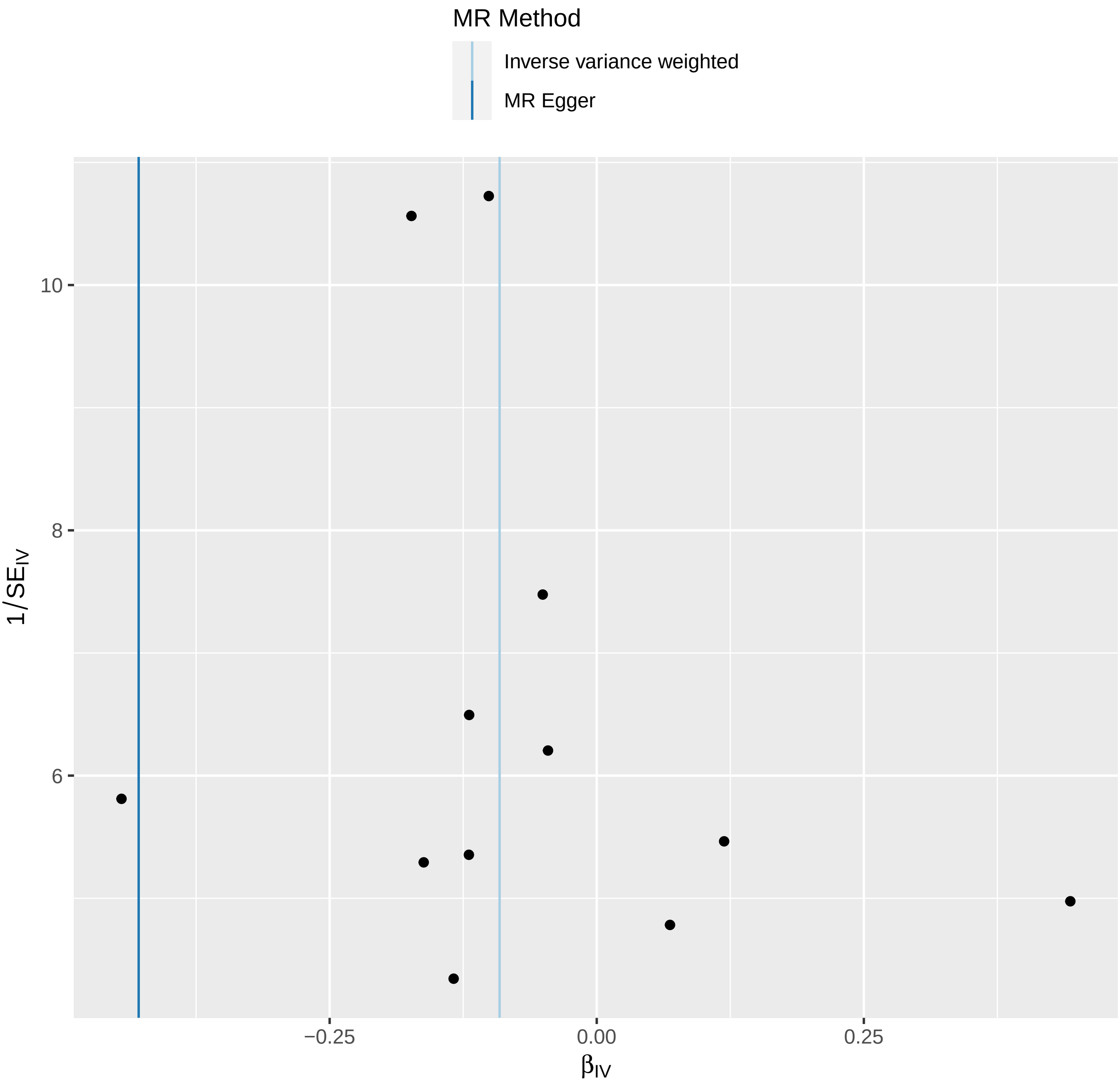

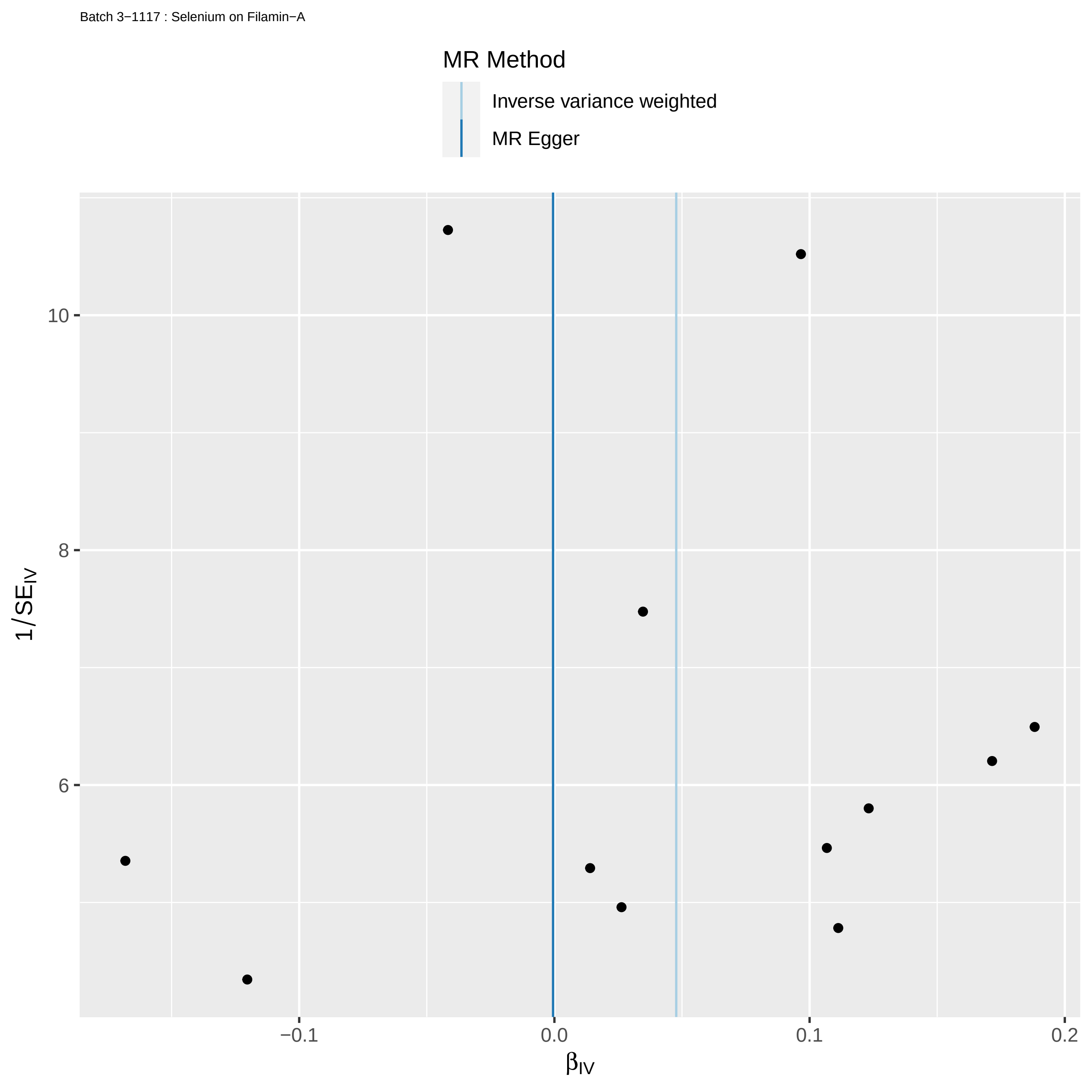
Batch 3−1117 : Selenium on Filamin−A
MR Method
Inverse variance weighted
MR Egger
10
8
6
1/SE_IV
−0.1
0.0
0.1
0.2
β_IV

Batch 3−1118 : Selenium on Filamin−A

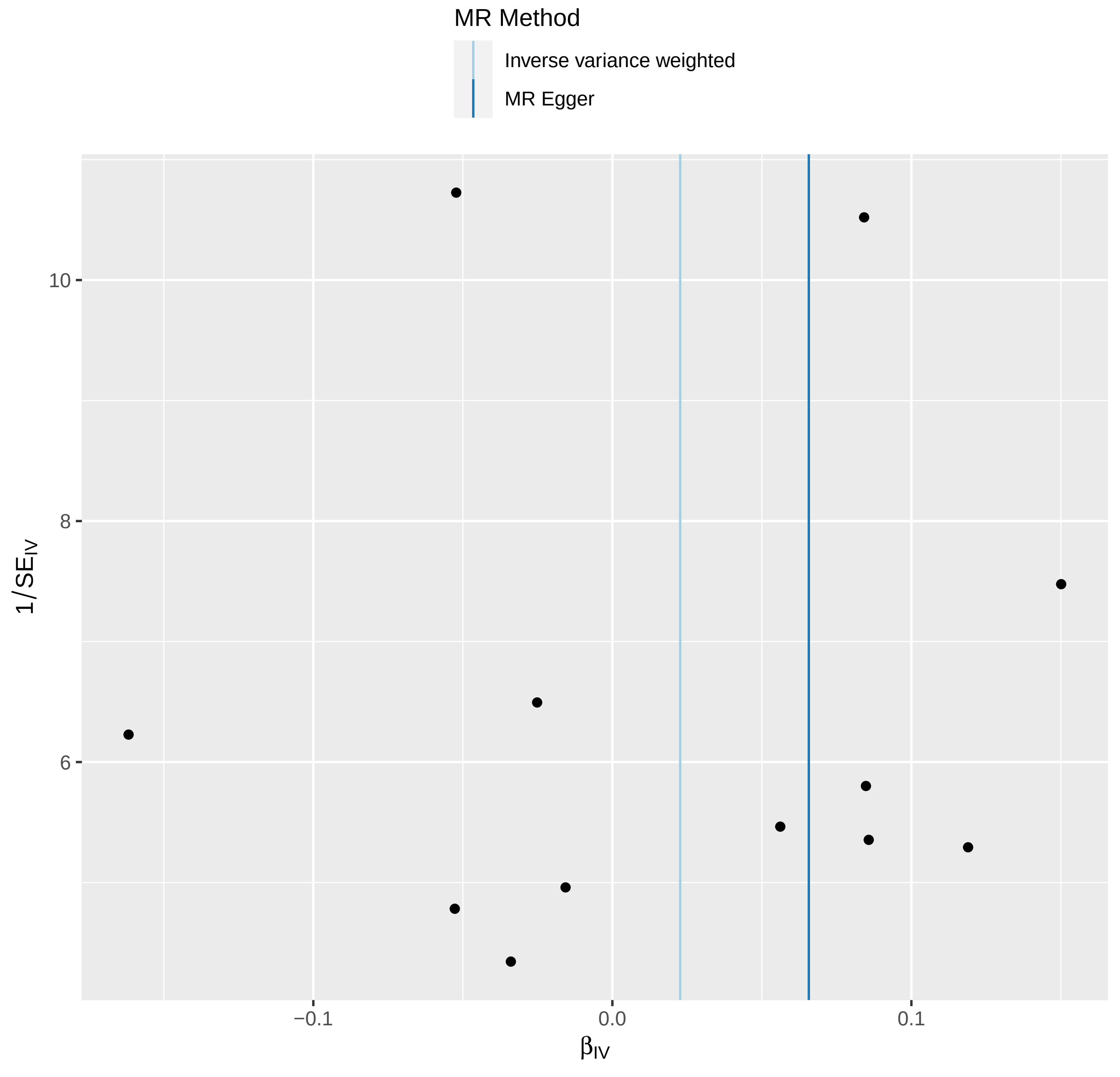

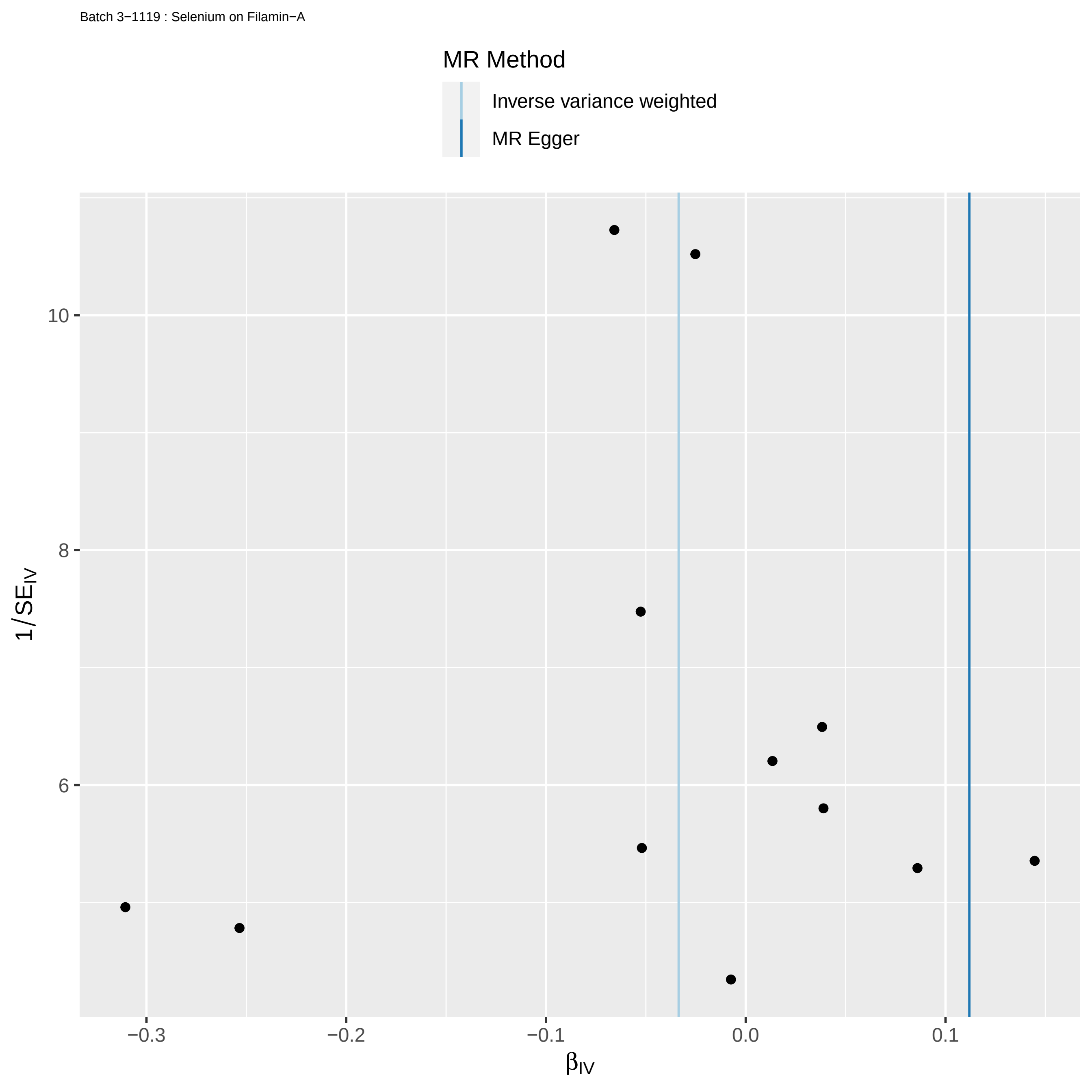

Batch 3−1119 : Selenium on Filamin−A
MR Method
Inverse variance weighted
MR Egger
10
8
6
−0.3
−0.2
−0.1
0.0
0.1
1/SE_IV
β_IV

Batch 3−1120 : Selenium on Filamin−A

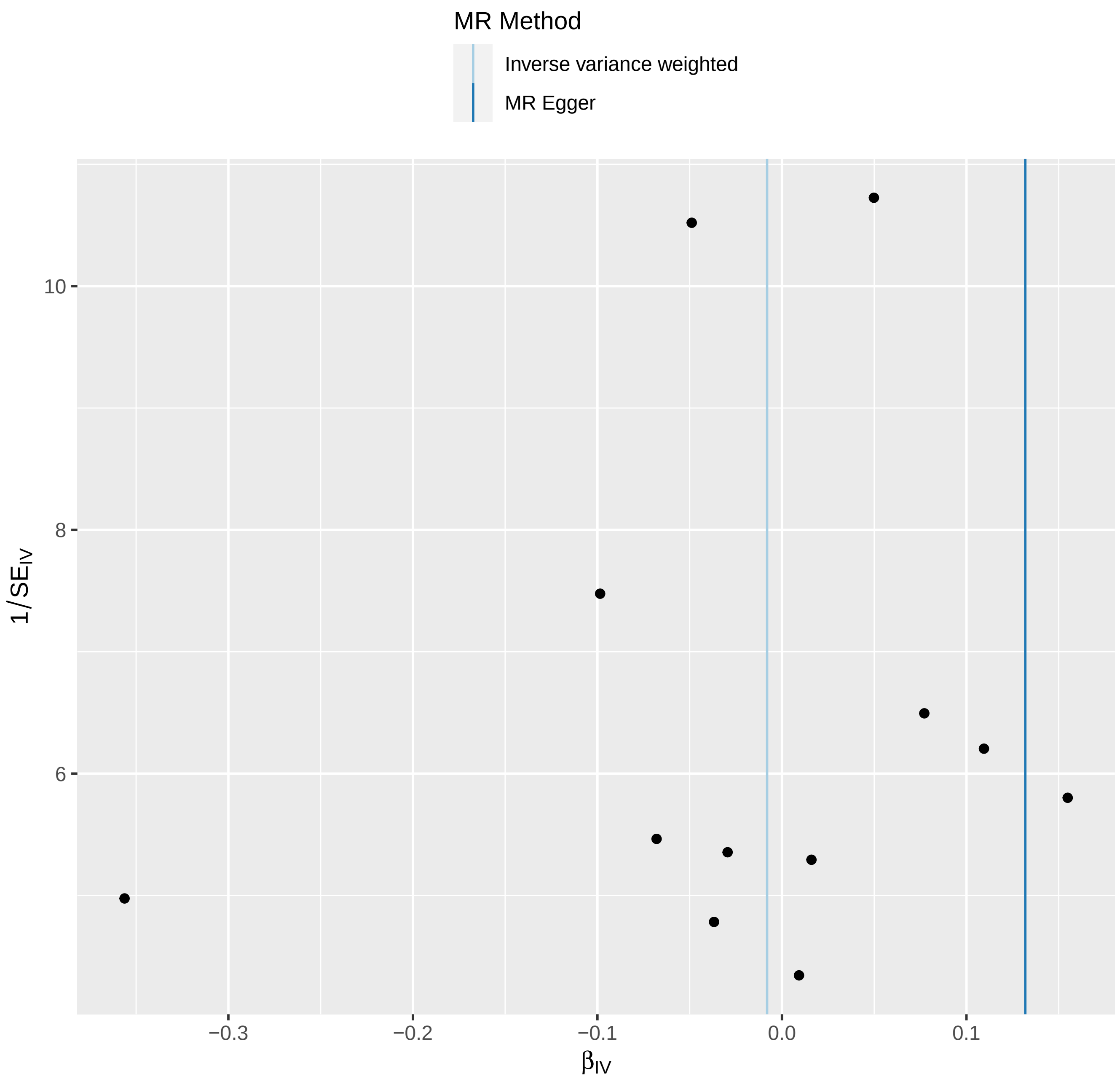

Batch 3−1121 : Selenium on Leucine−rich repeat transmembrane protein FLRT1

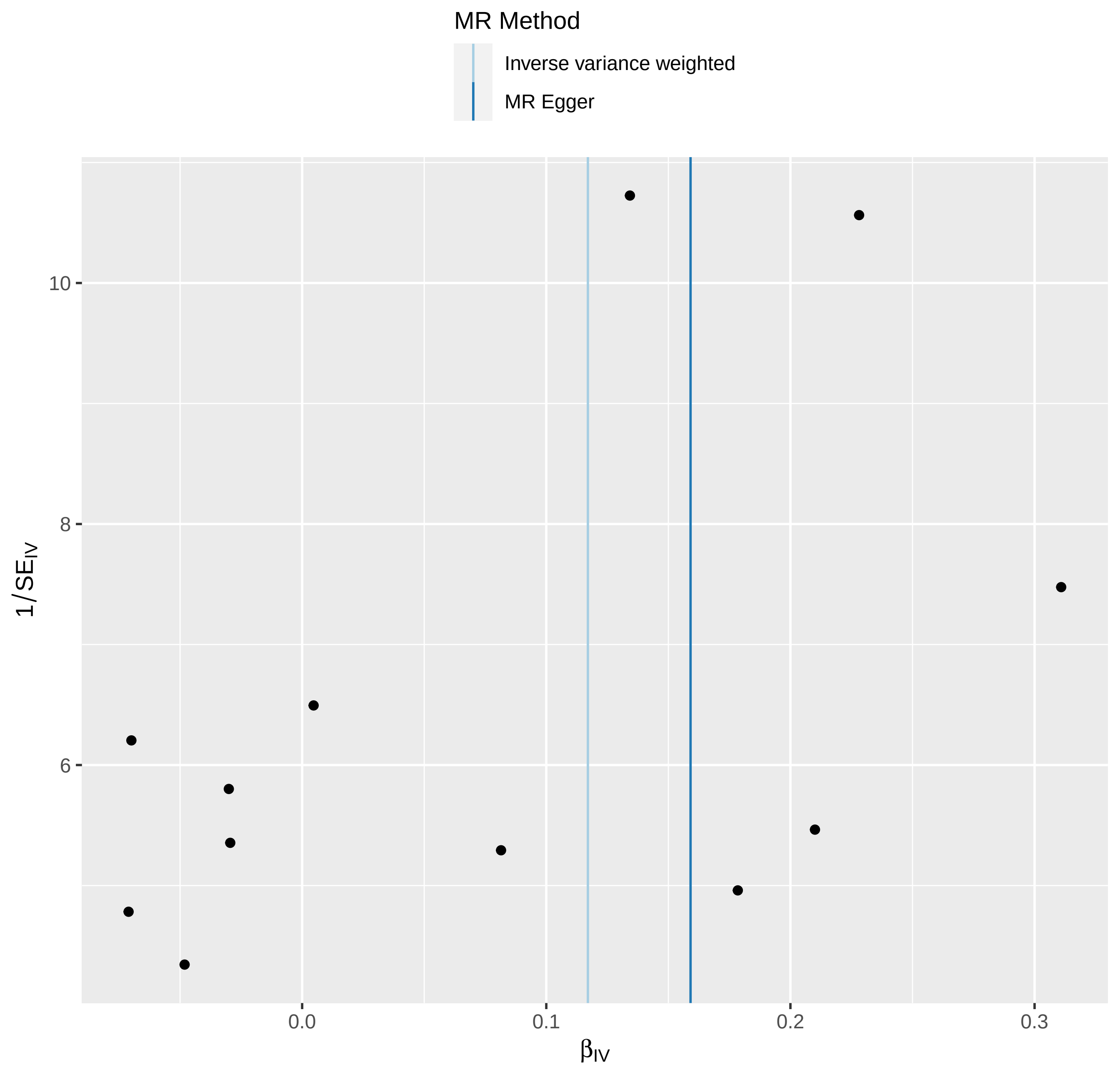

Batch 3−1122 : Selenium on Leucine−rich repeat transmembrane protein FLRT1

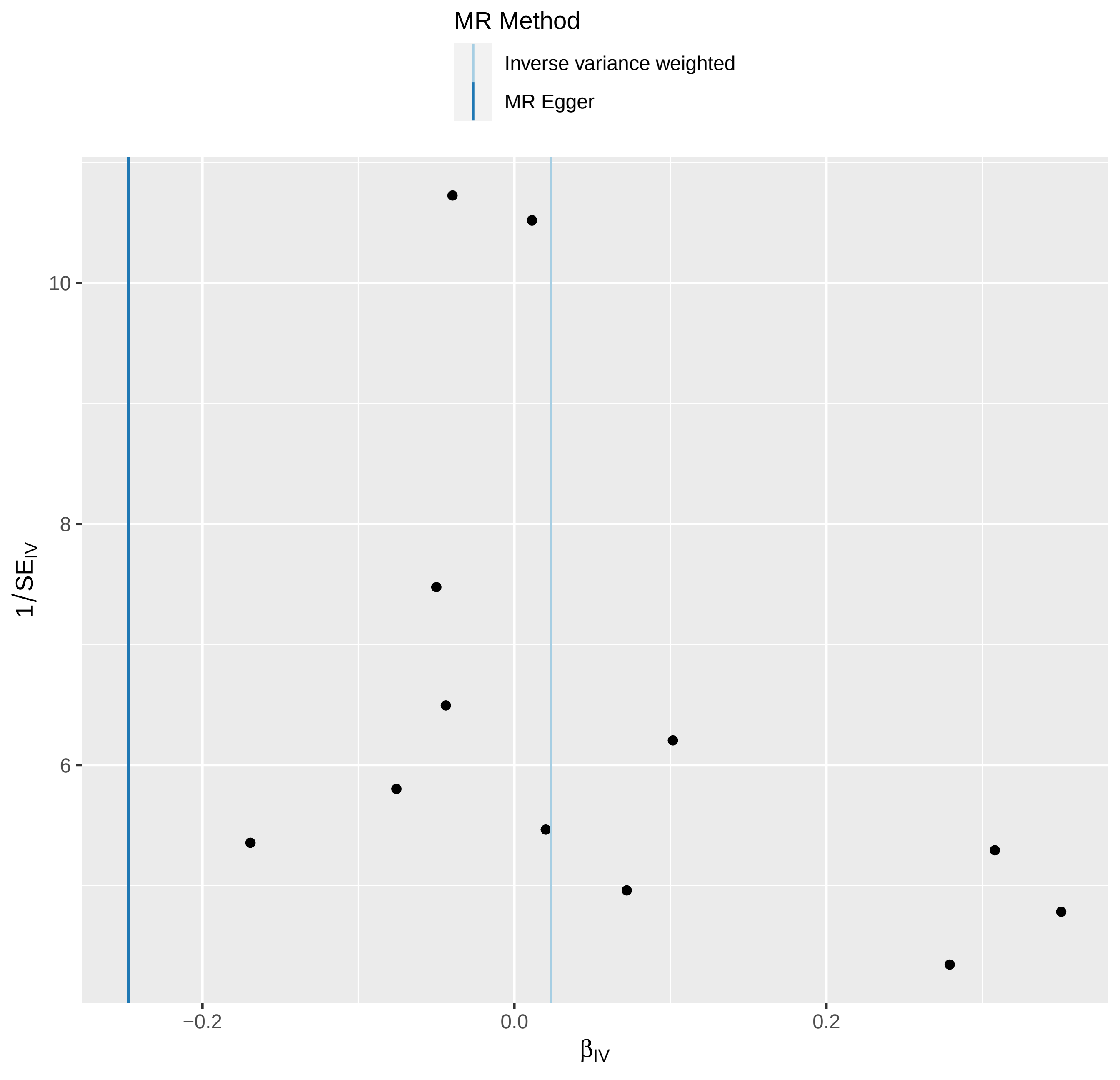

Batch 3−1123 : Selenium on Leucine−rich repeat transmembrane protein FLRT2

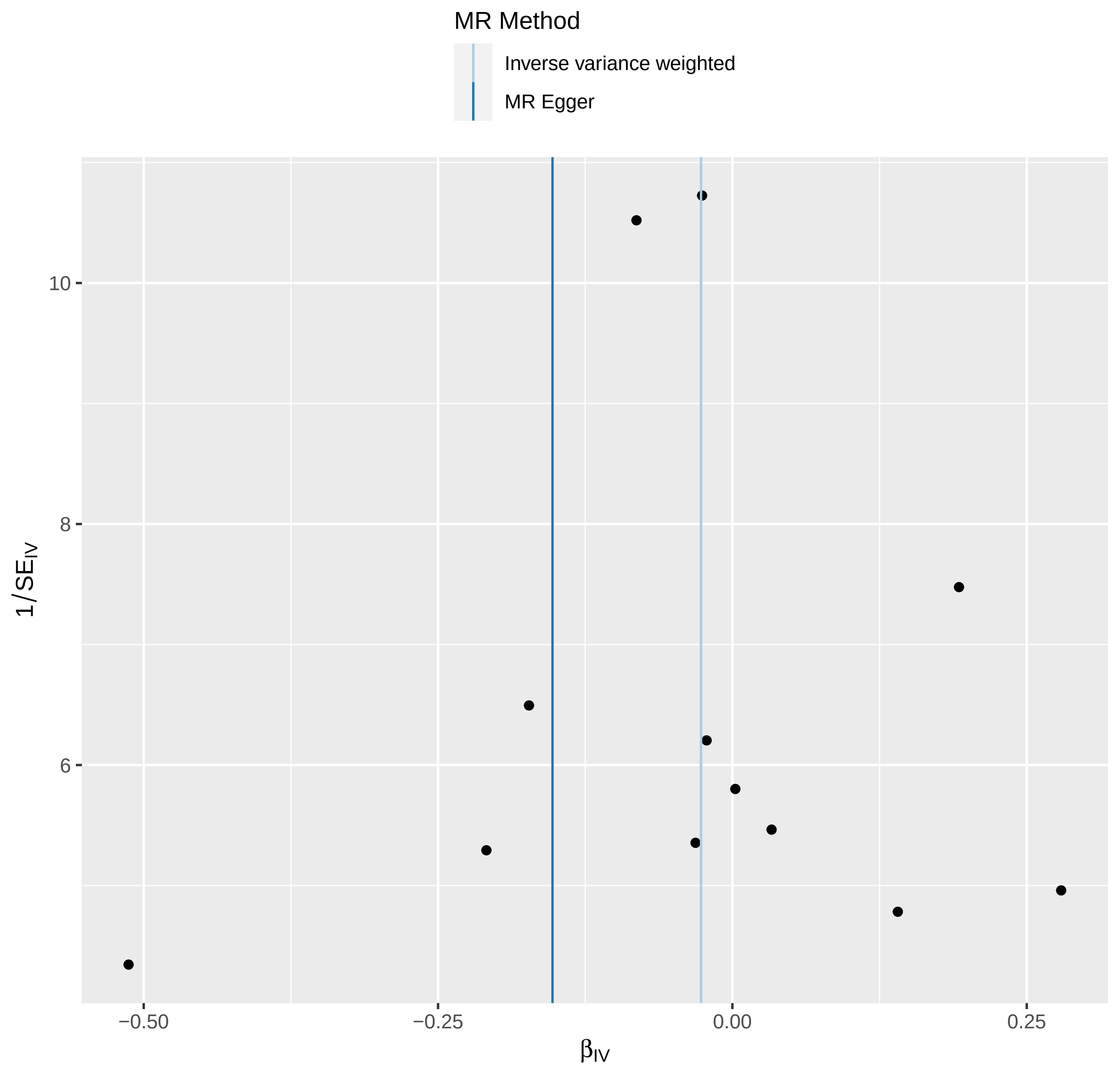

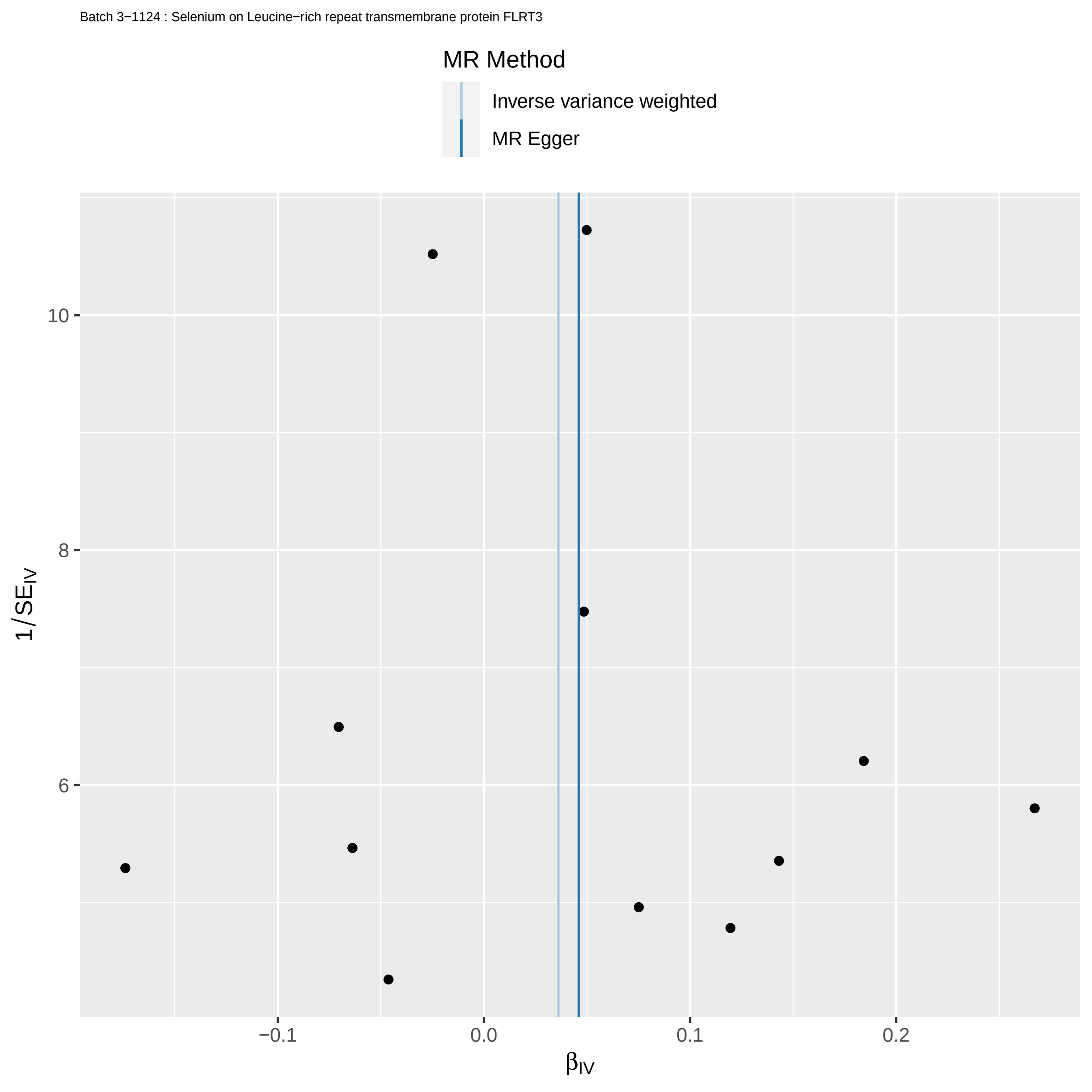
Batch 3−1124 : Selenium on Leucine−rich repeat transmembrane protein FLRT3
MR Method
Inverse variance weighted
MR Egger
10
8
6
1/SE_IV
−0.1
0.0
0.1
0.2
β_IV

Batch 3−1125 : Selenium on Leucine−rich repeat transmembrane protein FLRT3

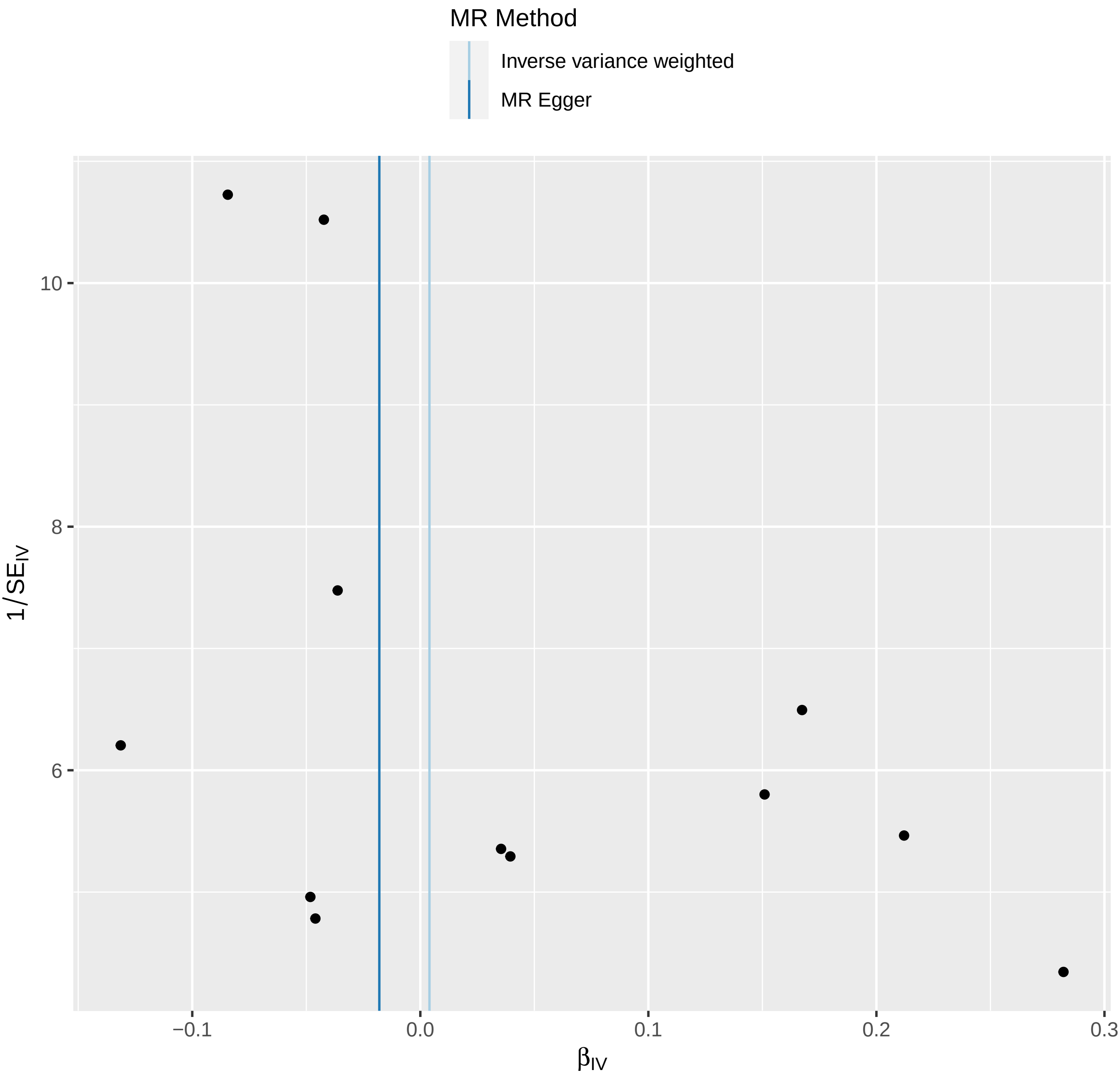

Batch 3−1126 : Selenium on Receptor−type tyrosine−protein kinase FLT3

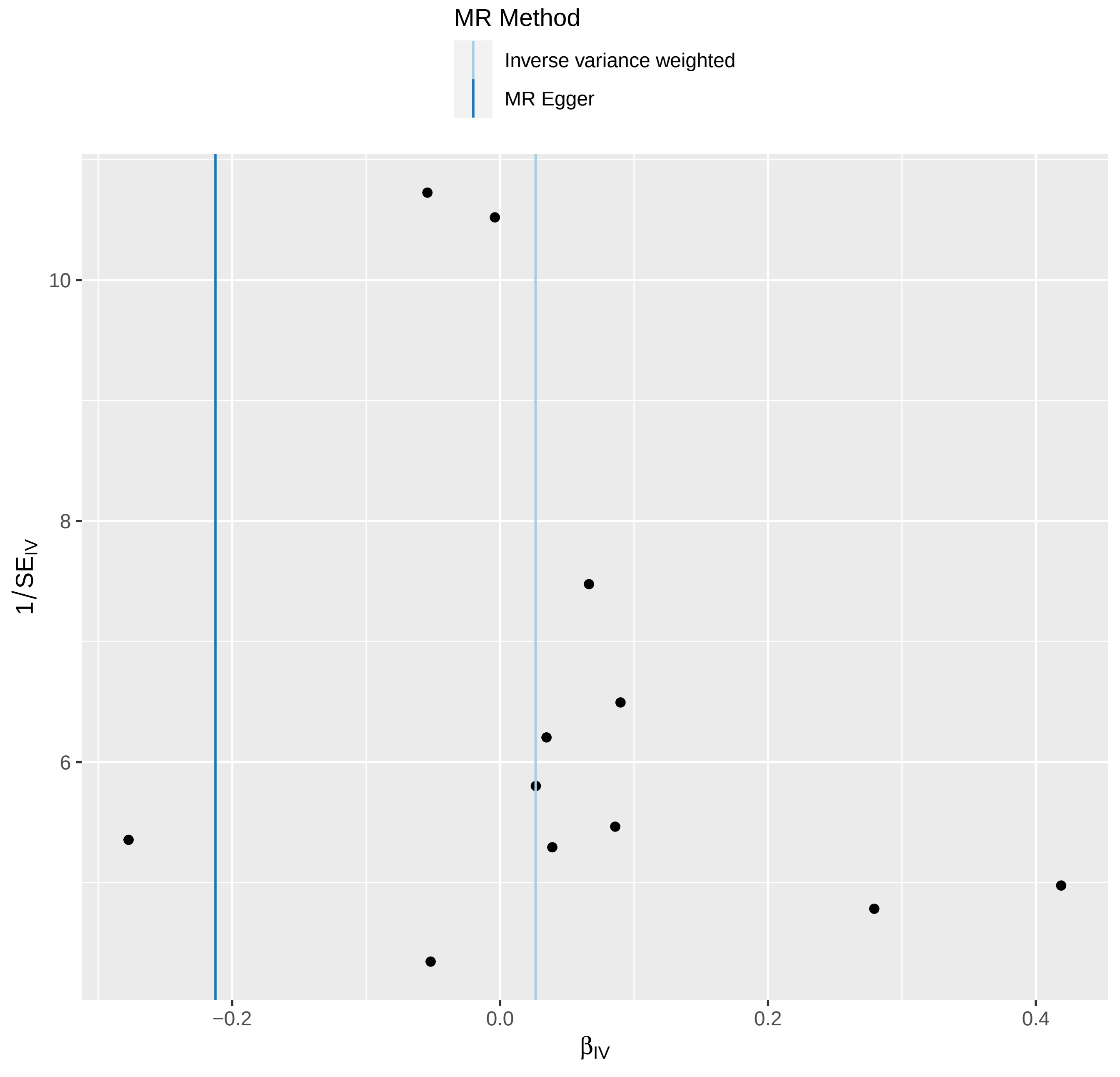

Batch 3−1127 : Selenium on Fms−related tyrosine kinase 3 ligand

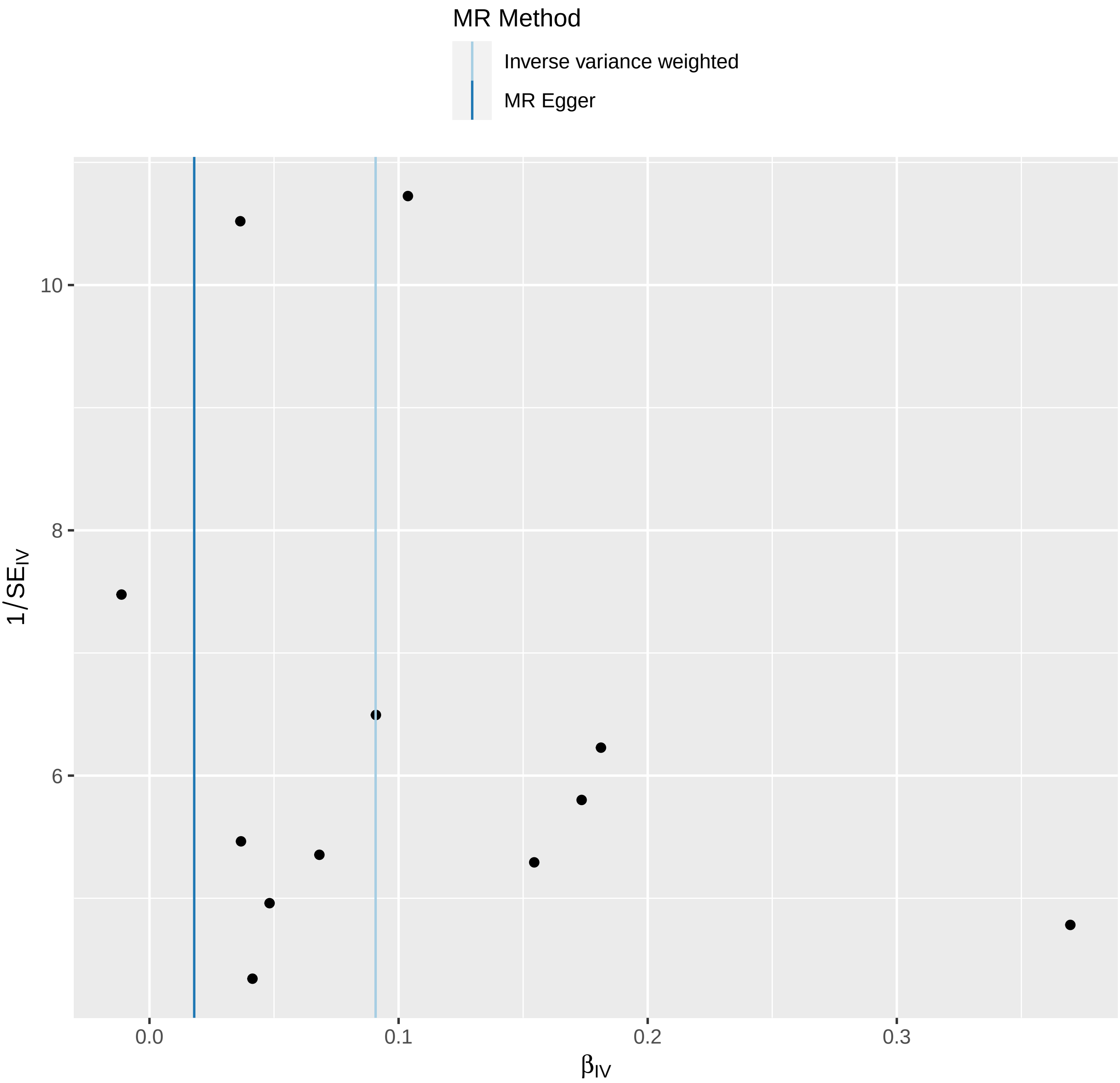

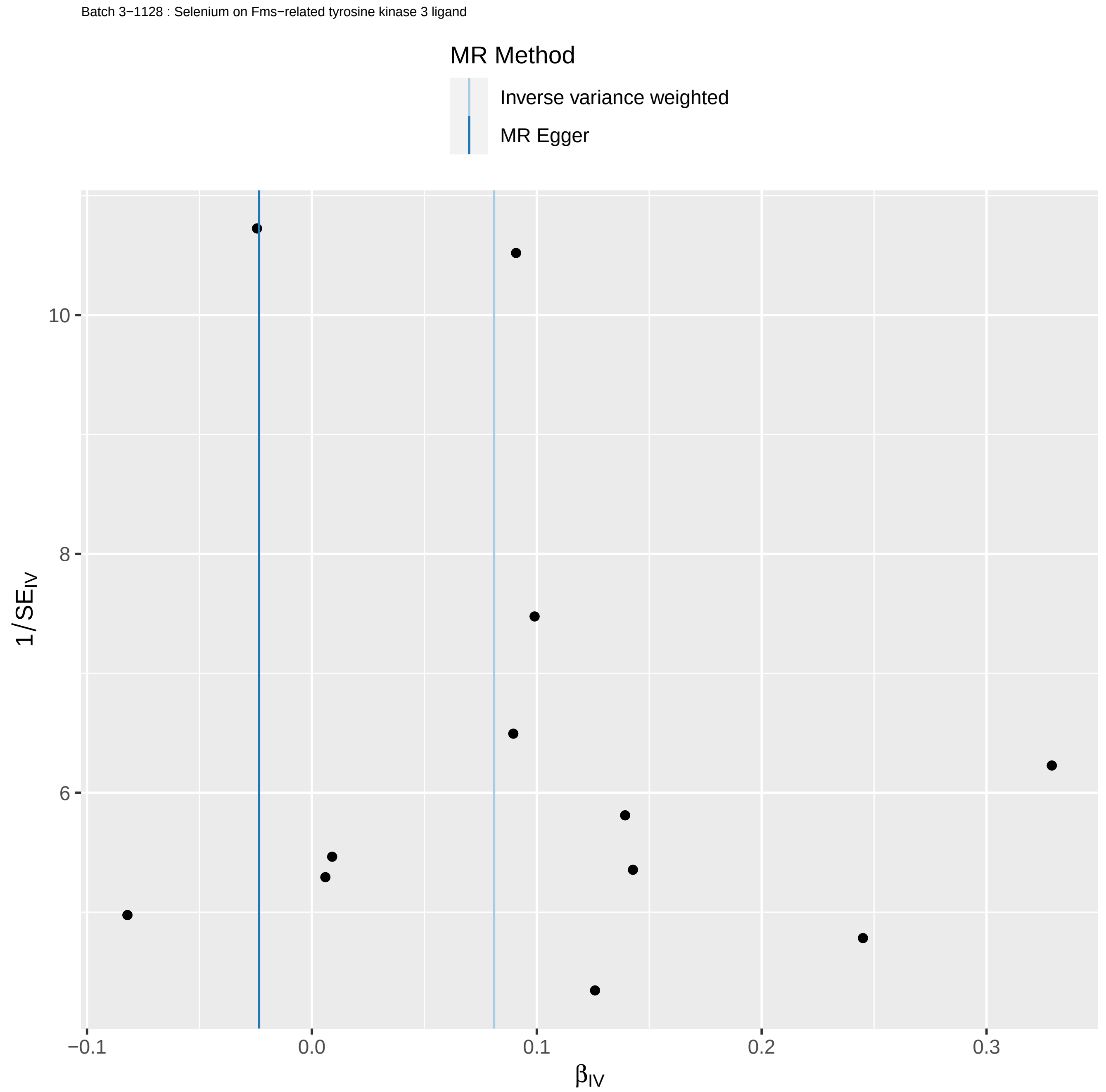
Batch 3−1128 : Selenium on Fms−related tyrosine kinase 3 ligand
MR Method
Inverse variance weighted
MR Egger
10
8
6
1/SE$_{IV}$
−0.1
0.0
0.1
0.2
0.3
β$_{IV}$

Batch 3−1129 : Selenium on Vascular endothelial growth factor receptor 3

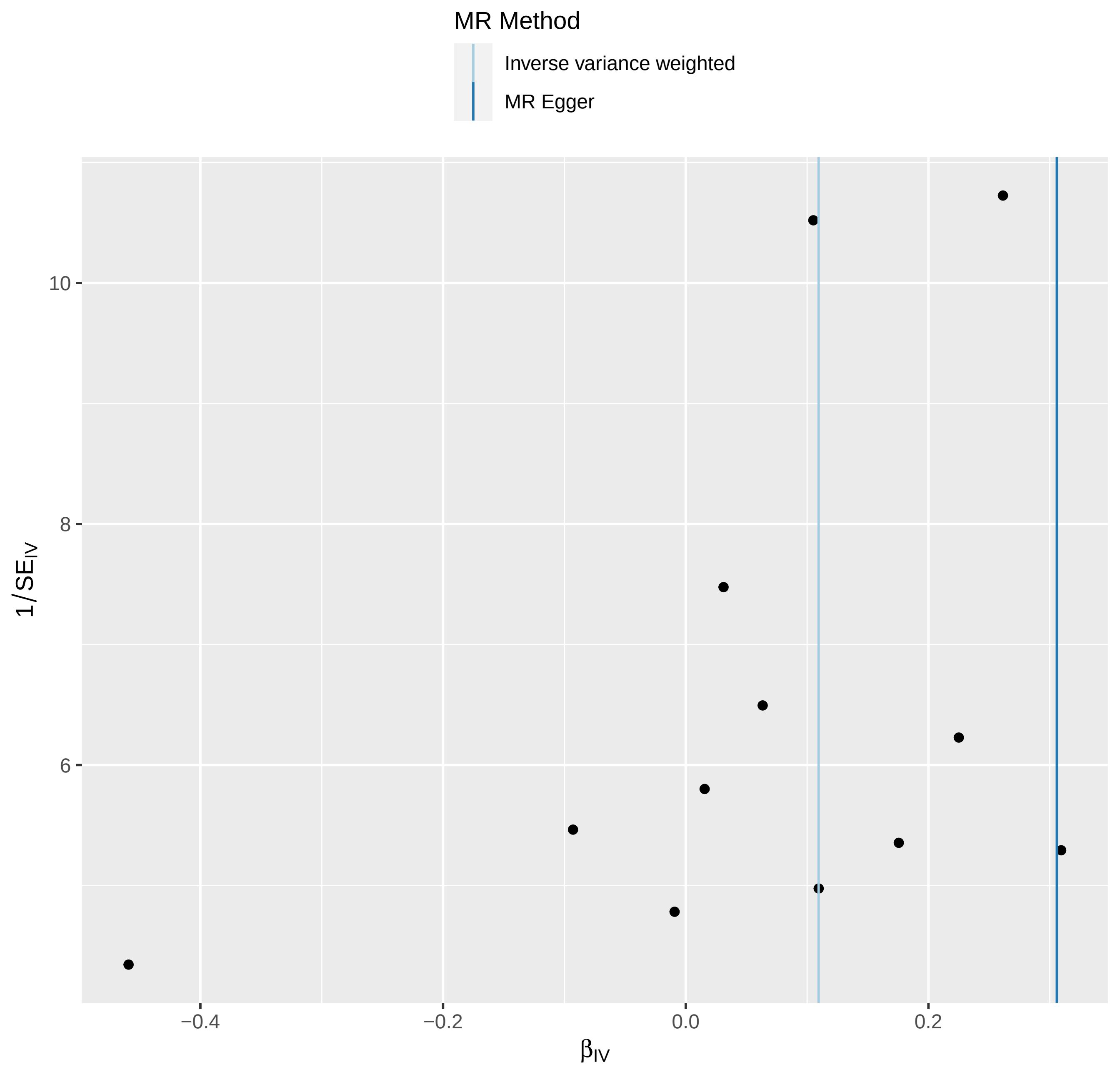

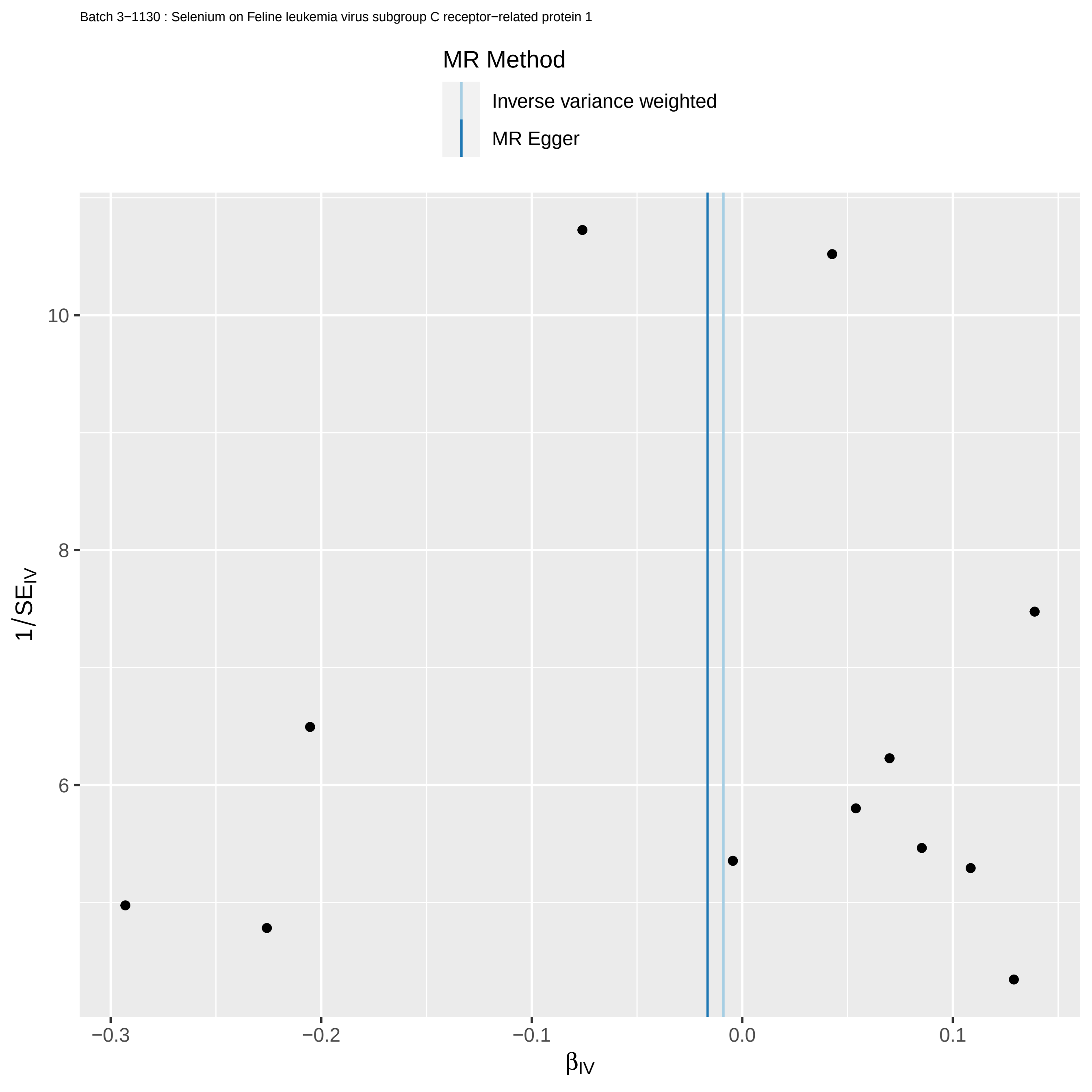

Batch 3−1130 : Selenium on Feline leukemia virus subgroup C receptor−related protein 1
MR Method
Inverse variance weighted
MR Egger
10
8
6
1/SE_IV
−0.3
−0.2
−0.1
0.0
0.1
β_IV

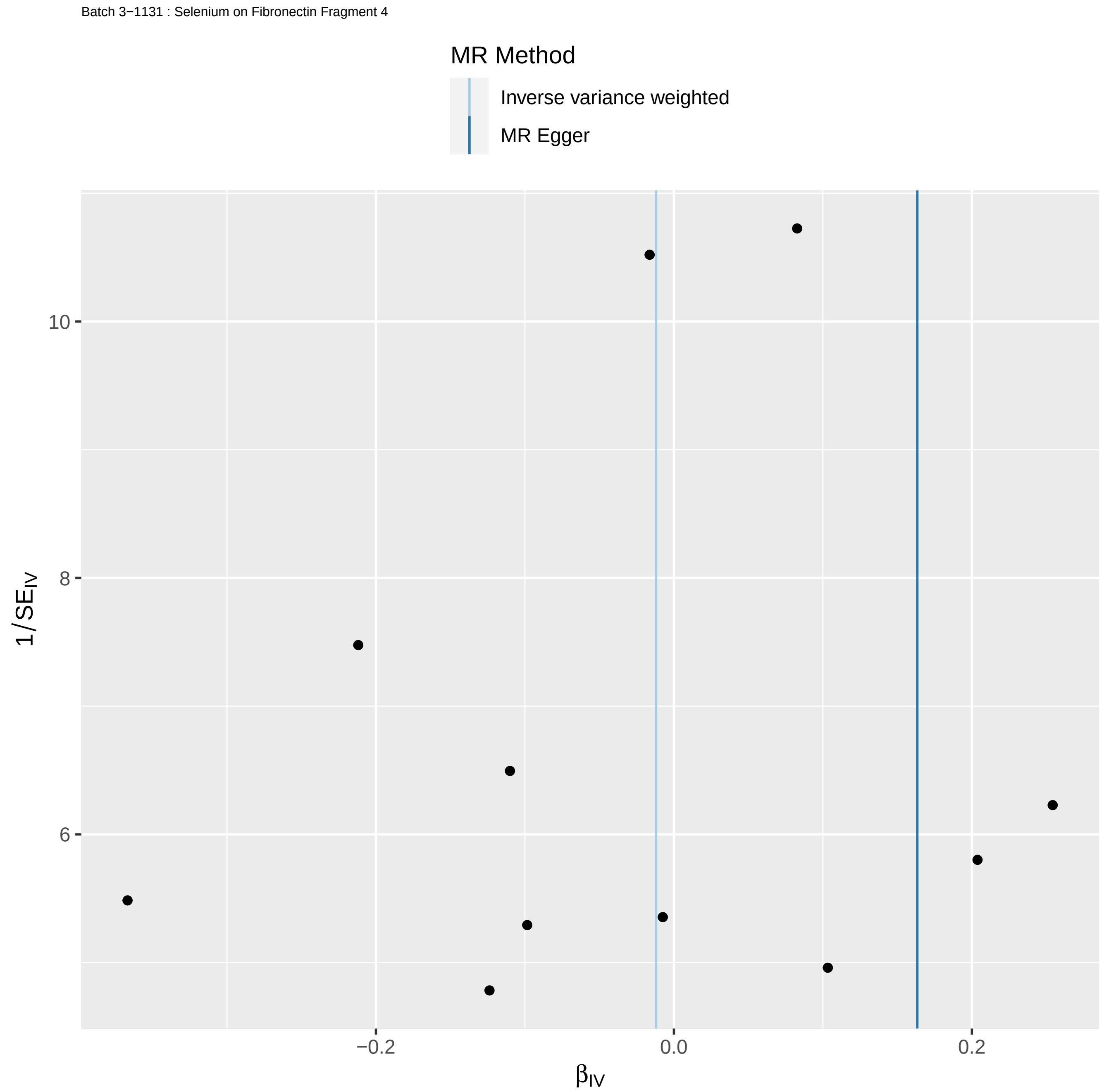

Batch 3−1131 : Selenium on Fibronectin Fragment 4
MR Method
Inverse variance weighted
MR Egger
10
8
6
1/SE_IV
−0.2
0.0
0.2
β_IV

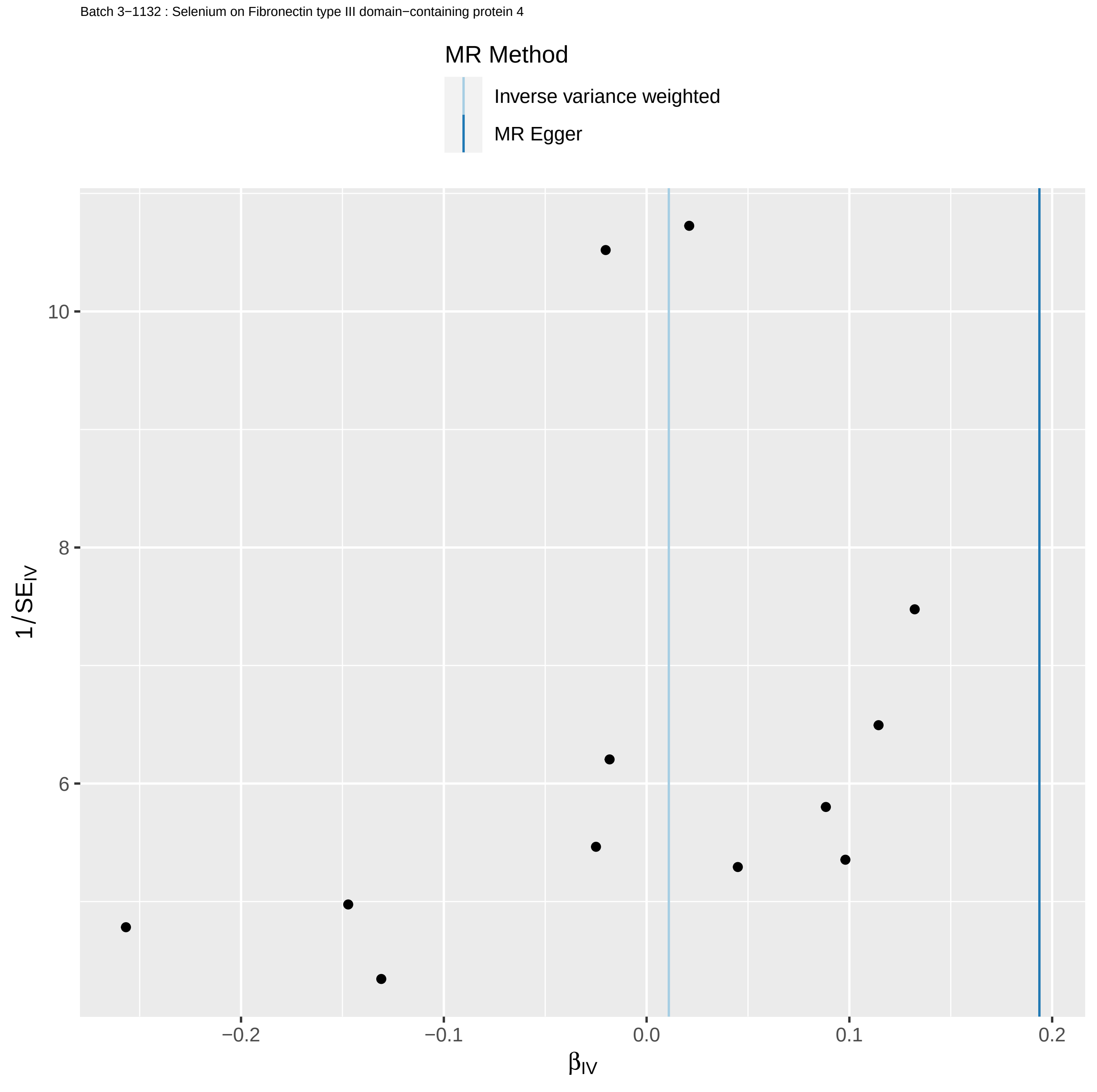

Batch 3−1132 : Selenium on Fibronectin type III domain−containing protein 4
MR Method
Inverse variance weighted
MR Egger
10
8
6
1/SE_IV
−0.2
−0.1
0.0
0.1
0.2
β_IV

Batch 3−1133 : Selenium on Fibronectin type III domain−containing protein 5

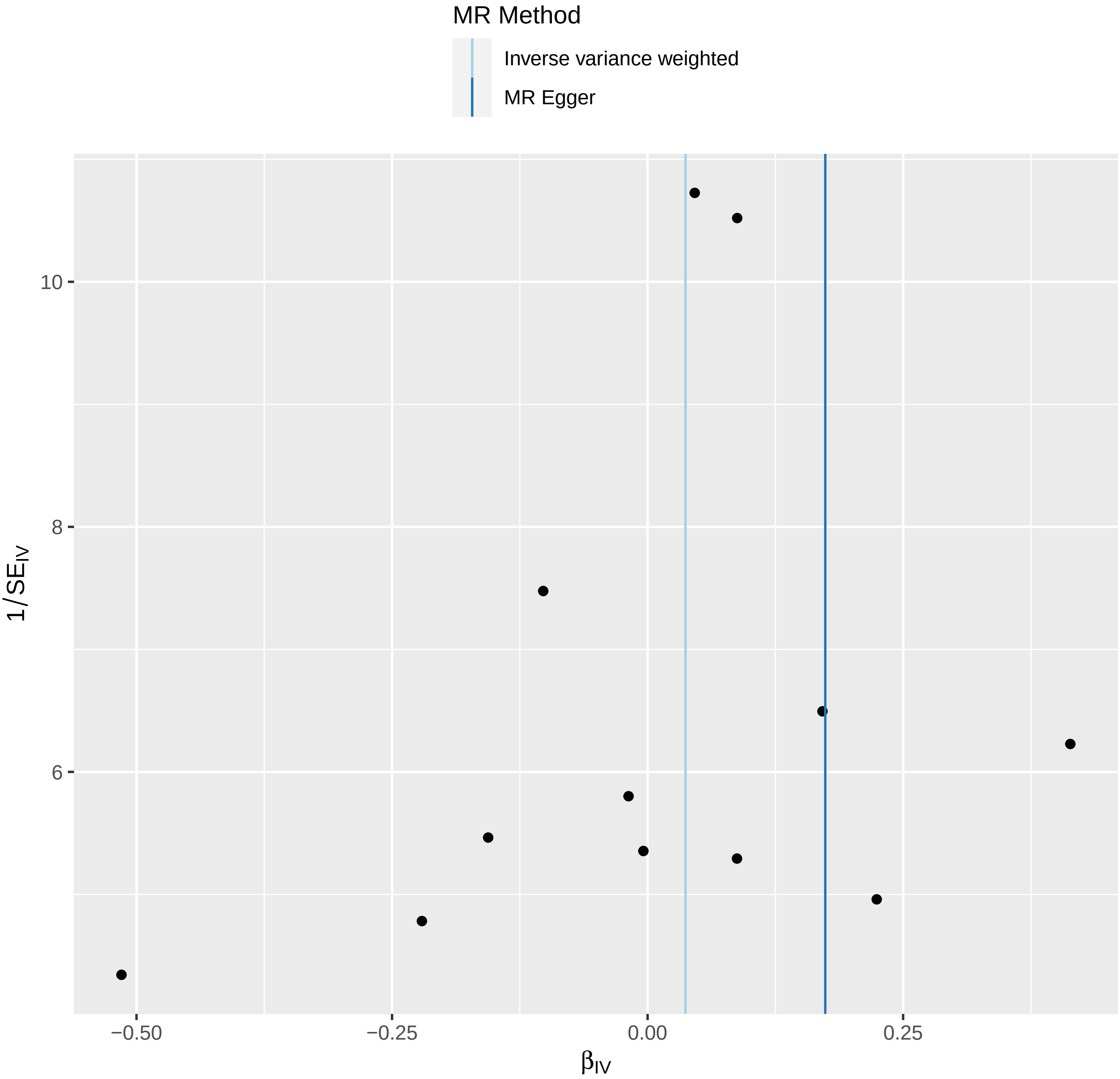

Batch 3−1134 : Selenium on Glutamate carboxypeptidase 2

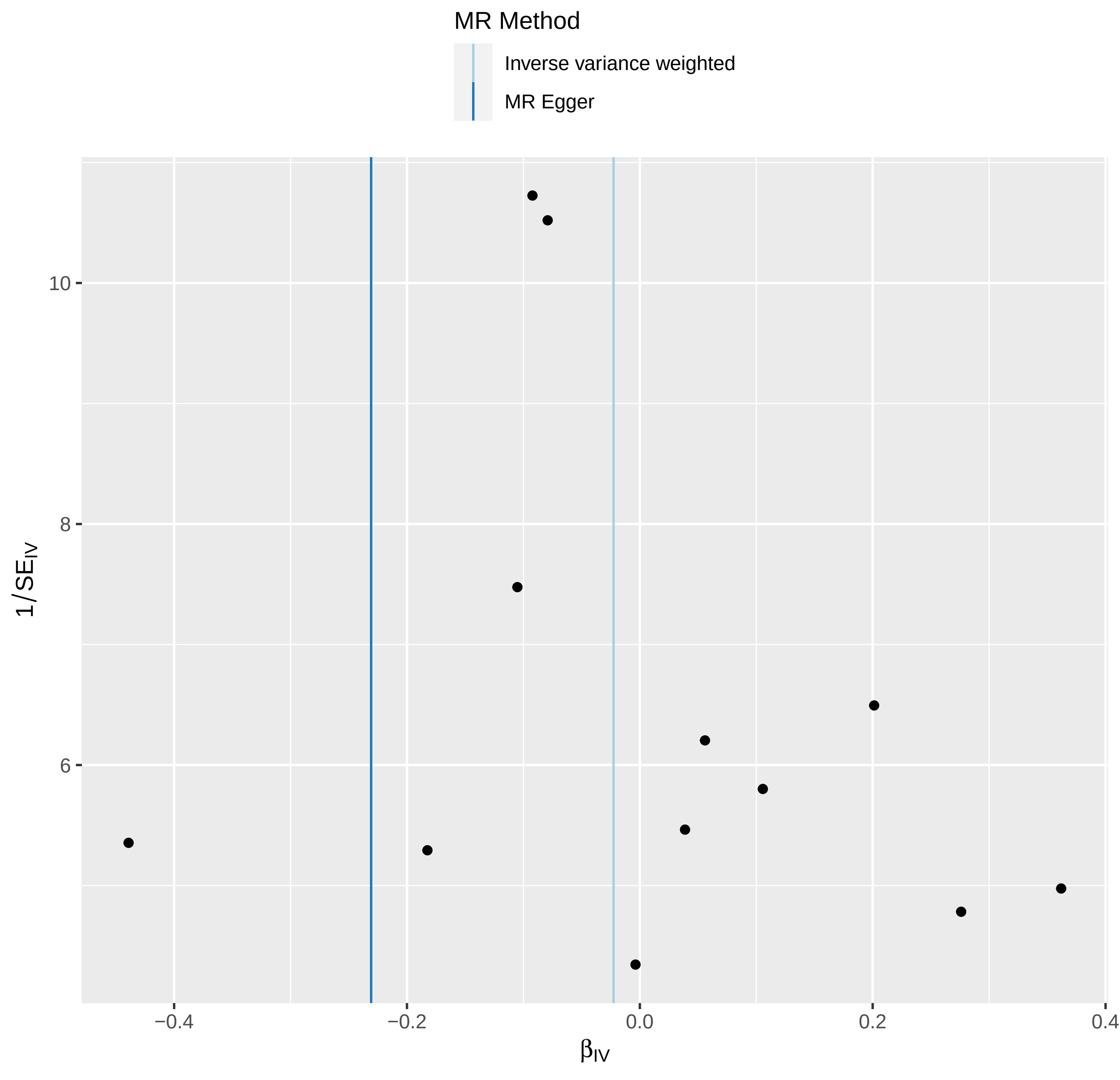

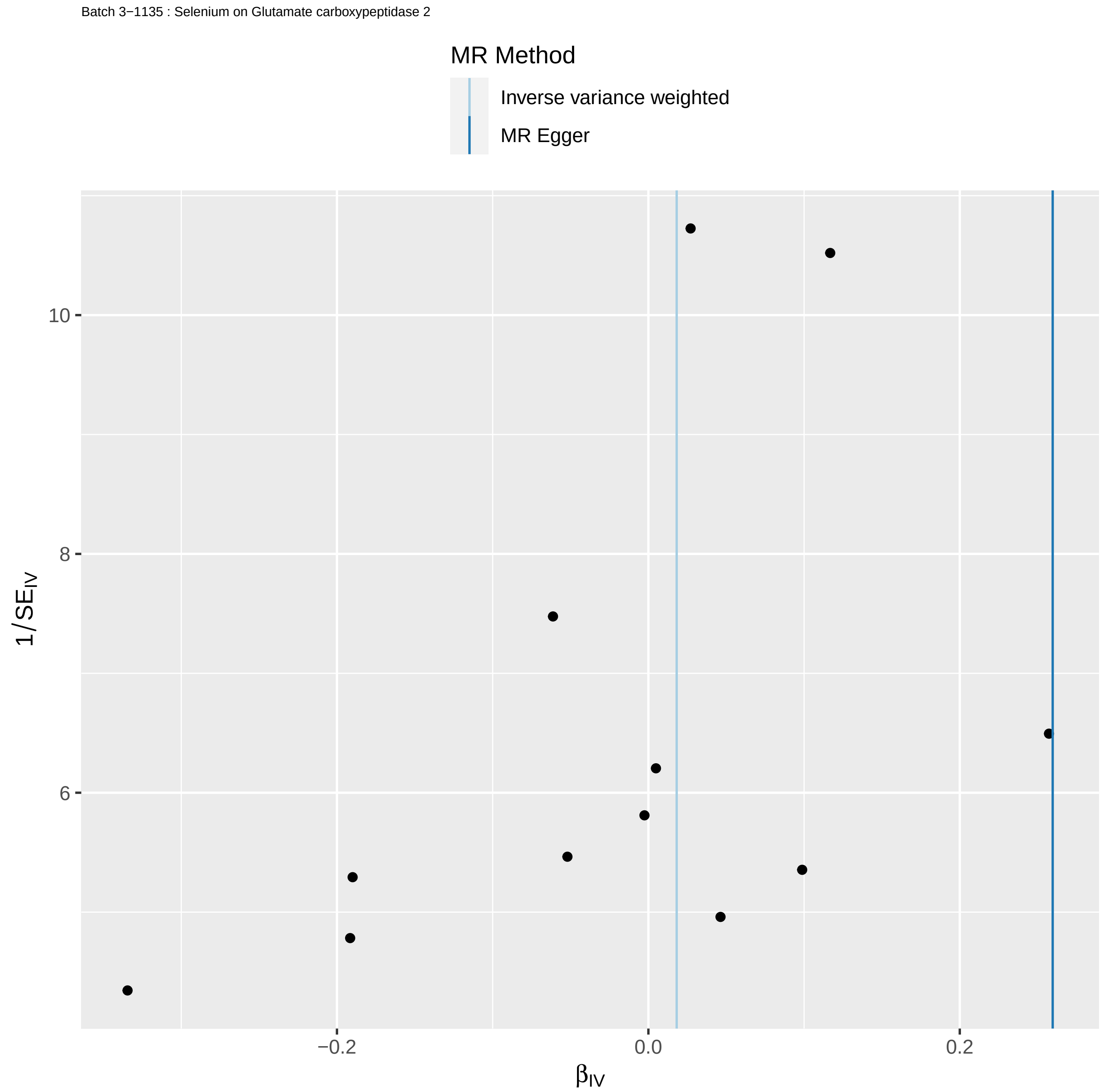
Batch 3−1135 : Selenium on Glutamate carboxypeptidase 2
MR Method
Inverse variance weighted
MR Egger
10
8
6
1/SE_IV
−0.2
0.0
0.2
β_IV

Batch 3−1136 : Selenium on Forkhead box protein C2

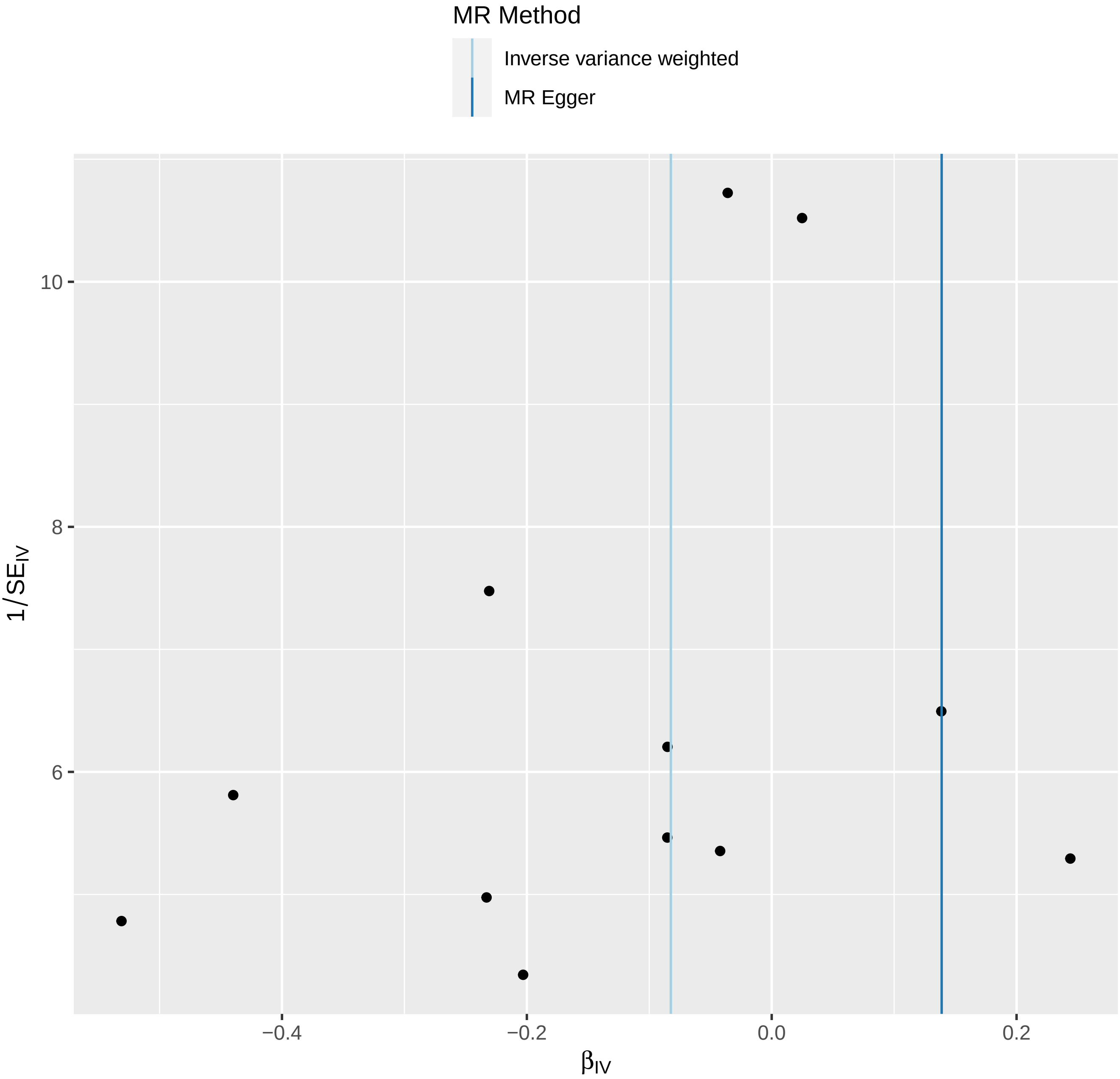

Batch 3−1137 : Selenium on Forkhead box protein J2

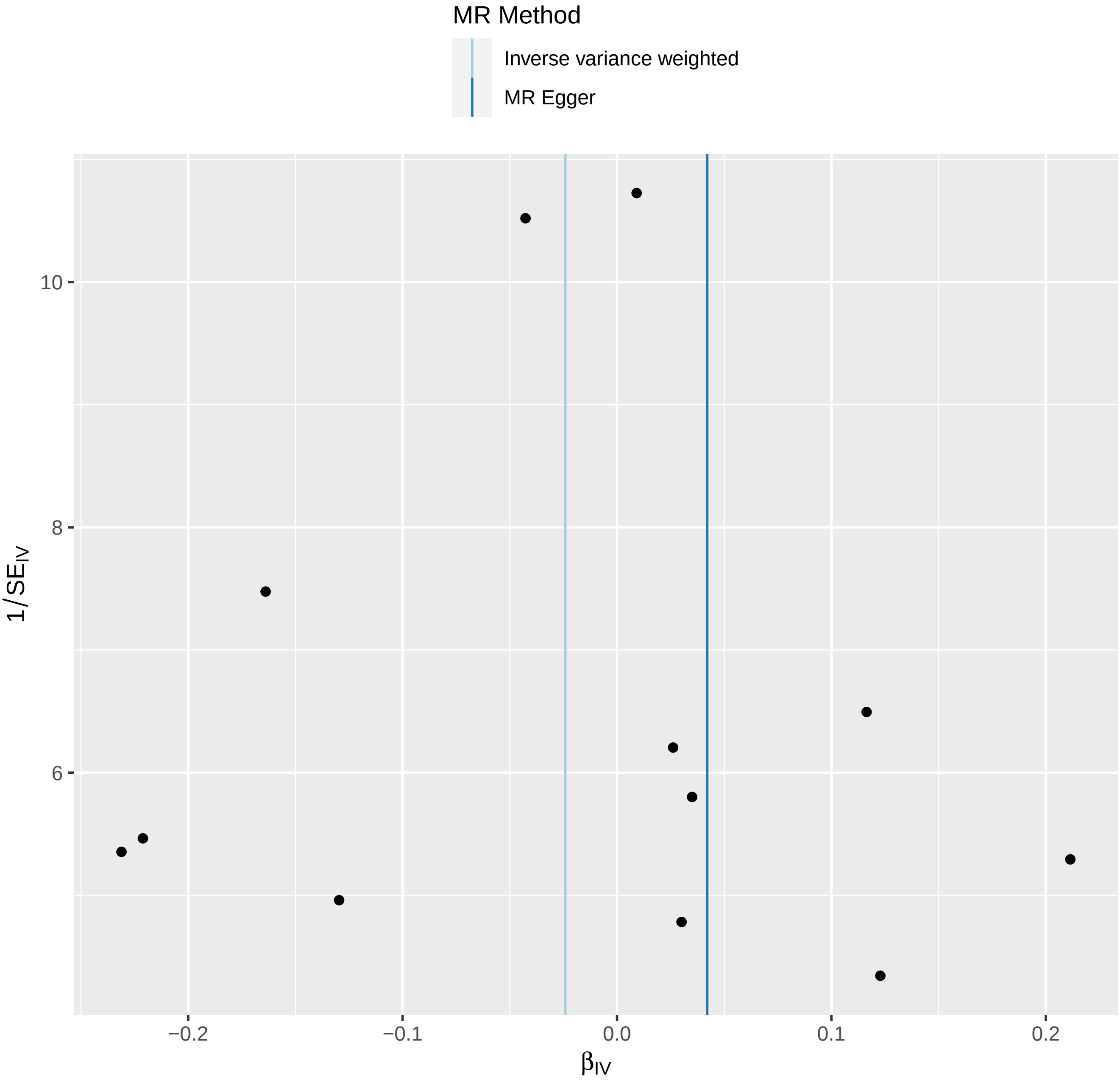

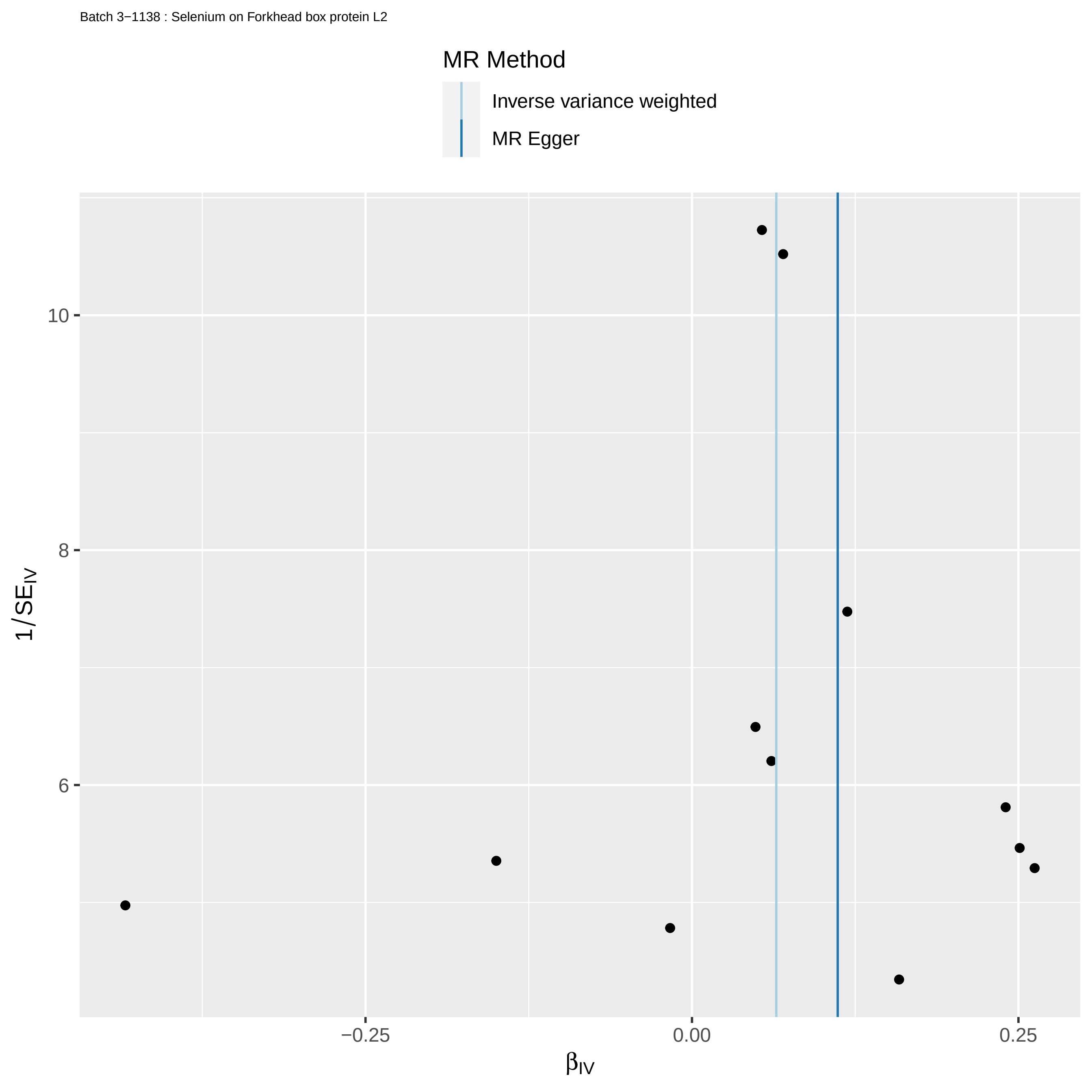

Batch 3−1138 : Selenium on Forkhead box protein L2
MR Method
Inverse variance weighted
MR Egger
10
8
6
1/SE_IV
−0.25
0.00
0.25
β_IV

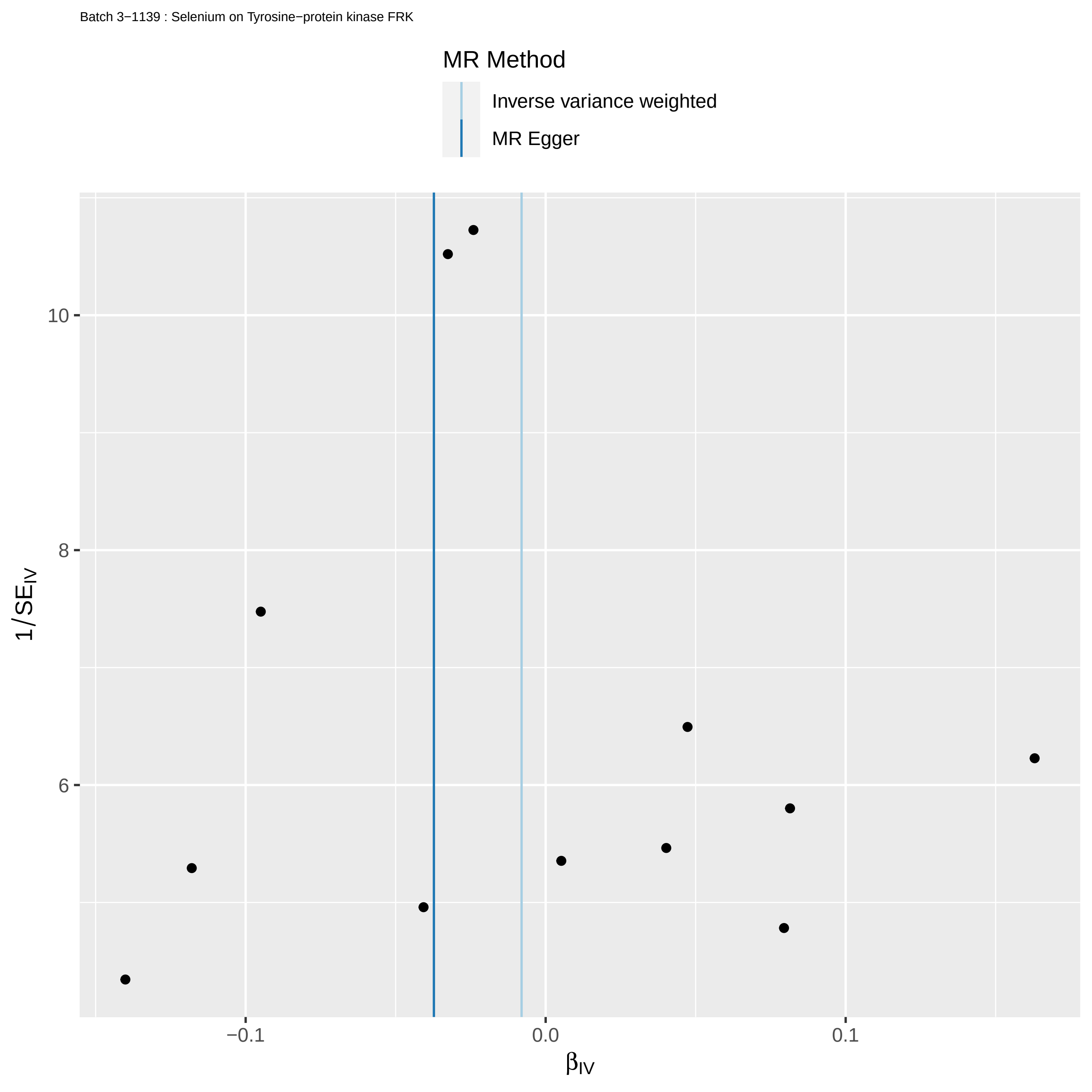
Batch 3−1139 : Selenium on Tyrosine−protein kinase FRK
MR Method
Inverse variance weighted
MR Egger
10
8
6
1/SE_IV
−0.1
0.0
0.1
β_IV

Batch 3−1140 : Selenium on Secreted frizzled−related protein 3

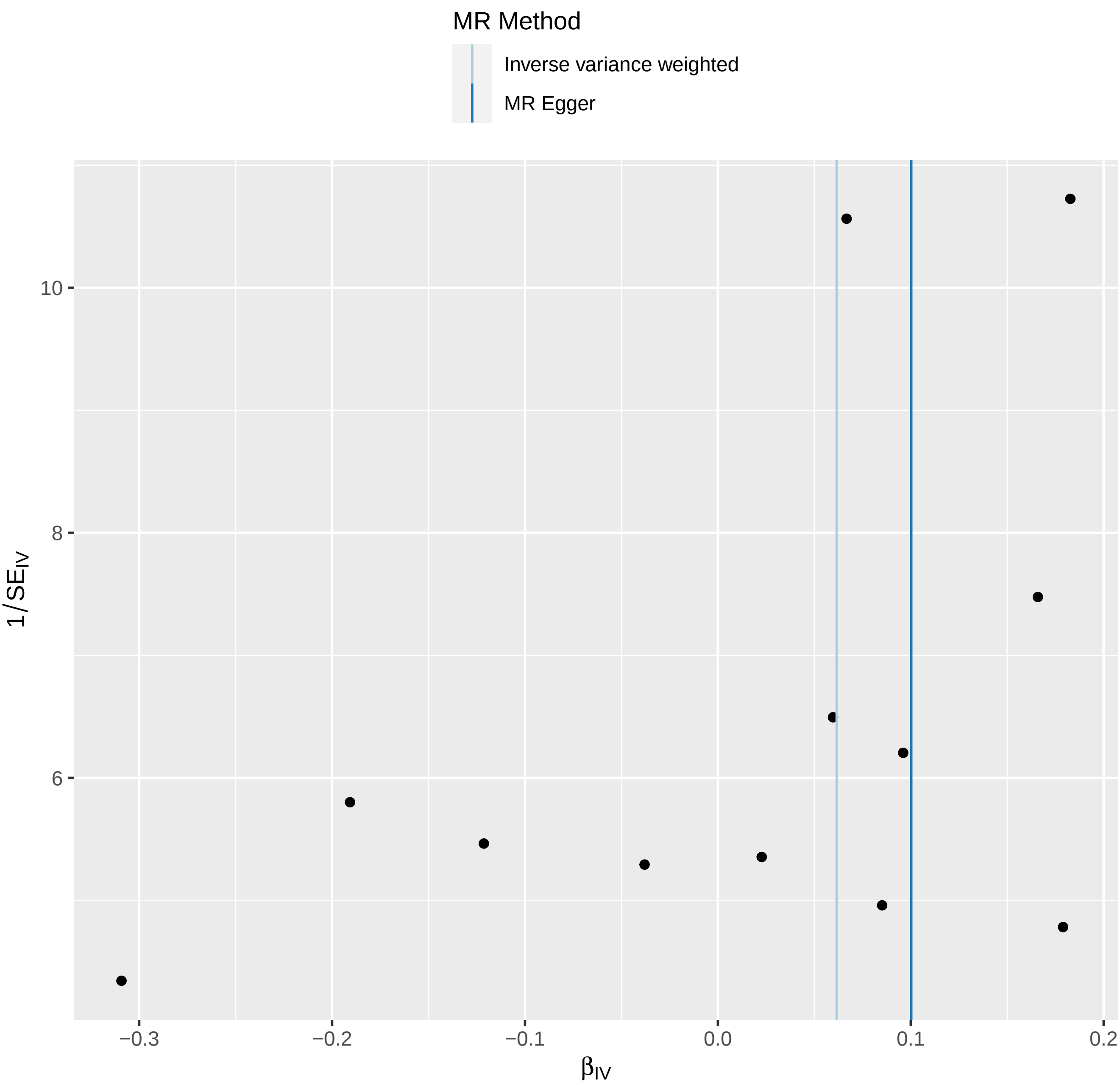

Batch 3−1141 : Selenium on Secreted frizzled−related protein 3

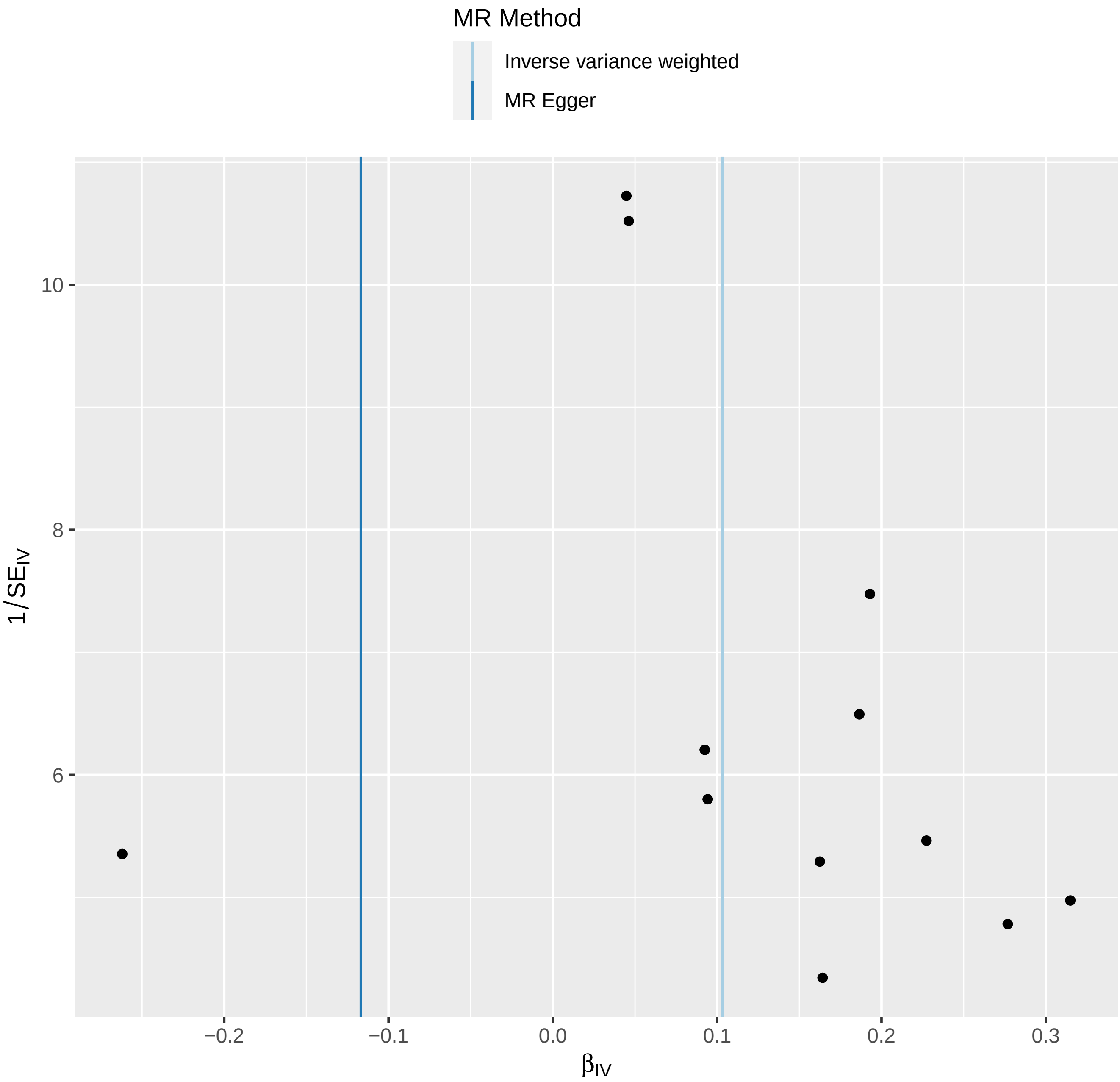

Batch 3−1142 : Selenium on Follistatin

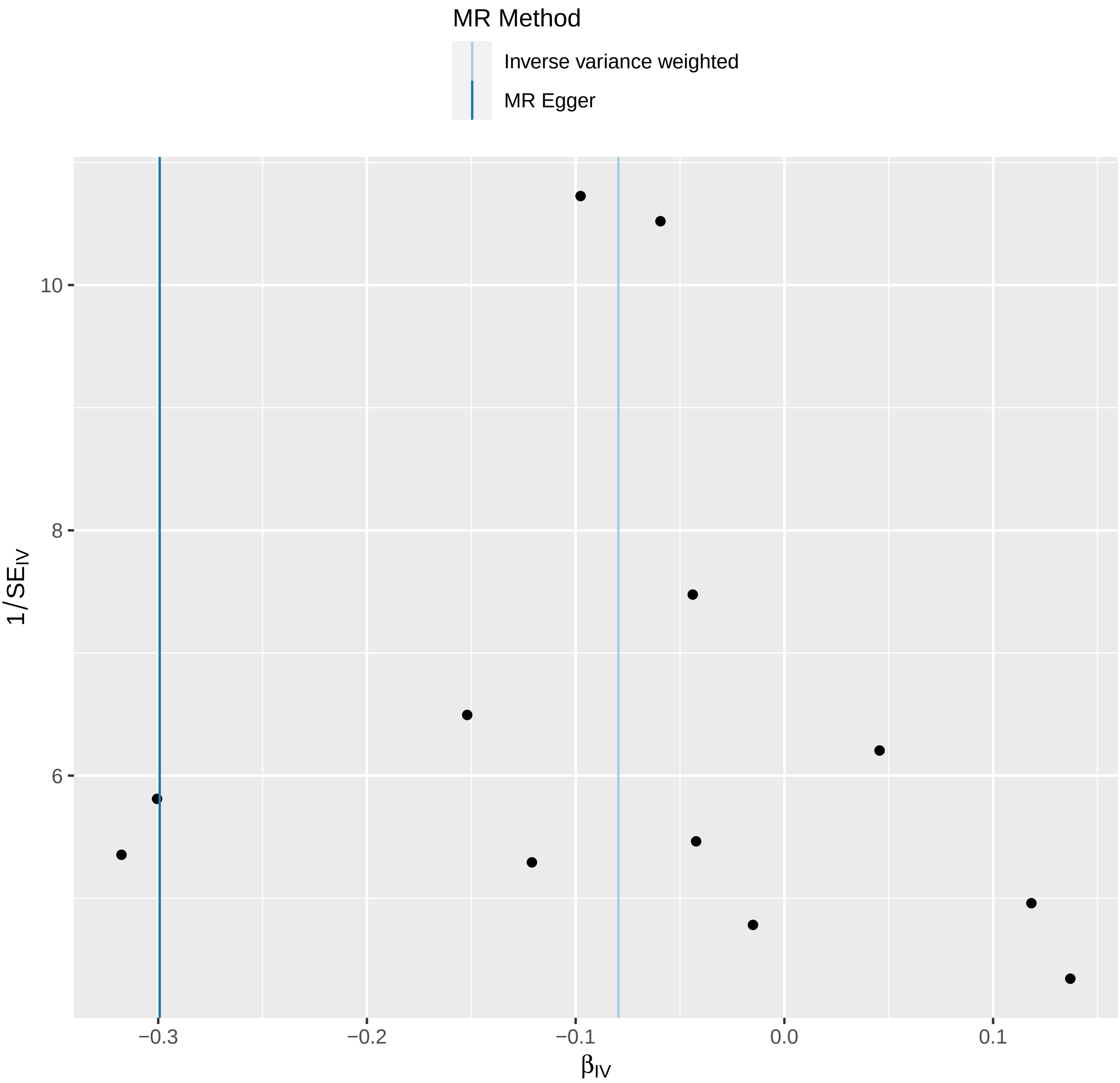

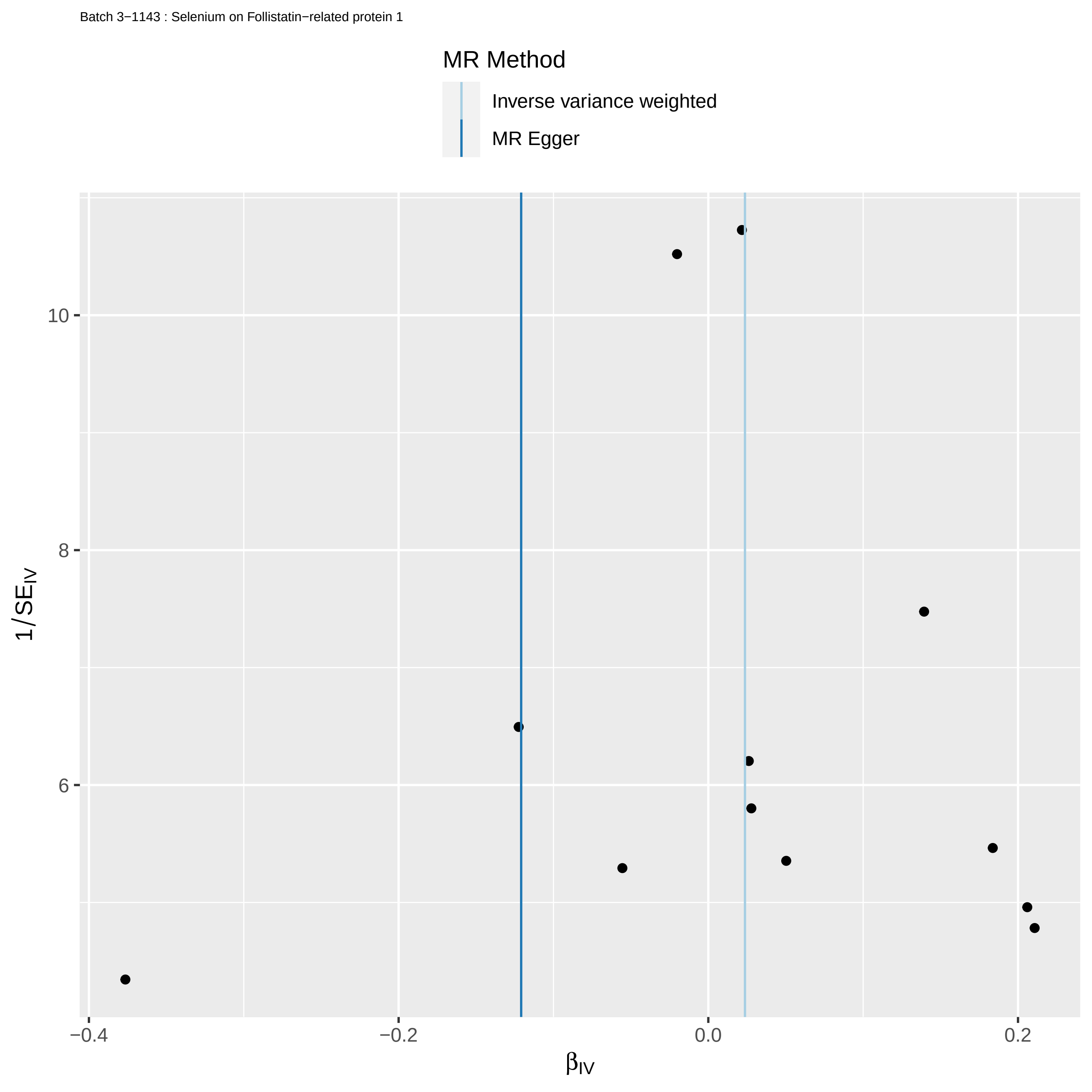

Batch 3−1143 : Selenium on Follistatin−related protein 1
MR Method
Inverse variance weighted
MR Egger
10
8
6
1/SE$_{IV}$
−0.4
−0.2
0.0
0.2
β$_{IV}$

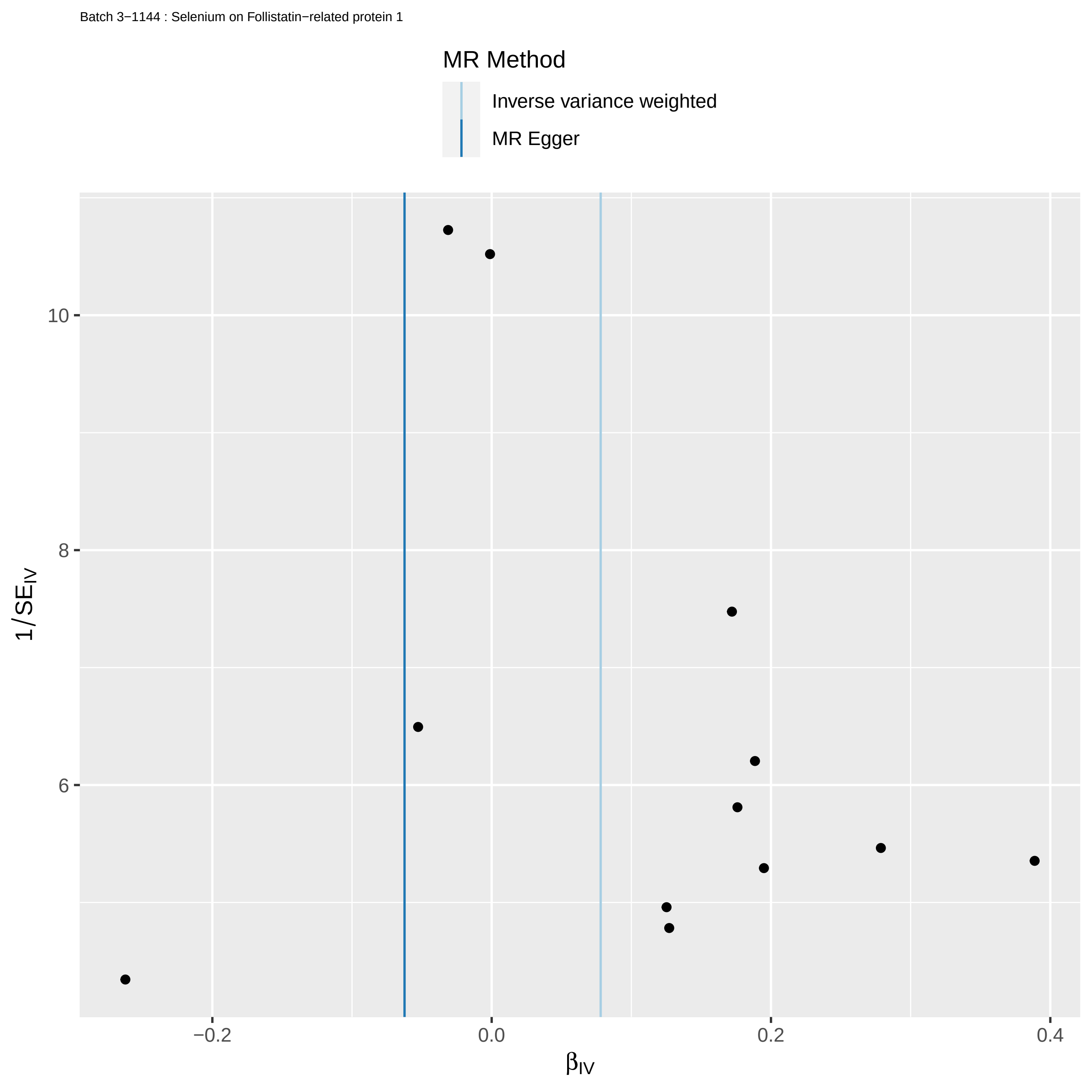

Batch 3−1144 : Selenium on Follistatin−related protein 1
MR Method
Inverse variance weighted
MR Egger
10
8
6
1/SE_IV
−0.2
0.0
0.2
0.4
β_IV

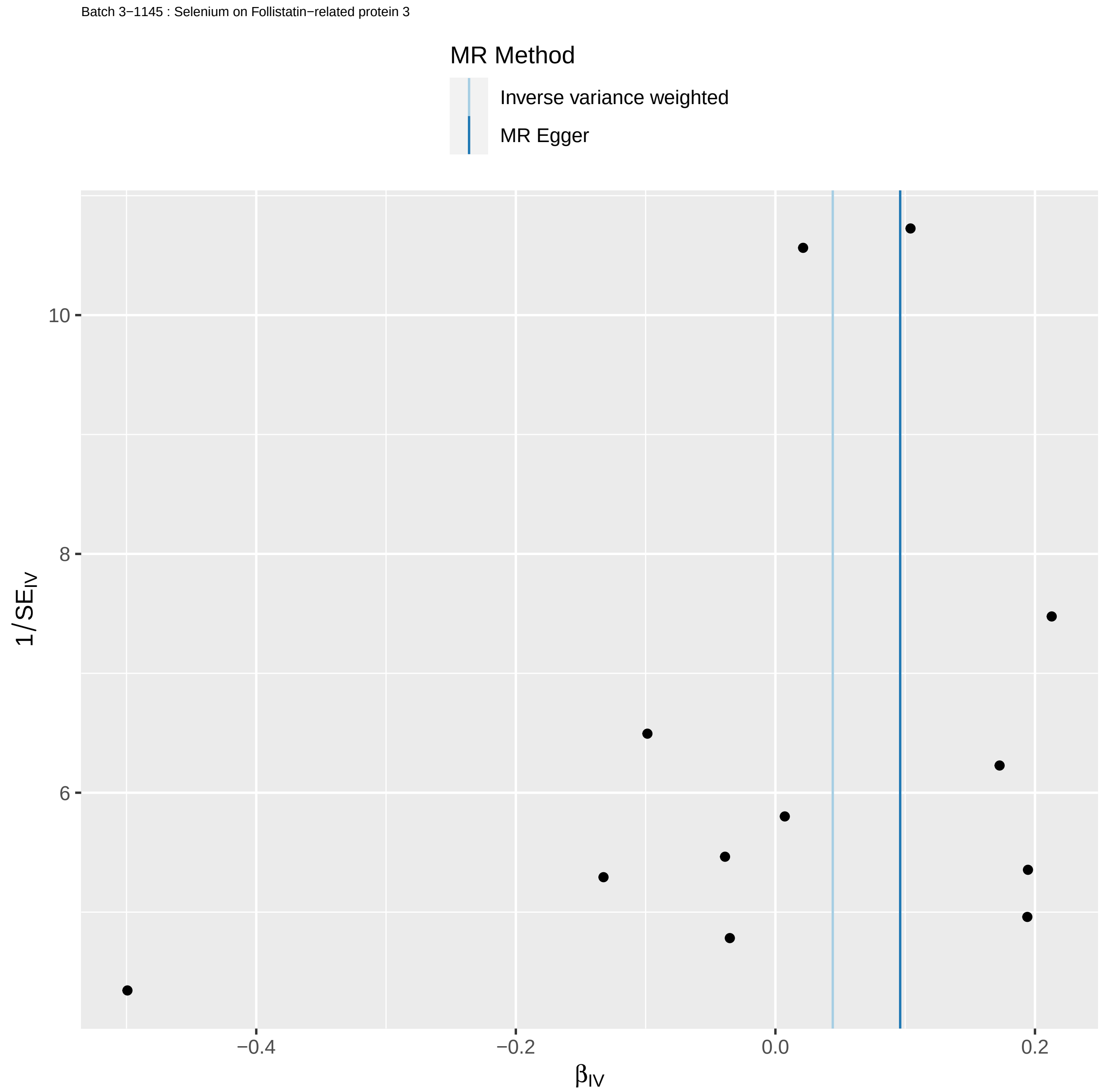
Batch 3−1145 : Selenium on Follistatin−related protein 3
MR Method
Inverse variance weighted
MR Egger
10
8
6
−0.4
−0.2
0.0
0.2
$1/SE_{IV}$
β_{IV}

Batch 3−1146 : Selenium on Follistatin−related protein 4

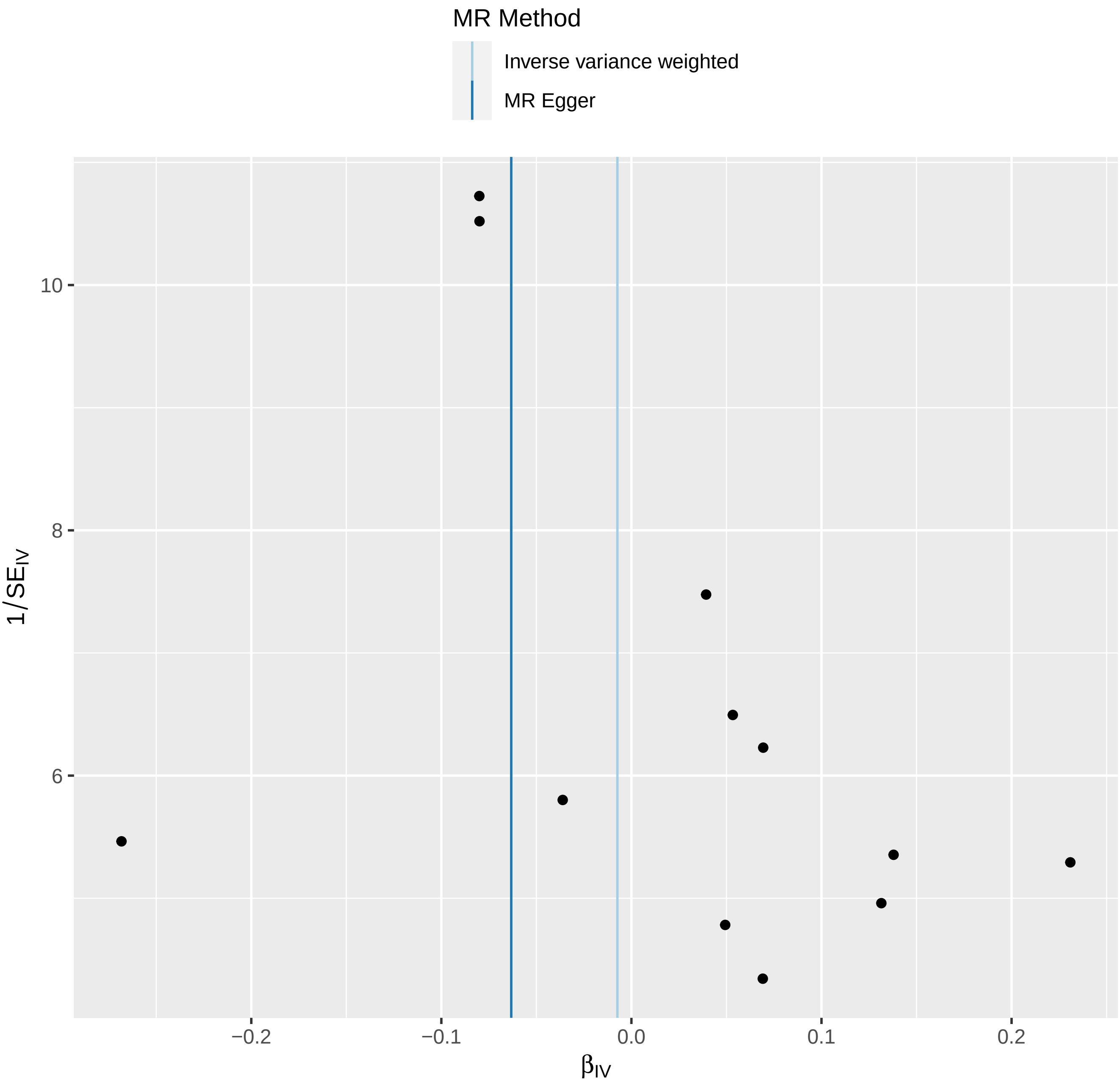

Batch 3−1147 : Selenium on Follistatin−related protein 5

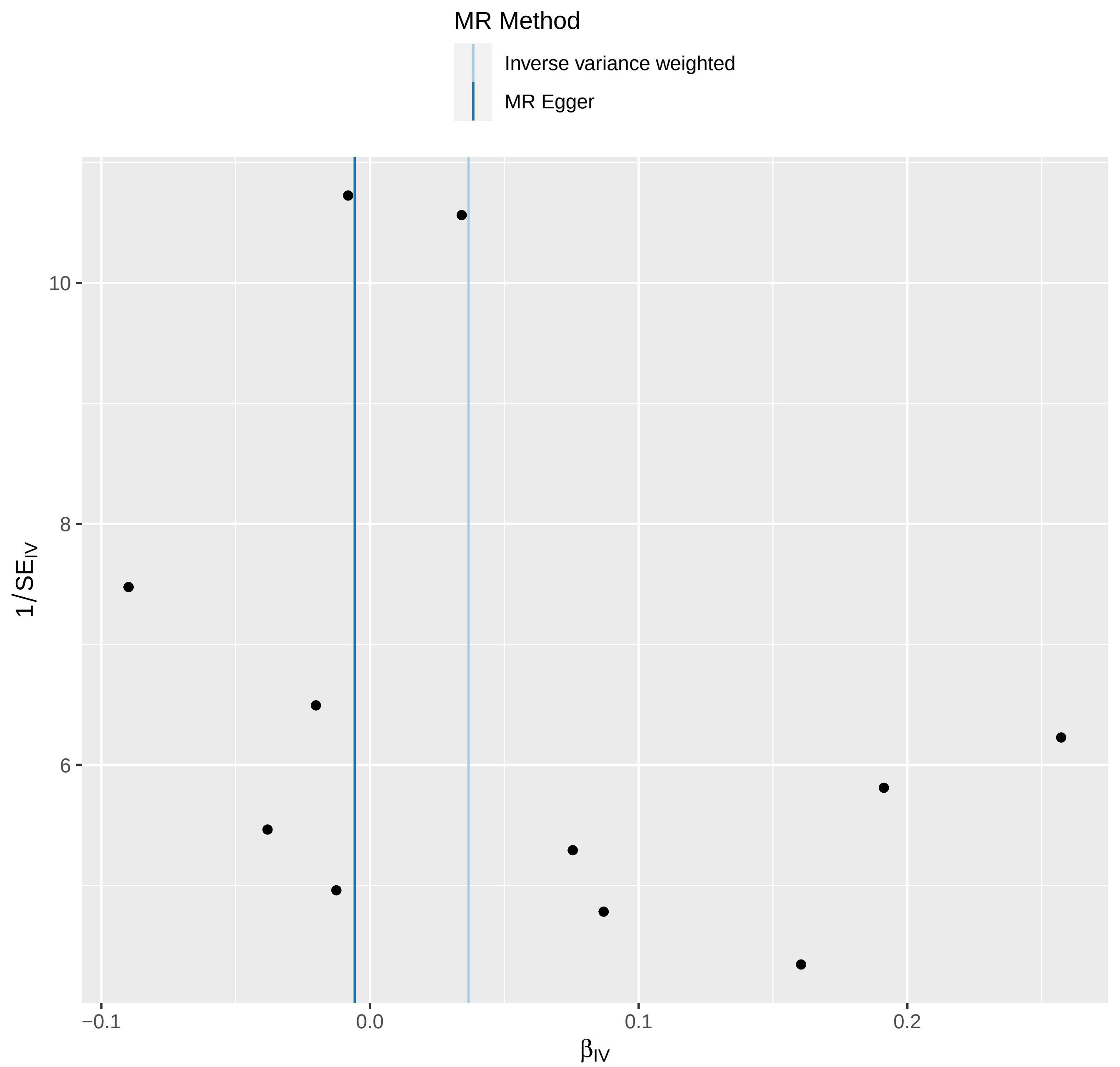

Batch 3−1148 : Selenium on Ferritin

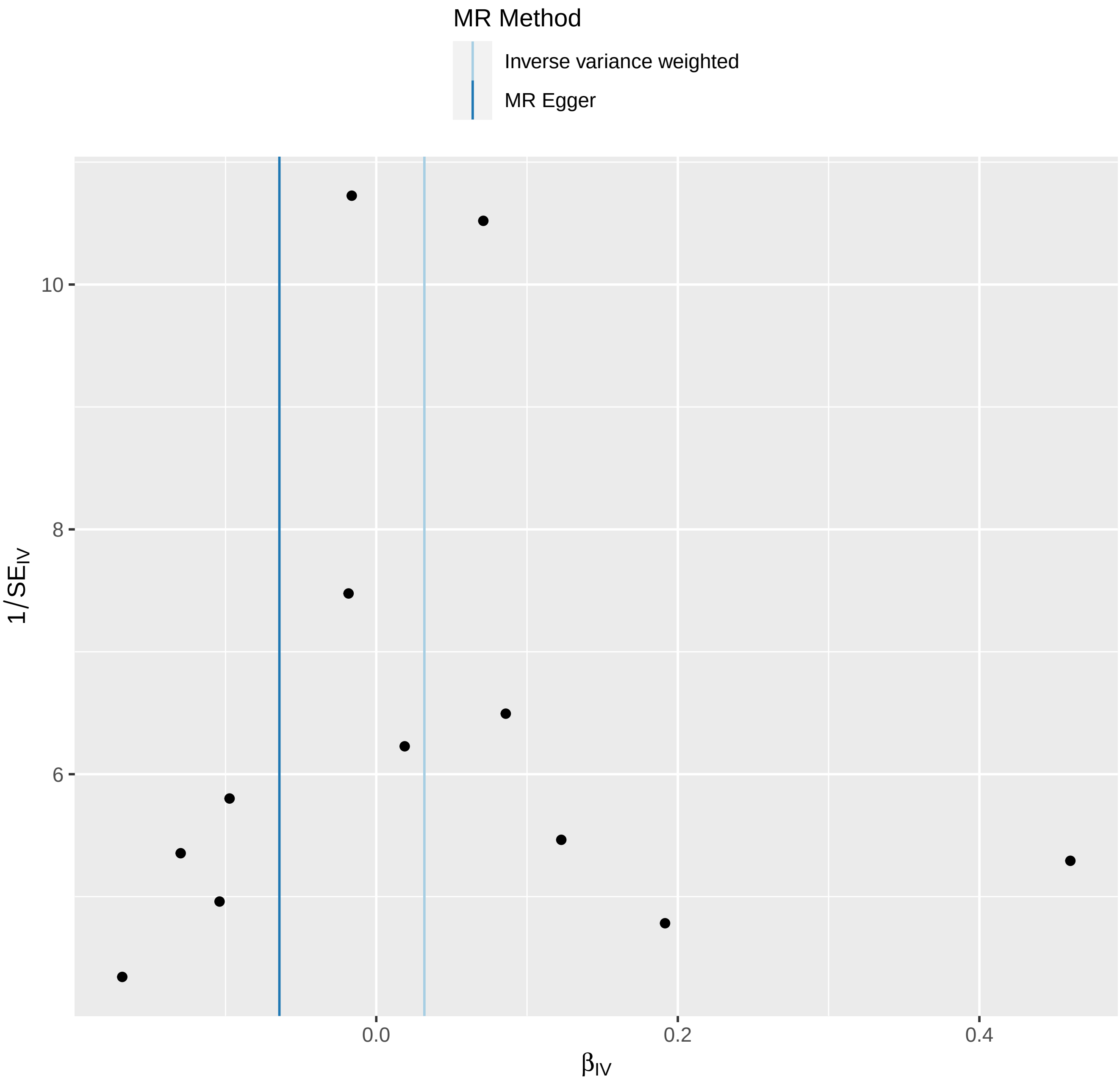

Batch 3−1149 : Selenium on Ferritin

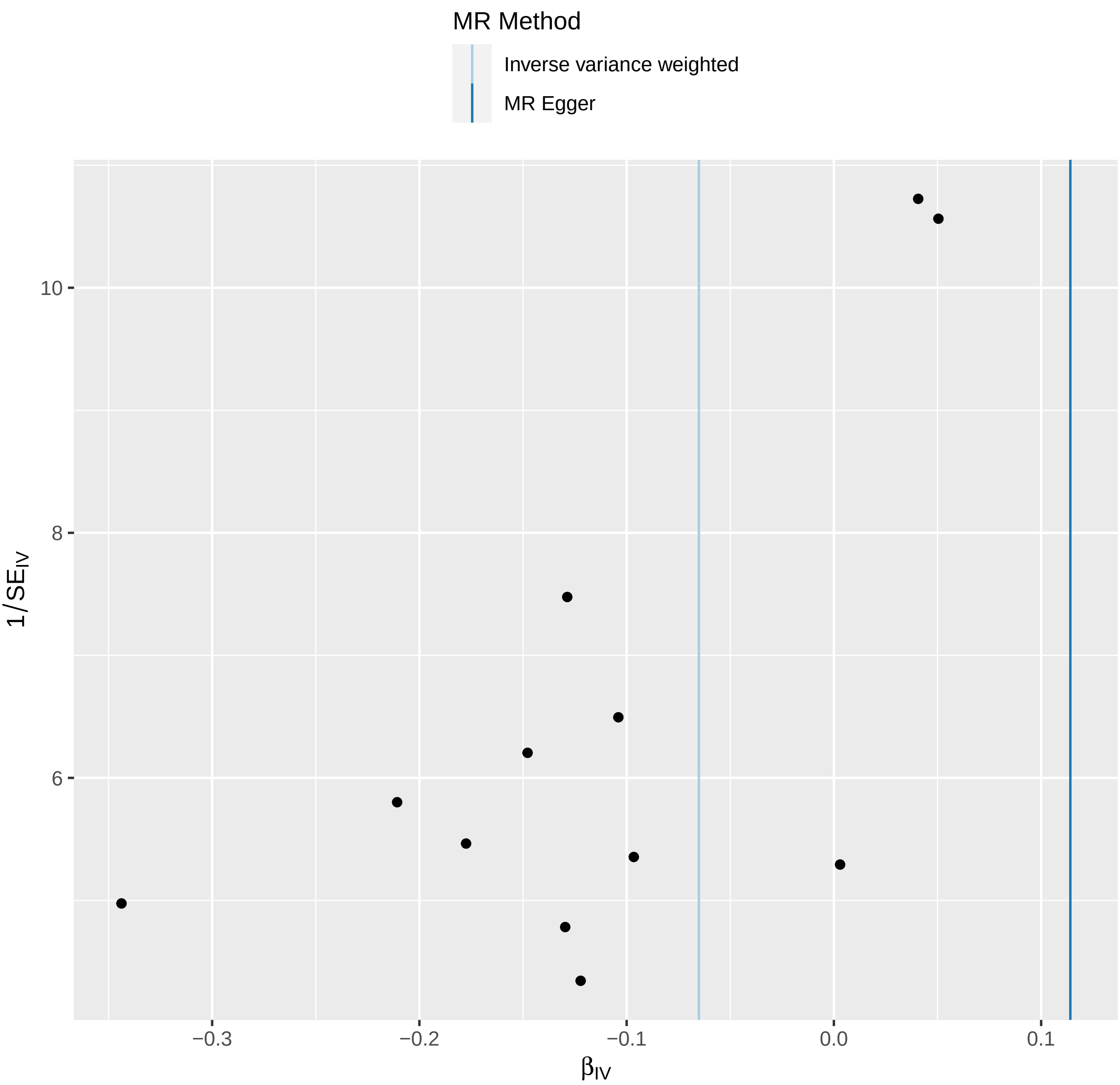

Batch 3−1150 : Selenium on Furin

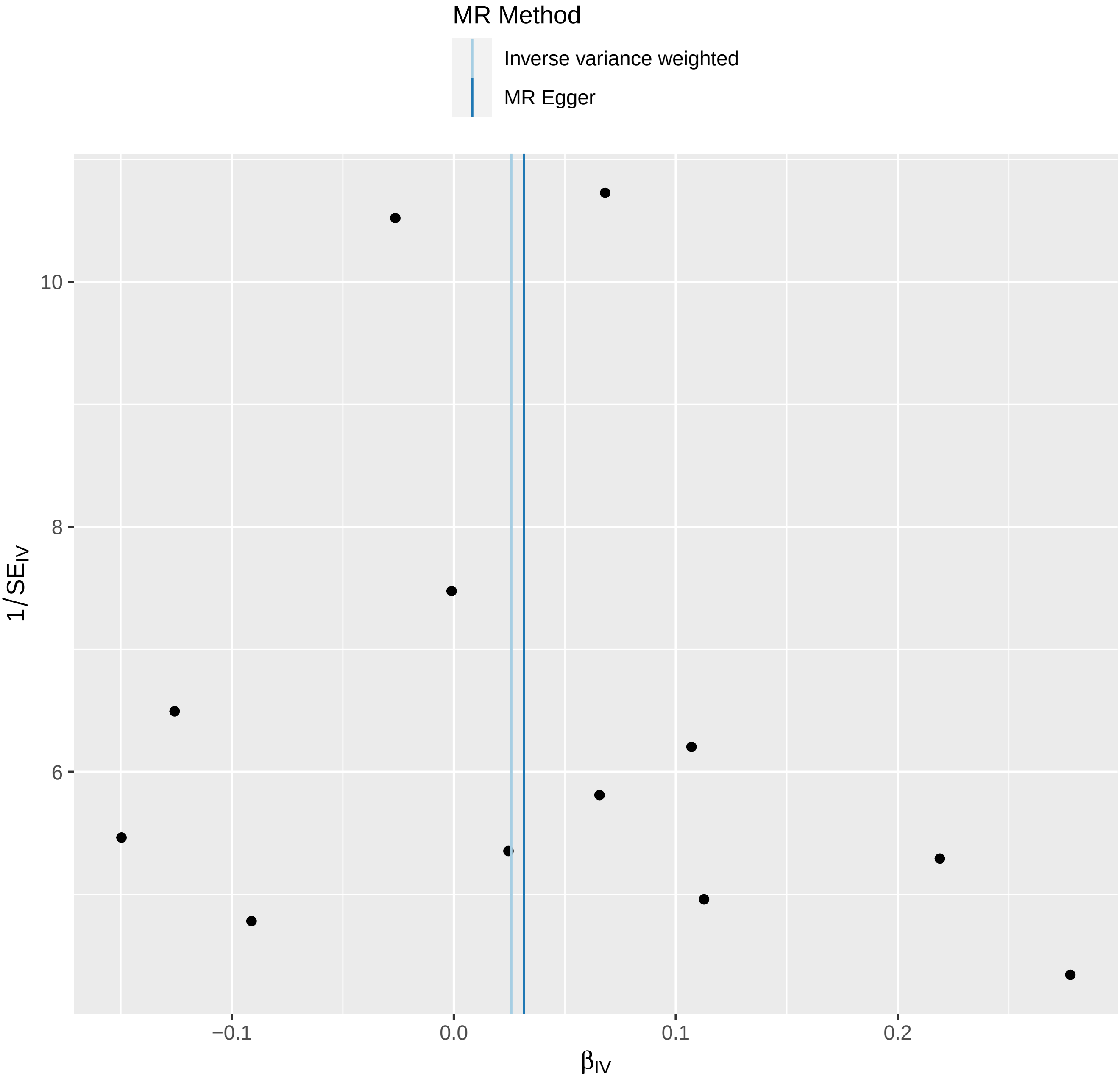

Batch 3−1151 : Selenium on Alpha−(1,3)−fucosyltransferase 10

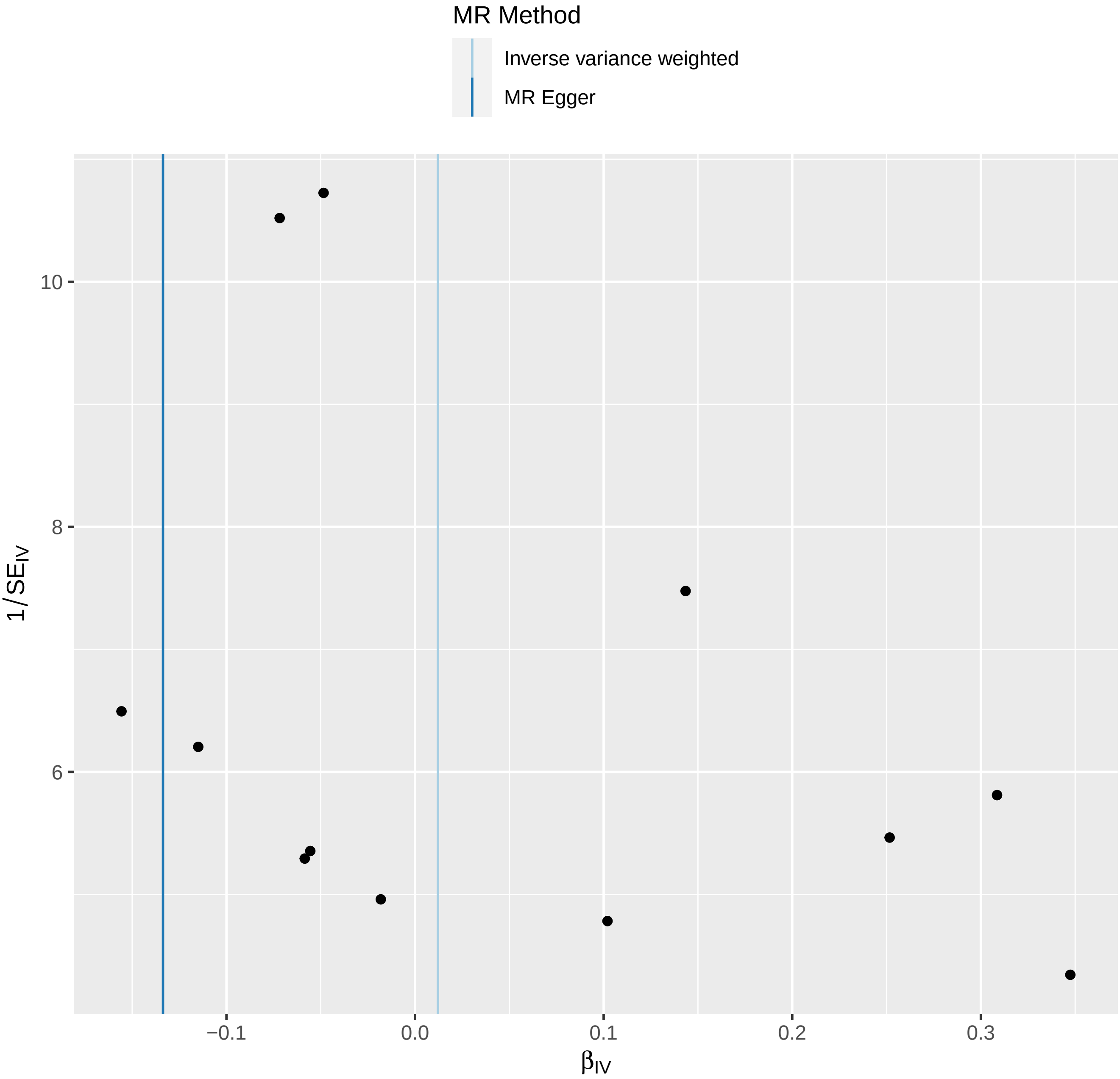

Batch 3−1152 : Selenium on Galactoside 3(4)−L−fucosyltransferase

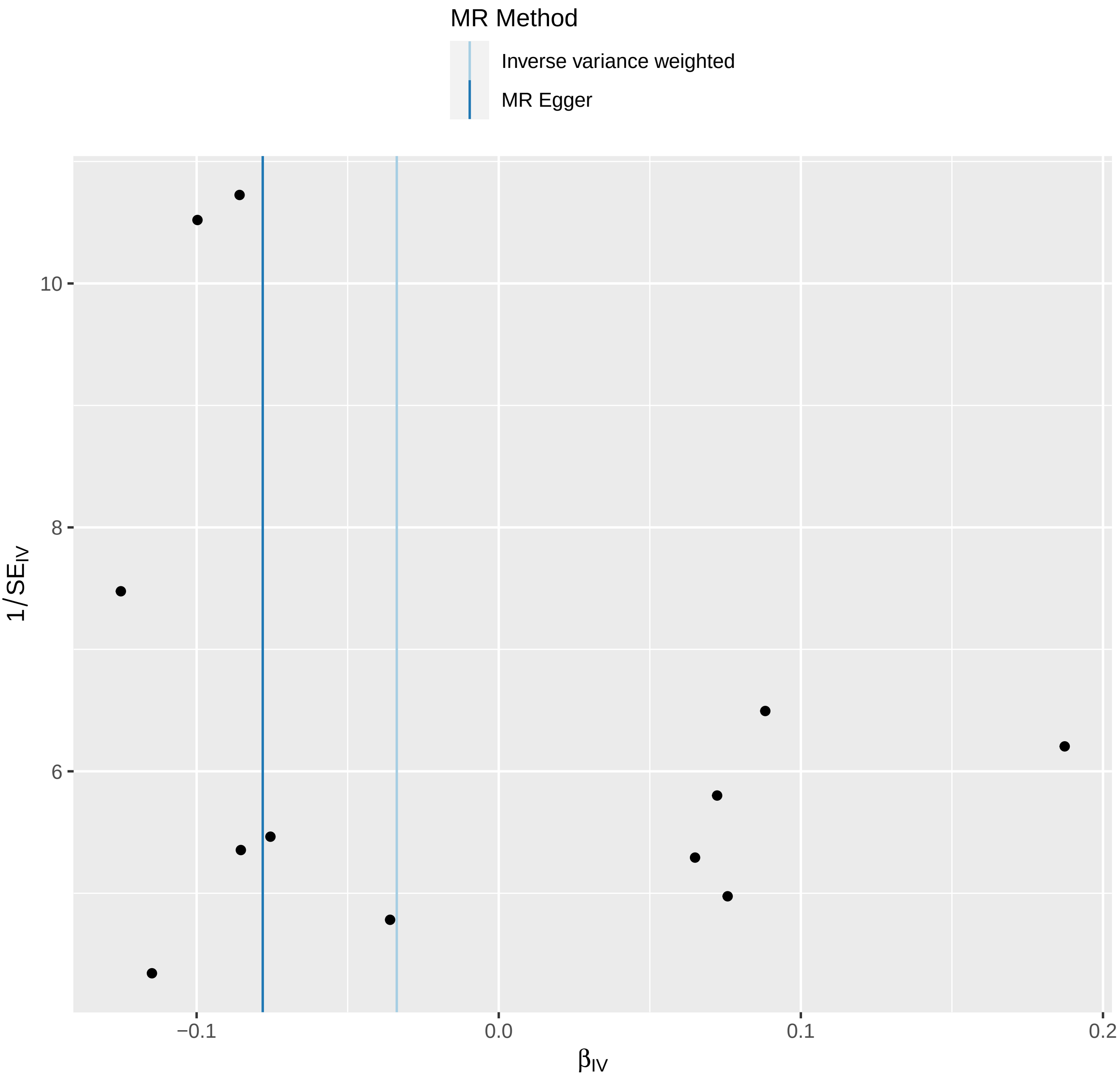

Batch 3−1153 : Selenium on Alpha−(1,3)−fucosyltransferase 5

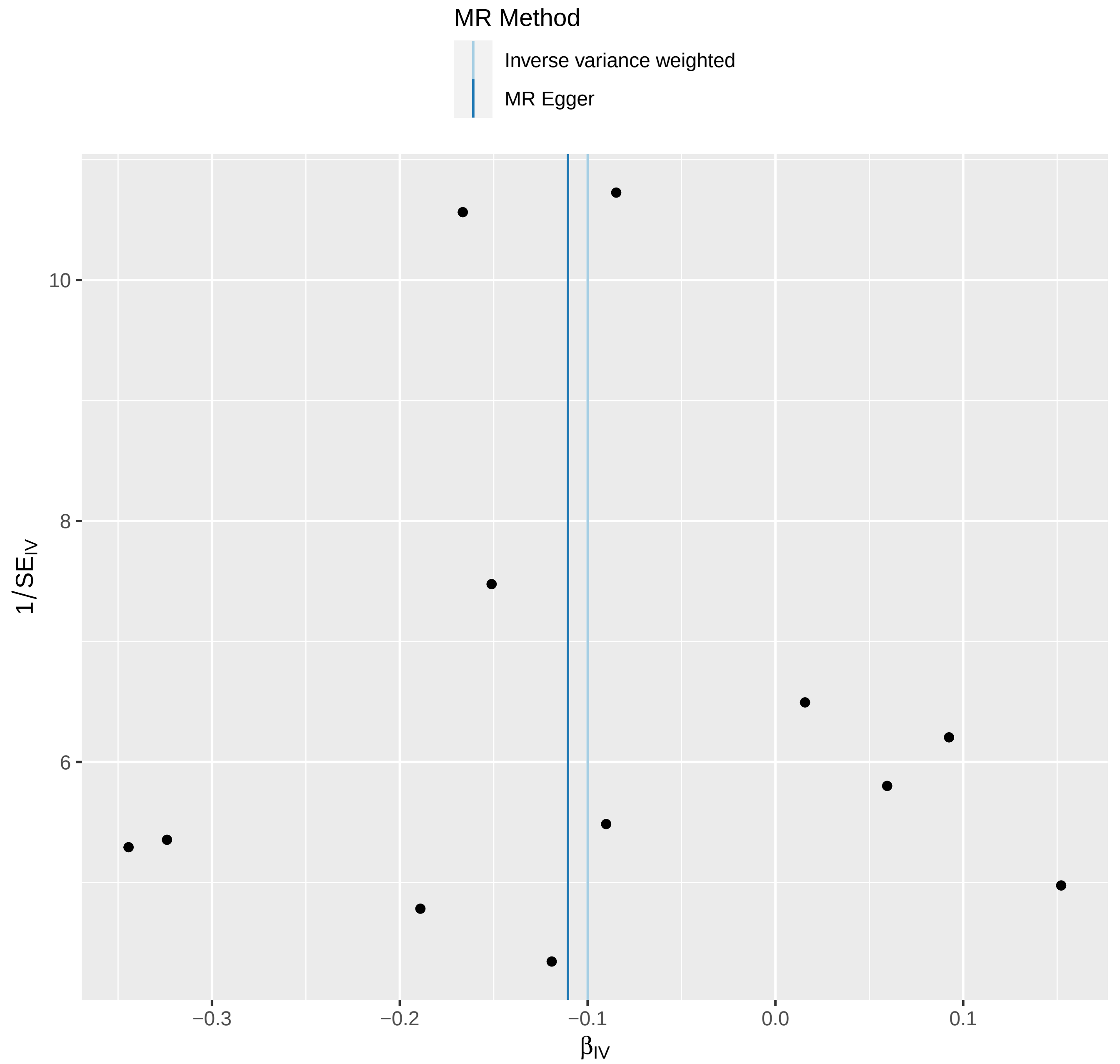

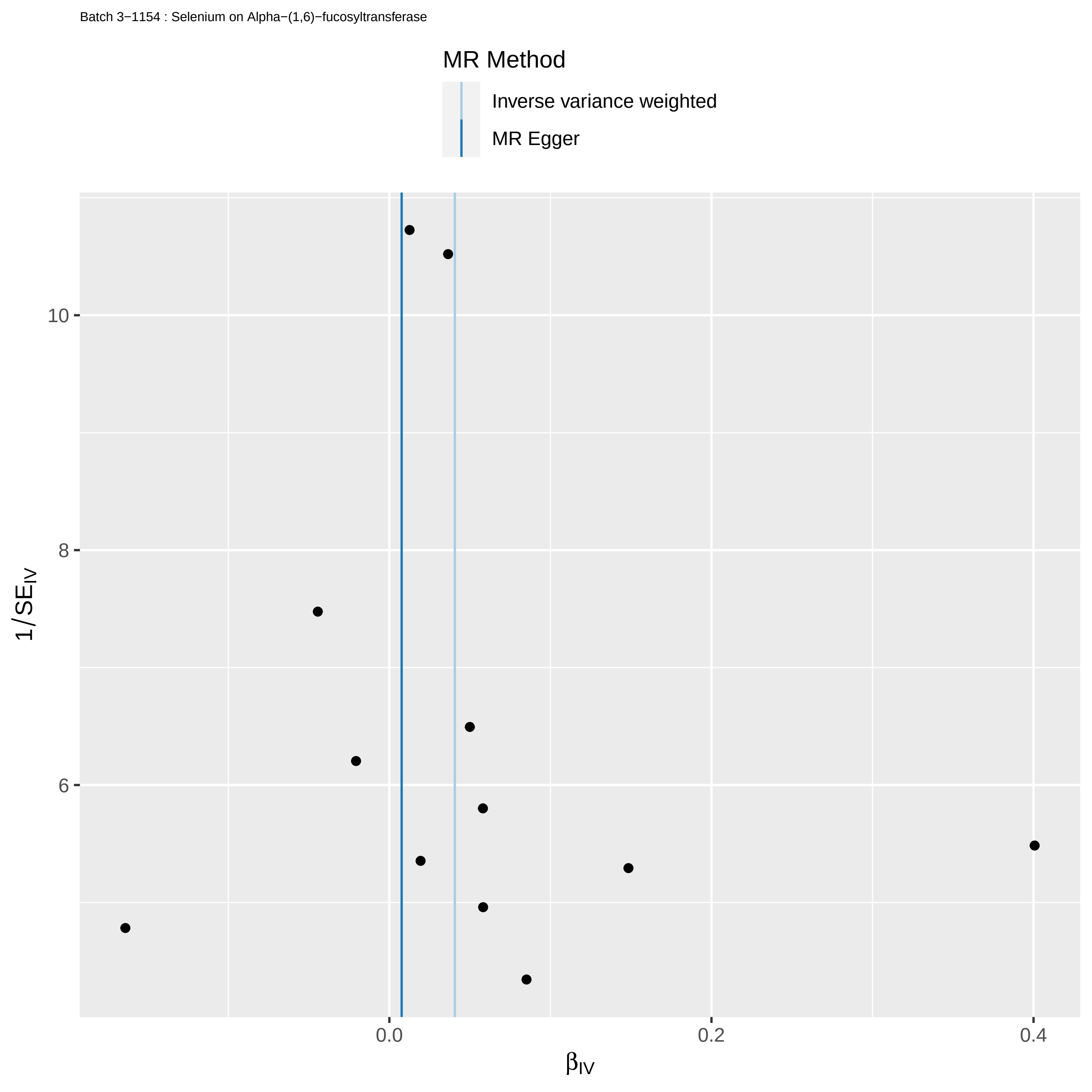
Batch 3−1154 : Selenium on Alpha−(1,6)−fucosyltransferase
MR Method
Inverse variance weighted
MR Egger
10
8
6
1/SE_IV
0.0
0.2
0.4
β_IV

Batch 3−1155 : Selenium on Alpha−(1,3)−fucosyltransferase 9

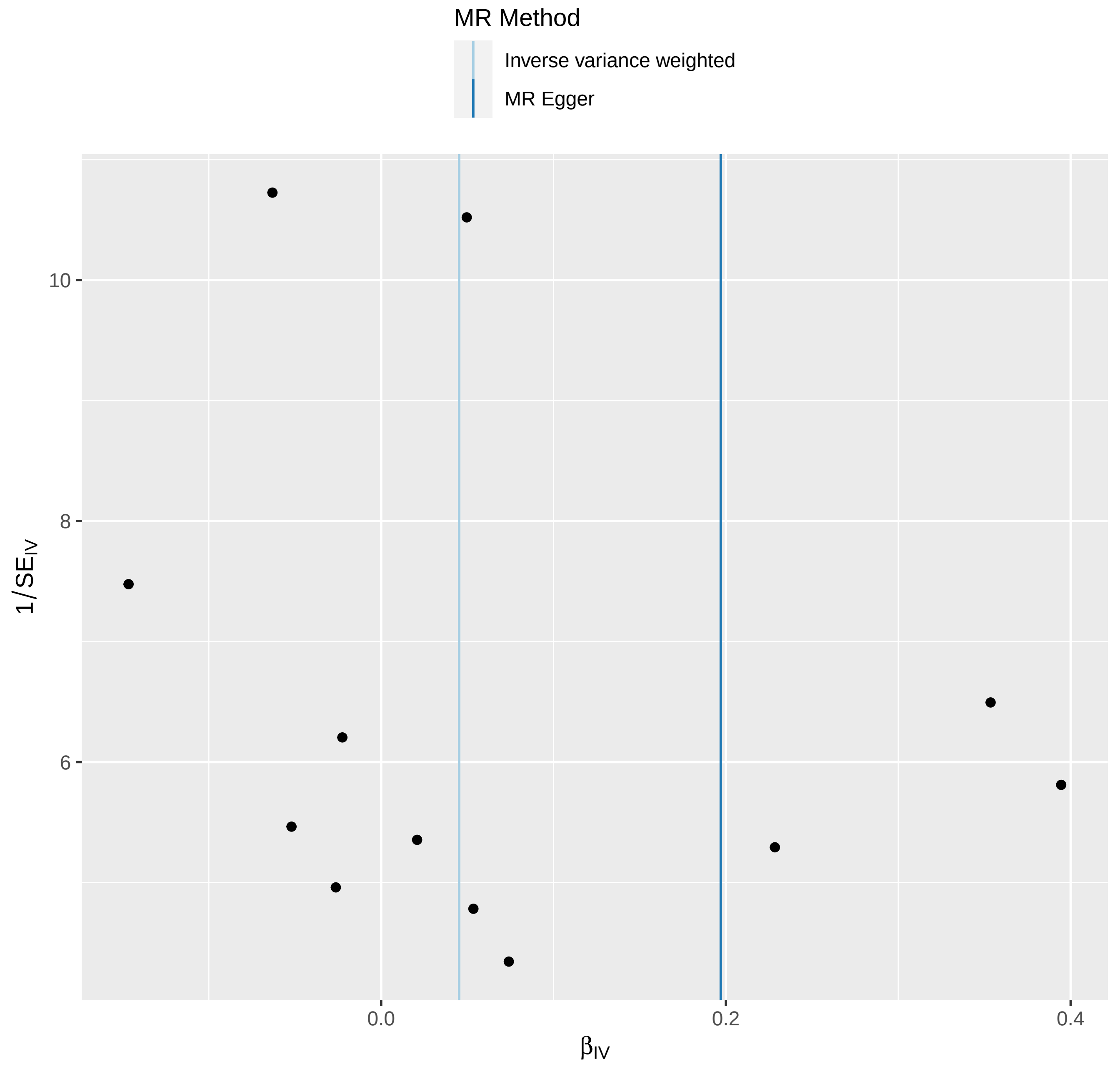

Batch 3−1156 : Selenium on Fragile X mental retardation syndrome−related protein 1

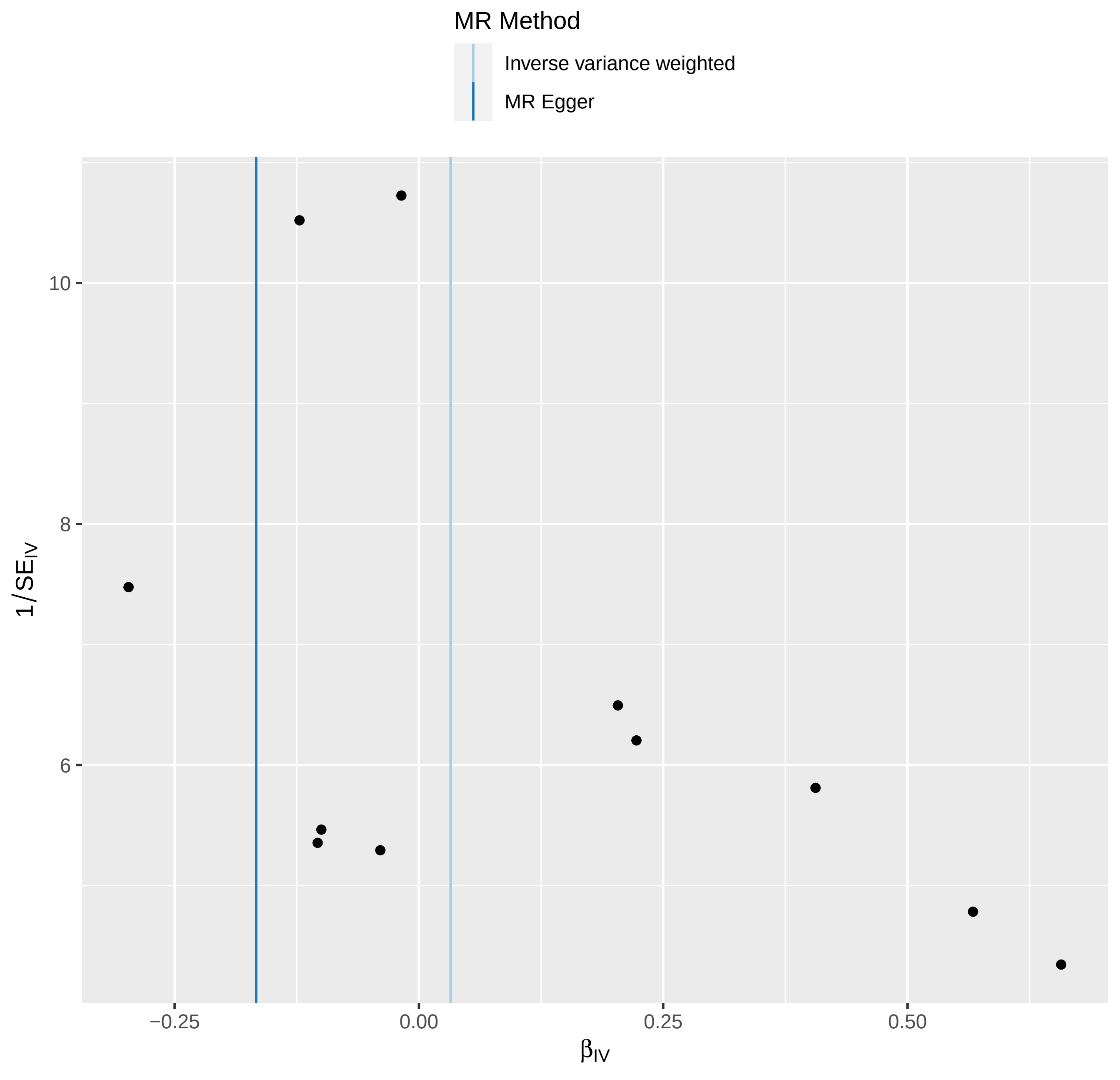

Batch 3−1157 : Selenium on Tyrosine−protein kinase Fyn

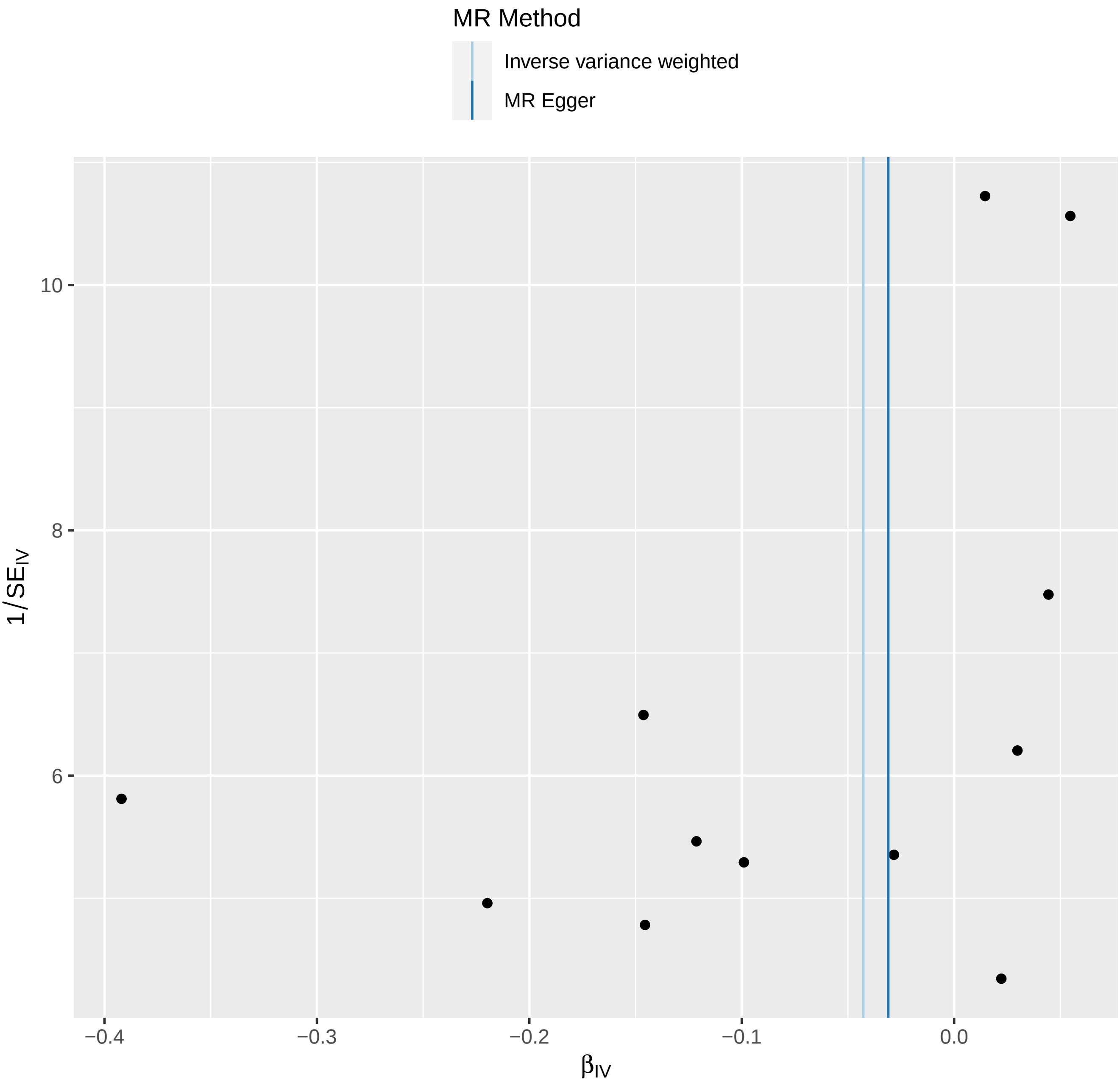

Batch 3−1158 : Selenium on G0/G1 switch protein 2

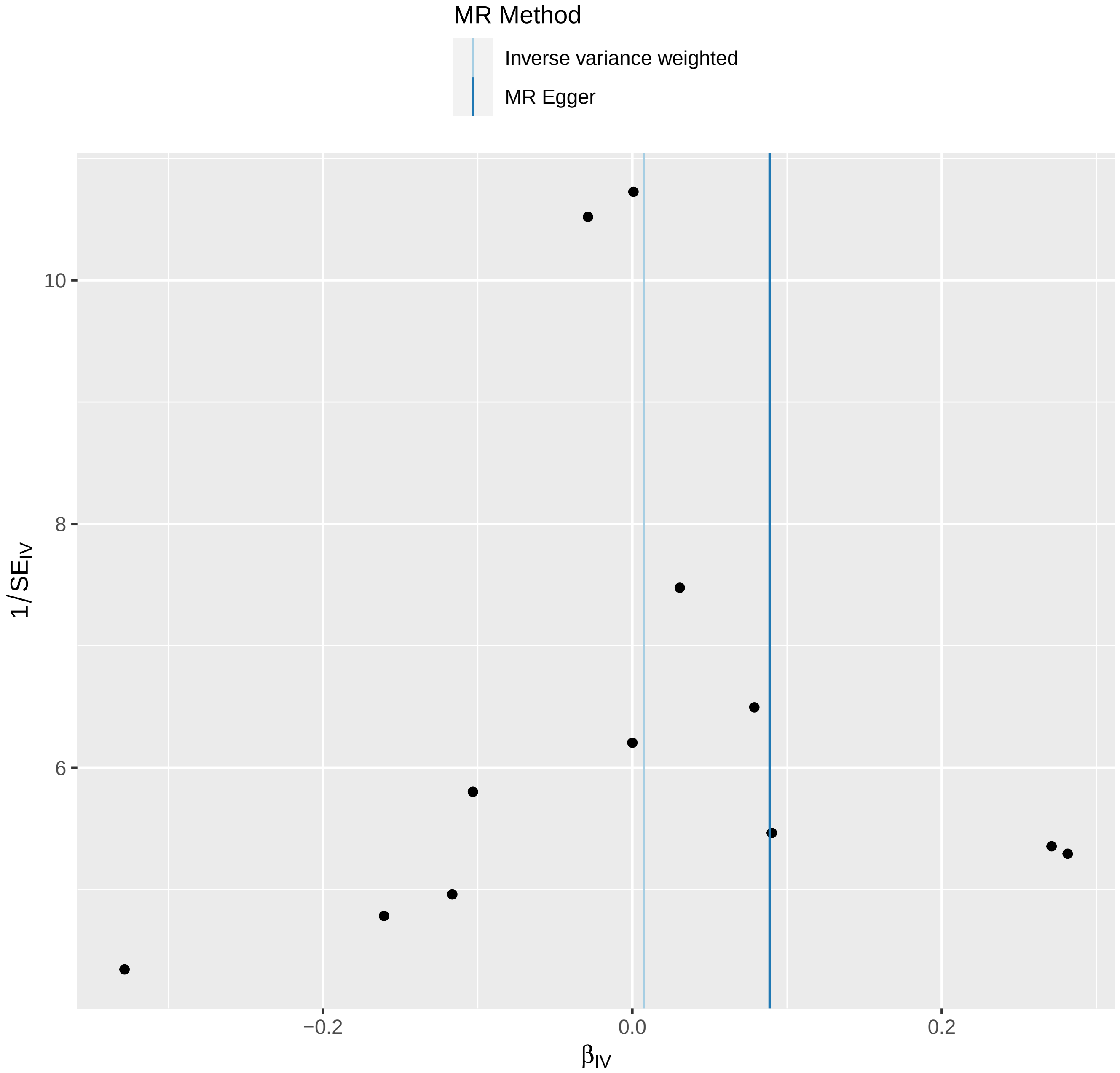

Batch 3−1159 : Selenium on Protein G6b

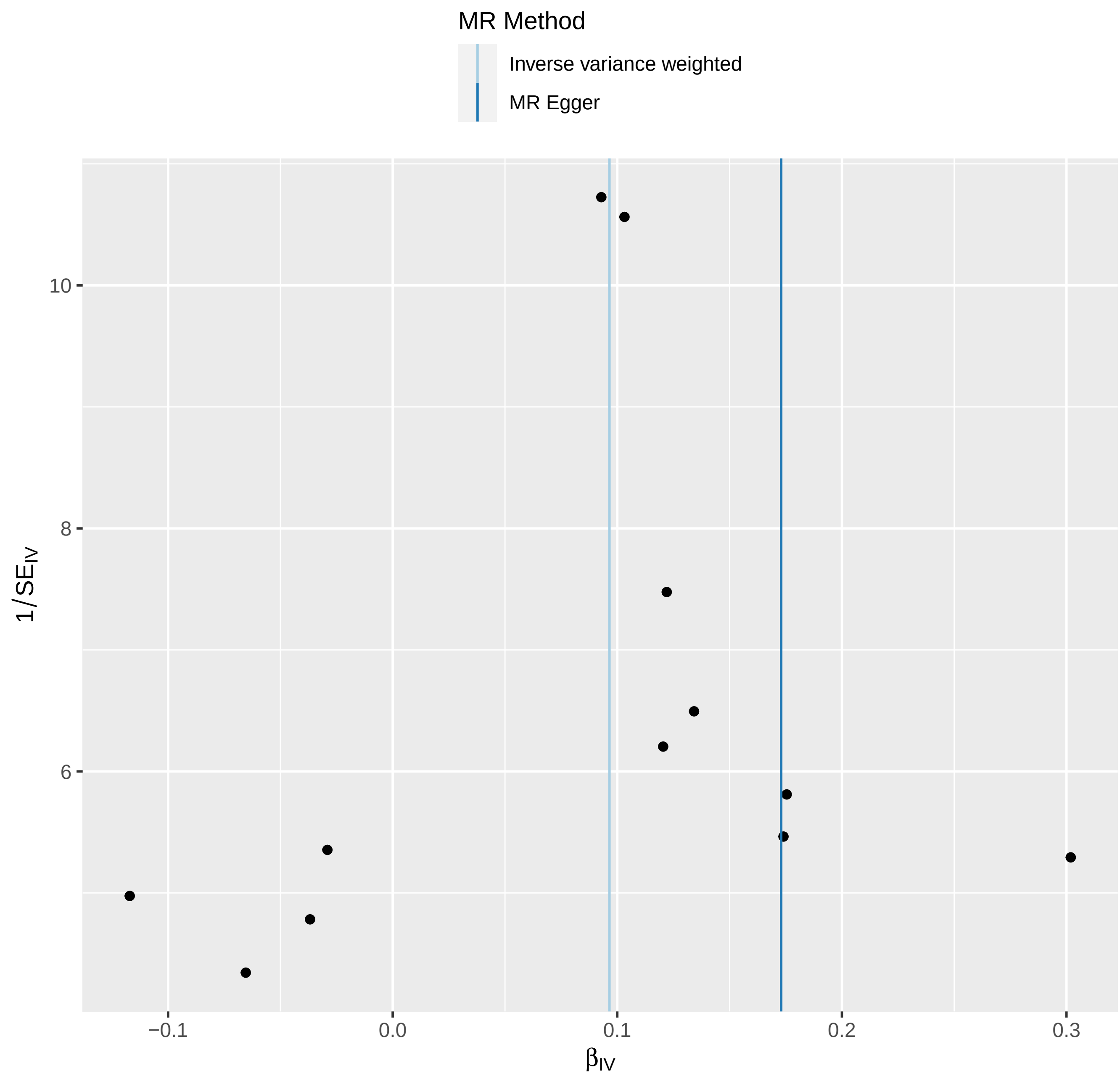

Batch 3−1160 : Selenium on Protein G6b

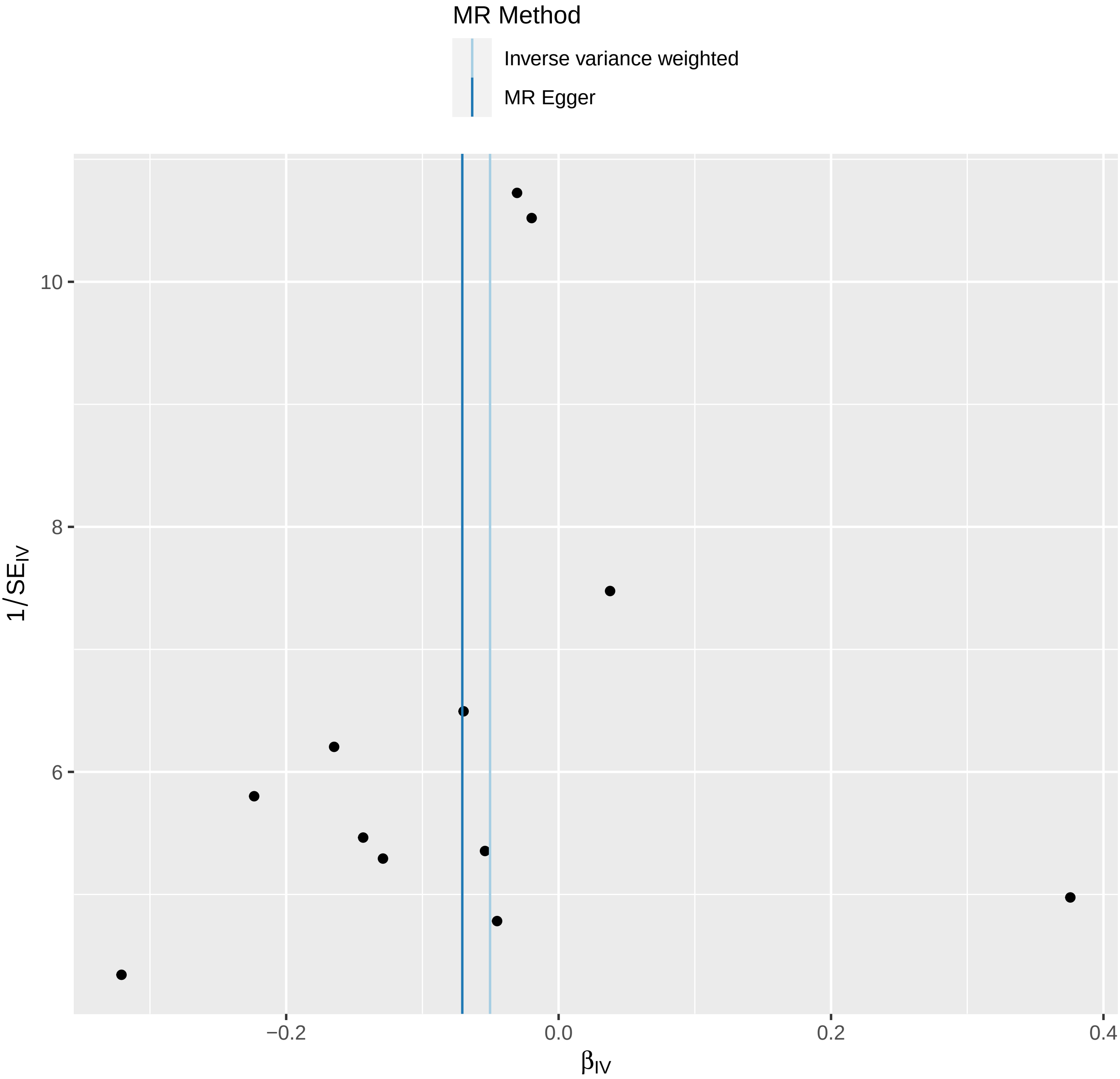

Batch 3−1161 : Selenium on Gamma−aminobutyric acid receptor−associated protein−like 1

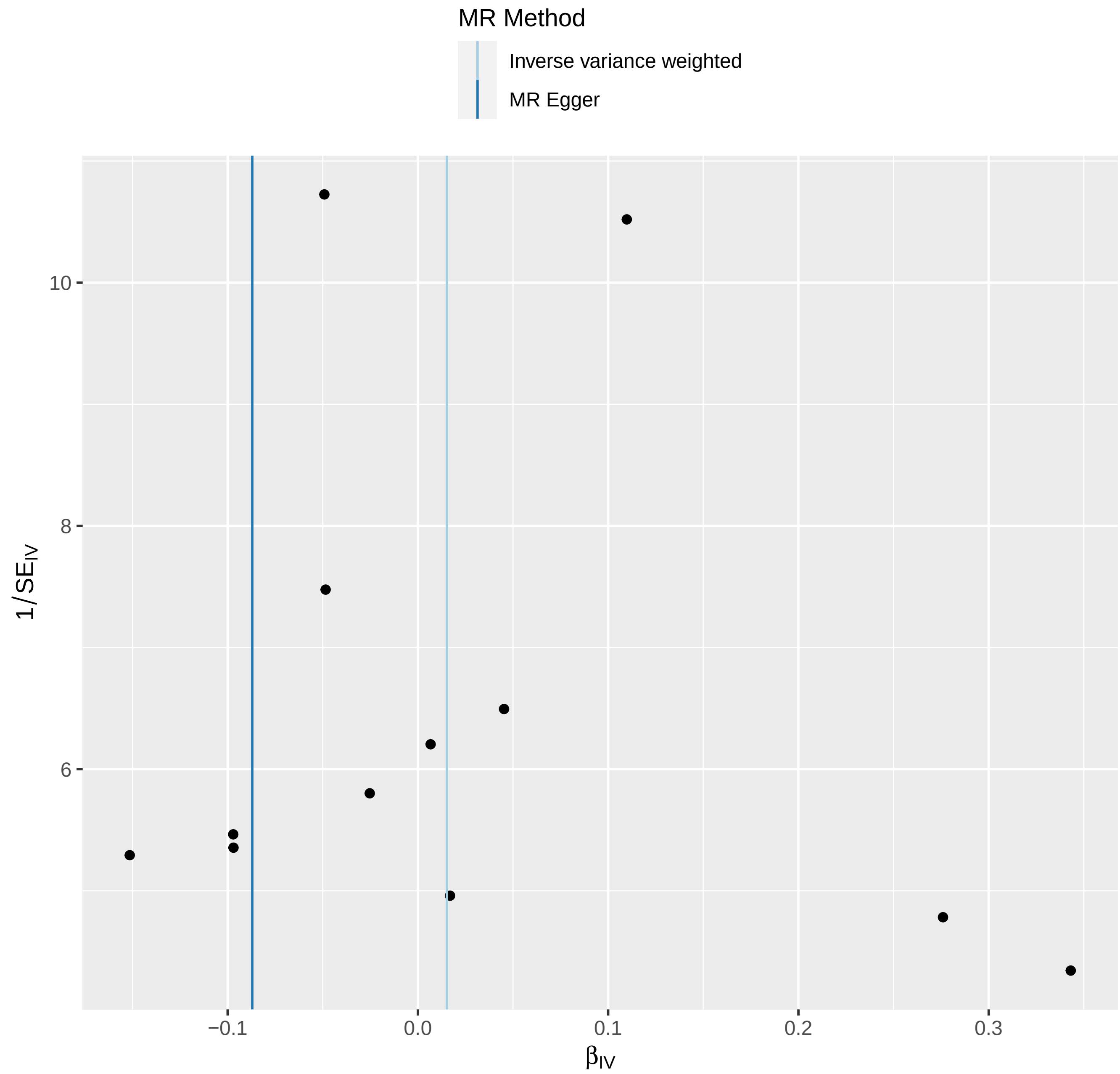

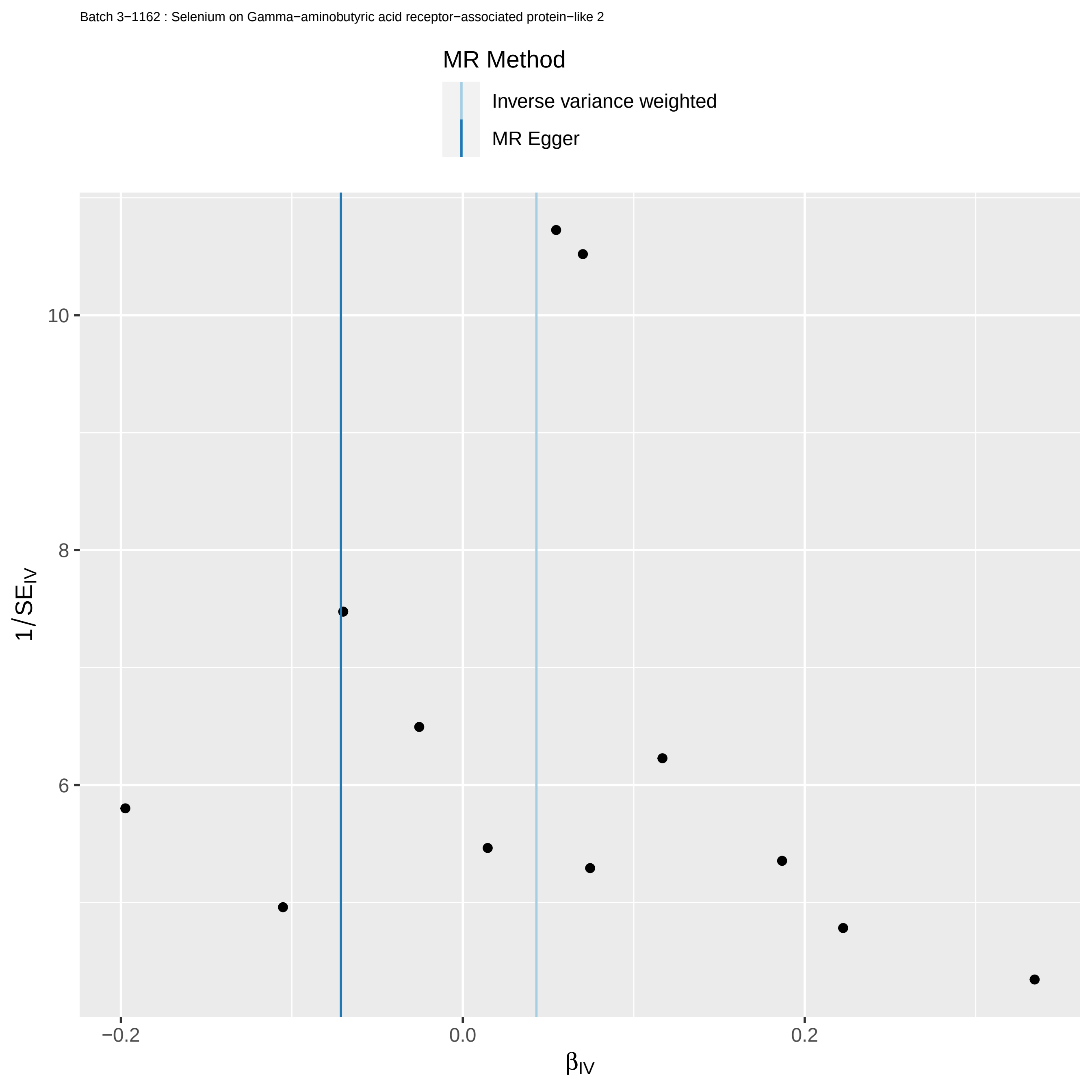
Batch 3−1162 : Selenium on Gamma−aminobutyric acid receptor−associated protein−like 2
MR Method
Inverse variance weighted
MR Egger
10
8
6
−0.2
0.0
0.2
$1/SE_{IV}$
β_{IV}

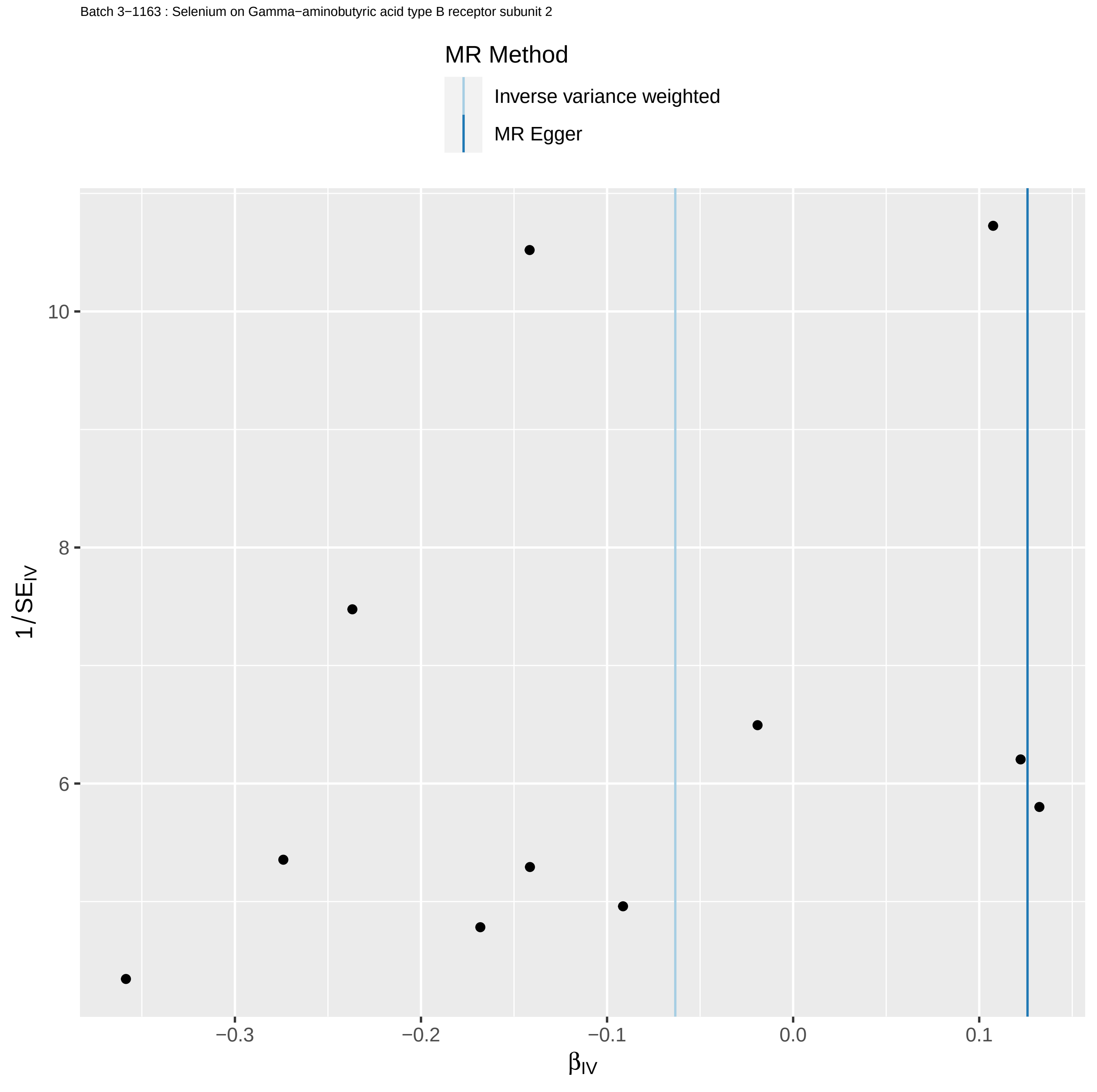
Batch 3−1163 : Selenium on Gamma−aminobutyric acid type B receptor subunit 2
MR Method
Inverse variance weighted
MR Egger
10
8
6
1/SE$_{IV}$
−0.3
−0.2
−0.1
0.0
0.1
β$_{IV}$

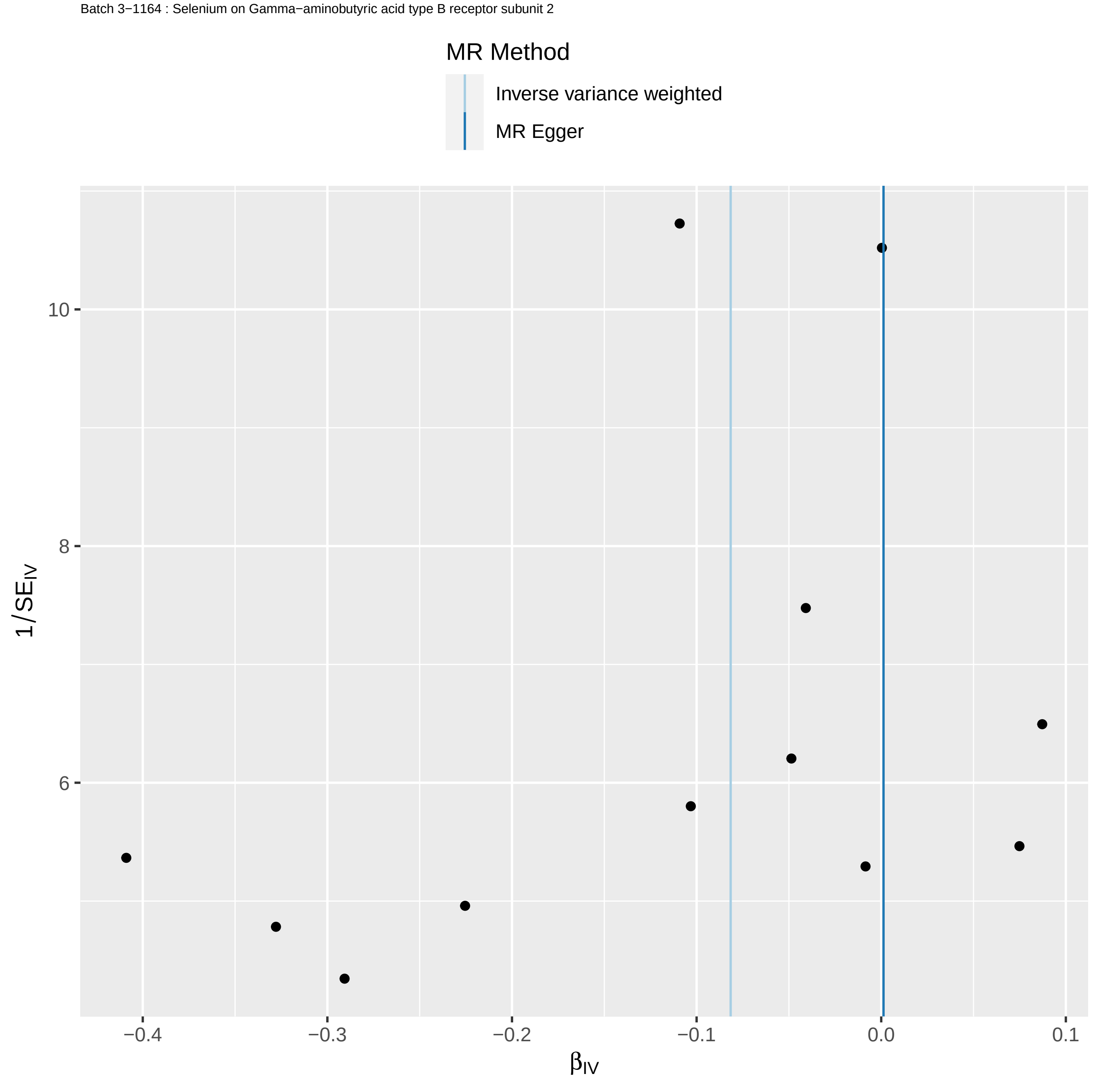
Batch 3−1164 : Selenium on Gamma−aminobutyric acid type B receptor subunit 2
MR Method
Inverse variance weighted
MR Egger
10
8
6
1/SE_IV
−0.4
−0.3
−0.2
−0.1
0.0
0.1
β_IV

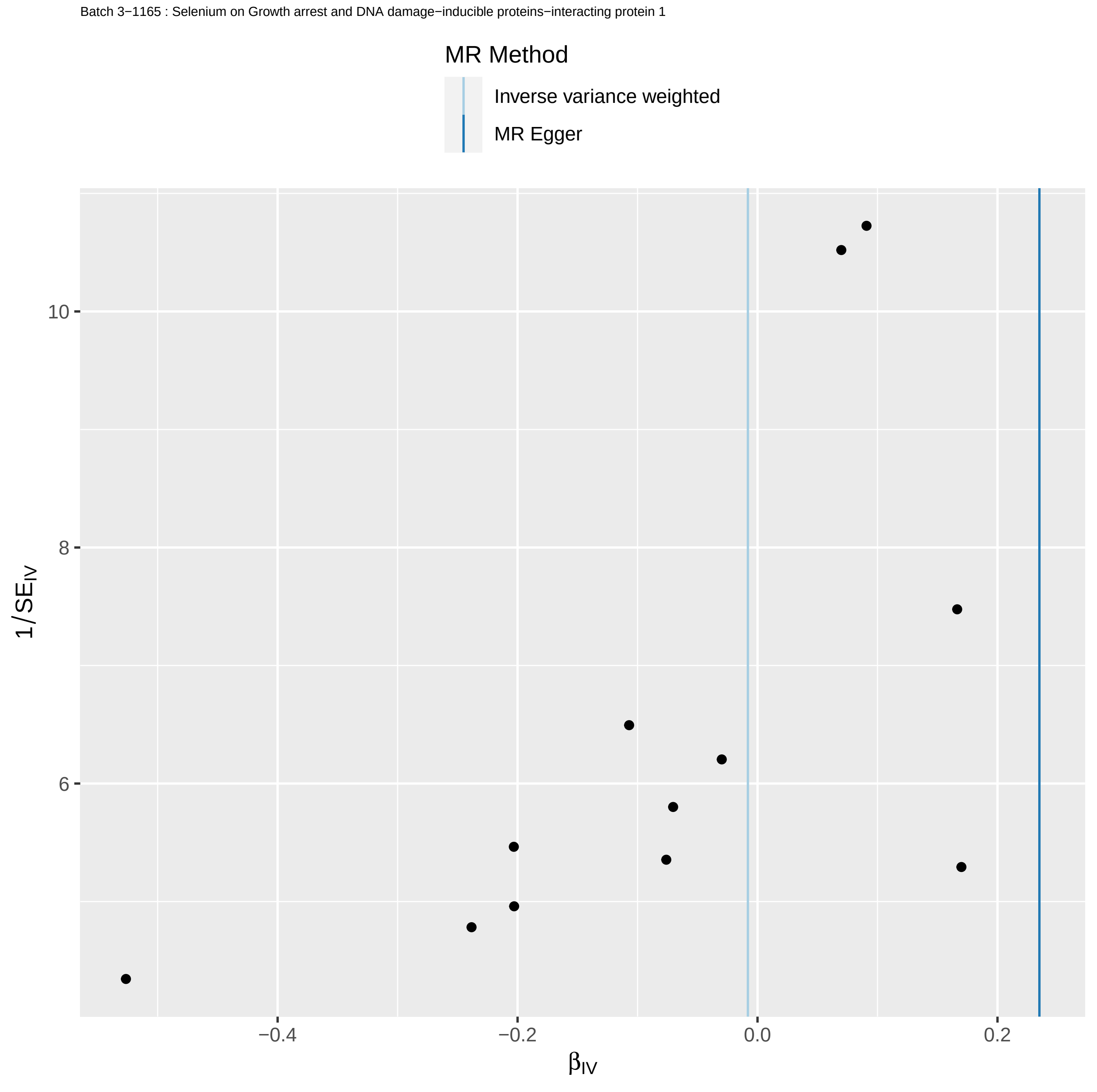

Batch 3−1165 : Selenium on Growth arrest and DNA damage−inducible proteins−interacting protein 1
MR Method
Inverse variance weighted
MR Egger
10
8
6
1/SE_IV
−0.4
−0.2
0.0
0.2
β_IV

Batch 3−1166 : Selenium on Galanin peptides

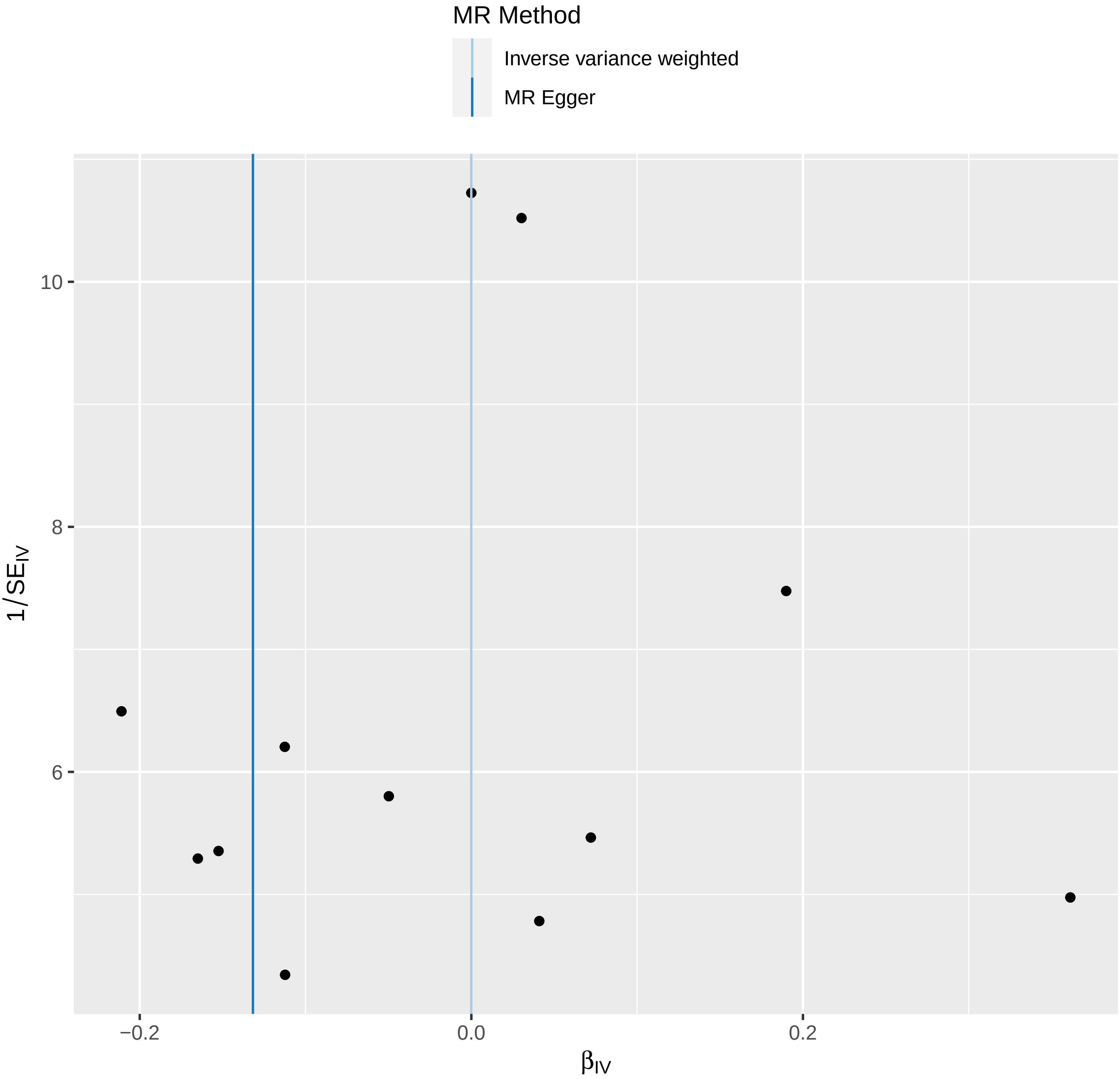

Batch 3−1167 : Selenium on Polypeptide N−acetylgalactosaminyltransferase 10

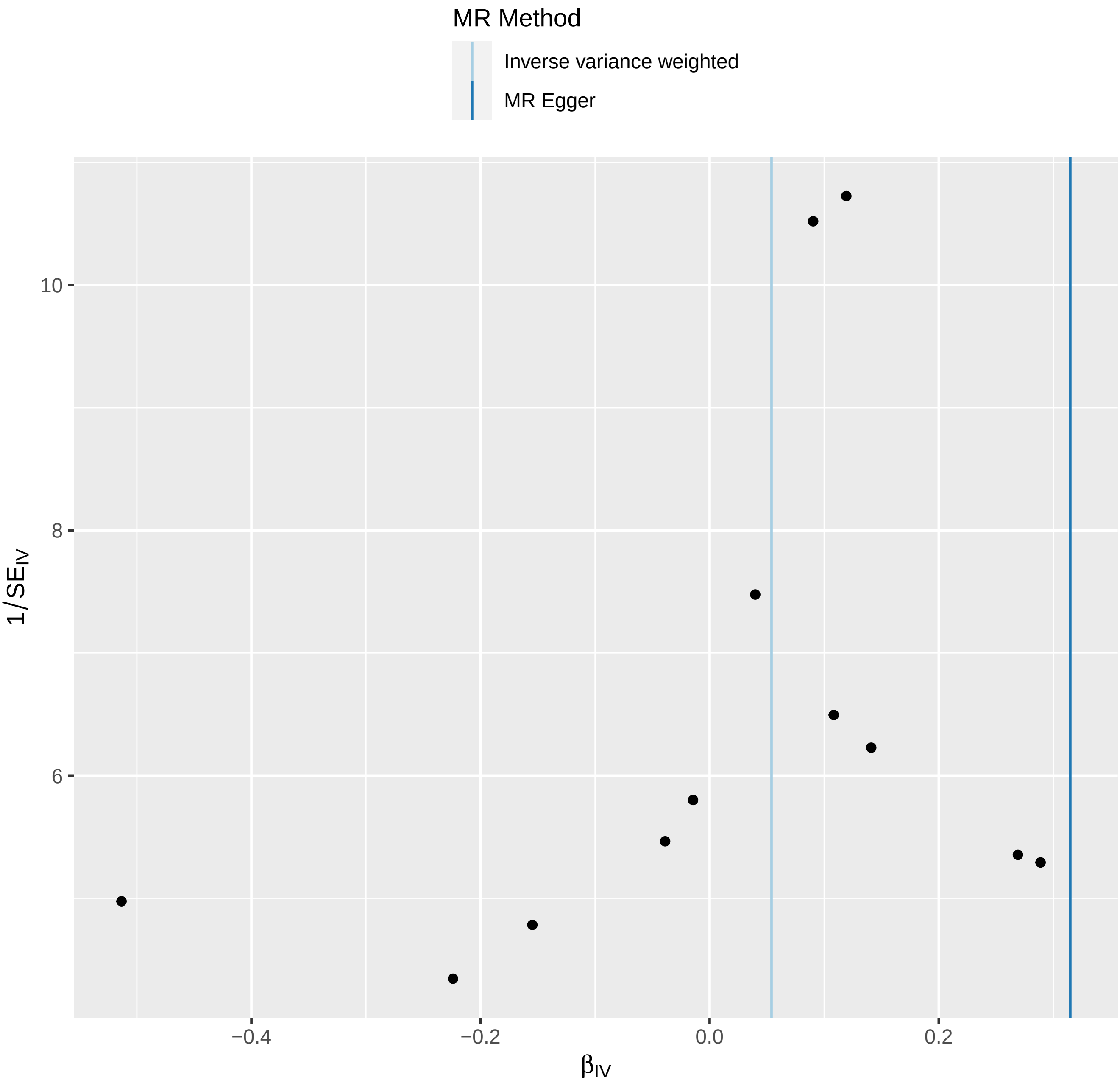

Batch 3−1168 : Selenium on Polypeptide N−acetylgalactosaminyltransferase 11

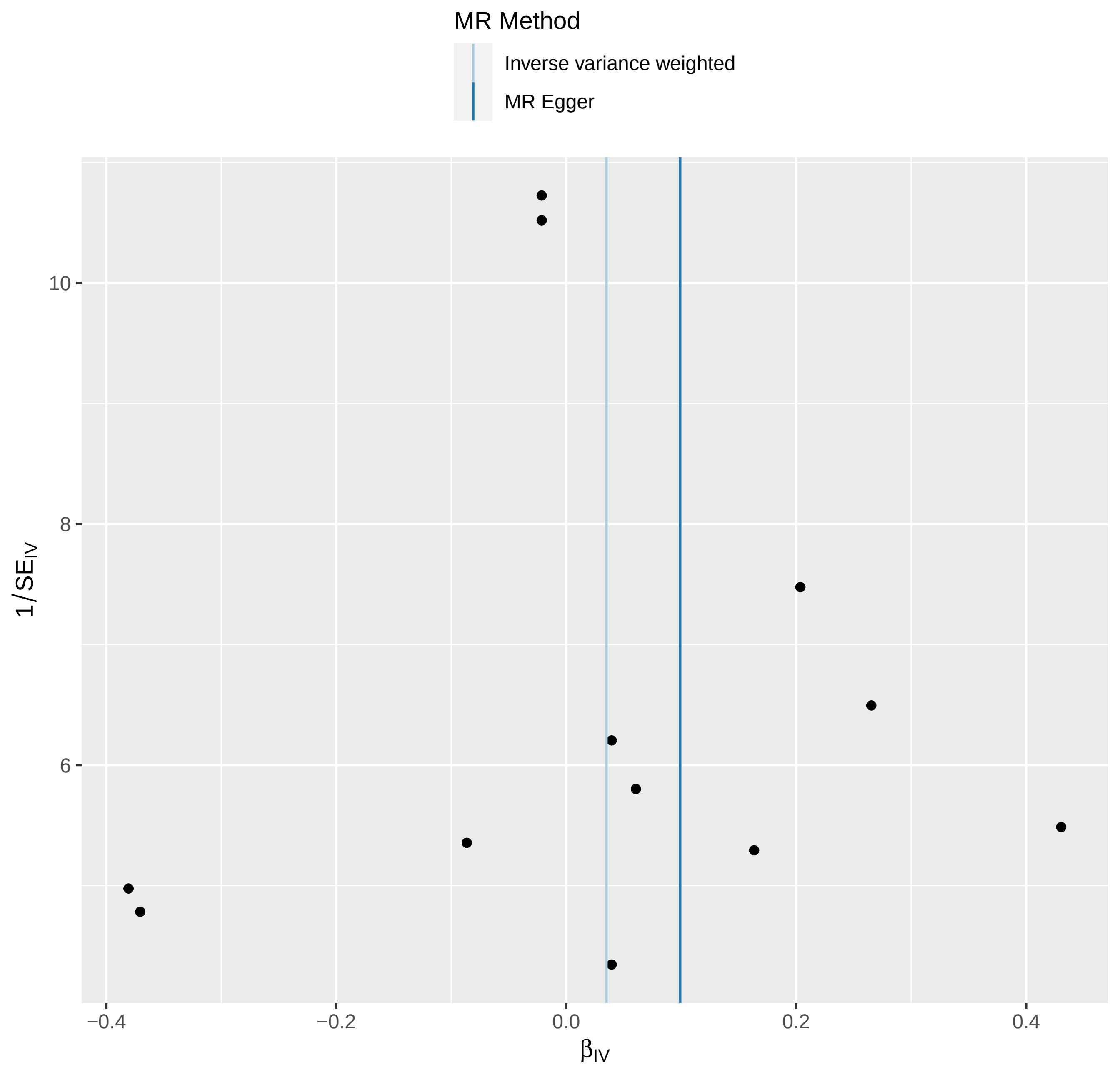

Batch 3−1169 : Selenium on Polypeptide N−acetylgalactosaminyltransferase 16

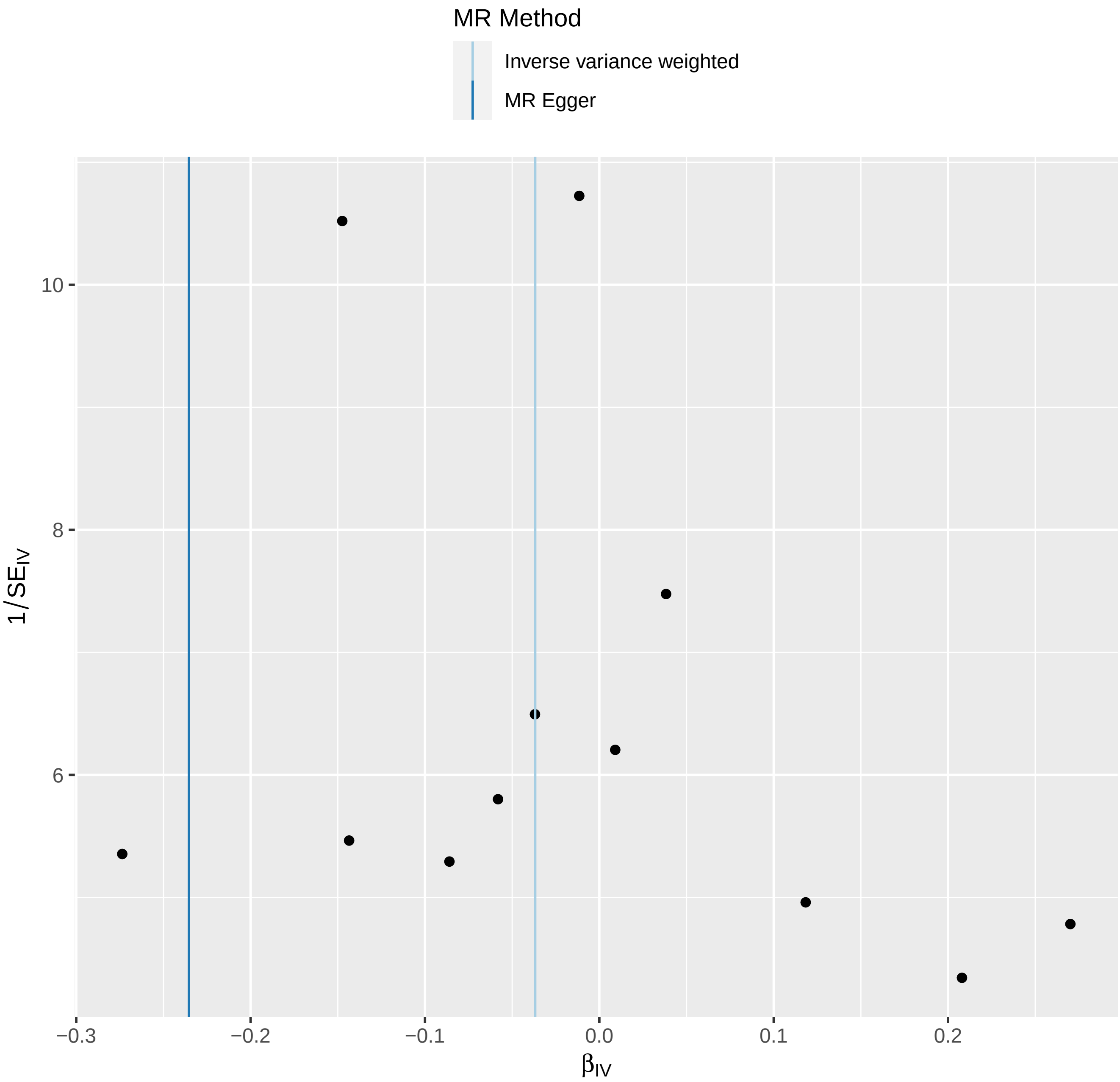

Batch 3−1170 : Selenium on Polypeptide N−acetylgalactosaminyltransferase 1

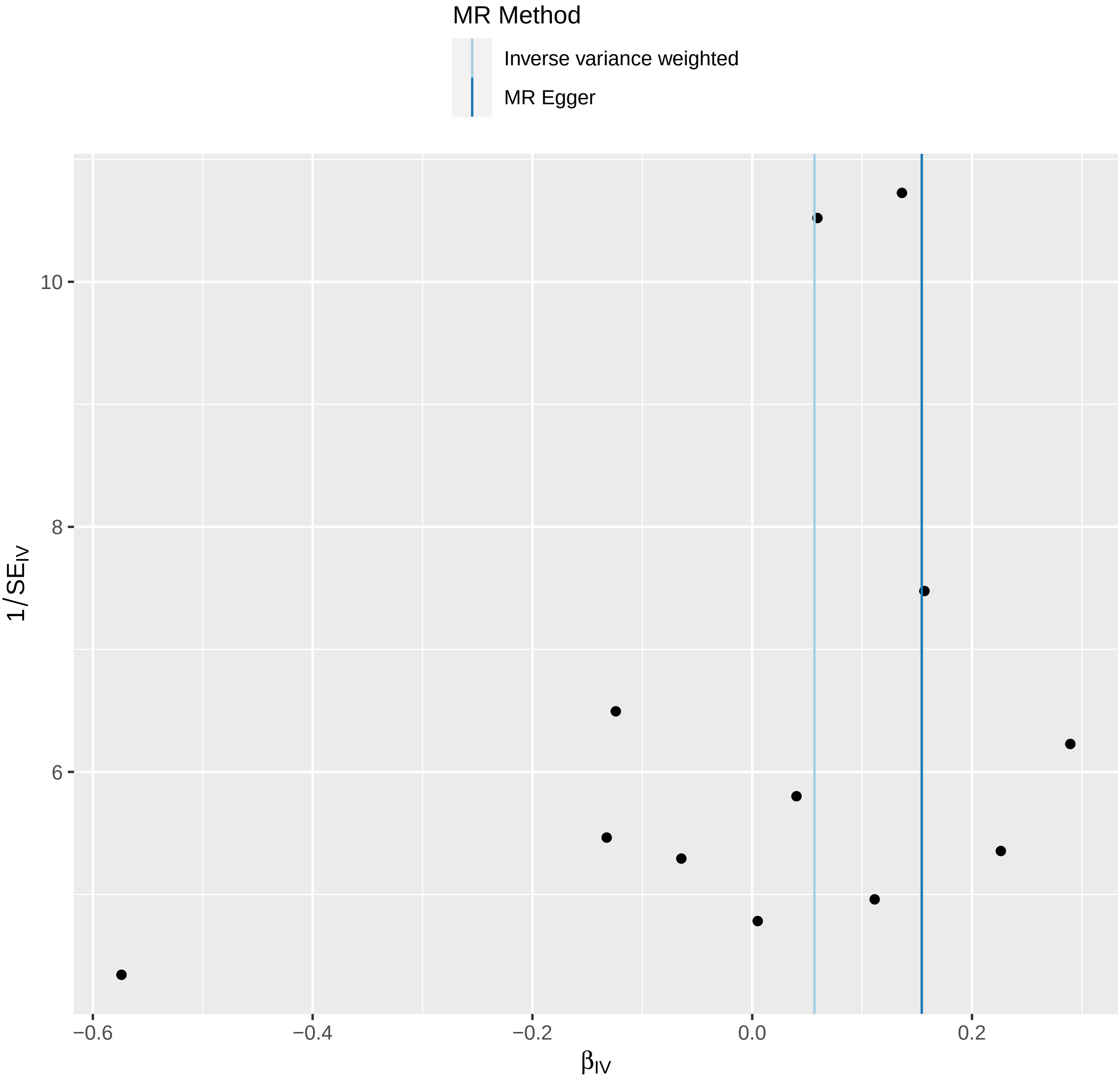

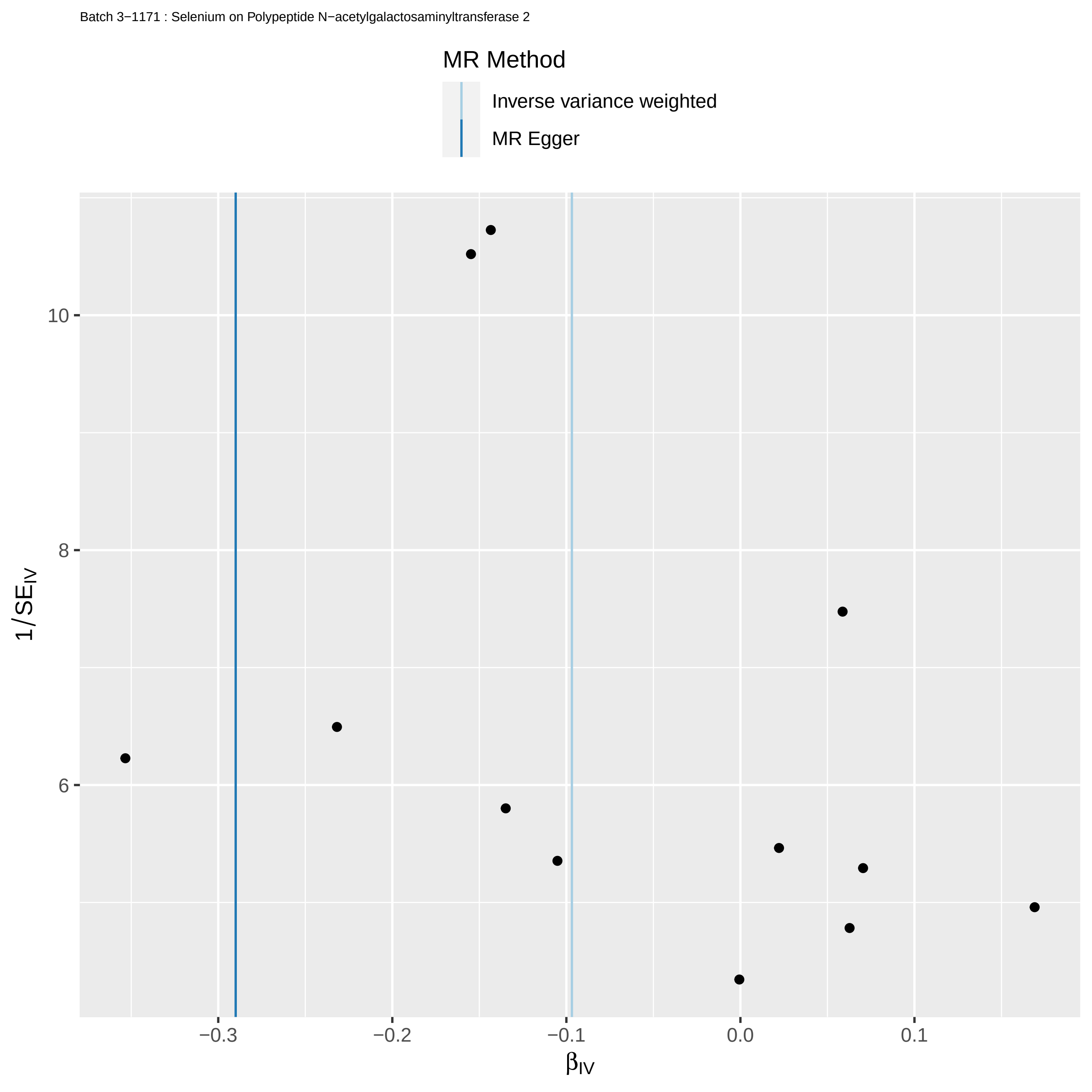

Batch 3−1171 : Selenium on Polypeptide N−acetylgalactosaminyltransferase 2
MR Method
Inverse variance weighted
MR Egger
10
8
6
1/SE_IV
−0.3
−0.2
−0.1
0.0
0.1
β_IV

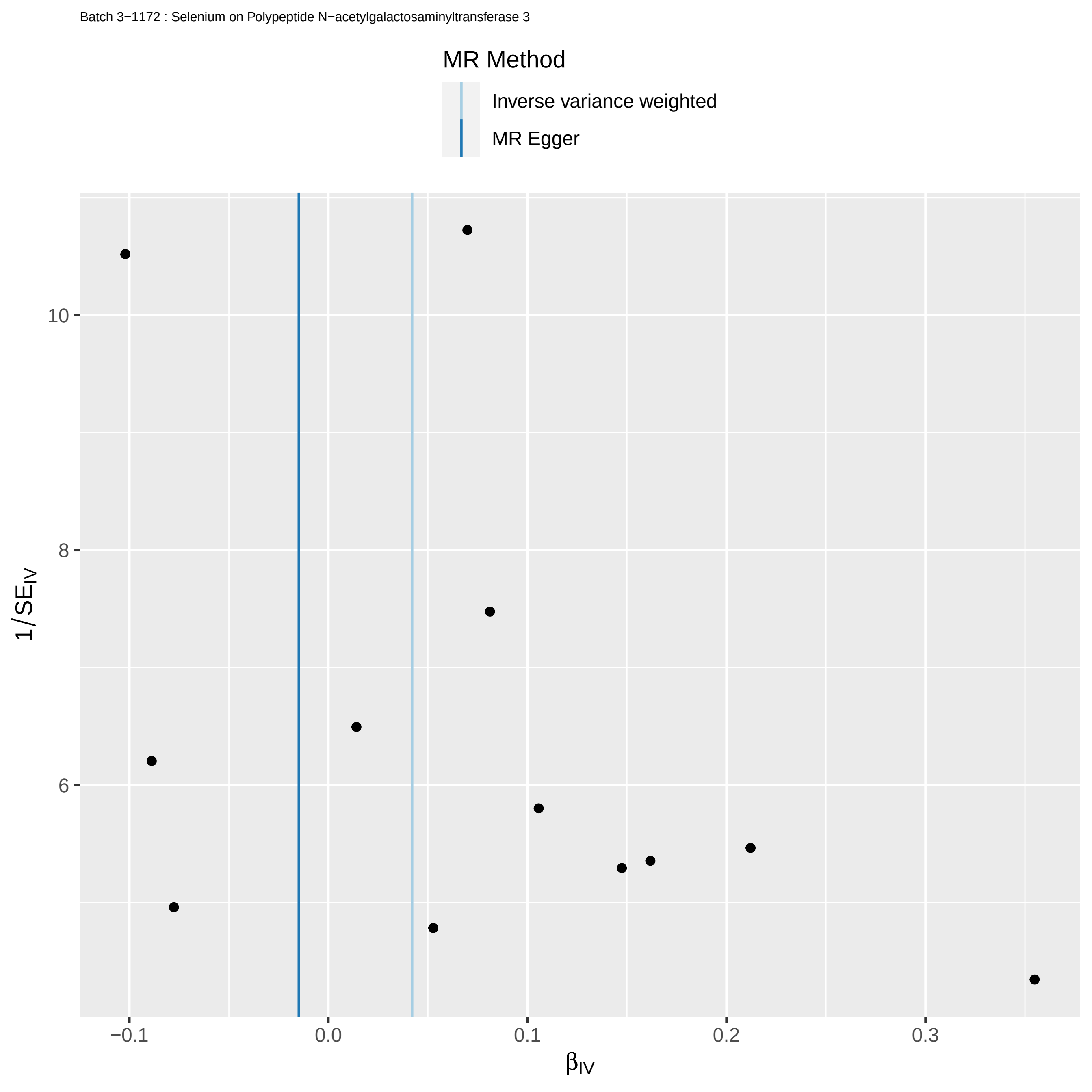

Batch 3−1172 : Selenium on Polypeptide N−acetylgalactosaminyltransferase 3
MR Method
Inverse variance weighted
MR Egger
10
8
6
1/SE_IV
−0.1
0.0
0.1
0.2
0.3
β_IV

Batch 3−1173 : Selenium on N−acetylgalactosaminyltransferase 7

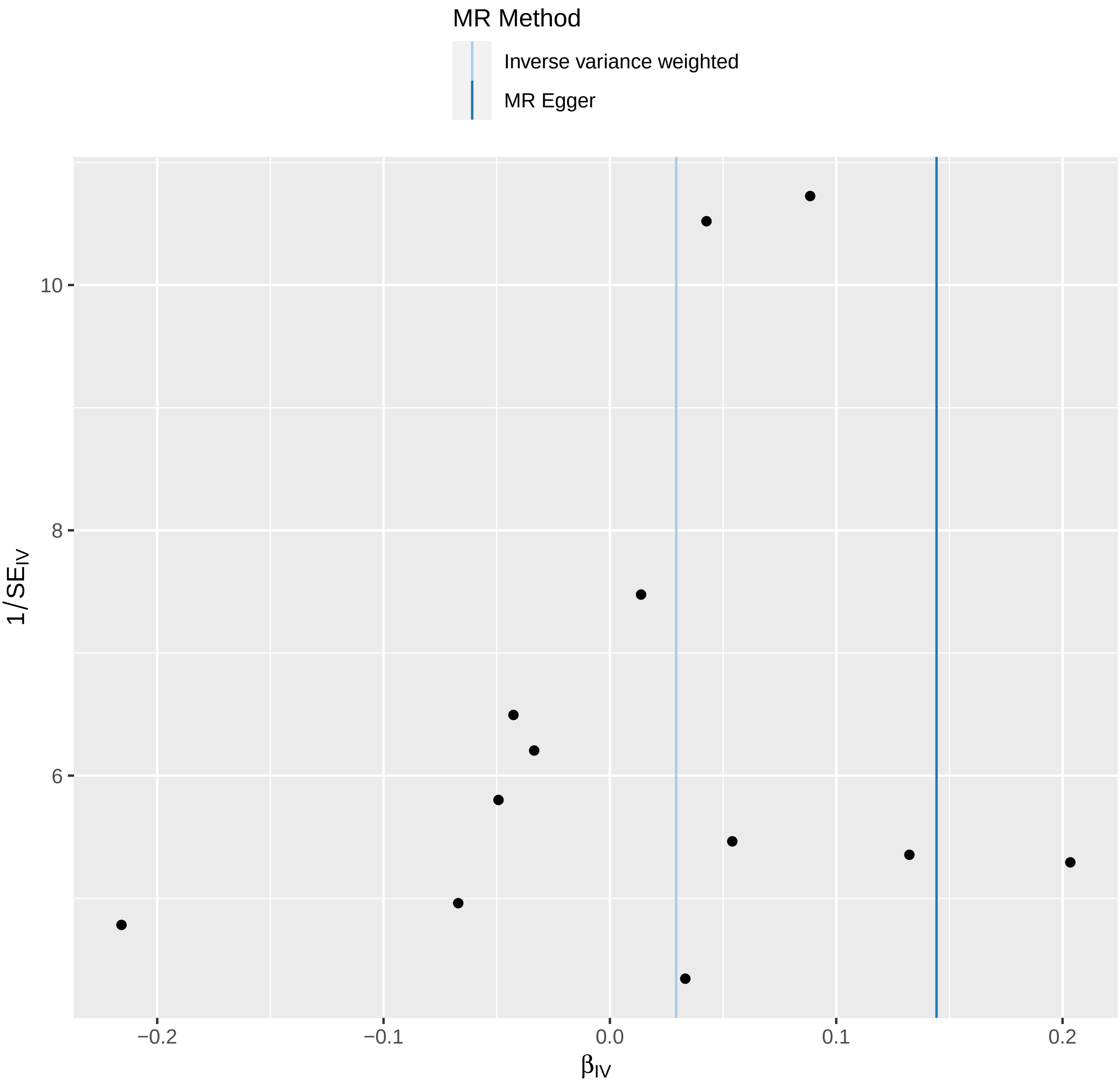

Batch 3−1174 : Selenium on Galanin−like peptide

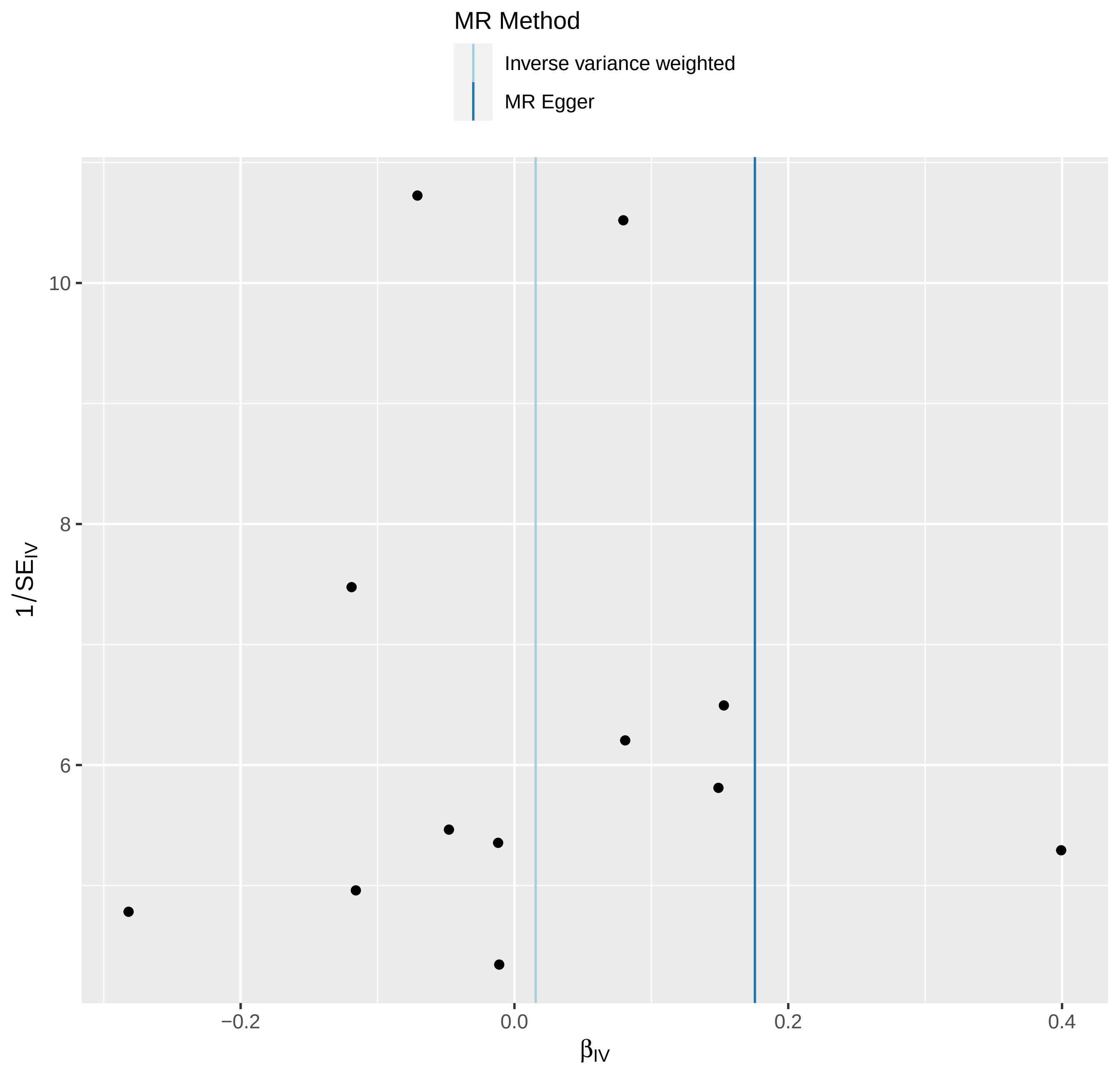

Batch 3−1175 : Selenium on Gigaxonin

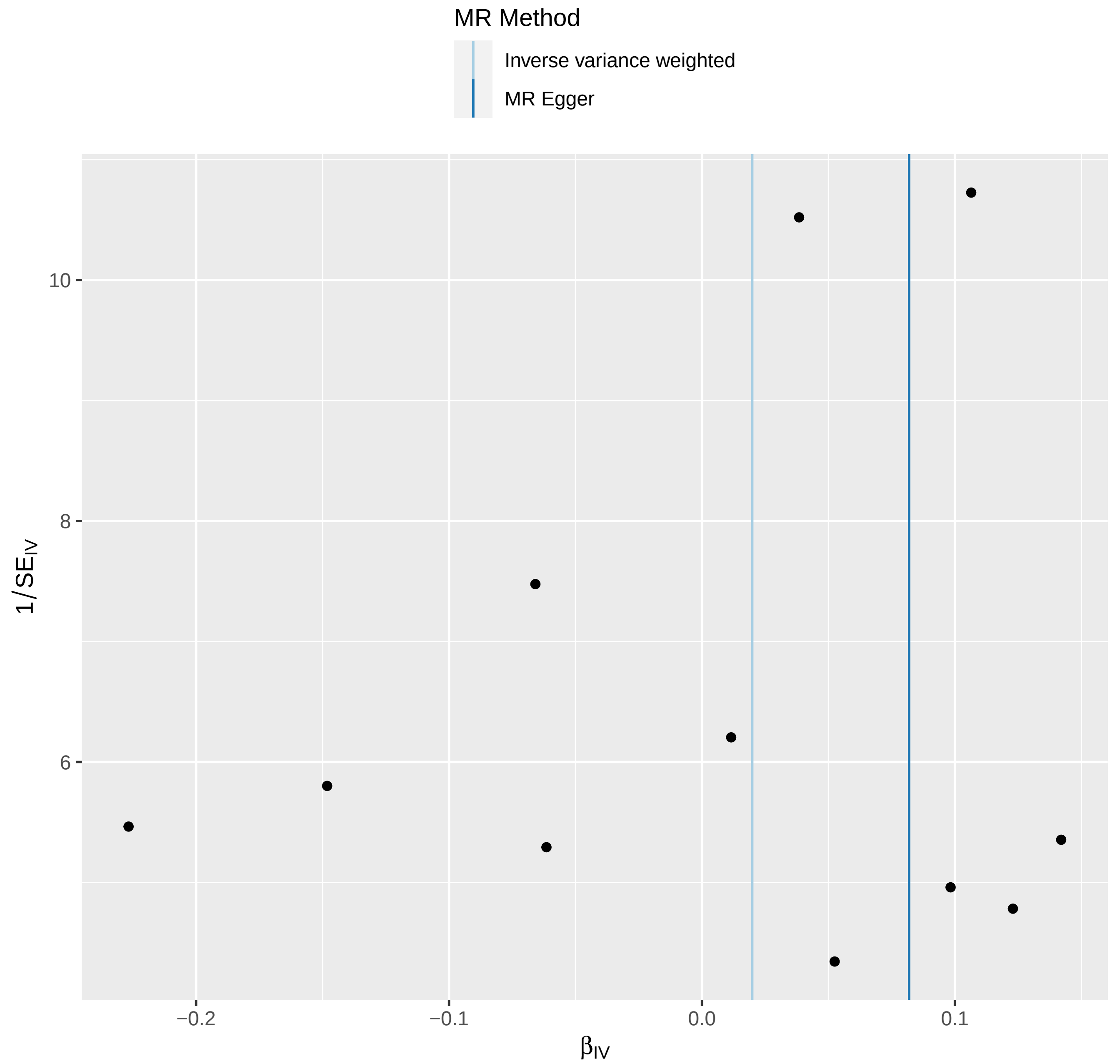

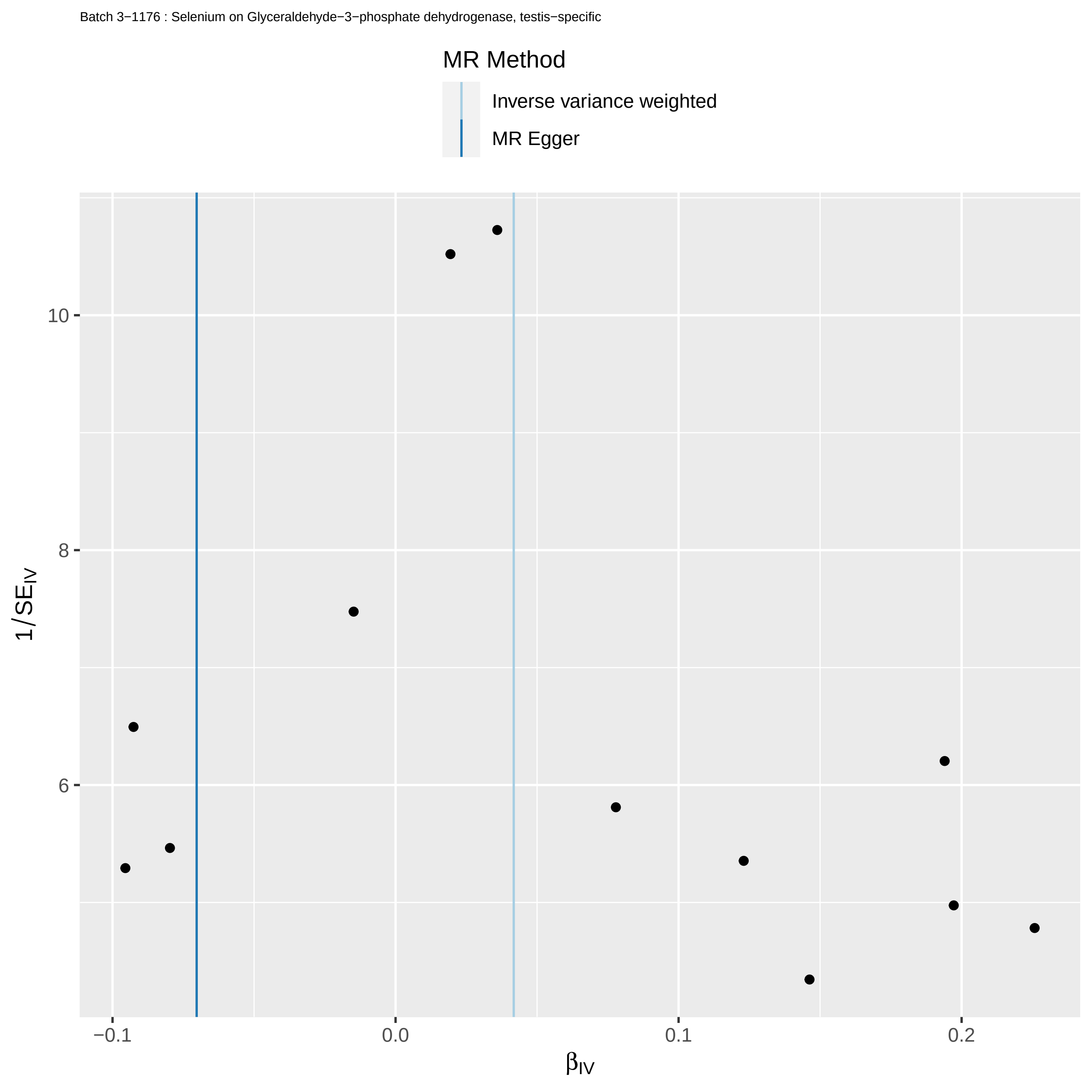

Batch 3−1176 : Selenium on Glyceraldehyde−3−phosphate dehydrogenase, testis−specific
MR Method
Inverse variance weighted
MR Egger
10
8
6
1/SE_IV
−0.1
0.0
0.1
0.2
β_IV

Batch 3−1177 : Selenium on Growth arrest−specific protein 1

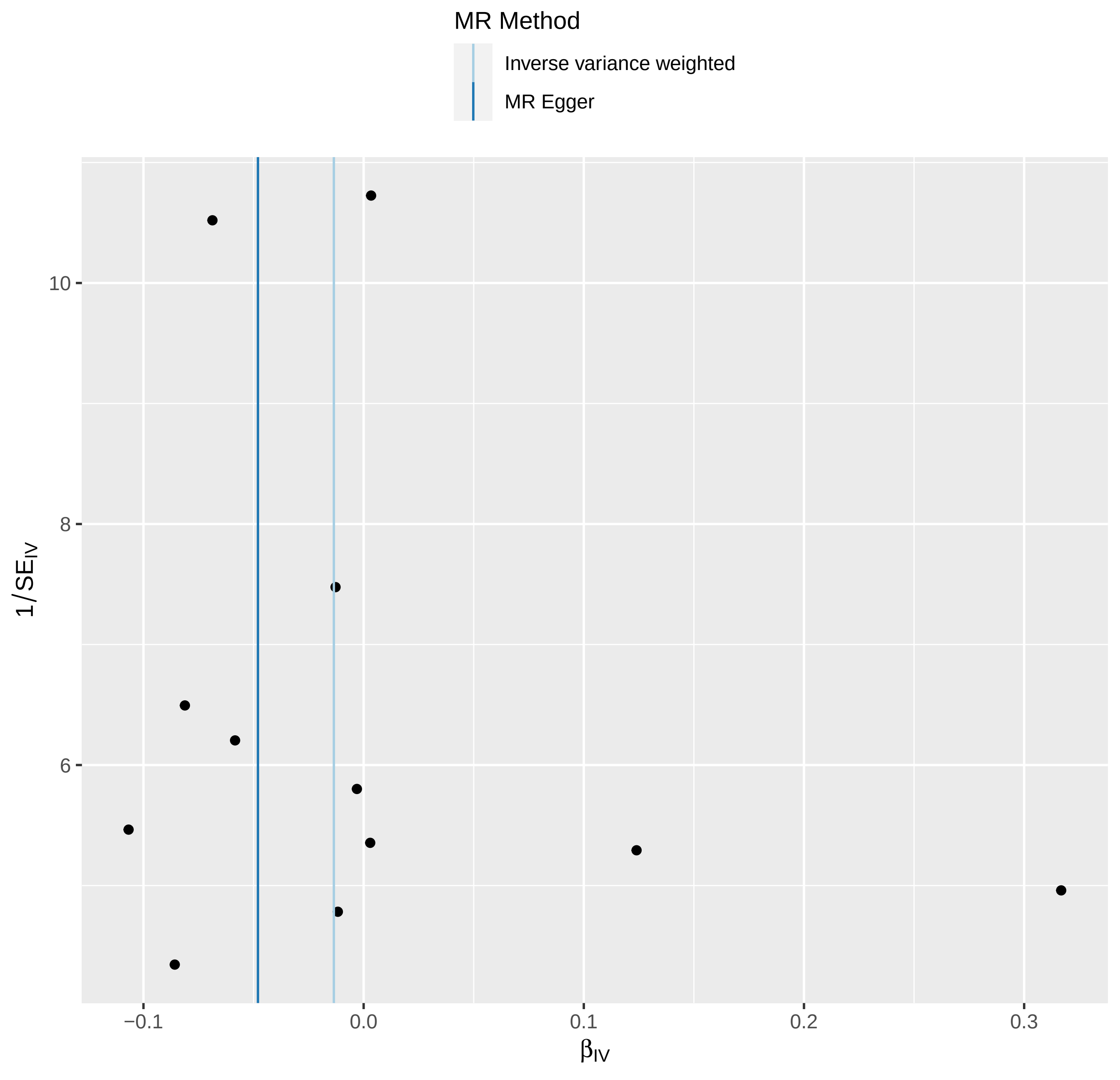

Batch 3−1178 : Selenium on Guanylate−binding protein 6

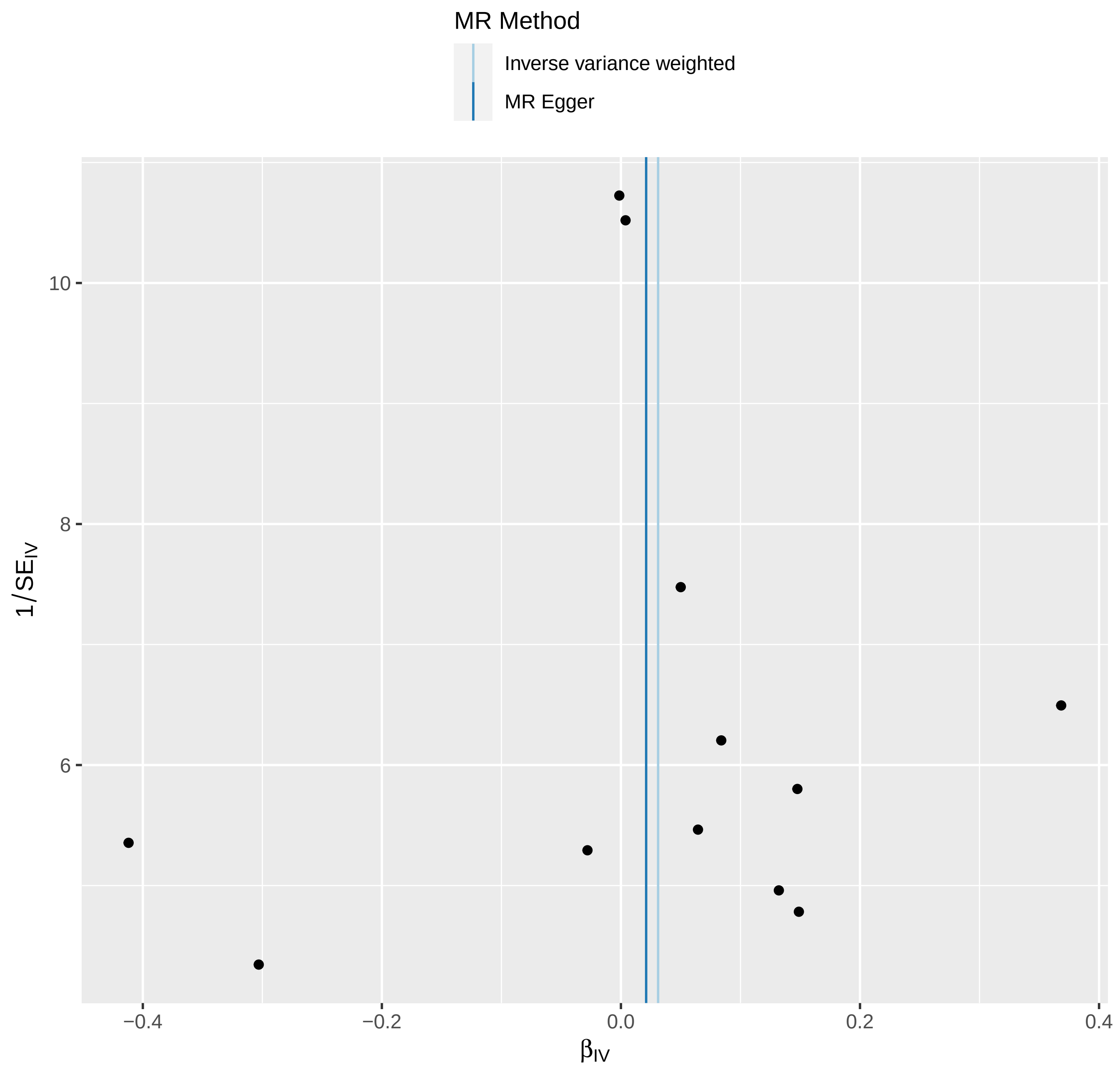

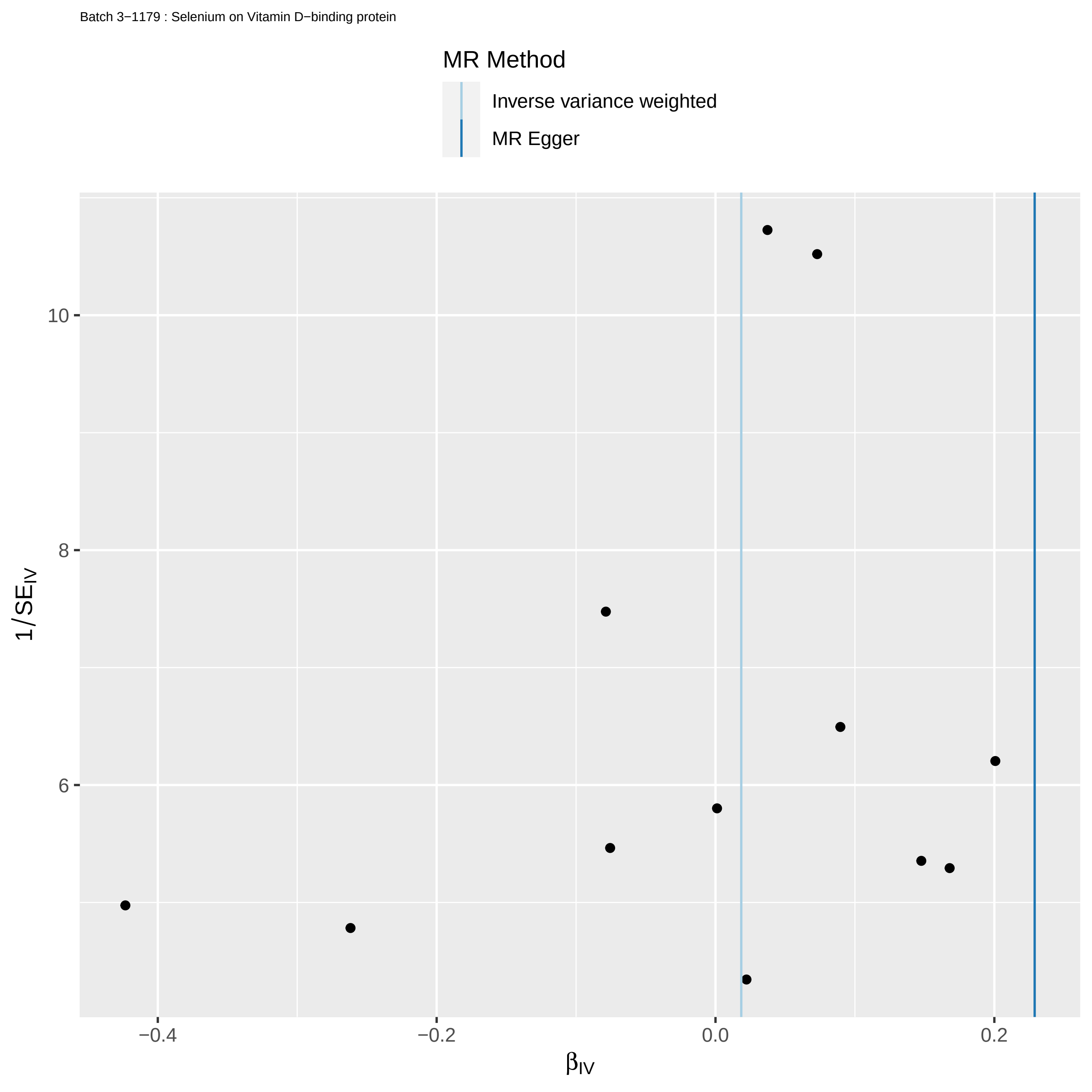
Batch 3−1179 : Selenium on Vitamin D−binding protein
MR Method
Inverse variance weighted
MR Egger
10
8
6
1/SE_IV
−0.4
−0.2
0.0
0.2
β_IV

Batch 3−1180 : Selenium on Grancalcin

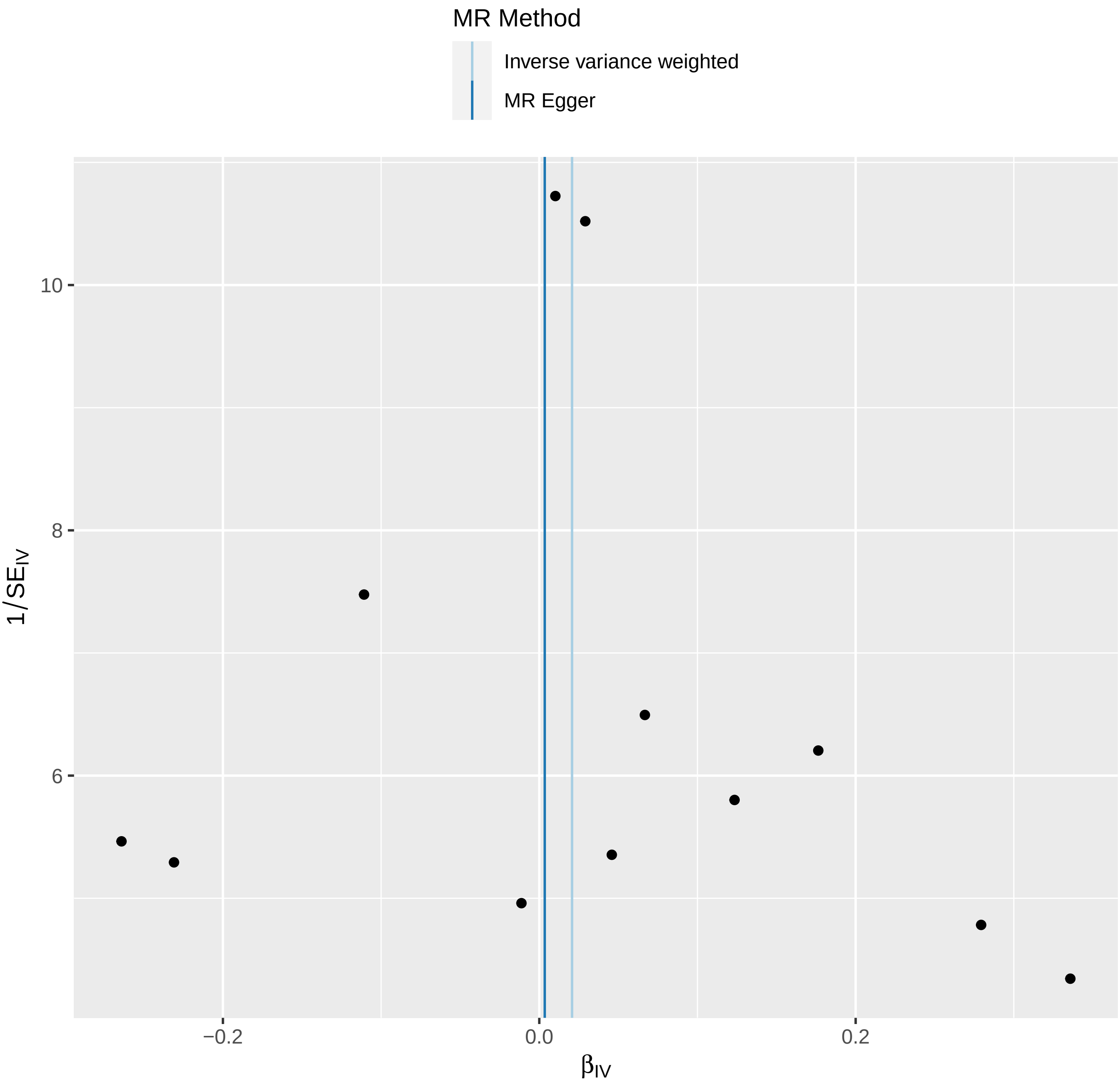

Batch 3−1181 : Selenium on Glucagon

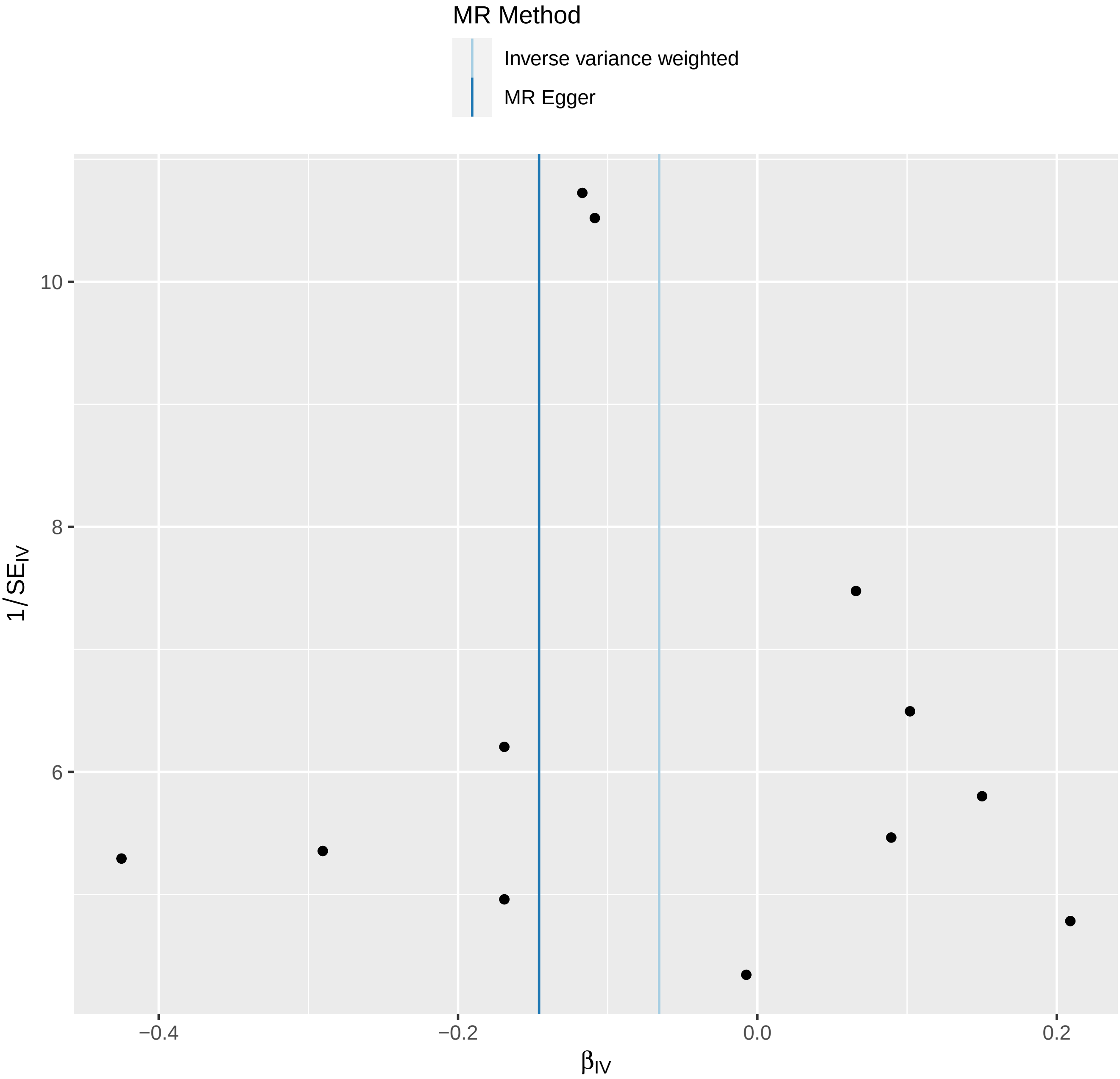

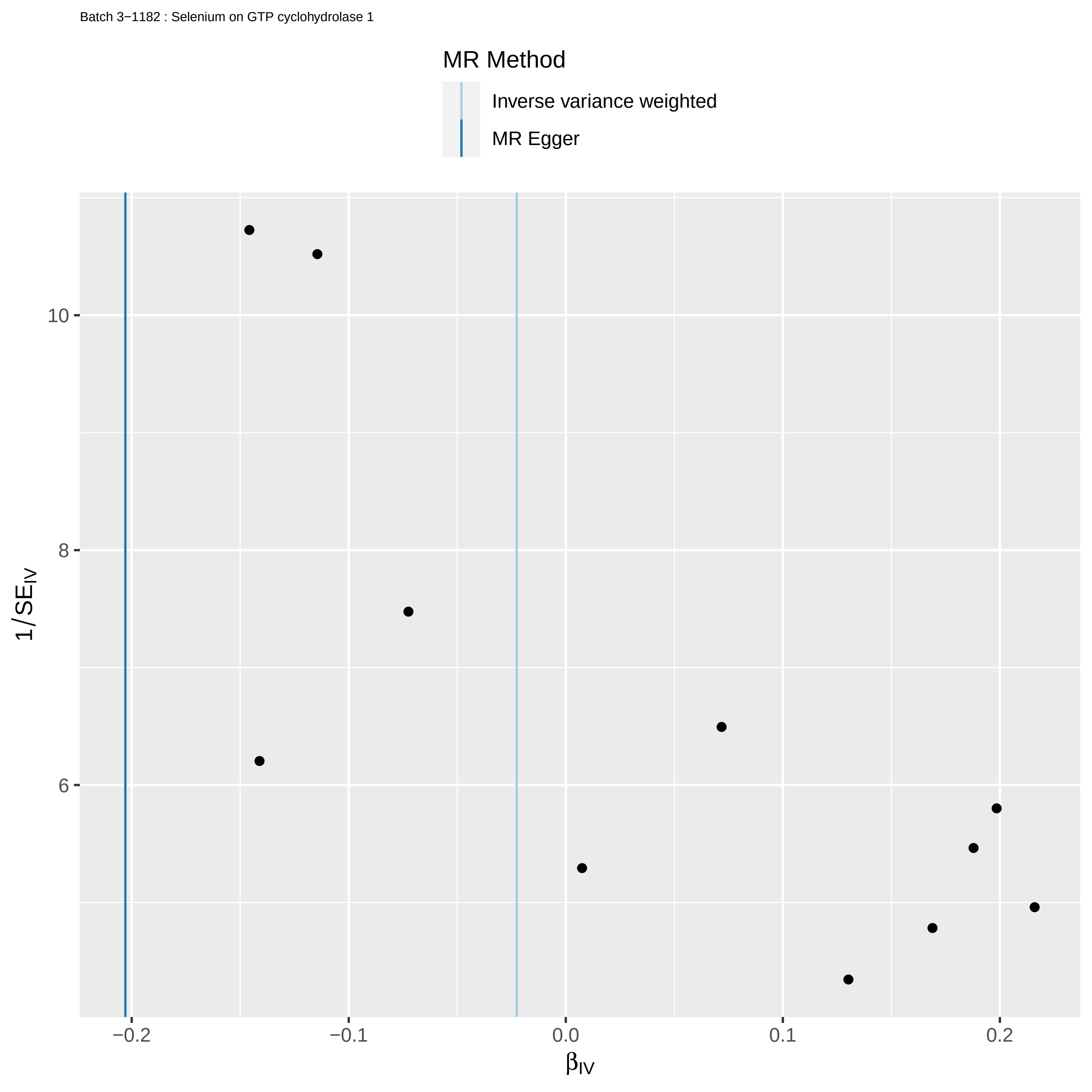

Batch 3−1182 : Selenium on GTP cyclohydrolase 1
MR Method
Inverse variance weighted
MR Egger
10
8
6
1/SE_IV
−0.2
−0.1
0.0
0.1
0.2
β_IV

Batch 3−1183 : Selenium on Glucokinase

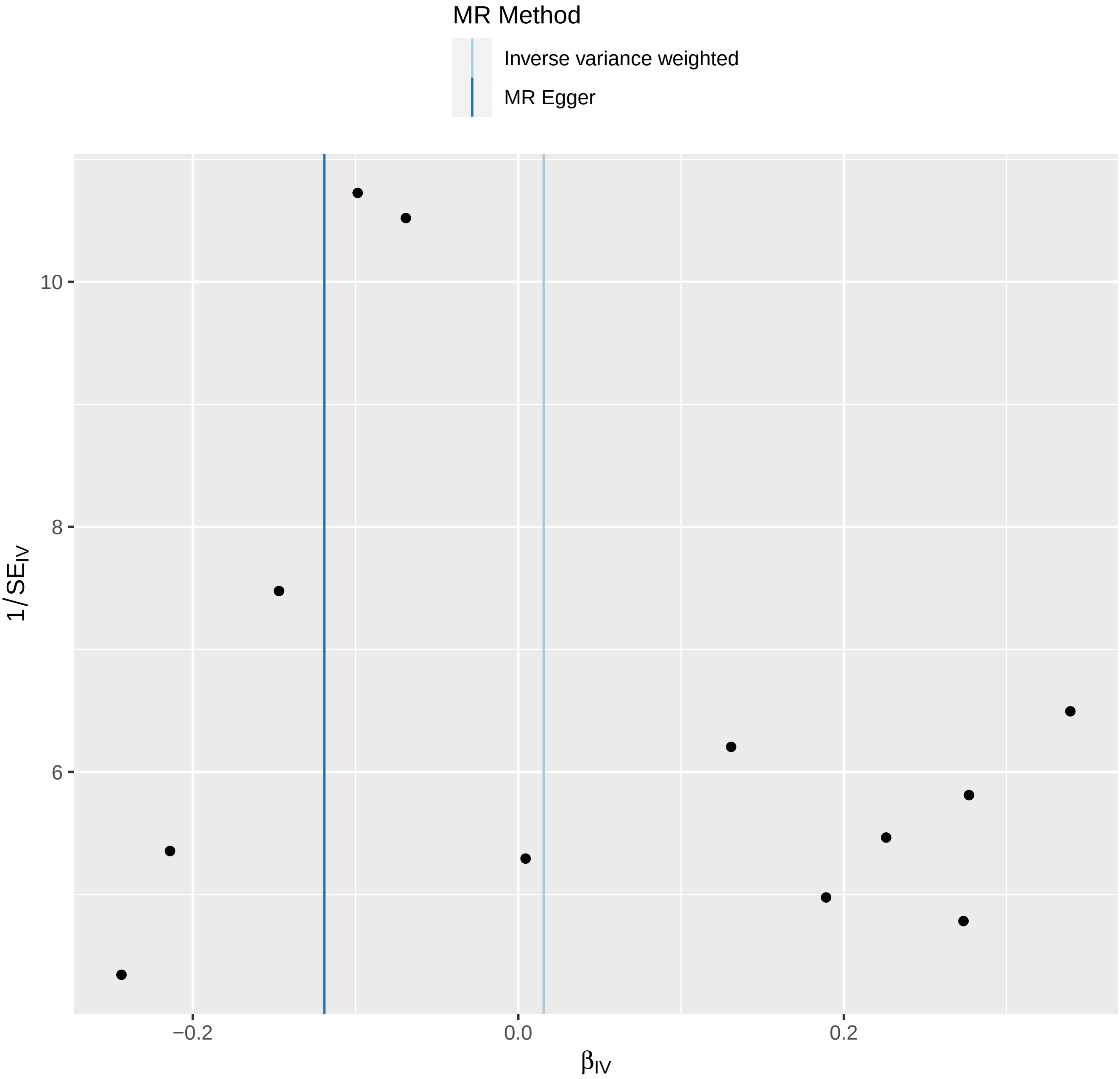

Batch 3−1184 : Selenium on Glucokinase regulatory protein

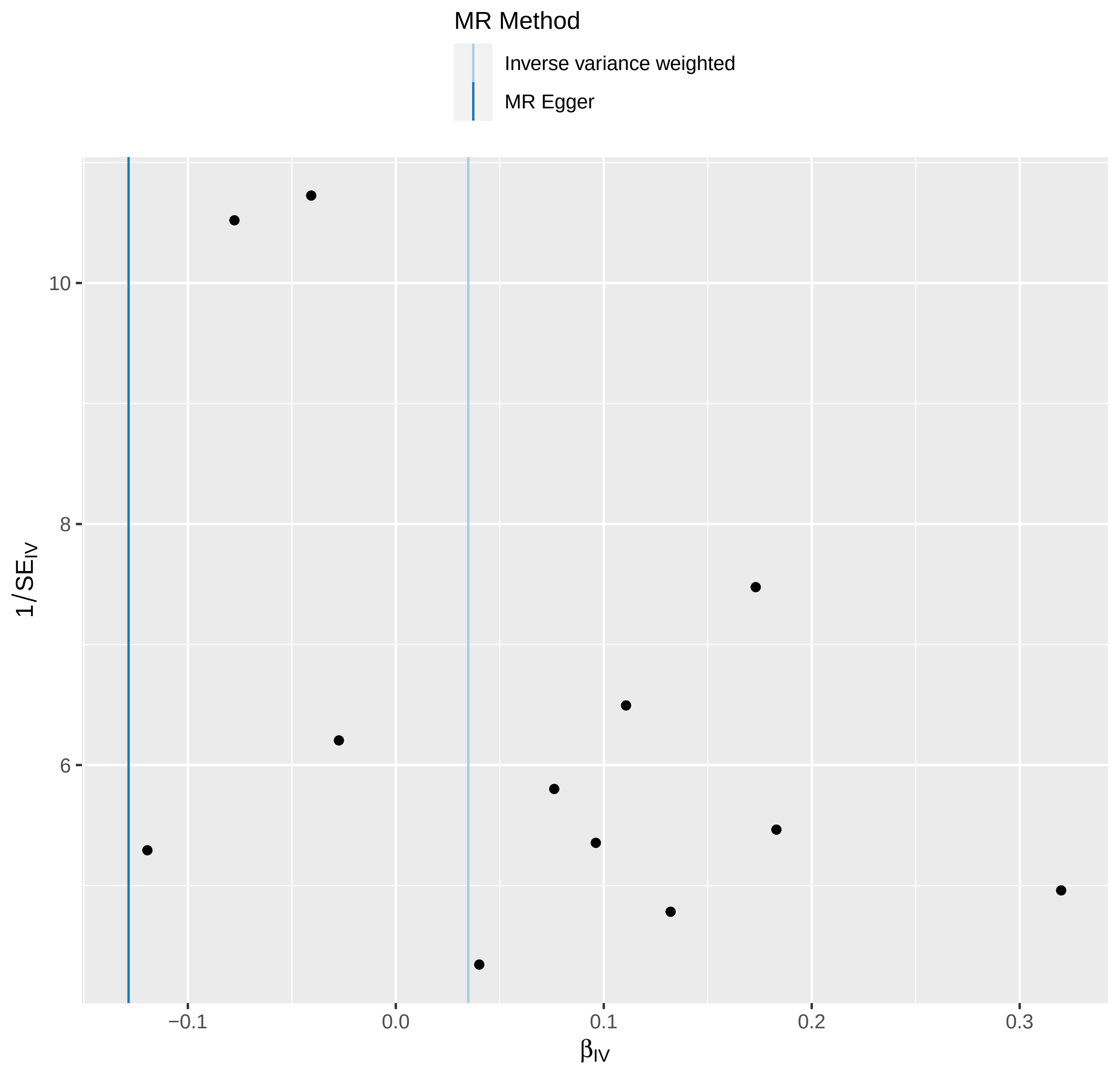

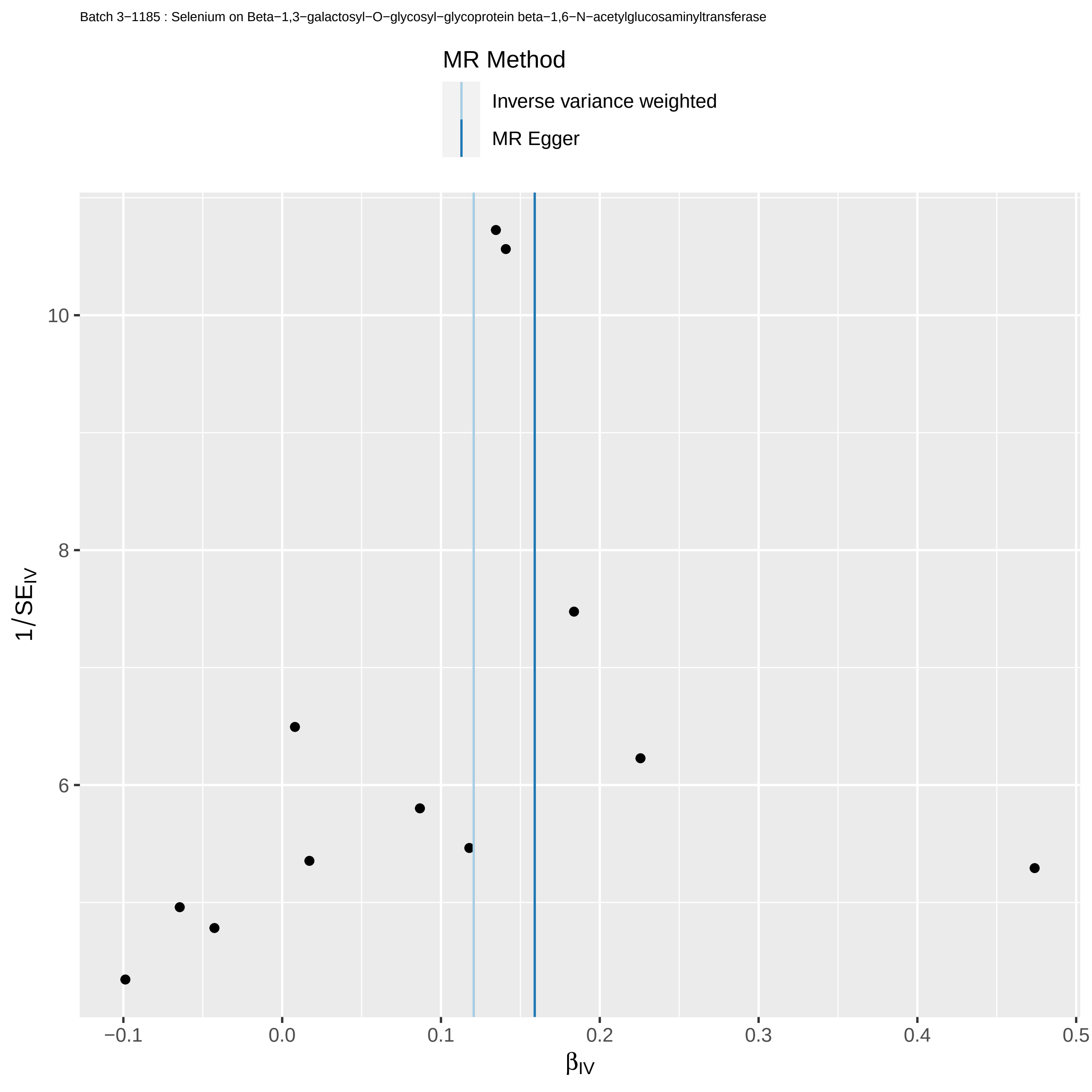
Batch 3−1185 : Selenium on Beta−1,3−galactosyl−O−glycosyl−glycoprotein beta−1,6−N−acetylglucosaminyltransferase
MR Method
Inverse variance weighted
MR Egger
10
8
6
1/SE_IV
−0.1
0.0
0.1
0.2
0.3
0.4
0.5
β_IV

Batch 3−1186 : Selenium on N−acetyllactosaminide beta−1,6−N−acetylglucosaminyl−transferase, isoform C

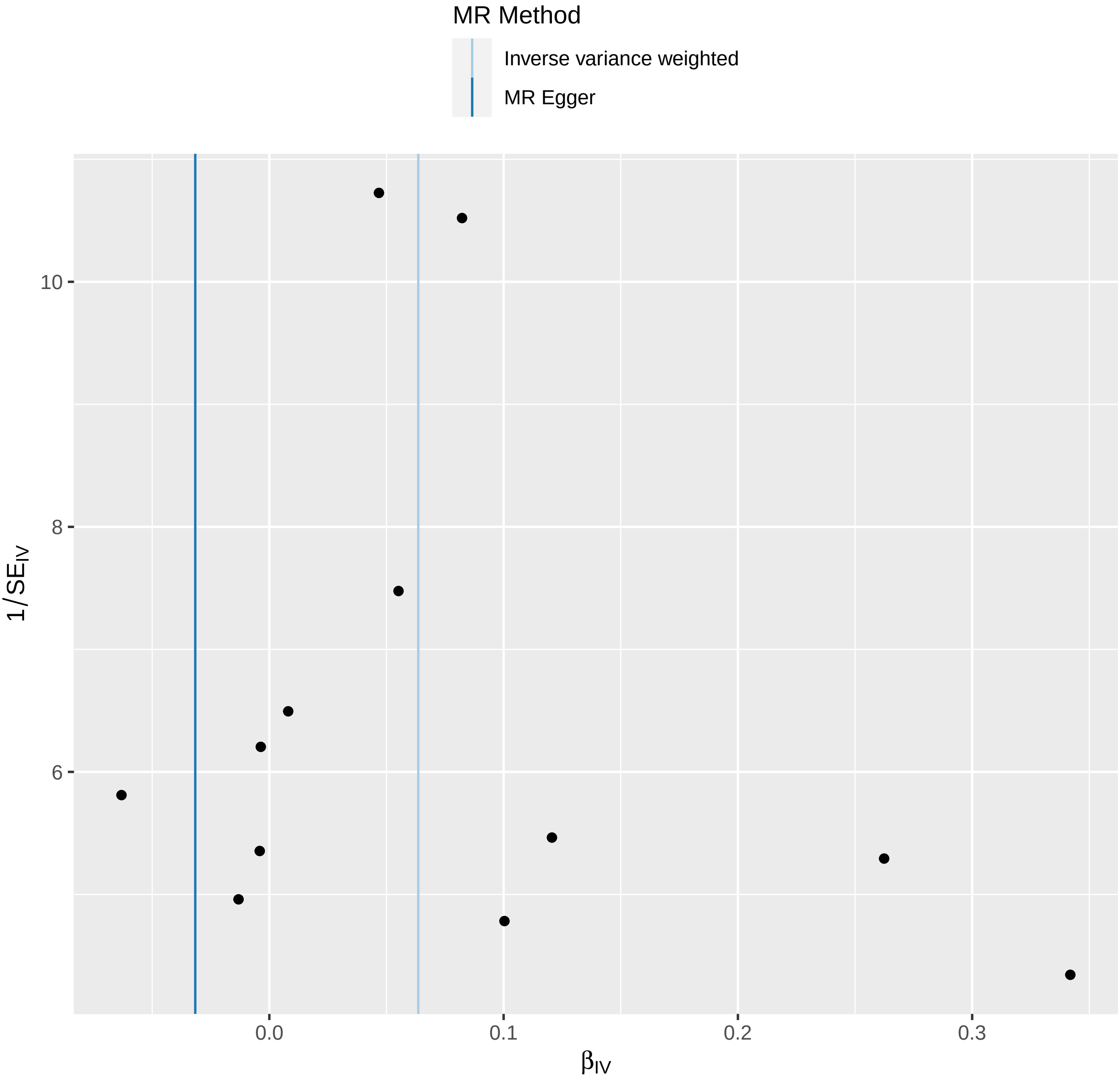

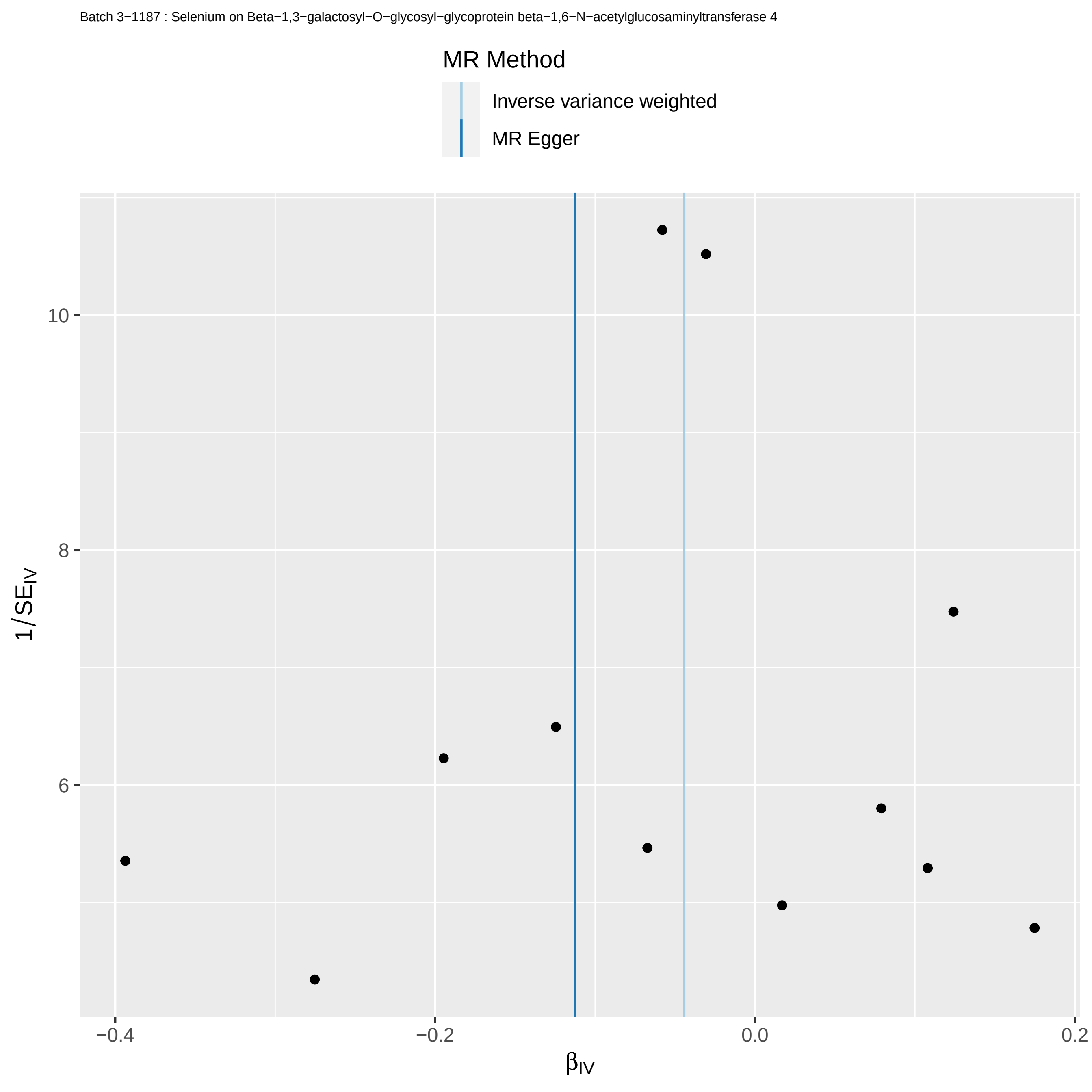
Batch 3−1187 : Selenium on Beta−1,3−galactosyl−O−glycosyl−glycoprotein beta−1,6−N−acetylglucosaminyltransferase 4
MR Method
Inverse variance weighted
MR Egger
10
8
6
1/SE$_{IV}$
−0.4
−0.2
0.0
0.2
β_{IV}

Batch 3−1188 : Selenium on Beta−1,3−galactosyl−O−glycosyl−glycoprotein beta−1,6−N−acetylglucosaminyltransferase 4

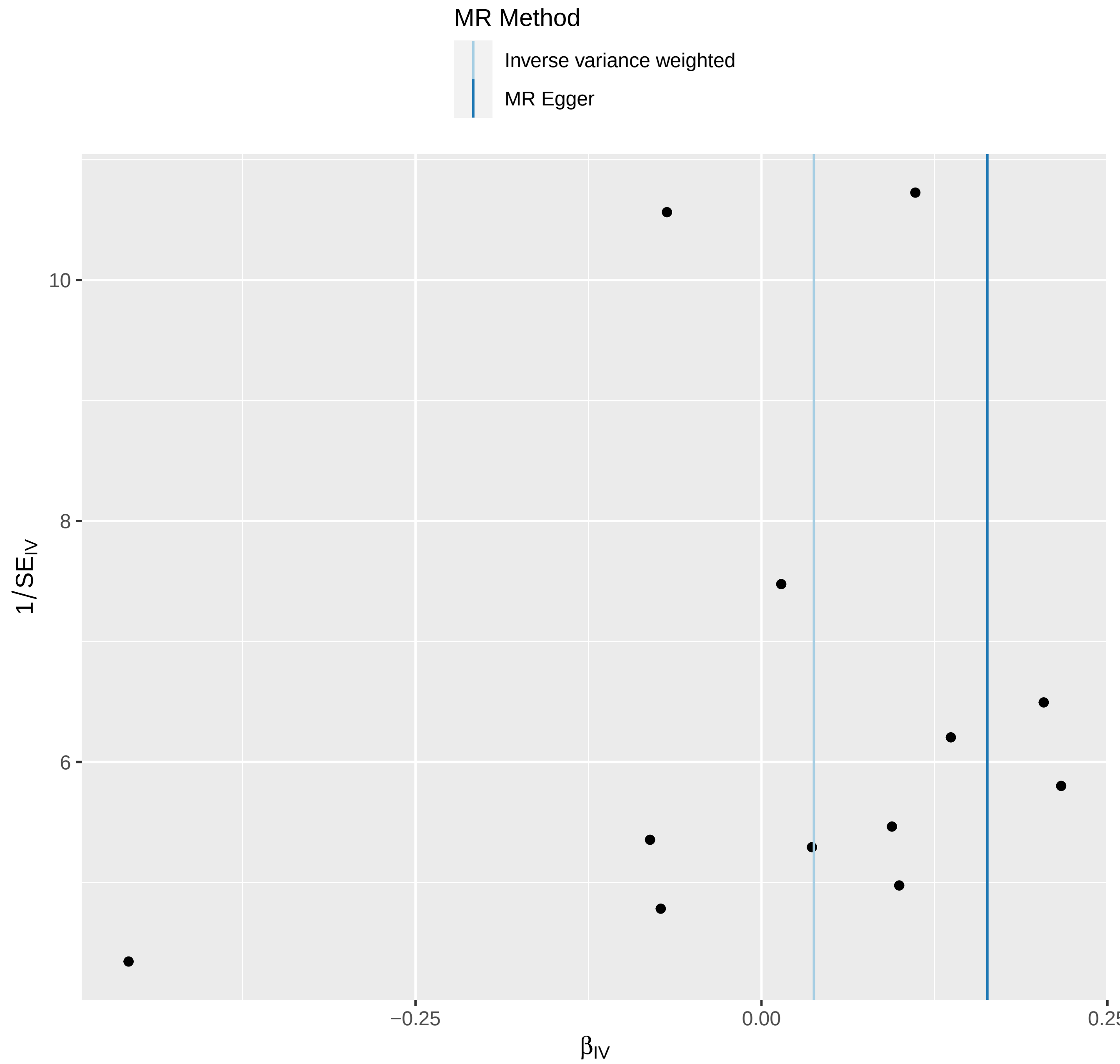

Batch 3−1189 : Selenium on Guanine deaminase

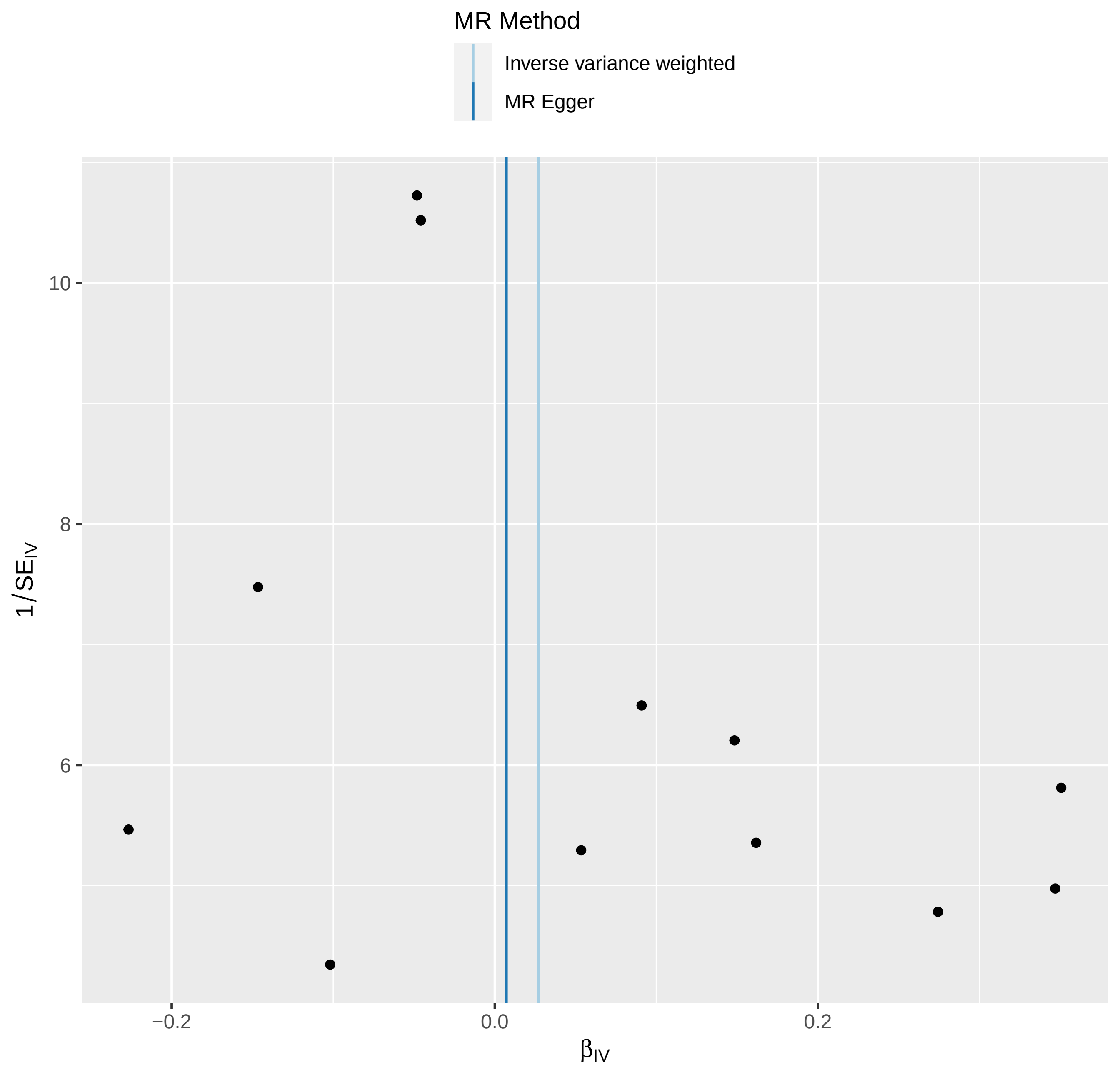

Batch 3−1190 : Selenium on Growth/differentiation factor 11

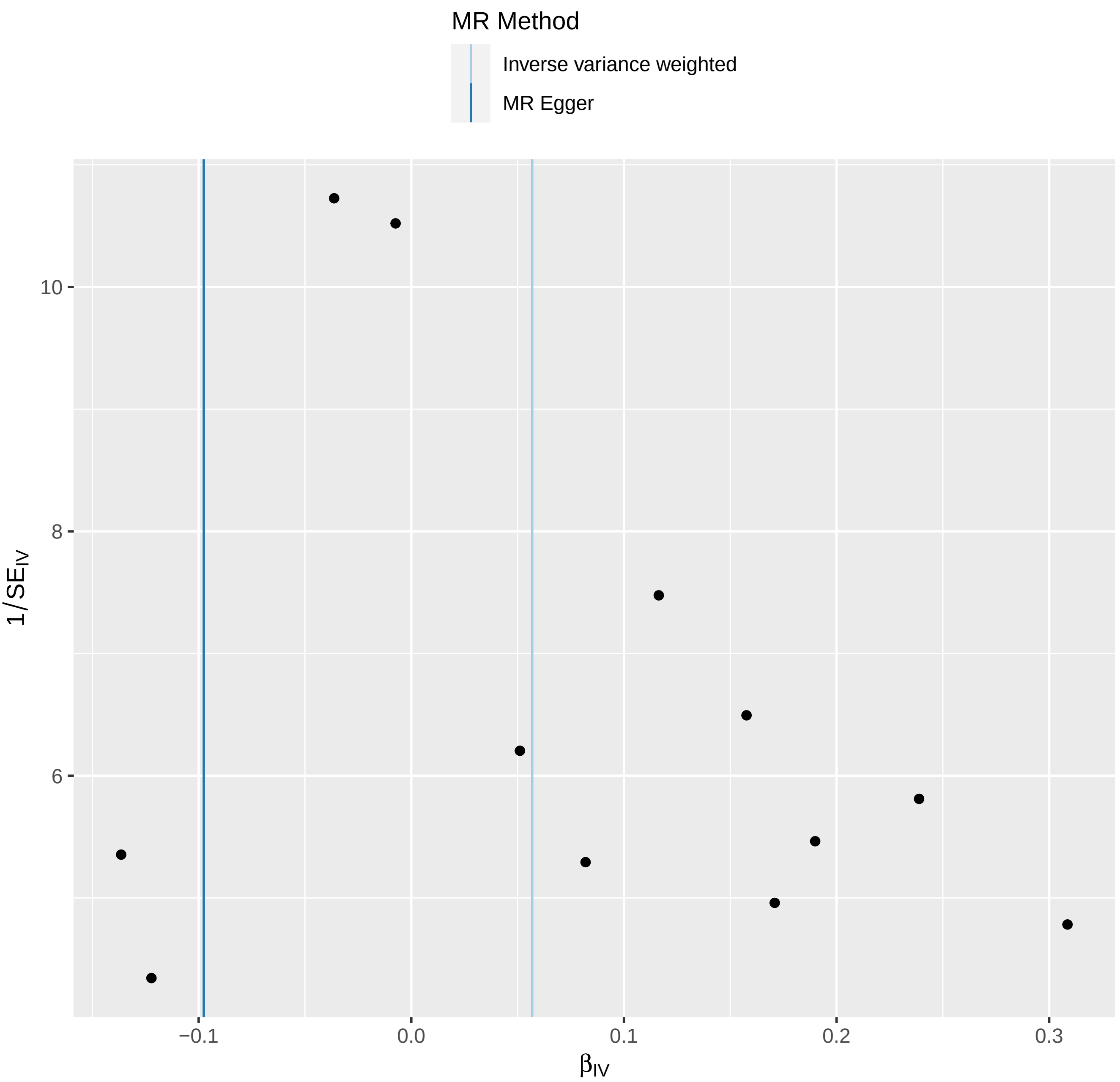

Batch 3−1191 : Selenium on Growth/differentiation factor 11

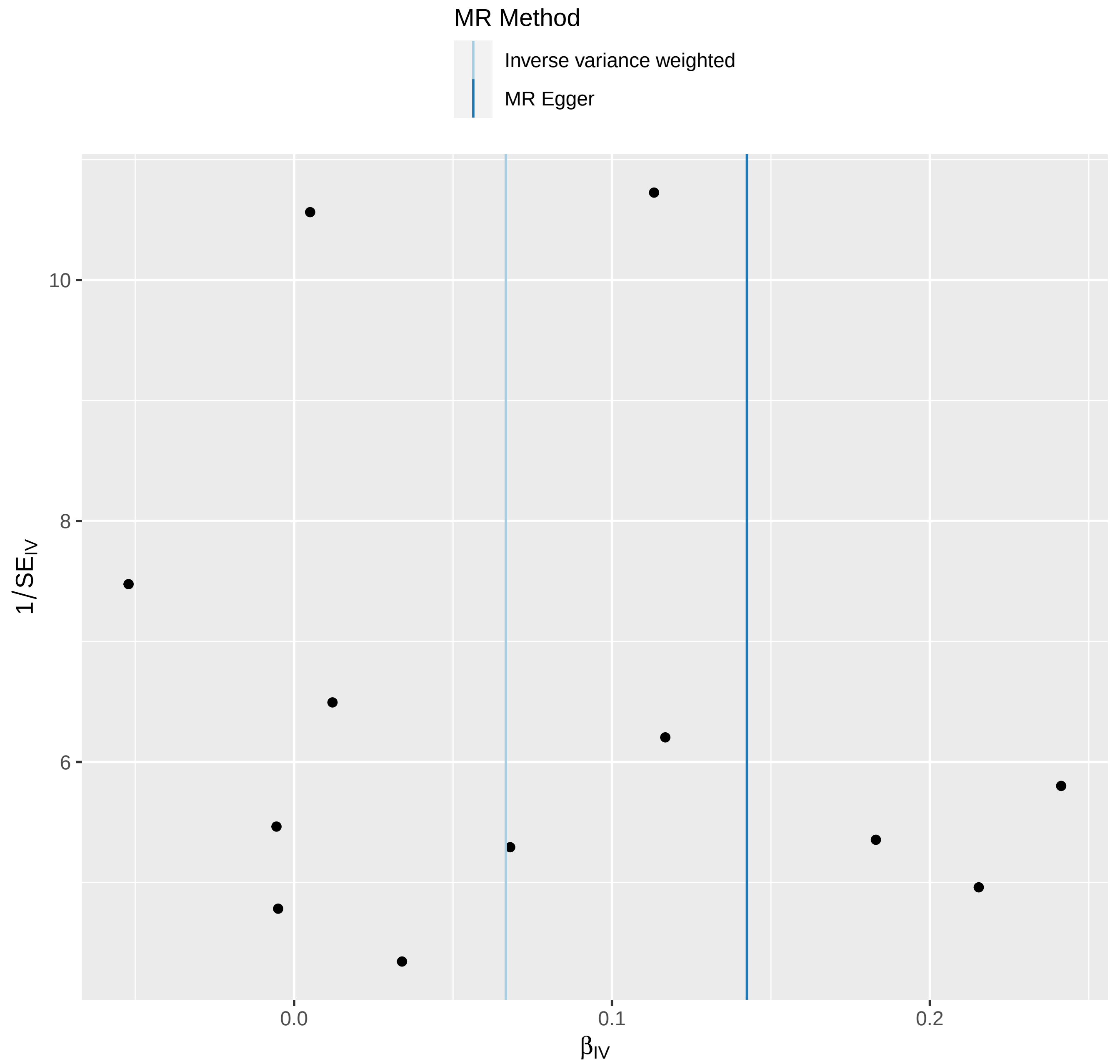

Batch 3−1192 : Selenium on Growth/differentiation factor 11

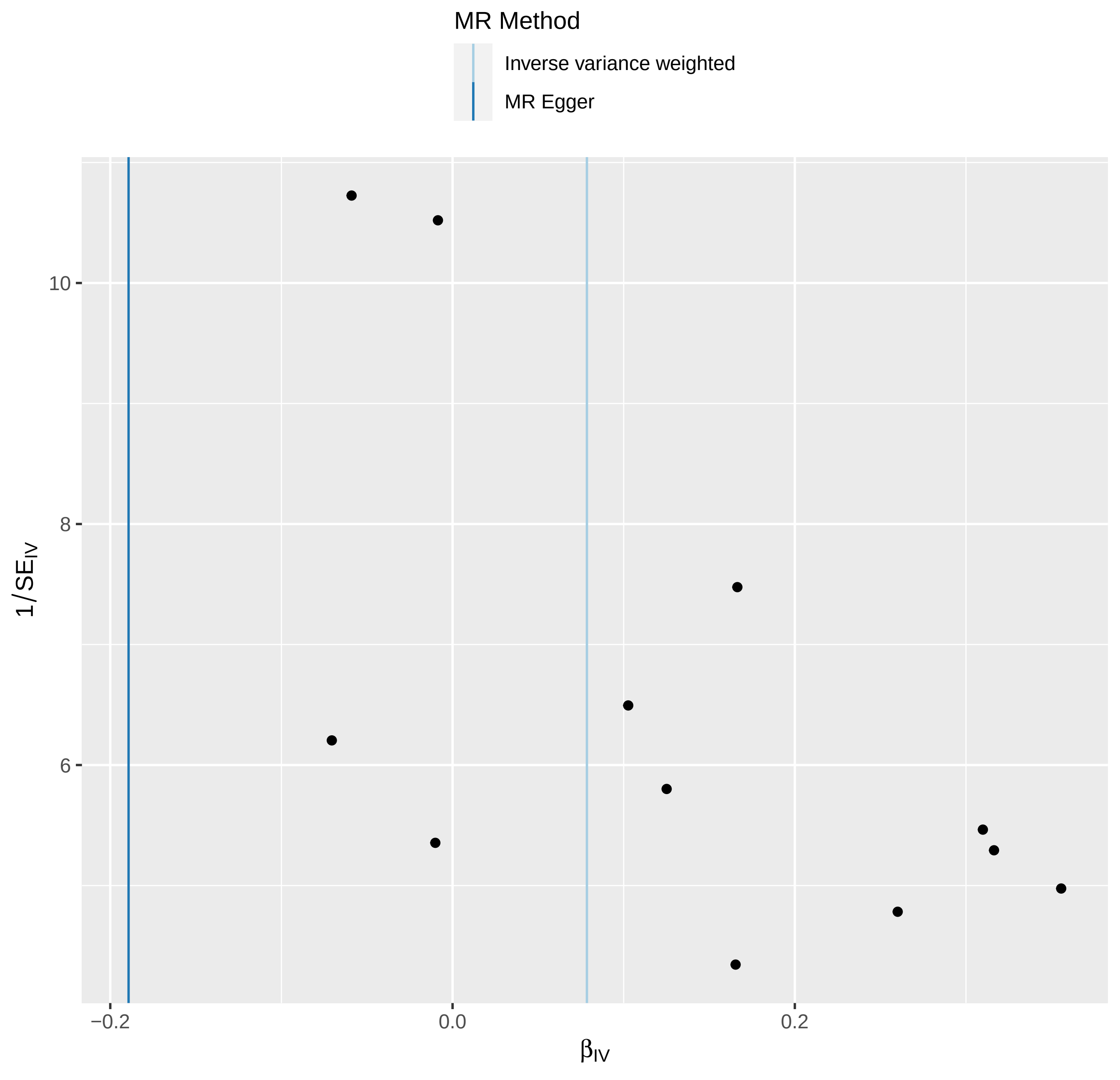

Batch 3−1193 : Selenium on Growth/differentiation factor 11

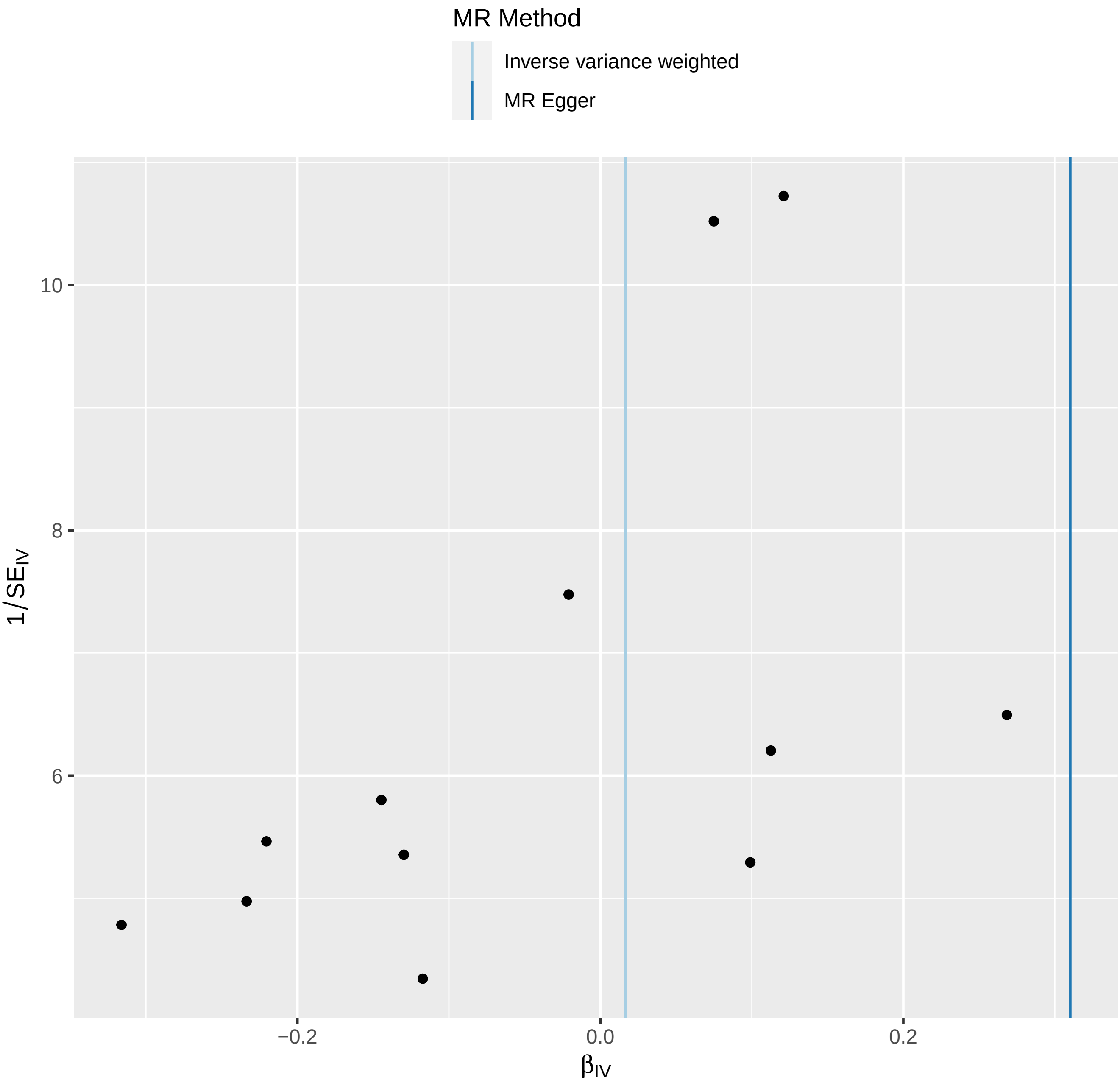

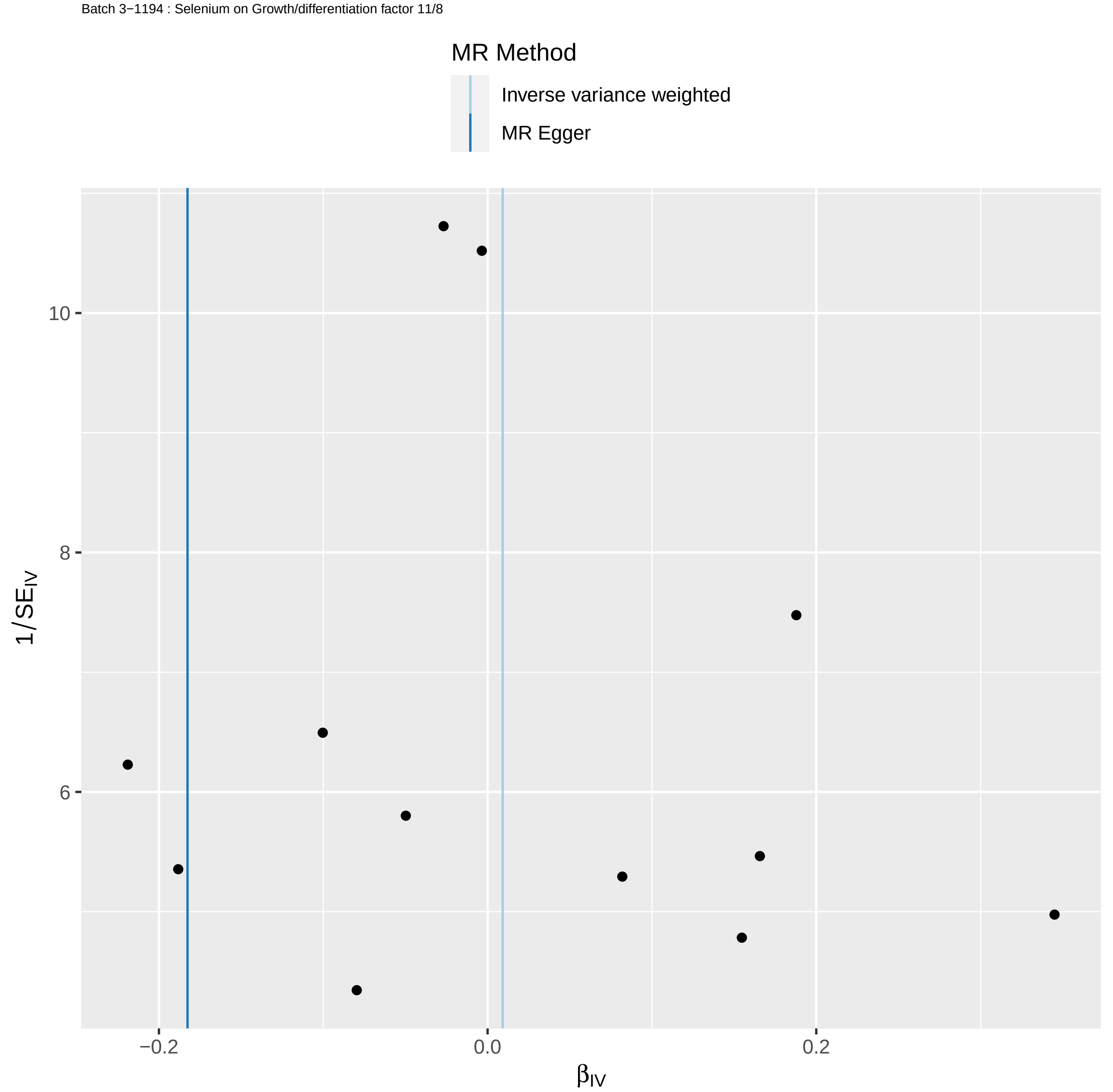
Batch 3−1194 : Selenium on Growth/differentiation factor 11/8
MR Method
Inverse variance weighted
MR Egger
10
8
6
1/SE$_{IV}$
−0.2
0.0
0.2
β$_{IV}$

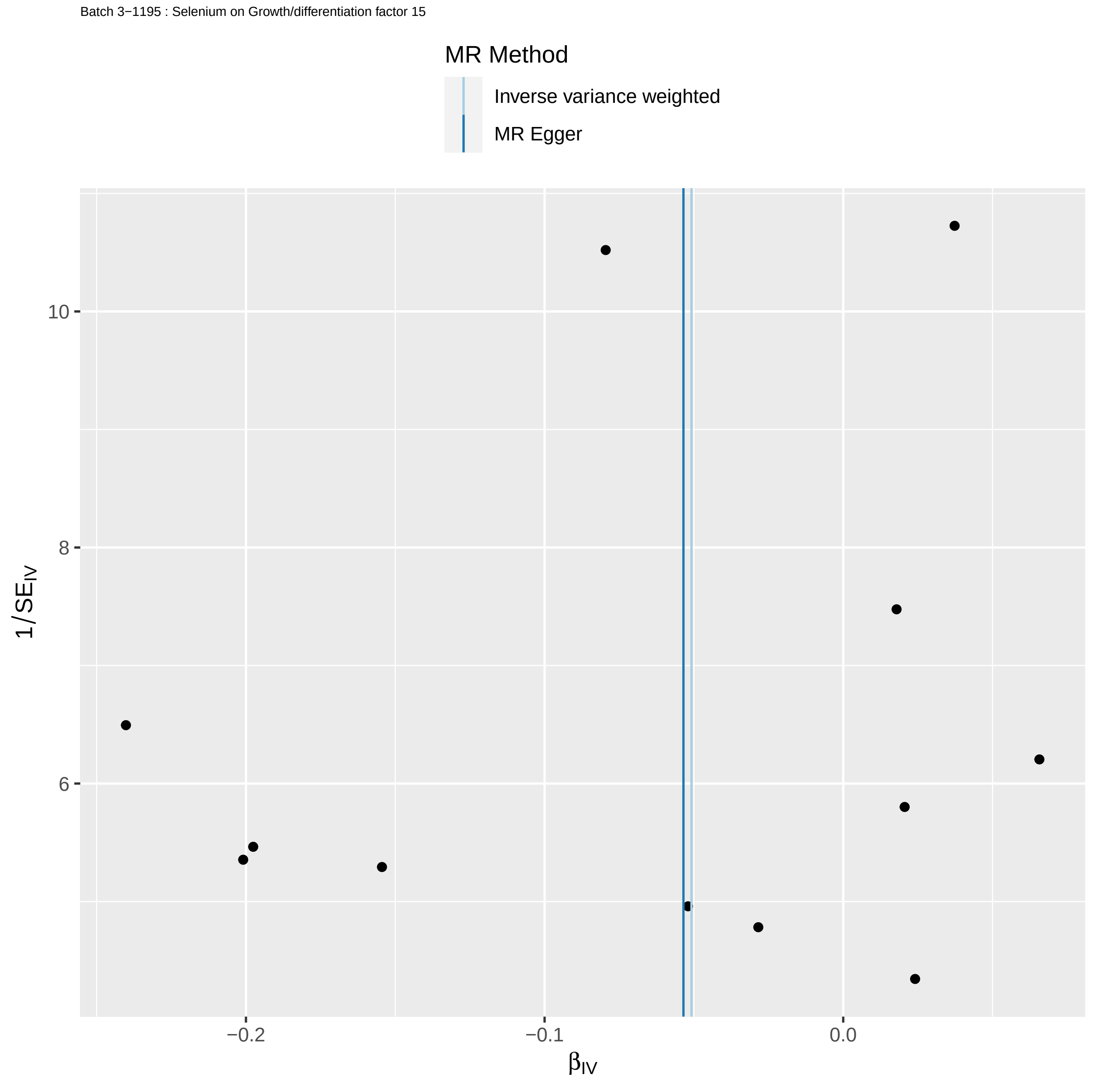
Batch 3−1195 : Selenium on Growth/differentiation factor 15
MR Method
Inverse variance weighted
MR Egger
10
8
6
1/SE_IV
−0.2
−0.1
0.0
β_IV

Batch 3−1196 : Selenium on Growth/differentiation factor 5

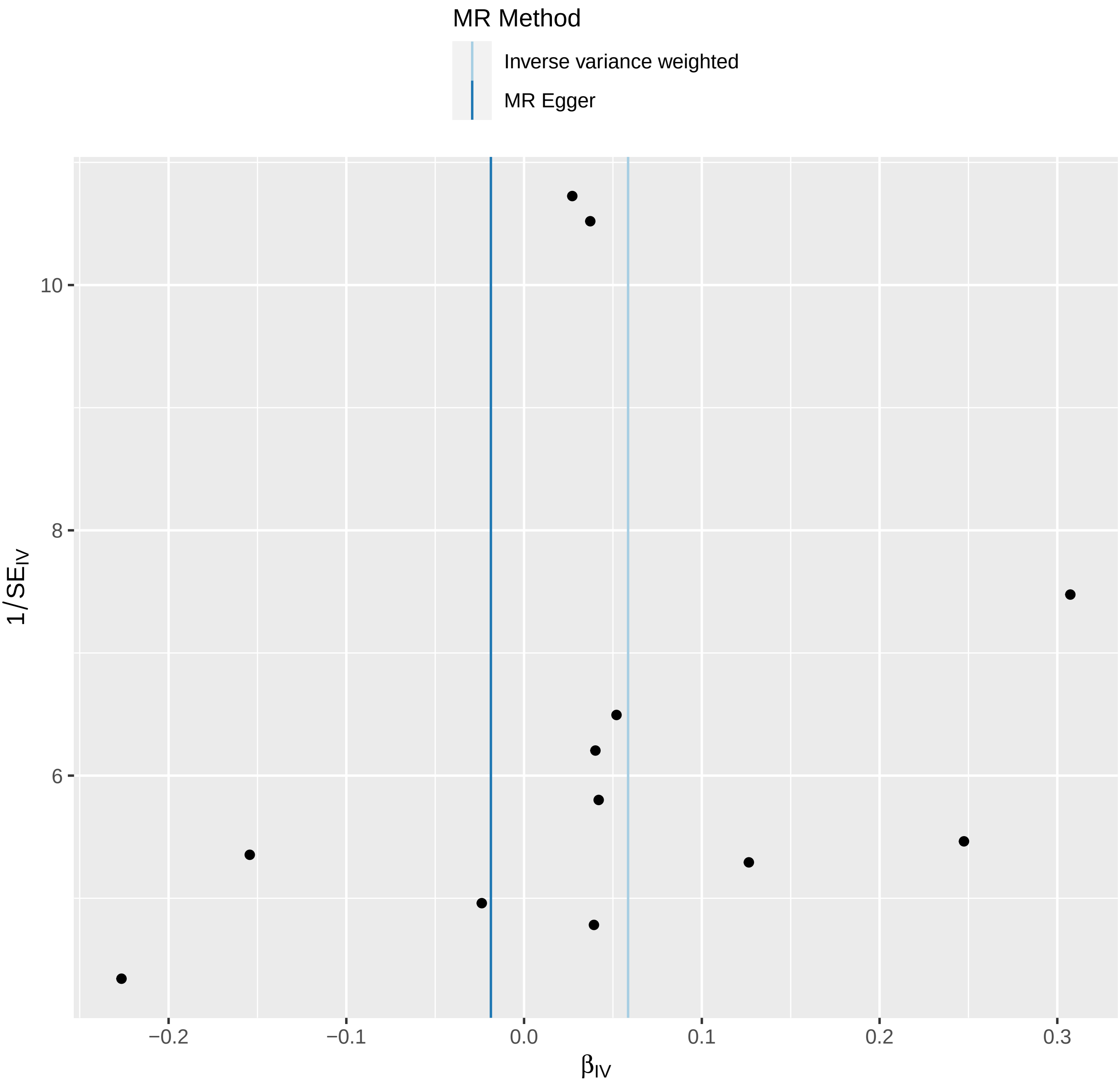

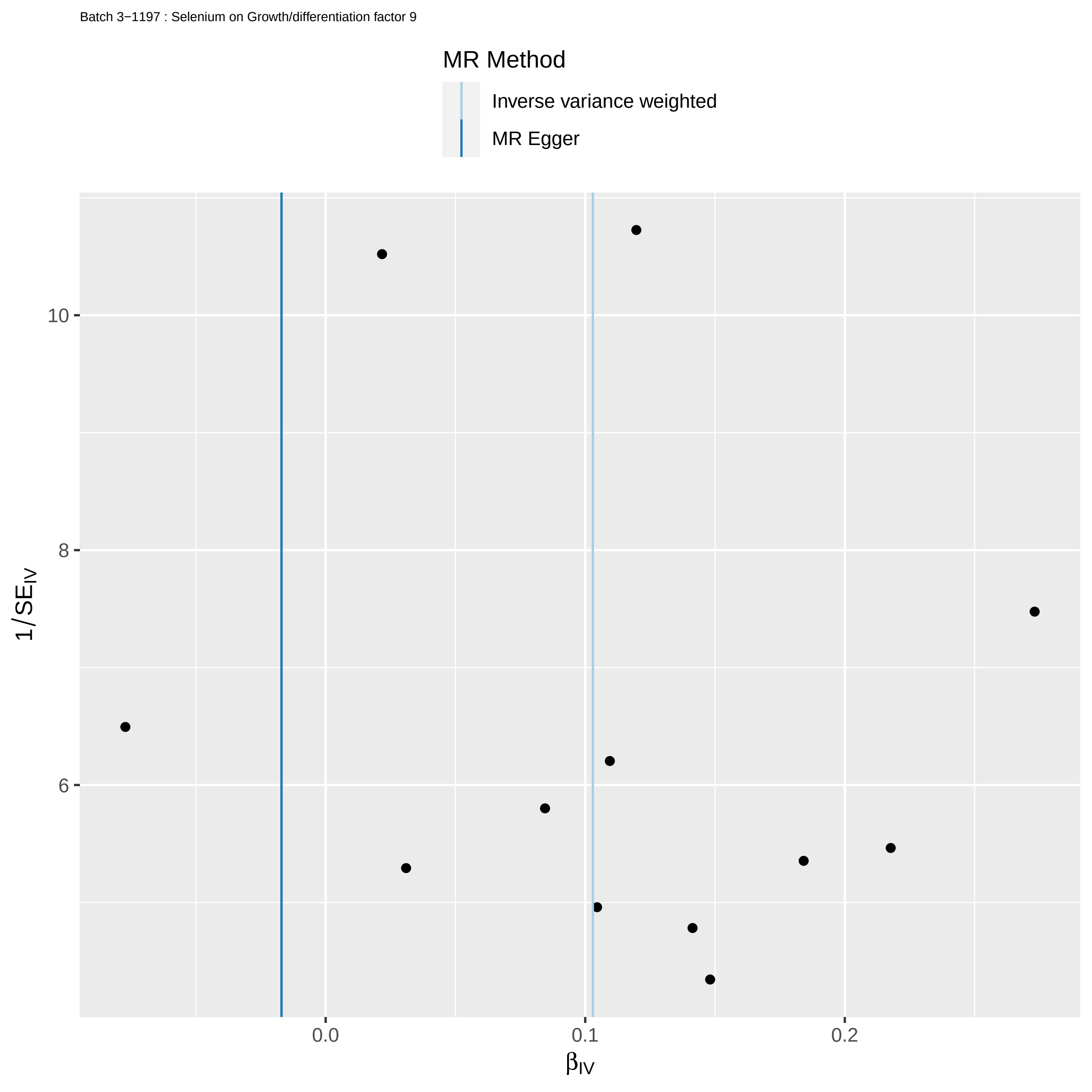

Batch 3−1197 : Selenium on Growth/differentiation factor 9
MR Method
Inverse variance weighted
MR Egger
10
8
6
1/SE_IV
0.0
0.1
0.2
β_IV

Batch 3−1198 : Selenium on Glial cell line−derived neurotrophic factor

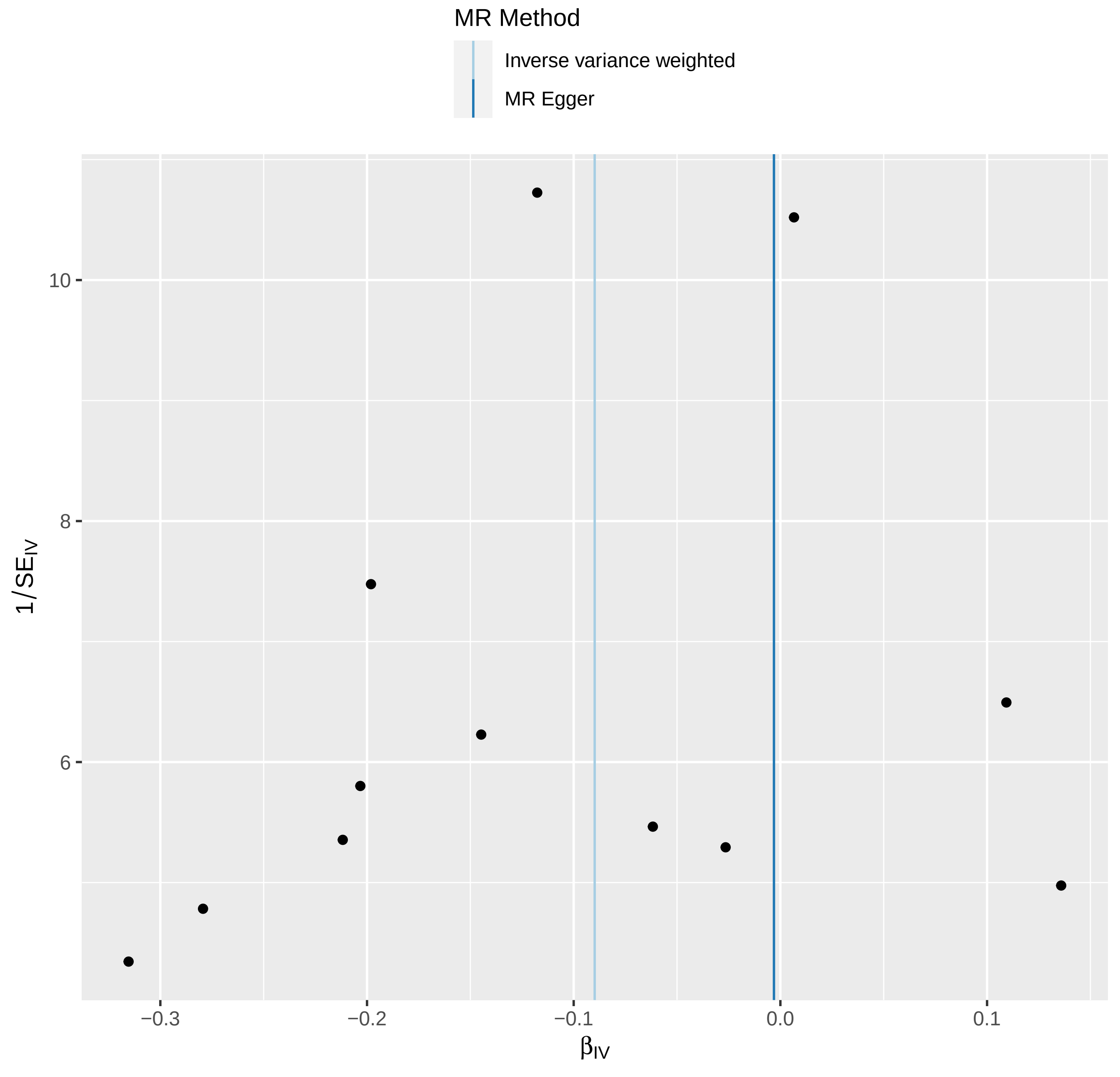

Batch 3−1199 : Selenium on GTP−binding protein GEM

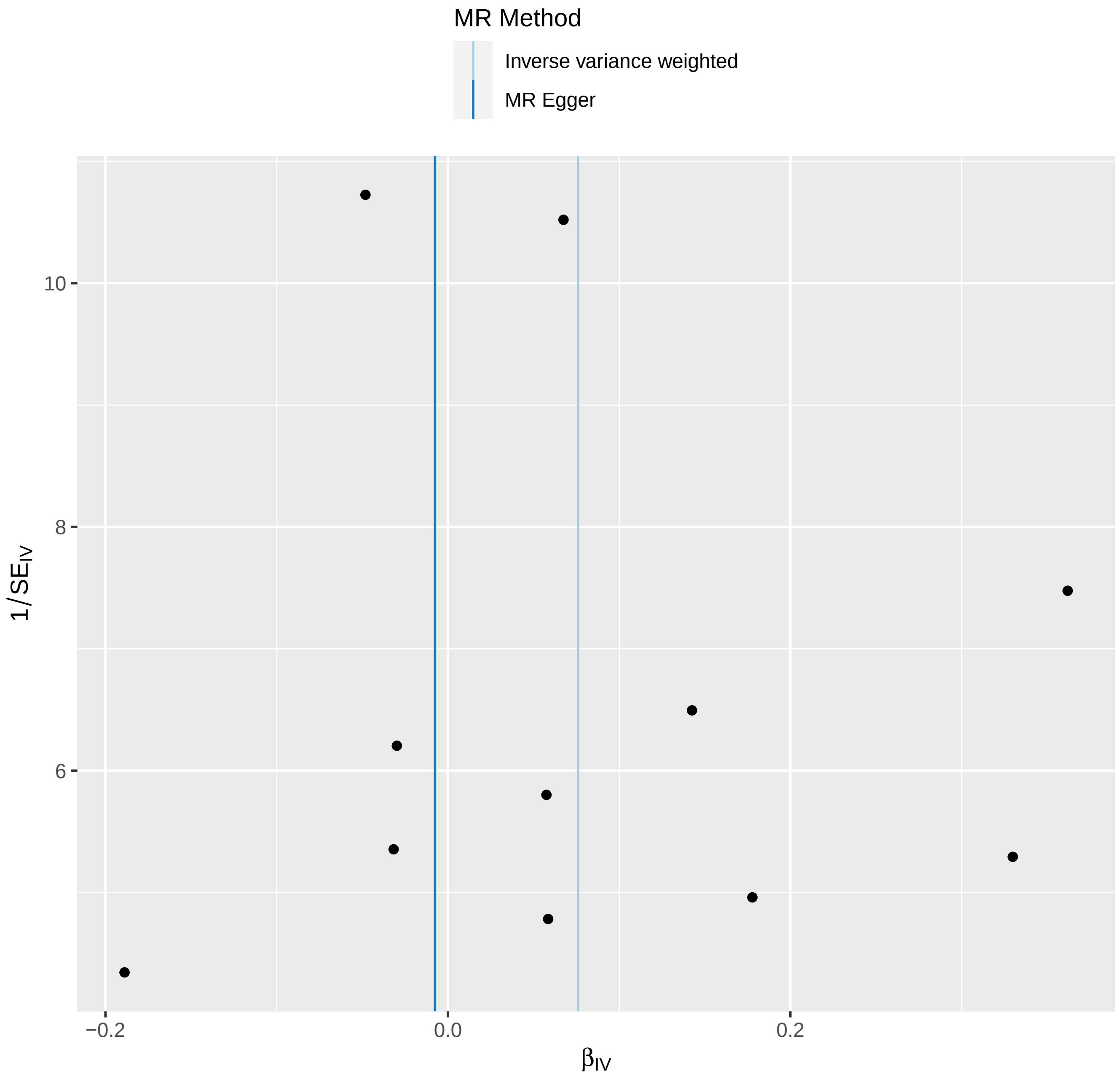

Batch 3−1200 : Selenium on Glial fibrillary acidic protein

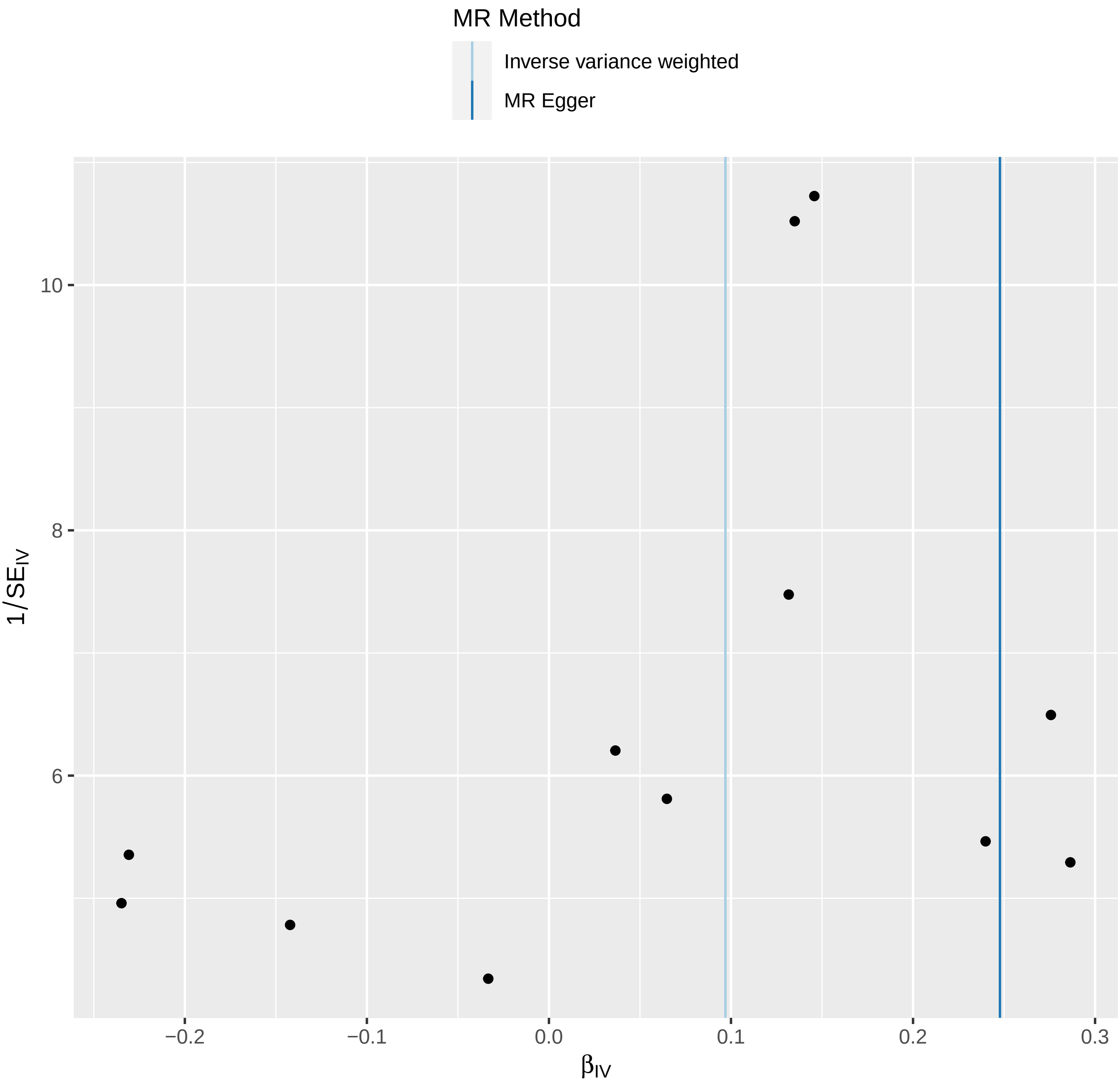

Batch 3−1201 : Selenium on GDNF family receptor alpha−1

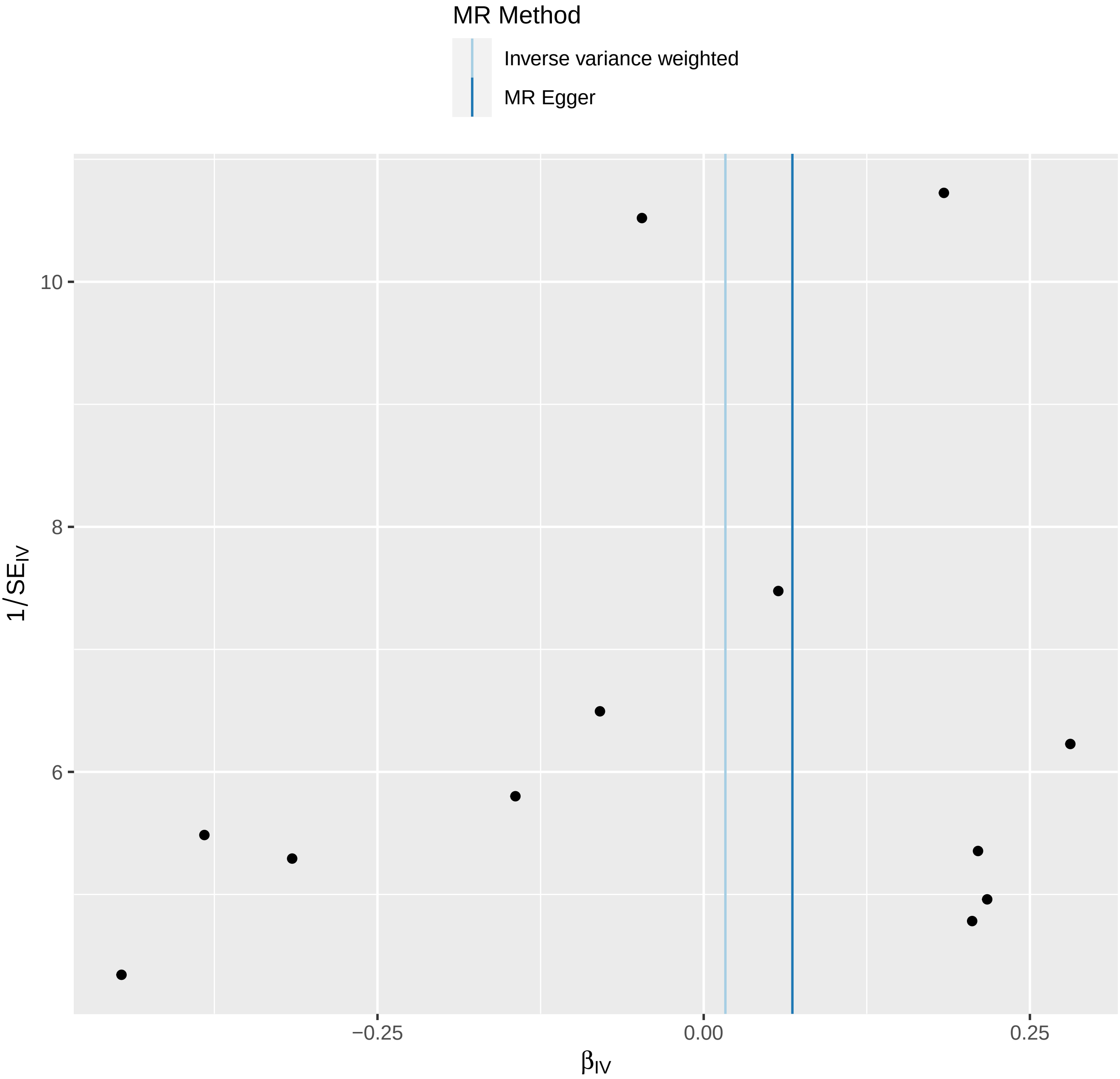

Batch 3−1202 : Selenium on GDNF family receptor alpha−2

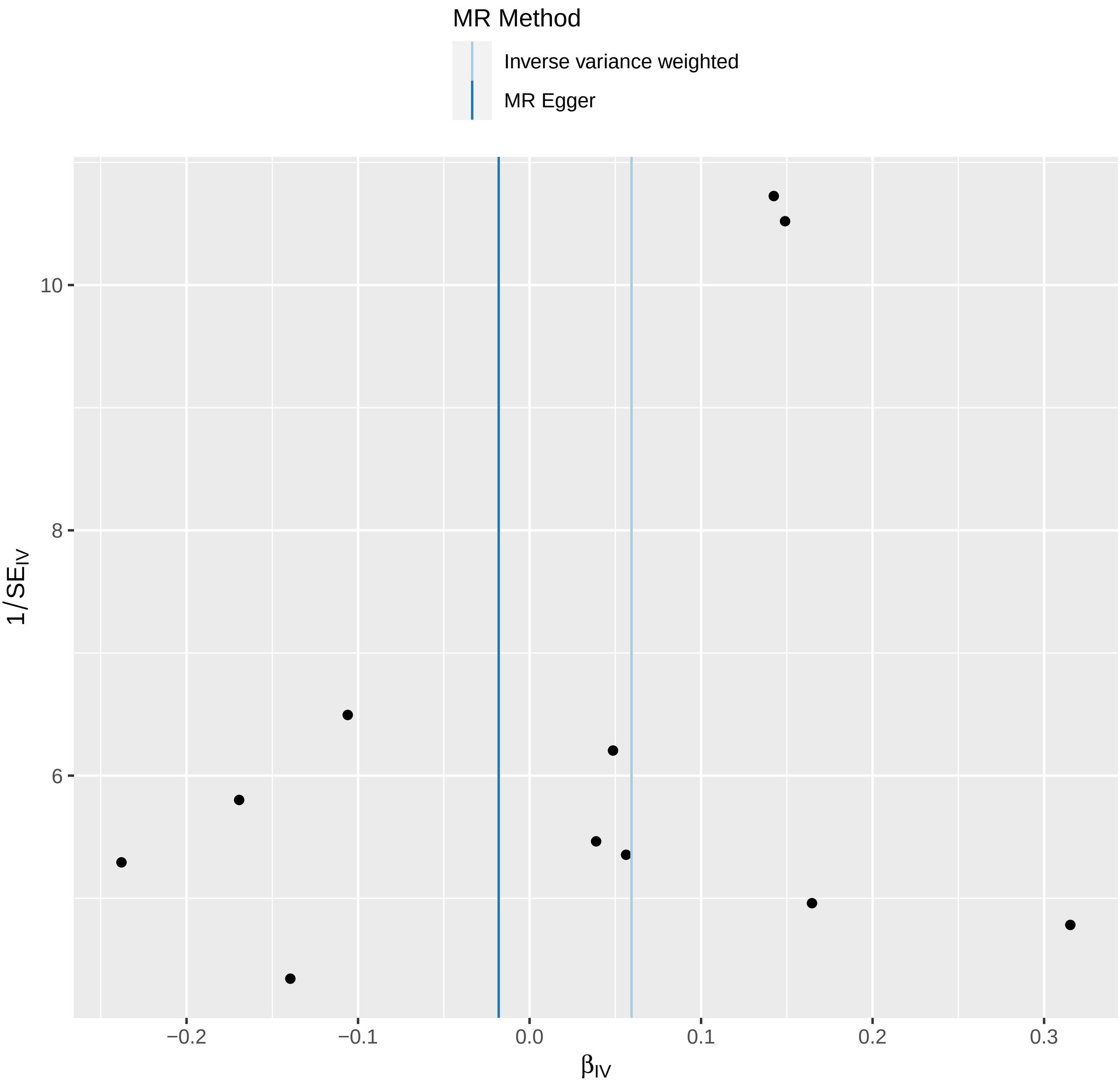

Batch 3−1203 : Selenium on GDNF family receptor alpha−3

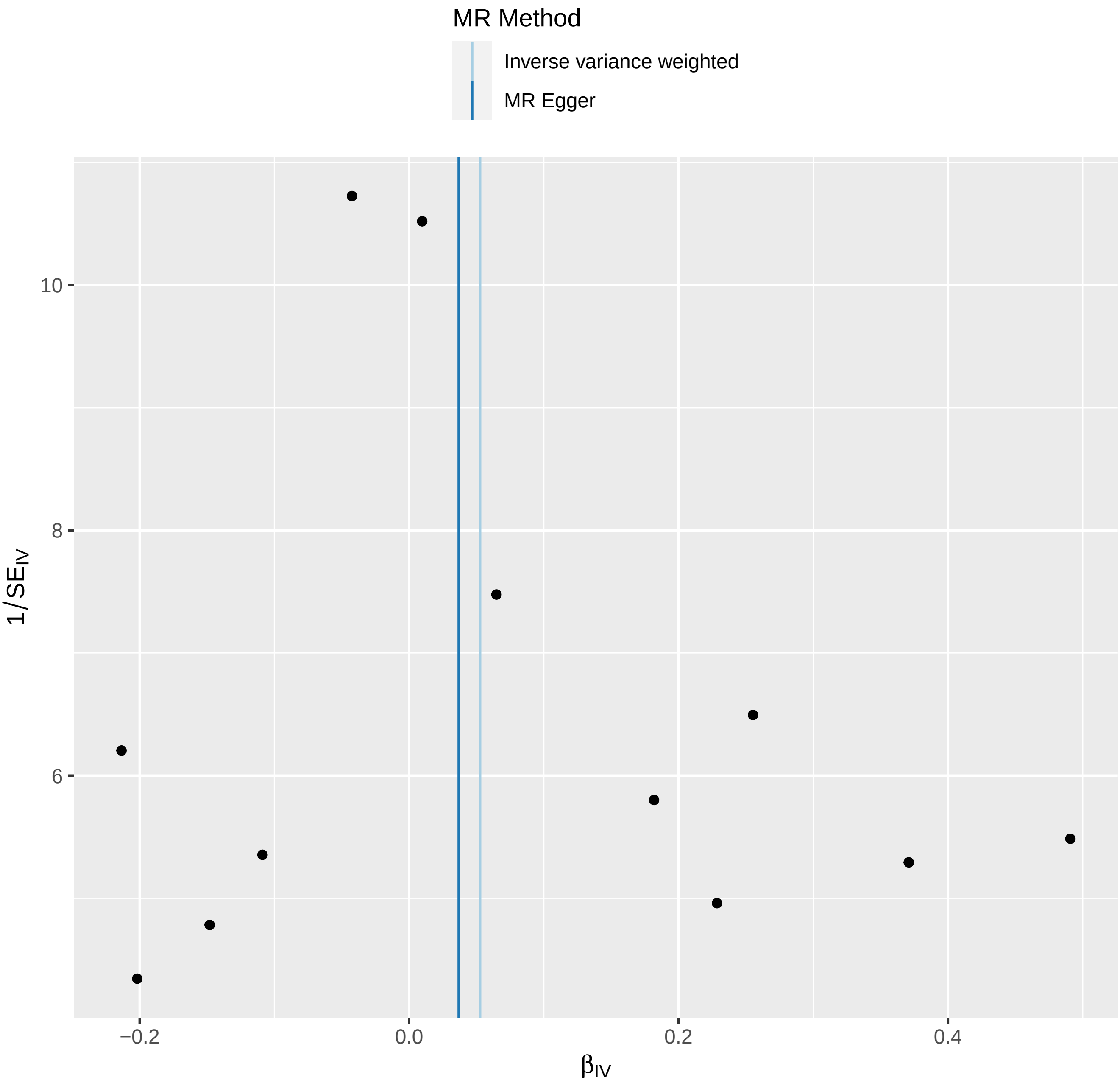

Batch 3−1204 : Selenium on GDNF family receptor alpha−like

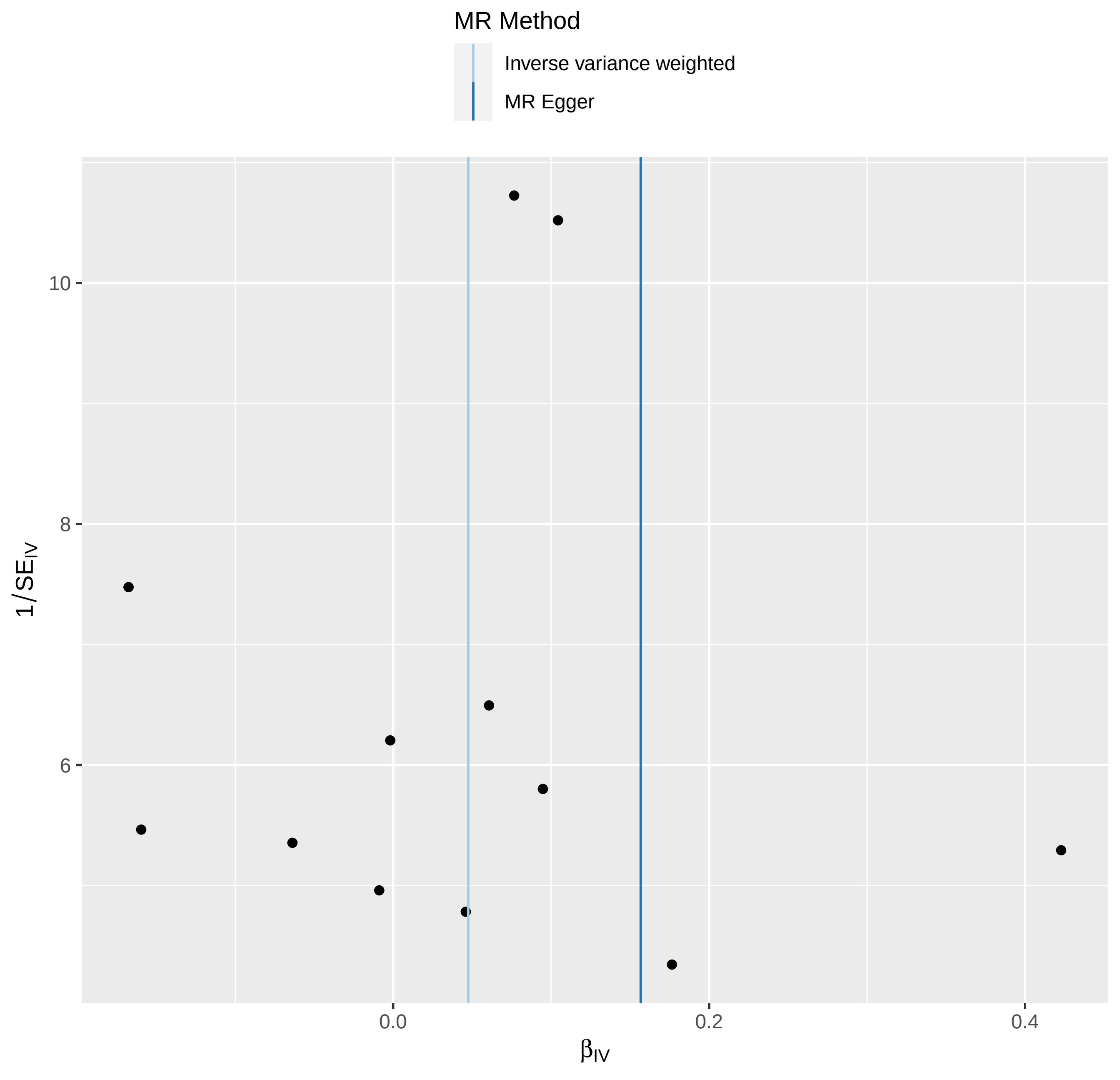

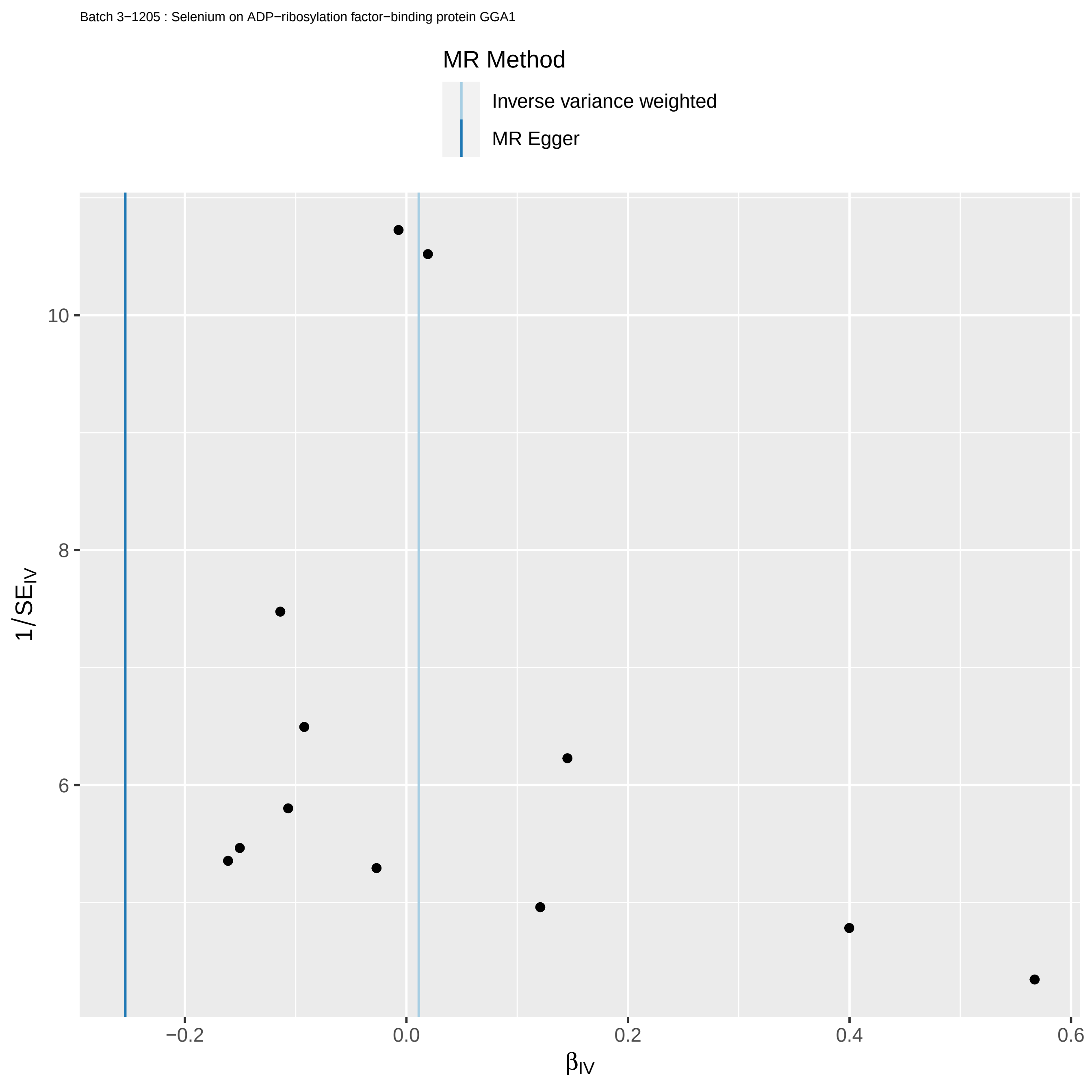
Batch 3−1205 : Selenium on ADP−ribosylation factor−binding protein GGA1
MR Method
Inverse variance weighted
MR Egger
10
8
6
1/SE_IV
−0.2
0.0
0.2
0.4
0.6
β_IV

Batch 3−1206 : Selenium on ADP−ribosylation factor−binding protein GGA3

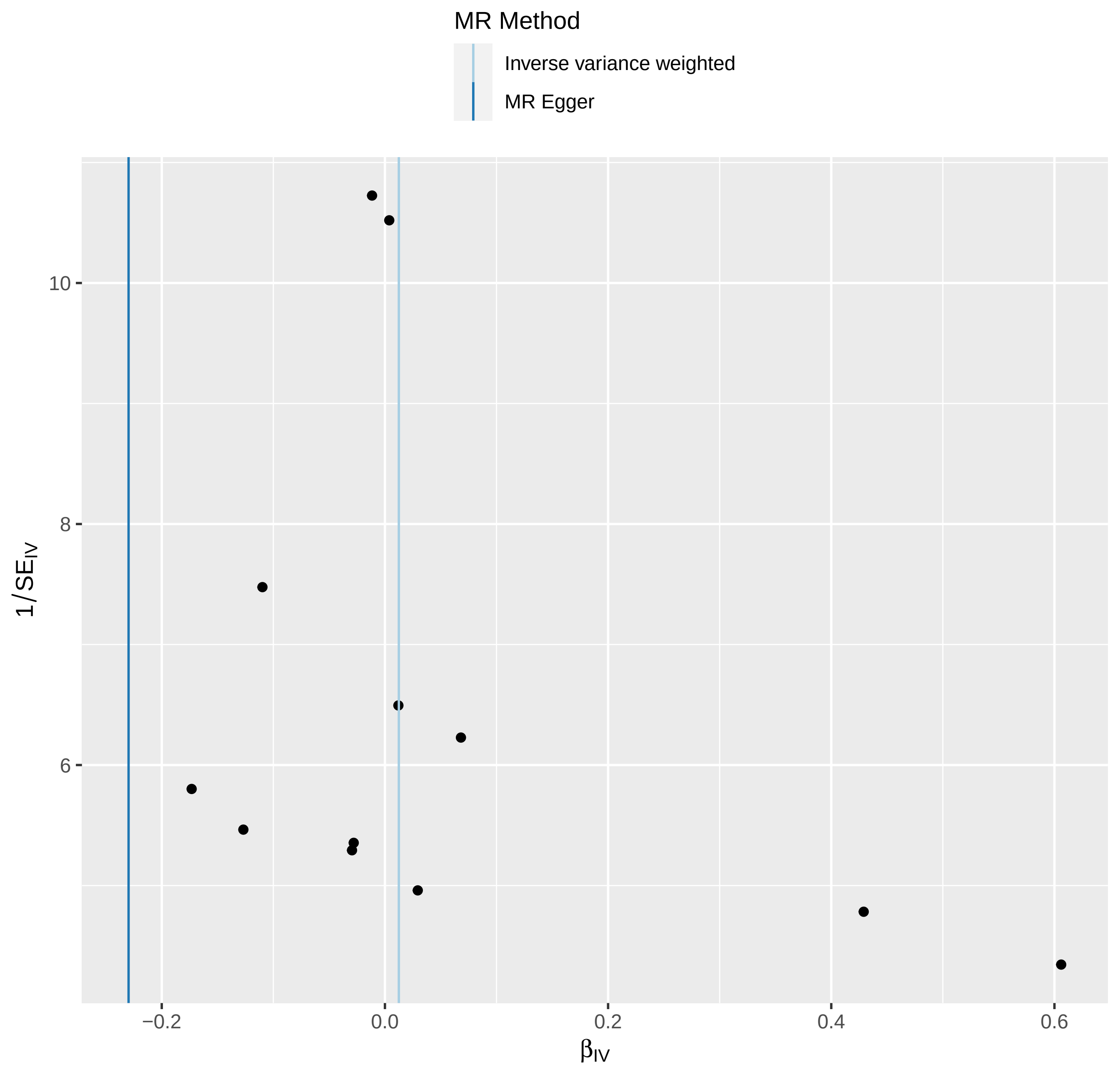

Batch 3−1207 : Selenium on Gamma−glutamyl hydrolase

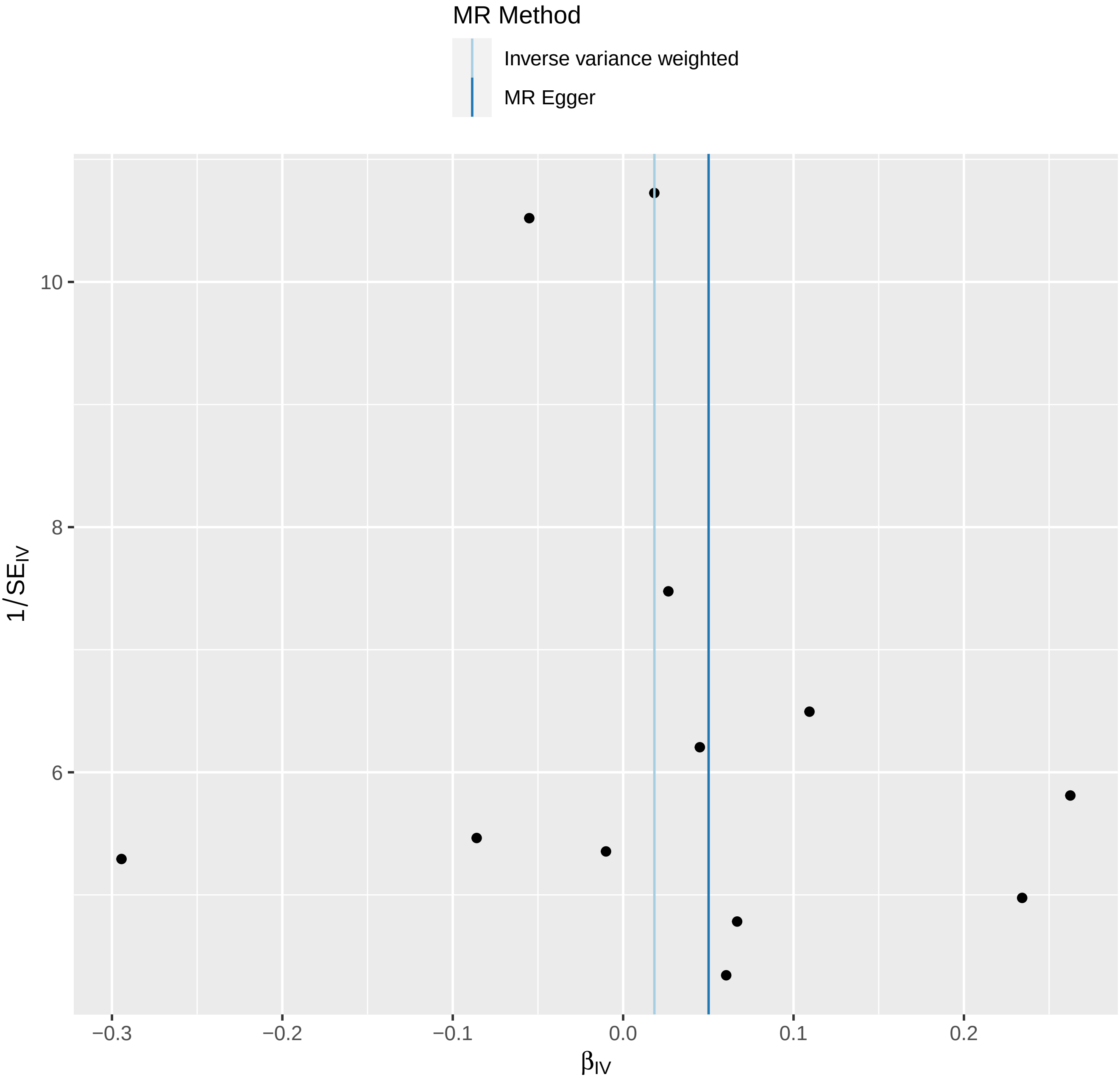

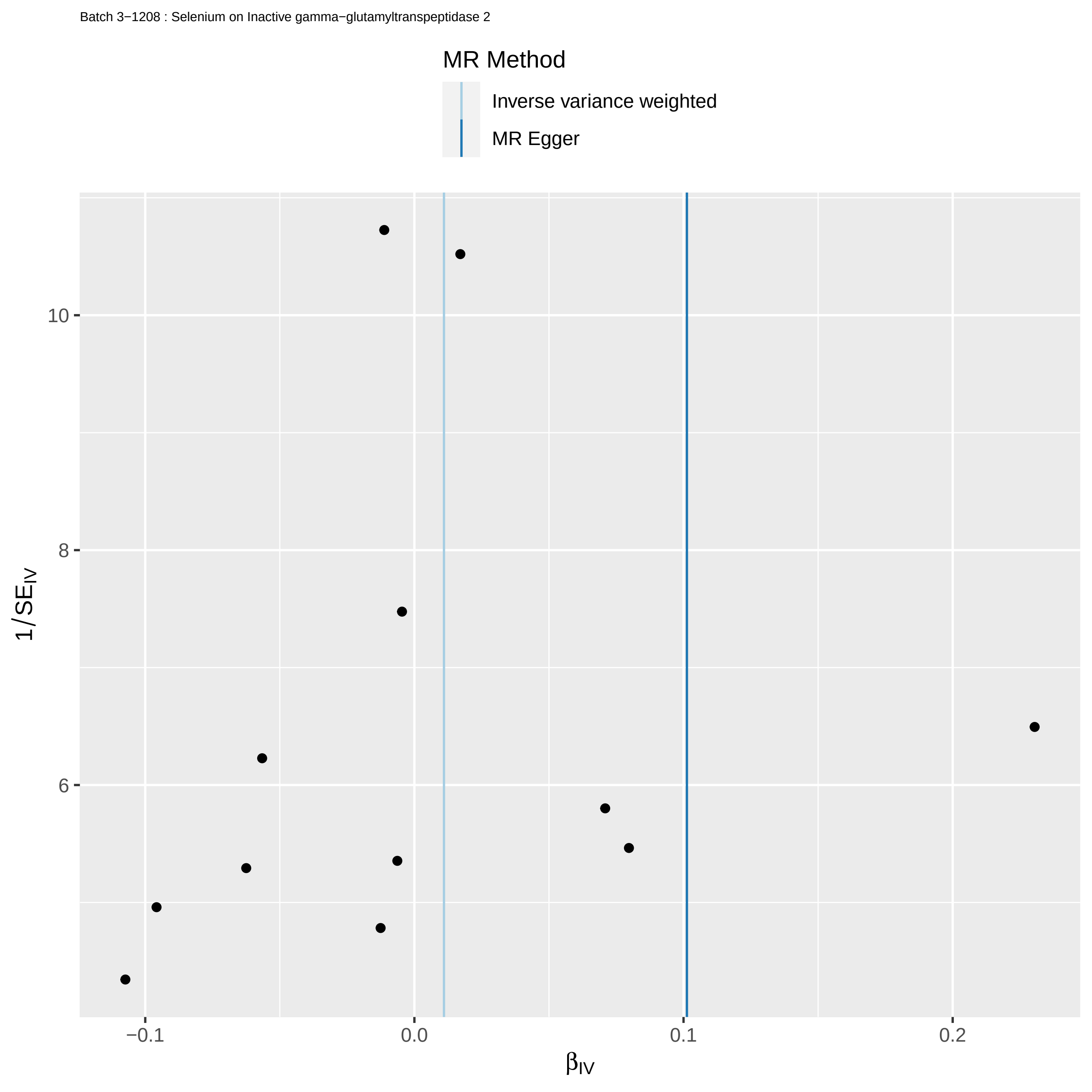

Batch 3−1208 : Selenium on Inactive gamma−glutamyltranspeptidase 2
MR Method
Inverse variance weighted
MR Egger
10
8
6
1/SE_IV
−0.1
0.0
0.1
0.2
β_IV

Batch 3−1209 : Selenium on Somatotropin

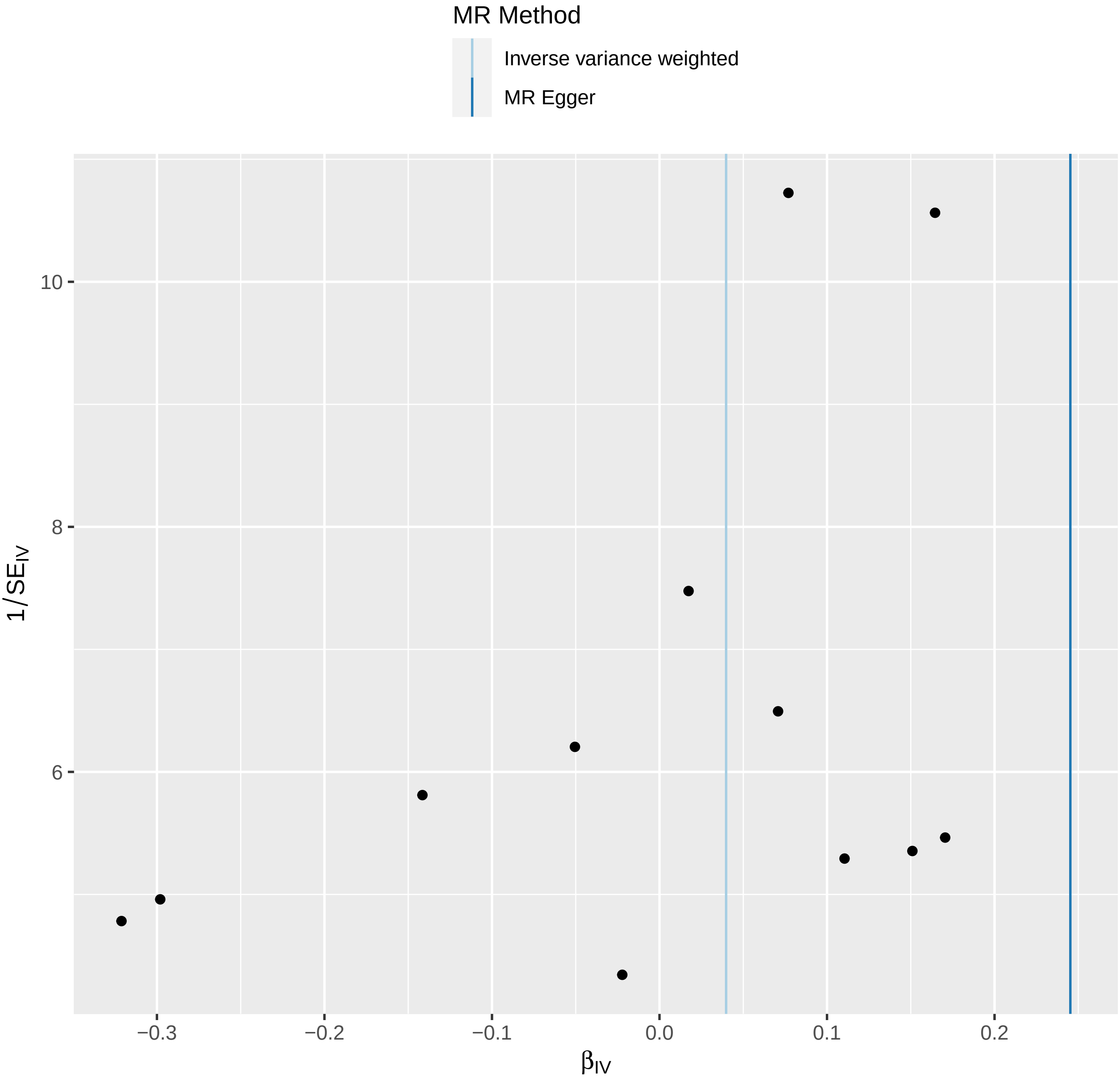

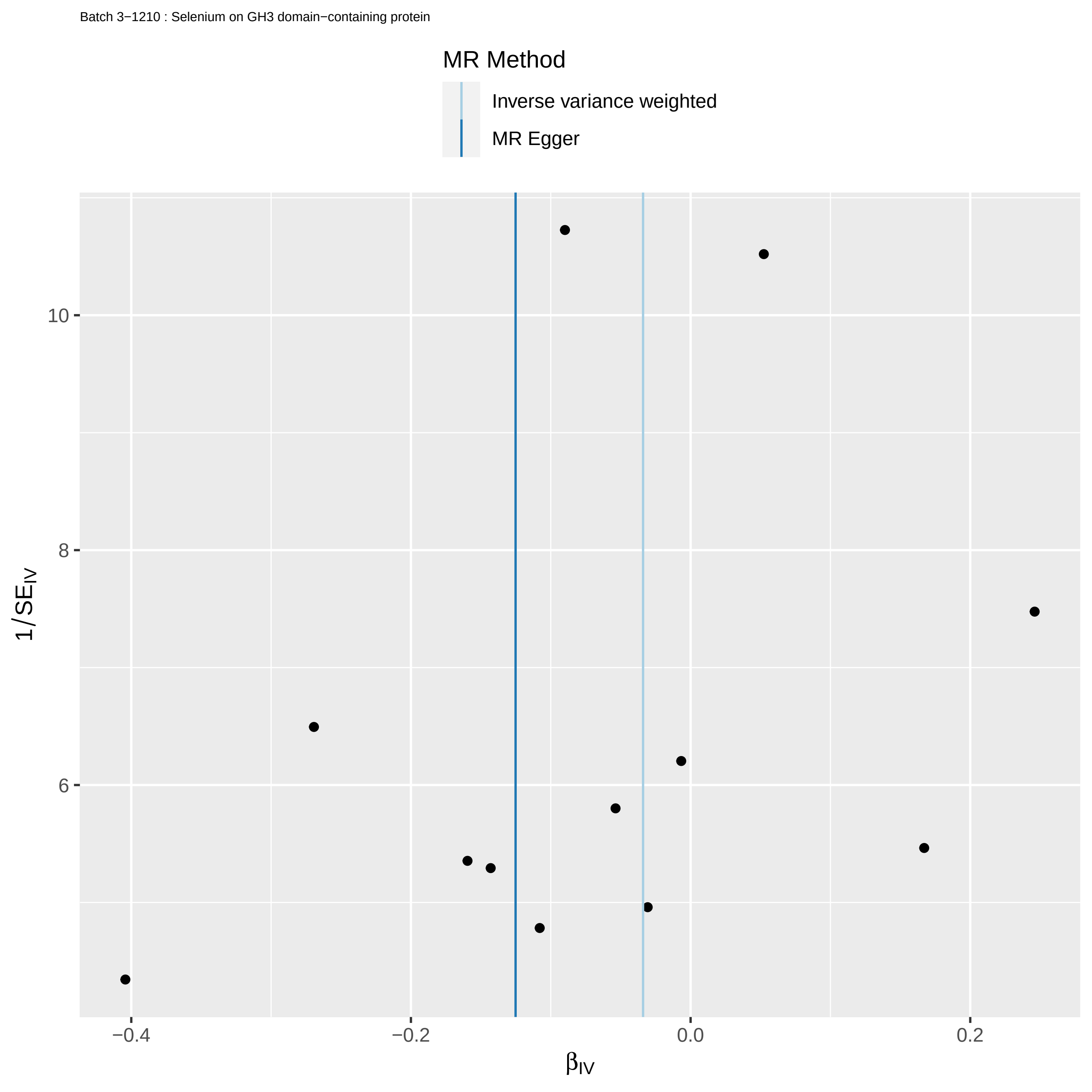
Batch 3−1210 : Selenium on GH3 domain−containing protein
MR Method
Inverse variance weighted
MR Egger
10
8
6
$1/SE_{IV}$
−0.4
−0.2
0.0
0.2
β_{IV}

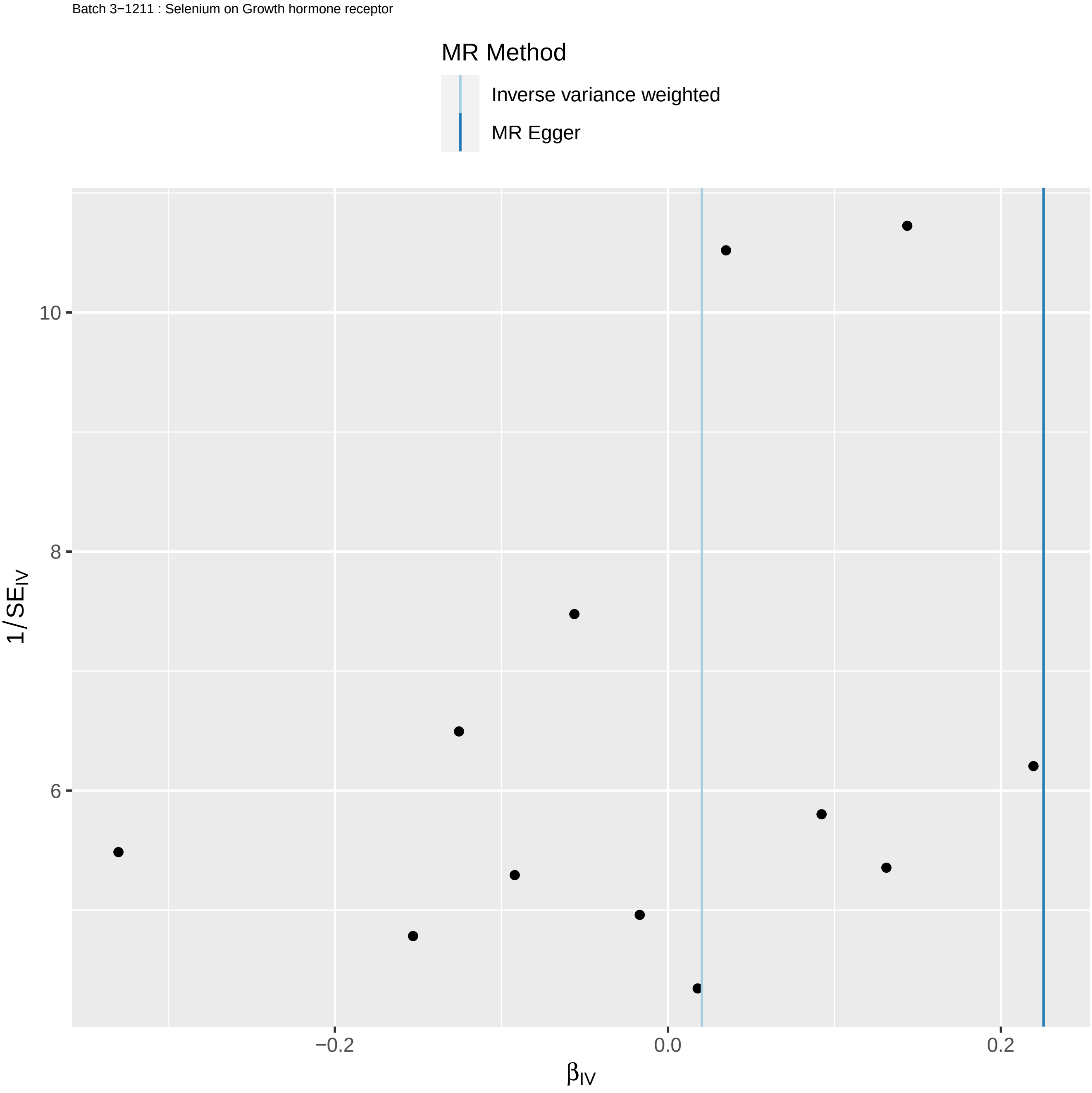

Batch 3−1211 : Selenium on Growth hormone receptor
MR Method
Inverse variance weighted
MR Egger
10
8
6
1/SE$_{IV}$
−0.2
0.0
0.2
β$_{IV}$

Batch 3−1212 : Selenium on Appetite−regulating hormone

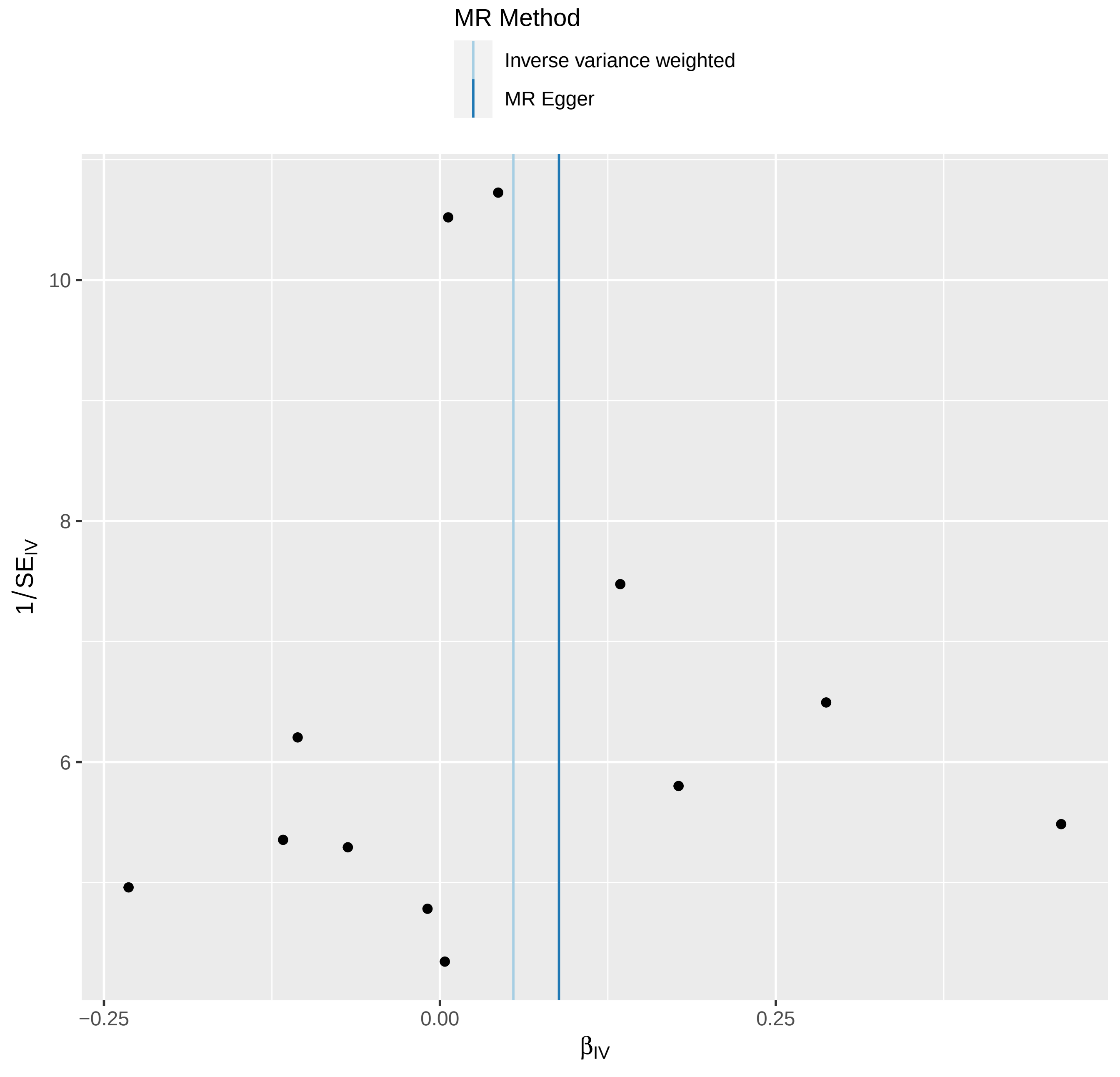

Batch 3−1213 : Selenium on Gap junction alpha−1 protein

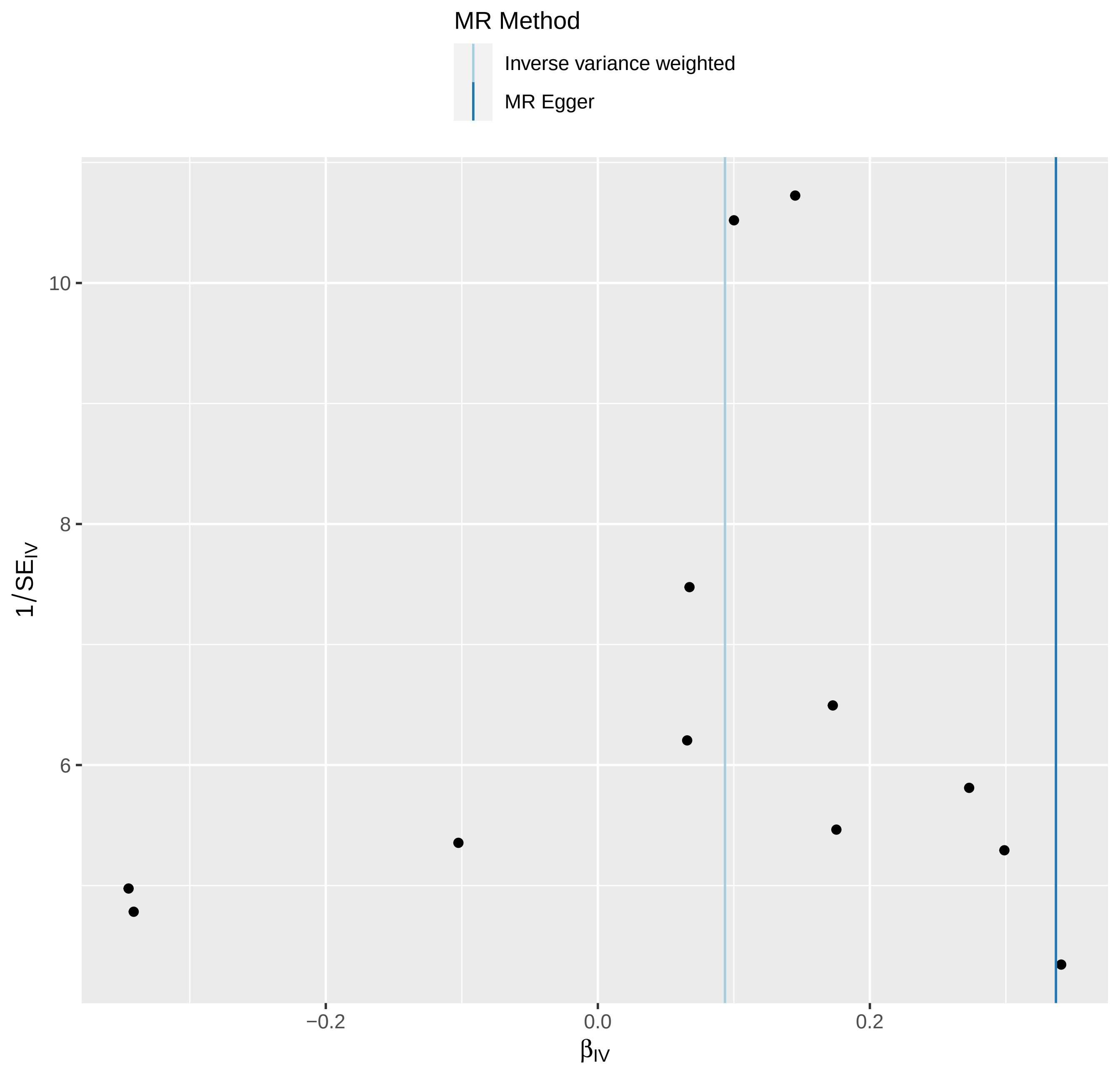

Batch 3−1214 : Selenium on Gap junction alpha−8 protein

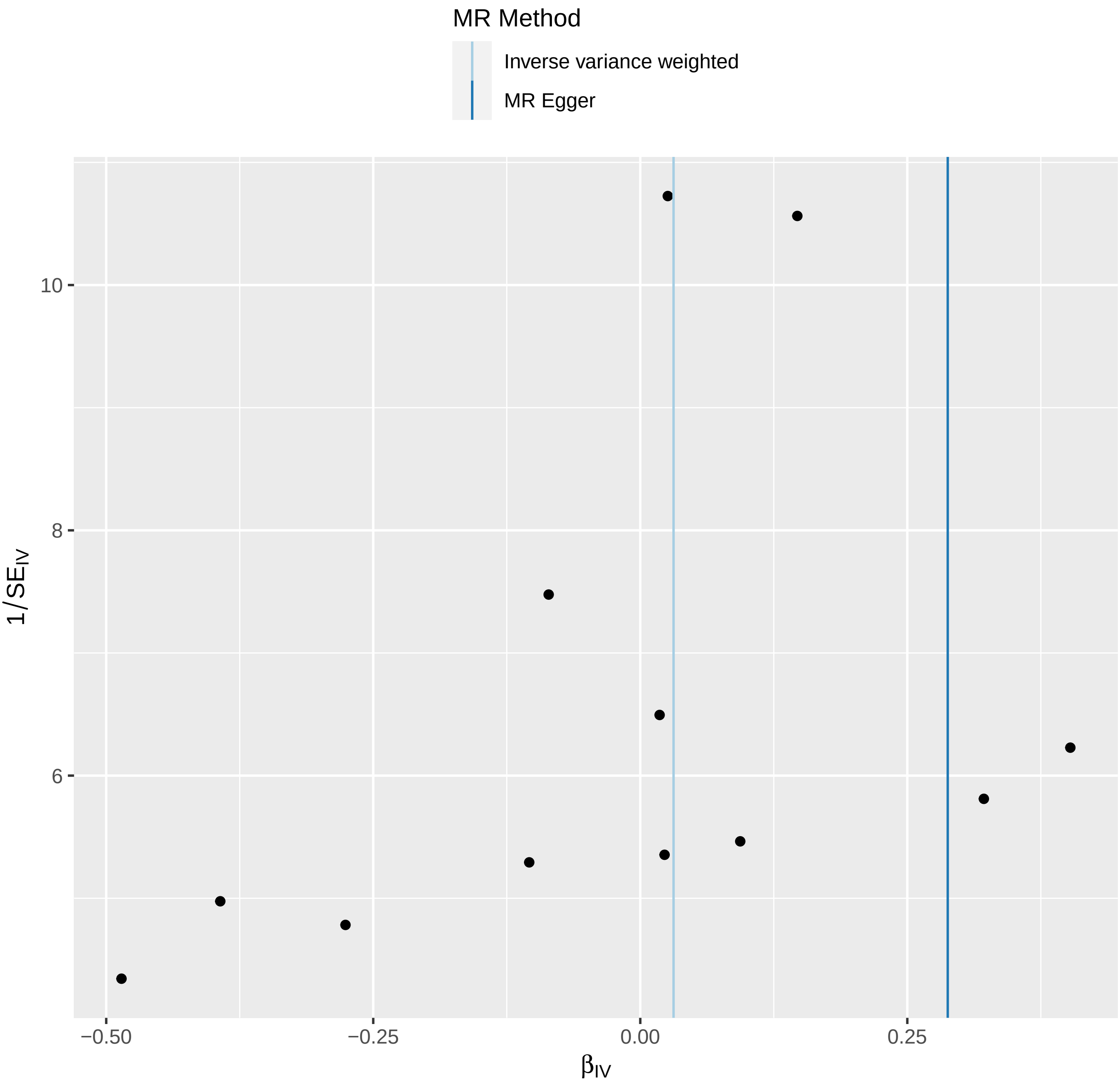

Batch 3−1215 : Selenium on Gap junction delta−2 protein

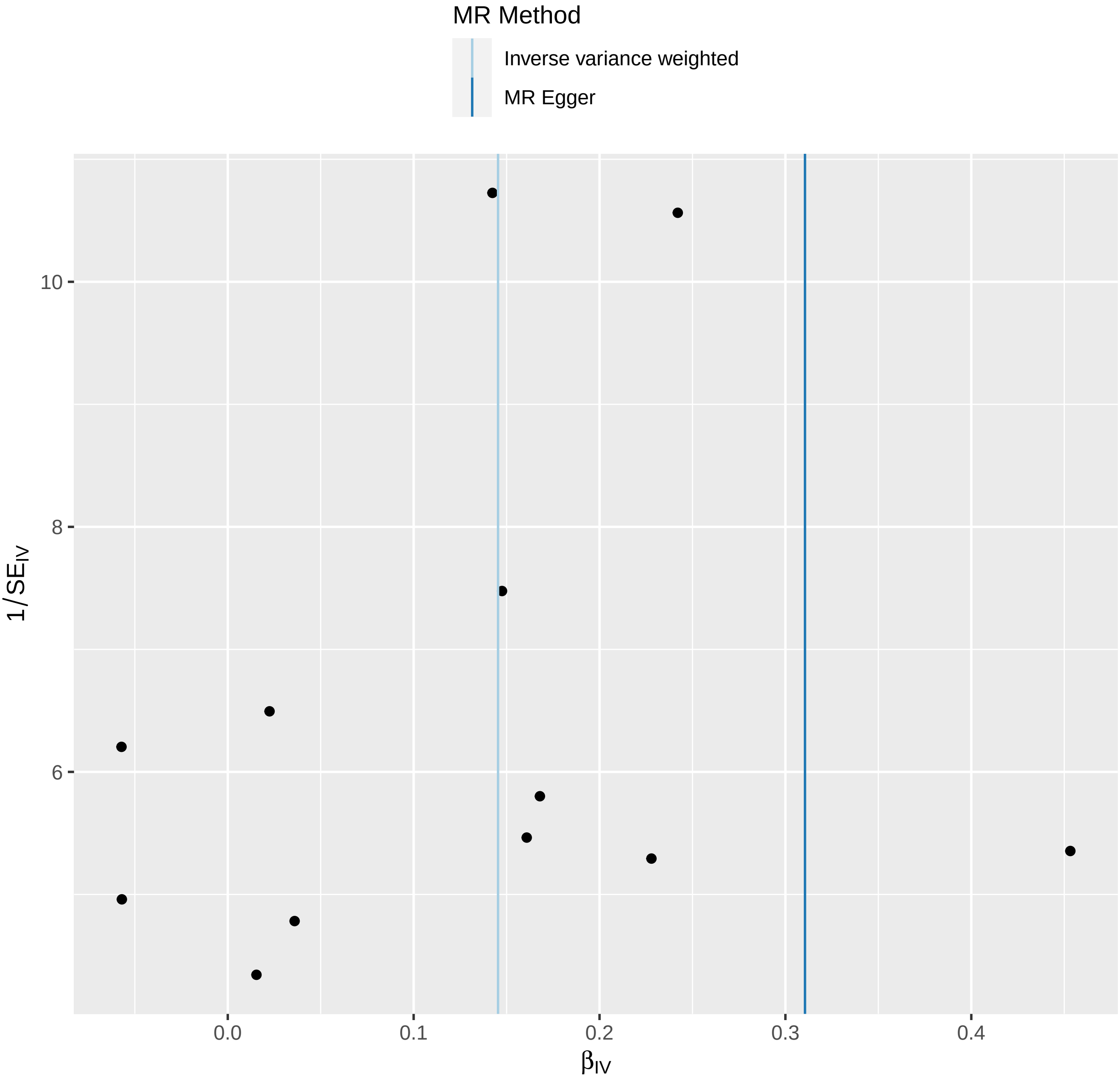

Batch 3−1216 : Selenium on D−glucuronyl C5−epimerase

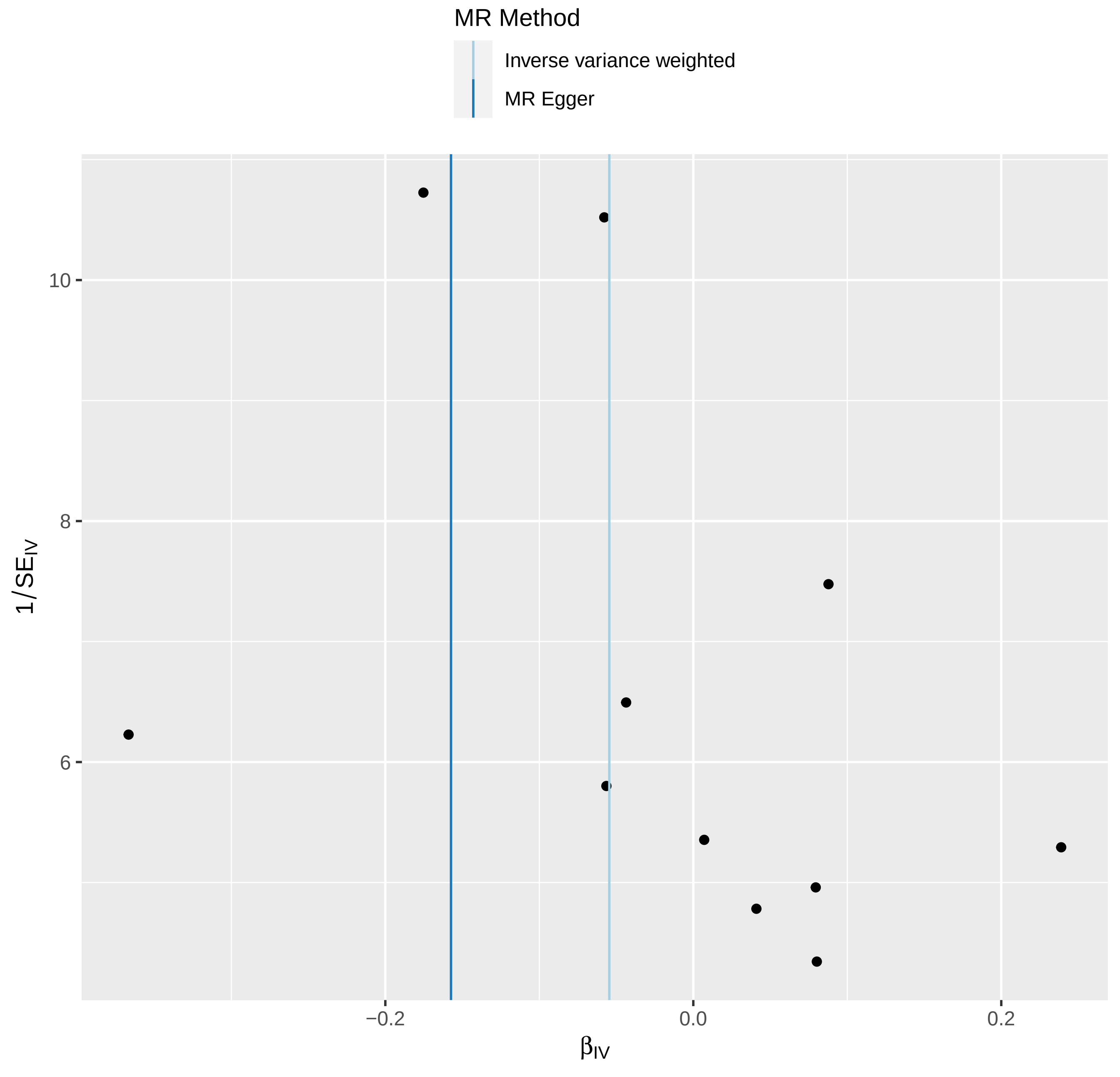

Batch 3−1217 : Selenium on Glioma pathogenesis−related protein 1

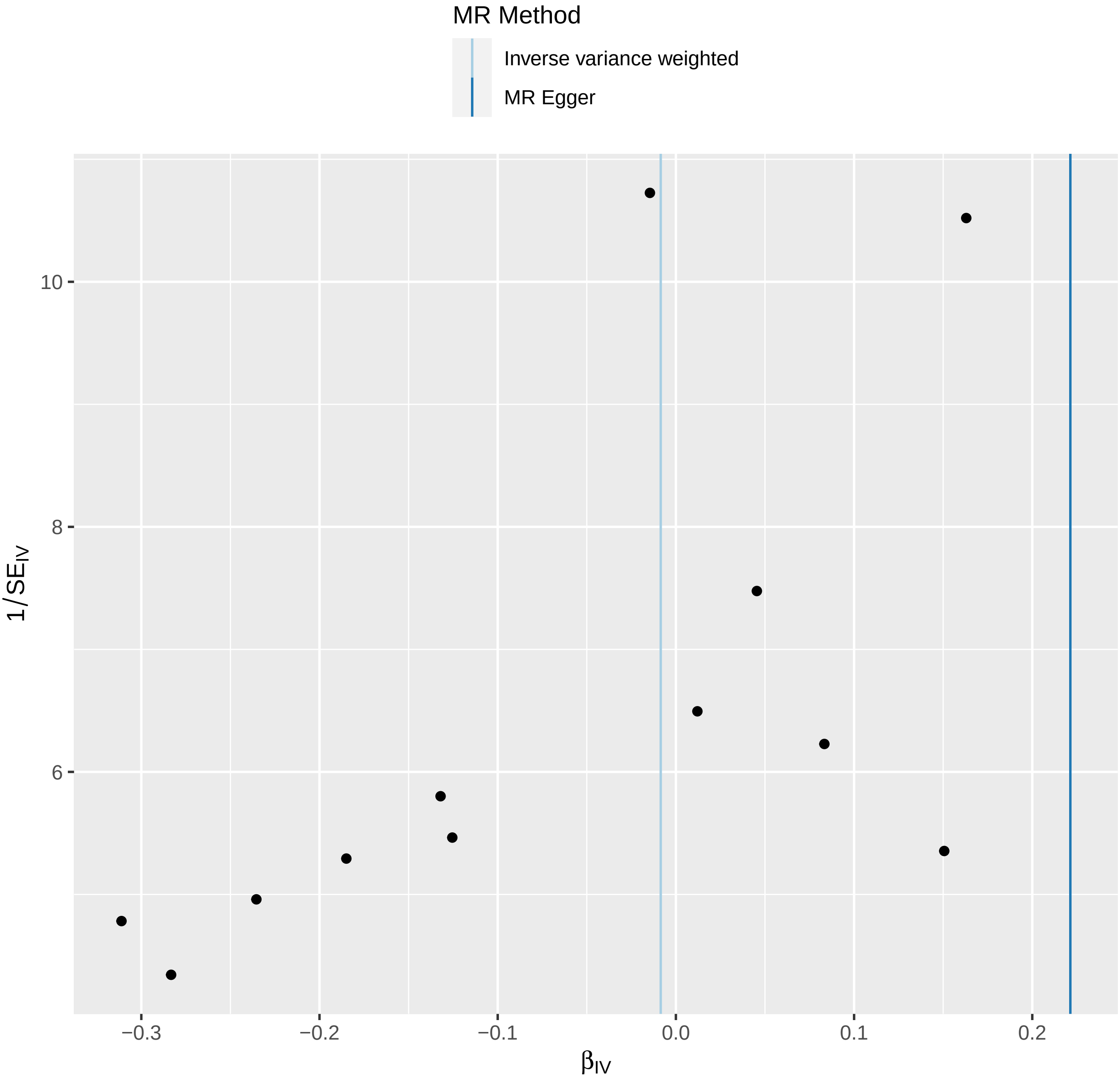

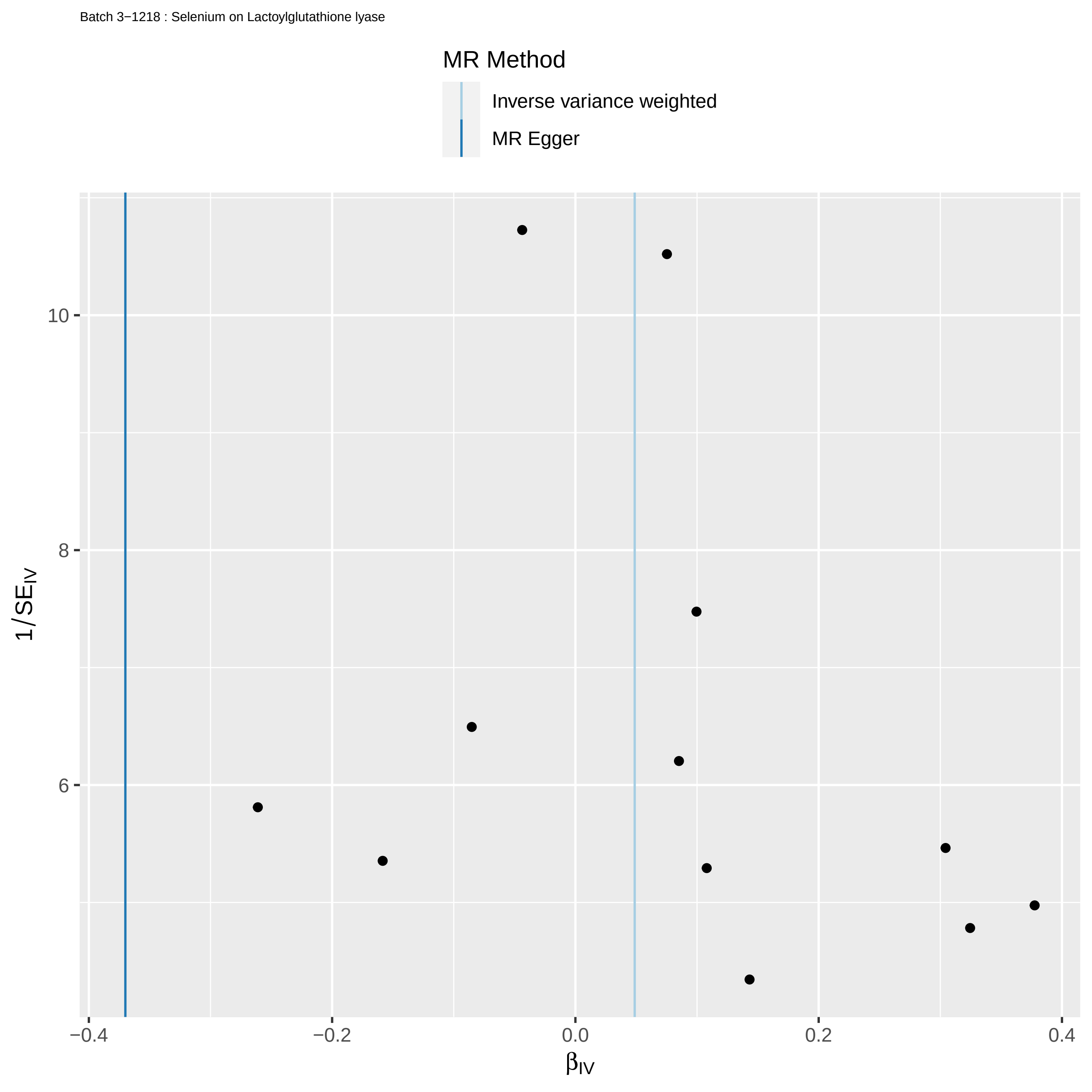
Batch 3−1218 : Selenium on Lactoylglutathione lyase
MR Method
Inverse variance weighted
MR Egger
10
8
6
1/SE$_{IV}$
−0.4
−0.2
0.0
0.2
0.4
β$_{IV}$

Batch 3−1219 : Selenium on Glucagon−like peptide 1 receptor

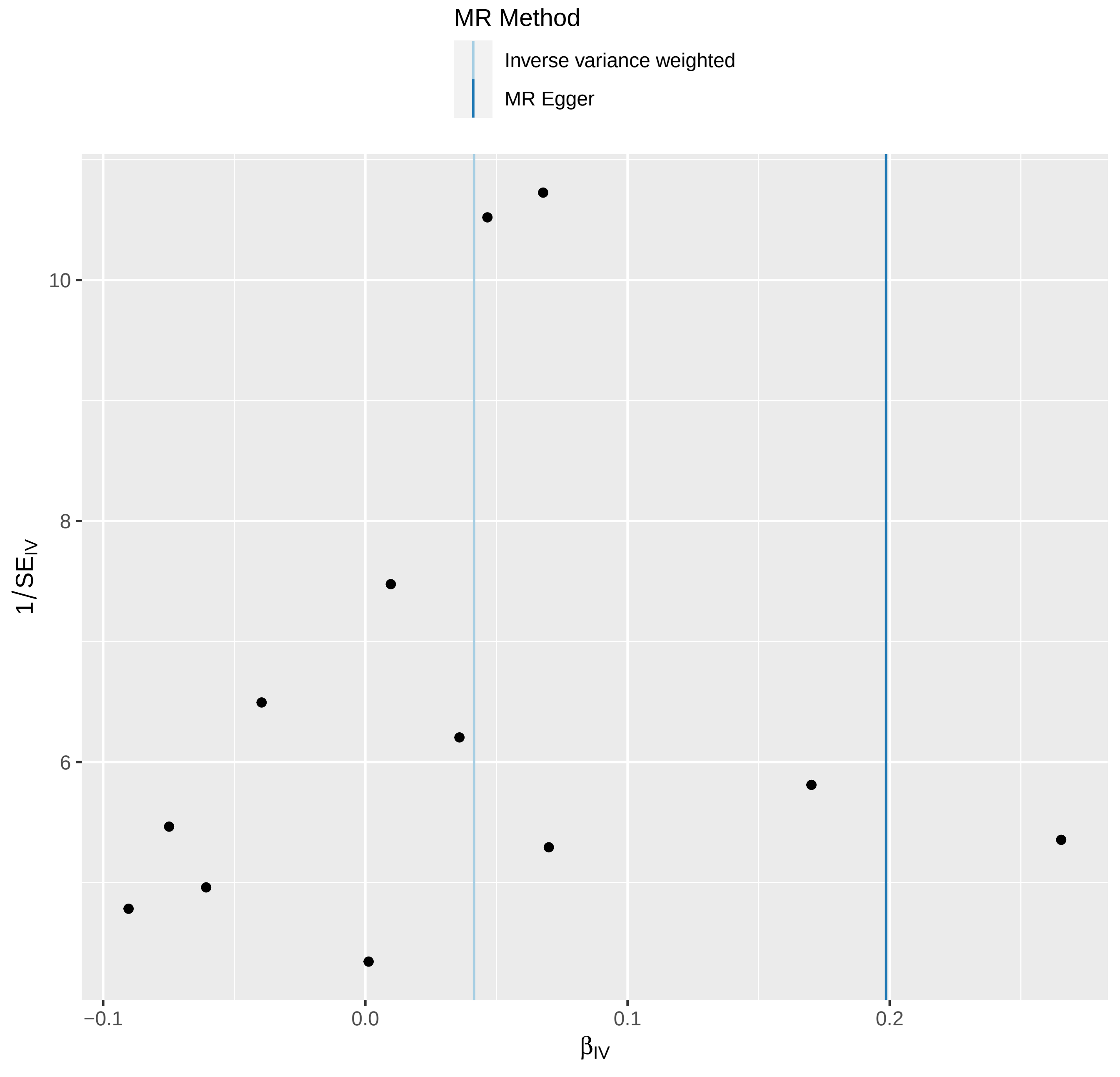

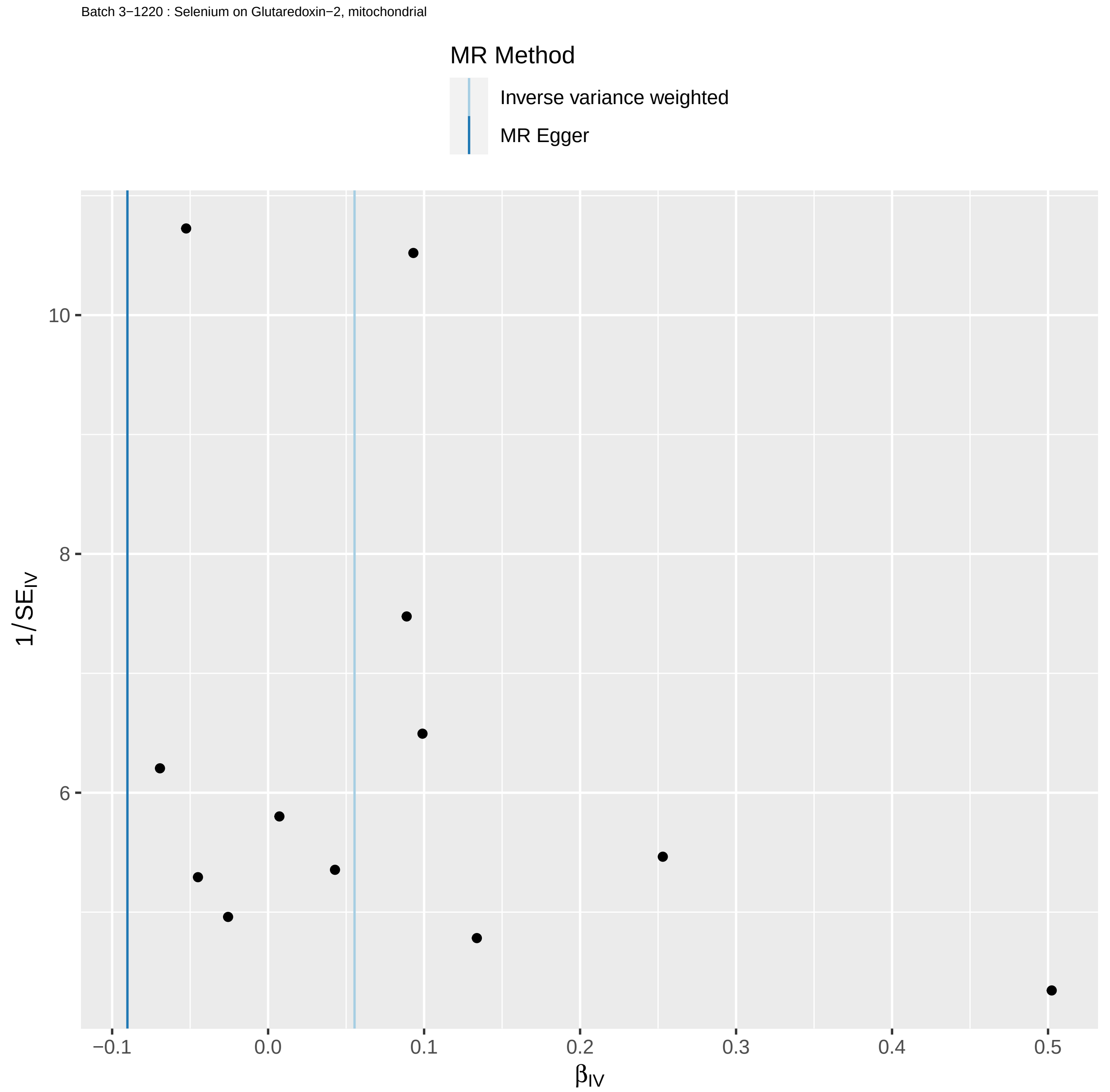

Batch 3−1220 : Selenium on Glutaredoxin−2, mitochondrial
MR Method
Inverse variance weighted
MR Egger
10
8
6
1/SE$_{IV}$
−0.1
0.0
0.1
0.2
0.3
0.4
0.5
β$_{IV}$

Batch 3−1221 : Selenium on Glycosyltransferase 8 domain−containing protein 1

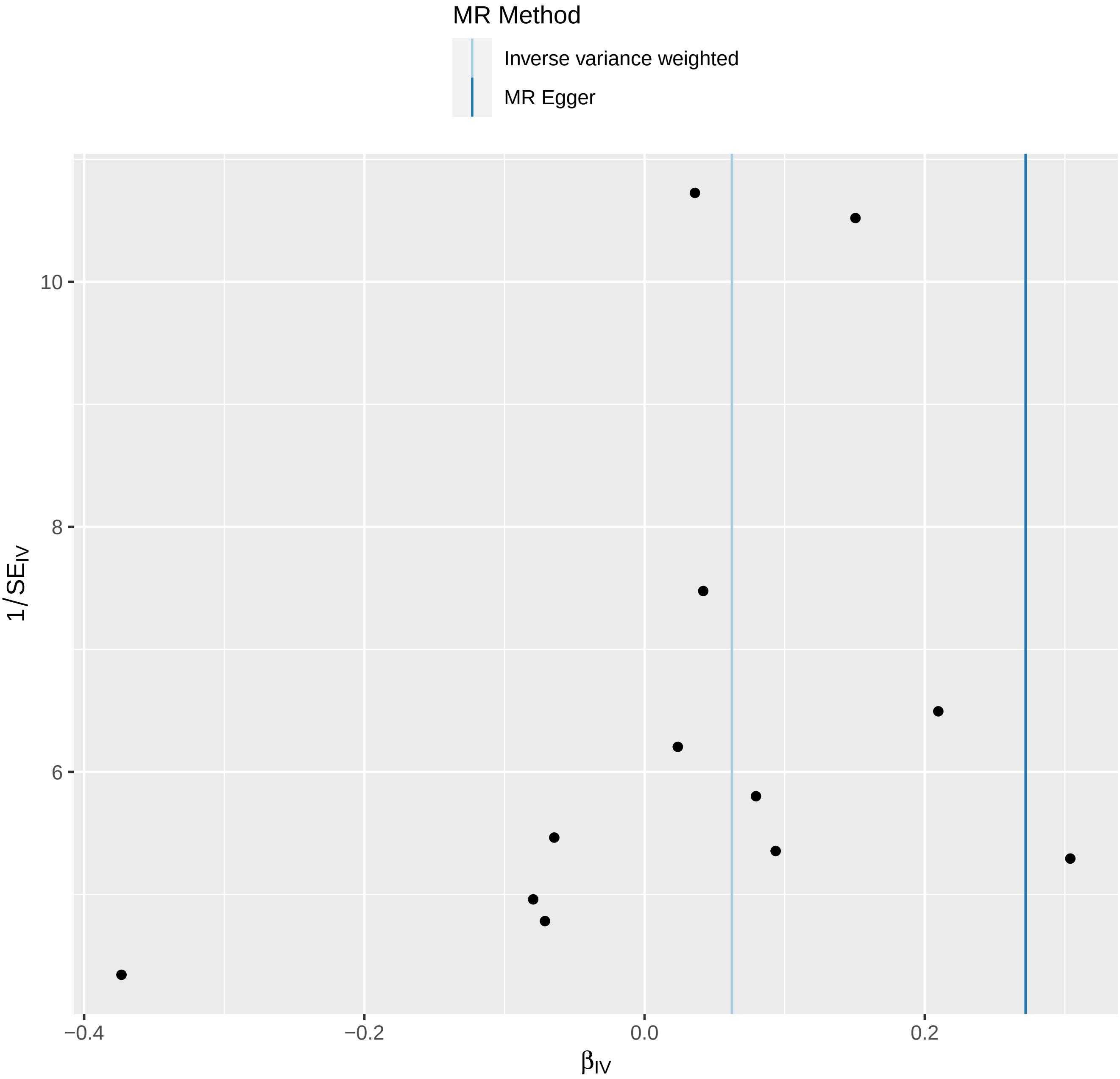

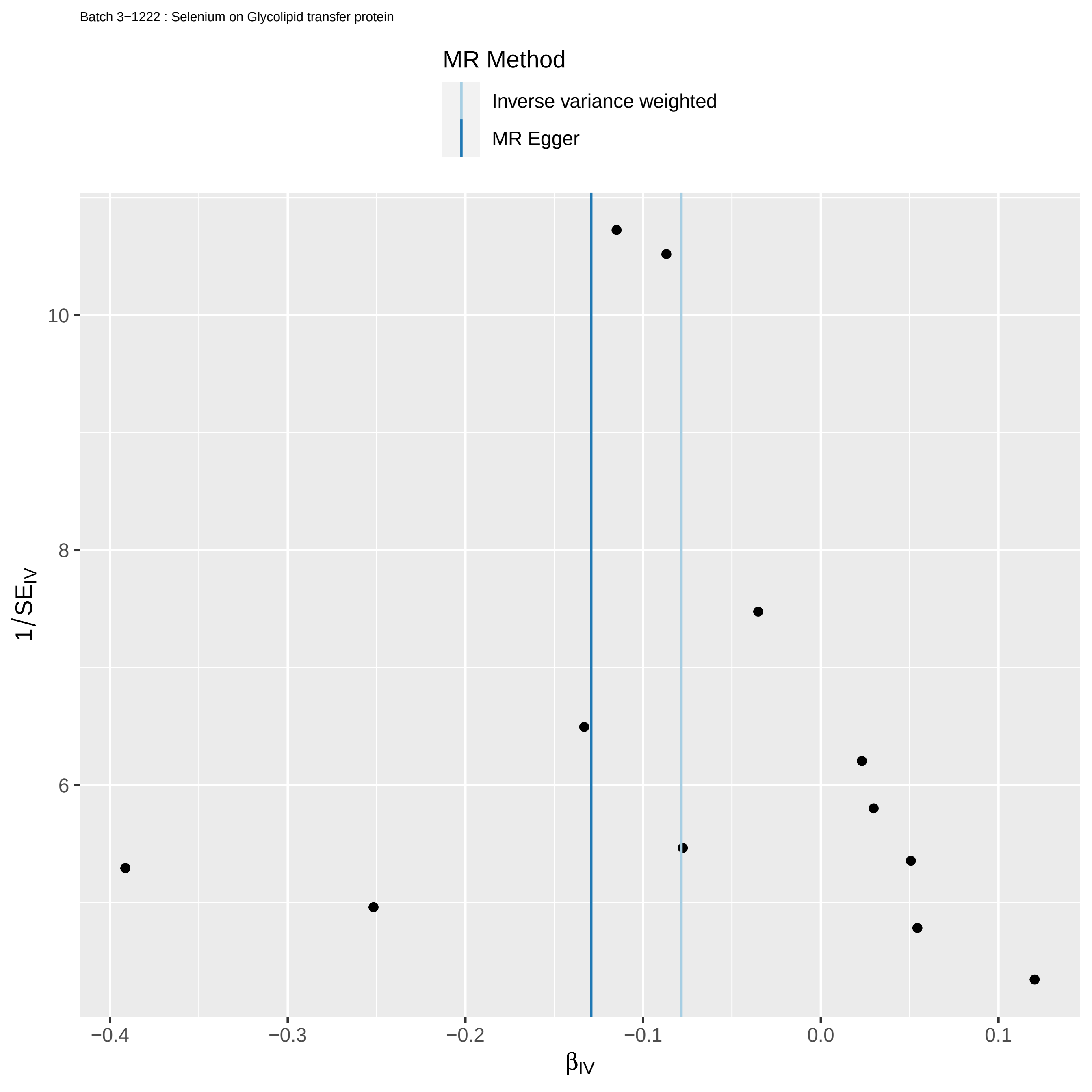

Batch 3−1222 : Selenium on Glycolipid transfer protein
MR Method
Inverse variance weighted
MR Egger
10
8
6
1/SE_IV
−0.4
−0.3
−0.2
−0.1
0.0
0.1
β_IV

Batch 3−1223 : Selenium on Glycolipid transfer protein domain−containing protein 2

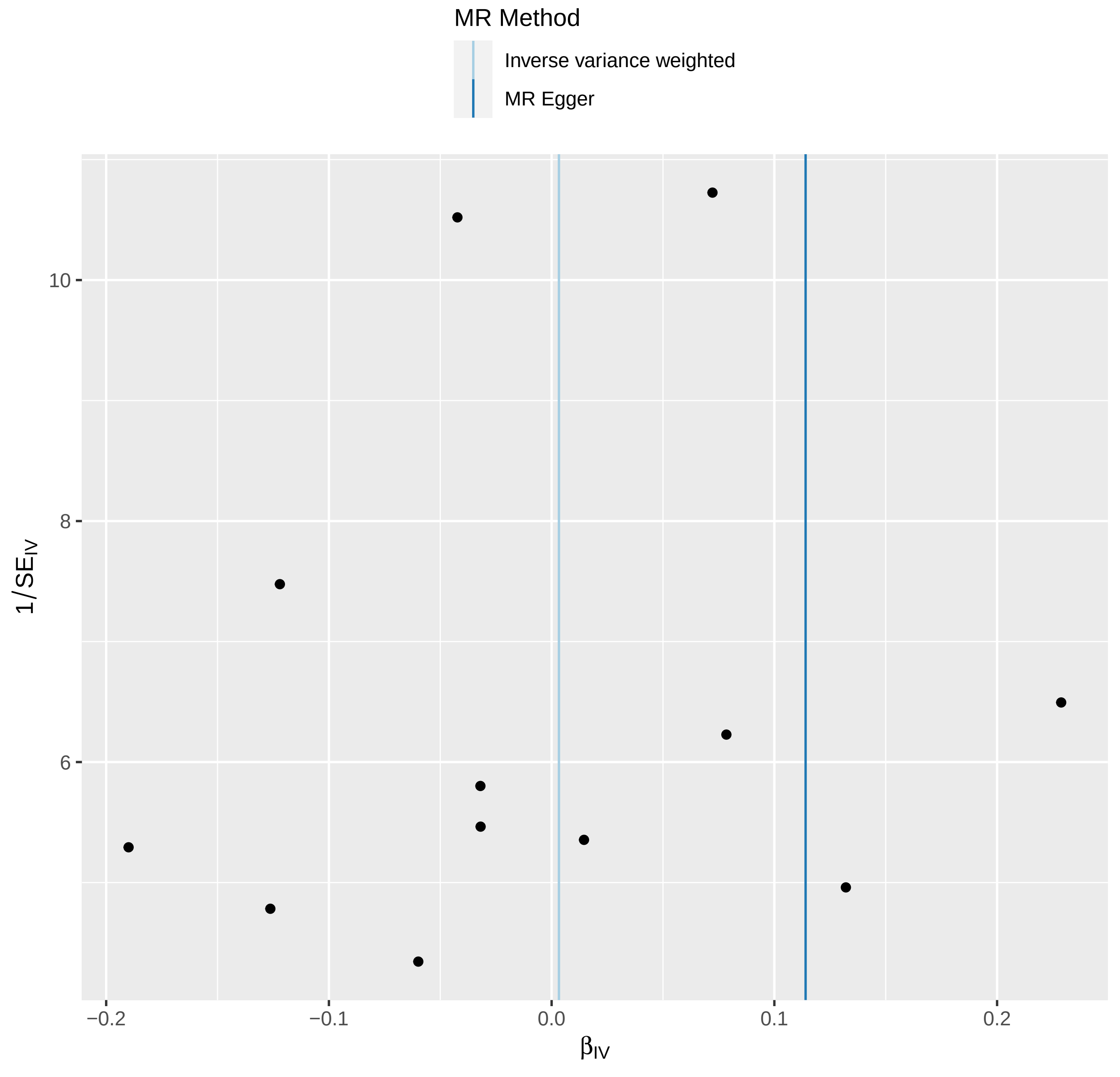

Batch 3−1224 : Selenium on Glucocorticoid modulatory element−binding protein 2

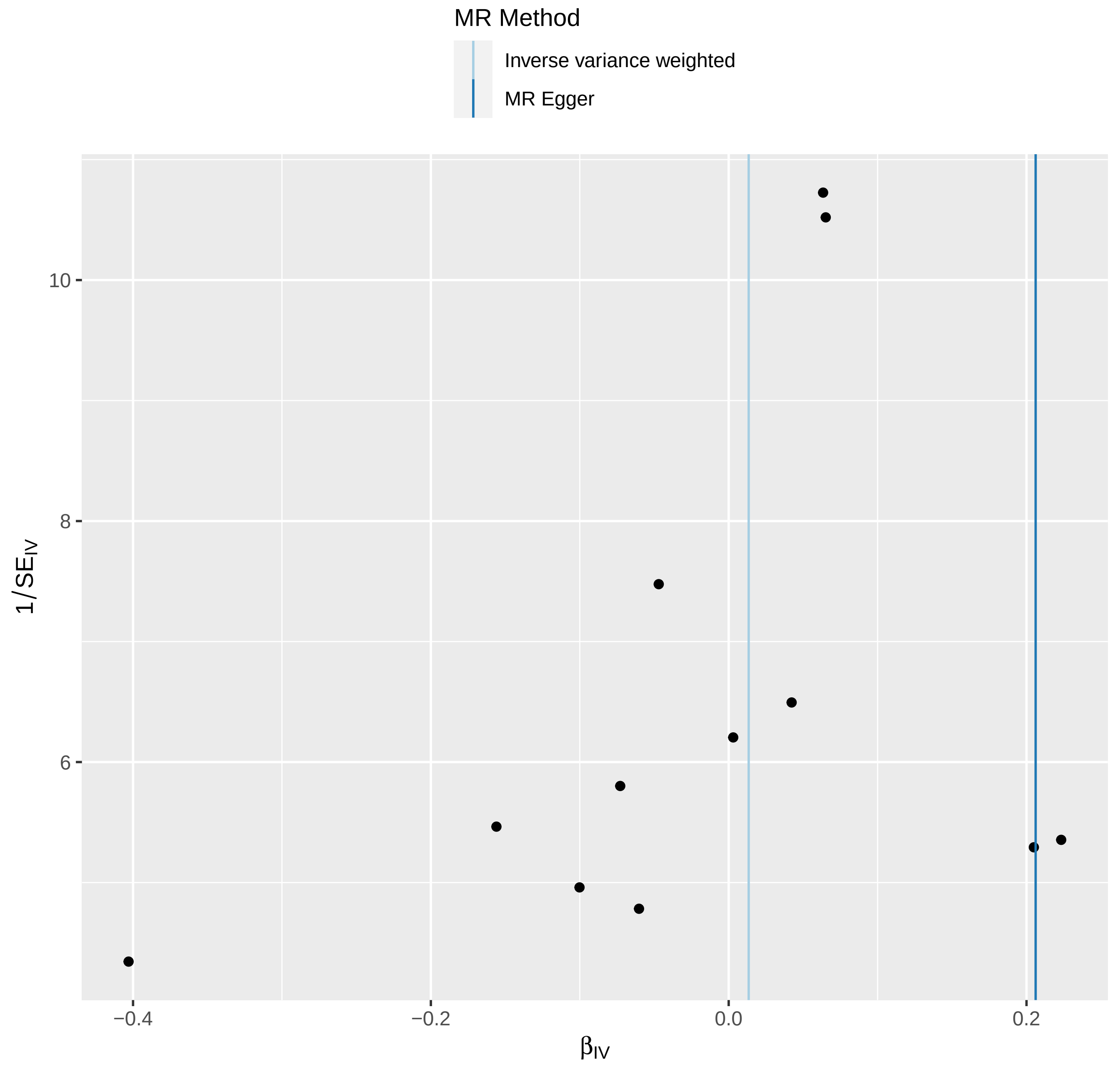

Batch 3−1225 : Selenium on Glia maturation factor gamma

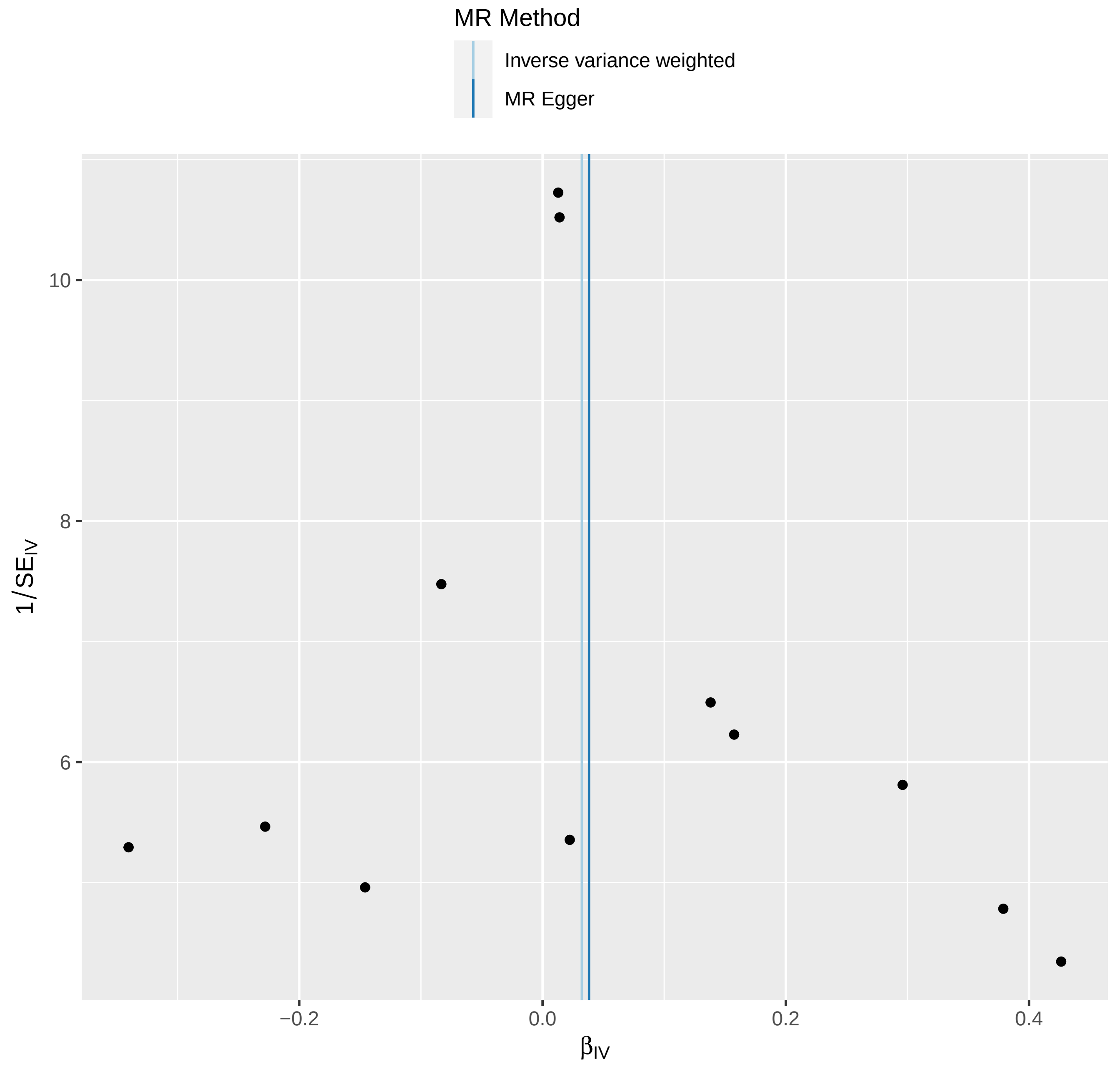

Batch 3−1226 : Selenium on Guanine nucleotide−binding protein G(k) subunit alpha

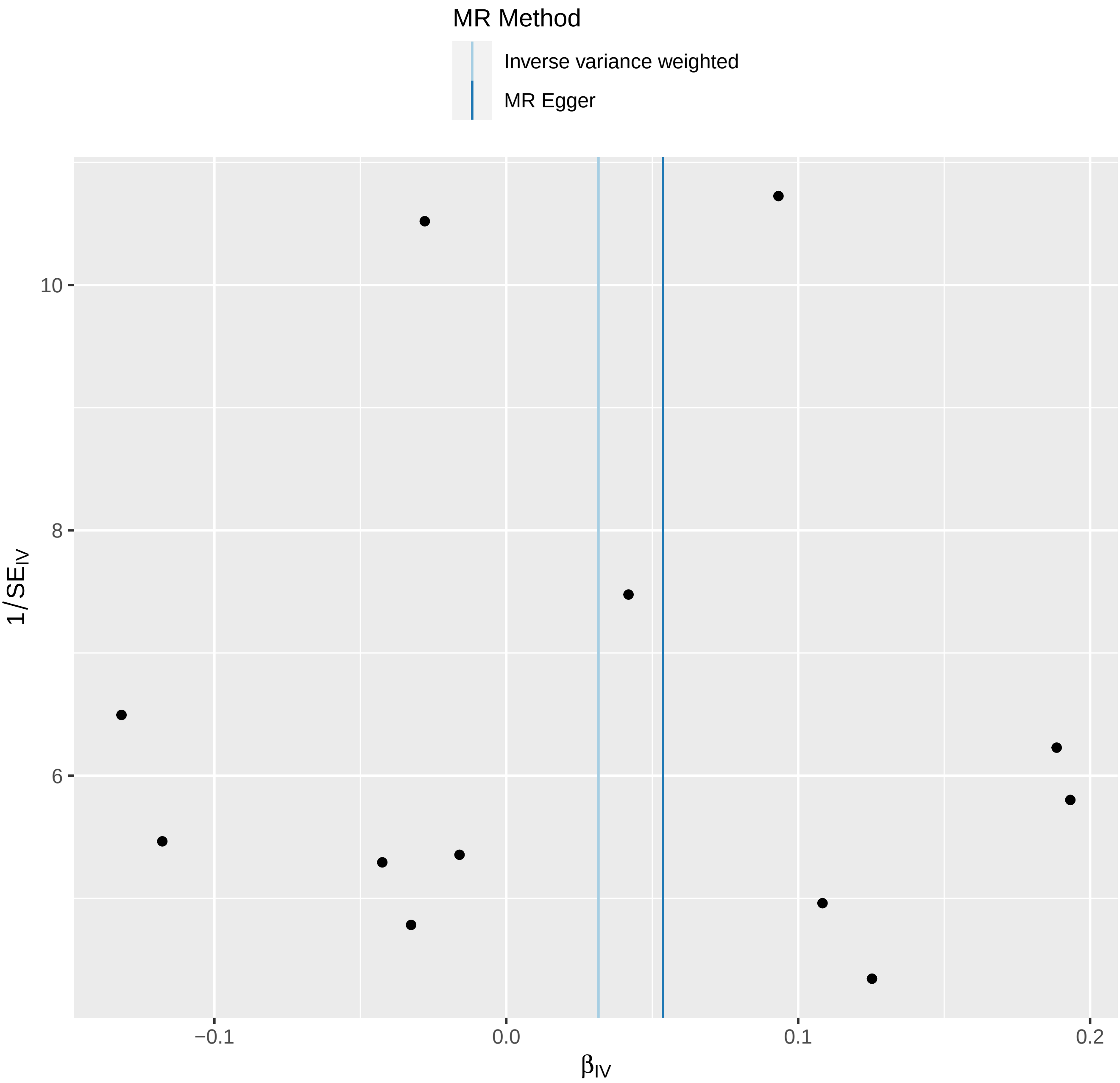

Batch 3−1227 : Selenium on Guanine nucleotide−binding protein G(I)/G(S)/G(O) subunit gamma−T2

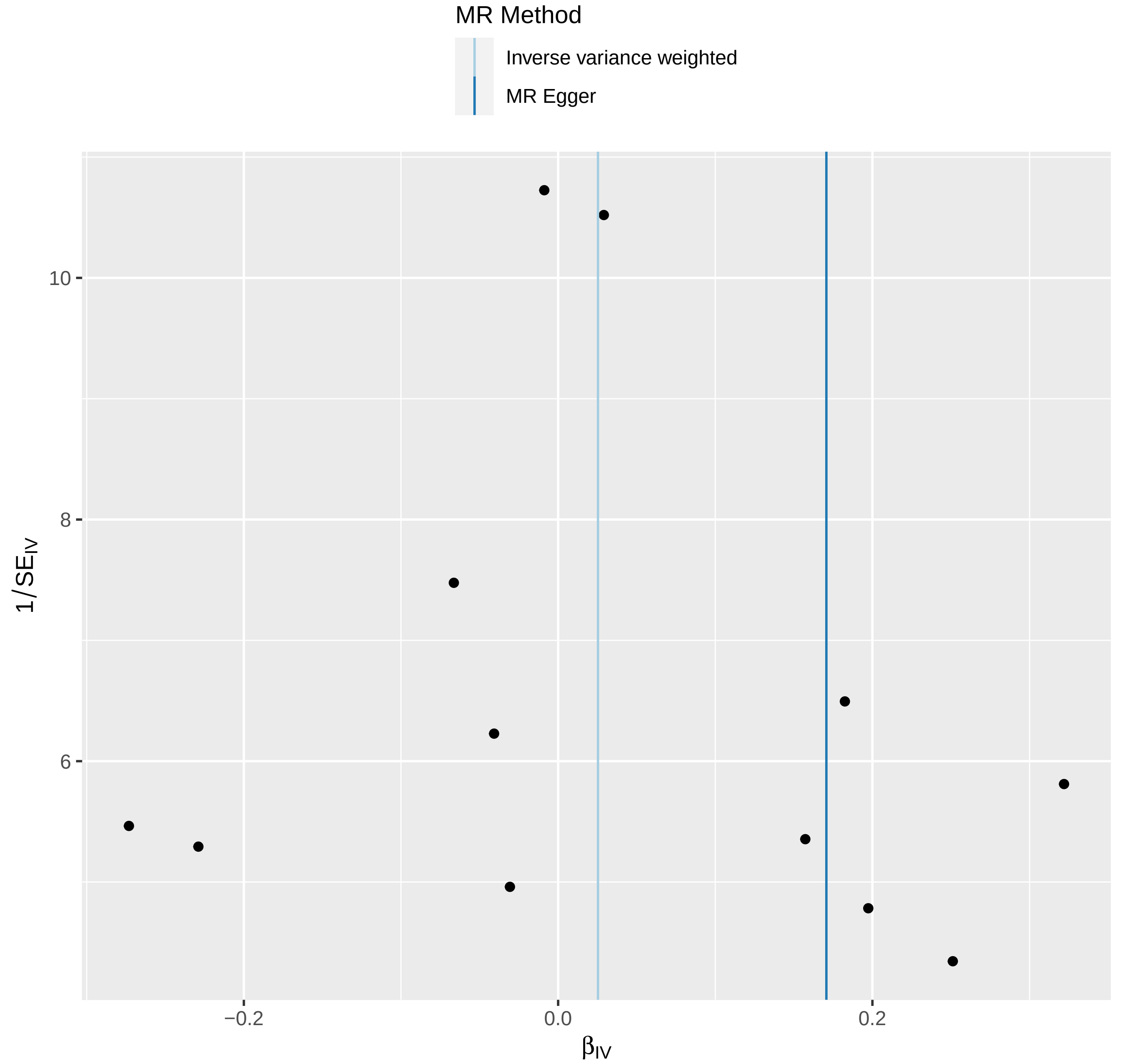

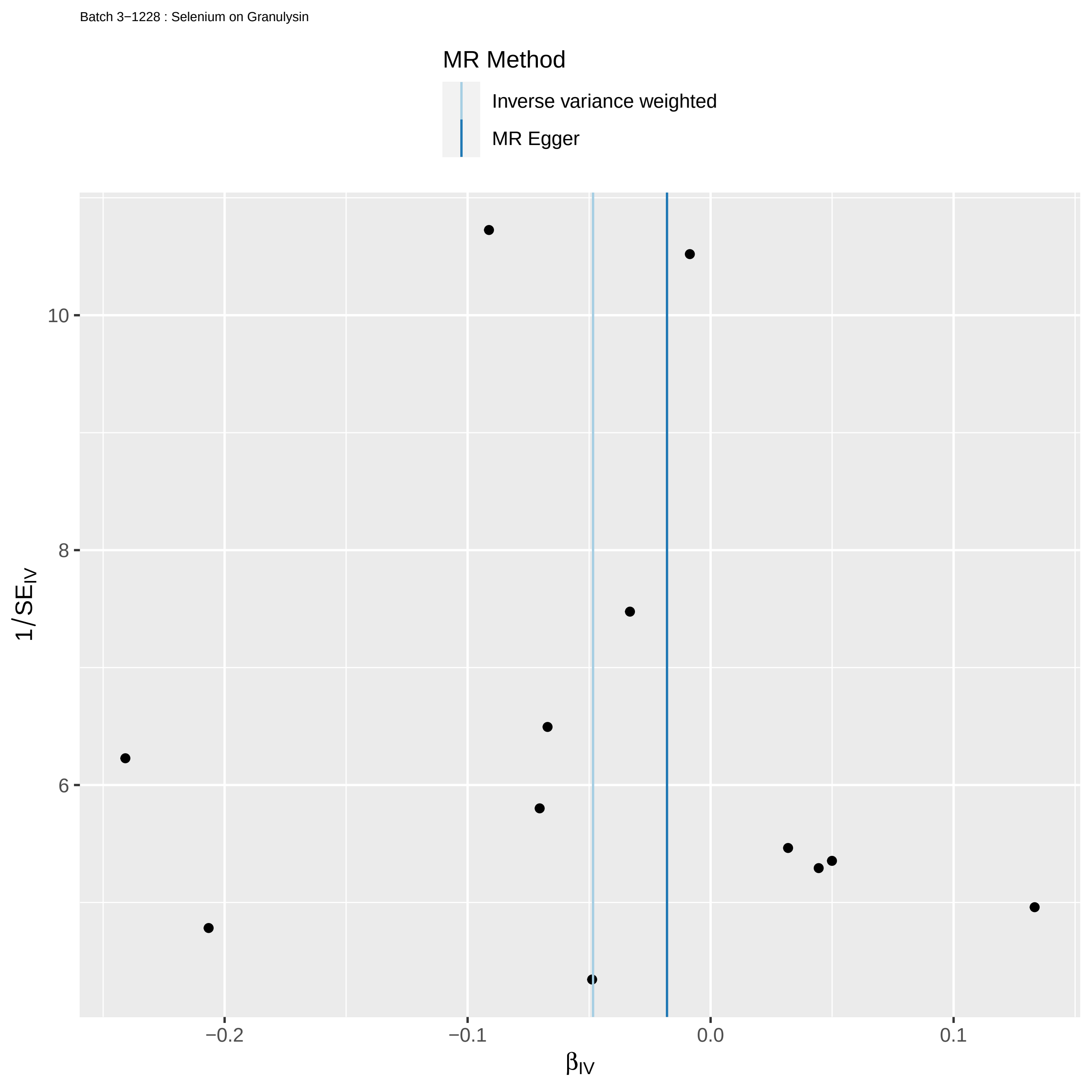

Batch 3−1228 : Selenium on Granulysin
MR Method
Inverse variance weighted
MR Egger
10
8
6
1/SE$_{IV}$
−0.2
−0.1
0.0
0.1
β$_{IV}$

Batch 3−1229 : Selenium on Glycine N−methyltransferase

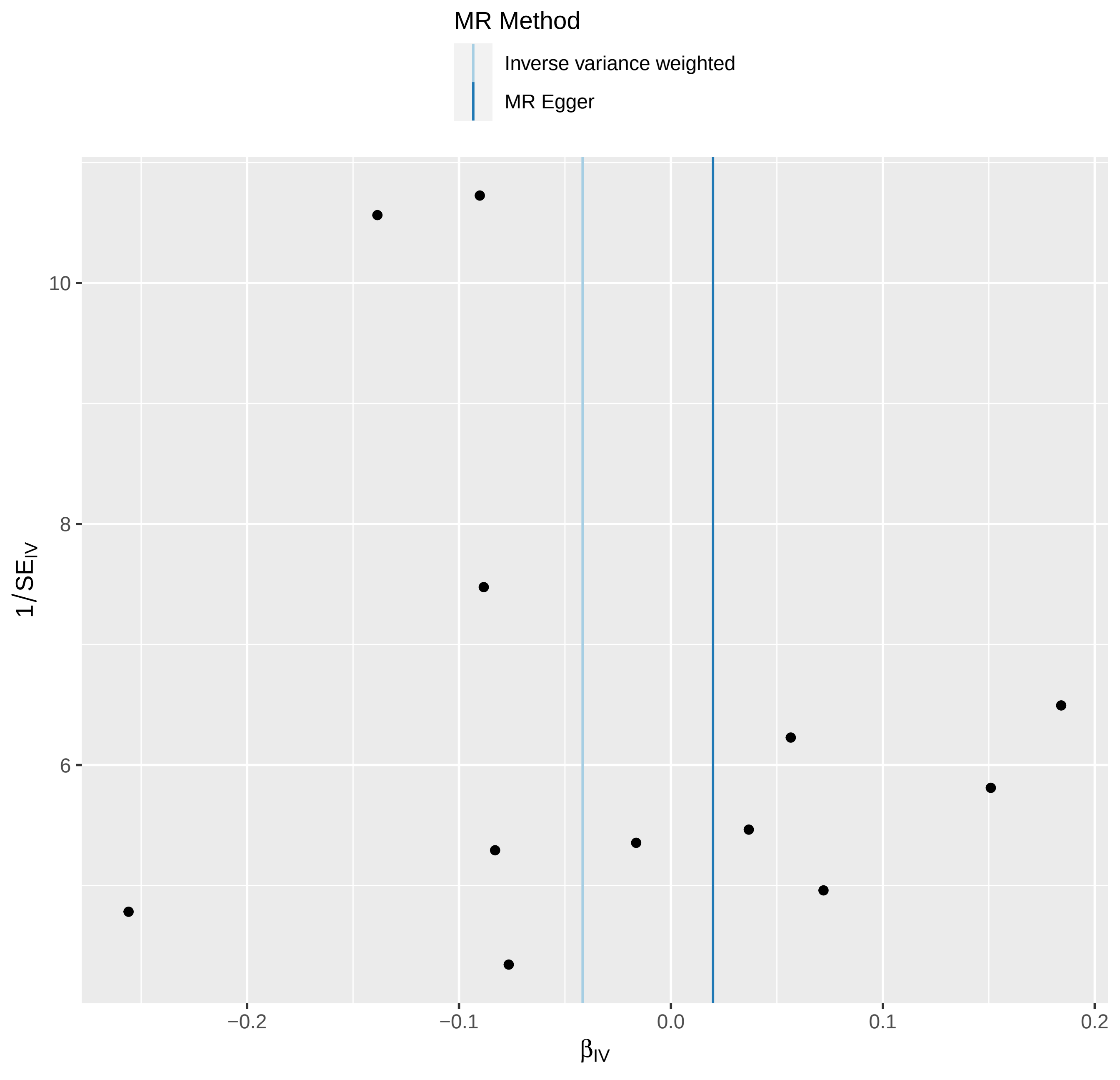

Batch 3−1230 : Selenium on Glucosamine−6−phosphate isomerase 1

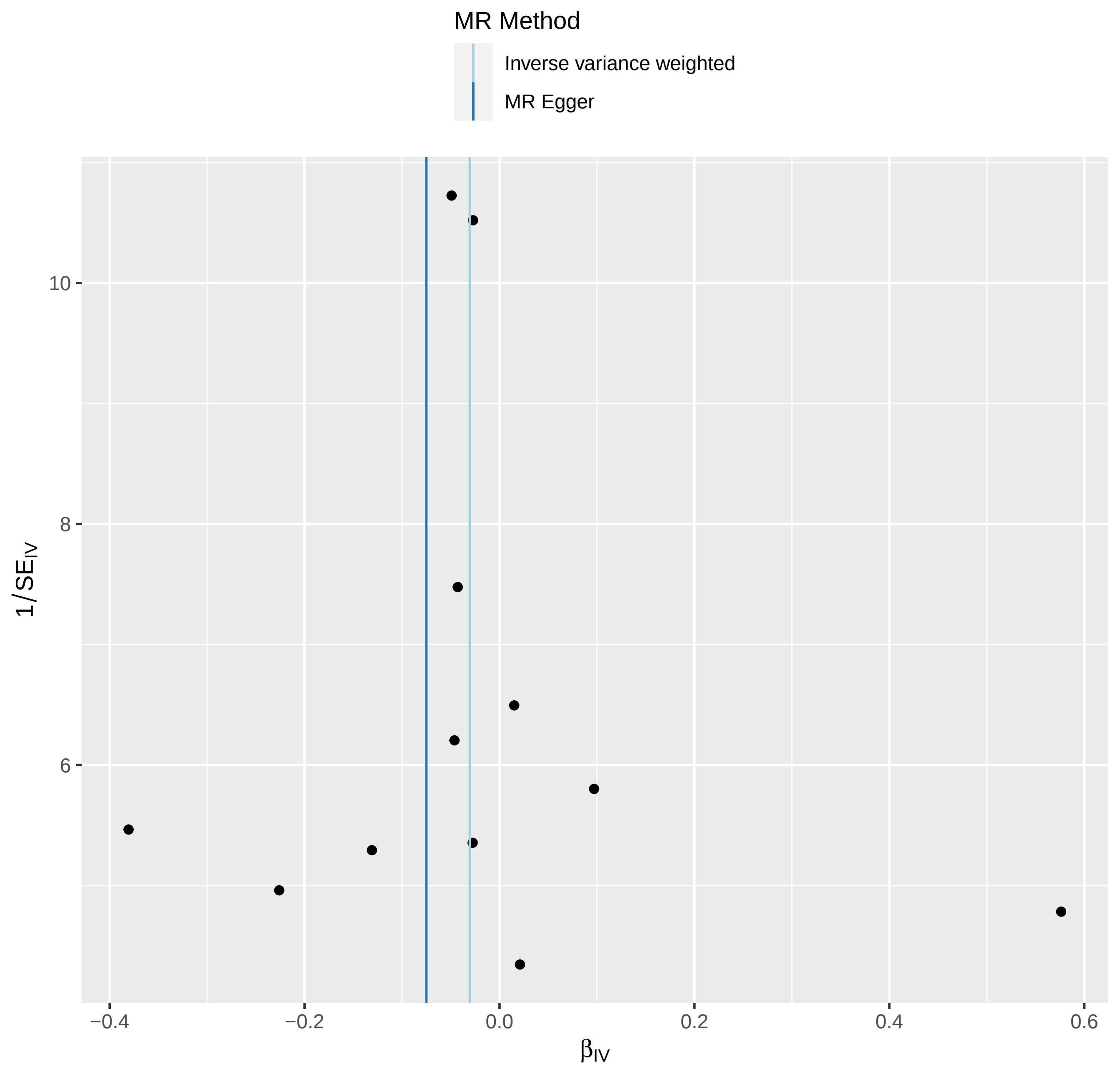

Batch 3−1231 : Selenium on Glucosamine 6−phosphate N−acetyltransferase

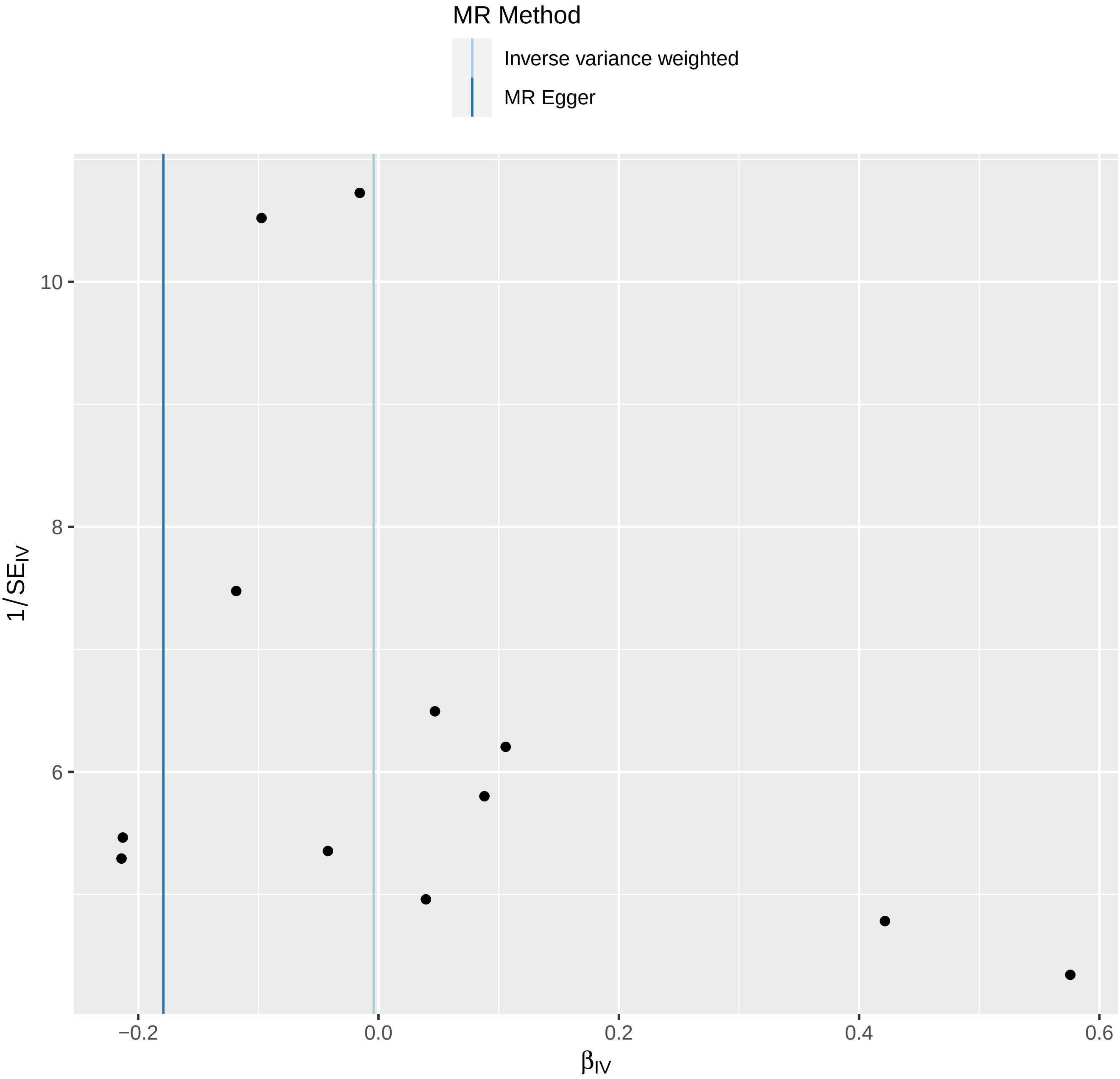

Batch 3−1232 : Selenium on N−acetylglucosamine−1−phosphotransferase subunit gamma

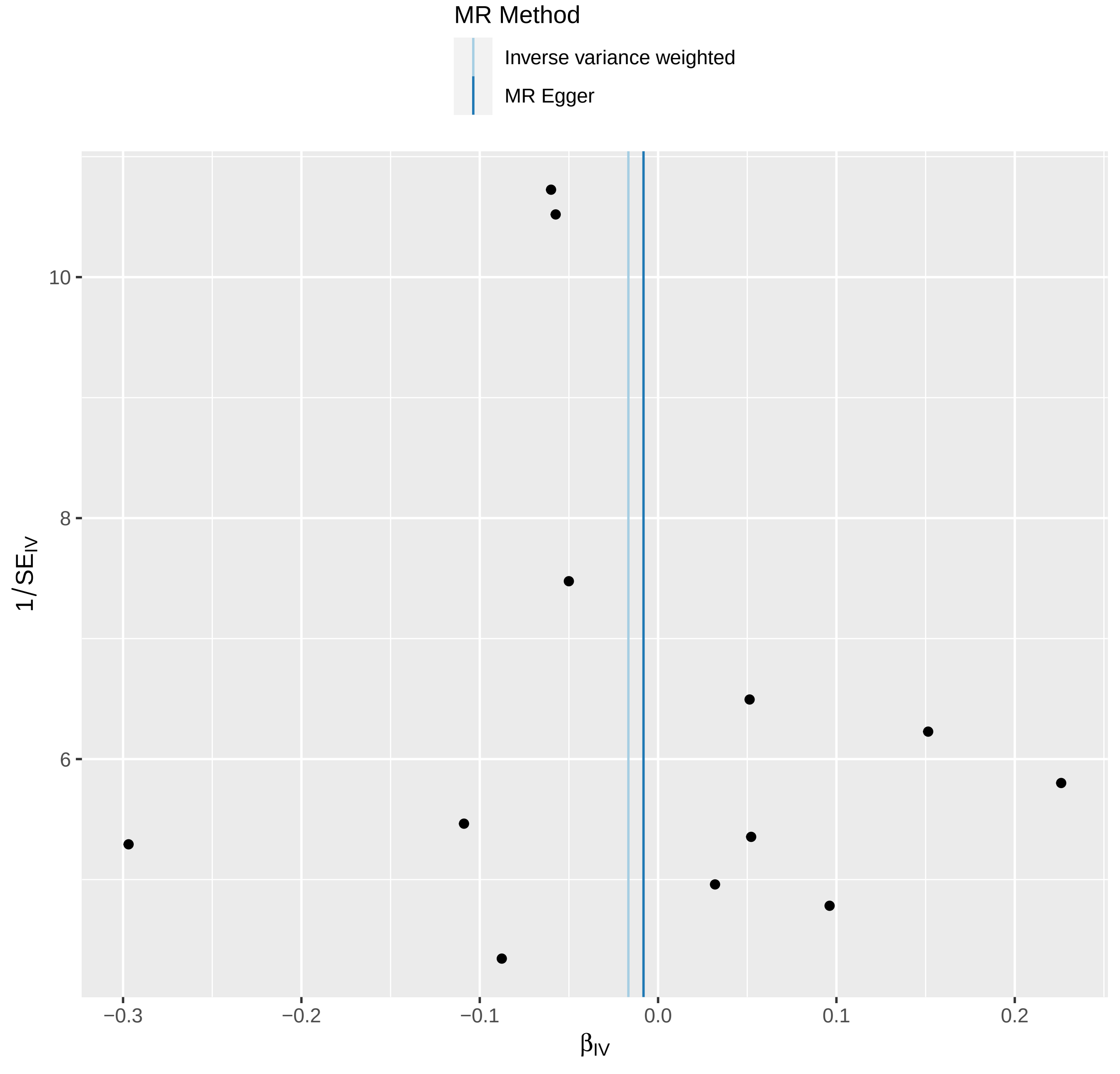

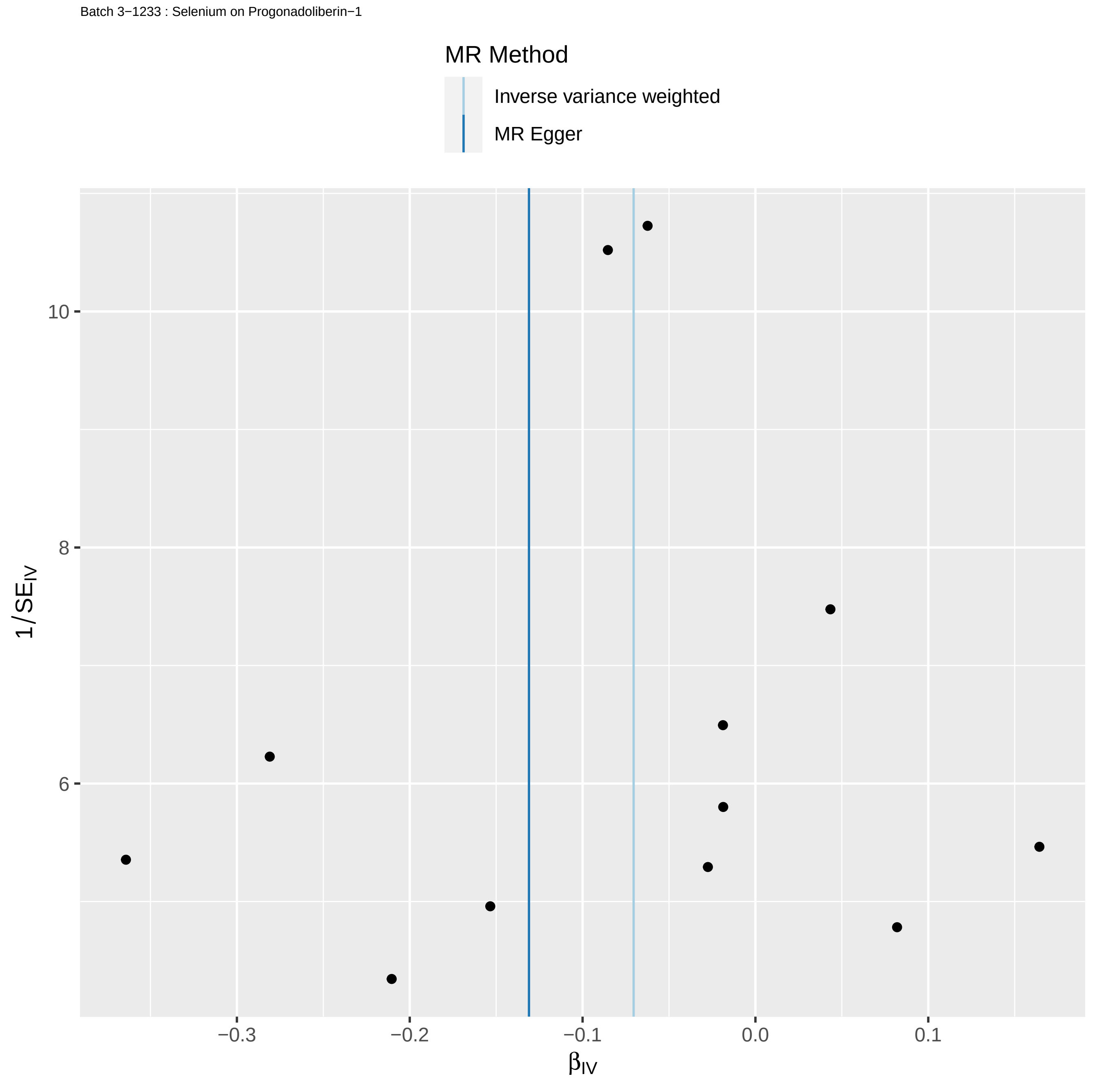
Batch 3−1233 : Selenium on Progonadoliberin−1
MR Method
Inverse variance weighted
MR Egger
10
8
6
1/SE$_{IV}$
−0.3
−0.2
−0.1
0.0
0.1
β$_{IV}$

Batch 3−1234 : Selenium on Progonadoliberin−2

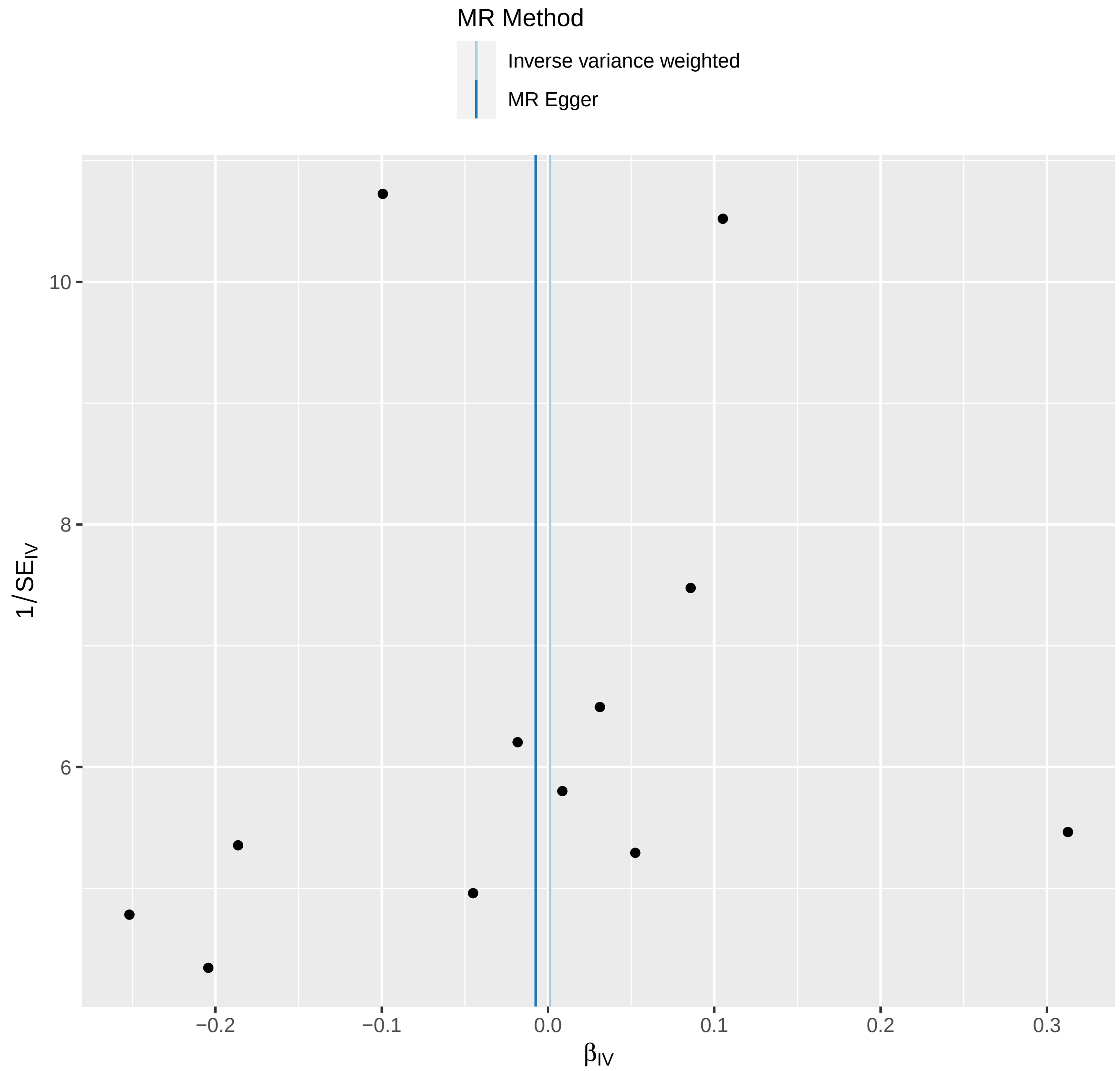

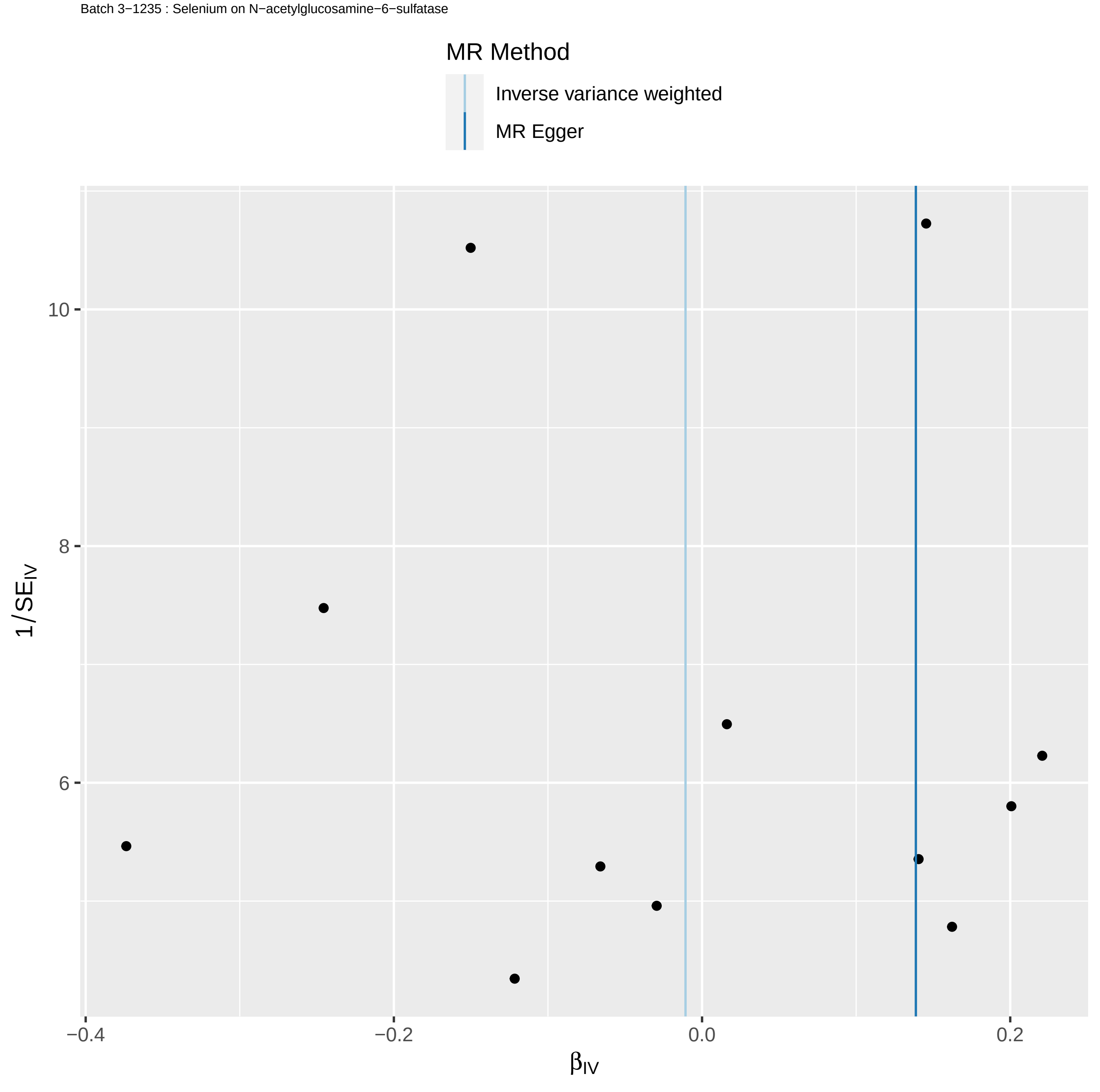
Batch 3−1235 : Selenium on N−acetylglucosamine−6−sulfatase
MR Method
Inverse variance weighted
MR Egger
1/SE$_{IV}$
10
8
6
−0.4
−0.2
0.0
0.2
β$_{IV}$

Batch 3−1236 : Selenium on N−acetylglucosamine−6−sulfatase

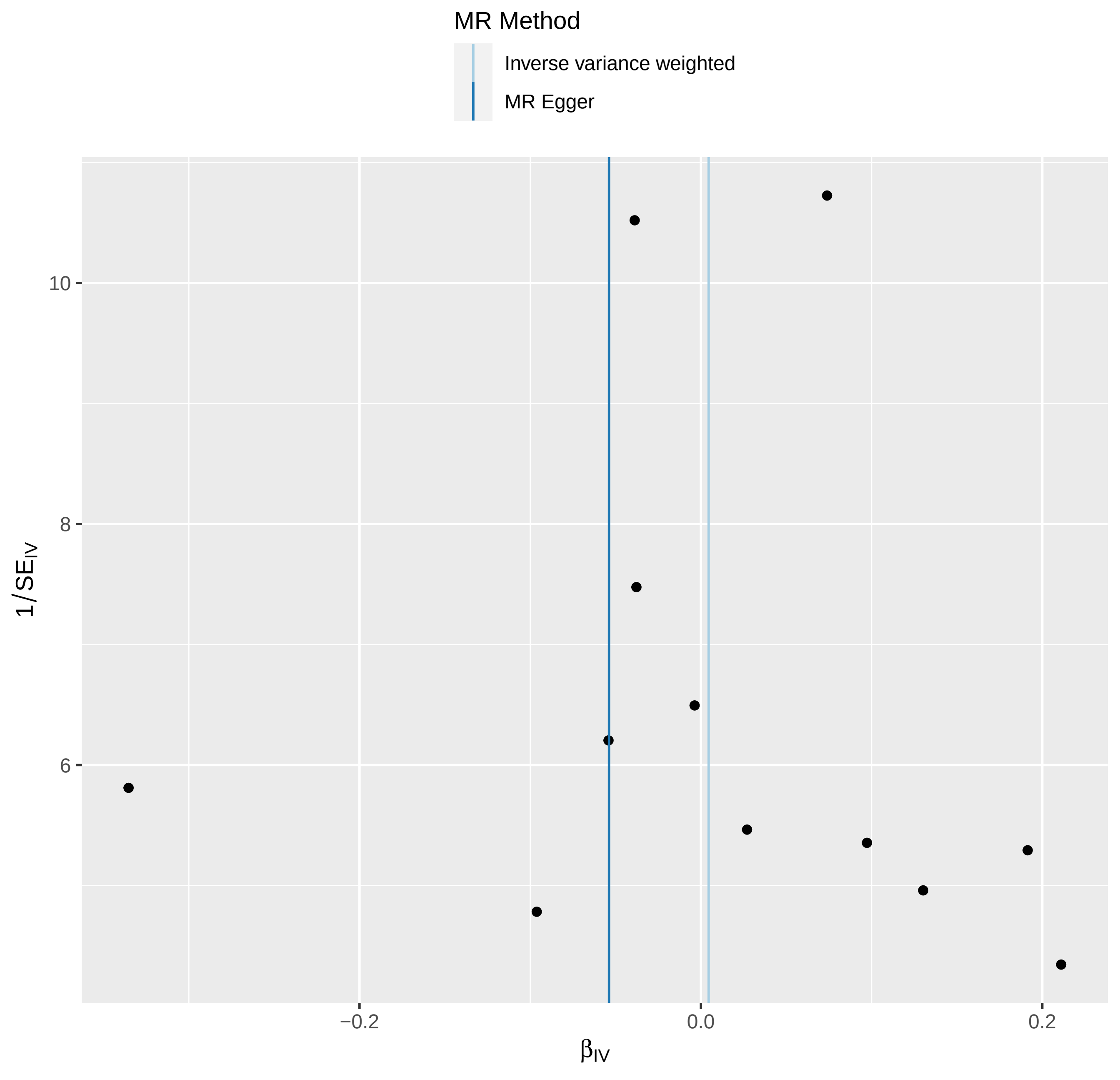

Batch 3−1237 : Selenium on Golgi membrane protein 1

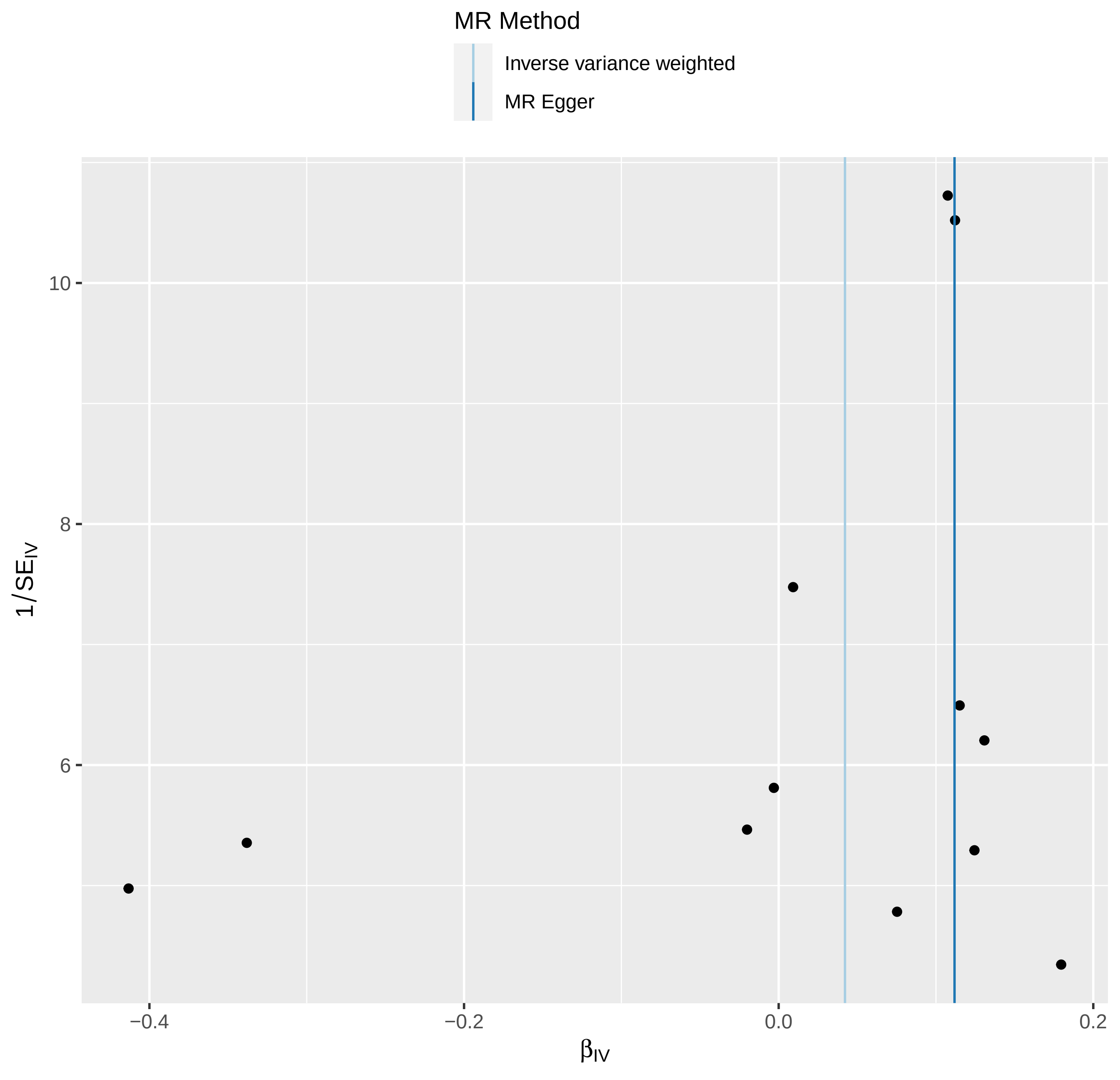

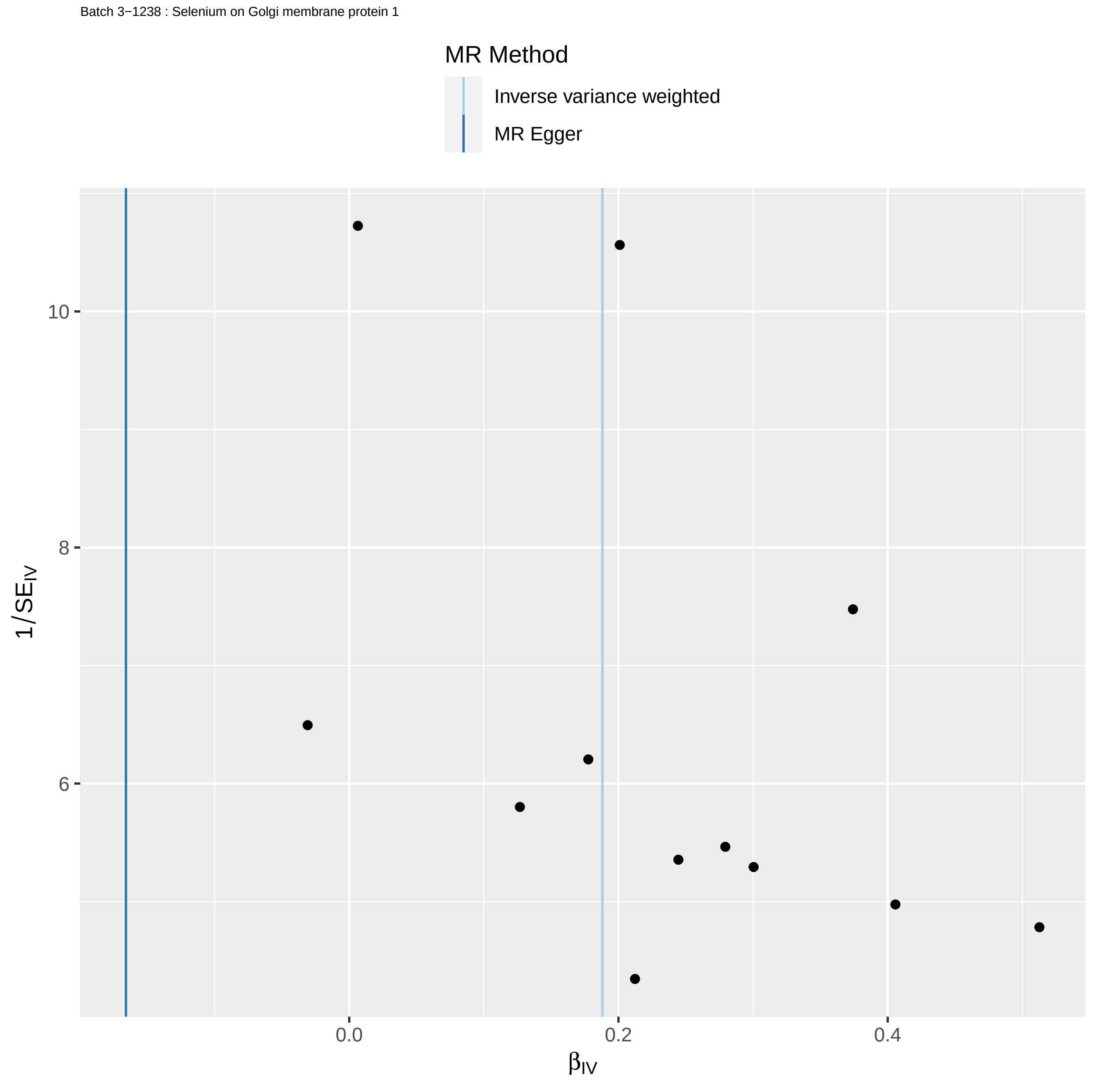
Batch 3−1238 : Selenium on Golgi membrane protein 1
MR Method
Inverse variance weighted
MR Egger
10
8
6
1/SE$_{IV}$
0.0
0.2
0.4
β$_{IV}$

Batch 3−1239 : Selenium on RAB6−interacting golgin

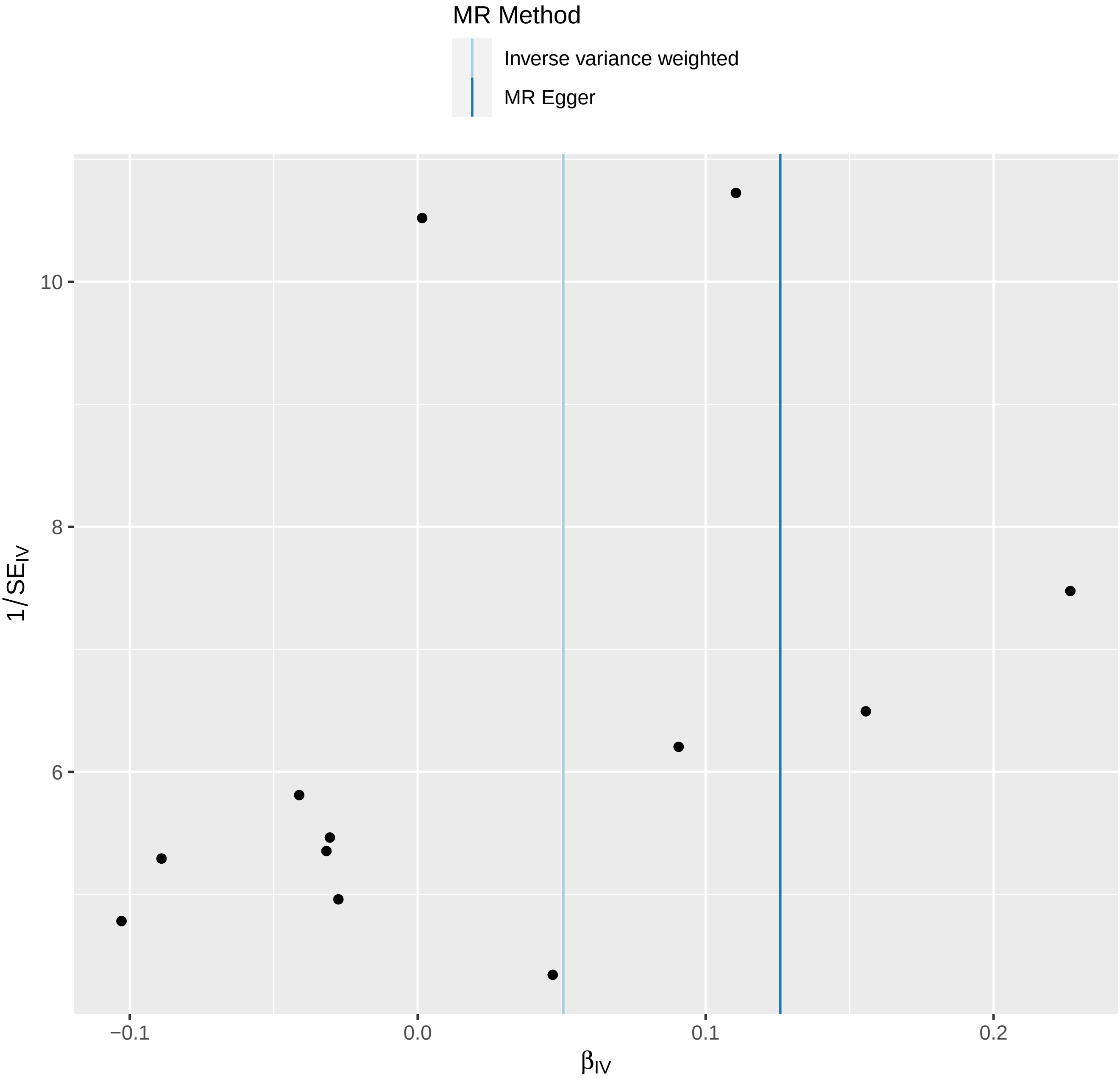

Batch 3−1240 : Selenium on Golgi SNAP receptor complex member 1

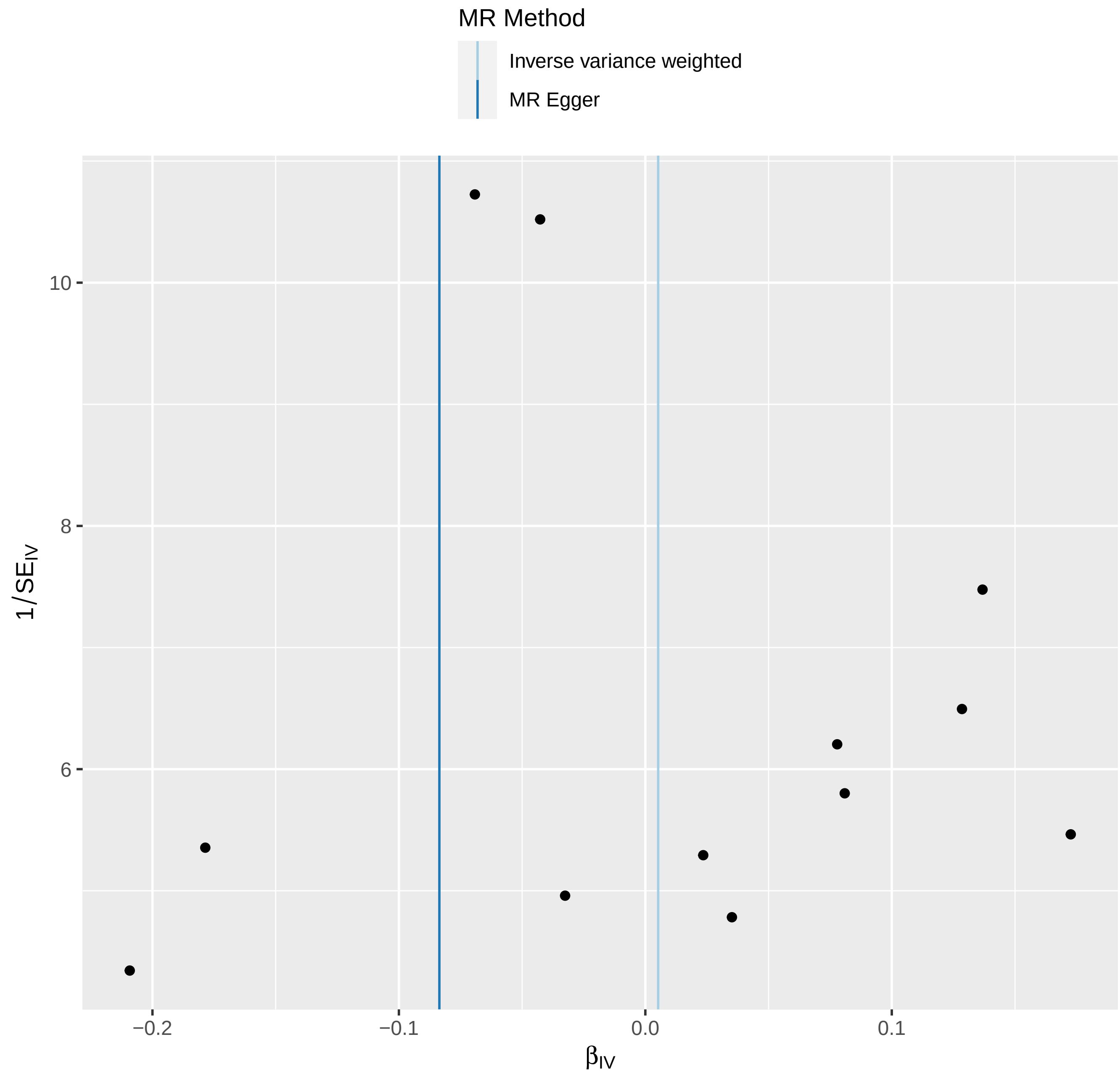

Batch 3−1241 : Selenium on Aspartate aminotransferase, cytoplasmic

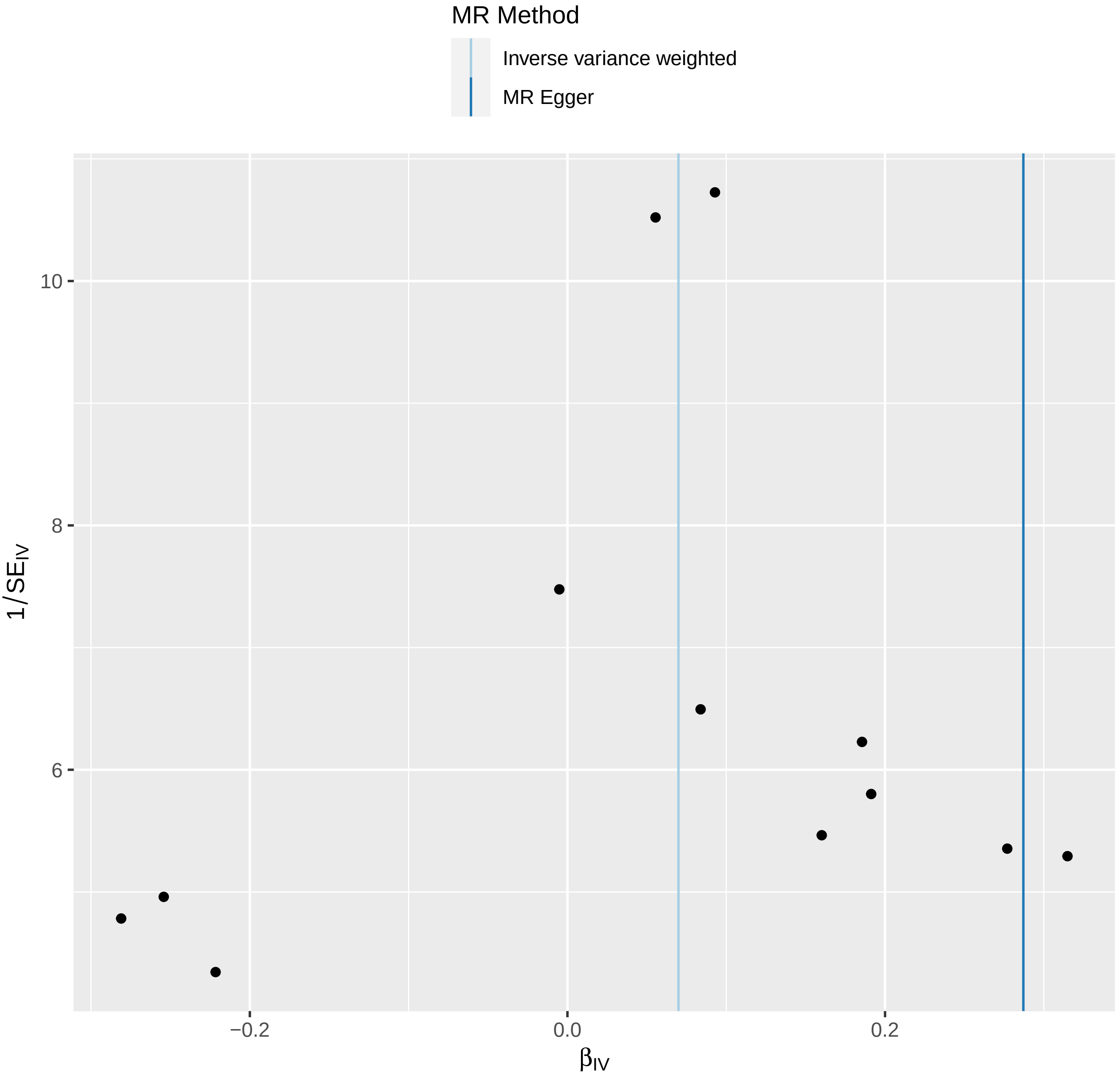

Batch 3−1242 : Selenium on Platelet glycoprotein Ib alpha chain

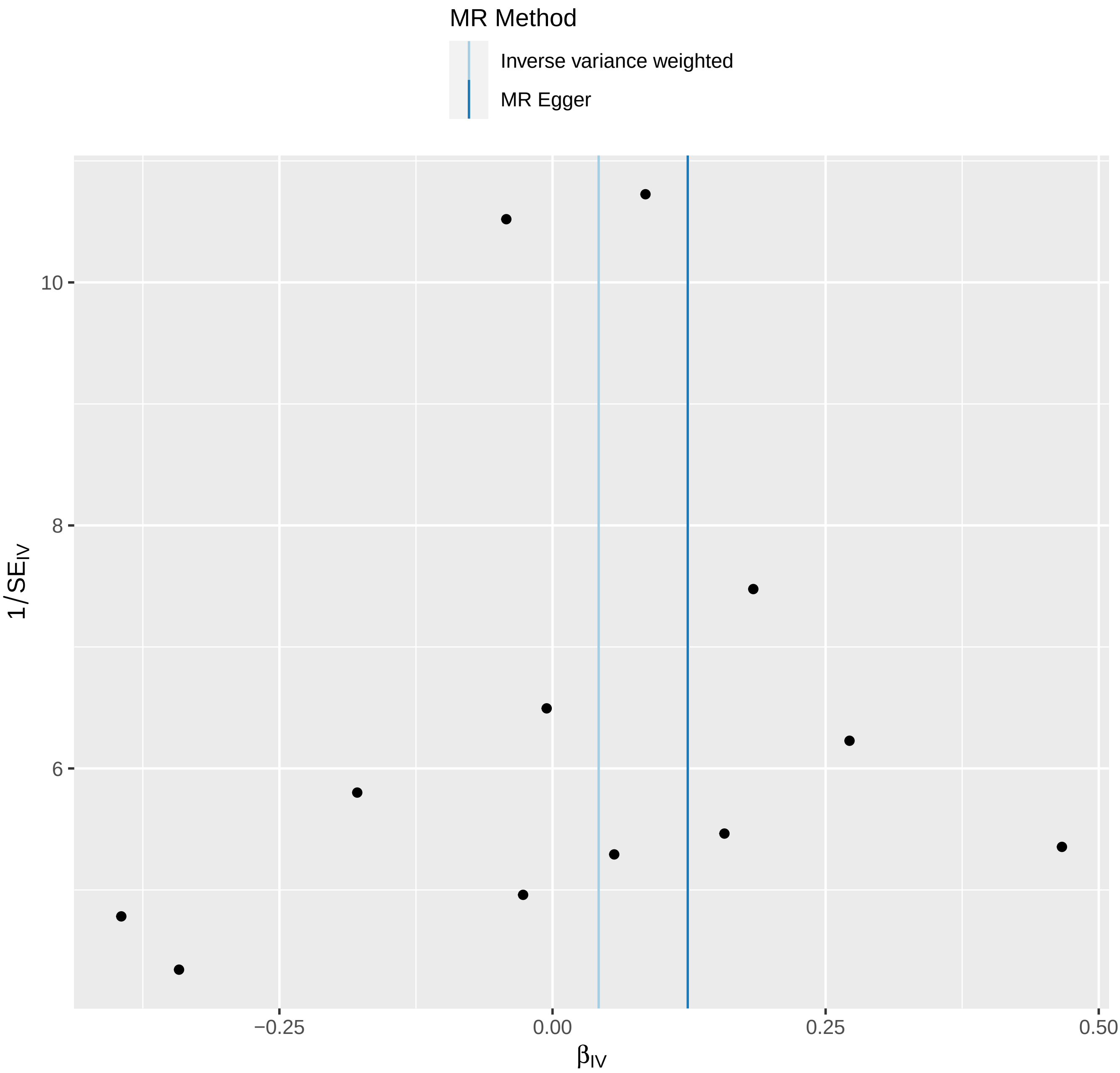

Batch 3−1243 : Selenium on Platelet glycoprotein V

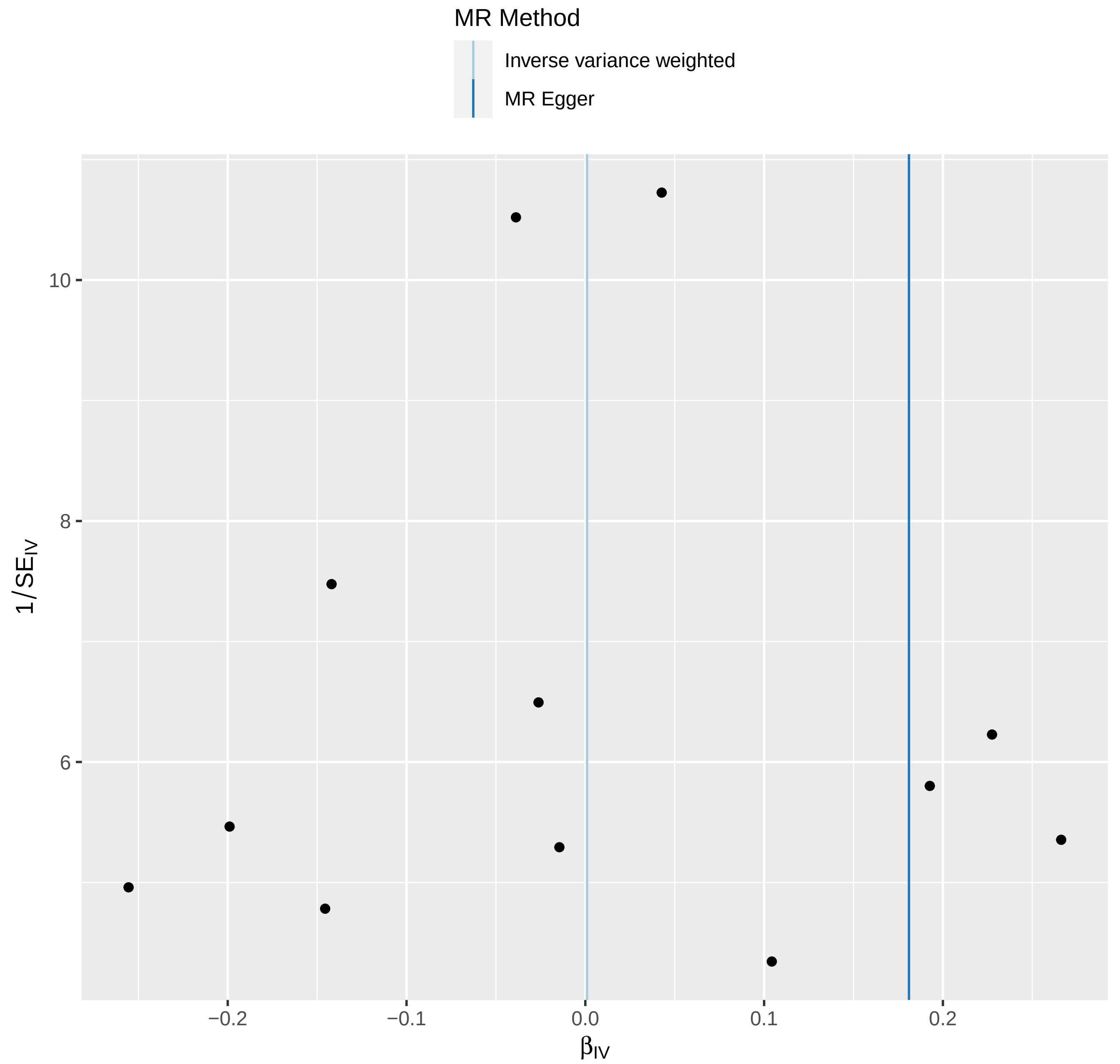

Batch 3−1244 : Selenium on Cell surface A33 antigen

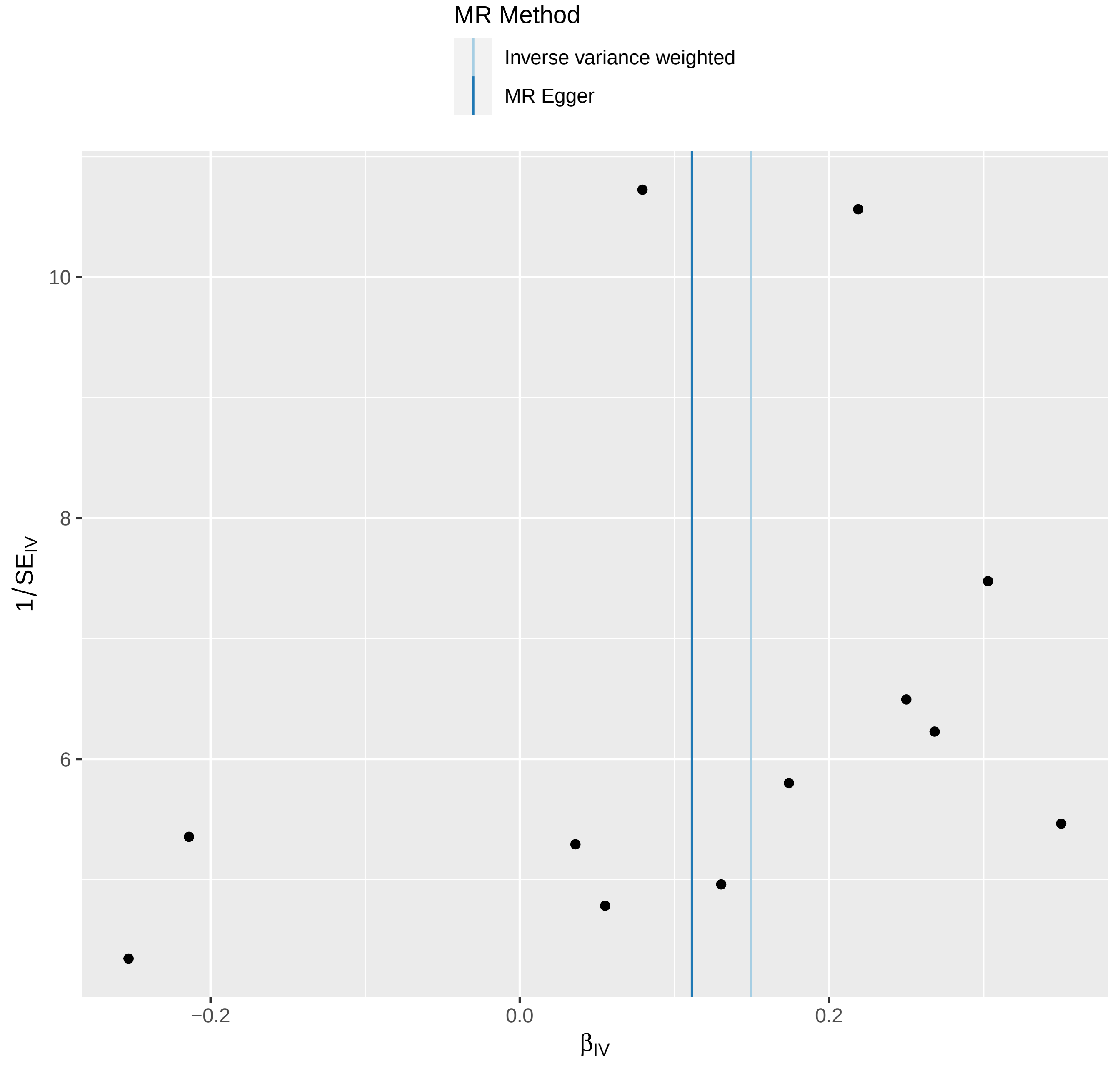

Batch 3−1245 : Selenium on Glypican−1

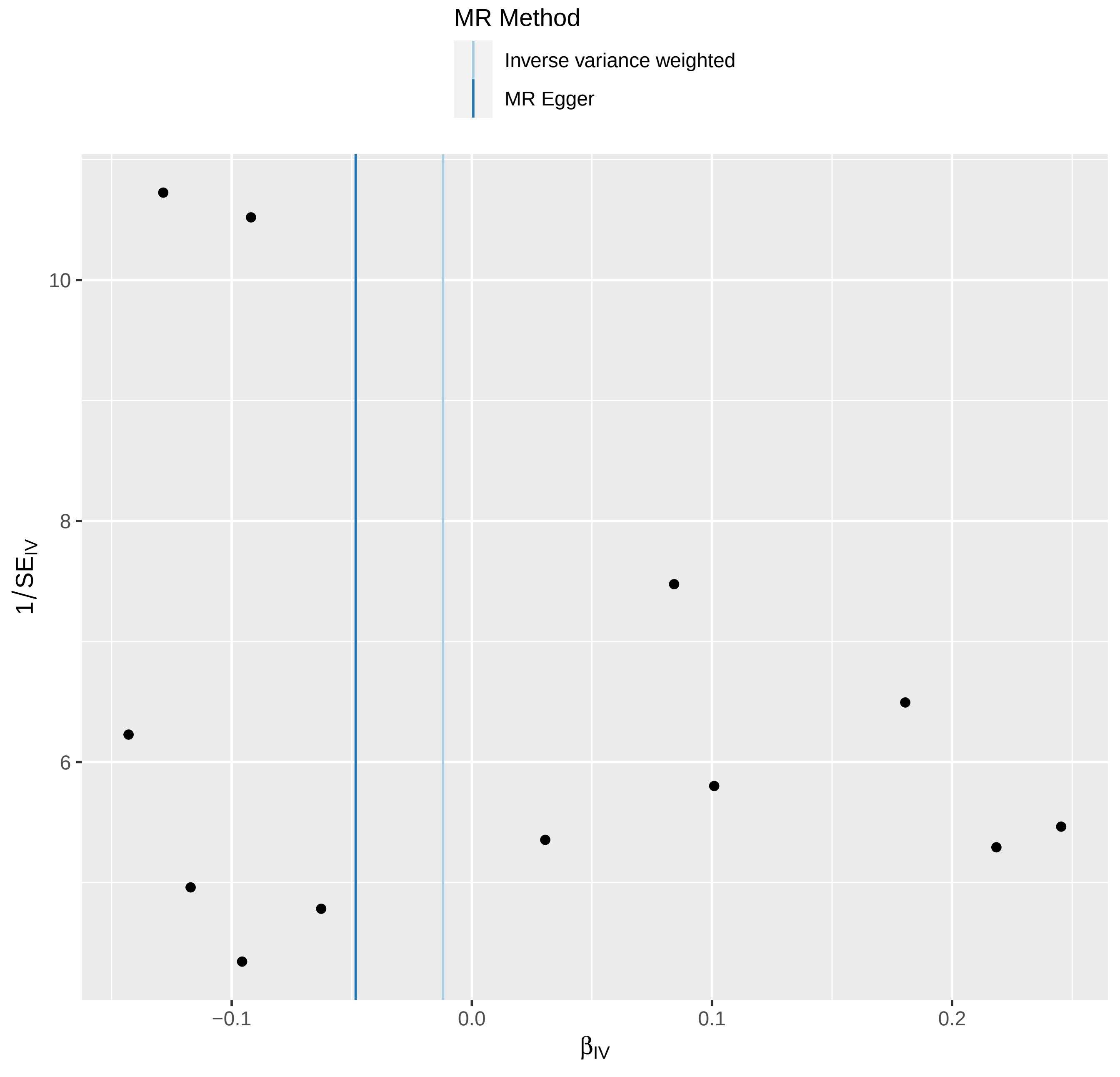

Batch 3−1246 : Selenium on Glypican−3

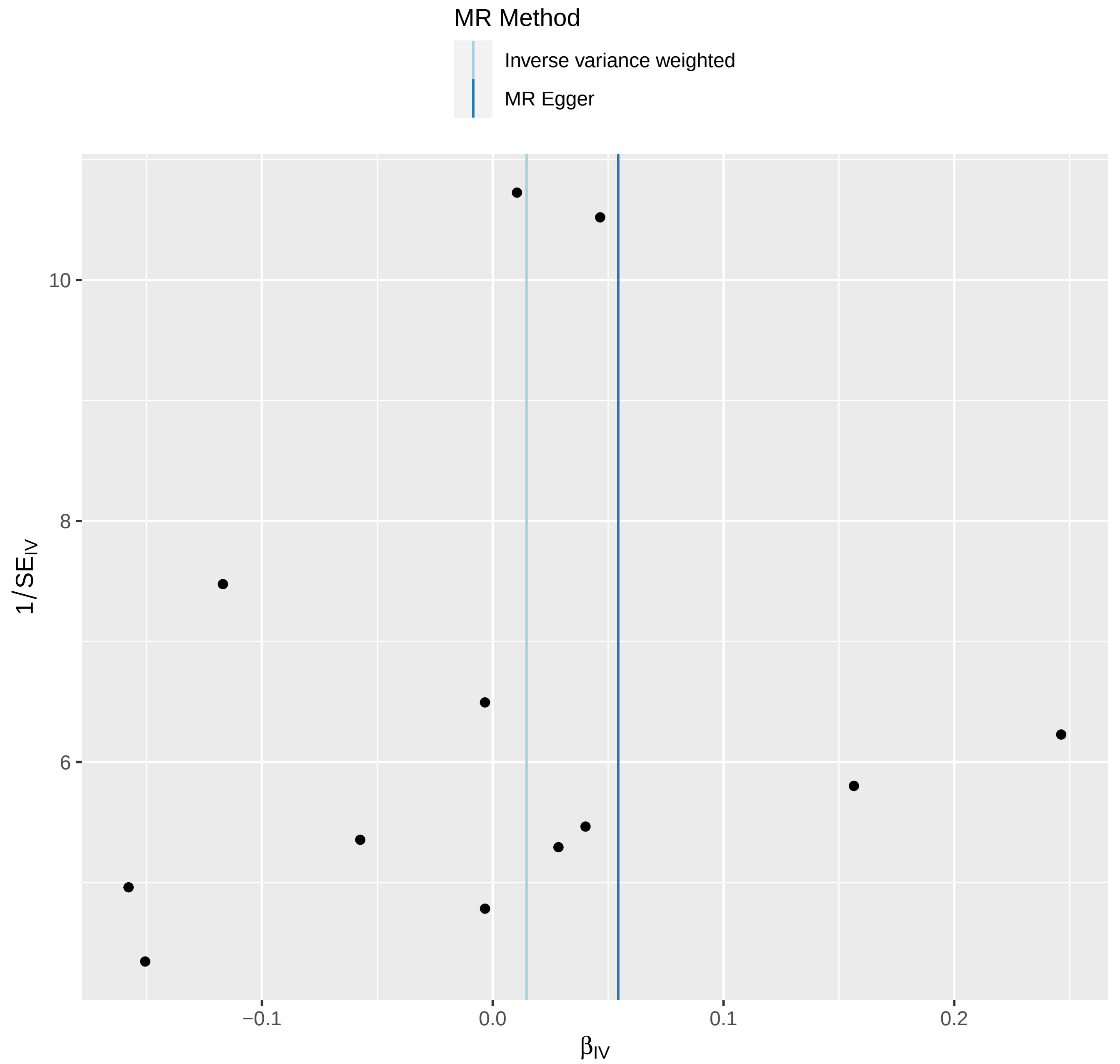

Batch 3−1247 : Selenium on Glypican−5

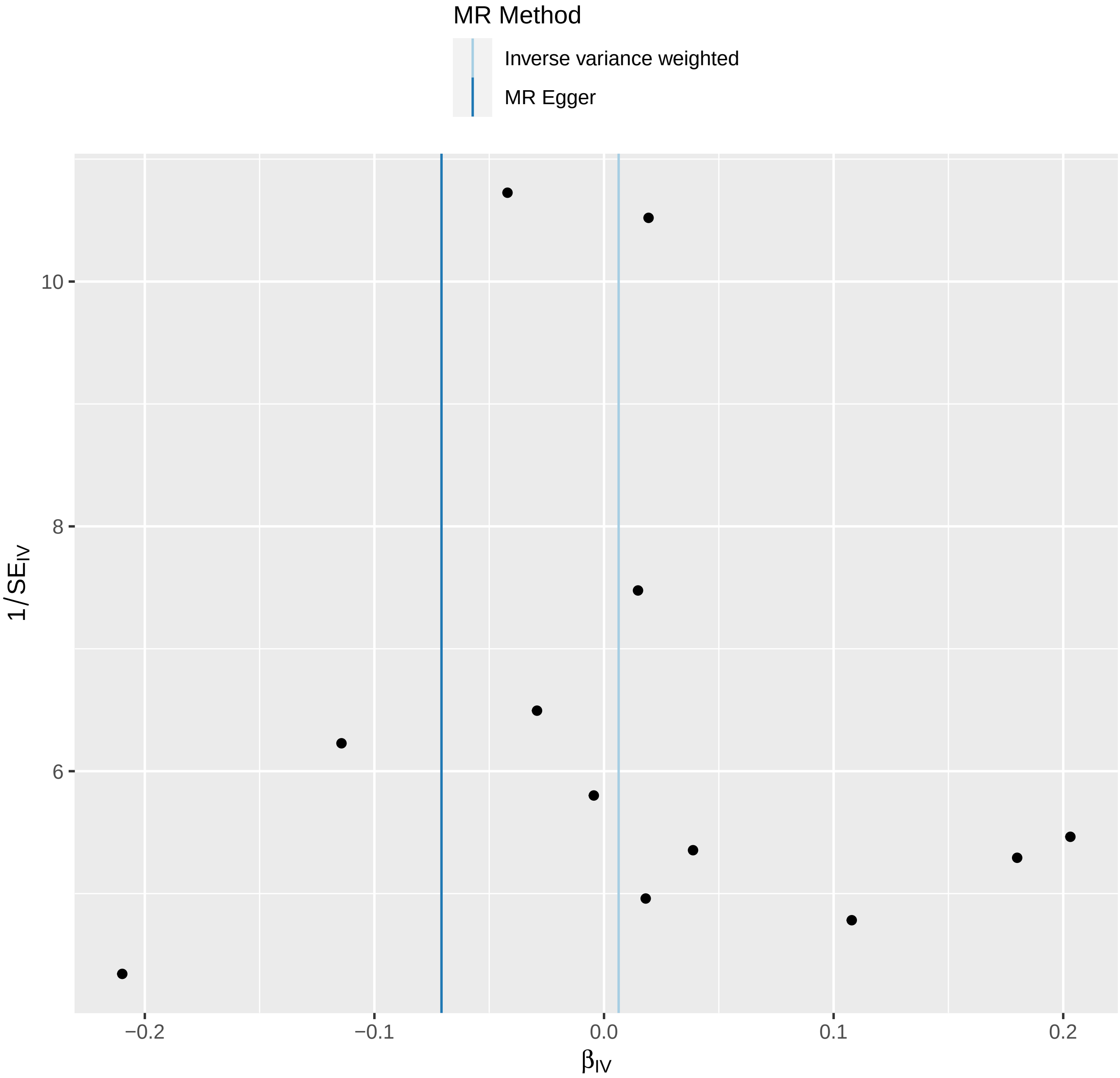

Batch 3−1248 : Selenium on Glypican−6

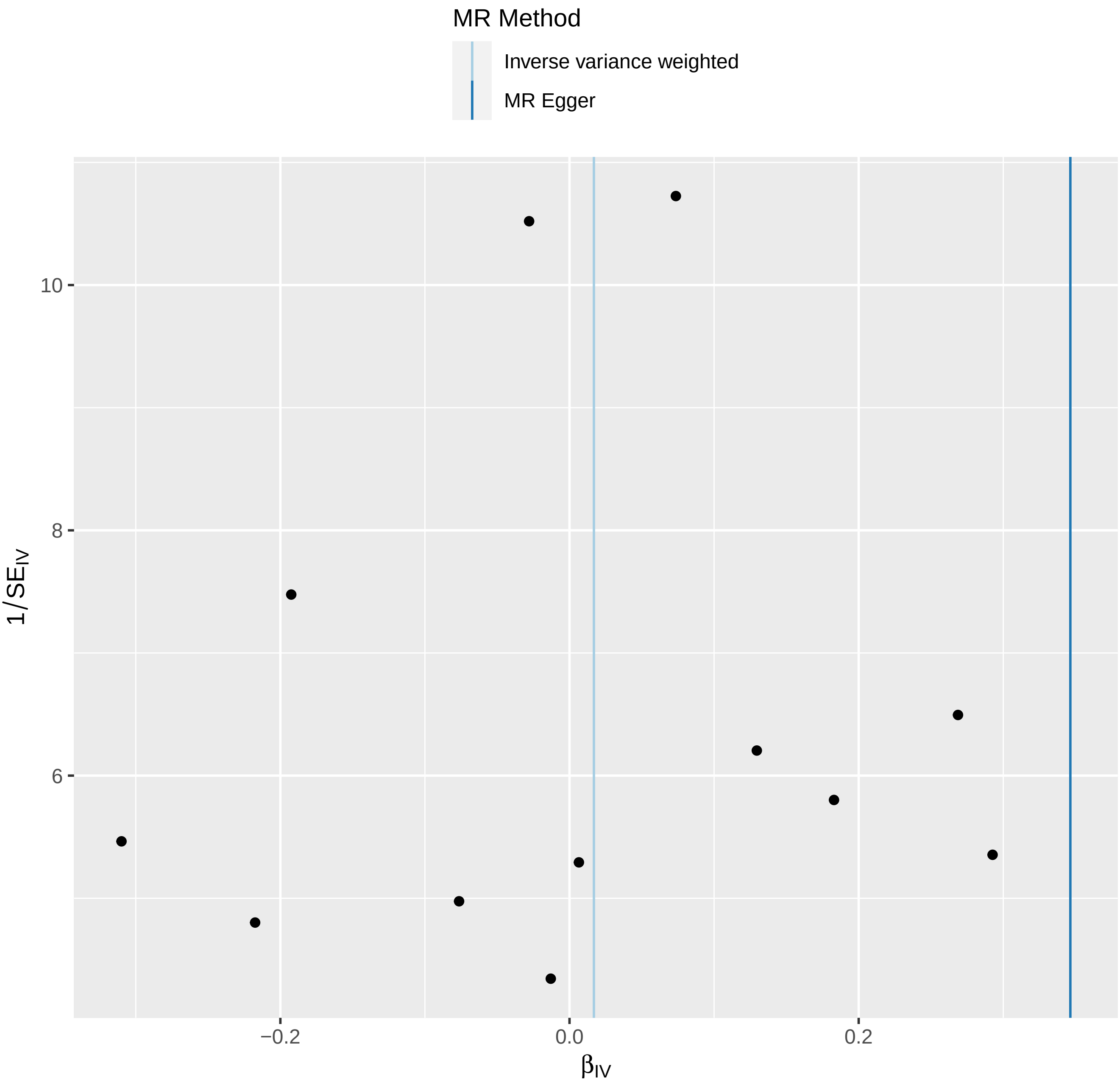

Batch 3−1249 : Selenium on Glycerophosphocholine phosphodiesterase GPCPD1

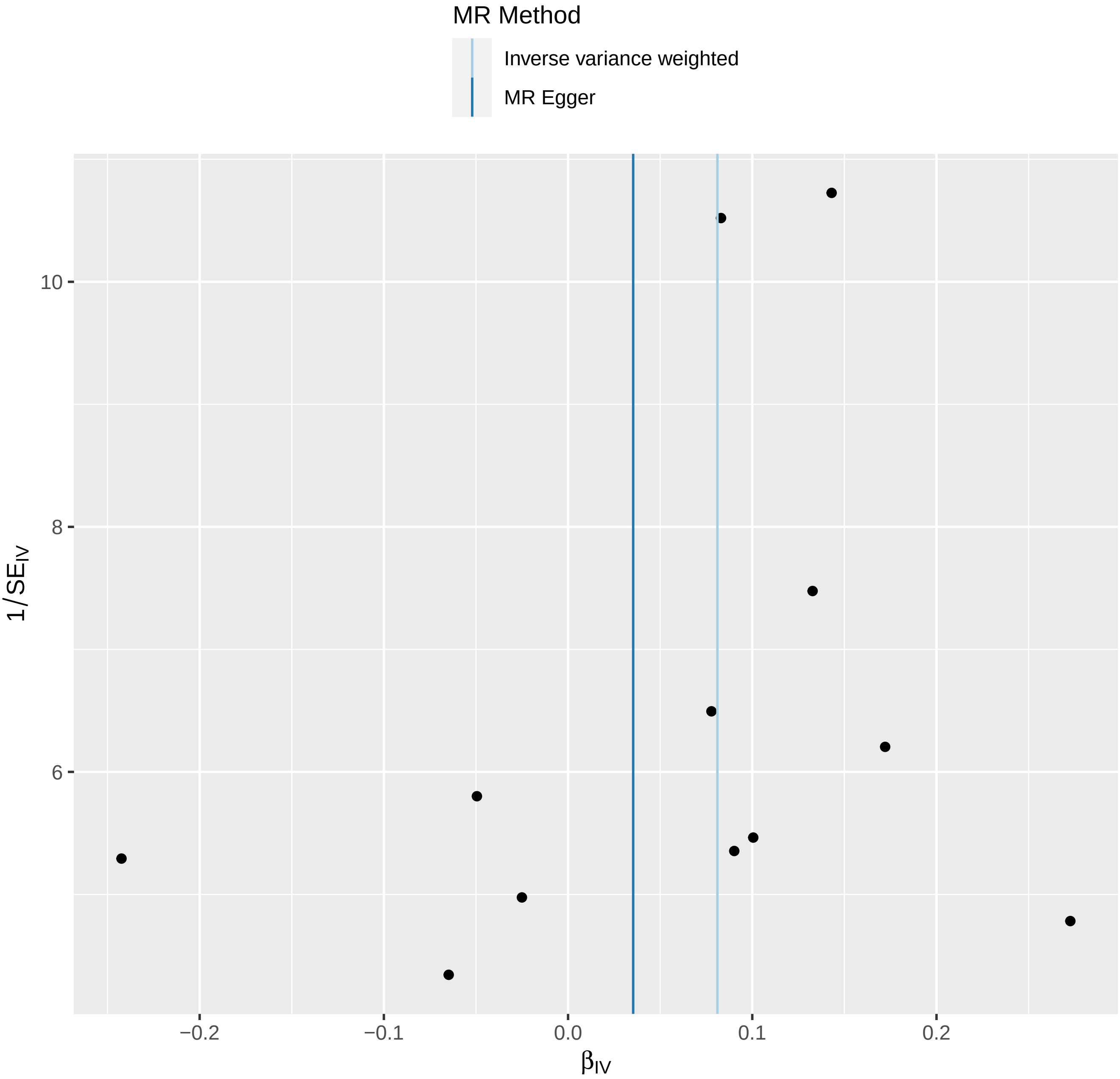

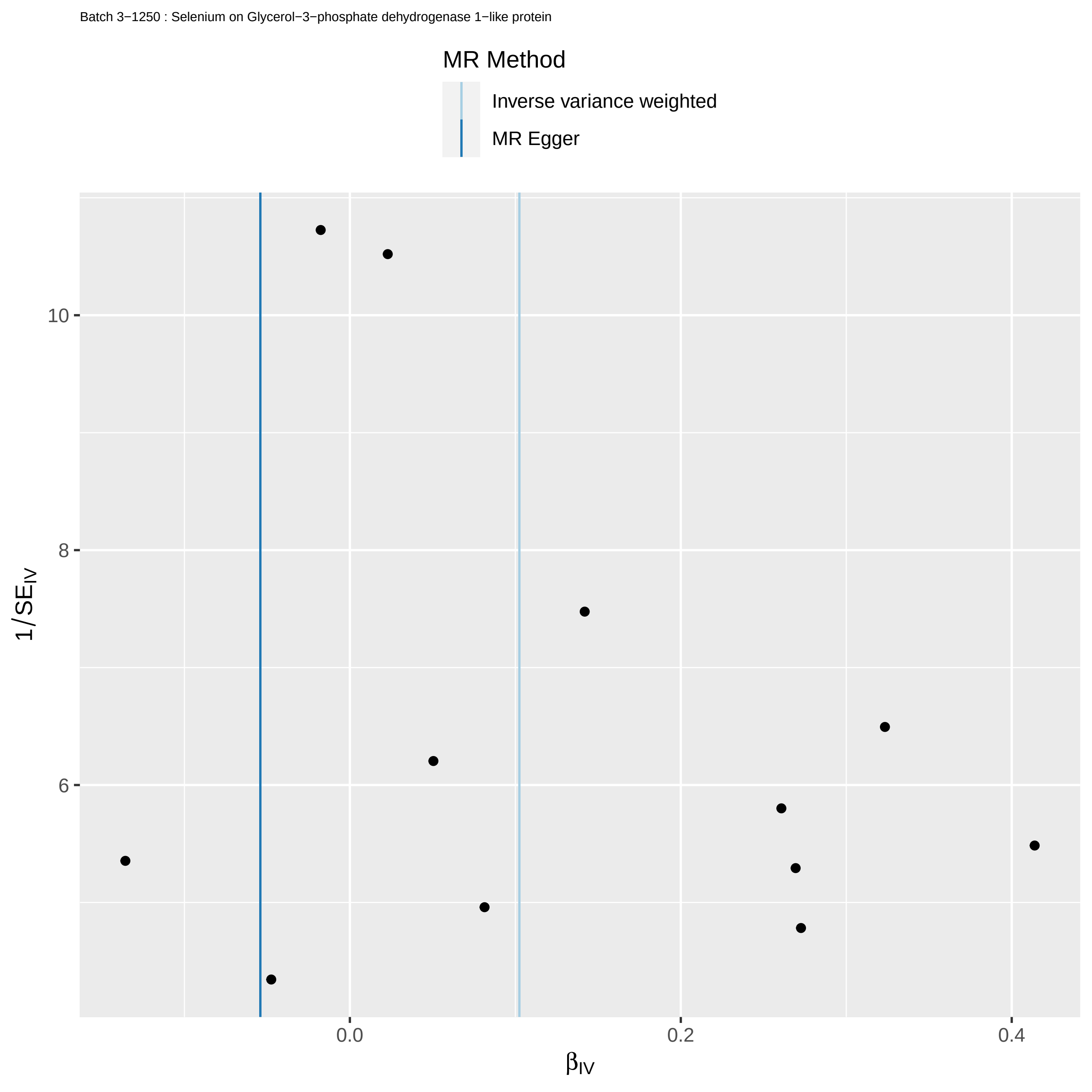

Batch 3−1250 : Selenium on Glycerol−3−phosphate dehydrogenase 1−like protein
MR Method
Inverse variance weighted
MR Egger
10
8
6
1/SE$_{IV}$
0.0
0.2
0.4
β$_{IV}$

Batch 3−1251 : Selenium on Glycoprotein hormone alpha−2

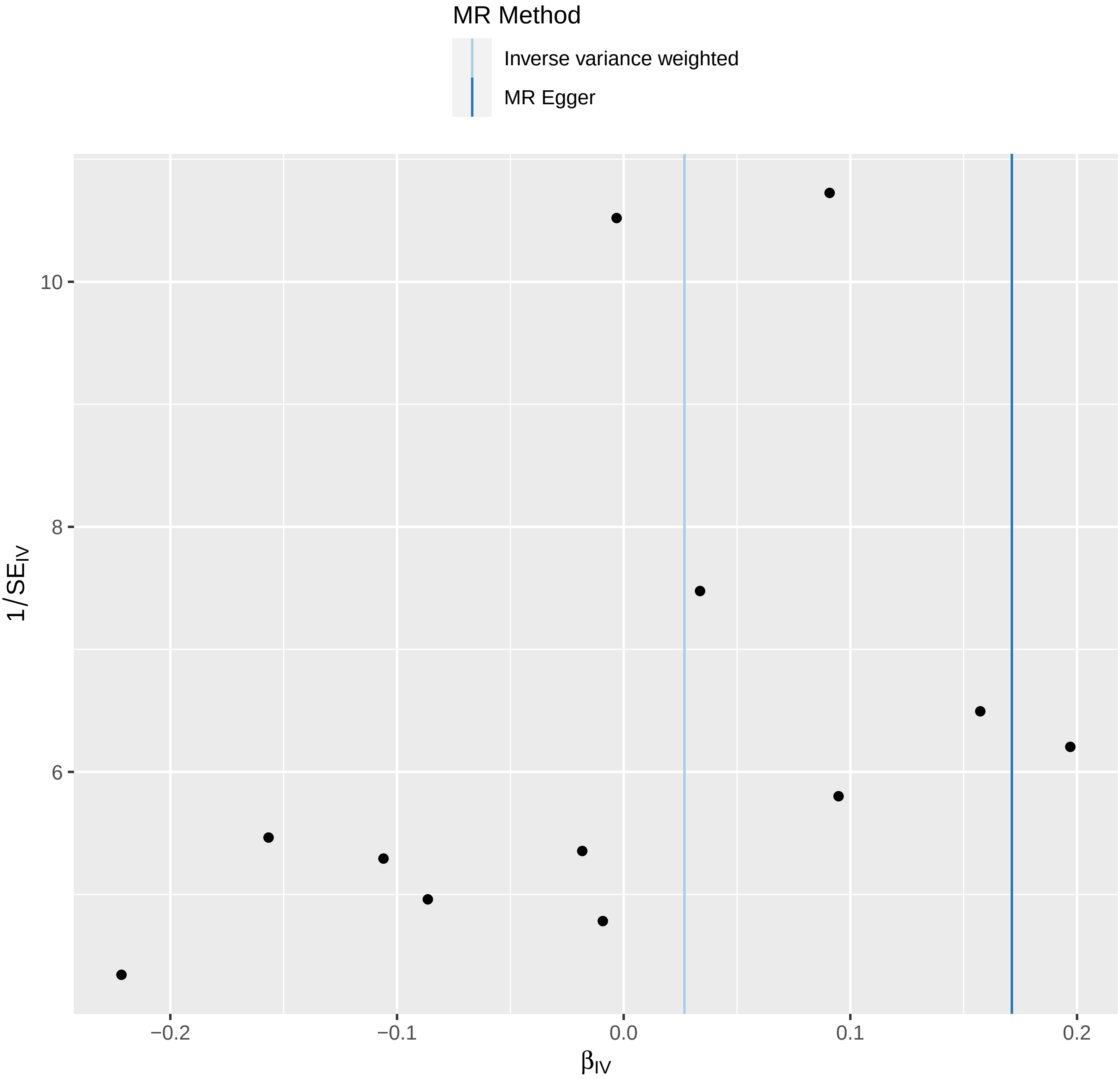

Batch 3−1252 : Selenium on Glucose−6−phosphate isomerase

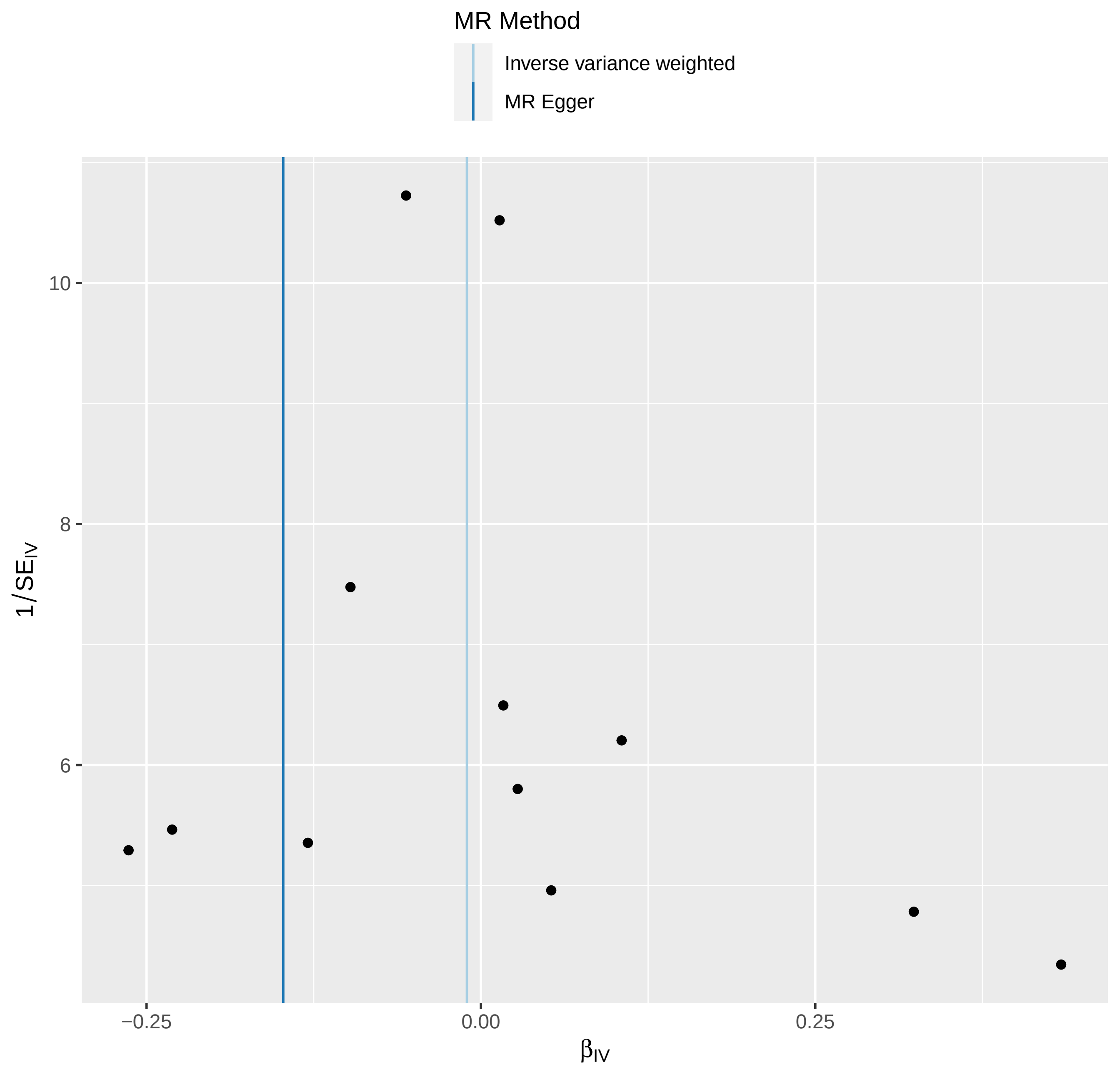

Batch 3−1253 : Selenium on Phosphatidylinositol−glycan−specific phospholipase D

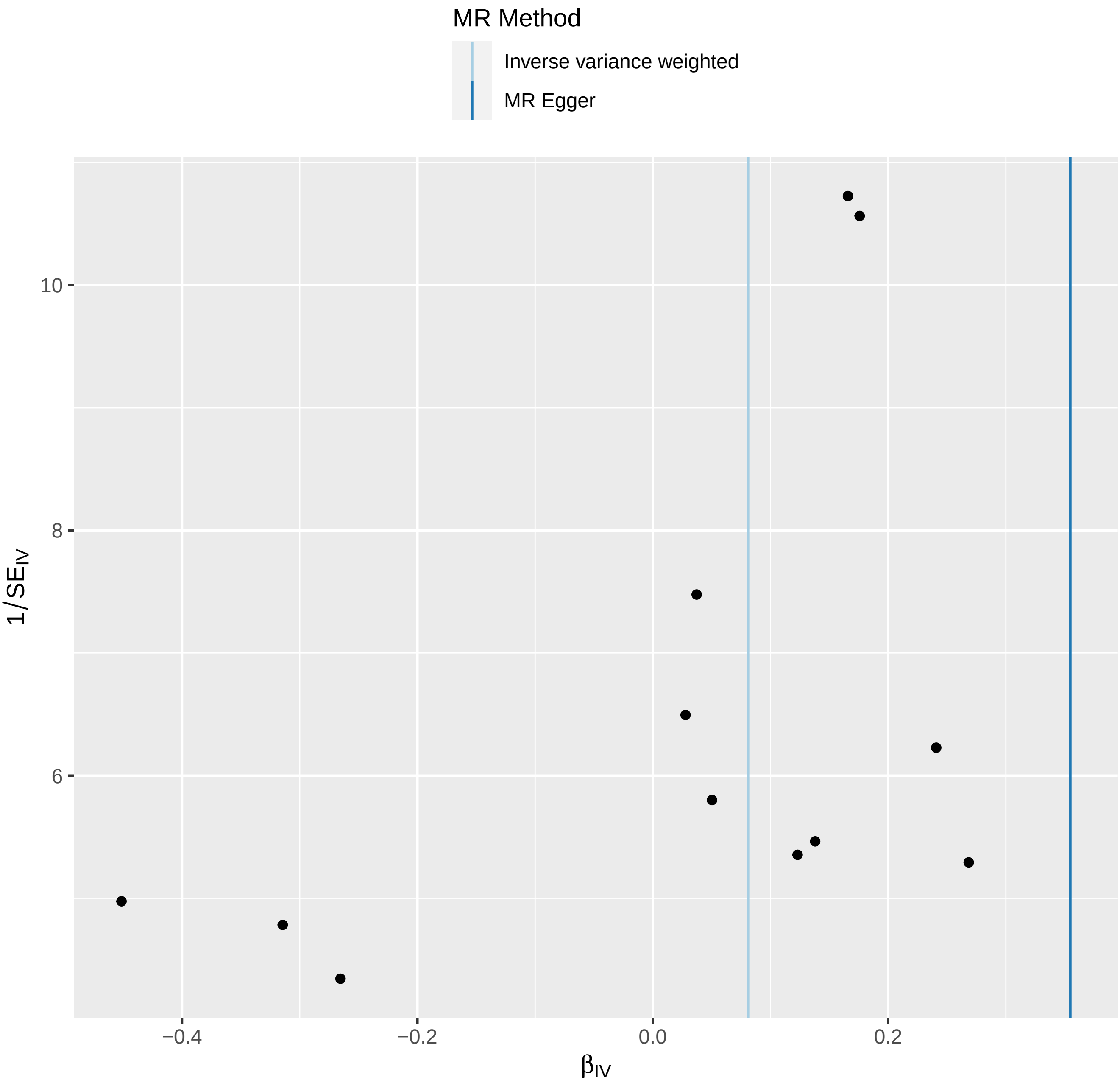

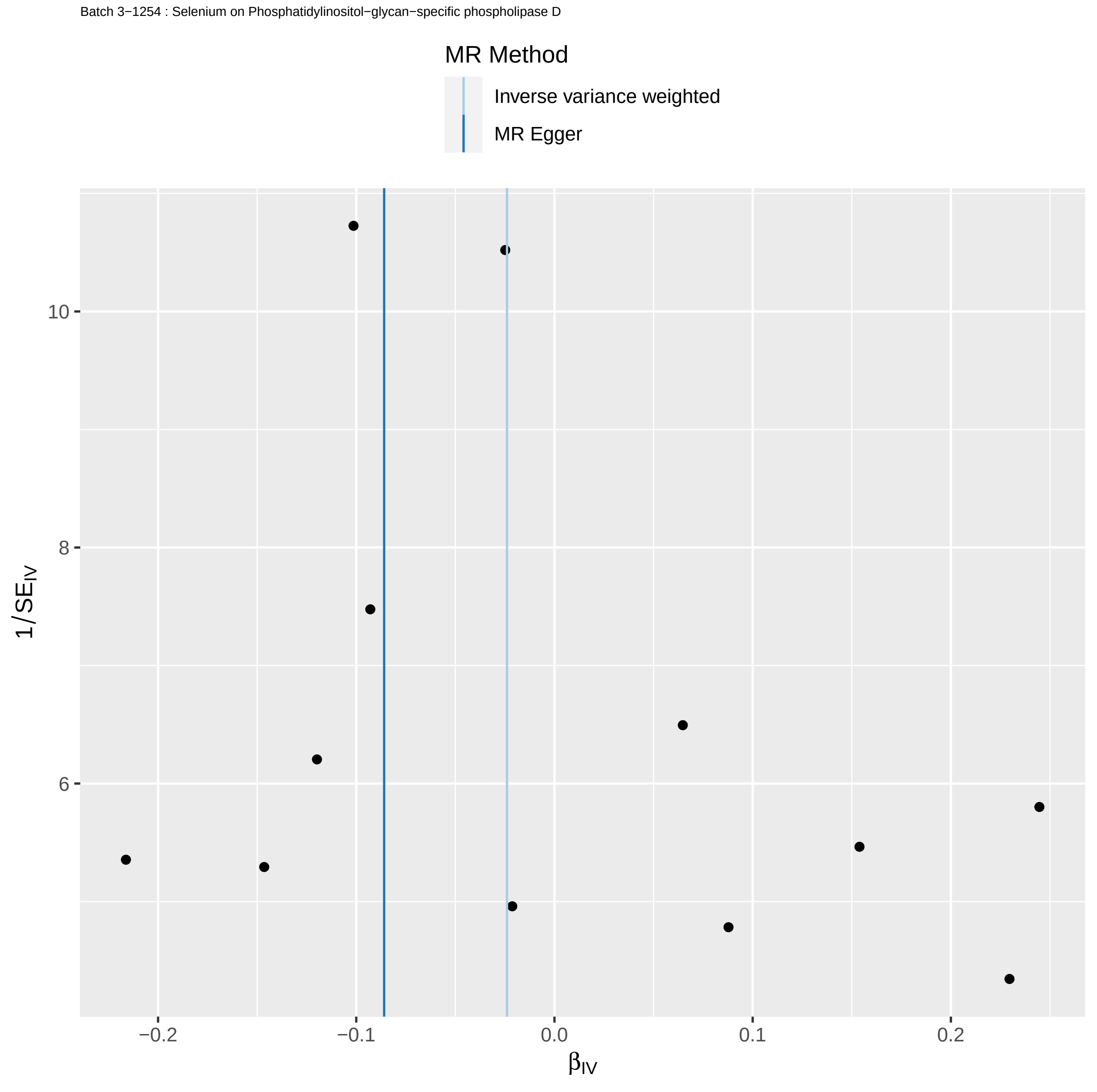
Batch 3−1254 : Selenium on Phosphatidylinositol−glycan−specific phospholipase D
MR Method
Inverse variance weighted
MR Egger
10
8
6
1/SE$_{IV}$
−0.2
−0.1
0.0
0.1
0.2
β$_{IV}$

Batch 3−1255 : Selenium on Transmembrane glycoprotein NMB

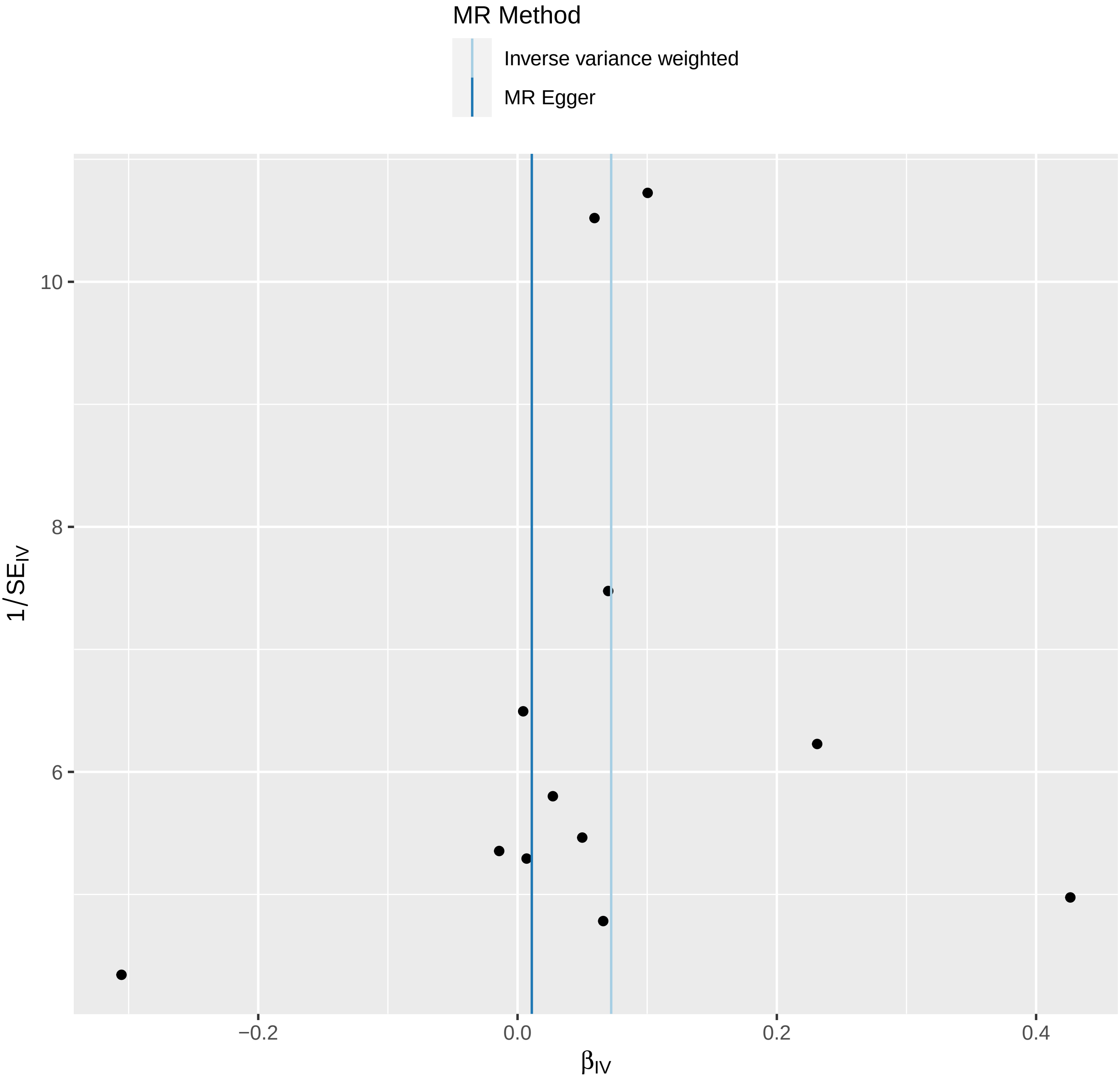

Batch 3−1256 : Selenium on Transmembrane glycoprotein NMB

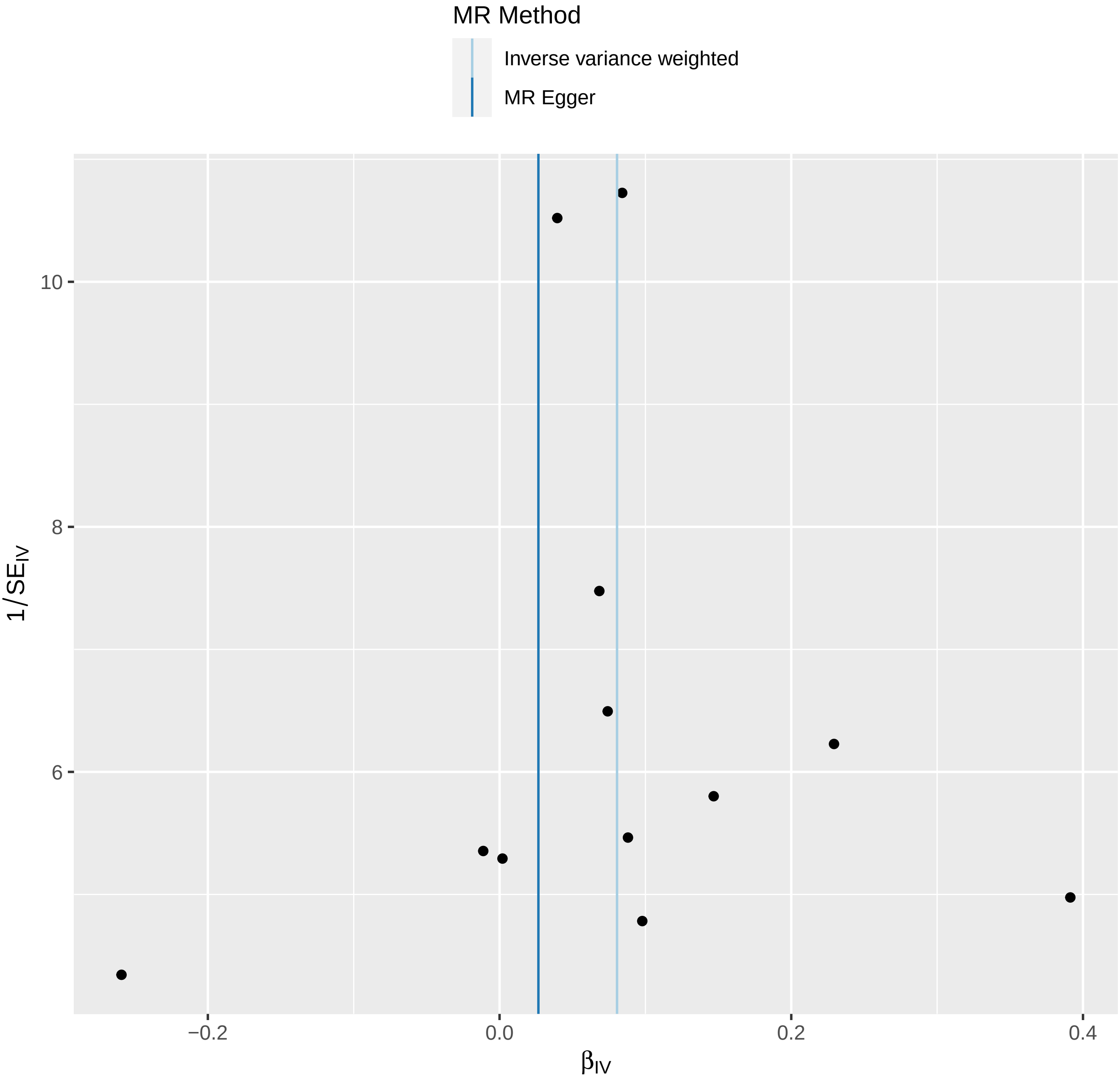

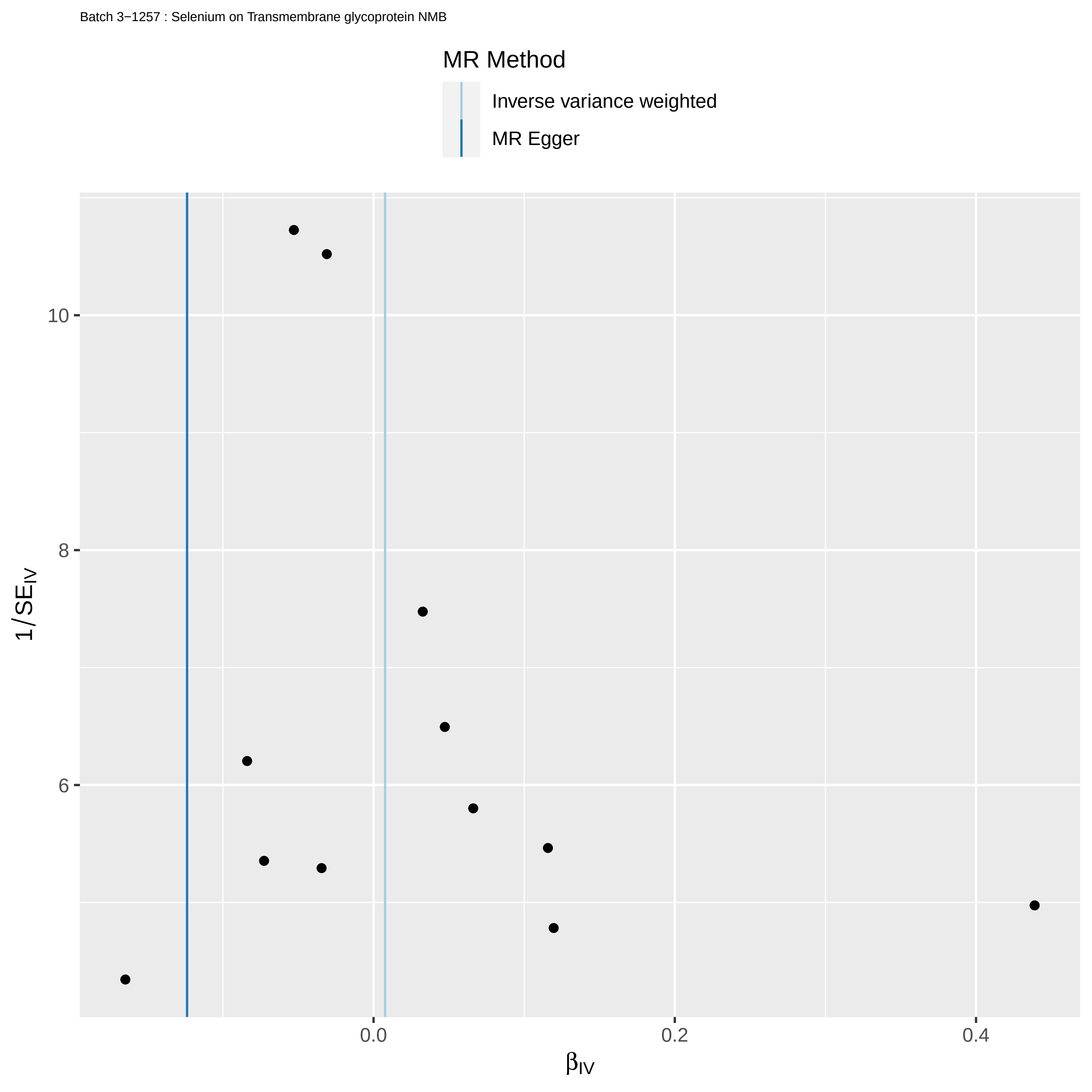

Batch 3−1257 : Selenium on Transmembrane glycoprotein NMB
MR Method
Inverse variance weighted
MR Egger
10
8
6
1/SE$_{IV}$
0.0
0.2
0.4
β$_{IV}$

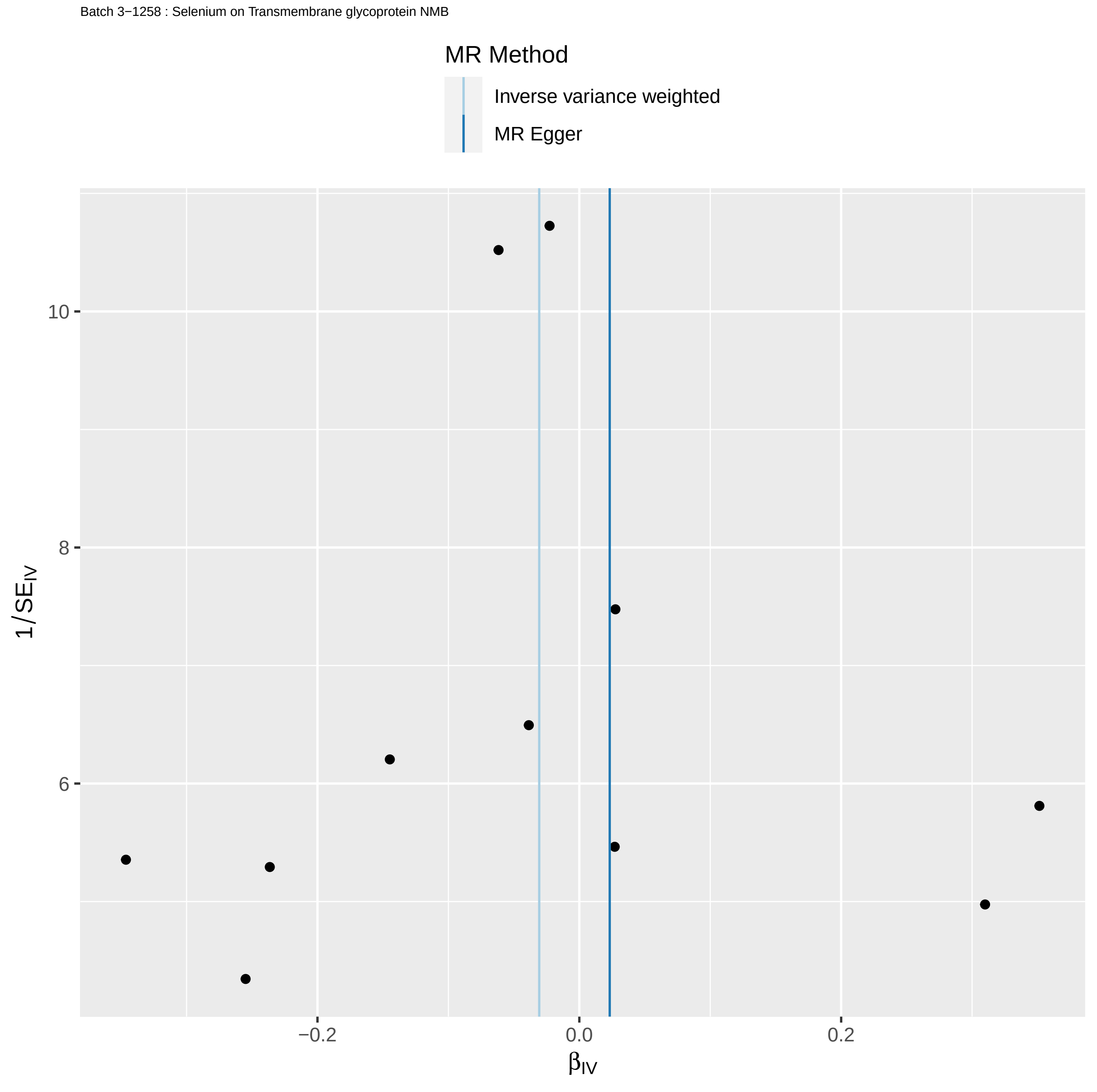

Batch 3−1258 : Selenium on Transmembrane glycoprotein NMB
MR Method
Inverse variance weighted
MR Egger
10
8
6
$1/SE_{IV}$
−0.2
0.0
0.2
β_{IV}

Batch 3−1259 : Selenium on Probable G−protein coupled receptor 101

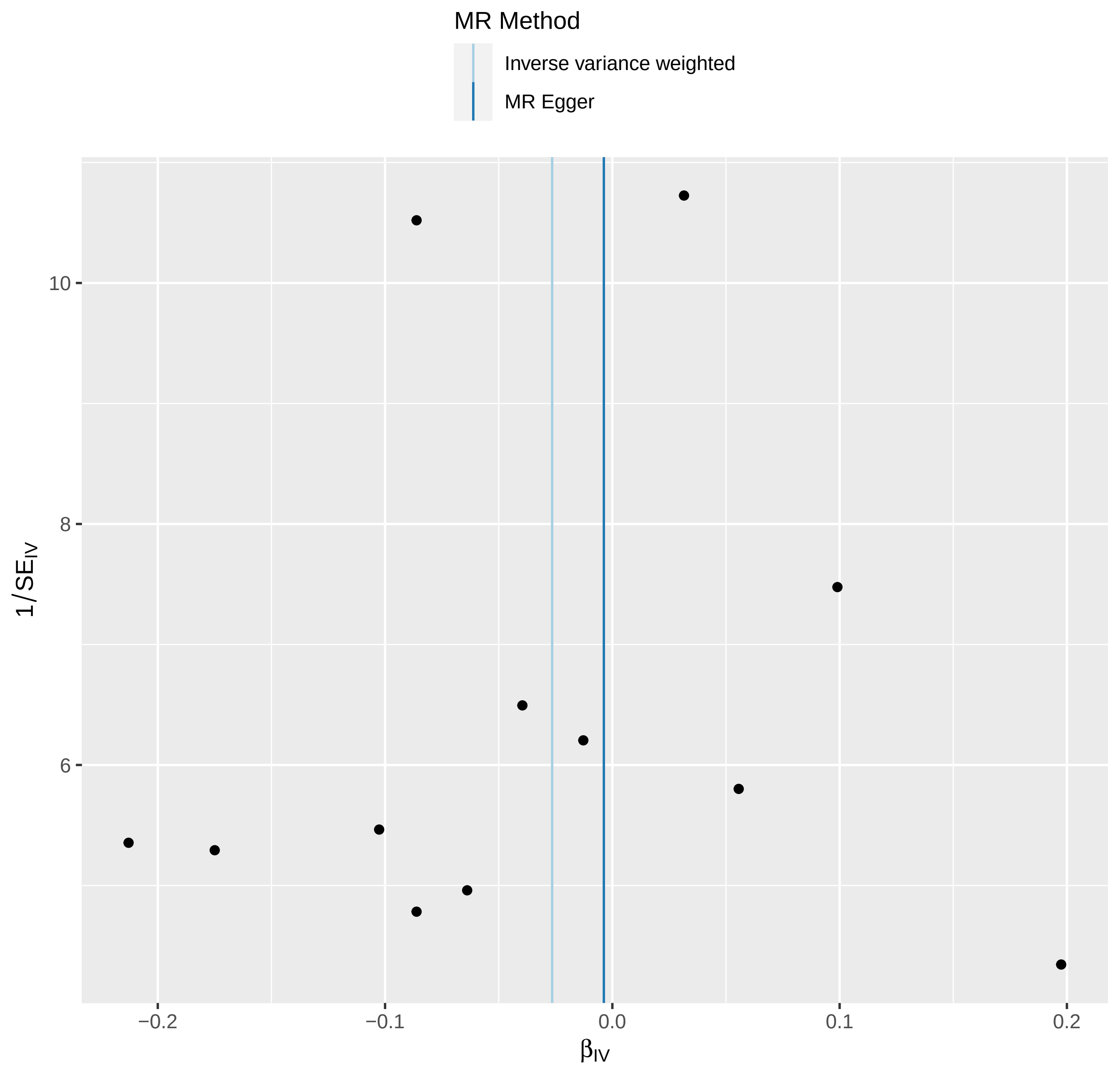

Batch 3−1260 : Selenium on Probable G−protein coupled receptor 101

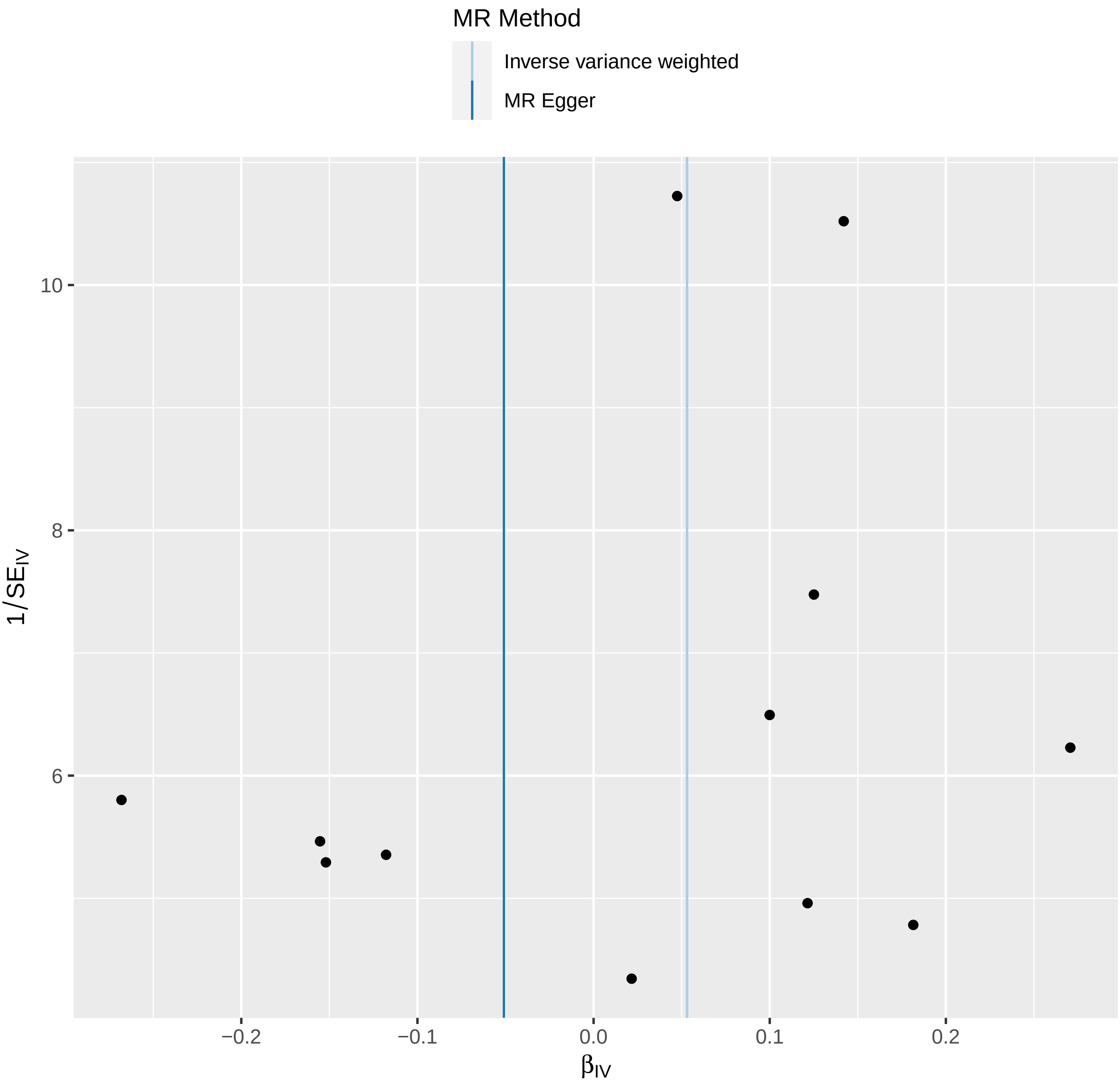

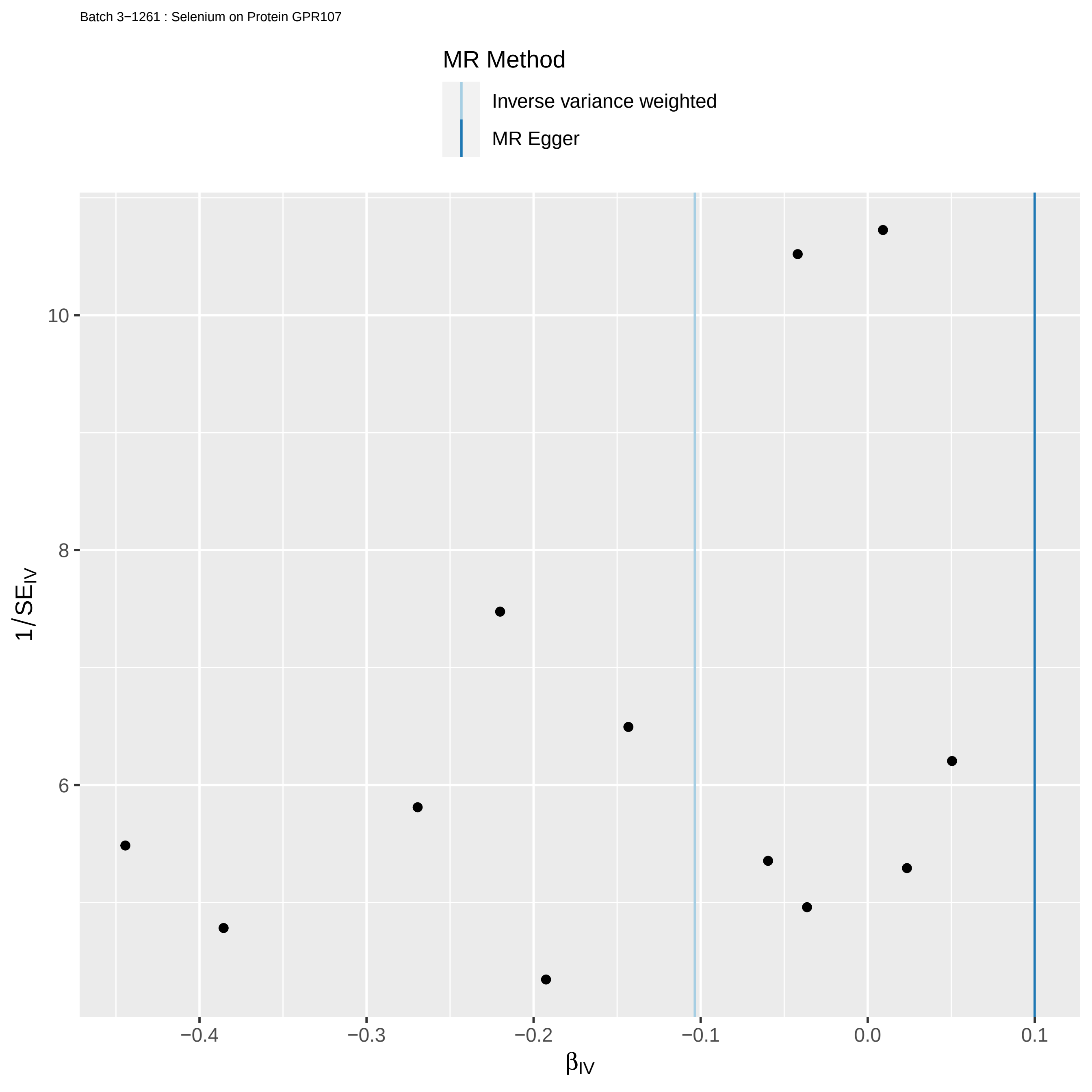

Batch 3−1261 : Selenium on Protein GPR107
MR Method
Inverse variance weighted
MR Egger
10
8
6
−0.4
−0.3
−0.2
−0.1
0.0
0.1
$1/SE_{IV}$
β_{IV}

Batch 3−1262 : Selenium on Probable G−protein coupled receptor 135

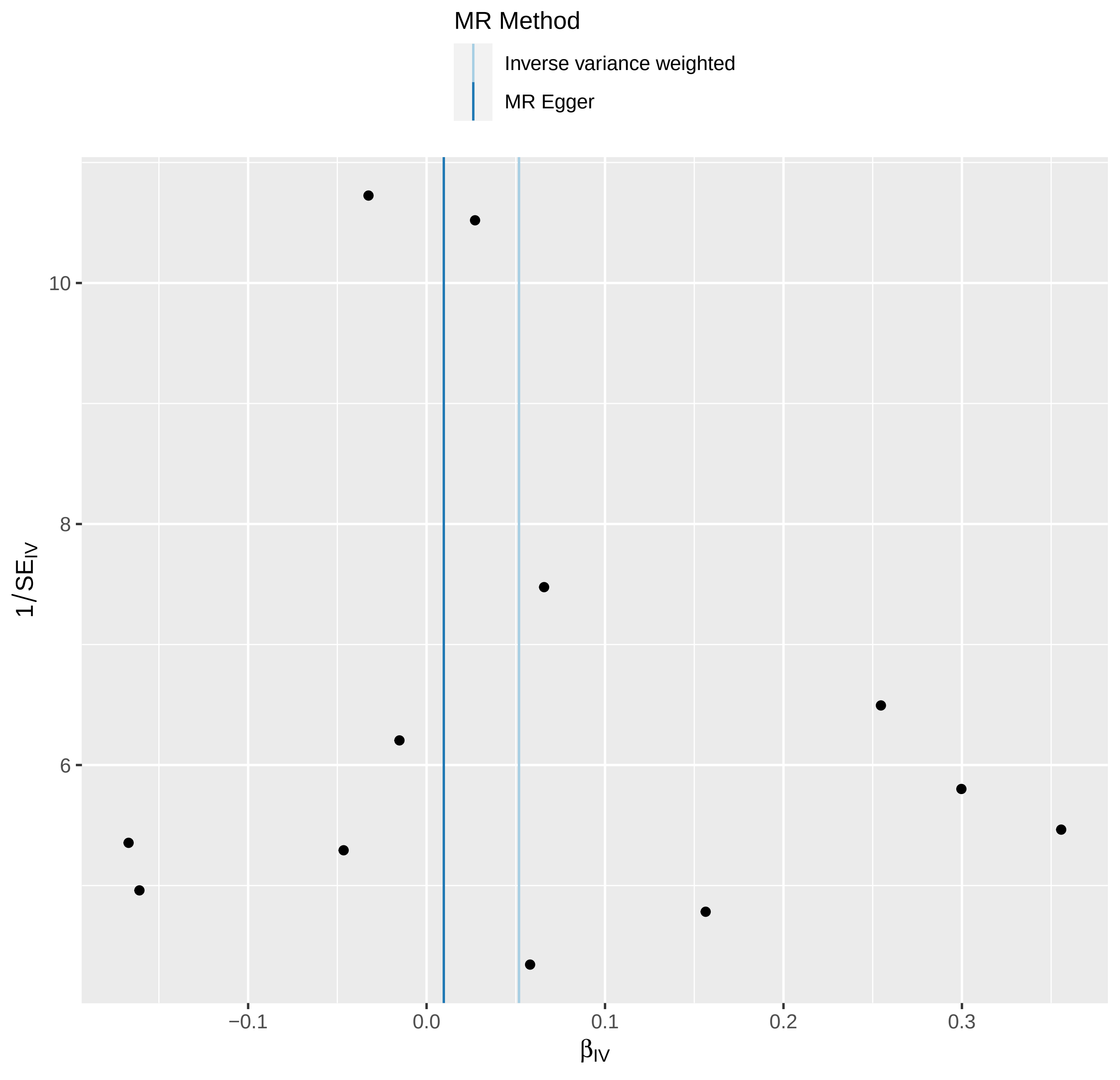

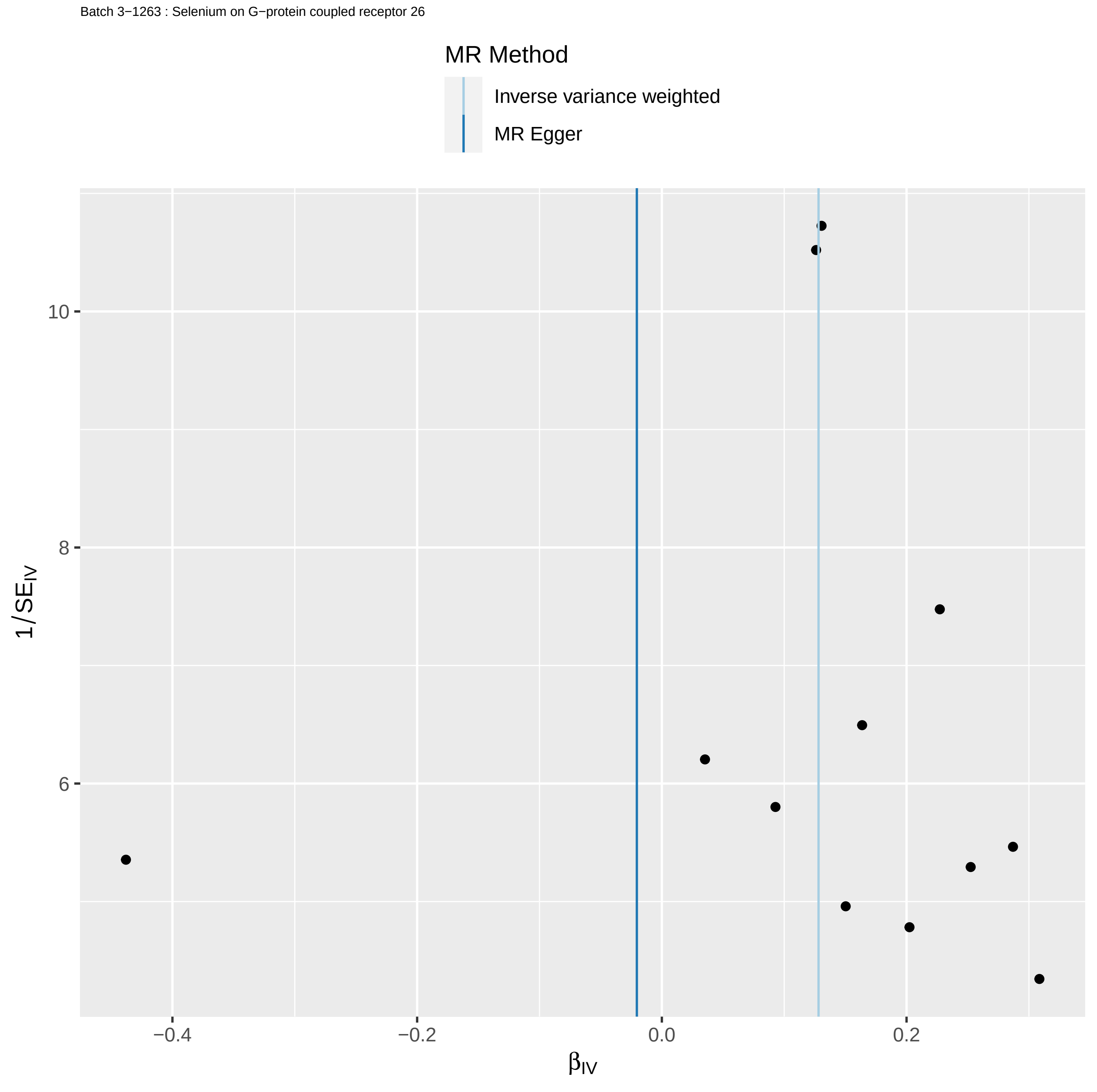

Batch 3−1263 : Selenium on G−protein coupled receptor 26
MR Method
Inverse variance weighted
MR Egger
10
8
6
1/SE$_{IV}$
−0.4
−0.2
0.0
0.2
β$_{IV}$

Batch 3−1264 : Selenium on Alanine aminotransferase 1

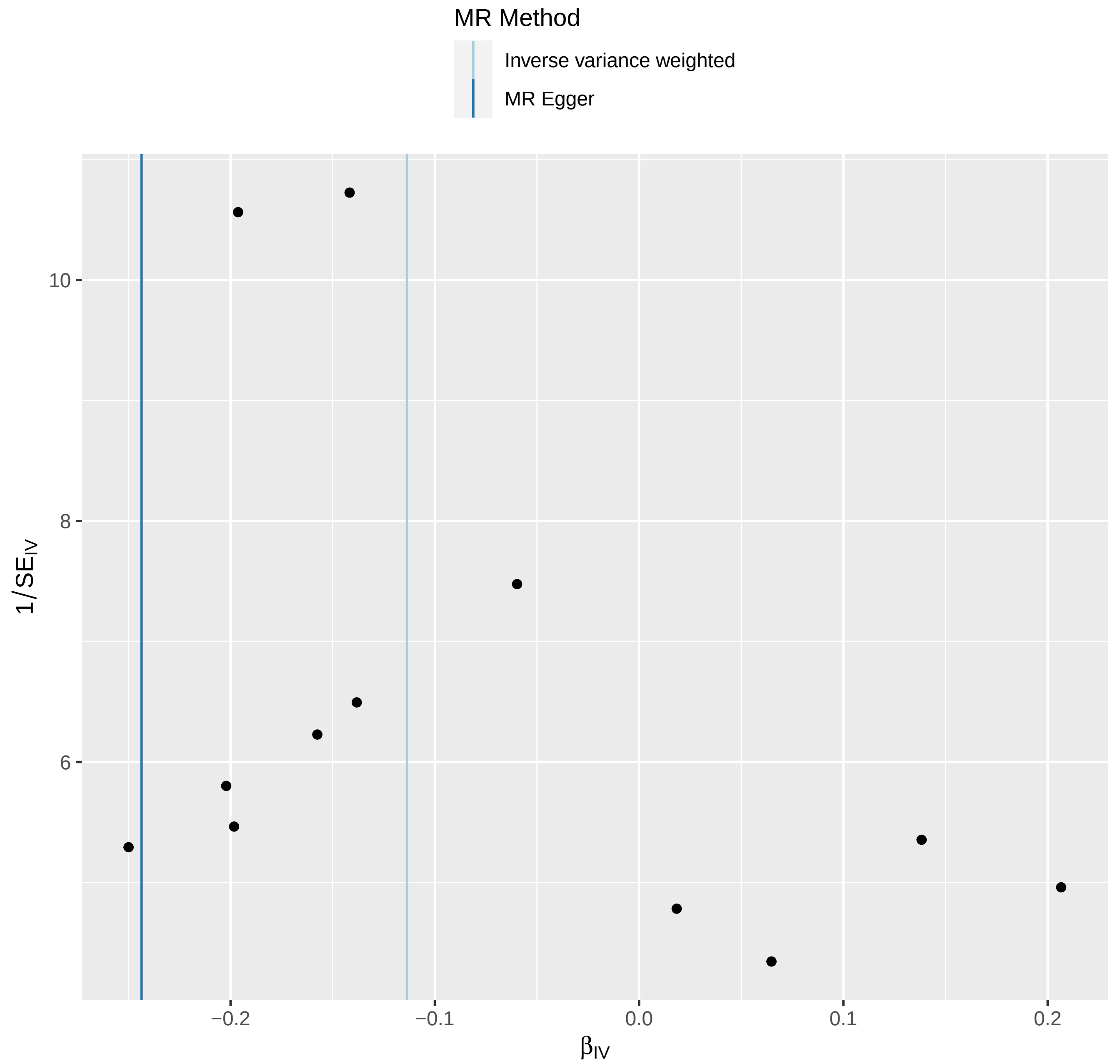

Batch 3−1265 : Selenium on Glutathione peroxidase 7

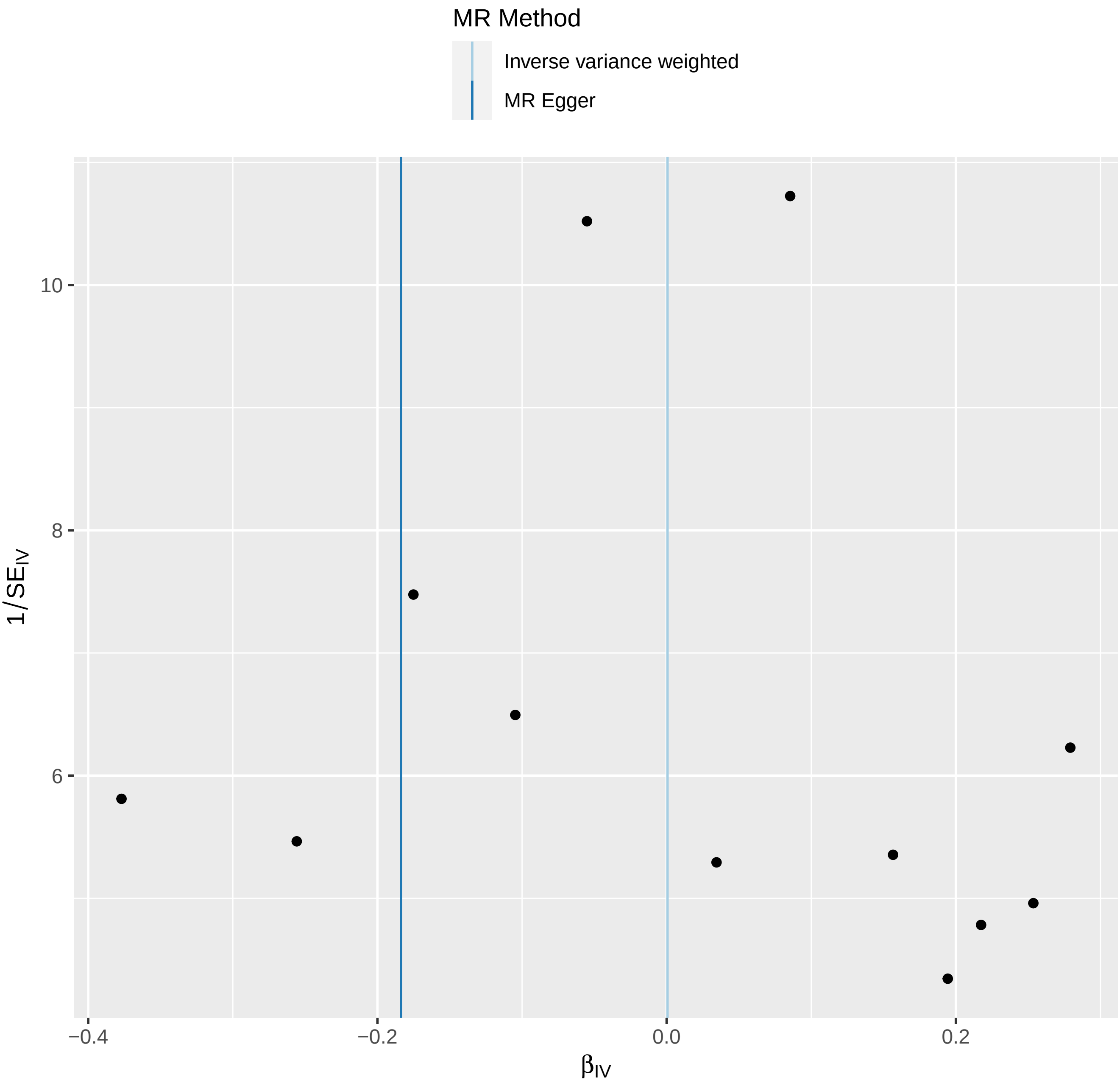

Batch 3−1266 : Selenium on GRAM domain−containing protein 1C

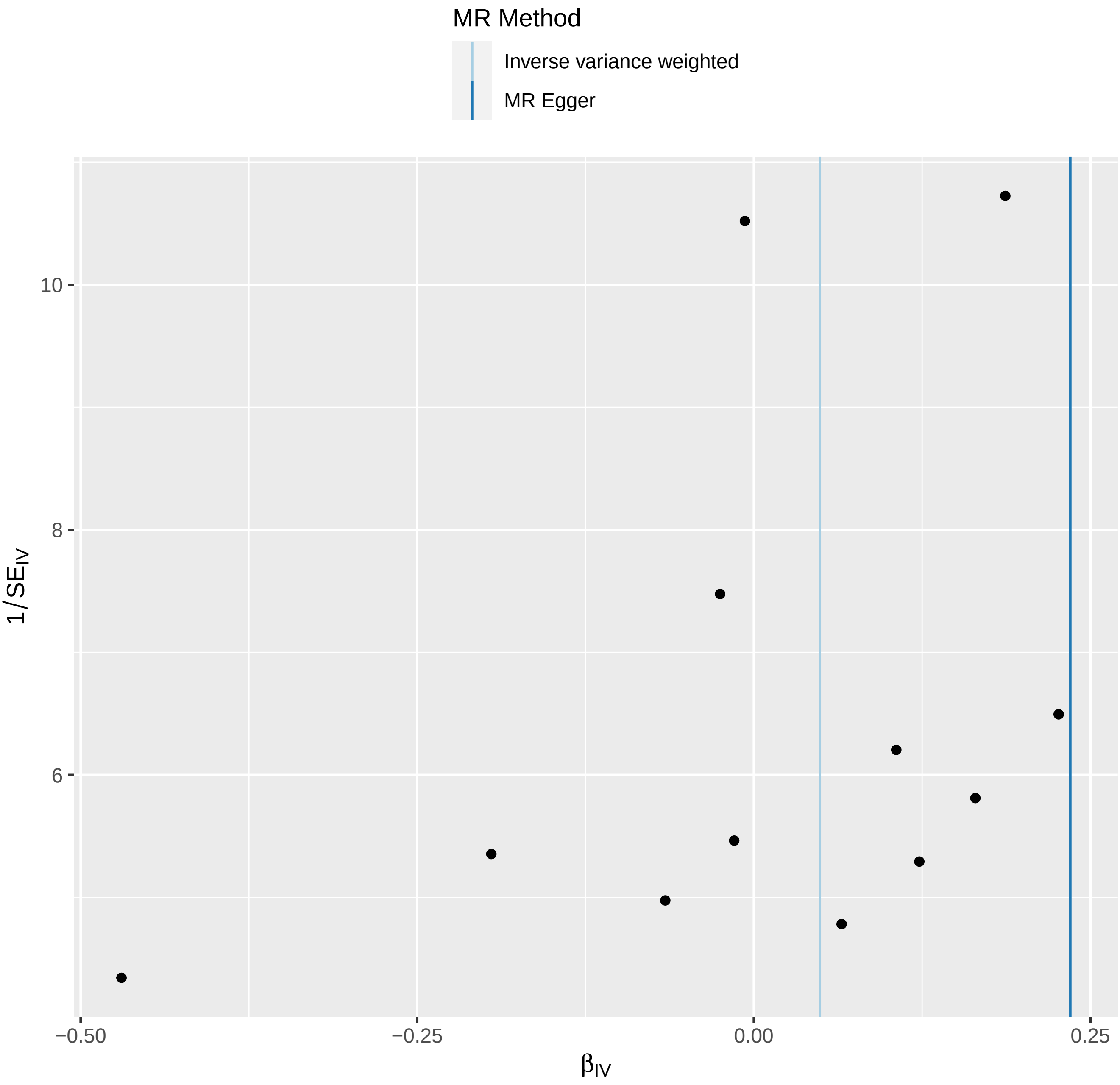

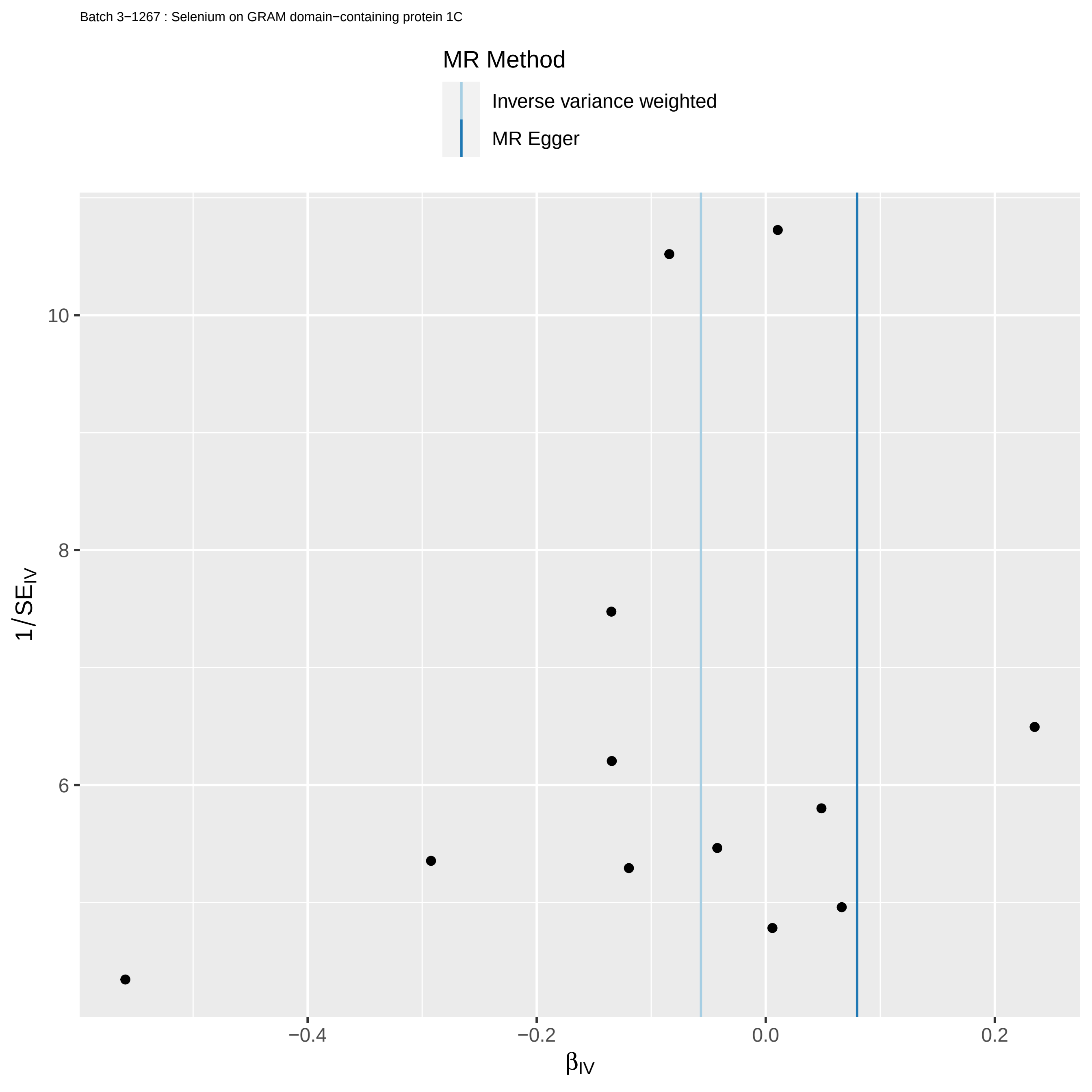

Batch 3−1267 : Selenium on GRAM domain−containing protein 1C
MR Method
Inverse variance weighted
MR Egger
10
8
6
1/SE$_{IV}$
−0.4
−0.2
0.0
0.2
β$_{IV}$

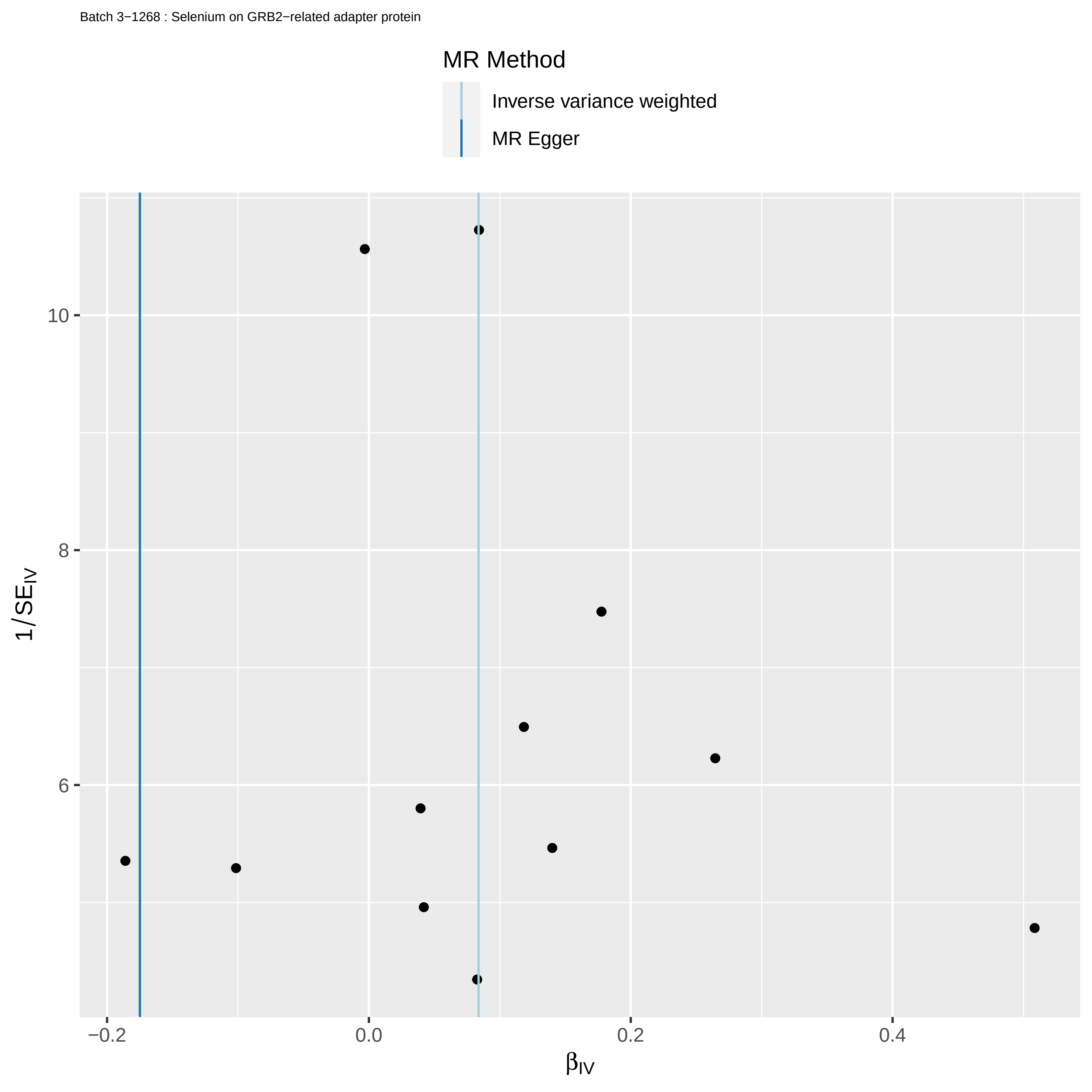
Batch 3−1268 : Selenium on GRB2−related adapter protein
MR Method
Inverse variance weighted
MR Egger
10
8
6
1/SE$_{IV}$
−0.2
0.0
0.2
0.4
β$_{IV}$

Batch 3−1269 : Selenium on GRB2−related adapter protein 2

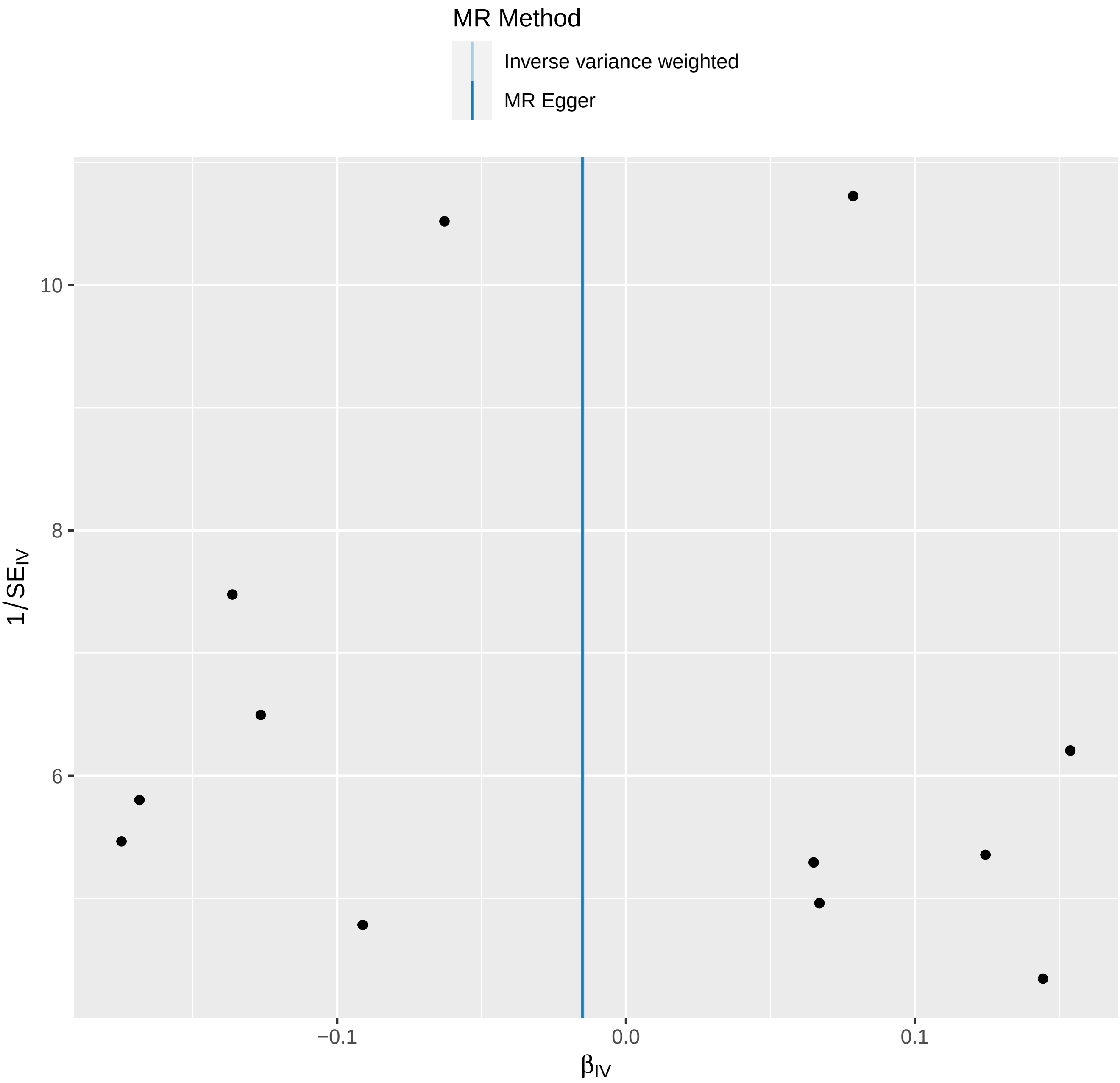

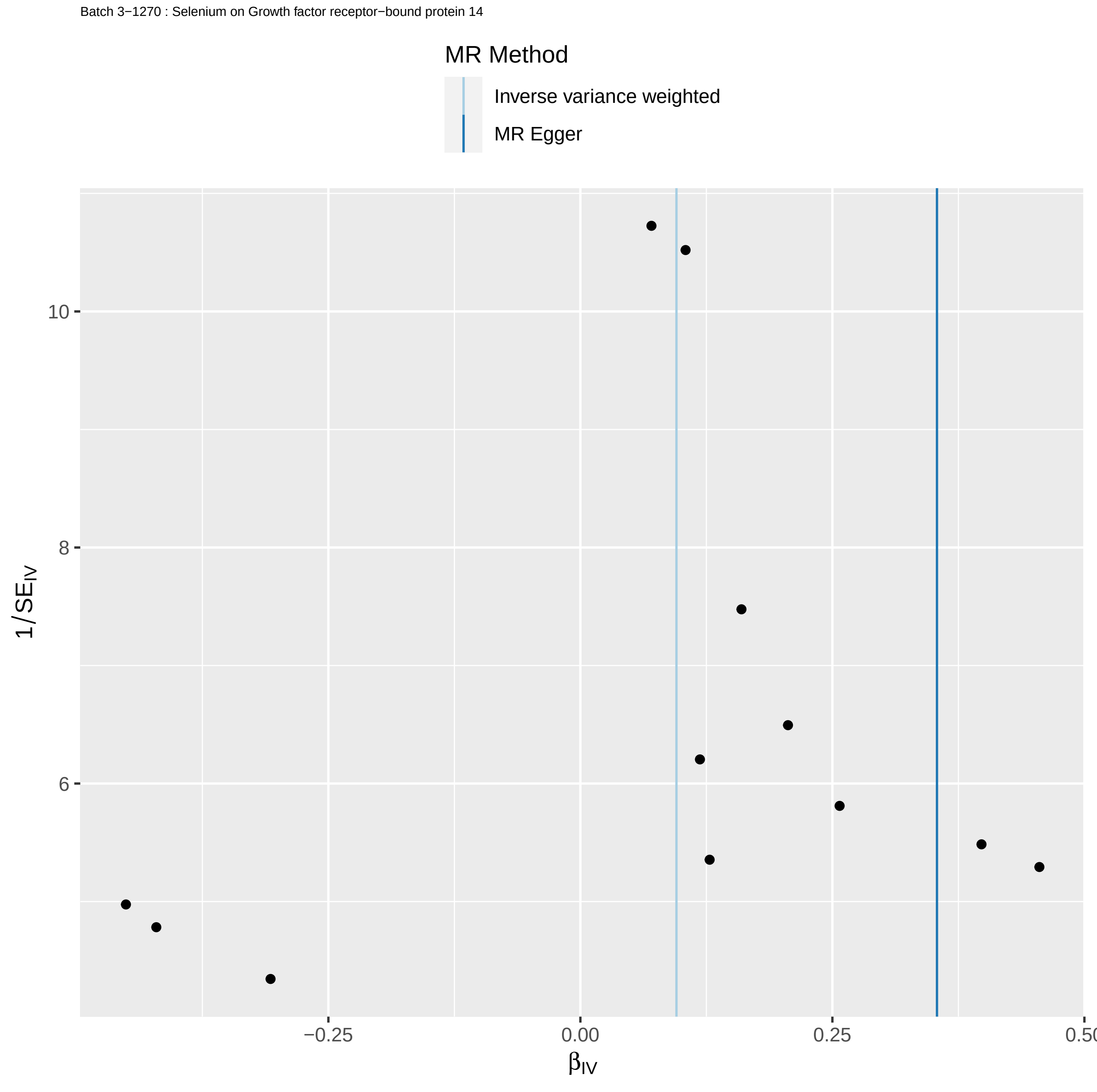

Batch 3−1270 : Selenium on Growth factor receptor−bound protein 14
MR Method
Inverse variance weighted
MR Egger
10
8
6
1/SE$_{IV}$
−0.25
0.00
0.25
0.50
β$_{IV}$

Batch 3−1271 : Selenium on Growth factor receptor−bound protein 2

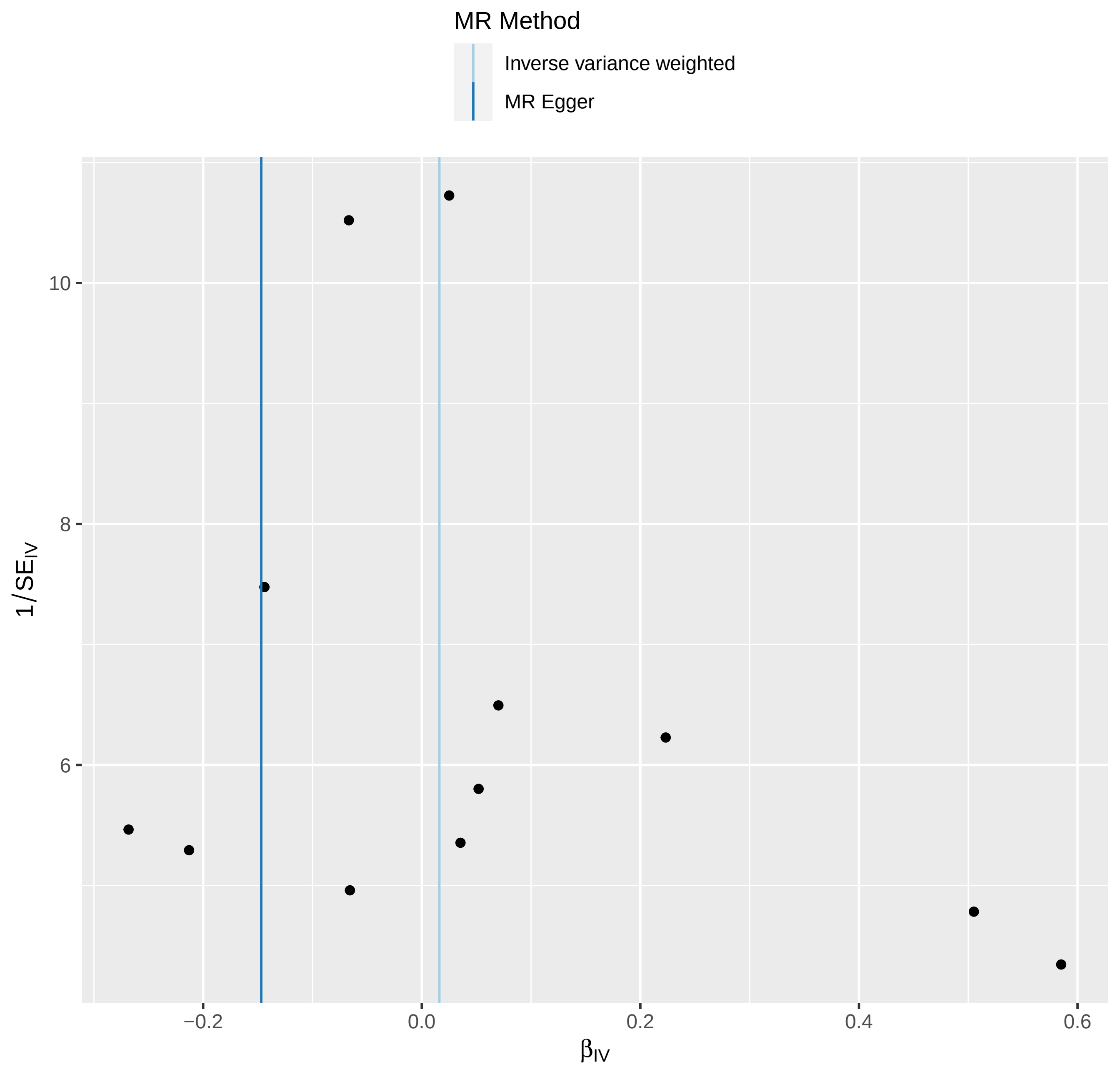

Batch 3−1272 : Selenium on Growth factor receptor−bound protein 7

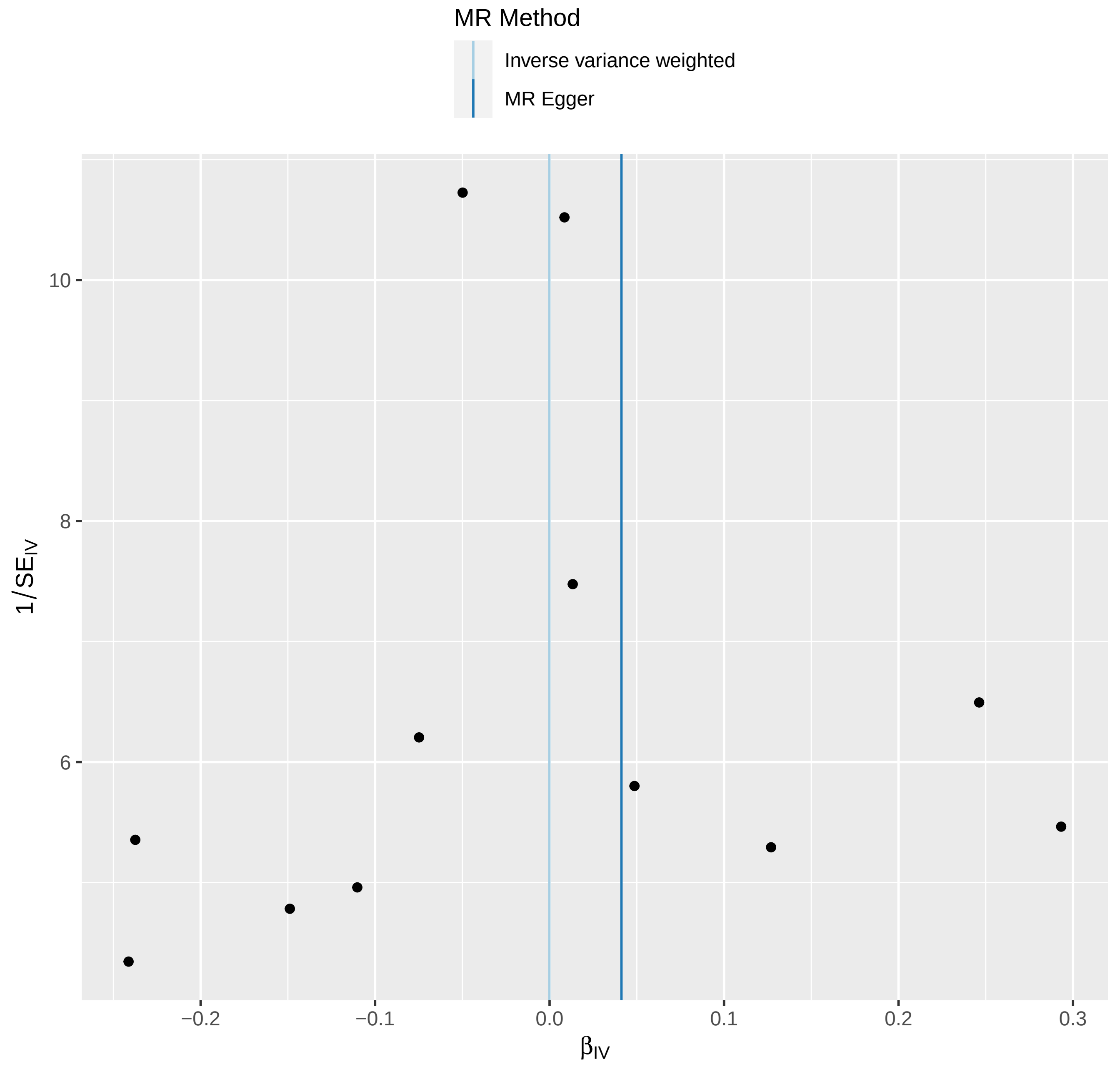

Batch 3−1273 : Selenium on Gremlin−1

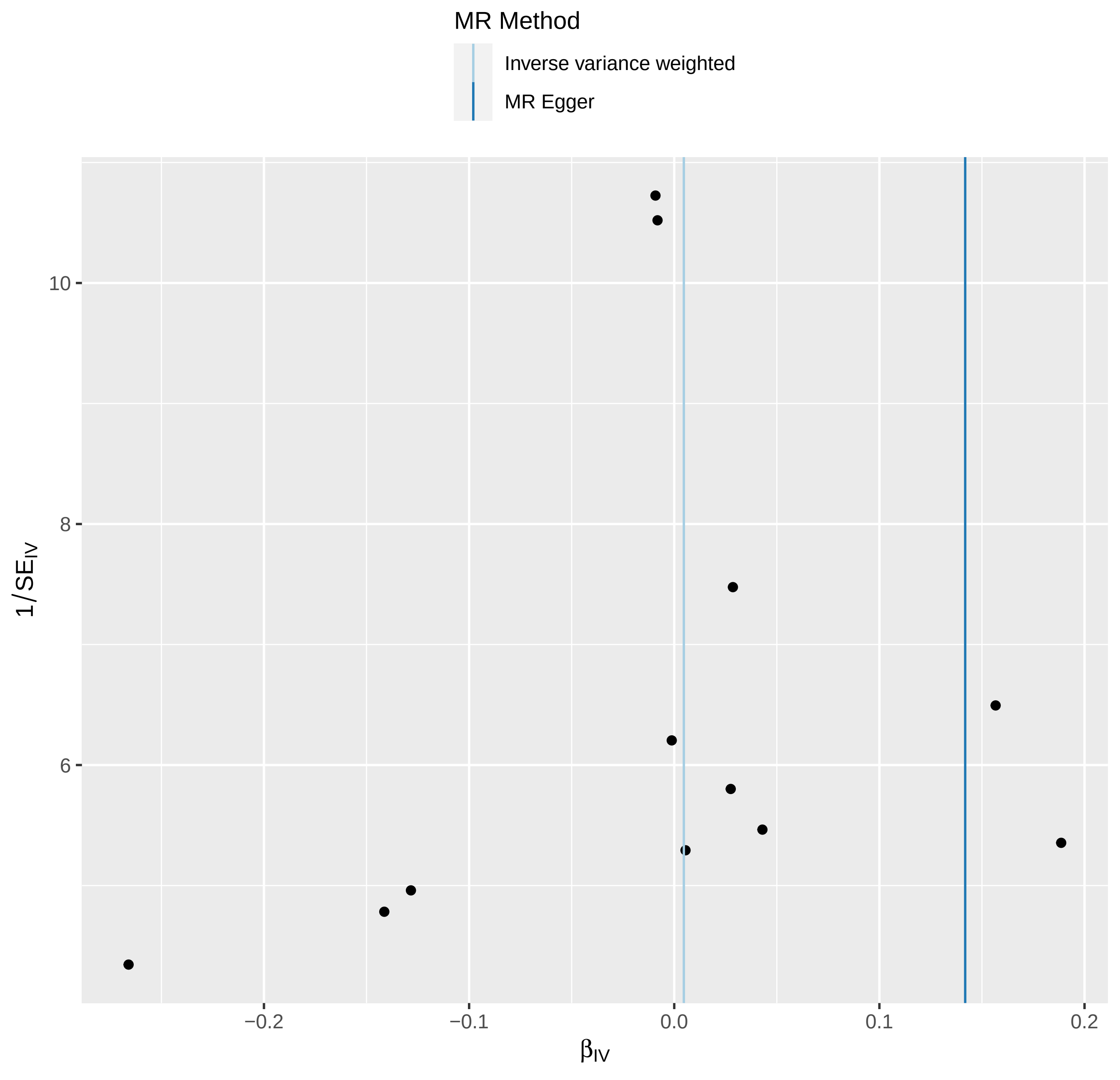

Batch 3−1274 : Selenium on Gremlin−2

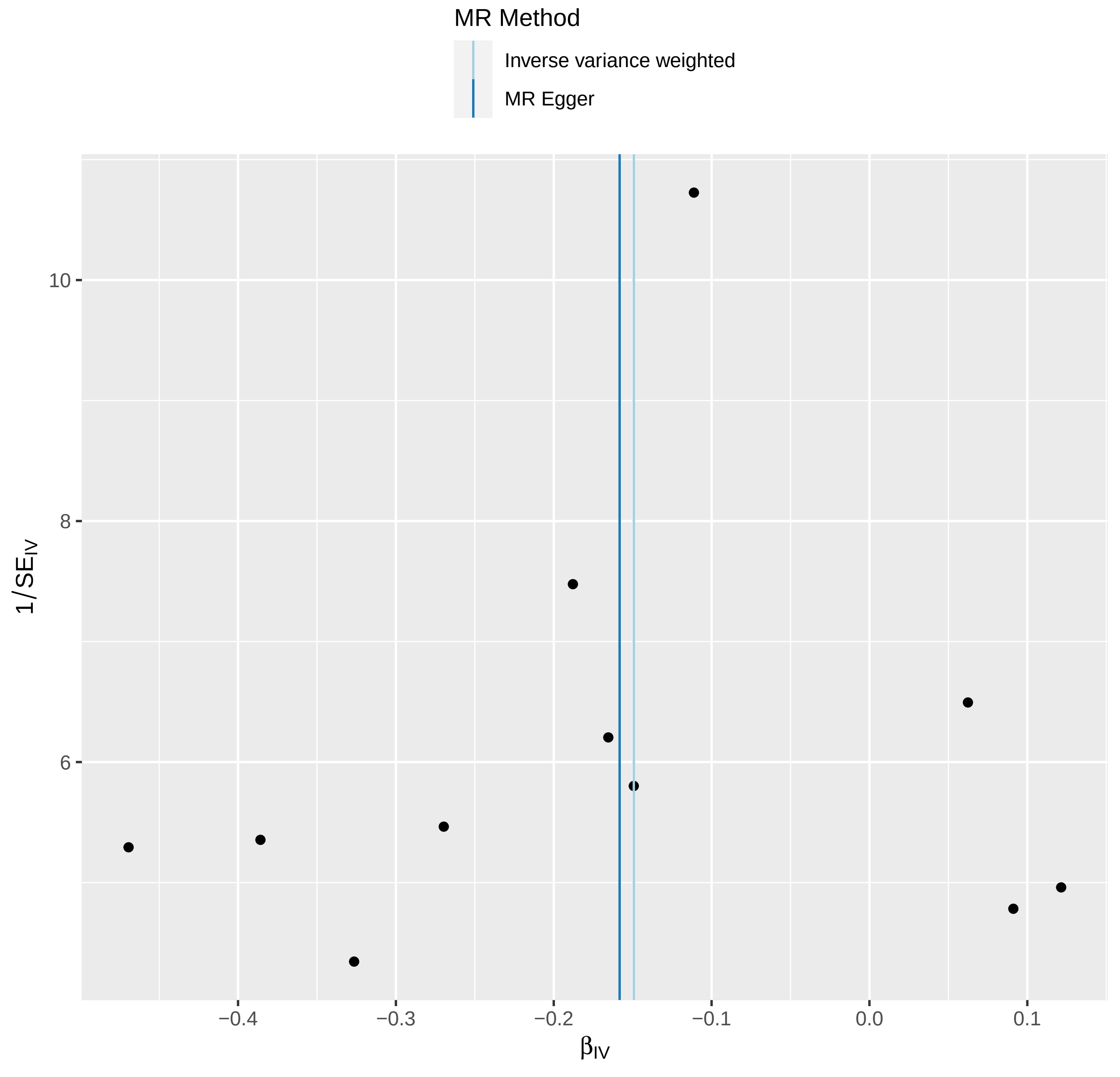

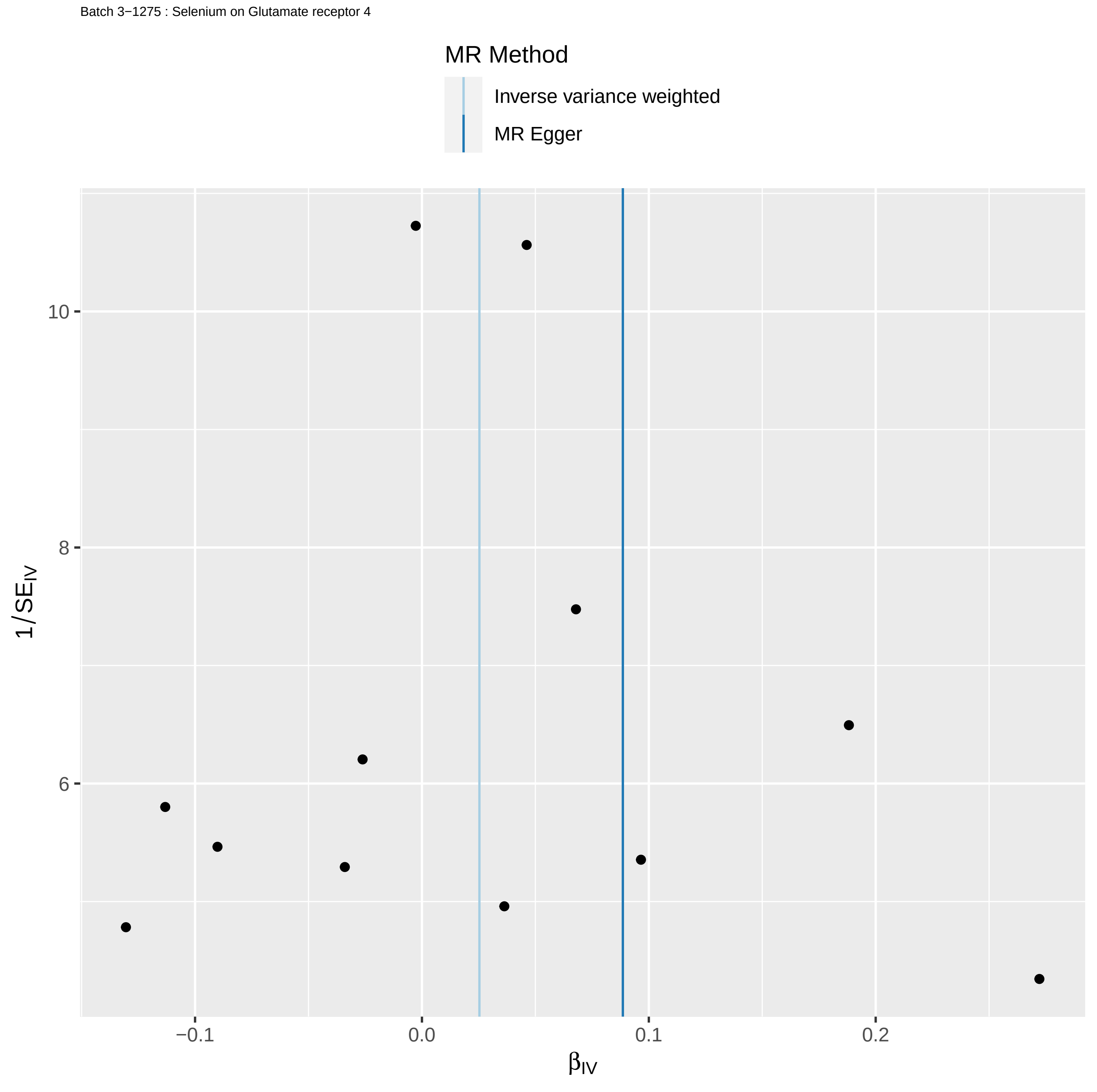

Batch 3−1275 : Selenium on Glutamate receptor 4
MR Method
Inverse variance weighted
MR Egger
10
8
6
1/SE_IV
−0.1
0.0
0.1
0.2
β_IV

Batch 3−1276 : Selenium on Glutamate receptor ionotropic, delta−2

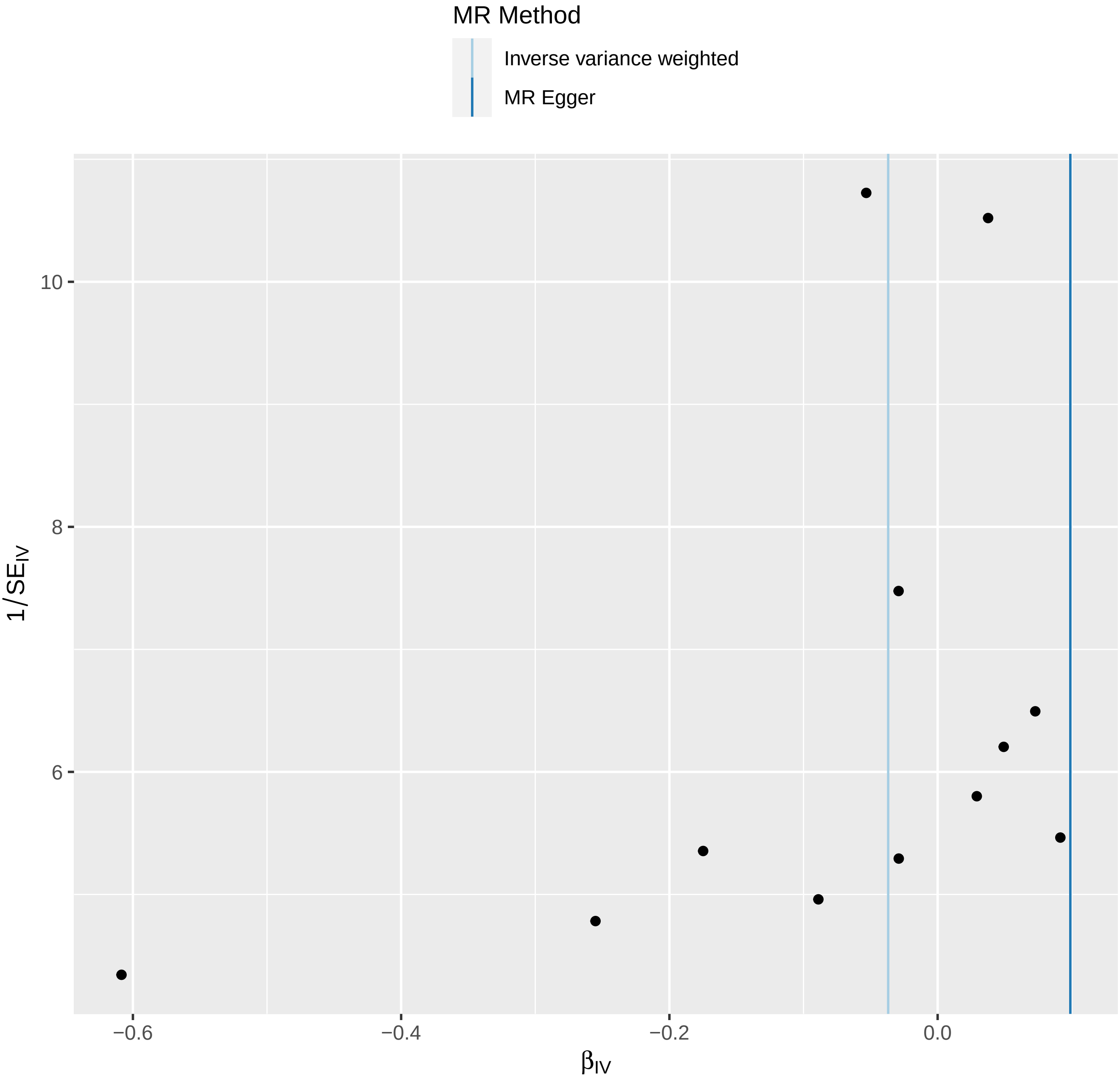

Batch 3−1277 : Selenium on Granulins

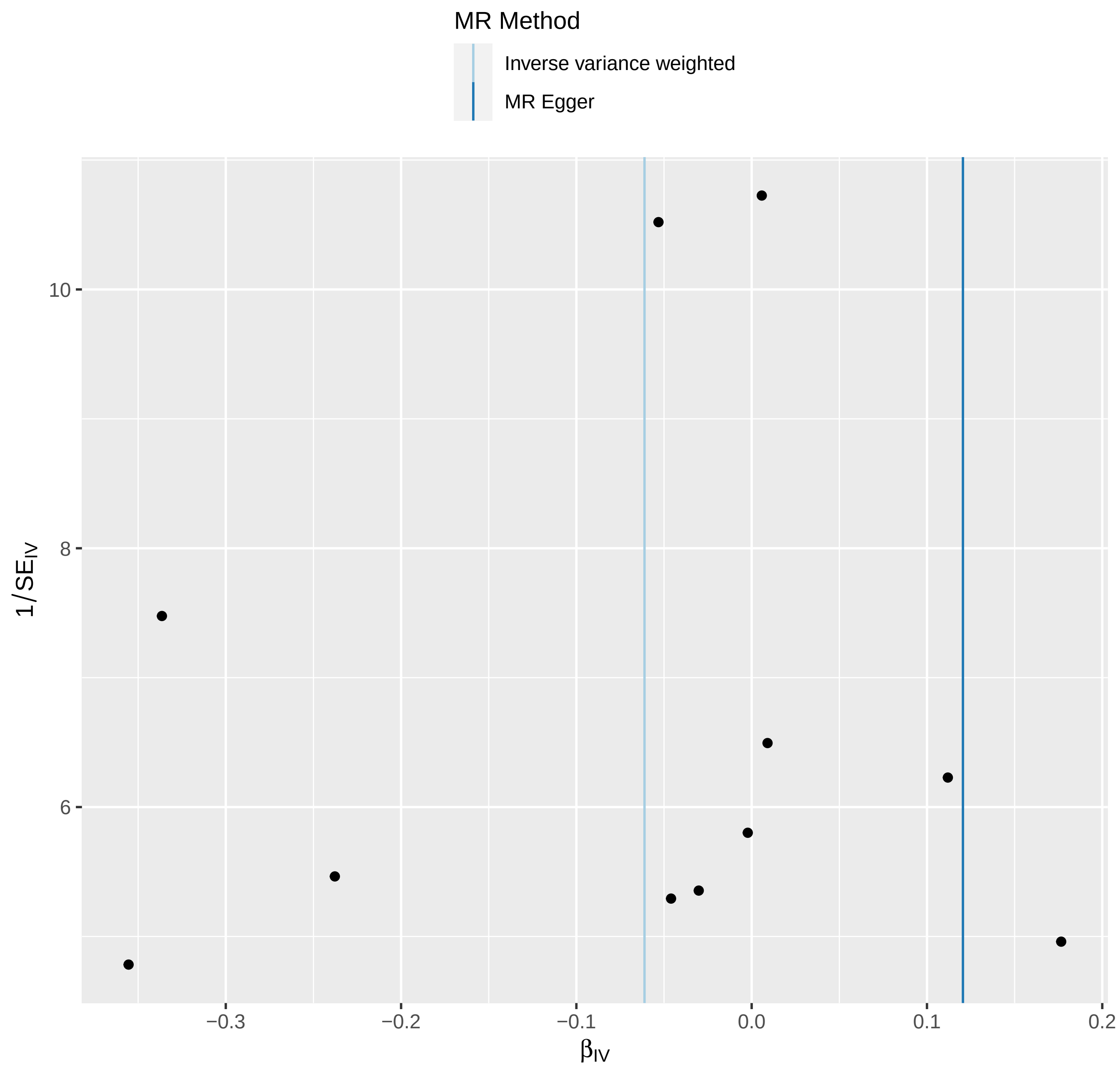

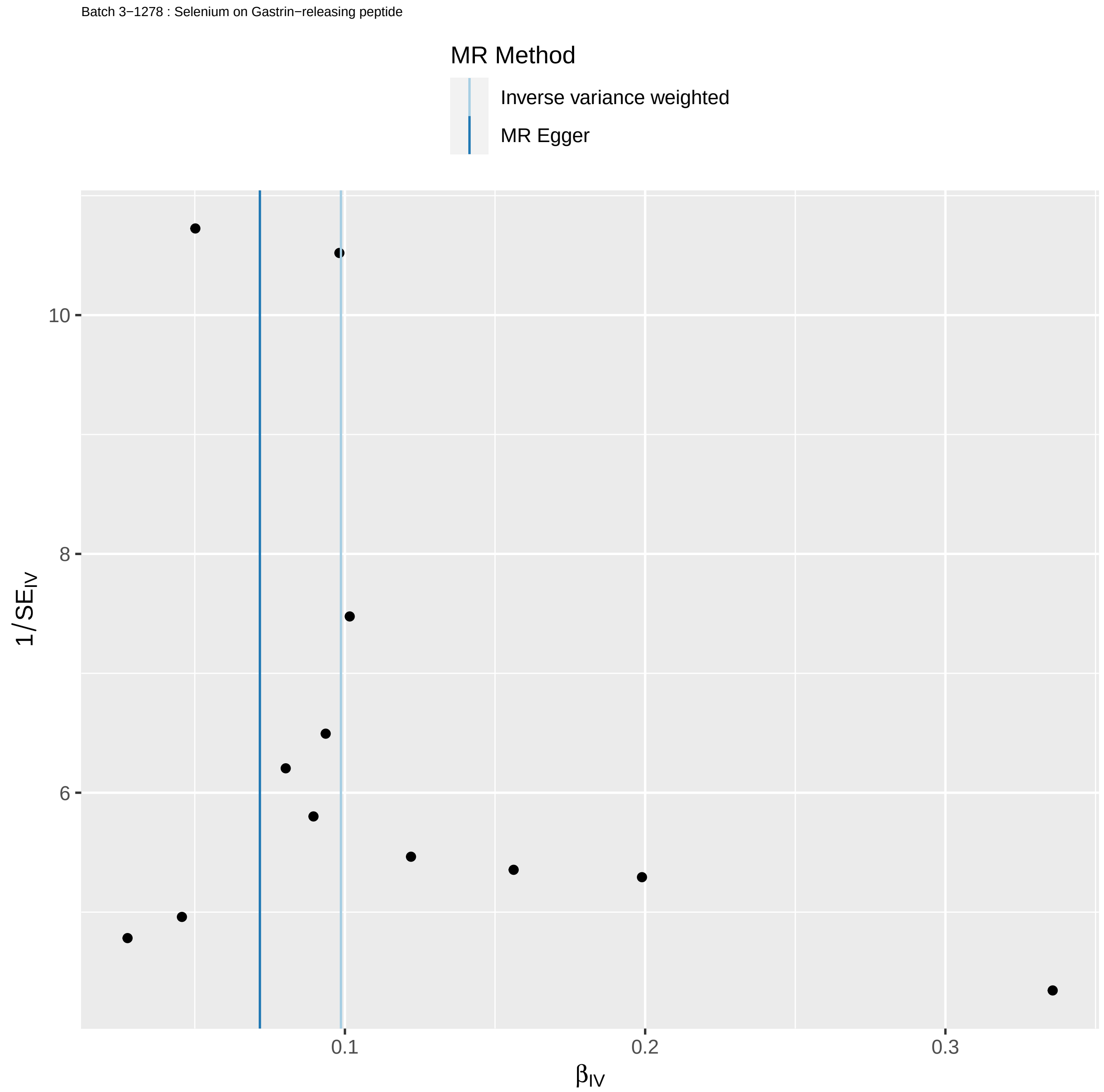

Batch 3−1278 : Selenium on Gastrin−releasing peptide
MR Method
Inverse variance weighted
MR Egger
10
8
6
1/SE_IV
0.1
0.2
0.3
β_IV

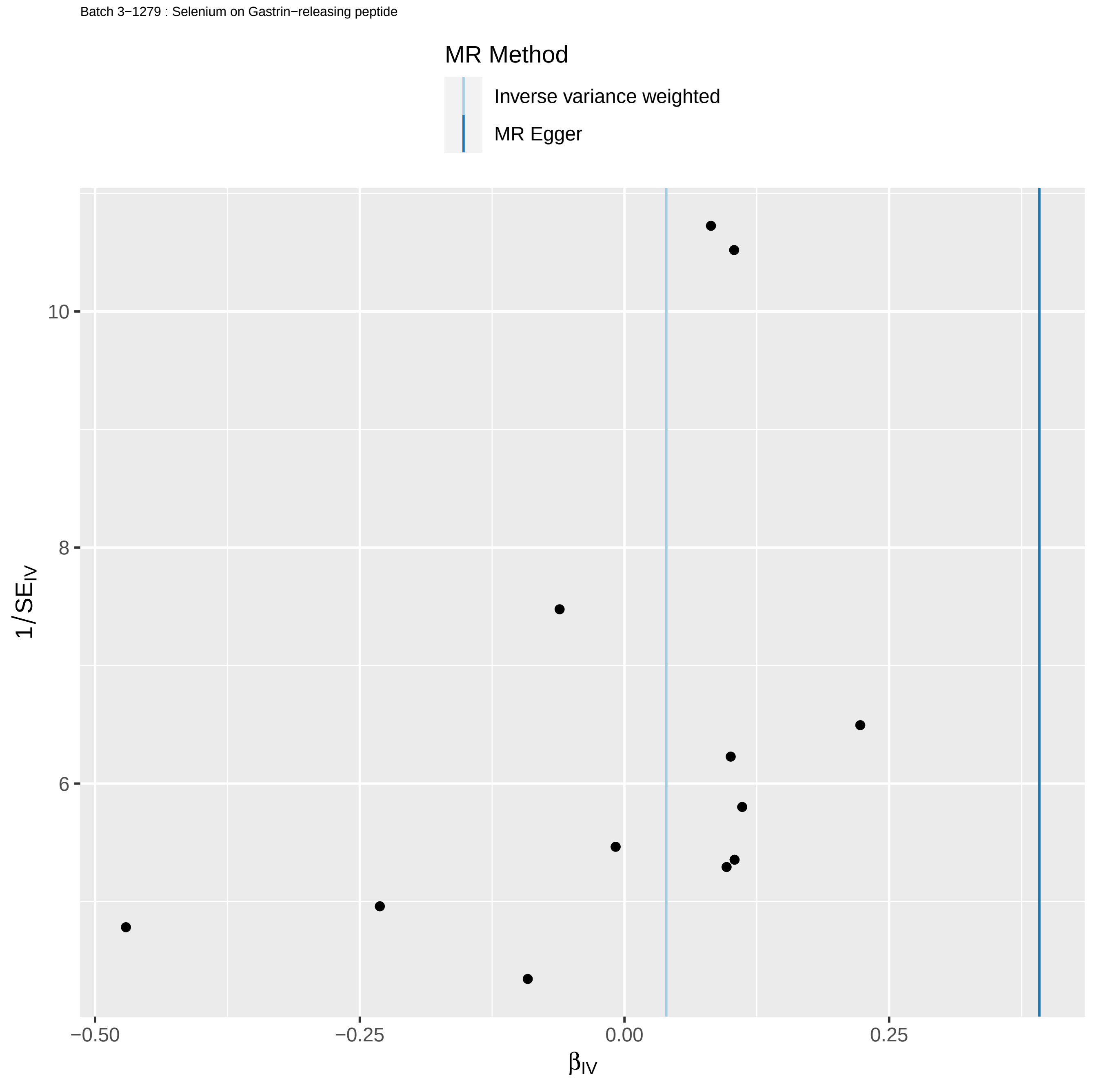

Batch 3−1279 : Selenium on Gastrin−releasing peptide
MR Method
Inverse variance weighted
MR Egger
10
8
6
1/SE_IV
−0.50
−0.25
0.00
0.25
β_IV

Batch 3−1280 : Selenium on Gastrin−releasing peptide

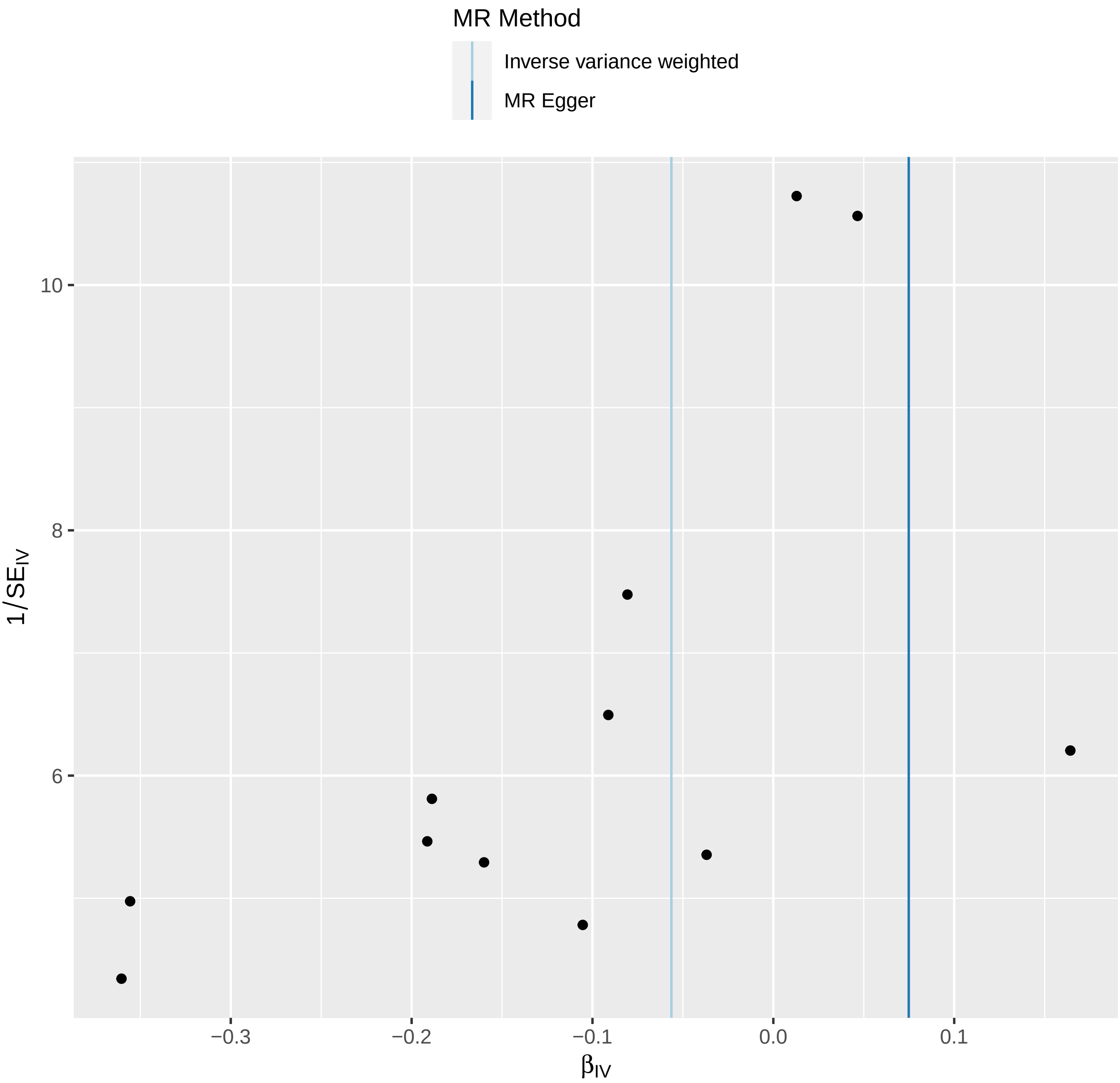

Batch 3−1281 : Selenium on GrpE protein homolog 1, mitochondrial

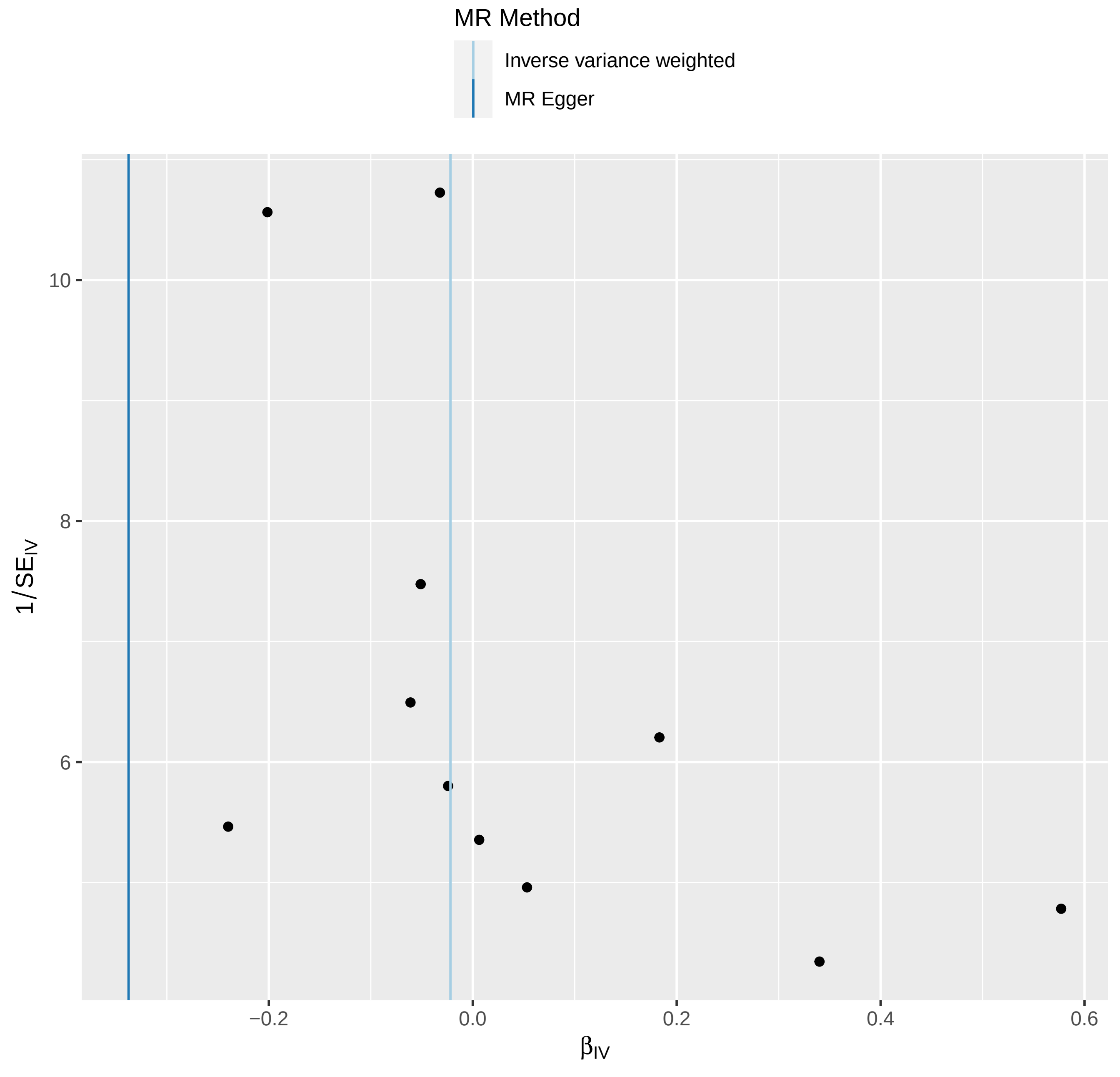

Batch 3−1282 : Selenium on GSK3−beta interaction protein

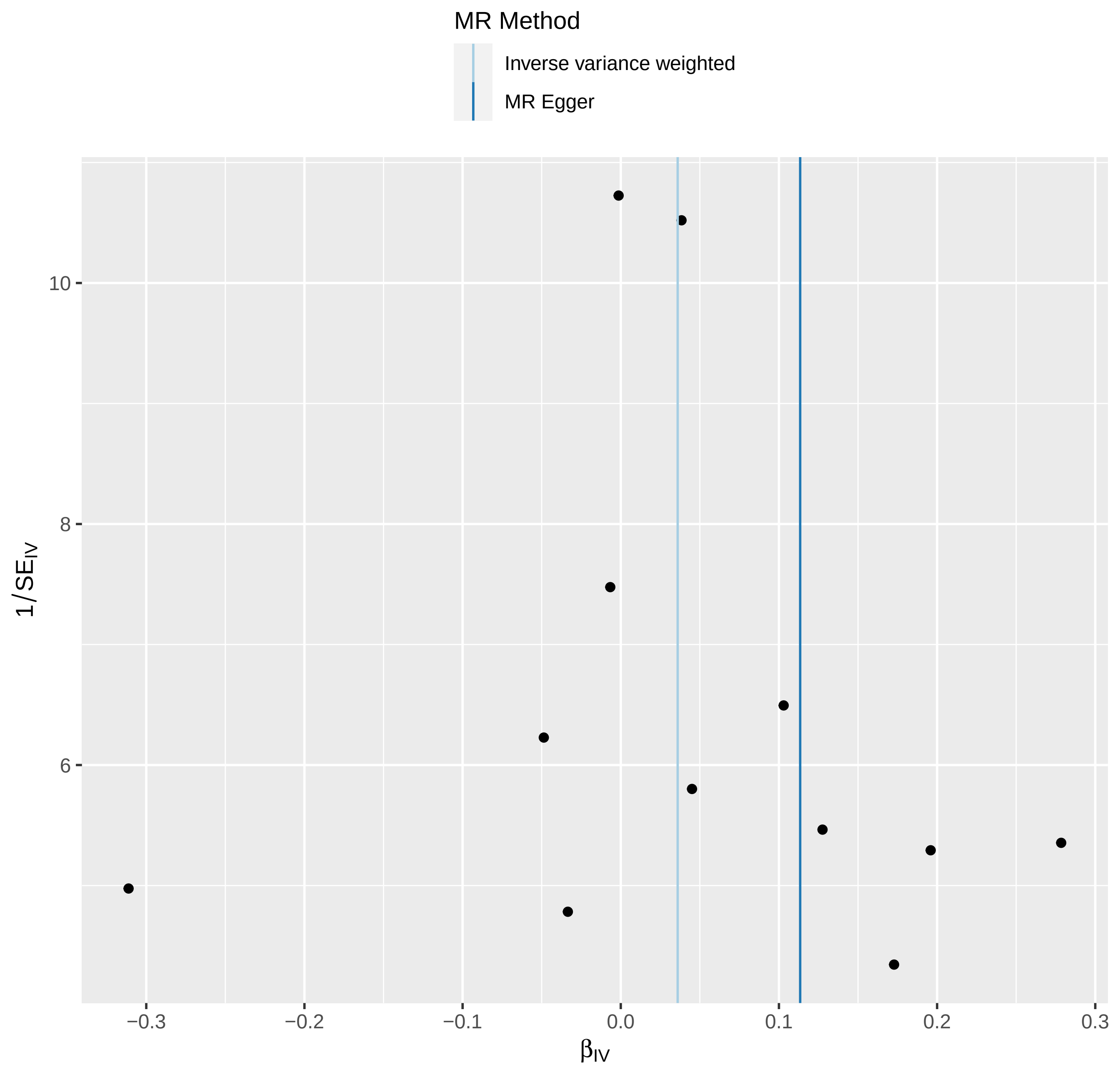

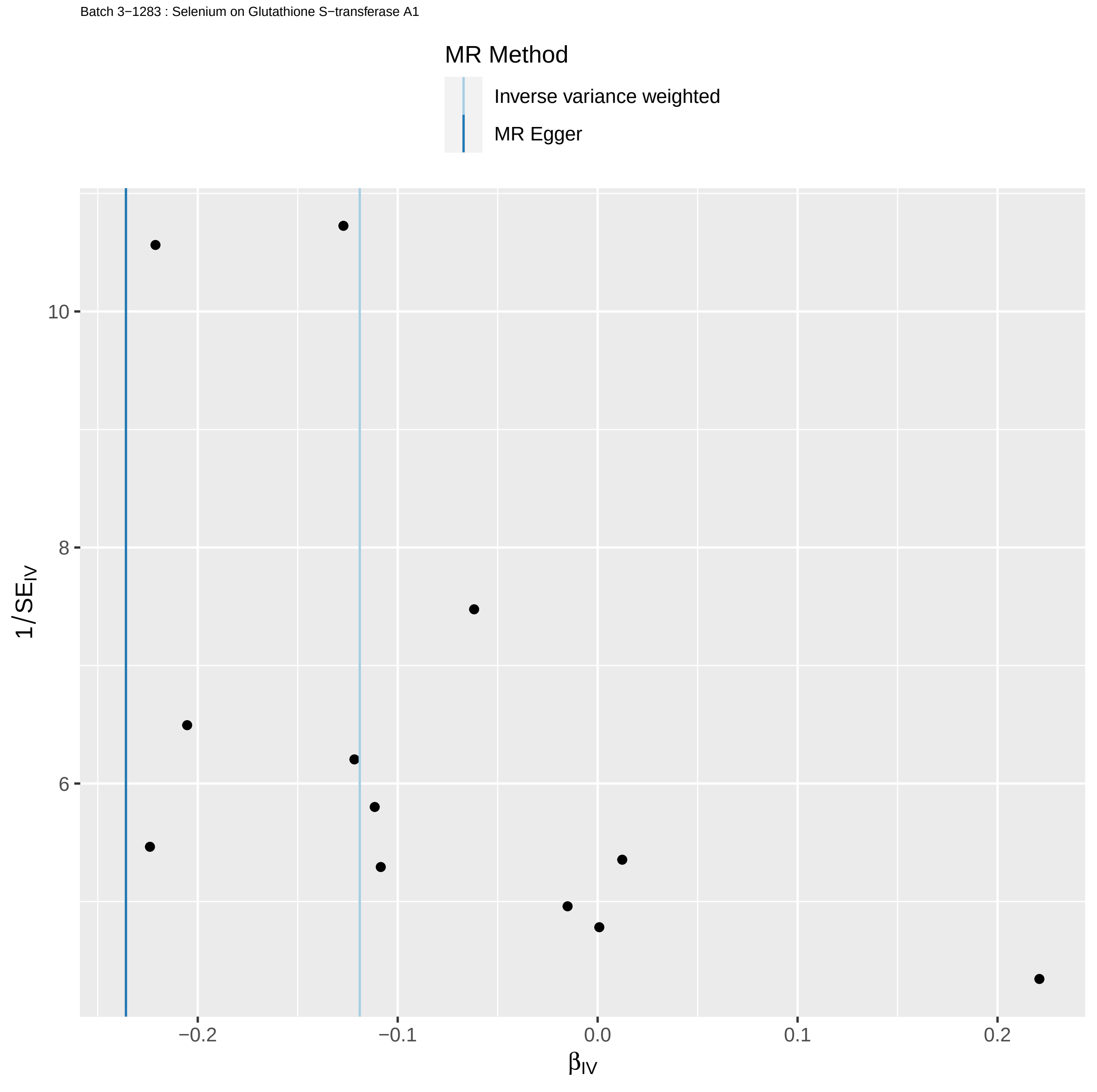
Batch 3−1283 : Selenium on Glutathione S−transferase A1
MR Method
Inverse variance weighted
MR Egger
10
8
6
$1/SE_{IV}$
−0.2
−0.1
0.0
0.1
0.2
β_{IV}

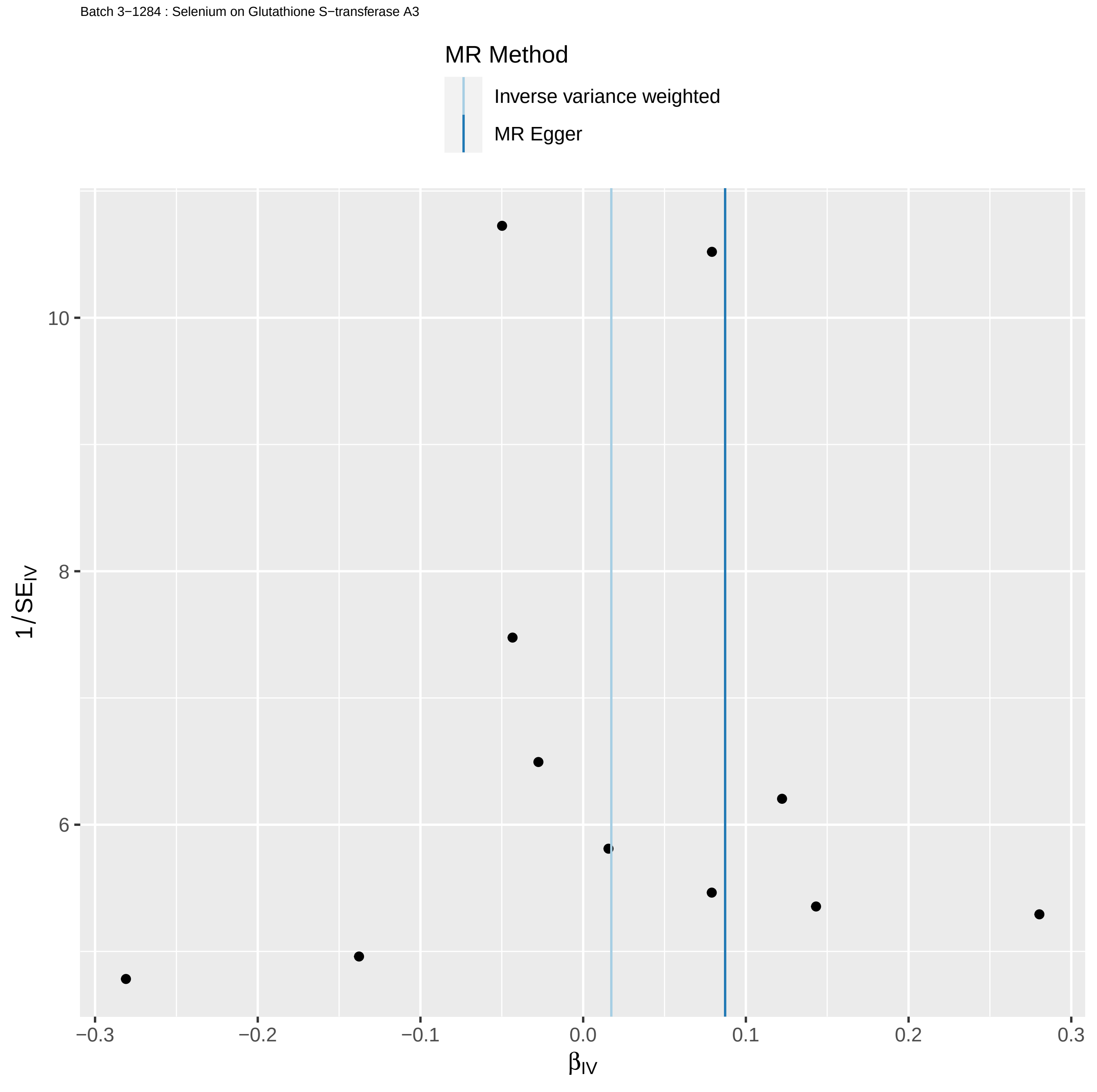

Batch 3−1284 : Selenium on Glutathione S−transferase A3
MR Method
Inverse variance weighted
MR Egger
10
8
6
1/SE_IV
−0.3
−0.2
−0.1
0.0
0.1
0.2
0.3
β_IV

Batch 3−1285 : Selenium on Glutathione S−transferase A4

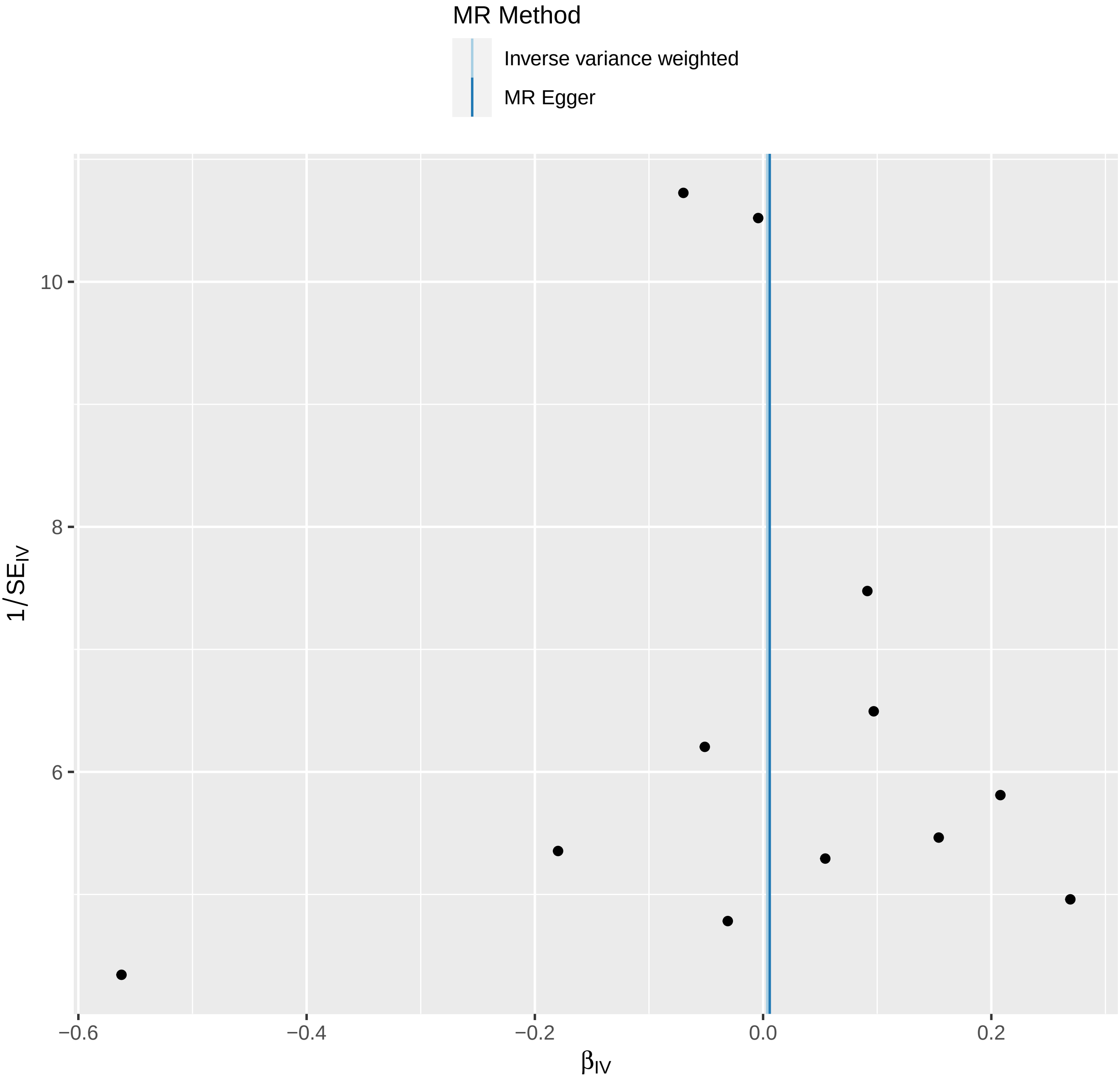

Batch 3−1286 : Selenium on Glutathione S−transferase kappa 1

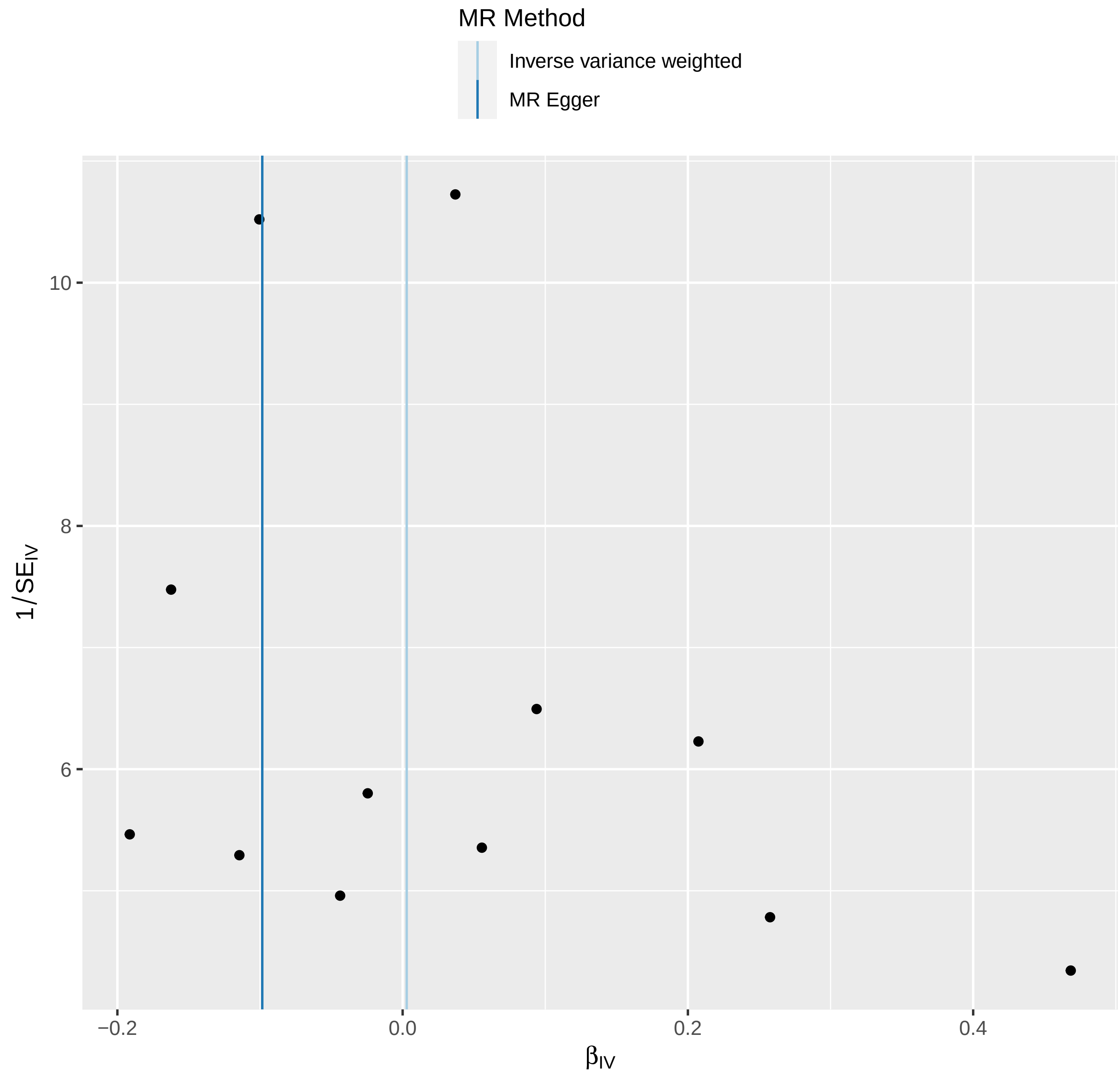

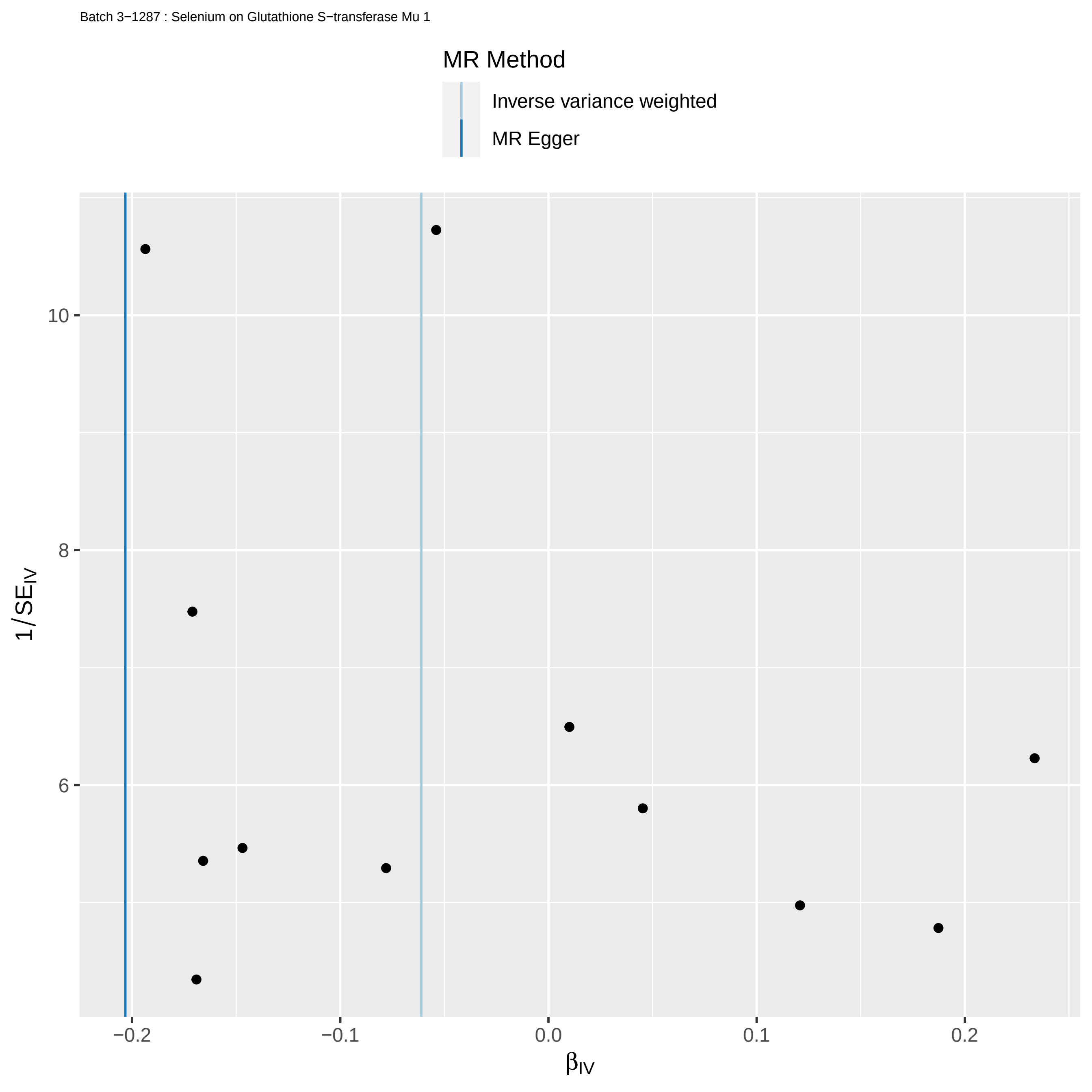
Batch 3−1287 : Selenium on Glutathione S−transferase Mu 1
MR Method
Inverse variance weighted
MR Egger
10
8
6
1/SE$_{IV}$
−0.2
−0.1
0.0
0.1
0.2
β$_{IV}$

Batch 3−1288 : Selenium on Glutathione S−transferase omega−1

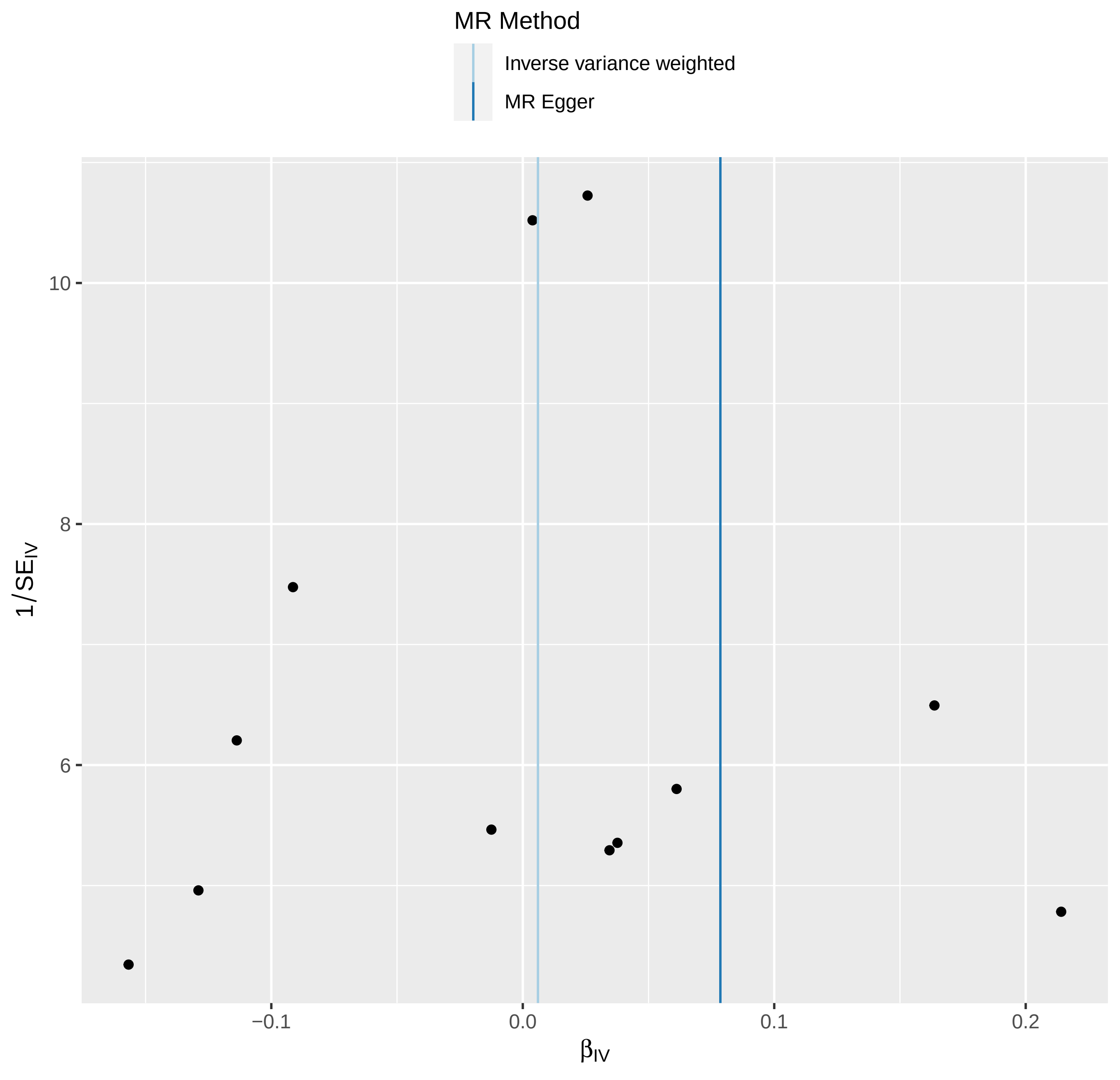

Batch 3−1289 : Selenium on Glutathione S−transferase P

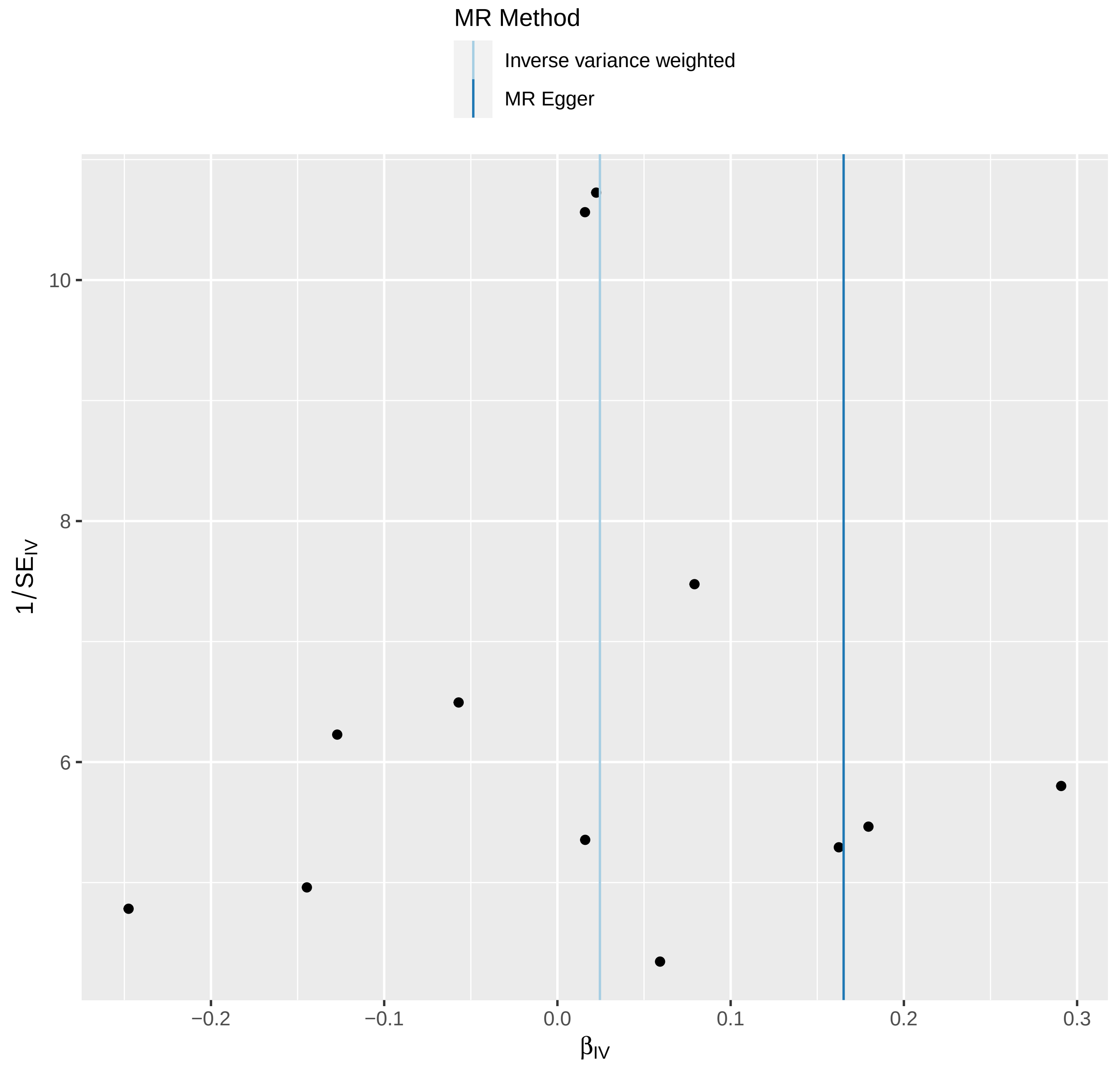

Batch 3−1290 : Selenium on Glutathione S−transferase P

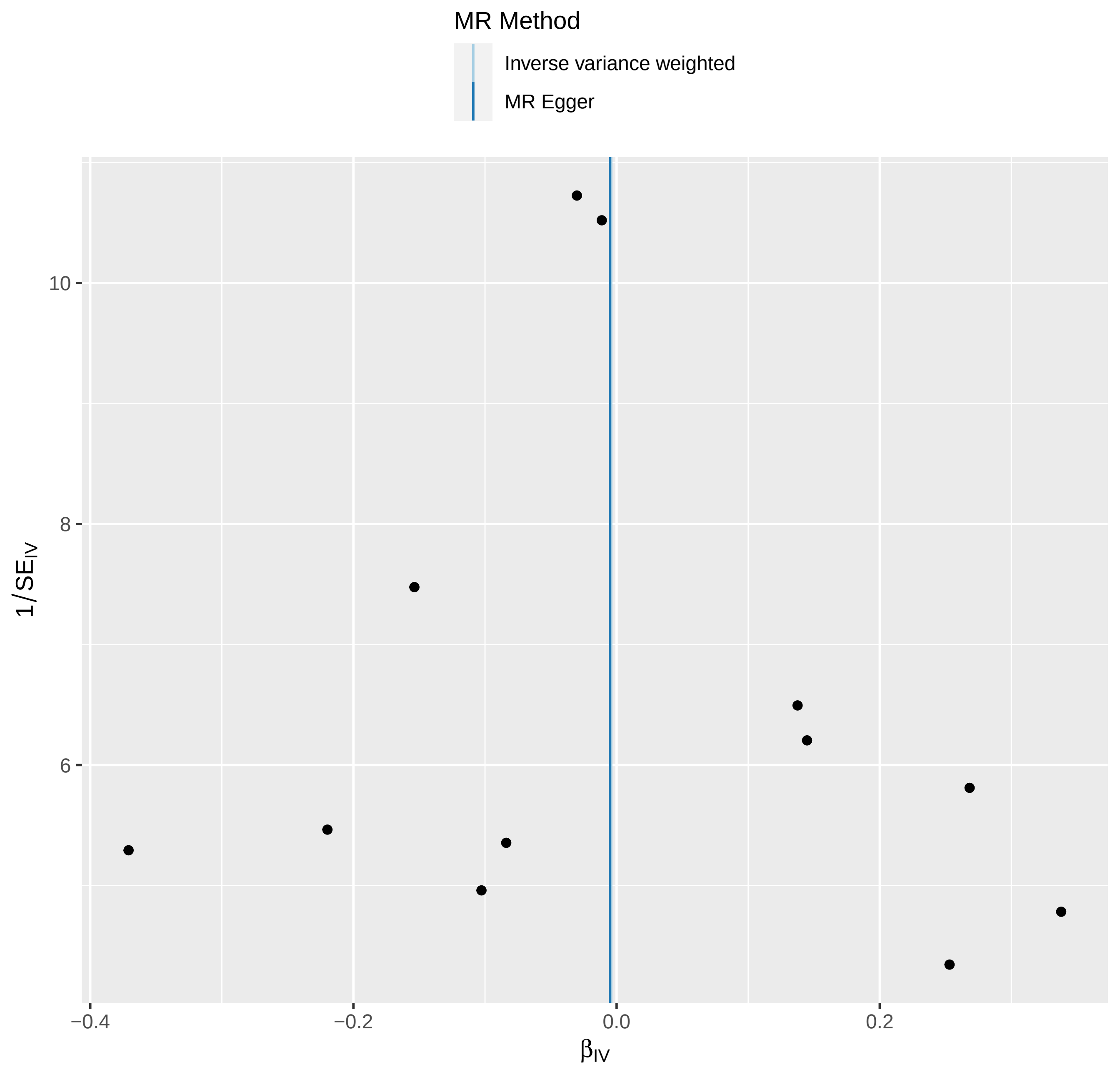

Batch 3−1291 : Selenium on Glutathione S−transferase theta−2B

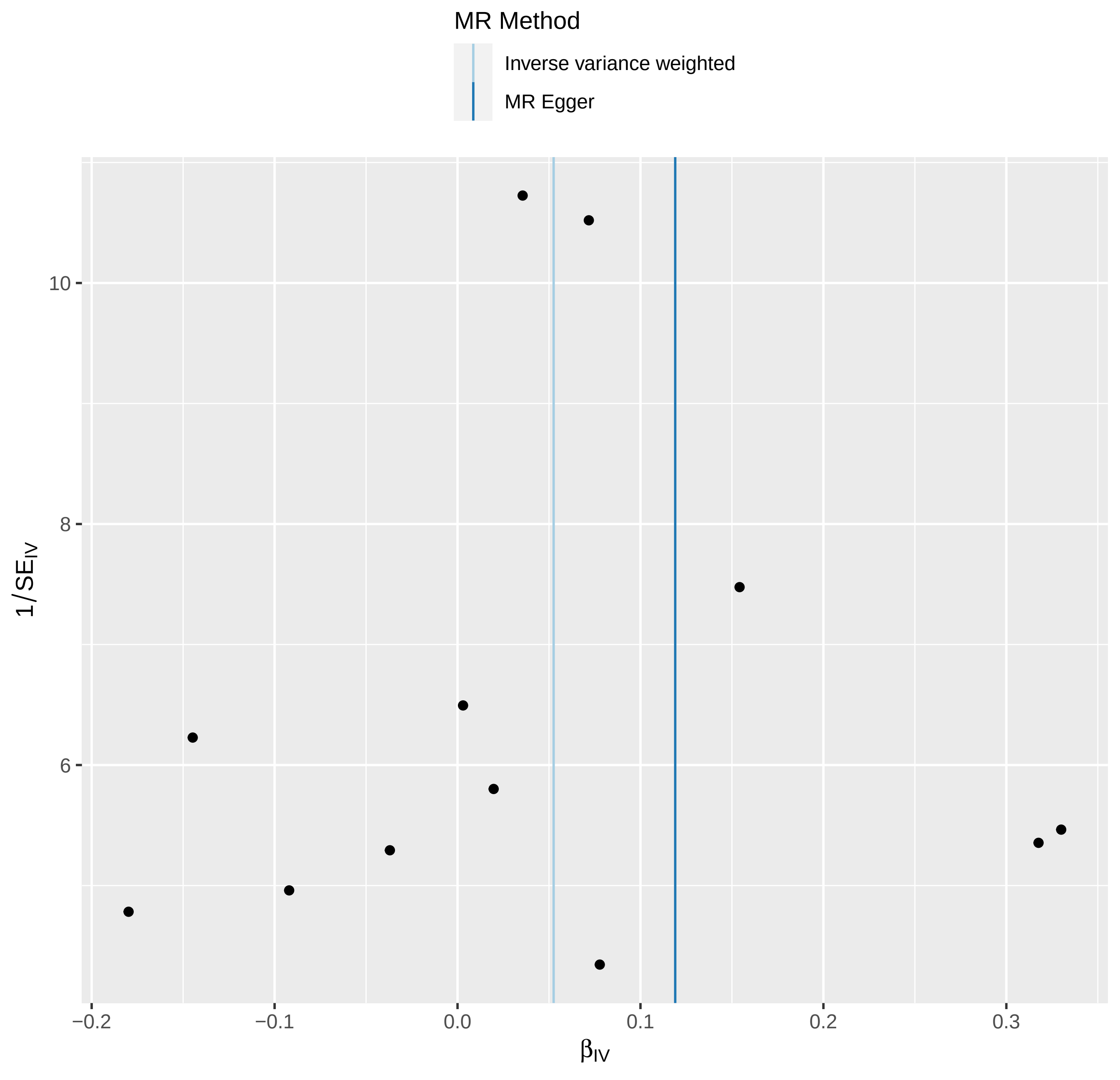

Batch 3−1292 : Selenium on General transcription factor II−I

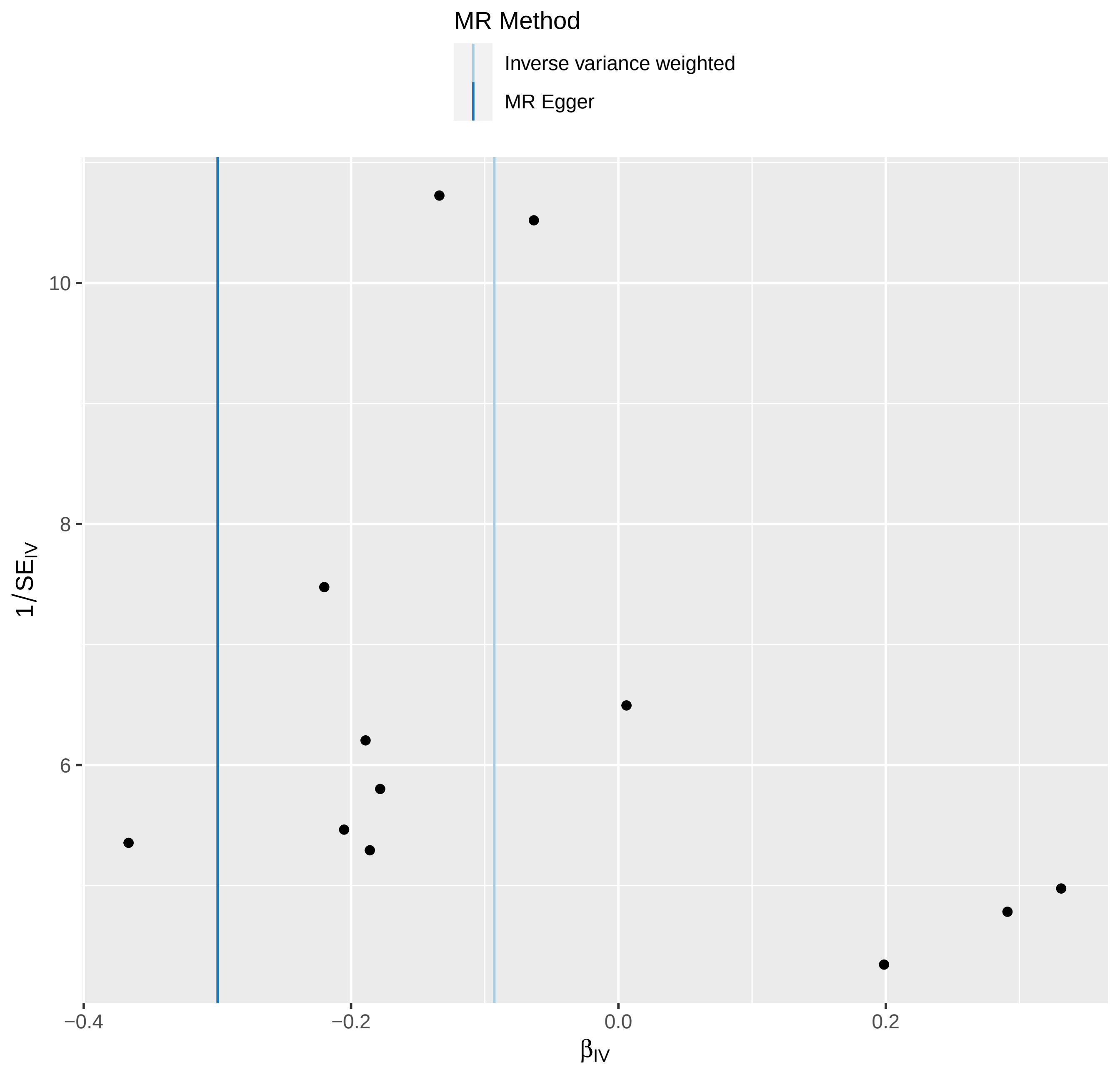

Batch 3−1293 : Selenium on Guanylyl cyclase−activating protein 1

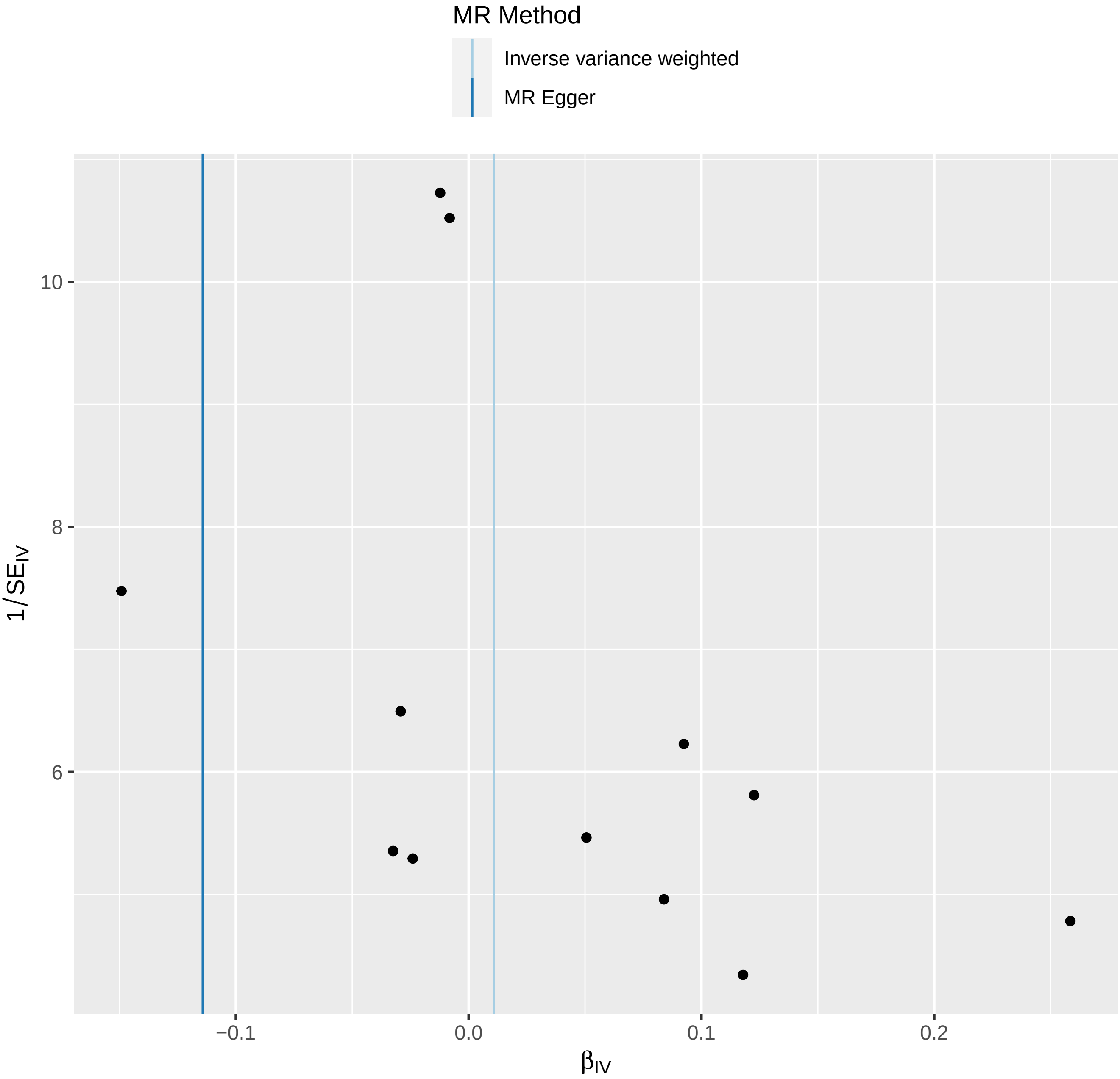

Batch 3−1294 : Selenium on Guanylate cyclase activator 2B

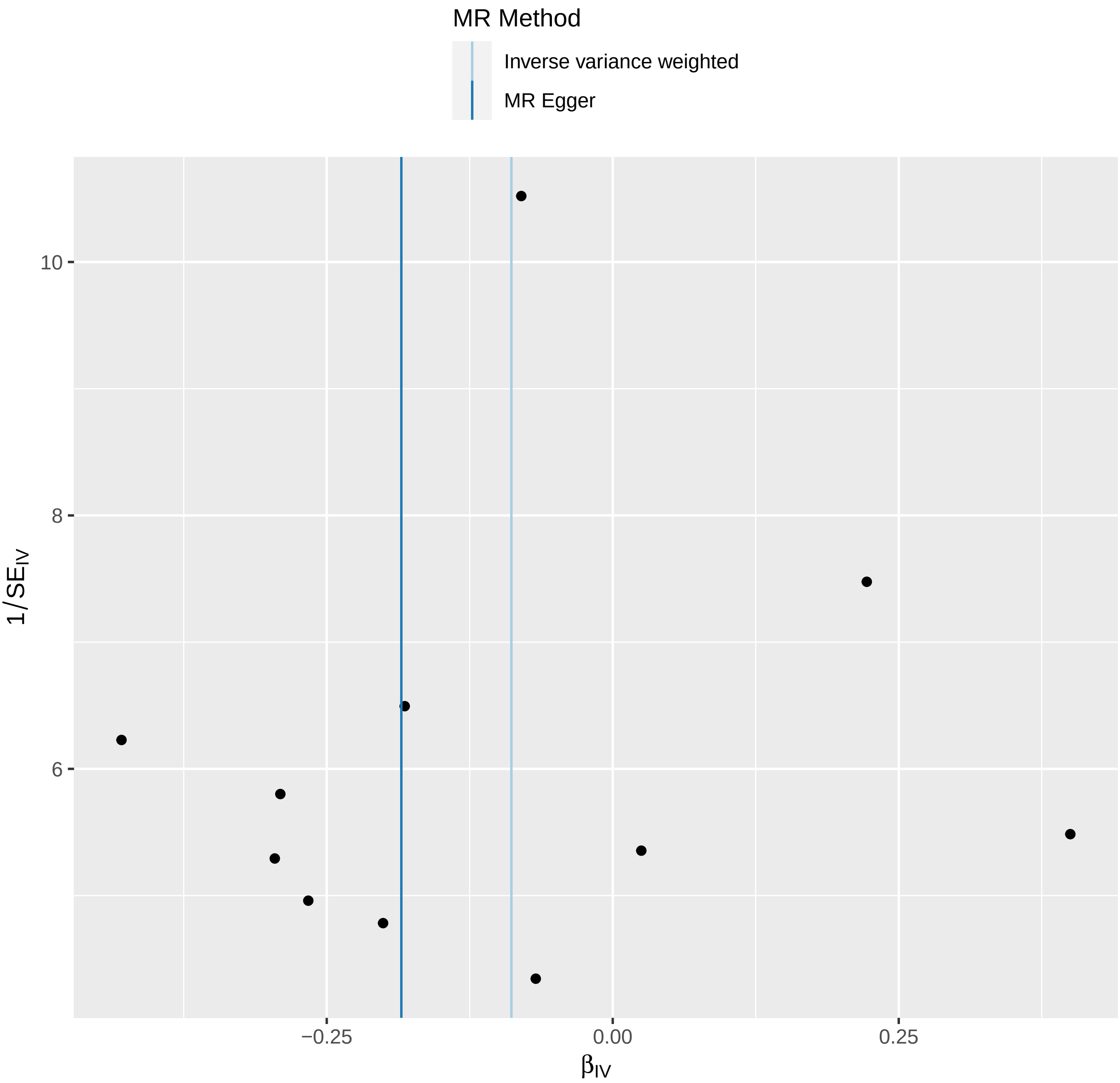

Batch 3−1295 : Selenium on Glucoside xylosyltransferase 1

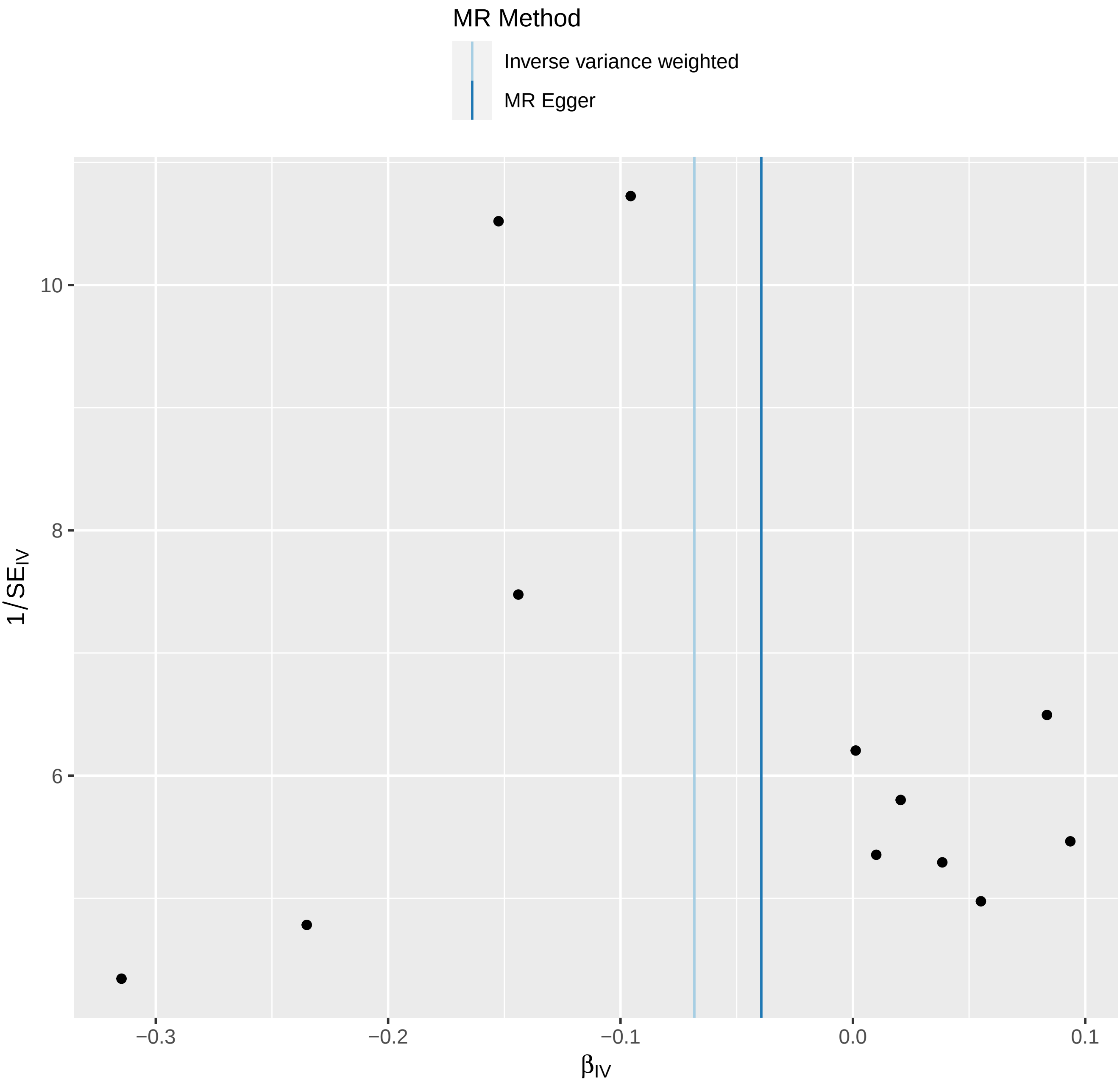

Batch 3−1296 : Selenium on Glycophorin−A

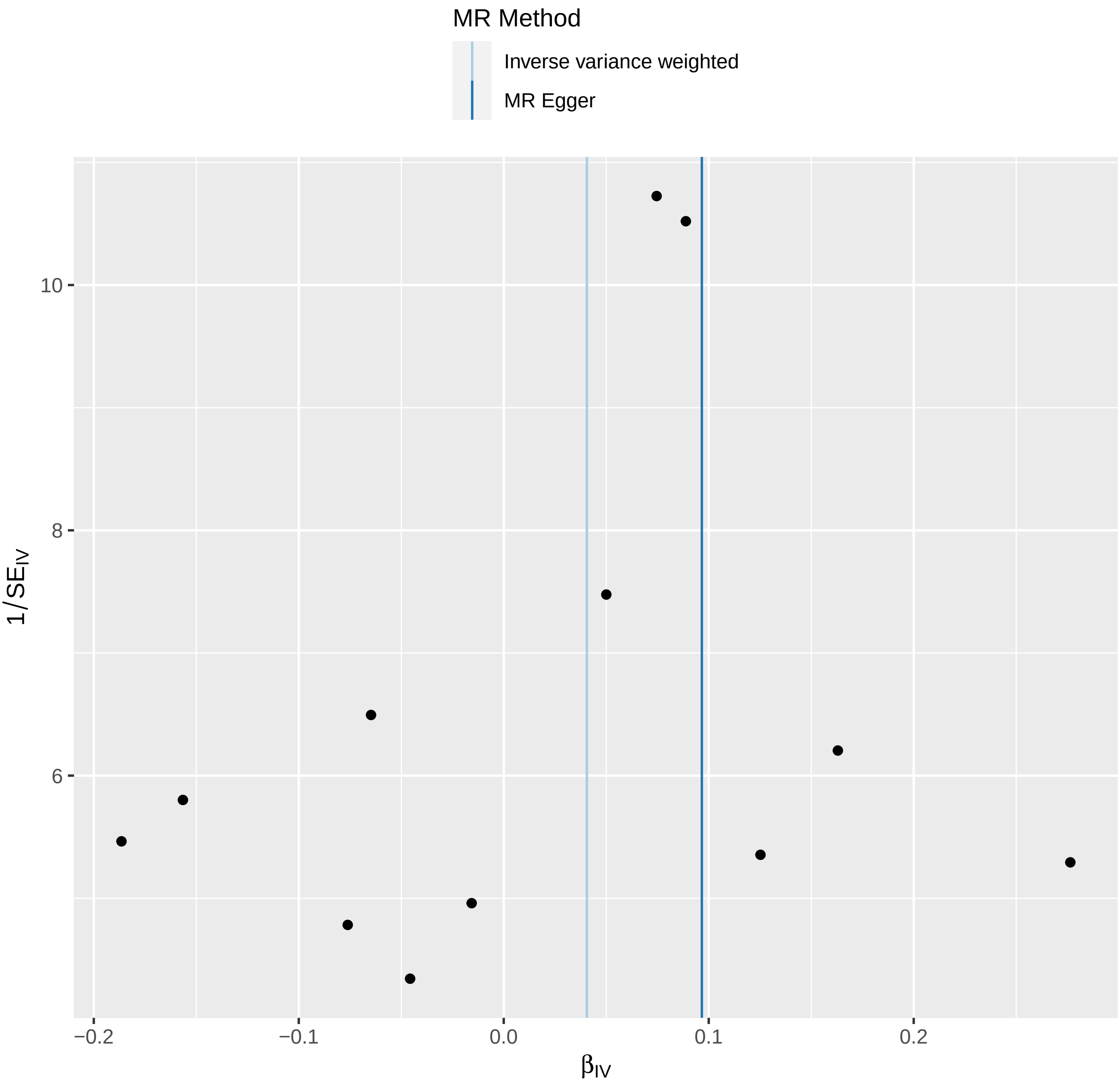

Batch 3−1297 : Selenium on Granzyme A

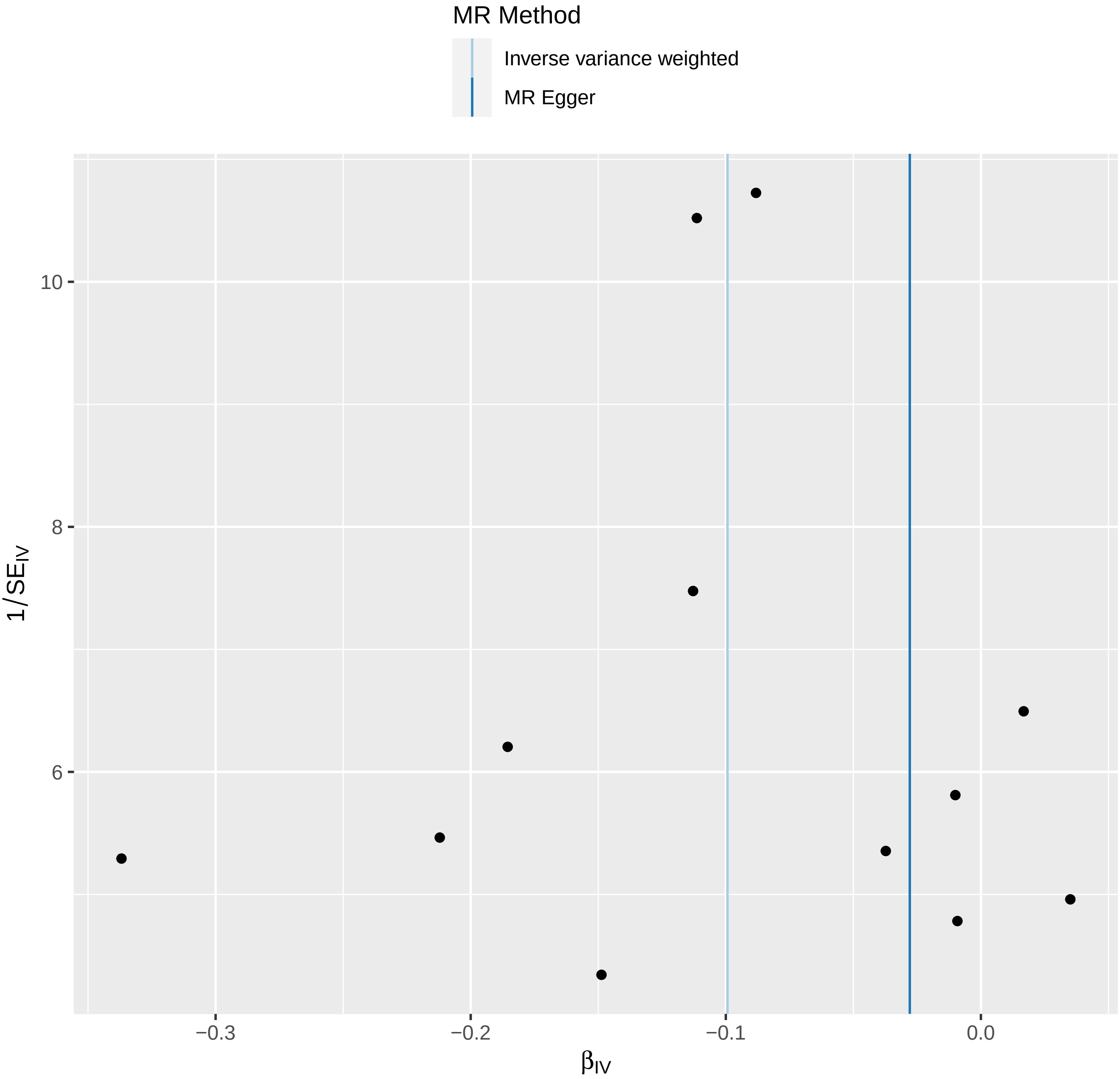

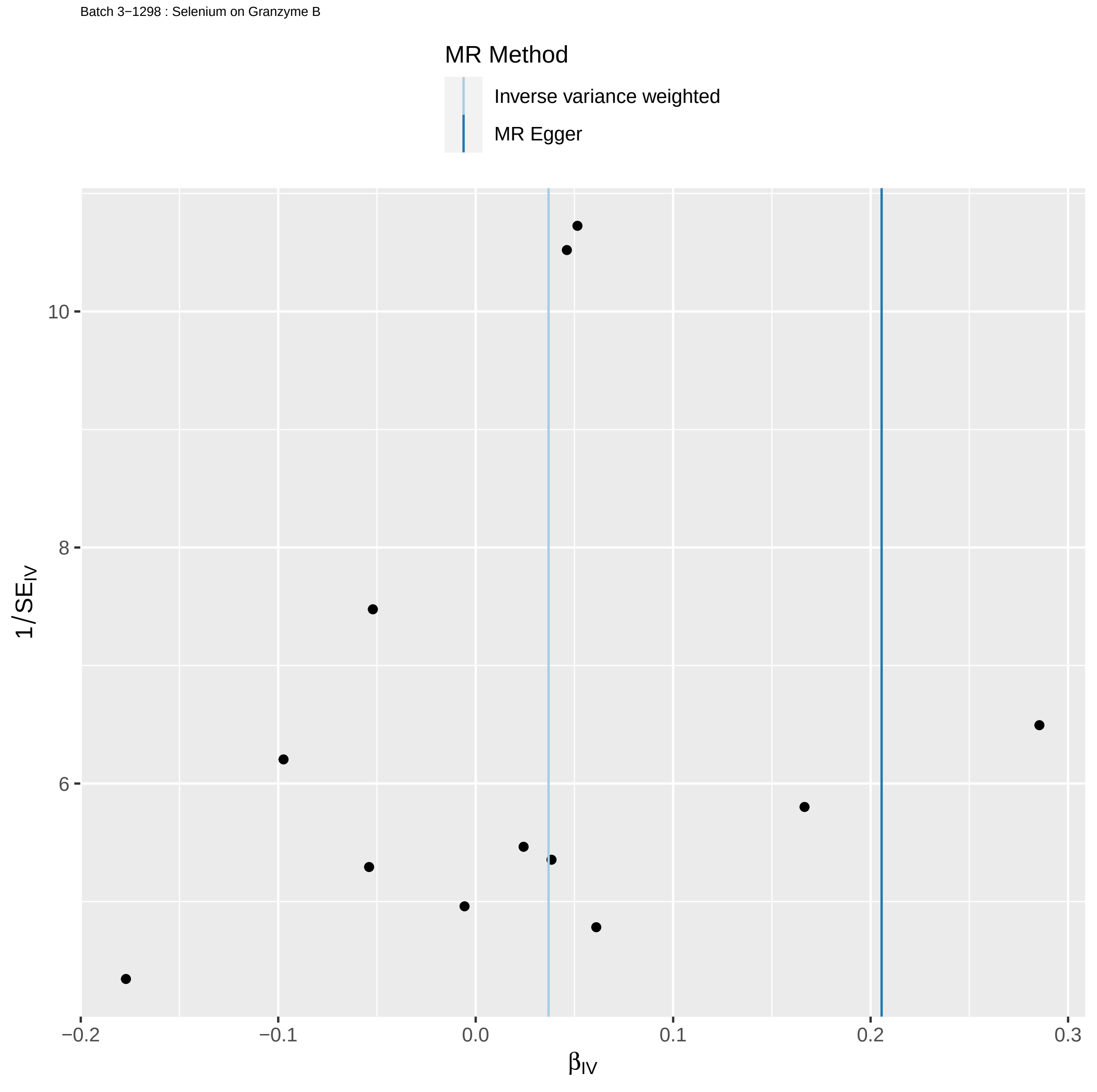
Batch 3−1298 : Selenium on Granzyme B
MR Method
Inverse variance weighted
MR Egger
10
8
6
1/SE_IV
−0.2
−0.1
0.0
0.1
0.2
0.3
β_IV

Batch 3−1299 : Selenium on Granzyme B

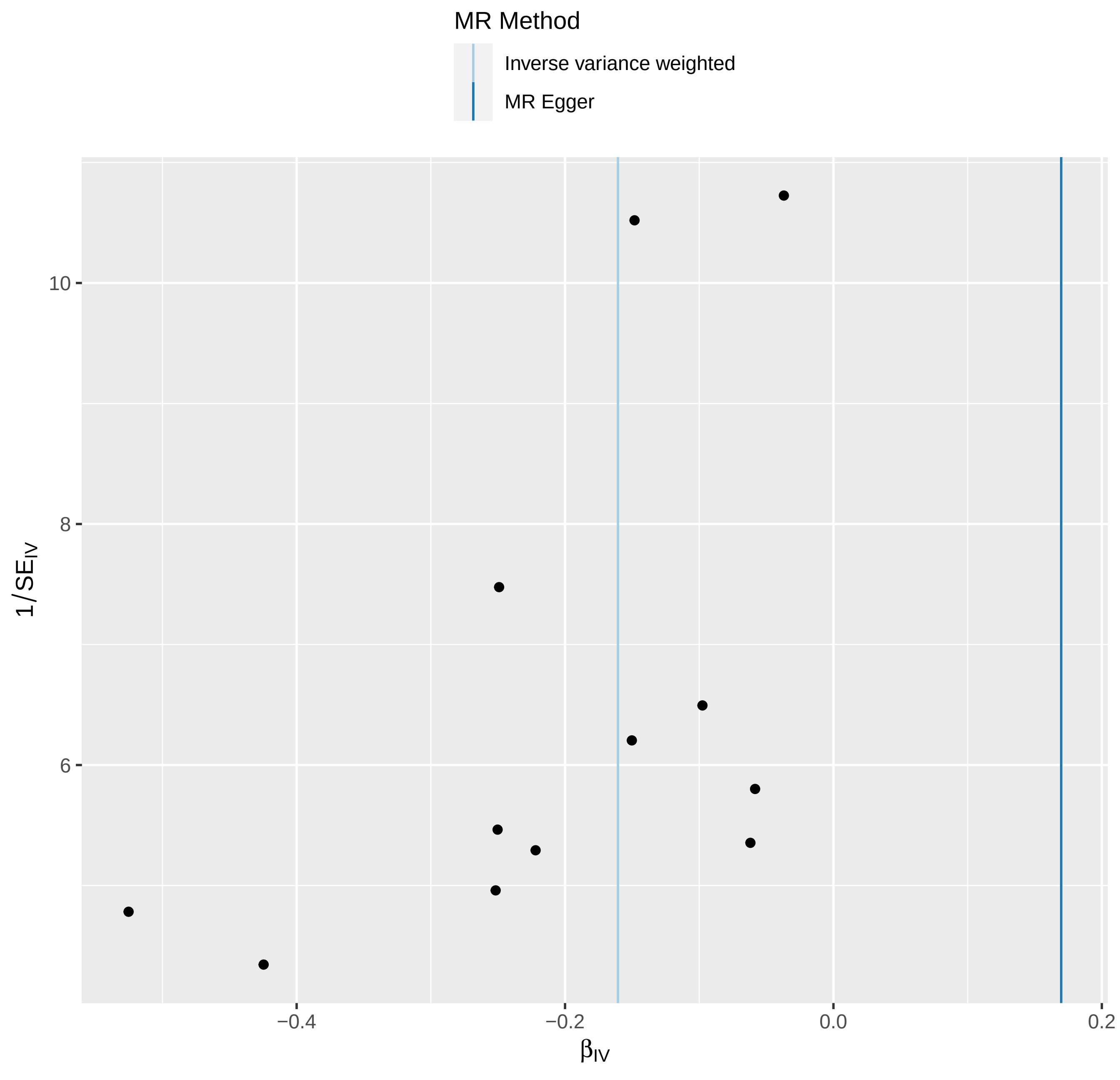

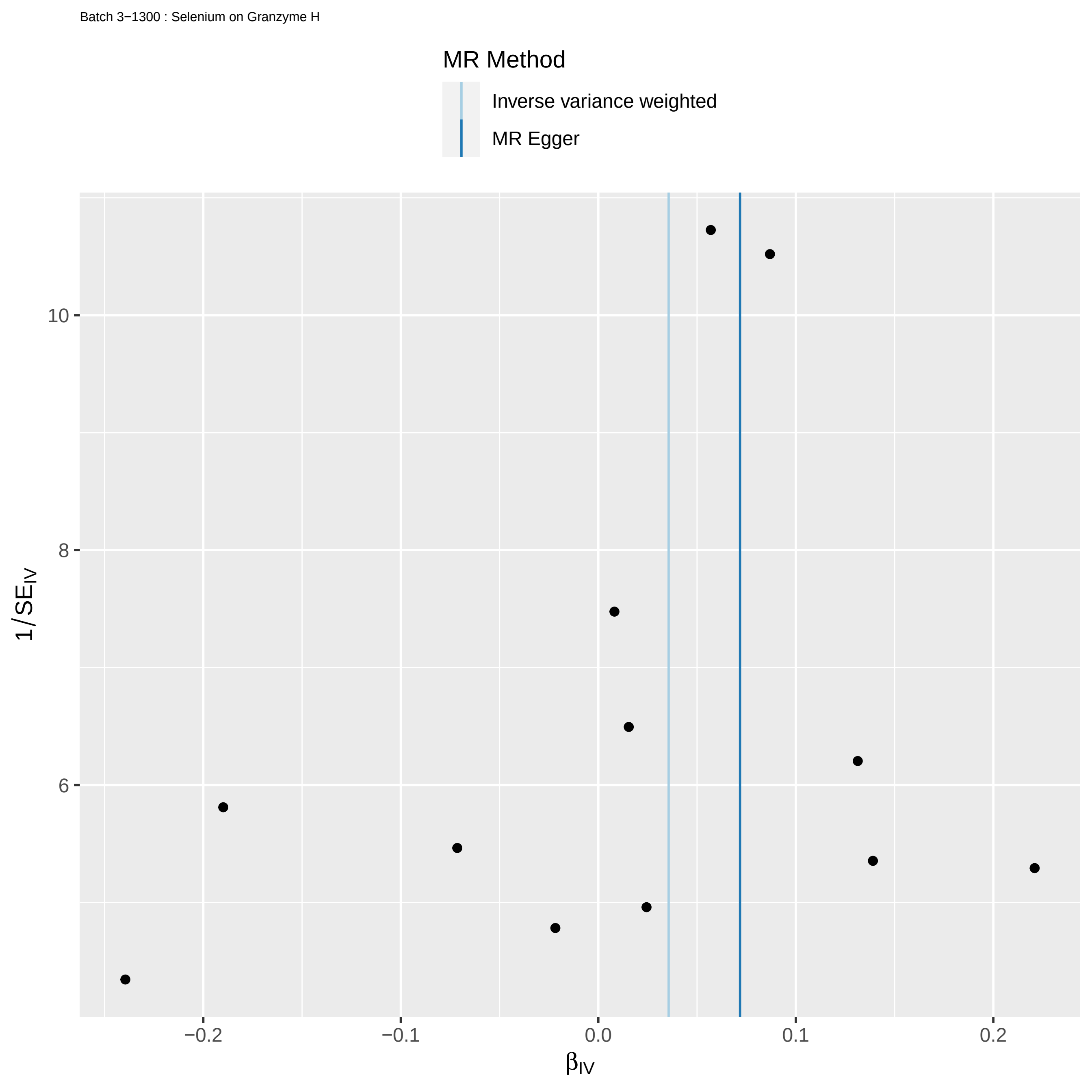
Batch 3−1300 : Selenium on Granzyme H
MR Method
Inverse variance weighted
MR Egger
10
8
6
1/SE_IV
−0.2
−0.1
0.0
0.1
0.2
β_IV

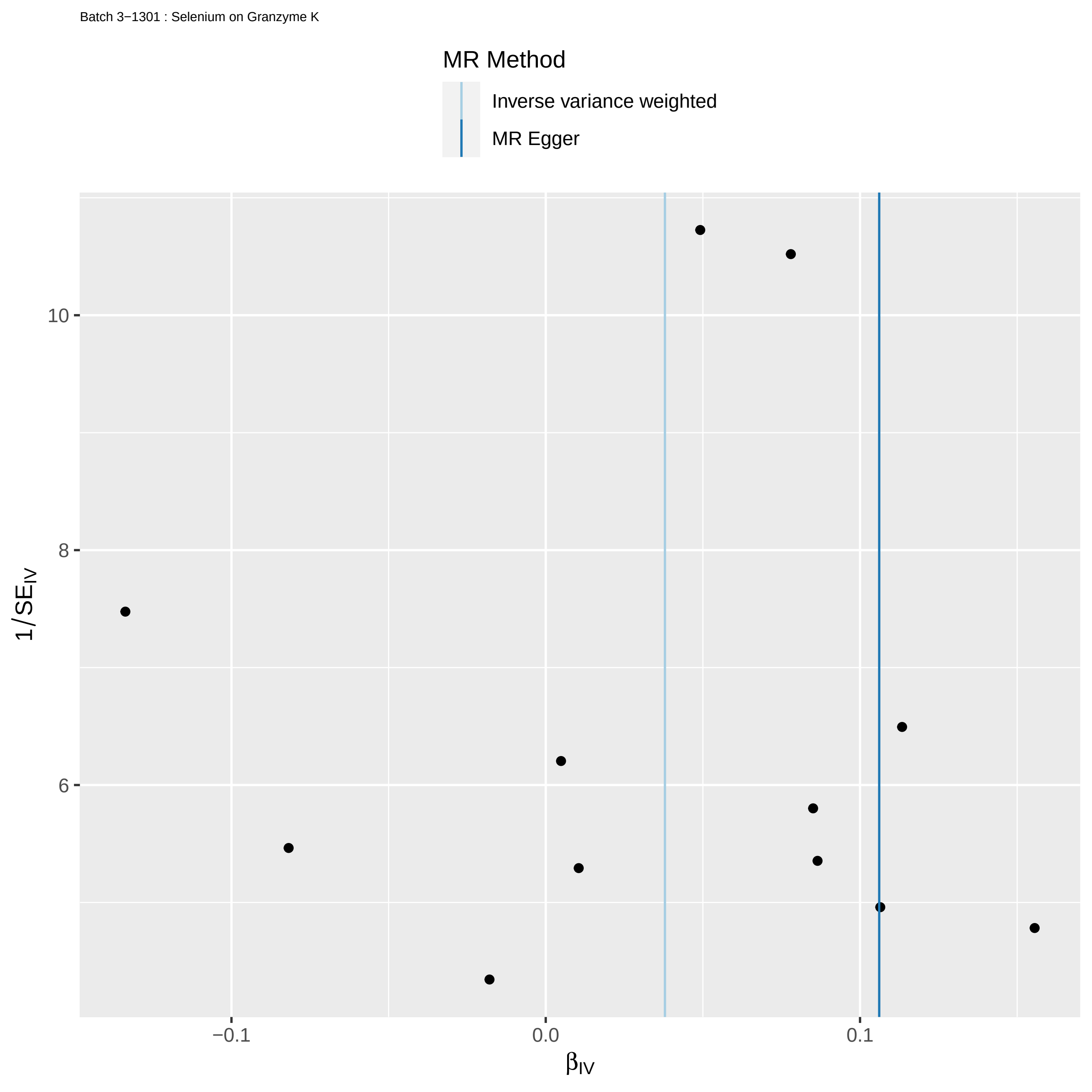
Batch 3−1301 : Selenium on Granzyme K
MR Method
Inverse variance weighted
MR Egger
10
8
6
1/SE_IV
−0.1
0.0
0.1
β_IV

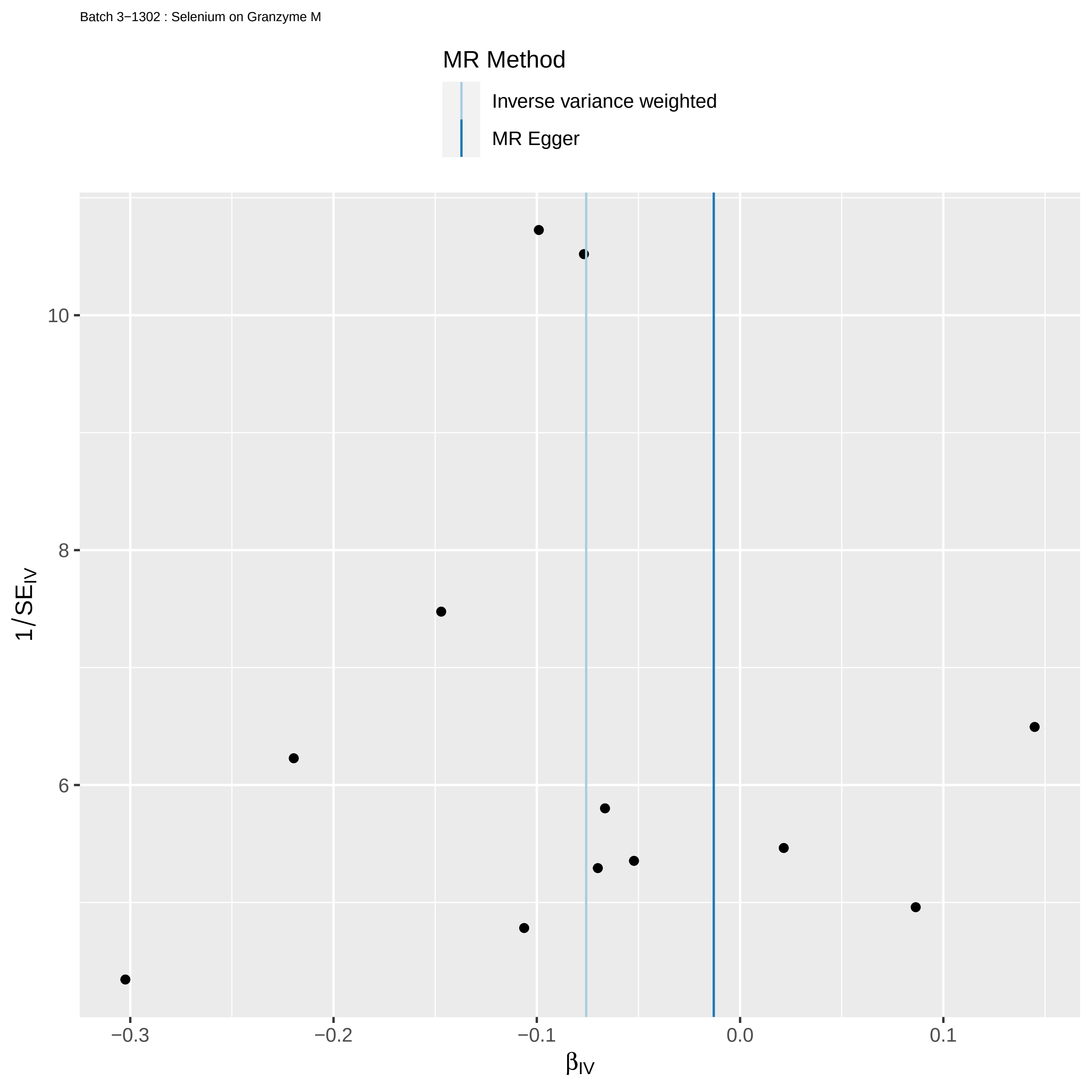

Batch 3−1302 : Selenium on Granzyme M
MR Method
Inverse variance weighted
MR Egger
1/SE_IV
10
8
6
−0.3
−0.2
−0.1
0.0
0.1
β_IV

Batch 3−1303 : Selenium on Histone H1x

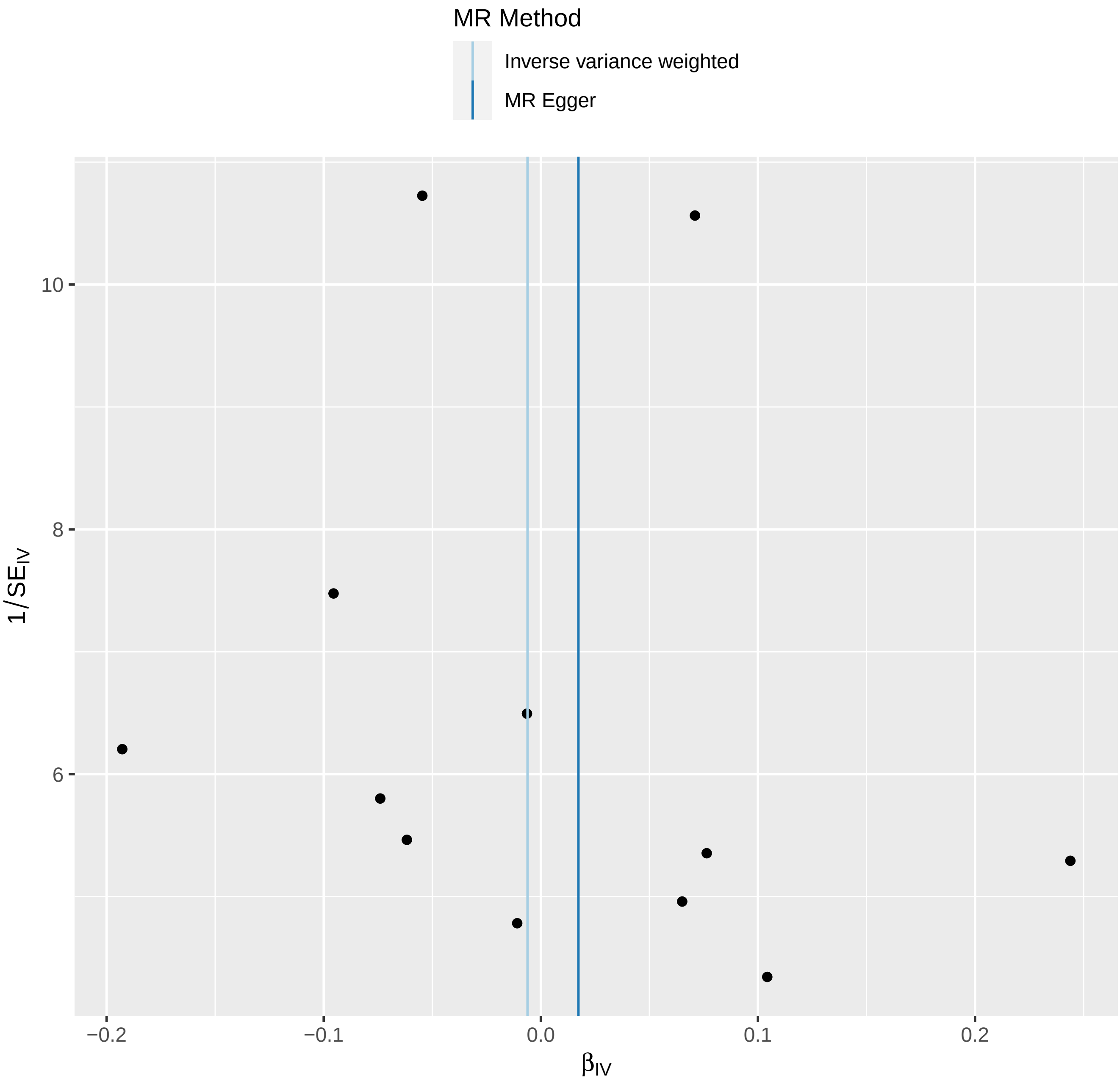

Batch 3−1304 : Selenium on Histone H2A.z

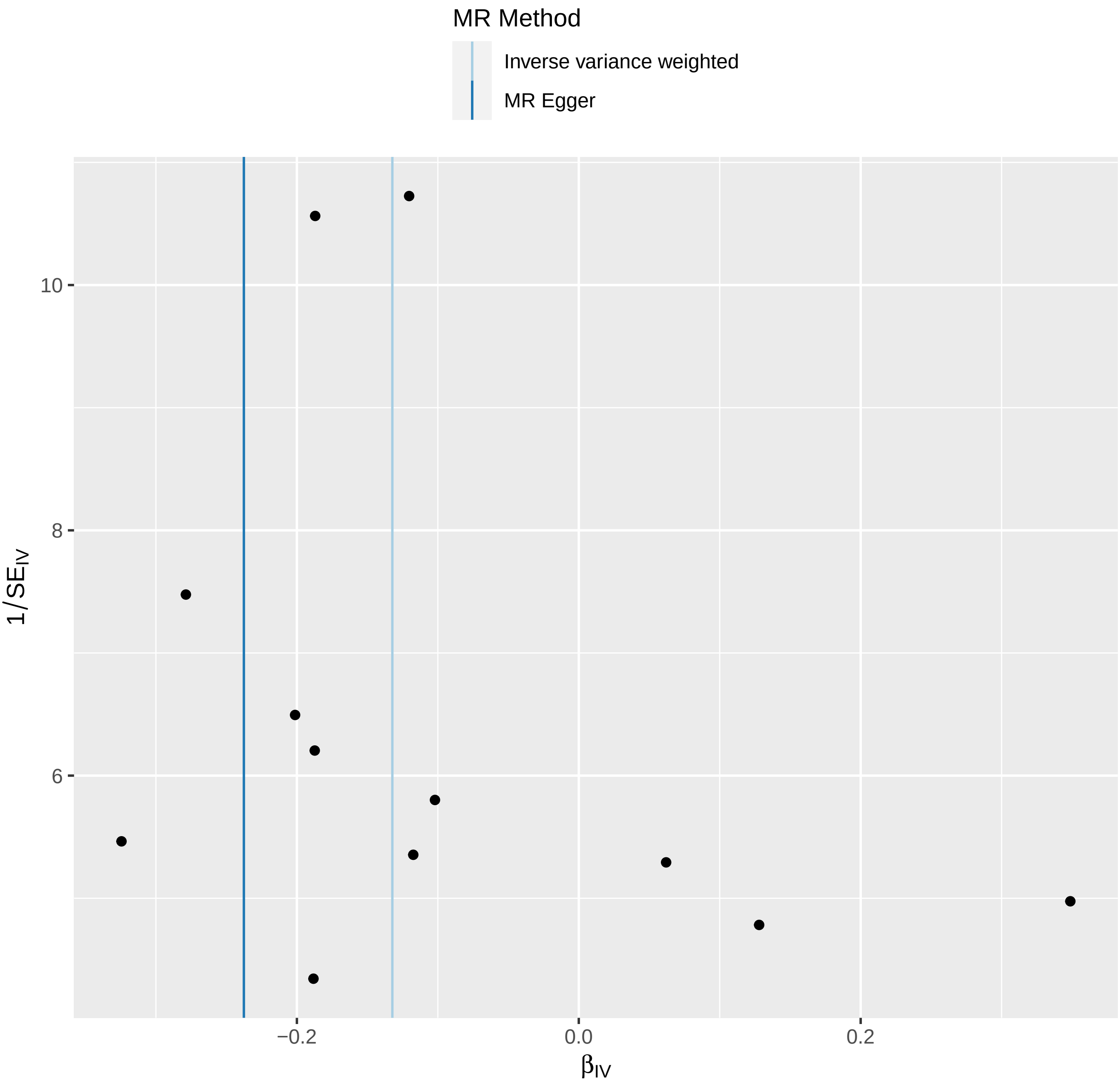

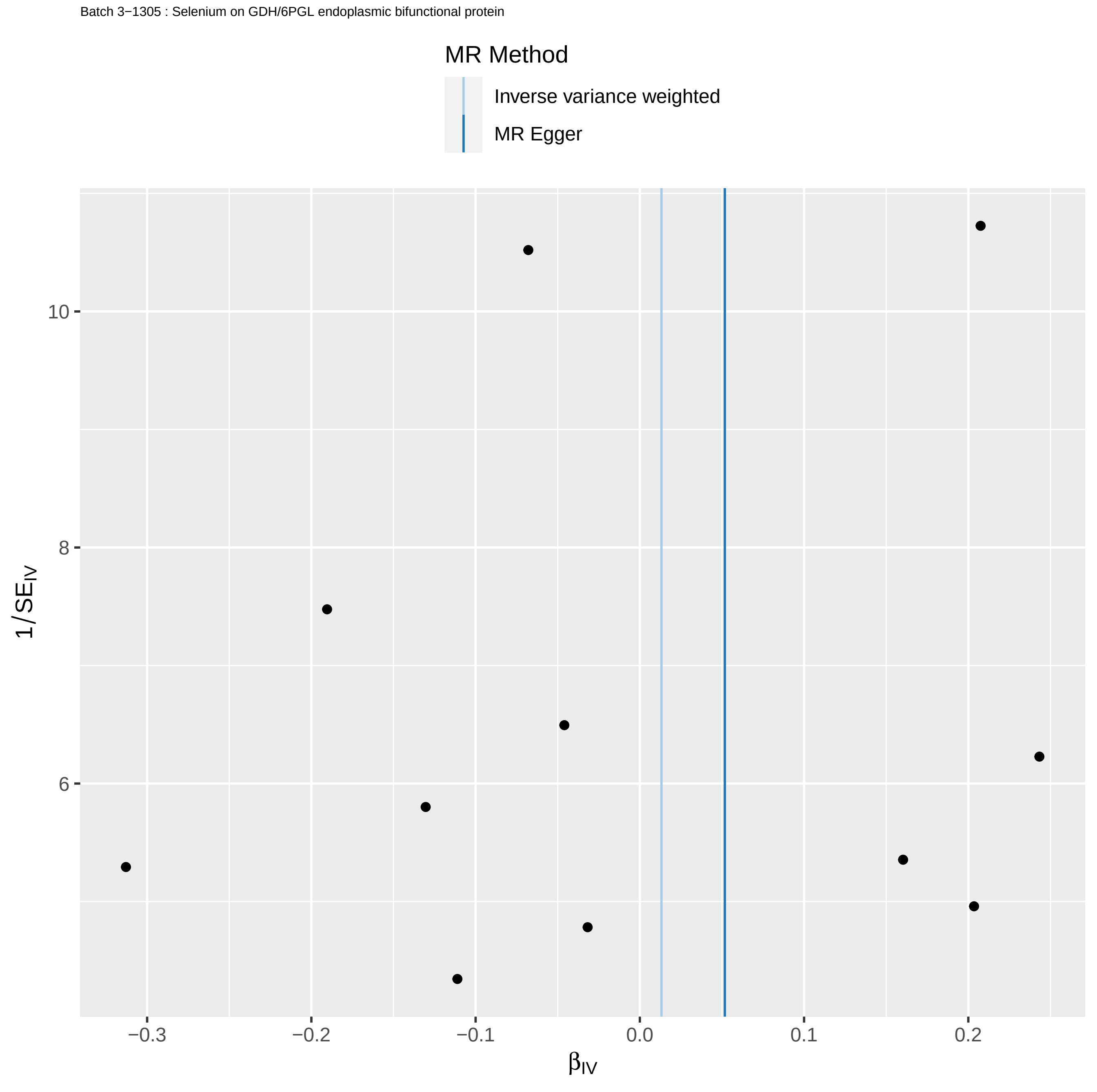

Batch 3−1305 : Selenium on GDH/6PGL endoplasmic bifunctional protein
MR Method
Inverse variance weighted
MR Egger
10
8
6
1/SE$_{IV}$
−0.3
−0.2
−0.1
0.0
0.1
0.2
β$_{IV}$

Batch 3−1306 : Selenium on 3−hydroxyanthranilate 3,4−dioxygenase

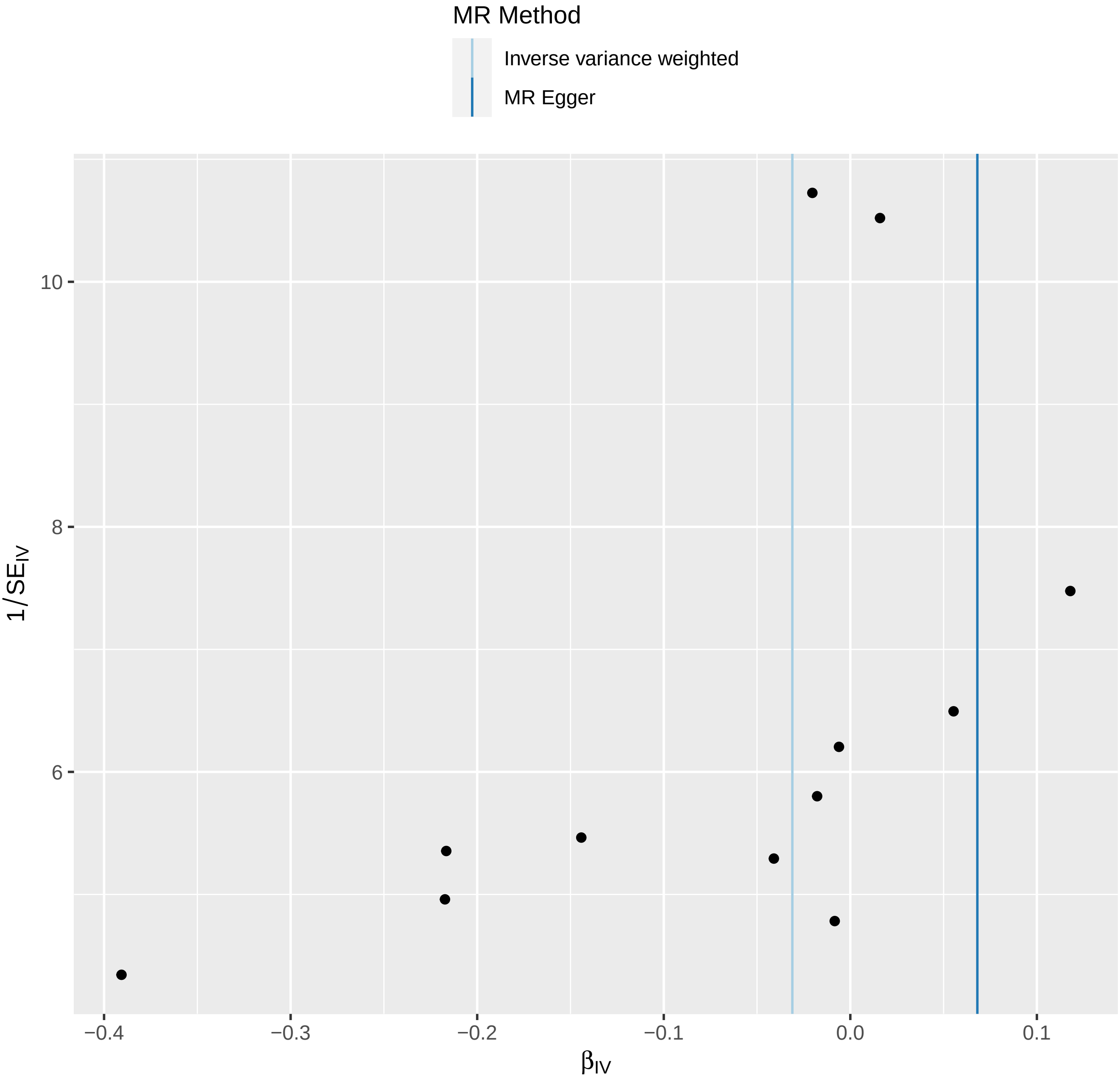

Batch 3−1307 : Selenium on Hepcidin

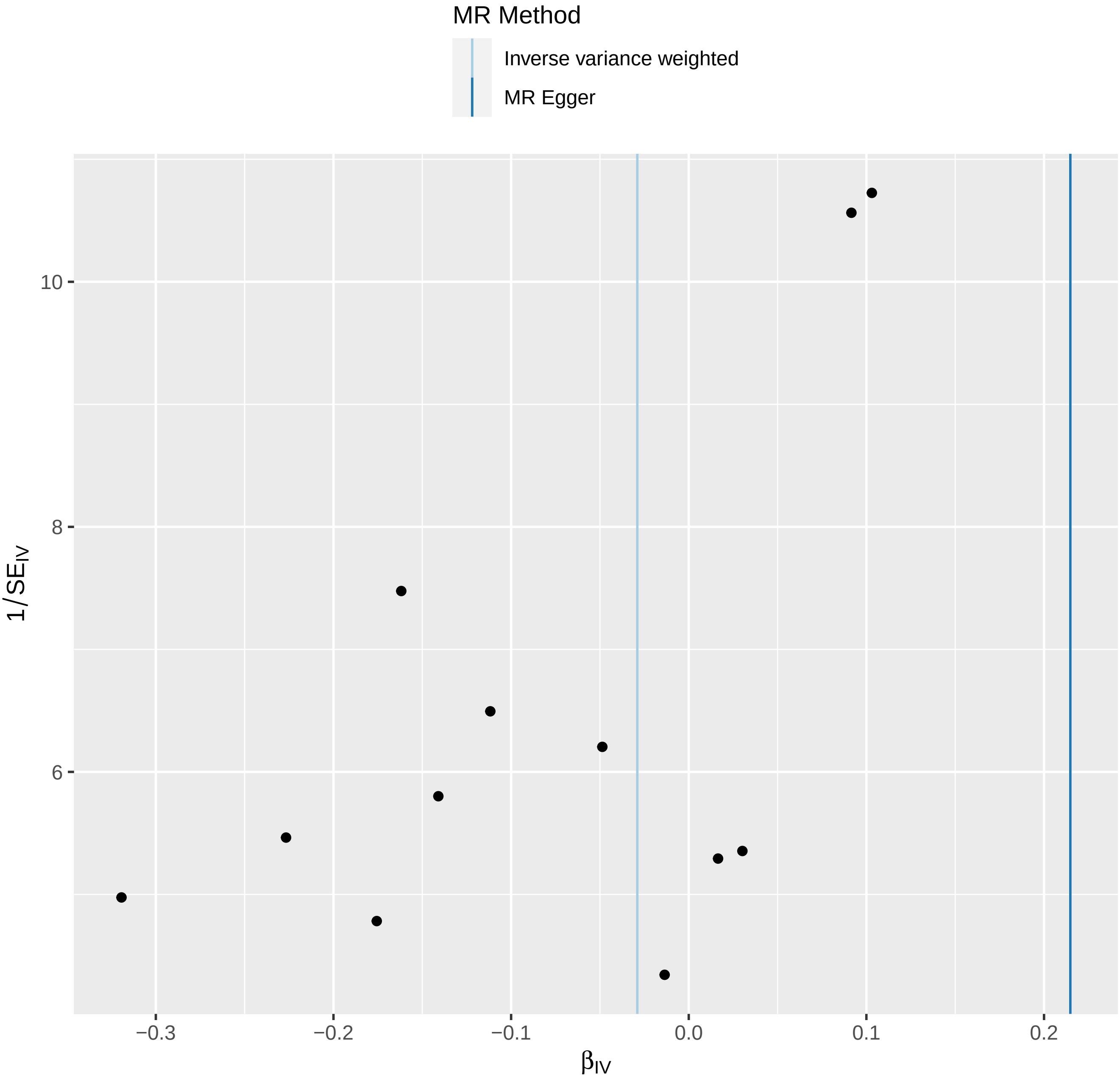

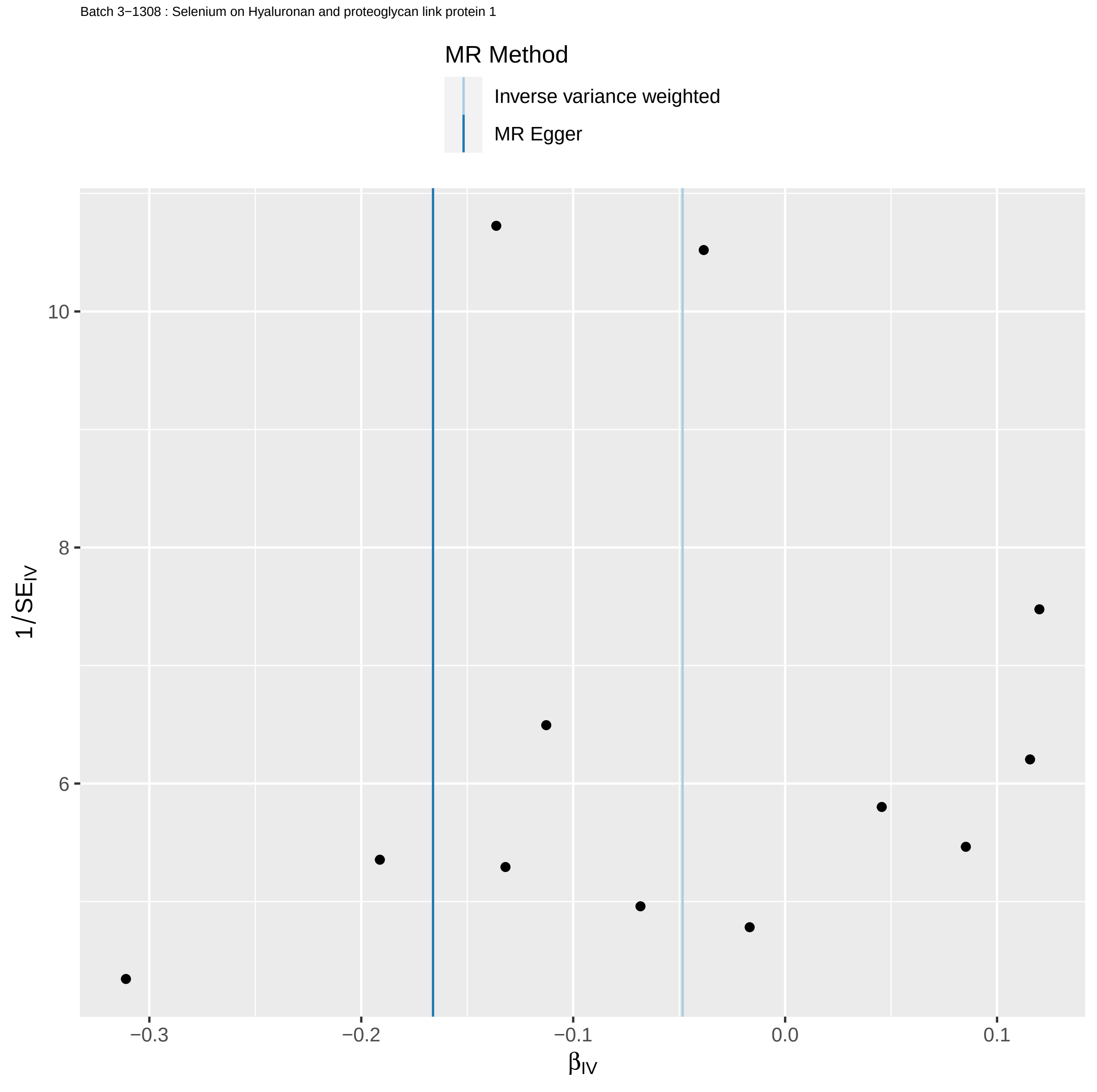

Batch 3−1308 : Selenium on Hyaluronan and proteoglycan link protein 1
MR Method
Inverse variance weighted
MR Egger
10
8
6
1/SE_IV
−0.3
−0.2
−0.1
0.0
0.1
β_IV

Batch 3−1309 : Selenium on Histone acetyltransferase type B catalytic subunit

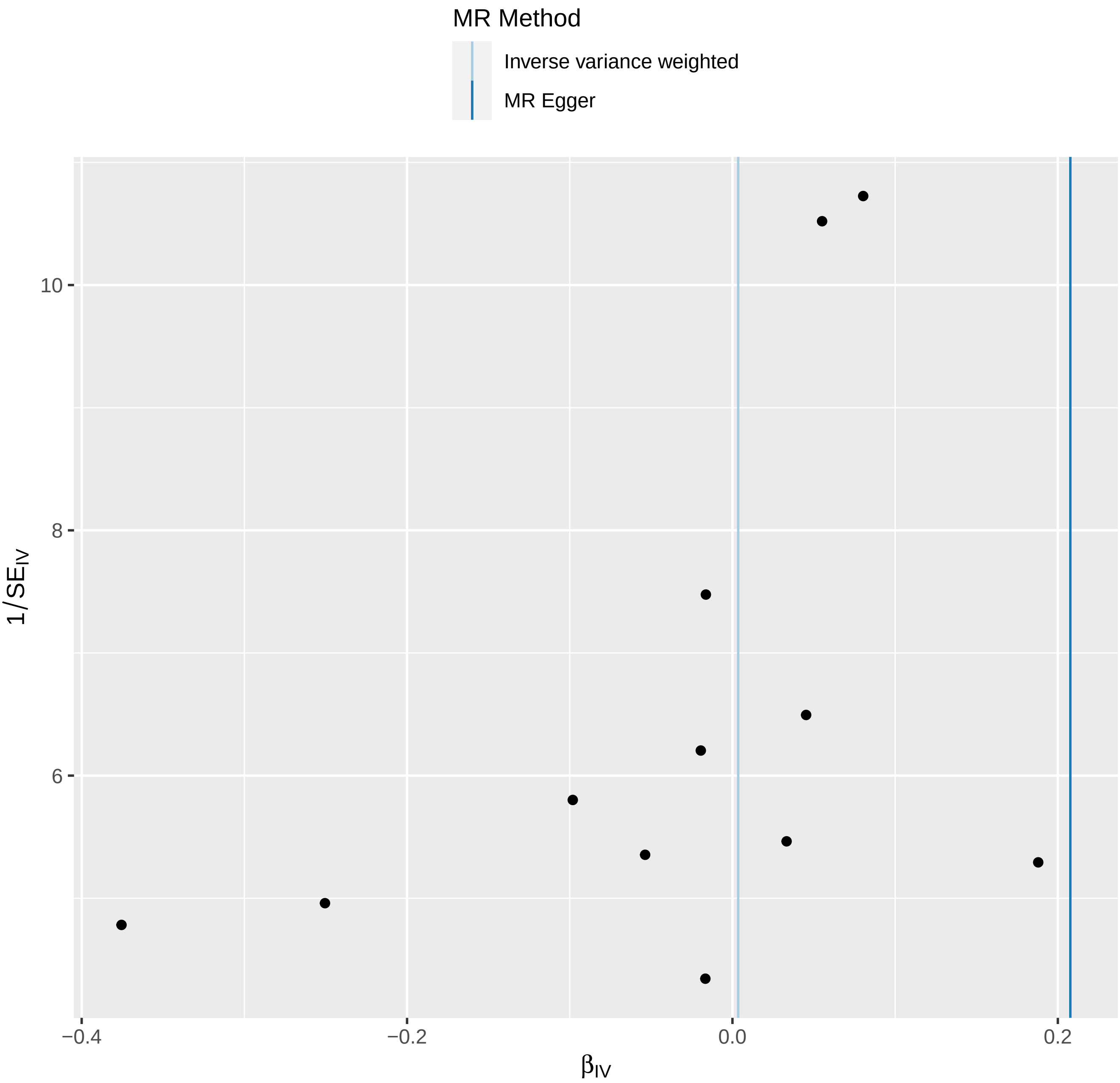

Batch 3−1310 : Selenium on Hepatitis A virus cellular receptor 1

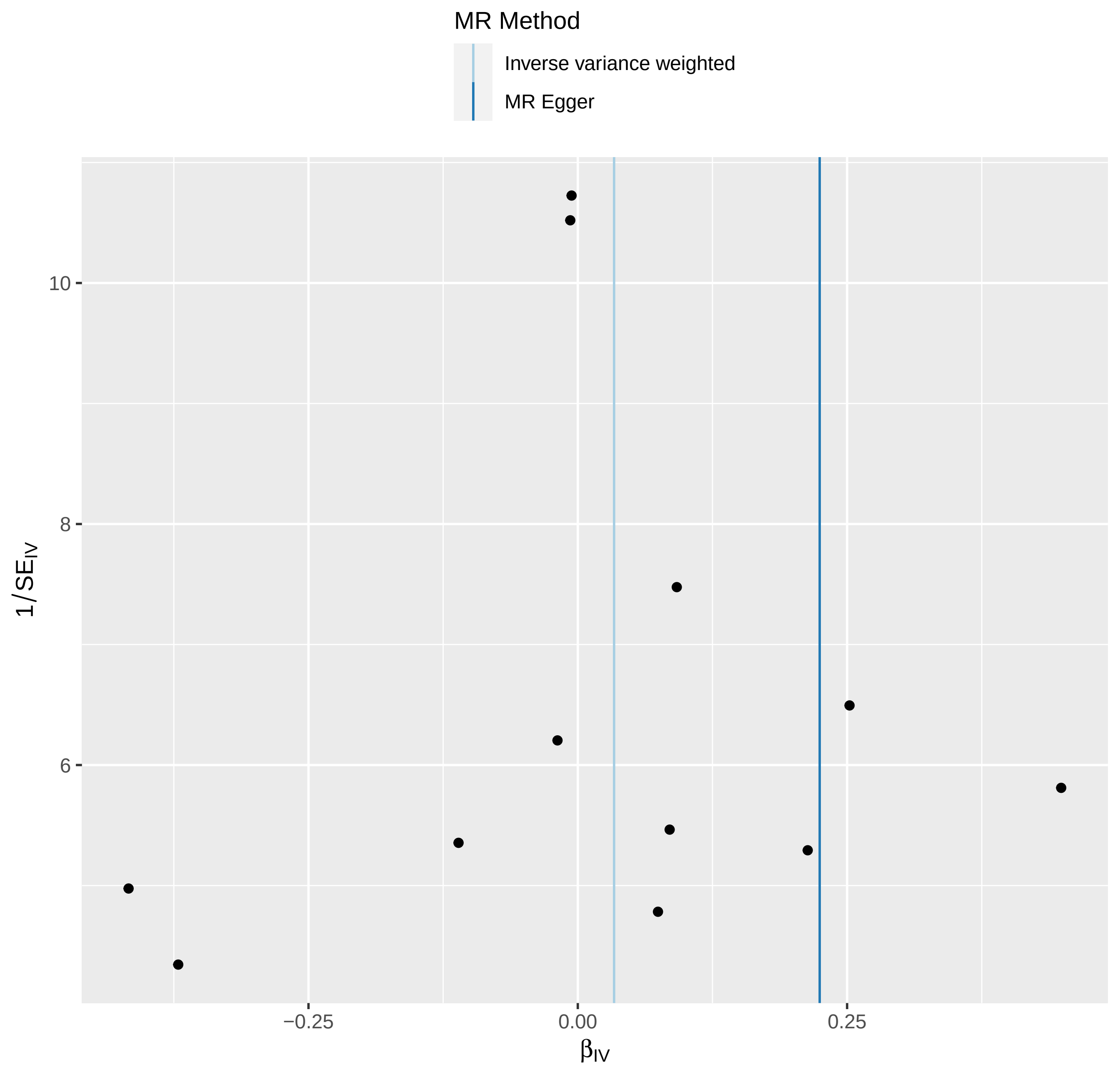

Batch 3−1311 : Selenium on Hepatitis A virus cellular receptor 2

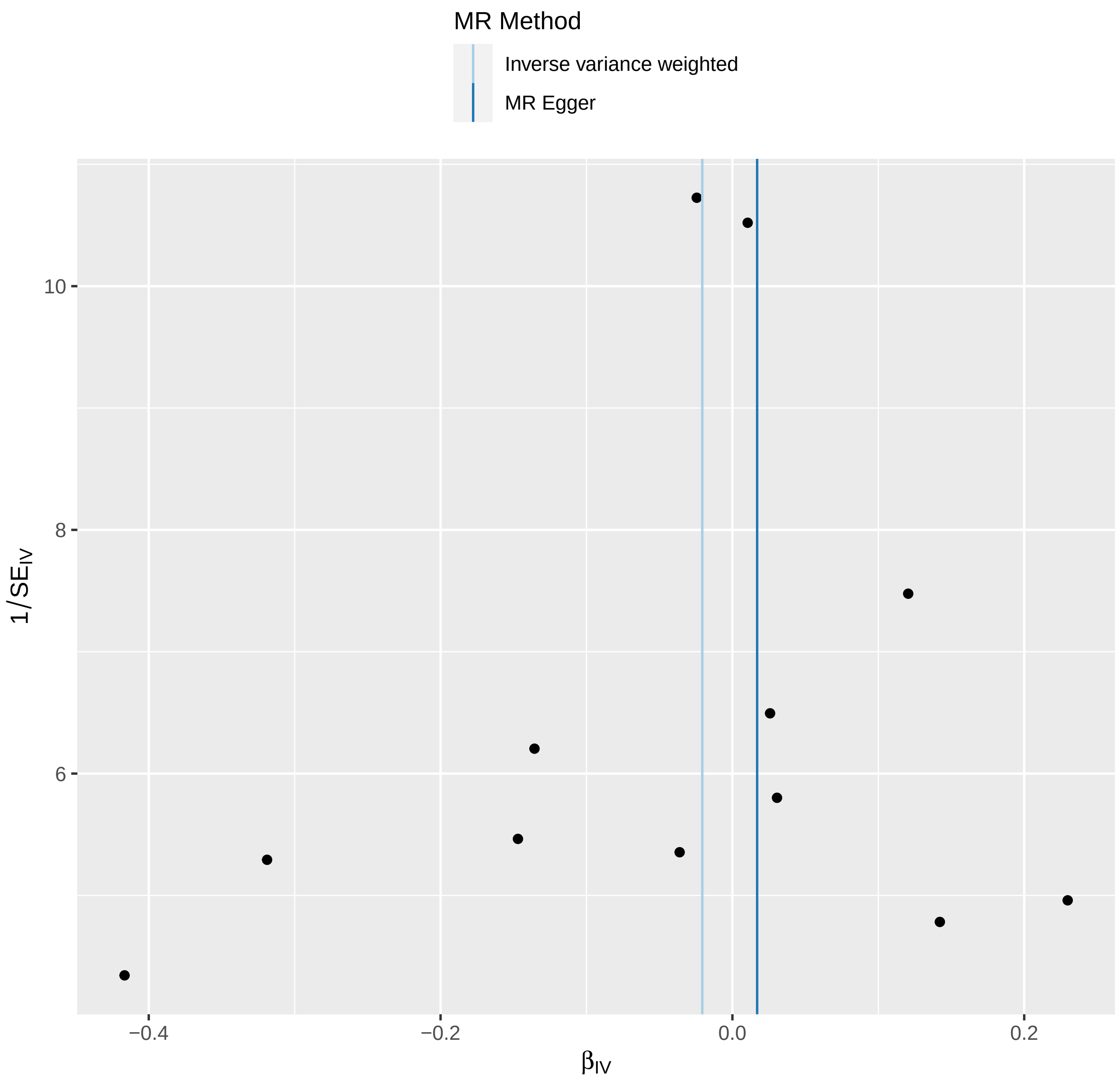

Batch 3−1312 : Selenium on Hepatitis A virus cellular receptor 2

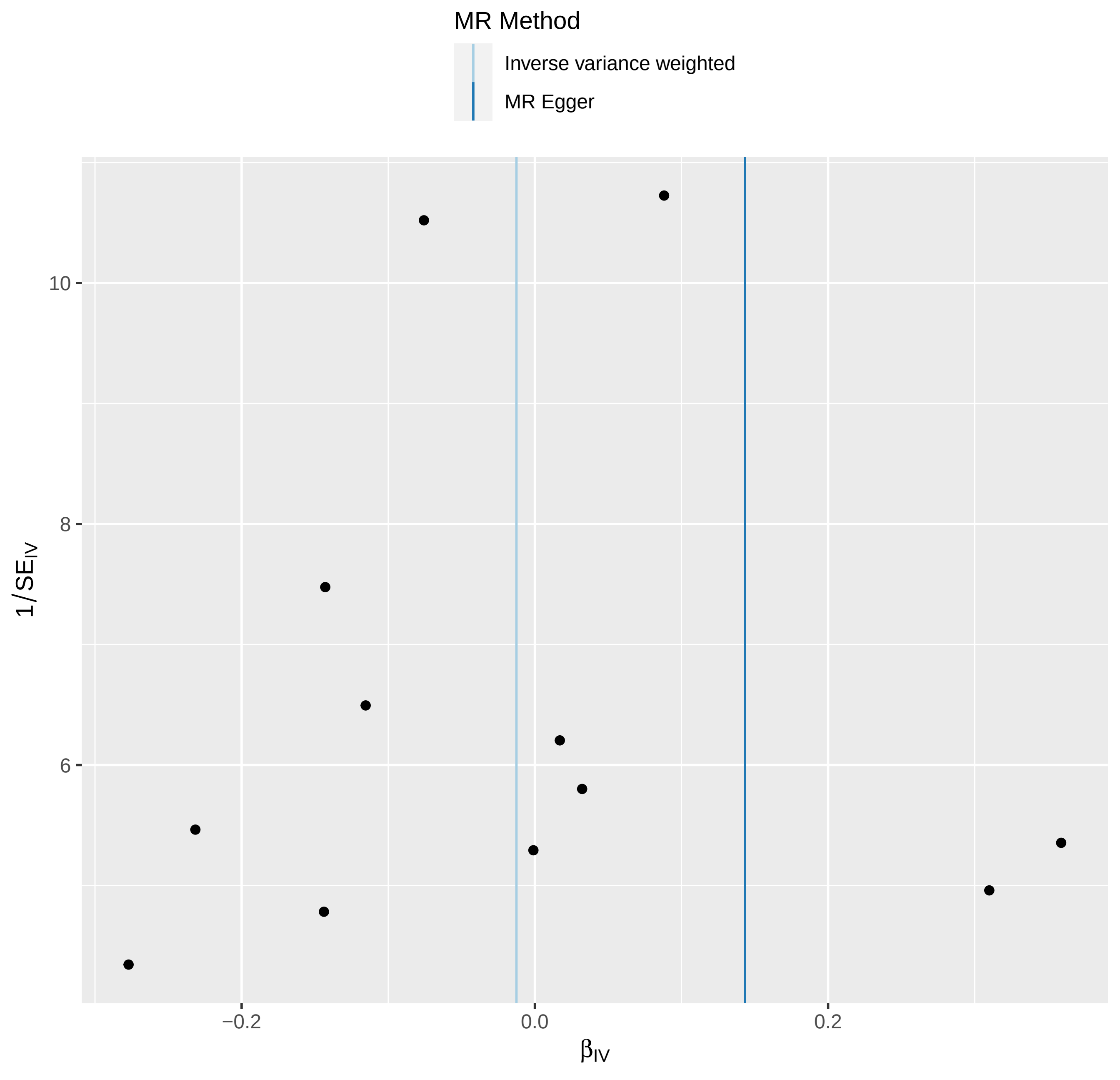

Batch 3−1313 : Selenium on Heparin−binding EGF−like growth factor

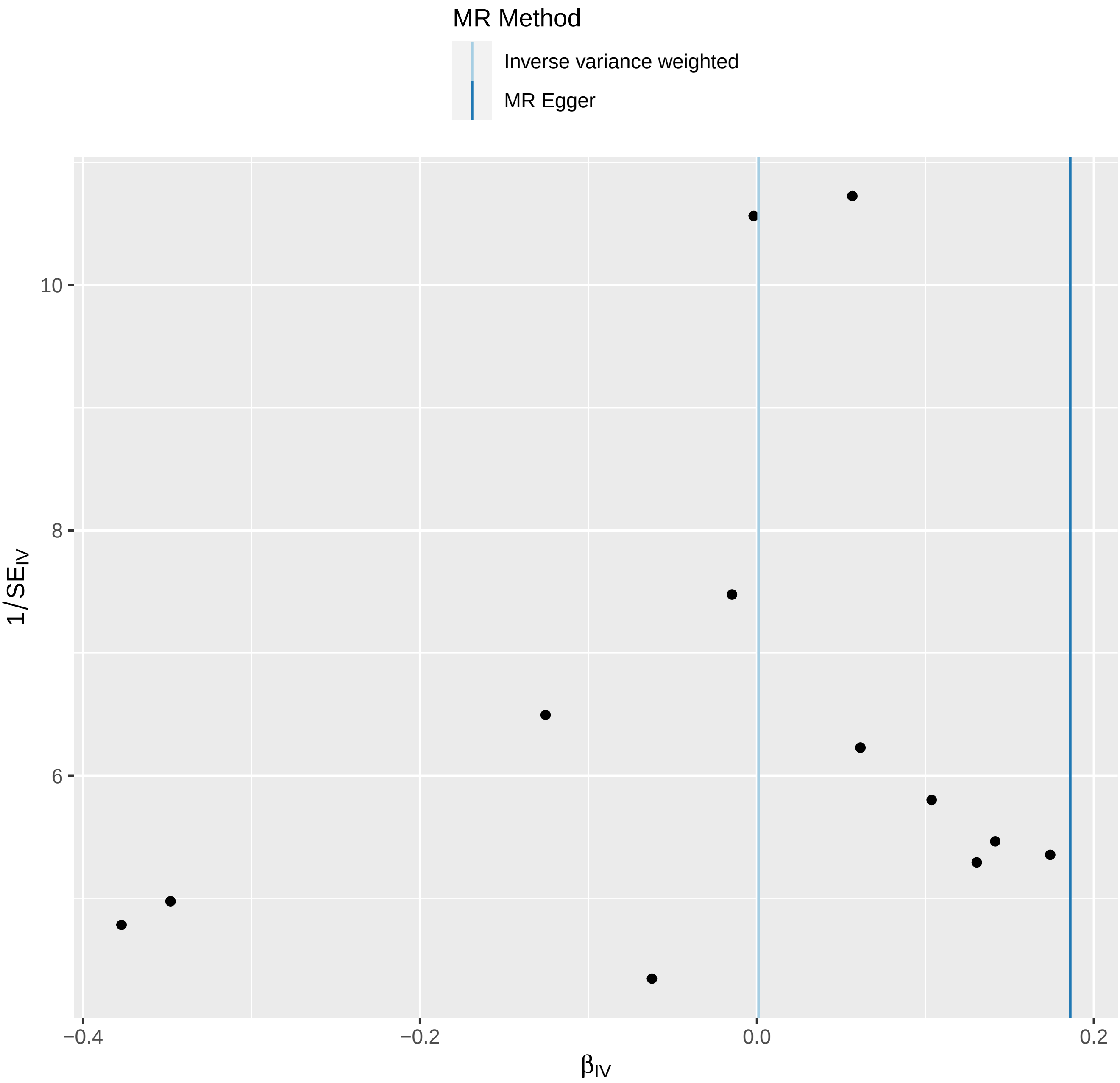

Batch 3−1314 : Selenium on Heparin−binding EGF−like growth factor

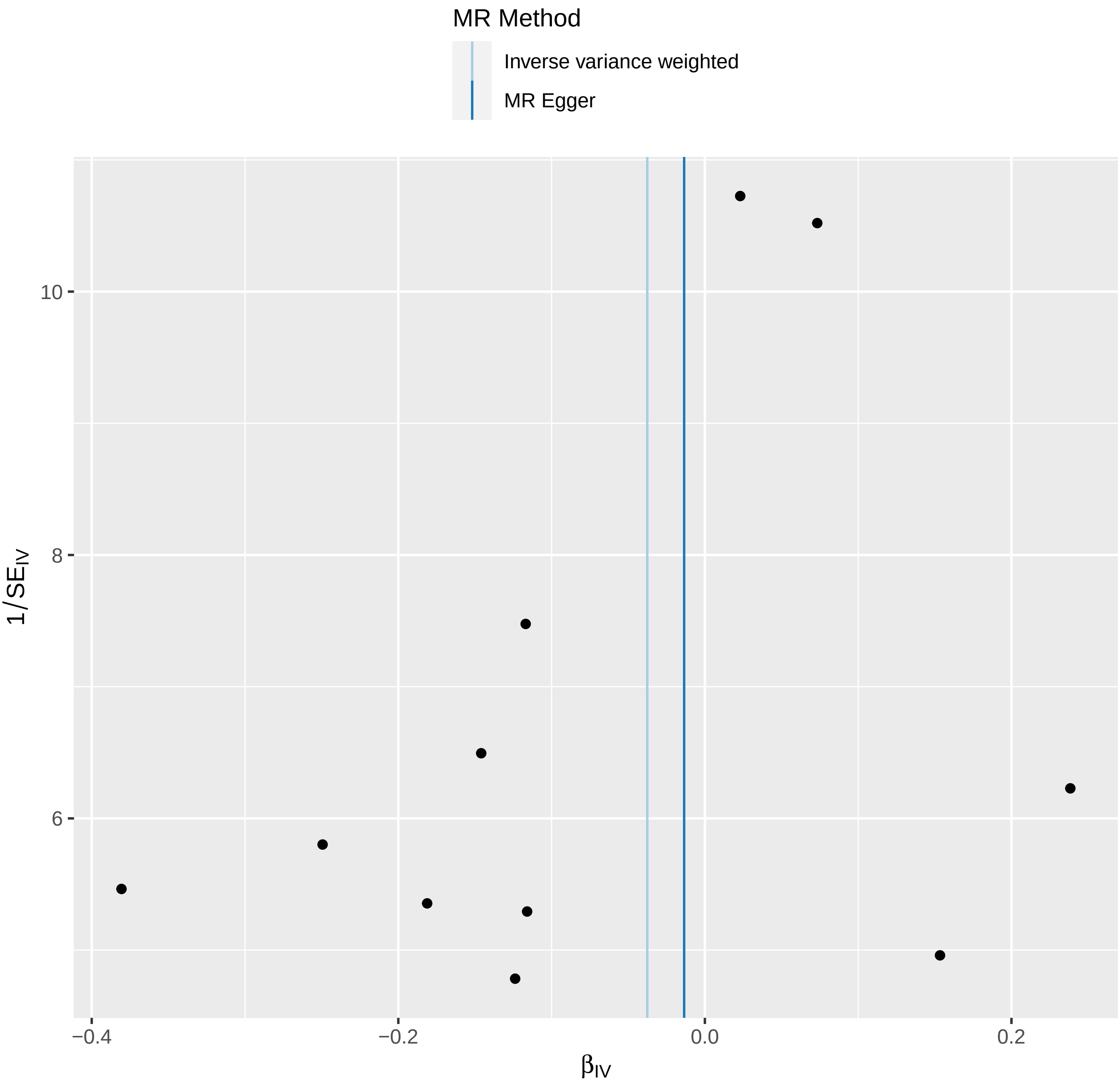

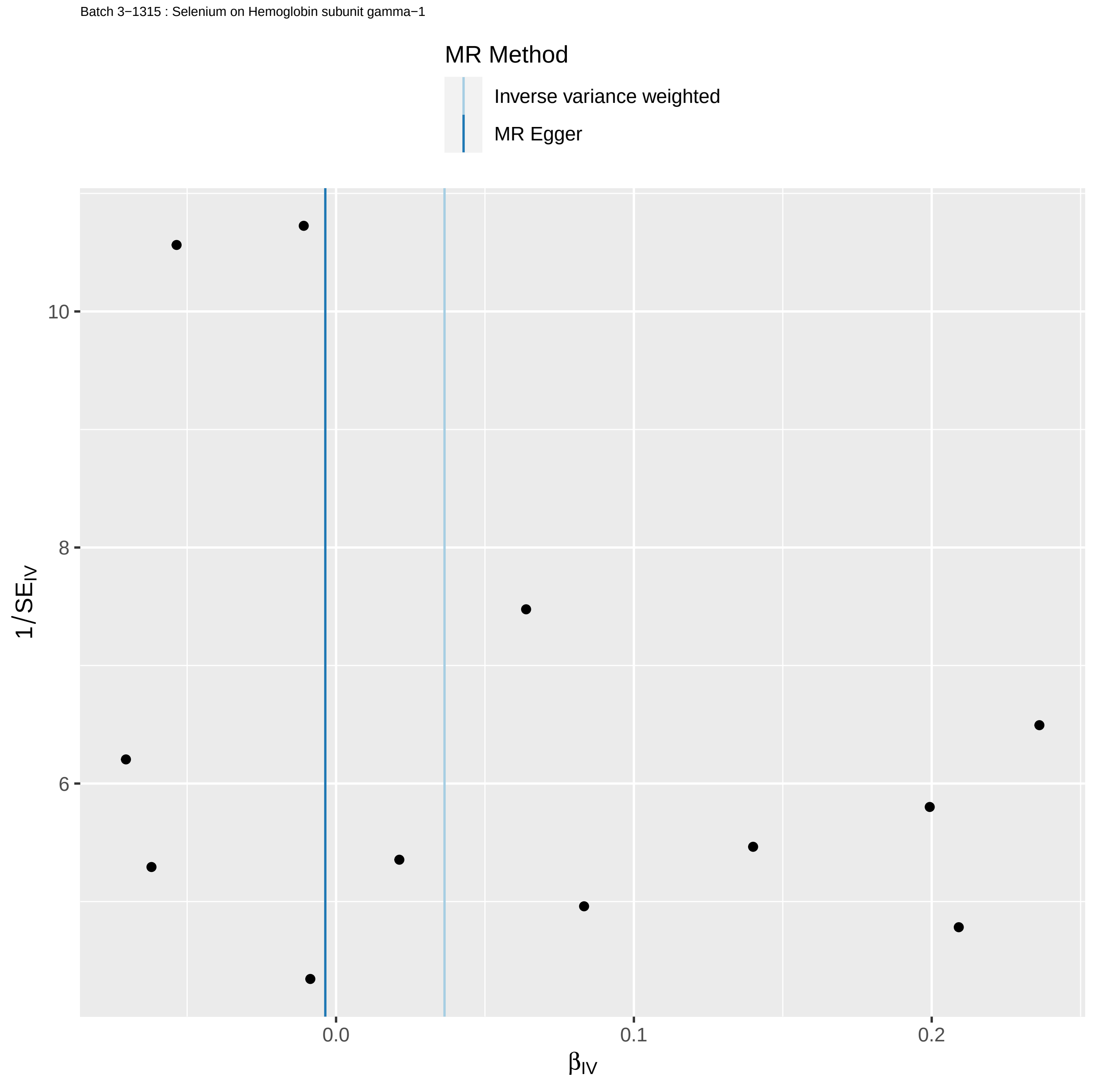

Batch 3−1315 : Selenium on Hemoglobin subunit gamma−1
MR Method
Inverse variance weighted
MR Egger
10
8
6
1/SE_IV
0.0
0.1
0.2
β_IV

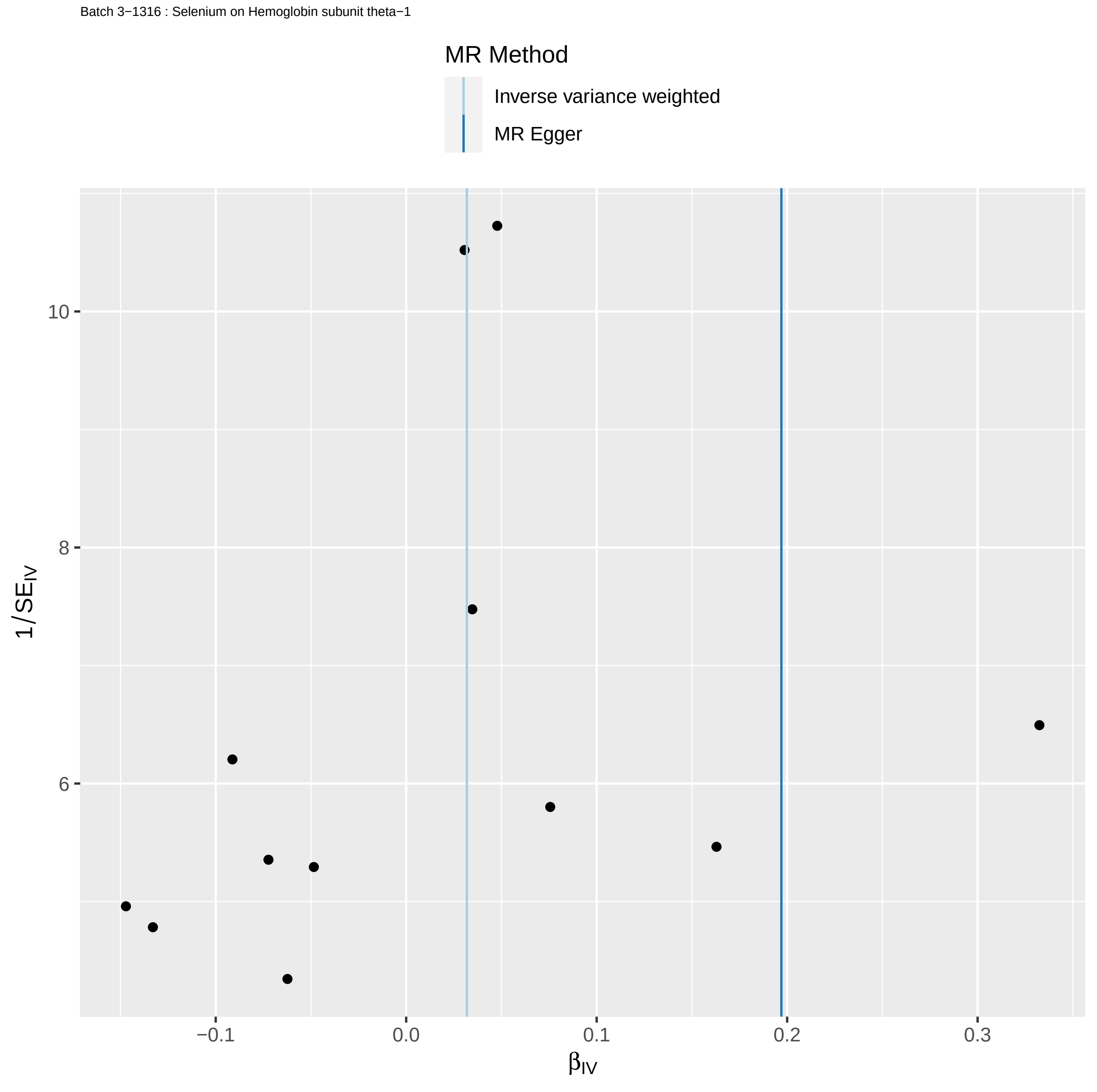

Batch 3−1316 : Selenium on Hemoglobin subunit theta−1
MR Method
Inverse variance weighted
MR Egger
10
8
6
1/SE$_{IV}$
−0.1
0.0
0.1
0.2
0.3
β$_{IV}$

Batch 3−1317 : Selenium on Hemoglobin subunit zeta

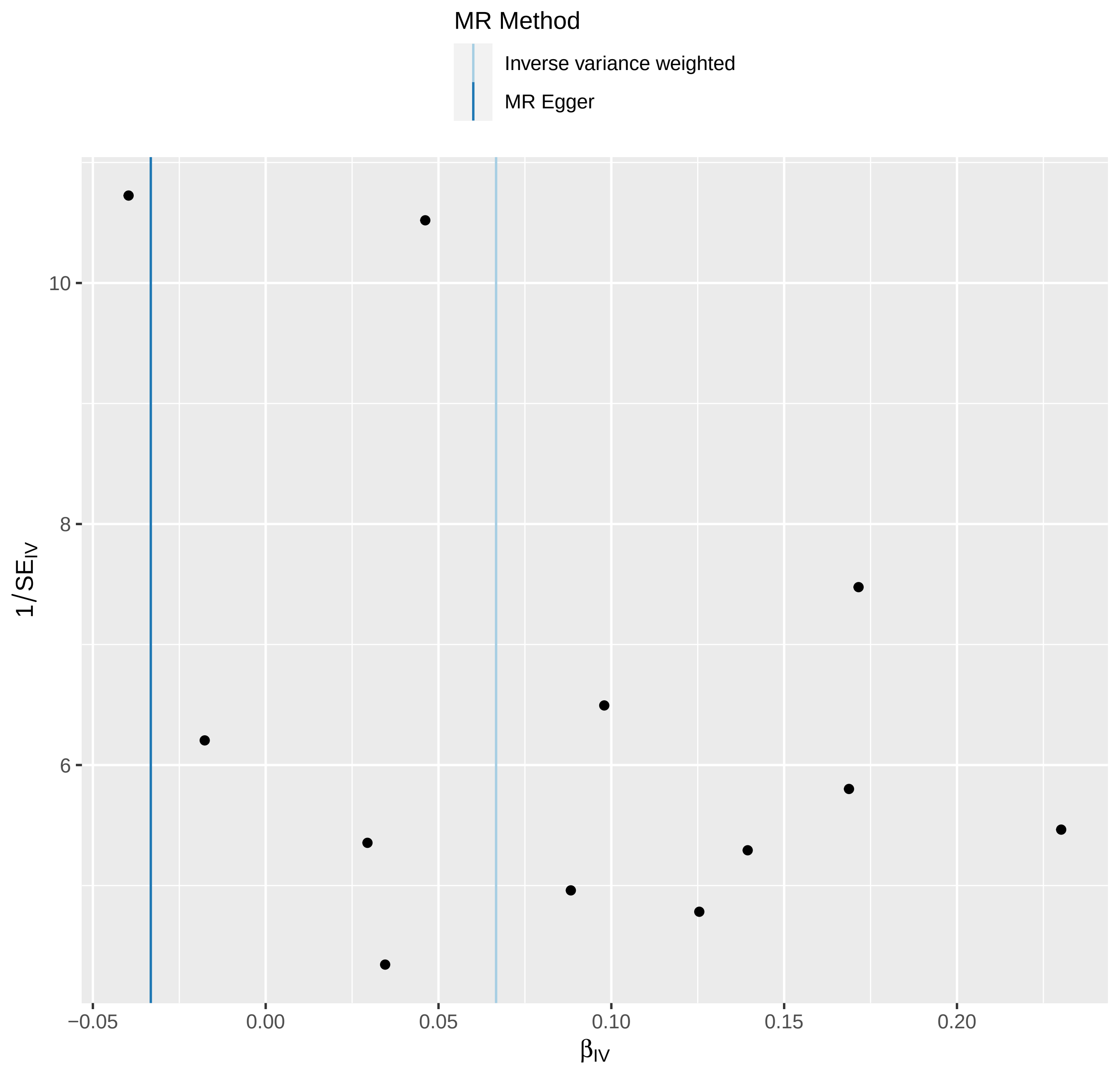

Batch 3−1318 : Selenium on Hydroxycarboxylic acid receptor 2

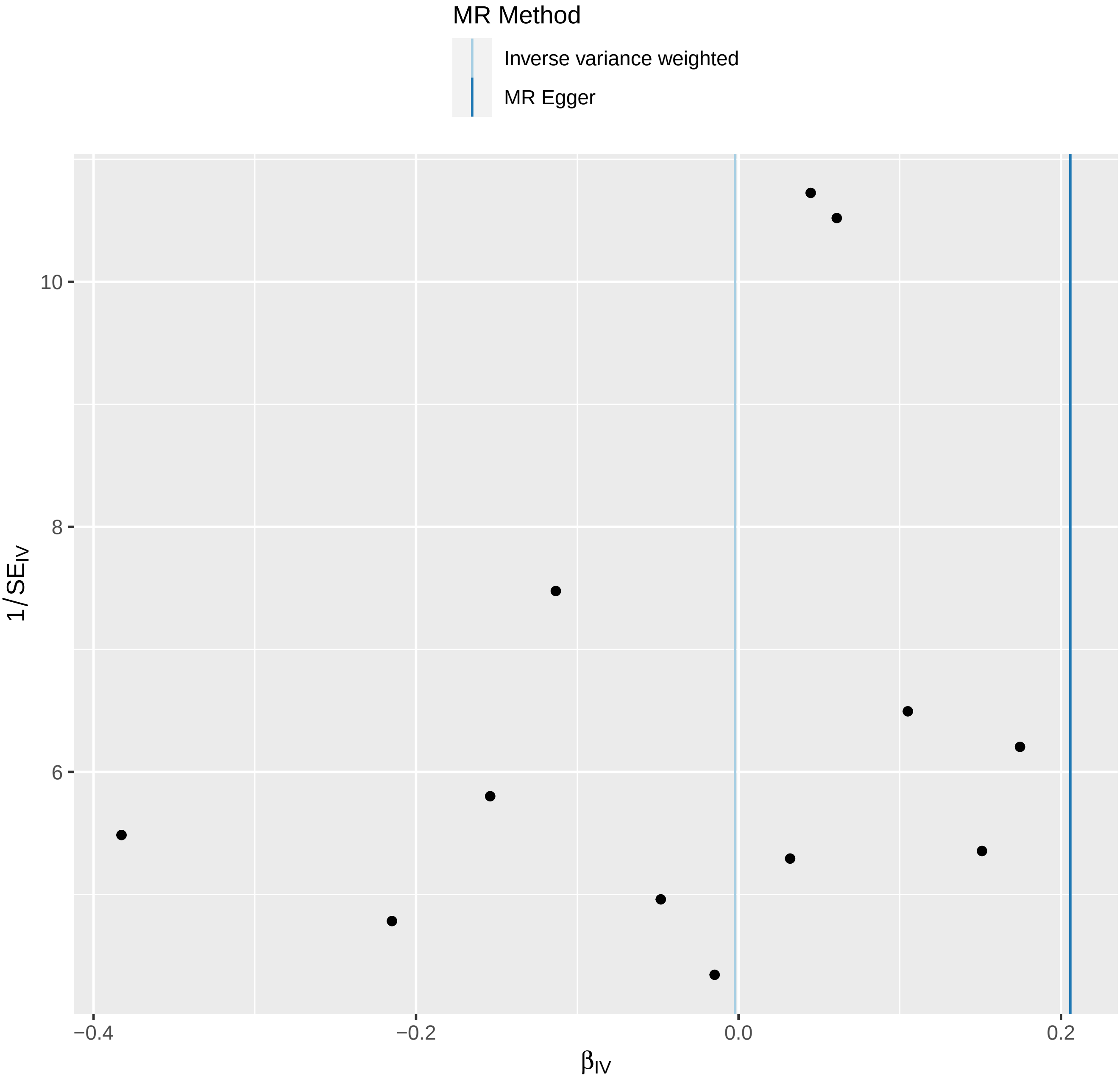

Batch 3−1319 : Selenium on Tyrosine−protein kinase HCK

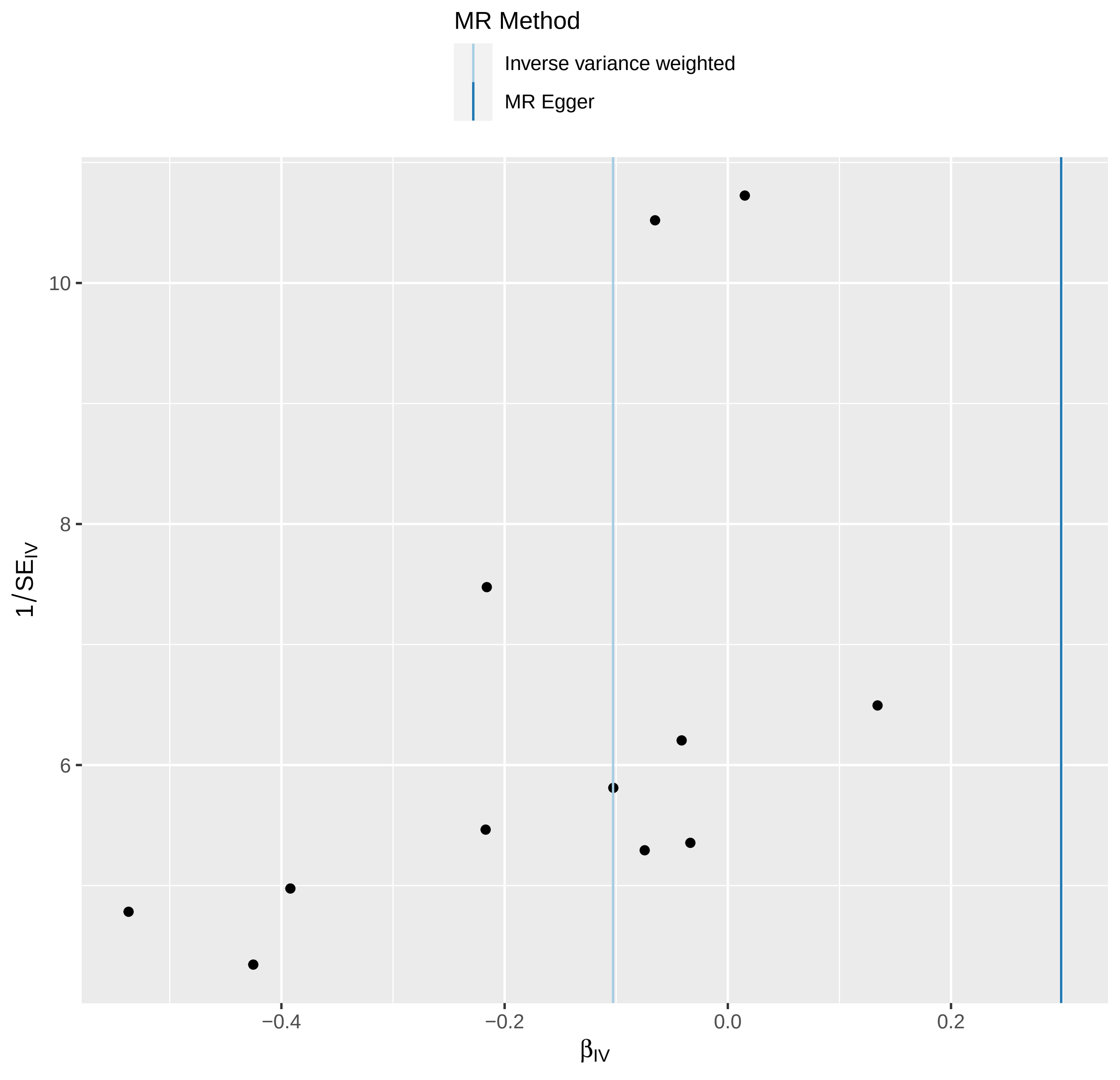

Batch 3−1320 : Selenium on Histone deacetylase 8

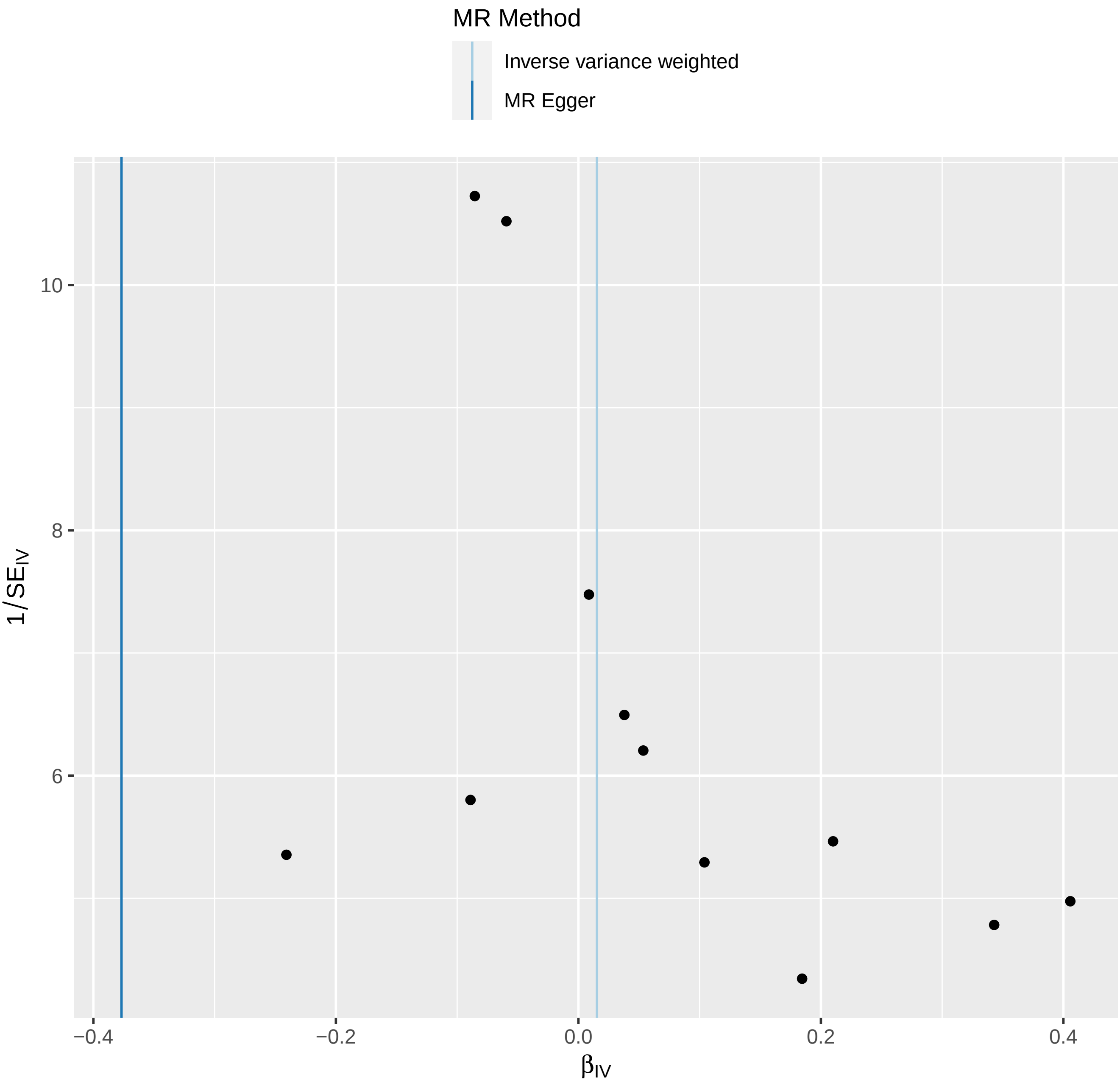

Batch 3−1321 : Selenium on Hepatoma−derived growth factor−related protein 2

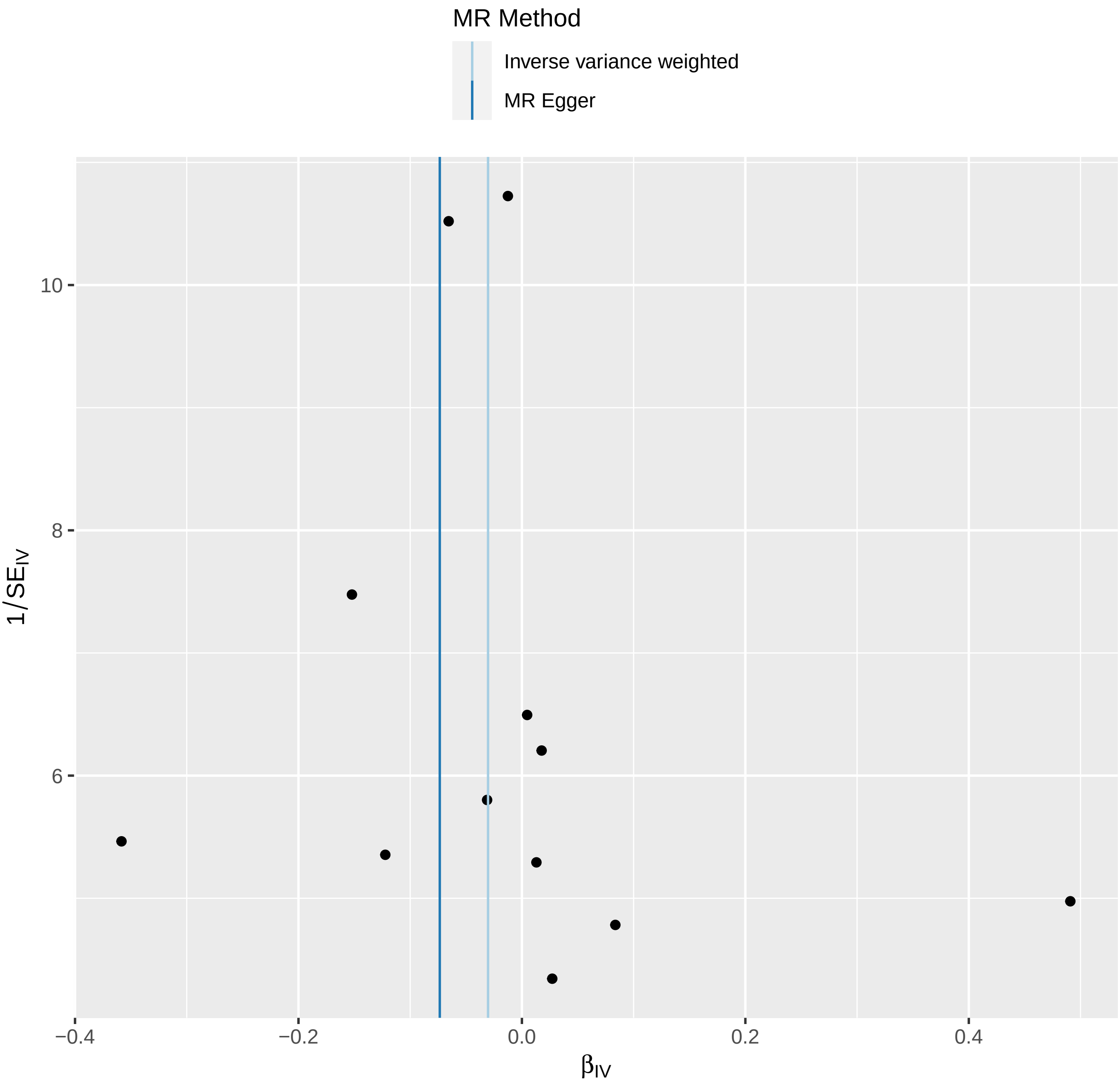

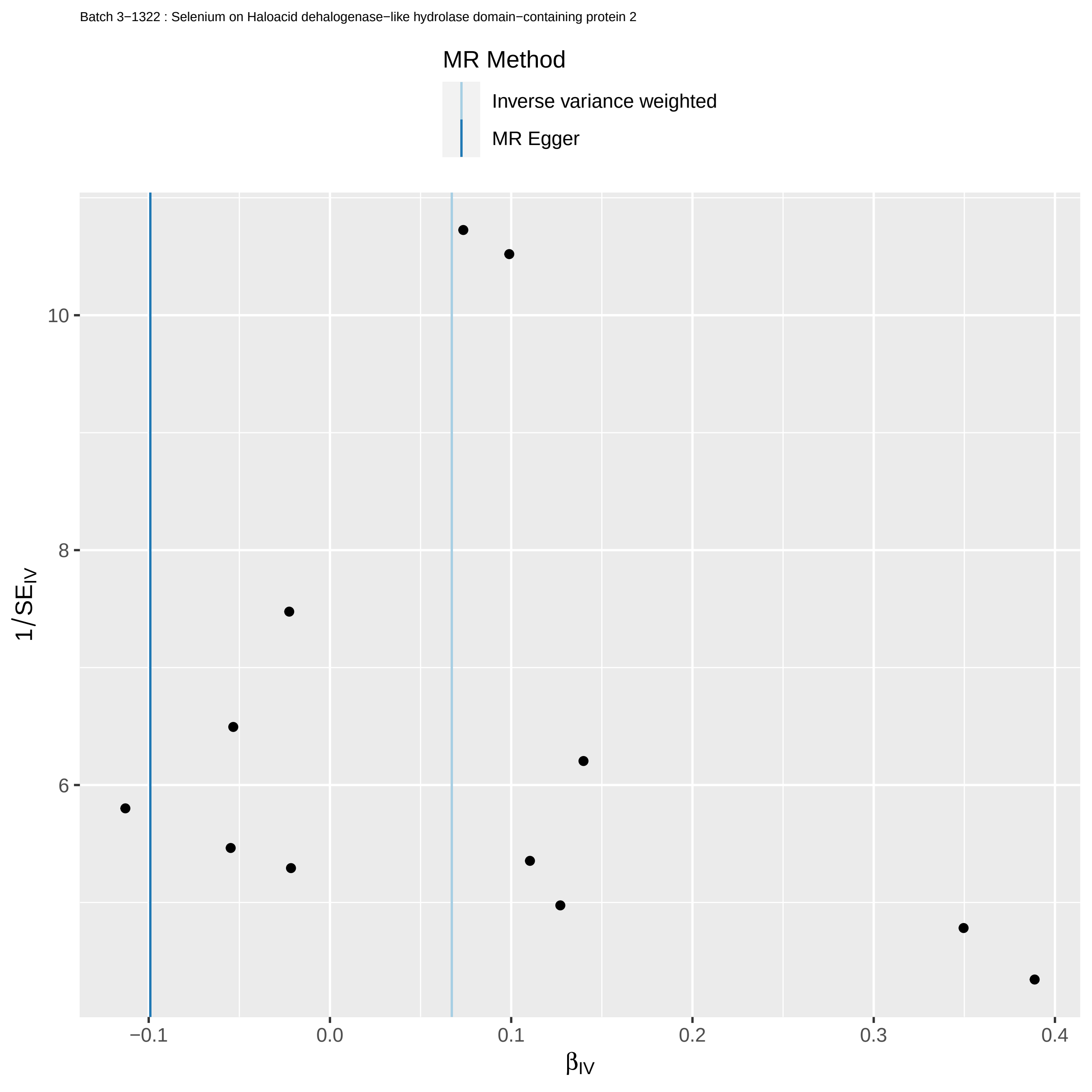

Batch 3−1322 : Selenium on Haloacid dehalogenase−like hydrolase domain−containing protein 2
MR Method
Inverse variance weighted
MR Egger
10
8
6
1/SE_IV
−0.1
0.0
0.1
0.2
0.3
0.4
β_IV

Batch 3−1323 : Selenium on Vigilin

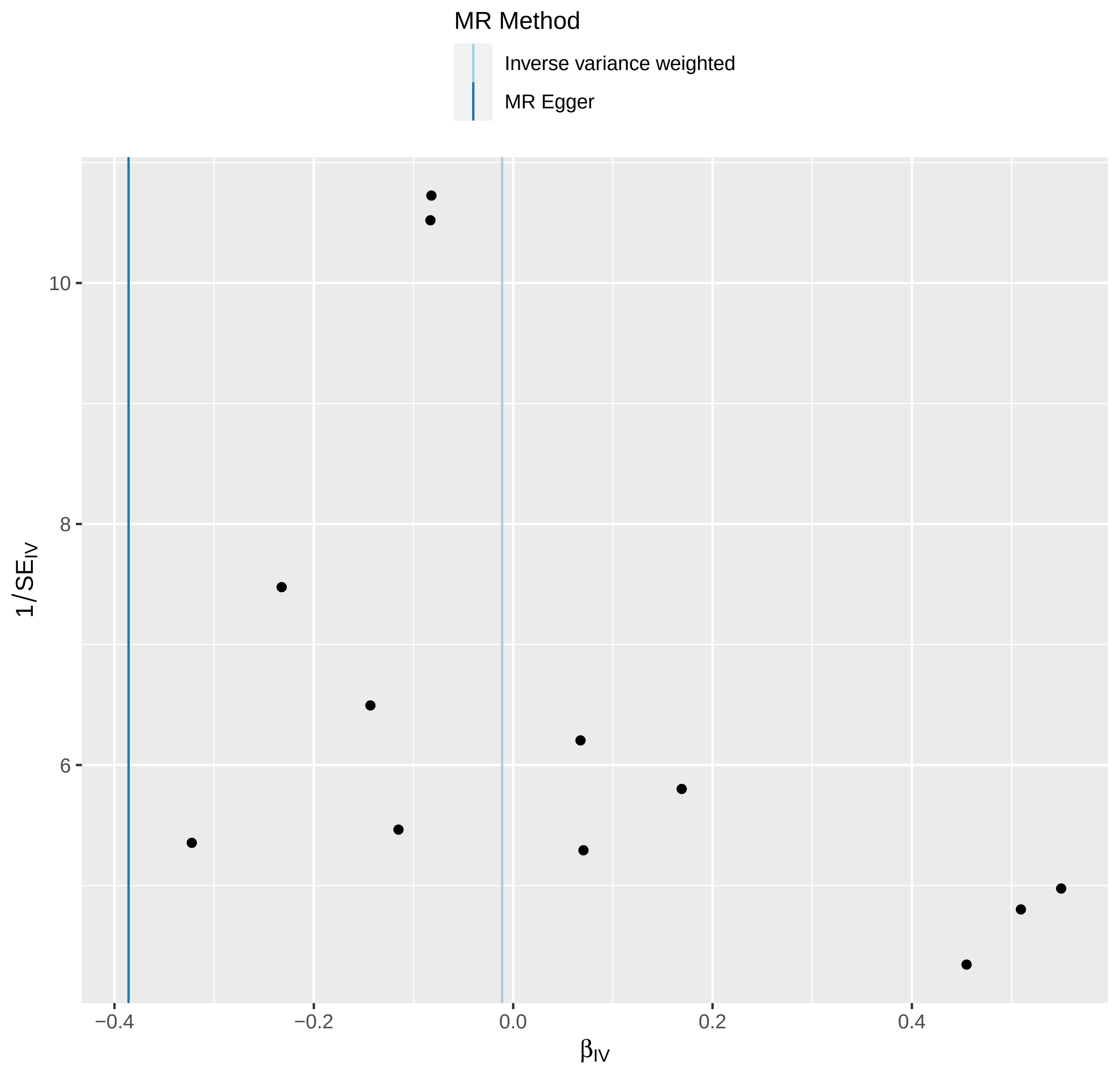

Batch 3−1324 : Selenium on E3 ubiquitin−protein ligase HECW1

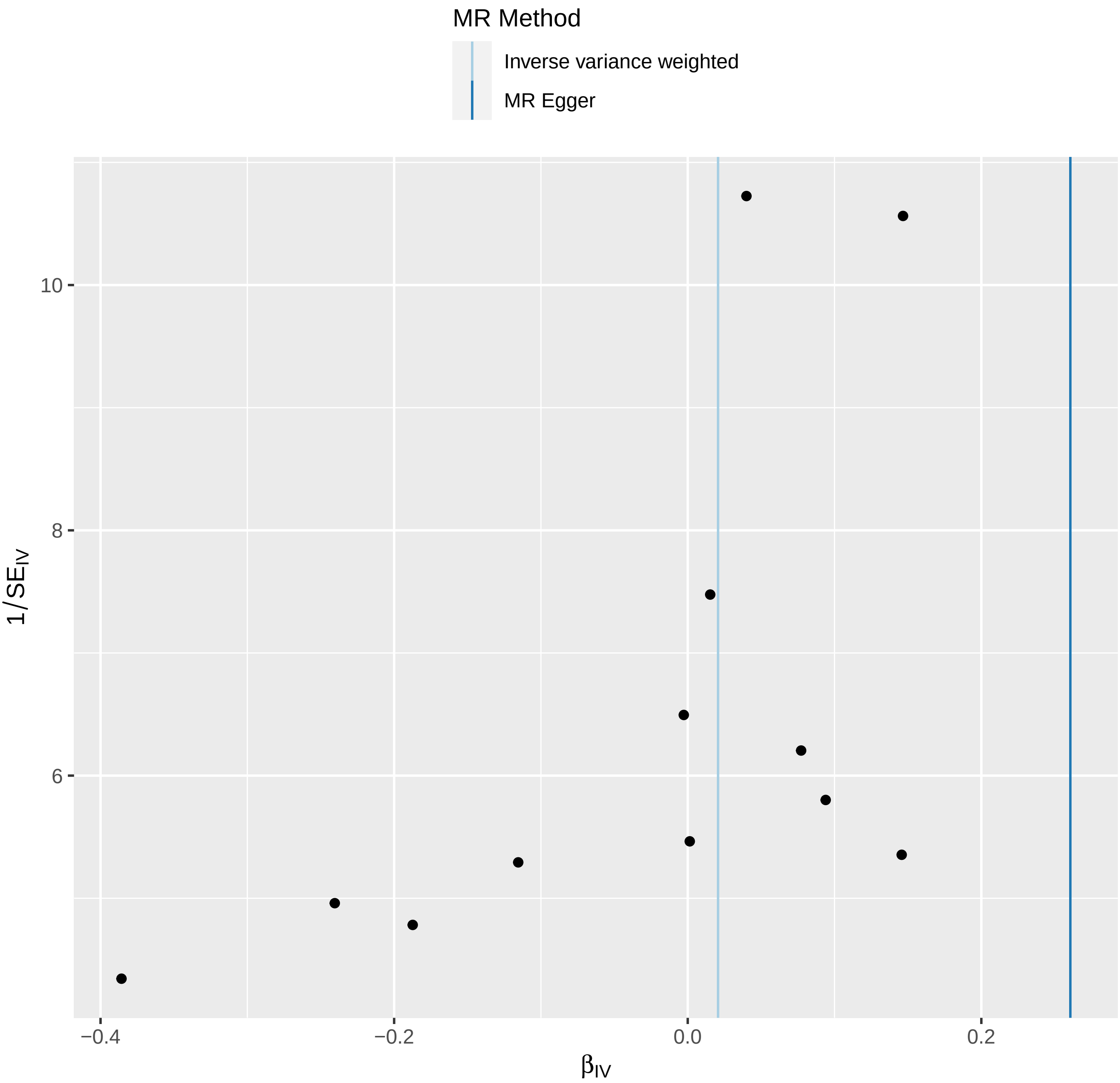

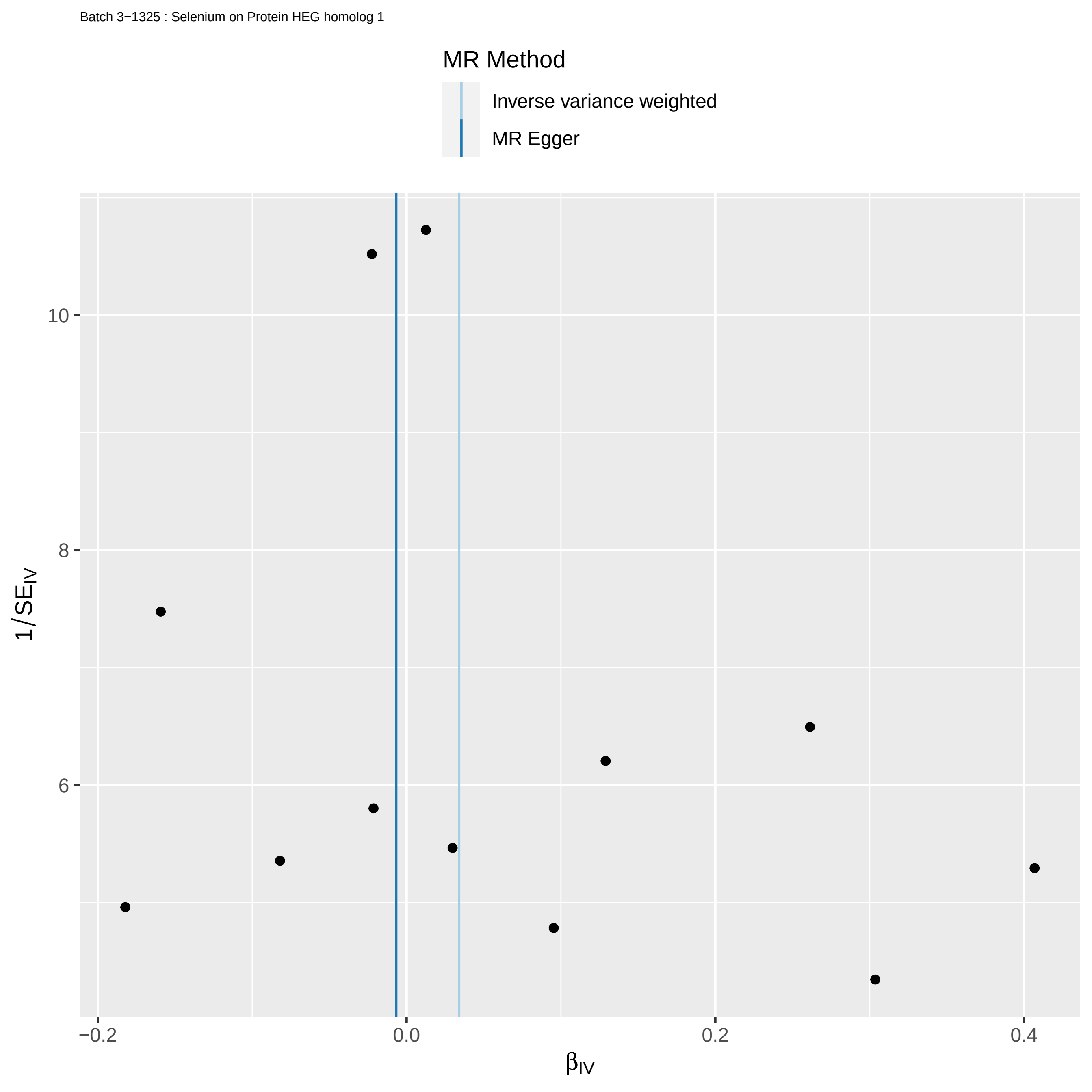
Batch 3−1325 : Selenium on Protein HEG homolog 1
MR Method
Inverse variance weighted
MR Egger
10
8
6
−0.2
0.0
0.2
0.4
$1/SE_{IV}$
β_{IV}

Batch 3−1326 : Selenium on HEPACAM family member 2

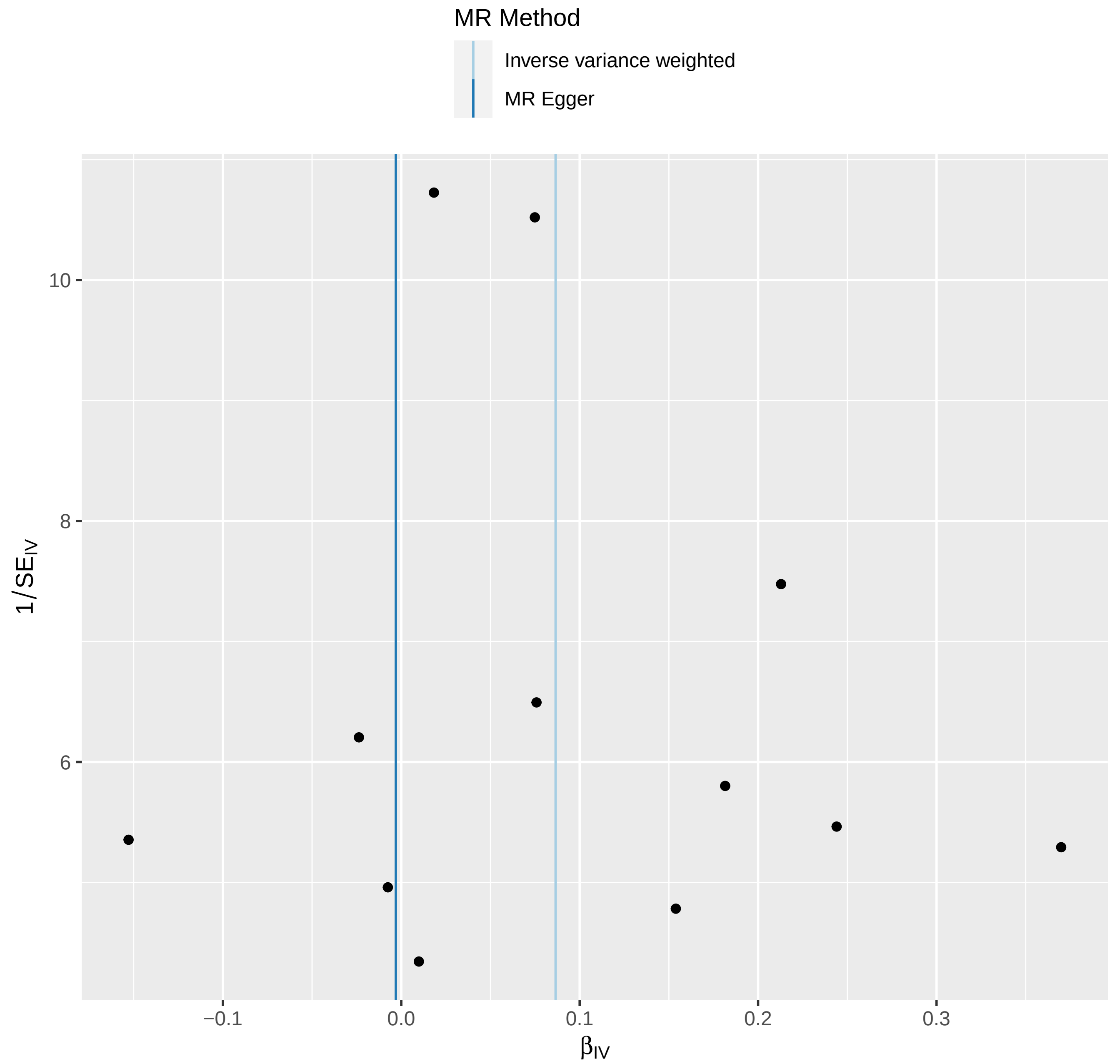

Batch 3−1327 : Selenium on HEPACAM family member 2

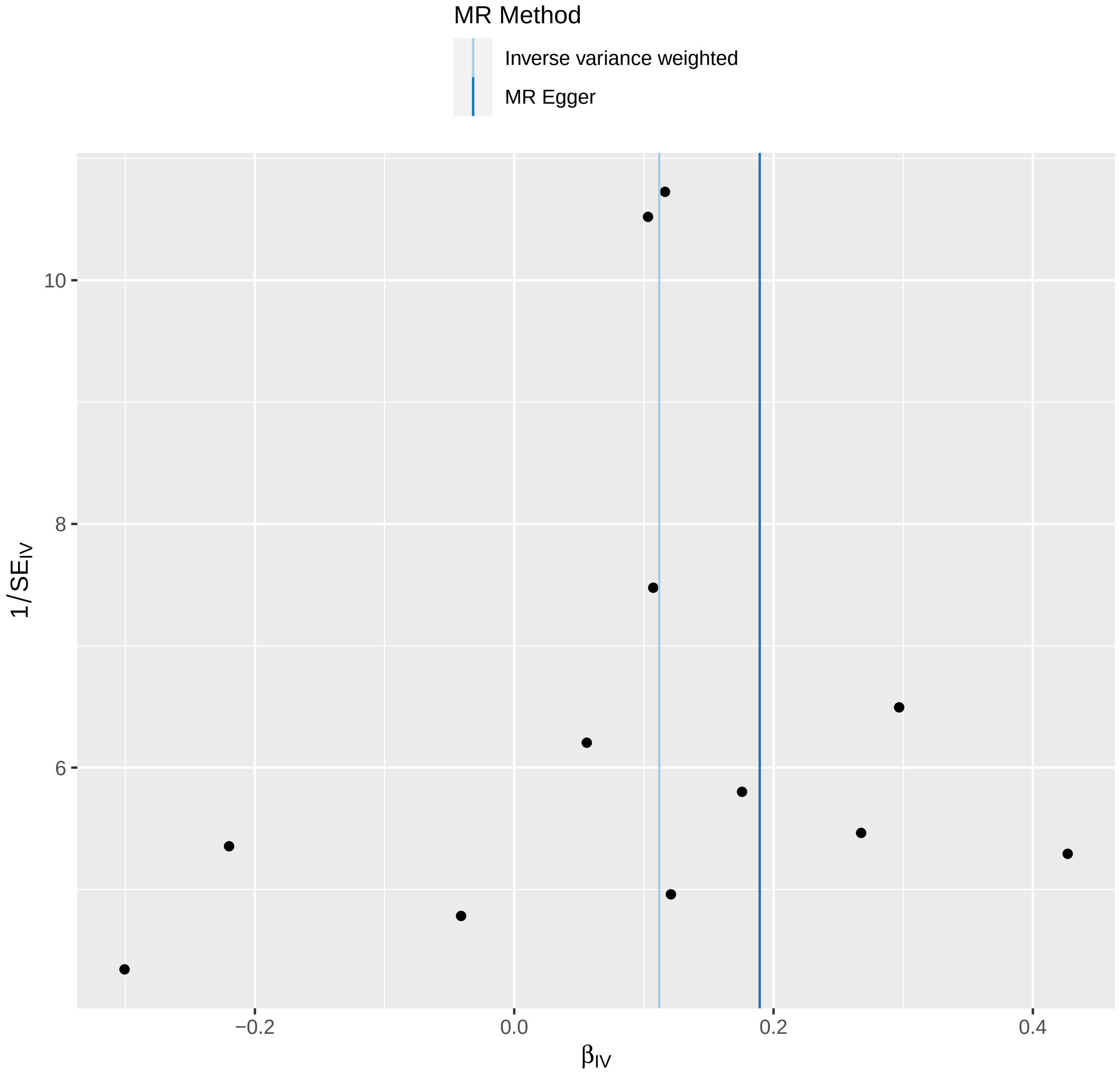

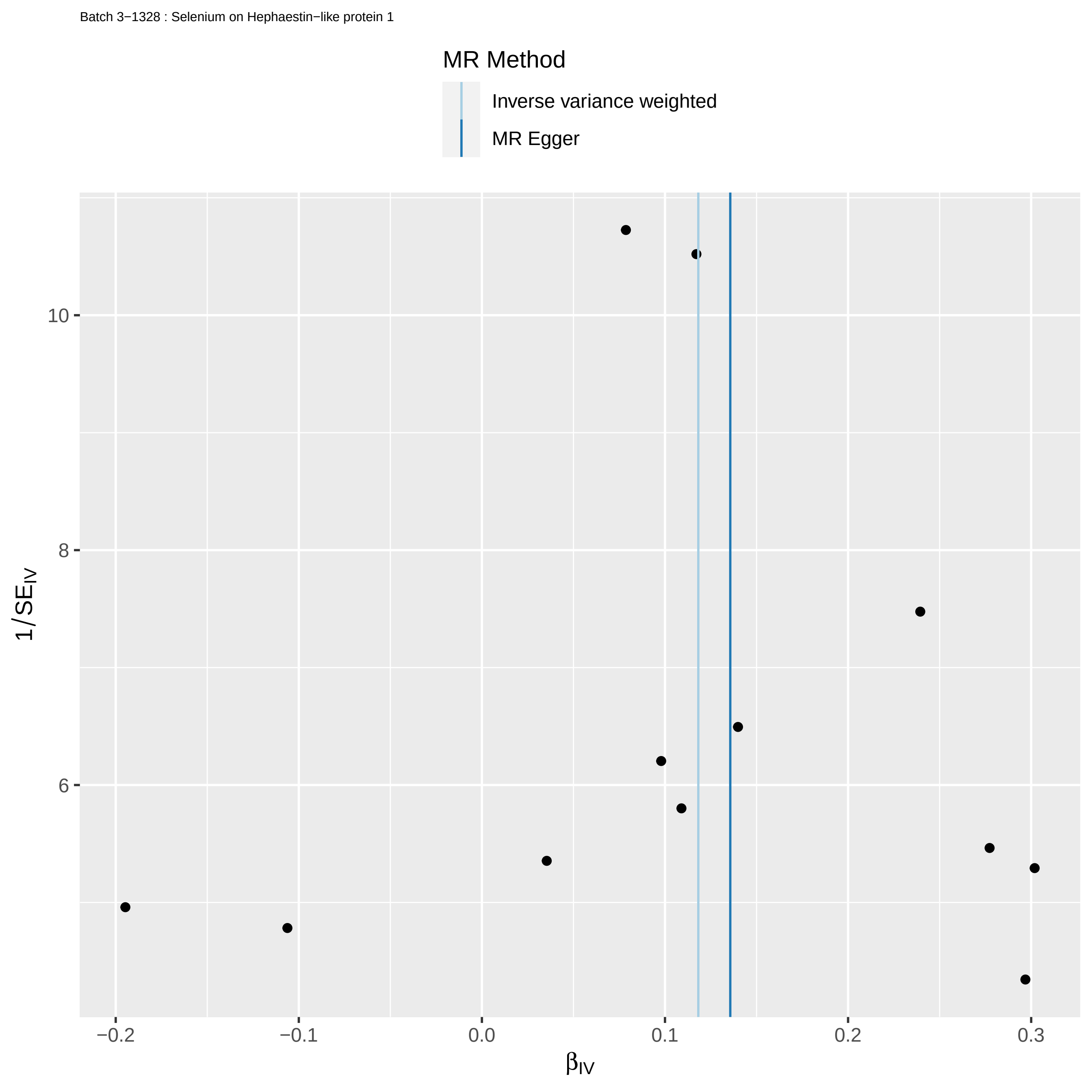

Batch 3−1328 : Selenium on Hephaestin−like protein 1
MR Method
Inverse variance weighted
MR Egger
10
8
6
1/SE$_{IV}$
−0.2
−0.1
0.0
0.1
0.2
0.3
β$_{IV}$

Batch 3−1329 : Selenium on Probable E3 ubiquitin−protein ligase HERC1

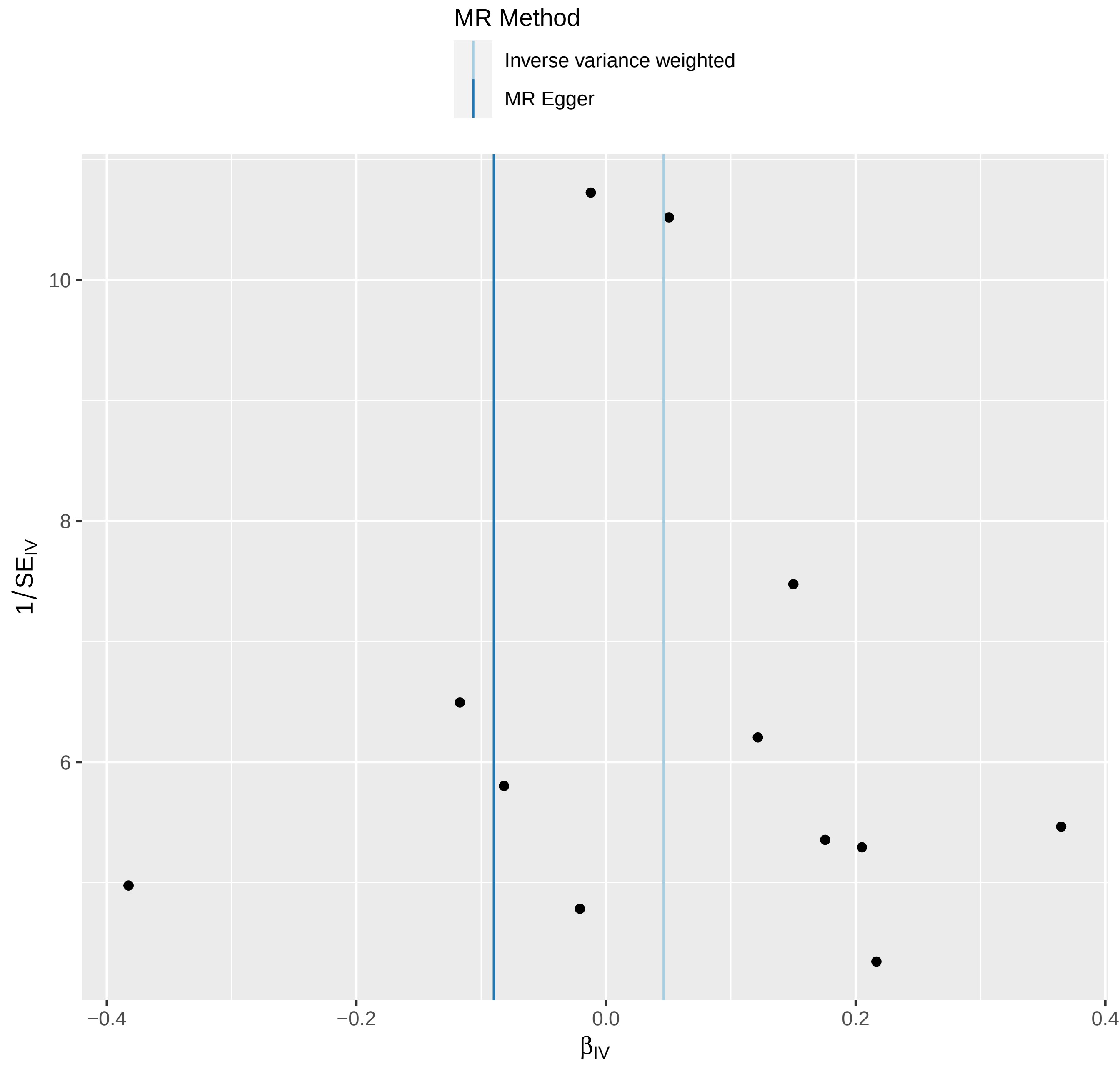

Batch 3−1330 : Selenium on Probable E3 ubiquitin−protein ligase HERC4

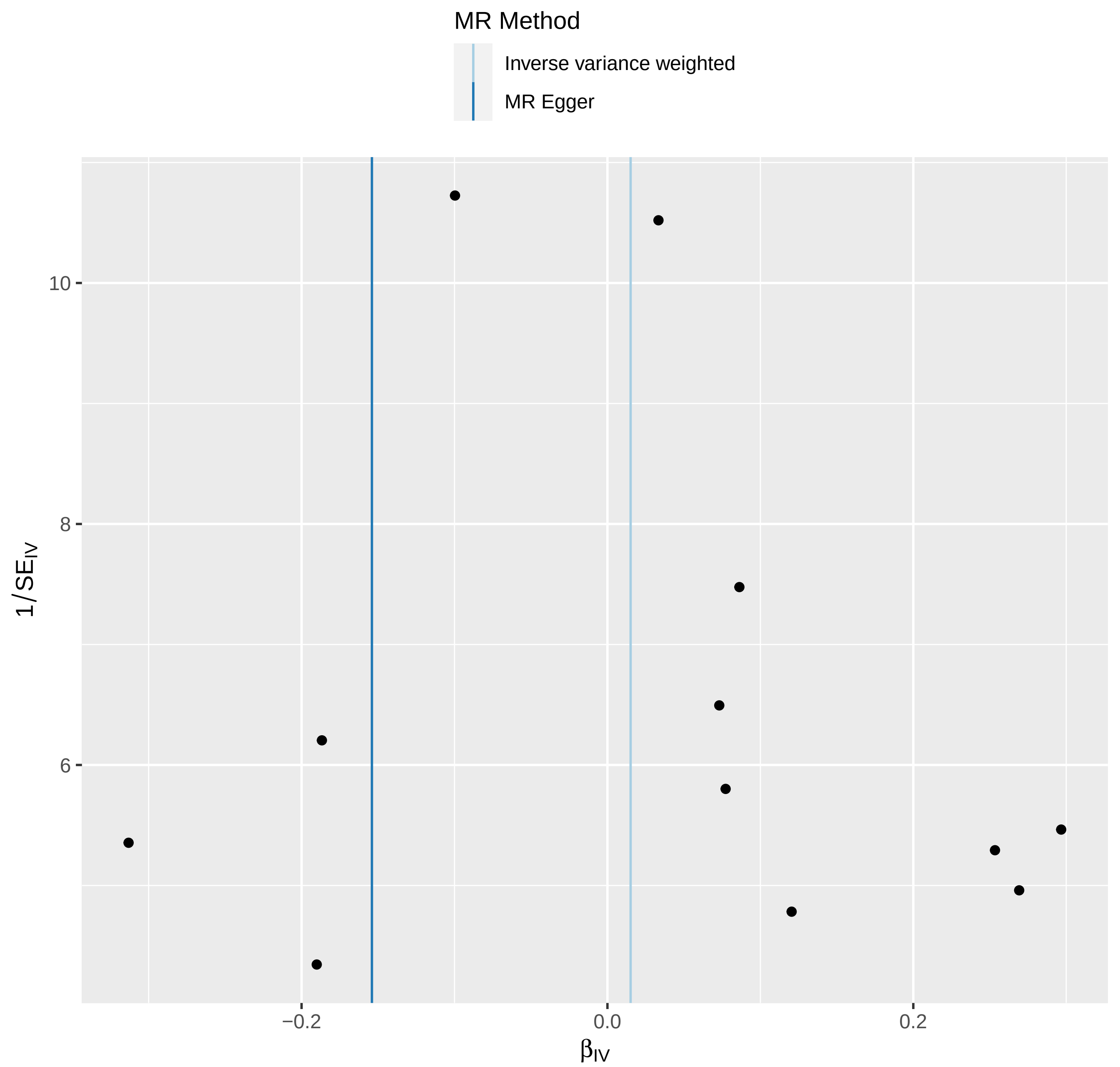

Batch 3−1331 : Selenium on E3 ISG15−−protein ligase HERC5

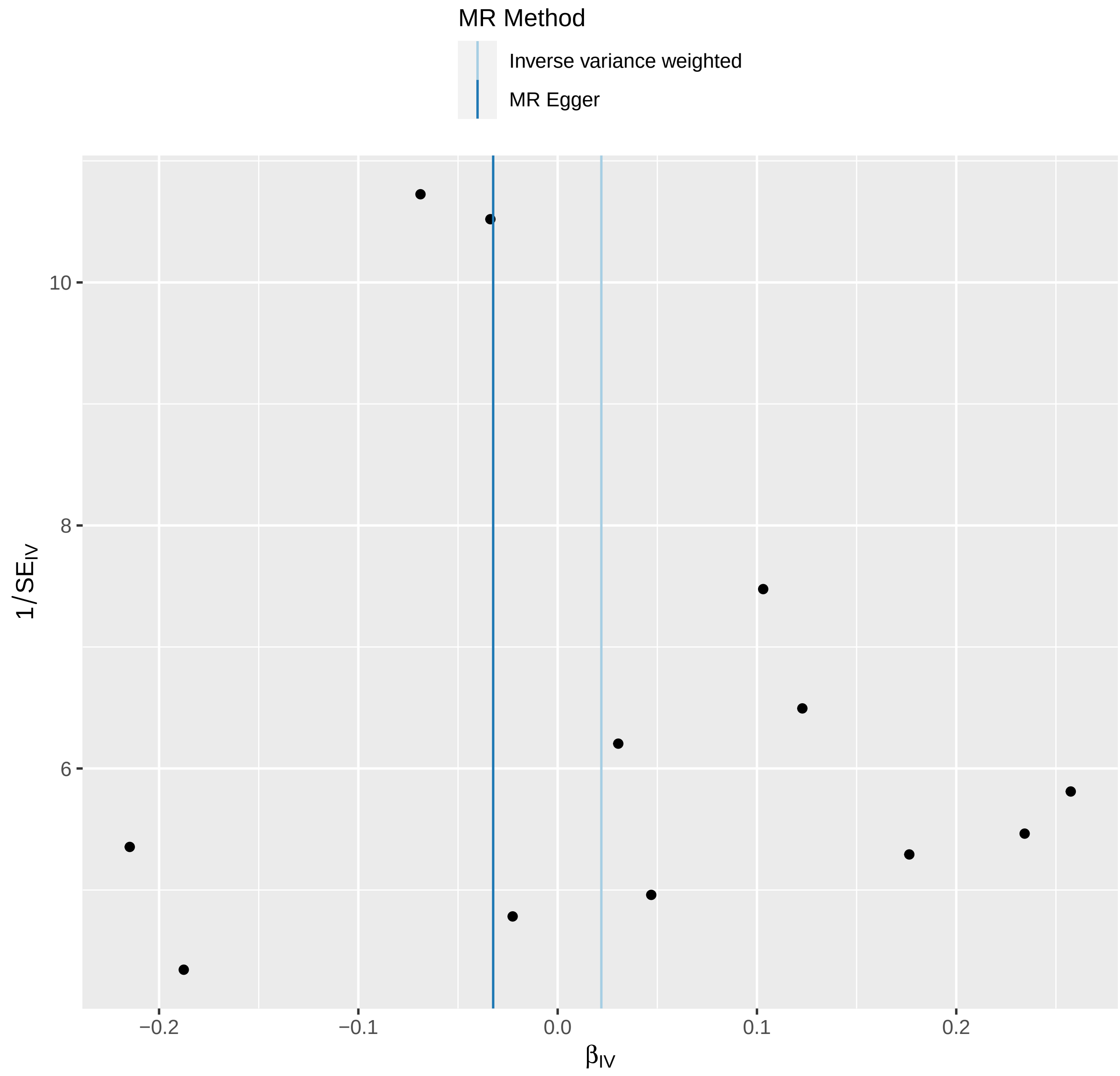

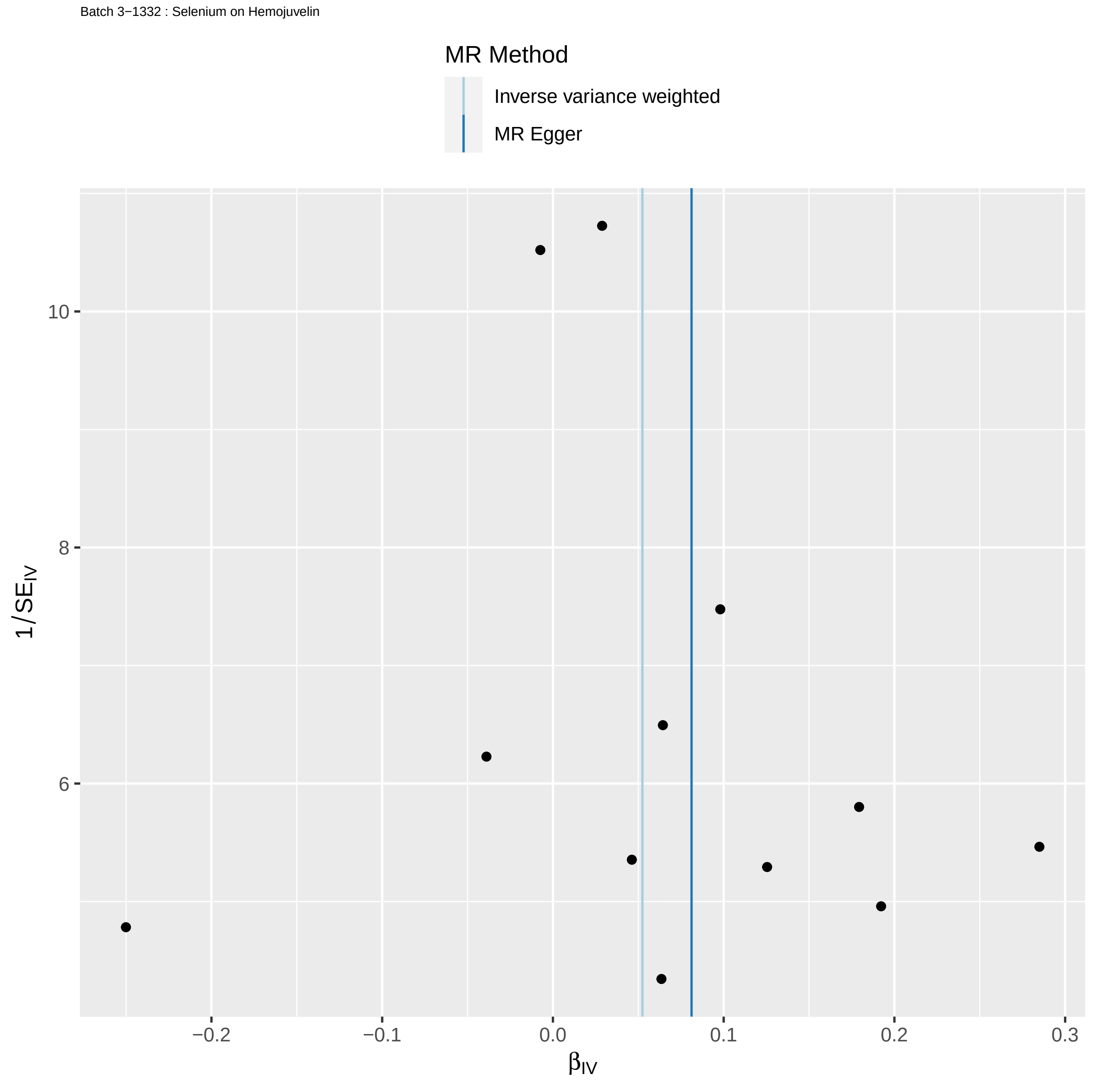
Batch 3−1332 : Selenium on Hemojuvelin
MR Method
Inverse variance weighted
MR Egger
10
8
6
1/SE$_{IV}$
−0.2
−0.1
0.0
0.1
0.2
0.3
β$_{IV}$

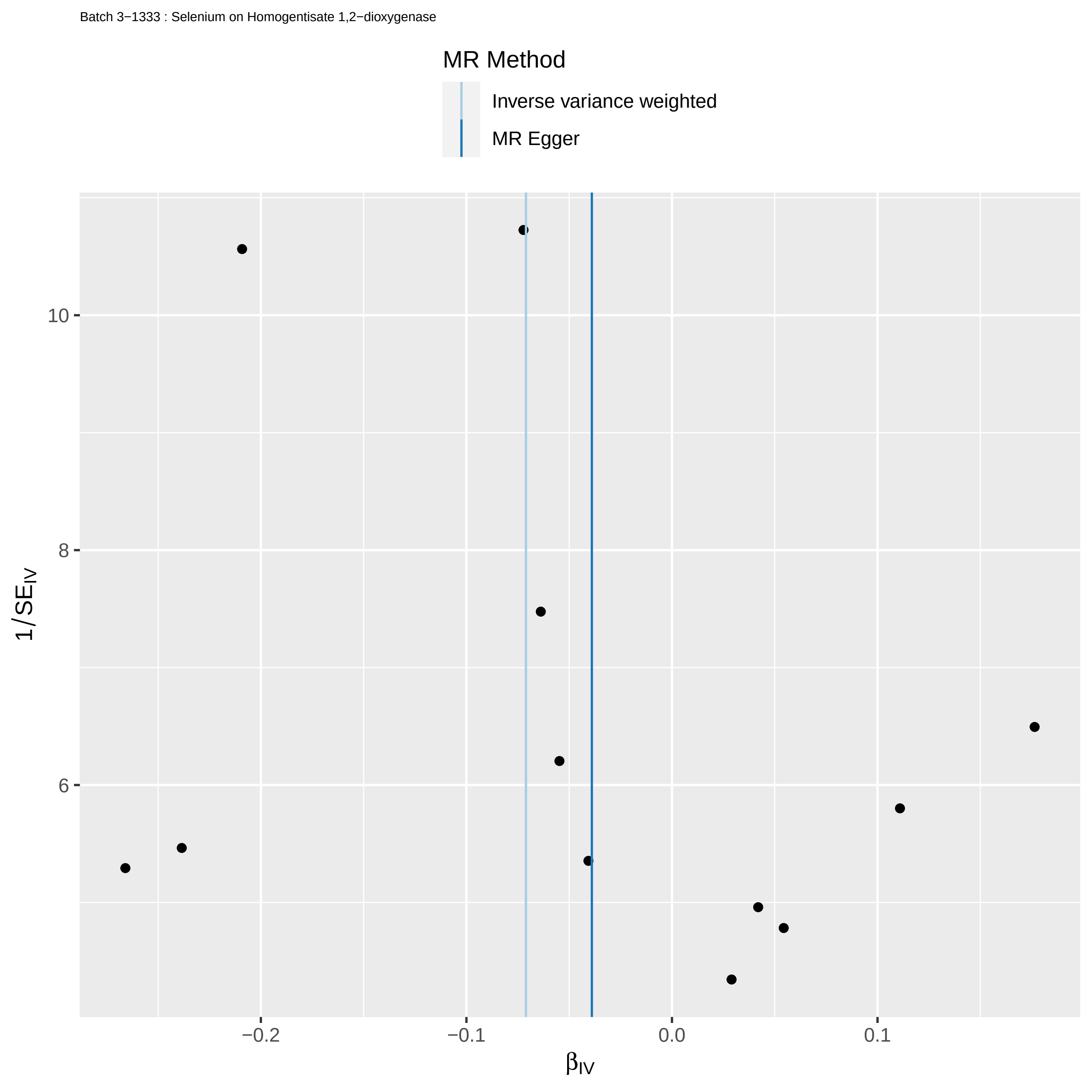

Batch 3−1333 : Selenium on Homogentisate 1,2−dioxygenase
MR Method
Inverse variance weighted
MR Egger
10
8
6
1/SE_IV
−0.2
−0.1
0.0
0.1
β_IV

Batch 3−1334 : Selenium on Hepatocyte growth factor

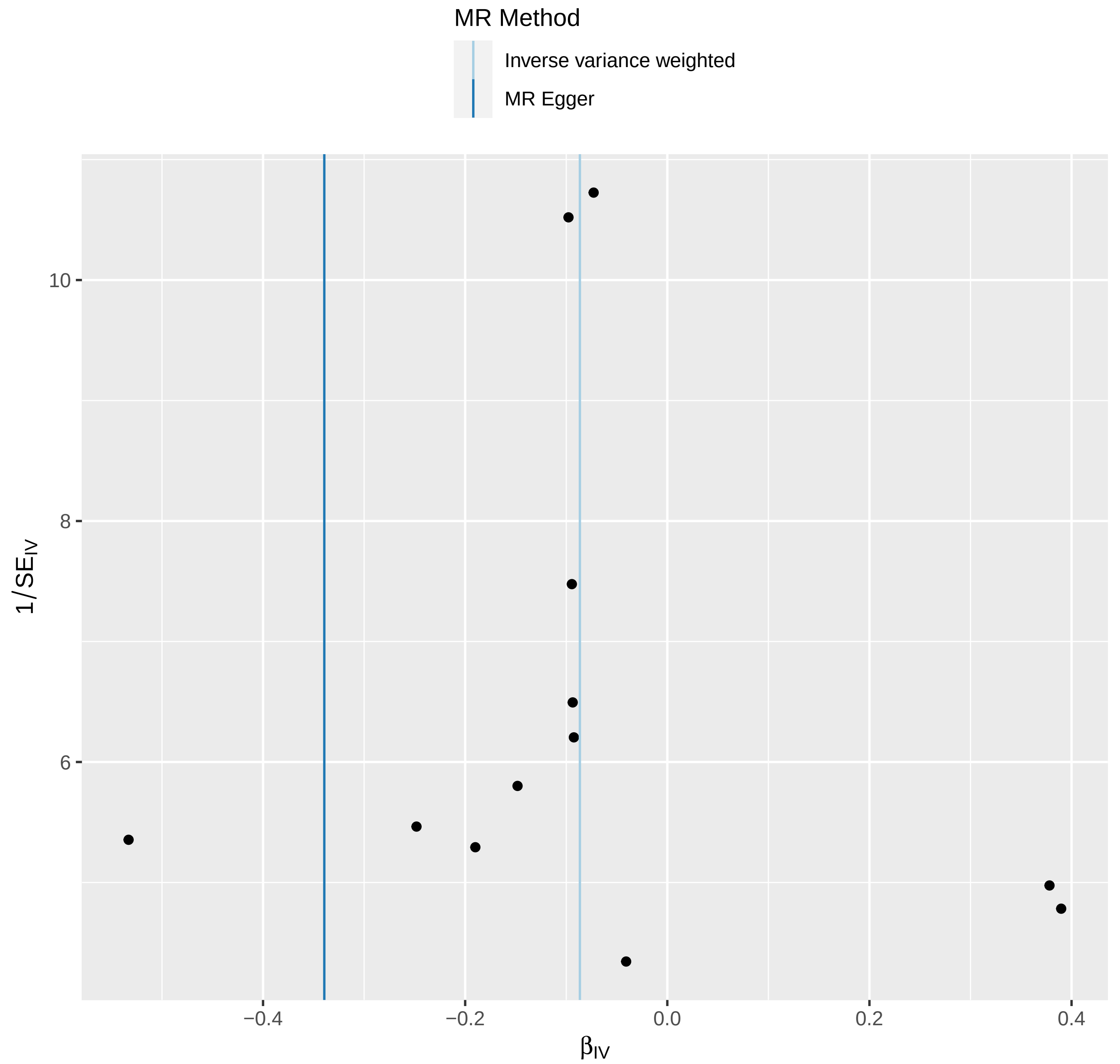

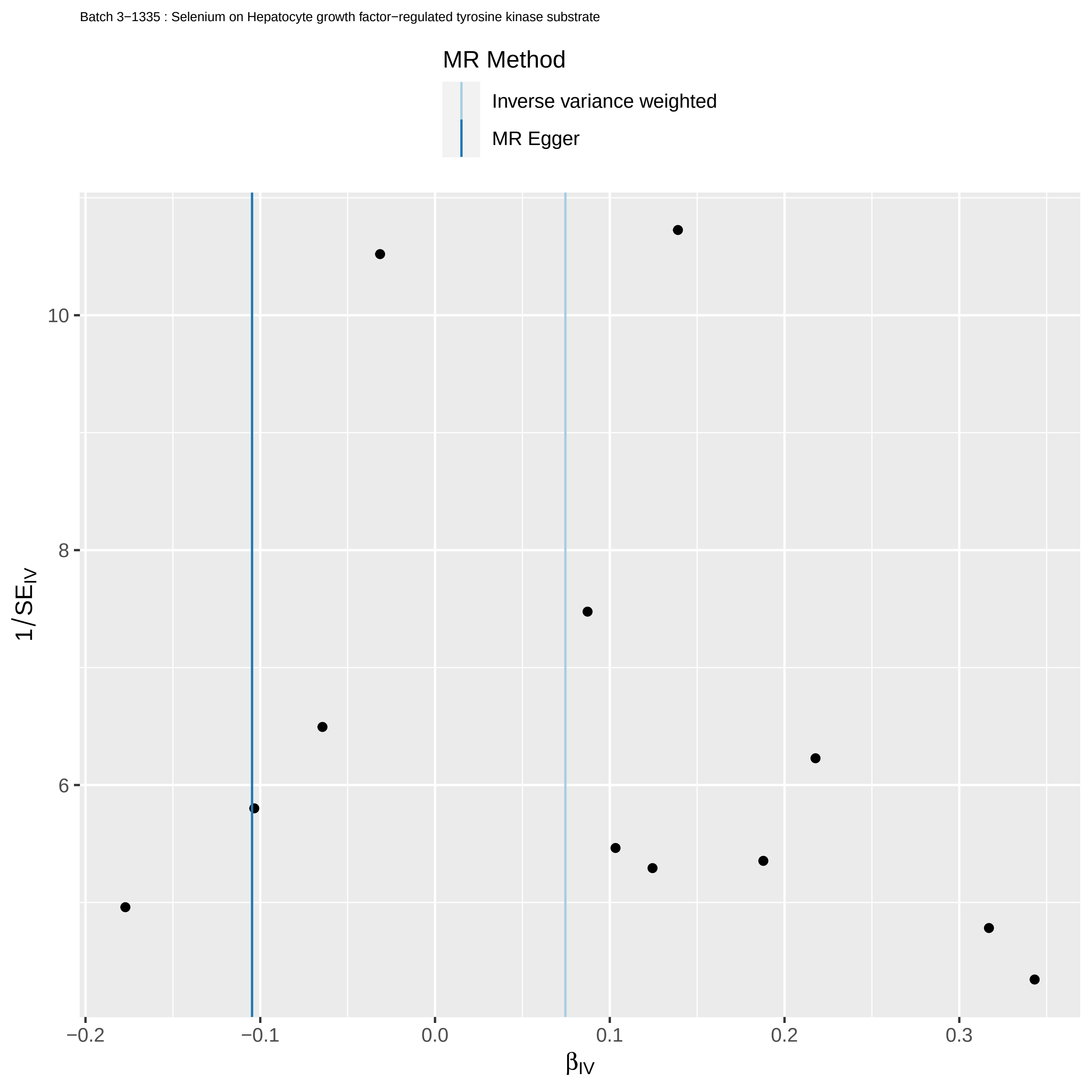
Batch 3−1335 : Selenium on Hepatocyte growth factor−regulated tyrosine kinase substrate
MR Method
Inverse variance weighted
MR Egger
10
8
6
1/SE_IV
−0.2
−0.1
0.0
0.1
0.2
0.3
β_IV

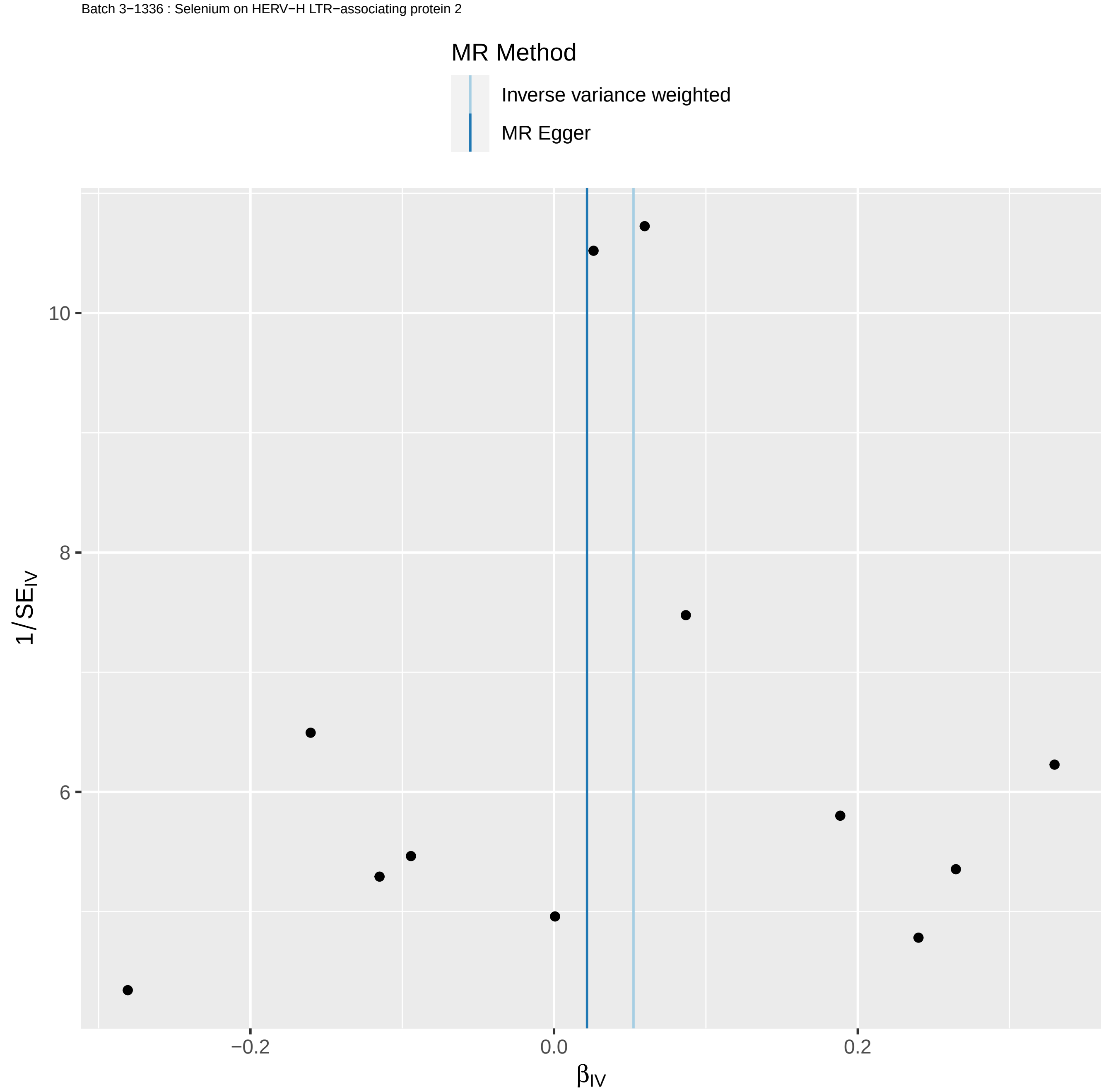
Batch 3−1336 : Selenium on HERV−H LTR−associating protein 2
MR Method
Inverse variance weighted
MR Egger
10
8
6
1/SE_IV
−0.2
0.0
0.2
β_IV

Batch 3−1337 : Selenium on Hypoxia−inducible factor 1−alpha

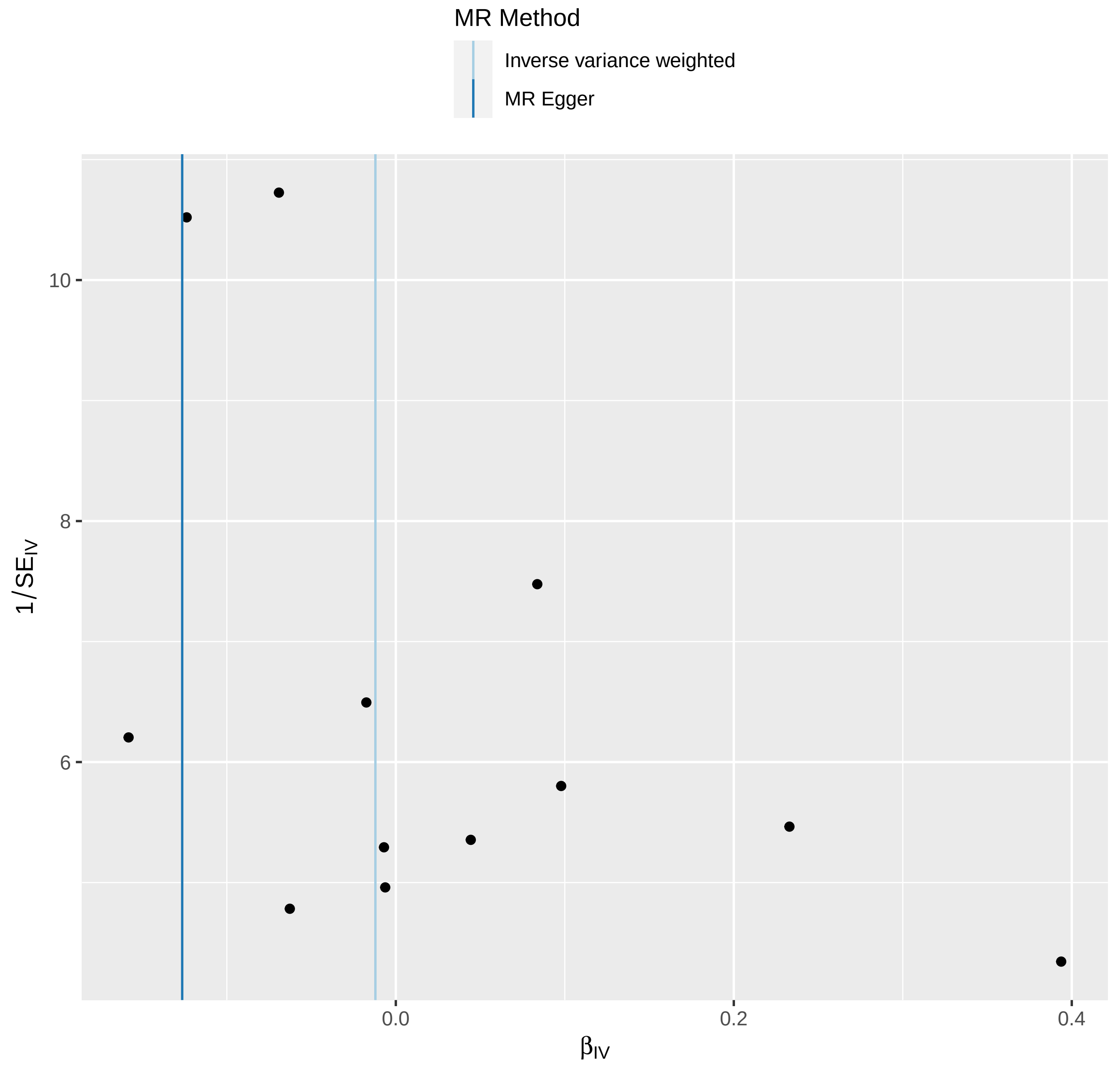

Batch 3−1338 : Selenium on Histidine triad nucleotide−binding protein 1

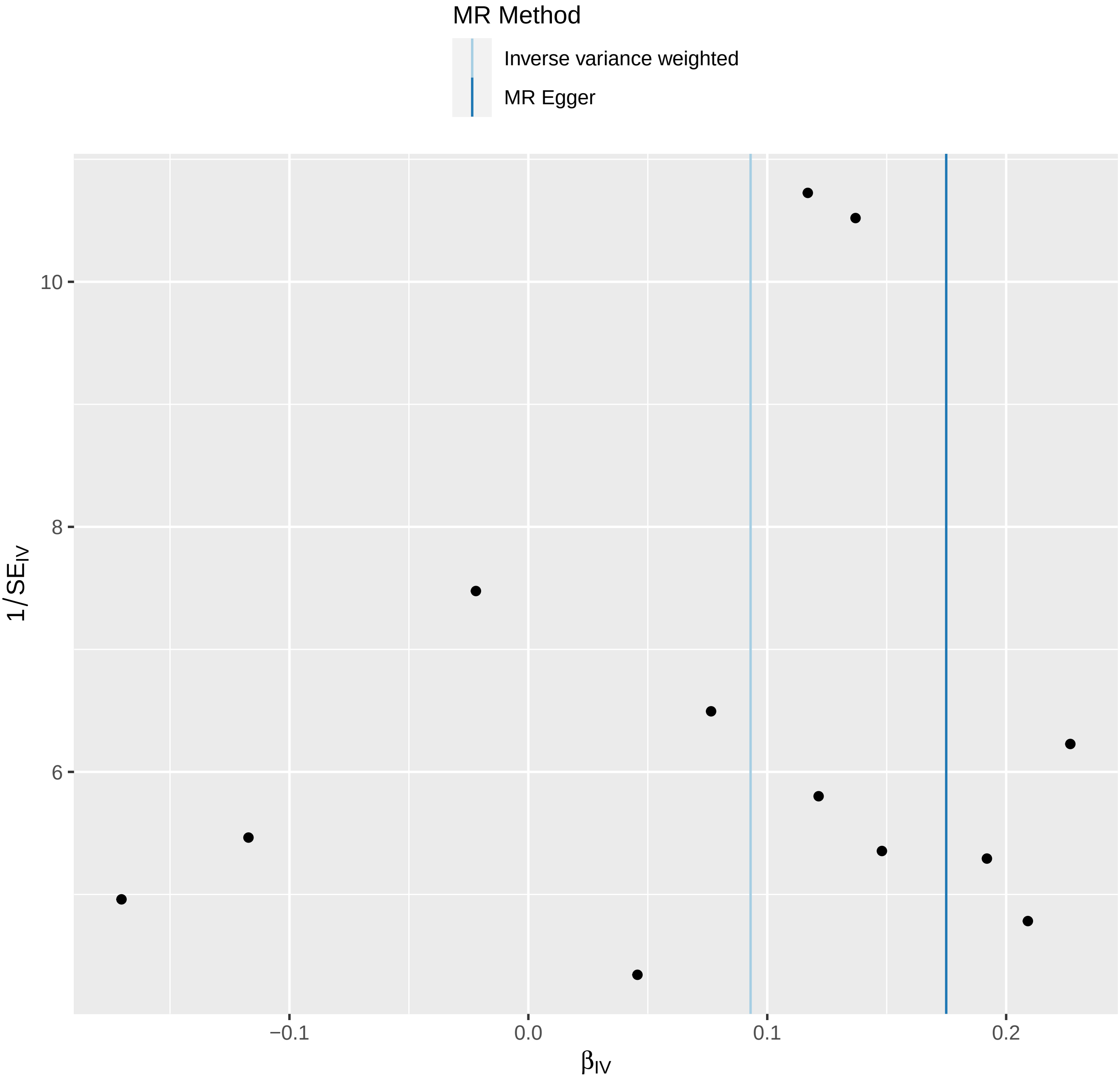

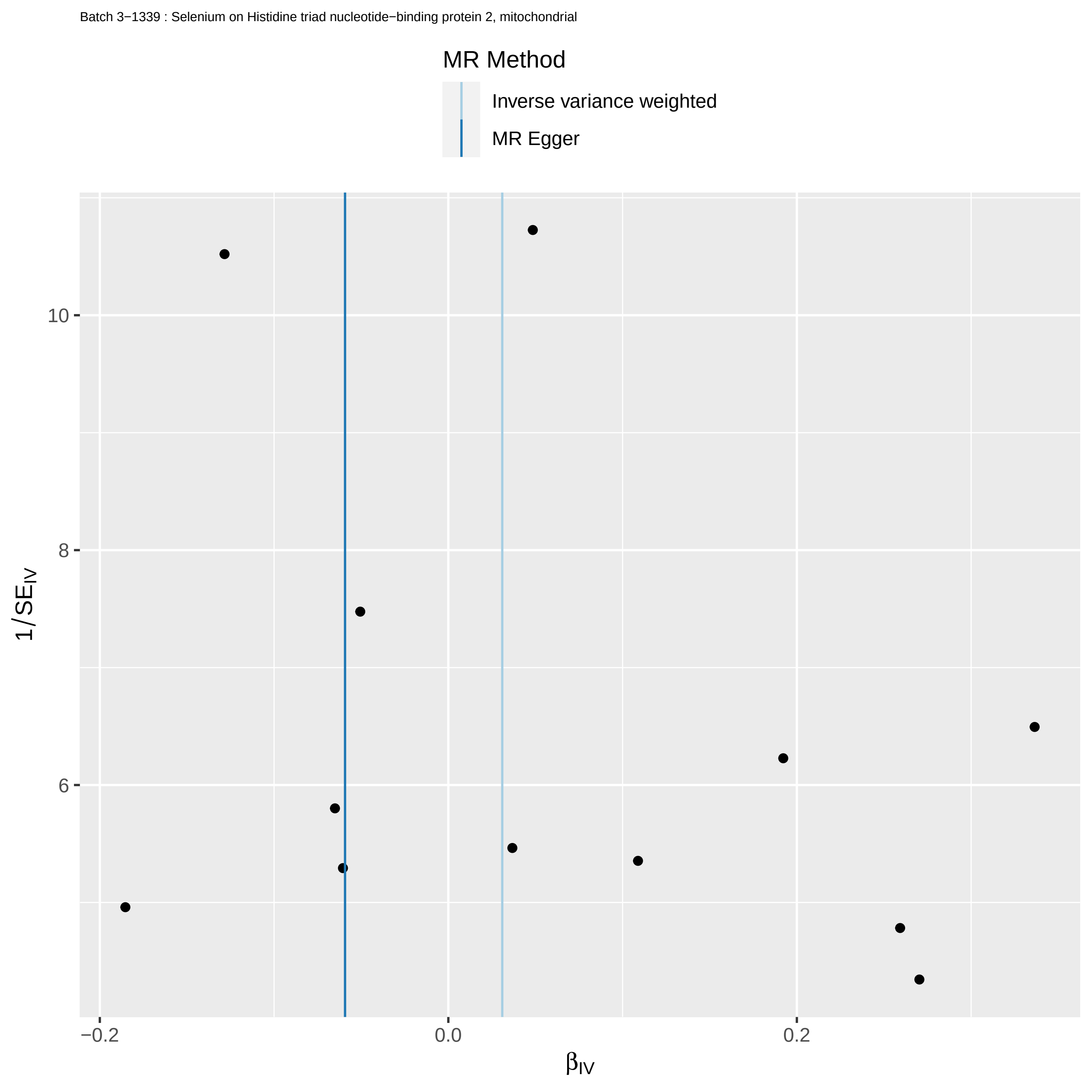

Batch 3−1339 : Selenium on Histidine triad nucleotide−binding protein 2, mitochondrial
MR Method
Inverse variance weighted
MR Egger
10
8
6
1/SE_IV
−0.2
0.0
0.2
β_IV

Batch 3−1340 : Selenium on Homeodomain−interacting protein kinase 3

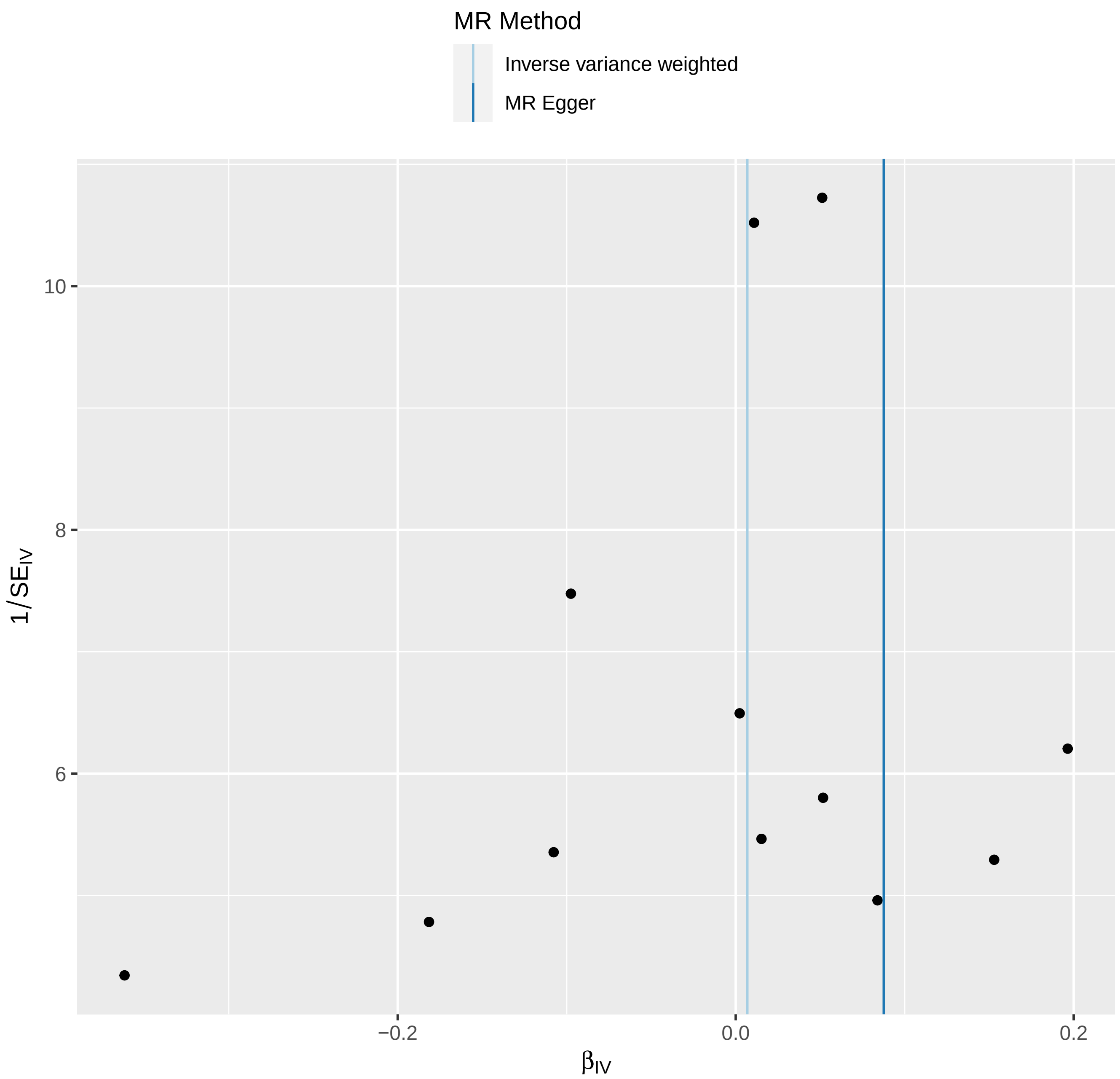

Batch 3−1341 : Selenium on Histone H1.2

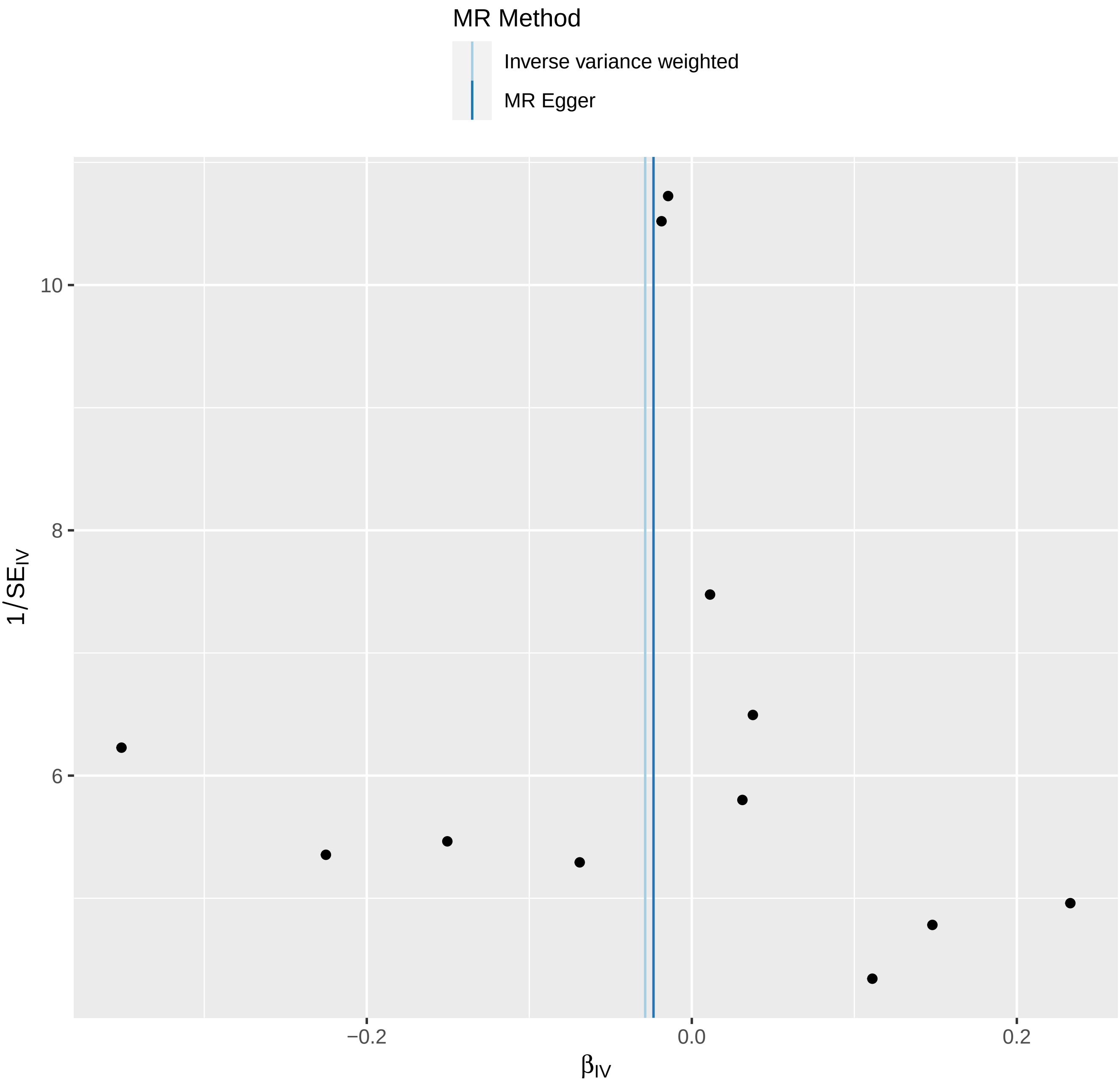

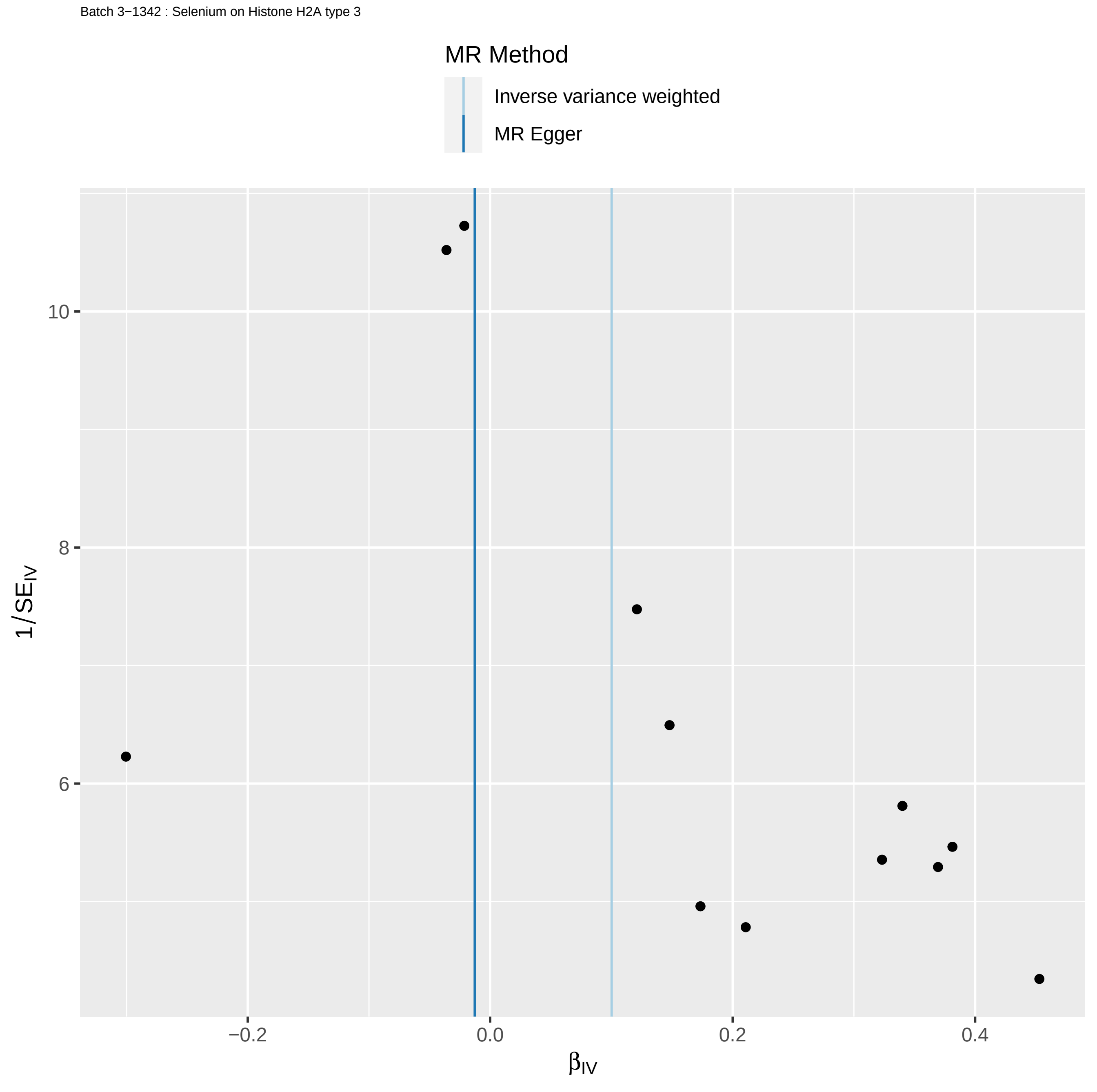
Batch 3−1342 : Selenium on Histone H2A type 3
MR Method
Inverse variance weighted
MR Egger
10
8
6
1/SE$_{IV}$
−0.2
0.0
0.2
0.4
β$_{IV}$

Batch 3−1343 : Selenium on Hexokinase−1

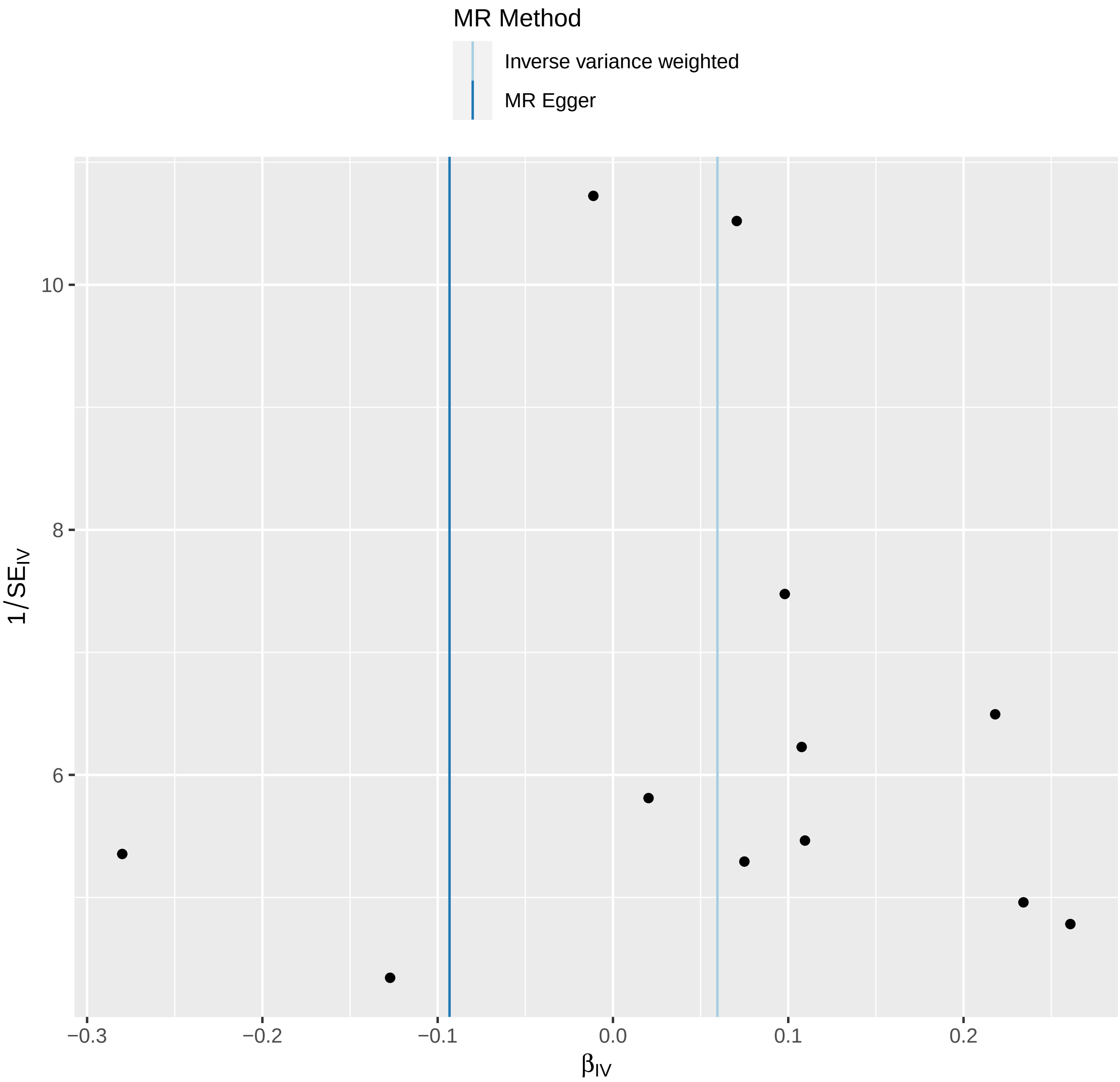

Batch 3−1344 : Selenium on Hexokinase−2

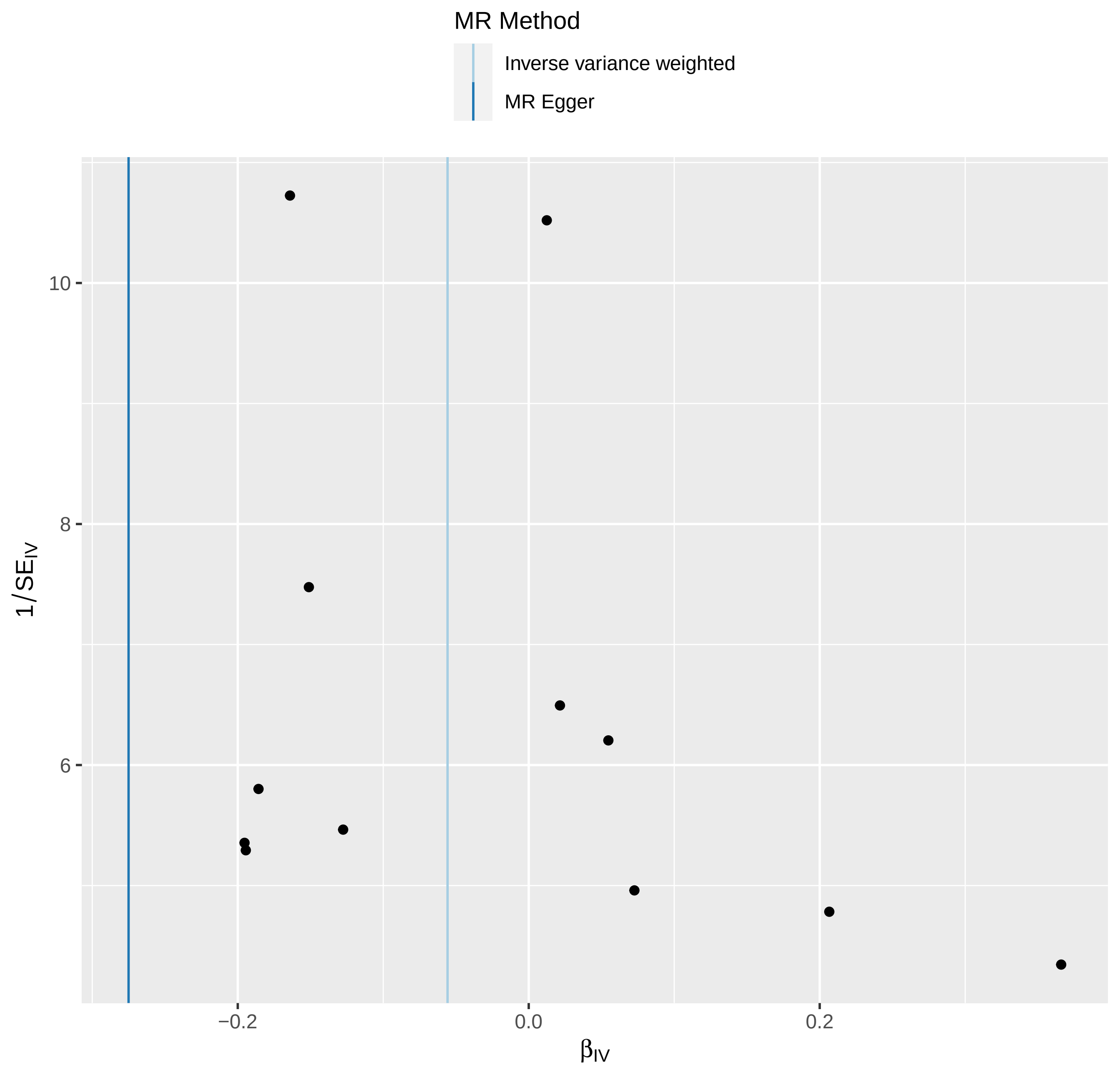

Batch 3−1345 : Selenium on HLA class II histocompatibility antigen, DM alpha chain

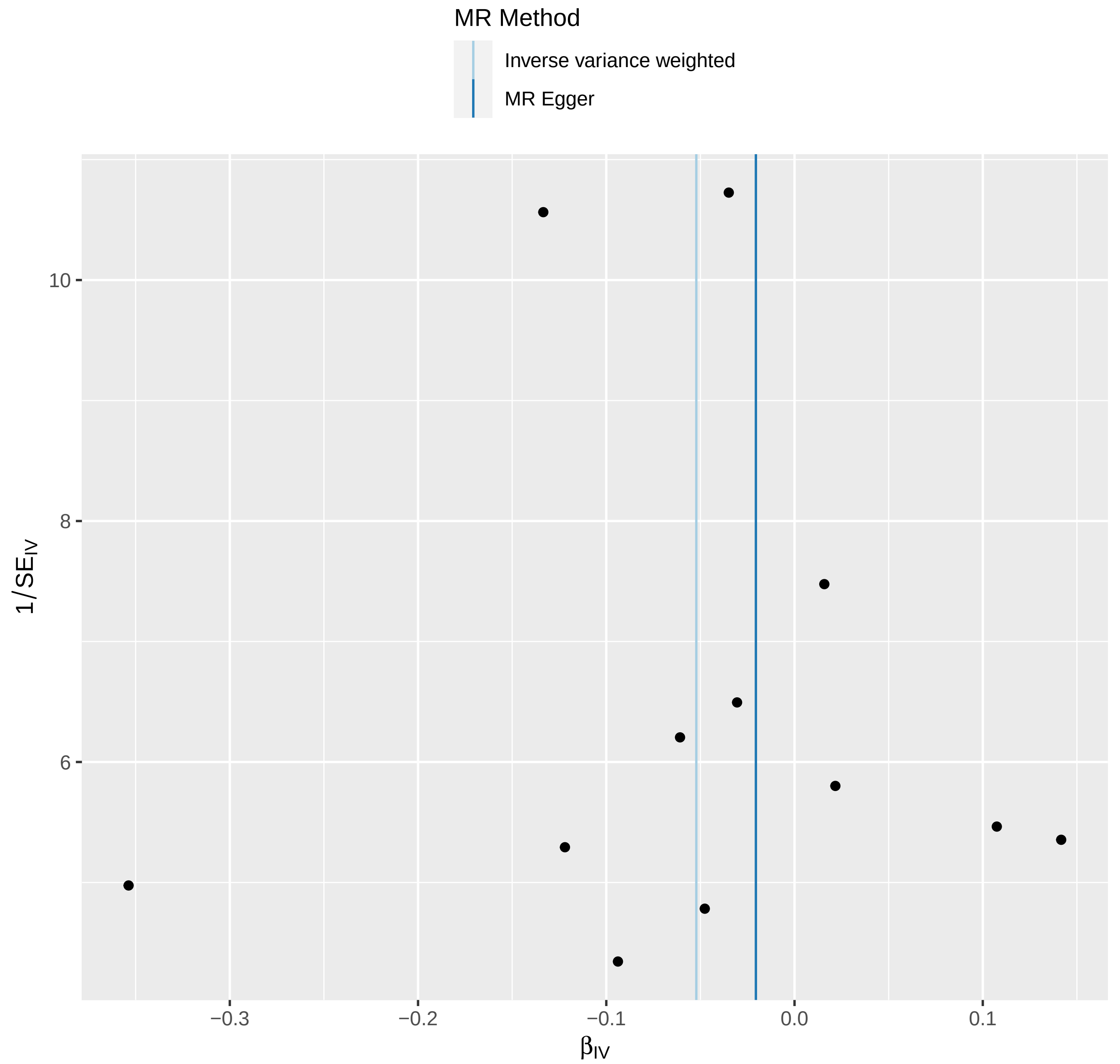

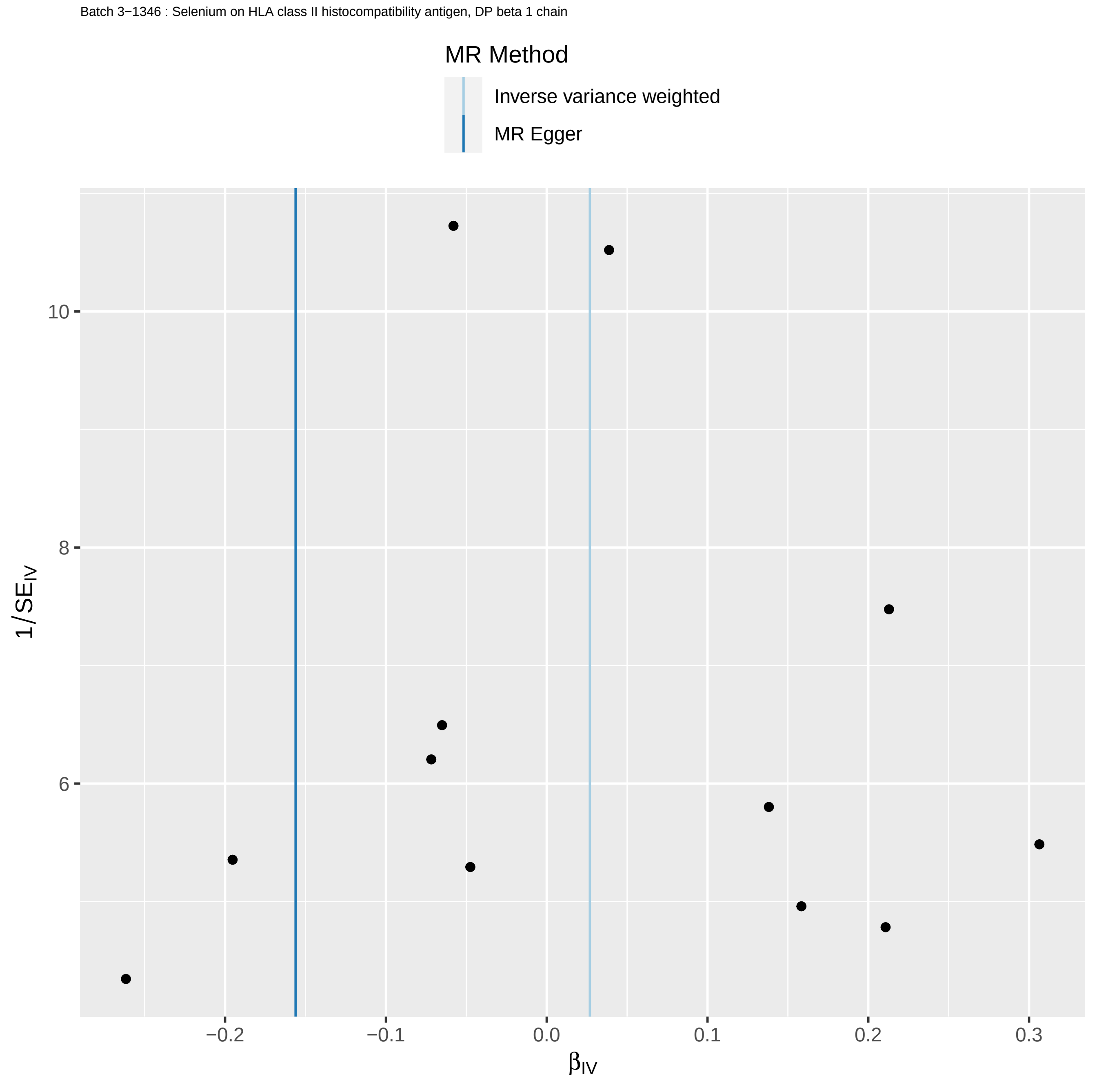
Batch 3−1346 : Selenium on HLA class II histocompatibility antigen, DP beta 1 chain
MR Method
Inverse variance weighted
MR Egger
10
8
6
1/SE_IV
−0.2
−0.1
0.0
0.1
0.2
0.3
β_IV

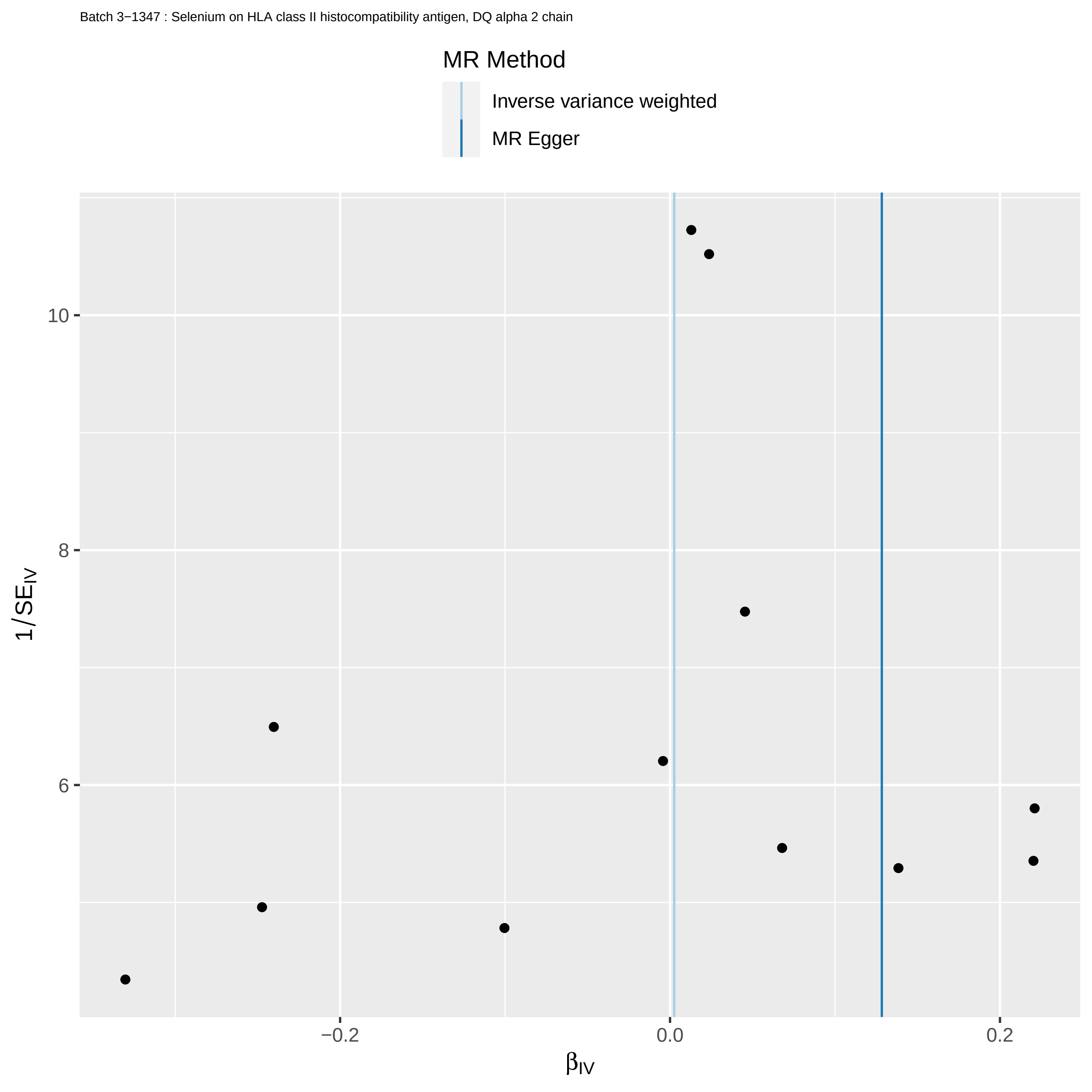

Batch 3−1347 : Selenium on HLA class II histocompatibility antigen, DQ alpha 2 chain
MR Method
Inverse variance weighted
MR Egger
10
8
6
1/SE_IV
−0.2
0.0
0.2
β_IV

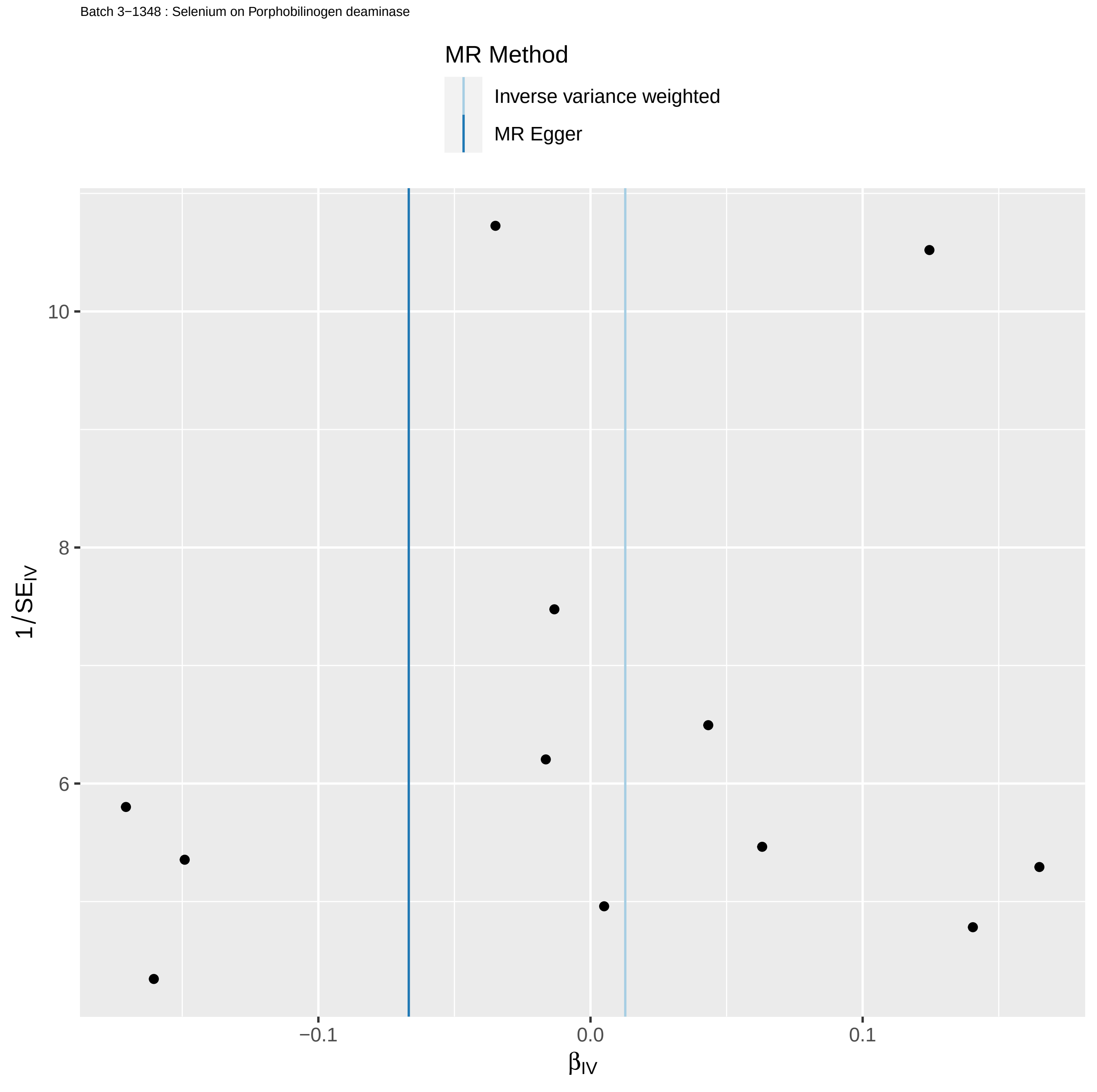

Batch 3−1348 : Selenium on Porphobilinogen deaminase
MR Method
Inverse variance weighted
MR Egger
10
8
6
1/SE_IV
−0.1
0.0
0.1
β_IV

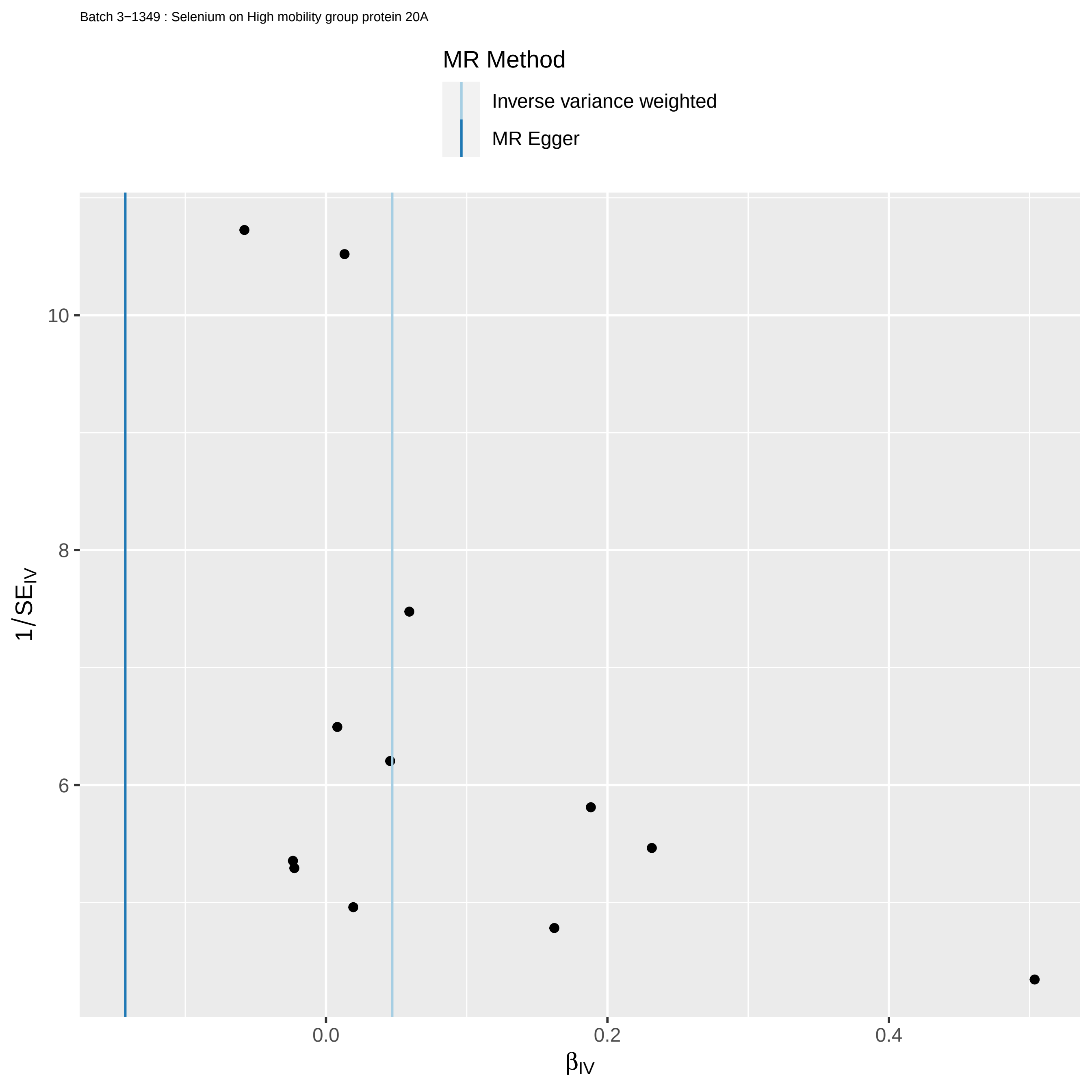
Batch 3−1349 : Selenium on High mobility group protein 20A
MR Method
Inverse variance weighted
MR Egger
10
8
6
$1/SE_{IV}$
0.0
0.2
0.4
β_{IV}

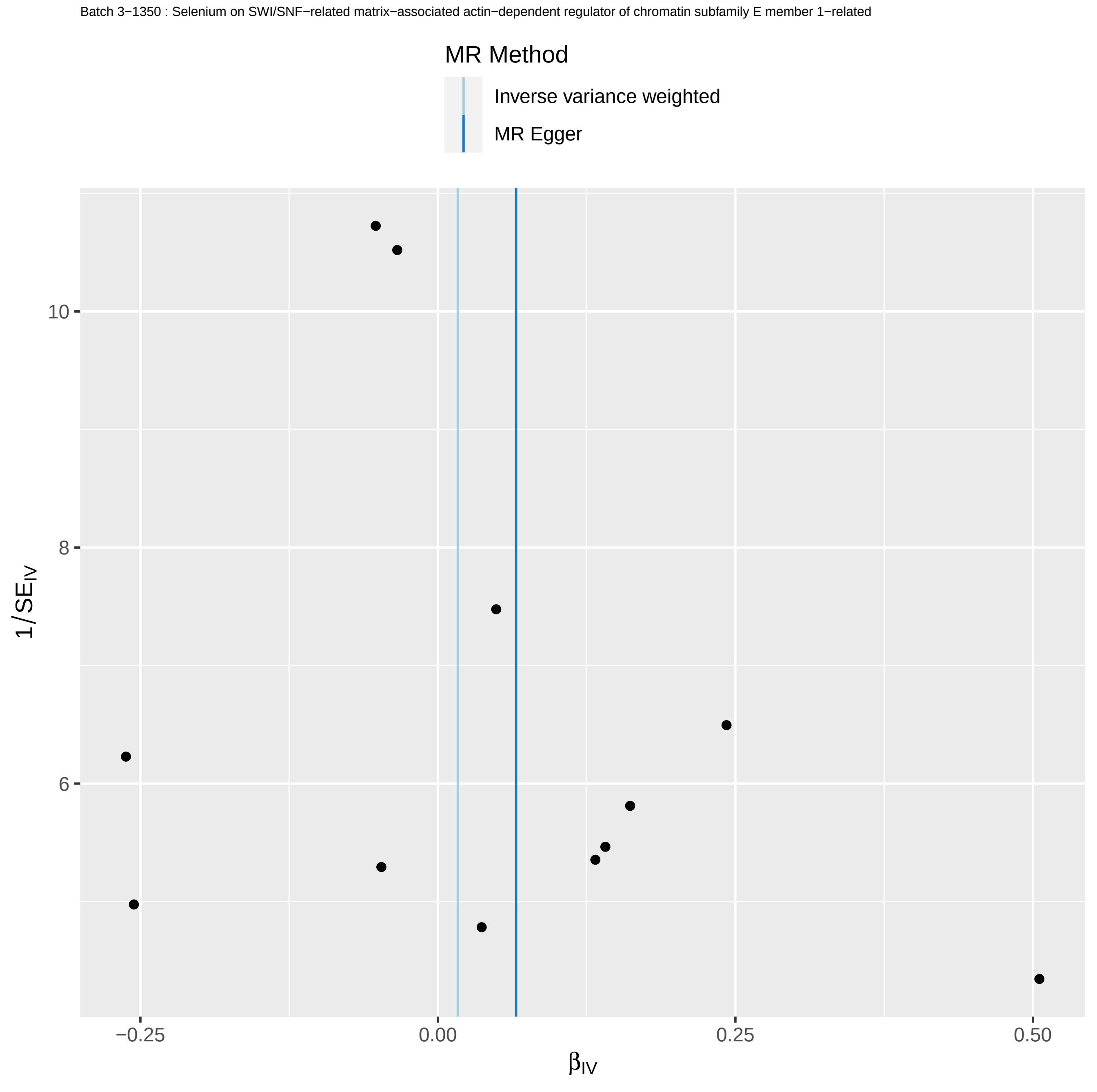

Batch 3−1350 : Selenium on SWI/SNF−related matrix−associated actin−dependent regulator of chromatin subfamily E member 1−related
MR Method
Inverse variance weighted
MR Egger
1/SE_IV
10
8
6
−0.25
0.00
0.25
0.50
β_IV

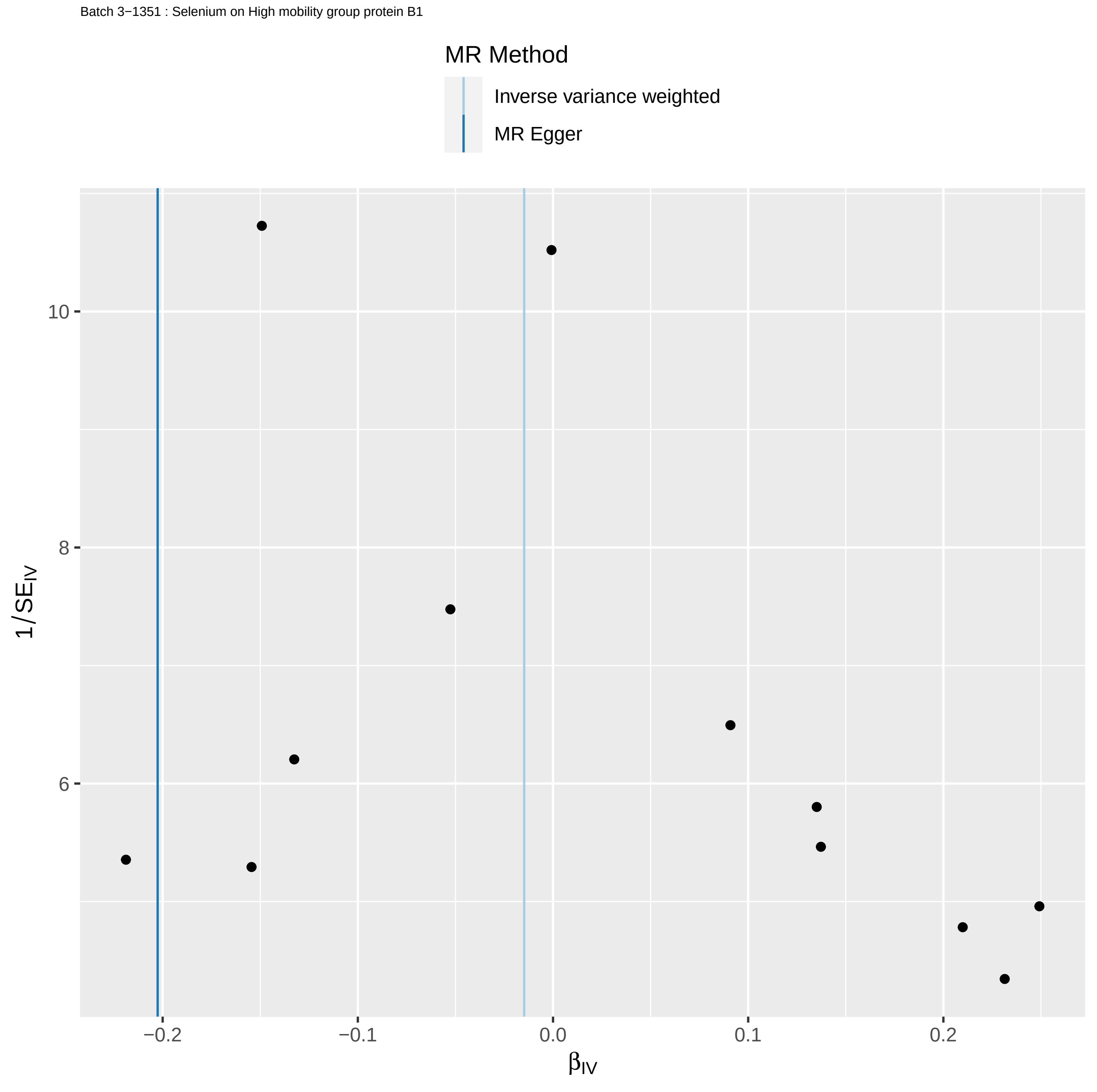

Batch 3−1351 : Selenium on High mobility group protein B1
MR Method
Inverse variance weighted
MR Egger
10
8
6
1/SE_IV
−0.2
−0.1
0.0
0.1
0.2
β_IV

Batch 3−1352 : Selenium on High mobility group protein B2

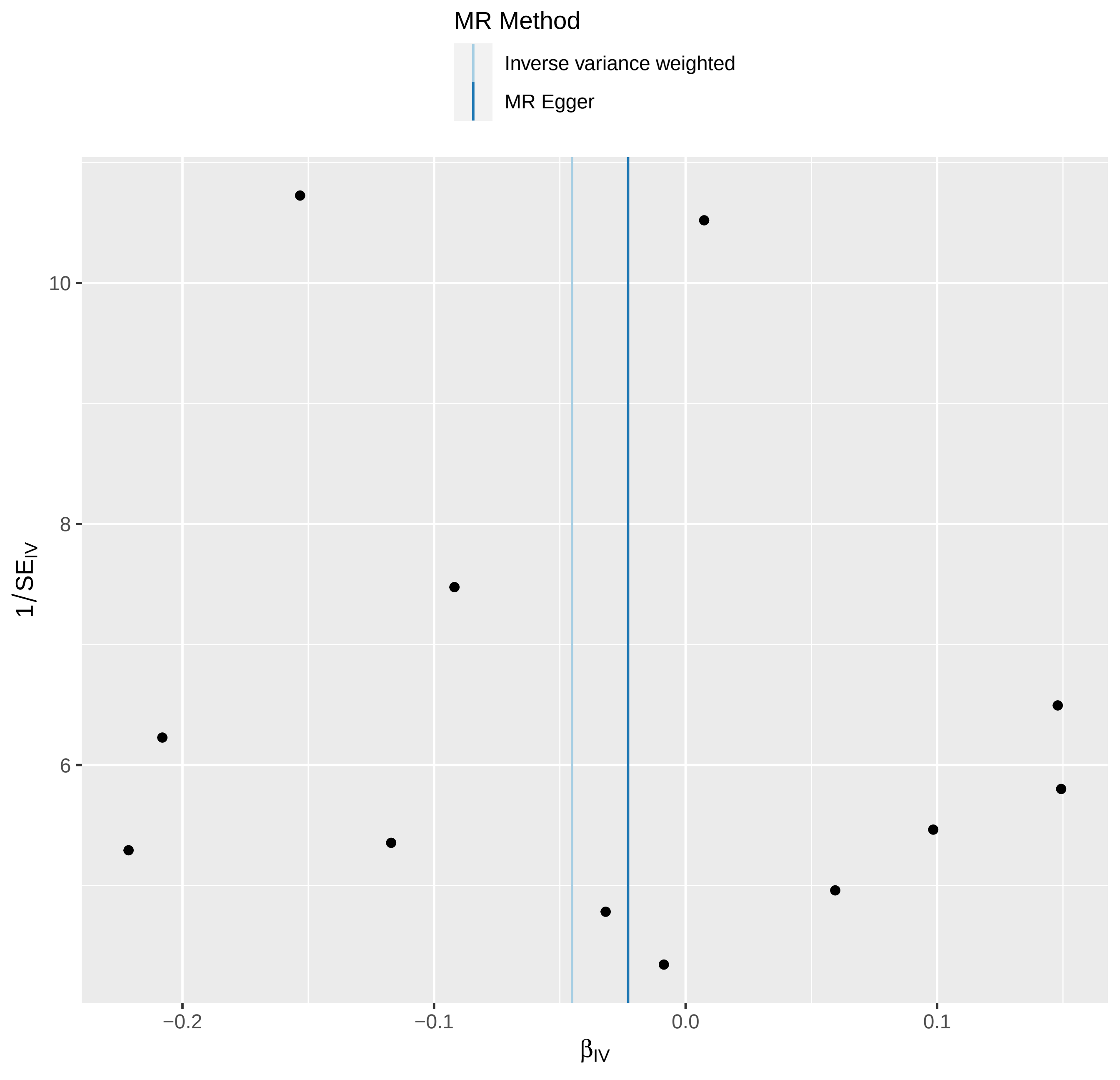

Batch 3−1353 : Selenium on High mobility group protein B3

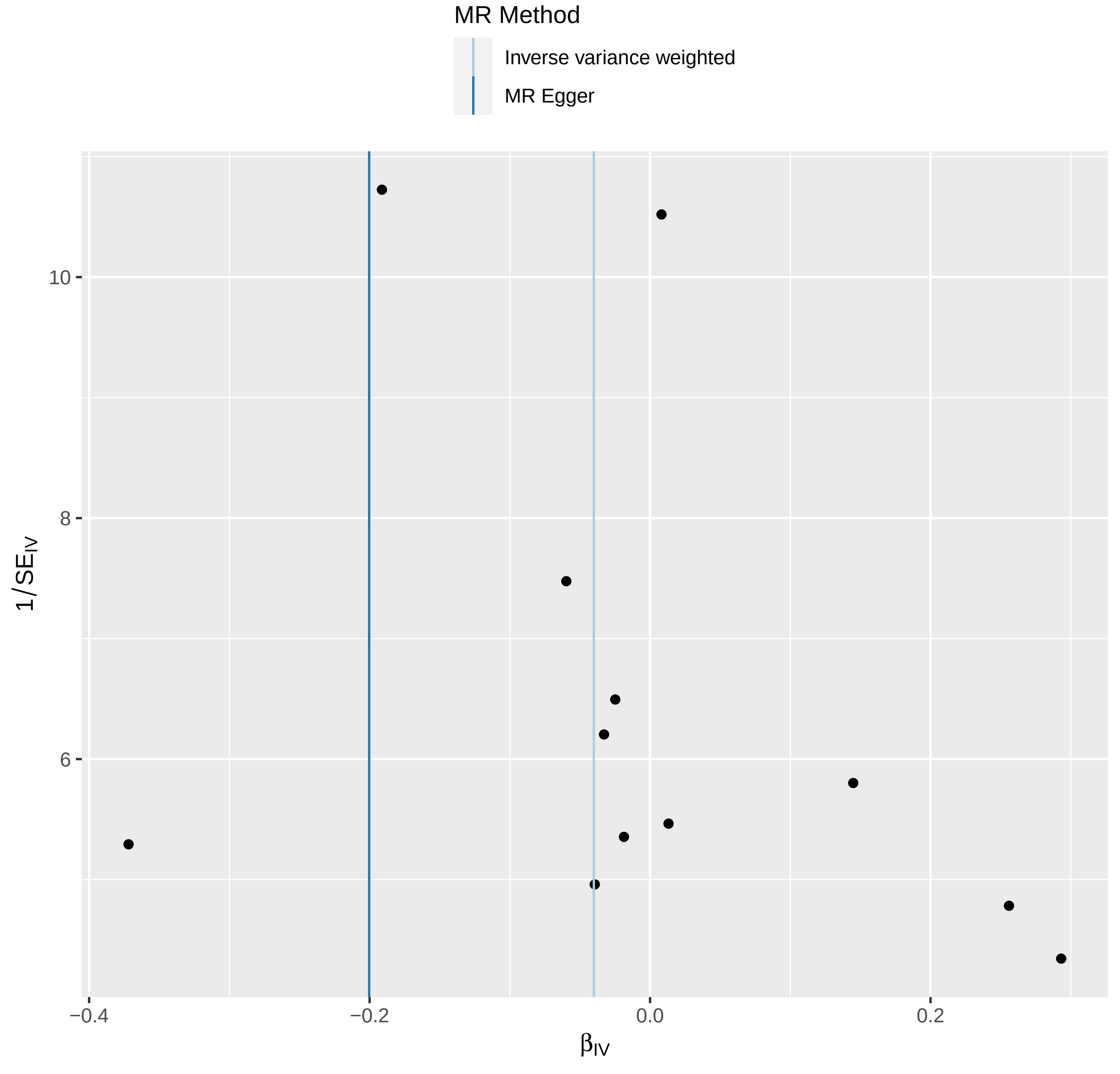

Batch 3−1354 : Selenium on 3−hydroxy−3−methylglutaryl−coenzyme A reductase

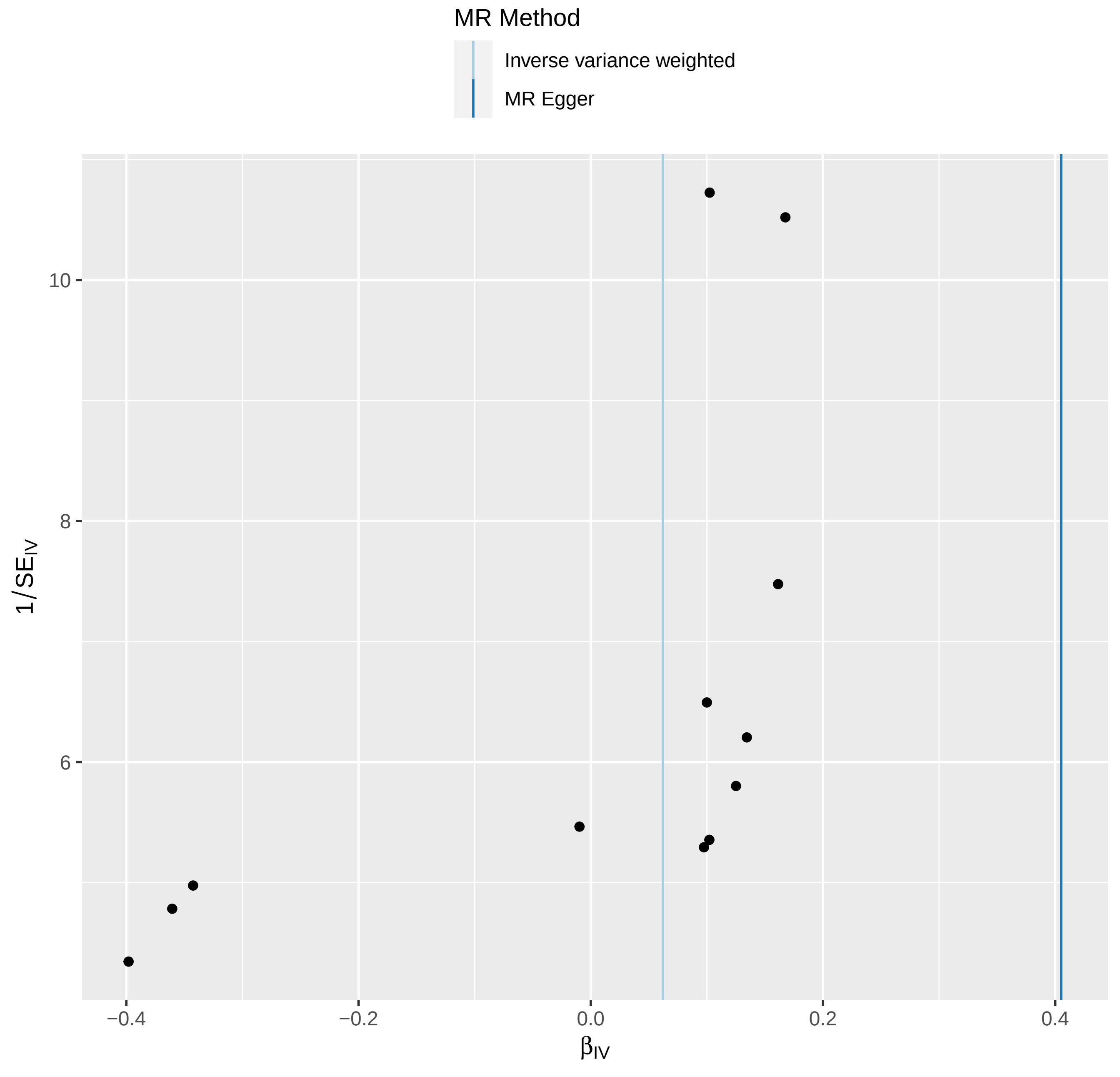

Batch 3−1355 : Selenium on Hydroxymethylglutaryl−CoA synthase, cytoplasmic

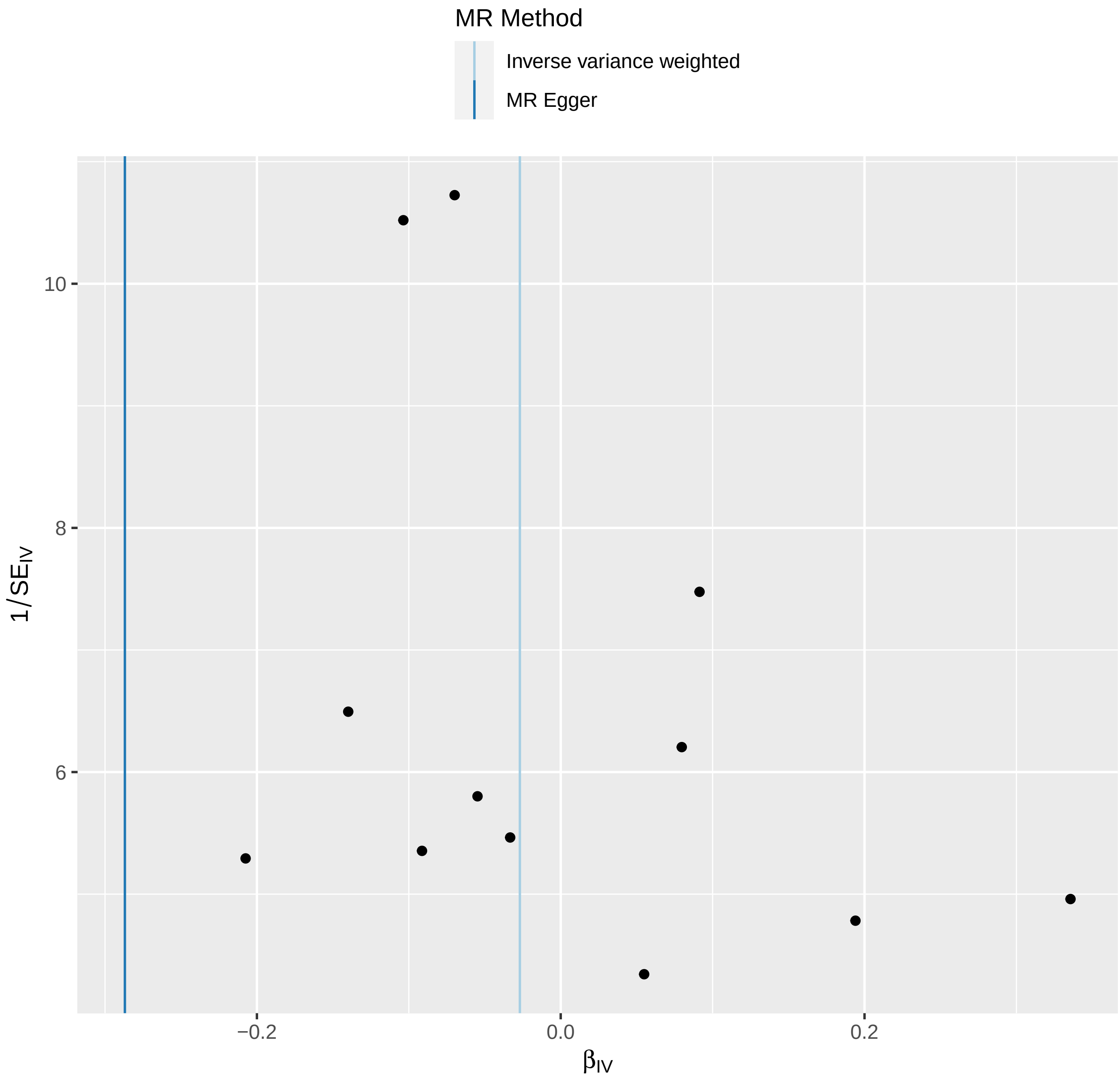

Batch 3−1356 : Selenium on Hydroxymethylglutaryl−CoA synthase, mitochondrial

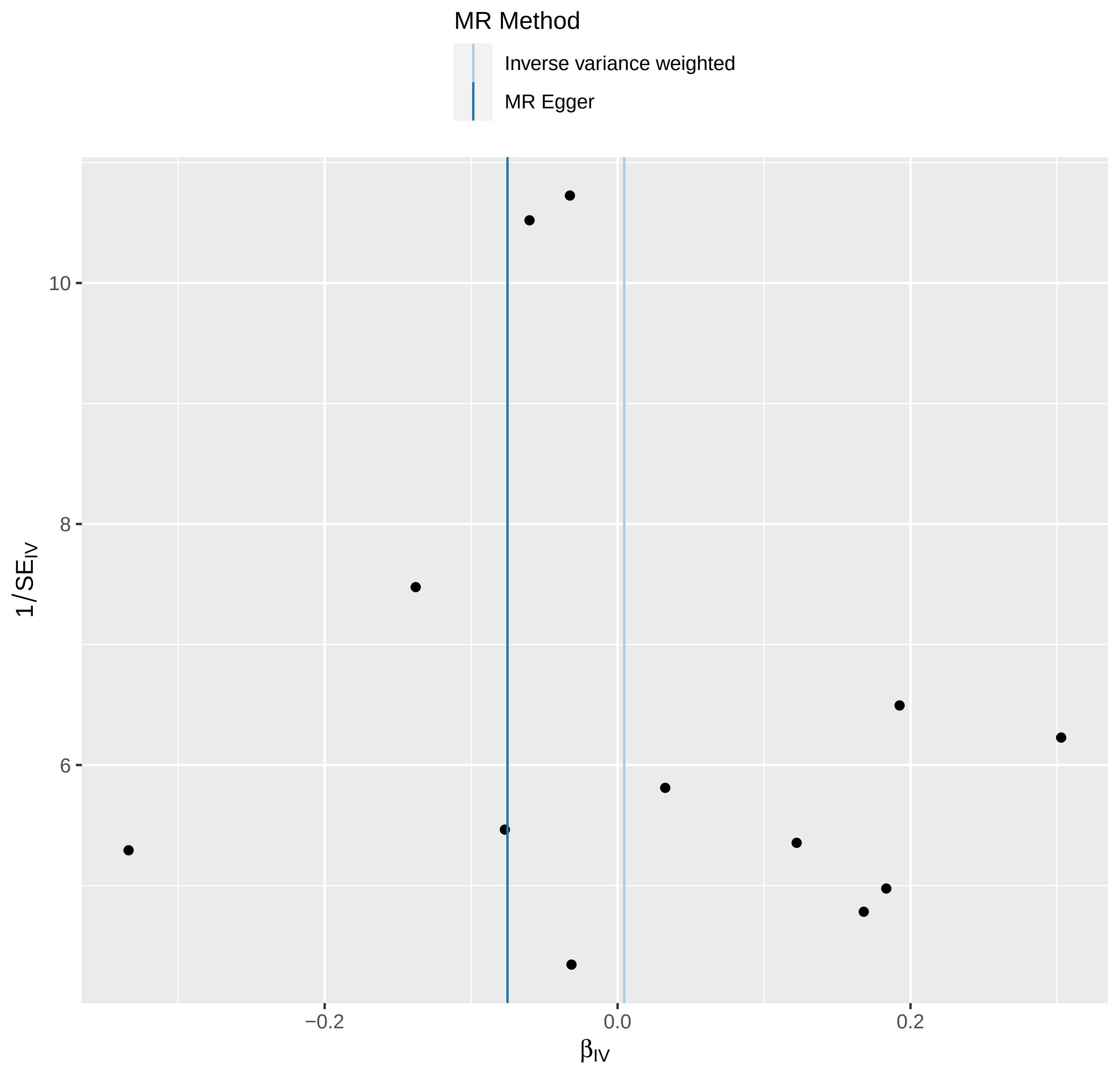

Batch 3−1357 : Selenium on Non−histone chromosomal protein HMG−14

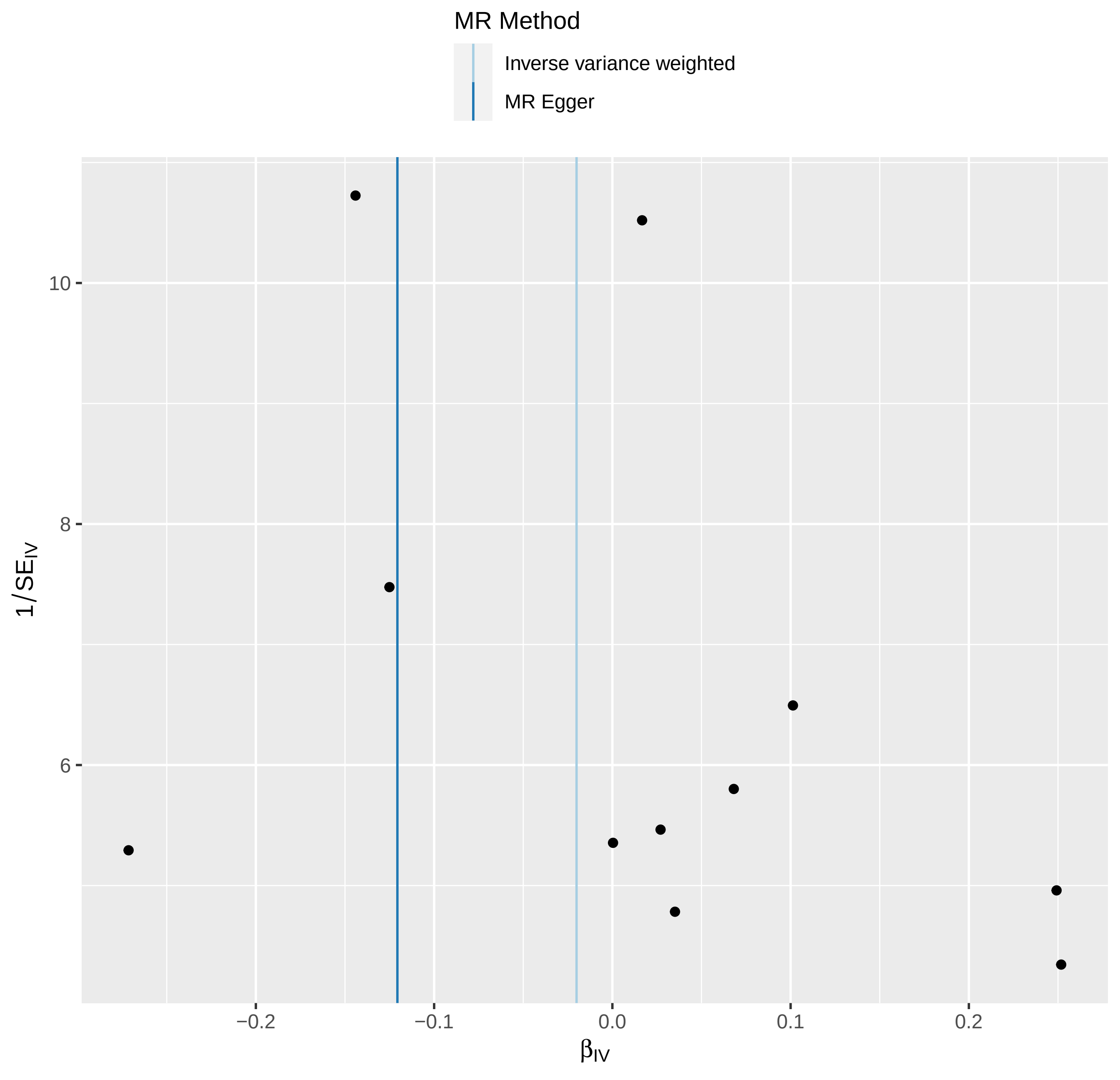

Batch 3−1358 : Selenium on Heme oxygenase 2

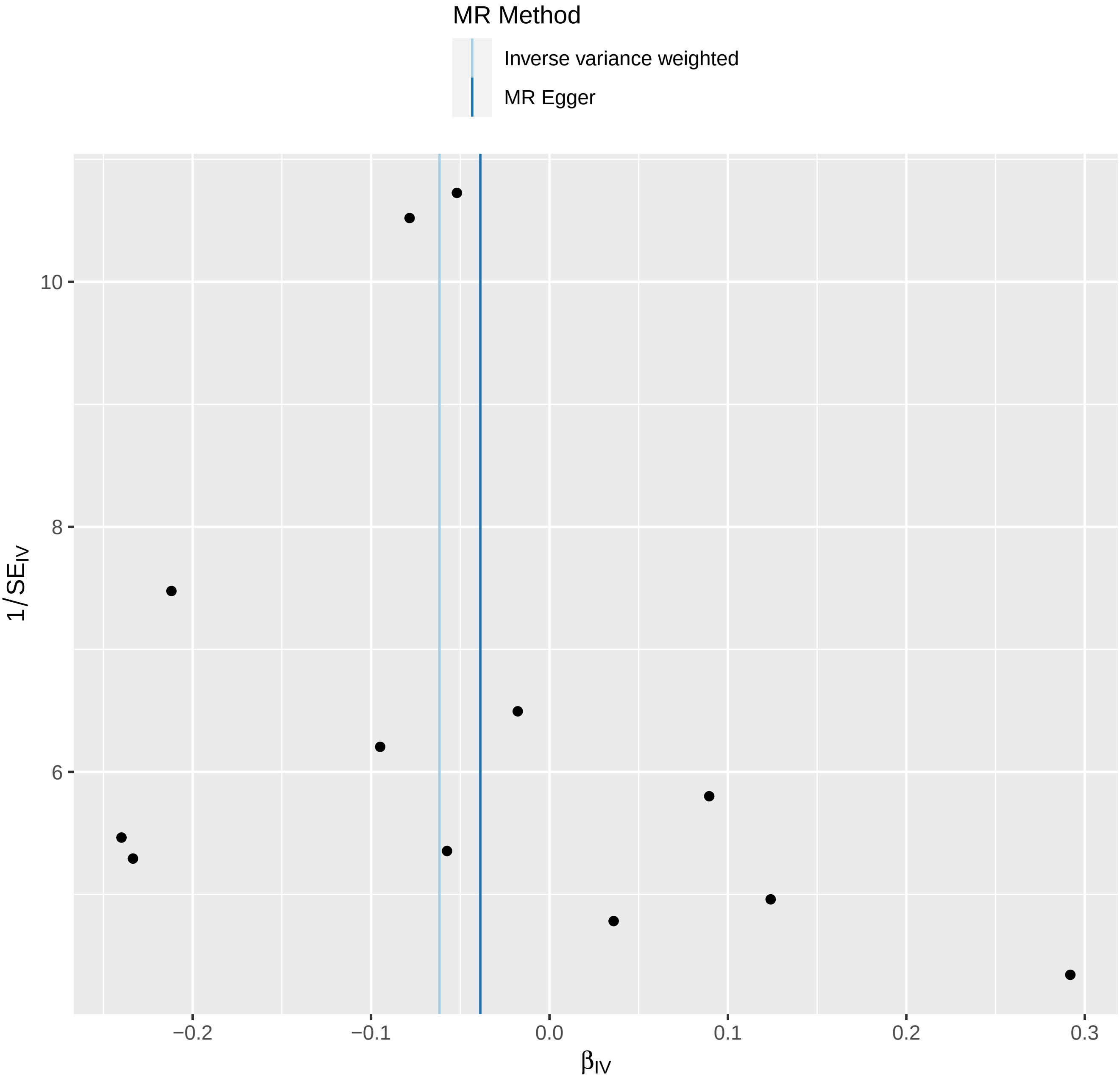

Batch 3−1359 : Selenium on Hepatocyte nuclear factor 1−alpha

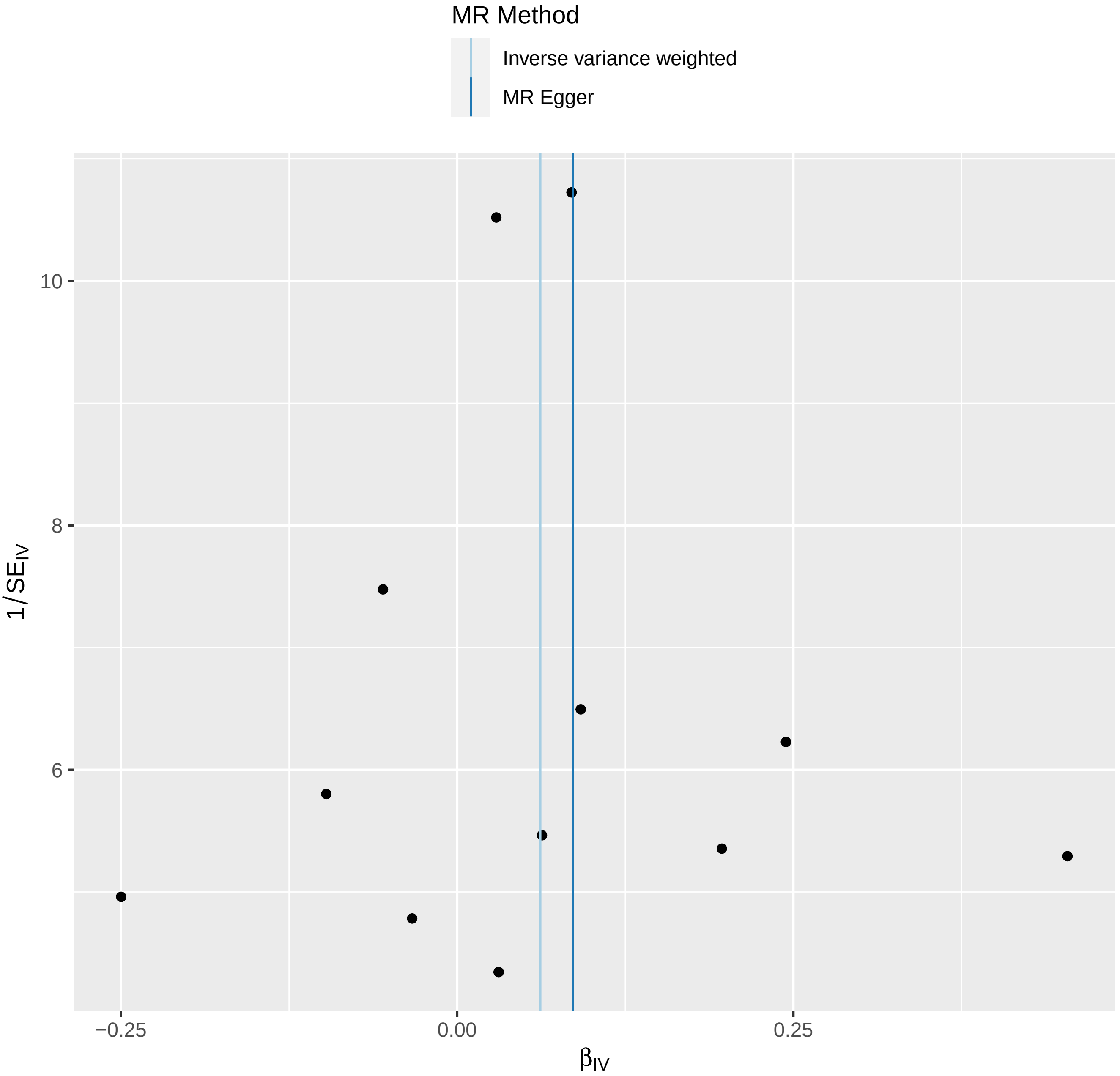

Batch 3−1360 : Selenium on Hepatocyte nuclear factor 4−alpha

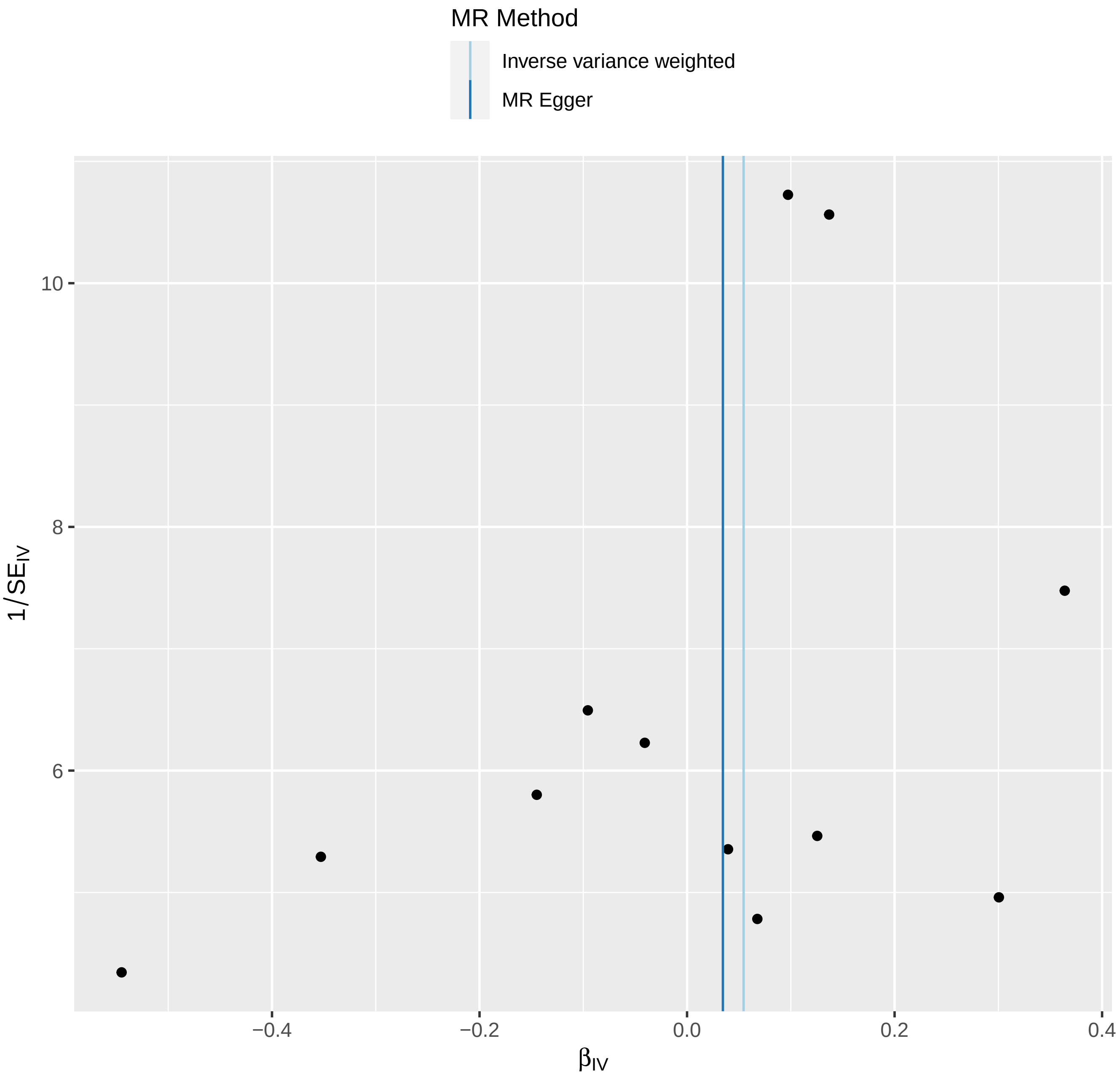

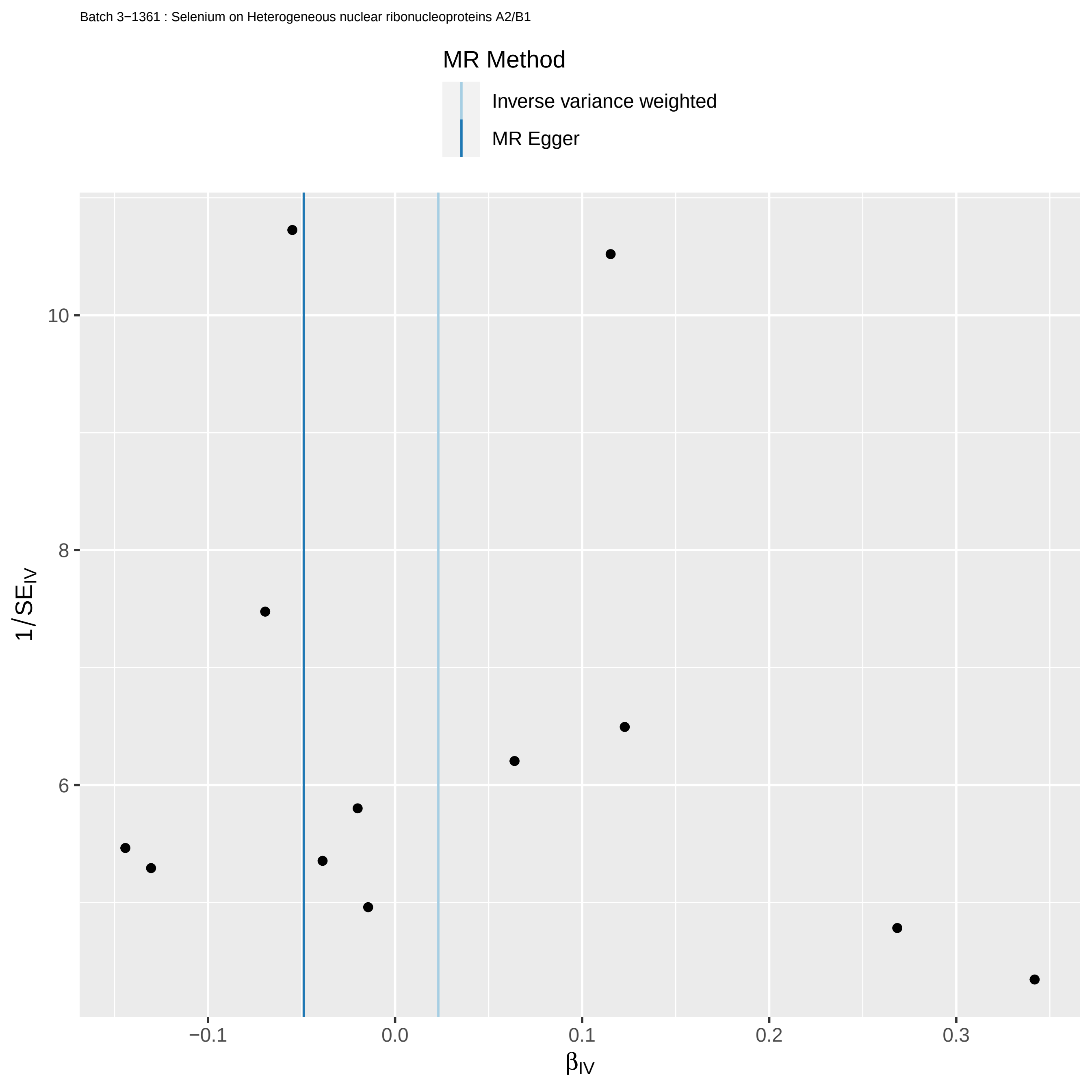
Batch 3−1361 : Selenium on Heterogeneous nuclear ribonucleoproteins A2/B1
MR Method
Inverse variance weighted
MR Egger
10
8
6
1/SE_IV
−0.1
0.0
0.1
0.2
0.3
β_IV

Batch 3−1362 : Selenium on Heterogeneous nuclear ribonucleoprotein A/B

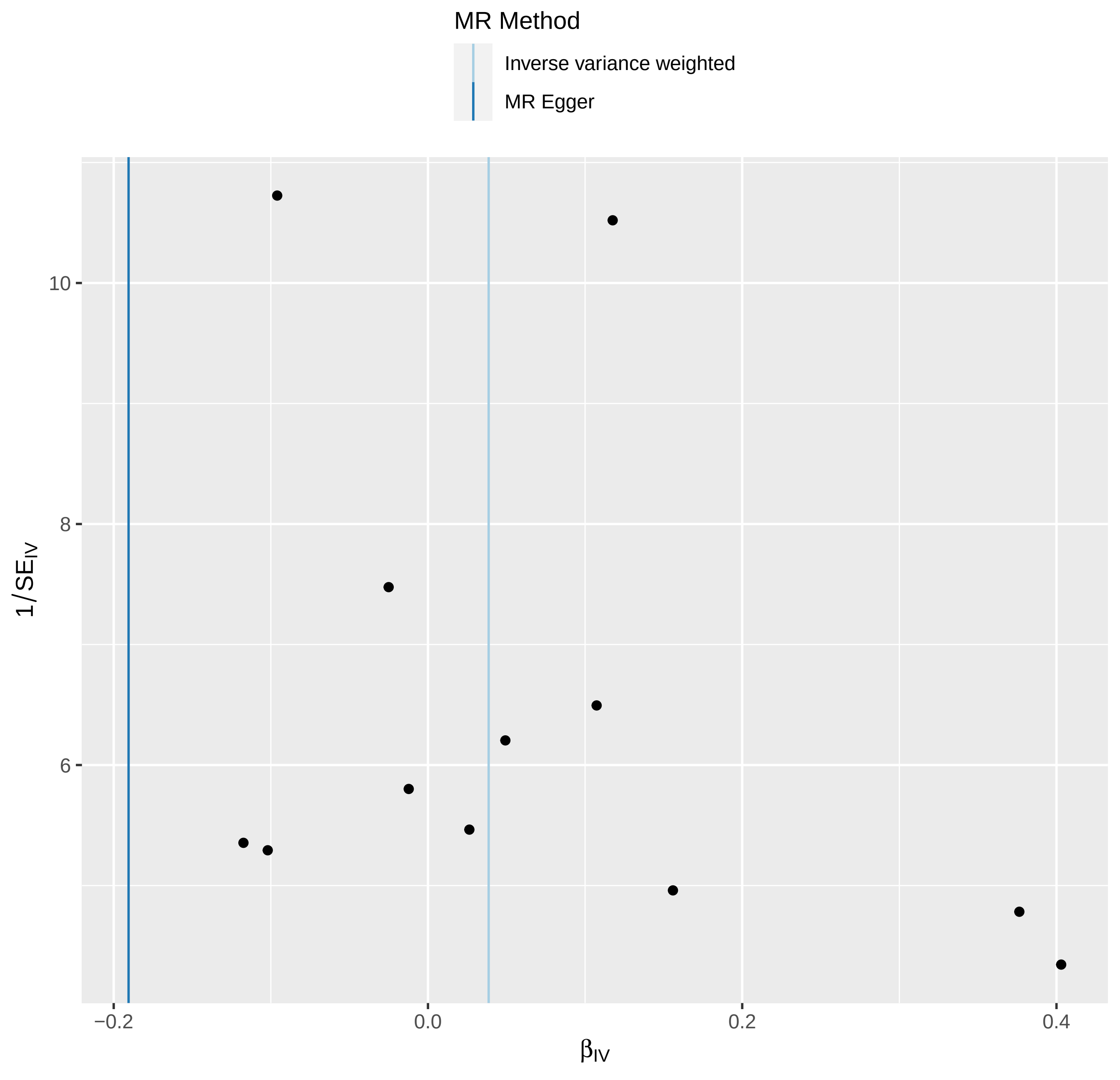

Batch 3−1363 : Selenium on Heterogeneous nuclear ribonucleoprotein A/B

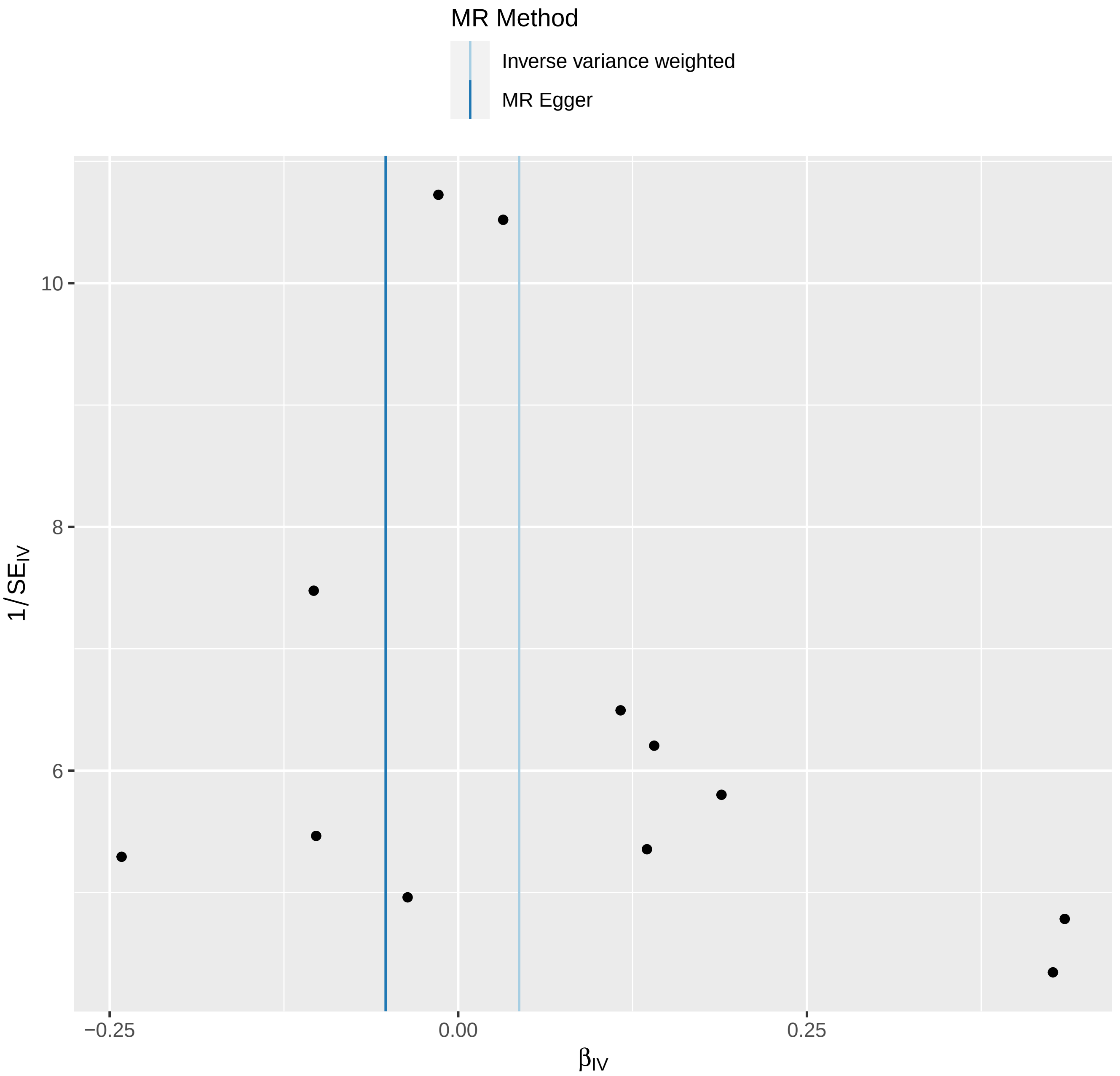

Batch 3−1364 : Selenium on Heterogeneous nuclear ribonucleoproteins C1/C2

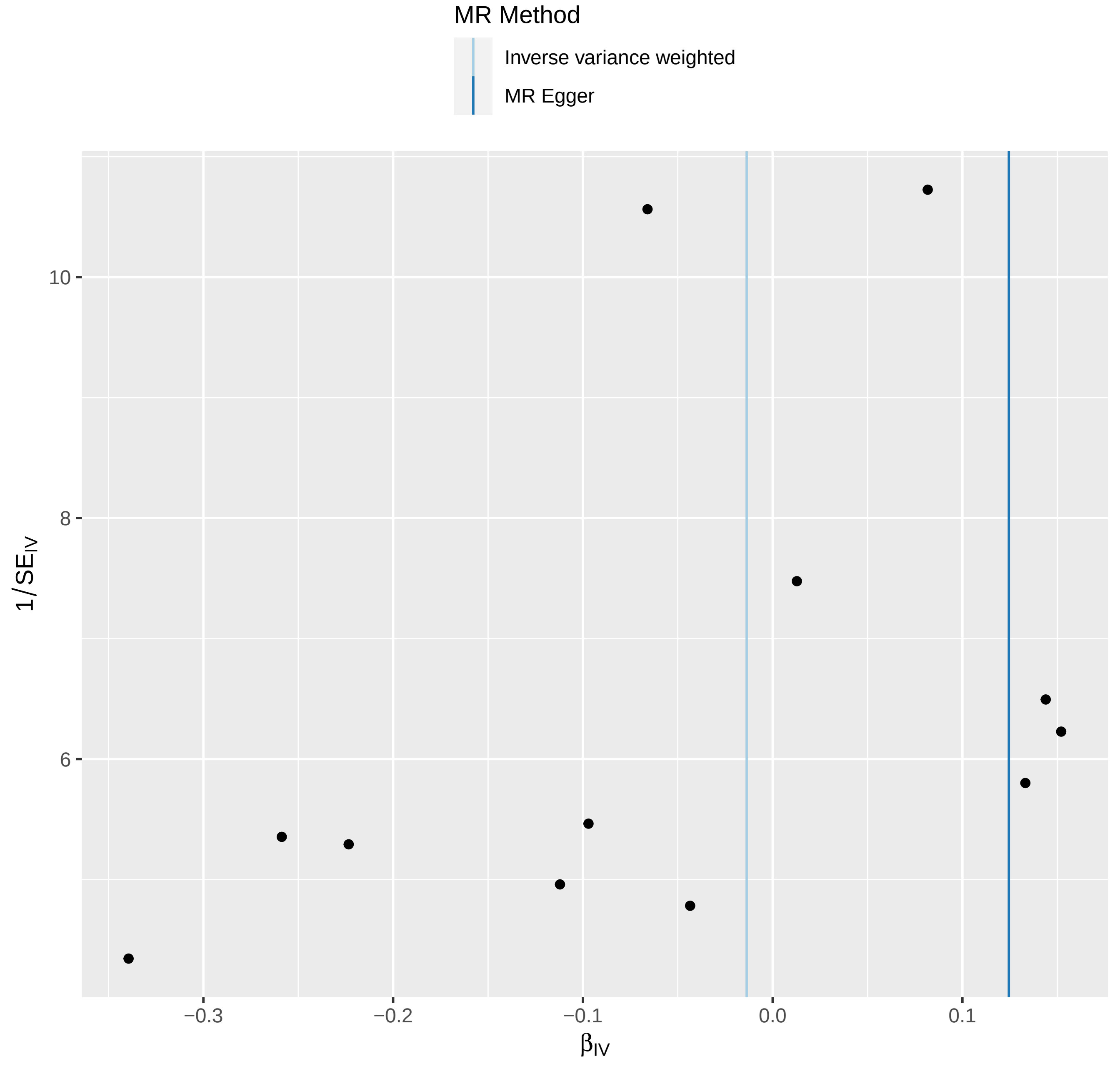

Batch 3−1365 : Selenium on Heterogeneous nuclear ribonucleoprotein D−like

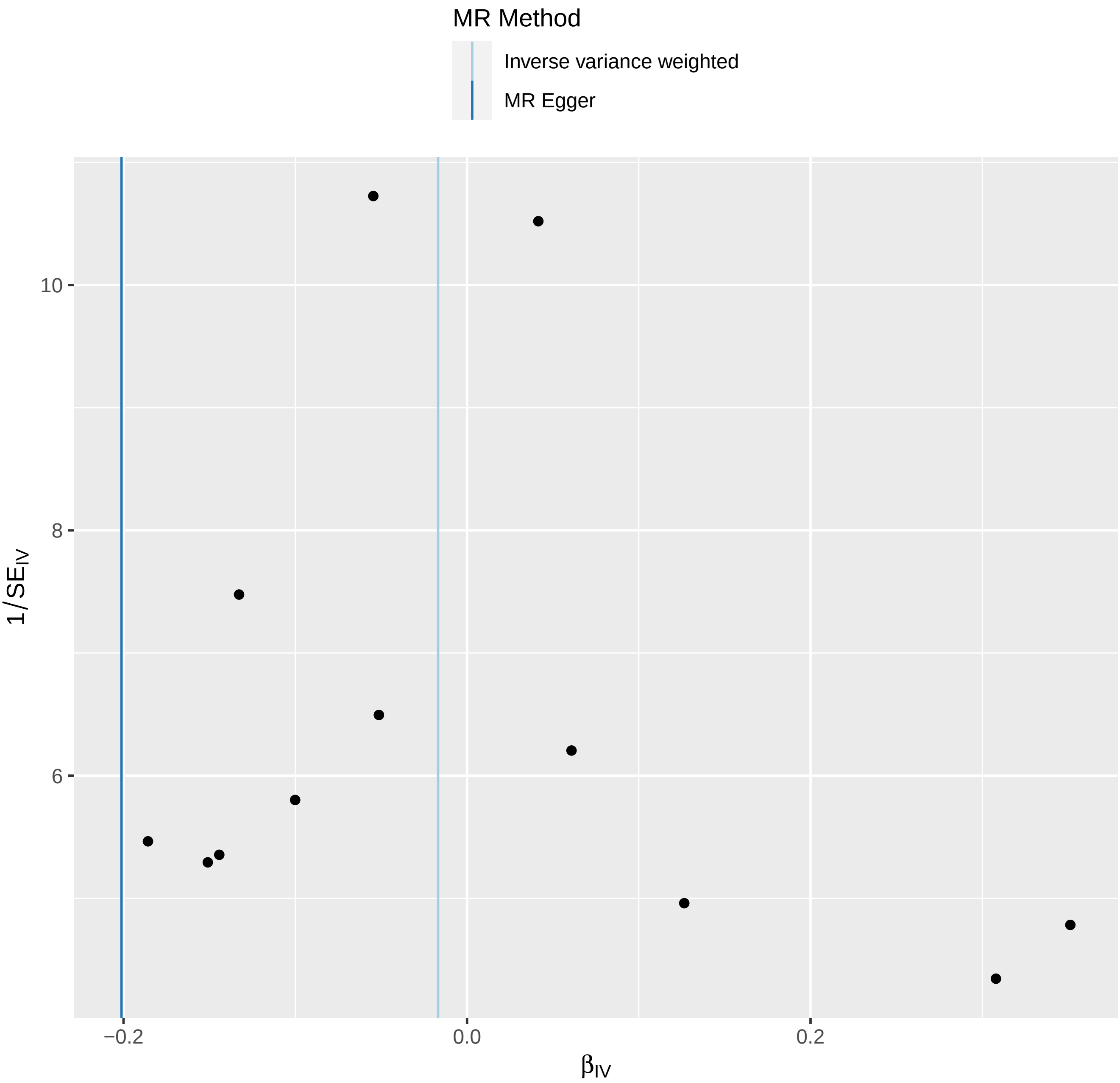

Batch 3−1366 : Selenium on Heterogeneous nuclear ribonucleoprotein K

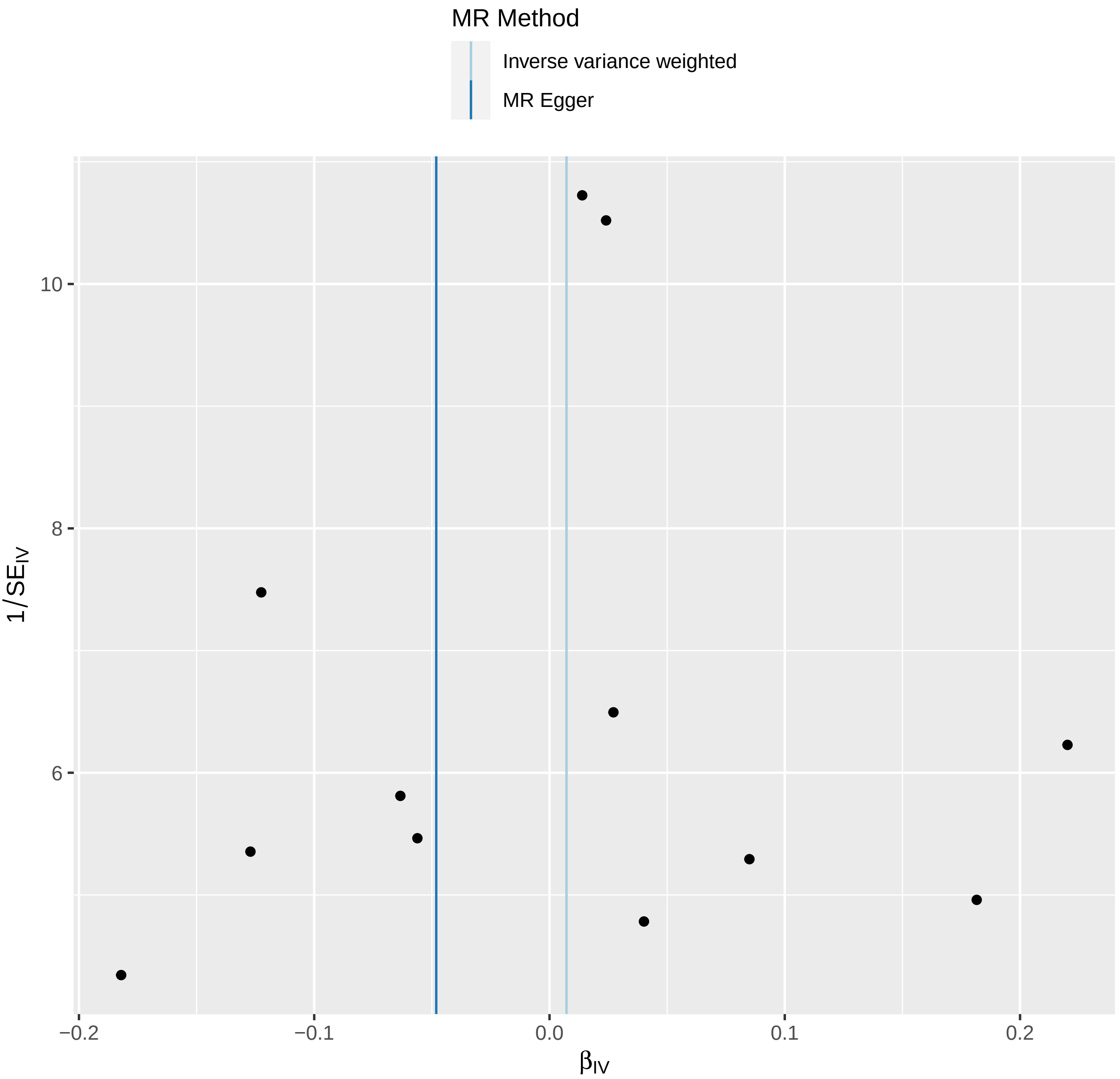

Batch 3−1367 : Selenium on Heterogeneous nuclear ribonucleoprotein M

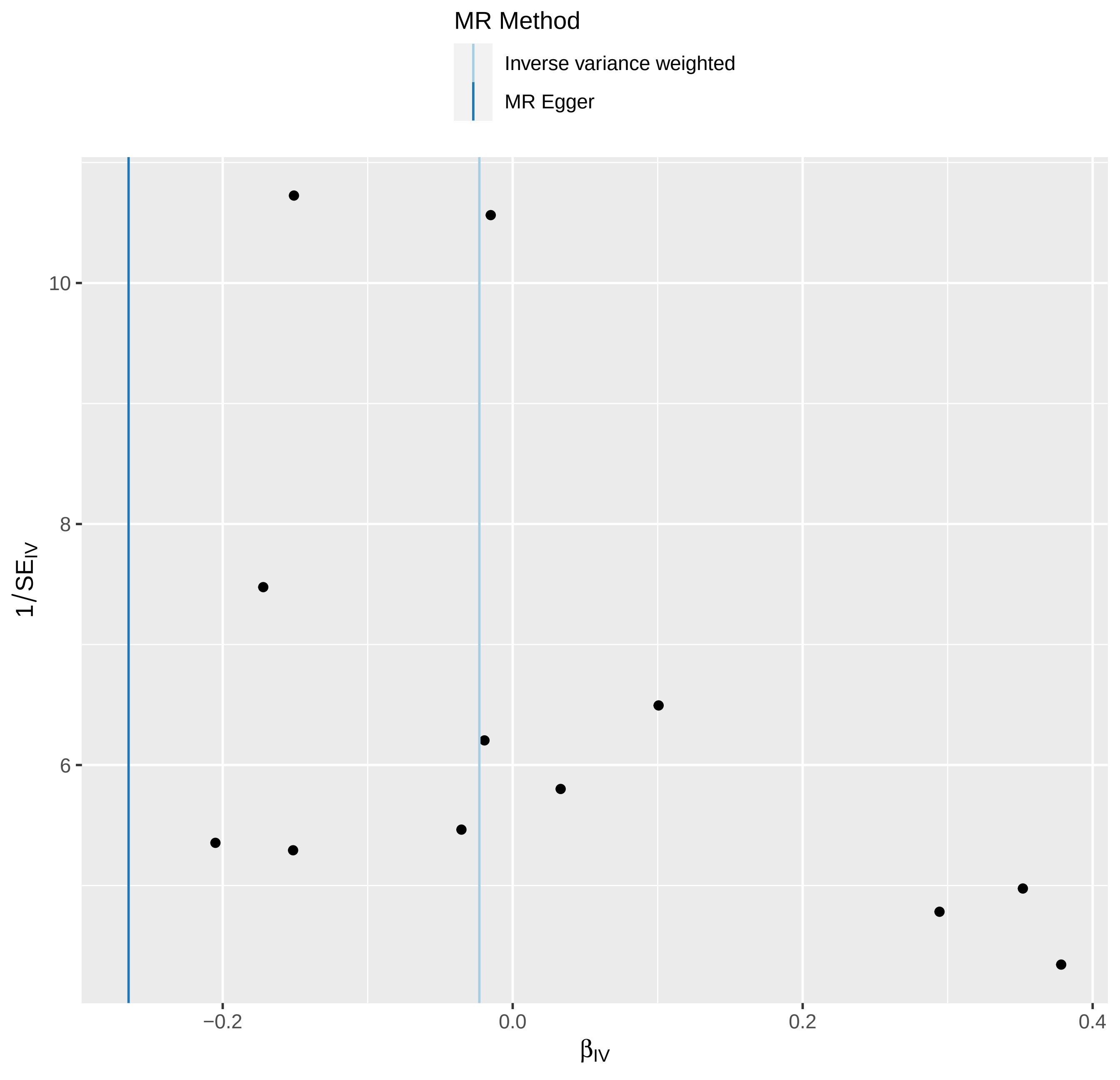

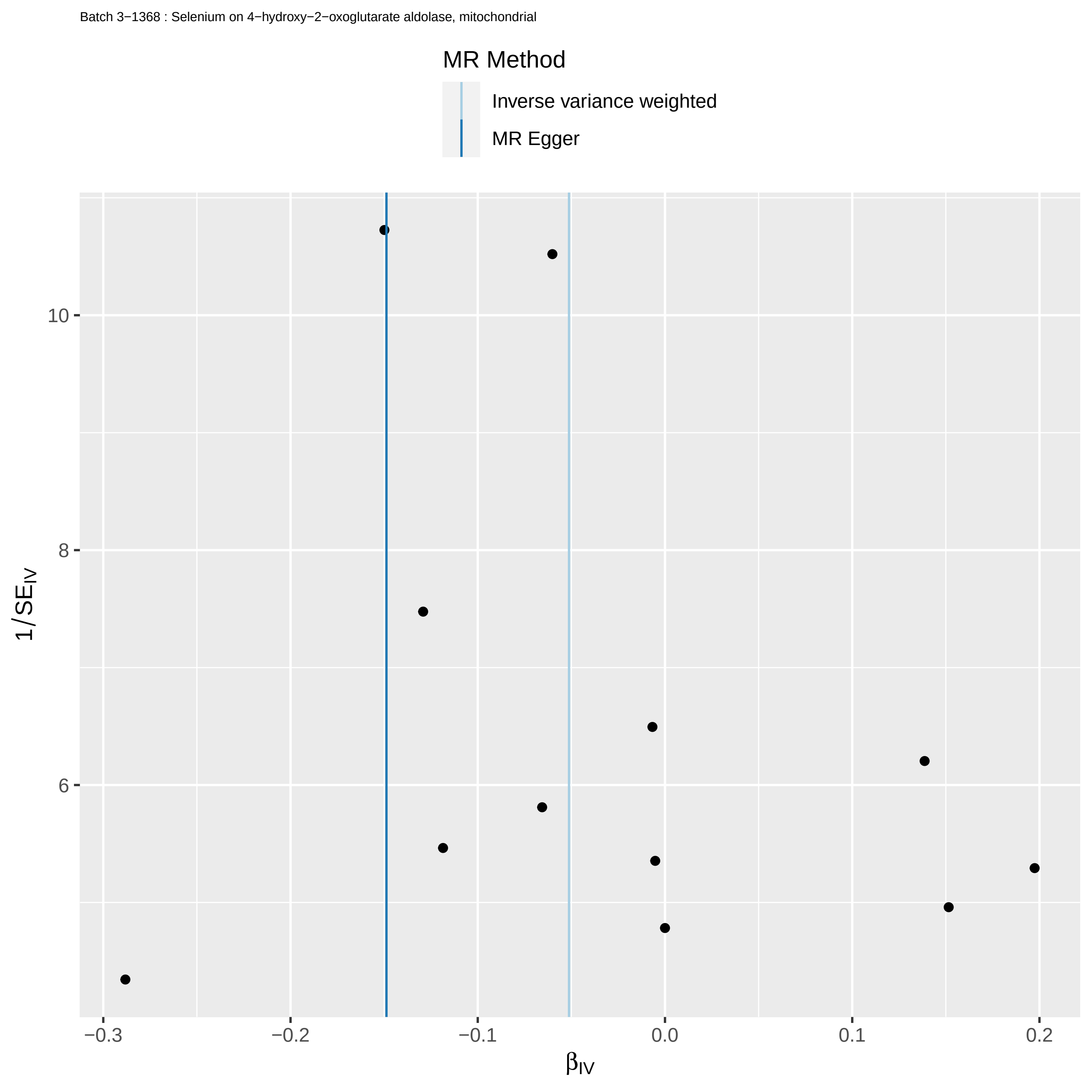
Batch 3−1368 : Selenium on 4−hydroxy−2−oxoglutarate aldolase, mitochondrial
MR Method
Inverse variance weighted
MR Egger
10
8
6
1/SE_IV
−0.3
−0.2
−0.1
0.0
0.1
0.2
β_IV

Batch 3−1369 : Selenium on Haptoglobin

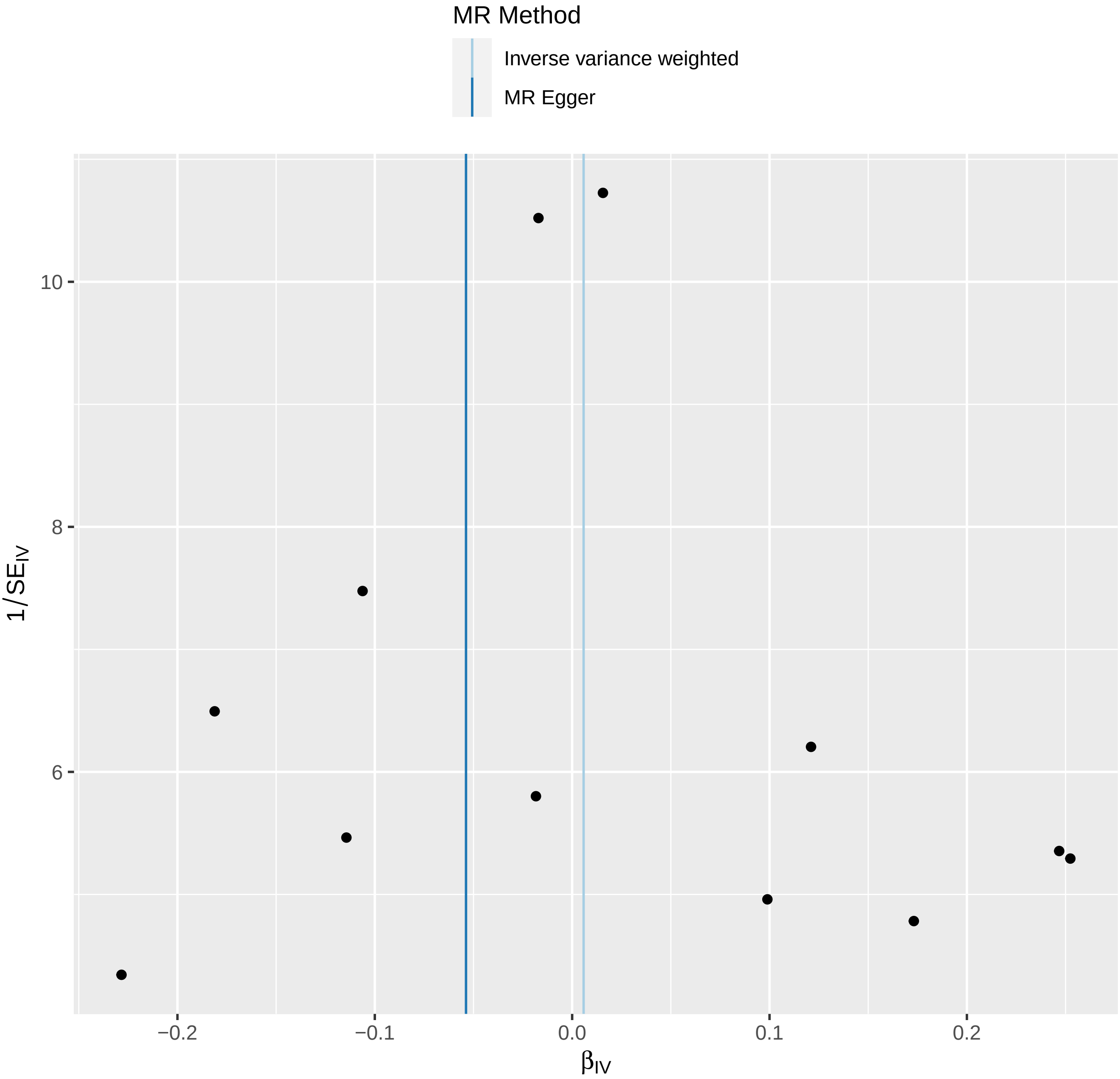

Batch 3−1370 : Selenium on 15−hydroxyprostaglandin dehydrogenase [NAD(+)]

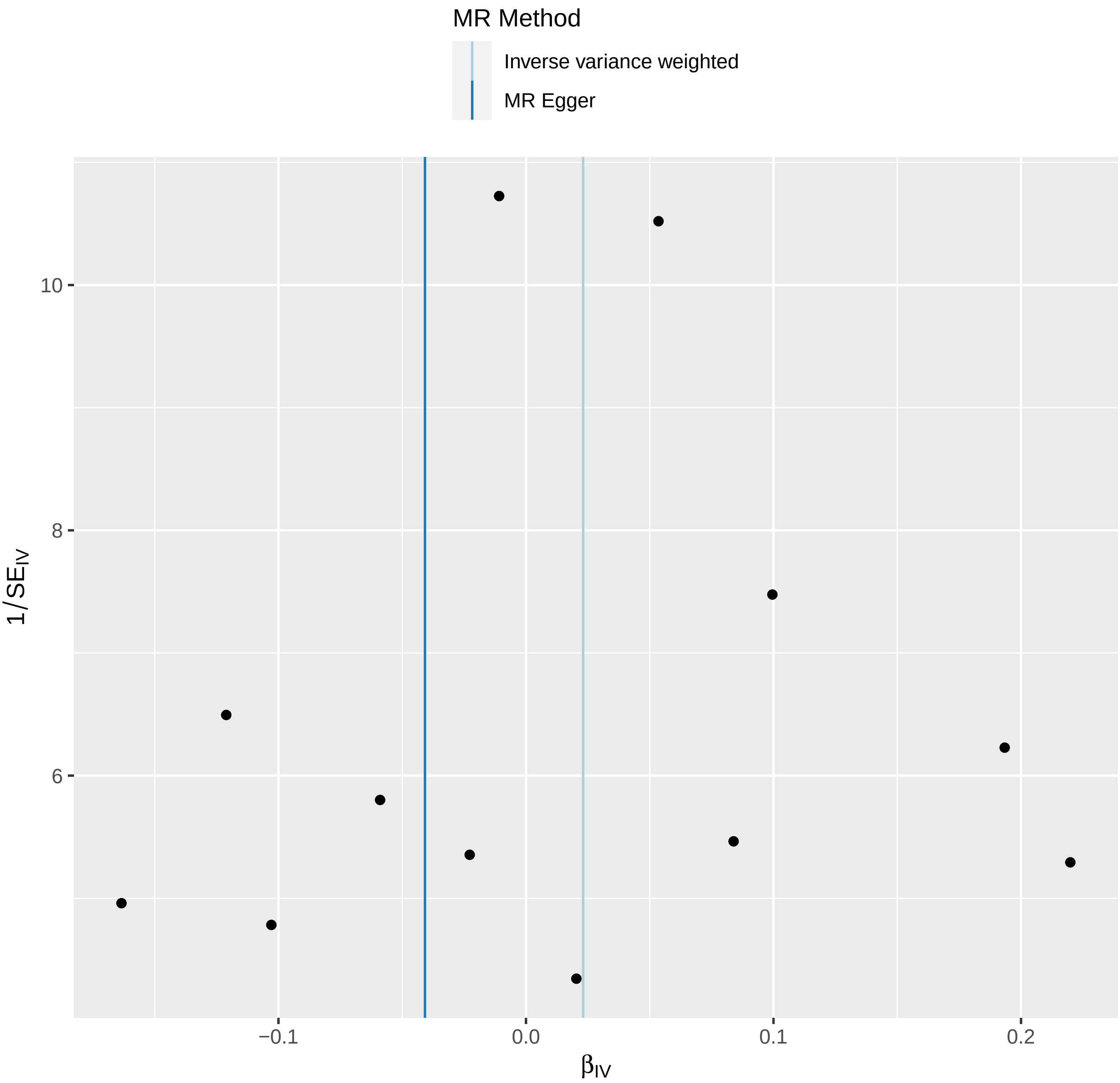

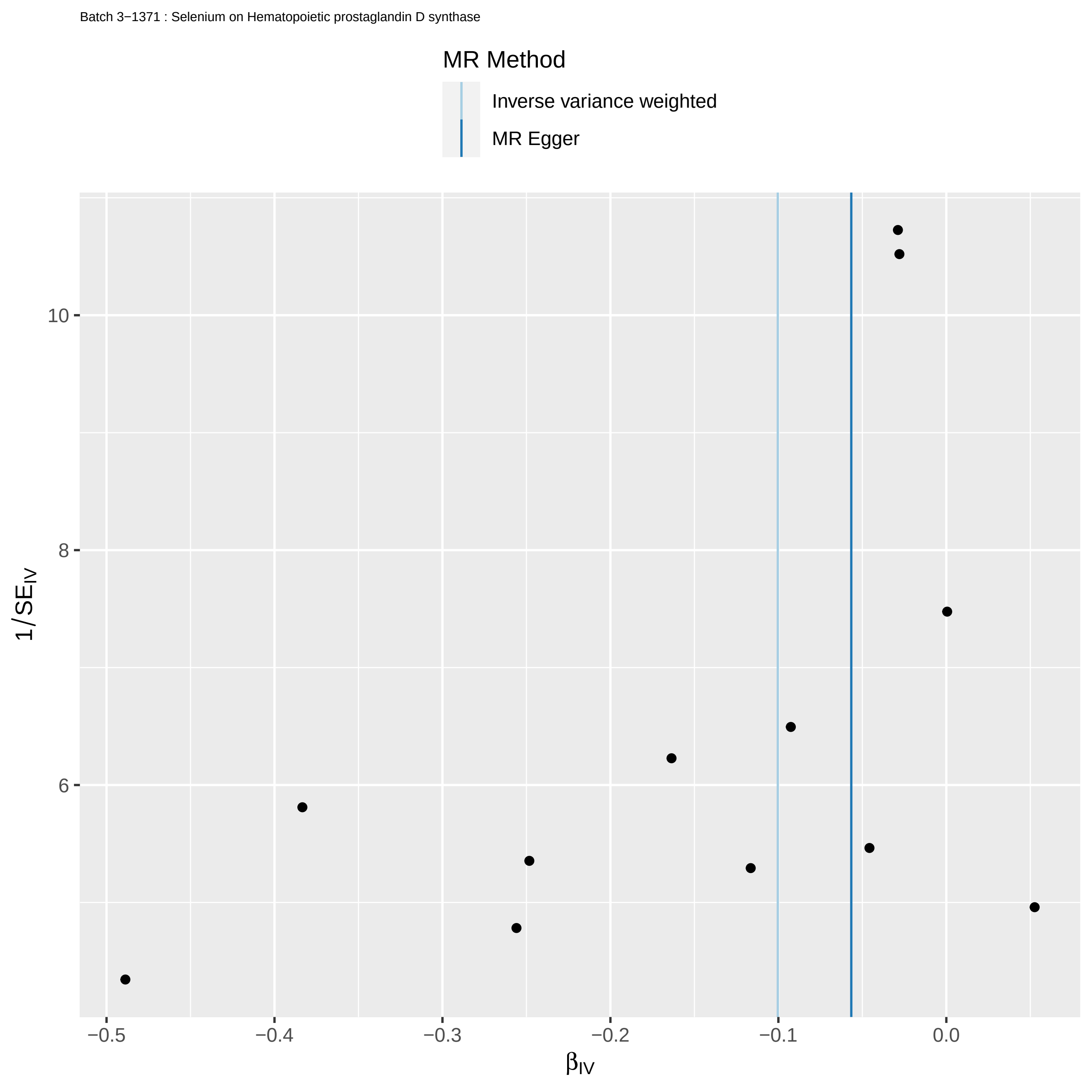

Batch 3−1371 : Selenium on Hematopoietic prostaglandin D synthase
MR Method
Inverse variance weighted
MR Egger
10
8
6
1/SE_IV
−0.5
−0.4
−0.3
−0.2
−0.1
0.0
β_IV

Batch 3−1372 : Selenium on HRAS−like suppressor 2

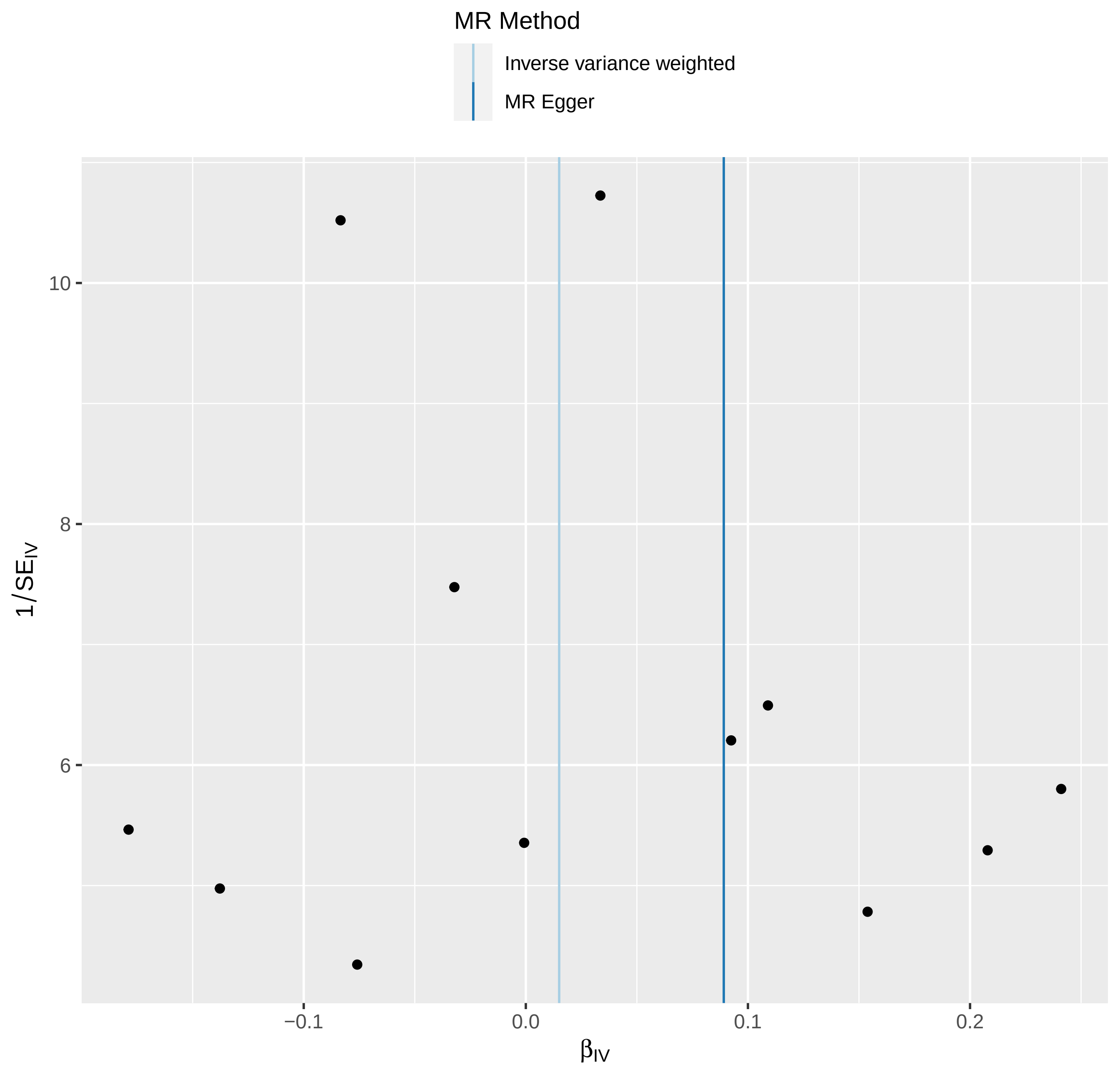

Batch 3−1373 : Selenium on Activator of apoptosis harakiri

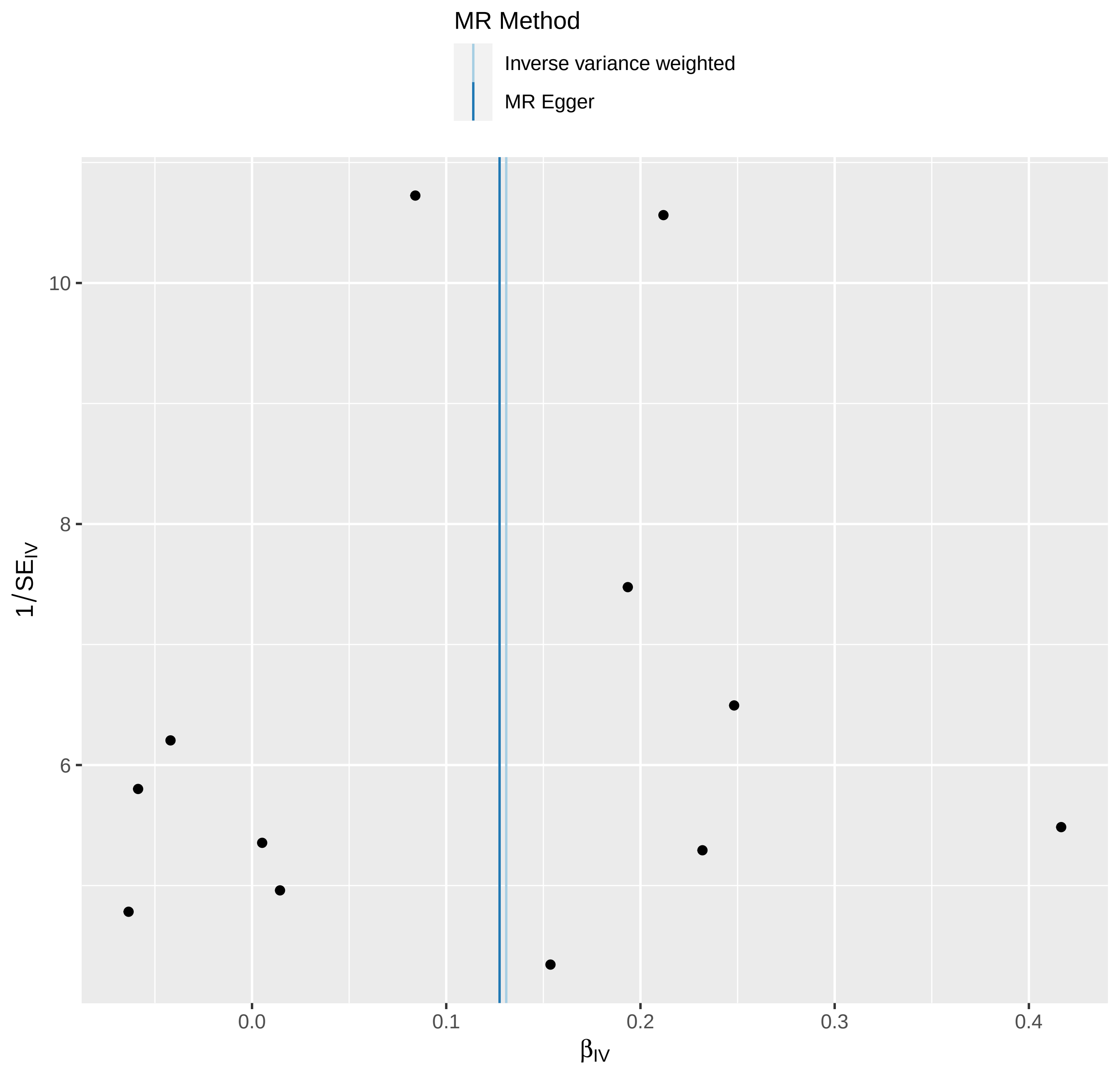

Batch 3−1374 : Selenium on Ribonuclease UK114

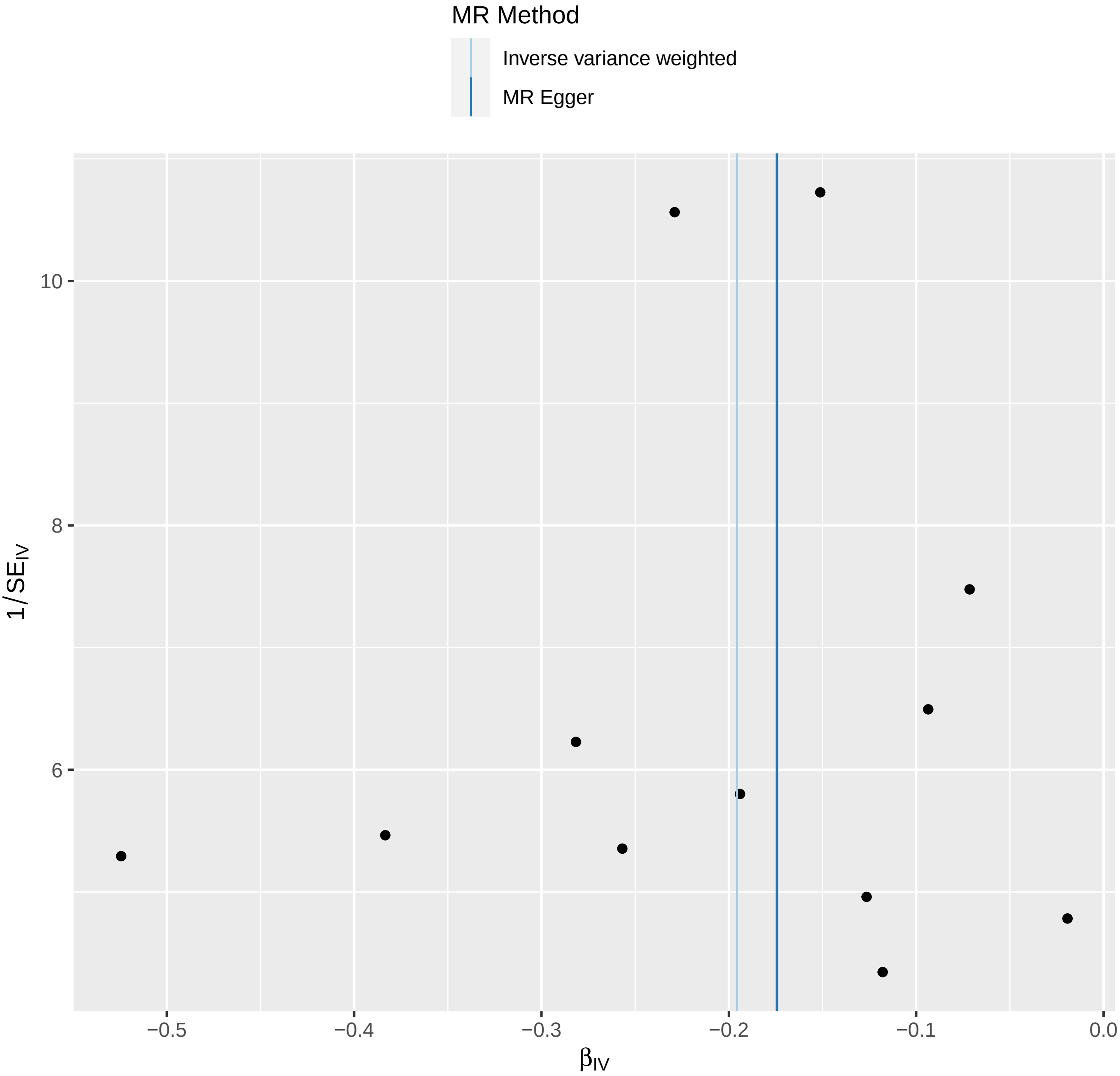

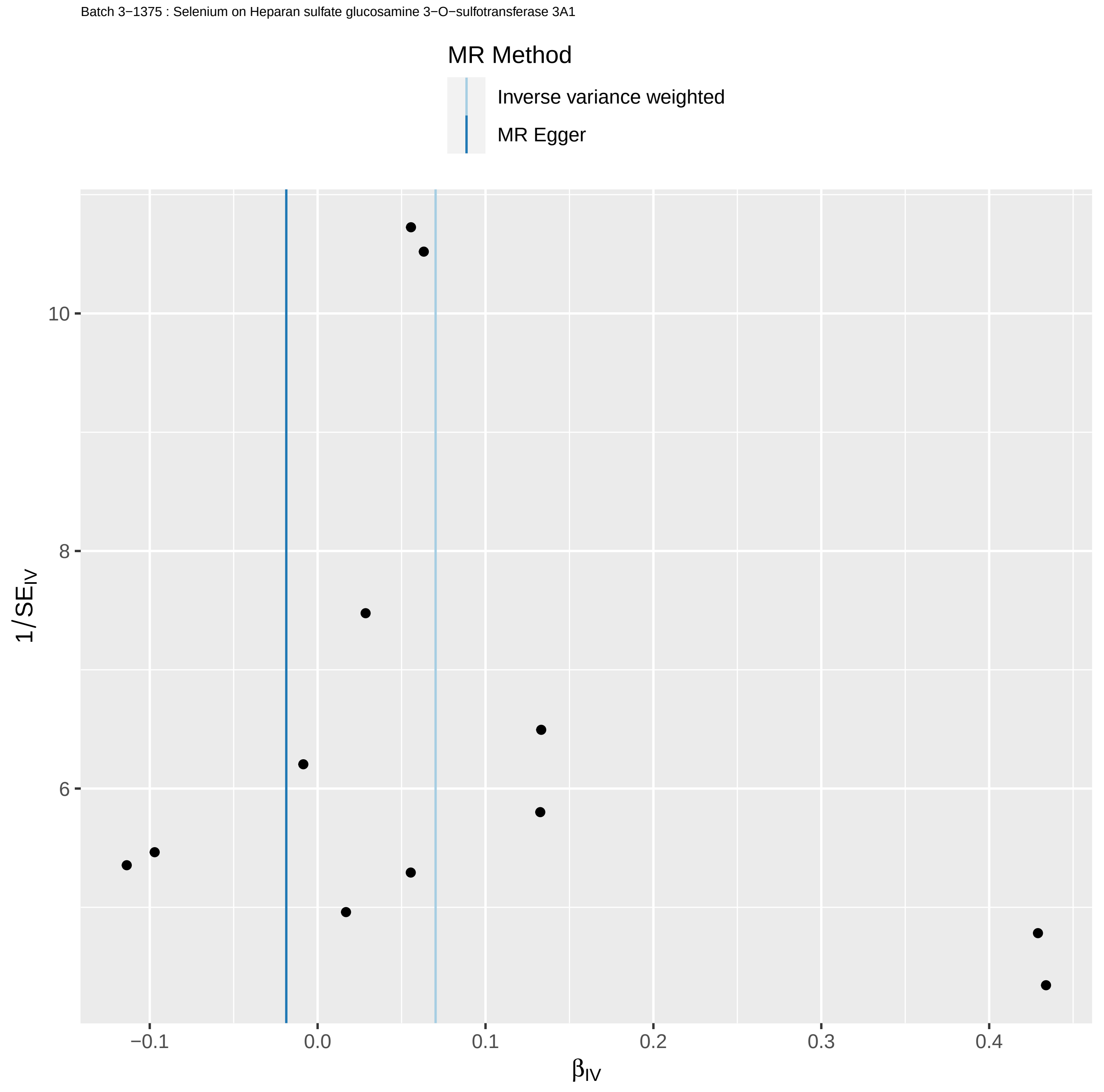
Batch 3−1375 : Selenium on Heparan sulfate glucosamine 3−O−sulfotransferase 3A1
MR Method
Inverse variance weighted
MR Egger
10
8
6
1/SE_IV
−0.1
0.0
0.1
0.2
0.3
0.4
β_IV

Batch 3−1376 : Selenium on Heparan sulfate glucosamine 3−O−sulfotransferase 3B1

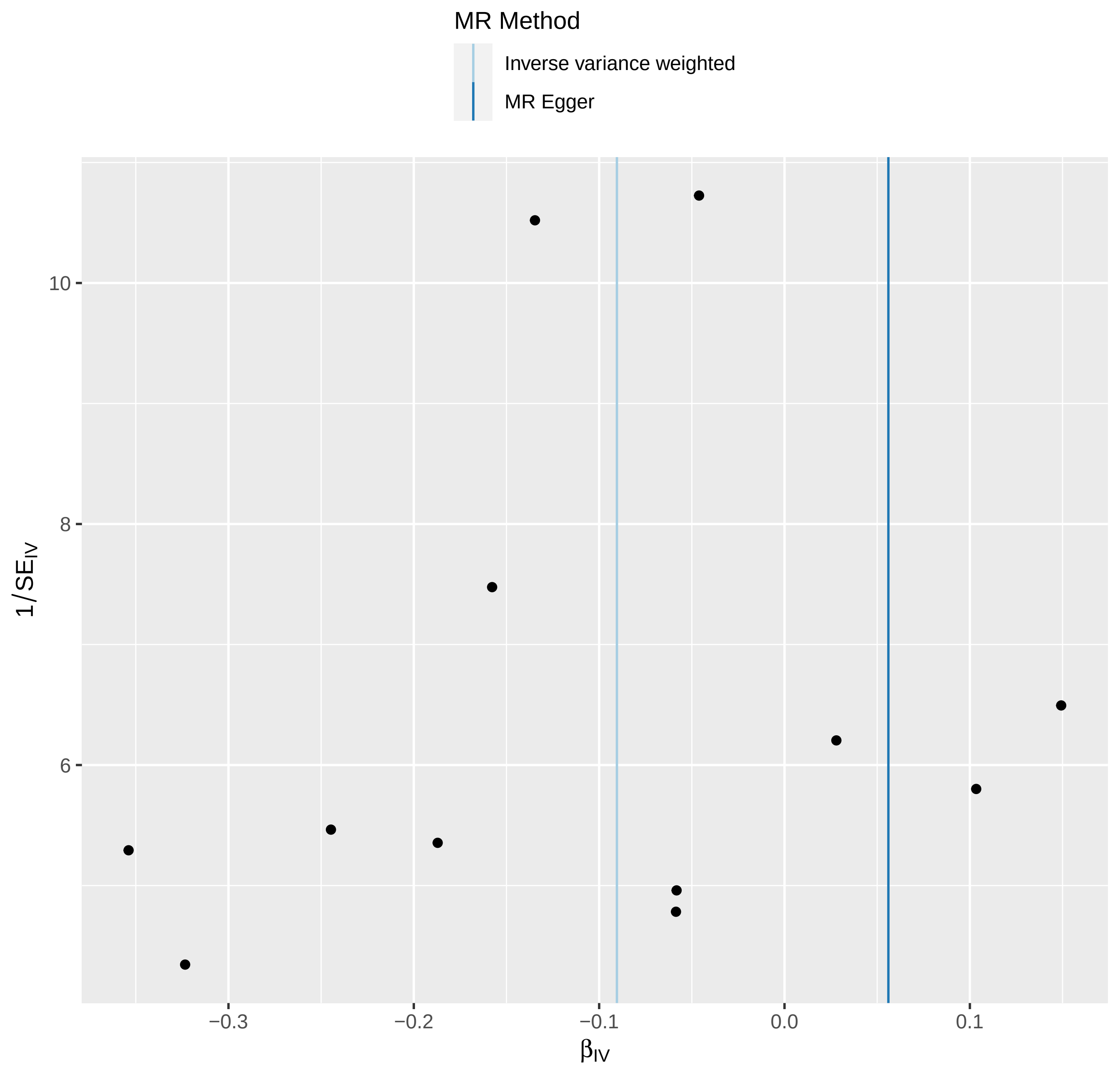

Batch 3−1377 : Selenium on Heparan sulfate glucosamine 3−O−sulfotransferase 4

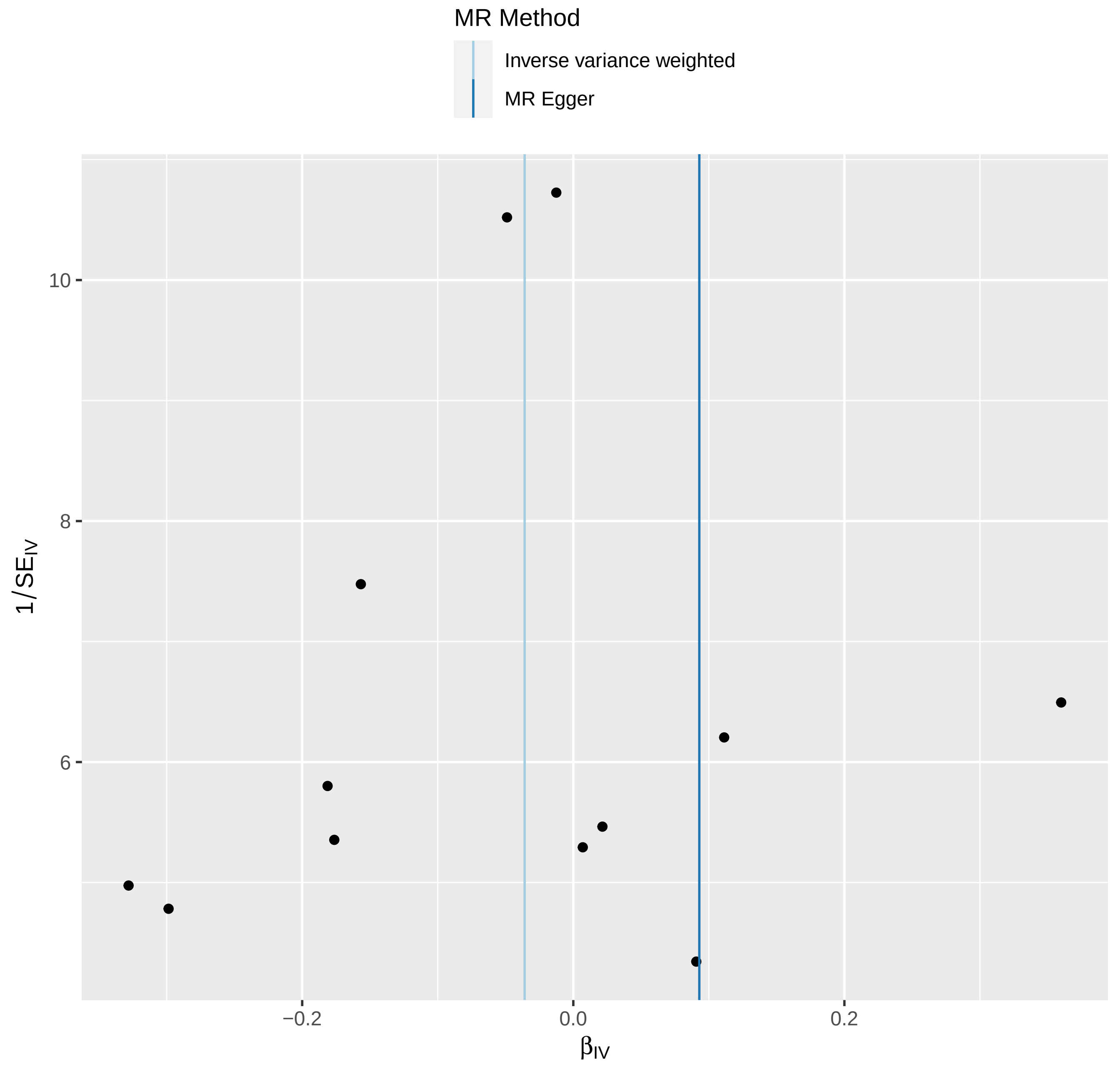

Batch 3−1378 : Selenium on Heparan−sulfate 6−O−sulfotransferase 1

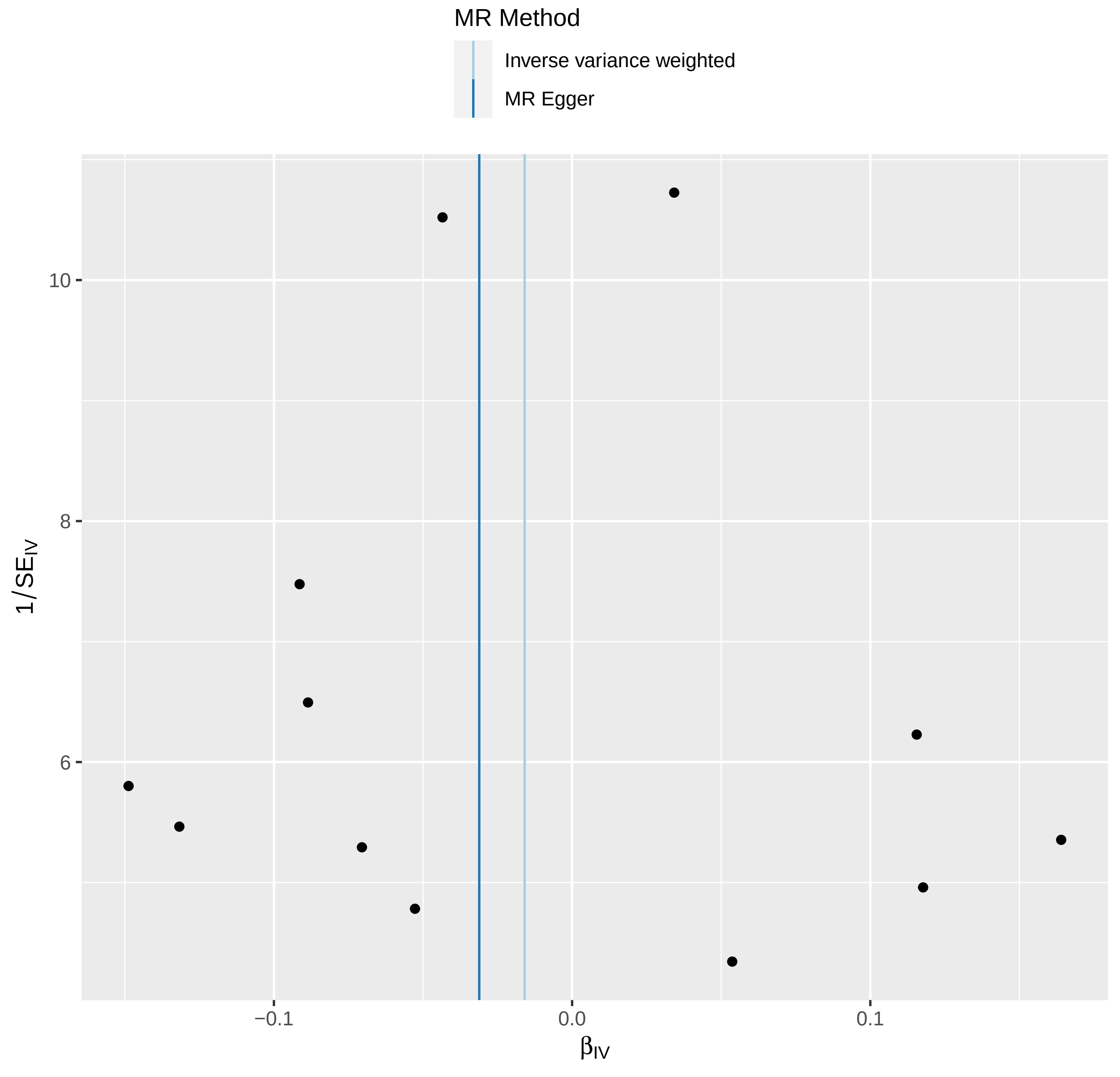

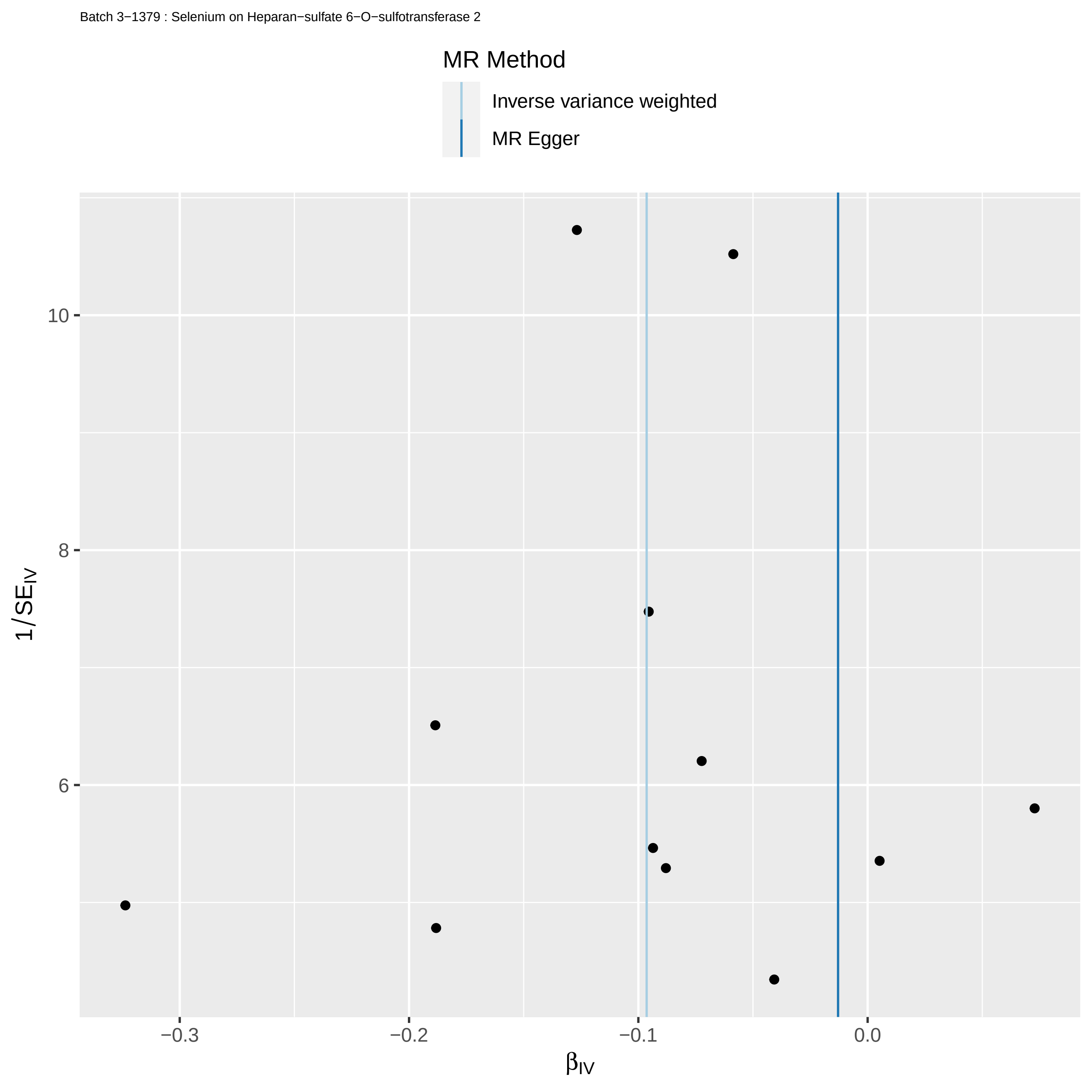
Batch 3−1379 : Selenium on Heparan−sulfate 6−O−sulfotransferase 2
MR Method
Inverse variance weighted
MR Egger
10
8
6
1/SE_IV
−0.3
−0.2
−0.1
0.0
β_IV

Batch 3−1380 : Selenium on 3−hydroxyacyl−CoA dehydrogenase type−2

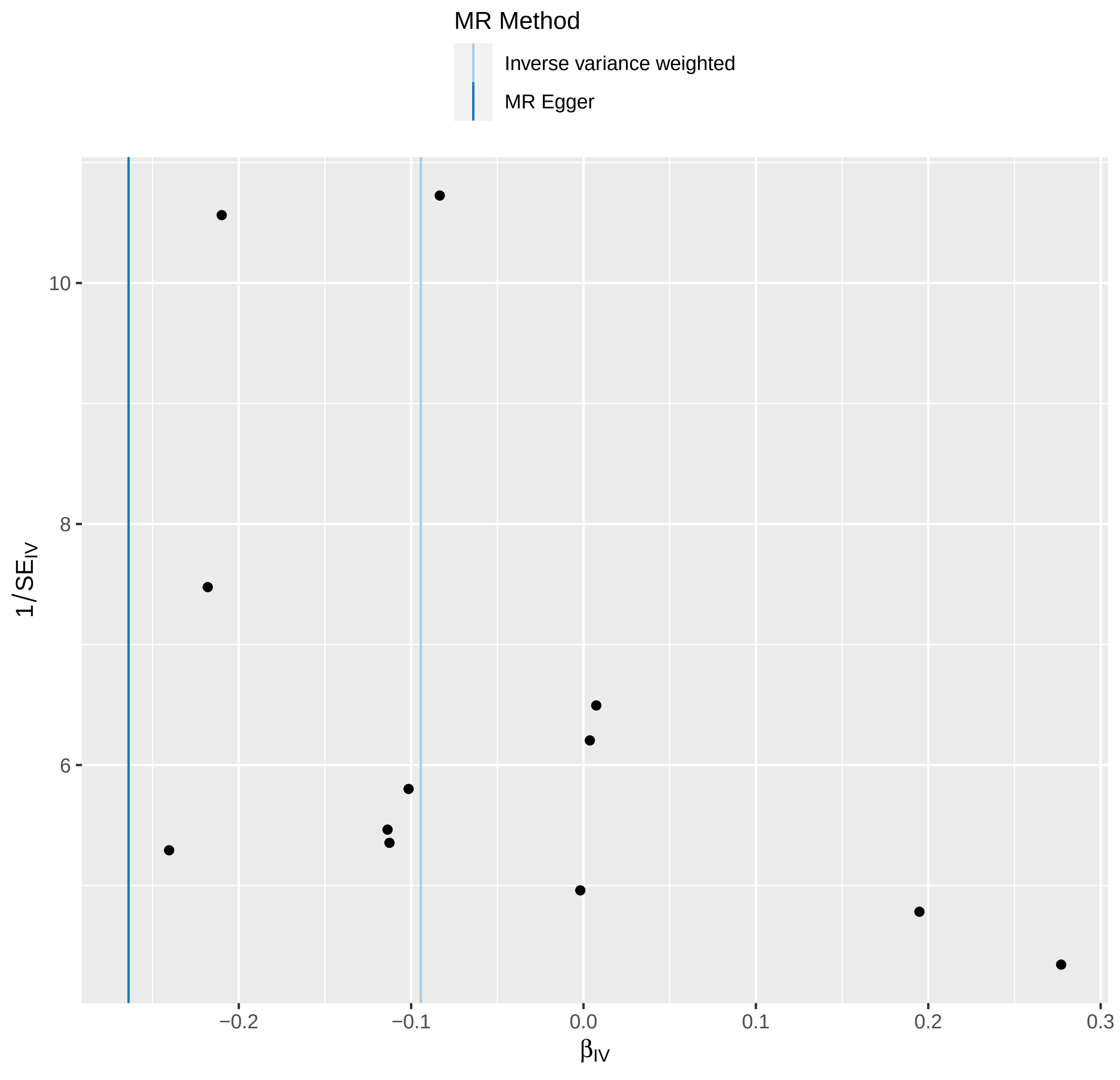

Batch 3−1381 : Selenium on 17−beta−hydroxysteroid dehydrogenase 14

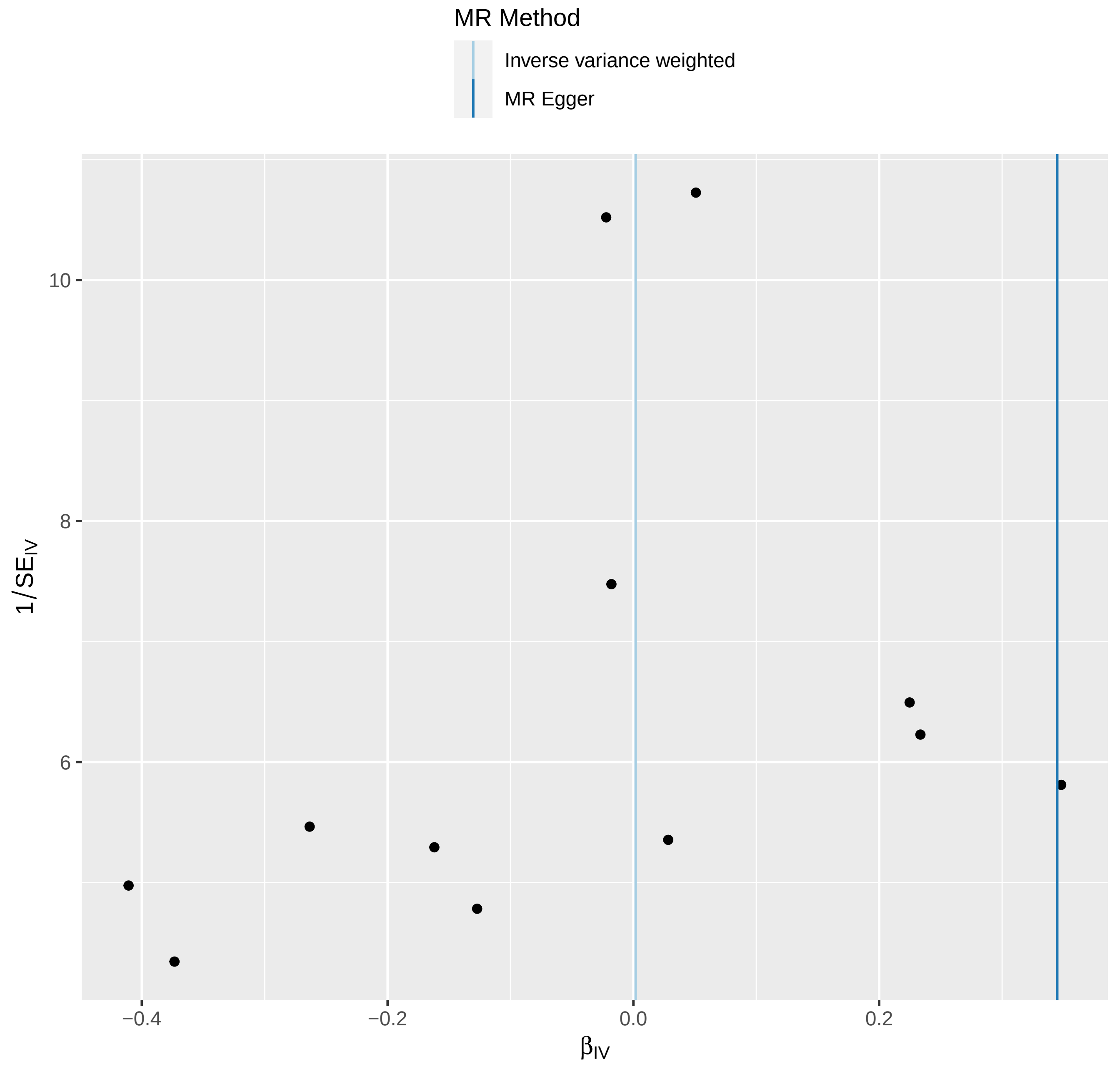

Batch 3−1382 : Selenium on Estradiol 17−beta−dehydrogenase 1

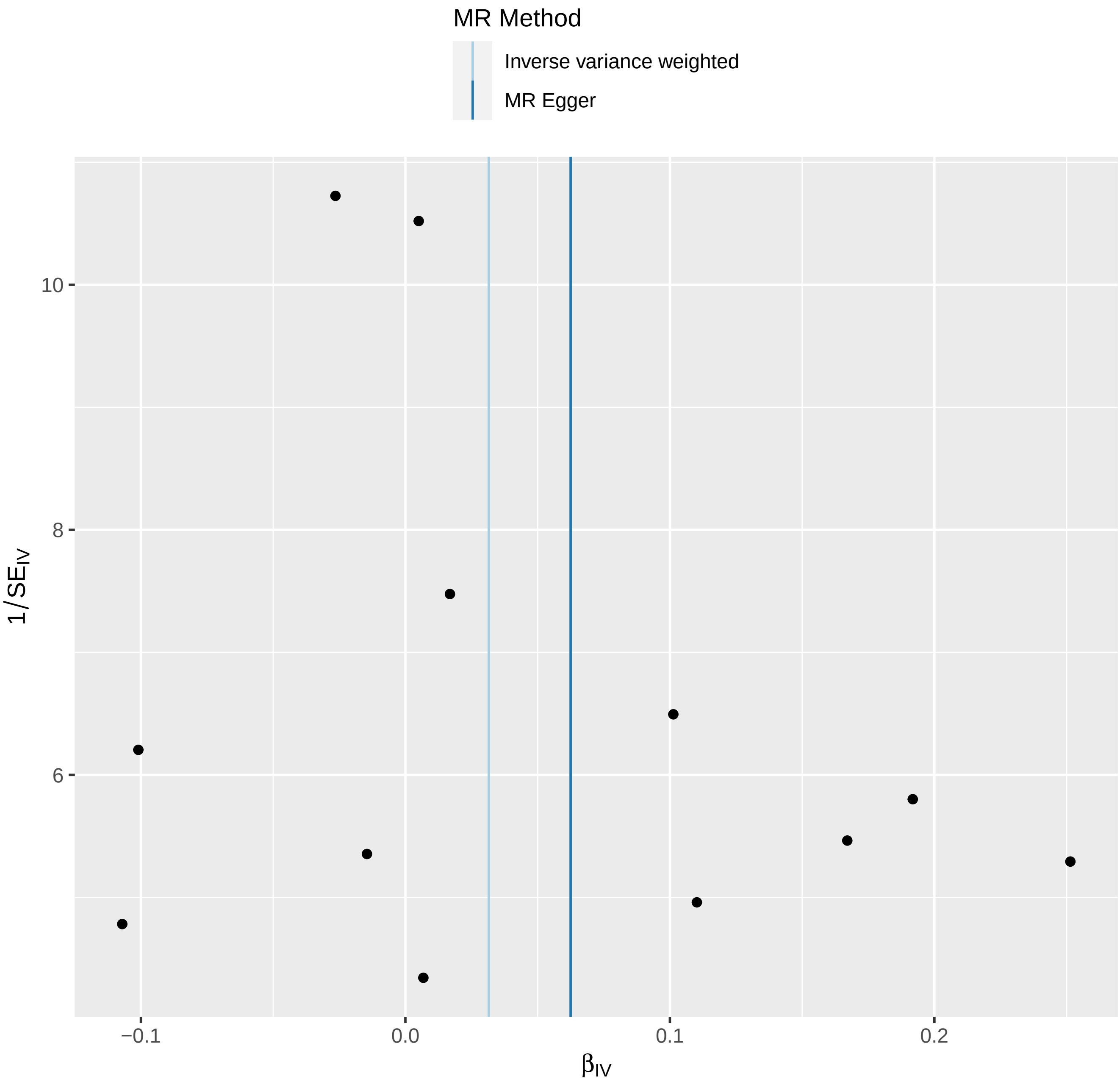

Batch 3−1383 : Selenium on Estradiol 17−beta−dehydrogenase 2

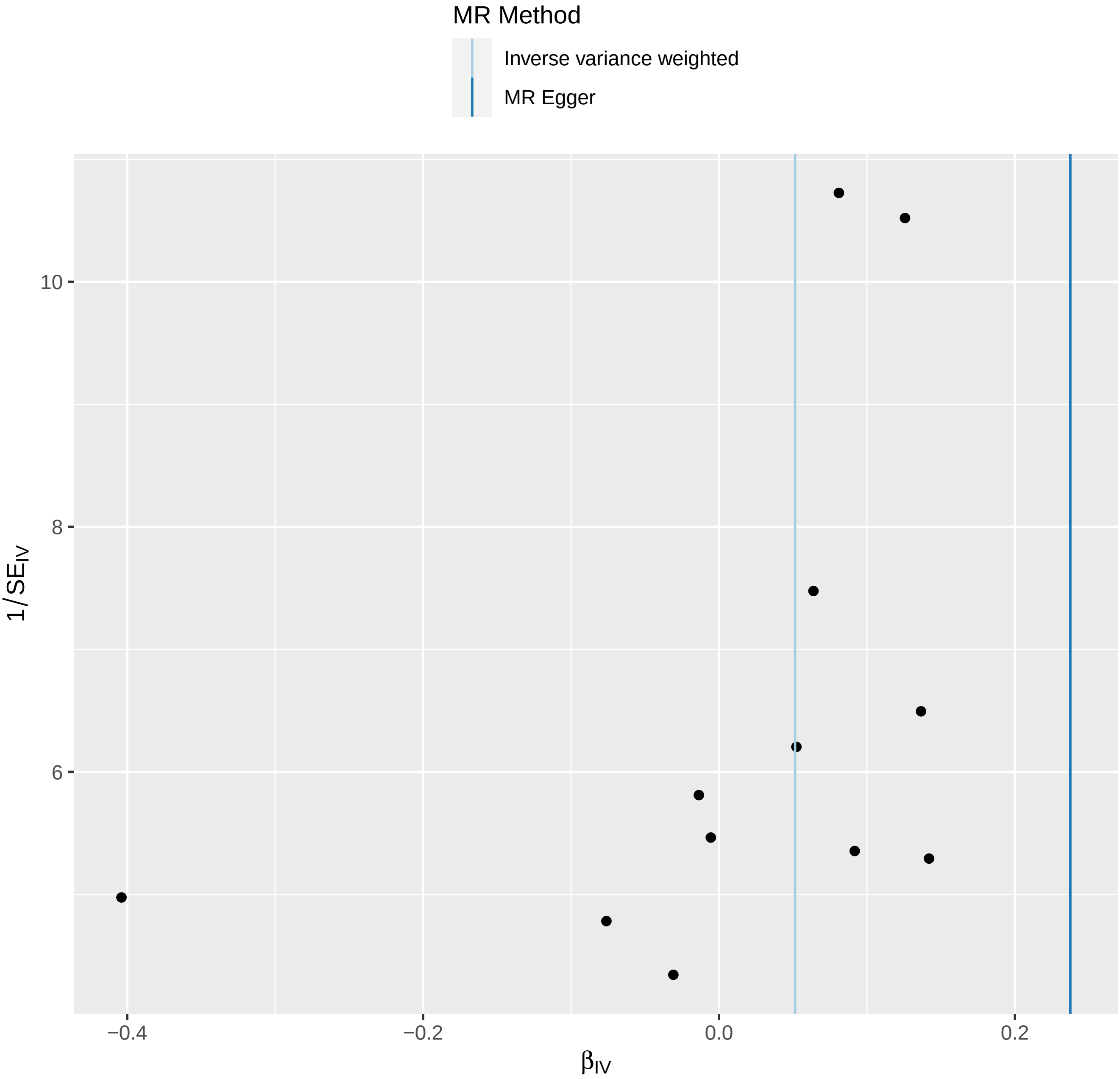

Batch 3−1384 : Selenium on Heat shock protein HSP 90−alpha/beta

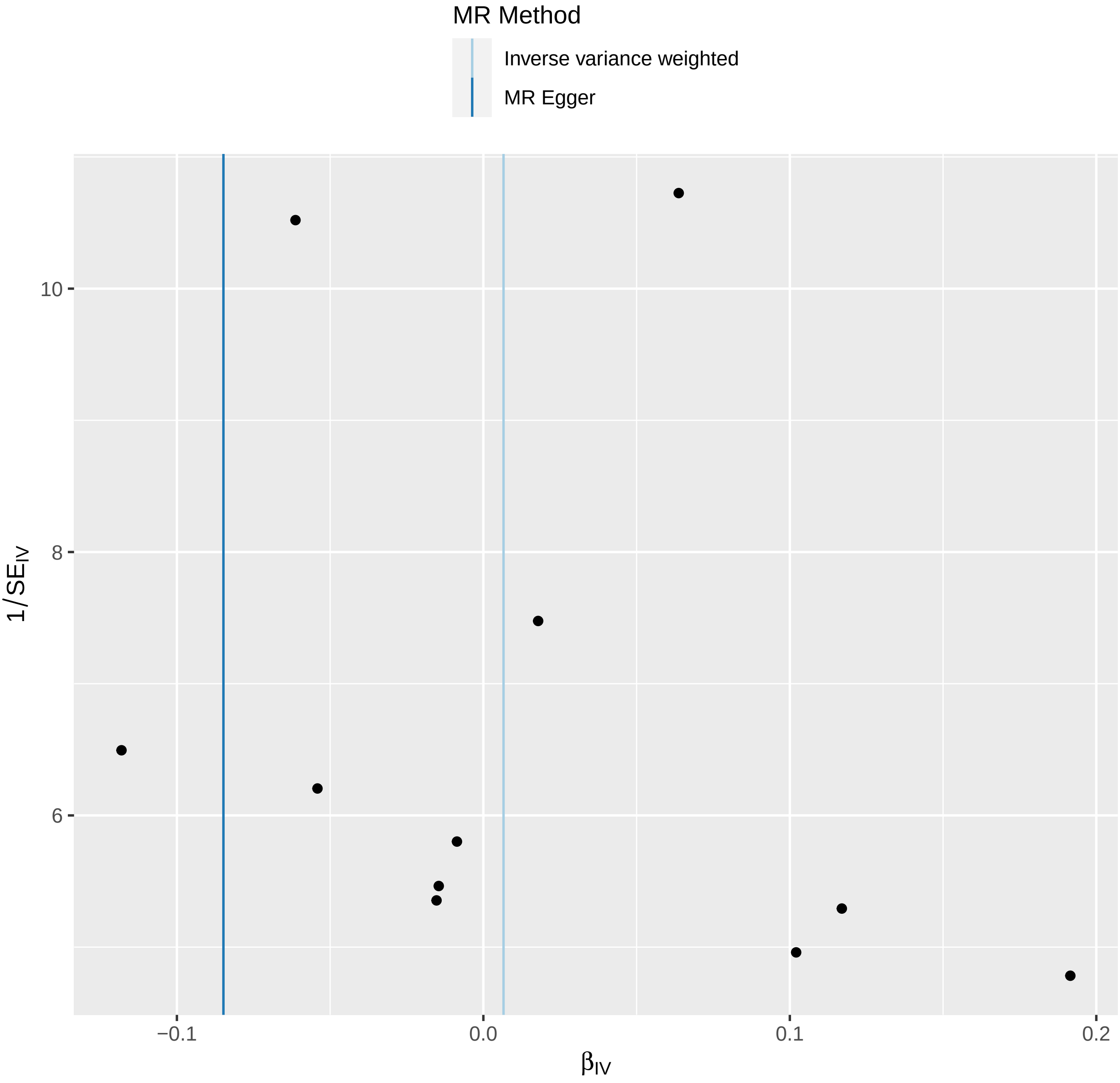

Batch 3−1385 : Selenium on Heat shock protein HSP 90−beta

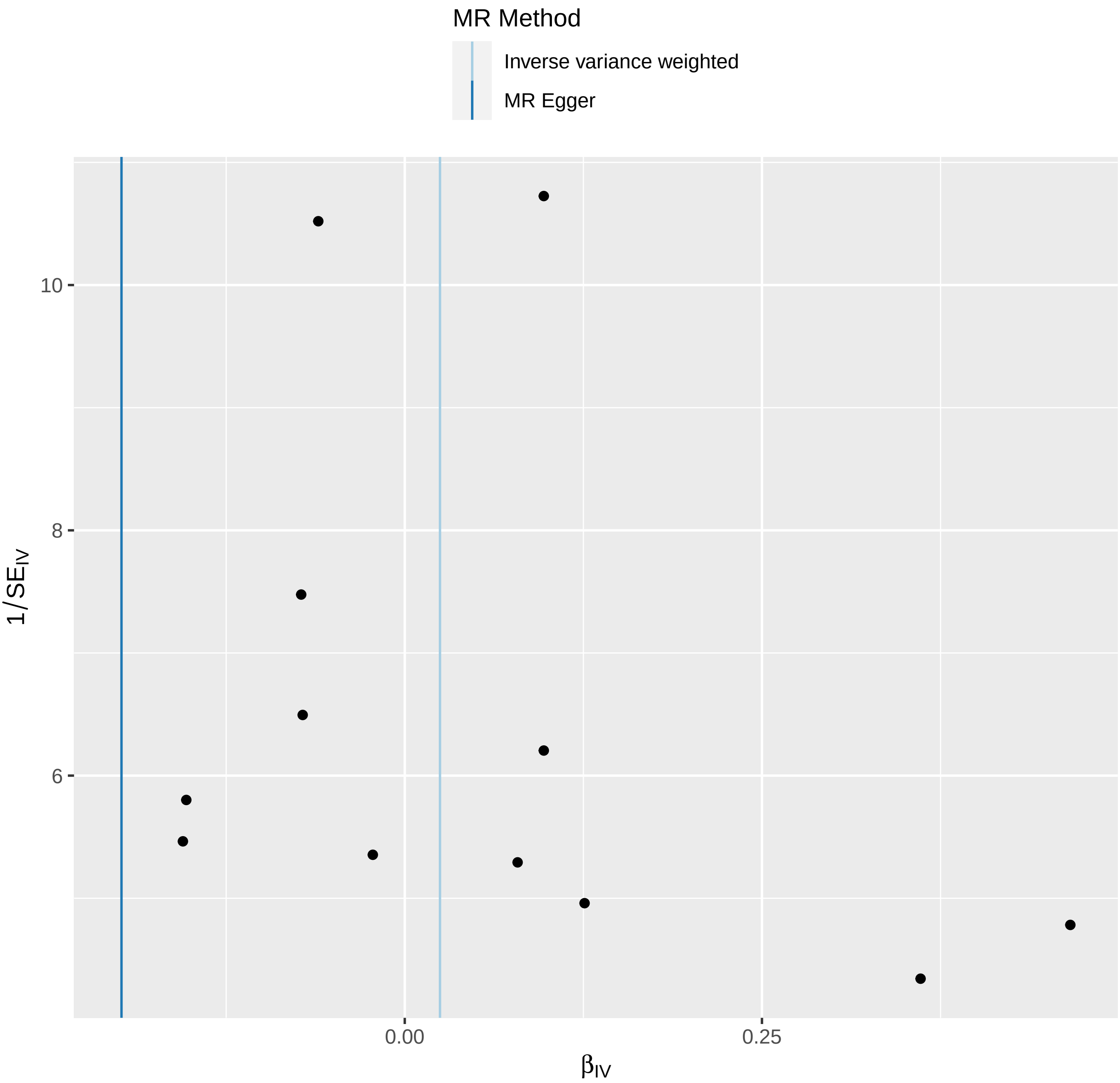

Batch 3−1386 : Selenium on Endoplasmin

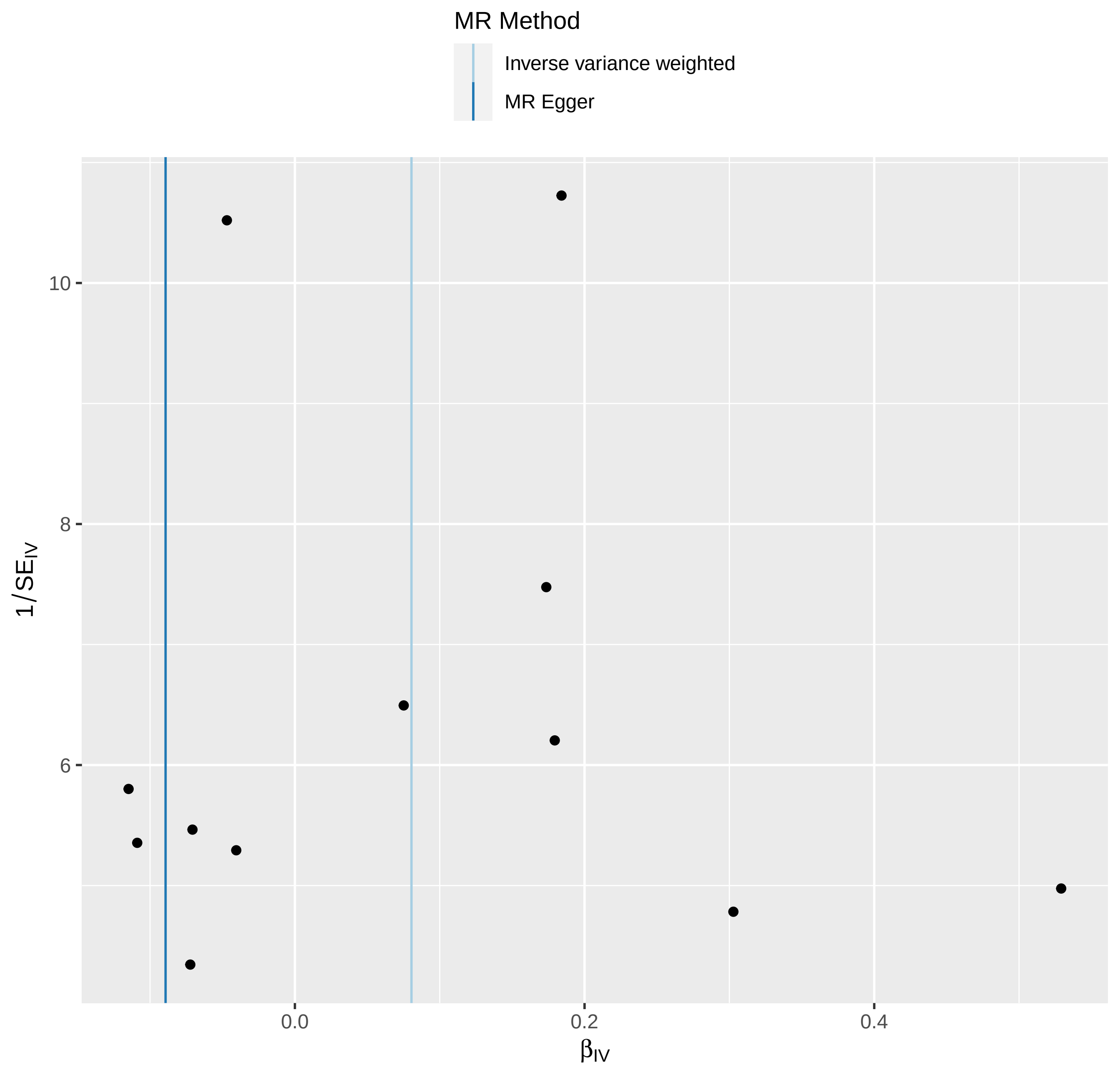

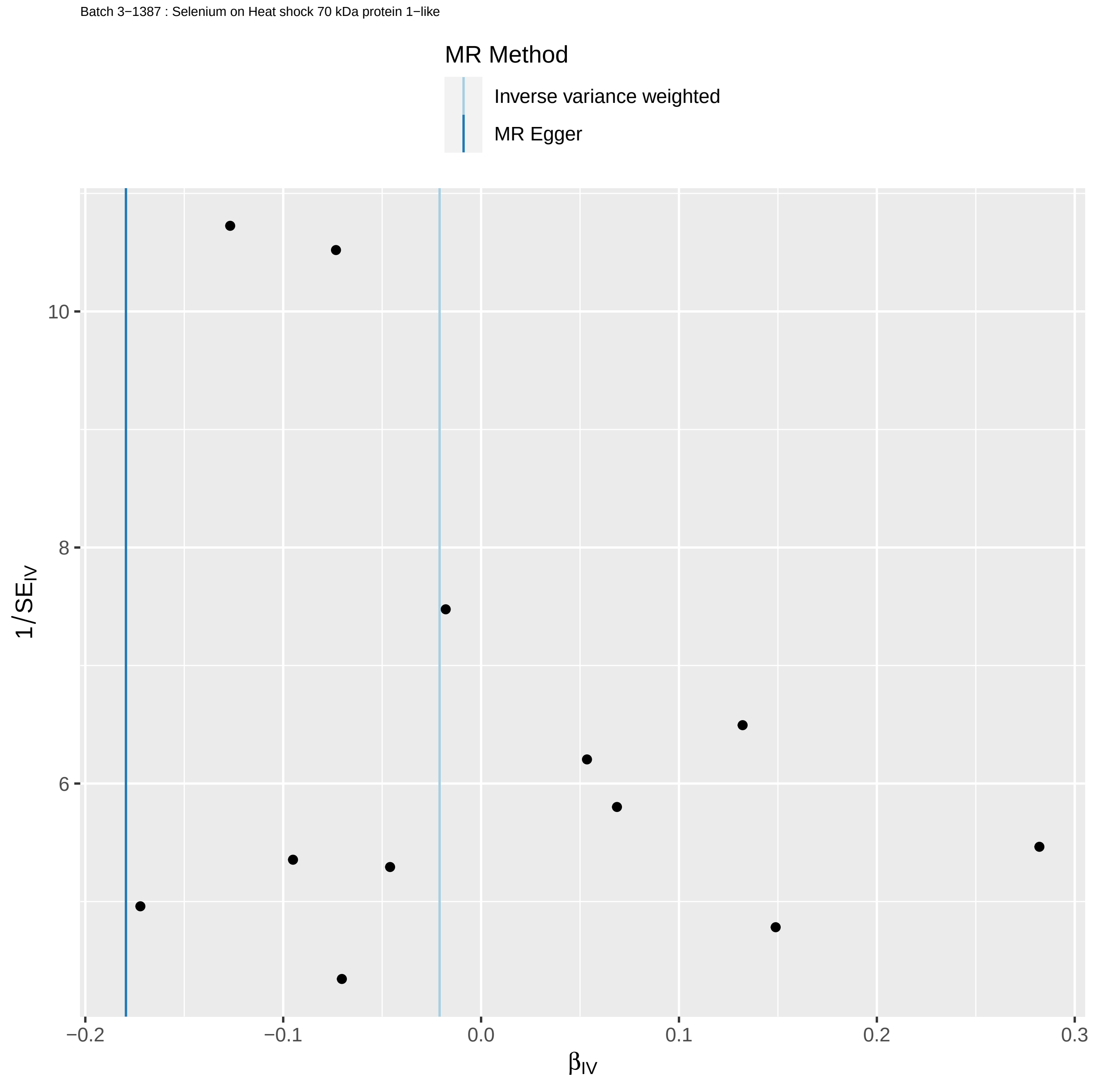
Batch 3−1387 : Selenium on Heat shock 70 kDa protein 1−like
MR Method
Inverse variance weighted
MR Egger
10
8
6
1/SEIV
−0.2
−0.1
0.0
0.1
0.2
0.3
βIV

Batch 3−1388 : Selenium on Heat shock 70 kDa protein 6

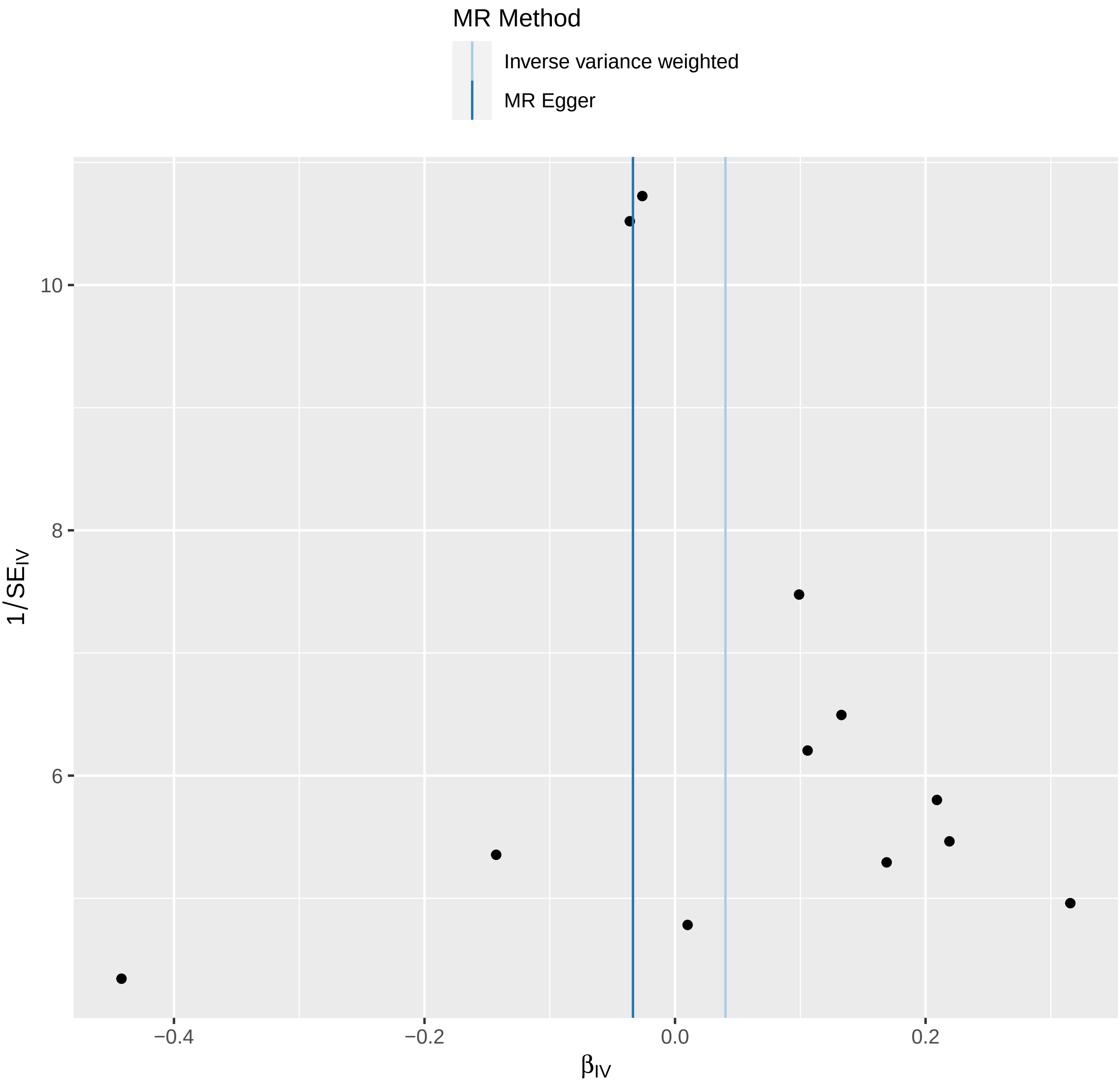

Batch 3−1389 : Selenium on Oxidoreductase HTATIP2

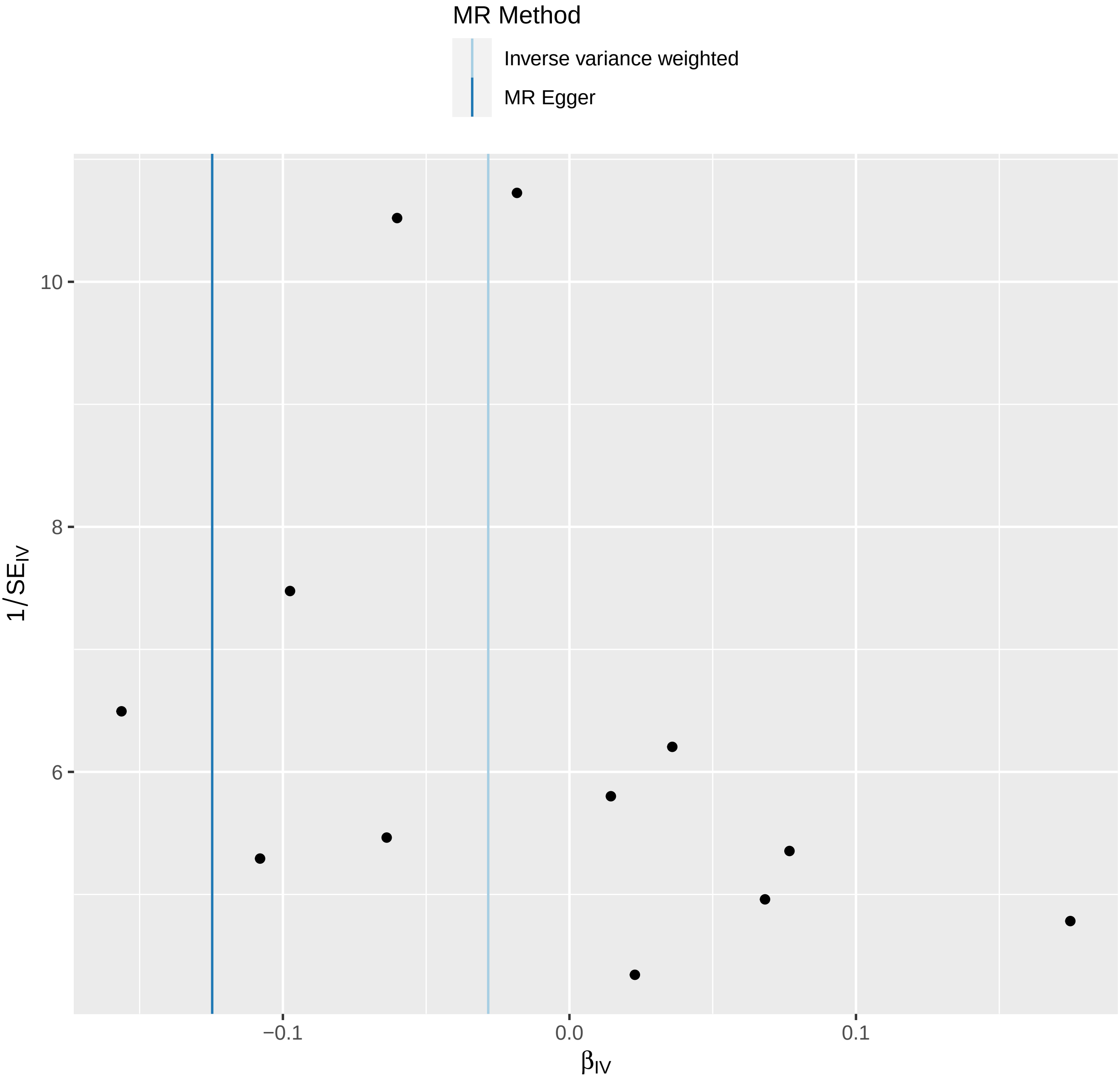

Batch 3−1390 : Selenium on Oxidoreductase HTATIP2

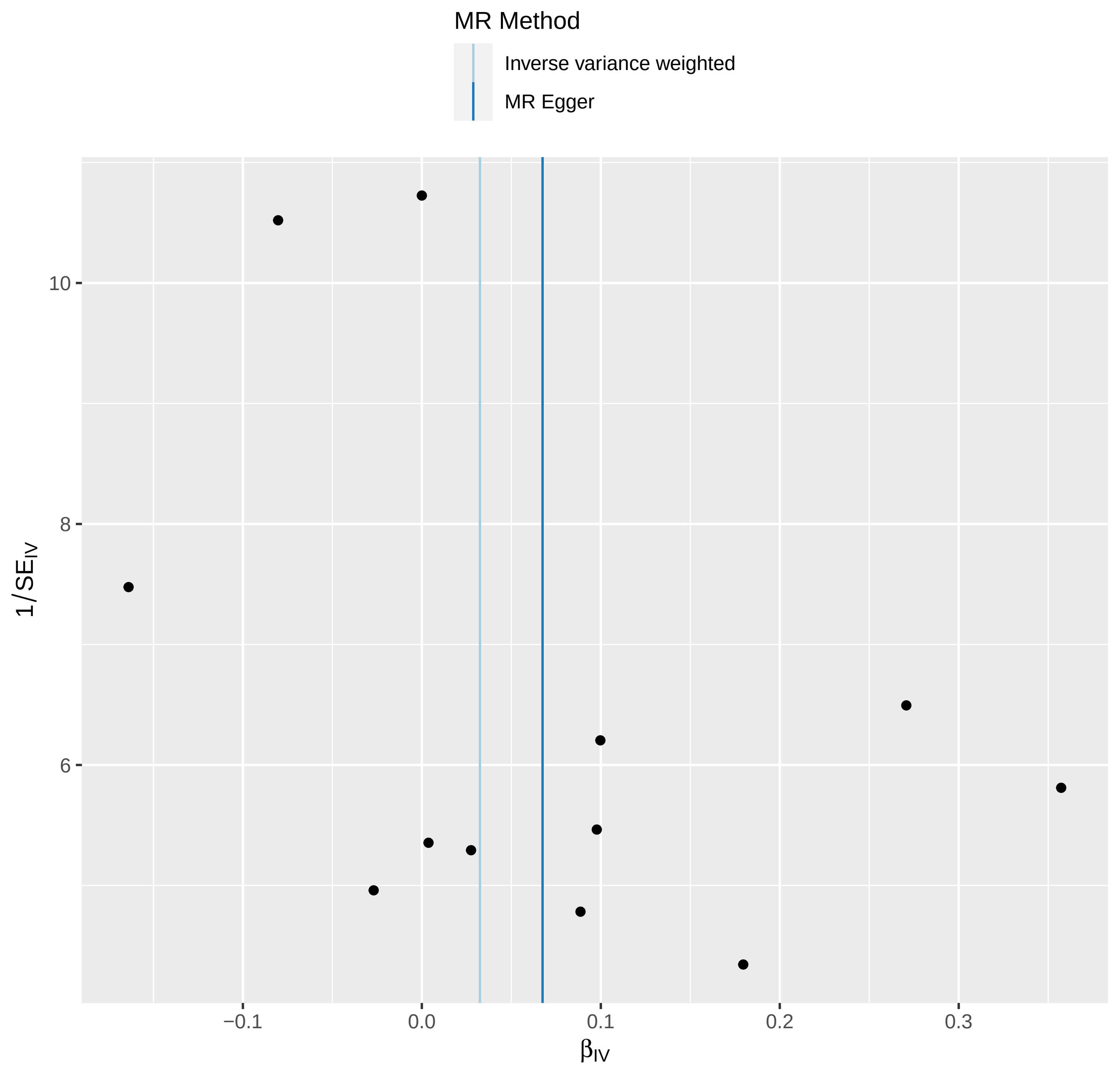

Batch 3−1391 : Selenium on Histatin−1

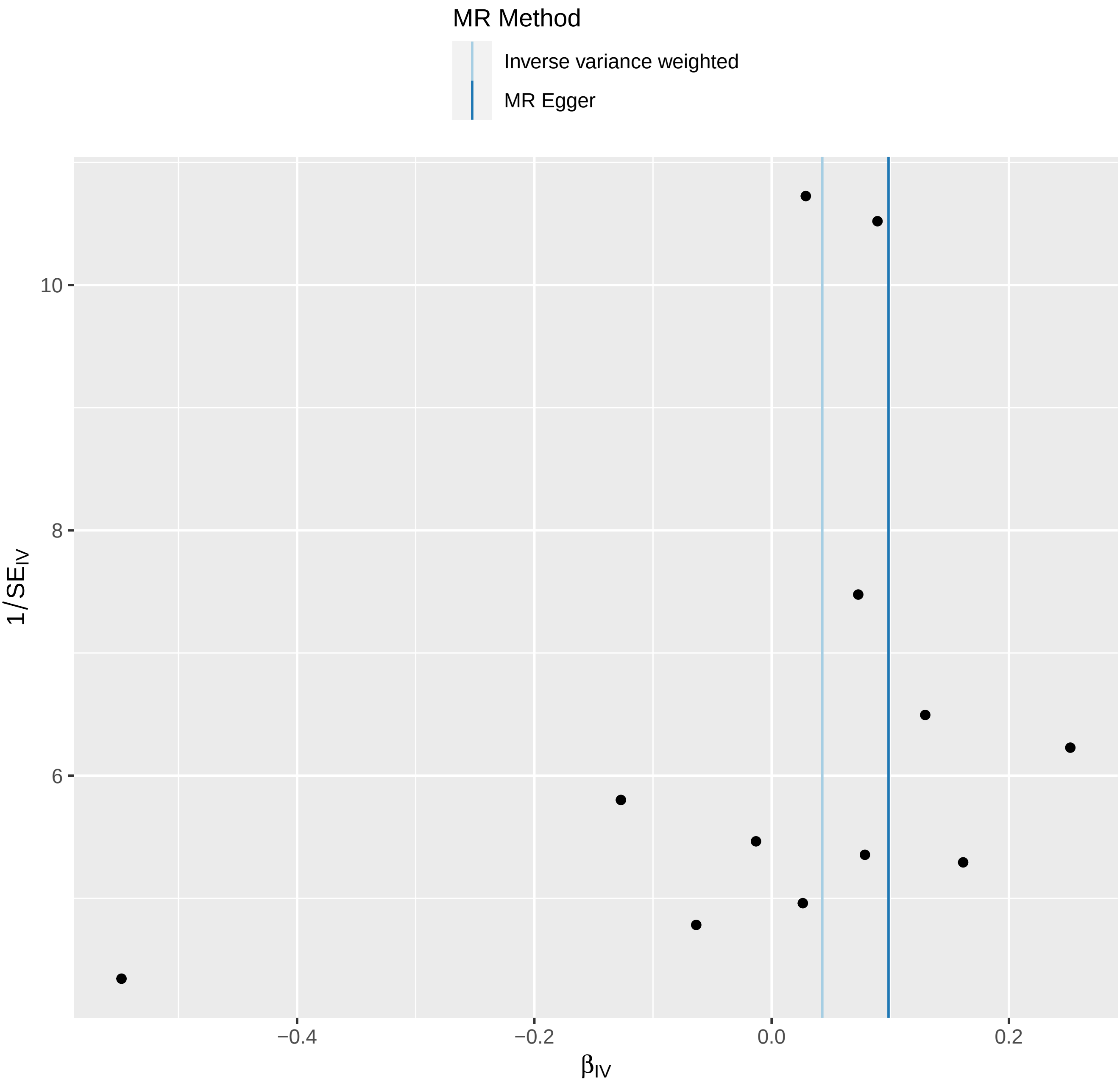

Batch 3−1392 : Selenium on Serine protease HTRA2, mitochondrial

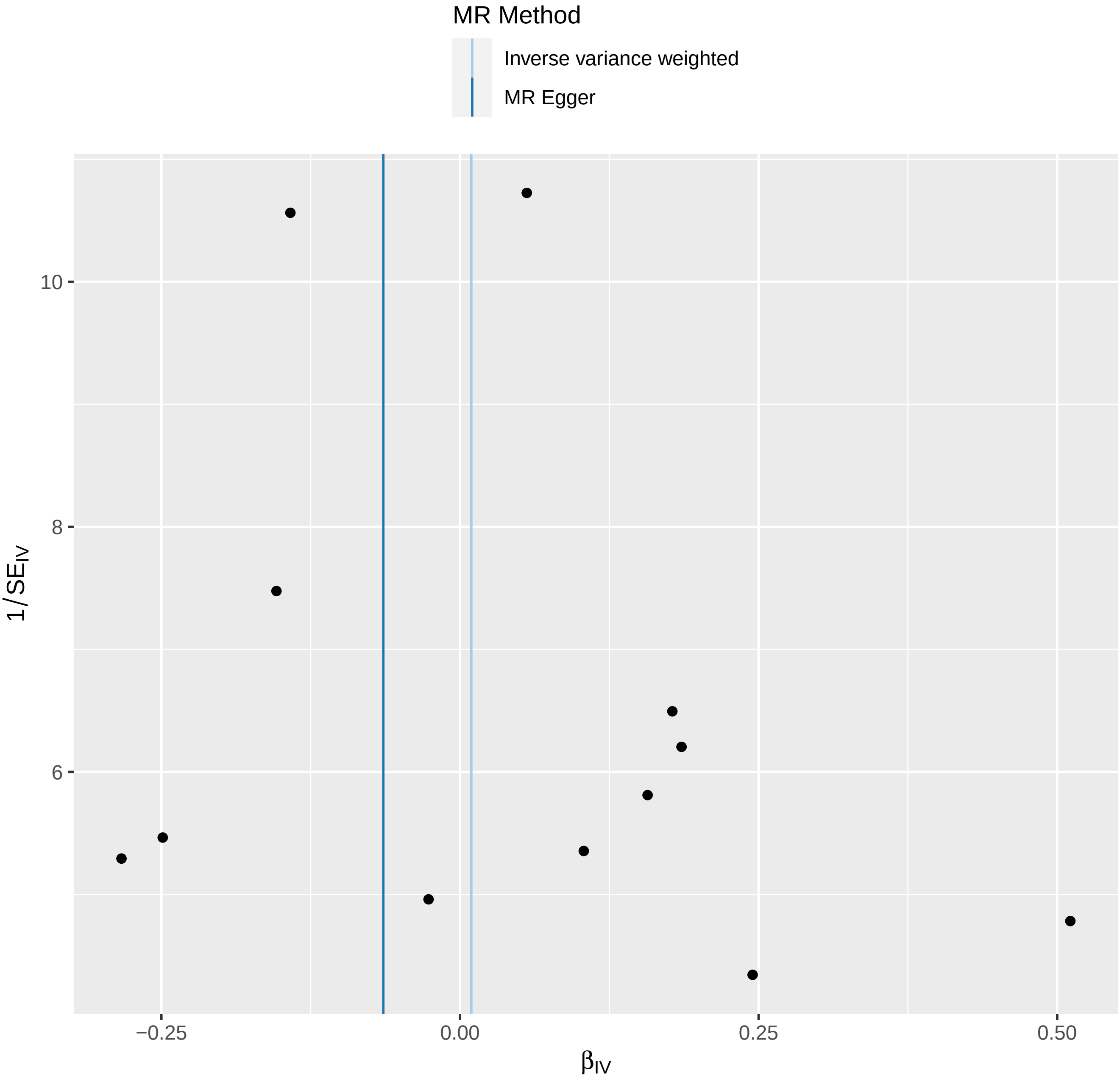

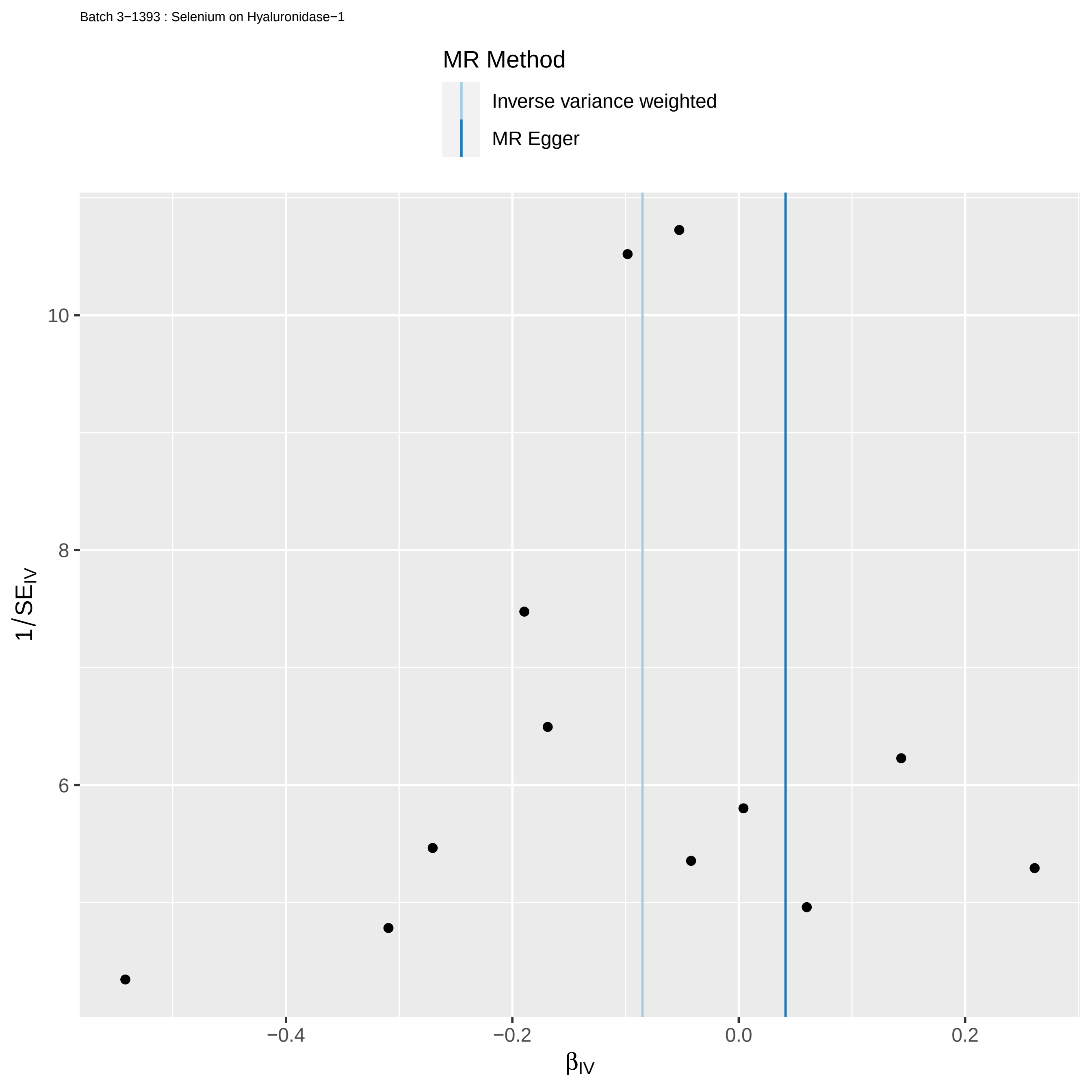
Batch 3−1393 : Selenium on Hyaluronidase−1
MR Method
Inverse variance weighted
MR Egger
10
8
6
1/SE_IV
−0.4
−0.2
0.0
0.2
β_IV

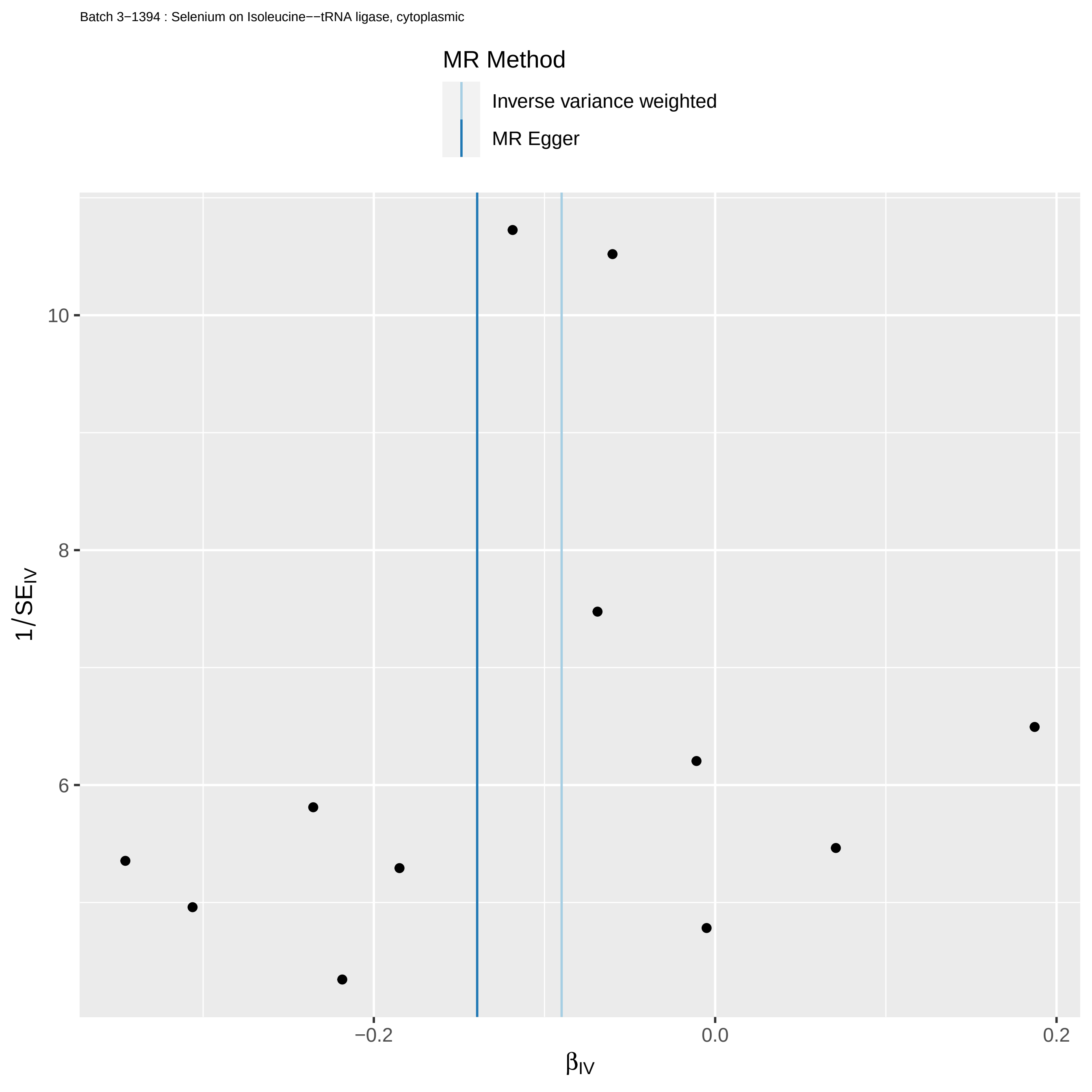

Batch 3−1394 : Selenium on Isoleucine−−tRNA ligase, cytoplasmic
MR Method
Inverse variance weighted
MR Egger
10
8
6
1/SE_IV
−0.2
0.0
0.2
β_IV

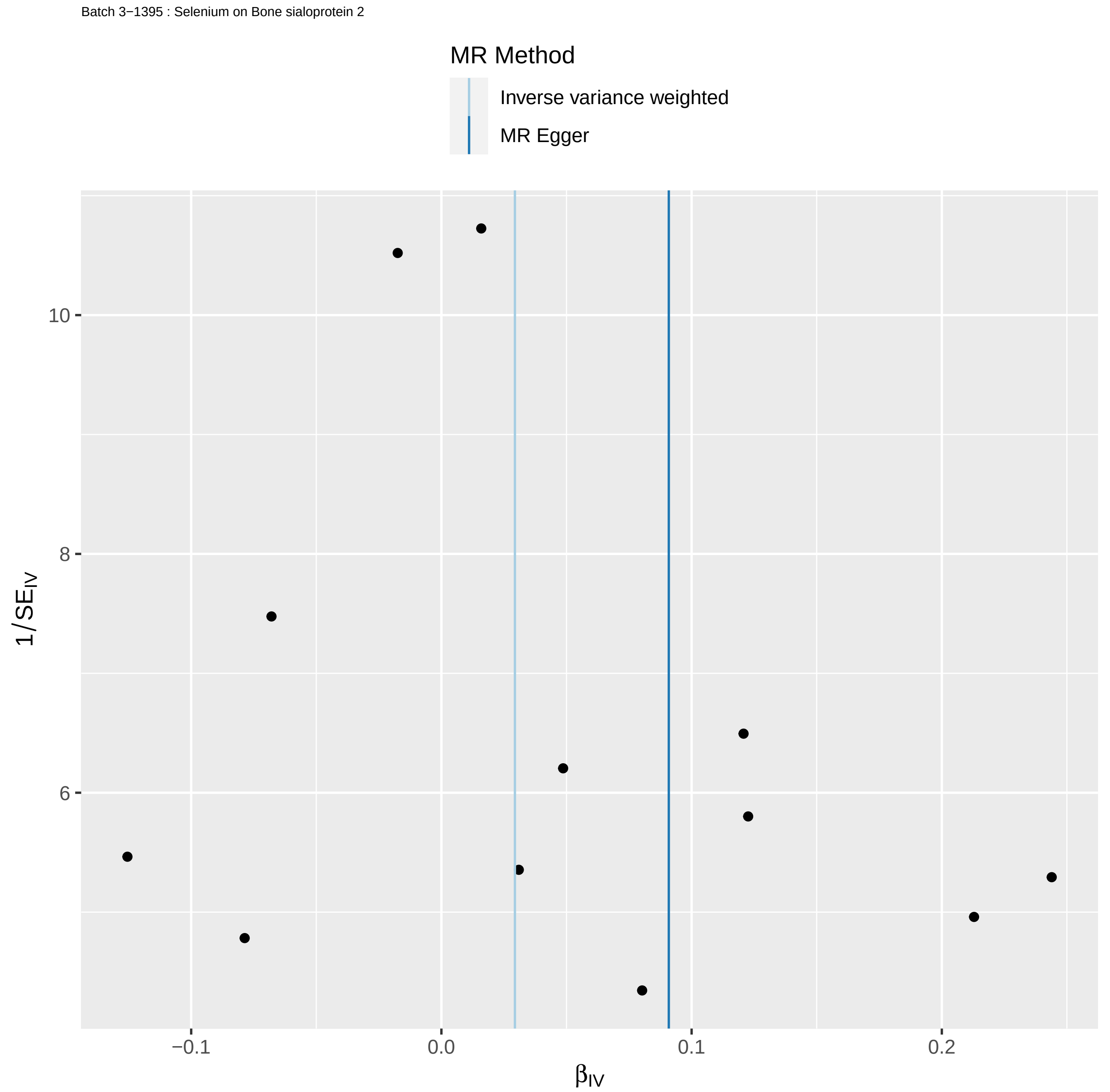

Batch 3−1395 : Selenium on Bone sialoprotein 2
MR Method
Inverse variance weighted
MR Egger
10
8
6
1/SE_IV
−0.1
0.0
0.1
0.2
β_IV

Batch 3−1396 : Selenium on Bone sialoprotein 2

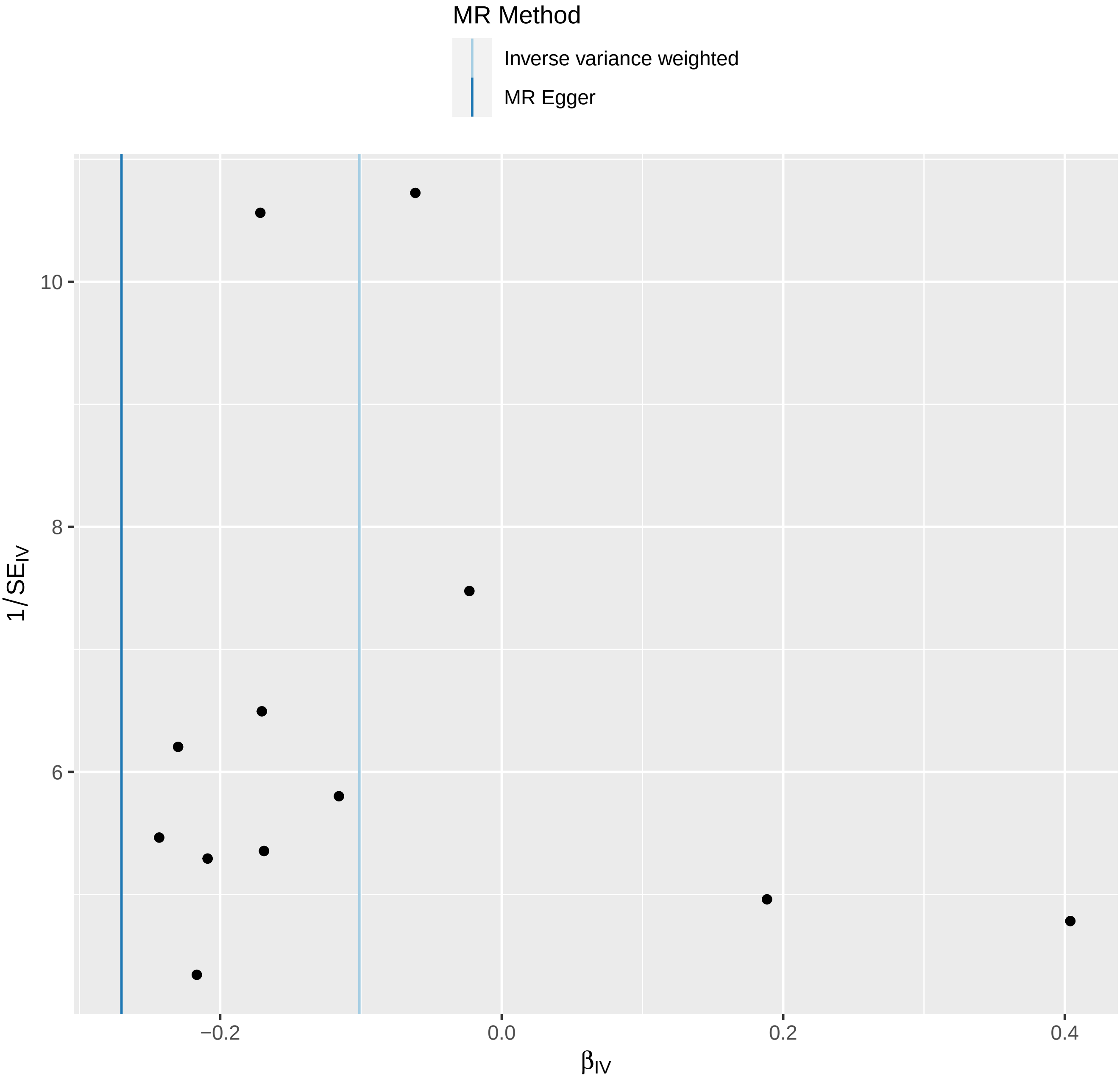

Batch 3−1397 : Selenium on Intercellular adhesion molecule 1

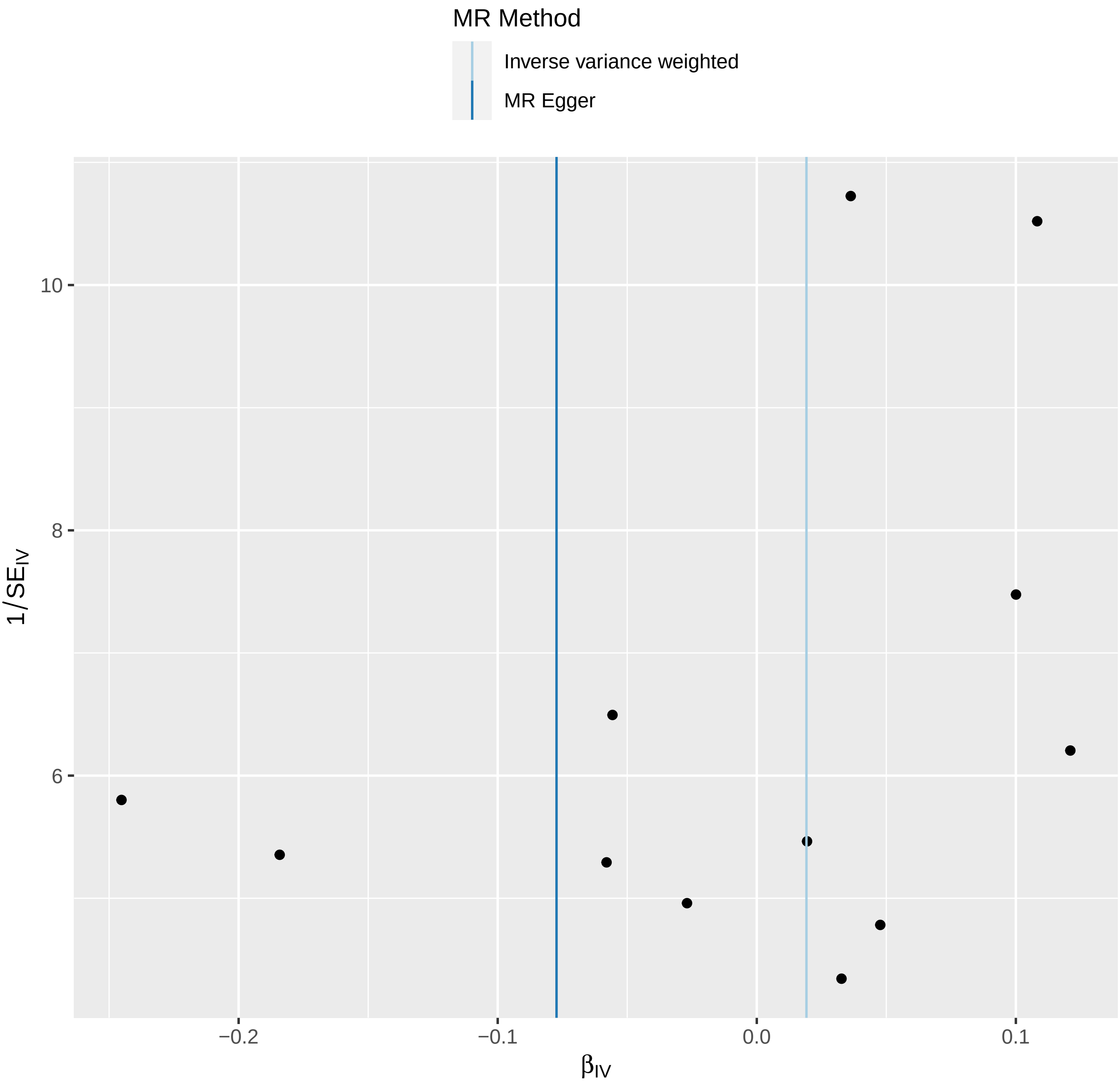

Batch 3−1398 : Selenium on Intercellular adhesion molecule 2

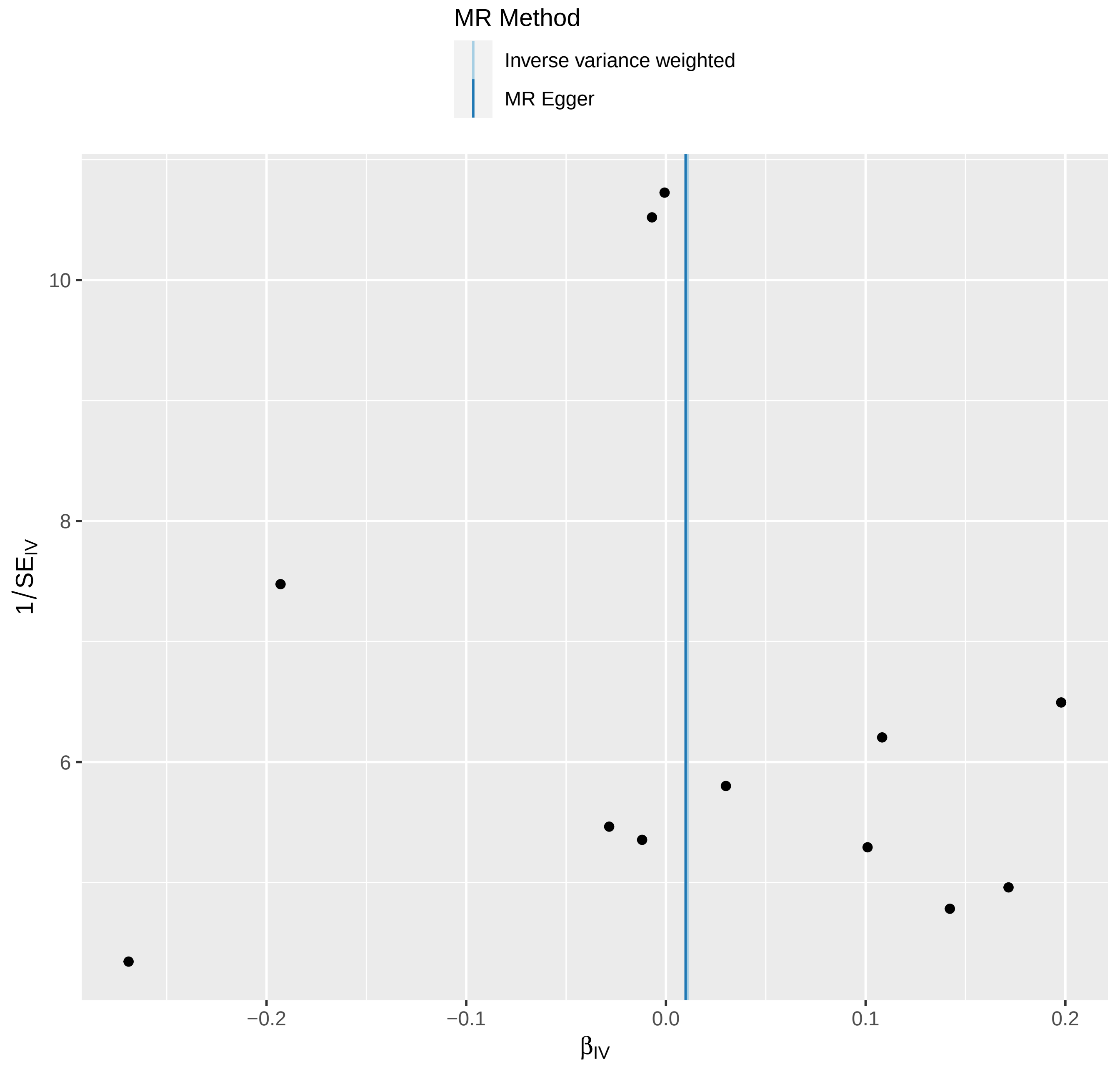

Batch 3−1399 : Selenium on Intercellular adhesion molecule 3

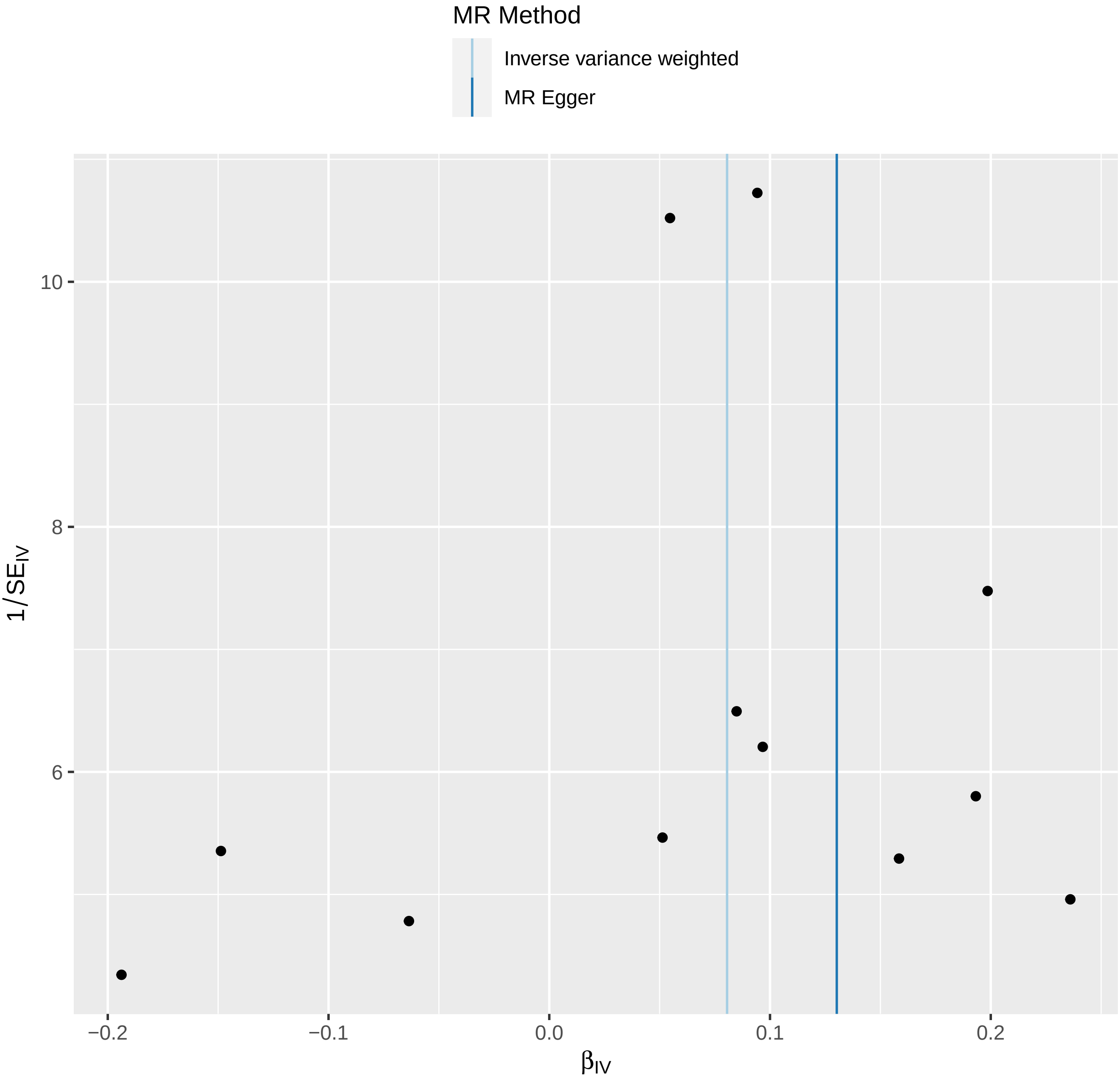

Batch 3−1400 : Selenium on Intercellular adhesion molecule 4

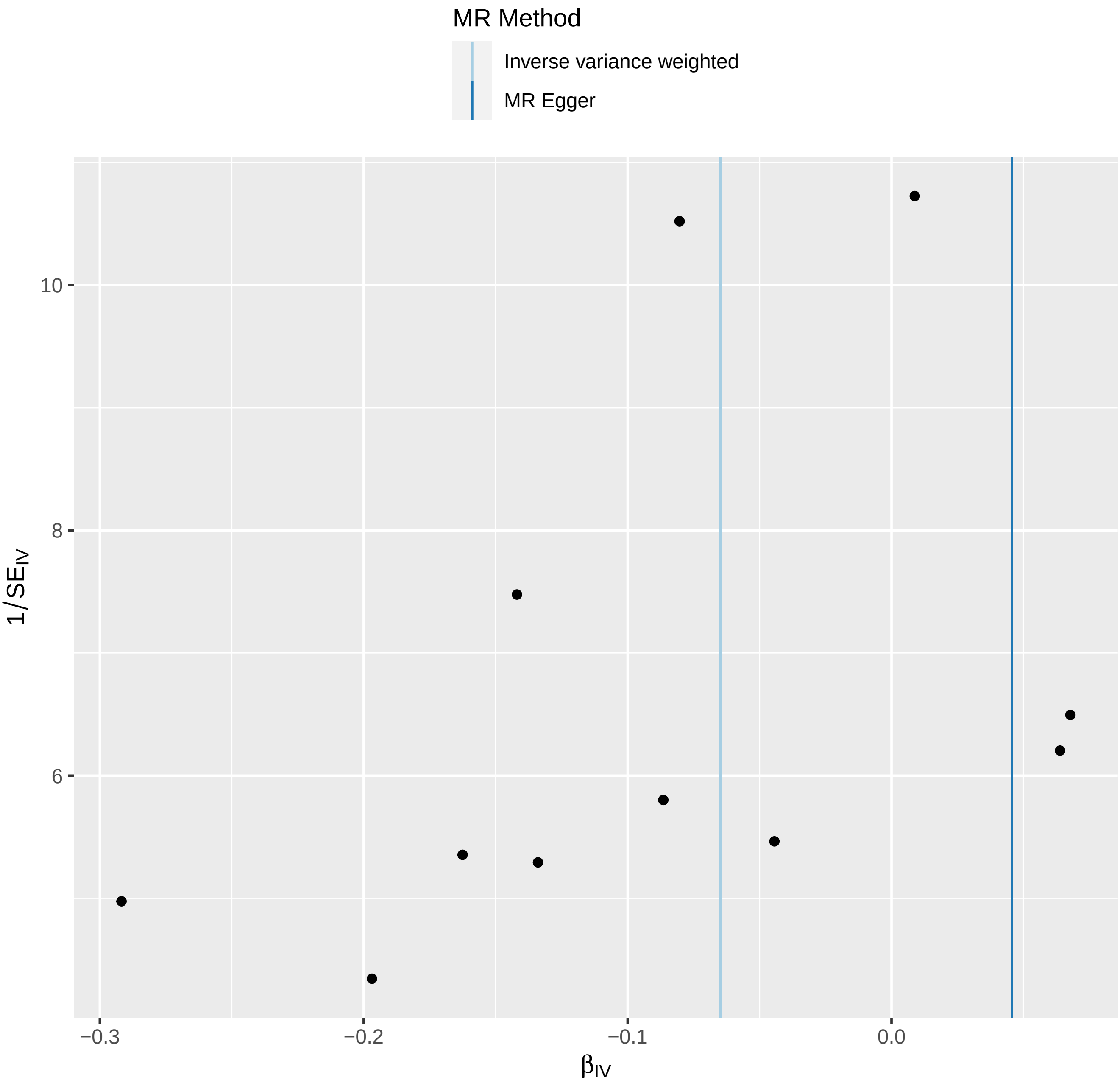

Batch 3−1401 : Selenium on Intercellular adhesion molecule 5

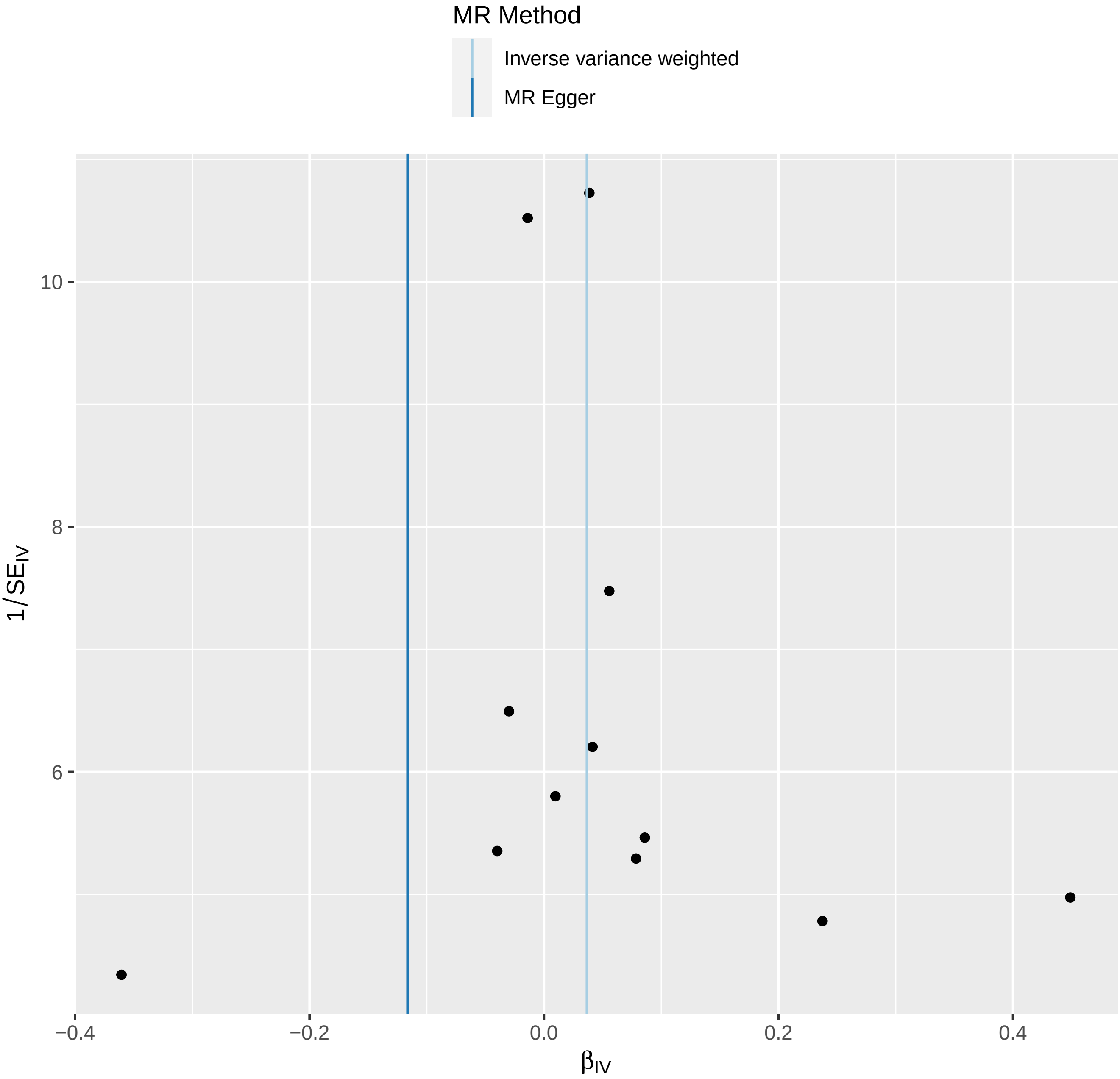

Batch 3−1402 : Selenium on Intercellular adhesion molecule 5

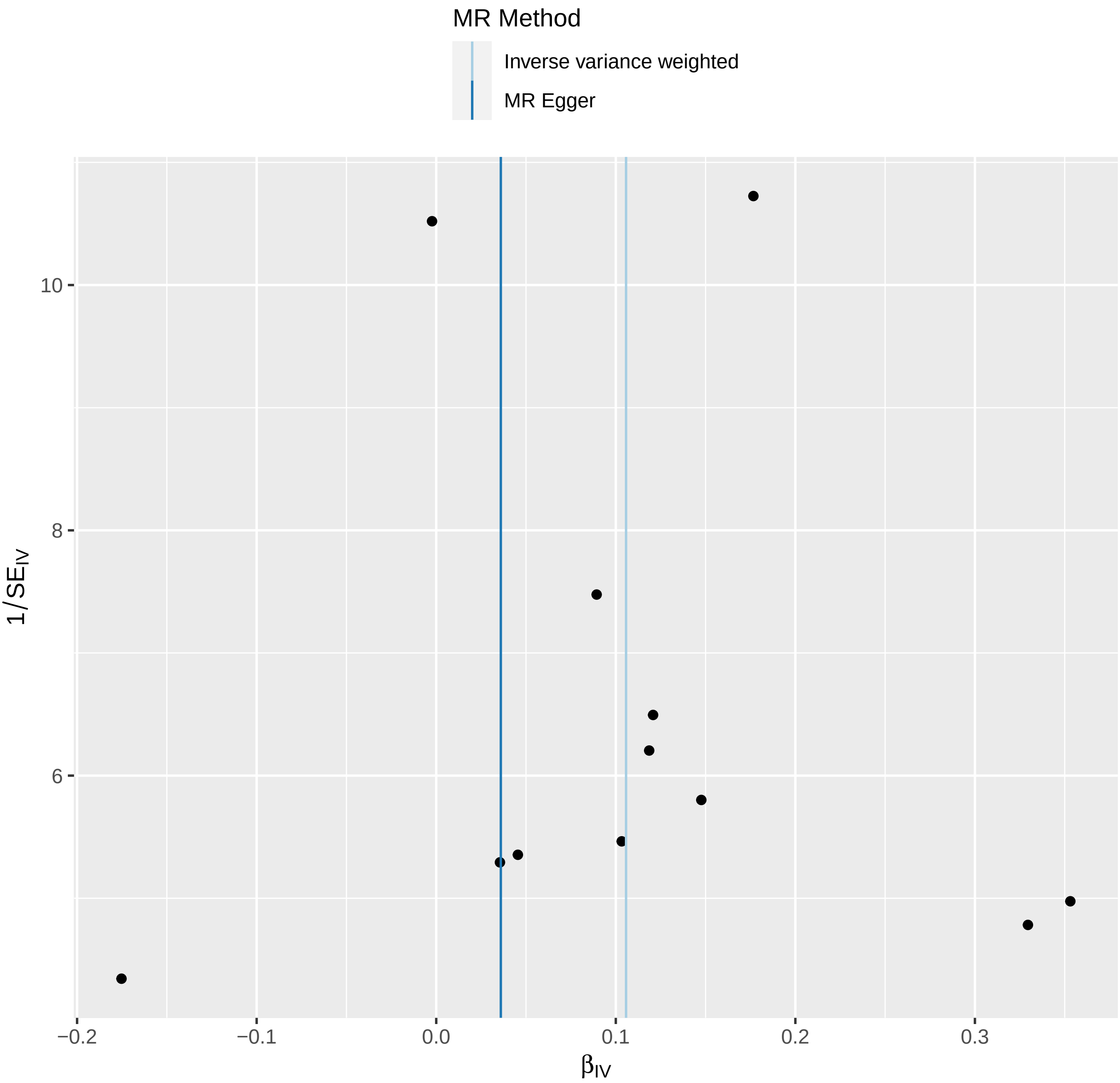

Batch 3−1403 : Selenium on Inducible T−cell costimulator

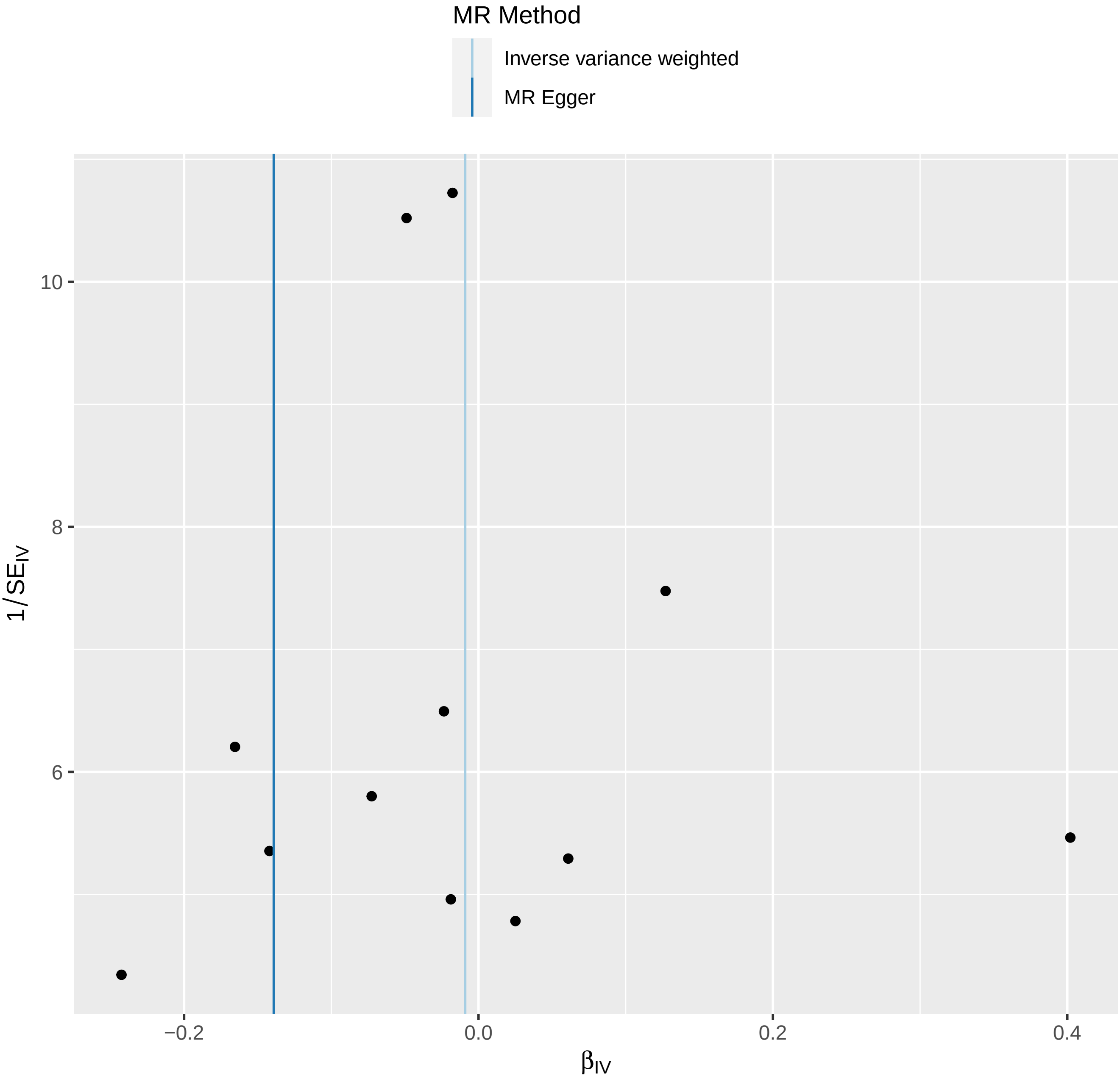

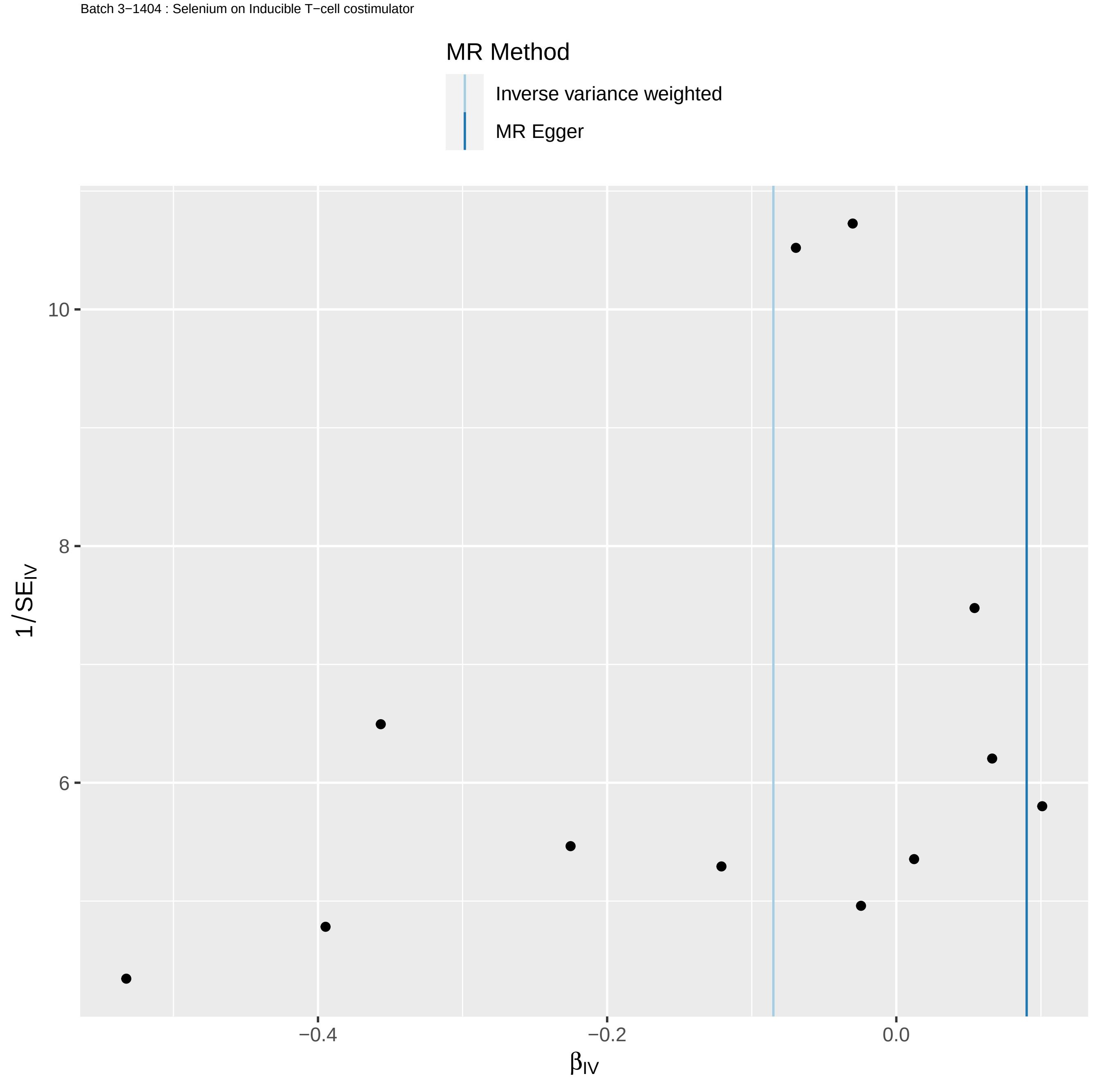

Batch 3−1404 : Selenium on Inducible T−cell costimulator
MR Method
Inverse variance weighted
MR Egger
10
8
6
1/SE$_{IV}$
−0.4
−0.2
0.0
β$_{IV}$

Batch 3−1405 : Selenium on ICOS ligand

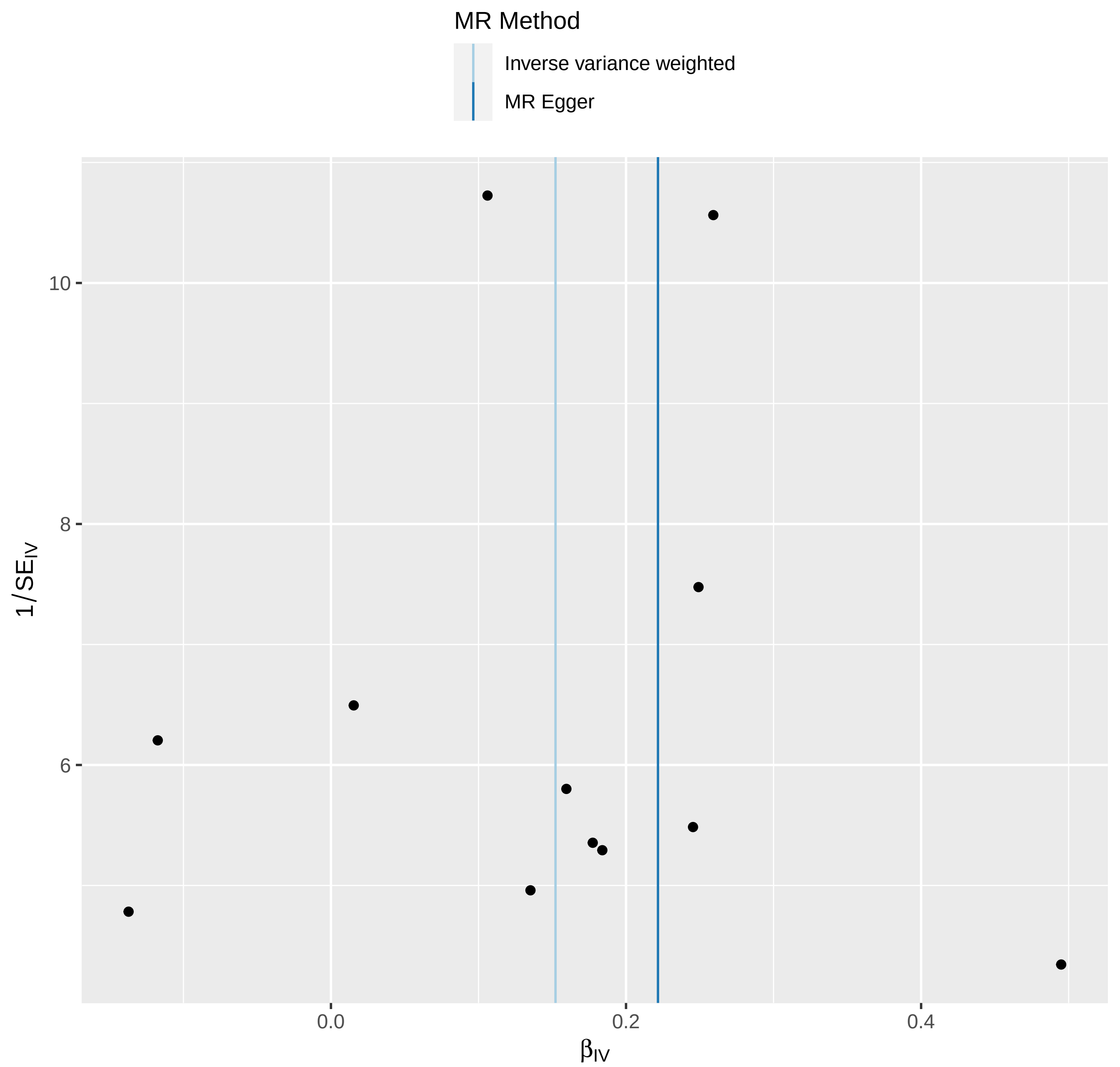

Batch 3−1406 : Selenium on ICOS ligand

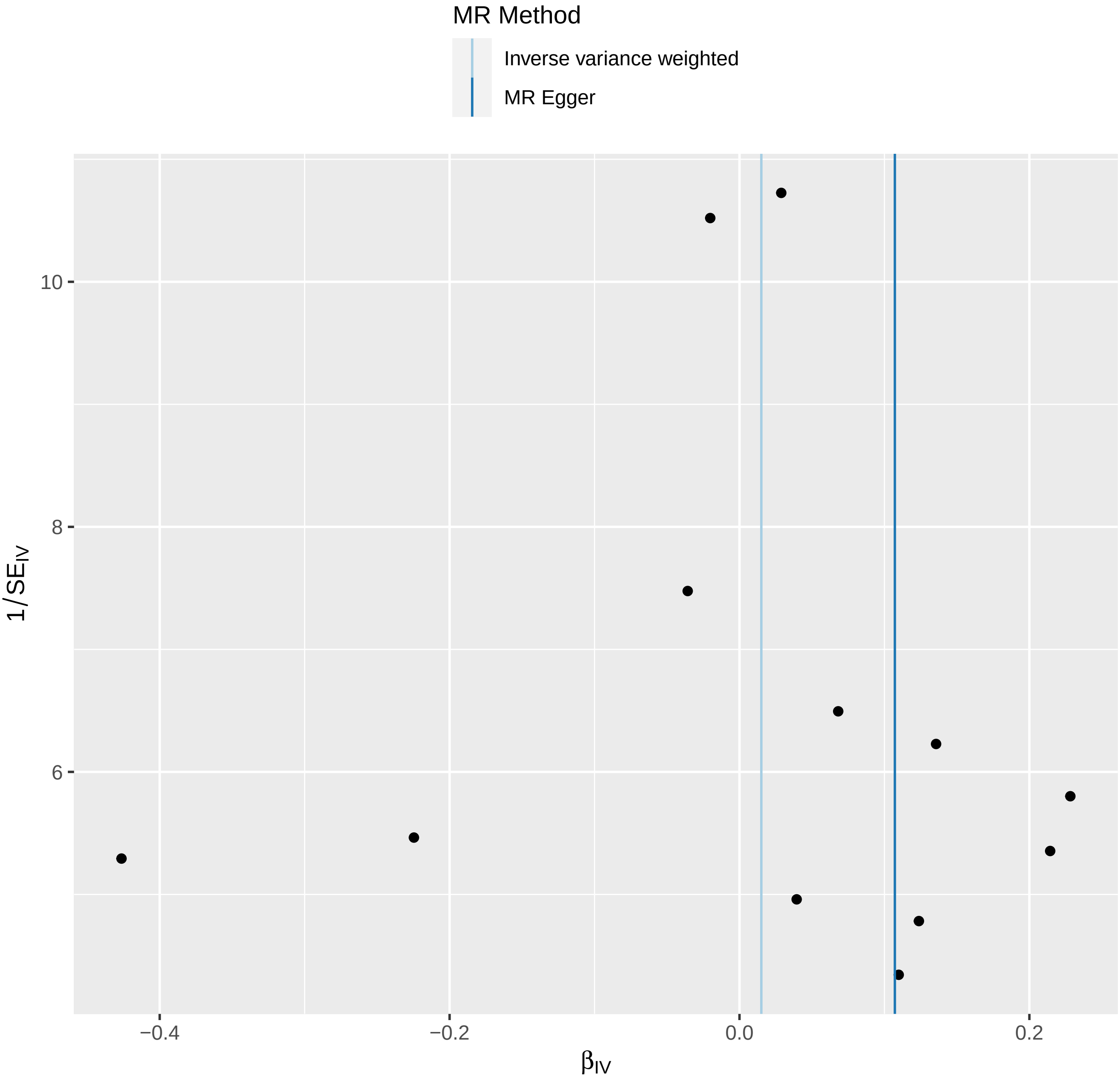

Batch 3−1407 : Selenium on DNA−binding protein inhibitor ID−1

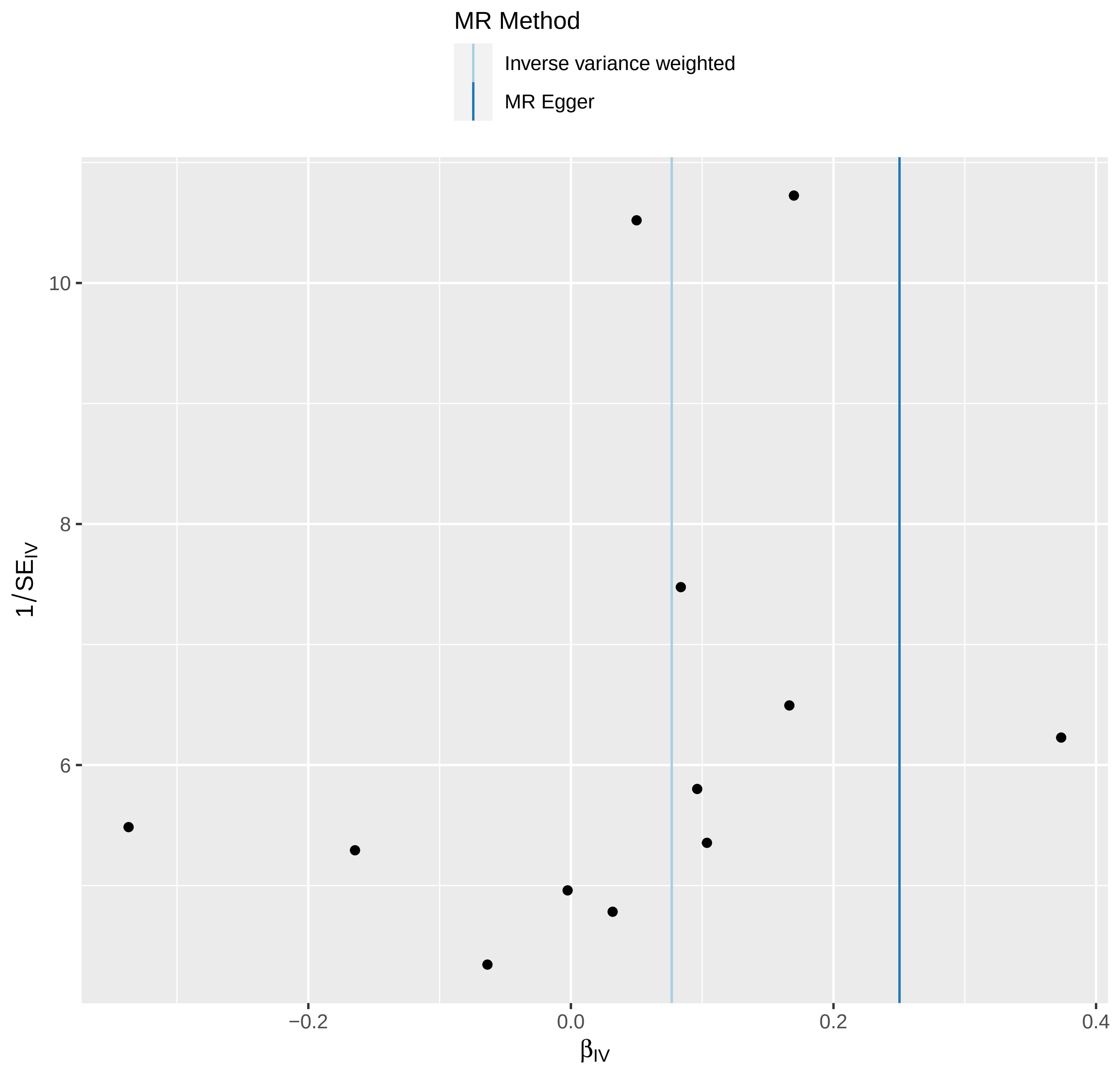

Batch 3−1408 : Selenium on DNA−binding protein inhibitor ID−2

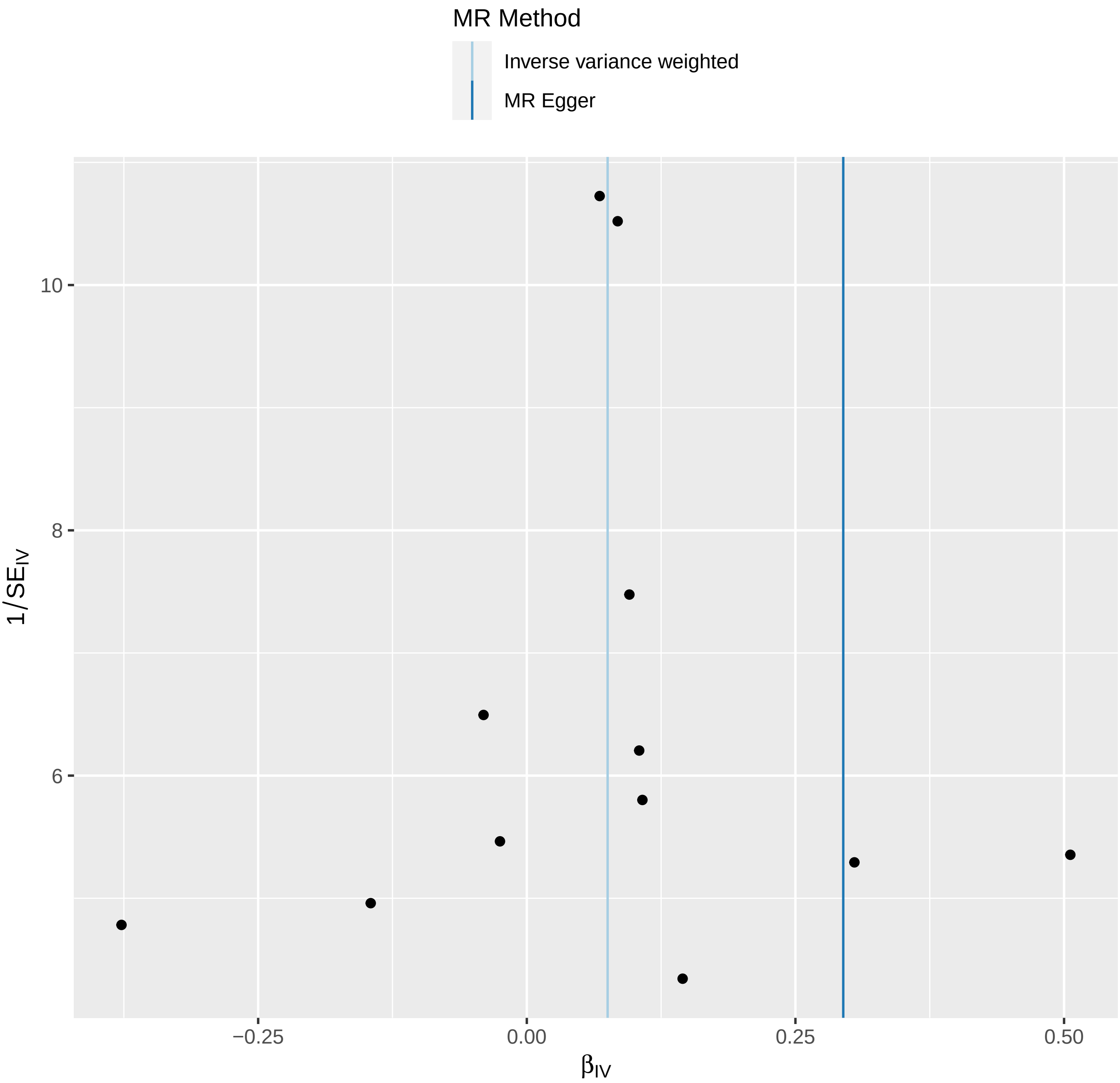

Batch 3−1409 : Selenium on Insulin−degrading enzyme

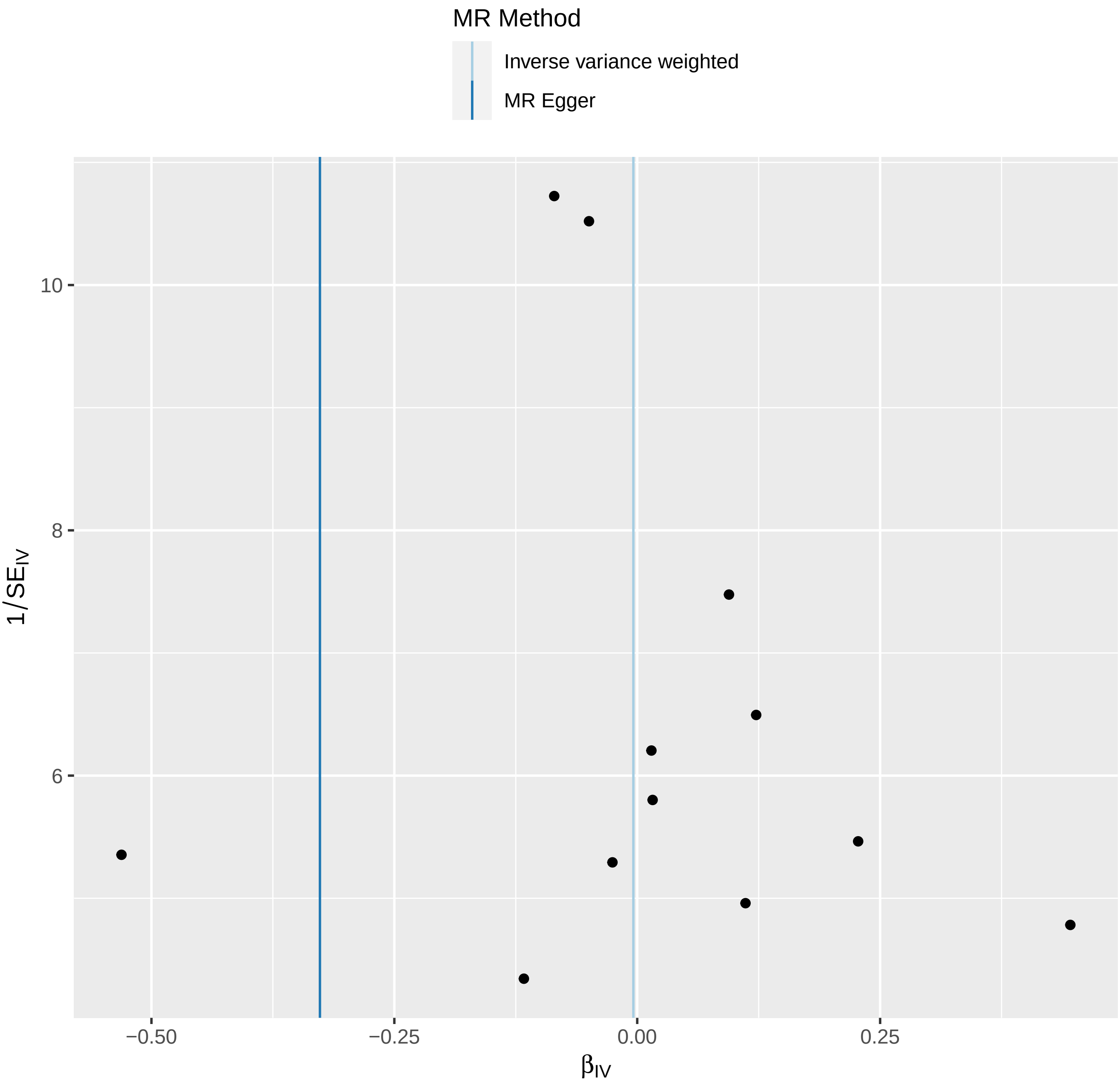

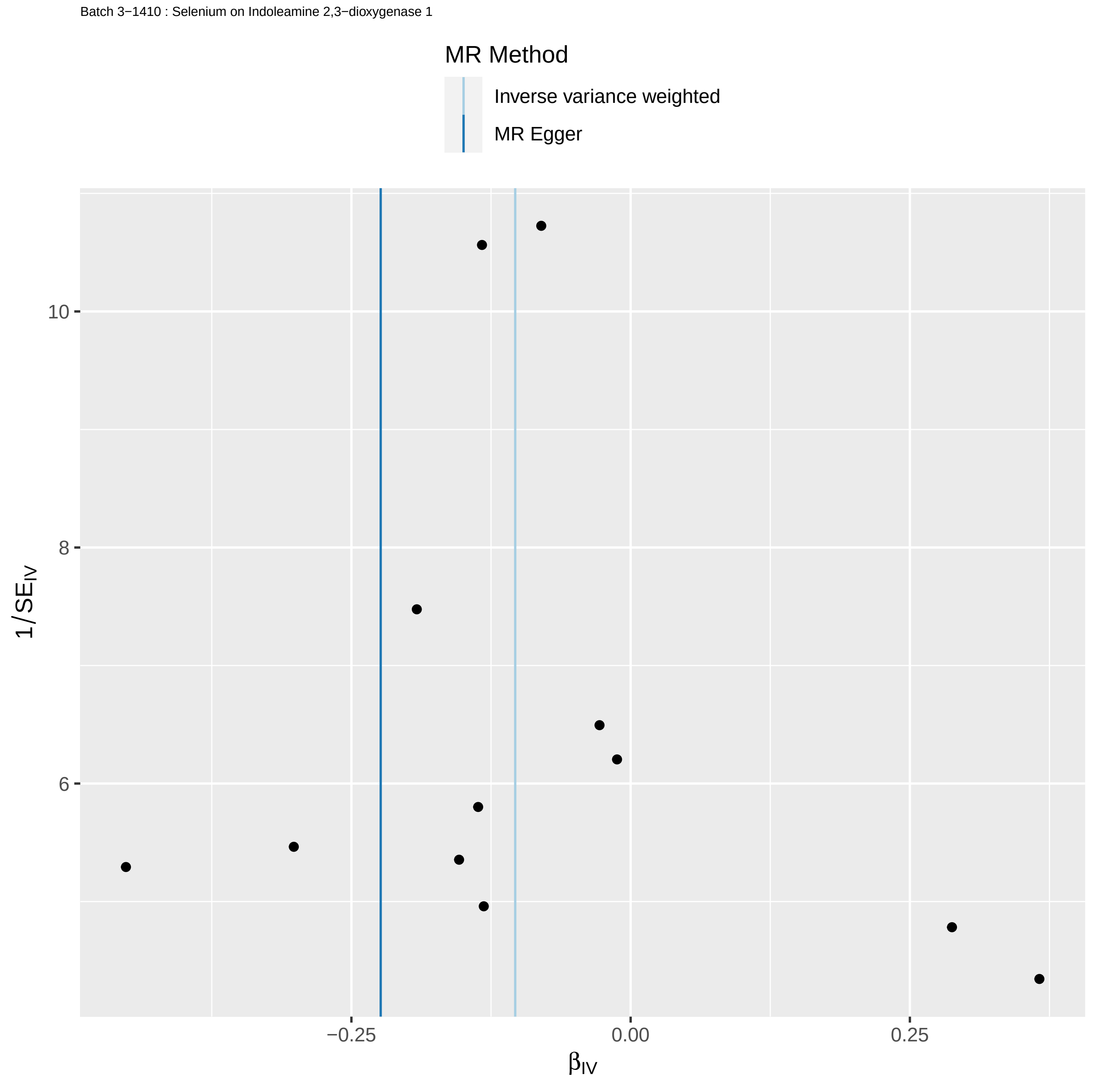

Batch 3−1410 : Selenium on Indoleamine 2,3−dioxygenase 1
MR Method
Inverse variance weighted
MR Egger
10
8
6
1/SE_IV
−0.25
0.00
0.25
β_IV

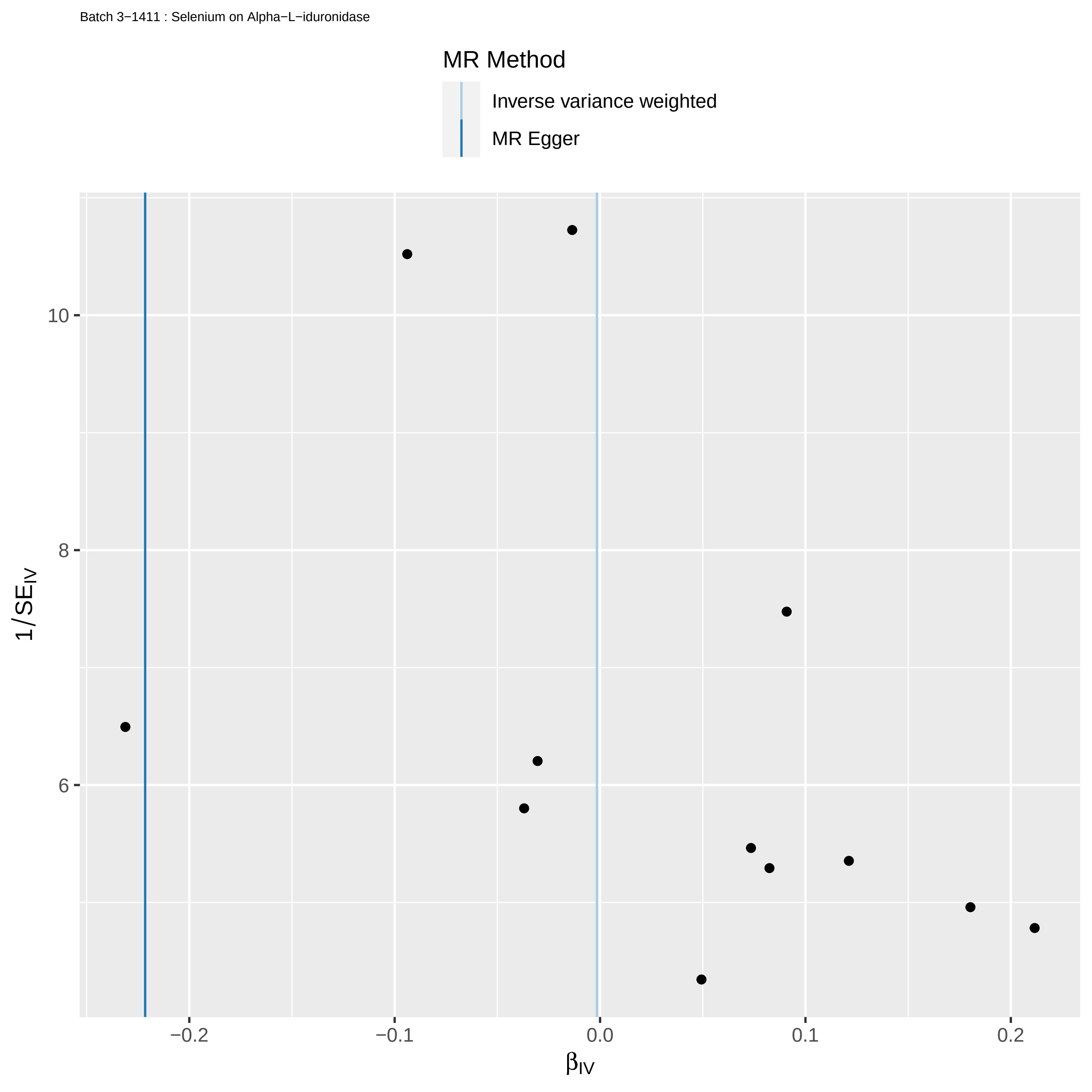

Batch 3−1411 : Selenium on Alpha−L−iduronidase
MR Method
Inverse variance weighted
MR Egger
10
8
6
1/SE_IV
−0.2
−0.1
0.0
0.1
0.2
β_IV

Batch 3−1412 : Selenium on Radiation−inducible immediate−early gene IEX−1

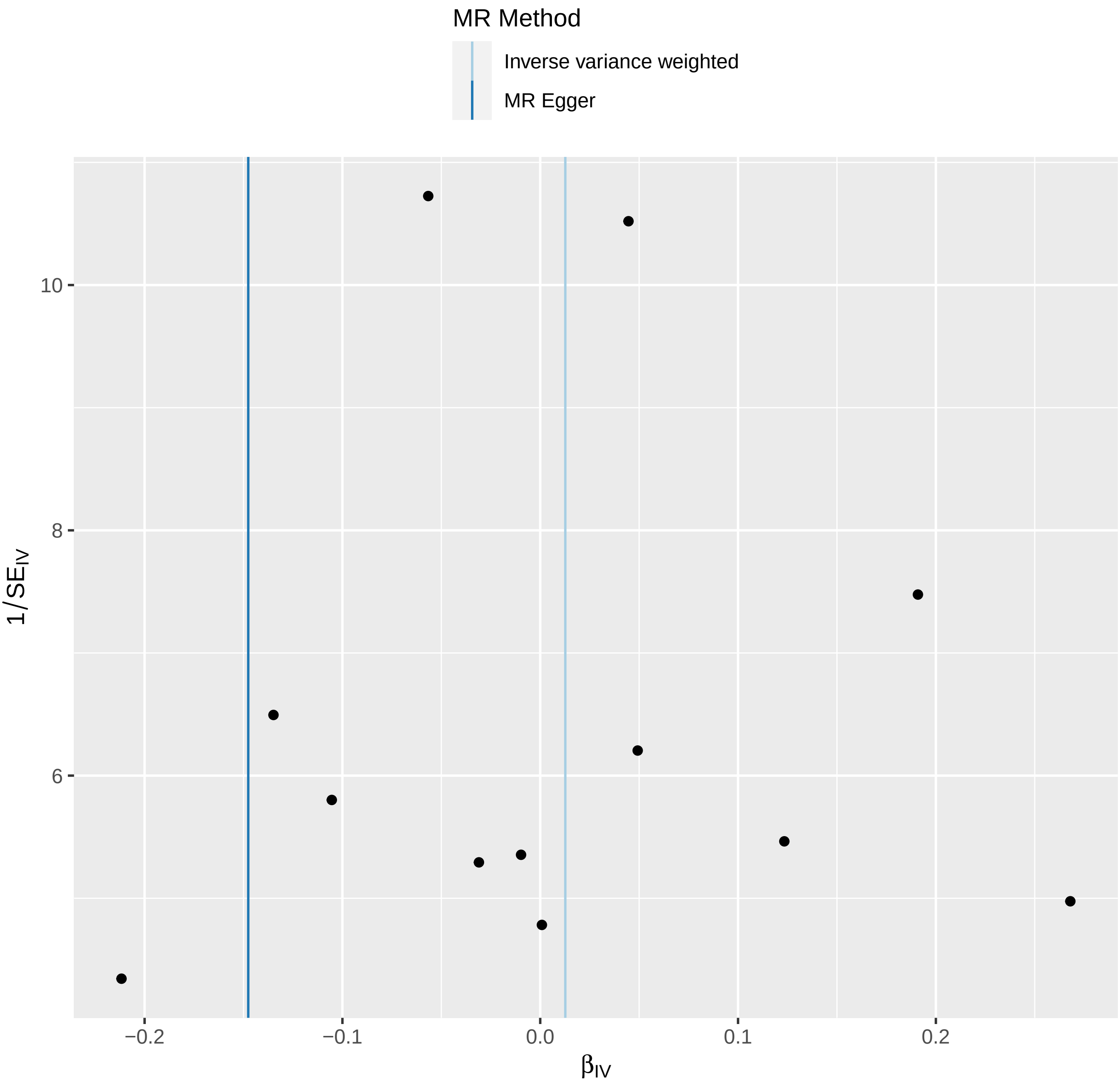

Batch 3−1413 : Selenium on Immediate early response 3−interacting protein 1

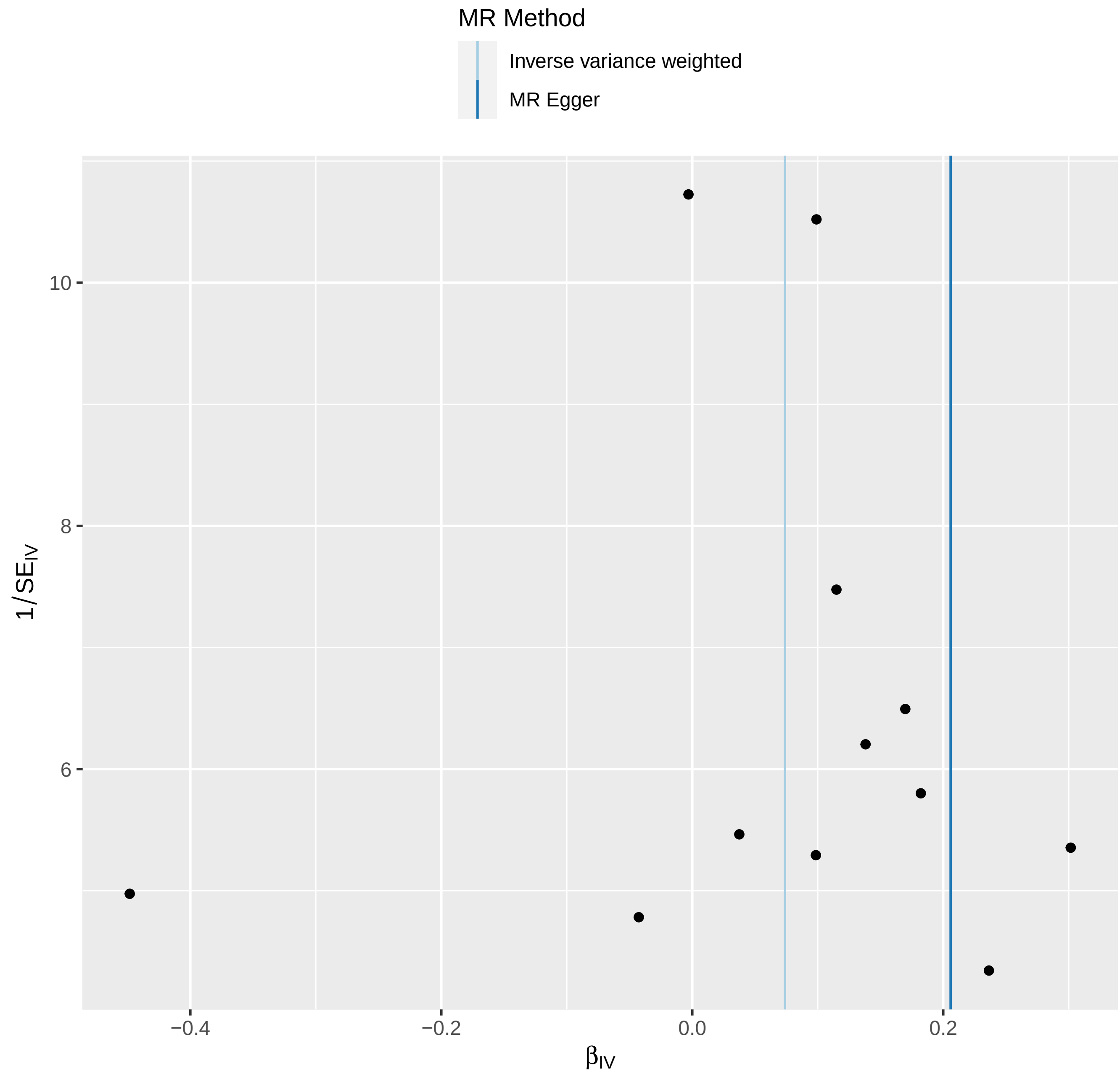

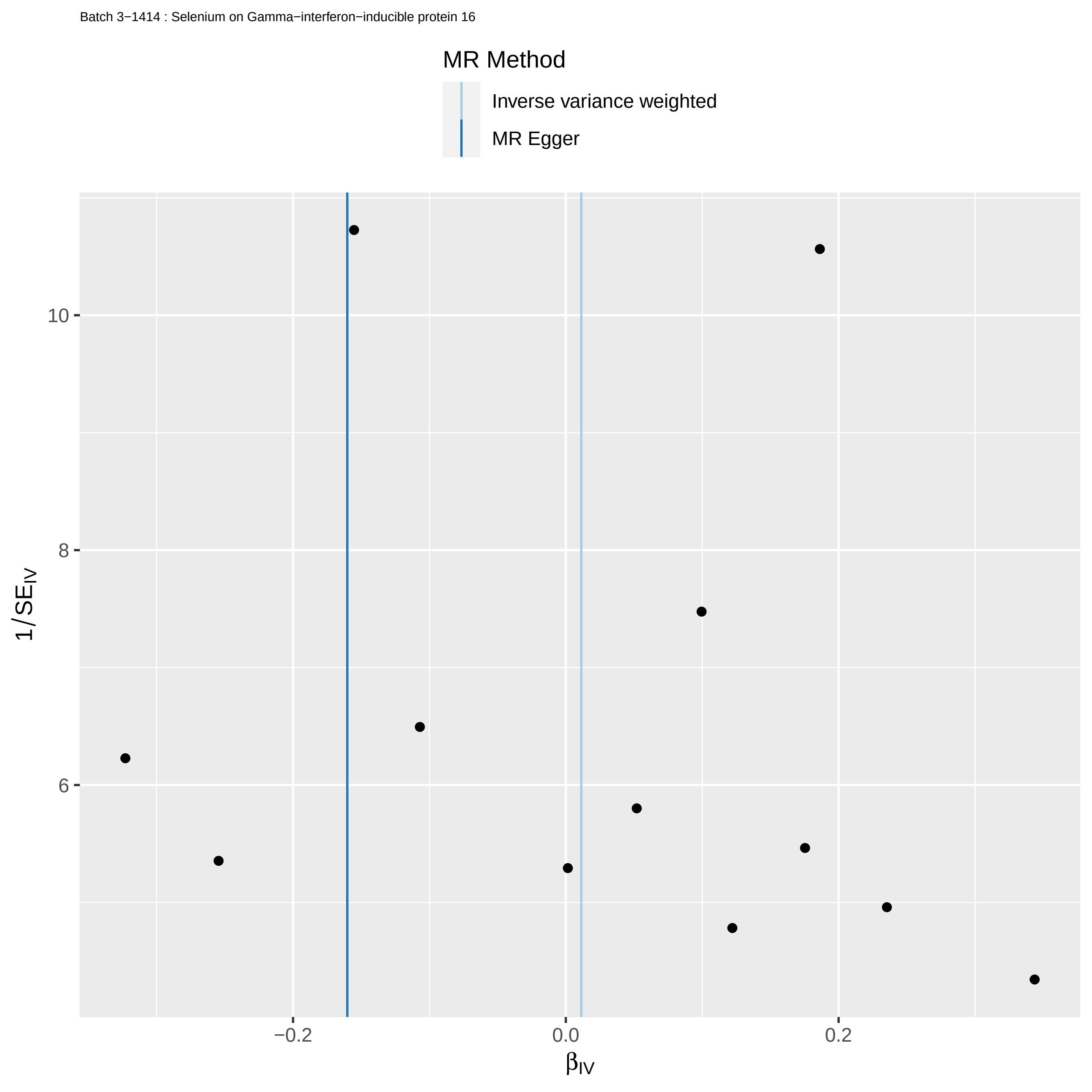
Batch 3−1414 : Selenium on Gamma−interferon−inducible protein 16
MR Method
Inverse variance weighted
MR Egger
10
8
6
1/SE$_{IV}$
−0.2
0.0
0.2
β$_{IV}$

Batch 3−1415 : Selenium on Gamma−interferon−inducible protein 16

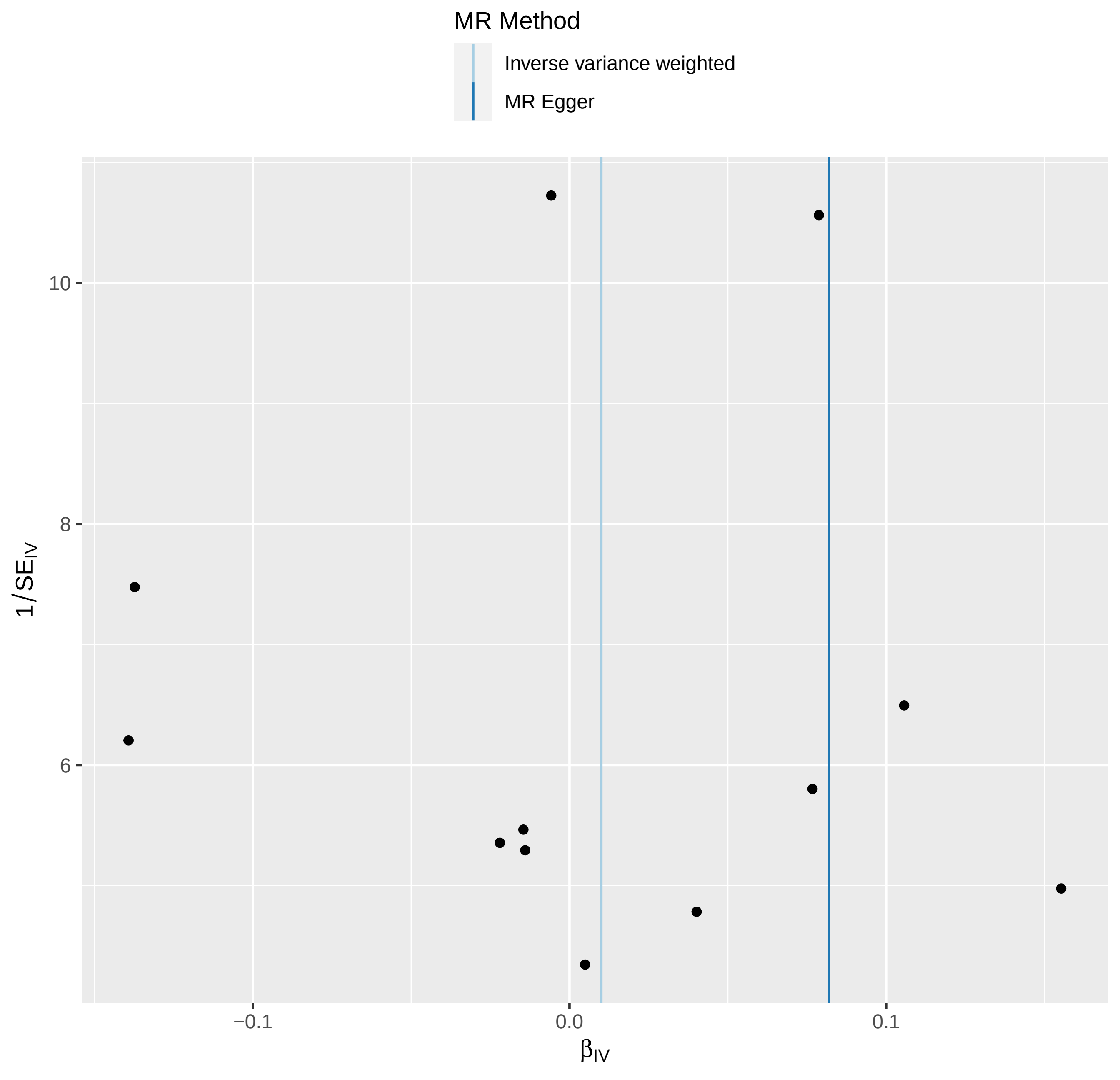

Batch 3−1416 : Selenium on Interferon−induced protein with tetratricopeptide repeats 2

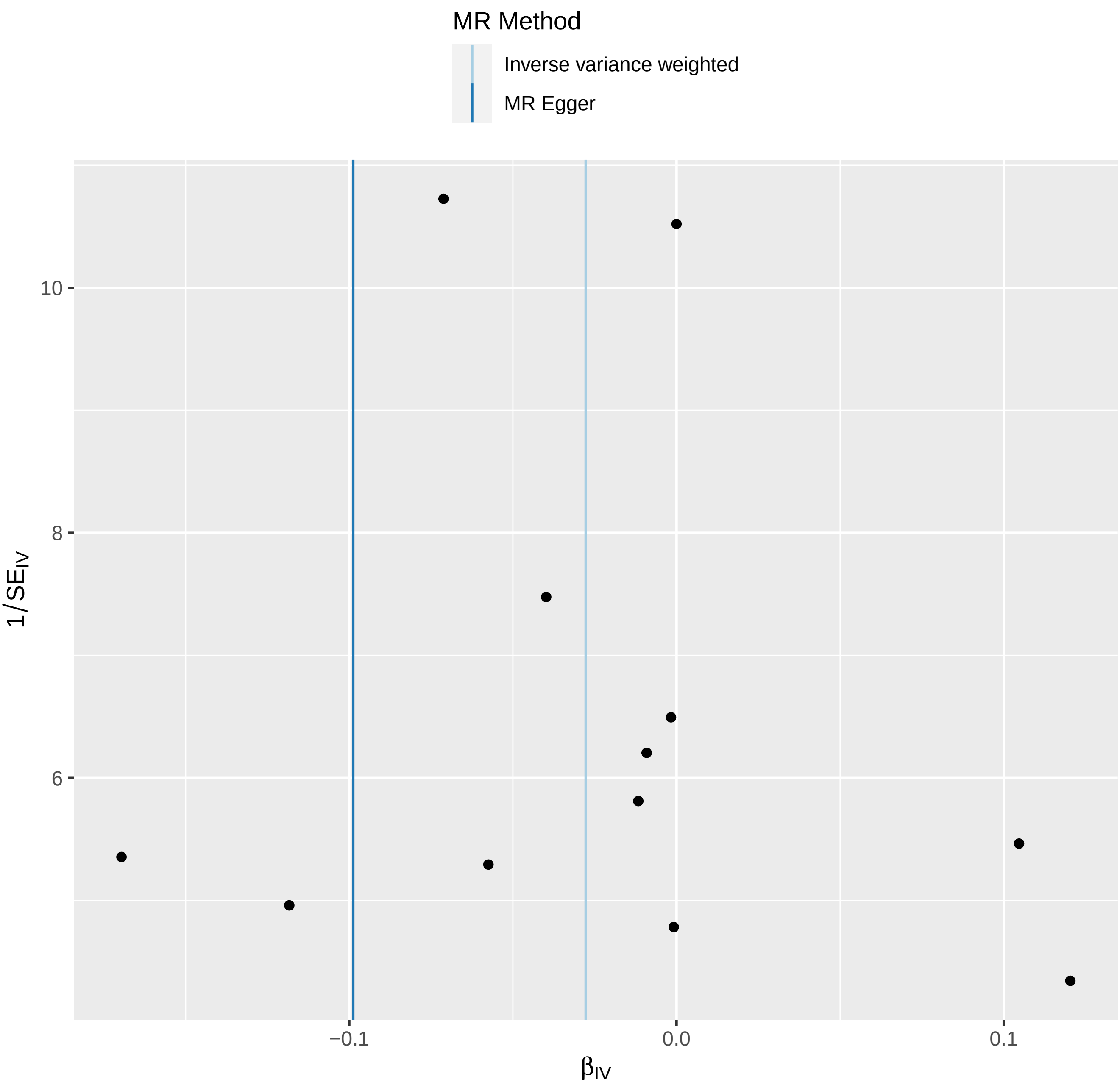

Batch 3−1417 : Selenium on Interferon−induced protein with tetratricopeptide repeats 3

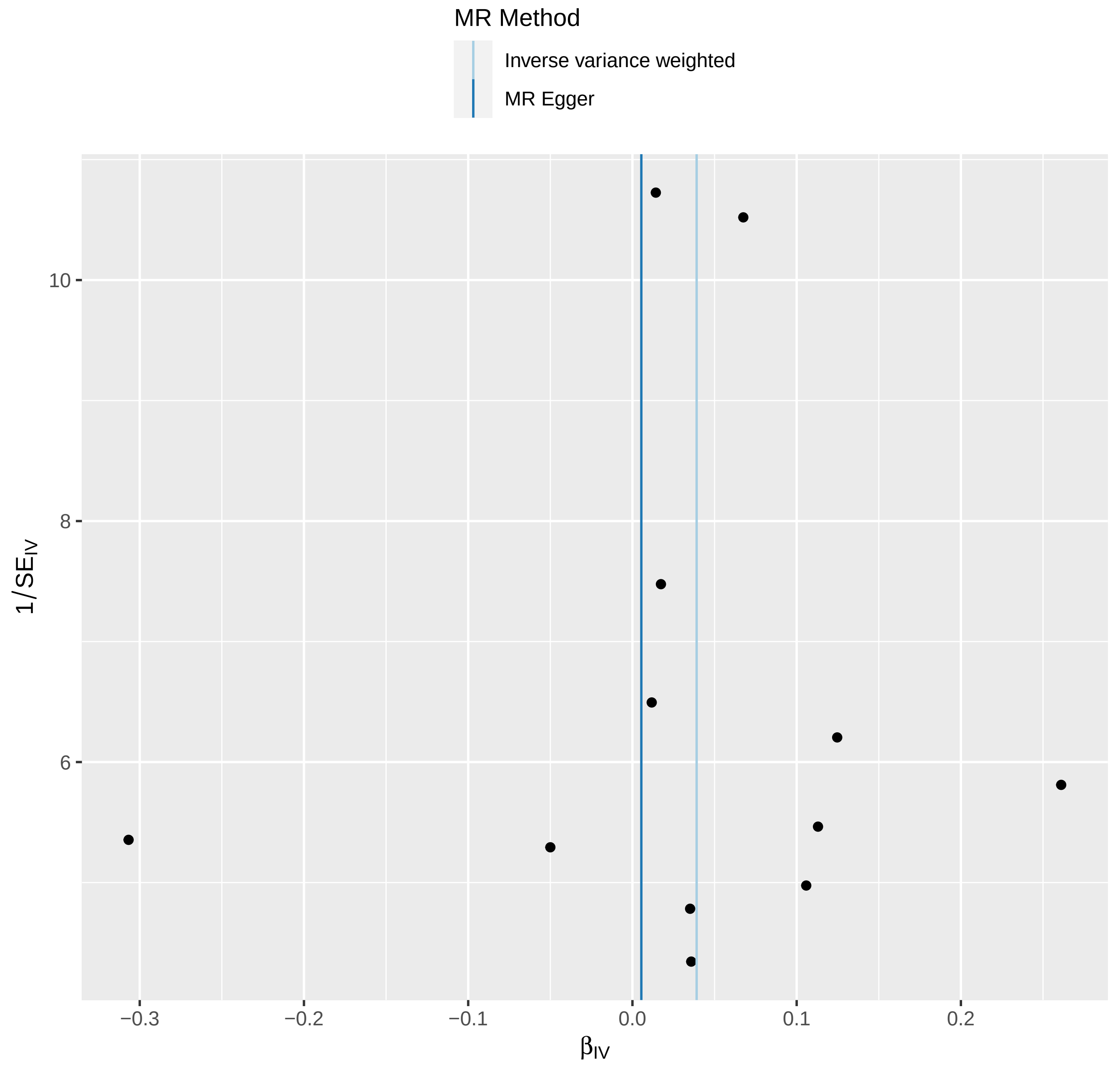

Batch 3−1418 : Selenium on Interferon alpha−10

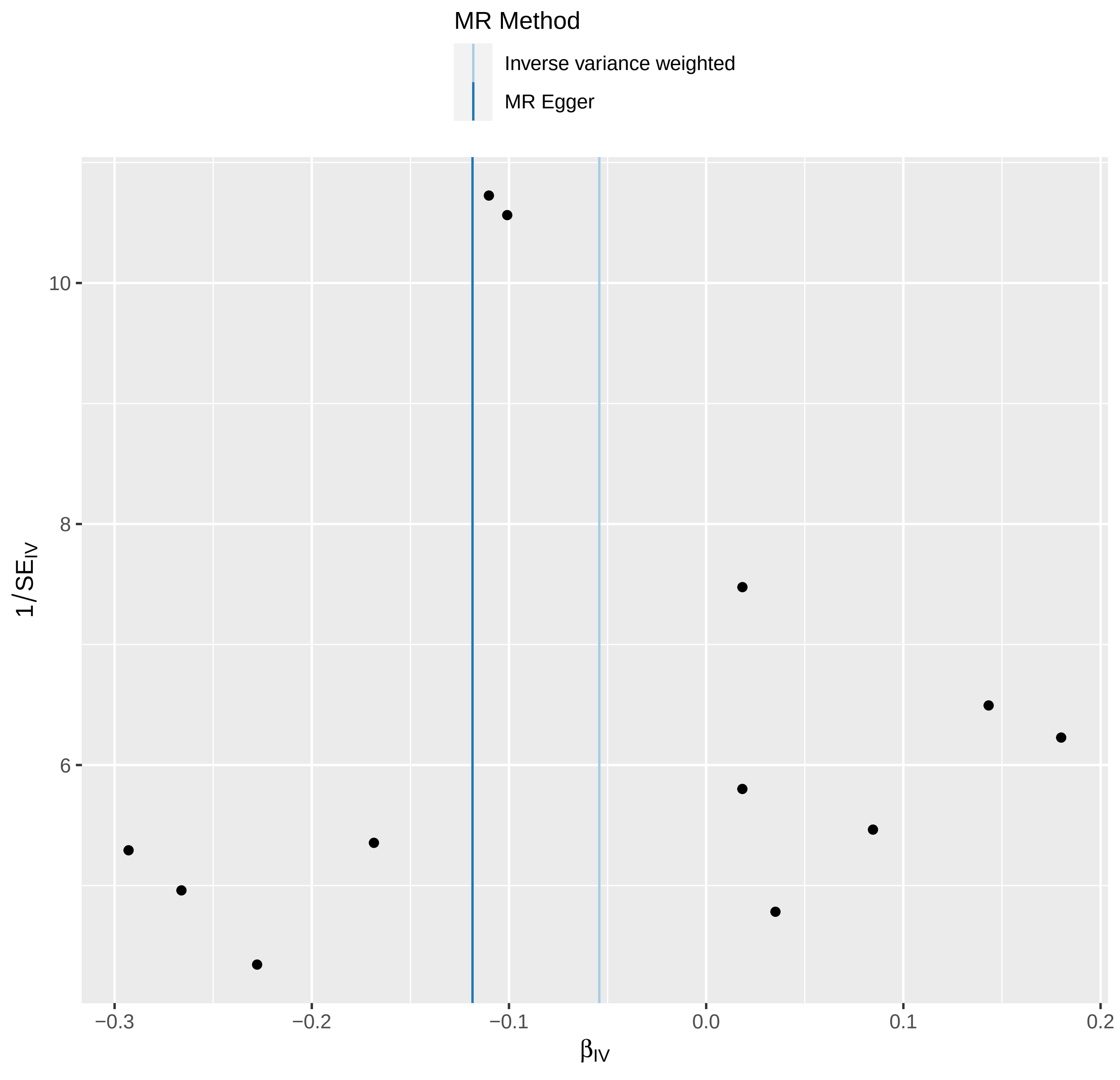

Batch 3−1419 : Selenium on Interferon alpha−10

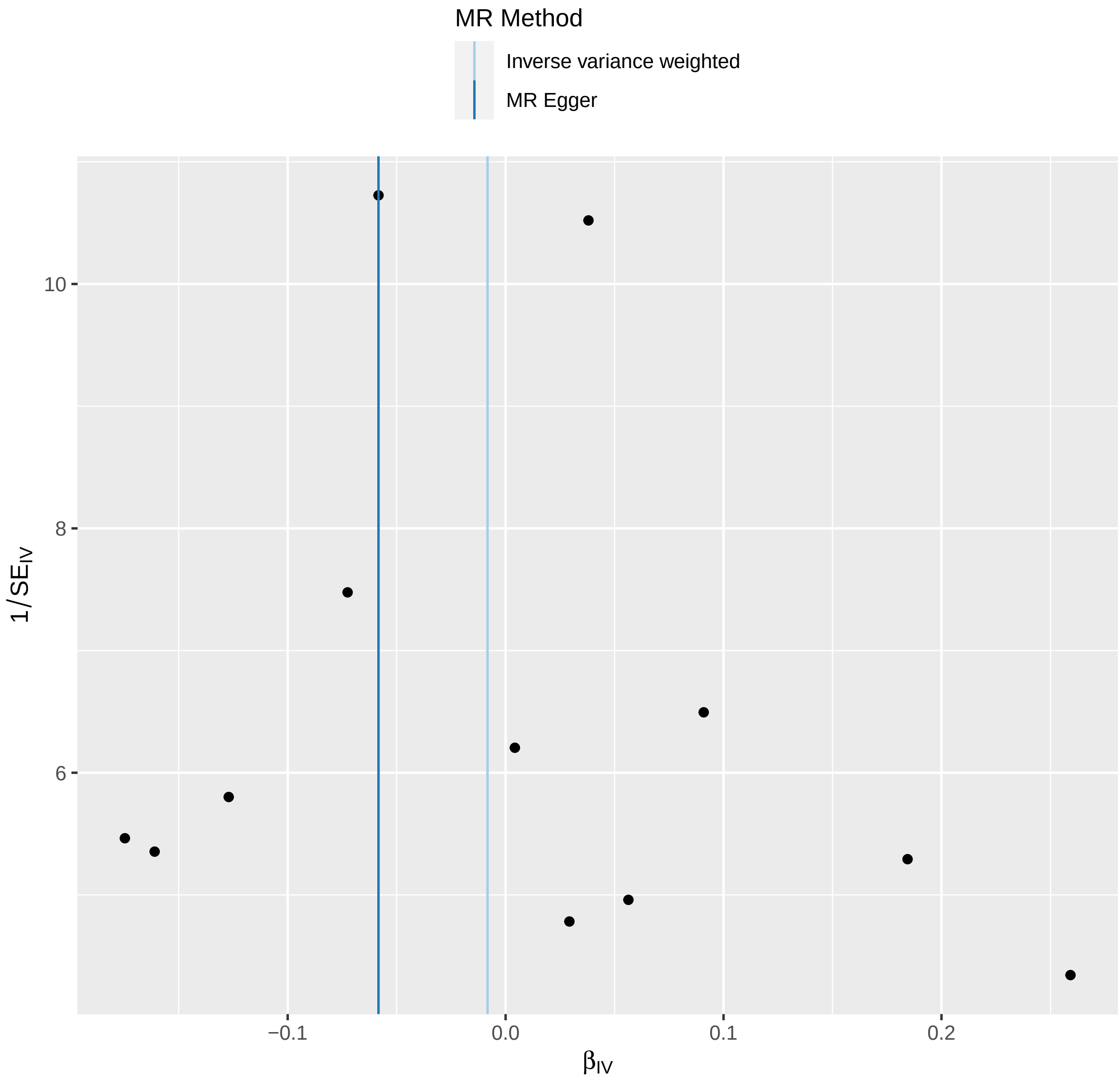

Batch 3−1420 : Selenium on Interferon alpha−14

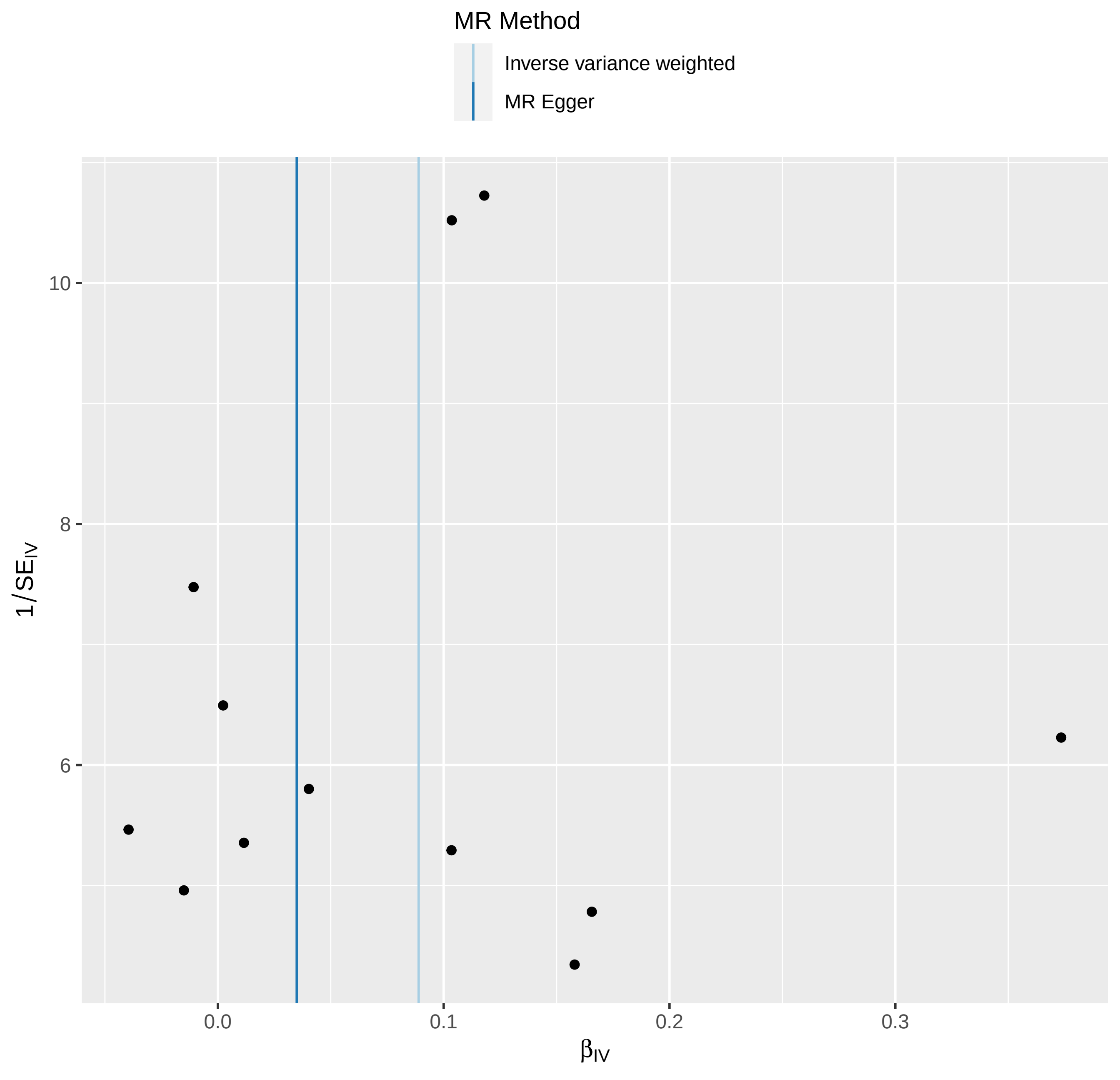

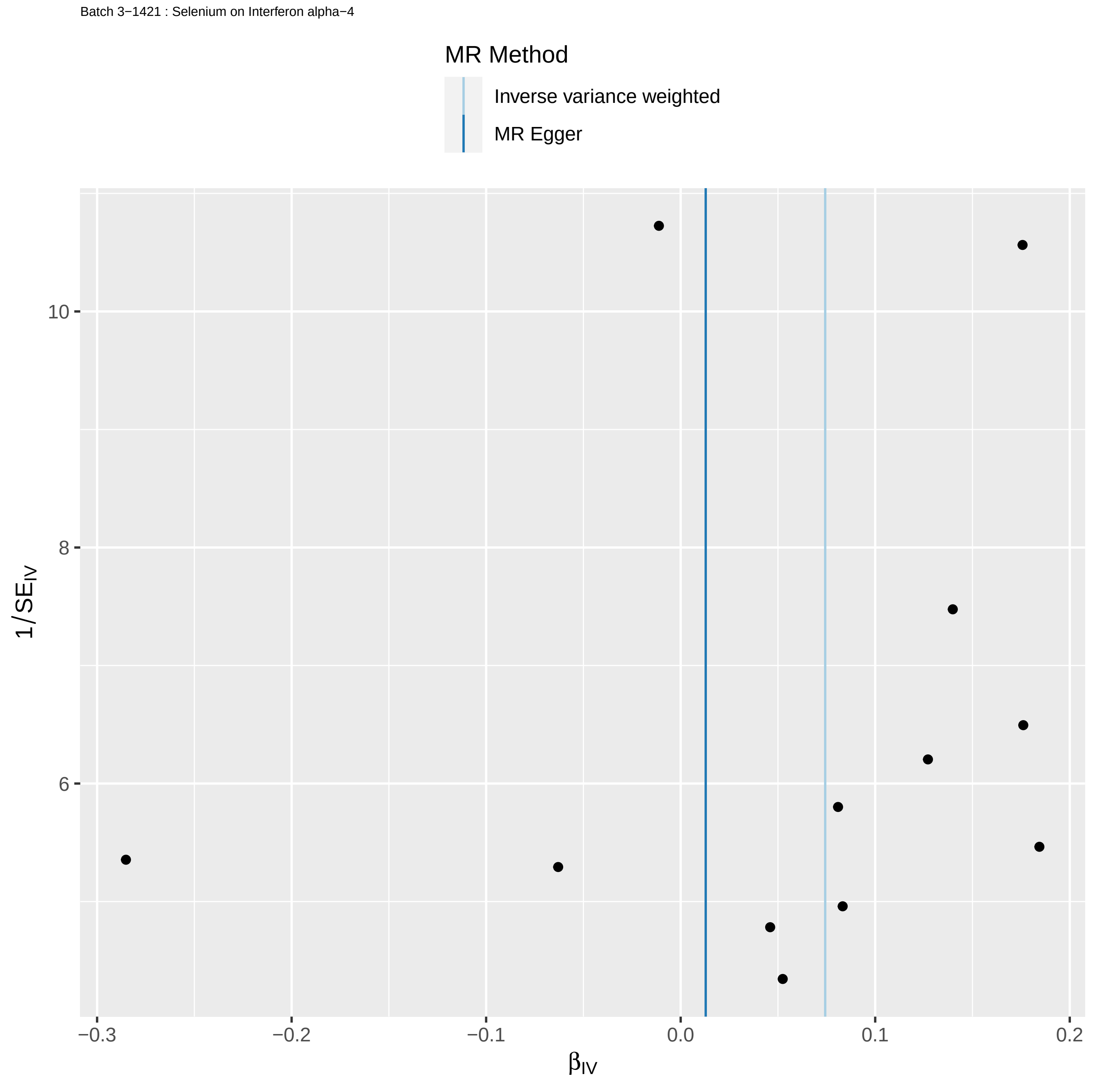
Batch 3−1421 : Selenium on Interferon alpha−4
MR Method
Inverse variance weighted
MR Egger
10
8
6
1/SE_IV
−0.3
−0.2
−0.1
0.0
0.1
0.2
β_IV

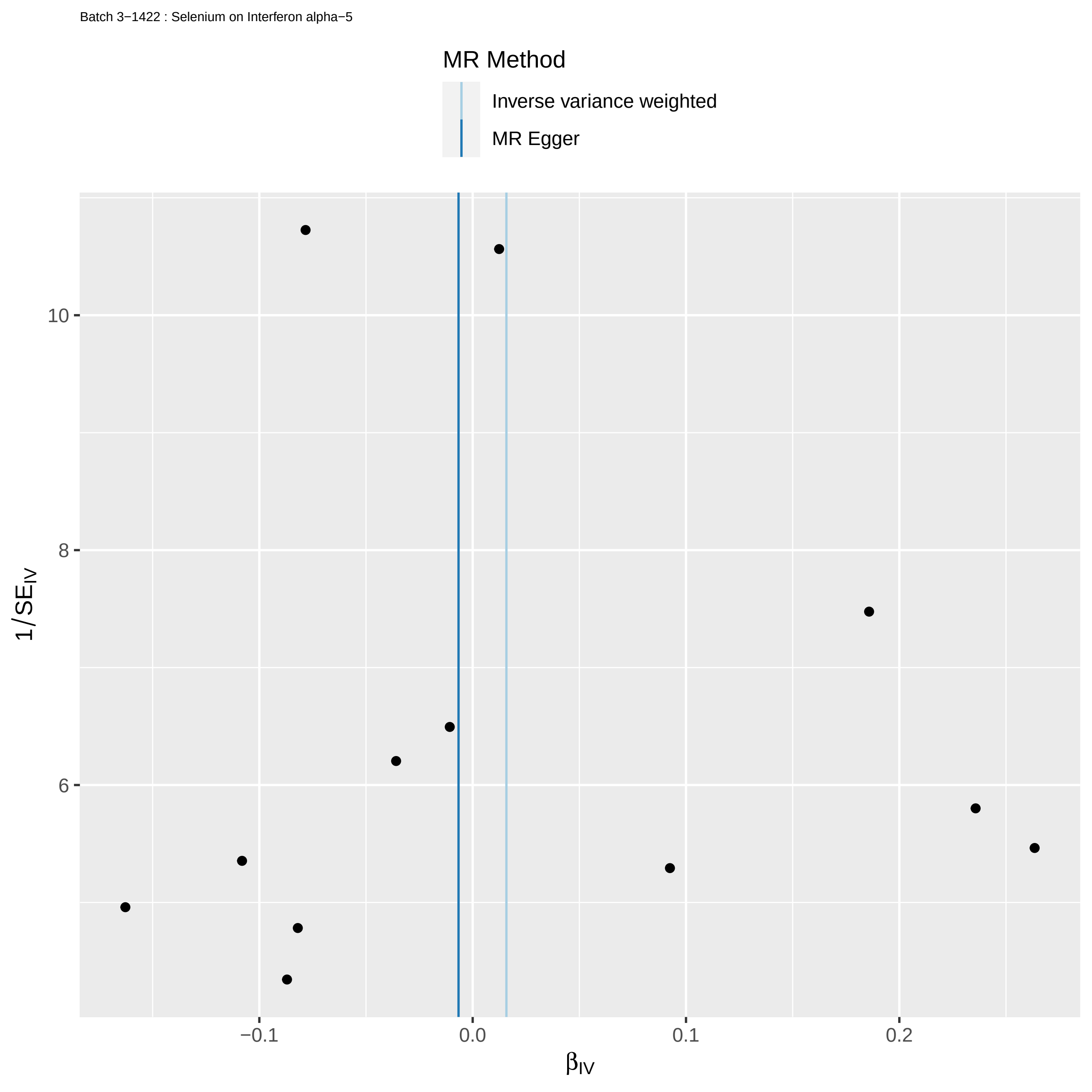

Batch 3−1422 : Selenium on Interferon alpha−5
MR Method
Inverse variance weighted
MR Egger
10
8
6
1/SE$_{IV}$
−0.1
0.0
0.1
0.2
β$_{IV}$

Batch 3−1423 : Selenium on Interferon alpha−6

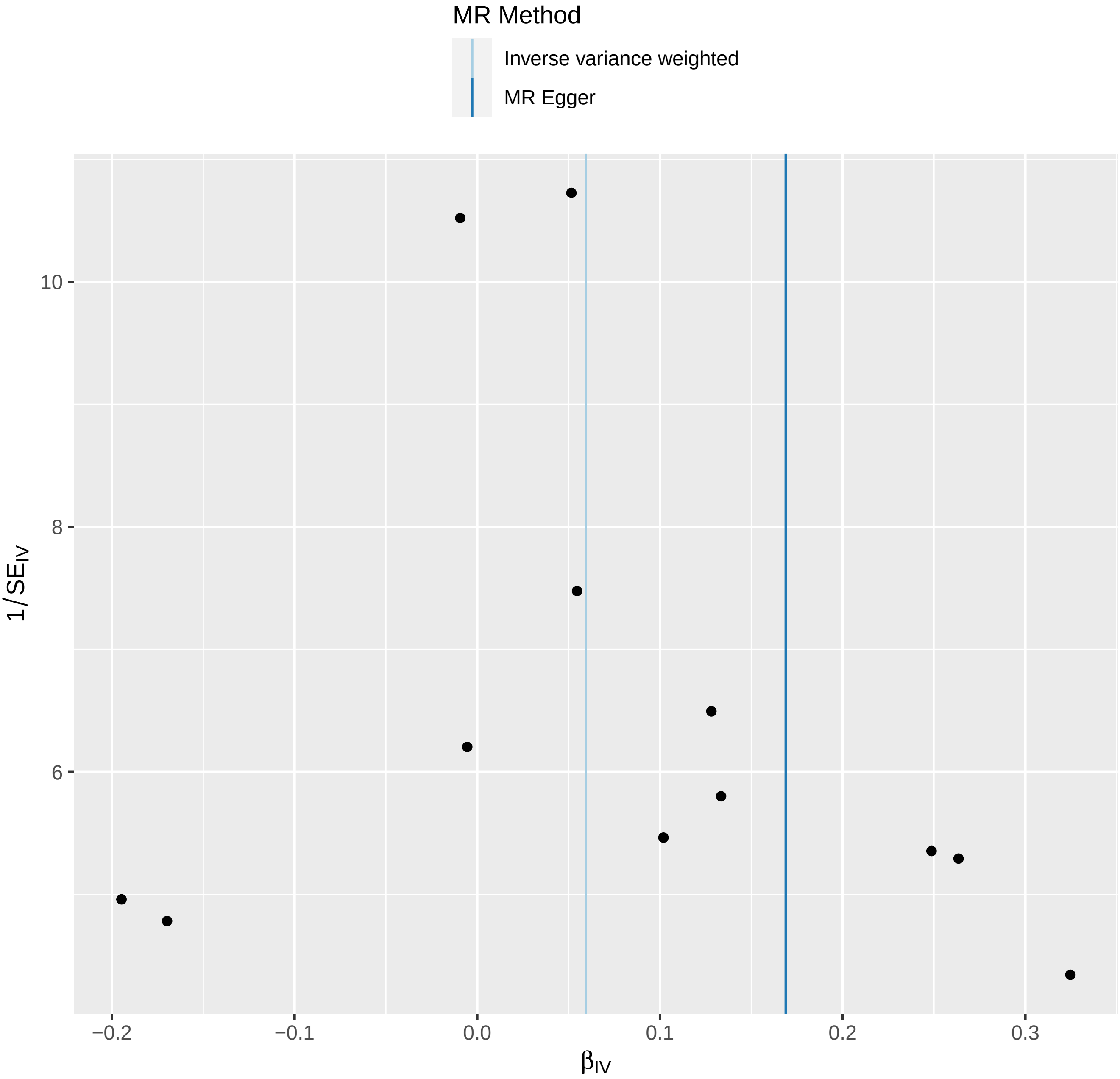

Batch 3−1424 : Selenium on Interferon alpha−7

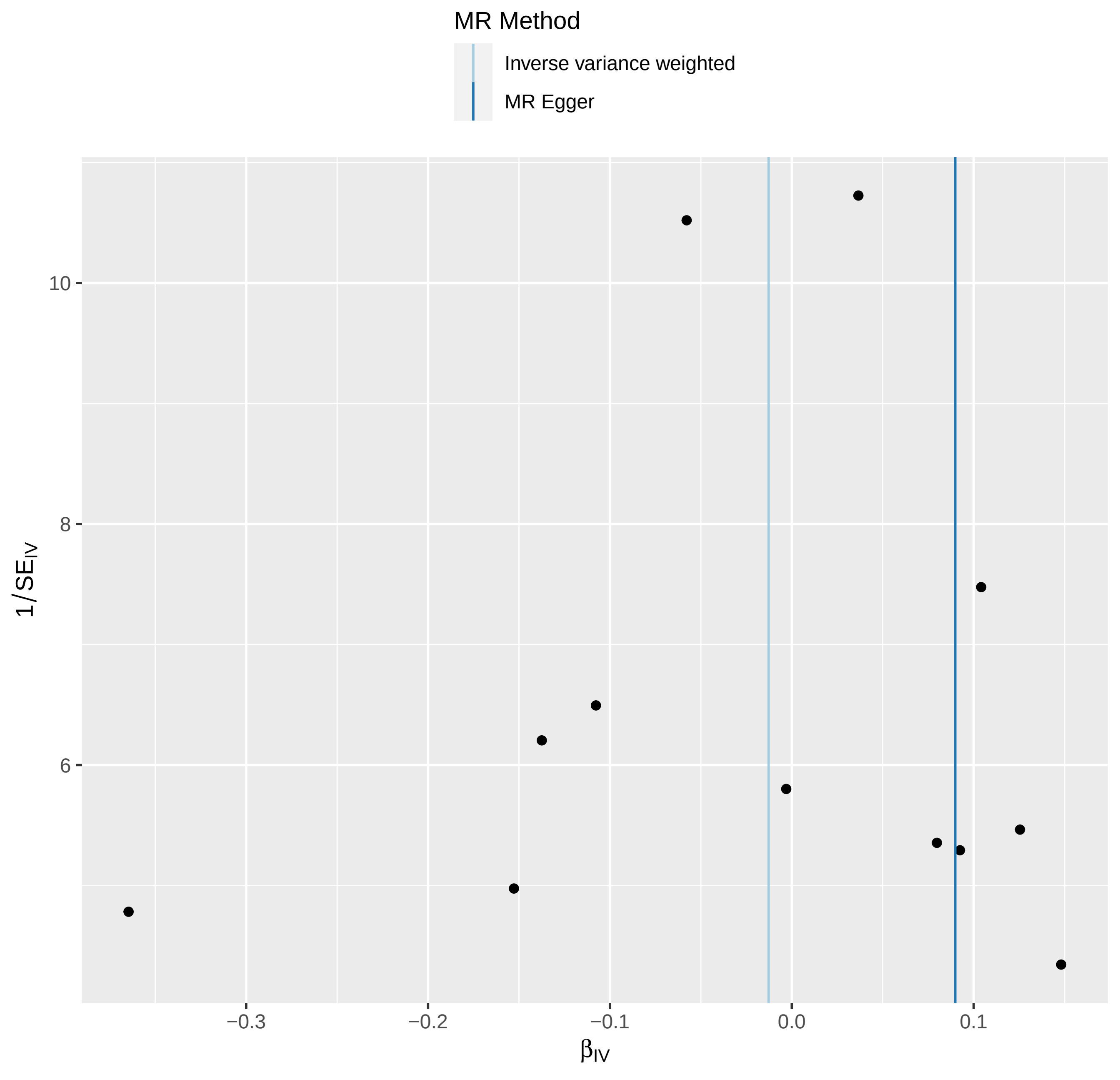

Batch 3−1425 : Selenium on Interferon alpha−8

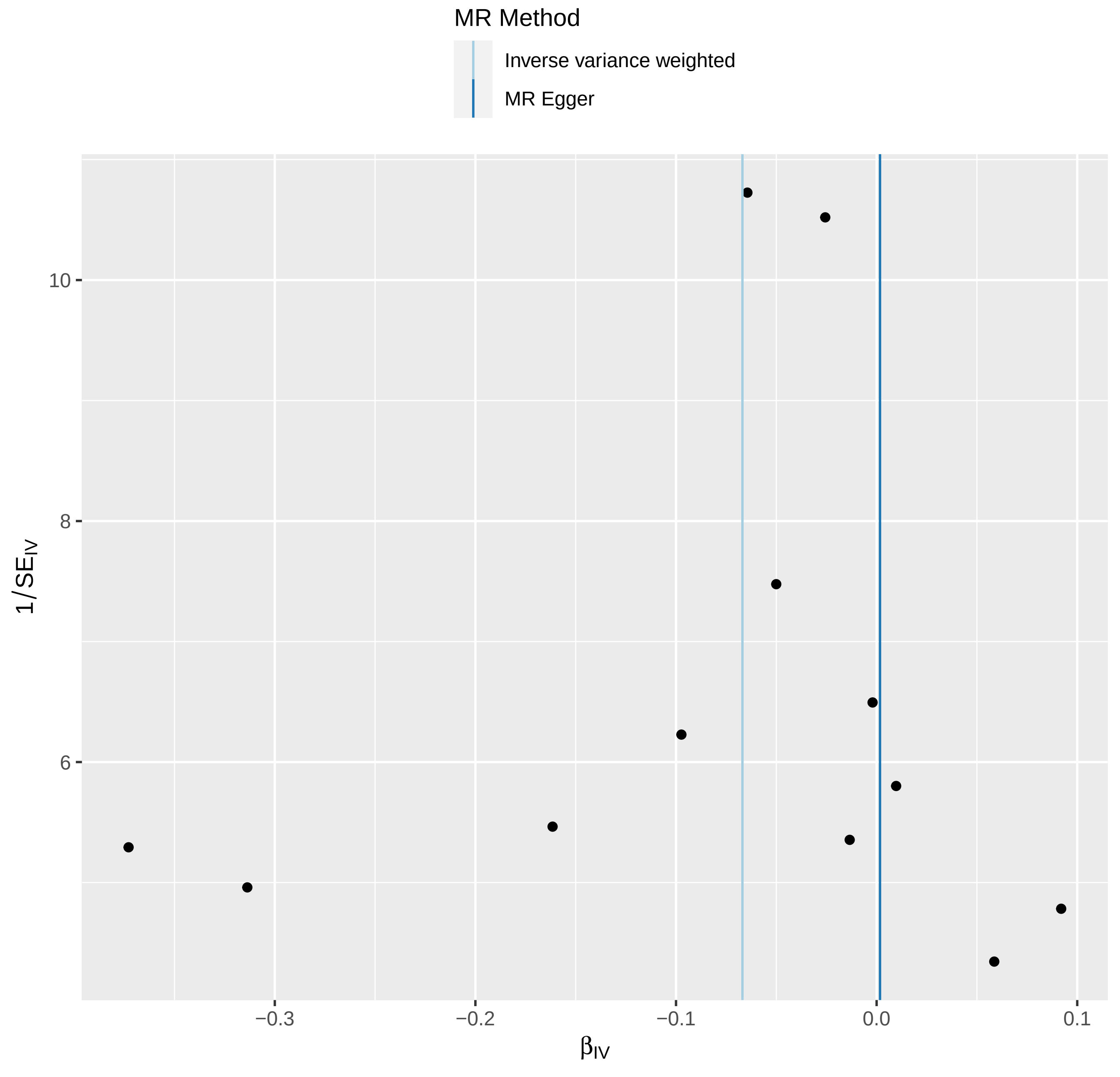

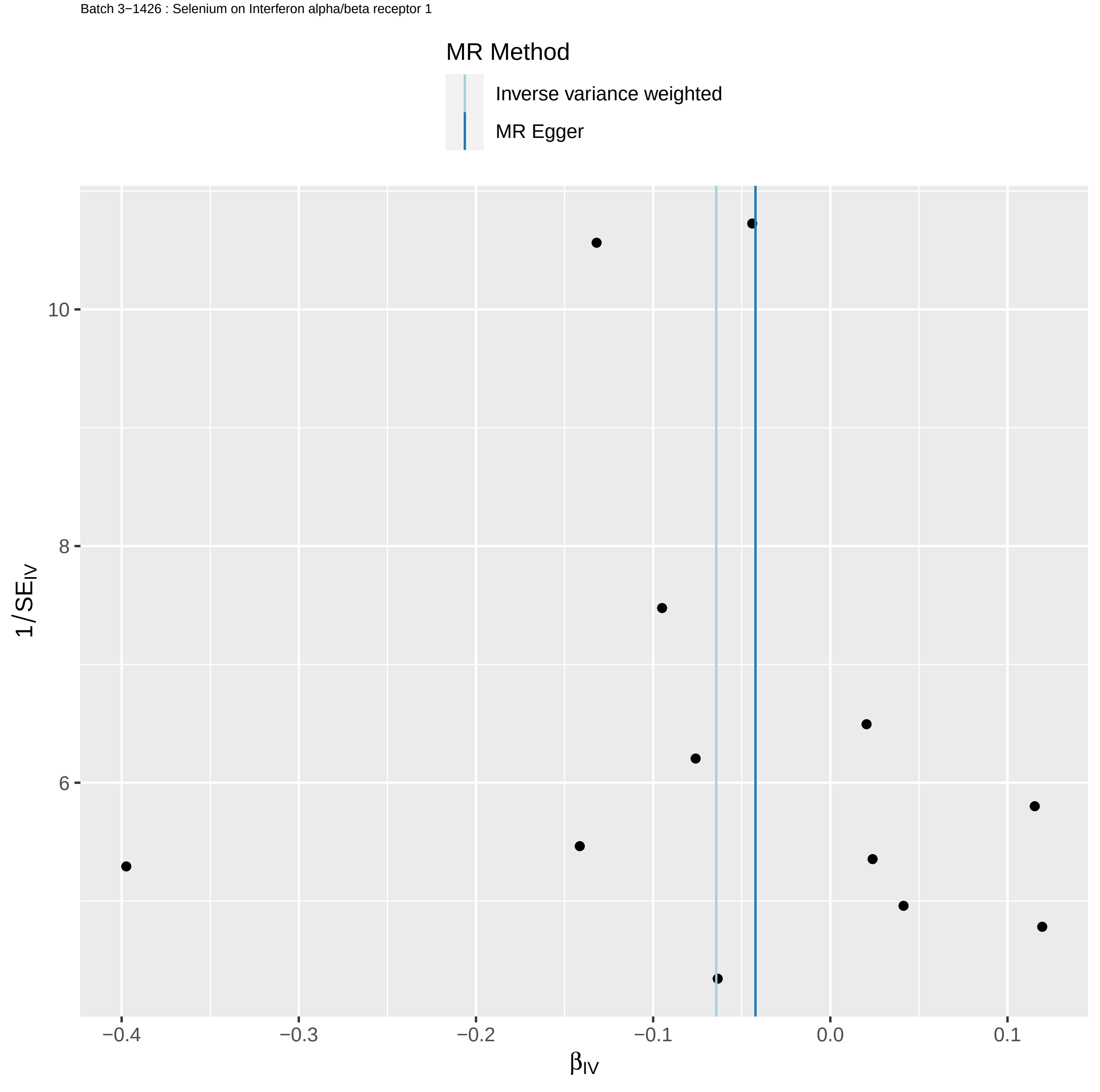

Batch 3−1426 : Selenium on Interferon alpha/beta receptor 1
MR Method
Inverse variance weighted
MR Egger
10
8
6
1/SE$_{IV}$
−0.4
−0.3
−0.2
−0.1
0.0
0.1
β$_{IV}$

Batch 3−1427 : Selenium on Interferon beta

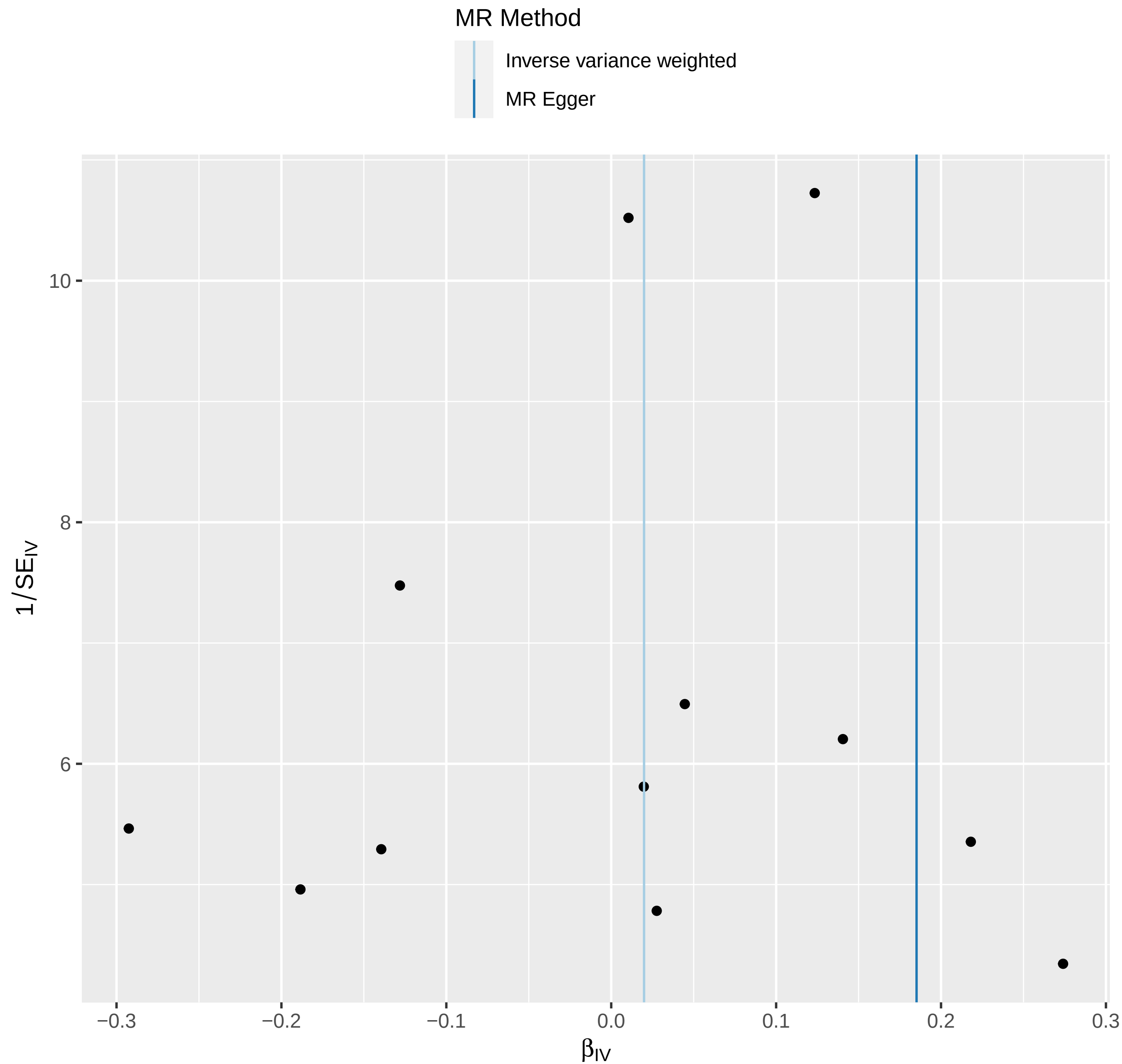

Batch 3−1428 : Selenium on Interferon gamma

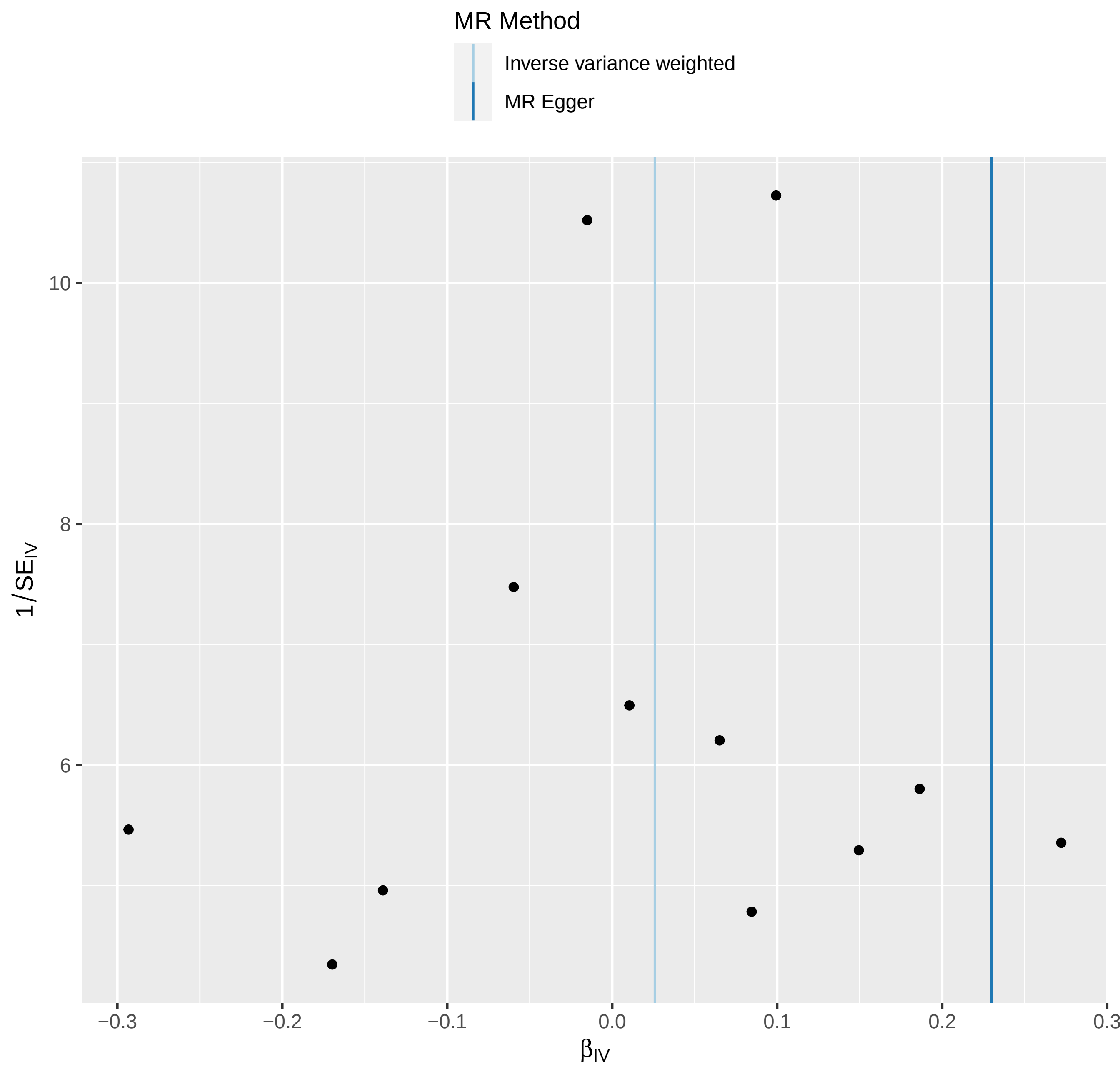

Batch 3−1429 : Selenium on Interferon gamma

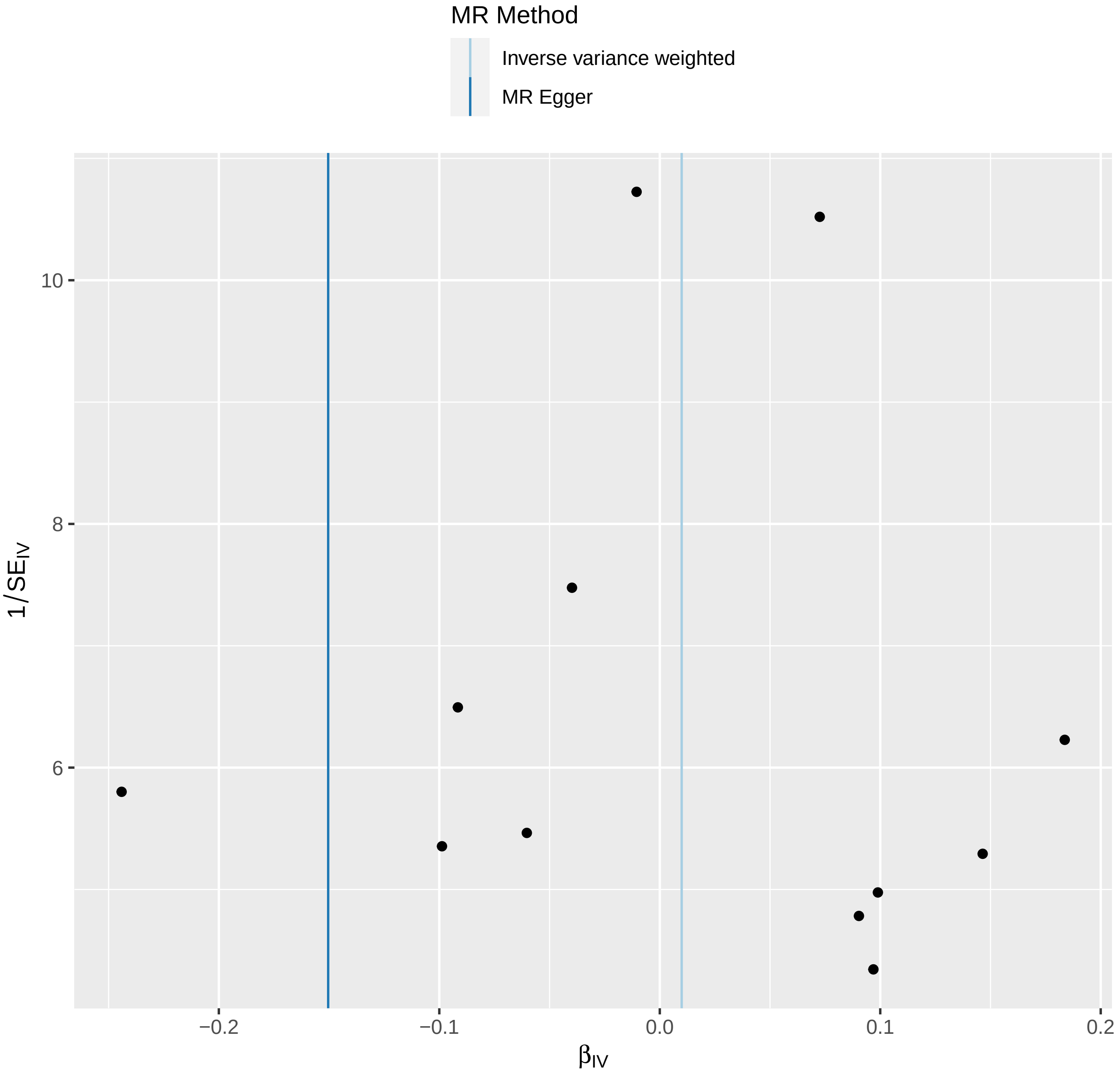

Batch 3−1430 : Selenium on Interferon gamma receptor 1

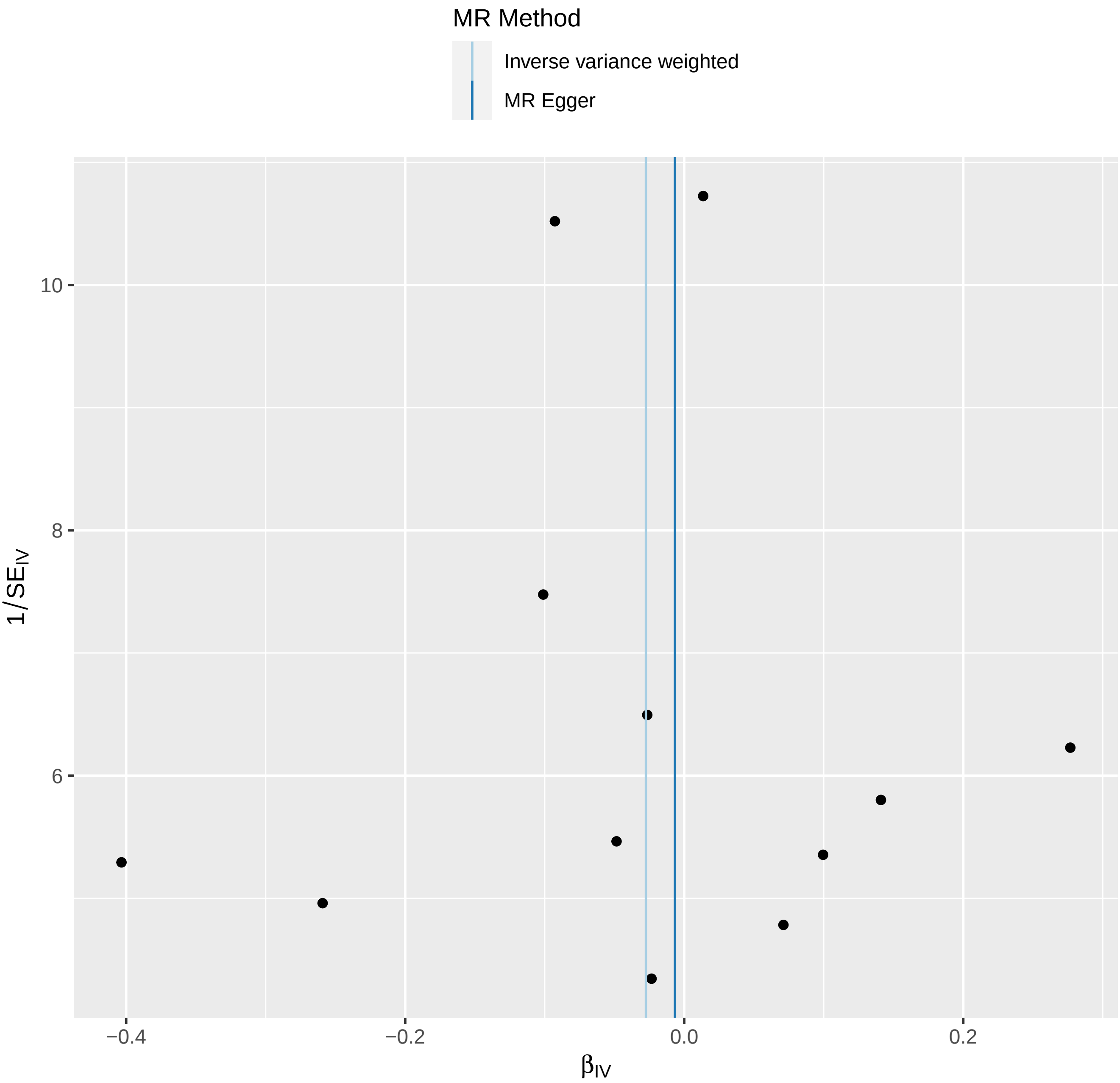

Batch 3−1431 : Selenium on Interferon gamma receptor 2

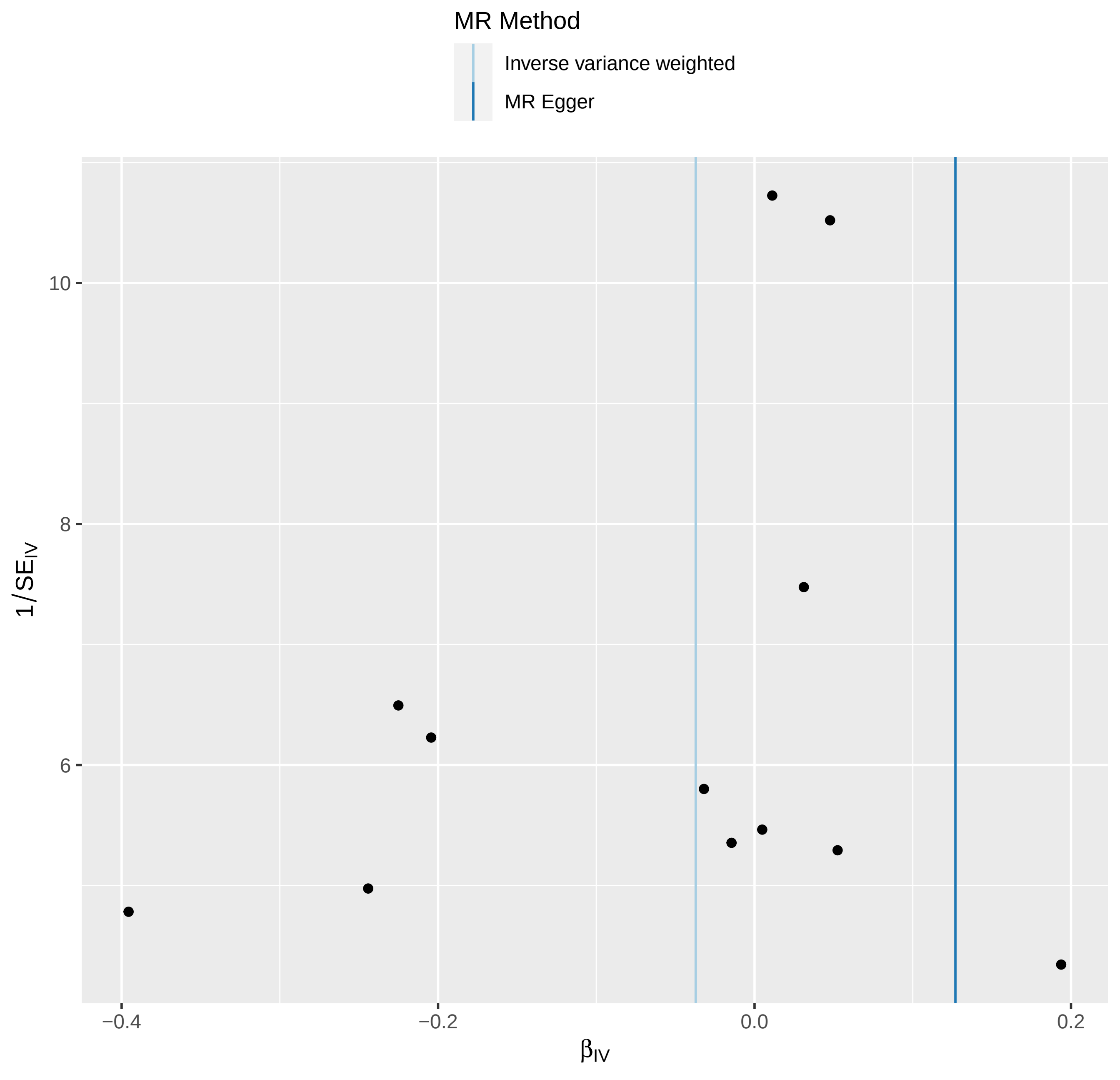

Batch 3−1432 : Selenium on Interferon gamma receptor 2

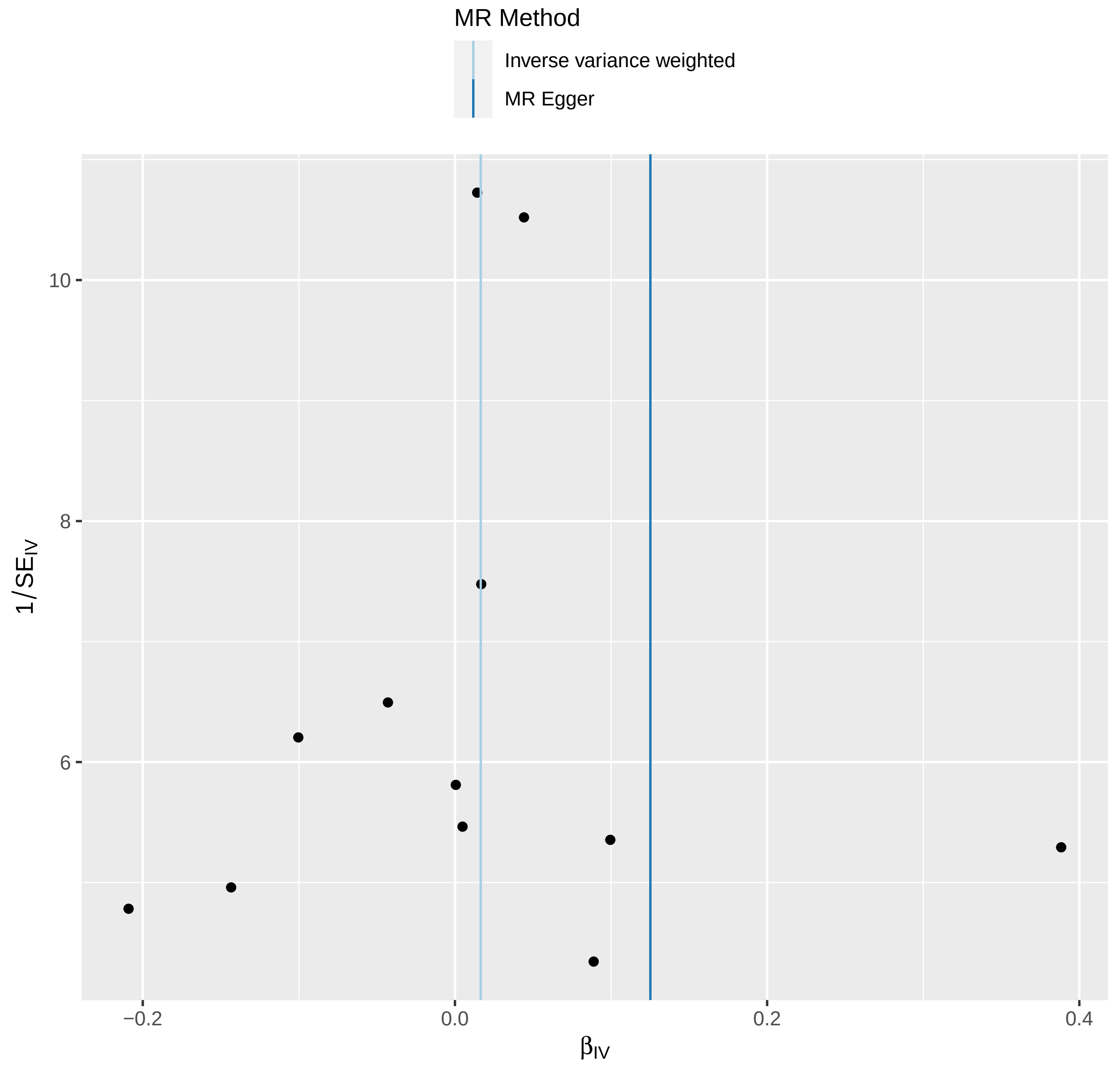

Batch 3−1433 : Selenium on Interferon gamma receptor 2

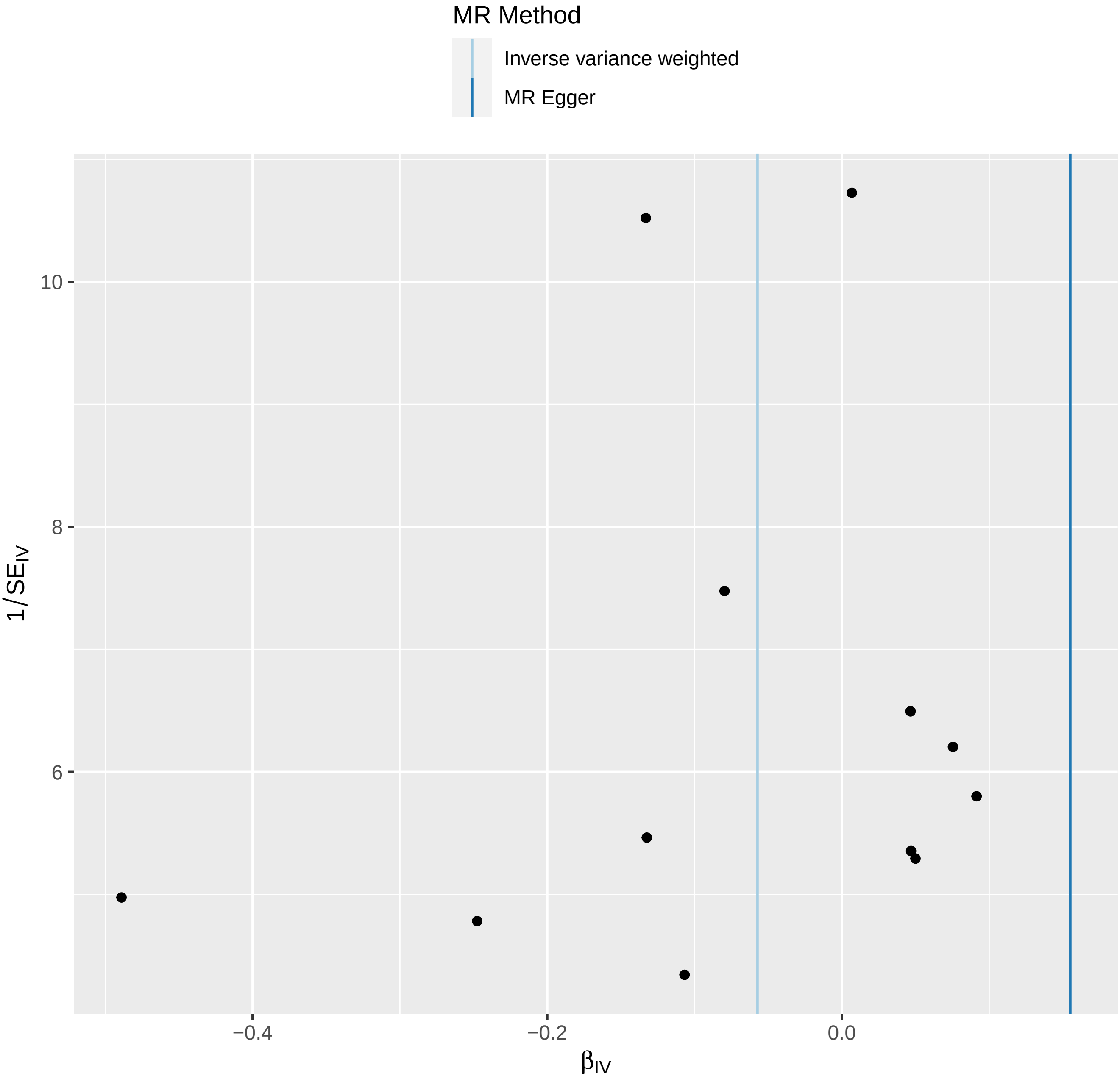

Batch 3−1434 : Selenium on Interferon lambda−1

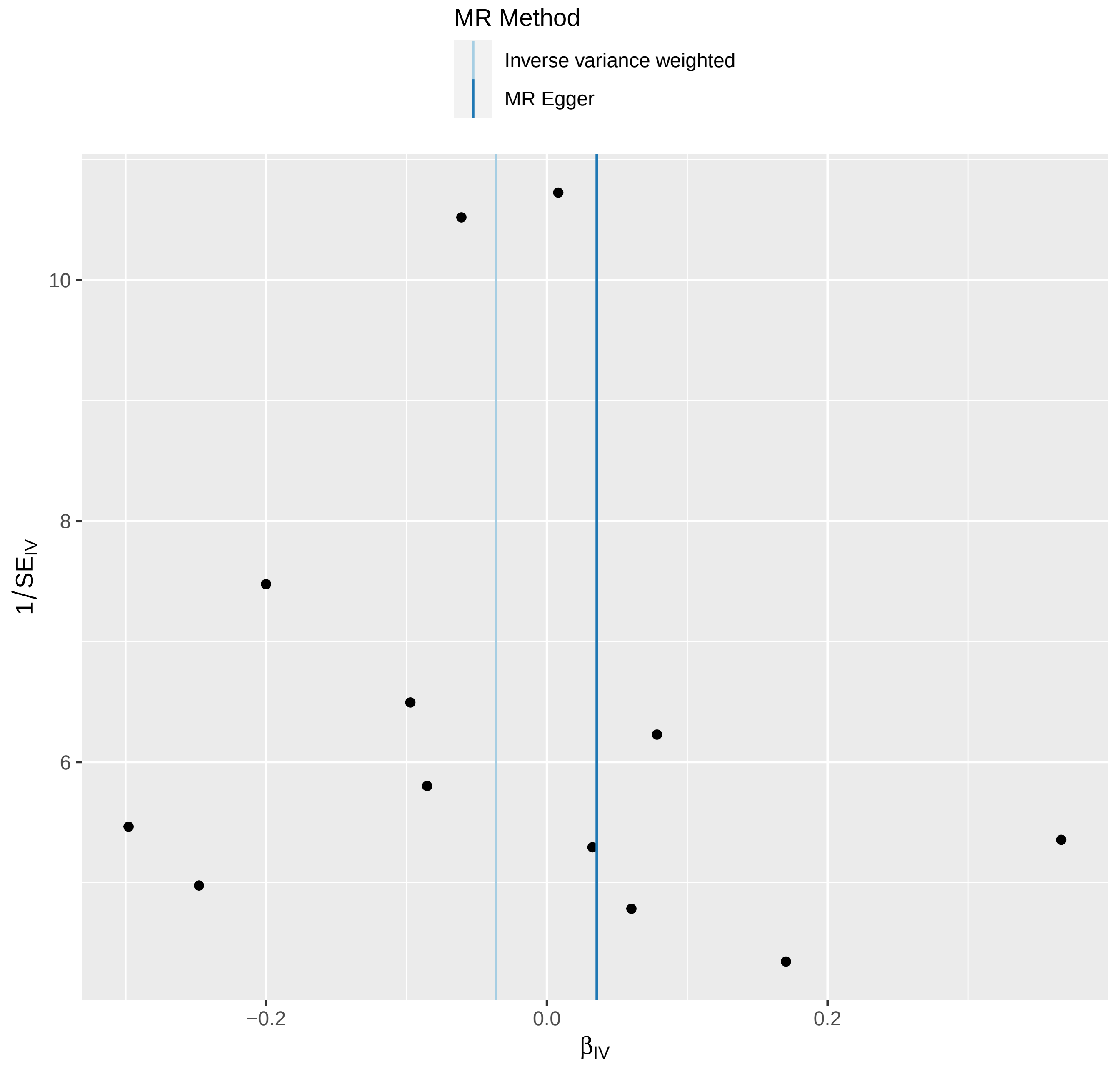

Batch 3−1435 : Selenium on Interferon lambda−1

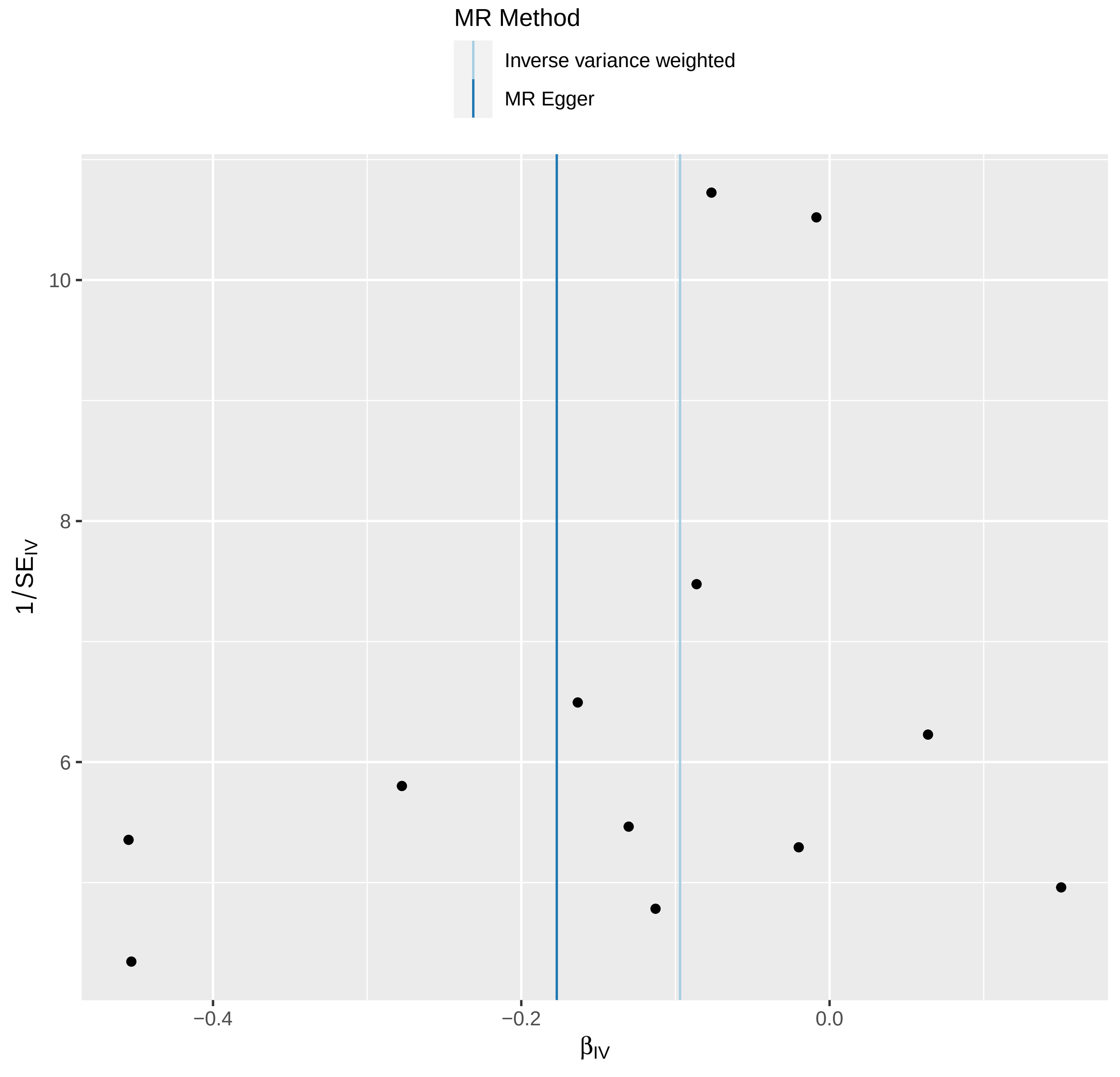

Batch 3−1436 : Selenium on Interferon lambda−2

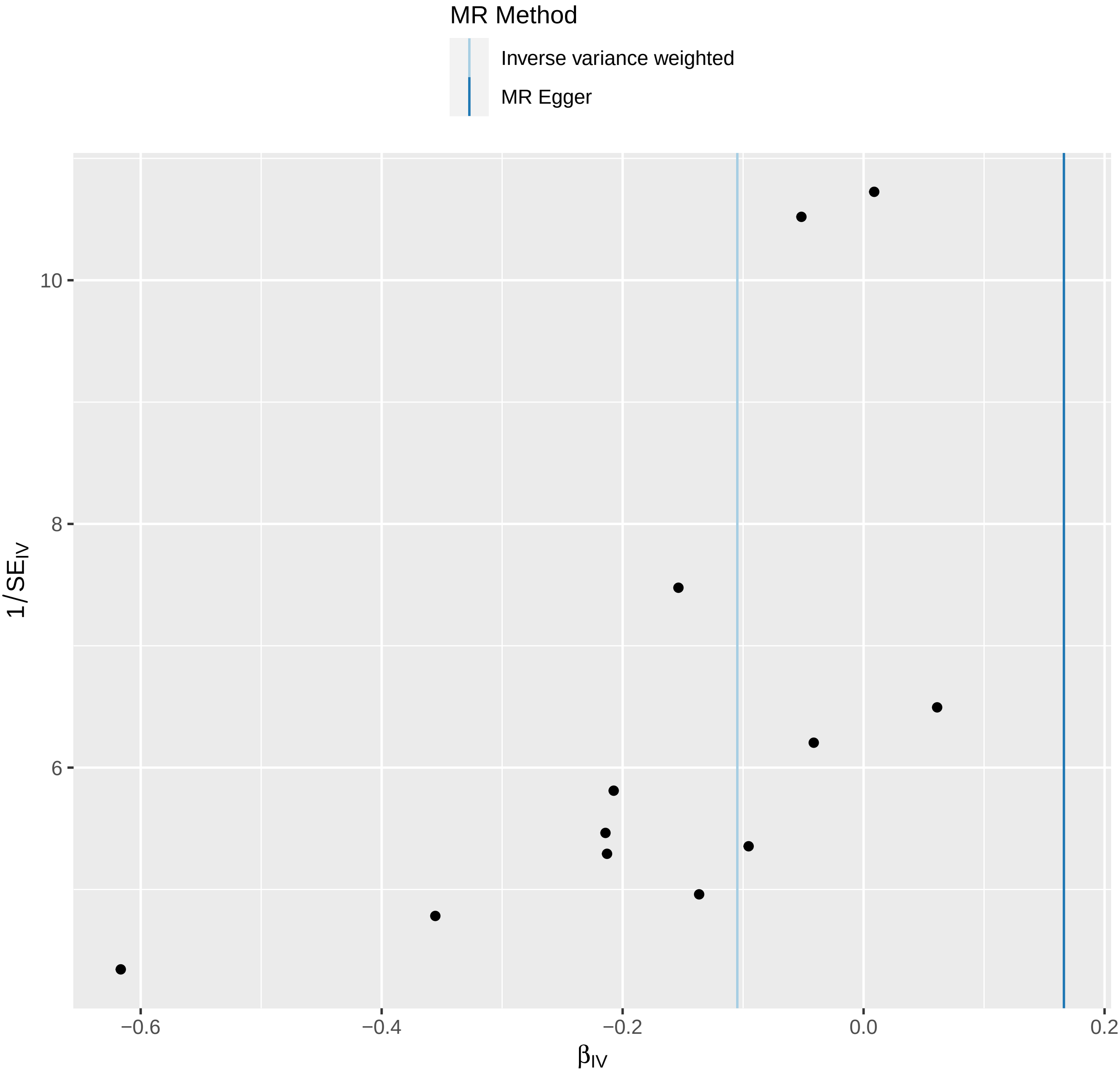

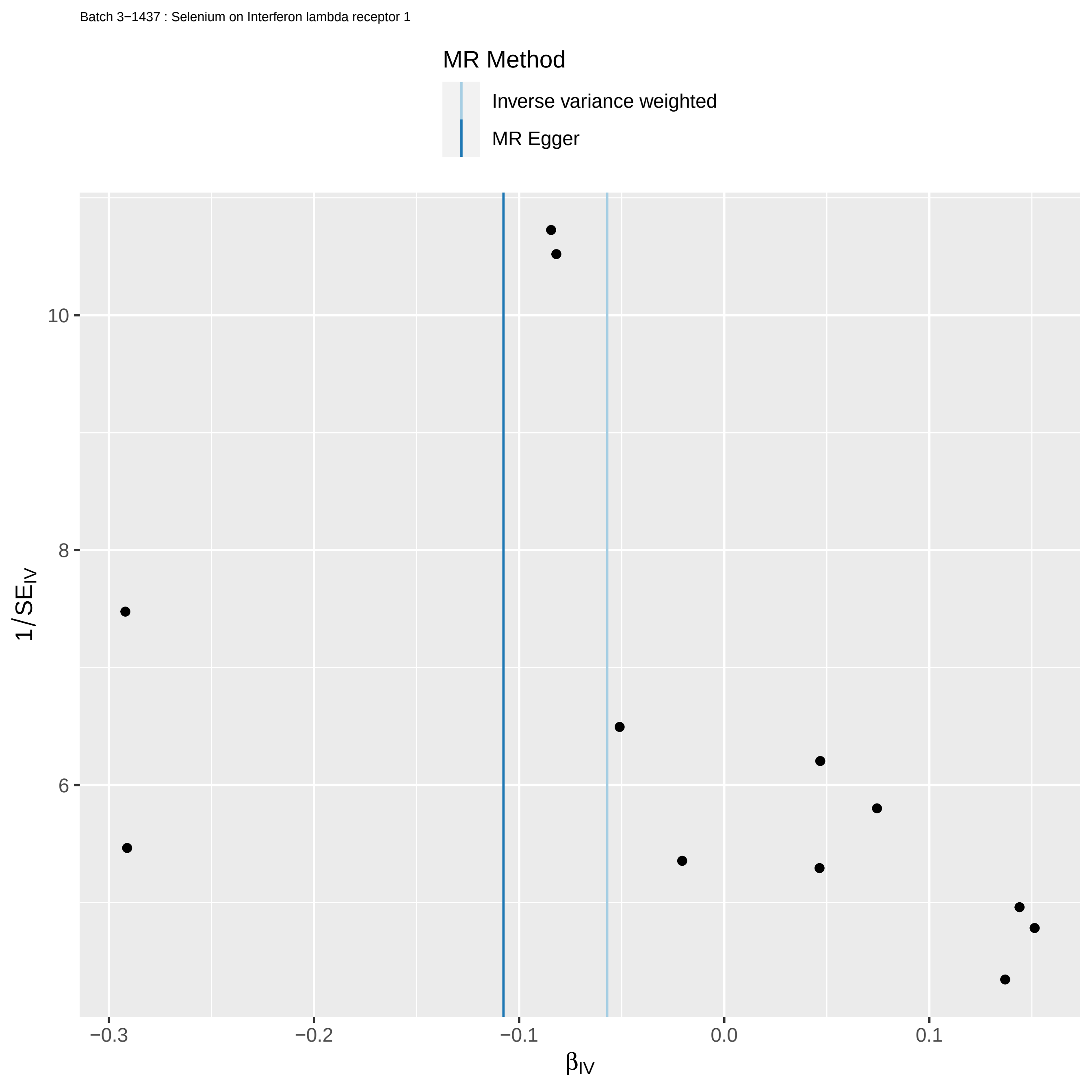
Batch 3−1437 : Selenium on Interferon lambda receptor 1
MR Method
Inverse variance weighted
MR Egger
10
8
6
1/SE_IV
−0.3
−0.2
−0.1
0.0
0.1
β_IV

Batch 3−1438 : Selenium on Interferon omega−1

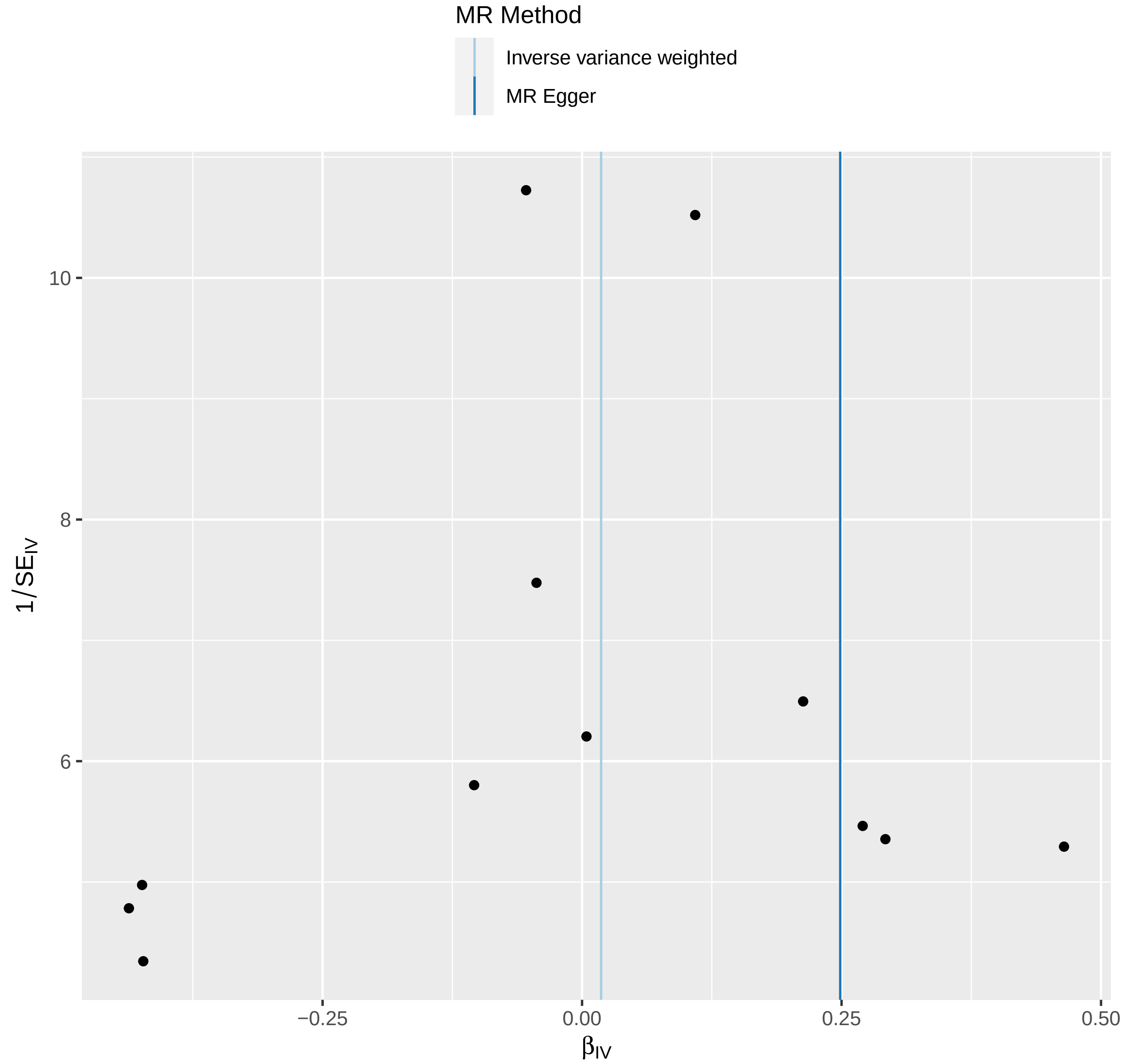

Batch 3−1439 : Selenium on Immunoglobulin−binding protein 1

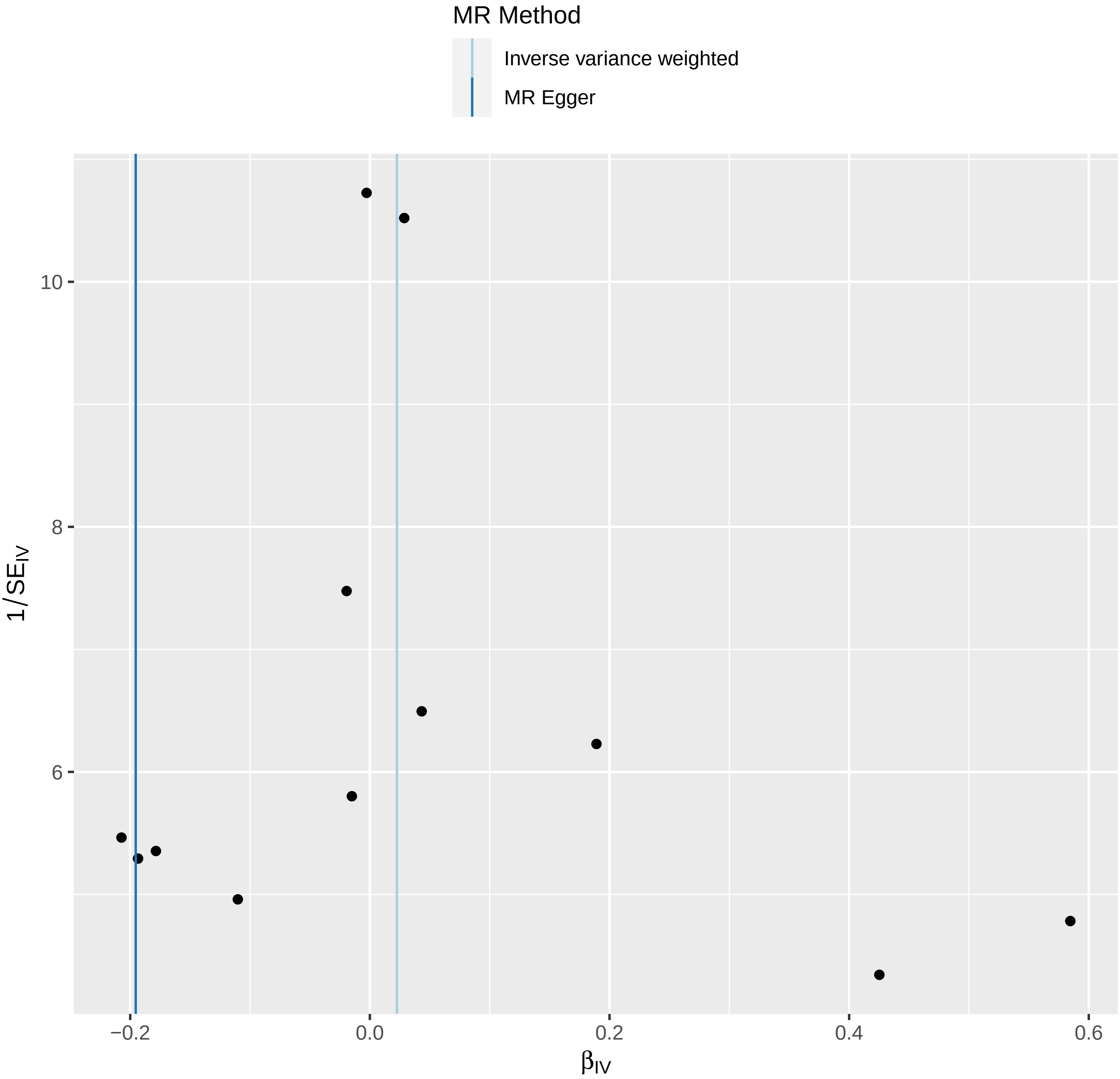

Batch 3−1440 : Selenium on Immunoglobulin superfamily DCC subclass member 3

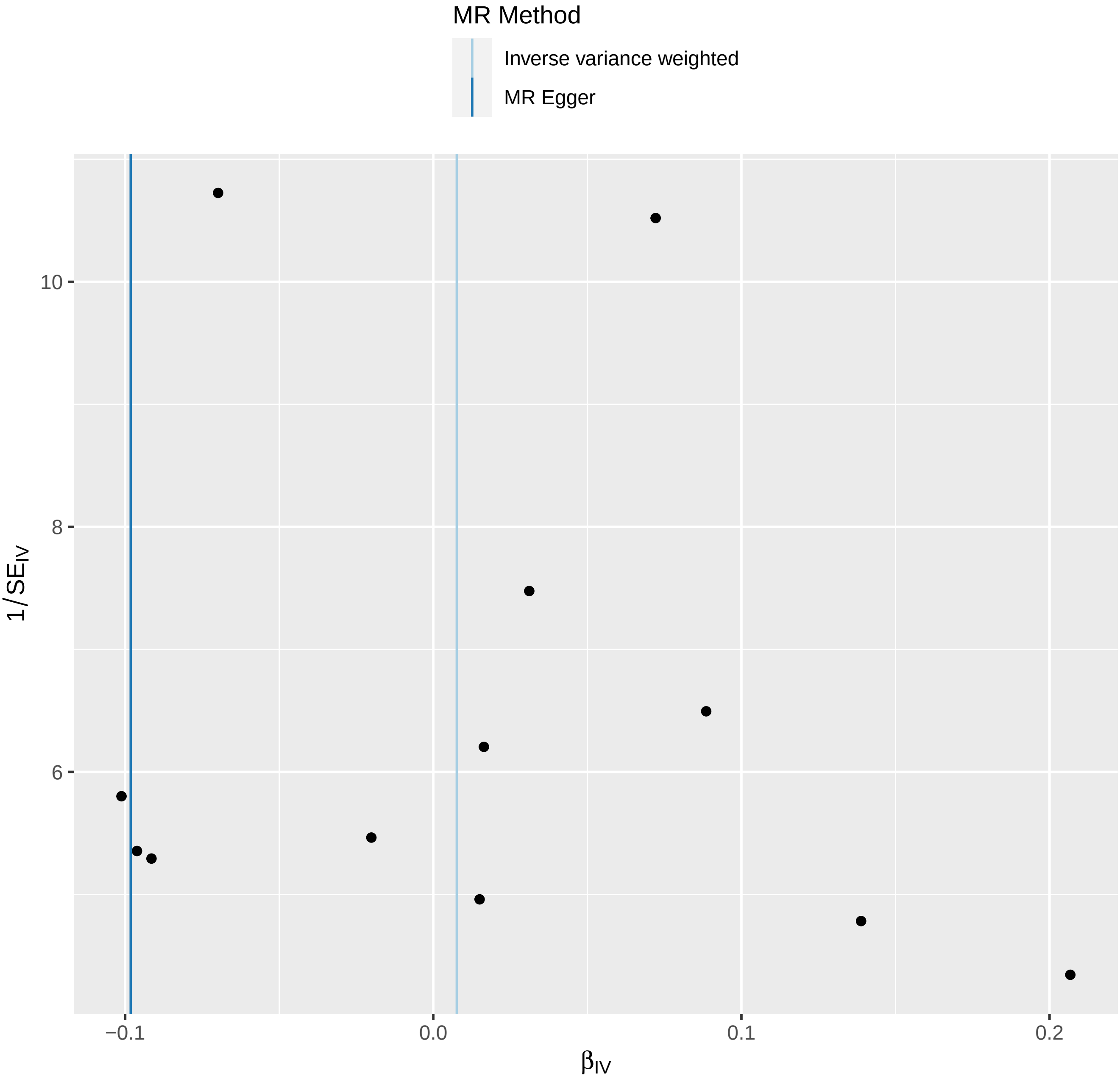

Batch 3−1441 : Selenium on Immunoglobulin superfamily DCC subclass member 3

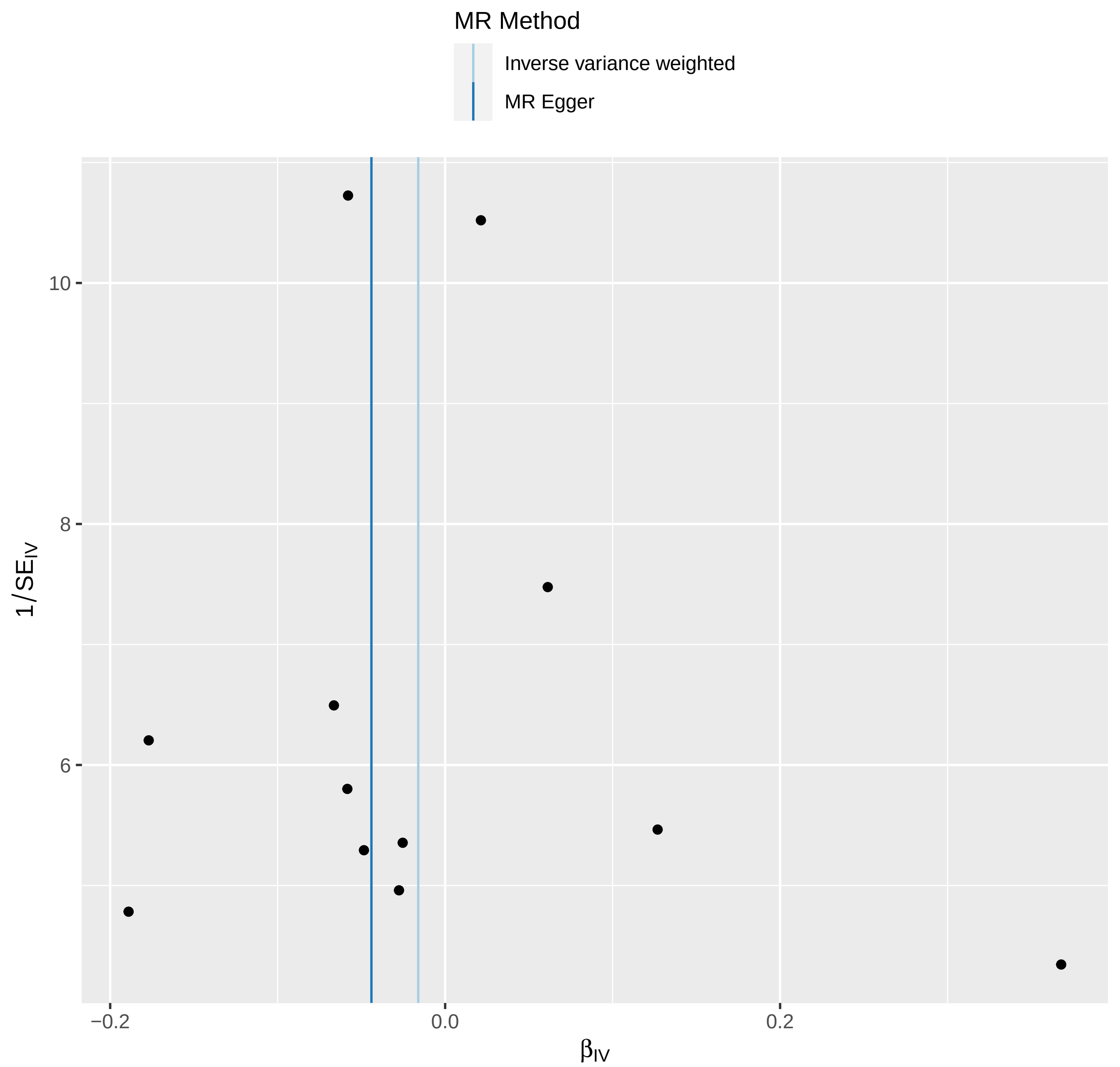

Batch 3−1442 : Selenium on Immunoglobulin superfamily DCC subclass member 4

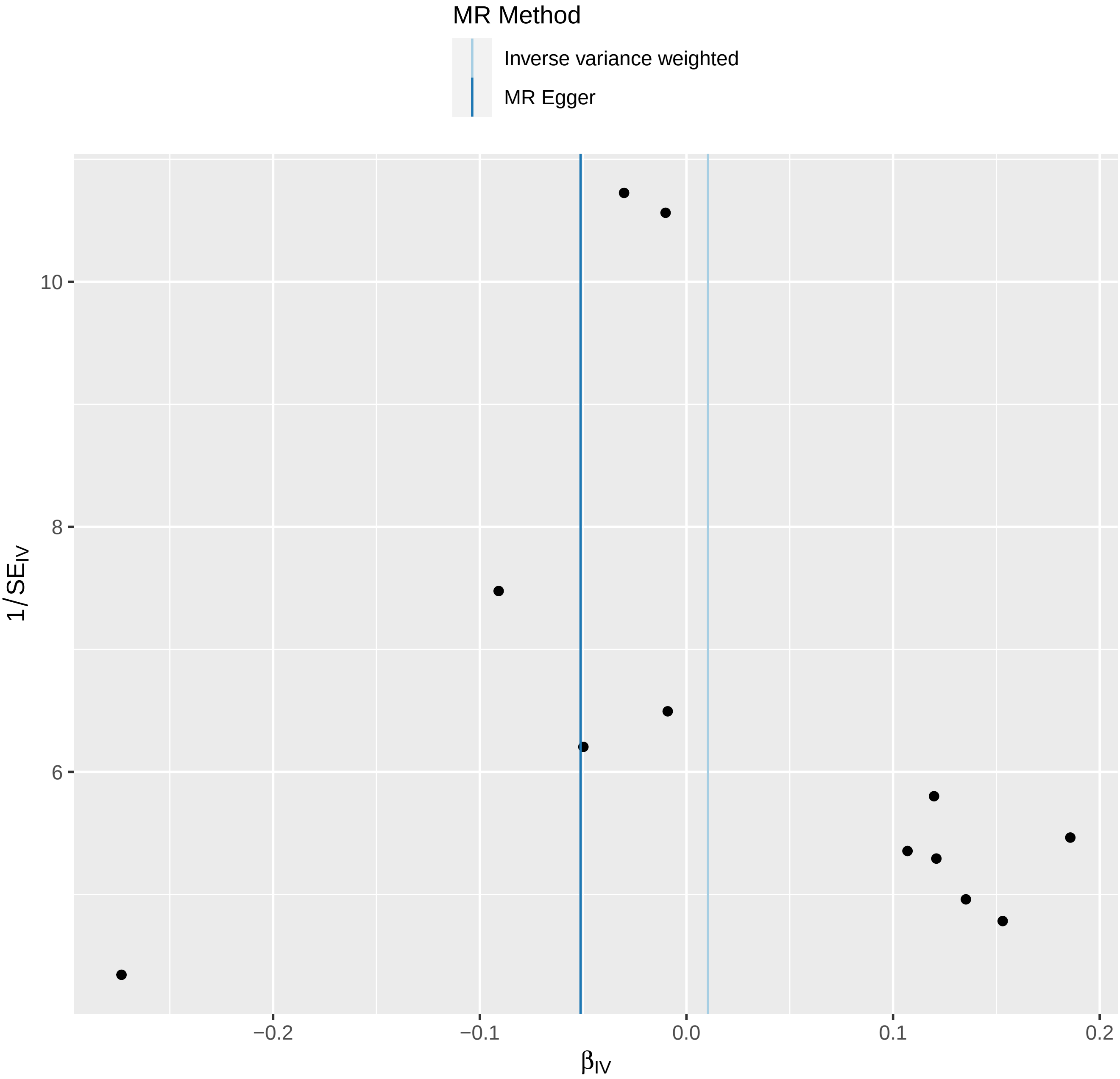

Batch 3−1443 : Selenium on Insulin−like growth factor I

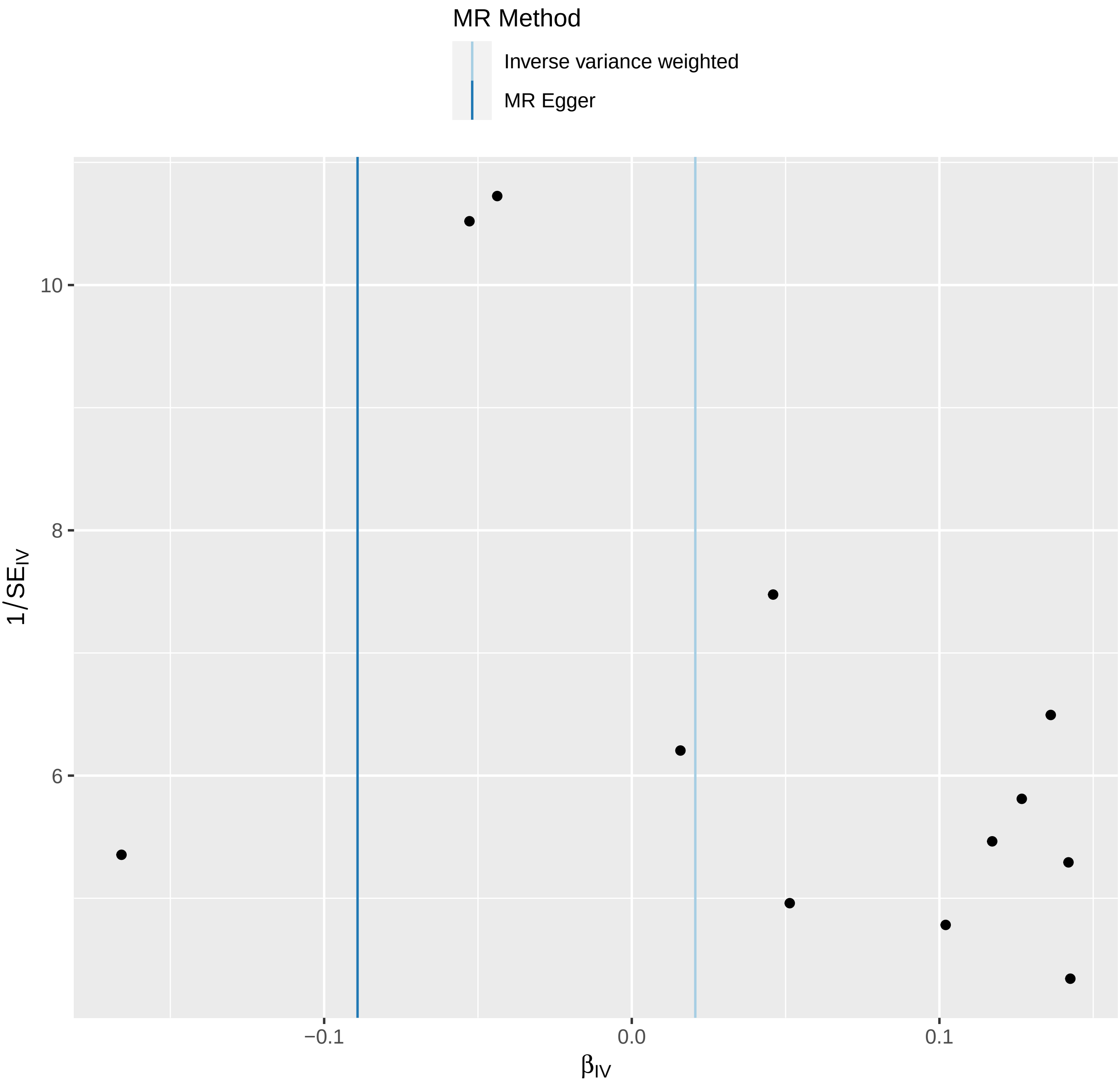

Batch 3−1444 : Selenium on Insulin−like growth factor 1 receptor

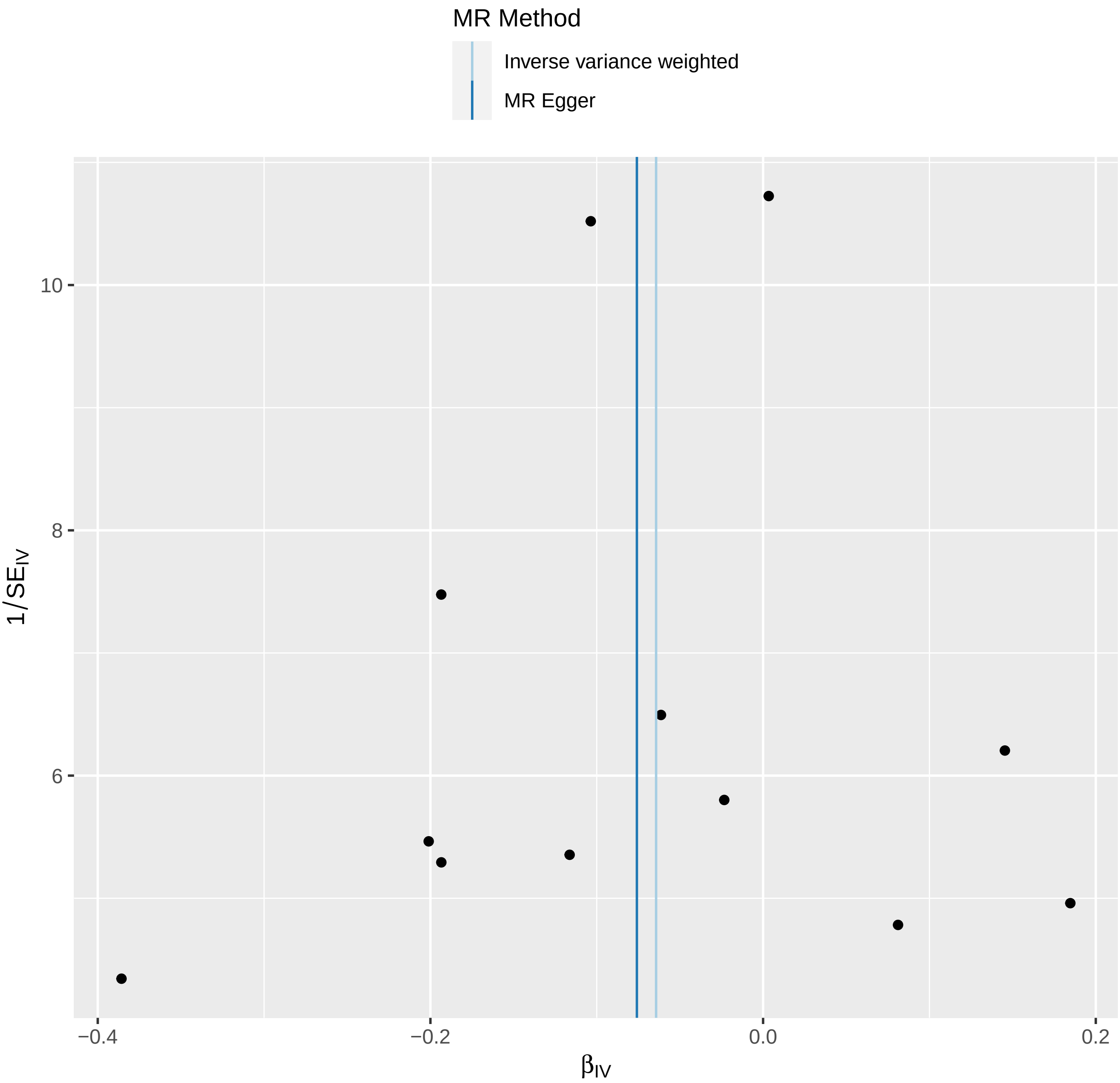

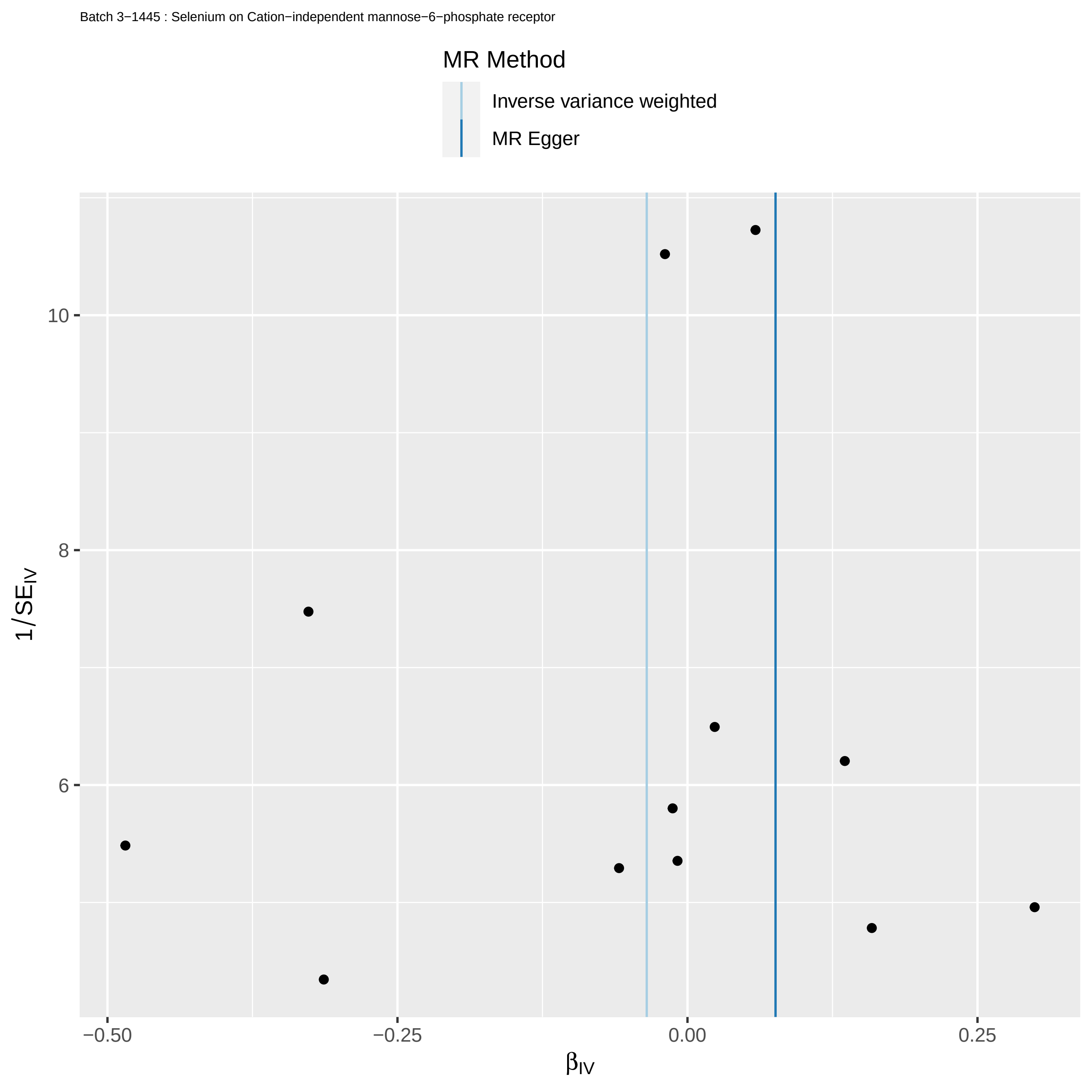

Batch 3−1445 : Selenium on Cation−independent mannose−6−phosphate receptor
MR Method
Inverse variance weighted
MR Egger
10
8
6
1/SE$_{IV}$
−0.50
−0.25
0.00
0.25
β$_{IV}$

Batch 3−1446 : Selenium on Insulin−like growth factor−binding protein 1

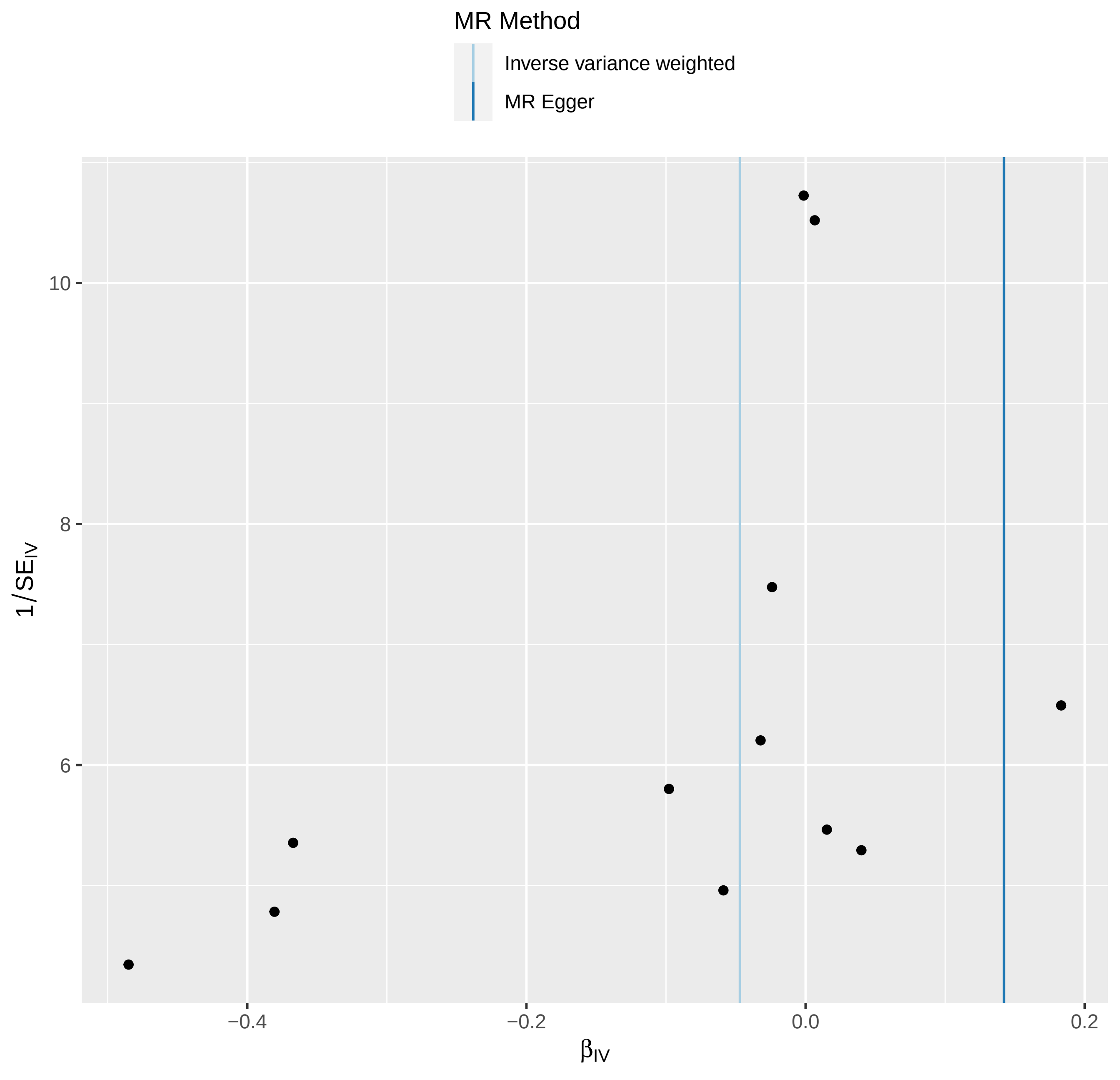

Batch 3−1447 : Selenium on Insulin−like growth factor−binding protein 1

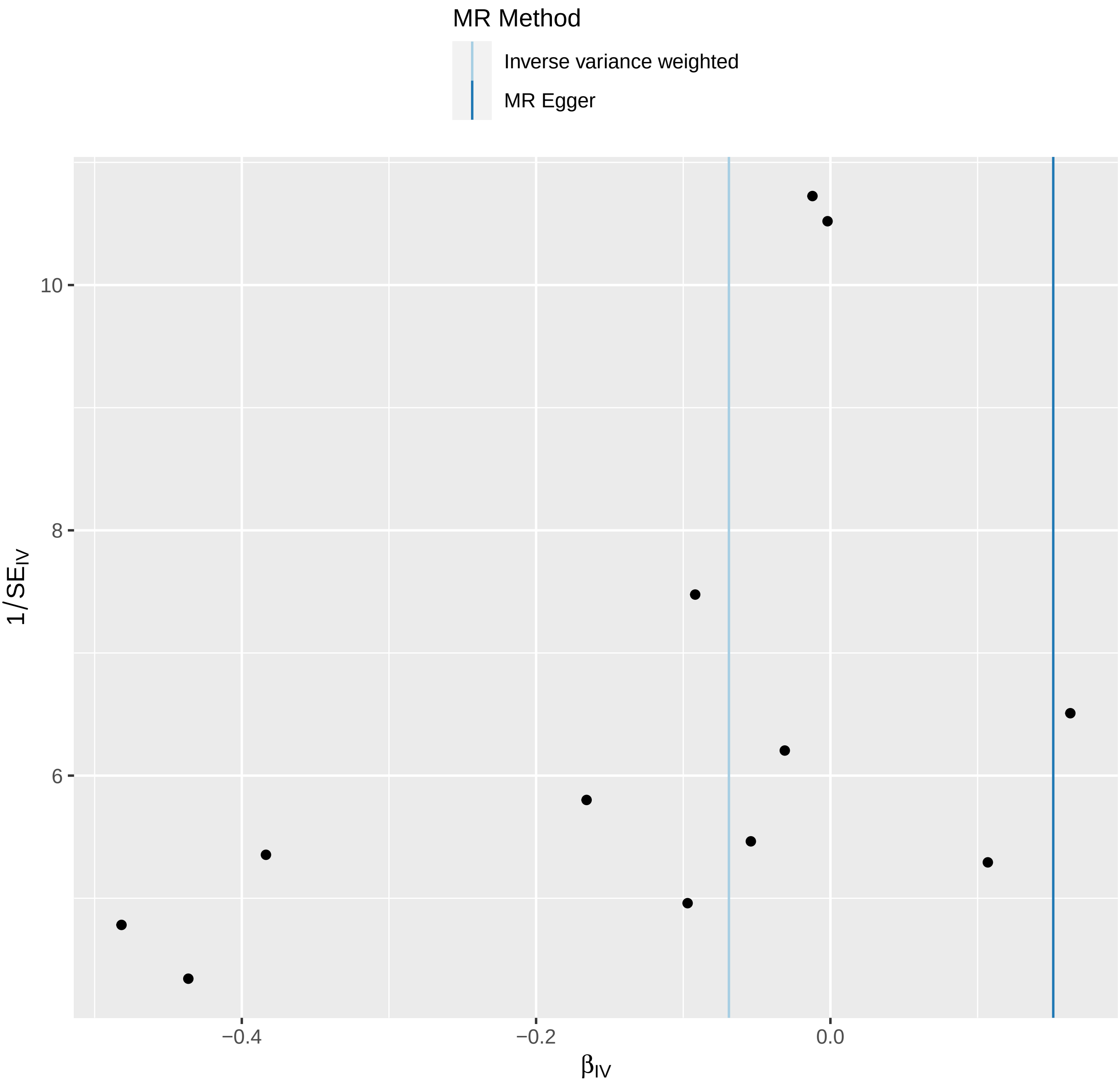

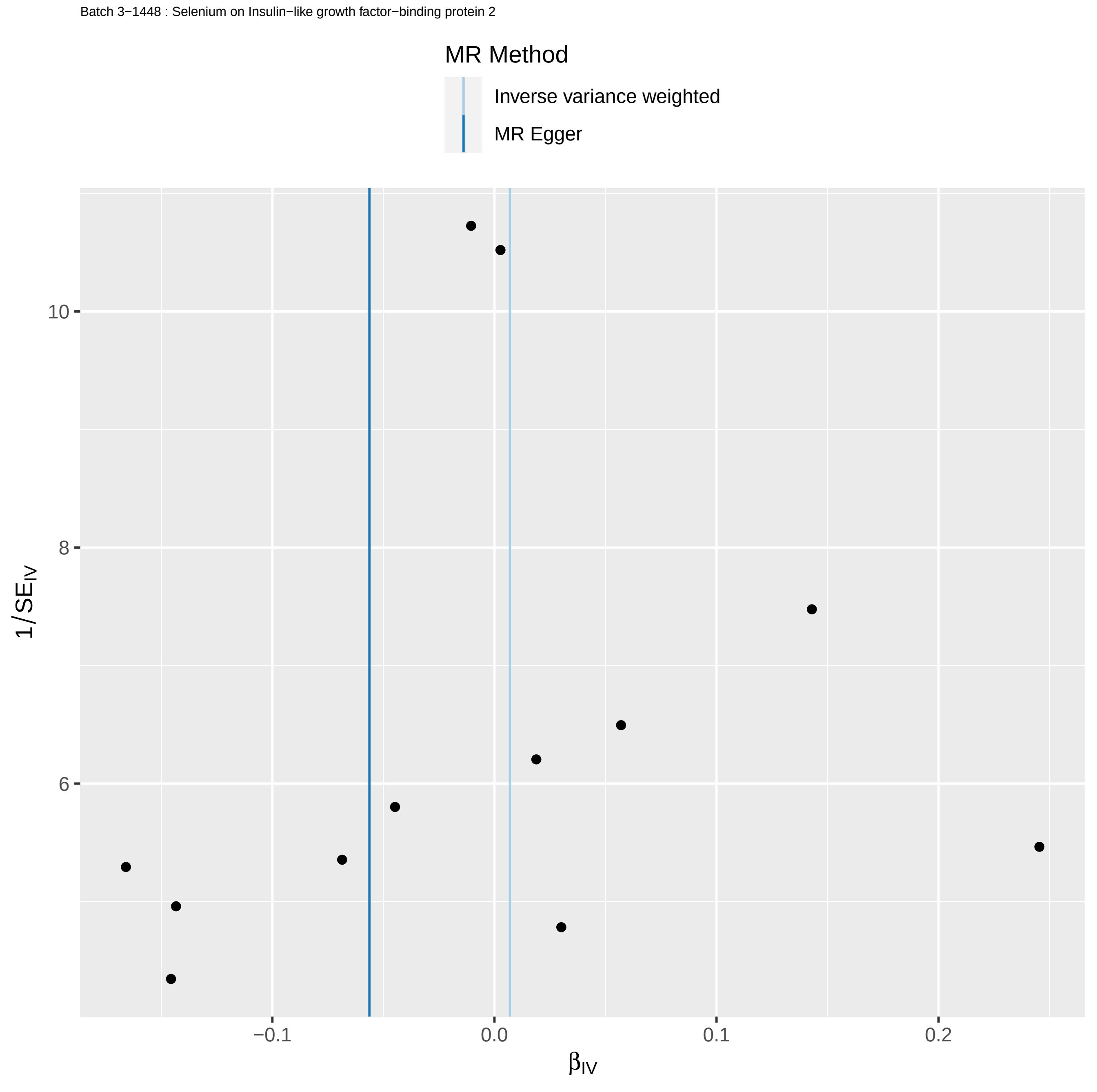
Batch 3−1448 : Selenium on Insulin−like growth factor−binding protein 2
MR Method
Inverse variance weighted
MR Egger
10
8
6
−0.1
0.0
0.1
0.2
$1/SE_{IV}$
β_{IV}

Batch 3−1449 : Selenium on Insulin−like growth factor−binding protein 3

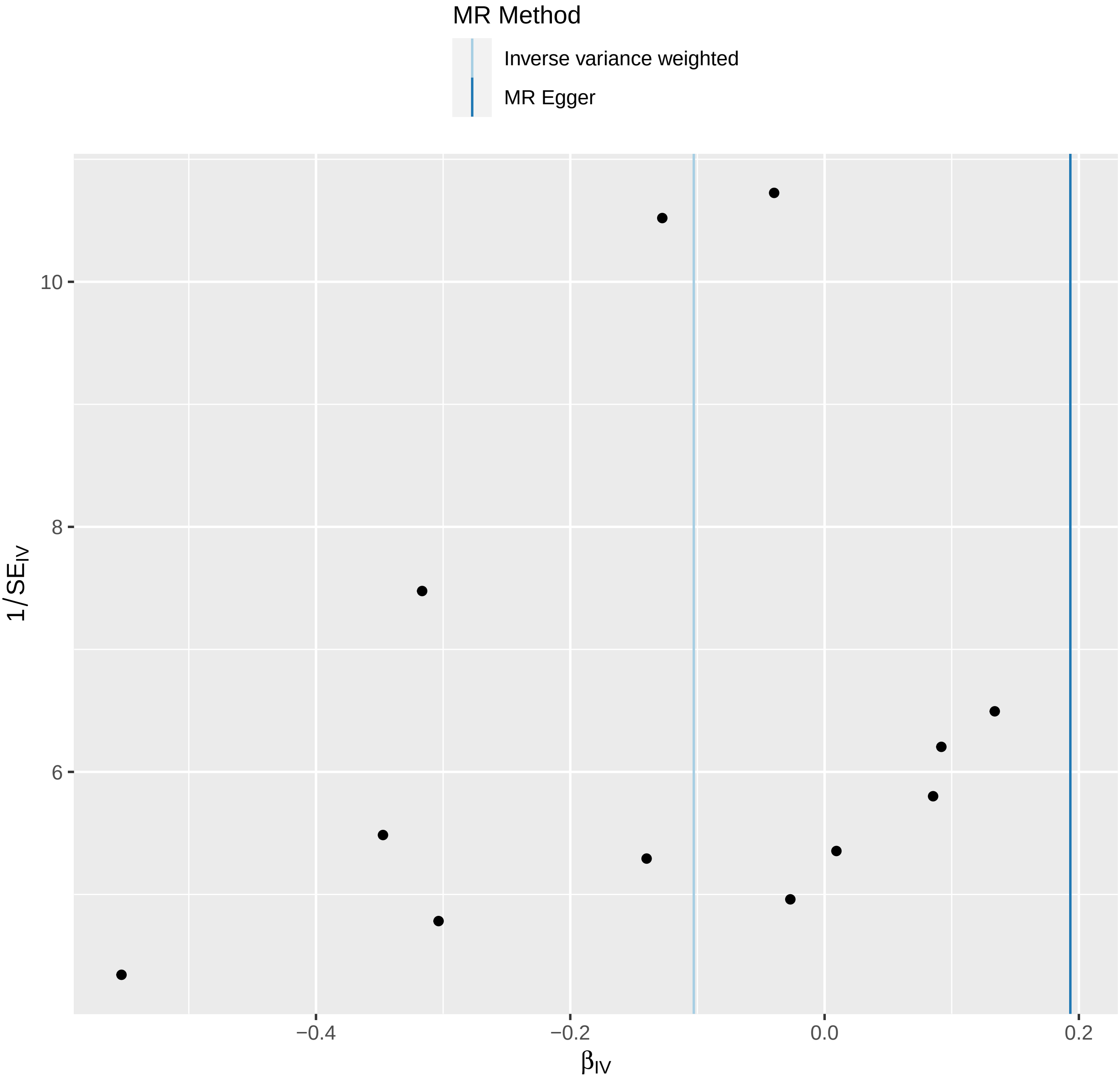

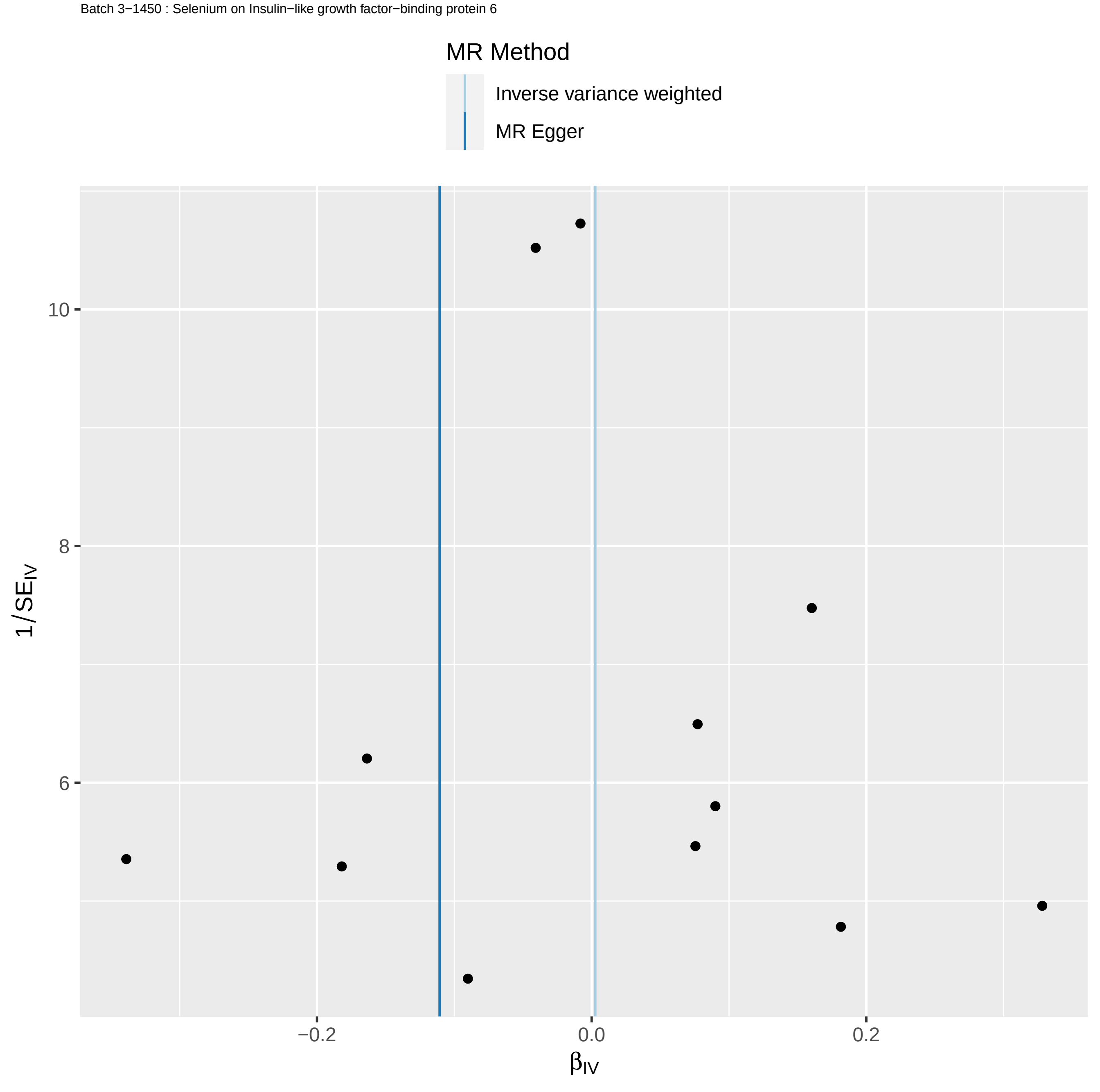
Batch 3−1450 : Selenium on Insulin−like growth factor−binding protein 6
MR Method
Inverse variance weighted
MR Egger
10
8
6
1/SE_IV
−0.2
0.0
0.2
β_IV

Batch 3−1451 : Selenium on Insulin−like growth factor−binding protein 7

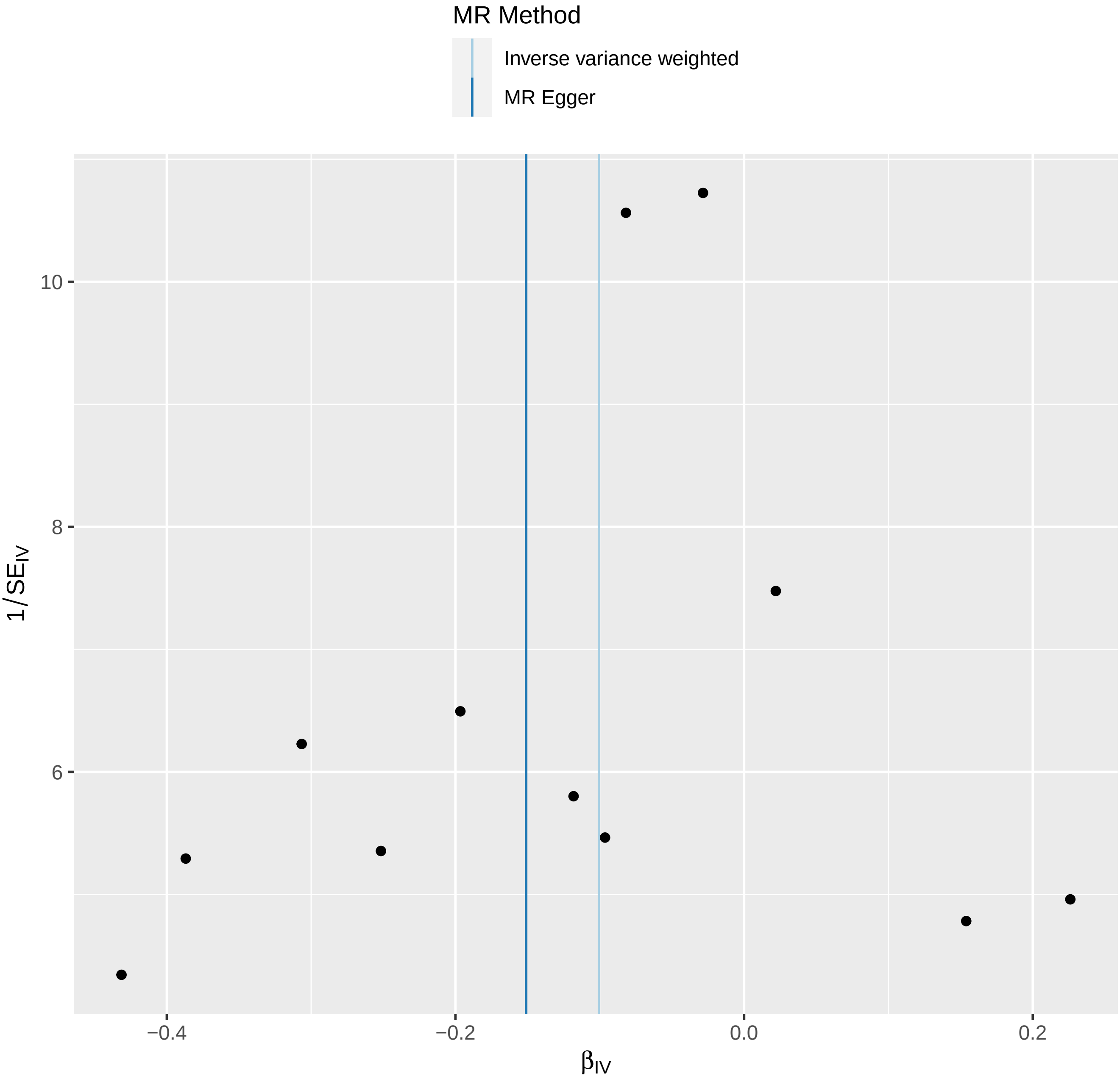

Batch 3−1452 : Selenium on Insulin−like growth factor−binding protein−like 1

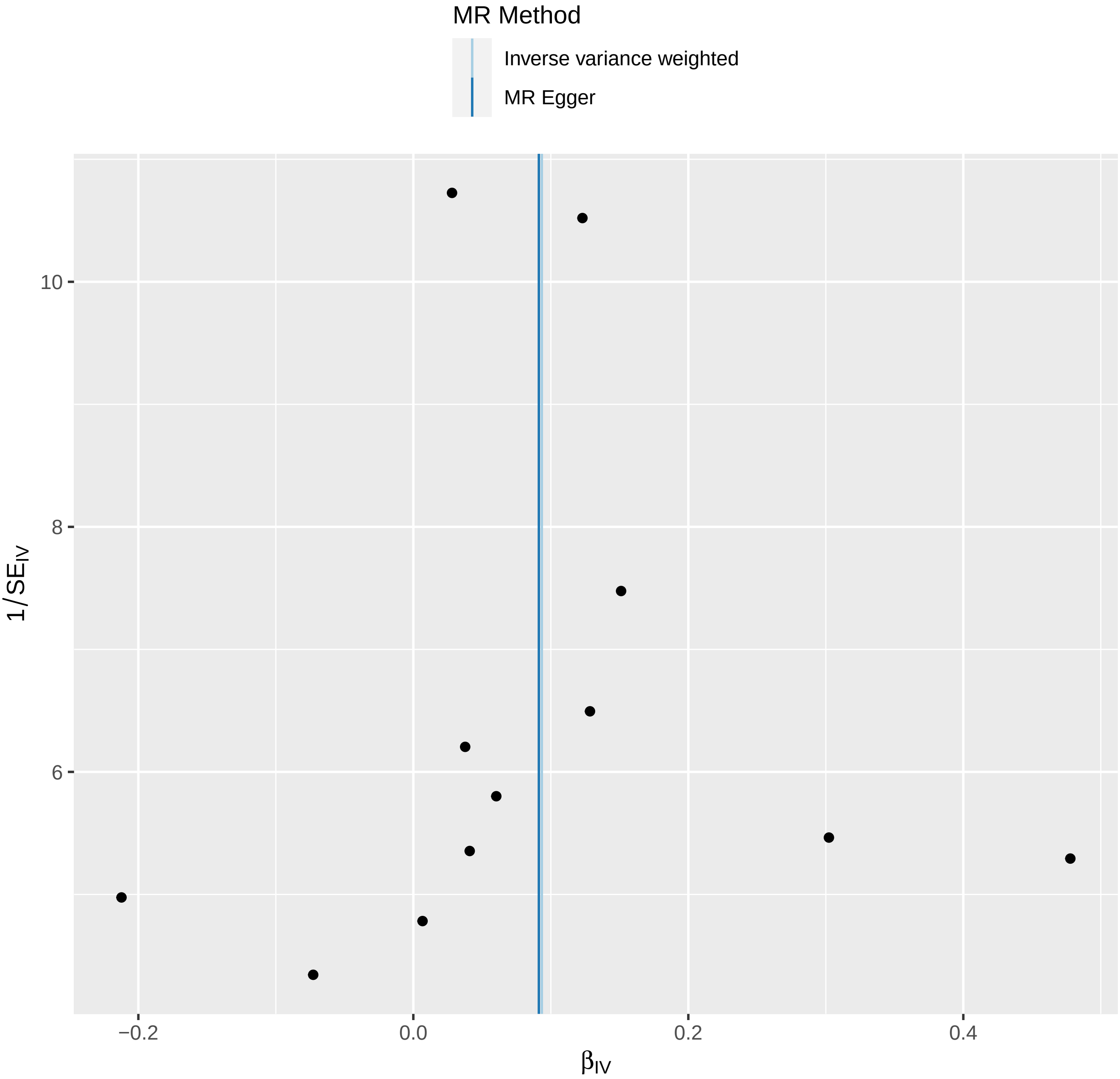

Batch 3−1453 : Selenium on Insulin growth factor−like family member 3

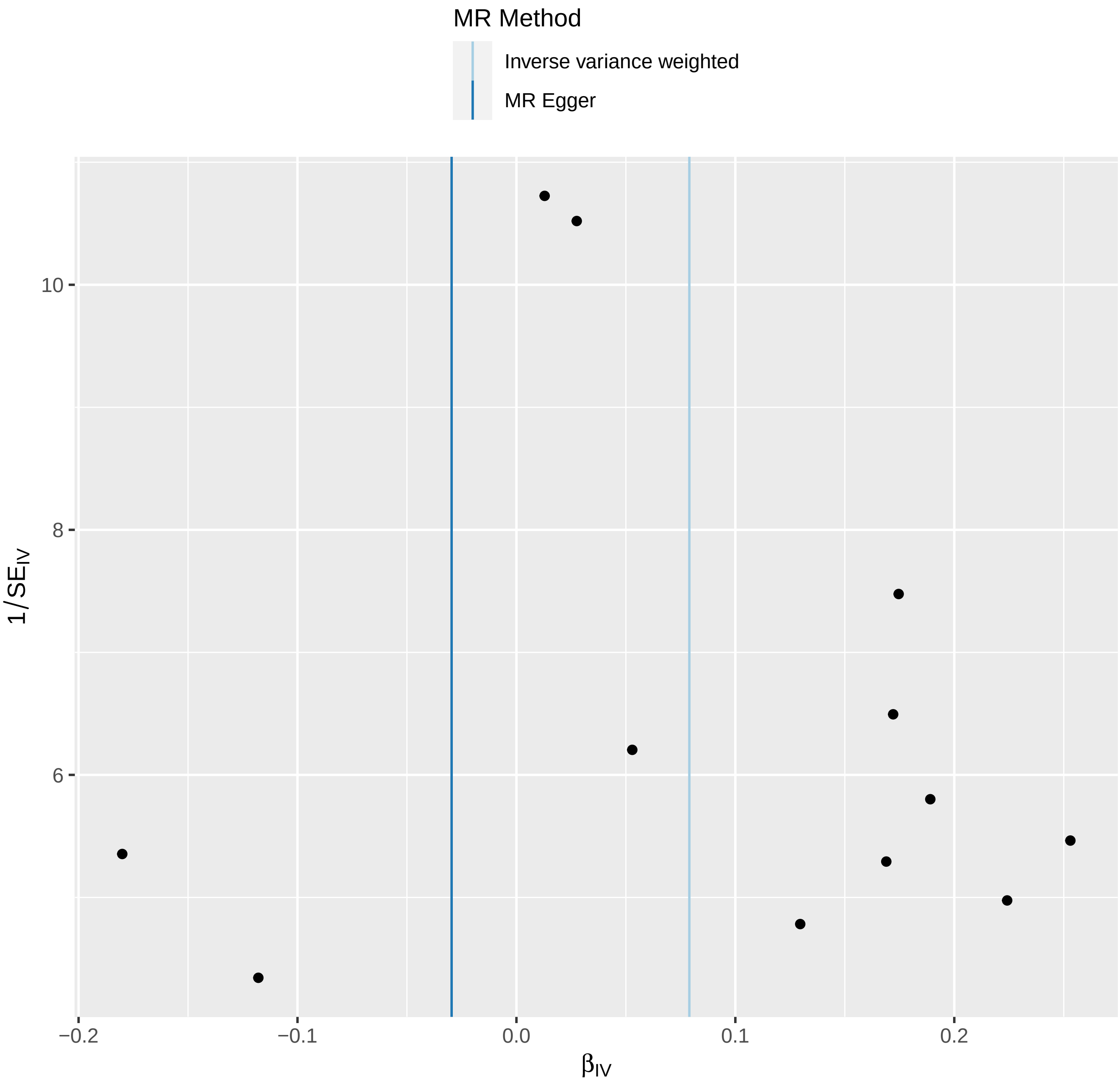

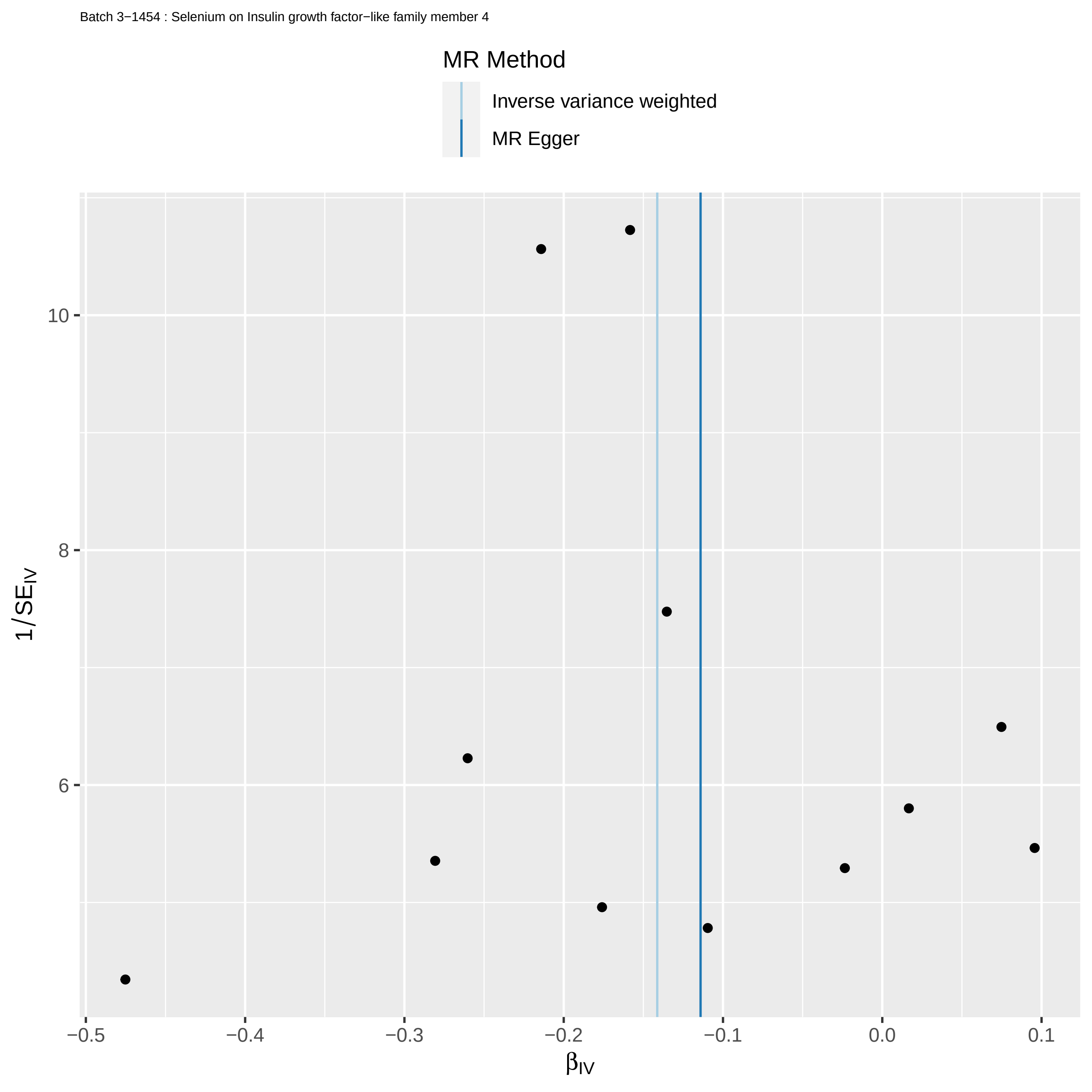
Batch 3−1454 : Selenium on Insulin growth factor−like family member 4
MR Method
Inverse variance weighted
MR Egger
10
8
6
1/SE$_{IV}$
−0.5
−0.4
−0.3
−0.2
−0.1
0.0
0.1
β$_{IV}$

Batch 3−1455 : Selenium on IGF−like family receptor 1

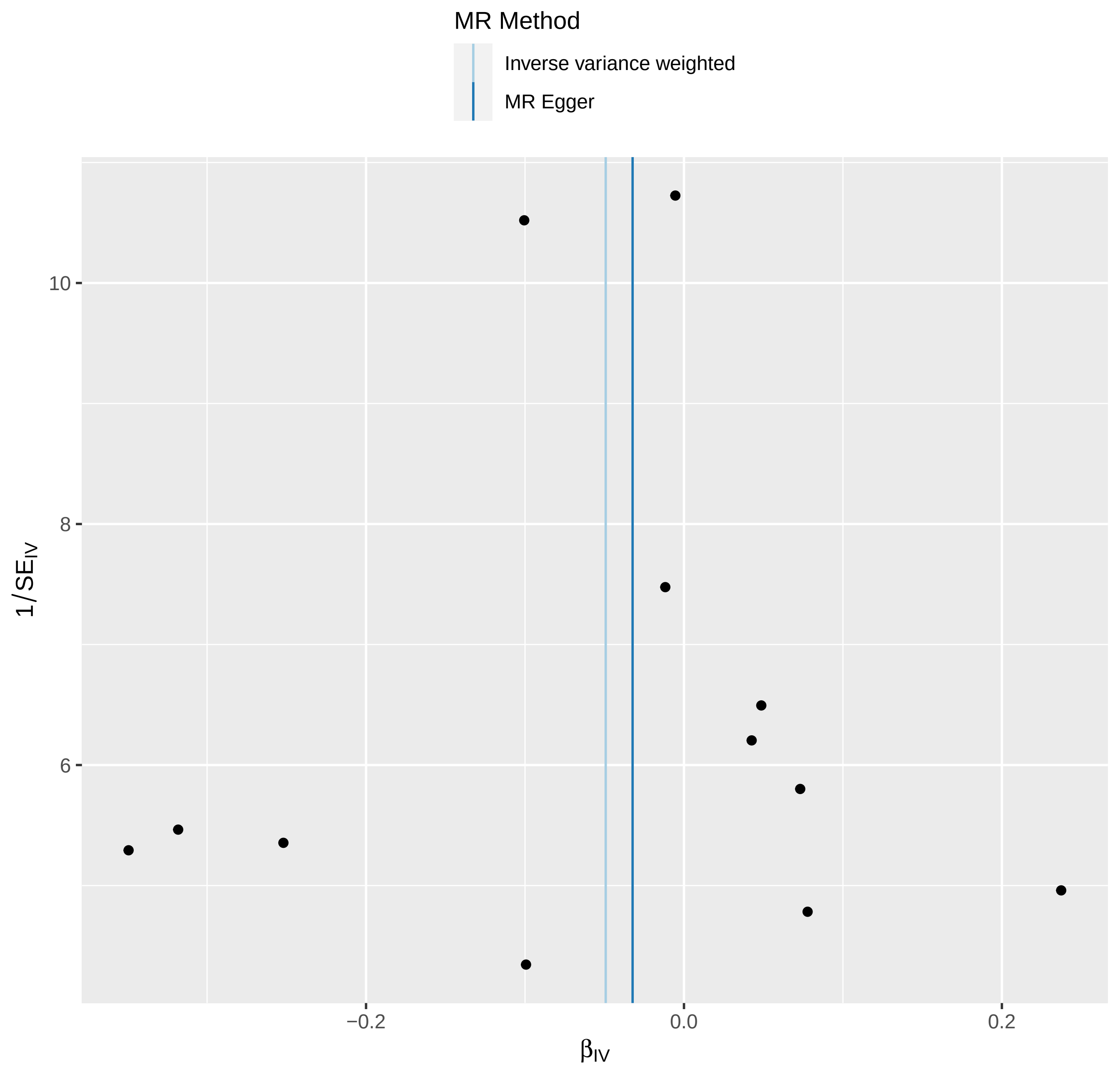

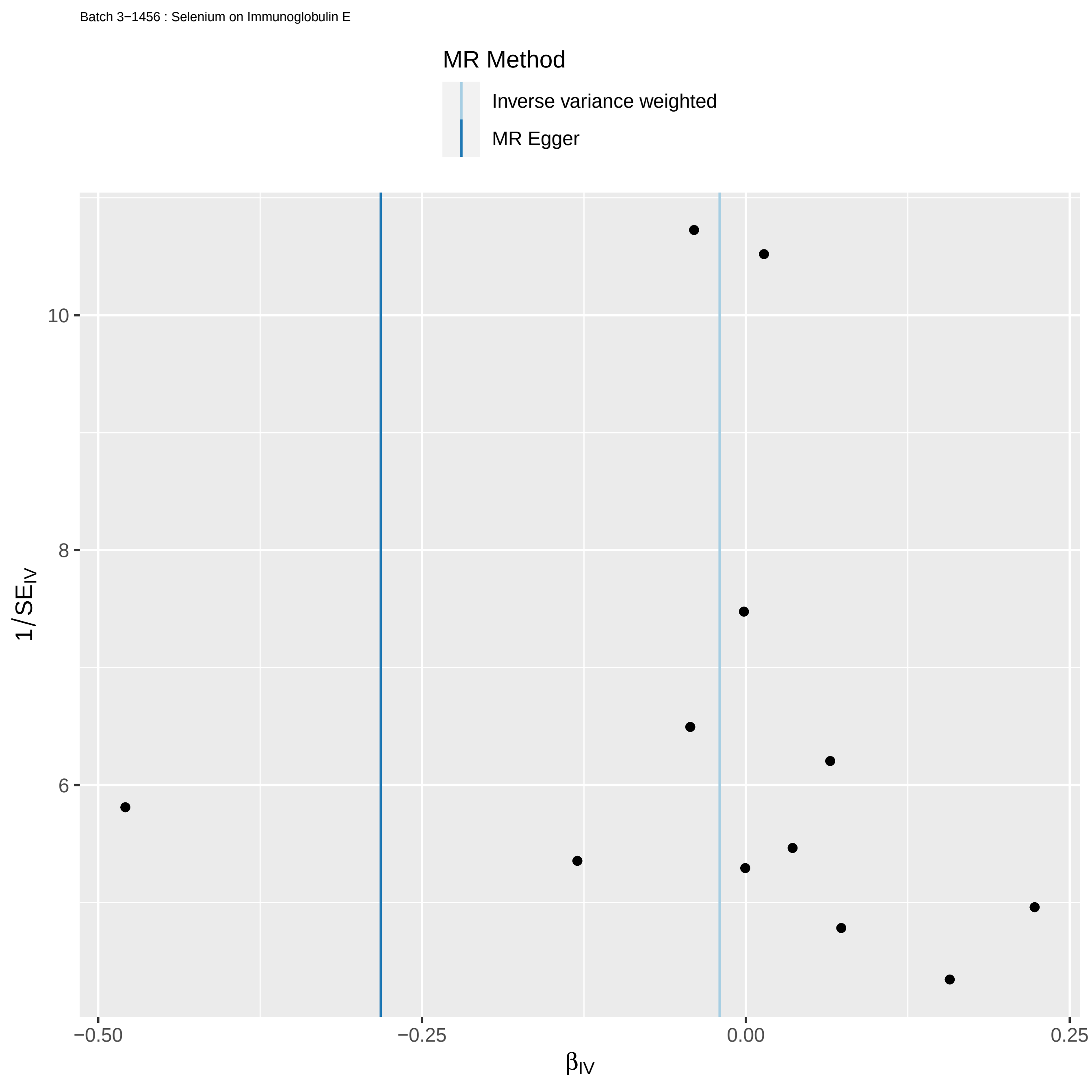

Batch 3−1456 : Selenium on Immunoglobulin E
MR Method
Inverse variance weighted
MR Egger
10
8
6
1/SE_IV
−0.50
−0.25
0.00
0.25
β_IV

Batch 3−1457 : Selenium on Immunoglobulin J chain

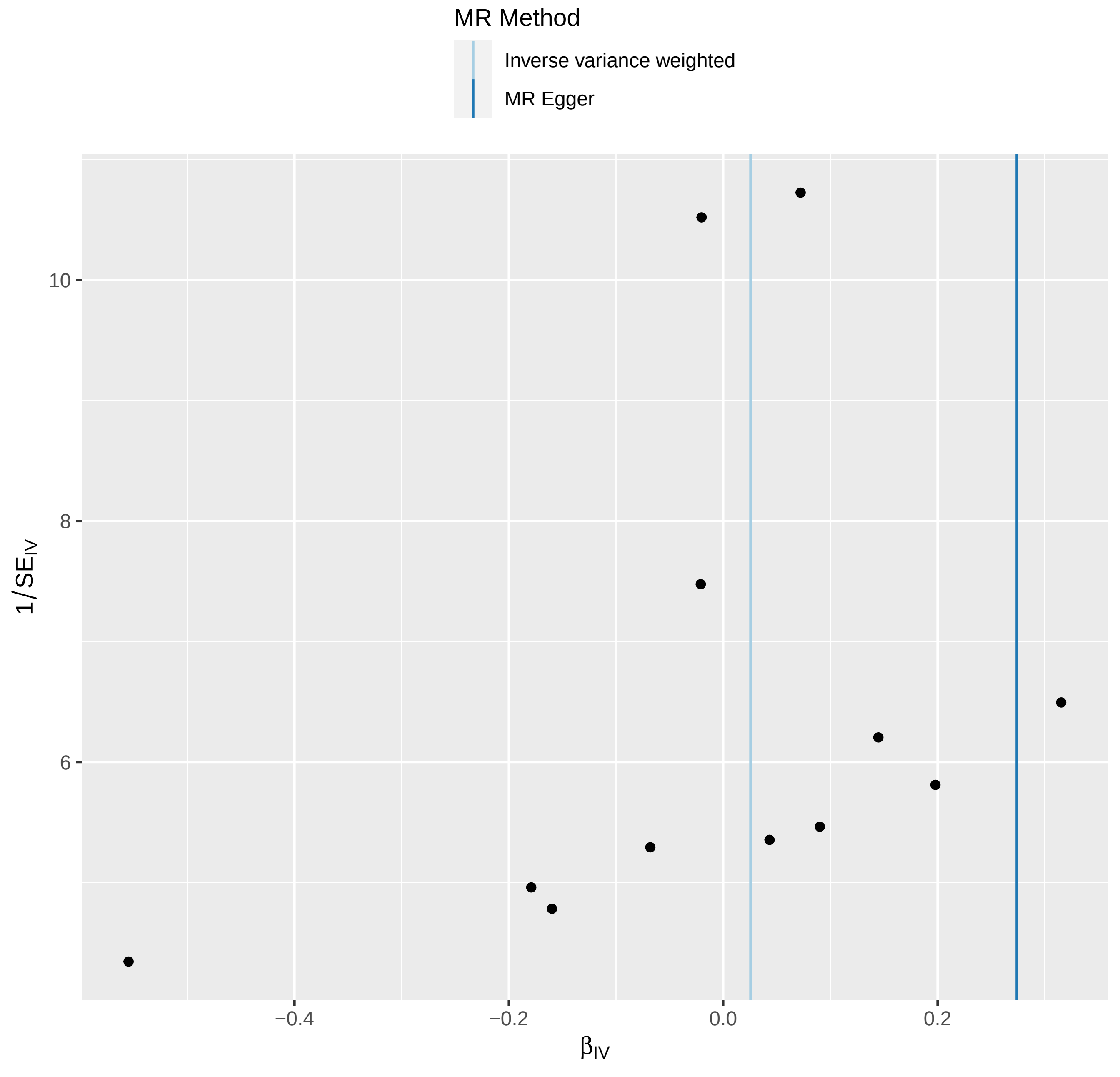

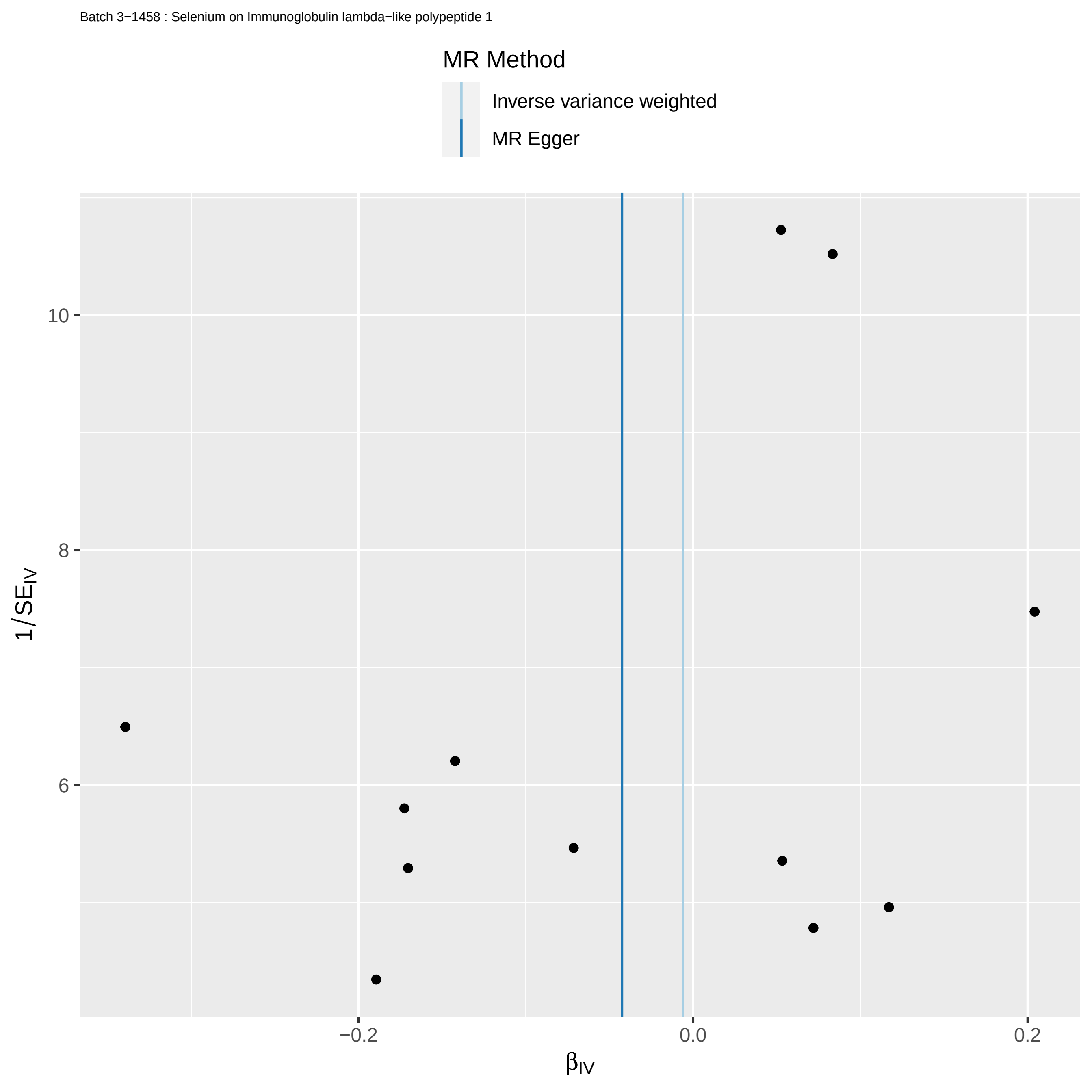
Batch 3−1458 : Selenium on Immunoglobulin lambda−like polypeptide 1
MR Method
Inverse variance weighted
MR Egger
10
8
6
1/SE_IV
−0.2
0.0
0.2
β_IV

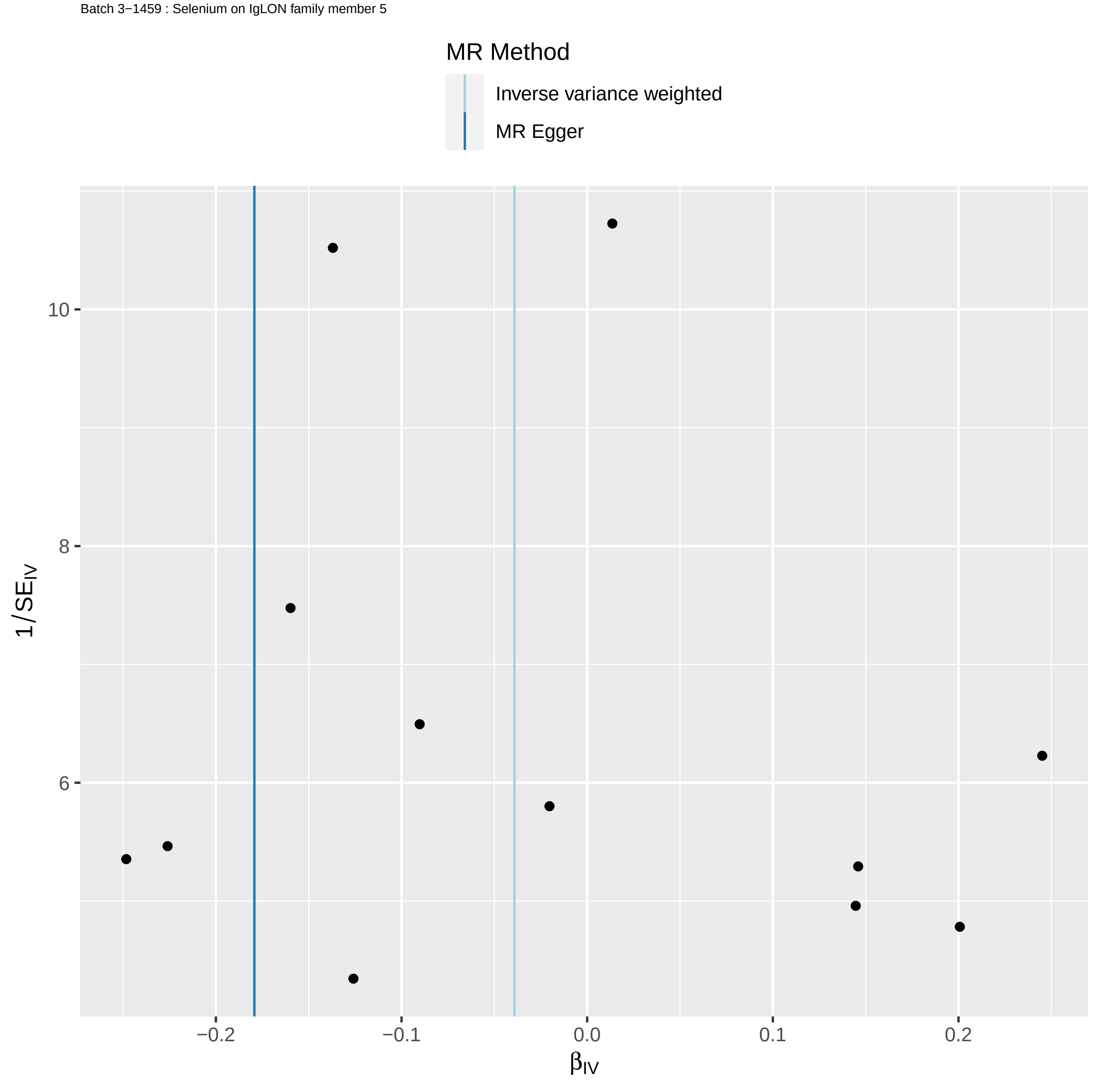
Batch 3−1459 : Selenium on IgLON family member 5
MR Method
Inverse variance weighted
MR Egger
10
8
6
1/SE$_{IV}$
−0.2
−0.1
0.0
0.1
0.2
β$_{IV}$

Batch 3−1460 : Selenium on Immunoglobulin superfamily member 11

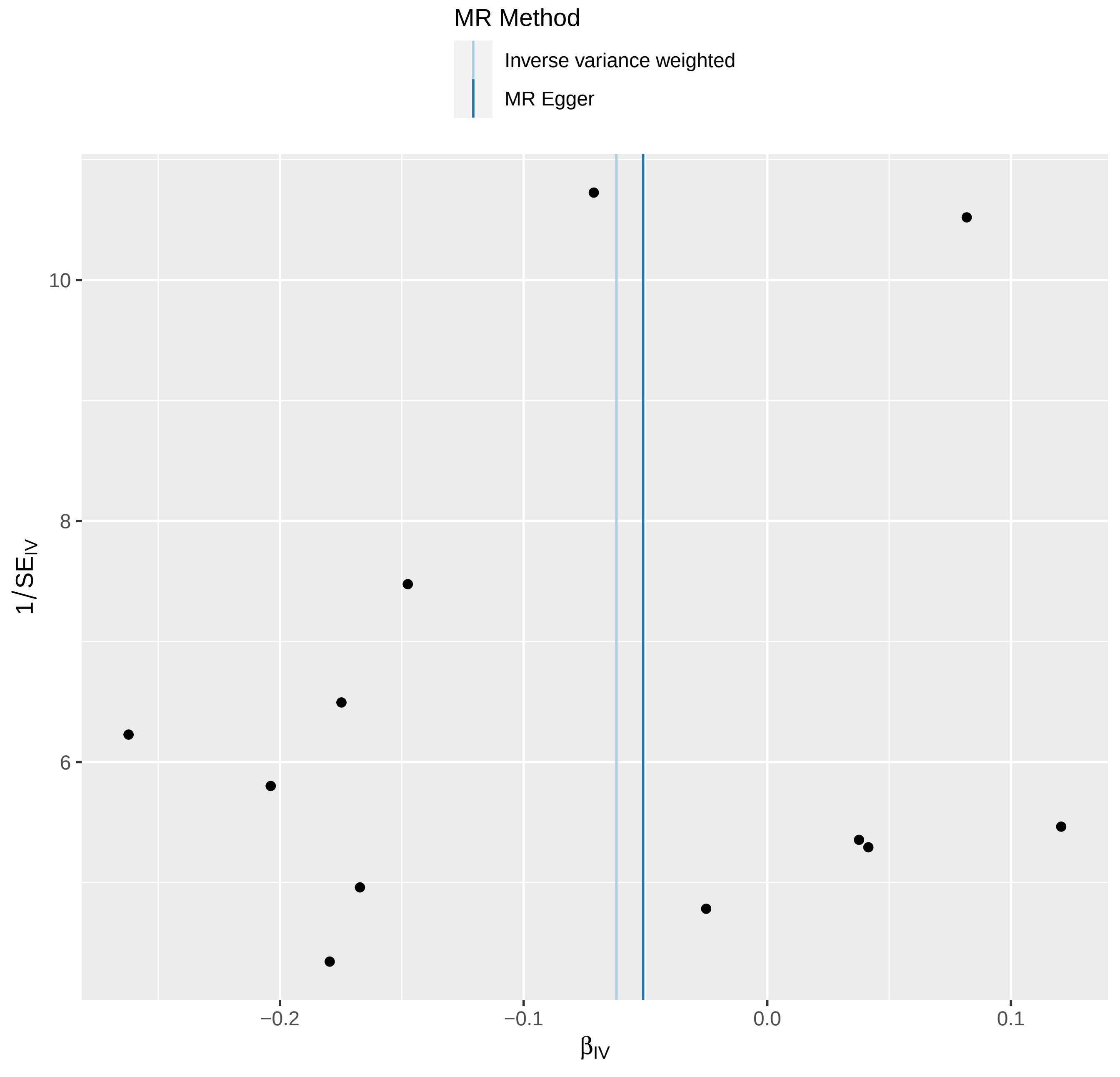

Batch 3−1461 : Selenium on Immunoglobulin superfamily member 11

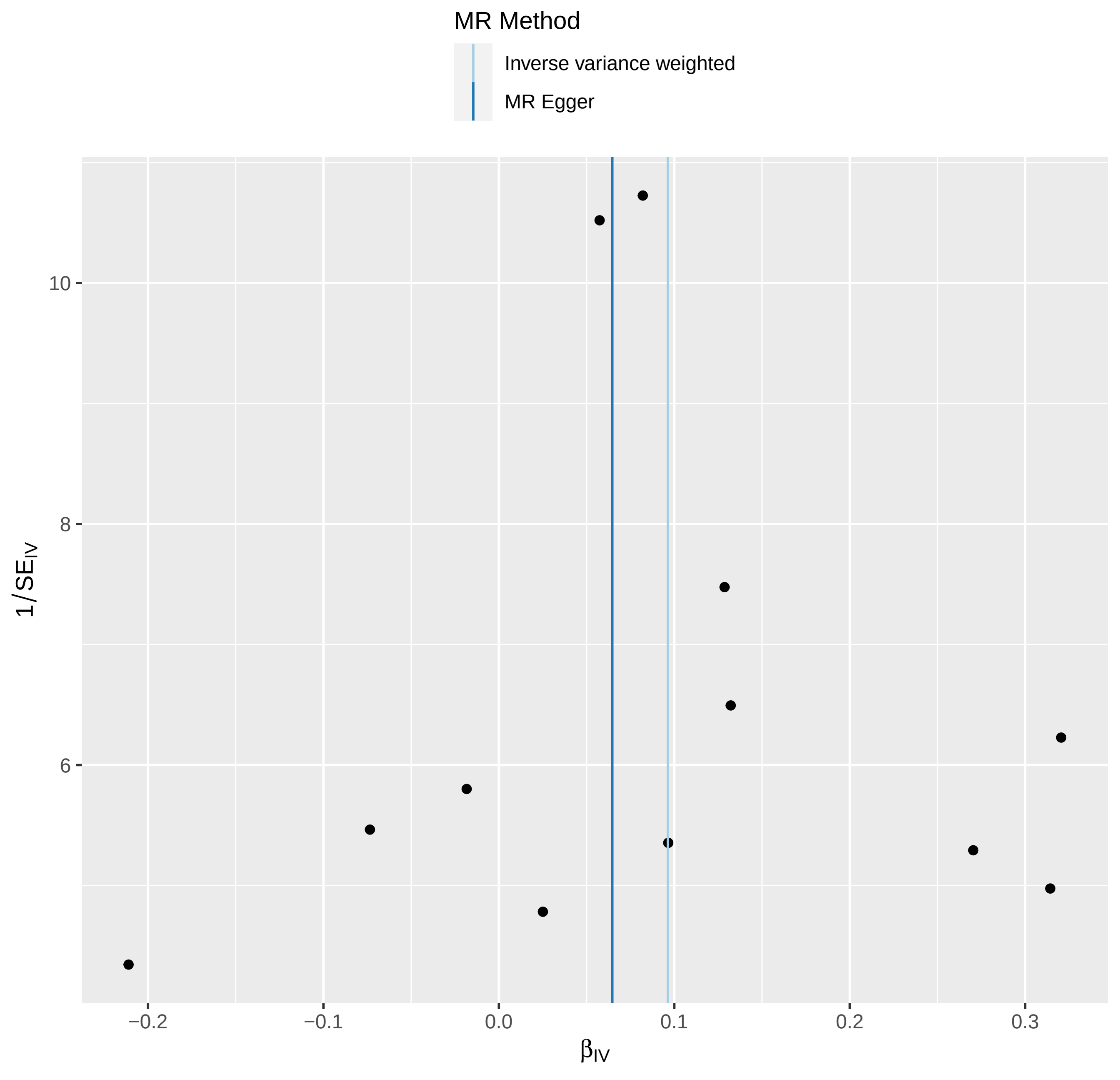

Batch 3−1462 : Selenium on Immunoglobulin superfamily member 8

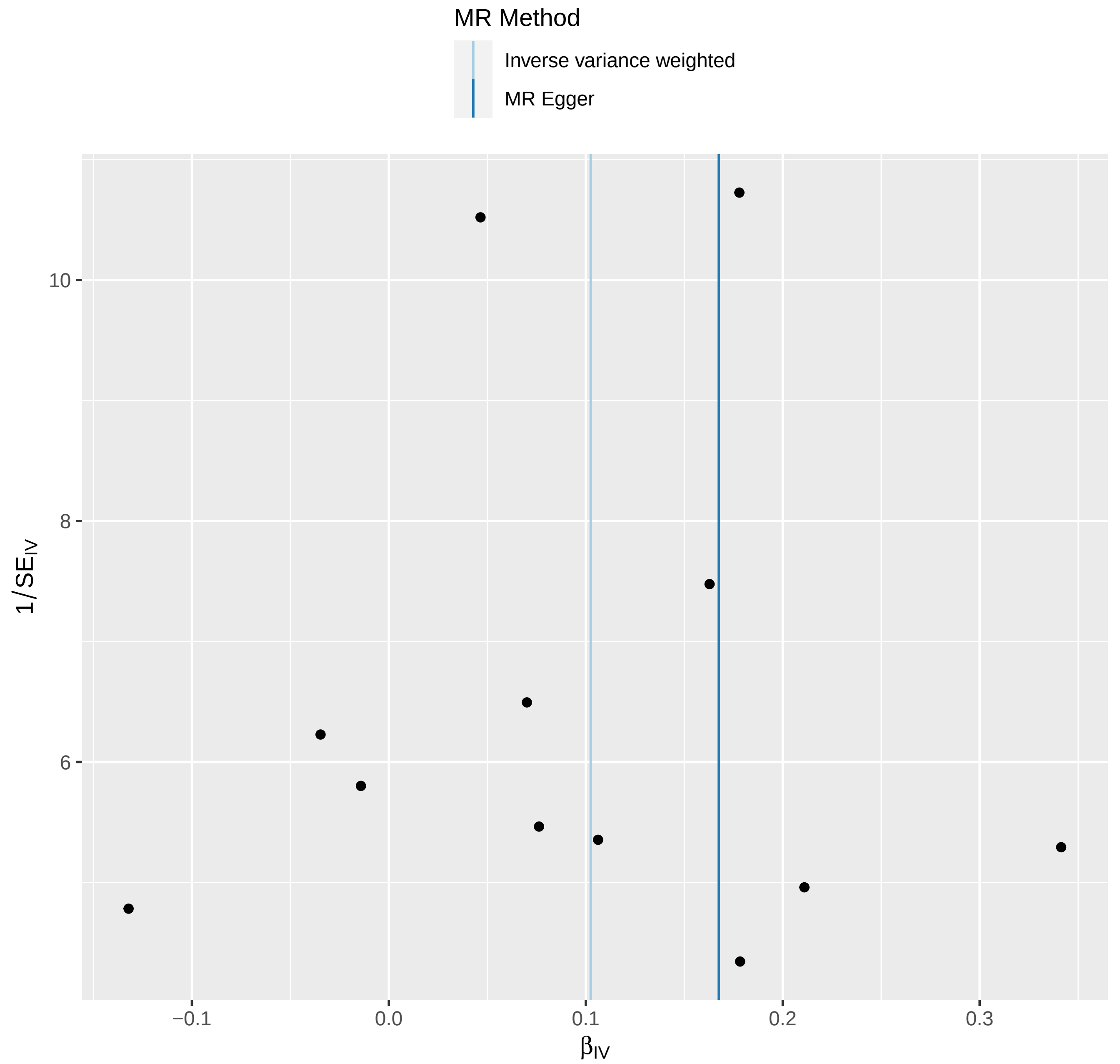

Batch 3−1463 : Selenium on Protein turtle homolog A

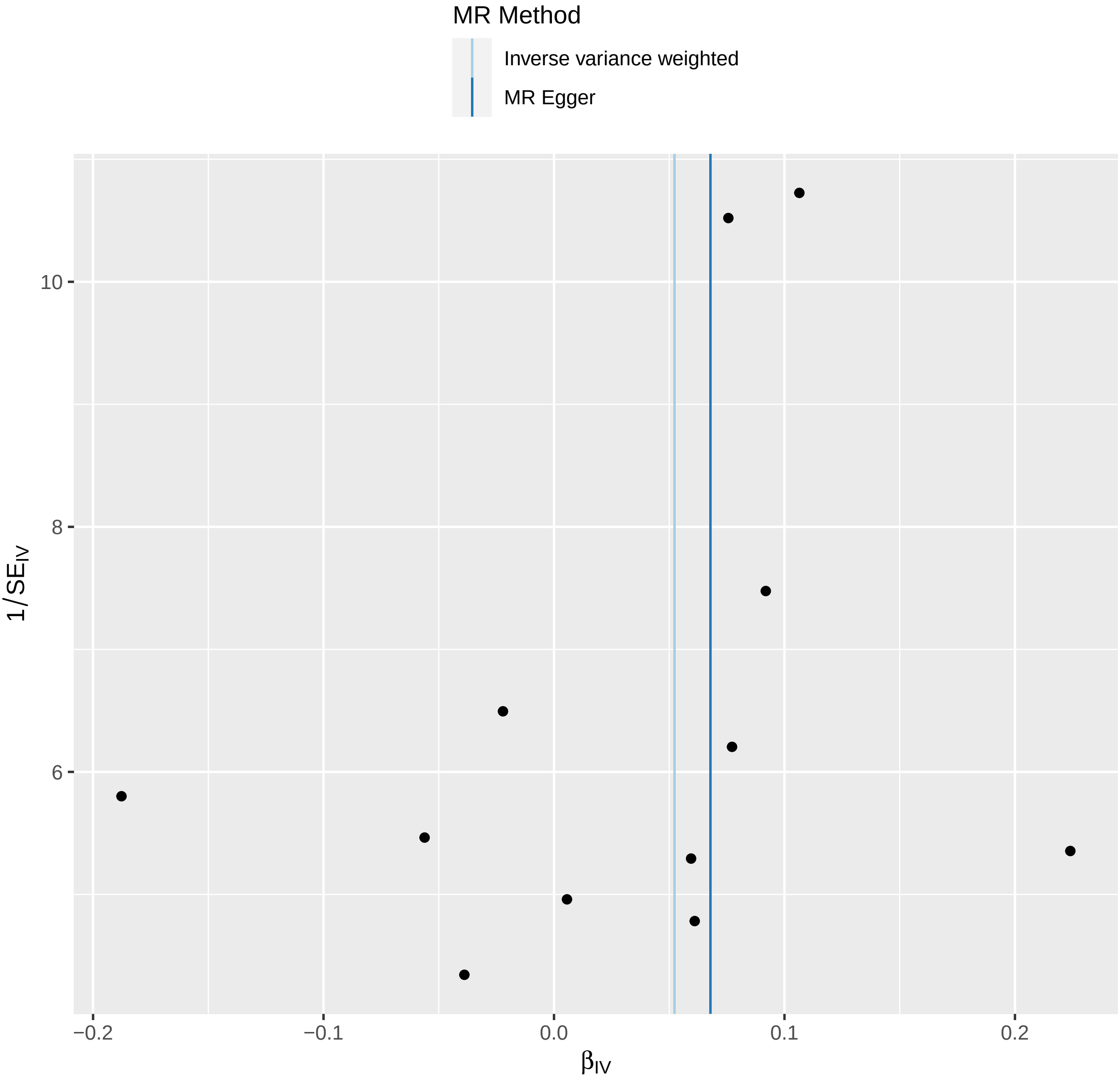

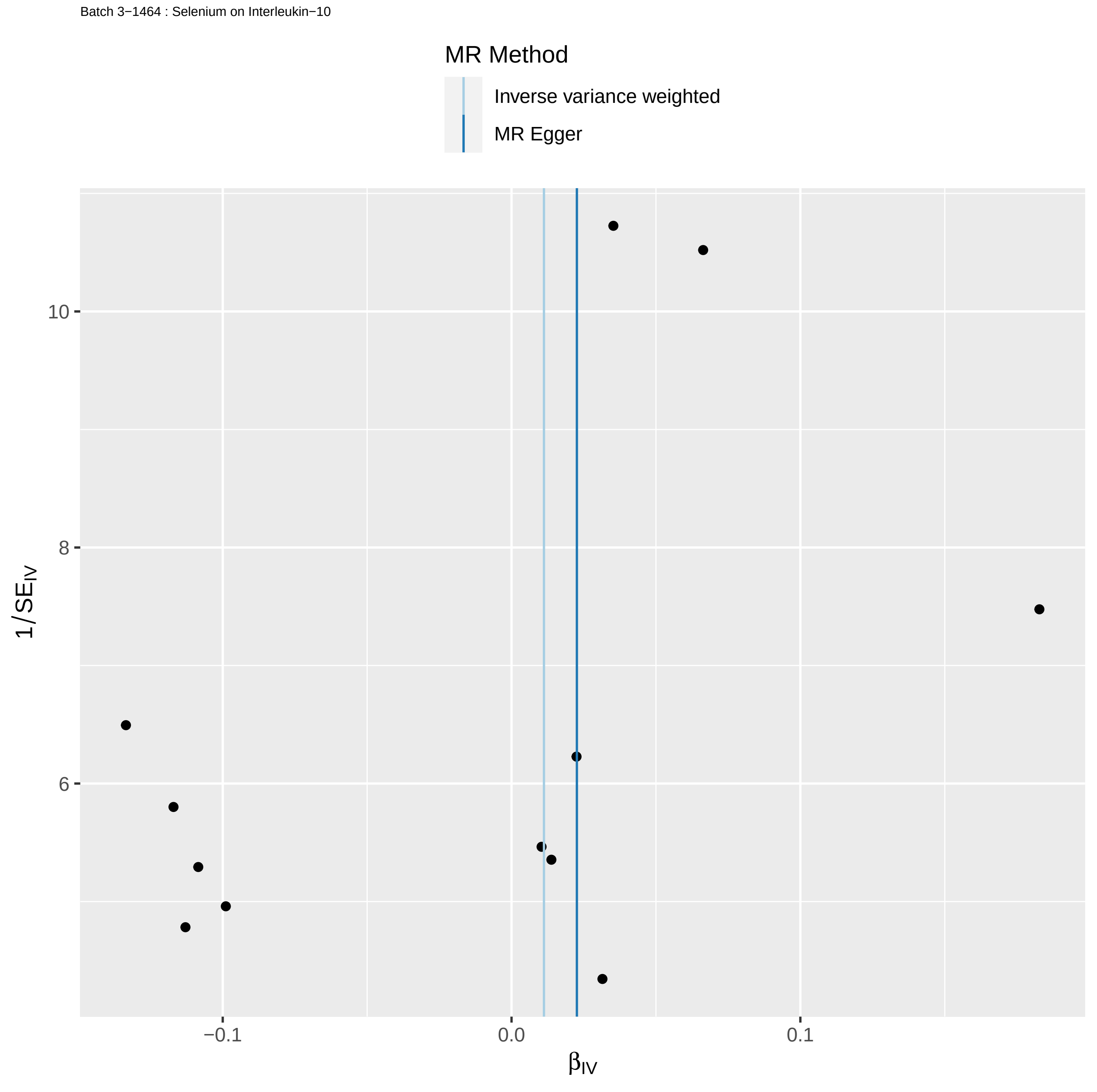

Batch 3−1464 : Selenium on Interleukin−10
MR Method
Inverse variance weighted
MR Egger
10
8
6
1/SE$_{IV}$
−0.1
0.0
0.1
β$_{IV}$

Batch 3−1465 : Selenium on Interleukin−10 receptor subunit beta

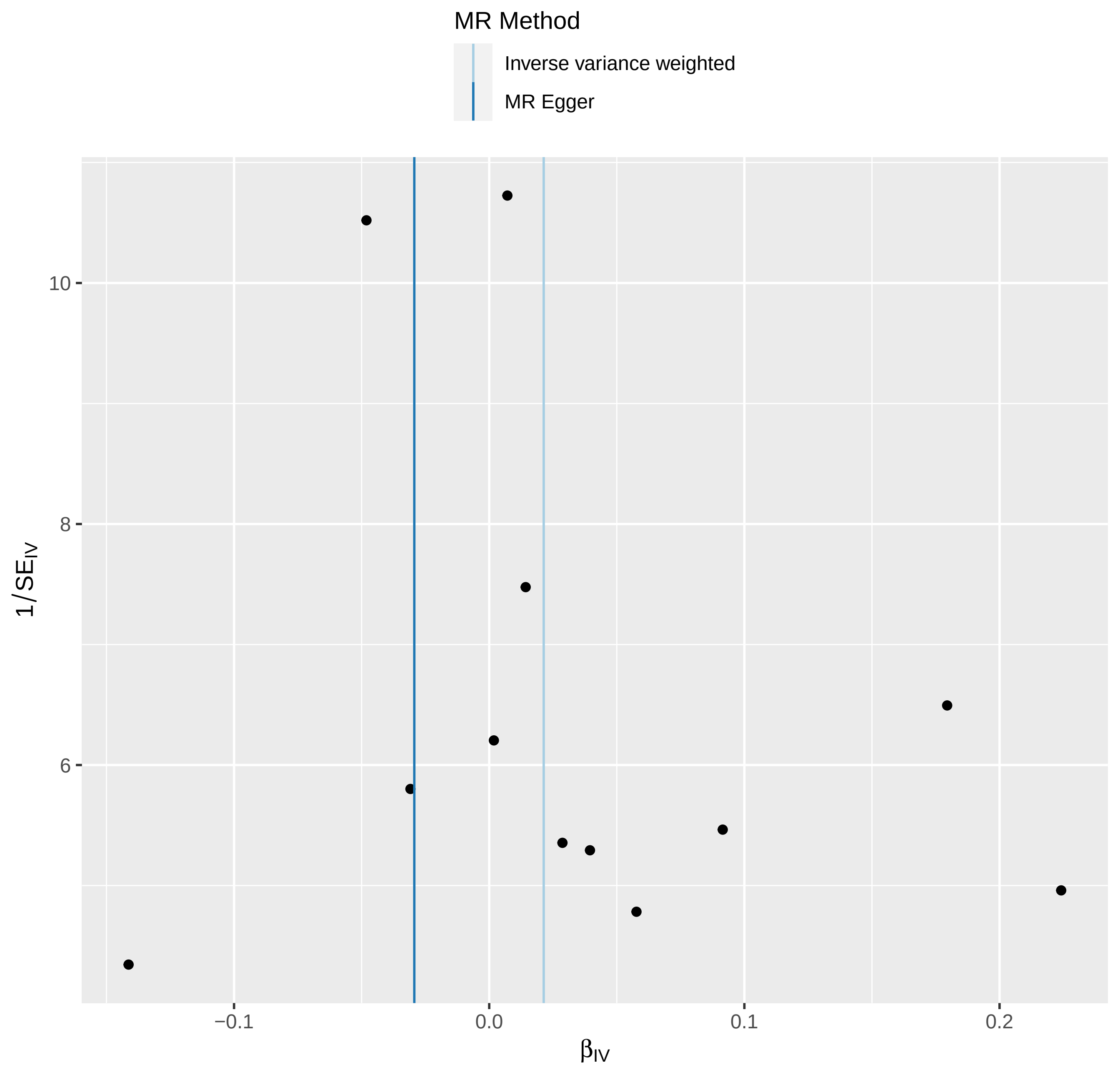

Batch 3−1466 : Selenium on Interleukin−11

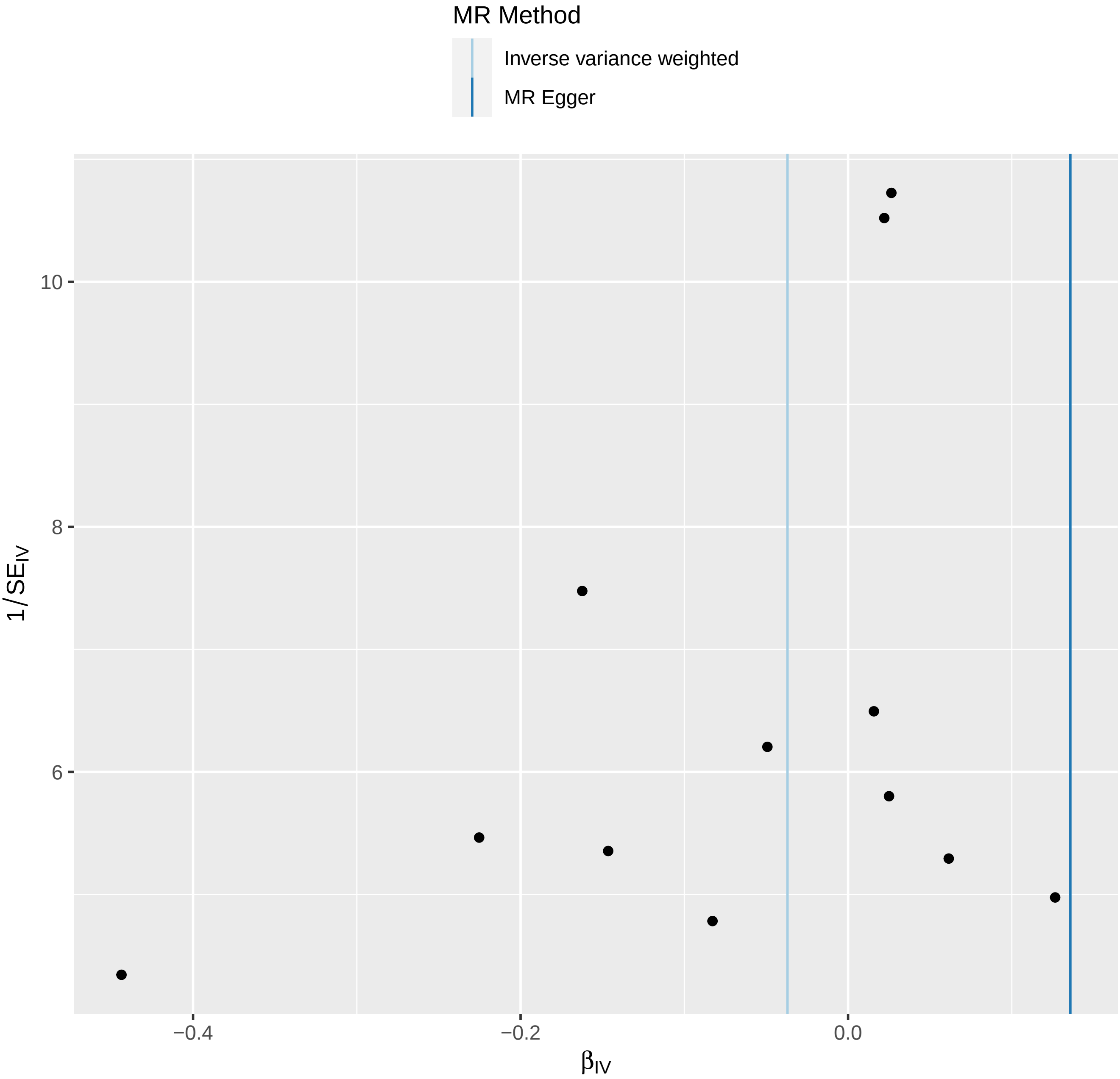

Batch 3−1467 : Selenium on Interleukin−11 receptor subunit alpha

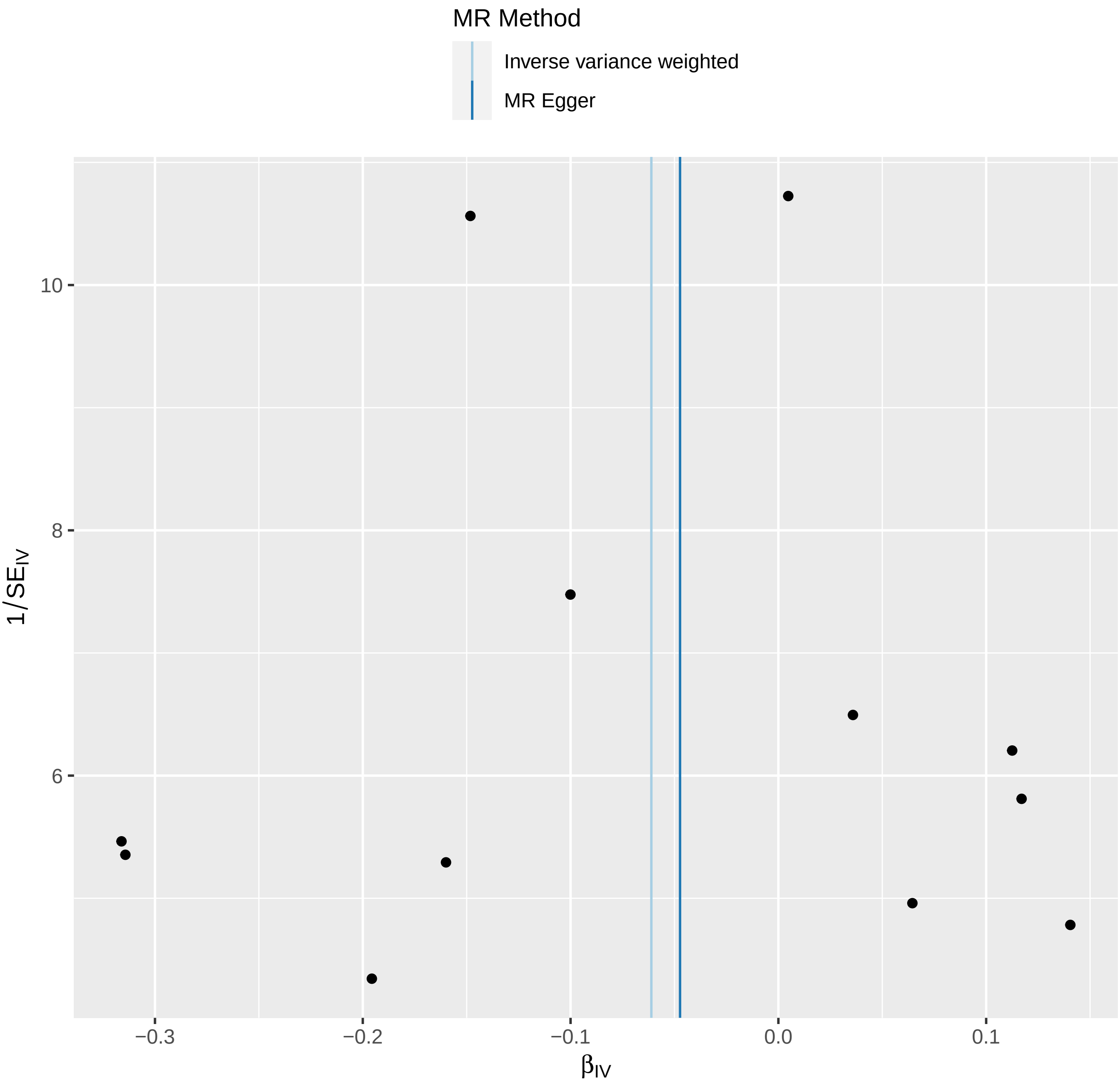

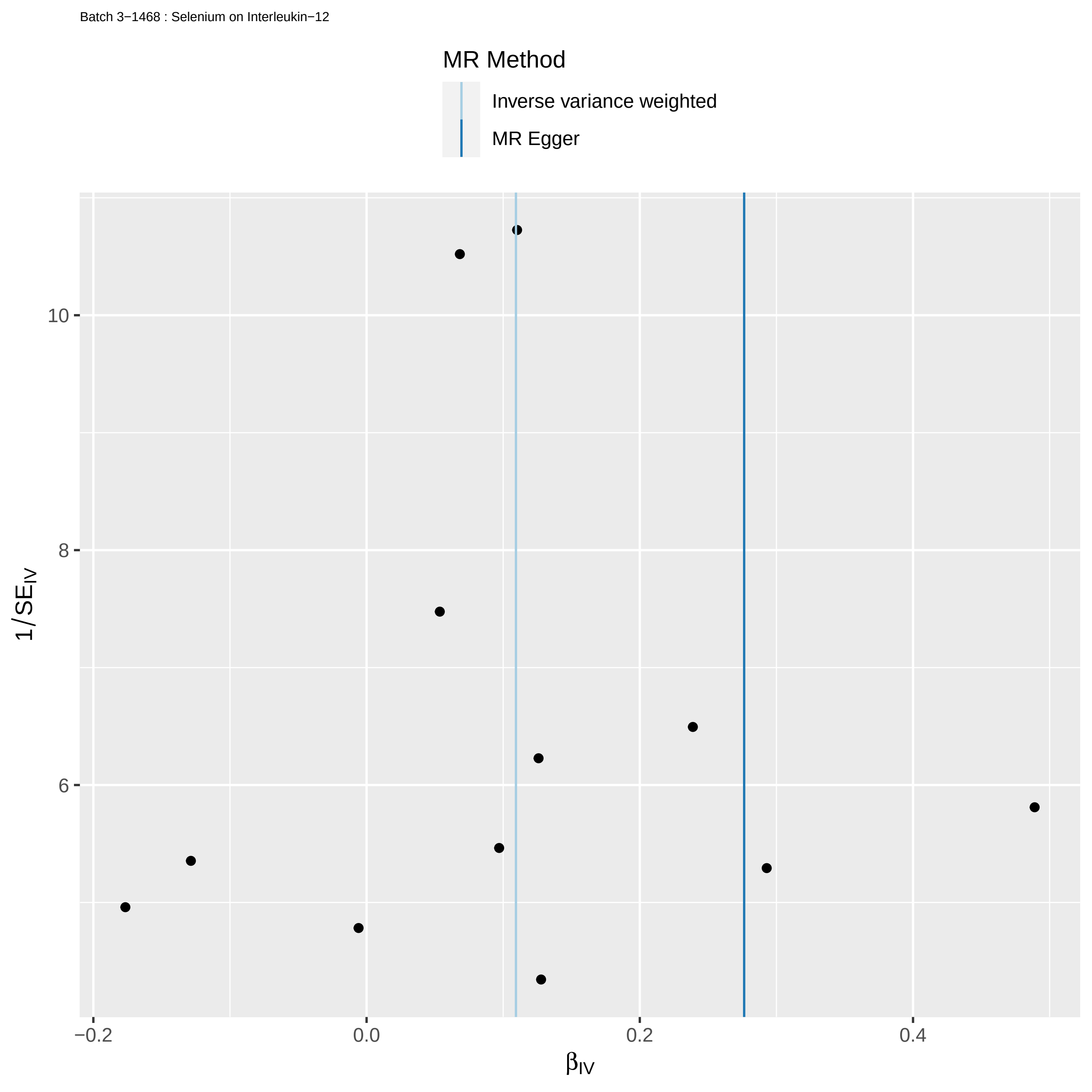

Batch 3−1468 : Selenium on Interleukin−12
MR Method
Inverse variance weighted
MR Egger
10
8
6
1/SE$_{IV}$
−0.2
0.0
0.2
0.4
β$_{IV}$

Batch 3−1469 : Selenium on Interleukin−12

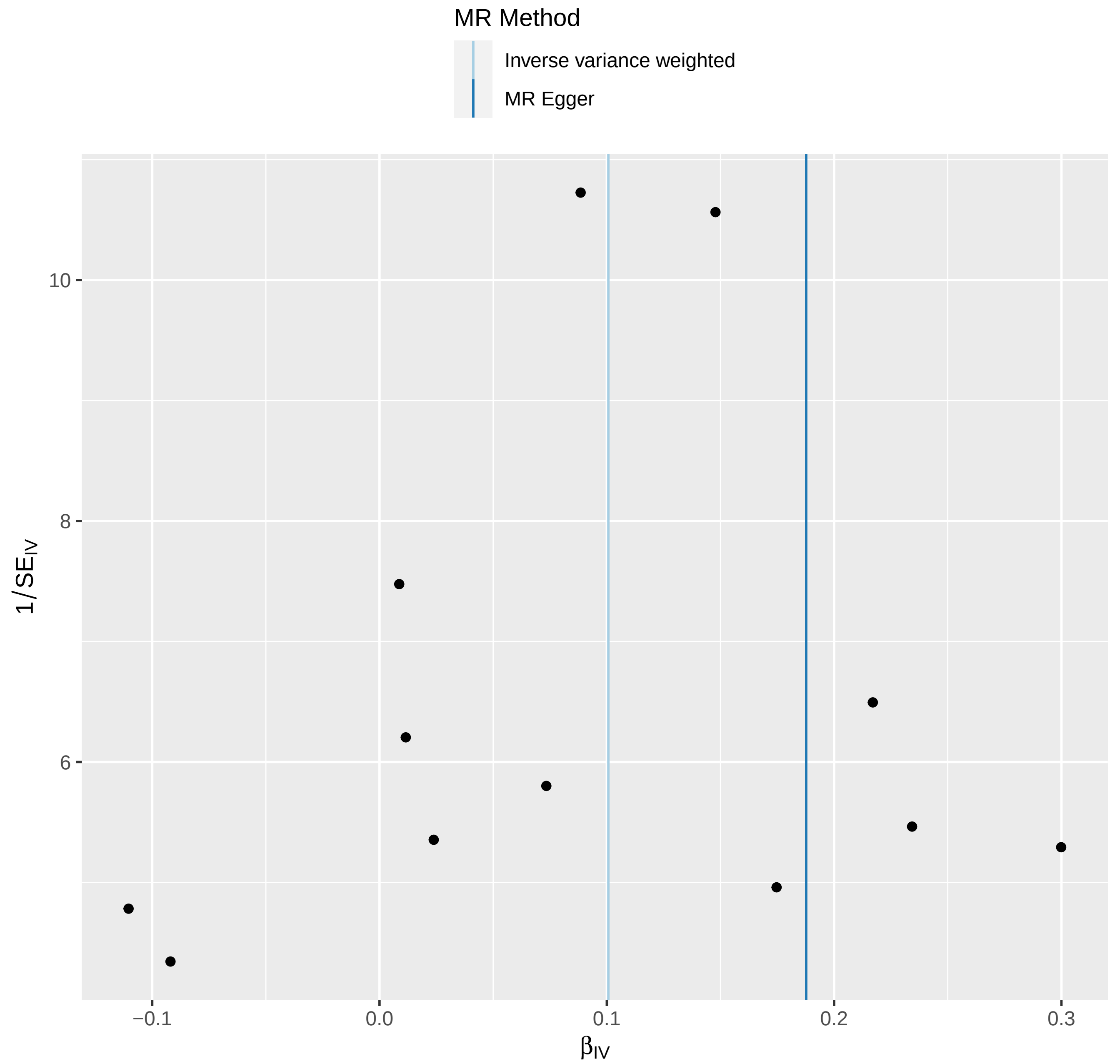

Batch 3−1470 : Selenium on Interleukin−12

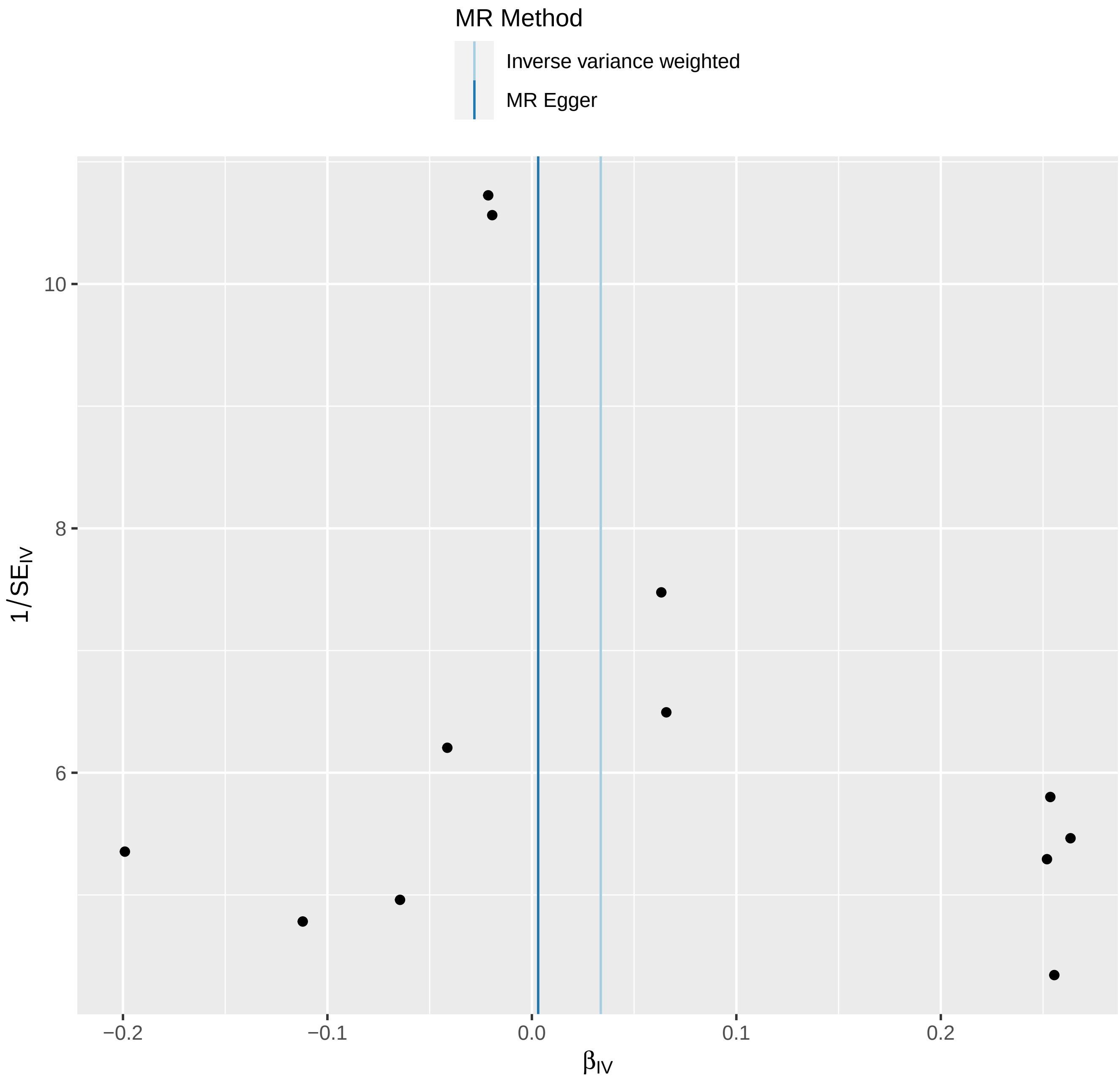

Batch 3−1471 : Selenium on Interleukin−23

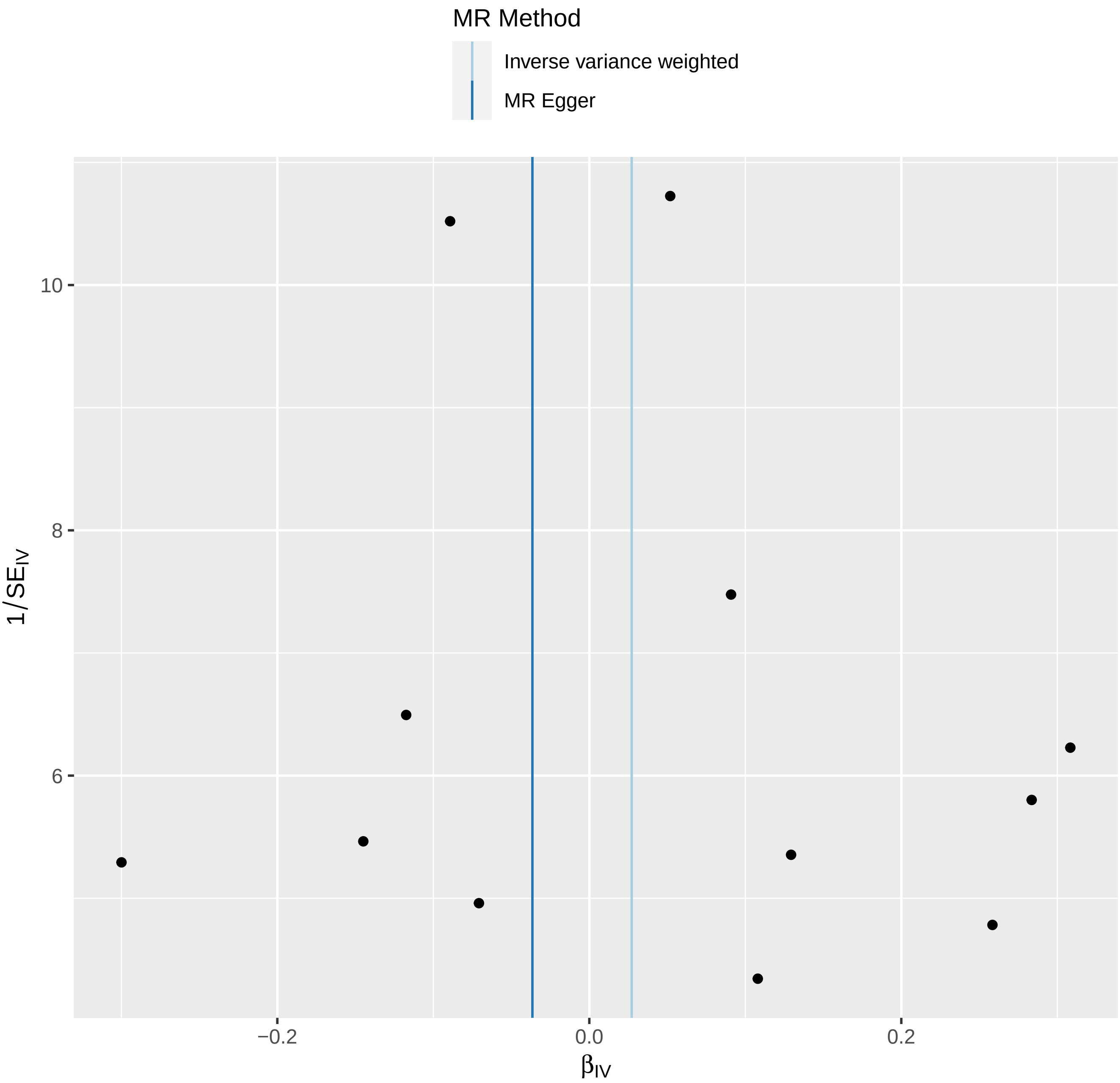

Batch 3−1472 : Selenium on Interleukin−23

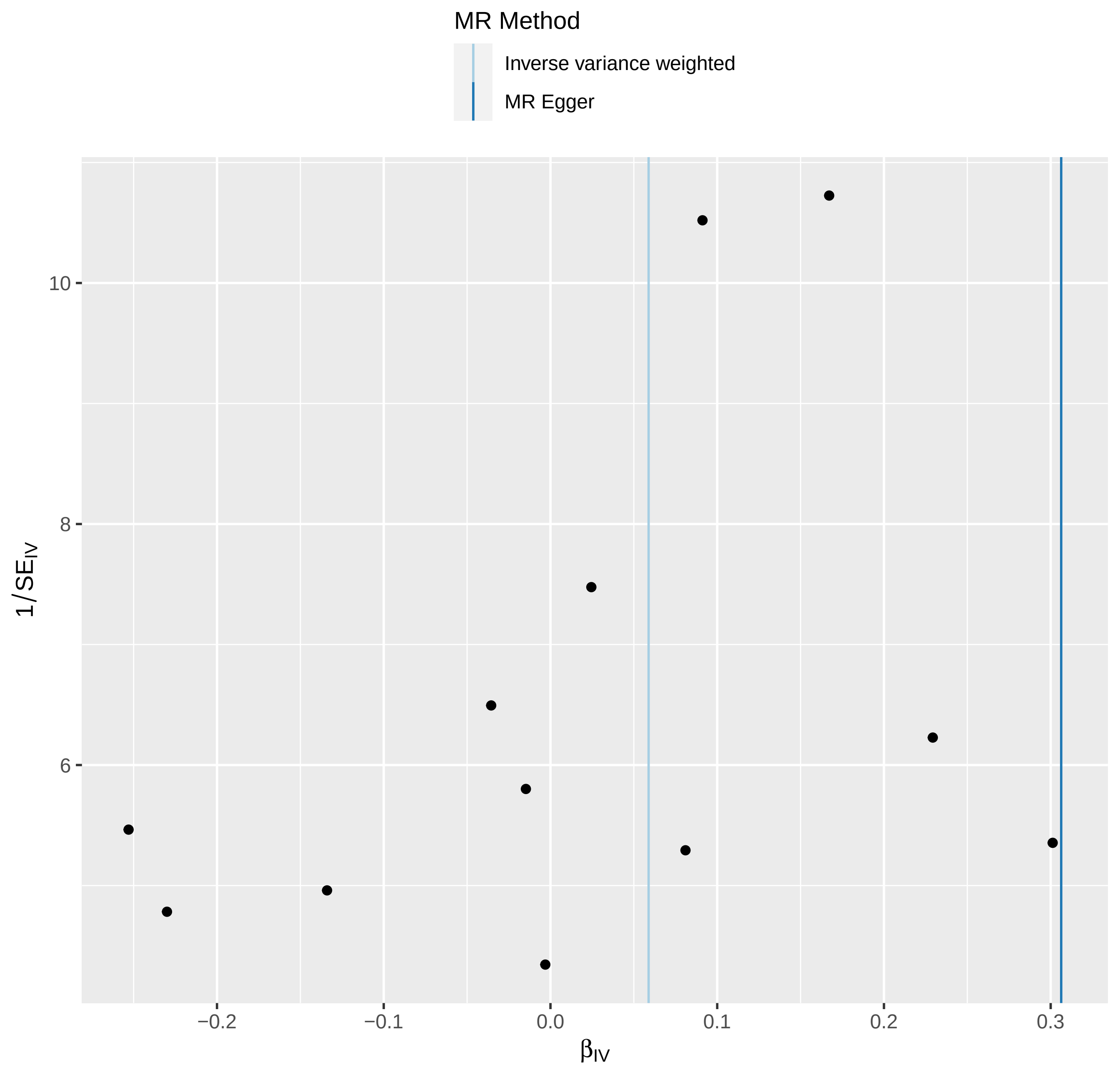

Batch 3−1473 : Selenium on Interleukin−12 receptor subunit beta−1

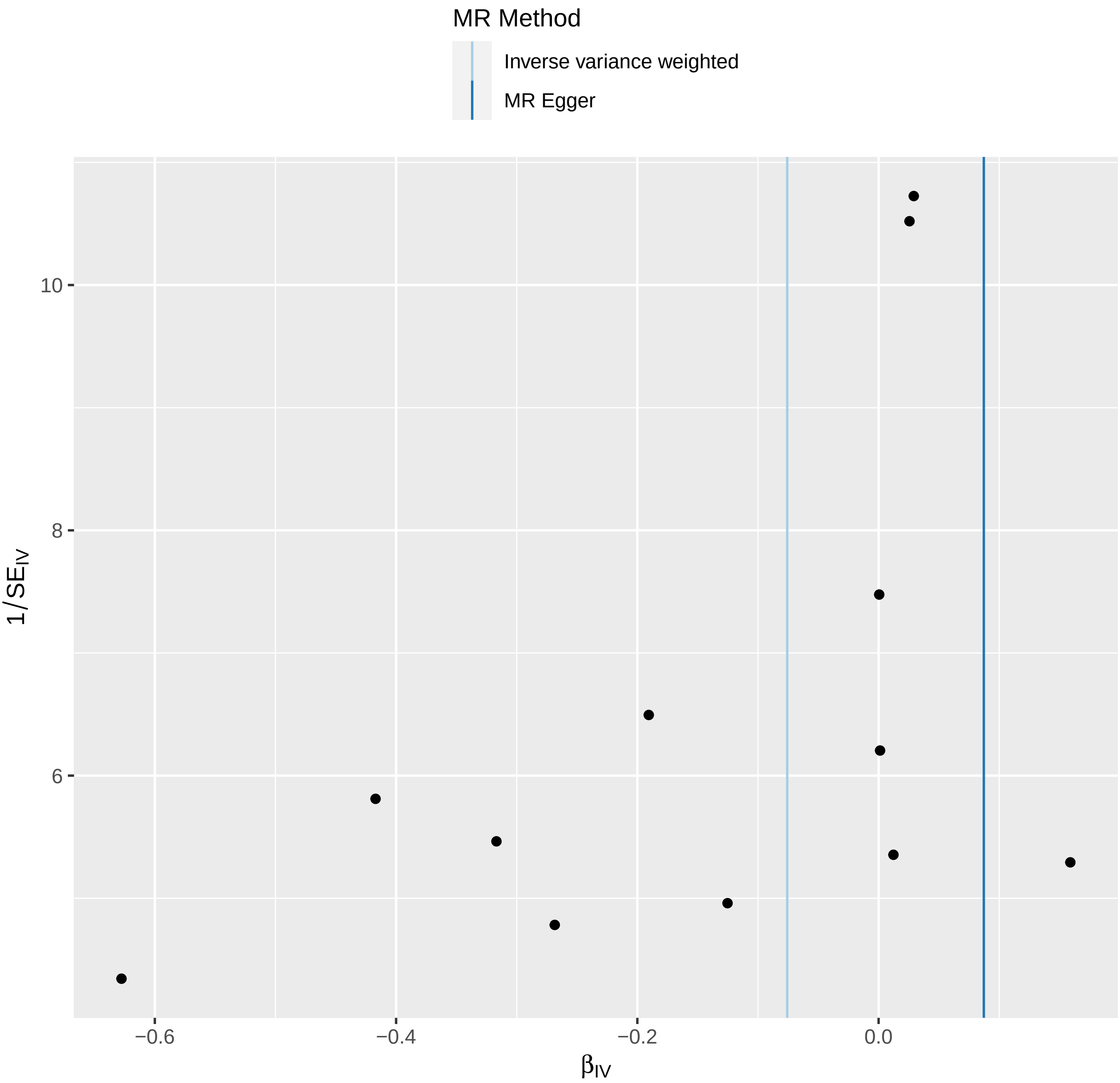

Batch 3−1474 : Selenium on Interleukin−12 receptor subunit beta−2

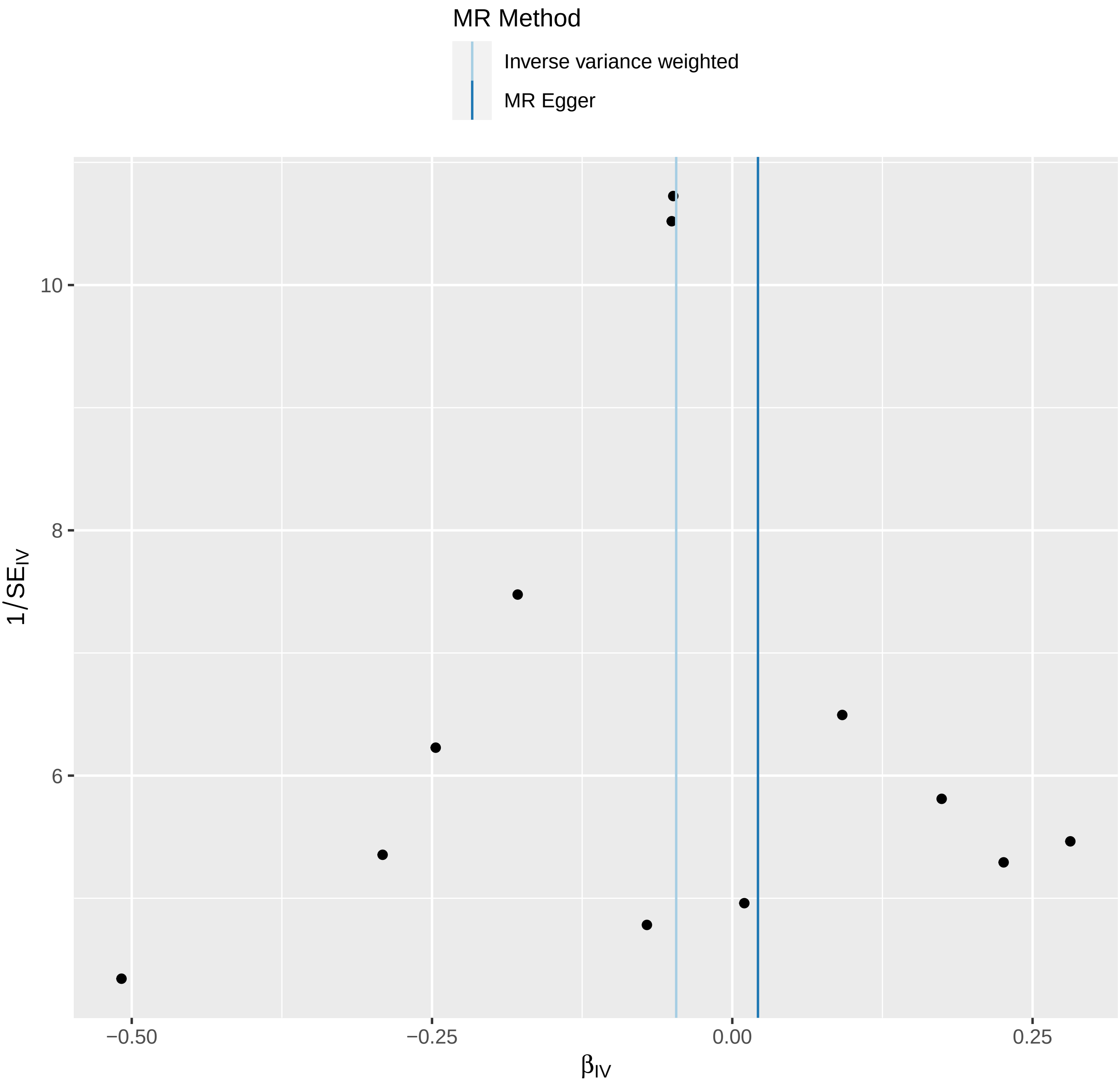

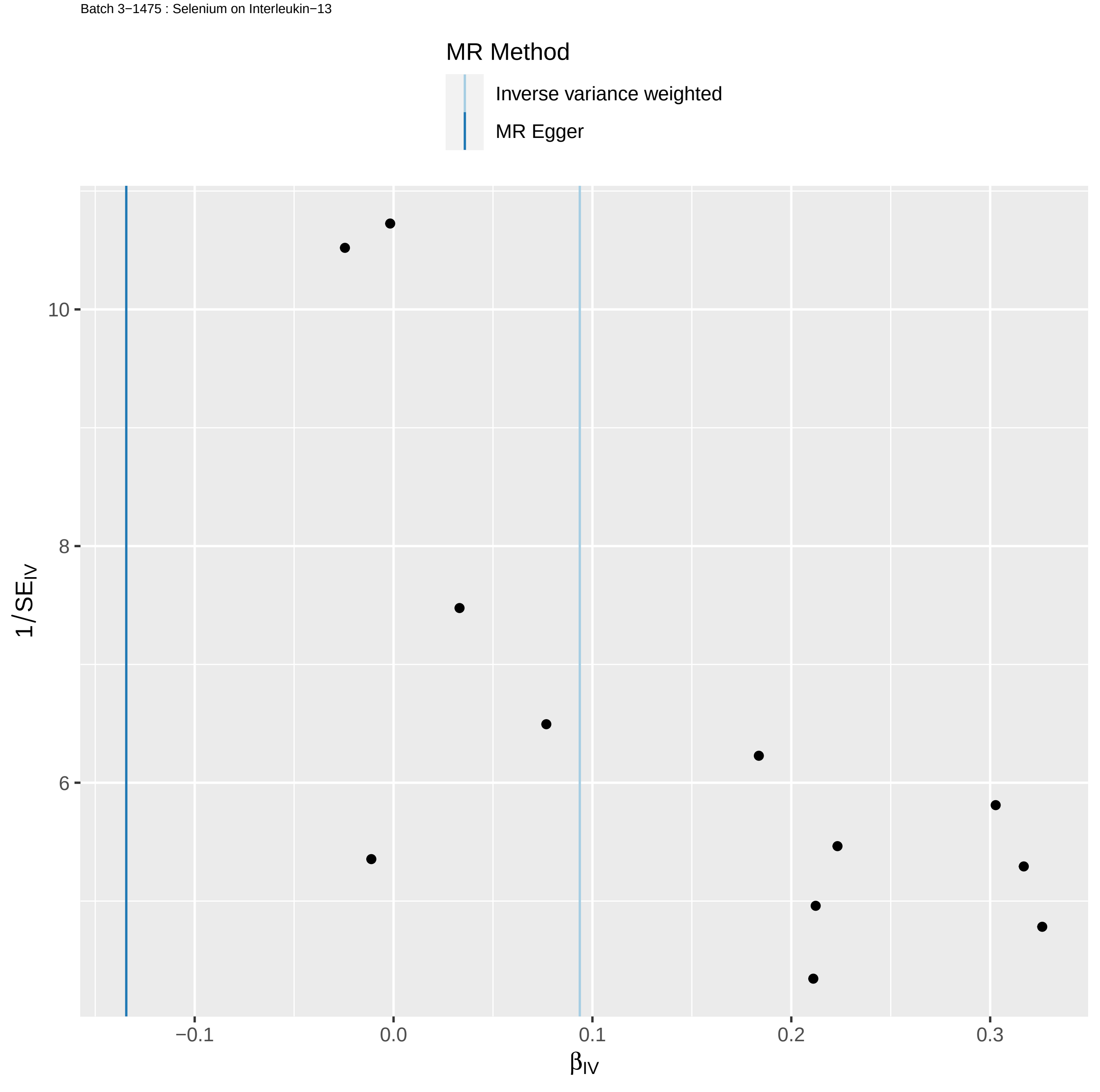

Batch 3−1475 : Selenium on Interleukin−13
MR Method
Inverse variance weighted
MR Egger
10
8
6
1/SE_IV
−0.1
0.0
0.1
0.2
0.3
β_IV

Batch 3−1476 : Selenium on Interleukin−13 receptor subunit alpha−1

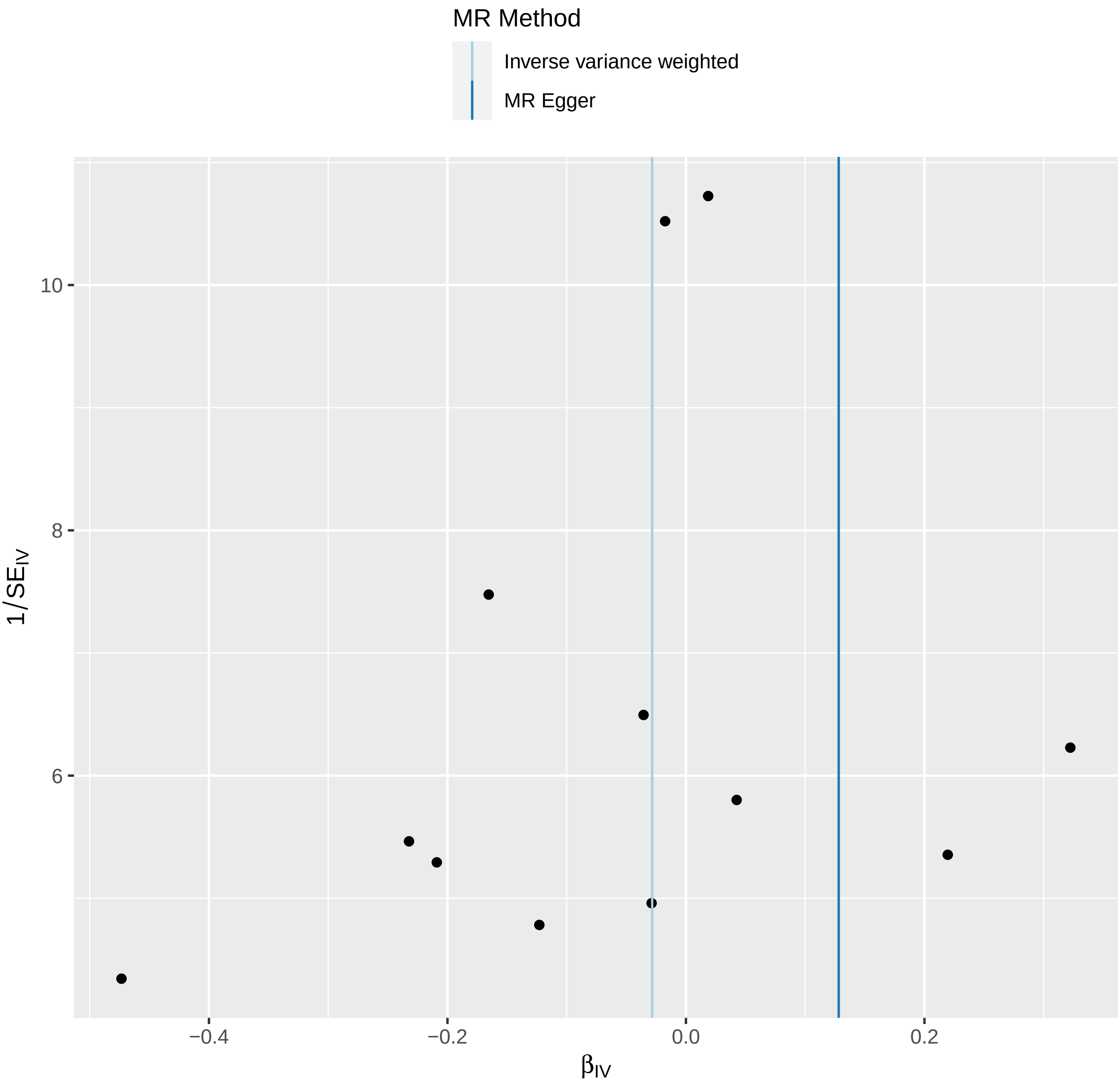

Batch 3−1477 : Selenium on Interleukin−15 receptor subunit alpha

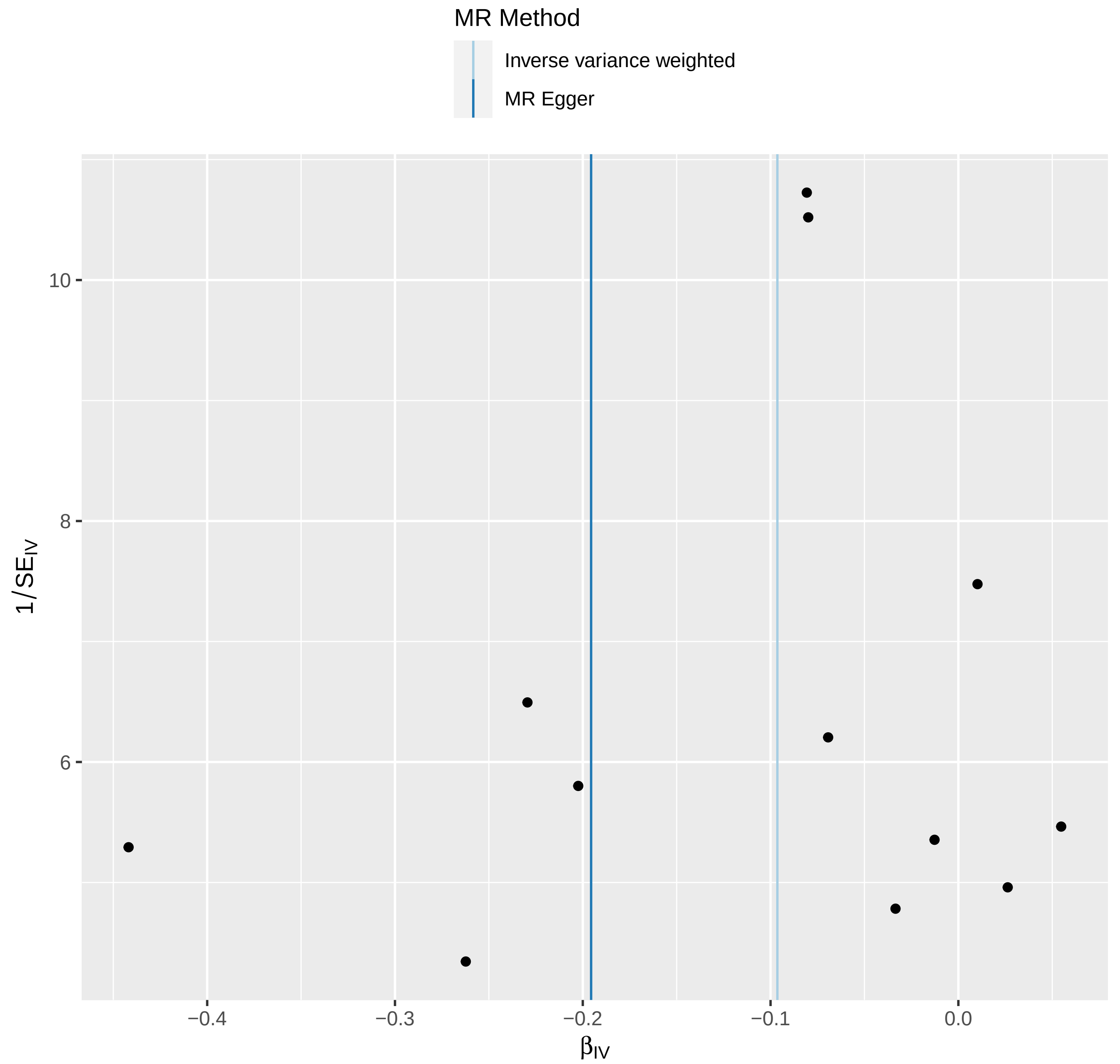

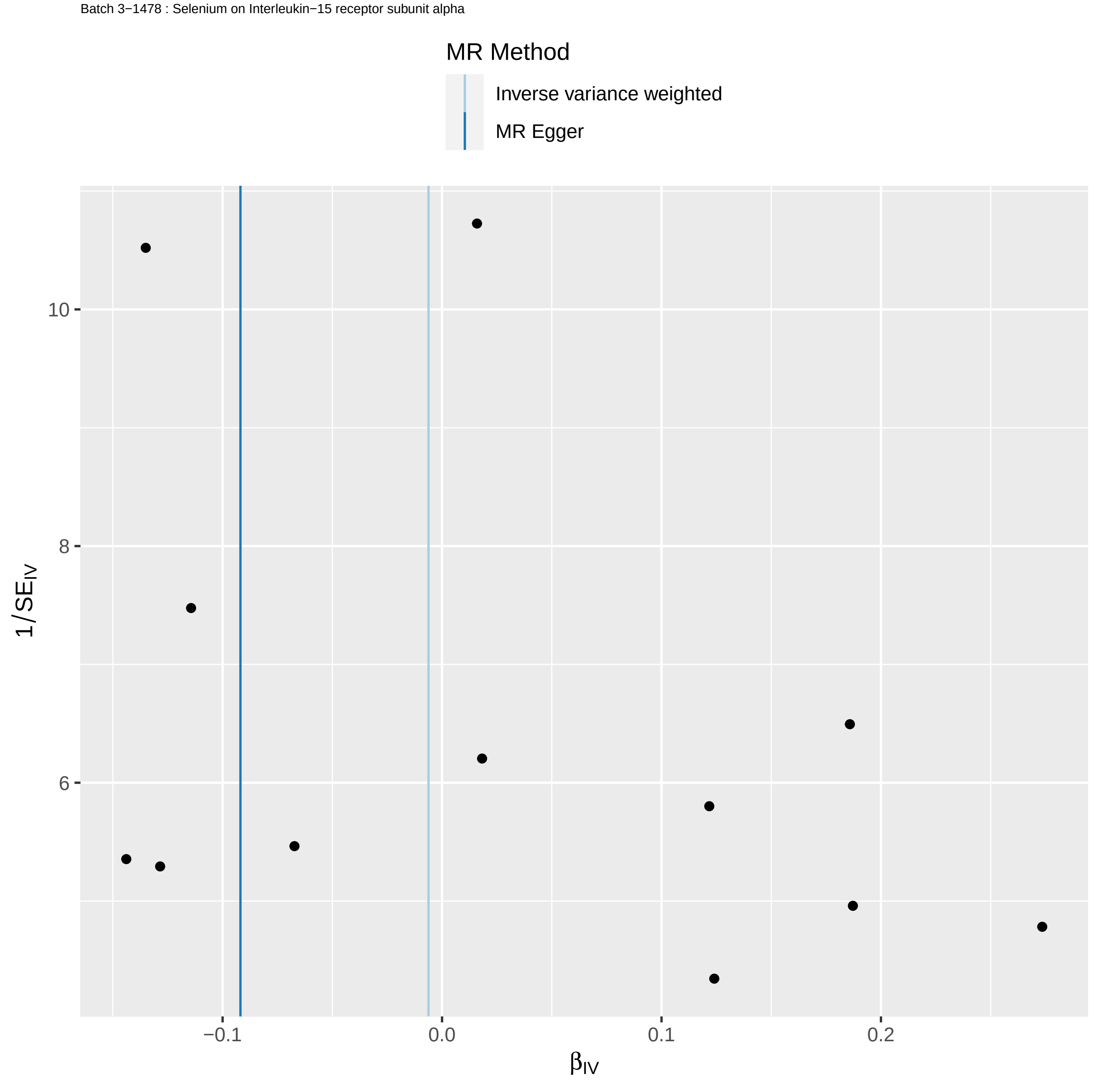
Batch 3−1478 : Selenium on Interleukin−15 receptor subunit alpha
MR Method
Inverse variance weighted
MR Egger
10
8
6
1/SE$_{IV}$
−0.1
0.0
0.1
0.2
β$_{IV}$

Batch 3−1479 : Selenium on Interleukin−16

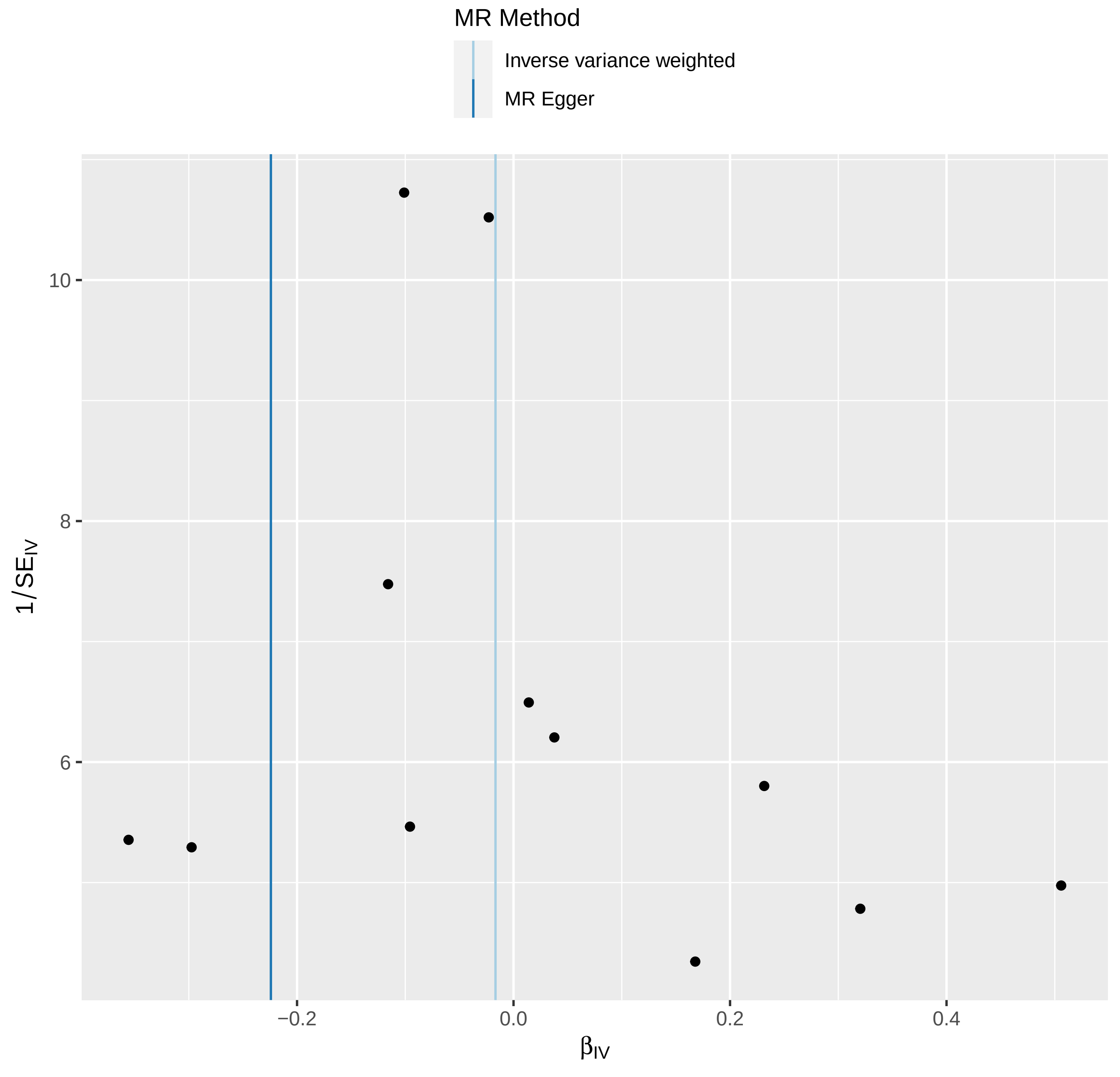

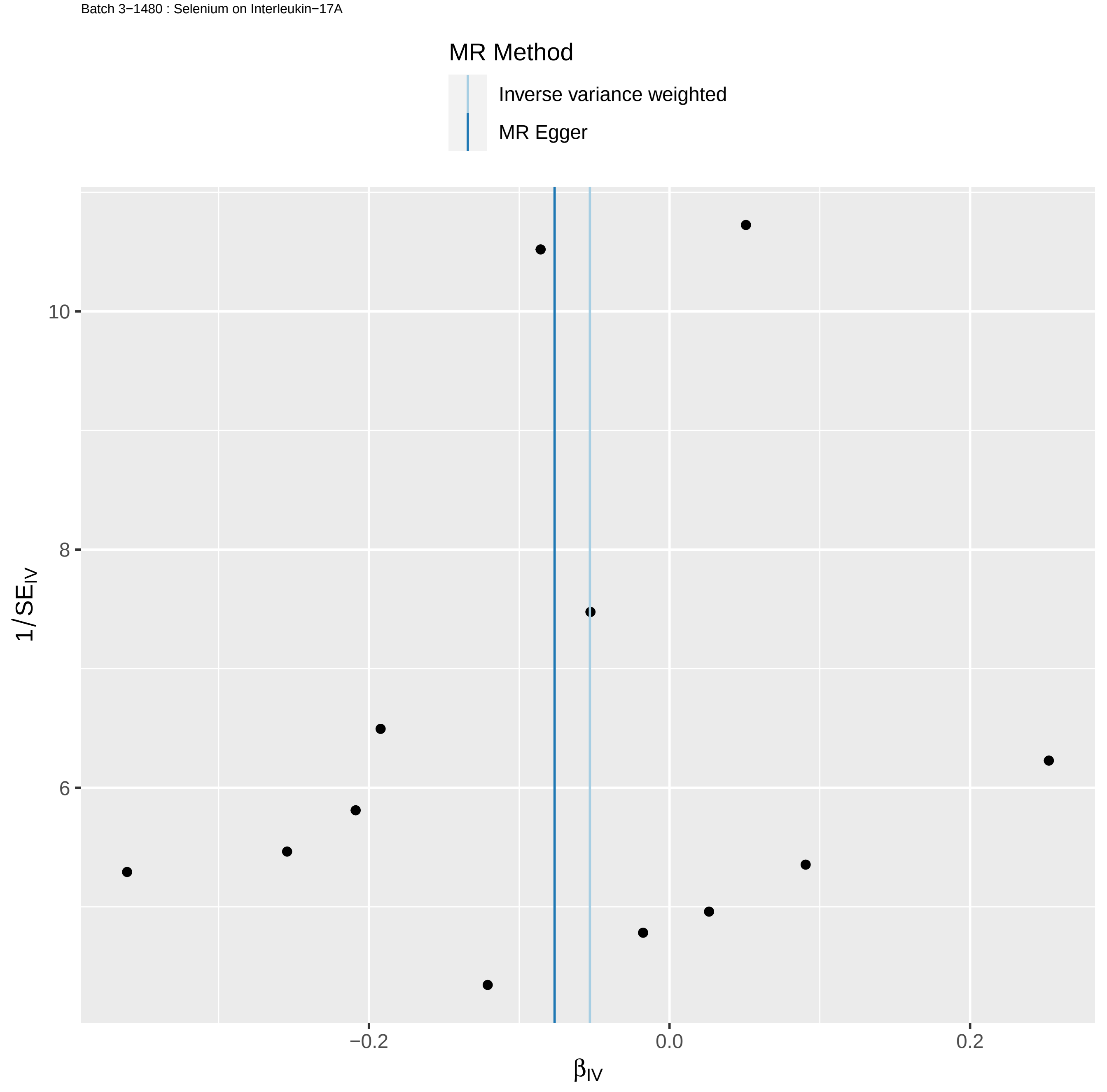

Batch 3−1480 : Selenium on Interleukin−17A
MR Method
Inverse variance weighted
MR Egger
10
8
6
1/SE_IV
−0.2
0.0
0.2
β_IV

Batch 3−1481 : Selenium on Interleukin−17A

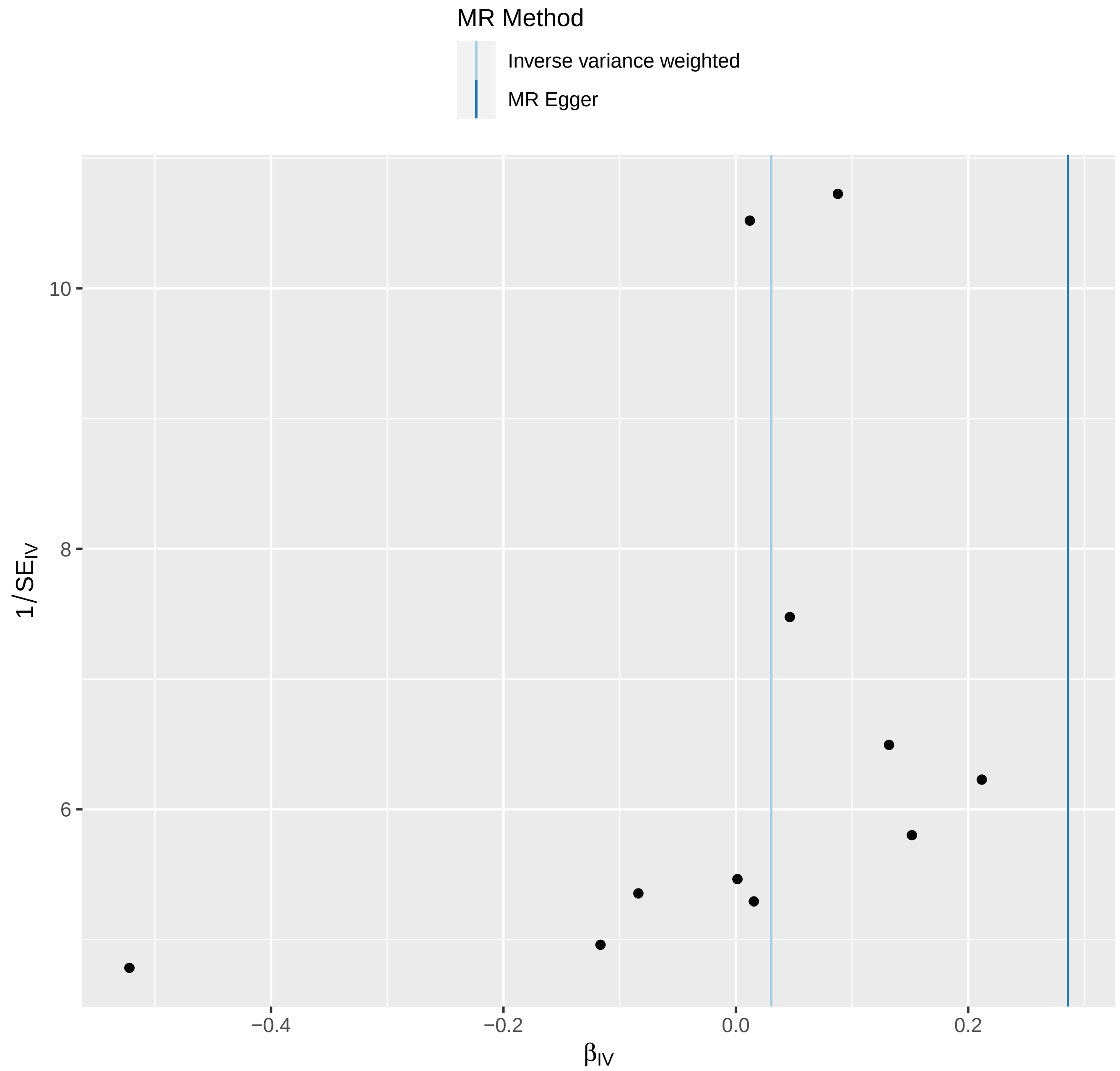

Batch 3−1482 : Selenium on Interleukin−17A

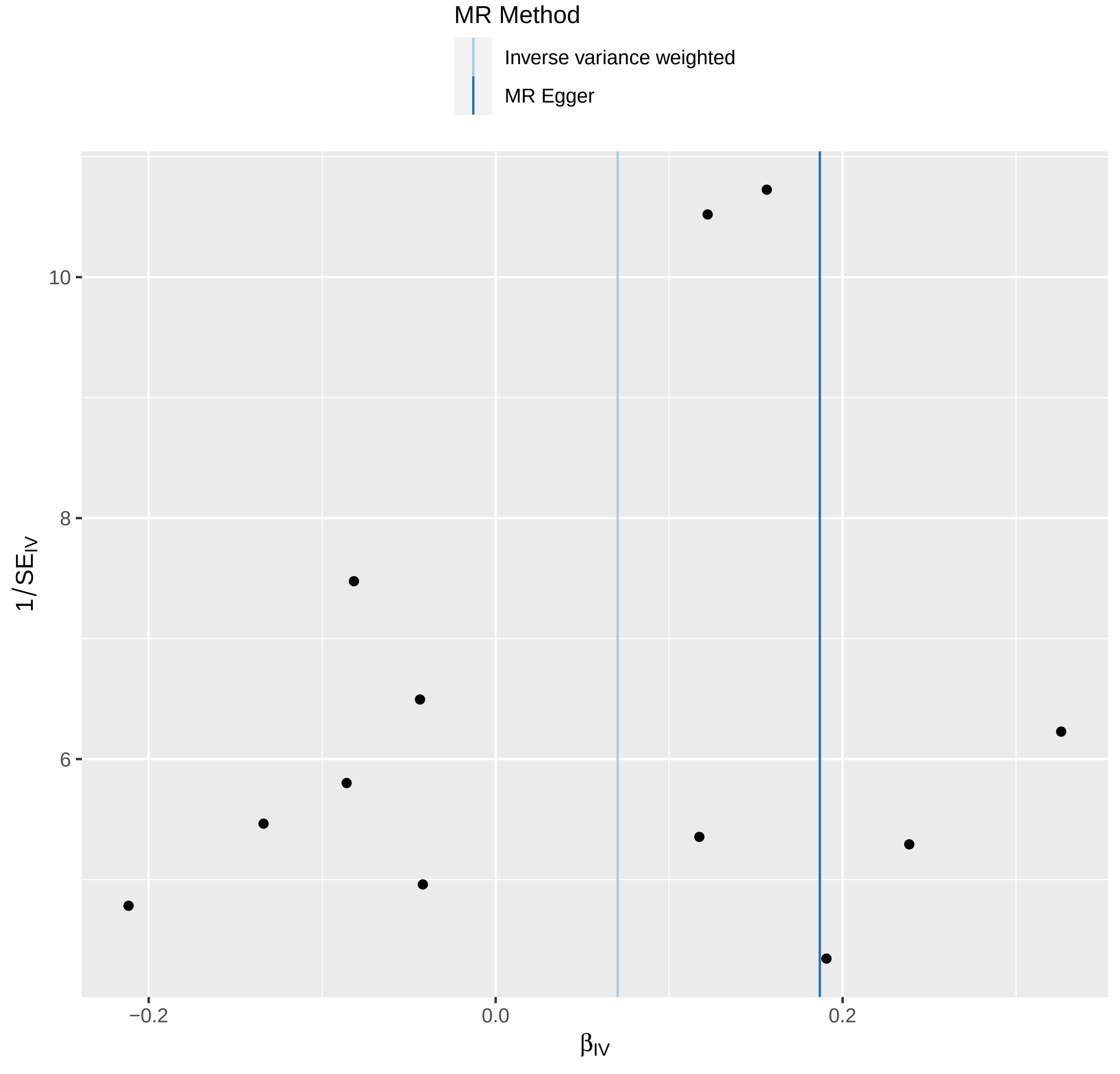

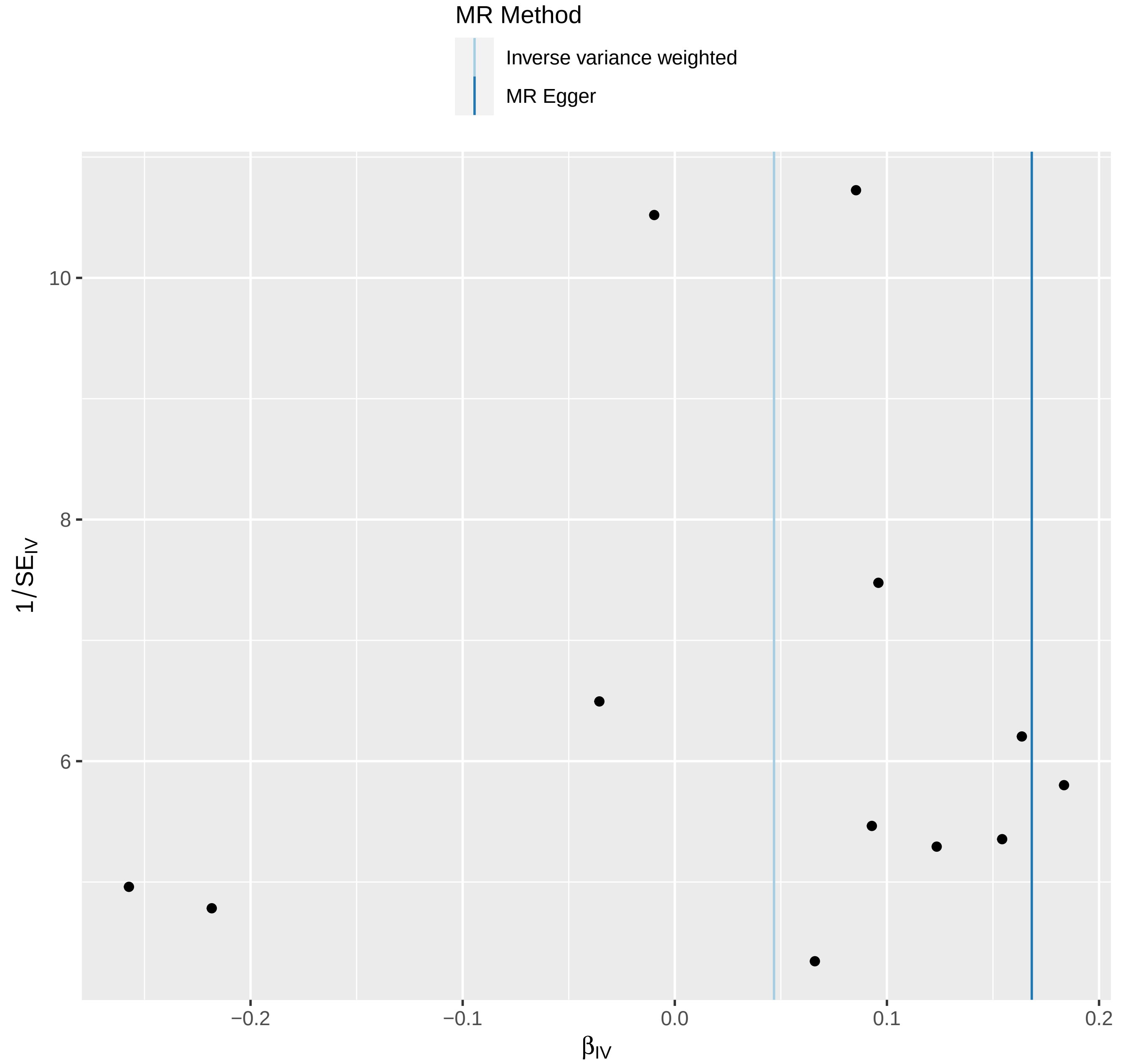
MR Method
Inverse variance weighted
MR Egger
10
8
6
1/SE$_{IV}$
−0.2
−0.1
0.0
0.1
0.2
β$_{IV}$

Batch 3−1484 : Selenium on Interleukin−17D

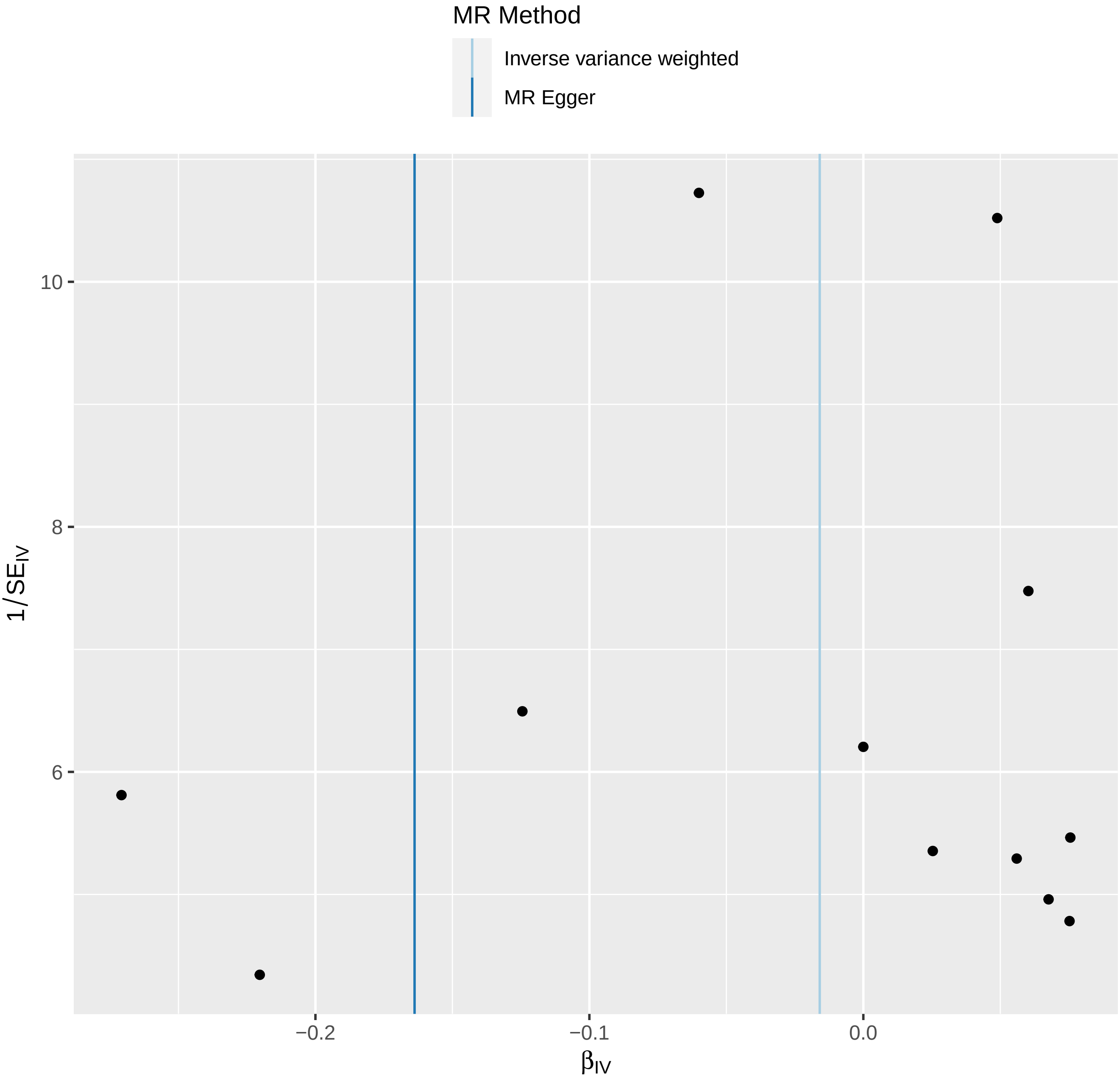

Batch 3−1485 : Selenium on Interleukin−17F

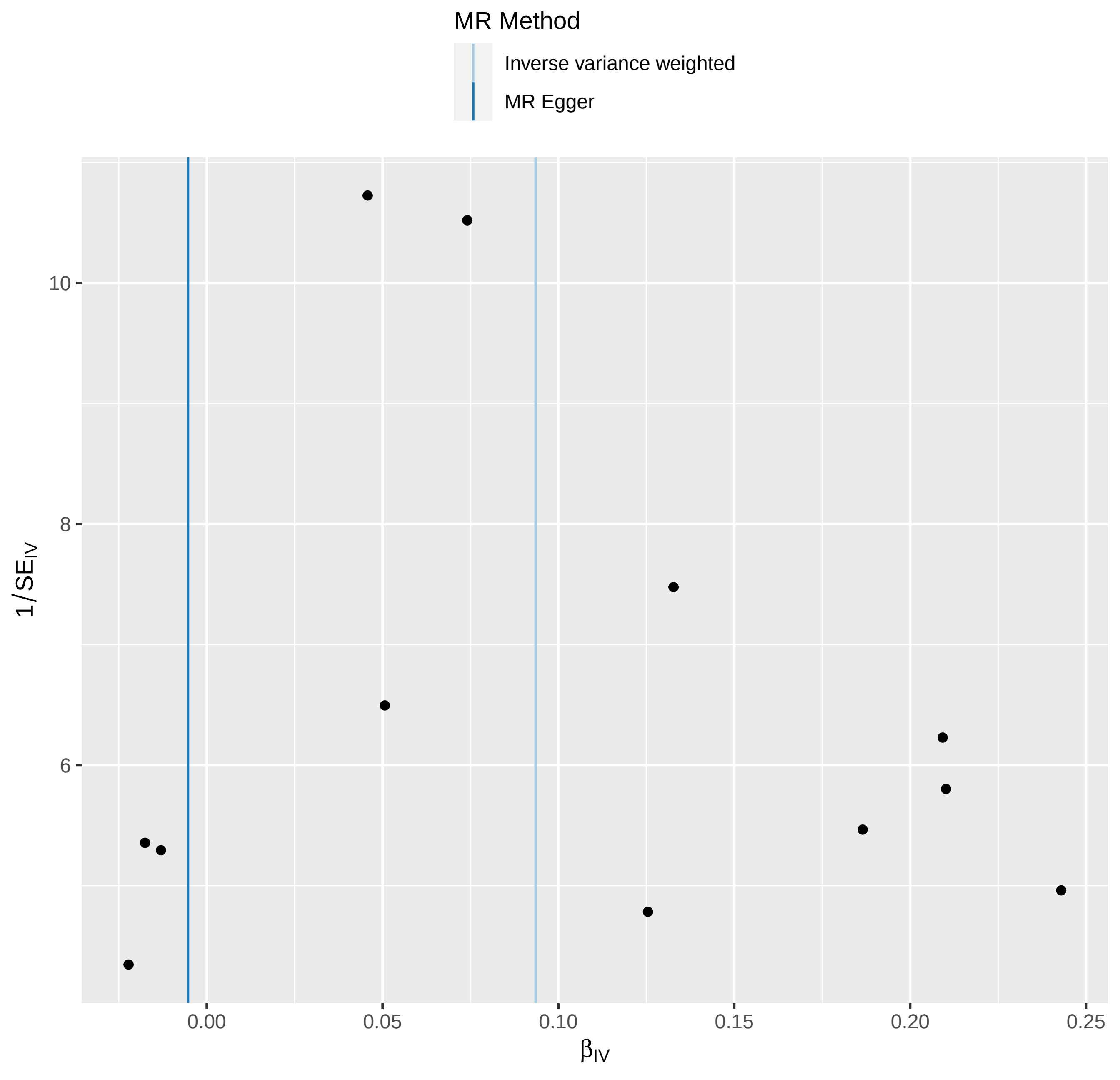

Batch 3−1486 : Selenium on Interleukin−17 receptor A

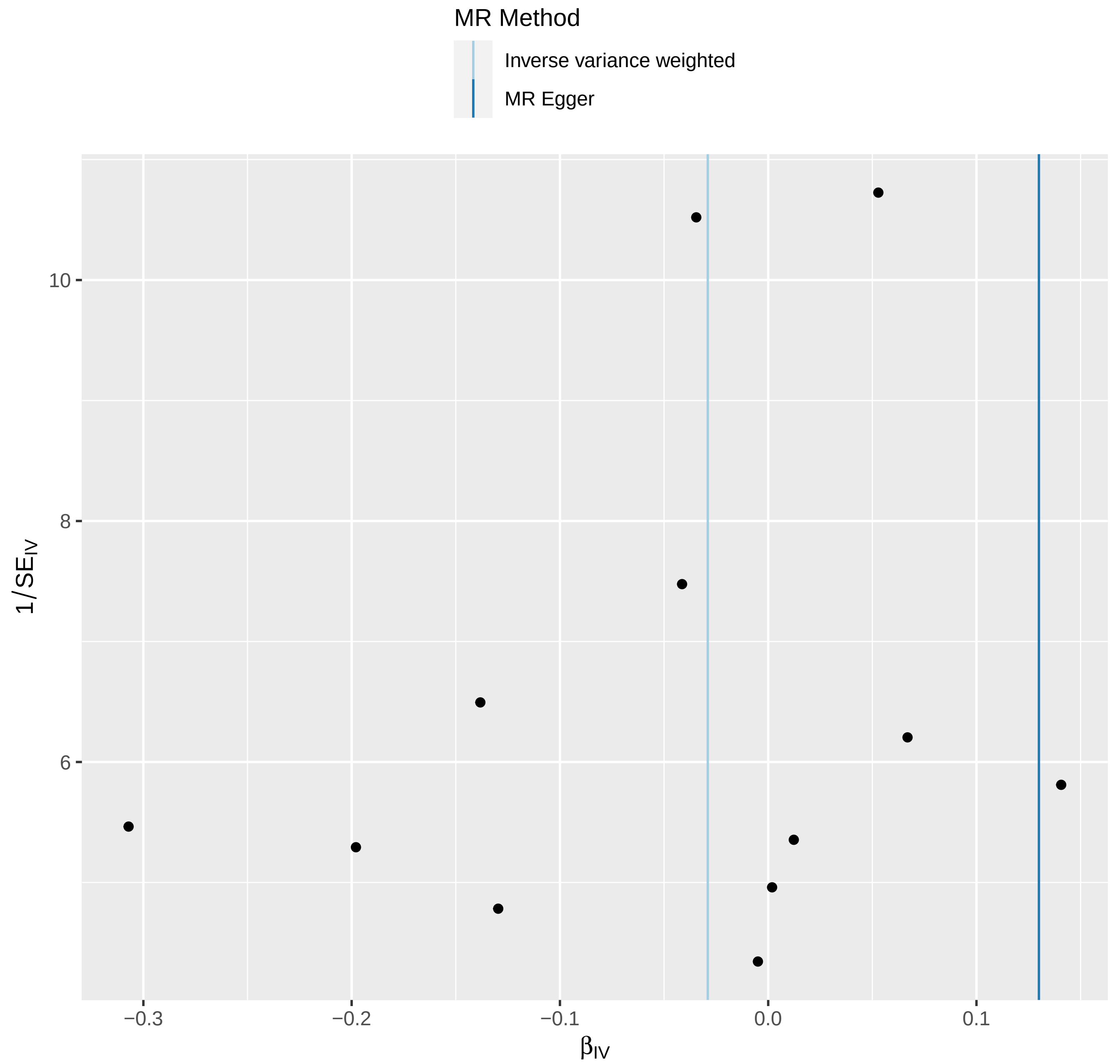

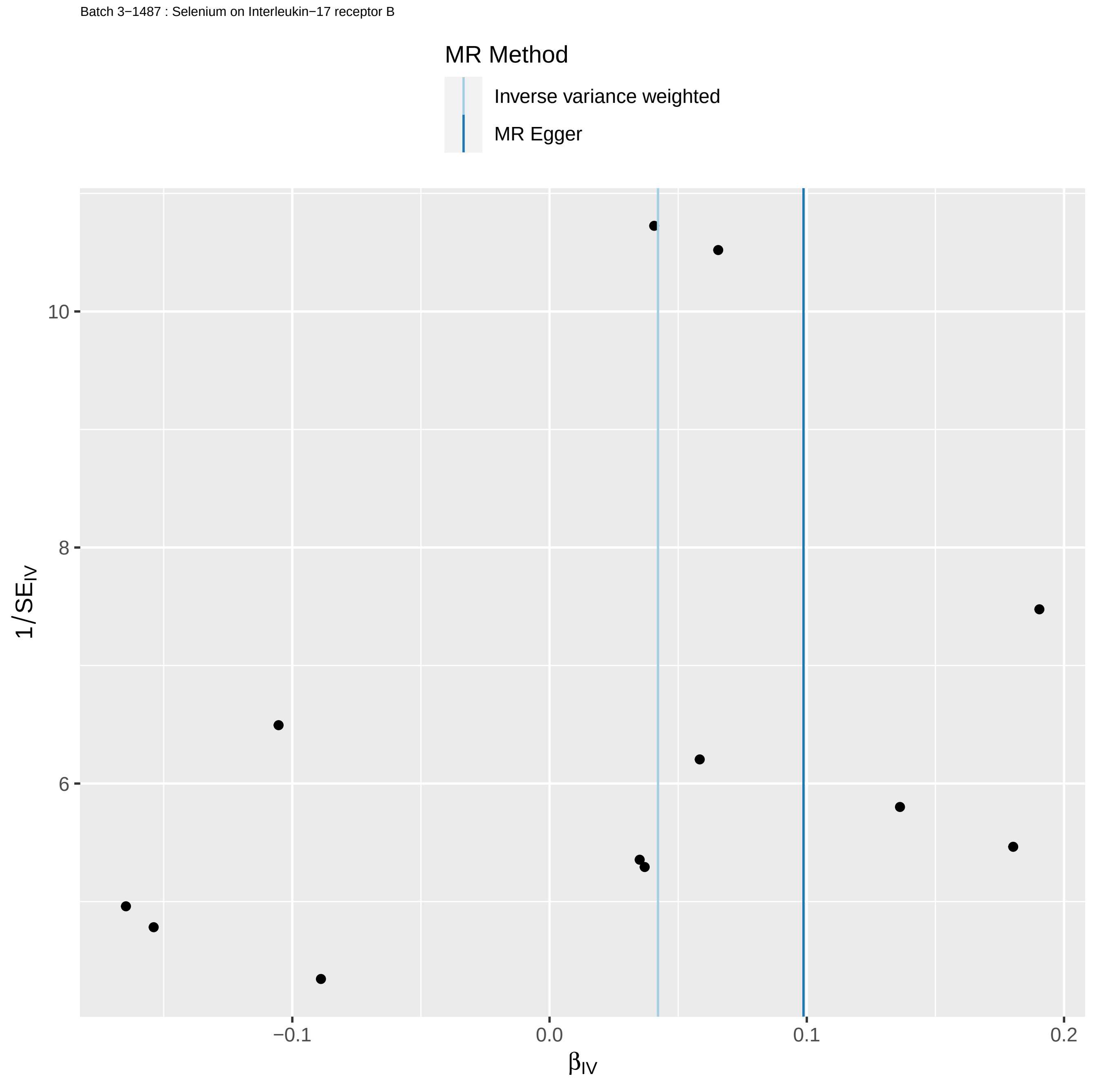
Batch 3−1487 : Selenium on Interleukin−17 receptor B
MR Method
Inverse variance weighted
MR Egger
10
8
6
1/SE$_{IV}$
−0.1
0.0
0.1
0.2
β$_{IV}$

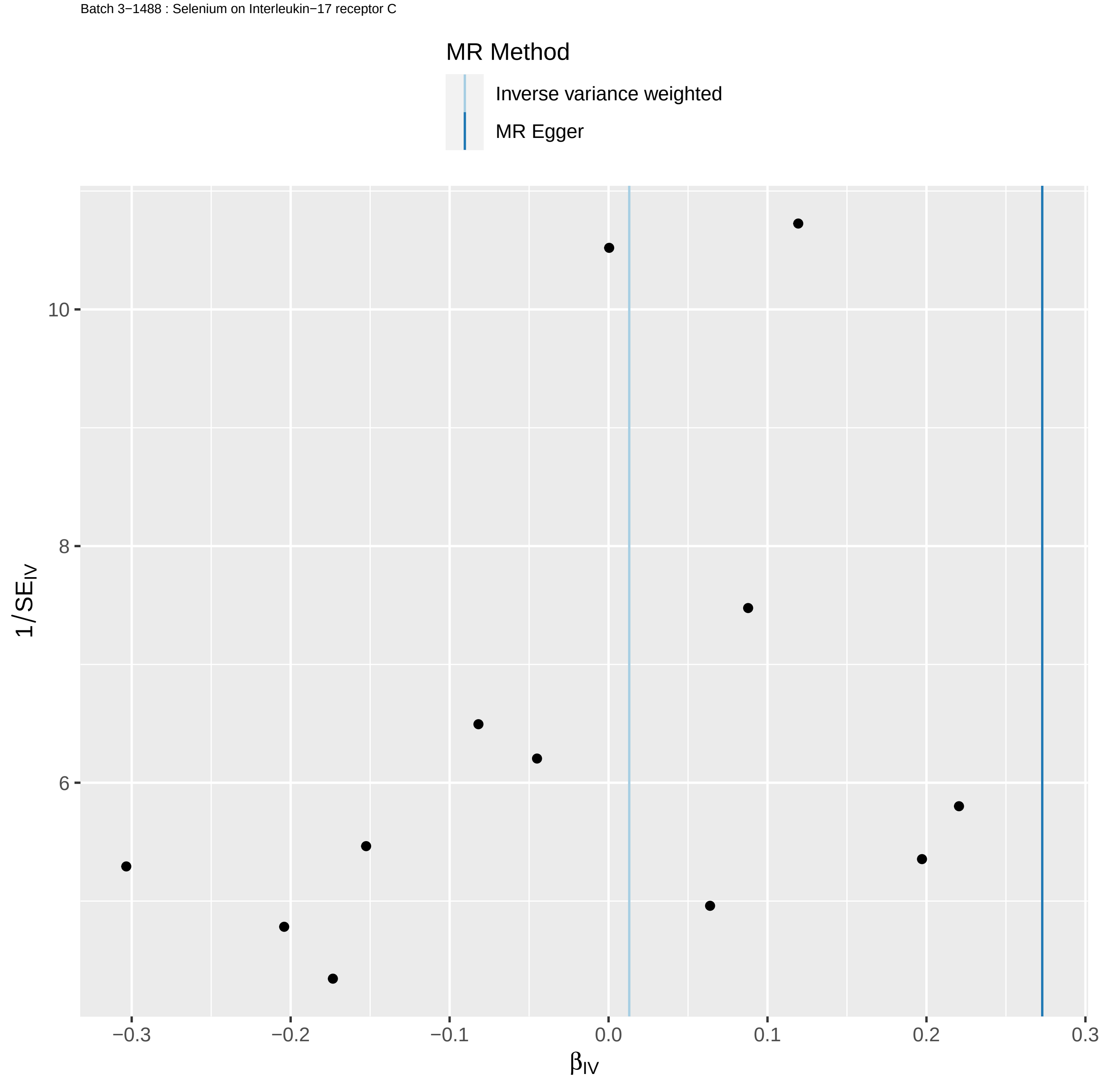
Batch 3−1488 : Selenium on Interleukin−17 receptor C
MR Method
Inverse variance weighted
MR Egger
10
8
6
1/SE_IV
−0.3
−0.2
−0.1
0.0
0.1
0.2
0.3
β_IV

Batch 3−1489 : Selenium on Interleukin−17 receptor D

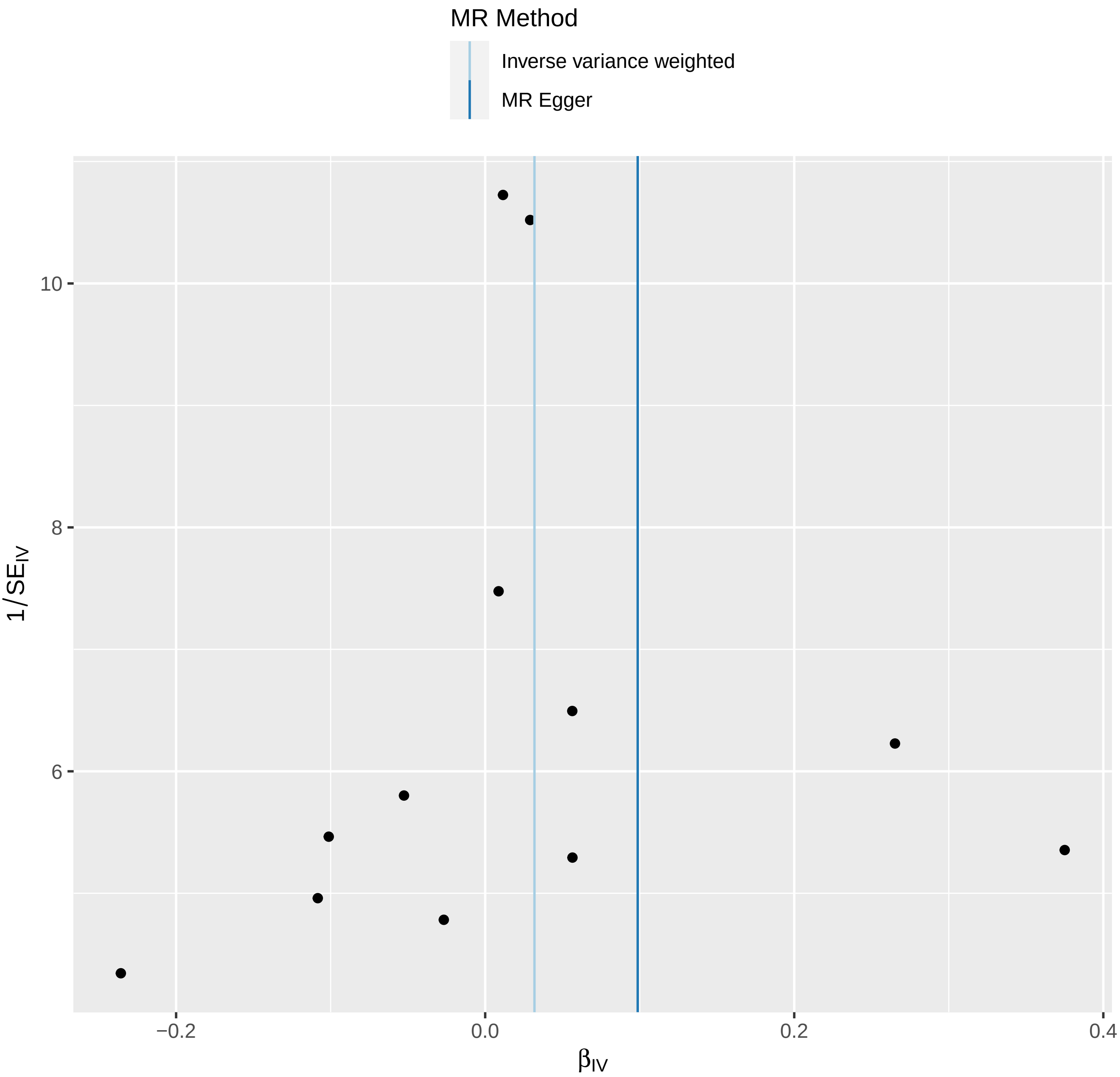

Batch 3−1490 : Selenium on Interleukin−18−binding protein

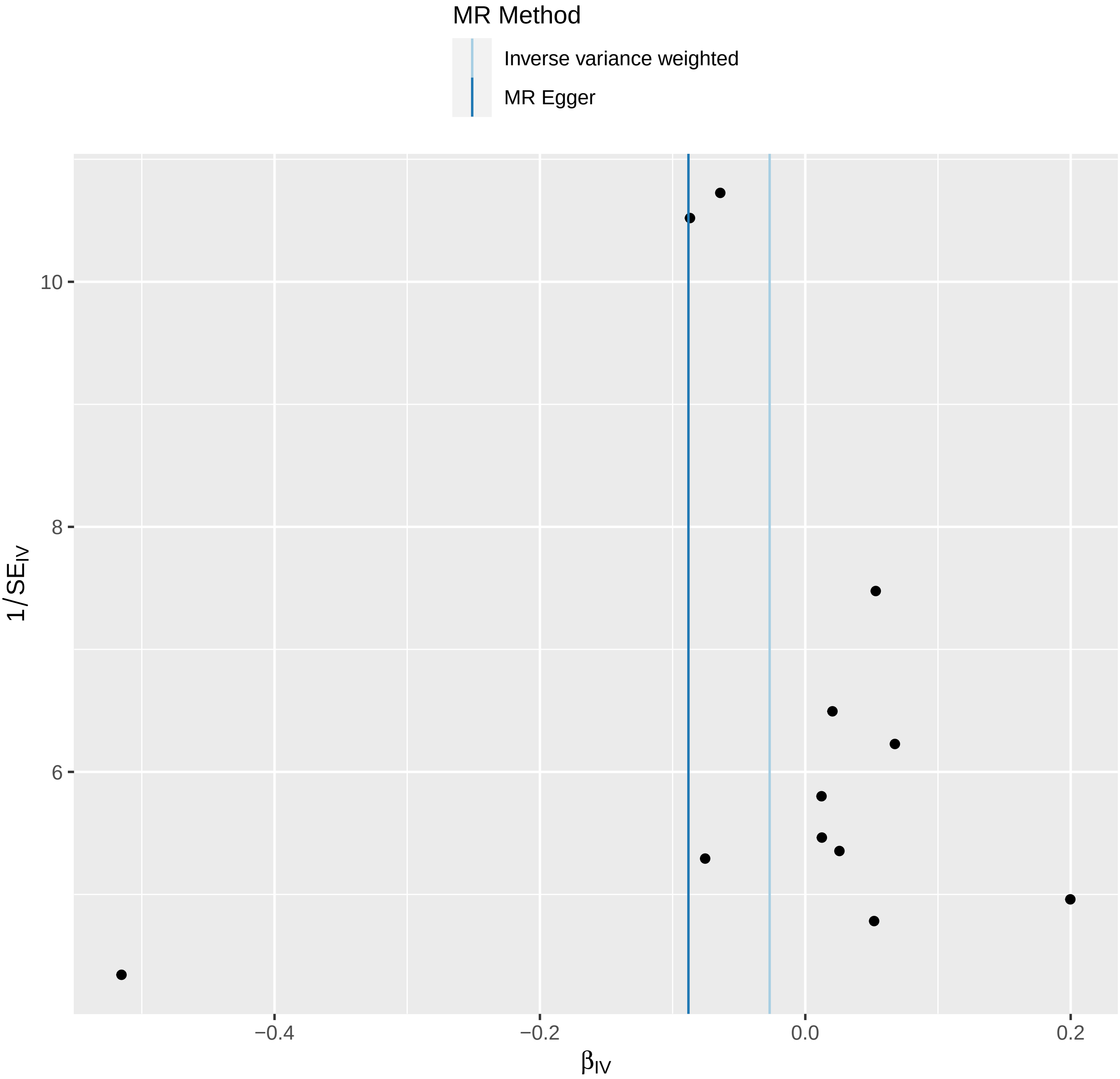

Batch 3−1491 : Selenium on Interleukin−18 receptor 1

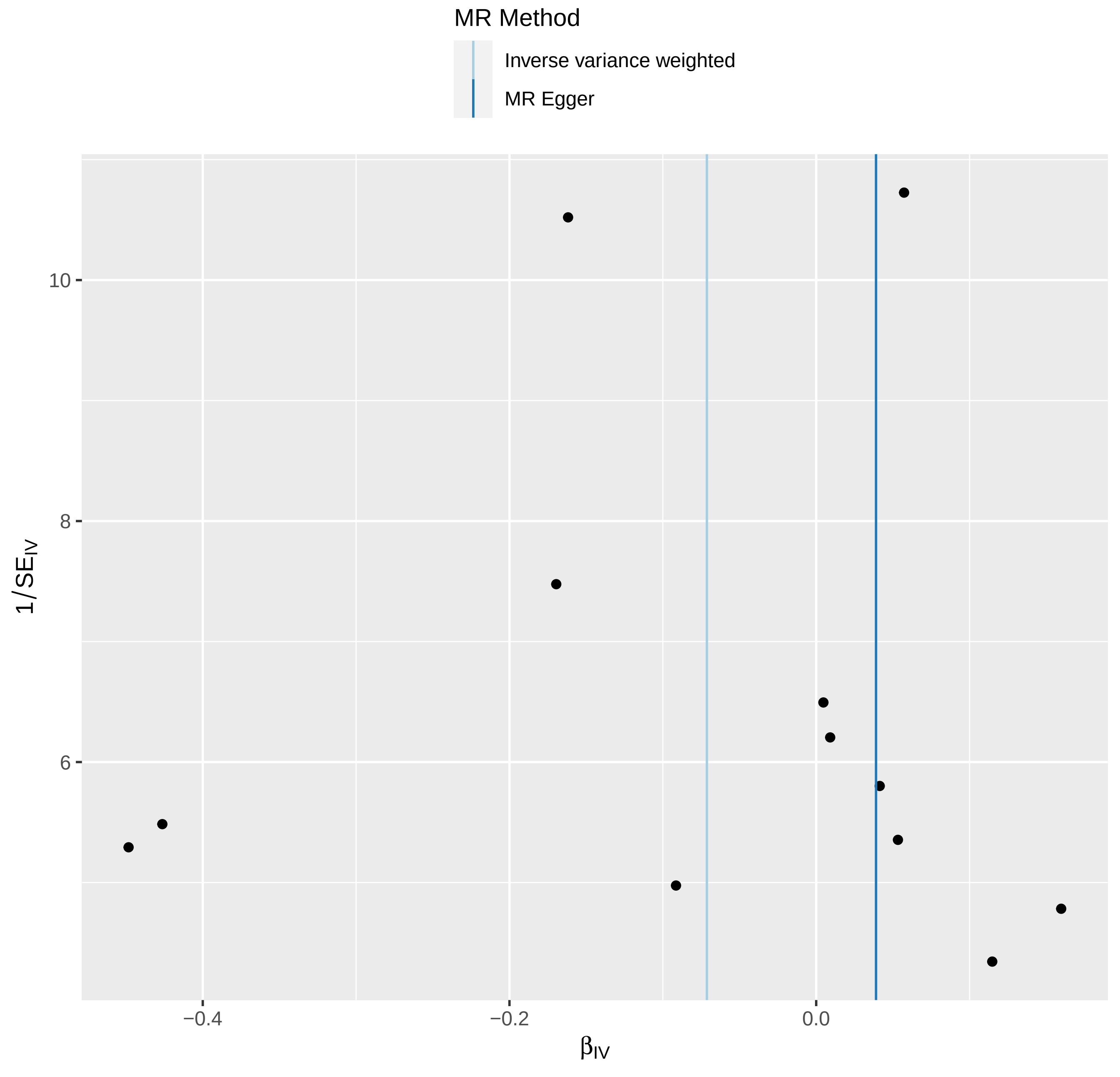

Batch 3−1492 : Selenium on Interleukin−18 receptor 1

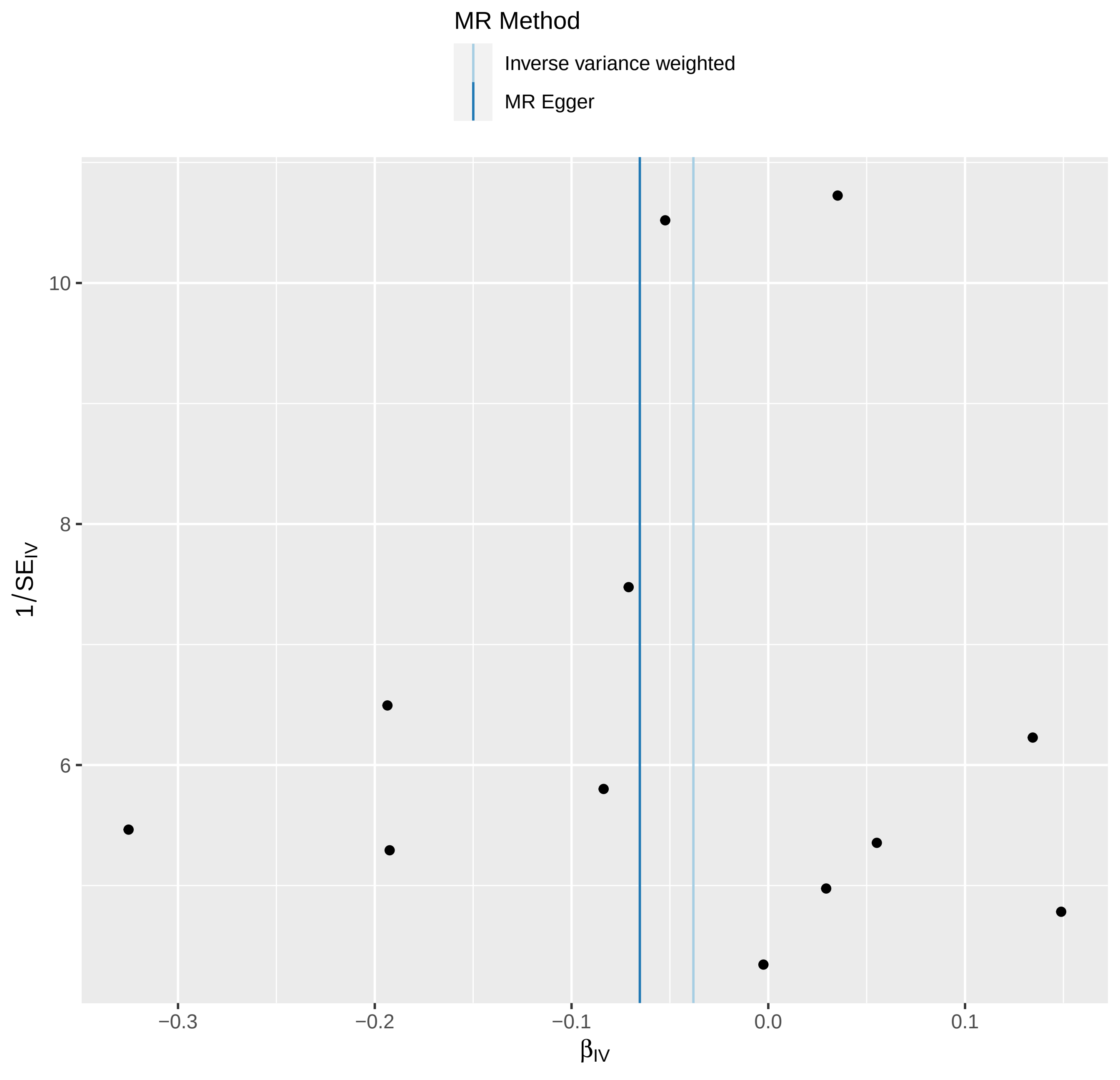

Batch 3−1493 : Selenium on Interleukin−19

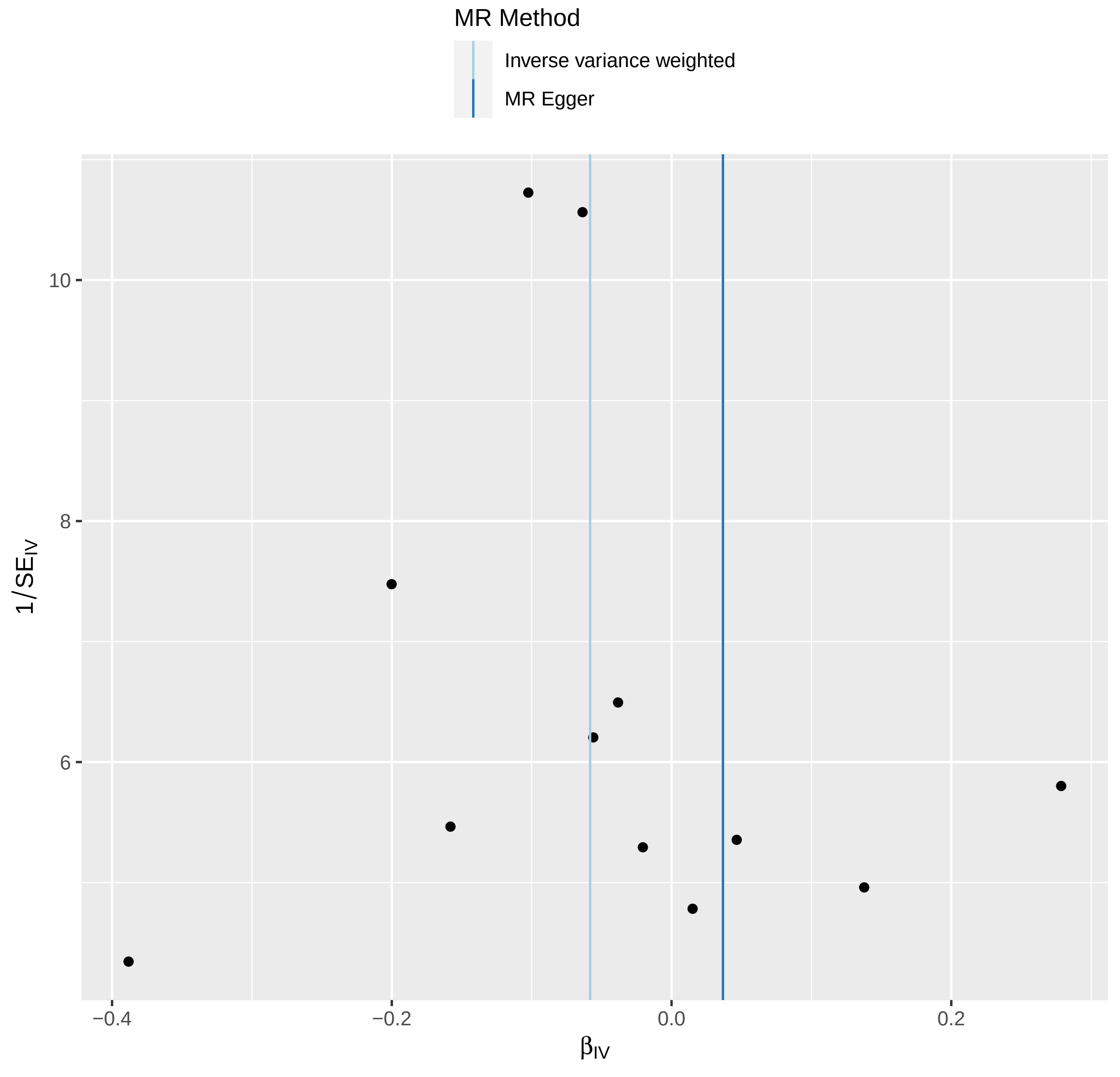

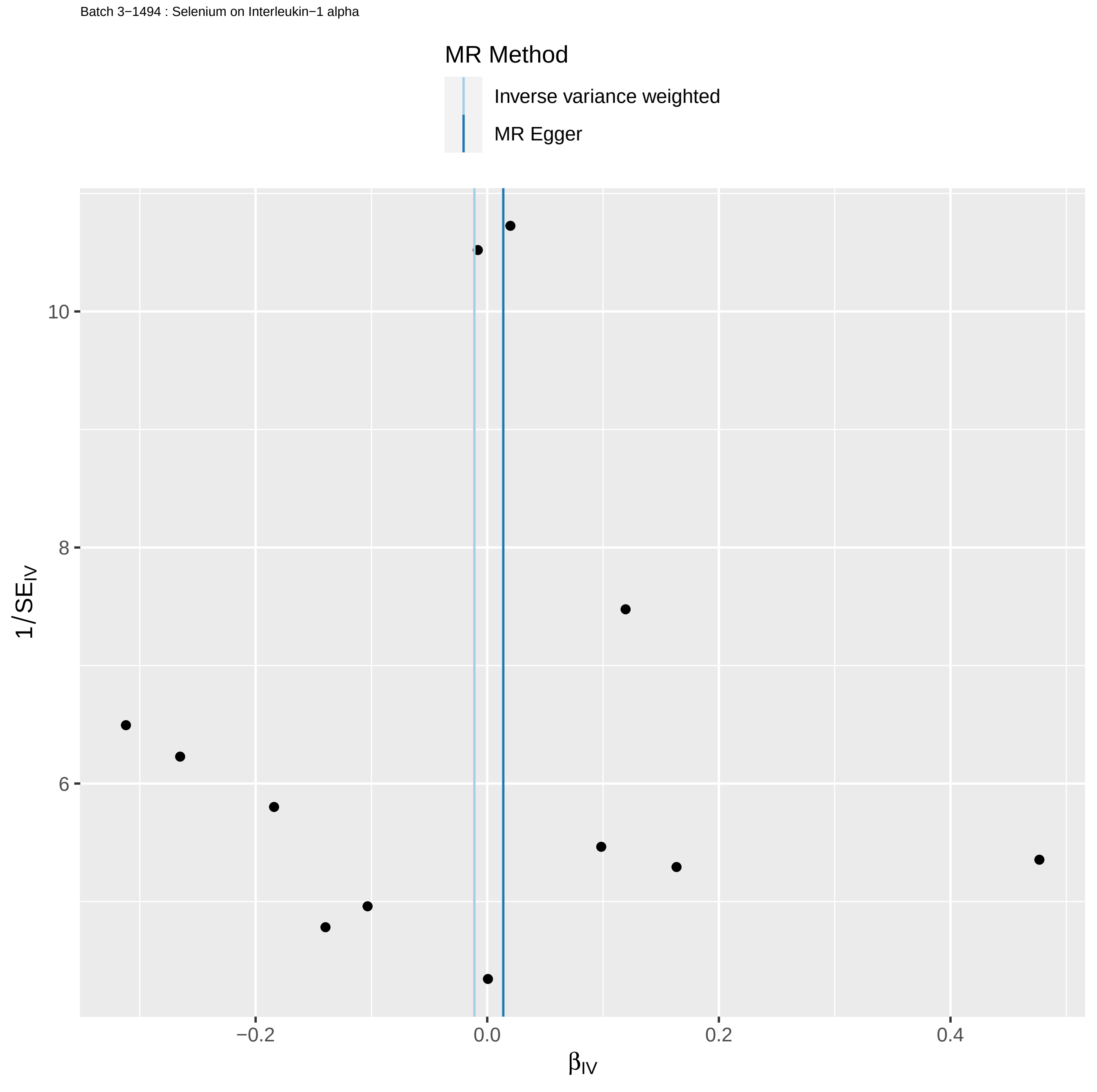

Batch 3−1494 : Selenium on Interleukin−1 alpha
MR Method
Inverse variance weighted
MR Egger
10
8
6
1/SE_IV
−0.2
0.0
0.2
0.4
β_IV

Batch 3−1495 : Selenium on Interleukin−1 beta

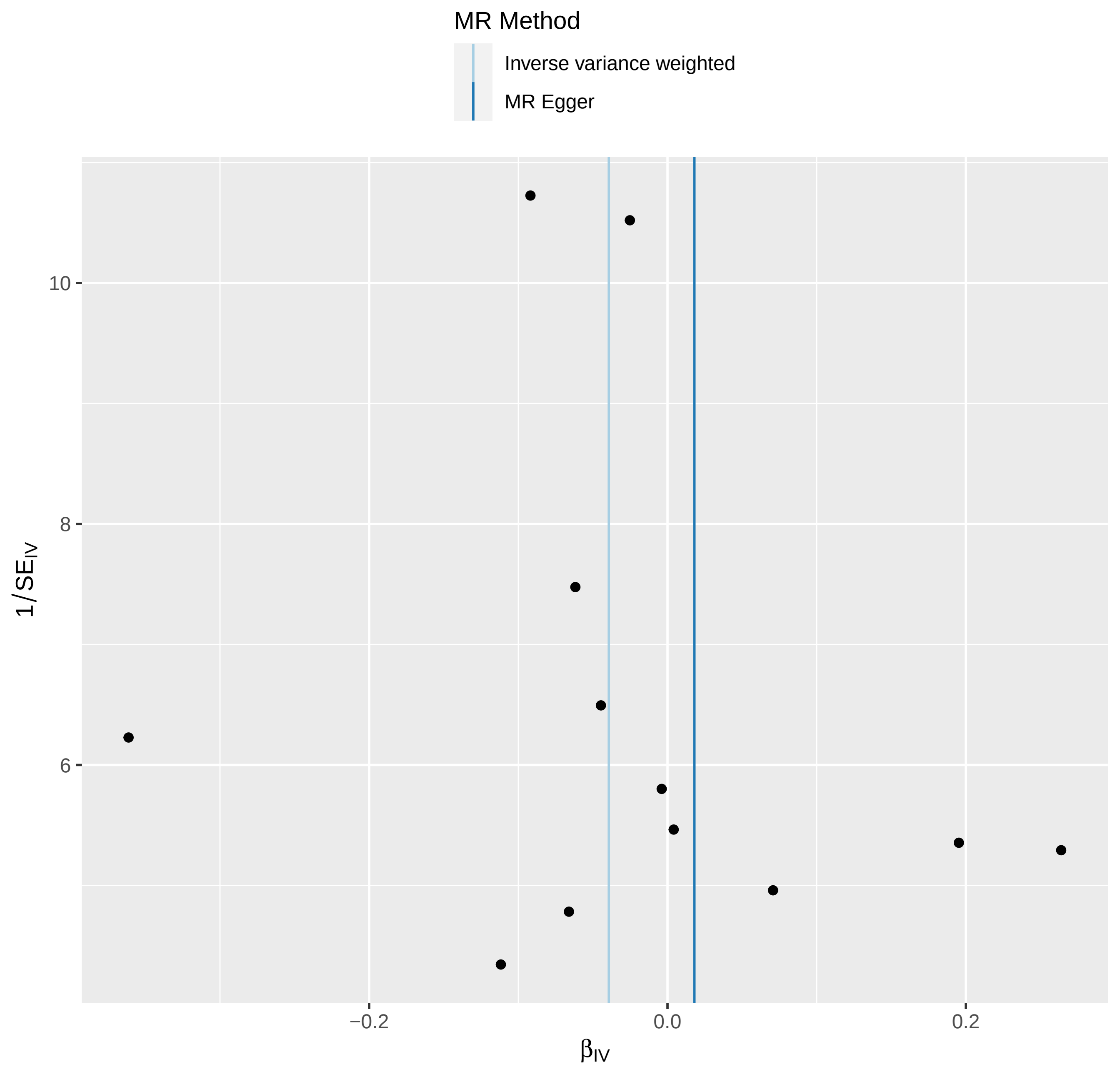

Batch 3−1496 : Selenium on Interleukin−1 receptor type 1

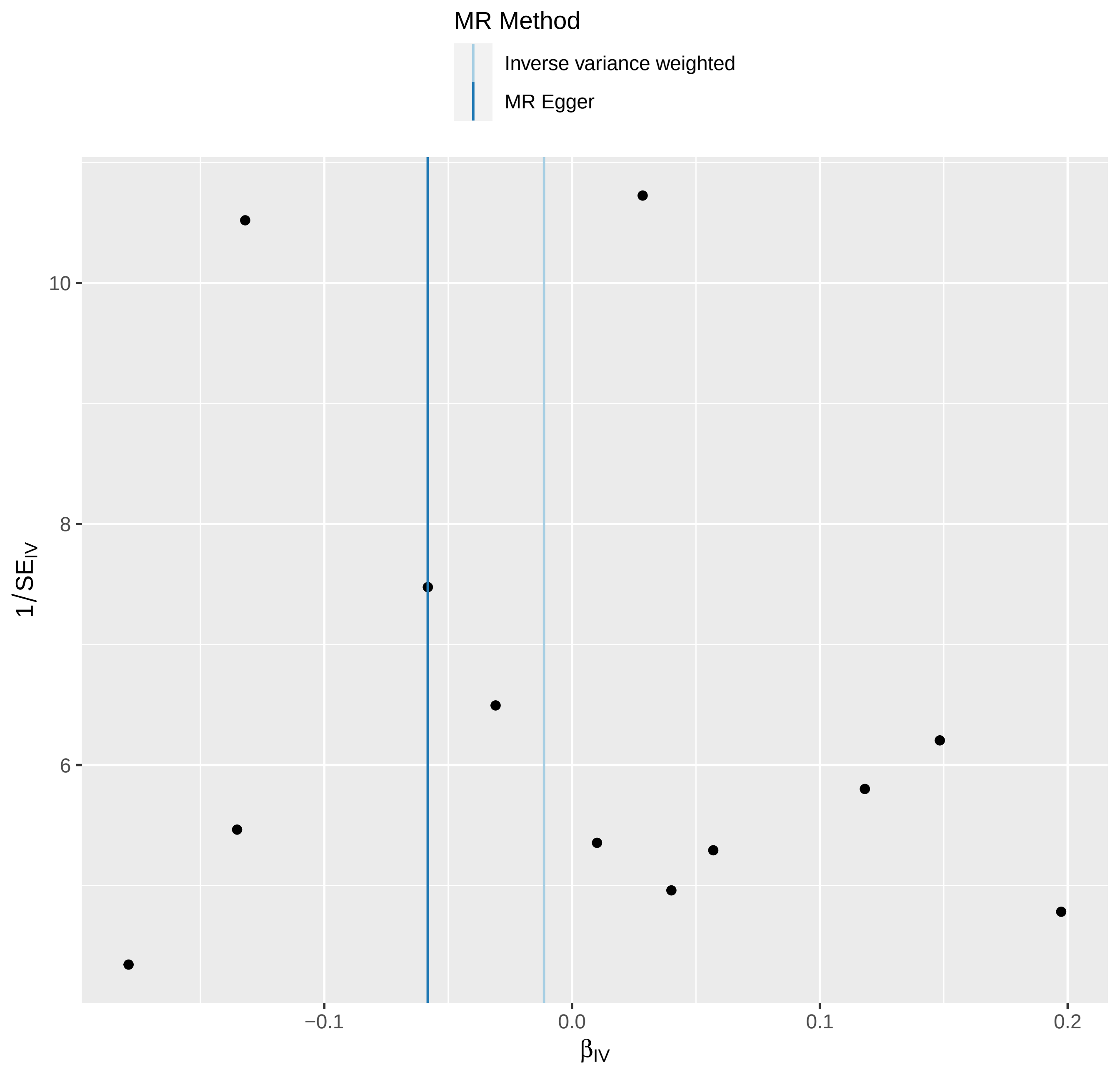

Batch 3−1497 : Selenium on Interleukin−1 receptor type 2

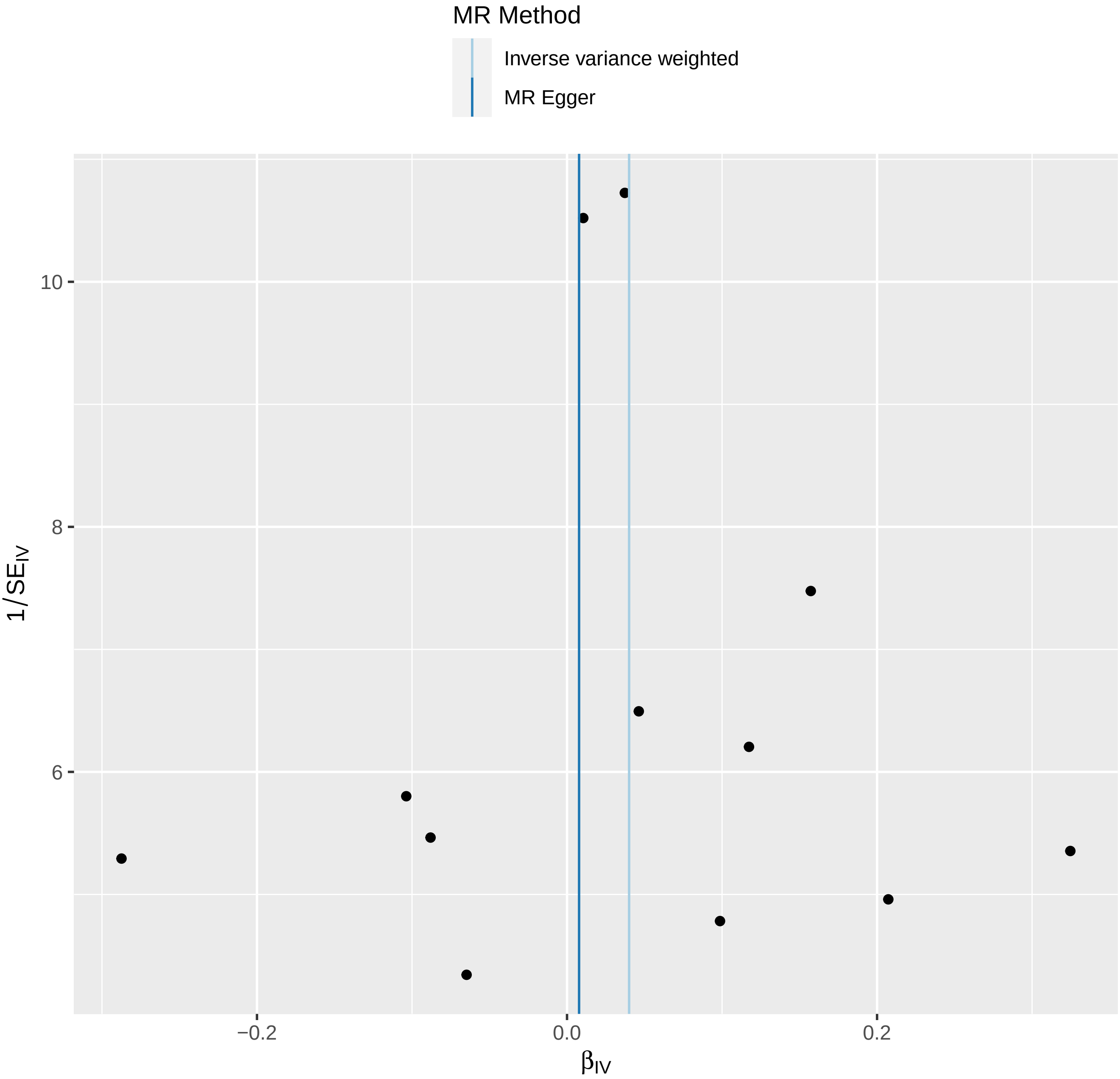

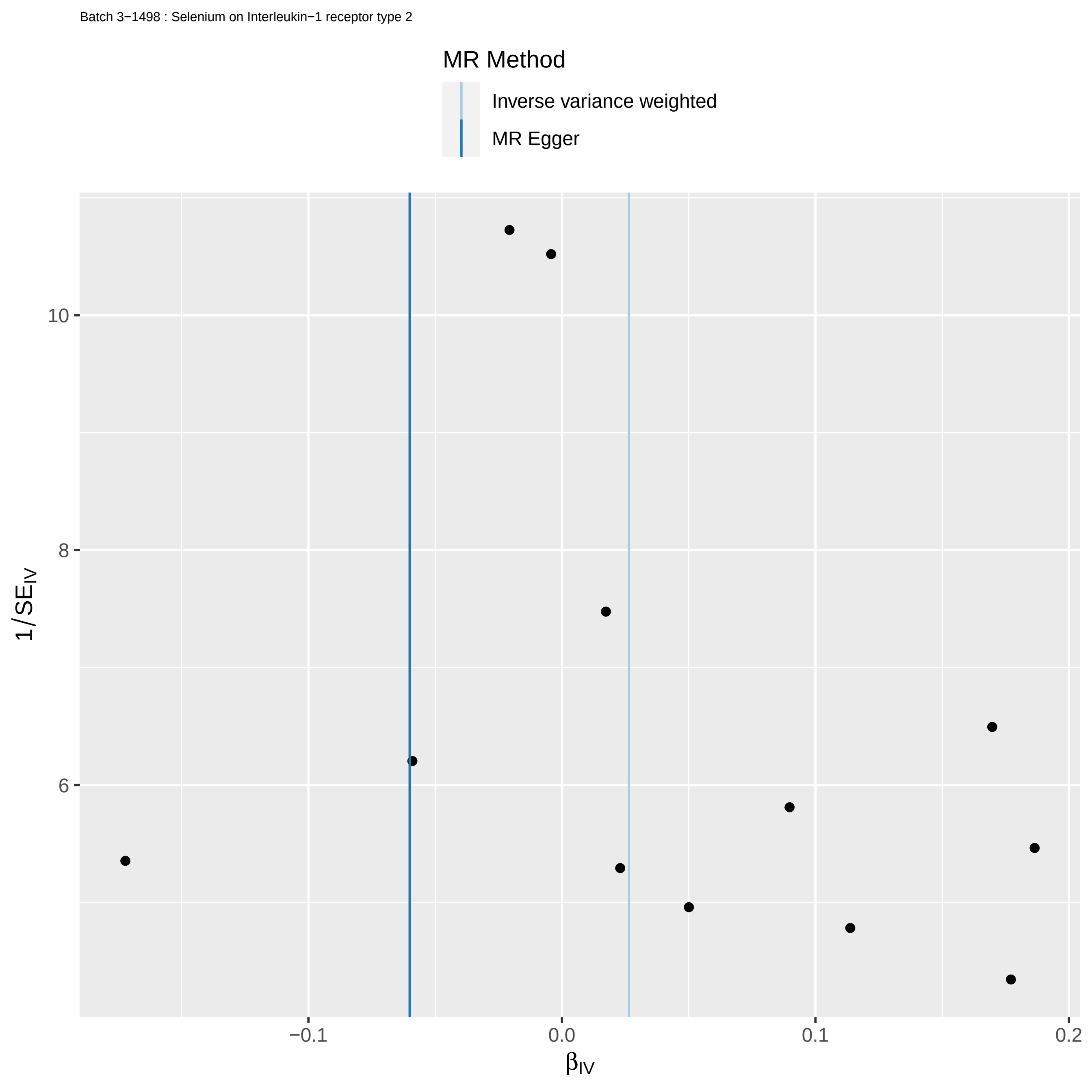
Batch 3−1498 : Selenium on Interleukin−1 receptor type 2
MR Method
Inverse variance weighted
MR Egger
10
8
6
1/SE$_{IV}$
−0.1
0.0
0.1
0.2
β$_{IV}$

Batch 3−1499 : Selenium on Interleukin−1 Receptor accessory protein

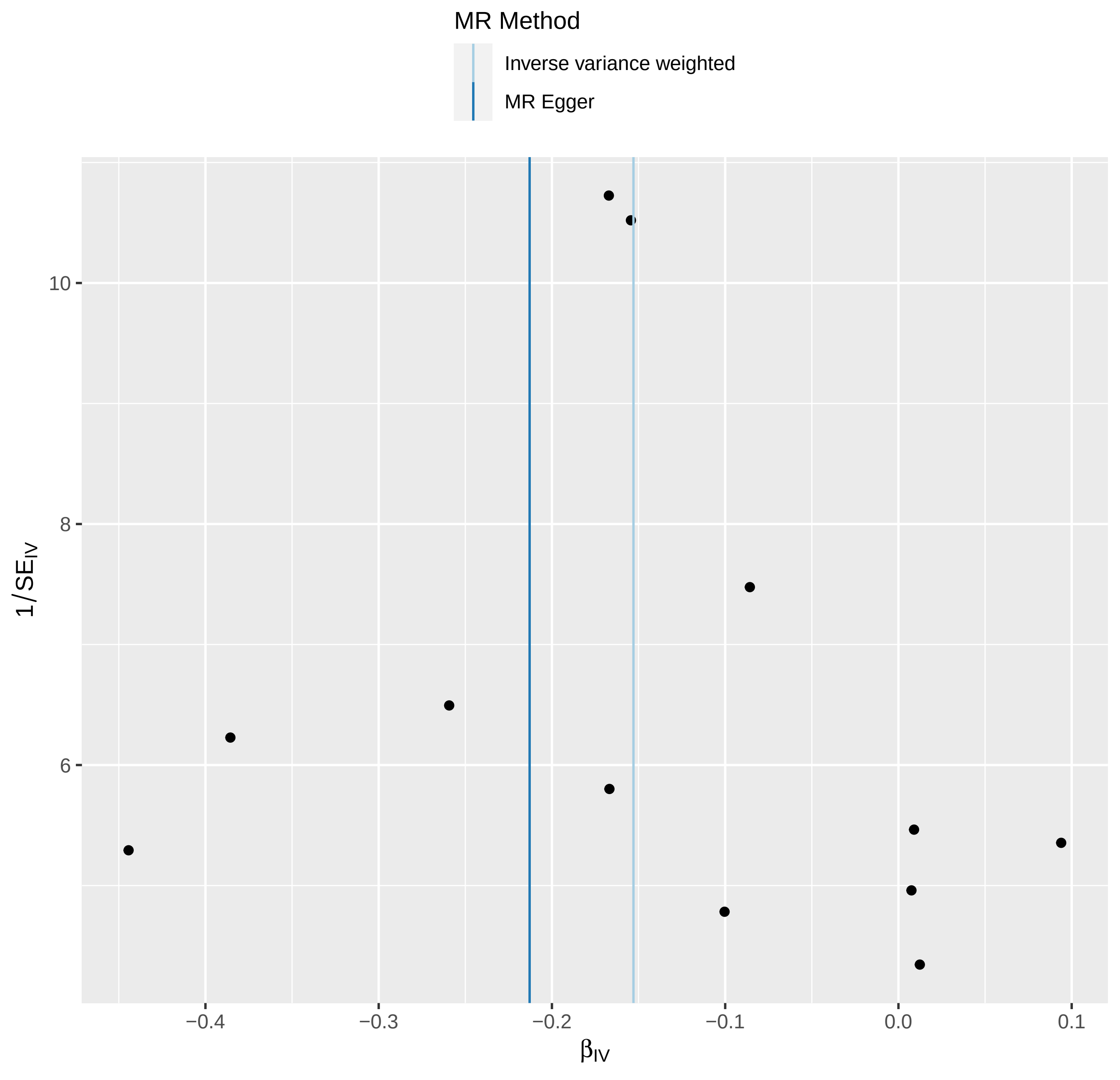

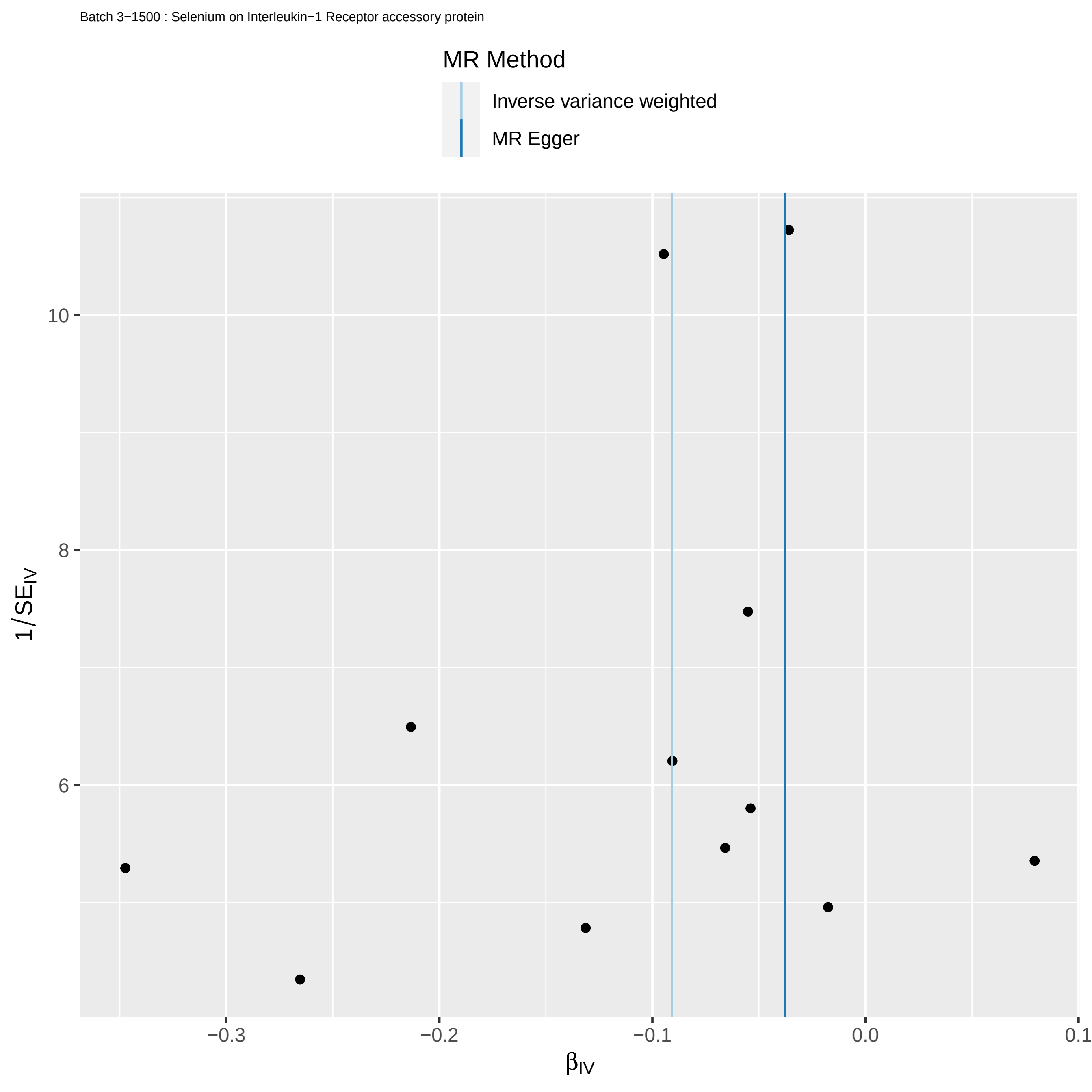
Batch 3−1500 : Selenium on Interleukin−1 Receptor accessory protein
MR Method
Inverse variance weighted
MR Egger
10
8
6
1/SE_IV
−0.3
−0.2
−0.1
0.0
0.1
β_IV

Batch 3−1501 : Selenium on X−linked interleukin−1 receptor accessory protein−like 2

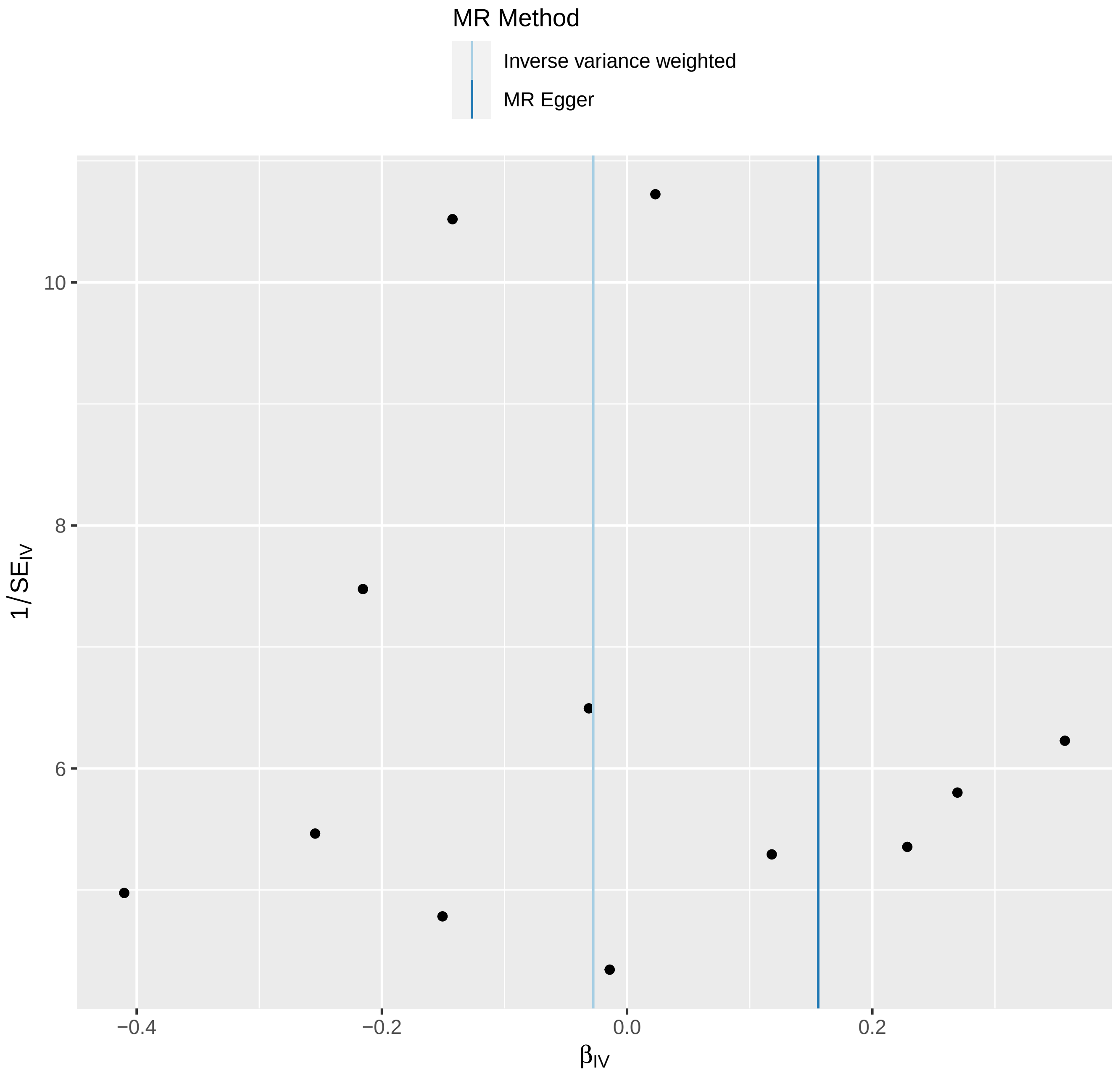

Batch 3−1502 : Selenium on Interleukin−1 receptor−like 1

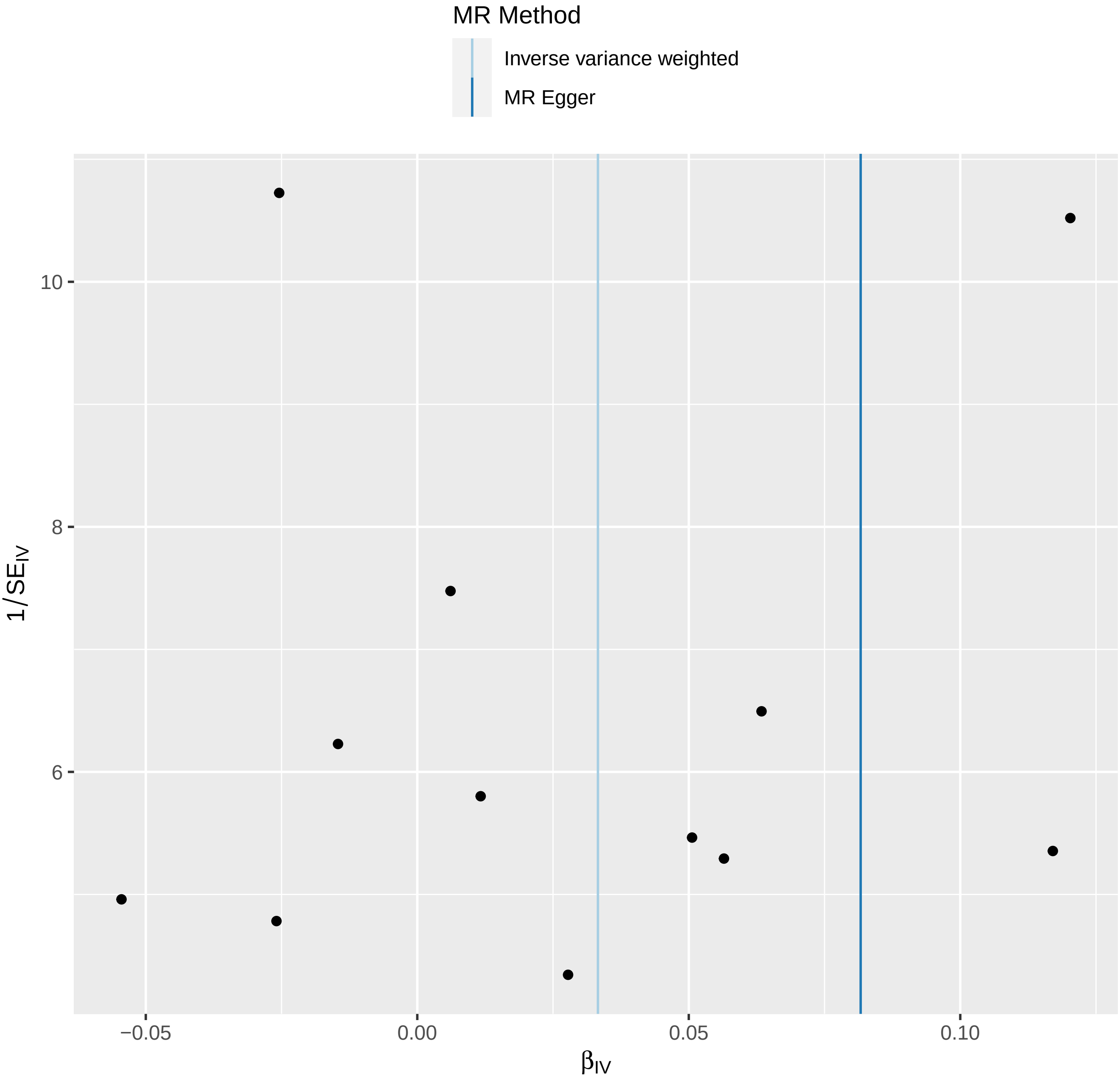

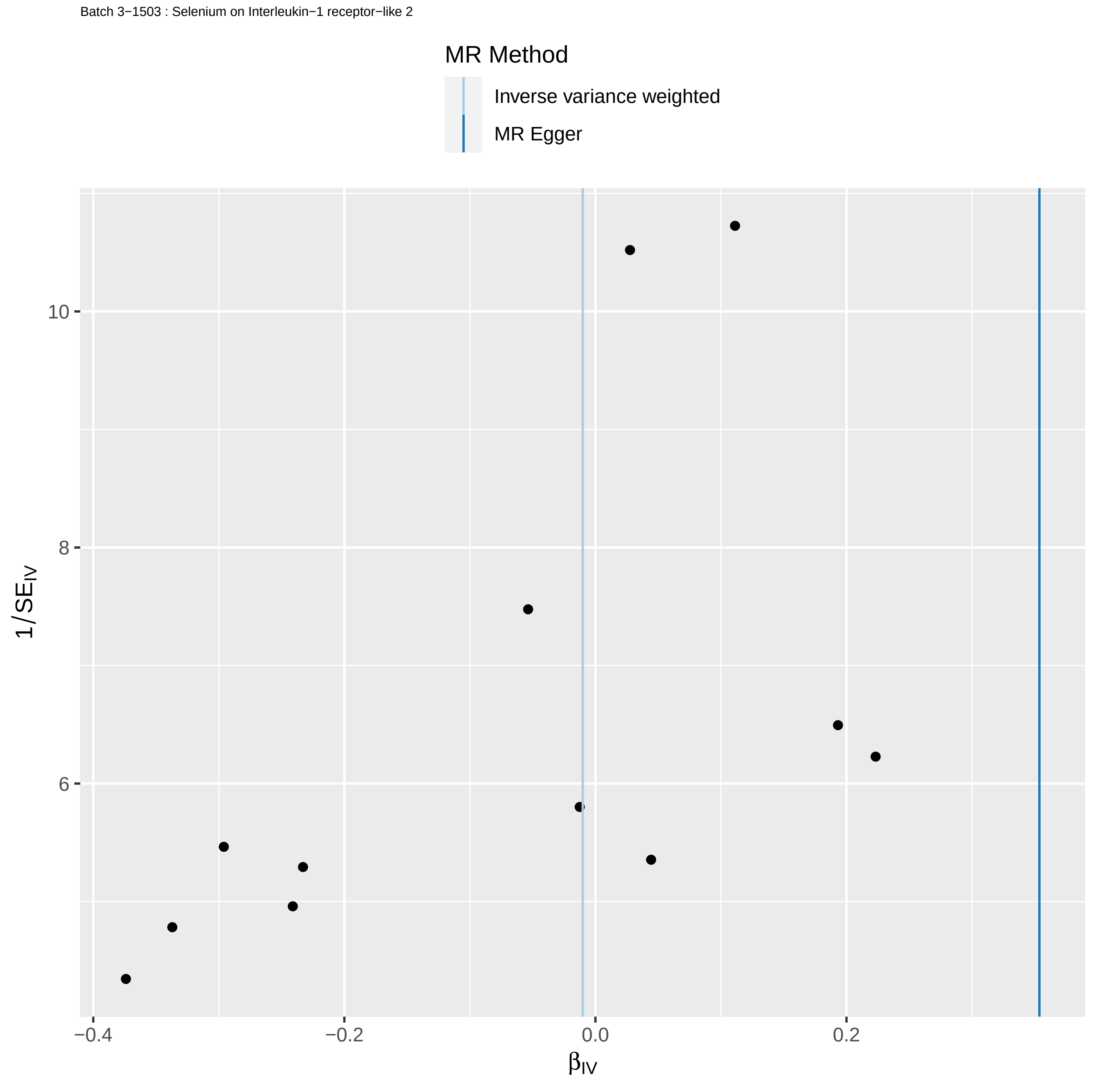

Batch 3−1503 : Selenium on Interleukin−1 receptor−like 2
MR Method
Inverse variance weighted
MR Egger
10
8
6
1/SE_IV
−0.4
−0.2
0.0
0.2
β_IV

Batch 3−1504 : Selenium on Interleukin−1 receptor antagonist protein

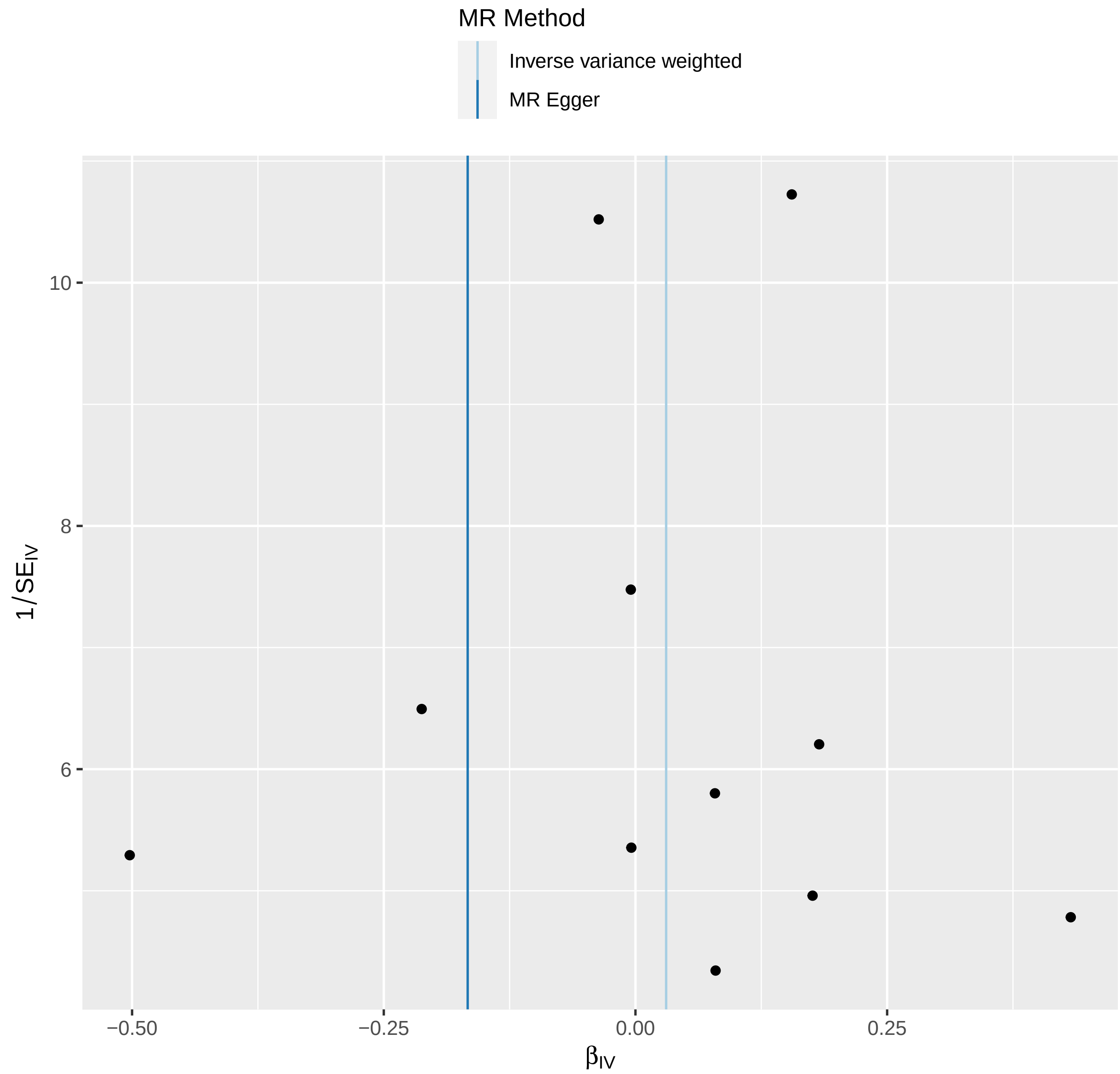

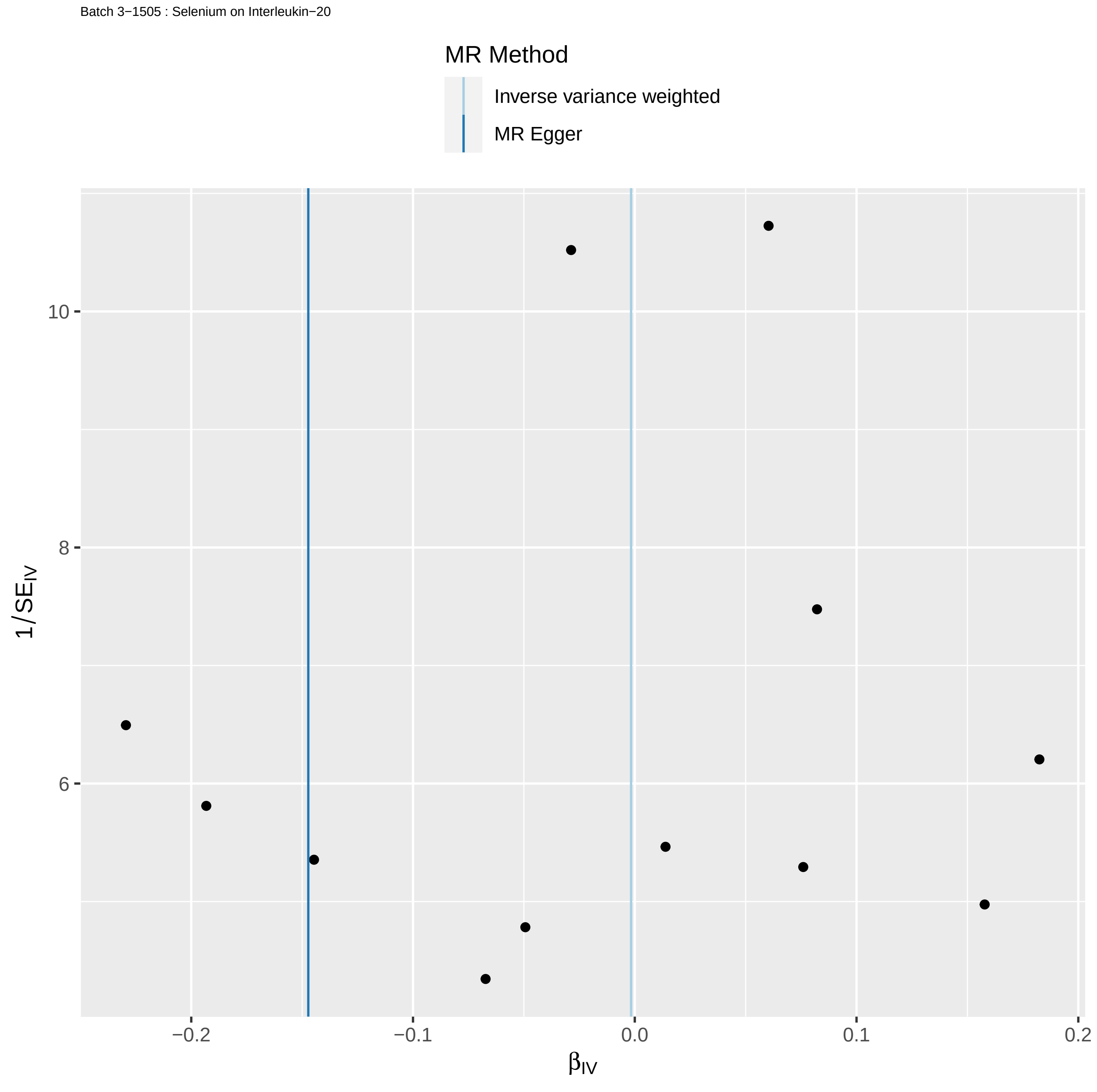

Batch 3−1505 : Selenium on Interleukin−20
MR Method
Inverse variance weighted
MR Egger
10
8
6
1/SE_IV
−0.2
−0.1
0.0
0.1
0.2
β_IV

Batch 3−1506 : Selenium on Interleukin−21

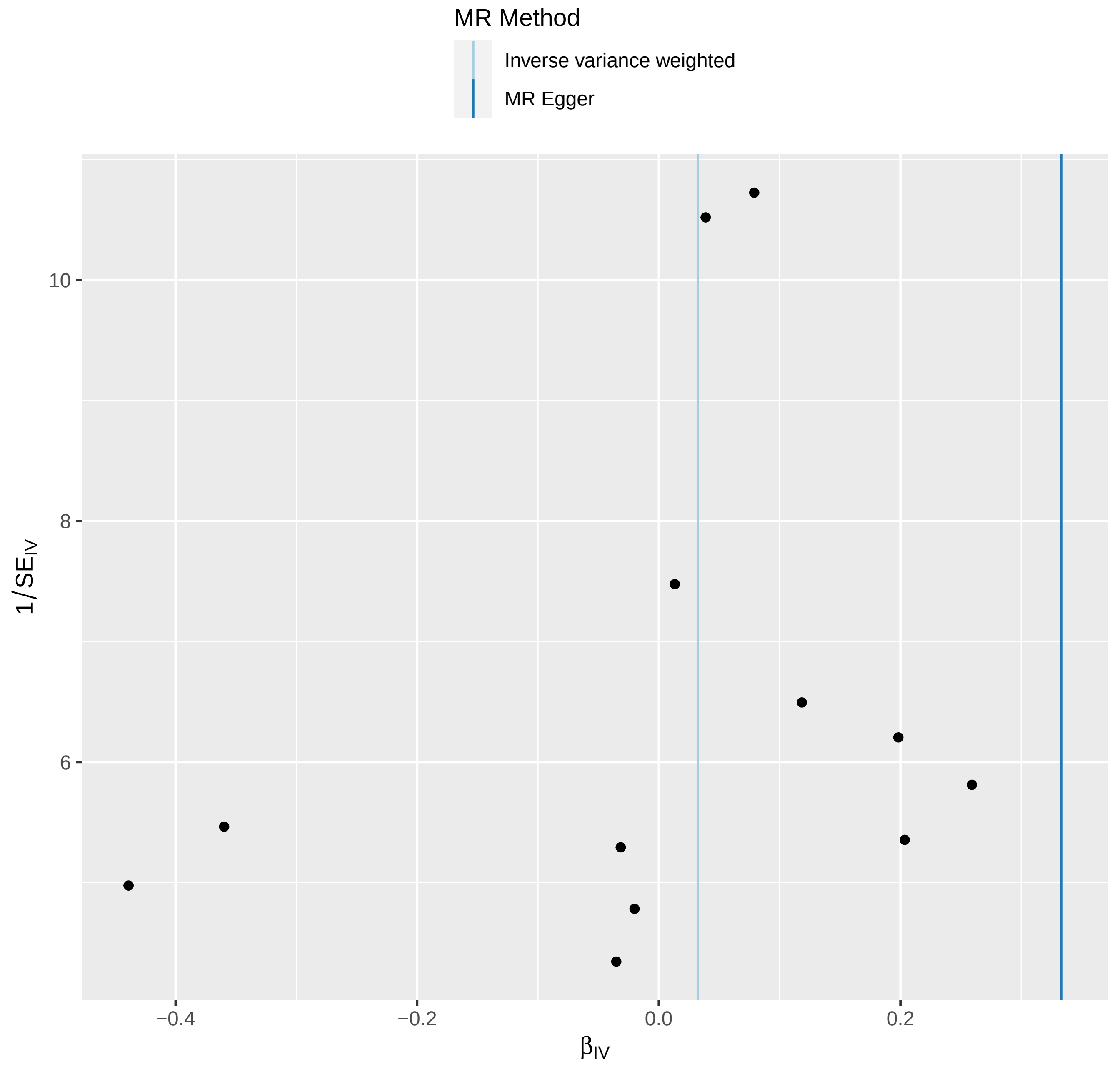

Batch 3−1507 : Selenium on Interleukin−22

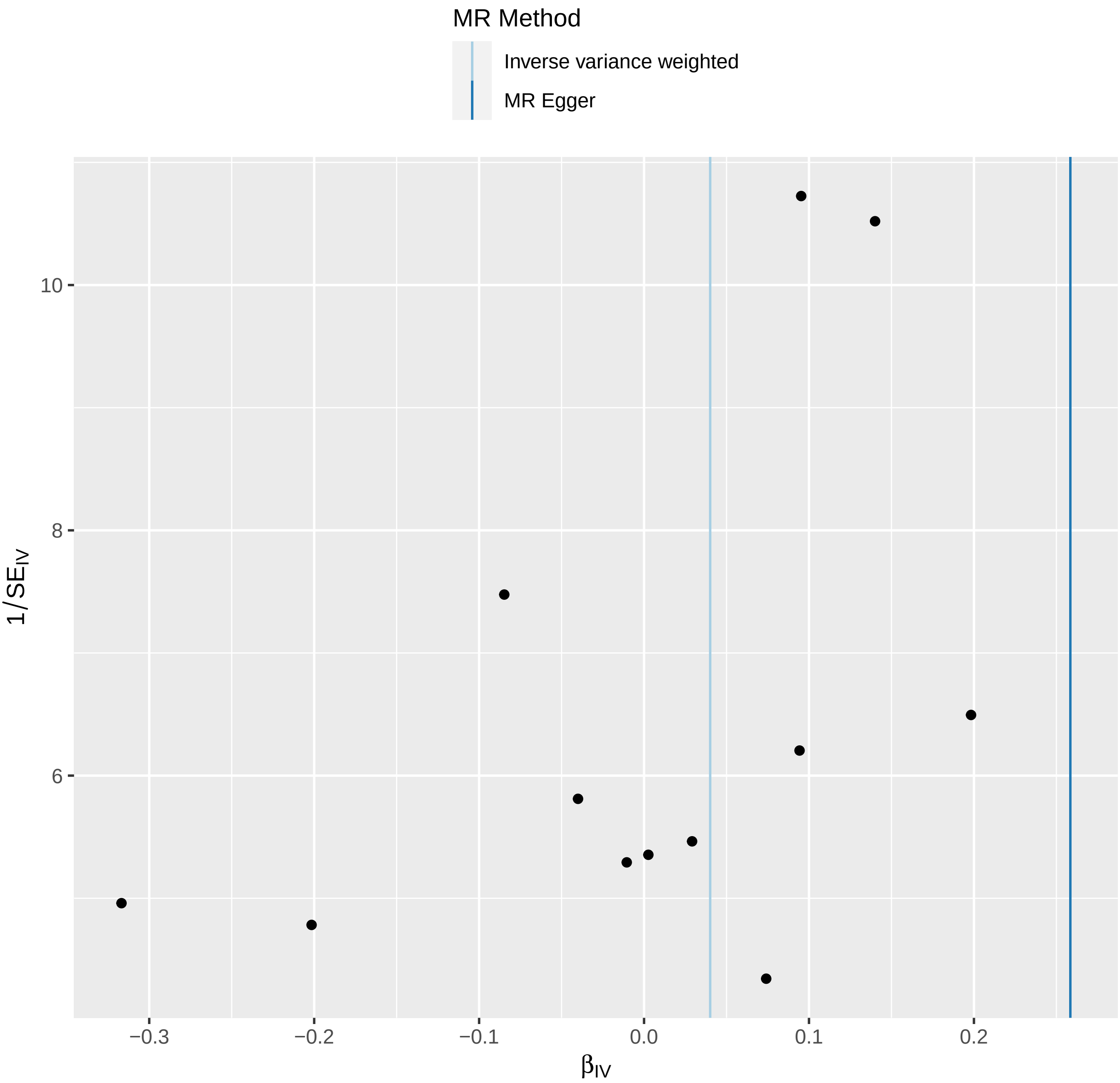

Batch 3−1508 : Selenium on Interleukin−22 receptor subunit alpha−1

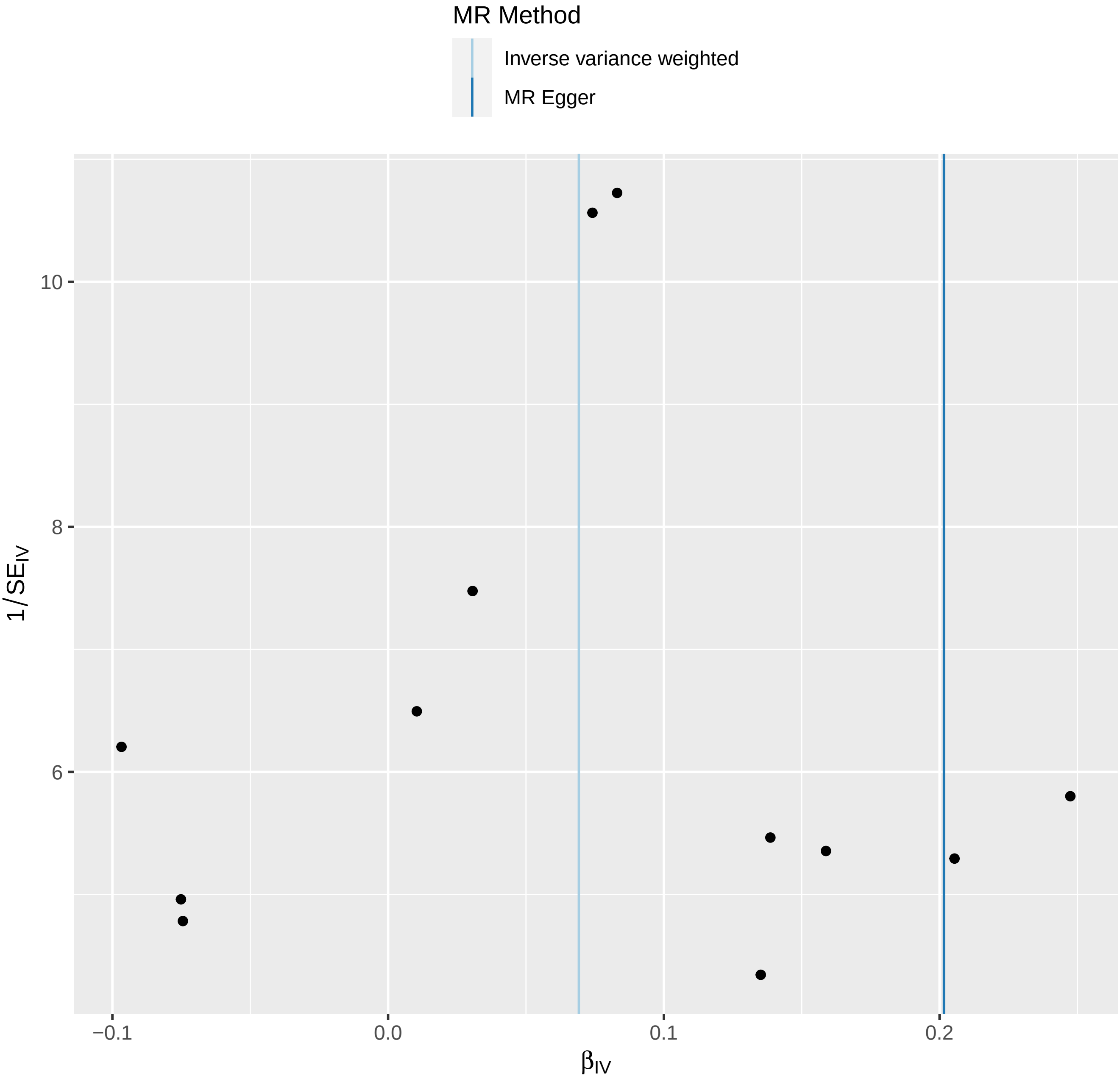

Batch 3−1509 : Selenium on Interleukin−22 receptor subunit alpha−1

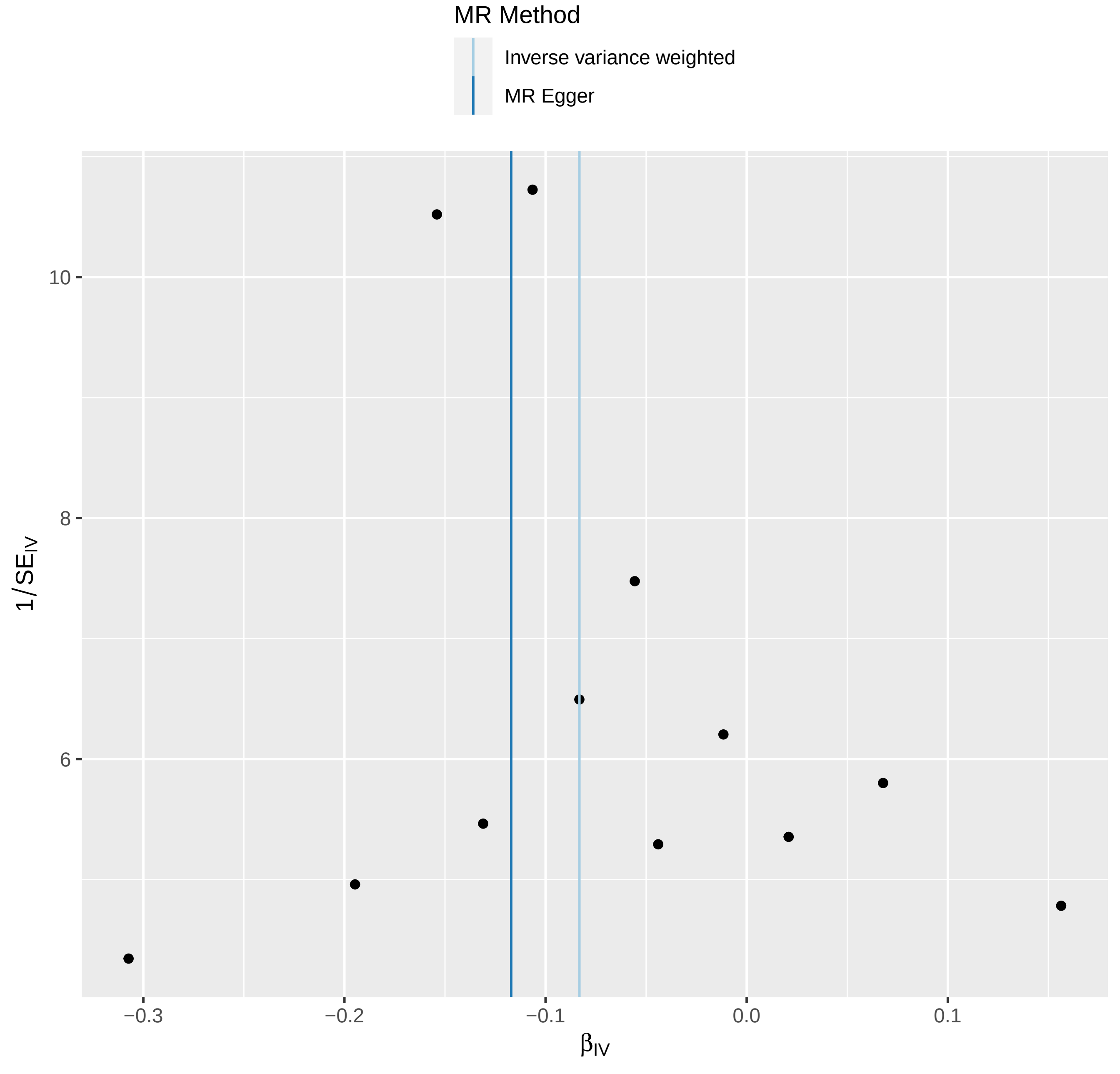

Batch 3−1510 : Selenium on Interleukin−22 receptor subunit alpha−2

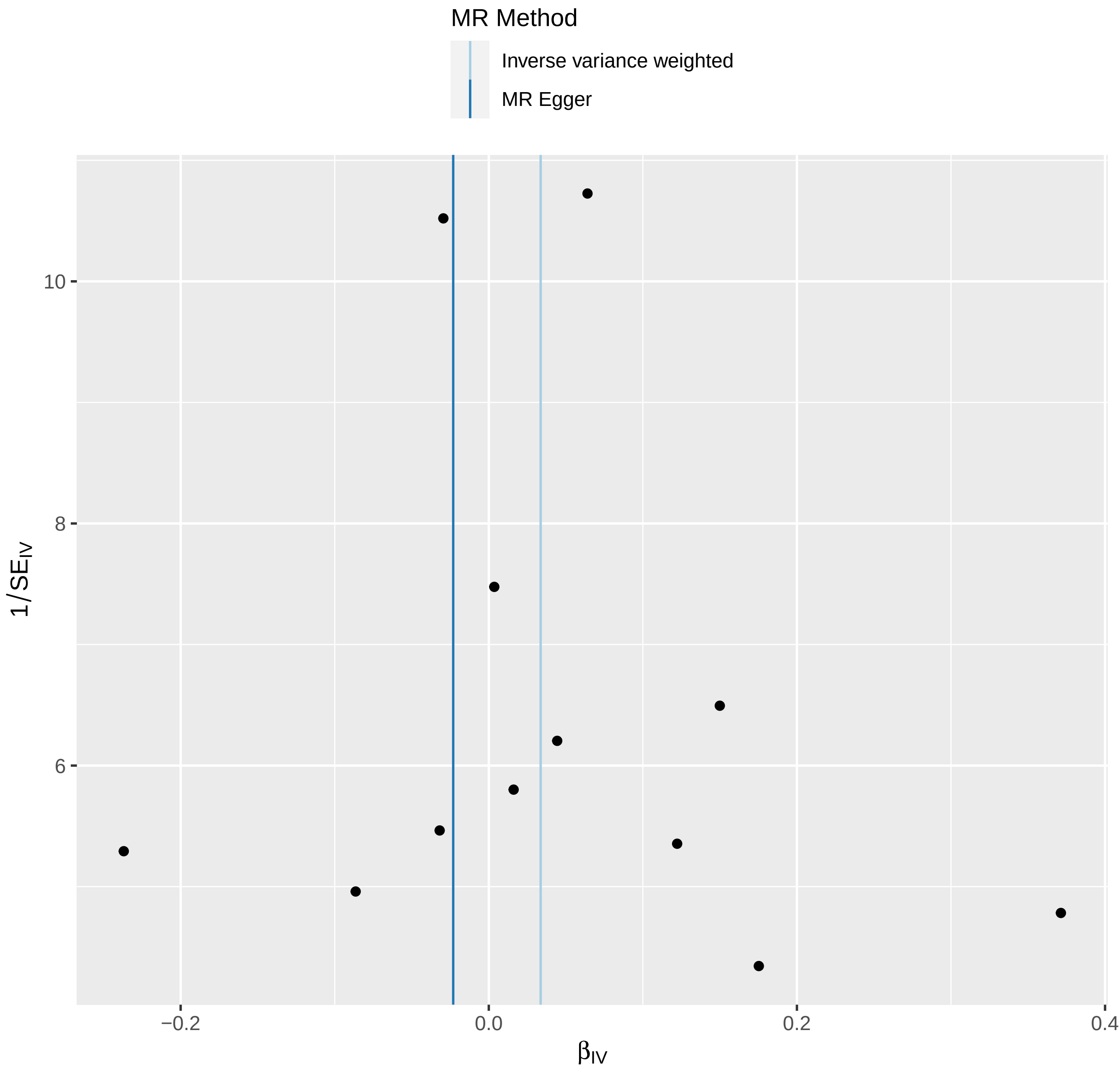

Batch 3−1511 : Selenium on Interleukin−22 receptor subunit alpha−2

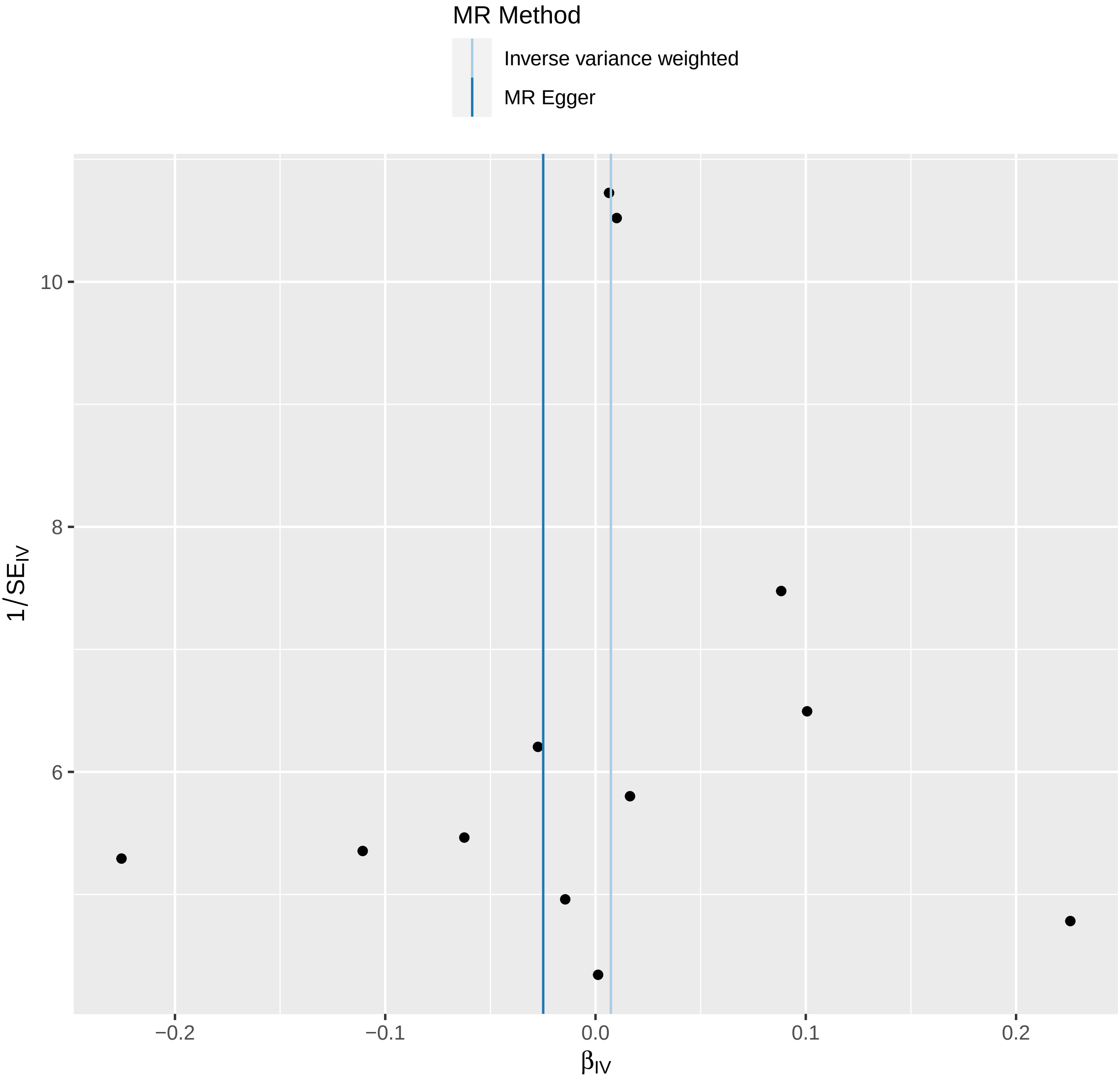

Batch 3−1512 : Selenium on Interleukin−2

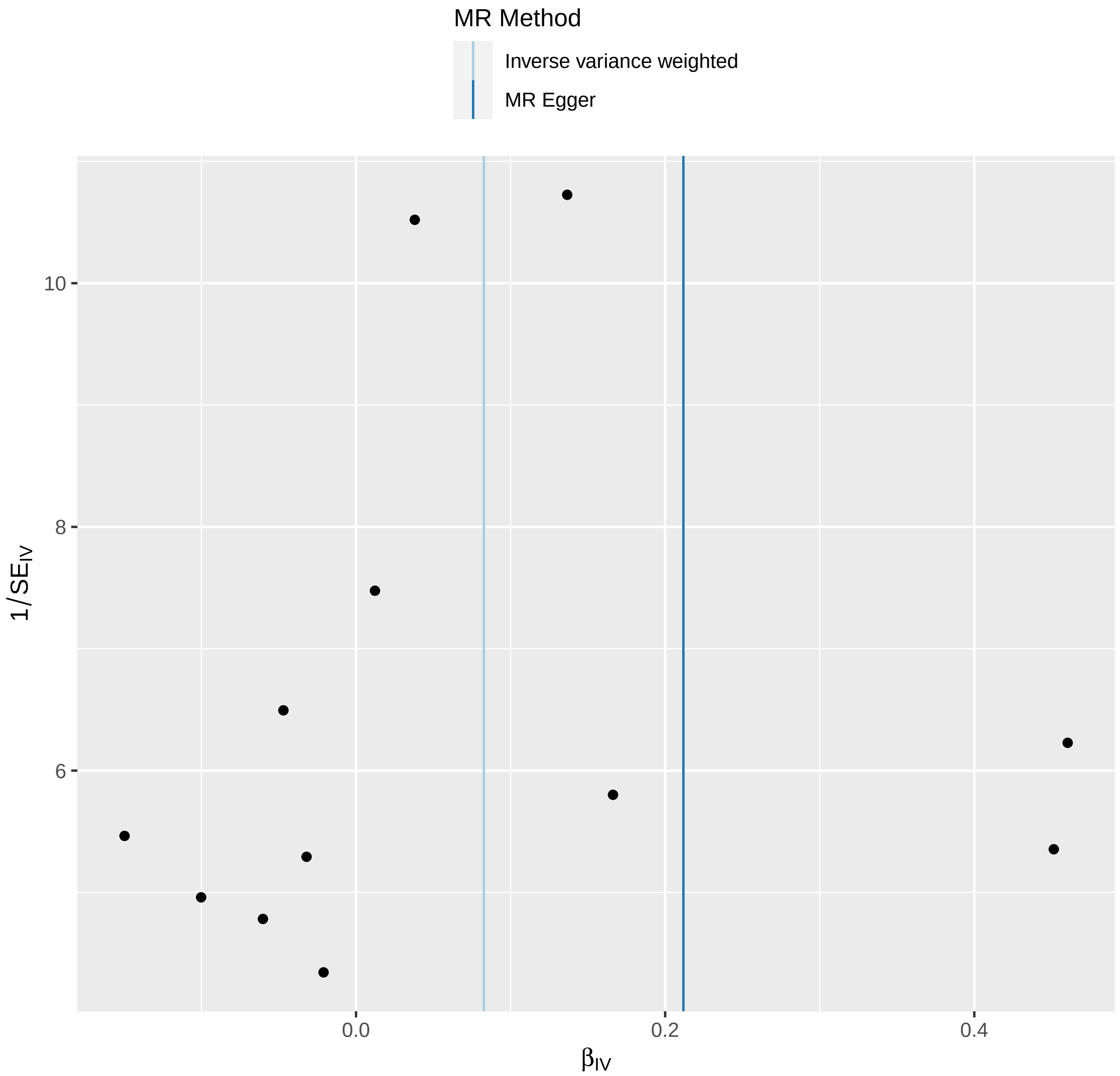

Batch 3−1513 : Selenium on Interleukin−23 receptor

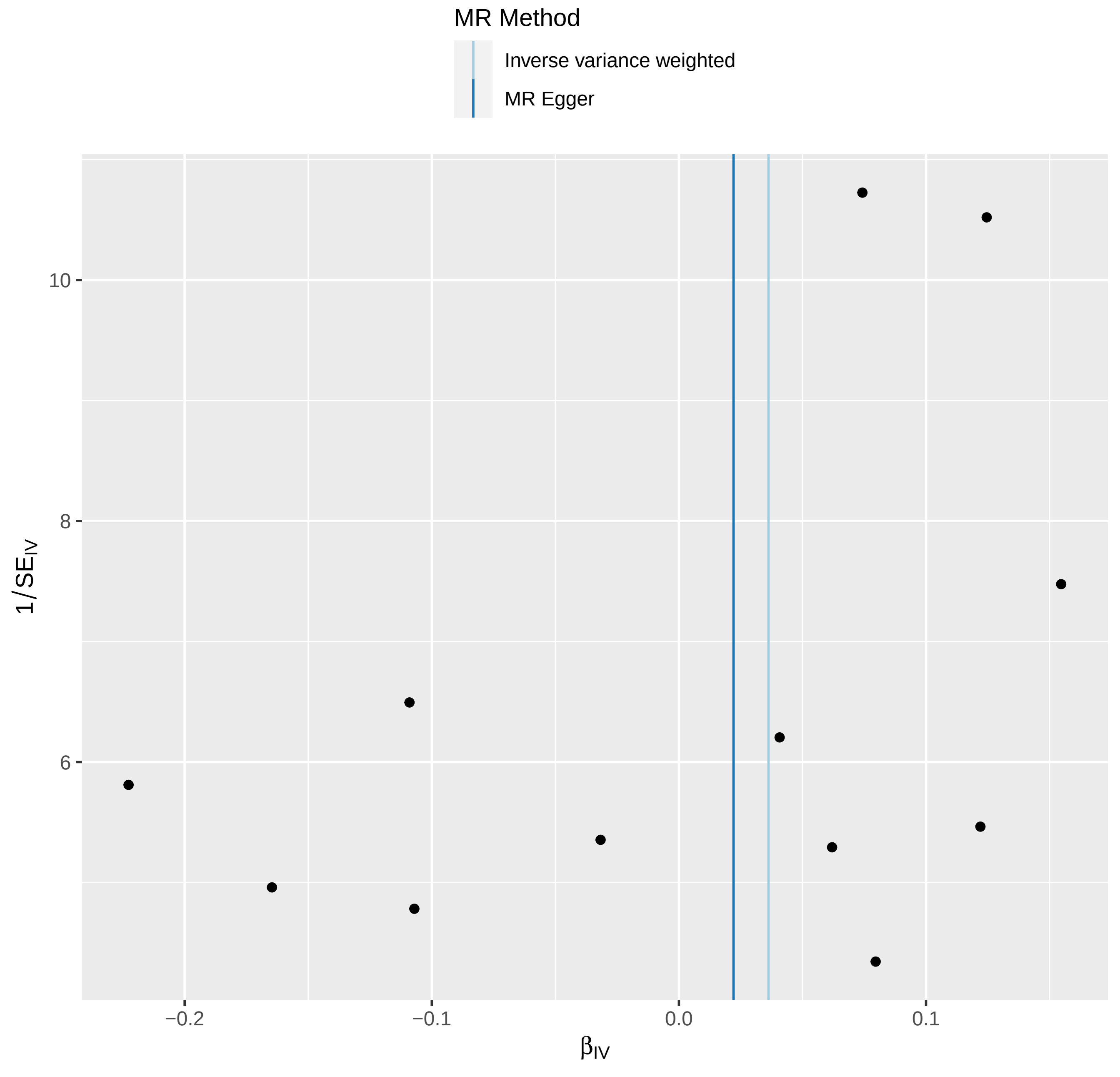

Batch 3−1514 : Selenium on Interleukin−24

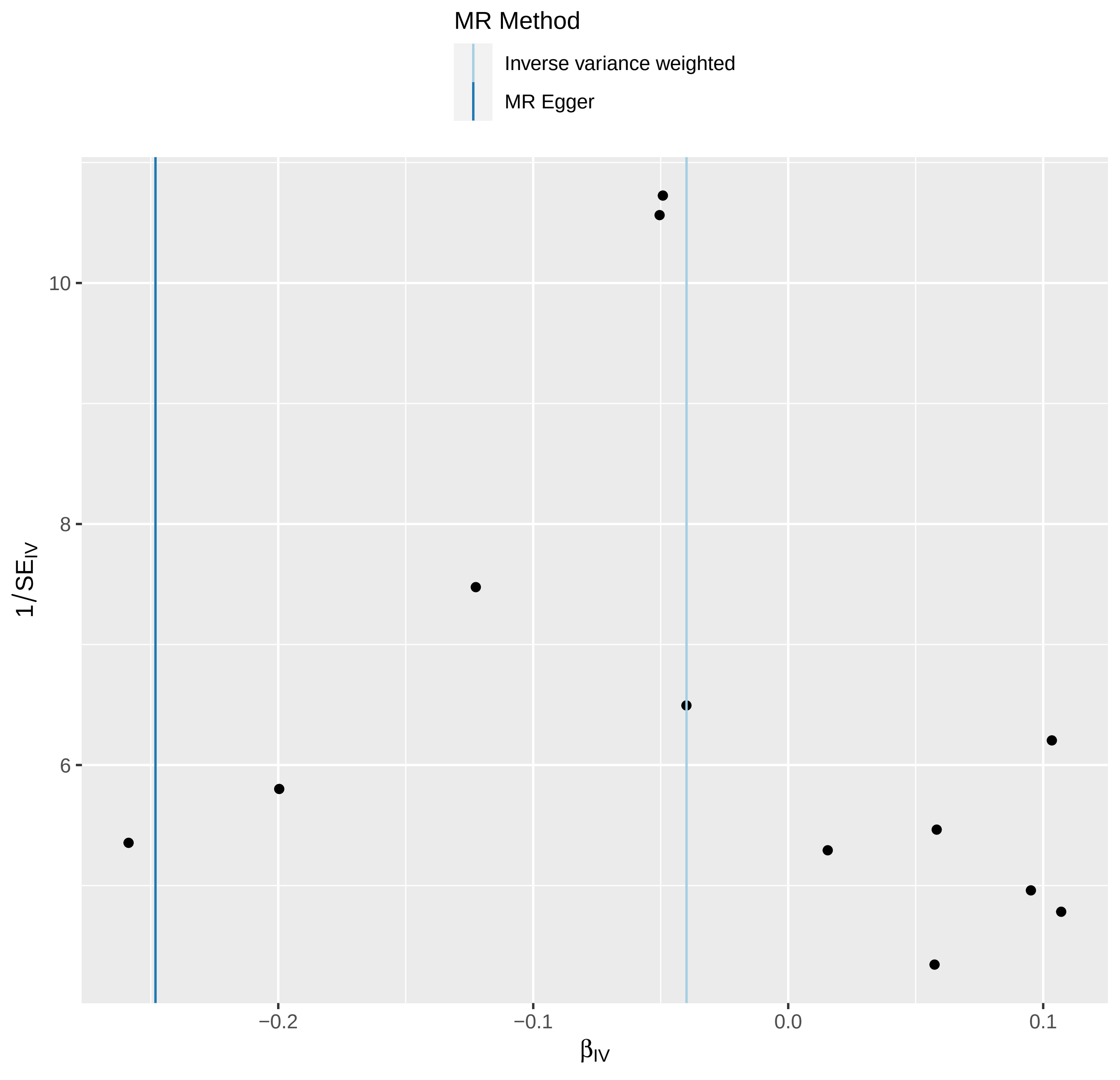

Batch 3−1515 : Selenium on Interleukin−25

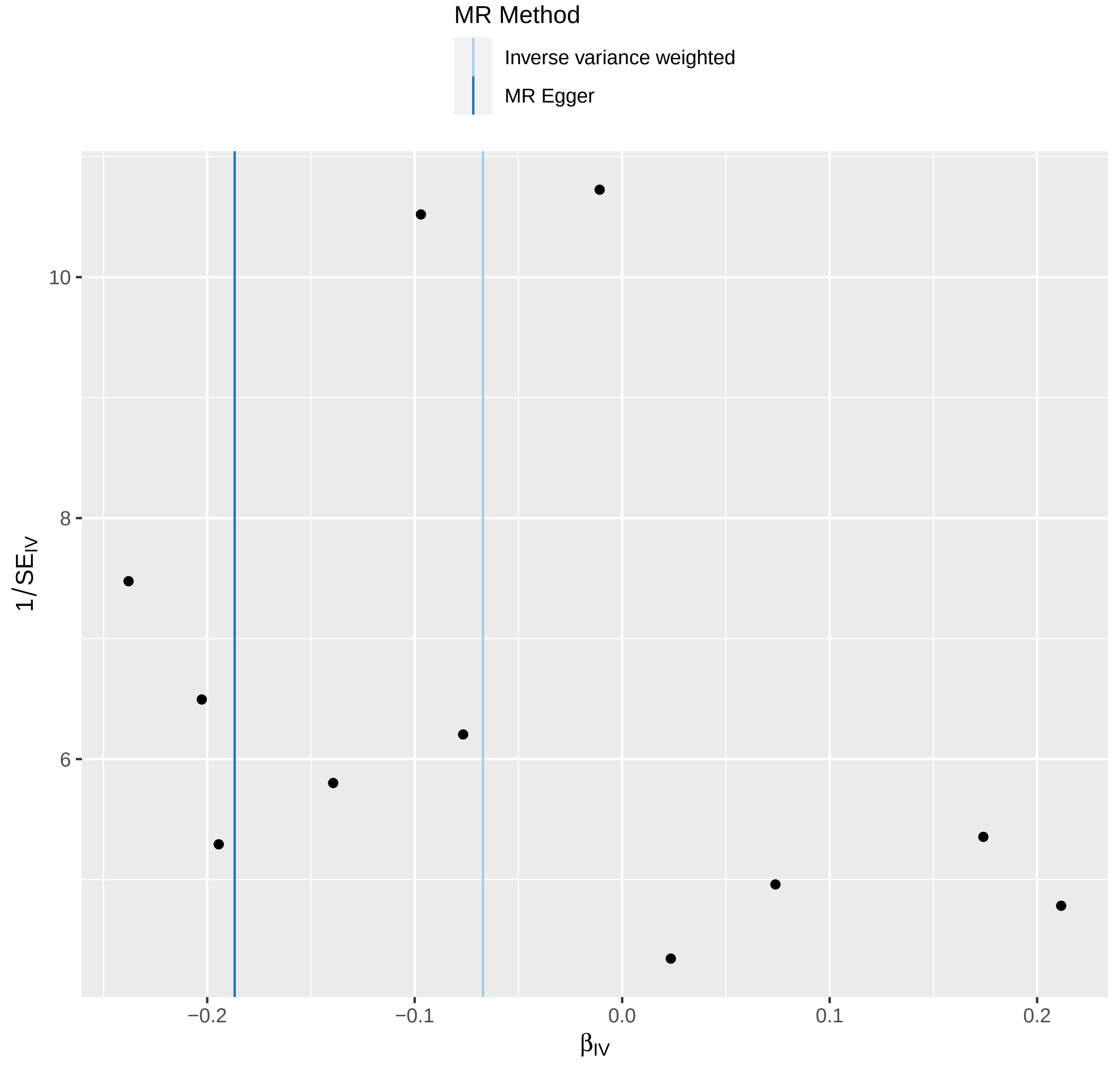

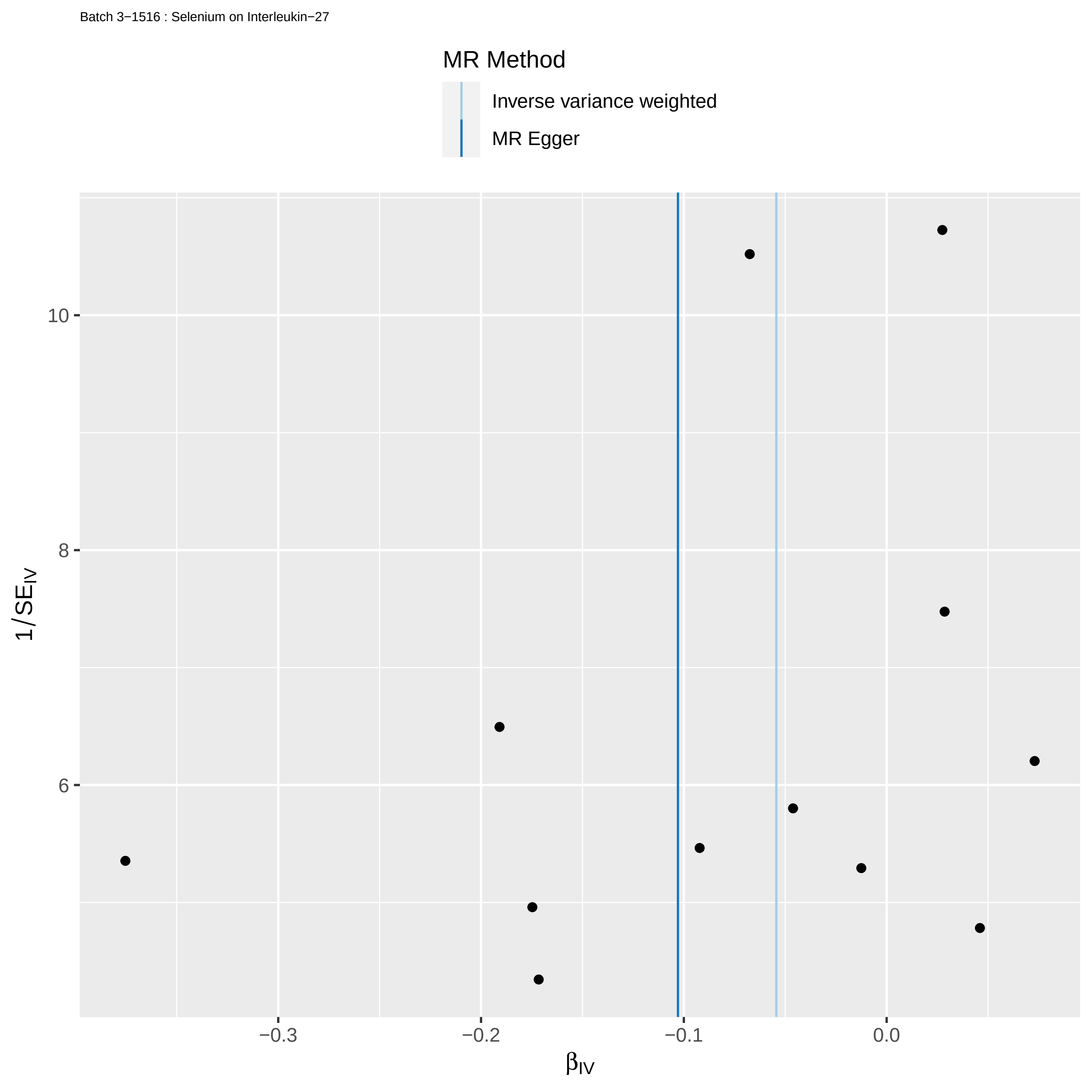
Batch 3−1516 : Selenium on Interleukin−27
MR Method
Inverse variance weighted
MR Egger
10
8
6
1/SE_IV
−0.3
−0.2
−0.1
0.0
β_IV

Batch 3−1517 : Selenium on Interleukin−27 receptor subunit alpha

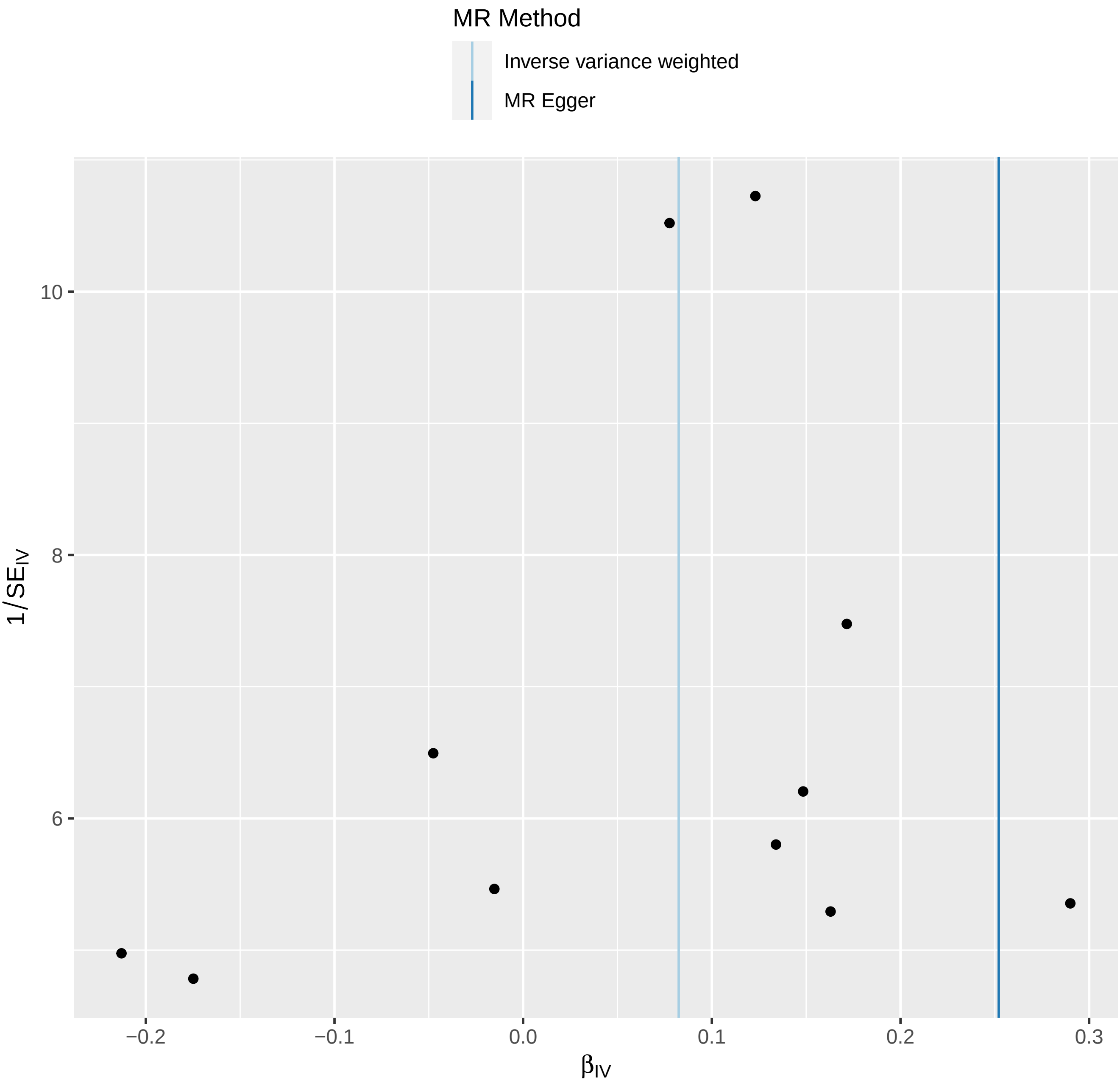

Batch 3−1518 : Selenium on Interleukin−2 receptor subunit alpha

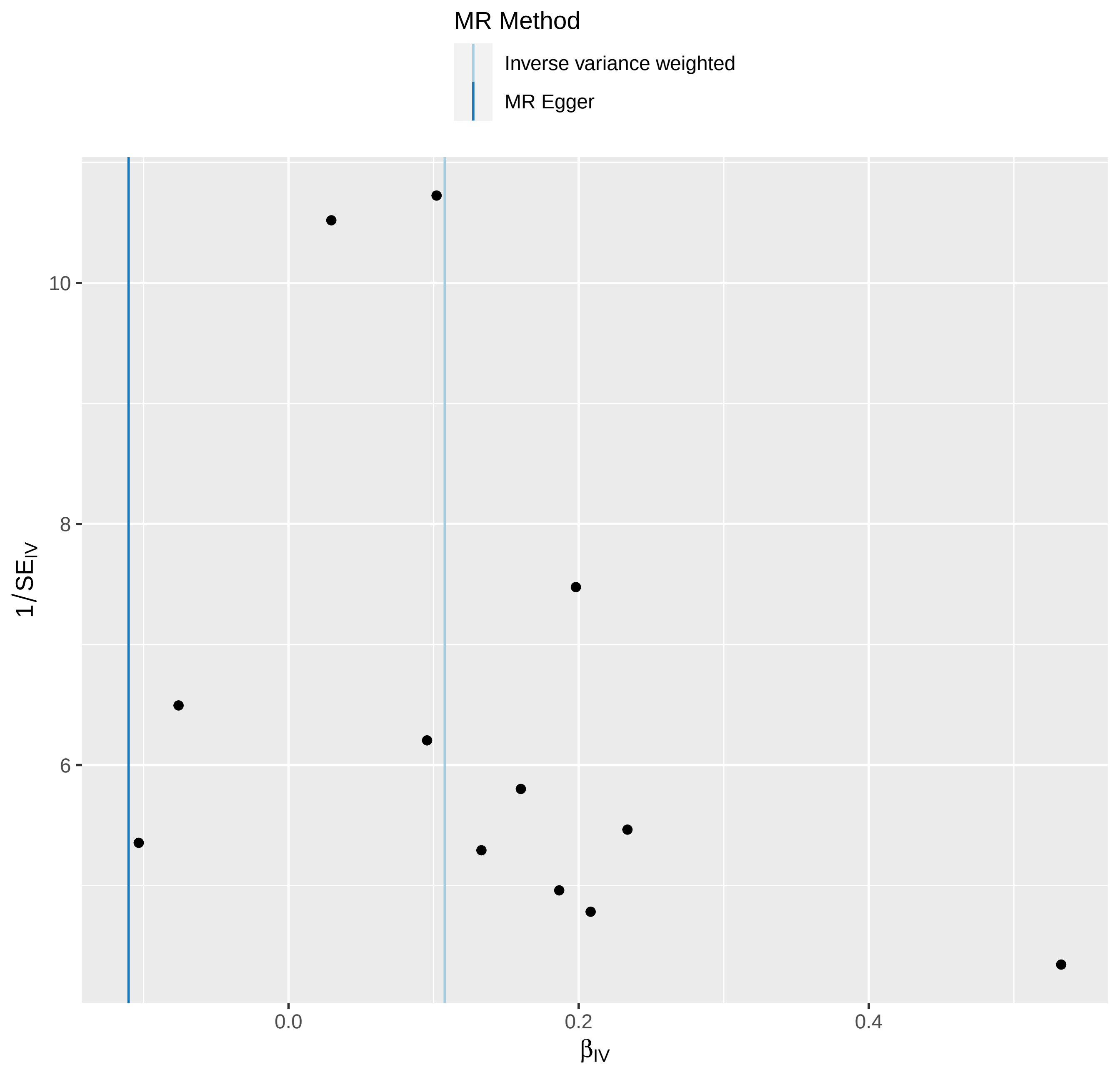

Batch 3−1519 : Selenium on Interleukin−2 receptor subunit beta

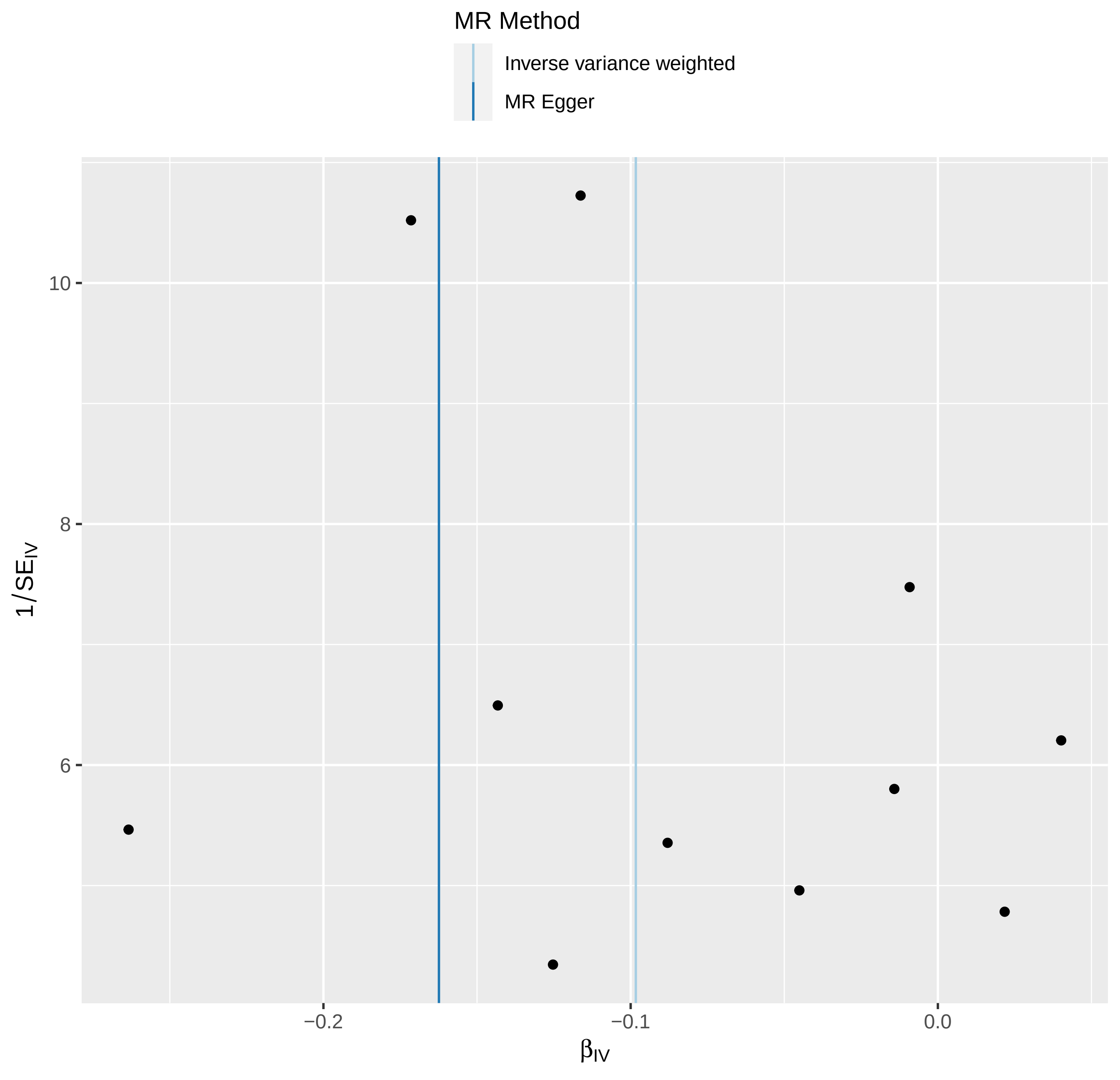

Batch 3−1520 : Selenium on Cytokine receptor common subunit gamma

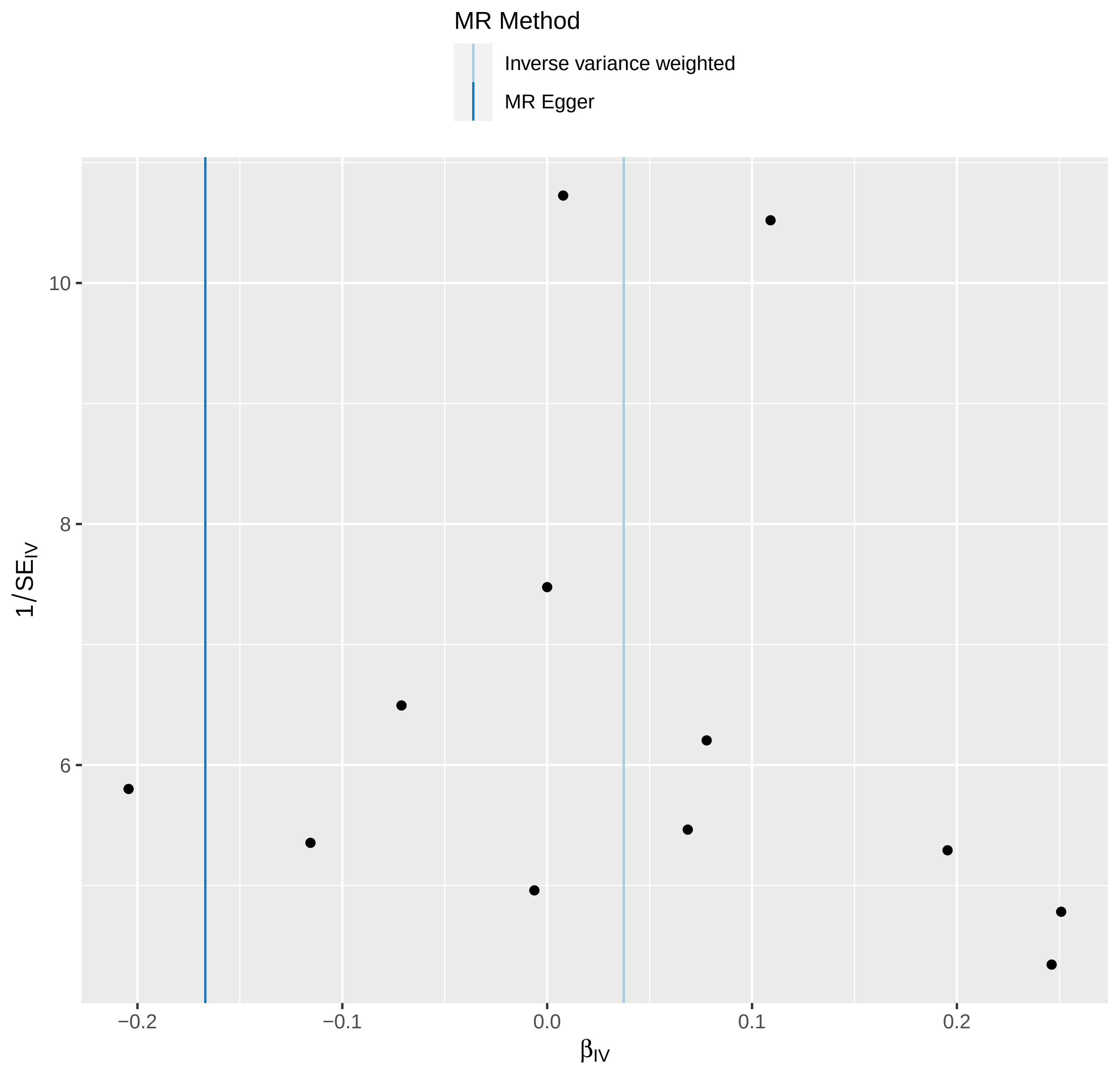

Batch 3−1521 : Selenium on Interleukin−31

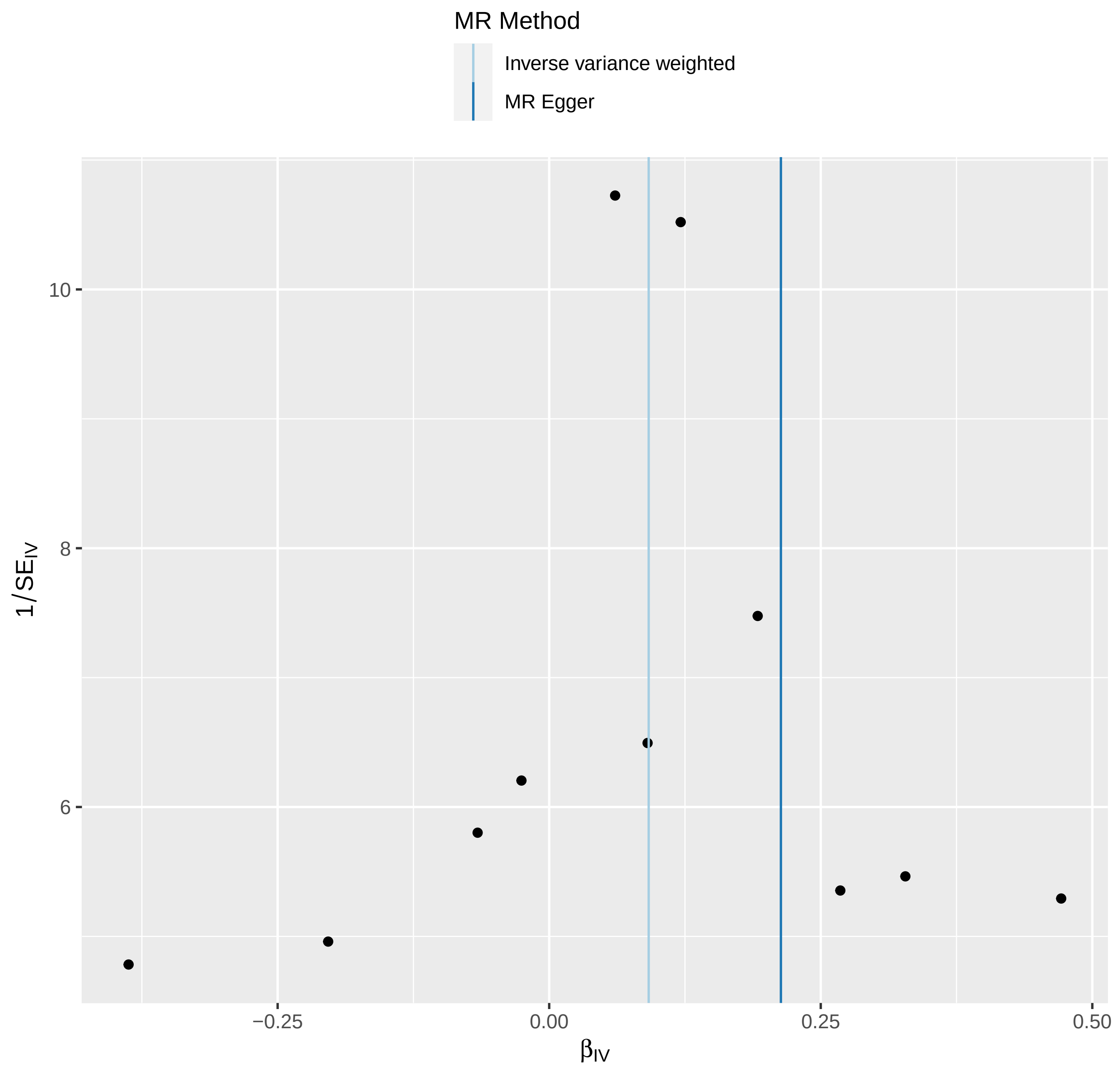

Batch 3−1522 : Selenium on Interleukin−31 receptor subunit alpha

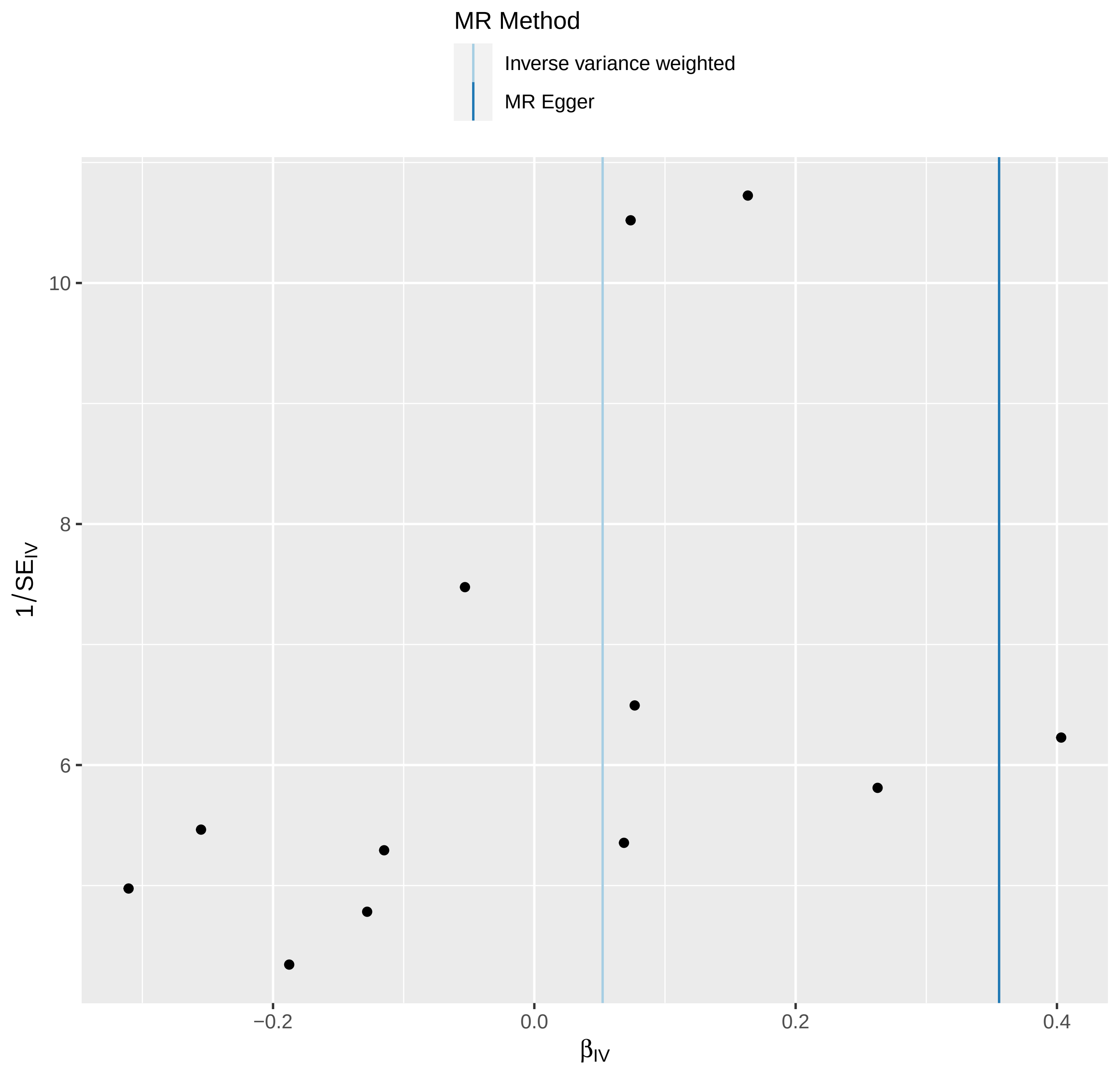

Batch 3−1523 : Selenium on Interleukin−32

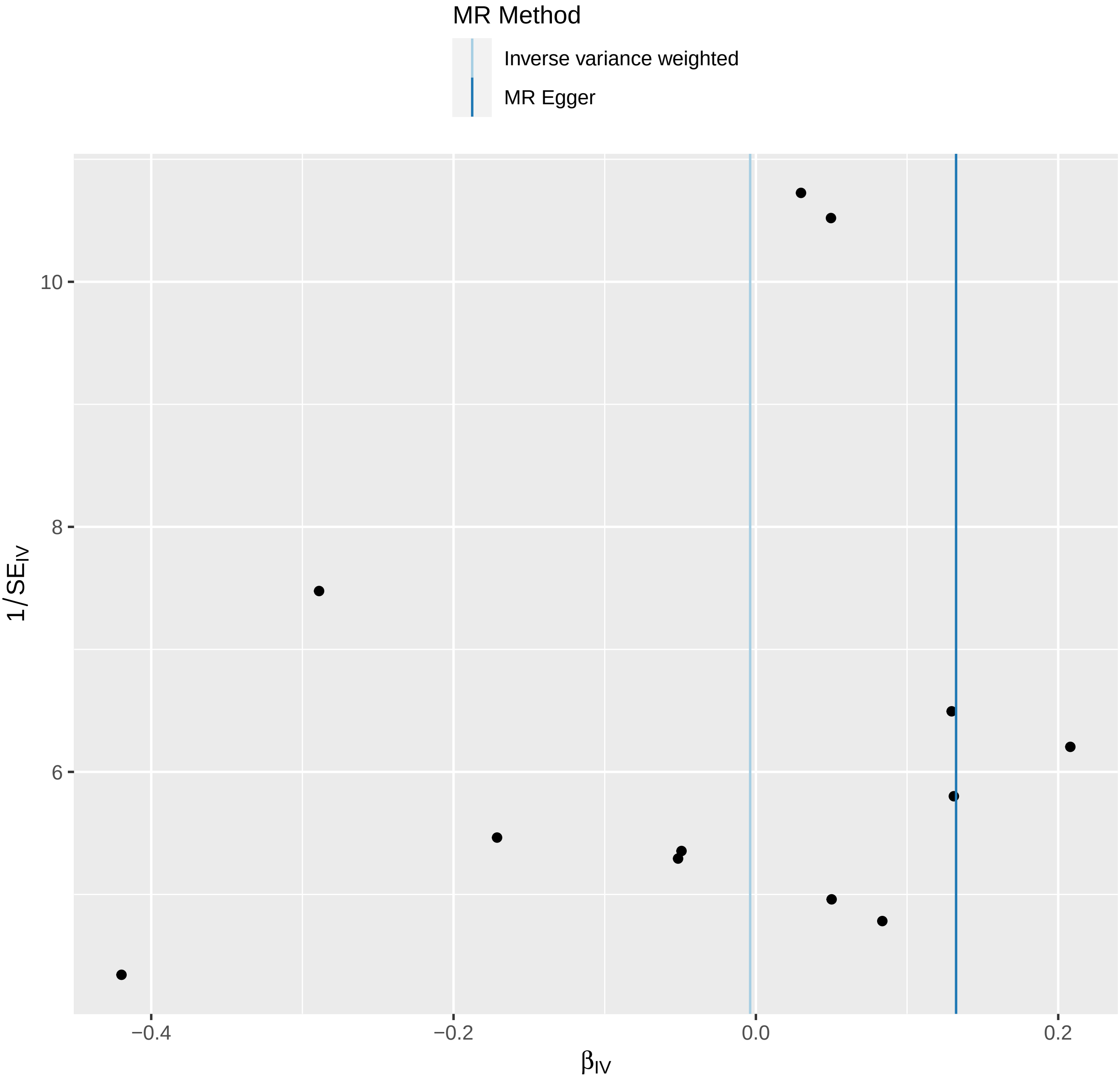

Batch 3−1524 : Selenium on Interleukin−34

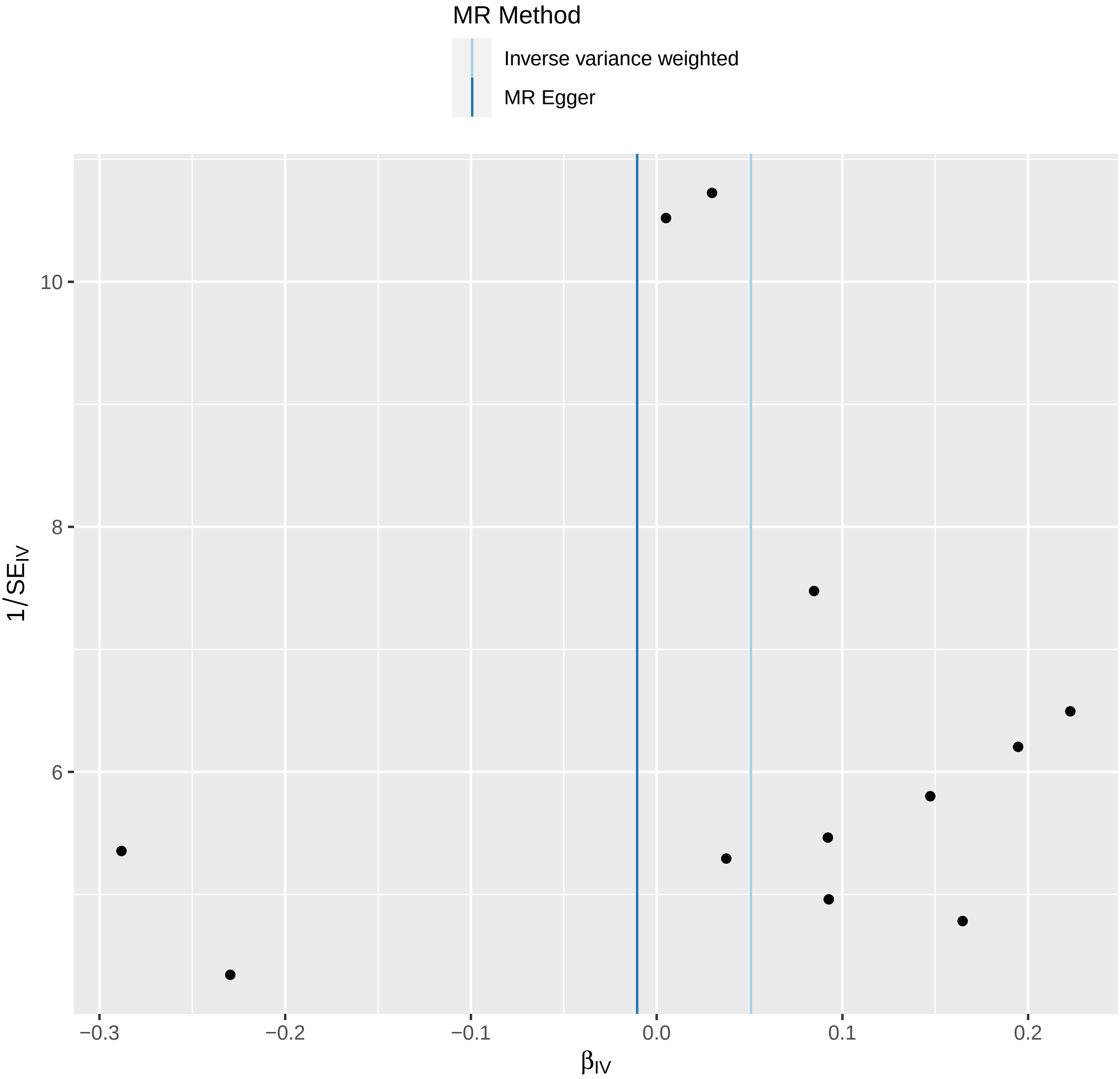

Batch 3−1525 : Selenium on Interleukin−3

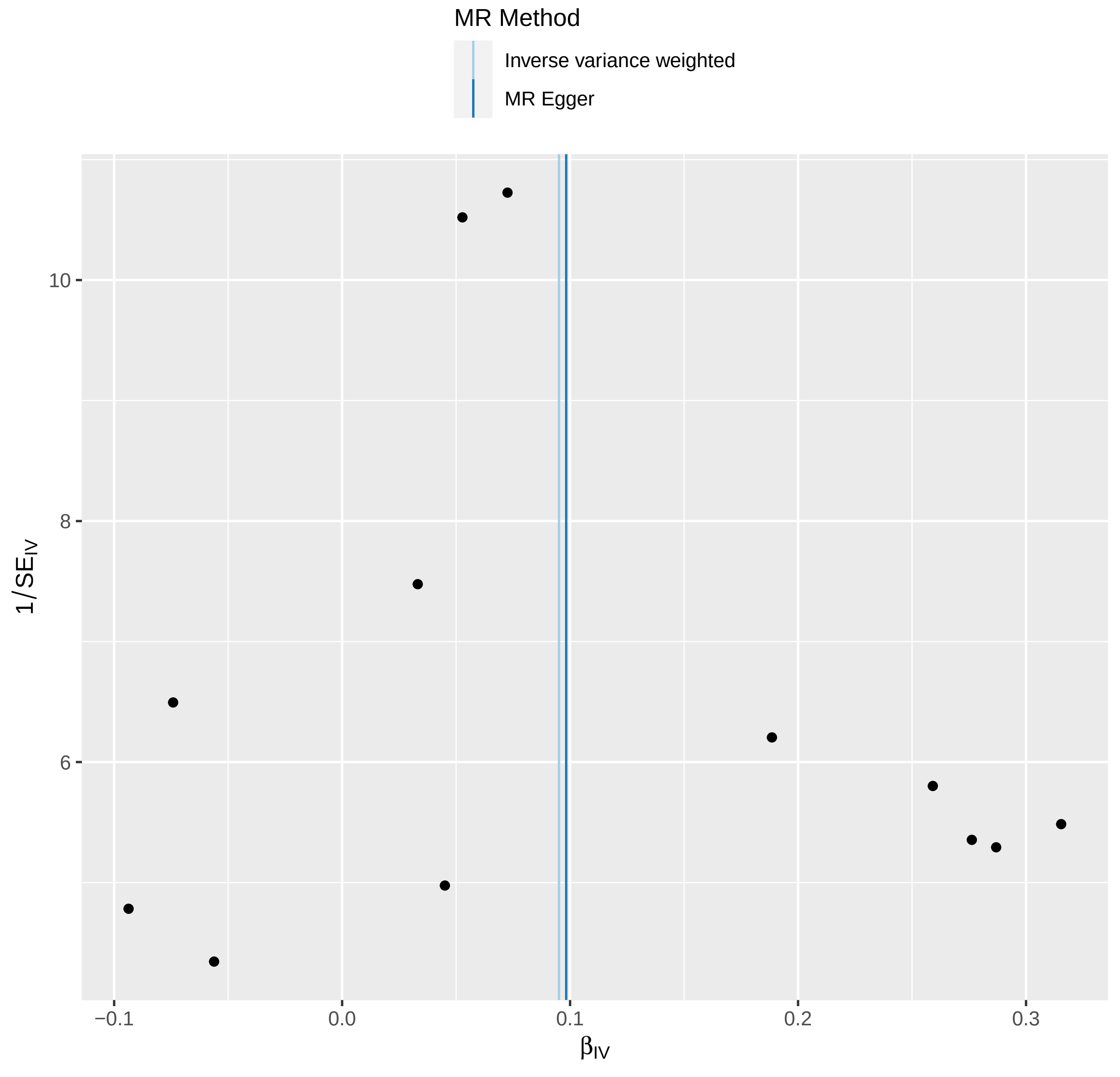

Batch 3−1526 : Selenium on Interleukin−36 alpha

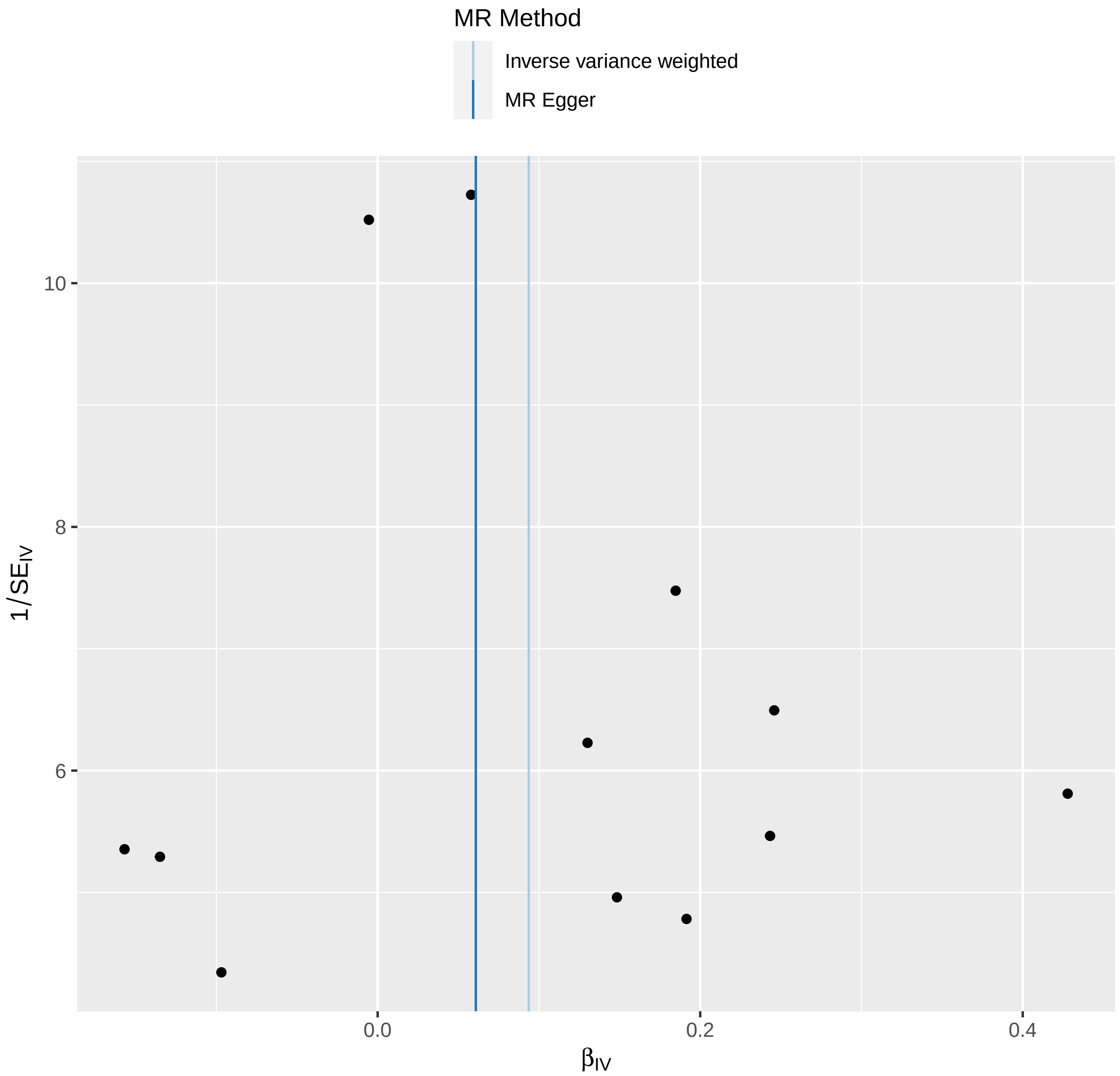

Batch 3−1527 : Selenium on Interleukin−36 beta

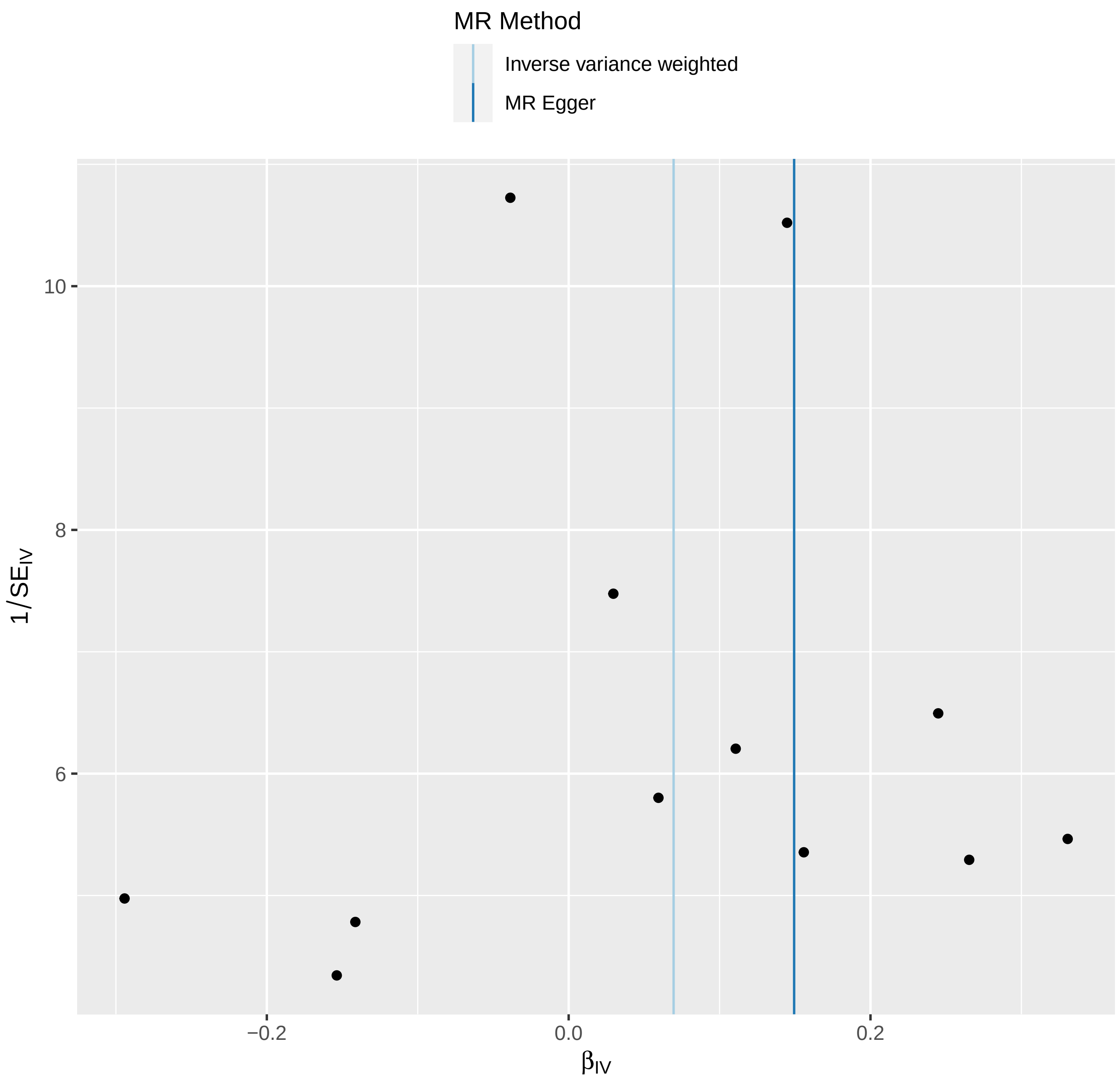

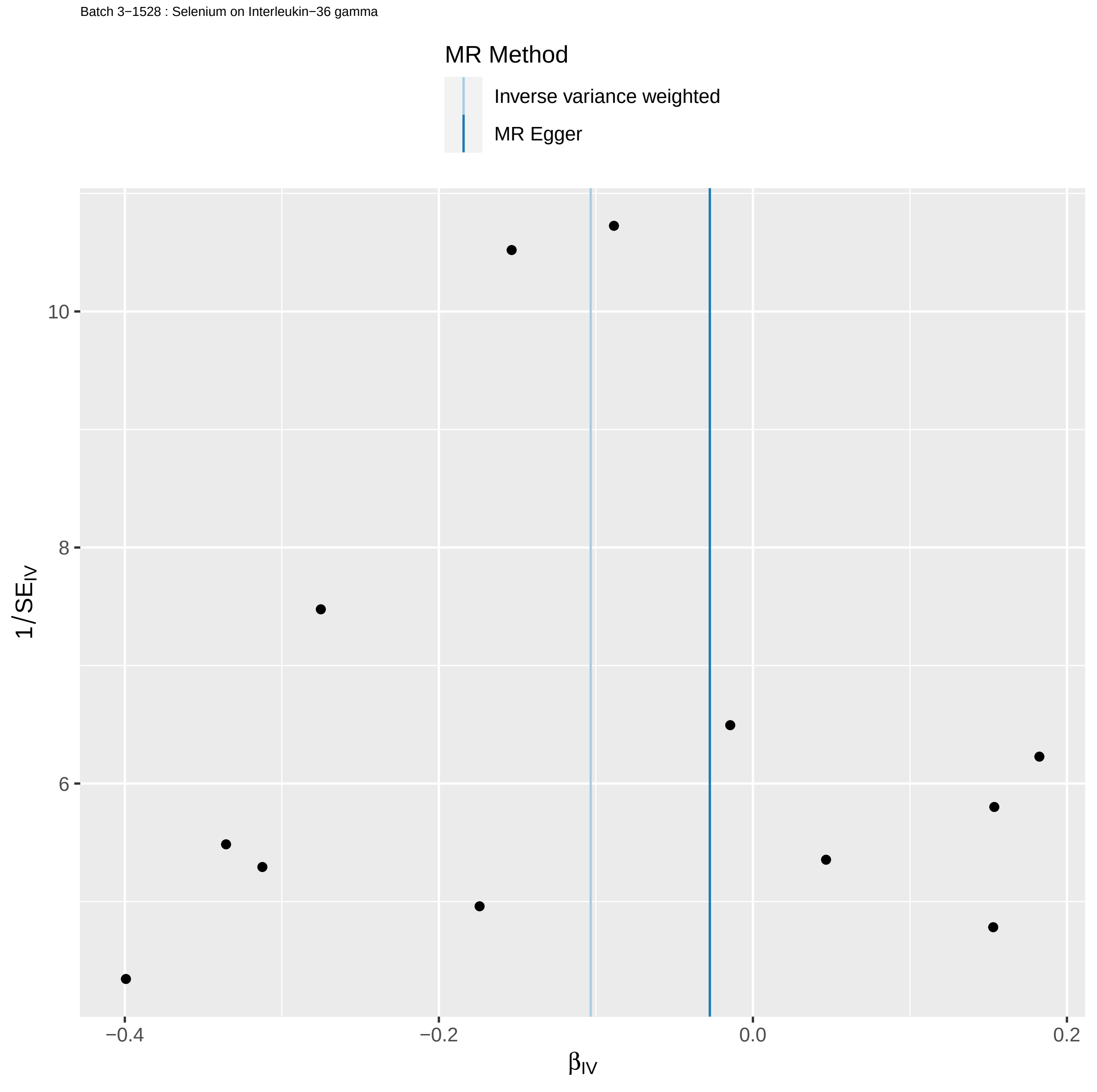

Batch 3−1528 : Selenium on Interleukin−36 gamma
MR Method
Inverse variance weighted
MR Egger
10
8
6
1/SE_IV
−0.4
−0.2
0.0
0.2
β_IV

Batch 3−1529 : Selenium on Interleukin−37

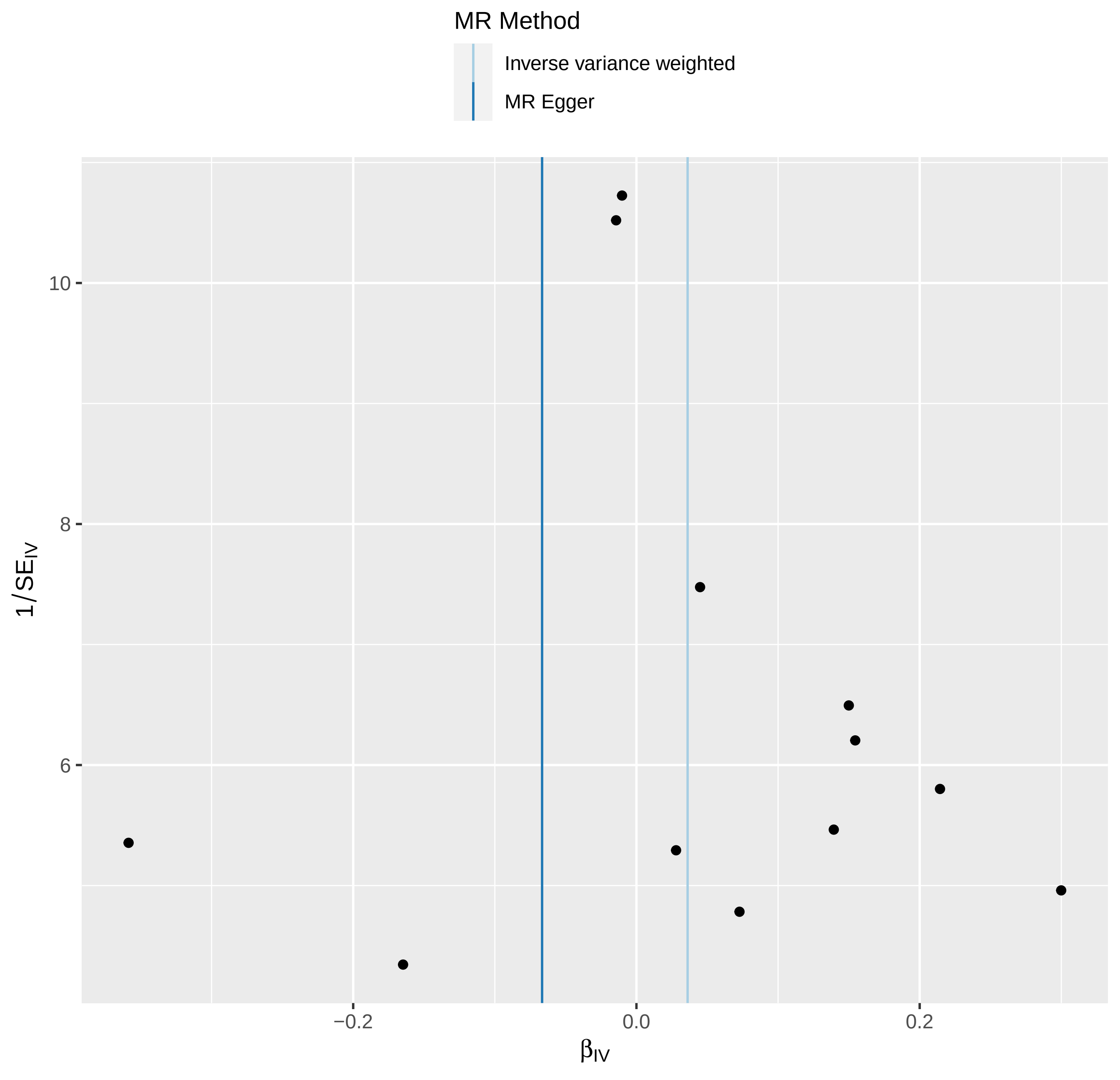

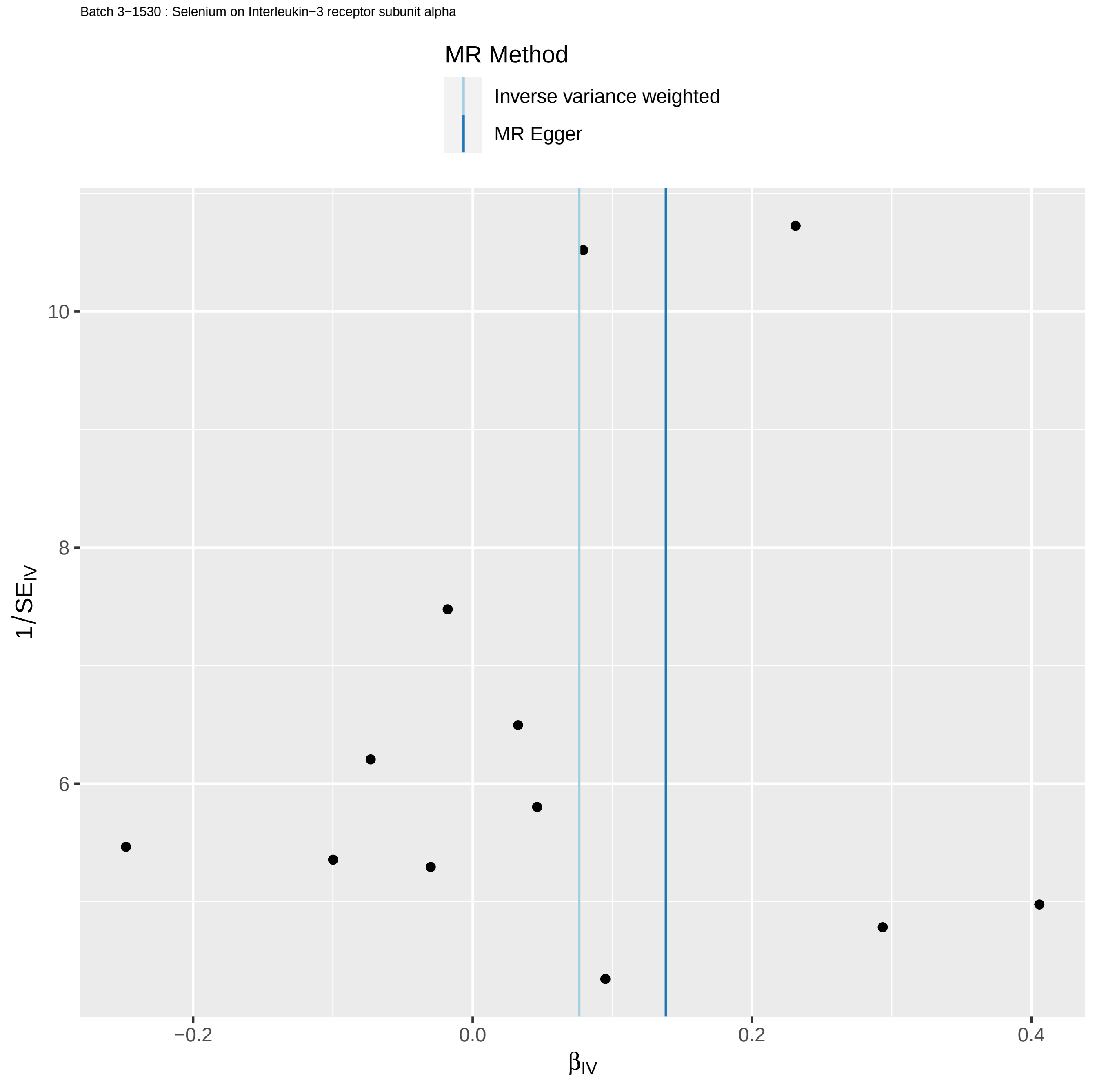

Batch 3−1530 : Selenium on Interleukin−3 receptor subunit alpha
MR Method
Inverse variance weighted
MR Egger
10
8
6
1/SE$_{IV}$
−0.2
0.0
0.2
0.4
β_{IV}

Batch 3−1531 : Selenium on Interleukin−3 receptor subunit alpha

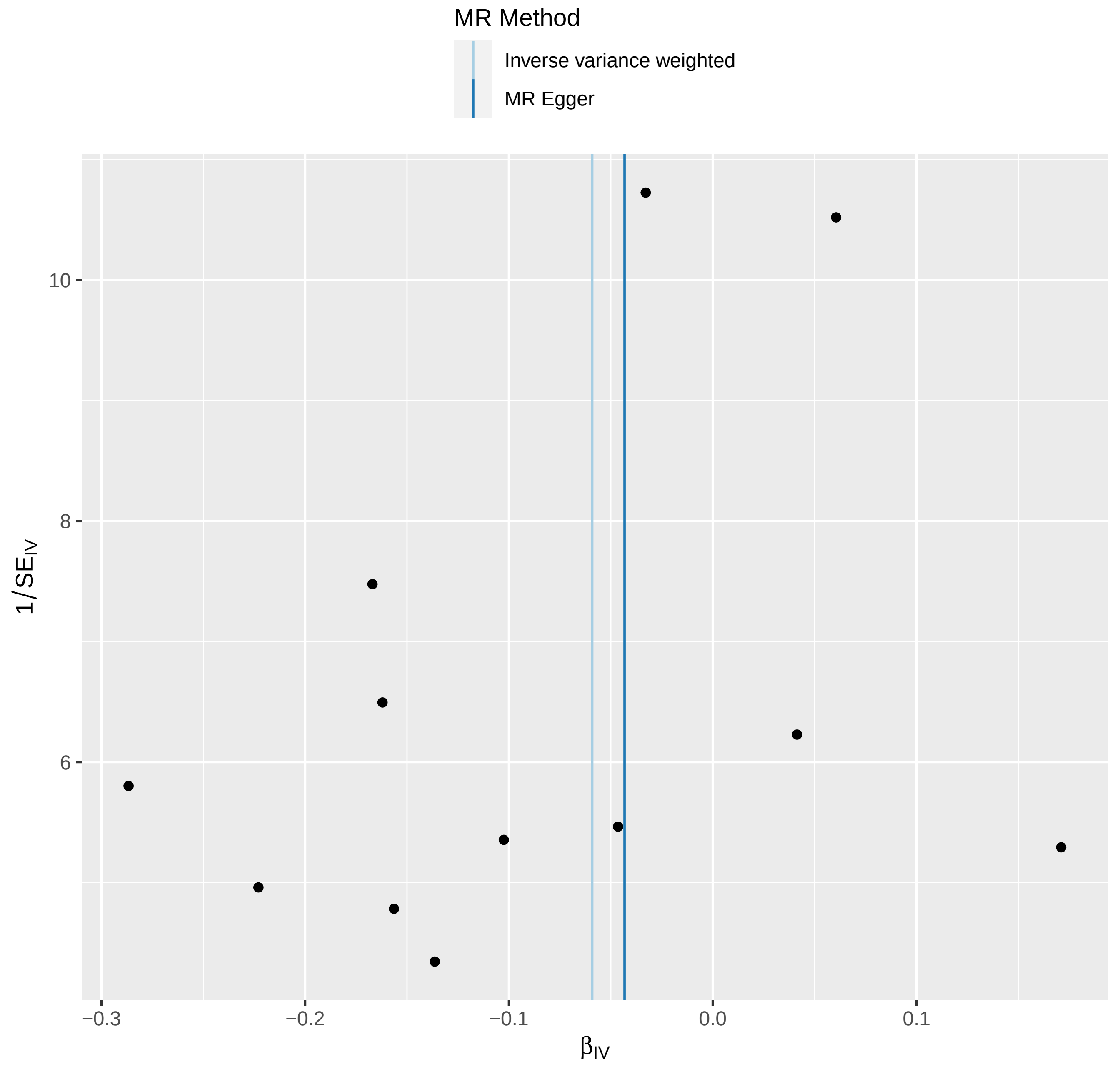

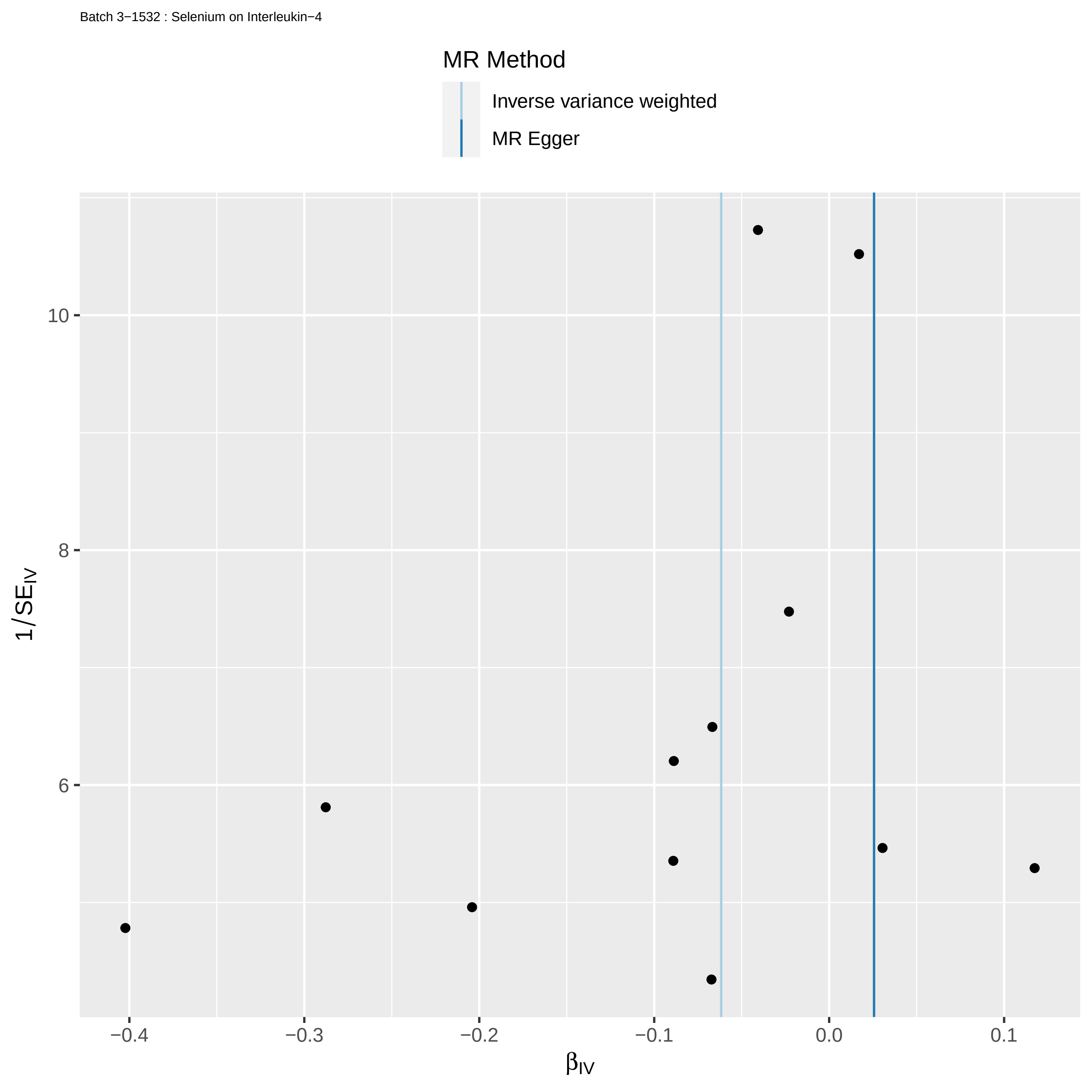

Batch 3−1532 : Selenium on Interleukin−4
MR Method
Inverse variance weighted
MR Egger
10
8
6
1/SE$_{IV}$
−0.4
−0.3
−0.2
−0.1
0.0
0.1
β$_{IV}$

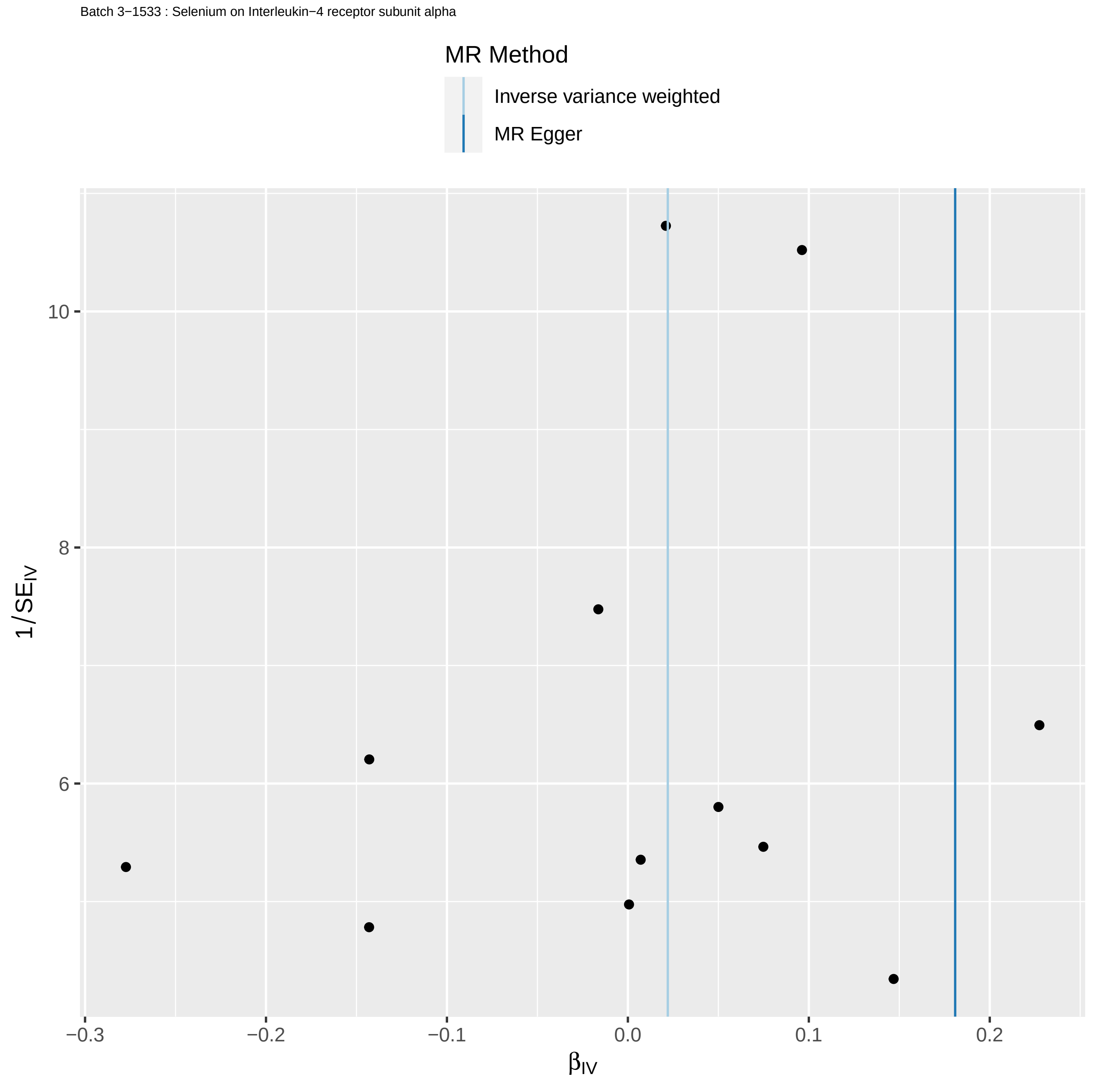

Batch 3−1533 : Selenium on Interleukin−4 receptor subunit alpha
MR Method
Inverse variance weighted
MR Egger
10
8
6
1/SE_IV
−0.3
−0.2
−0.1
0.0
0.1
0.2
β_IV

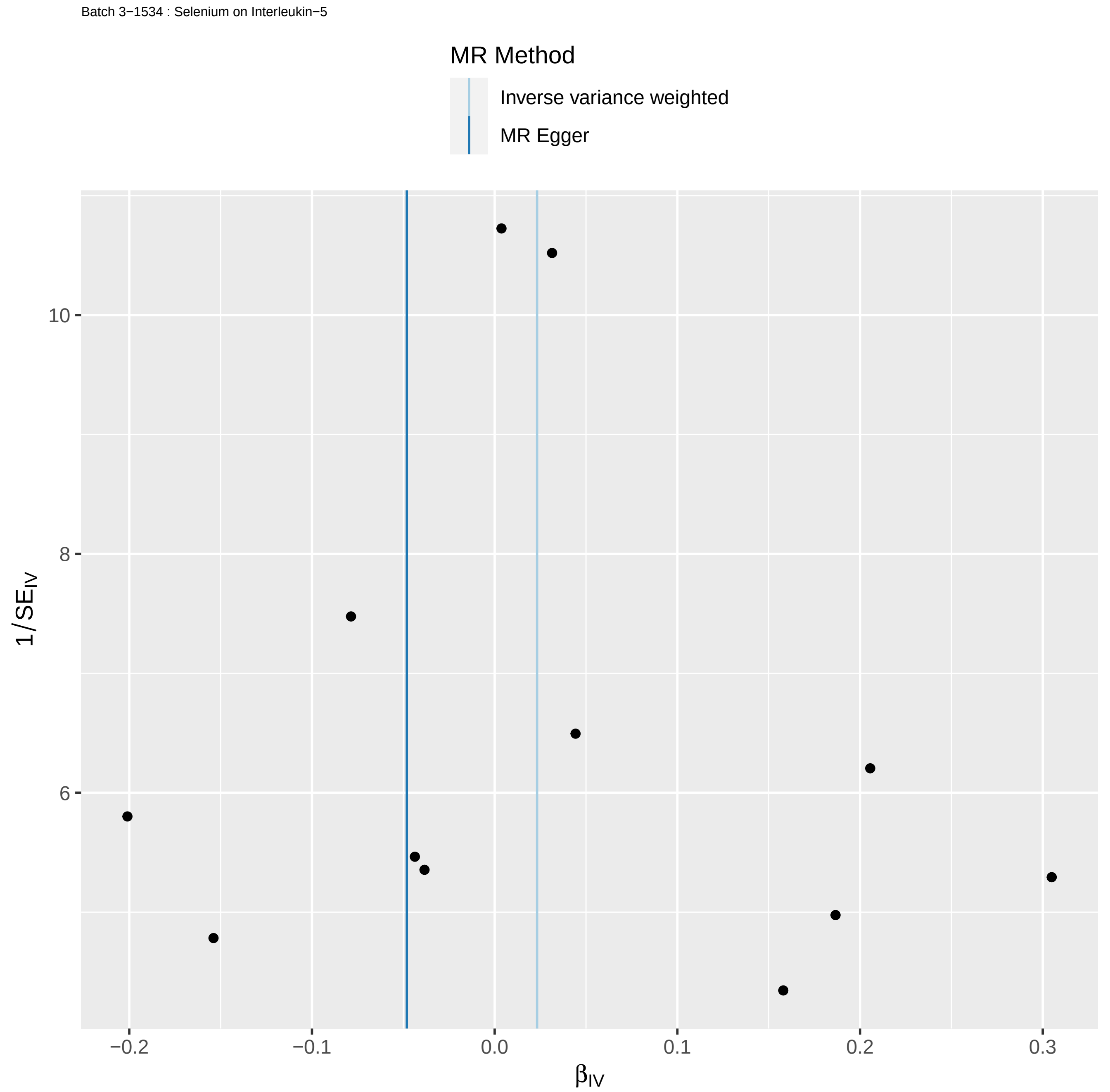

Batch 3−1534 : Selenium on Interleukin−5
MR Method
Inverse variance weighted
MR Egger
10
8
6
1/SE$_{IV}$
−0.2
−0.1
0.0
0.1
0.2
0.3
β$_{IV}$

Batch 3−1535 : Selenium on Interleukin−5

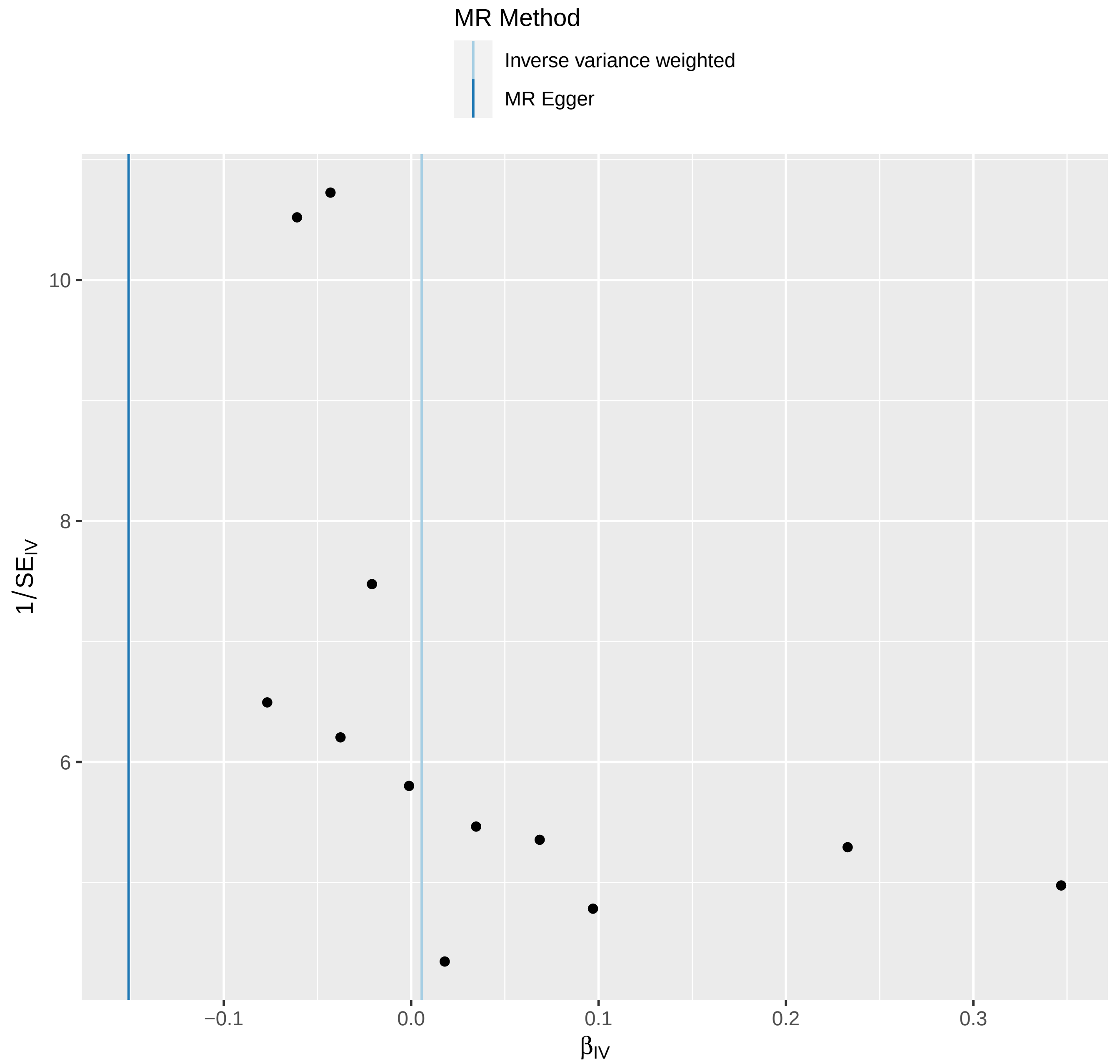

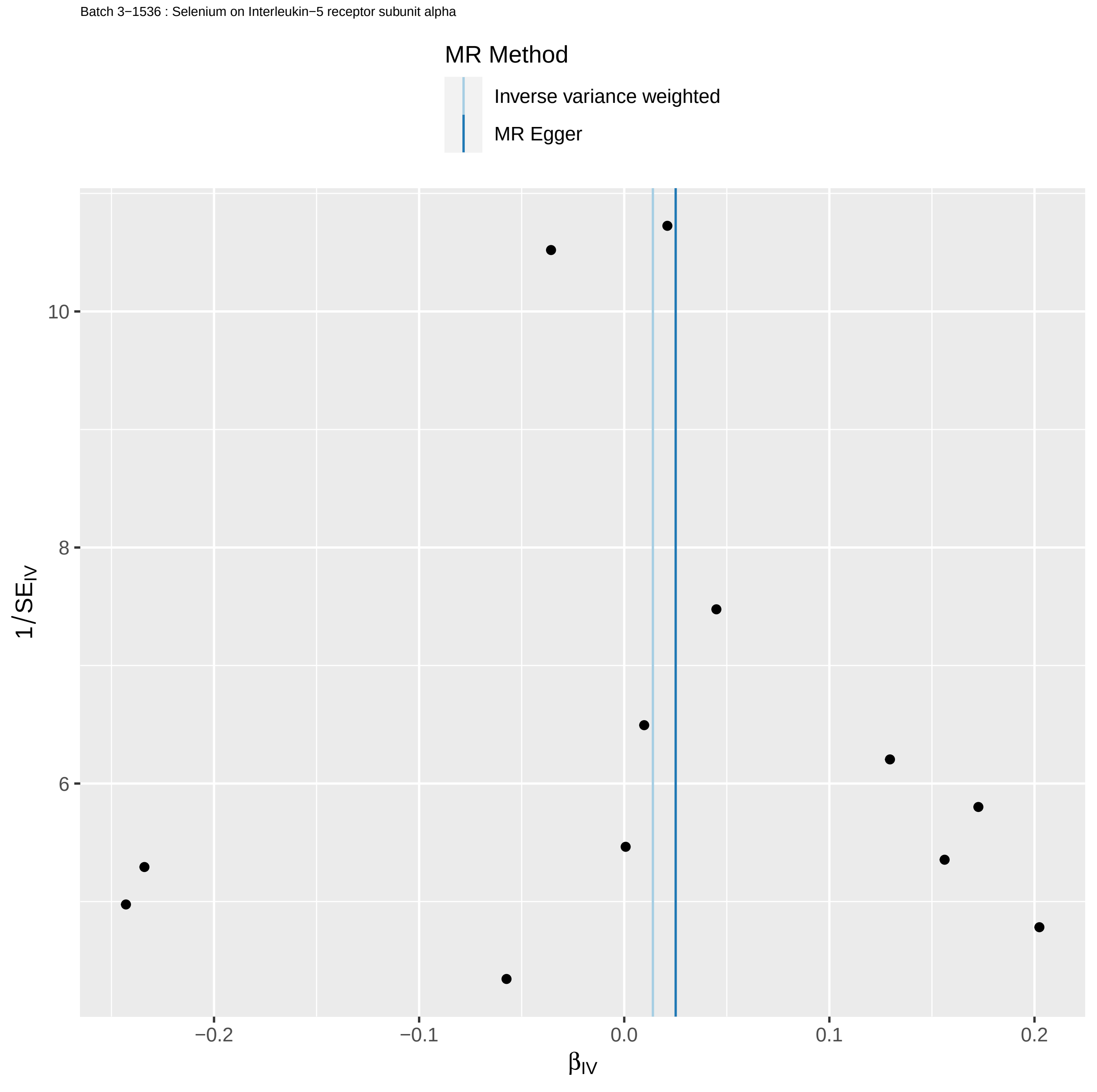
Batch 3−1536 : Selenium on Interleukin−5 receptor subunit alpha
MR Method
Inverse variance weighted
MR Egger
10
8
6
1/SE$_{IV}$
−0.2
−0.1
0.0
0.1
0.2
β$_{IV}$

Batch 3−1537 : Selenium on Interleukin−5 receptor subunit alpha

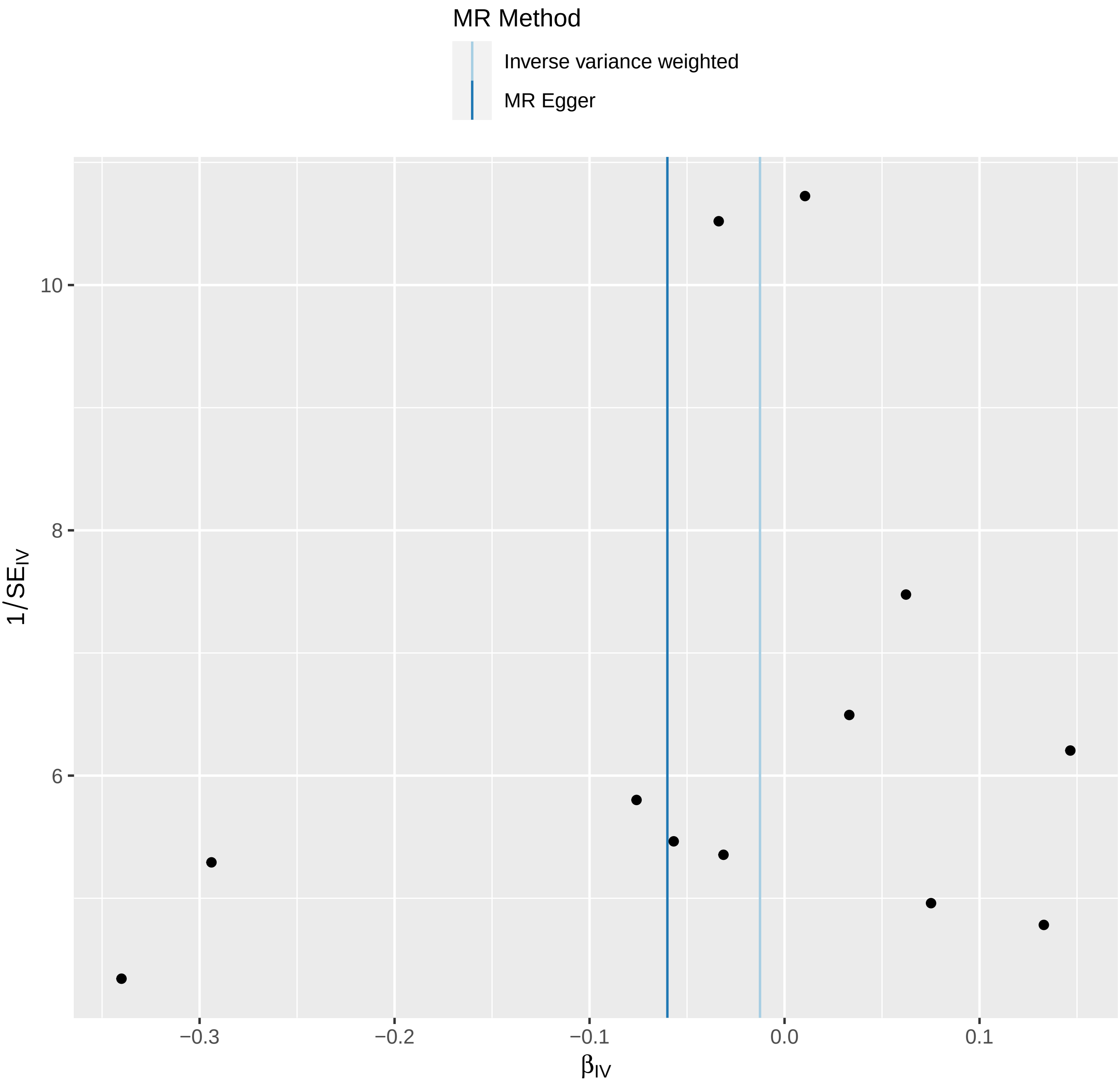

Batch 3−1538 : Selenium on Interleukin−6

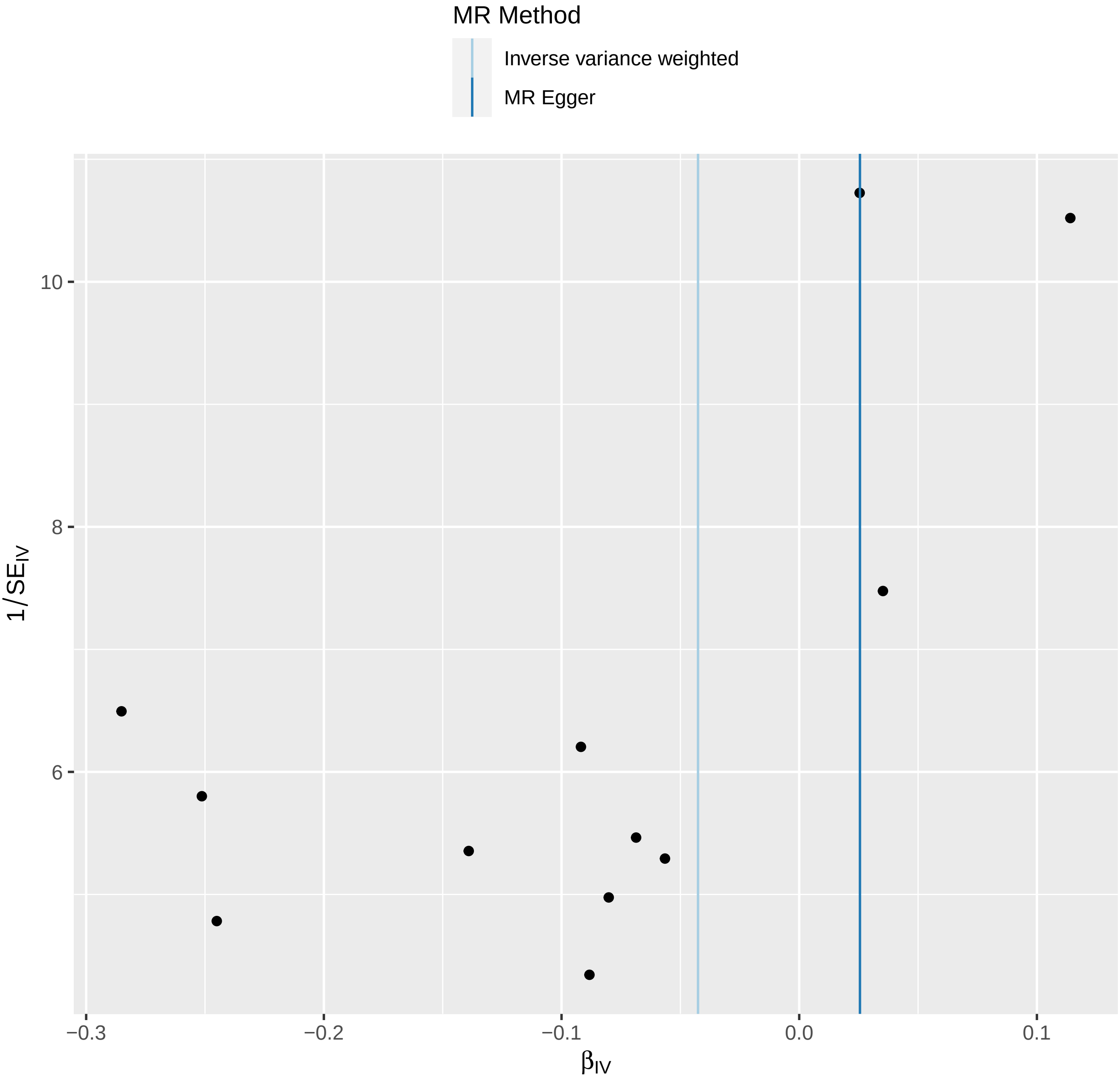

Batch 3−1539 : Selenium on Interleukin−6

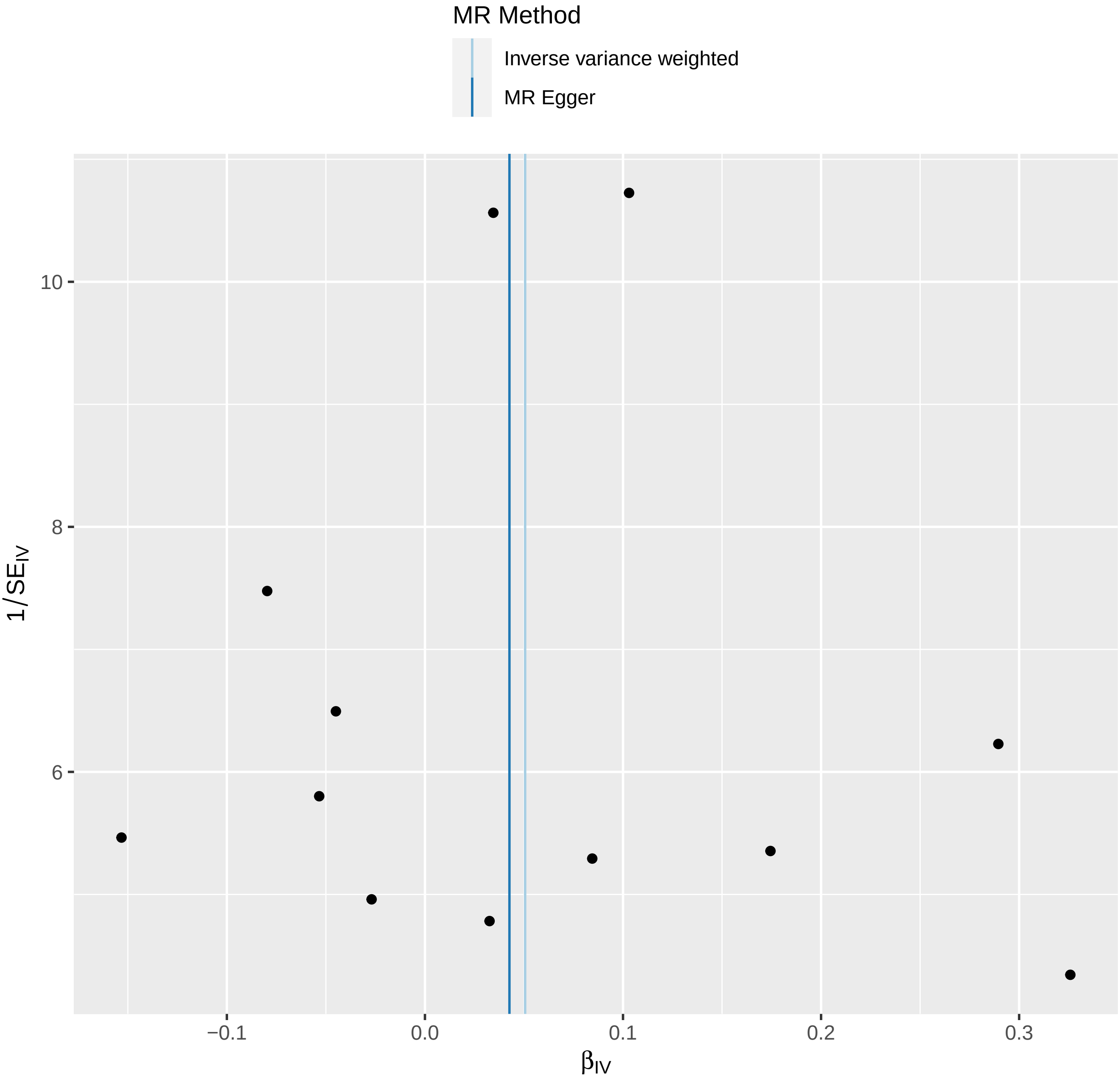

Batch 3−1540 : Selenium on Interleukin−6 receptor subunit alpha

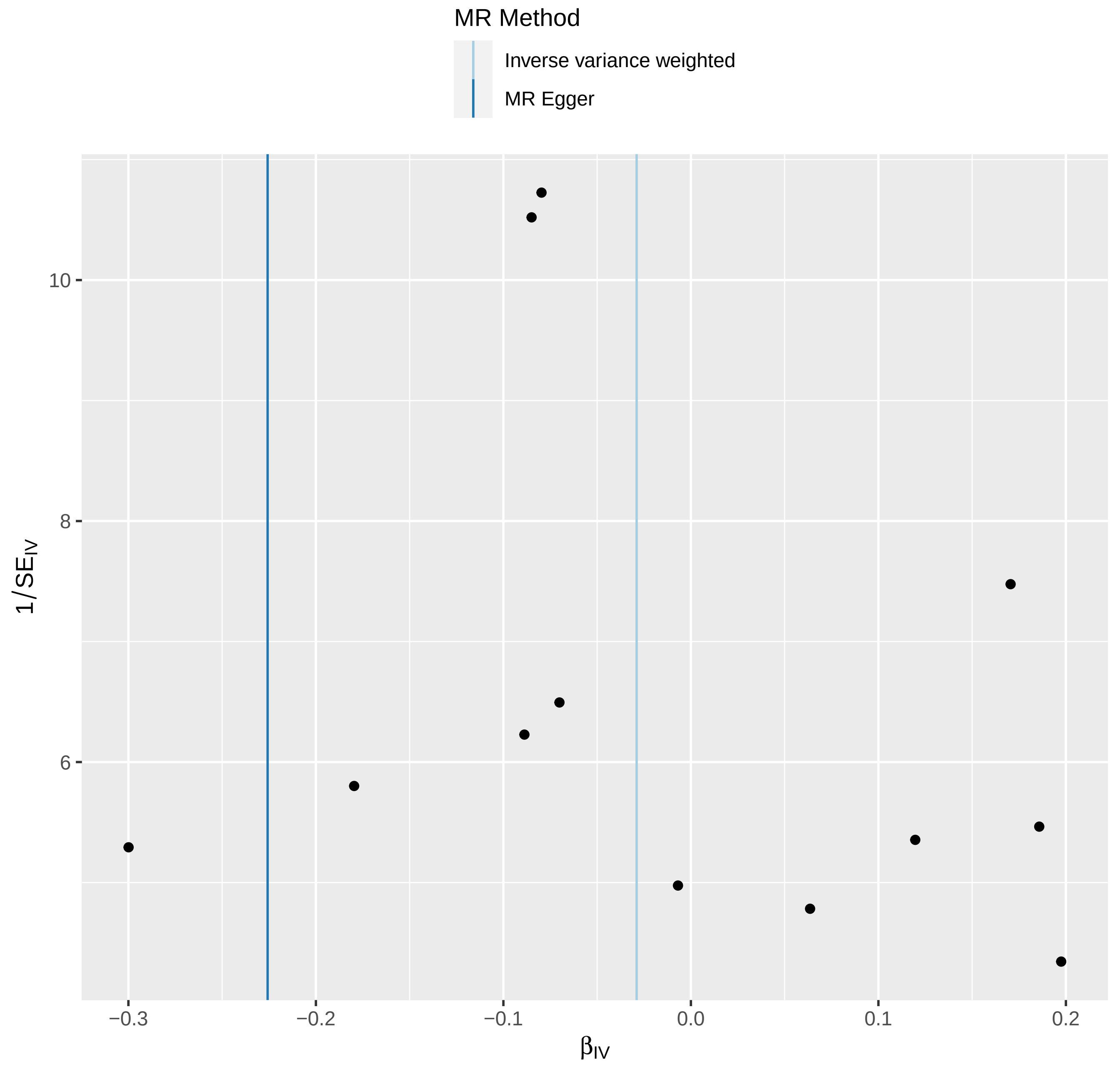

Batch 3−1541 : Selenium on Interleukin−6 receptor subunit alpha

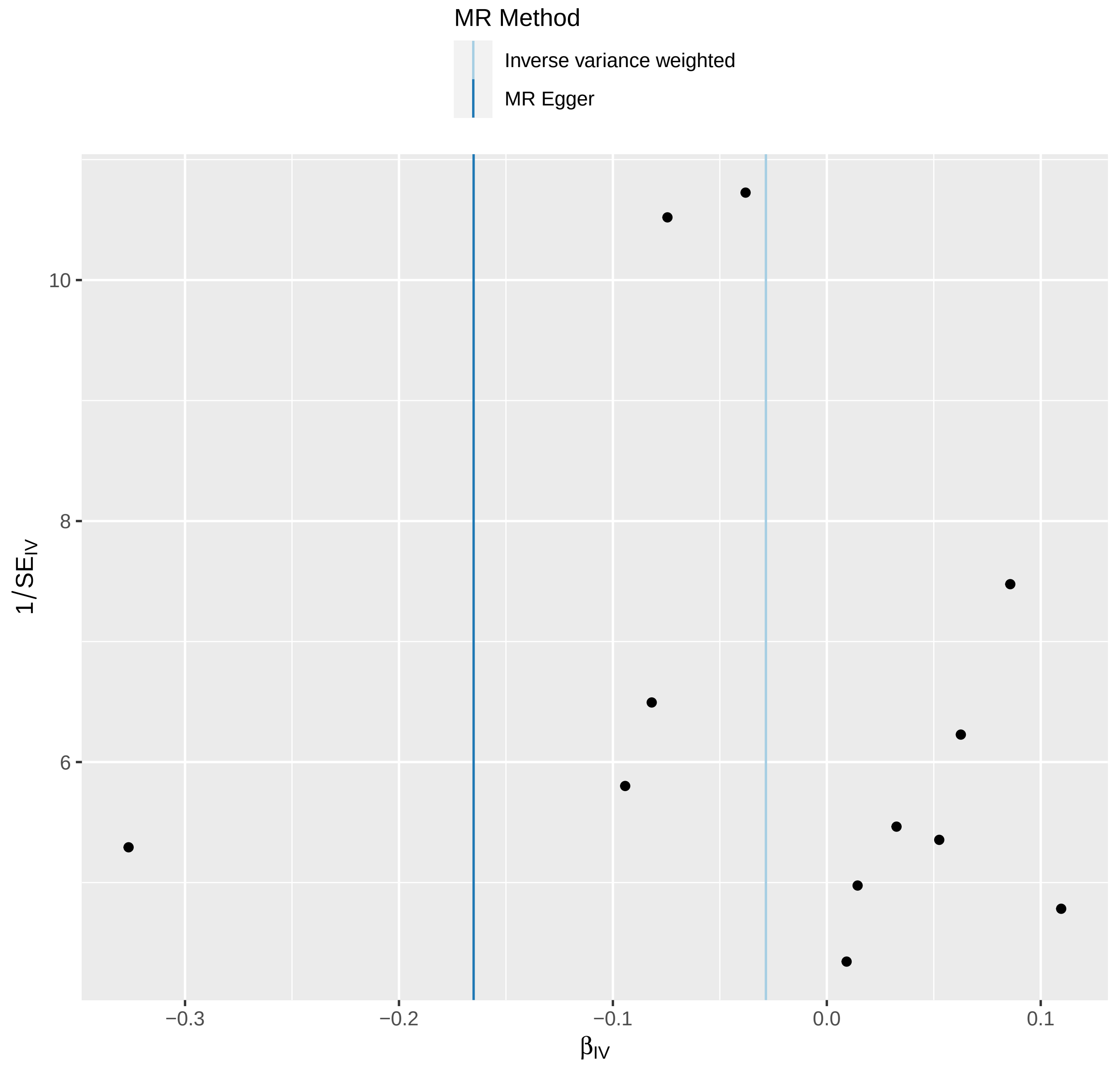

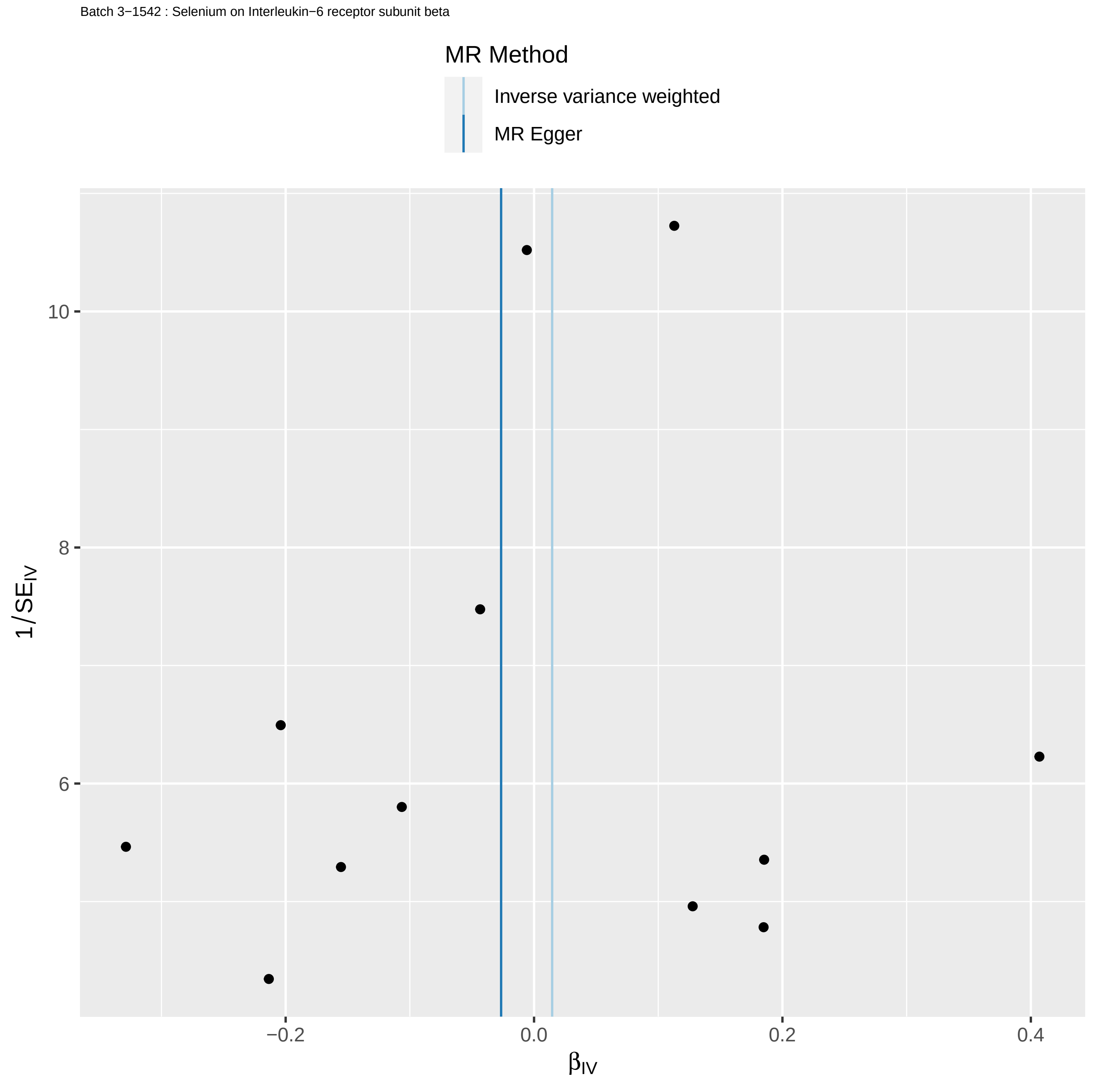

Batch 3−1542 : Selenium on Interleukin−6 receptor subunit beta
MR Method
Inverse variance weighted
MR Egger
10
8
6
1/SE_IV
−0.2
0.0
0.2
0.4
β_IV

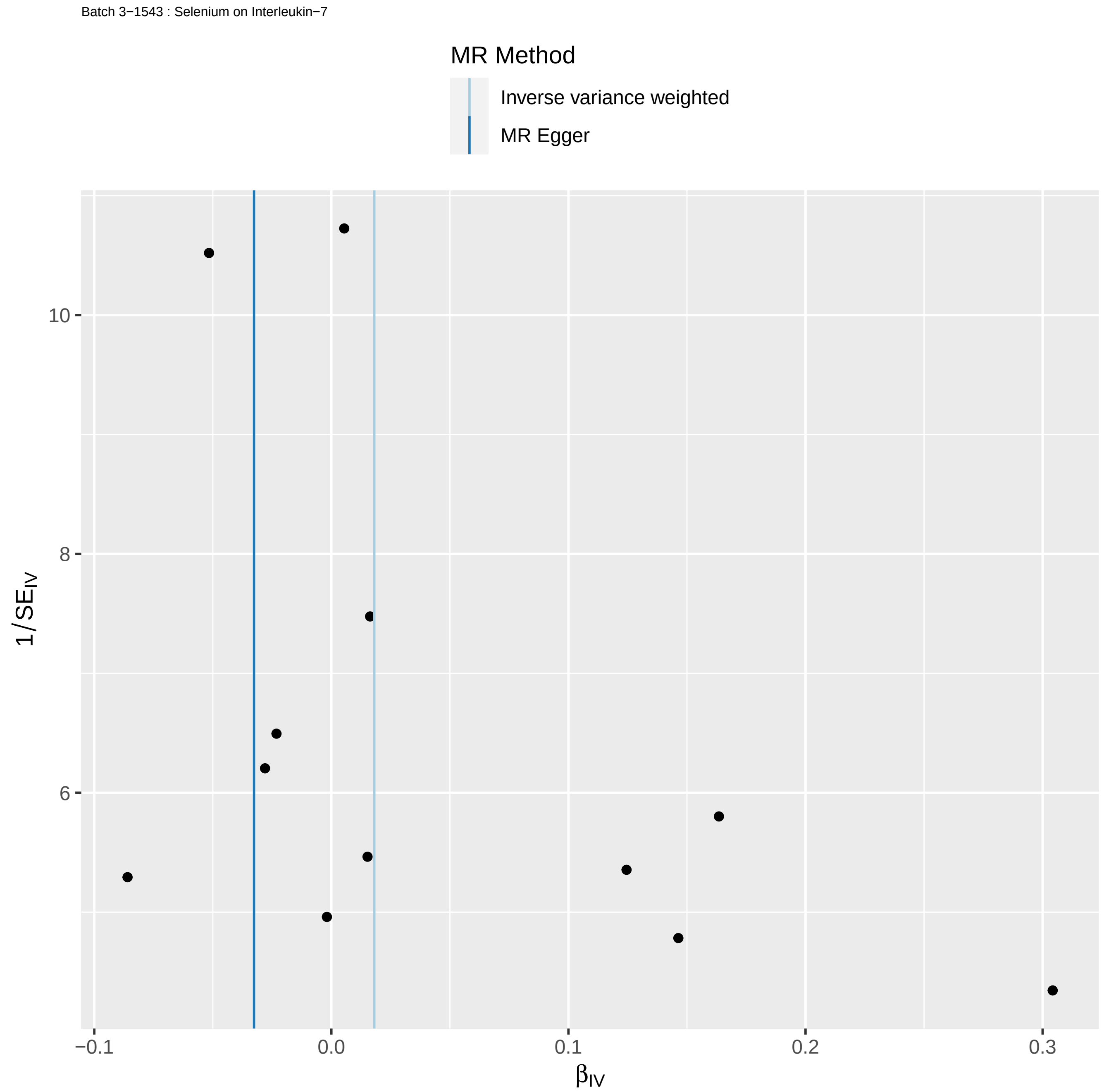

Batch 3−1543 : Selenium on Interleukin−7
MR Method
Inverse variance weighted
MR Egger
10
8
6
1/SE$_{IV}$
−0.1
0.0
0.1
0.2
0.3
β$_{IV}$

Batch 3−1544 : Selenium on Interleukin−7 receptor subunit alpha

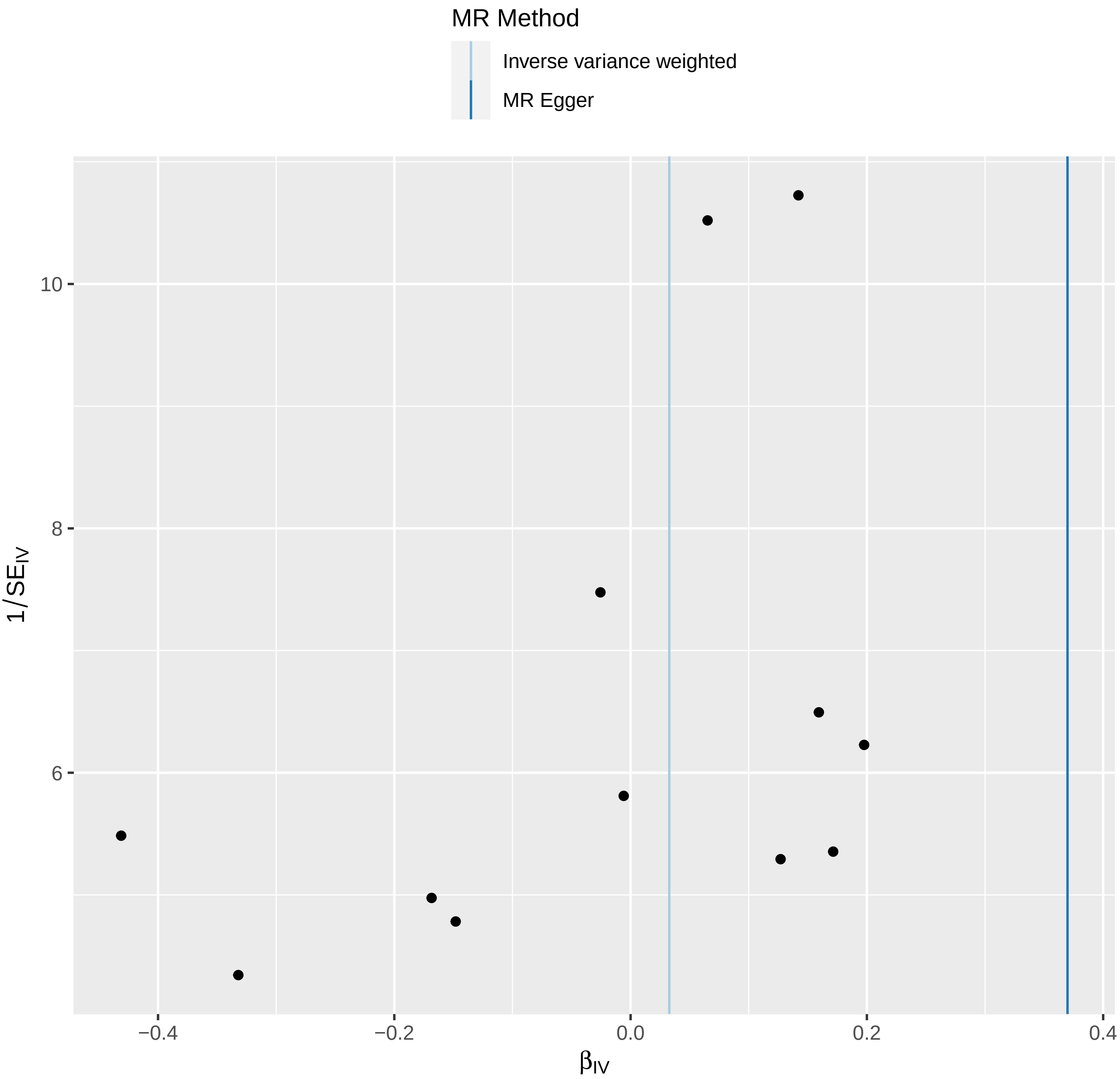

Batch 3−1545 : Selenium on Interleukin−9

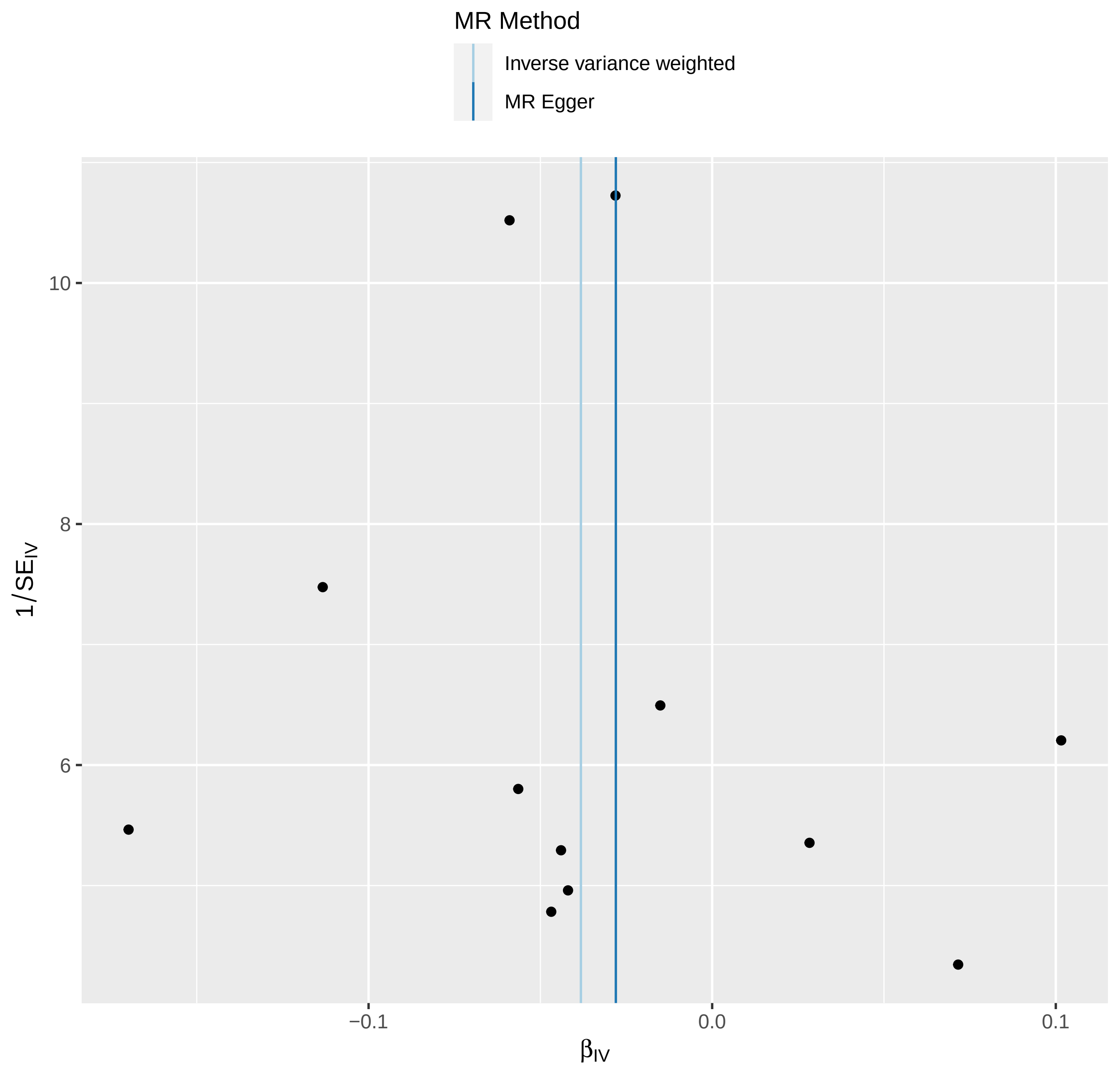

Batch 3−1546 : Selenium on Interleukin−9

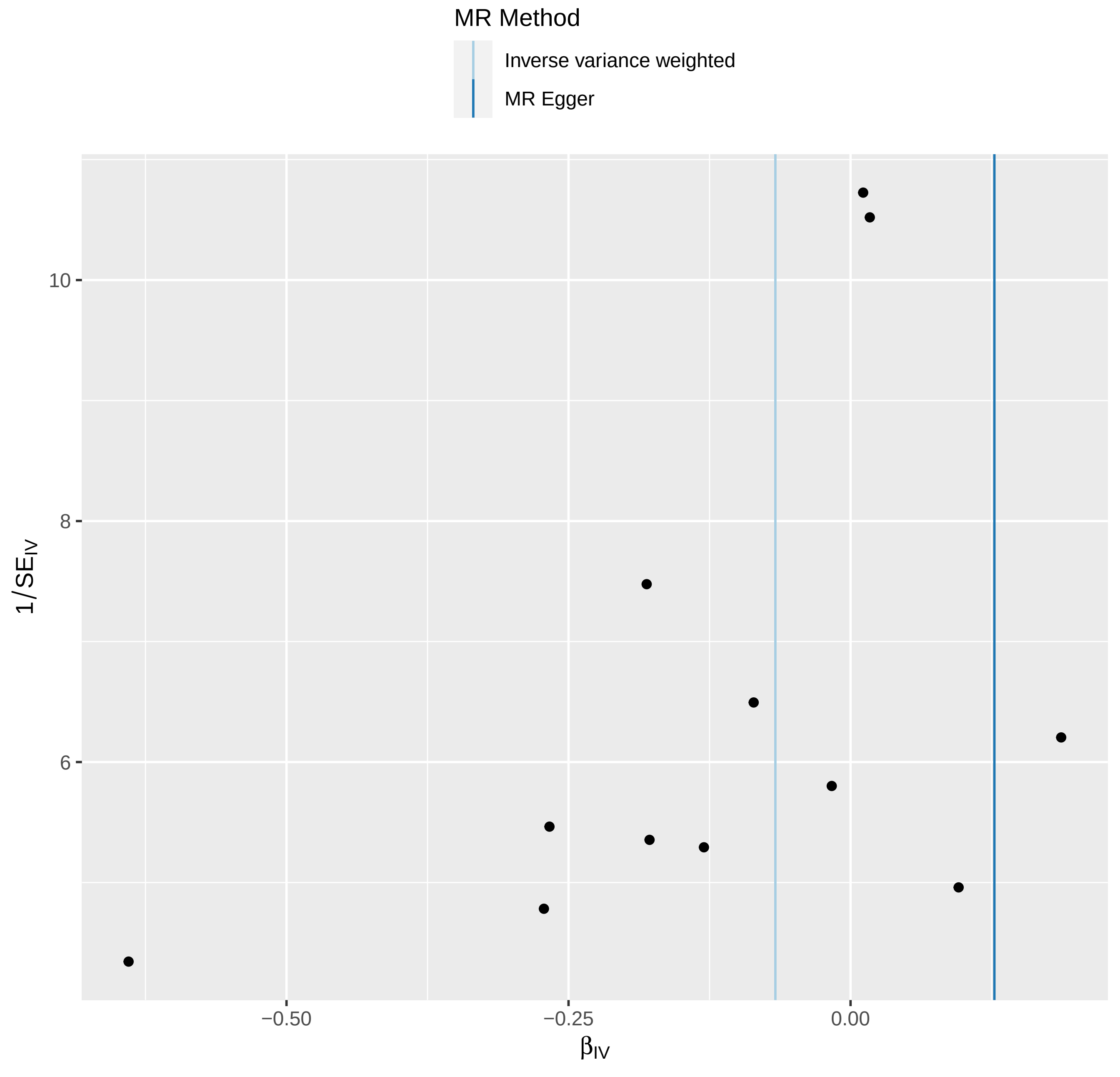

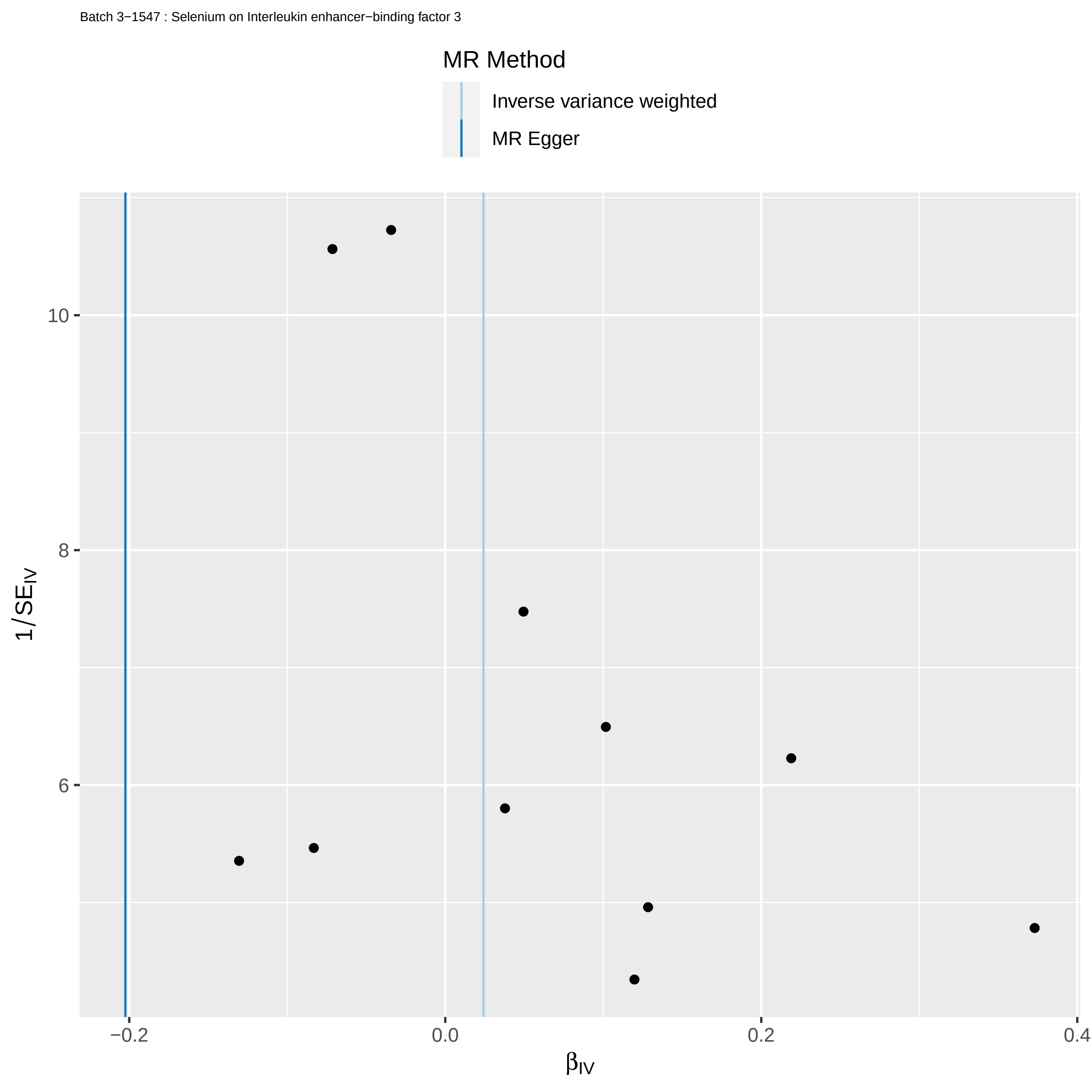

Batch 3−1547 : Selenium on Interleukin enhancer−binding factor 3
MR Method
Inverse variance weighted
MR Egger
10
8
6
1/SE_IV
−0.2
0.0
0.2
0.4
β_IV

Batch 3−1548 : Selenium on Inositol monophosphatase 3

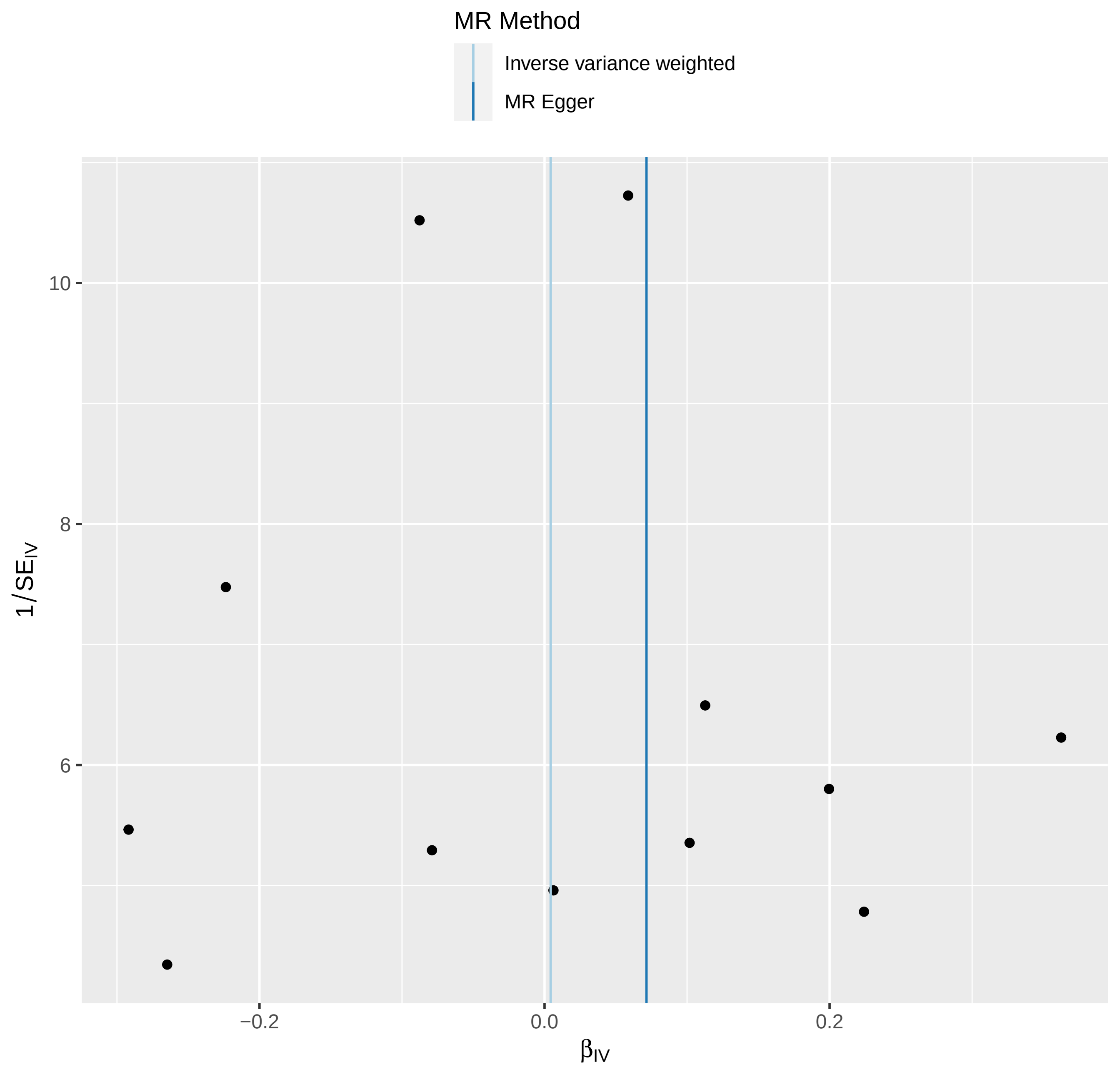

Batch 3−1549 : Selenium on Inosine−5'−monophosphate dehydrogenase 1

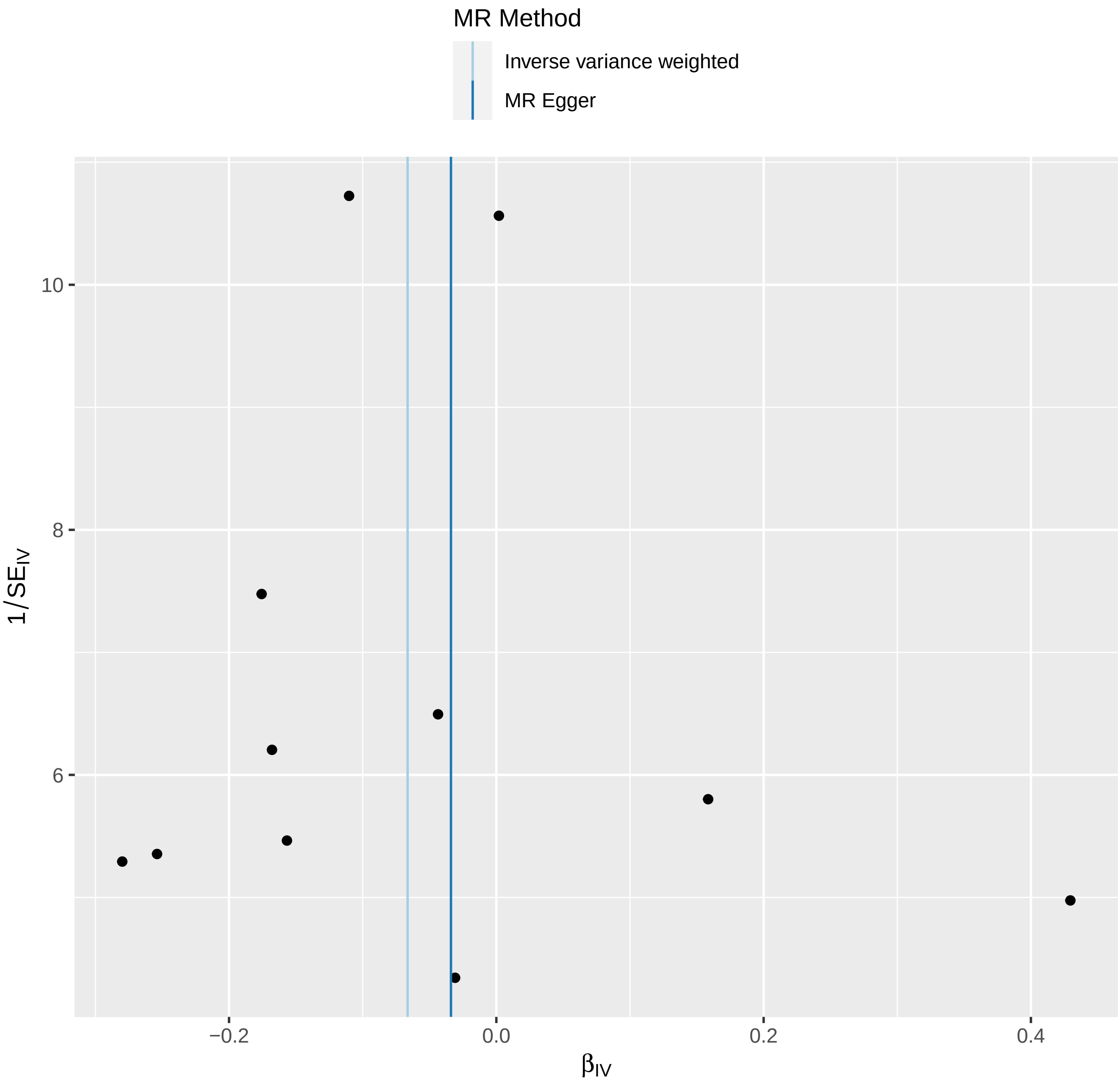

Batch 3−1550 : Selenium on Inosine−5'−monophosphate dehydrogenase 2

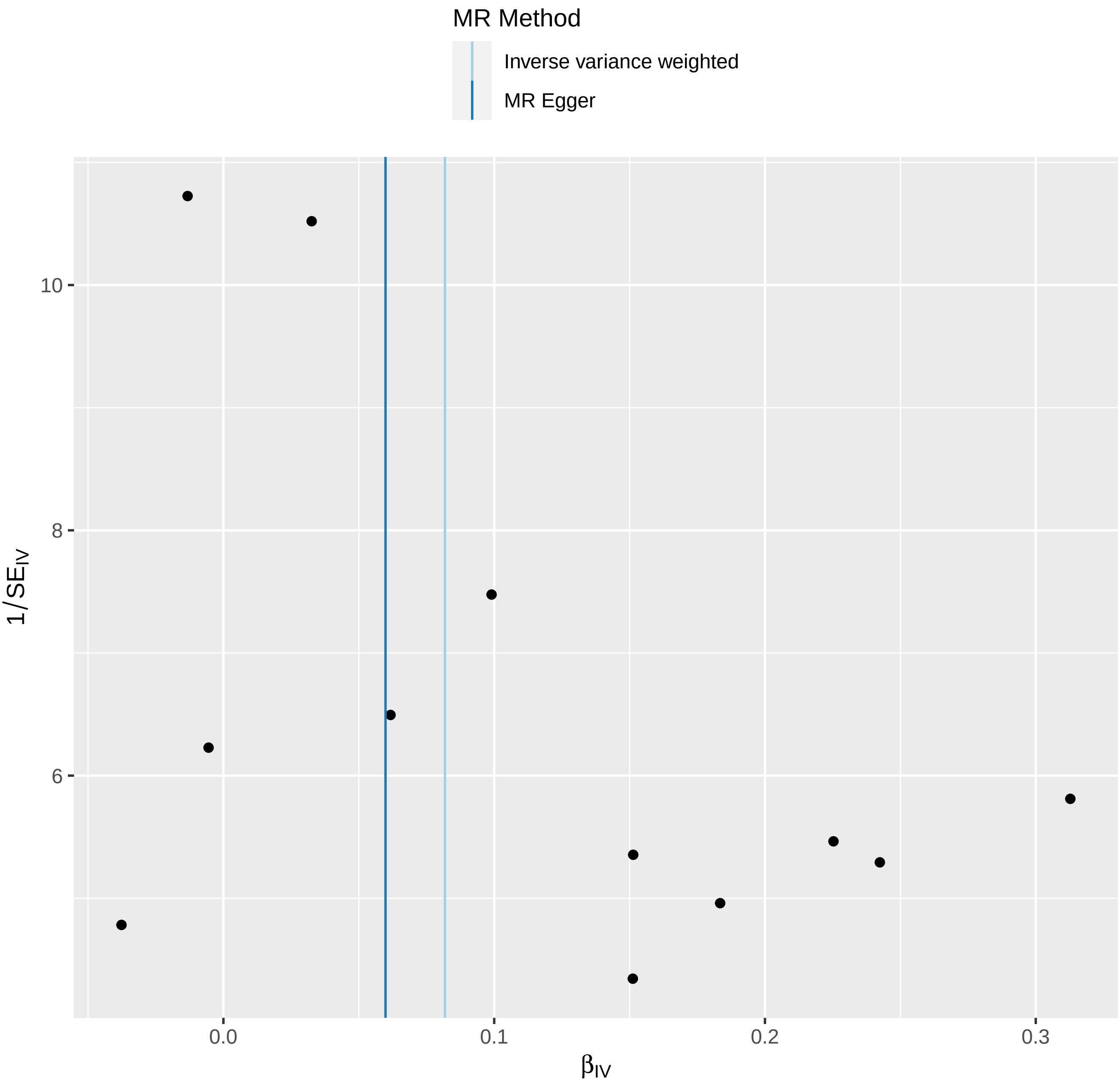

Batch 3−1551 : Selenium on Alpha−internexin

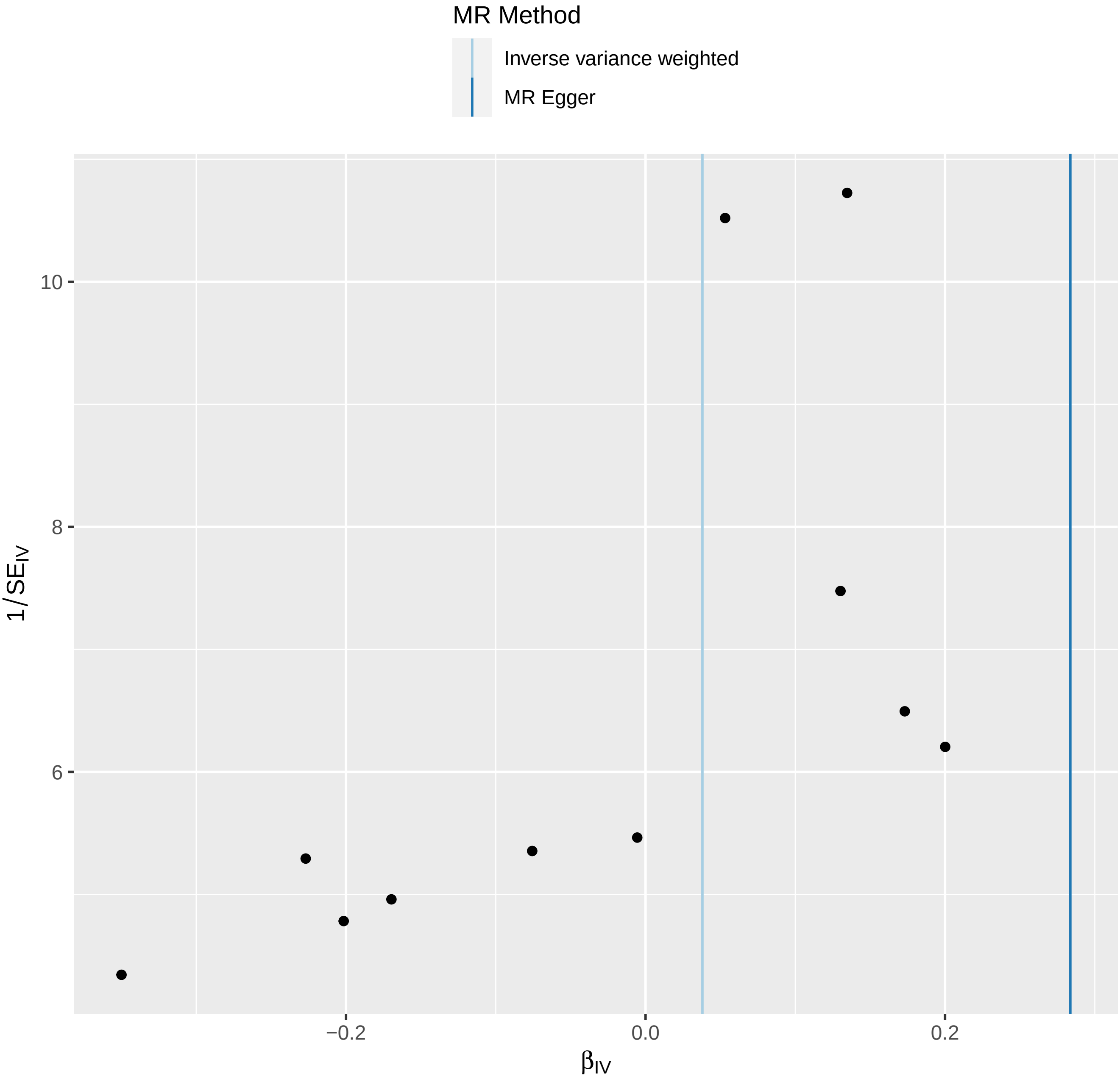

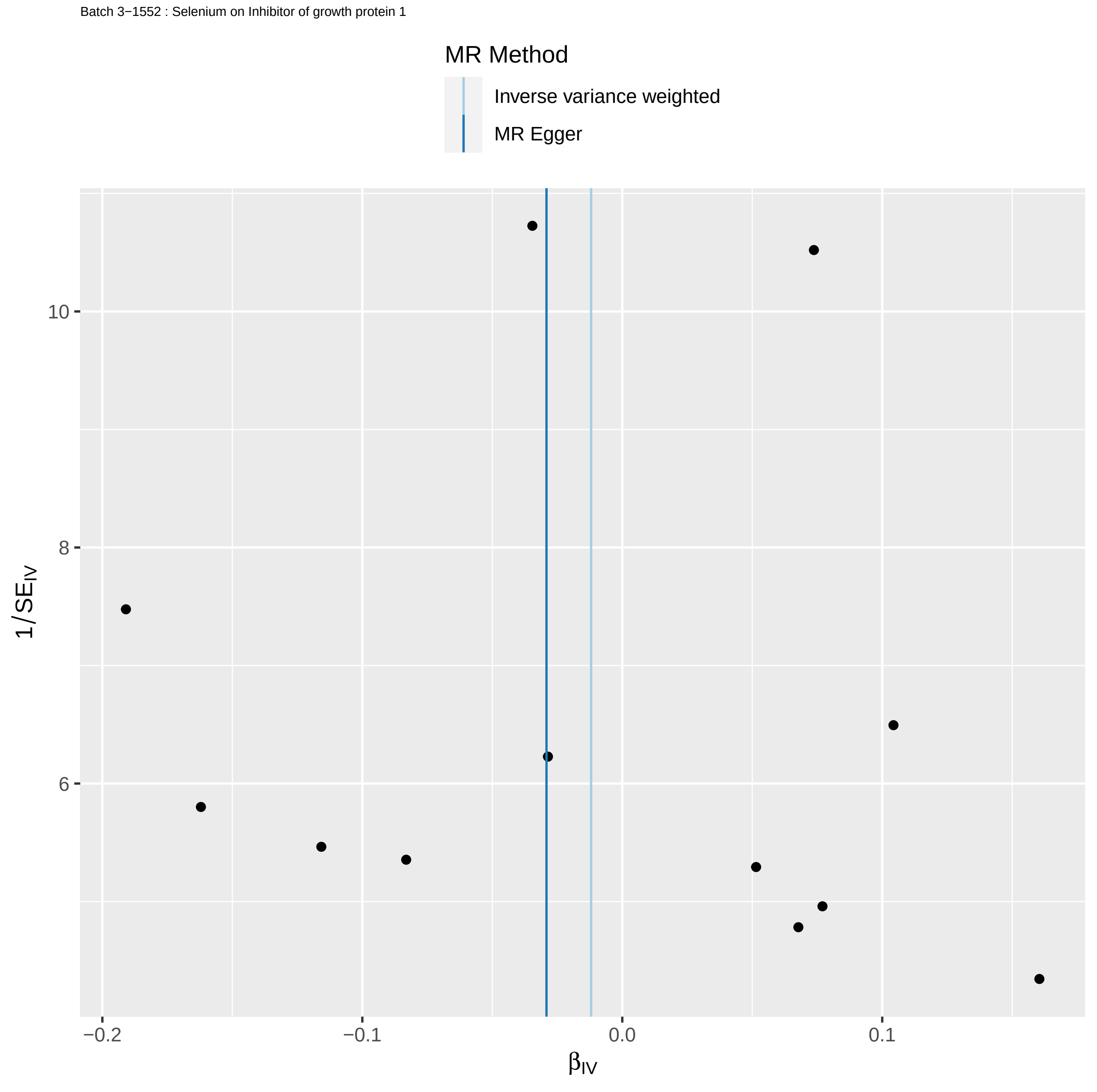
Batch 3−1552 : Selenium on Inhibitor of growth protein 1
MR Method
Inverse variance weighted
MR Egger
10
8
6
1/SE_IV
−0.2
−0.1
0.0
0.1
β_IV

Batch 3−1553 : Selenium on Inhibin beta A chain

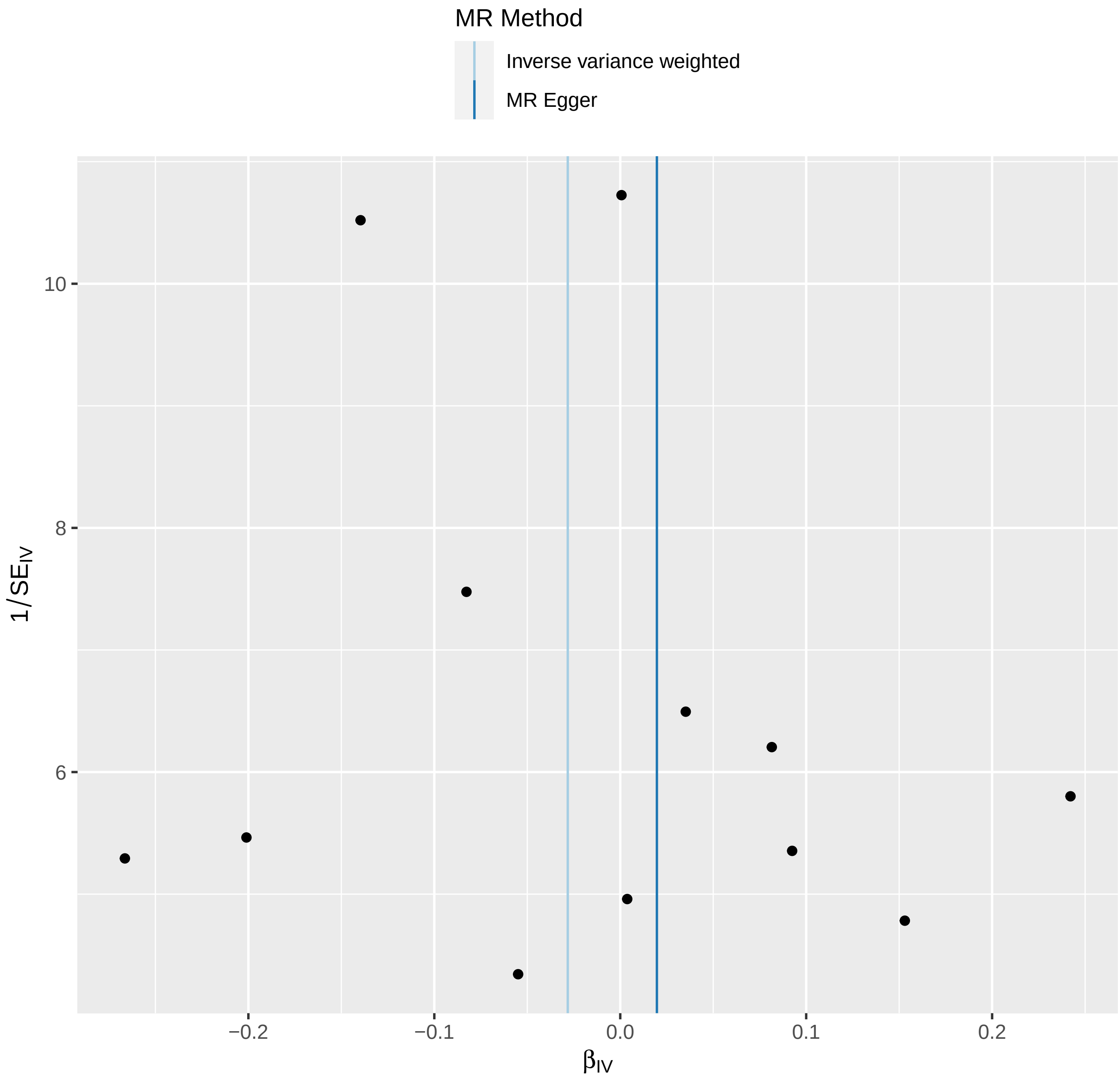

Batch 3−1554 : Selenium on Inhibin beta A chain:Inhibin beta B chain heterodimer

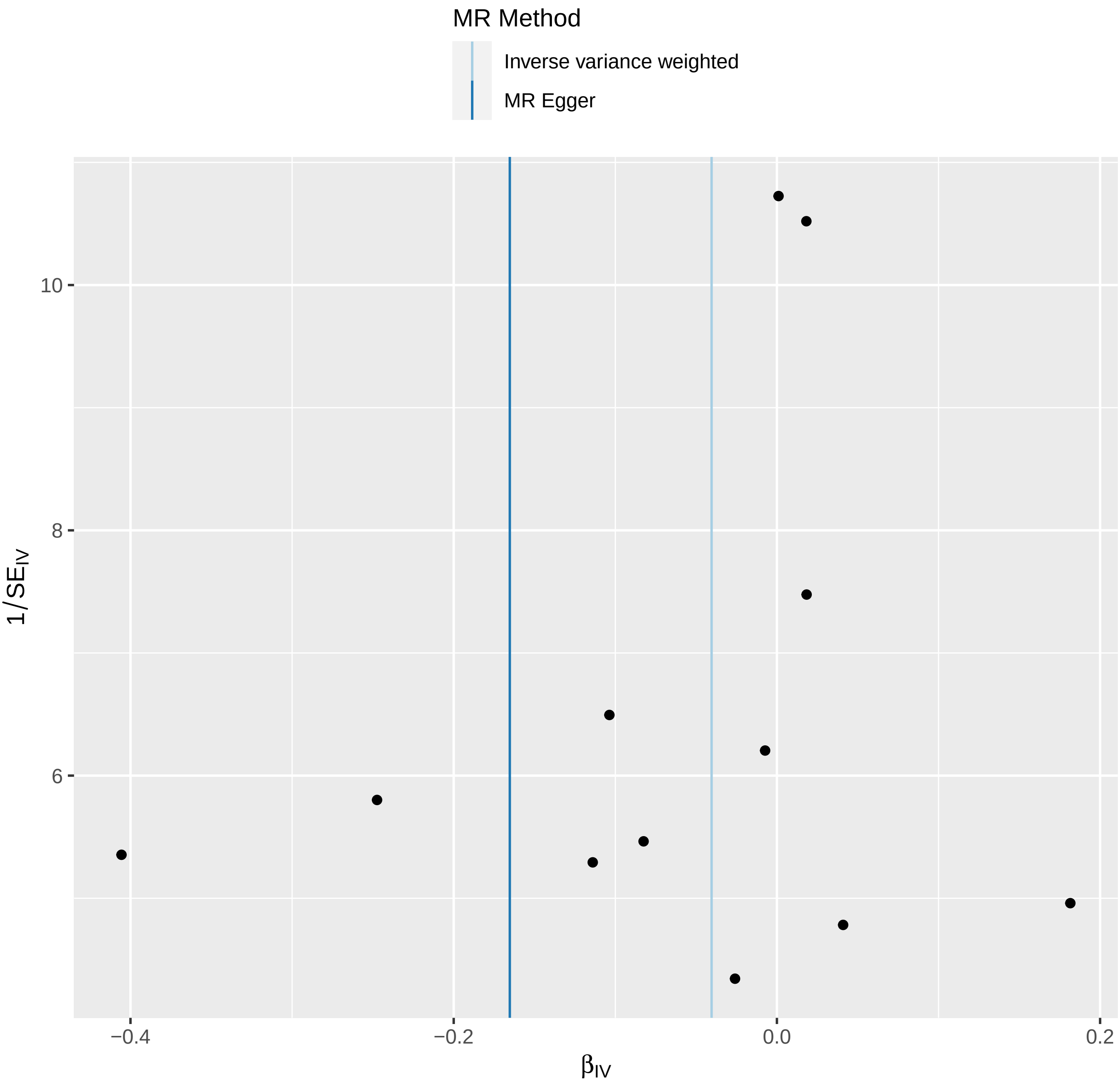

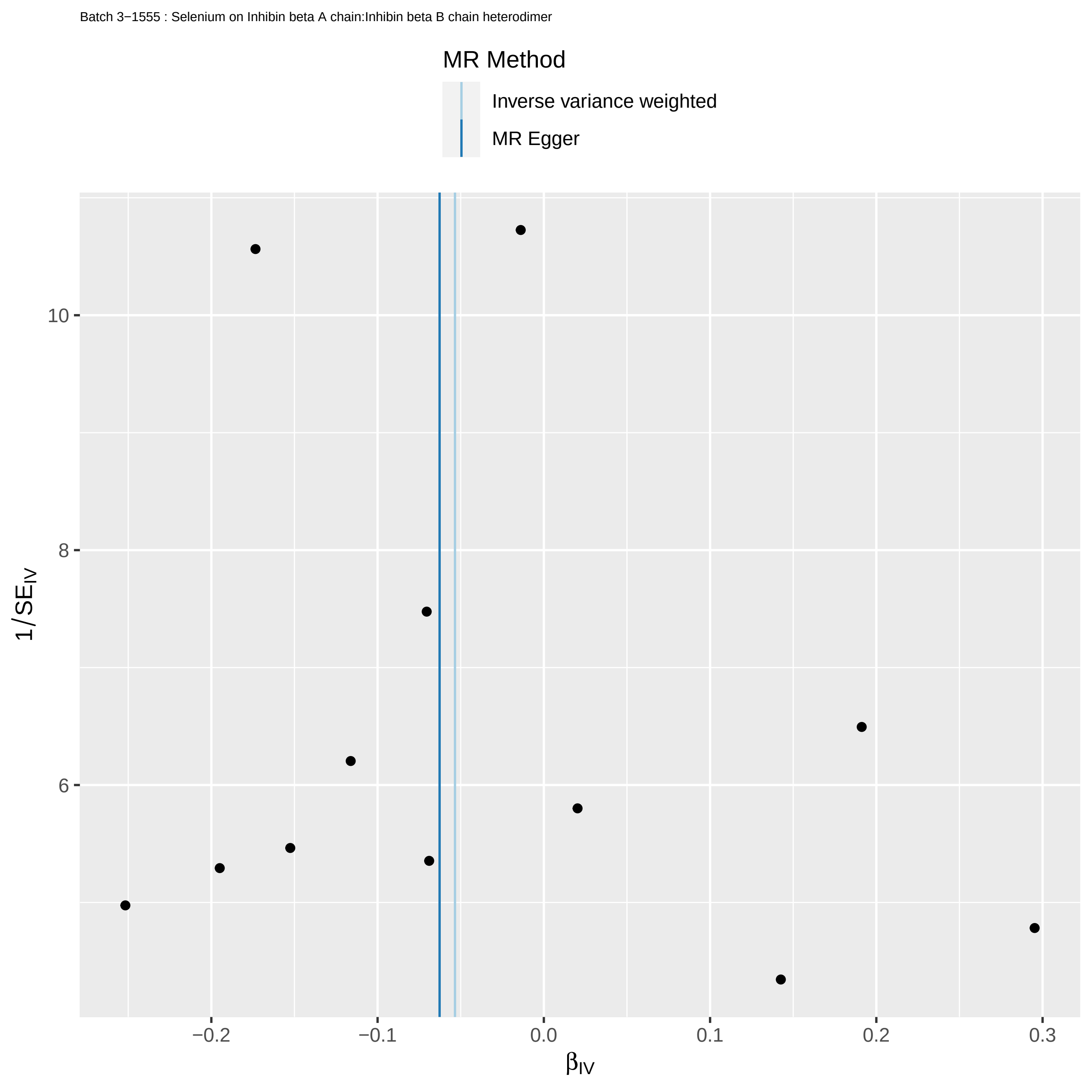

Batch 3−1555 : Selenium on Inhibin beta A chain:Inhibin beta B chain heterodimer
MR Method
Inverse variance weighted
MR Egger
10
8
6
1/SE_IV
−0.2
−0.1
0.0
0.1
0.2
0.3
β_IV

Batch 3−1556 : Selenium on Inhibin beta C chain

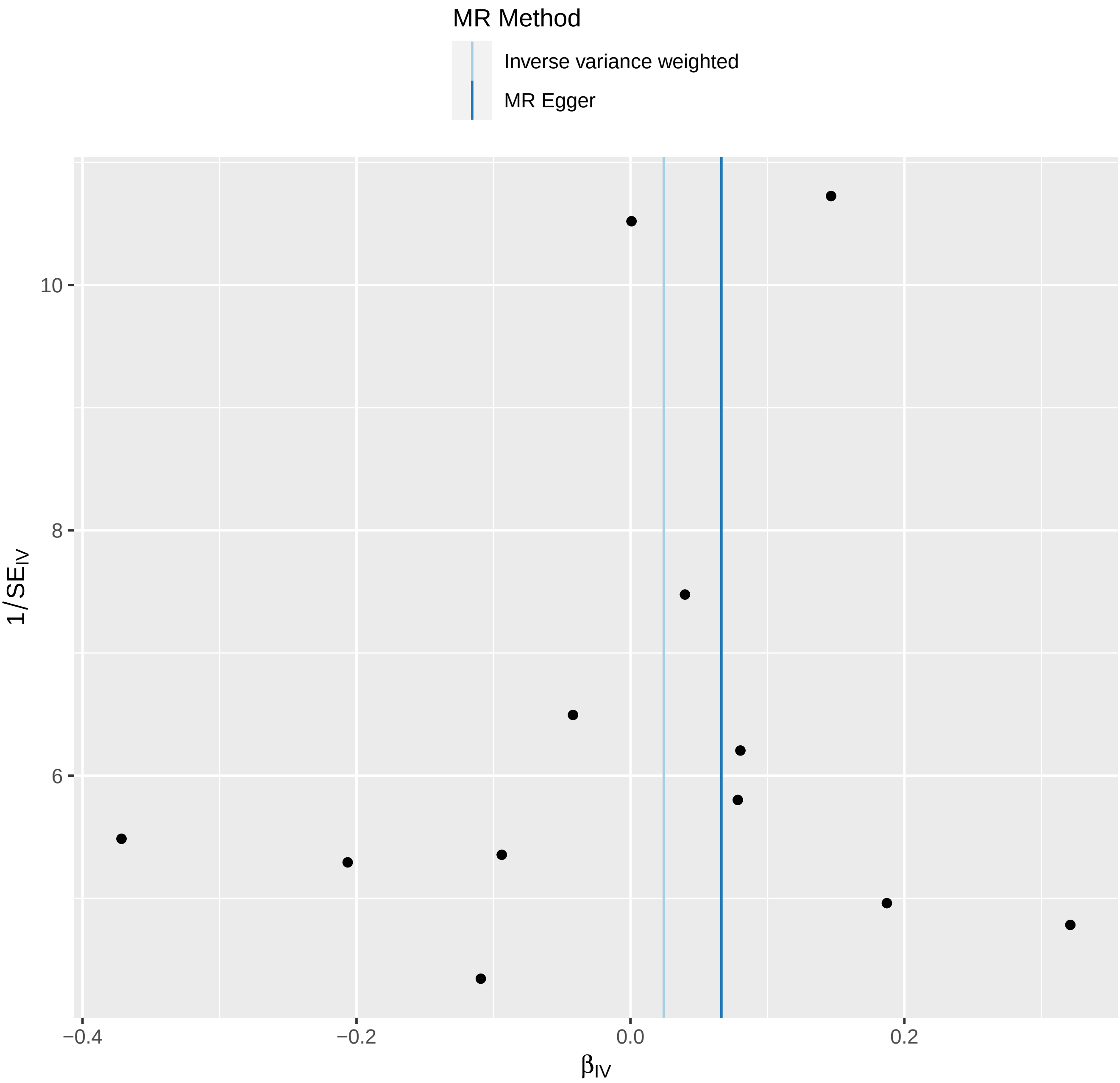

Batch 3−1557 : Selenium on 72 kDa inositol polyphosphate 5−phosphatase

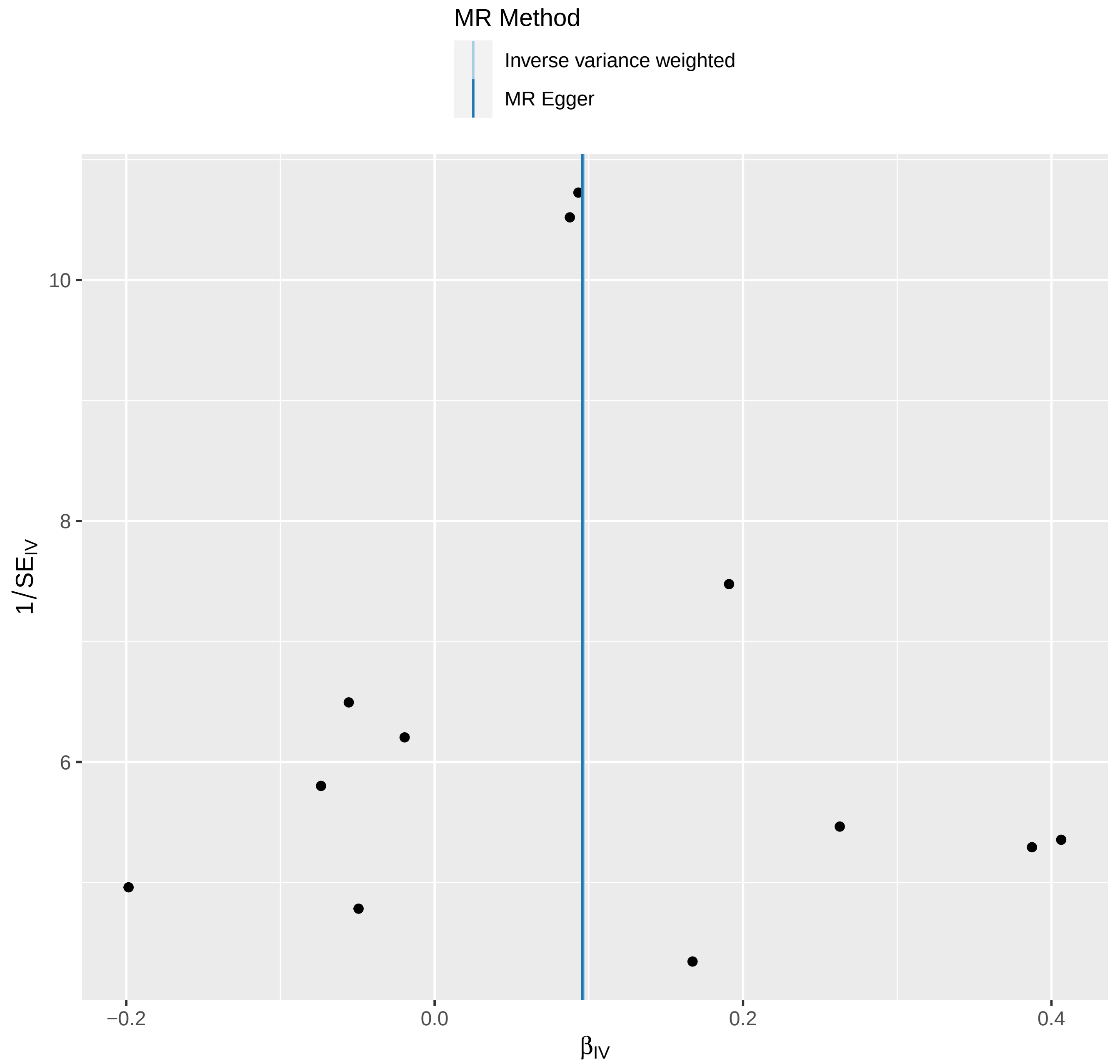

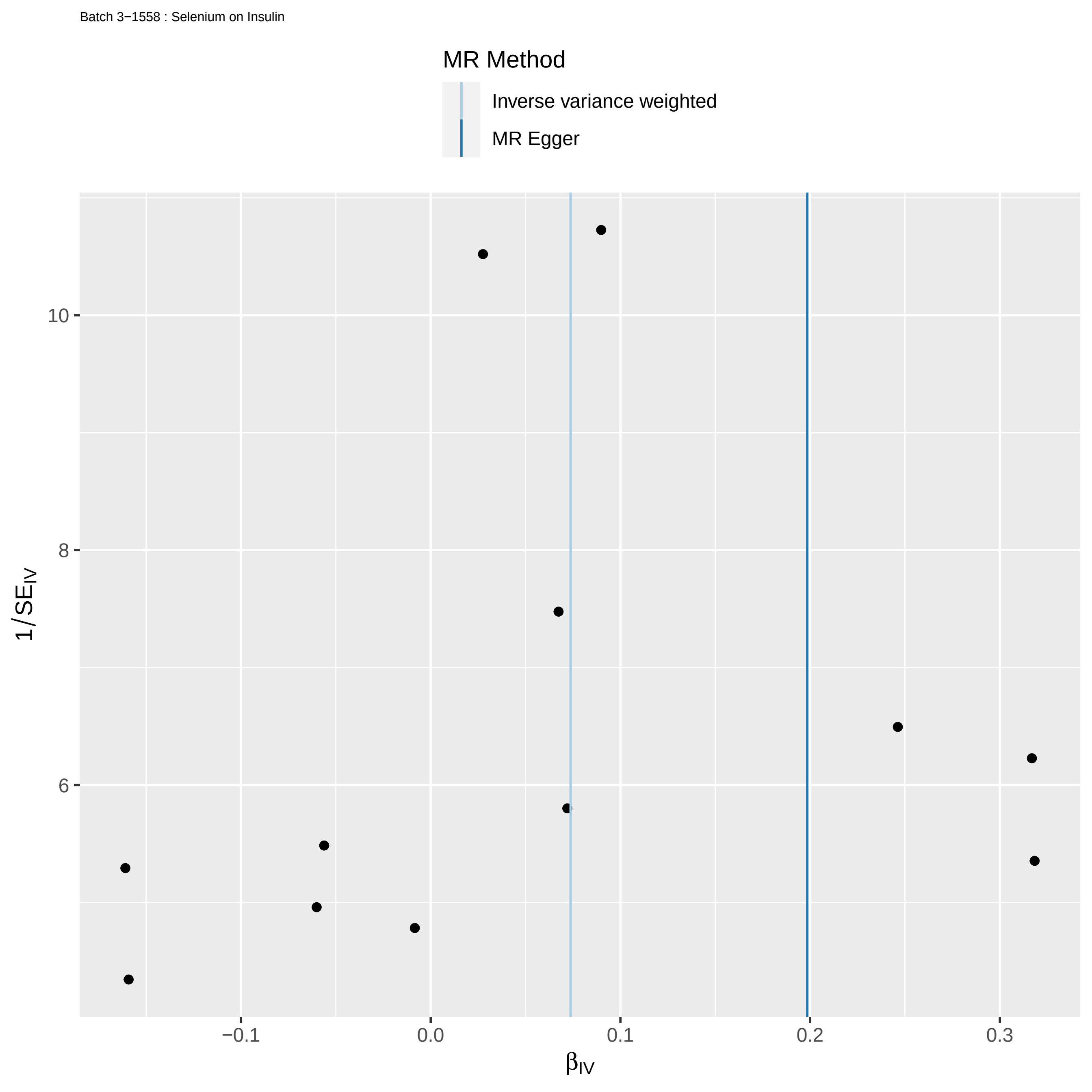

Batch 3−1558 : Selenium on Insulin
MR Method
Inverse variance weighted
MR Egger
10
8
6
1/SE_IV
−0.1
0.0
0.1
0.2
0.3
β_IV

Batch 3−1559 : Selenium on Insulin−induced gene 1 protein

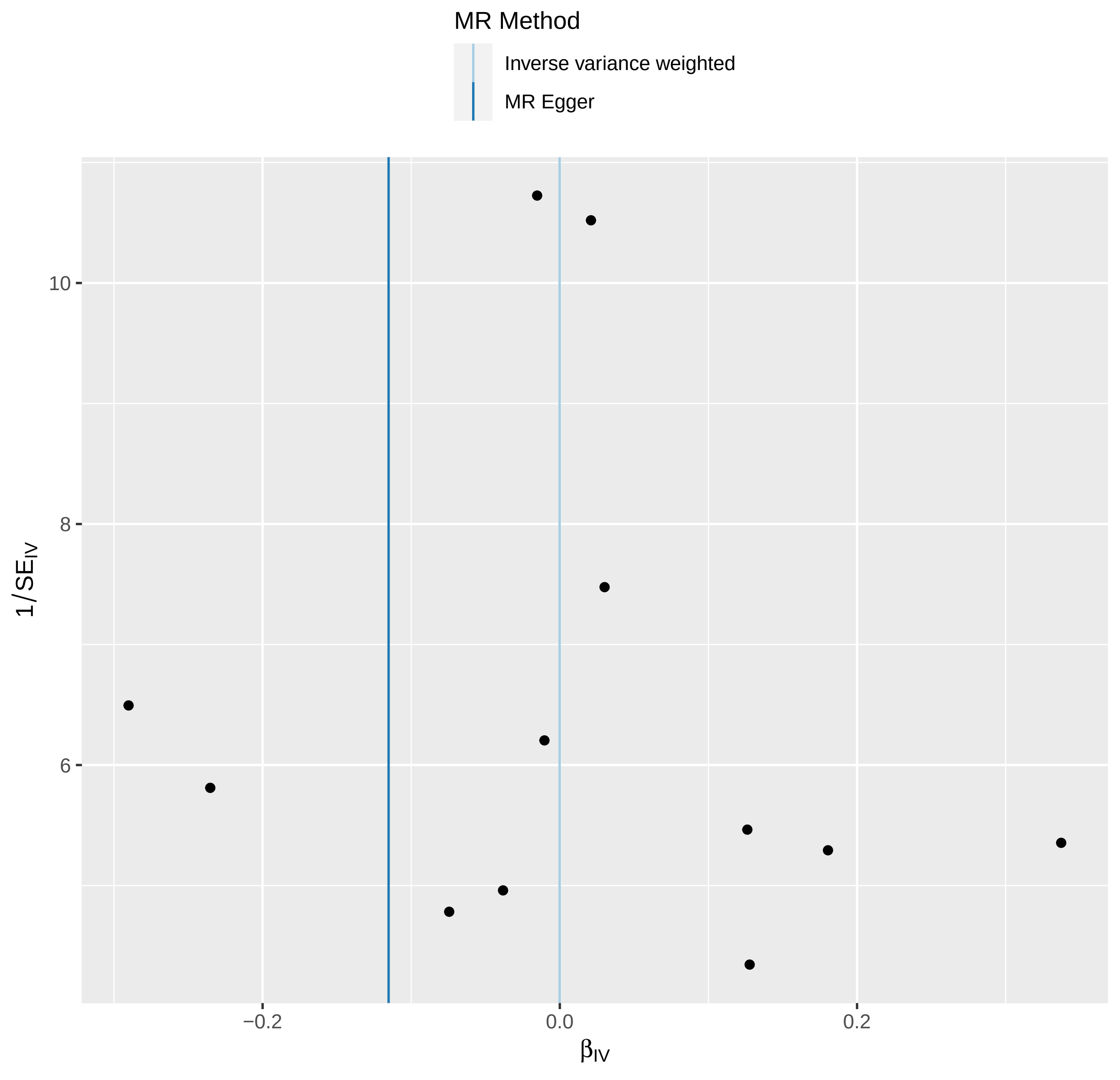

Batch 3−1560 : Selenium on Insulin−like 3

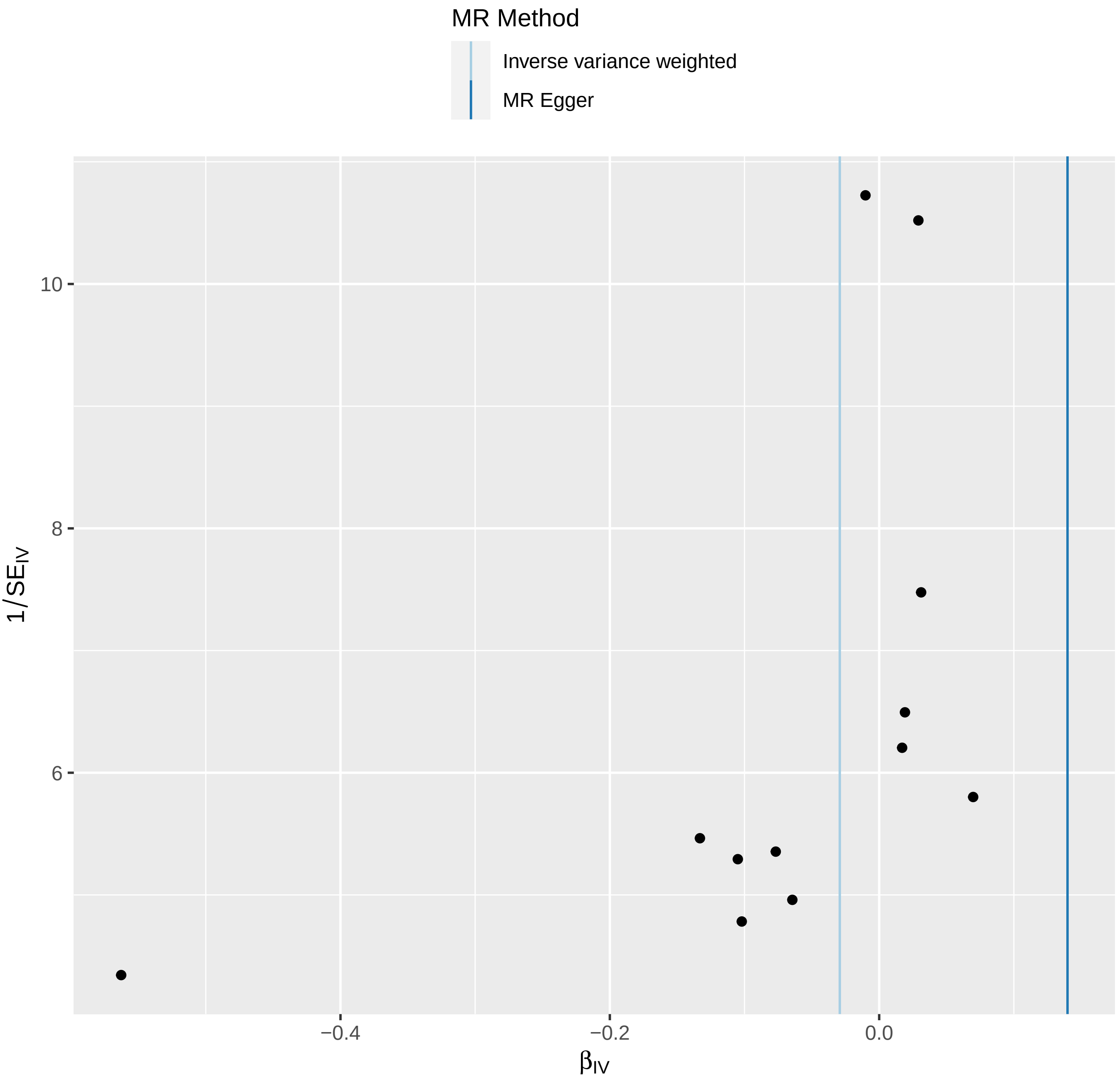

Batch 3−1561 : Selenium on Early placenta insulin−like peptide

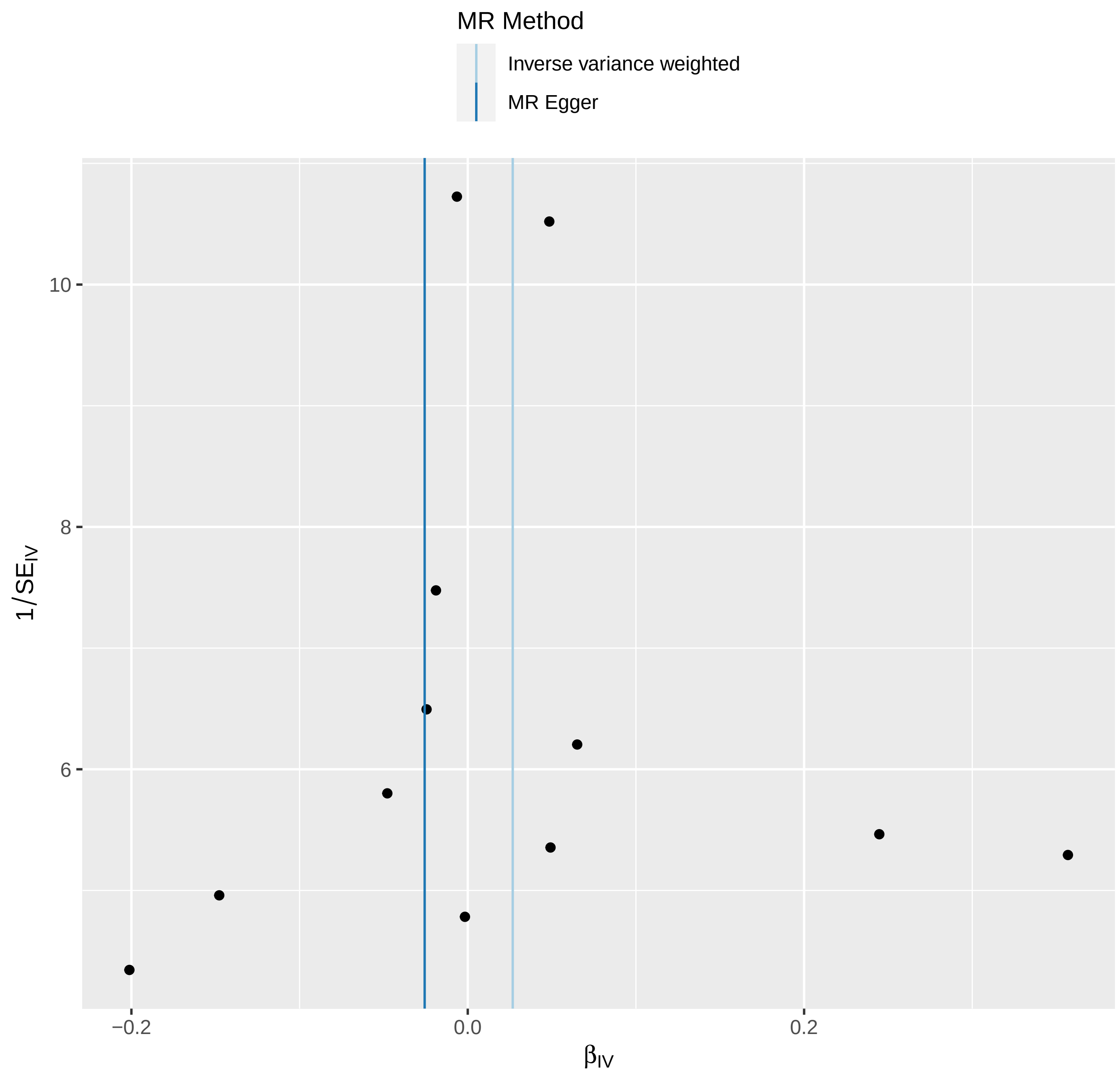

Batch 3−1562 : Selenium on Insulin−like peptide INSL5

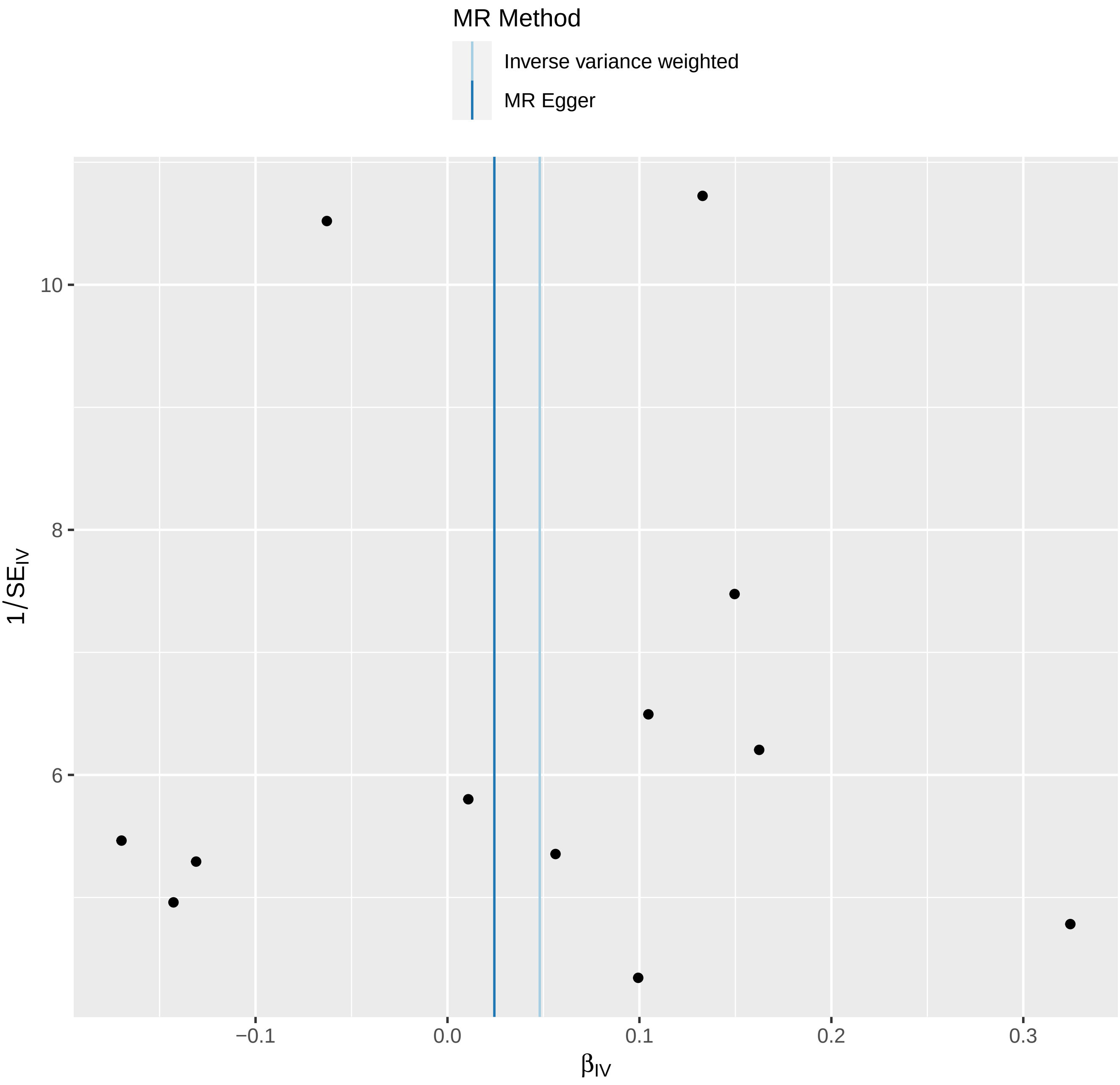

Batch 3−1563 : Selenium on Insulin−like peptide INSL6

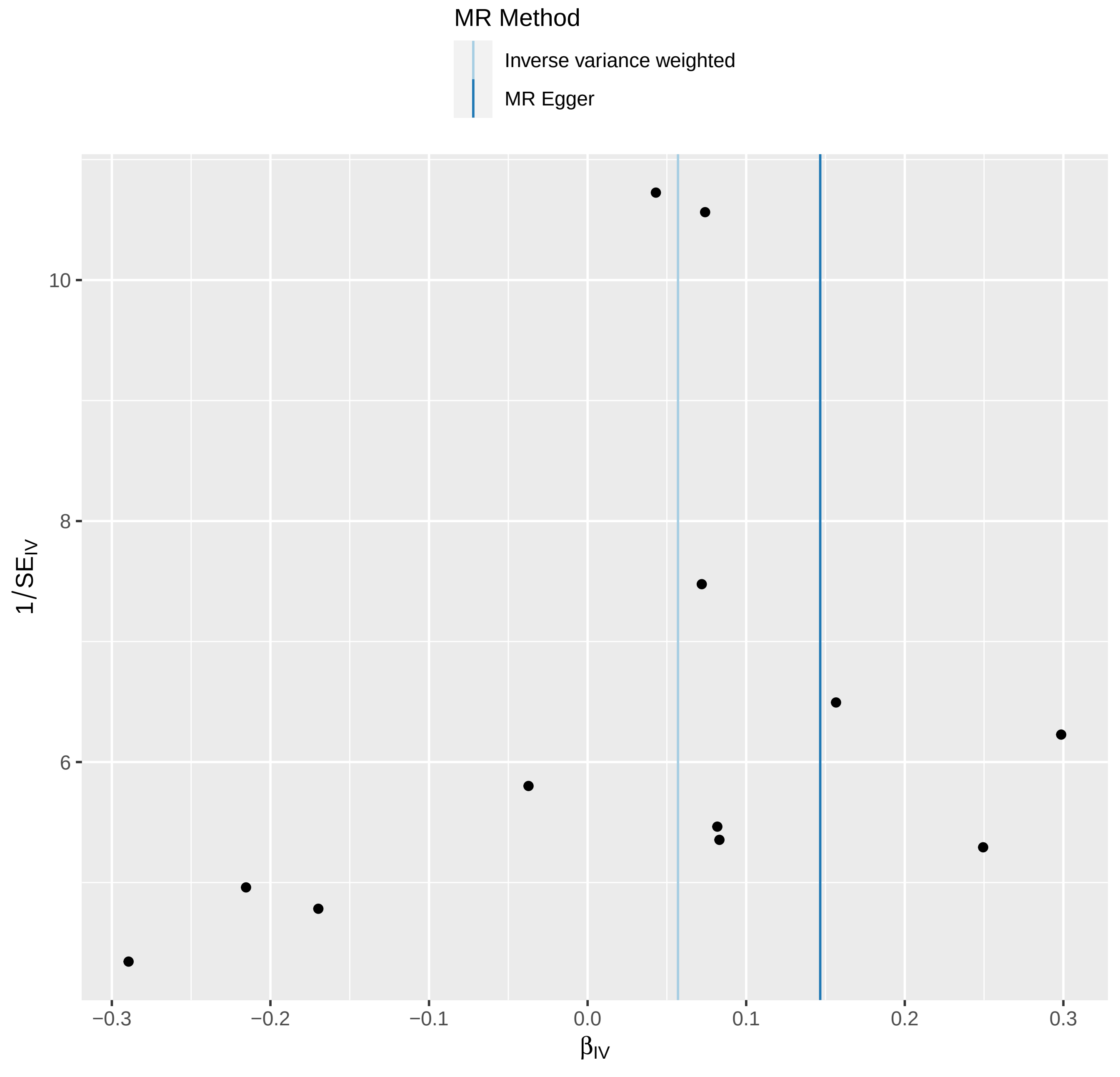

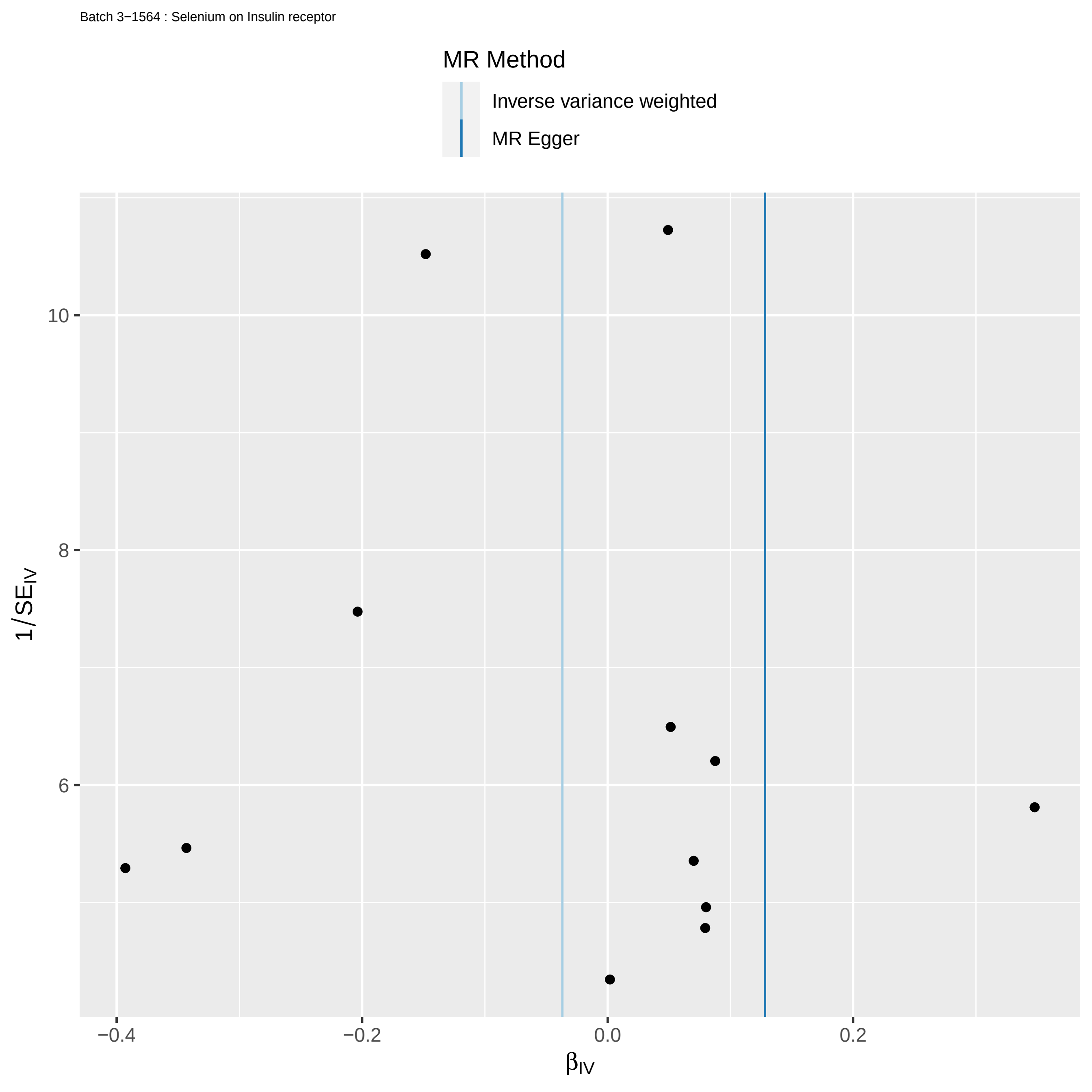
Batch 3−1564 : Selenium on Insulin receptor
MR Method
Inverse variance weighted
MR Egger
10
8
6
1/SE_IV
−0.4
−0.2
0.0
0.2
β_IV

Batch 3−1565 : Selenium on IQ domain−containing protein F1

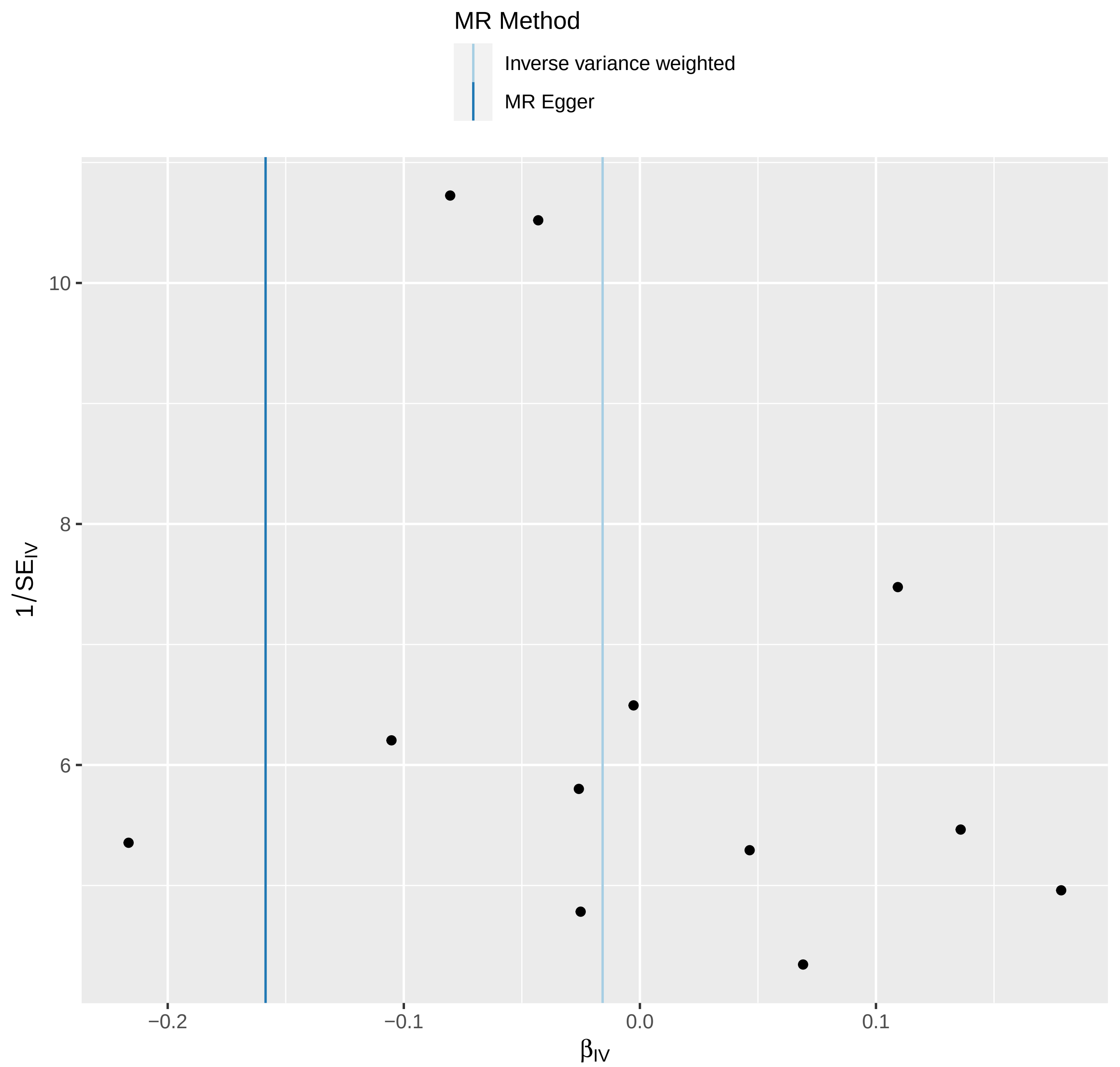

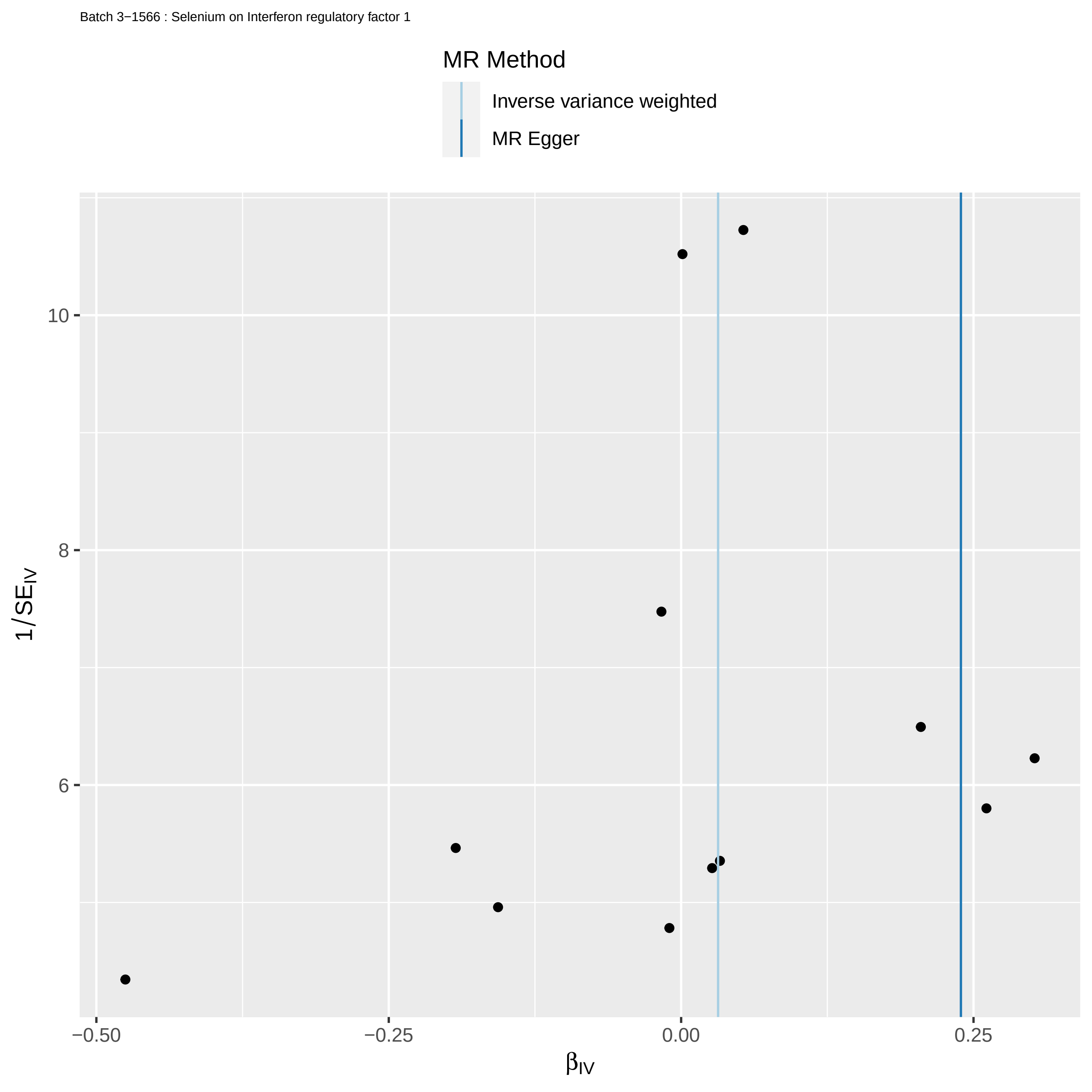
Batch 3−1566 : Selenium on Interferon regulatory factor 1
MR Method
Inverse variance weighted
MR Egger
10
8
6
1/SE_IV
−0.50
−0.25
0.00
0.25
β_IV

Batch 3−1567 : Selenium on Interferon regulatory factor 1

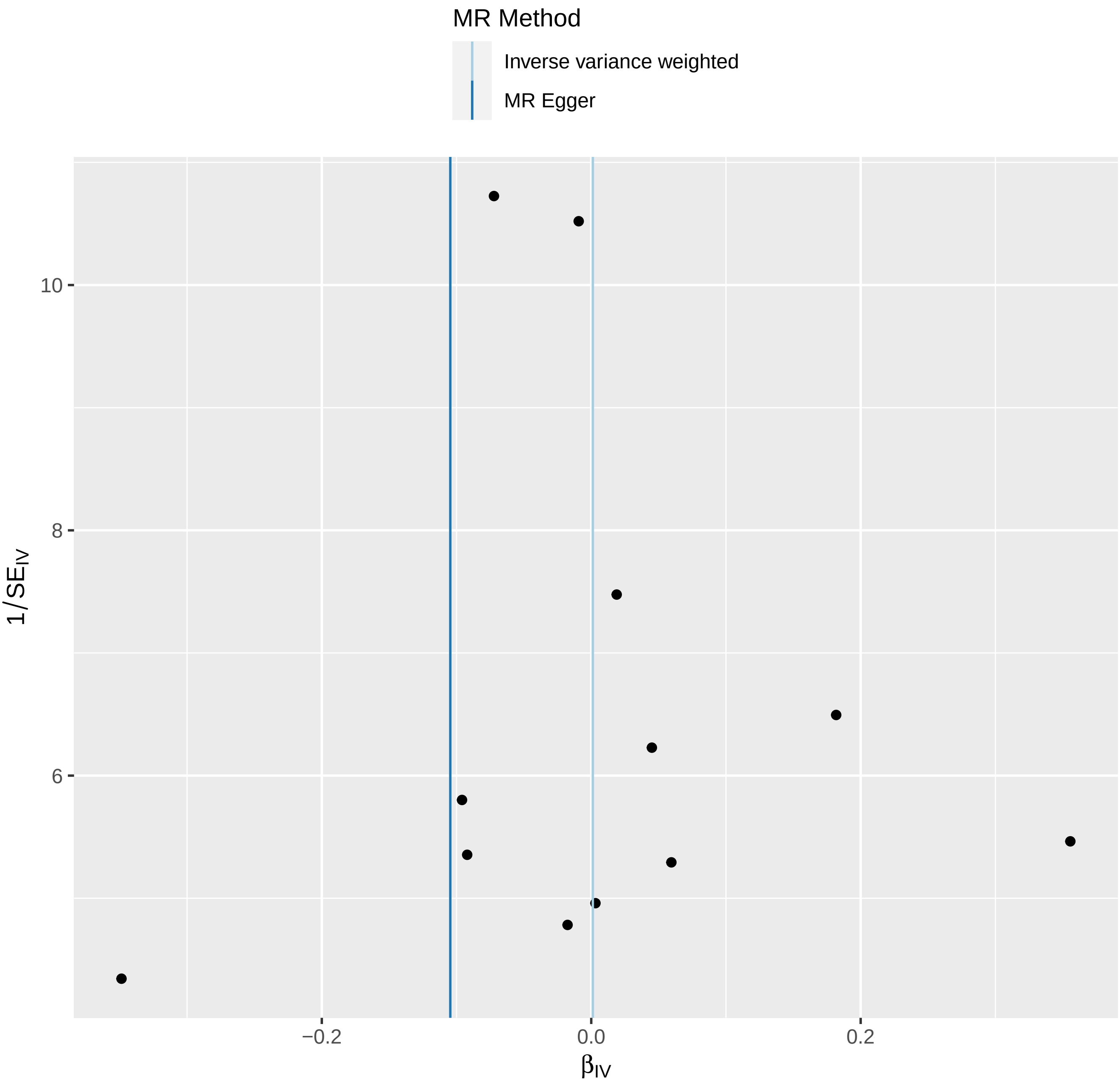

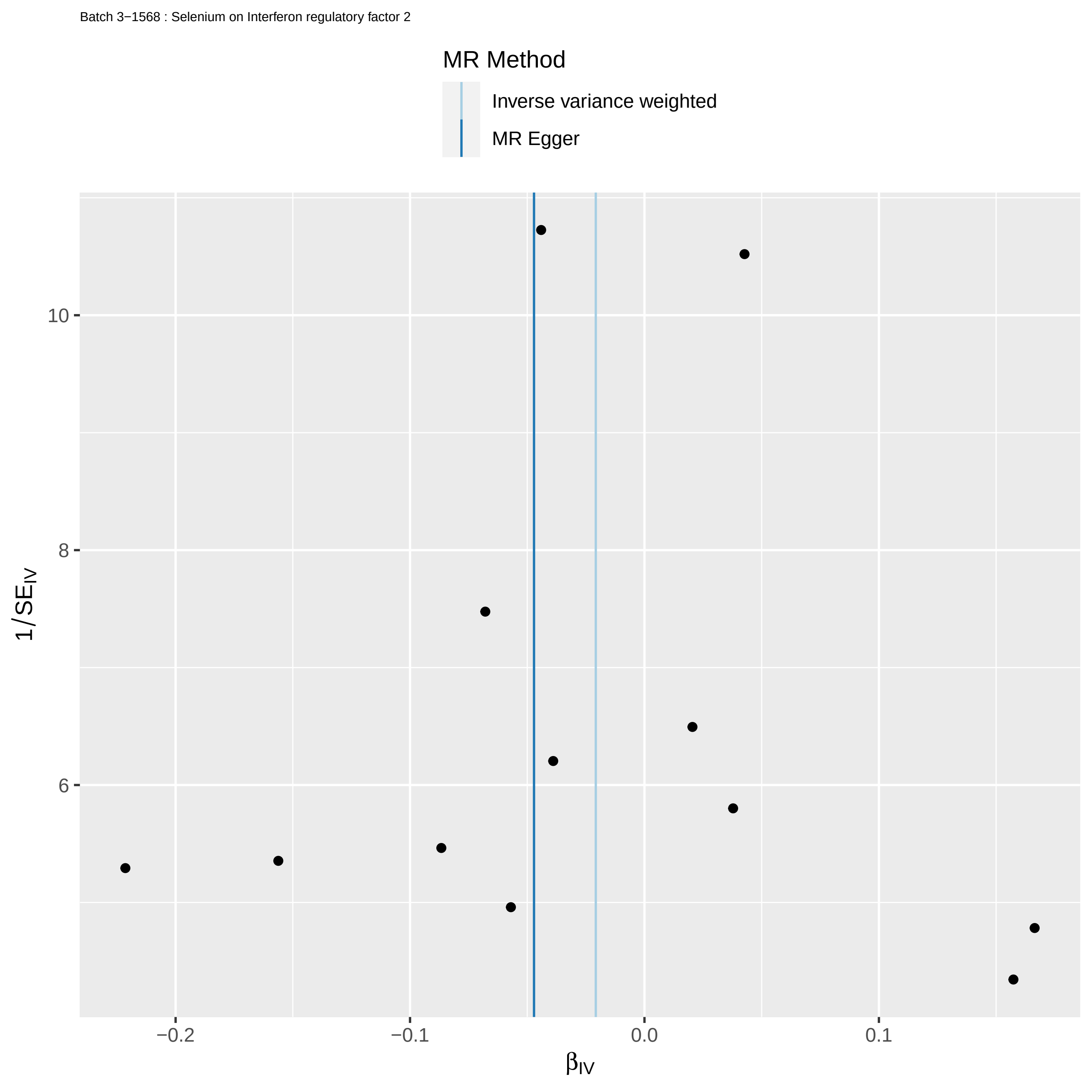

Batch 3−1568 : Selenium on Interferon regulatory factor 2
MR Method
Inverse variance weighted
MR Egger
10
8
6
1/SE$_{IV}$
−0.2
−0.1
0.0
0.1
β$_{IV}$

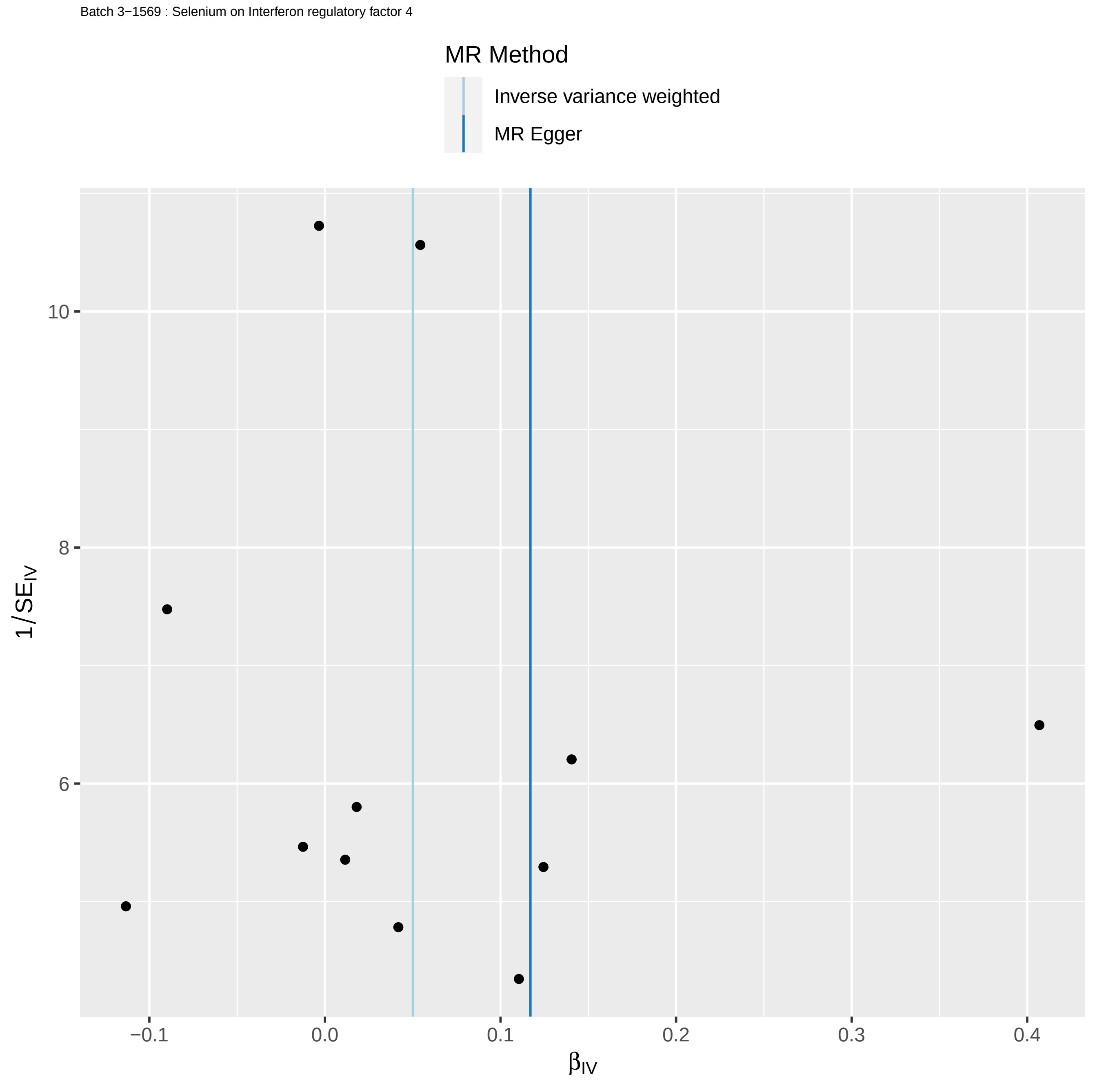
Batch 3−1569 : Selenium on Interferon regulatory factor 4
MR Method
Inverse variance weighted
MR Egger
10
8
6
1/SE$_{IV}$
−0.1
0.0
0.1
0.2
0.3
0.4
β$_{IV}$

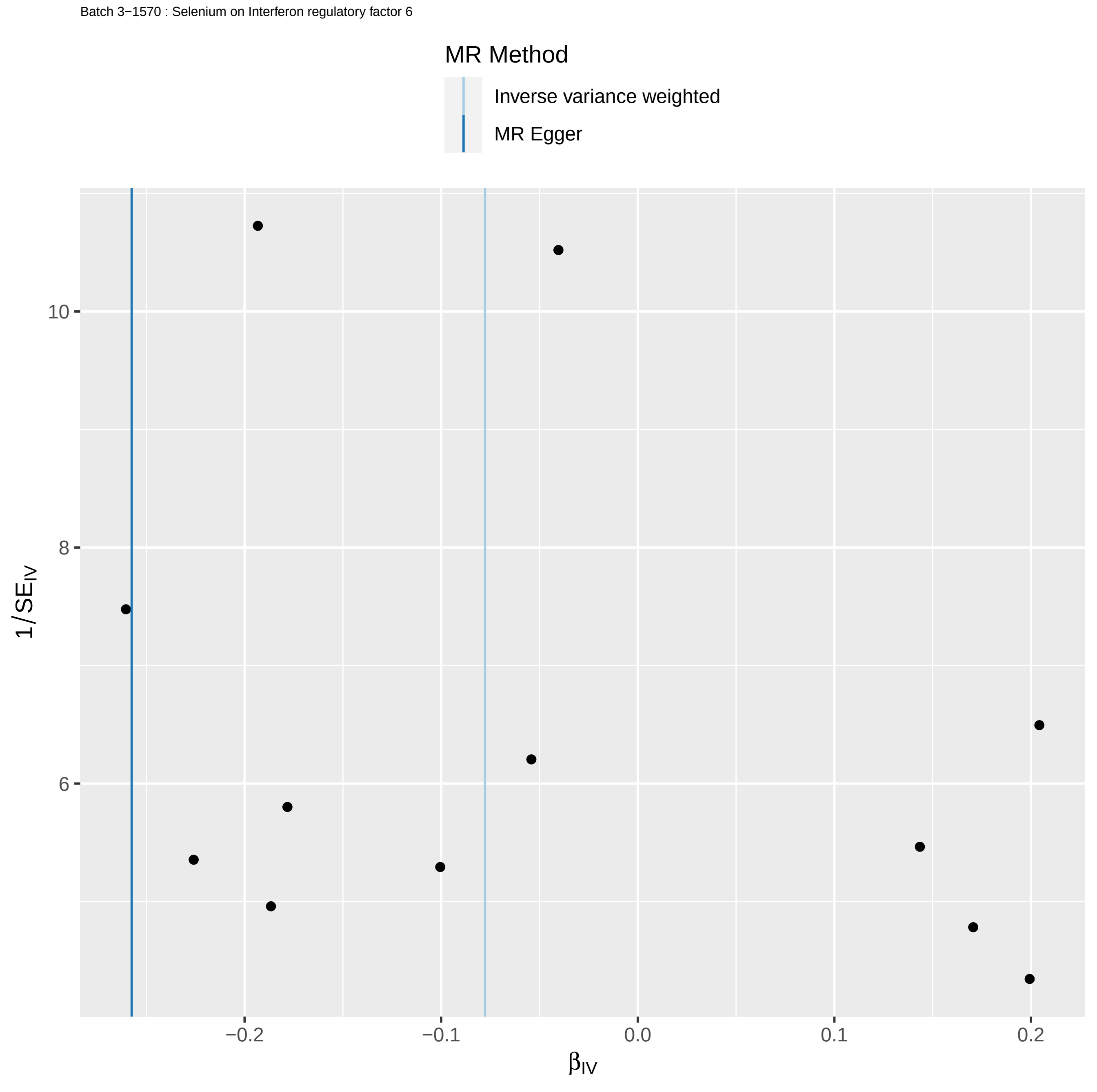
Batch 3−1570 : Selenium on Interferon regulatory factor 6
MR Method
Inverse variance weighted
MR Egger
10
8
6
1/SE$_{IV}$
−0.2
−0.1
0.0
0.1
0.2
β_{IV}

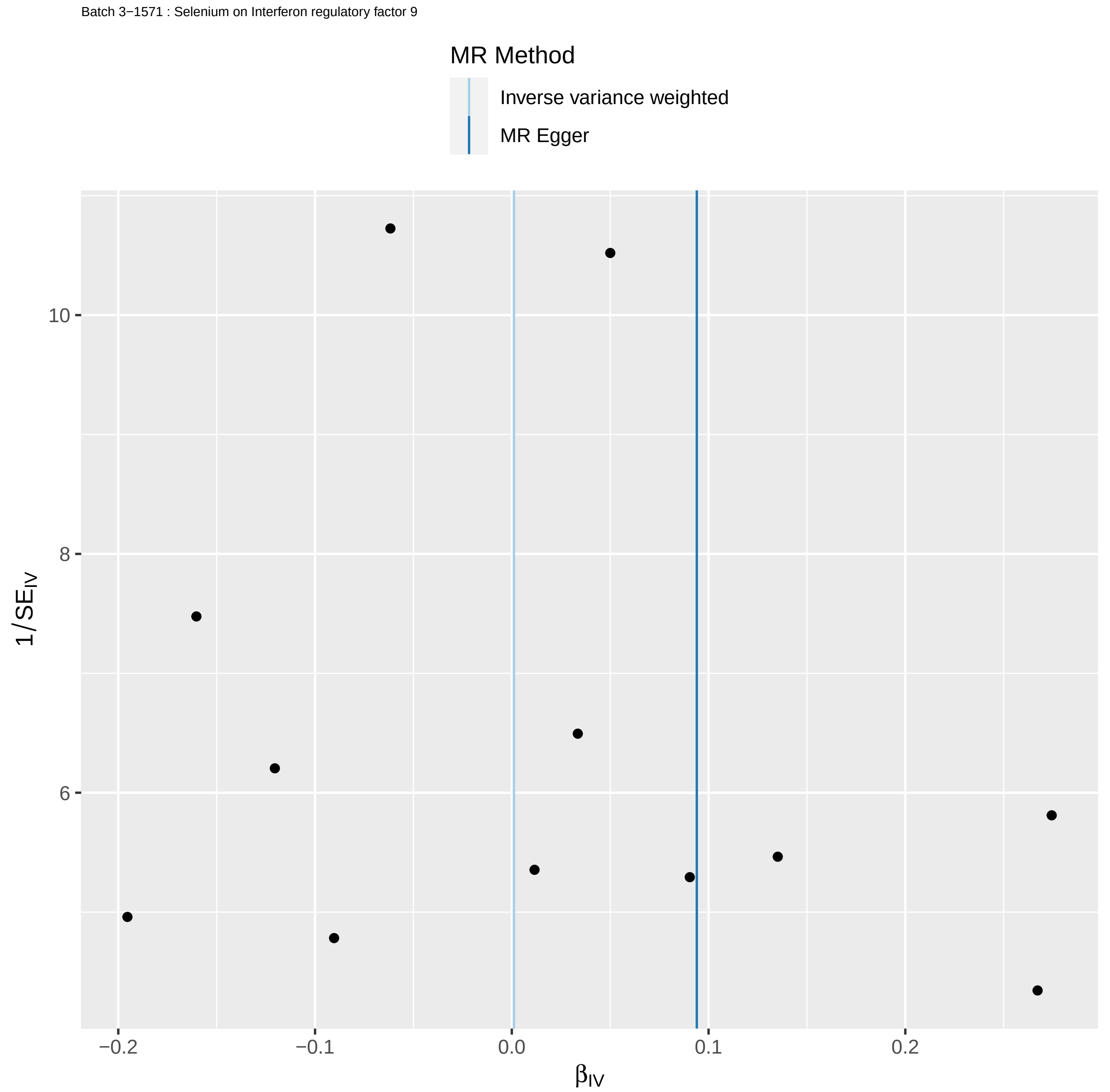

Batch 3−1571 : Selenium on Interferon regulatory factor 9
MR Method
Inverse variance weighted
MR Egger
10
8
6
1/SE_IV
−0.2
−0.1
0.0
0.1
0.2
β_IV

Batch 3−1572 : Selenium on Iron−sulfur cluster assembly enzyme ISCU, mitochondrial

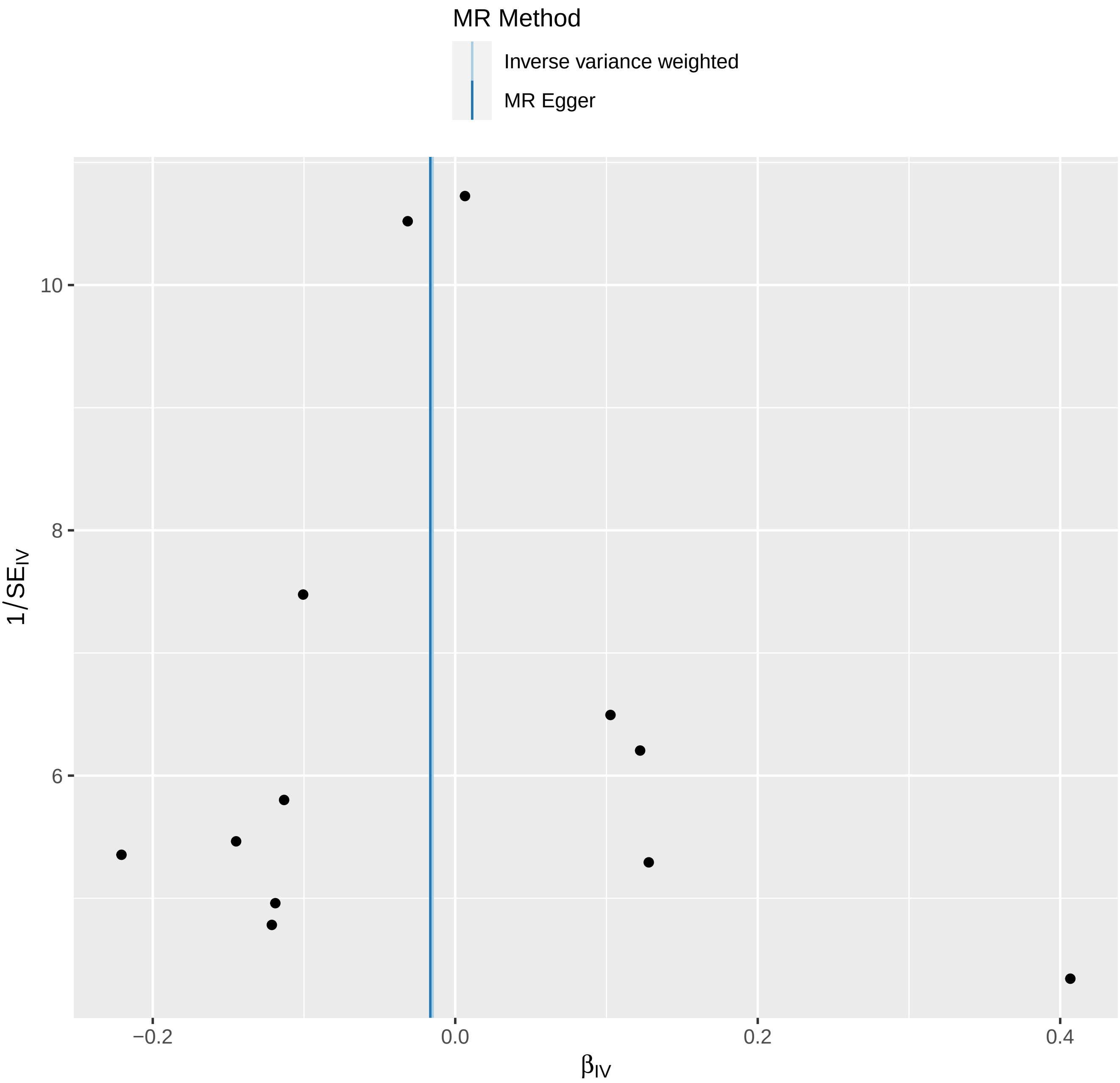

Batch 3−1573 : Selenium on Ubiquitin−like protein ISG15

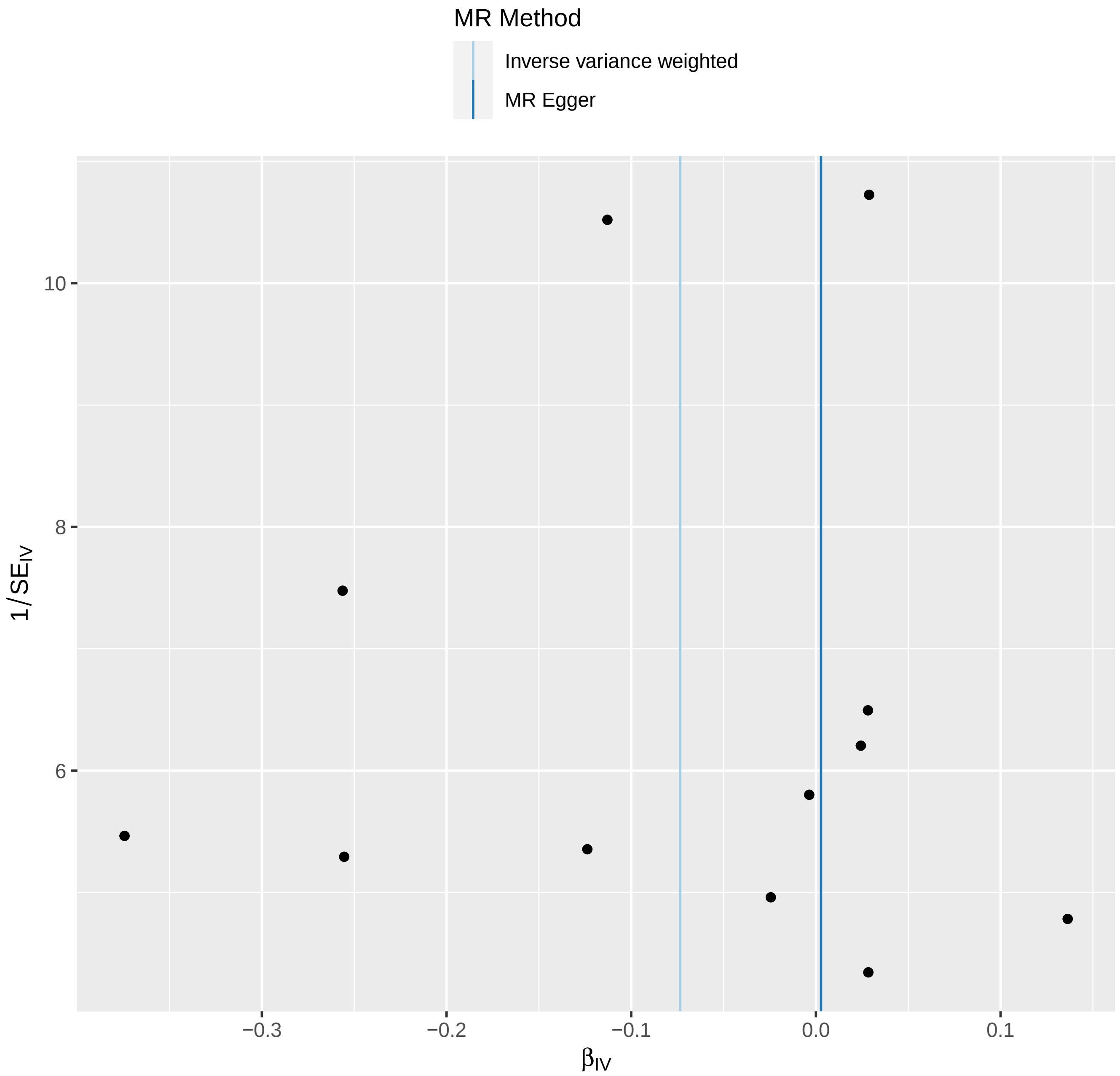

Batch 3−1574 : Selenium on Ubiquitin−like protein ISG15

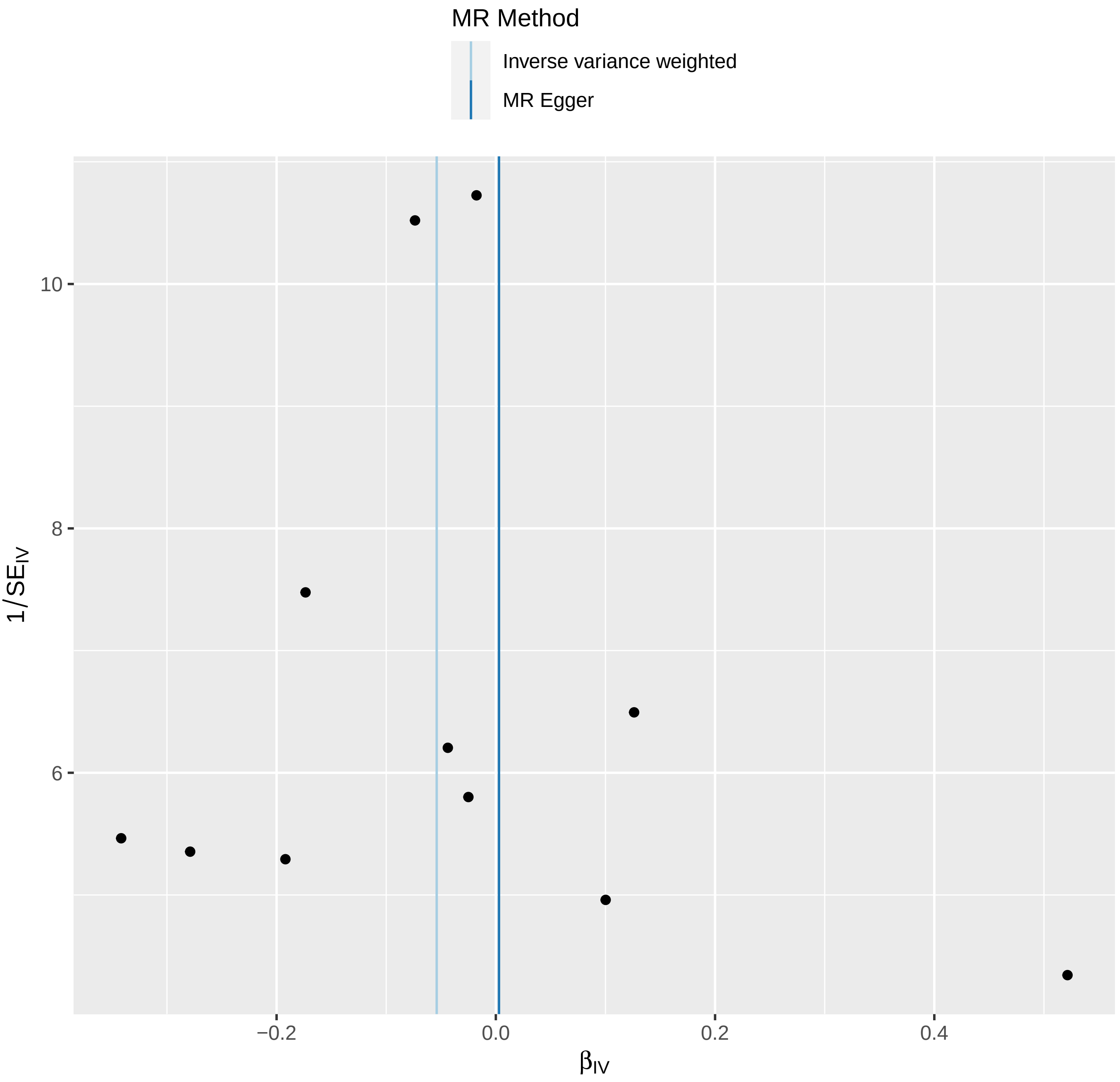

Batch 3−1575 : Selenium on Insulin gene enhancer protein ISL−1

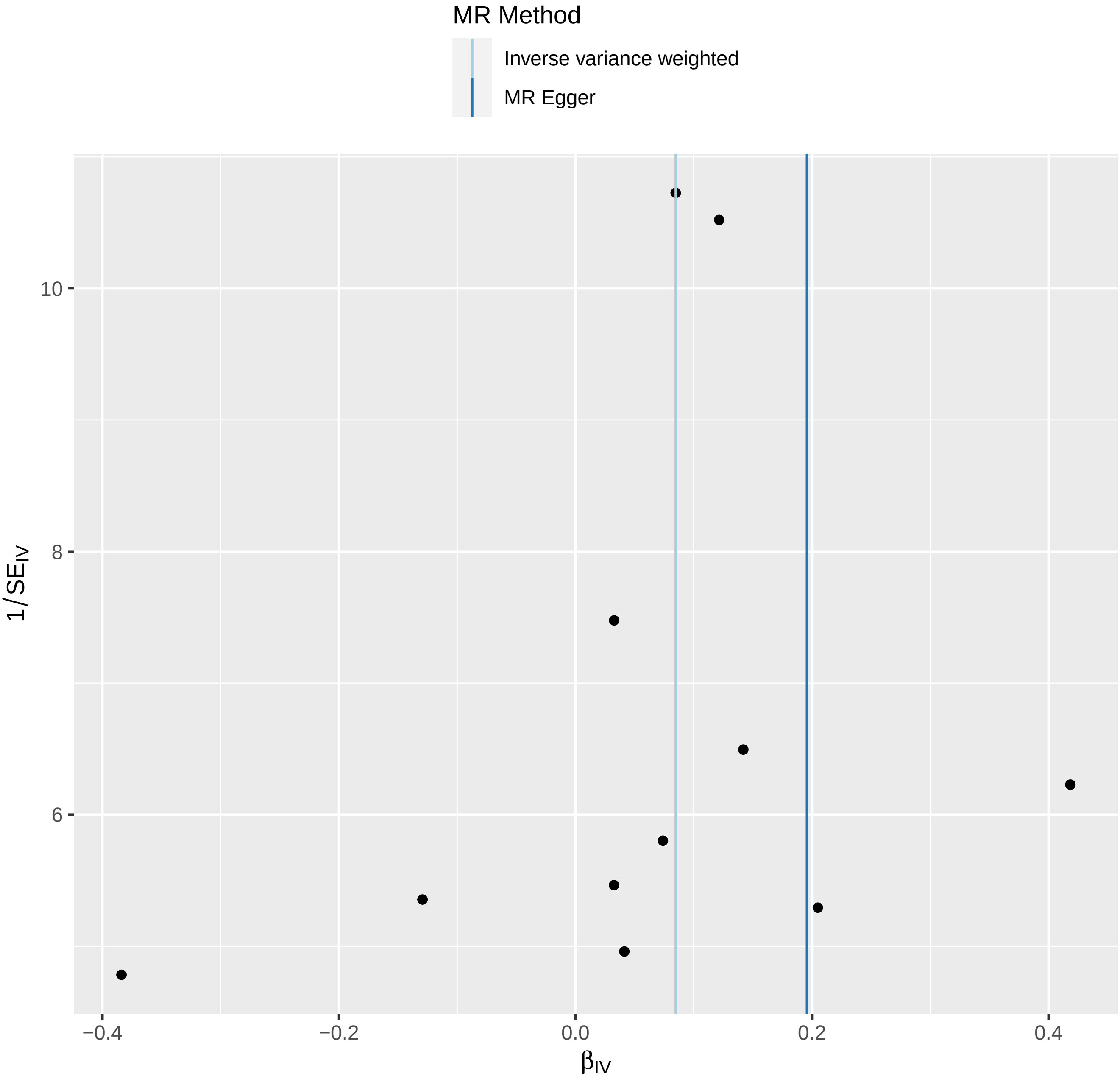

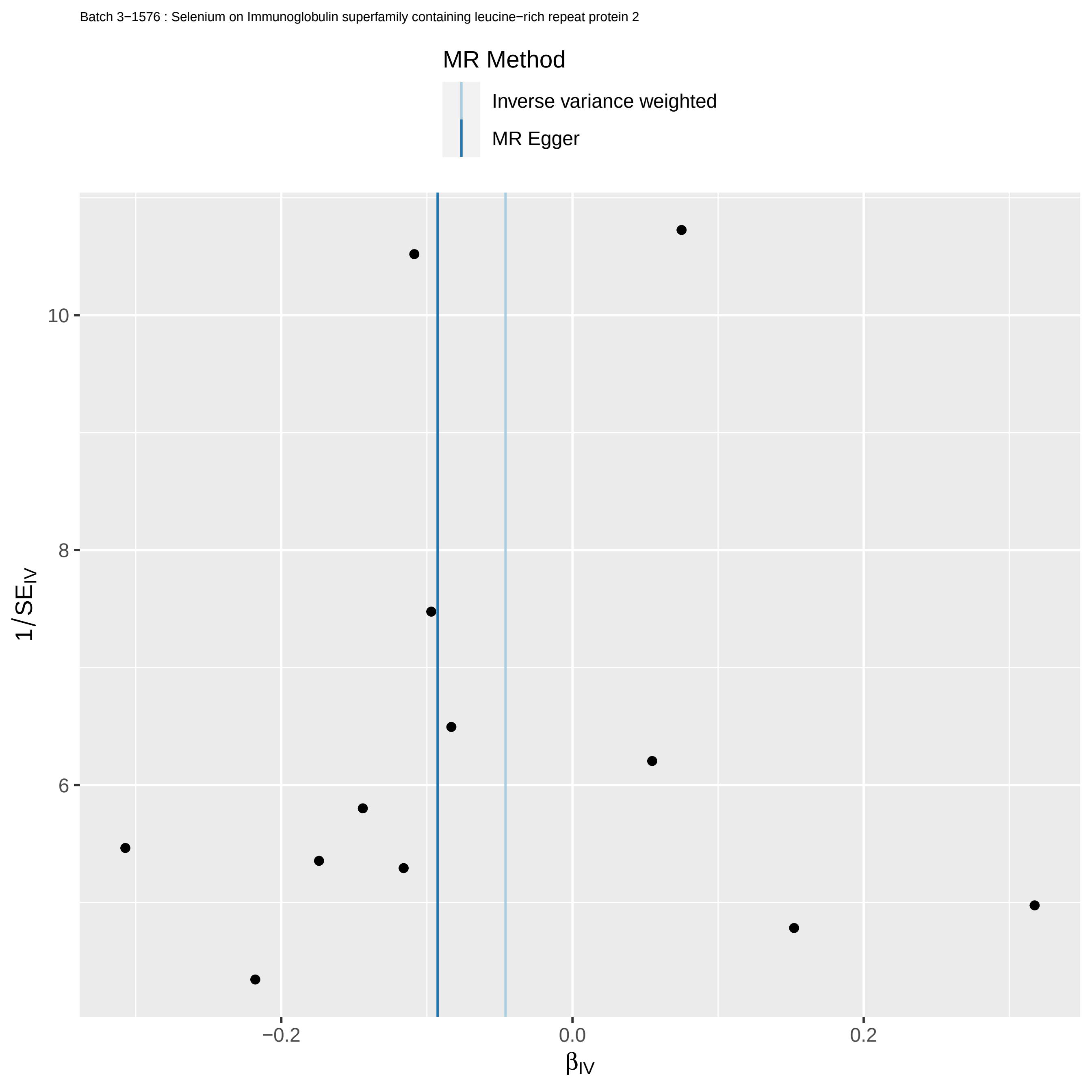

Batch 3−1576 : Selenium on Immunoglobulin superfamily containing leucine−rich repeat protein 2
MR Method
Inverse variance weighted
MR Egger
10
8
6
1/SE$_{IV}$
−0.2
0.0
0.2
β$_{IV}$

Batch 3−1577 : Selenium on Isthmin−1

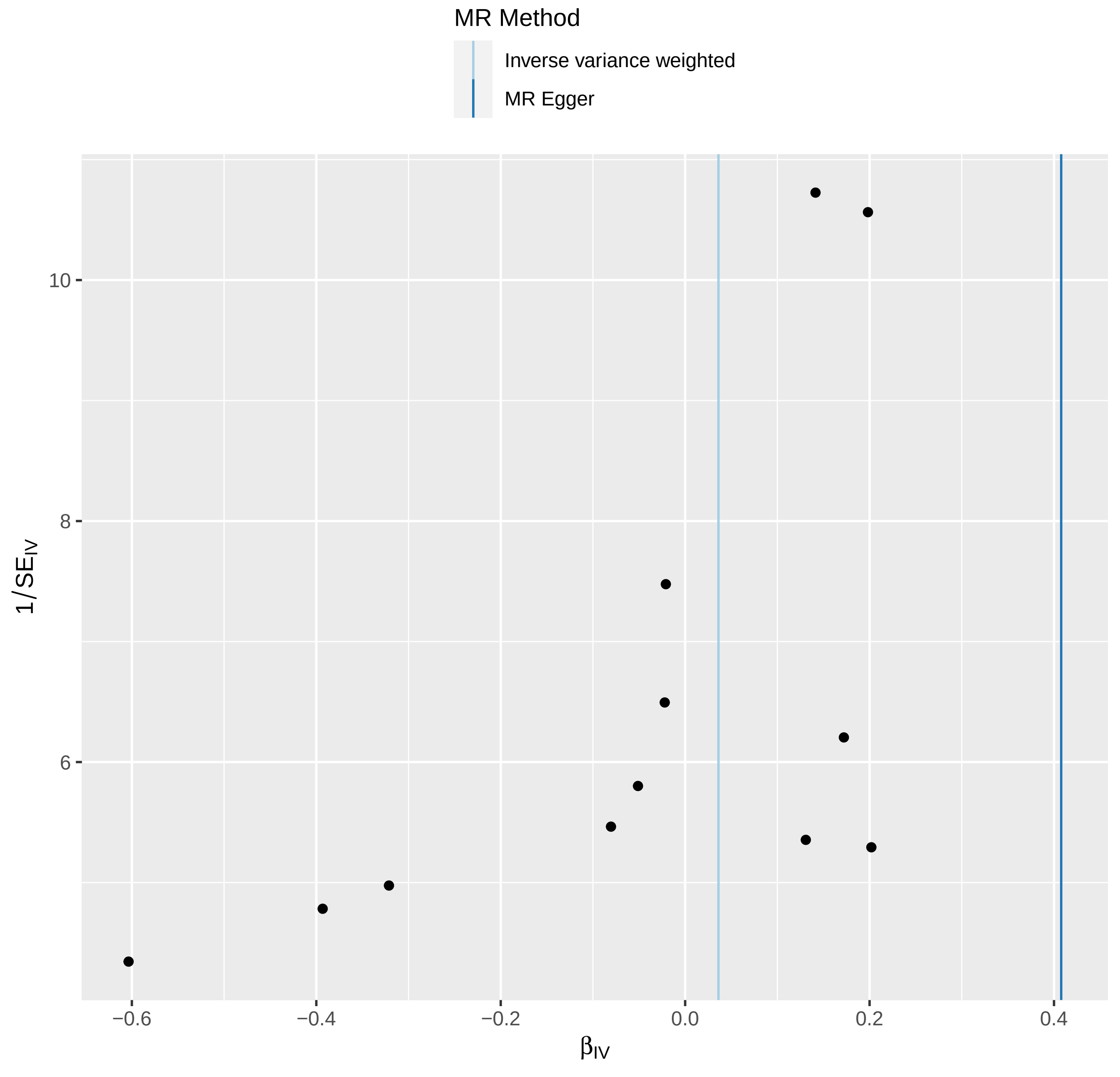

Batch 3−1578 : Selenium on Isochorismatase domain−containing protein 1

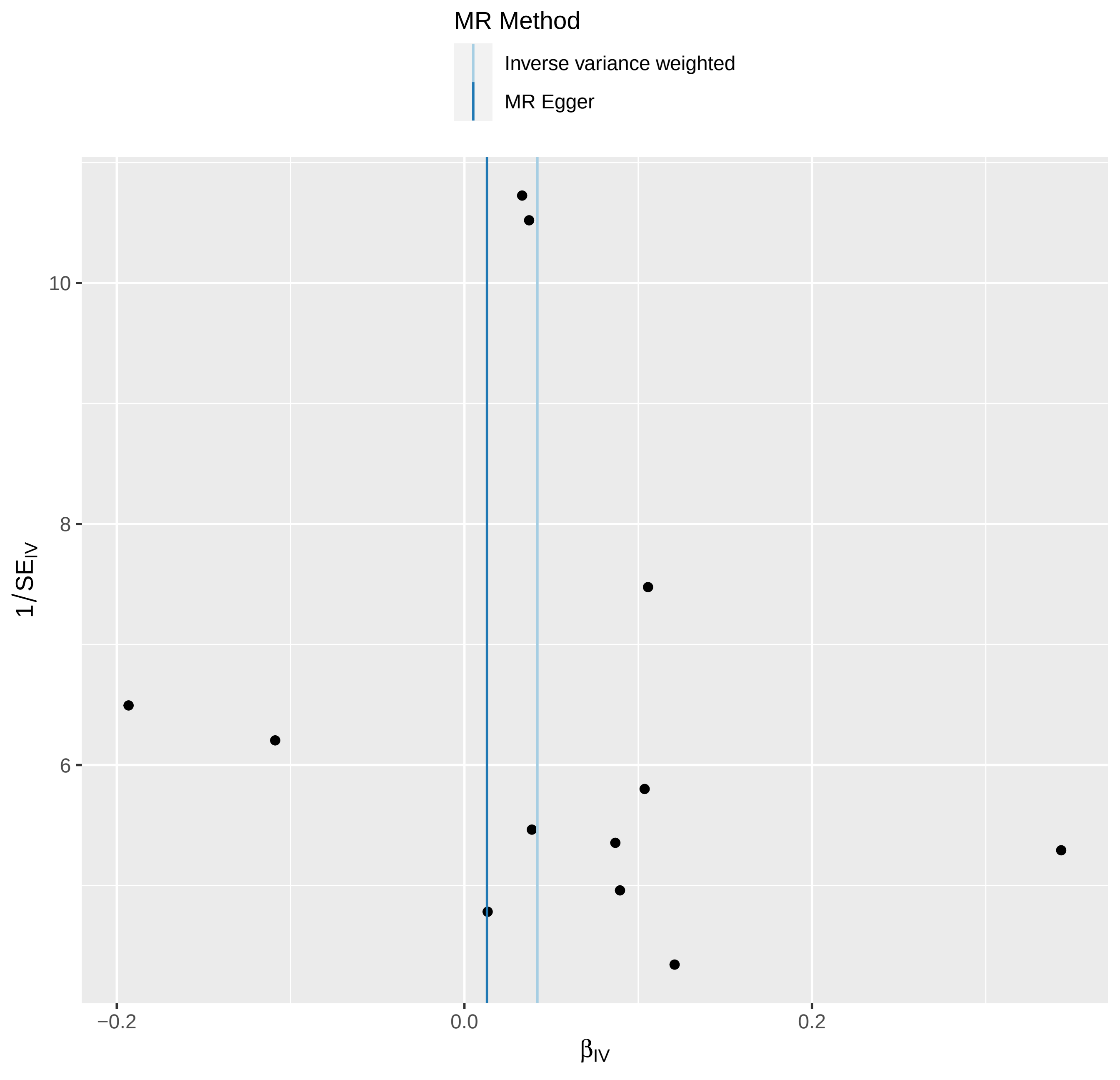

Batch 3−1579 : Selenium on IST1 homolog

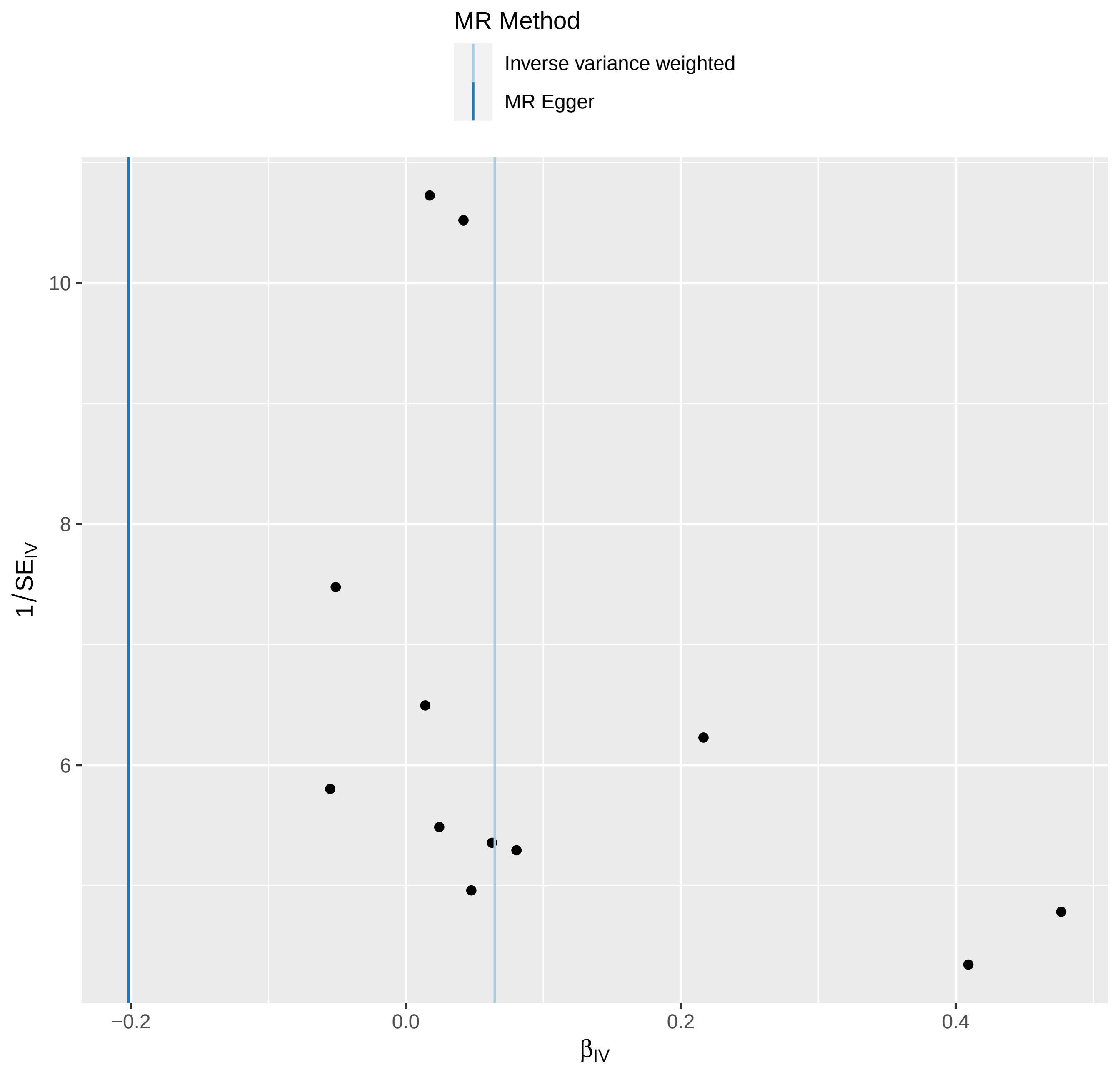

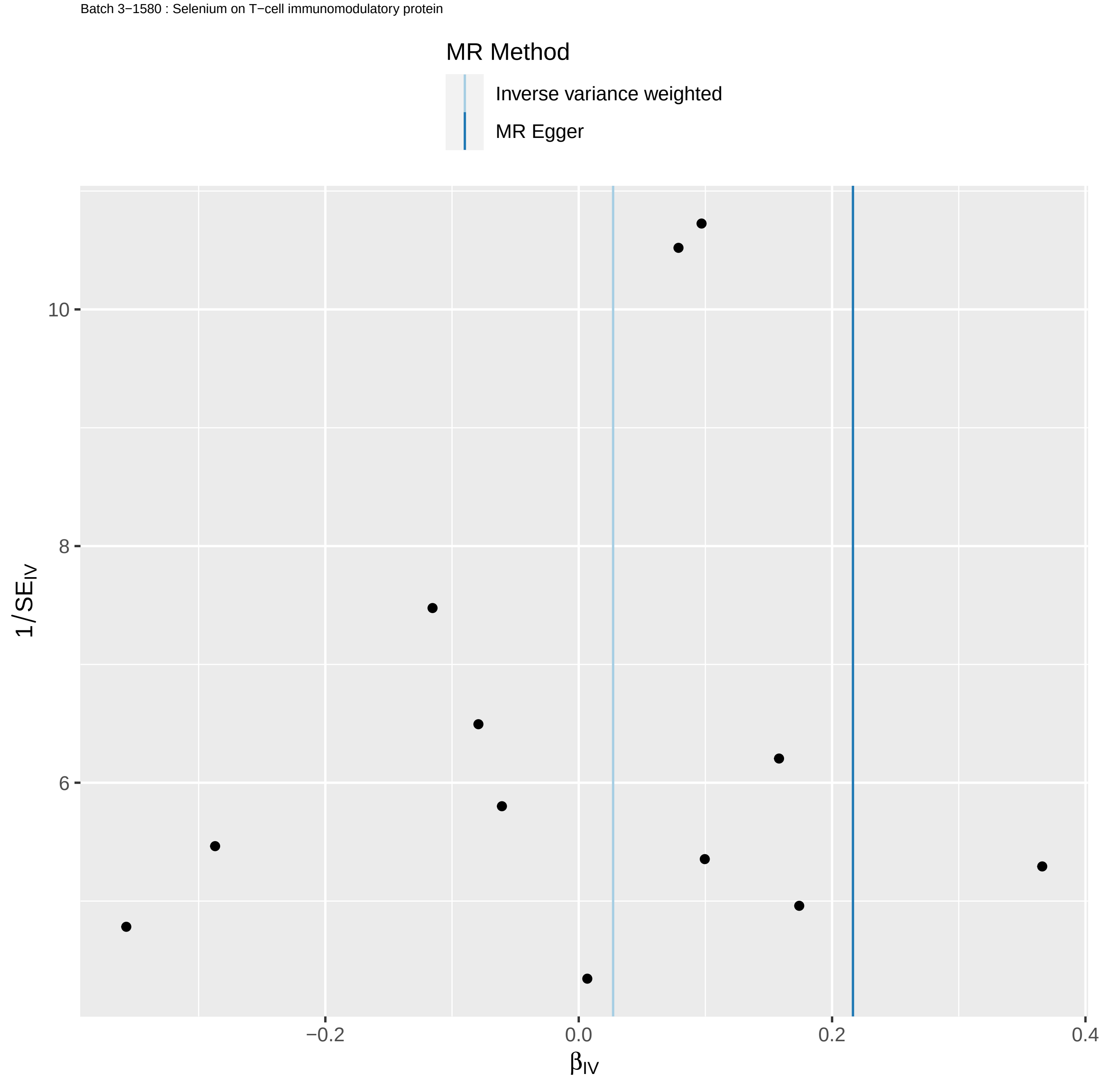

Batch 3−1580 : Selenium on T−cell immunomodulatory protein
MR Method
Inverse variance weighted
MR Egger
10
8
6
1/SE$_{IV}$
−0.2
0.0
0.2
0.4
β$_{IV}$

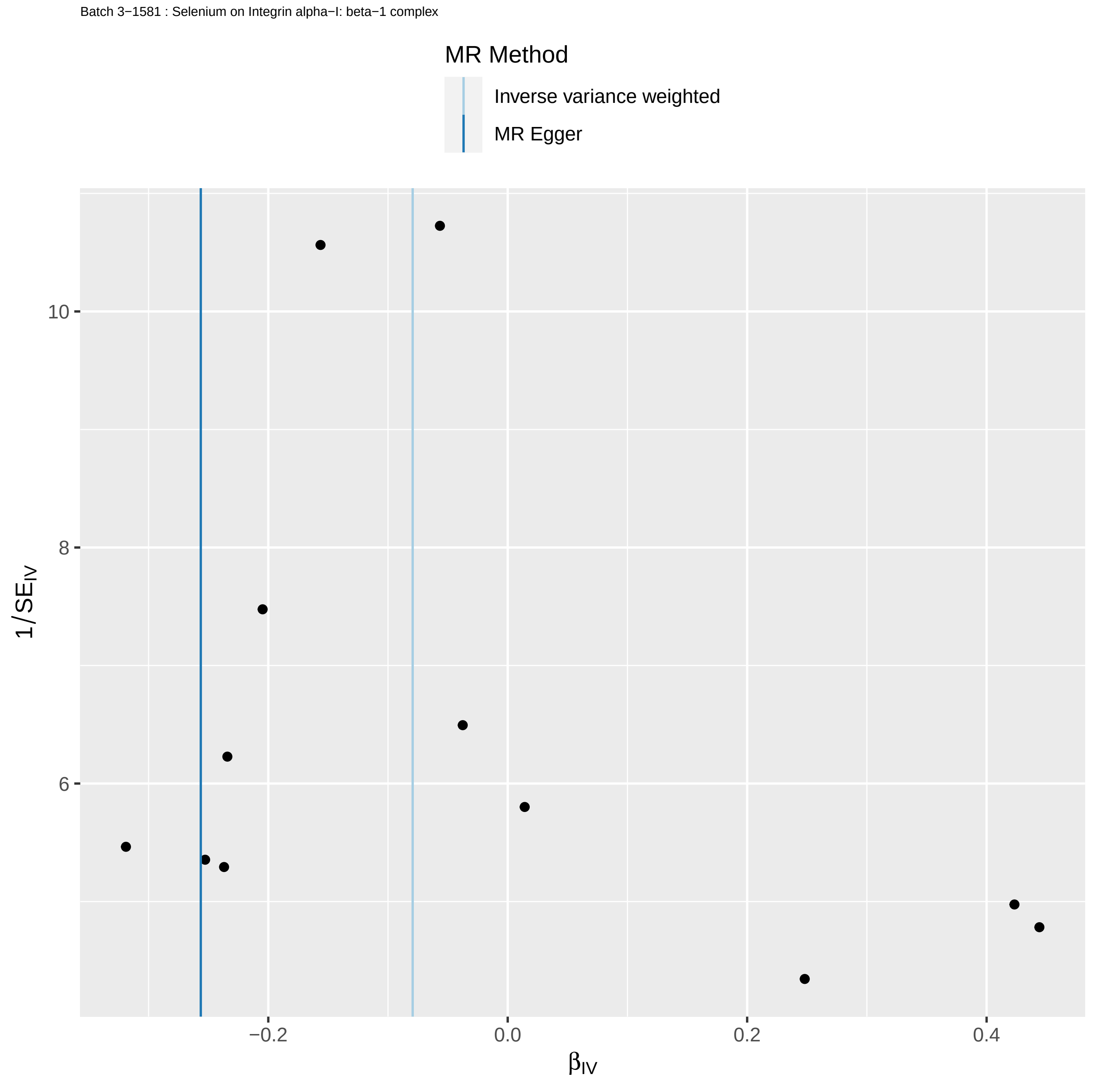
Batch 3−1581 : Selenium on Integrin alpha−I: beta−1 complex
MR Method
Inverse variance weighted
MR Egger
10
8
6
1/SE$_{IV}$
−0.2
0.0
0.2
0.4
β$_{IV}$

Batch 3−1582 : Selenium on Integrin alpha−5

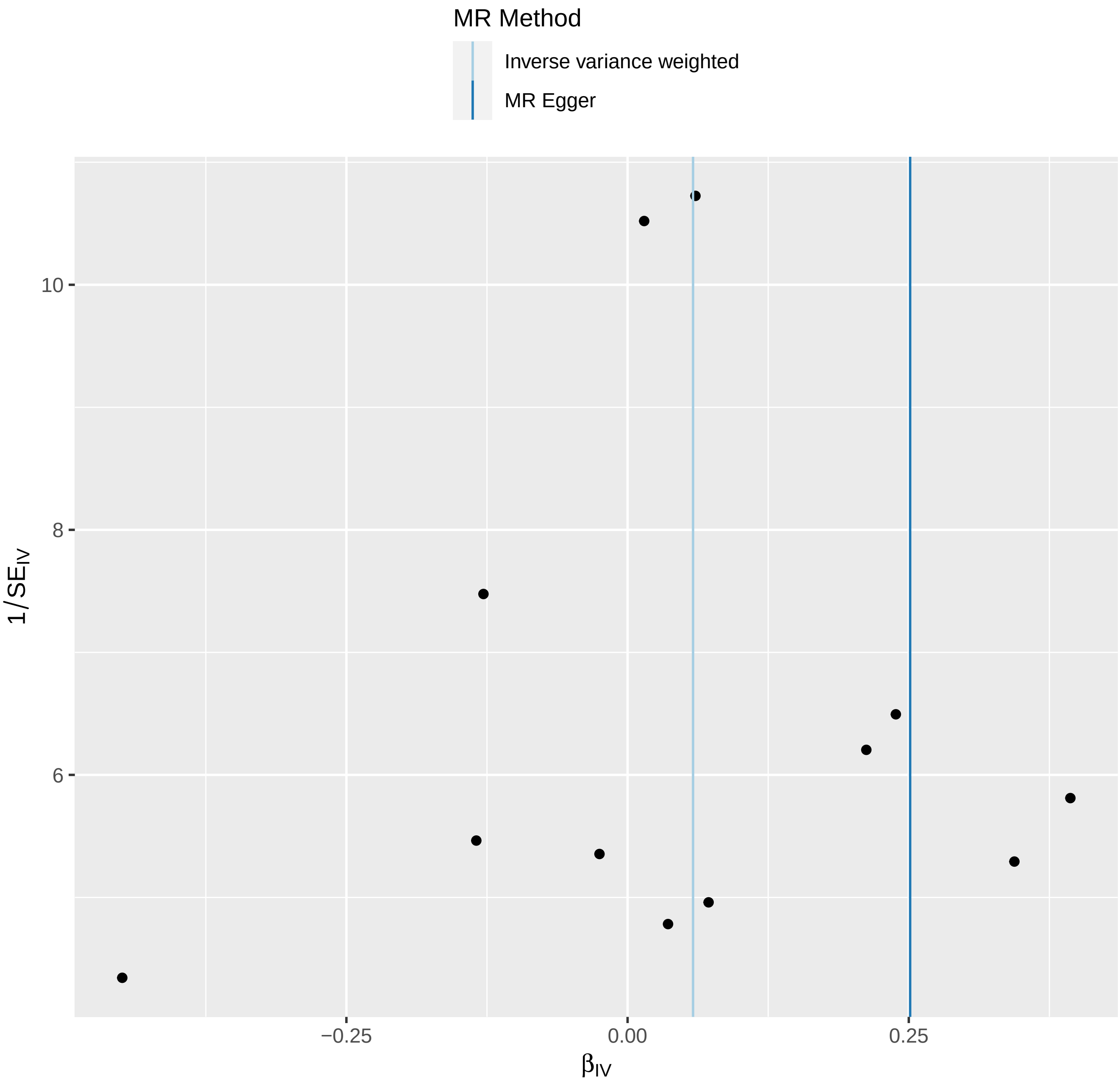

Batch 3−1583 : Selenium on Integrin alpha−L

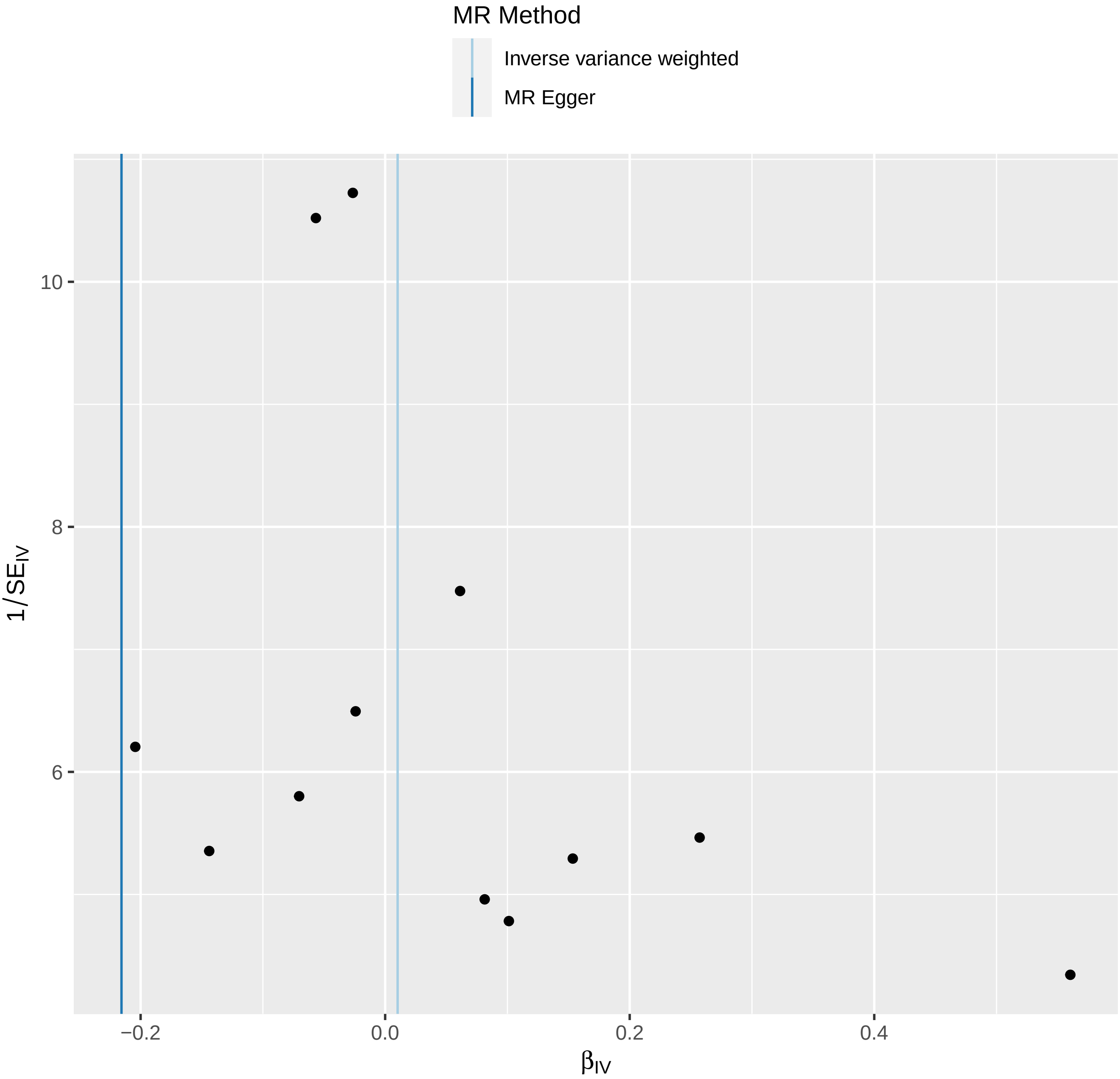

Batch 3−1584 : Selenium on Integrin alpha−V: beta−5 complex

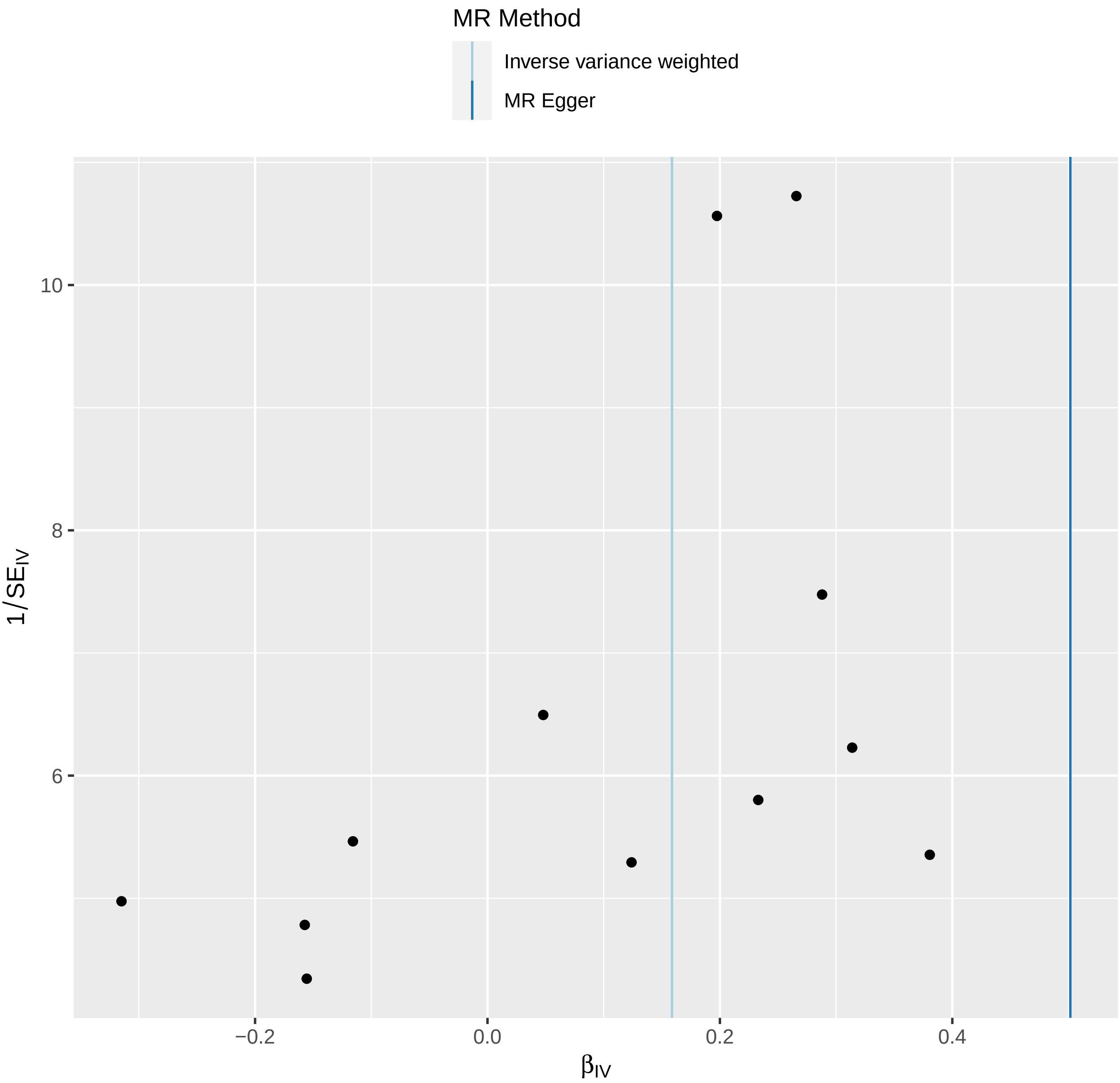

Batch 3−1585 : Selenium on Integrin beta−7

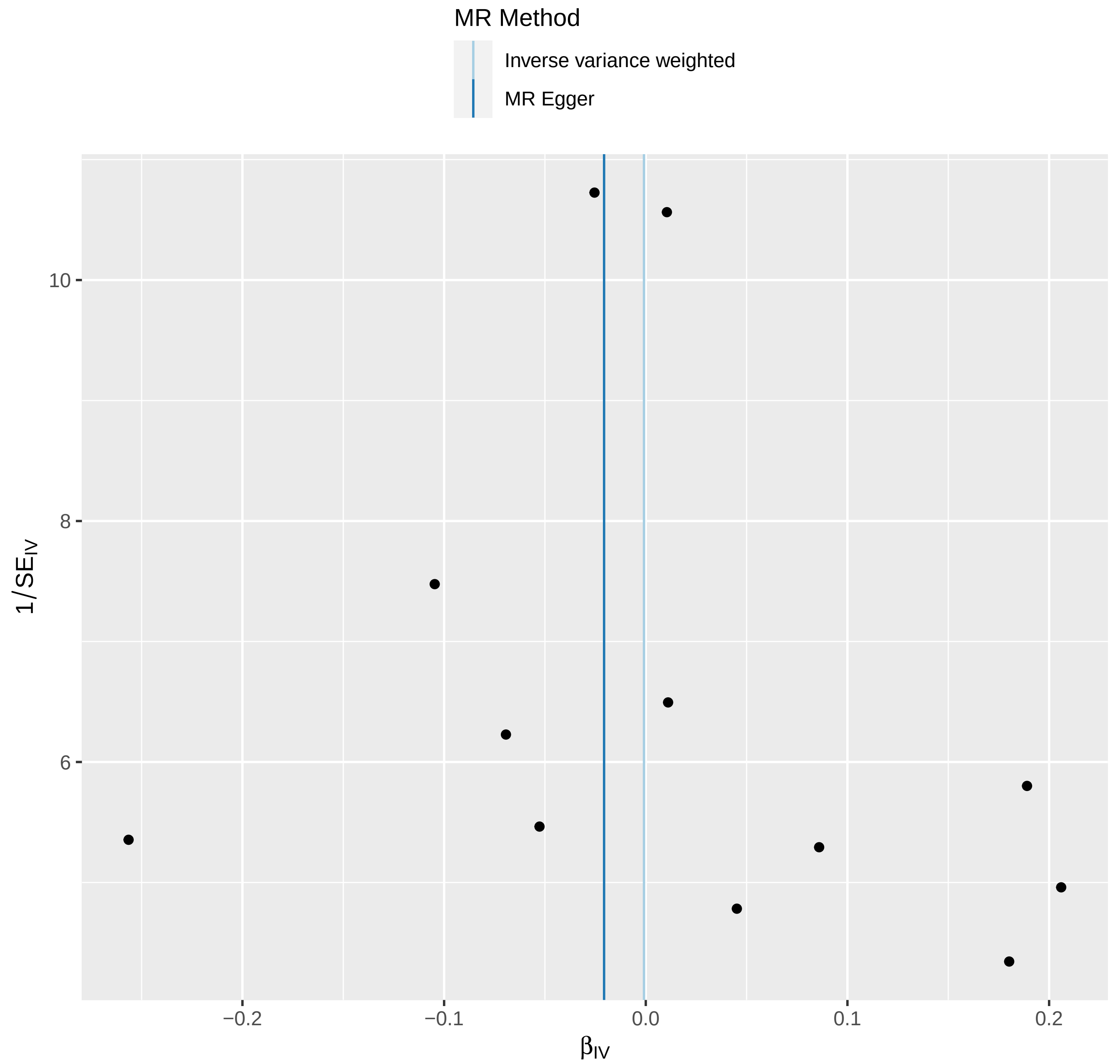

Batch 3−1586 : Selenium on Inter−alpha−trypsin inhibitor heavy chain H1

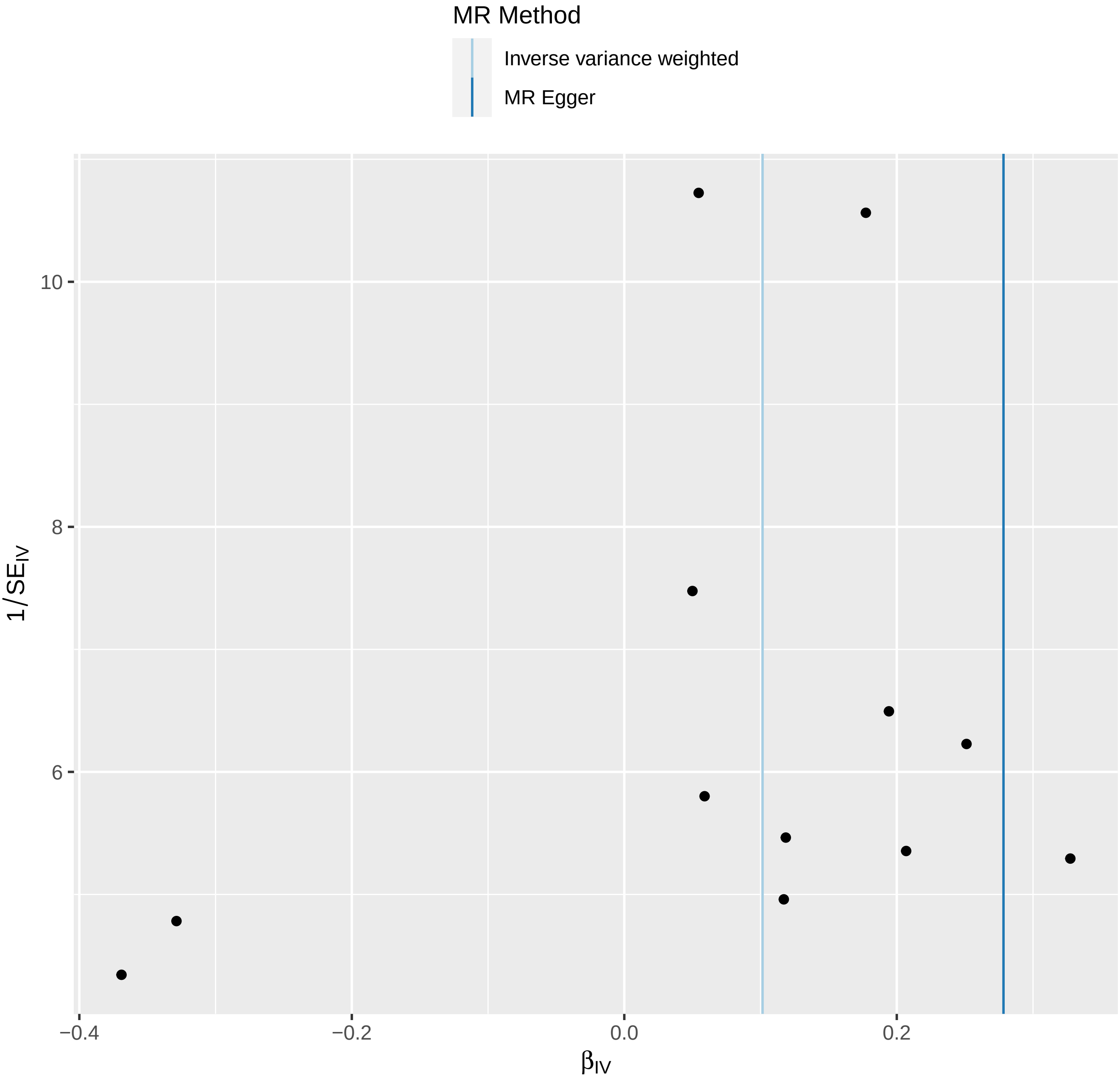

Batch 3−1587 : Selenium on Inter−alpha−trypsin inhibitor heavy chain H5

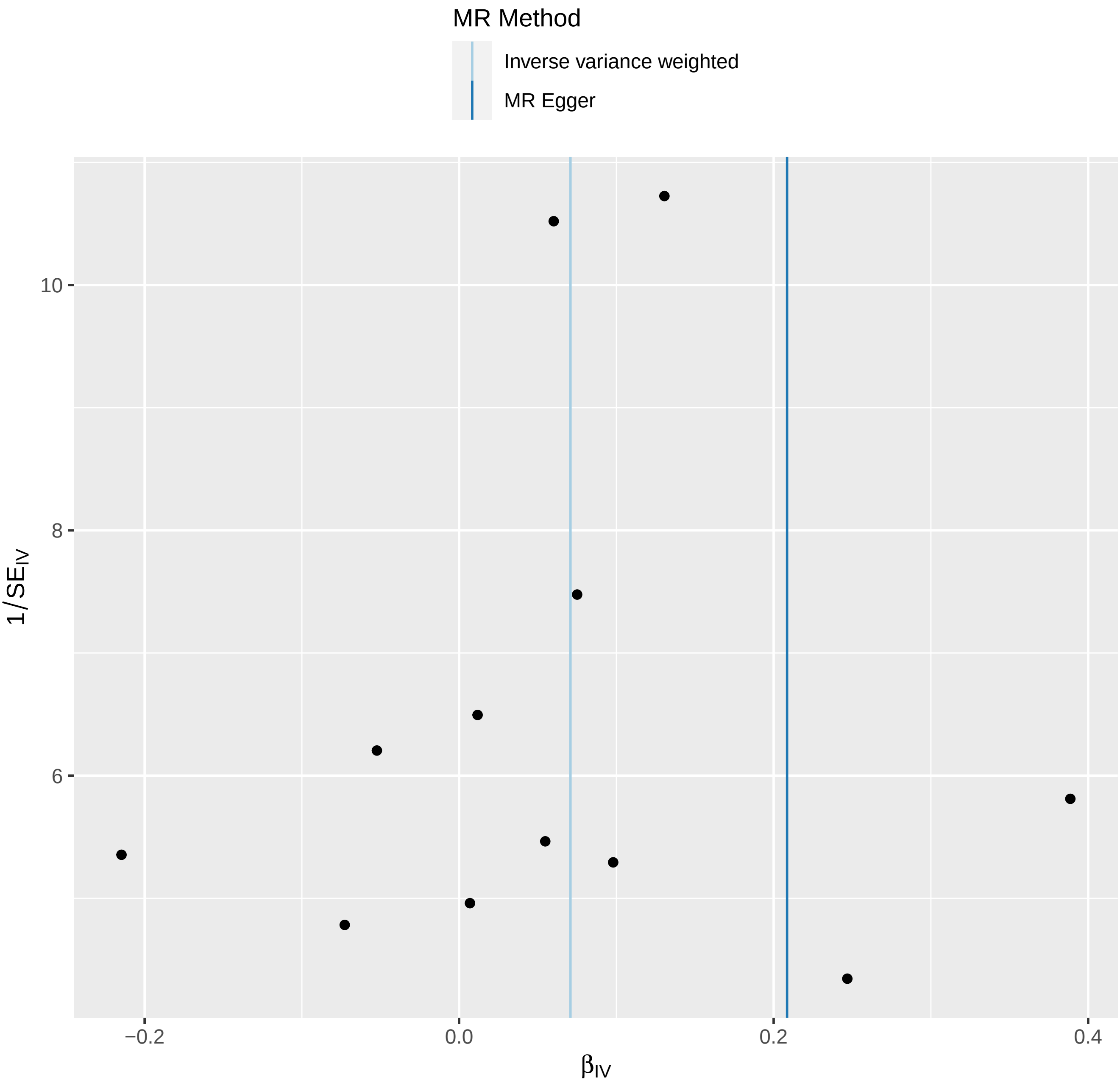

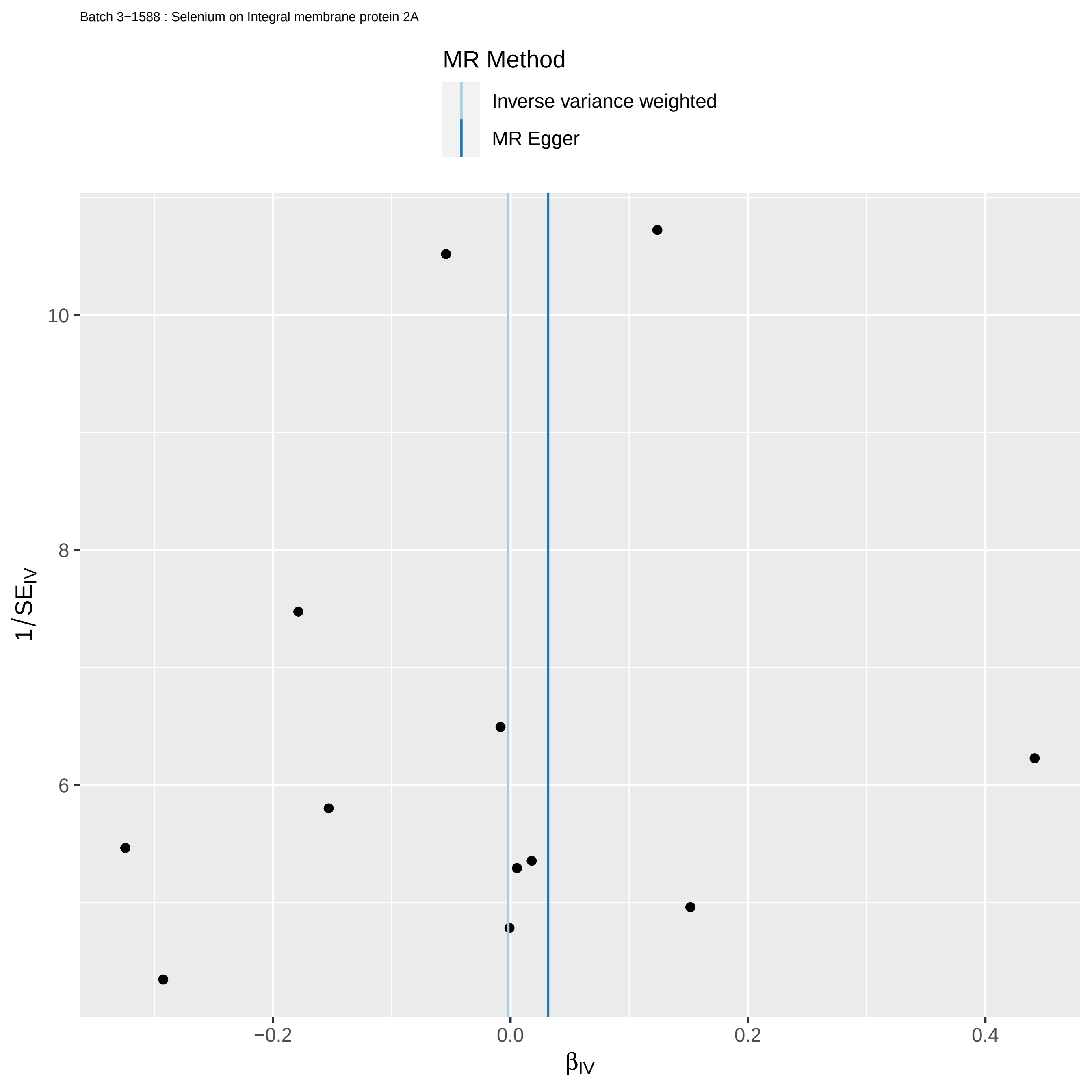
Batch 3−1588 : Selenium on Integral membrane protein 2A
MR Method
Inverse variance weighted
MR Egger
10
8
6
1/SE_IV
−0.2
0.0
0.2
0.4
β_IV

Batch 3−1589 : Selenium on Integral membrane protein 2A

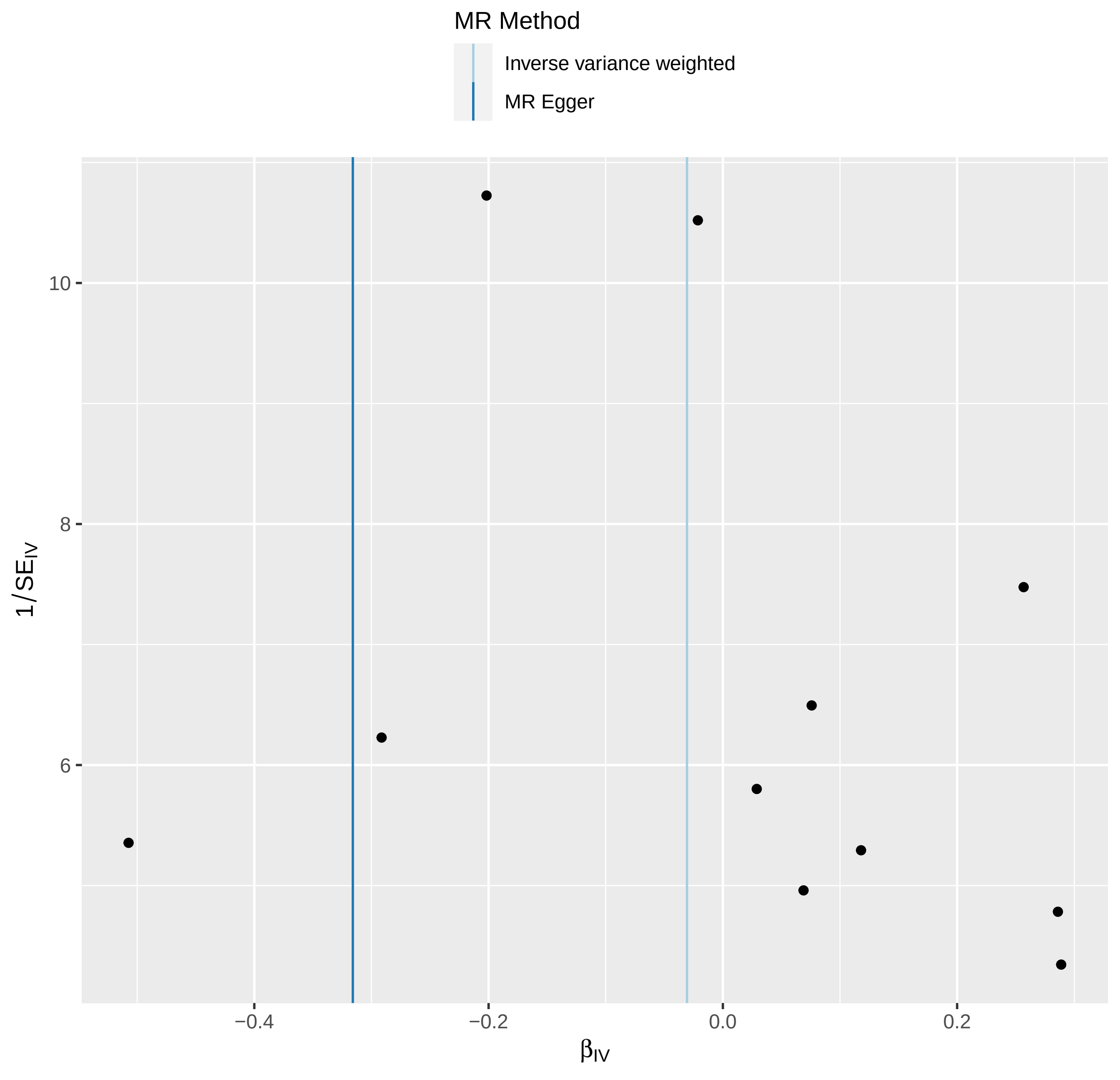

Batch 3−1590 : Selenium on Integral membrane protein 2B

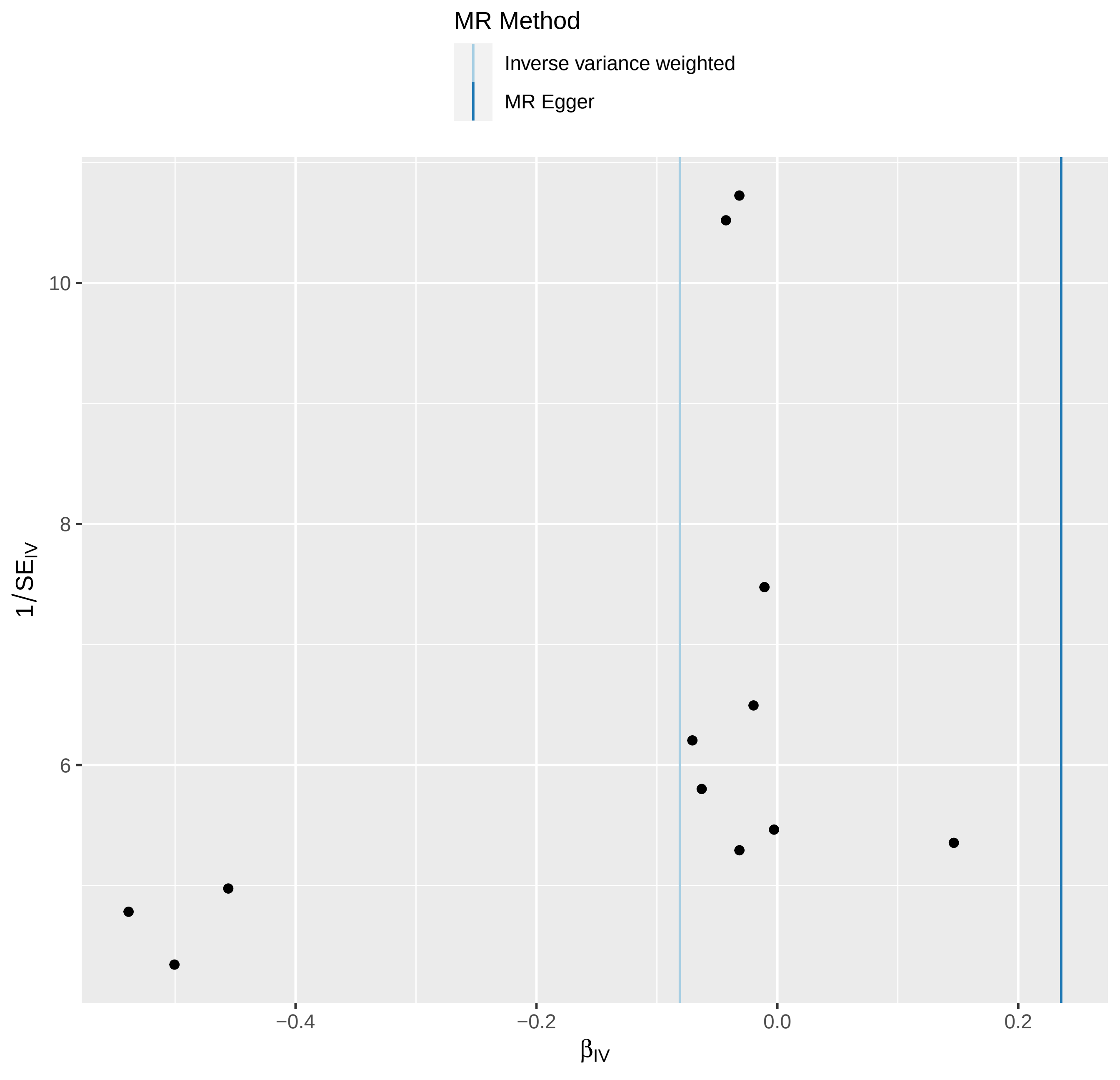

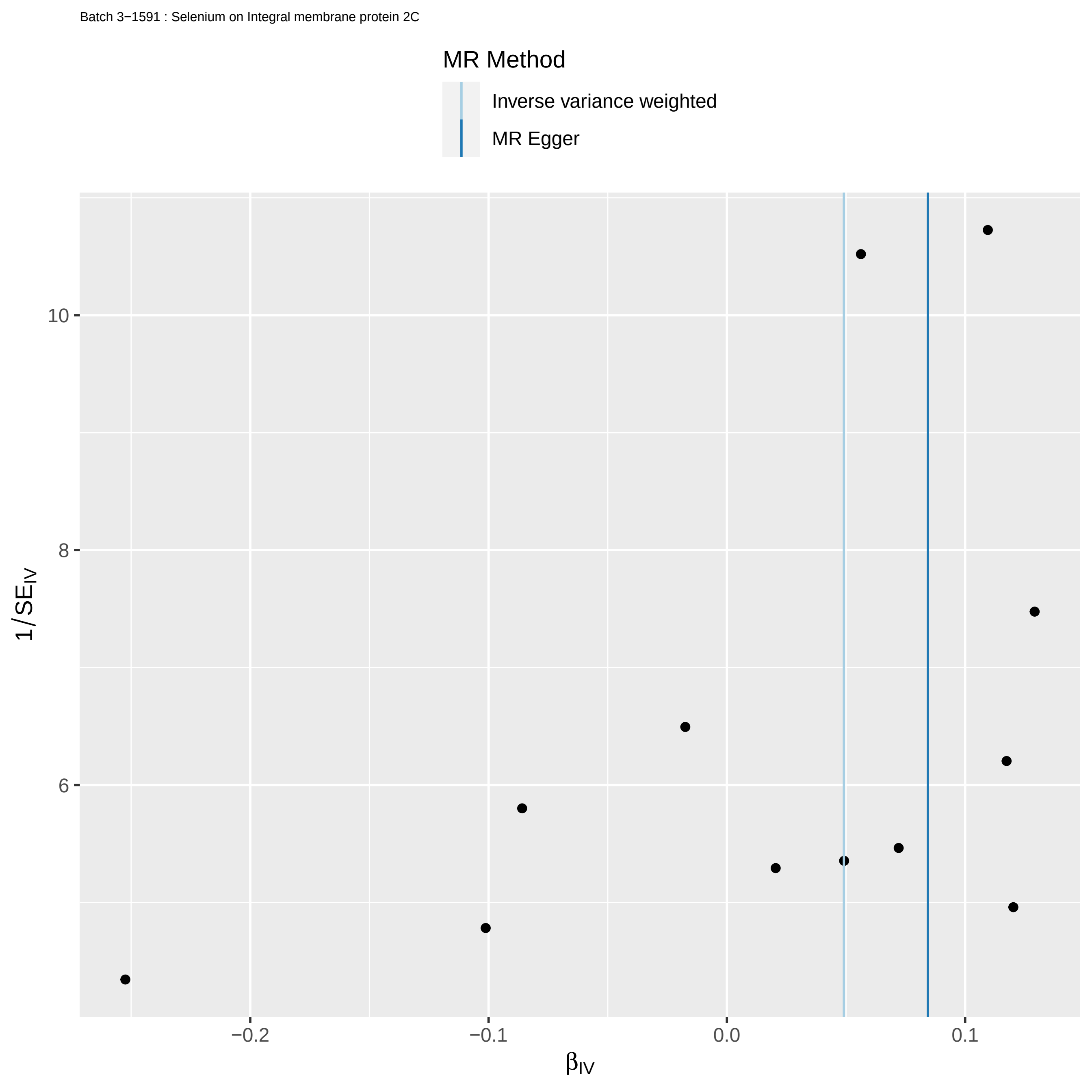

Batch 3−1591 : Selenium on Integral membrane protein 2C
MR Method
Inverse variance weighted
MR Egger
10
8
6
1/SE_IV
−0.2
−0.1
0.0
0.1
β_IV

Batch 3−1592 : Selenium on Integral membrane protein 2C

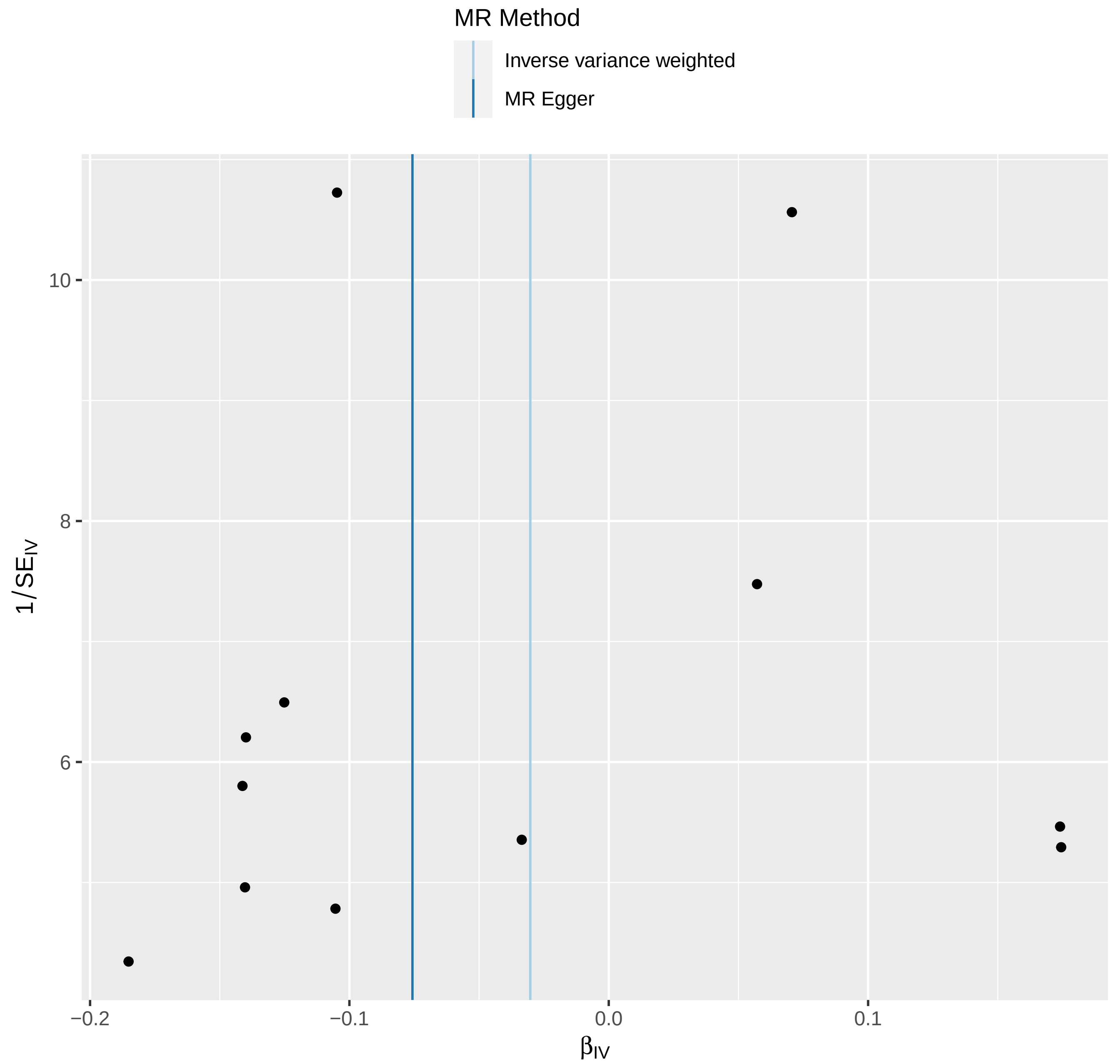

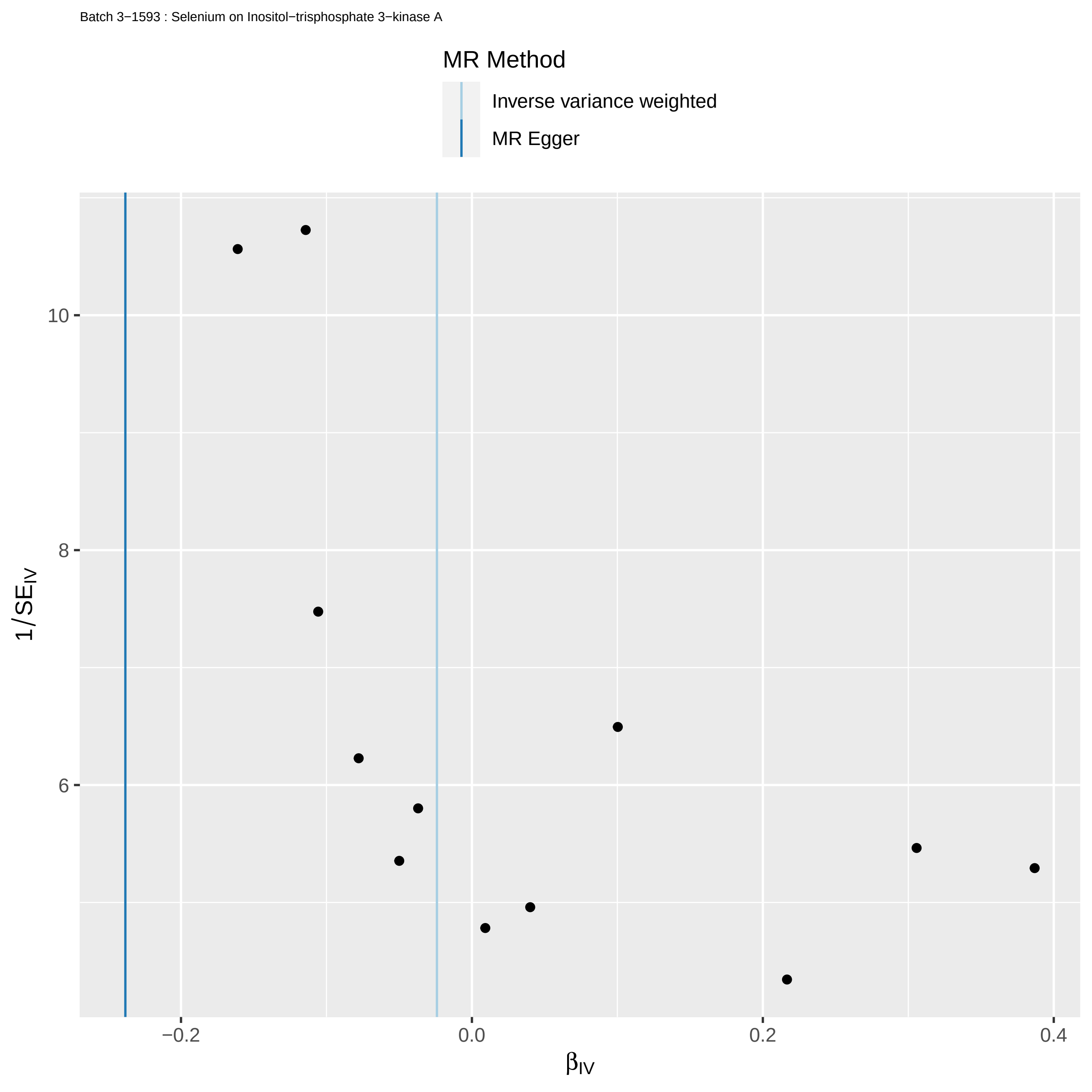

Batch 3−1593 : Selenium on Inositol−trisphosphate 3−kinase A
MR Method
Inverse variance weighted
MR Egger
10
8
6
1/SE$_{IV}$
−0.2
0.0
0.2
0.4
β$_{IV}$

Batch 3−1594 : Selenium on Inositol 1,4,5−trisphosphate receptor−interacting protein−like 1

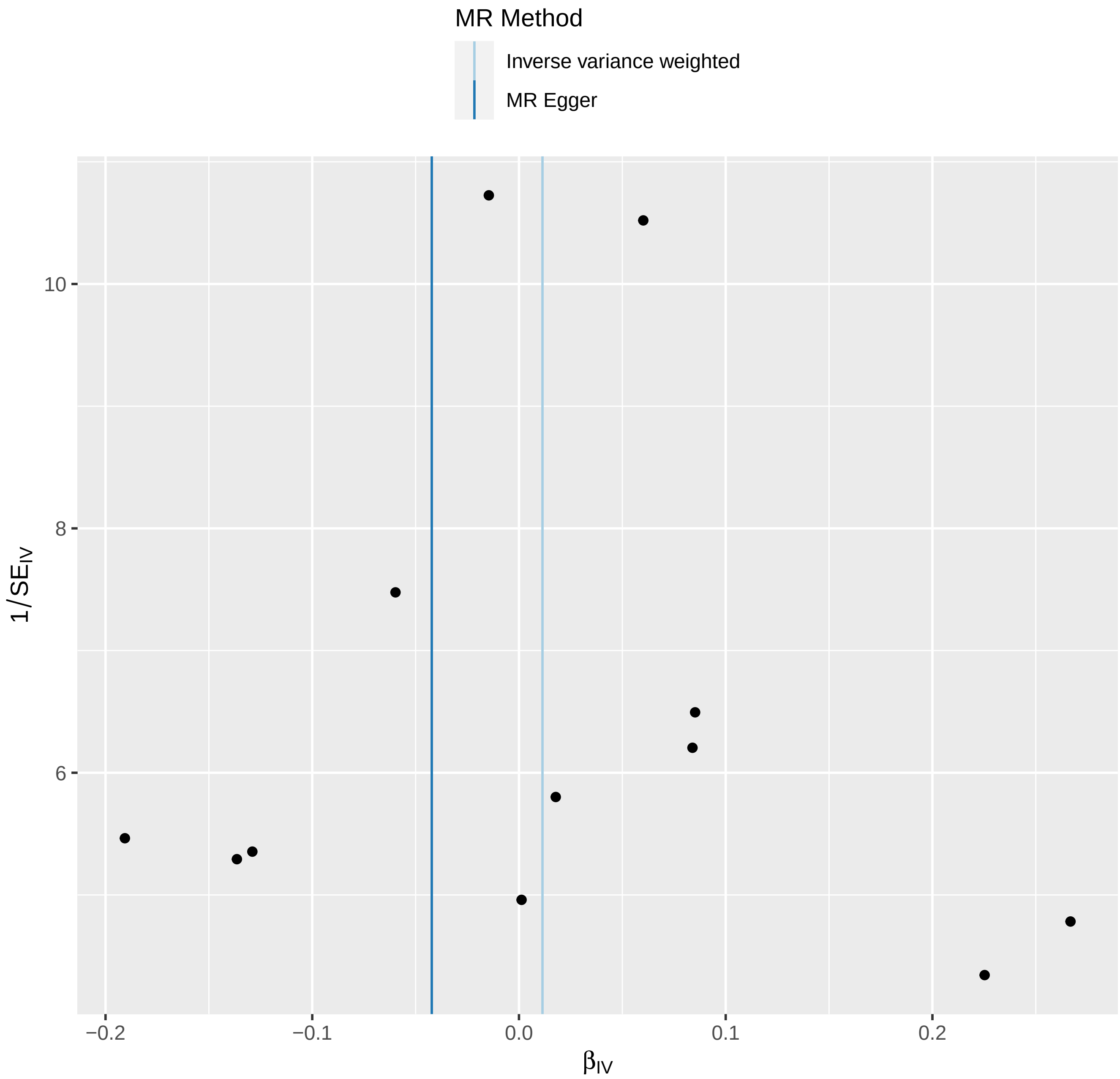

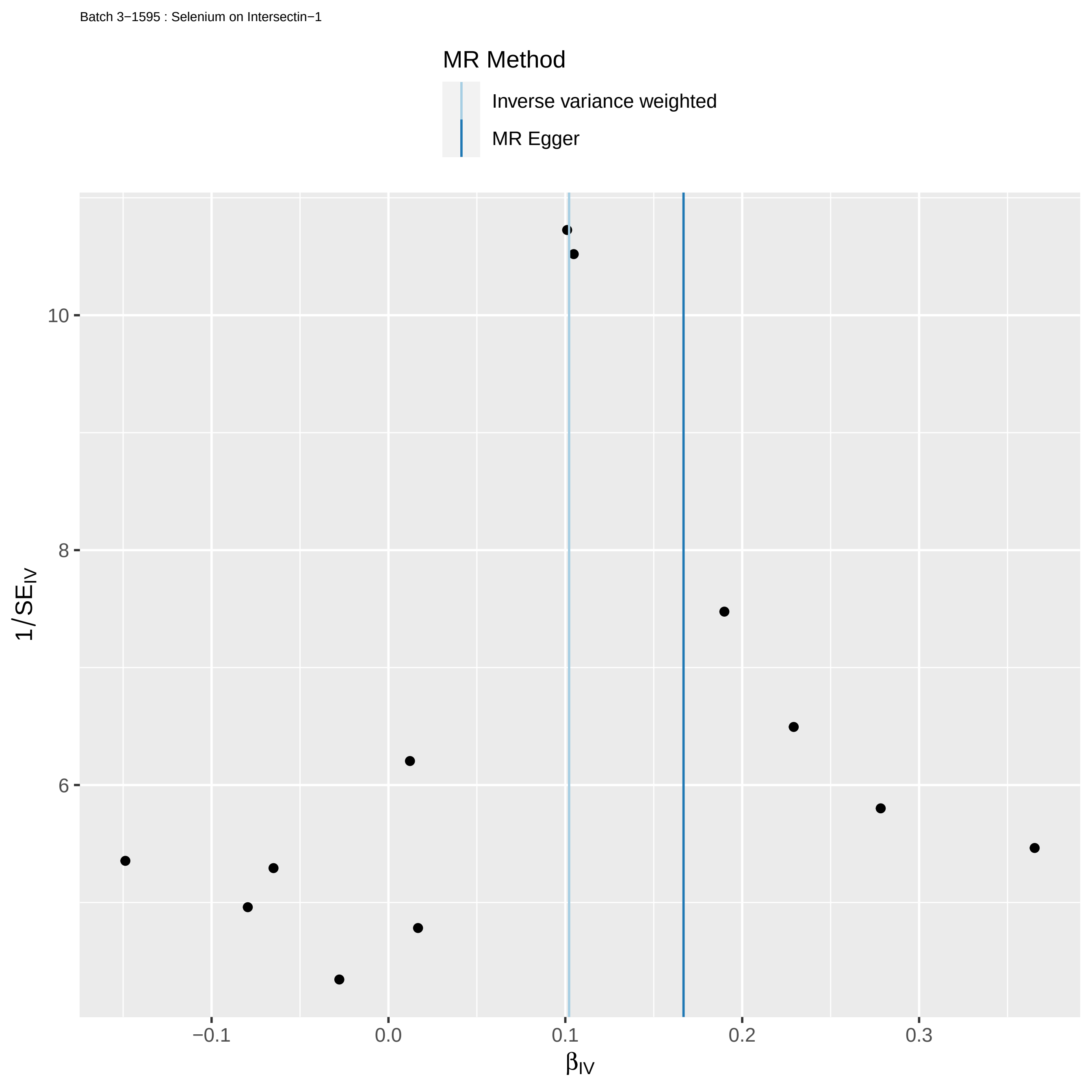
Batch 3−1595 : Selenium on Intersectin−1
MR Method
Inverse variance weighted
MR Egger
10
8
6
1/SE_IV
−0.1
0.0
0.1
0.2
0.3
β_IV

Batch 3−1596 : Selenium on Protein jagged−1

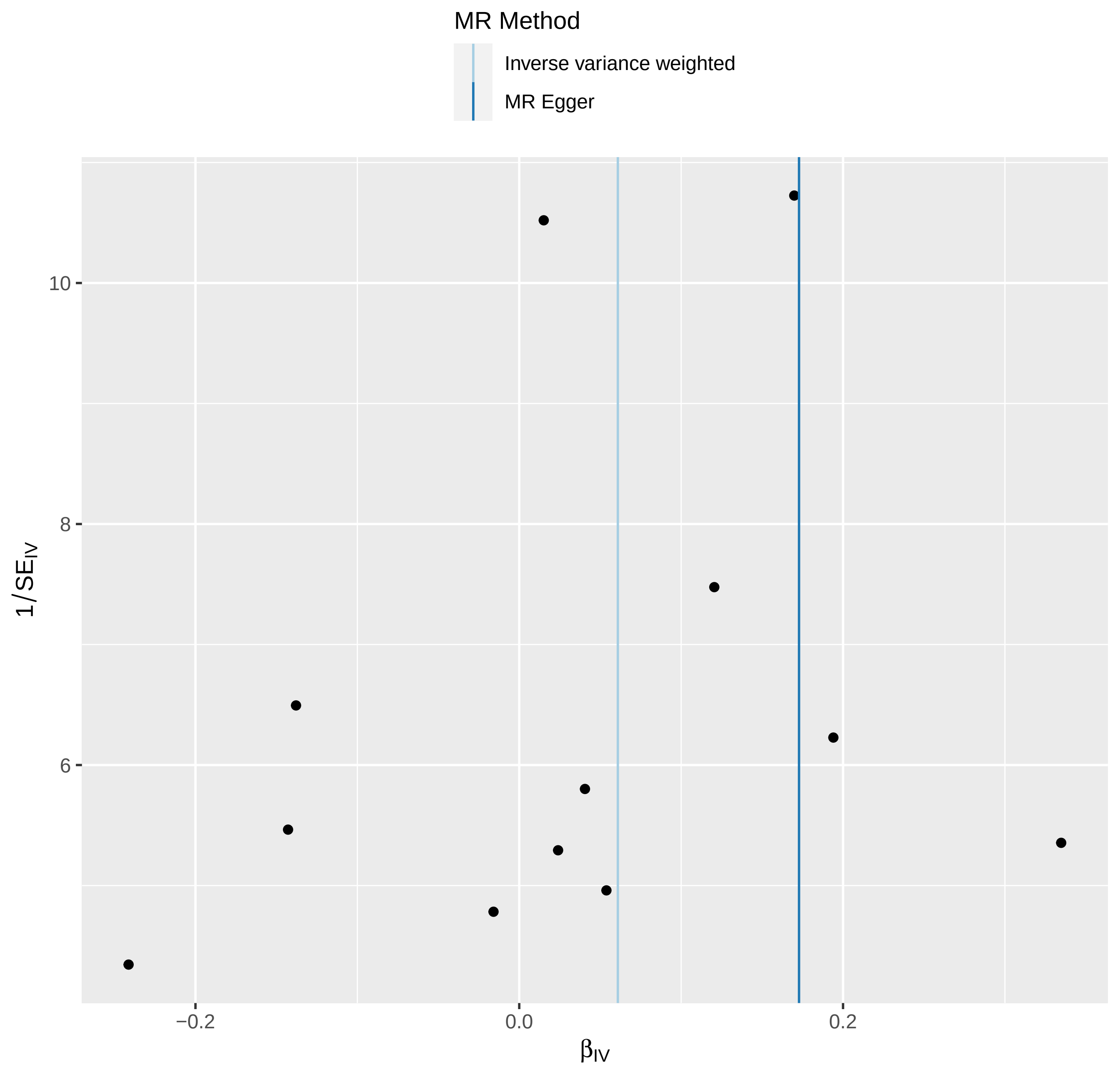

Batch 3−1597 : Selenium on Protein jagged−2

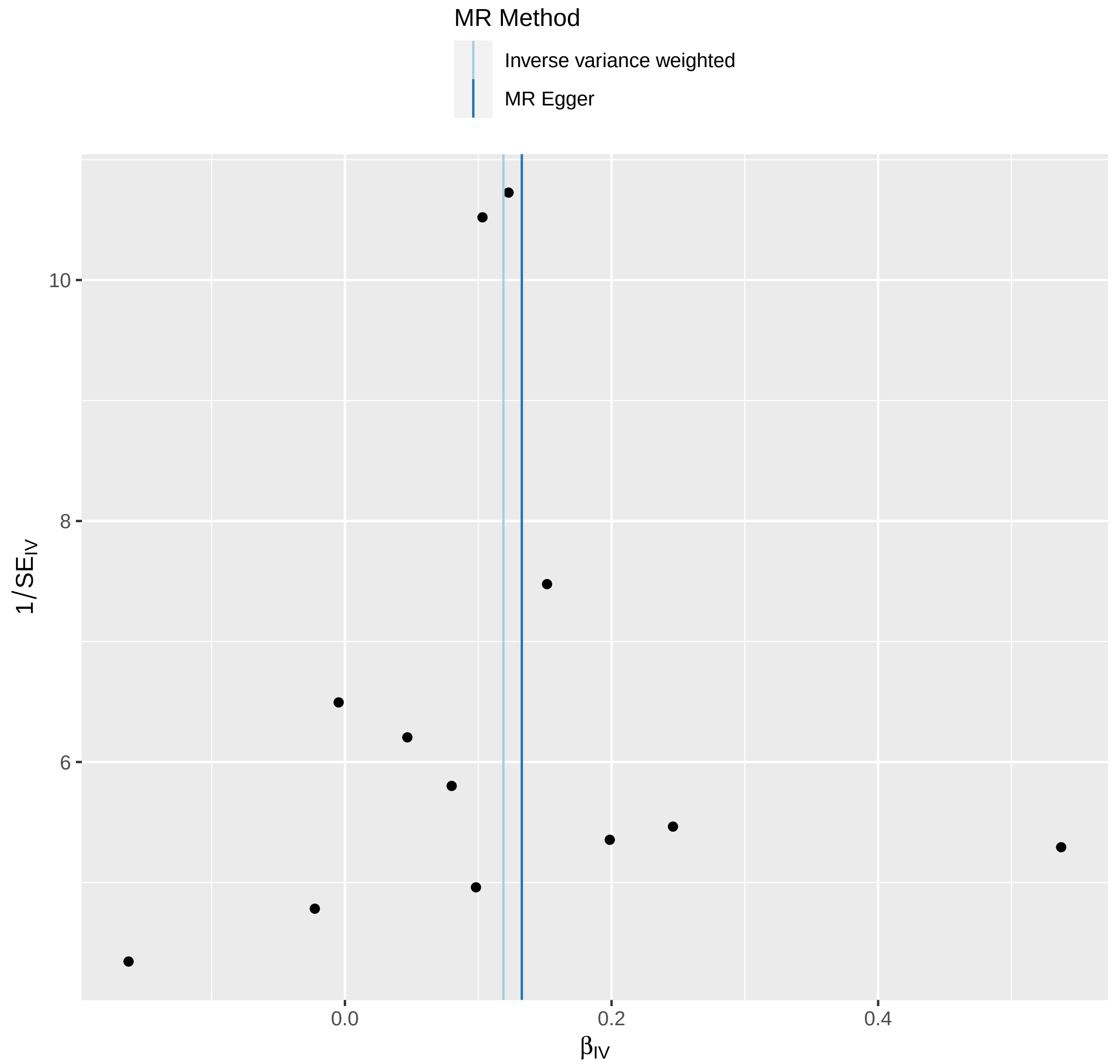

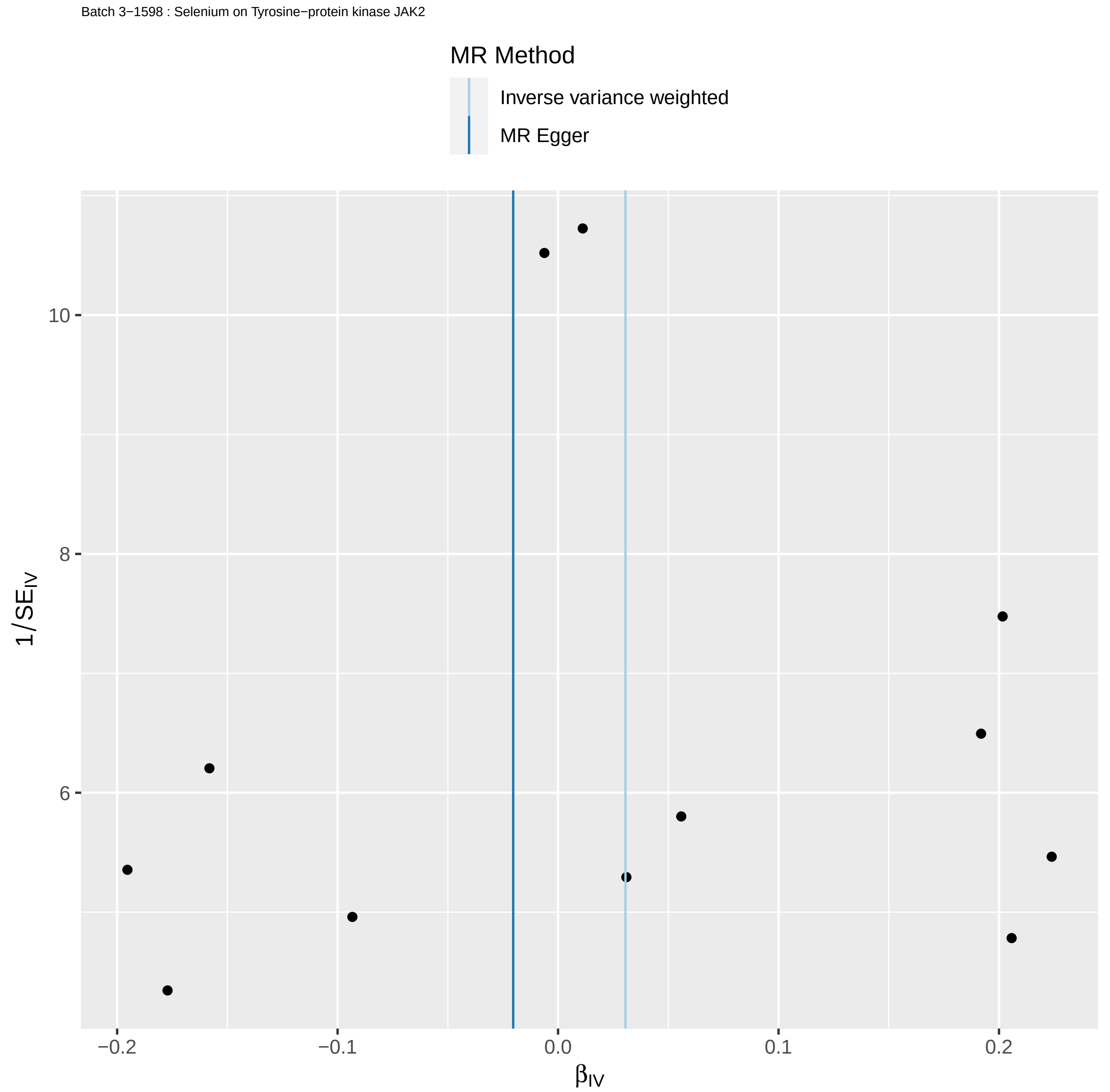
Batch 3−1598 : Selenium on Tyrosine−protein kinase JAK2
MR Method
Inverse variance weighted
MR Egger
10
8
6
1/SE_IV
−0.2
−0.1
0.0
0.1
0.2
β_IV

Batch 3−1599 : Selenium on Janus kinase and microtubule−interacting protein 3

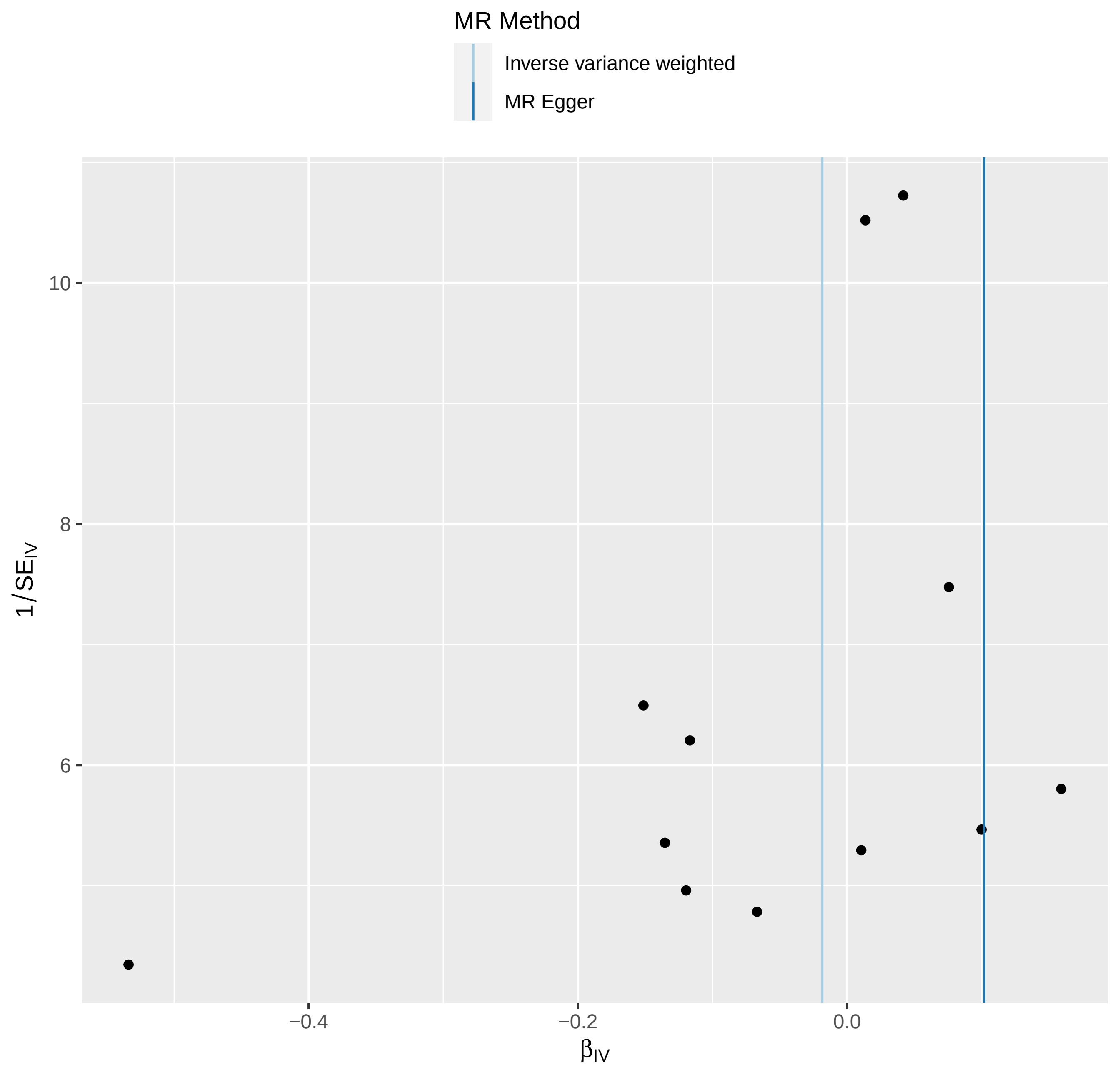

Batch 3−1600 : Selenium on Junctional adhesion molecule B

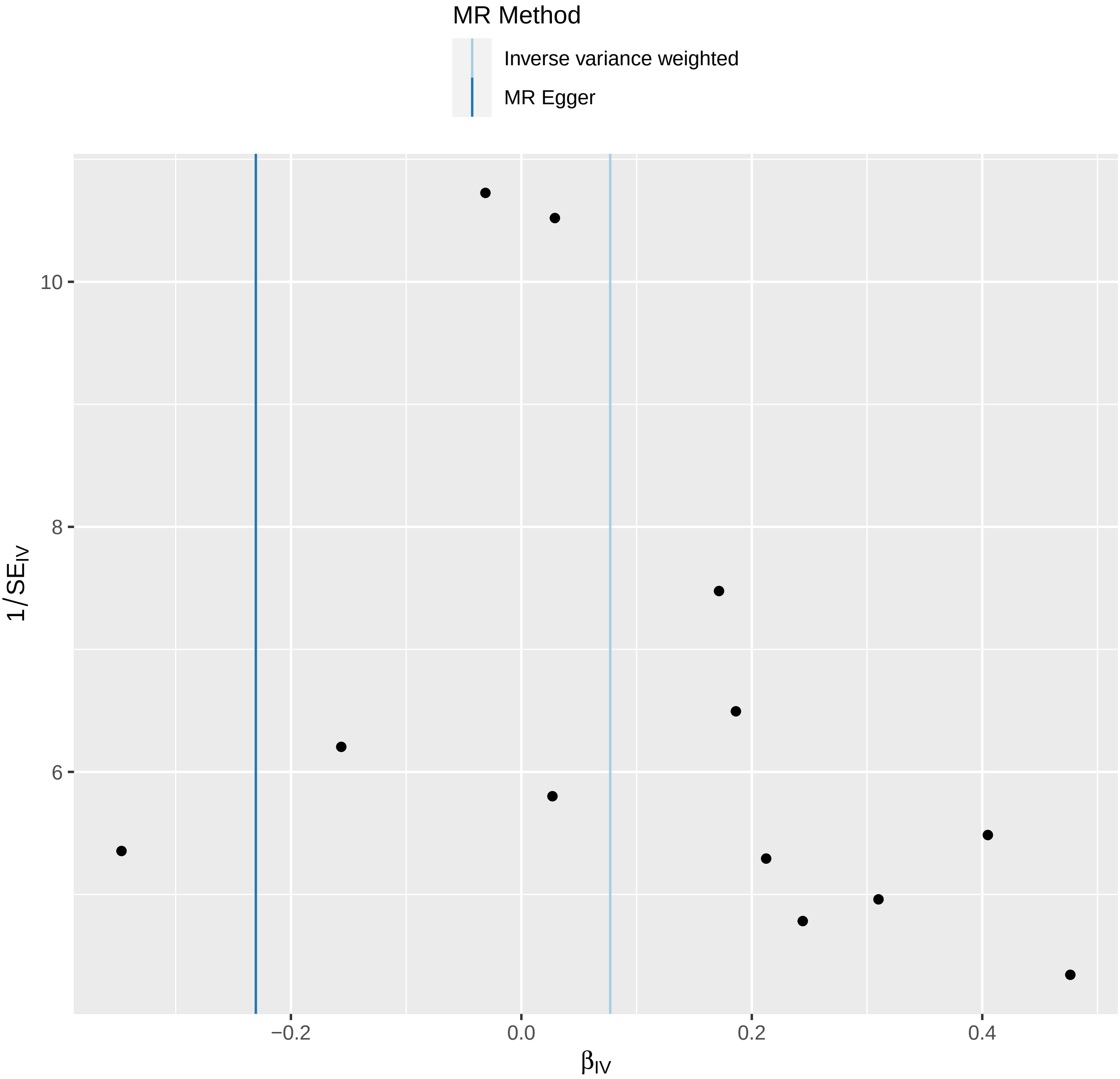

Batch 3−1601 : Selenium on Junctional adhesion molecule C

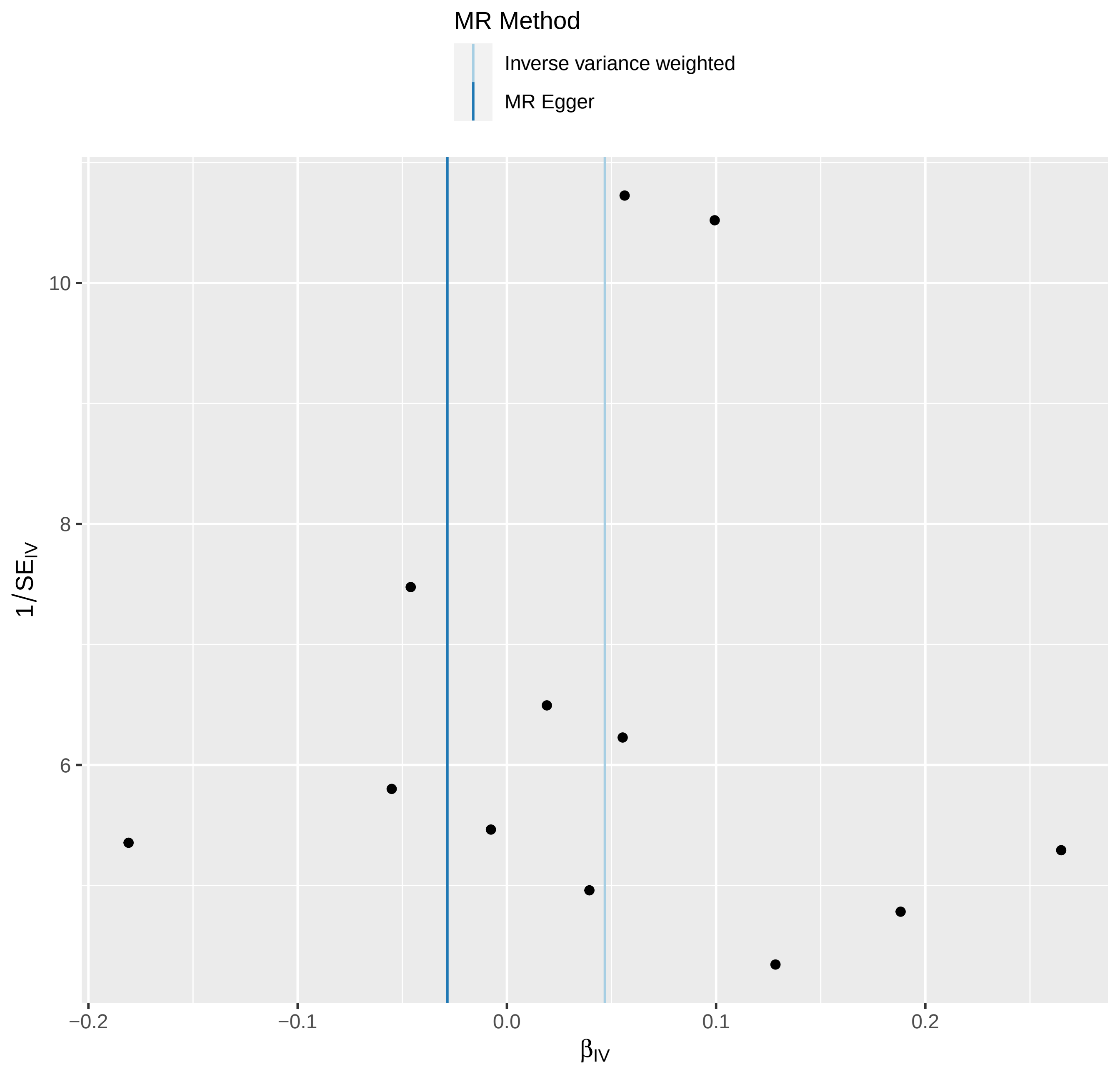

Batch 3−1602 : Selenium on Junctophilin−1

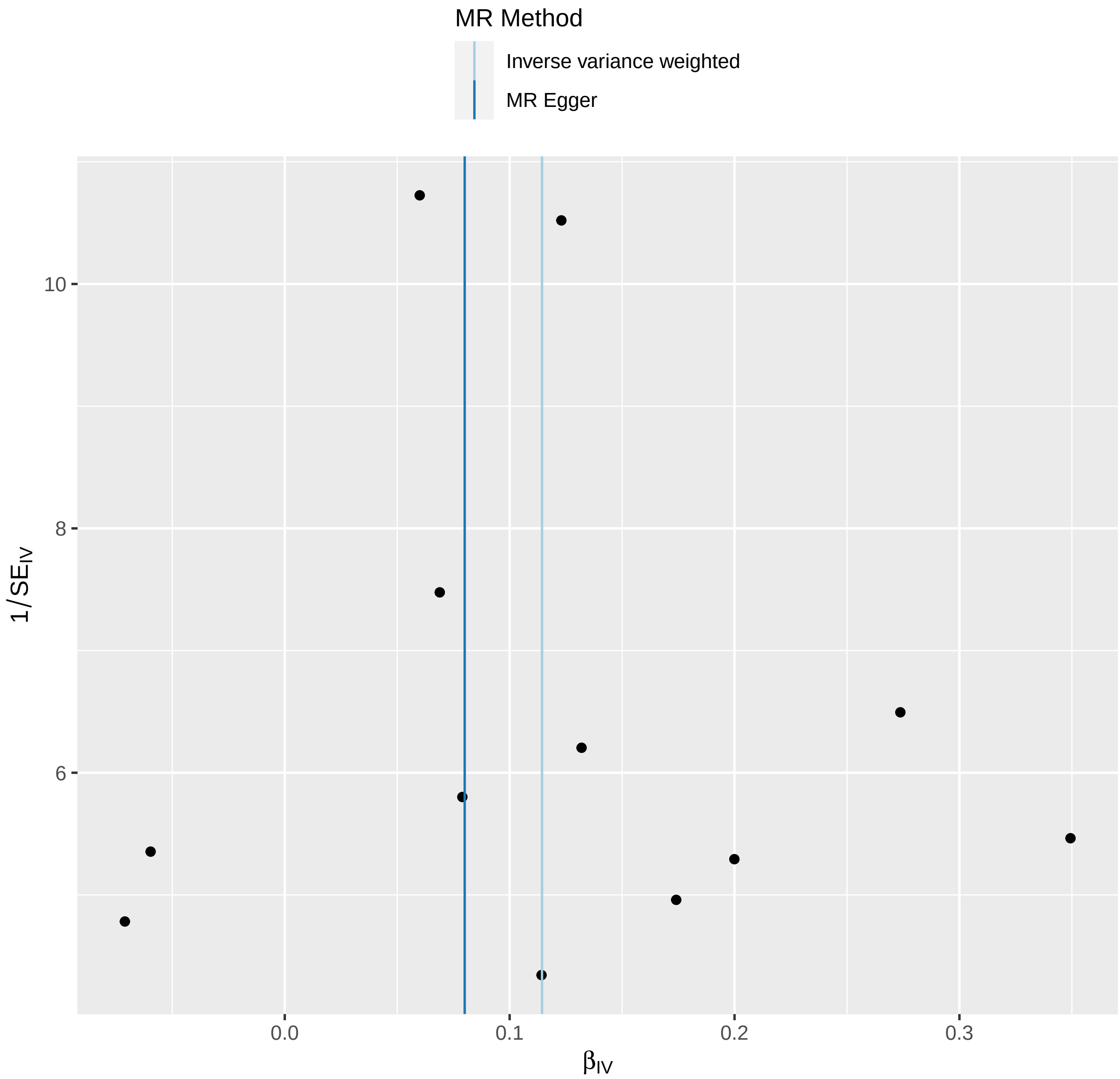

Batch 3−1603 : Selenium on Junctophilin−3

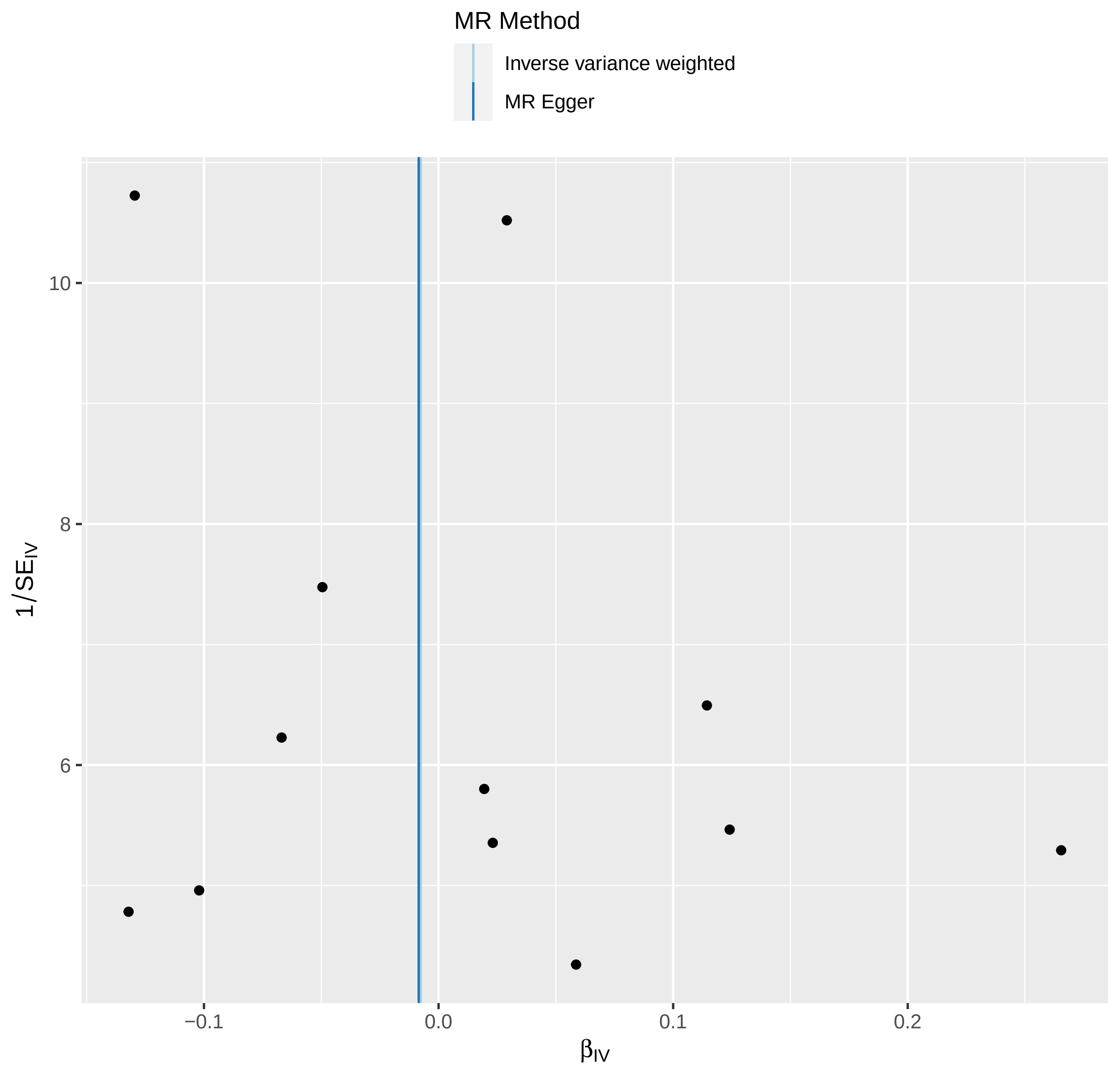

Batch 3−1604 : Selenium on Protein JTB

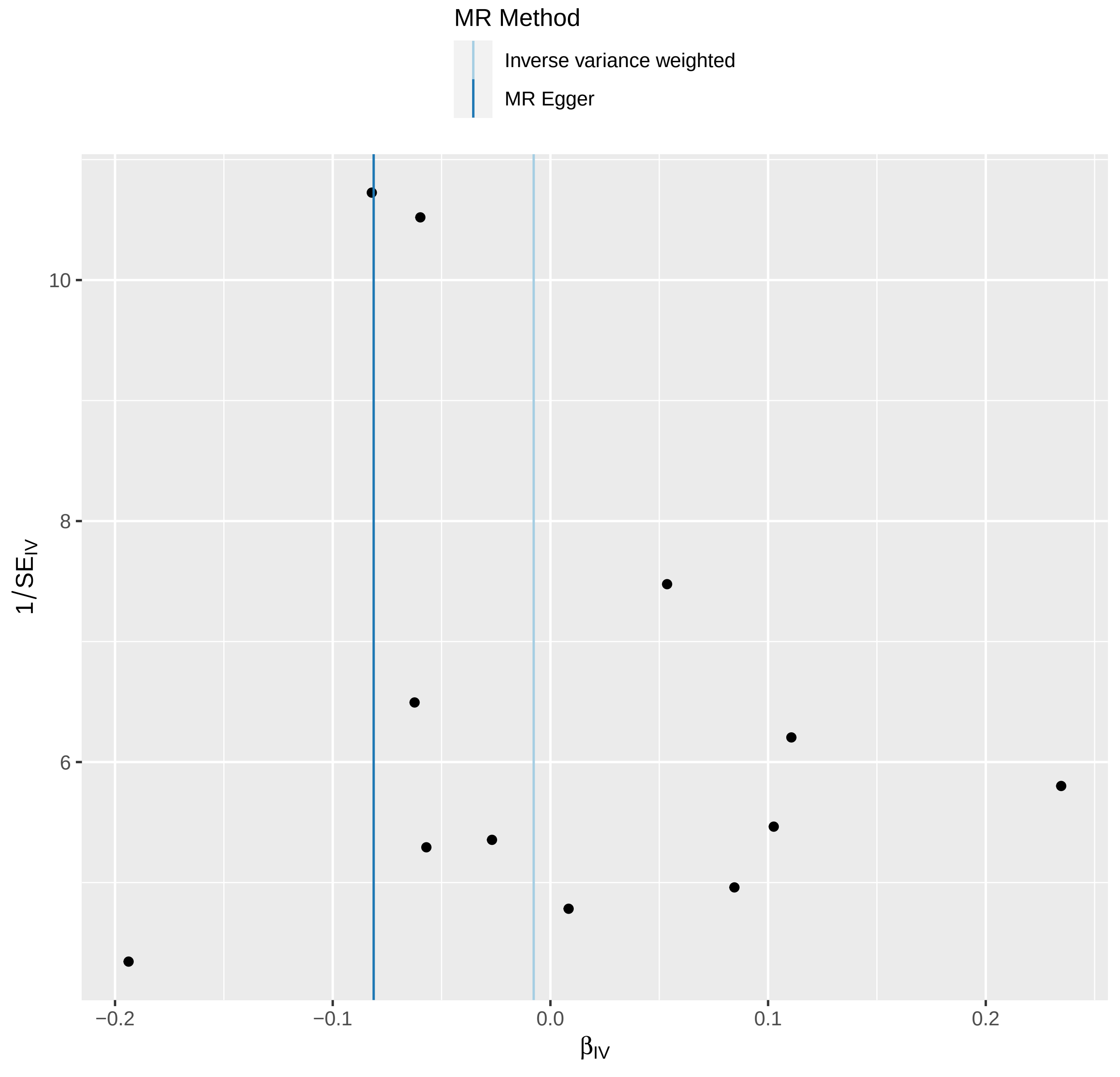

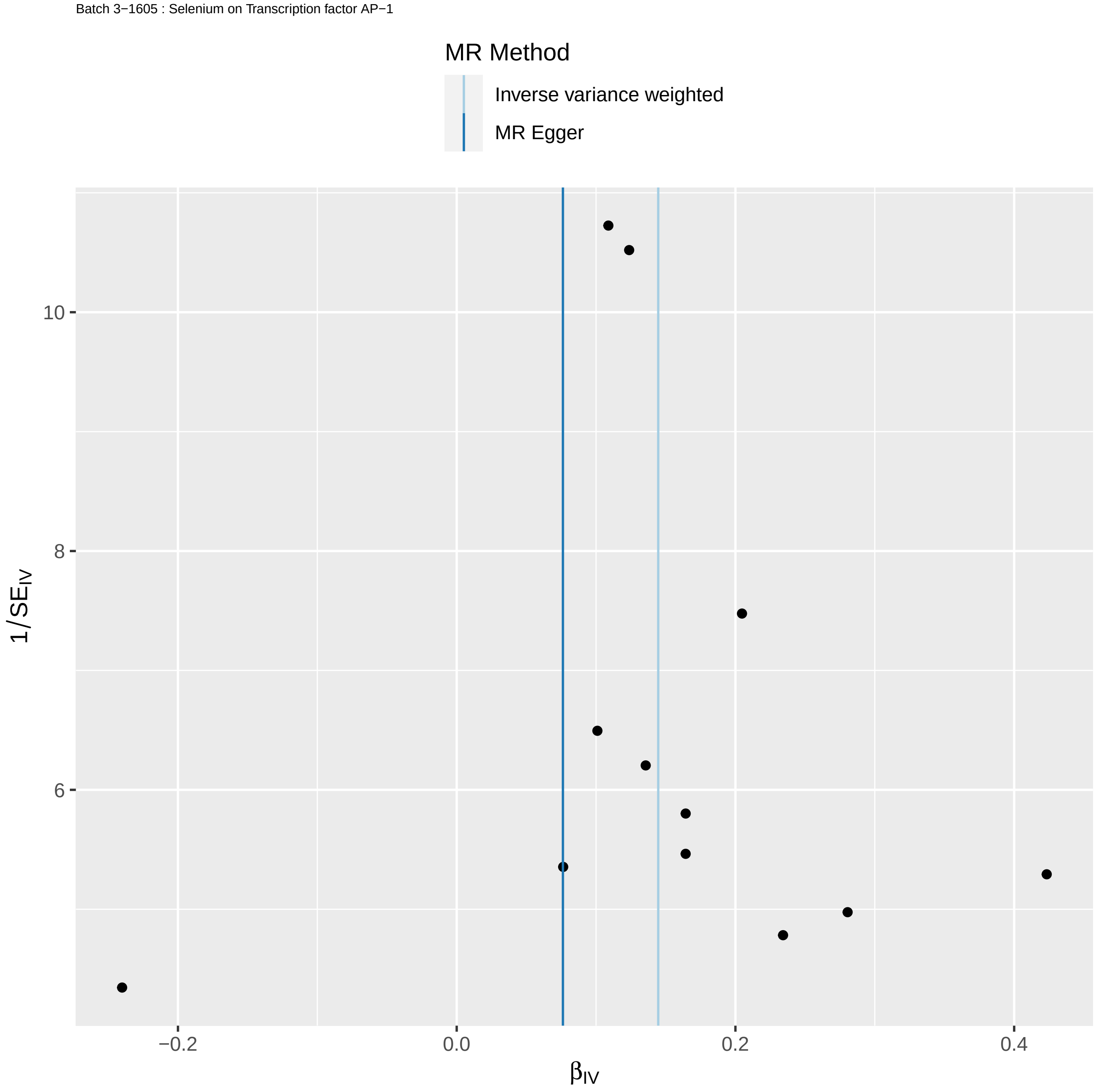
Batch 3−1605 : Selenium on Transcription factor AP−1
MR Method
Inverse variance weighted
MR Egger
10
8
6
1/SE_IV
−0.2
0.0
0.2
0.4
β_IV

Batch 3−1606 : Selenium on Anosmin−1

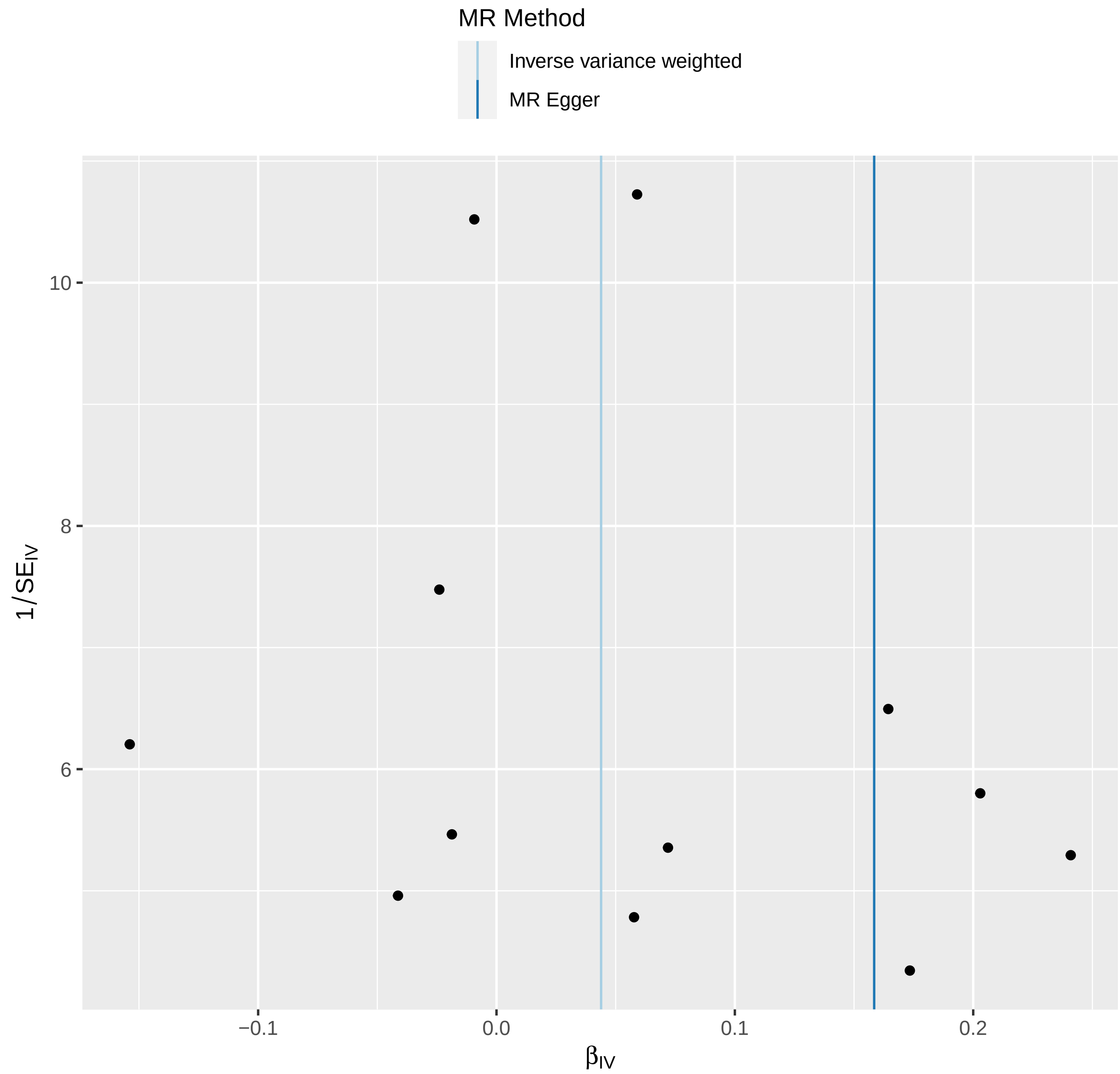

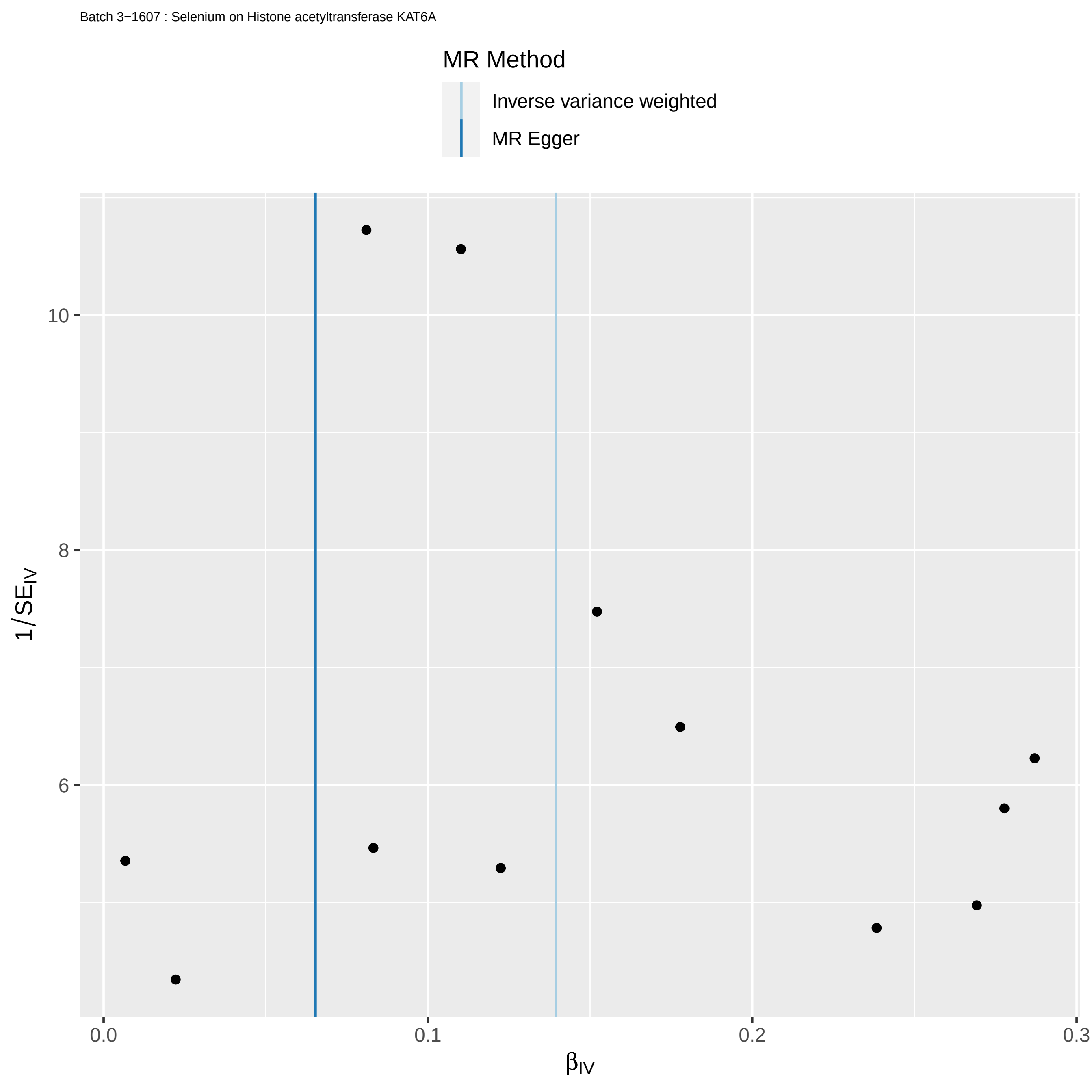

Batch 3−1607 : Selenium on Histone acetyltransferase KAT6A
MR Method
Inverse variance weighted
MR Egger
10
8
6
$1/SE_{IV}$
0.0
0.1
0.2
0.3
β_{IV}

Batch 3−1608 : Selenium on Potassium voltage−gated channel subfamily A member 10

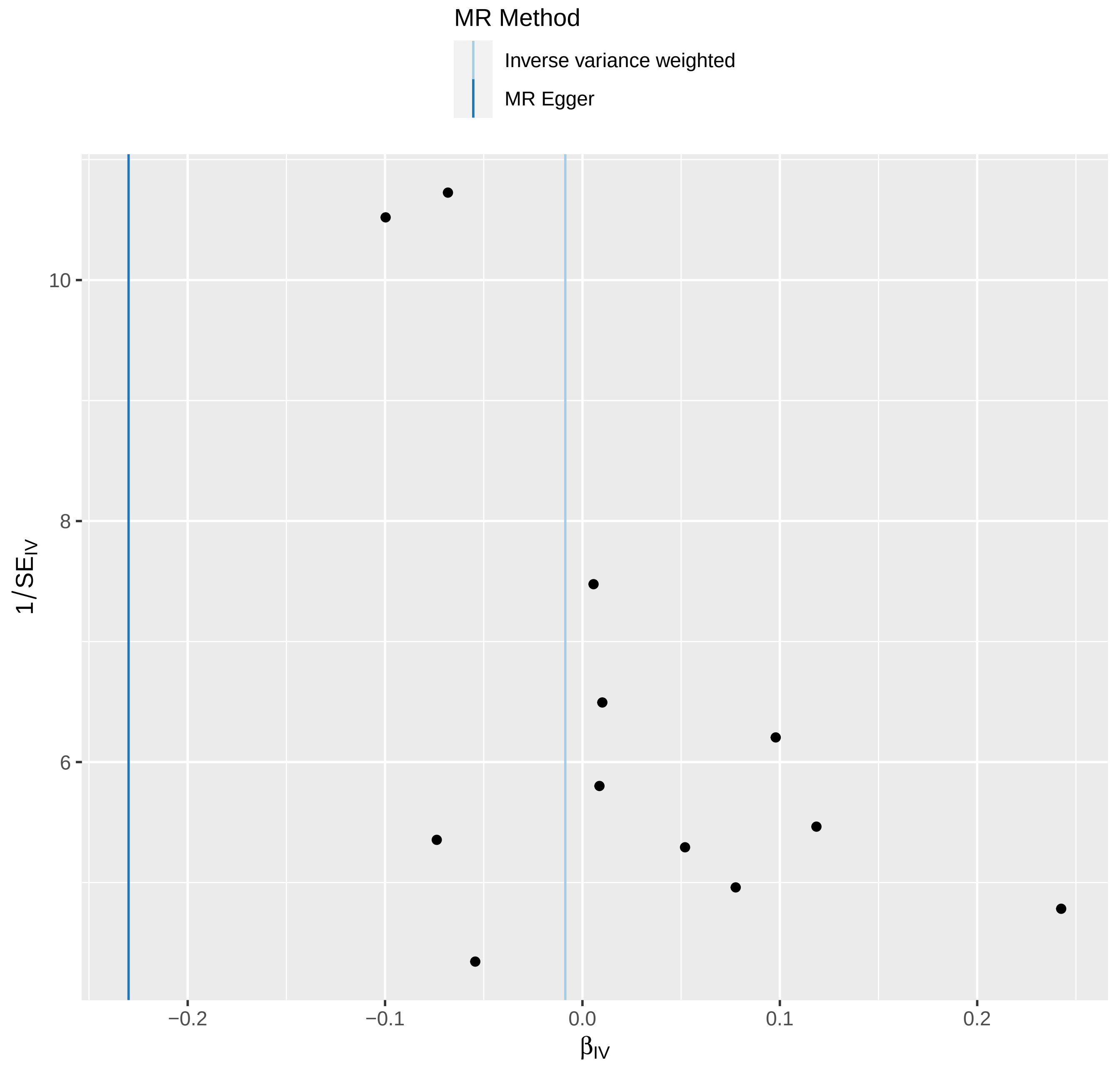

Batch 3−1609 : Selenium on Voltage−gated potassium channel subunit beta−2

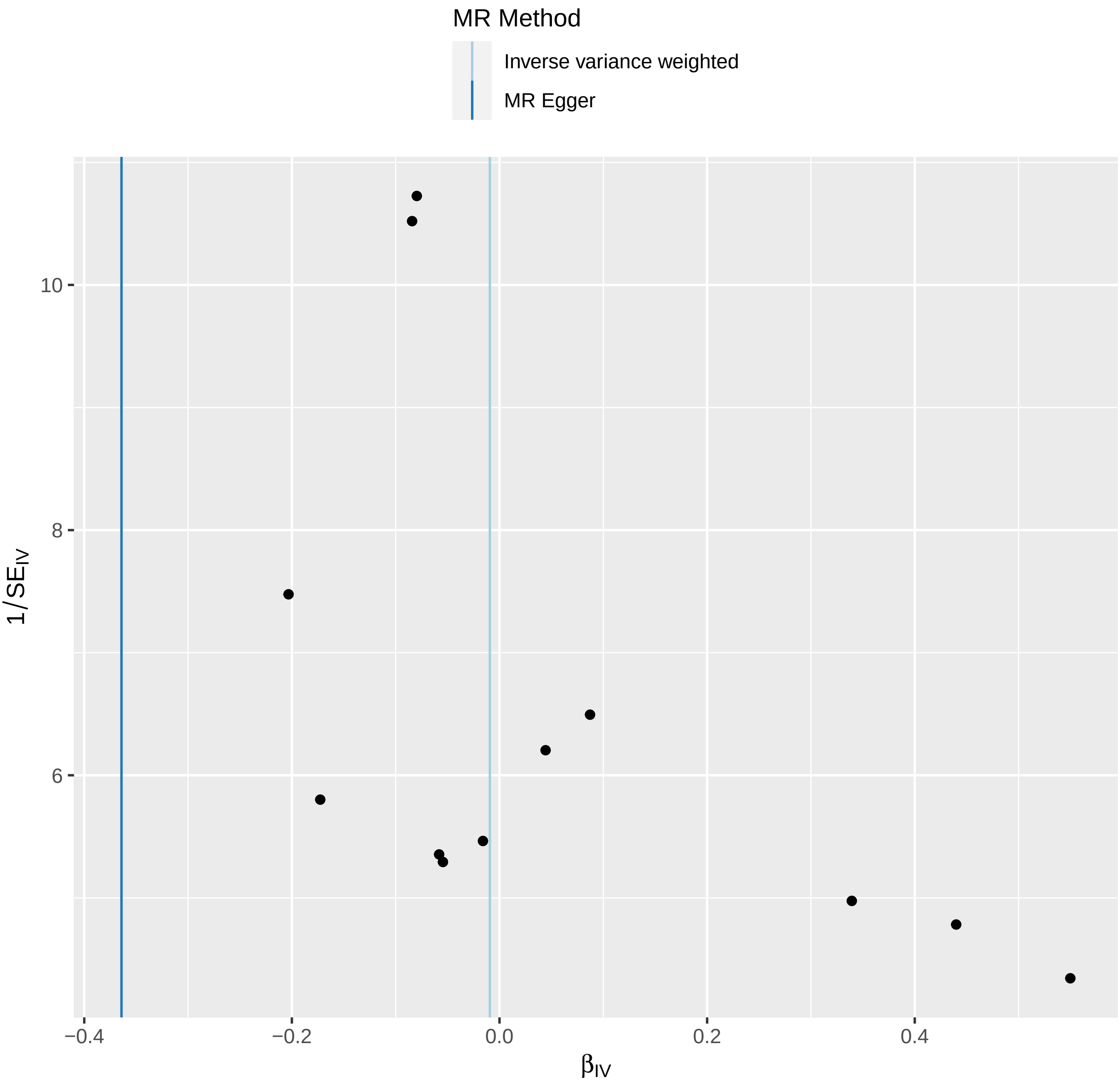

Batch 3−1610 : Selenium on Potassium voltage−gated channel subfamily E member 2

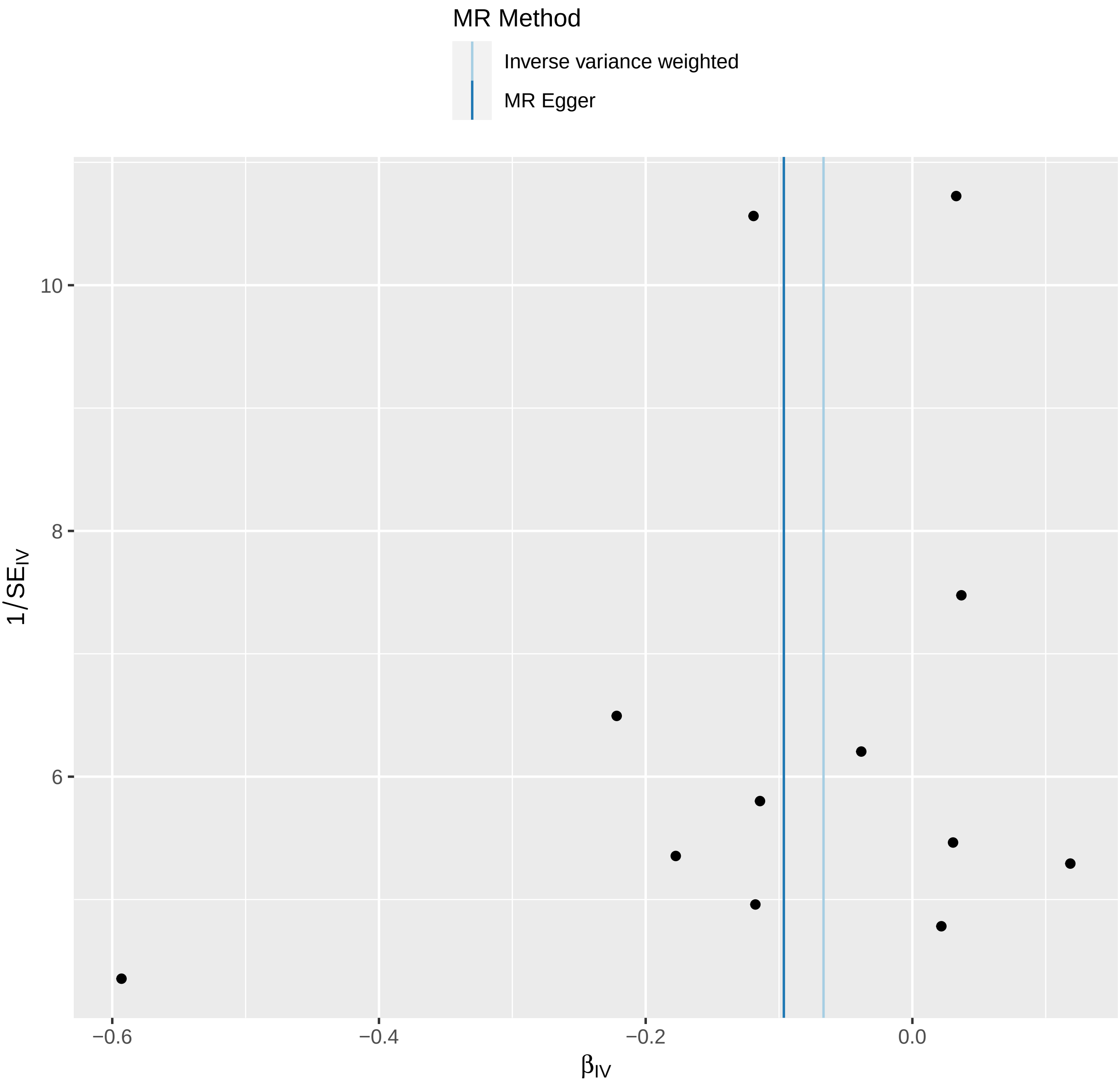

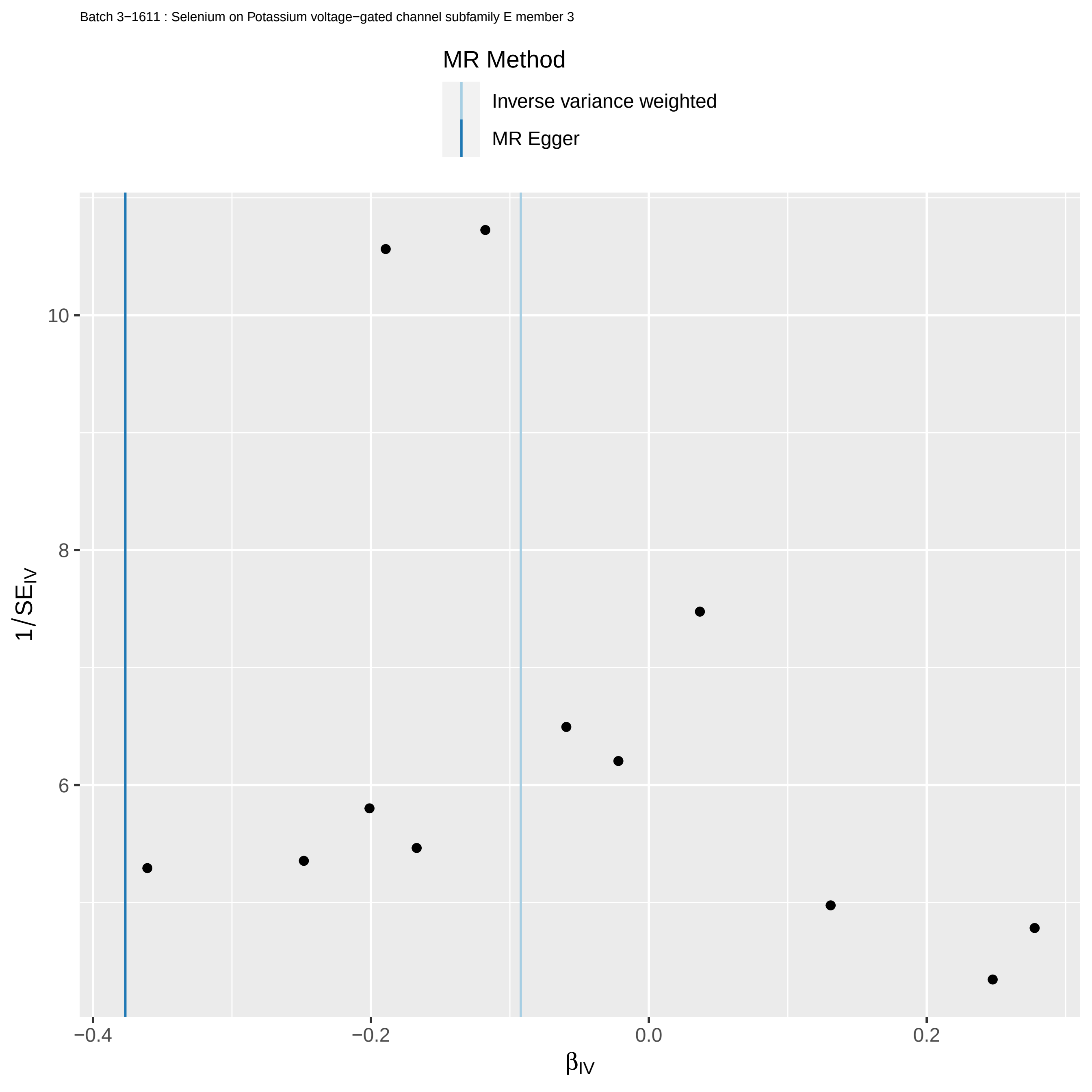

Batch 3−1611 : Selenium on Potassium voltage−gated channel subfamily E member 3
MR Method
Inverse variance weighted
MR Egger
10
8
6
1/SE_IV
−0.4
−0.2
0.0
0.2
β_IV

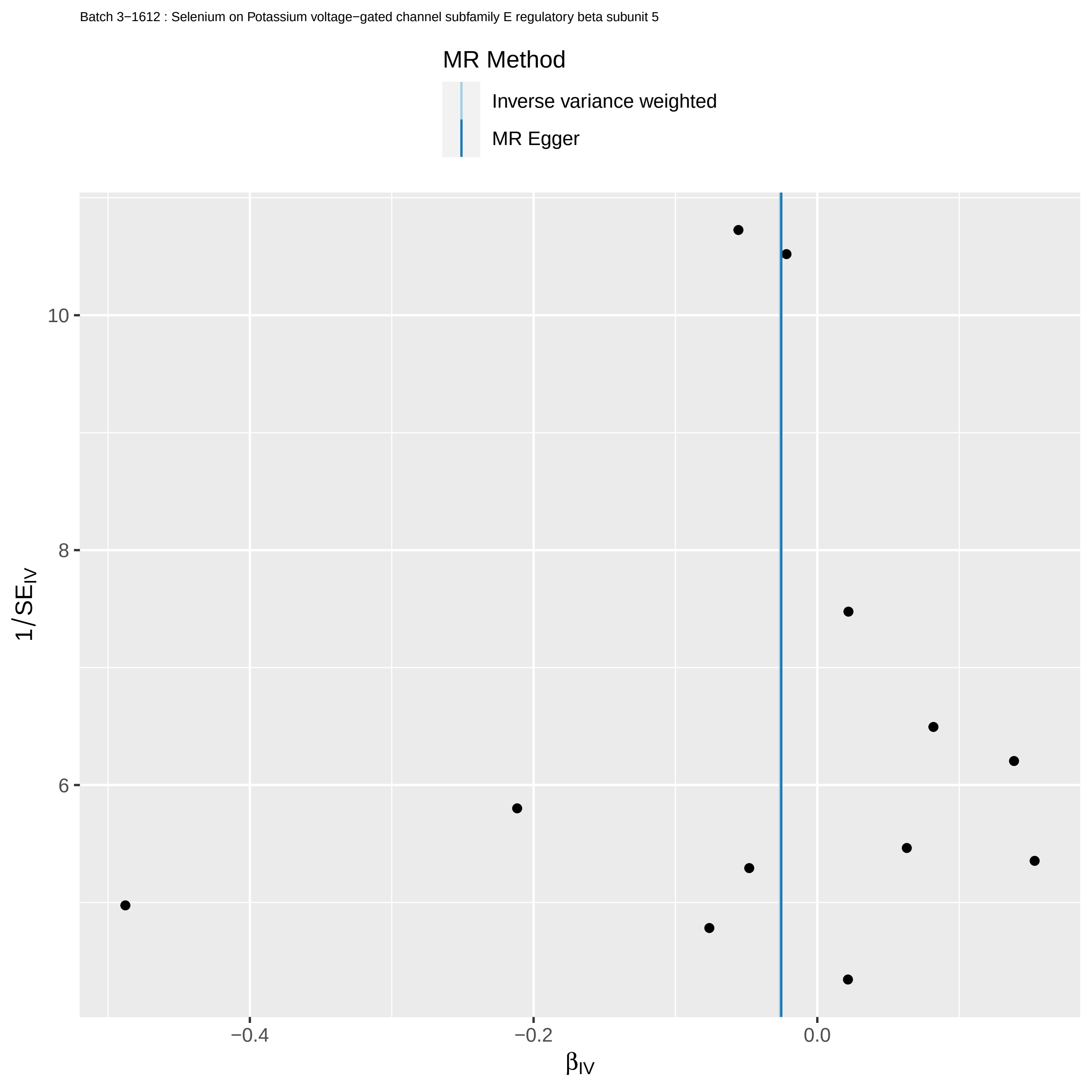

Batch 3−1612 : Selenium on Potassium voltage−gated channel subfamily E regulatory beta subunit 5
MR Method
Inverse variance weighted
MR Egger
10
8
6
1/SE_IV
−0.4
−0.2
0.0
β_IV

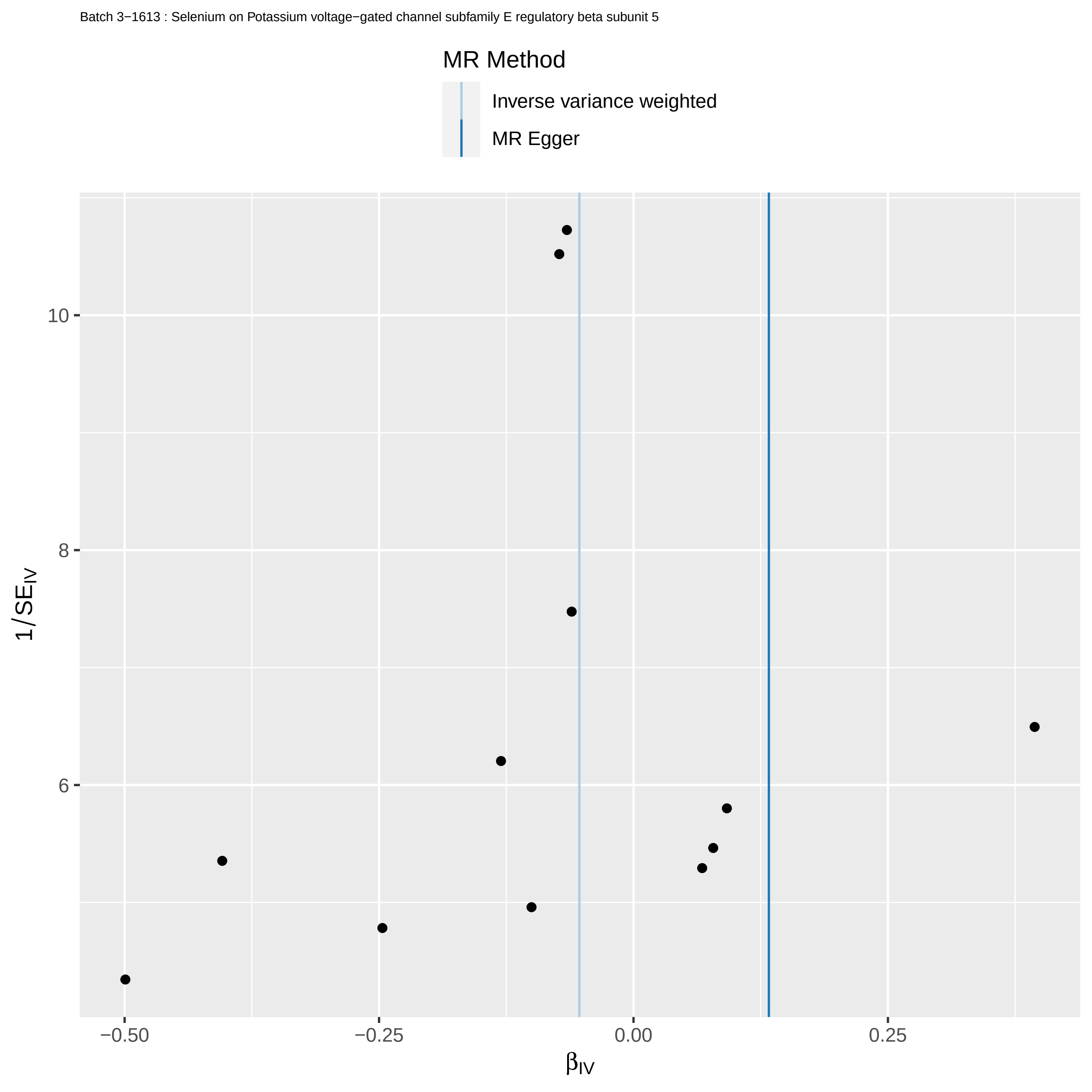
Batch 3−1613 : Selenium on Potassium voltage−gated channel subfamily E regulatory beta subunit 5
MR Method
Inverse variance weighted
MR Egger
10
8
6
1/SE_IV
−0.50
−0.25
0.00
0.25
β_IV

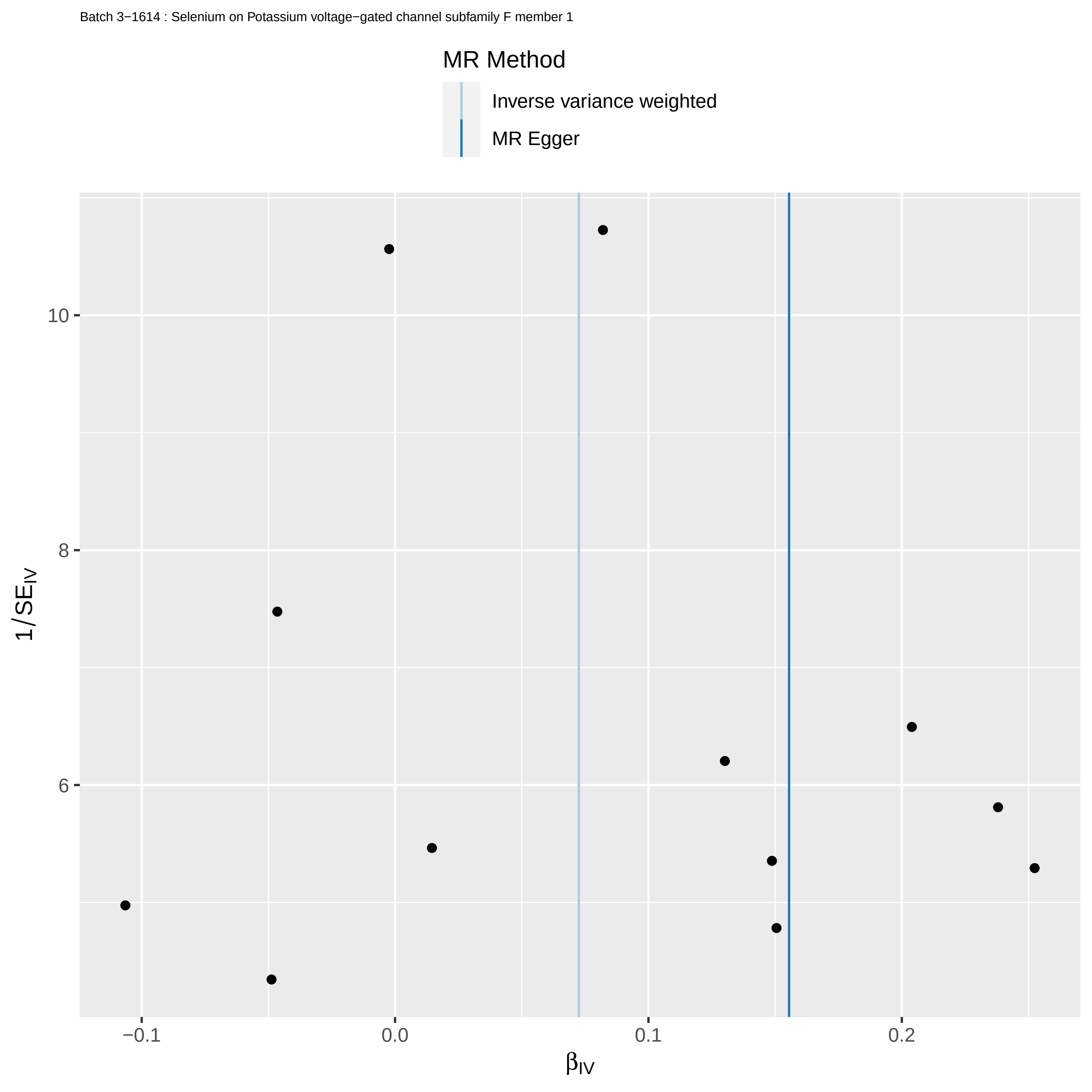

Batch 3−1614 : Selenium on Potassium voltage−gated channel subfamily F member 1
MR Method
Inverse variance weighted
MR Egger
10
8
6
1/SE_IV
−0.1
0.0
0.1
0.2
β_IV

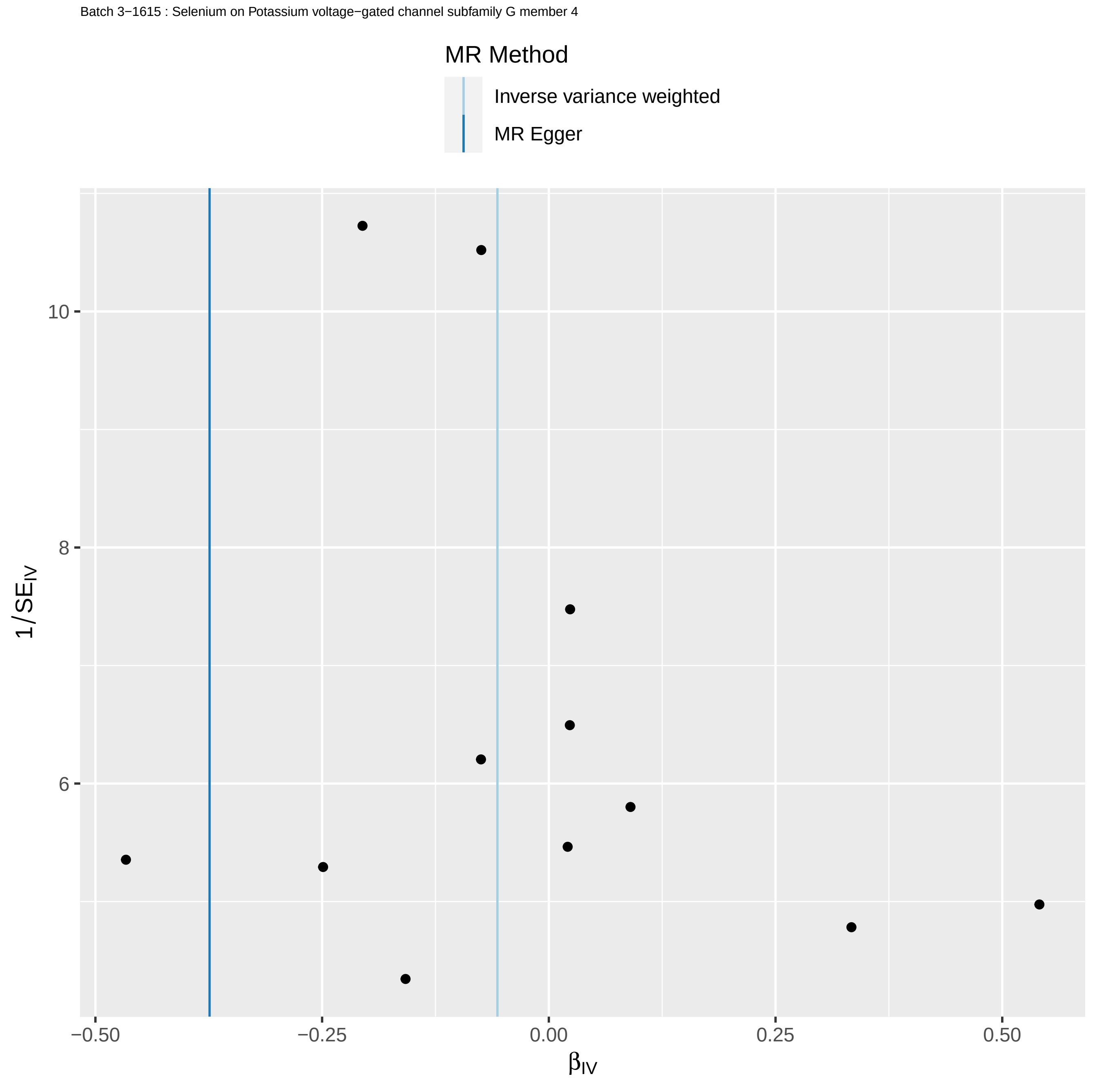

Batch 3−1615 : Selenium on Potassium voltage−gated channel subfamily G member 4
MR Method
Inverse variance weighted
MR Egger
10
8
6
$1/SE_{IV}$
−0.50
−0.25
0.00
0.25
0.50
β_{IV}

Batch 3−1616 : Selenium on Kv channel−interacting protein 1

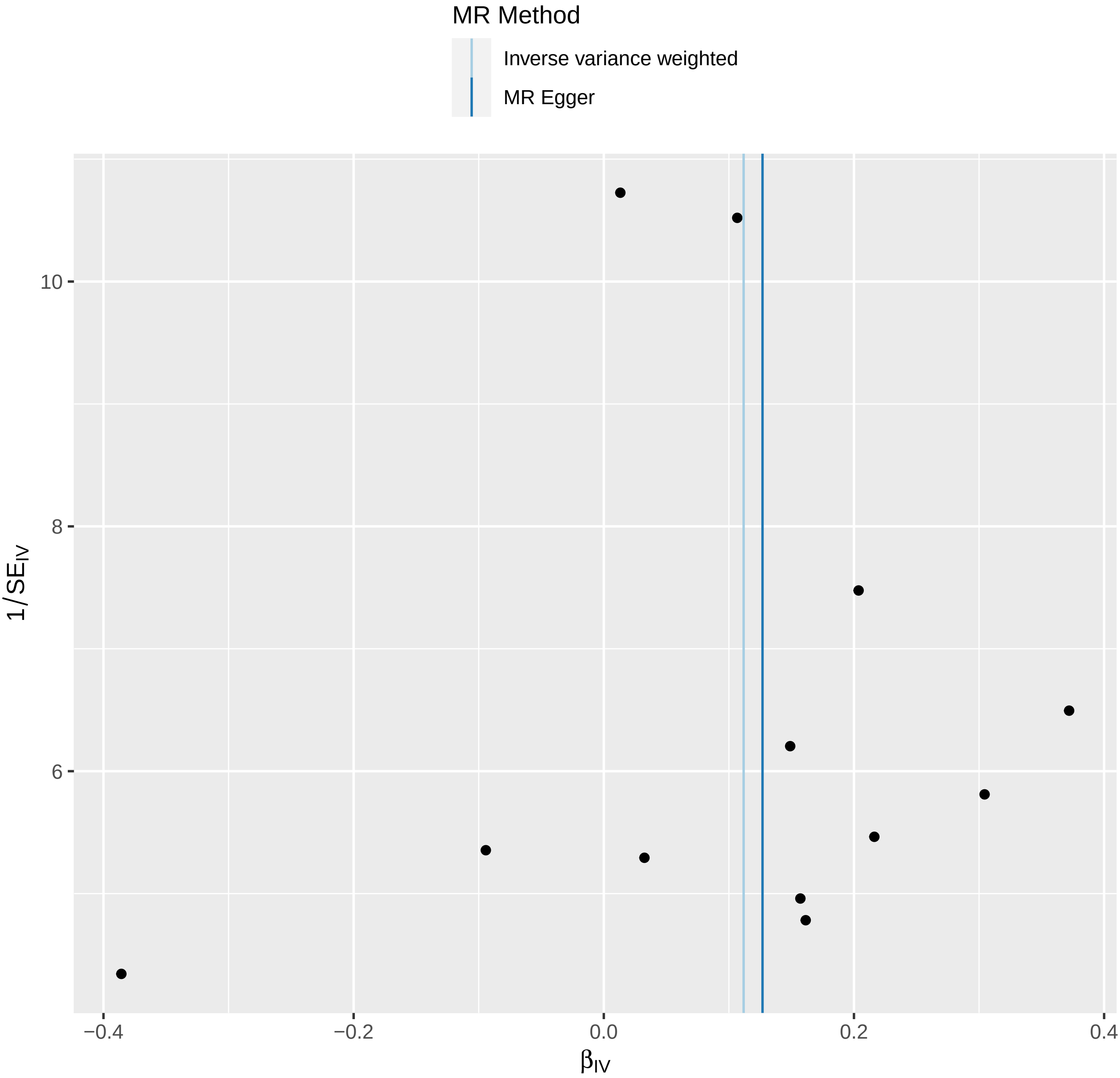

Batch 3−1617 : Selenium on Calsenilin

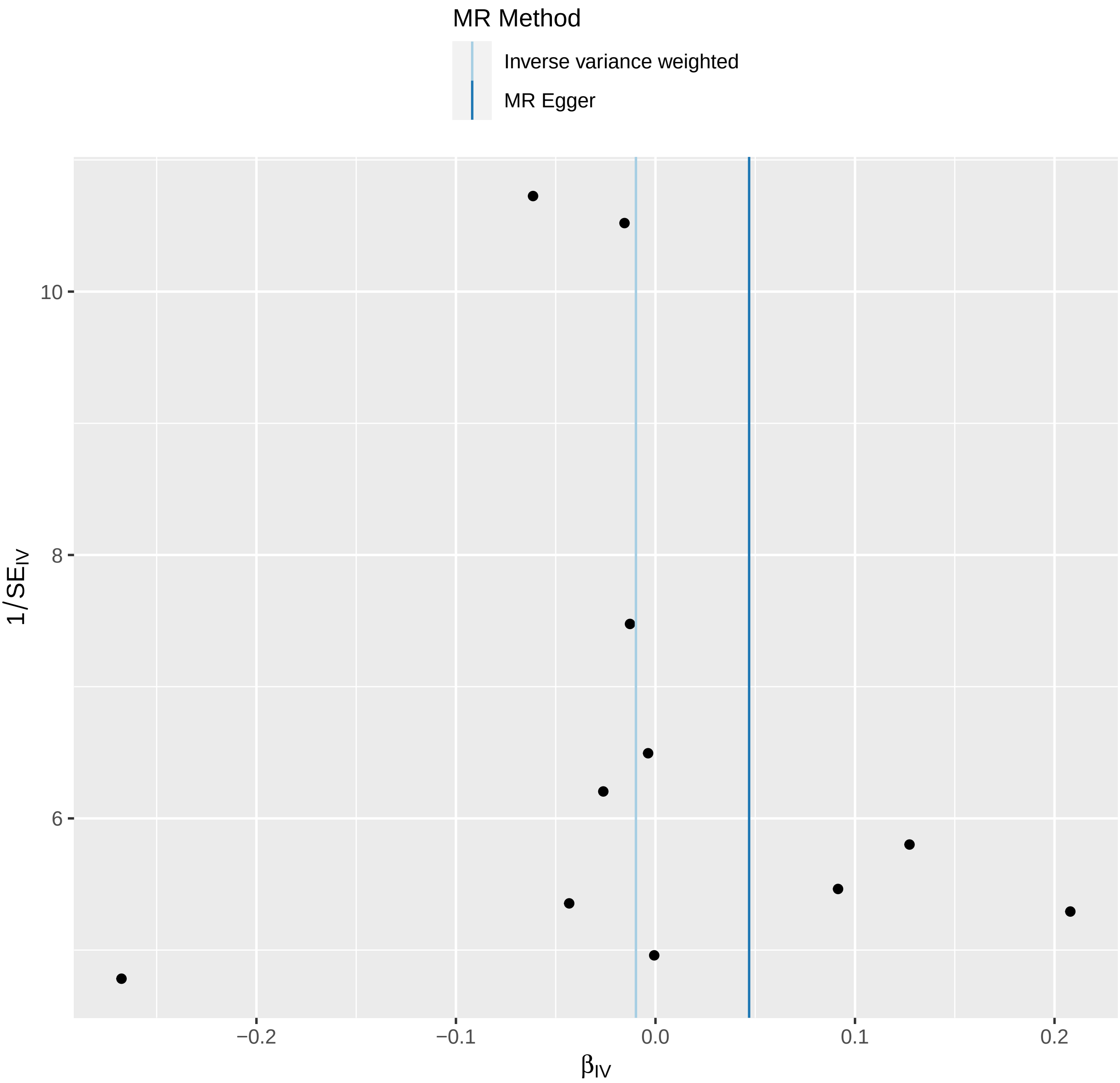

Batch 3−1618 : Selenium on Small conductance calcium−activated potassium channel protein 1

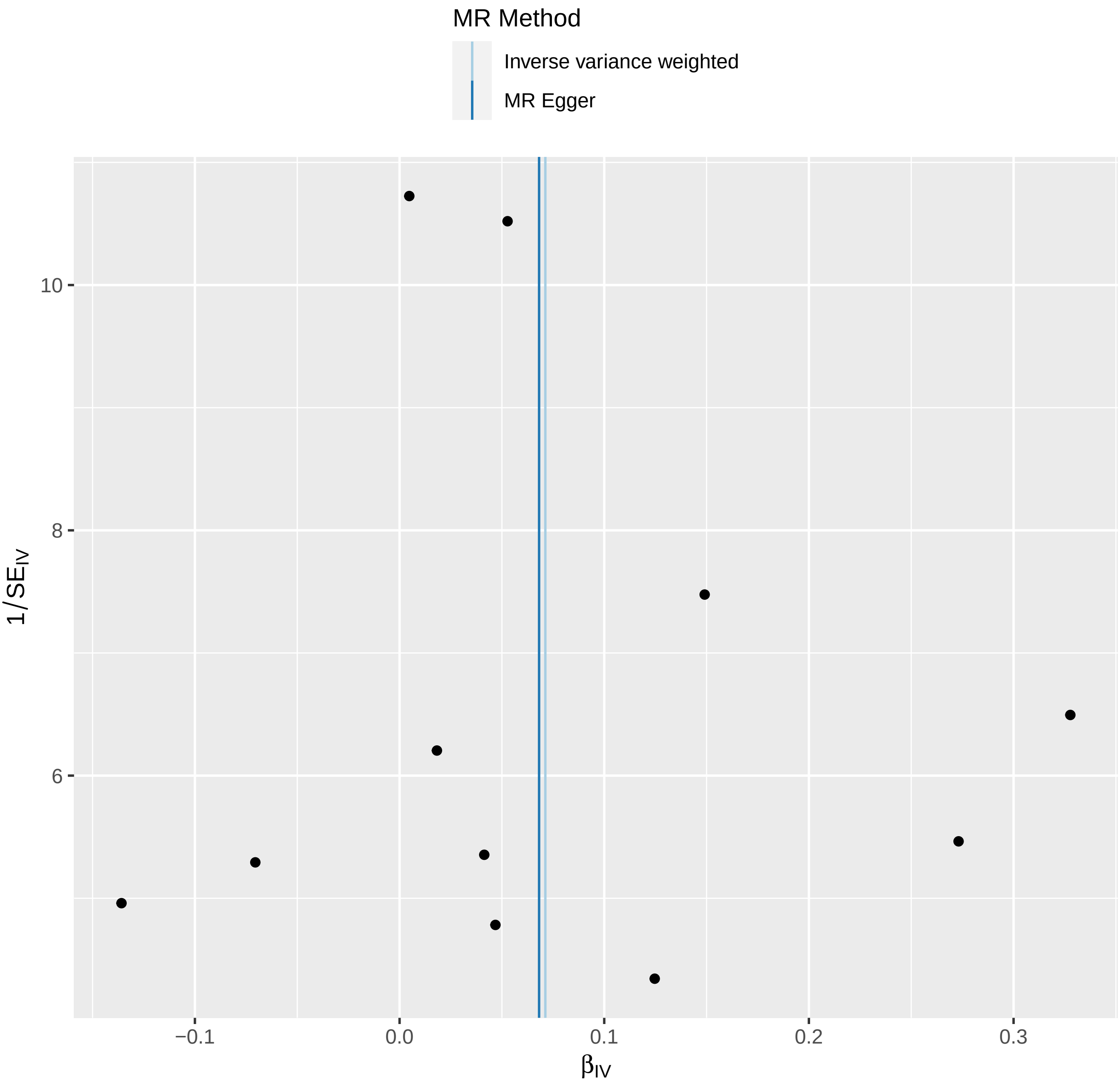

Batch 3−1619 : Selenium on Keratinocyte−associated transmembrane protein 2

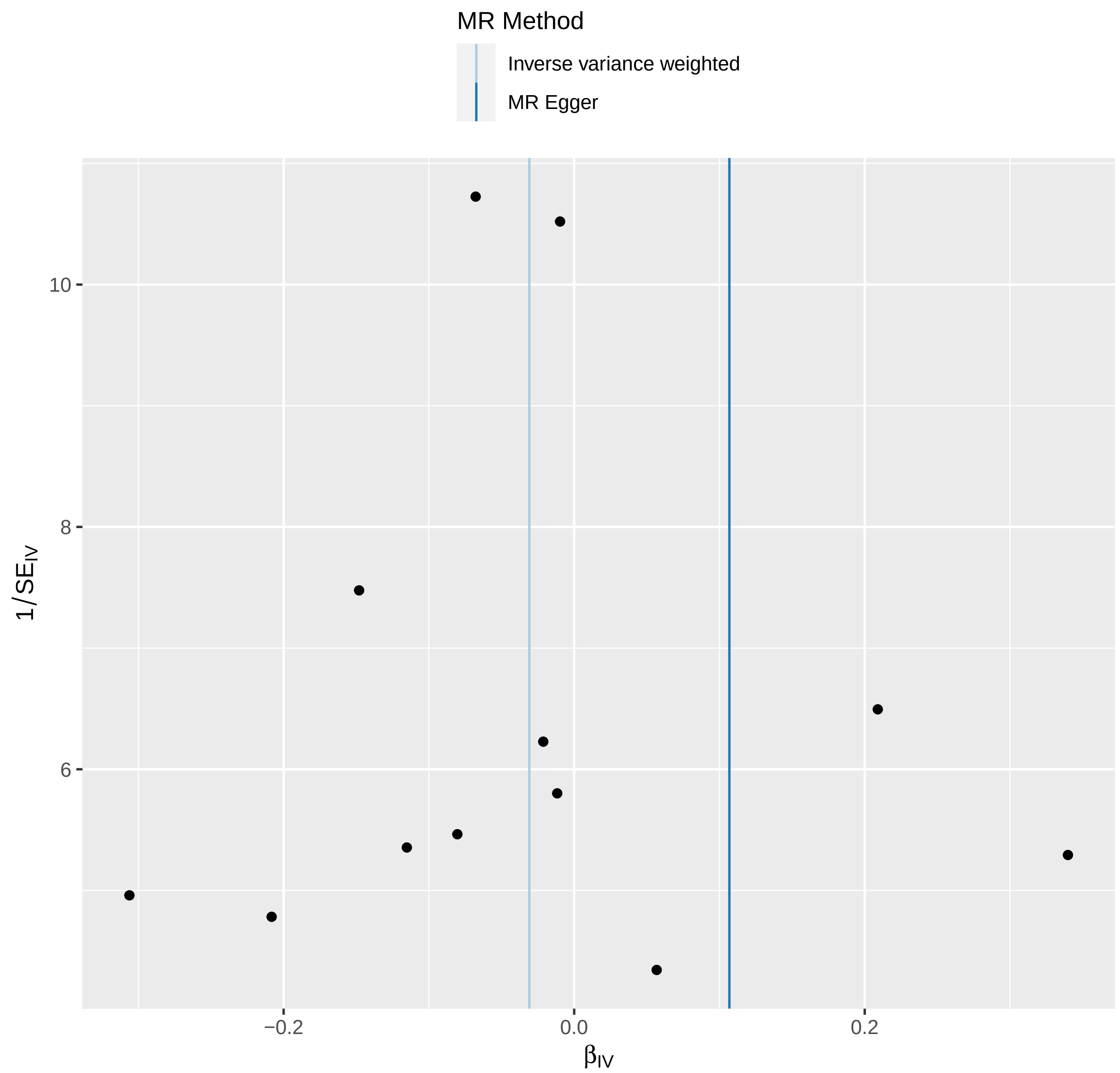

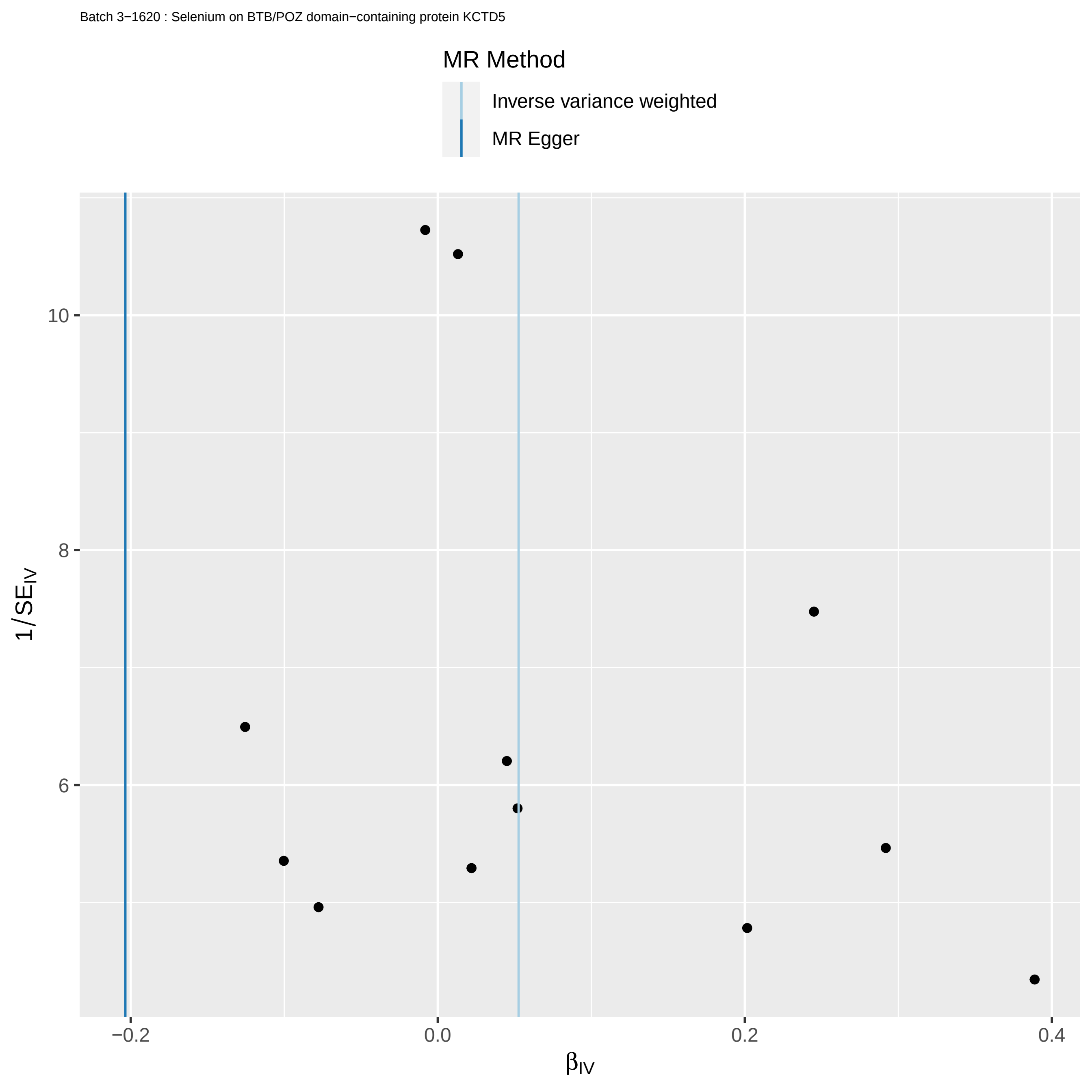
Batch 3−1620 : Selenium on BTB/POZ domain−containing protein KCTD5
MR Method
Inverse variance weighted
MR Egger
10
8
6
1/SE$_{IV}$
−0.2
0.0
0.2
0.4
β$_{IV}$

Batch 3−1621 : Selenium on KDEL motif−containing protein 2

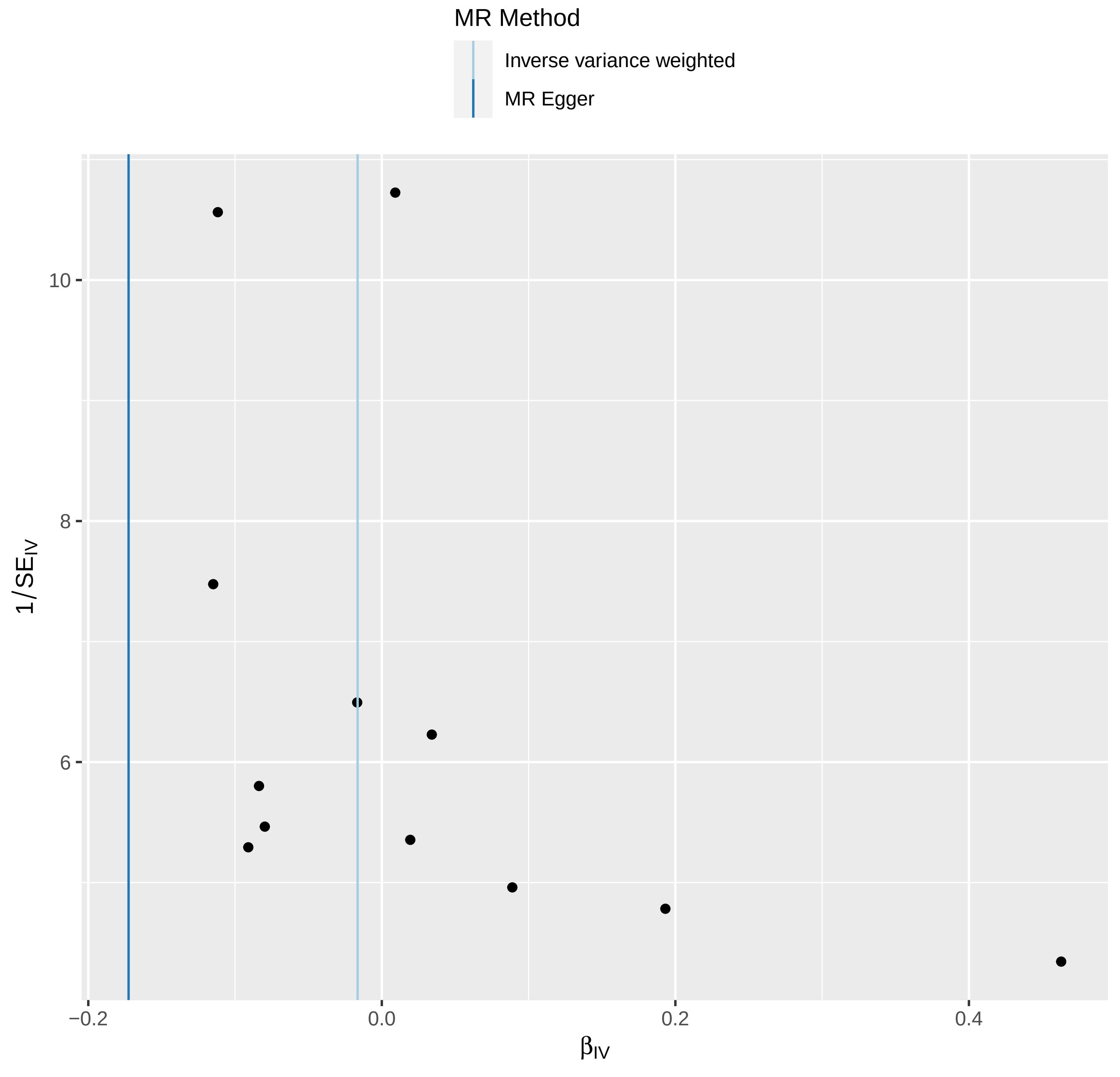

Batch 3−1622 : Selenium on Vascular endothelial growth factor receptor 2

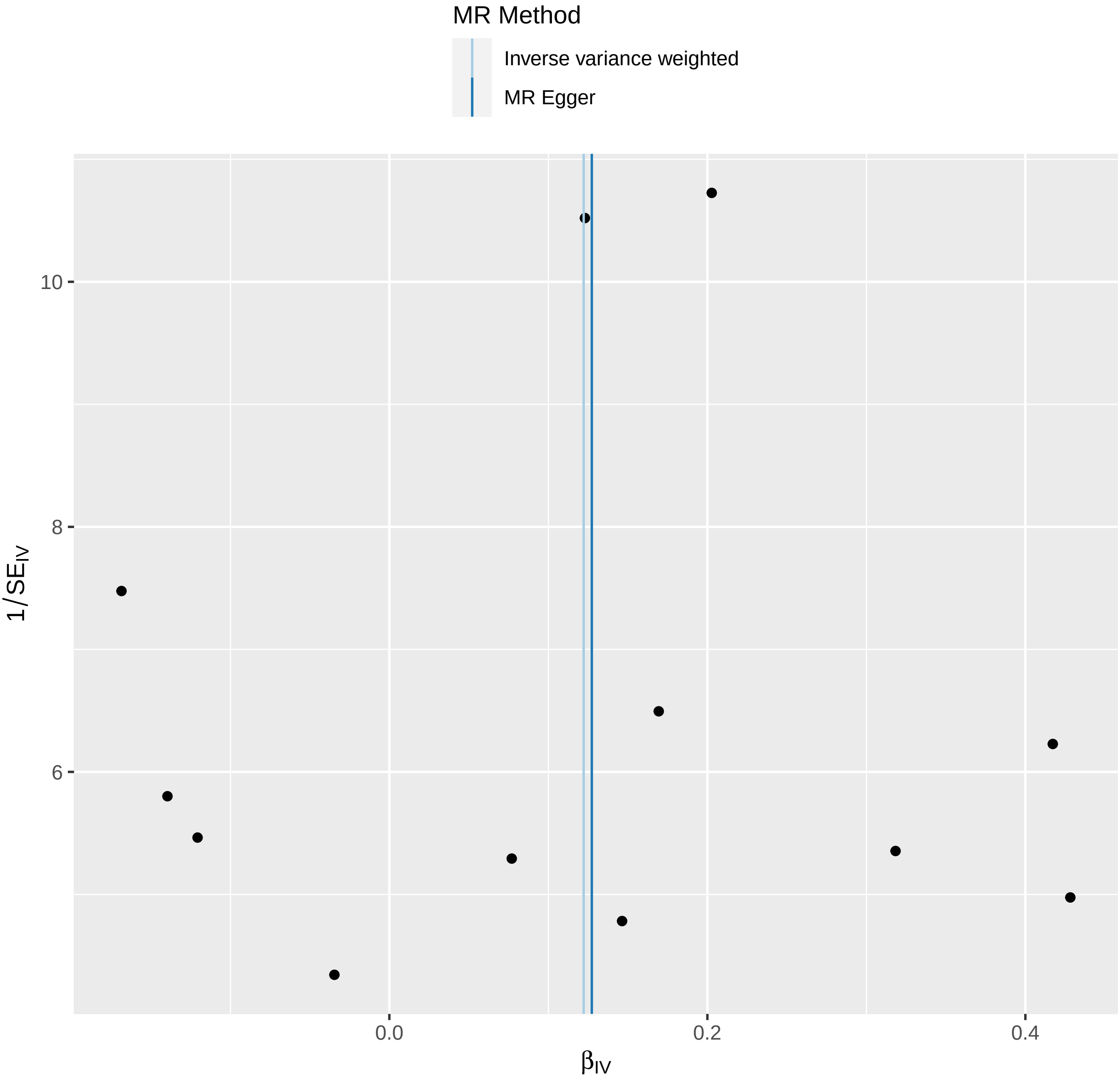

Batch 3−1623 : Selenium on Kell blood group glycoprotein

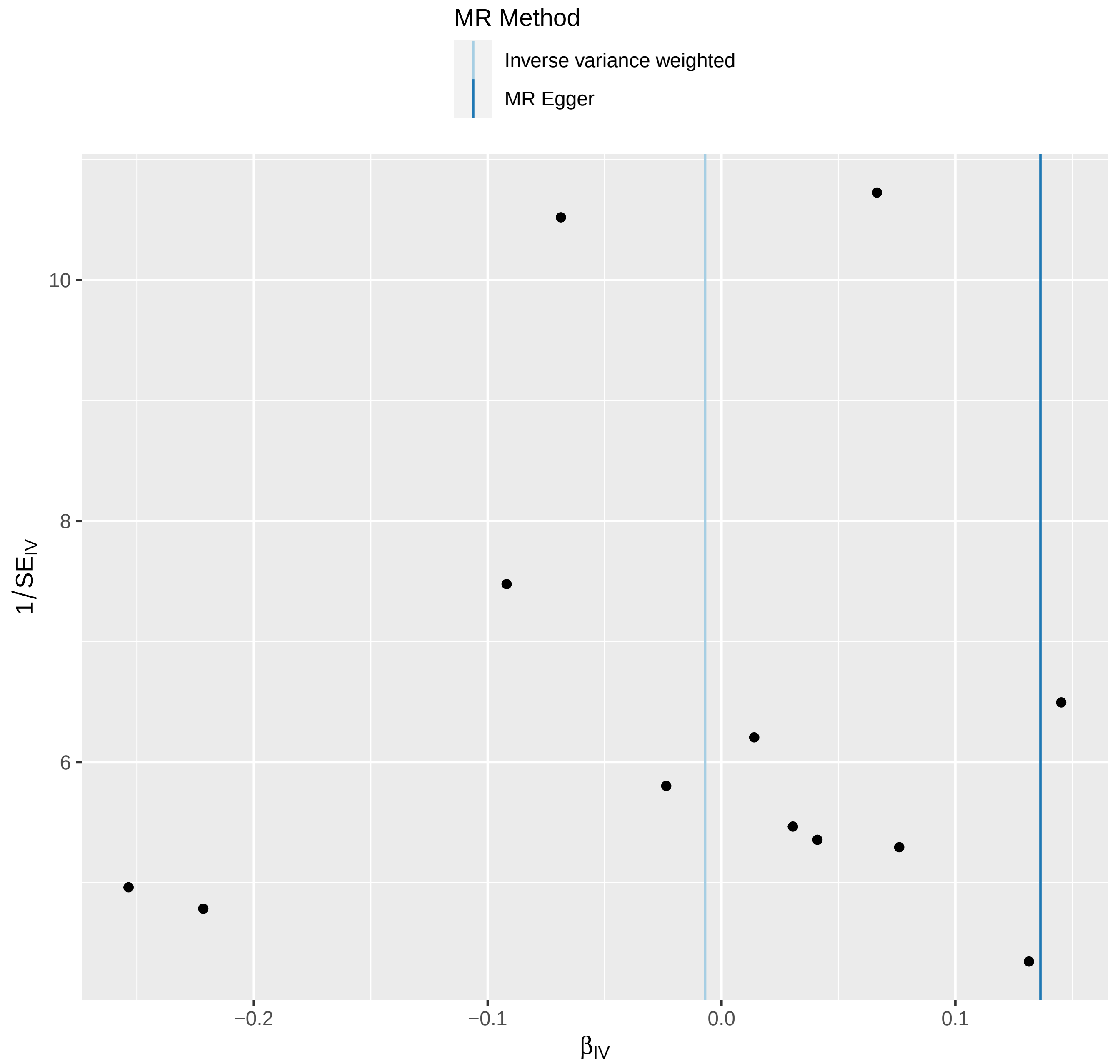

Batch 3−1624 : Selenium on Uncharacterized protein KIAA0040

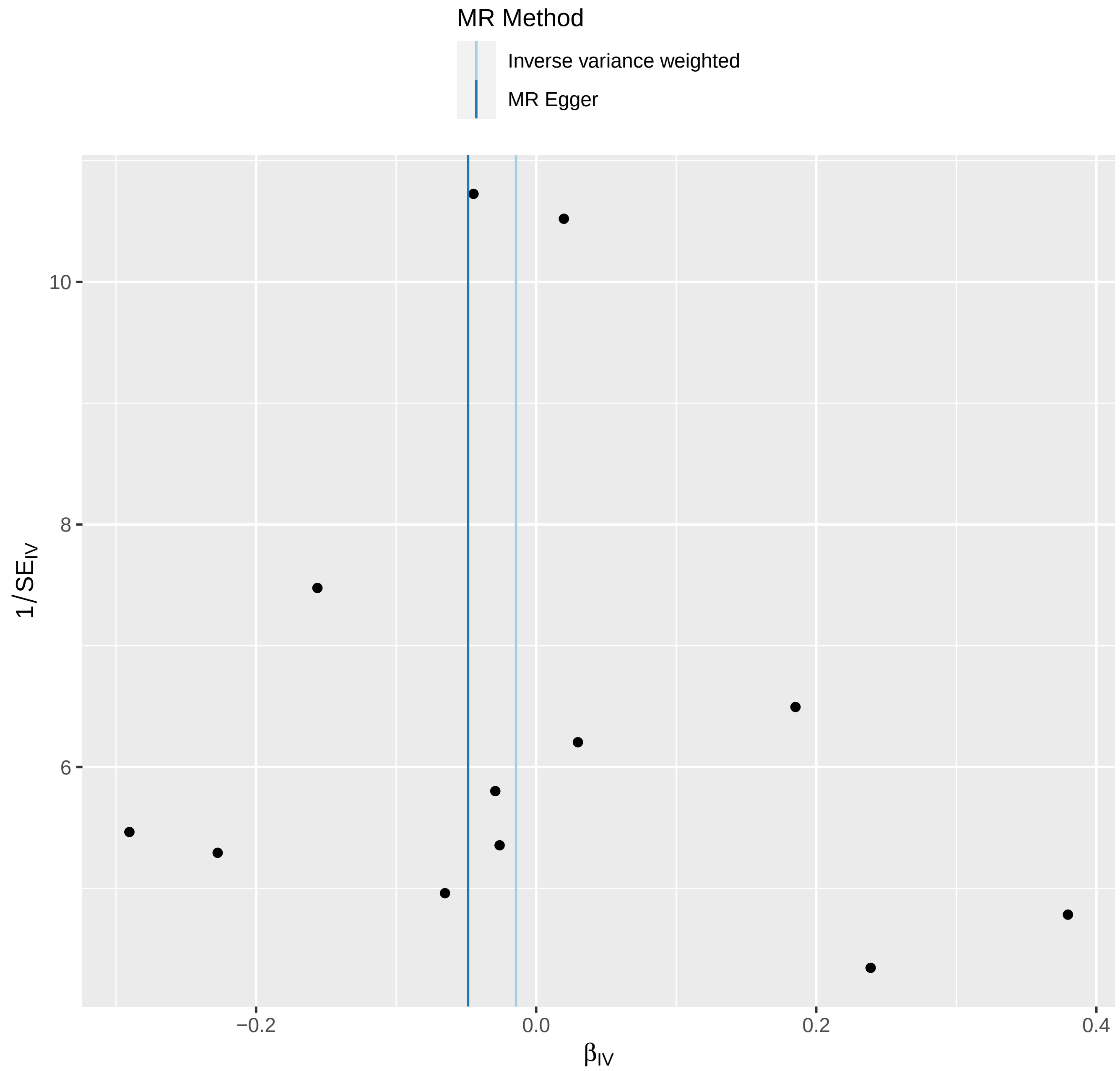

Batch 3−1625 : Selenium on Dyslexia−associated protein KIAA0319

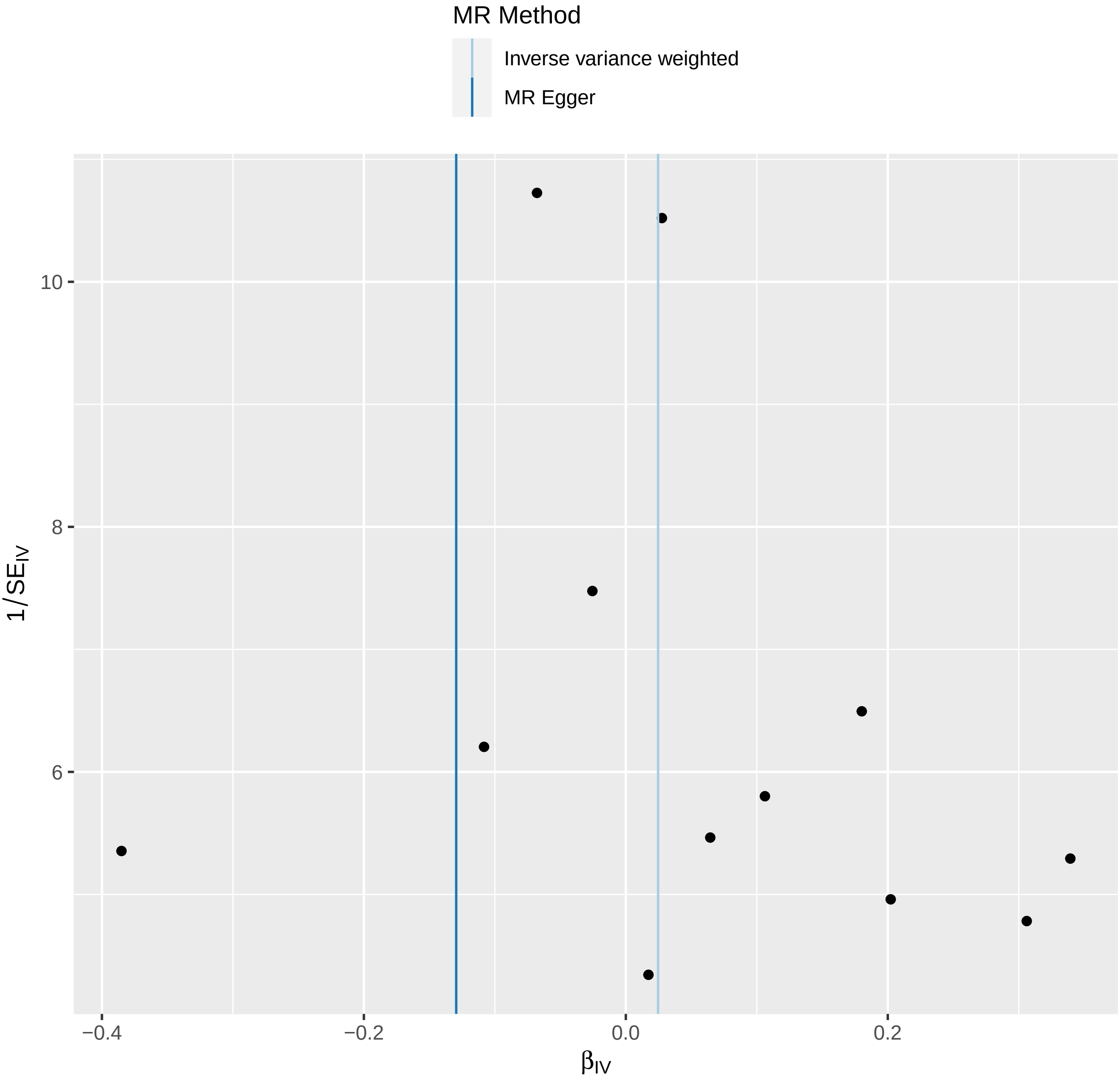

Batch 3−1626 : Selenium on Dyslexia−associated protein KIAA0319−like protein

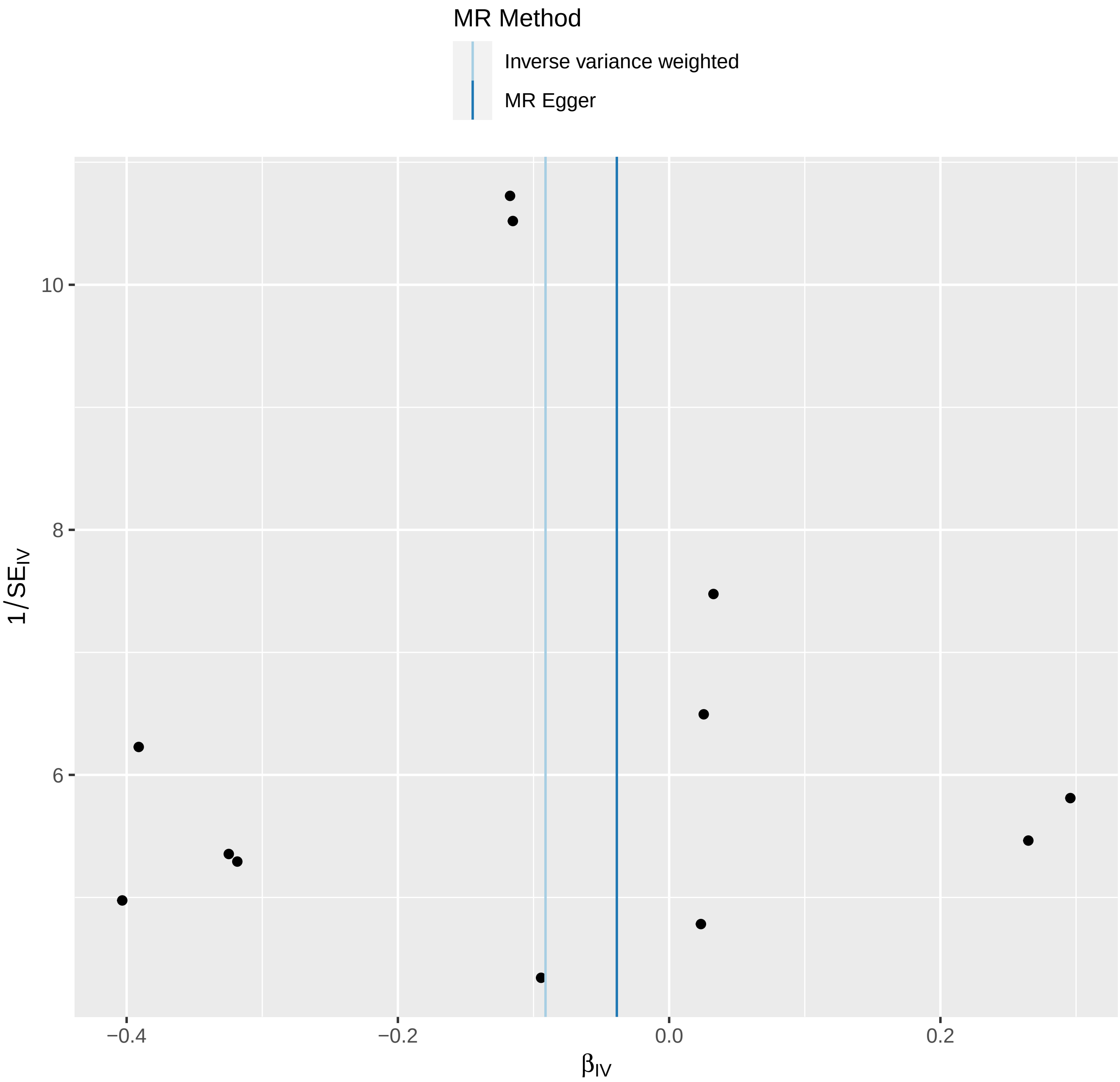

Batch 3−1627 : Selenium on UPF0258 protein KIAA1024

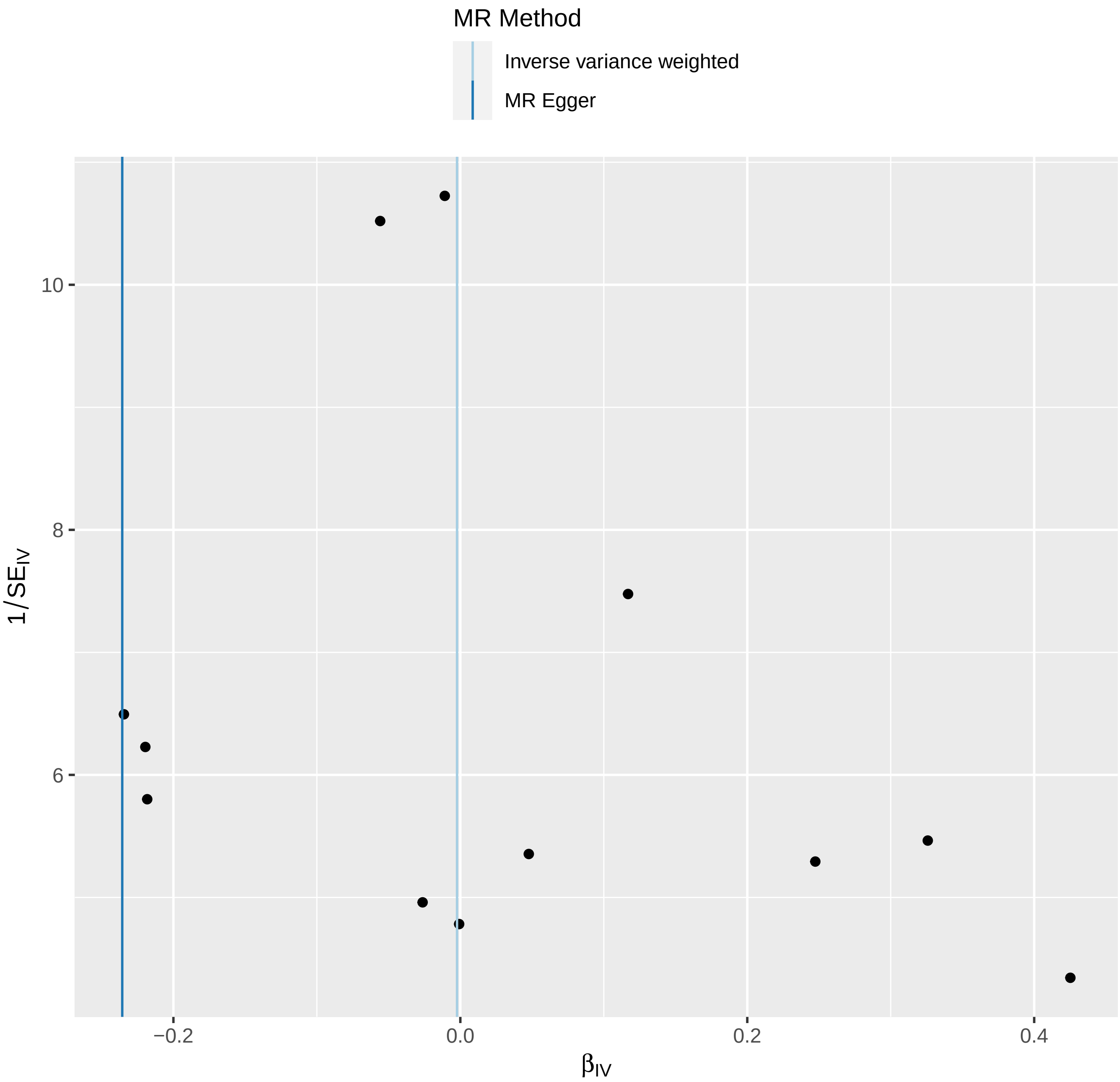

Batch 3−1628 : Selenium on Uncharacterized family 31 glucosidase KIAA1161

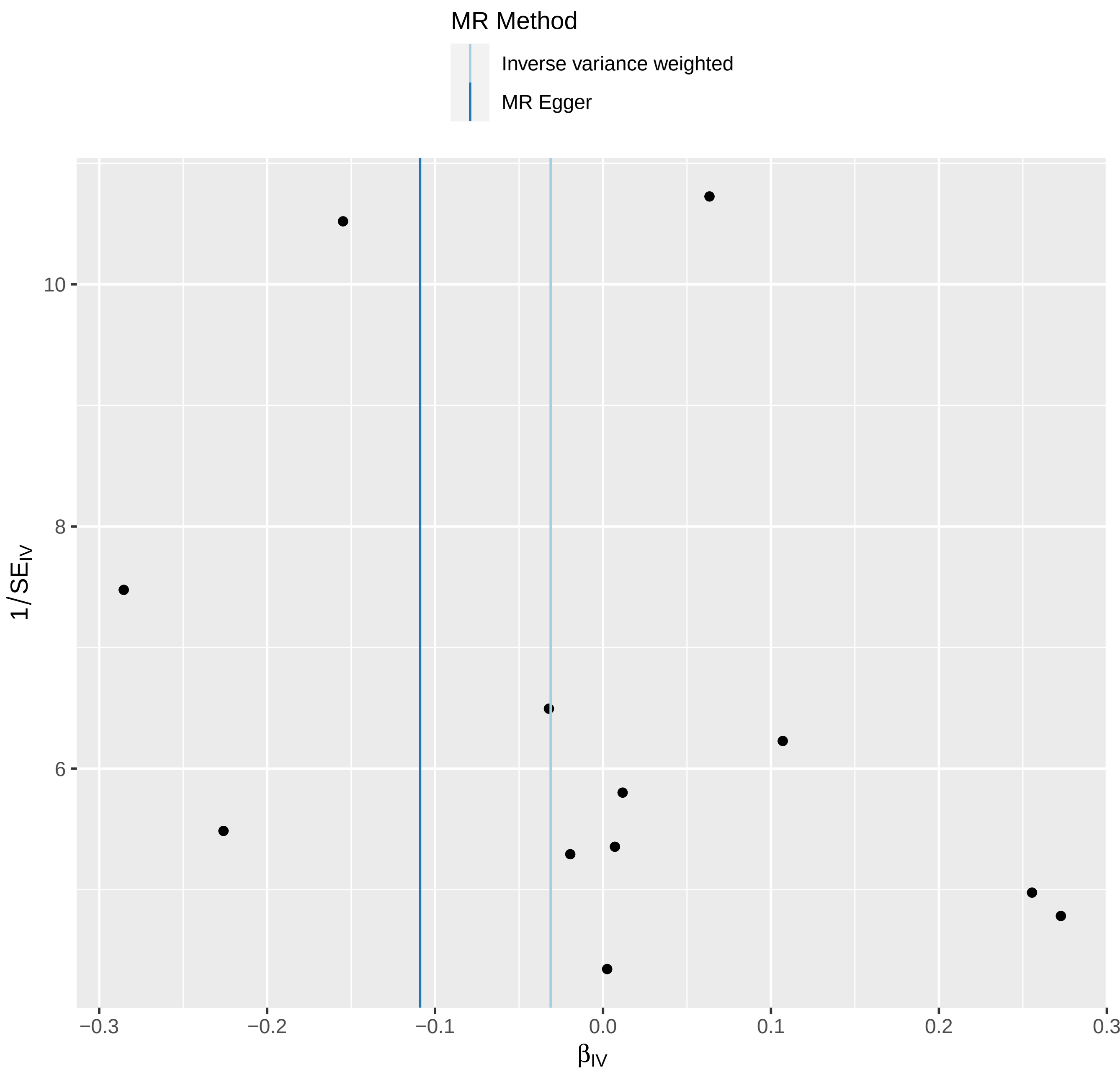

Batch 3−1629 : Selenium on Uncharacterized family 31 glucosidase KIAA1161

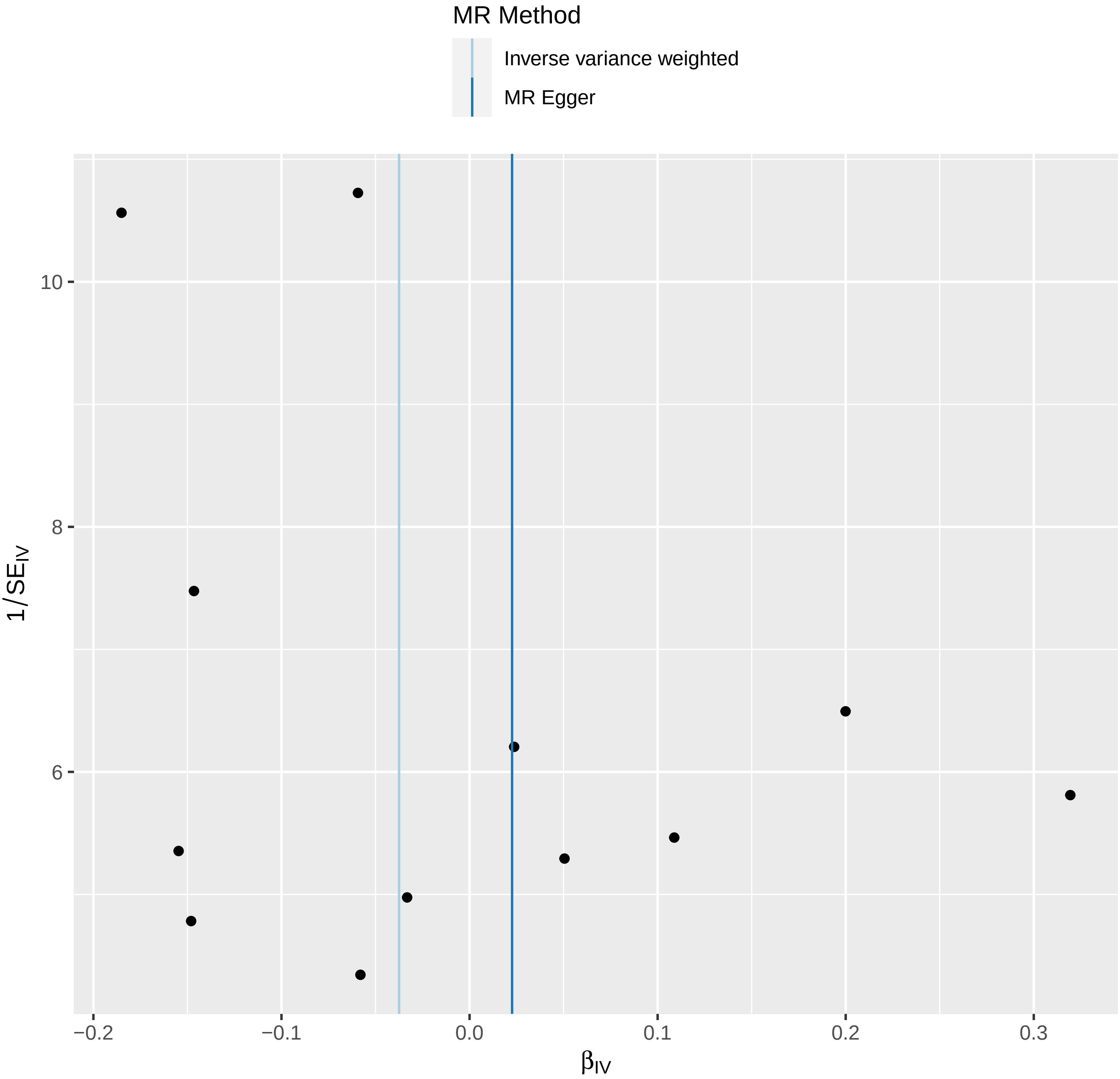

Batch 3−1630 : Selenium on UPF0577 protein KIAA1324

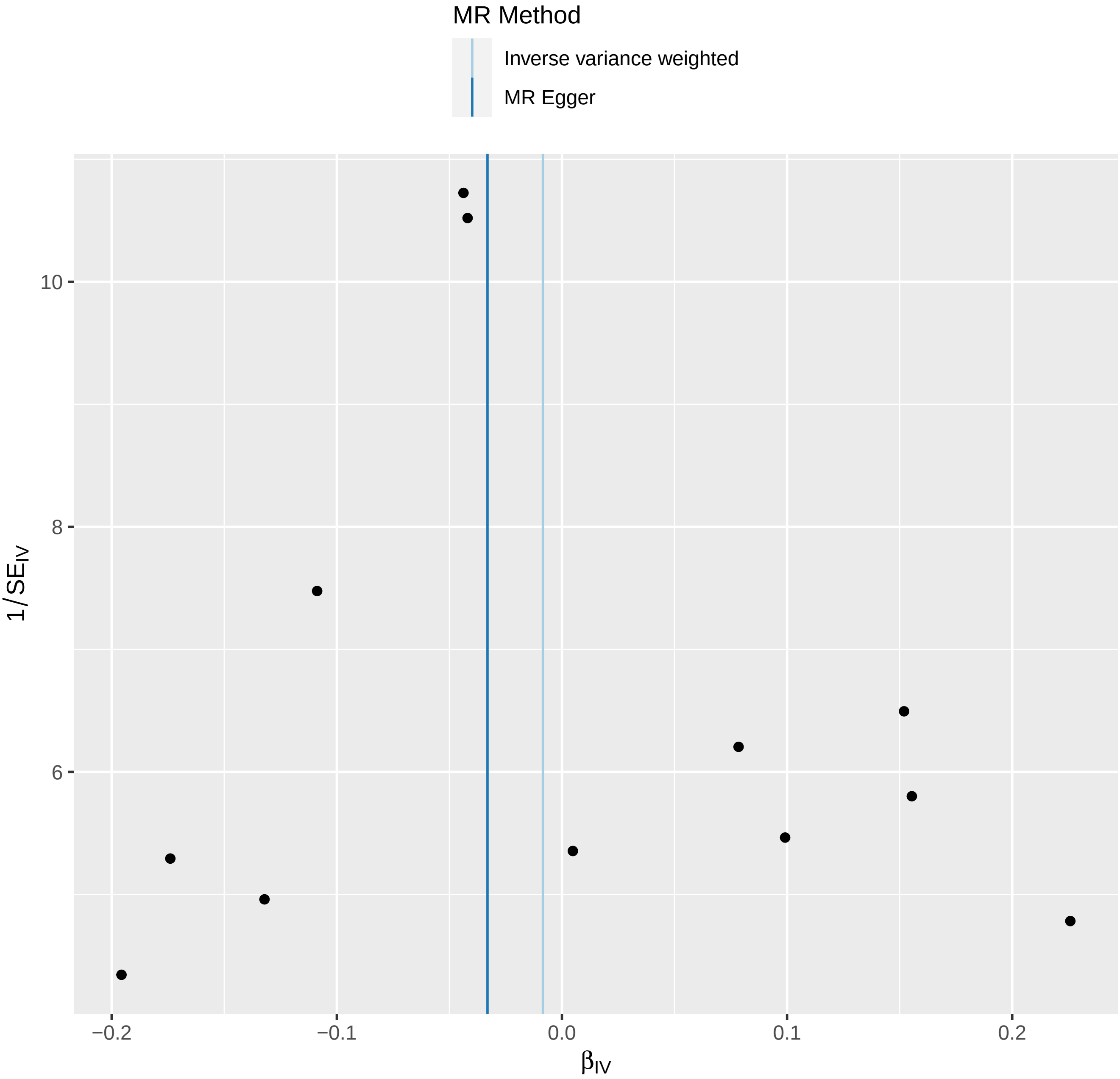

Batch 3−1631 : Selenium on UPF0577 protein KIAA1324

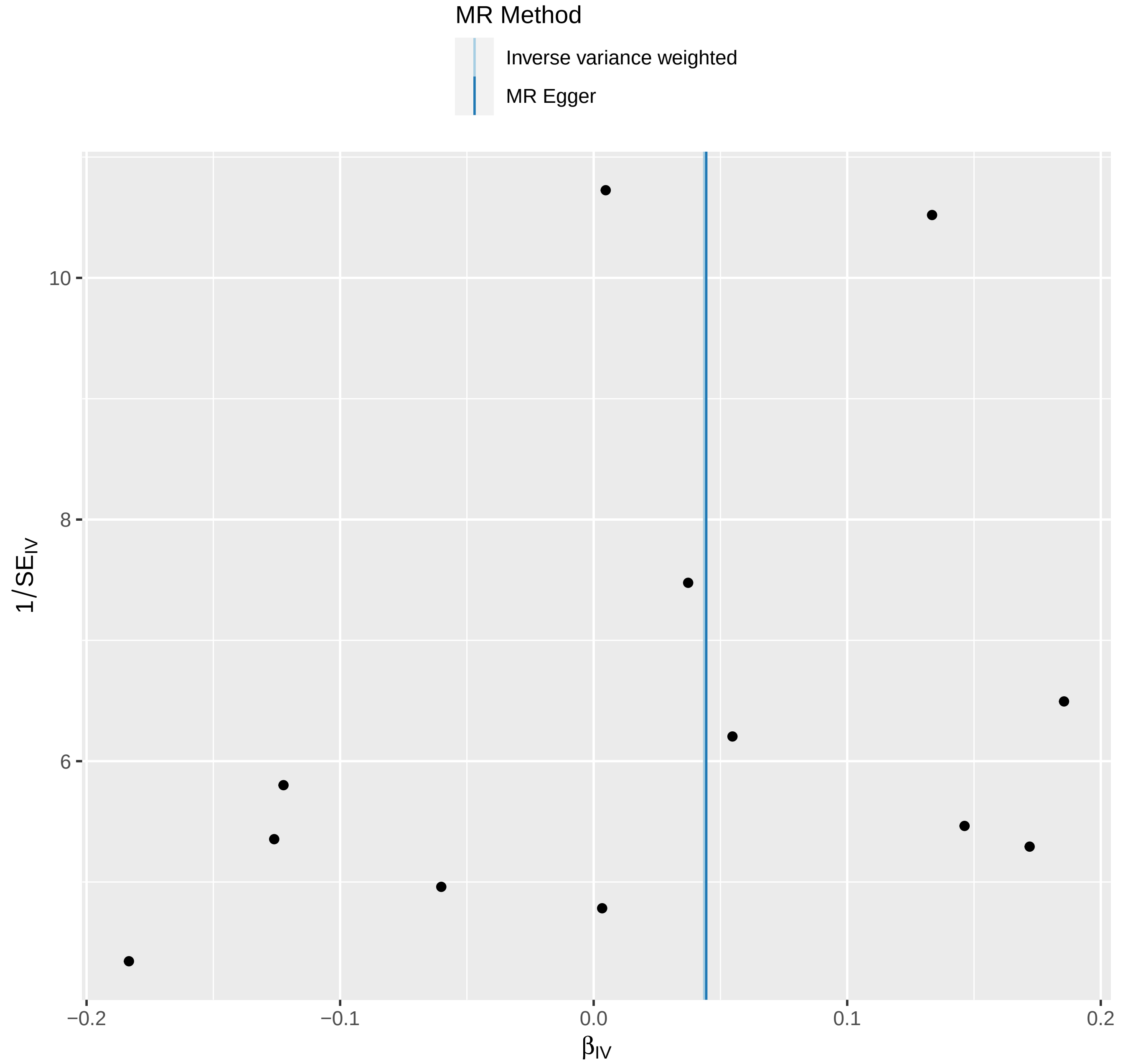

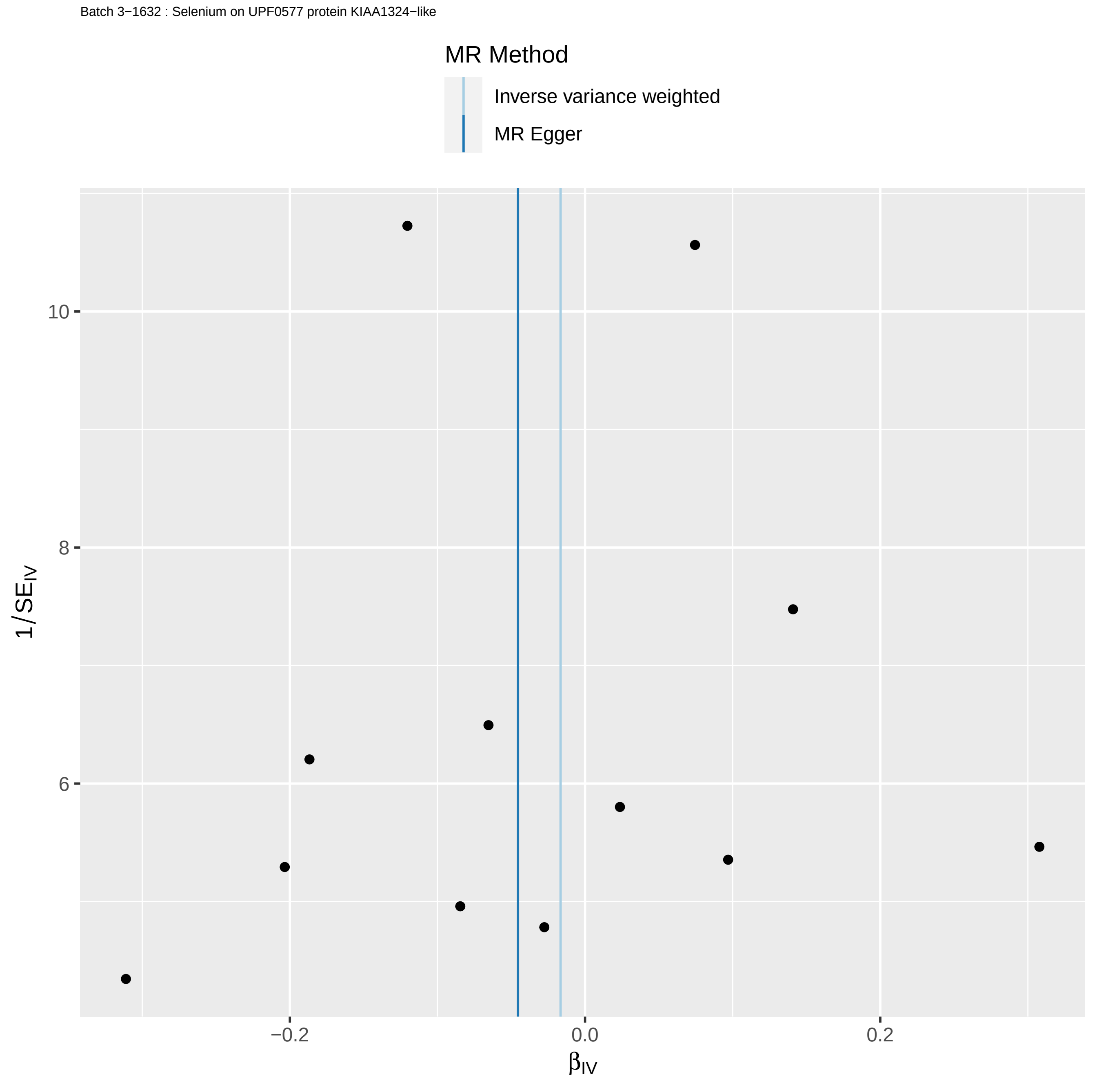

Batch 3−1632 : Selenium on UPF0577 protein KIAA1324−like
MR Method
Inverse variance weighted
MR Egger
10
8
6
1/SE$_{IV}$
−0.2
0.0
0.2
β$_{IV}$

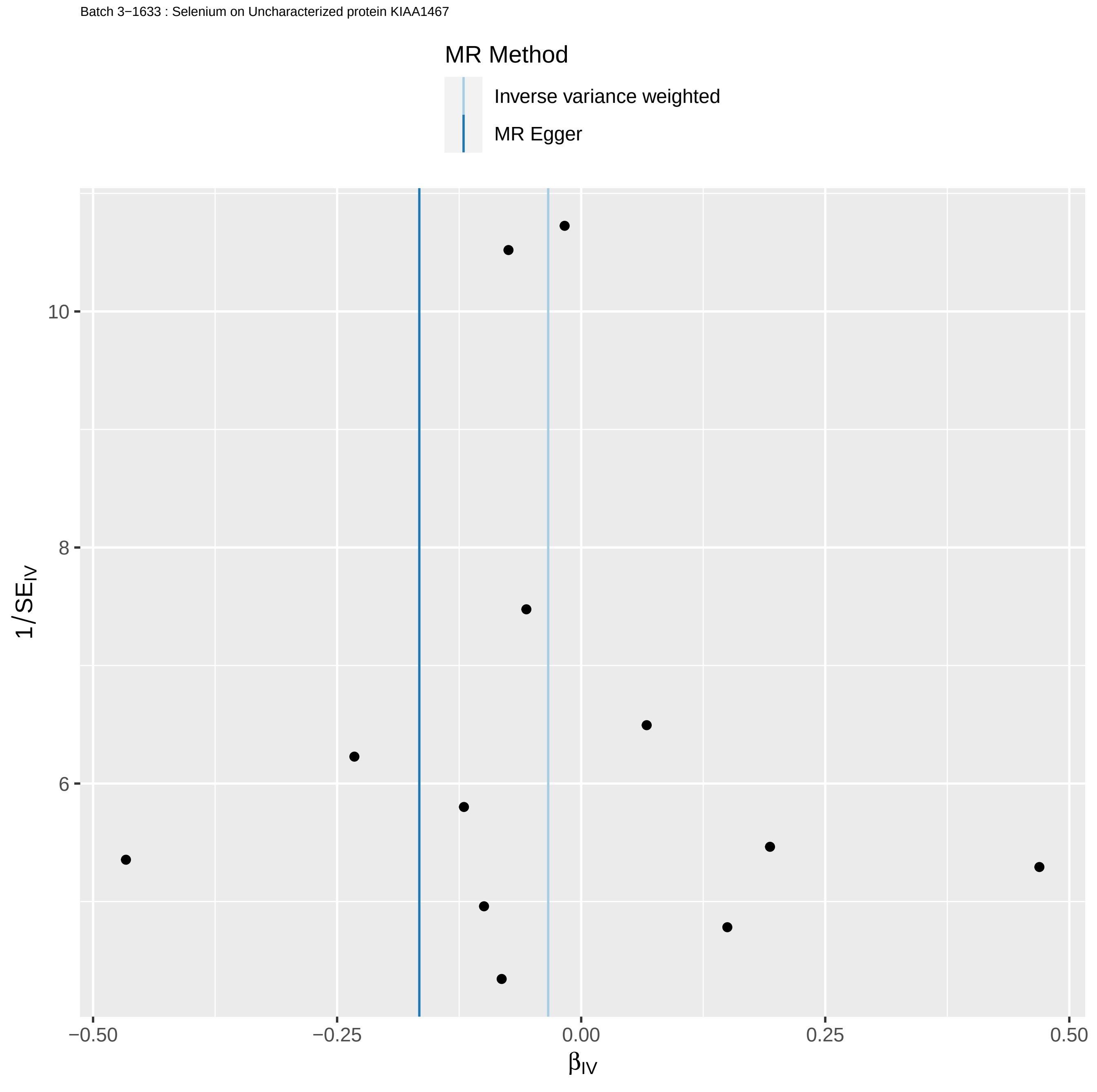
Batch 3−1633 : Selenium on Uncharacterized protein KIAA1467
MR Method
Inverse variance weighted
MR Egger
10
8
6
$1/SE_{IV}$
−0.50
−0.25
0.00
0.25
0.50
β_{IV}

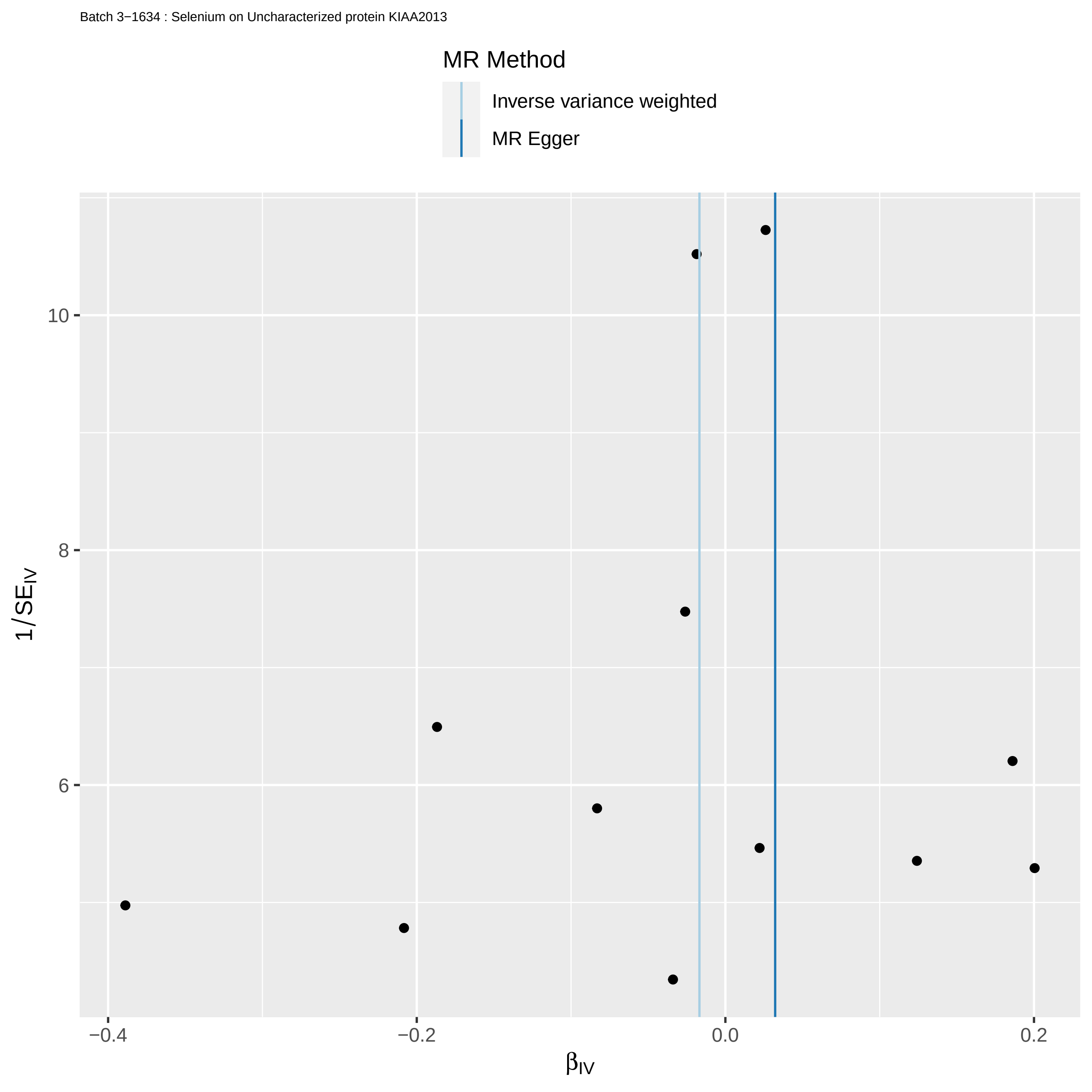

Batch 3−1634 : Selenium on Uncharacterized protein KIAA2013
MR Method
Inverse variance weighted
MR Egger
10
8
6
1/SE$_{IV}$
−0.4
−0.2
0.0
0.2
β$_{IV}$

Batch 3−1635 : Selenium on Kinesin−like protein KIF16B

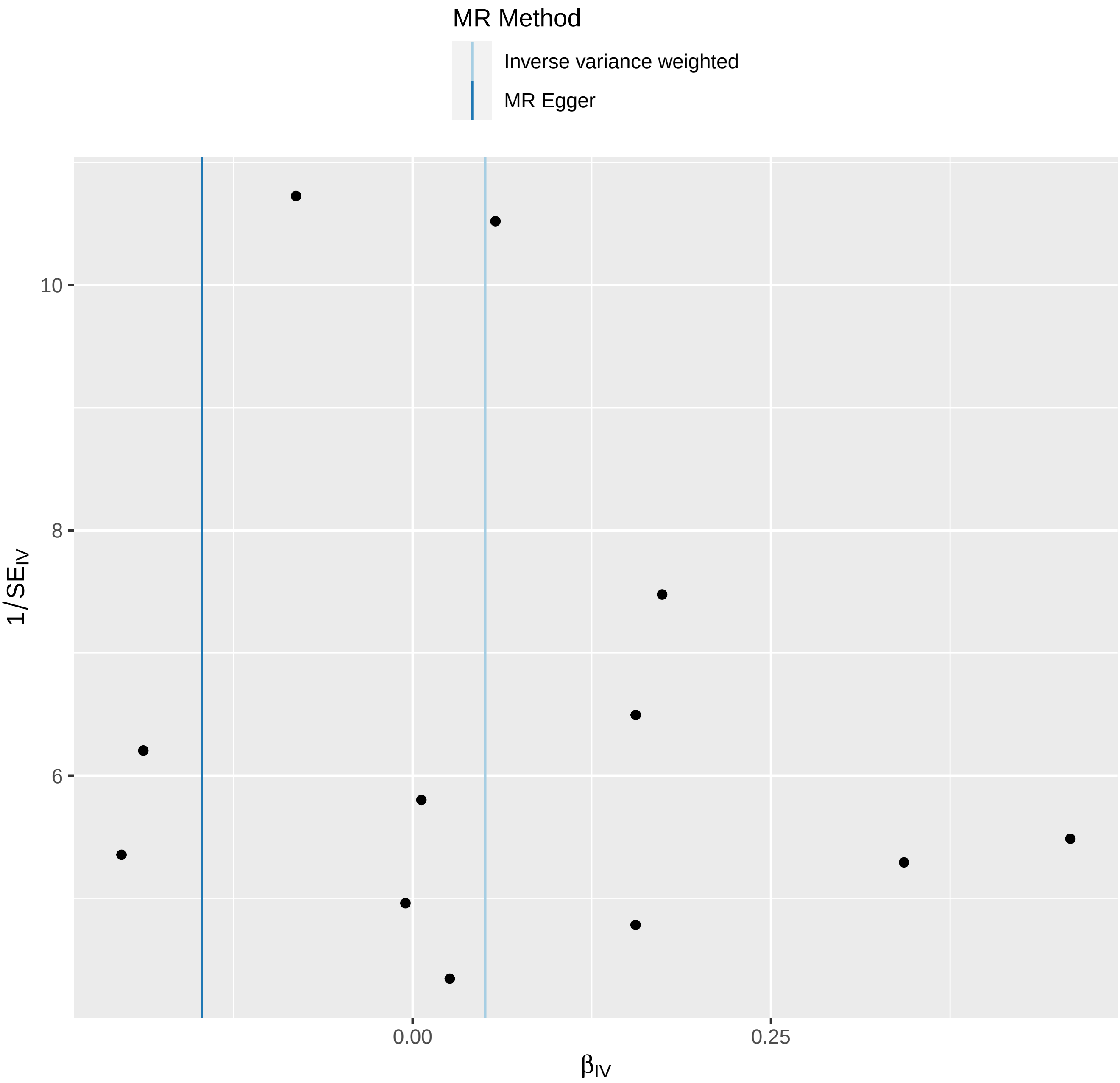

Batch 3−1636 : Selenium on Kinesin−like protein KIF1C

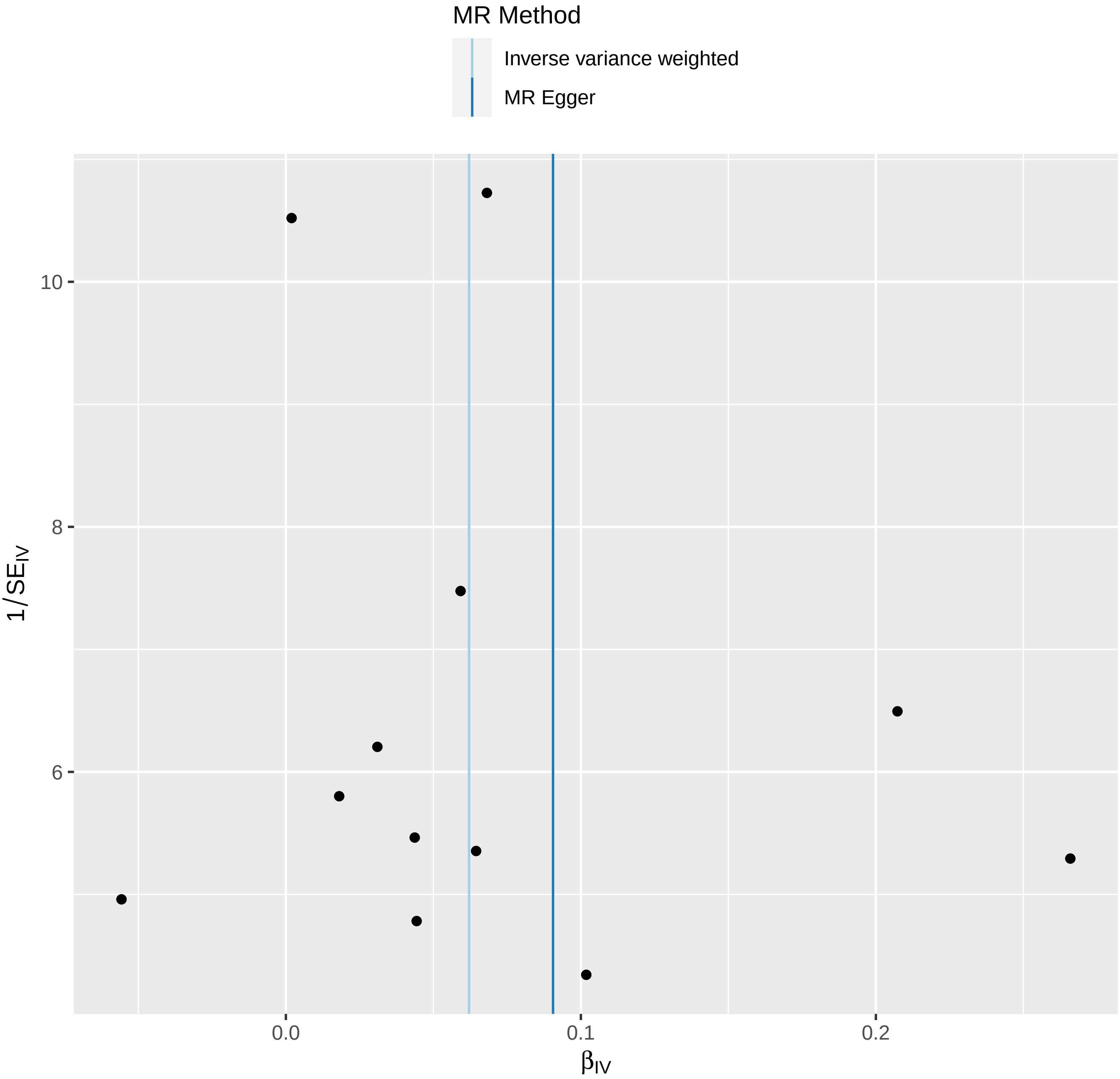

Batch 3−1637 : Selenium on Kinesin−like protein KIF23

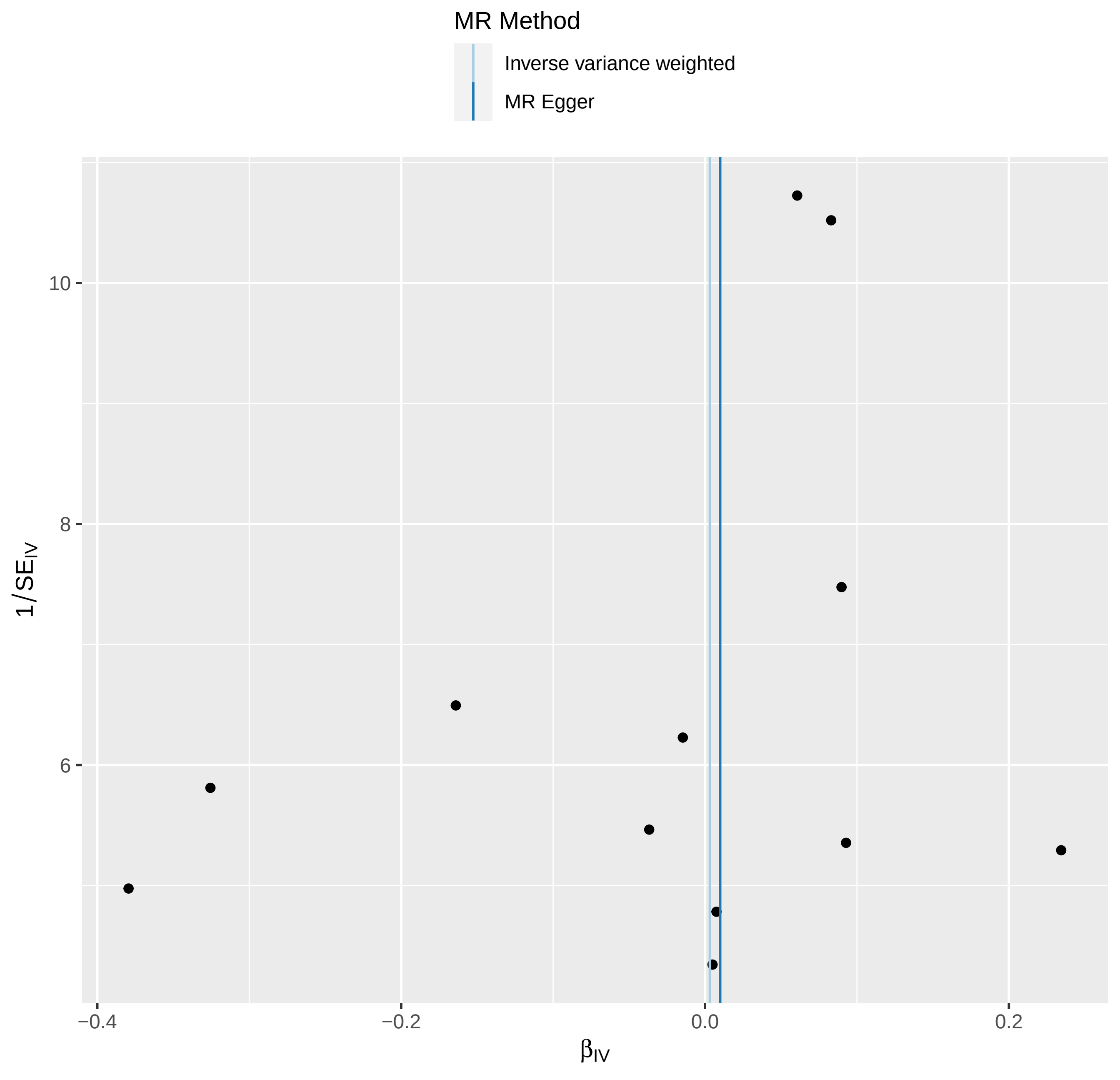

Batch 3−1638 : Selenium on DNA/RNA−binding protein KIN17

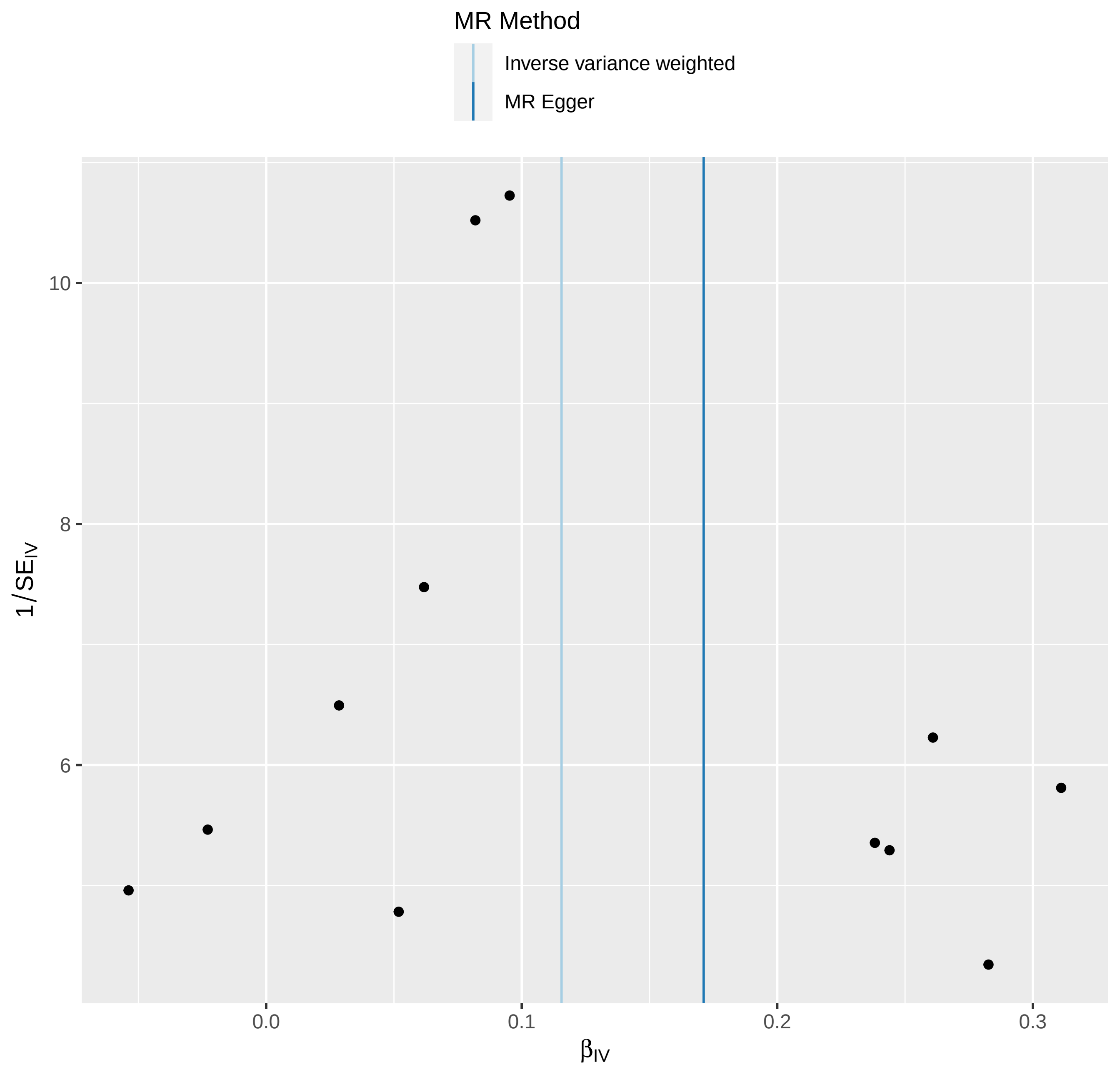

Batch 3−1639 : Selenium on DNA/RNA−binding protein KIN17

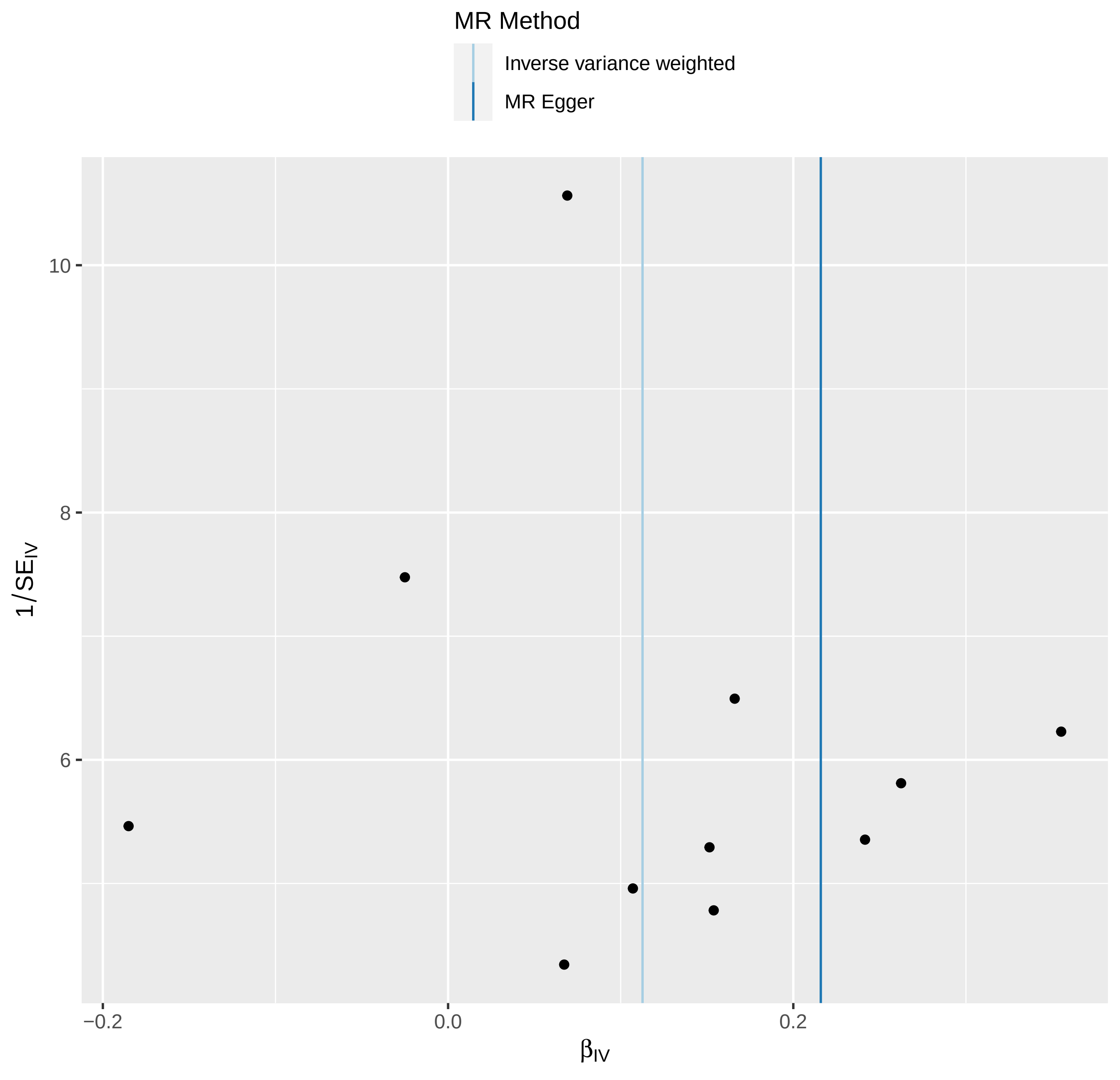

Batch 3−1640 : Selenium on Killer cell immunoglobulin−like receptor 2DL2

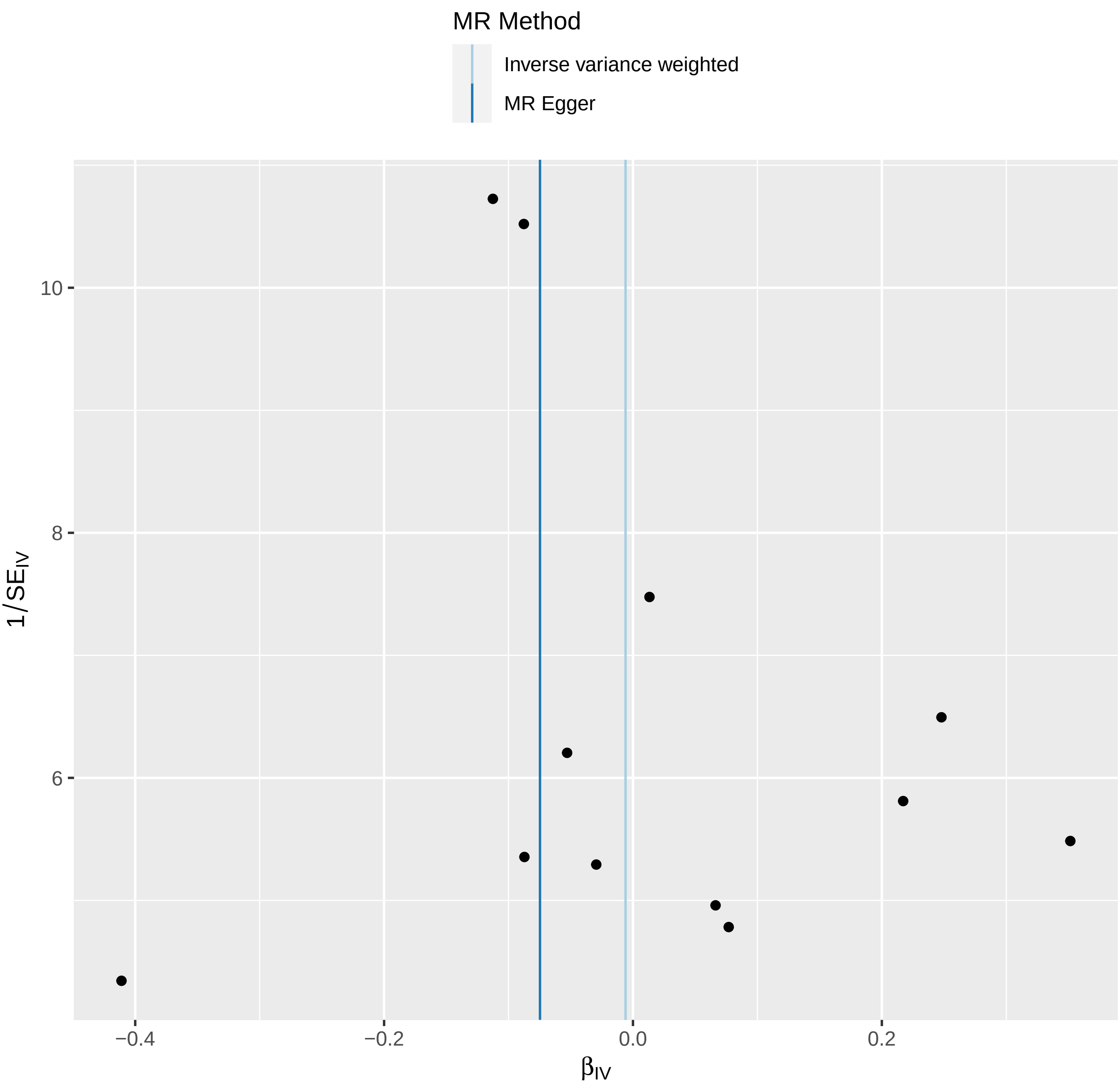

Batch 3−1641 : Selenium on Killer cell immunoglobulin−like receptor 2DL4

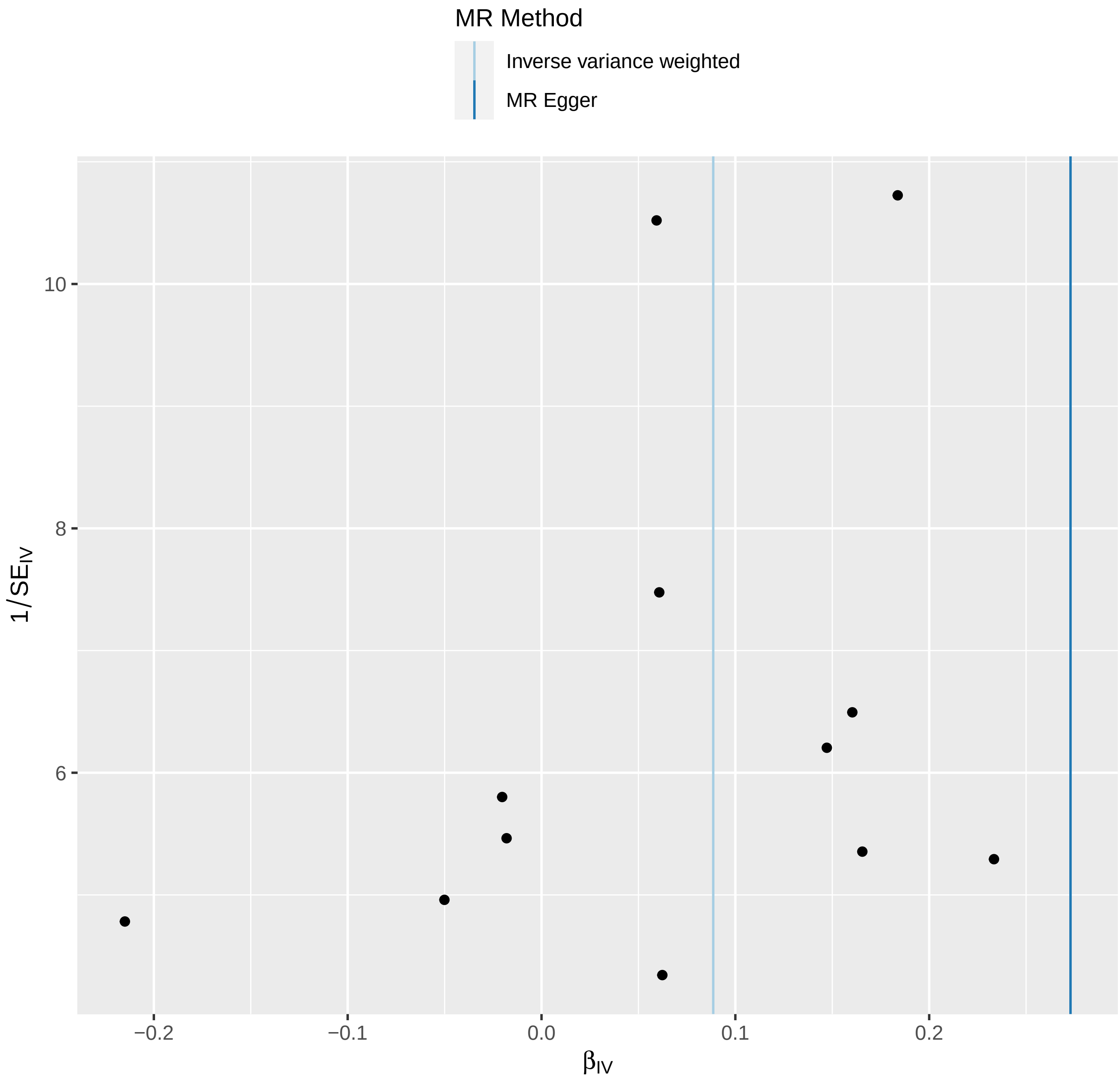

Batch 3−1642 : Selenium on Killer cell immunoglobulin−like receptor 2DL4

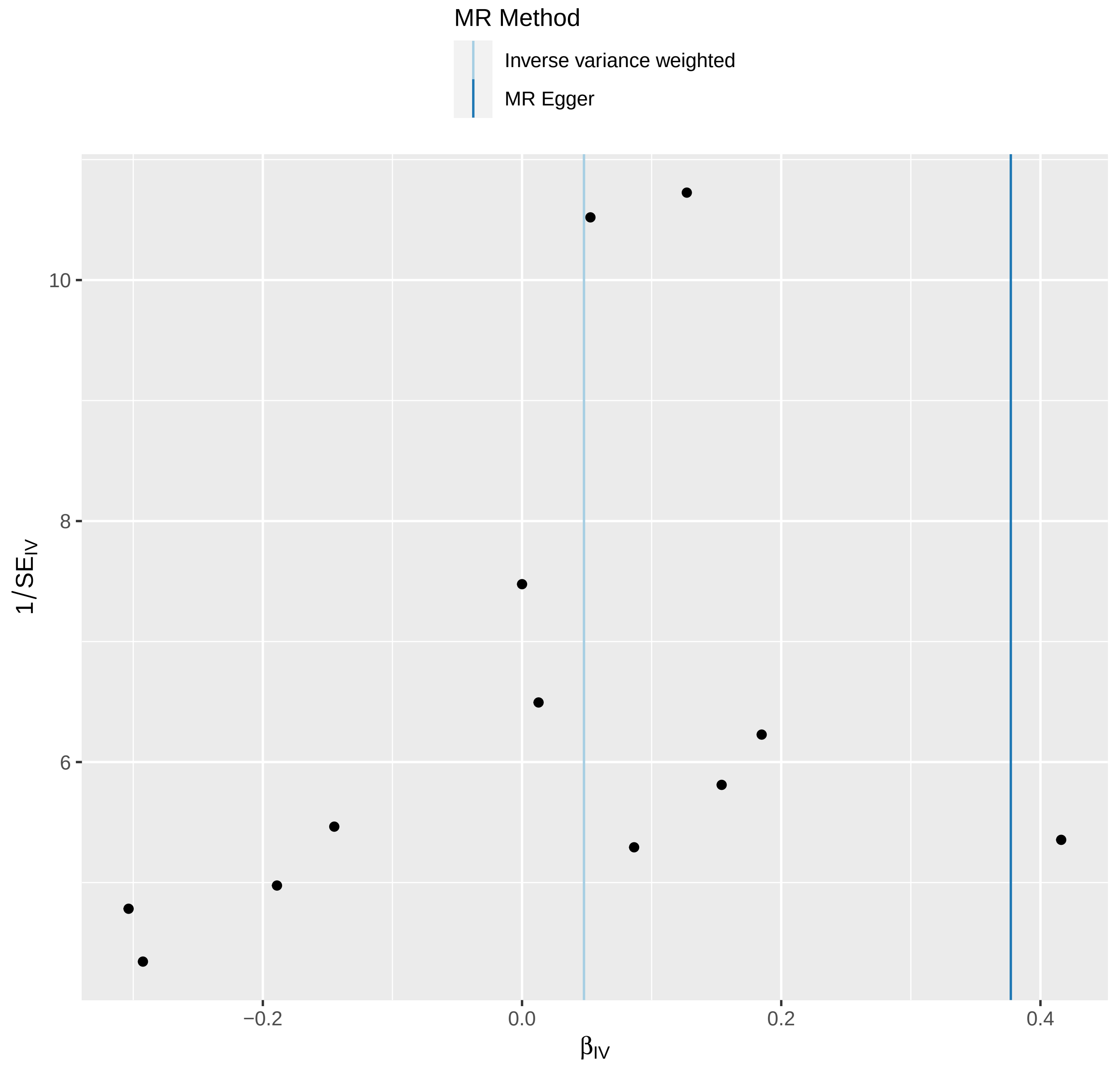

Batch 3−1643 : Selenium on Killer cell immunoglobulin−like receptor 2DL5A

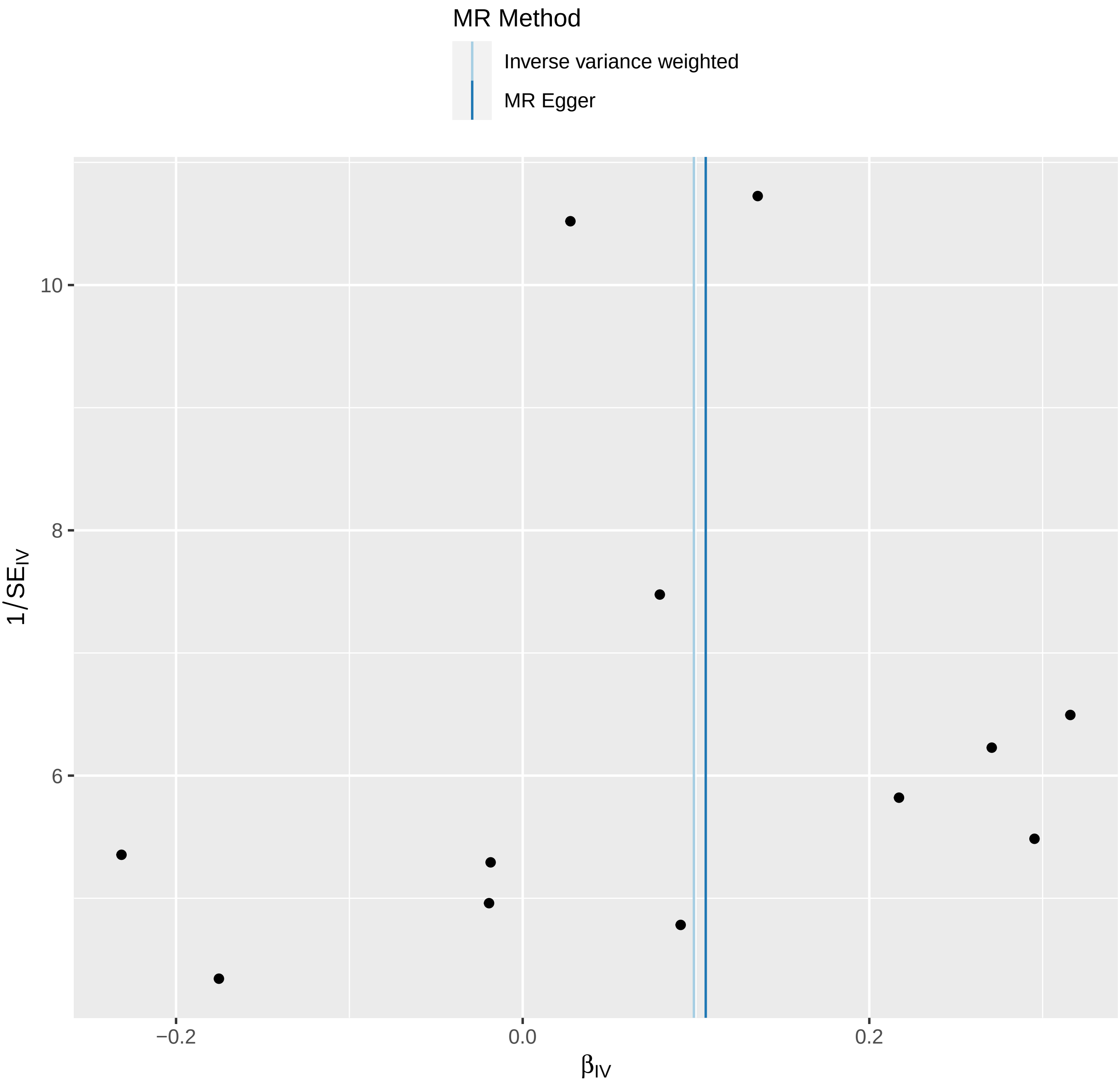

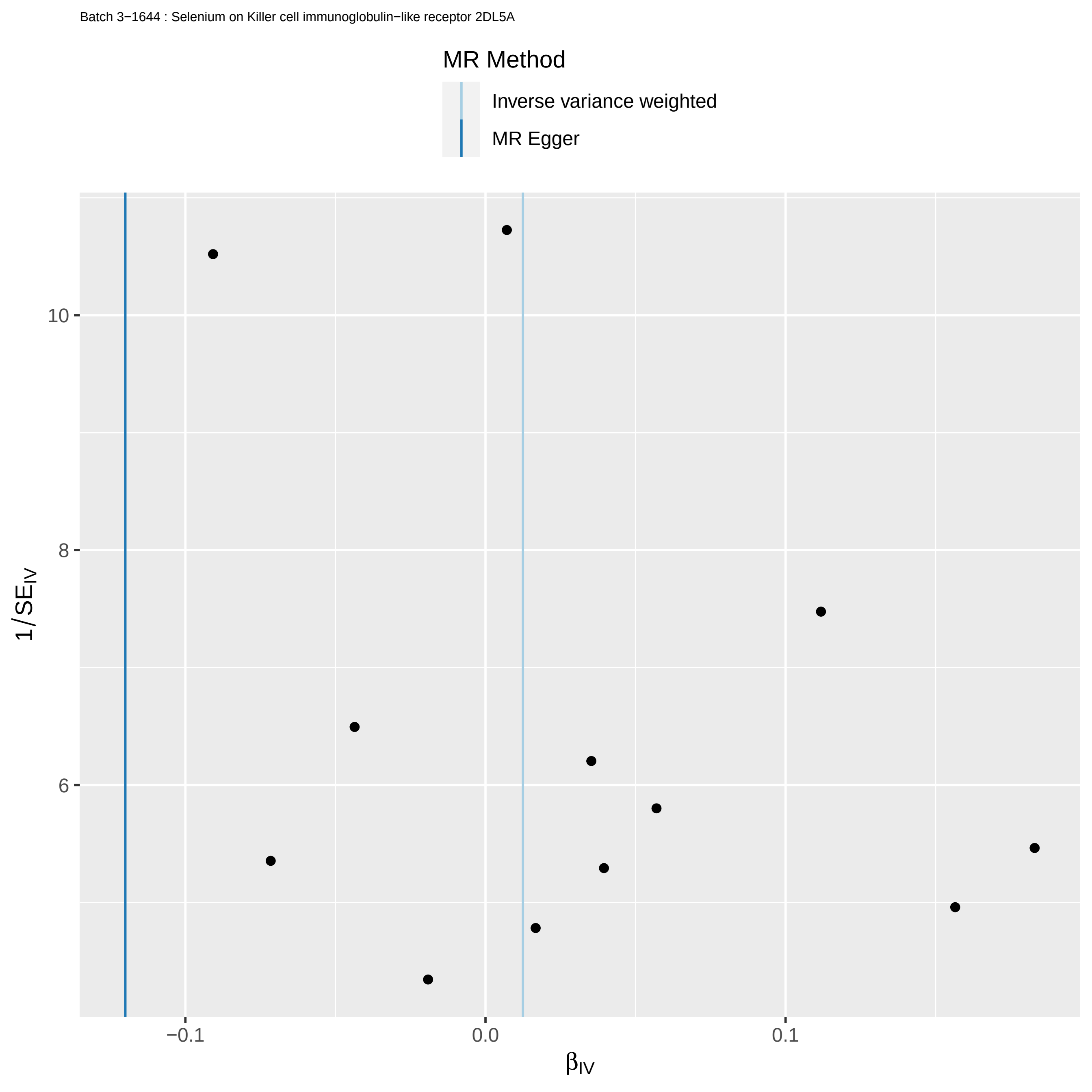
Batch 3−1644 : Selenium on Killer cell immunoglobulin−like receptor 2DL5A
MR Method
Inverse variance weighted
MR Egger
10
8
6
1/SE_IV
−0.1
0.0
0.1
β_IV

Batch 3−1645 : Selenium on Killer cell immunoglobulin−like receptor 2DS2

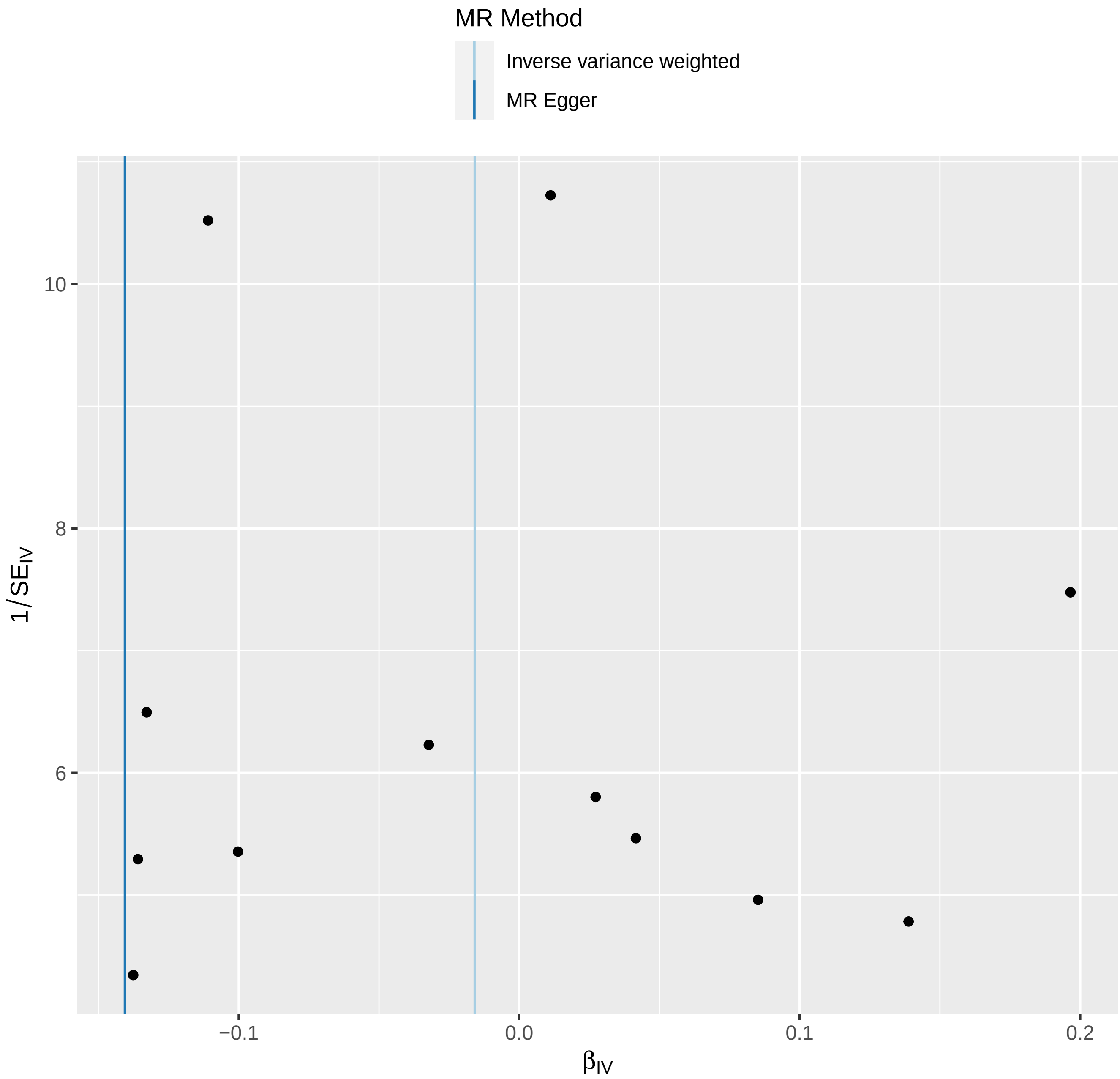

Batch 3−1646 : Selenium on Killer cell immunoglobulin−like receptor 3DL2

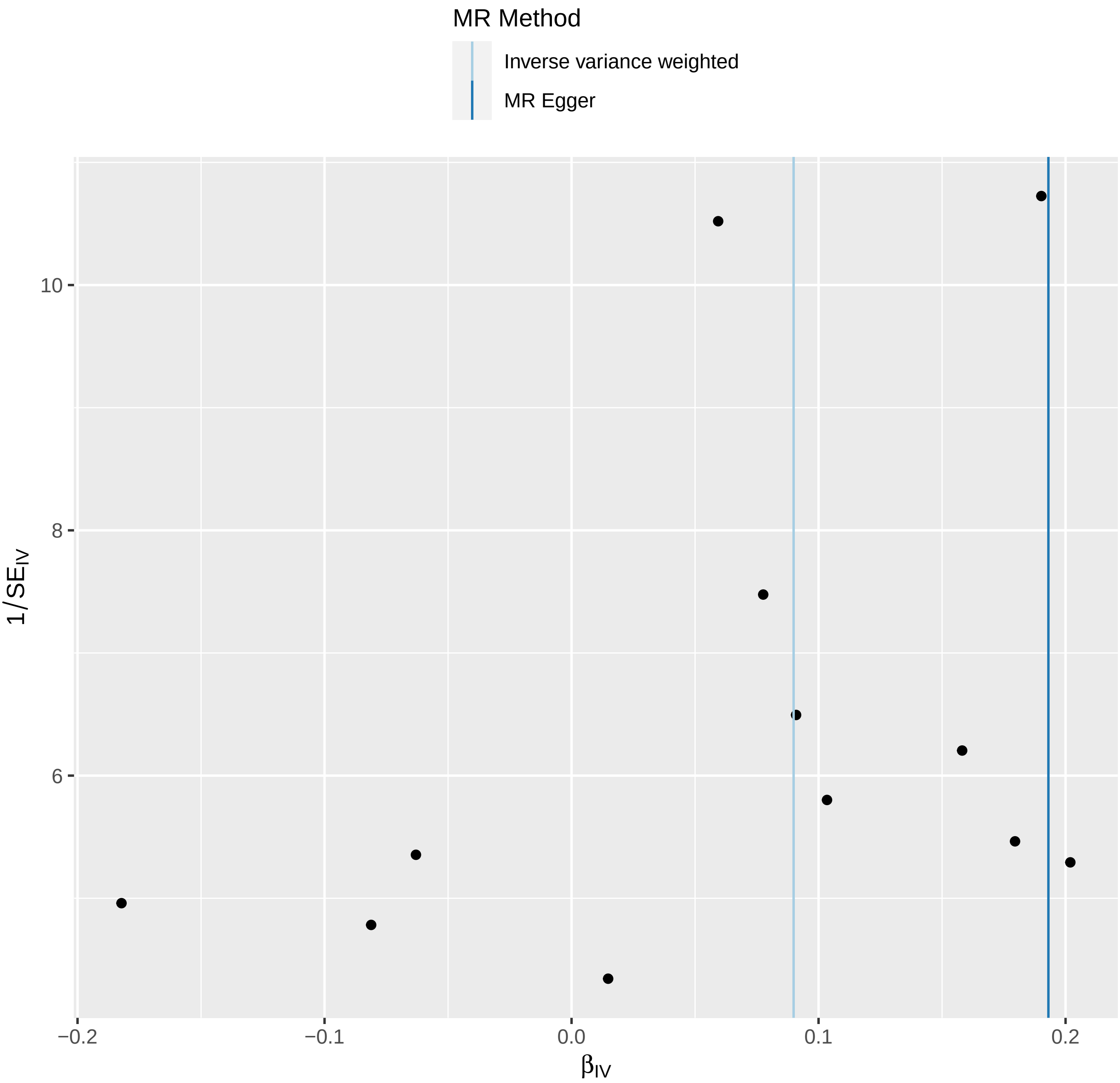

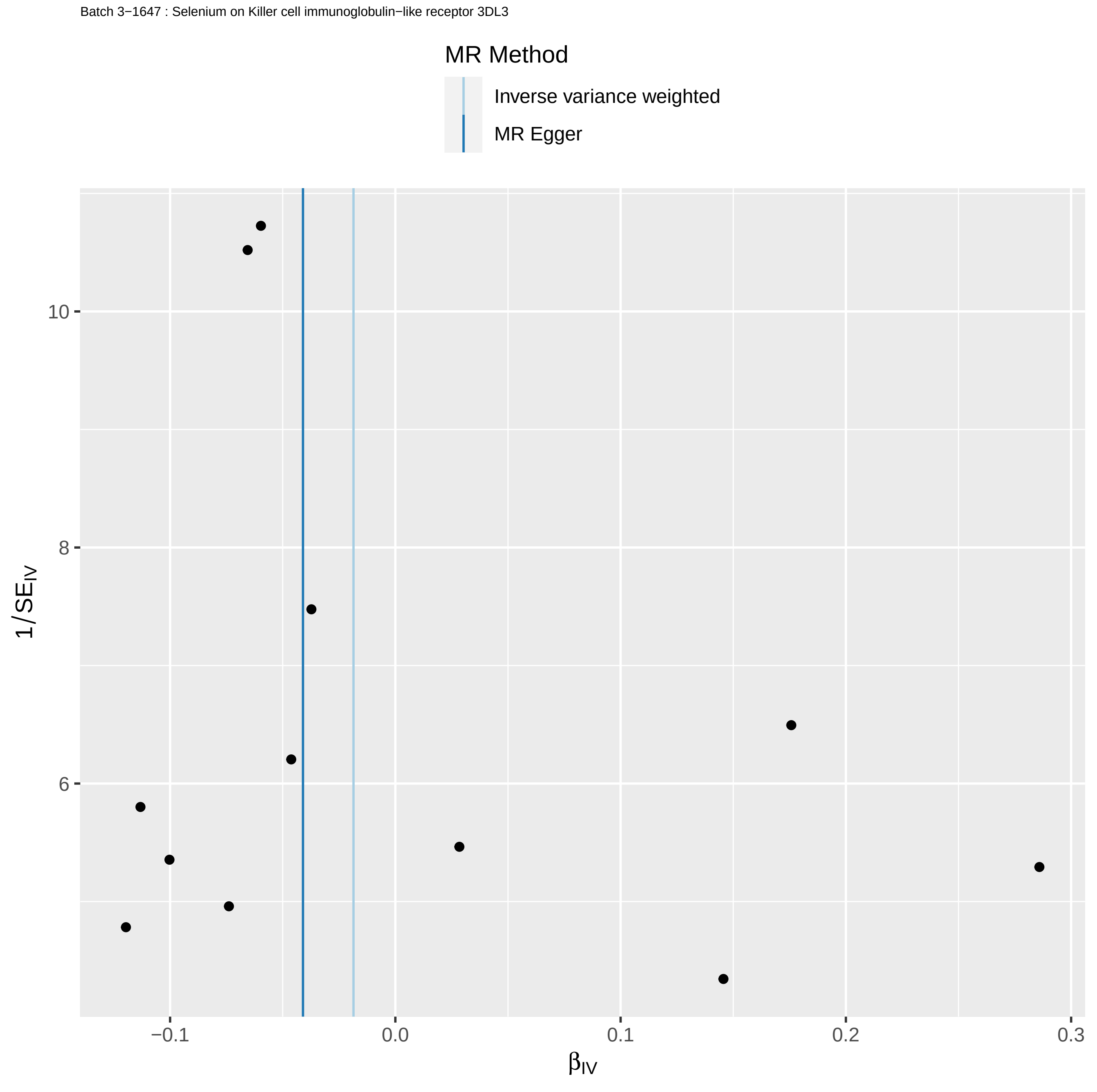

Batch 3−1647 : Selenium on Killer cell immunoglobulin−like receptor 3DL3
MR Method
Inverse variance weighted
MR Egger
10
8
6
1/SE$_{IV}$
−0.1
0.0
0.1
0.2
0.3
β$_{IV}$

Batch 3−1648 : Selenium on Killer cell immunoglobulin−like receptor 3DS1

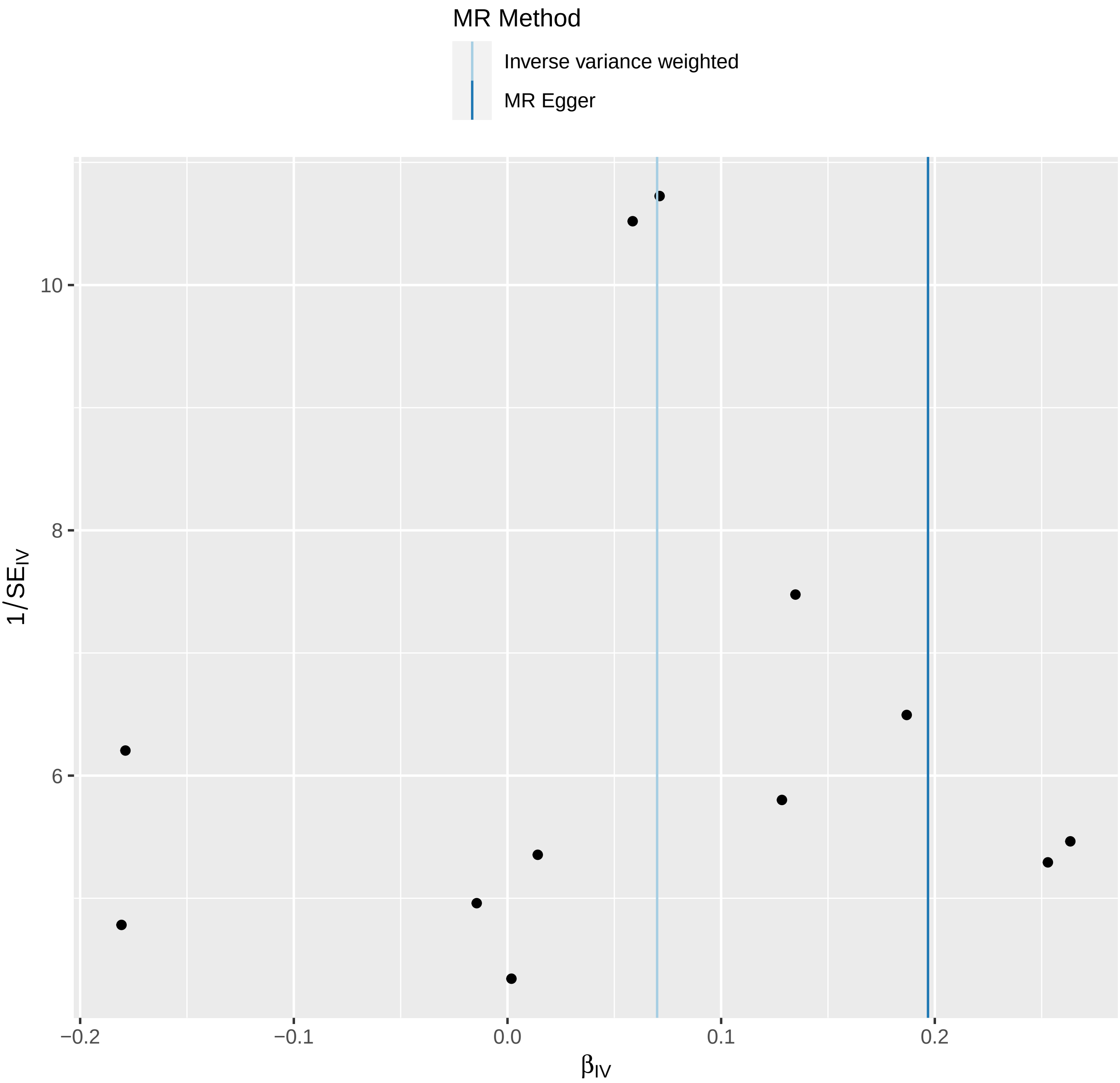

Batch 3−1649 : Selenium on Kin of IRRE−like protein 2

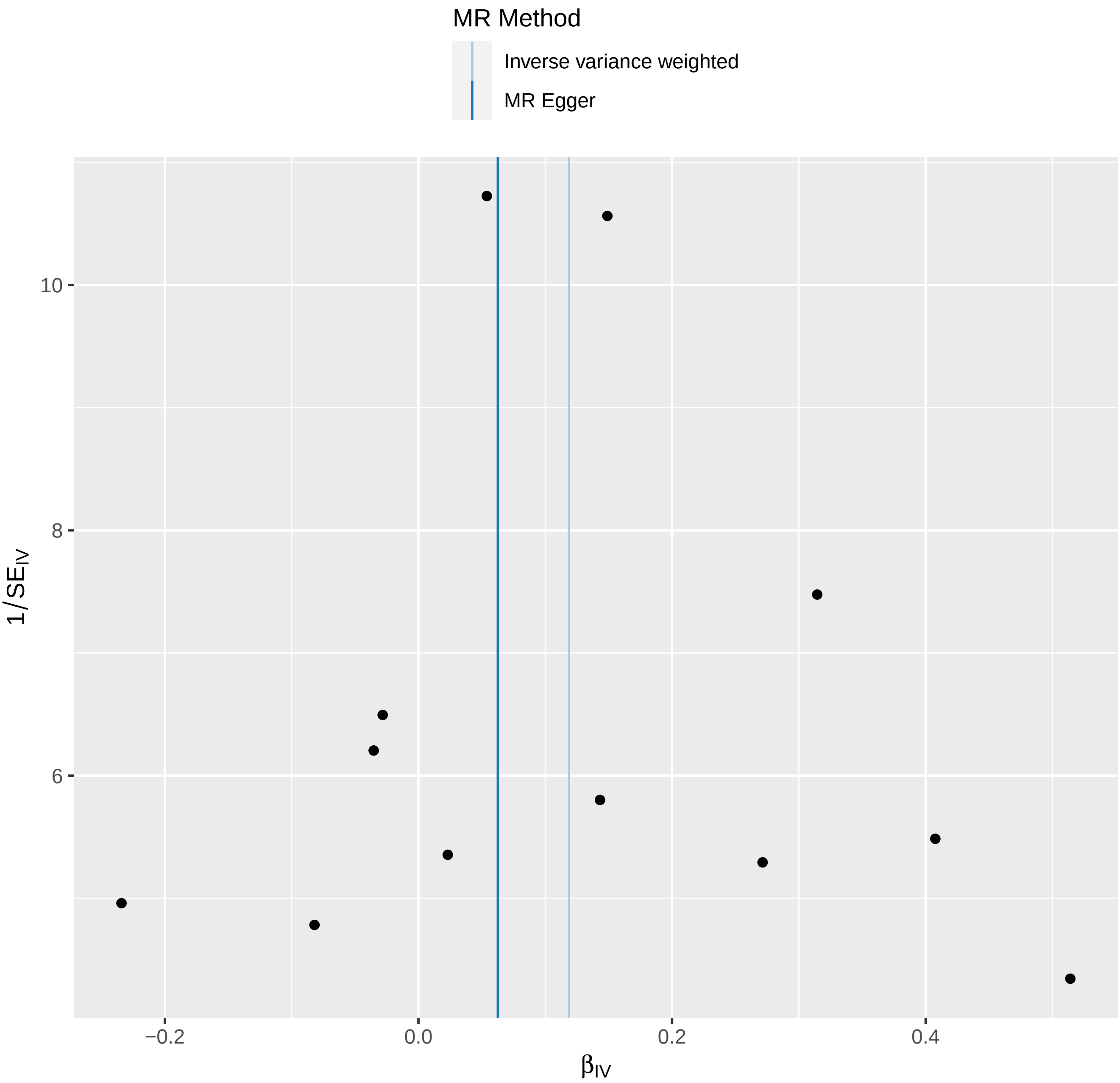

Batch 3−1650 : Selenium on Kin of IRRE−like protein 2

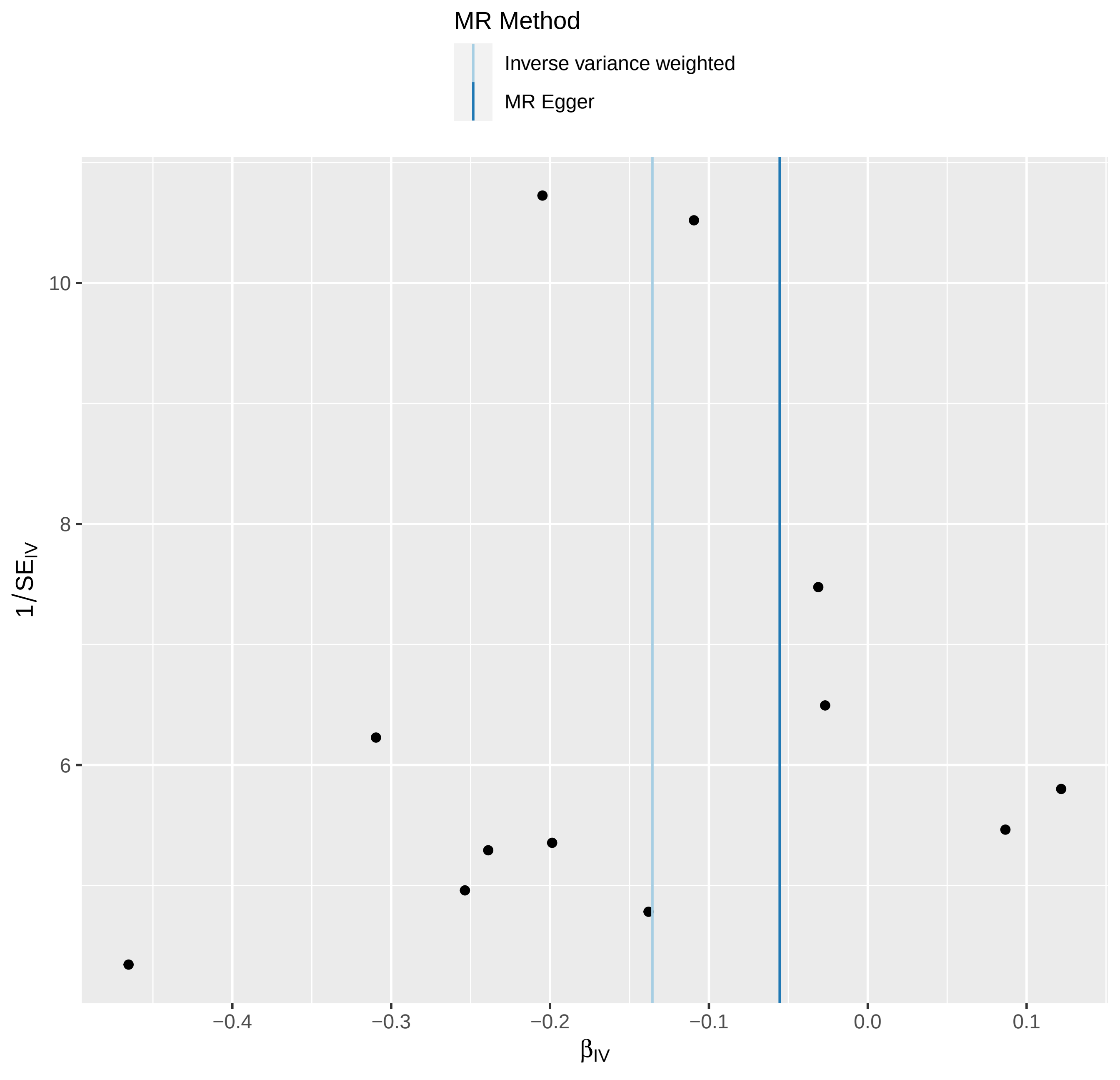

Batch 3−1651 : Selenium on Kin of IRRE−like protein 3

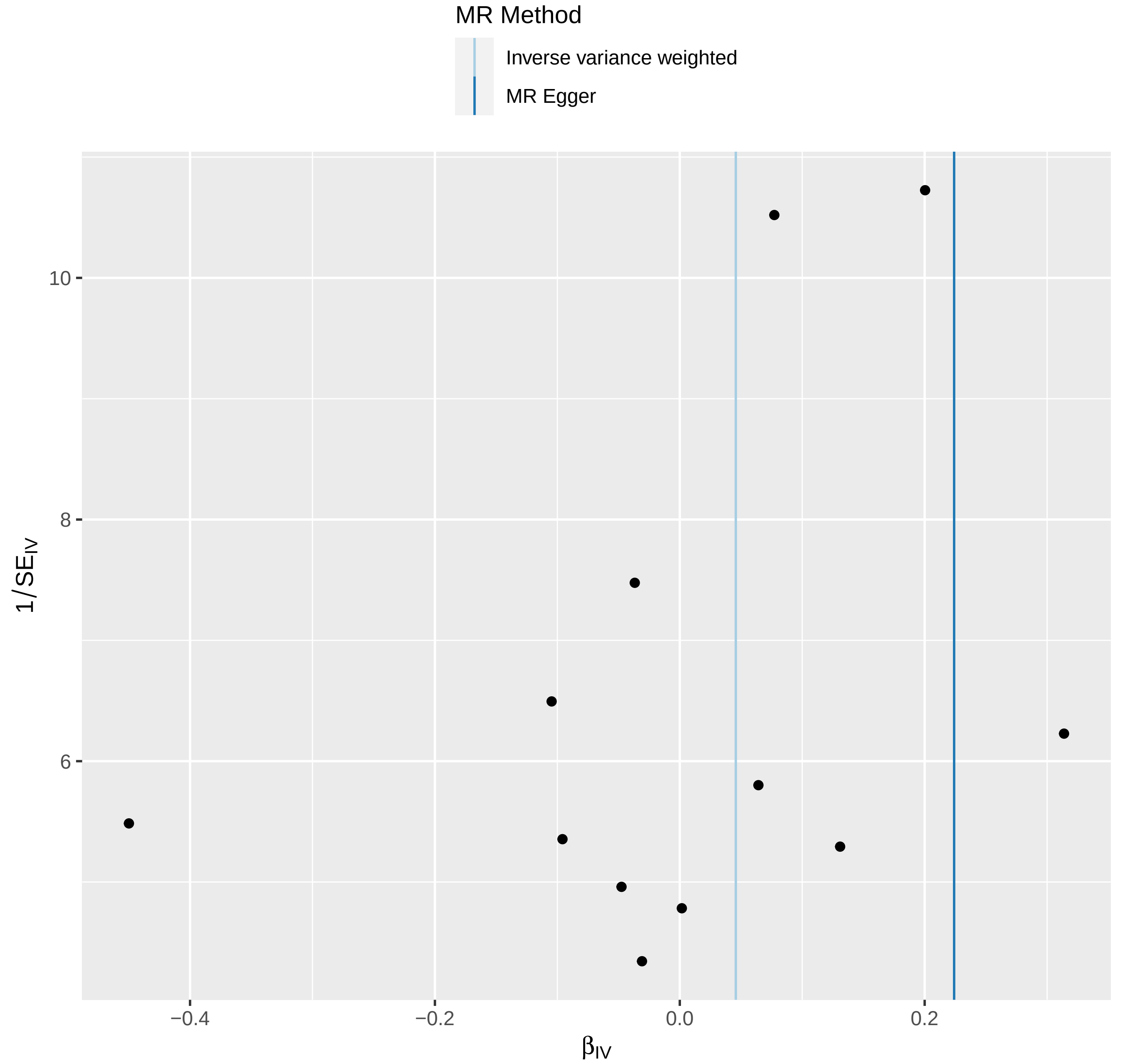

Batch 3−1652 : Selenium on Mast/stem cell growth factor receptor Kit

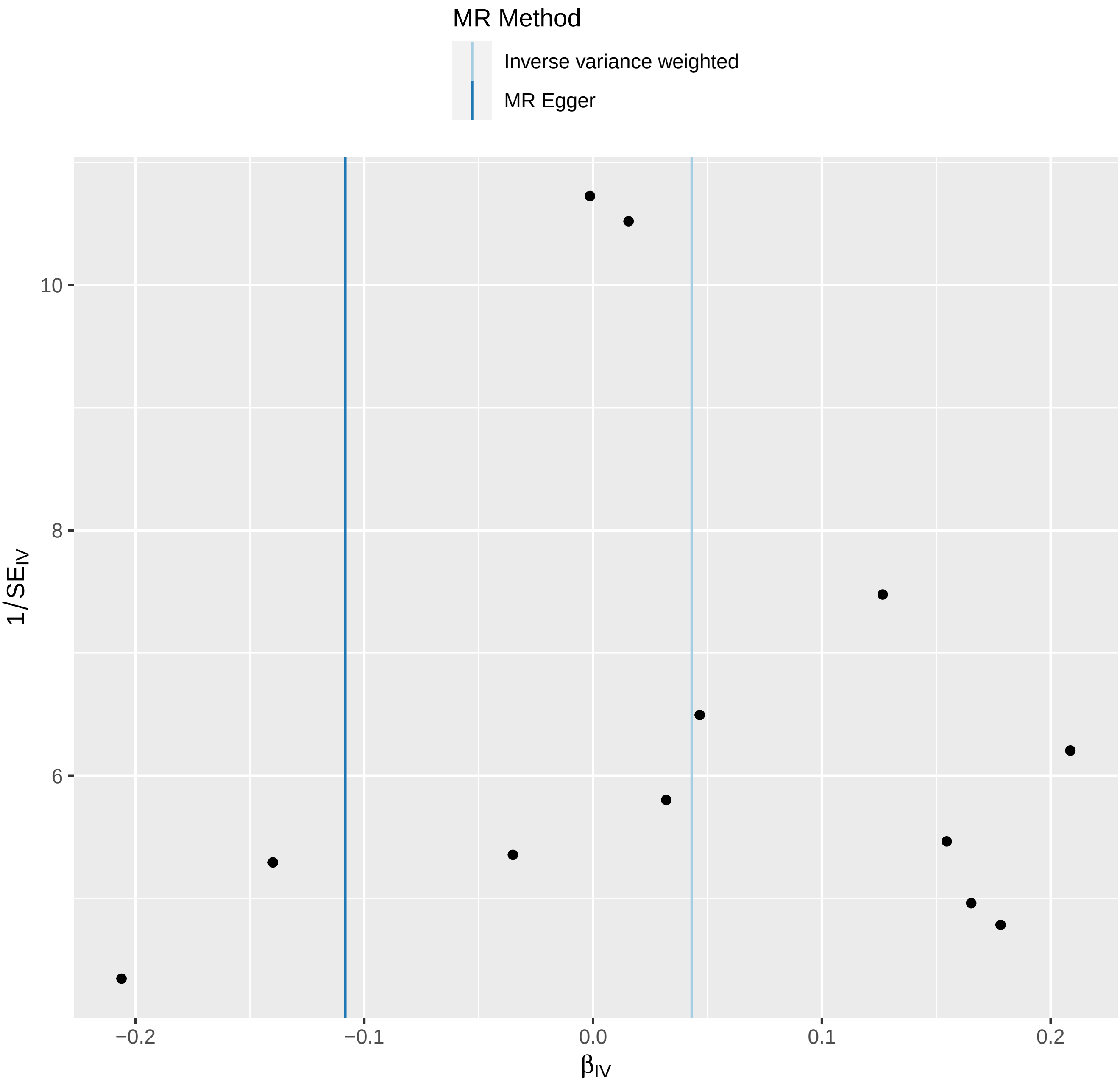

Batch 3−1653 : Selenium on Kit ligand

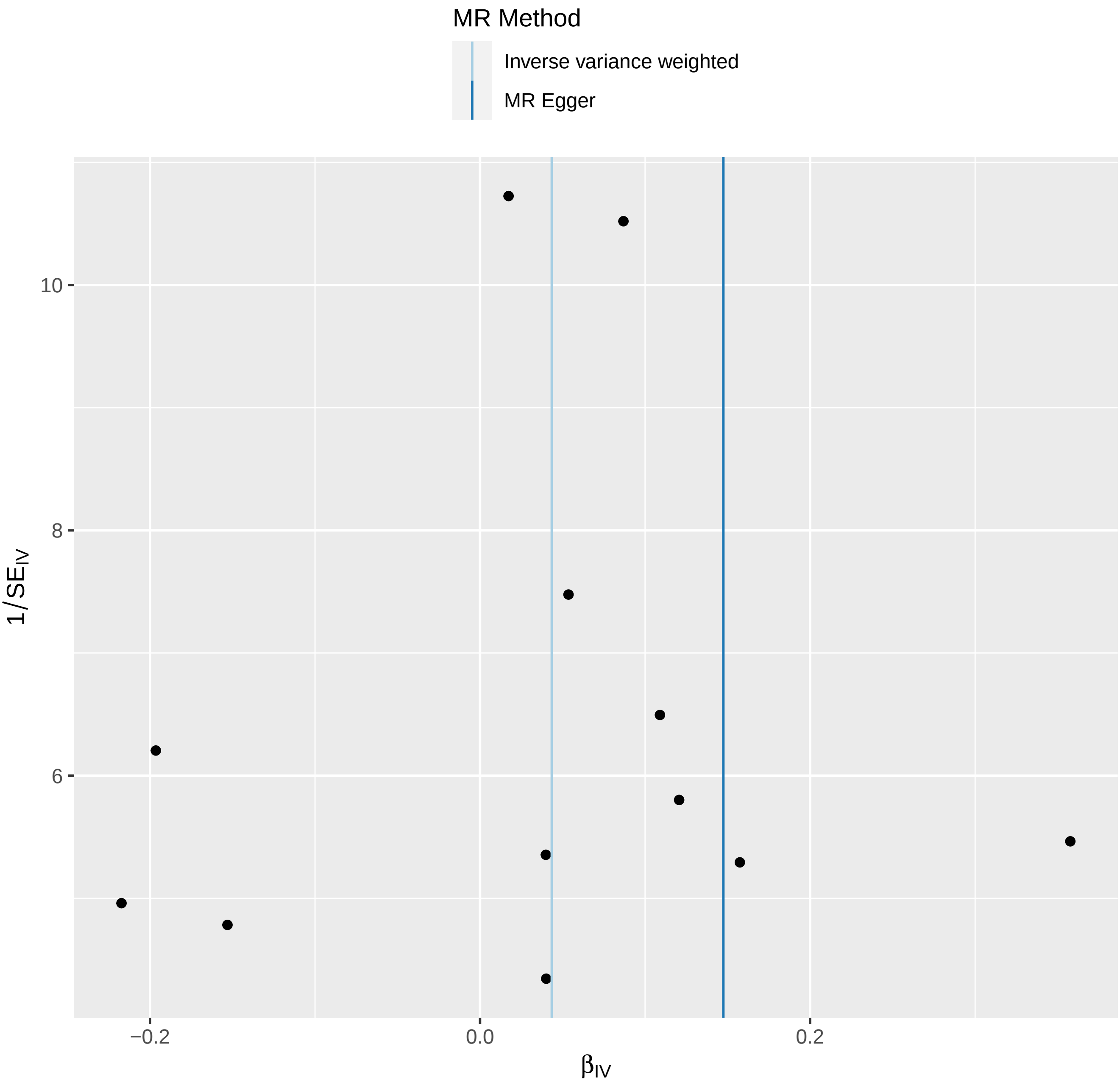

Batch 3−1654 : Selenium on Kelch−like protein 12

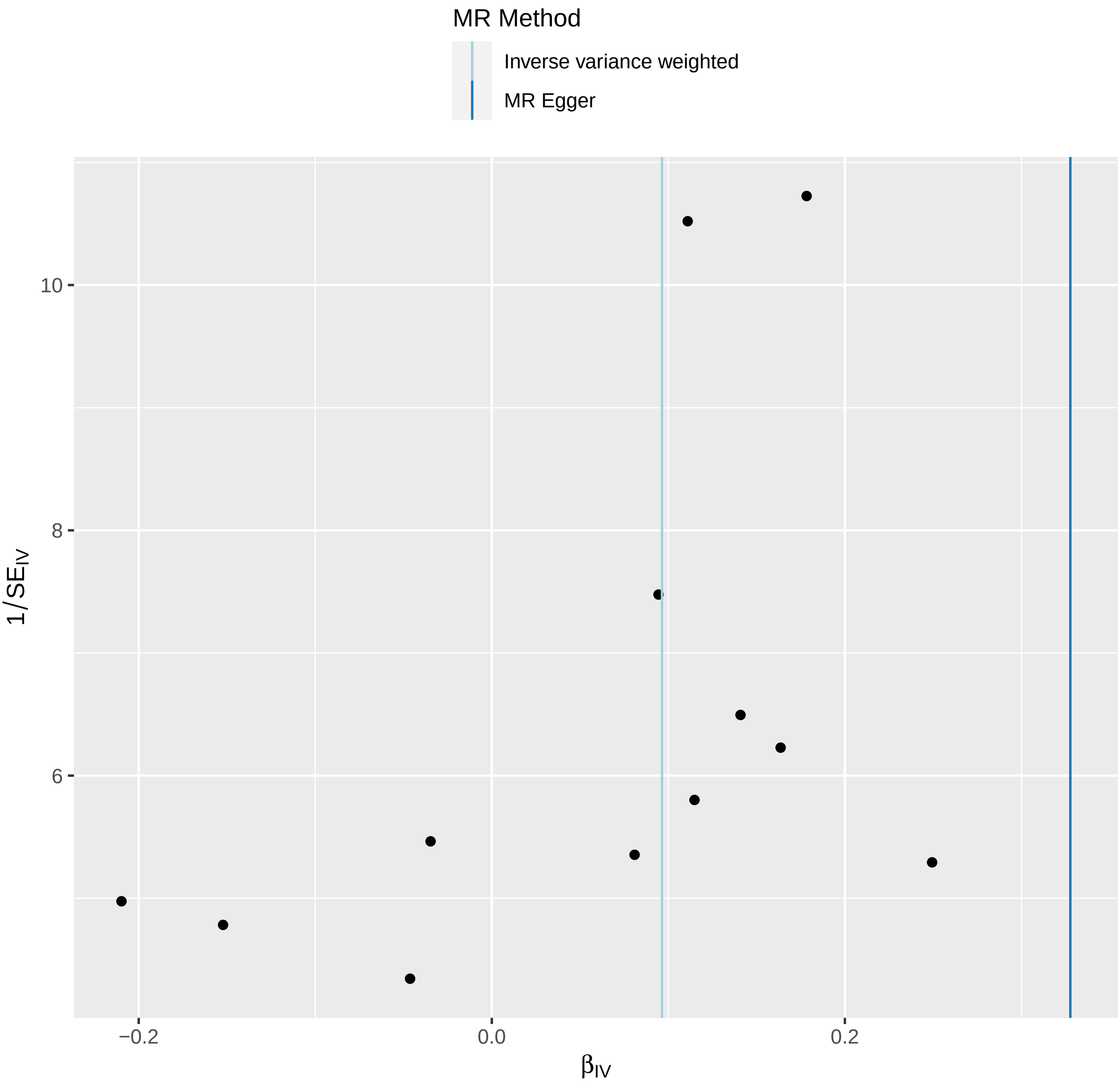

Batch 3−1655 : Selenium on Kelch−like protein 13

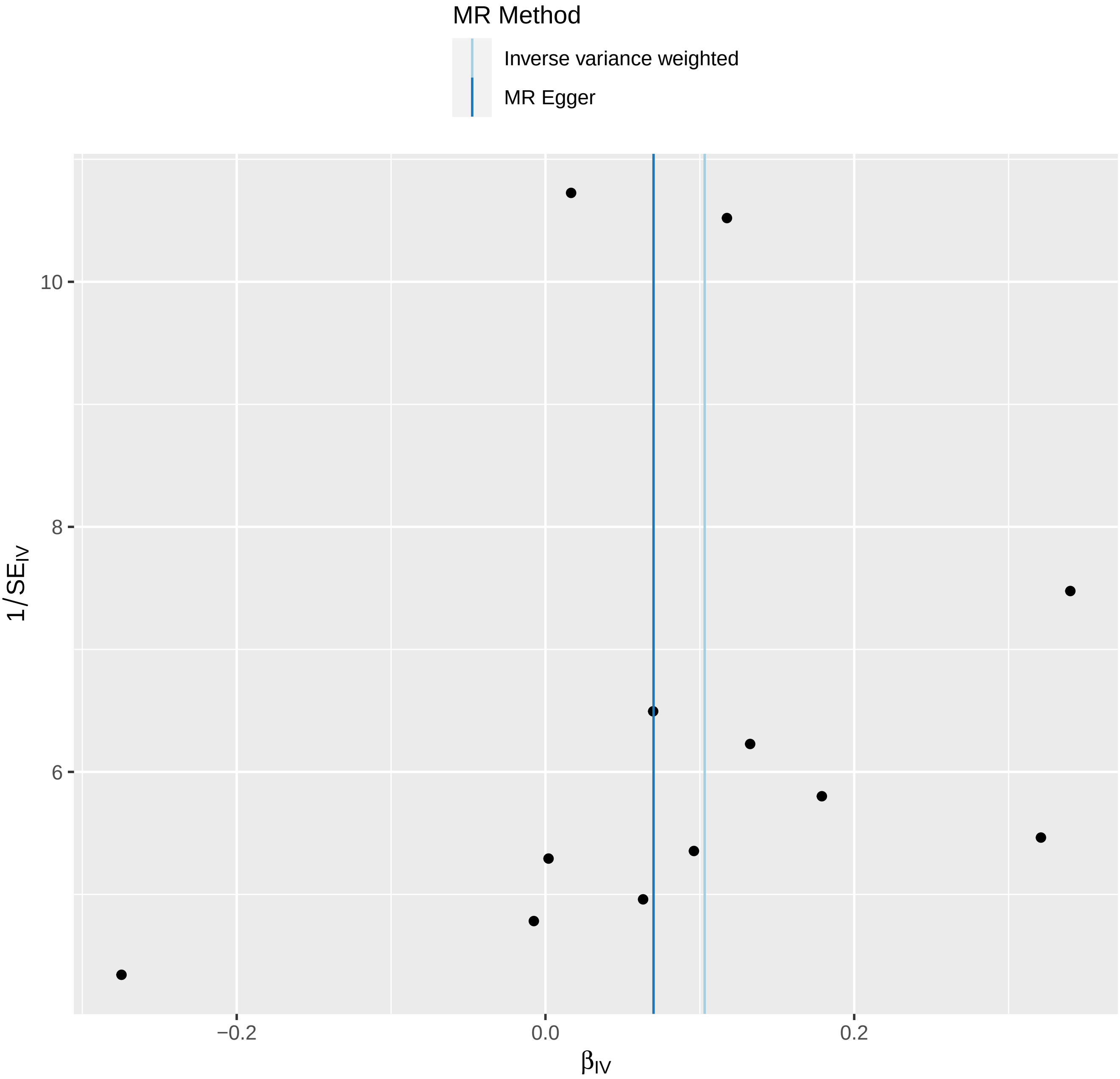

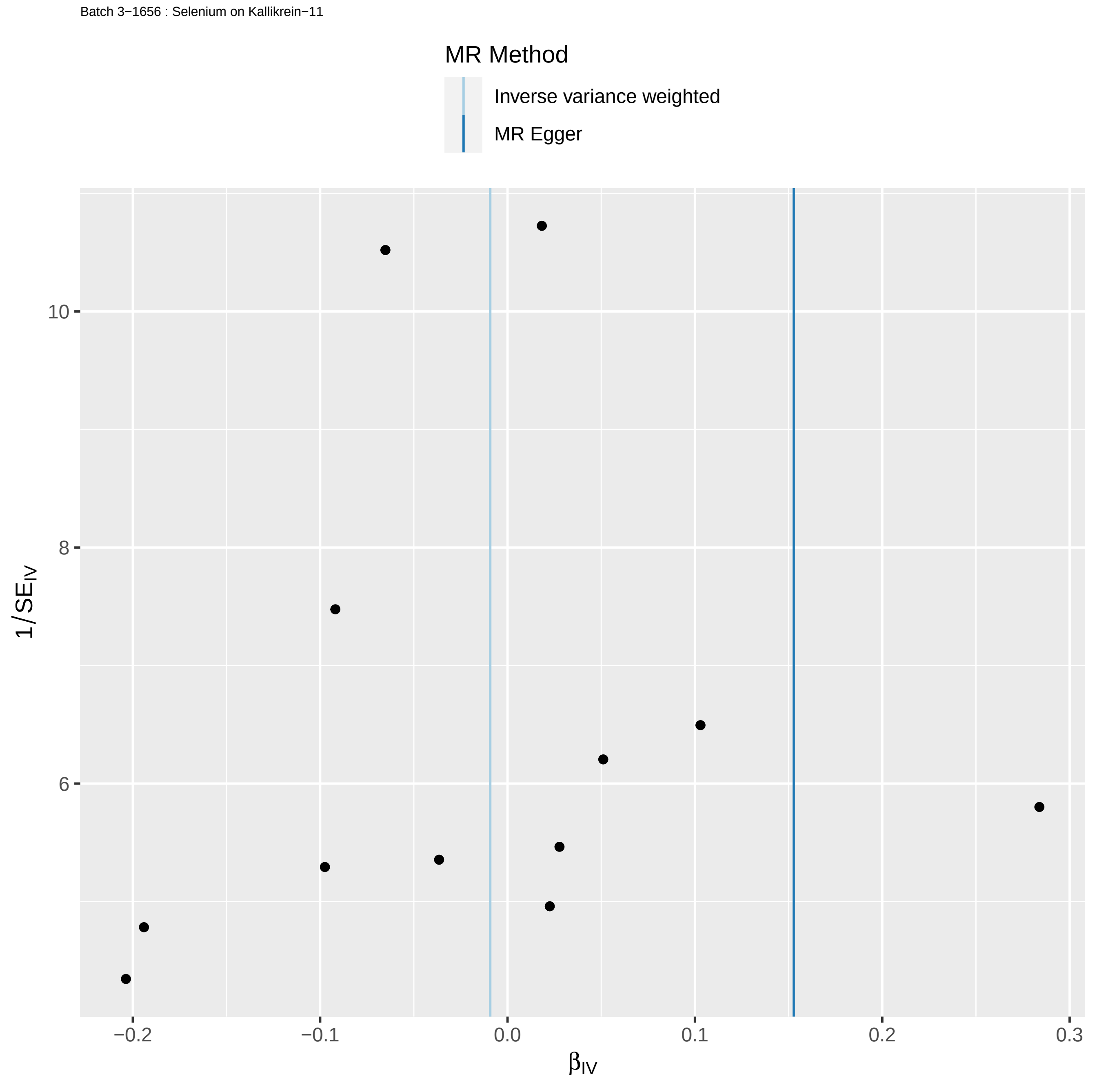
Batch 3−1656 : Selenium on Kallikrein−11
MR Method
Inverse variance weighted
MR Egger
10
8
6
1/SE$_{IV}$
−0.2
−0.1
0.0
0.1
0.2
0.3
β$_{IV}$

Batch 3−1657 : Selenium on Kallikrein−12

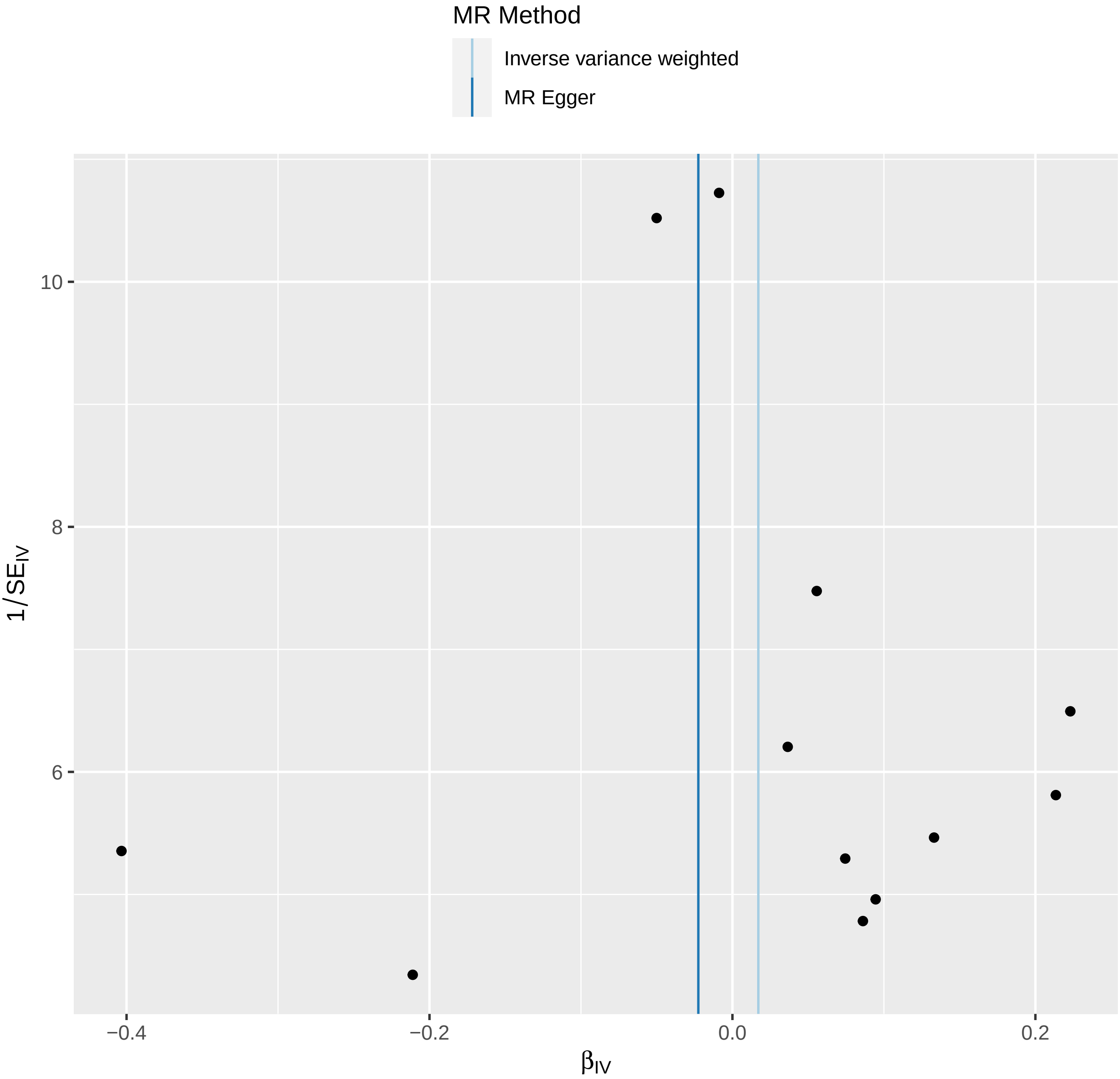

Batch 3−1658 : Selenium on Kallikrein−14

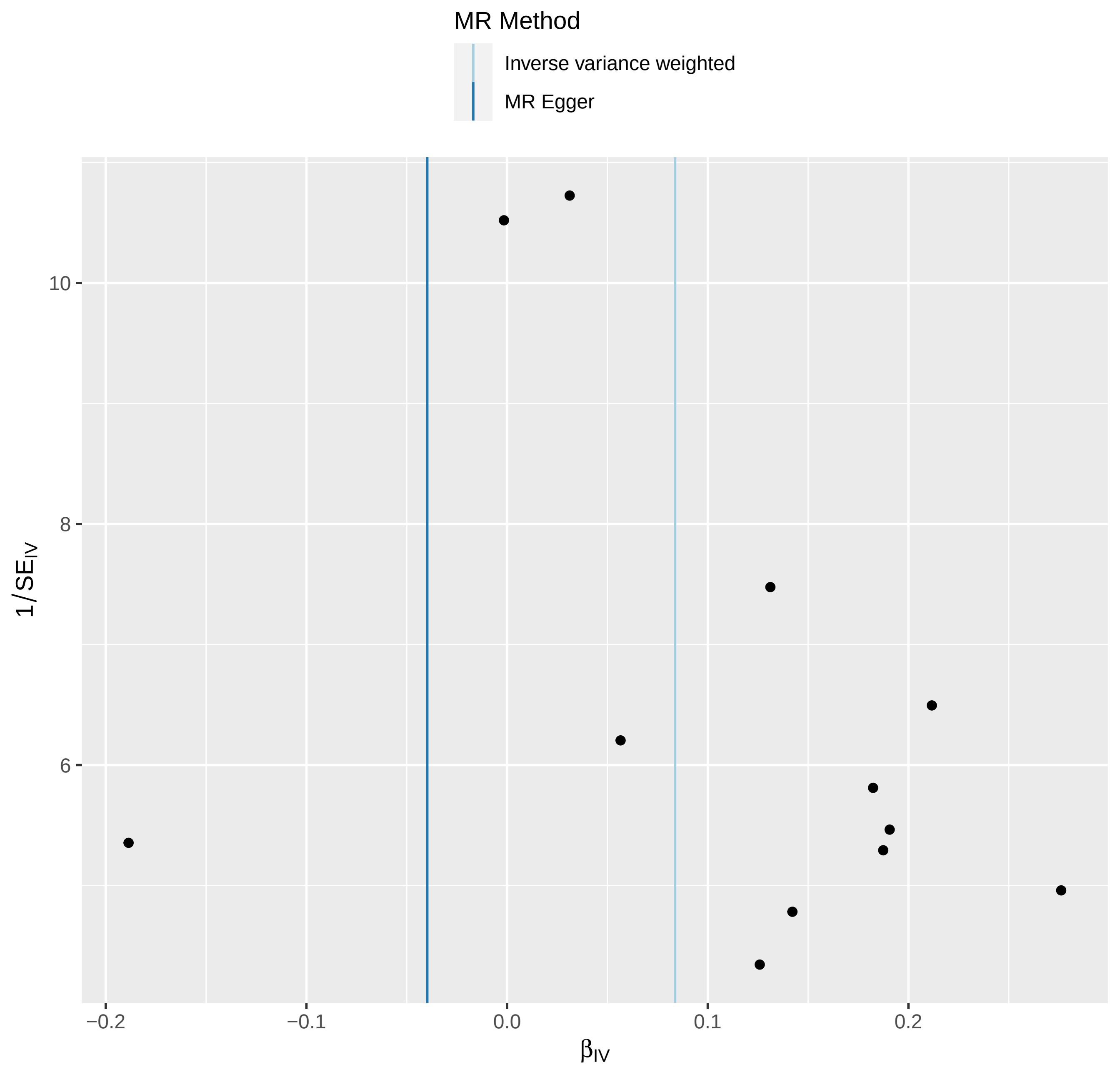

Batch 3−1659 : Selenium on Kallikrein−14

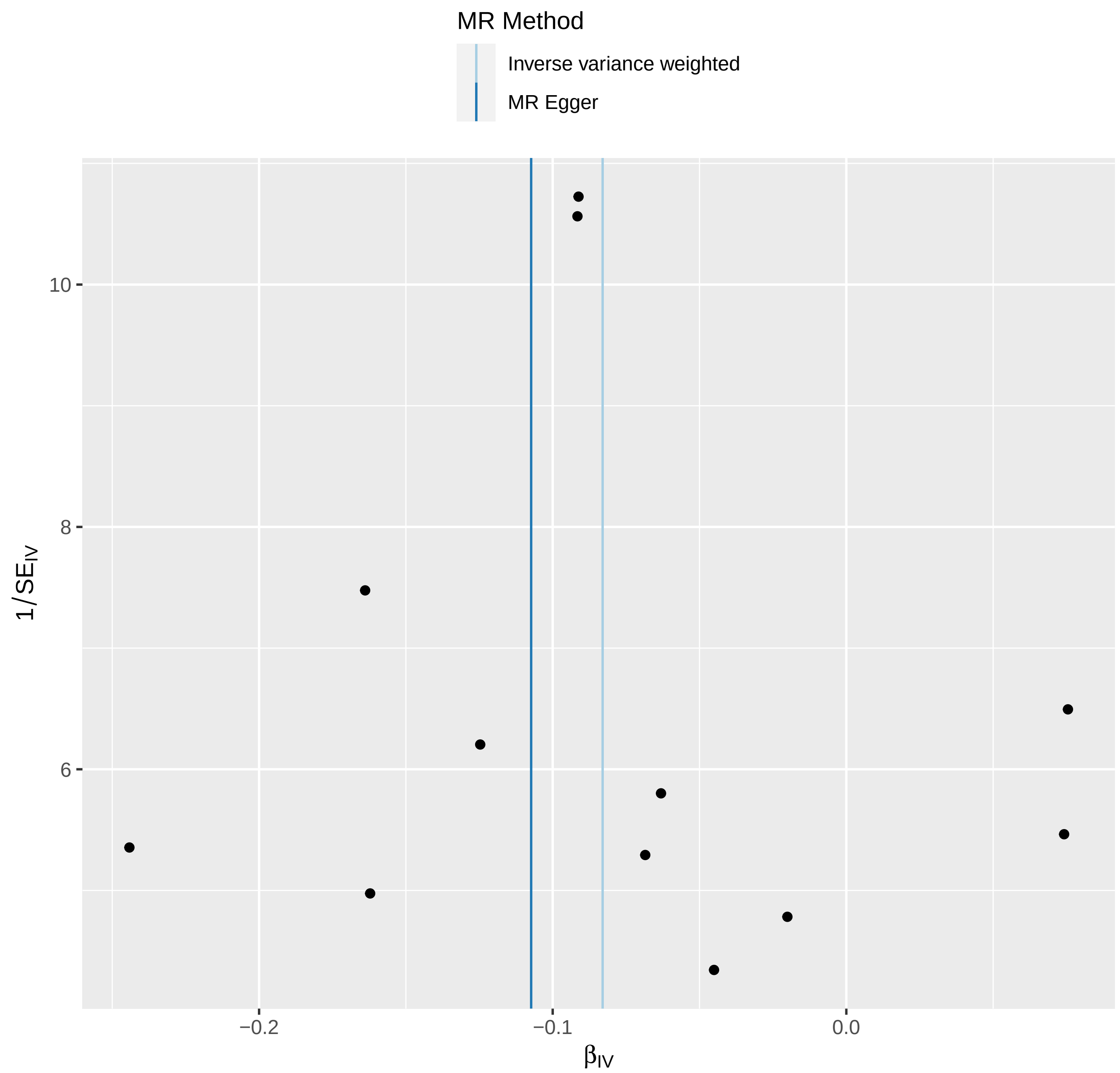

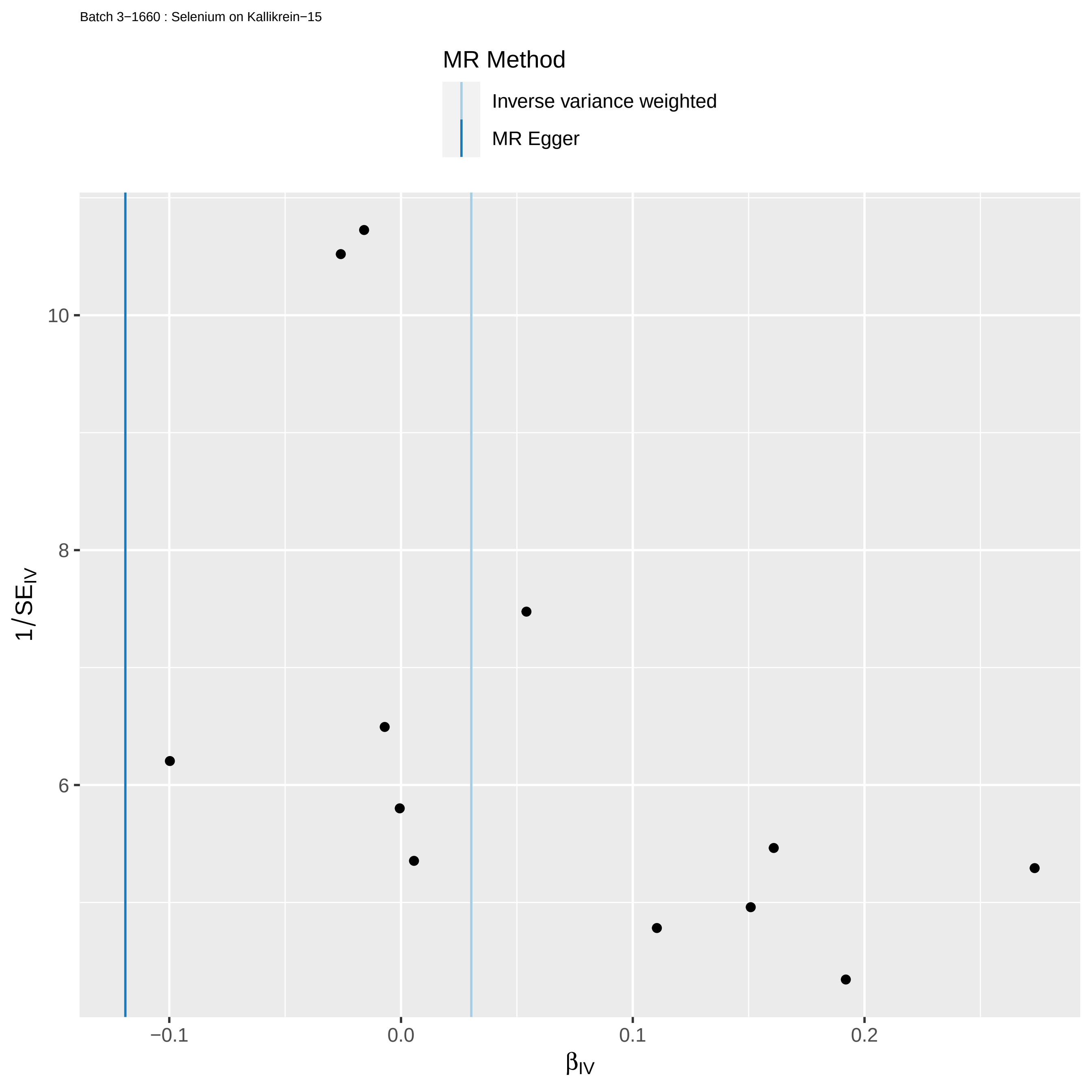
Batch 3−1660 : Selenium on Kallikrein−15
MR Method
Inverse variance weighted
MR Egger
10
8
6
−0.1
0.0
0.1
0.2
1/SE_IV
β_IV

Batch 3−1661 : Selenium on Prostate−specific antigen

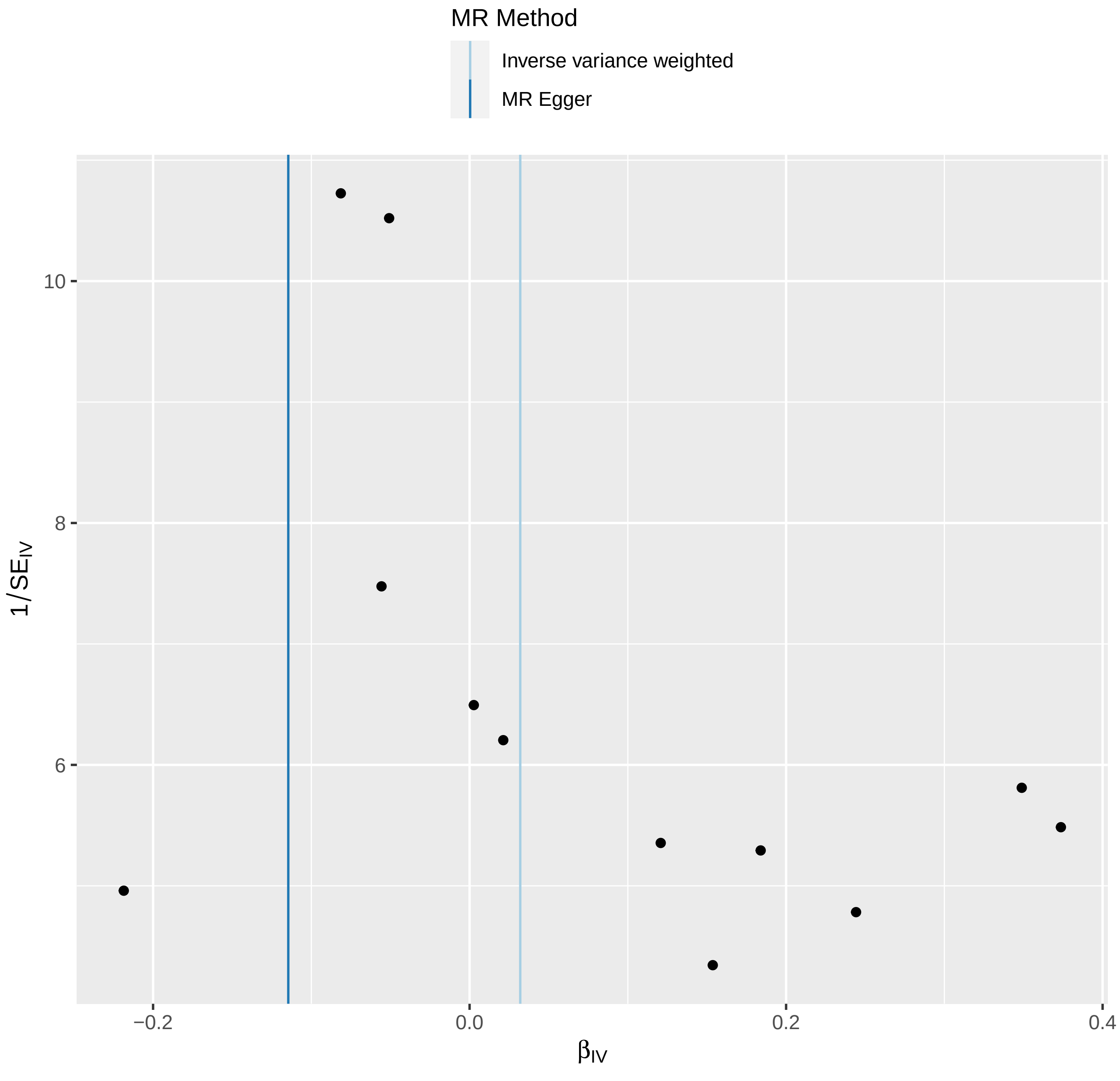

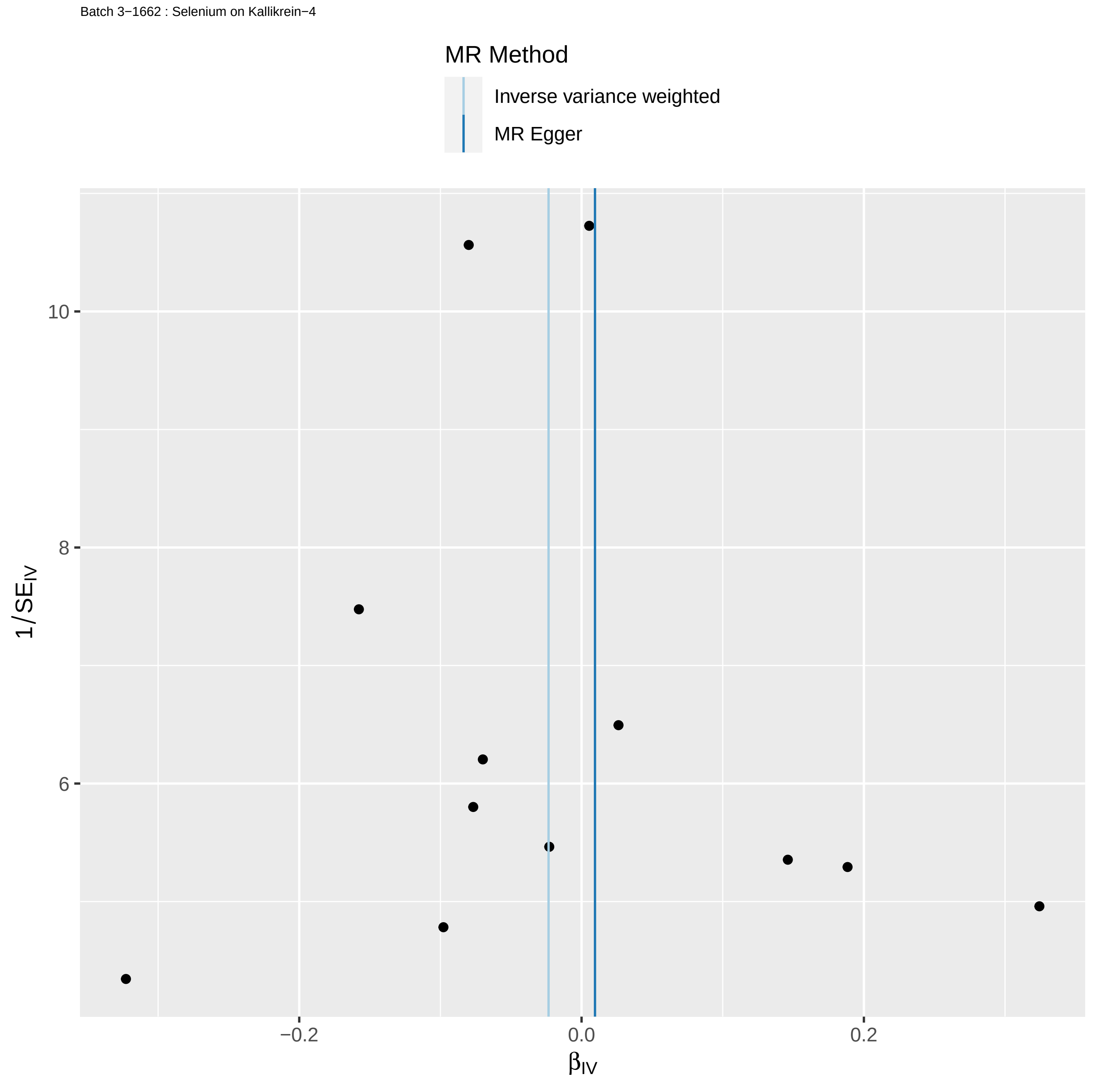
Batch 3−1662 : Selenium on Kallikrein−4
MR Method
Inverse variance weighted
MR Egger
10
8
6
1/SE_IV
−0.2
0.0
0.2
β_IV

Batch 3−1663 : Selenium on Kallikrein−5

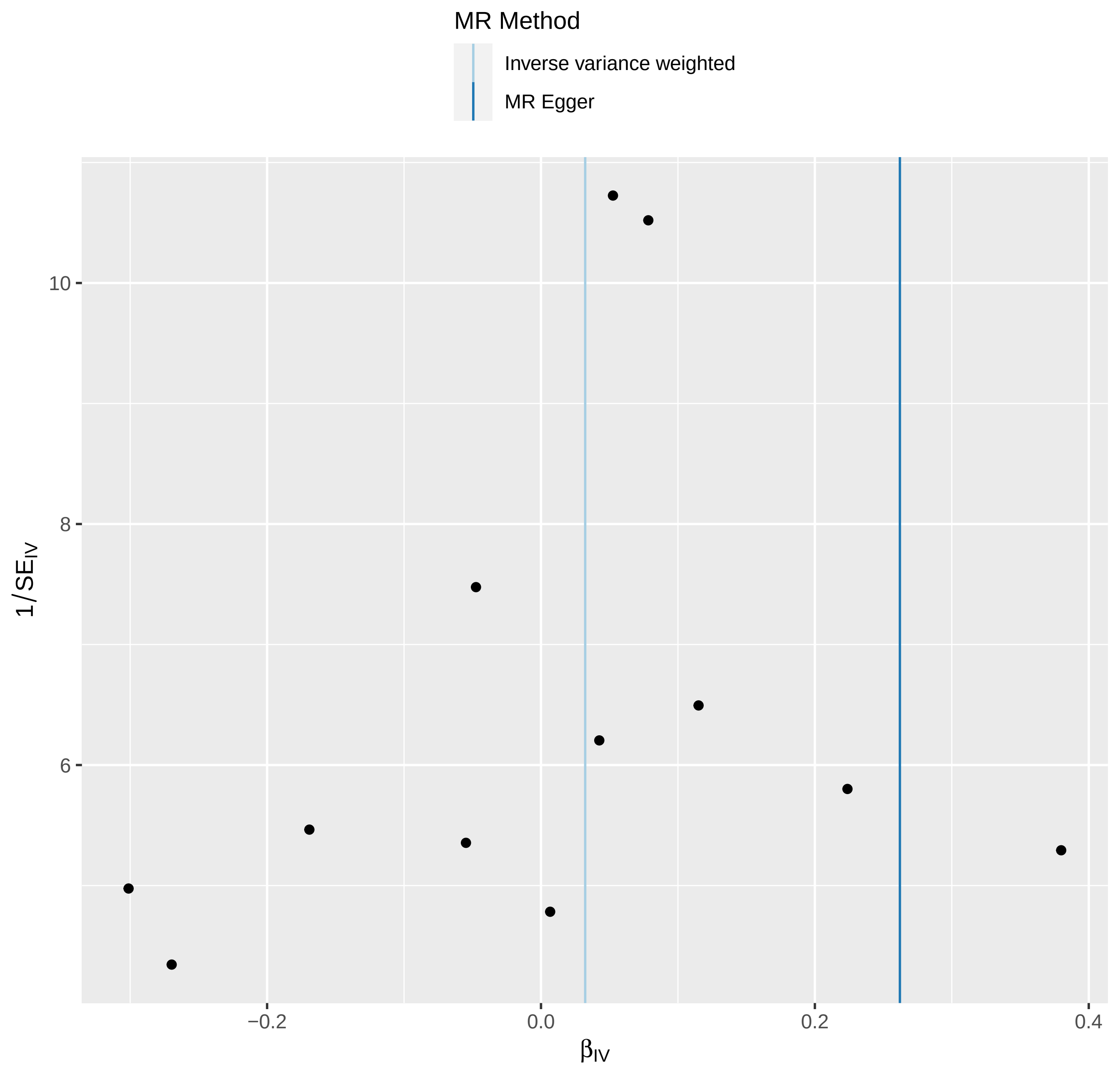

Batch 3−1664 : Selenium on Kallikrein−5

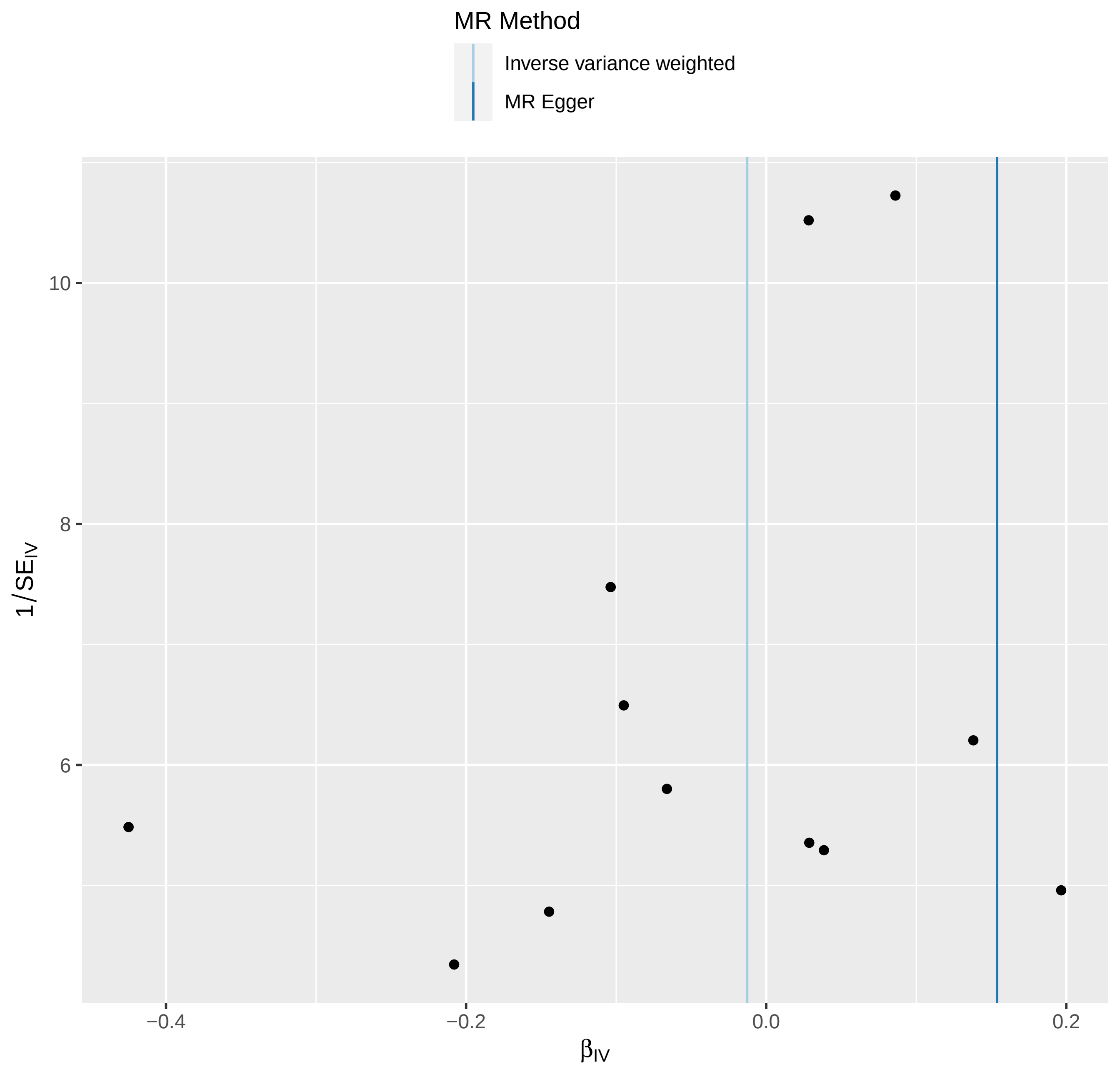

Batch 3−1665 : Selenium on Kallikrein−6

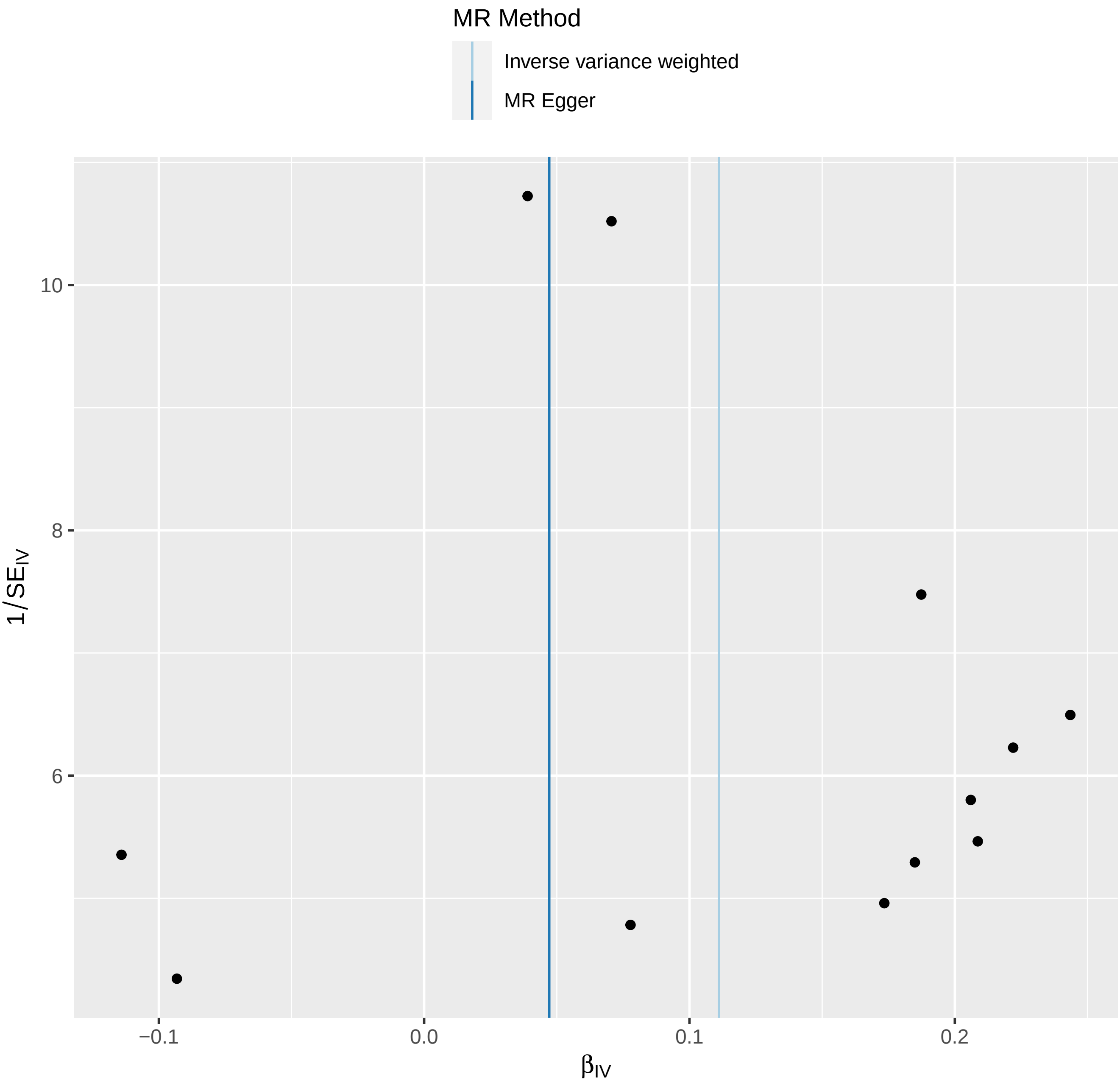

Batch 3−1666 : Selenium on Kallikrein−7

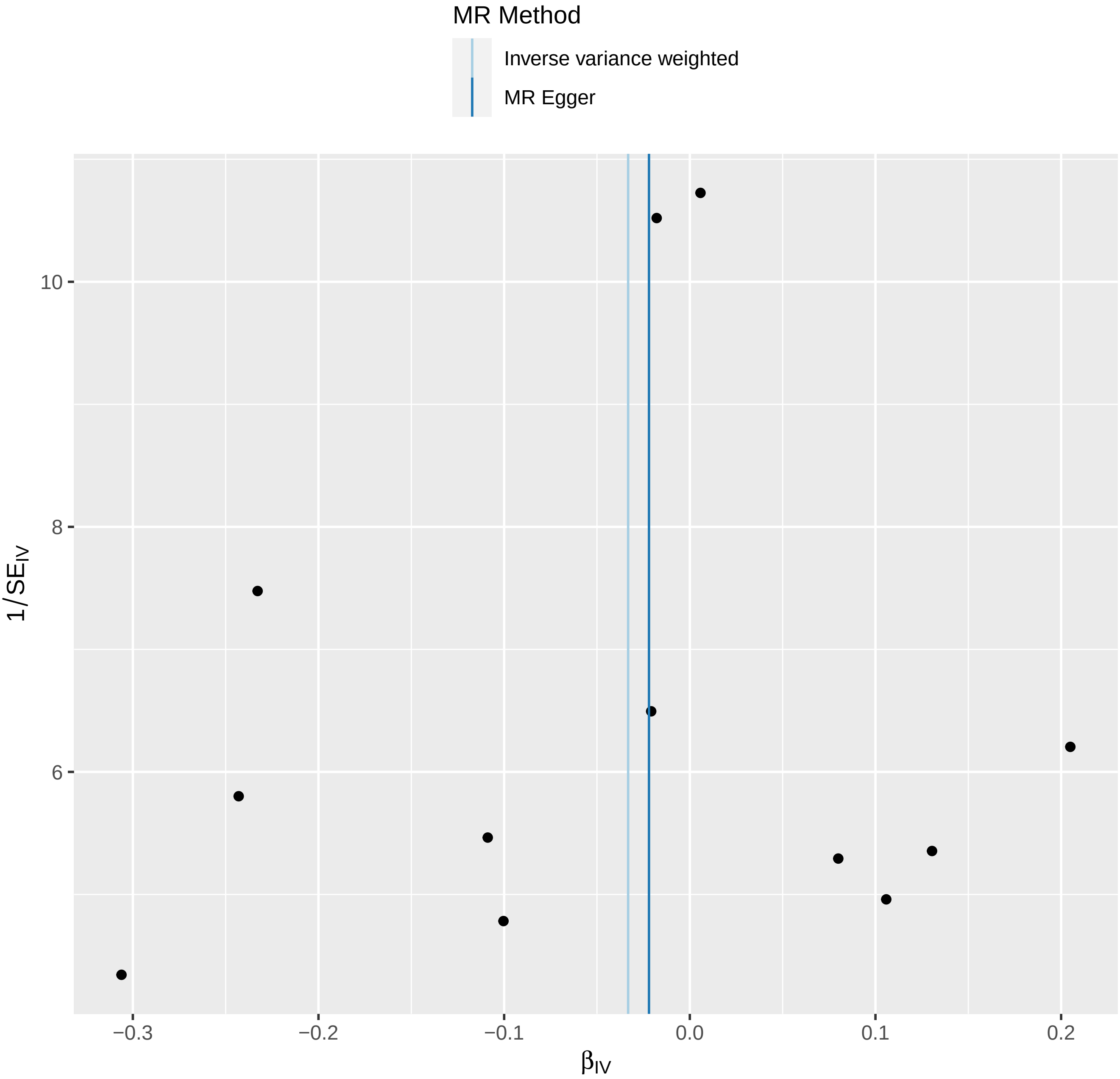

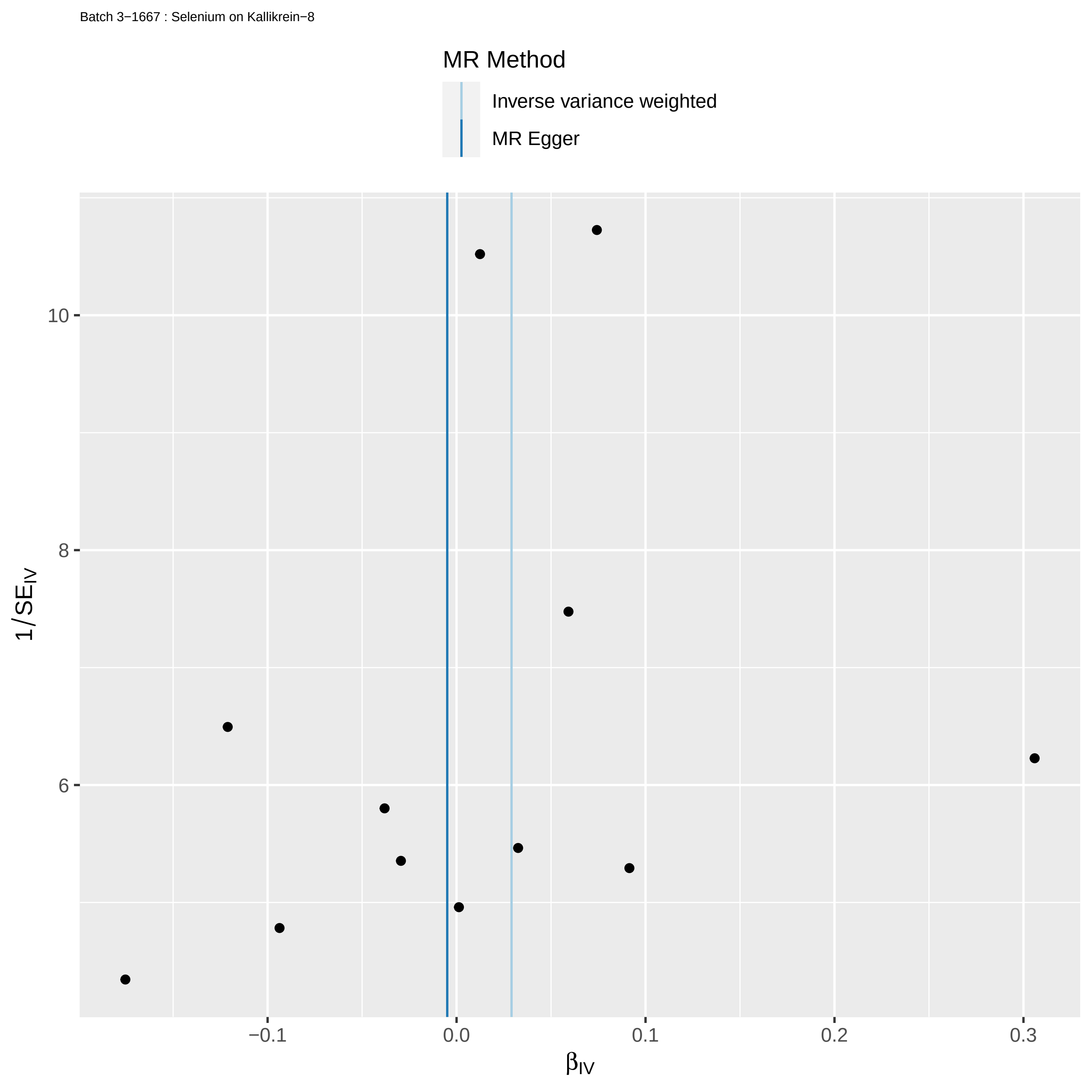

Batch 3−1667 : Selenium on Kallikrein−8
MR Method
Inverse variance weighted
MR Egger
10
8
6
1/SE_IV
−0.1
0.0
0.1
0.2
0.3
β_IV

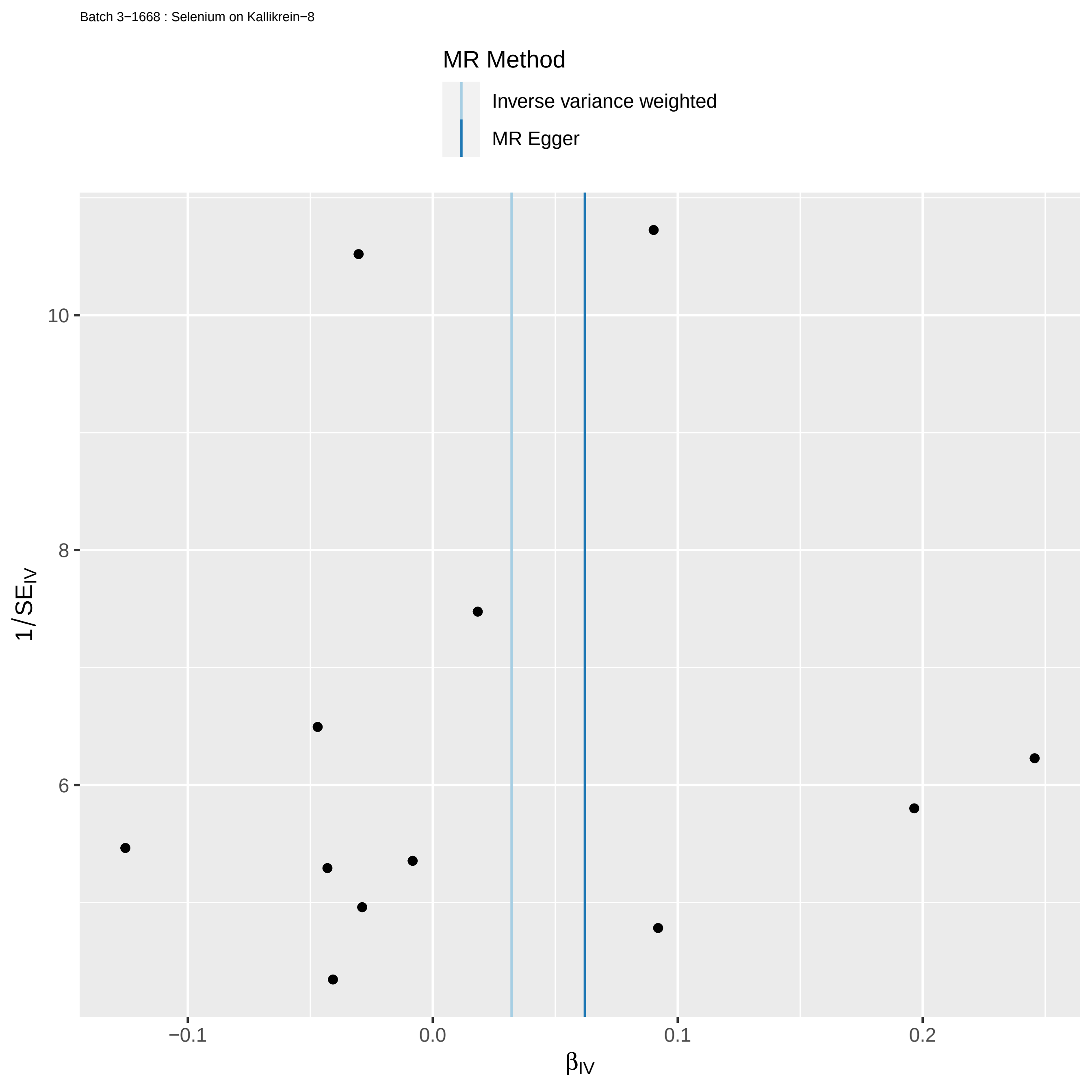
Batch 3−1668 : Selenium on Kallikrein−8
MR Method
Inverse variance weighted
MR Egger
10
8
6
$1/SE_{IV}$
−0.1
0.0
0.1
0.2
β_{IV}

Batch 3−1669 : Selenium on NKG2−A/NKG2−B type II integral membrane protein

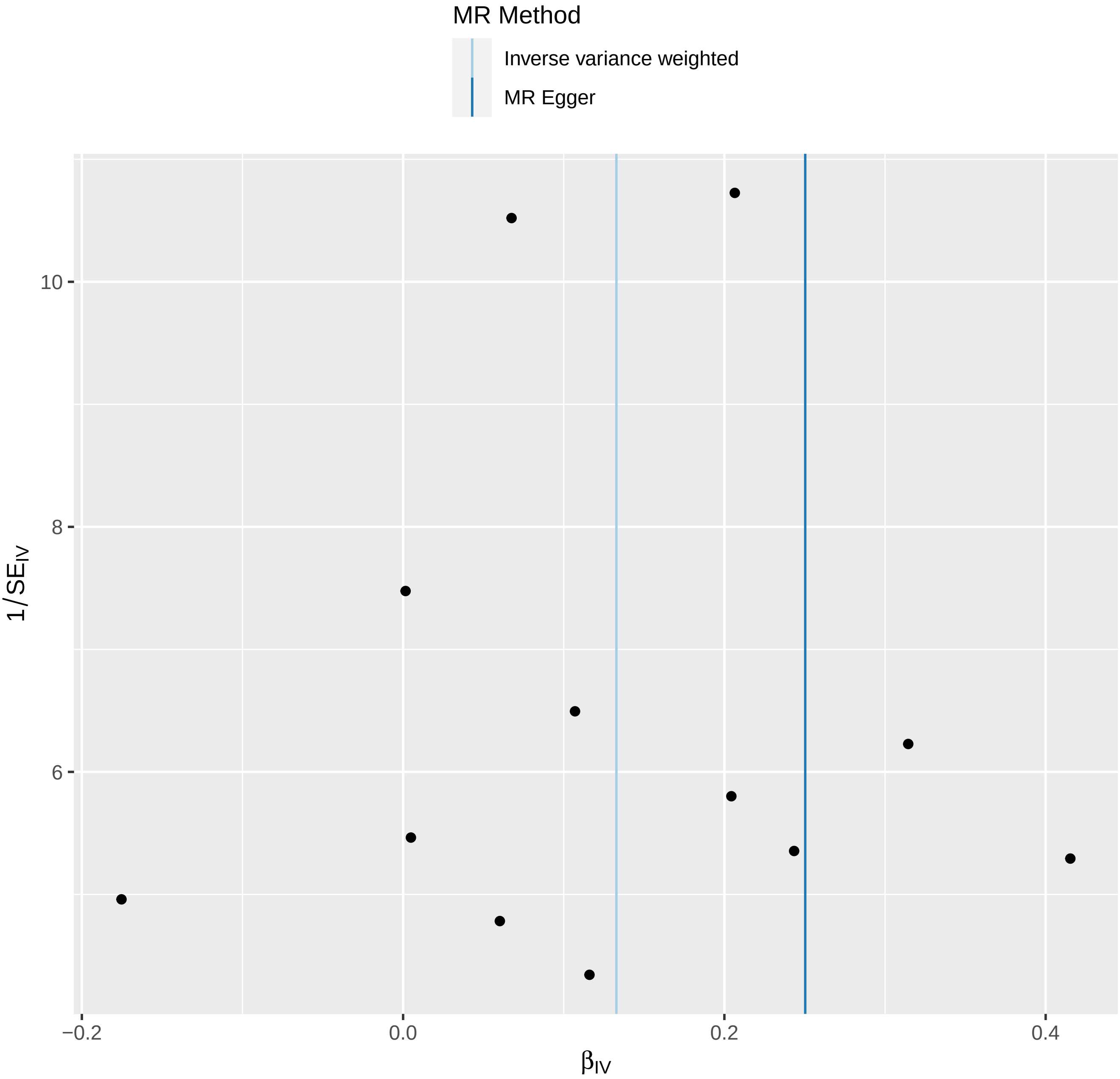

Batch 3−1670 : Selenium on NKG2−E type II integral membrane protein

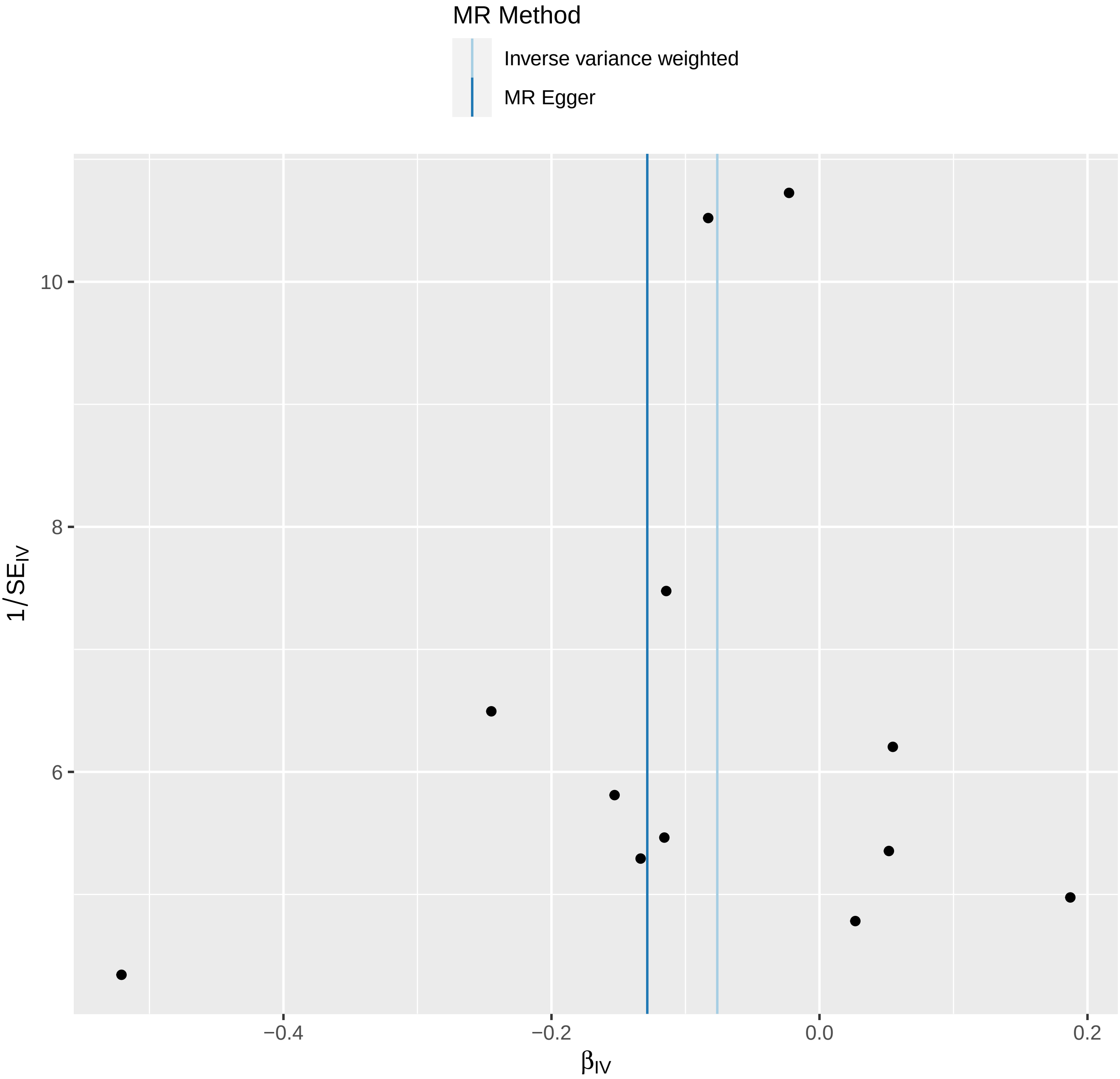

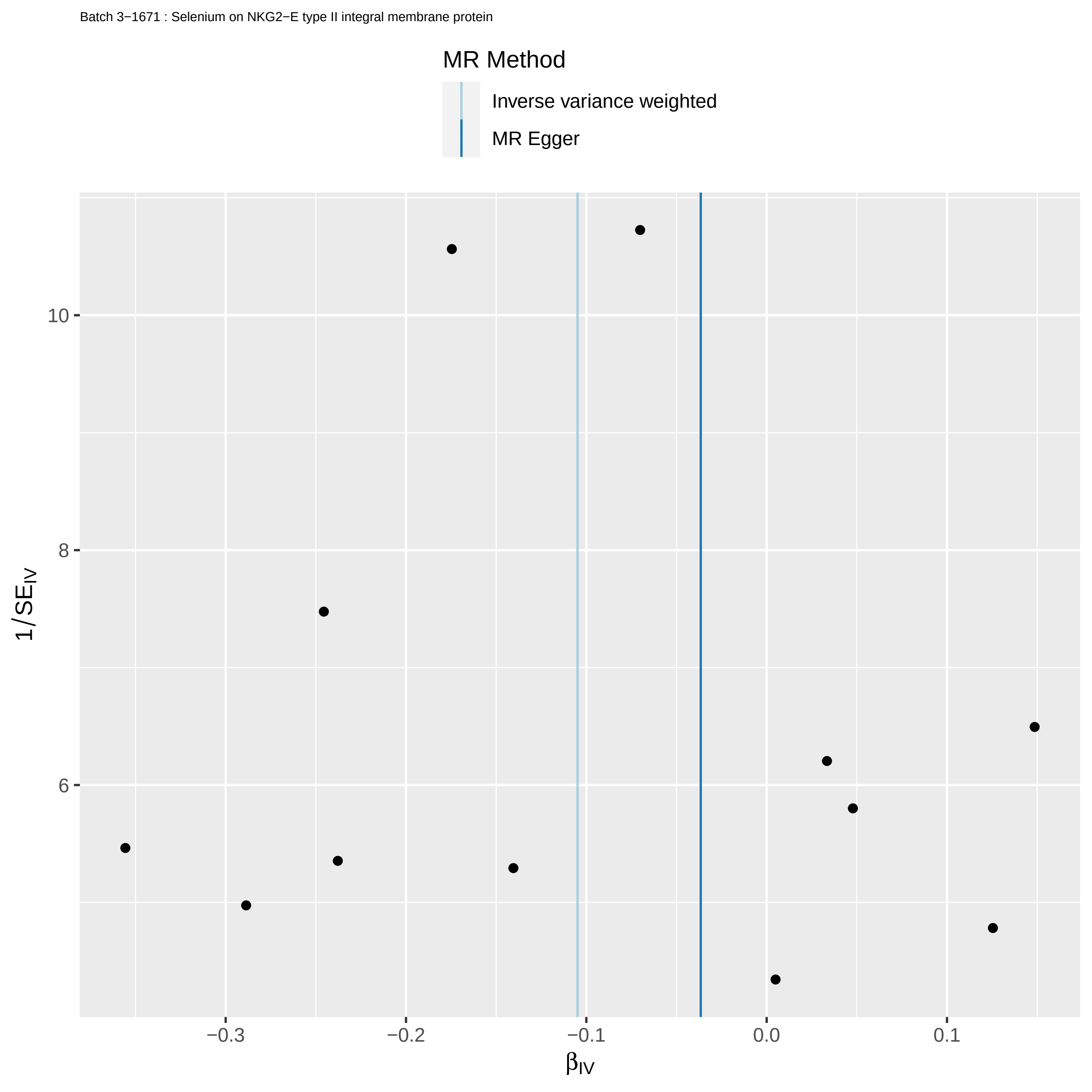
Batch 3−1671 : Selenium on NKG2−E type II integral membrane protein
MR Method
Inverse variance weighted
MR Egger
10
8
6
1/SE_IV
−0.3
−0.2
−0.1
0.0
0.1
β_IV

Batch 3−1672 : Selenium on Killer cell lectin−like receptor subfamily F member 1

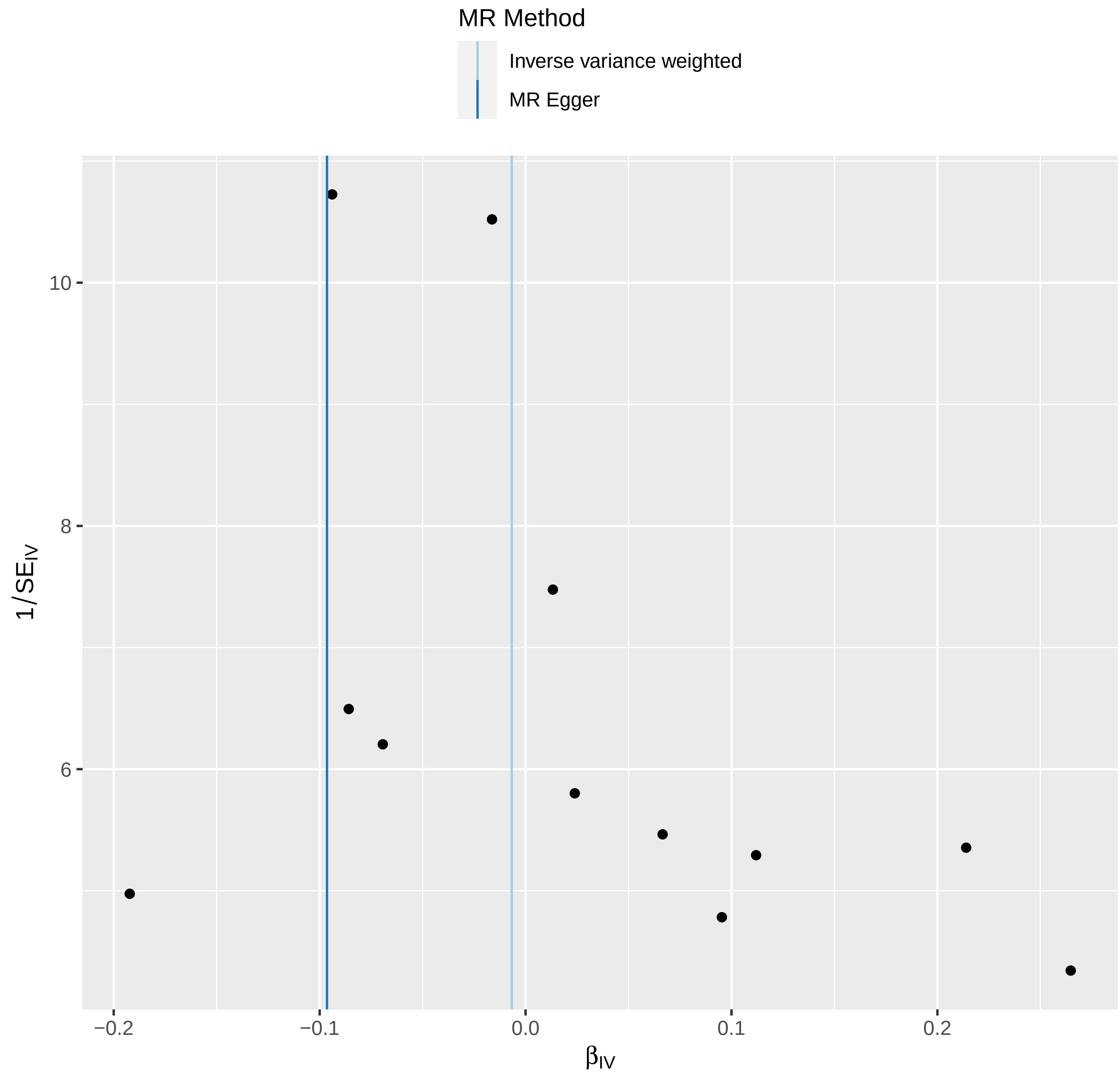

Batch 3−1673 : Selenium on Killer cell lectin−like receptor subfamily F member 1

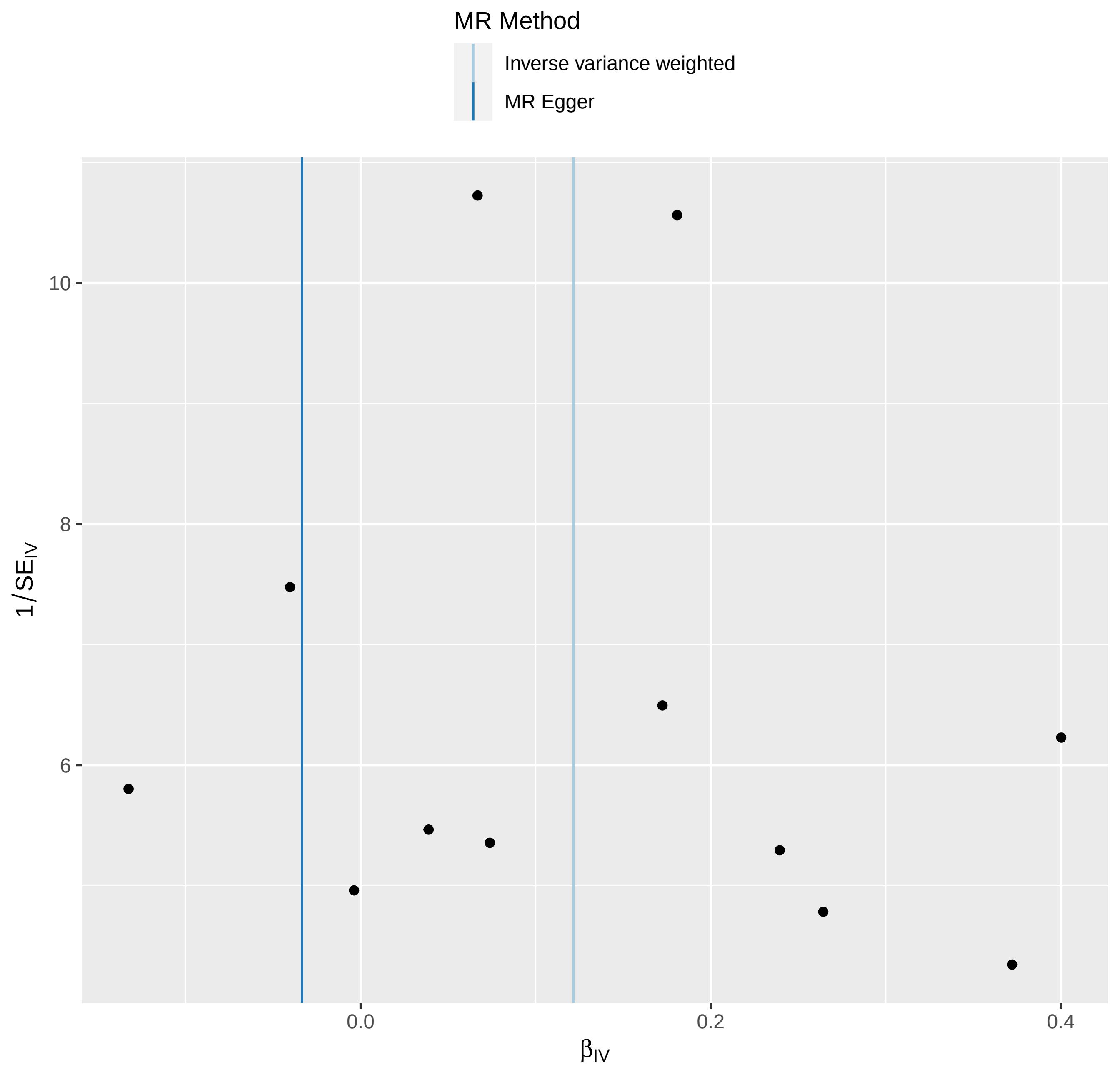

Batch 3−1674 : Selenium on NKG2−D type II integral membrane protein

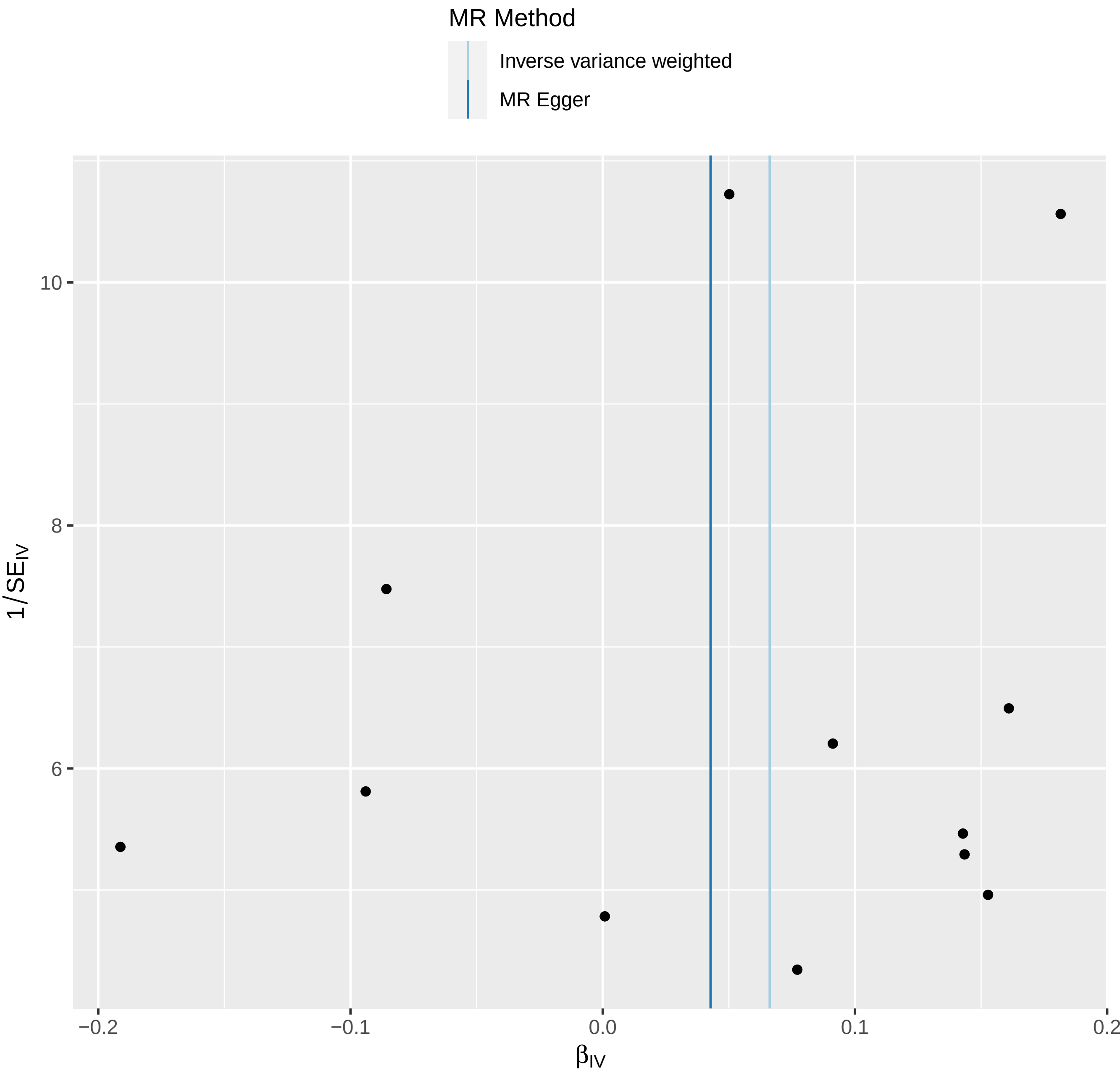

Batch 3−1675 : Selenium on NKG2−D type II integral membrane protein

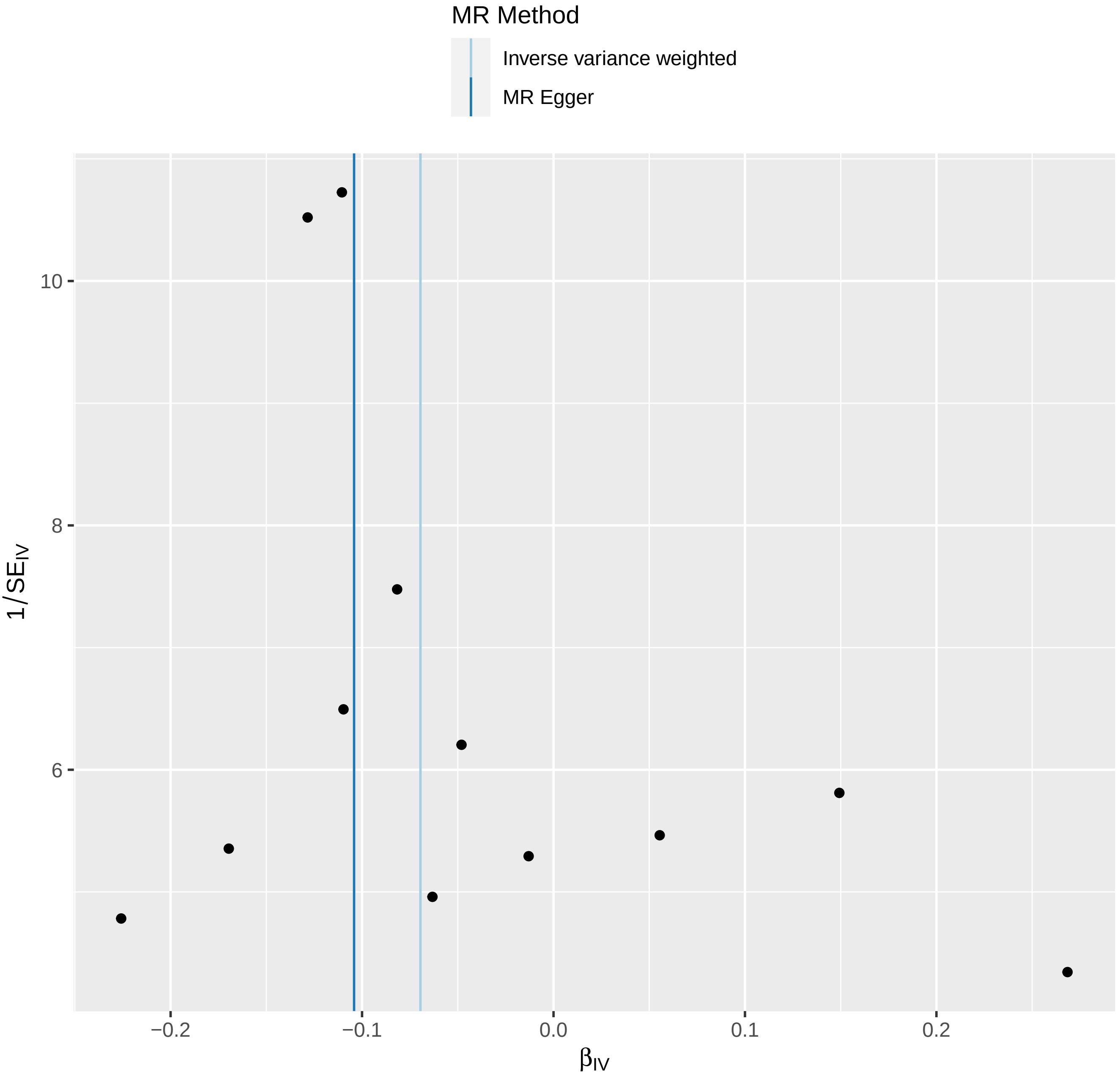

Batch 3−1676 : Selenium on Histone−lysine N−methyltransferase 2C

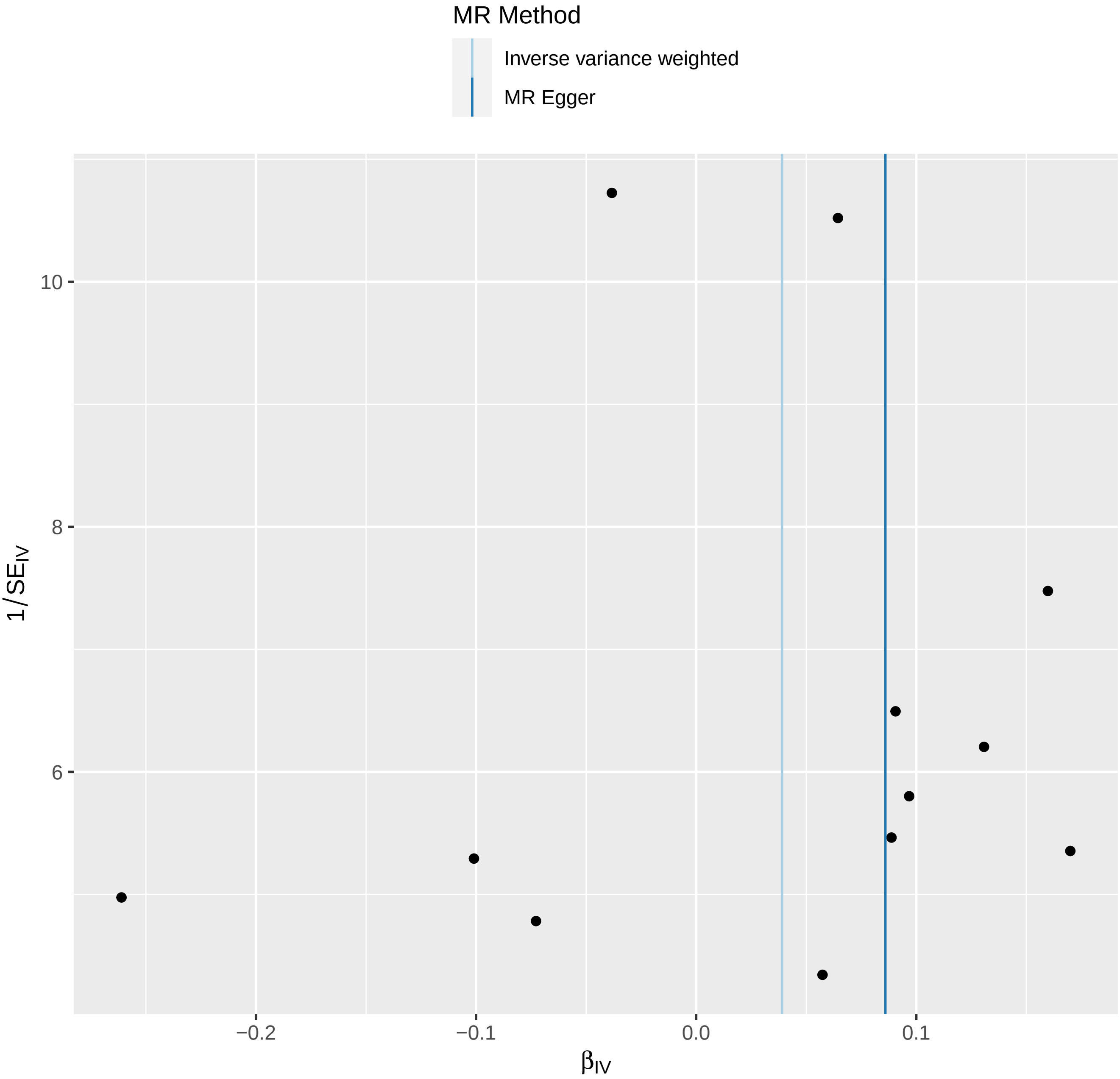

Batch 3−1677 : Selenium on Importin subunit alpha−1

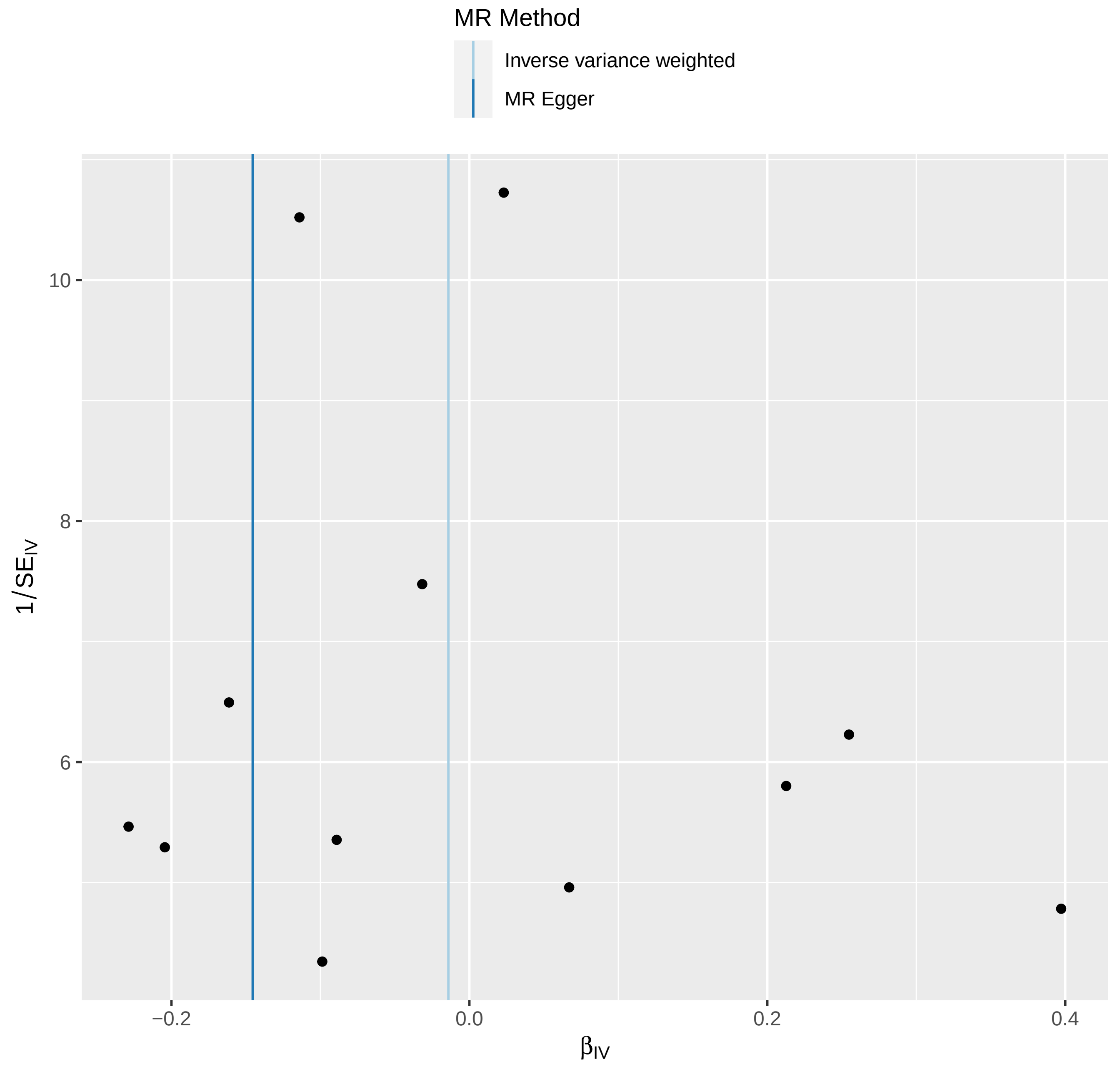

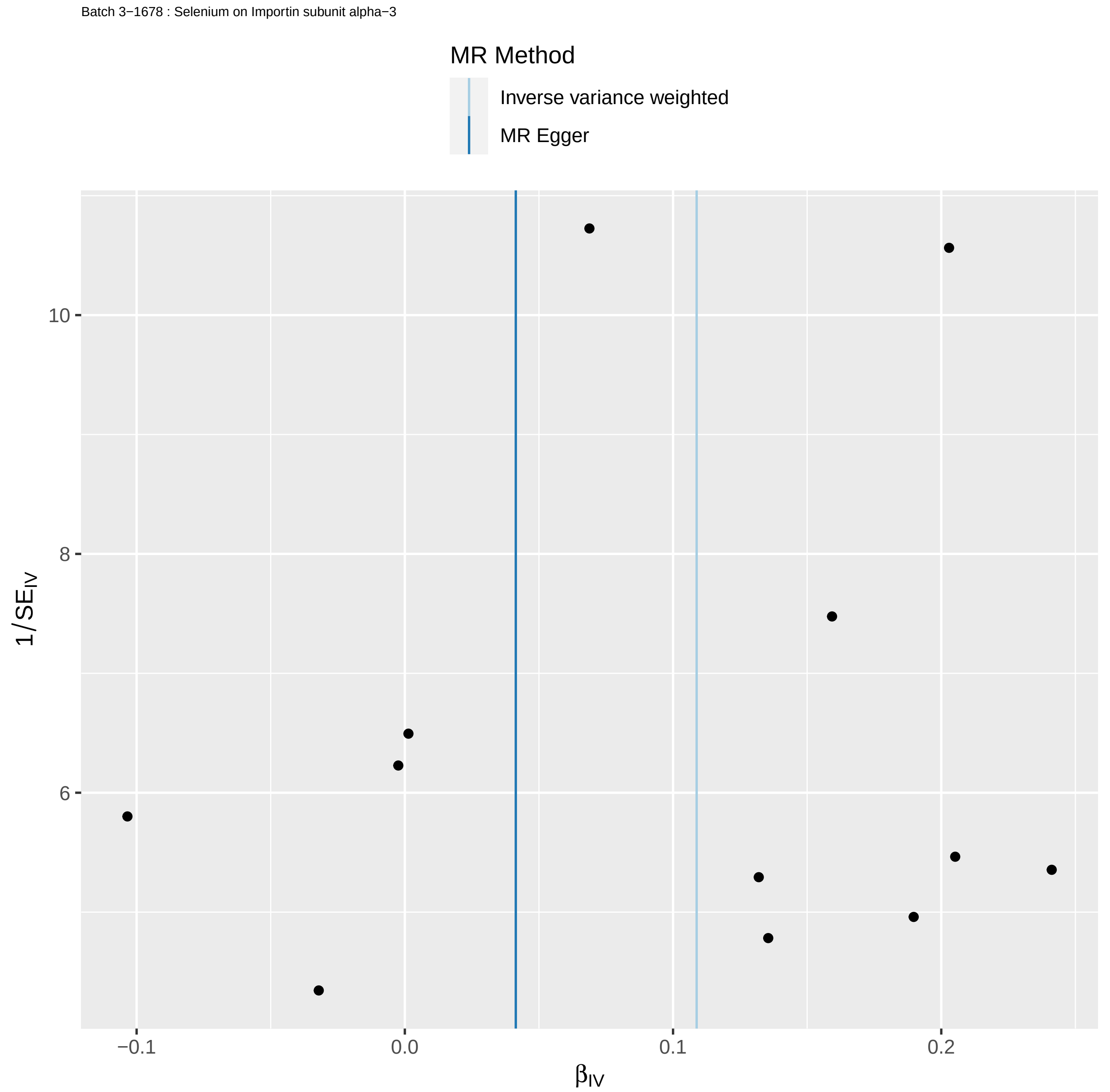

Batch 3−1678 : Selenium on Importin subunit alpha−3
MR Method
Inverse variance weighted
MR Egger
10
8
6
$1/SE_{IV}$
−0.1
0.0
0.1
0.2
β_{IV}

Batch 3−1679 : Selenium on GTPase KRas

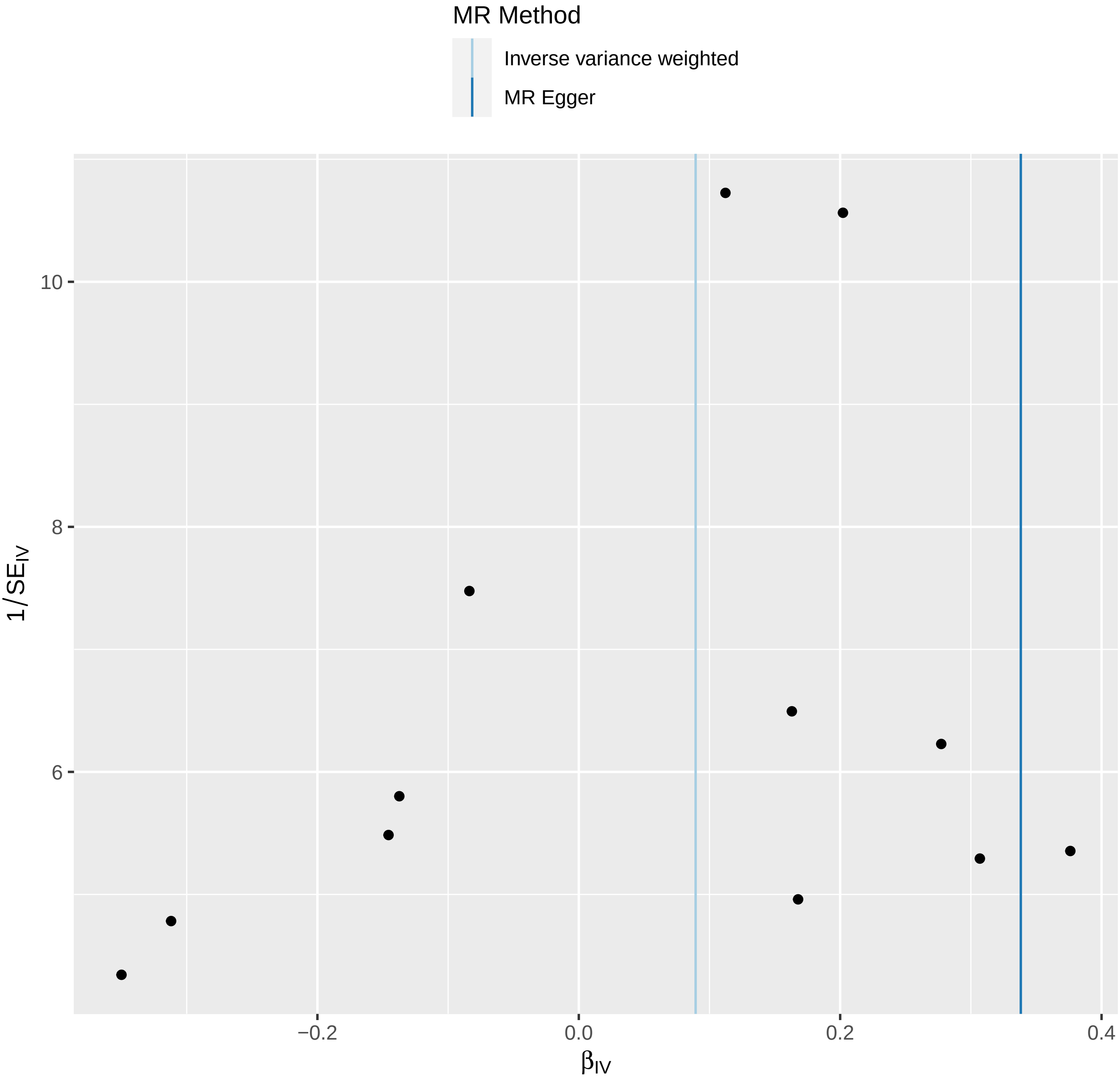

Batch 3−1680 : Selenium on Kremen protein 1

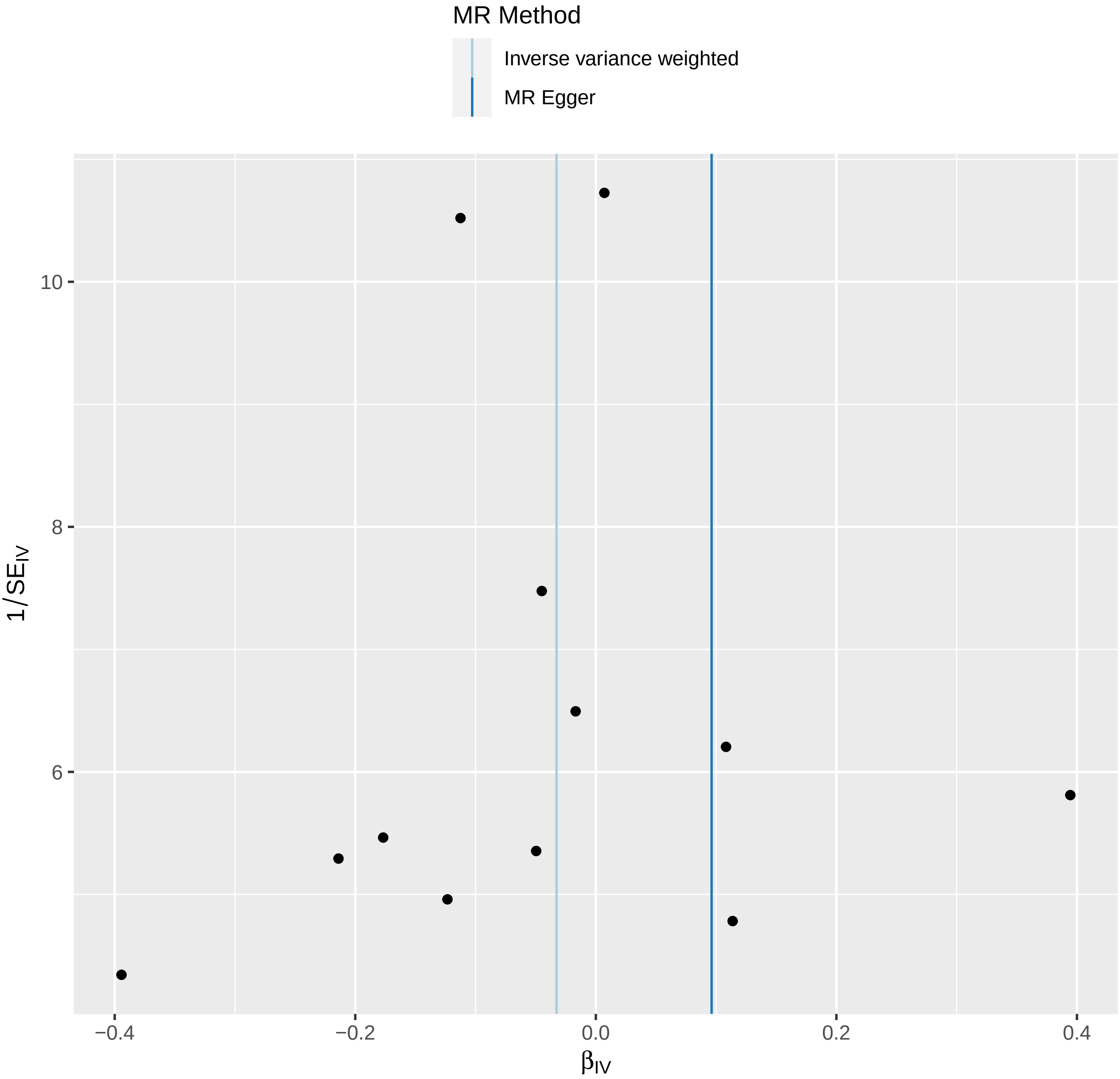

Batch 3−1681 : Selenium on Kremen protein 2

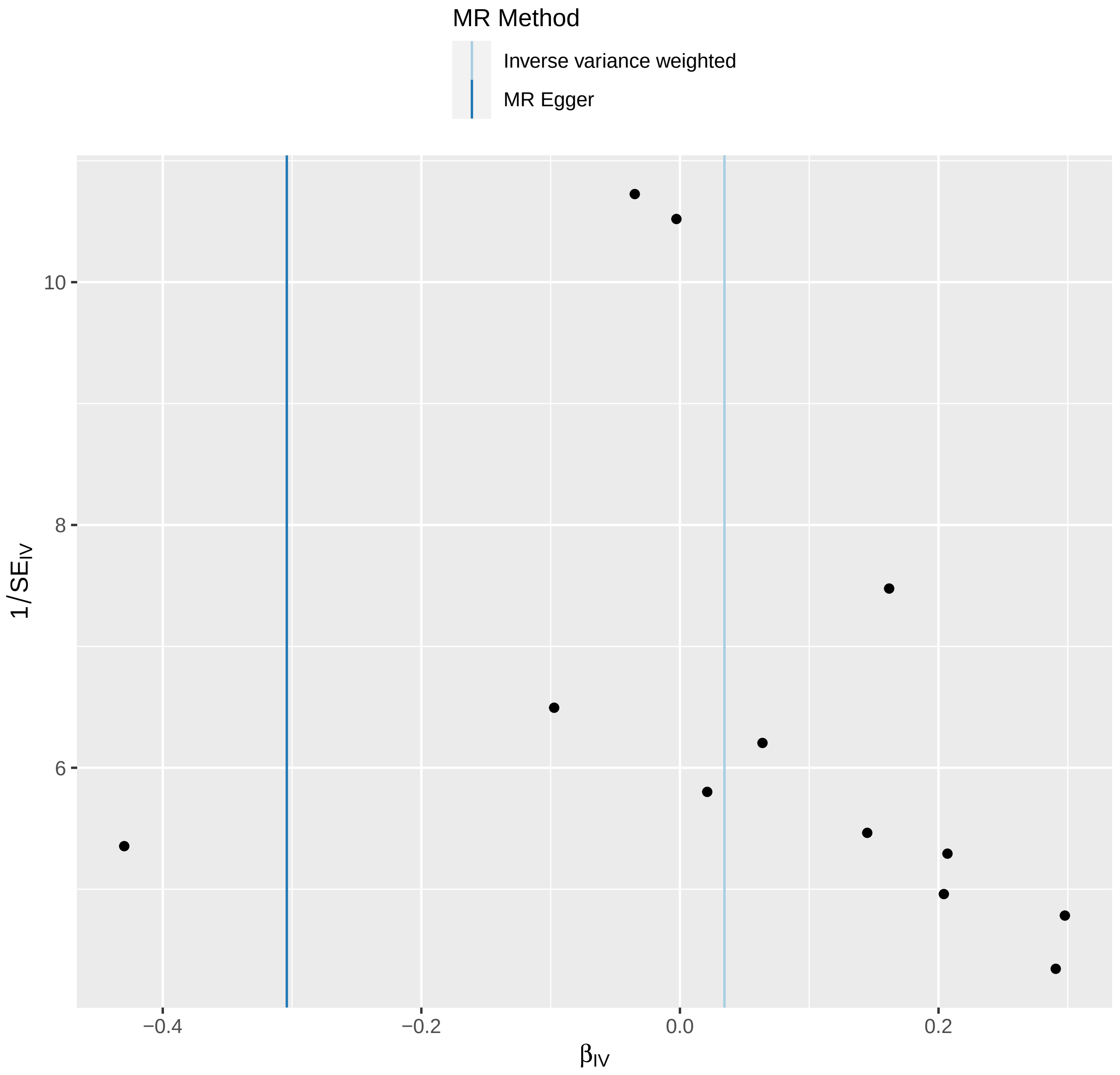

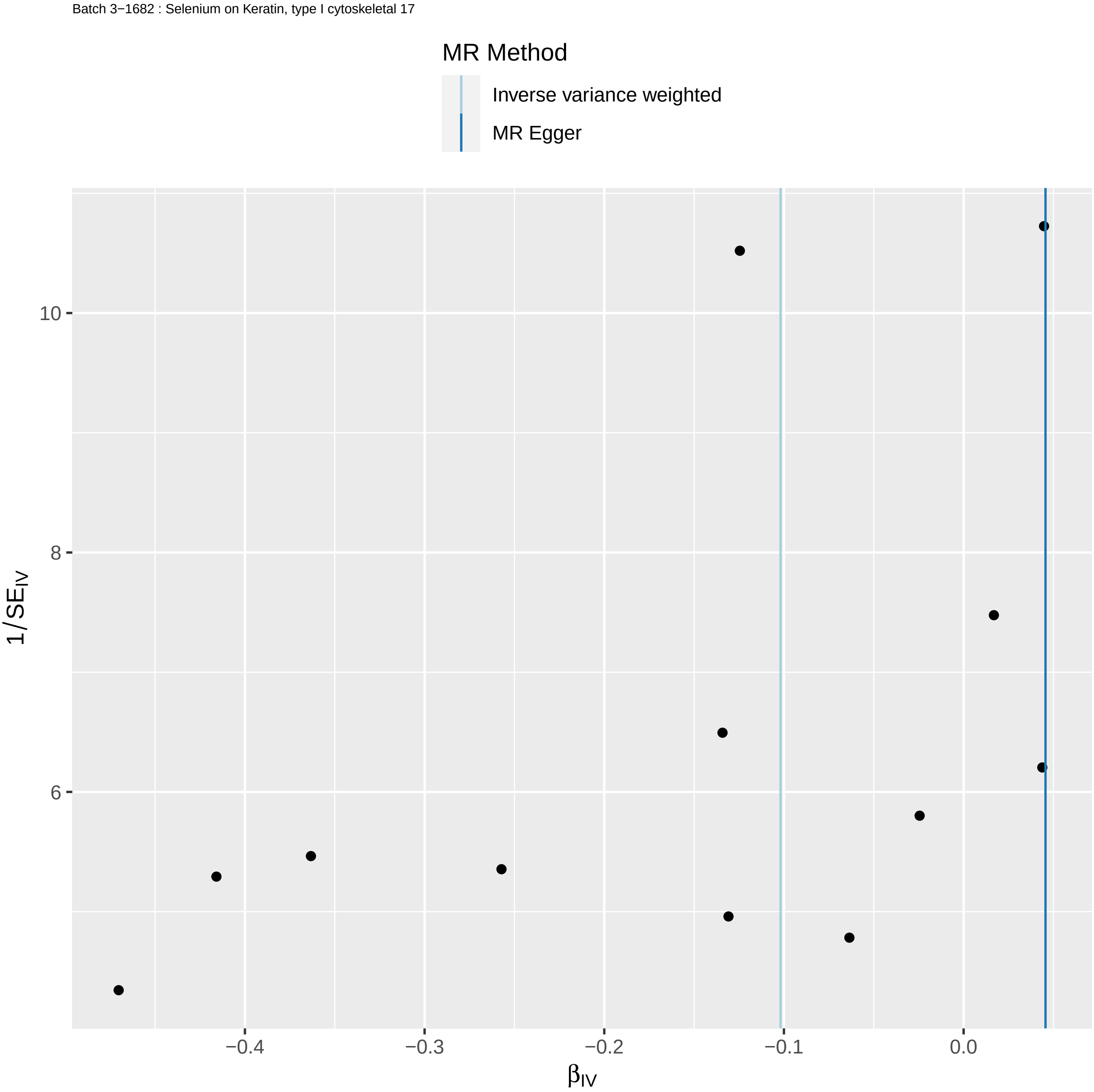

Batch 3−1682 : Selenium on Keratin, type I cytoskeletal 17
MR Method
Inverse variance weighted
MR Egger
10
8
6
1/SE_IV
−0.4
−0.3
−0.2
−0.1
0.0
β_IV

Batch 3−1683 : Selenium on Keratin, type I cytoskeletal 18

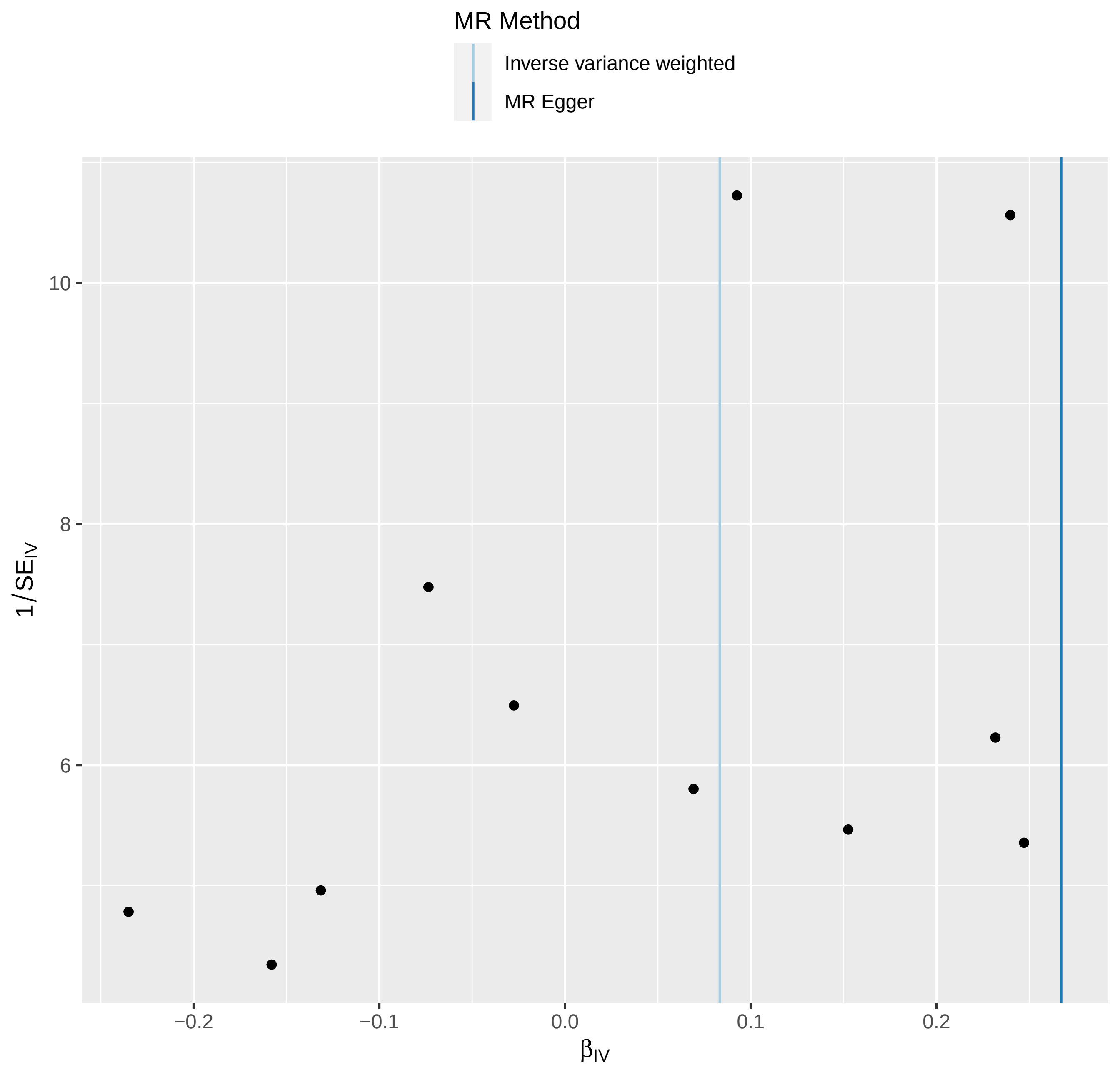

Batch 3−1684 : Selenium on Keratin, type I cytoskeletal 20

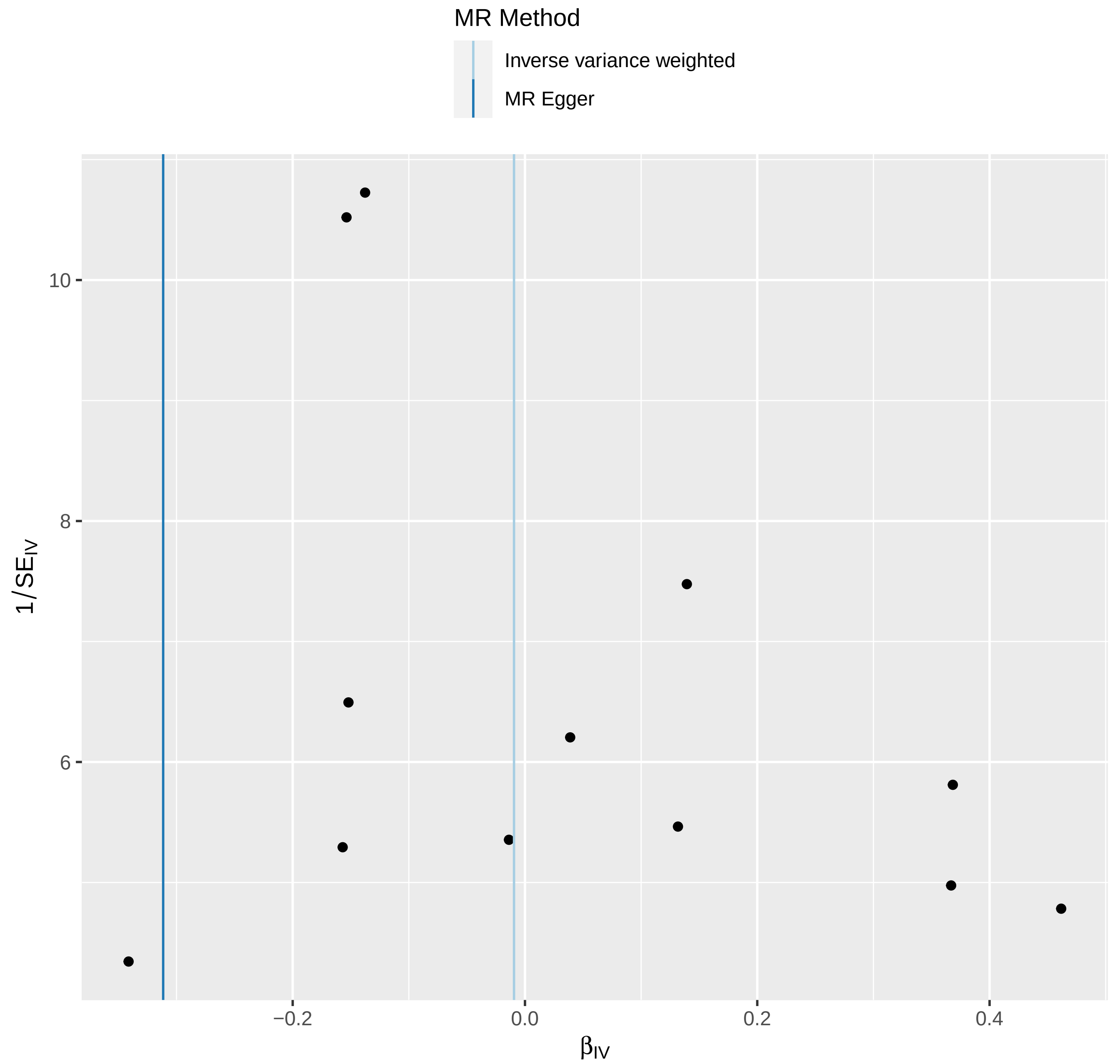

Batch 3−1685 : Selenium on Keratin, type II cytoskeletal 5

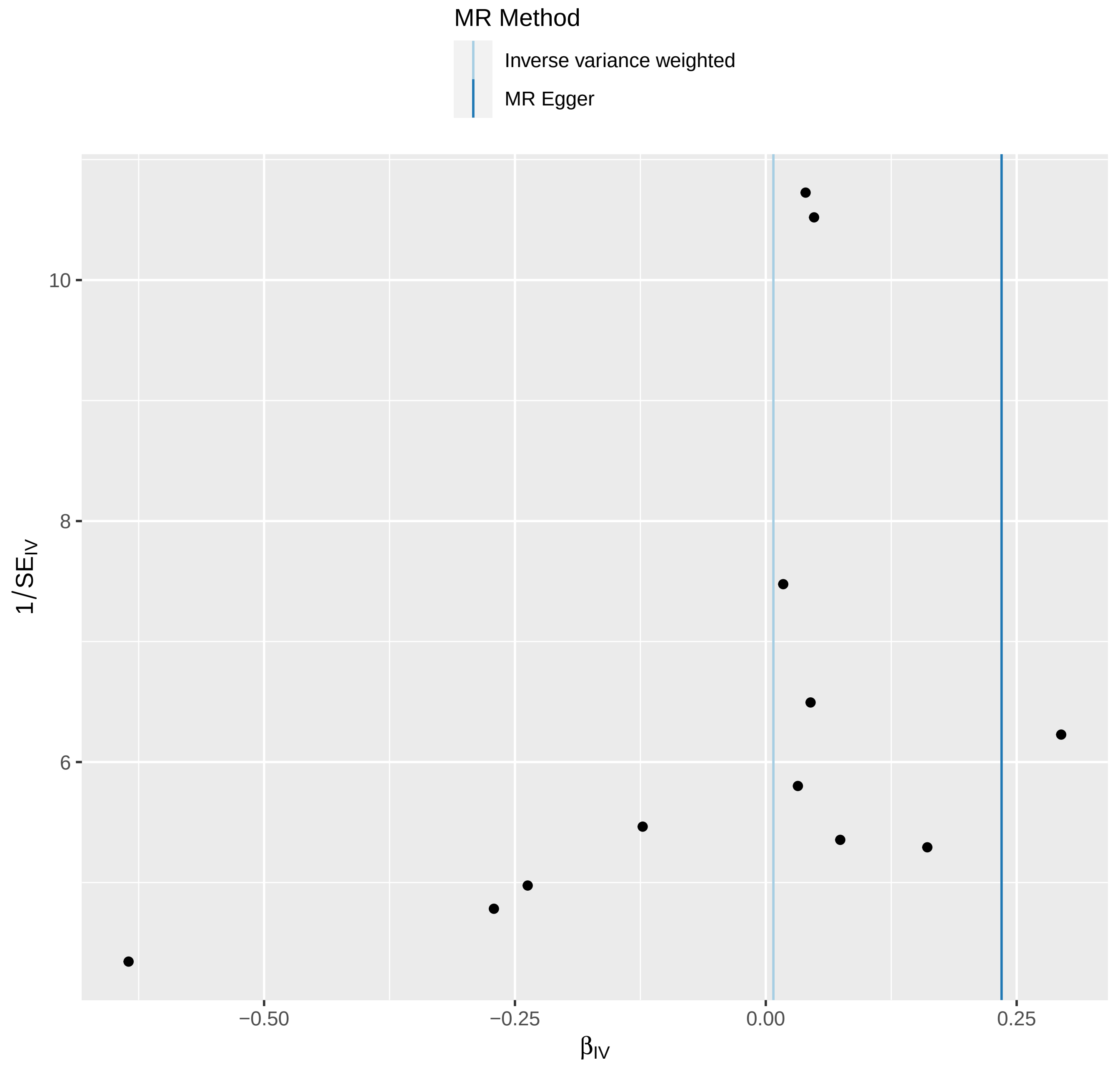

Batch 3−1686 : Selenium on Keratin, type II cytoskeletal 7

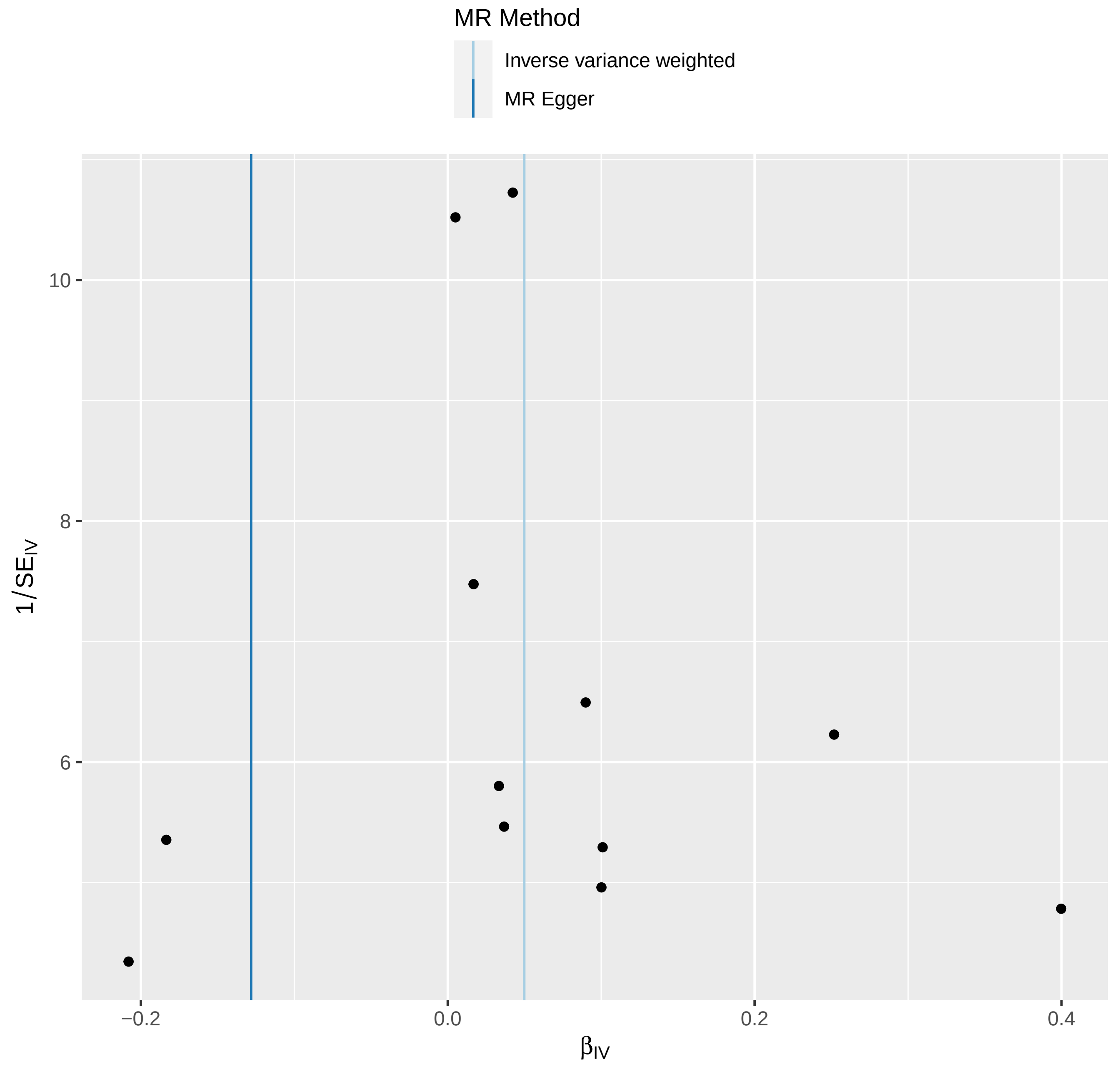

Batch 3−1687 : Selenium on Keratin−associated protein 2−4

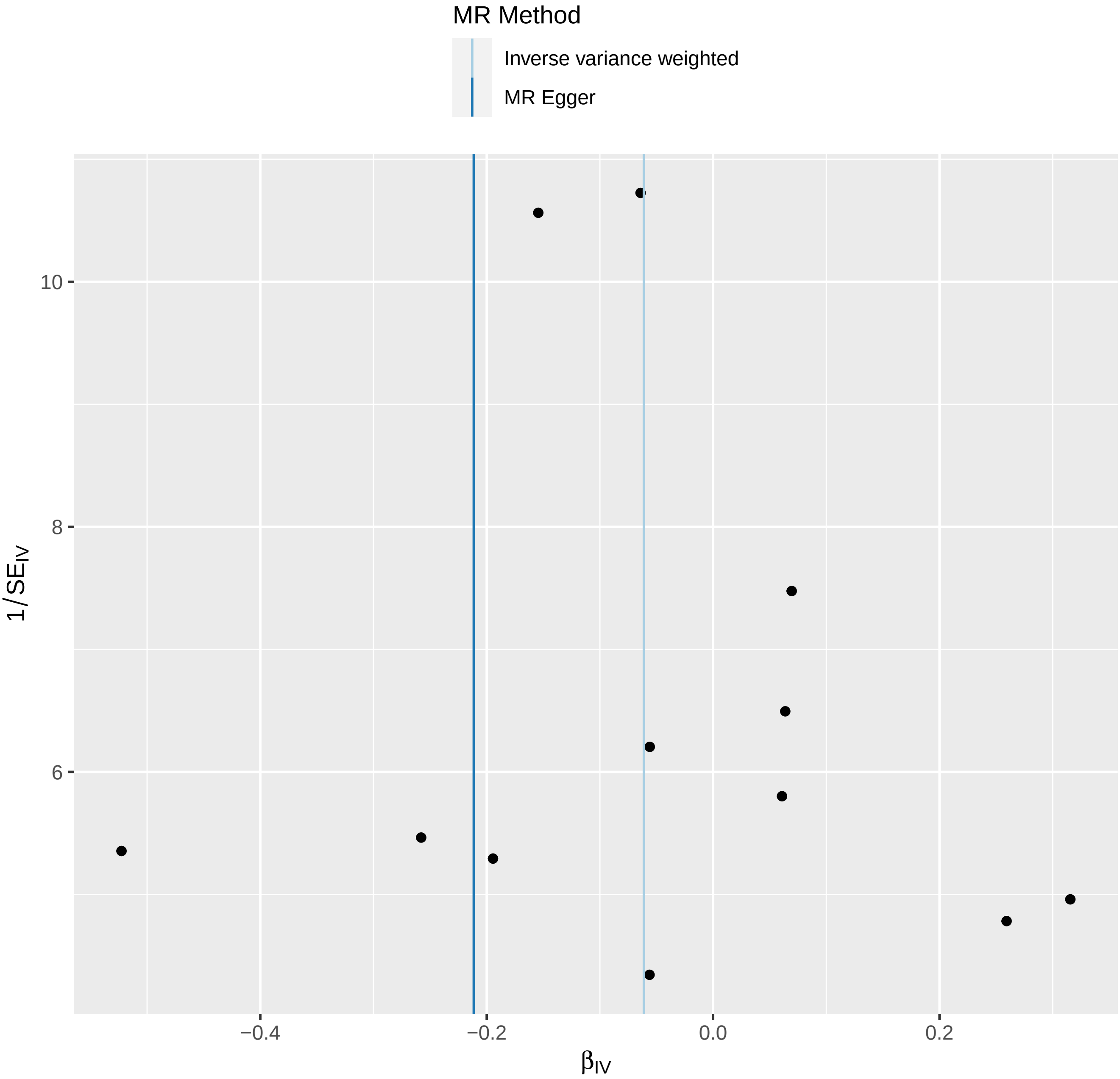

Batch 3−1688 : Selenium on Keratinocyte differentiation−associated protein

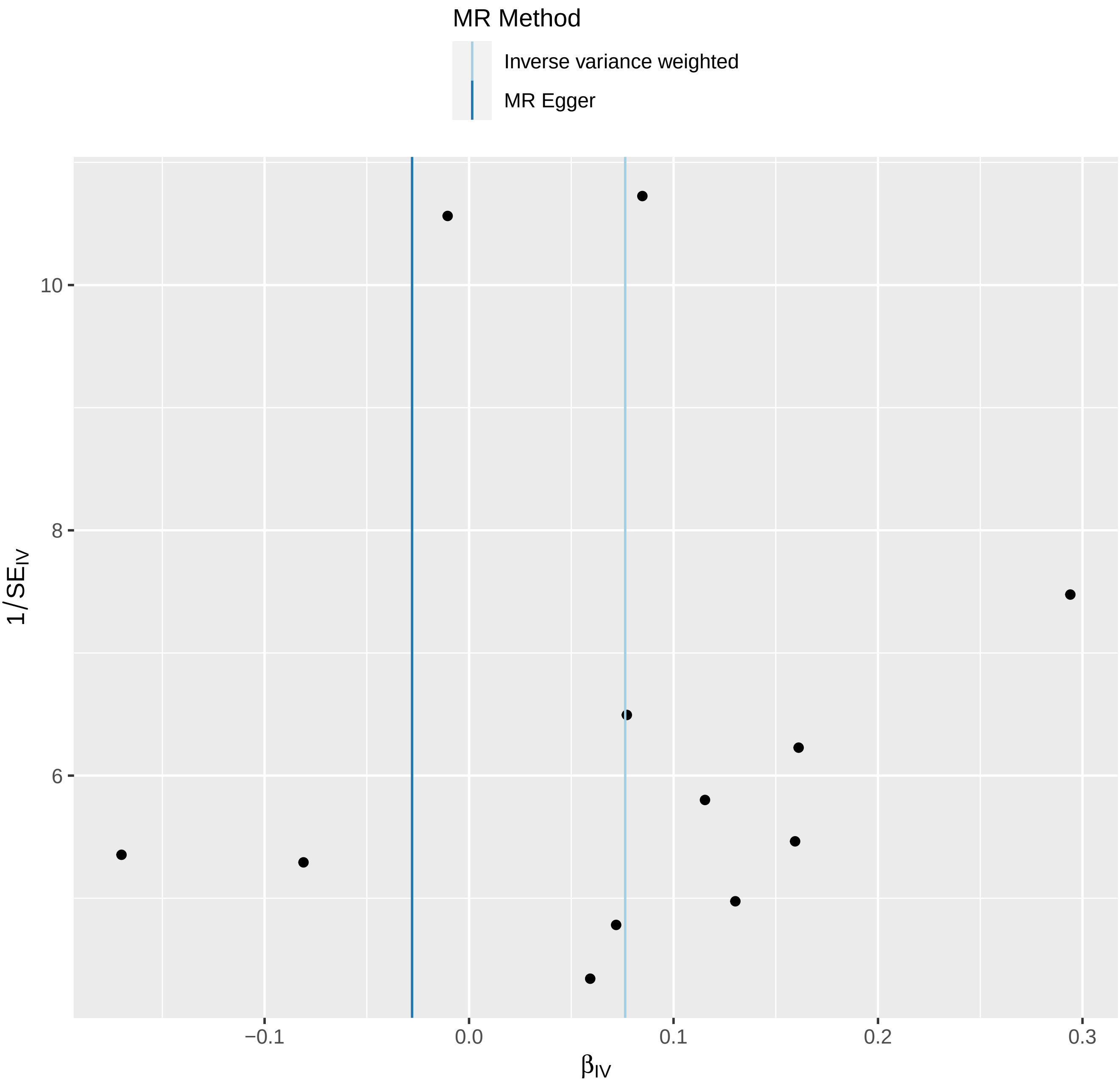

Batch 3−1689 : Selenium on Kynureninase

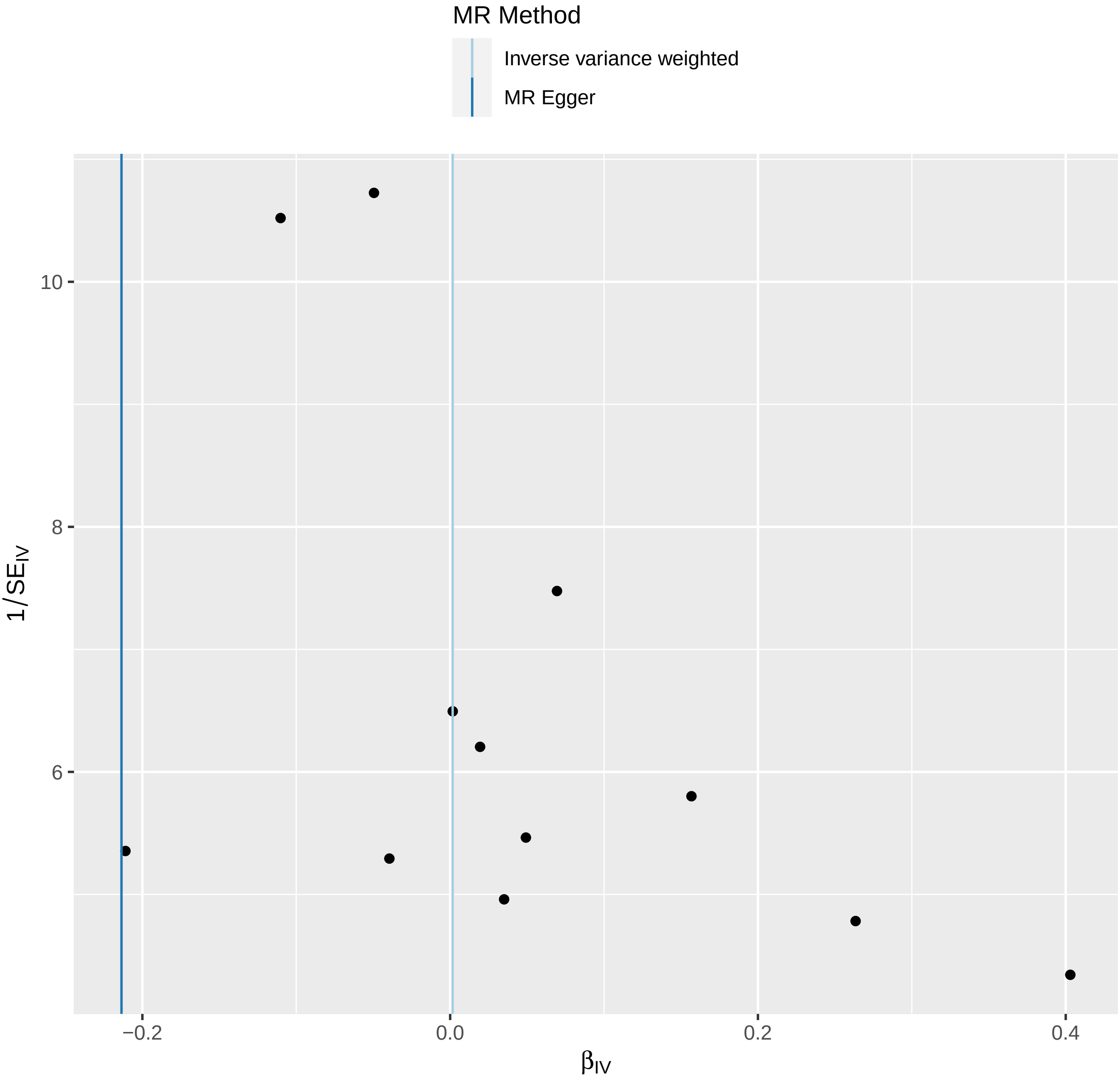

Batch 3−1690 : Selenium on Neural cell adhesion molecule L1

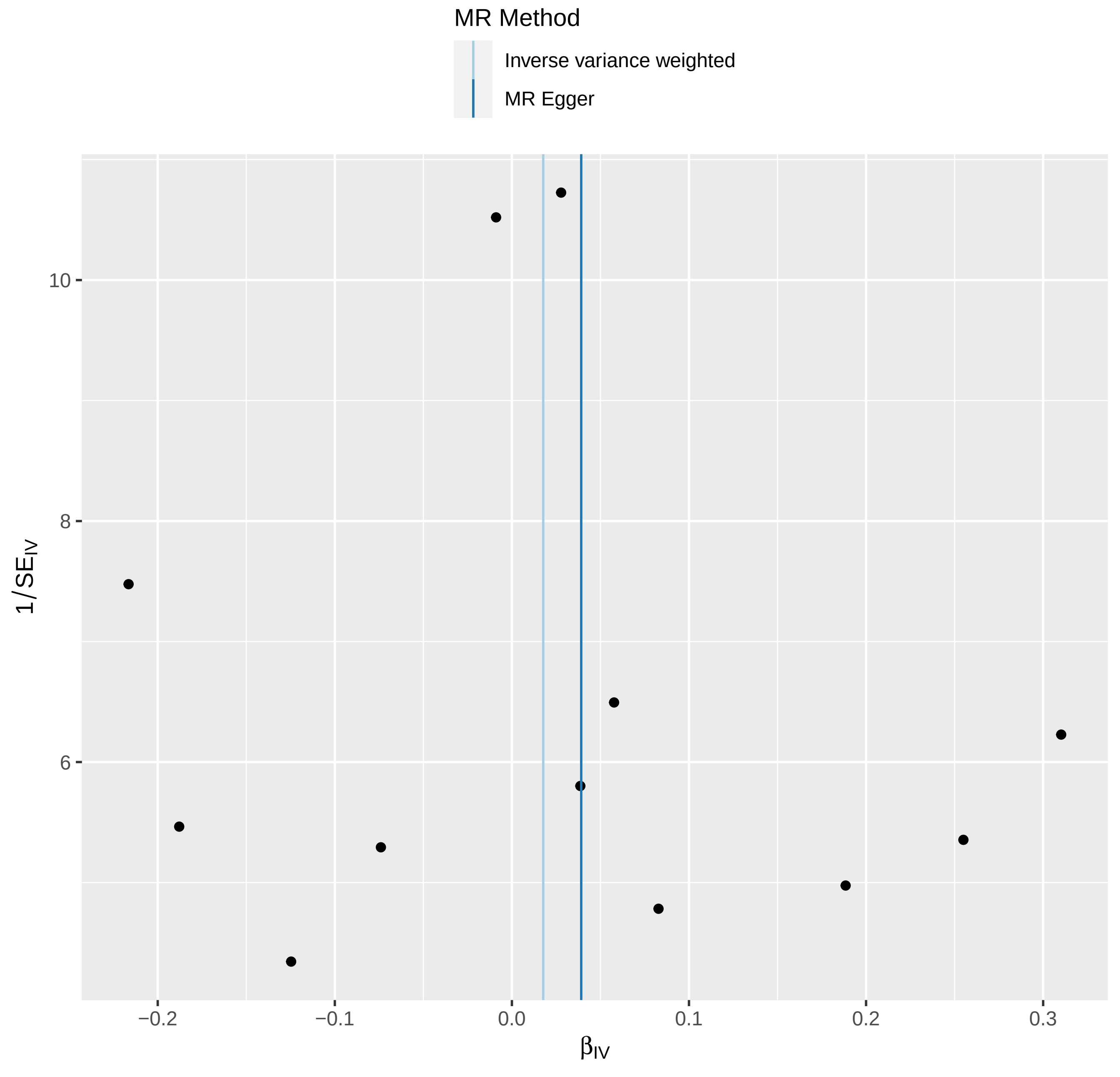

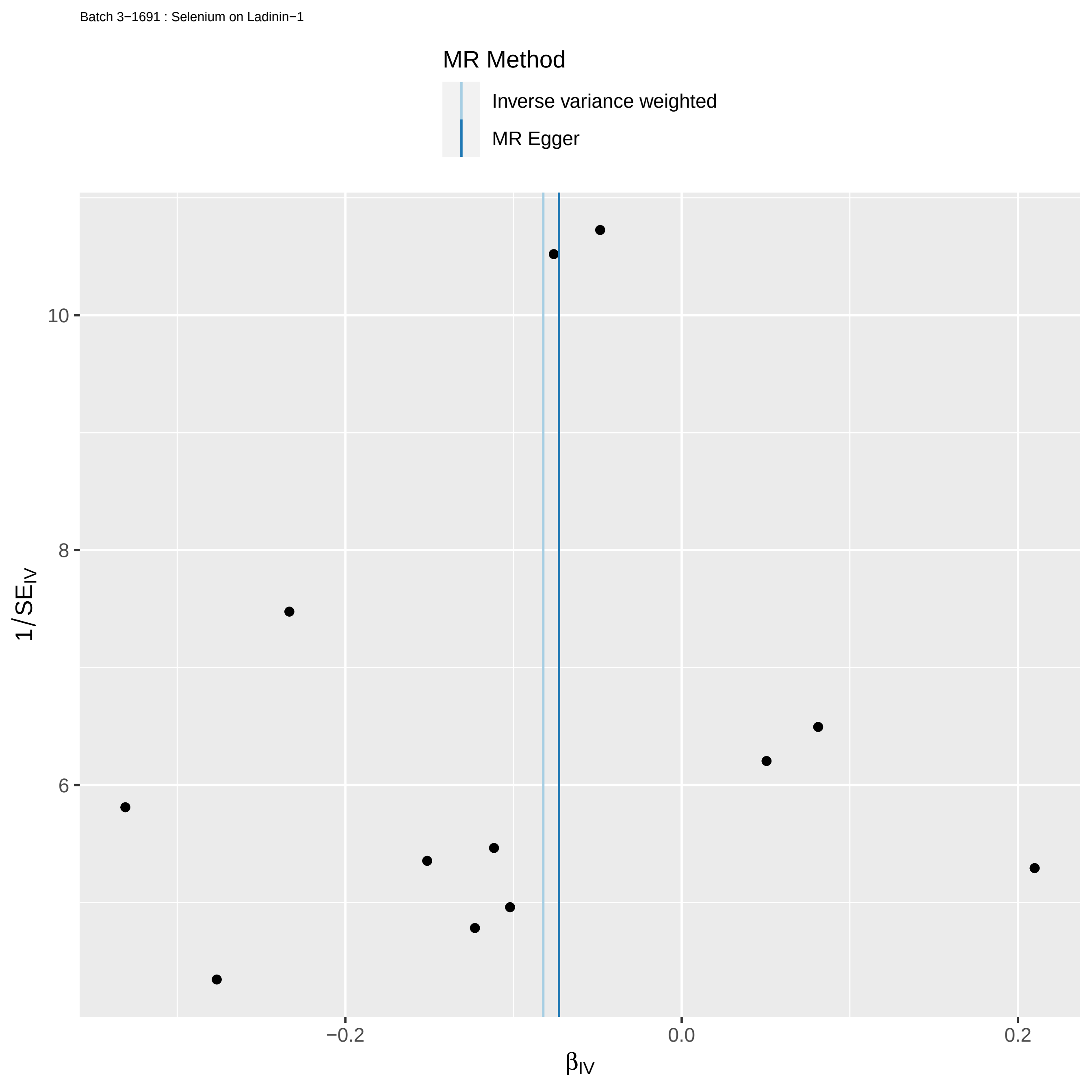

Batch 3−1691 : Selenium on Ladinin−1
MR Method
Inverse variance weighted
MR Egger
10
8
6
1/SE_IV
−0.2
0.0
0.2
β_IV

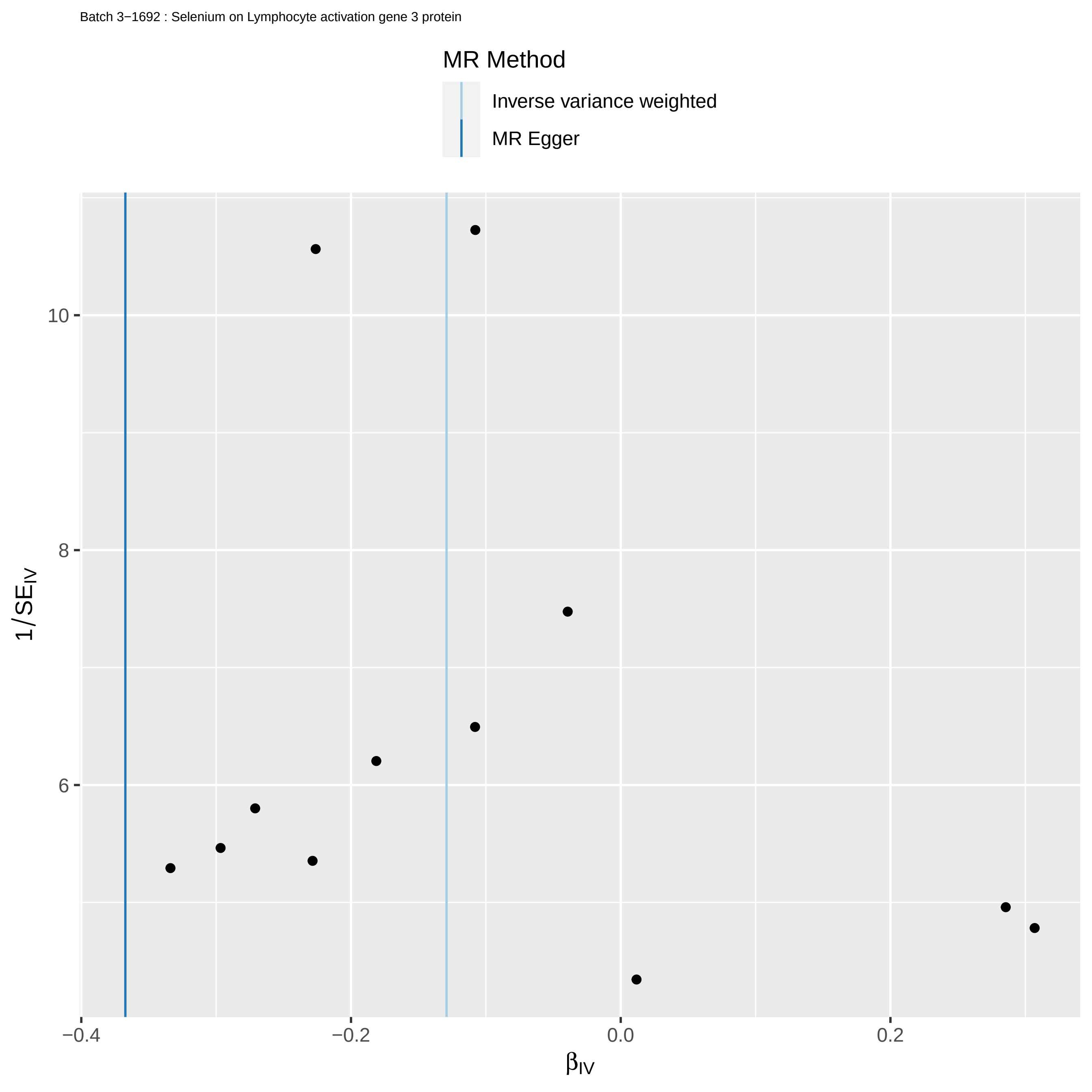
Batch 3−1692 : Selenium on Lymphocyte activation gene 3 protein
MR Method
Inverse variance weighted
MR Egger
10
8
6
1/SE_IV
−0.4
−0.2
0.0
0.2
β_IV

Batch 3−1693 : Selenium on Lymphocyte activation gene 3 protein

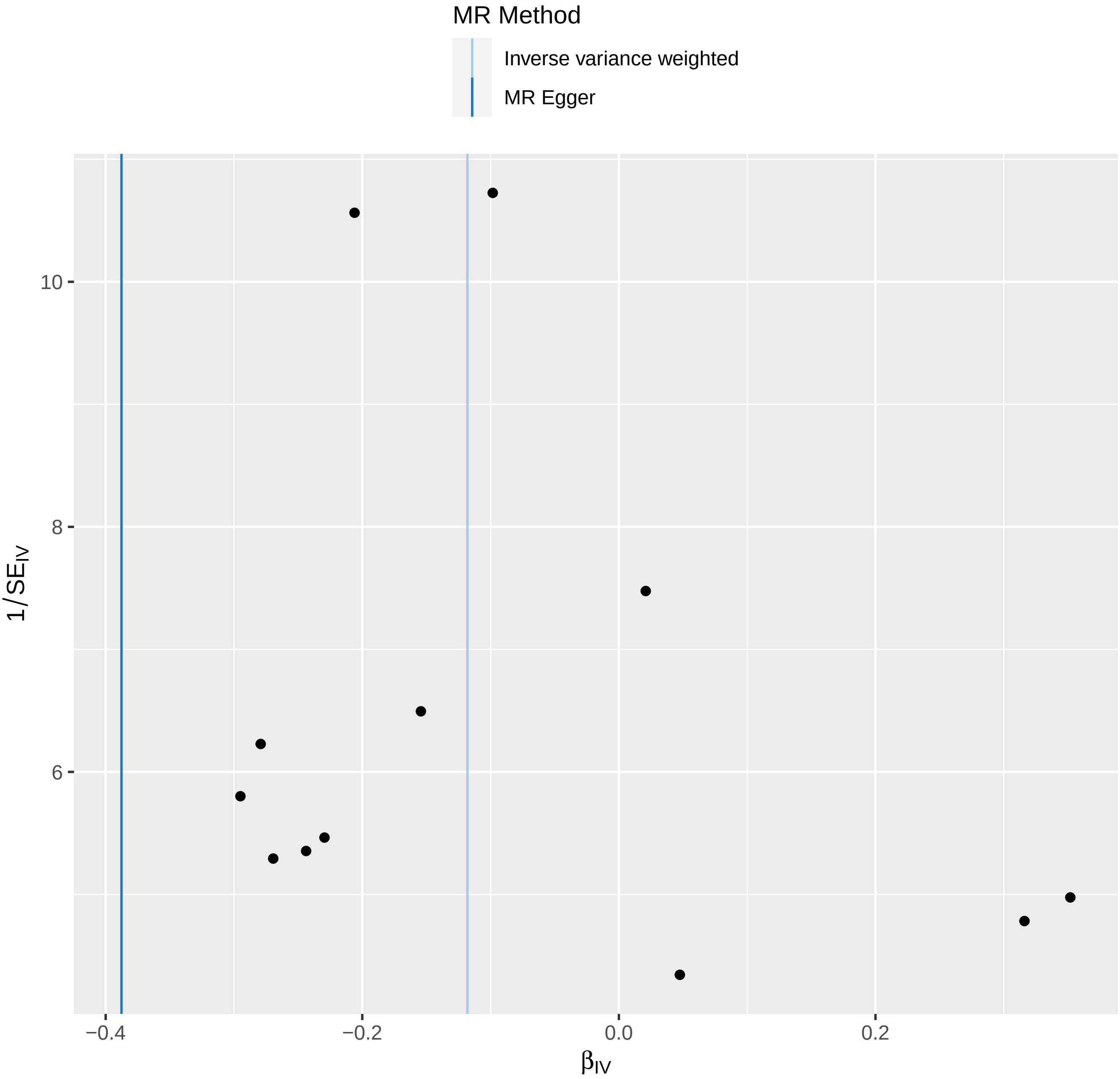

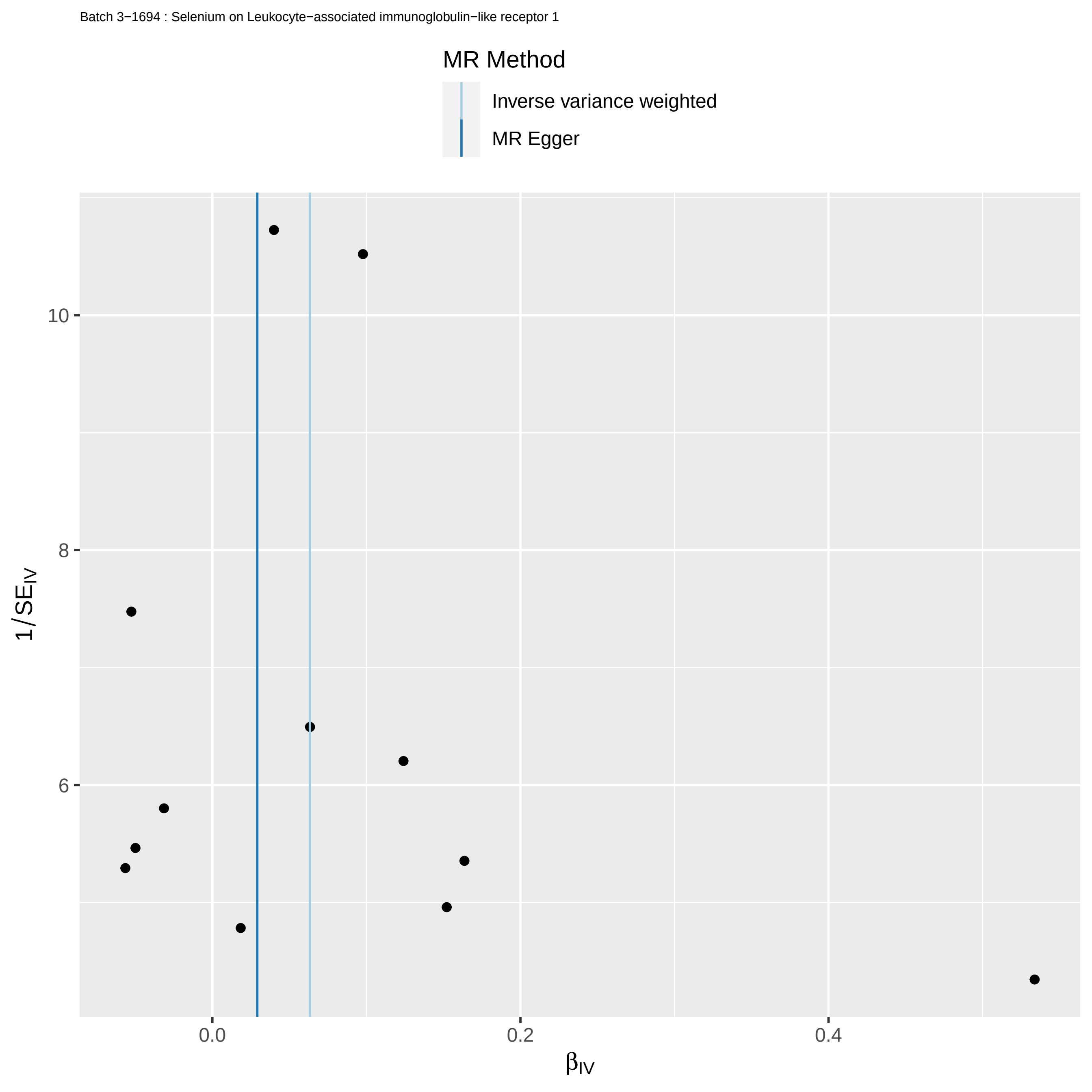
Batch 3−1694 : Selenium on Leukocyte−associated immunoglobulin−like receptor 1
MR Method
Inverse variance weighted
MR Egger
10
8
6
0.0
0.2
0.4
$1/SE_{IV}$
β_{IV}

Batch 3−1695 : Selenium on Laminin

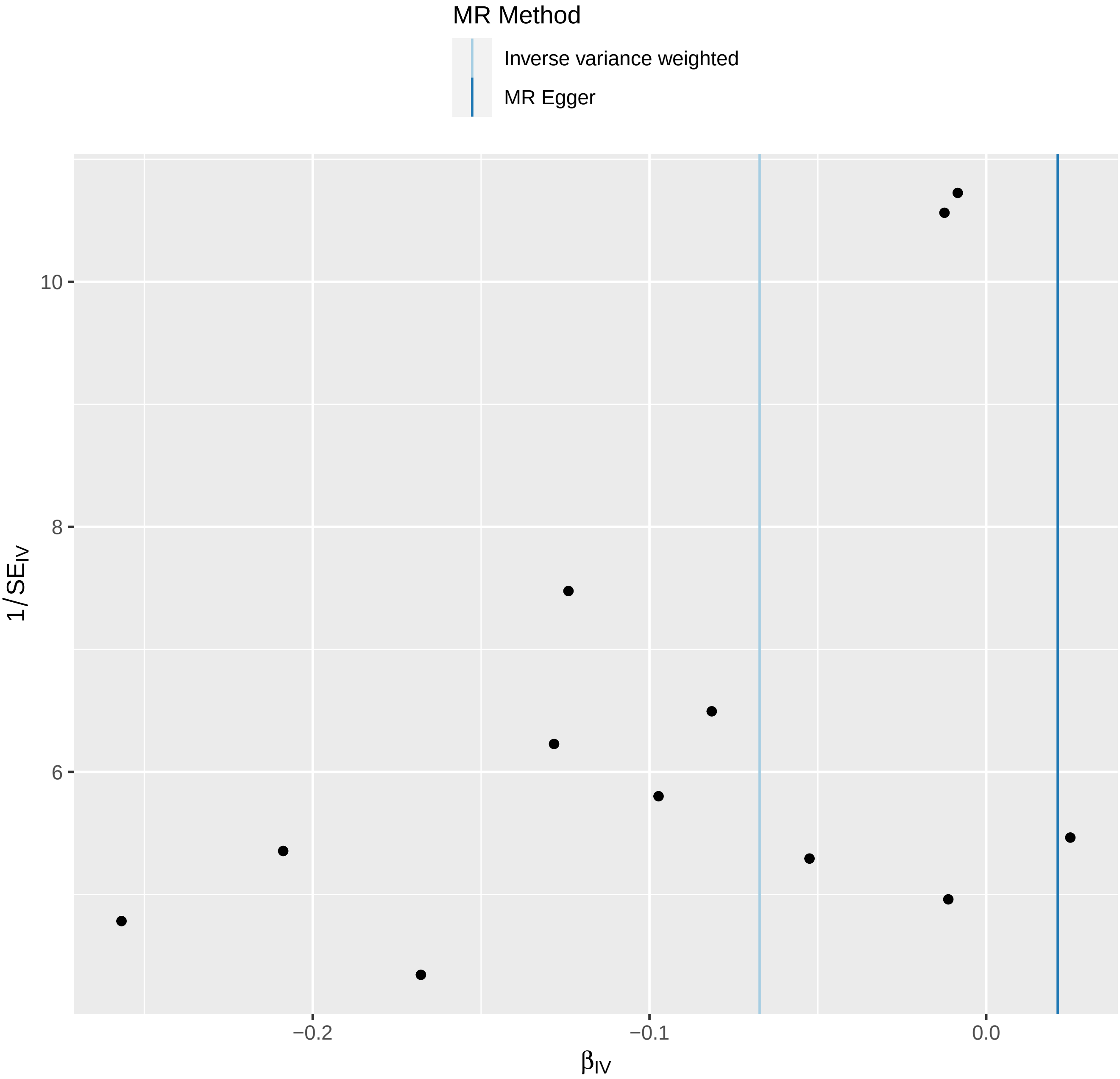

Batch 3−1696 : Selenium on Laminin subunit alpha−4

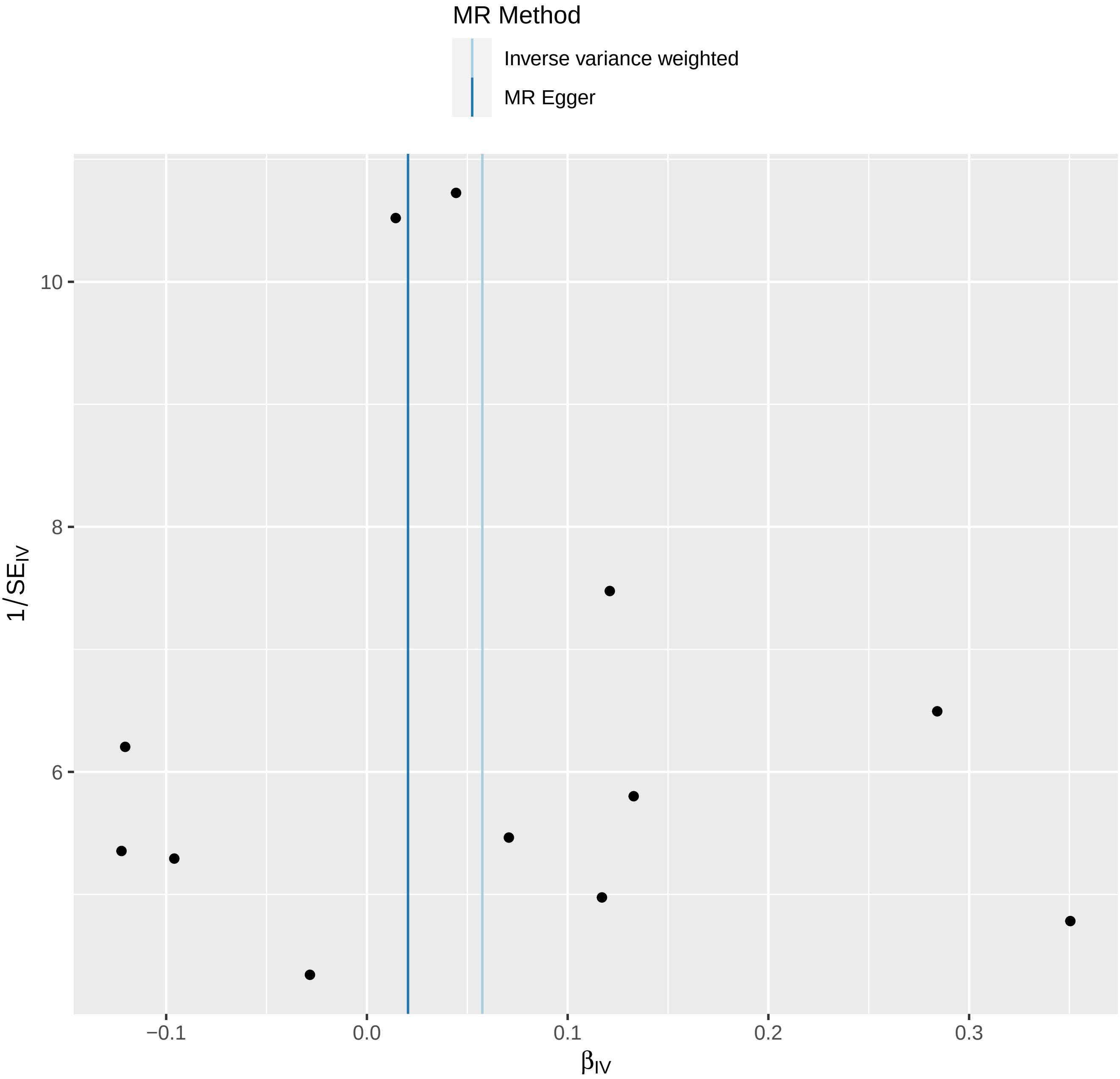

Batch 3−1697 : Selenium on Laminin subunit gamma−2

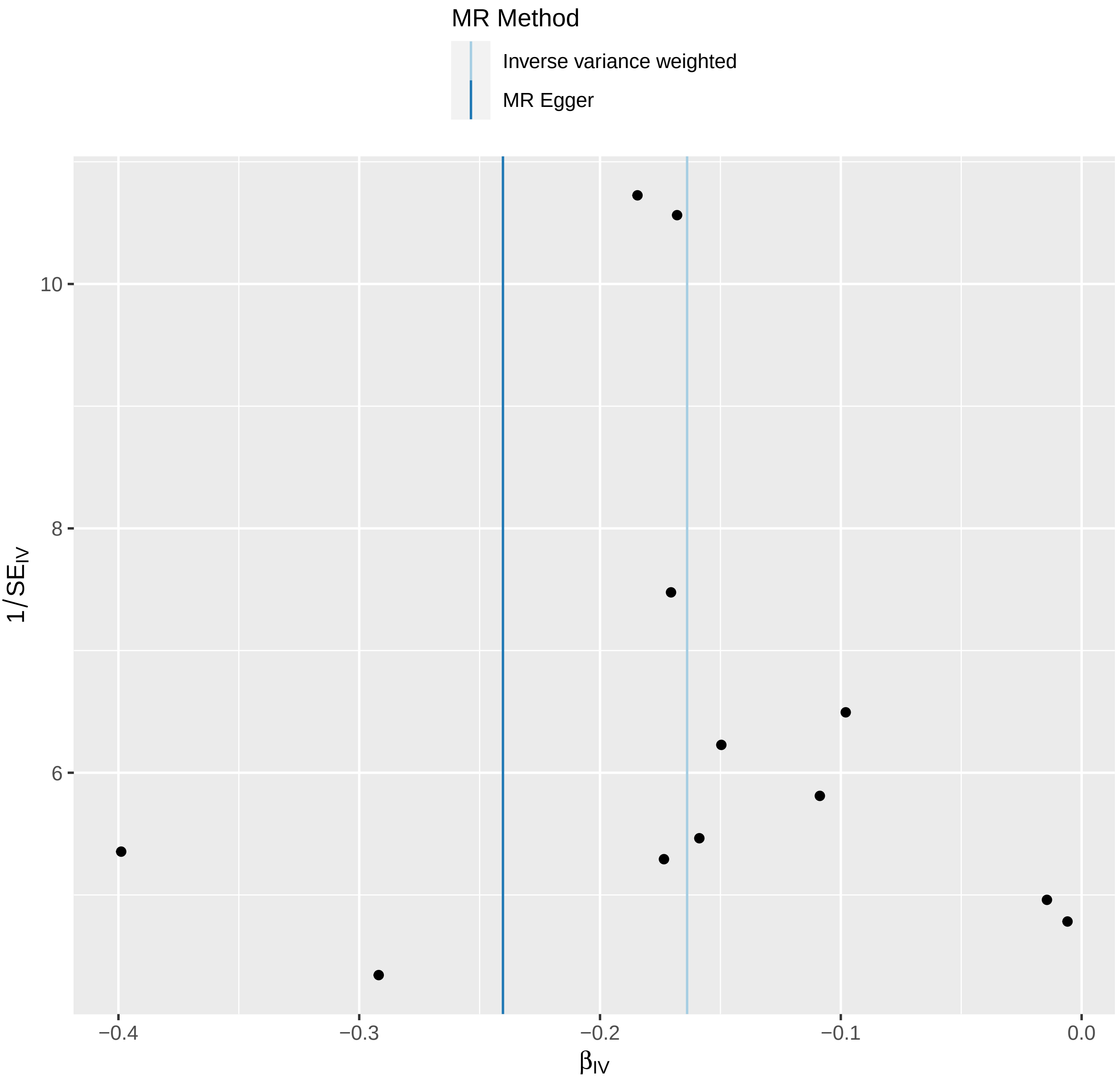

Batch 3−1698 : Selenium on Lysosome−associated membrane glycoprotein 3

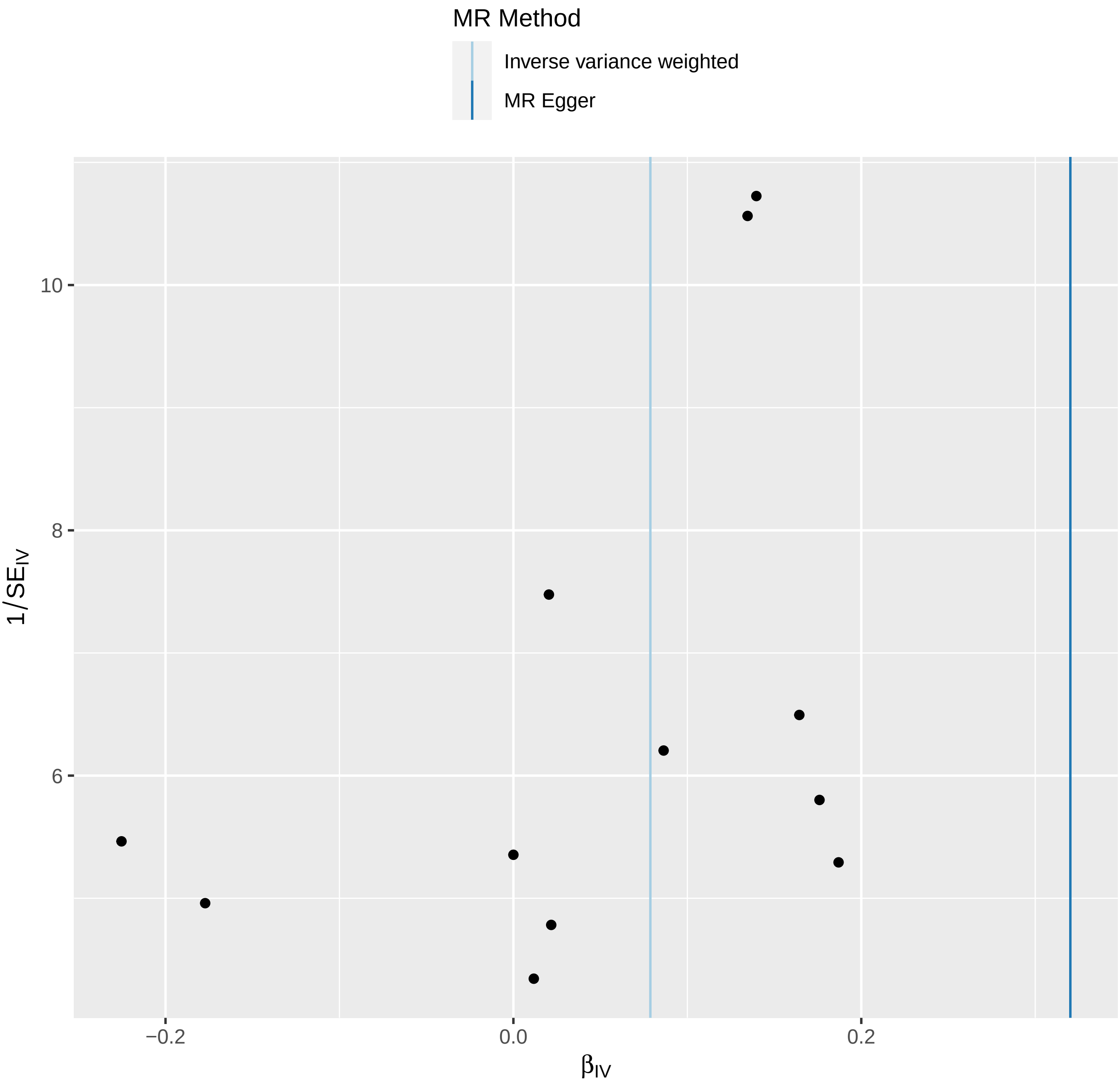

Batch 3−1699 : Selenium on Ragulator complex protein LAMTOR3

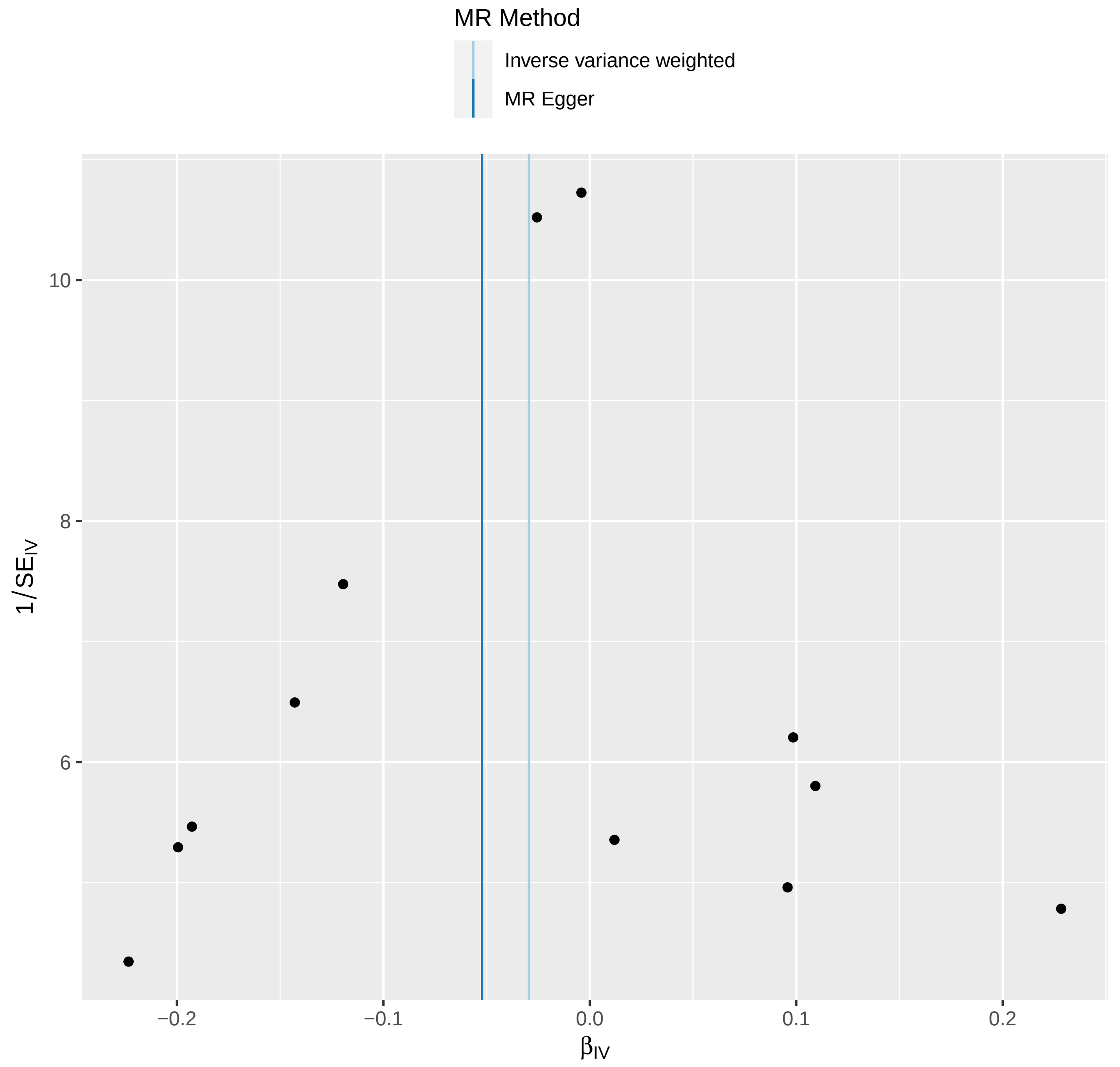

Batch 3−1700 : Selenium on Glycosyltransferase−like protein LARGE1

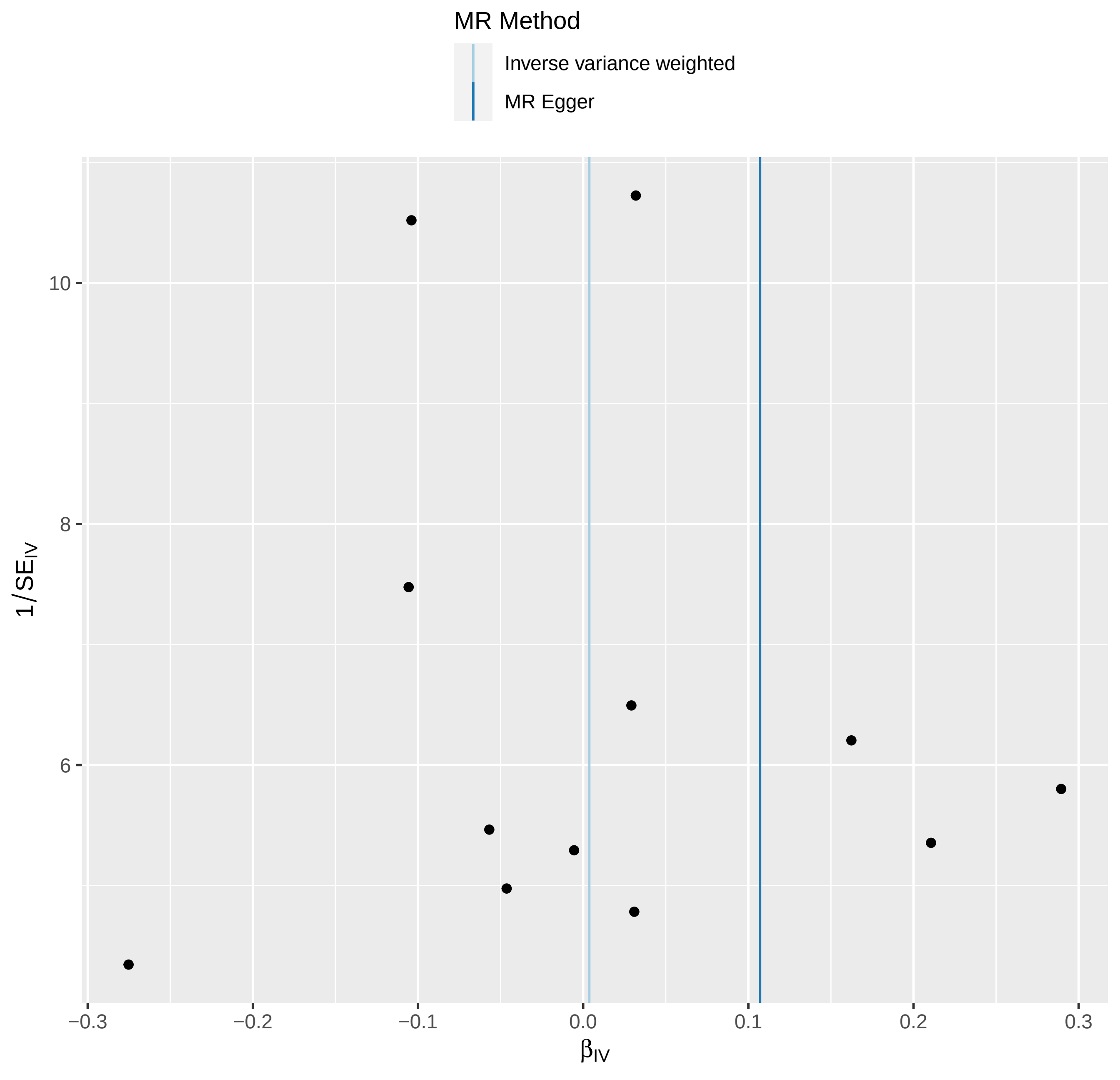

Batch 3−1701 : Selenium on Linker for activation of T−cells family member 1

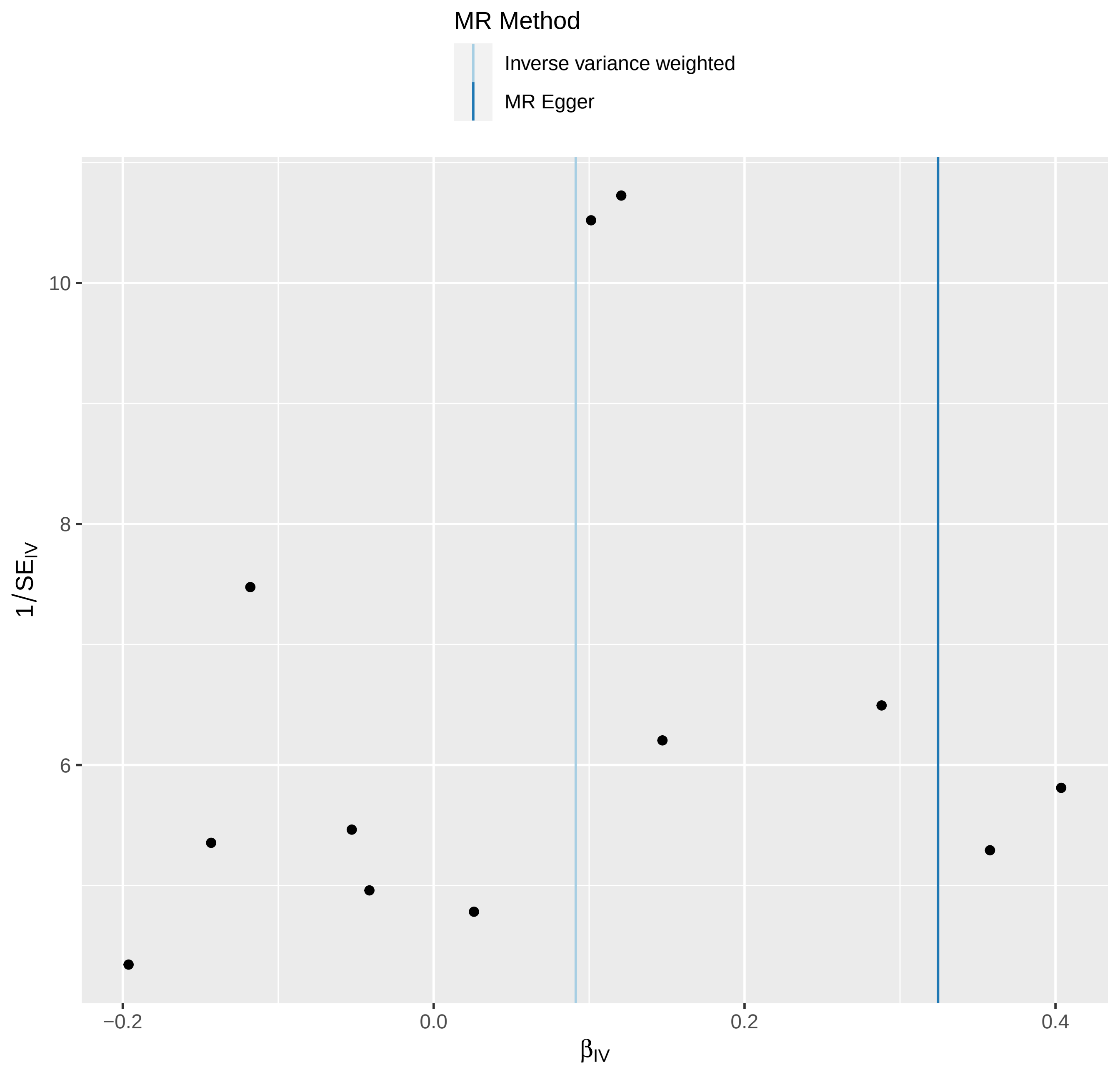

Batch 3−1702 : Selenium on Linker for activation of T−cells family member 2

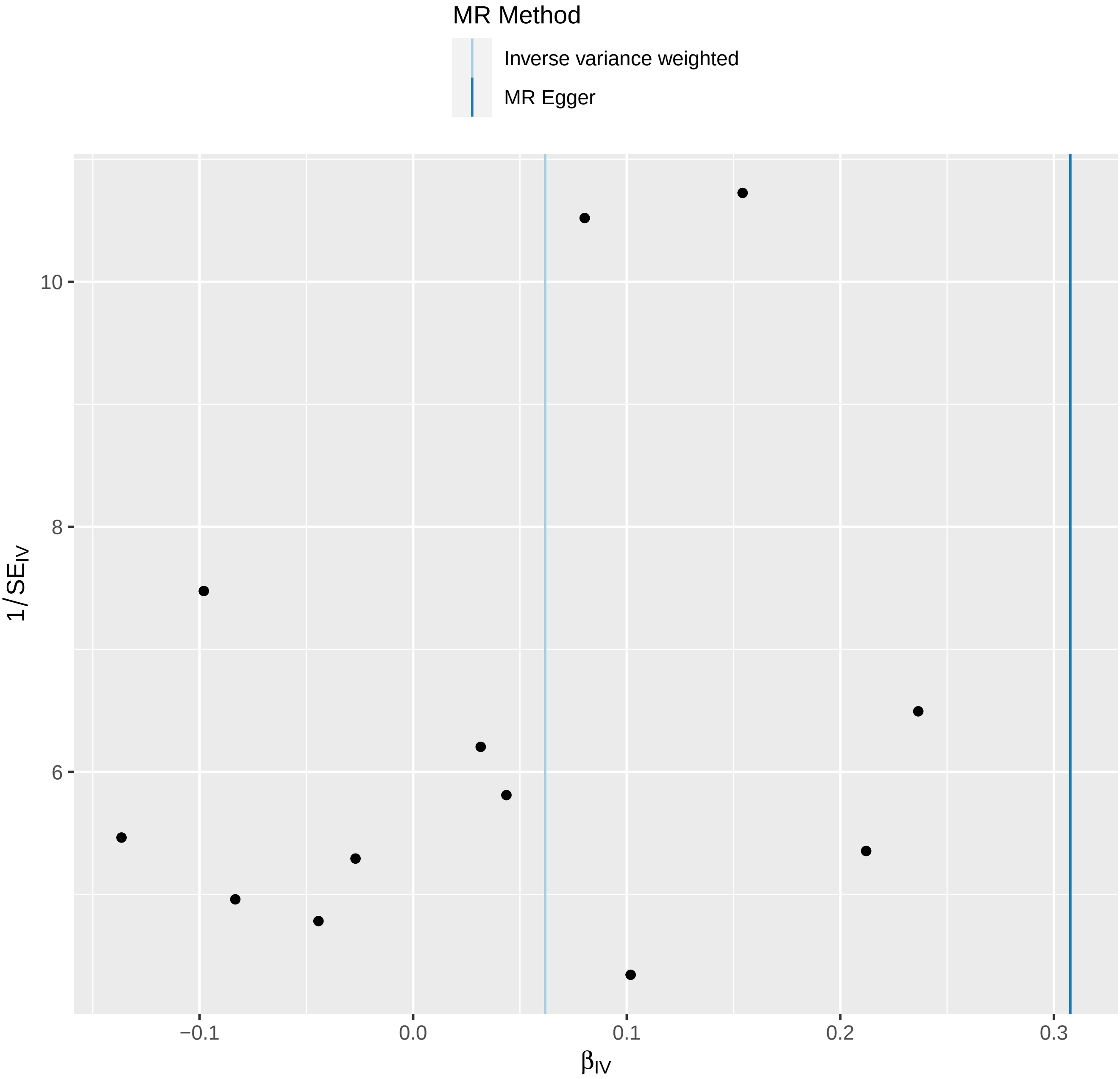

Batch 3−1703 : Selenium on Layilin

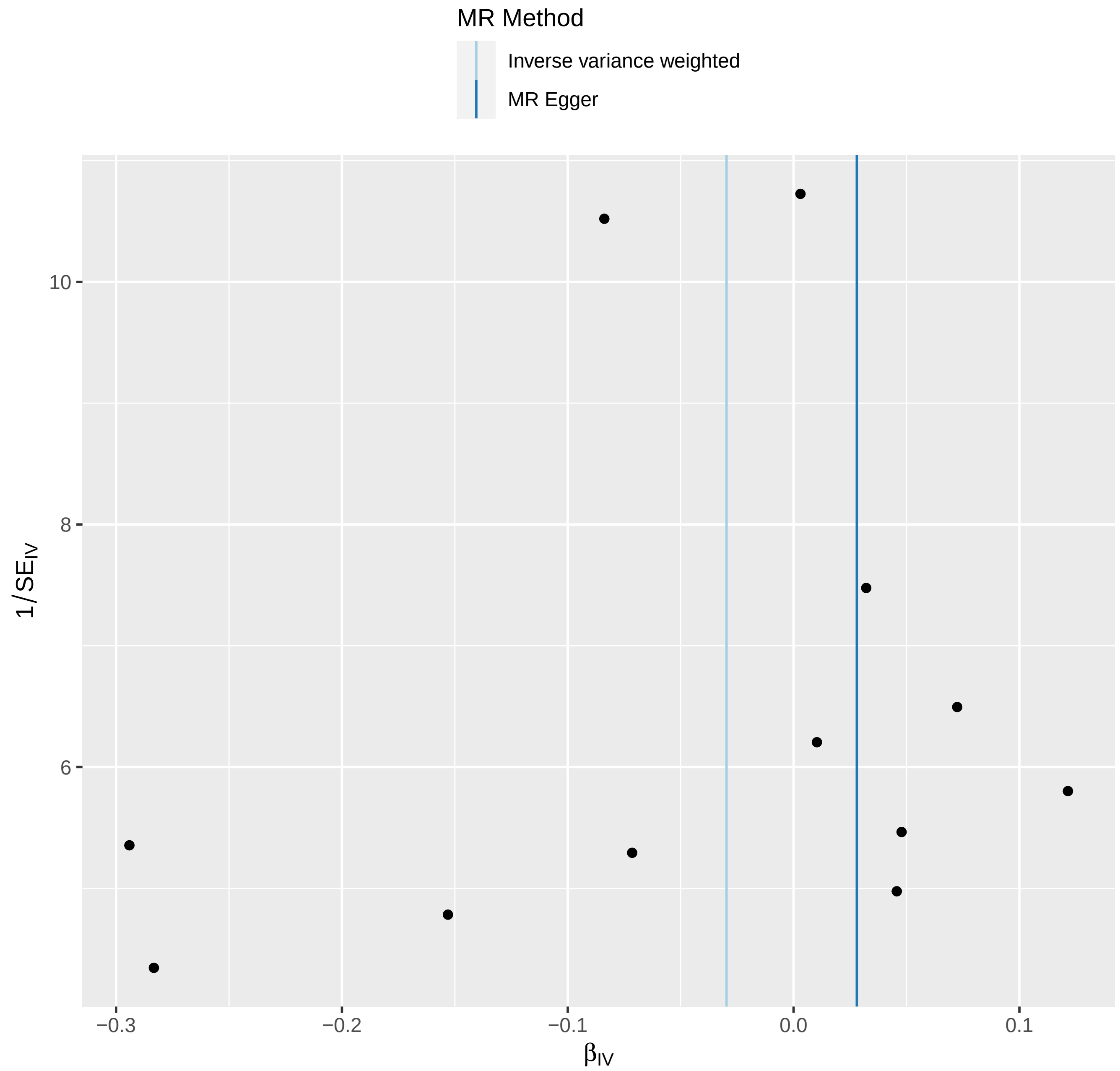

Batch 3−1704 : Selenium on Tyrosine−protein kinase Lck

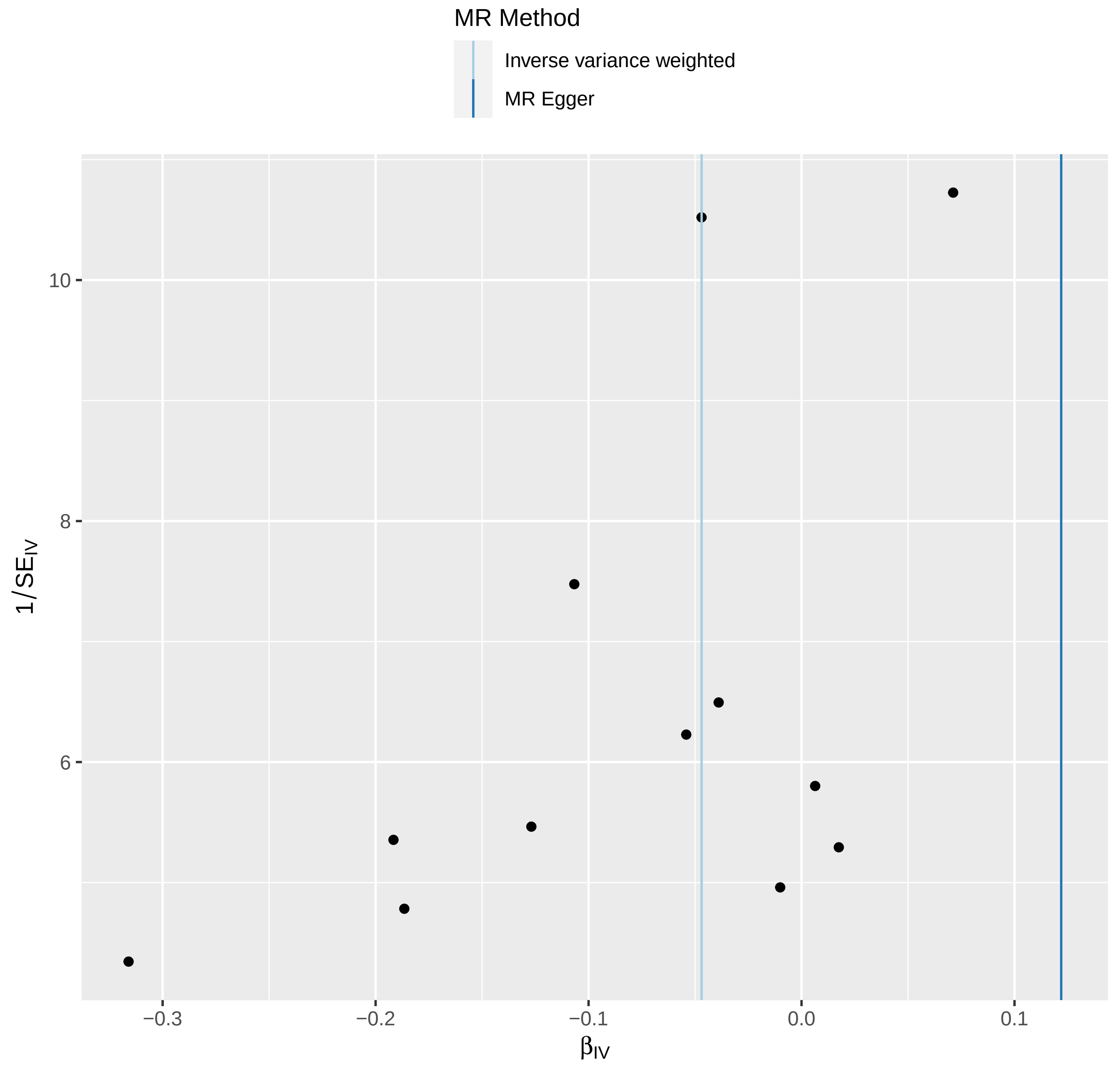

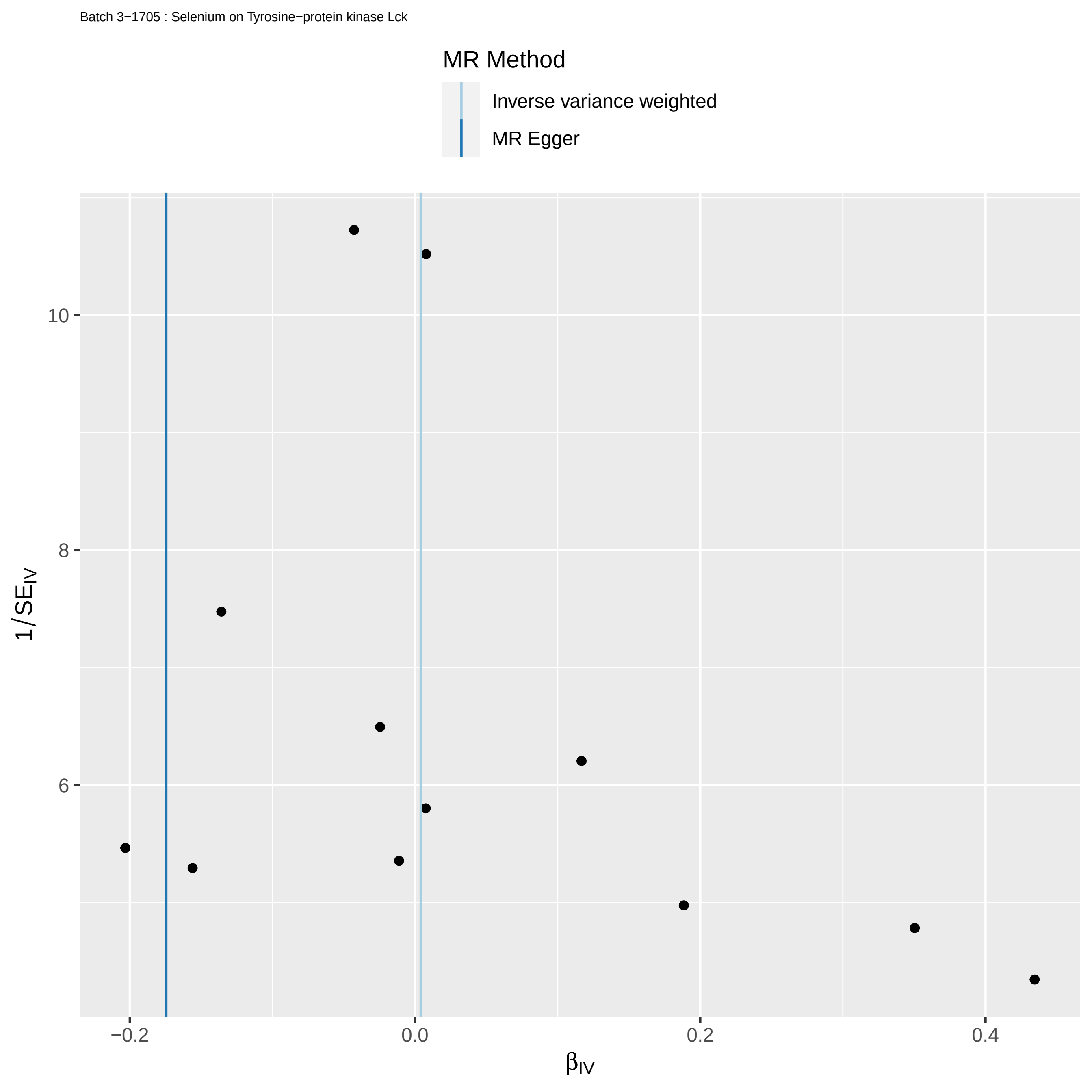

Batch 3−1705 : Selenium on Tyrosine−protein kinase Lck
MR Method
Inverse variance weighted
MR Egger
10
8
6
1/SE_IV
−0.2
0.0
0.2
0.4
β_IV

Batch 3−1706 : Selenium on Leucine carboxyl methyltransferase 1

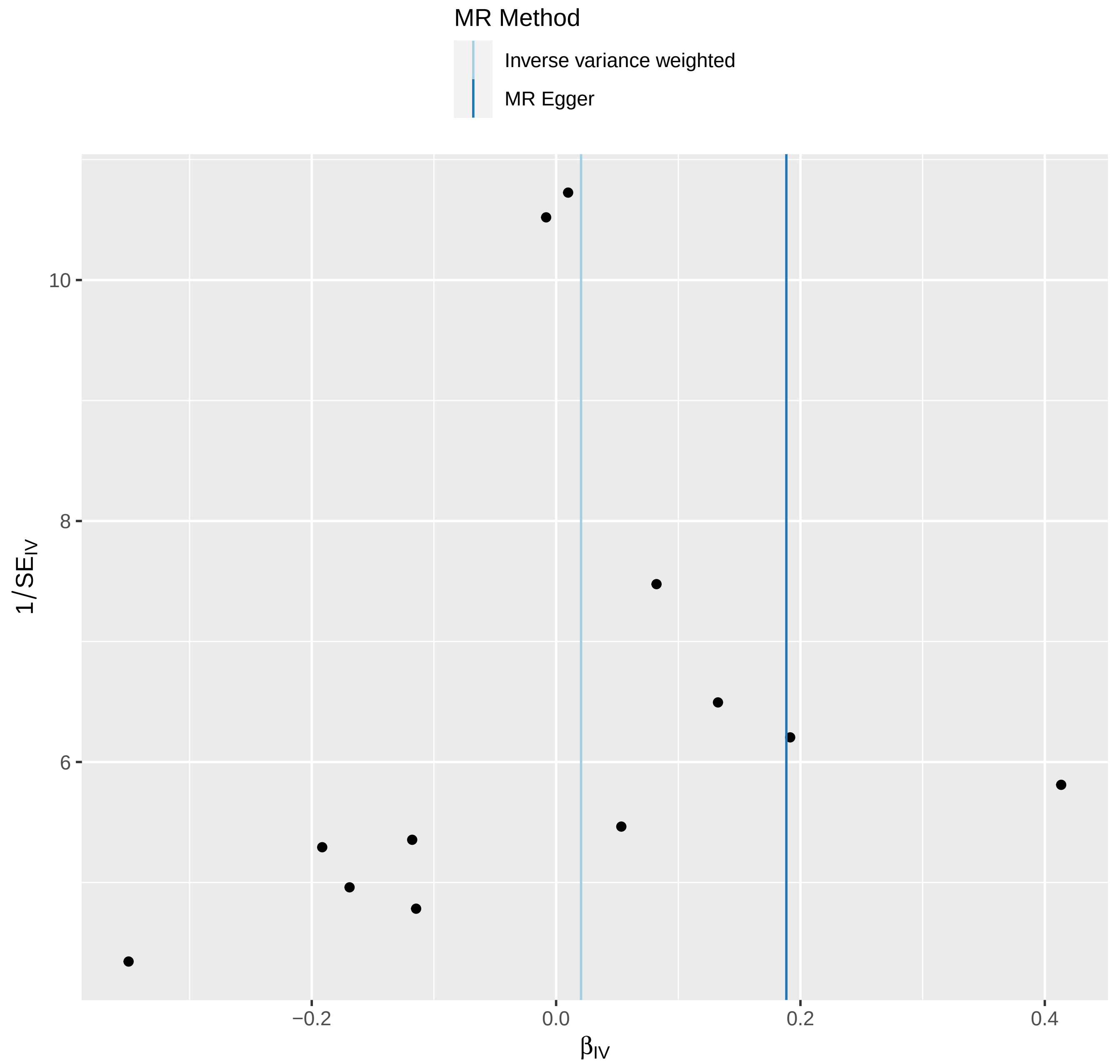

Batch 3−1707 : Selenium on Epididymal−specific lipocalin−10

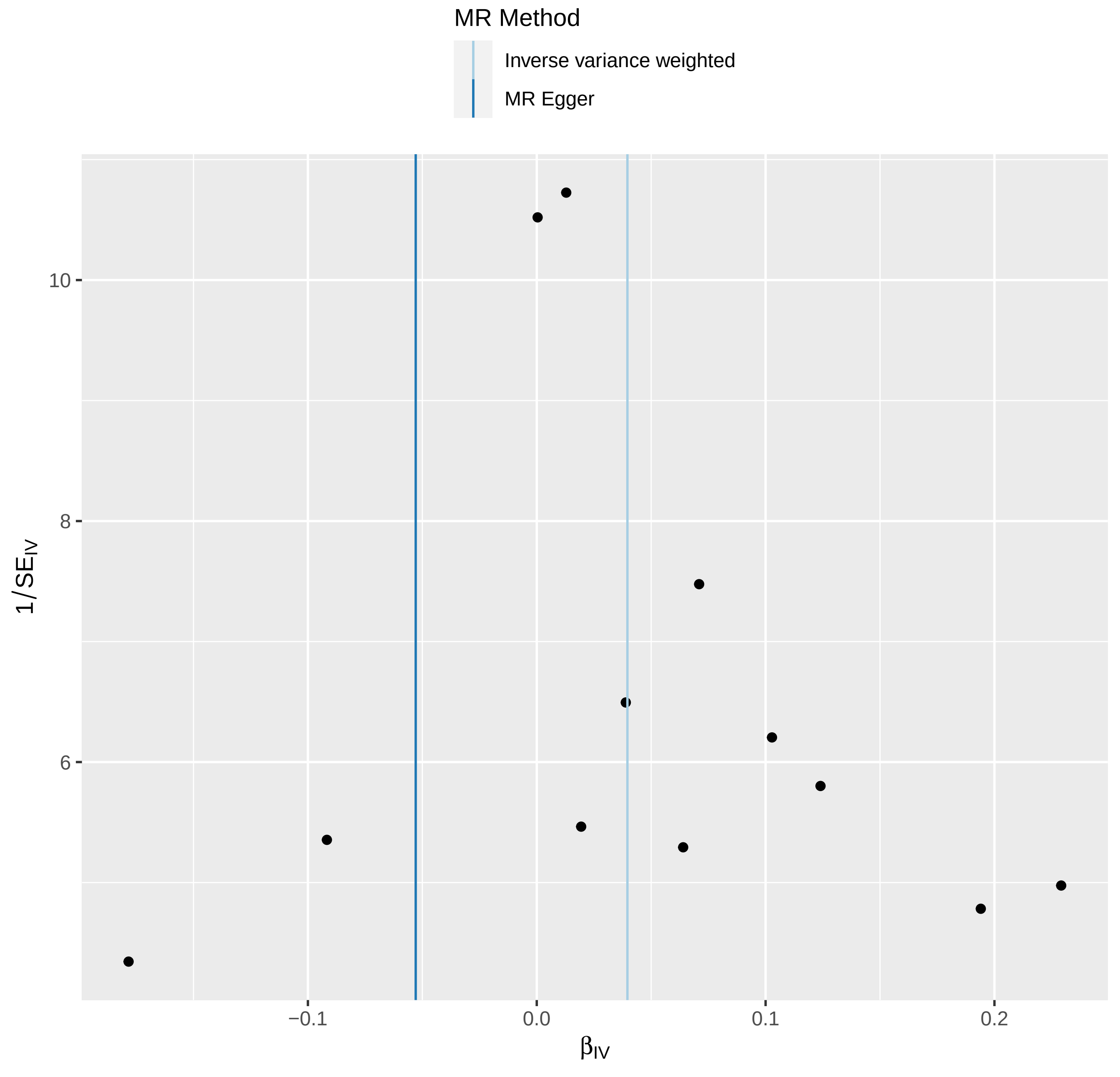

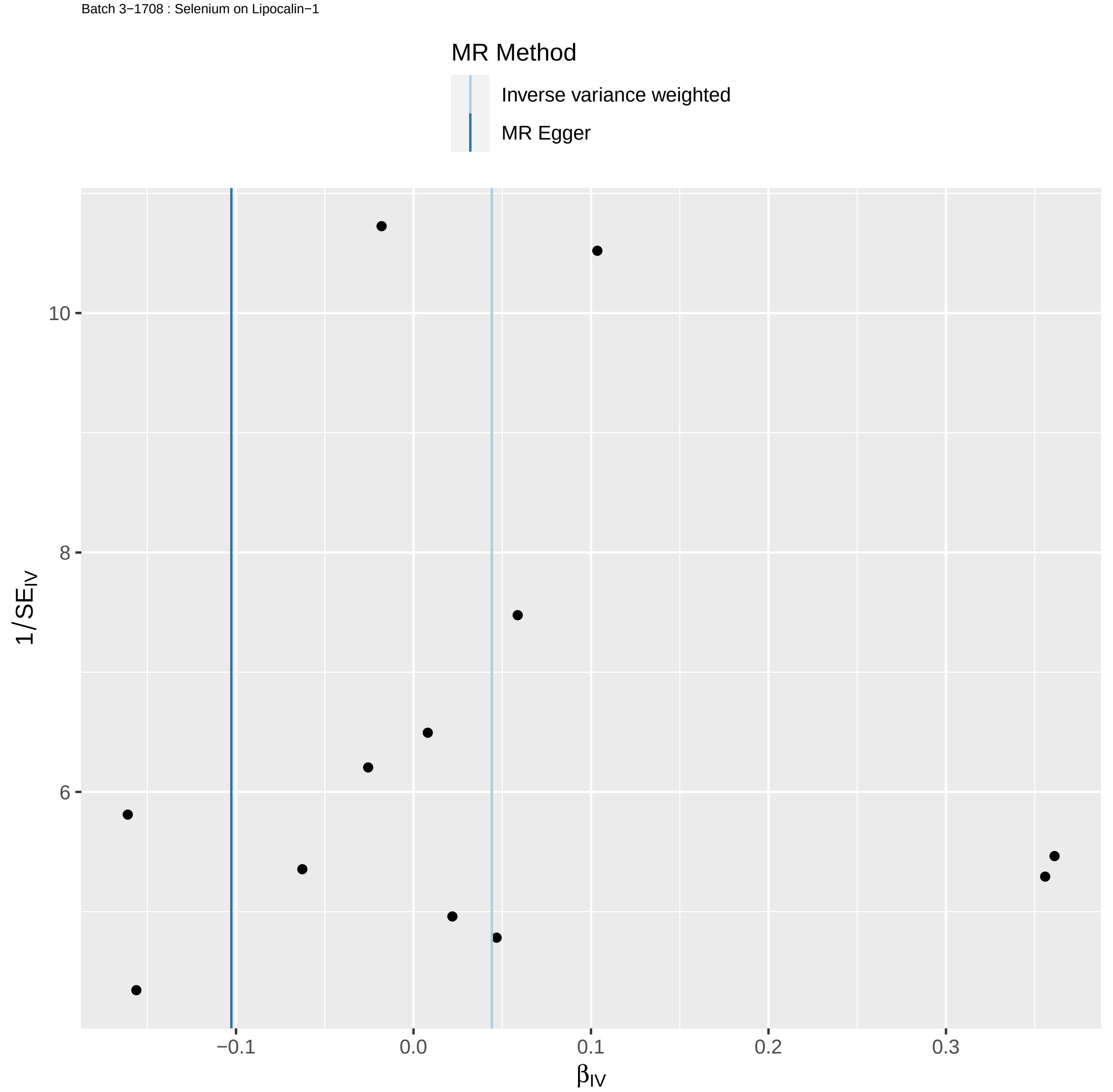
Batch 3−1708 : Selenium on Lipocalin−1
MR Method
Inverse variance weighted
MR Egger
10
8
6
1/SE$_{IV}$
−0.1
0.0
0.1
0.2
0.3
β$_{IV}$

Batch 3−1709 : Selenium on Neutrophil gelatinase−associated lipocalin

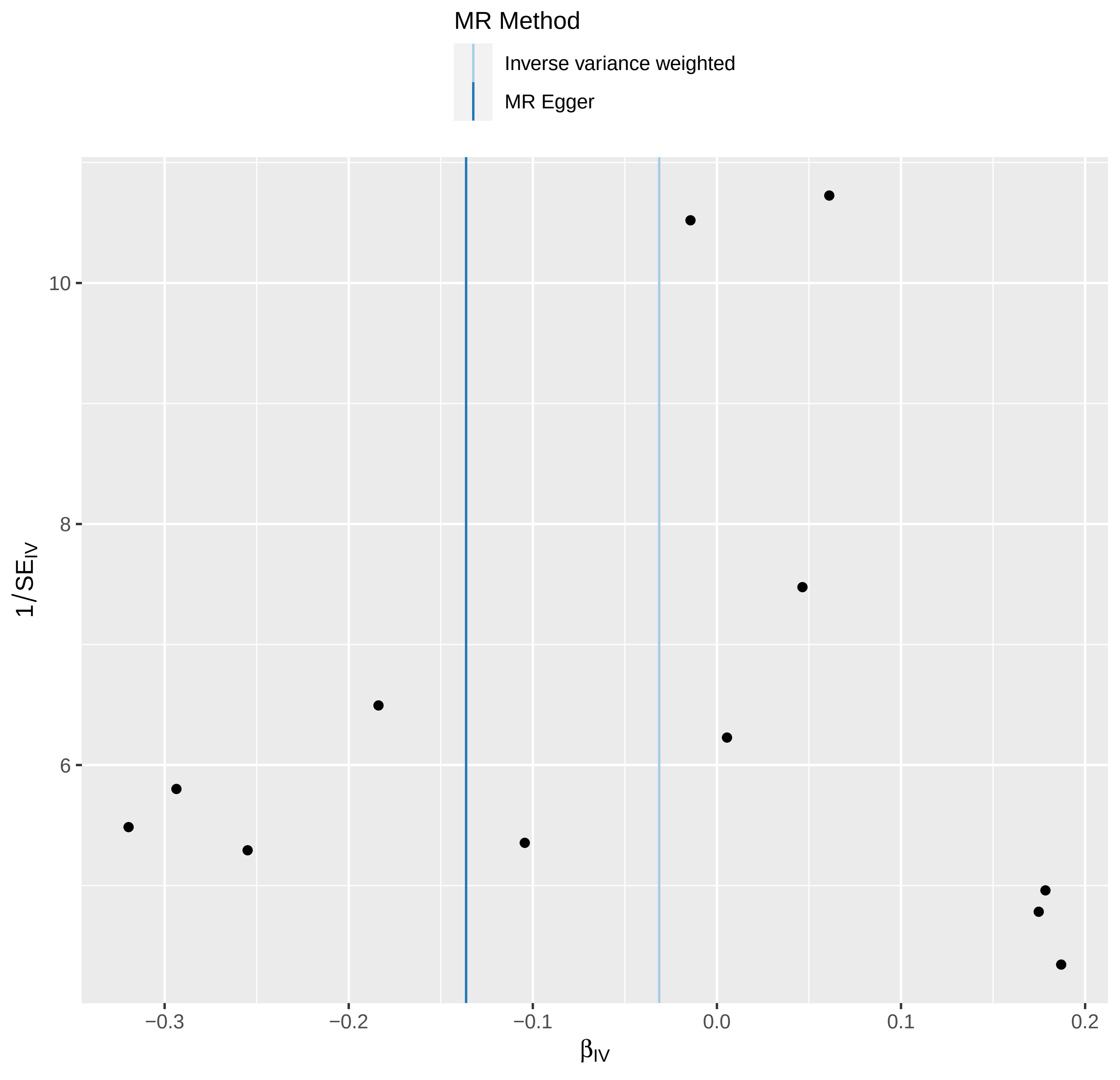

Batch 3−1710 : Selenium on Epididymal−specific lipocalin−8

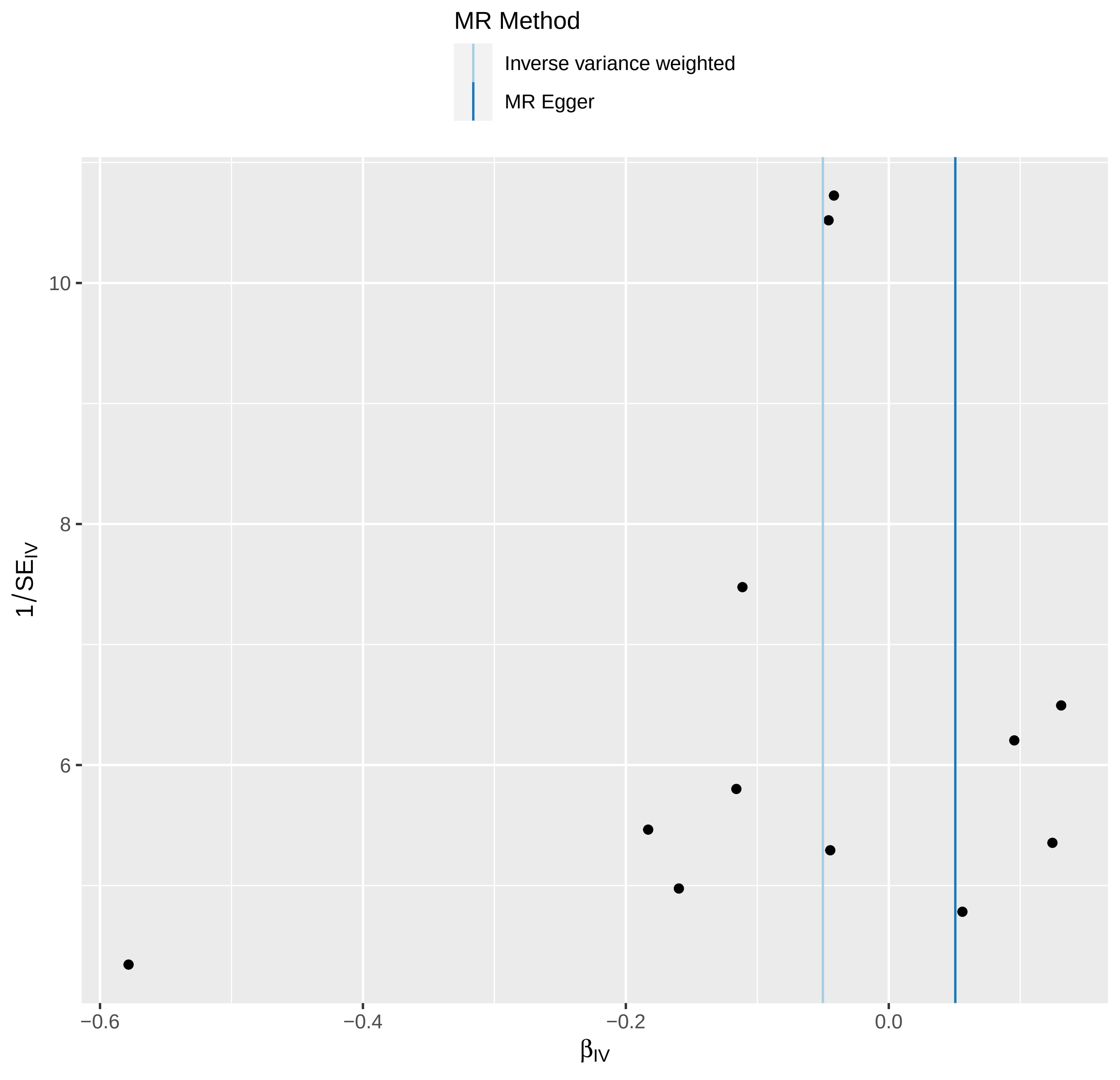

Batch 3−1711 : Selenium on Ligand−dependent nuclear receptor corepressor−like protein

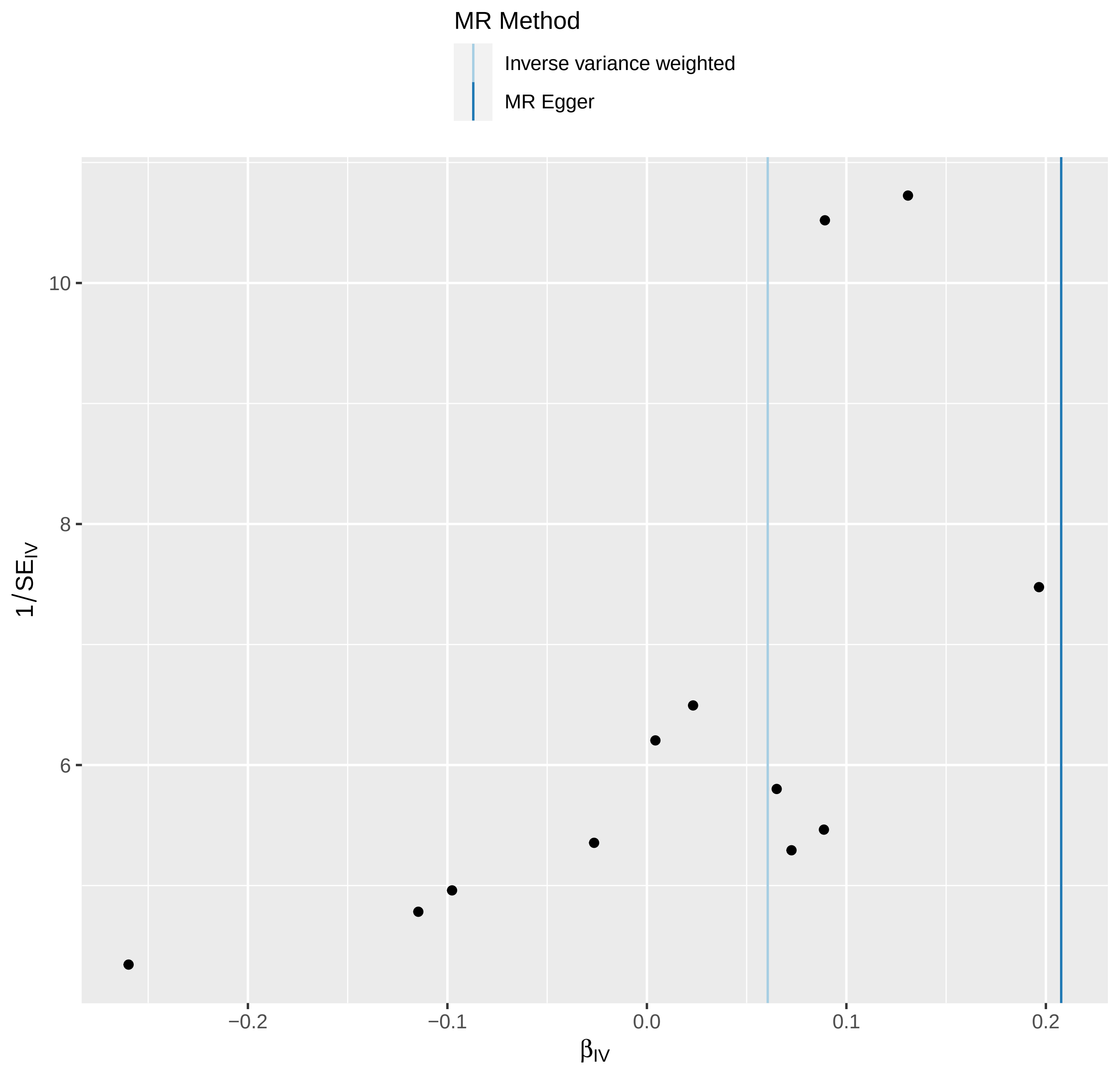

Batch 3−1712 : Selenium on Plastin−2

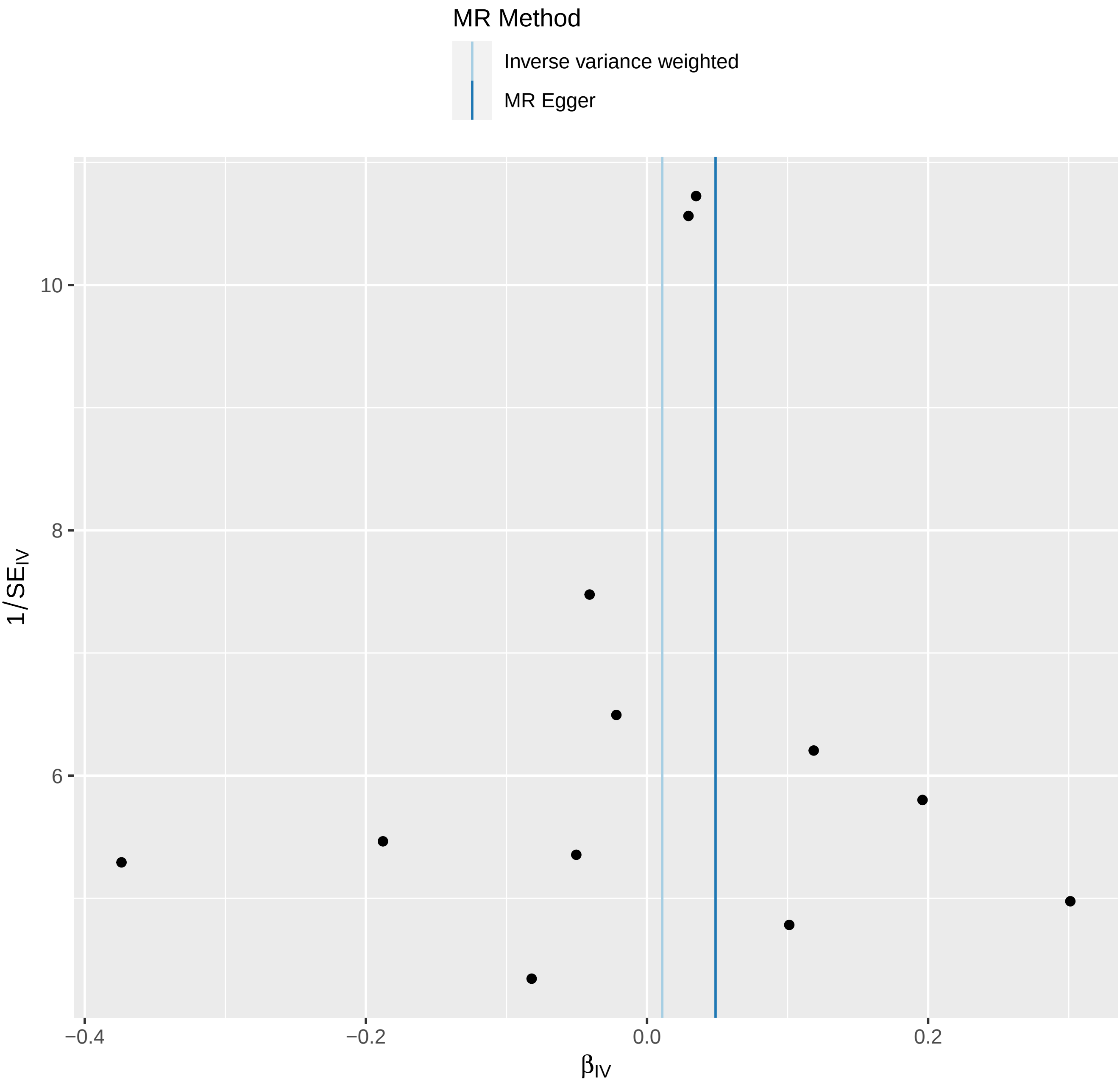

Batch 3−1713 : Selenium on Lactase−phlorizin hydrolase

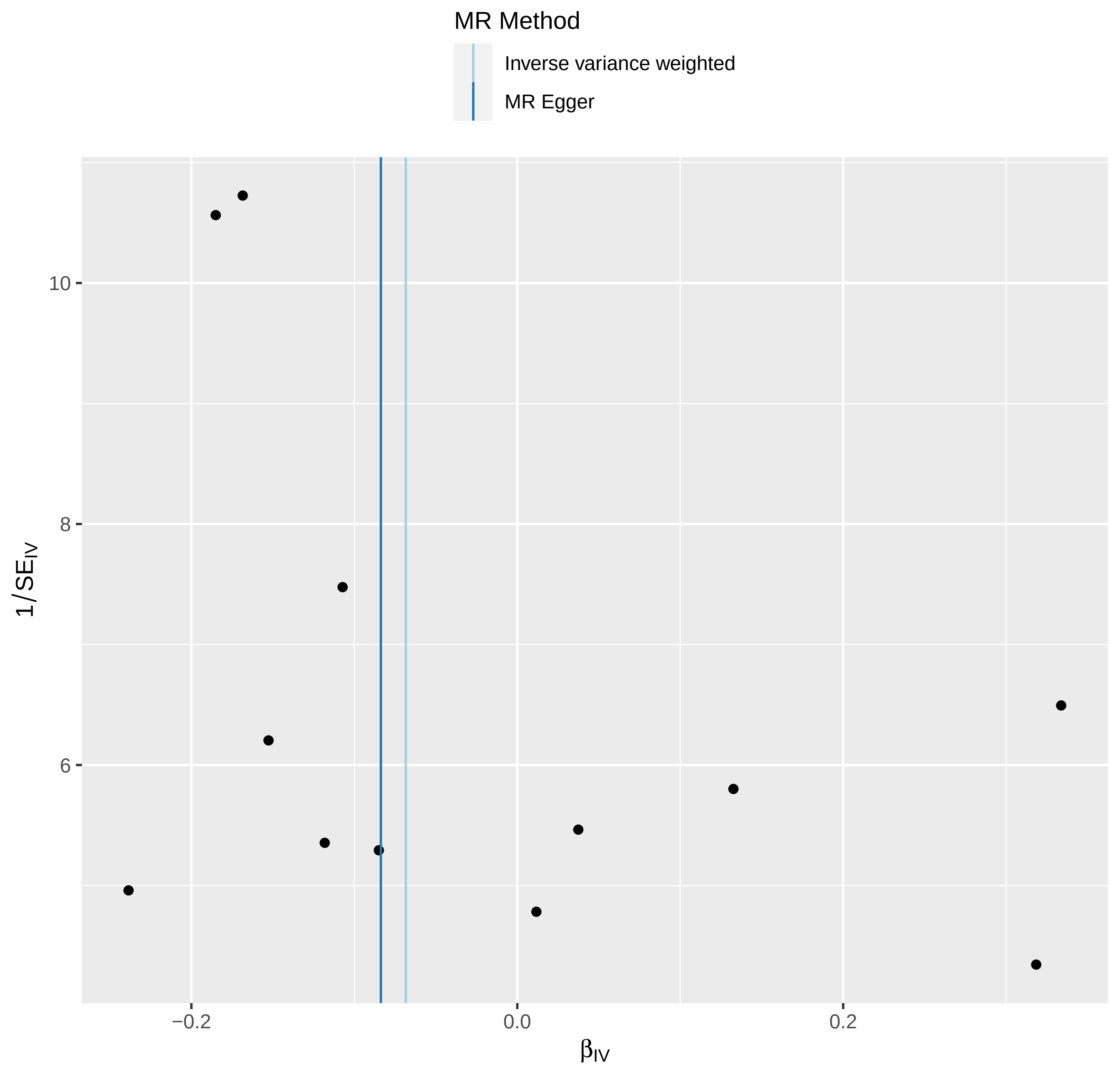

Batch 3−1714 : Selenium on Lactase−like protein

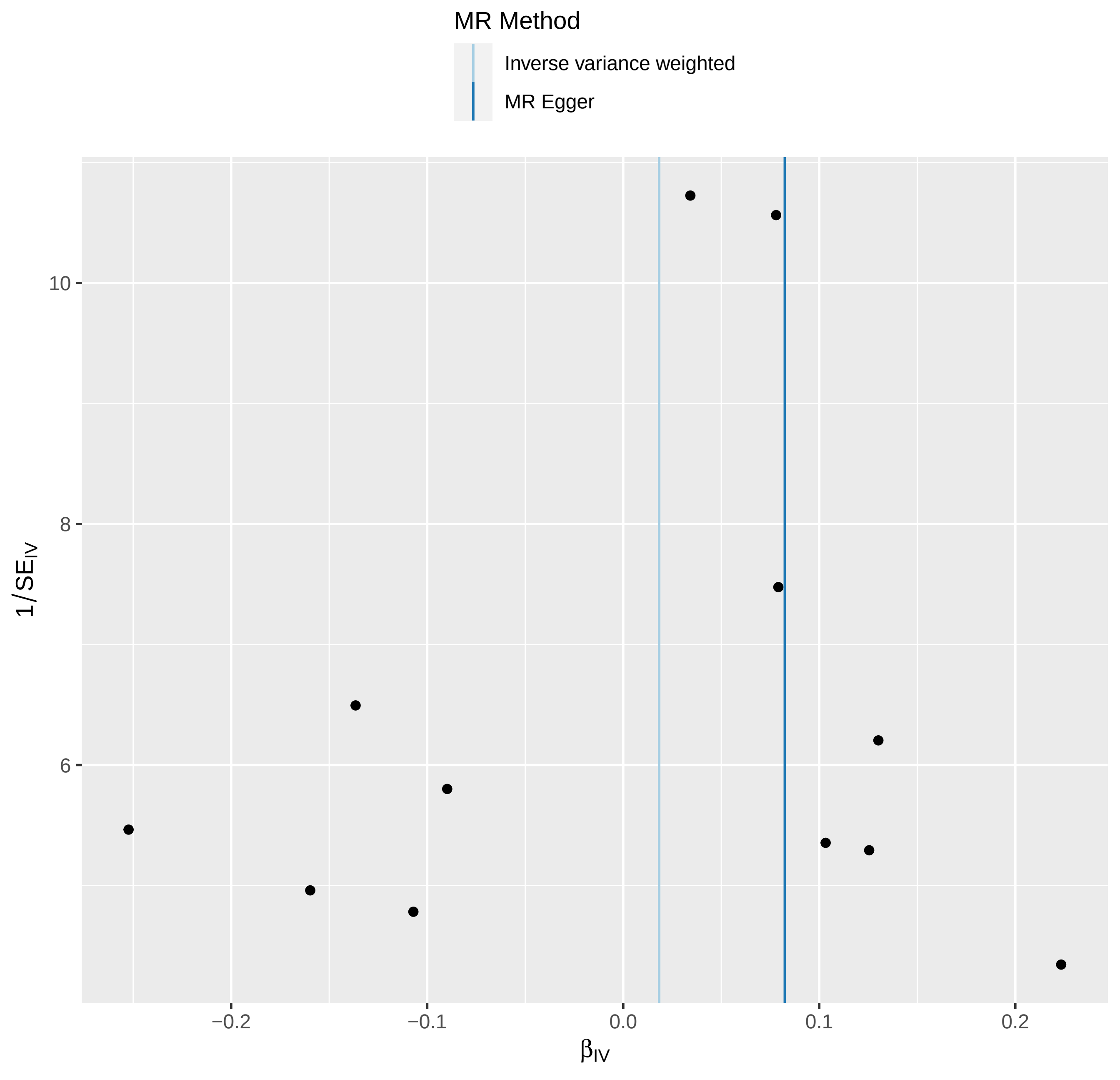

Batch 3−1715 : Selenium on L−lactate dehydrogenase B chain

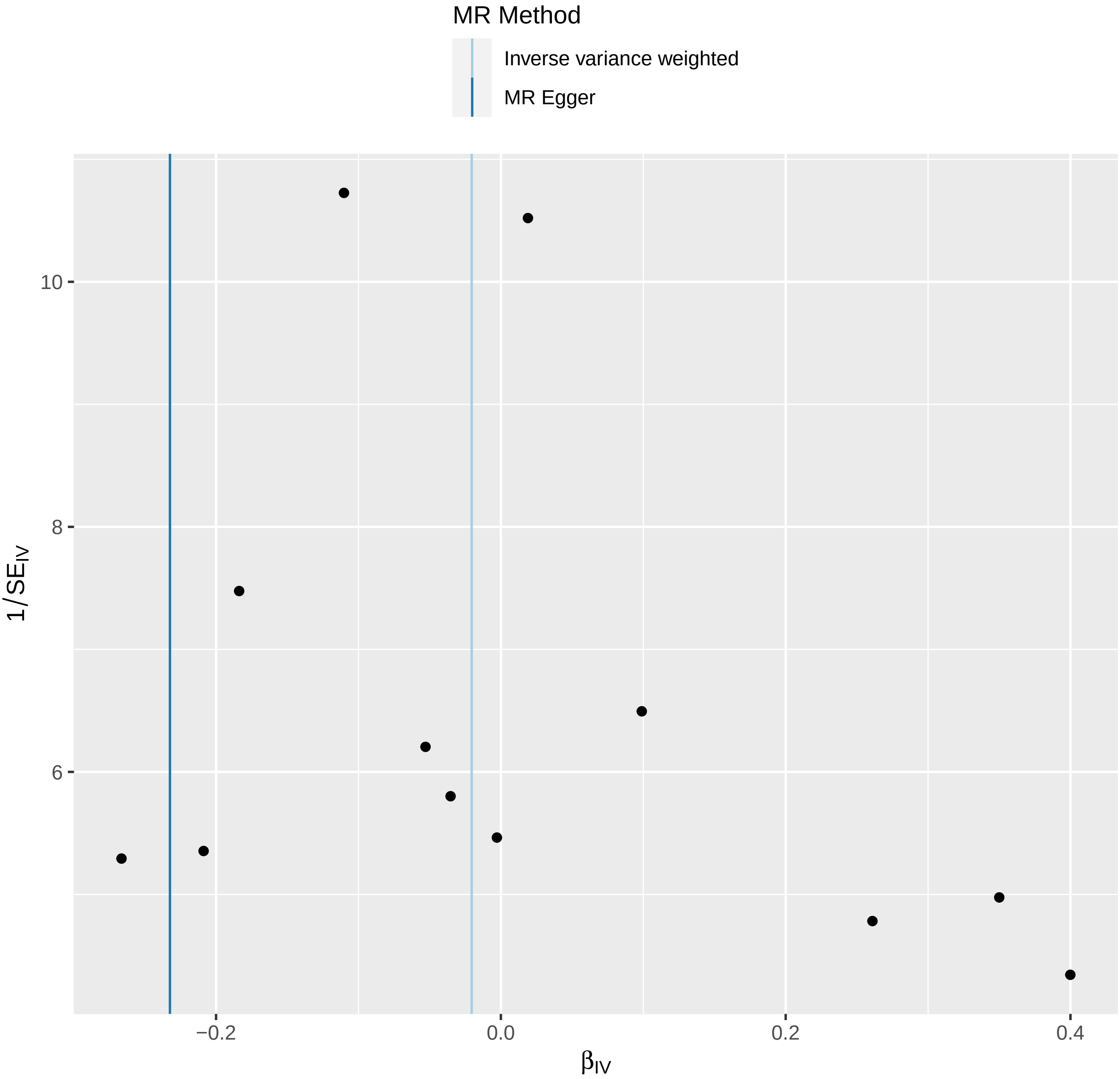

Batch 3−1716 : Selenium on L−lactate dehydrogenase C chain

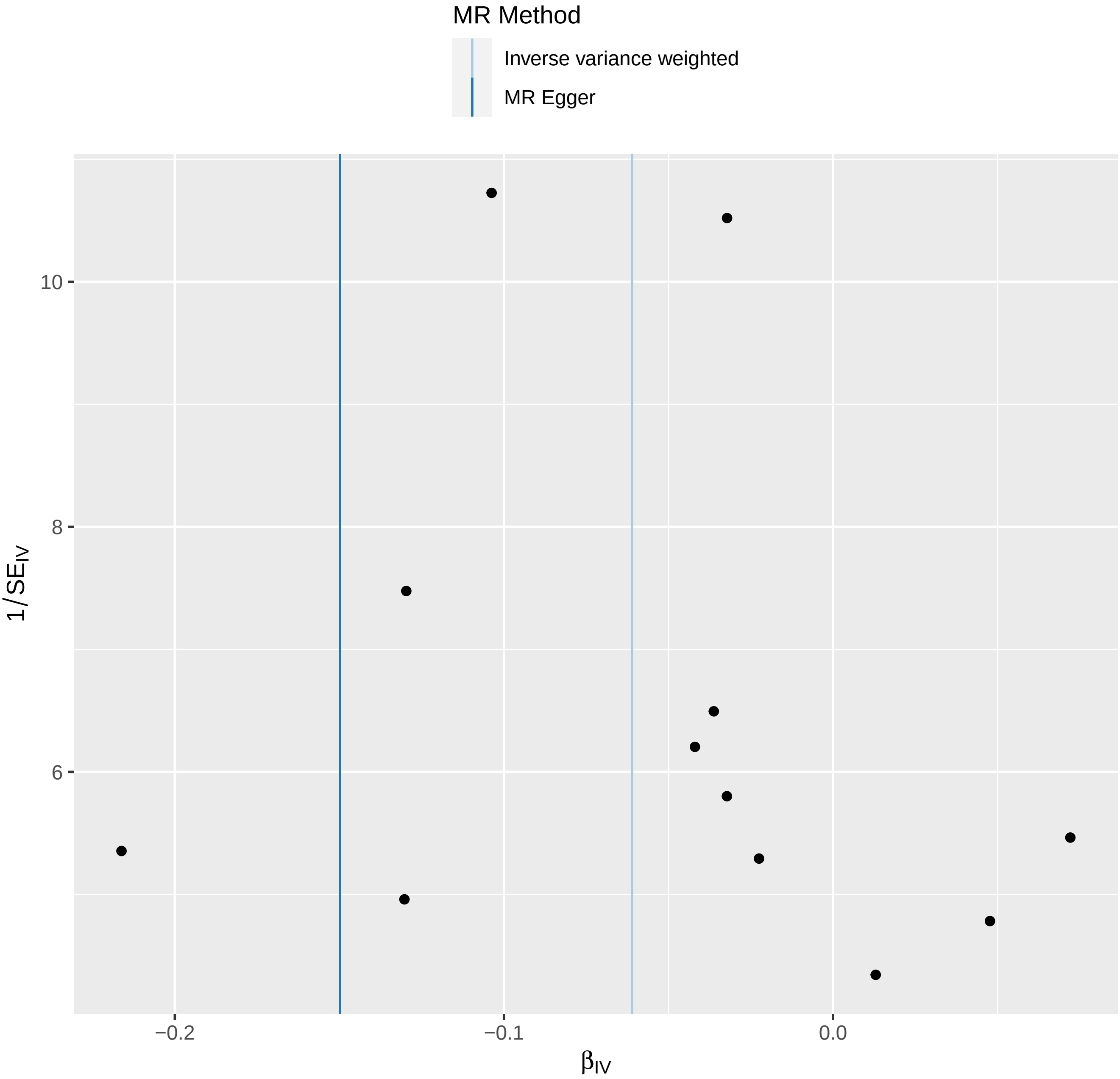

Batch 3−1717 : Selenium on Low−density lipoprotein receptor

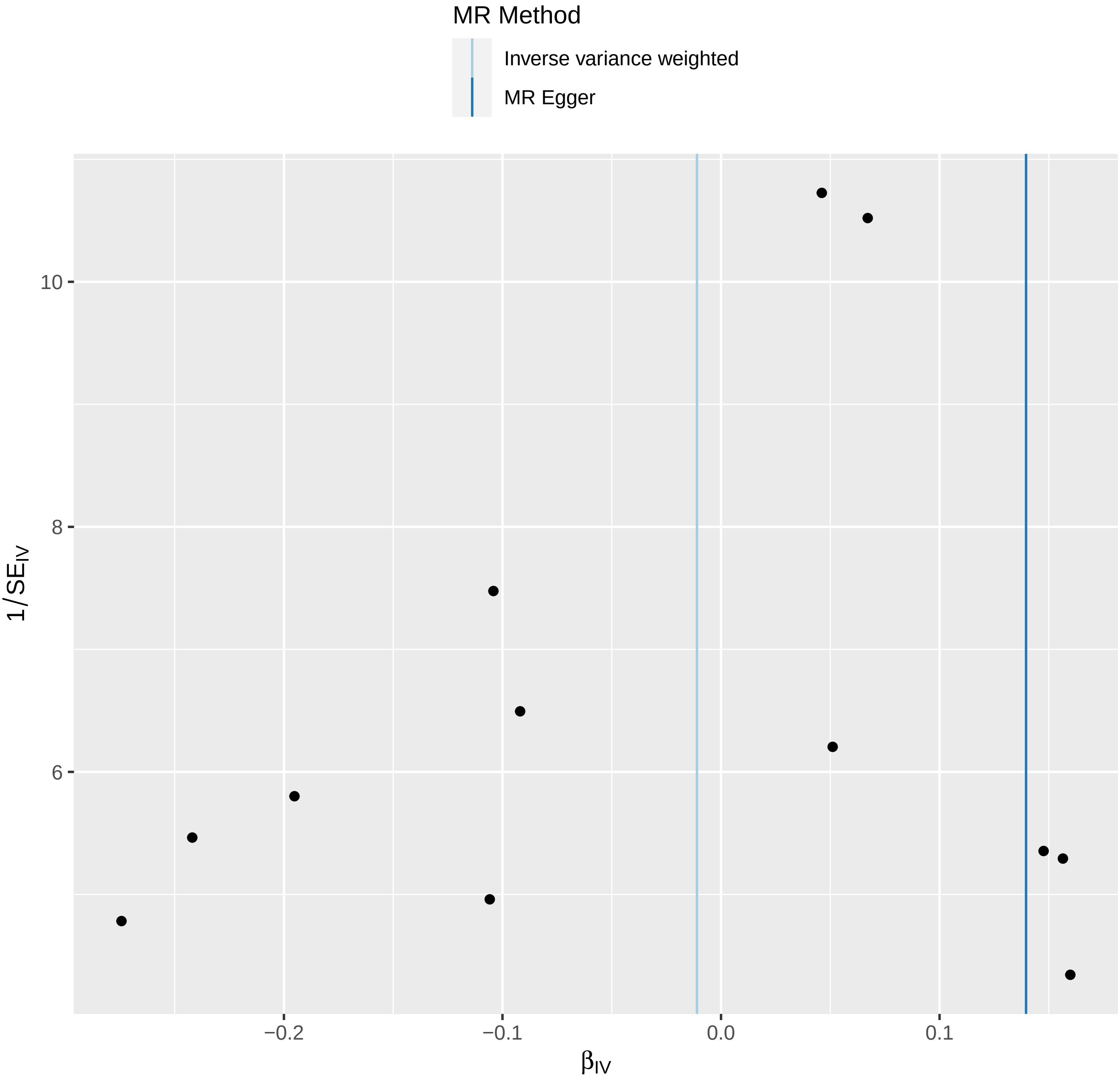

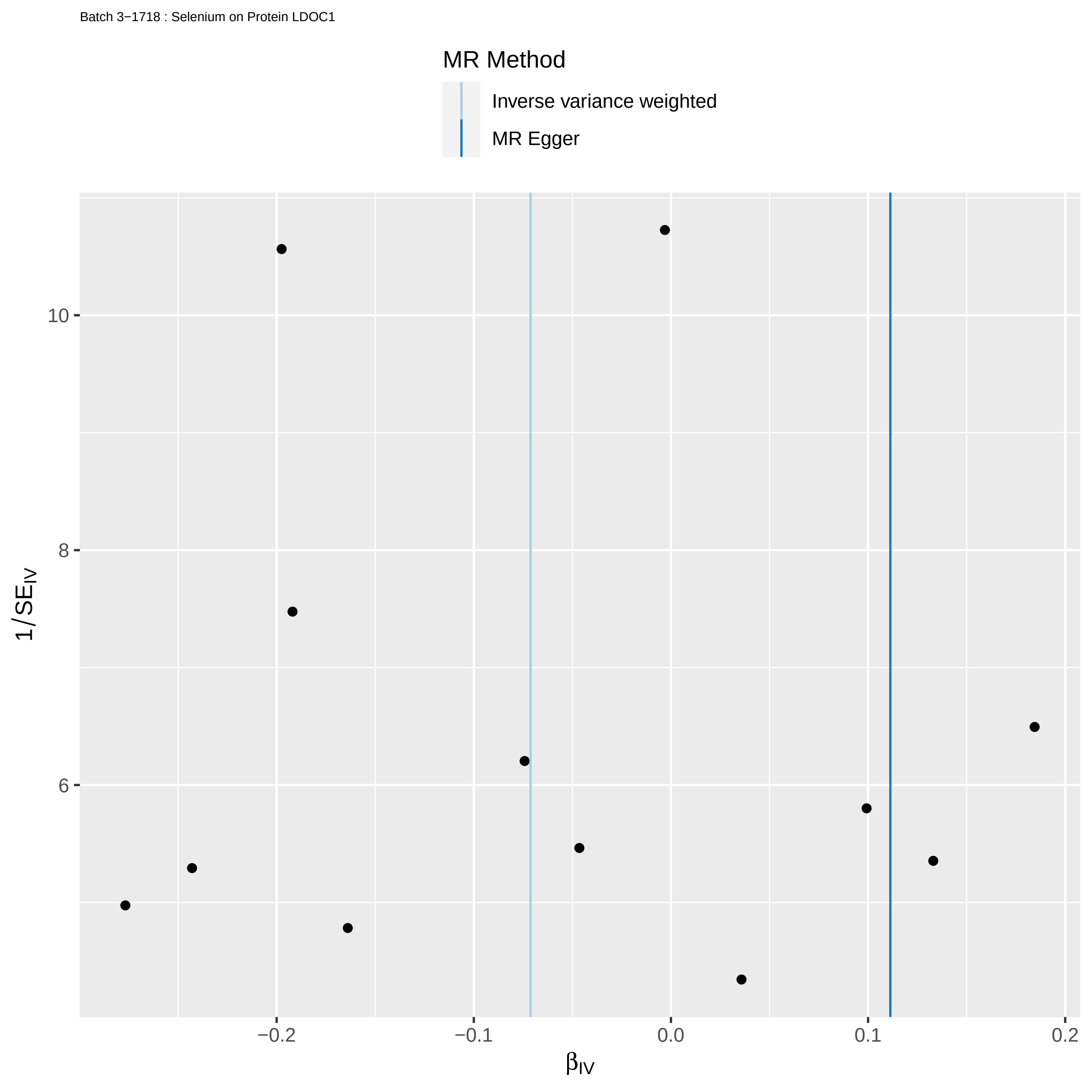

Batch 3−1718 : Selenium on Protein LDOC1
MR Method
Inverse variance weighted
MR Egger
10
8
6
1/SE_IV
−0.2
−0.1
0.0
0.1
0.2
β_IV

Batch 3−1719 : Selenium on Leukocyte cell−derived chemotaxin 1

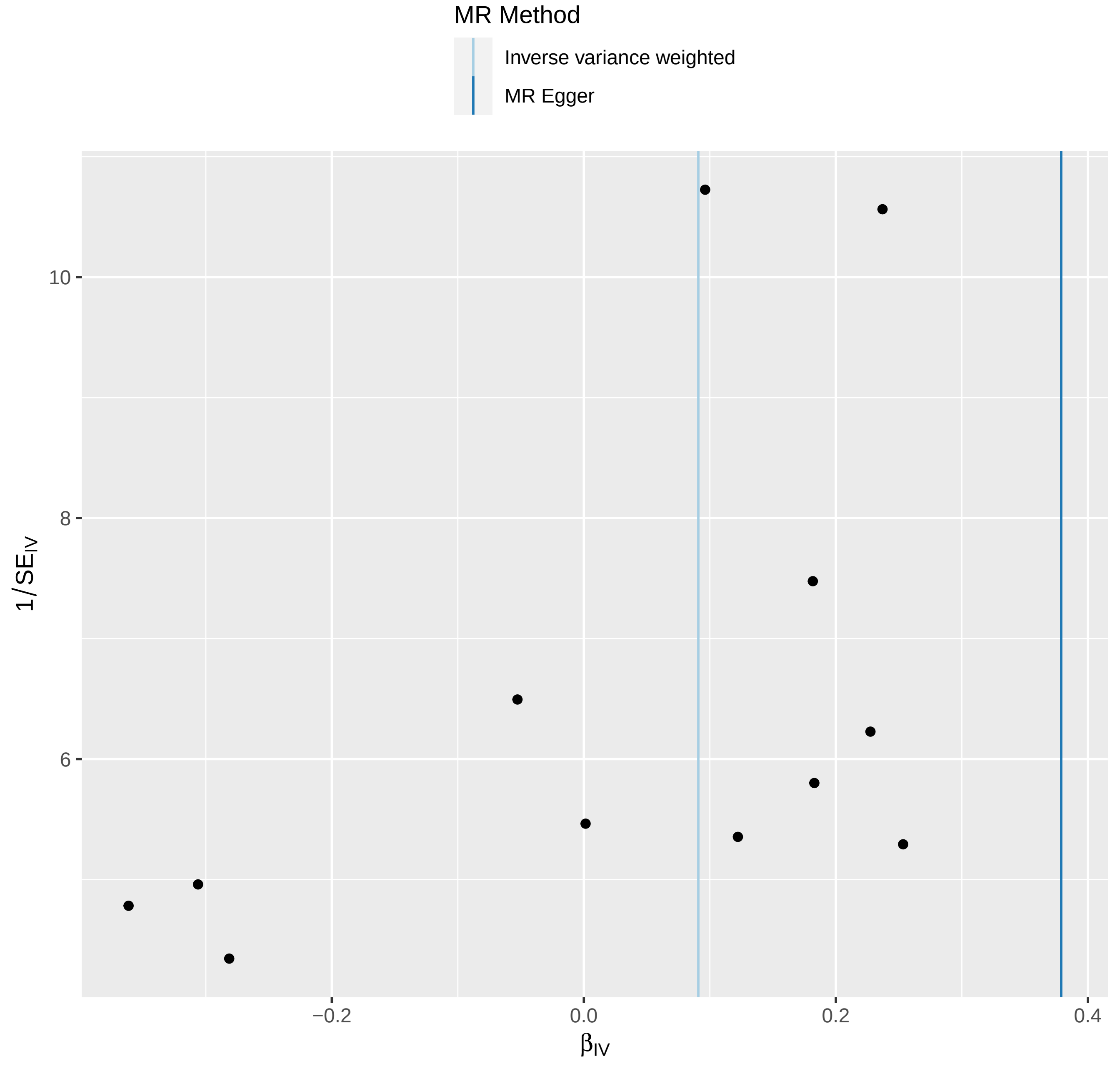

Batch 3−1720 : Selenium on LEM domain−containing protein 1

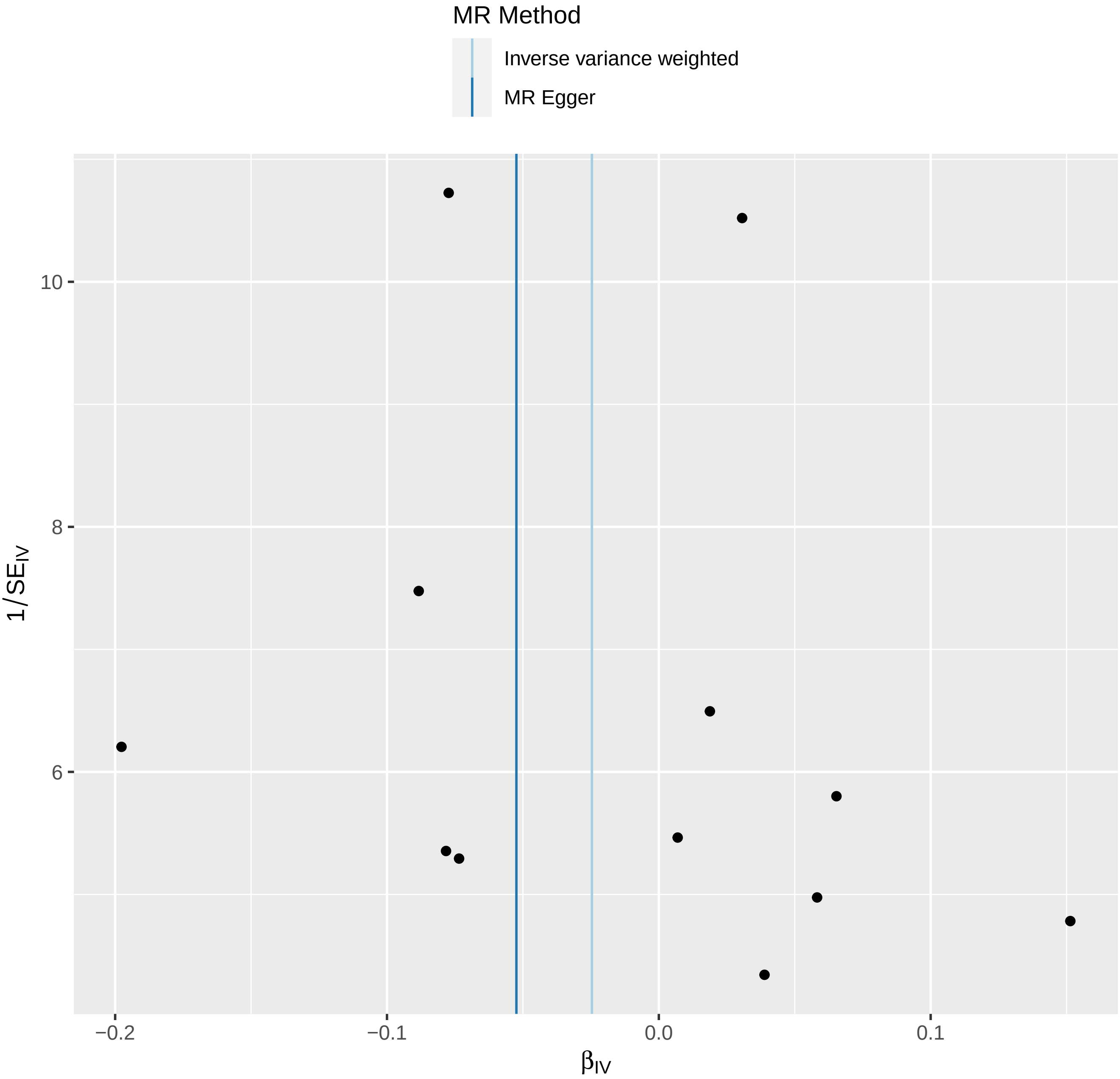

Batch 3−1721 : Selenium on Leptin

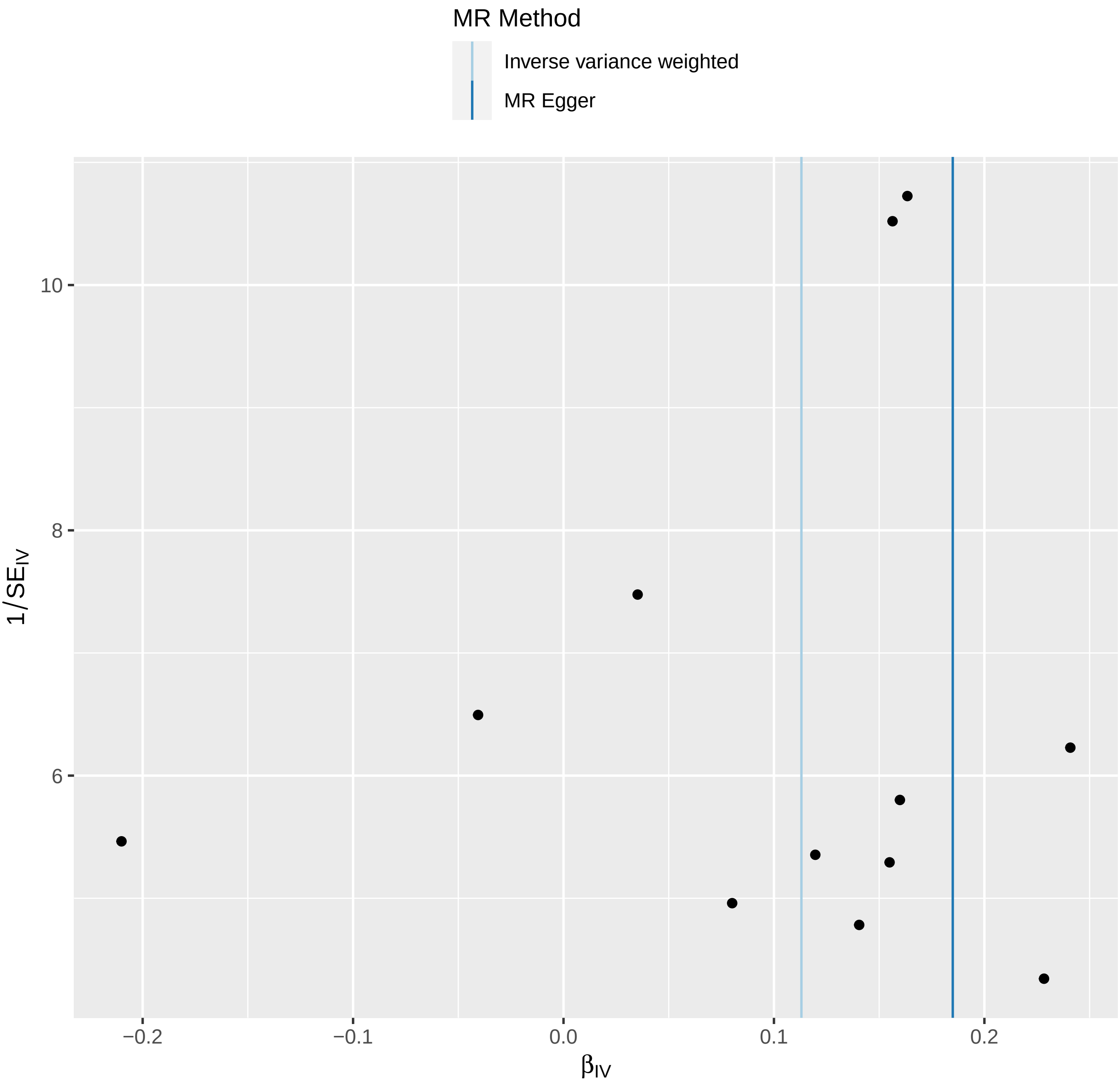

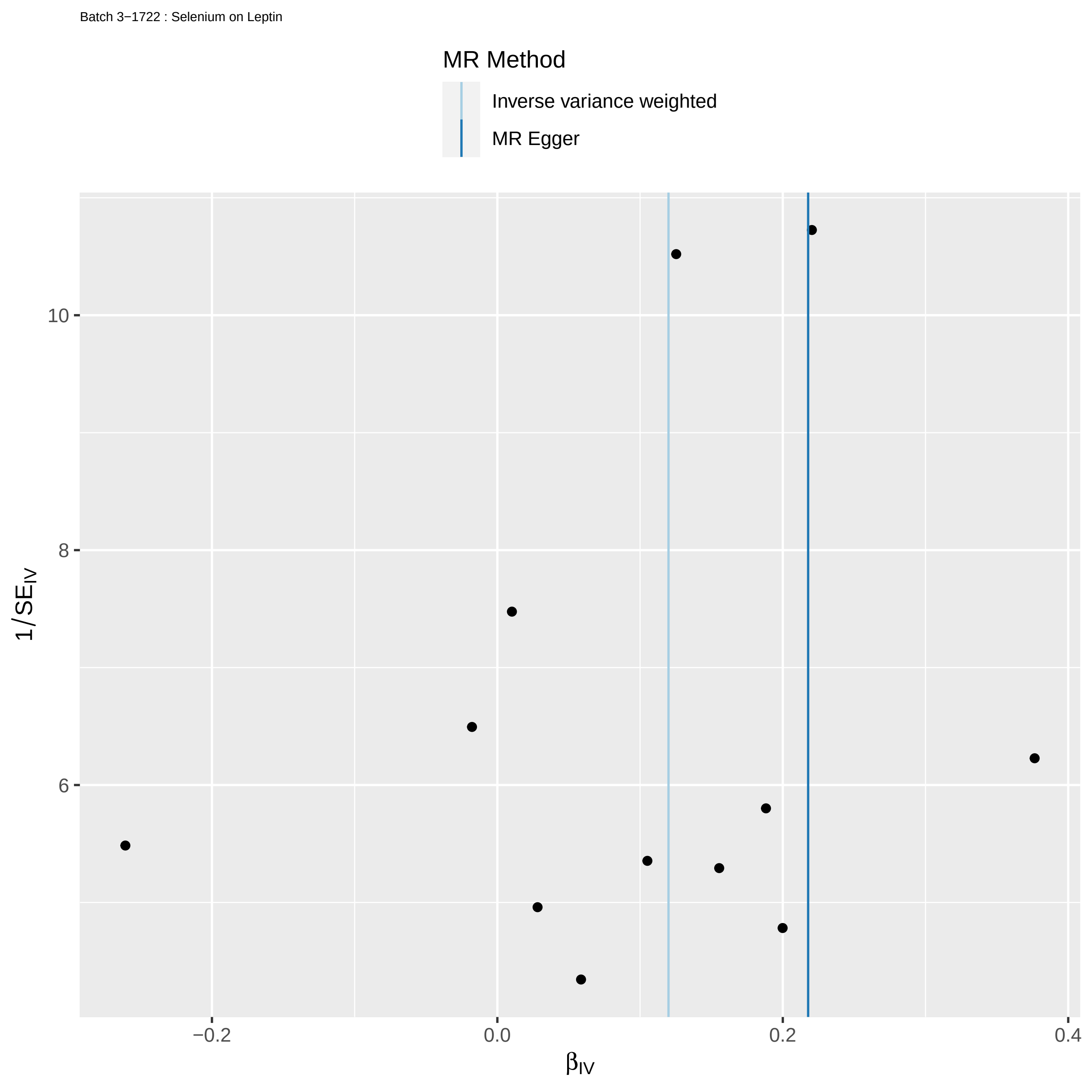

Batch 3−1722 : Selenium on Leptin
MR Method
Inverse variance weighted
MR Egger
10
8
6
1/SE_IV
−0.2
0.0
0.2
0.4
β_IV

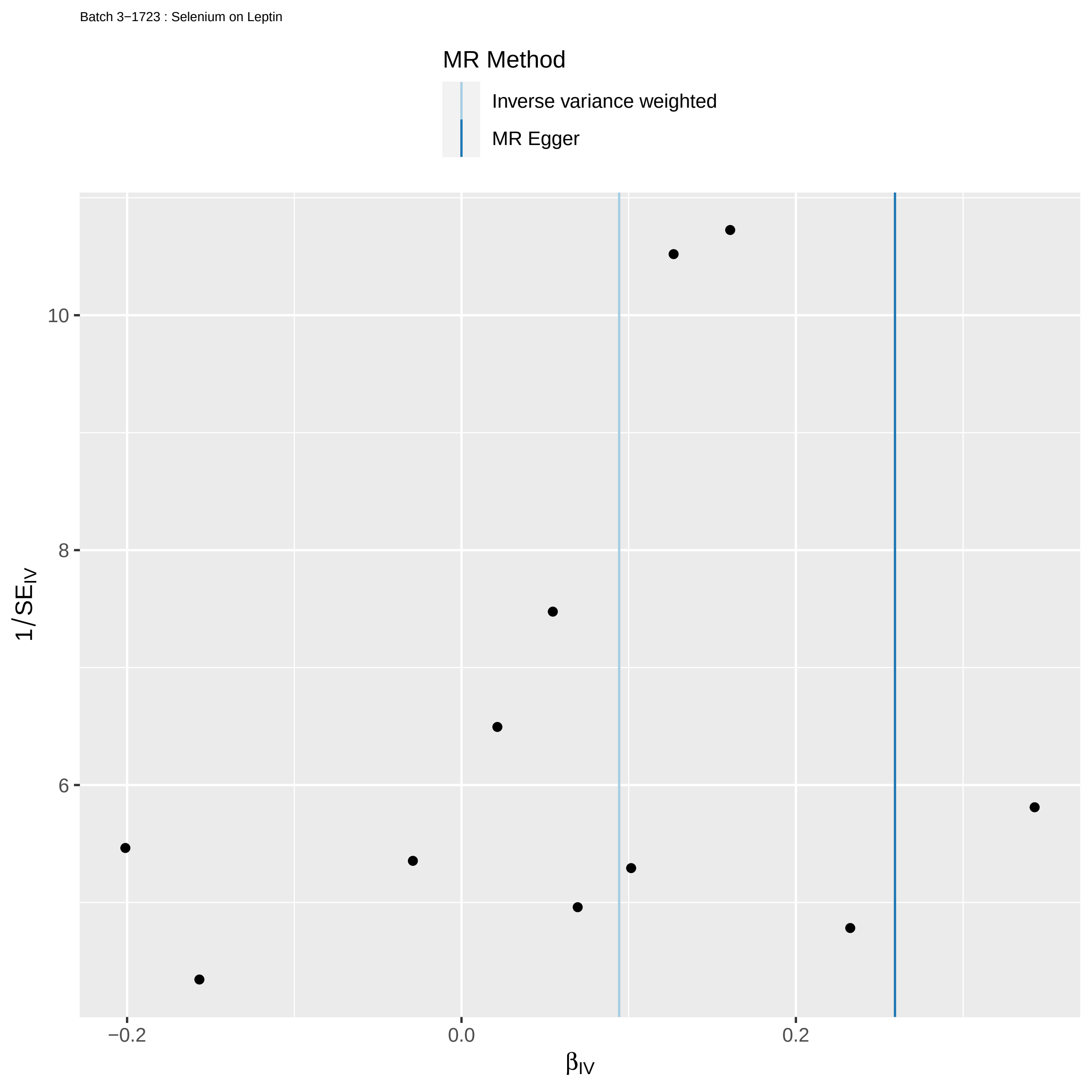

Batch 3−1723 : Selenium on Leptin
MR Method
Inverse variance weighted
MR Egger
10
8
6
1/SE_IV
−0.2
0.0
0.2
β_IV

Batch 3−1724 : Selenium on Leptin receptor

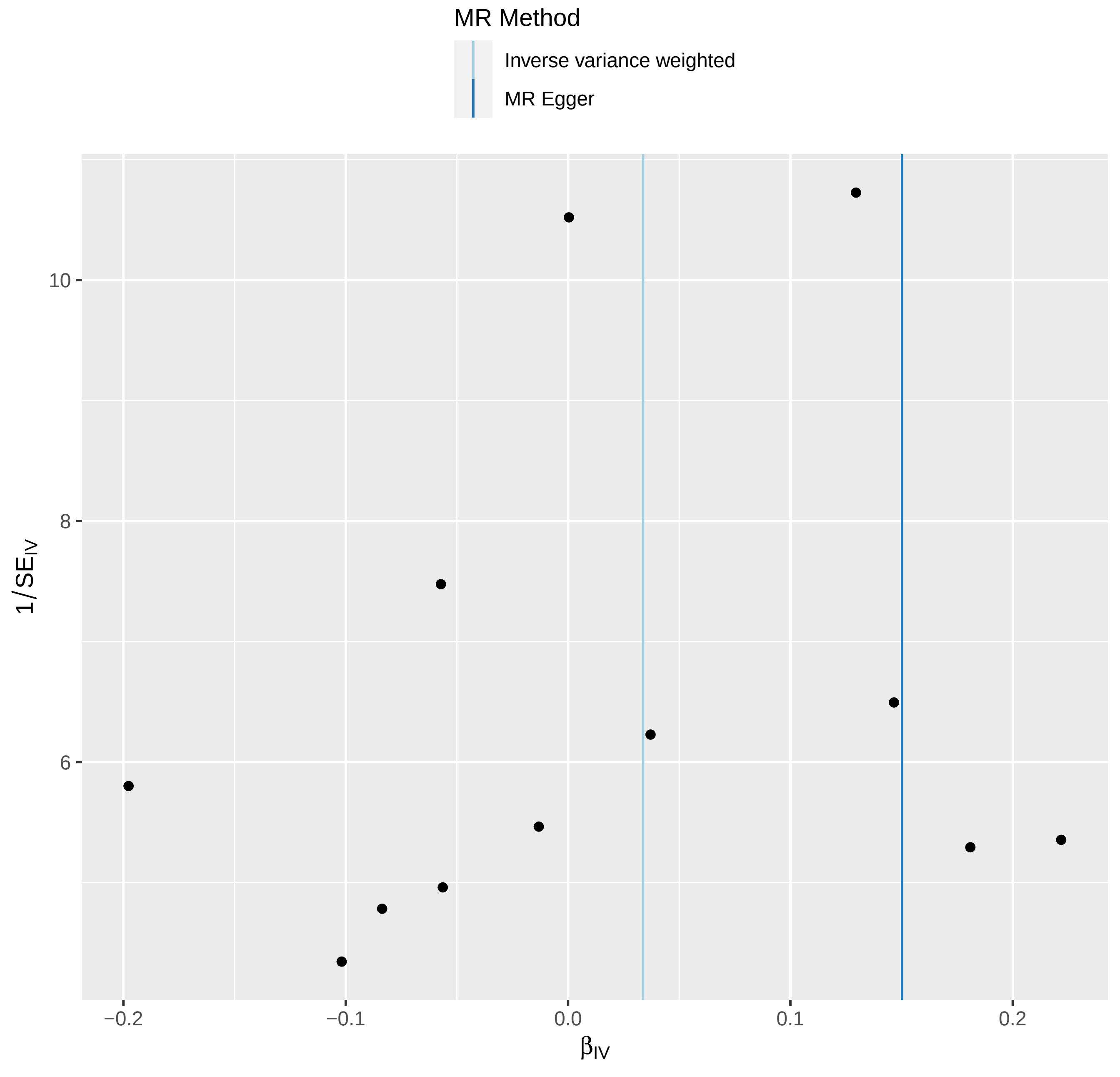

Batch 3−1725 : Selenium on Leptin receptor

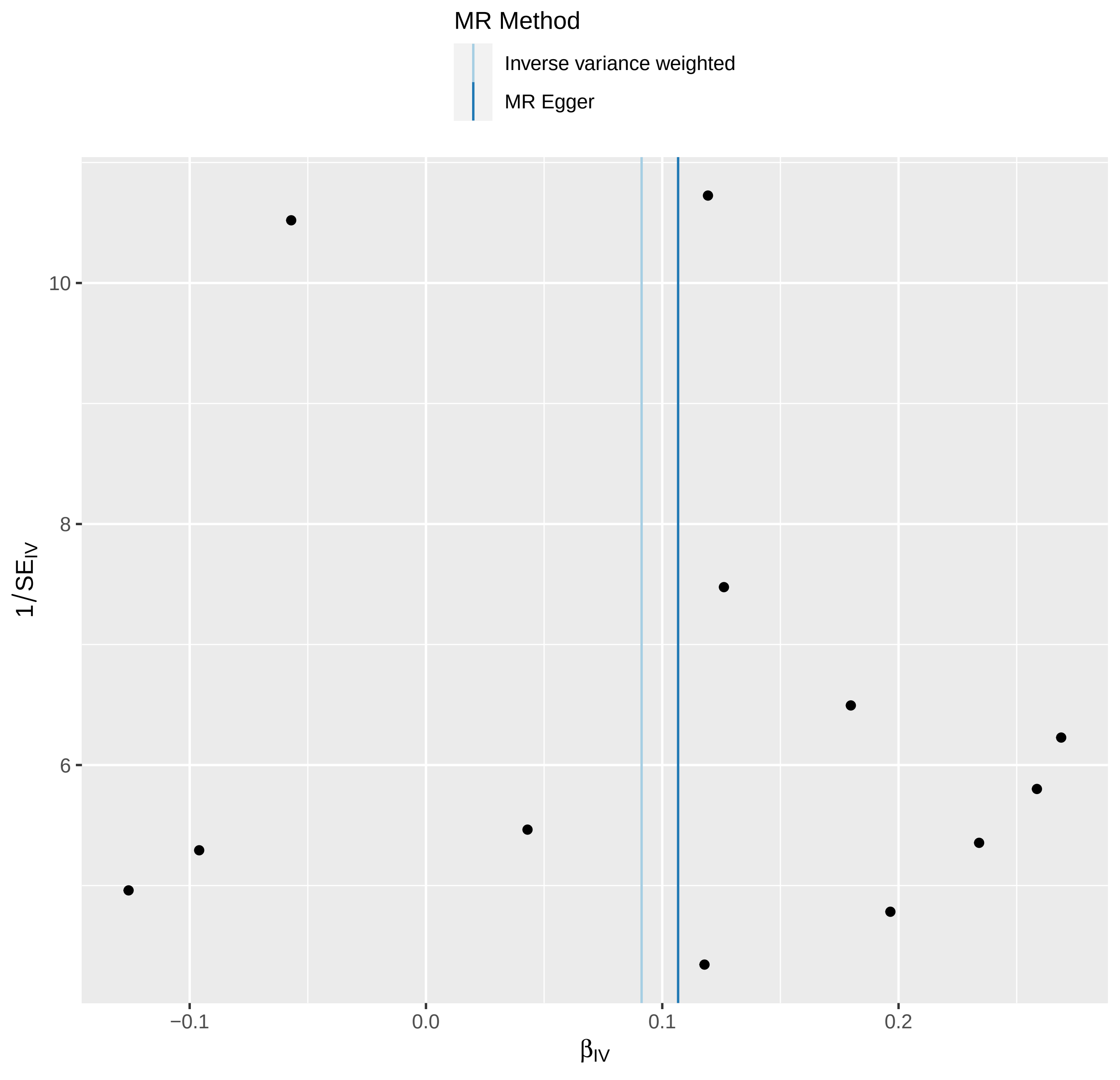

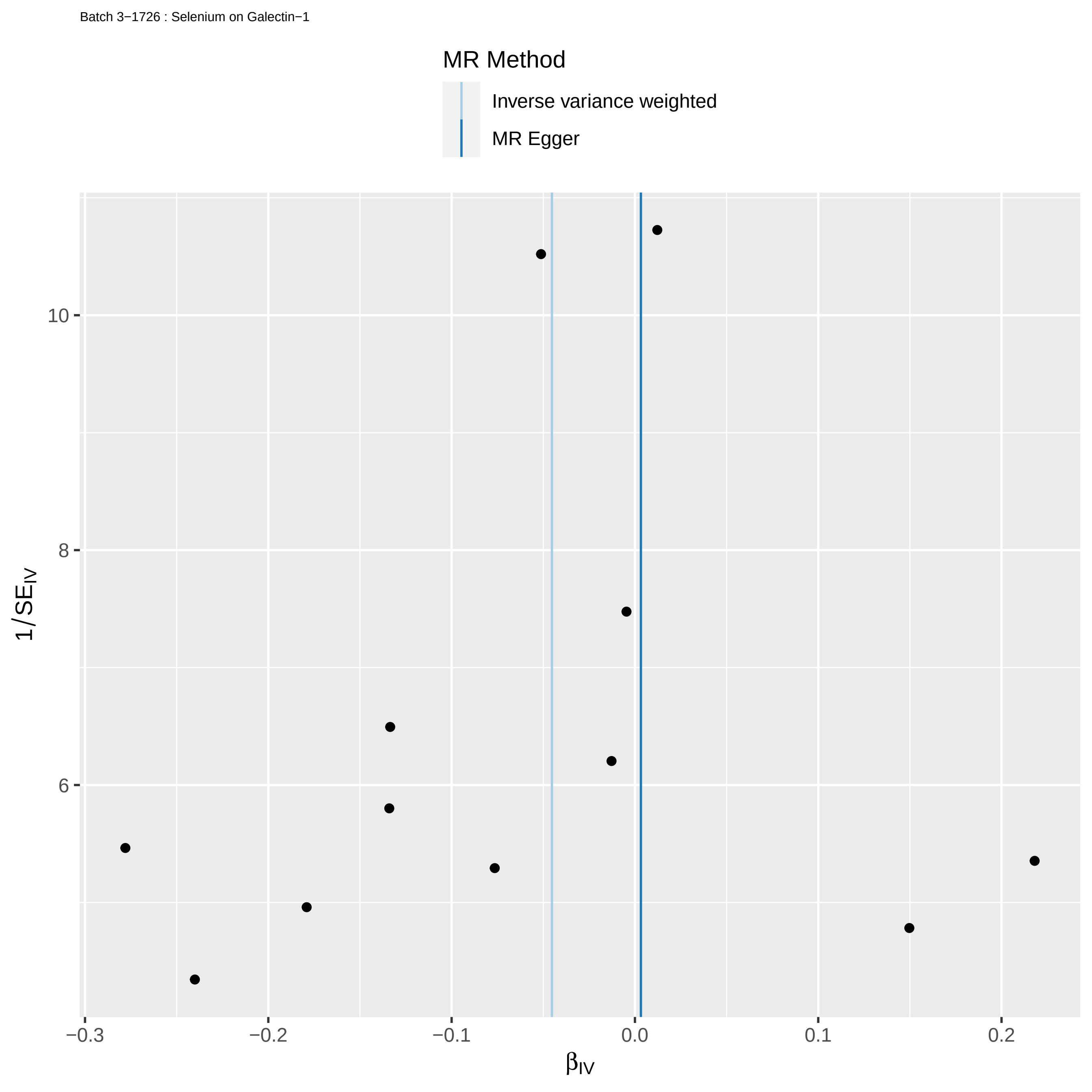

Batch 3−1726 : Selenium on Galectin−1
MR Method
Inverse variance weighted
MR Egger
10
8
6
1/SE_IV
−0.3
−0.2
−0.1
0.0
0.1
0.2
β_IV

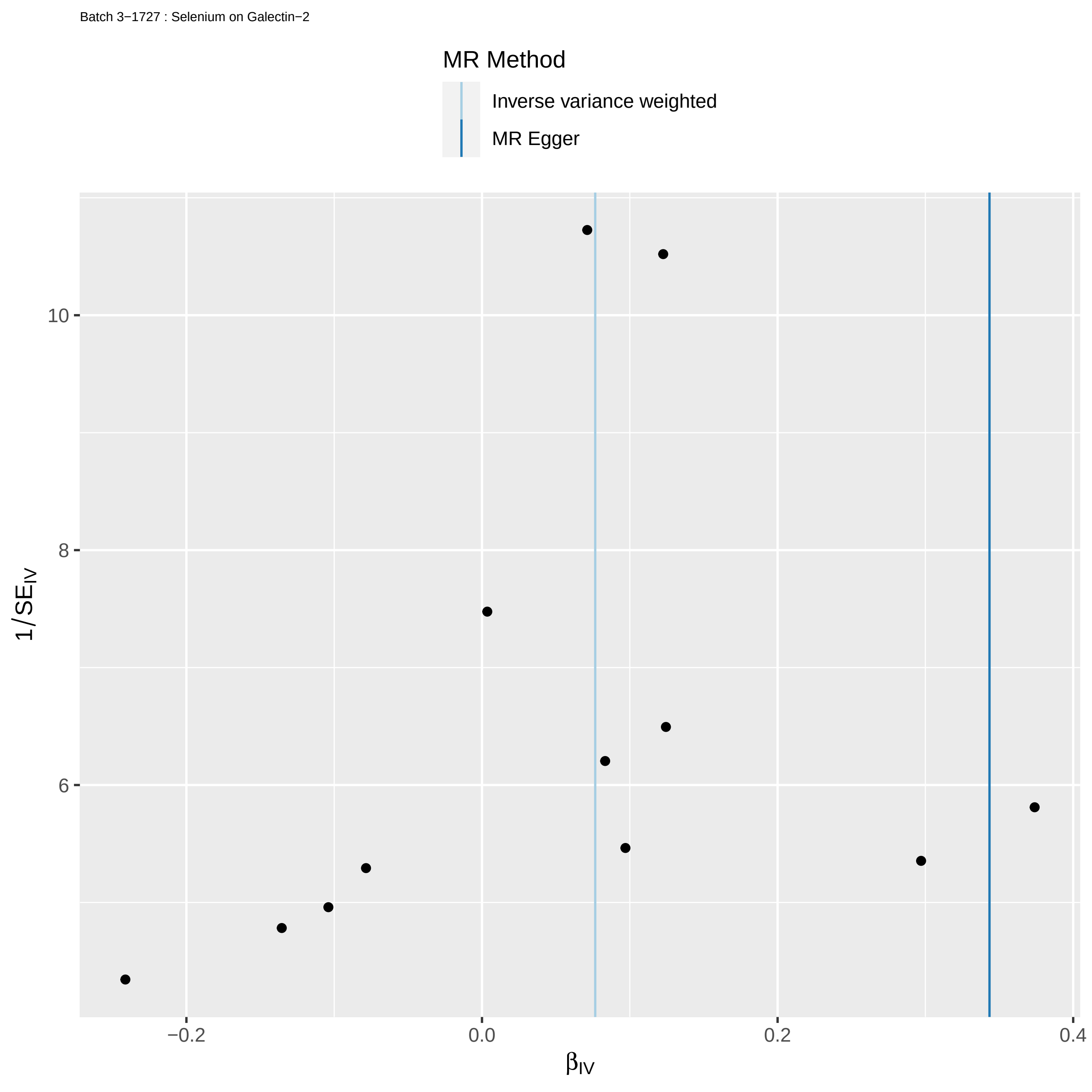

Batch 3−1727 : Selenium on Galectin−2
MR Method
Inverse variance weighted
MR Egger
10
8
6
1/SE$_{IV}$
−0.2
0.0
0.2
0.4
β$_{IV}$

Batch 3−1728 : Selenium on Galectin−4

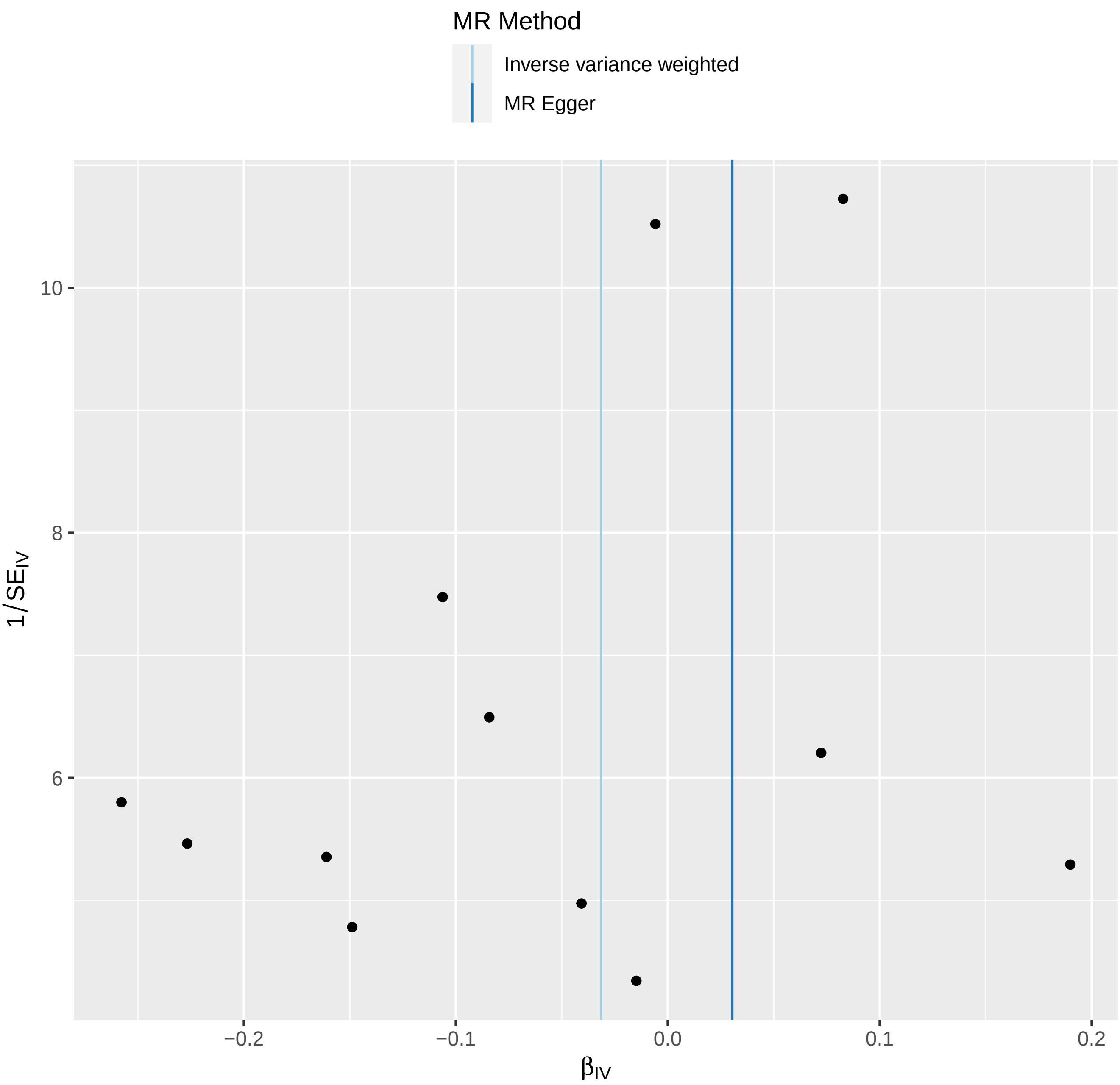

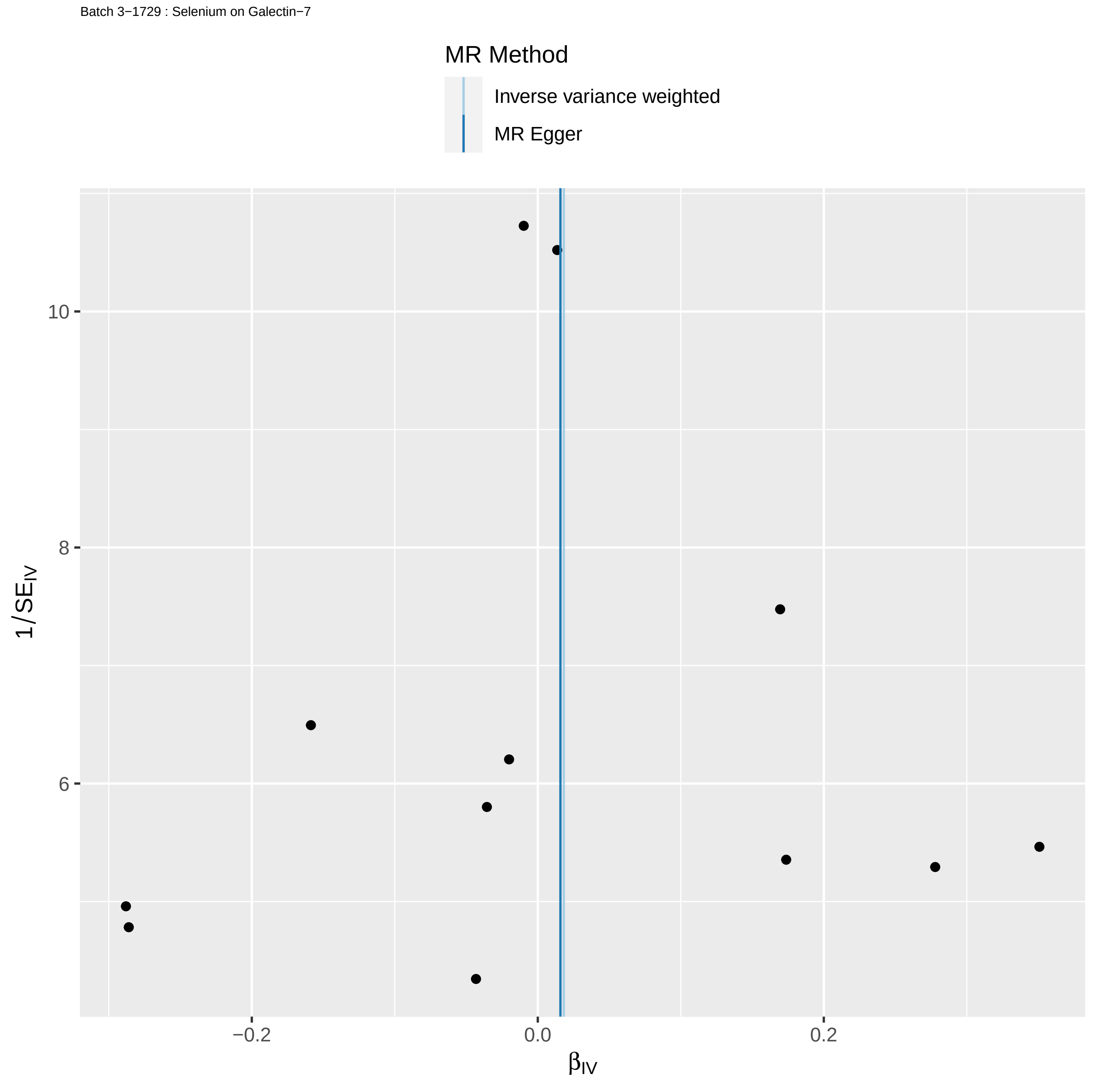

Batch 3−1729 : Selenium on Galectin−7
MR Method
Inverse variance weighted
MR Egger
10
8
6
1/SE_IV
−0.2
0.0
0.2
β_IV

Batch 3−1730 : Selenium on Galectin−7

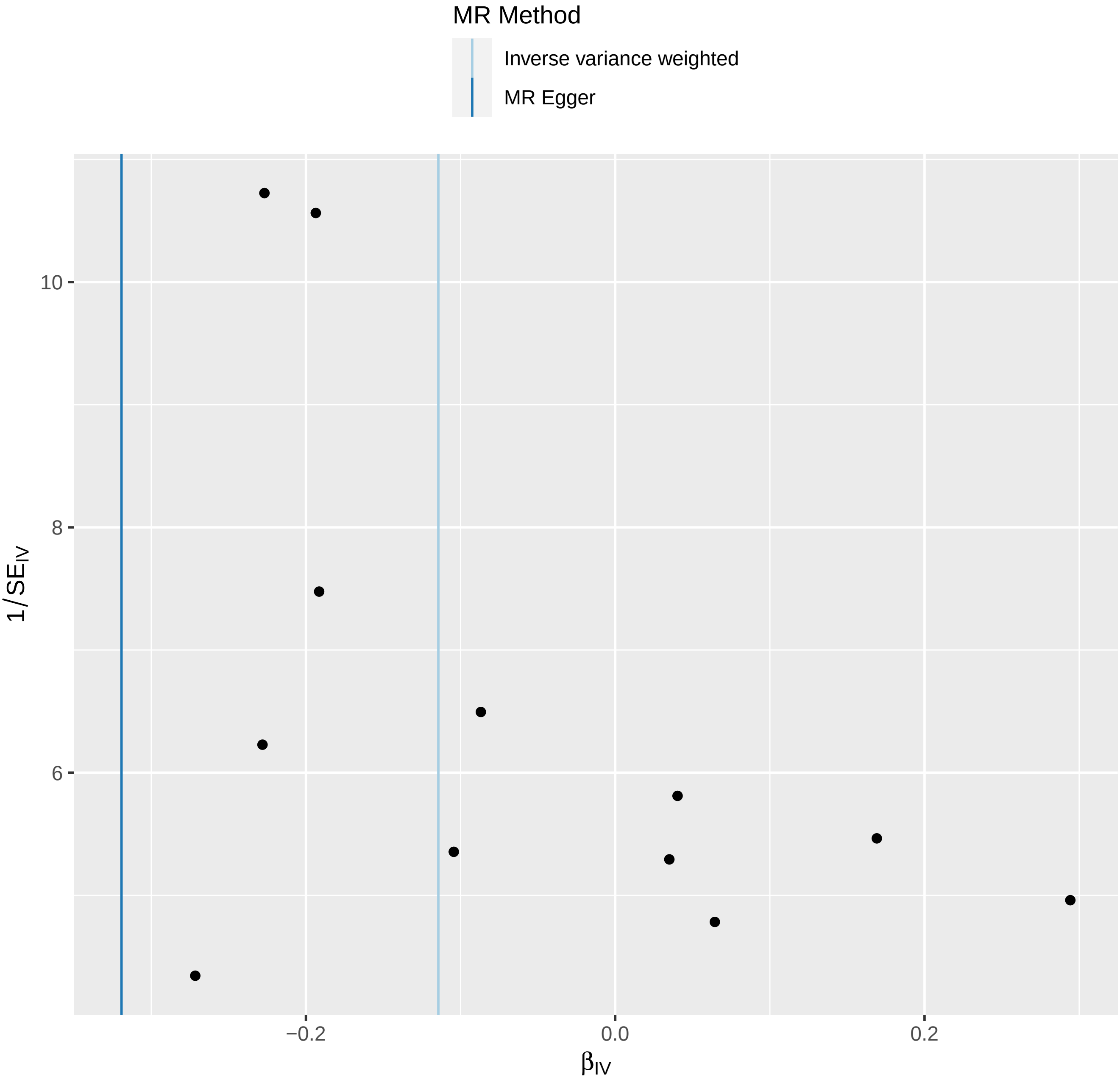

Batch 3−1731 : Selenium on Galectin−8

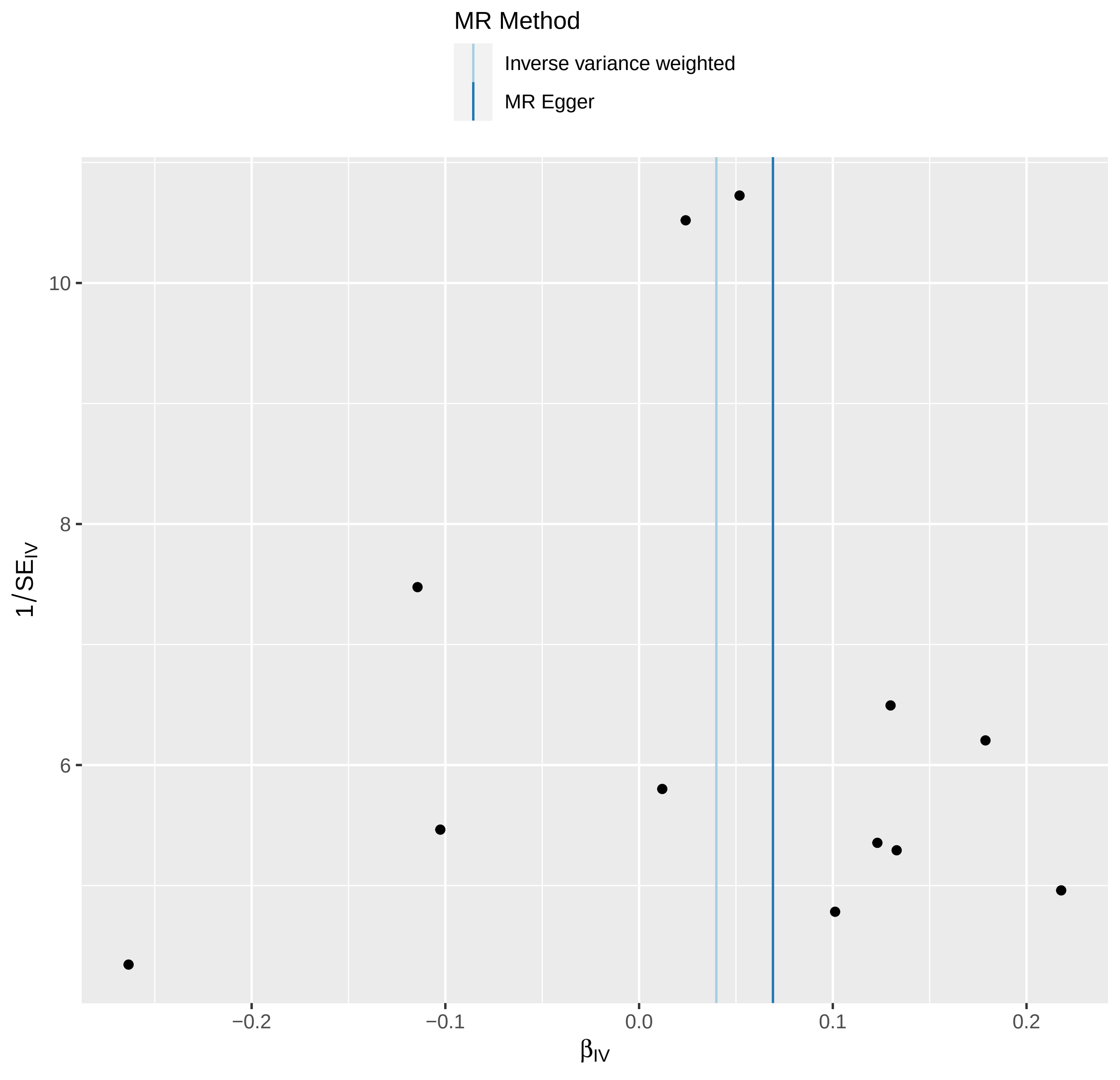

Batch 3−1732 : Selenium on Galectin−9

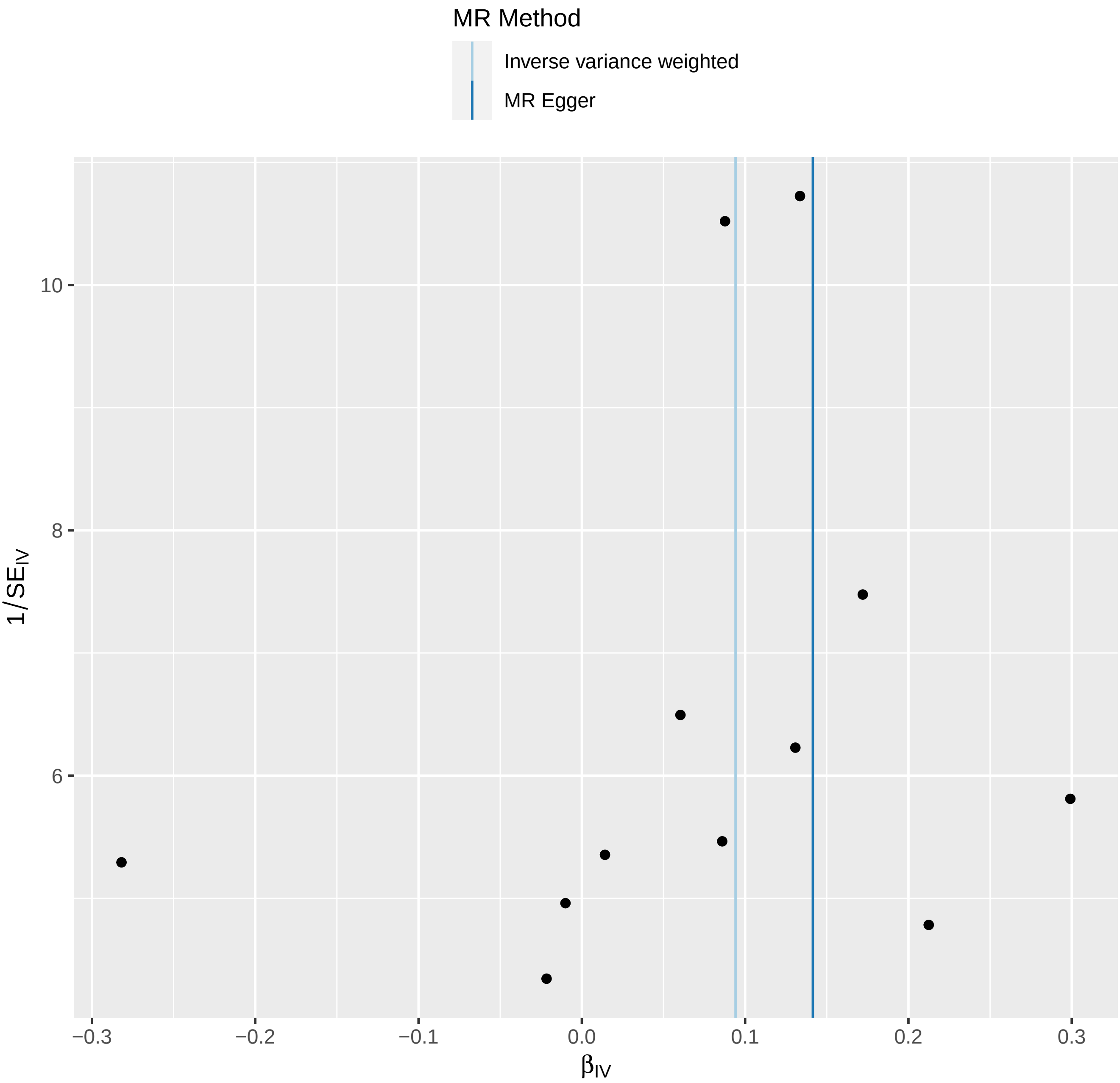

Batch 3−1733 : Selenium on Leucine−rich repeat LGI family member 3

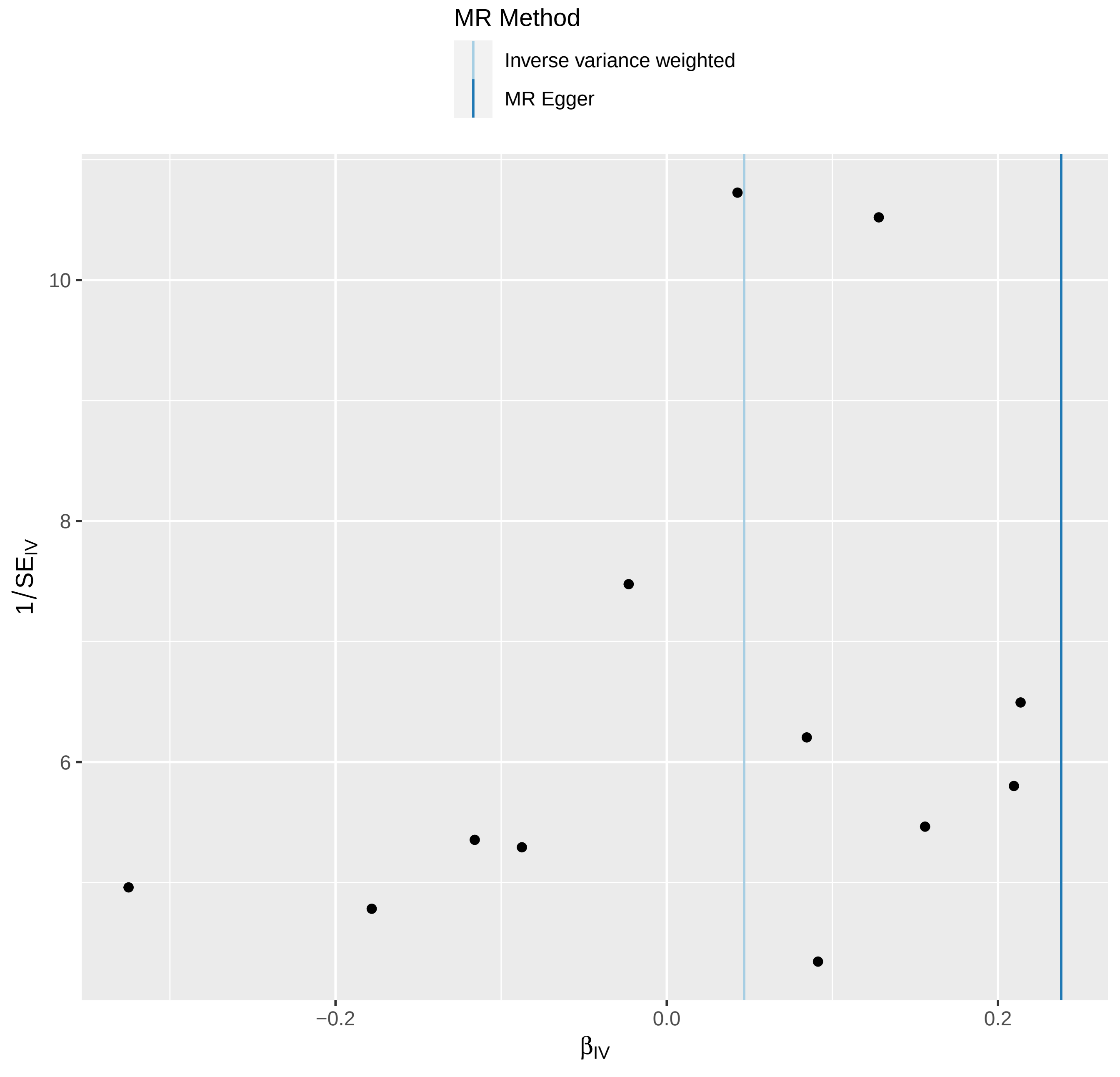

Batch 3−1734 : Selenium on Legumain

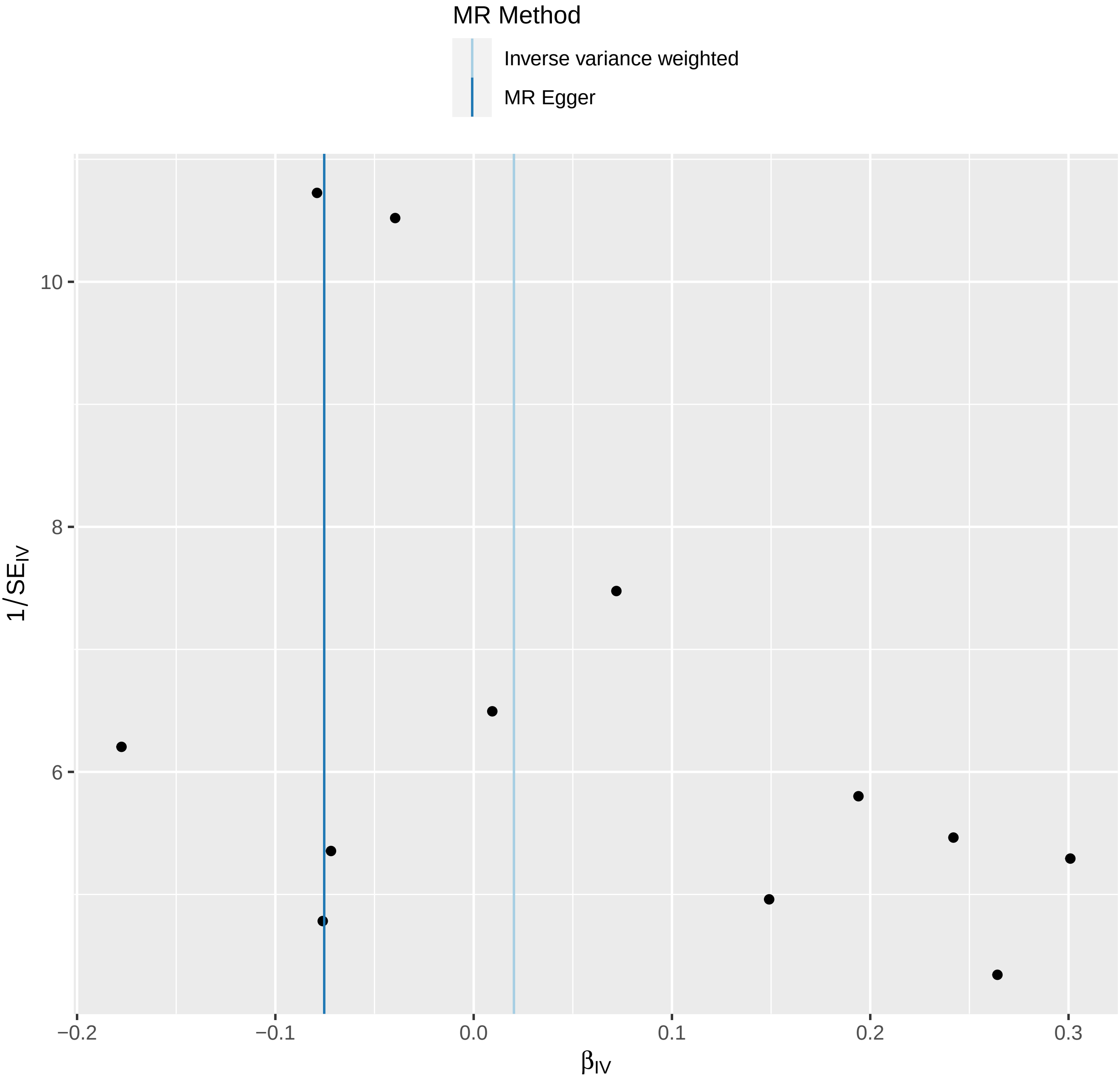

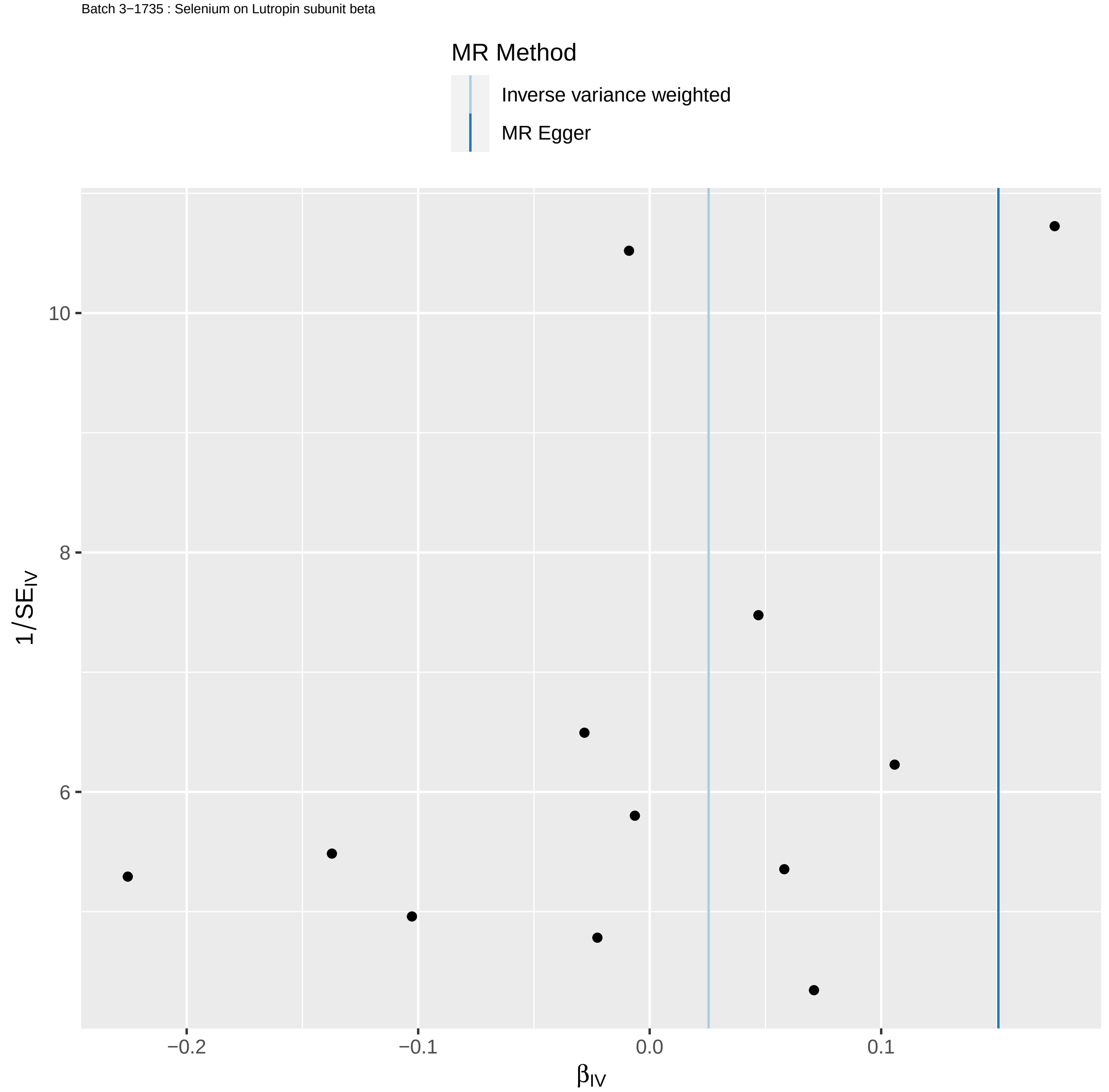

Batch 3−1735 : Selenium on Lutropin subunit beta
MR Method
Inverse variance weighted
MR Egger
10
8
6
1/SE$_{IV}$
−0.2
−0.1
0.0
0.1
β$_{IV}$

Batch 3−1736 : Selenium on Leukemia inhibitory factor

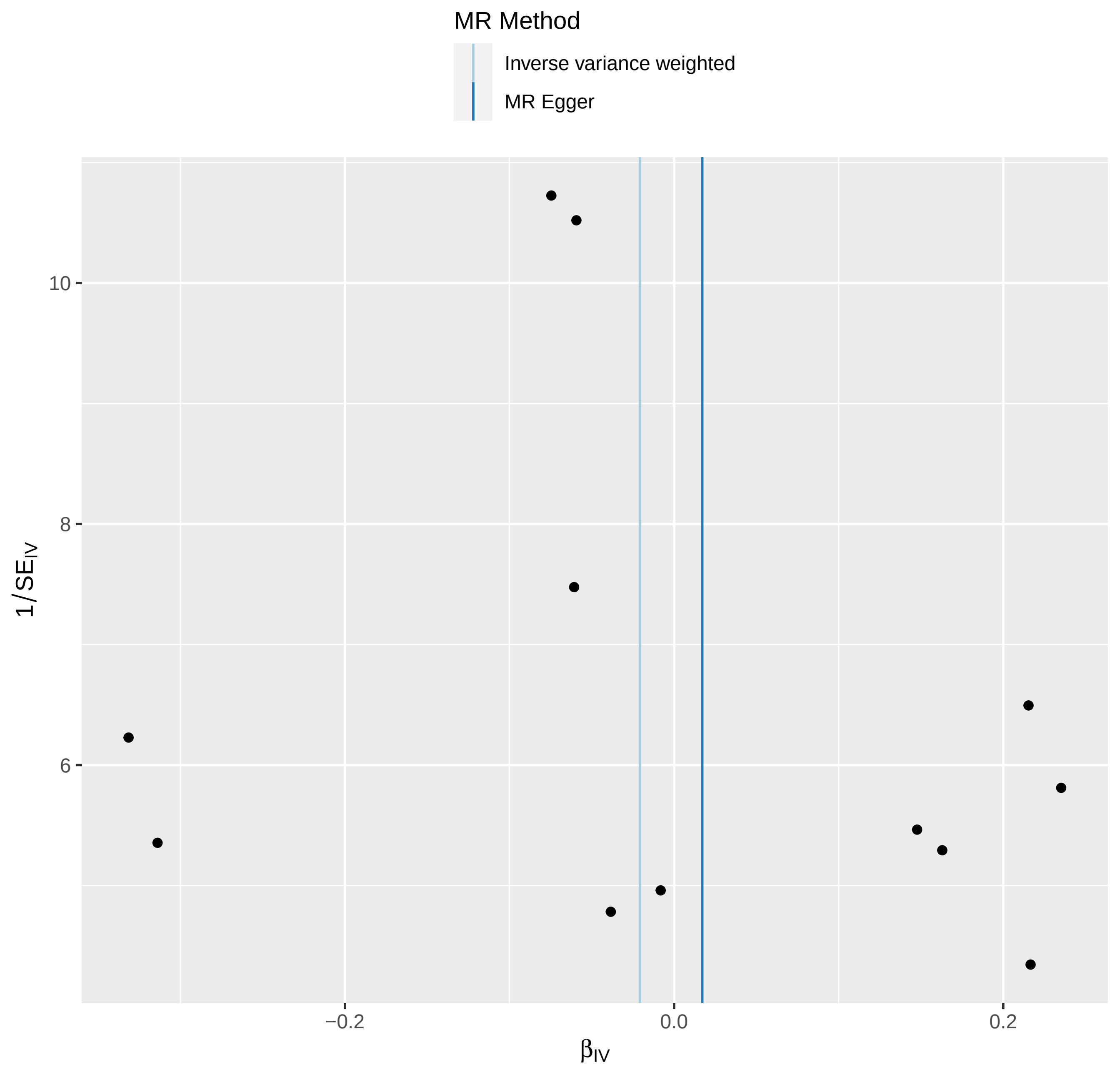

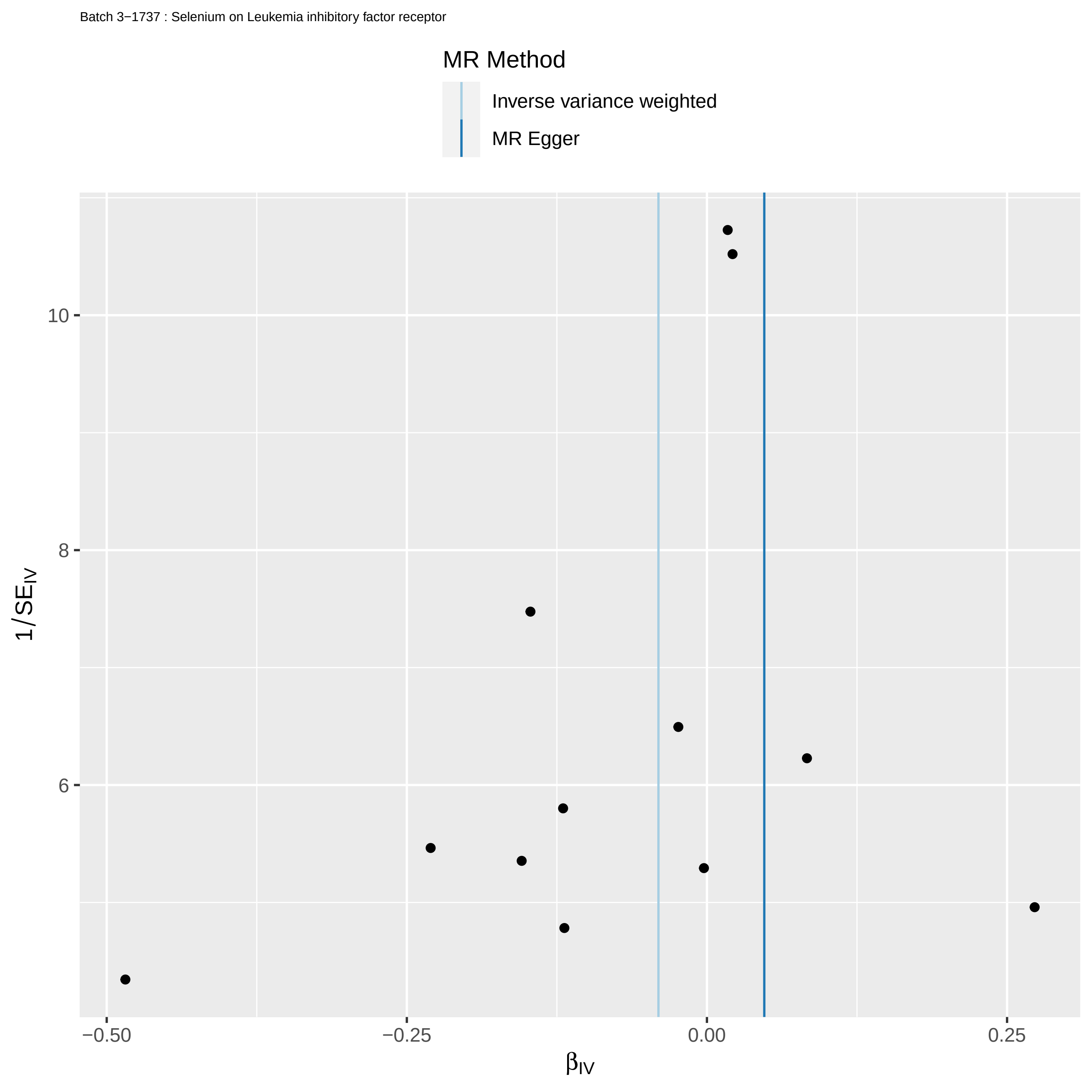

Batch 3−1737 : Selenium on Leukemia inhibitory factor receptor
MR Method
Inverse variance weighted
MR Egger
10
8
6
1/SE$_{IV}$
−0.50
−0.25
0.00
0.25
β$_{IV}$

Batch 3−1738 : Selenium on Leukocyte immunoglobulin−like receptor subfamily A member 4

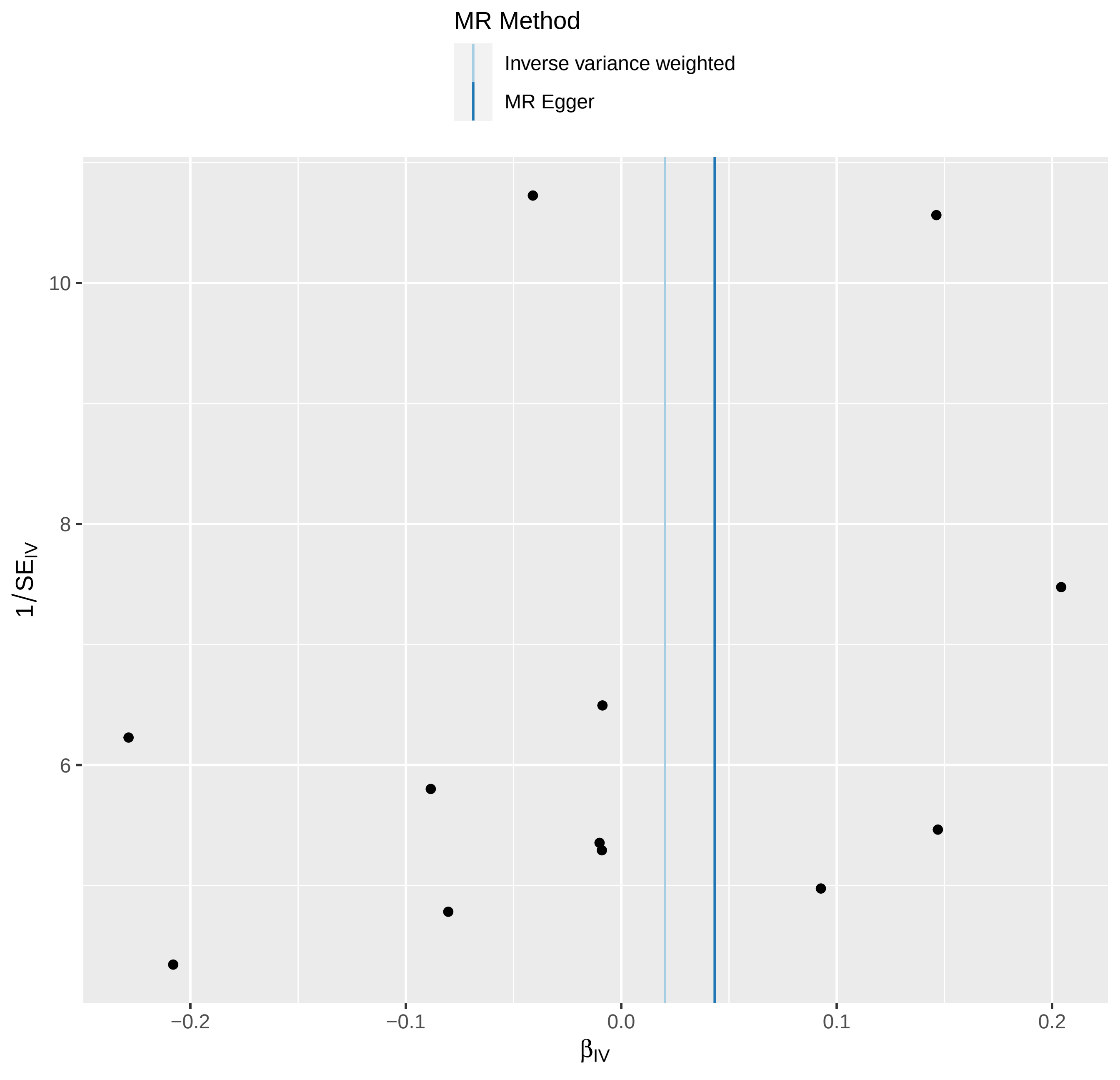

Batch 3−1739 : Selenium on Leukocyte immunoglobulin−like receptor subfamily A member 5

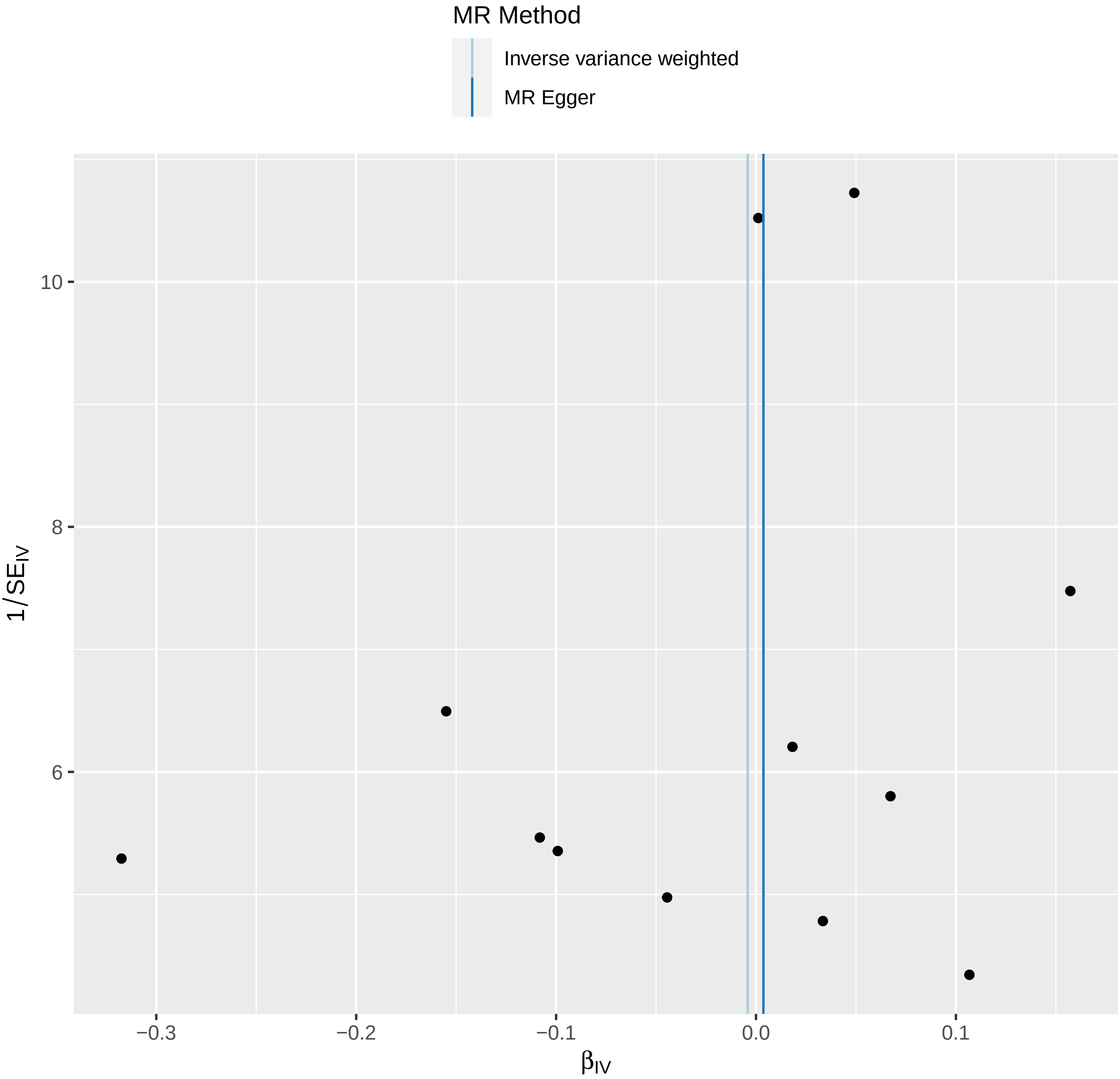

Batch 3−1740 : Selenium on Leukocyte immunoglobulin−like receptor subfamily A member 5

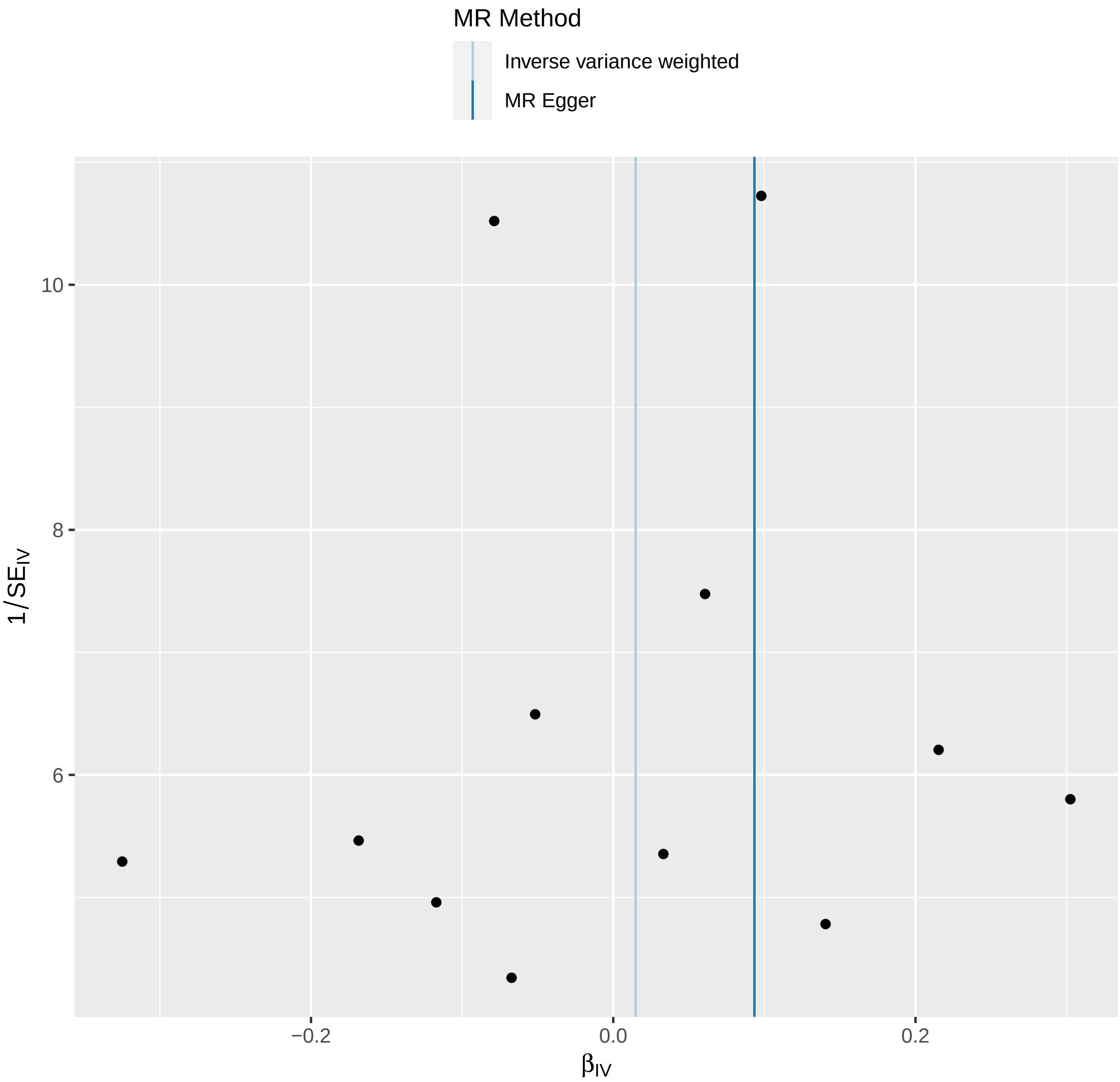

Batch 3−1741 : Selenium on Leukocyte immunoglobulin−like receptor subfamily A member 6

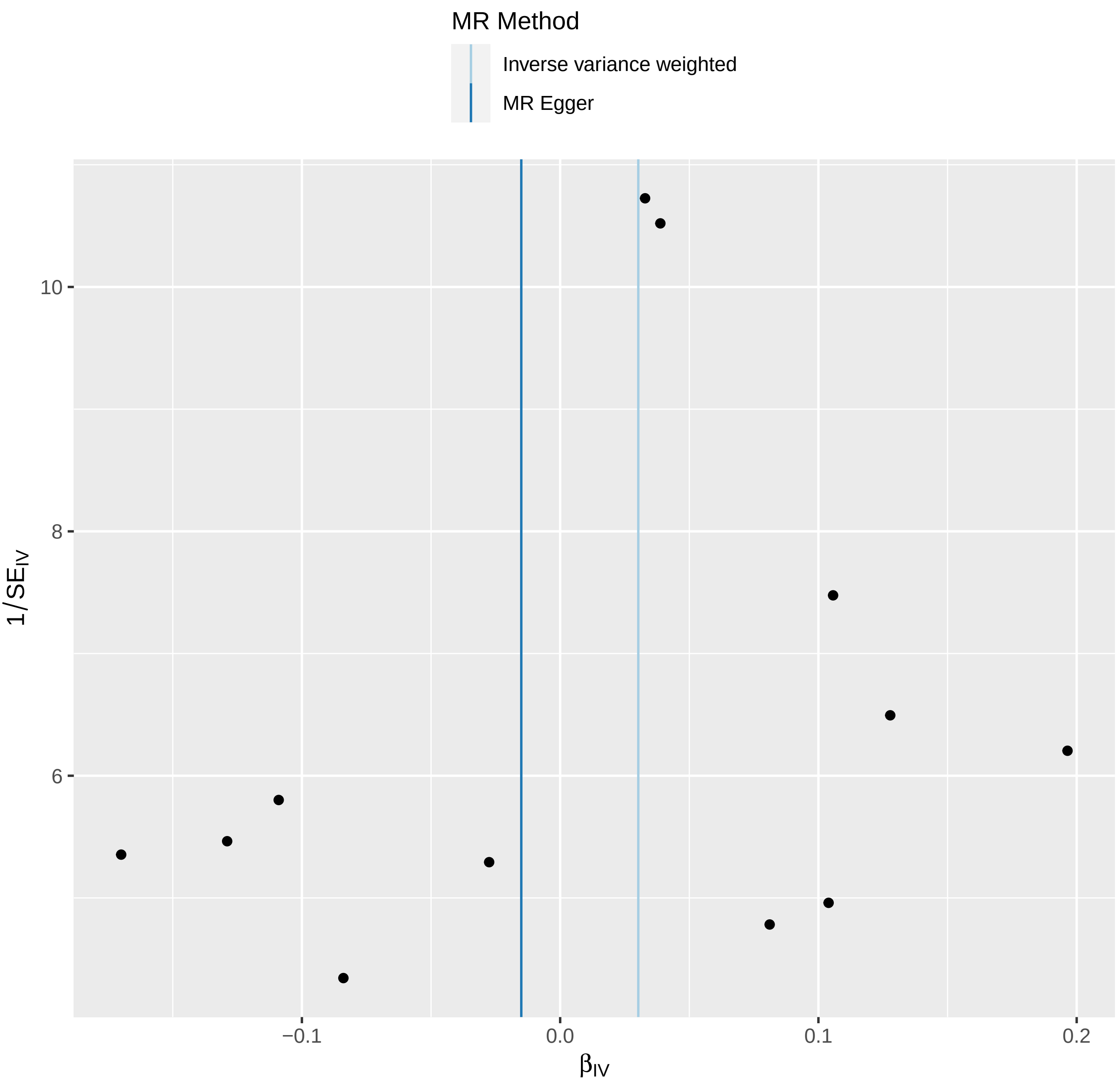

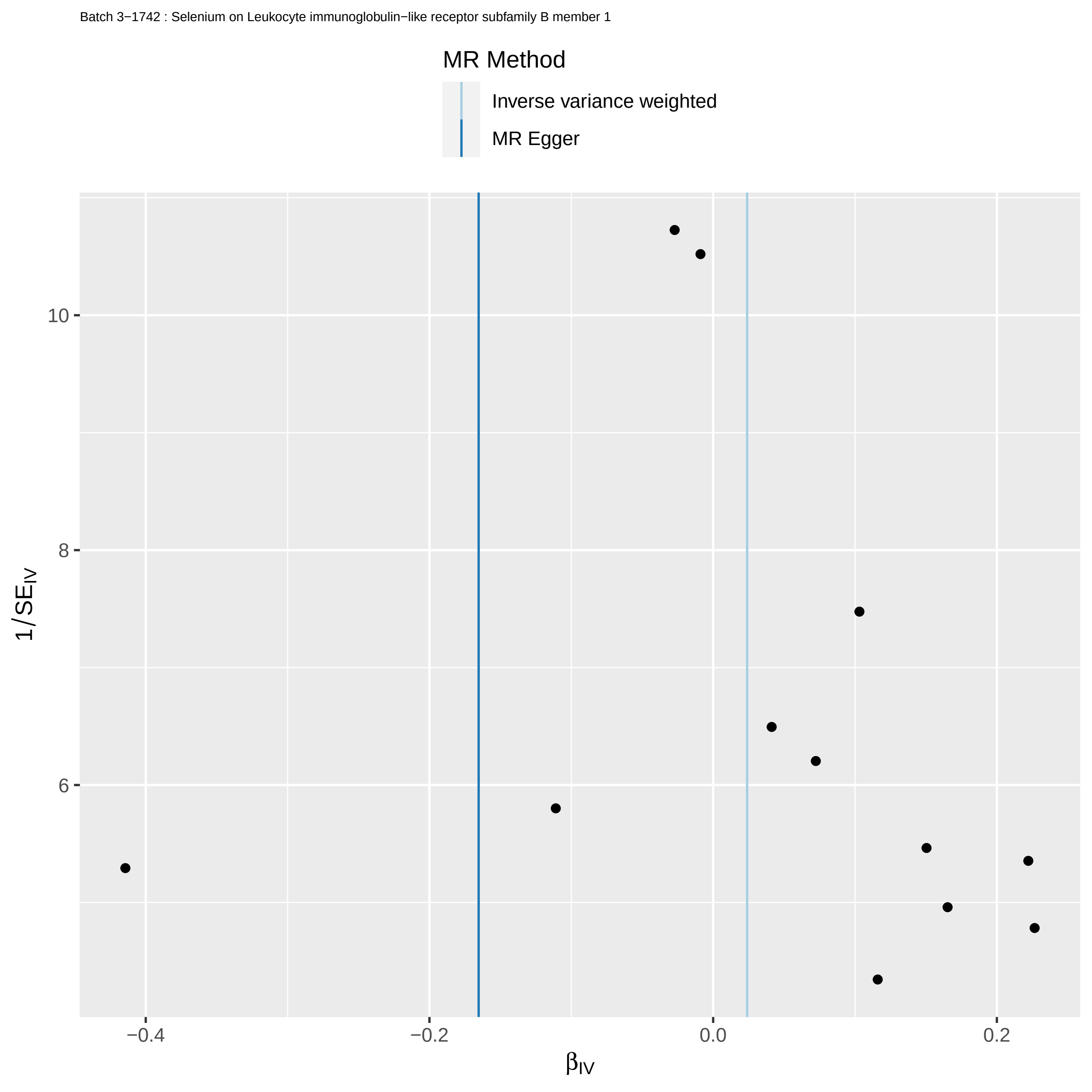

Batch 3−1742 : Selenium on Leukocyte immunoglobulin−like receptor subfamily B member 1
MR Method
Inverse variance weighted
MR Egger
10
8
6
1/SE$_{IV}$
−0.4
−0.2
0.0
0.2
β$_{IV}$

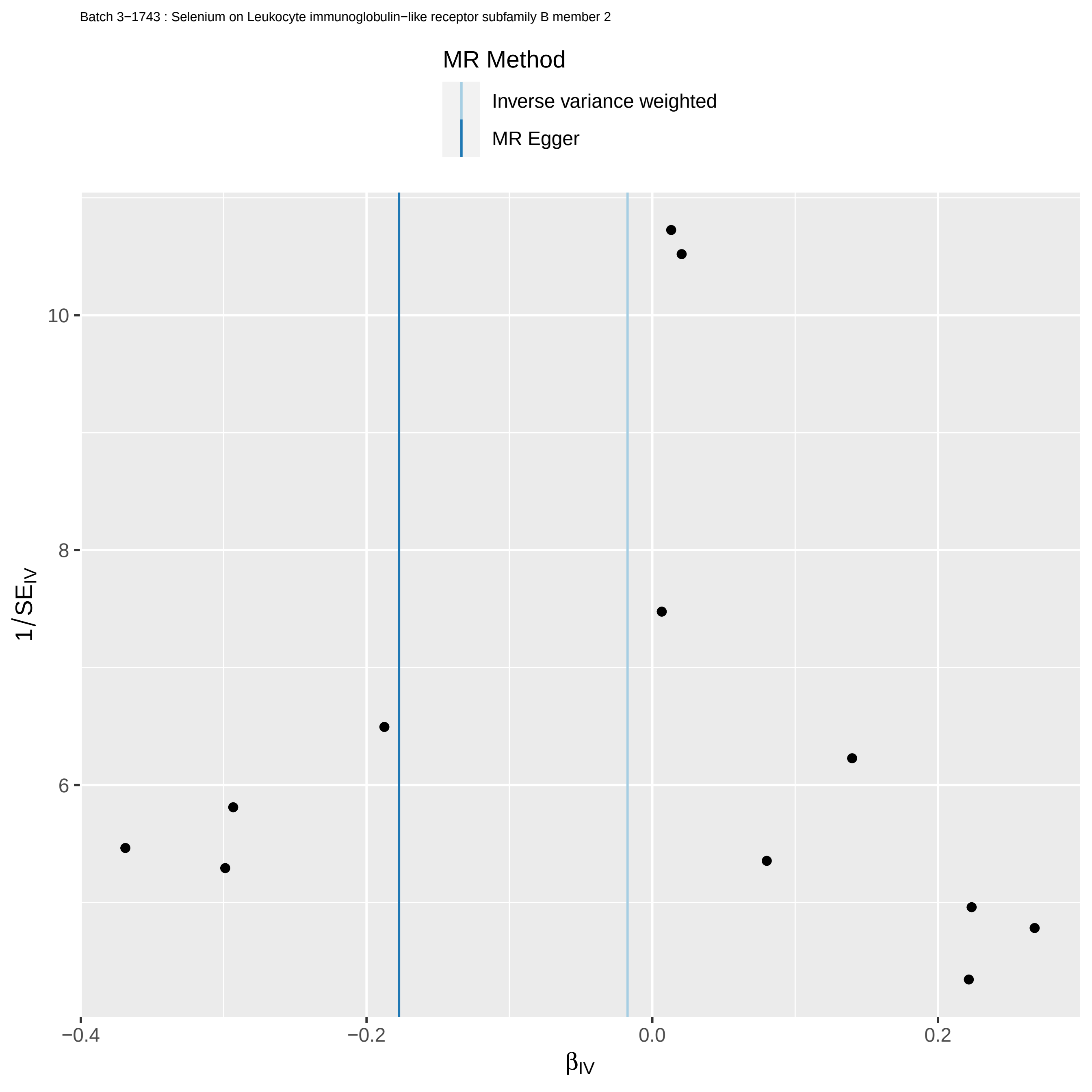

Batch 3−1743 : Selenium on Leukocyte immunoglobulin−like receptor subfamily B member 2
MR Method
Inverse variance weighted
MR Egger
10
8
6
1/SE_IV
−0.4
−0.2
0.0
0.2
β_IV

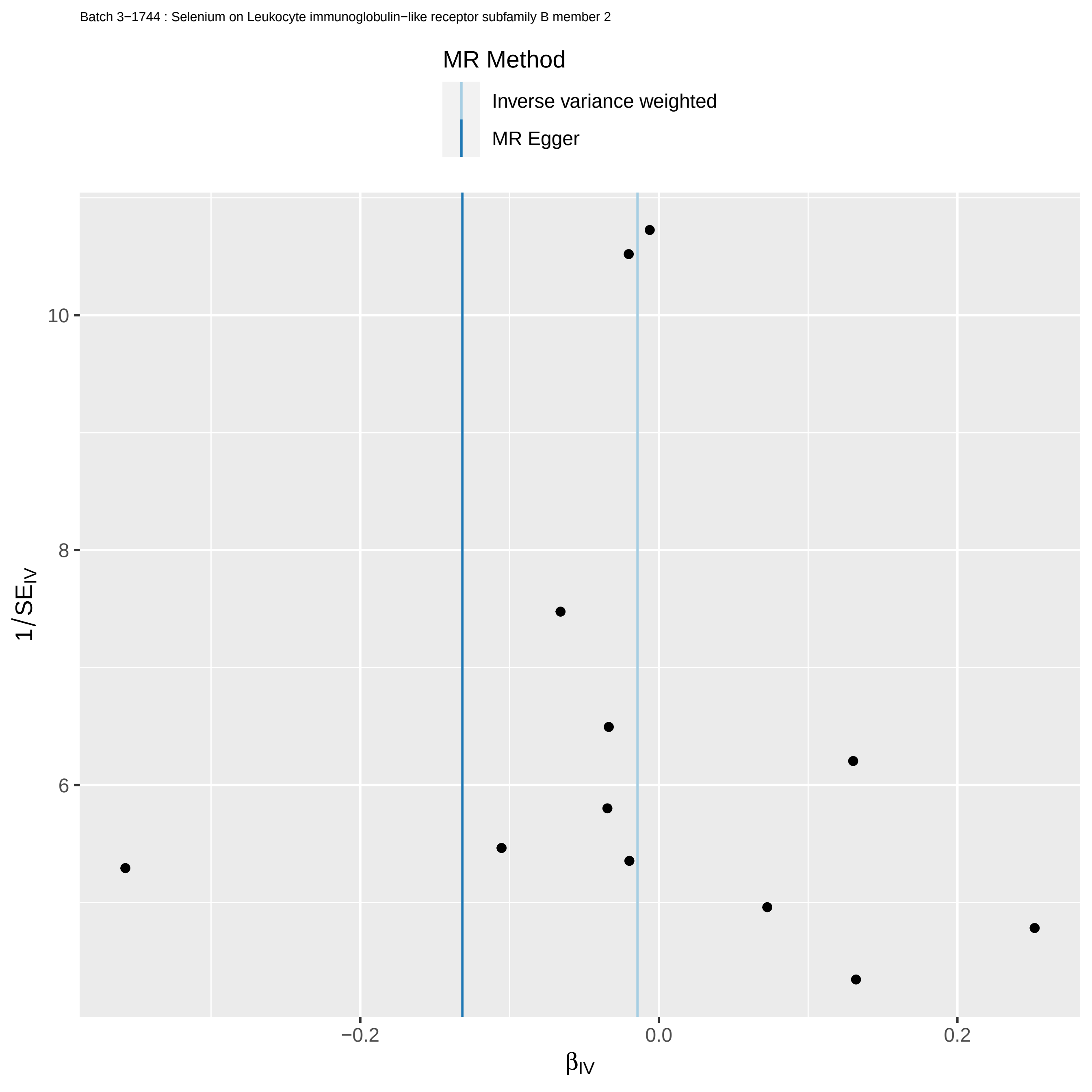
Batch 3−1744 : Selenium on Leukocyte immunoglobulin−like receptor subfamily B member 2
MR Method
Inverse variance weighted
MR Egger
10
8
6
1/SE$_{IV}$
−0.2
0.0
0.2
β$_{IV}$

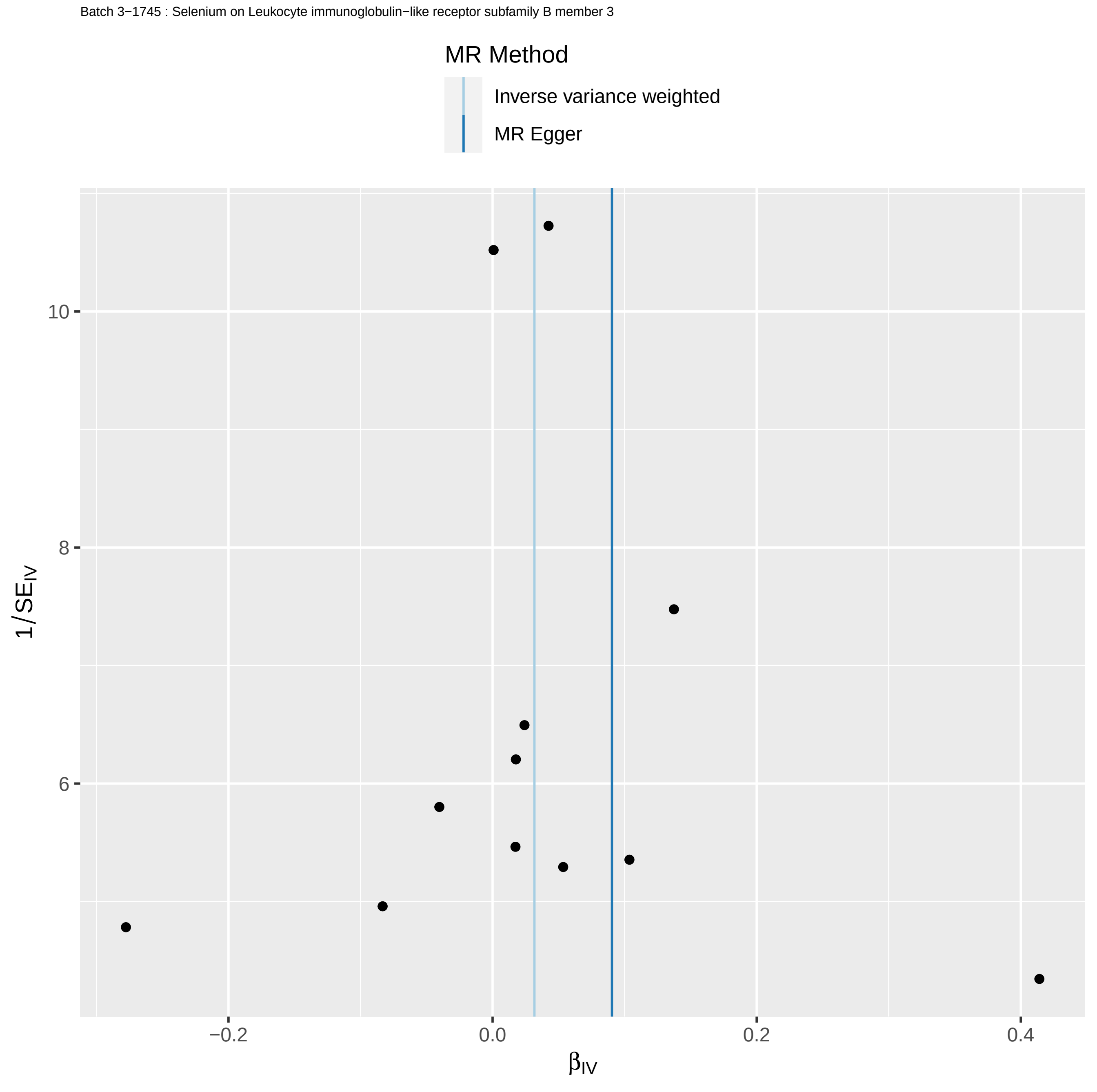
Batch 3−1745 : Selenium on Leukocyte immunoglobulin−like receptor subfamily B member 3
MR Method
Inverse variance weighted
MR Egger
10
8
6
1/SE_IV
−0.2
0.0
0.2
0.4
β_IV

Batch 3−1746 : Selenium on Leukocyte immunoglobulin−like receptor subfamily B member 4

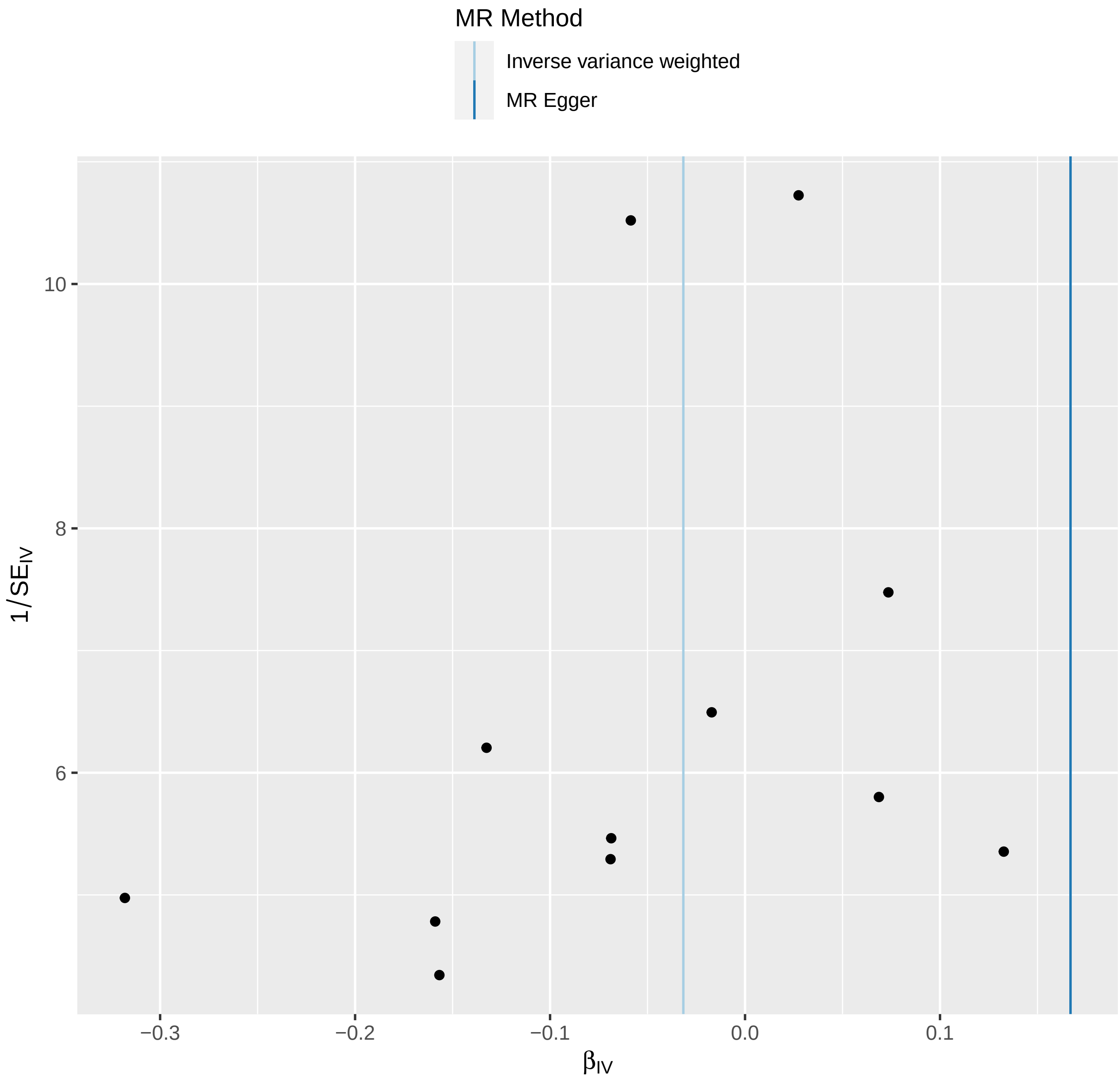

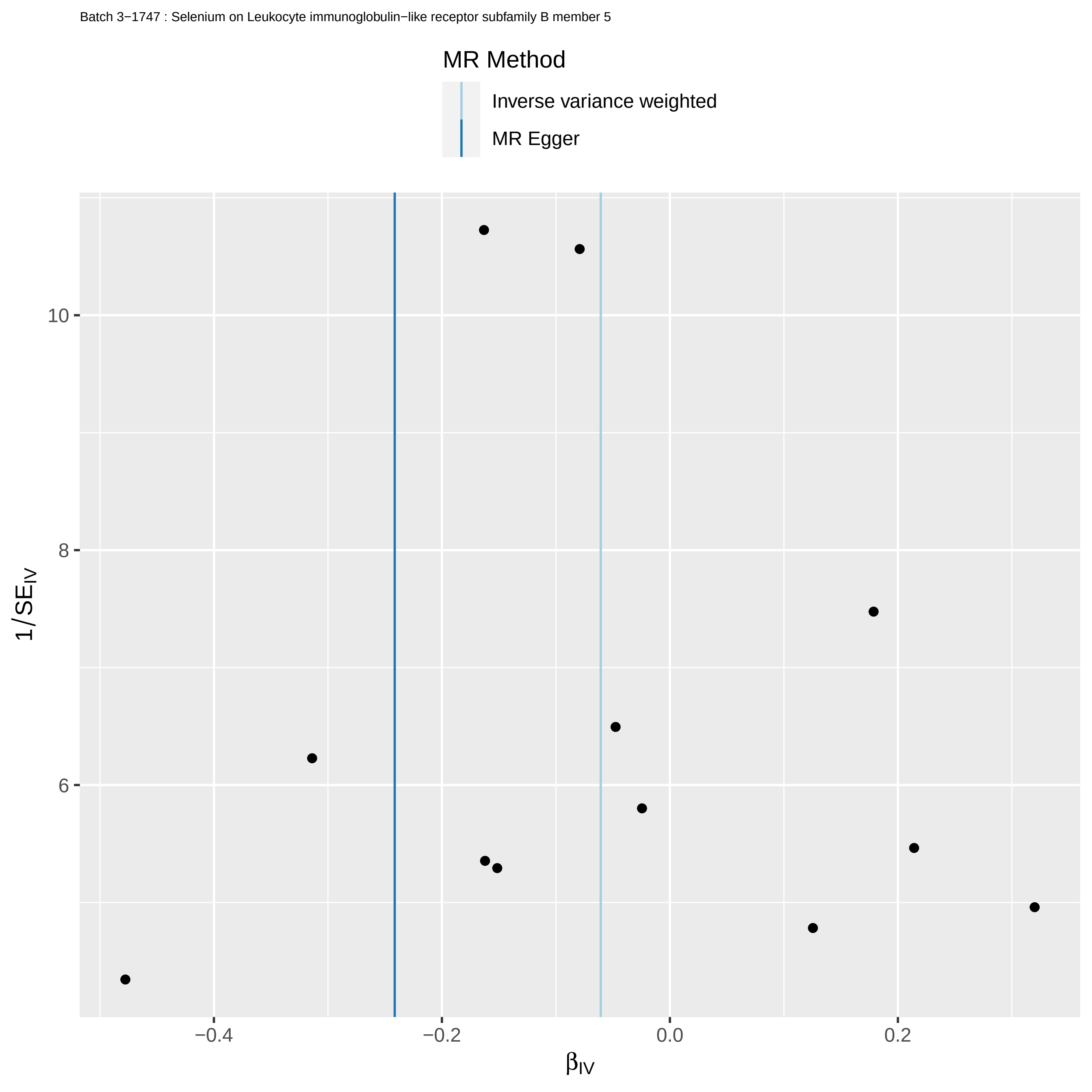

Batch 3−1747 : Selenium on Leukocyte immunoglobulin−like receptor subfamily B member 5
MR Method
Inverse variance weighted
MR Egger
10
8
6
1/SE_IV
−0.4
−0.2
0.0
0.2
β_IV

Batch 3−1748 : Selenium on Protein lin−7 homolog B

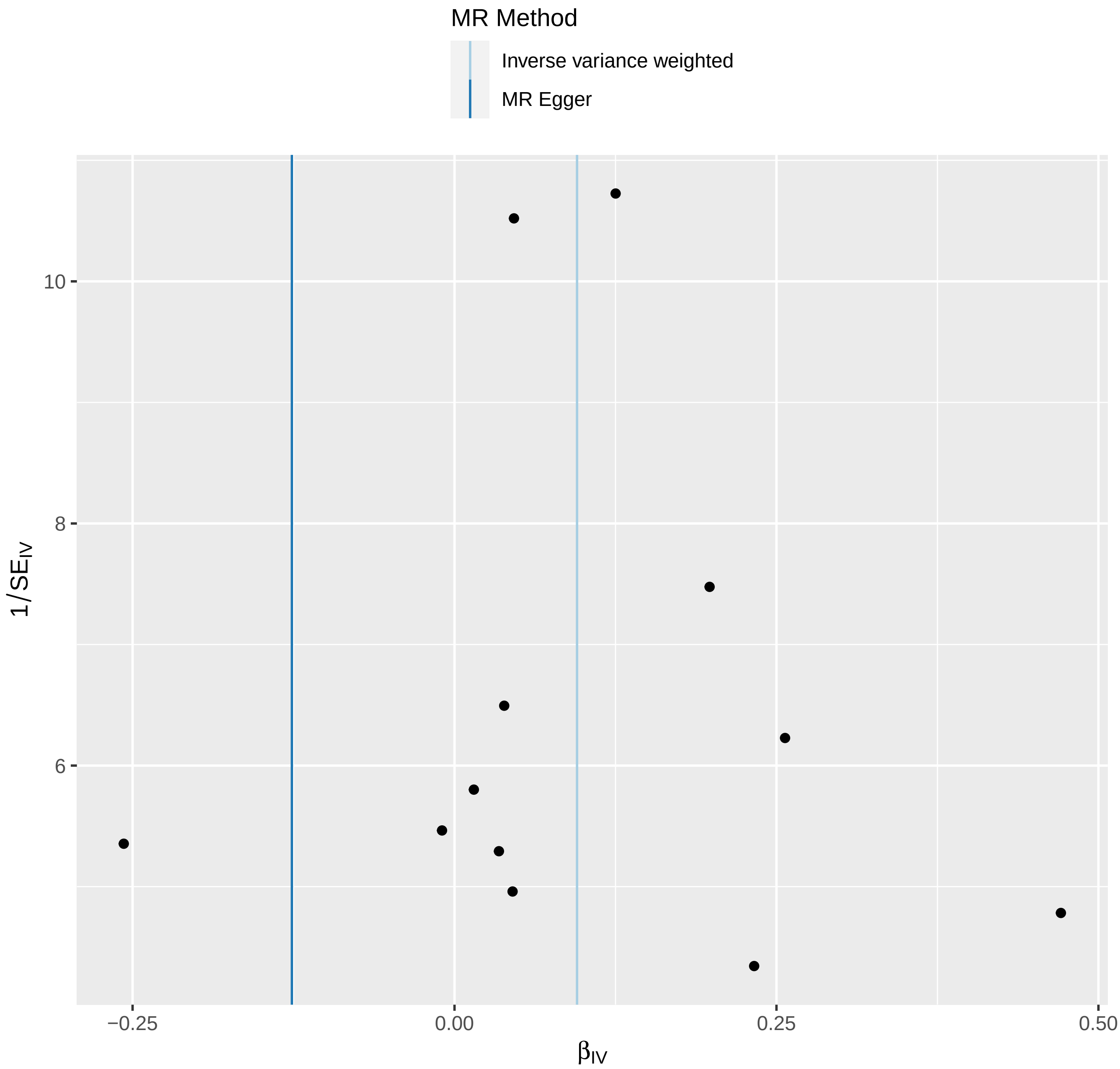

Batch 3−1749 : Selenium on Leucine−rich repeat and immunoglobulin−like domain−containing nogo receptor−interacting protein 1

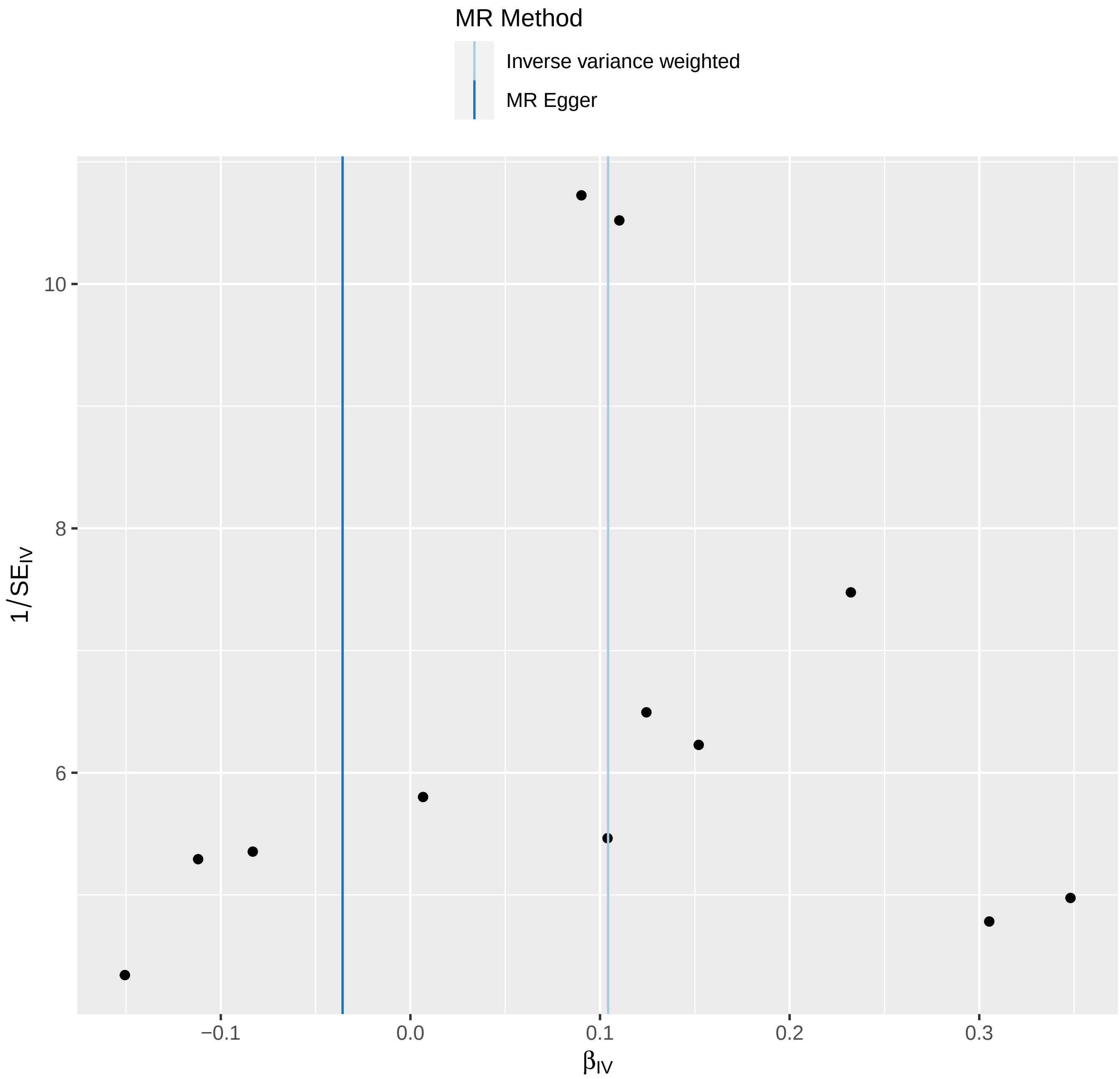

Batch 3−1750 : Selenium on Leucine−rich repeat and immunoglobulin−like domain−containing nogo receptor−interacting protein 3

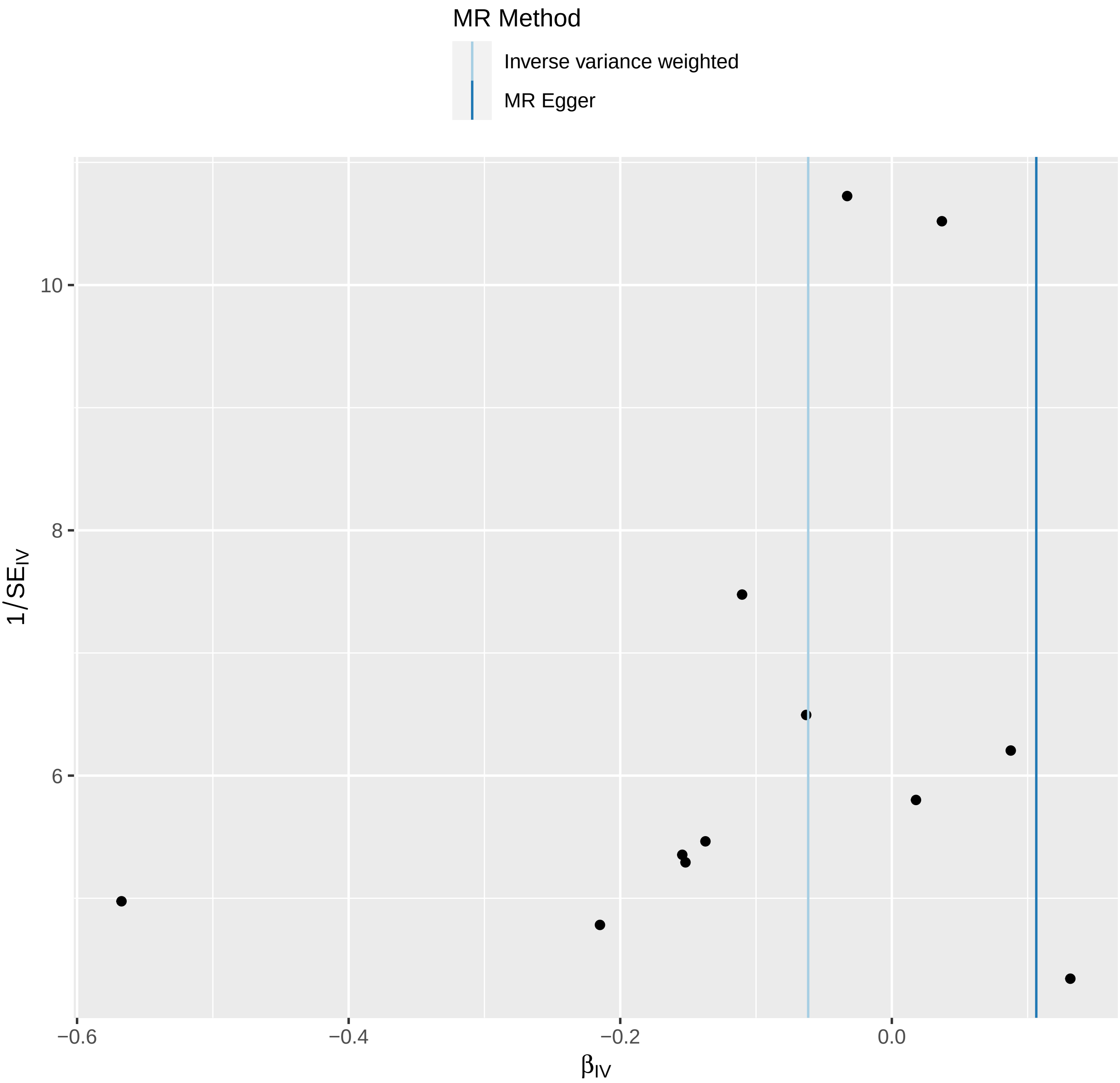

Batch 3−1751 : Selenium on Gastric triacylglycerol lipase

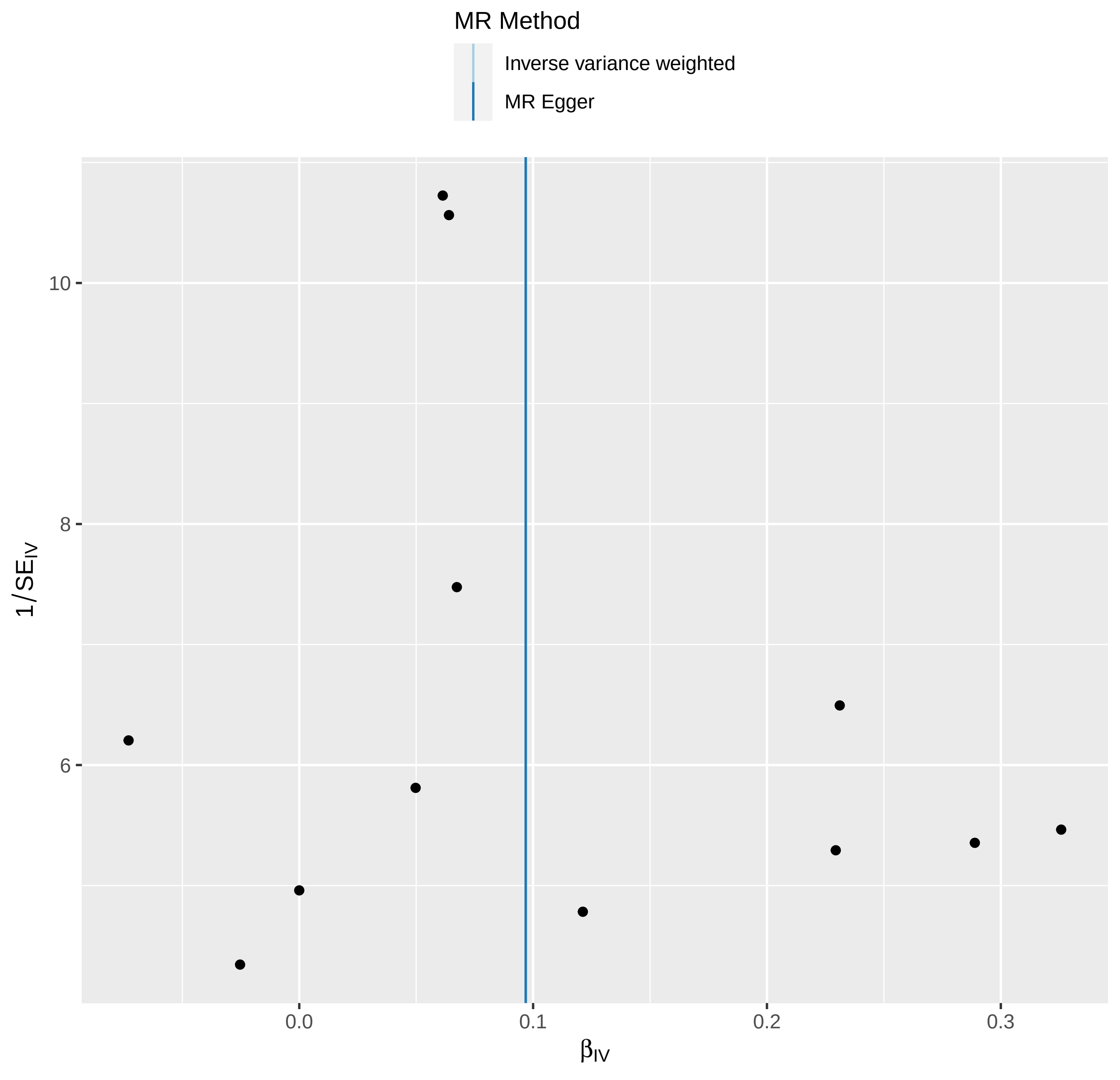

Batch 3−1752 : Selenium on Lipase member K

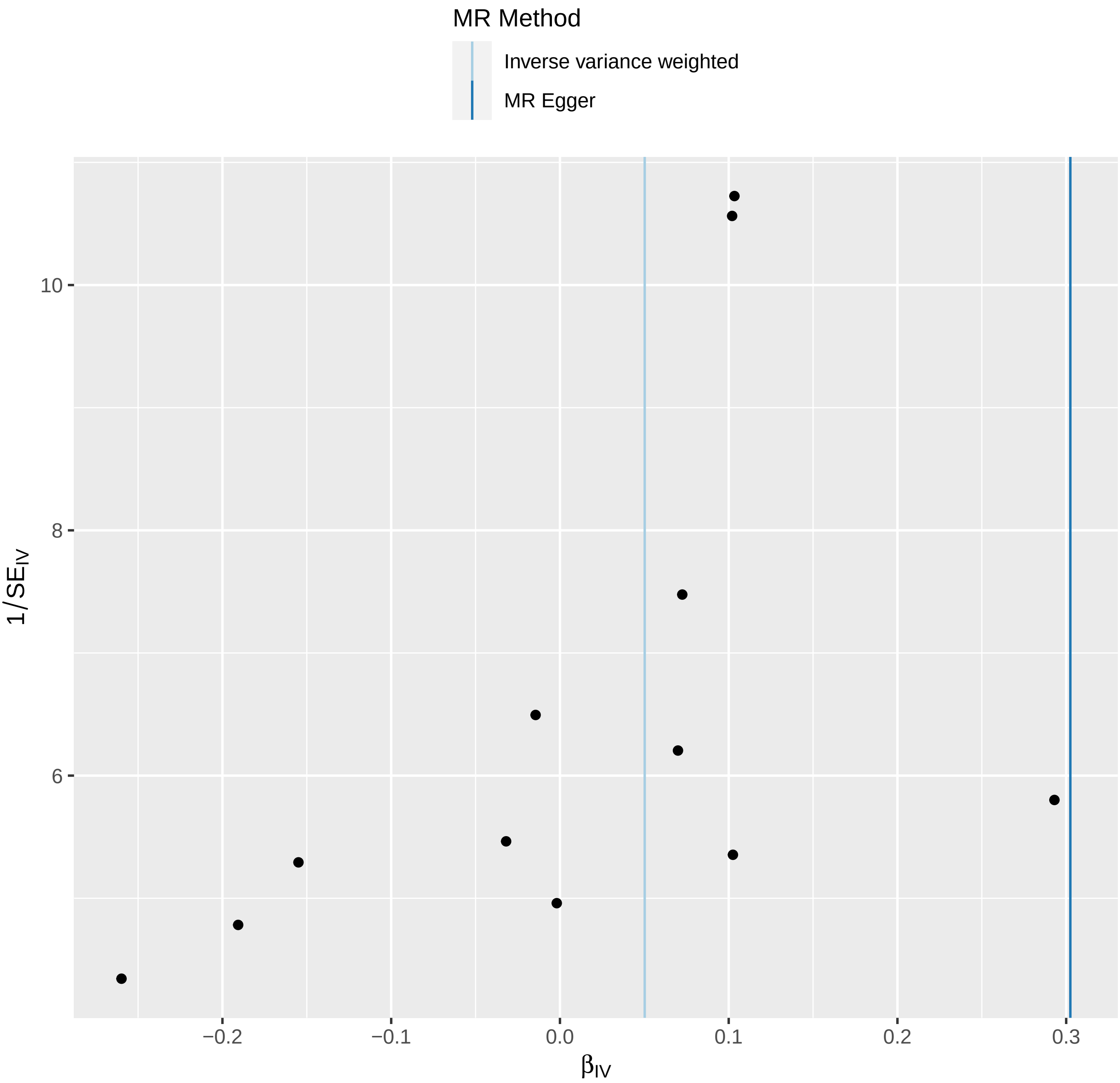

Batch 3−1753 : Selenium on Lipase member N

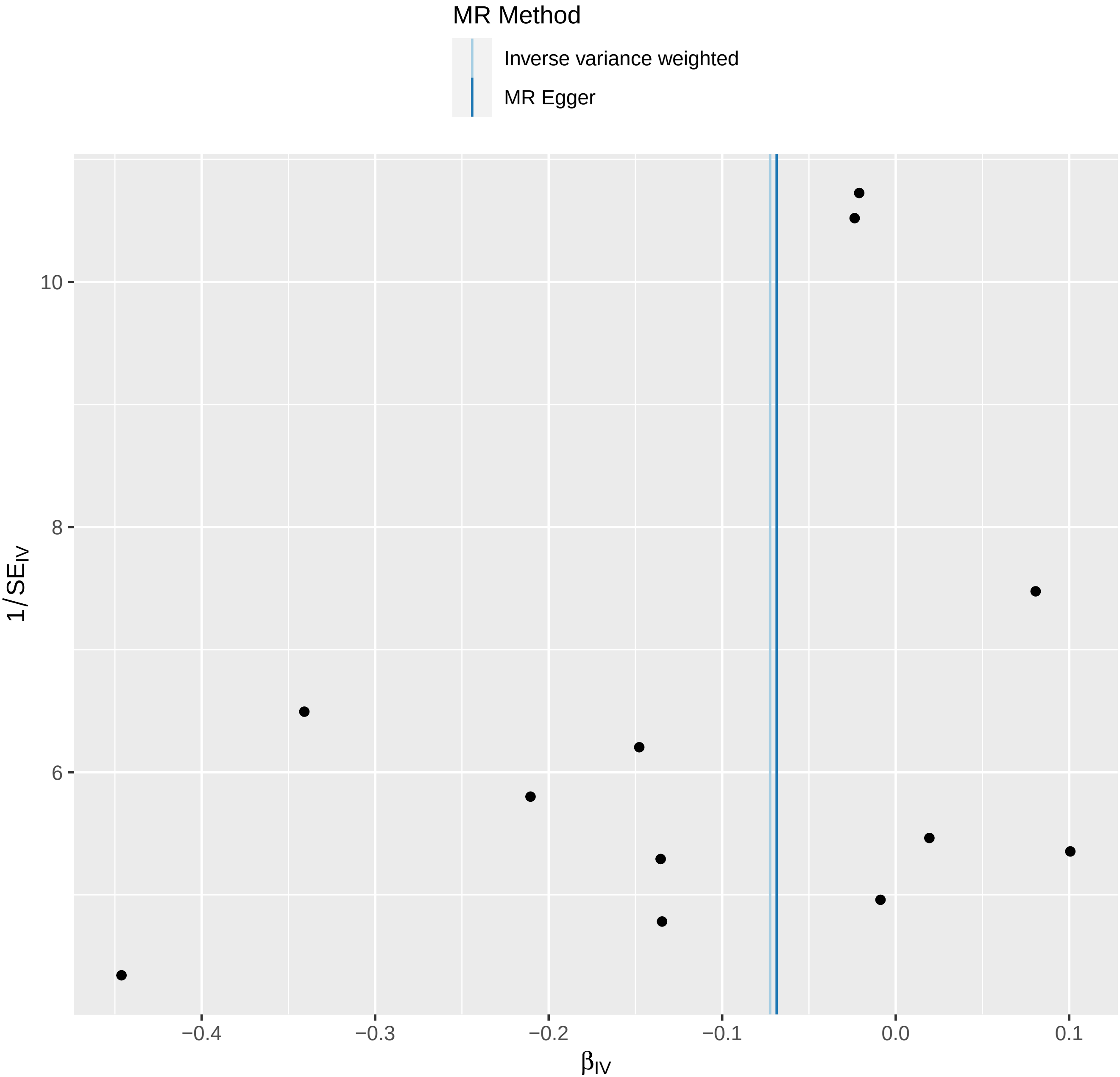

Batch 3−1754 : Selenium on Vesicular integral−membrane protein VIP36

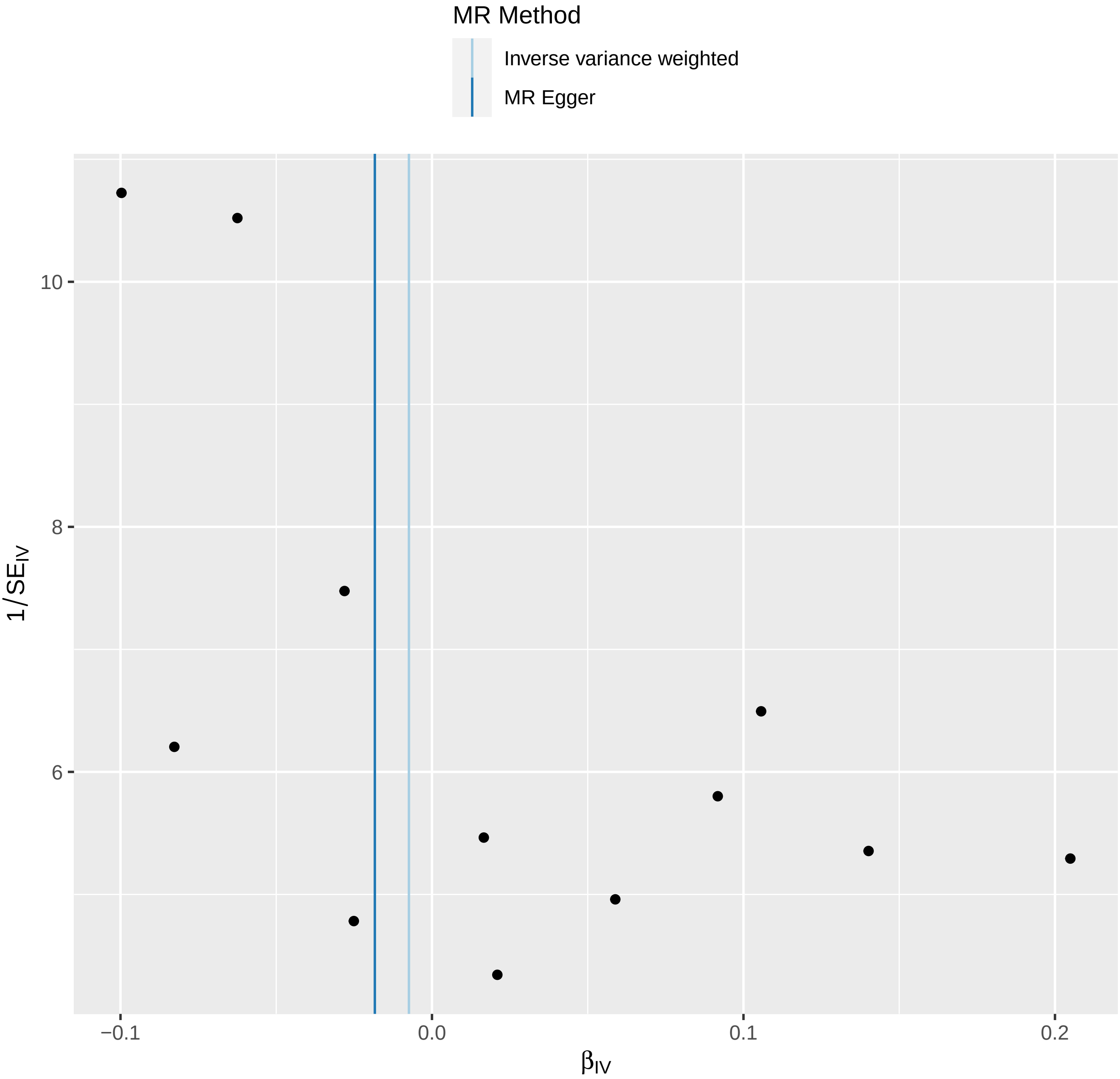

Batch 3−1755 : Selenium on Vesicular integral−membrane protein VIP36

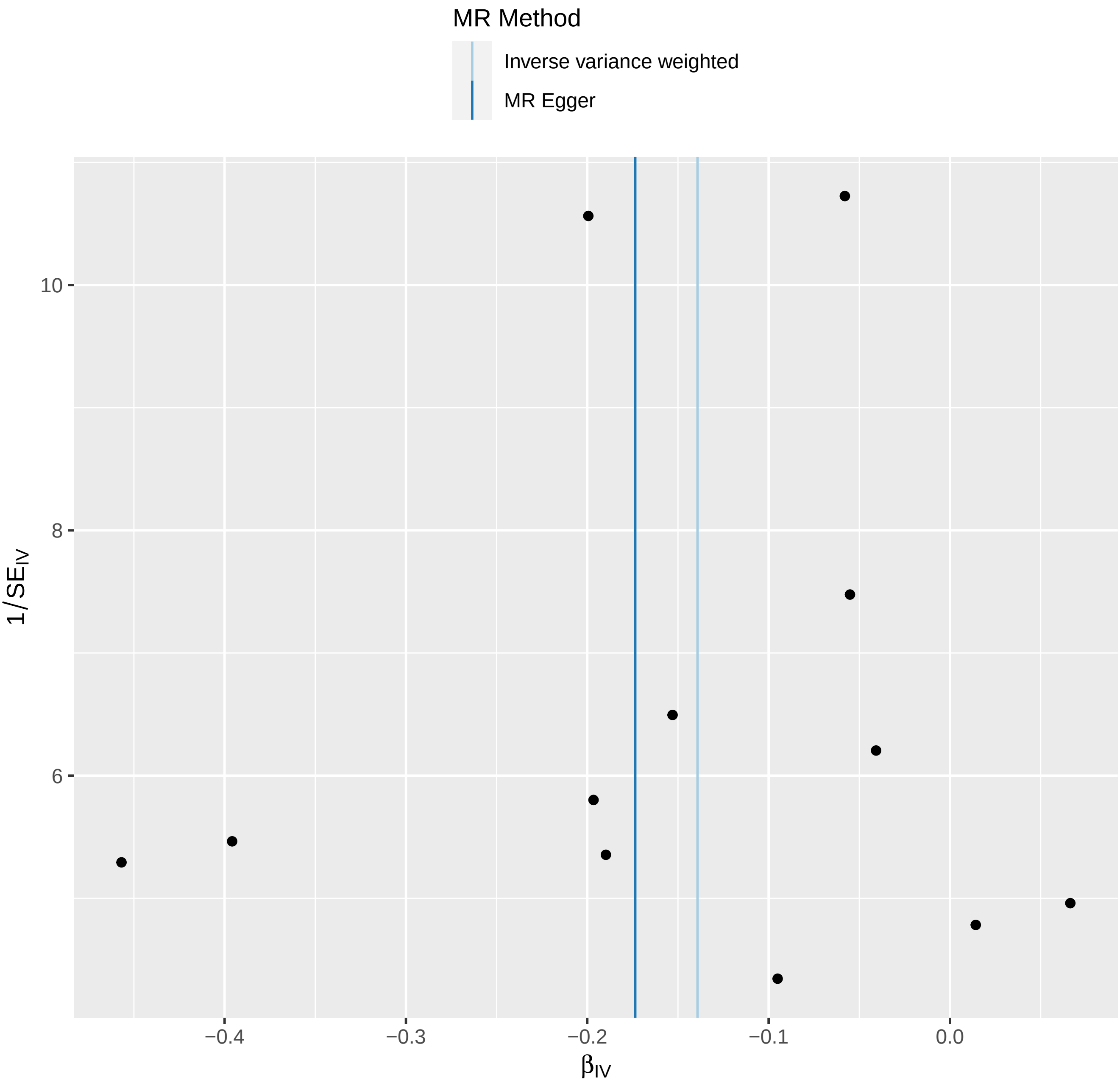

Batch 3−1756 : Selenium on VIP36−like protein

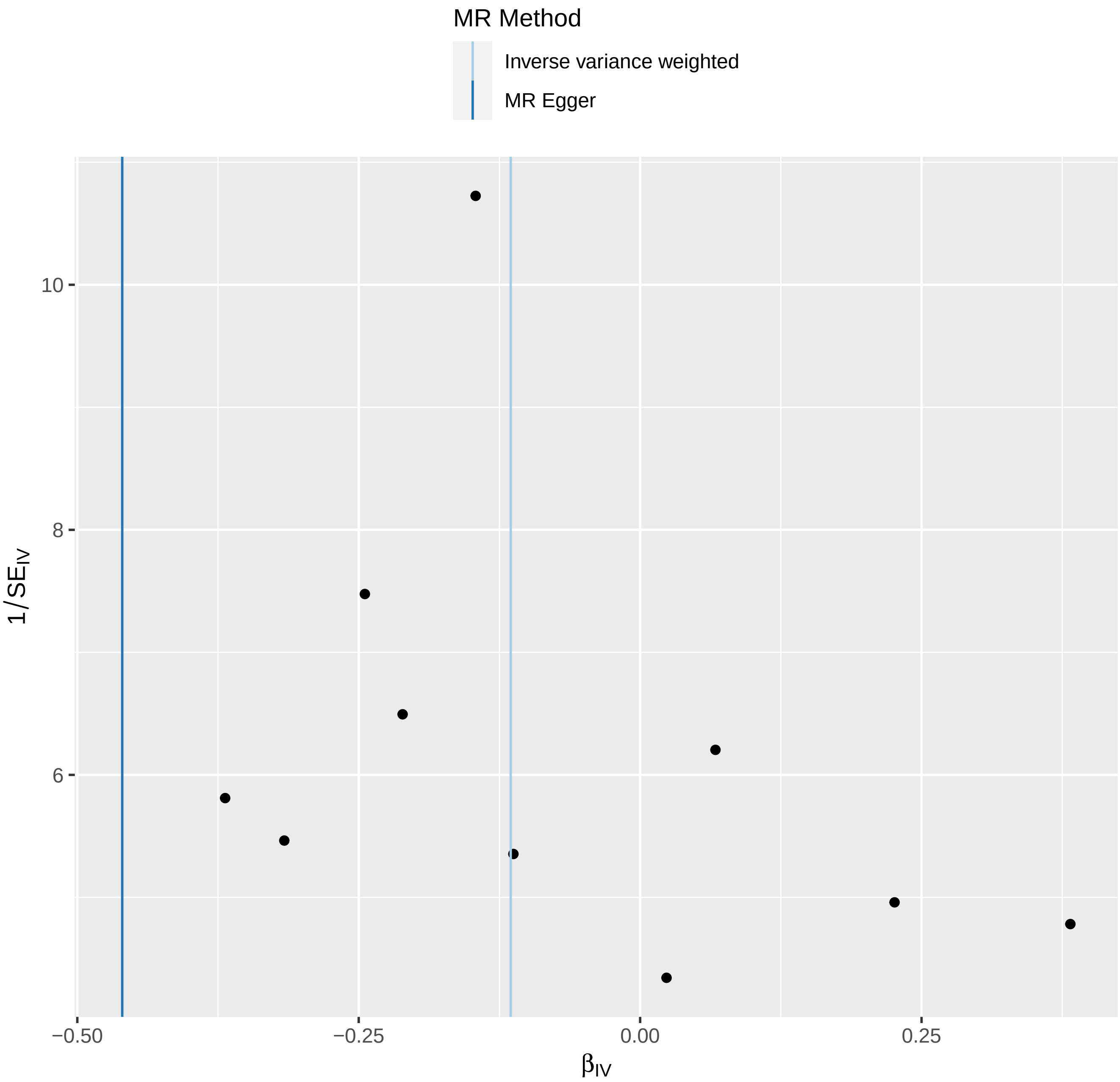

Batch 3−1757 : Selenium on LIM and cysteine−rich domains protein 1

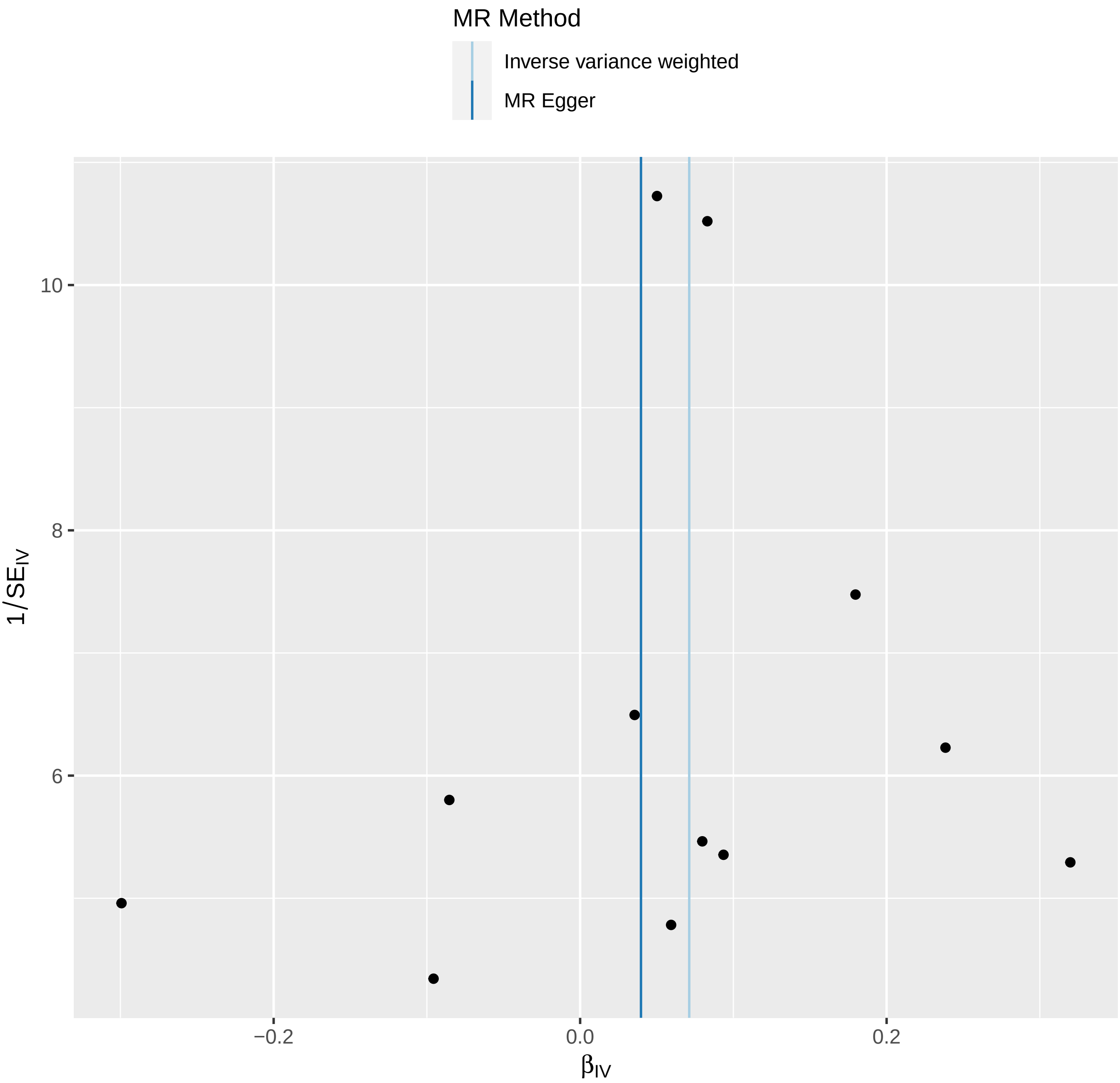

Batch 3−1758 : Selenium on Lamin−B1

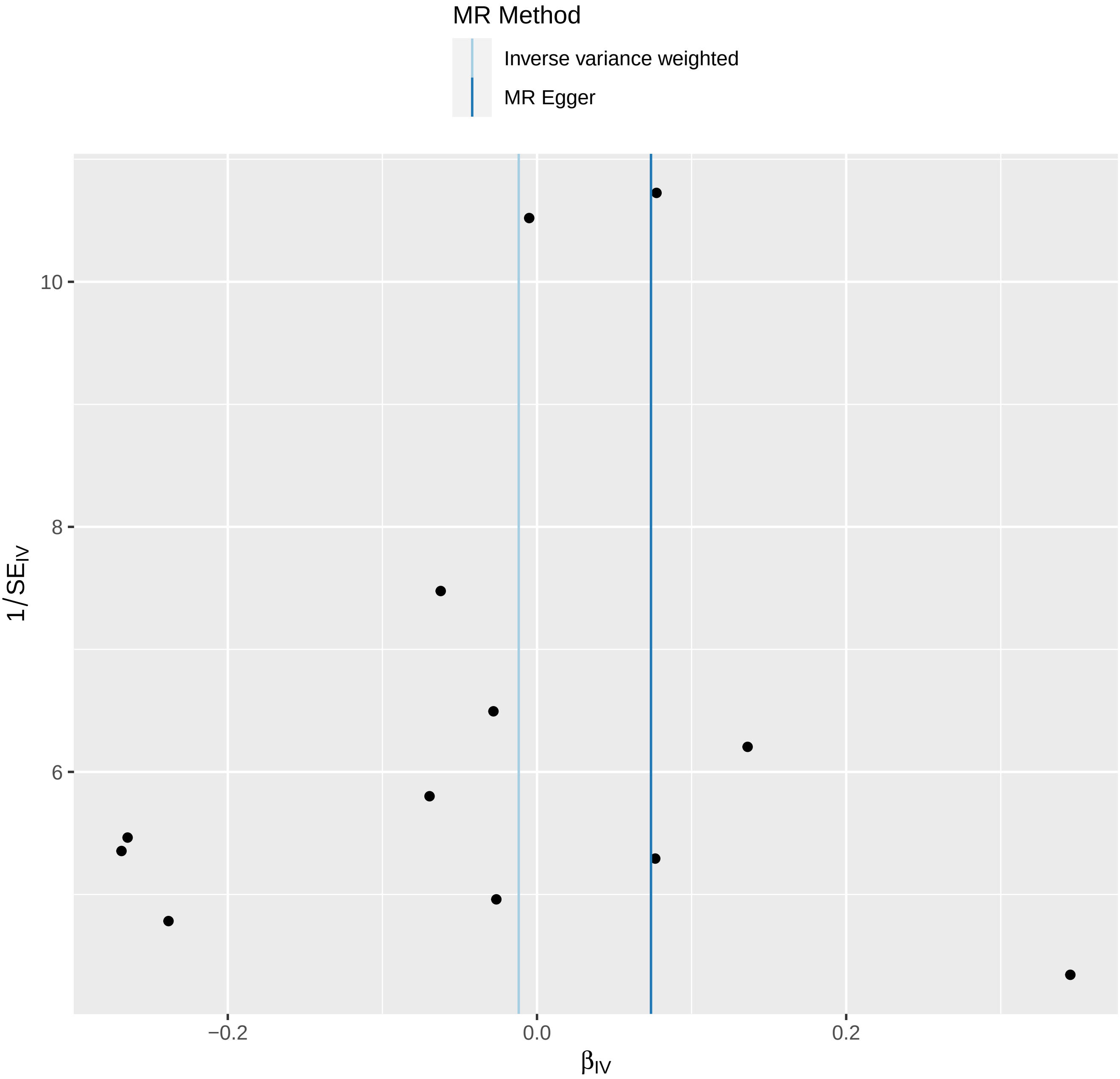

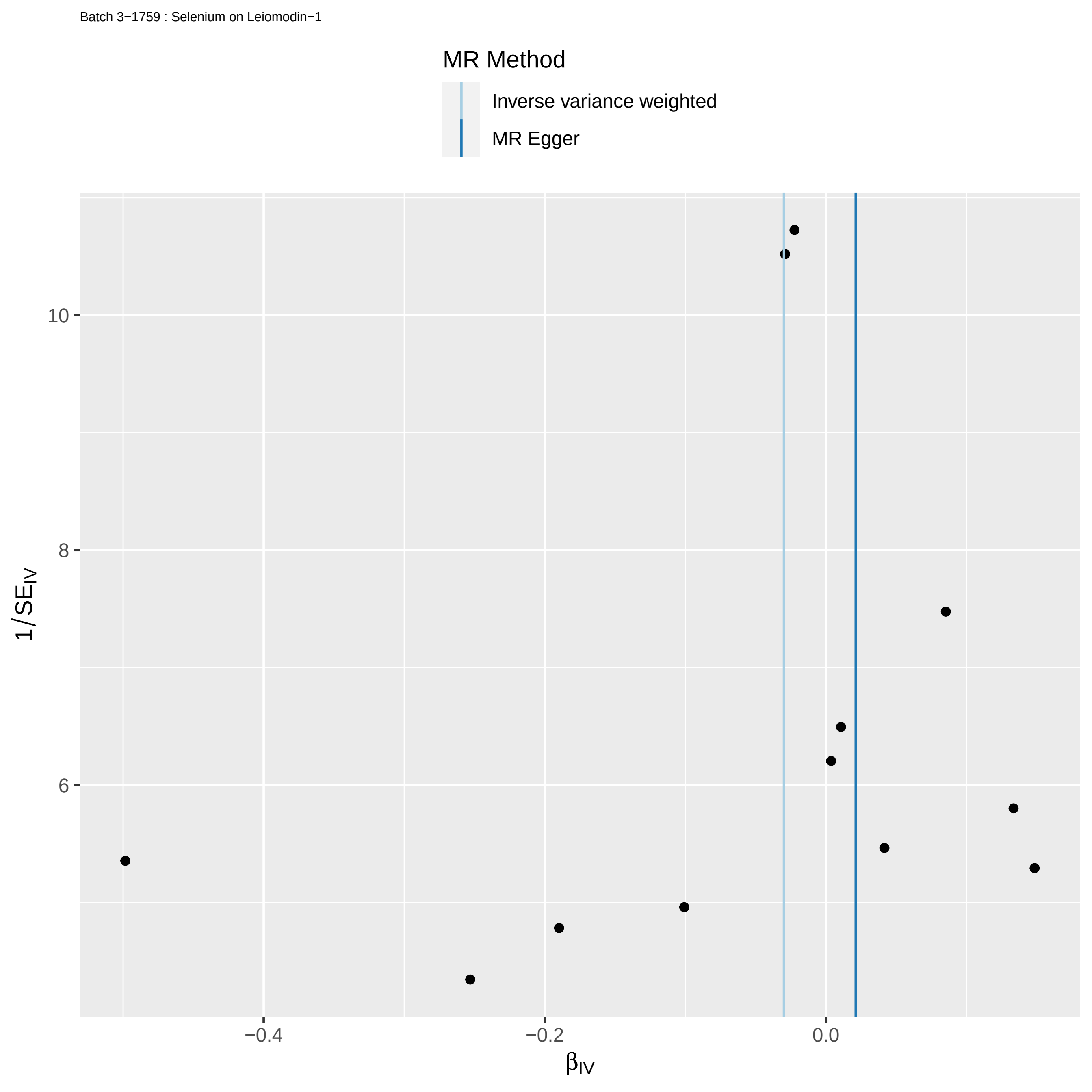

Batch 3−1759 : Selenium on Leiomodin−1
MR Method
Inverse variance weighted
MR Egger
10
8
6
1/SE$_{IV}$
−0.4
−0.2
0.0
β$_{IV}$

Batch 3−1760 : Selenium on Ig Kappa chain V−I region HK102− like

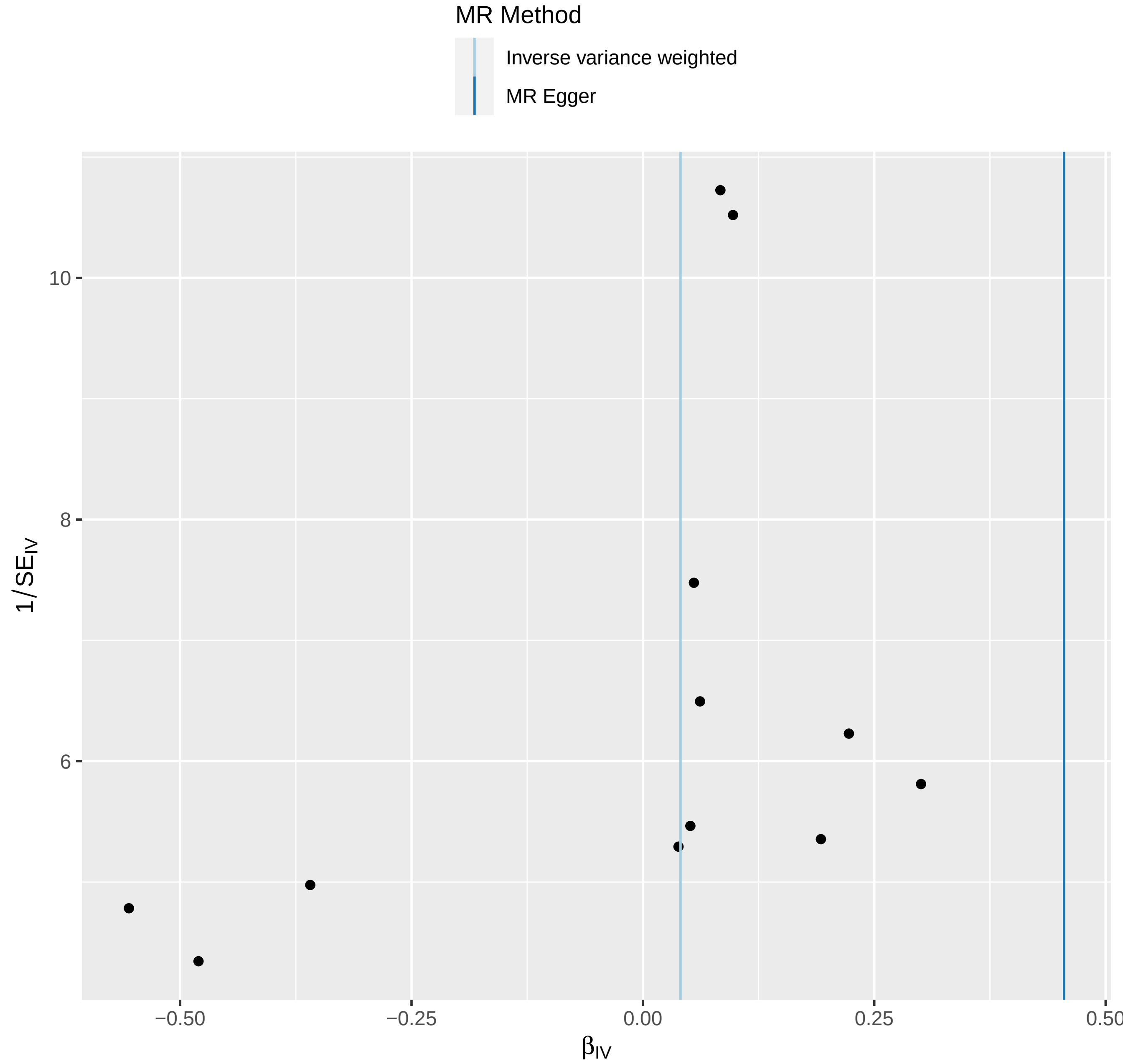

Batch 3−1761 : Selenium on Lon protease homolog, mitochondrial

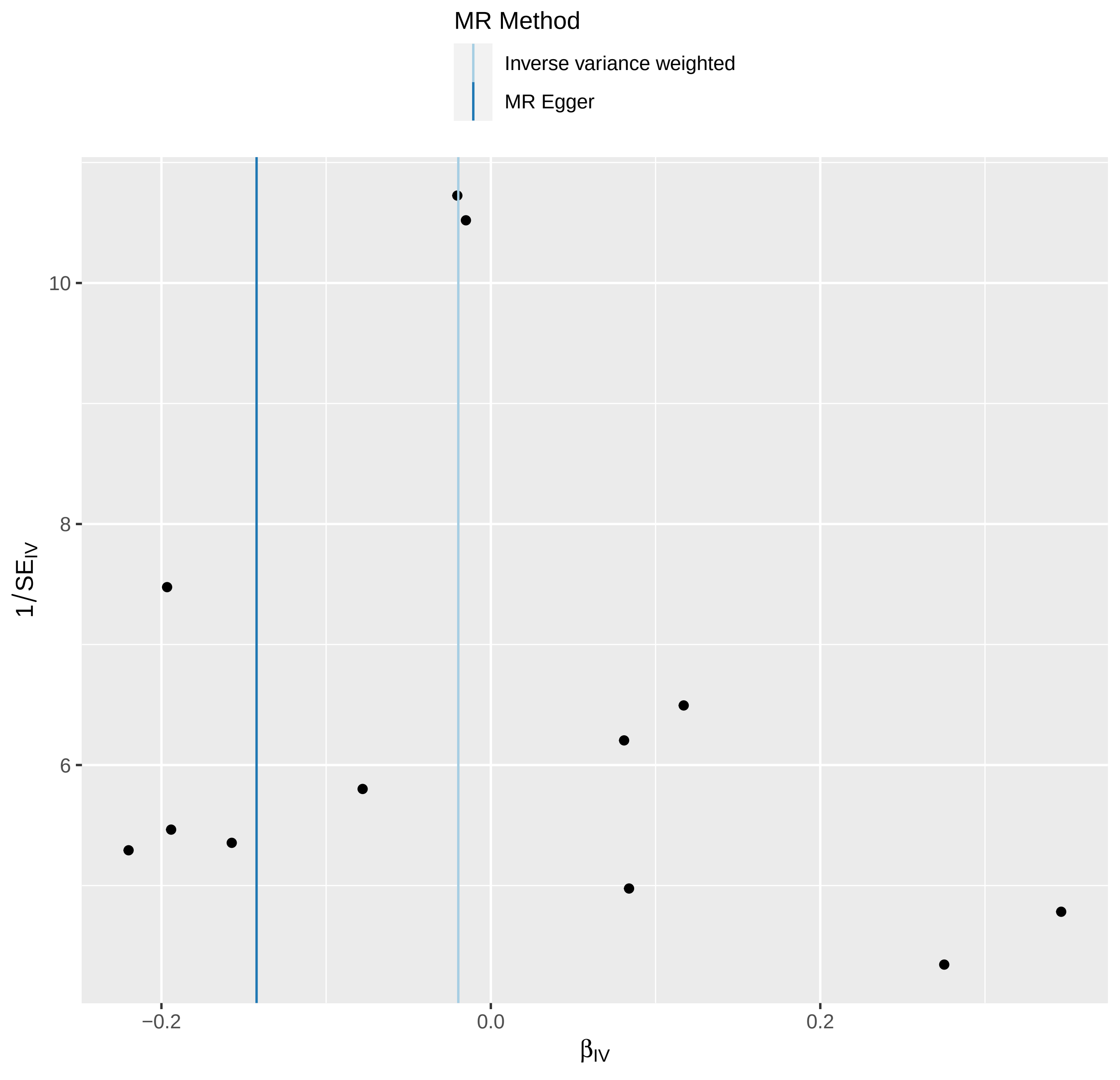

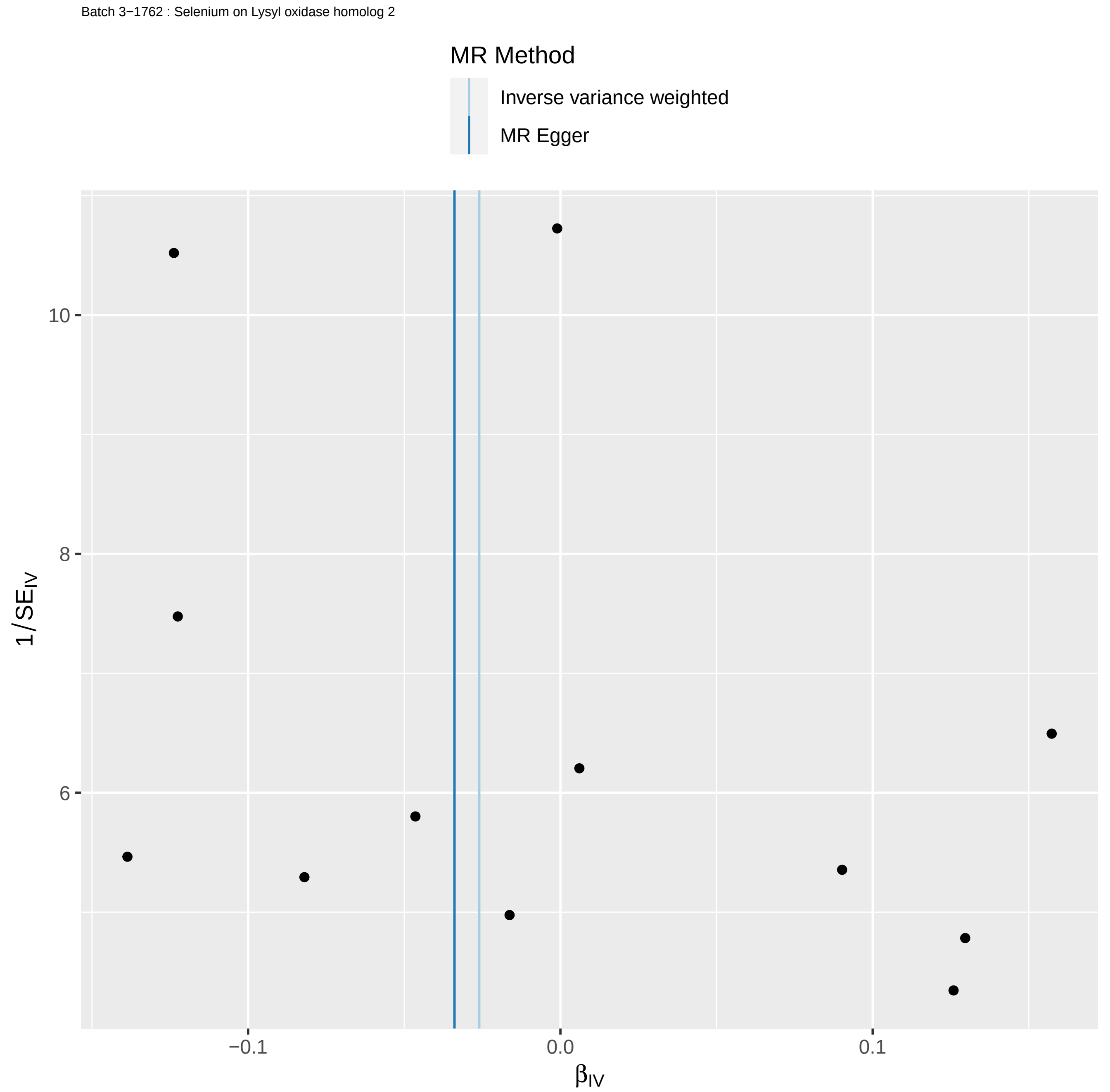

Batch 3−1762 : Selenium on Lysyl oxidase homolog 2
MR Method
Inverse variance weighted
MR Egger
10
8
6
1/SE$_{IV}$
−0.1
0.0
0.1
β$_{IV}$

Batch 3−1763 : Selenium on Putative apolipoprotein(a)−like protein 2

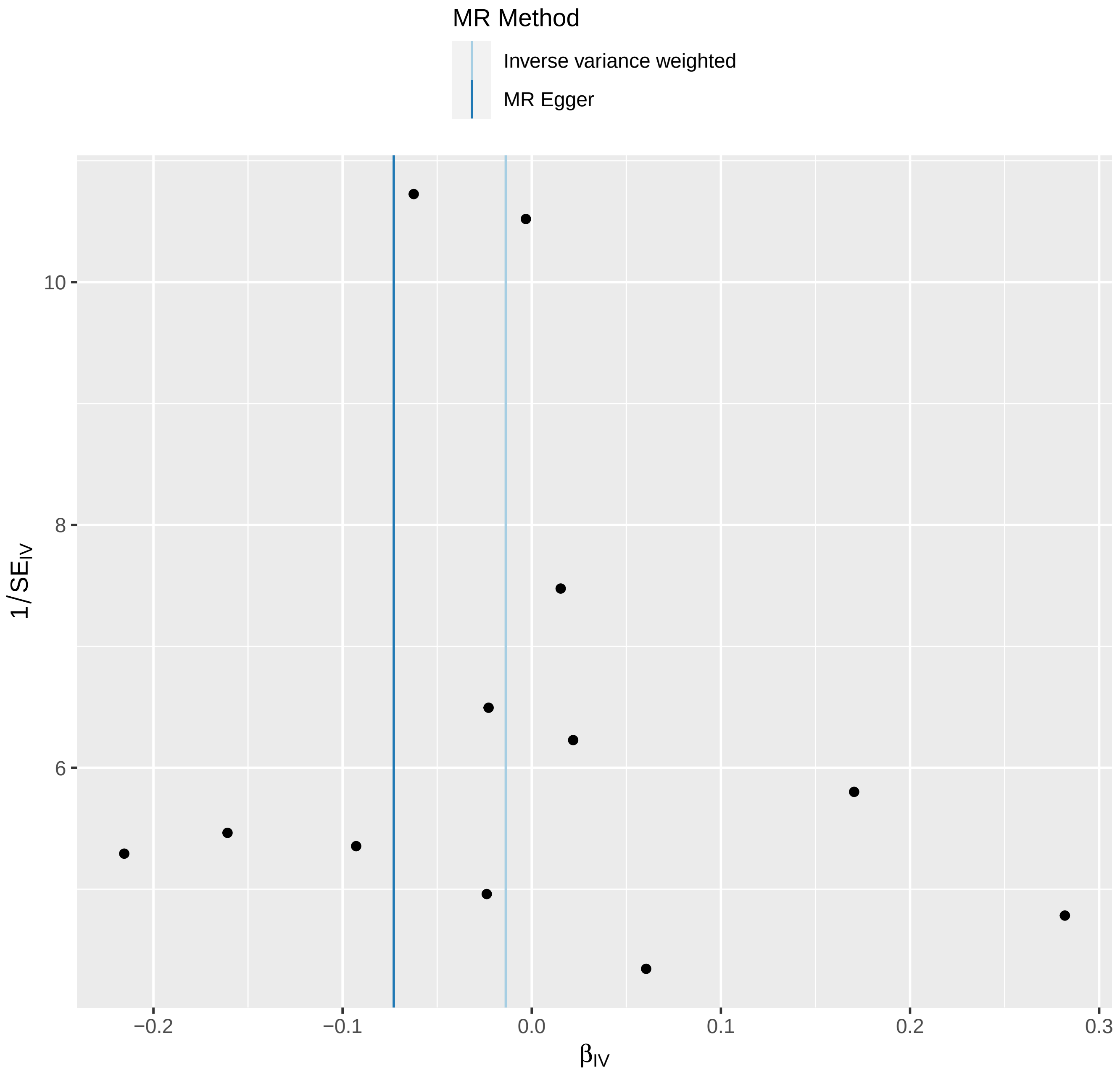

Batch 3−1764 : Selenium on Lysophosphatidylcholine acyltransferase 2

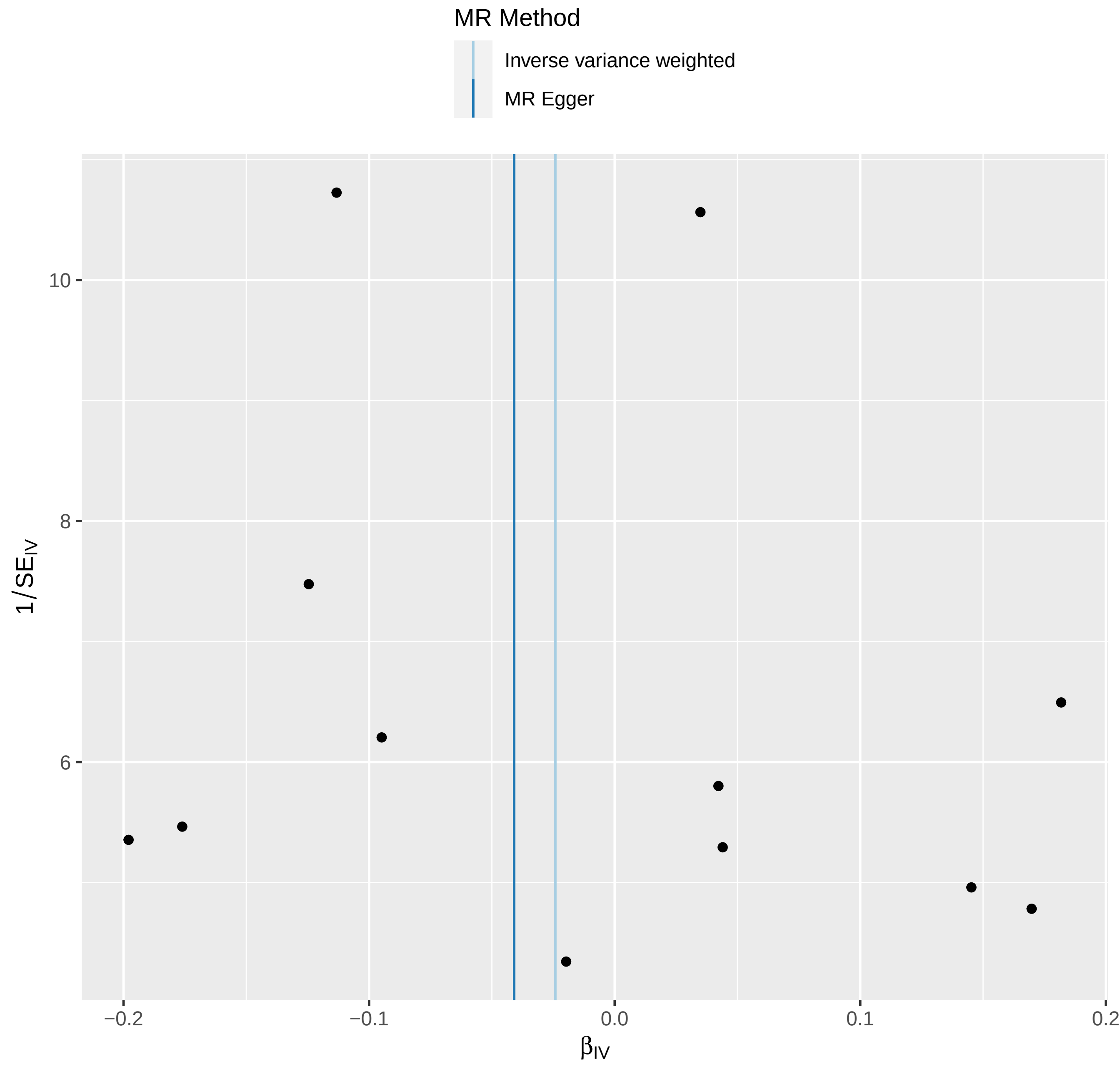

Batch 3−1765 : Selenium on Lactoperoxidase

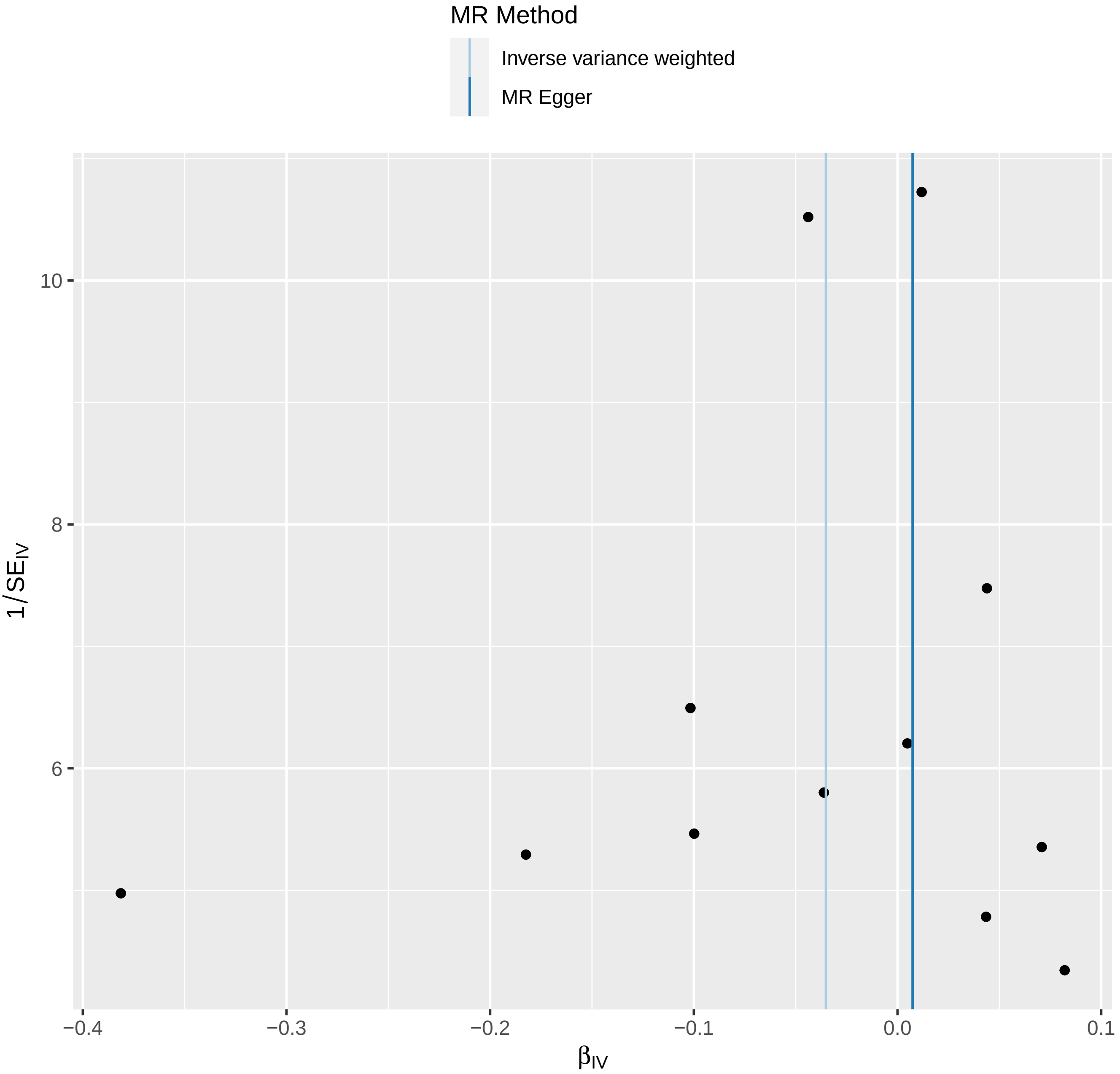

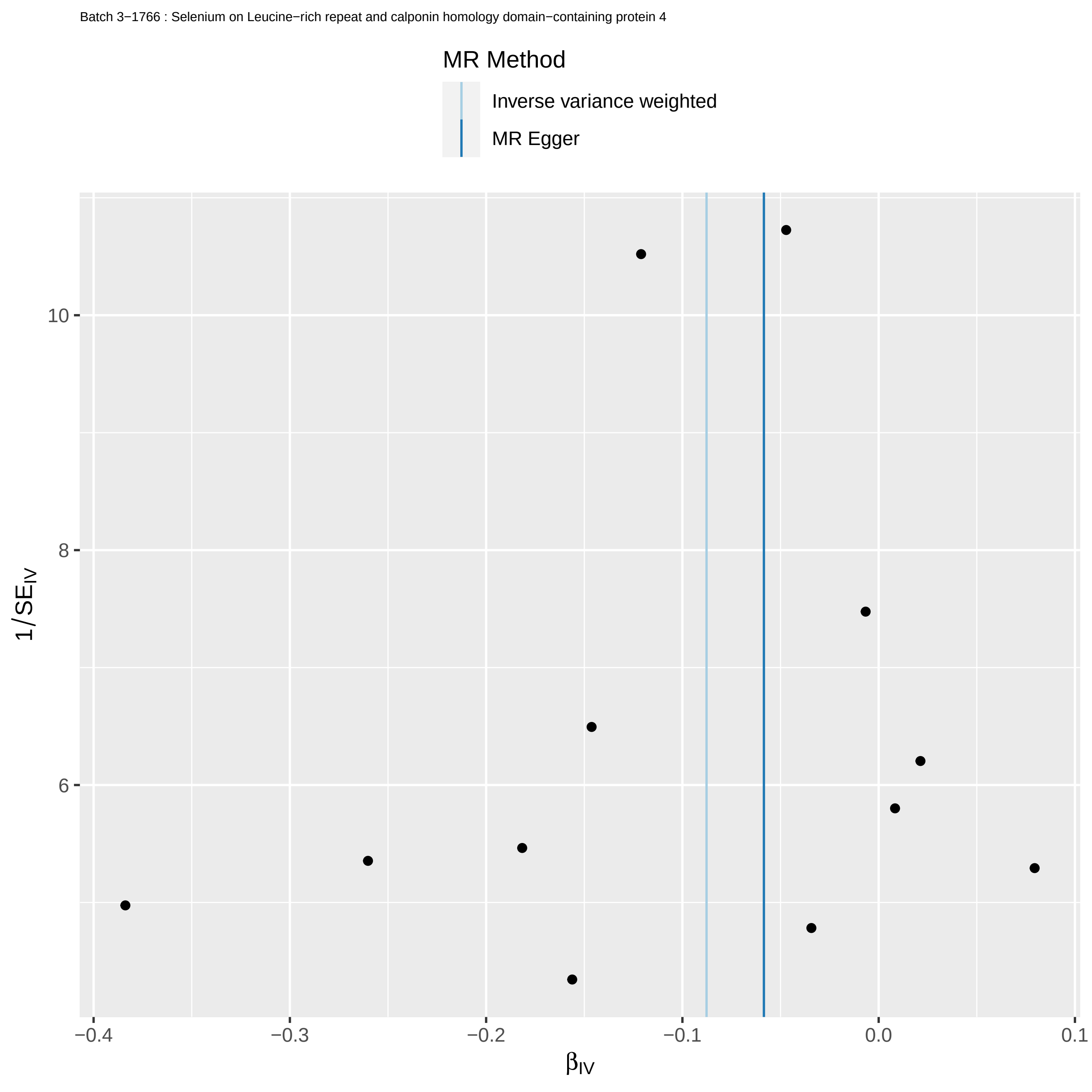
Batch 3−1766 : Selenium on Leucine−rich repeat and calponin homology domain−containing protein 4
MR Method
Inverse variance weighted
MR Egger
10
8
6
1/SE_IV
−0.4
−0.3
−0.2
−0.1
0.0
0.1
β_IV

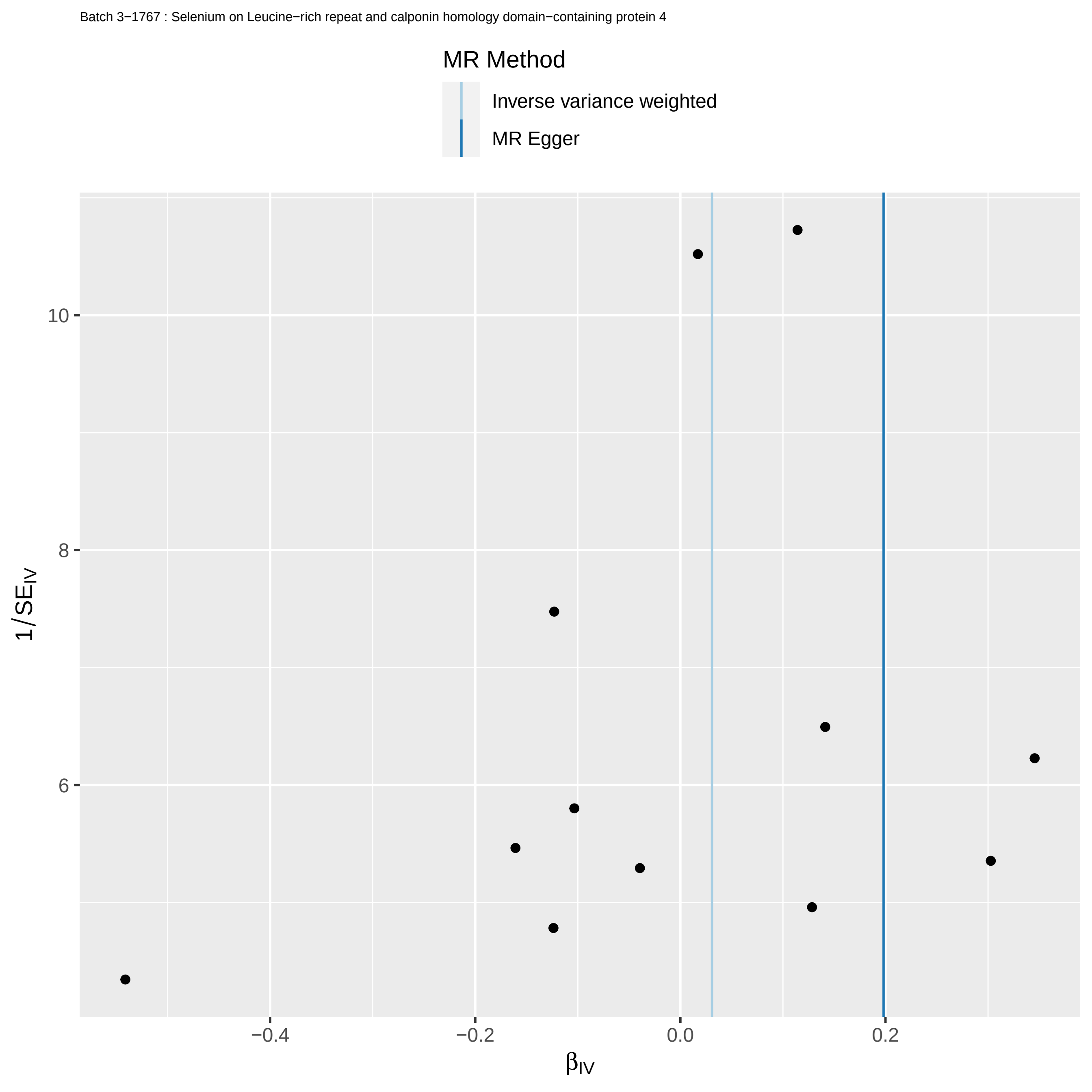

Batch 3−1767 : Selenium on Leucine−rich repeat and calponin homology domain−containing protein 4
MR Method
Inverse variance weighted
MR Egger
10
8
6
1/SE_IV
−0.4
−0.2
0.0
0.2
β_IV

Batch 3−1768 : Selenium on Leucine−rich repeat and fibronectin type III domain−containing protein 1

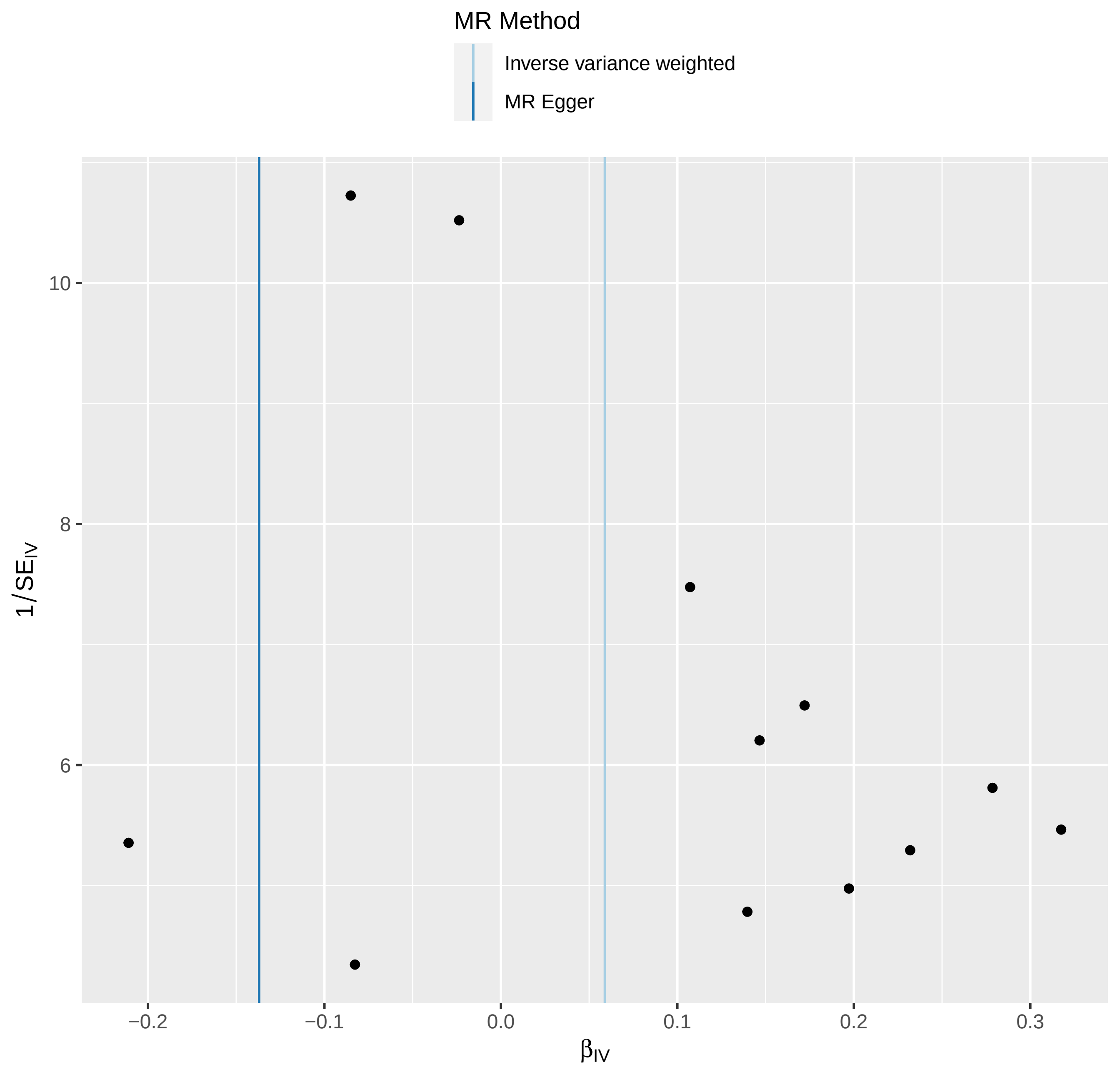

Batch 3−1769 : Selenium on Leucine−rich repeat and fibronectin type−III domain−containing protein 2

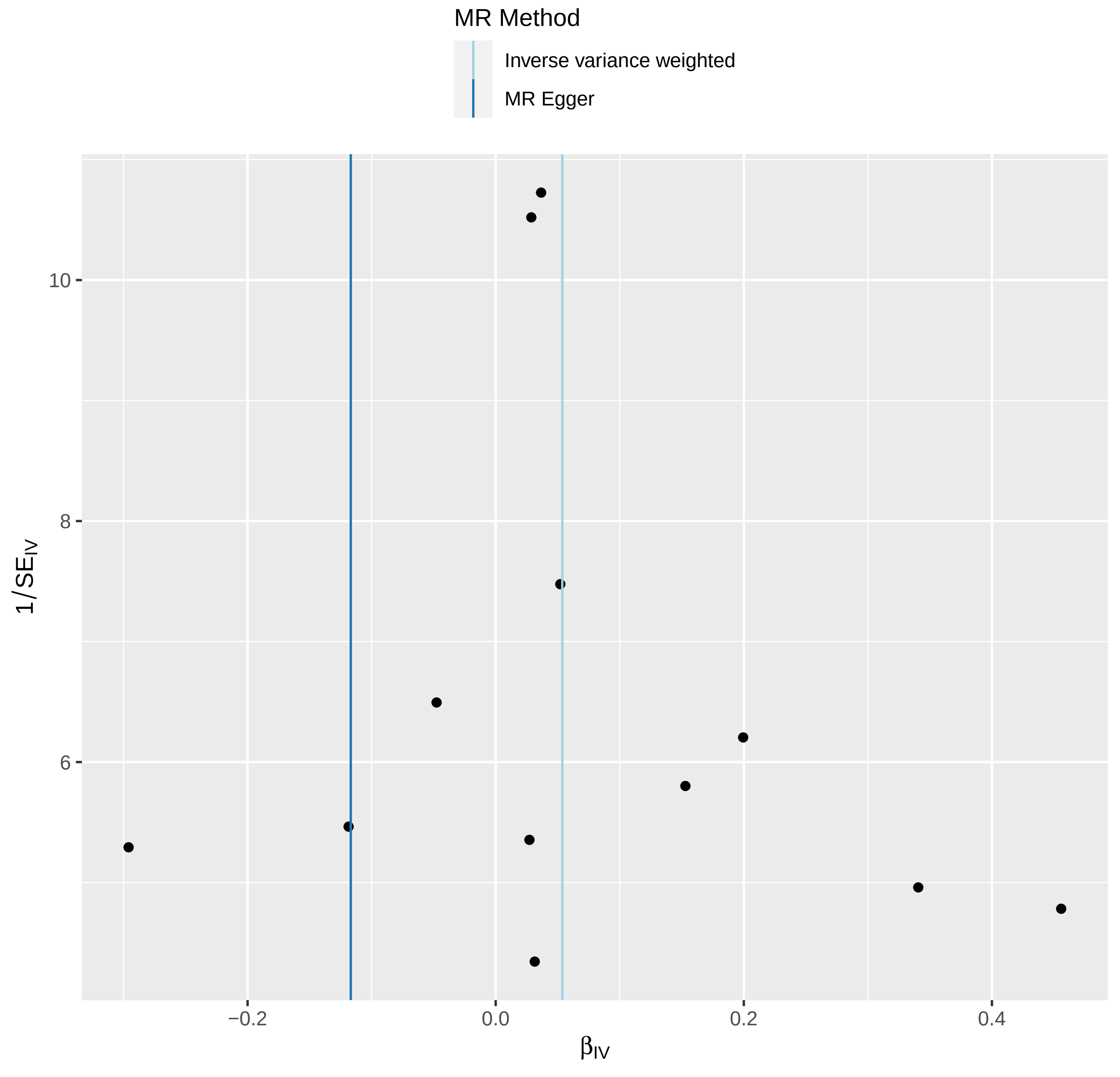

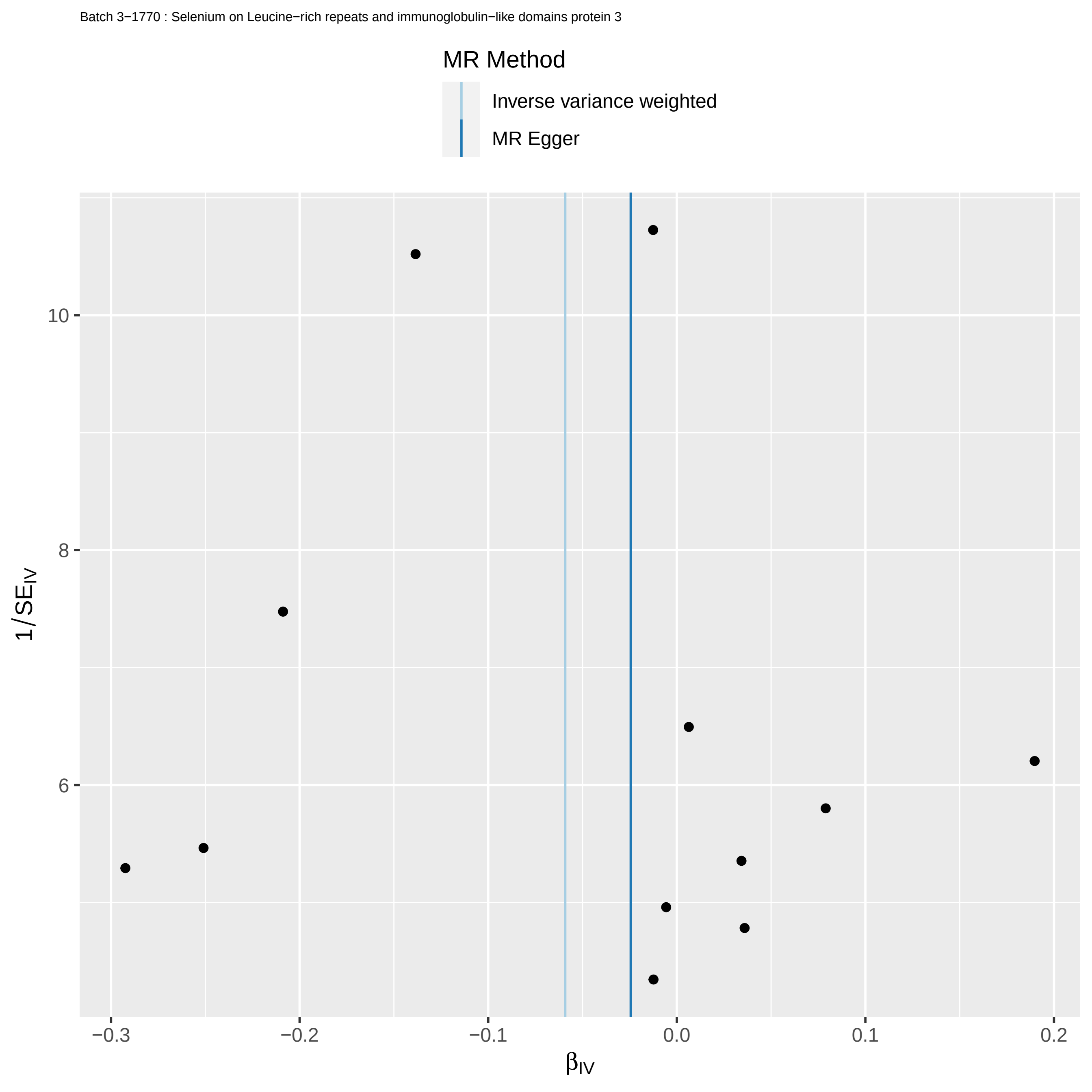

Batch 3−1770 : Selenium on Leucine−rich repeats and immunoglobulin−like domains protein 3
MR Method
Inverse variance weighted
MR Egger
10
8
6
1/SE_IV
−0.3
−0.2
−0.1
0.0
0.1
0.2
β_IV

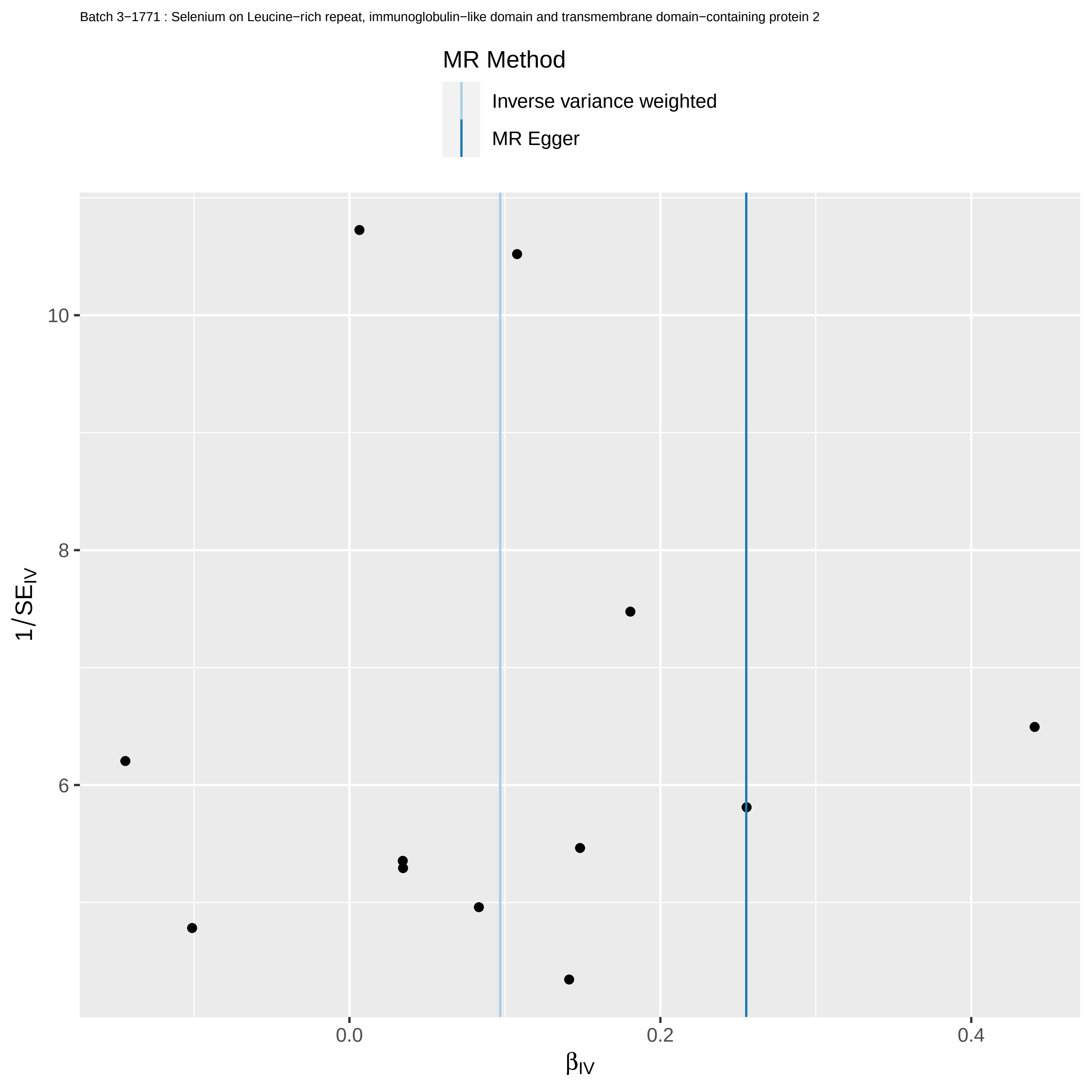
Batch 3−1771 : Selenium on Leucine−rich repeat, immunoglobulin−like domain and transmembrane domain−containing protein 2
MR Method
Inverse variance weighted
MR Egger
10
8
6
1/SE$_{IV}$
0.0
0.2
0.4
β_{IV}

Batch 3−1772 : Selenium on Leucine−rich repeat, immunoglobulin−like domain and transmembrane domain−containing protein 3

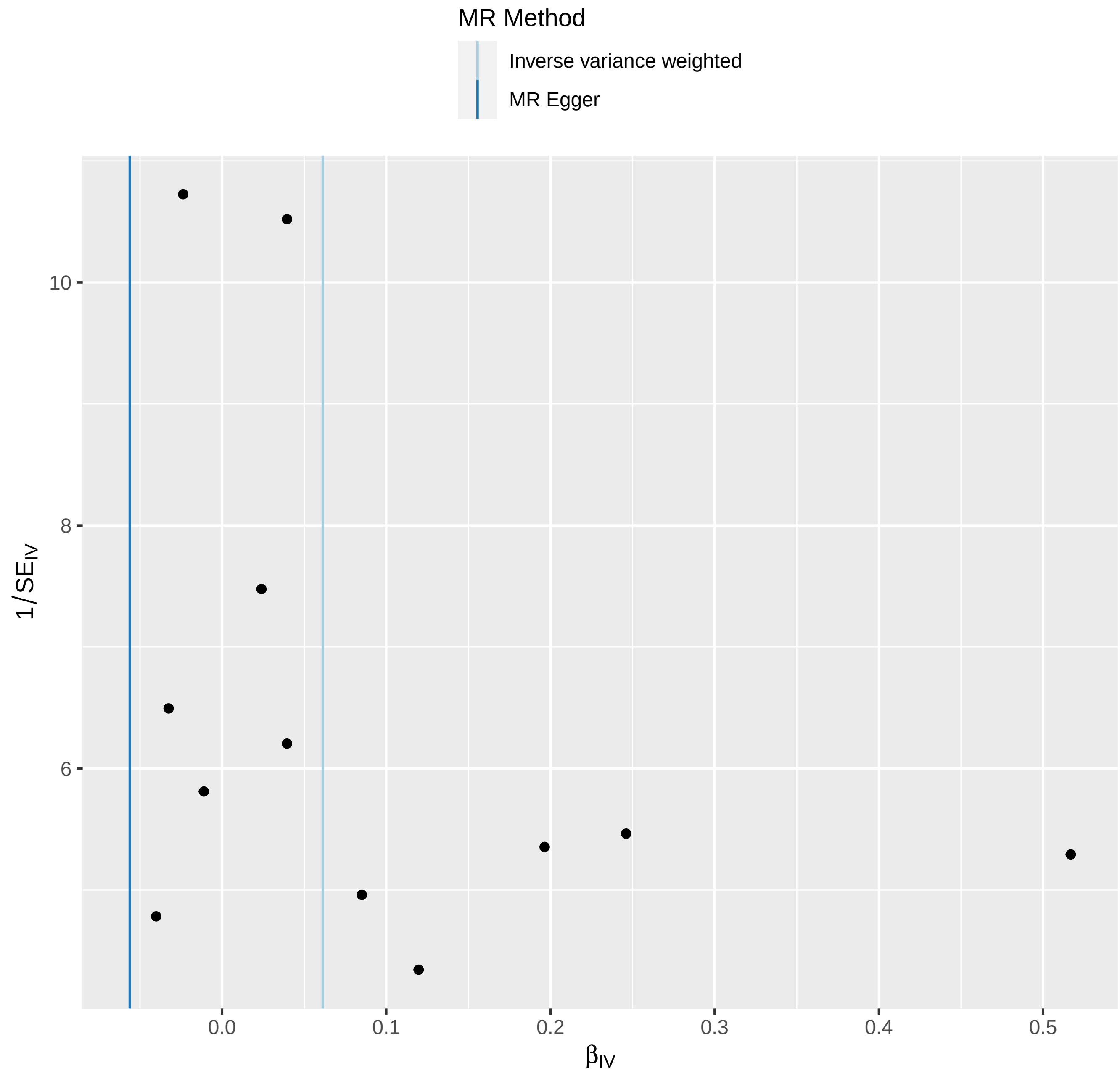

Batch 3−1773 : Selenium on Leucine−rich repeat, immunoglobulin−like domain and transmembrane domain−containing protein 3

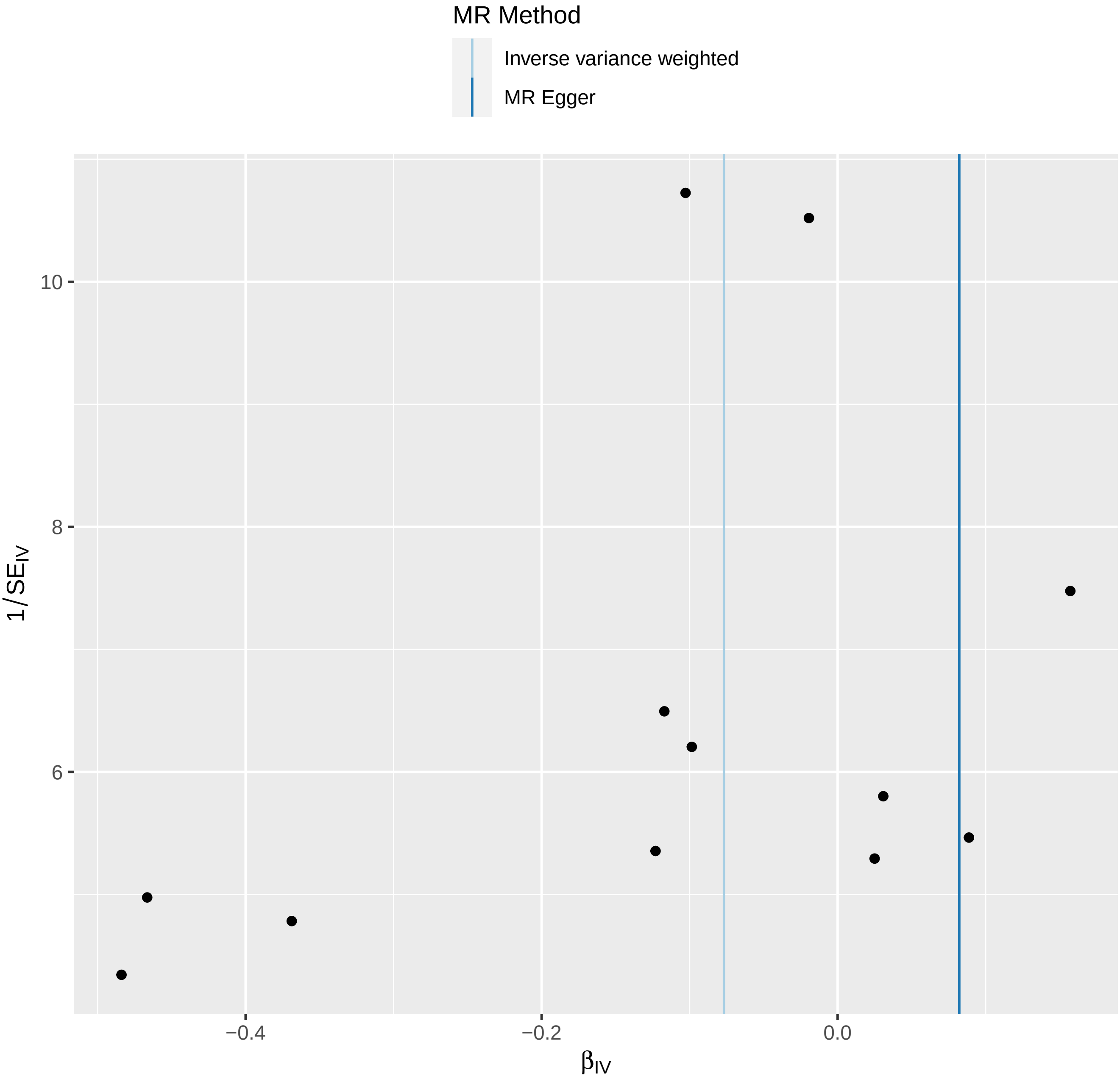

Batch 3−1774 : Selenium on Lymphoid−restricted membrane protein

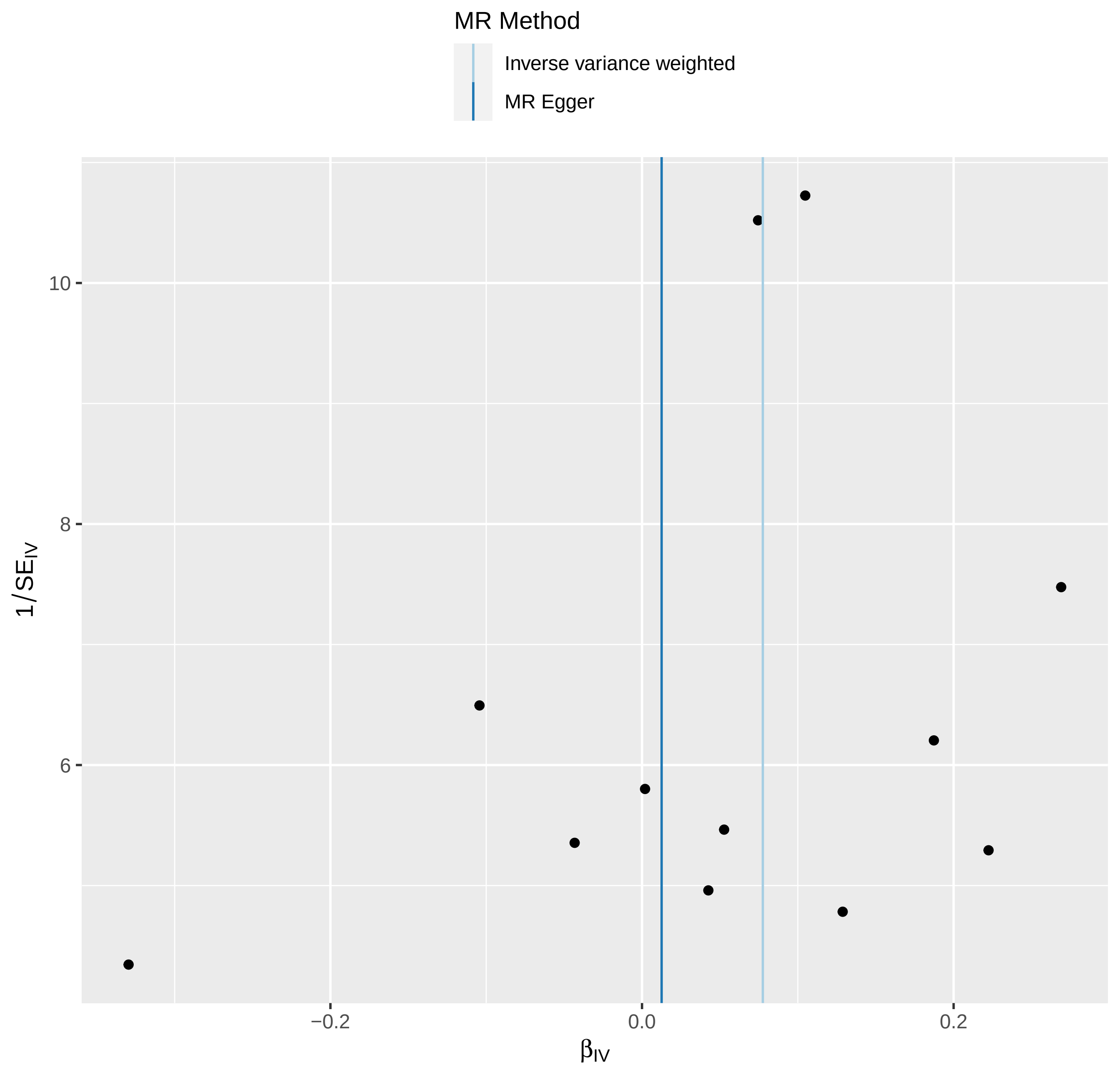

Batch 3−1775 : Selenium on Prolow−density lipoprotein receptor−related protein 1

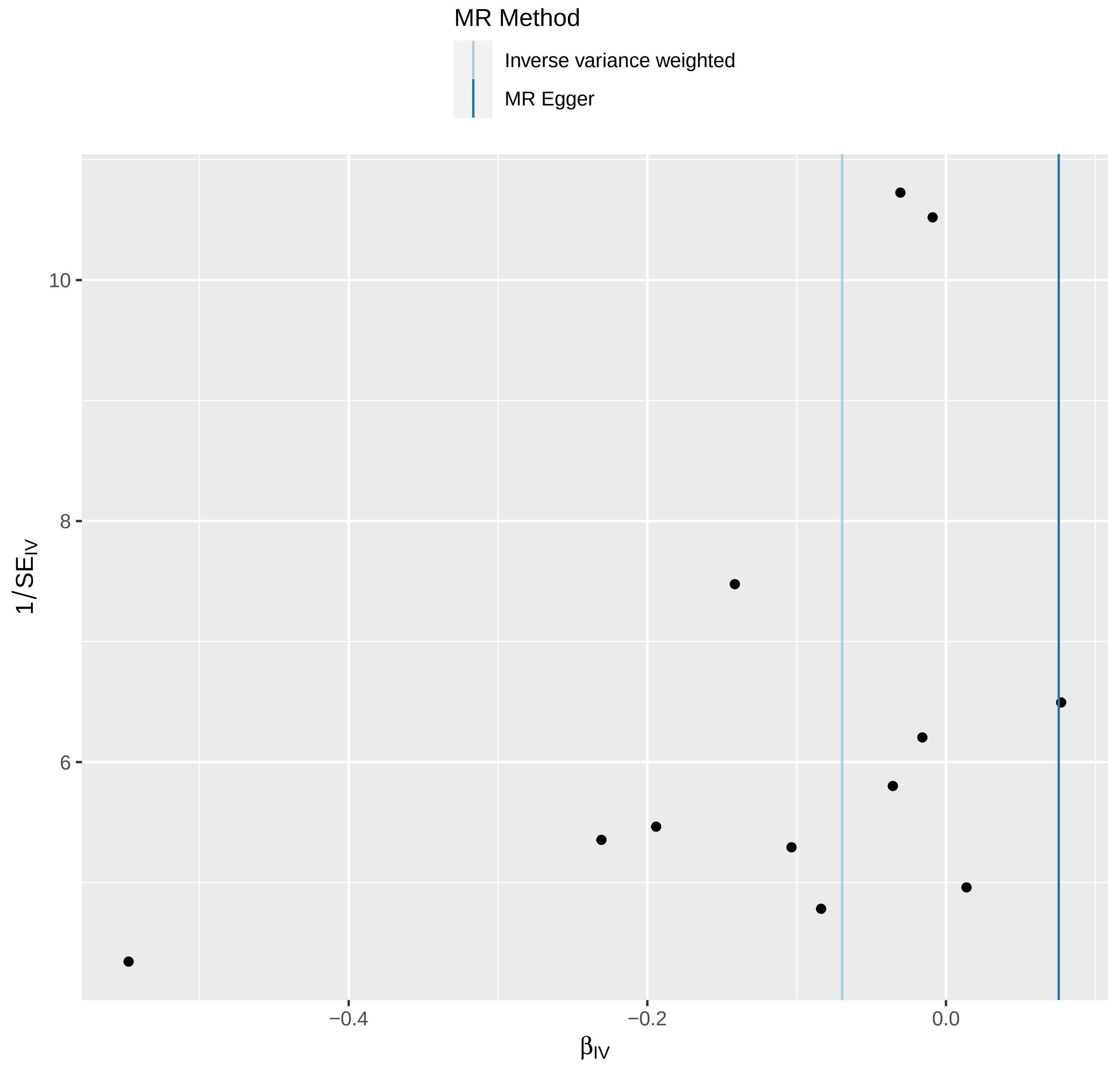

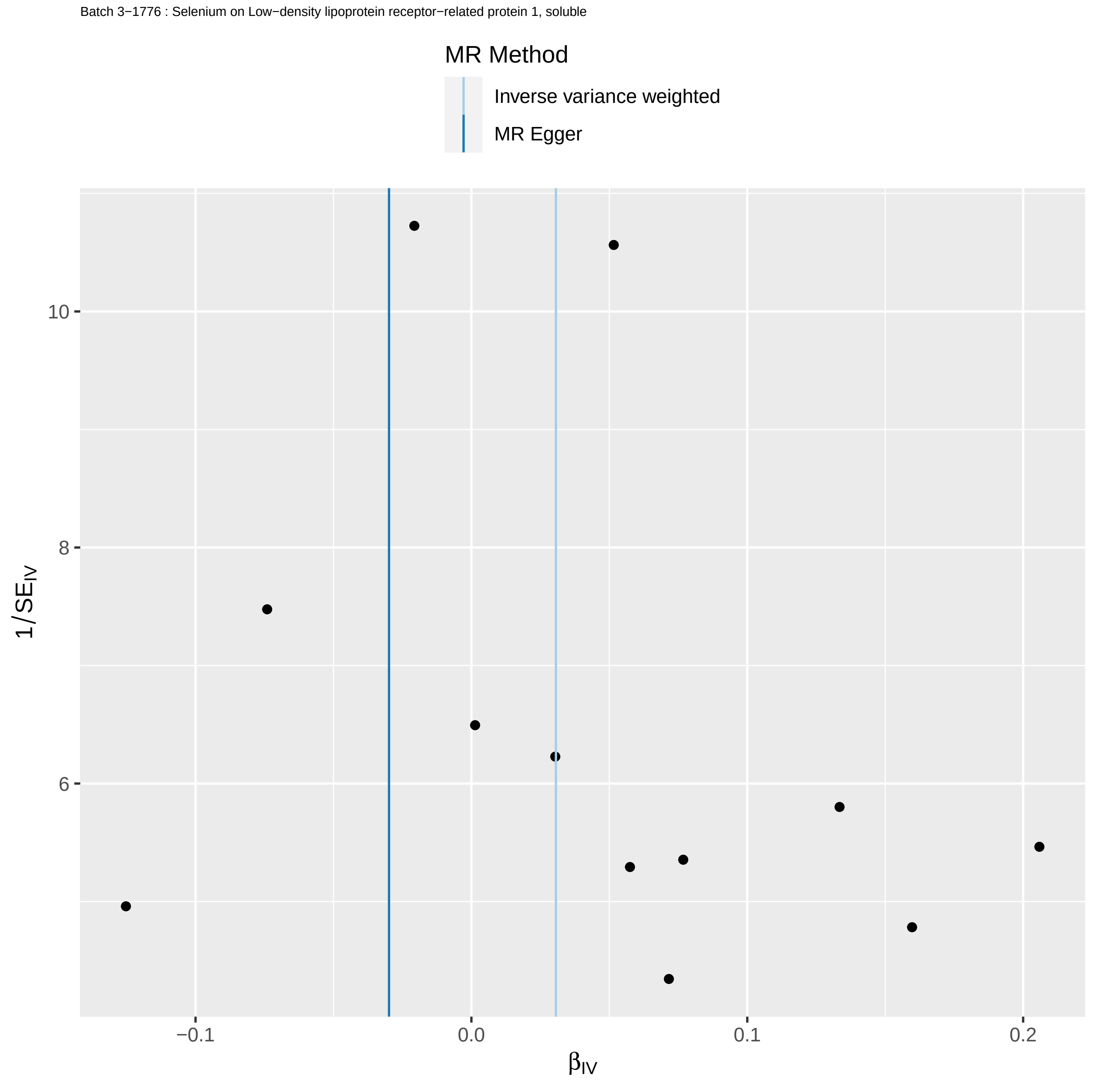

Batch 3−1776 : Selenium on Low−density lipoprotein receptor−related protein 1, soluble
MR Method
Inverse variance weighted
MR Egger
10
8
6
1/SE_IV
−0.1
0.0
0.1
0.2
β_IV

Batch 3−1777 : Selenium on Low−density lipoprotein receptor−related protein 1, soluble

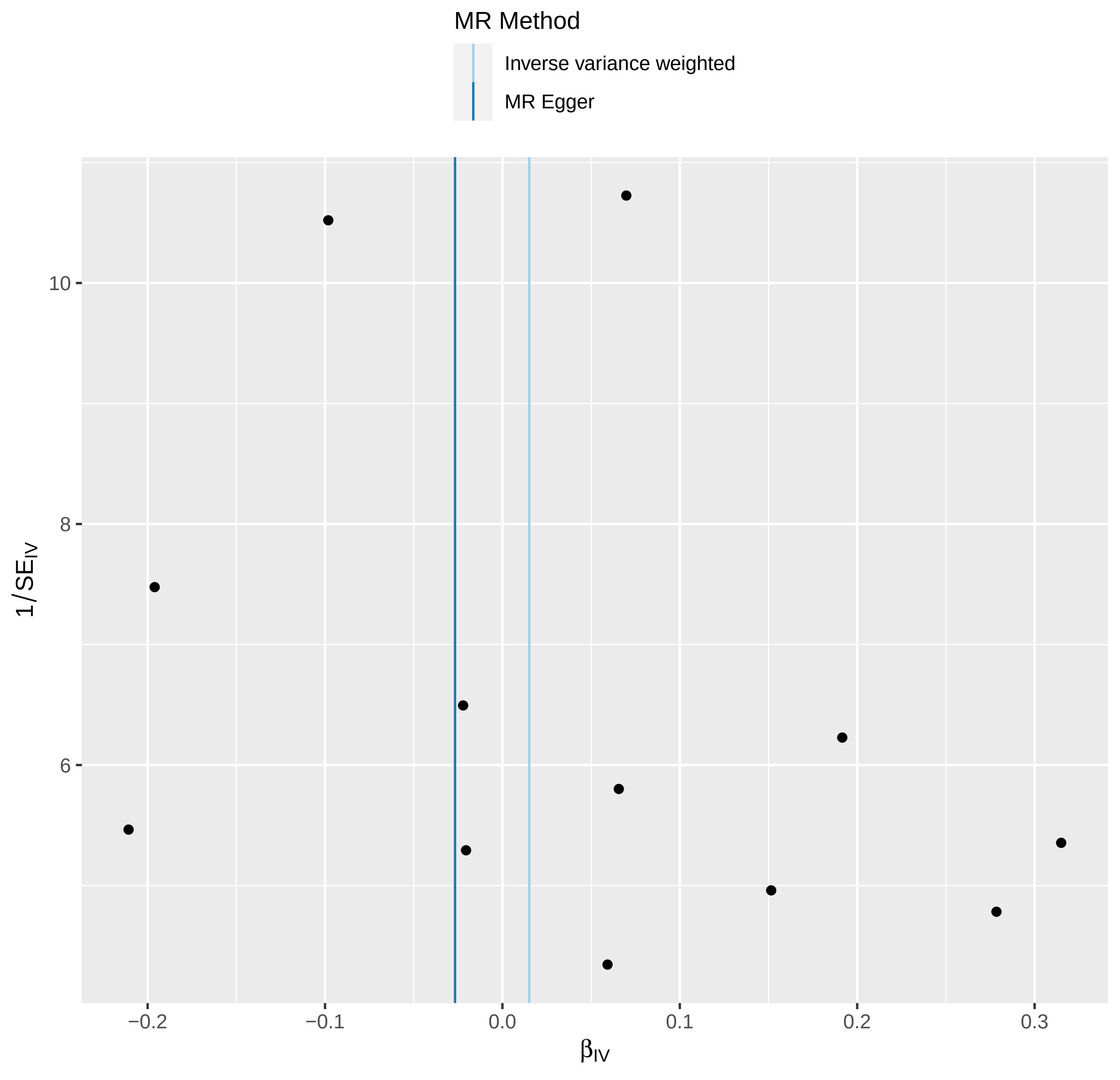

Batch 3−1778 : Selenium on Low−density lipoprotein receptor−related protein 1B

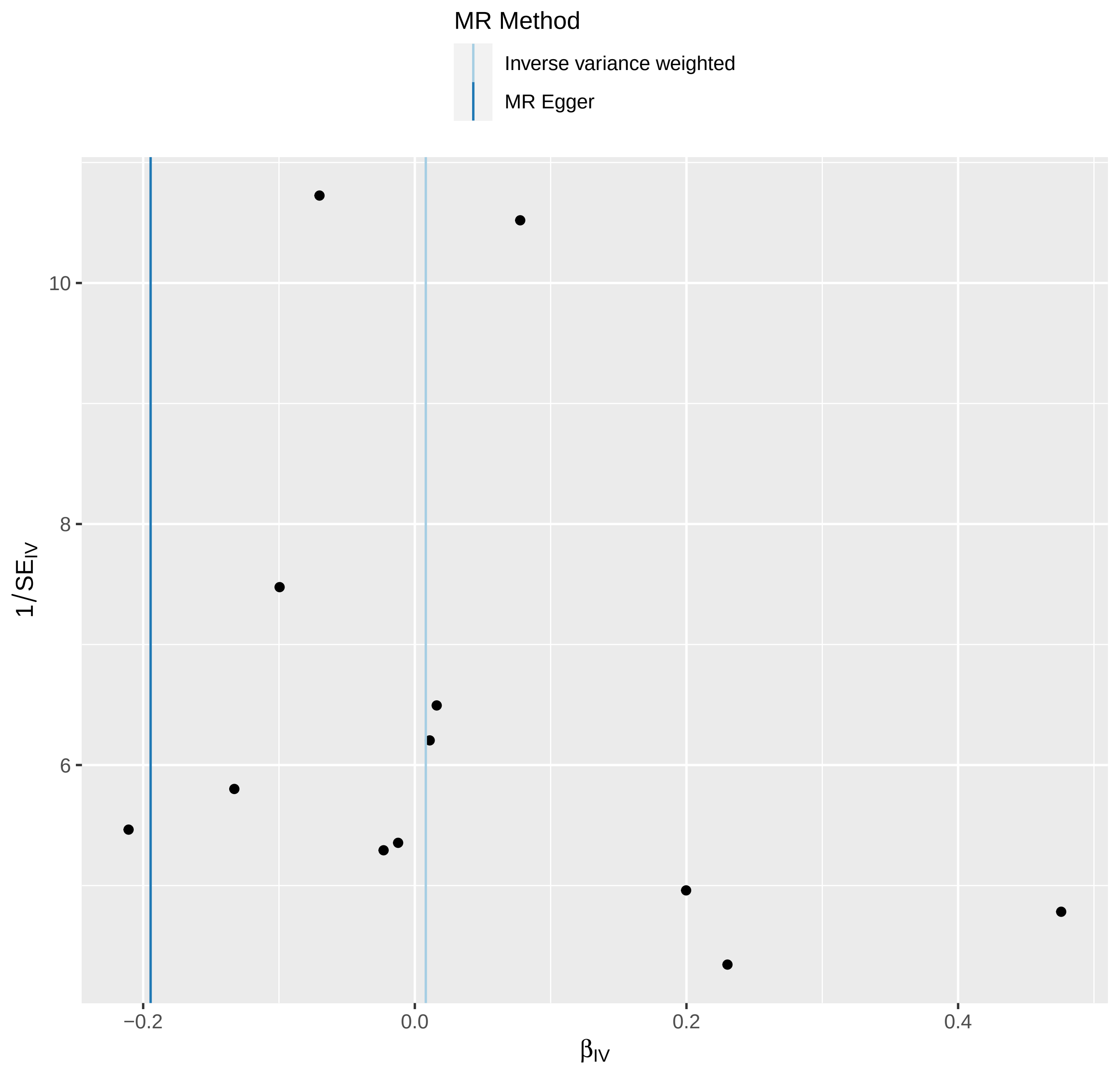

Batch 3−1779 : Selenium on Low−density lipoprotein receptor−related protein 1B

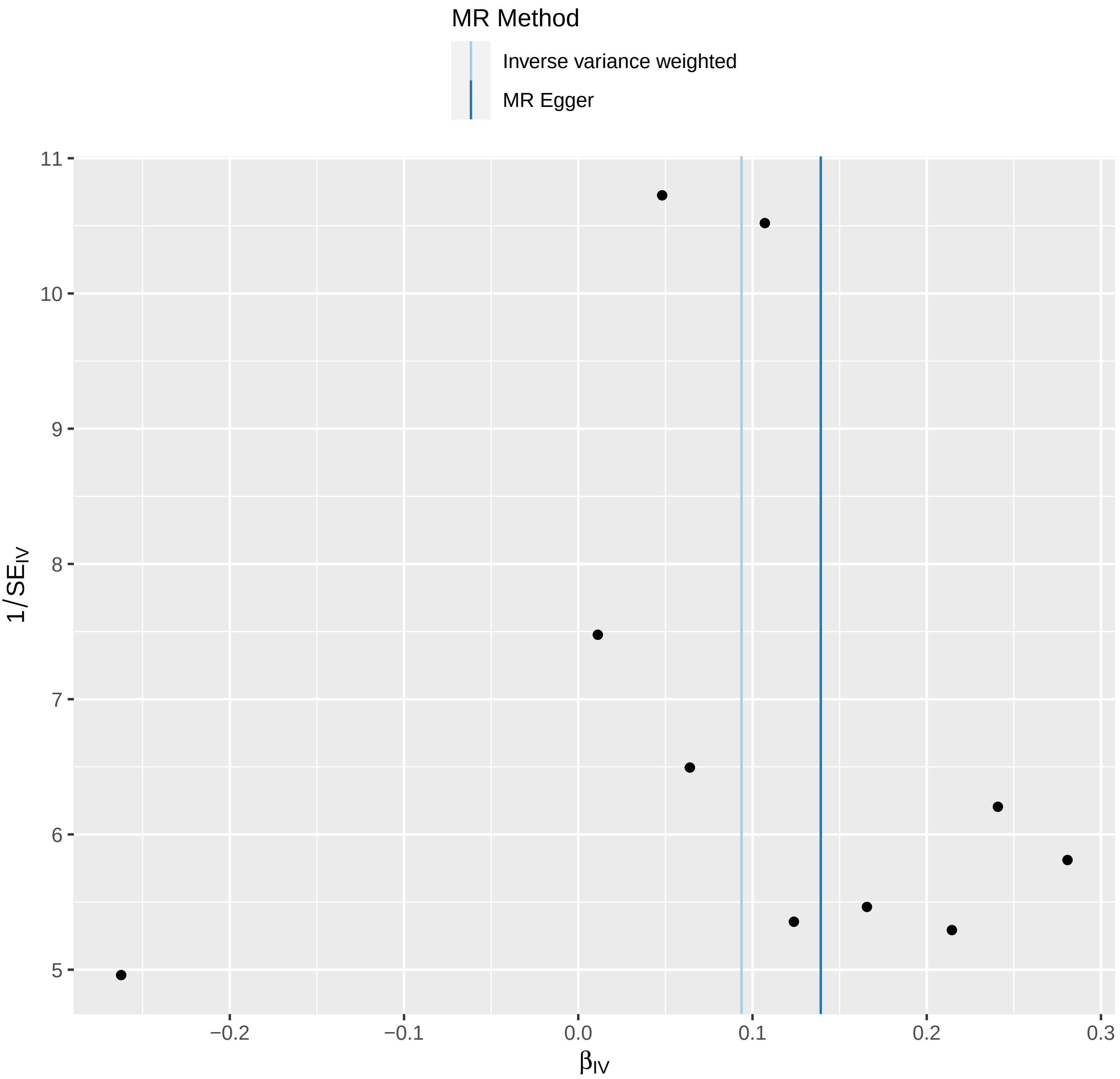

Batch 3−1780 : Selenium on Low−density lipoprotein receptor−related protein 8

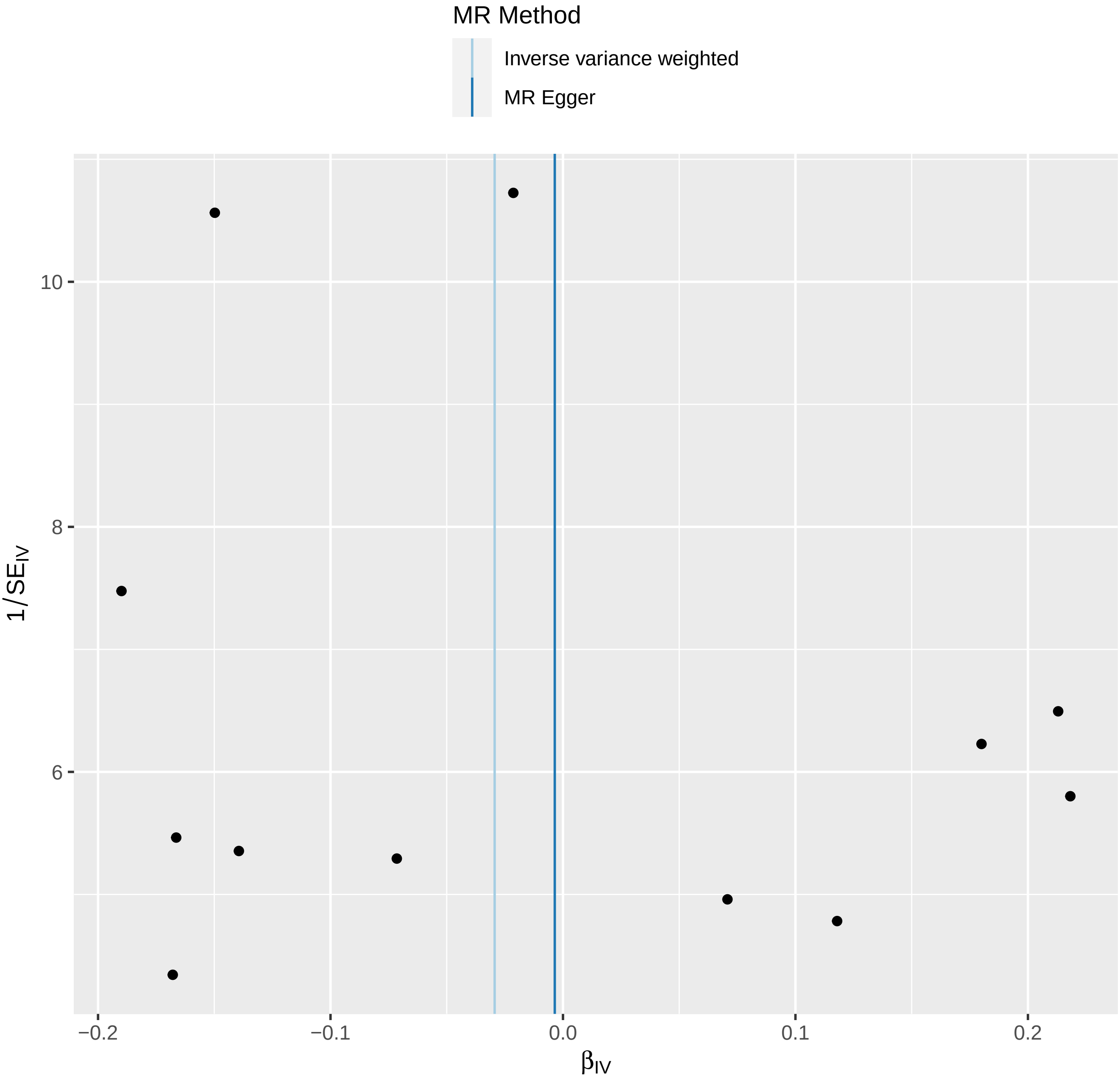

Batch 3−1781 : Selenium on alpha−2−macroglobulin receptor−associated protein

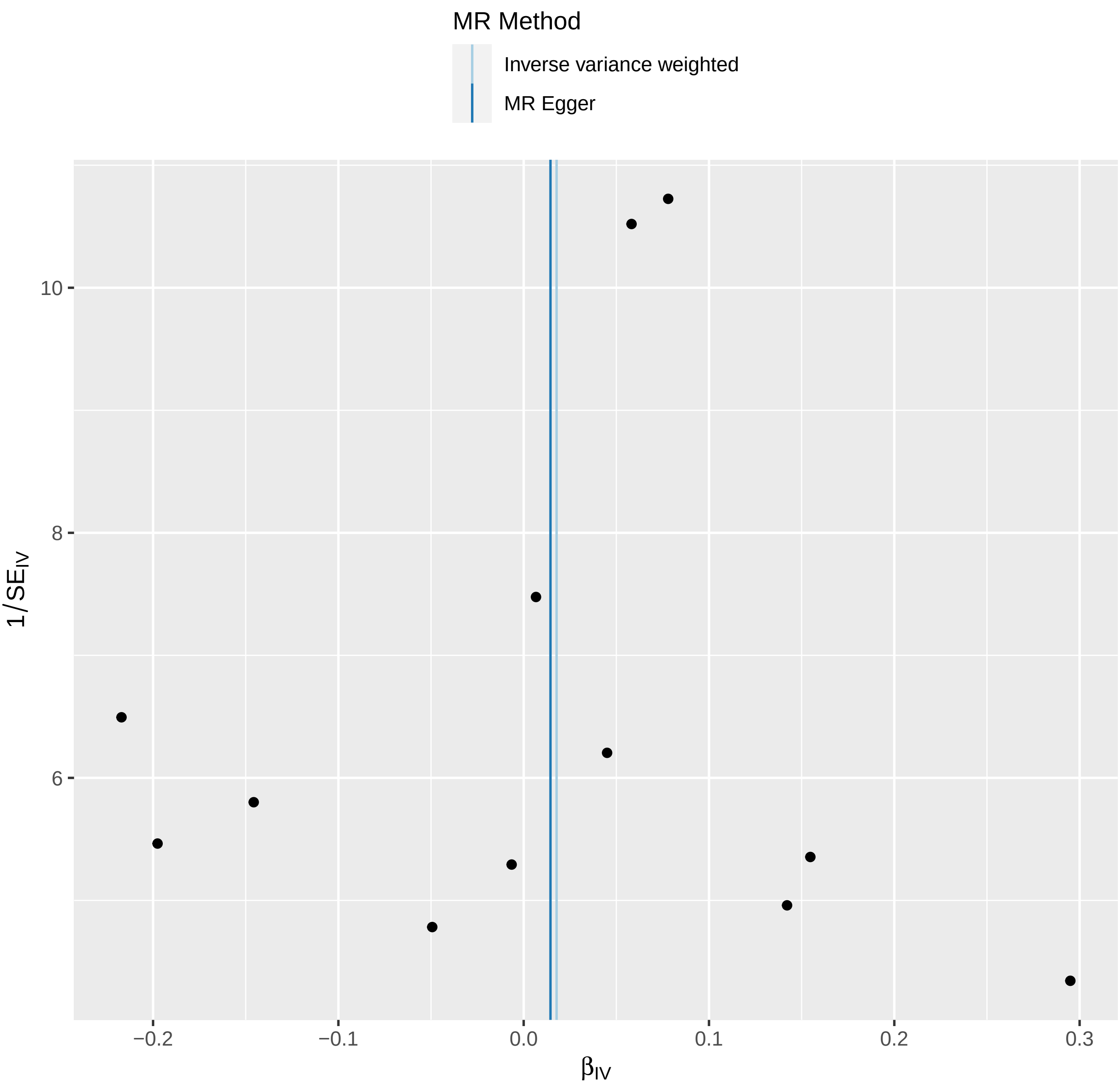

Batch 3−1782 : Selenium on alpha−2−macroglobulin receptor−associated protein

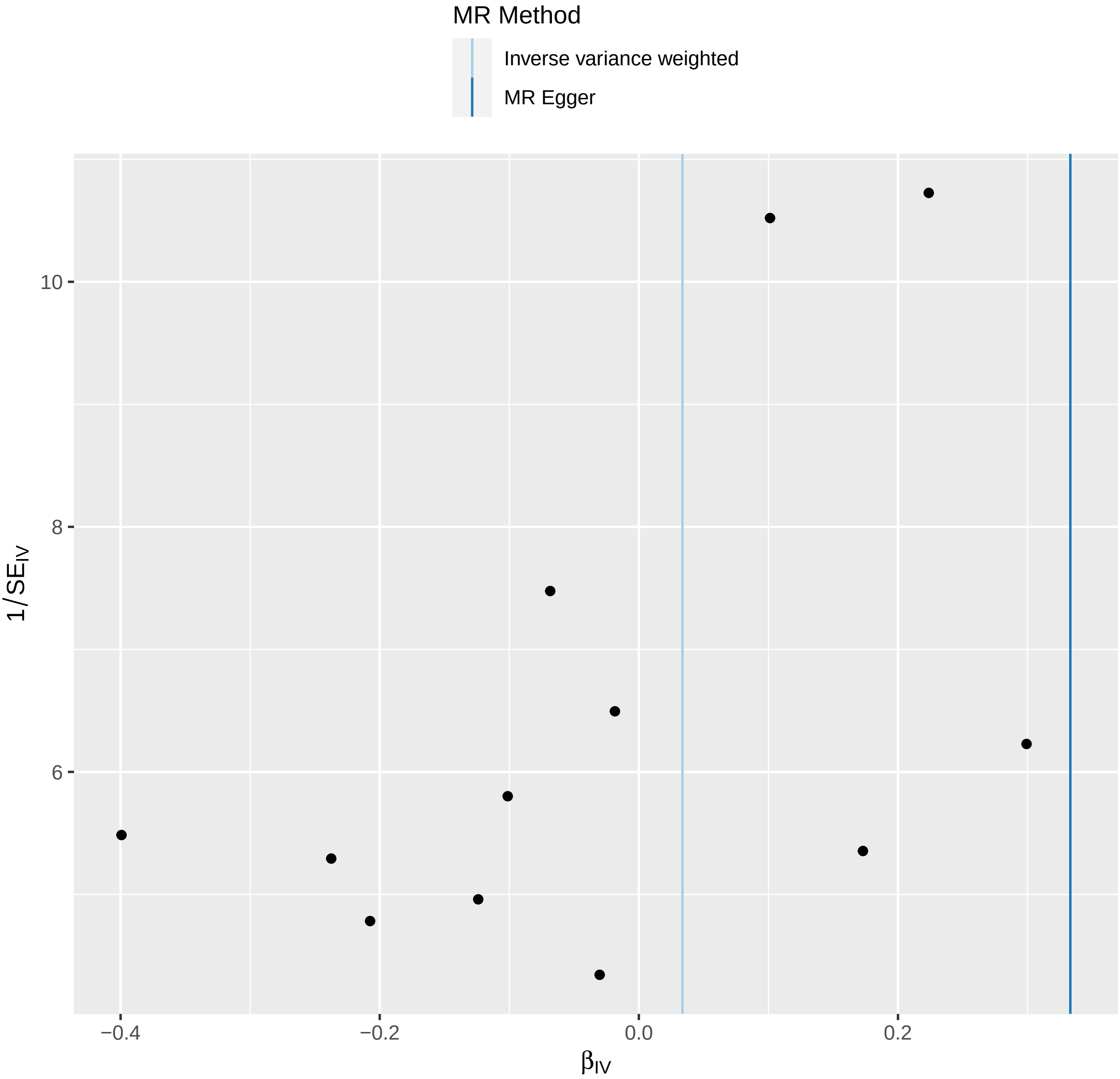

Batch 3−1783 : Selenium on Leucine−rich PPR motif−containing protein, mitochondrial

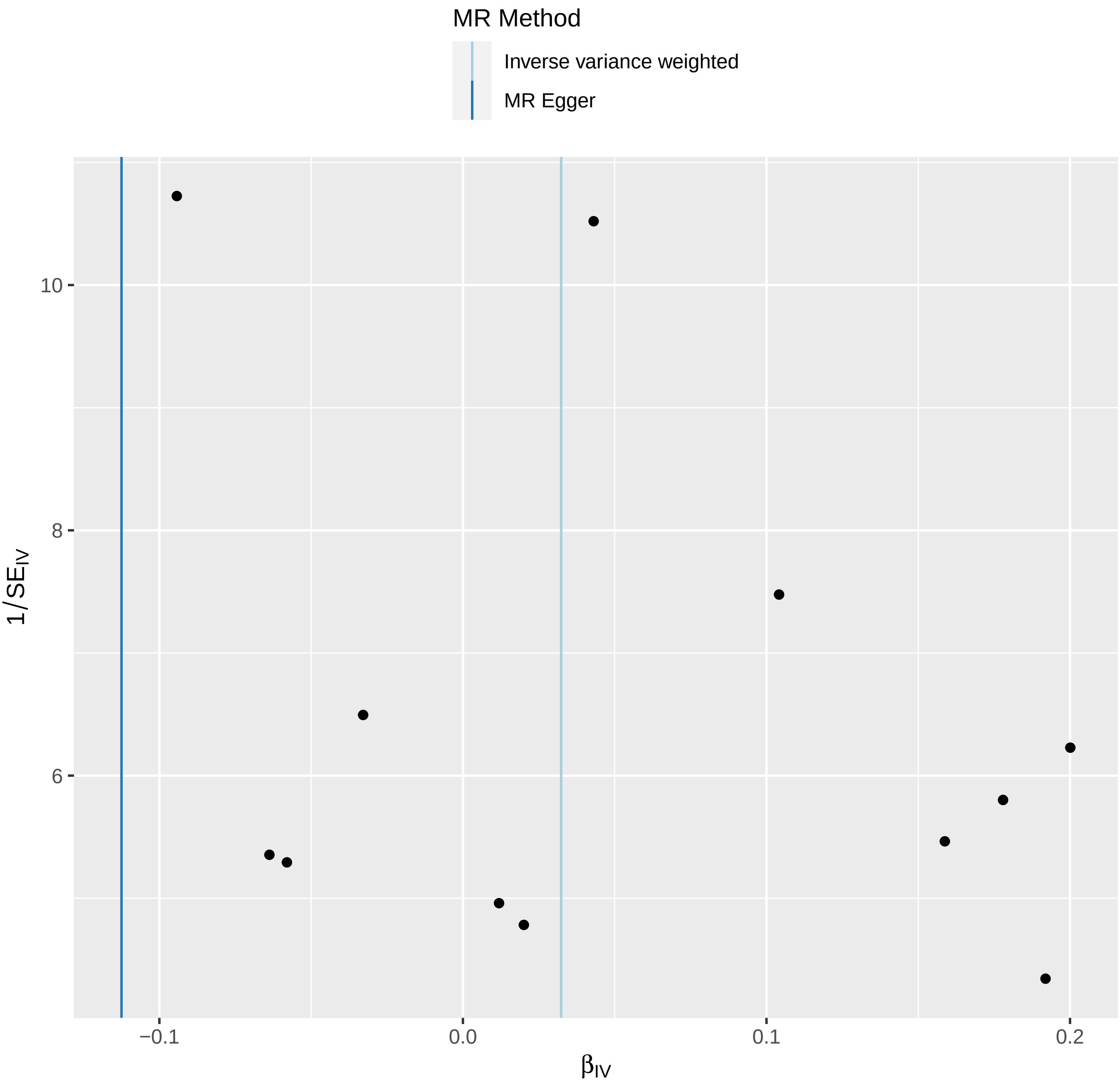

Batch 3−1784 : Selenium on Leucine−rich repeat−containing protein 15

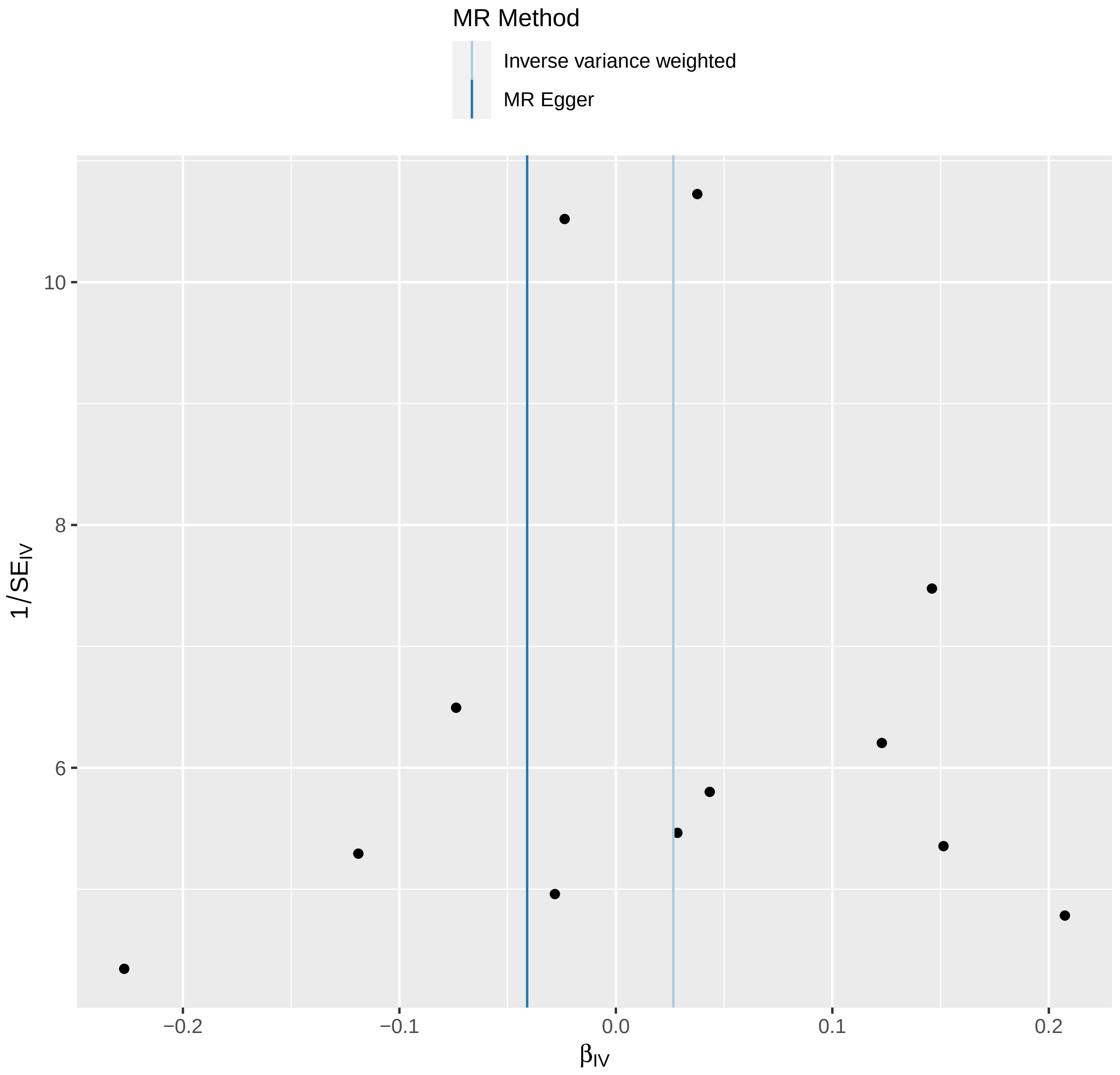

Batch 3−1785 : Selenium on Leucine−rich repeat−containing protein 19

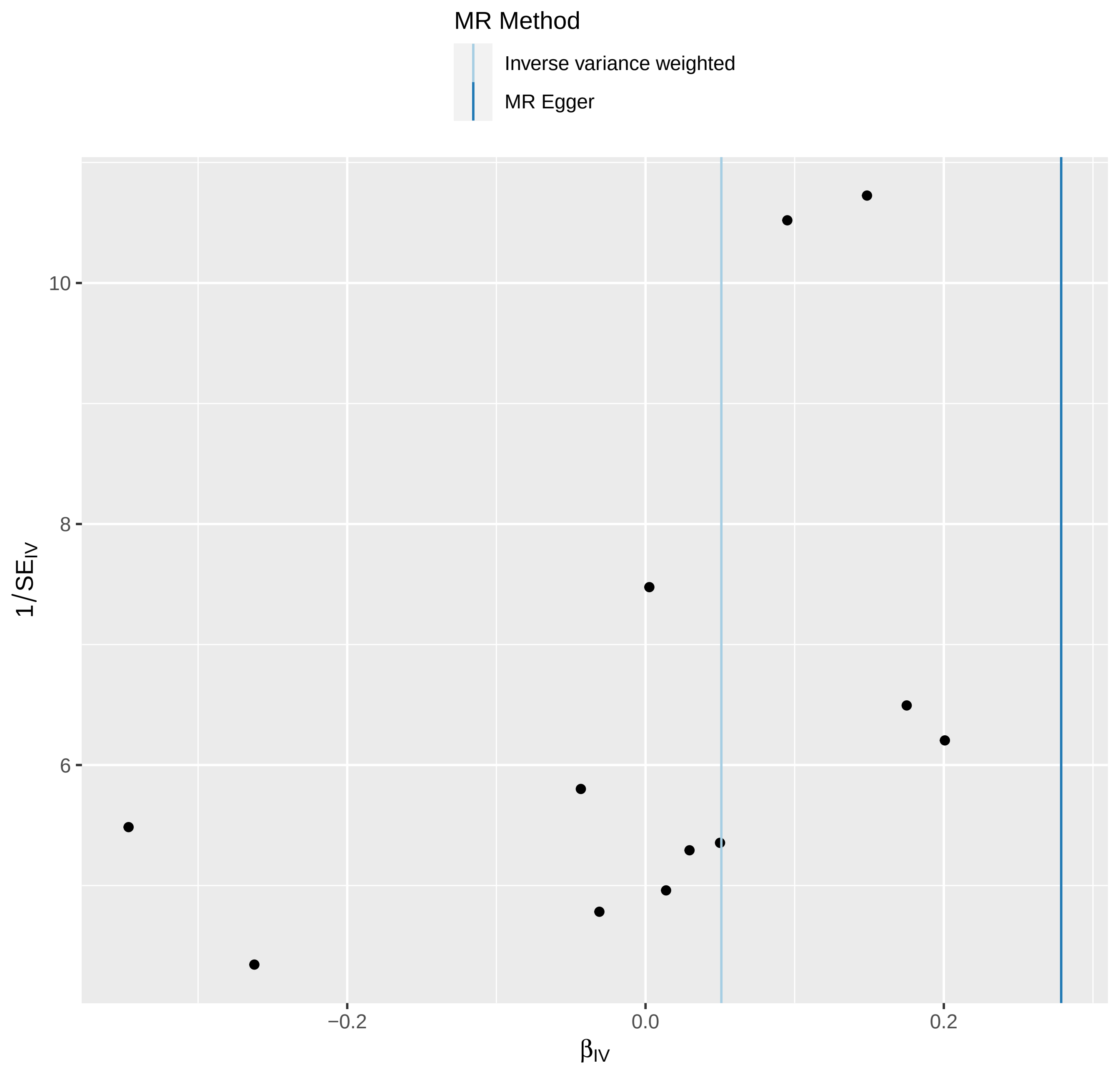

Batch 3−1786 : Selenium on Leucine−rich repeat−containing protein 3

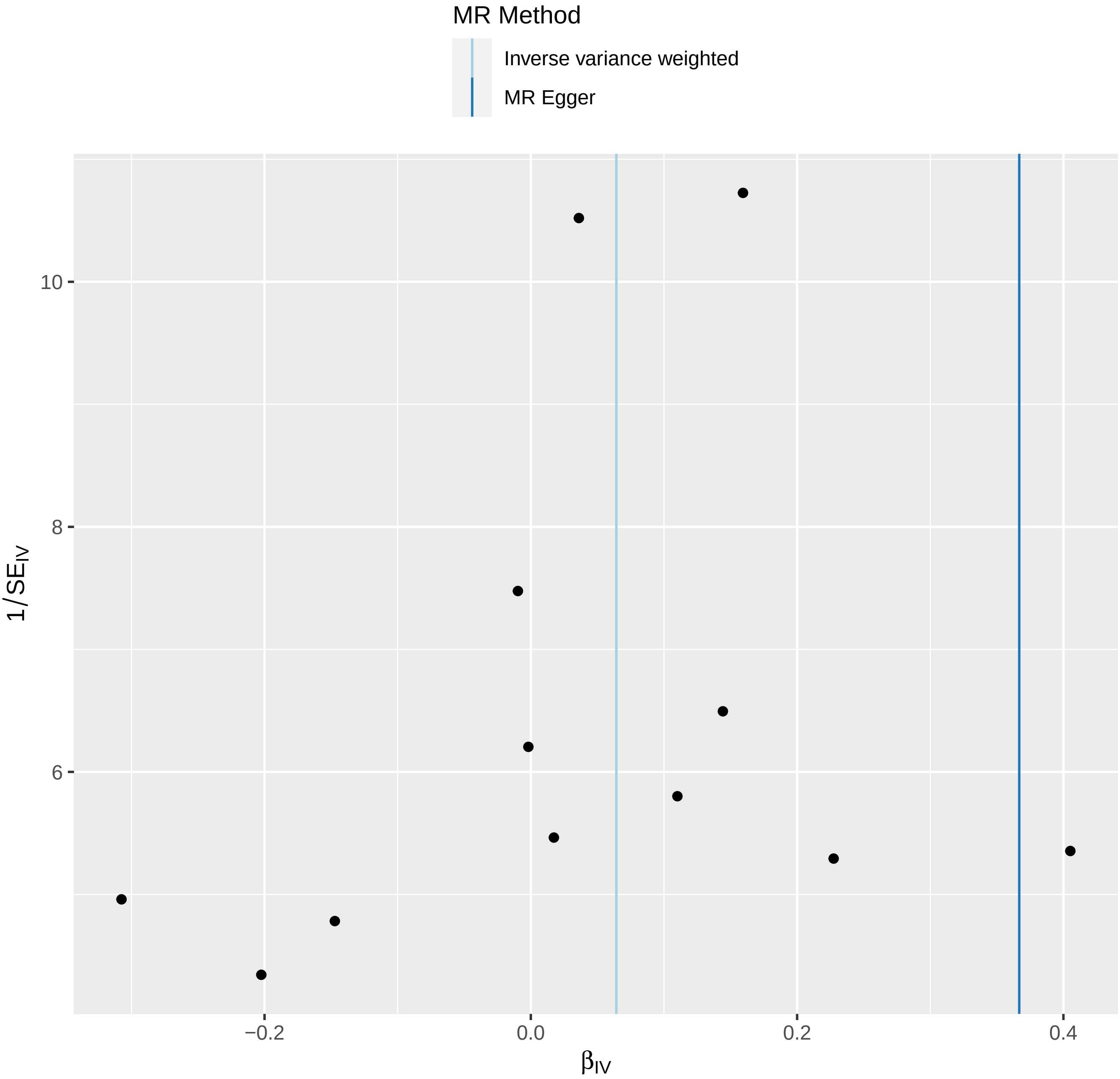

Batch 3−1787 : Selenium on Leucine−rich repeat−containing protein 37A2

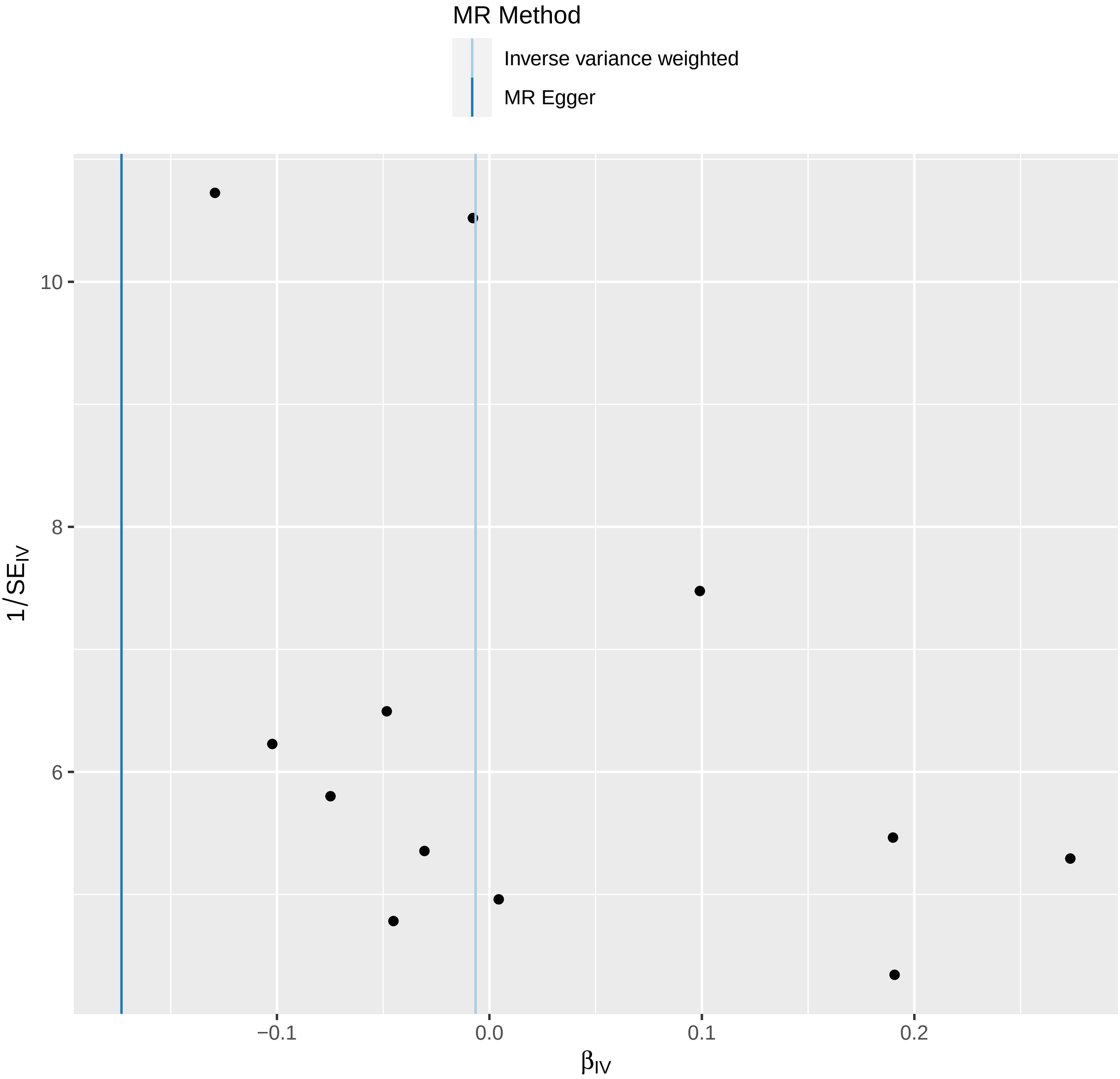

Batch 3−1788 : Selenium on Leucine−rich repeat−containing protein 4B

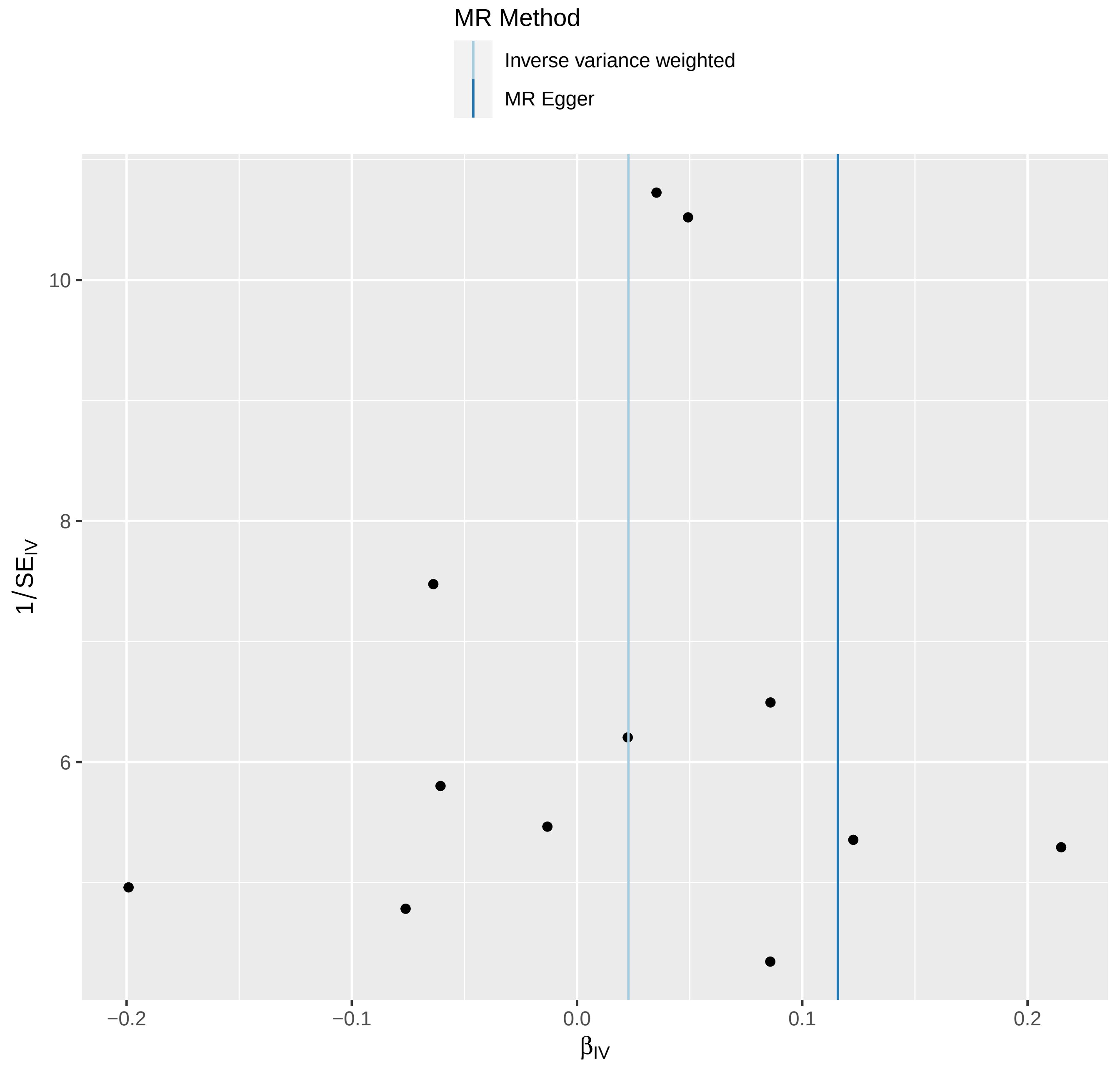

Batch 3−1789 : Selenium on Leucine−rich repeat−containing protein 4C

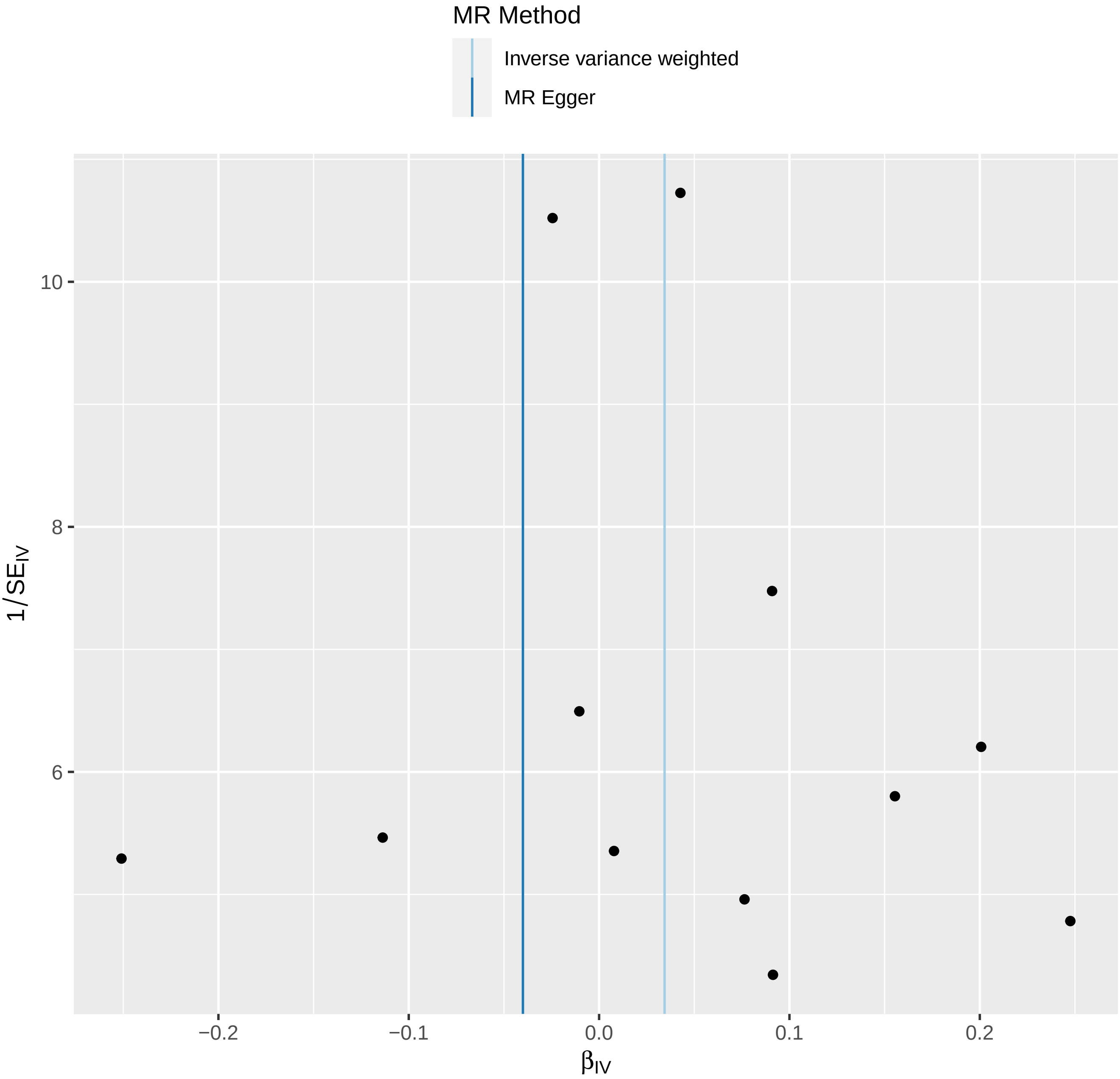

Batch 3−1790 : Selenium on Leucine−rich repeat−containing protein 74A

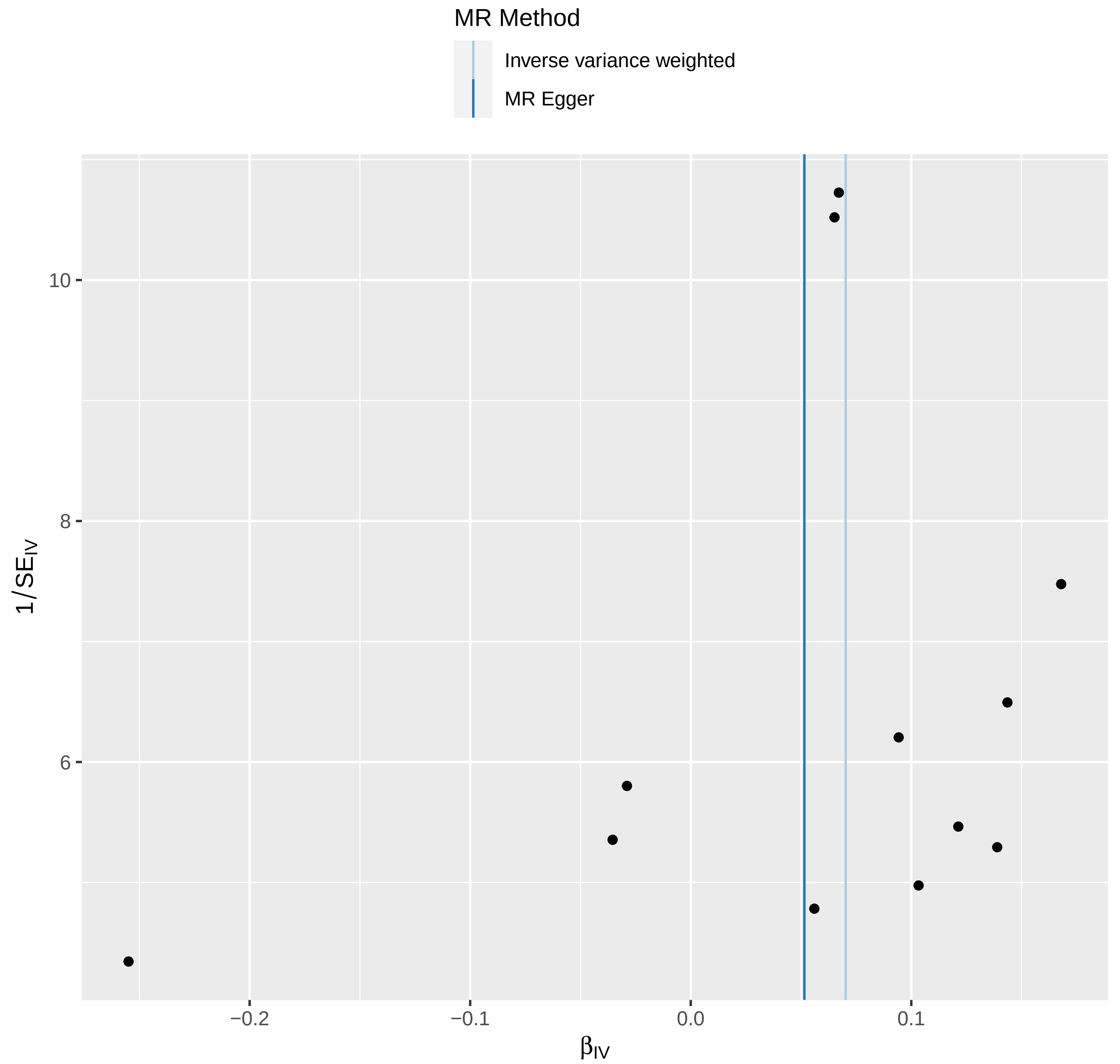

Batch 3−1791 : Selenium on Leucine−rich repeat serine/threonine−protein kinase 2

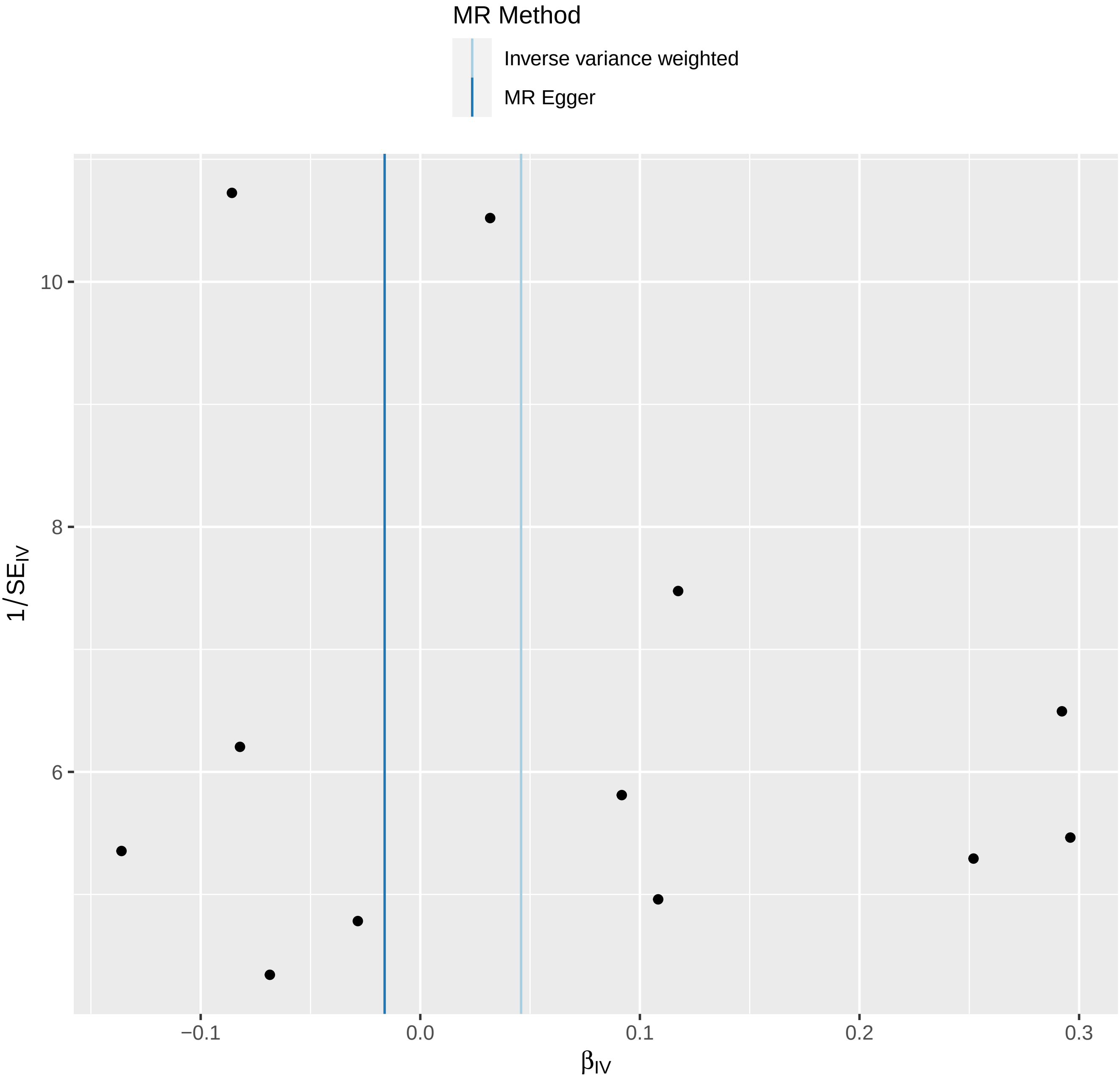

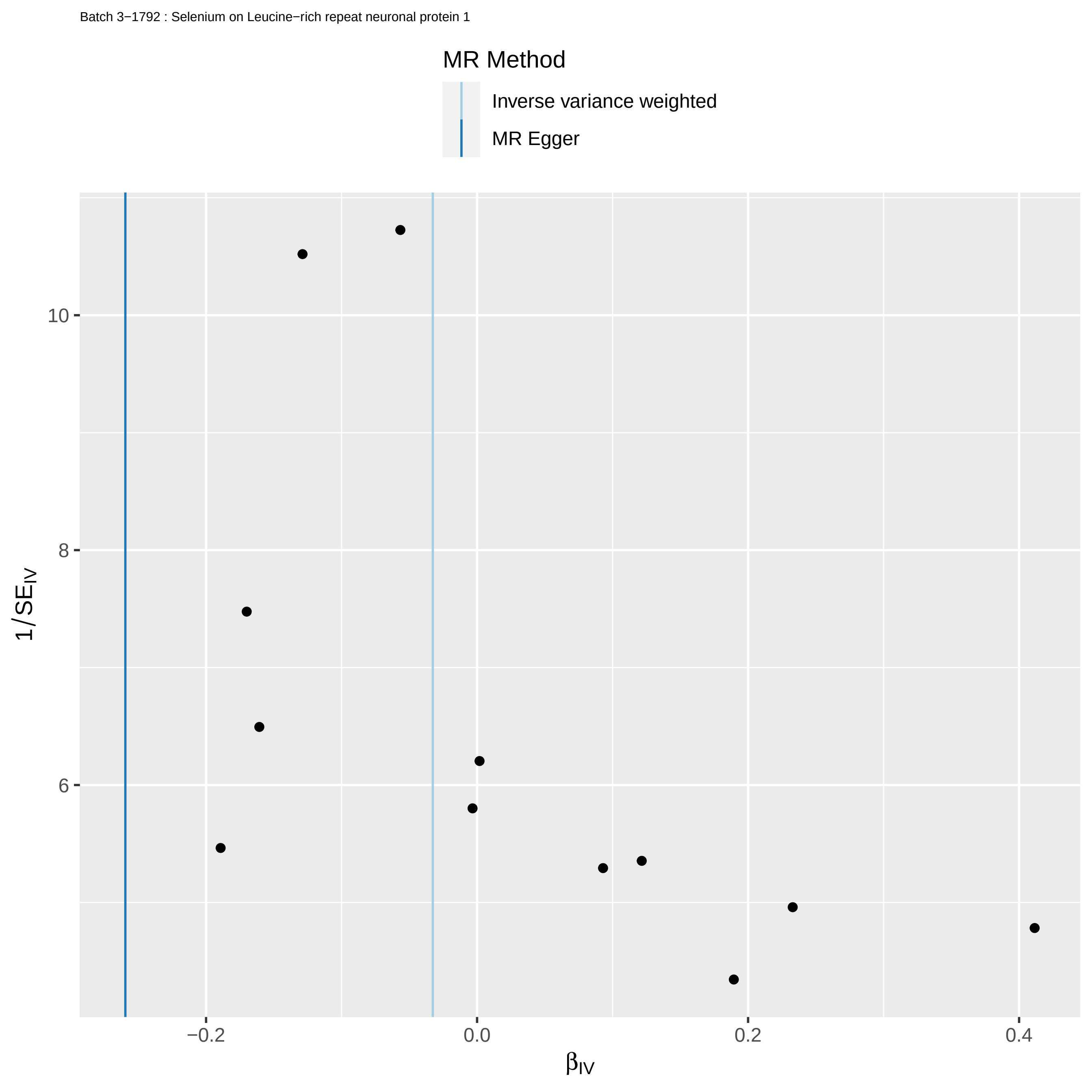

Batch 3−1792 : Selenium on Leucine−rich repeat neuronal protein 1
MR Method
Inverse variance weighted
MR Egger
10
8
6
−0.2
0.0
0.2
0.4
1/SE_IV
β_IV

Batch 3−1793 : Selenium on Leucine−rich repeat neuronal protein 1

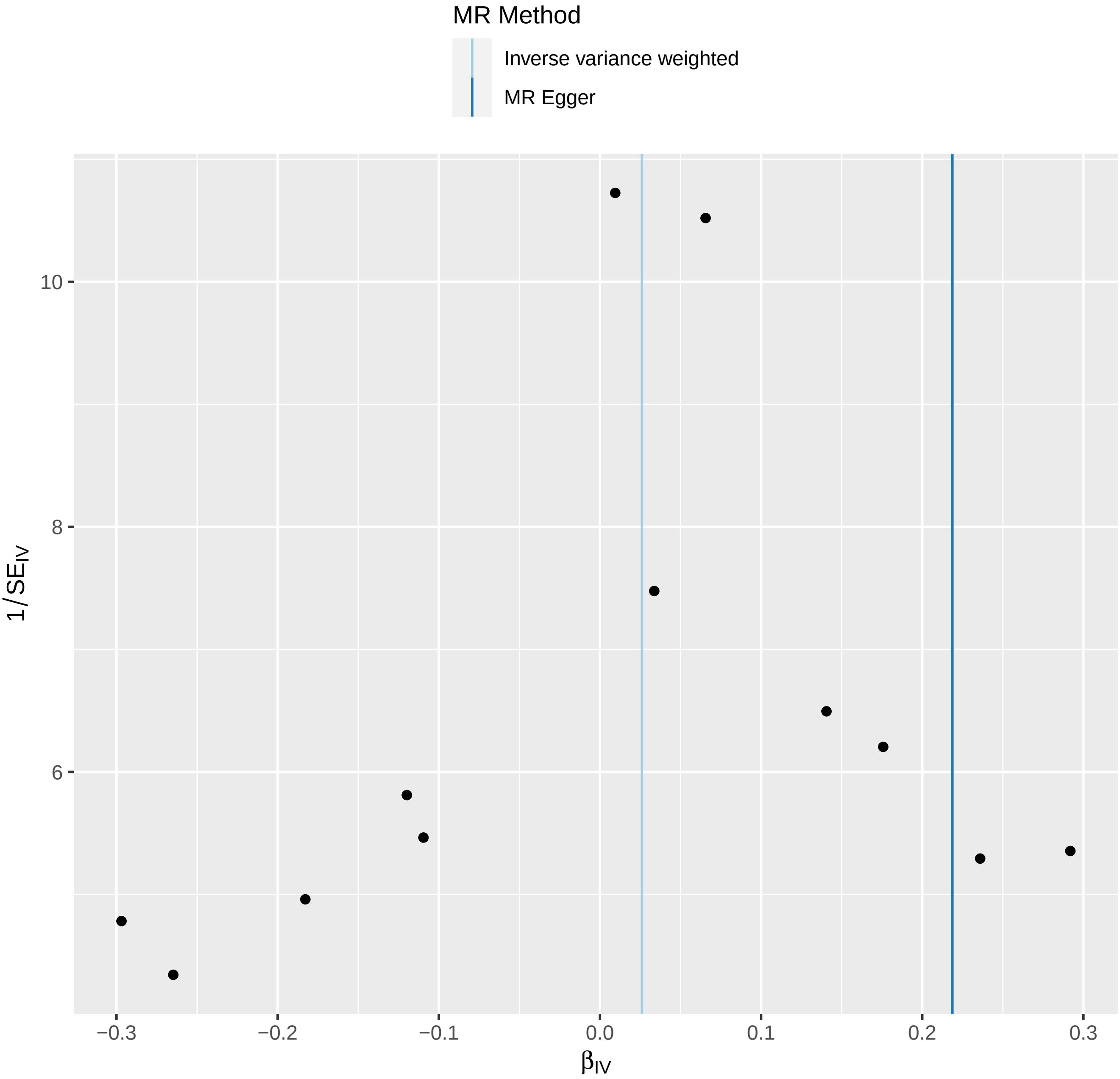

Batch 3−1794 : Selenium on Leucine−rich repeat neuronal protein 3

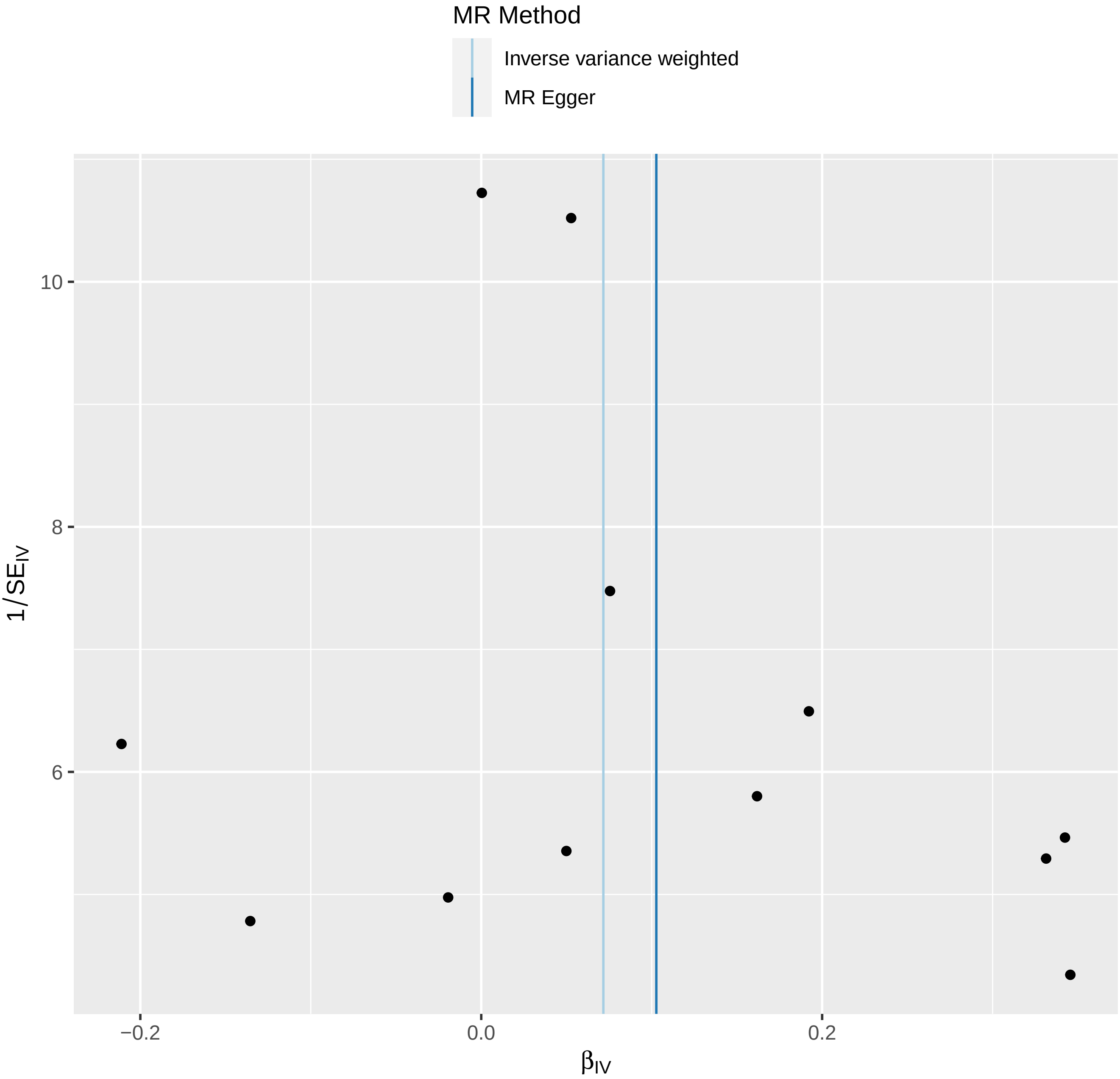

Batch 3−1795 : Selenium on Leucine−rich repeat transmembrane neuronal protein 1

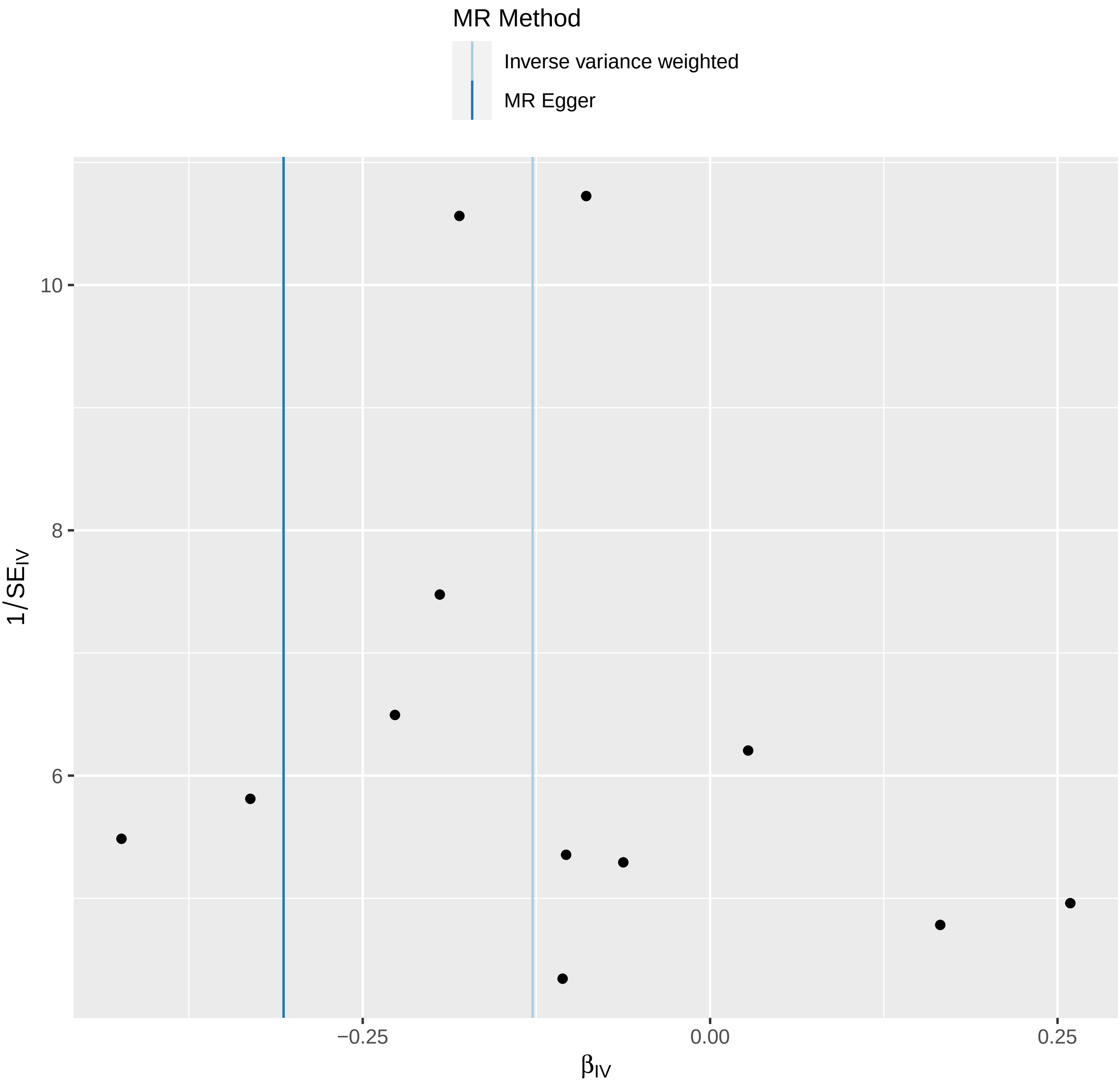

Batch 3−1796 : Selenium on Leucine−rich repeat transmembrane neuronal protein 2

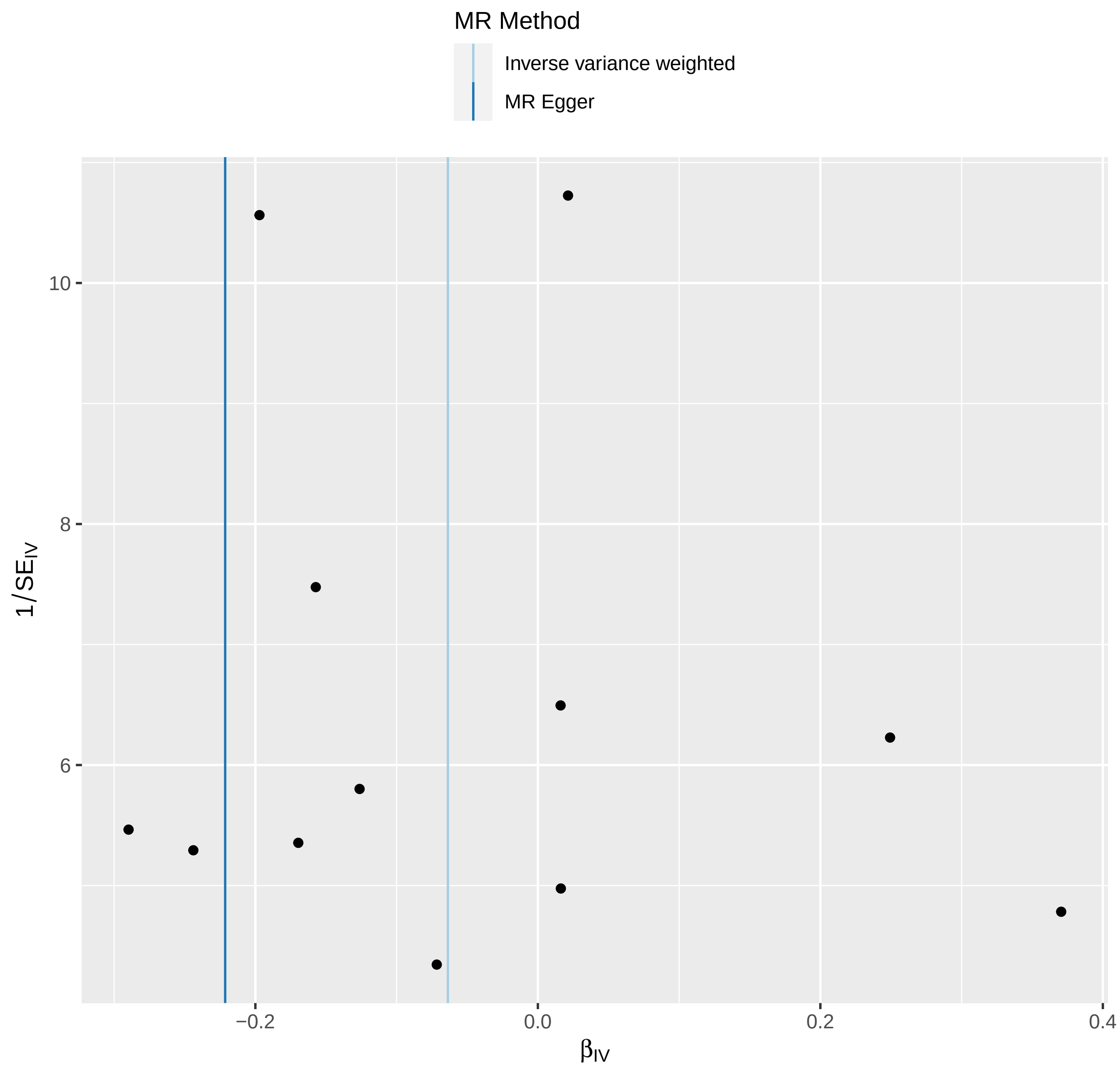

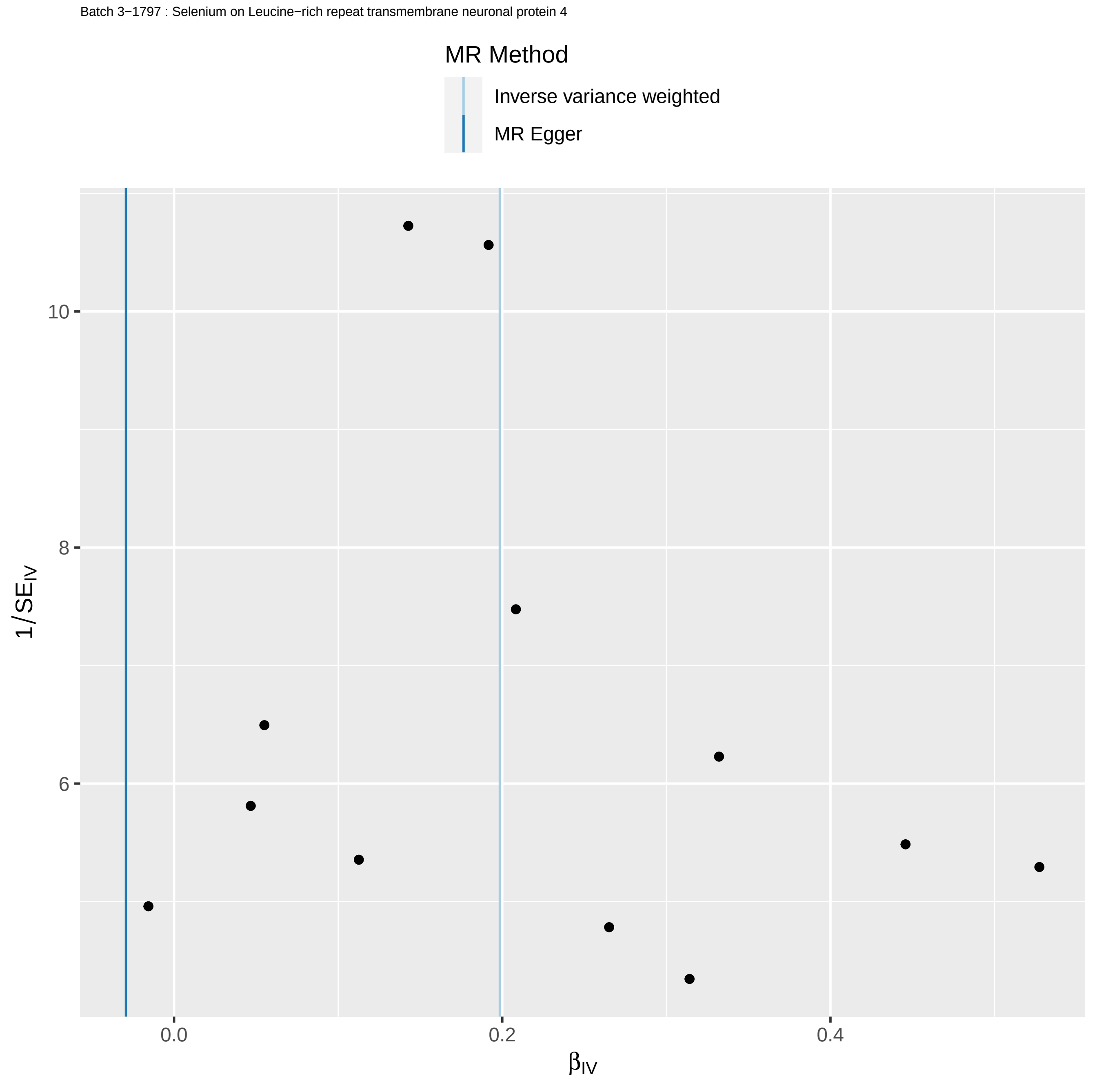

Batch 3−1797 : Selenium on Leucine−rich repeat transmembrane neuronal protein 4
MR Method
Inverse variance weighted
MR Egger
10
8
6
0.0
0.2
0.4
1/SE$_{IV}$
β$_{IV}$

Batch 3−1798 : Selenium on Leucine−rich repeat transmembrane neuronal protein 4

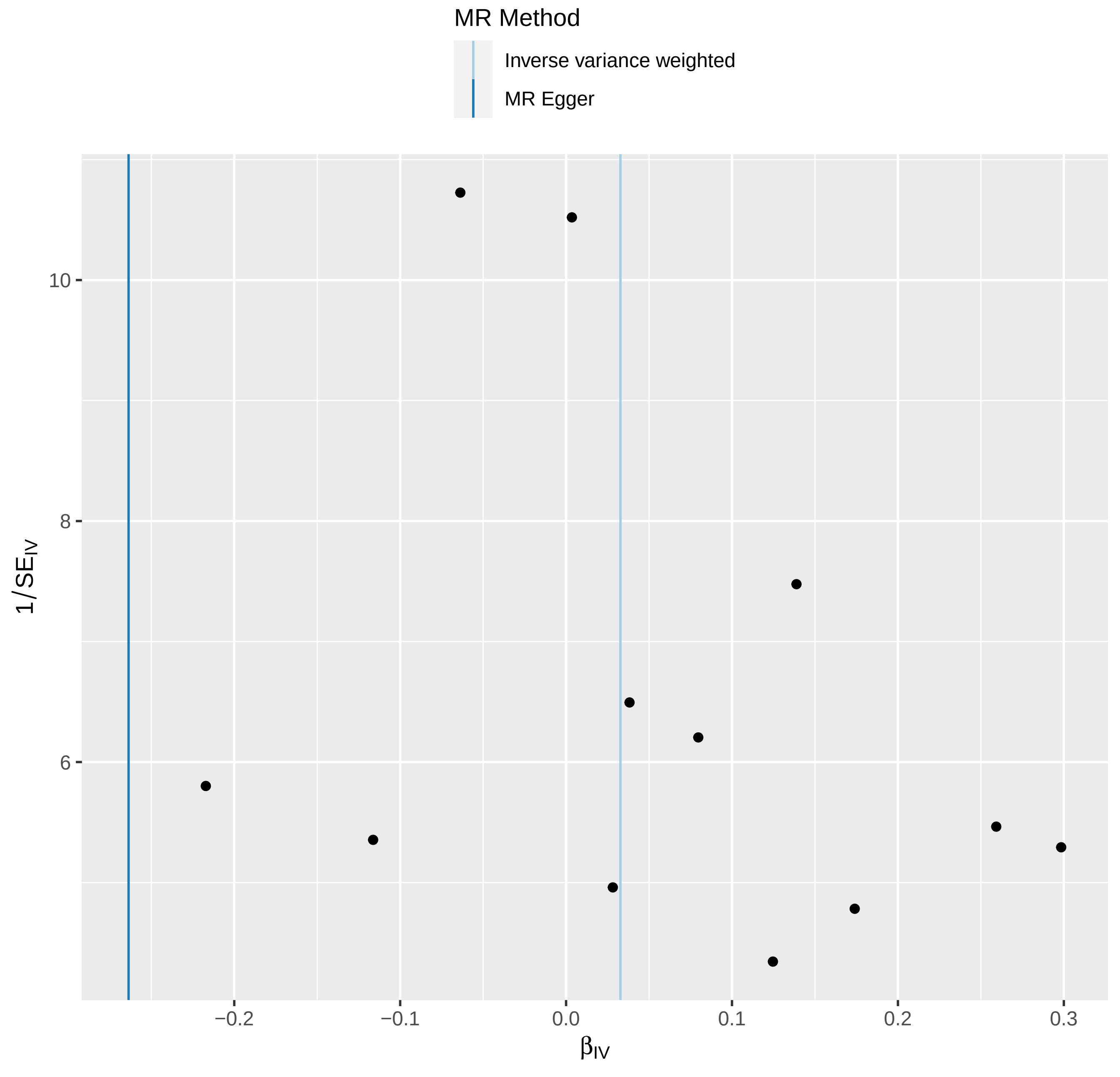

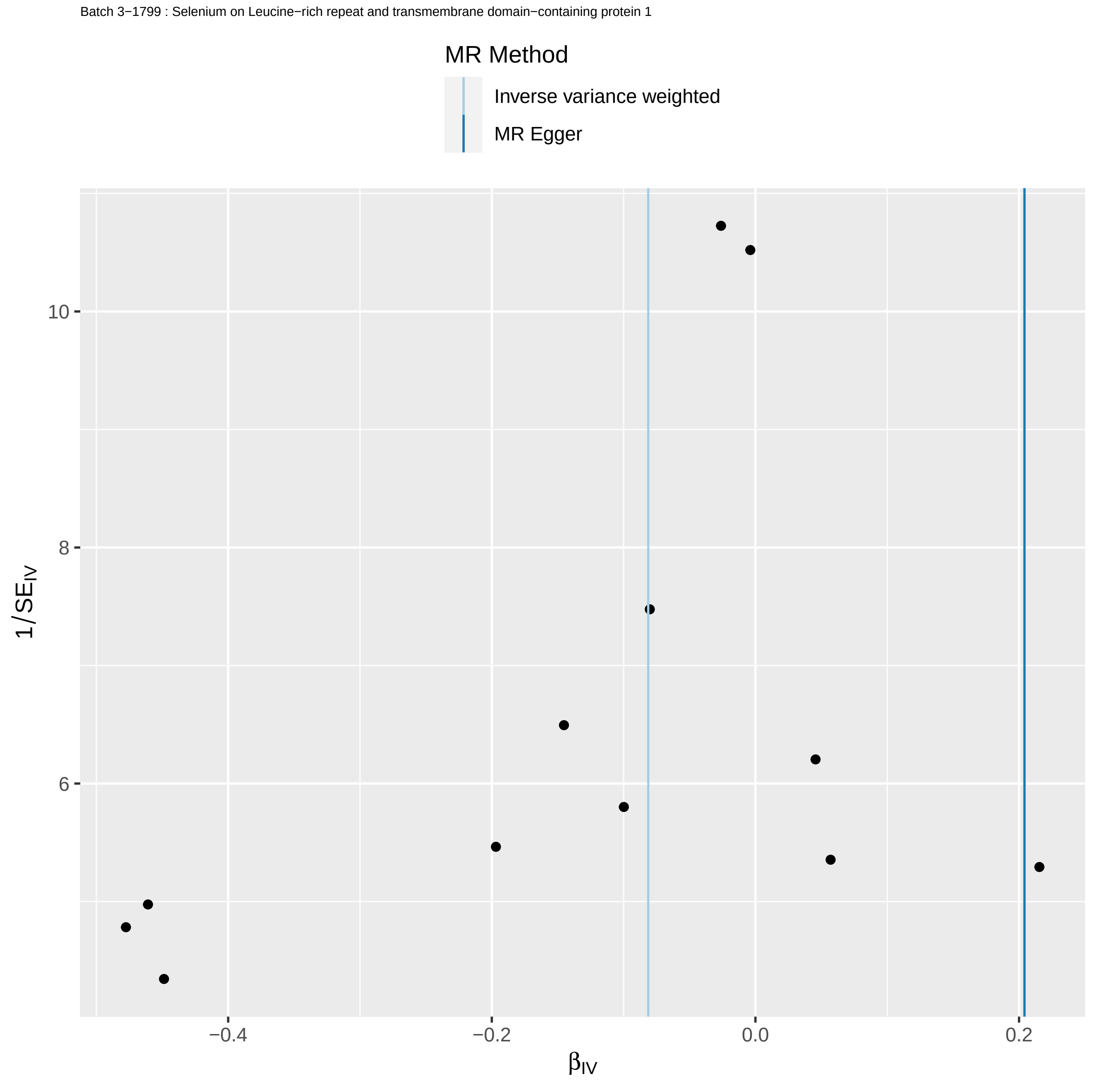

Batch 3−1799 : Selenium on Leucine−rich repeat and transmembrane domain−containing protein 1
MR Method
Inverse variance weighted
MR Egger
10
8
6
1/SE_IV
−0.4
−0.2
0.0
0.2
β_IV

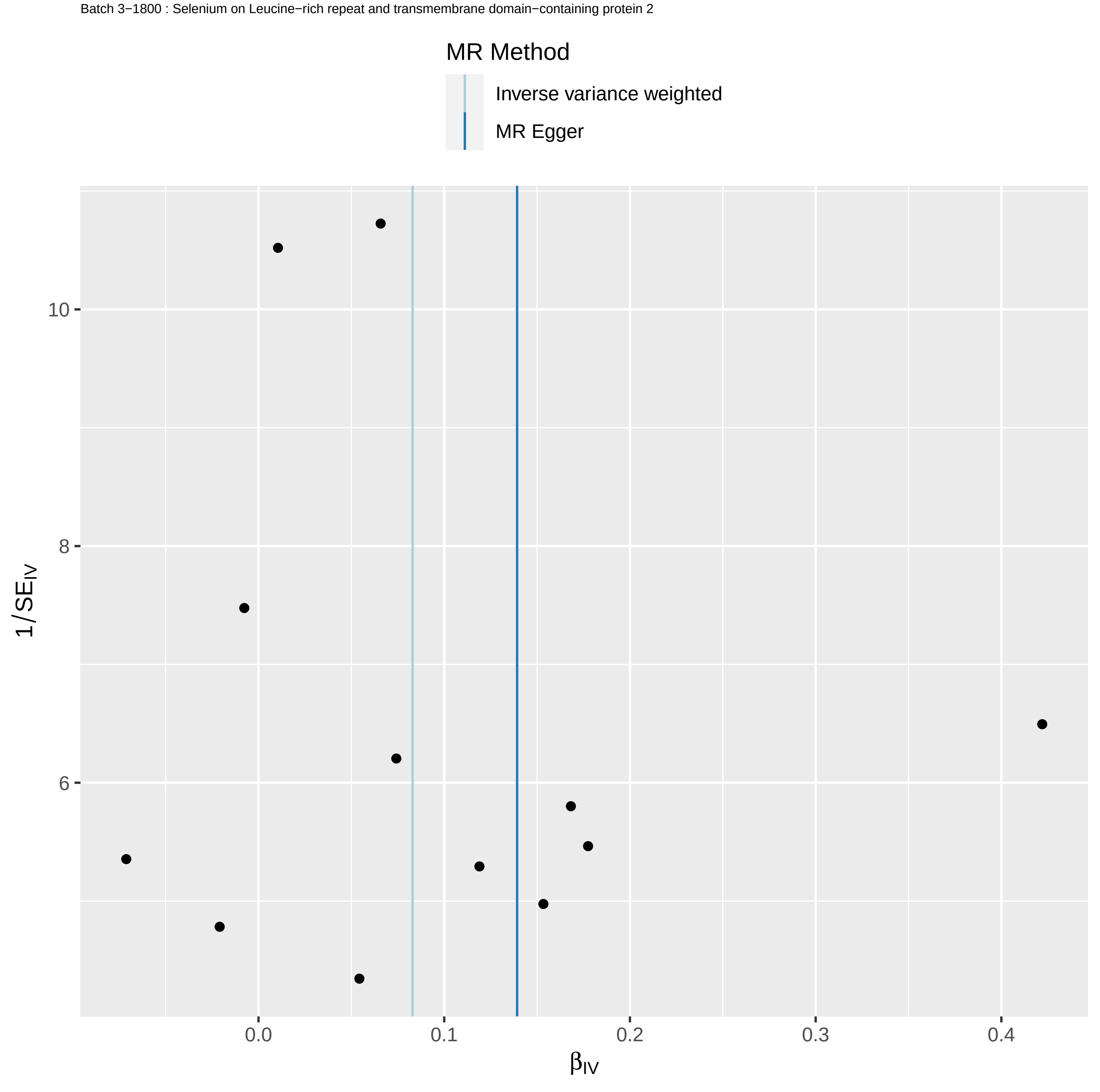

Batch 3−1800 : Selenium on Leucine−rich repeat and transmembrane domain−containing protein 2
MR Method
Inverse variance weighted
MR Egger
10
8
6
1/SE$_{IV}$
0.0
0.1
0.2
0.3
0.4
β$_{IV}$

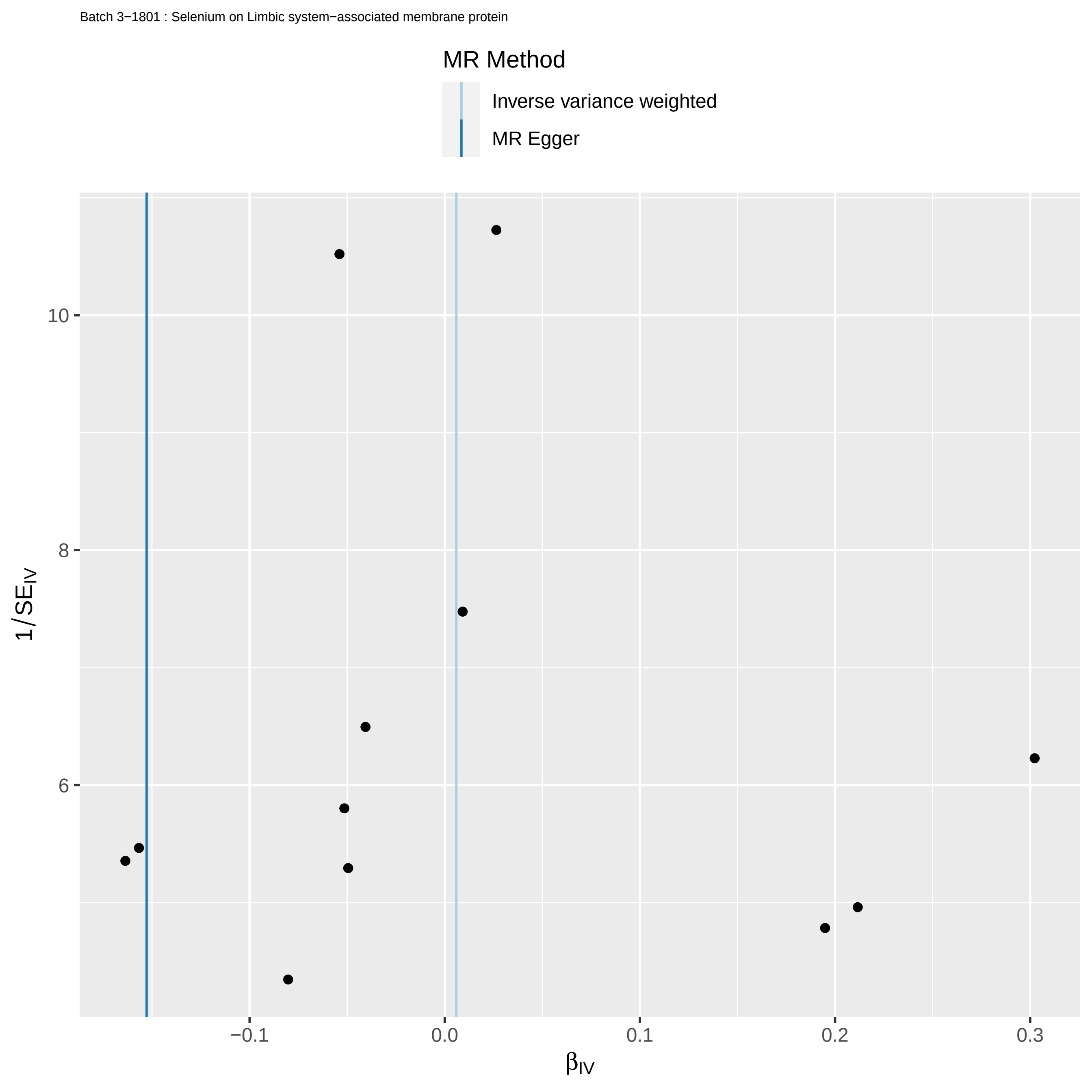
Batch 3−1801 : Selenium on Limbic system−associated membrane protein
MR Method
Inverse variance weighted
MR Egger
10
8
6
1/SE_IV
−0.1
0.0
0.1
0.2
0.3
β_IV

Batch 3−1802 : Selenium on Leukocyte−specific transcript 1 protein

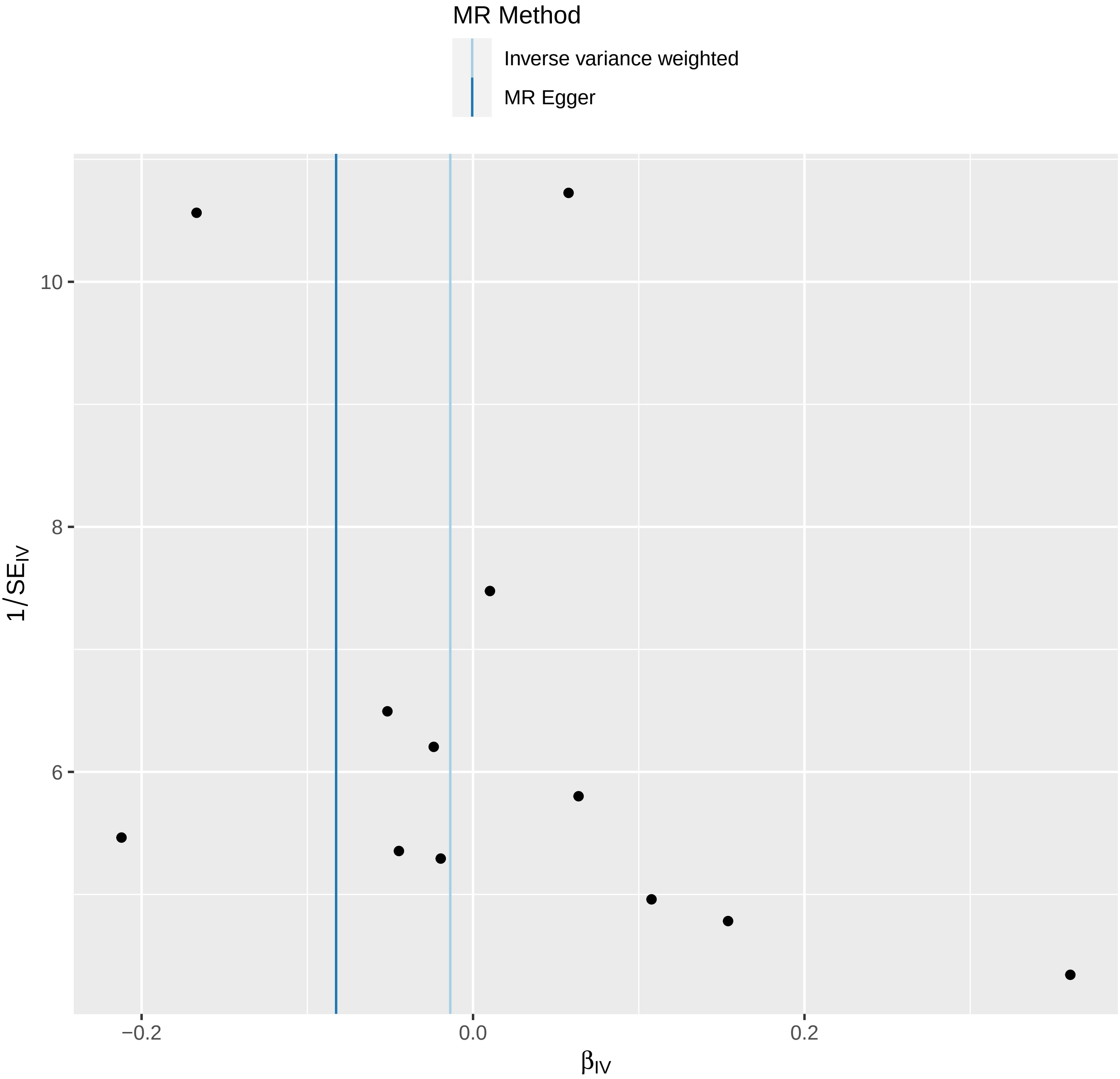

Batch 3−1803 : Selenium on Lymphotoxin−alpha

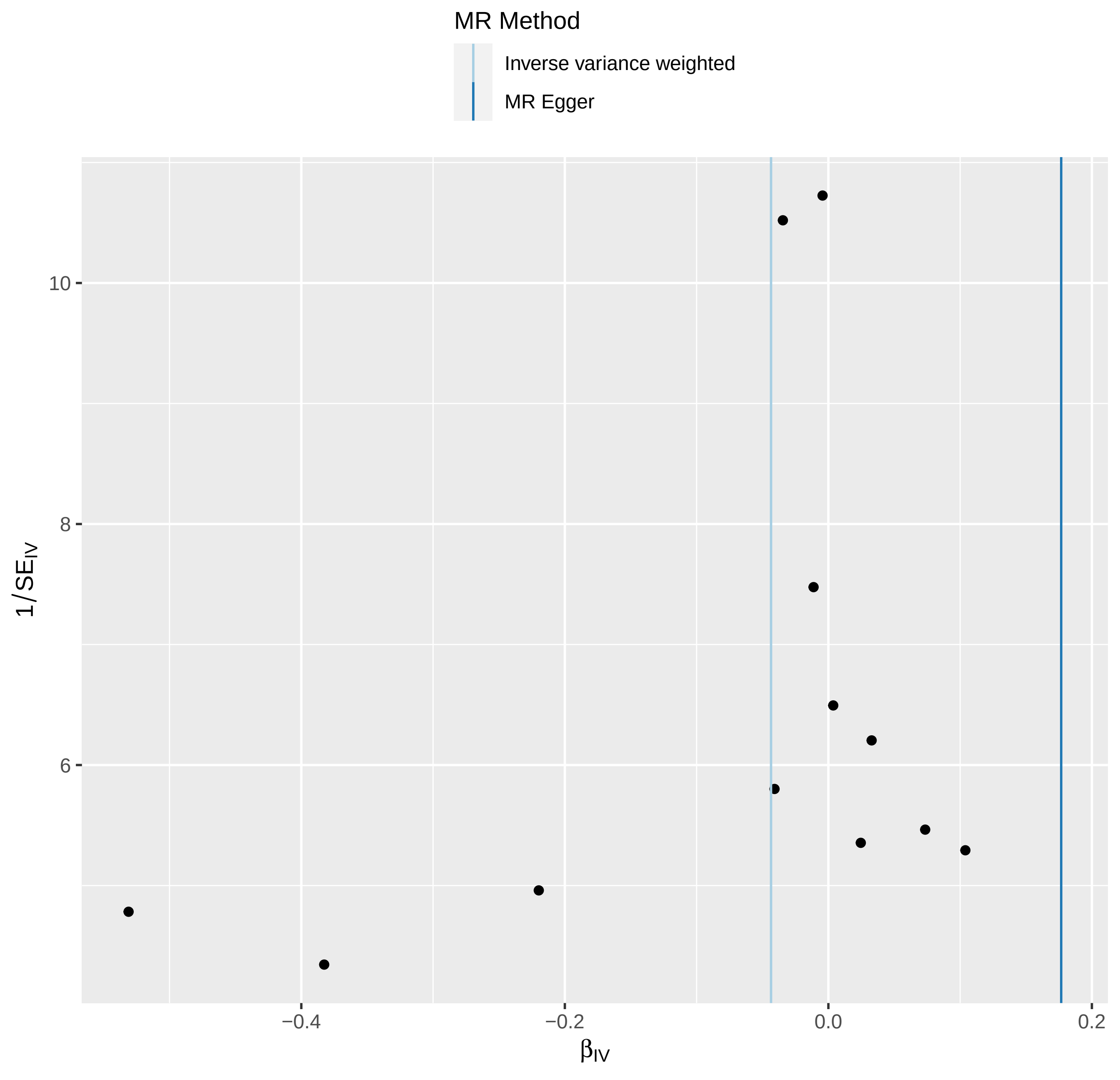

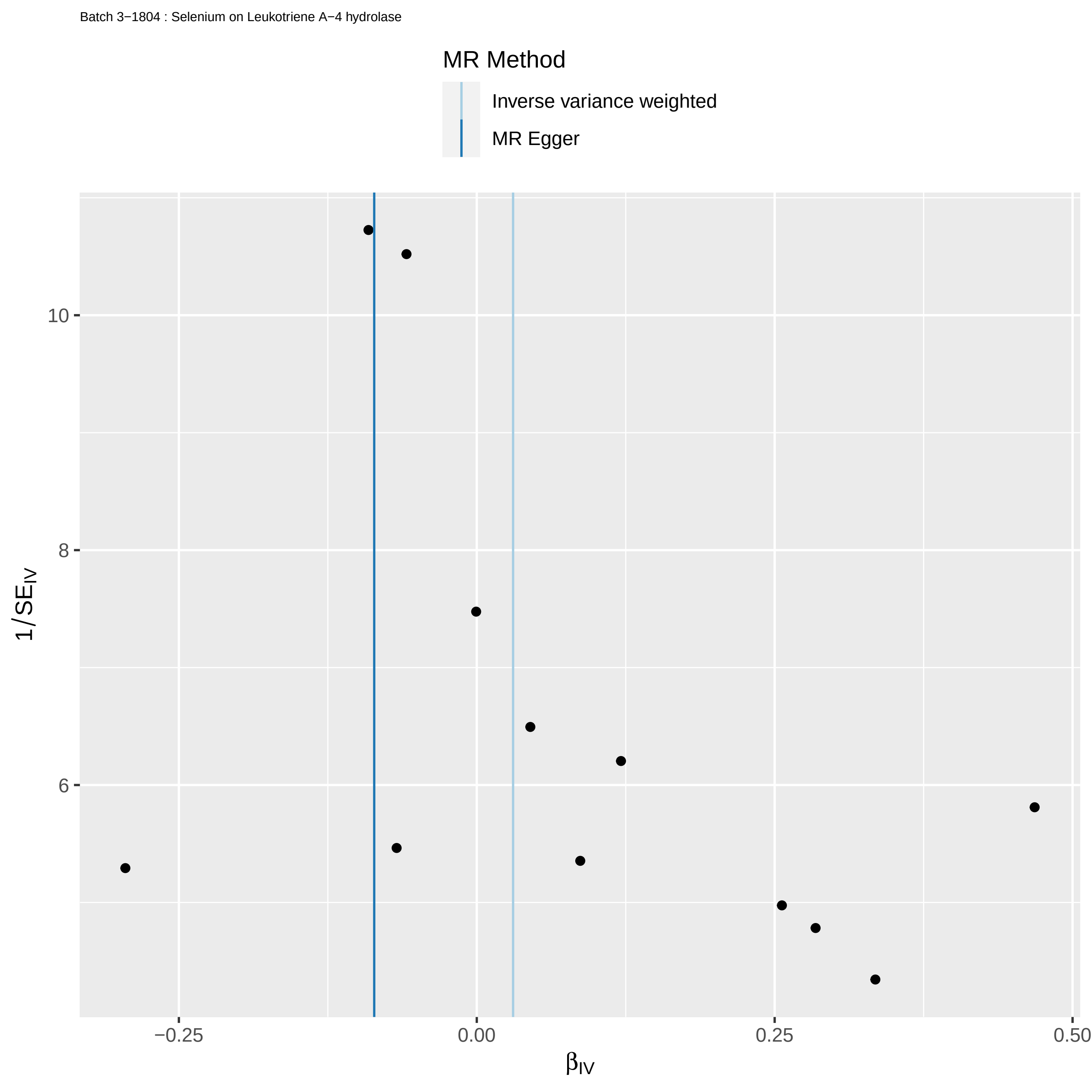
Batch 3−1804 : Selenium on Leukotriene A−4 hydrolase
MR Method
Inverse variance weighted
MR Egger
10
8
6
1/SE_IV
−0.25
0.00
0.25
0.50
β_IV

Batch 3−1805 : Selenium on Lymphotoxin alpha2:beta1

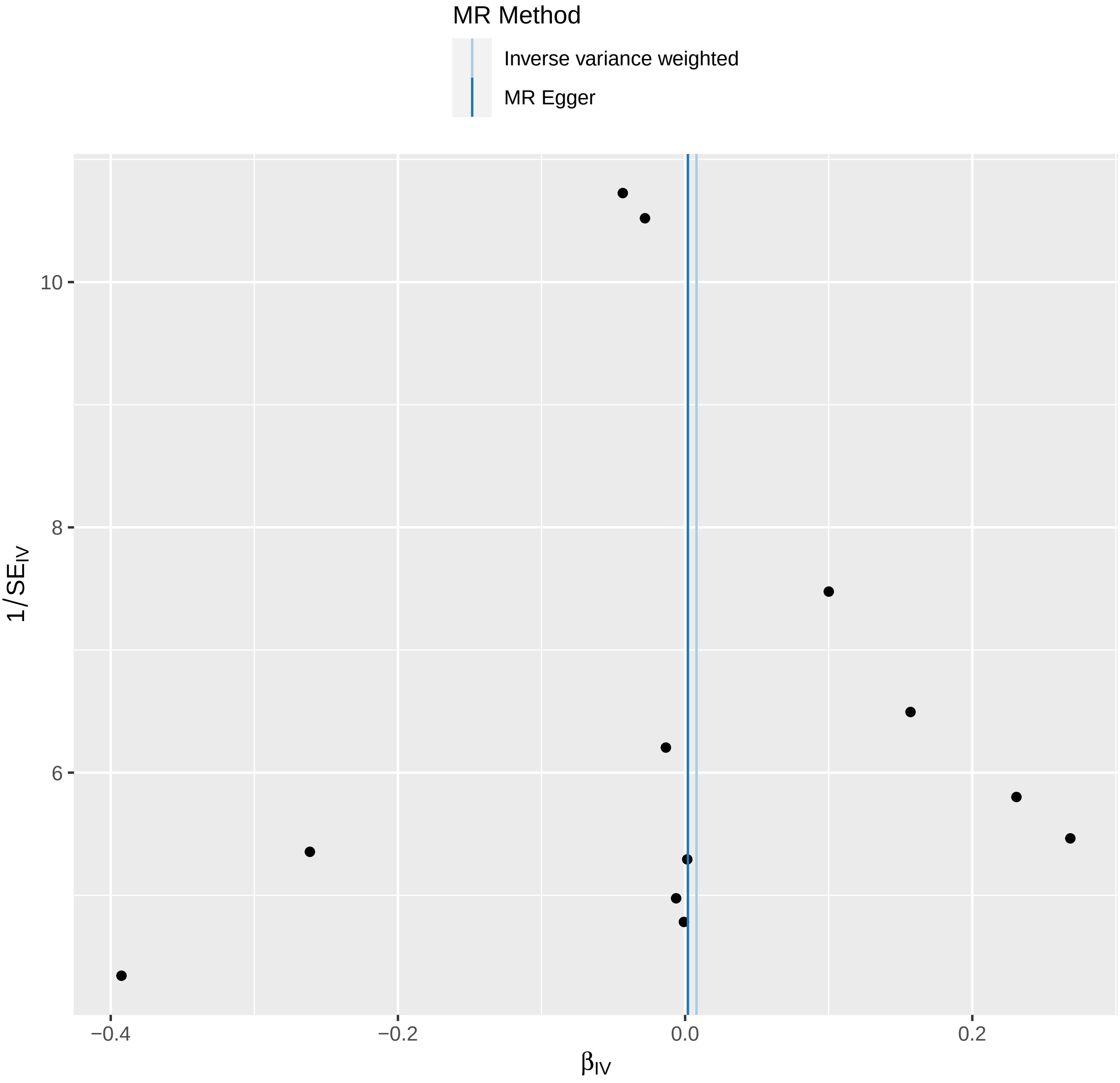

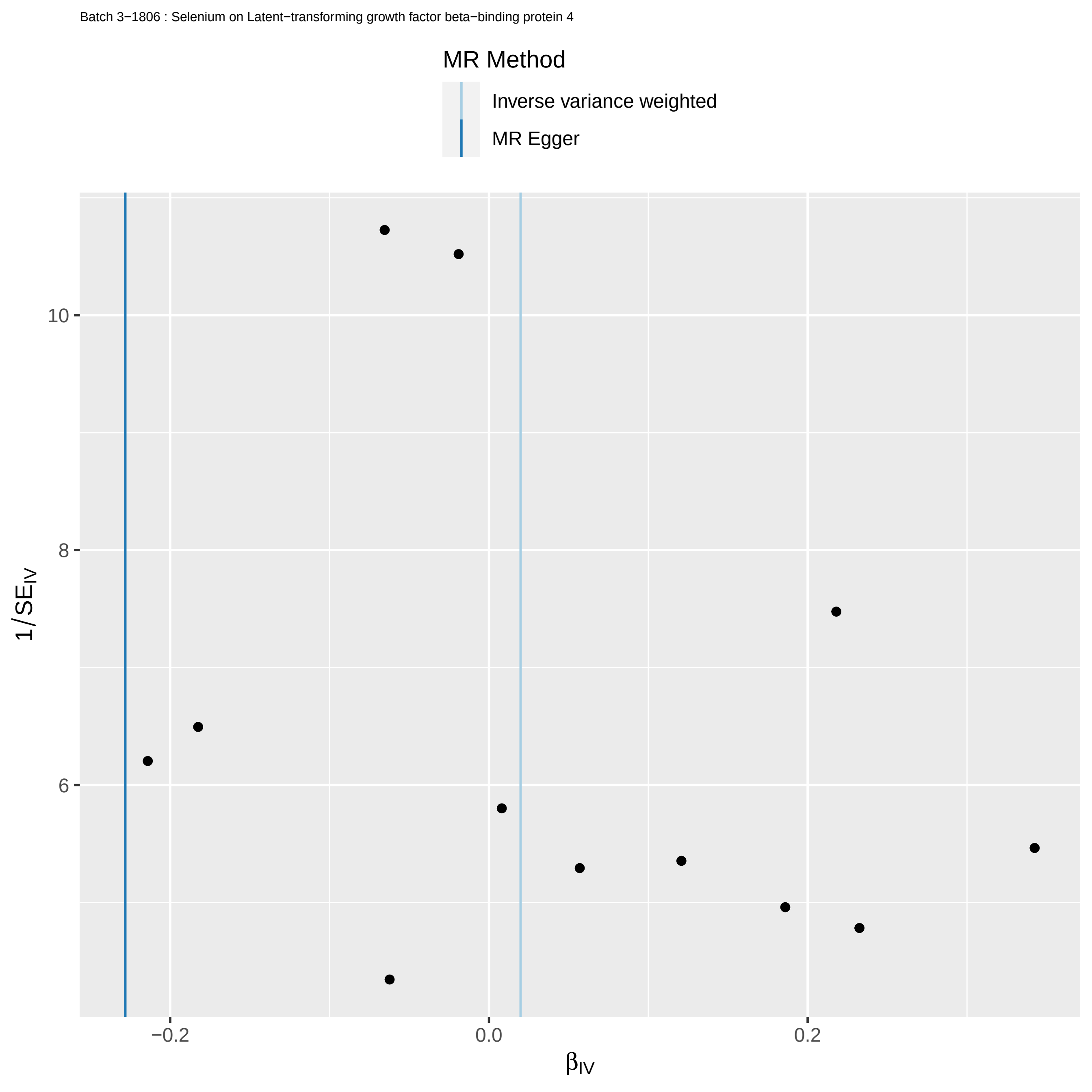

Batch 3−1806 : Selenium on Latent−transforming growth factor beta−binding protein 4
MR Method
Inverse variance weighted
MR Egger
10
8
6
1/SE$_{IV}$
−0.2
0.0
0.2
β$_{IV}$

Batch 3−1807 : Selenium on Tumor necrosis factor receptor superfamily member 3

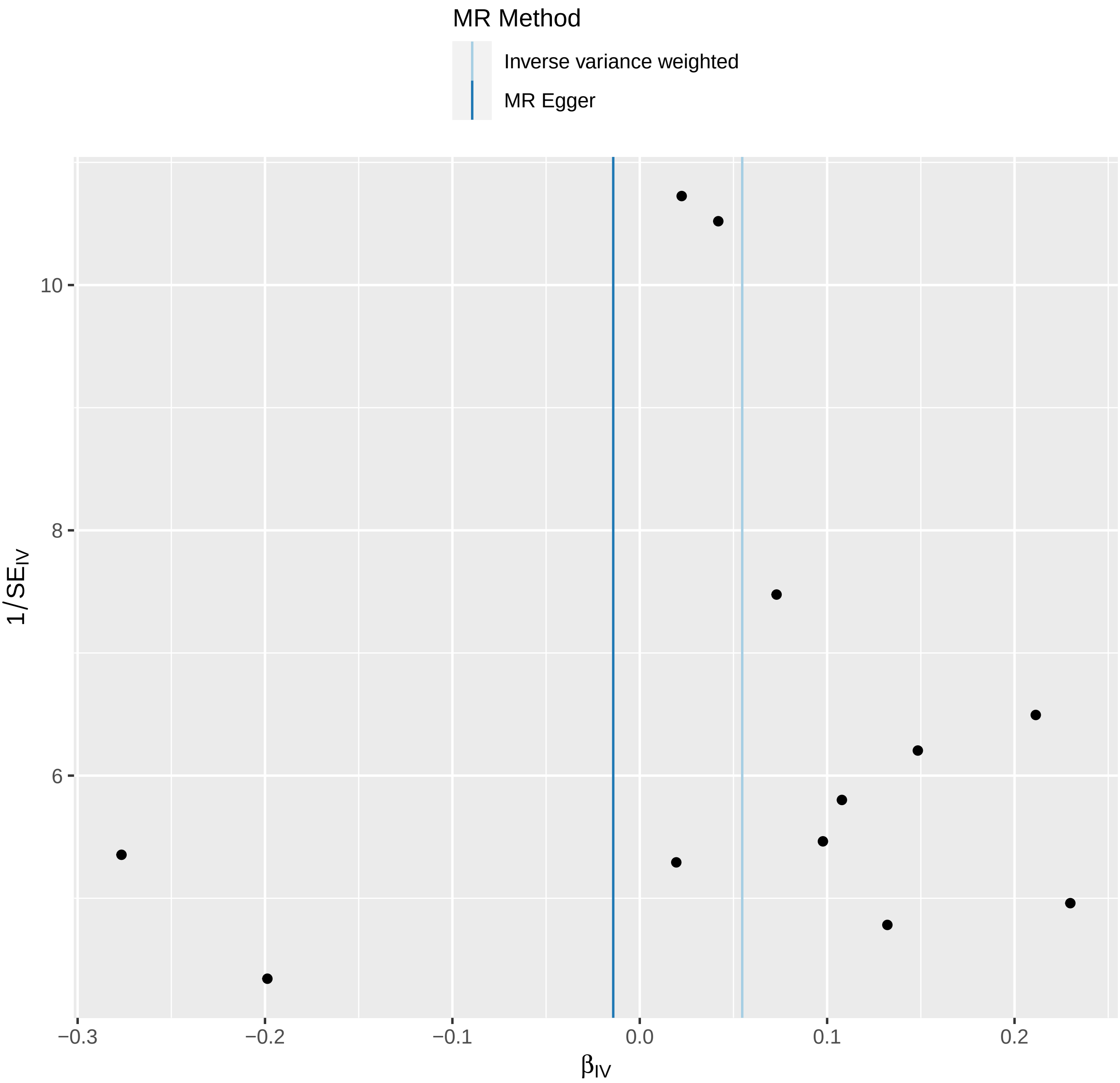

Batch 3−1808 : Selenium on Lactotransferrin

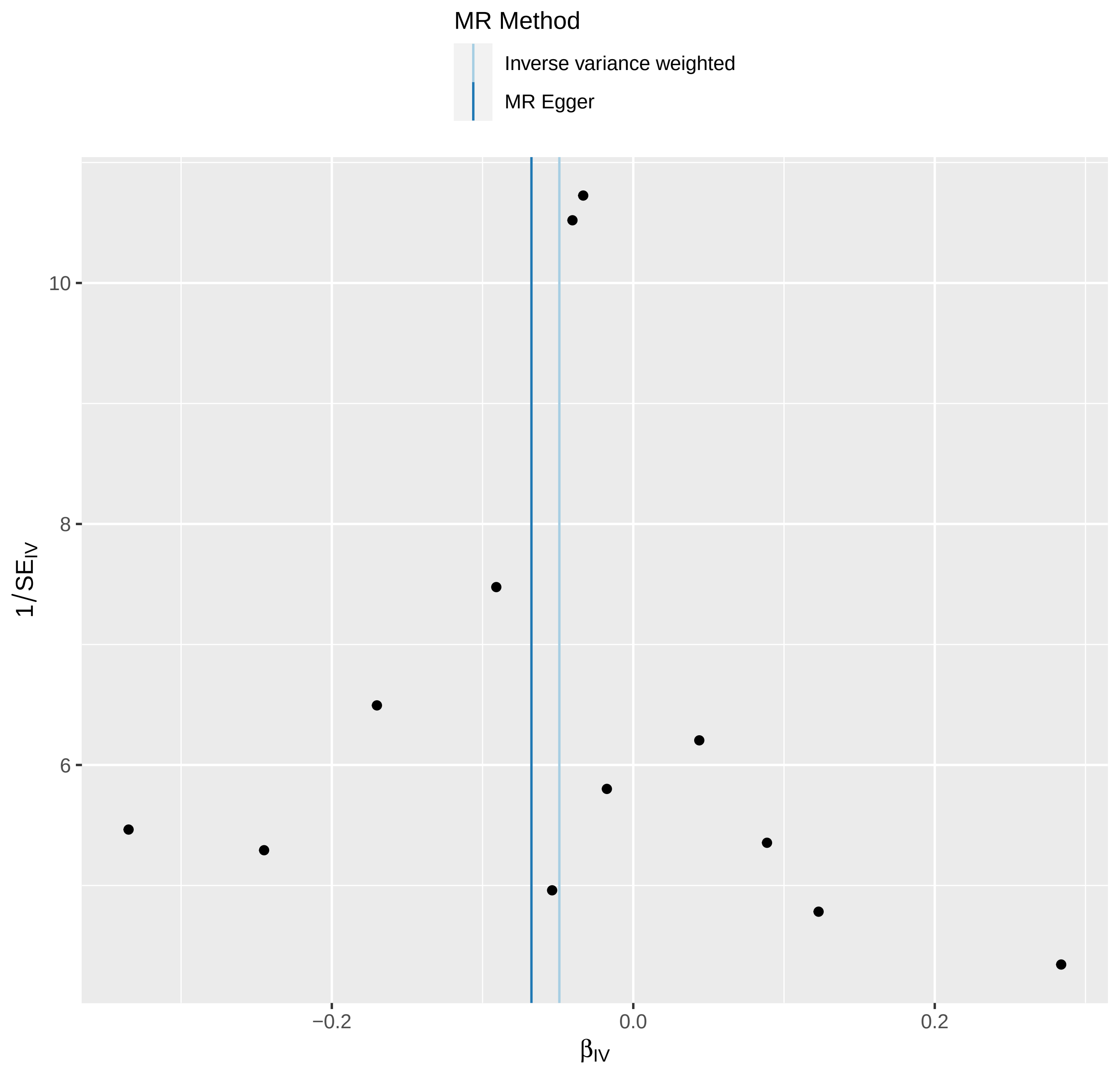

Batch 3−1809 : Selenium on Lactotransferrin

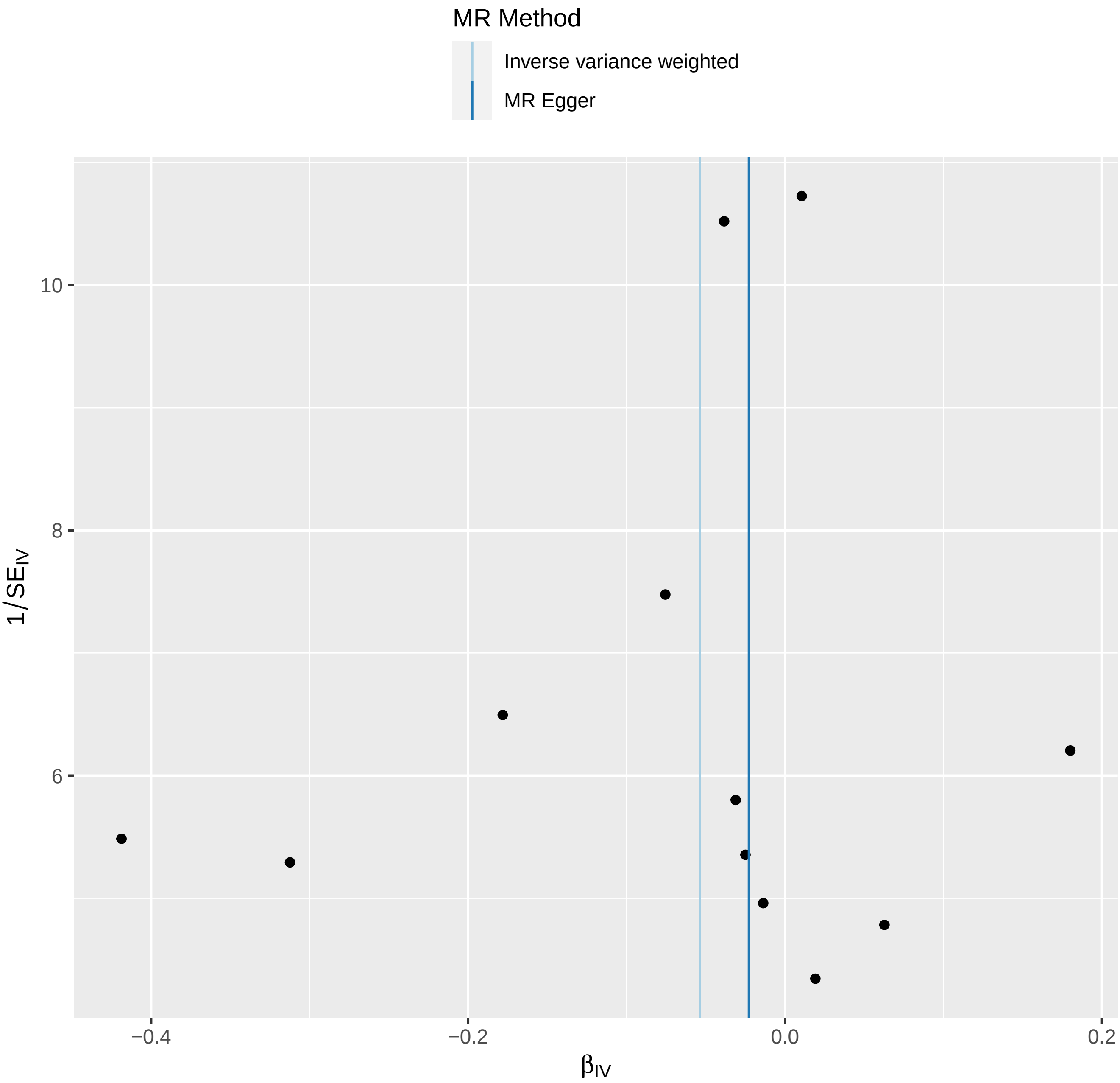

Batch 3−1810 : Selenium on Lymphocyte antigen 6 complex locus protein G6c

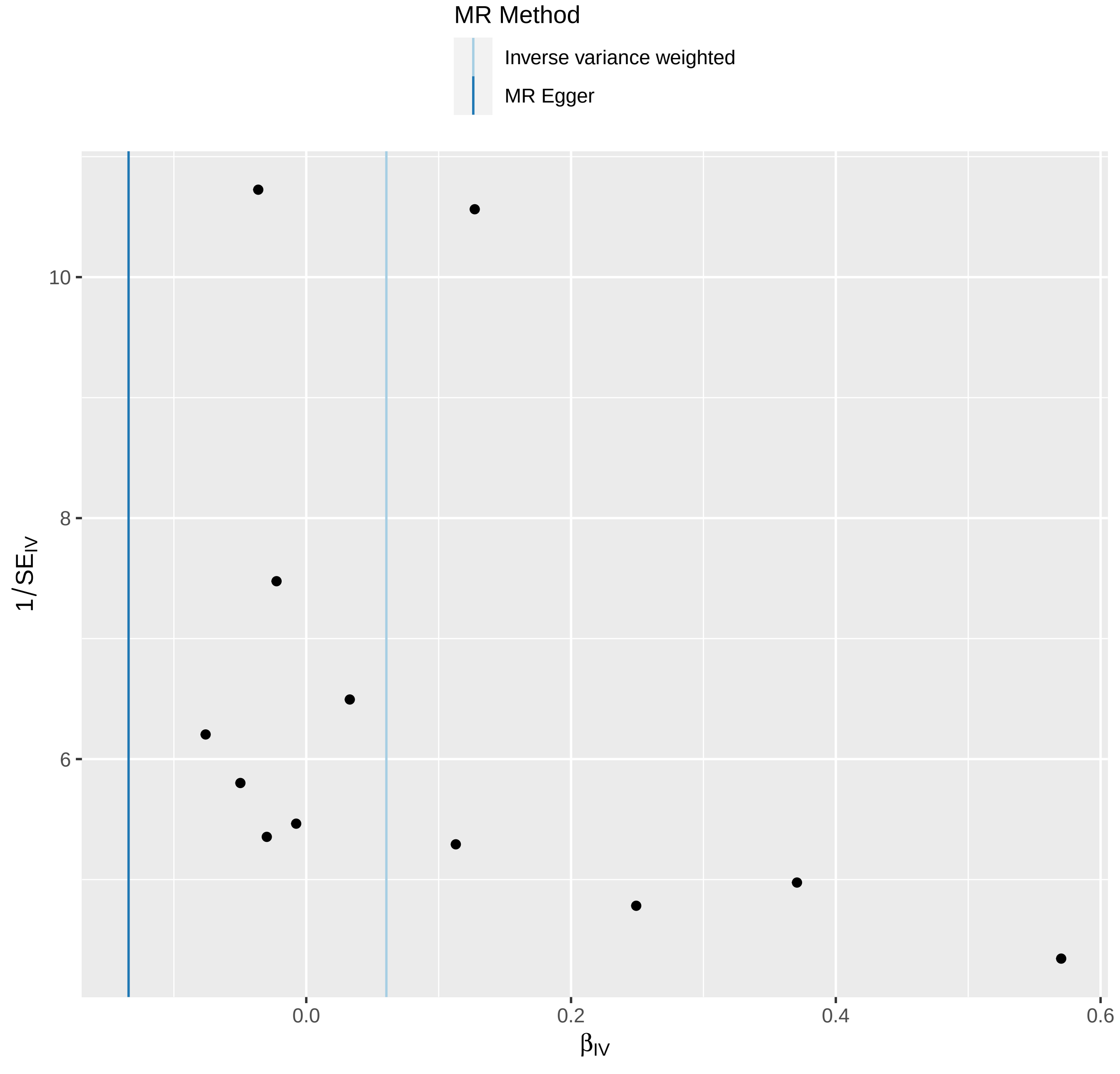

Batch 3−1811 : Selenium on Lymphocyte antigen 6 complex locus protein G6d

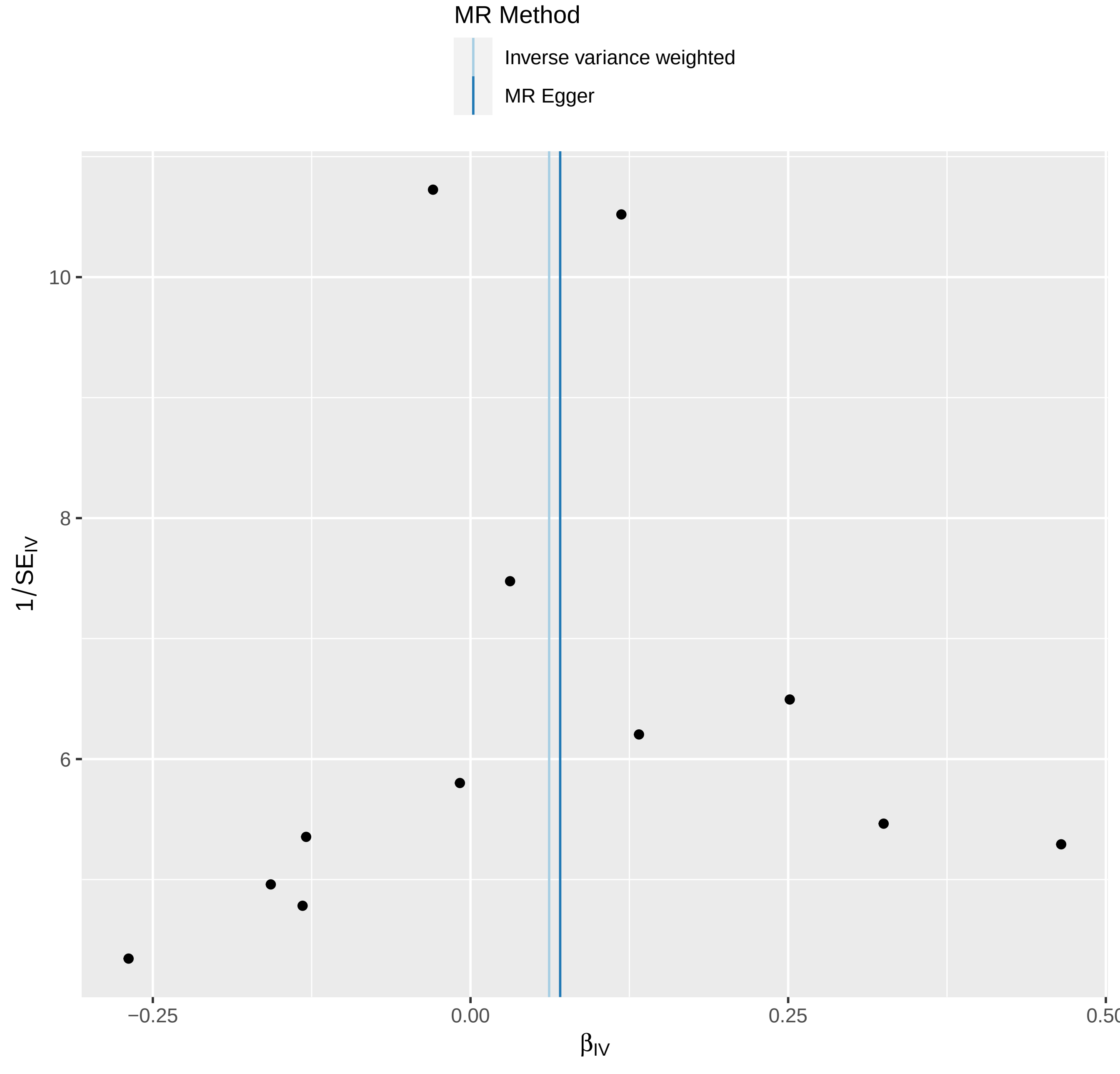

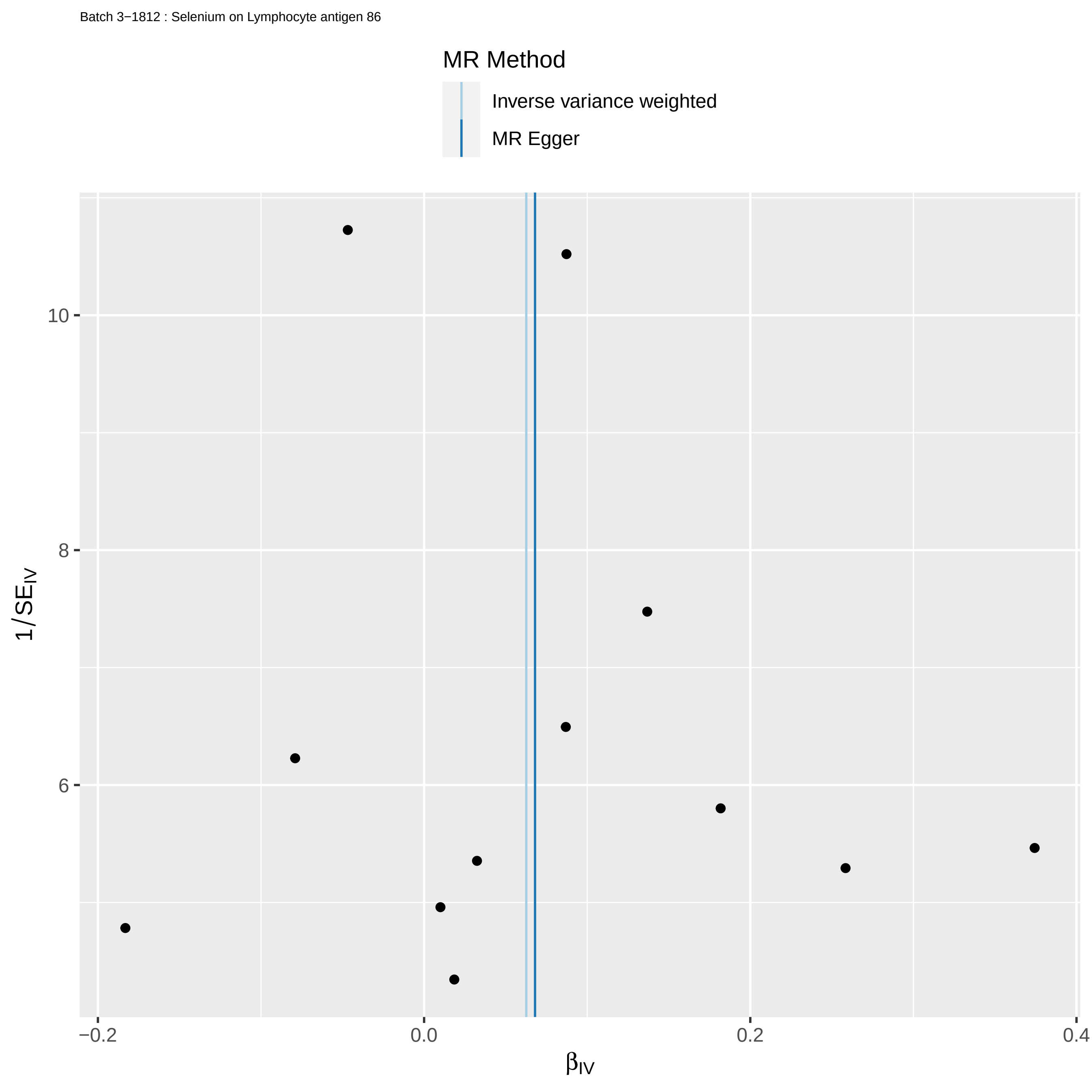

Batch 3−1812 : Selenium on Lymphocyte antigen 86
MR Method
Inverse variance weighted
MR Egger
10
8
6
1/SE$_{IV}$
−0.2
0.0
0.2
0.4
β$_{IV}$

Batch 3−1813 : Selenium on T−lymphocyte surface antigen Ly−9

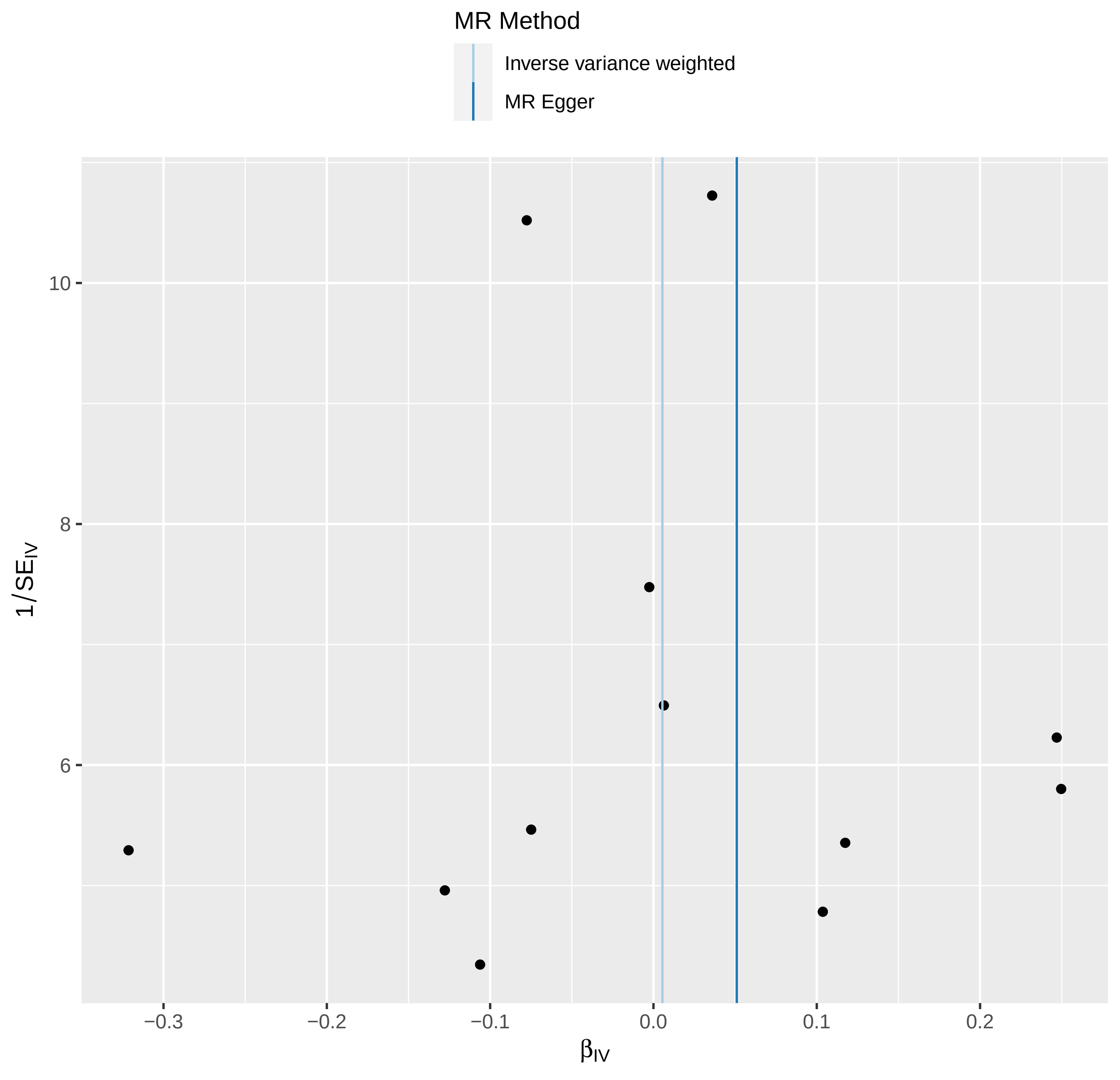

Batch 3−1814 : Selenium on Lysozyme g−like protein 1

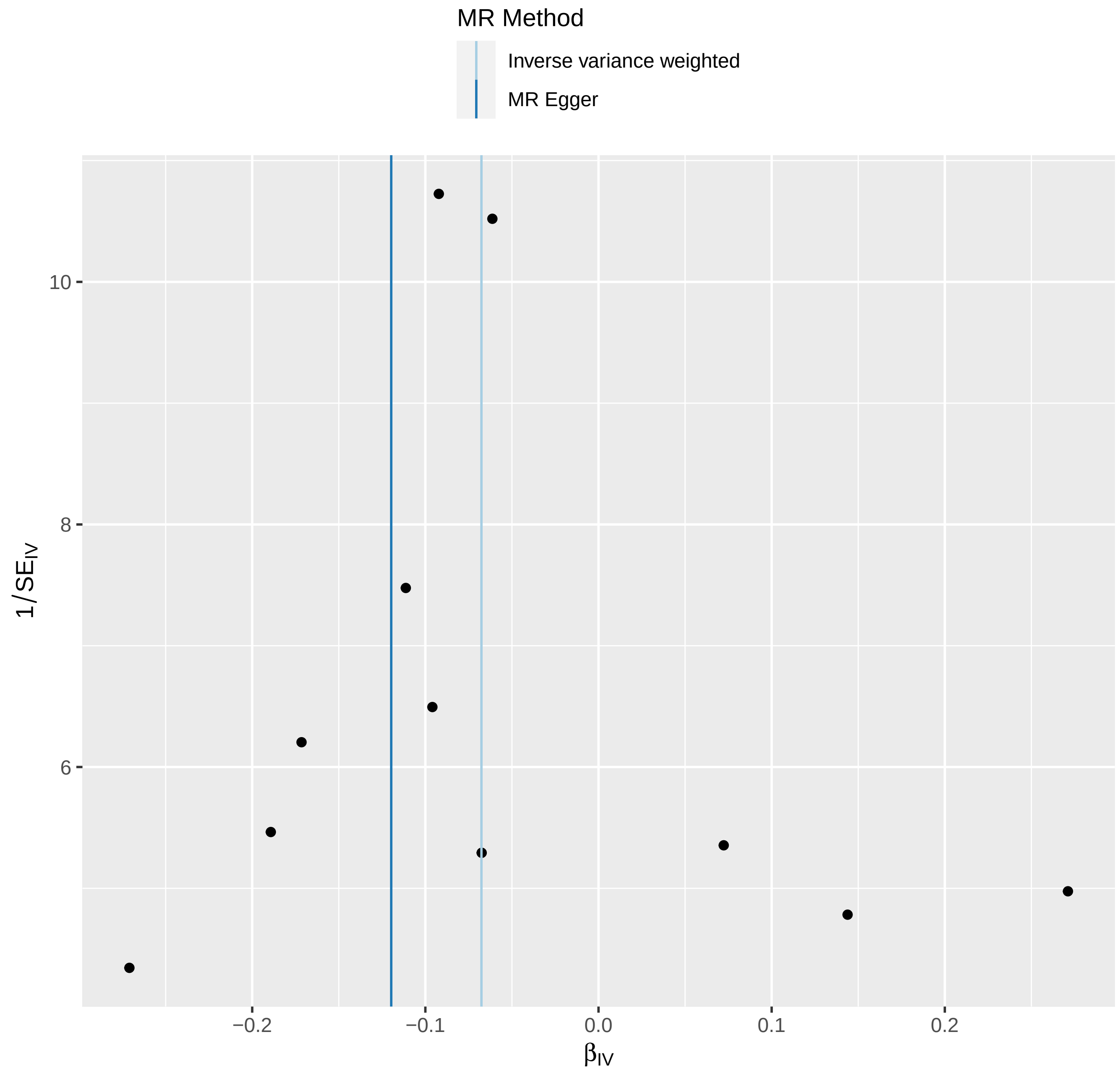

Batch 3−1815 : Selenium on Tyrosine−protein kinase Lyn, isoform B

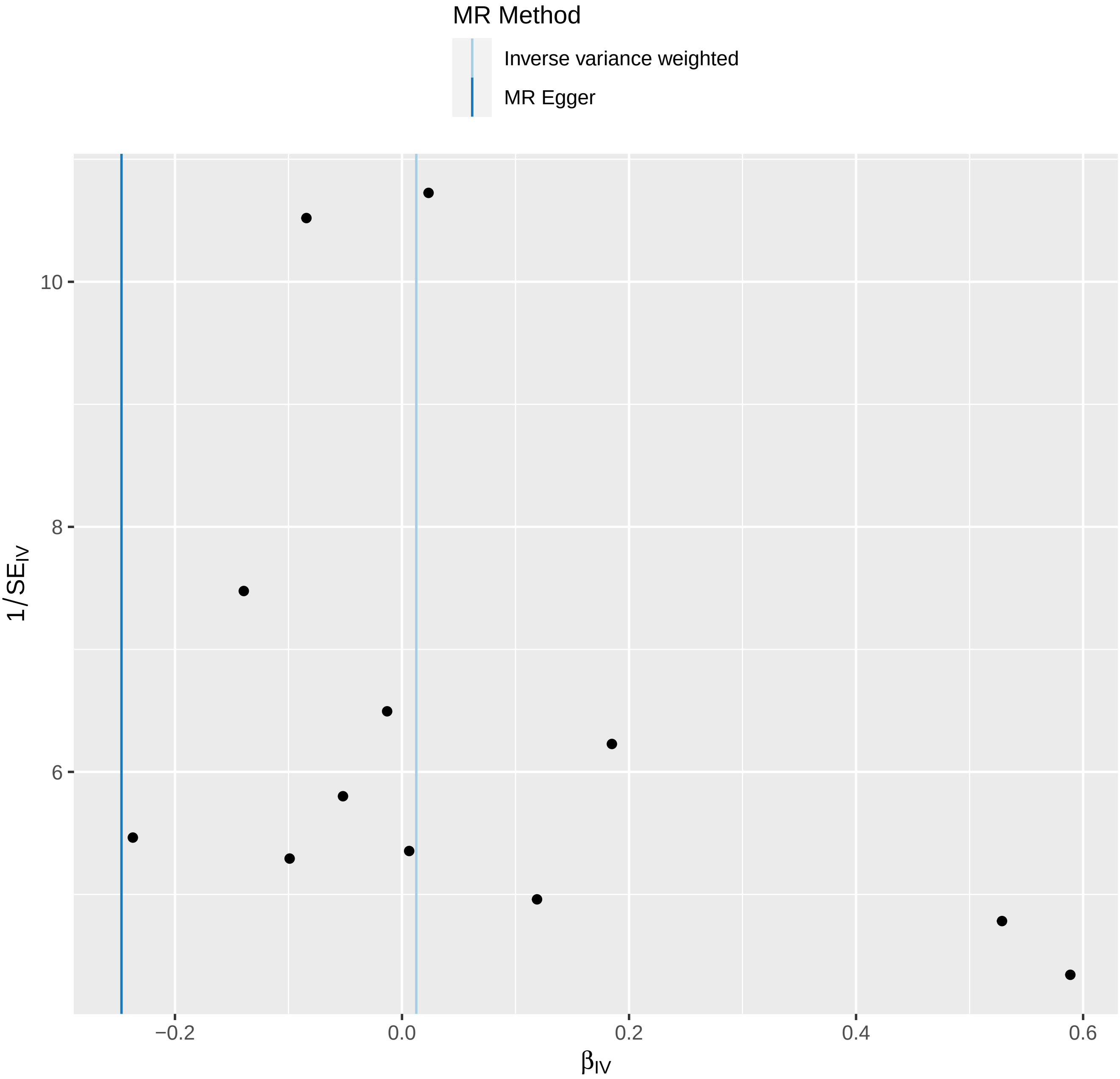

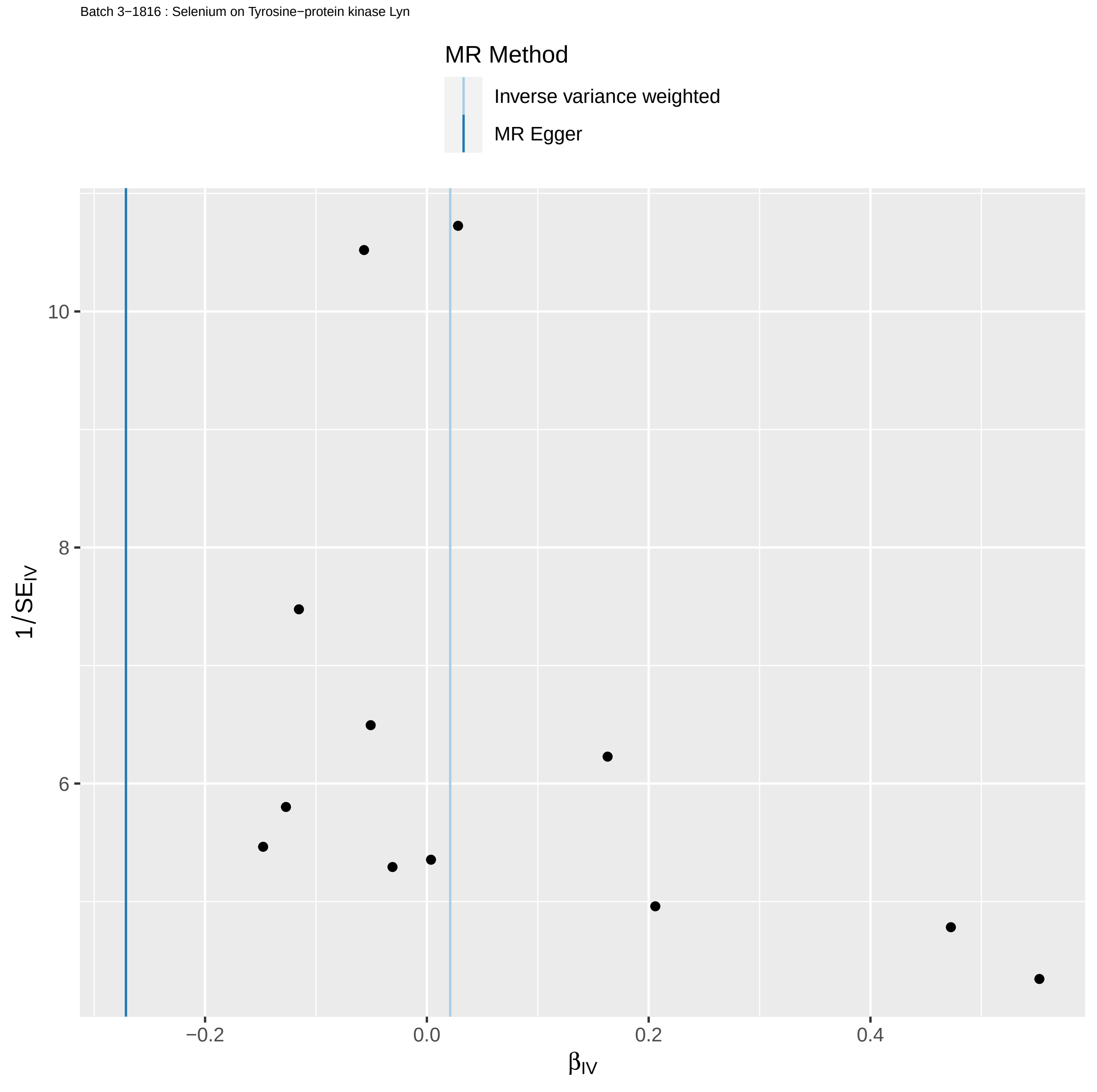
Batch 3−1816 : Selenium on Tyrosine−protein kinase Lyn
MR Method
Inverse variance weighted
MR Egger
10
8
6
1/SE_IV
−0.2
0.0
0.2
0.4
β_IV

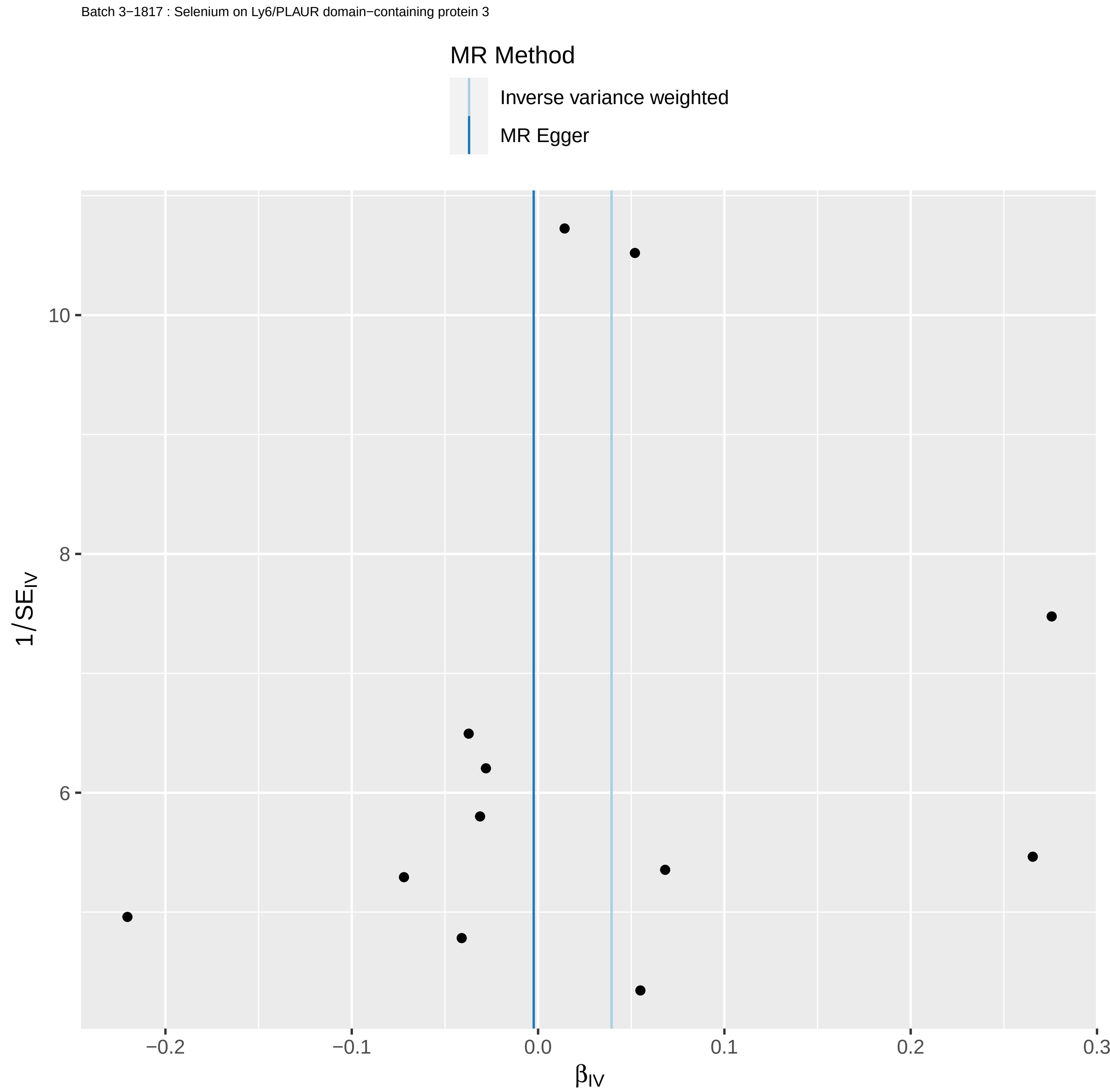

Batch 3−1817 : Selenium on Ly6/PLAUR domain−containing protein 3
MR Method
Inverse variance weighted
MR Egger
10
8
6
1/SE$_{IV}$
−0.2
−0.1
0.0
0.1
0.2
0.3
β$_{IV}$

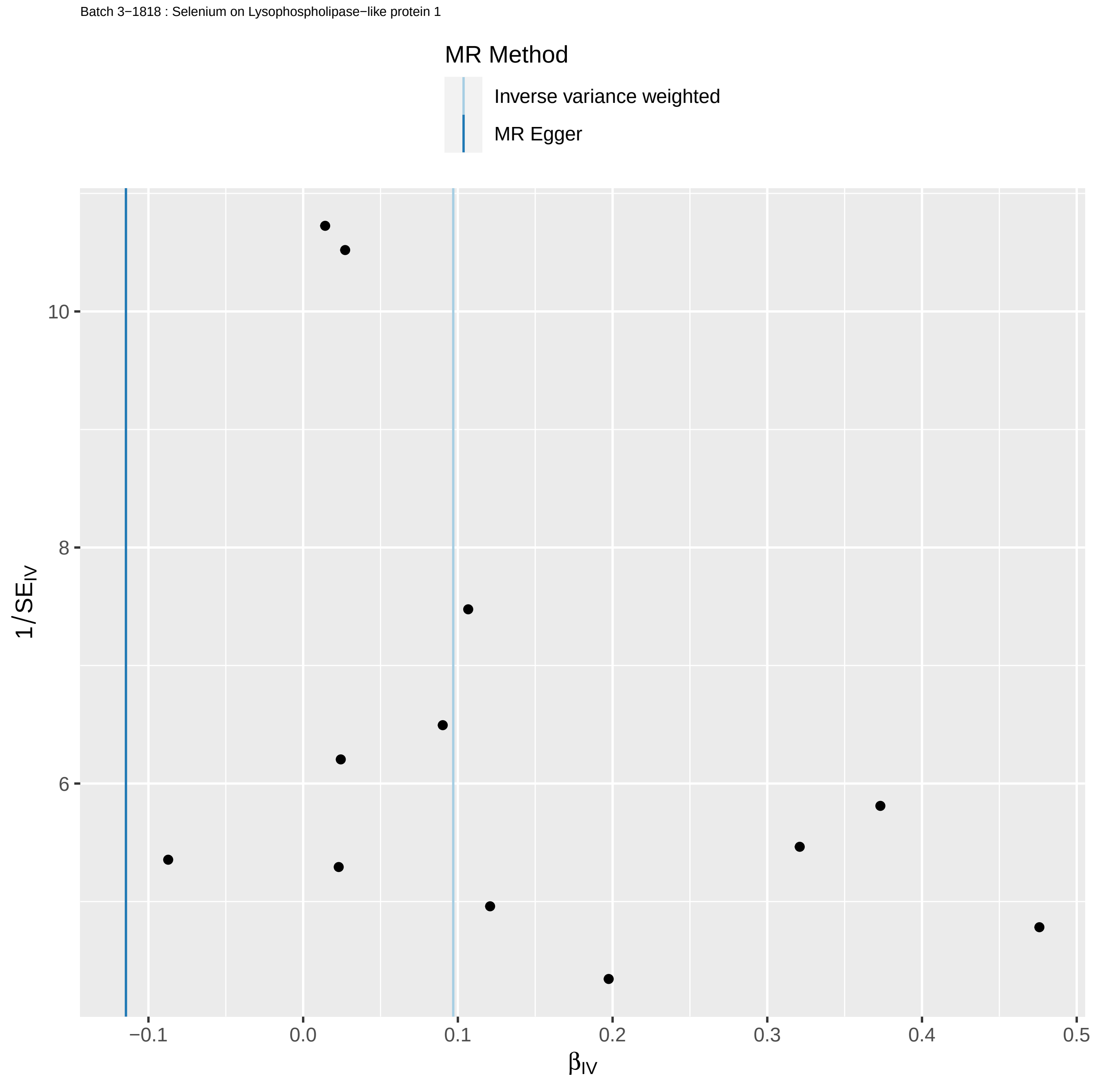

Batch 3−1818 : Selenium on Lysophospholipase−like protein 1
MR Method
Inverse variance weighted
MR Egger
10
8
6
1/SE$_{IV}$
−0.1
0.0
0.1
0.2
0.3
0.4
0.5
β$_{IV}$

Batch 3−1819 : Selenium on LysM and putative peptidoglycan−binding domain−containing protein 3

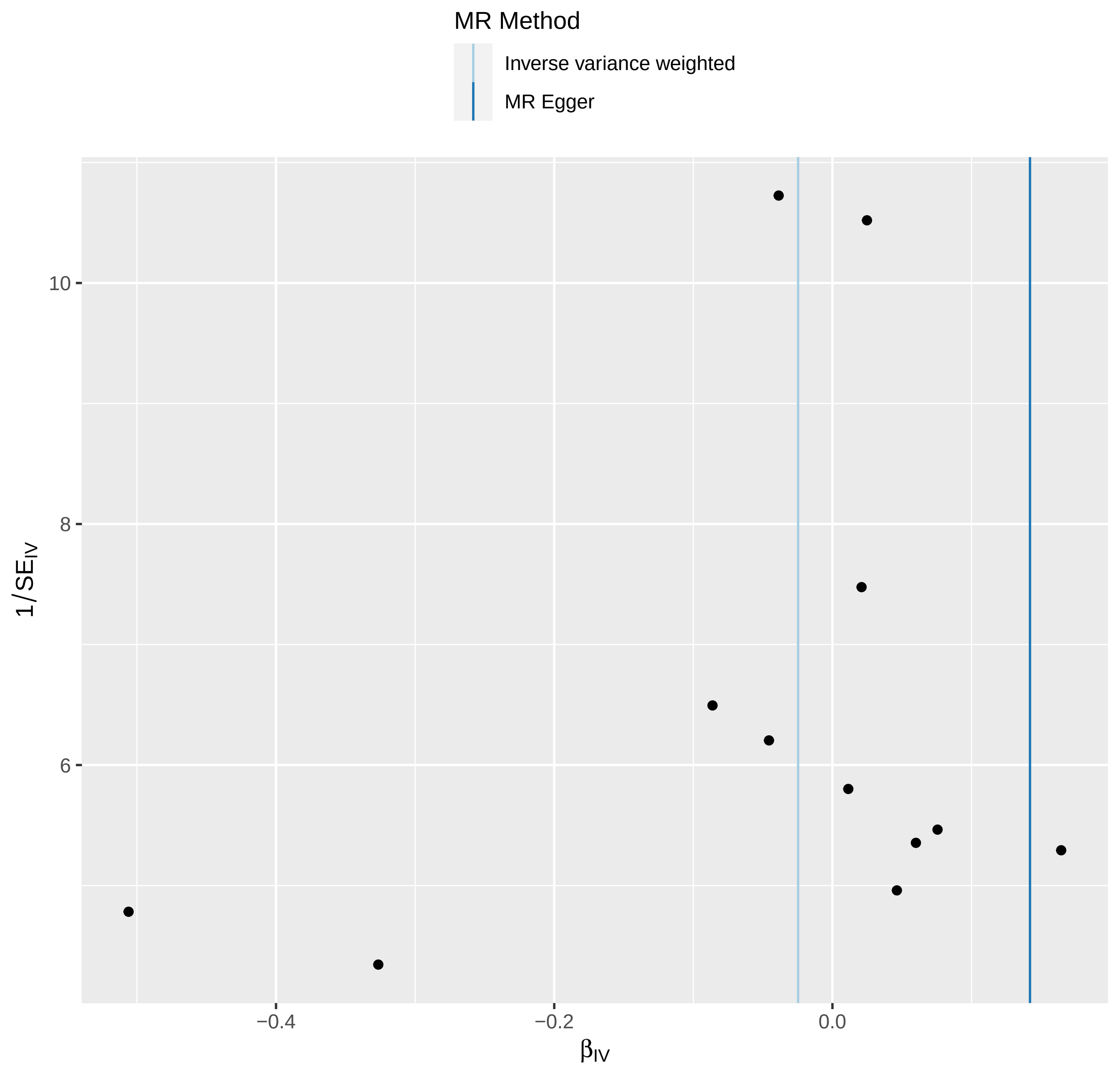

Batch 3−1820 : Selenium on LysM and putative peptidoglycan−binding domain−containing protein 4

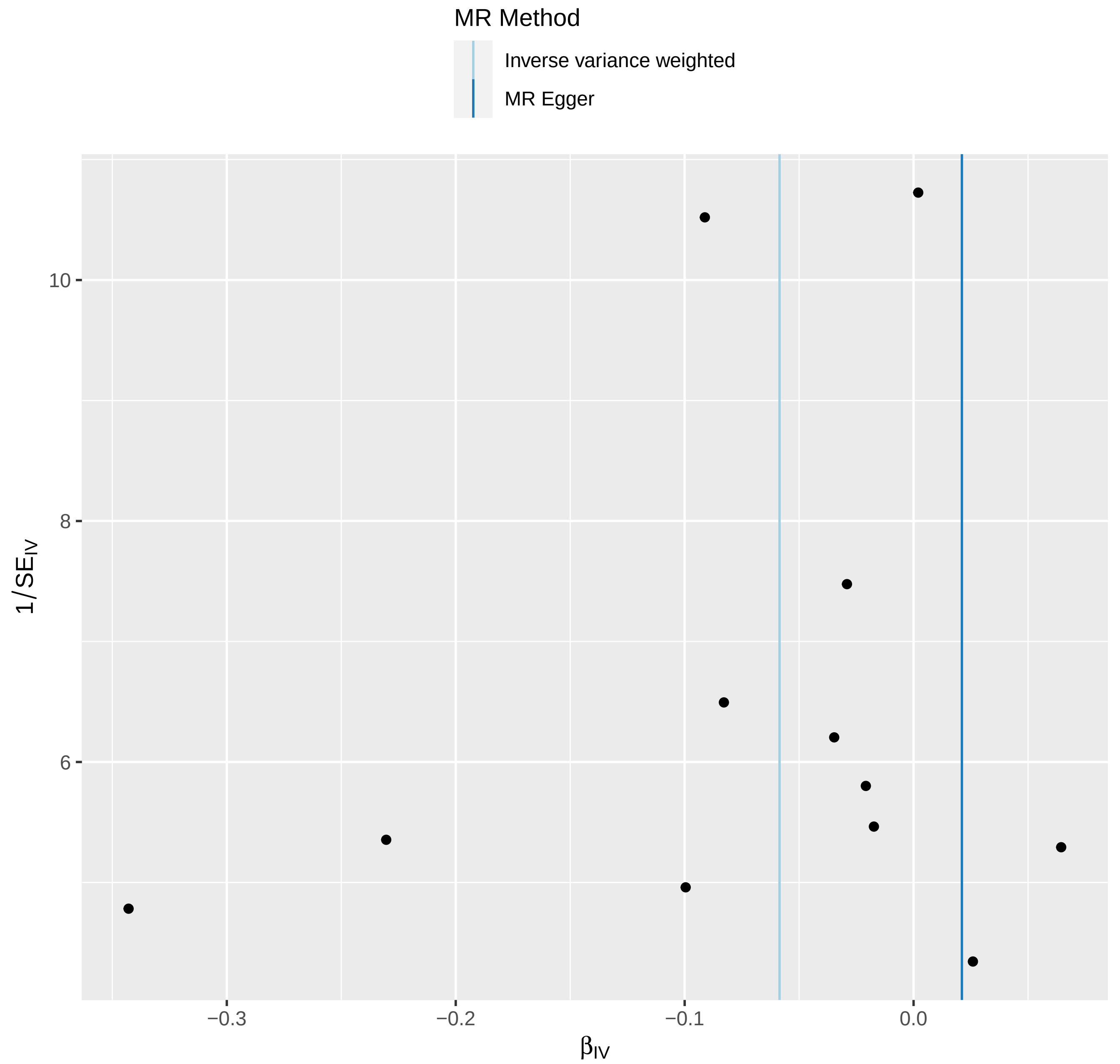

Batch 3−1821 : Selenium on Lysozyme C

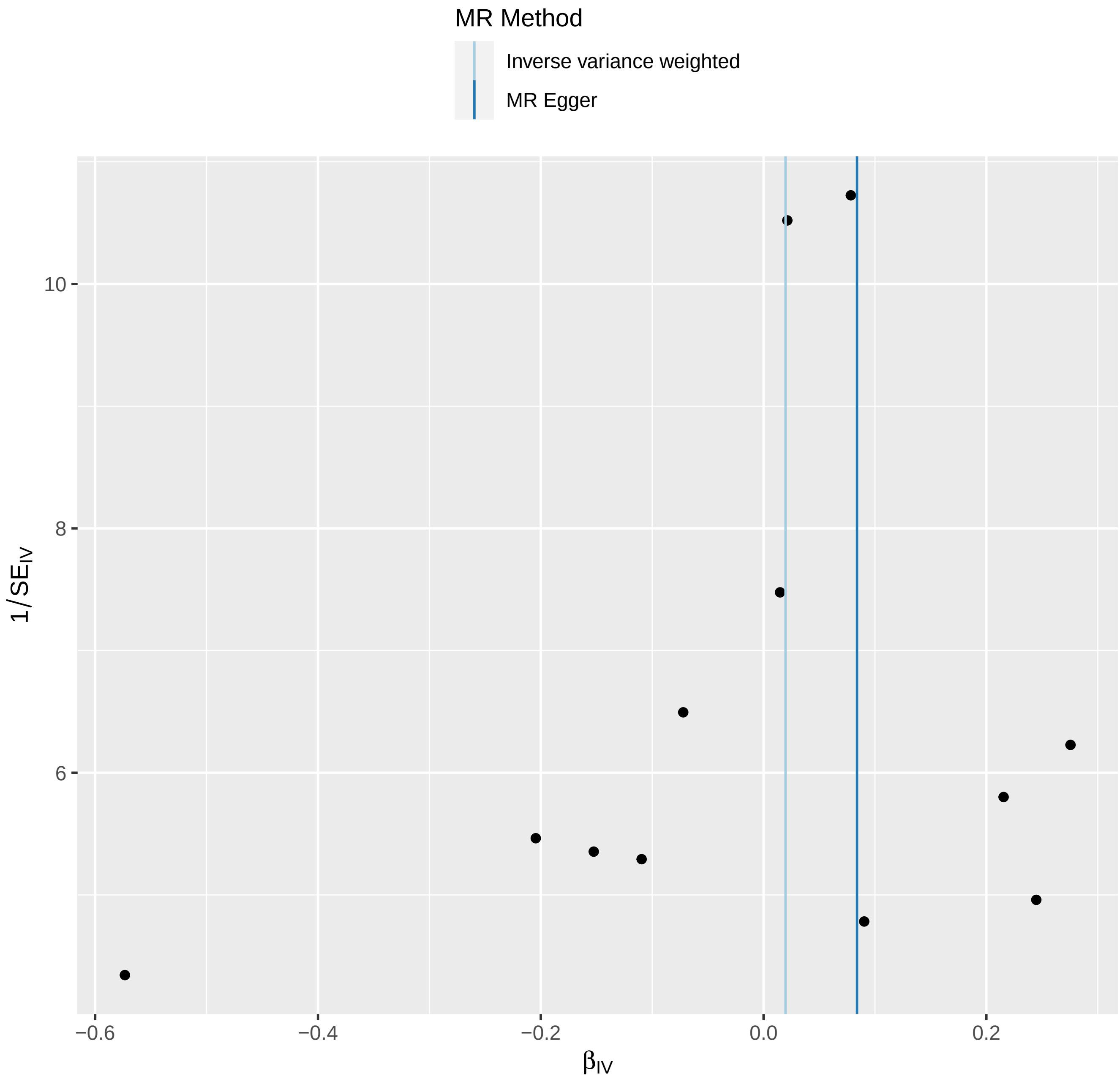

Batch 3−1822 : Selenium on Lysozyme−like protein 2

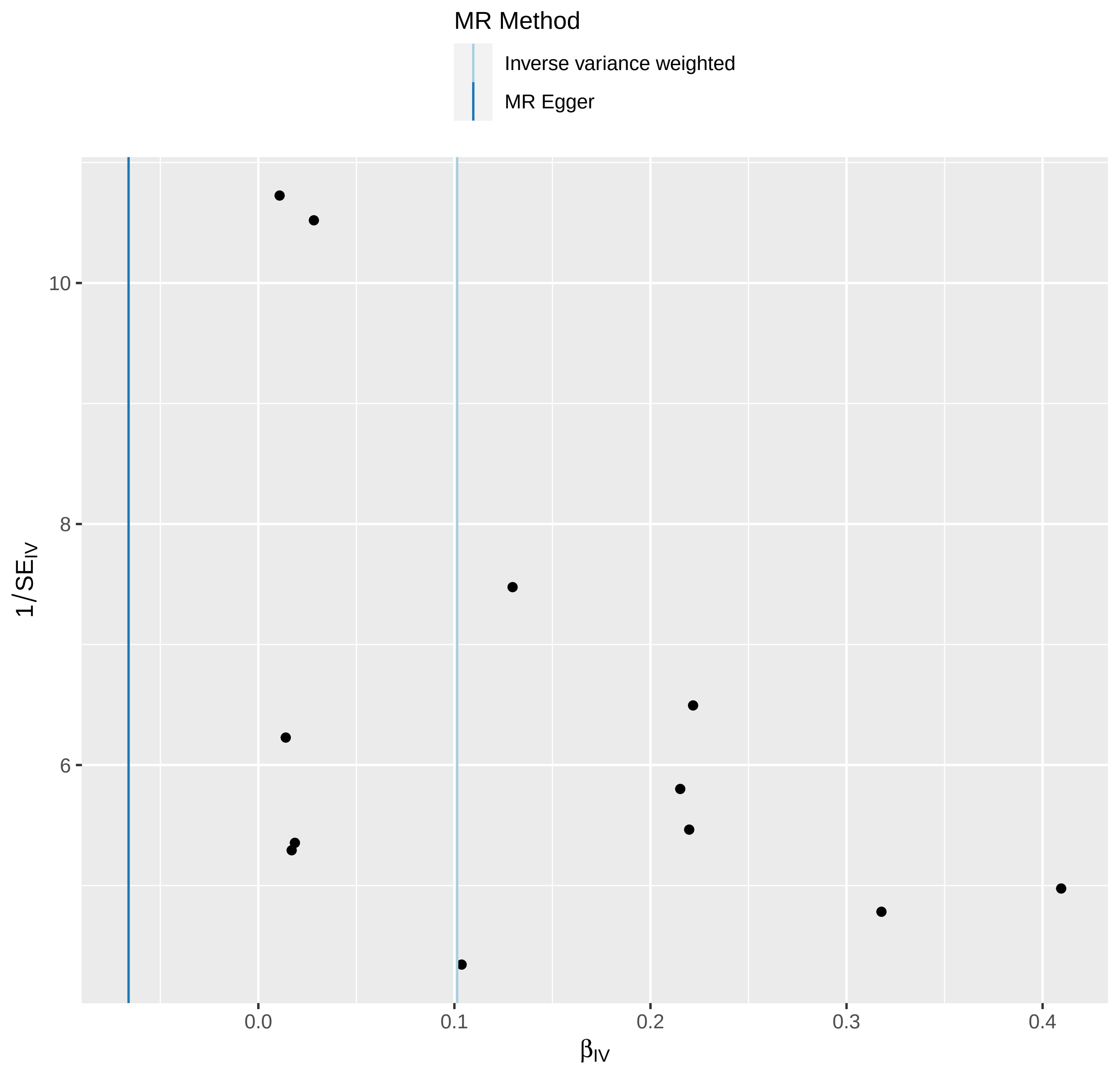

Batch 3−1823 : Selenium on Cation−dependent mannose−6−phosphate receptor

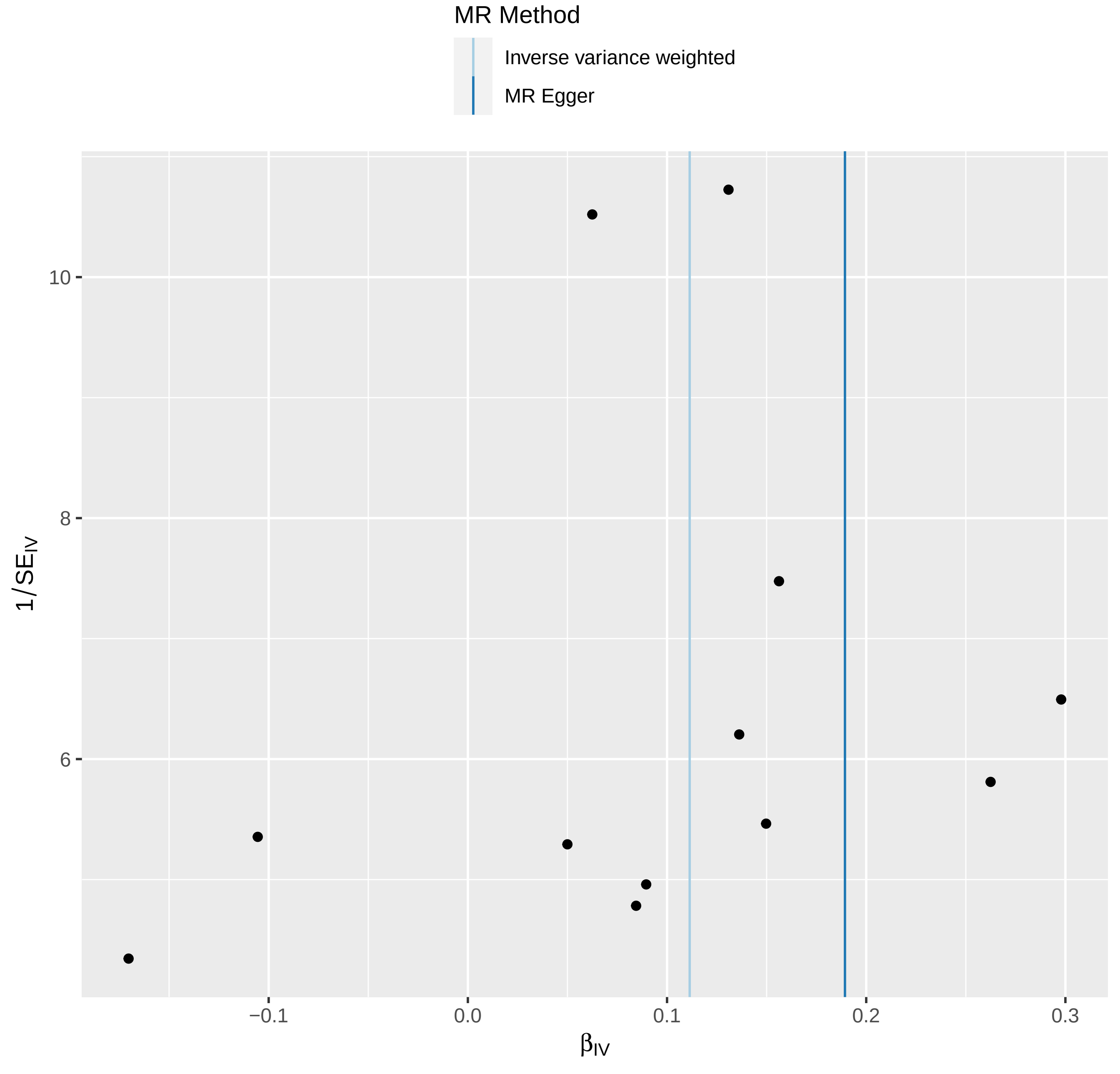

Batch 3−1824 : Selenium on O−acetyl−ADP−ribose deacetylase MACROD1

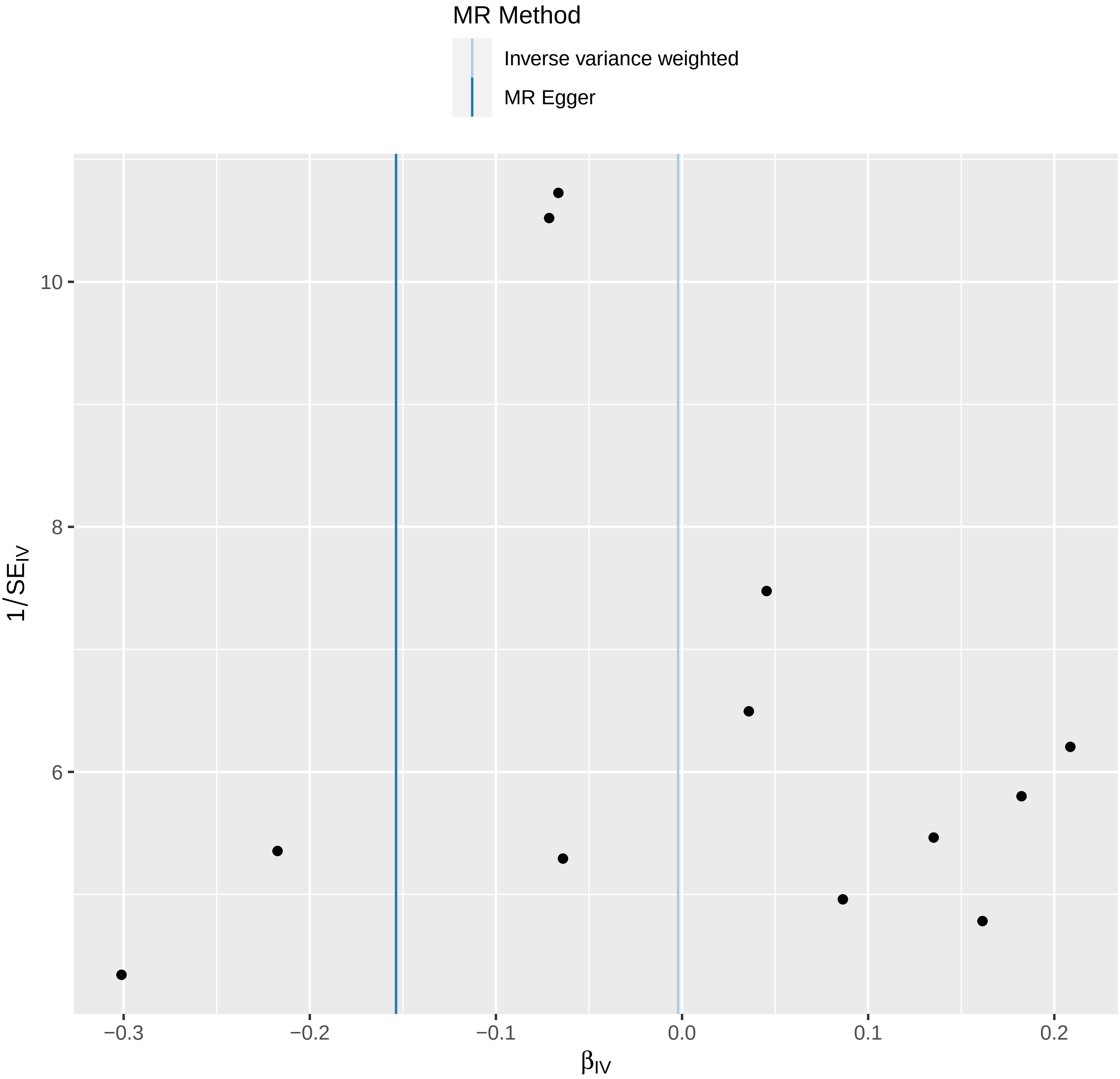

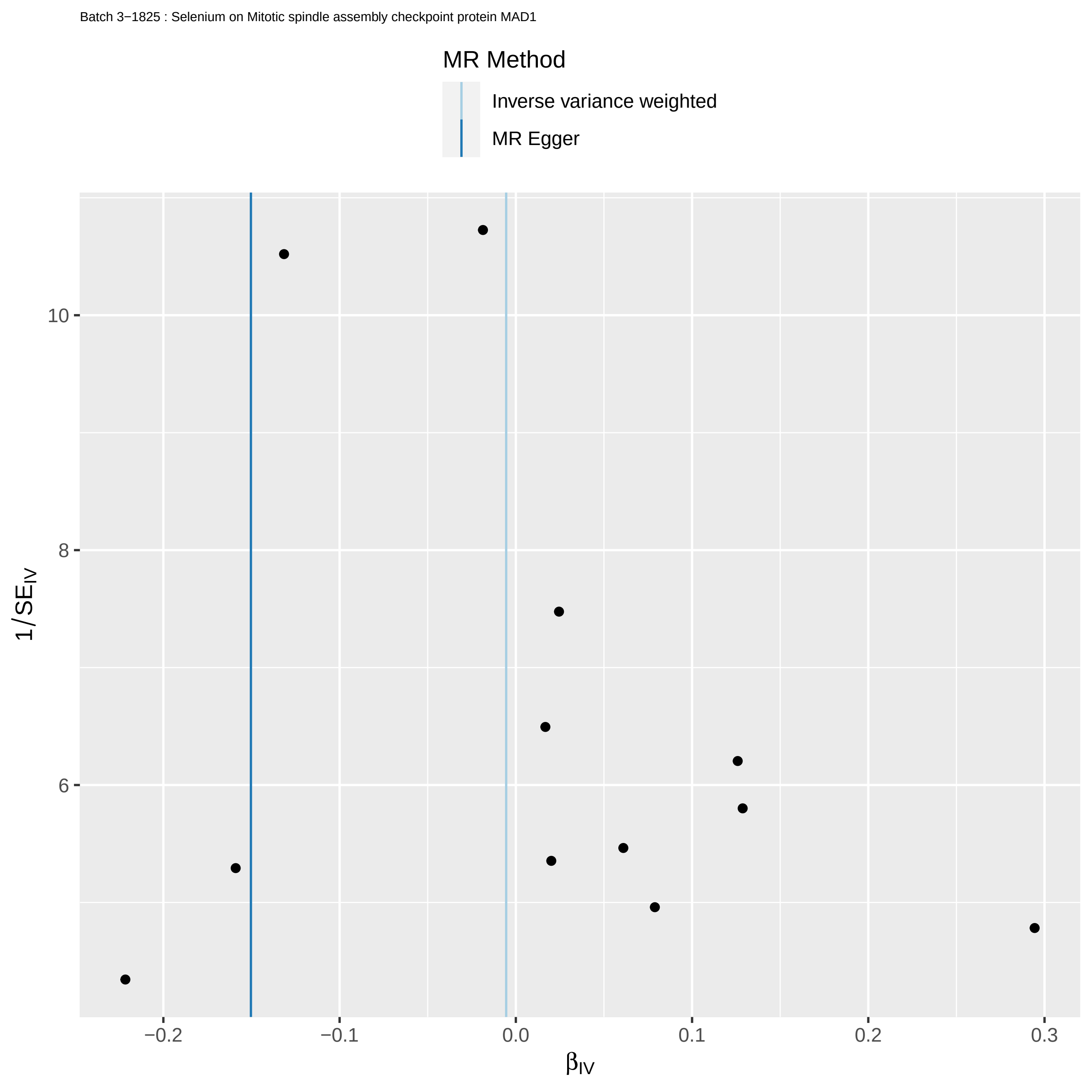
Batch 3−1825 : Selenium on Mitotic spindle assembly checkpoint protein MAD1
MR Method
Inverse variance weighted
MR Egger
10
8
6
1/SE_IV
−0.2
−0.1
0.0
0.1
0.2
0.3
β_IV

Batch 3−1826 : Selenium on Mucosal addressin cell adhesion molecule 1

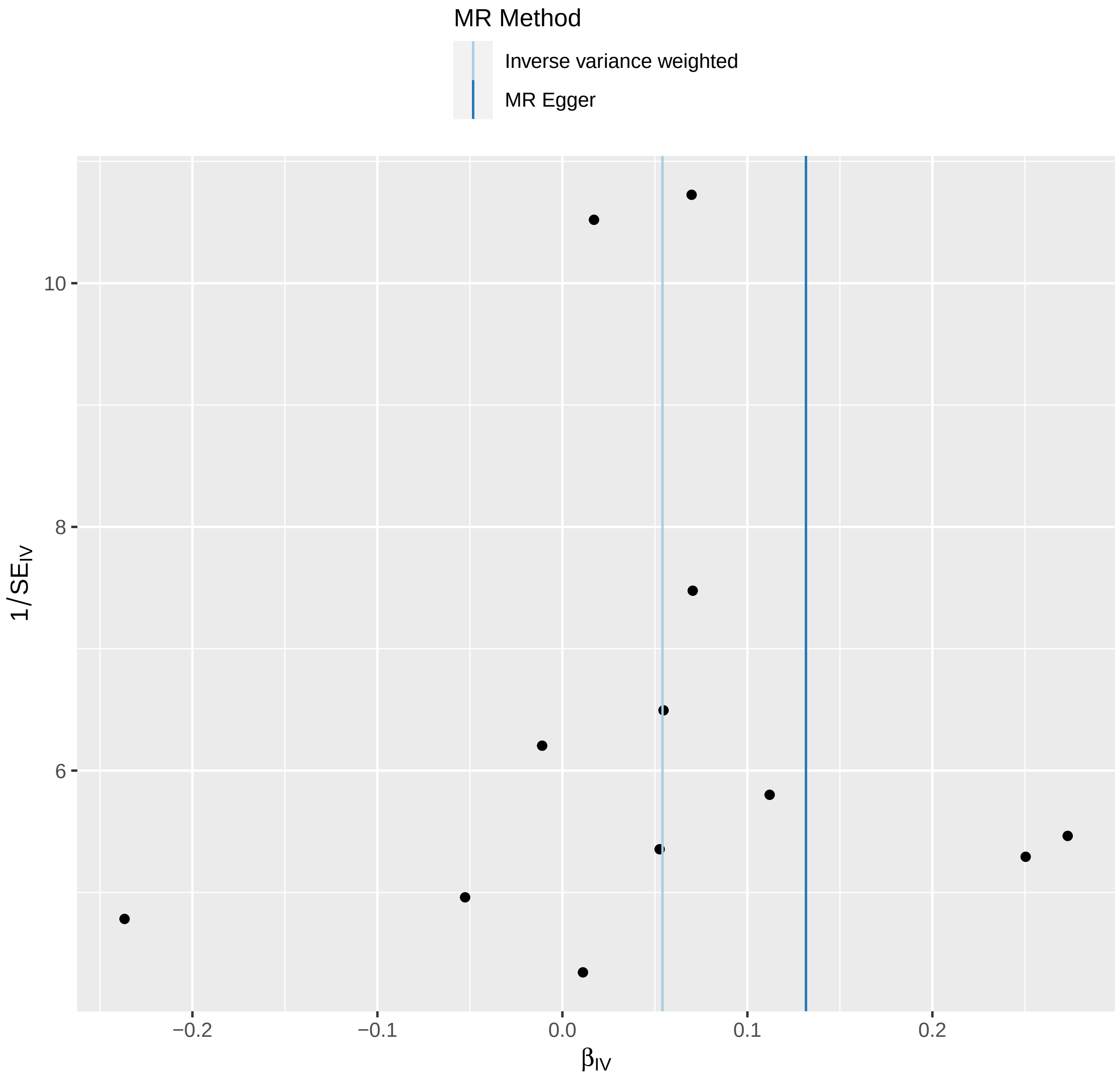

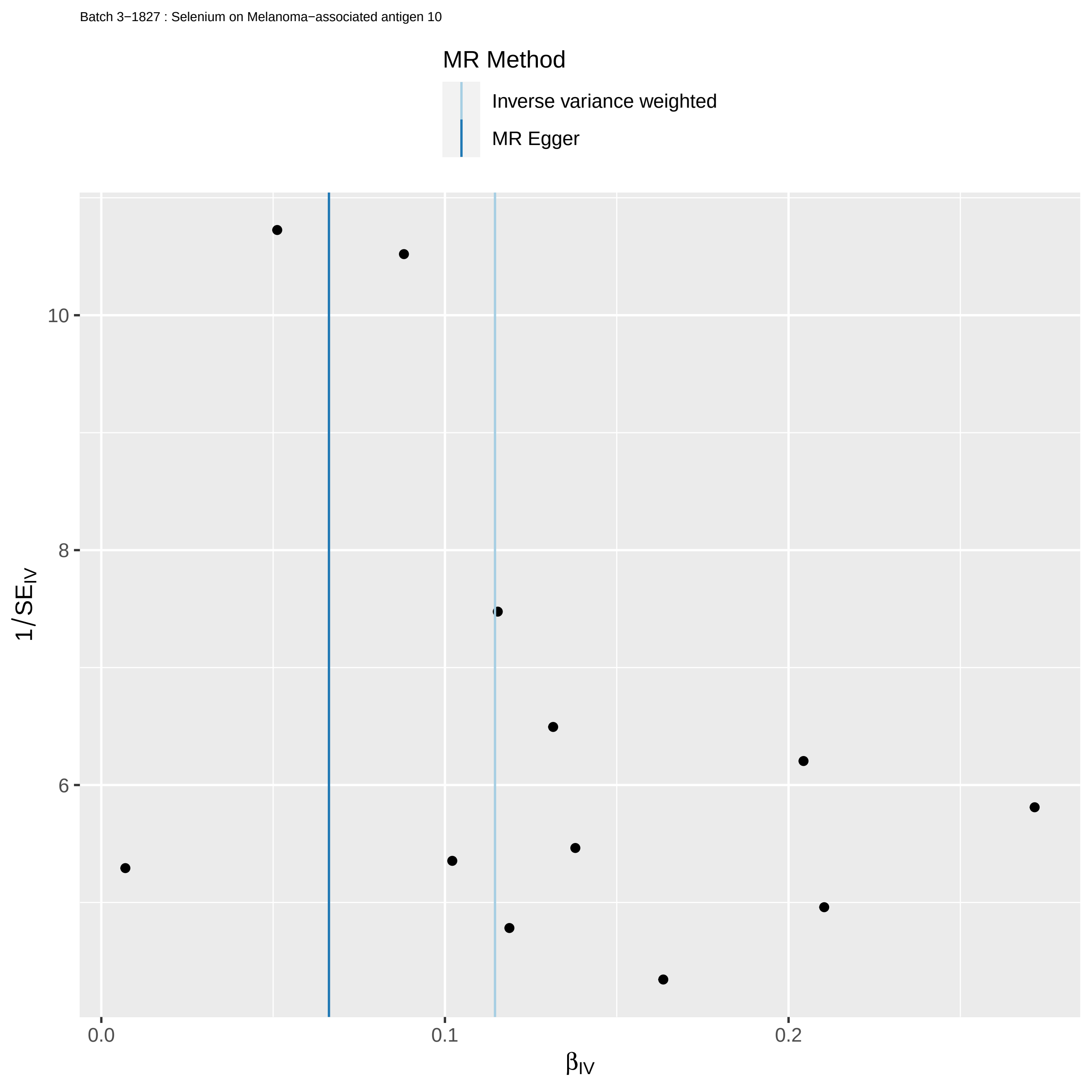
Batch 3−1827 : Selenium on Melanoma−associated antigen 10
MR Method
Inverse variance weighted
MR Egger
10
8
6
1/SE$_{IV}$
0.0
0.1
0.2
β$_{IV}$

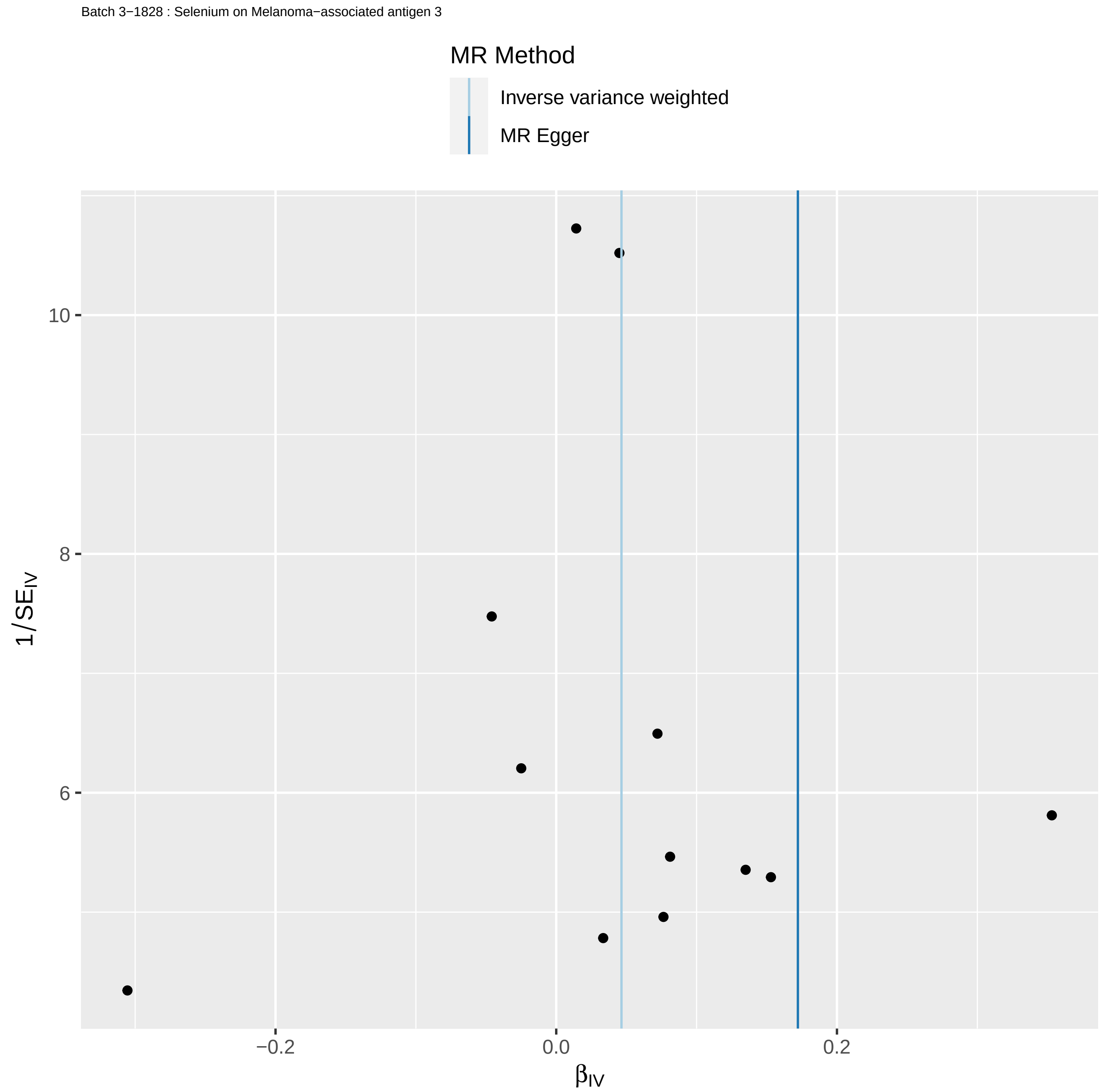
Batch 3−1828 : Selenium on Melanoma−associated antigen 3
MR Method
Inverse variance weighted
MR Egger
10
8
6
1/SE$_{IV}$
−0.2
0.0
0.2
β$_{IV}$

Batch 3−1829 : Selenium on Melanoma−associated antigen B10

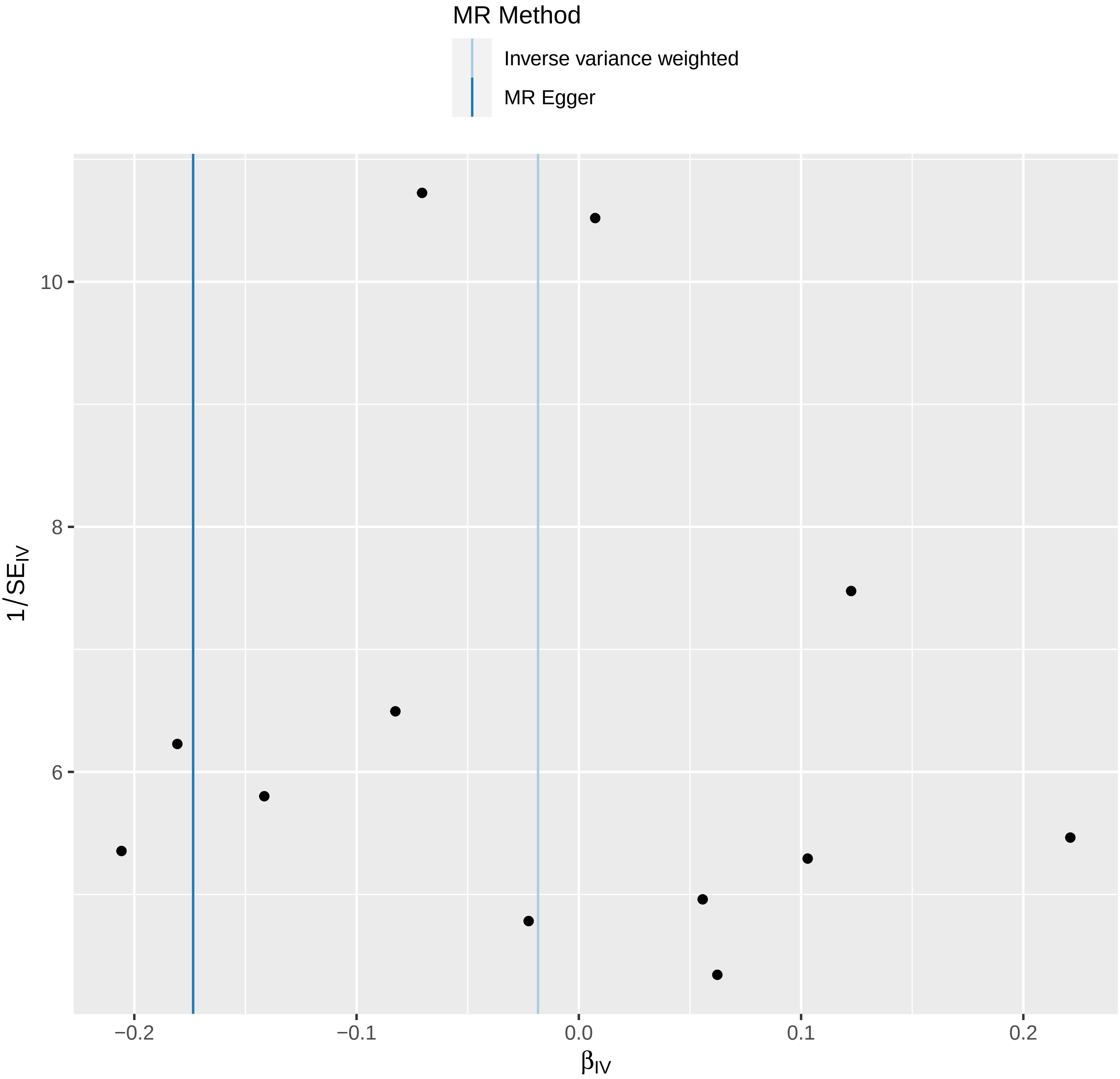

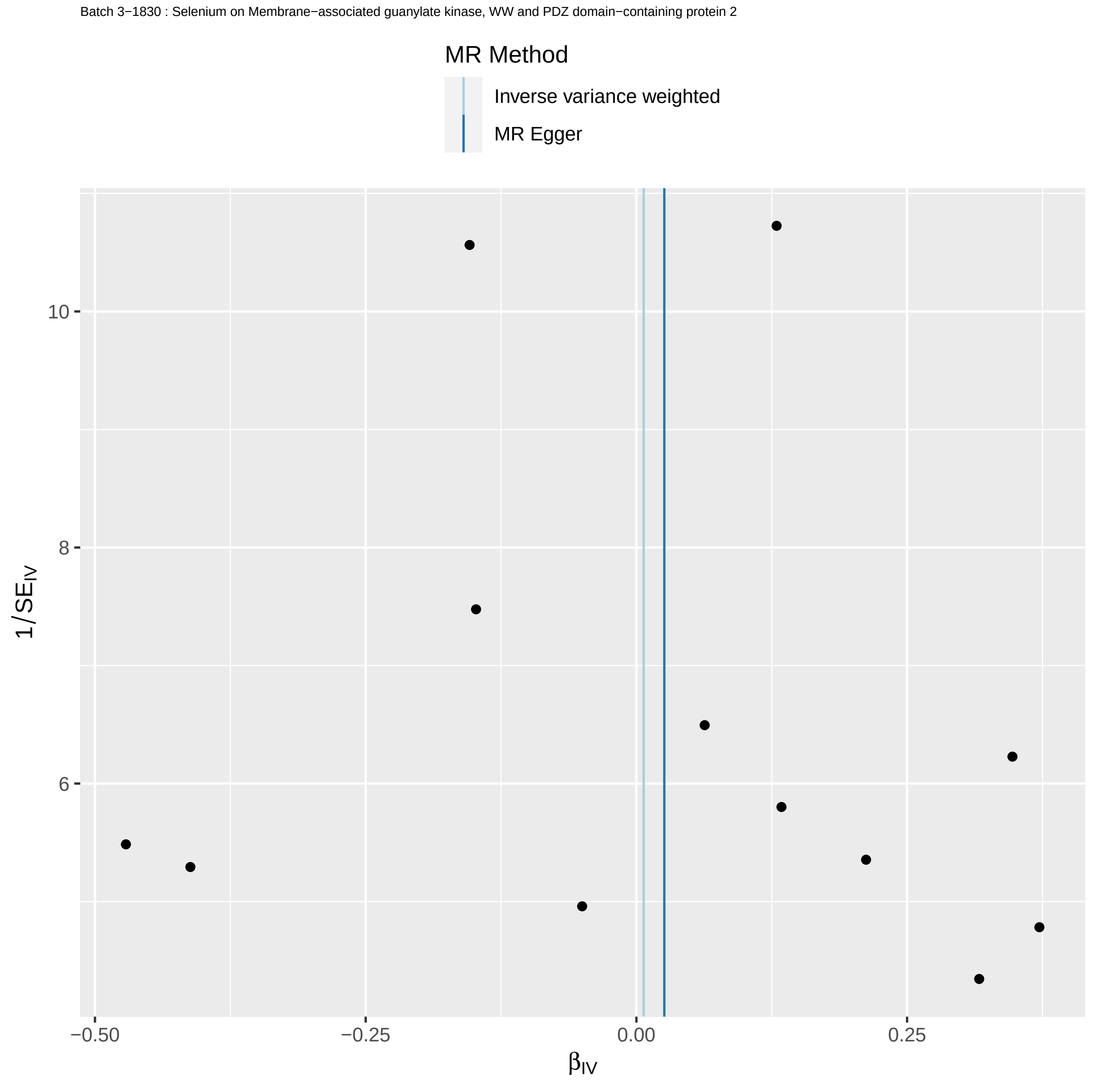
Batch 3−1830 : Selenium on Membrane−associated guanylate kinase, WW and PDZ domain−containing protein 2
MR Method
Inverse variance weighted
MR Egger
10
8
6
1/SE$_{IV}$
−0.50
−0.25
0.00
0.25
β$_{IV}$

Batch 3−1831 : Selenium on MAM domain−containing protein 2

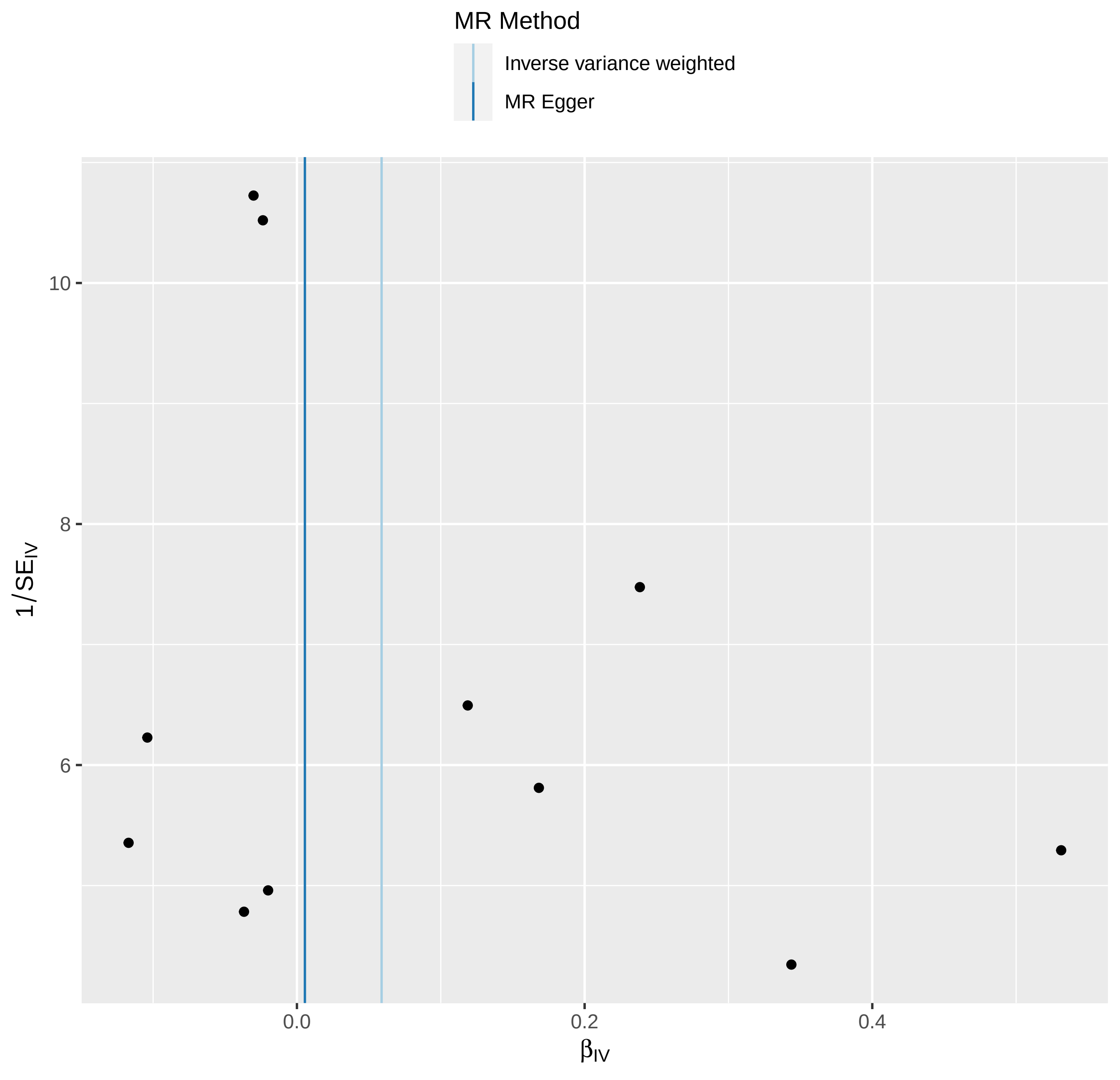

Batch 3−1832 : Selenium on Mannosyl−oligosaccharide 1,2−alpha−mannosidase IA

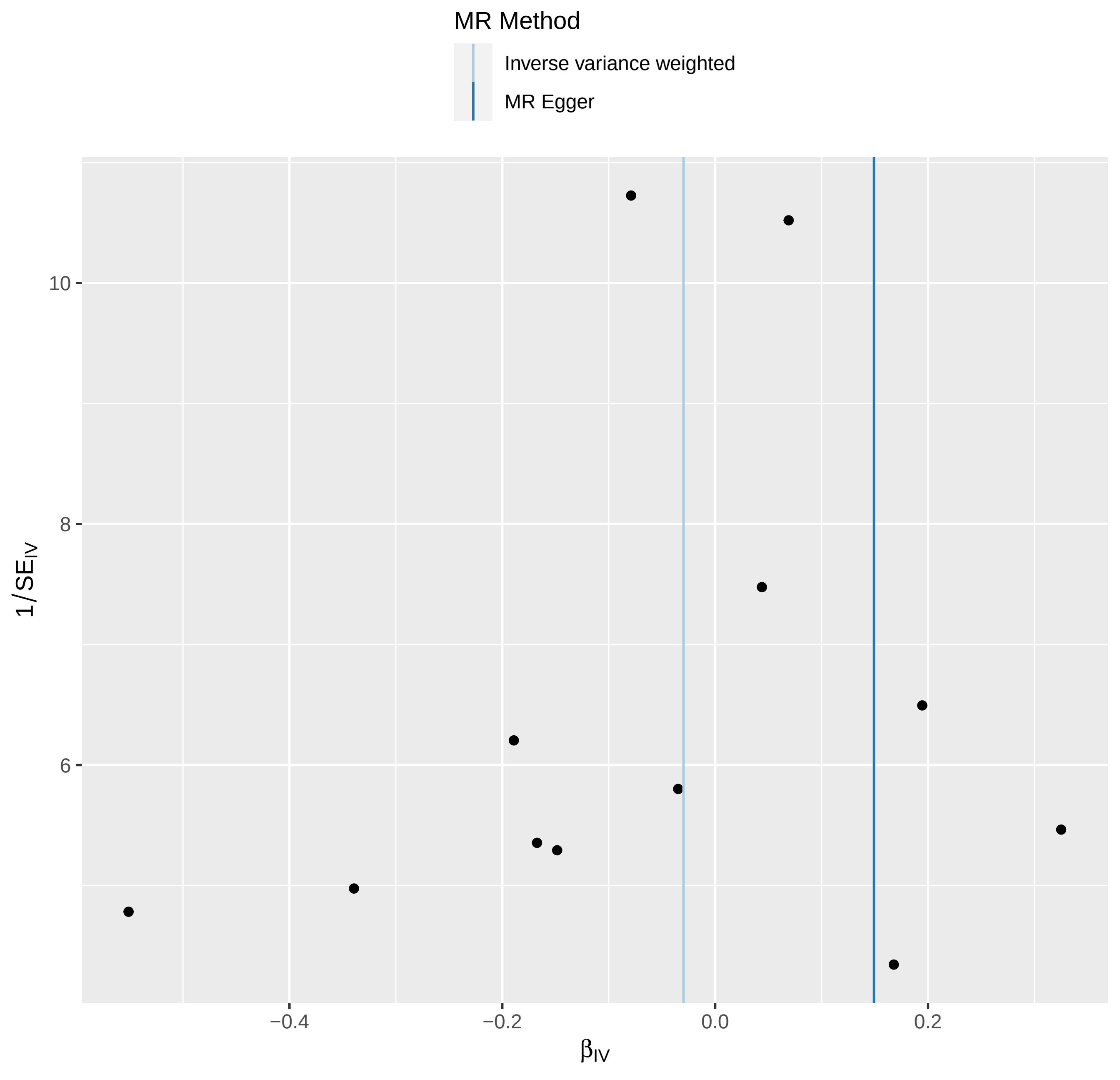

Batch 3−1833 : Selenium on Mannosyl−oligosaccharide 1,2−alpha−mannosidase IB

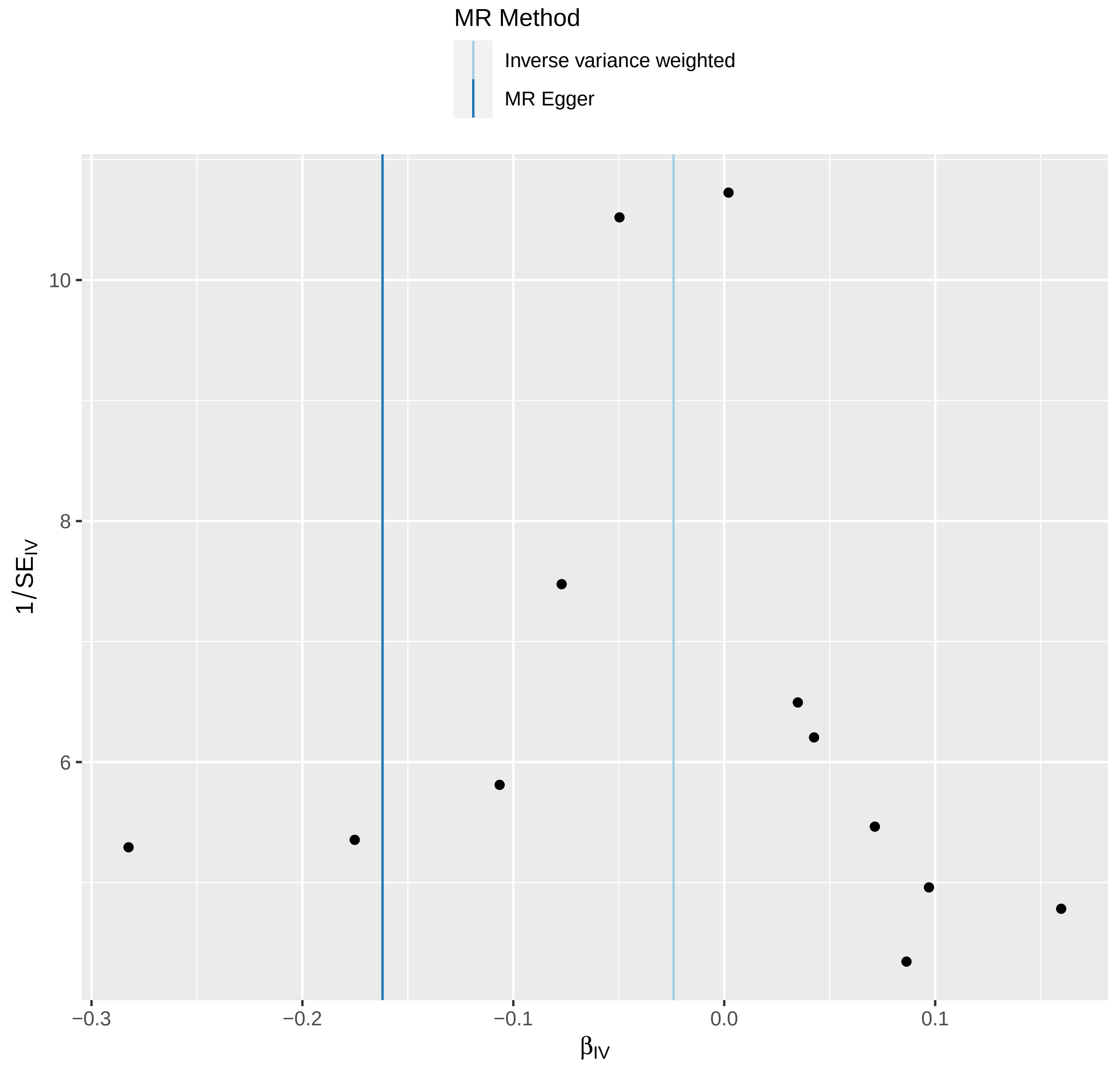

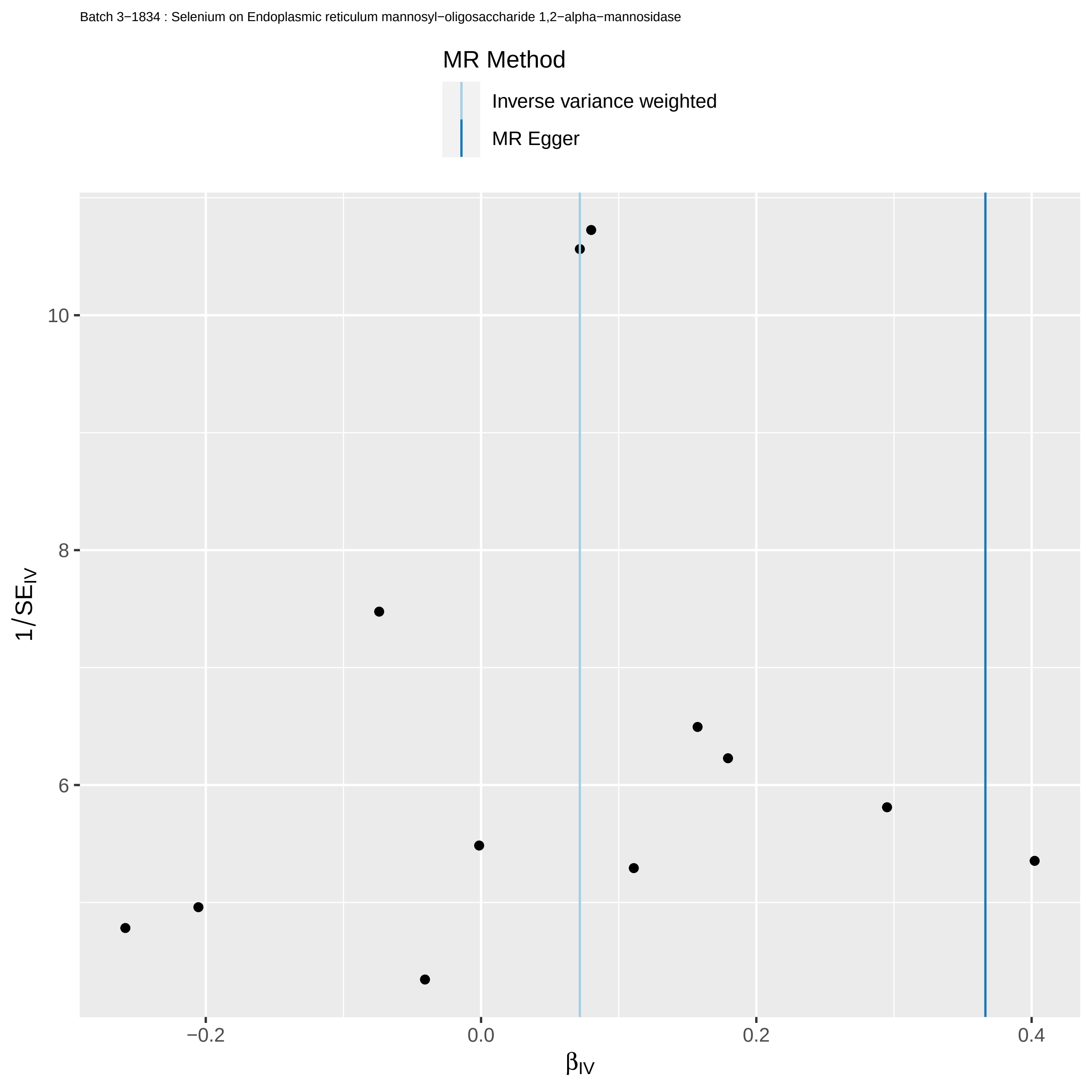
Batch 3−1834 : Selenium on Endoplasmic reticulum mannosyl−oligosaccharide 1,2−alpha−mannosidase
MR Method
Inverse variance weighted
MR Egger
10
8
6
1/SE$_{IV}$
−0.2
0.0
0.2
0.4
β$_{IV}$

Batch 3−1835 : Selenium on Epididymis−specific alpha−mannosidase

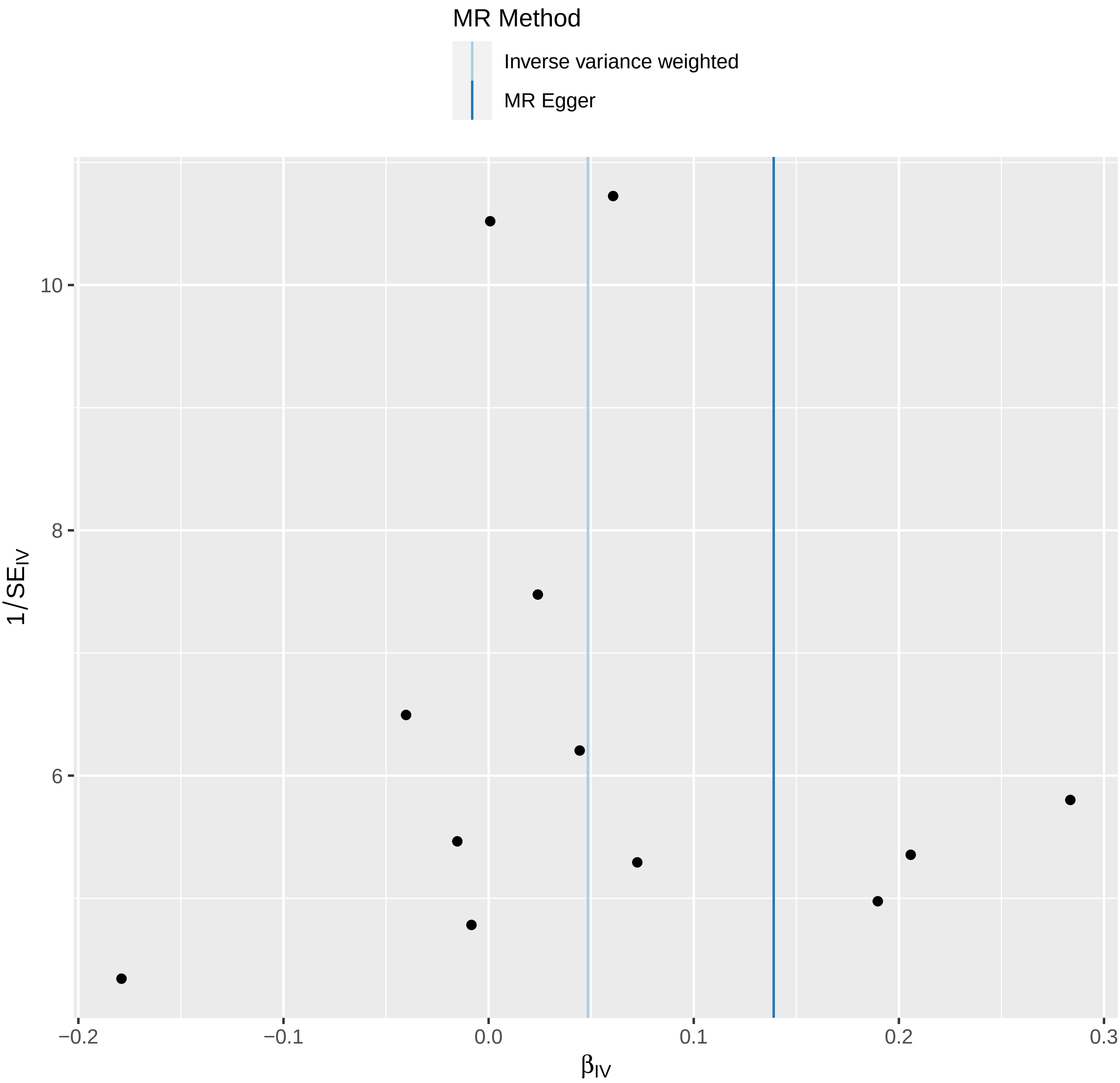

Batch 3−1836 : Selenium on Beta−mannosidase

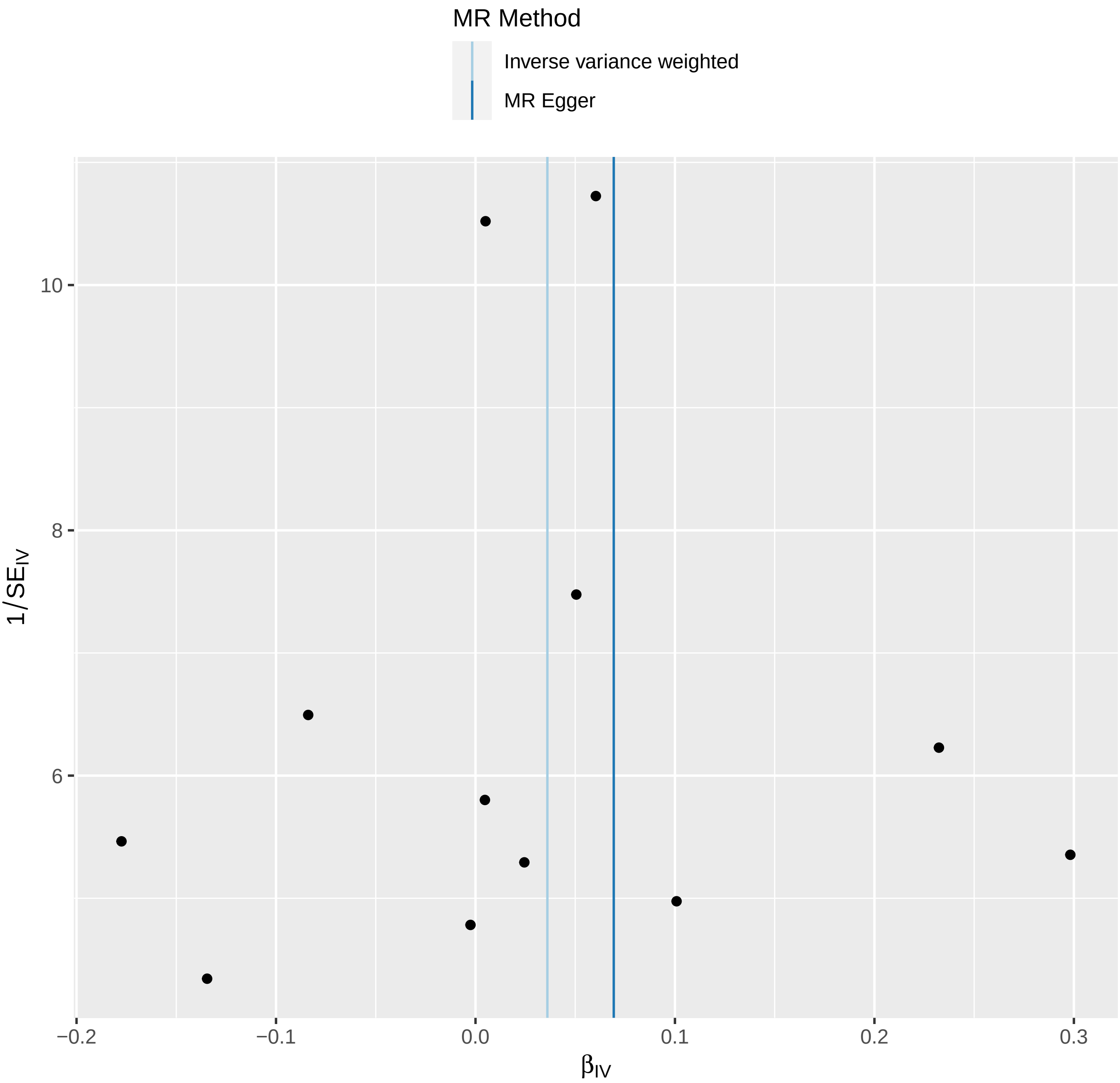

Batch 3−1837 : Selenium on Glycoprotein endo−alpha−1,2−mannosidase

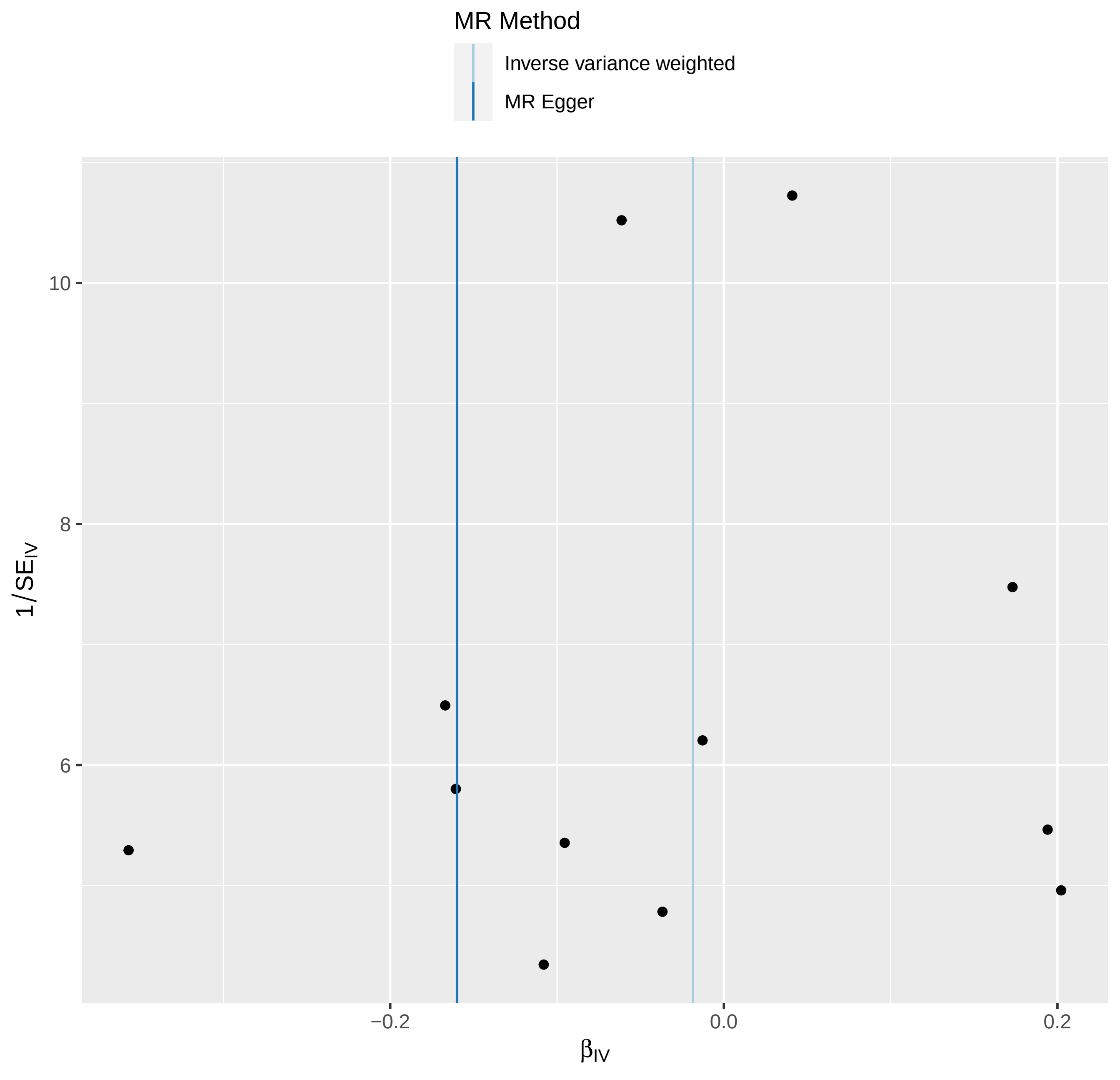

Batch 3−1838 : Selenium on MANSC domain−containing protein 1

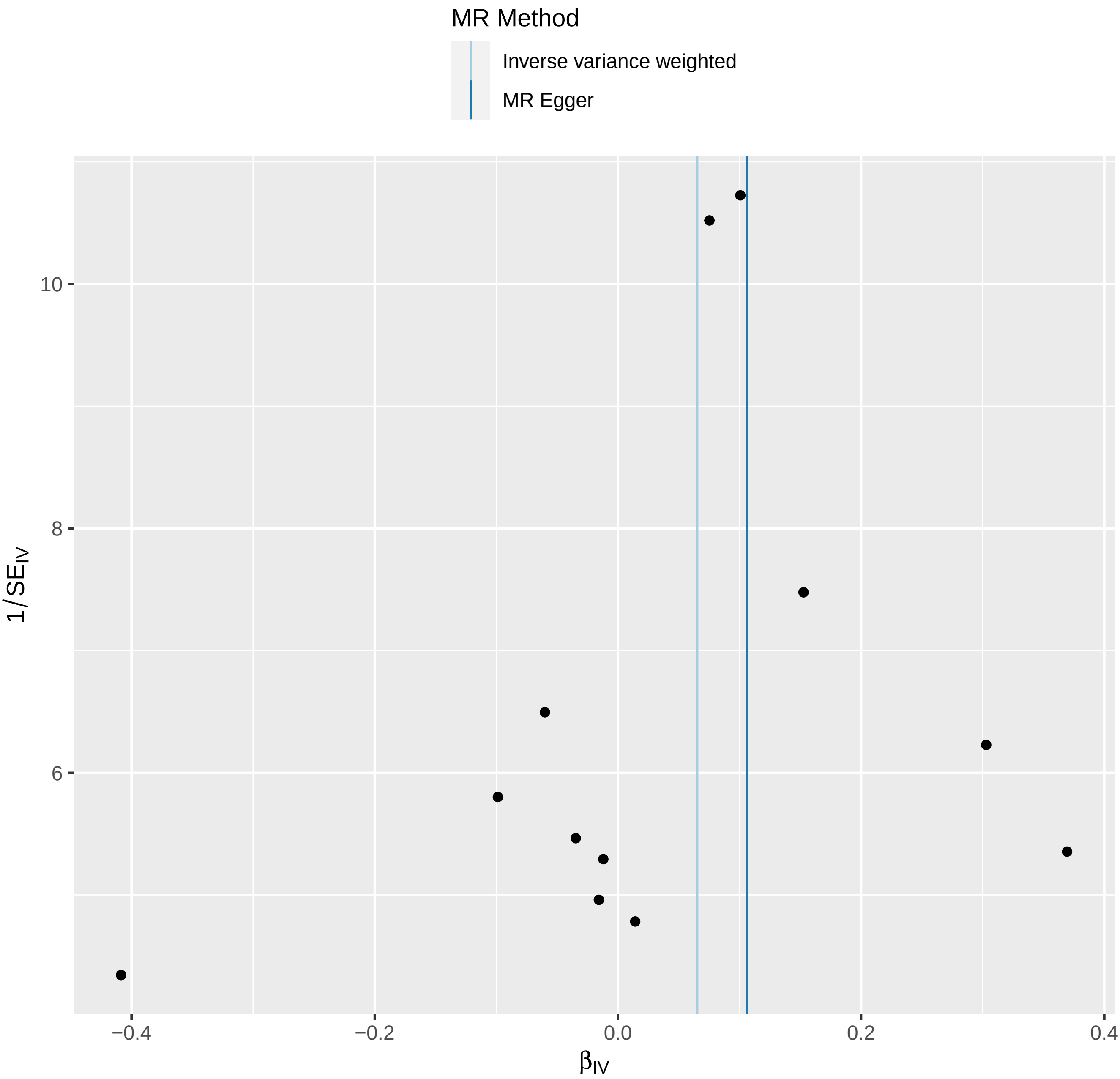

Batch 3−1839 : Selenium on MANSC domain−containing protein 4

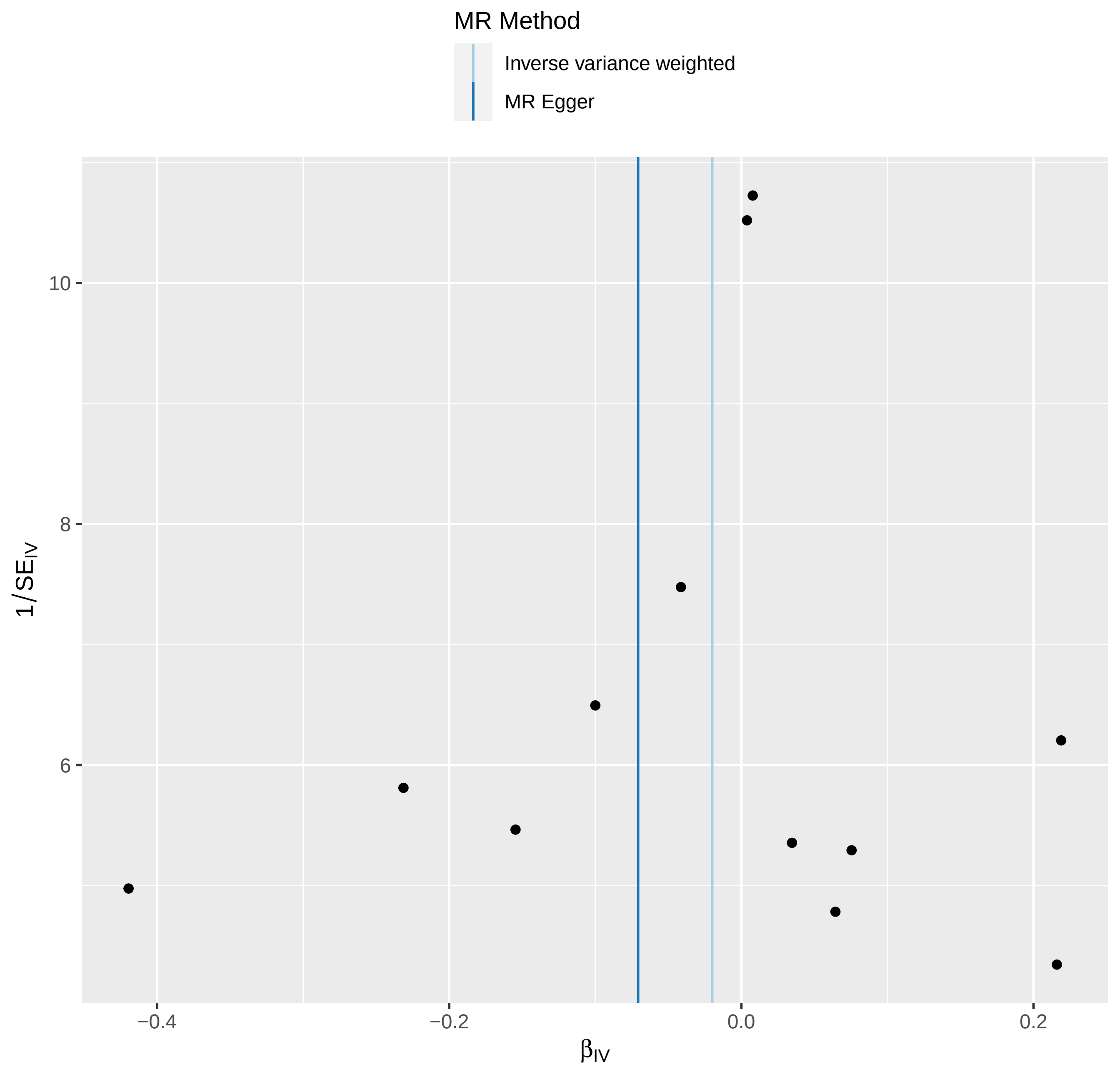

Batch 3−1840 : Selenium on Dual specificity mitogen−activated protein kinase kinase 1

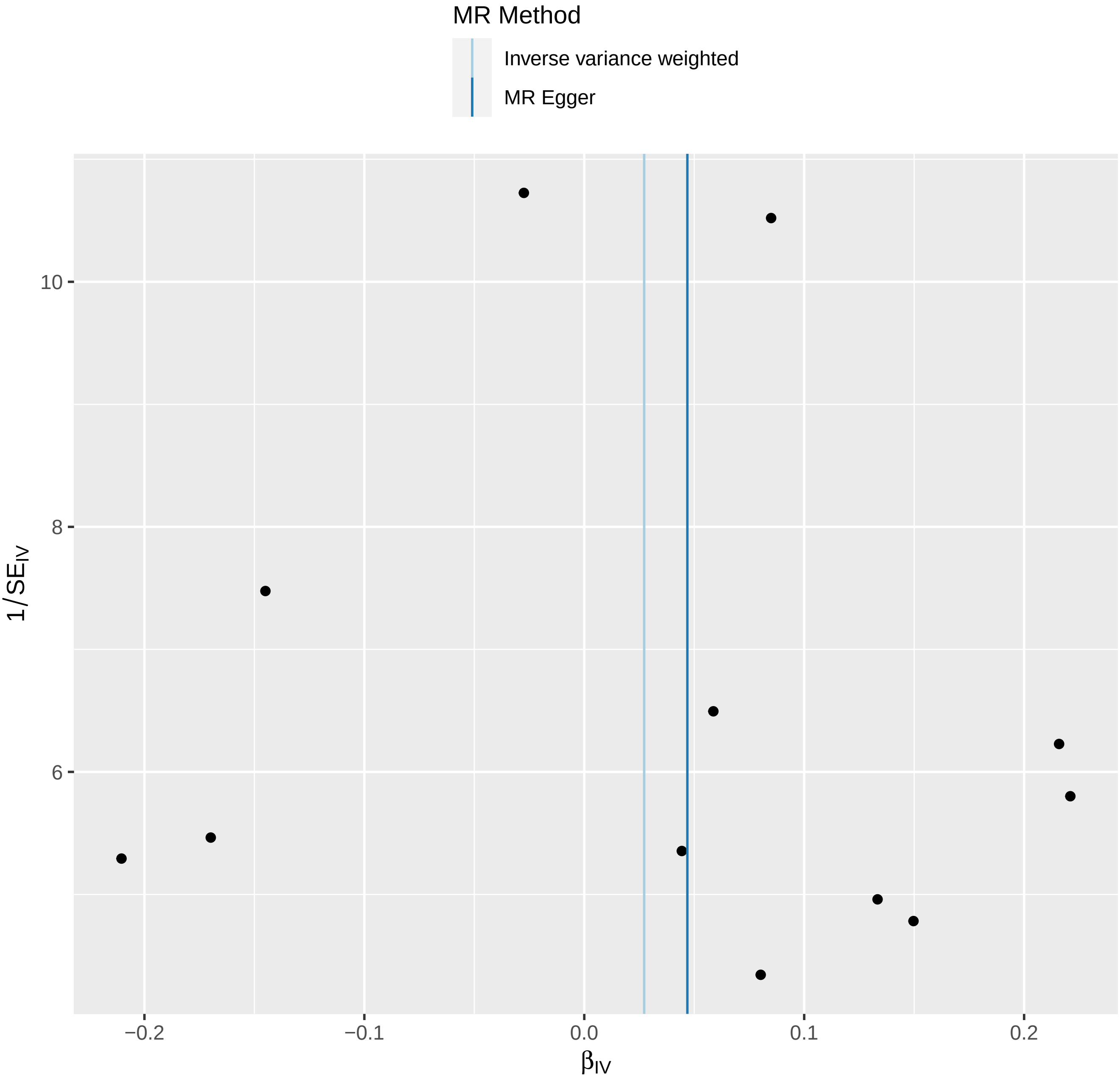

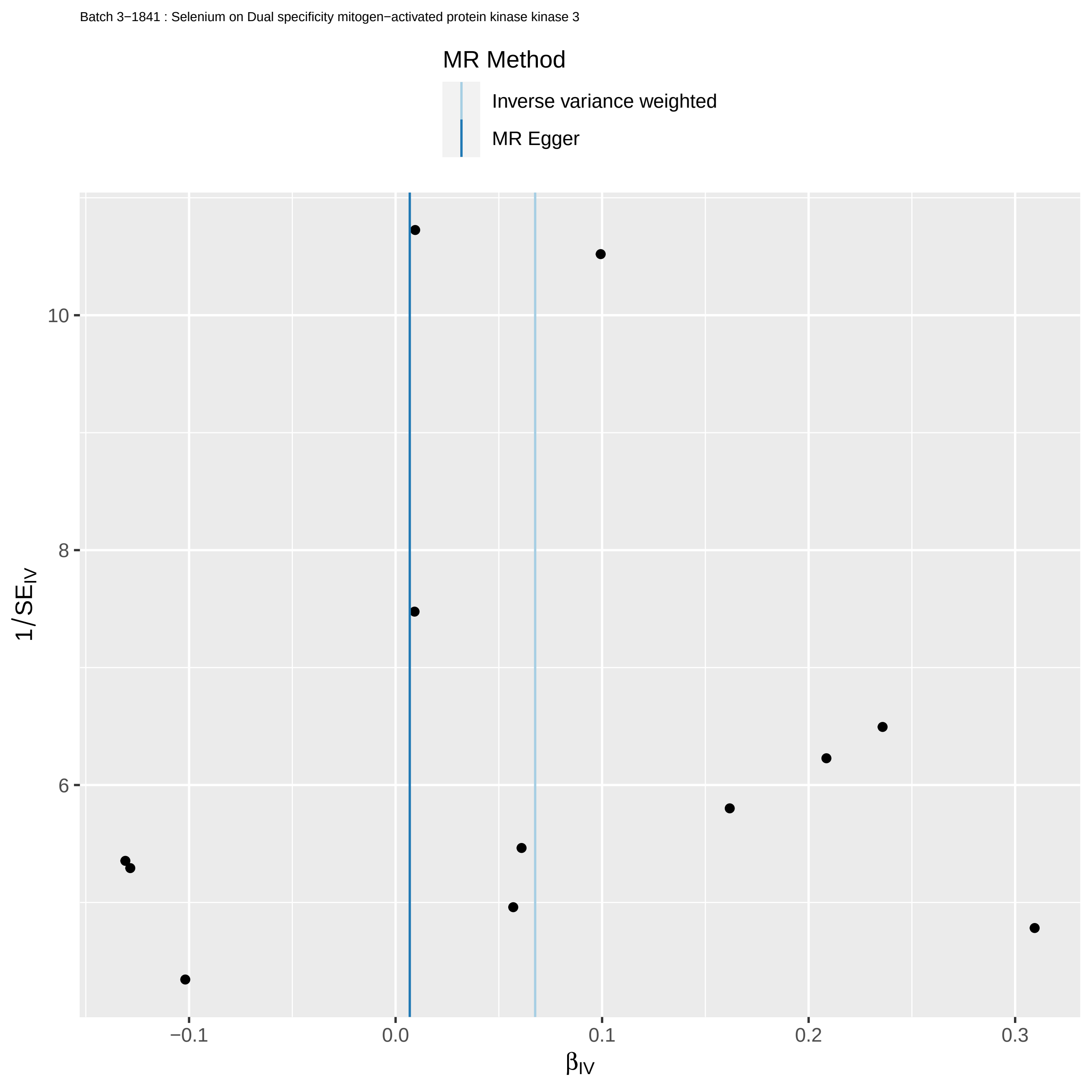

Batch 3−1841 : Selenium on Dual specificity mitogen−activated protein kinase kinase 3
MR Method
Inverse variance weighted
MR Egger
10
8
6
1/SE_IV
−0.1
0.0
0.1
0.2
0.3
β_IV

Batch 3−1842 : Selenium on Dual specificity mitogen−activated protein kinase kinase 4

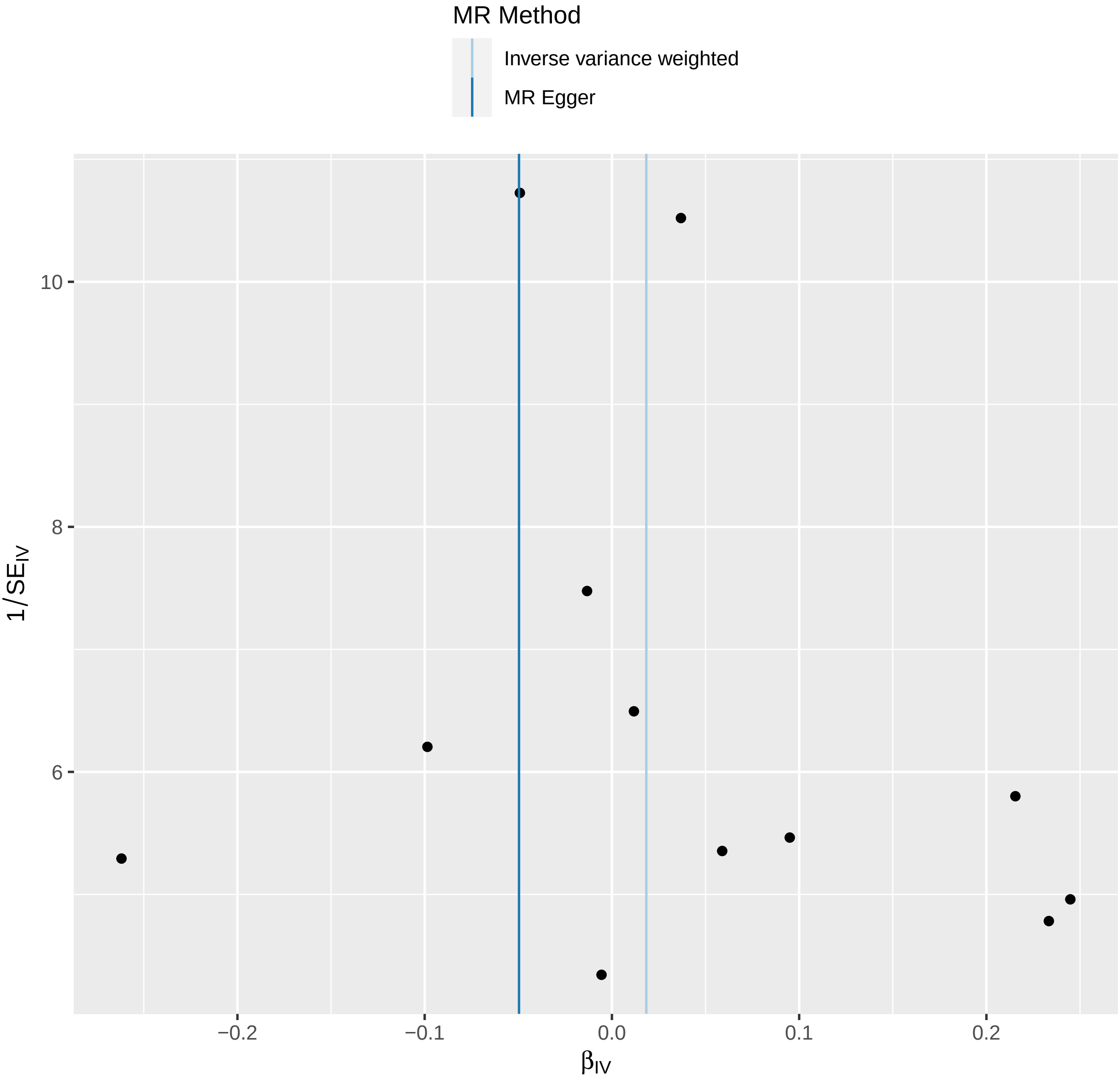

Batch 3−1843 : Selenium on Mitogen−activated protein kinase kinase kinase 3

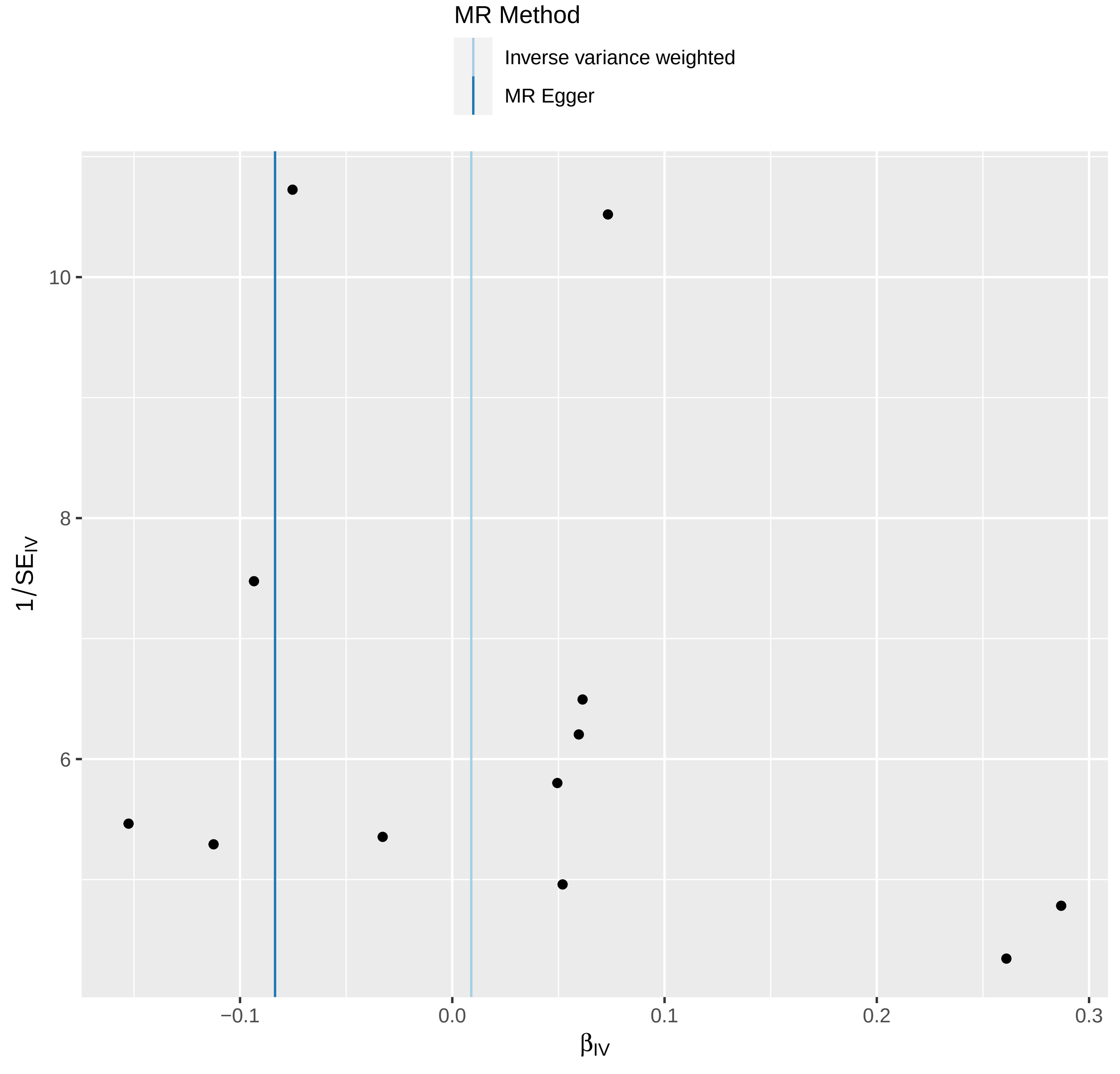

Batch 3−1844 : Selenium on Mitogen−activated protein kinase kinase kinase 7:TGF−beta−activated kinase 1 and MAP3K7−binding protein 1 fusion

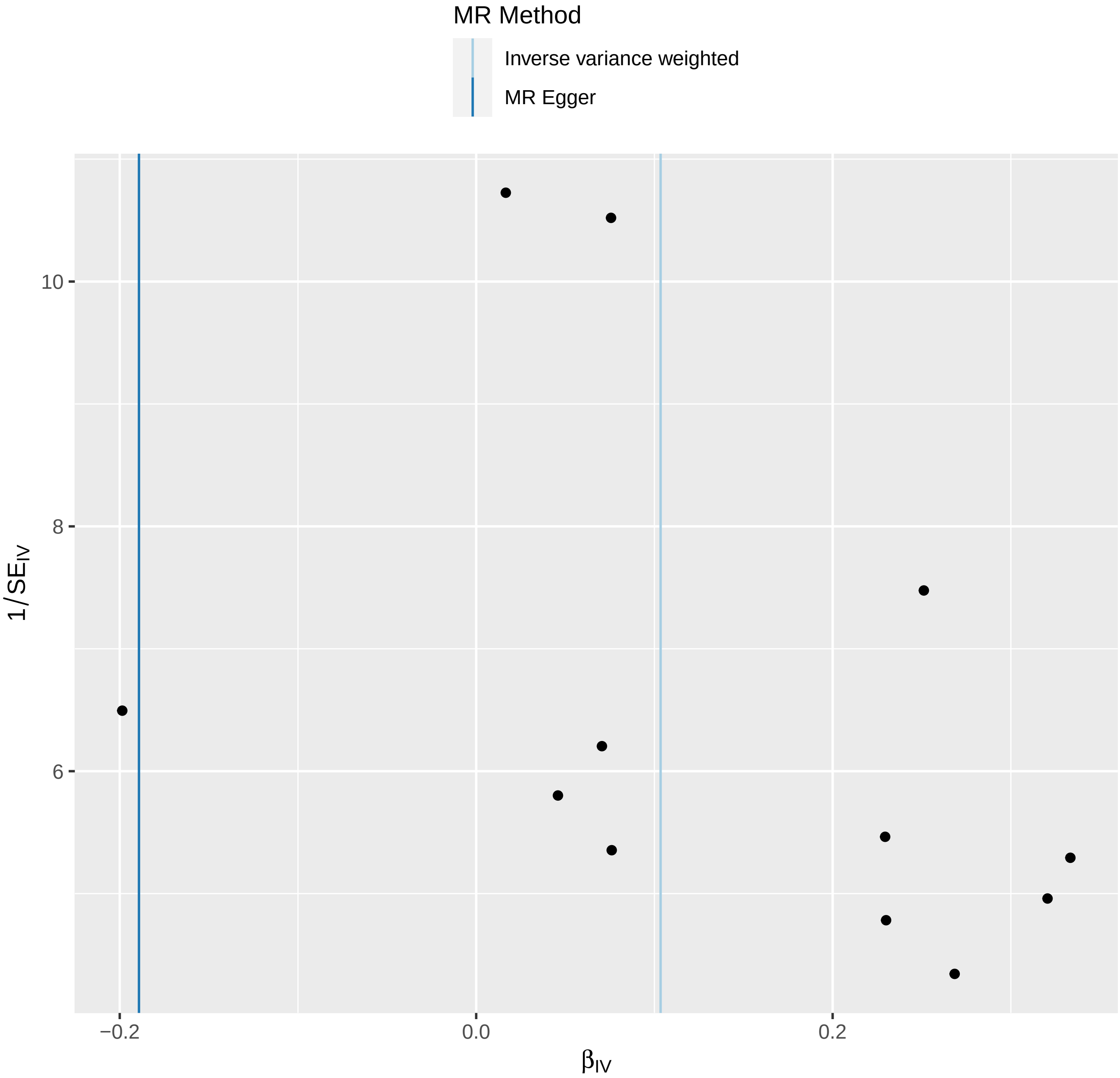

Batch 3−1845 : Selenium on Mitogen−activated protein kinase 1

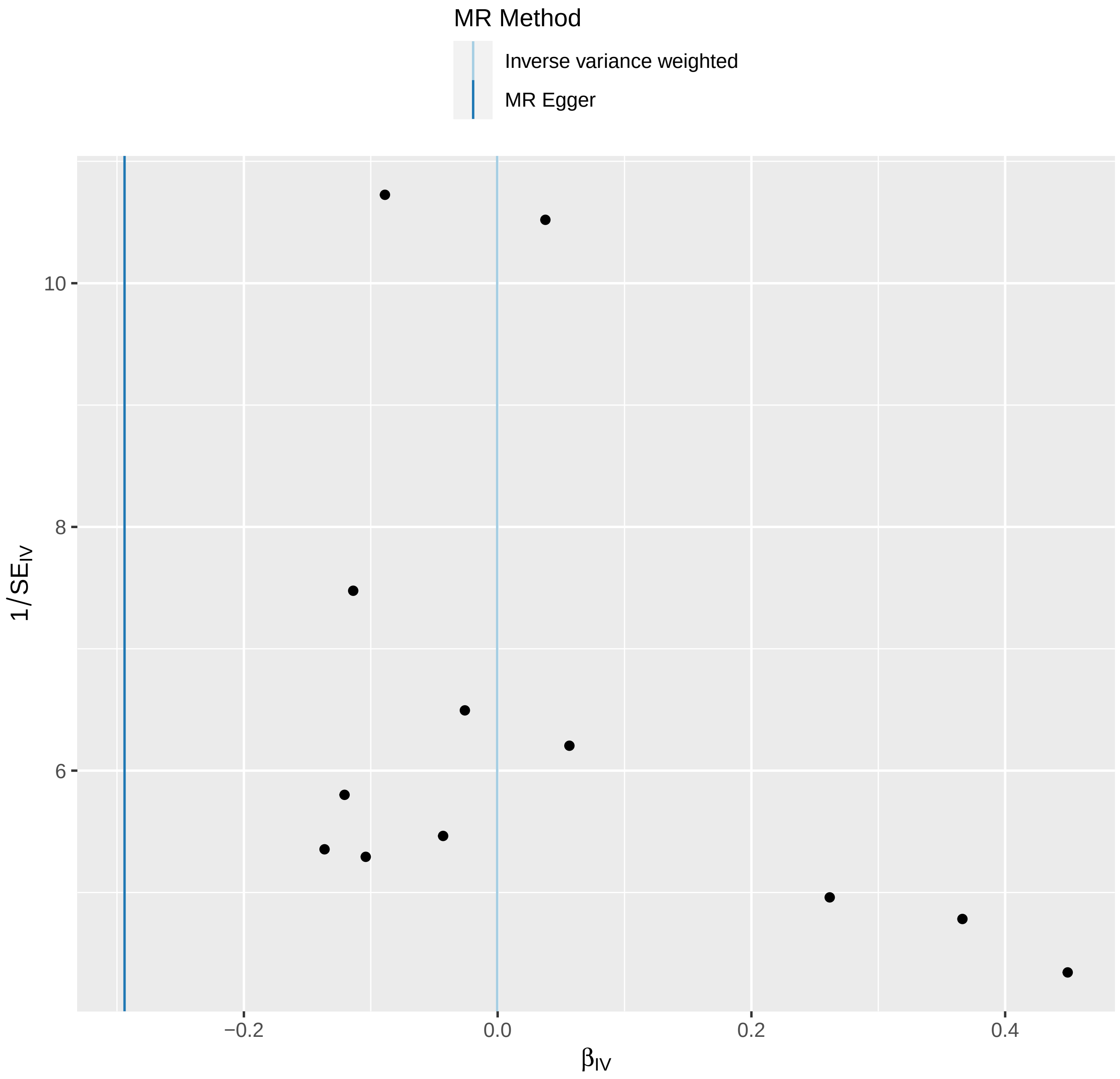

Batch 3−1846 : Selenium on Mitogen−activated protein kinase 13

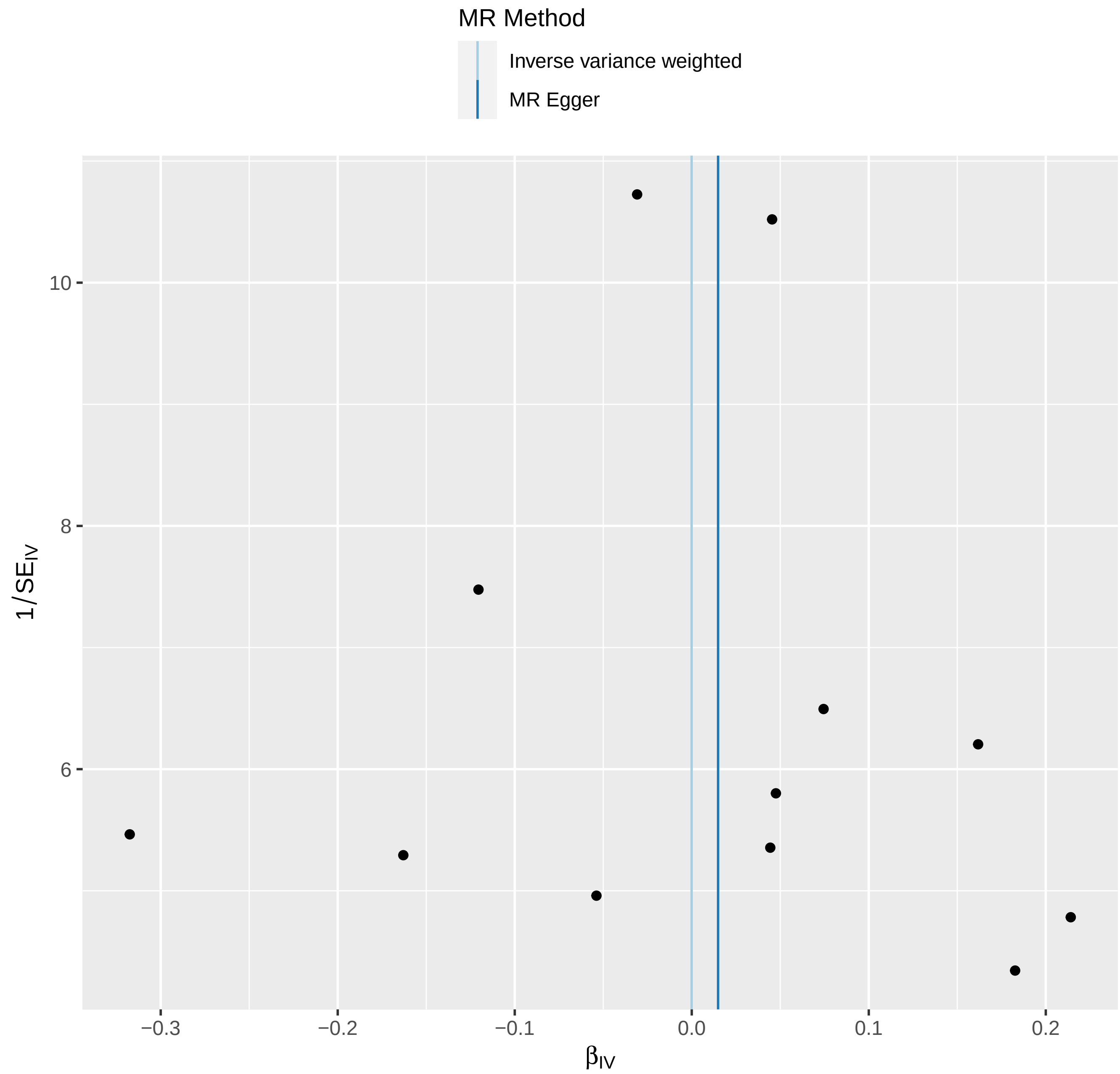

Batch 3−1847 : Selenium on Mitogen−activated protein kinase 14

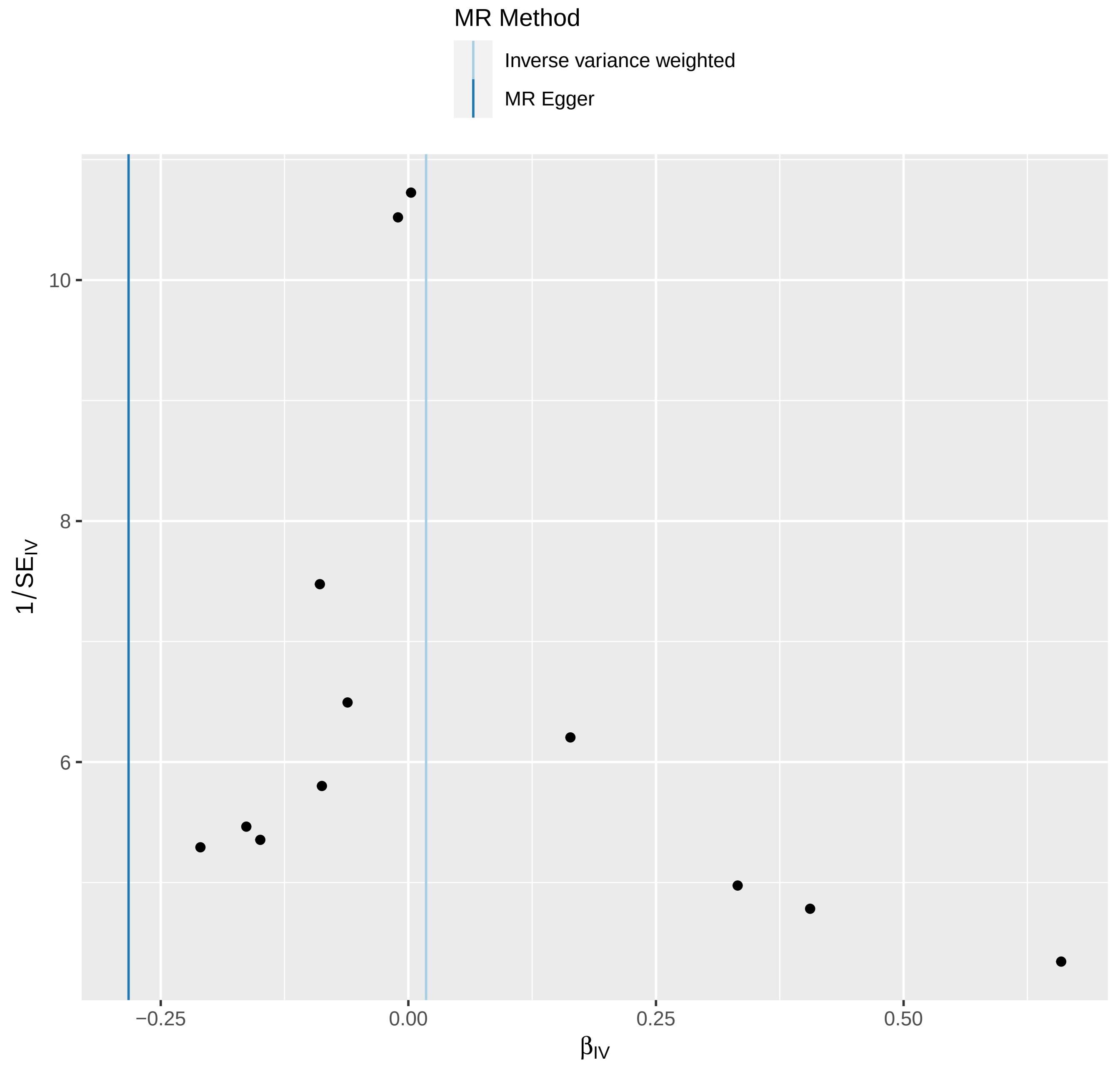

Batch 3−1848 : Selenium on Mitogen−activated protein kinase 3

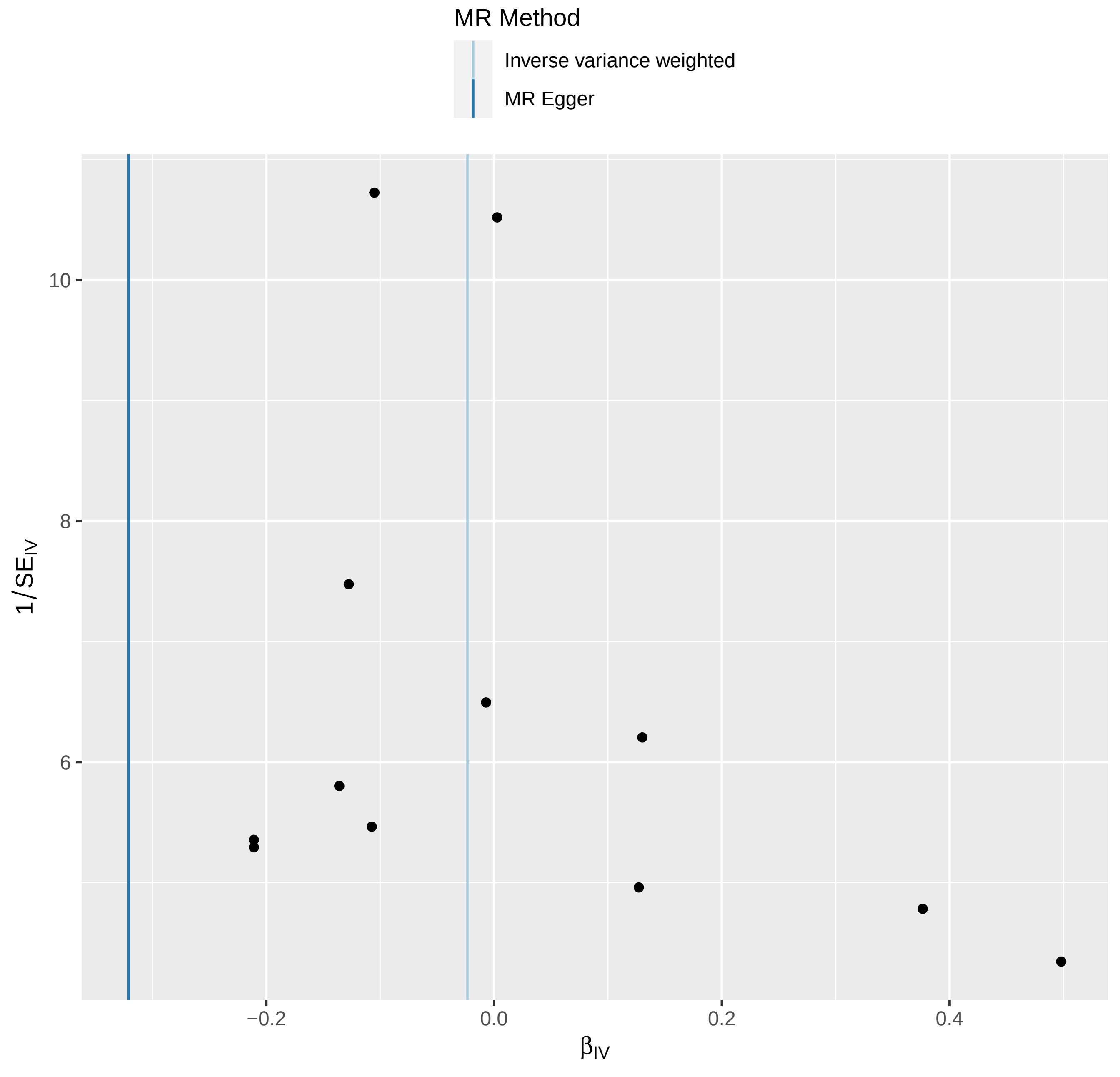

Batch 3−1849 : Selenium on Mitogen−activated protein kinase 8

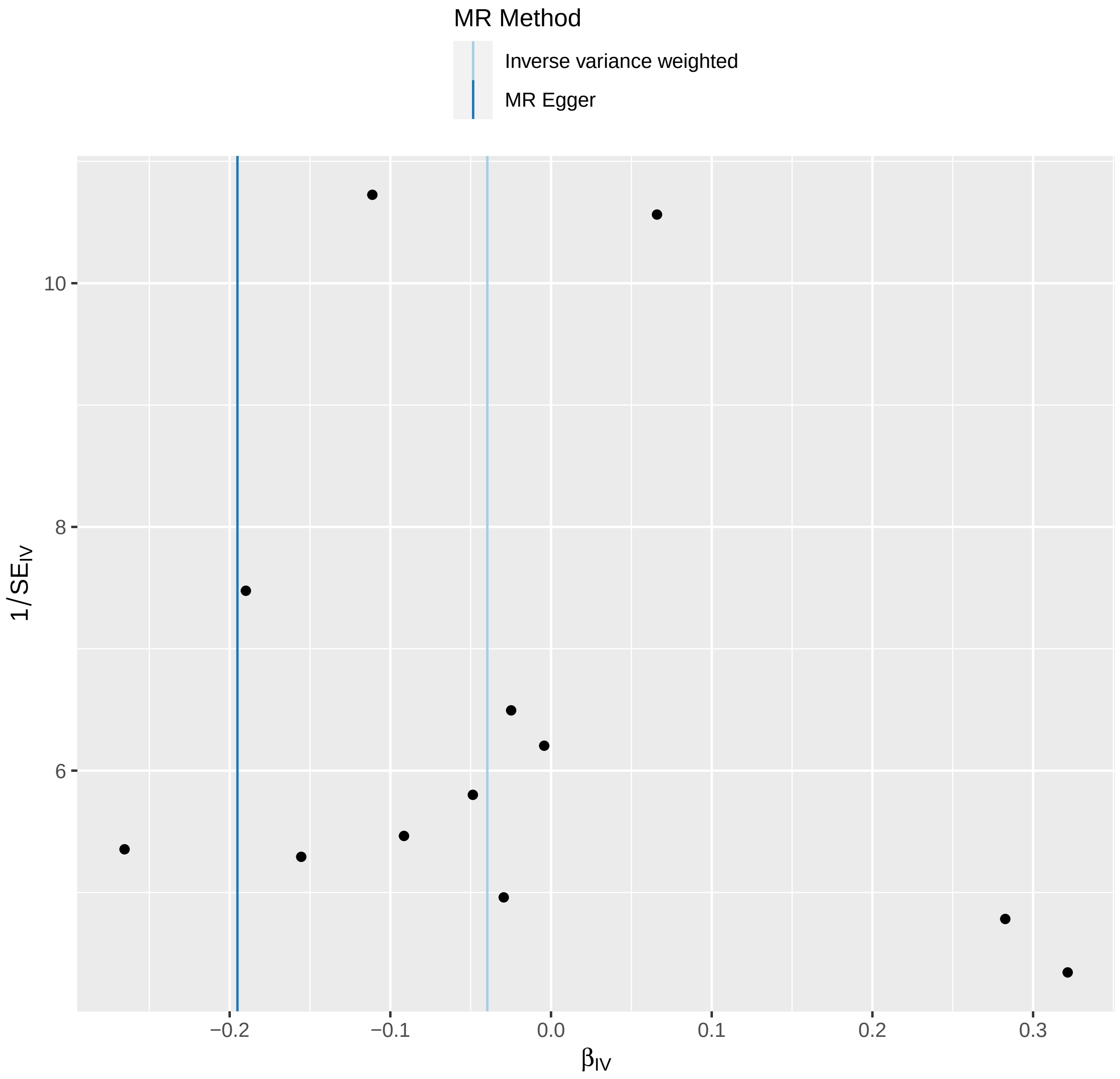

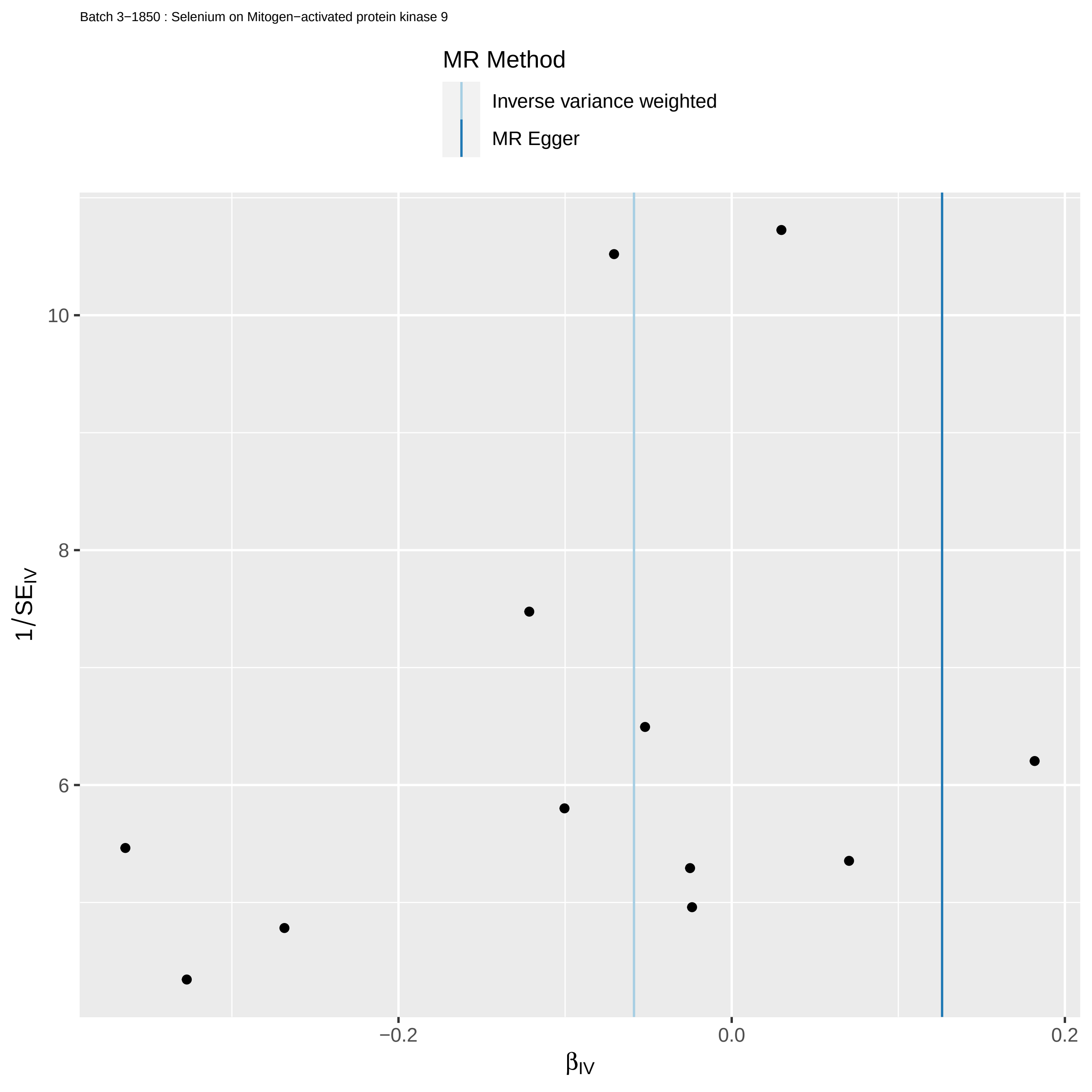
Batch 3−1850 : Selenium on Mitogen−activated protein kinase 9
MR Method
Inverse variance weighted
MR Egger
10
8
6
1/SE_IV
−0.2
0.0
0.2
β_IV

Batch 3−1851 : Selenium on MAP kinase−activated protein kinase 2

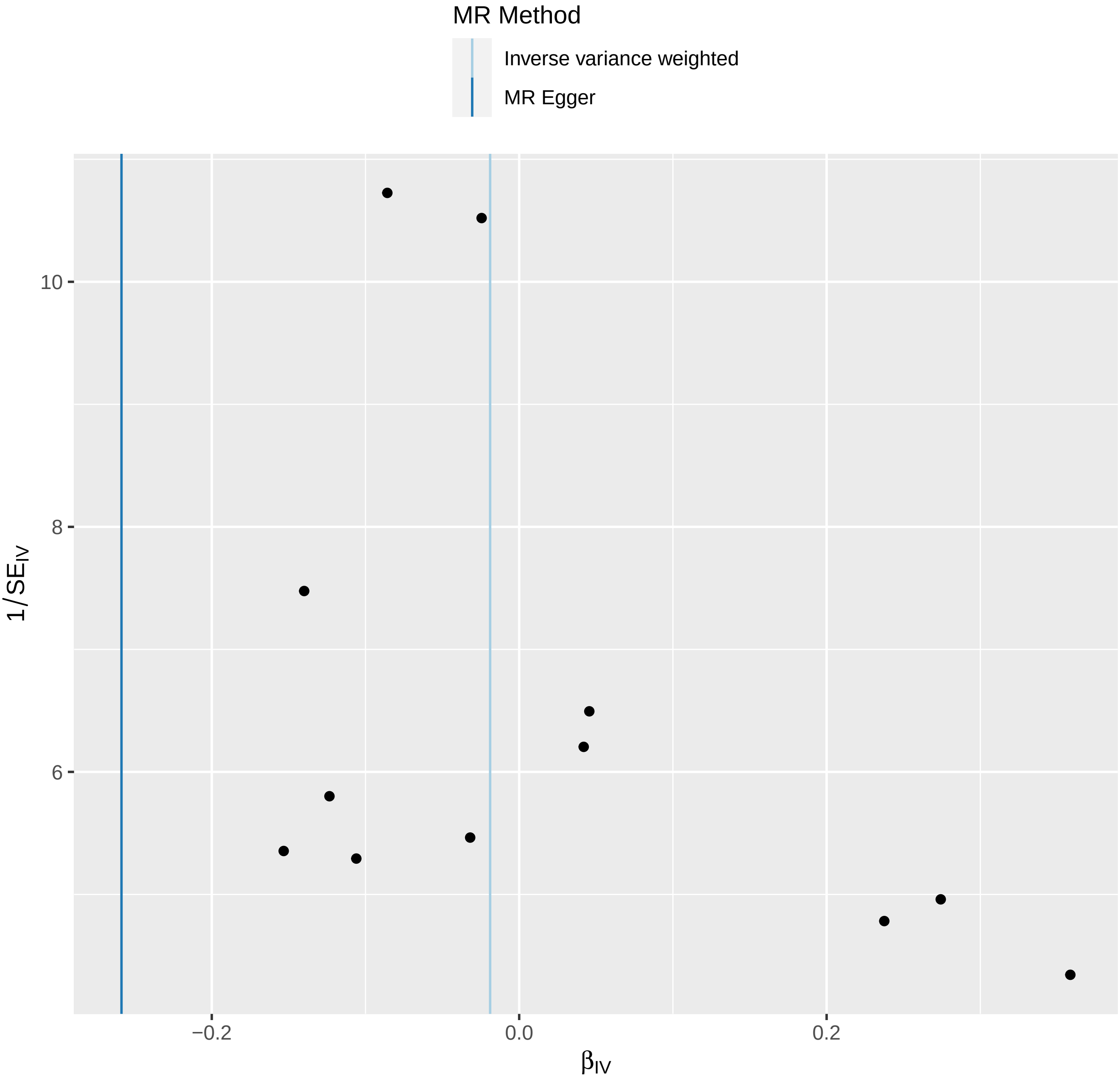

Batch 3−1852 : Selenium on MAP kinase−activated protein kinase 3

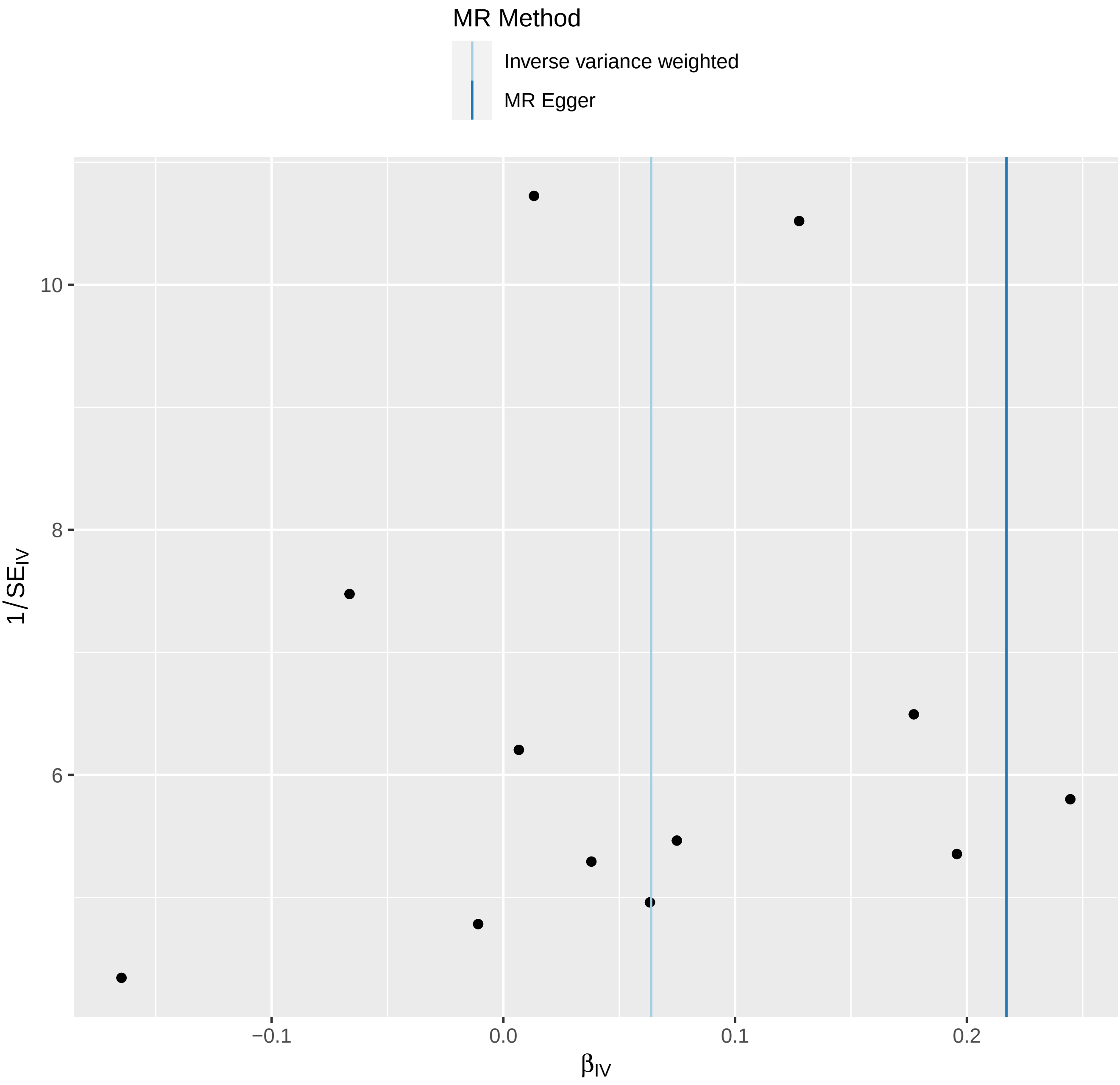

Batch 3−1853 : Selenium on MAP kinase−activated protein kinase 5

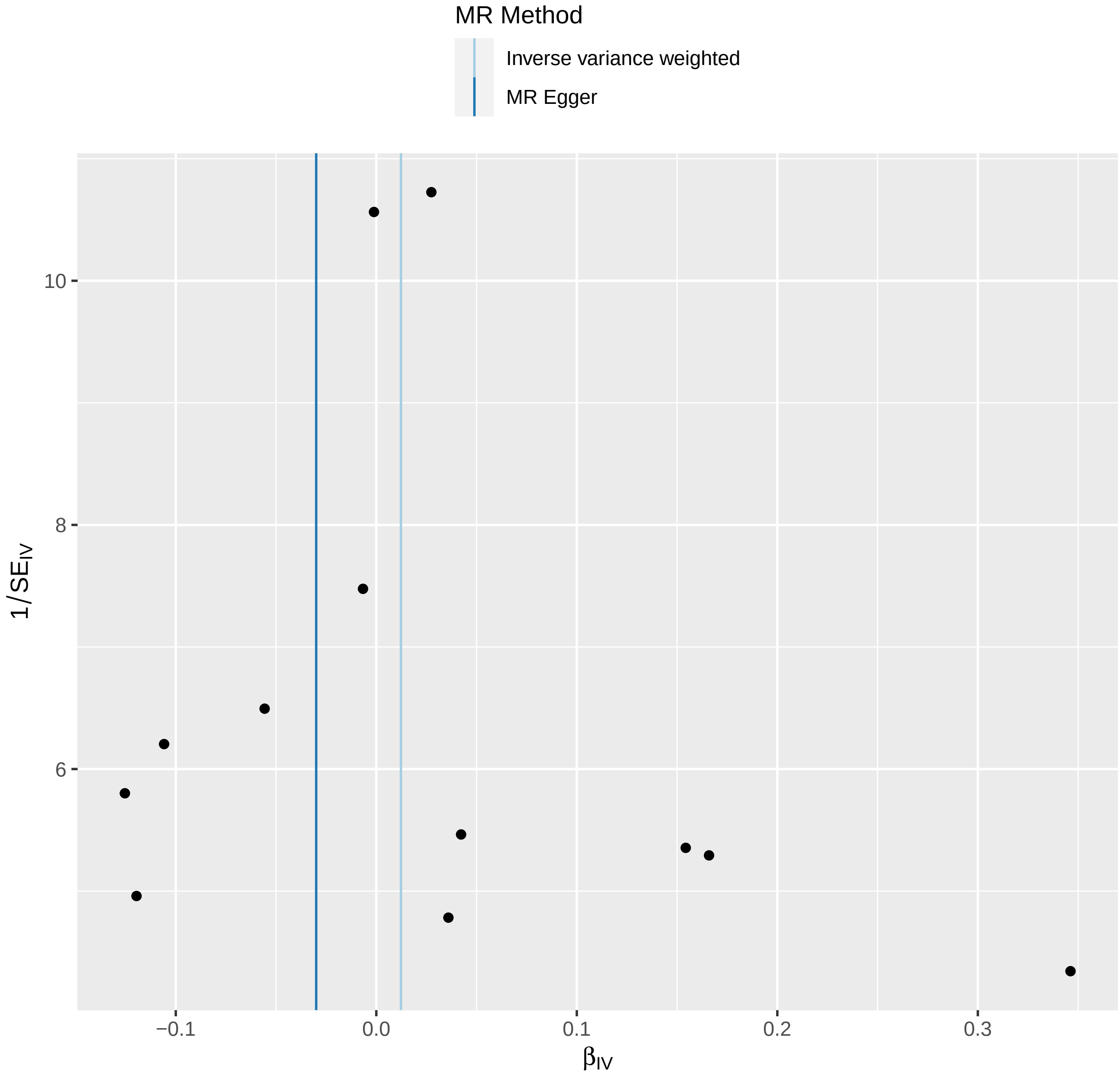

Batch 3−1854 : Selenium on MAP kinase−activated protein kinase 5

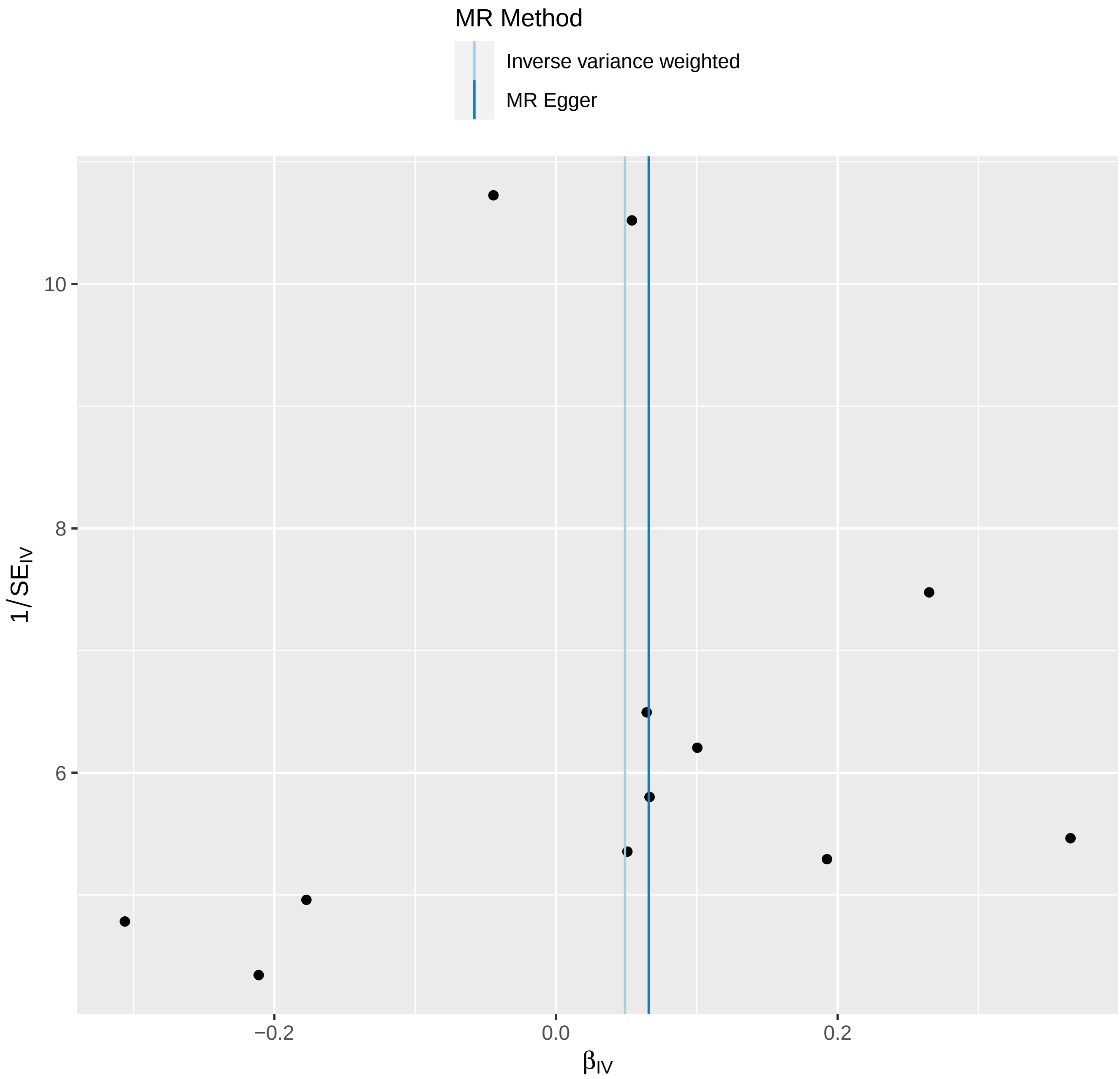

Batch 3−1855 : Selenium on Mannan−binding lectin serine protease 1

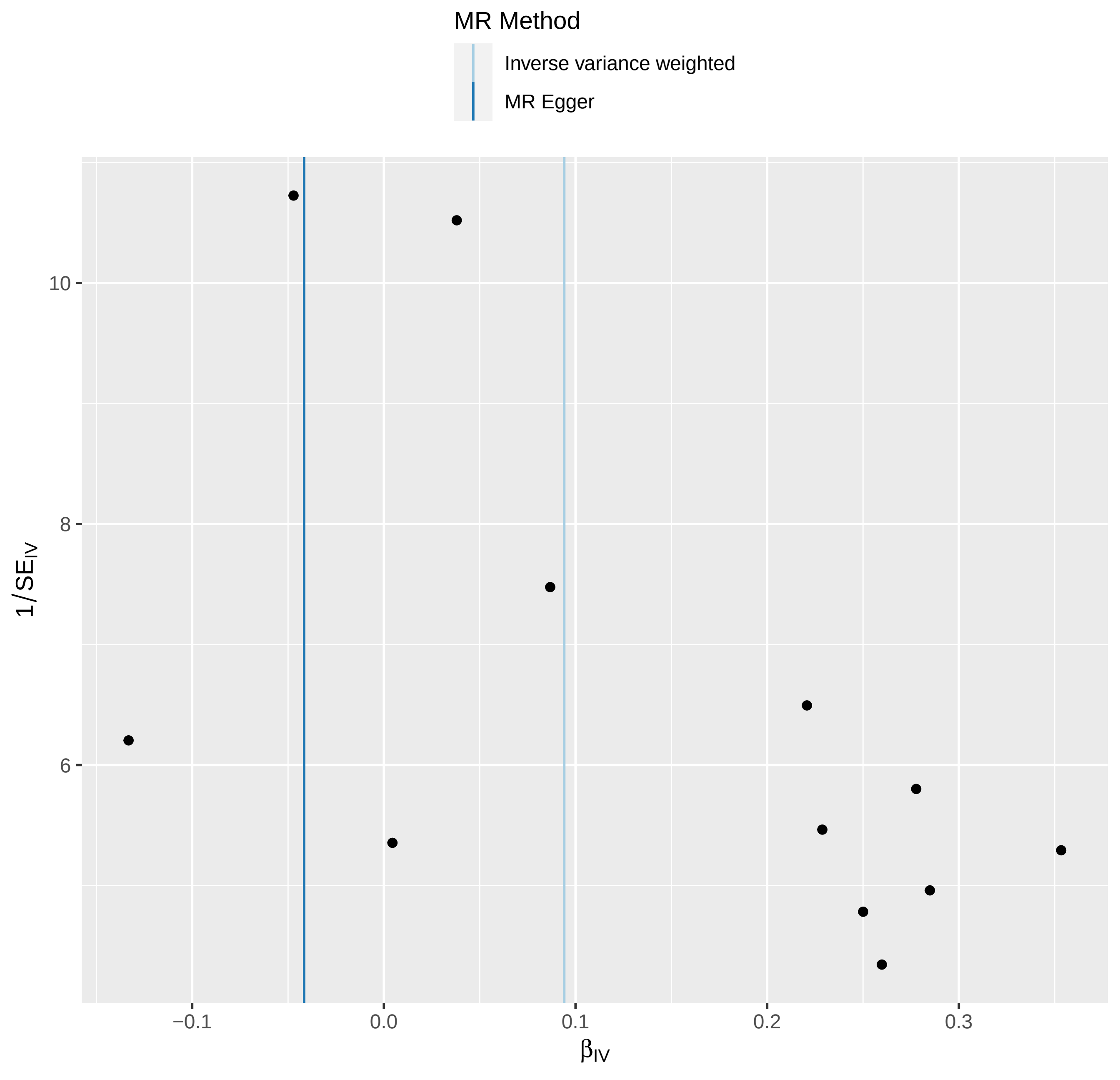

Batch 3−1856 : Selenium on Megakaryocyte−associated tyrosine−protein kinase

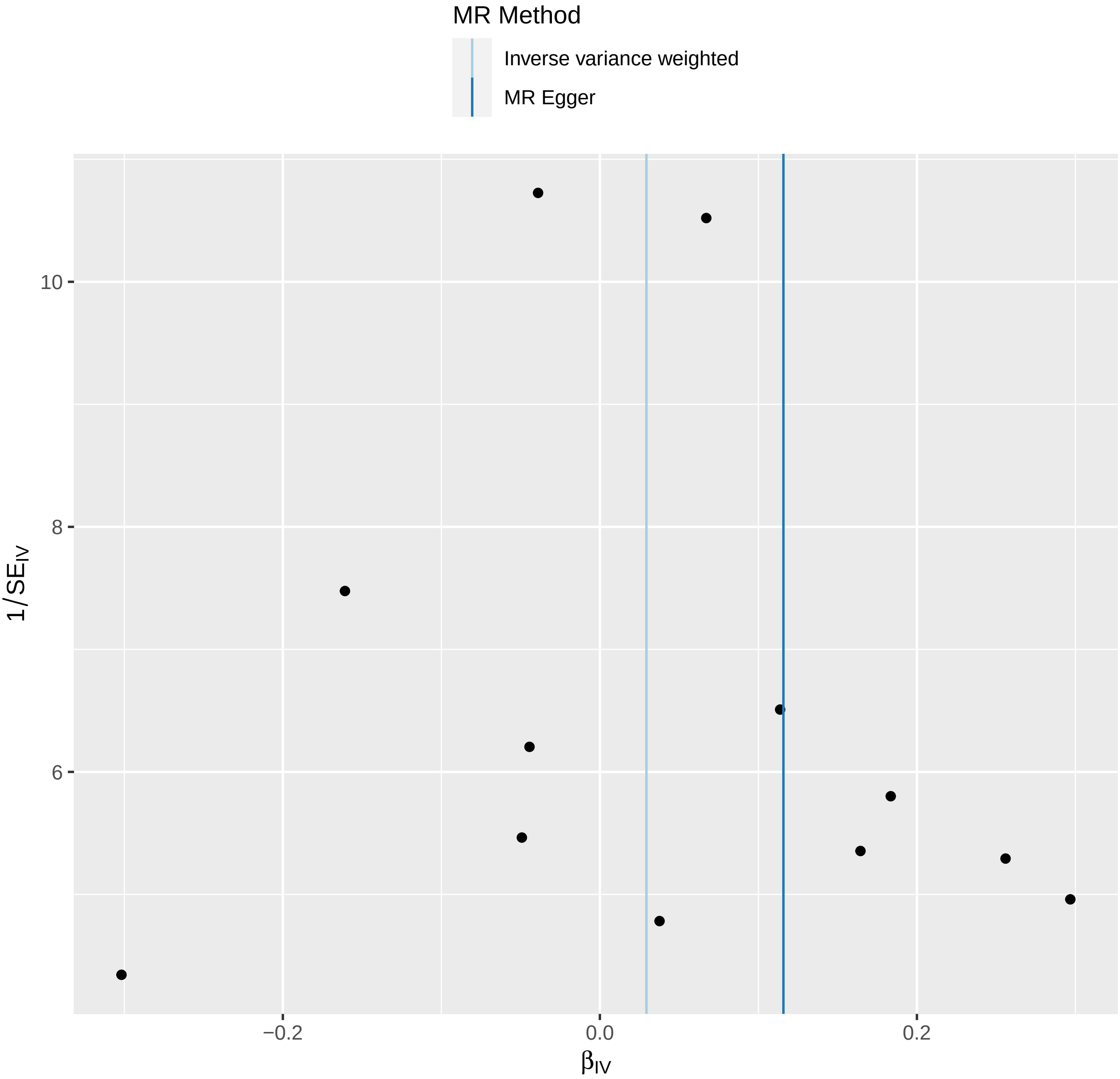

Batch 3−1857 : Selenium on Matrilin−2

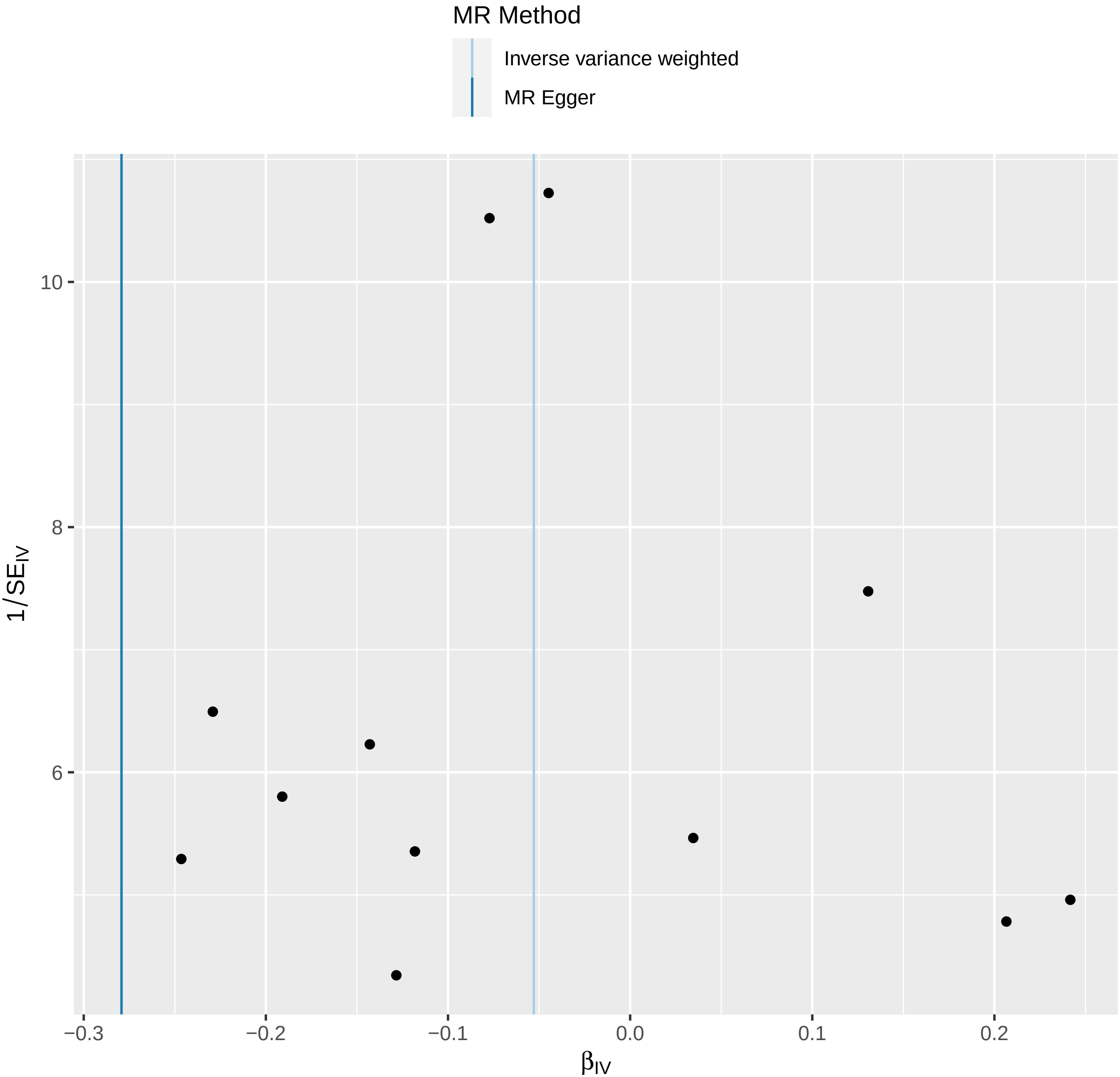

Batch 3−1858 : Selenium on Matrilin−3

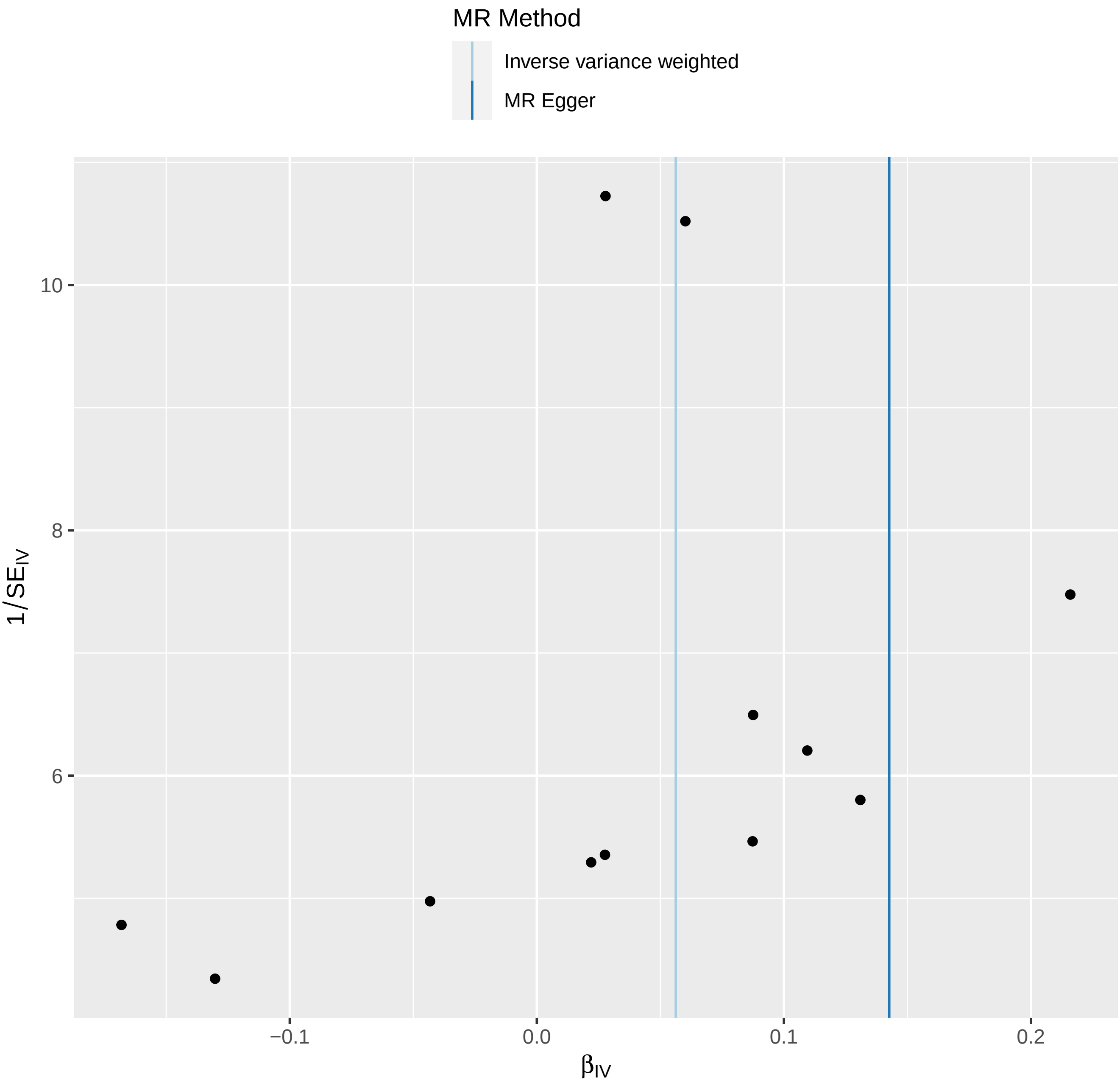

Batch 3−1859 : Selenium on Matrilin−4

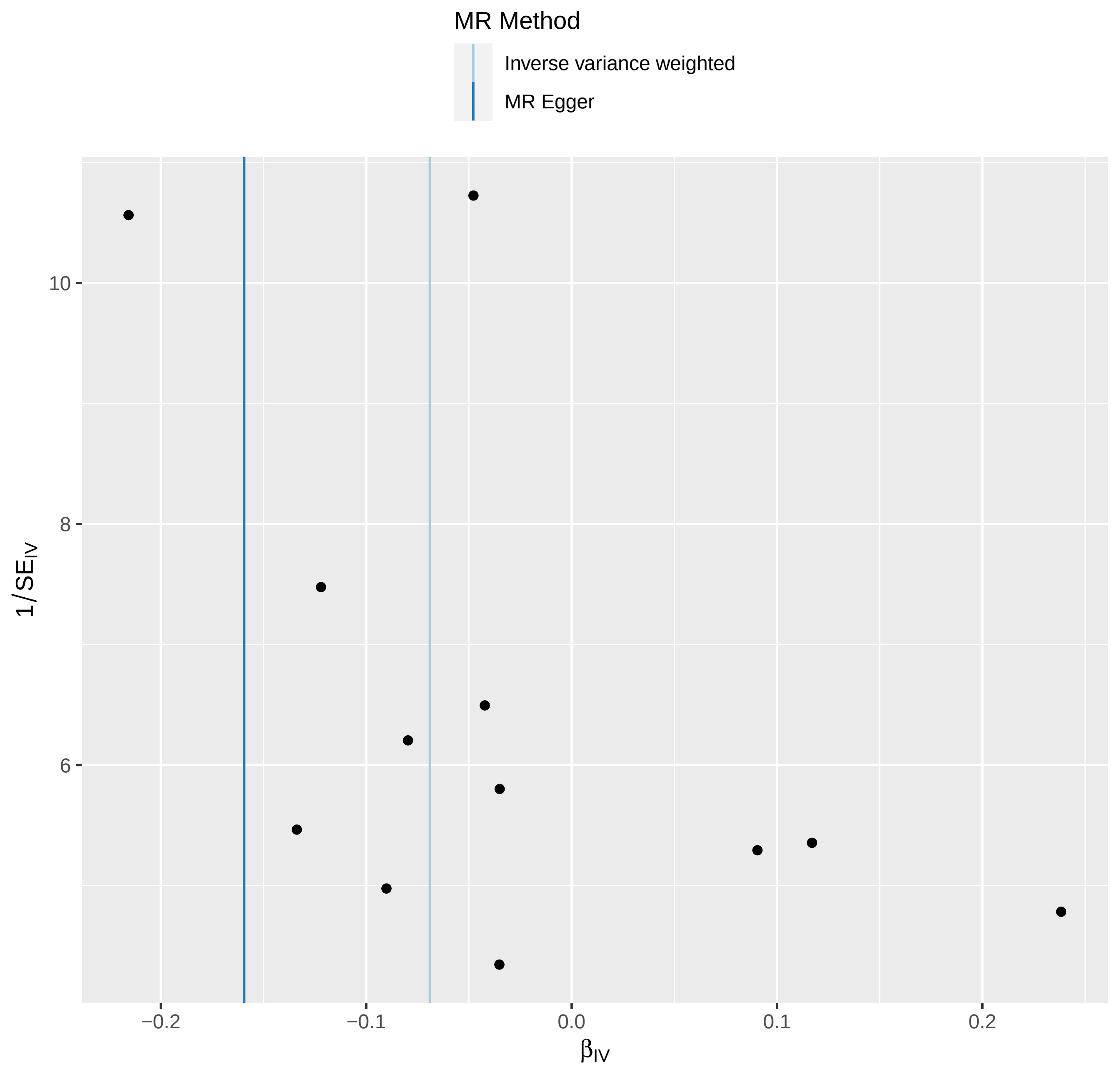

Batch 3−1860 : Selenium on Myc−associated zinc finger protein

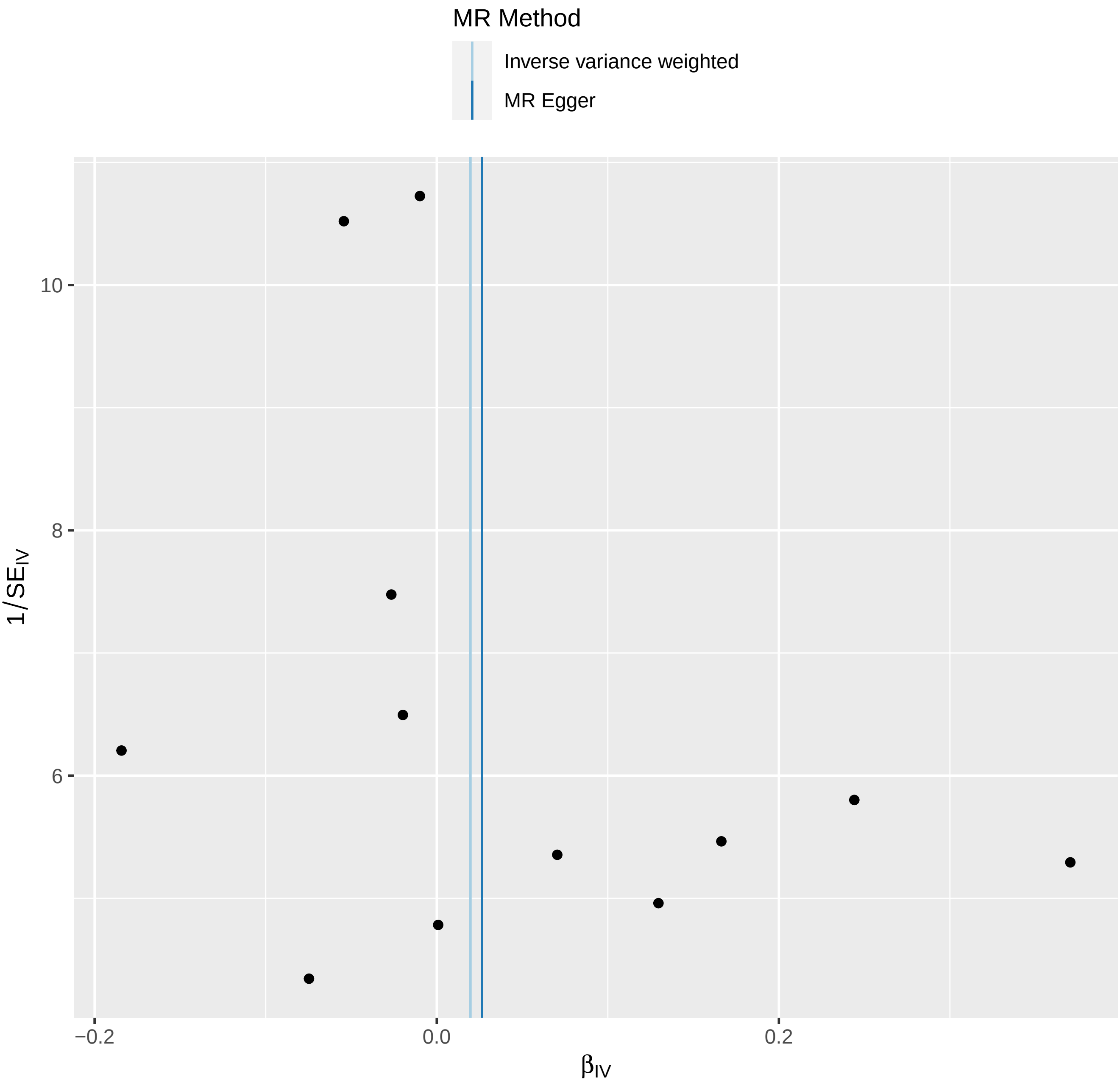

Batch 3−1861 : Selenium on Myoglobin

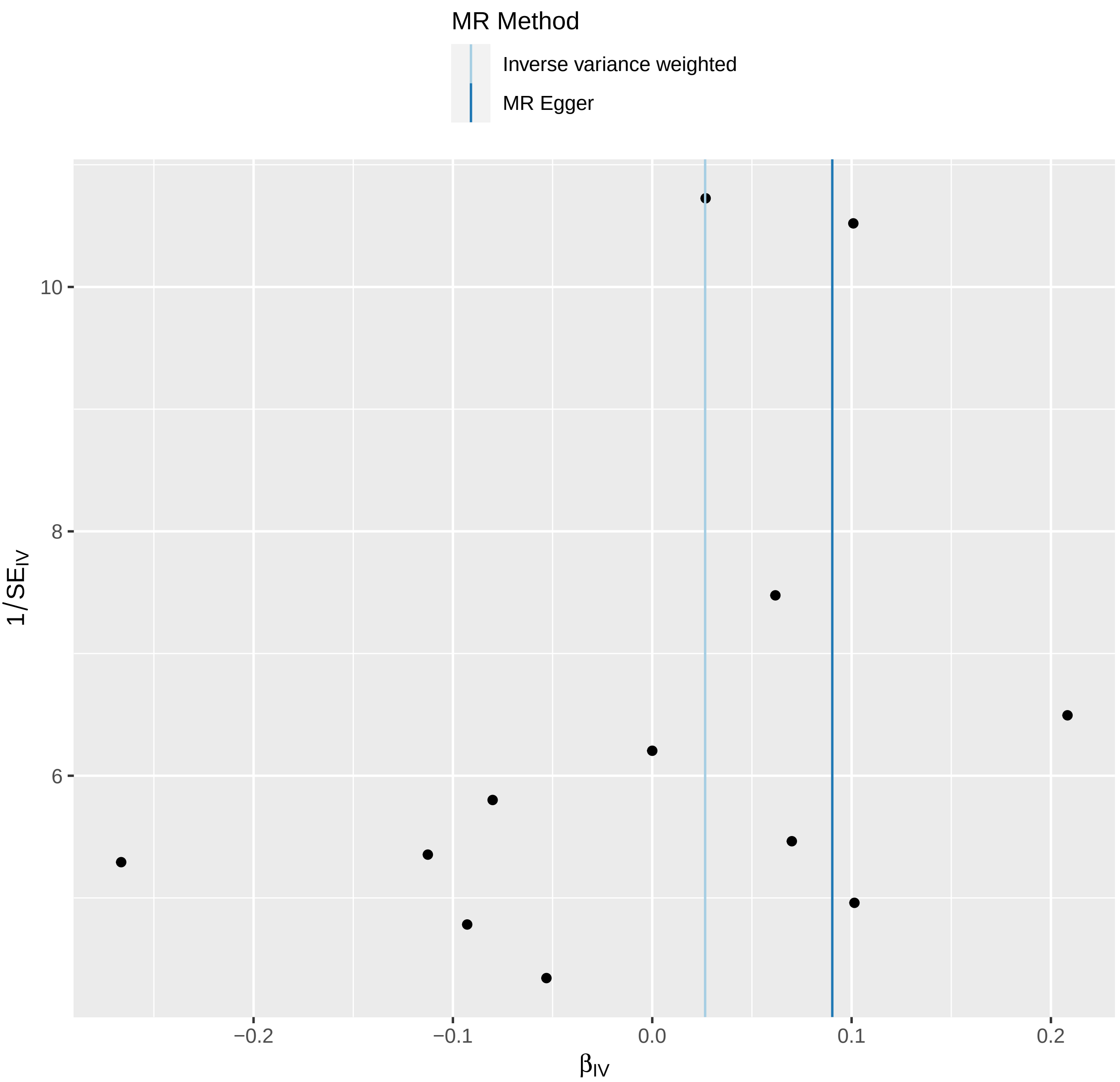

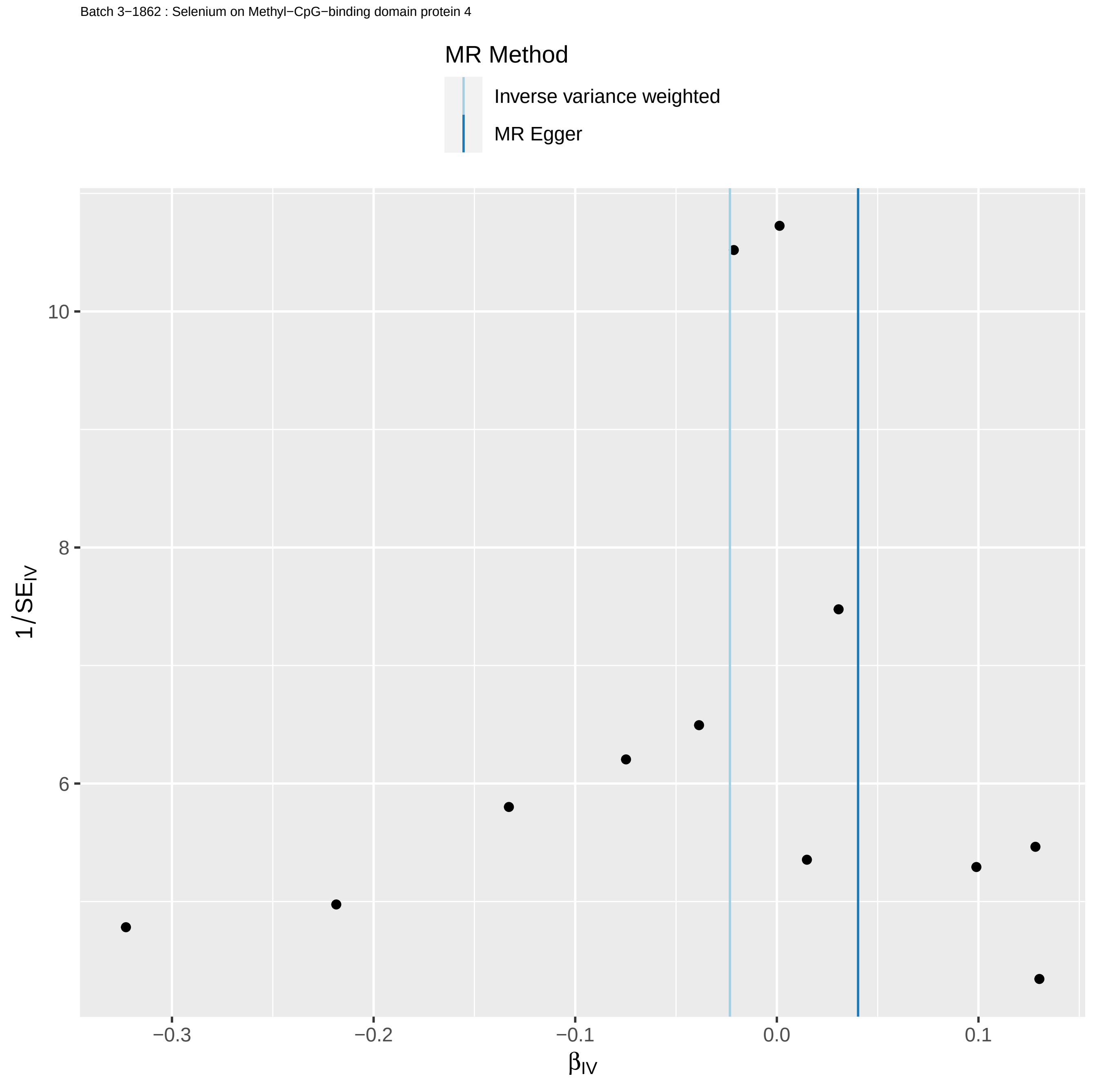

Batch 3−1862 : Selenium on Methyl−CpG−binding domain protein 4
MR Method
Inverse variance weighted
MR Egger
10
8
6
1/SE$_{IV}$
−0.3
−0.2
−0.1
0.0
0.1
β_{IV}

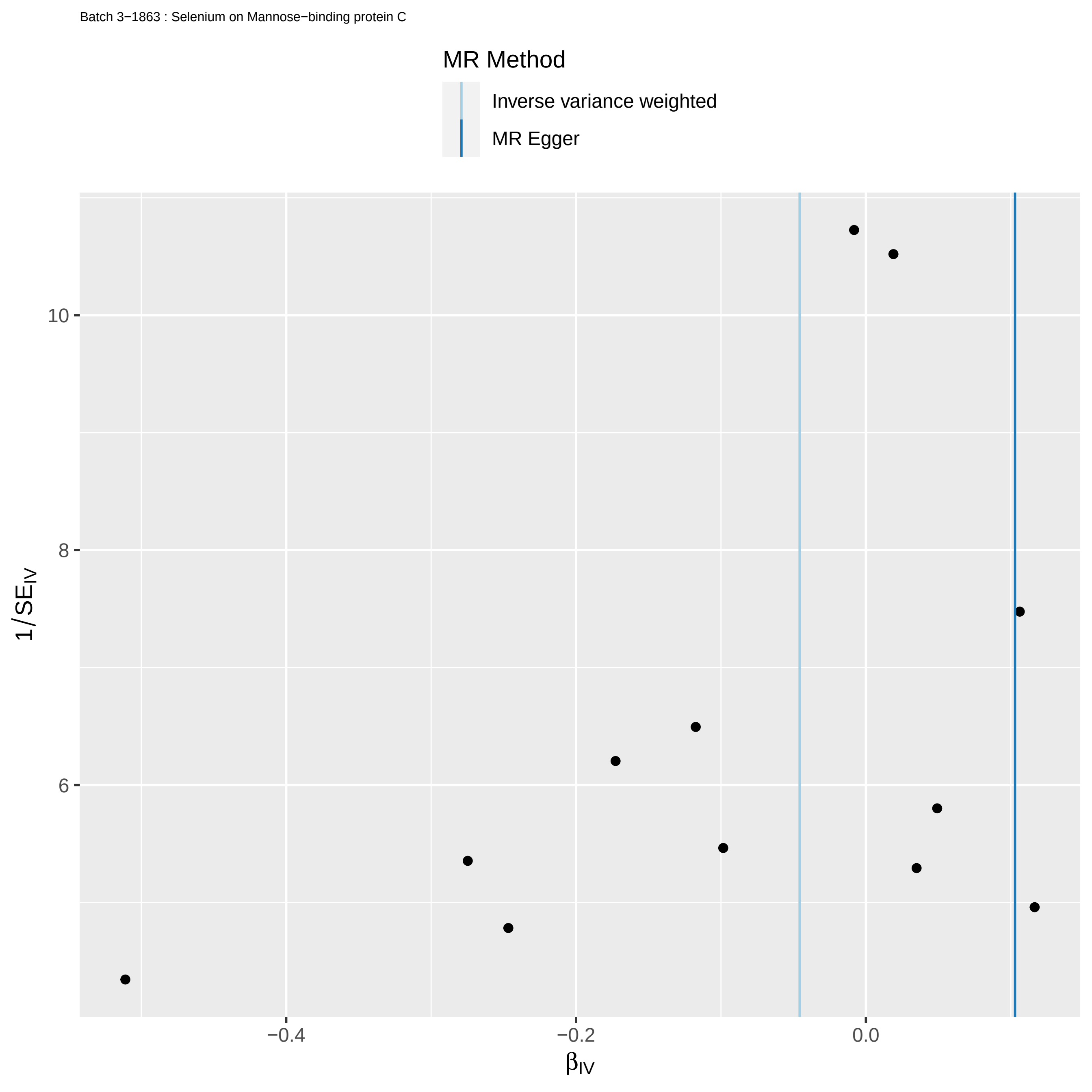

Batch 3−1863 : Selenium on Mannose−binding protein C
MR Method
Inverse variance weighted
MR Egger
10
8
6
1/SE$_{IV}$
−0.4
−0.2
0.0
β$_{IV}$

Batch 3−1864 : Selenium on Methylmalonyl−CoA epimerase, mitochondrial

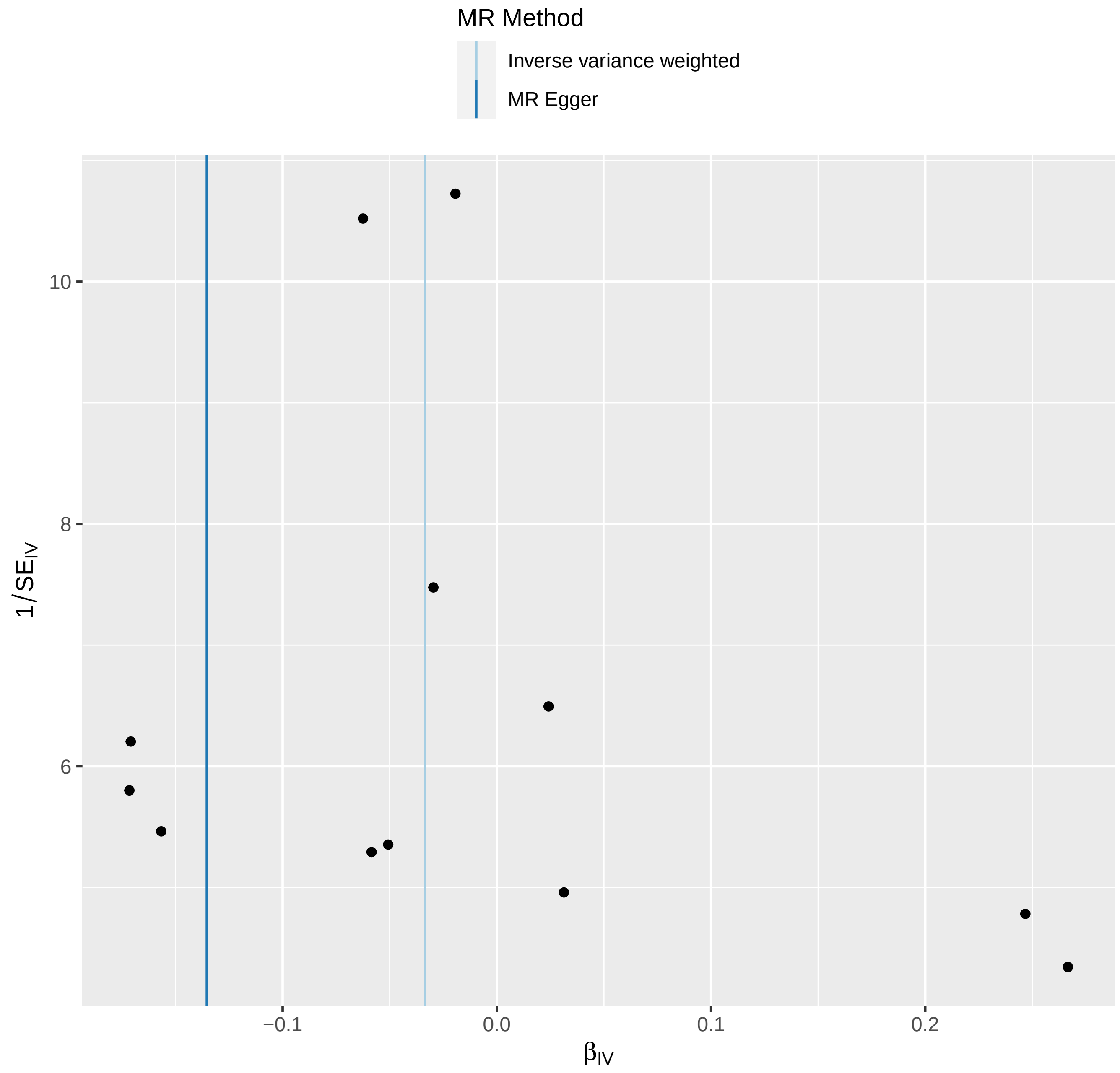

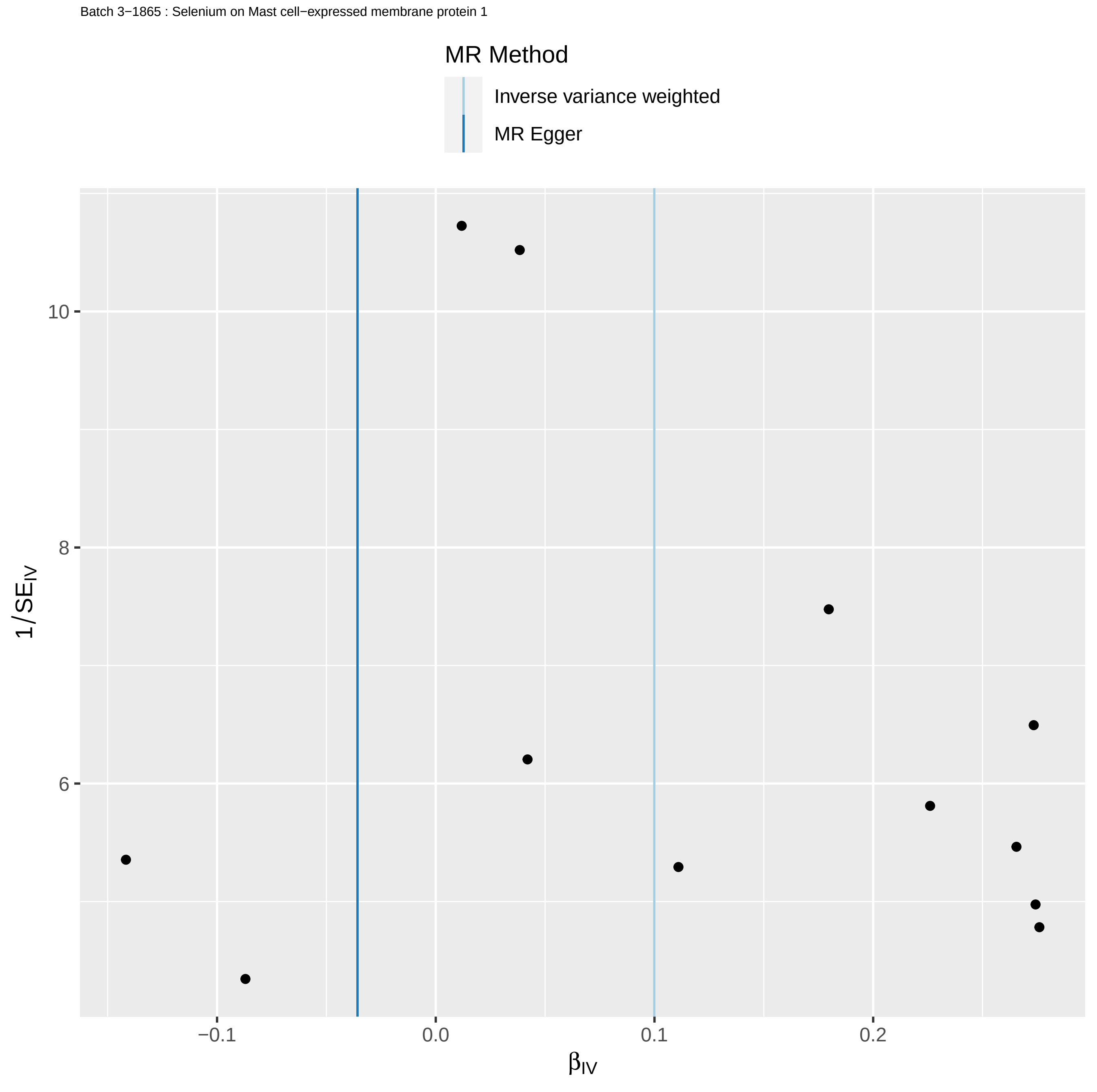

Batch 3−1865 : Selenium on Mast cell−expressed membrane protein 1
MR Method
Inverse variance weighted
MR Egger
10
8
6
1/SE$_{IV}$
−0.1
0.0
0.1
0.2
β$_{IV}$

Batch 3−1866 : Selenium on Guanine nucleotide exchange factor DBS

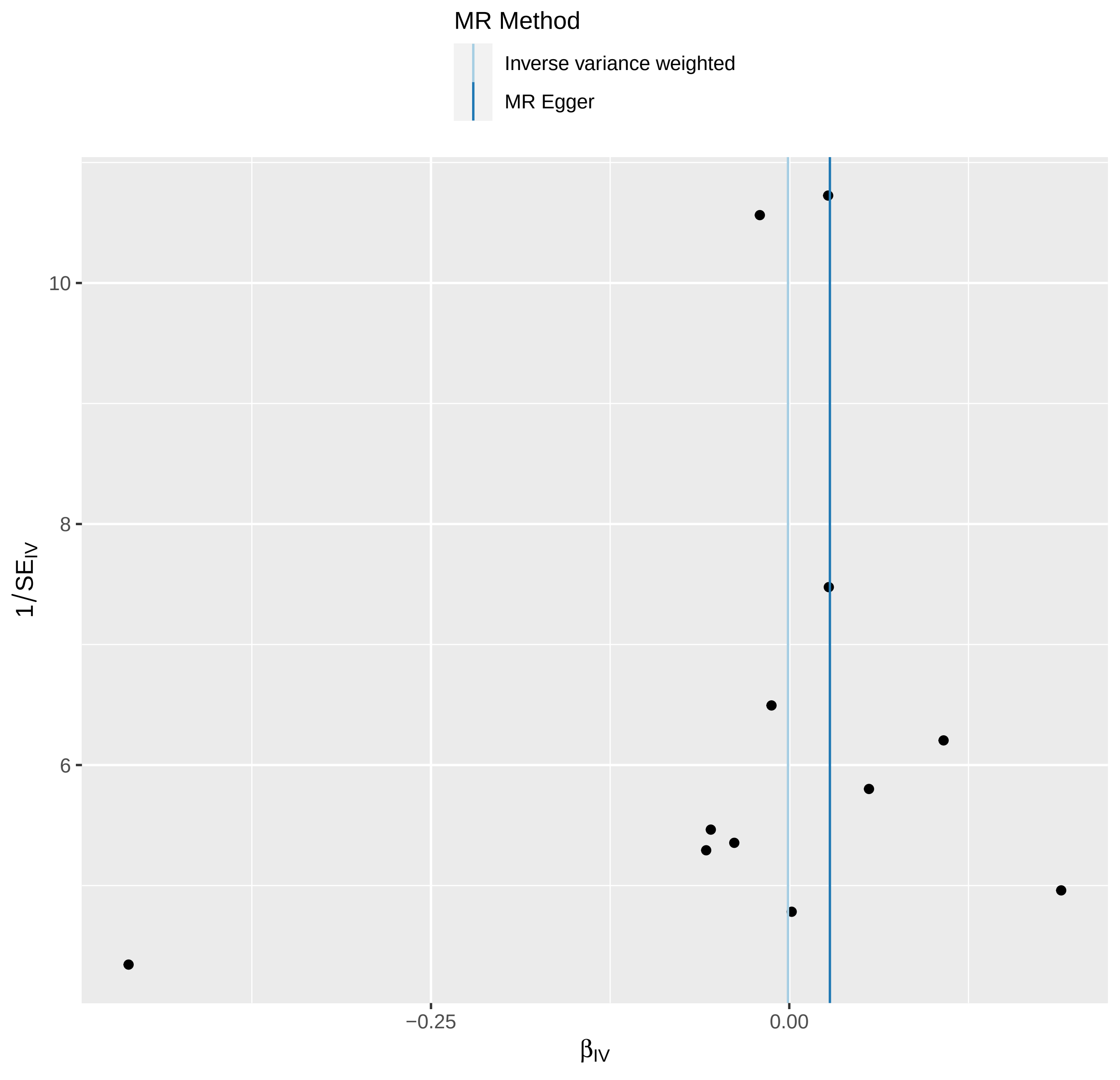

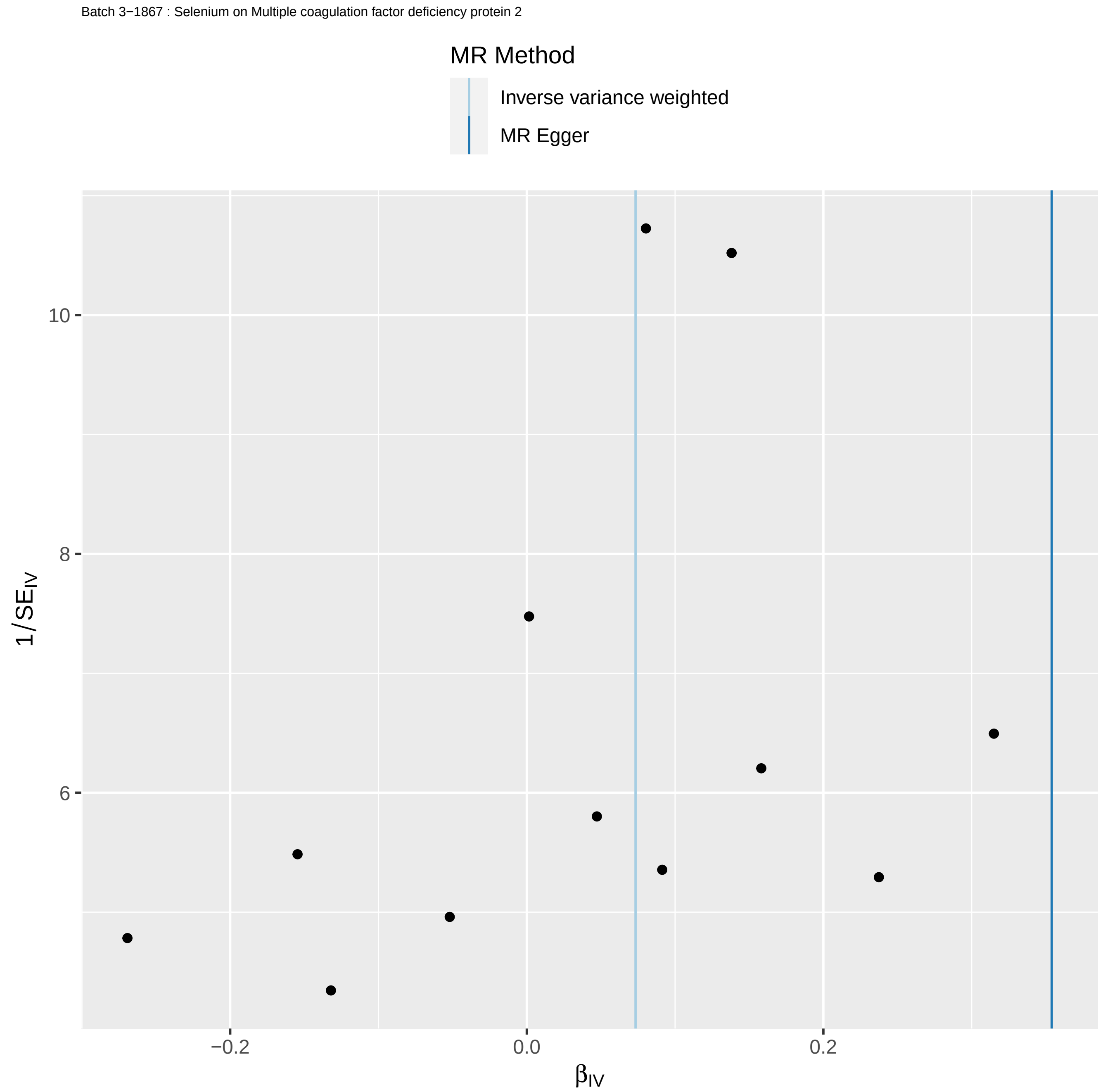
Batch 3−1867 : Selenium on Multiple coagulation factor deficiency protein 2
MR Method
Inverse variance weighted
MR Egger
10
8
6
−0.2
0.0
0.2
1/SE_IV
β_IV

Batch 3−1868 : Selenium on Induced myeloid leukemia cell differentiation protein Mcl−1

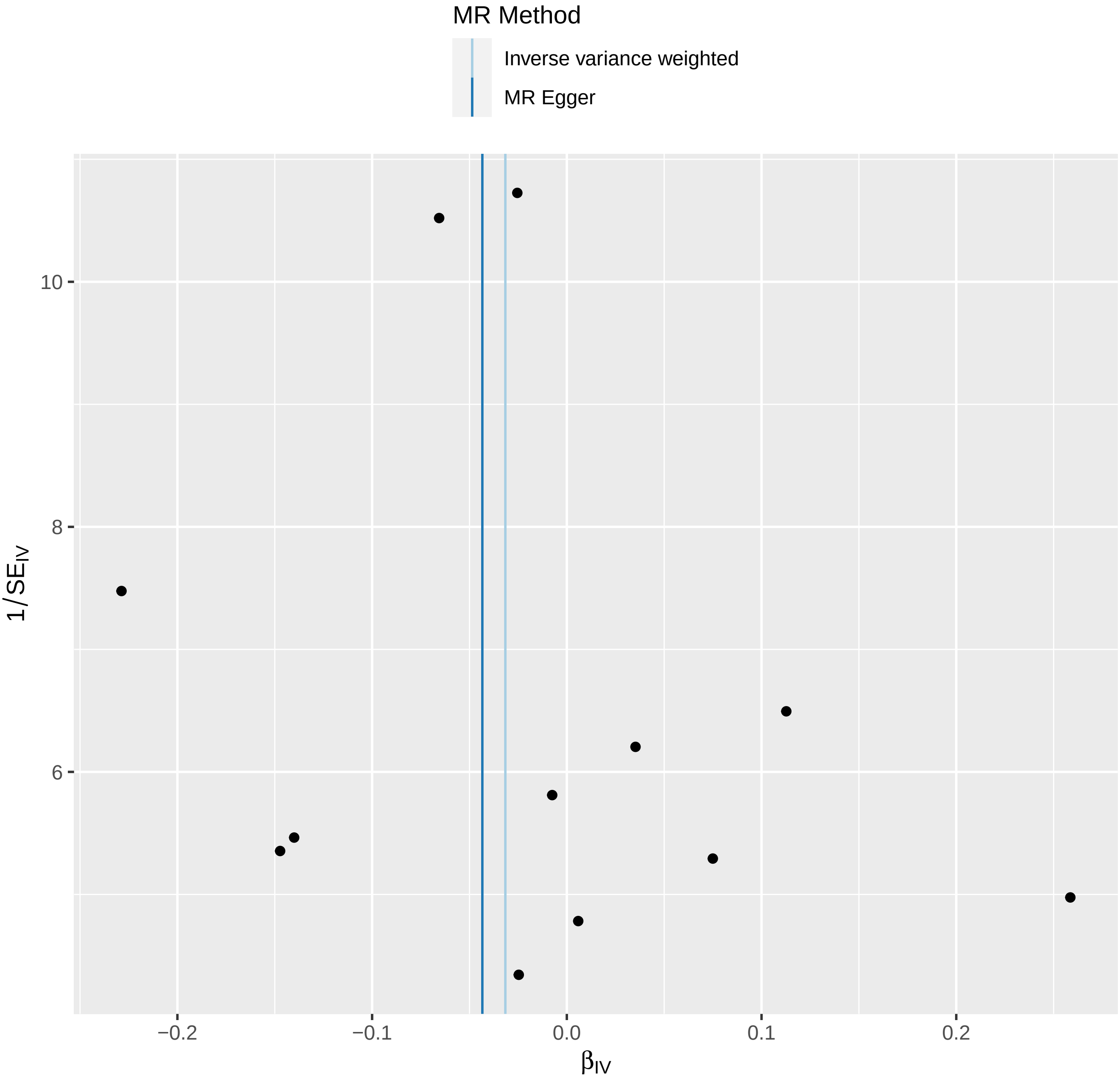

Batch 3−1869 : Selenium on Malignant T−cell−amplified sequence 1

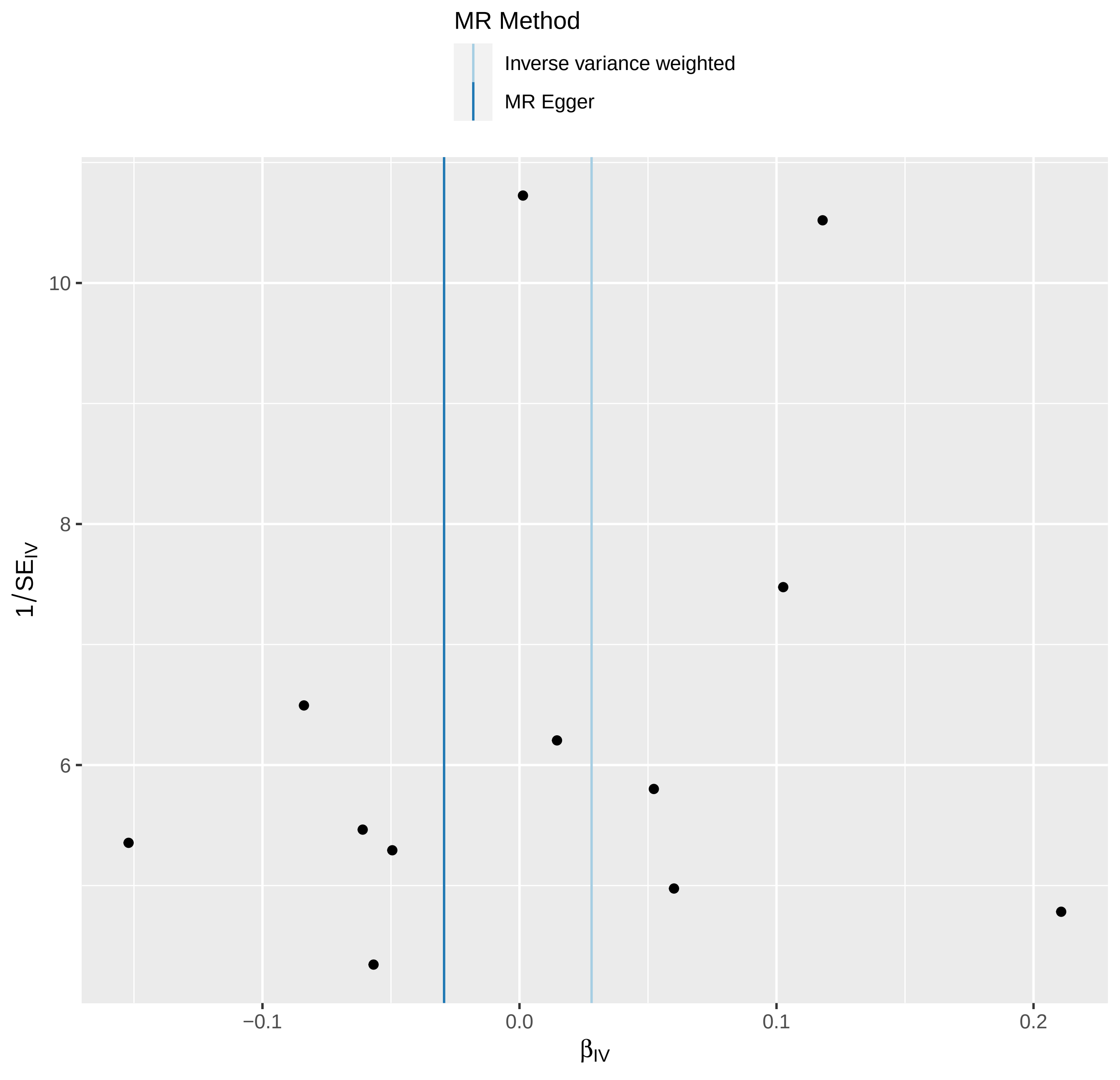

Batch 3−1870 : Selenium on Malate dehydrogenase, cytoplasmic

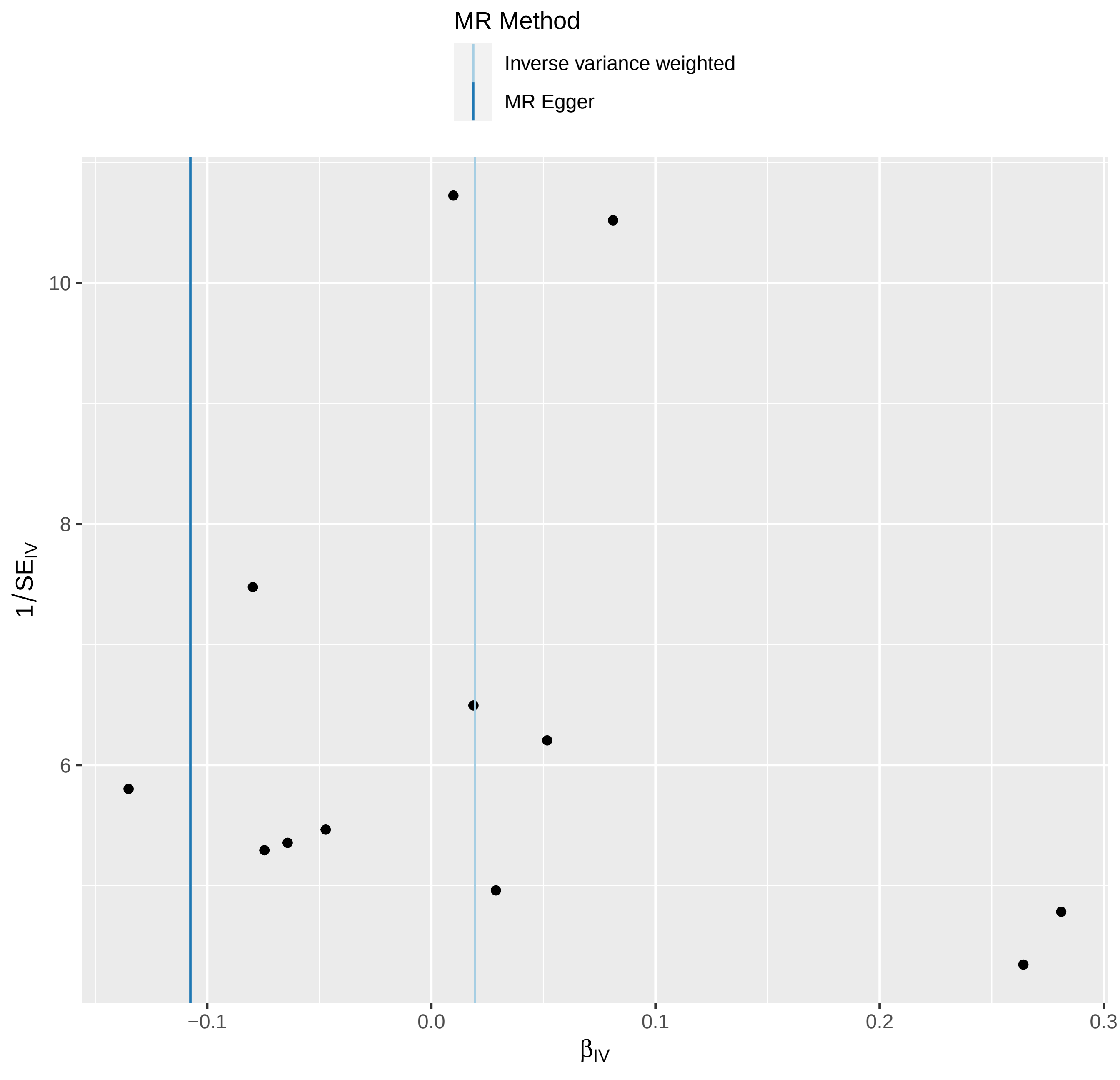

Batch 3−1871 : Selenium on Midkine

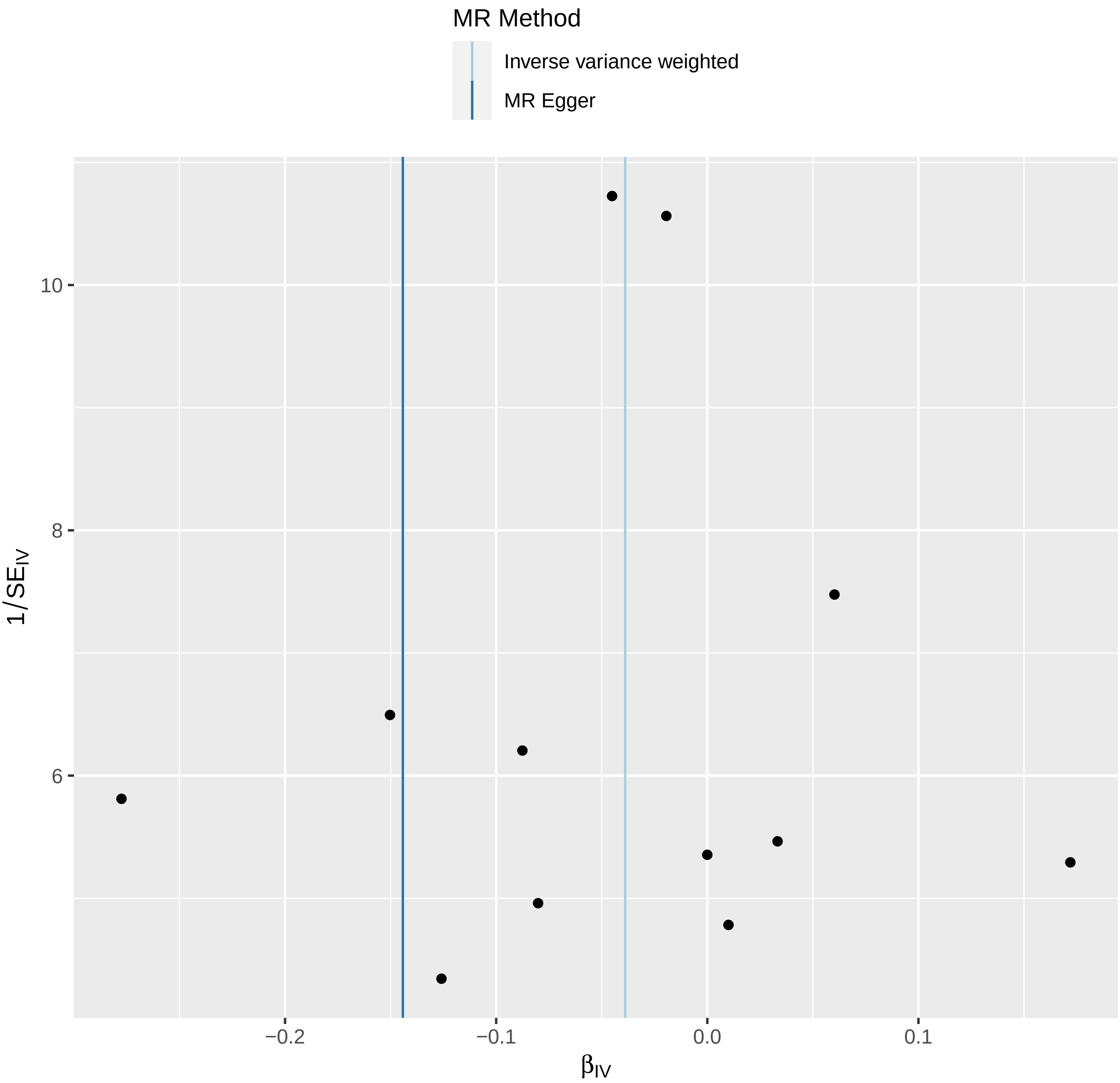

Batch 3−1872 : Selenium on Nuclear protein MDM1

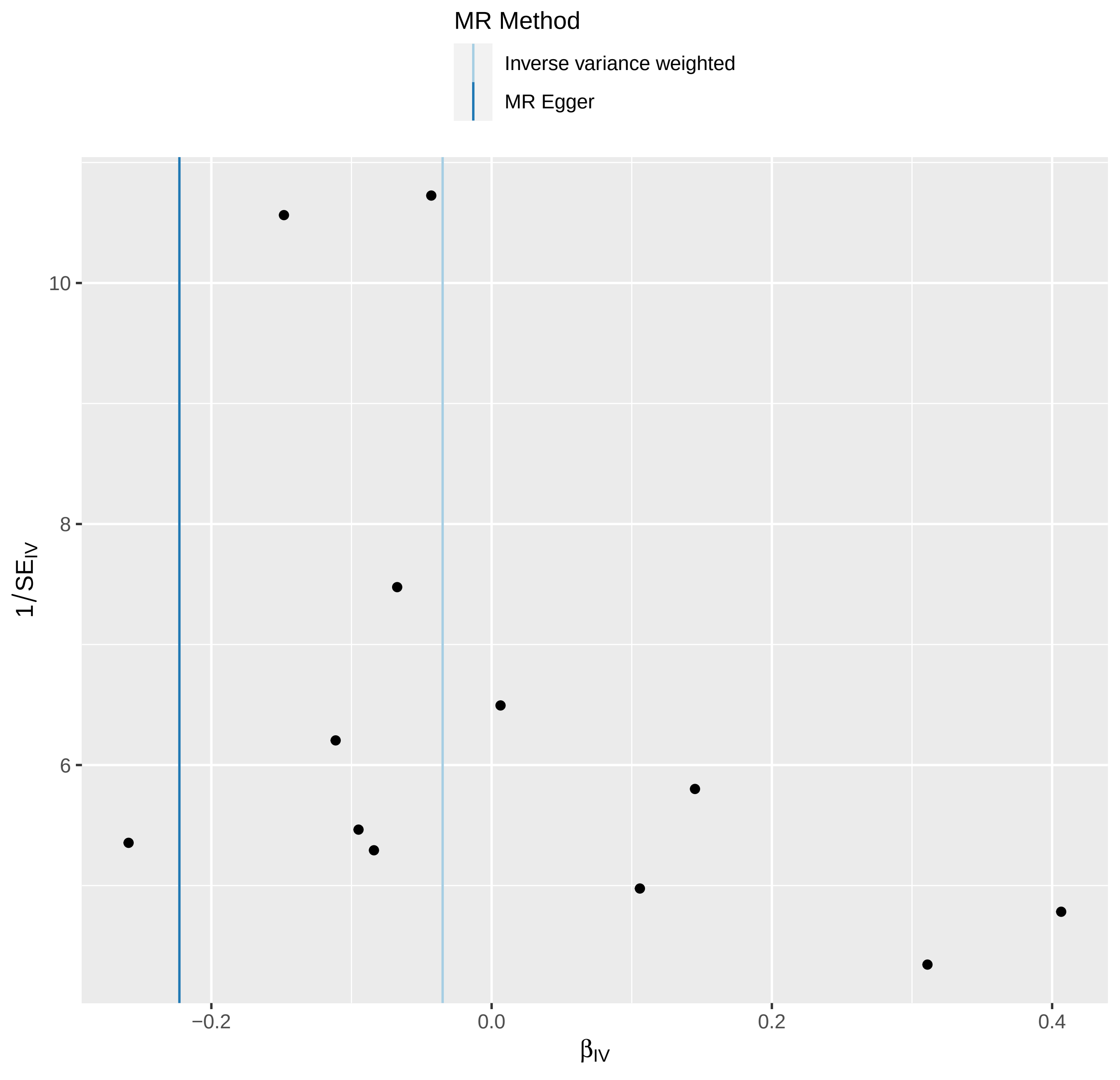

Batch 3−1873 : Selenium on E3 ubiquitin−protein ligase Mdm2

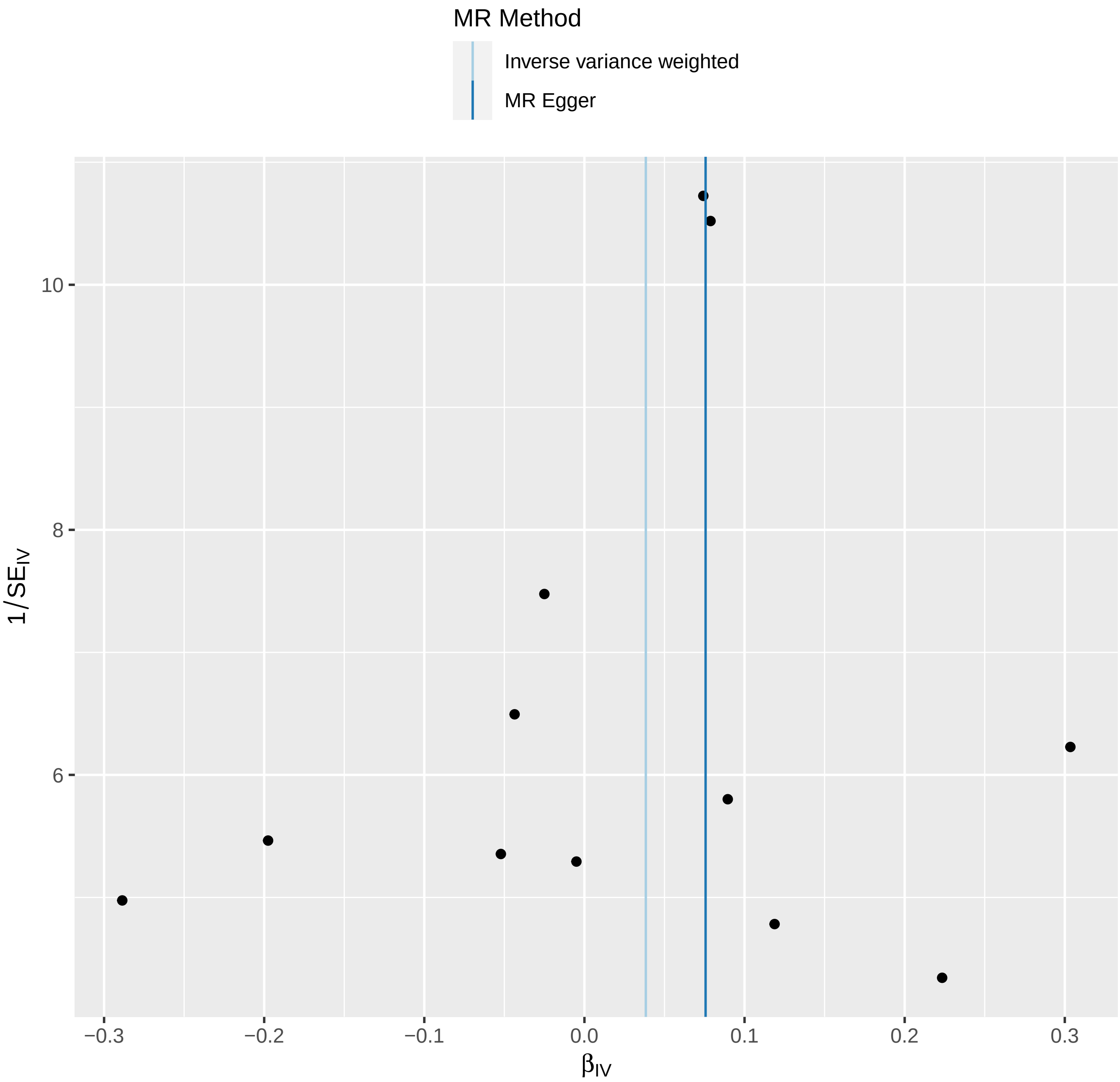

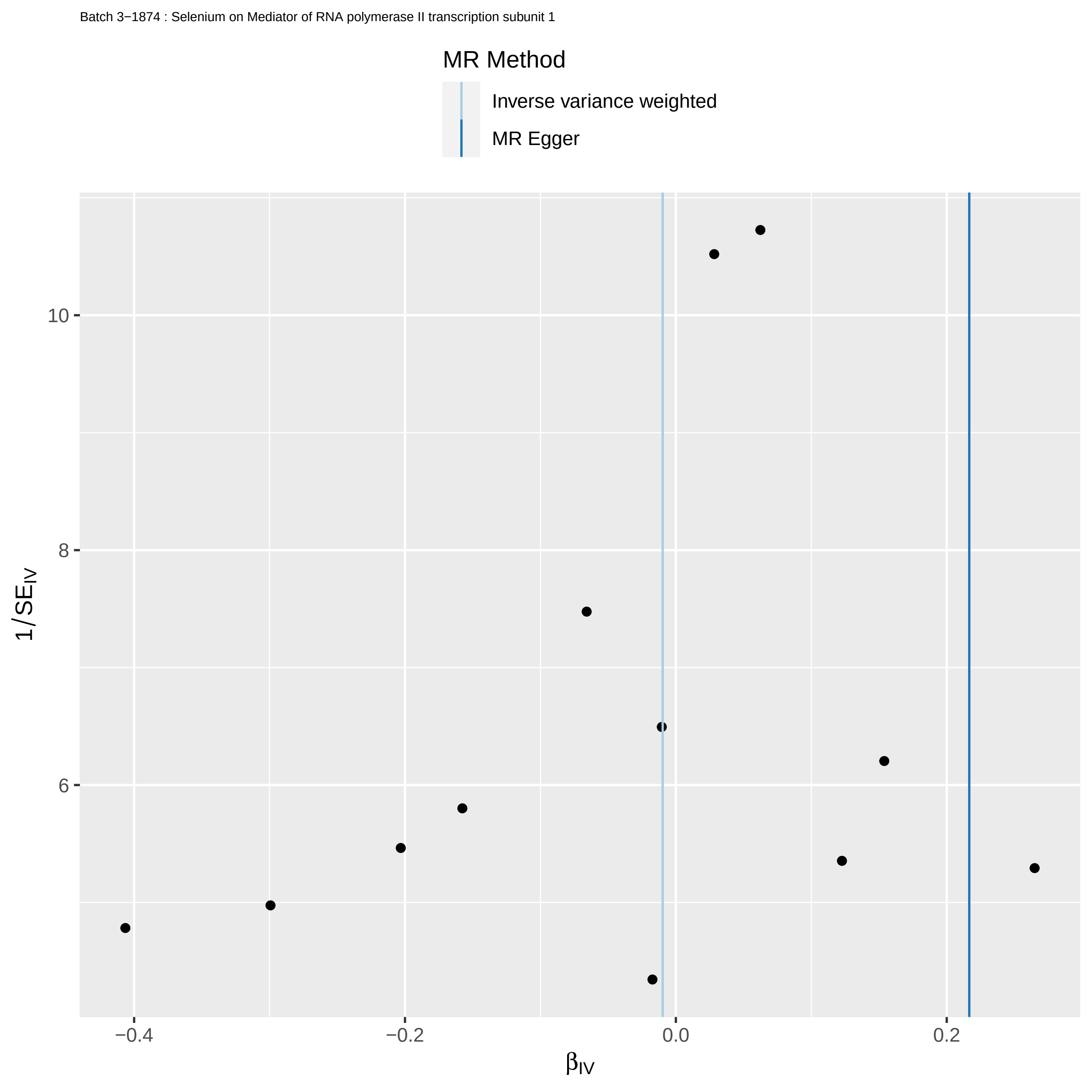

Batch 3−1874 : Selenium on Mediator of RNA polymerase II transcription subunit 1
MR Method
Inverse variance weighted
MR Egger
10
8
6
−0.4
−0.2
0.0
0.2
1/SE_IV
β_IV

Batch 3−1875 : Selenium on Mediator of RNA polymerase II transcription subunit 1

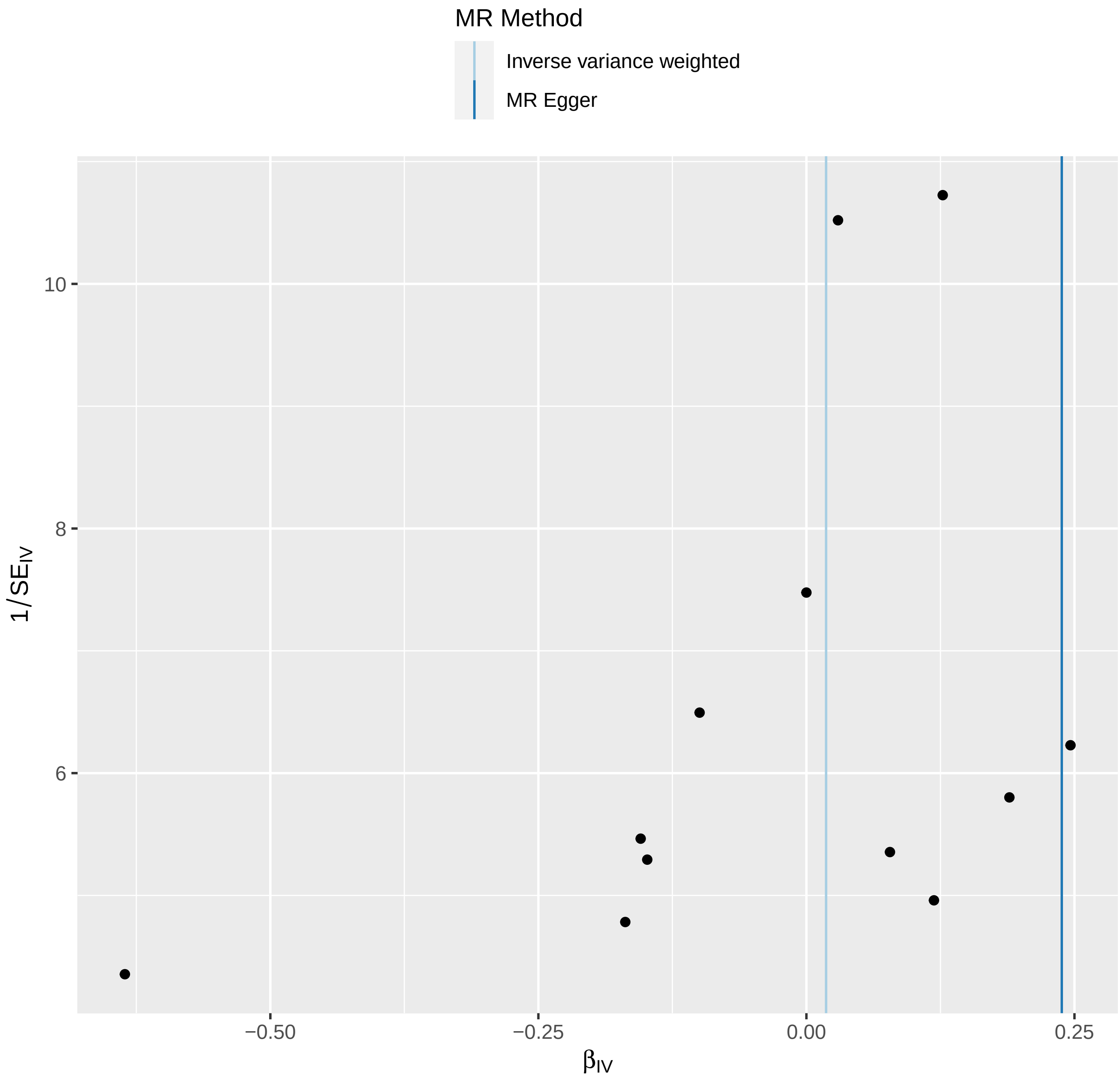

Batch 3−1876 : Selenium on Mediator of RNA polymerase II transcription subunit 4

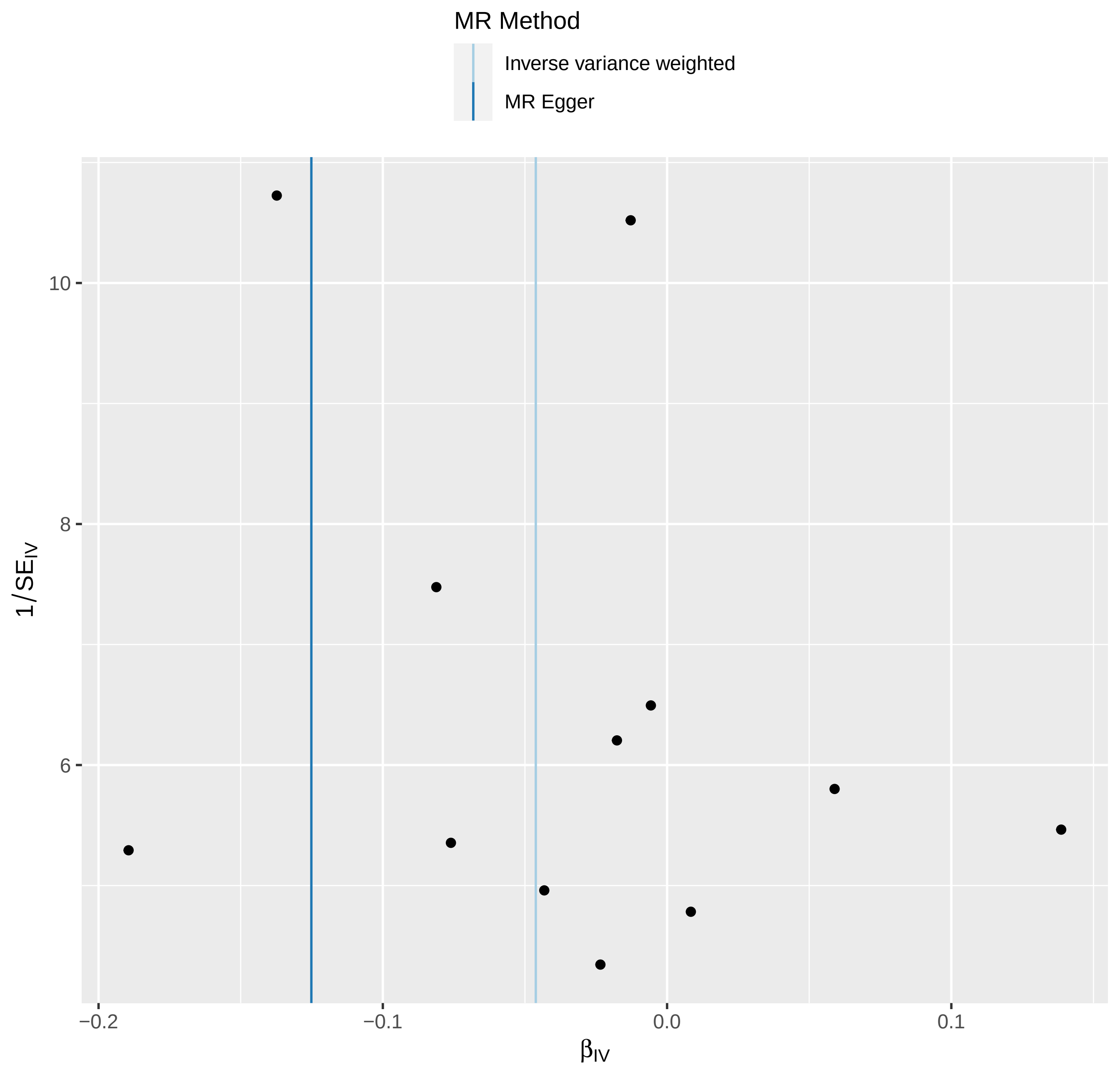

Batch 3−1877 : Selenium on Myocyte−specific enhancer factor 2C

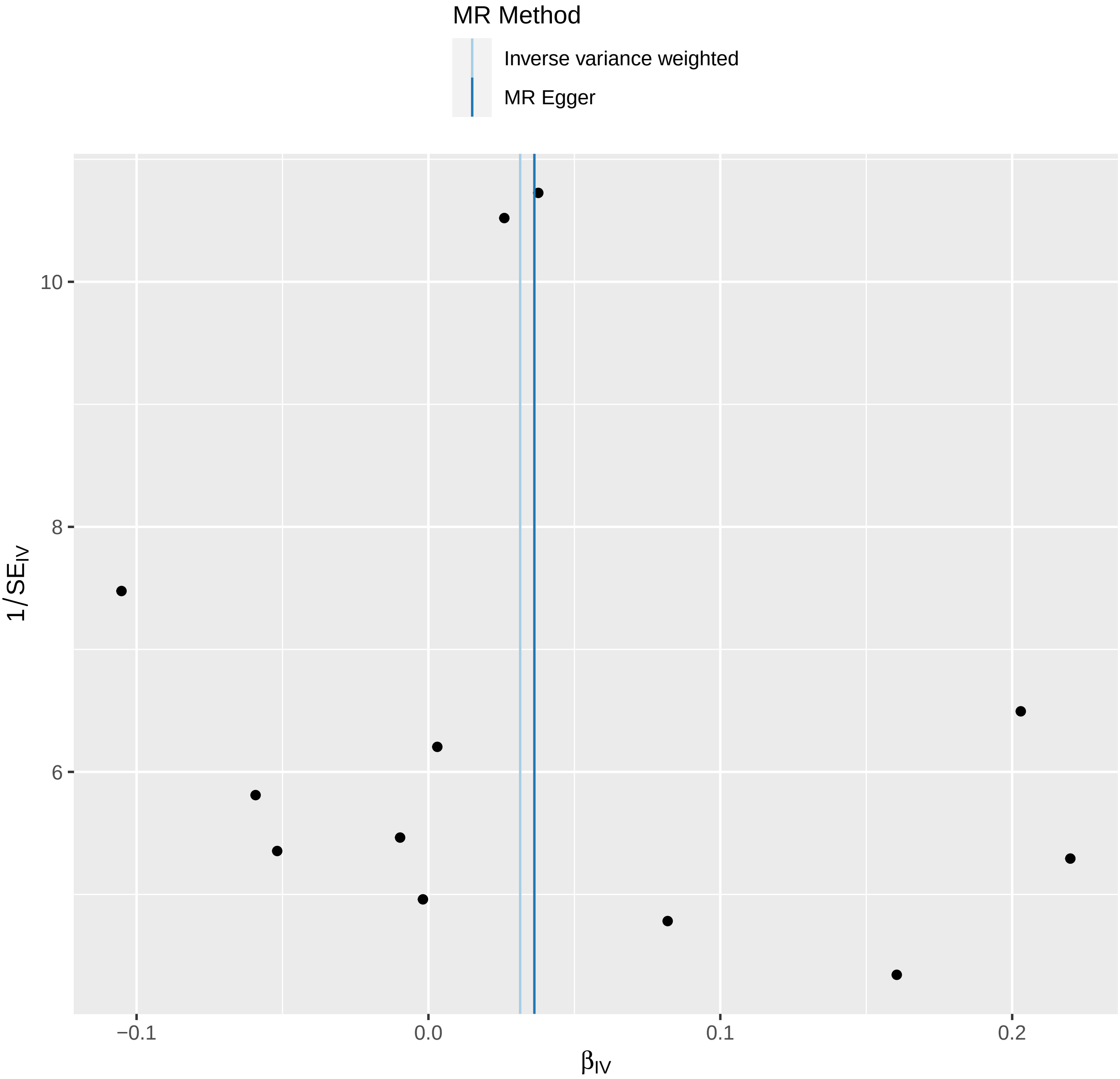

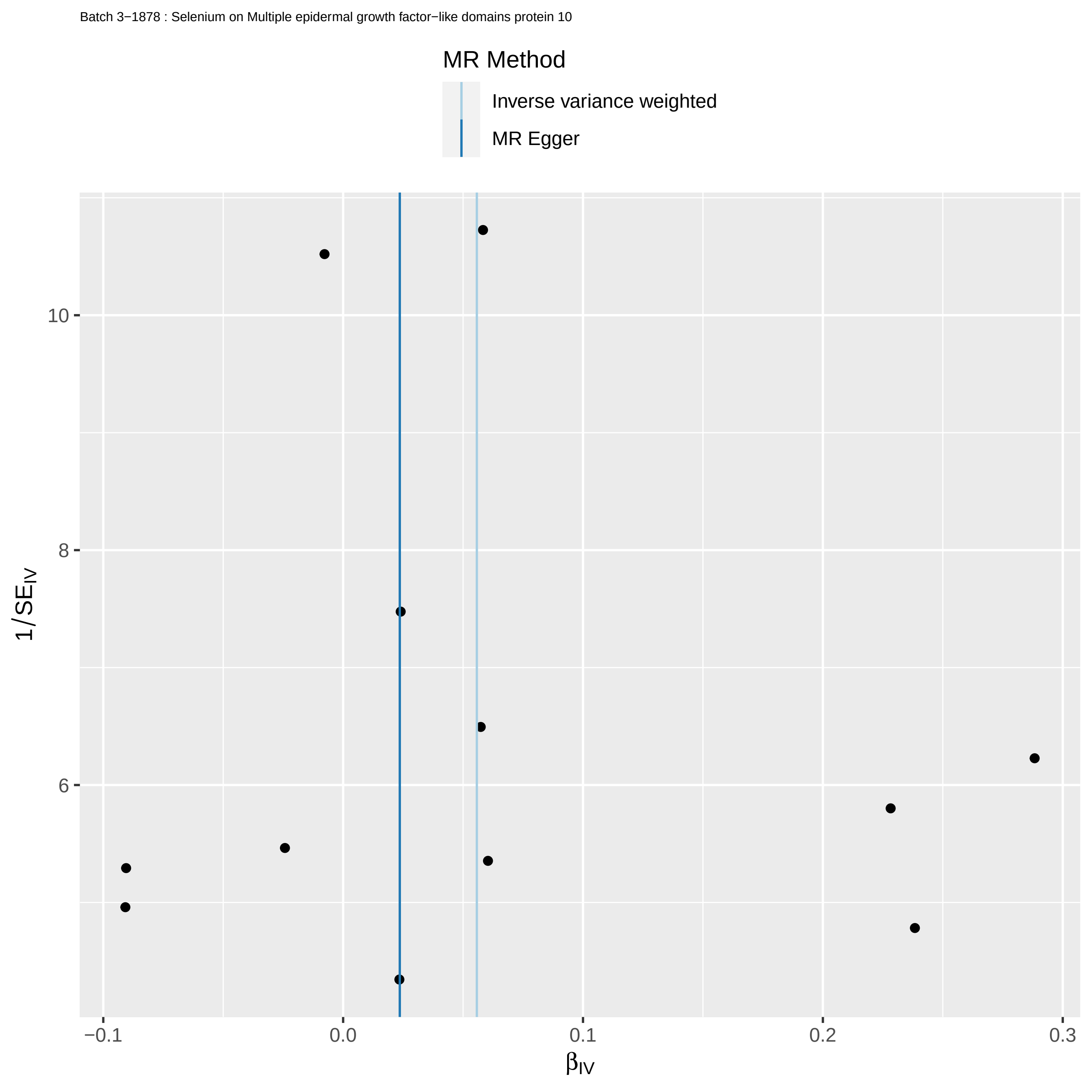
Batch 3−1878 : Selenium on Multiple epidermal growth factor−like domains protein 10
MR Method
Inverse variance weighted
MR Egger
10
8
6
1/SE_IV
−0.1
0.0
0.1
0.2
0.3
β_IV

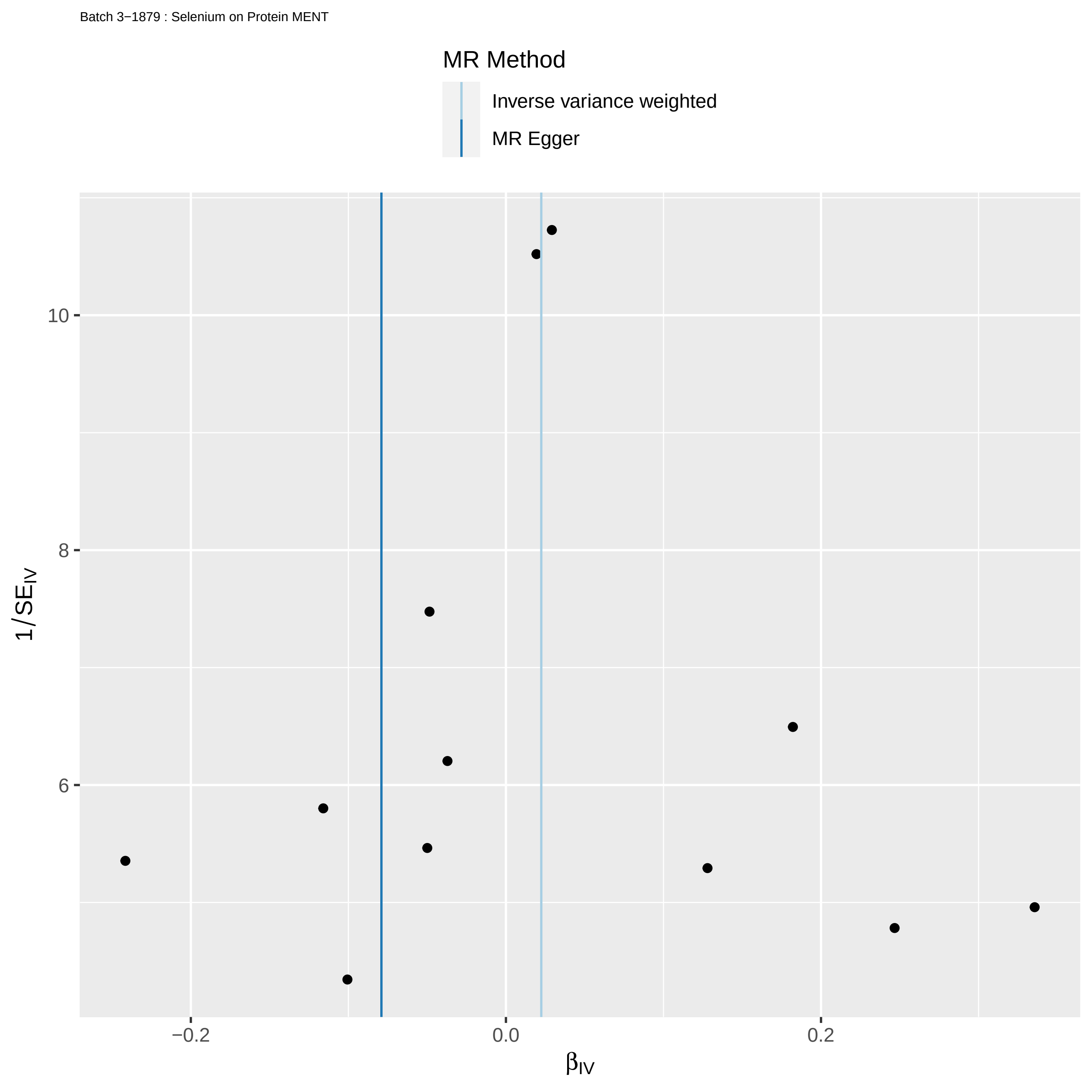
Batch 3−1879 : Selenium on Protein MENT
MR Method
Inverse variance weighted
MR Egger
10
8
6
1/SE_IV
−0.2
0.0
0.2
β_IV

Batch 3−1880 : Selenium on Matrix extracellular phosphoglycoprotein

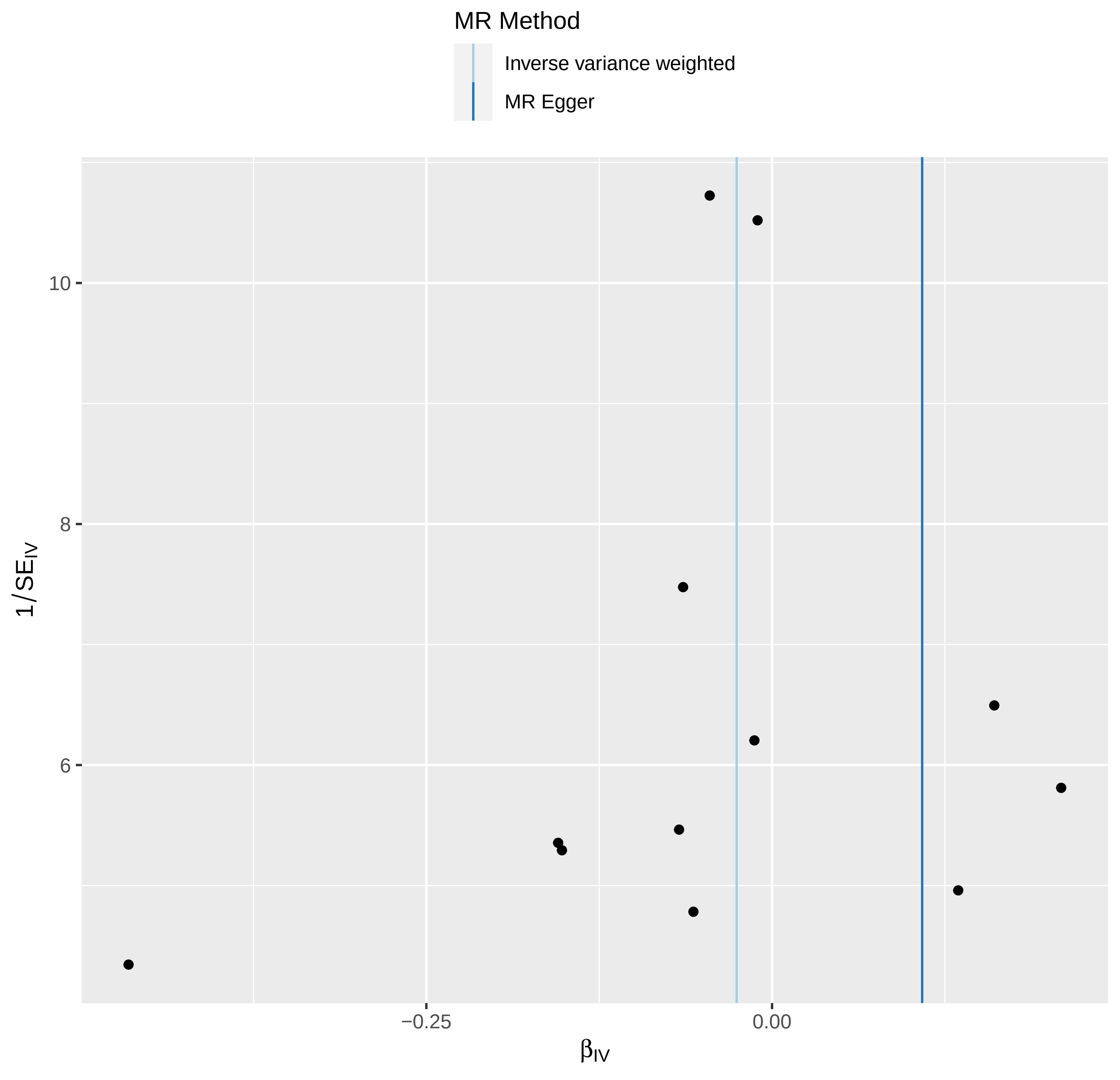

Batch 3−1881 : Selenium on LDLR chaperone MESD

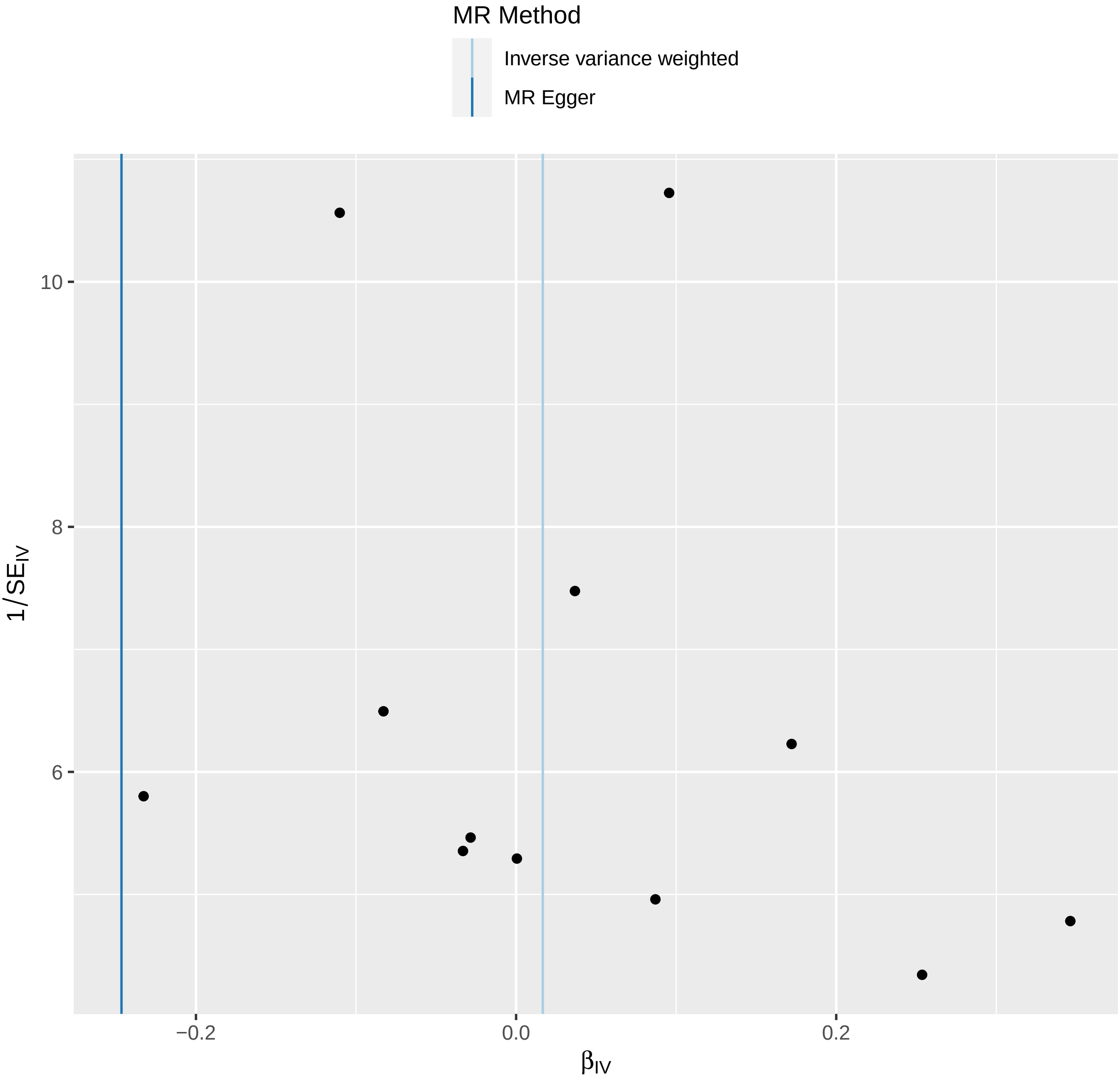

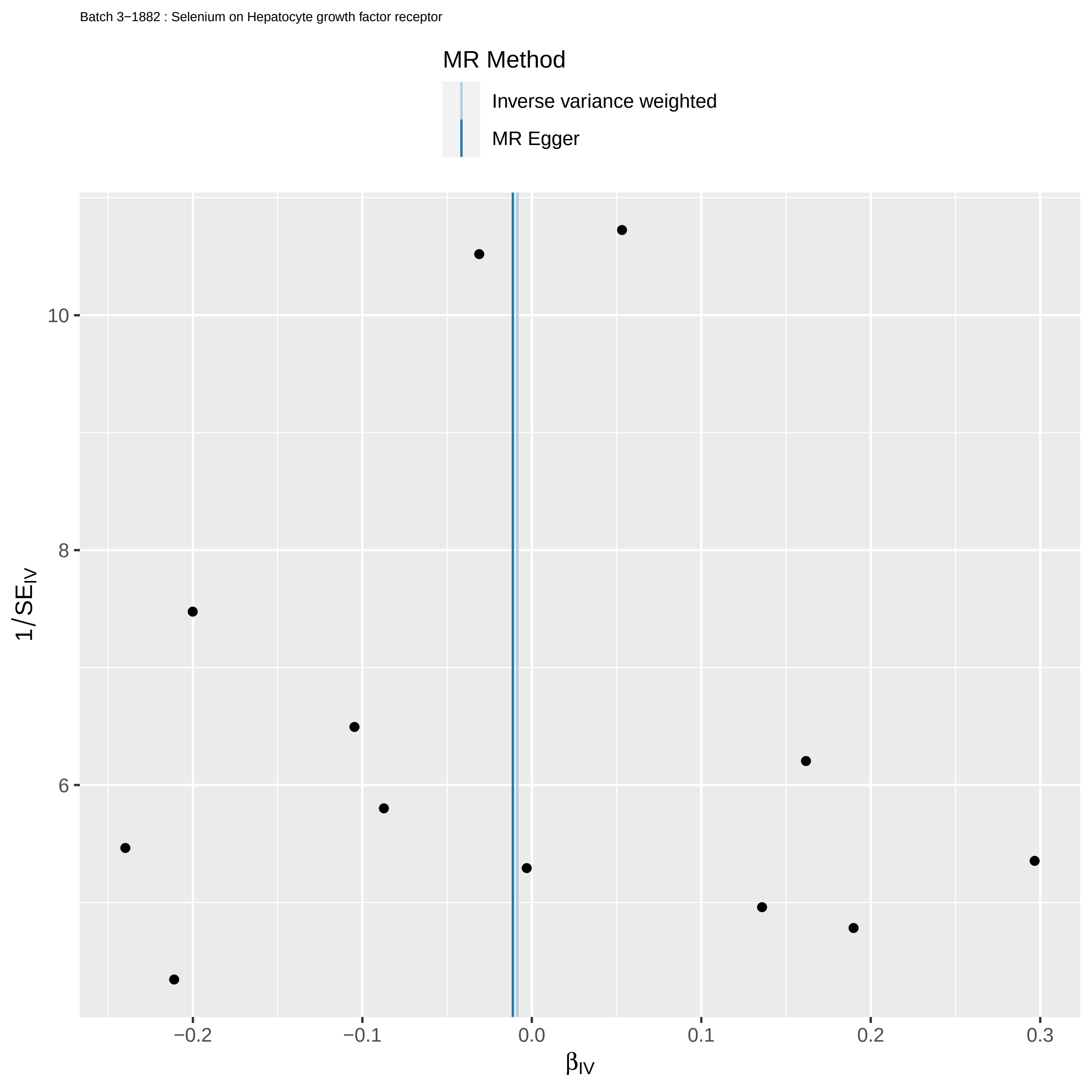

Batch 3−1882 : Selenium on Hepatocyte growth factor receptor
MR Method
Inverse variance weighted
MR Egger
10
8
6
1/SE_IV
−0.2
−0.1
0.0
0.1
0.2
0.3
β_IV

Batch 3−1883 : Selenium on tRNA (guanine−N(7)−)−methyltransferase

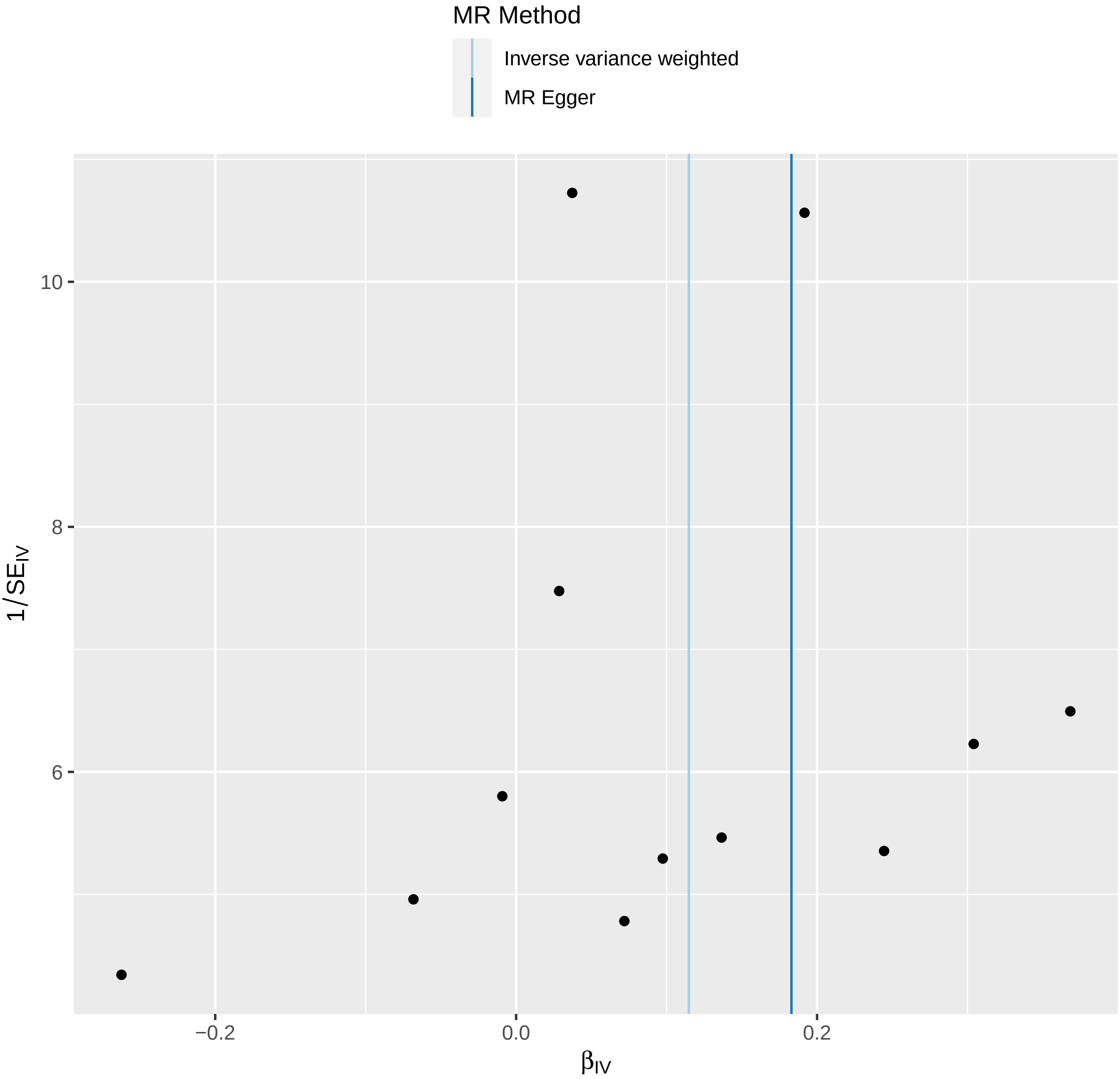

Batch 3−1884 : Selenium on Microfibrillar−associated protein 1

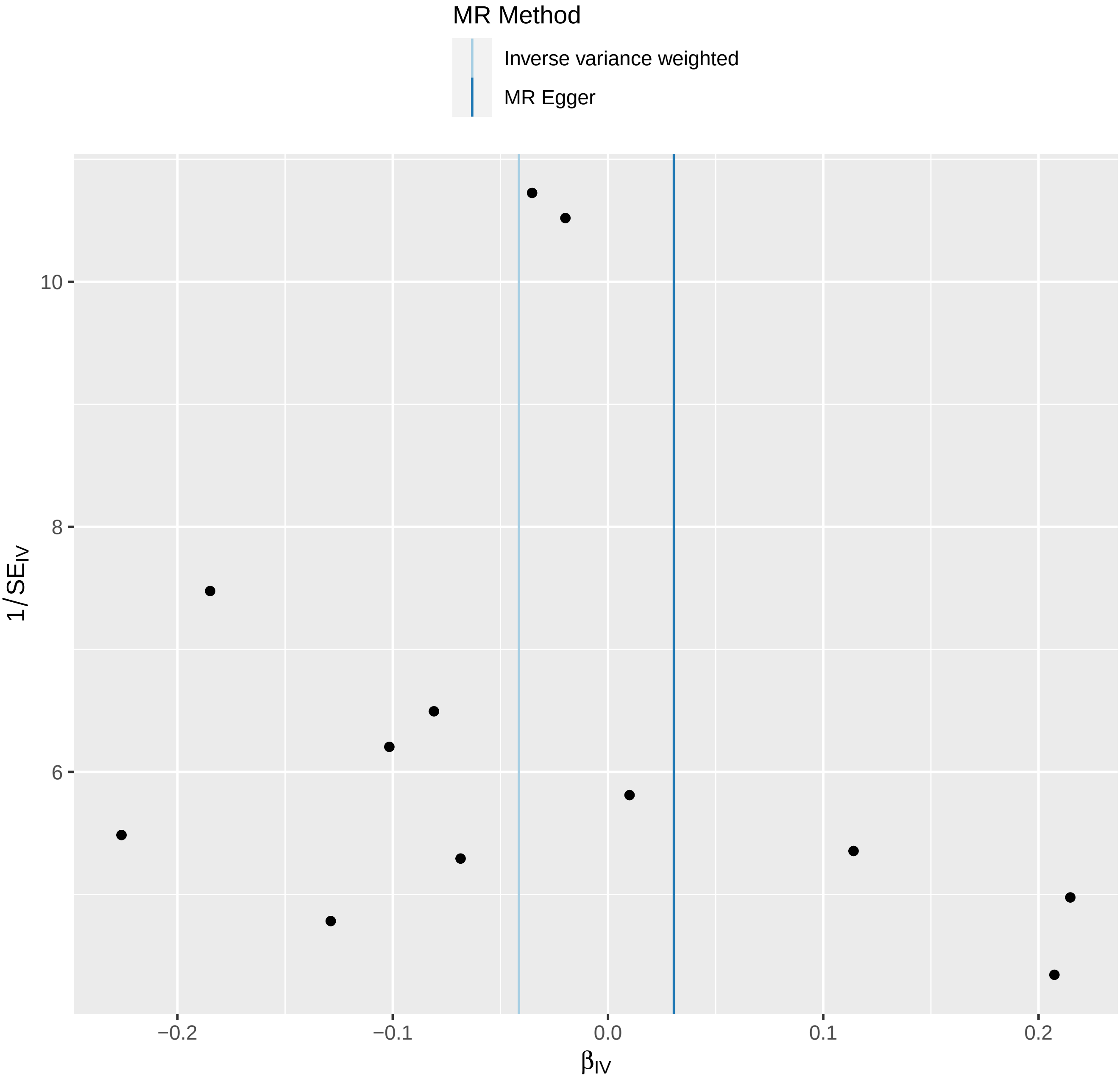

Batch 3−1885 : Selenium on Microfibrillar−associated protein 2

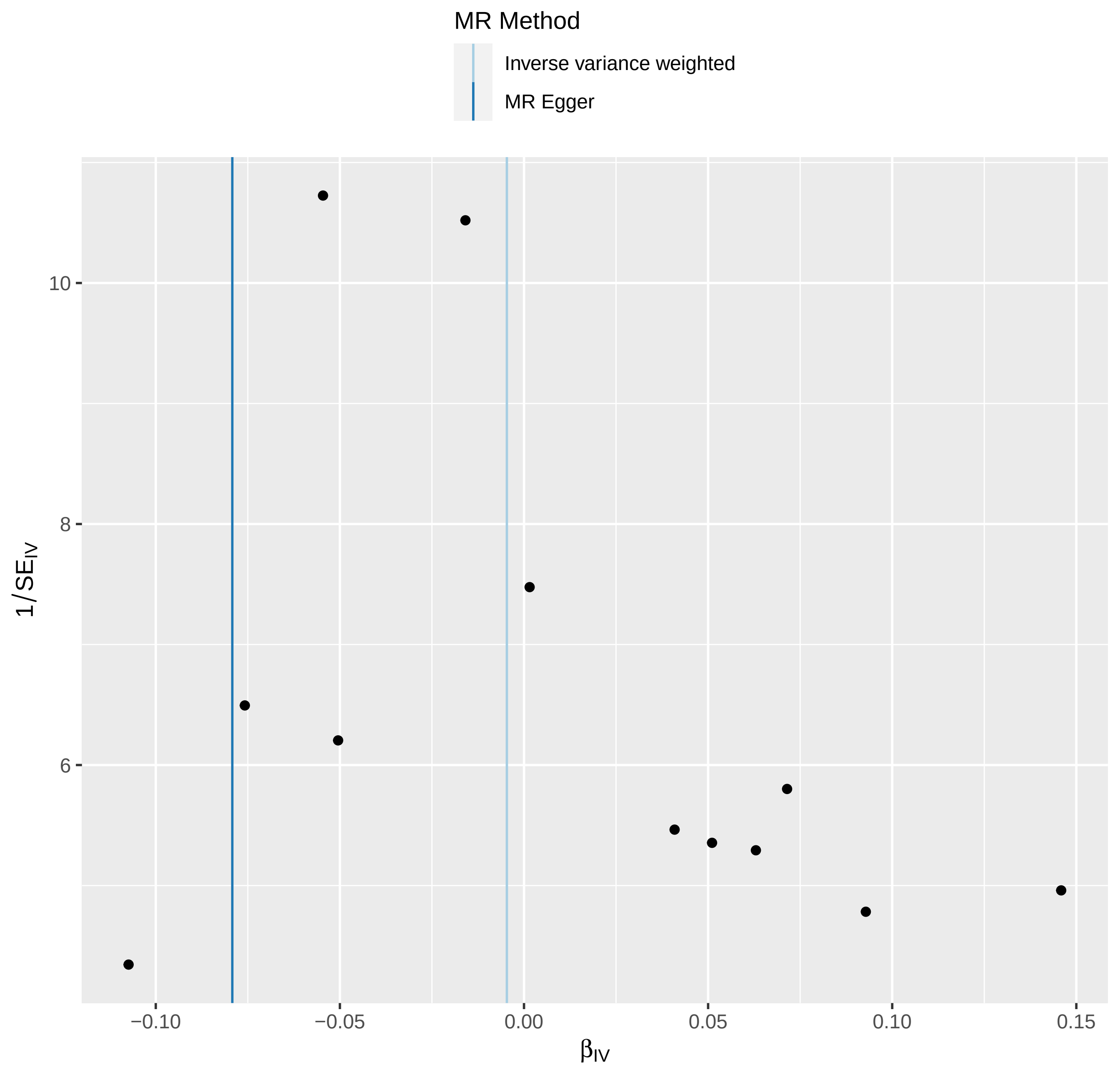

Batch 3−1886 : Selenium on Microfibril−associated glycoprotein 4

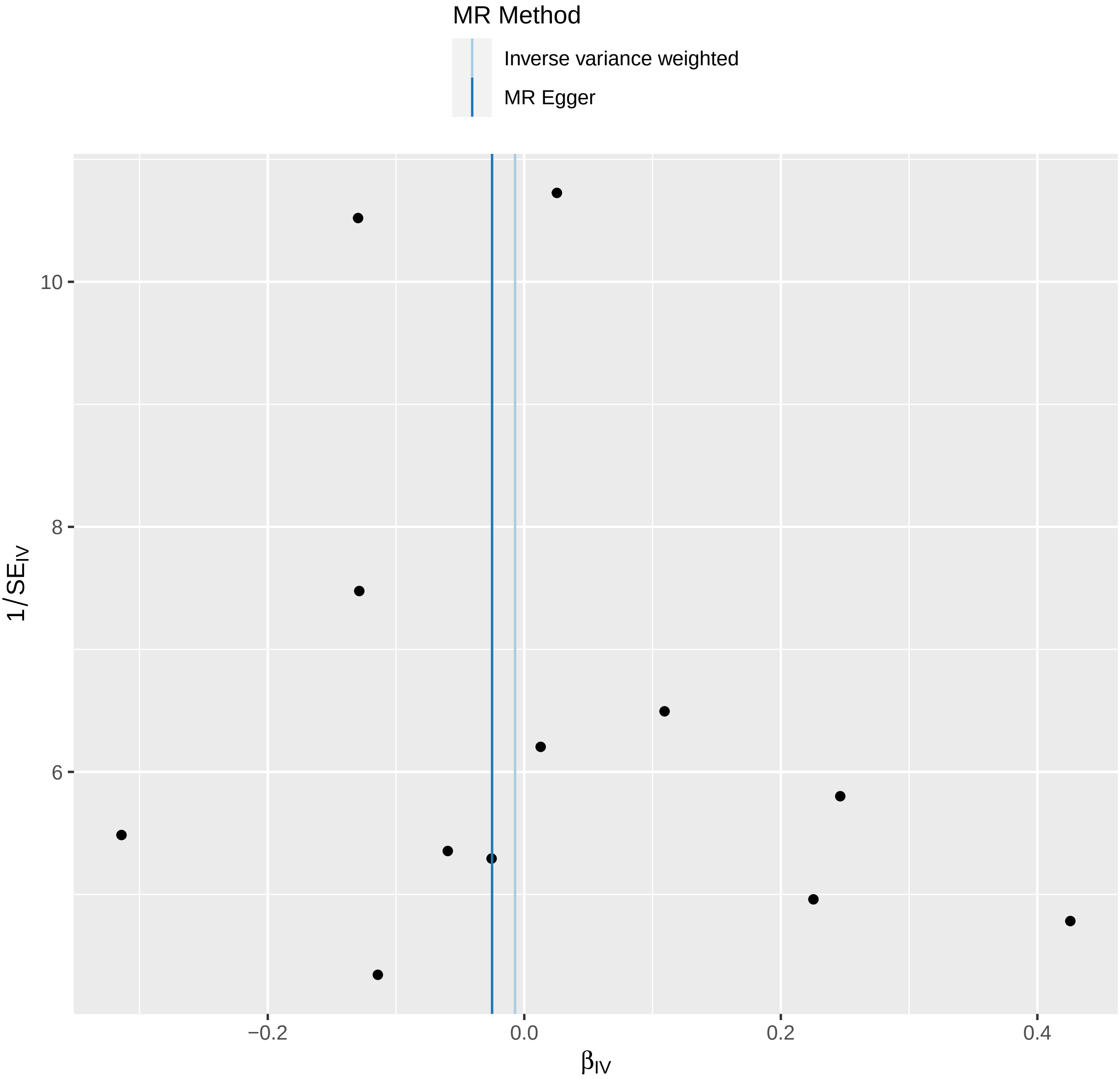

Batch 3−1887 : Selenium on Microfibrillar−associated protein 5

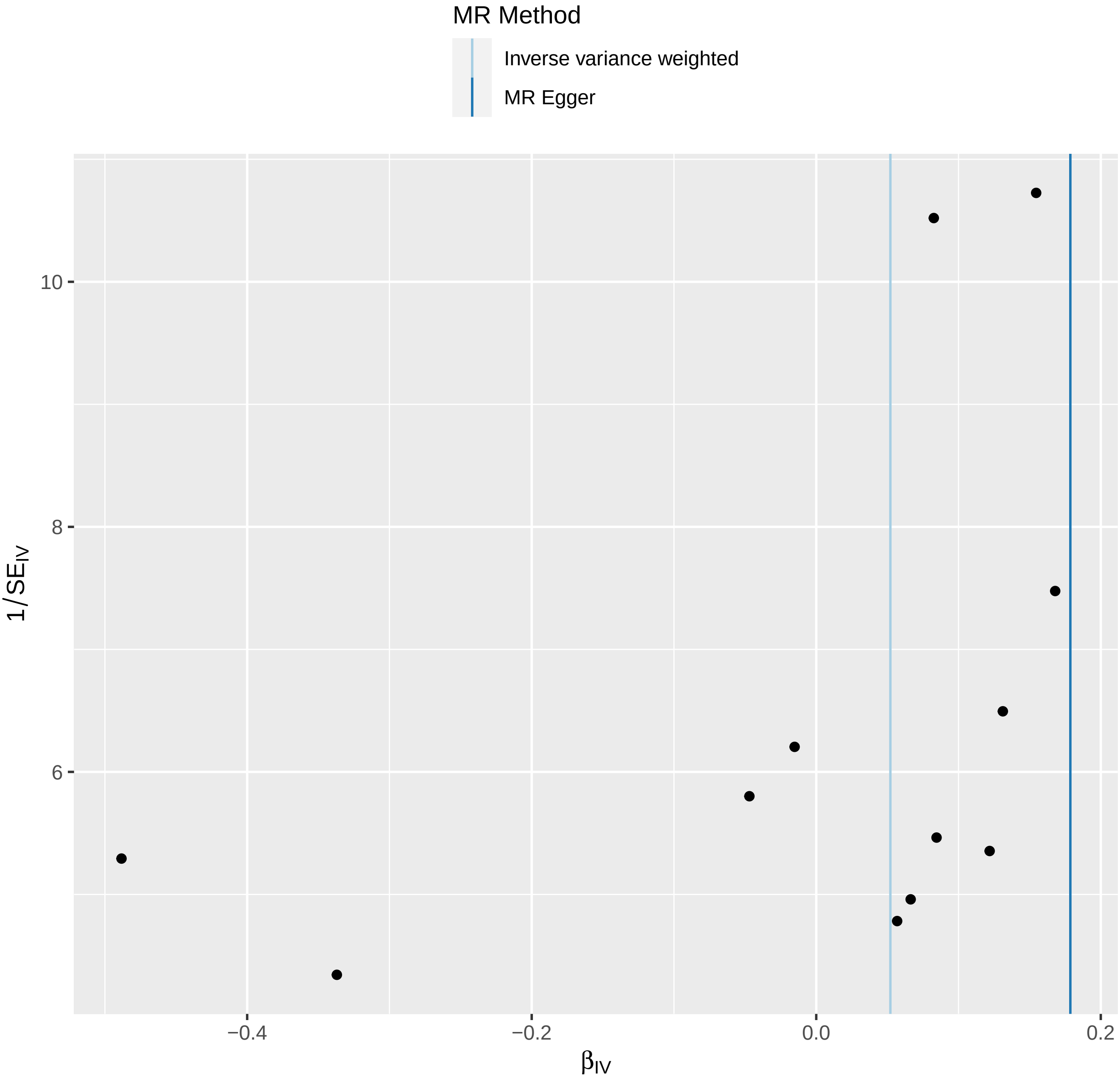

Batch 3−1888 : Selenium on Lactadherin

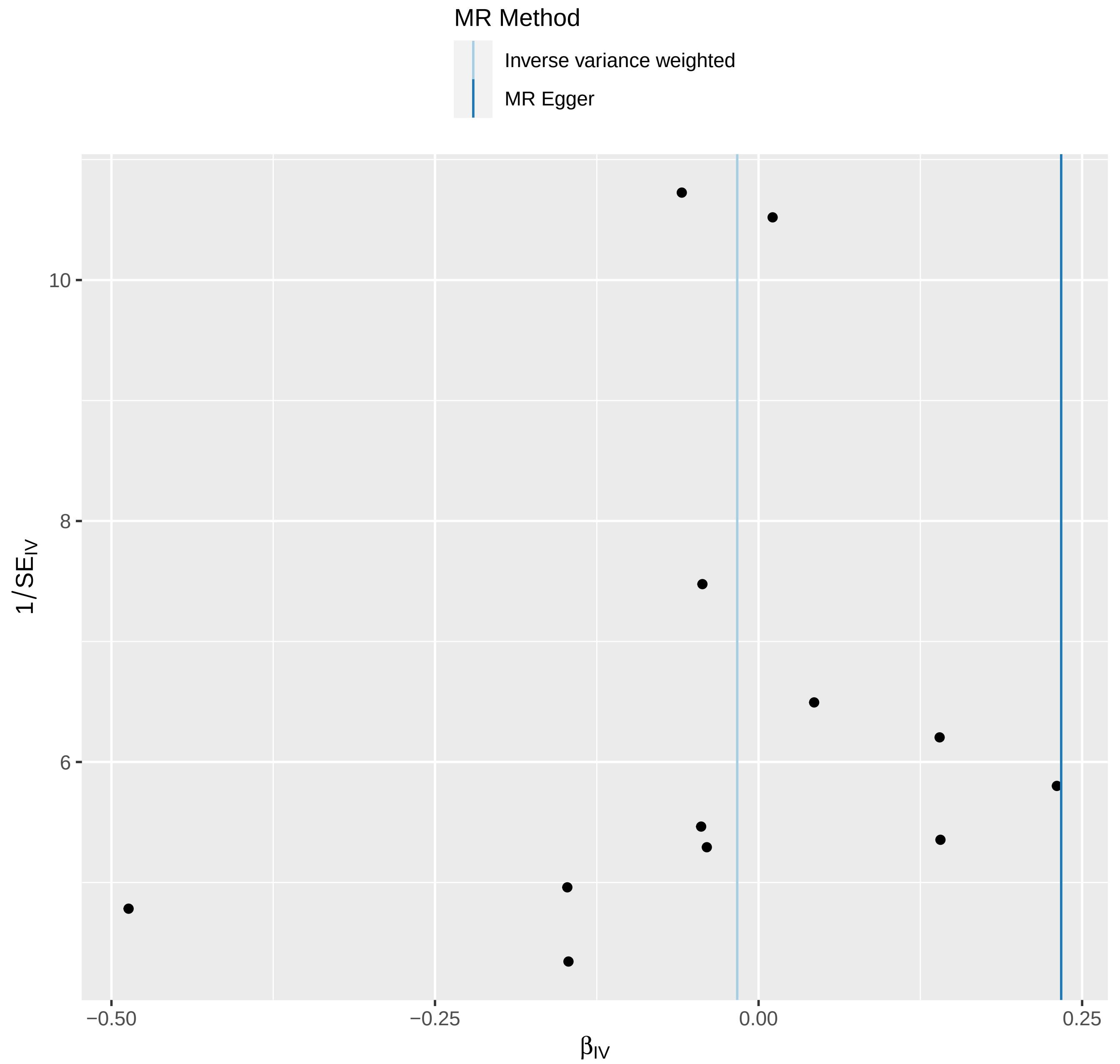

Batch 3−1889 : Selenium on Mitofusin−1

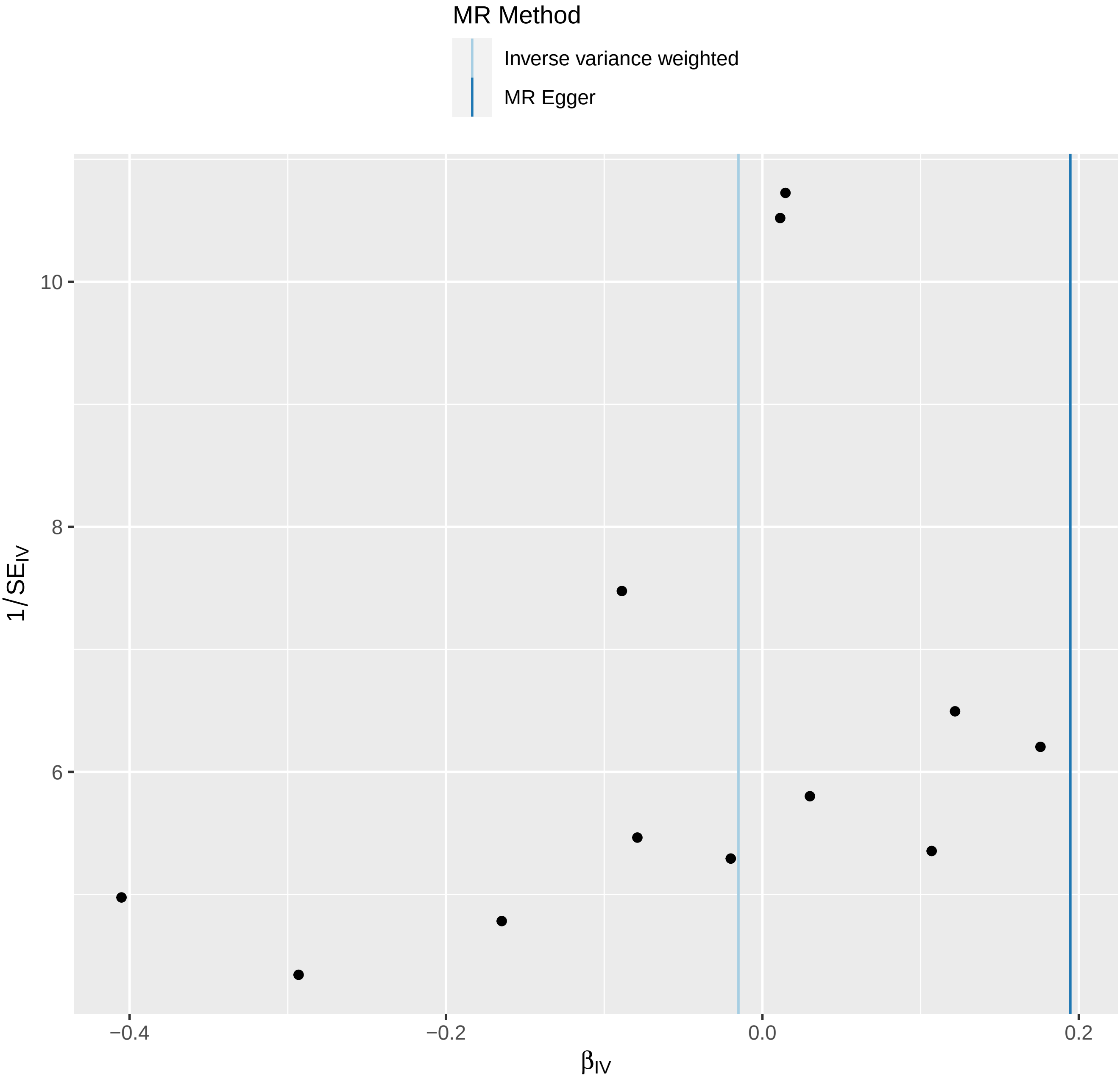

Batch 3−1890 : Selenium on Beta−1,3−N−acetylglucosaminyltransferase manic fringe

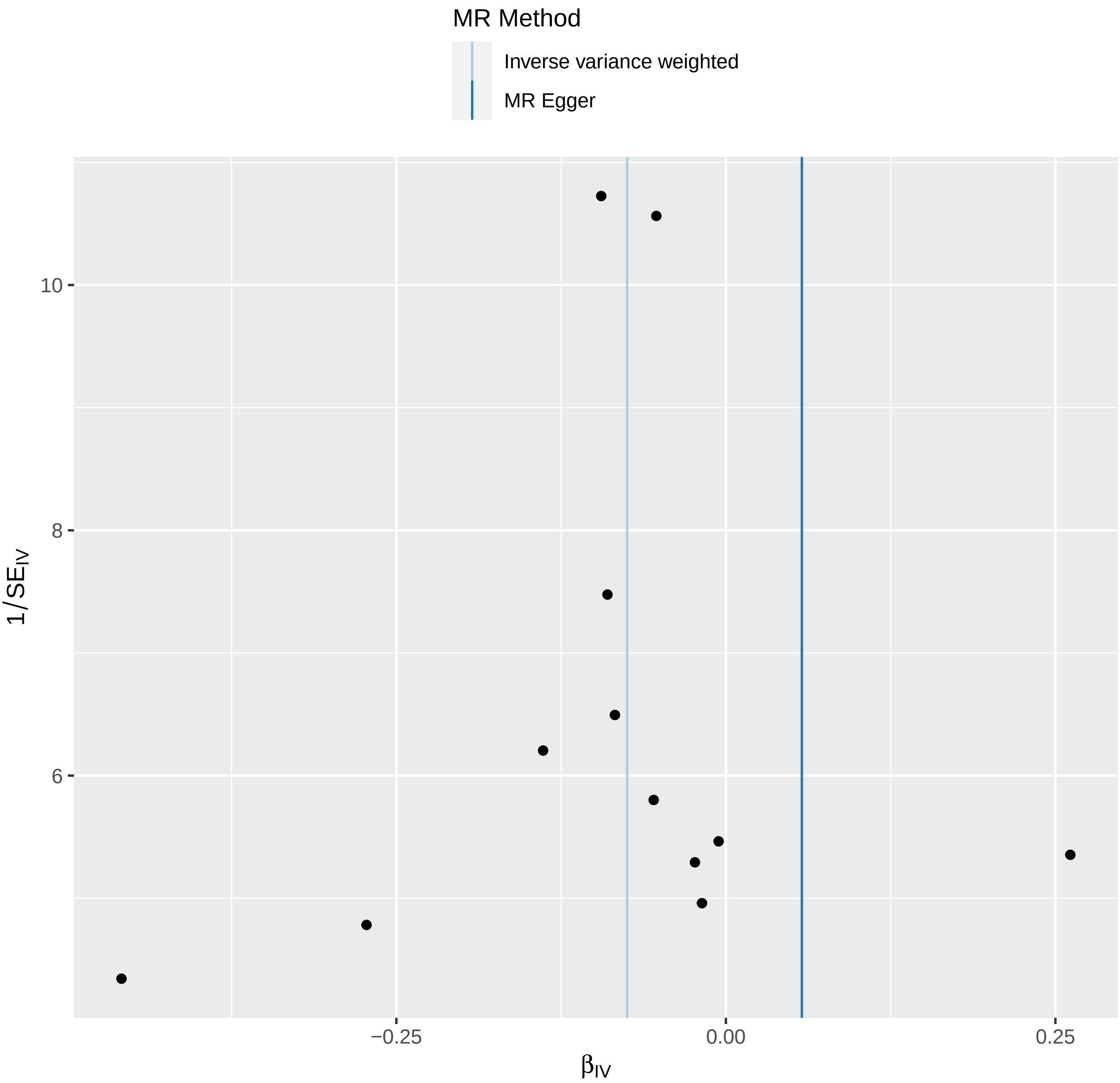

Batch 3−1891 : Selenium on MAX gene−associated protein

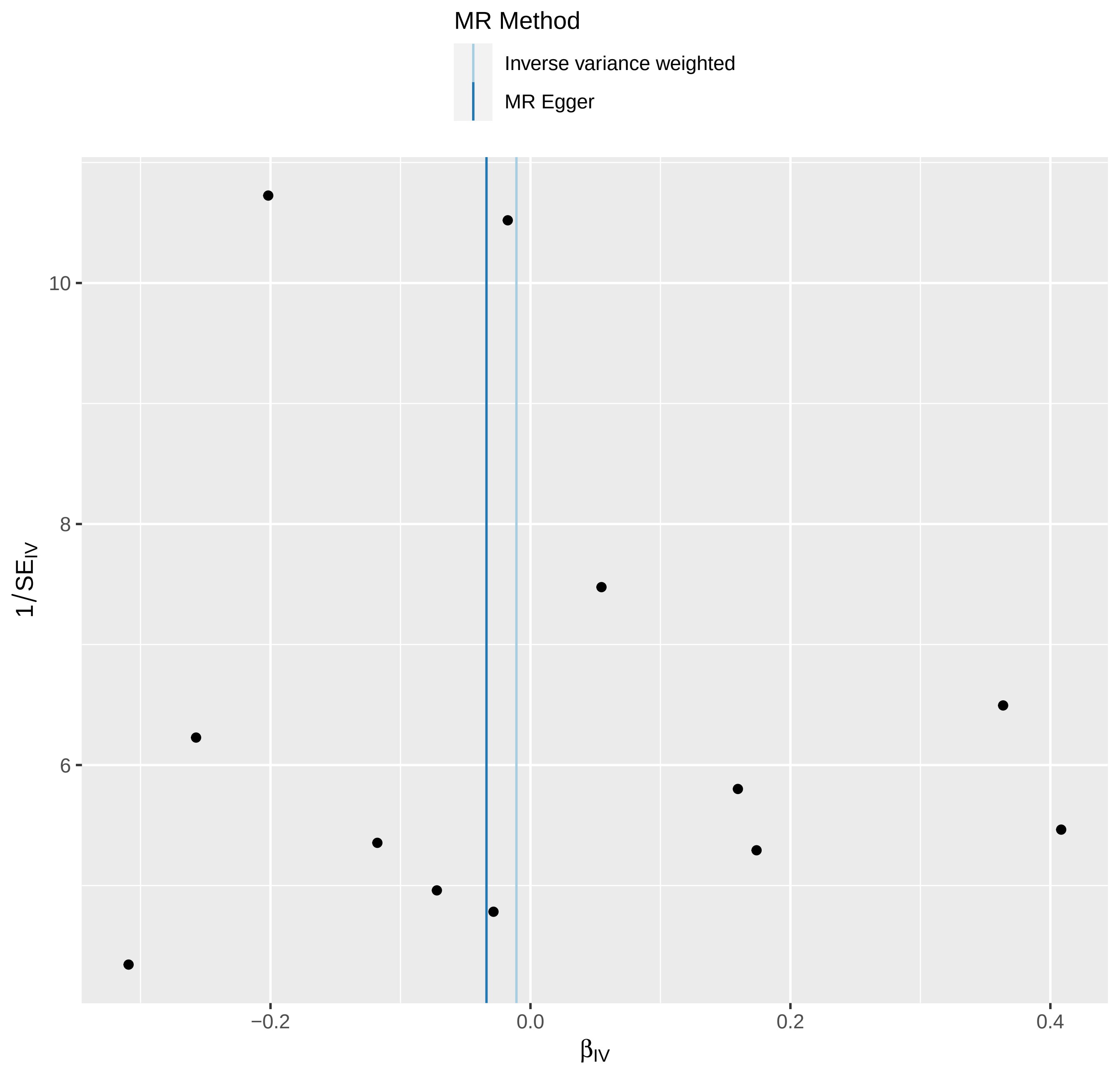

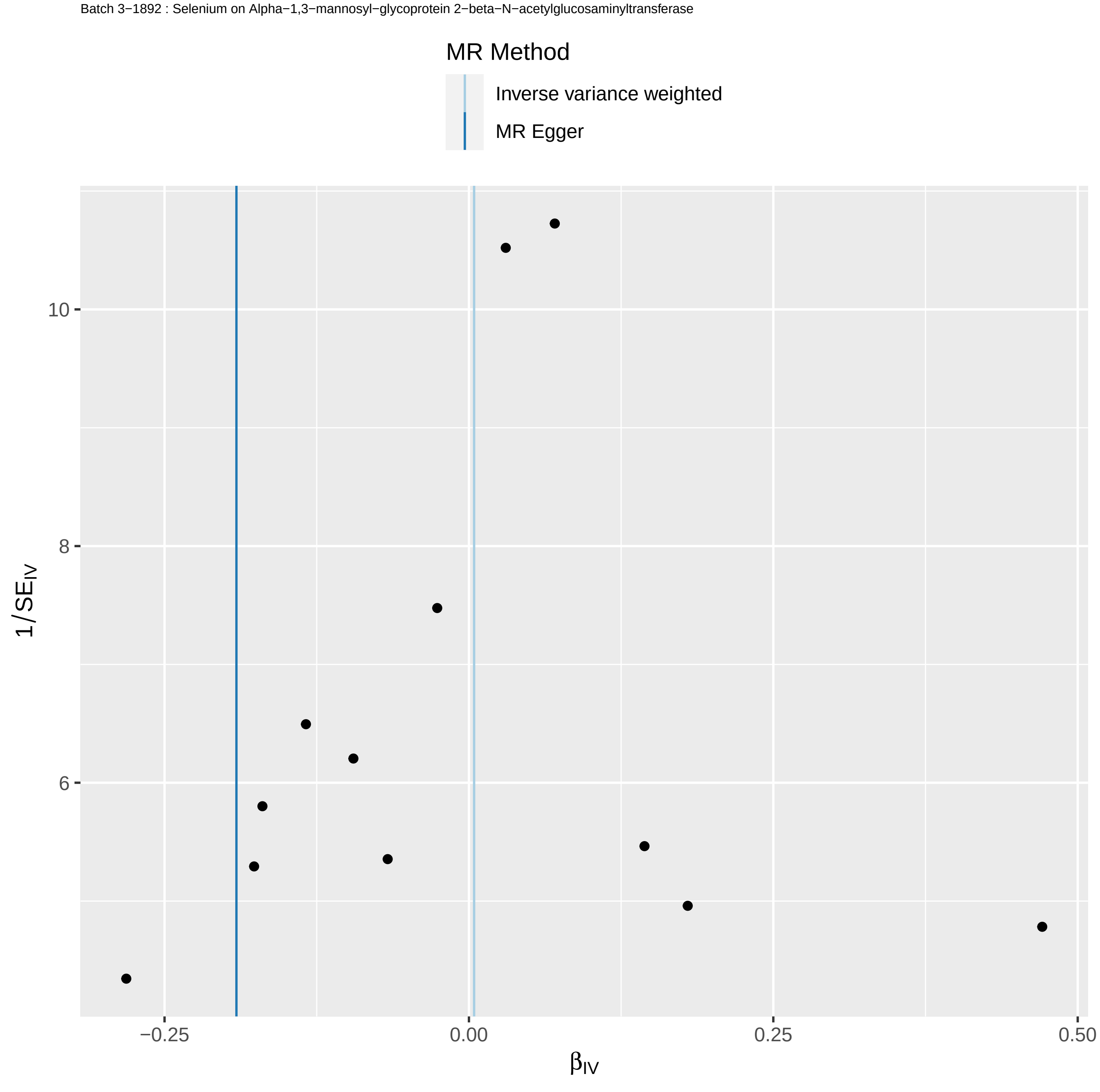

Batch 3−1892 : Selenium on Alpha−1,3−mannosyl−glycoprotein 2−beta−N−acetylglucosaminyltransferase
MR Method
Inverse variance weighted
MR Egger
10
8
6
1/SE$_{IV}$
−0.25
0.00
0.25
0.50
β$_{IV}$

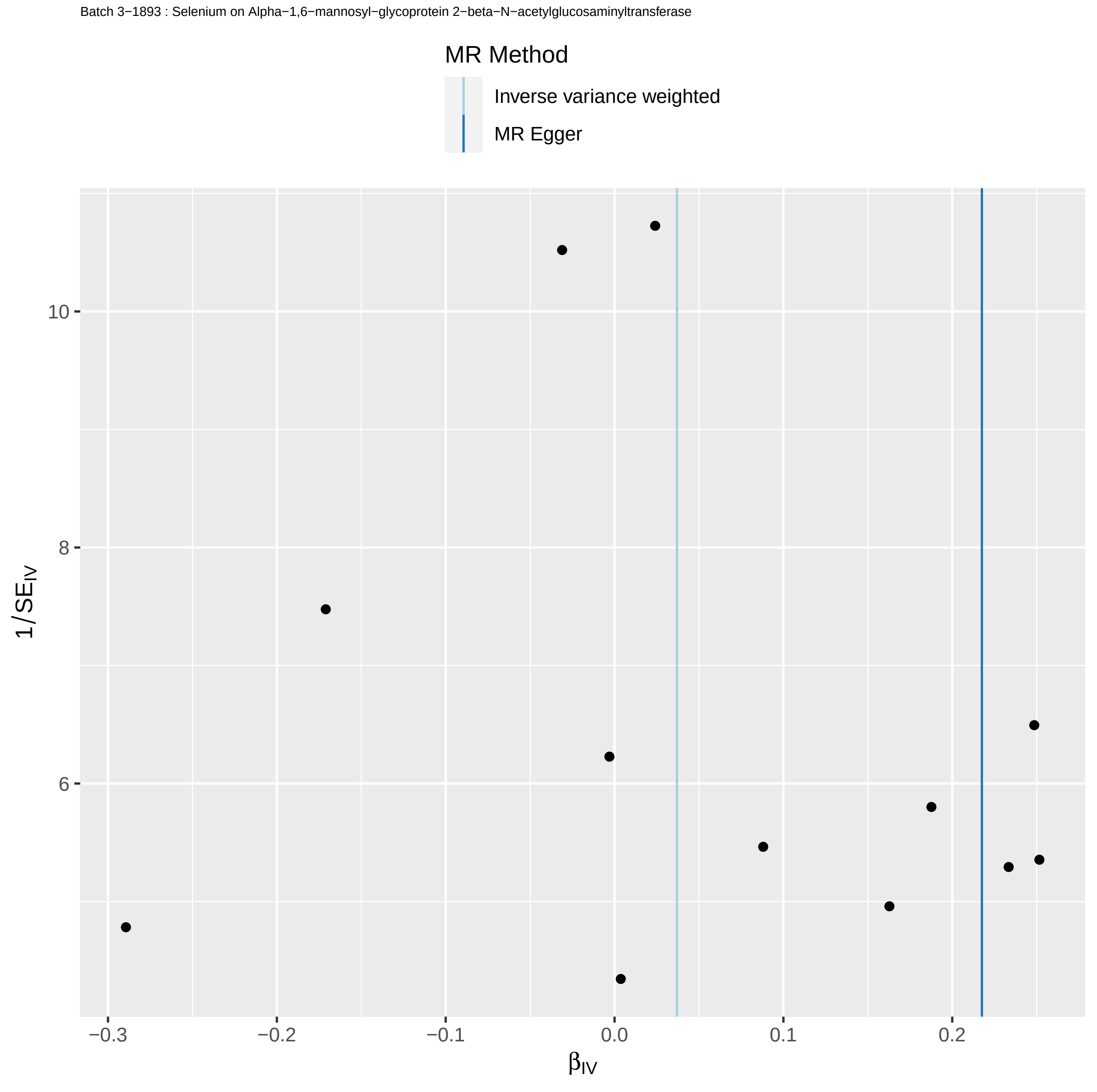
Batch 3−1893 : Selenium on Alpha−1,6−mannosyl−glycoprotein 2−beta−N−acetylglucosaminyltransferase
MR Method
Inverse variance weighted
MR Egger
10
8
6
1/SE_IV
−0.3
−0.2
−0.1
0.0
0.1
0.2
β_IV

Batch 3−1894 : Selenium on Alpha−1,3−mannosyl−glycoprotein 4−beta−N−acetylglucosaminyltransferase B

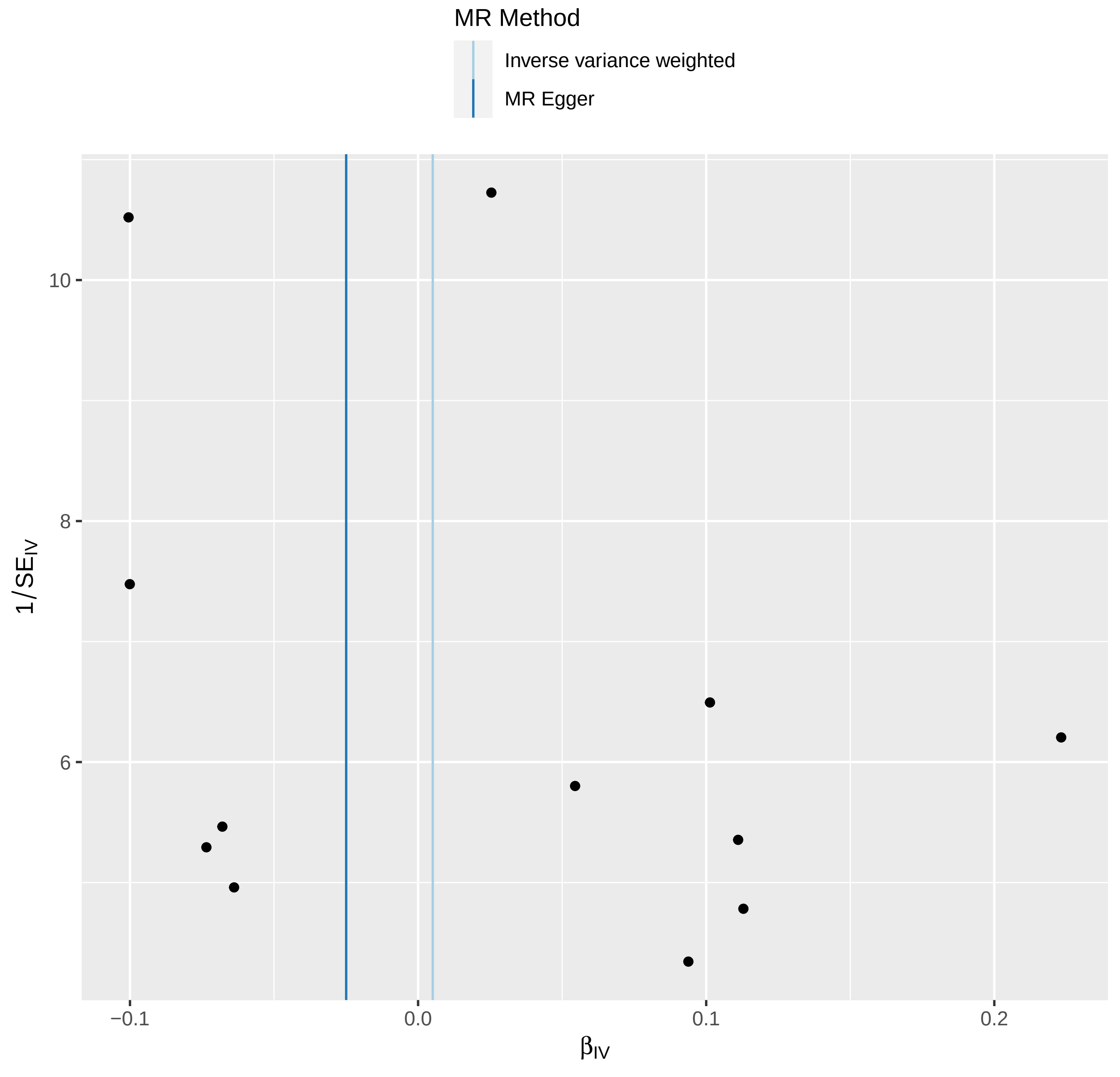

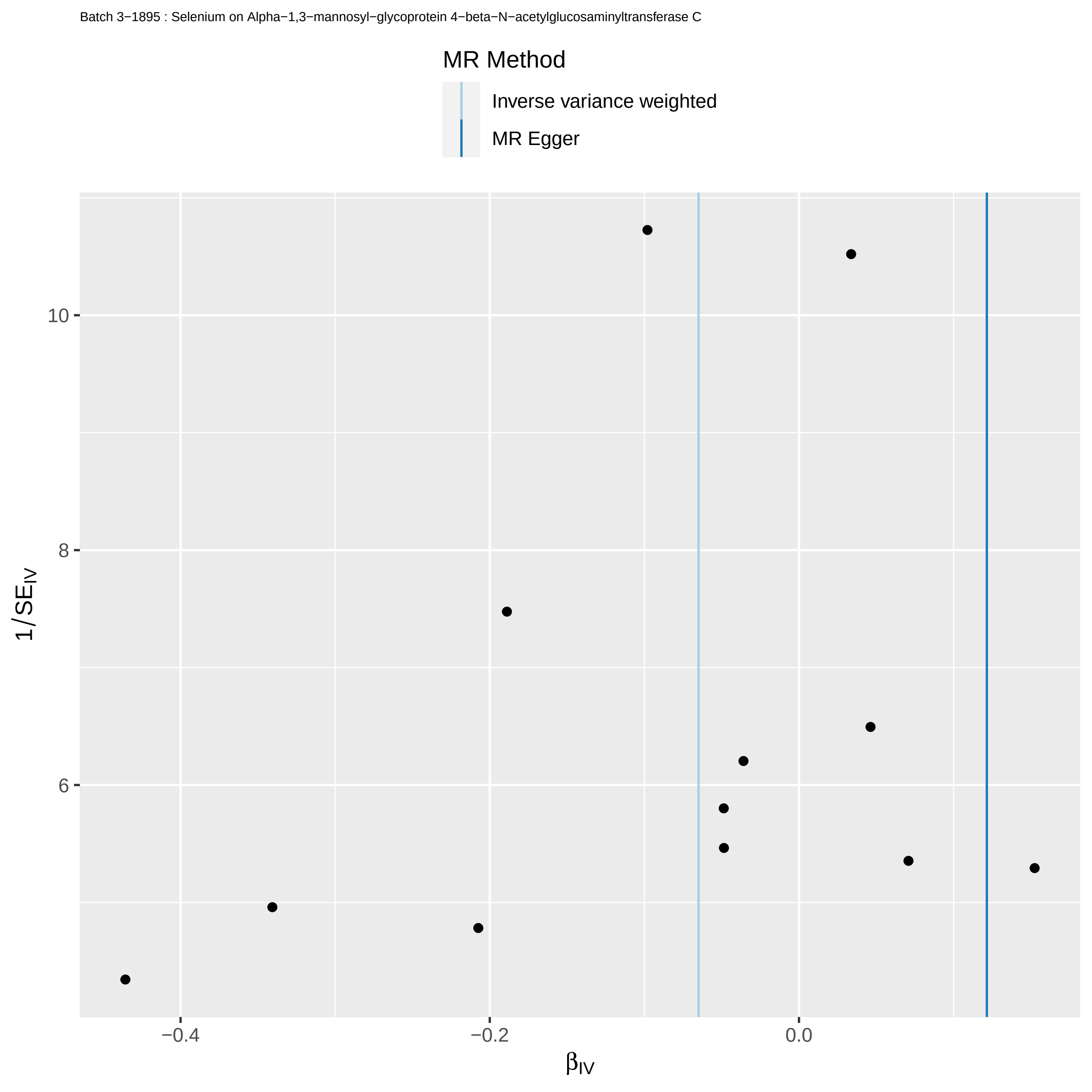

Batch 3−1895 : Selenium on Alpha−1,3−mannosyl−glycoprotein 4−beta−N−acetylglucosaminyltransferase C
MR Method
Inverse variance weighted
MR Egger
10
8
6
1/SE_IV
−0.4
−0.2
0.0
β_IV

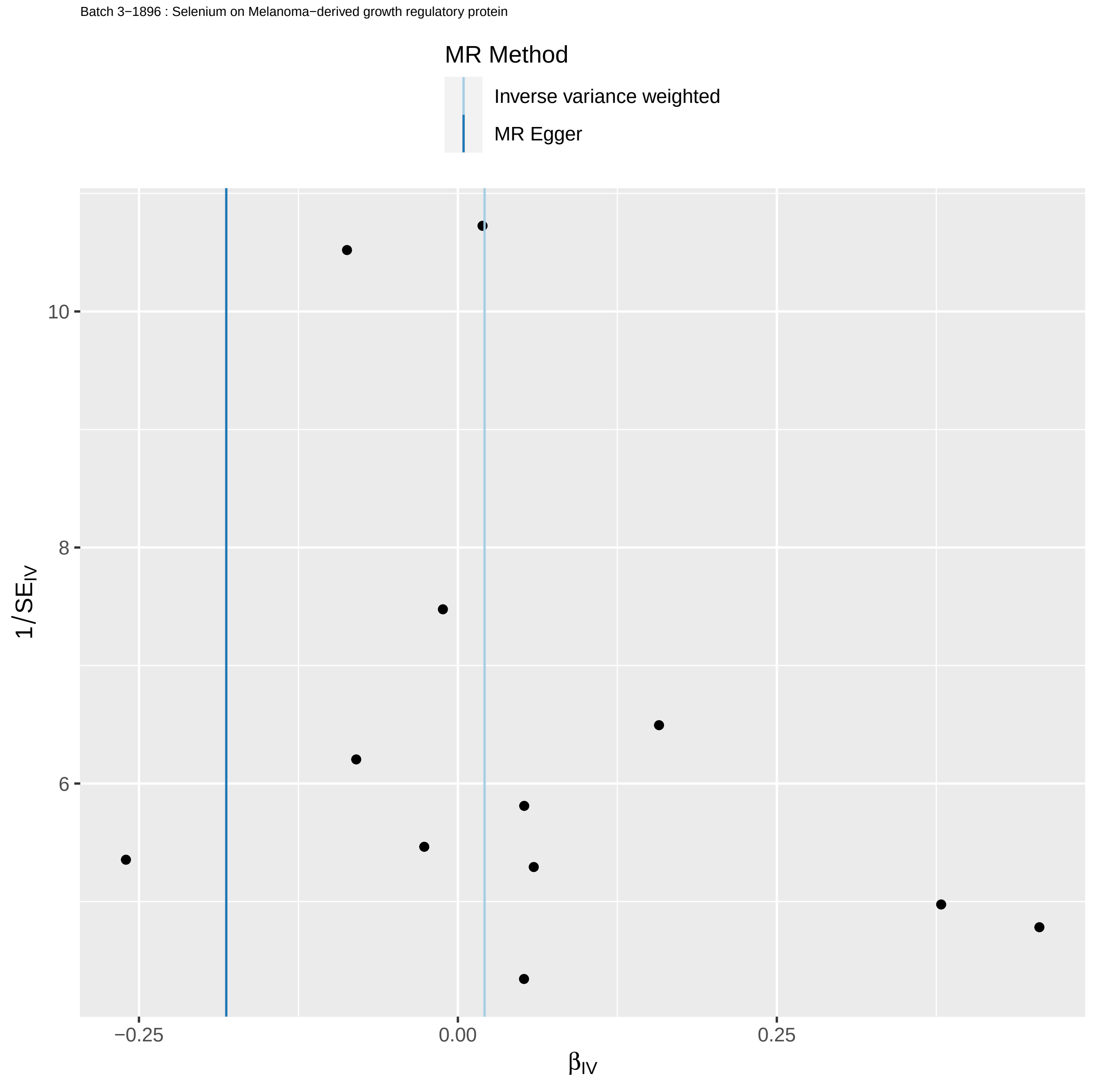
Batch 3−1896 : Selenium on Melanoma−derived growth regulatory protein
MR Method
Inverse variance weighted
MR Egger
10
8
6
1/SE_IV
−0.25
0.00
0.25
β_IV

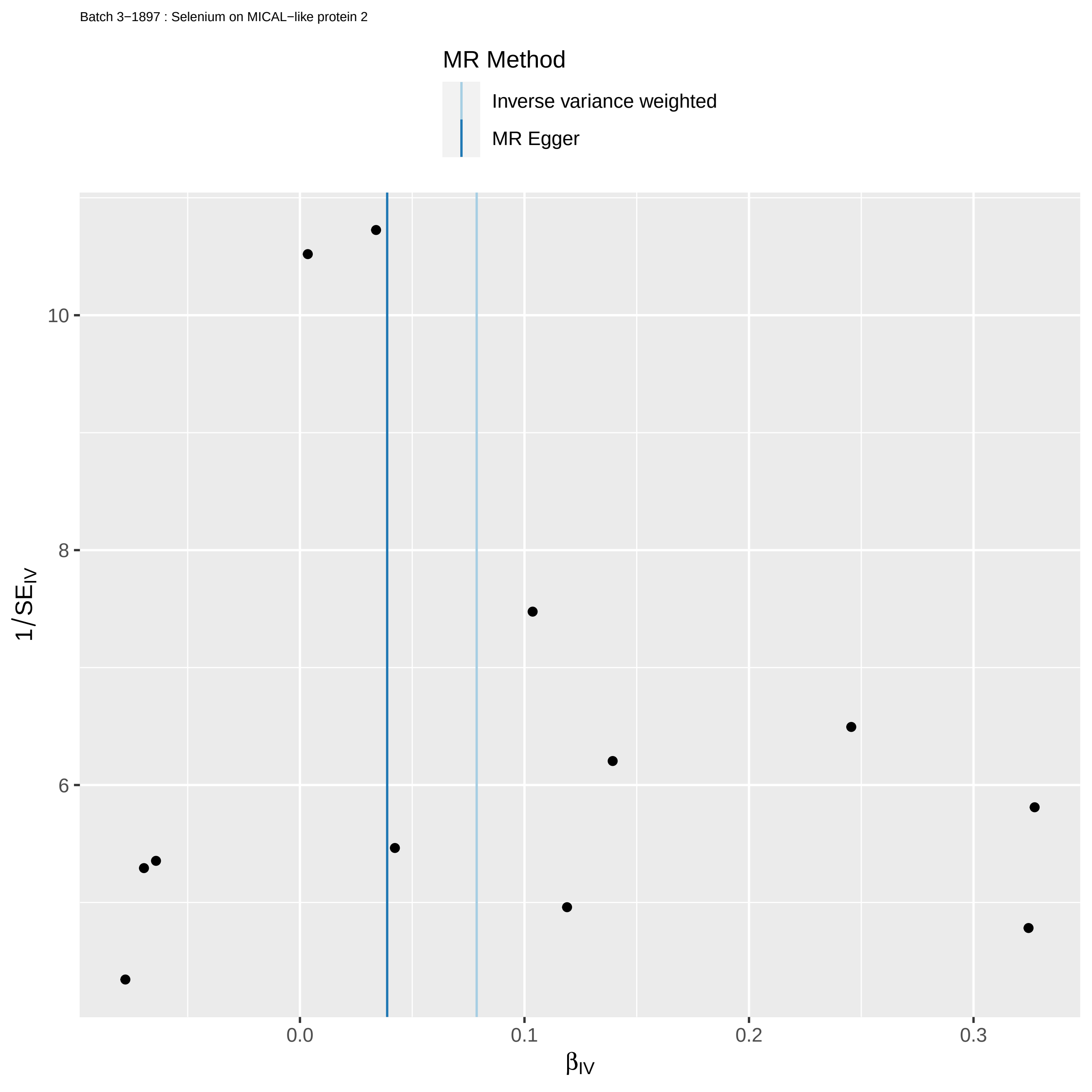

Batch 3−1897 : Selenium on MICAL−like protein 2
MR Method
Inverse variance weighted
MR Egger
10
8
6
0.0
0.1
0.2
0.3
$1/SE_{IV}$
β_{IV}

Batch 3−1898 : Selenium on MHC class I polypeptide−related sequence B

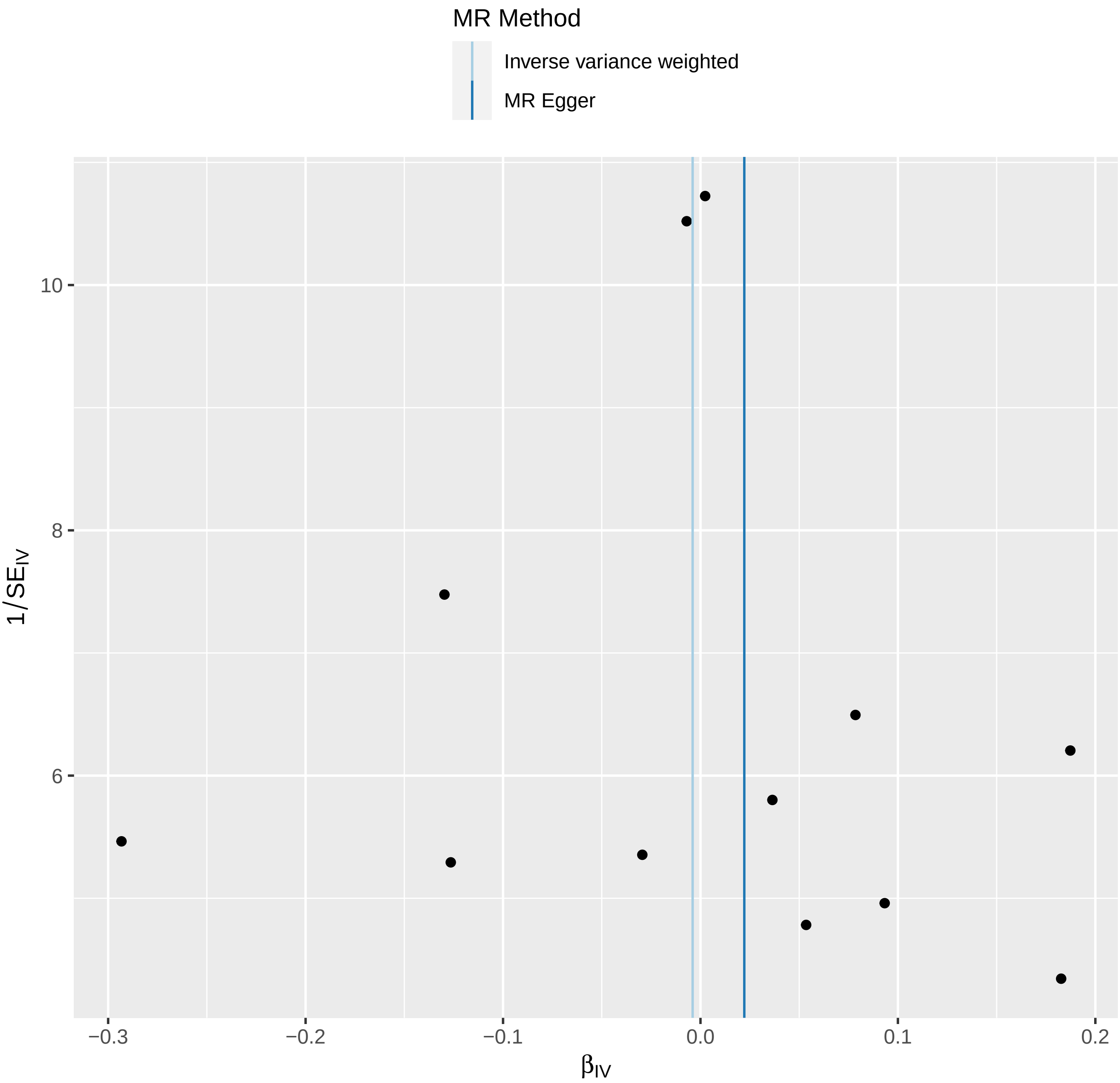

Batch 3−1899 : Selenium on Probable E3 ubiquitin−protein ligase MID2

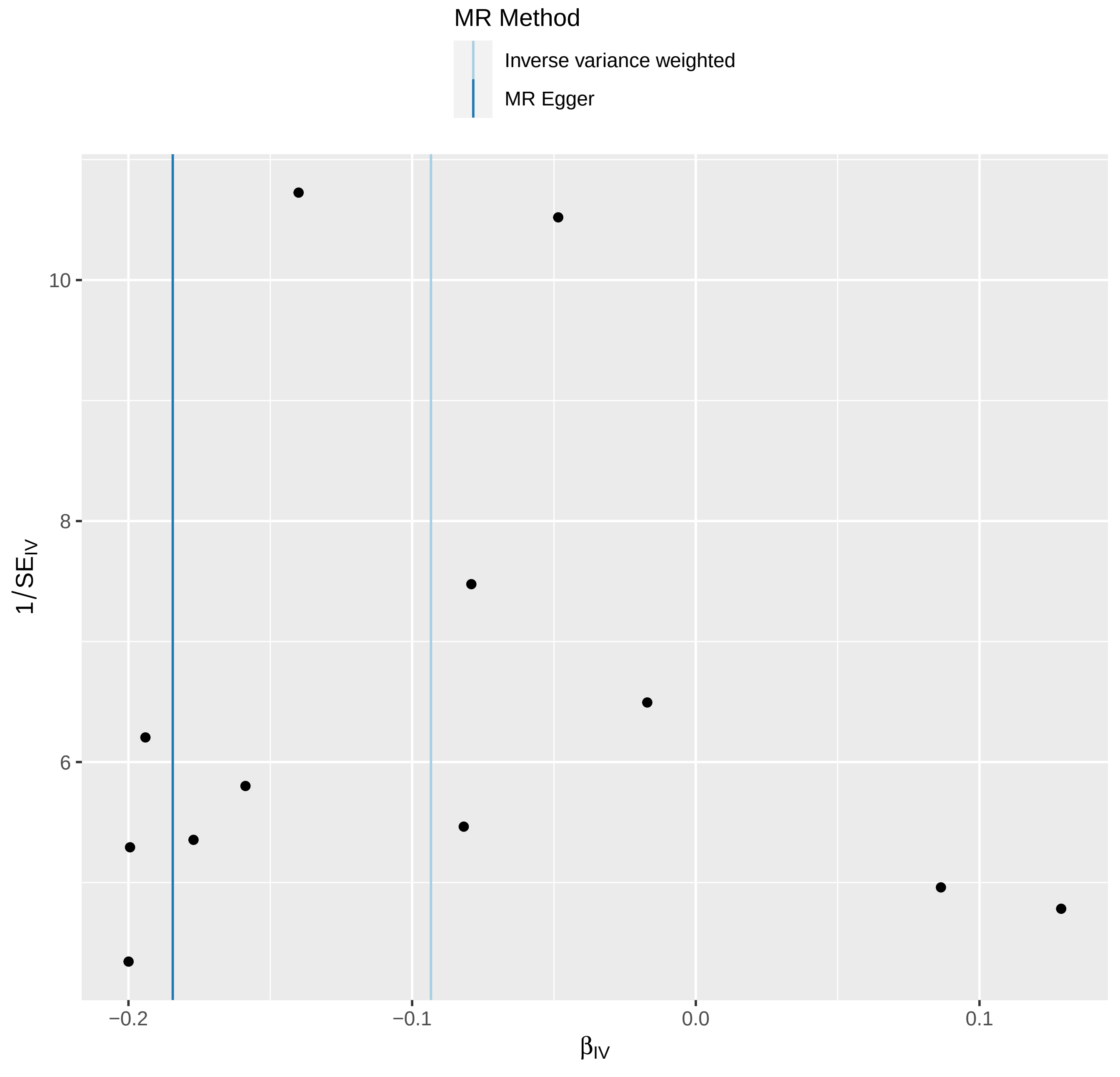

Batch 3−1900 : Selenium on Macrophage migration inhibitory factor

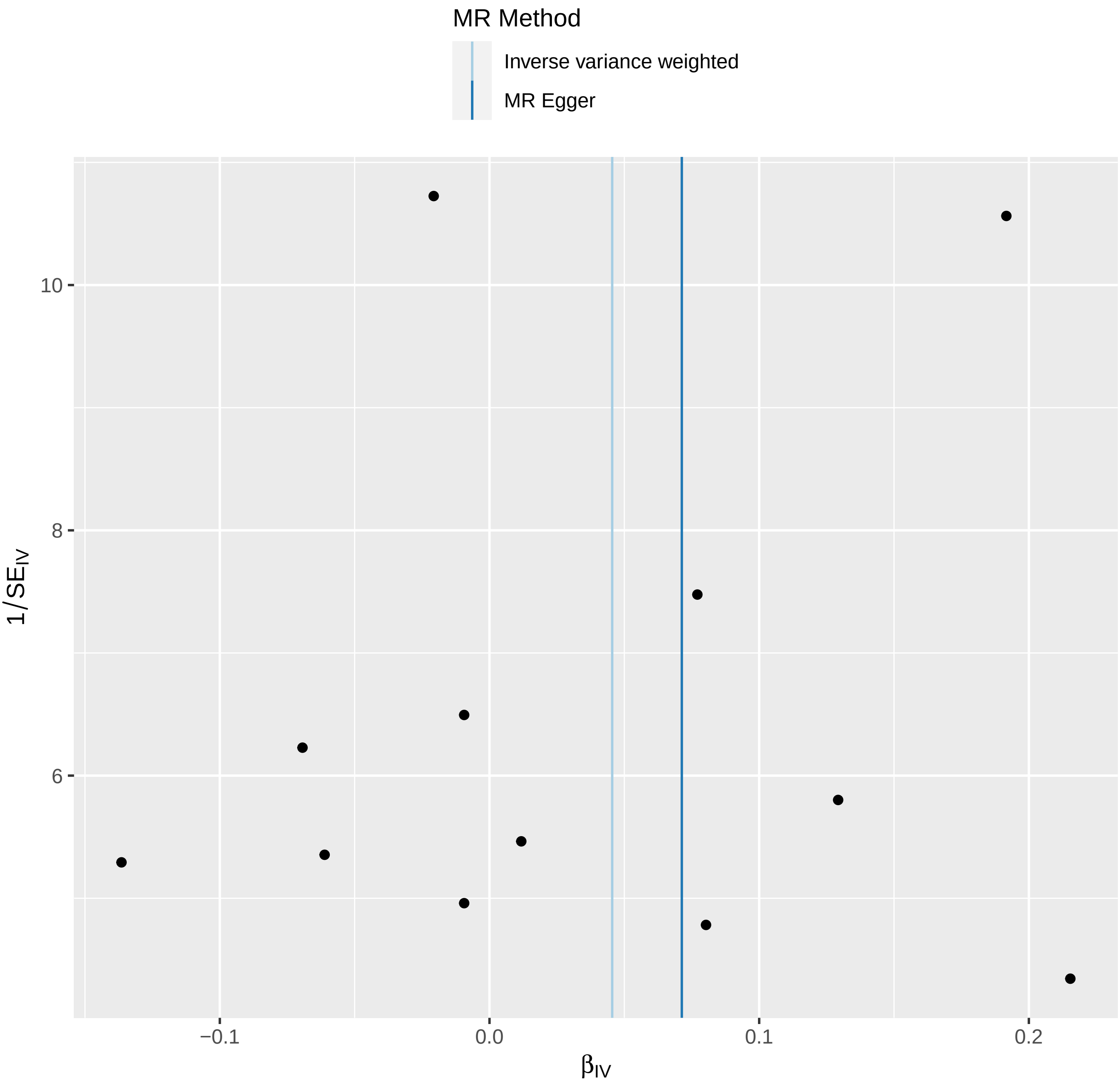

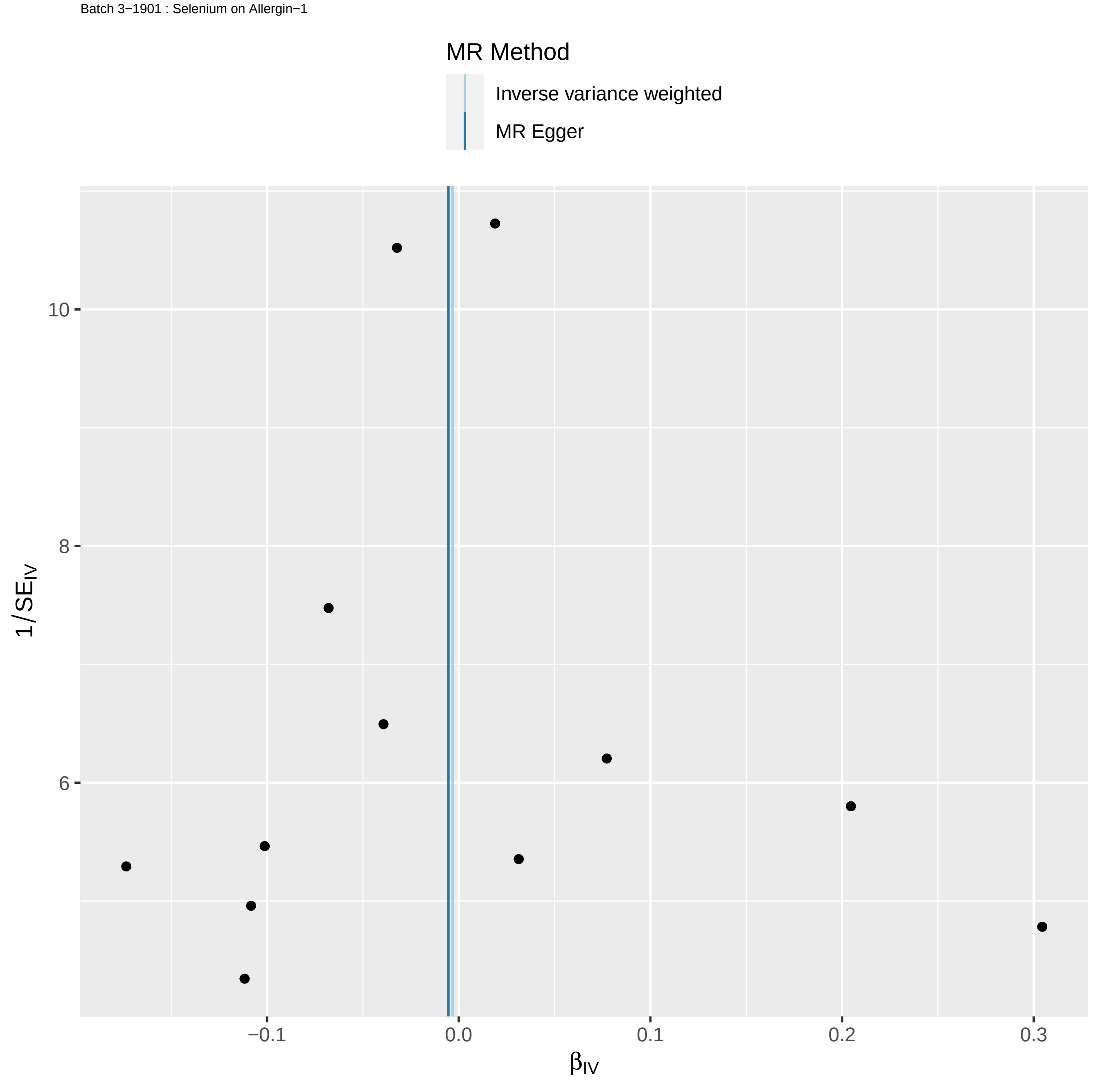

Batch 3−1901 : Selenium on Allergin−1
MR Method
Inverse variance weighted
MR Egger
10
8
6
1/SE$_{IV}$
−0.1
0.0
0.1
0.2
0.3
β$_{IV}$

Batch 3−1902 : Selenium on MICOS complex subunit MIC10

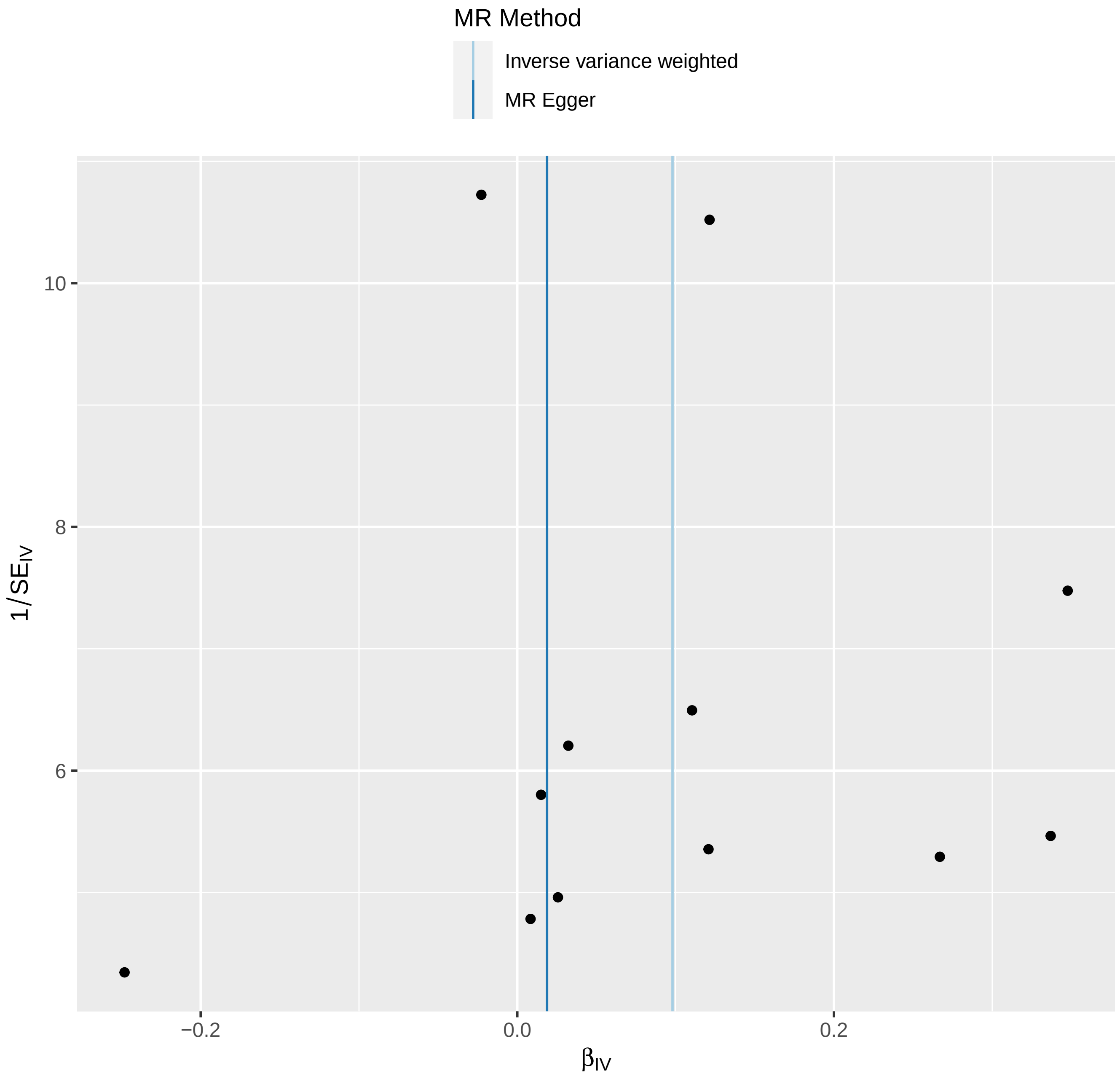

Batch 3−1903 : Selenium on Multiple inositol polyphosphate phosphatase 1

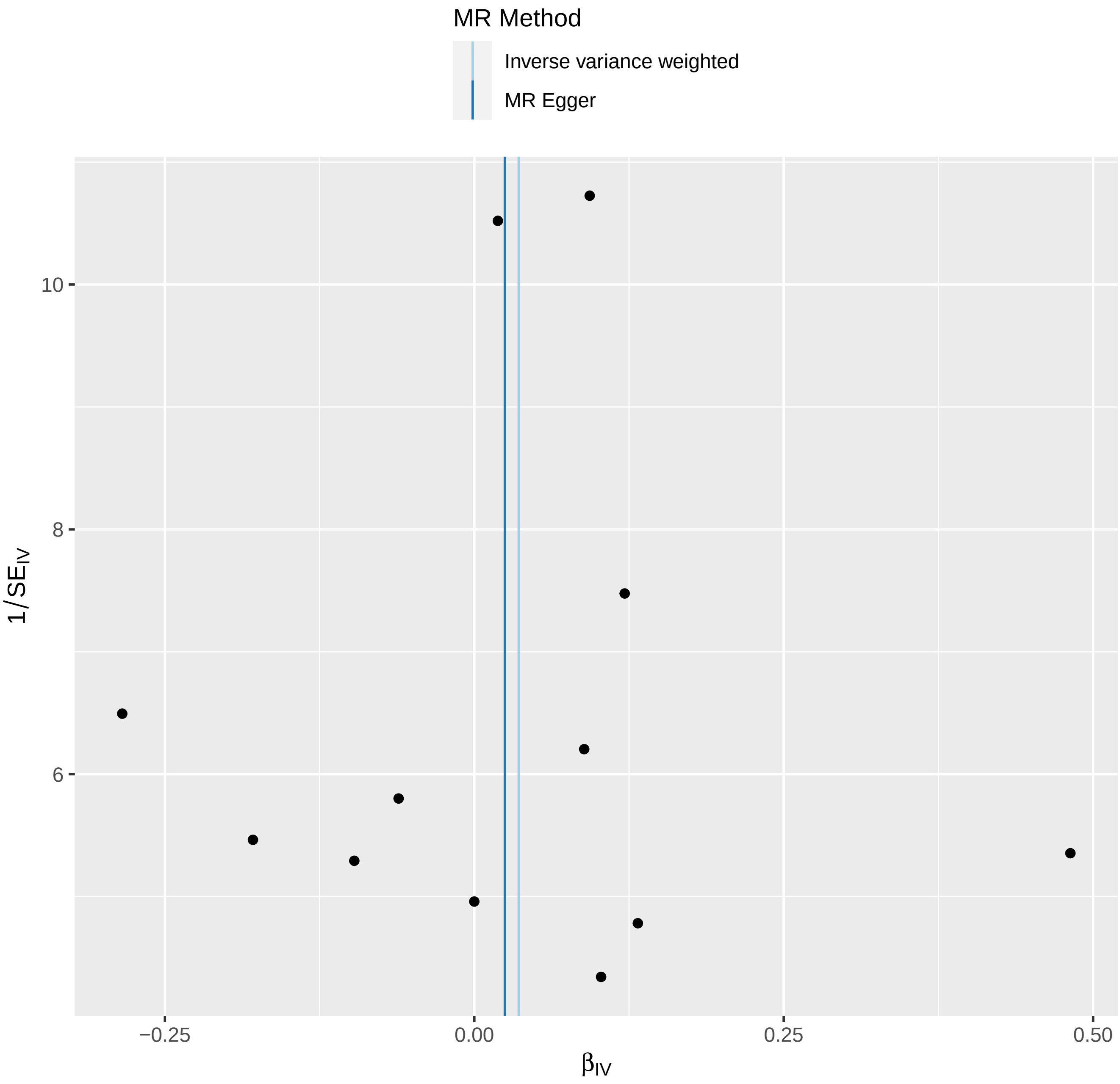

Batch 3−1904 : Selenium on Malectin

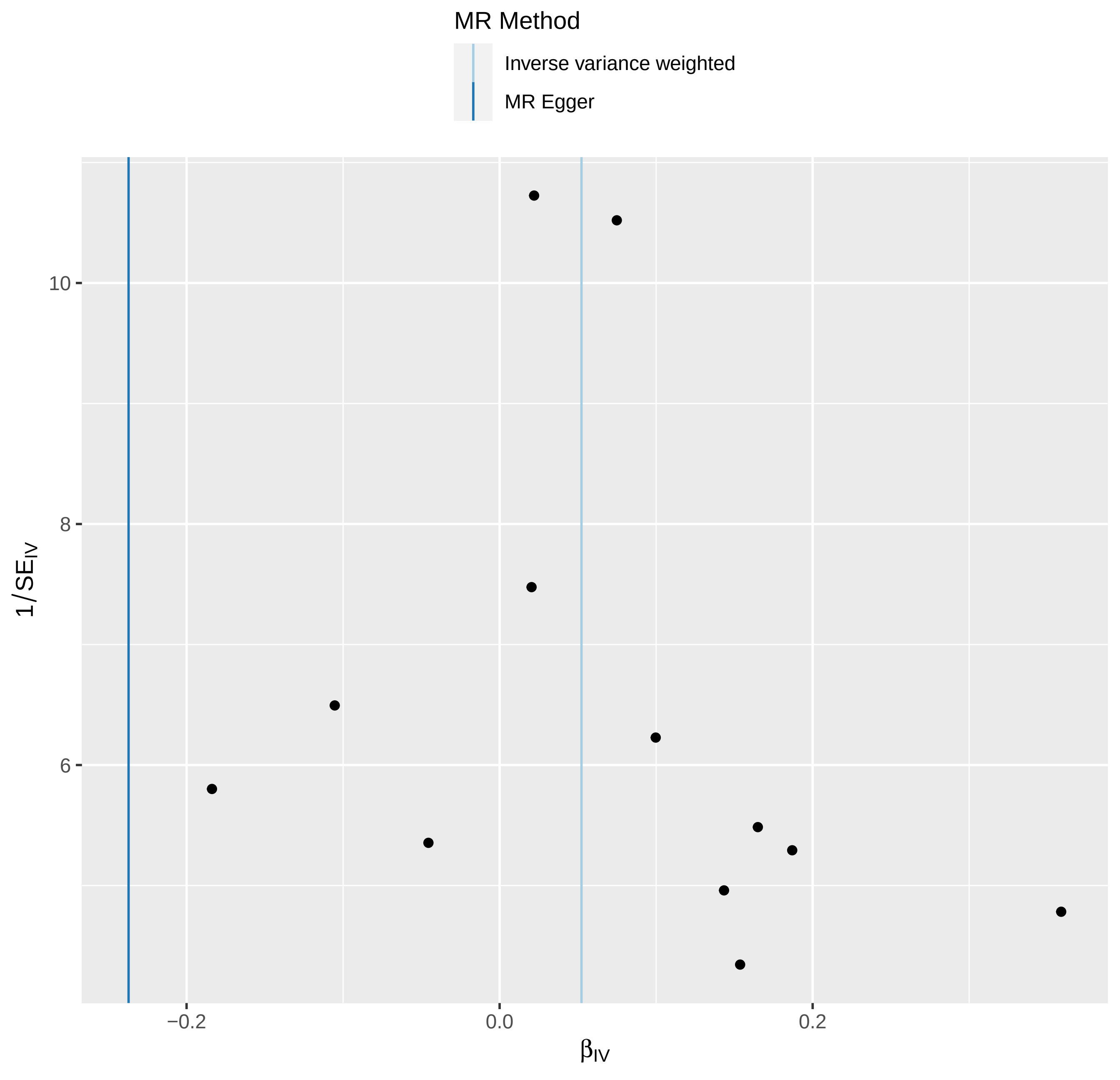

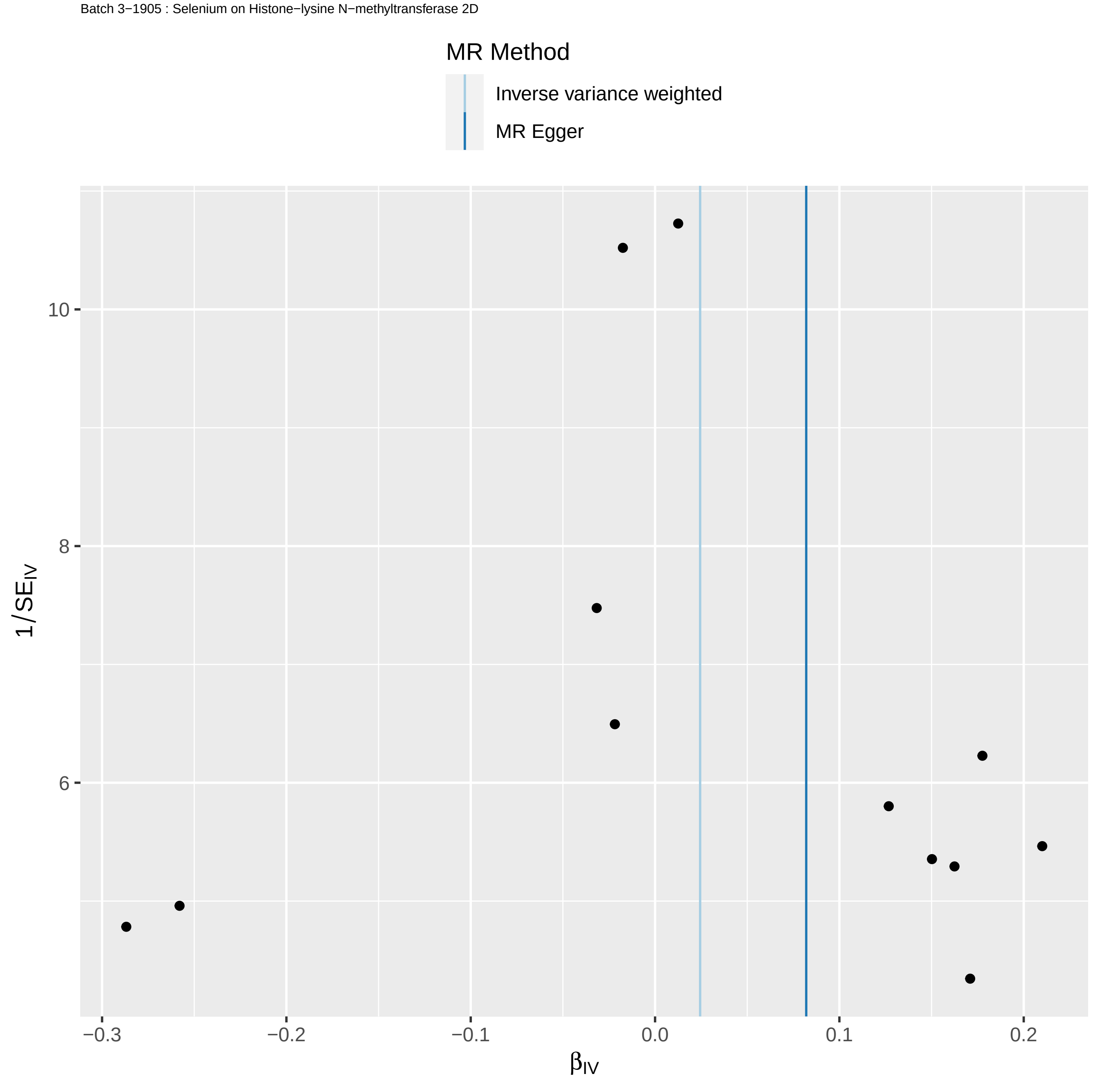

Batch 3−1905 : Selenium on Histone−lysine N−methyltransferase 2D
MR Method
Inverse variance weighted
MR Egger
10
8
6
$1/SE_{IV}$
−0.3
−0.2
−0.1
0.0
0.1
0.2
β_{IV}

Batch 3−1906 : Selenium on Promotilin

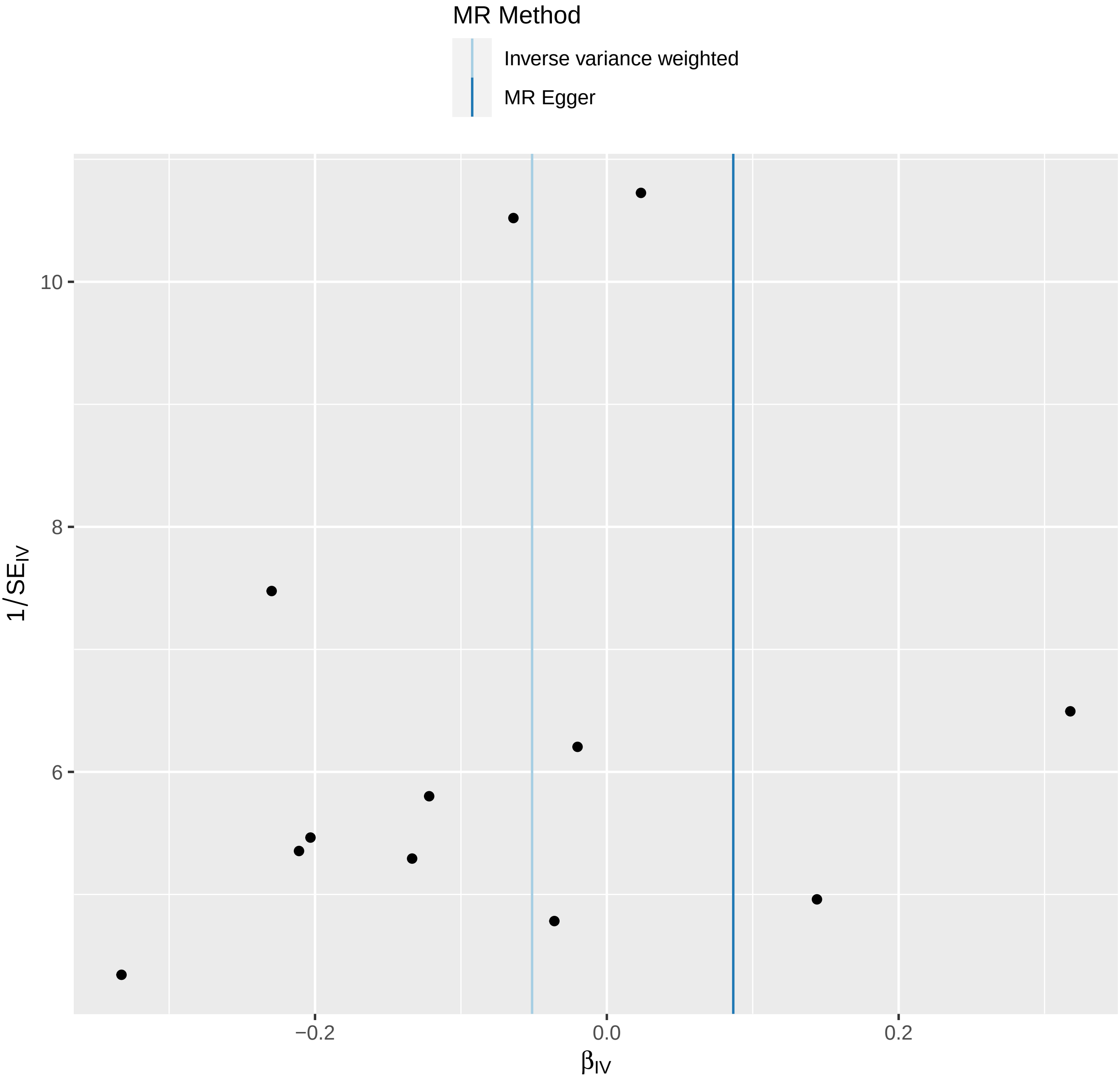

Batch 3−1907 : Selenium on Malonyl−CoA decarboxylase, mitochondrial

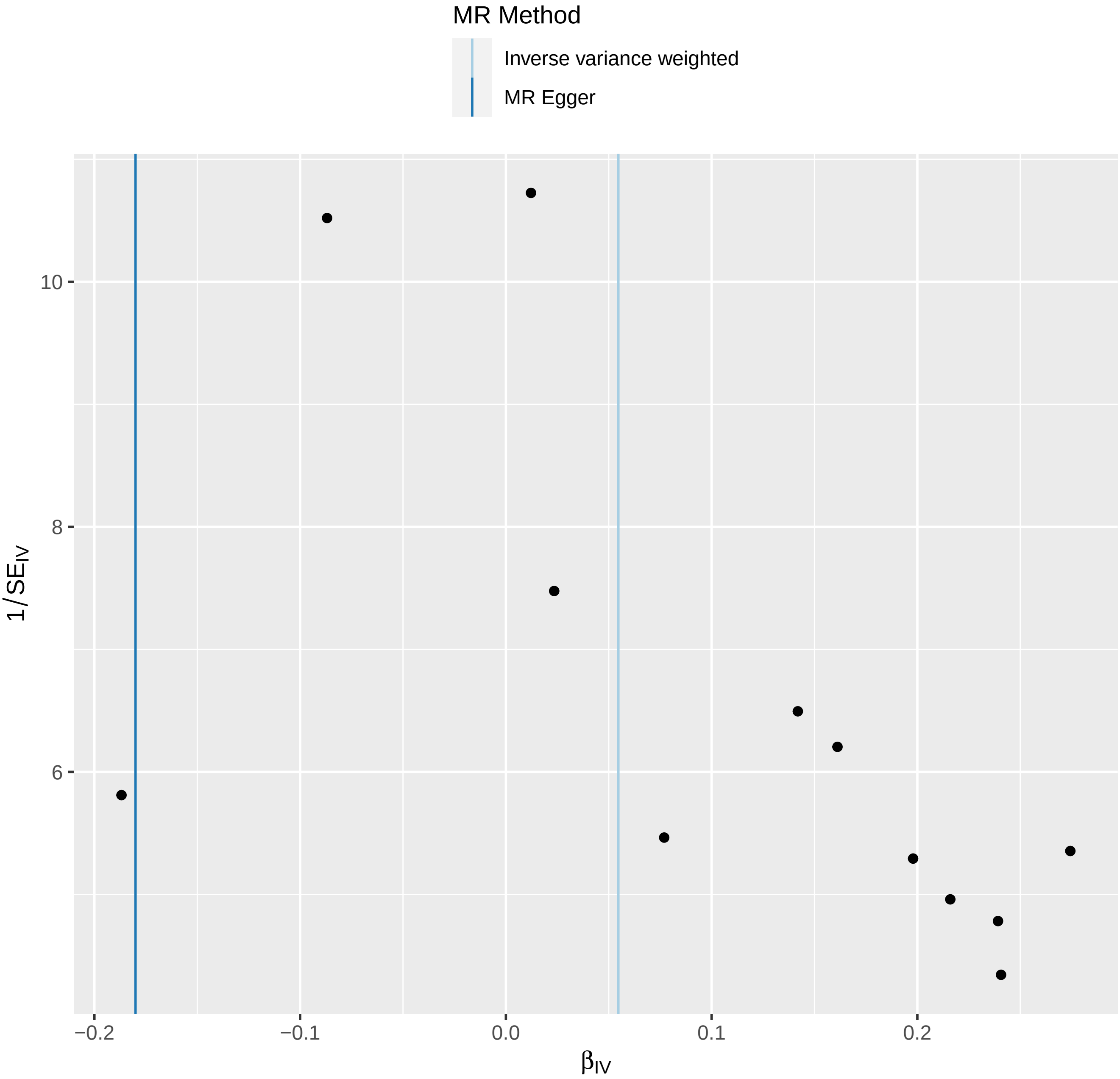

Batch 3−1908 : Selenium on Membrane metallo−endopeptidase−like 1

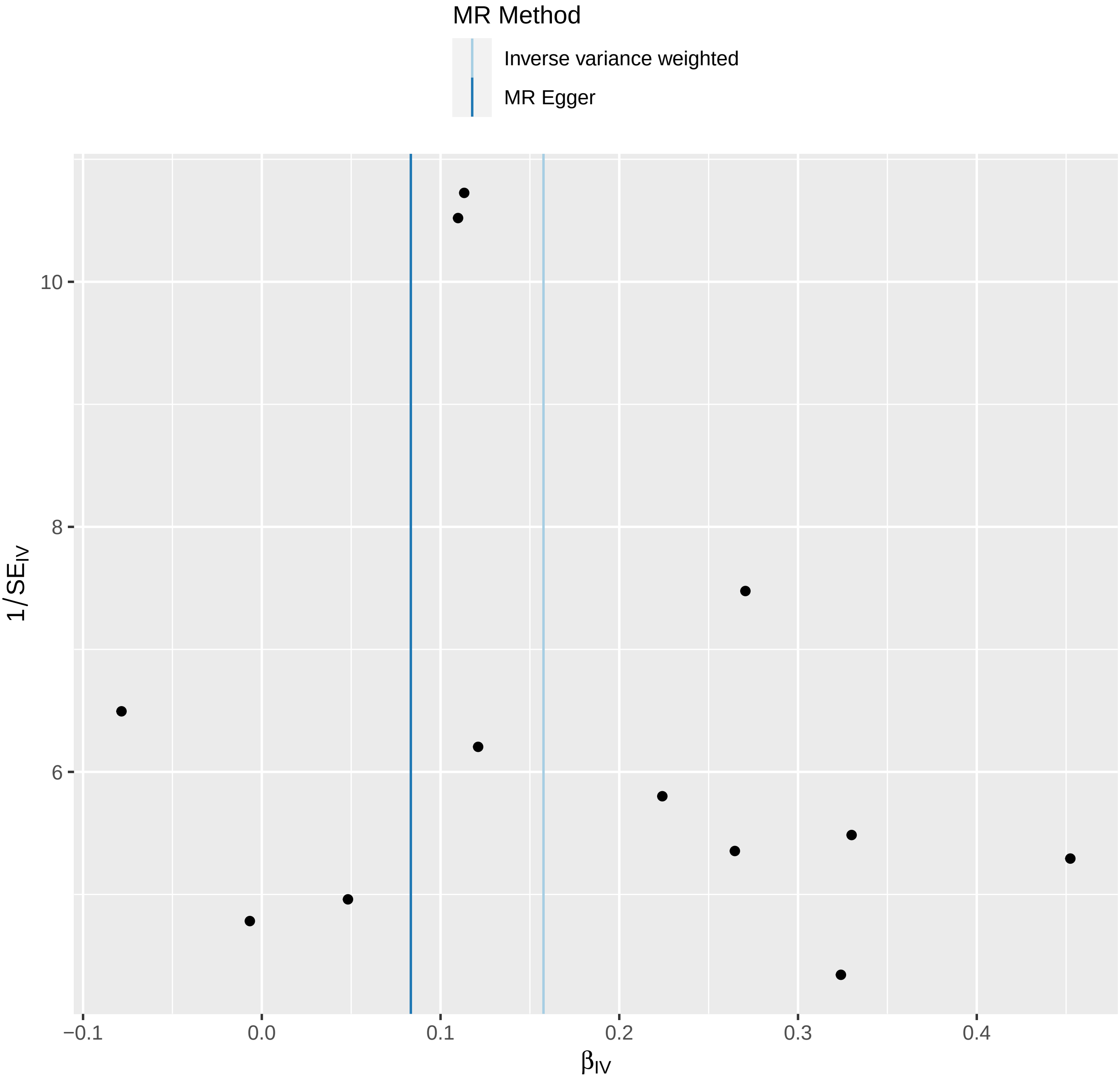

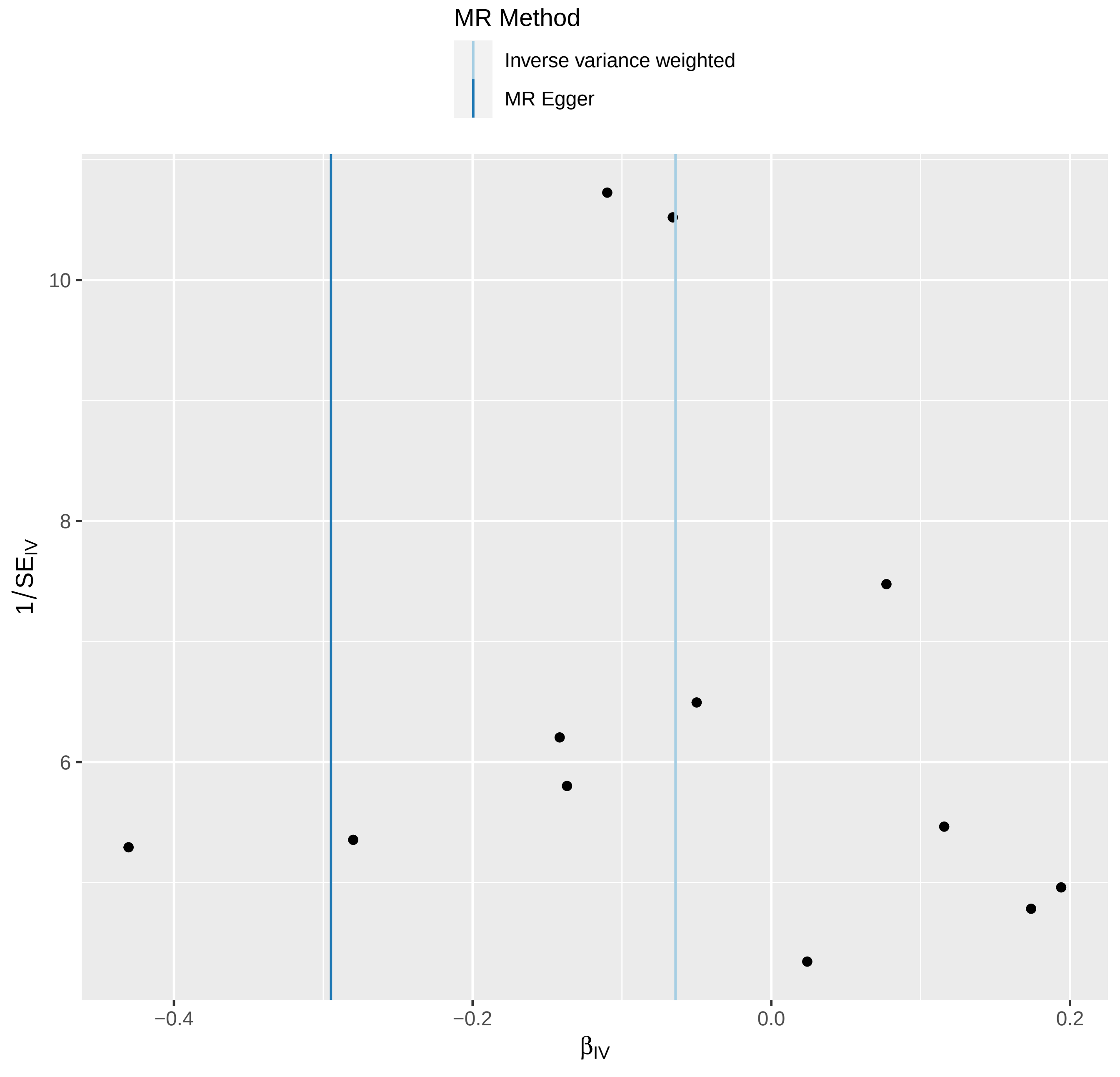
MR Method
Inverse variance weighted
MR Egger
10
8
6
1/SE$_{IV}$
−0.4
−0.2
0.0
0.2
β$_{IV}$

Batch 3−1910 : Selenium on Stromelysin−2

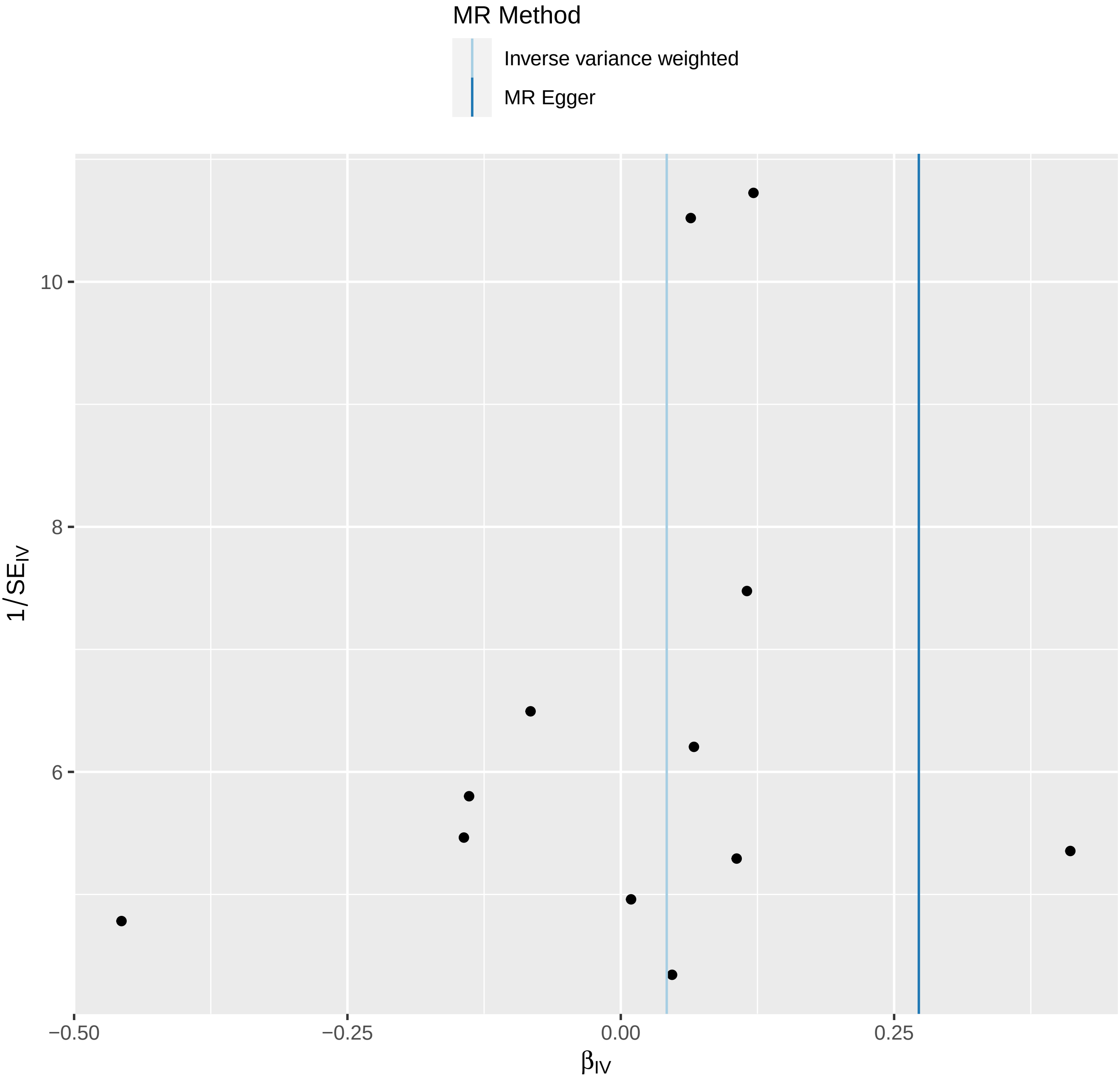

Batch 3−1911 : Selenium on Stromelysin−2

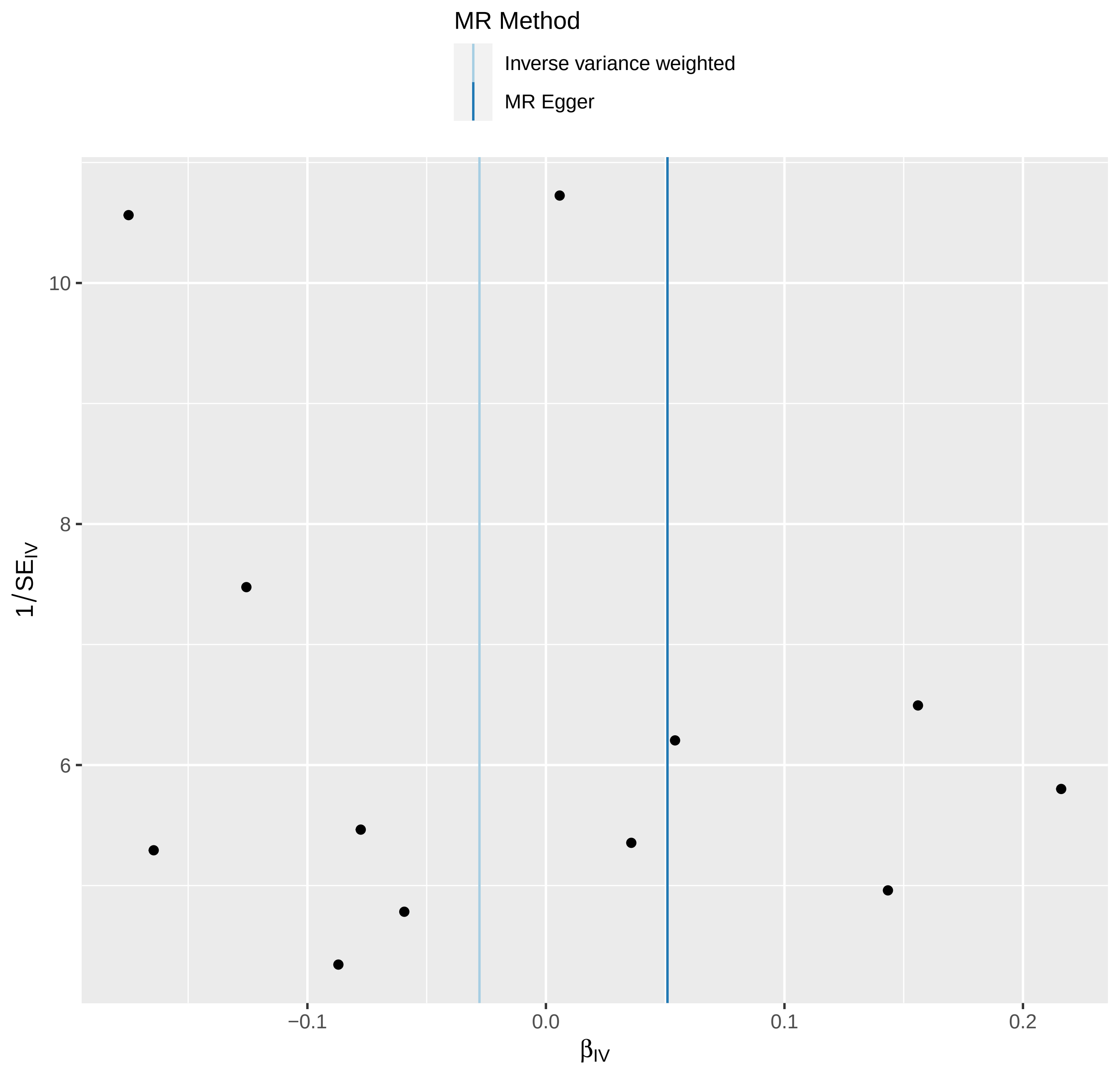

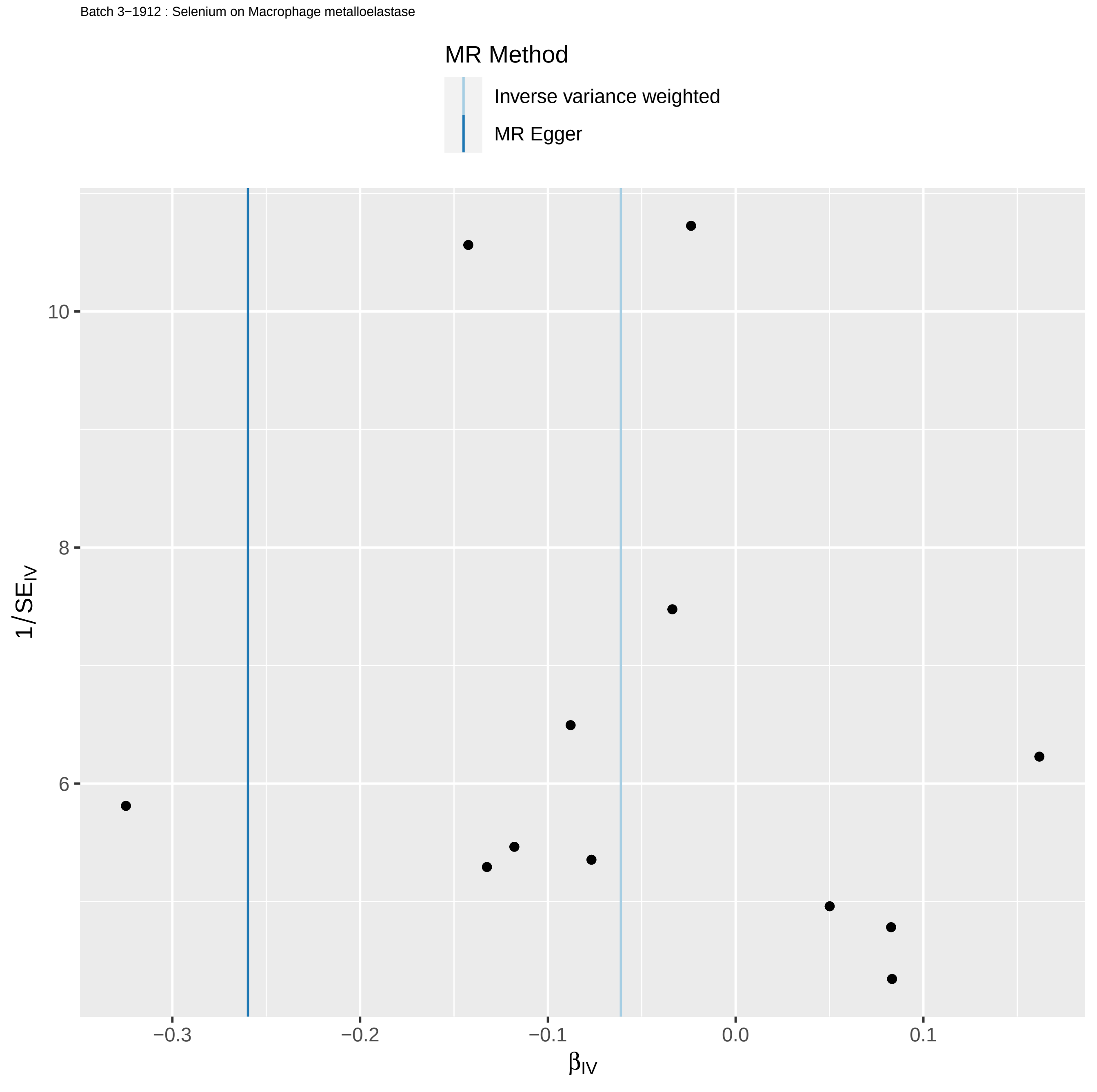

Batch 3−1912 : Selenium on Macrophage metalloelastase
MR Method
Inverse variance weighted
MR Egger
10
8
6
1/SE$_{IV}$
−0.3
−0.2
−0.1
0.0
0.1
β$_{IV}$

Batch 3−1913 : Selenium on Collagenase 3

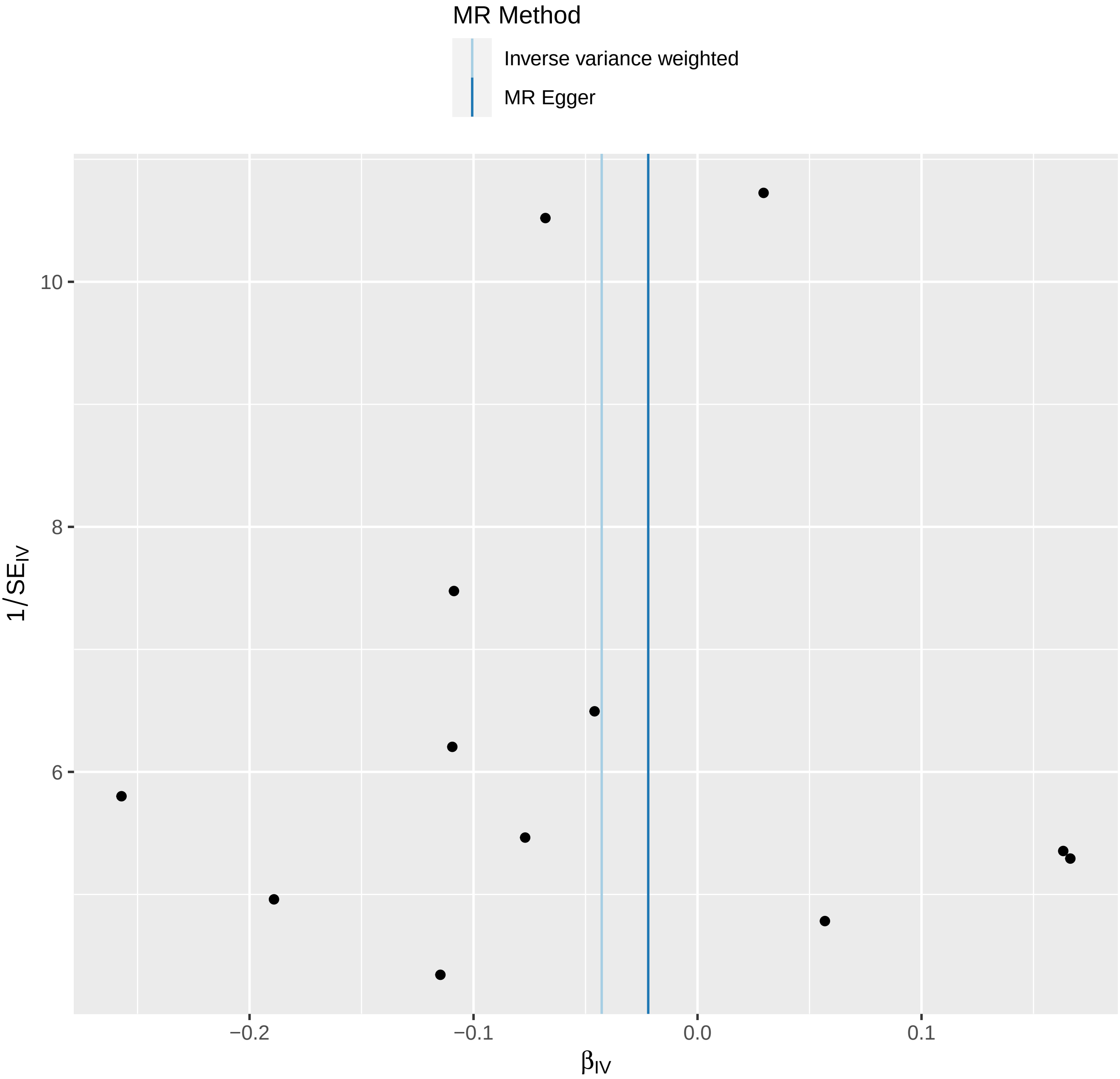

Batch 3−1914 : Selenium on Matrix metalloproteinase−14

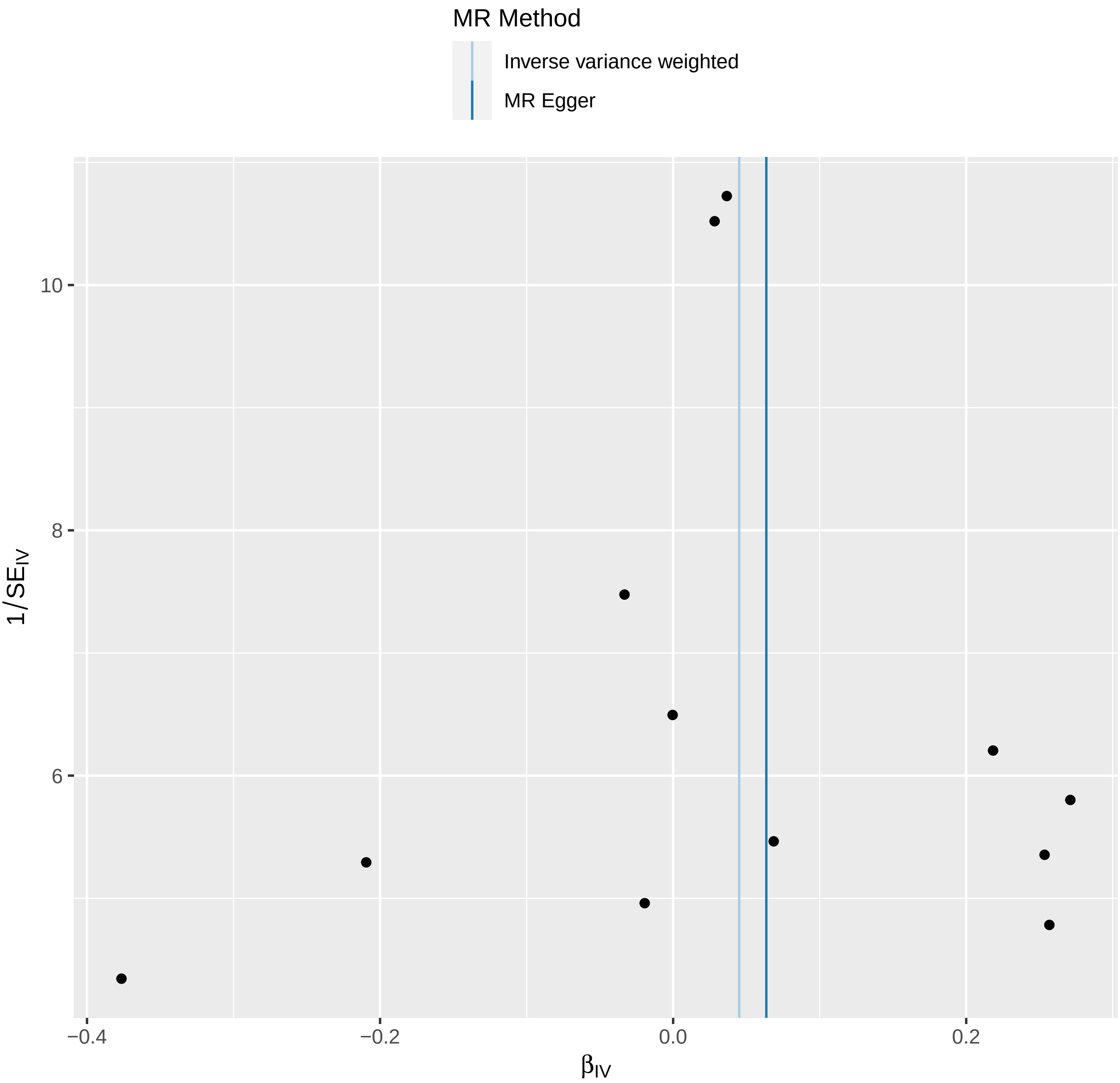

Batch 3−1915 : Selenium on Matrix metalloproteinase−16

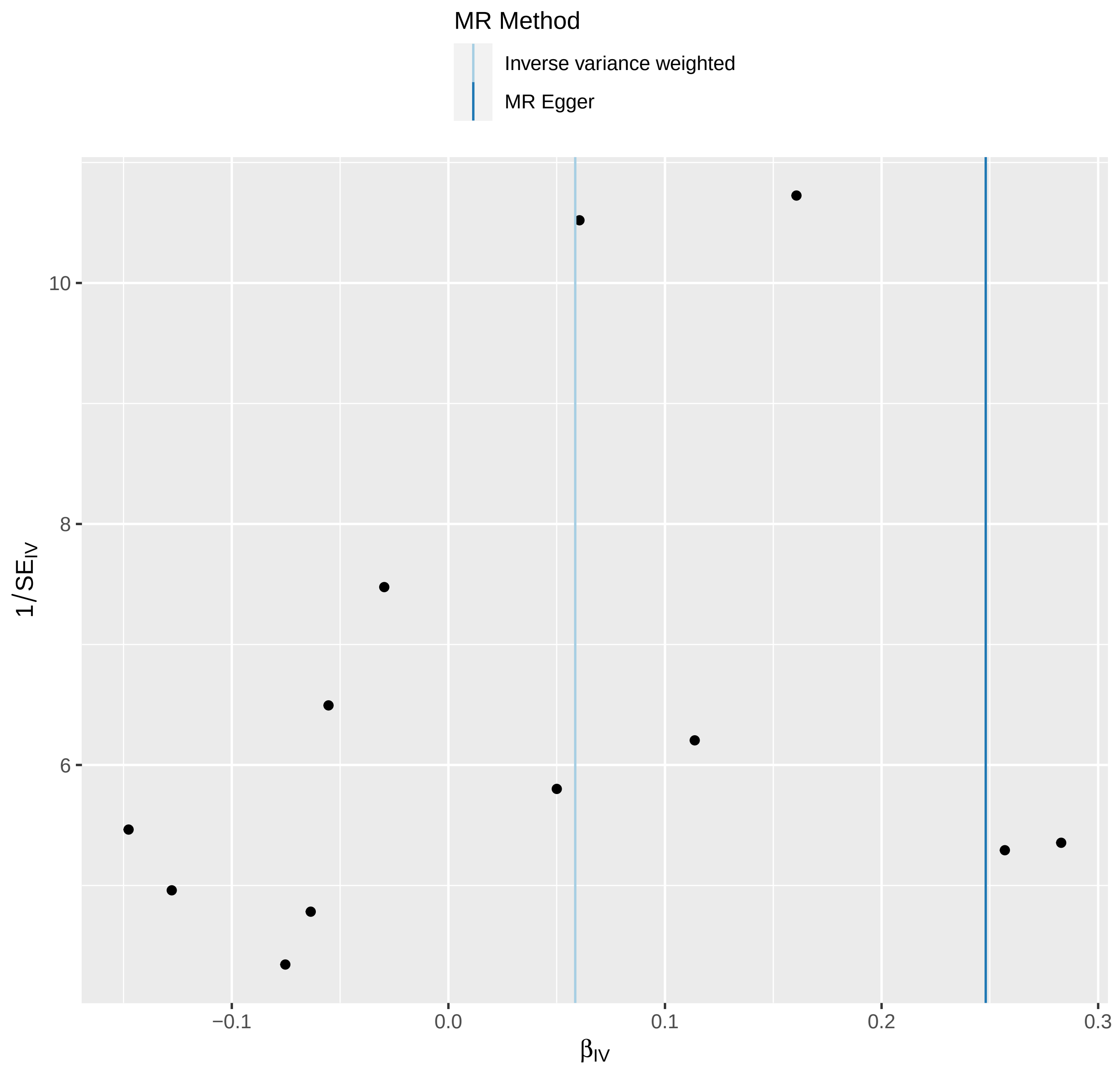

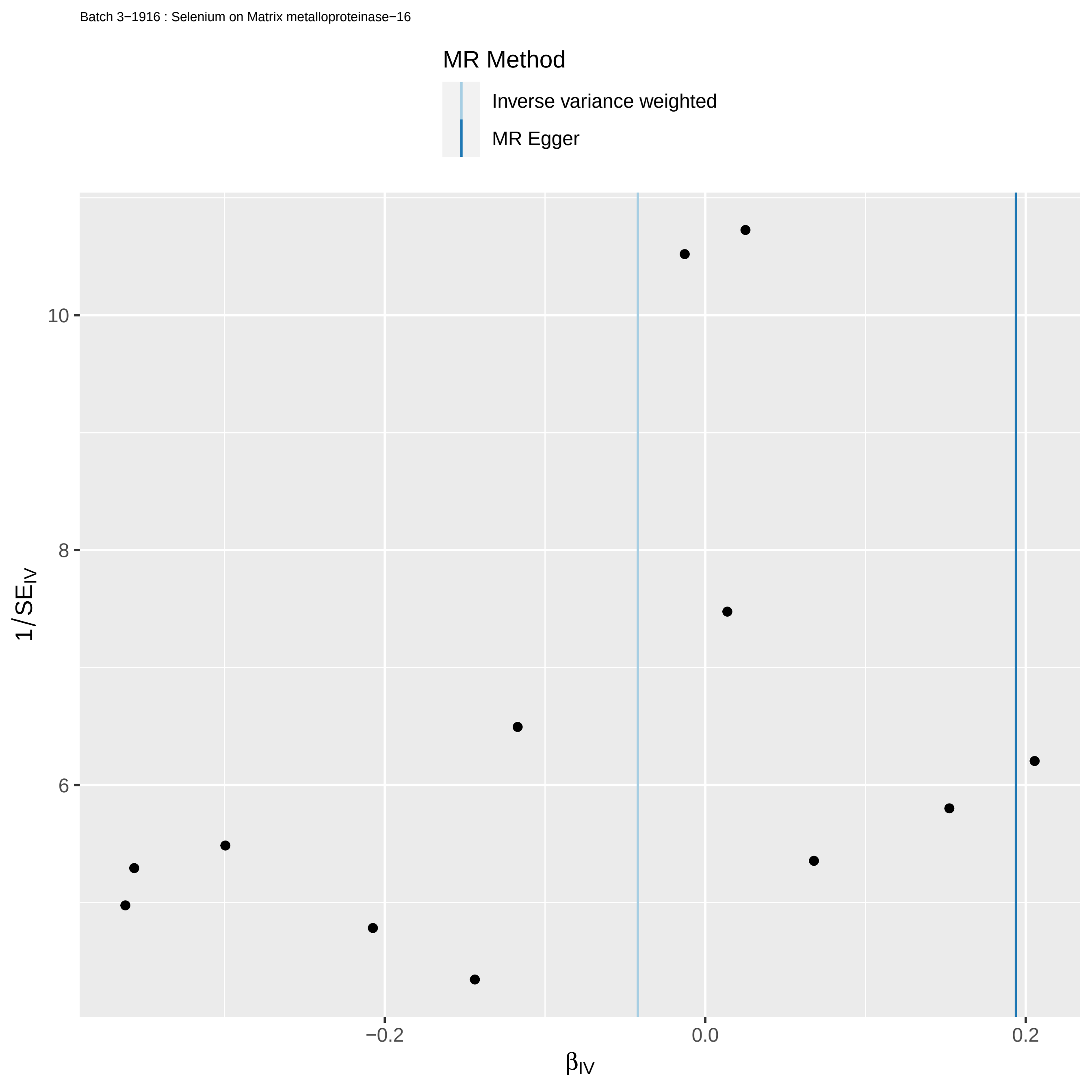
Batch 3−1916 : Selenium on Matrix metalloproteinase−16
MR Method
Inverse variance weighted
MR Egger
10
8
6
1/SE_IV
−0.2
0.0
0.2
β_IV

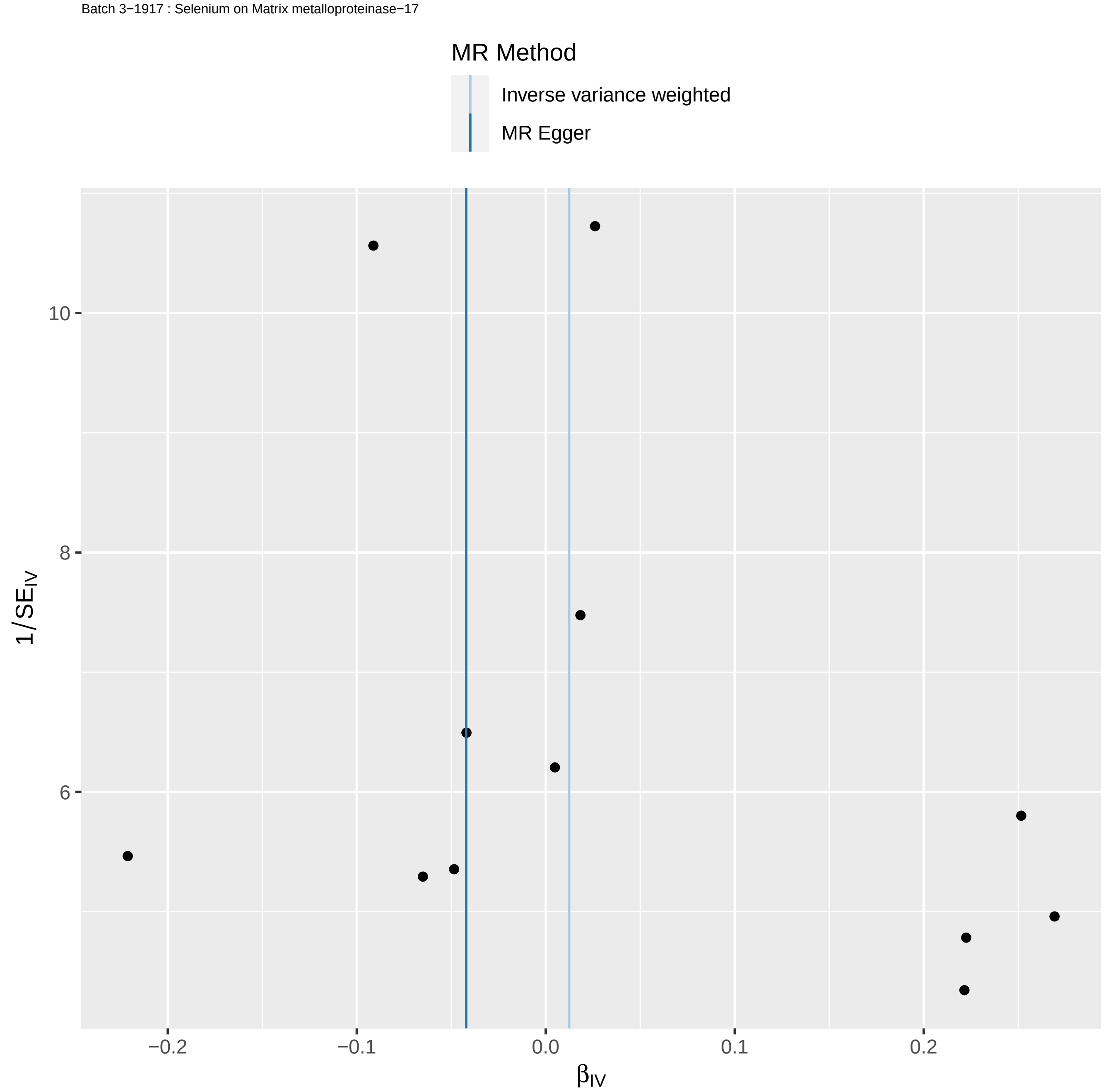
Batch 3−1917 : Selenium on Matrix metalloproteinase−17
MR Method
Inverse variance weighted
MR Egger
10
8
6
1/SE_IV
−0.2
−0.1
0.0
0.1
0.2
β_IV

Batch 3−1918 : Selenium on 72 kDa type IV collagenase

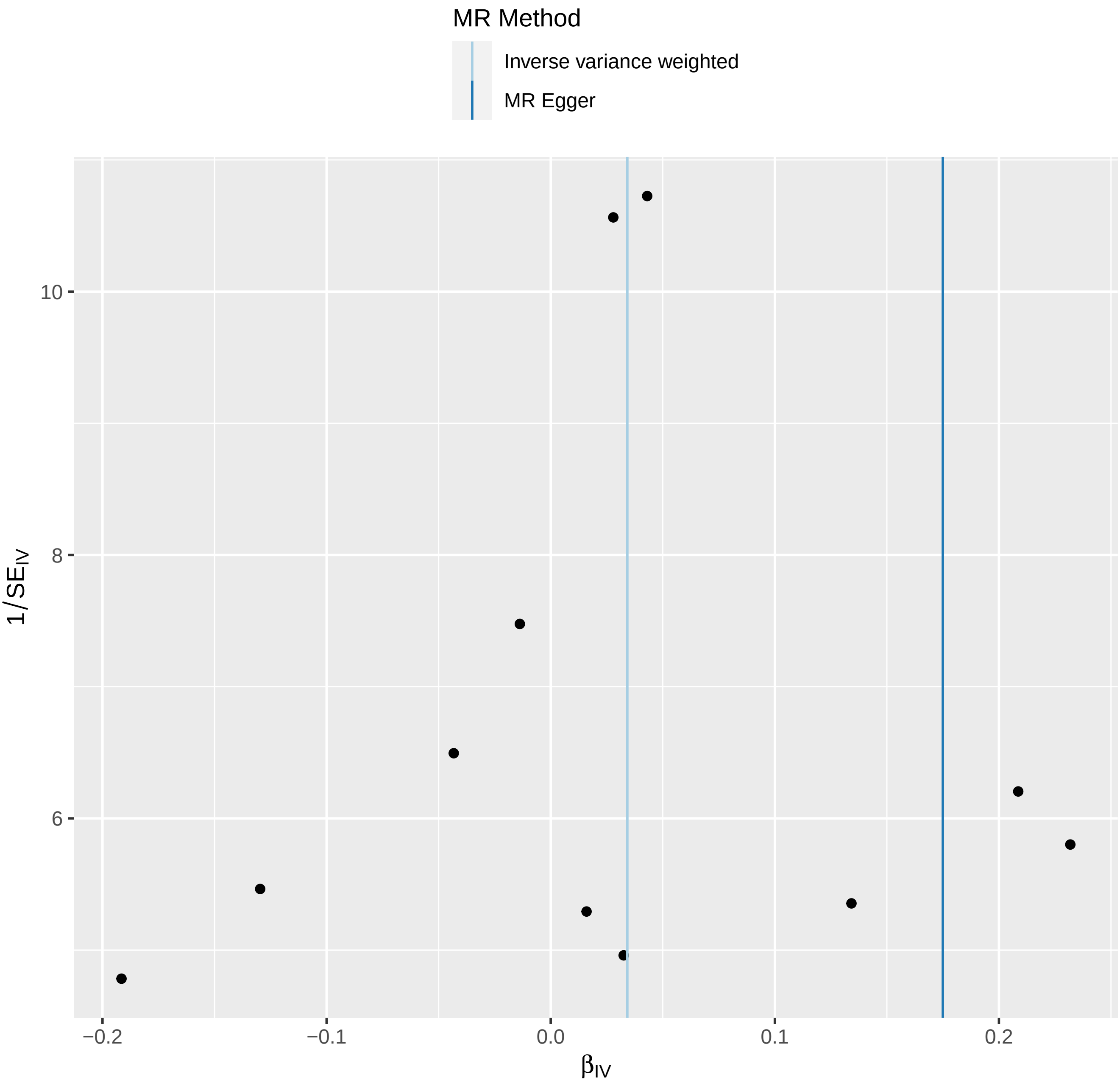

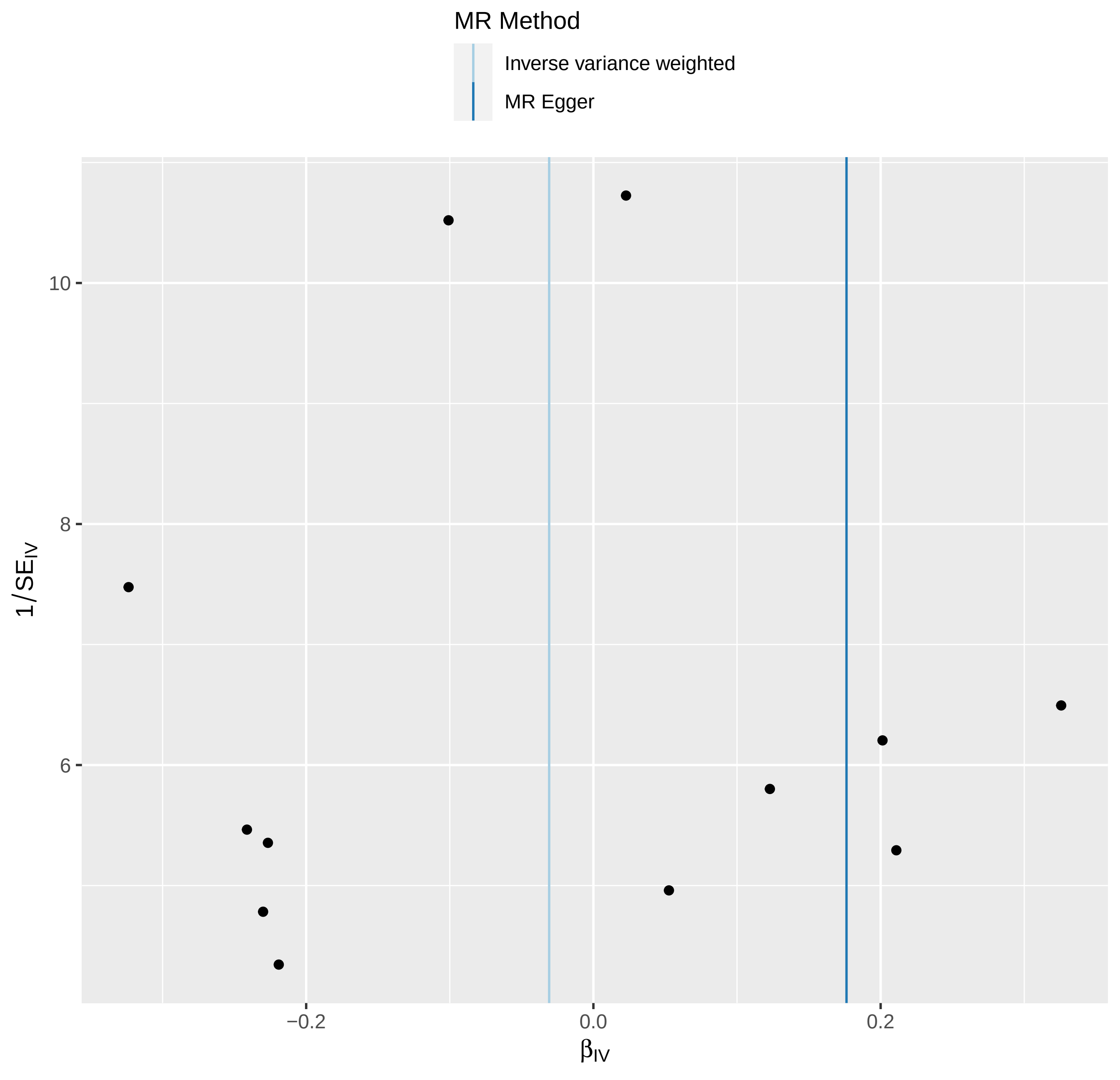

MR Method
Inverse variance weighted
MR Egger
10
8
6
$1/SE_{IV}$
−0.2
0.0
0.2
β_{IV}

Batch 3−1920 : Selenium on Neutrophil collagenase

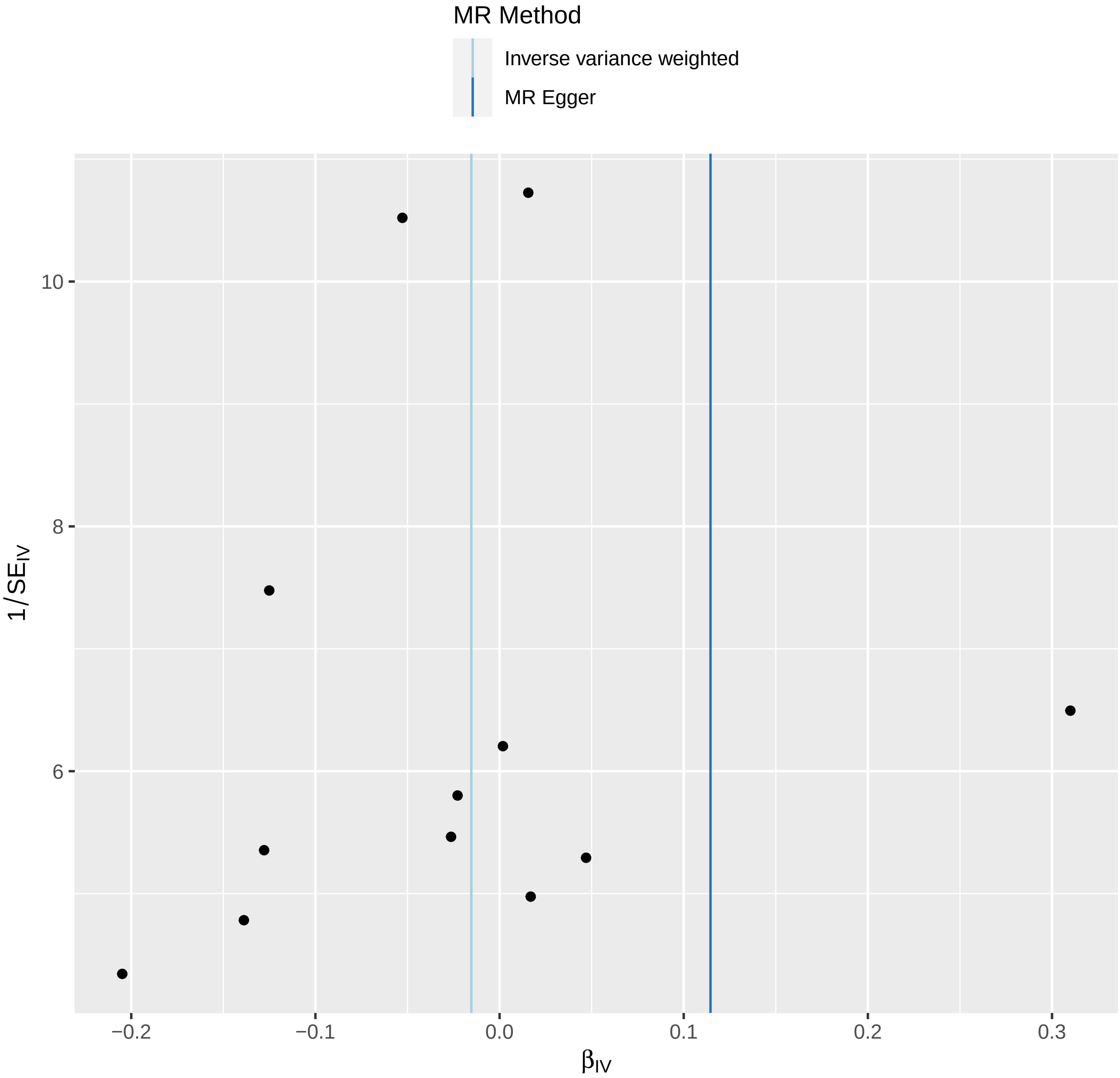

Batch 3−1921 : Selenium on Matrix metalloproteinase−9

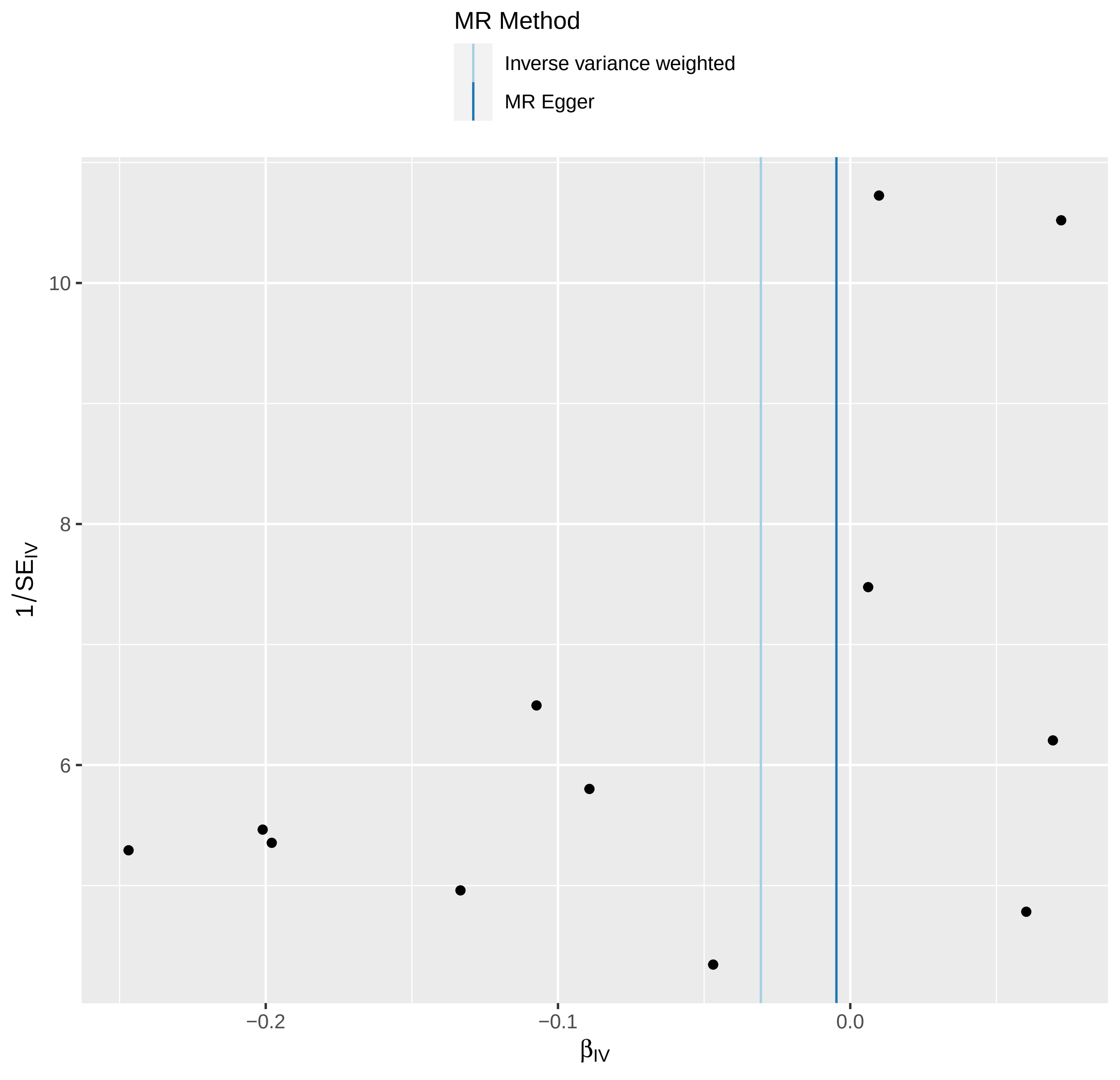

Batch 3−1922 : Selenium on Multimerin−2

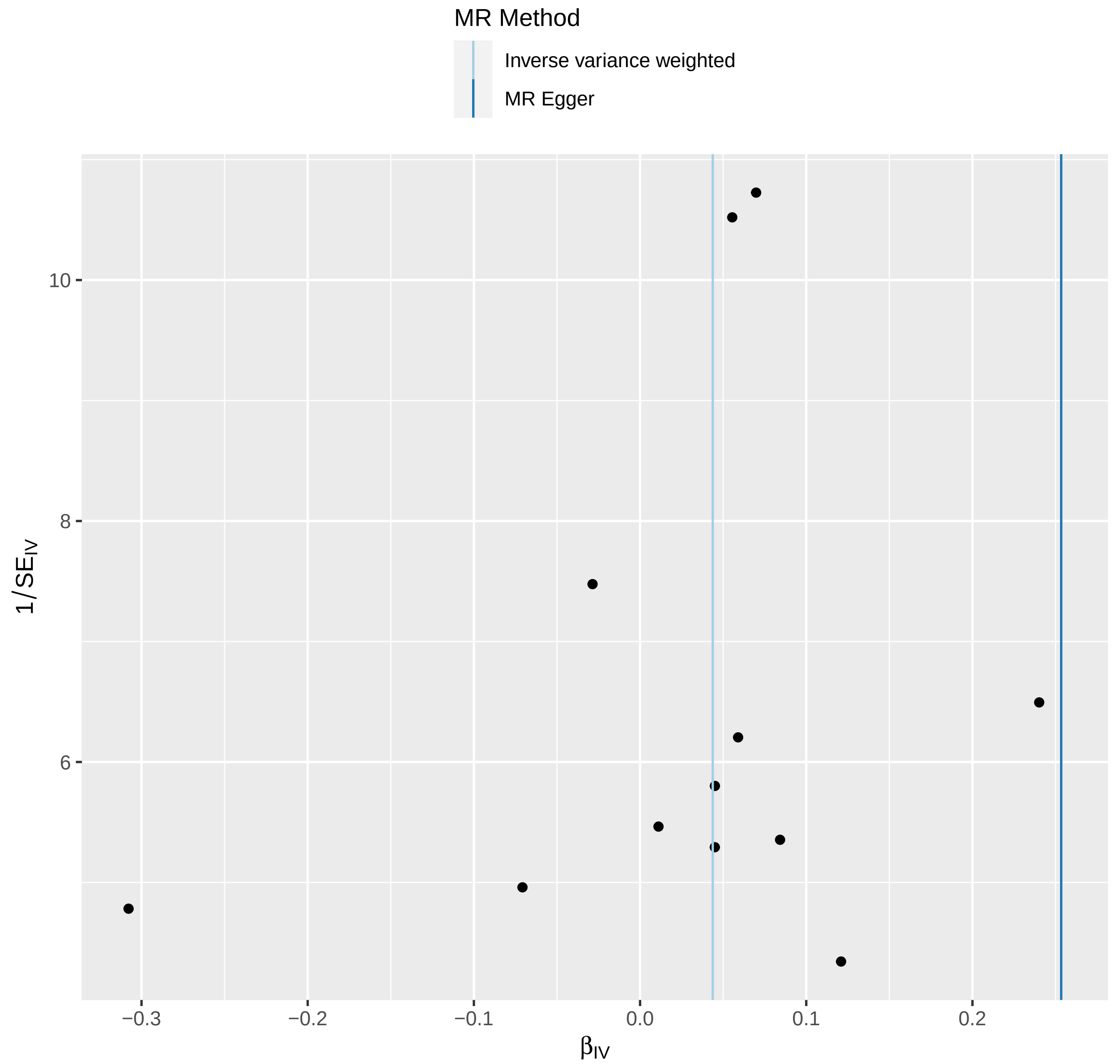

Batch 3−1923 : Selenium on Multimerin−2

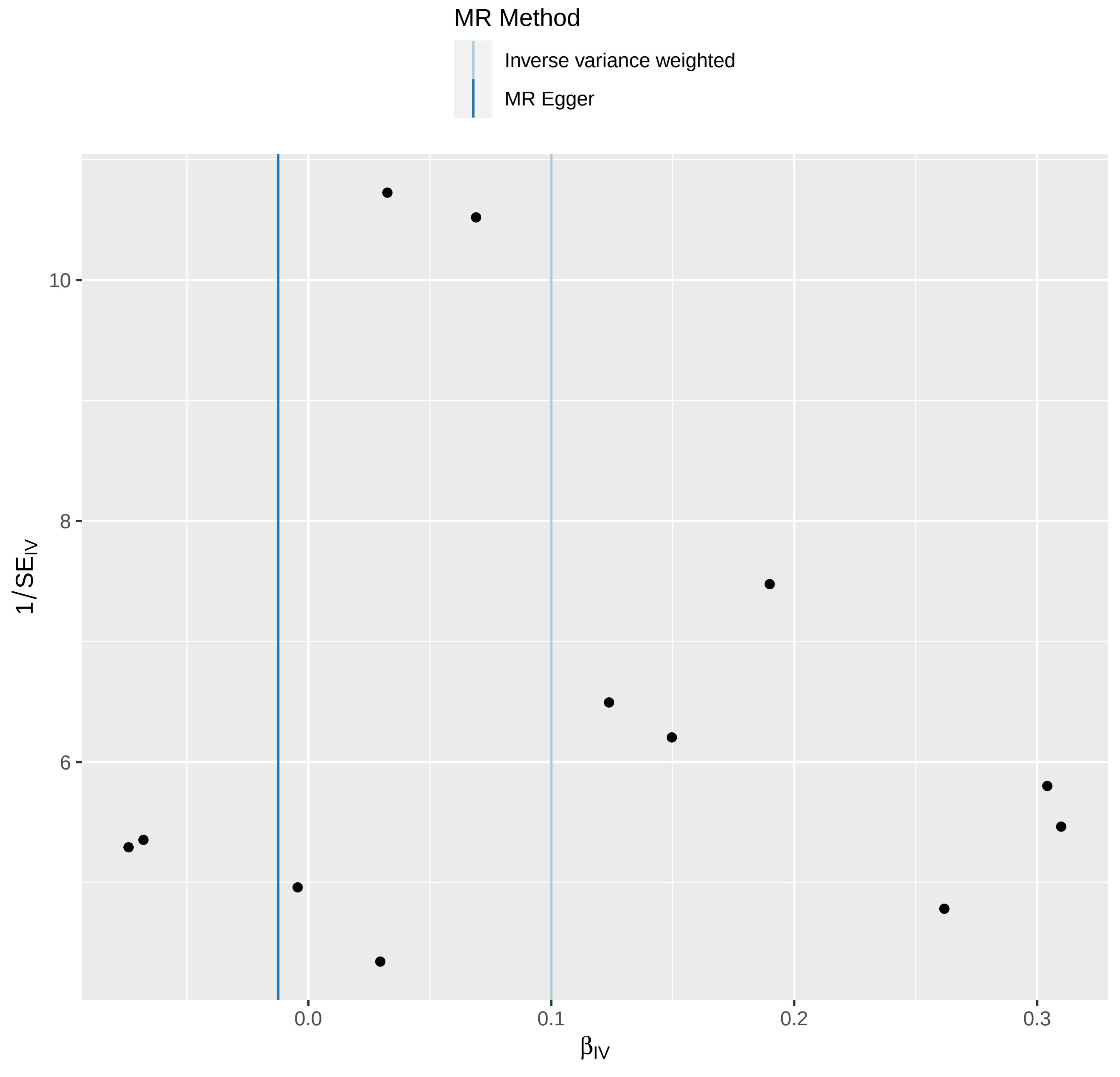

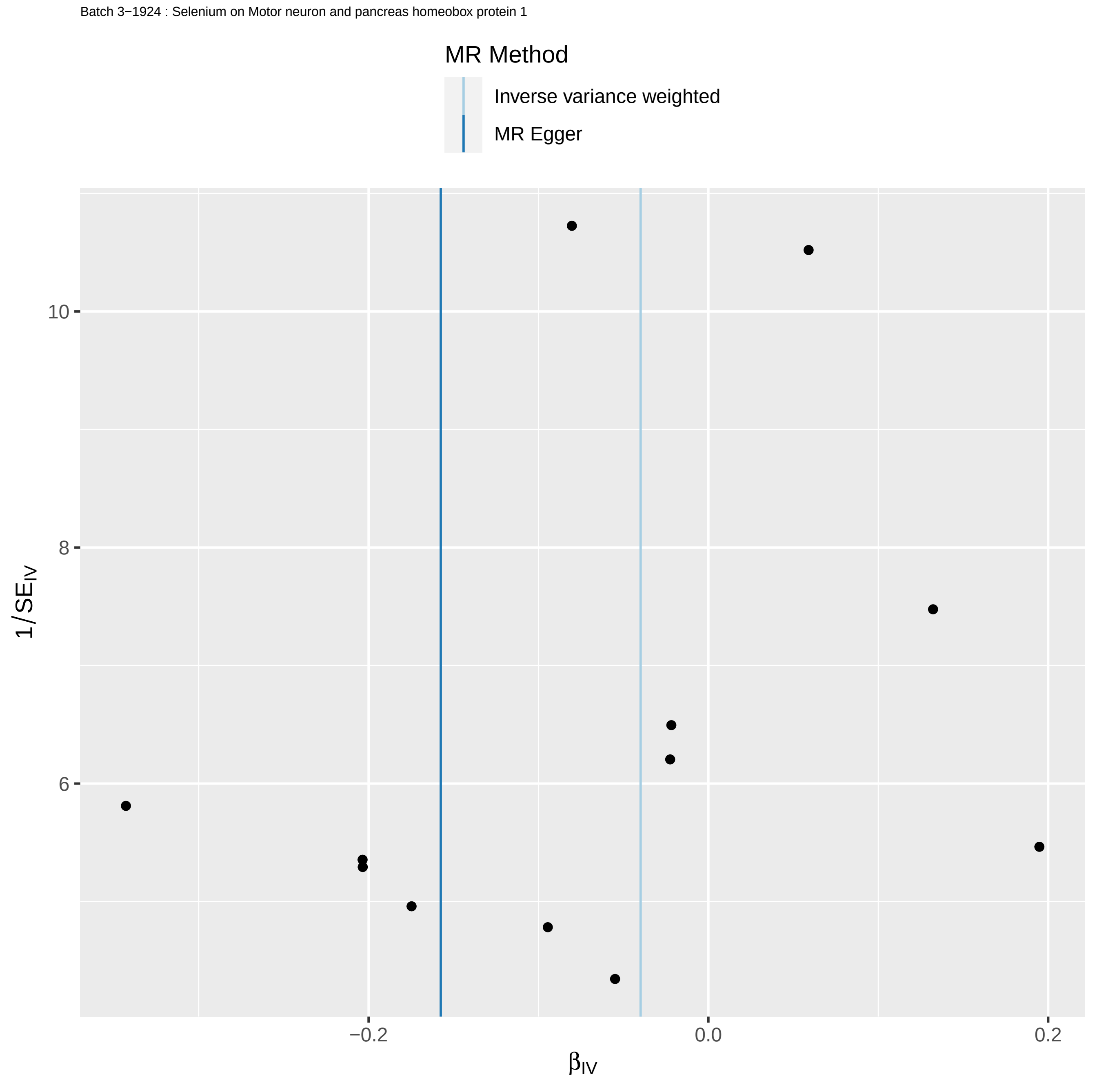
Batch 3−1924 : Selenium on Motor neuron and pancreas homeobox protein 1
MR Method
Inverse variance weighted
MR Egger
10
8
6
$1/SE_{IV}$
−0.2
0.0
0.2
β_{IV}

Batch 3−1925 : Selenium on MOB kinase activator 1A

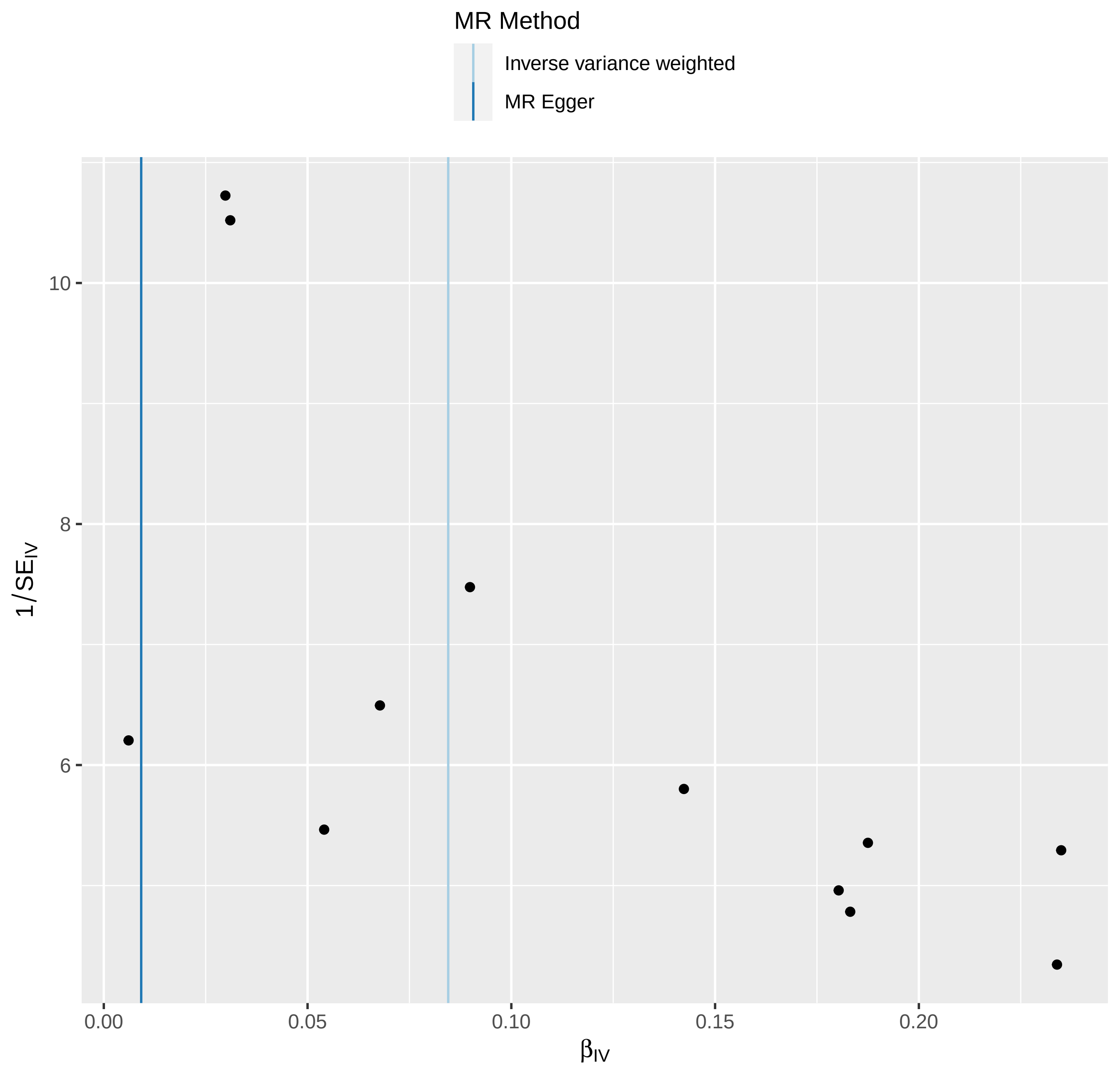

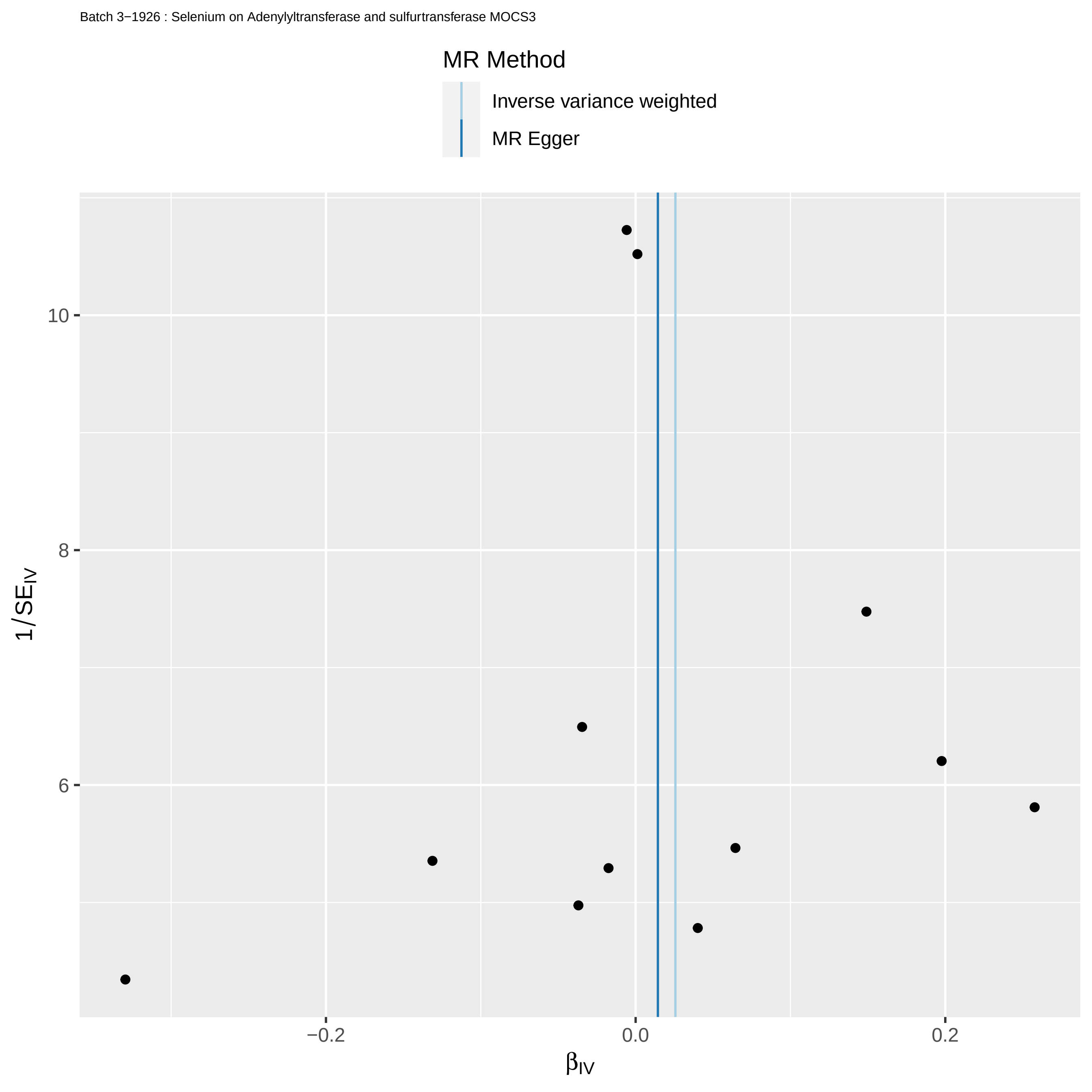

Batch 3−1926 : Selenium on Adenylyltransferase and sulfurtransferase MOCS3
MR Method
Inverse variance weighted
MR Egger
10
8
6
1/SE$_{IV}$
−0.2
0.0
0.2
β$_{IV}$

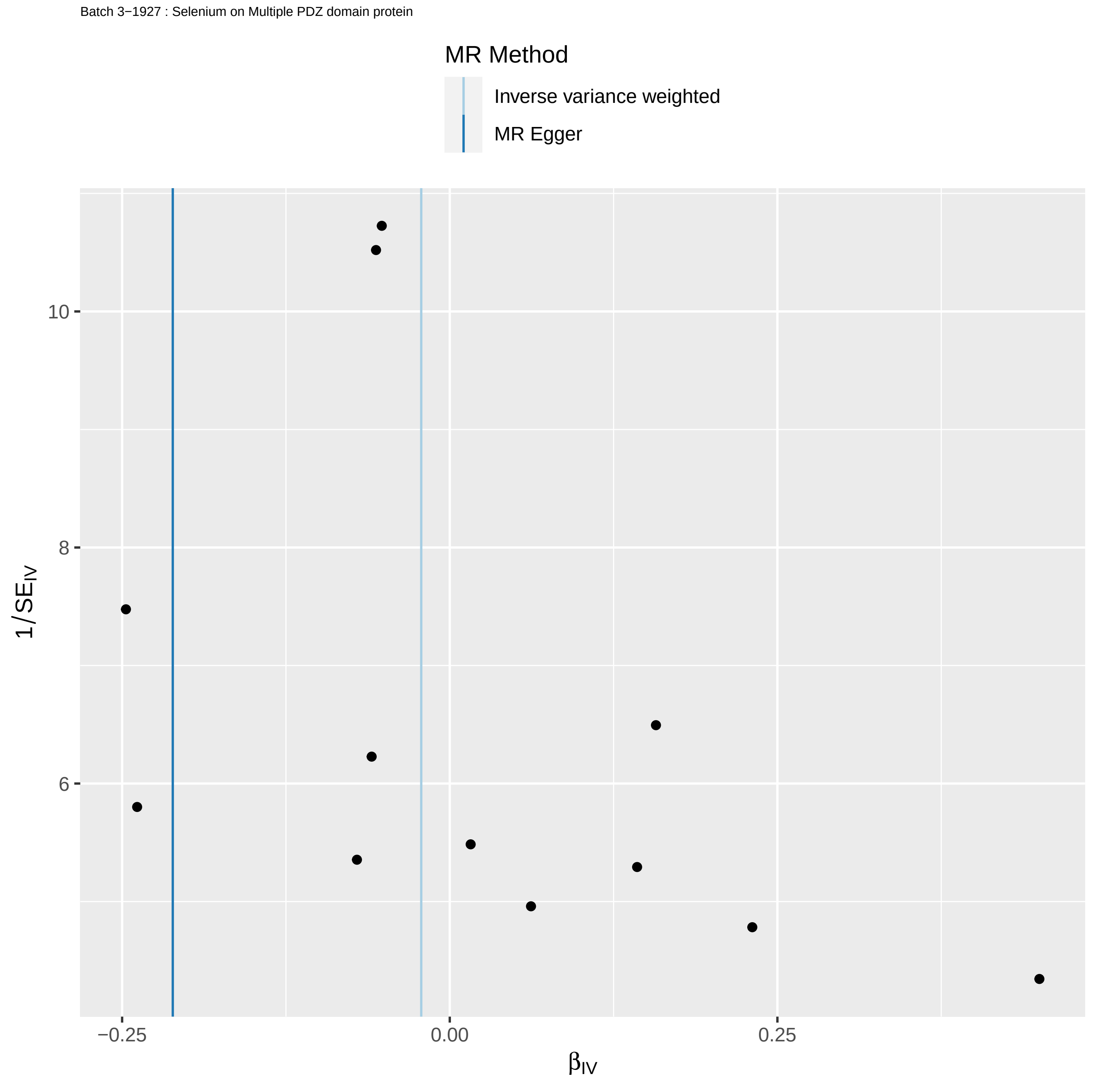

Batch 3−1927 : Selenium on Multiple PDZ domain protein
MR Method
Inverse variance weighted
MR Egger
10
8
6
1/SE$_{IV}$
−0.25
0.00
0.25
β$_{IV}$

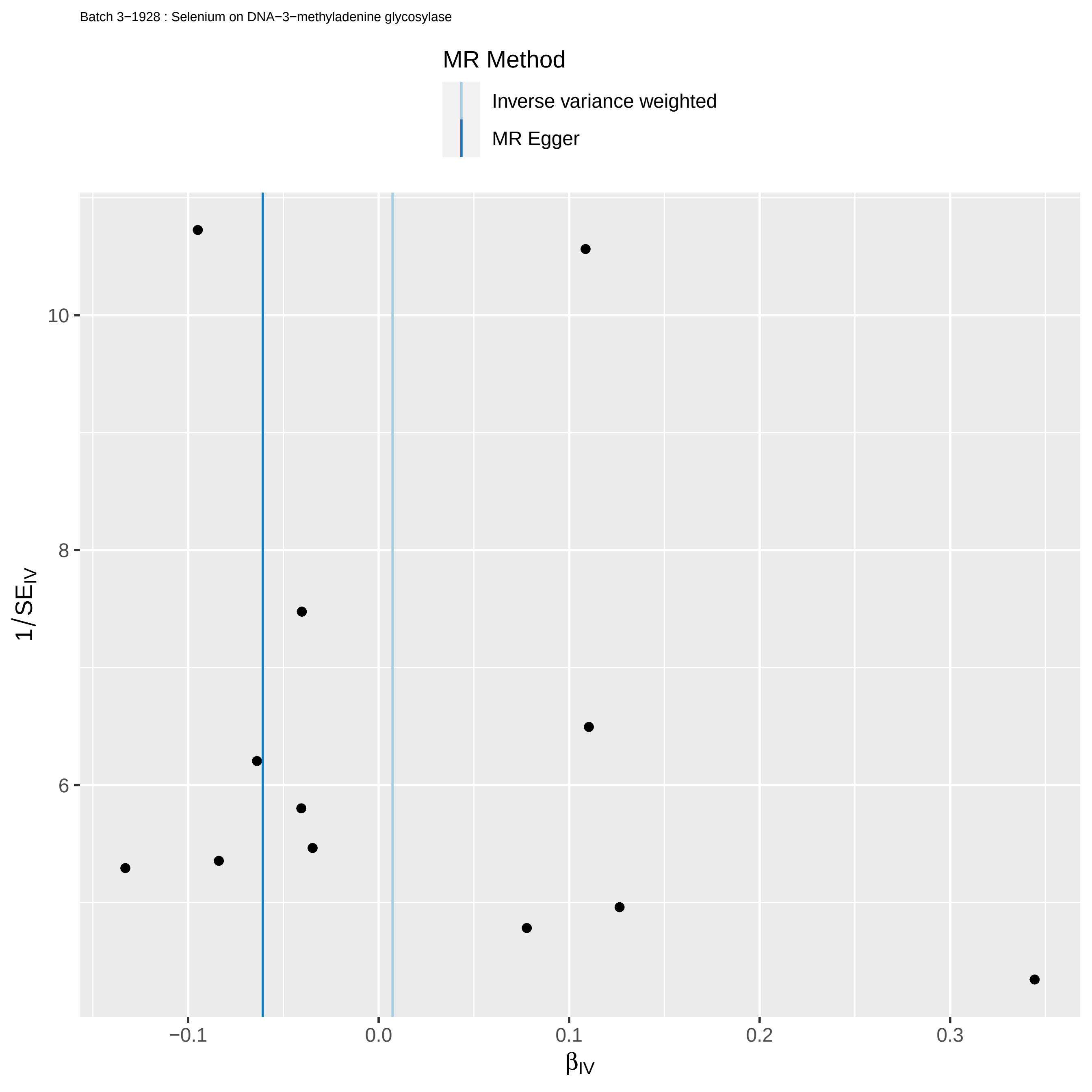
Batch 3−1928 : Selenium on DNA−3−methyladenine glycosylase
MR Method
Inverse variance weighted
MR Egger
10
8
6
1/SE$_{IV}$
−0.1
0.0
0.1
0.2
0.3
β$_{IV}$

Batch 3−1929 : Selenium on Thrombopoietin Receptor

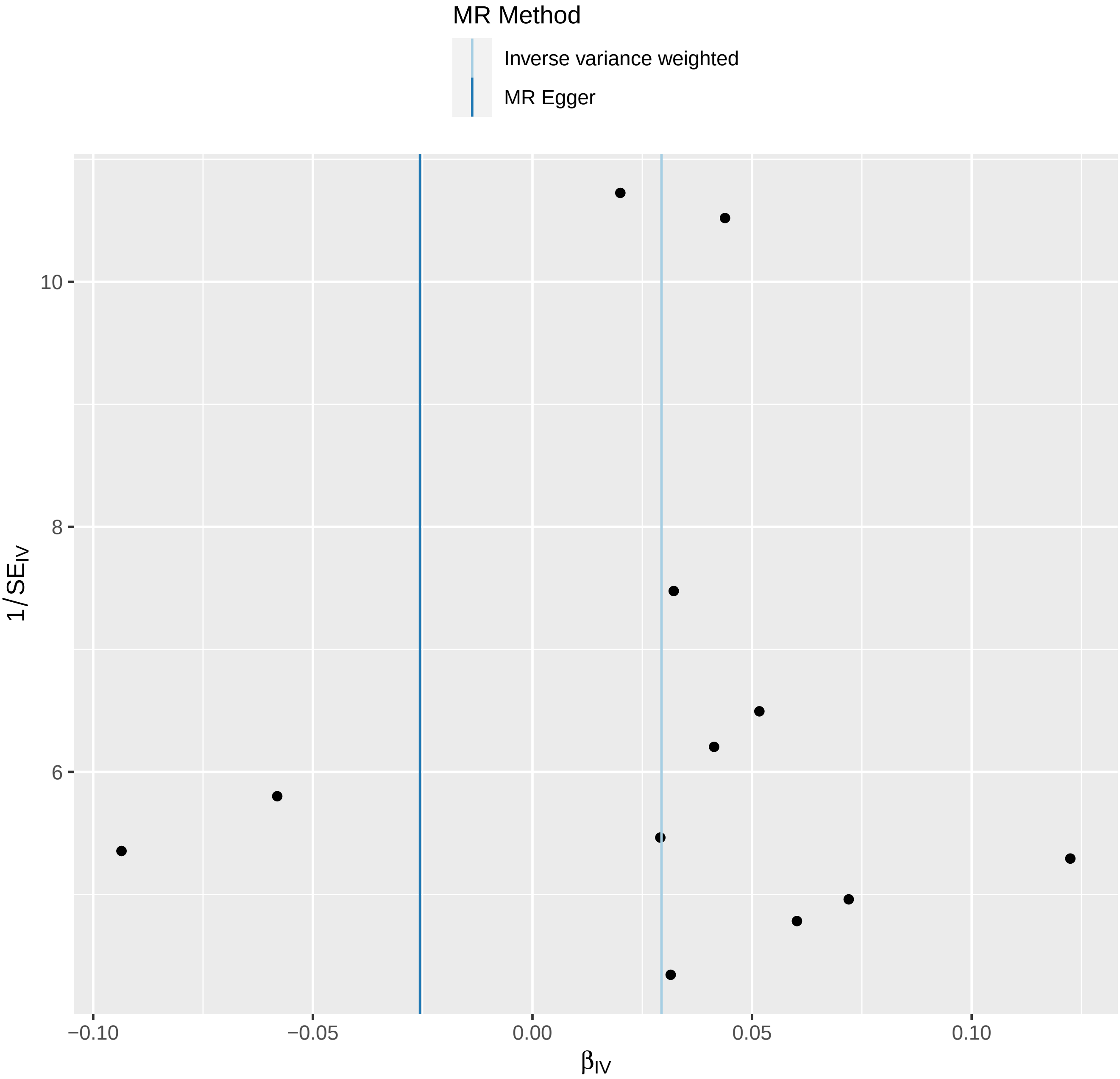

Batch 3−1930 : Selenium on Myeloperoxidase

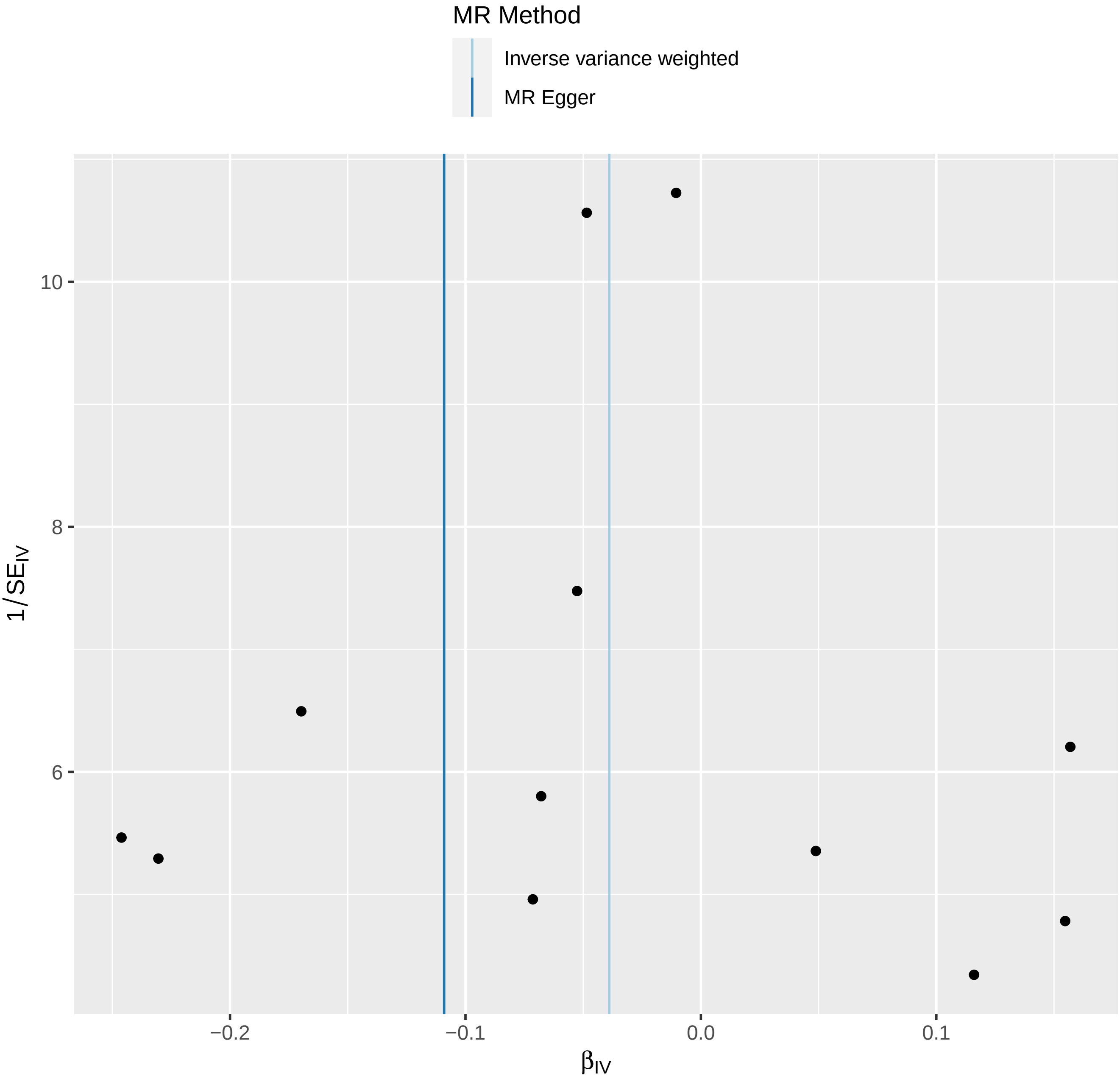

Batch 3−1931 : Selenium on MAGUK p55 subfamily member 6

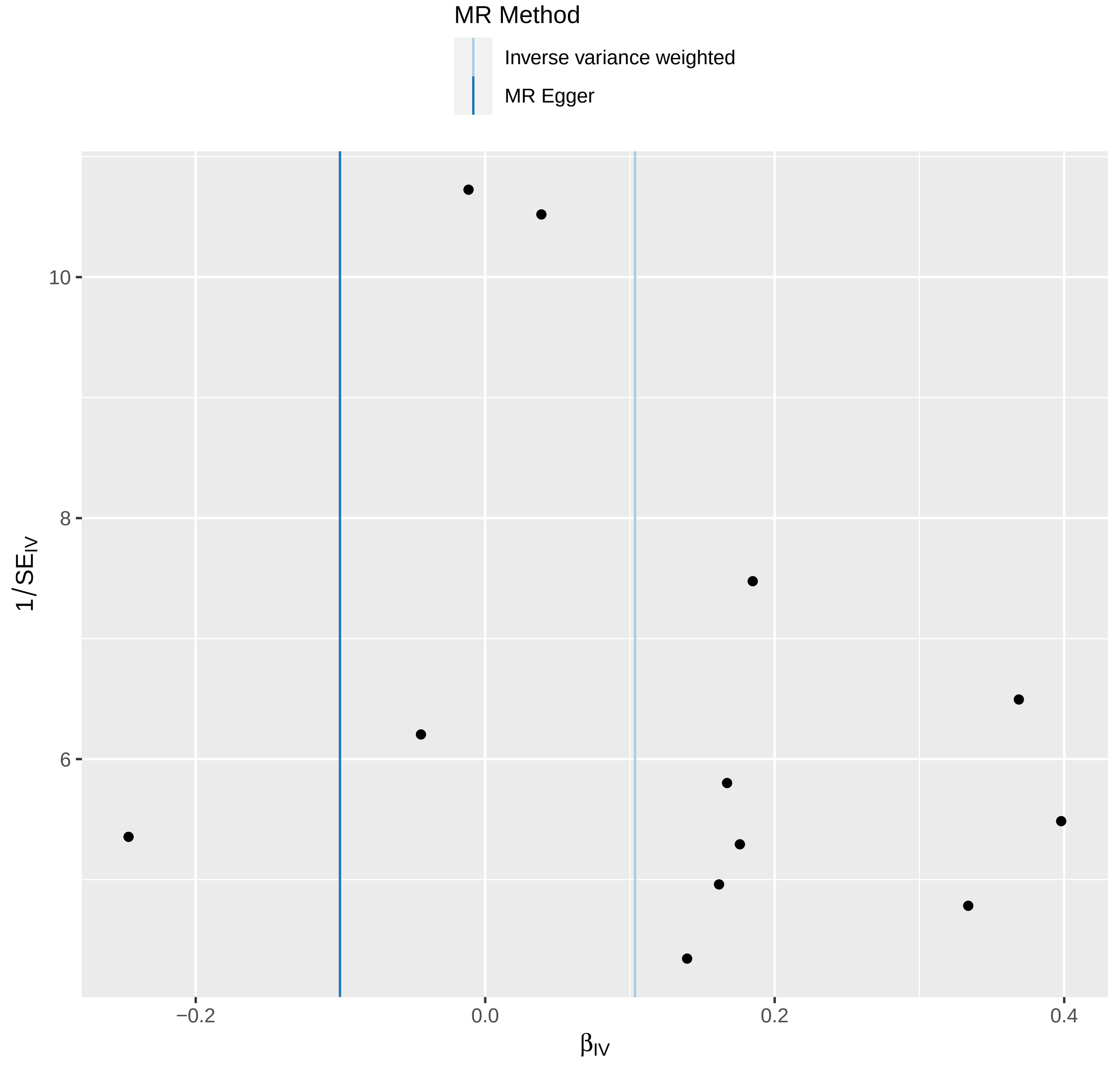

Batch 3−1932 : Selenium on MAGUK p55 subfamily member 7

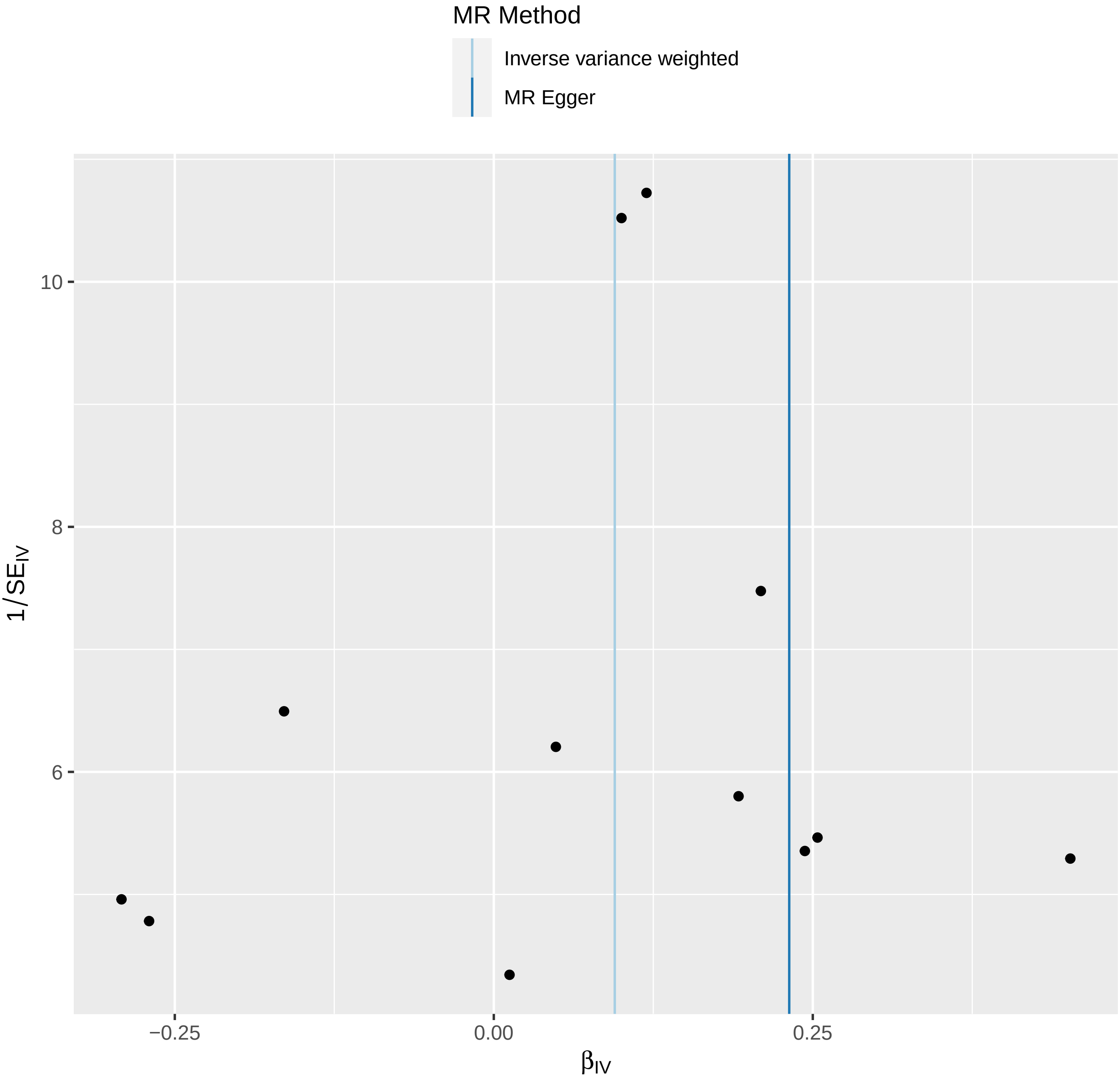

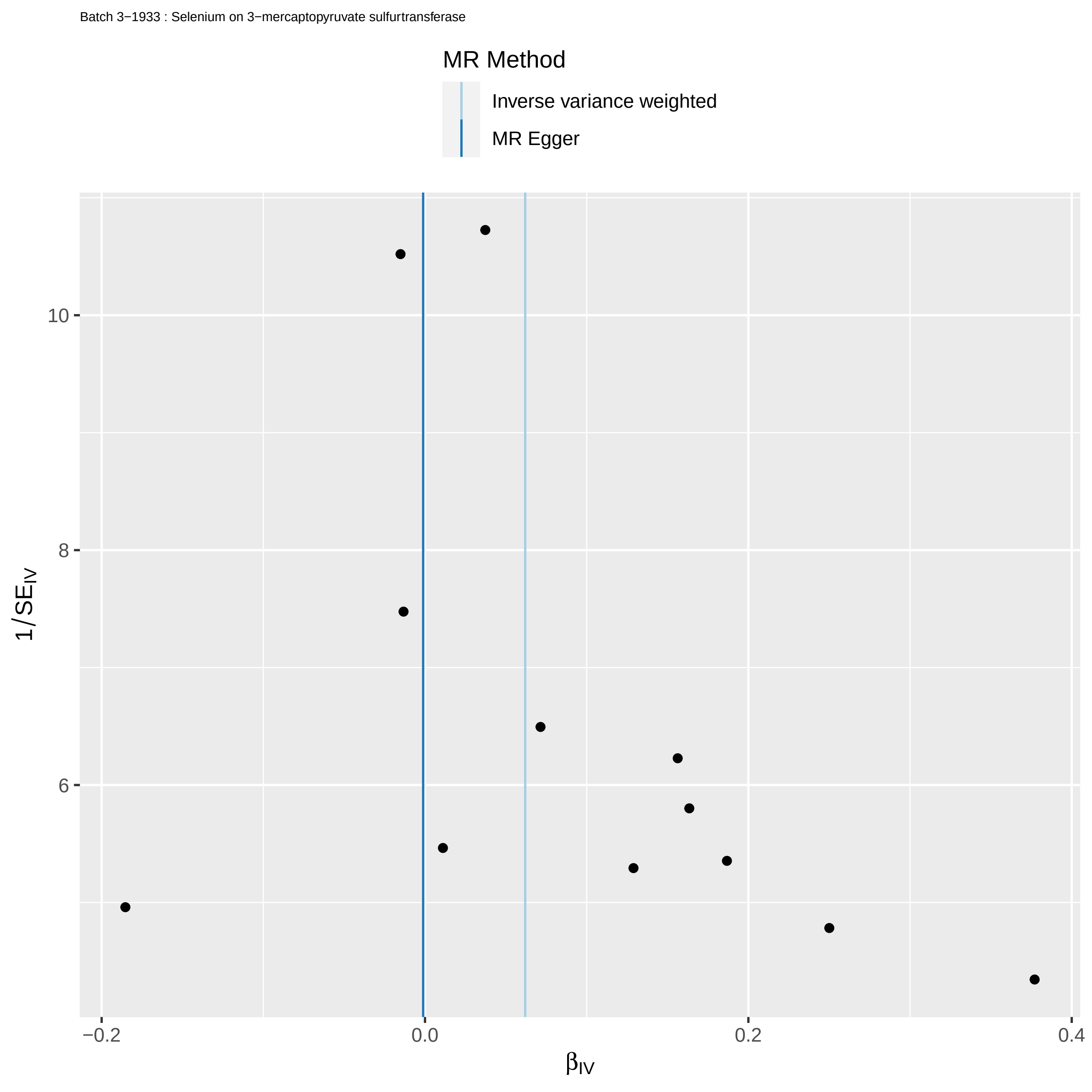

Batch 3−1933 : Selenium on 3−mercaptopyruvate sulfurtransferase
MR Method
Inverse variance weighted
MR Egger
10
8
6
1/SE$_{IV}$
−0.2
0.0
0.2
0.4
β$_{IV}$

Batch 3−1934 : Selenium on Myelin protein P0

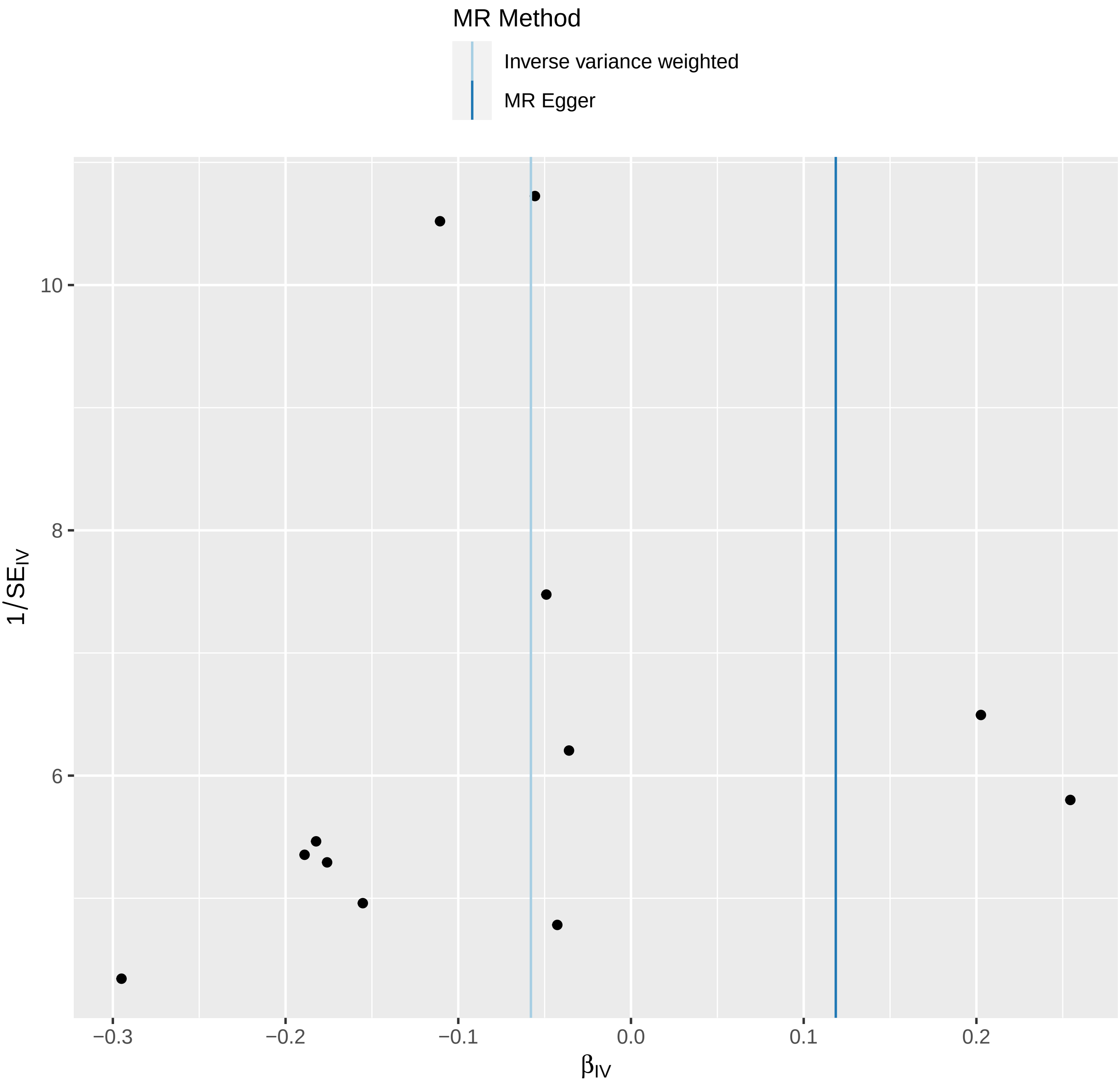

Batch 3−1935 : Selenium on Melanocortin−2 receptor accessory protein 2

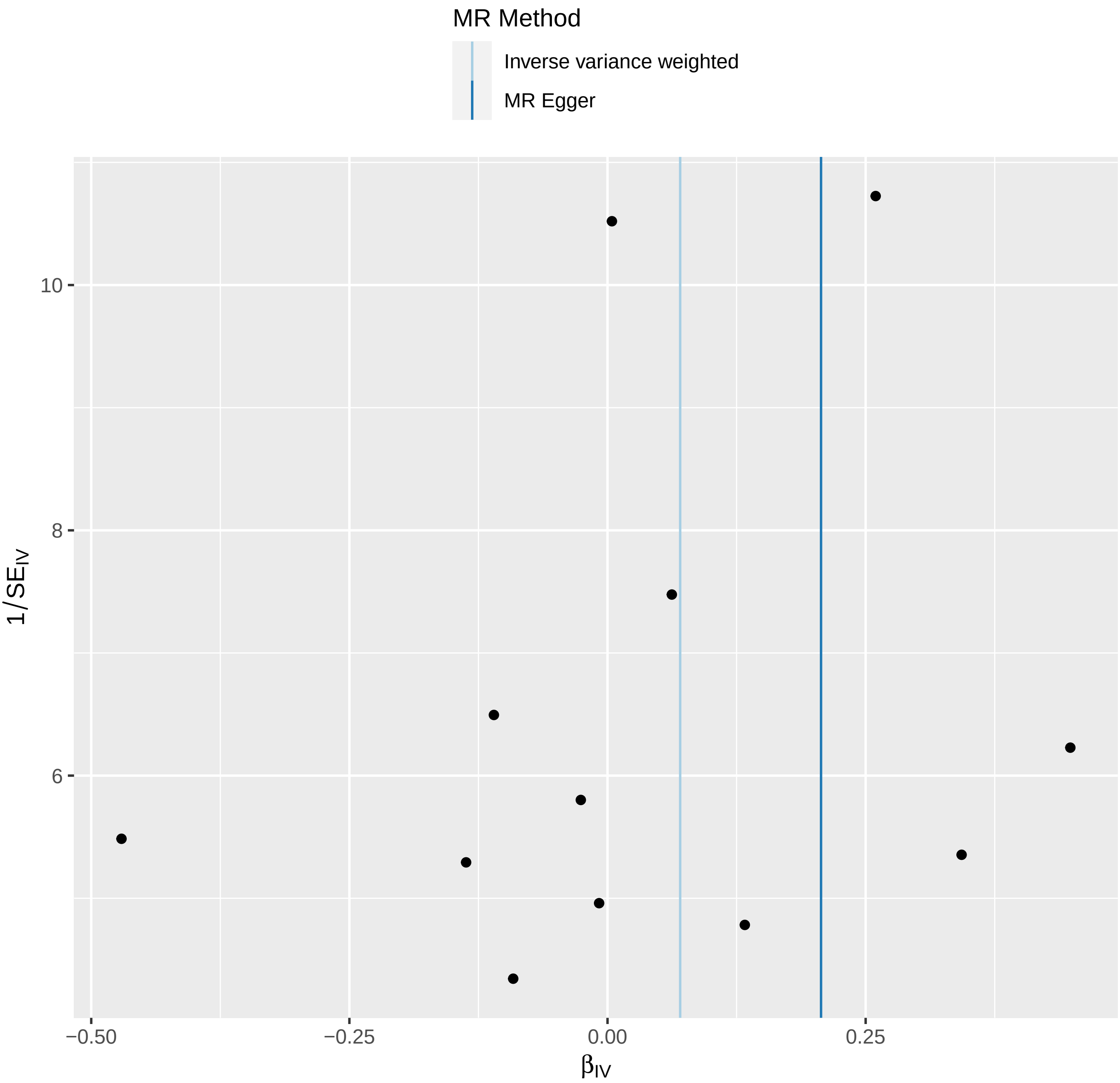

Batch 3−1936 : Selenium on Melanocortin−2 receptor accessory protein

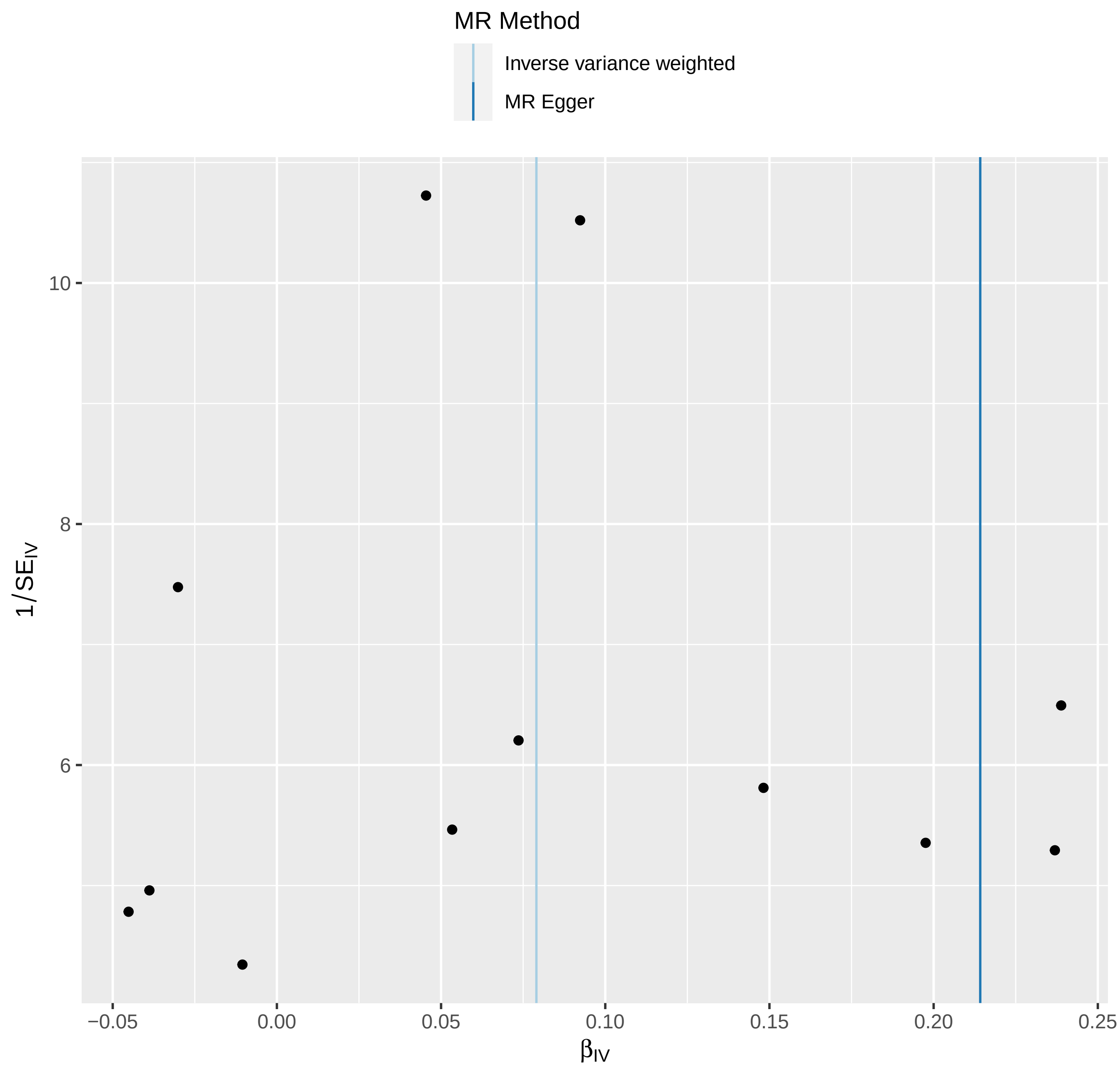

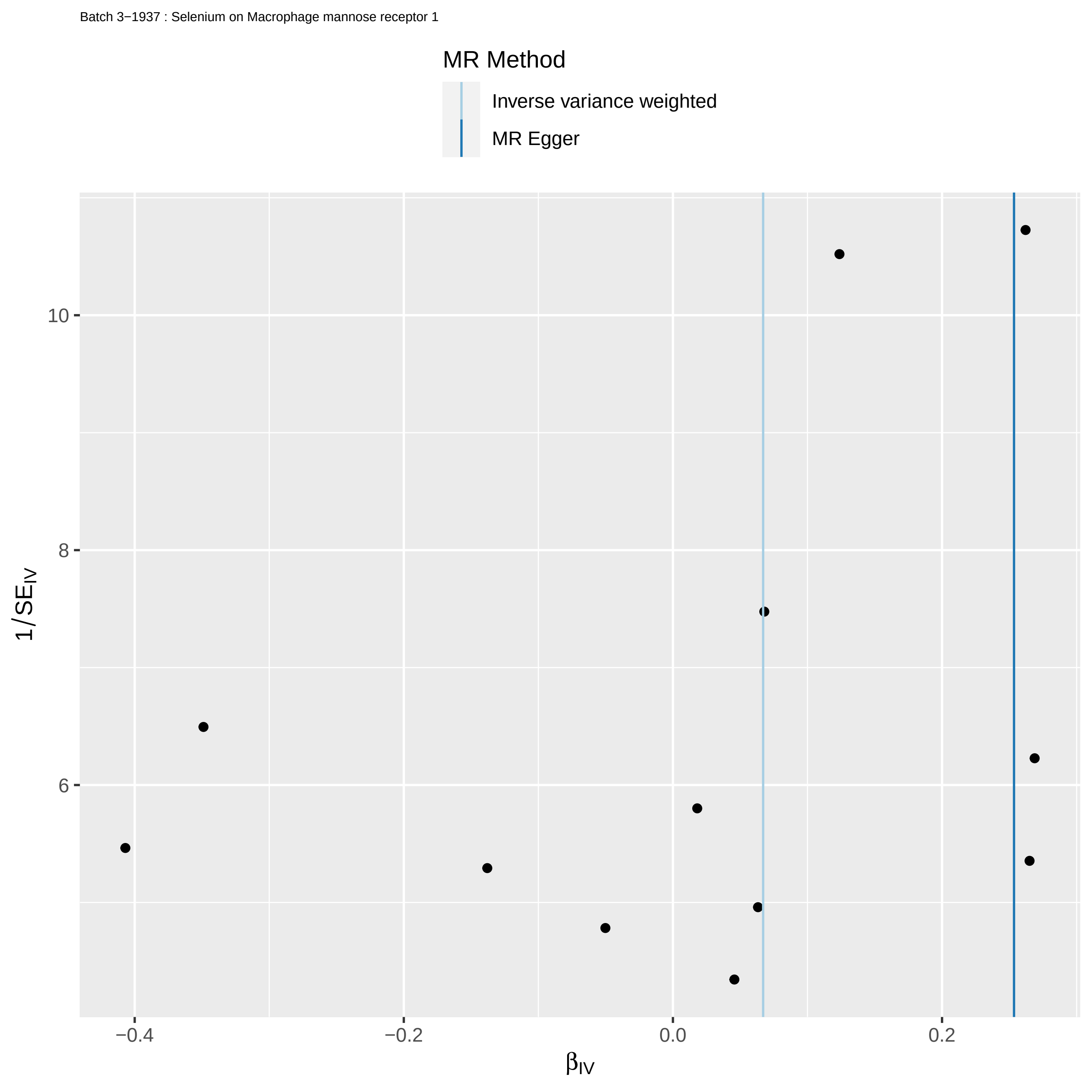
Batch 3−1937 : Selenium on Macrophage mannose receptor 1
MR Method
Inverse variance weighted
MR Egger
10
8
6
1/SE_IV
−0.4
−0.2
0.0
0.2
β_IV

Batch 3−1938 : Selenium on C−type mannose receptor 2

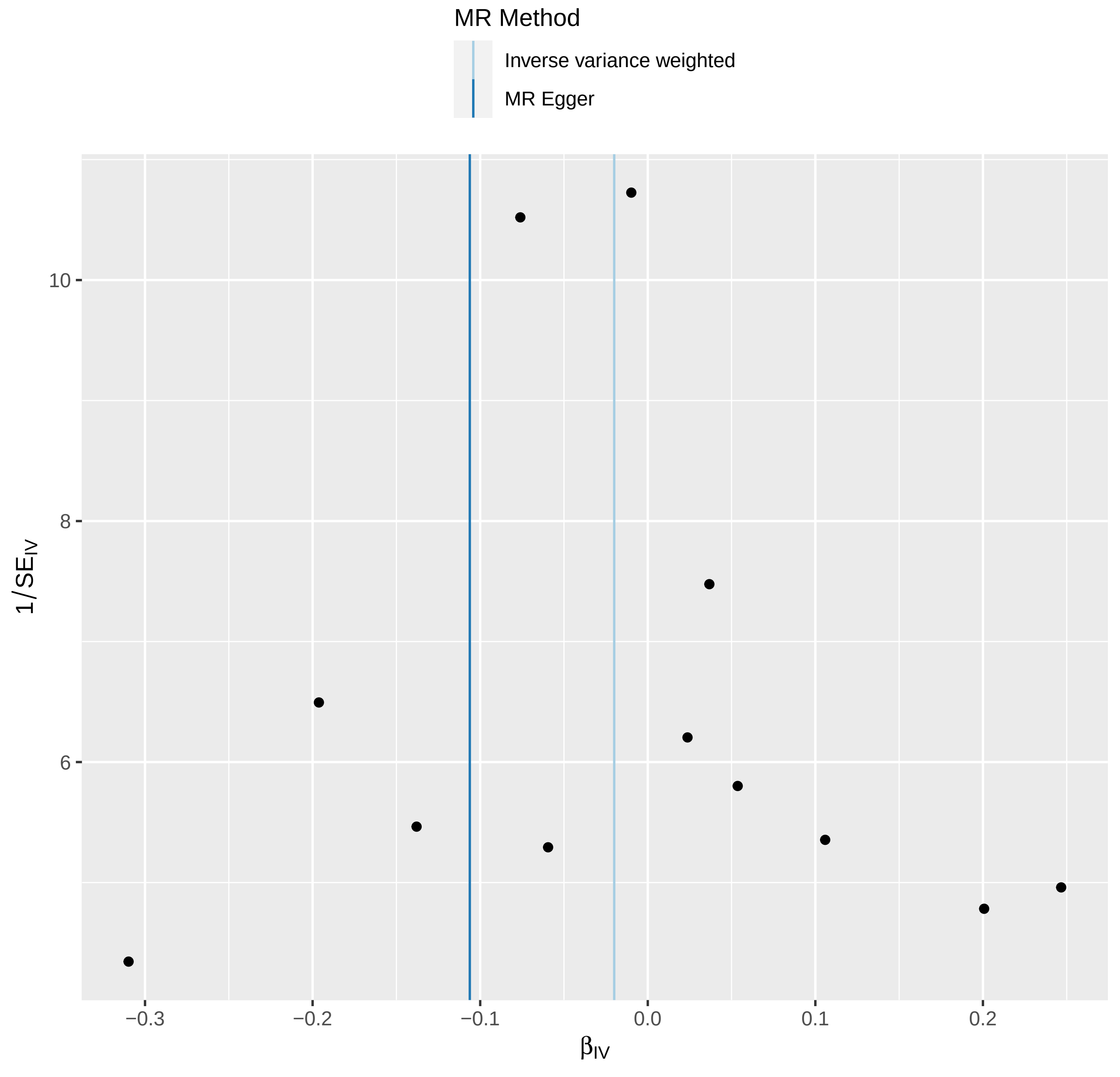

Batch 3−1939 : Selenium on Double−strand break repair protein MRE11A

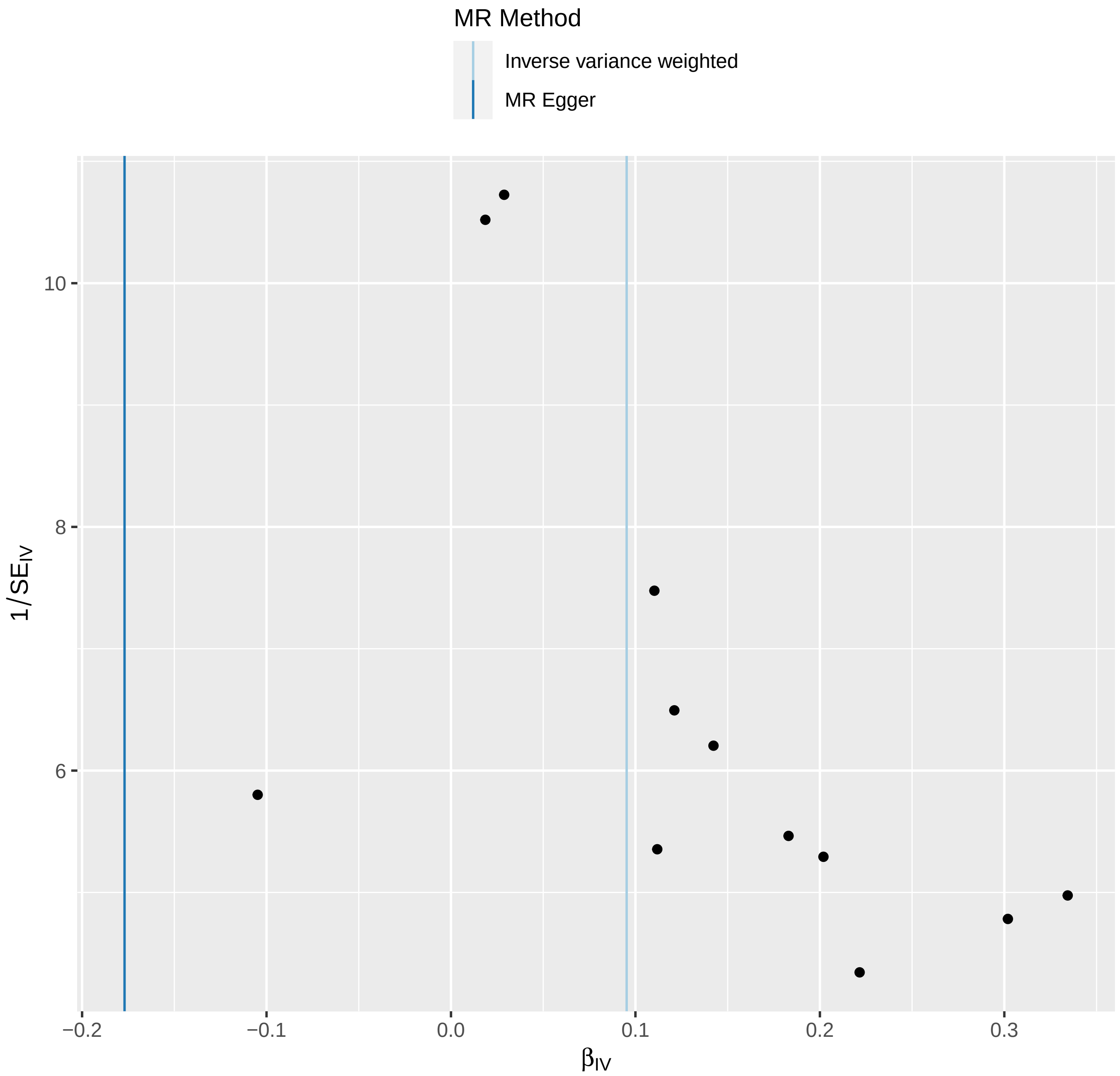

Batch 3−1940 : Selenium on 39S ribosomal protein L14, mitochondrial

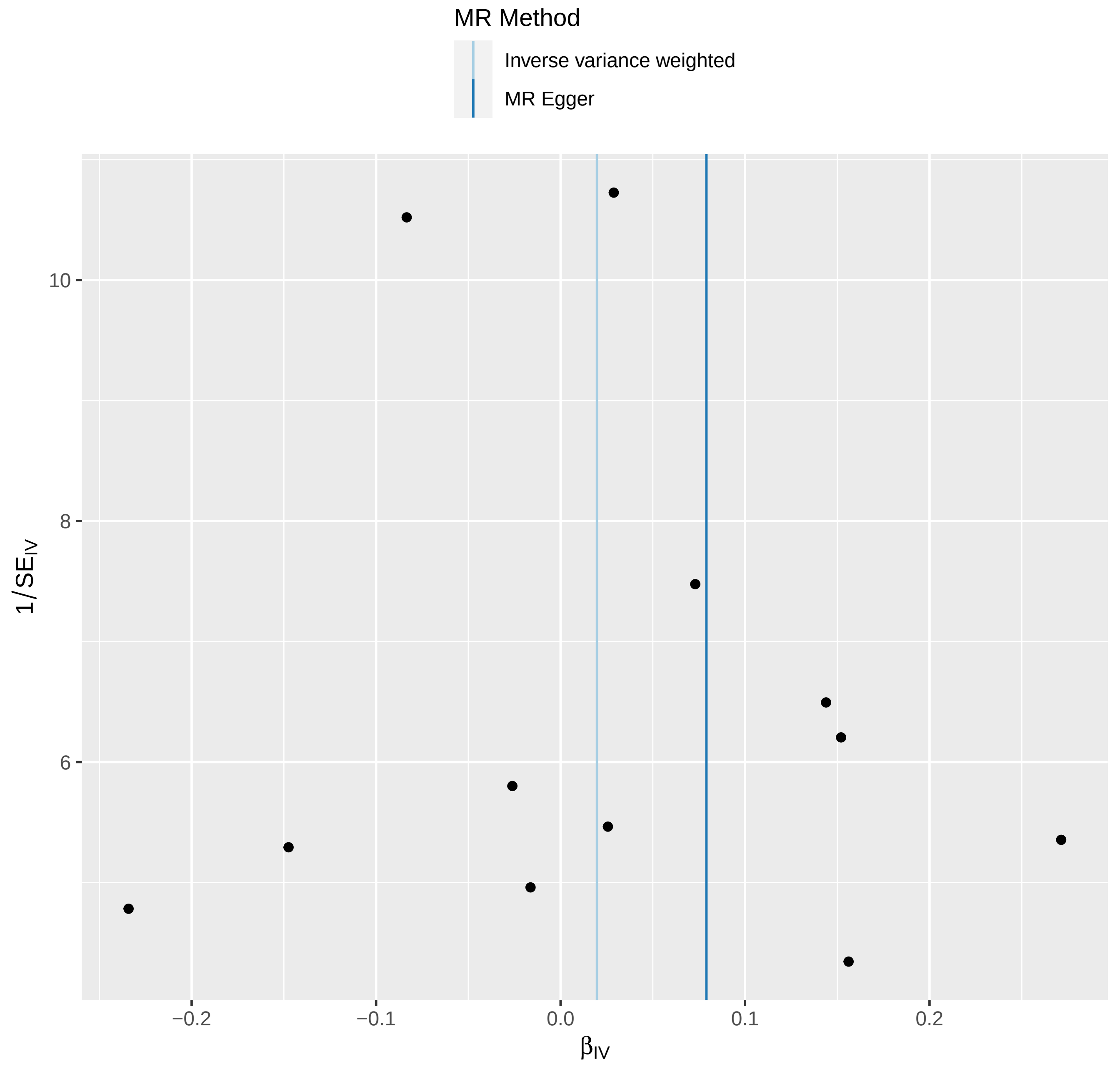

Batch 3−1941 : Selenium on 39S ribosomal protein L32, mitochondrial

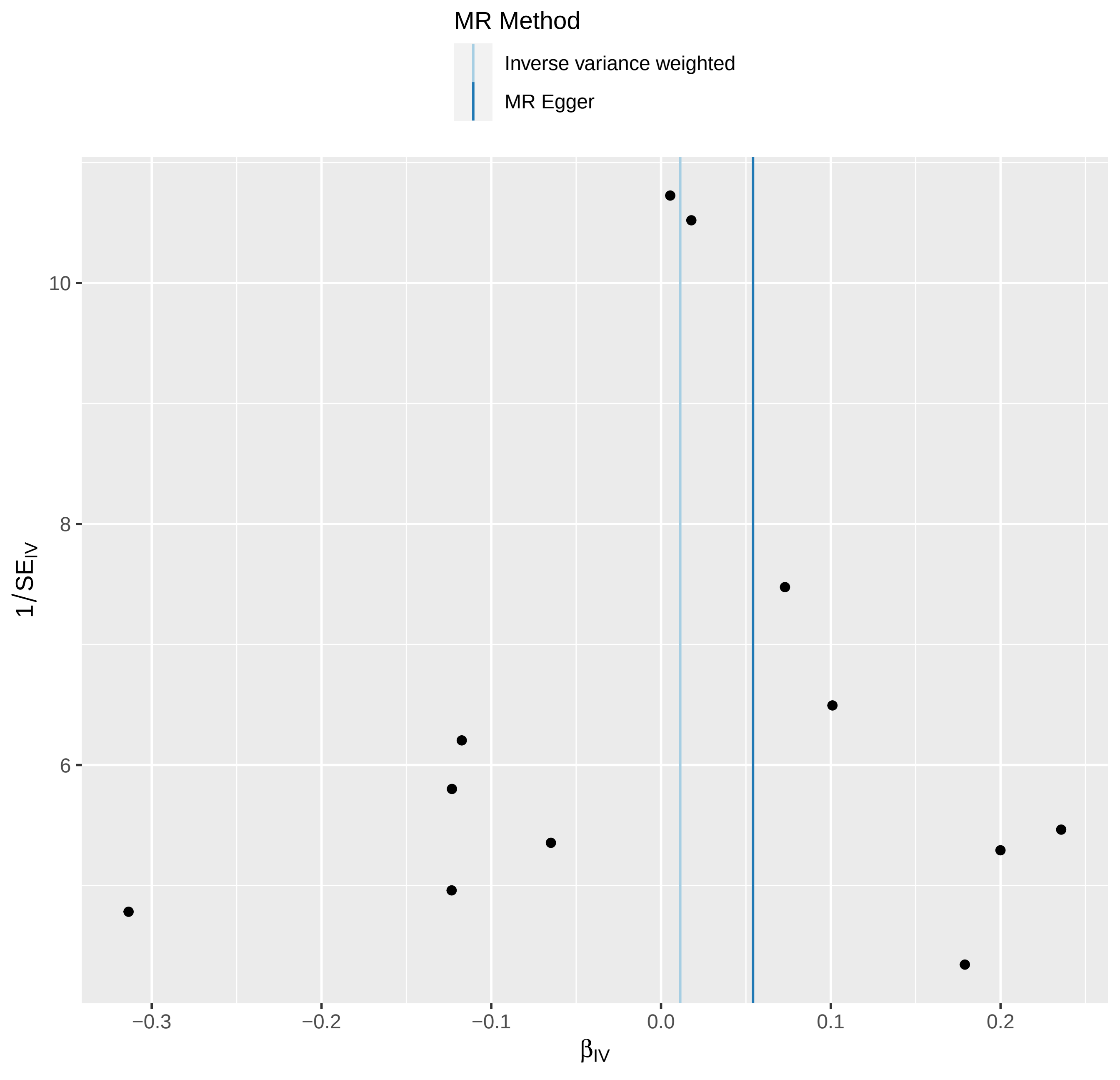

Batch 3−1942 : Selenium on 39S ribosomal protein L33, mitochondrial

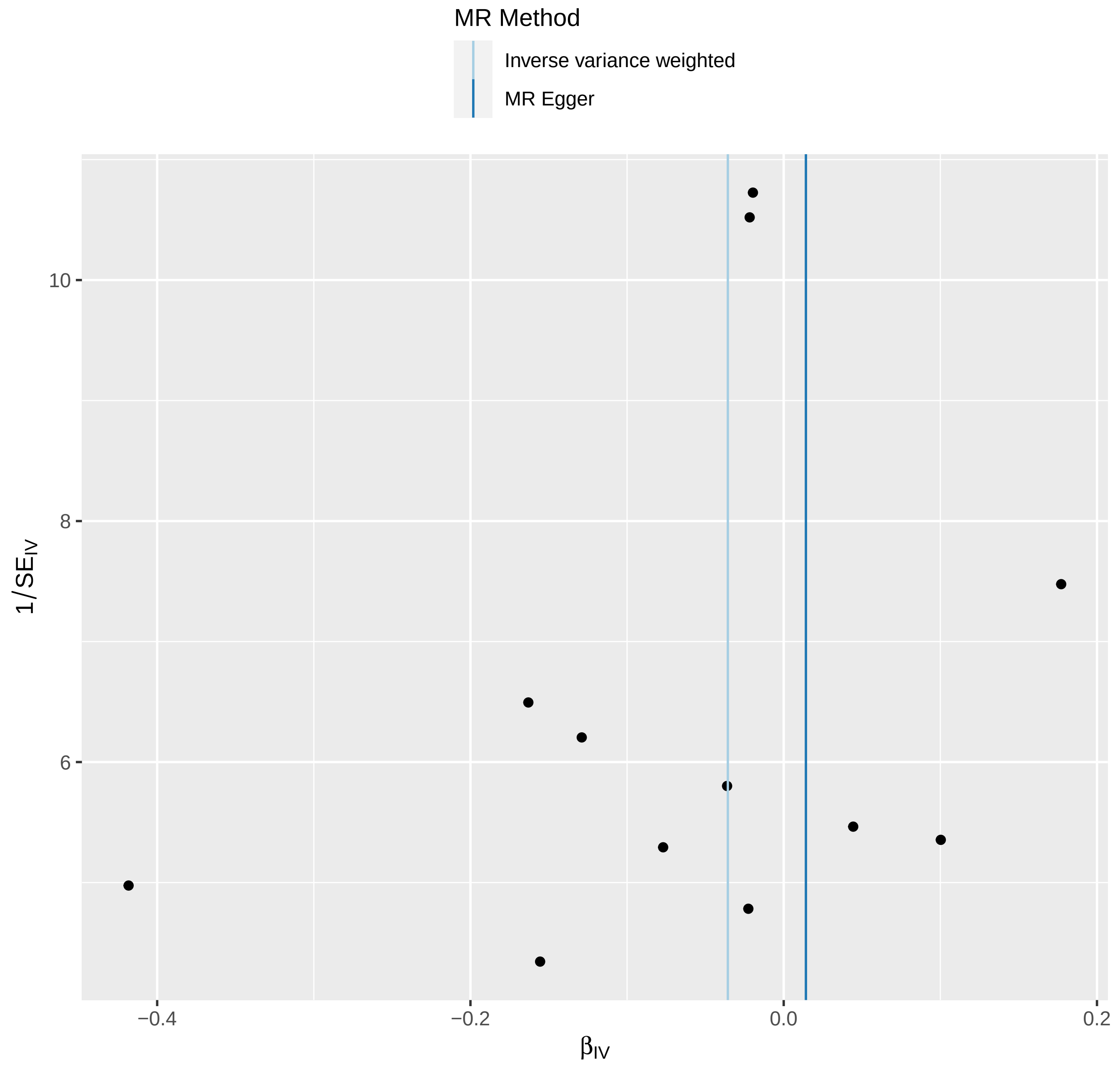

Batch 3−1943 : Selenium on 39S ribosomal protein L34, mitochondrial

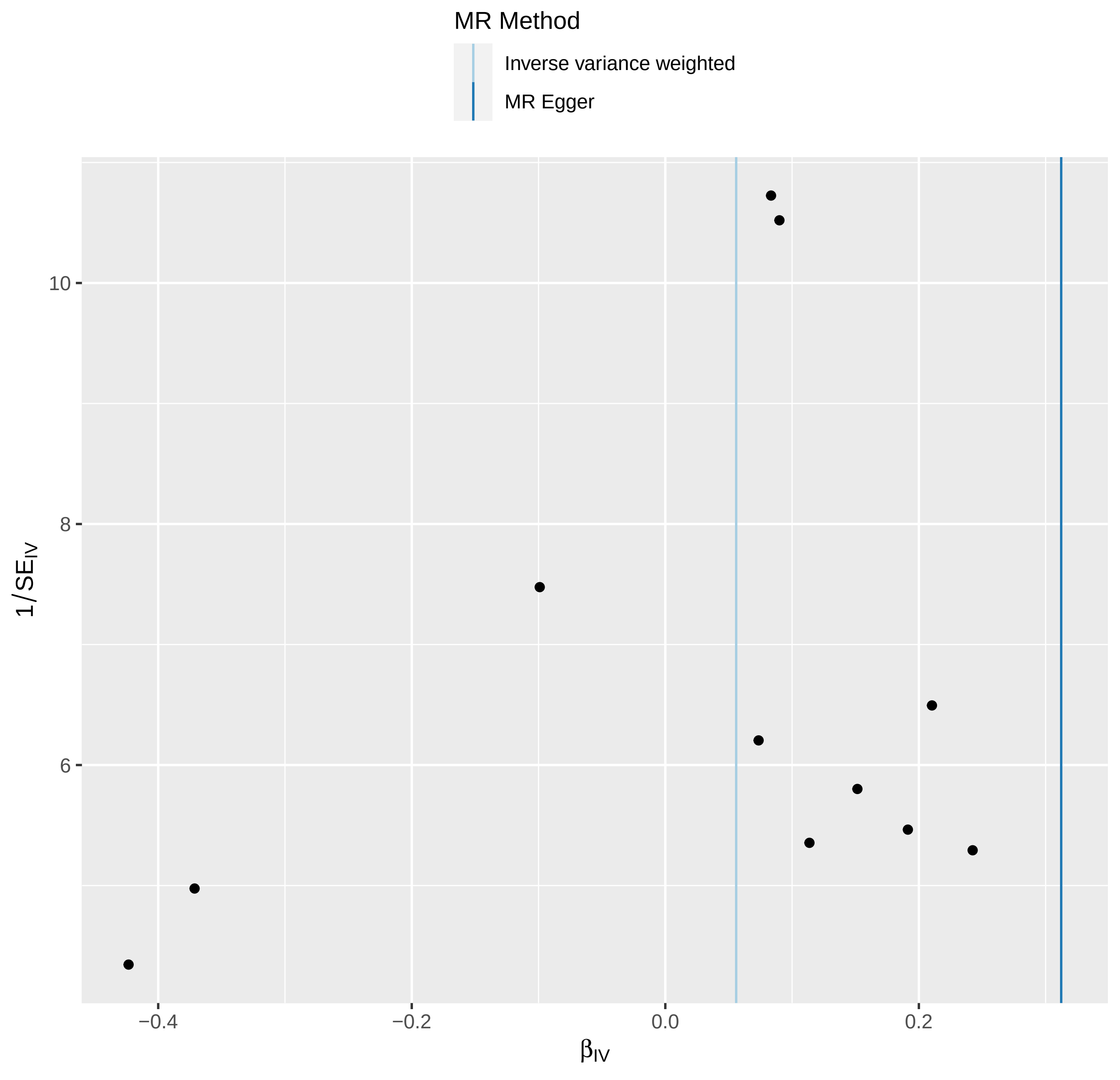

Batch 3−1944 : Selenium on 39S ribosomal protein L52, mitochondrial

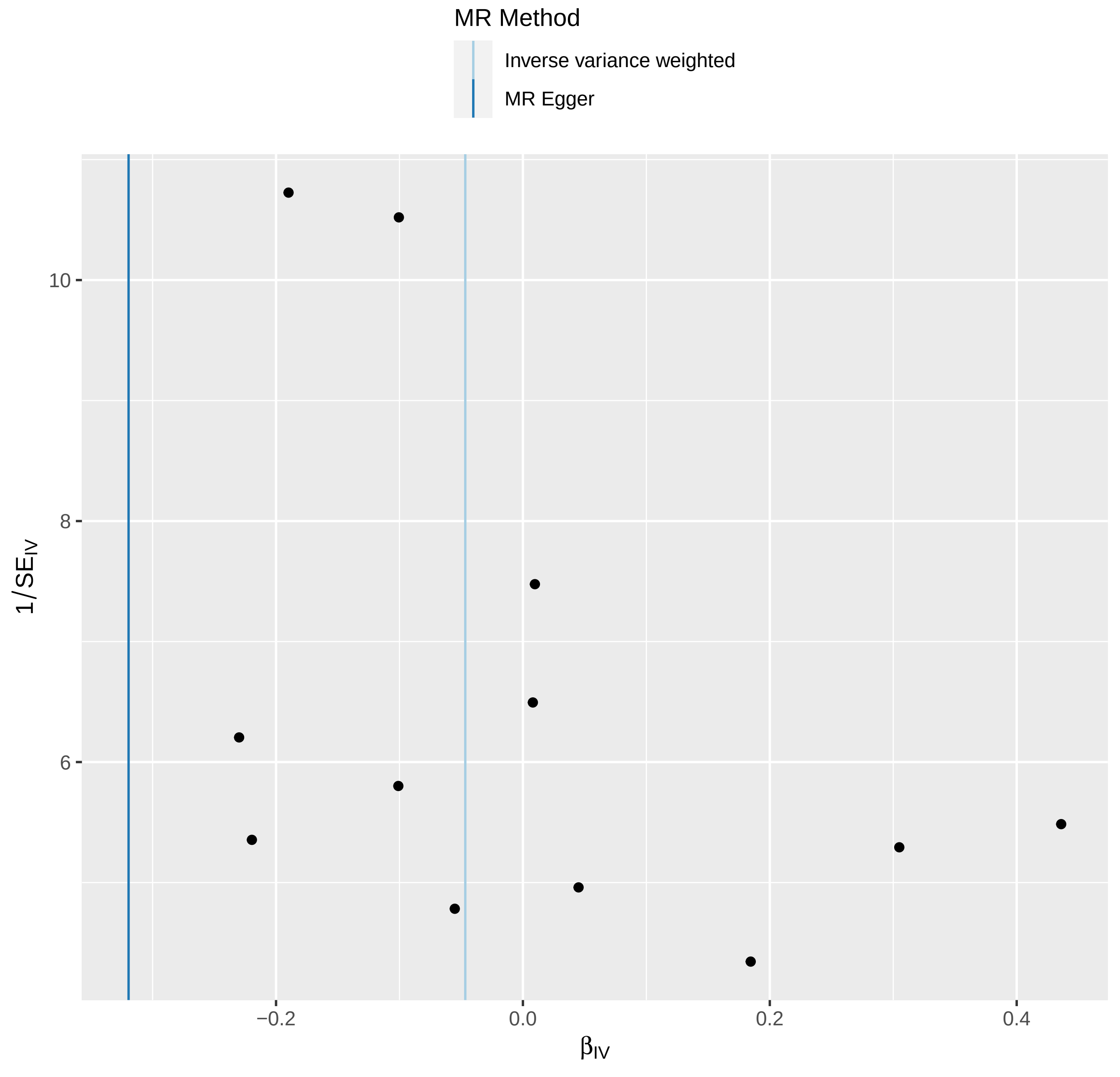

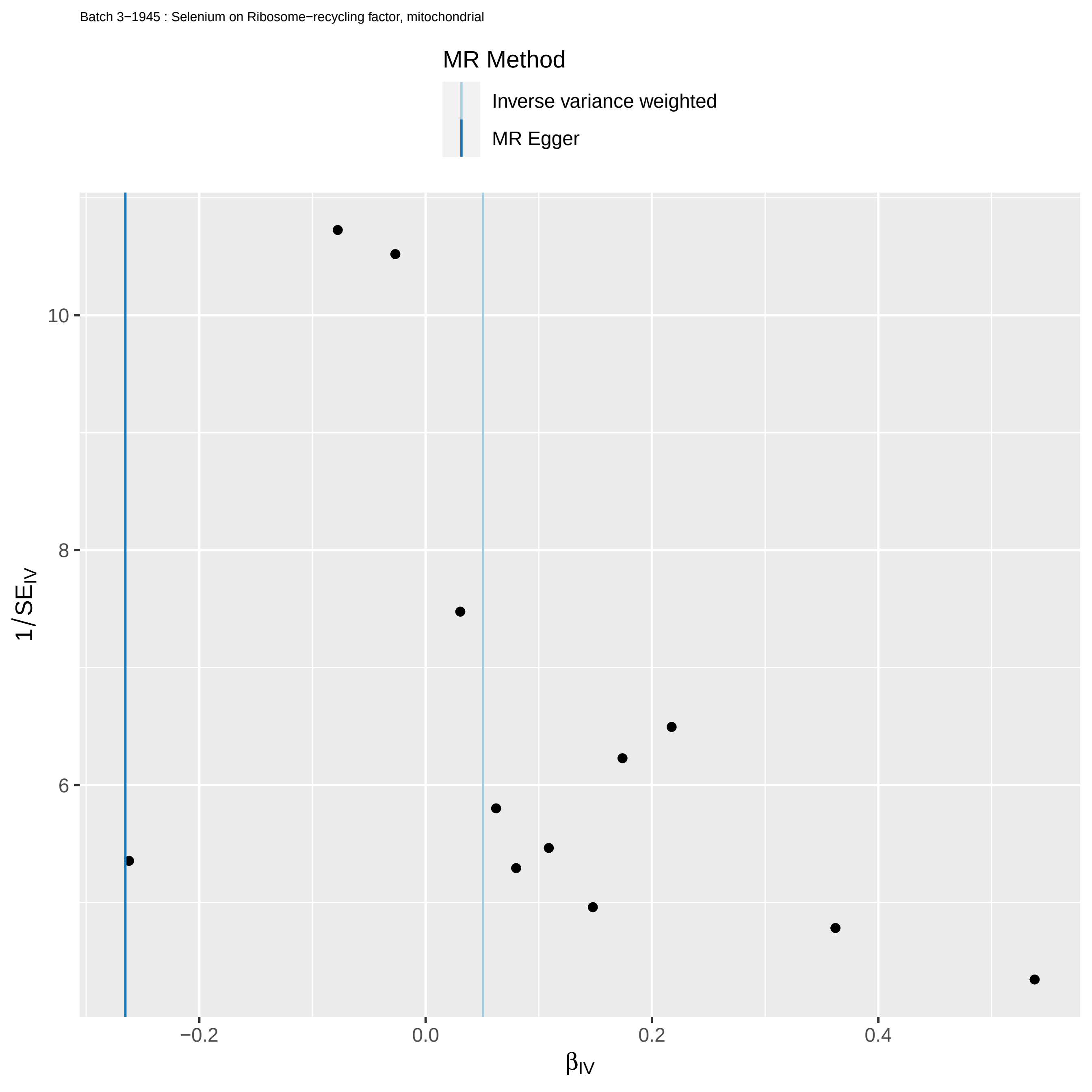

Batch 3−1945 : Selenium on Ribosome−recycling factor, mitochondrial
MR Method
Inverse variance weighted
MR Egger
10
8
6
1/SE$_{IV}$
−0.2
0.0
0.2
0.4
β$_{IV}$

Batch 3−1946 : Selenium on Protein MRVI1

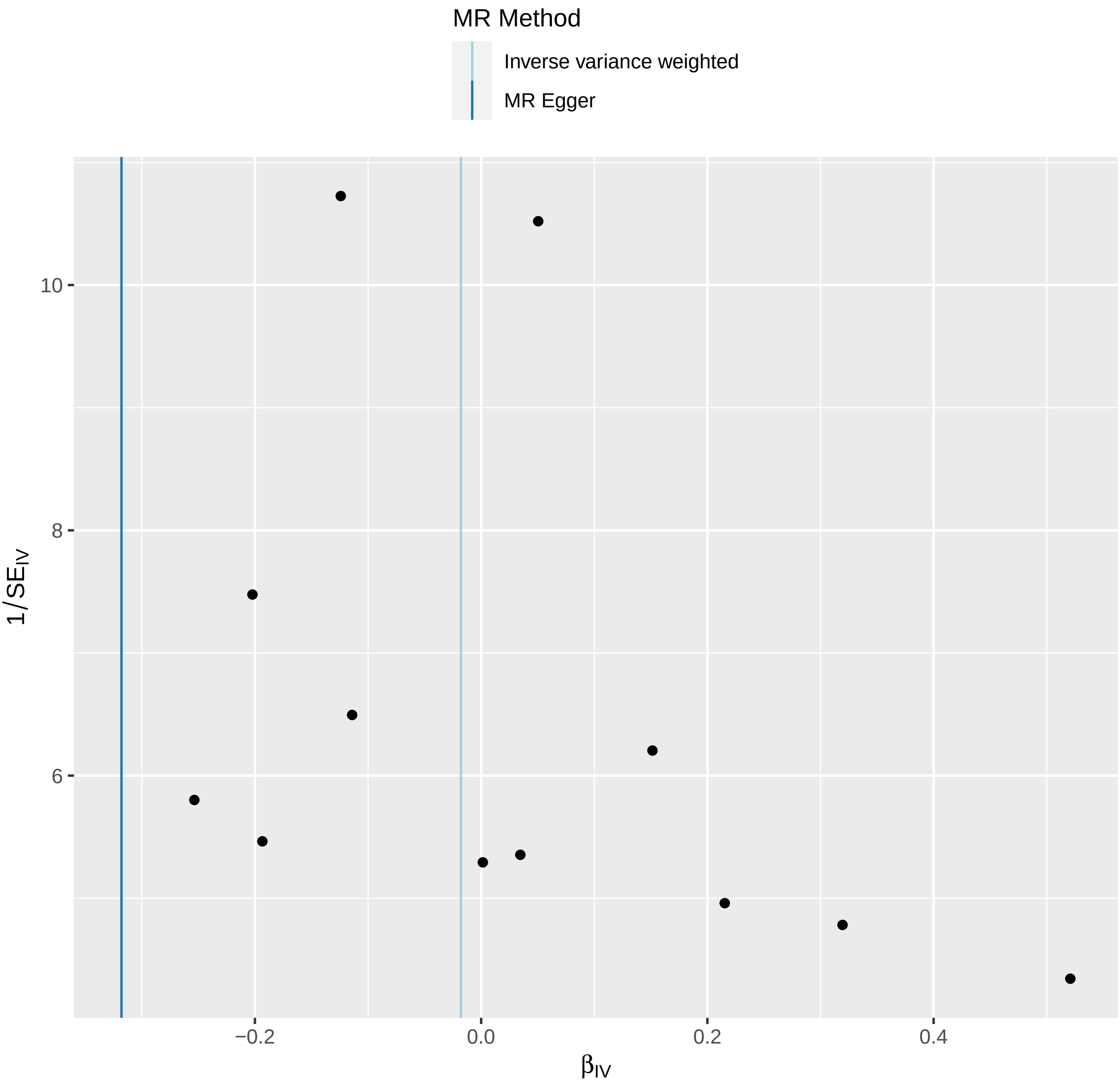

Batch 3−1947 : Selenium on Mesothelin

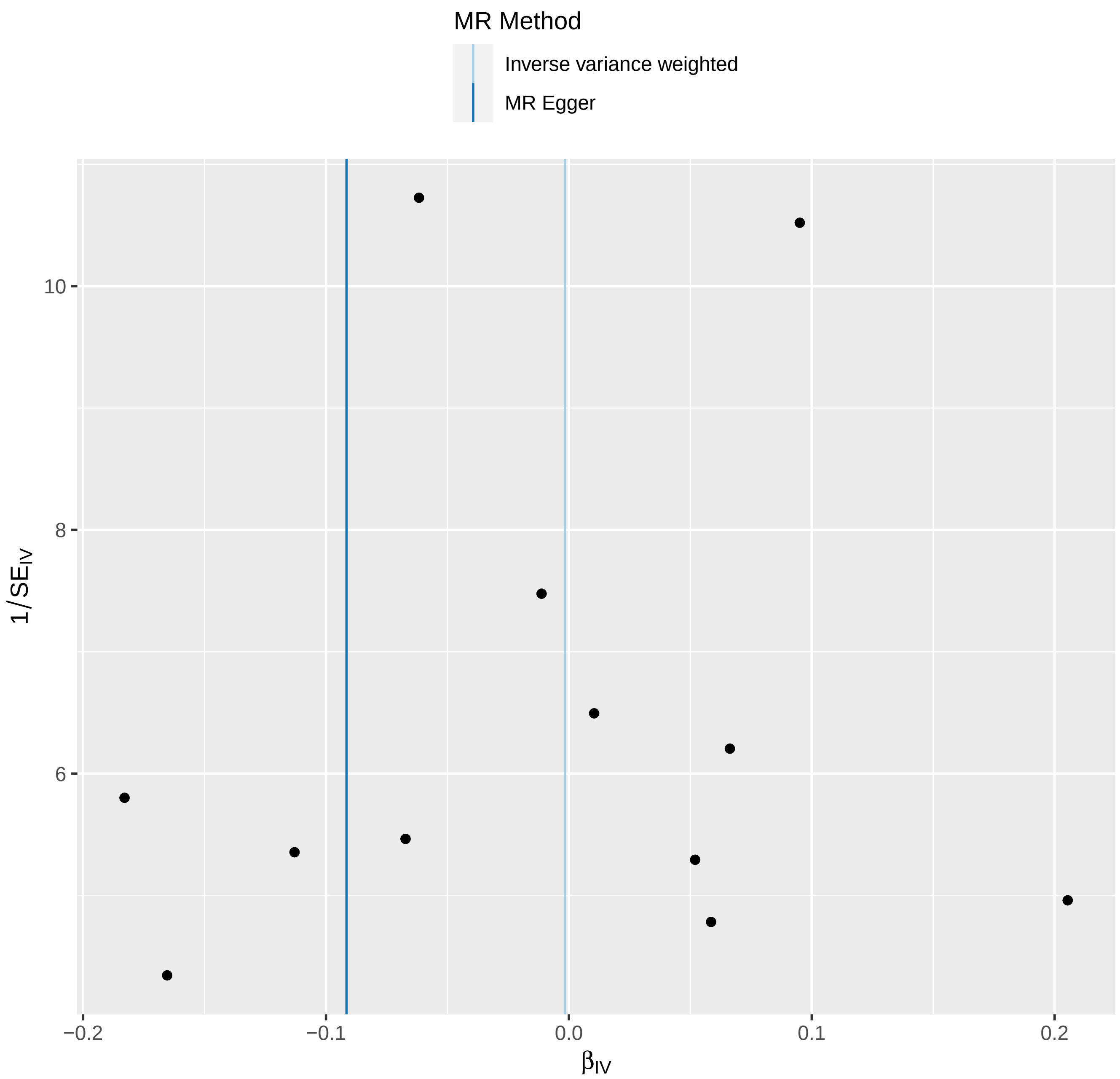

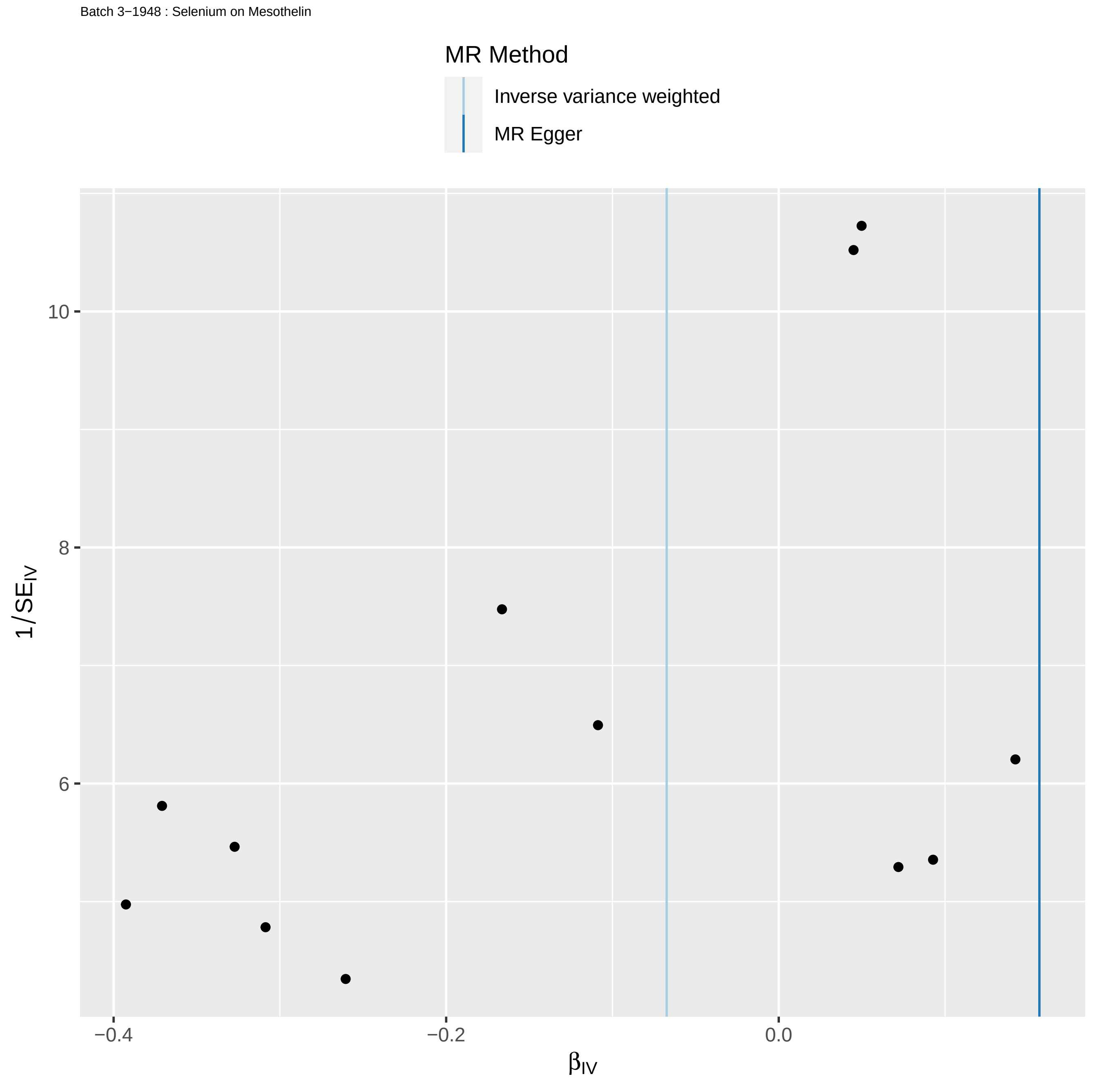
Batch 3−1948 : Selenium on Mesothelin
MR Method
Inverse variance weighted
MR Egger
10
8
6
1/SE_IV
−0.4
−0.2
0.0
β_IV

Batch 3−1949 : Selenium on Beta−microseminoprotein

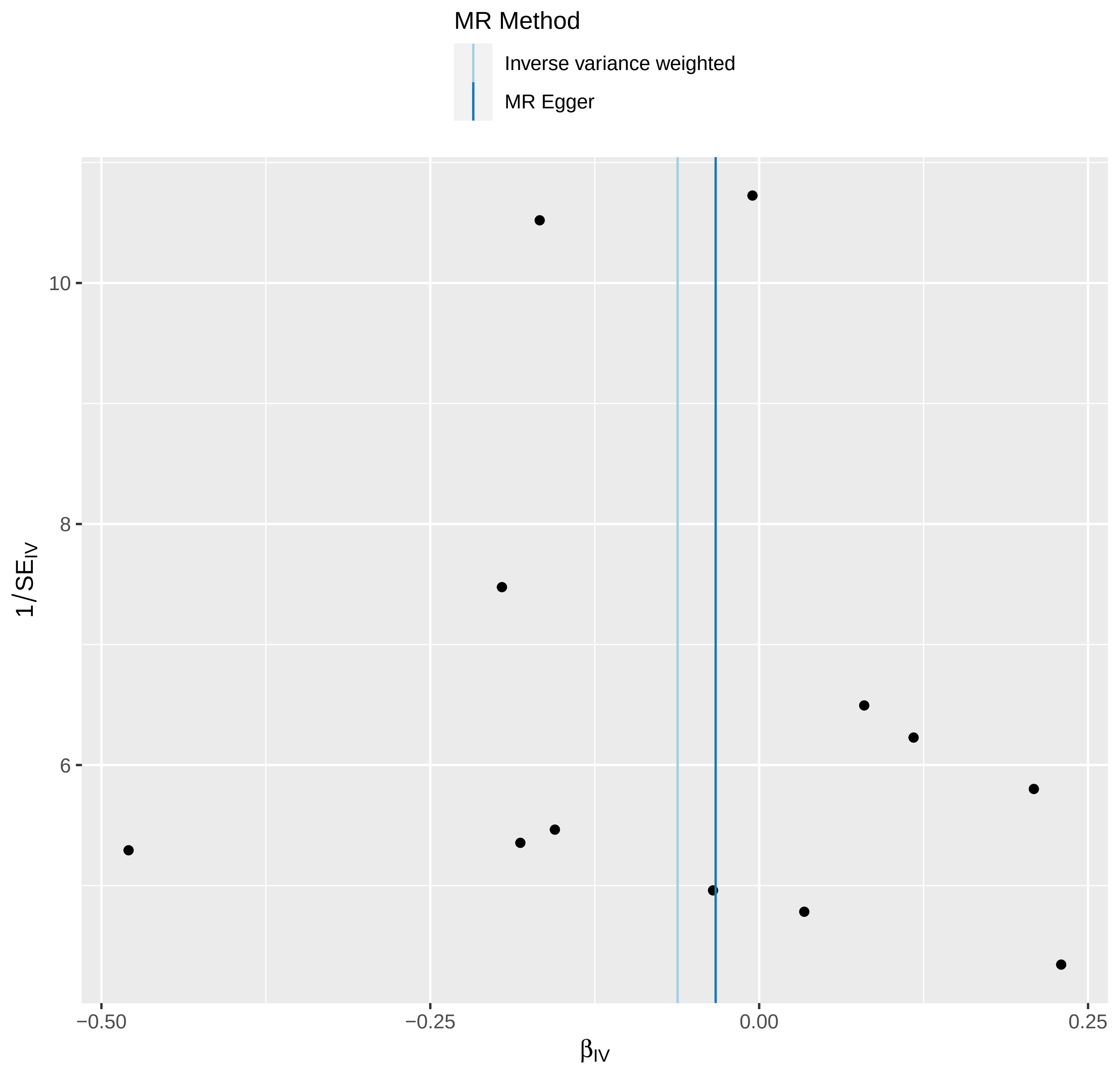

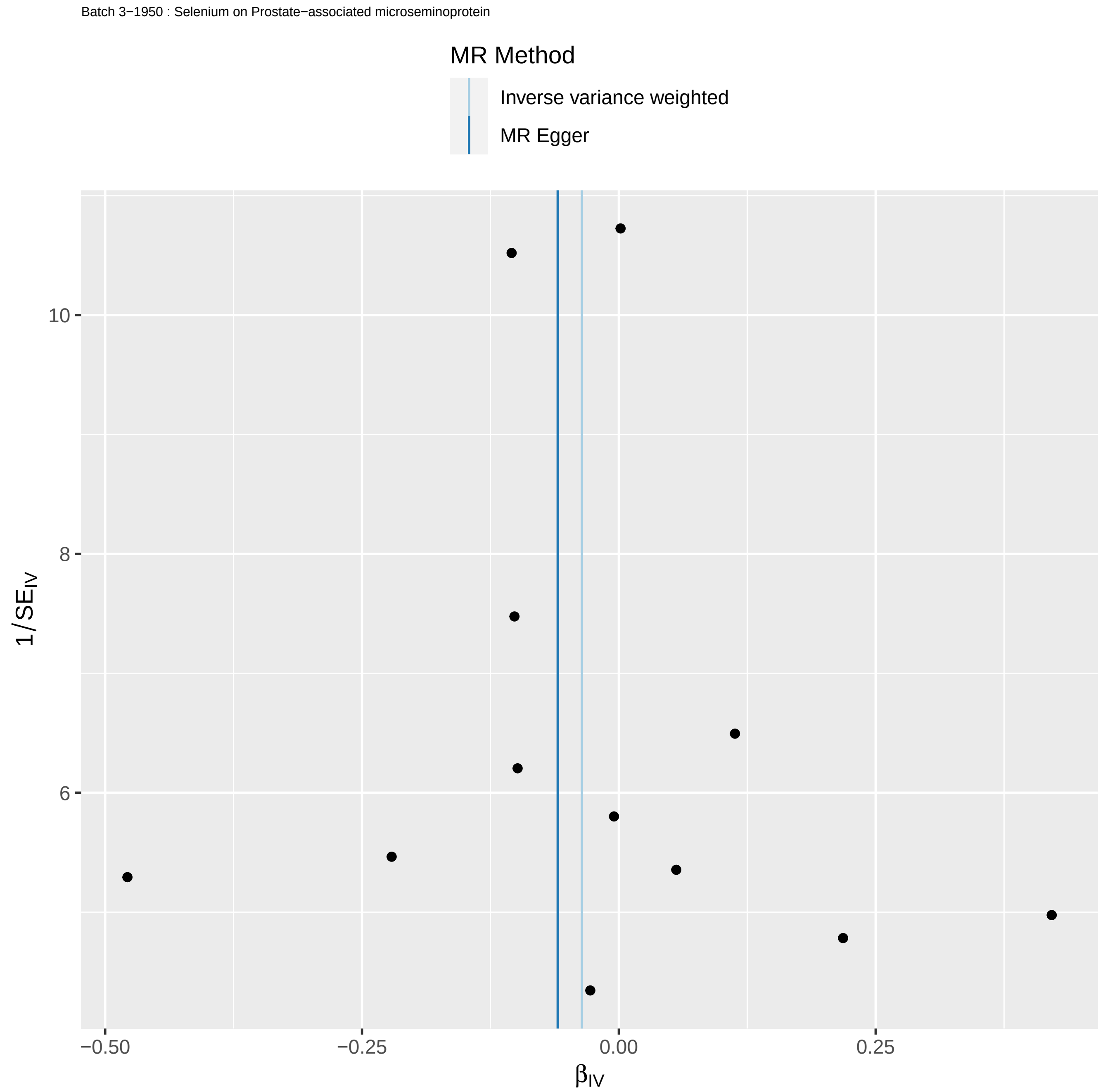

Batch 3−1950 : Selenium on Prostate−associated microseminoprotein
MR Method
Inverse variance weighted
MR Egger
10
8
6
1/SE$_{IV}$
−0.50
−0.25
0.00
0.25
β$_{IV}$

Batch 3−1951 : Selenium on Macrophage scavenger receptor types I and II

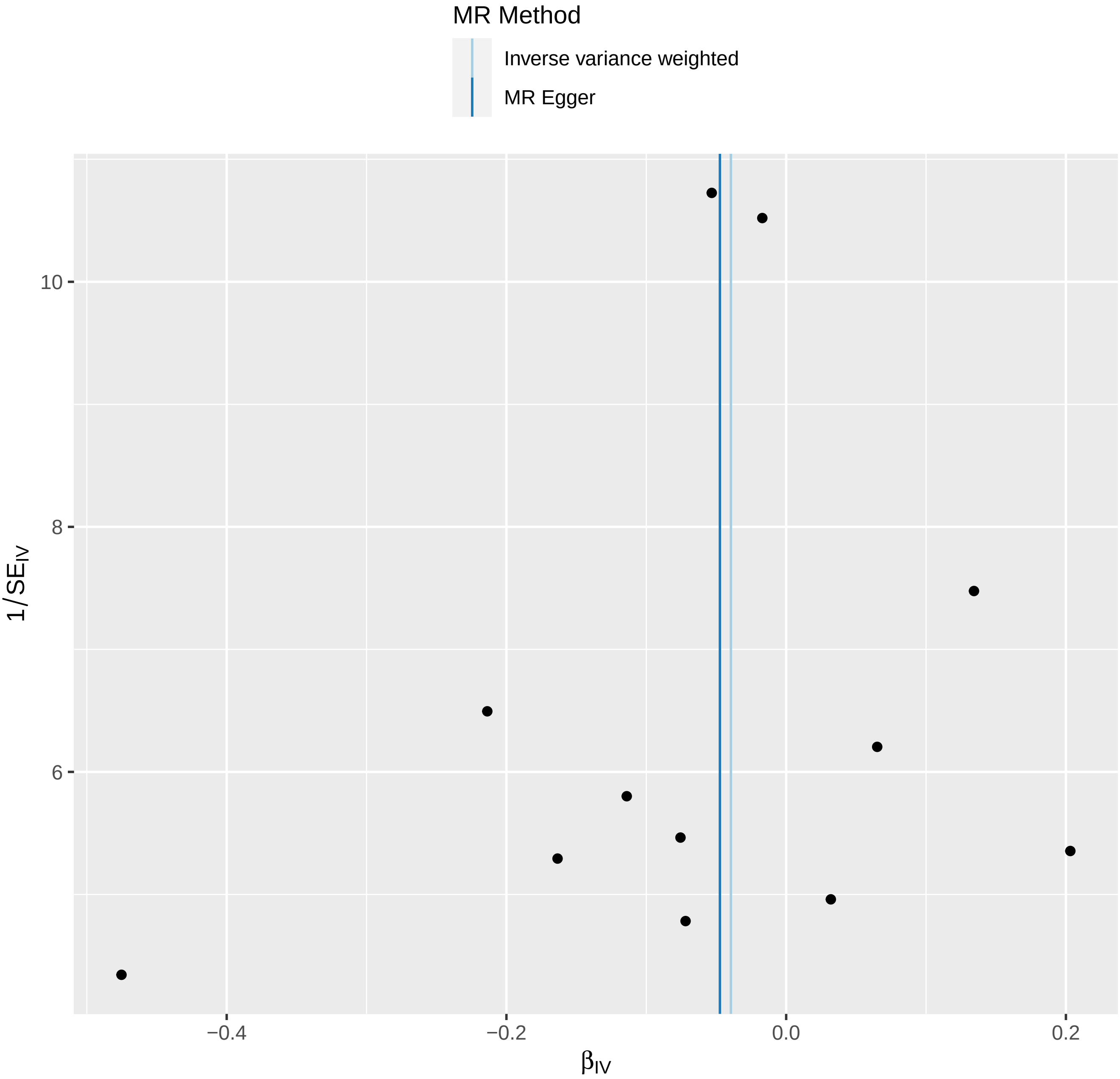

Batch 3−1952 : Selenium on Macrophage scavenger receptor types I and II

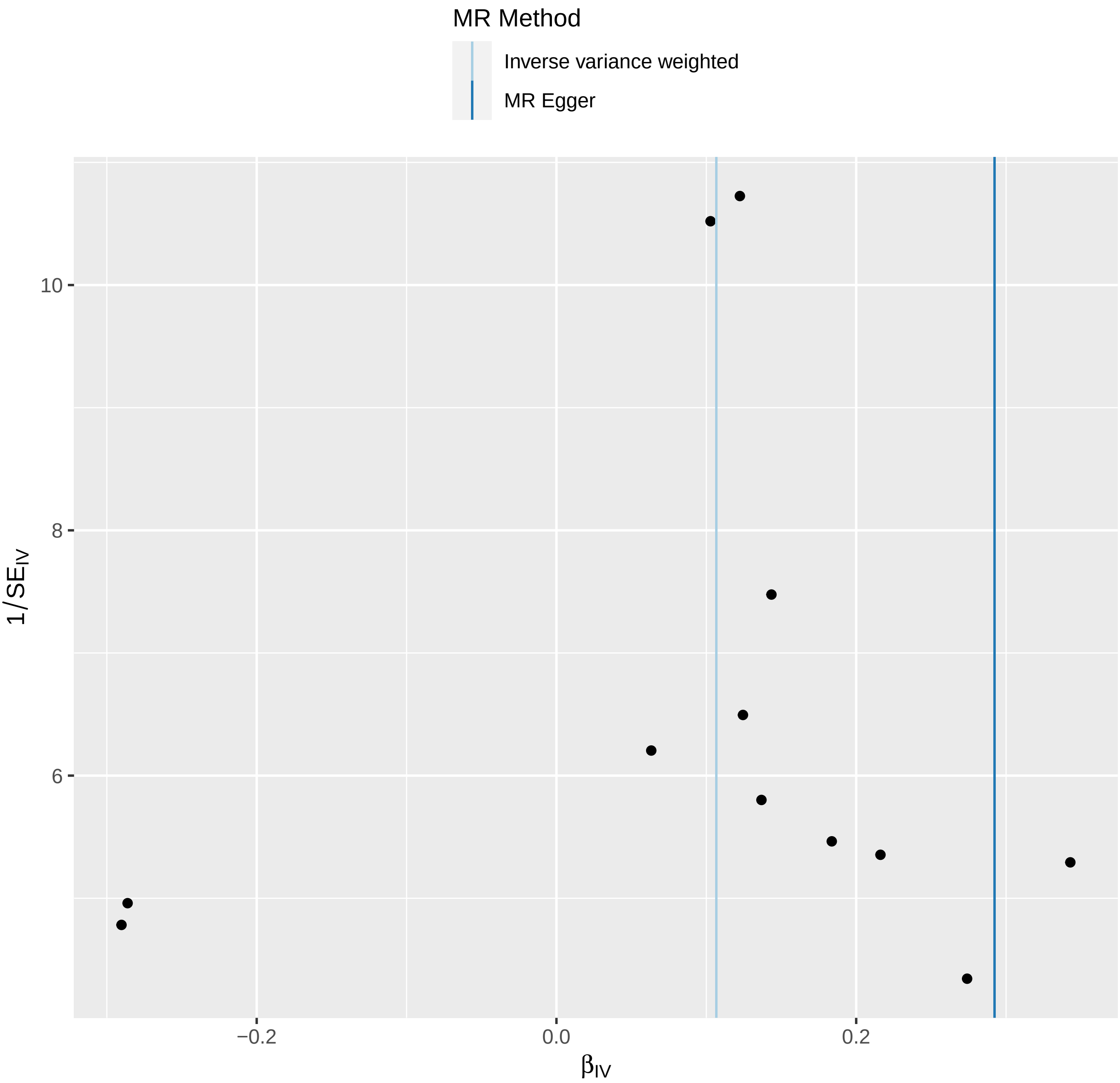

Batch 3−1953 : Selenium on Mitochondrial peptide methionine sulfoxide reductase

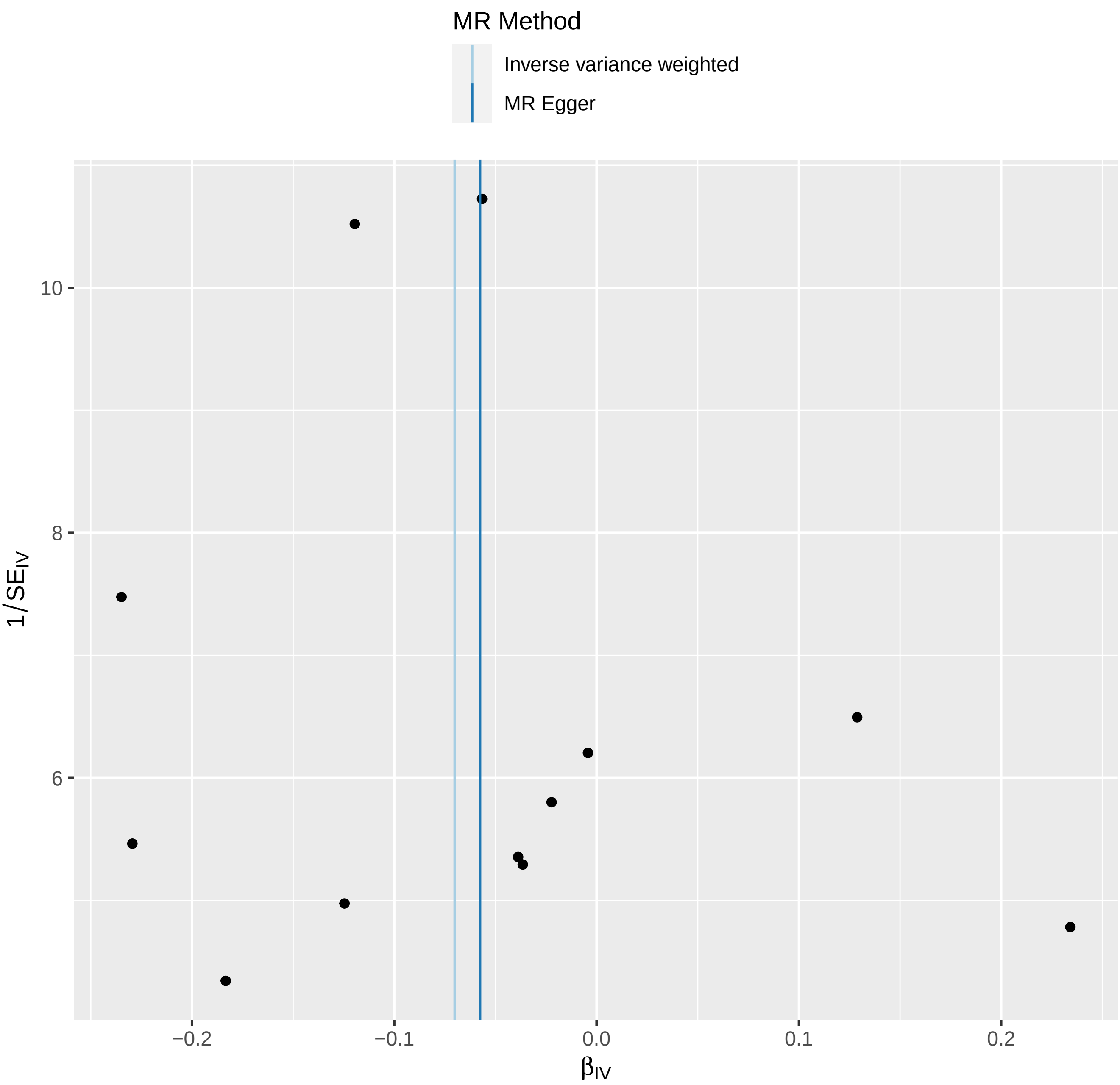

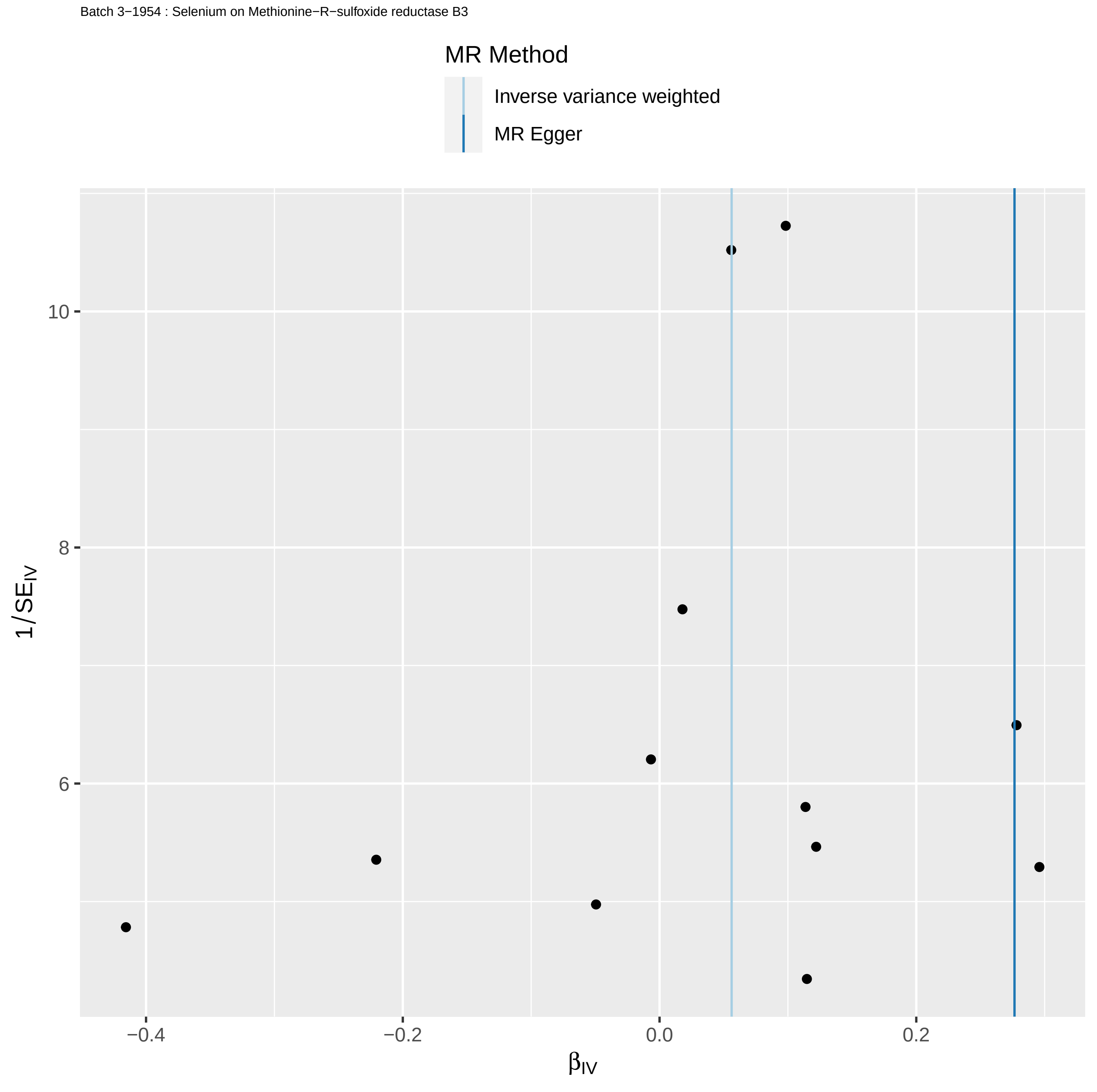

Batch 3−1954 : Selenium on Methionine−R−sulfoxide reductase B3
MR Method
Inverse variance weighted
MR Egger
10
8
6
1/SE_IV
−0.4
−0.2
0.0
0.2
β_IV

Batch 3−1955 : Selenium on Macrophage−stimulating protein receptor

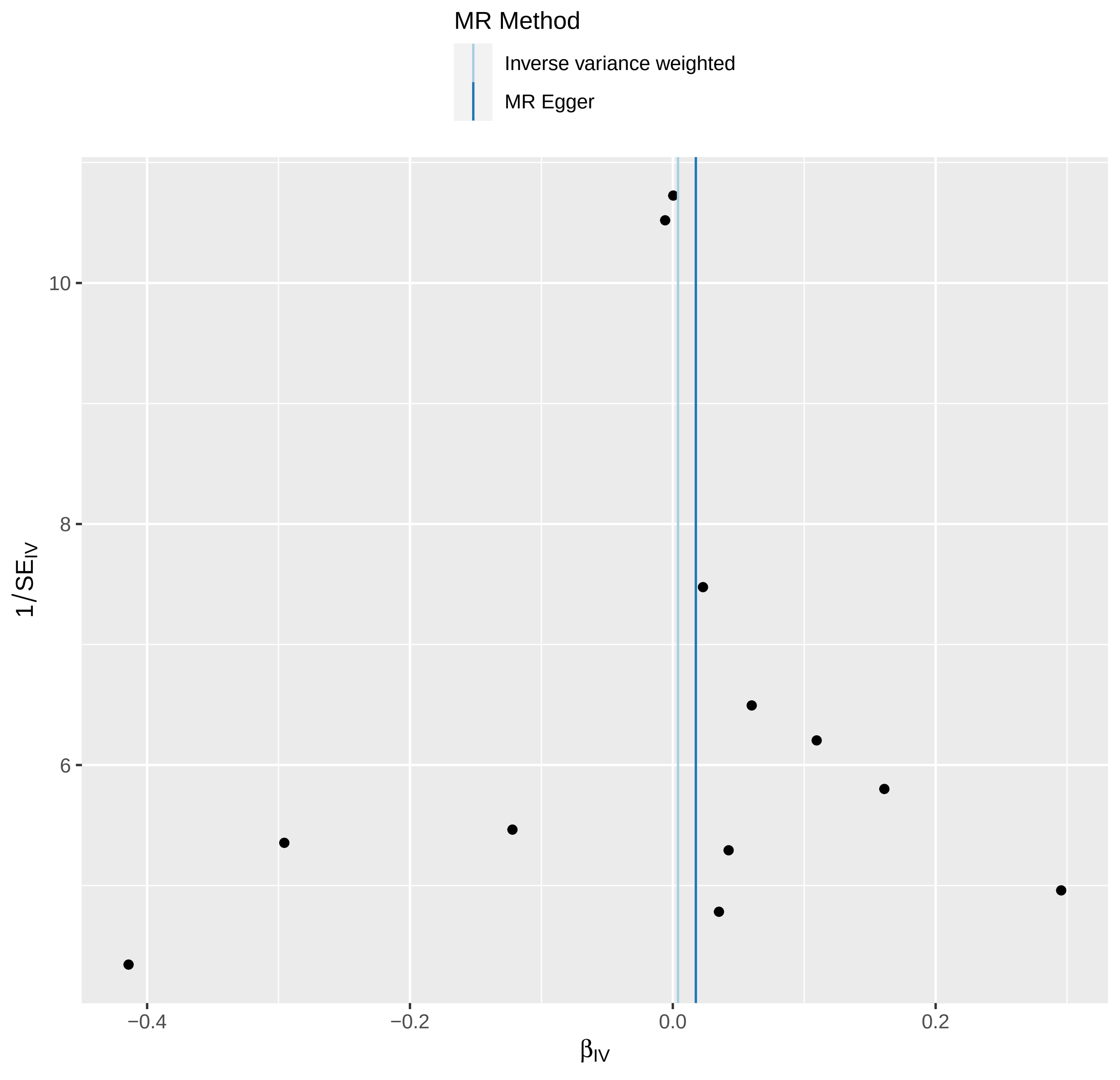

Batch 3−1956 : Selenium on Growth/differentiation factor 8

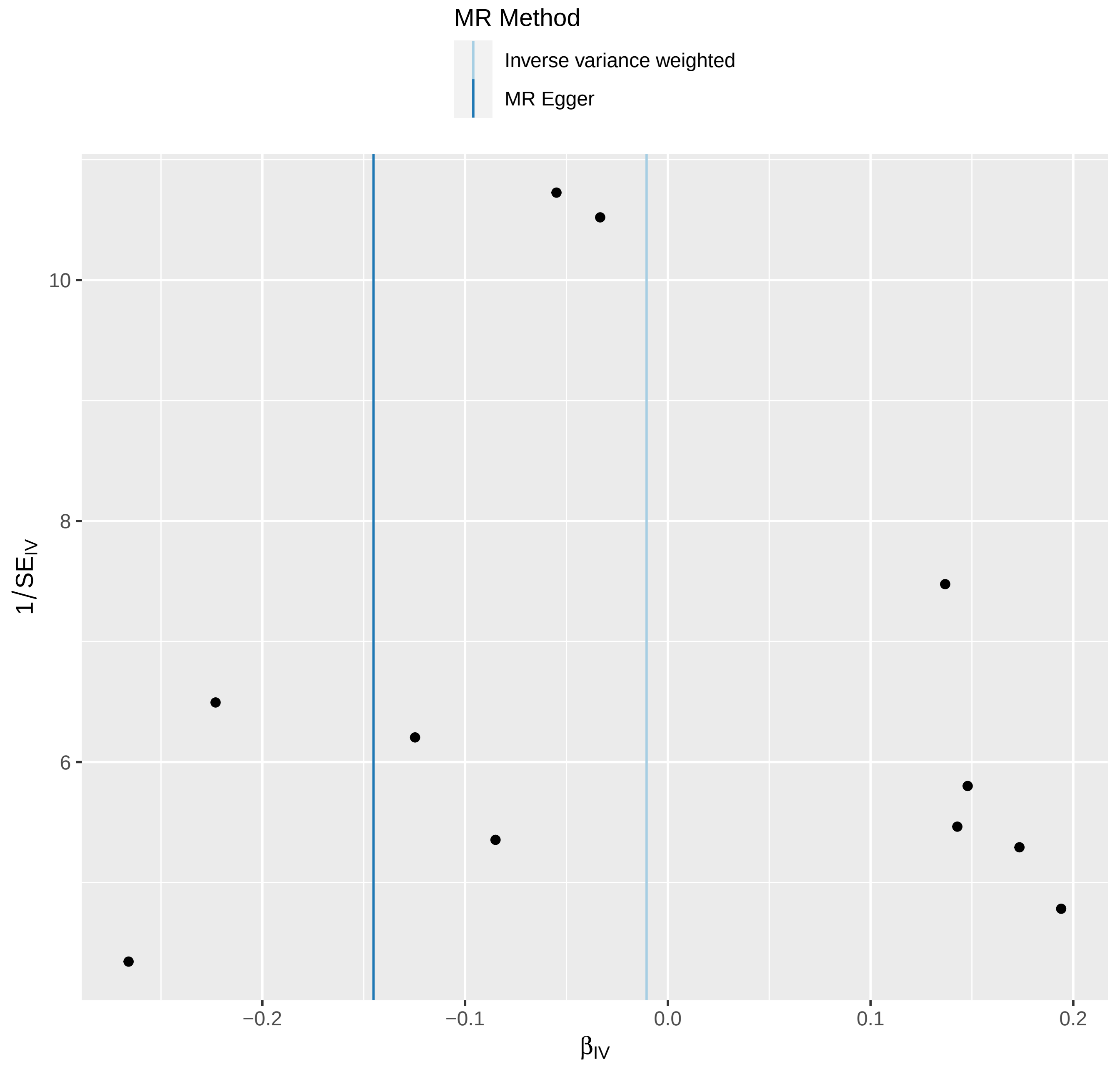

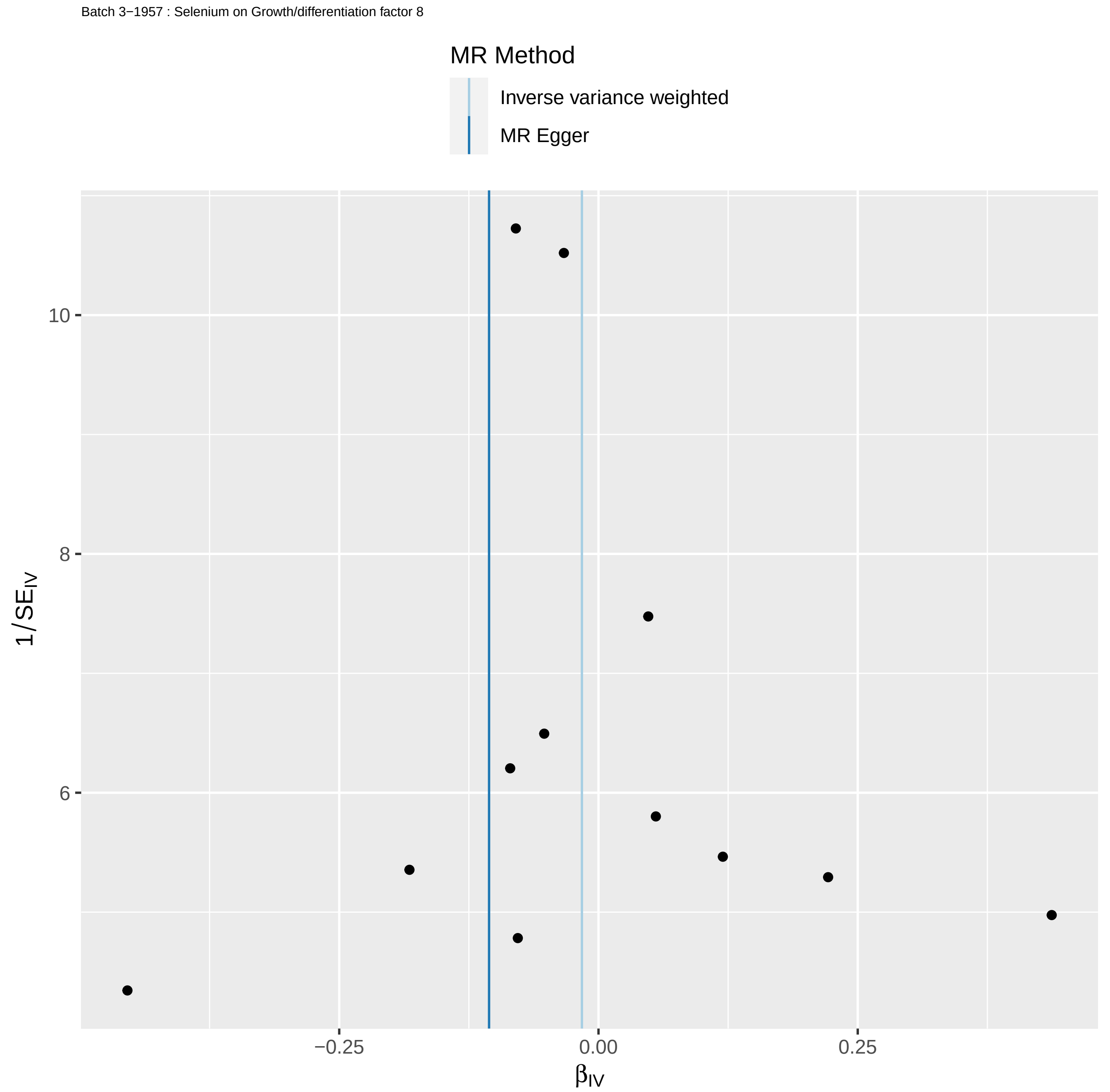
Batch 3−1957 : Selenium on Growth/differentiation factor 8
MR Method
Inverse variance weighted
MR Egger
10
8
6
1/SE_IV
−0.25
0.00
0.25
β_IV

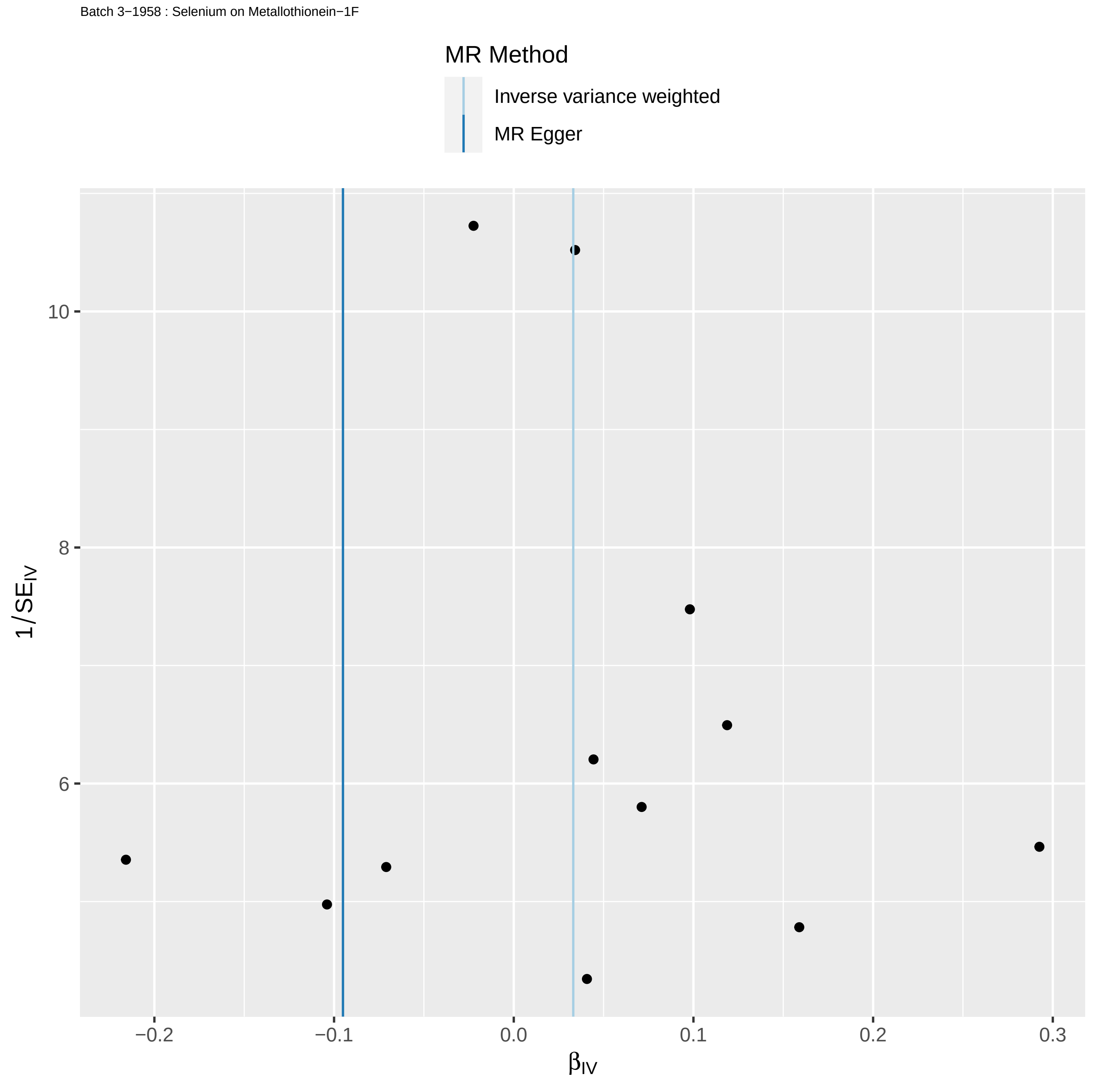

Batch 3−1958 : Selenium on Metallothionein−1F
MR Method
Inverse variance weighted
MR Egger
1/SE$_{IV}$
10
8
6
−0.2
−0.1
0.0
0.1
0.2
0.3
β$_{IV}$

Batch 3−1959 : Selenium on S−methyl−5'−thioadenosine phosphorylase

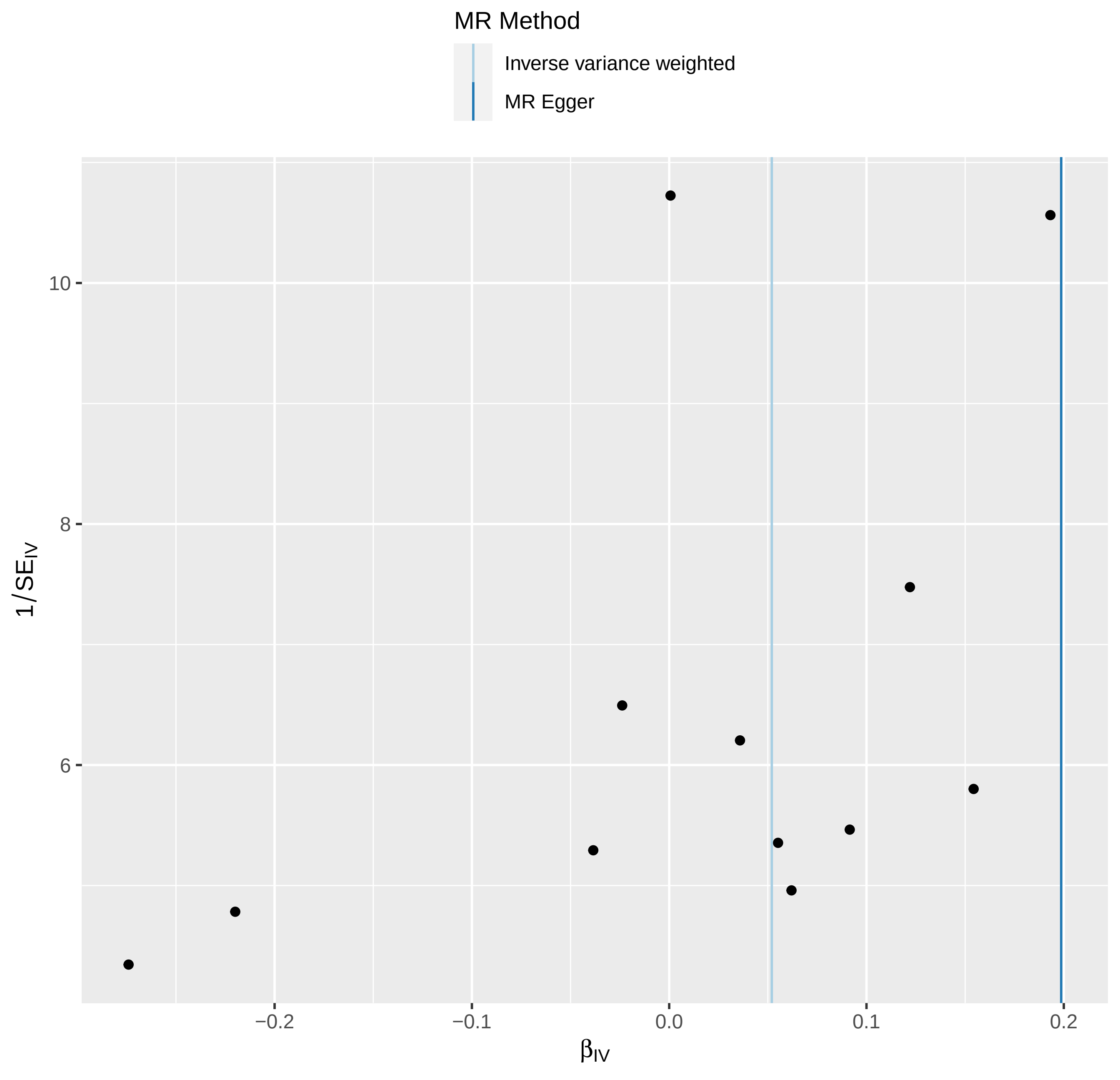

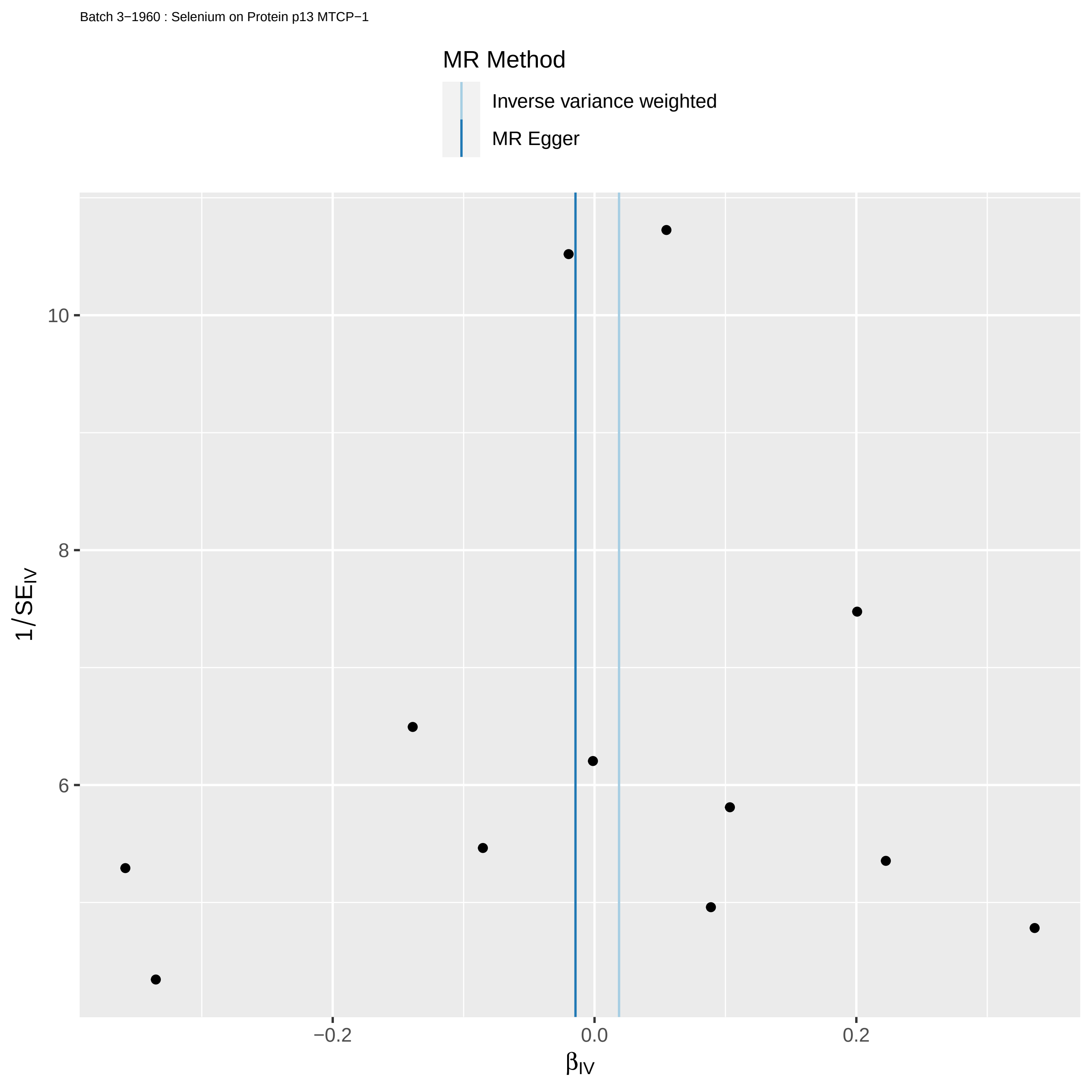

Batch 3−1960 : Selenium on Protein p13 MTCP−1
MR Method
Inverse variance weighted
MR Egger
10
8
6
1/SE_IV
−0.2
0.0
0.2
β_IV

Batch 3−1961 : Selenium on Mitochondrial fission regulator 1

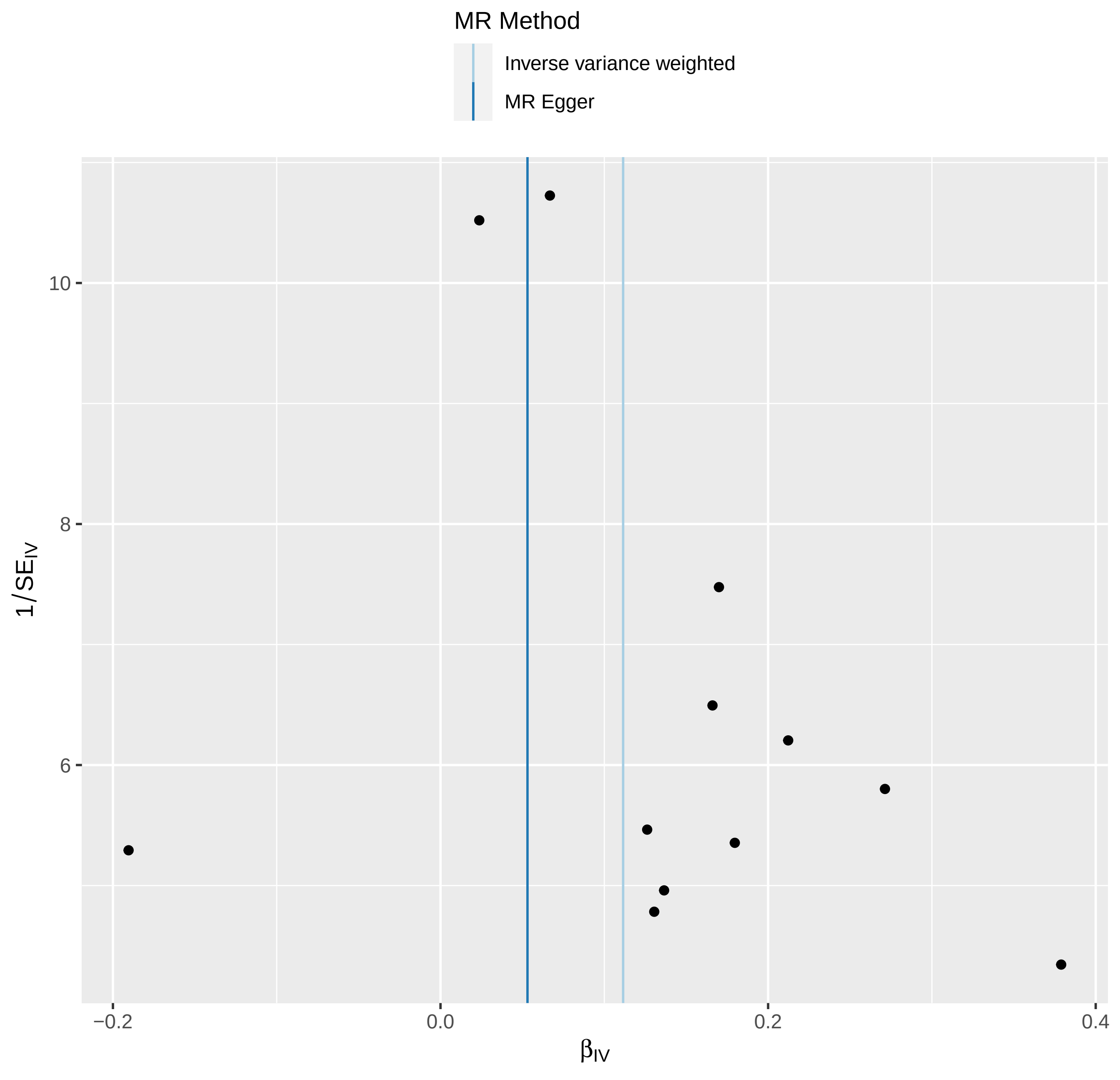

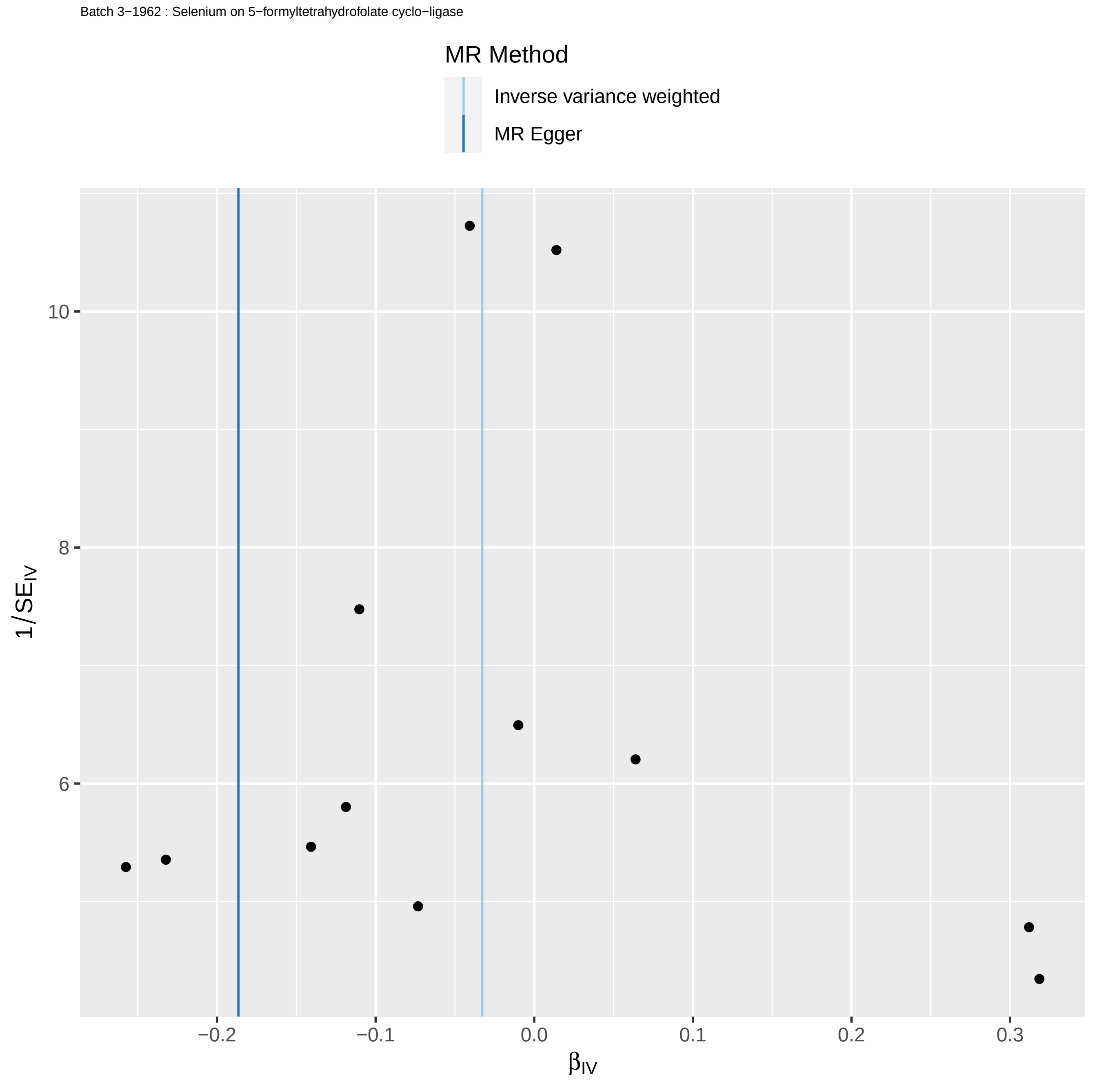
Batch 3−1962 : Selenium on 5−formyltetrahydrofolate cyclo−ligase
MR Method
Inverse variance weighted
MR Egger
10
8
6
1/SE_IV
−0.2
−0.1
0.0
0.1
0.2
0.3
β_IV

Batch 3−1963 : Selenium on Myotubularin−related protein 1

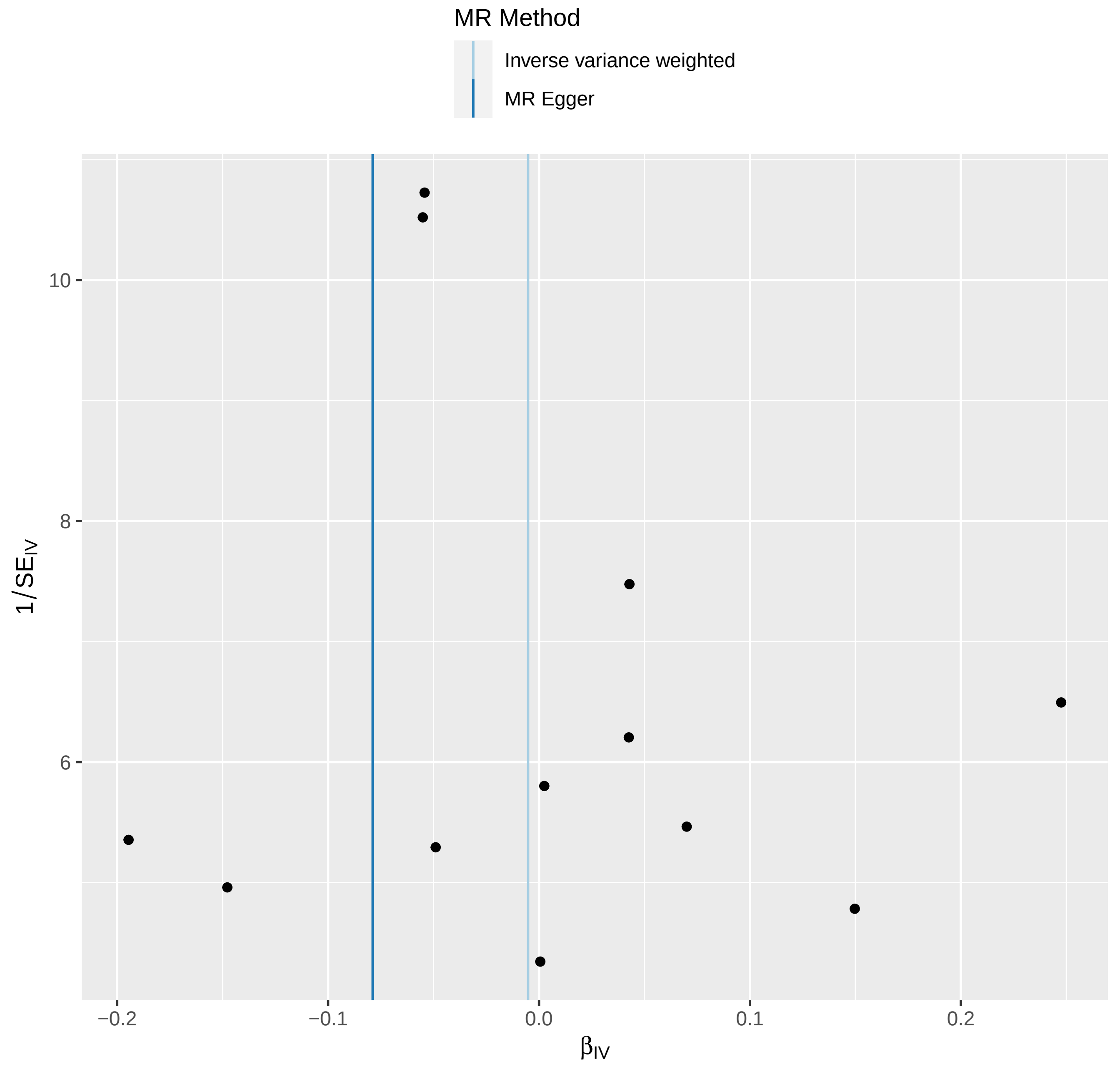

Batch 3−1964 : Selenium on Poly(A) RNA polymerase, mitochondrial

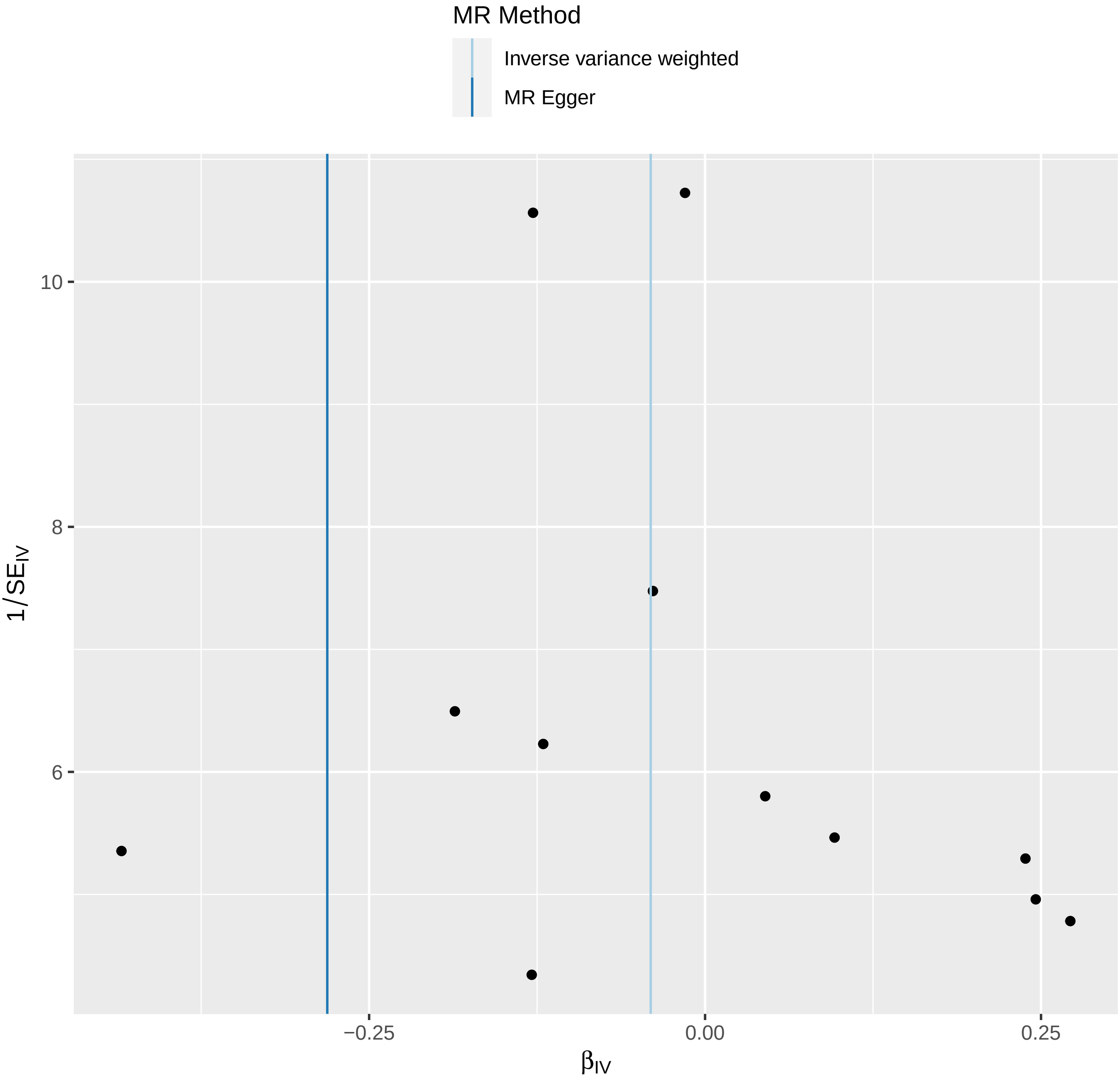

Batch 3−1965 : Selenium on Peptide chain release factor 1−like, mitochondrial

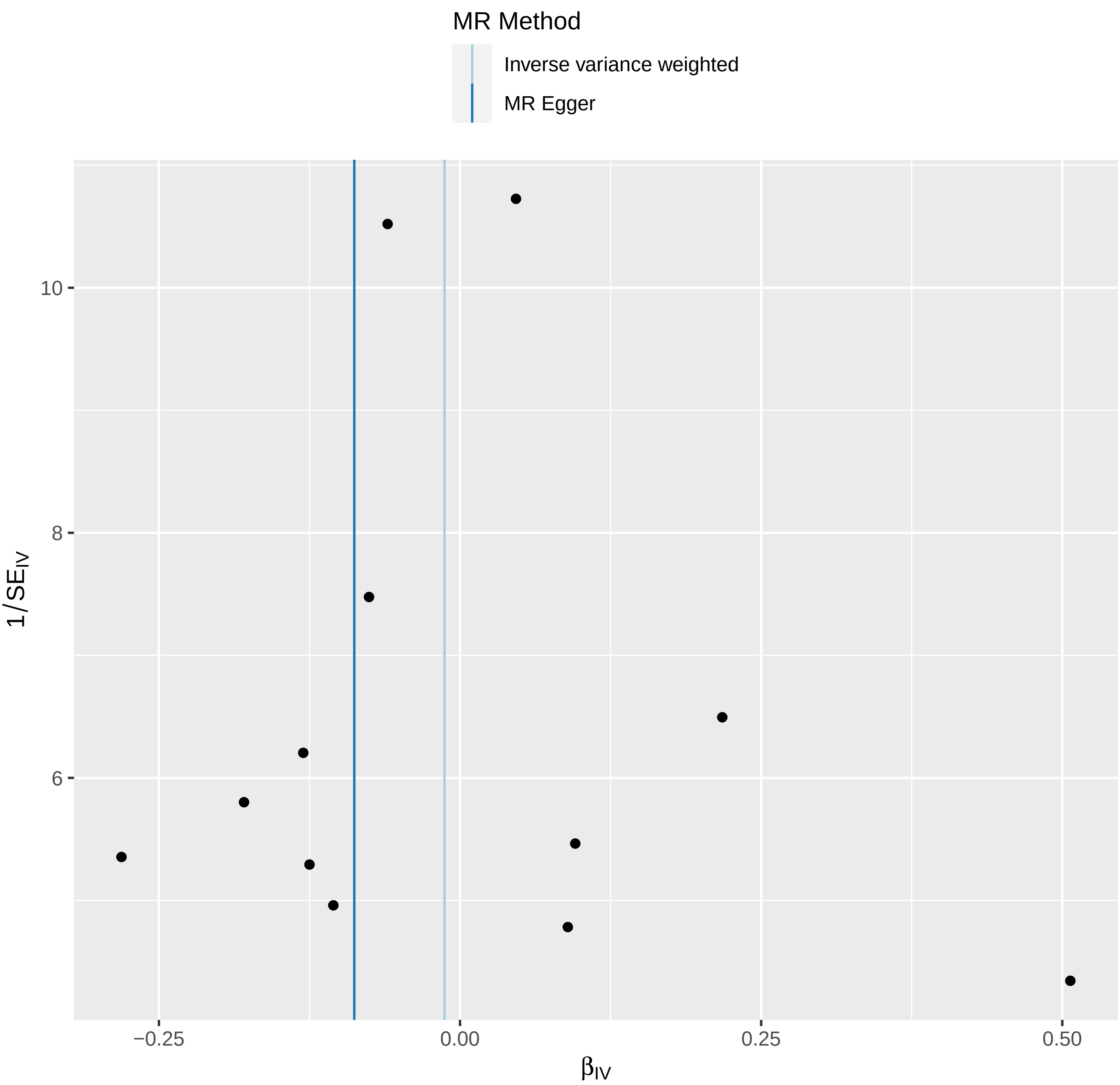

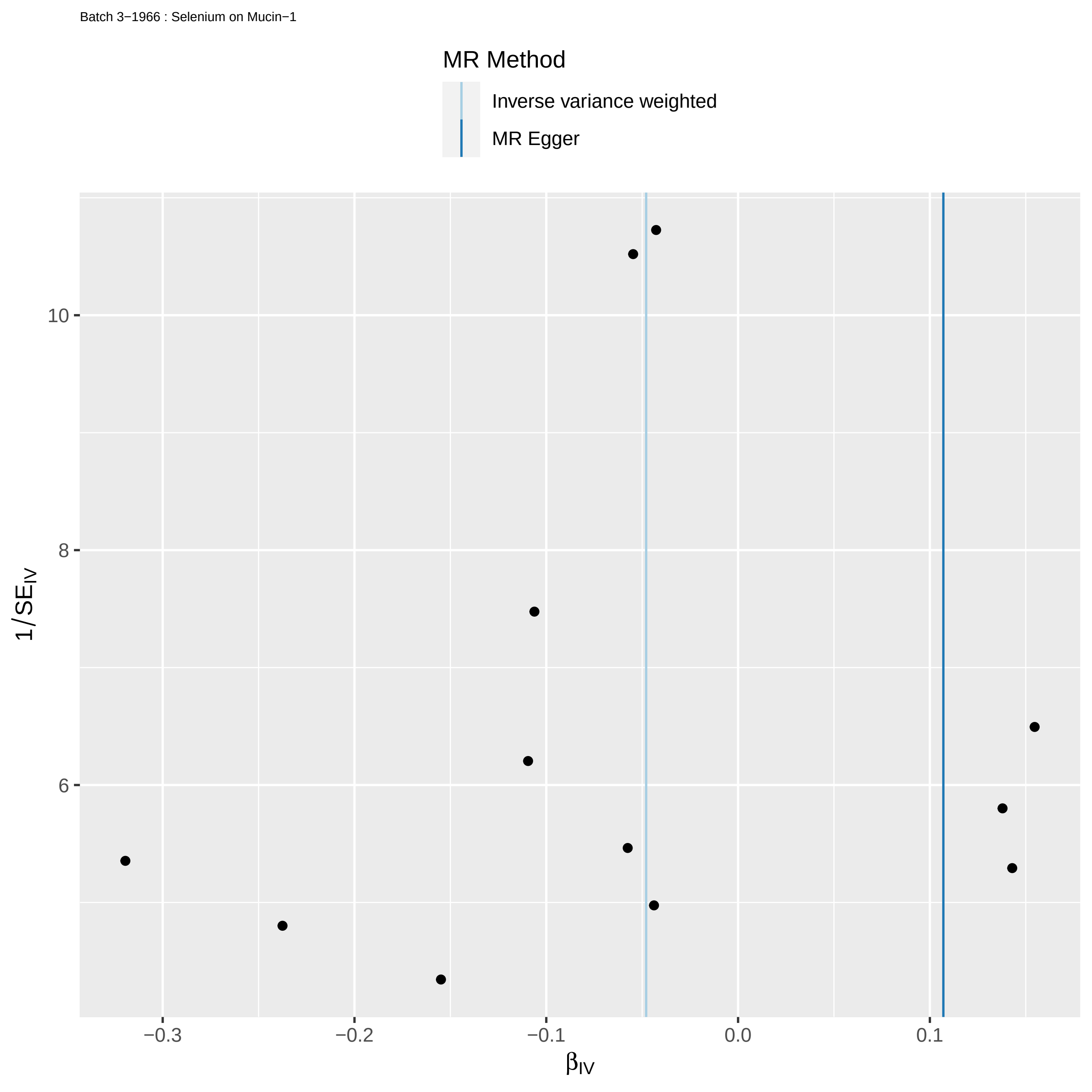
Batch 3−1966 : Selenium on Mucin−1
MR Method
Inverse variance weighted
MR Egger
10
8
6
1/SE_IV
−0.3
−0.2
−0.1
0.0
0.1
β_IV

Batch 3−1967 : Selenium on Mucin−1

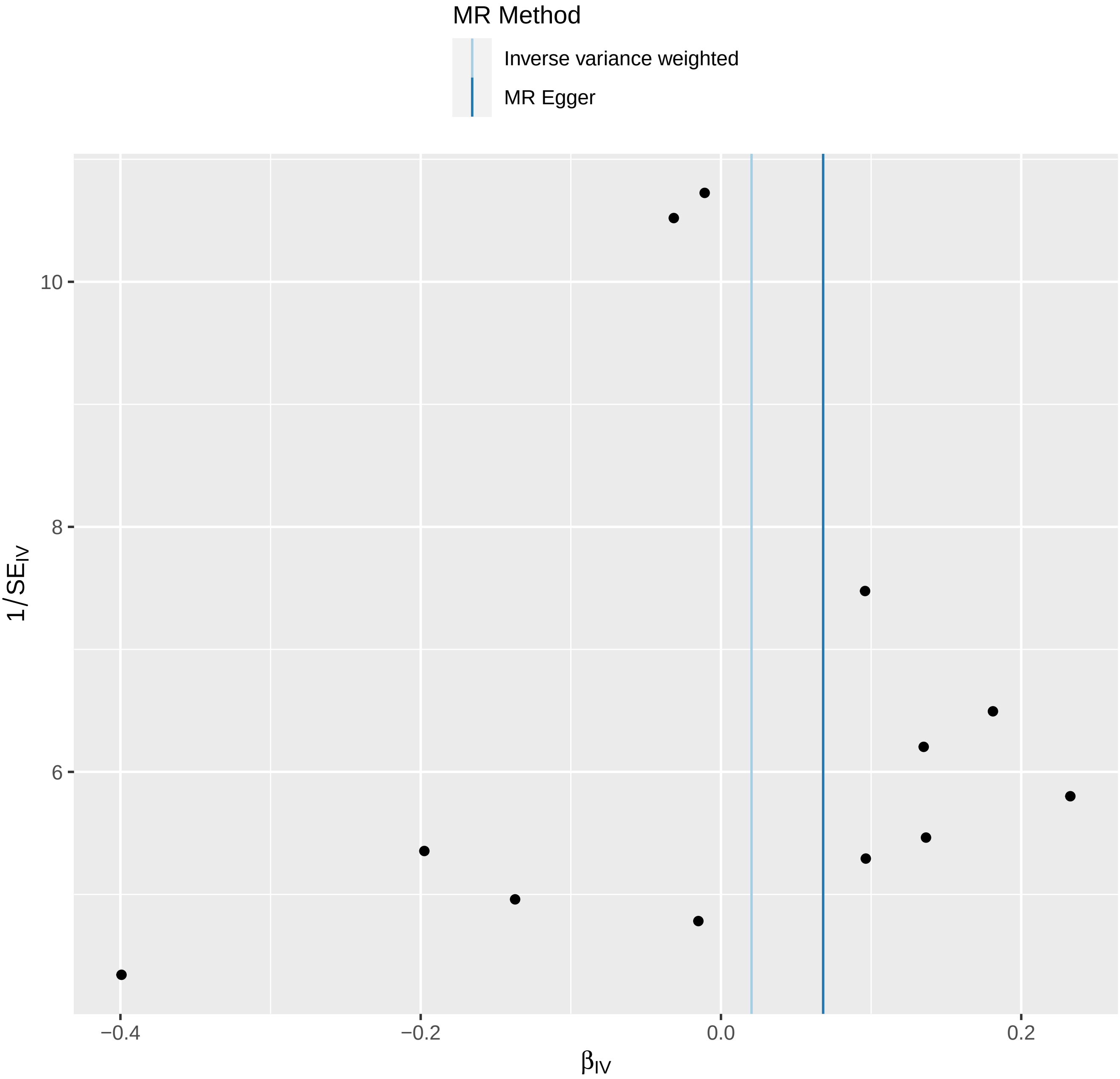

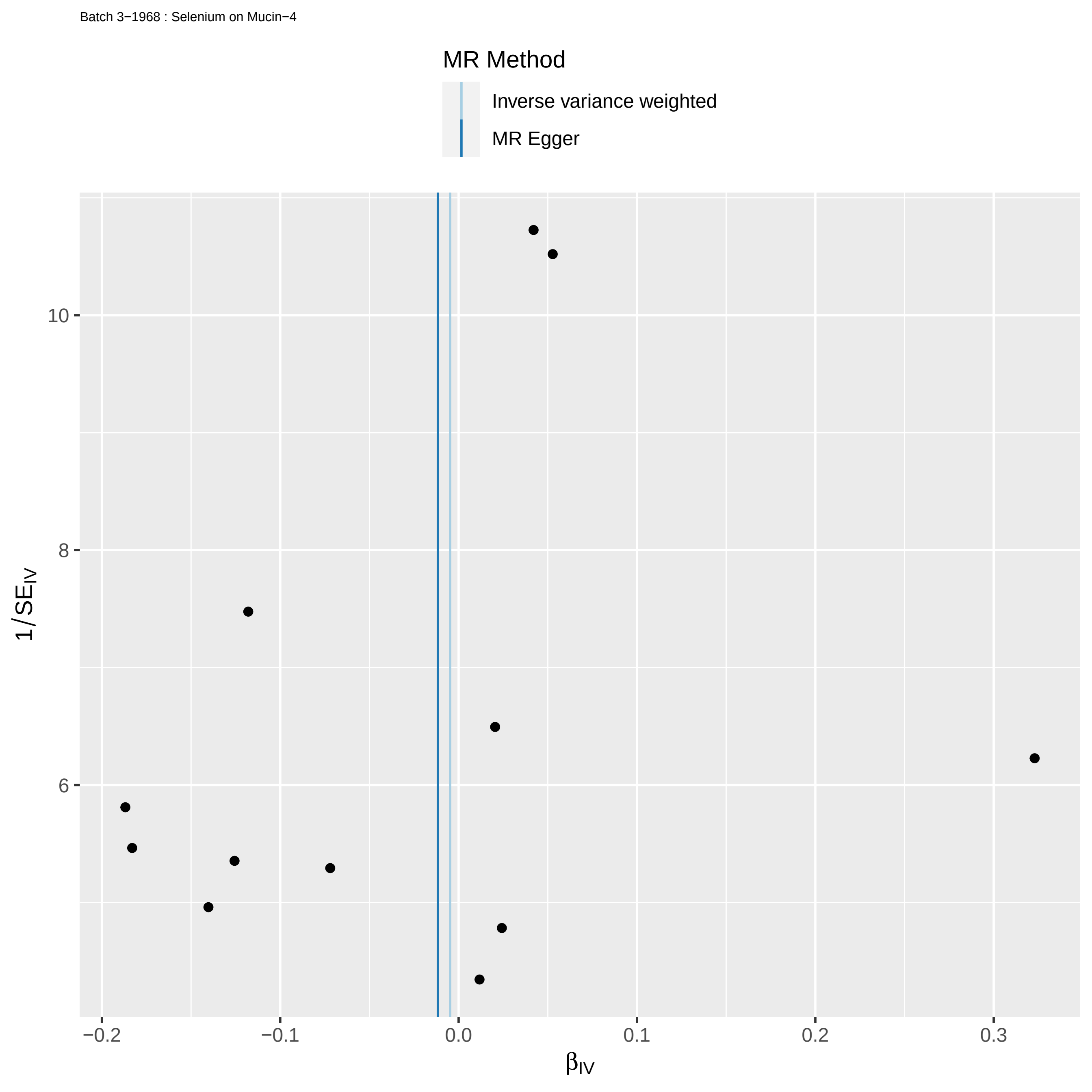
Batch 3−1968 : Selenium on Mucin−4
MR Method
Inverse variance weighted
MR Egger
10
8
6
1/SE$_{IV}$
−0.2
−0.1
0.0
0.1
0.2
0.3
β$_{IV}$

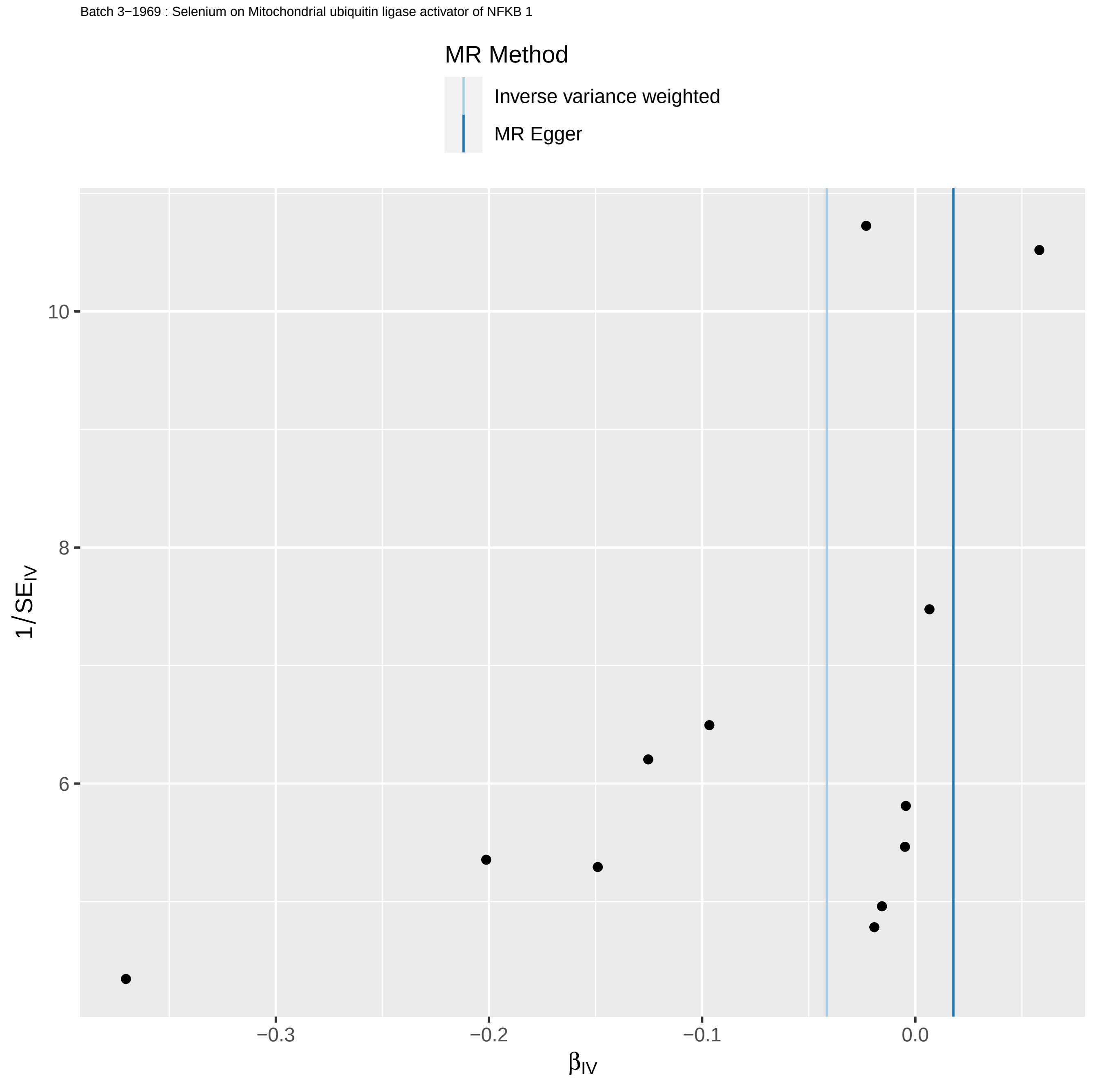
Batch 3−1969 : Selenium on Mitochondrial ubiquitin ligase activator of NFKB 1
MR Method
Inverse variance weighted
MR Egger
10
8
6
1/SE$_{IV}$
−0.3
−0.2
−0.1
0.0
β$_{IV}$

Batch 3−1970 : Selenium on Mitochondrial ubiquitin ligase activator of NFKB 1

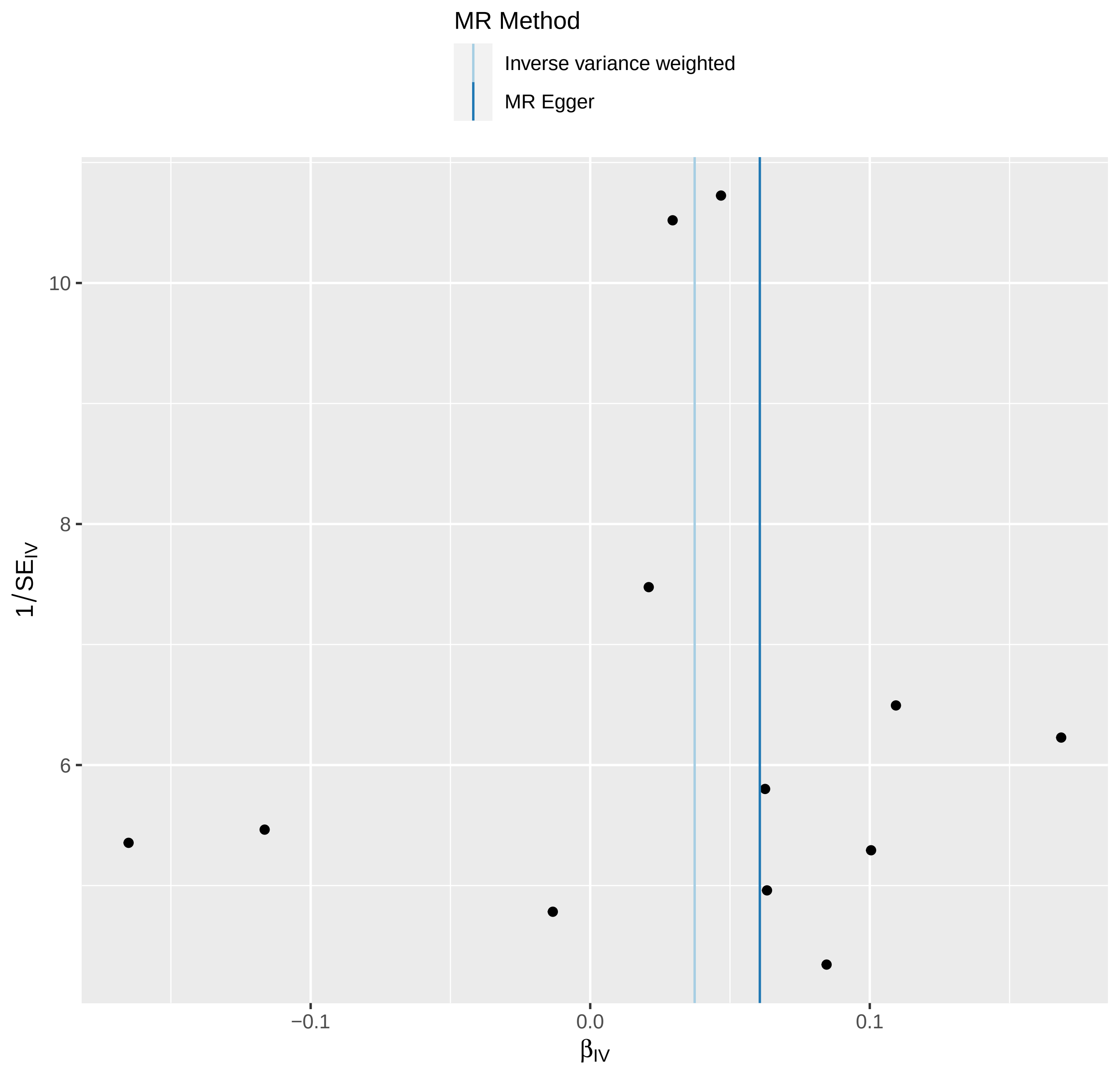

Batch 3−1971 : Selenium on Muscle, skeletal receptor tyrosine−protein kinase

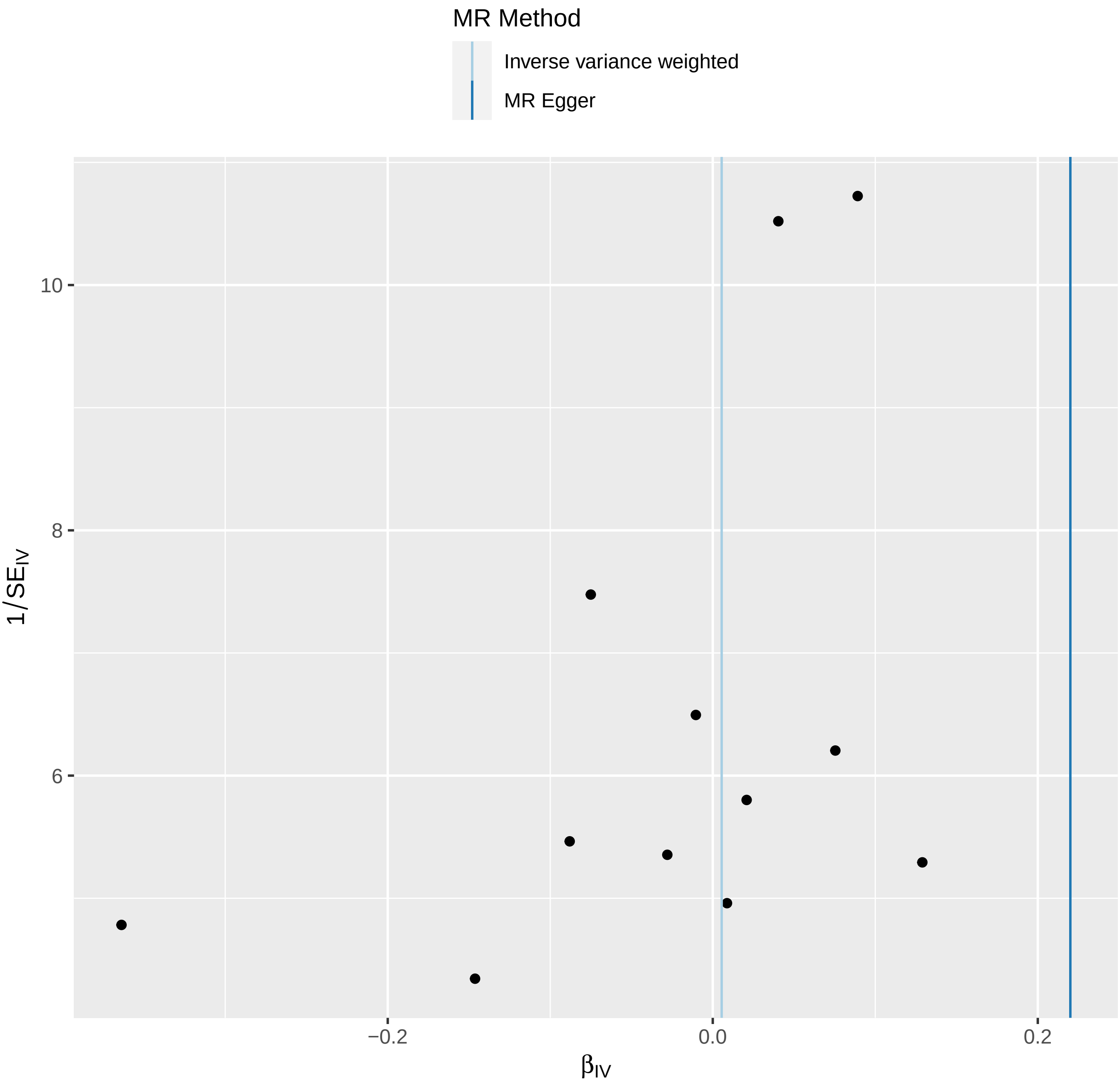

Batch 3−1972 : Selenium on A/G−specific adenine DNA glycosylase

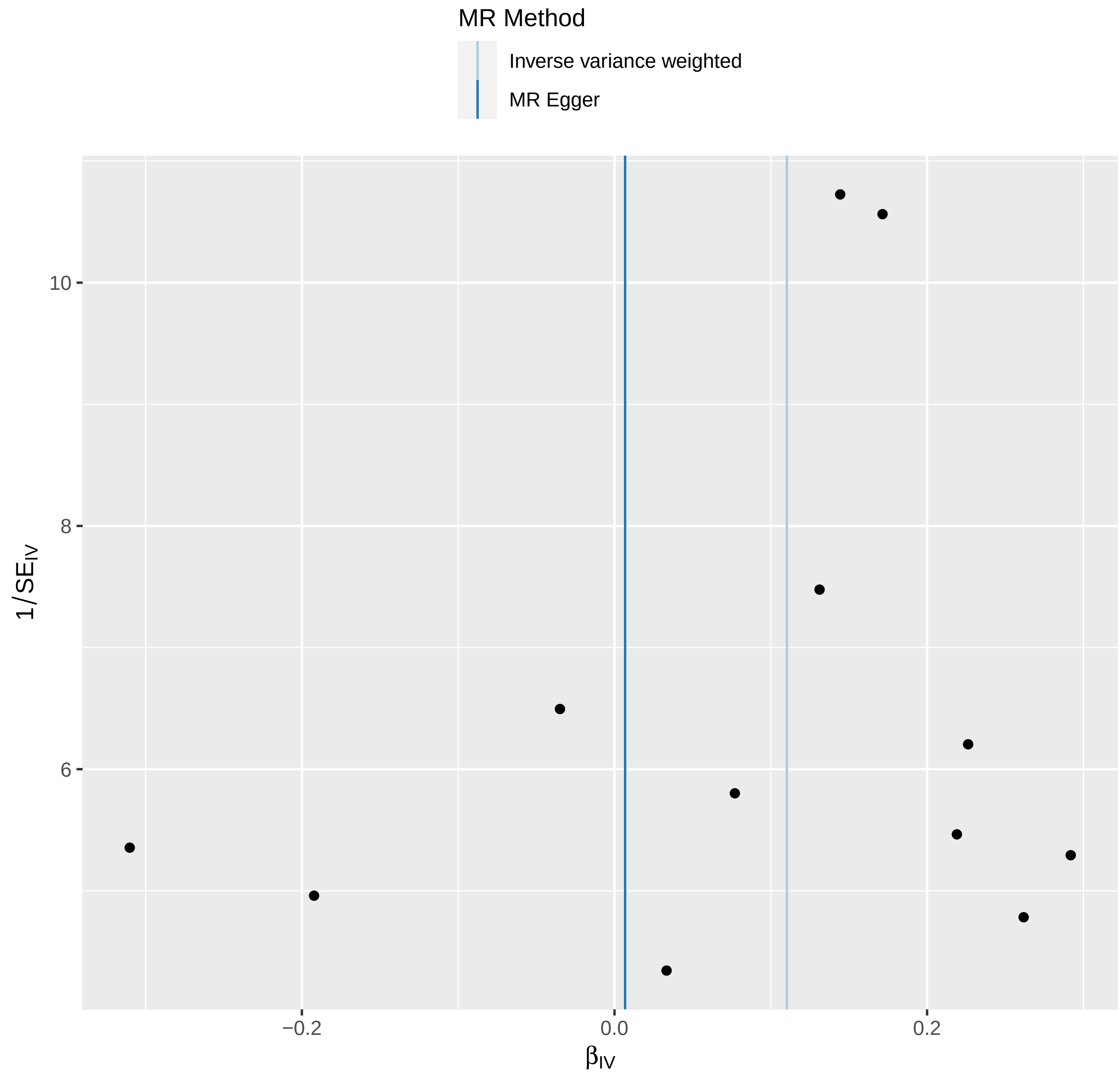

Batch 3−1973 : Selenium on MAX−interacting protein 1

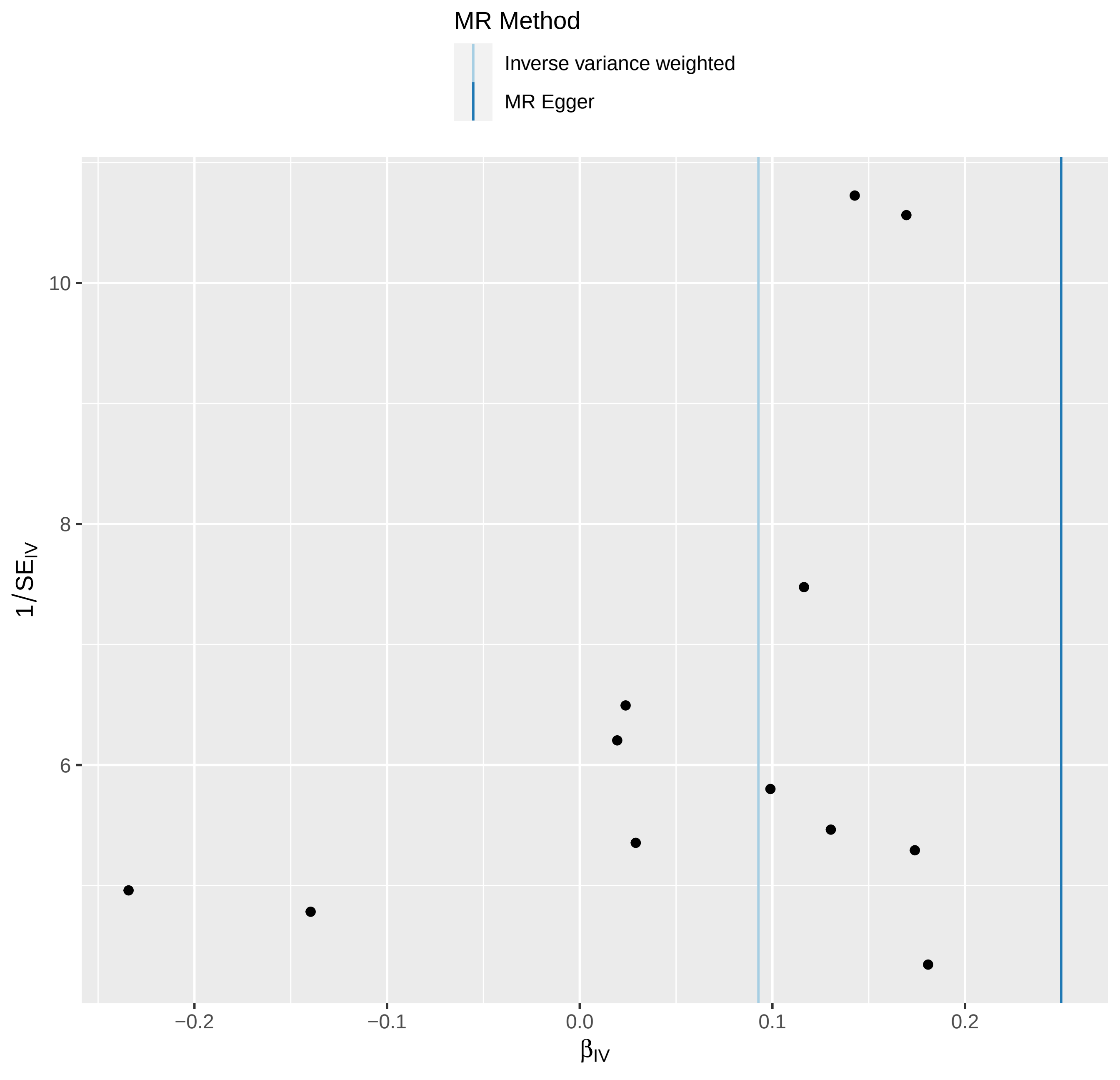

Batch 3−1974 : Selenium on Matrix−remodeling−associated protein 7

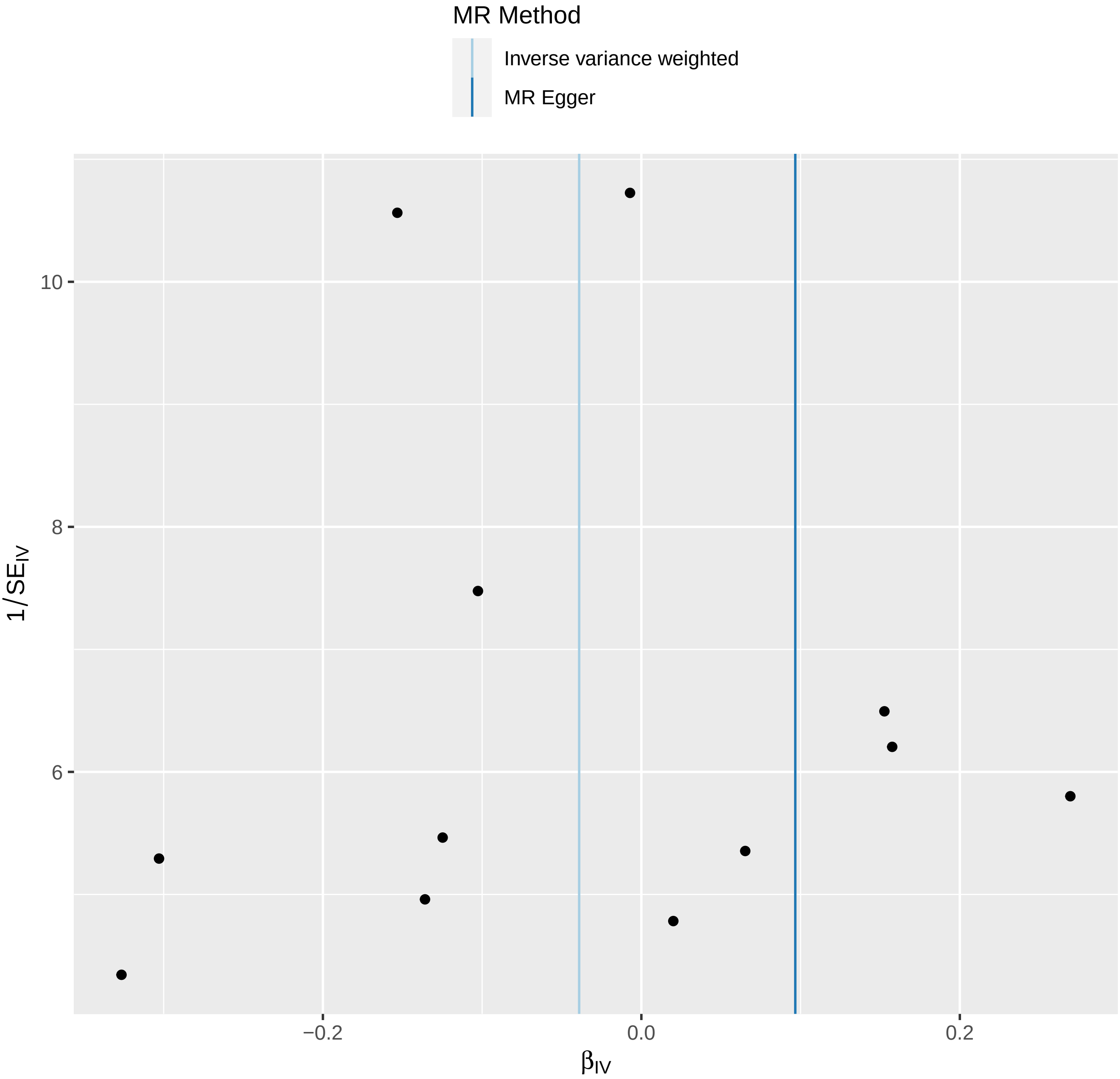

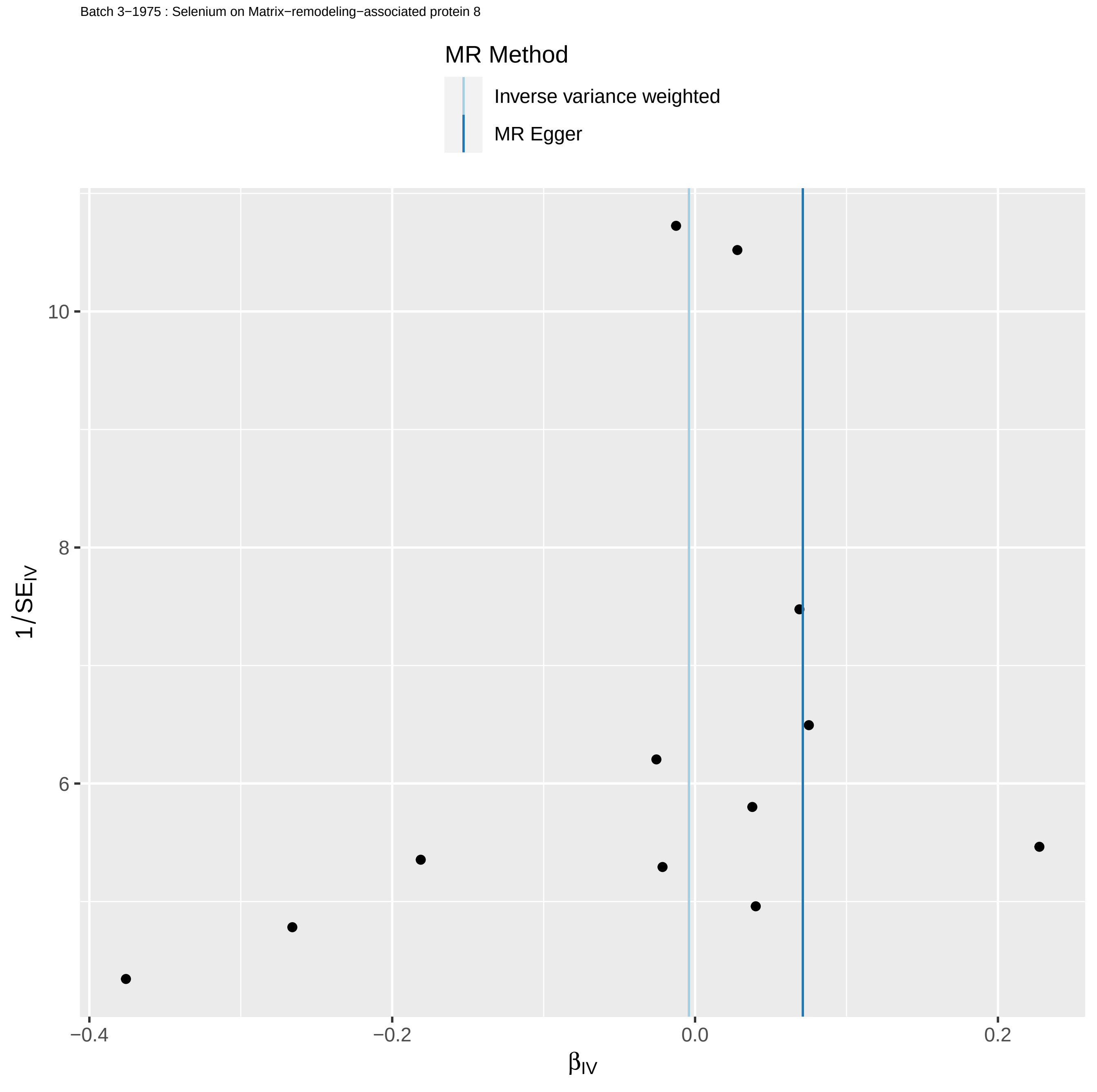

Batch 3−1975 : Selenium on Matrix−remodeling−associated protein 8
MR Method
Inverse variance weighted
MR Egger
10
8
6
−0.4
−0.2
0.0
0.2
1/SE_IV
β_IV

Batch 3−1976 : Selenium on Matrix−remodeling−associated protein 8

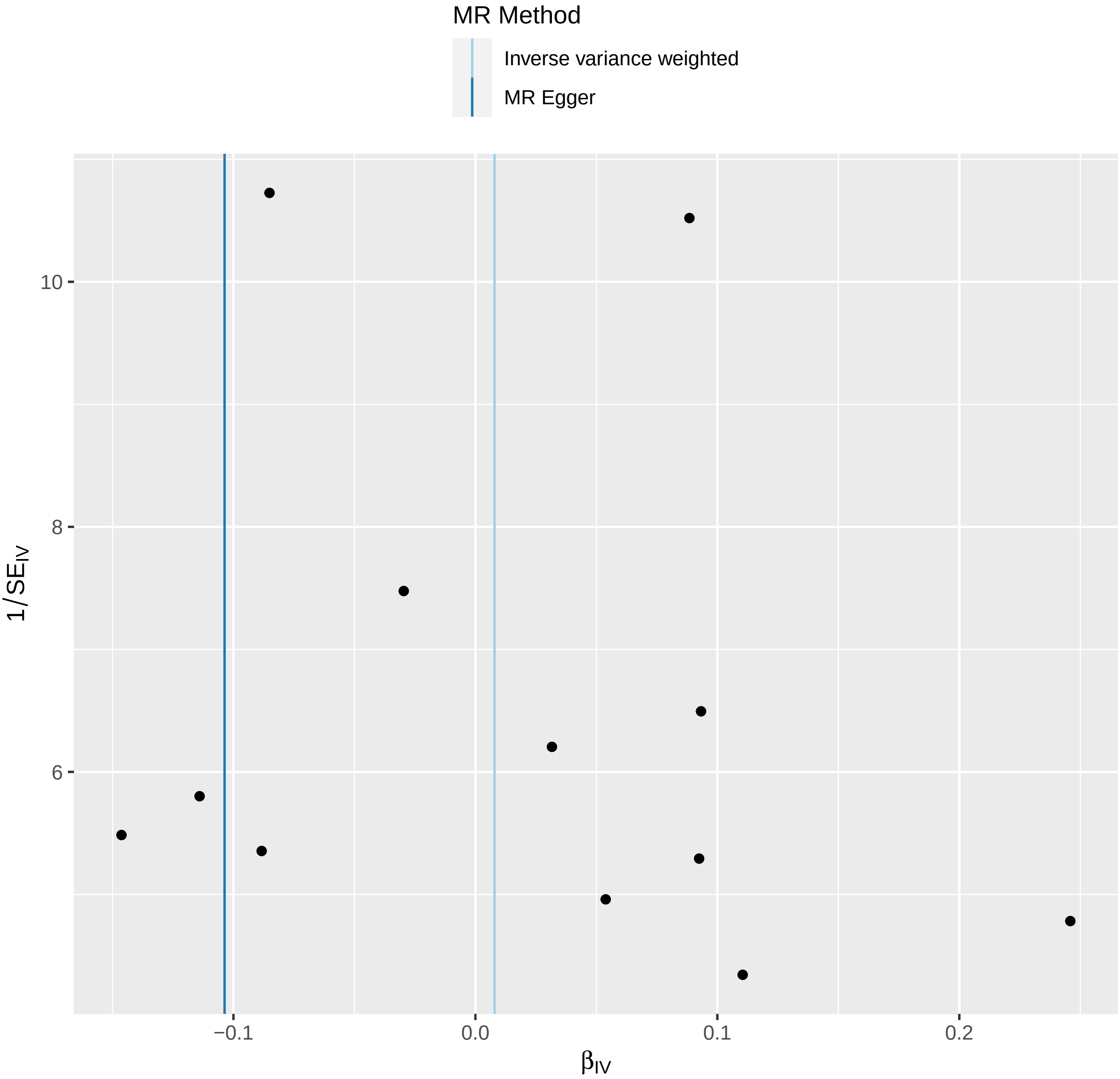

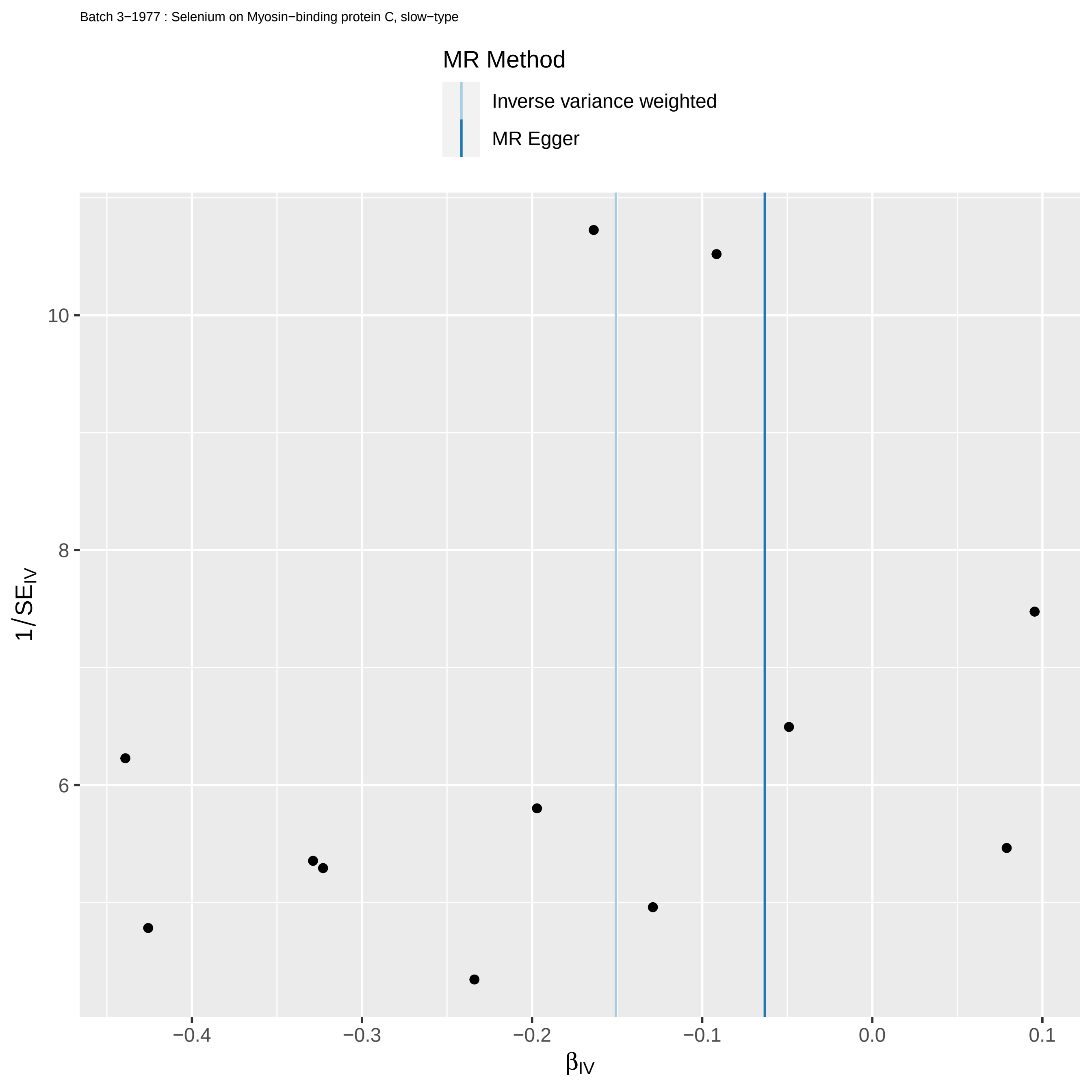
Batch 3−1977 : Selenium on Myosin−binding protein C, slow−type
MR Method
Inverse variance weighted
MR Egger
10
8
6
1/SE$_{IV}$
−0.4
−0.3
−0.2
−0.1
0.0
0.1
β$_{IV}$

Batch 3−1978 : Selenium on Myc proto−oncogene protein

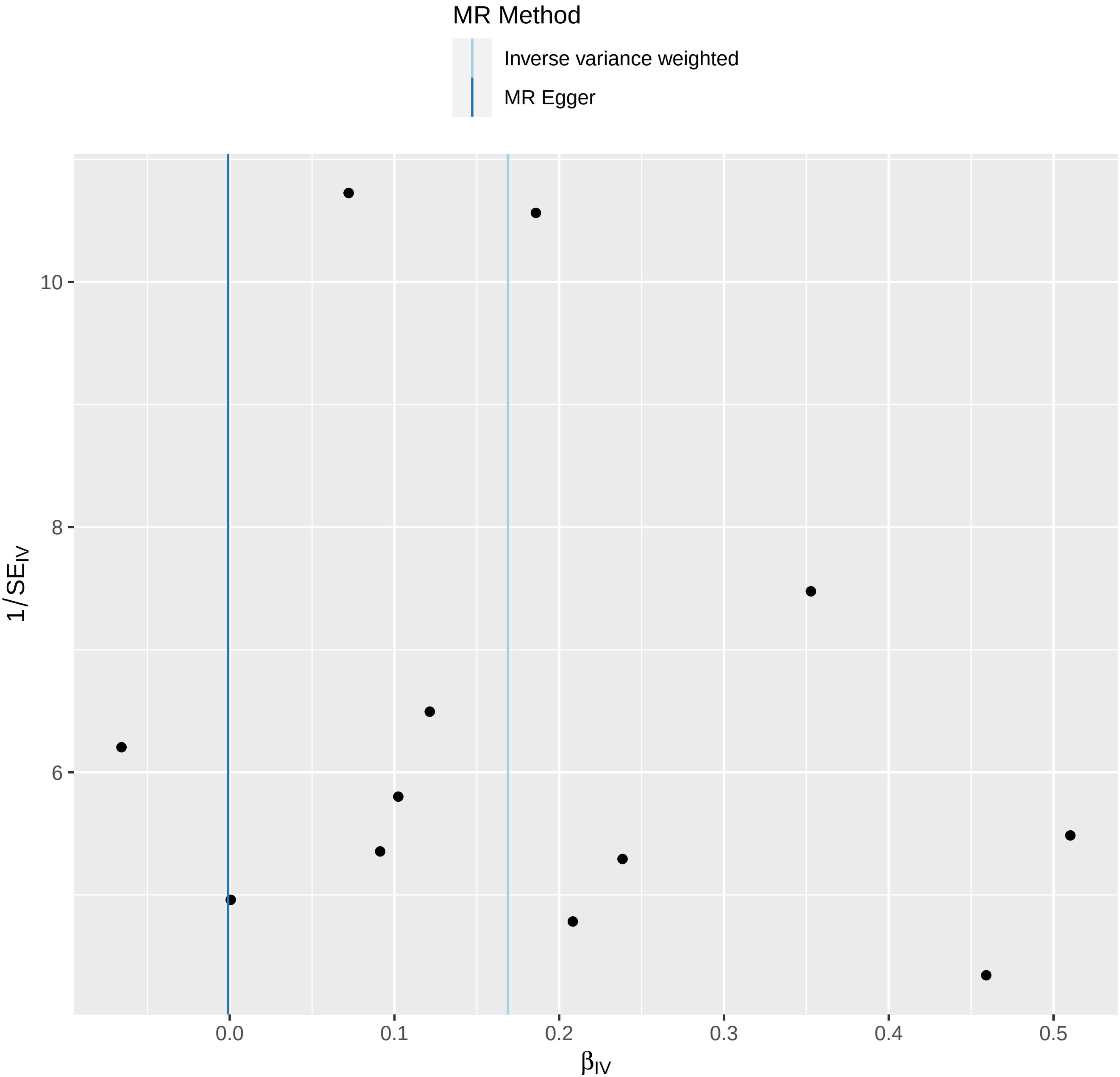

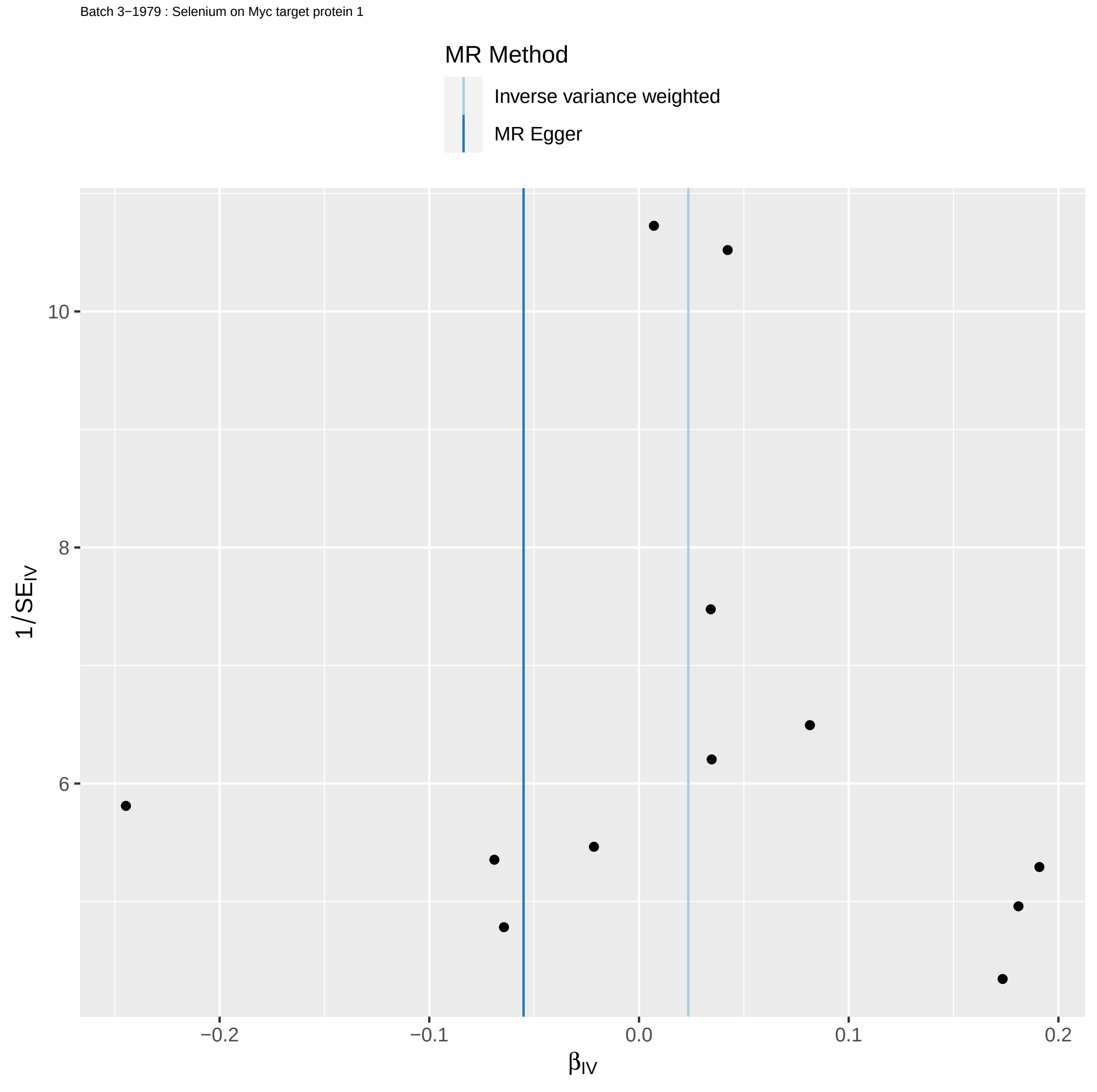

Batch 3−1979 : Selenium on Myc target protein 1
MR Method
Inverse variance weighted
MR Egger
10
8
6
1/SE$_{IV}$
−0.2
−0.1
0.0
0.1
0.2
β$_{IV}$

Batch 3−1980 : Selenium on Unconventional myosin−VI

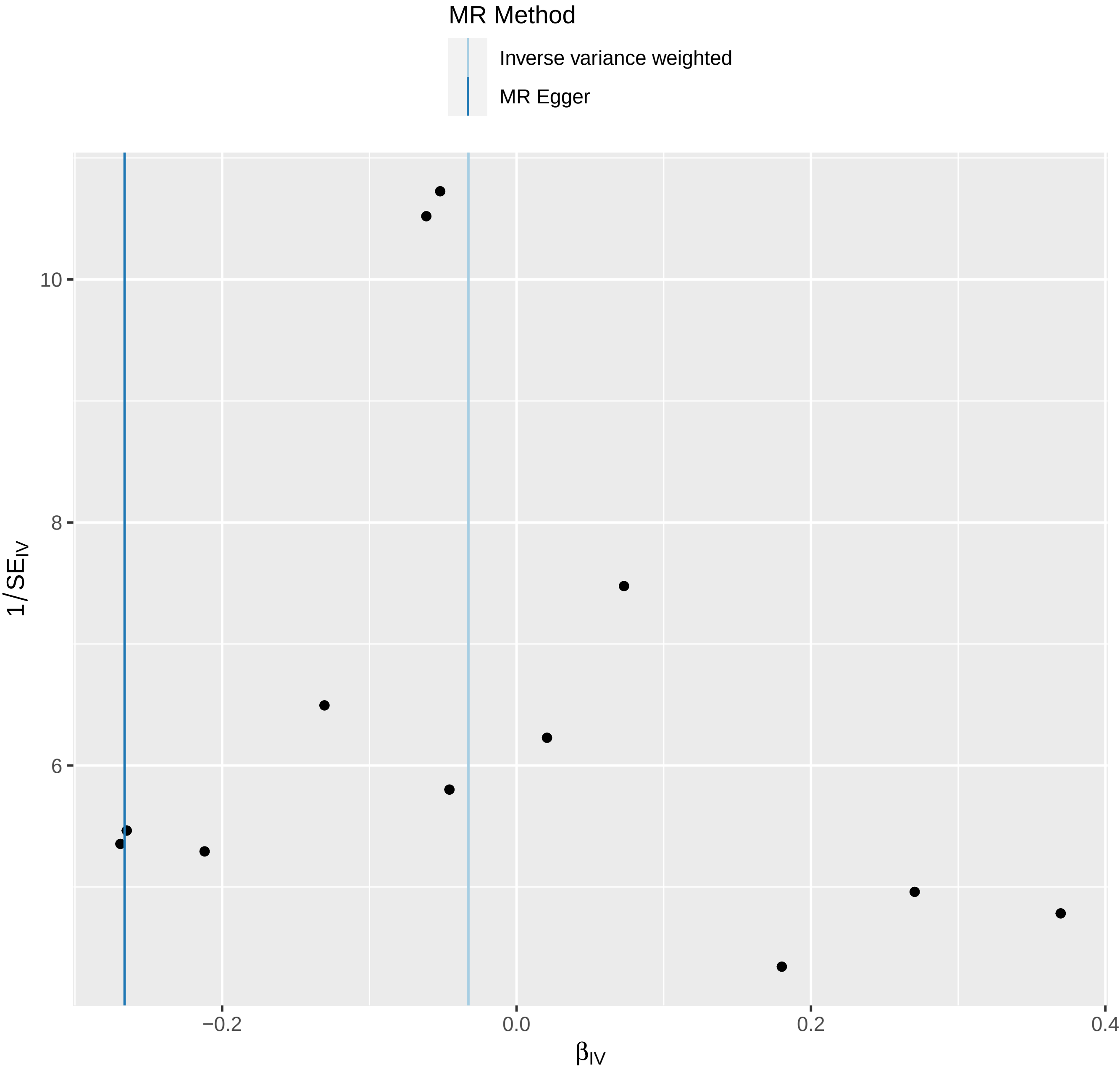

Batch 3−1981 : Selenium on Myomesin−2

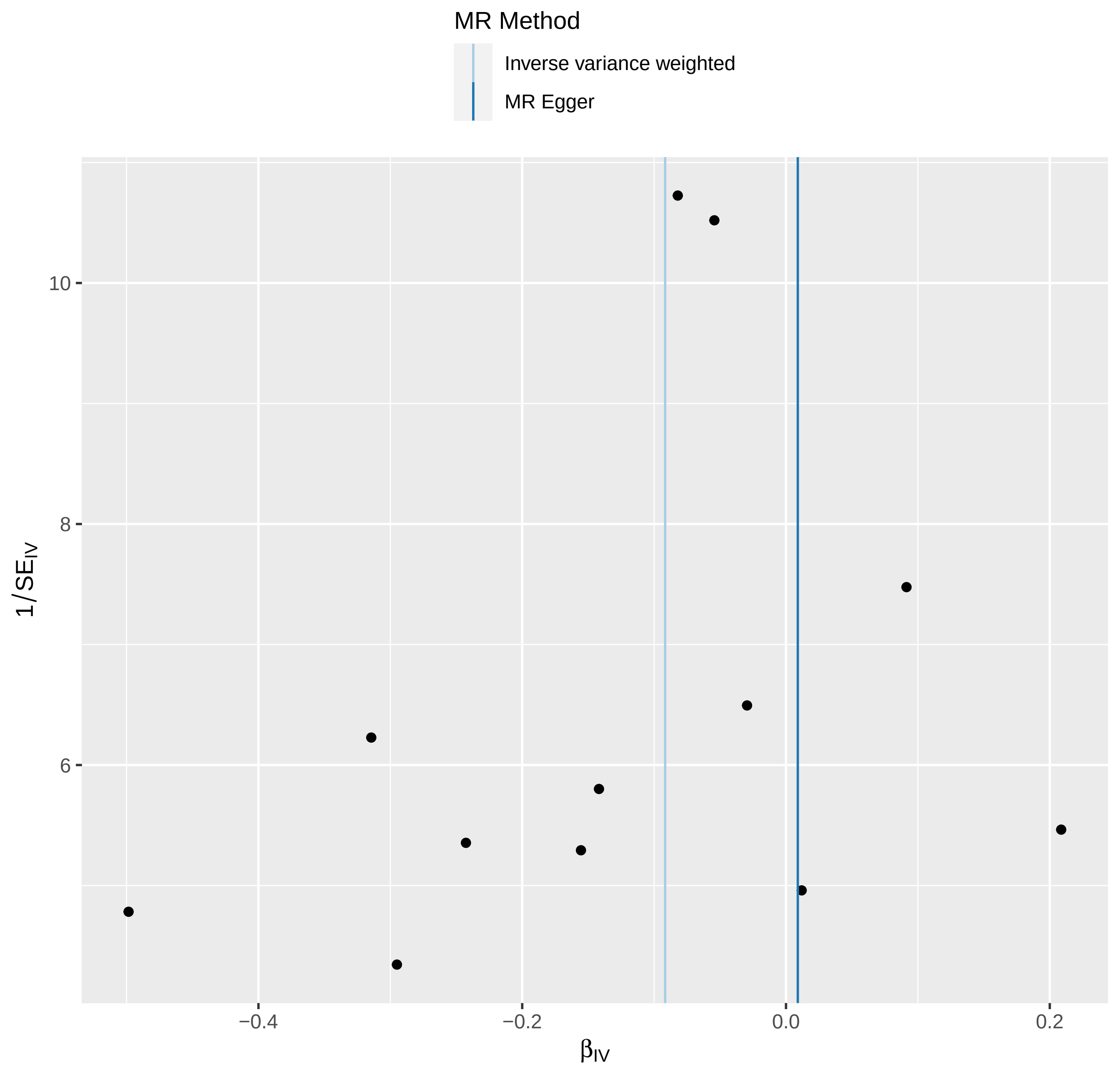

Batch 3−1982 : Selenium on Myelin regulatory factor

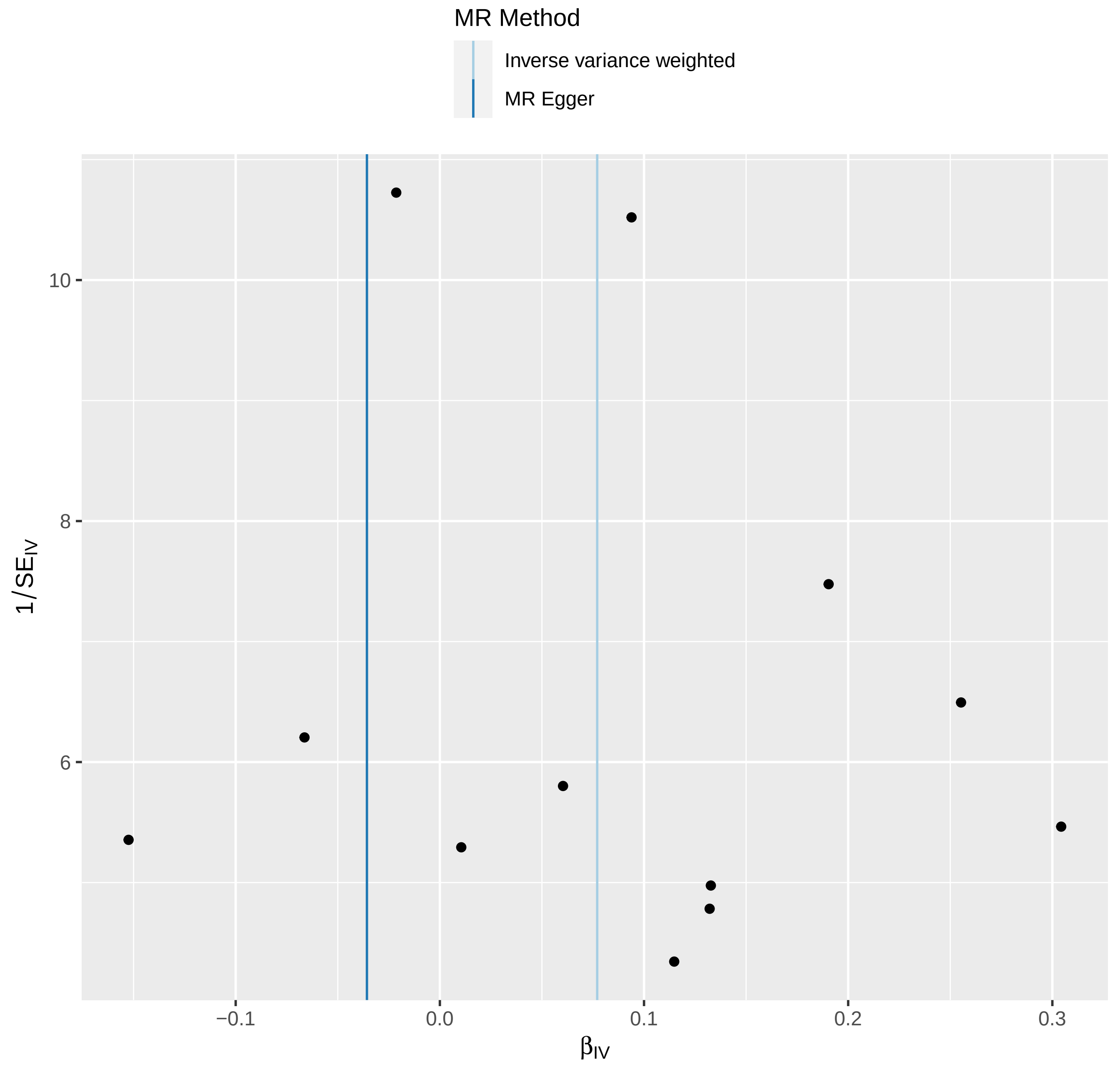

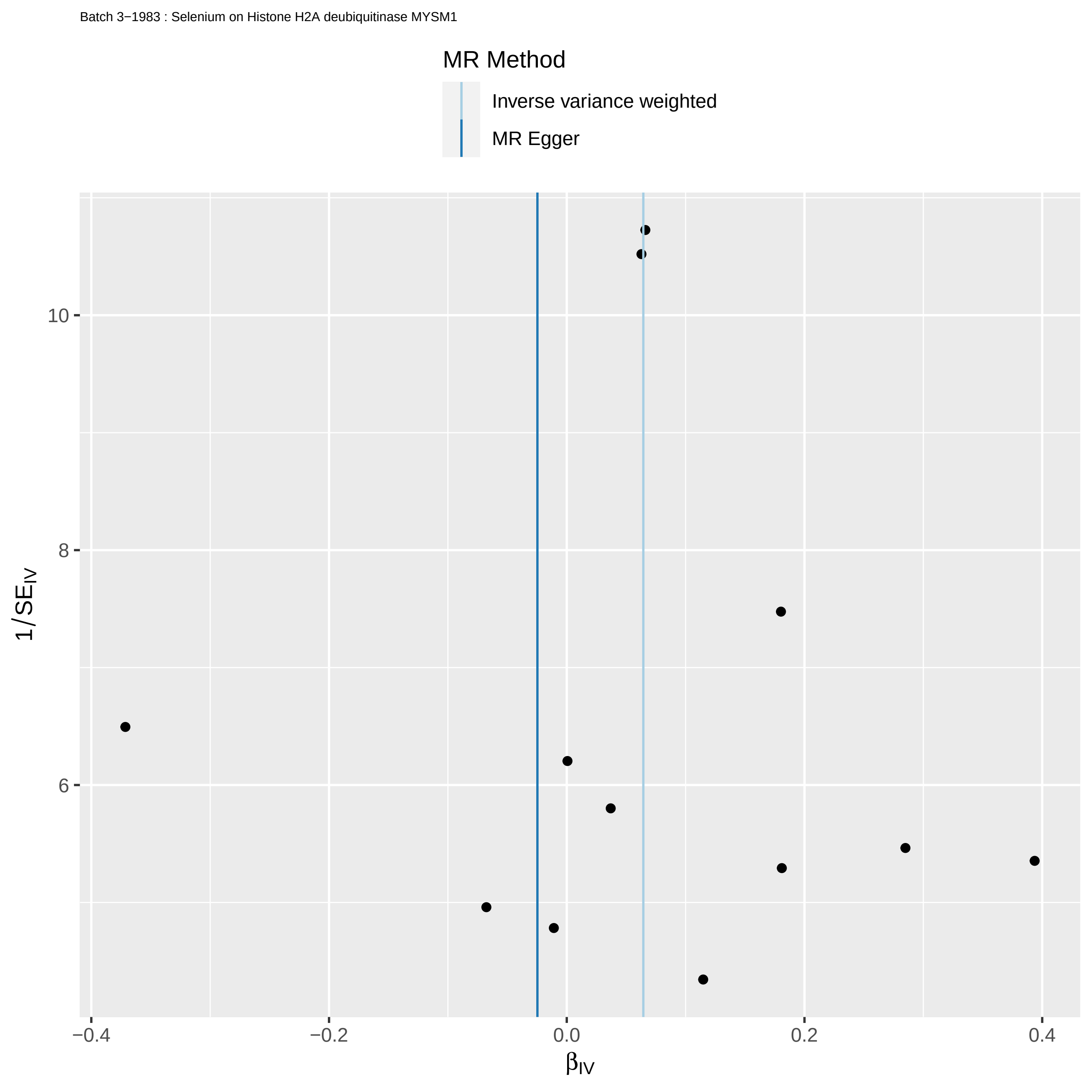
Batch 3−1983 : Selenium on Histone H2A deubiquitinase MYSM1
MR Method
Inverse variance weighted
MR Egger
10
8
6
1/SE_IV
−0.4
−0.2
0.0
0.2
0.4
β_IV

Batch 3−1984 : Selenium on Myocardial zonula adherens protein

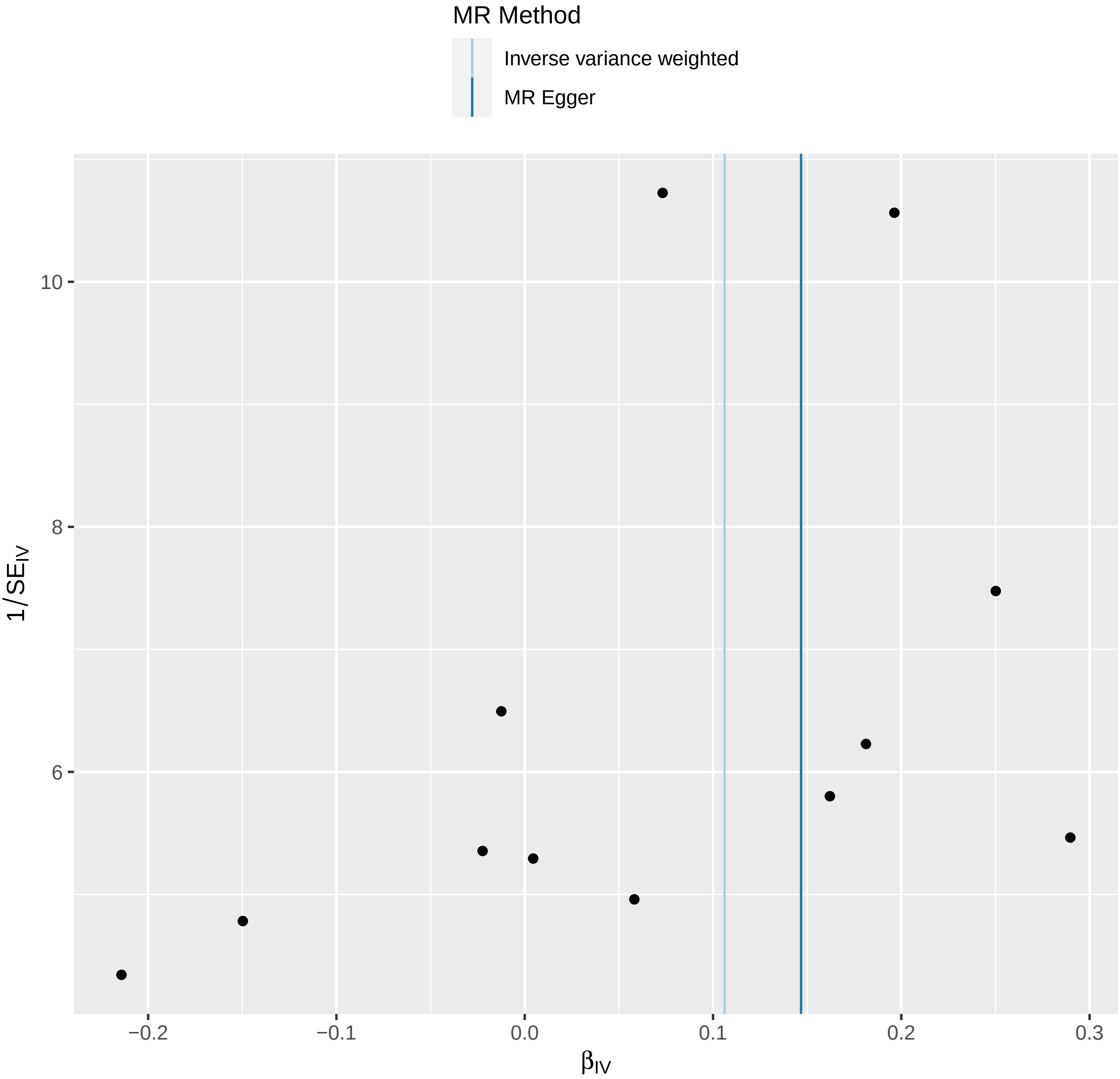

Batch 3−1985 : Selenium on Myocardial zonula adherens protein

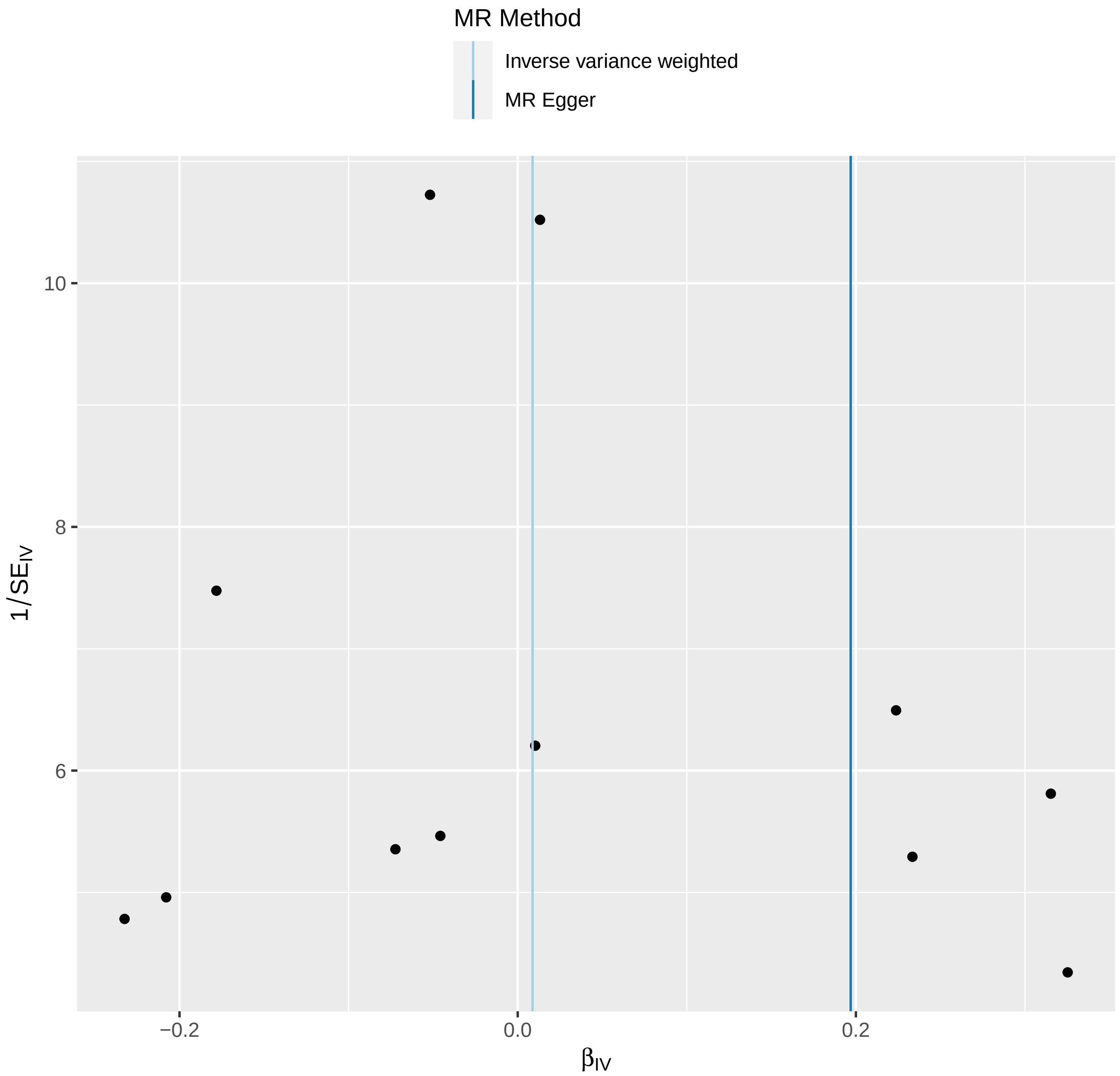

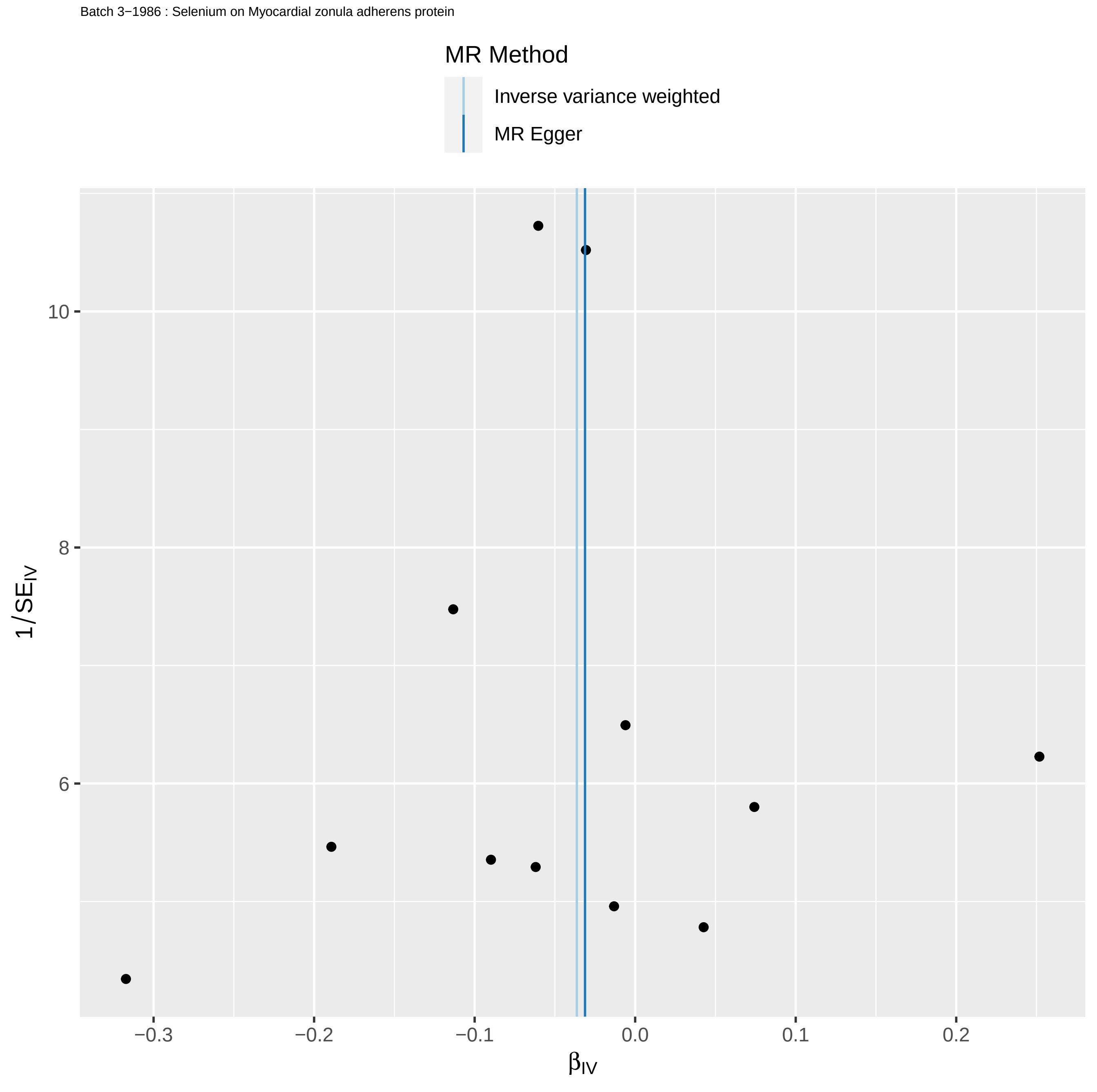
Batch 3−1986 : Selenium on Myocardial zonula adherens protein
MR Method
Inverse variance weighted
MR Egger
10
8
6
1/SE$_{IV}$
−0.3
−0.2
−0.1
0.0
0.1
0.2
β_{IV}

Batch 3−1987 : Selenium on Myeloid zinc finger 1

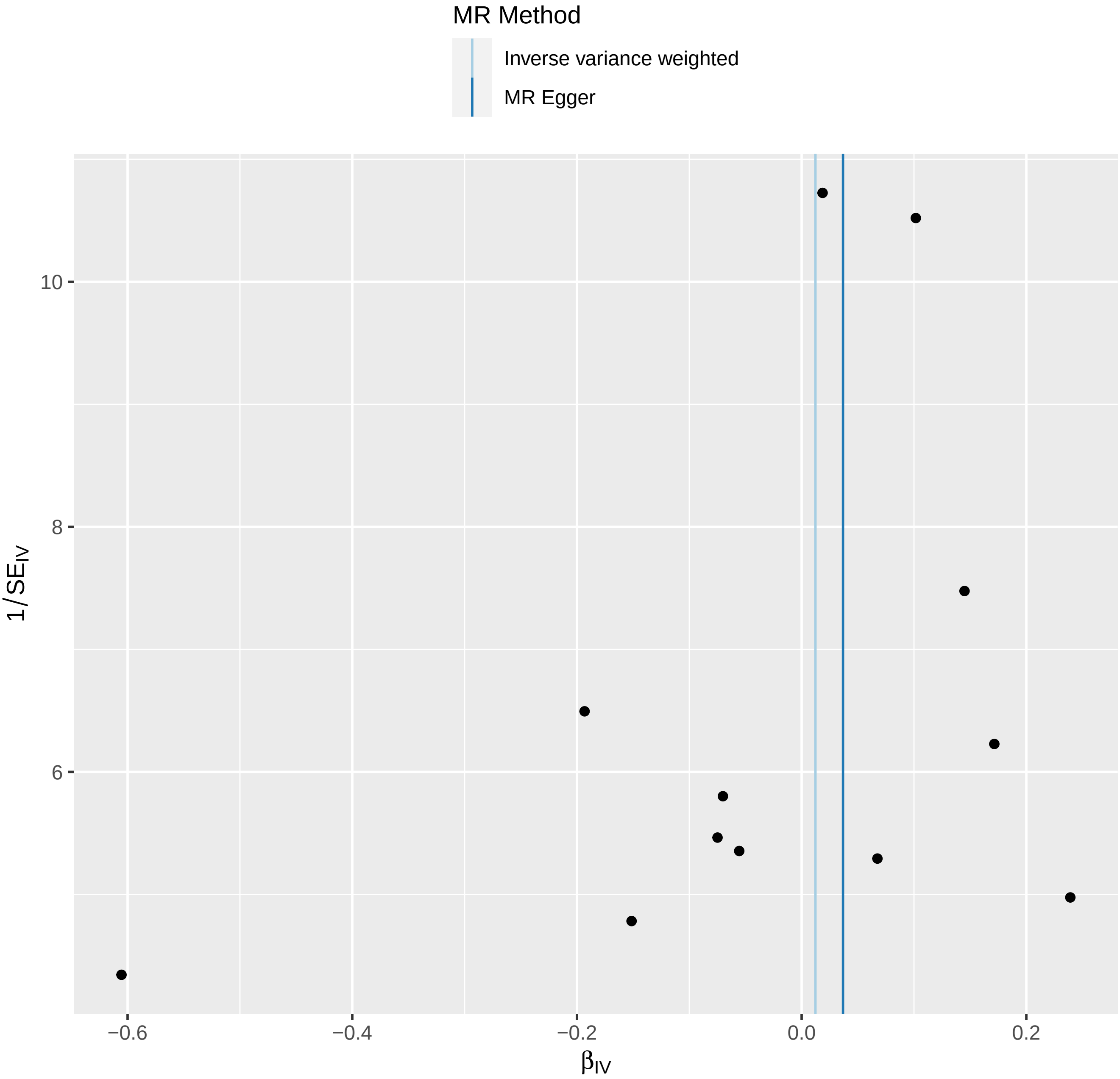

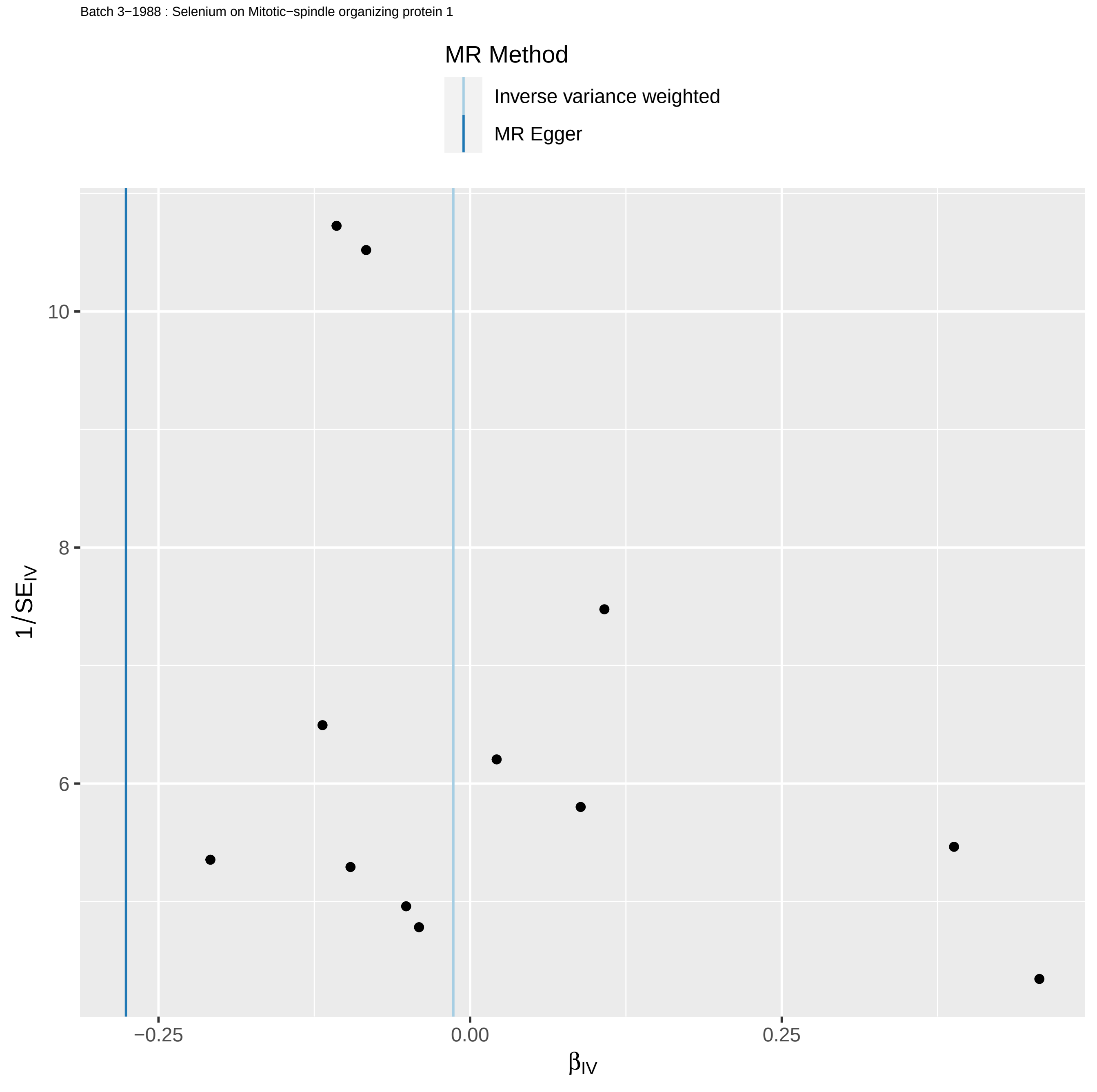
Batch 3−1988 : Selenium on Mitotic−spindle organizing protein 1
MR Method
Inverse variance weighted
MR Egger
10
8
6
1/SE_IV
−0.25
0.00
0.25
β_IV

Batch 3−1989 : Selenium on HemK methyltransferase family member 2

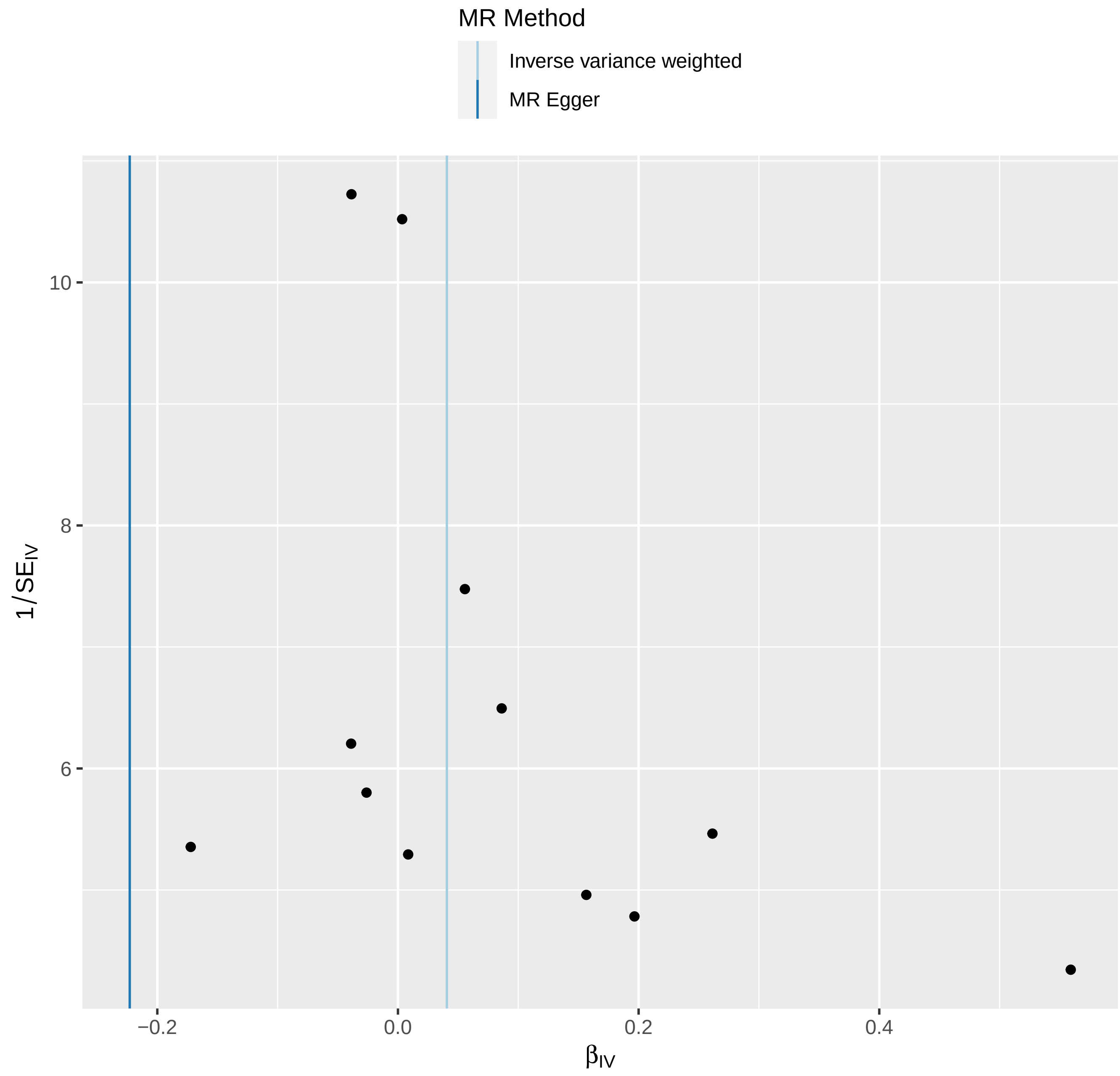

Batch 3−1990 : Selenium on N−acylethanolamine−hydrolyzing acid amidase

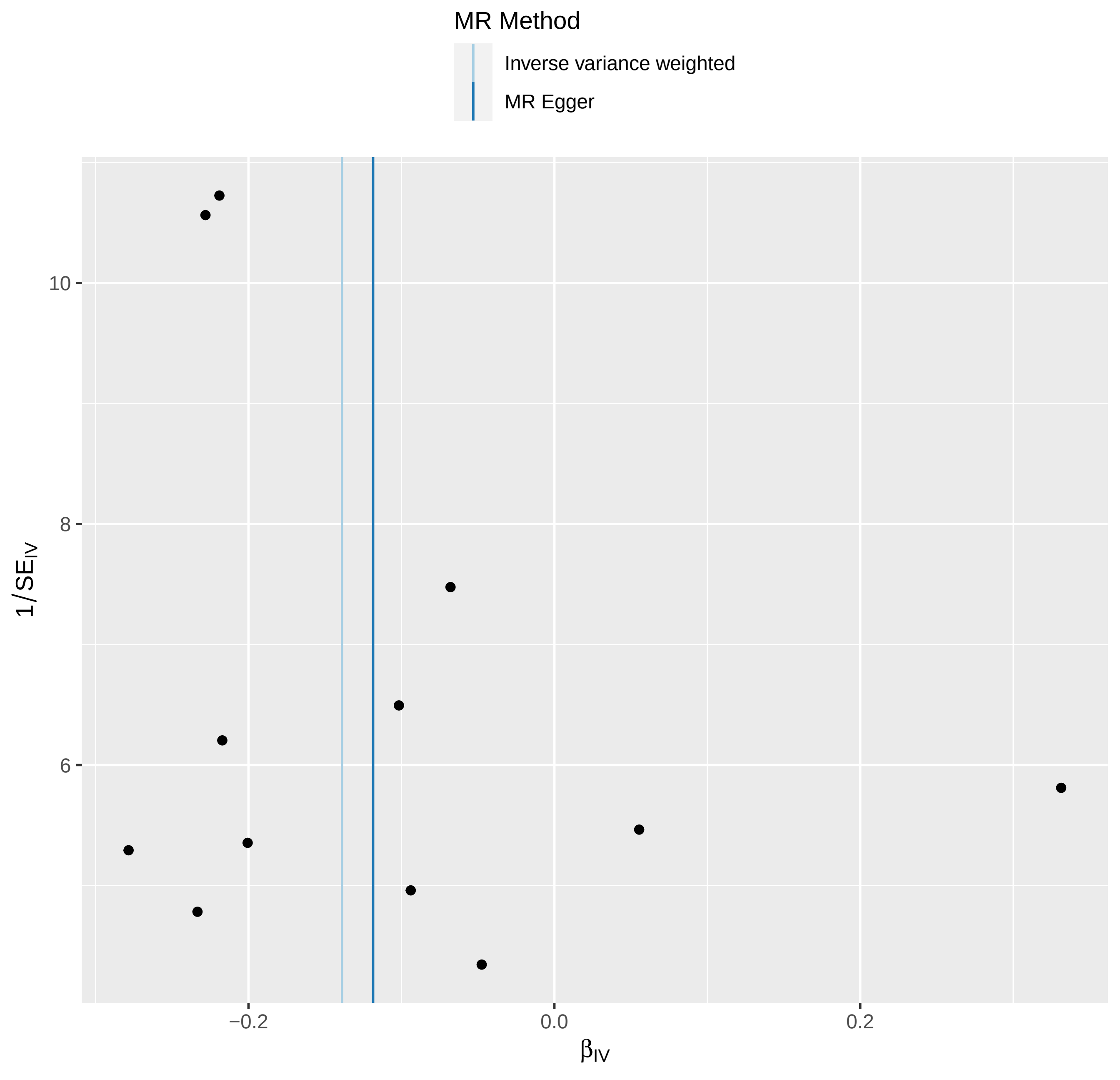

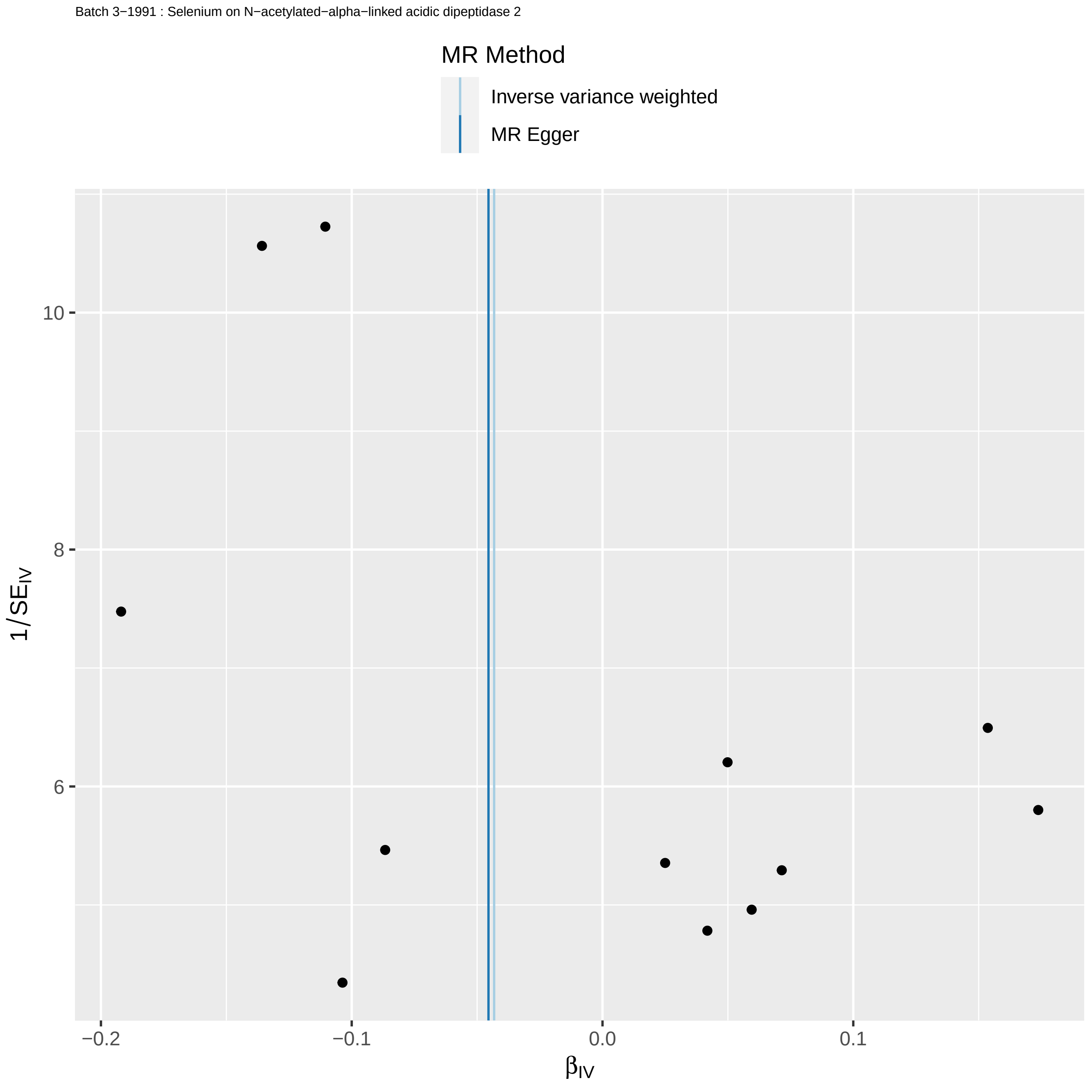
Batch 3−1991 : Selenium on N−acetylated−alpha−linked acidic dipeptidase 2
MR Method
Inverse variance weighted
MR Egger
10
8
6
1/SE$_{IV}$
−0.2
−0.1
0.0
0.1
β$_{IV}$

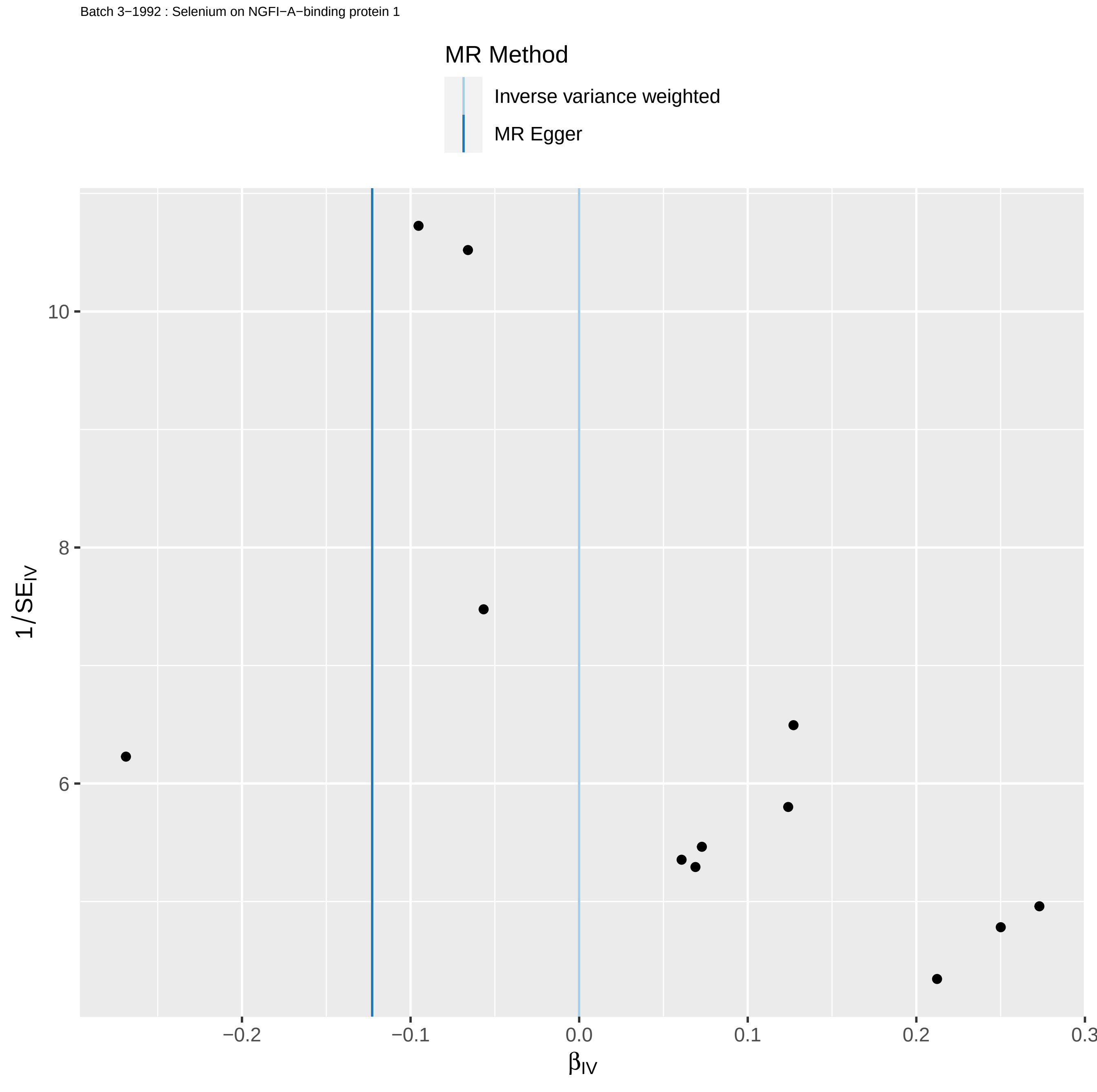

Batch 3−1992 : Selenium on NGFI−A−binding protein 1
MR Method
Inverse variance weighted
MR Egger
10
8
6
1/SE_IV
−0.2
−0.1
0.0
0.1
0.2
0.3
β_IV

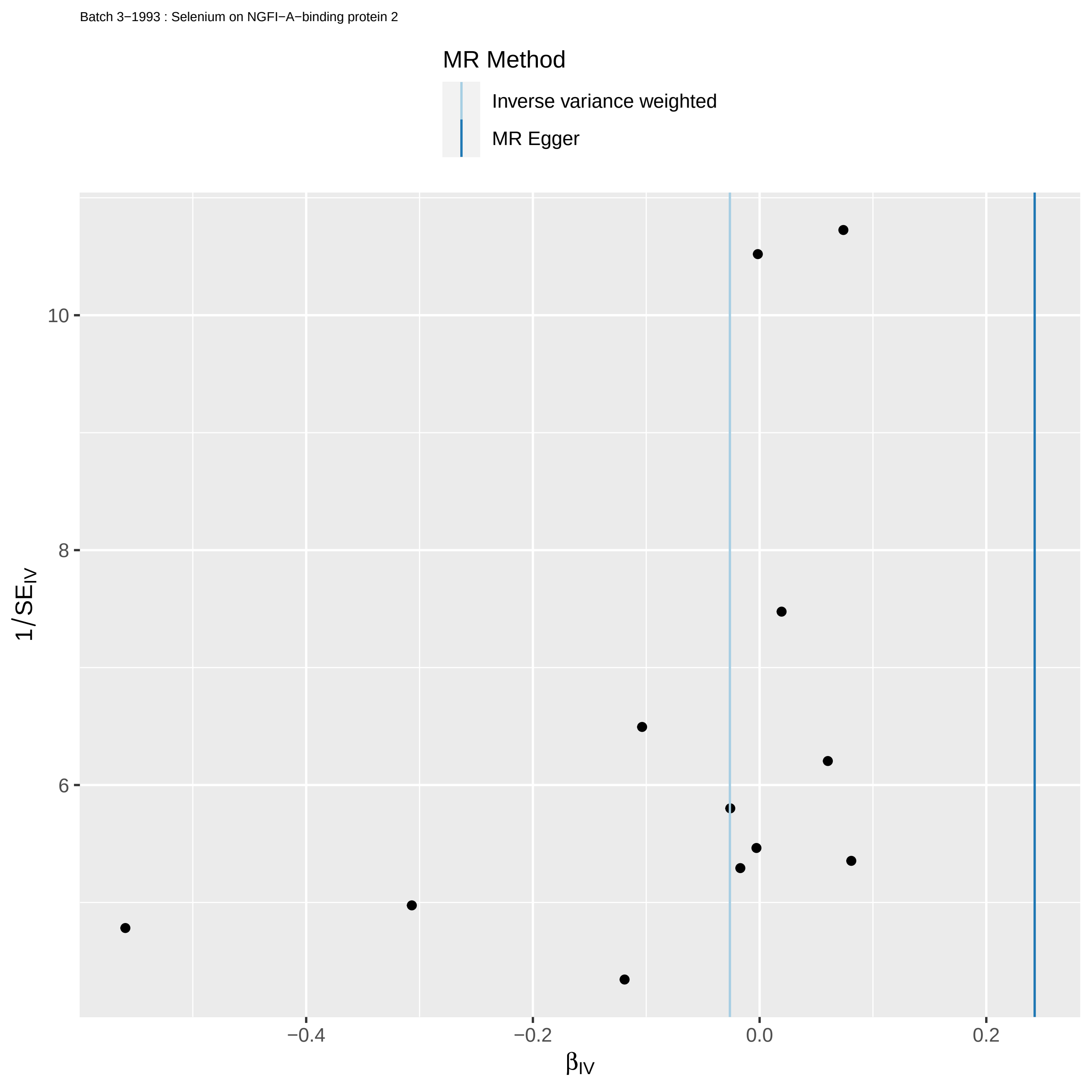

Batch 3−1993 : Selenium on NGFI−A−binding protein 2
MR Method
Inverse variance weighted
MR Egger
10
8
6
1/SE_IV
−0.4
−0.2
0.0
0.2
β_IV

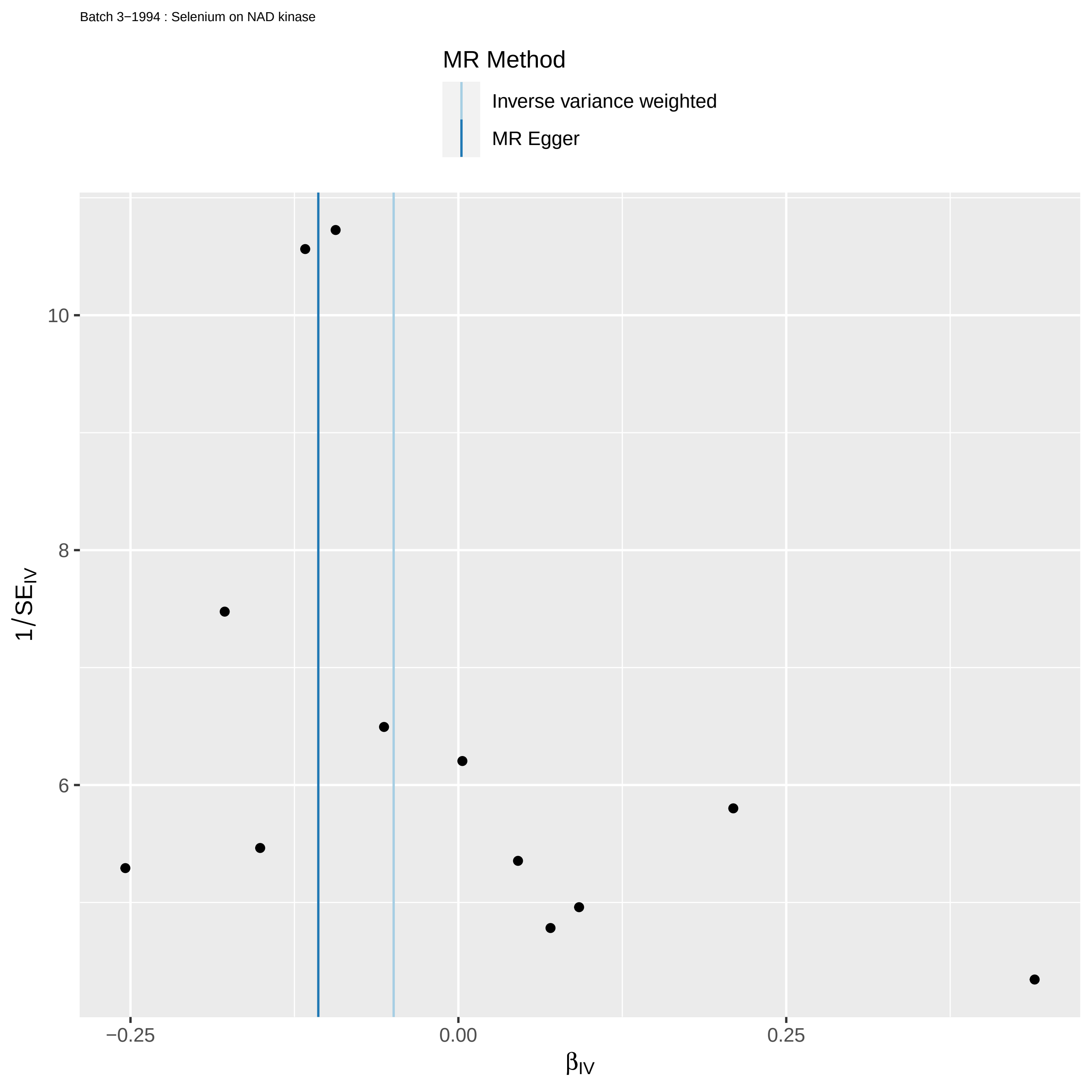
Batch 3−1994 : Selenium on NAD kinase
MR Method
Inverse variance weighted
MR Egger
10
8
6
1/SE_IV
−0.25
0.00
0.25
β_IV

Batch 3−1995 : Selenium on N−acetyl−D−glucosamine kinase

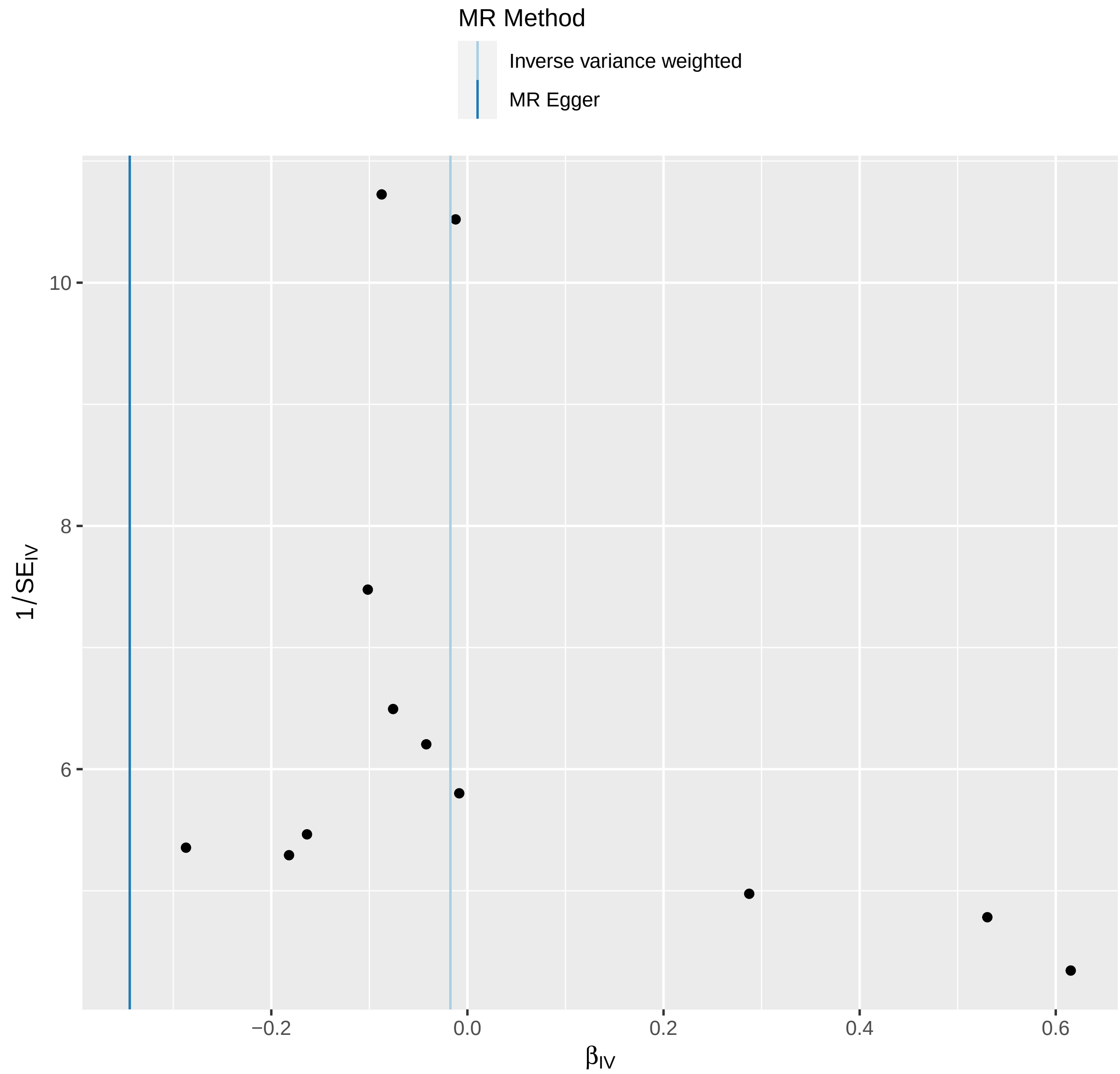

Batch 3−1996 : Selenium on N−acetylglucosamine−1−phosphodiester alpha−N−acetylglucosaminidase

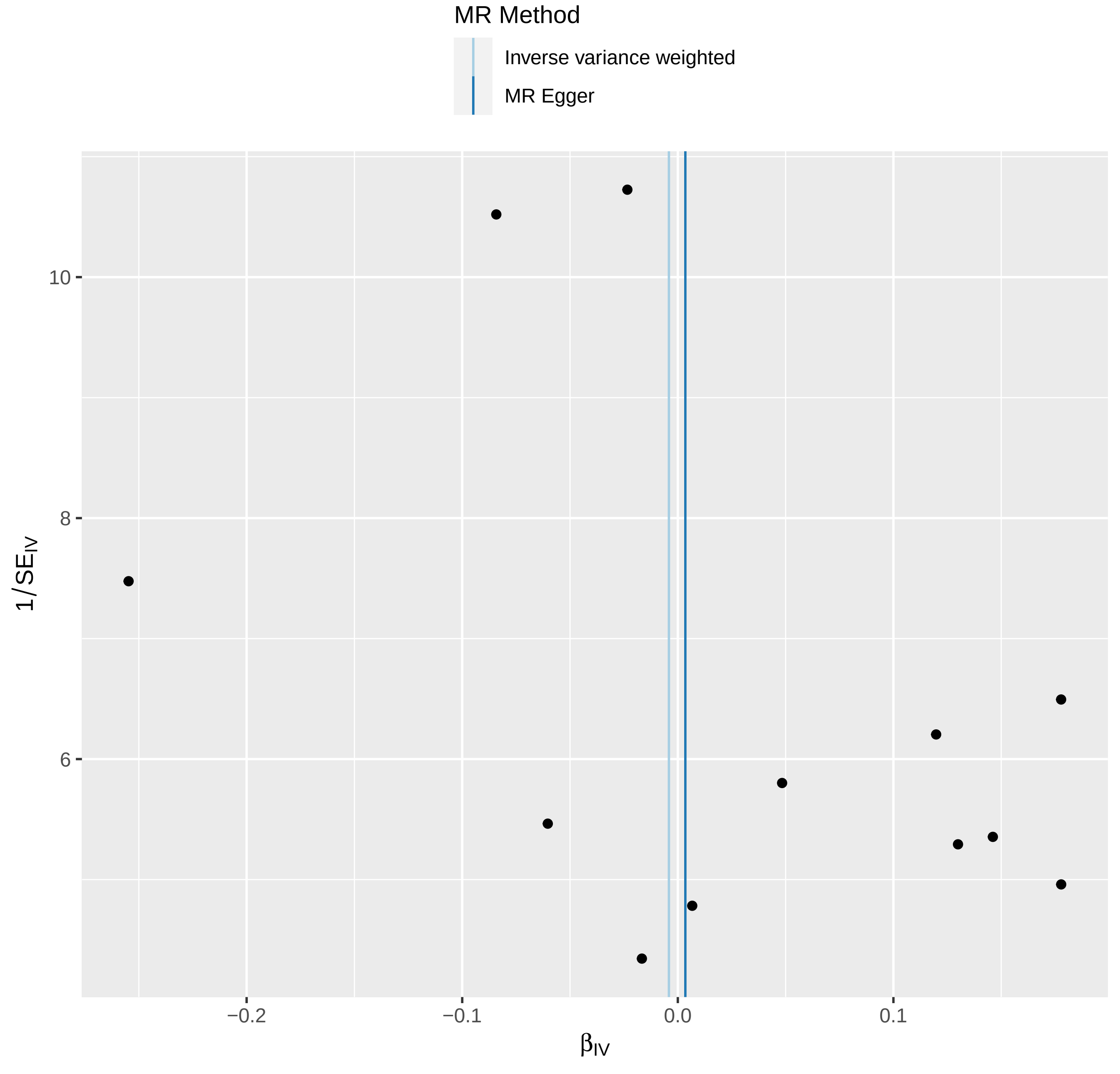

Batch 3−1997 : Selenium on N−acetylglutamate synthase, mitochondrial

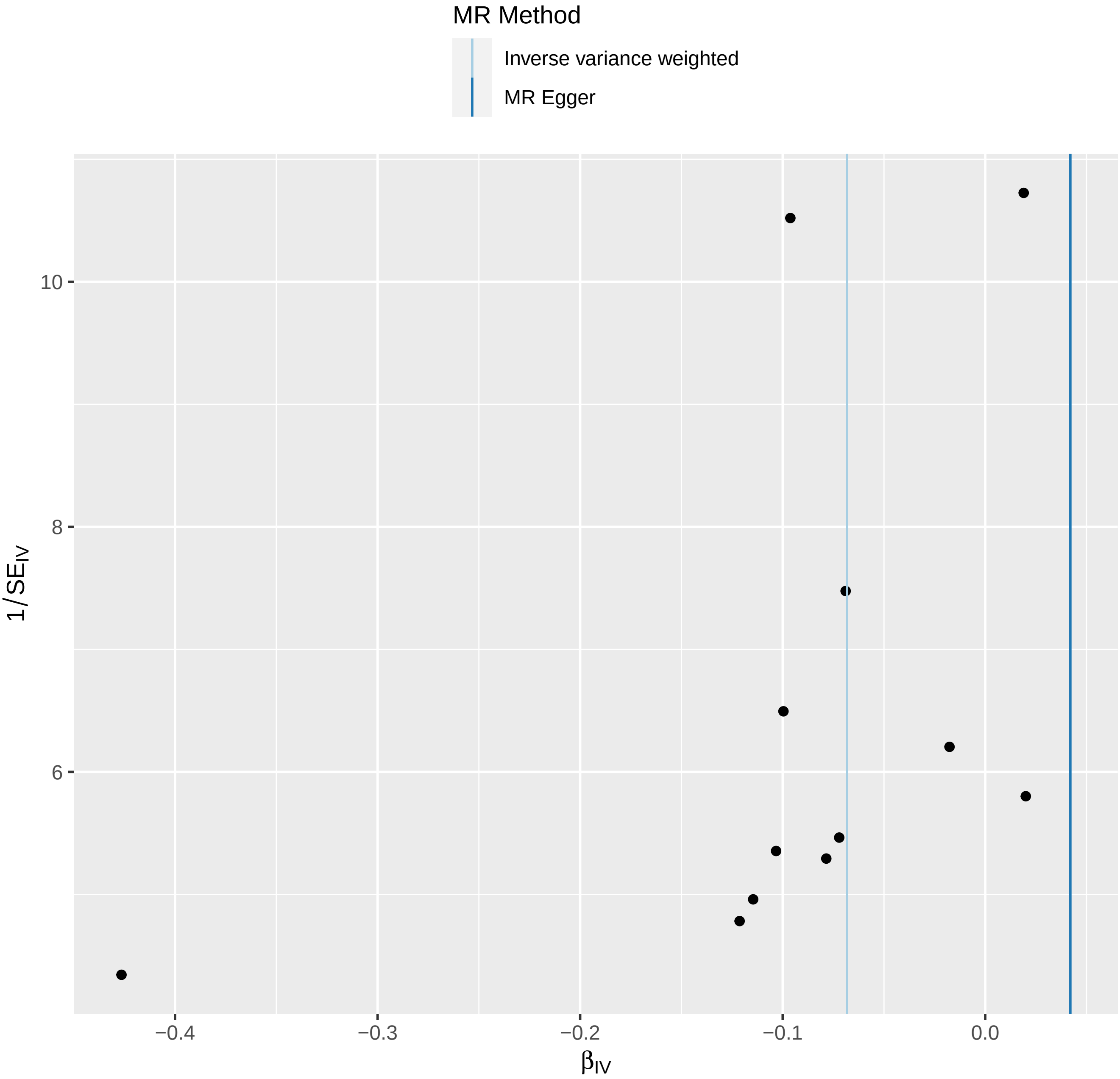

Batch 3−1998 : Selenium on Nicotinamide phosphoribosyltransferase

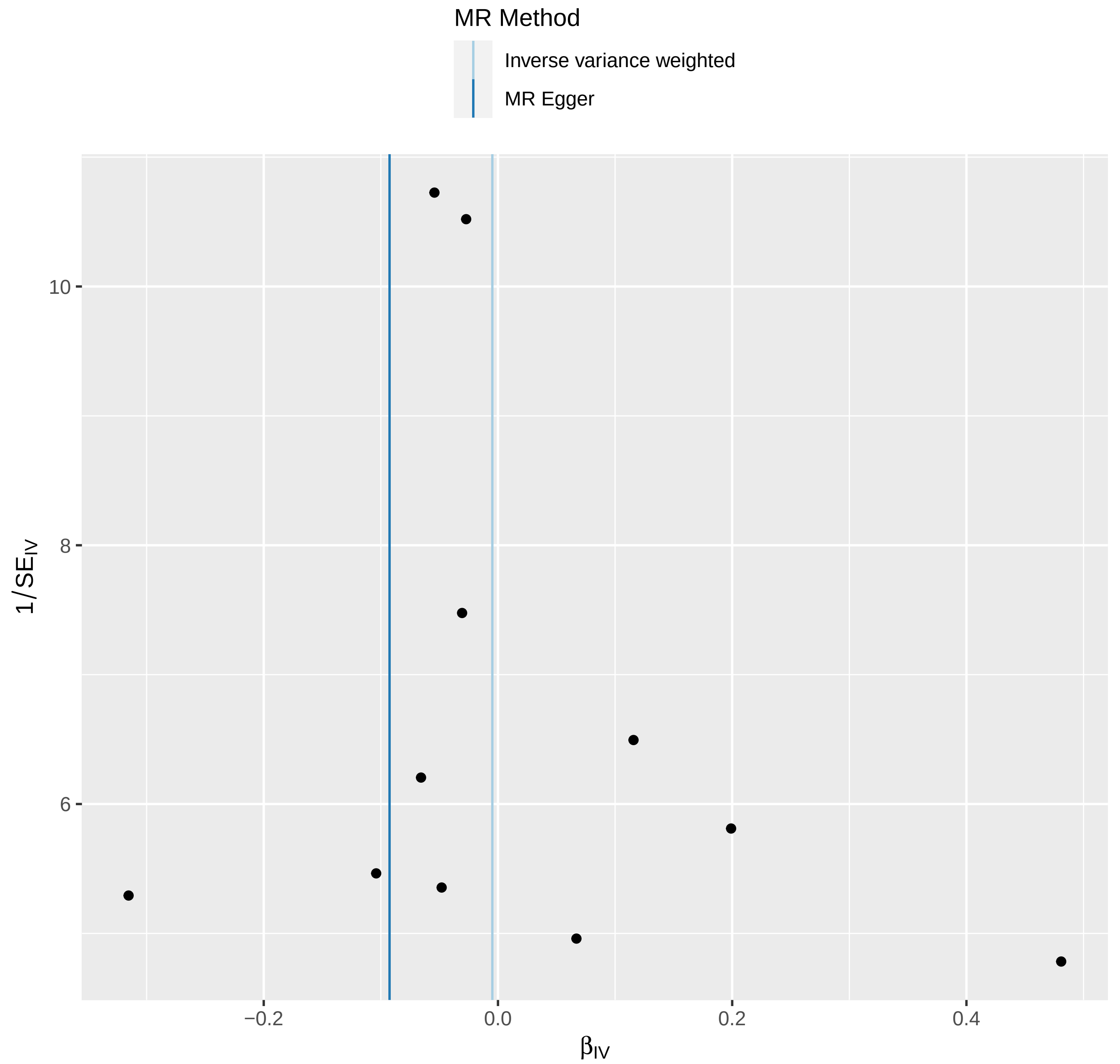

Batch 3−1999 : Selenium on Nucleosome assembly protein 1−like 2

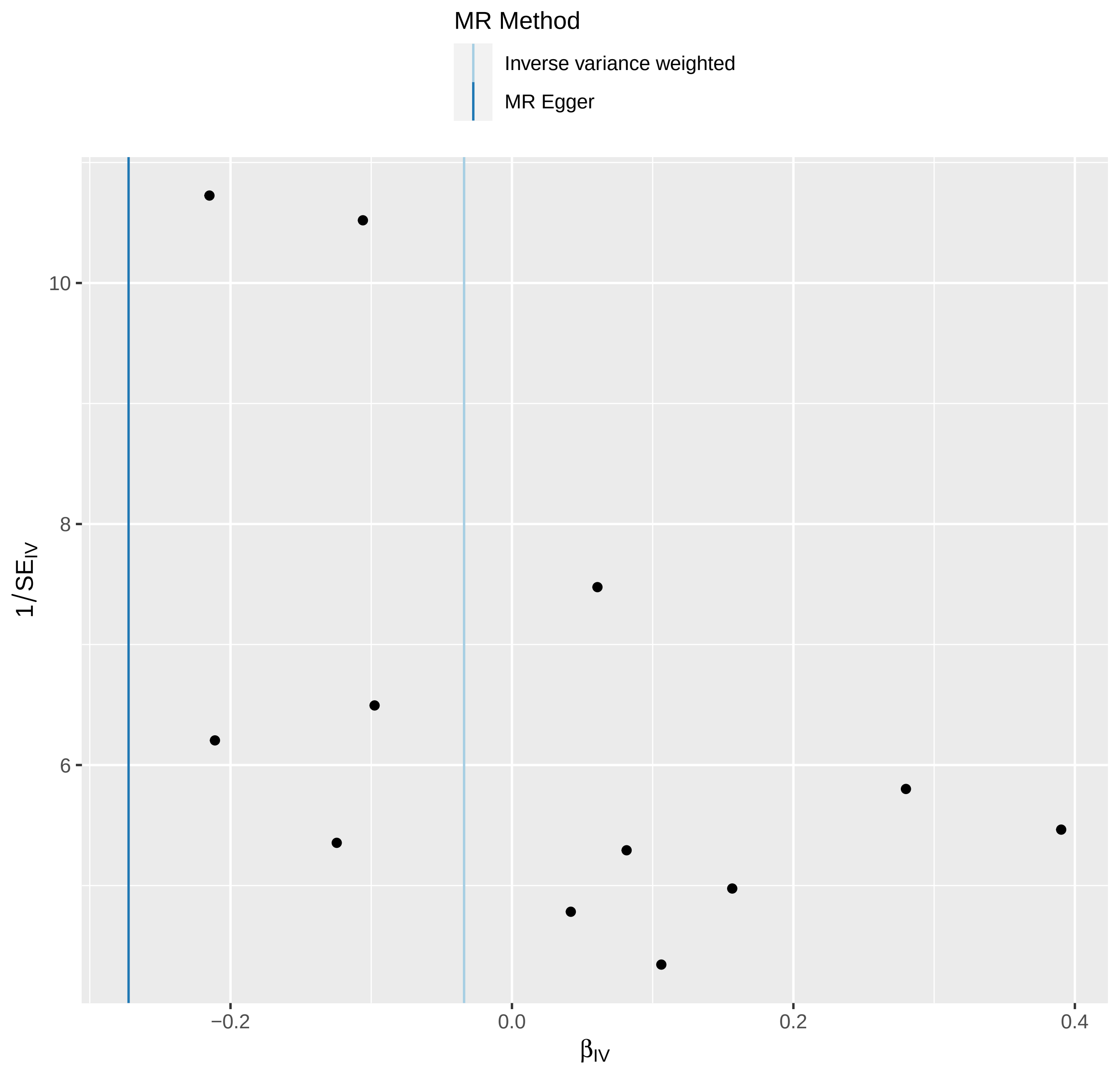

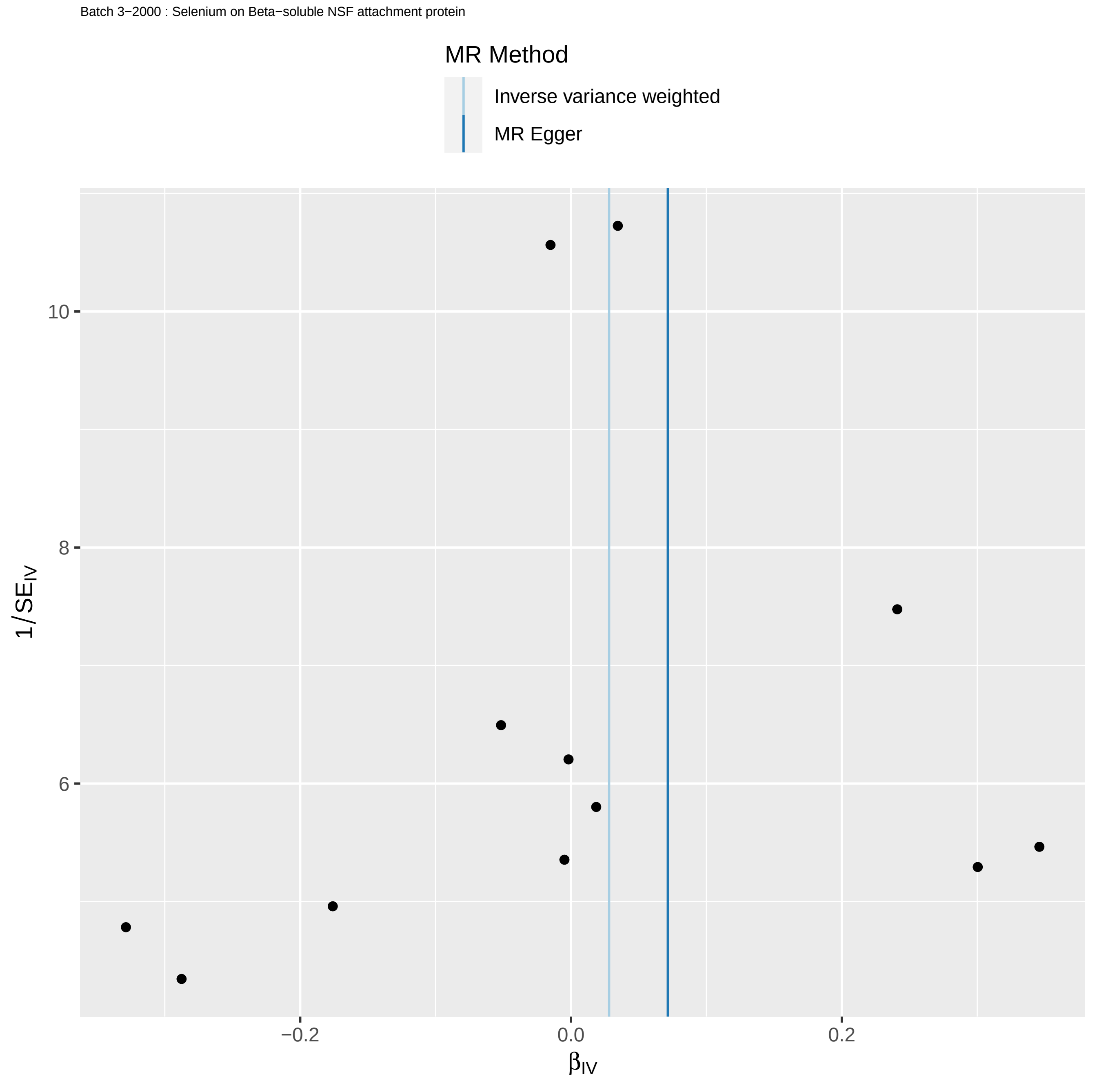

Batch 3−2000 : Selenium on Beta−soluble NSF attachment protein
MR Method
Inverse variance weighted
MR Egger
10
8
6
1/SE_IV
−0.2
0.0
0.2
β_IV

Batch 3−2001 : Selenium on Arylamine N−acetyltransferase 1

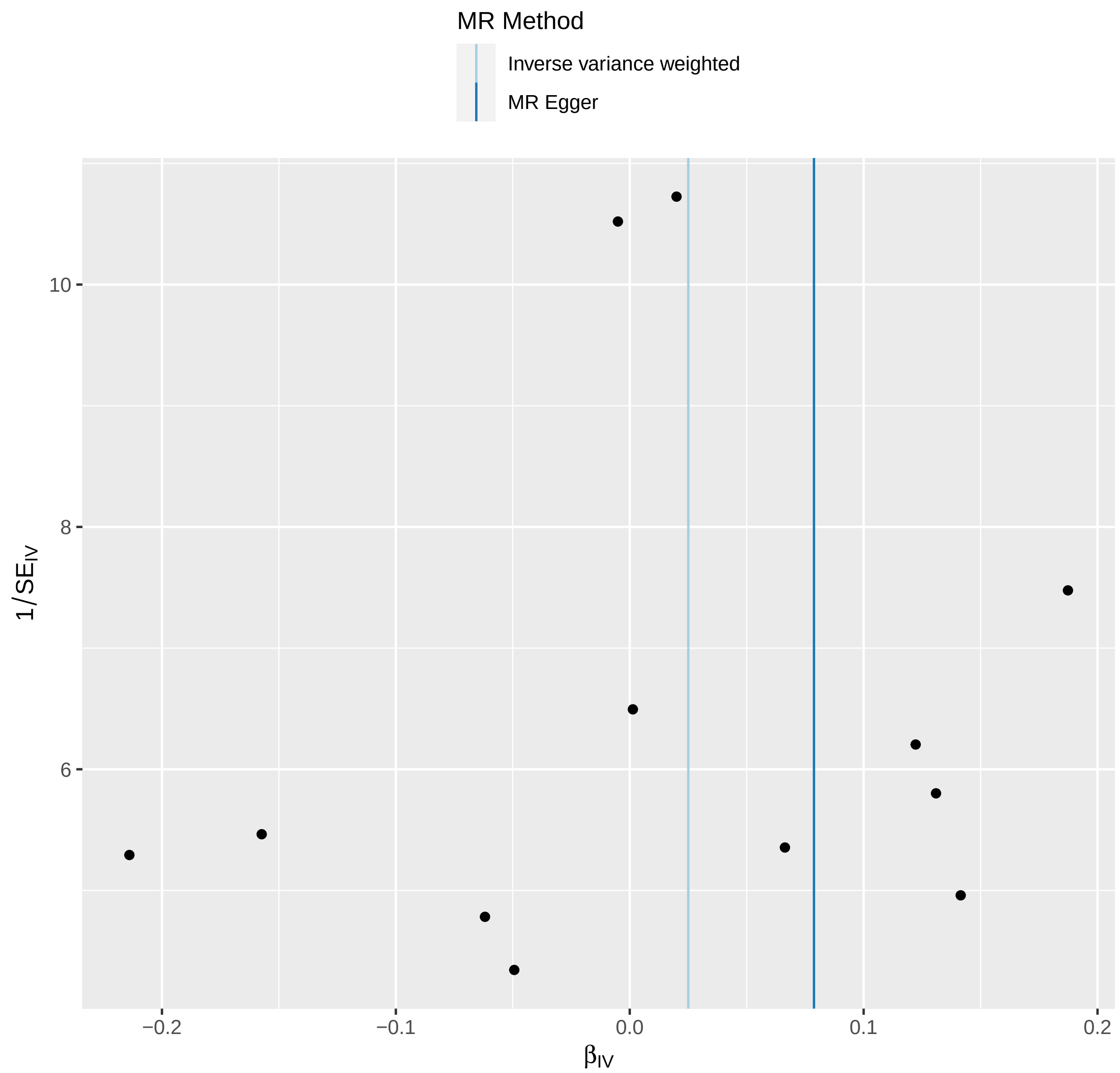

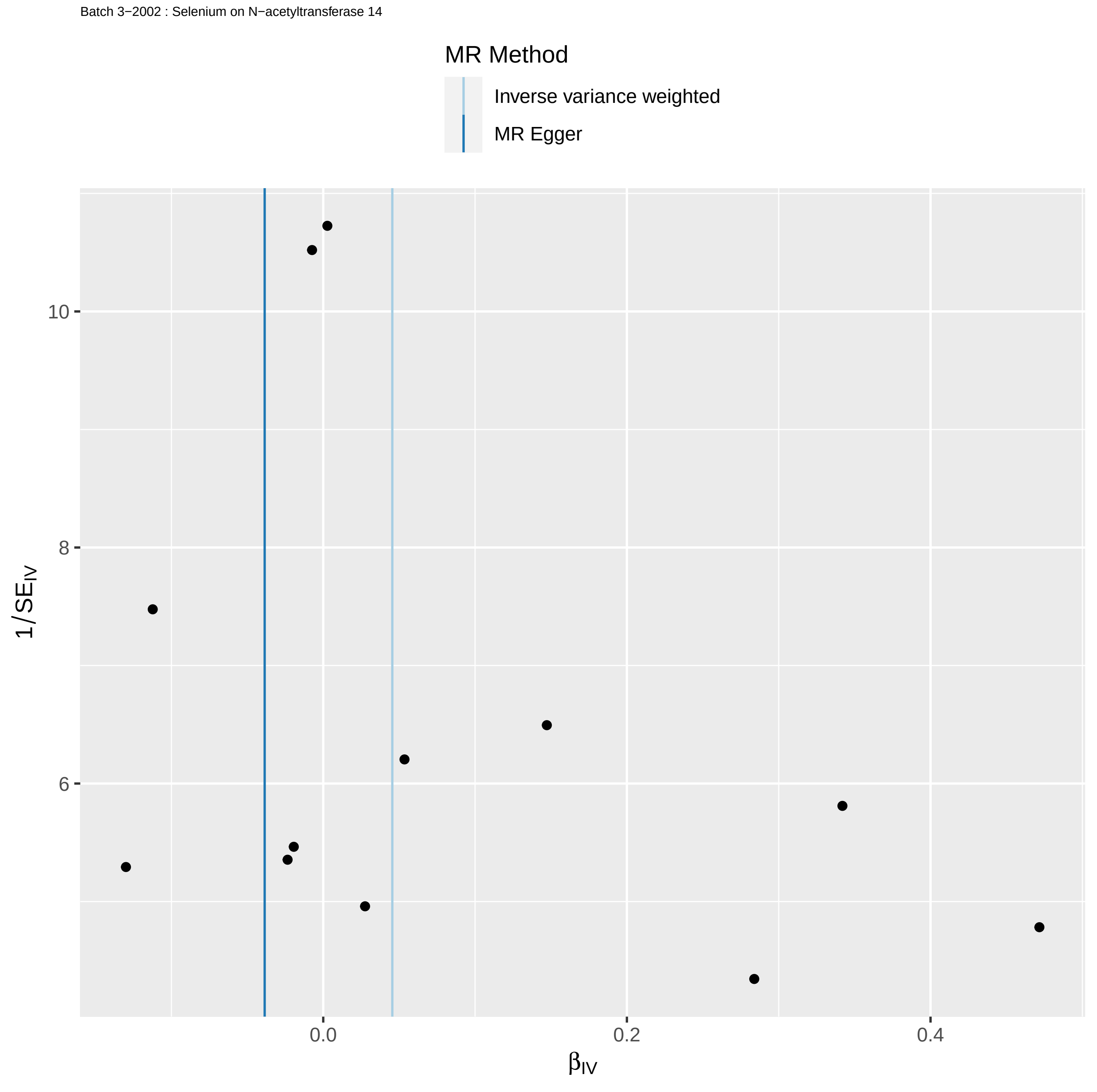

Batch 3−2002 : Selenium on N−acetyltransferase 14
MR Method
Inverse variance weighted
MR Egger
10
8
6
1/SE$_{IV}$
0.0
0.2
0.4
β$_{IV}$

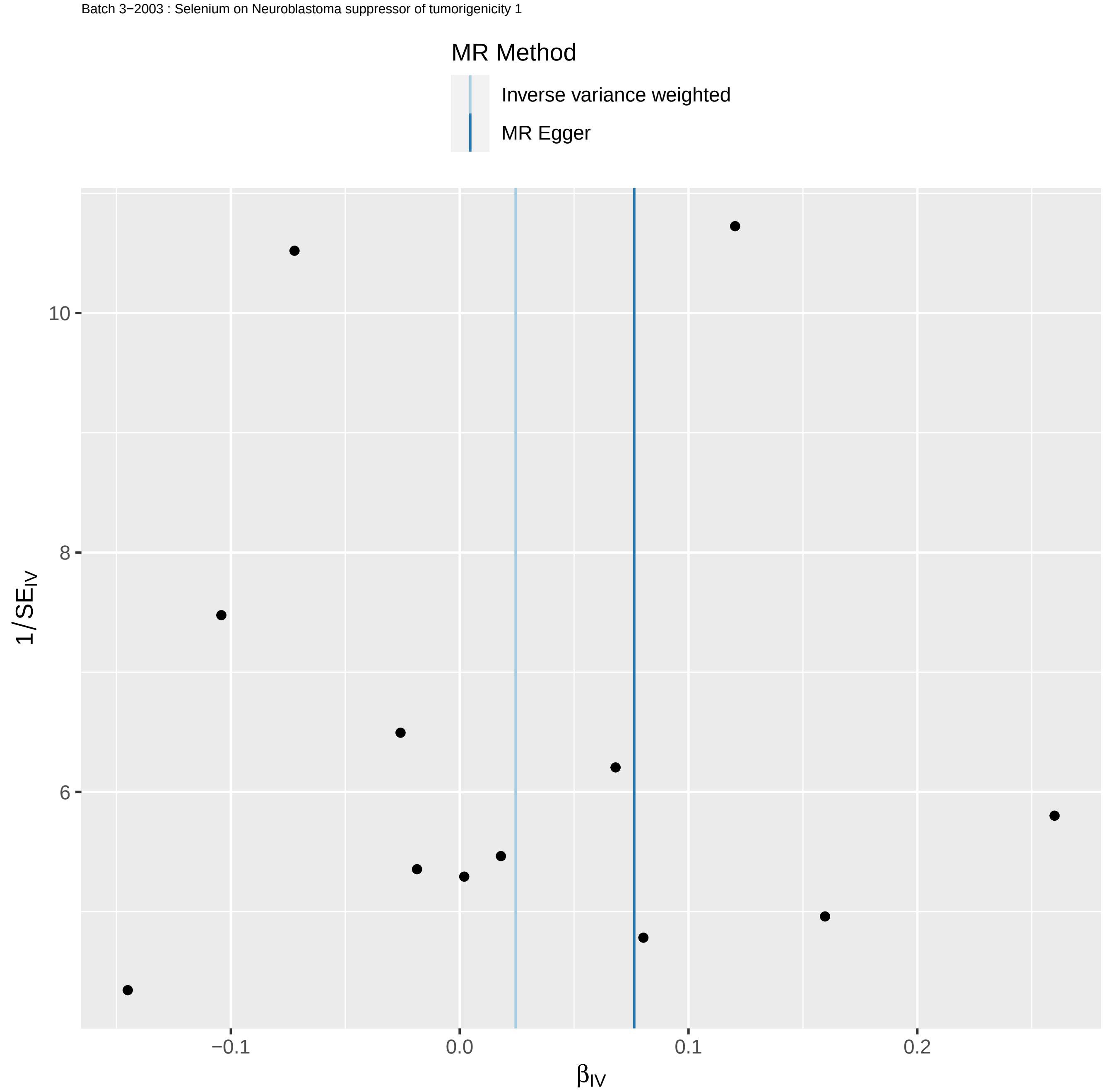
Batch 3−2003 : Selenium on Neuroblastoma suppressor of tumorigenicity 1
MR Method
Inverse variance weighted
MR Egger
10
8
6
1/SE_IV
−0.1
0.0
0.1
0.2
β_IV

Batch 3−2004 : Selenium on Next to BRCA1 gene 1 protein

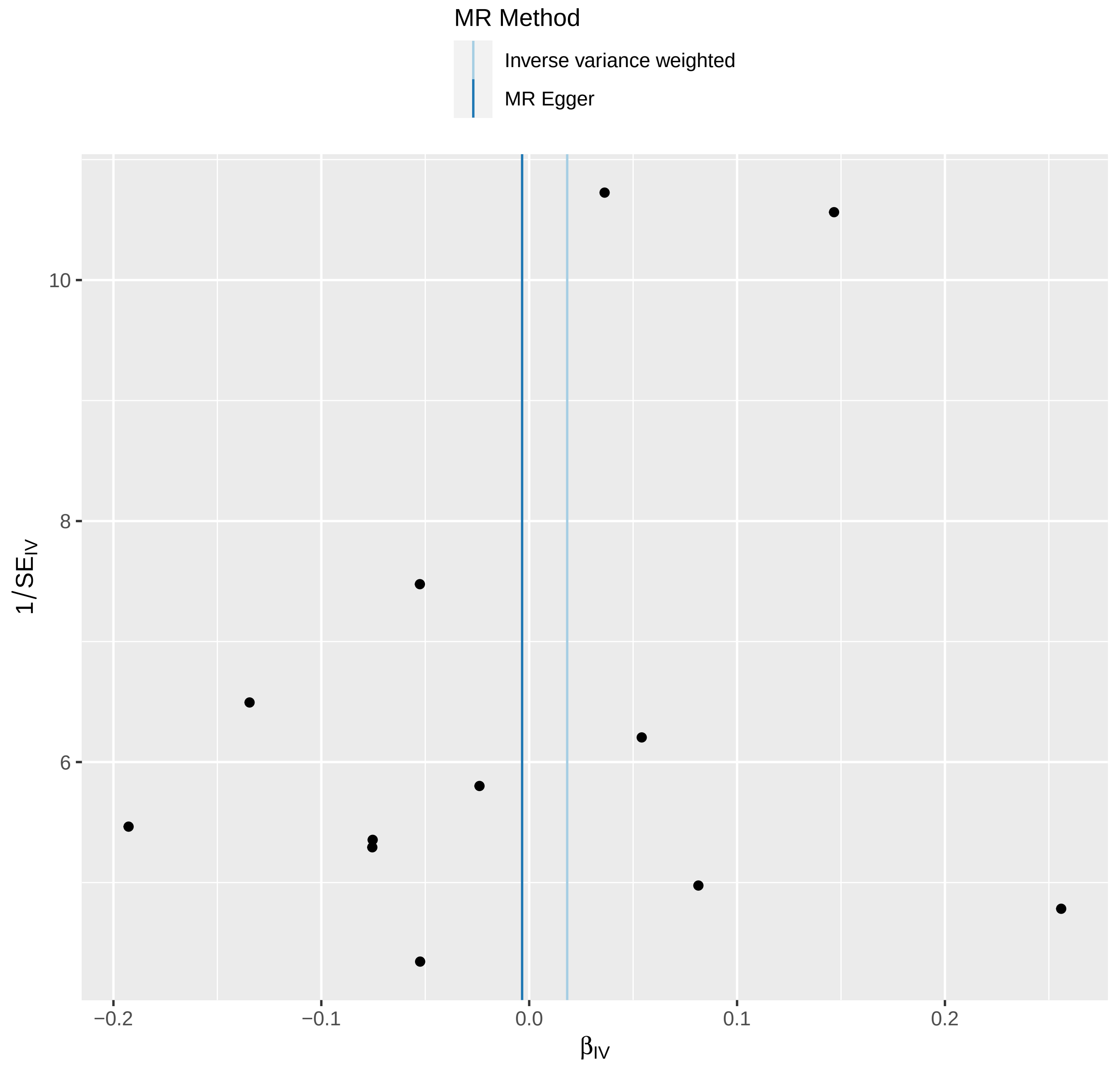

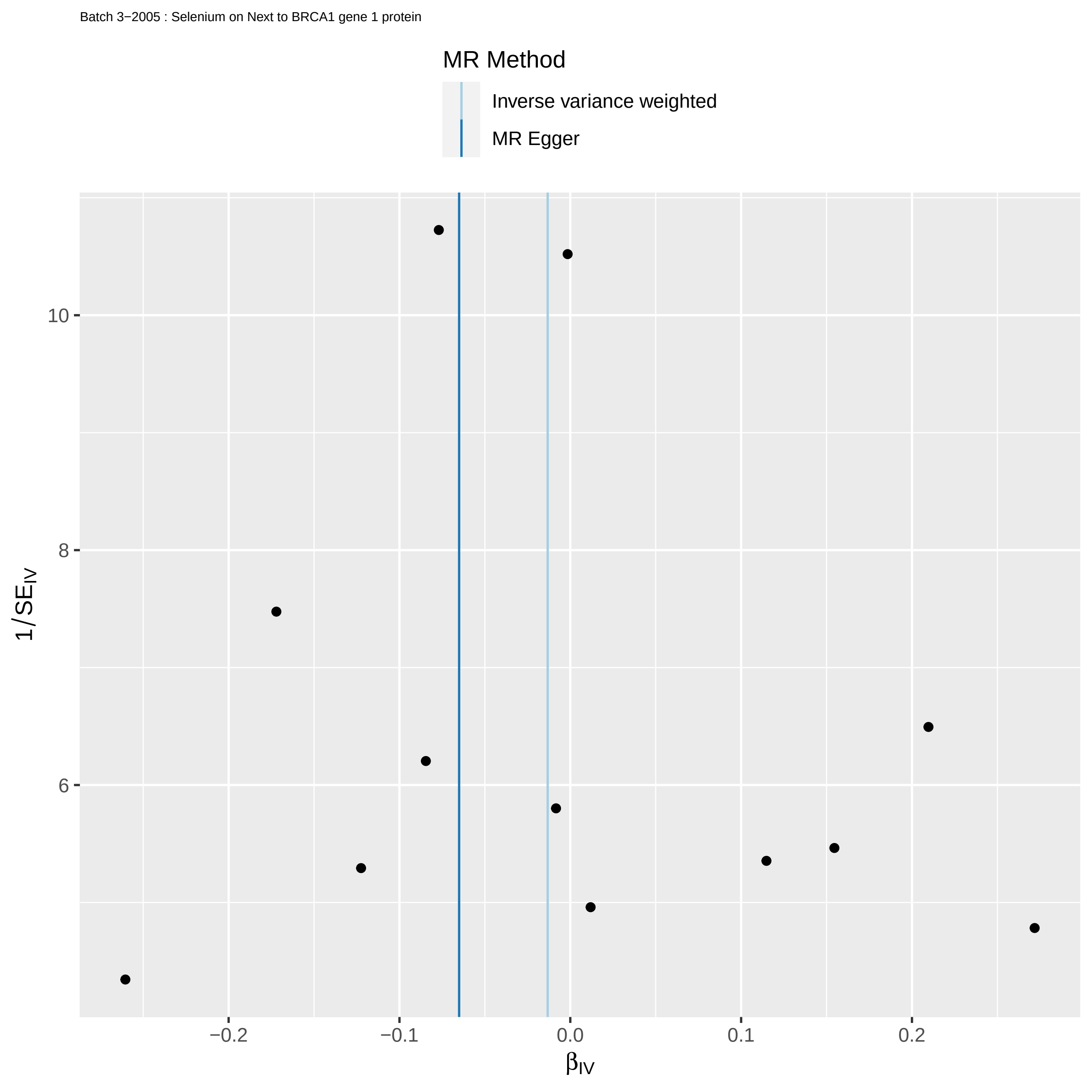
Batch 3−2005 : Selenium on Next to BRCA1 gene 1 protein
MR Method
Inverse variance weighted
MR Egger
10
8
6
$1/SE_{IV}$
−0.2
−0.1
0.0
0.1
0.2
β_{IV}

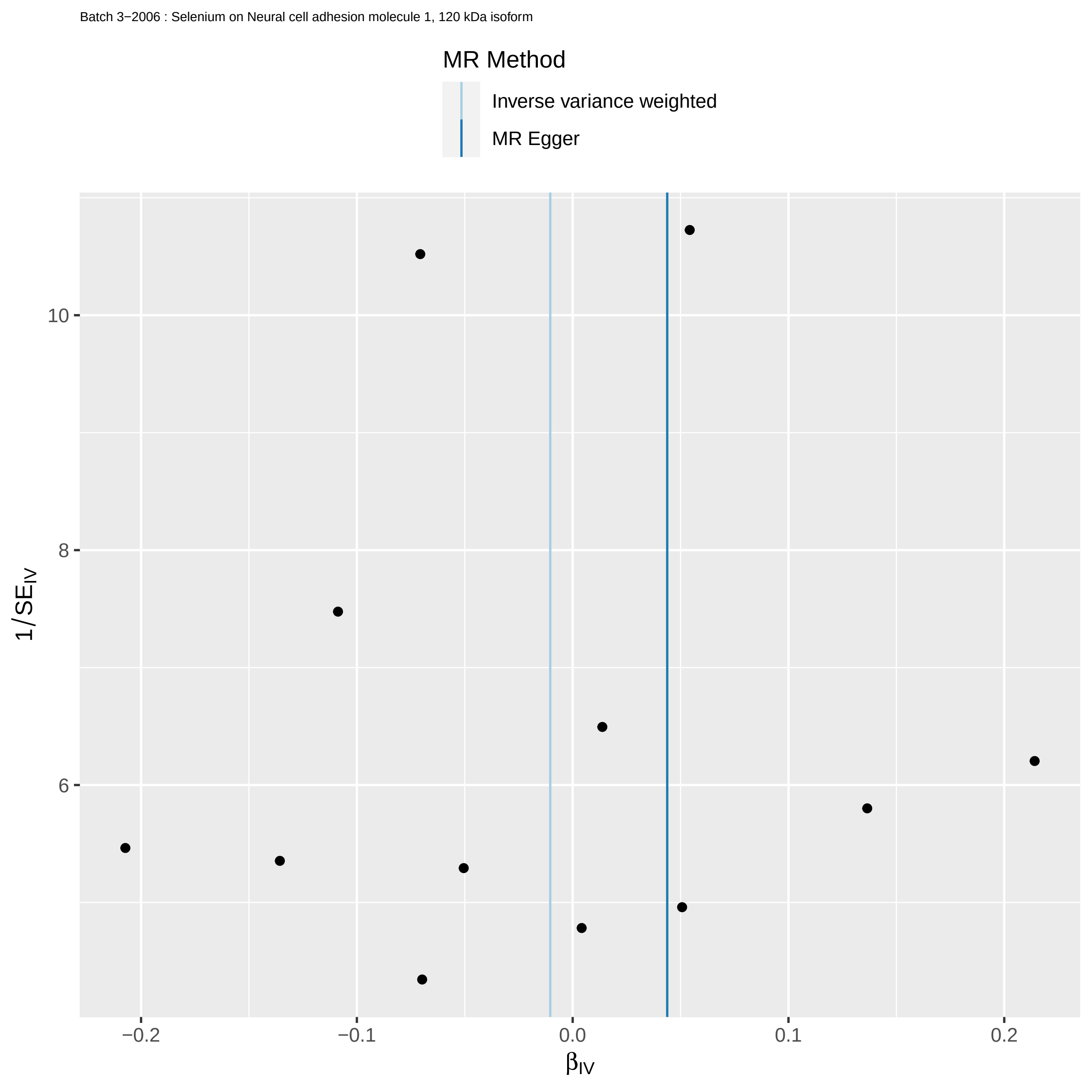
Batch 3−2006 : Selenium on Neural cell adhesion molecule 1, 120 kDa isoform
MR Method
Inverse variance weighted
MR Egger
10
8
6
1/SE_IV
−0.2
−0.1
0.0
0.1
0.2
β_IV

Batch 3−2007 : Selenium on Neural cell adhesion molecule 1

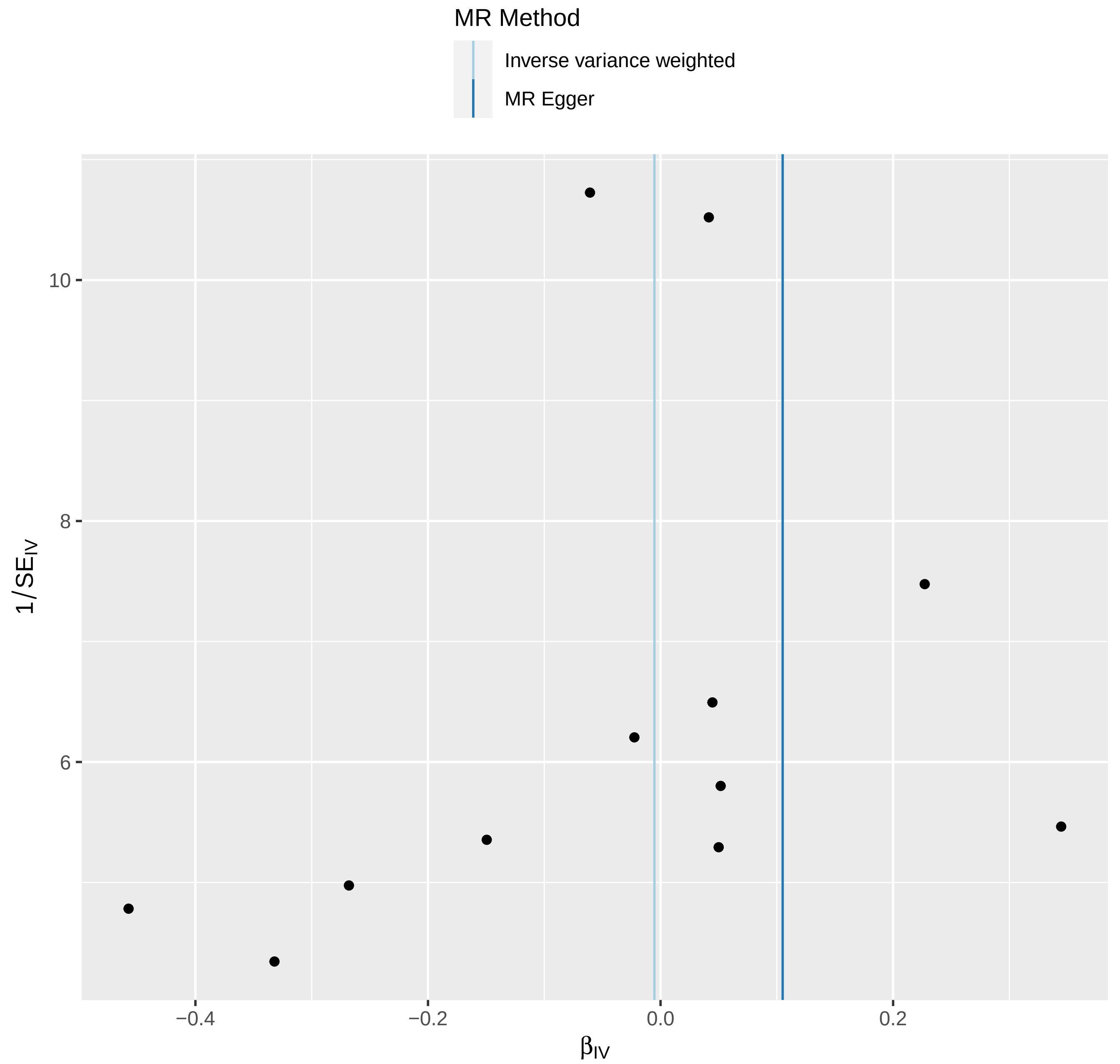

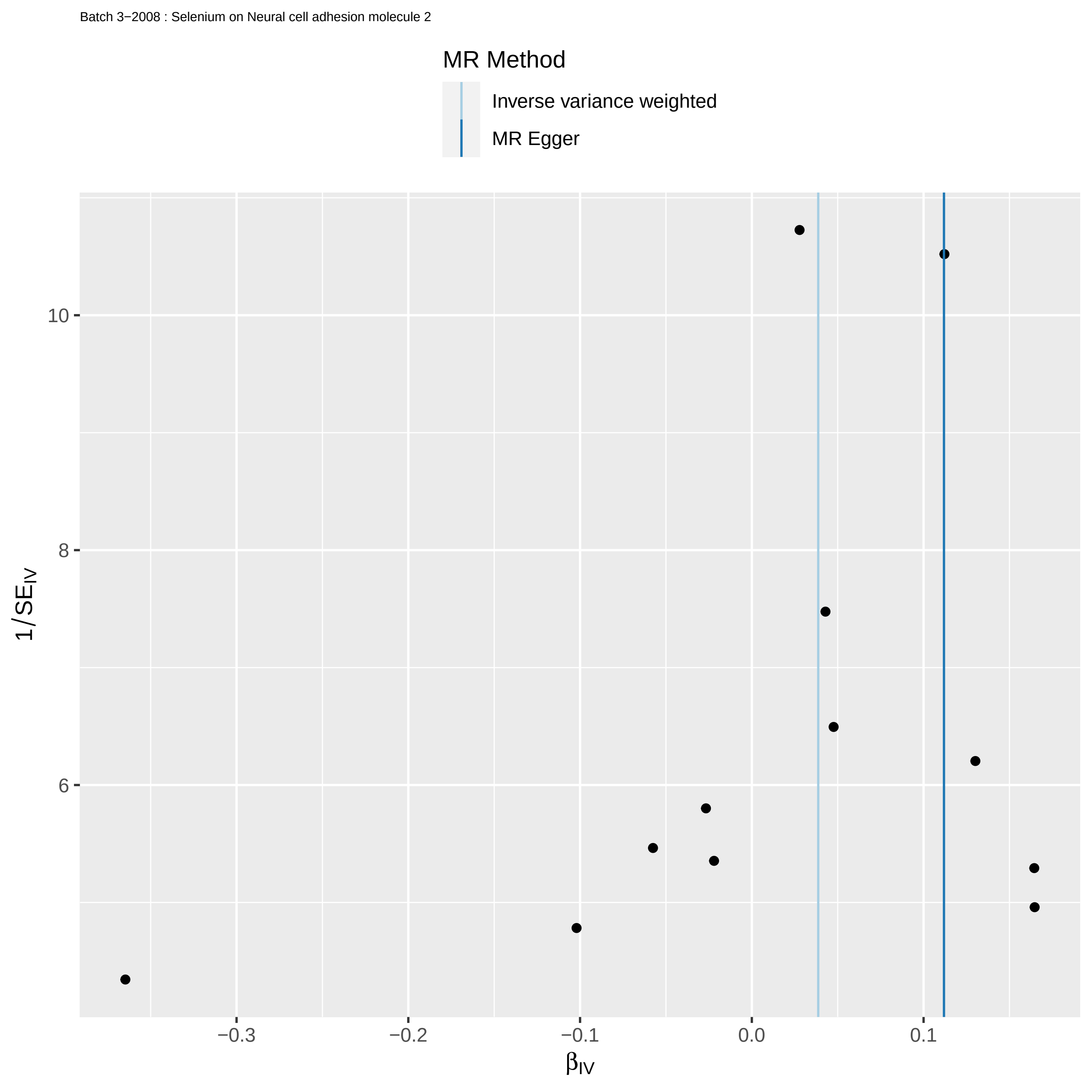

Batch 3−2008 : Selenium on Neural cell adhesion molecule 2
MR Method
Inverse variance weighted
MR Egger
10
8
6
1/SE$_{IV}$
−0.3
−0.2
−0.1
0.0
0.1
β$_{IV}$

Batch 3−2009 : Selenium on Neutrophil cytosol factor 2

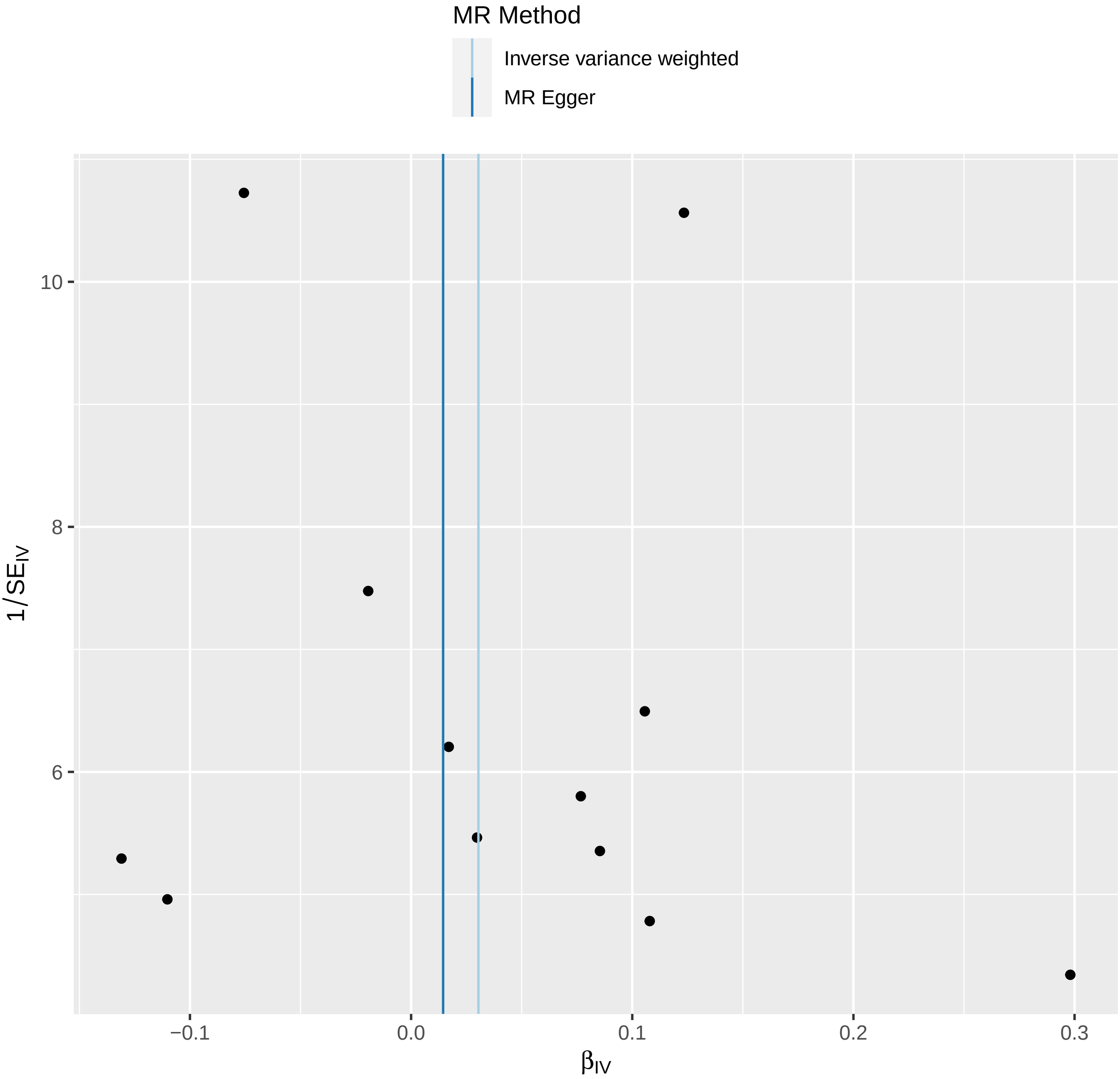

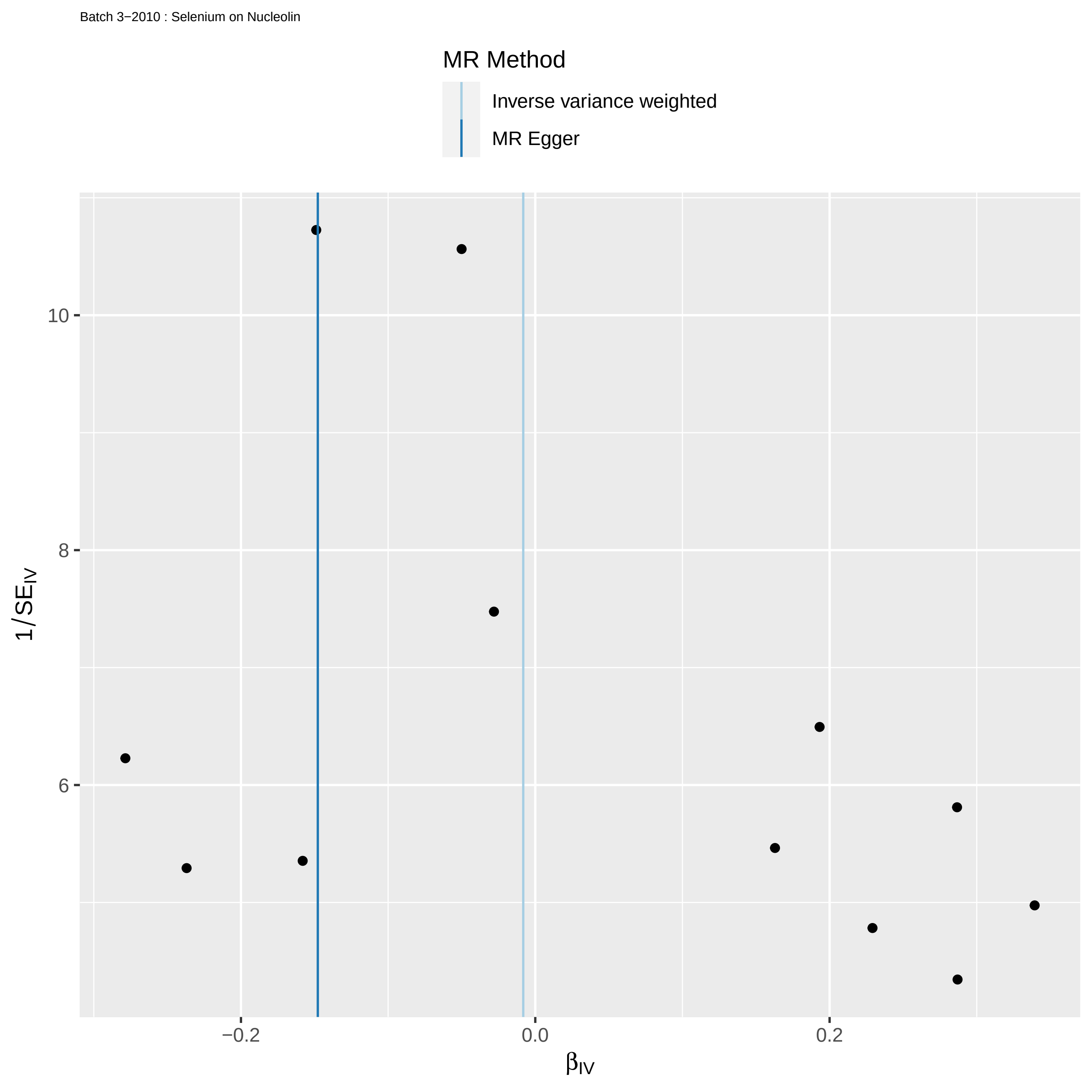
Batch 3−2010 : Selenium on Nucleolin
MR Method
Inverse variance weighted
MR Egger
10
8
6
$1/SE_{IV}$
−0.2
0.0
0.2
β_{IV}

Batch 3−2011 : Selenium on Noncompact myelin−associated protein

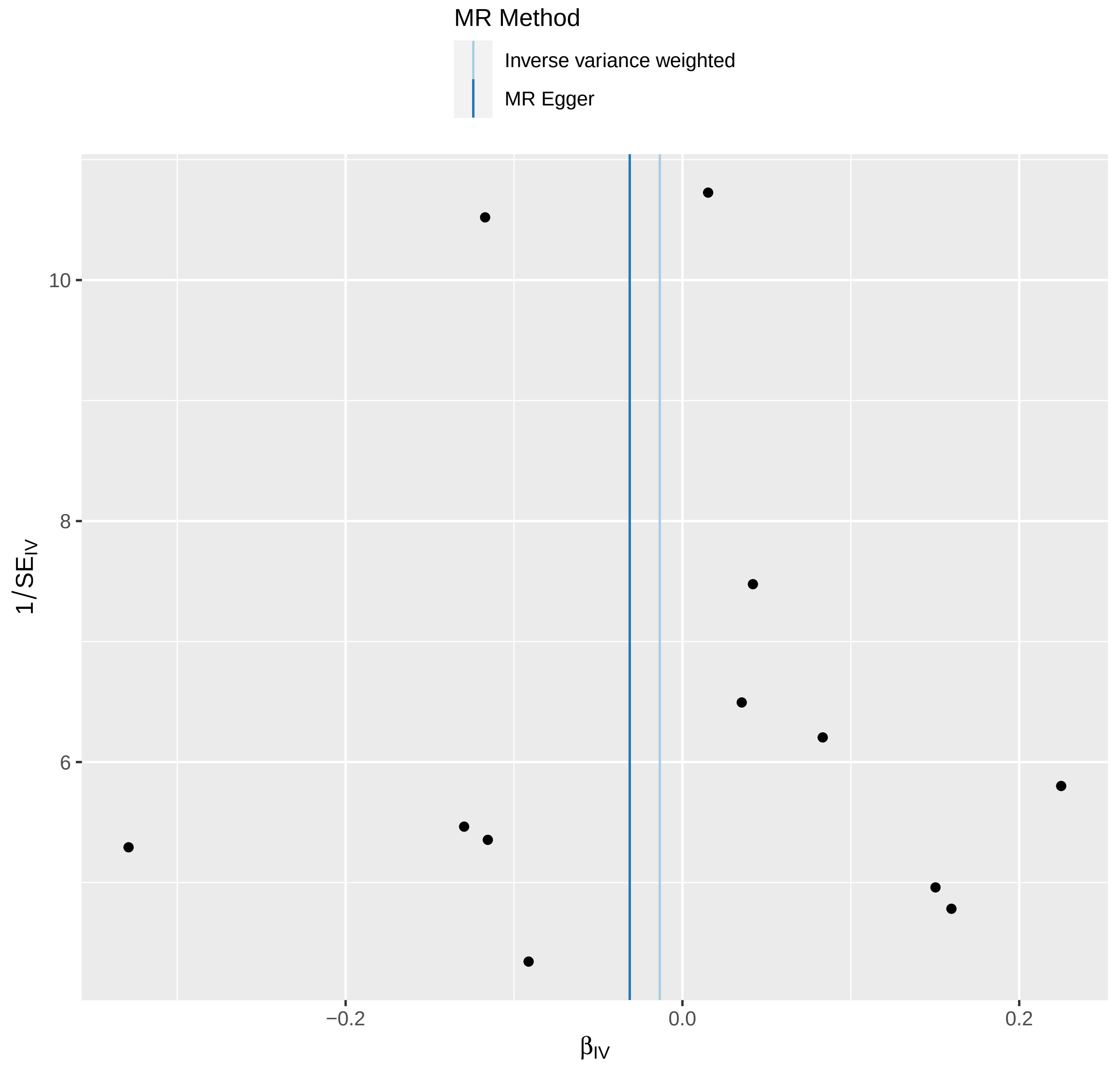

Batch 3−2012 : Selenium on Nuclear receptor coactivator 2

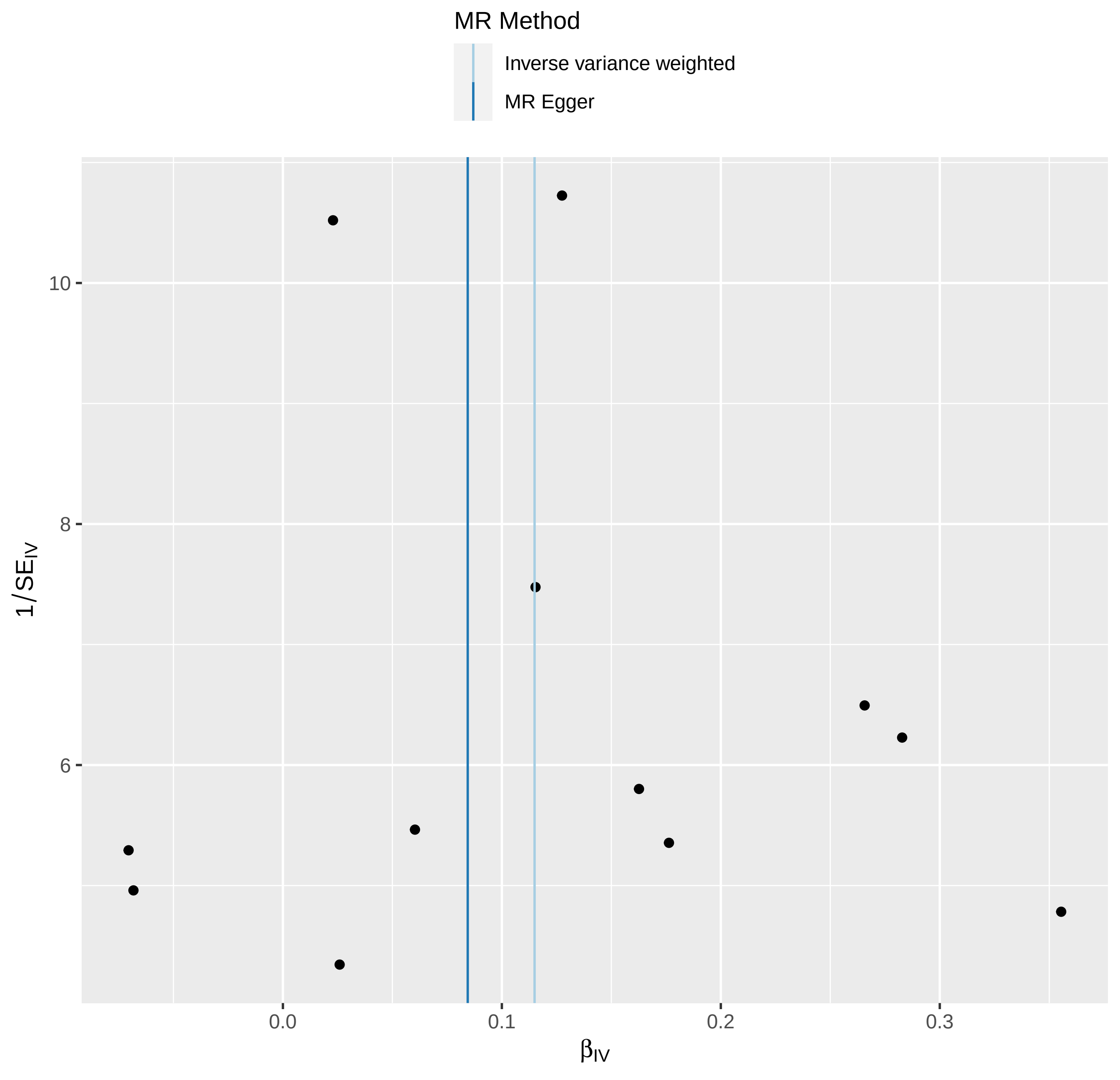

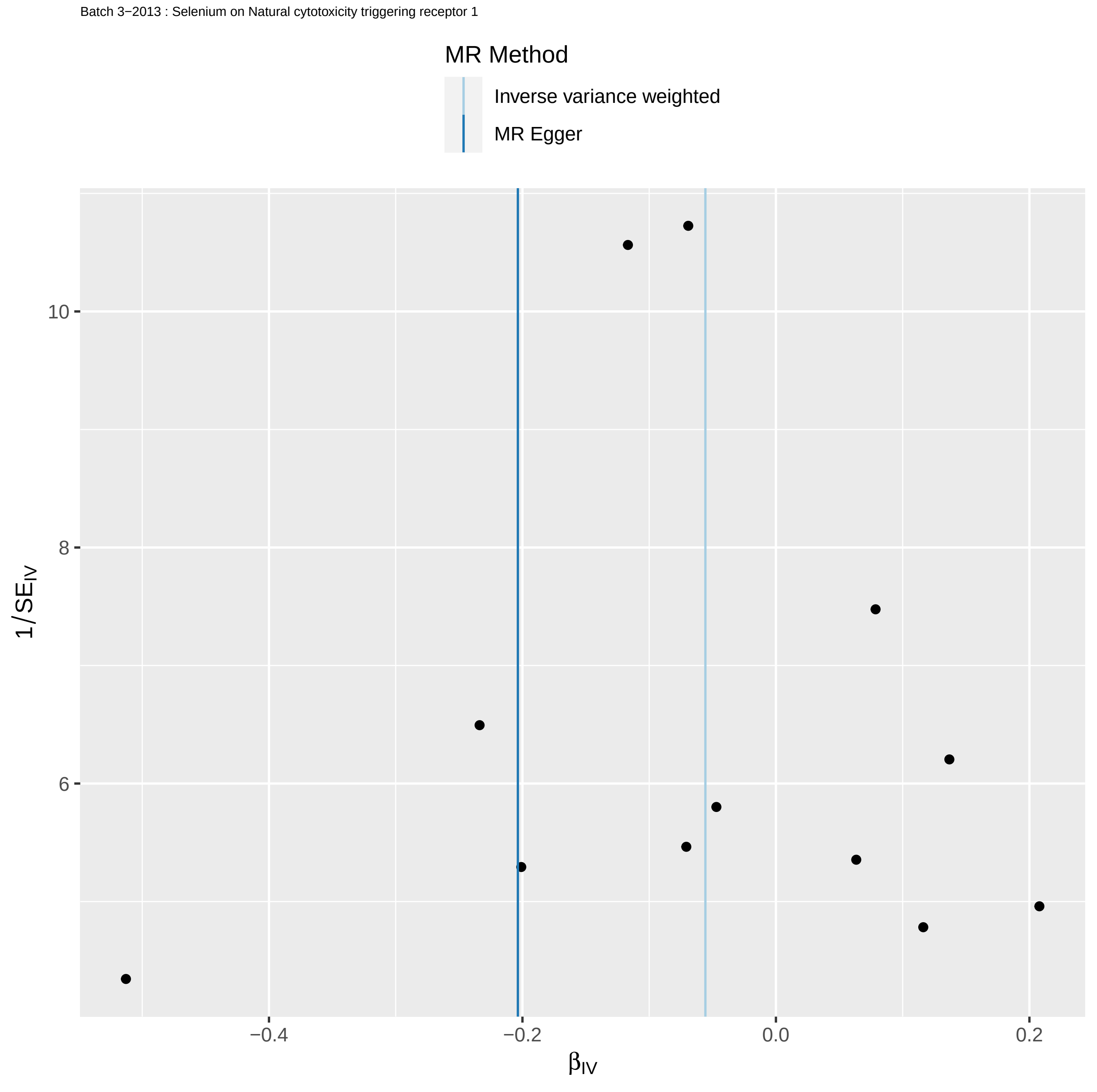
Batch 3−2013 : Selenium on Natural cytotoxicity triggering receptor 1
MR Method
Inverse variance weighted
MR Egger
10
8
6
1/SE_IV
−0.4
−0.2
0.0
0.2
β_IV

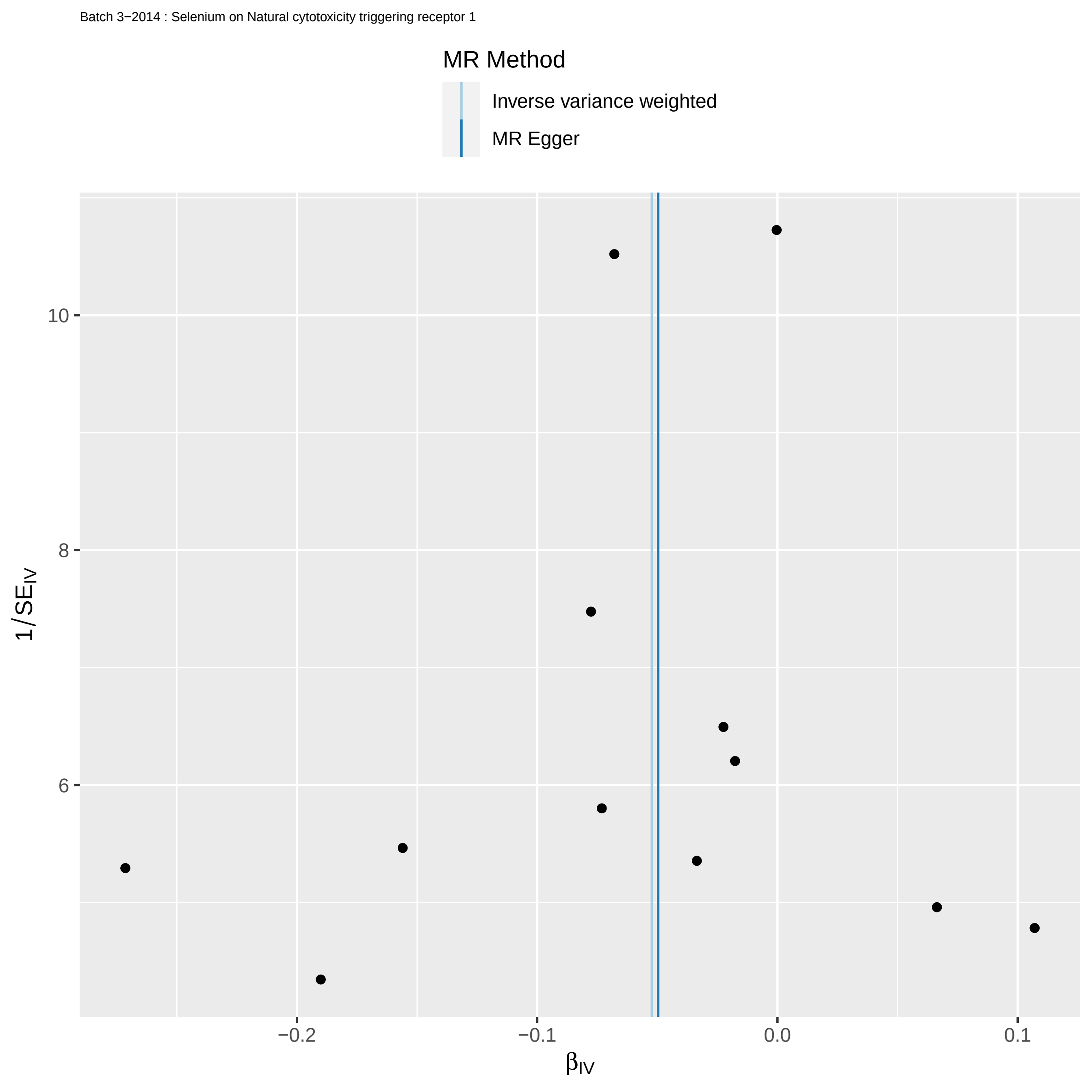

Batch 3−2014 : Selenium on Natural cytotoxicity triggering receptor 1
MR Method
Inverse variance weighted
MR Egger
10
8
6
1/SE$_{IV}$
−0.2
−0.1
0.0
0.1
β$_{IV}$

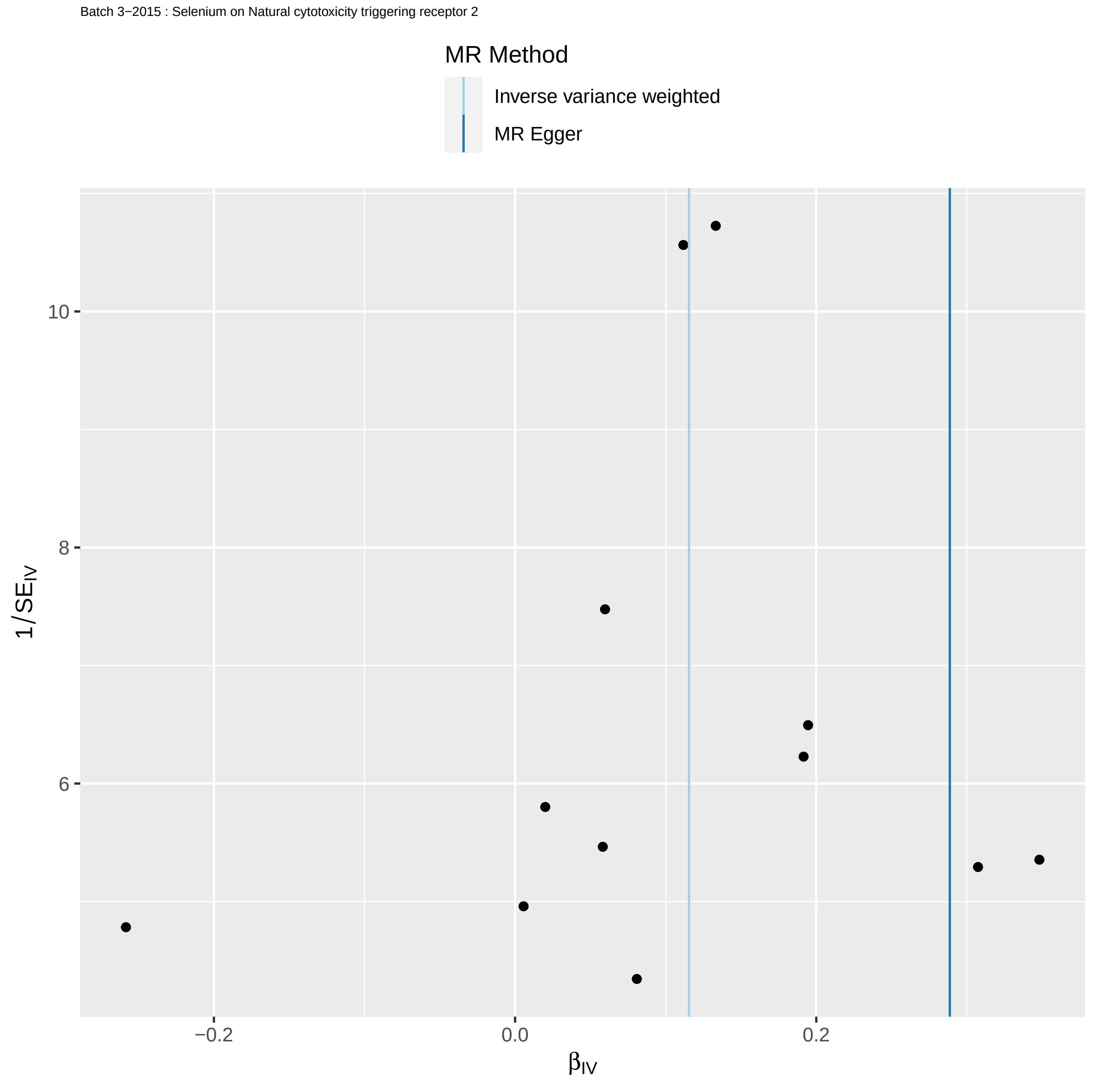

Batch 3−2015 : Selenium on Natural cytotoxicity triggering receptor 2
MR Method
Inverse variance weighted
MR Egger
10
8
6
1/SE_IV
−0.2
0.0
0.2
β_IV

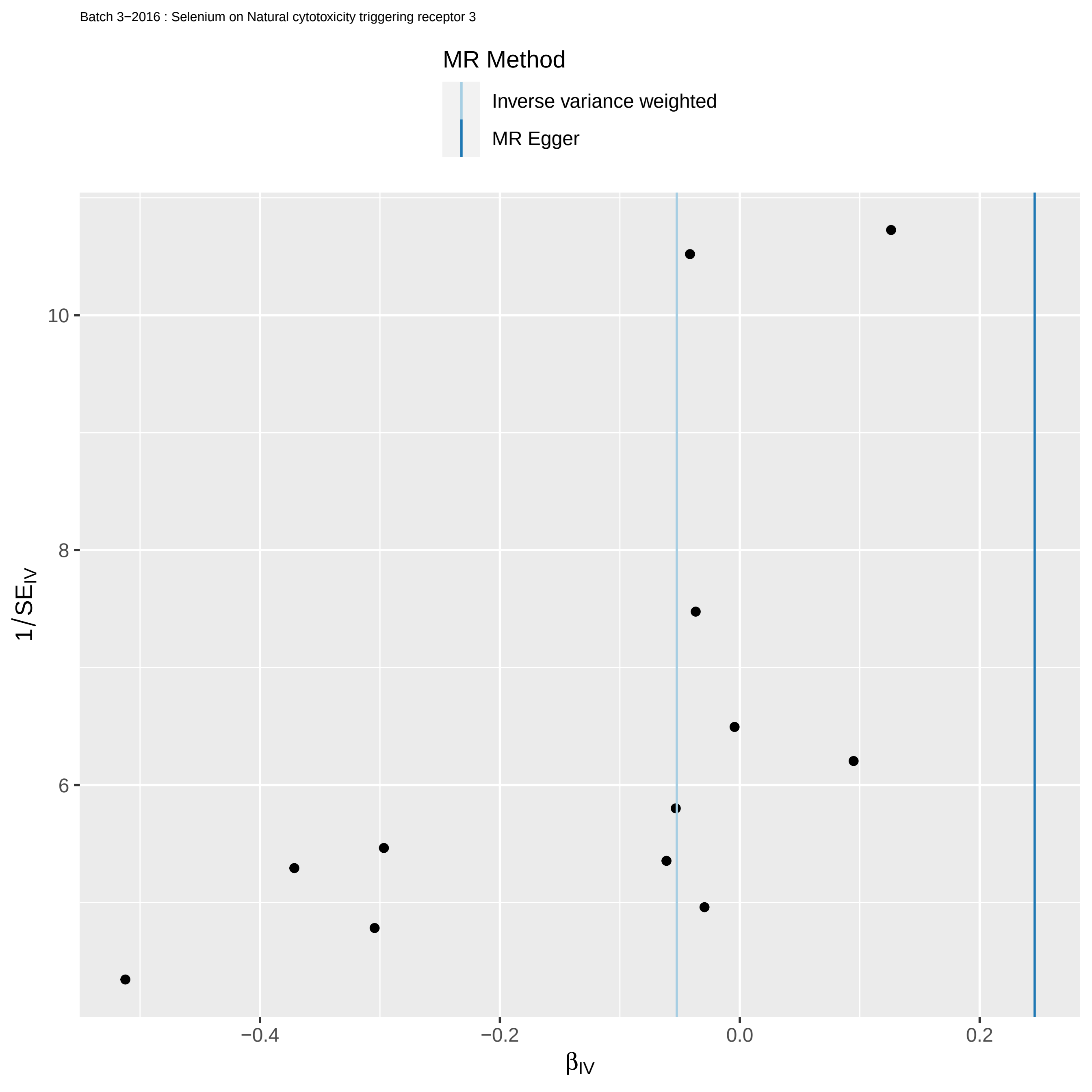

Batch 3−2016 : Selenium on Natural cytotoxicity triggering receptor 3
MR Method
Inverse variance weighted
MR Egger
10
8
6
1/SE$_{IV}$
−0.4
−0.2
0.0
0.2
β$_{IV}$

Batch 3−2017 : Selenium on Natural cytotoxicity triggering receptor 3 ligand 1

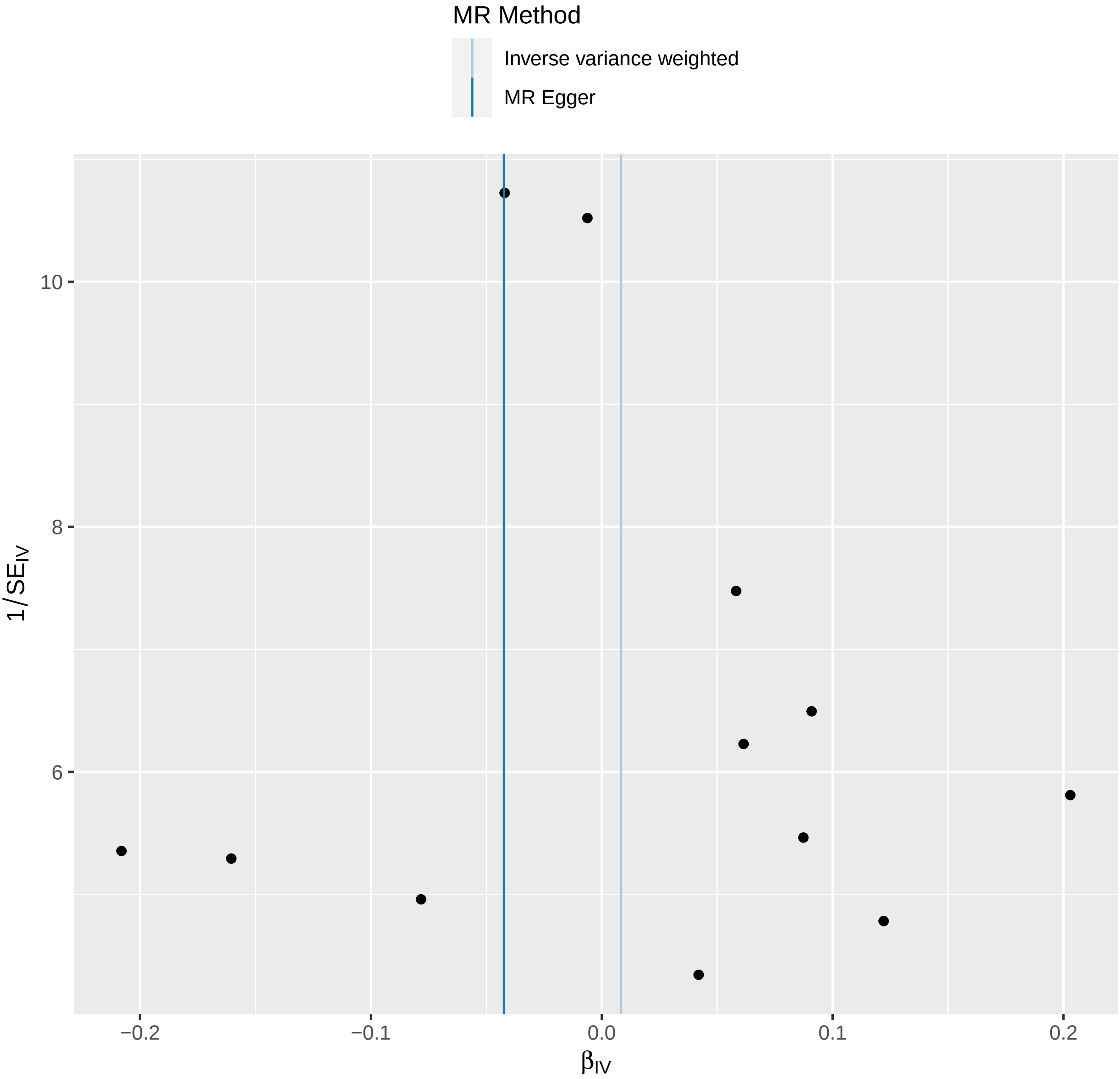

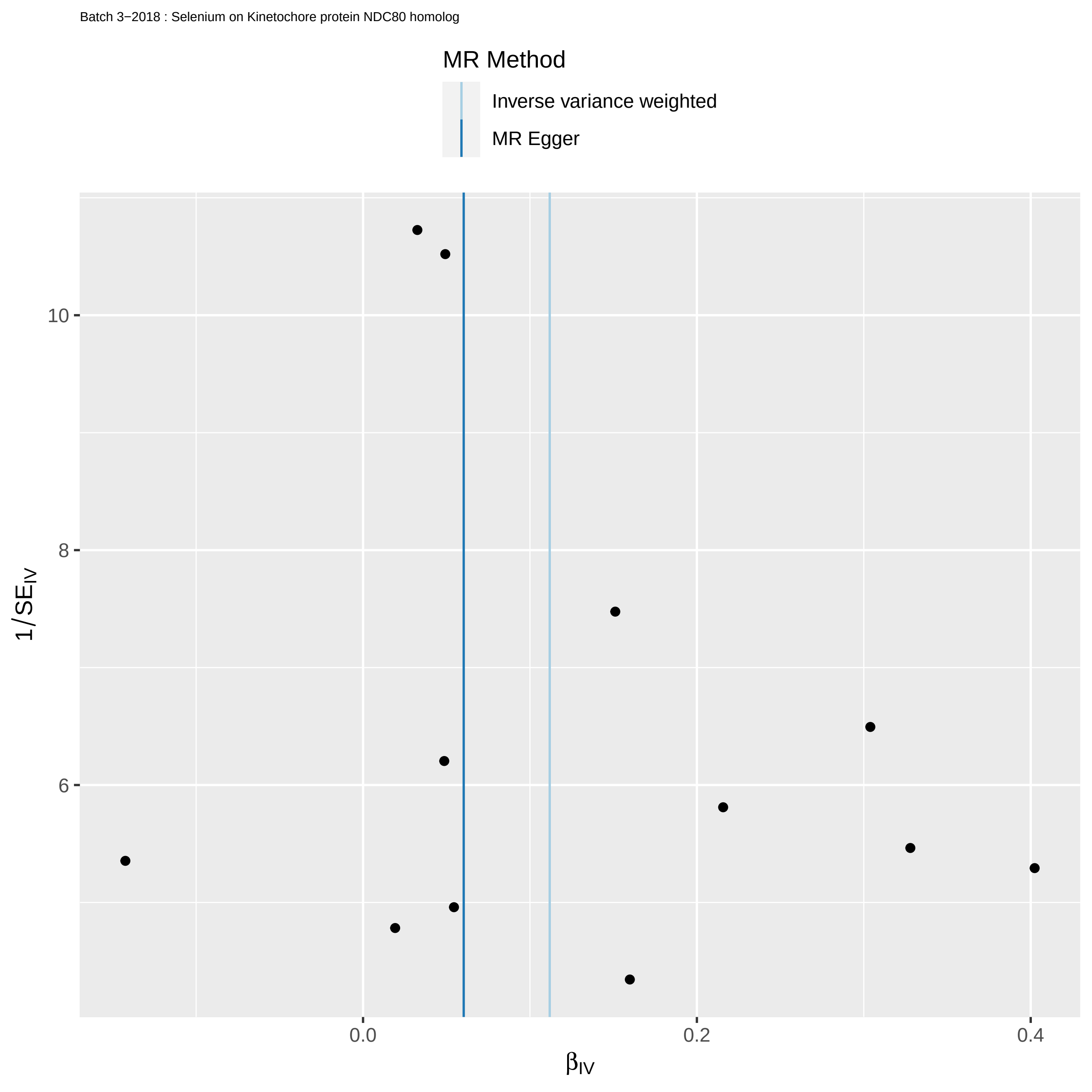

Batch 3−2018 : Selenium on Kinetochore protein NDC80 homolog
MR Method
Inverse variance weighted
MR Egger
10
8
6
1/SE$_{IV}$
0.0
0.2
0.4
β_{IV}

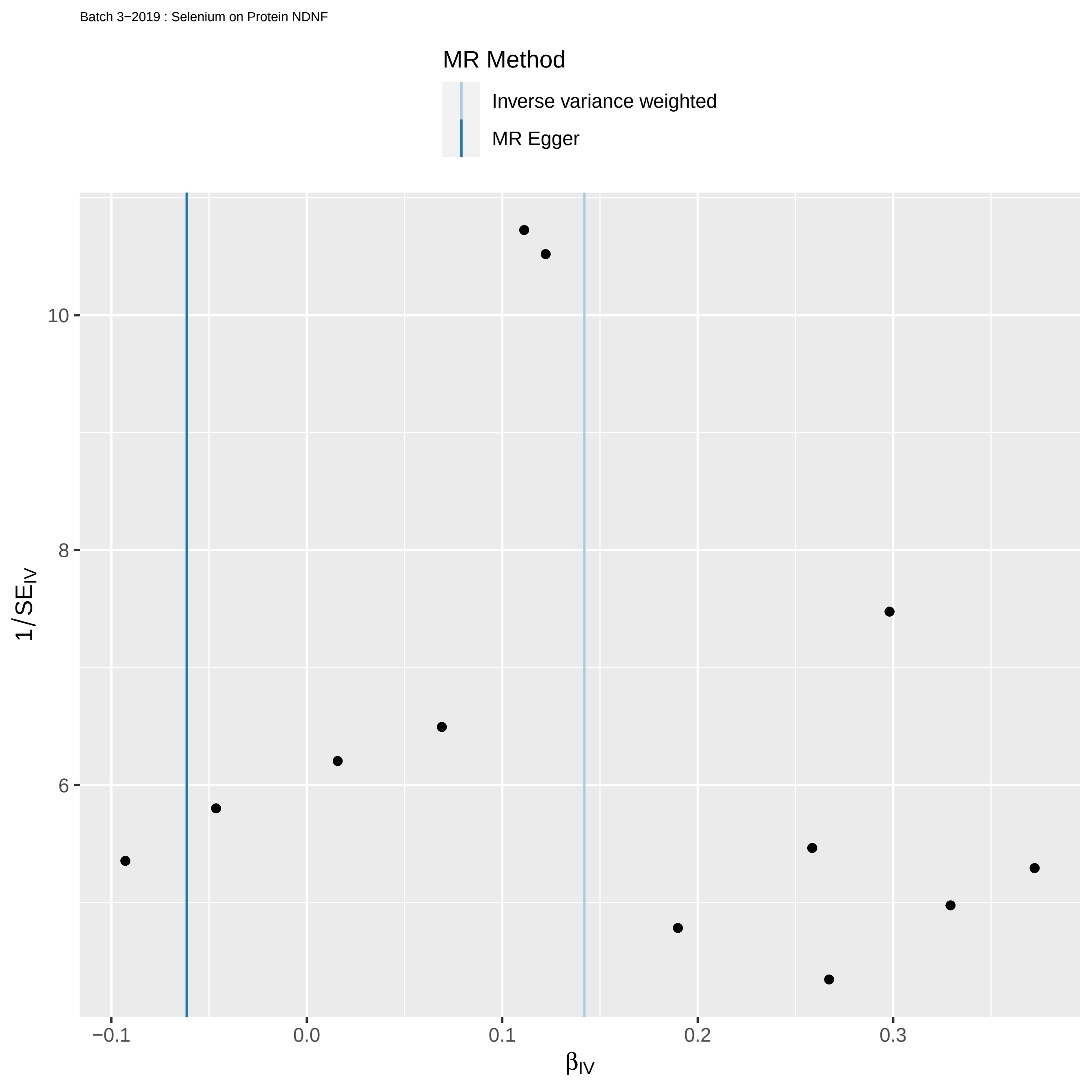
Batch 3−2019 : Selenium on Protein NDNF
MR Method
Inverse variance weighted
MR Egger
10
8
6
1/SE_IV
−0.1
0.0
0.1
0.2
0.3
β_IV

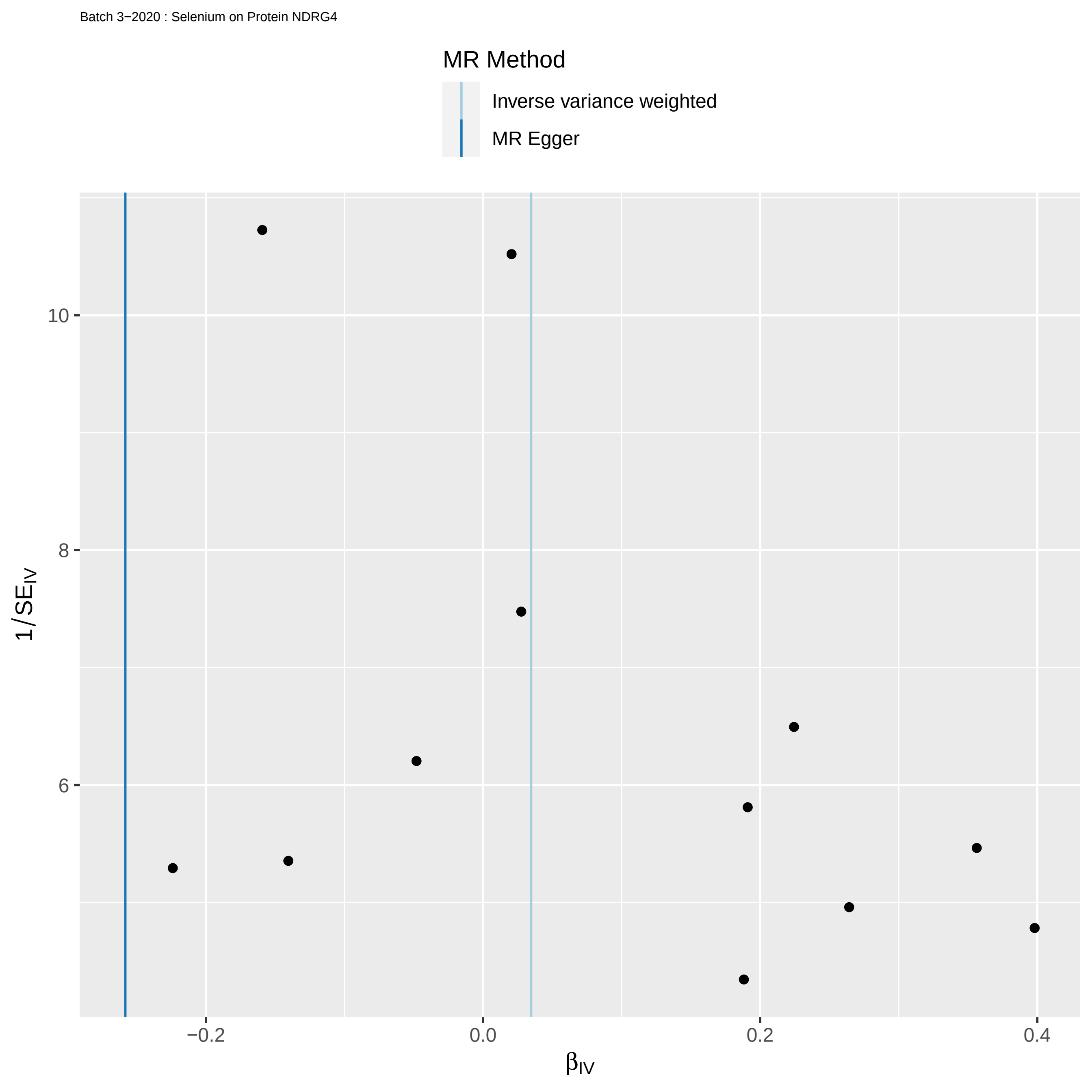
Batch 3−2020 : Selenium on Protein NDRG4
MR Method
Inverse variance weighted
MR Egger
10
8
6
1/SE_IV
−0.2
0.0
0.2
0.4
β_IV

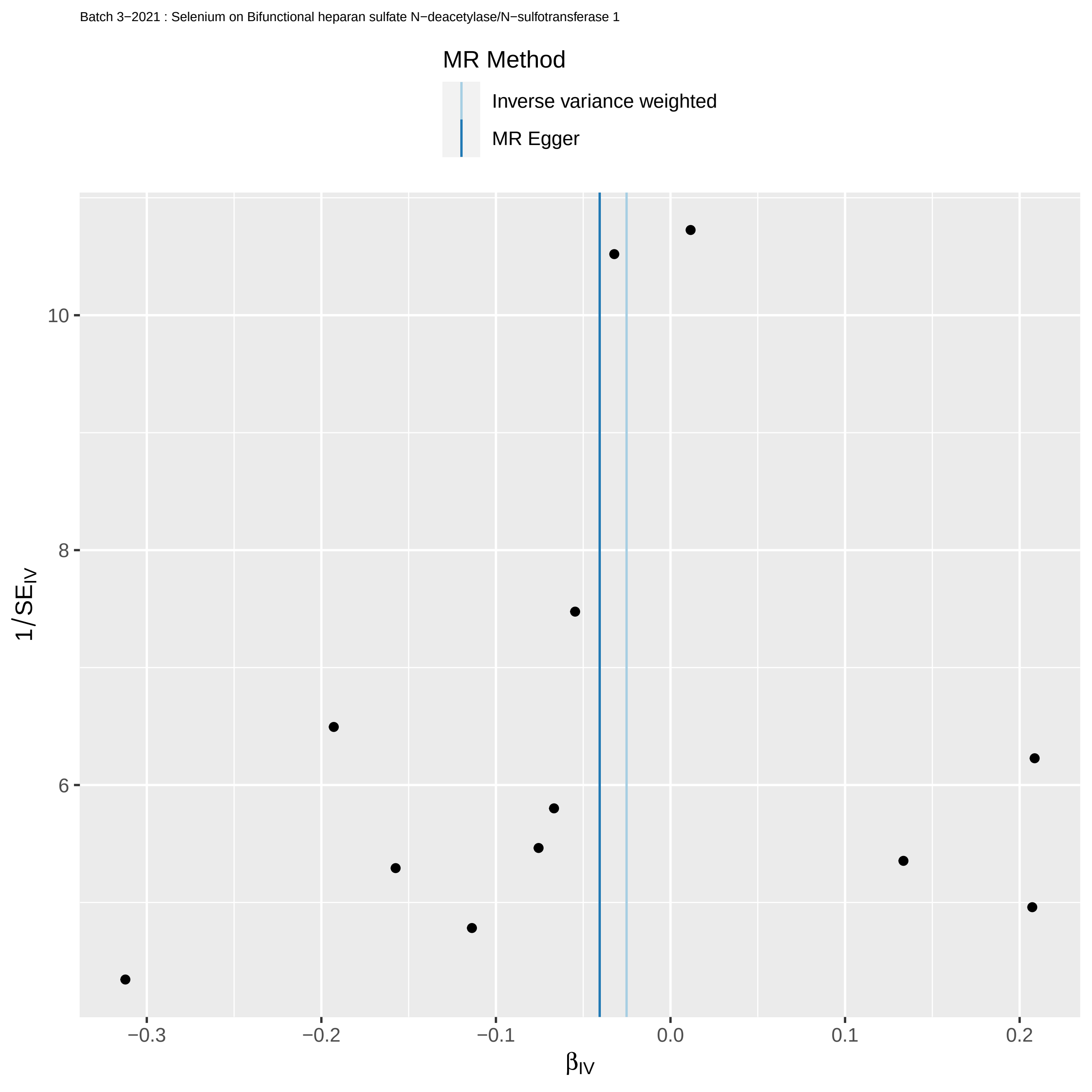
Batch 3−2021 : Selenium on Bifunctional heparan sulfate N−deacetylase/N−sulfotransferase 1
MR Method
Inverse variance weighted
MR Egger
10
8
6
1/SE_IV
−0.3
−0.2
−0.1
0.0
0.1
0.2
β_IV

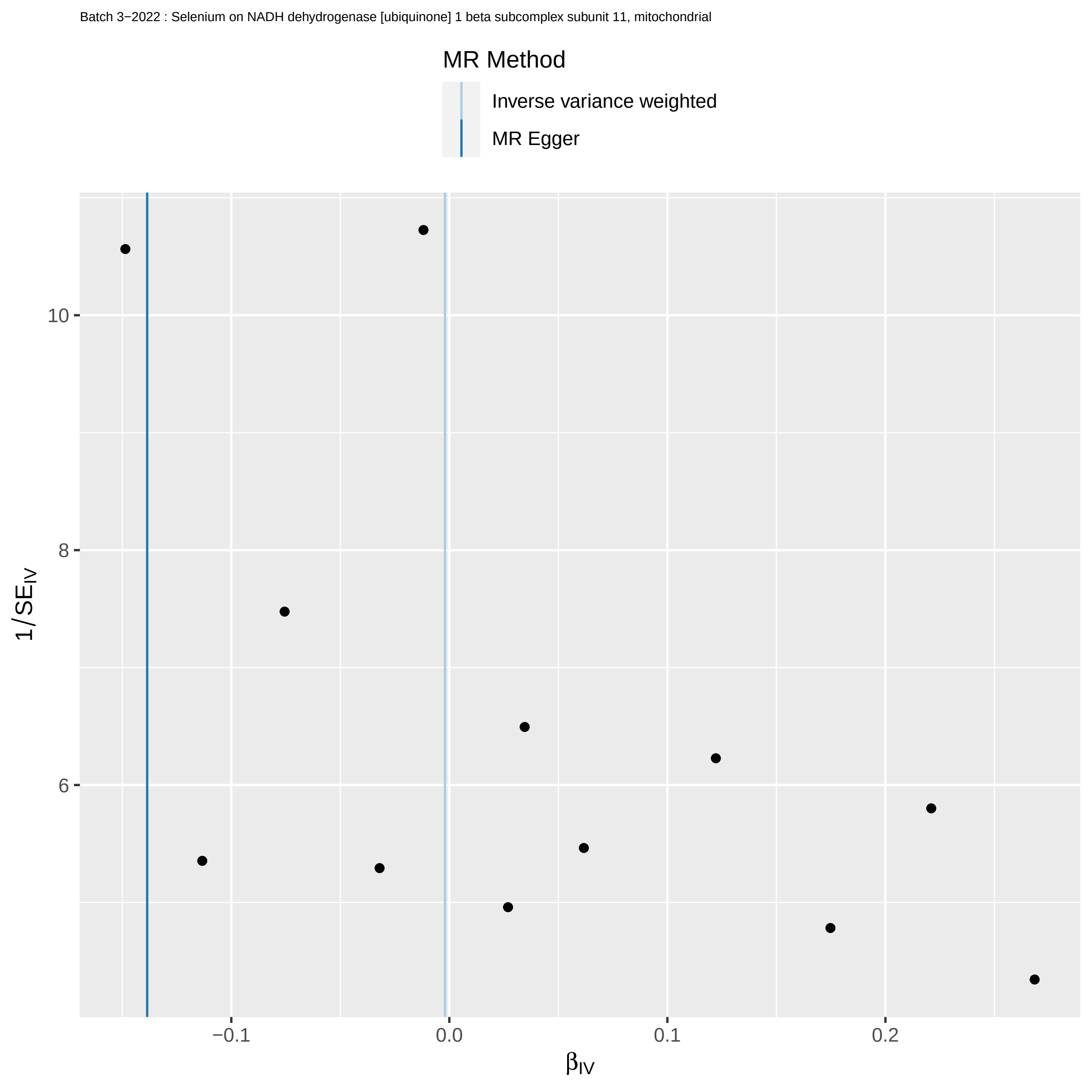

Batch 3−2022 : Selenium on NADH dehydrogenase [ubiquinone] 1 beta subcomplex subunit 11, mitochondrial
MR Method
Inverse variance weighted
MR Egger
10
8
6
1/SE$_{IV}$
−0.1
0.0
0.1
0.2
β$_{IV}$

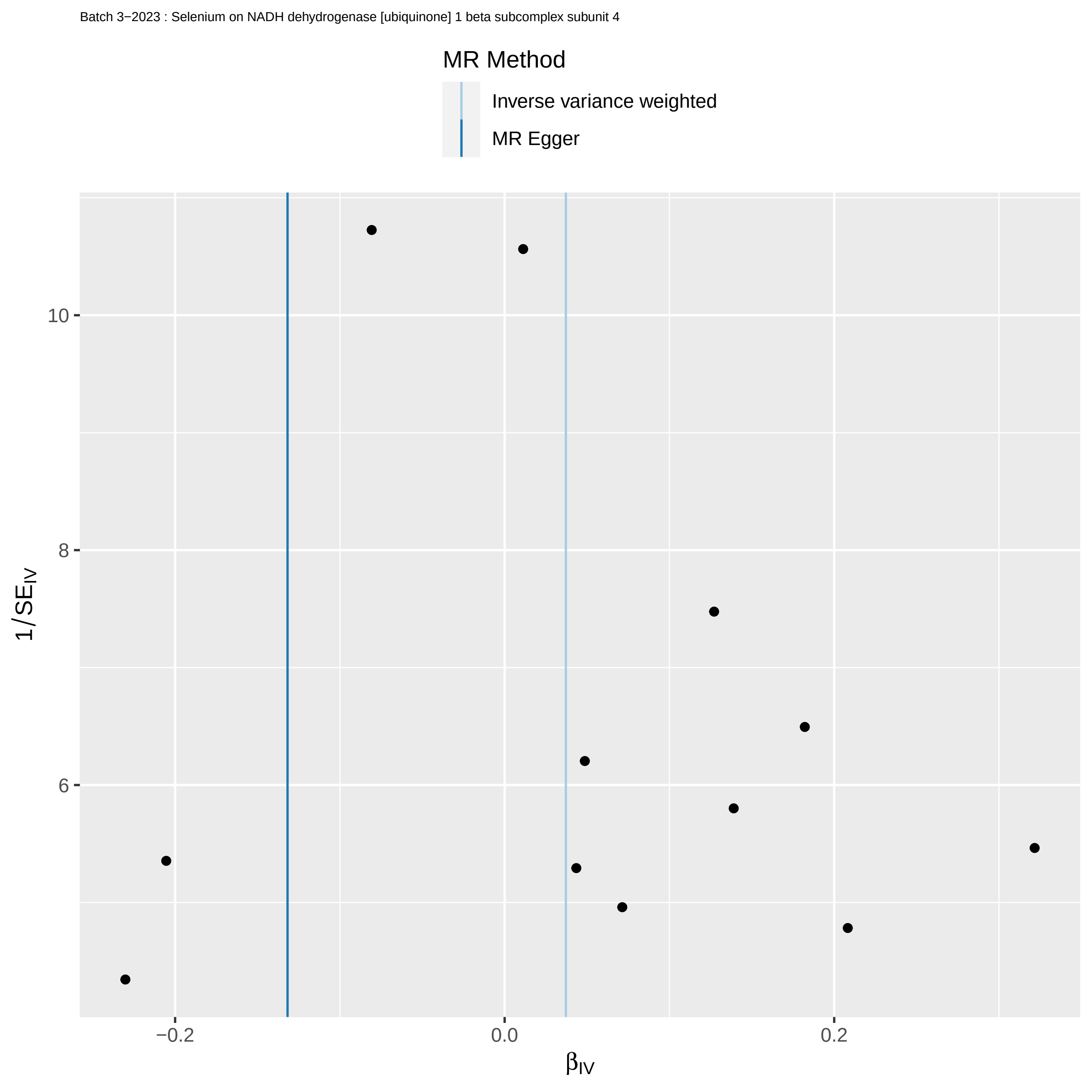

Batch 3−2023 : Selenium on NADH dehydrogenase [ubiquinone] 1 beta subcomplex subunit 4
MR Method
Inverse variance weighted
MR Egger
10
8
6
1/SE$_{IV}$
−0.2
0.0
0.2
β$_{IV}$

Batch 3−2024 : Selenium on NADH dehydrogenase [ubiquinone] 1 beta subcomplex subunit 8, mitochondrial

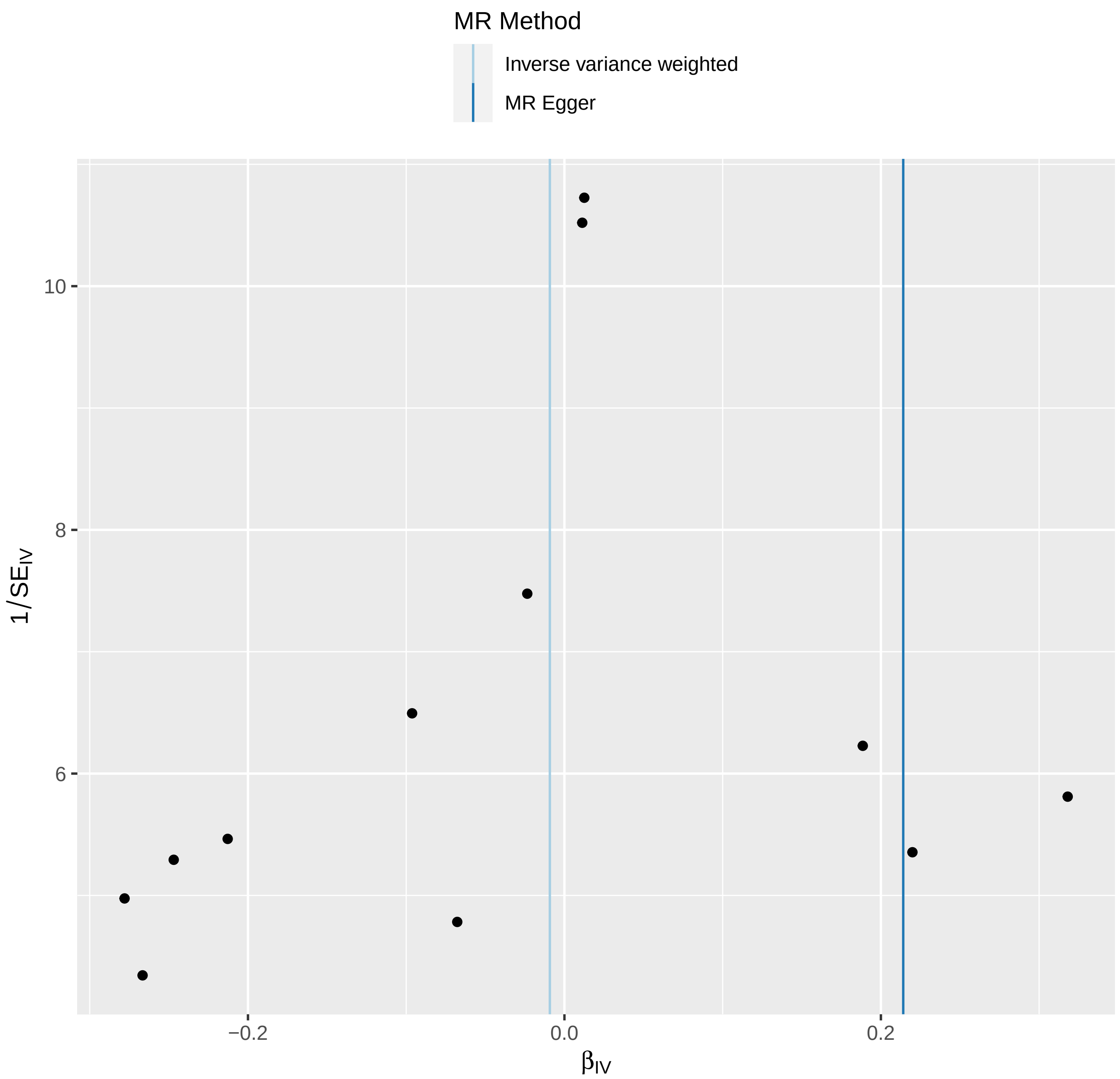

Batch 3−2025 : Selenium on NADH dehydrogenase [ubiquinone] iron−sulfur protein 4, mitochondrial

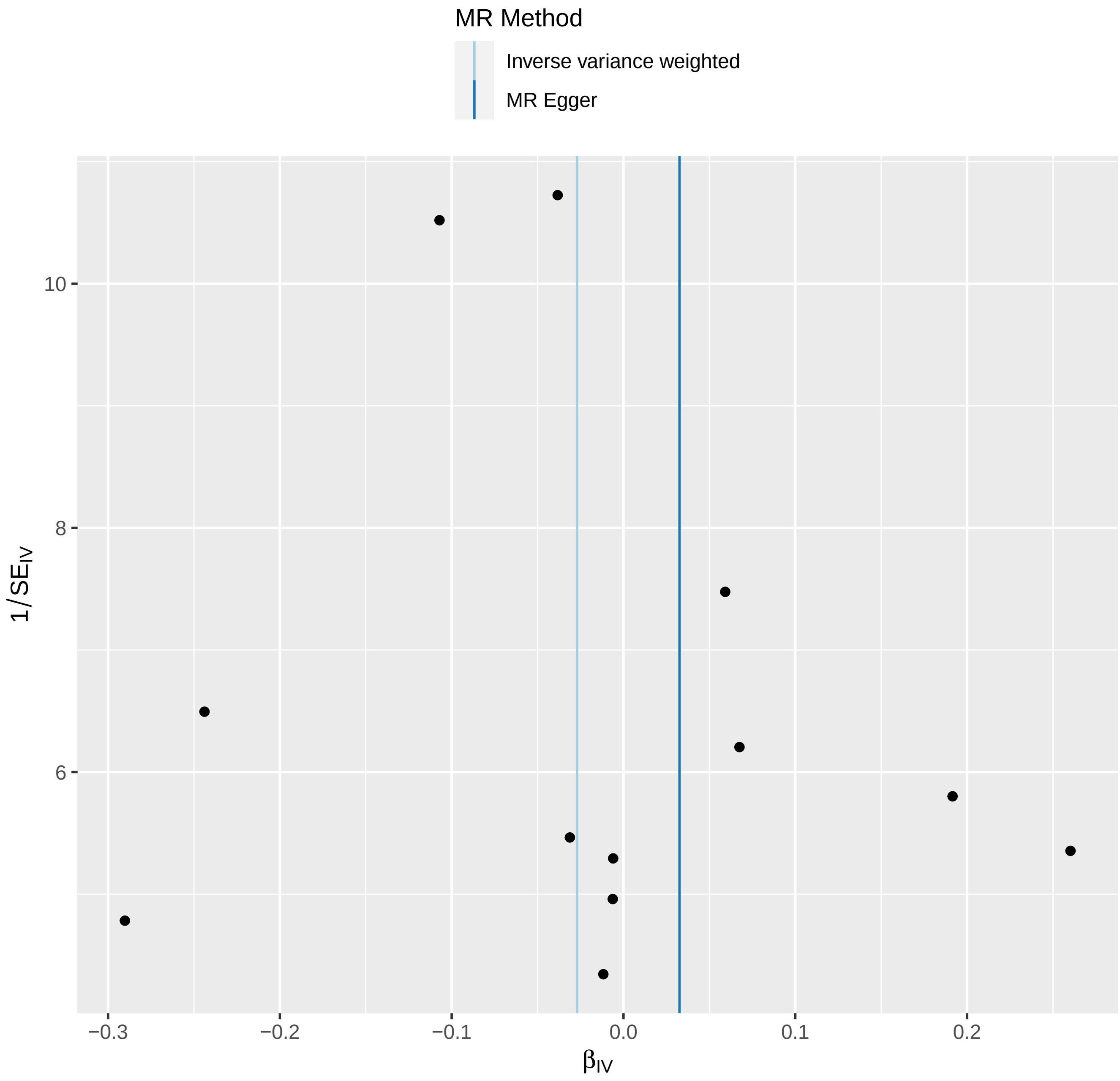

Batch 3−2026 : Selenium on NADH dehydrogenase [ubiquinone] flavoprotein 2, mitochondrial

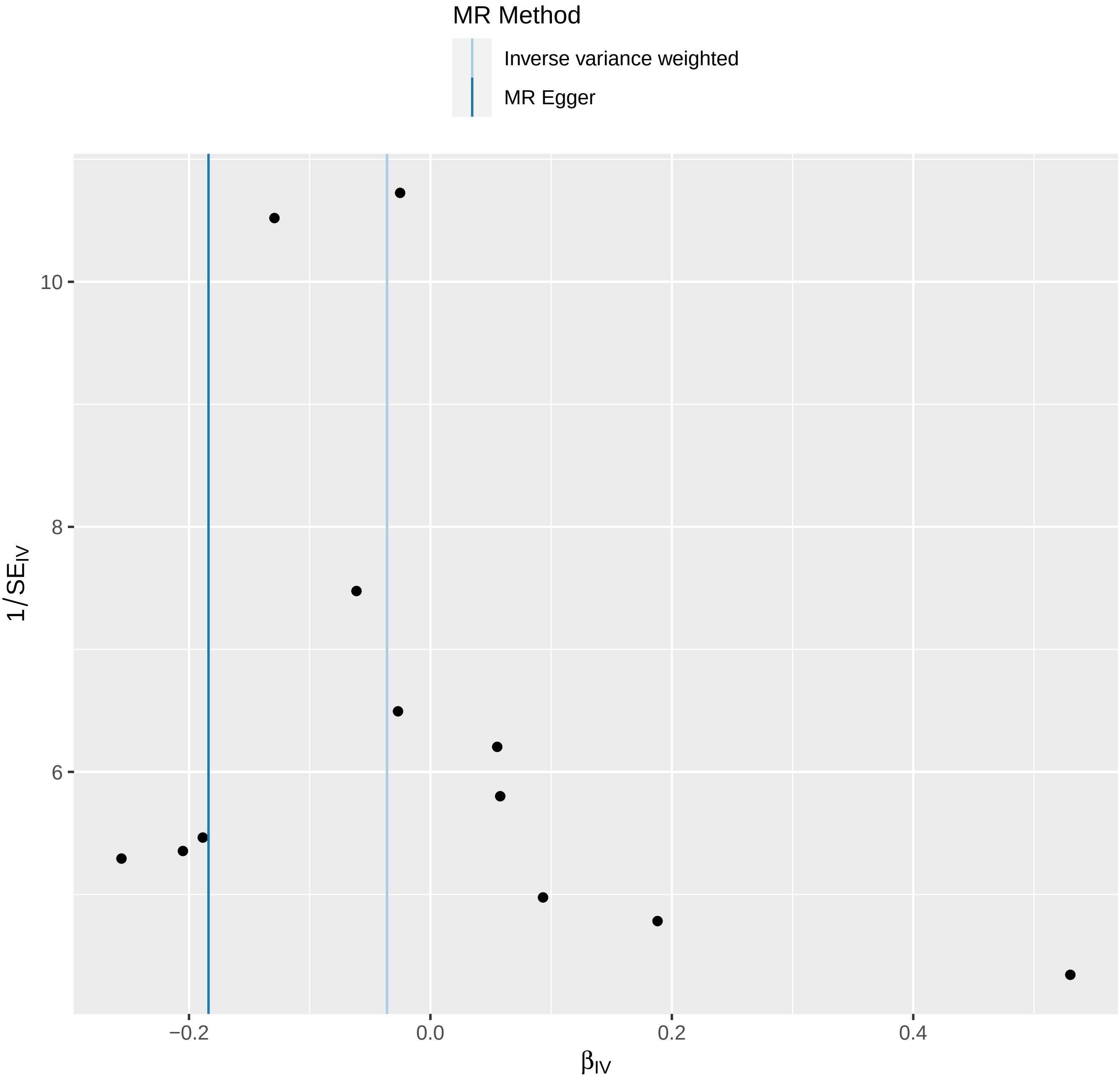

Batch 3−2027 : Selenium on Neurofilament heavy polypeptide

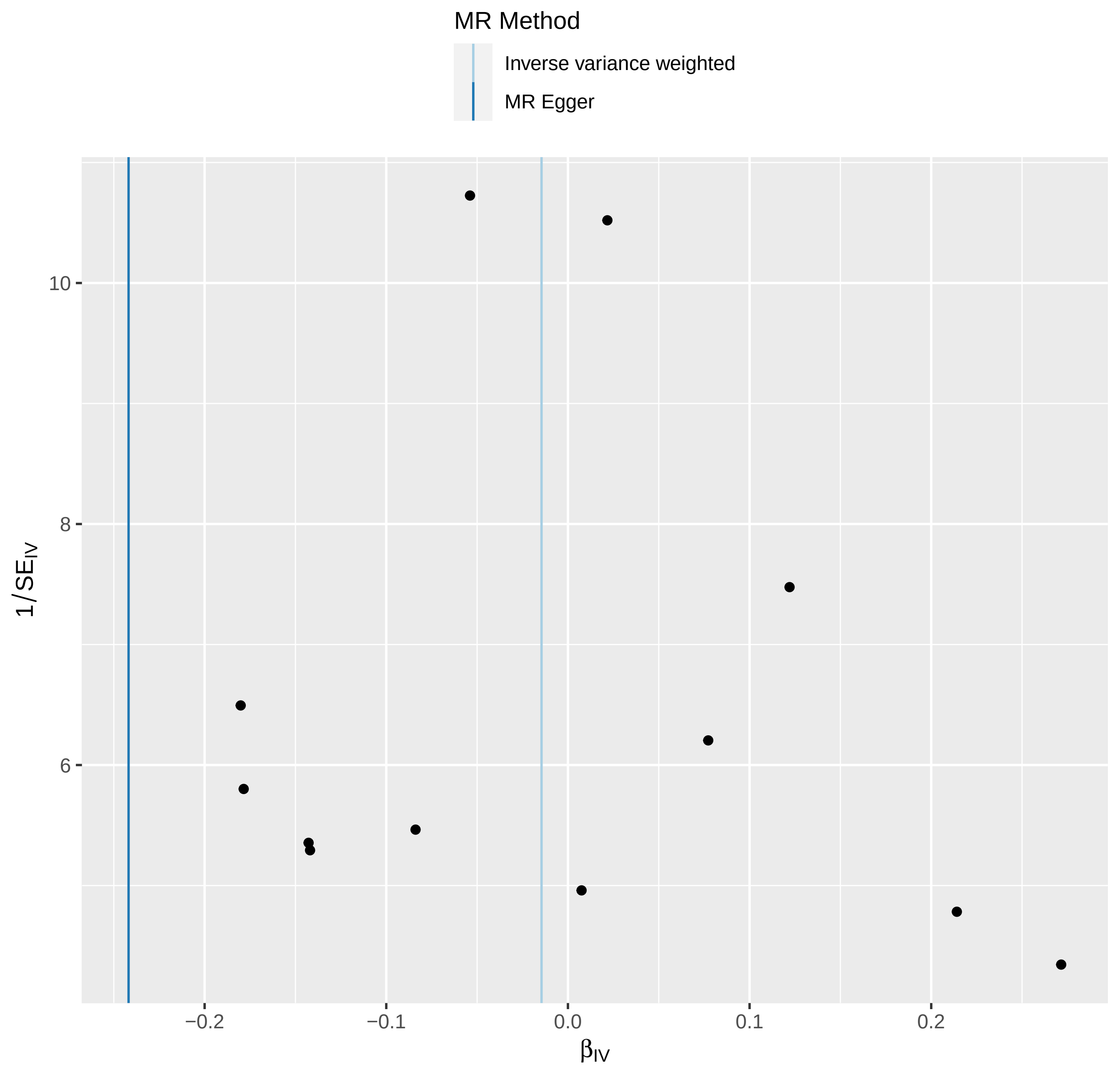

Batch 3−2028 : Selenium on Neuronal growth regulator 1

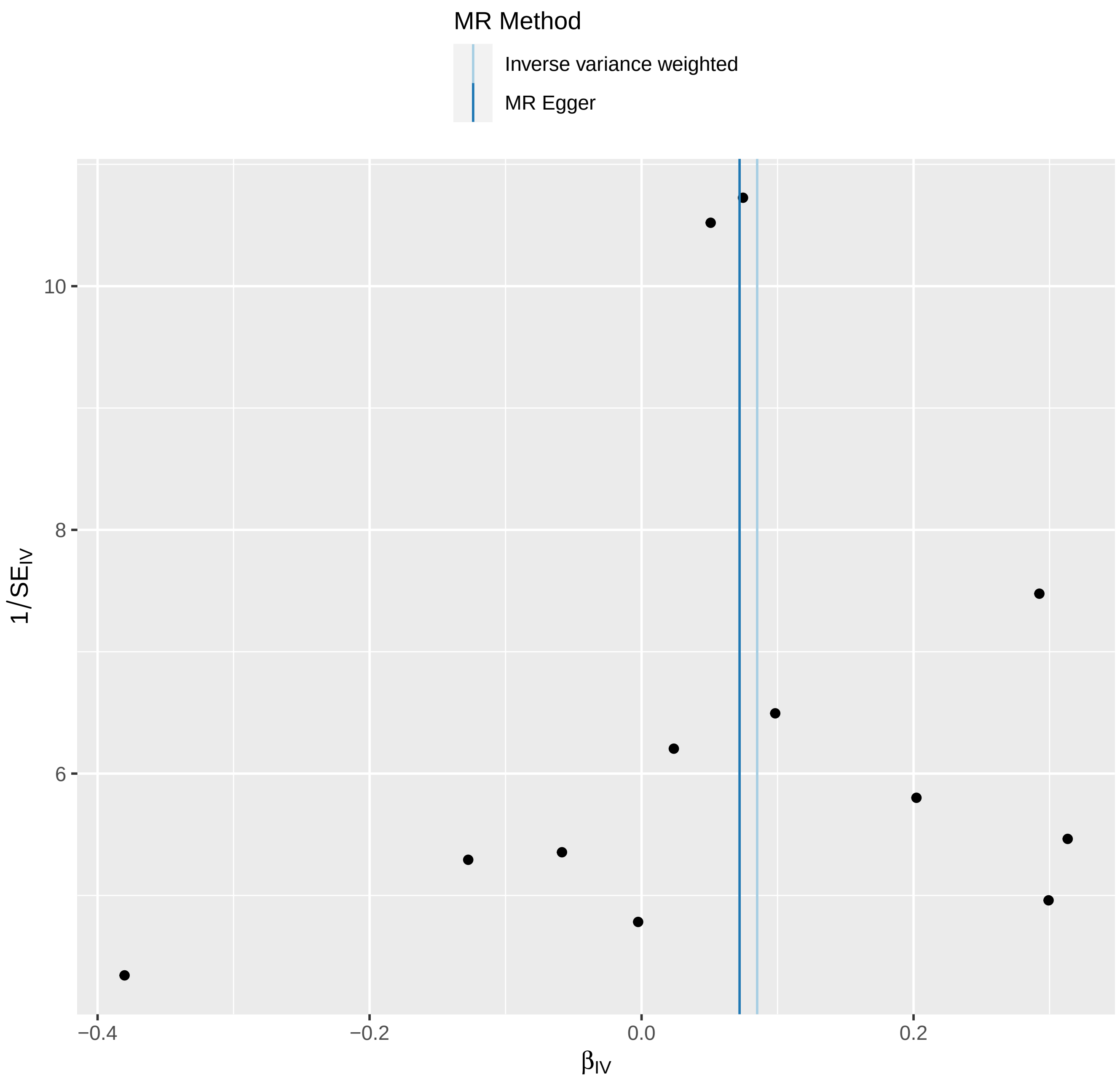

Batch 3−2029 : Selenium on Neuronal growth regulator 1

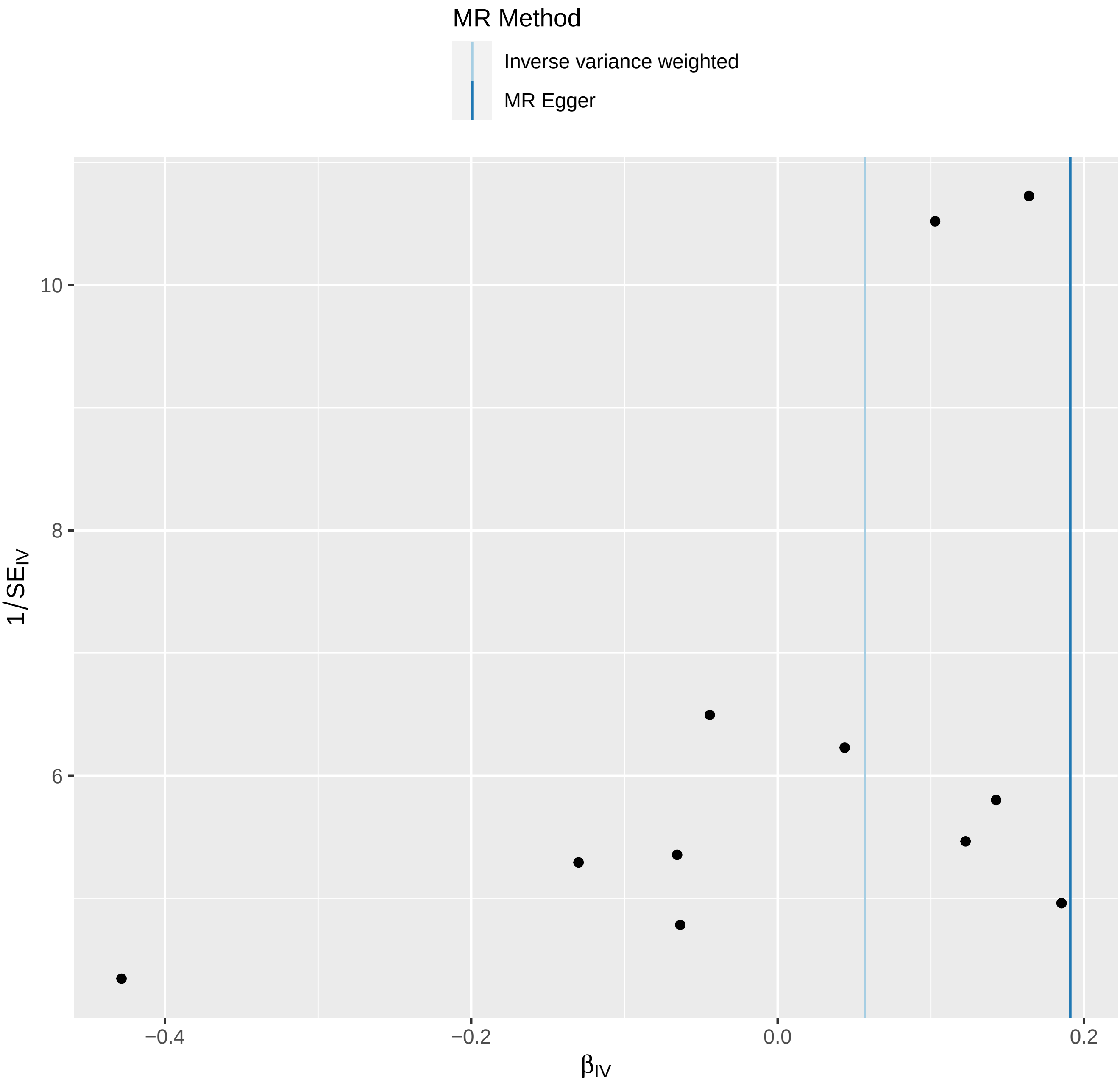

Batch 3−2030 : Selenium on Protein kinase C−binding protein NELL1

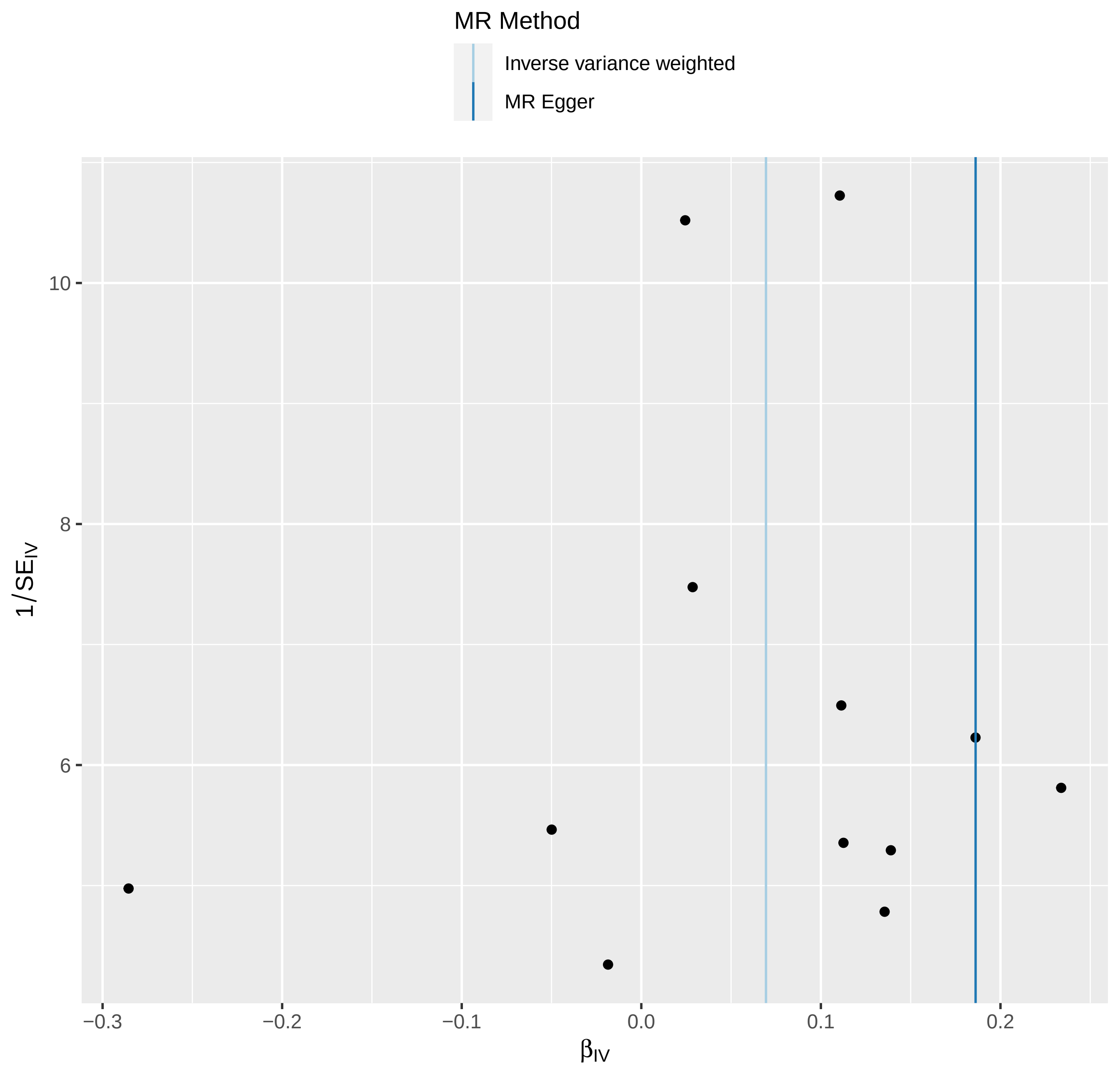

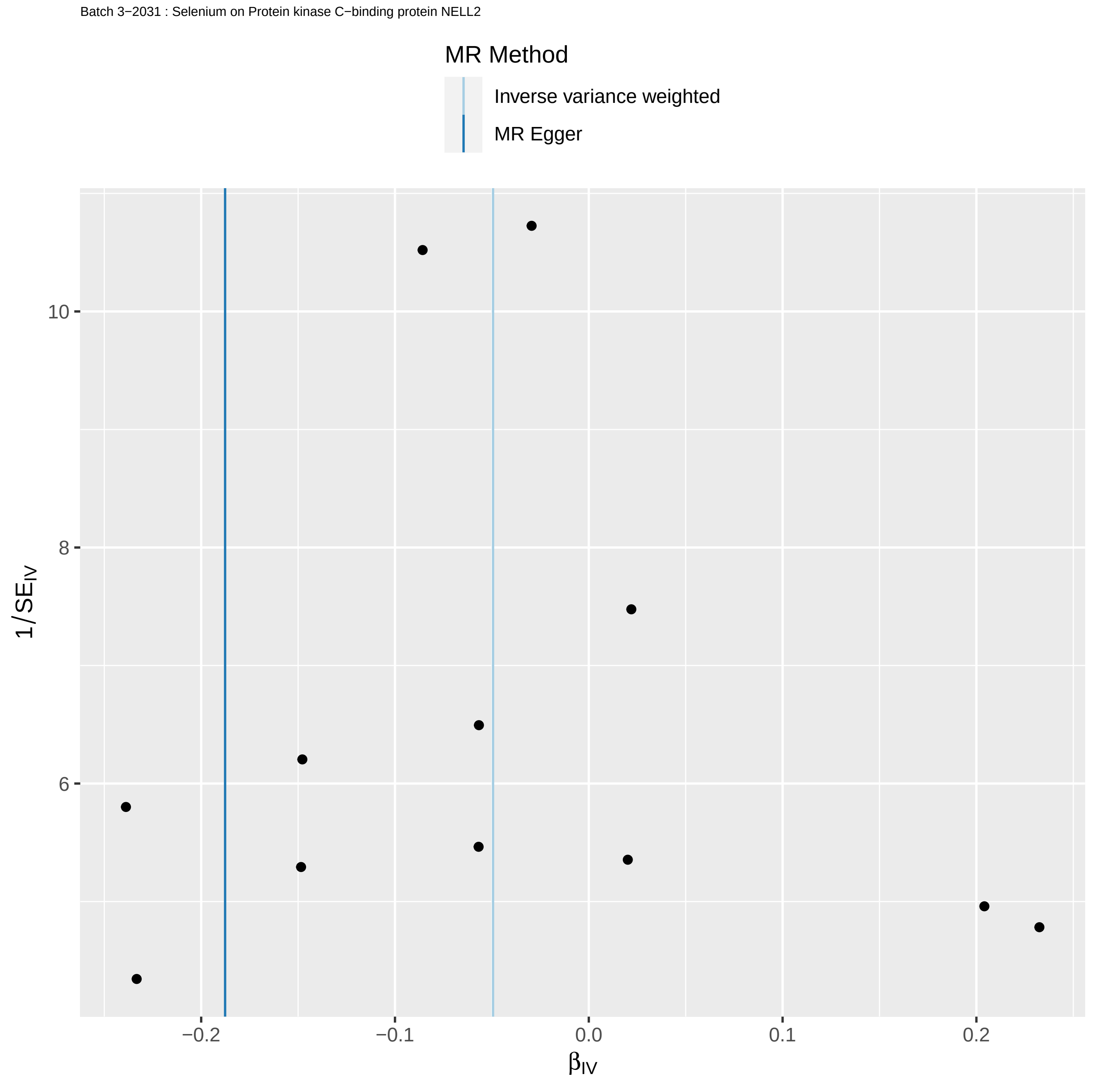

Batch 3−2031 : Selenium on Protein kinase C−binding protein NELL2
MR Method
Inverse variance weighted
MR Egger
10
8
6
1/SE_IV
−0.2
−0.1
0.0
0.1
0.2
β_IV

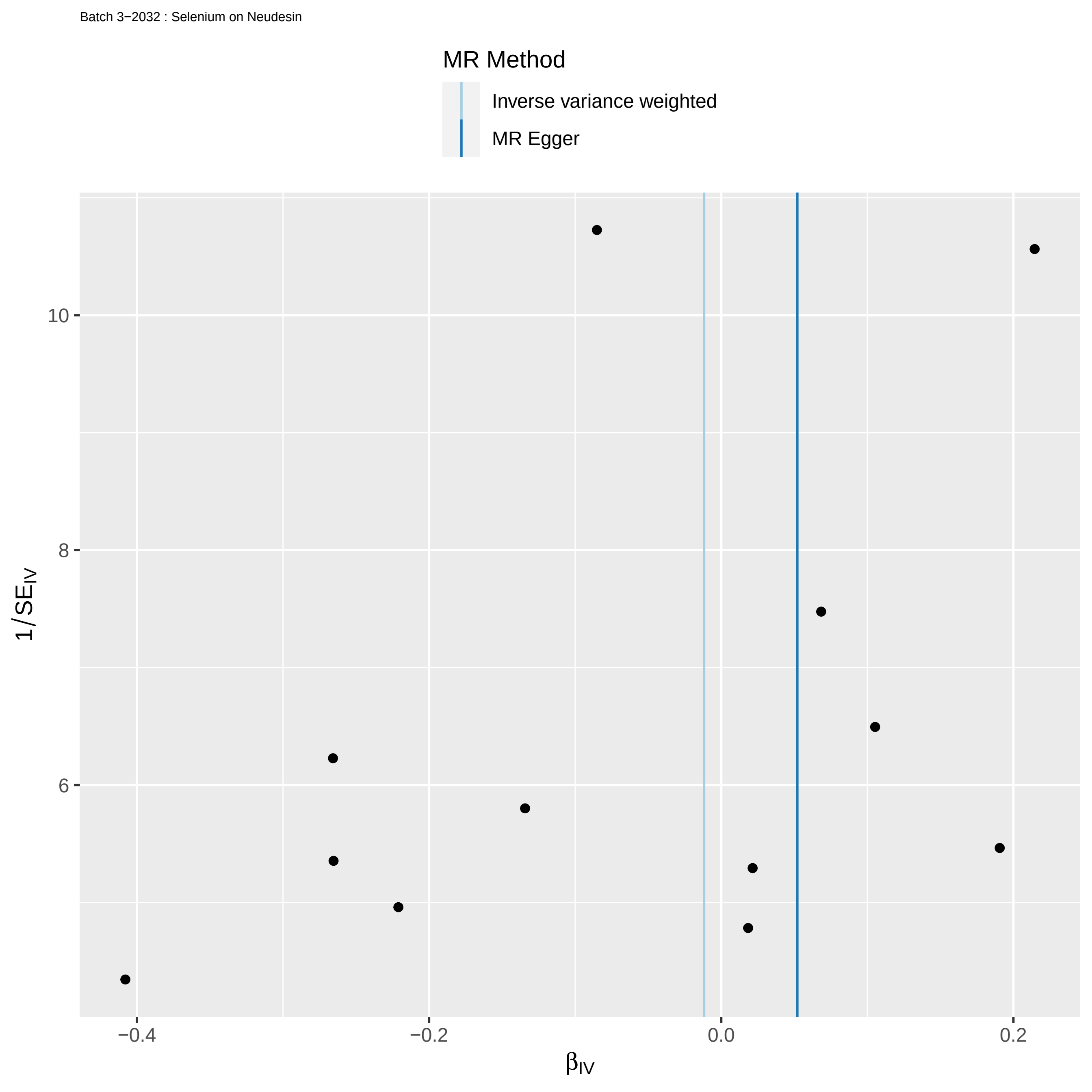

Batch 3−2032 : Selenium on Neudesin
MR Method
Inverse variance weighted
MR Egger
10
8
6
1/SE$_{IV}$
−0.4
−0.2
0.0
0.2
β$_{IV}$

Batch 3−2033 : Selenium on Neogenin

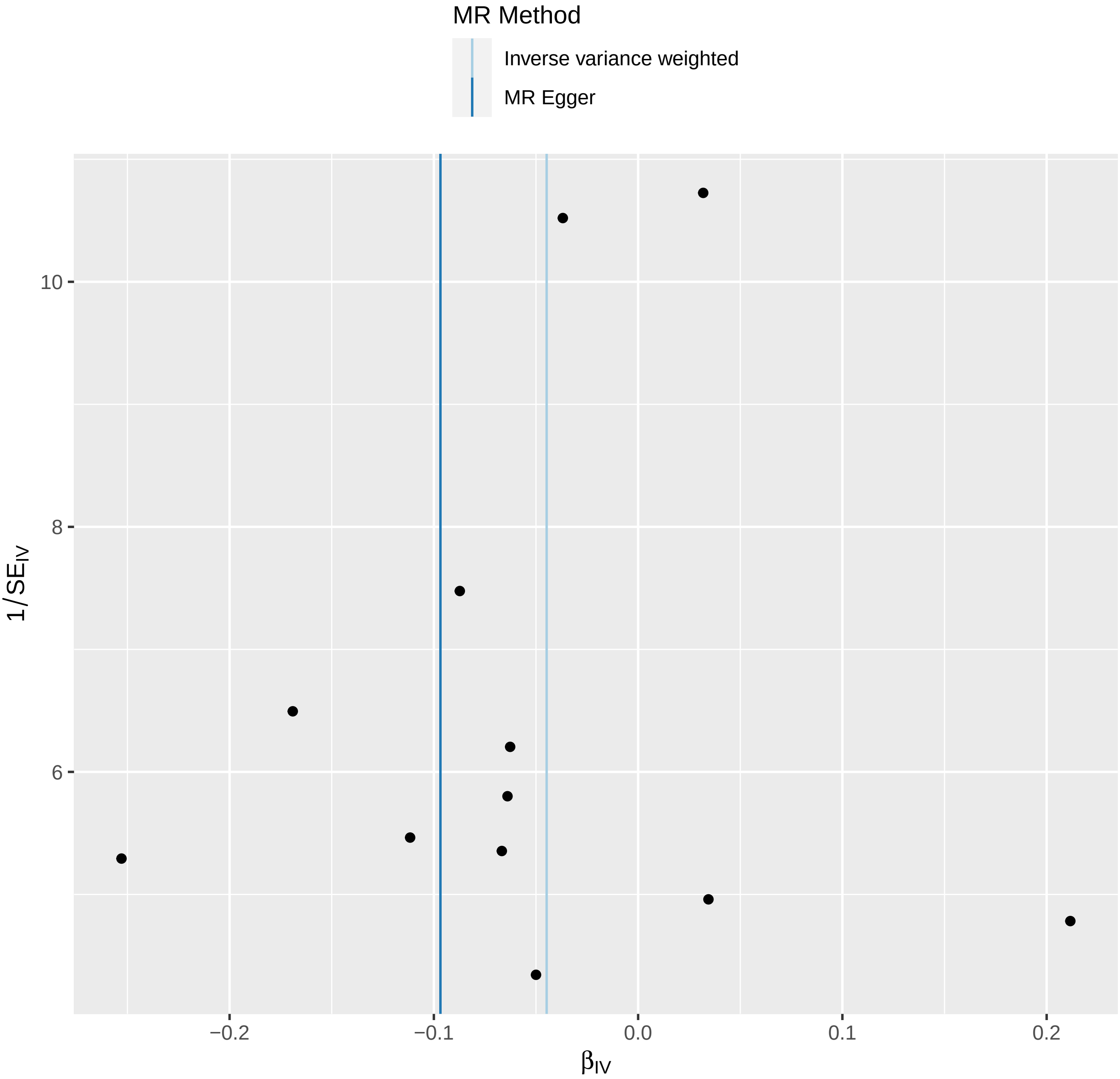

Batch 3−2034 : Selenium on Neuroepithelial cell−transforming gene 1 protein

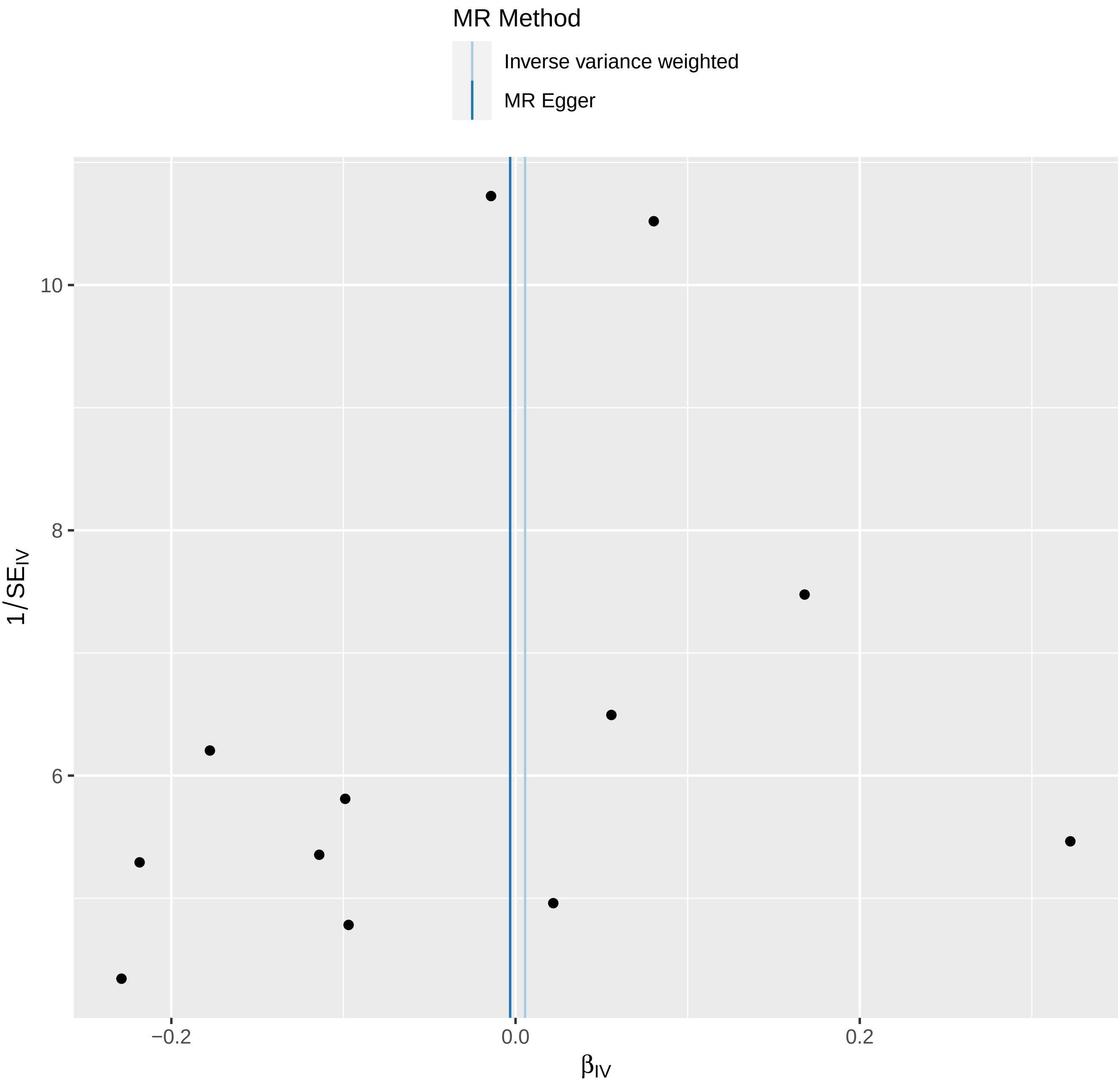

Batch 3−2035 : Selenium on Neuropilin and tolloid−like protein 1

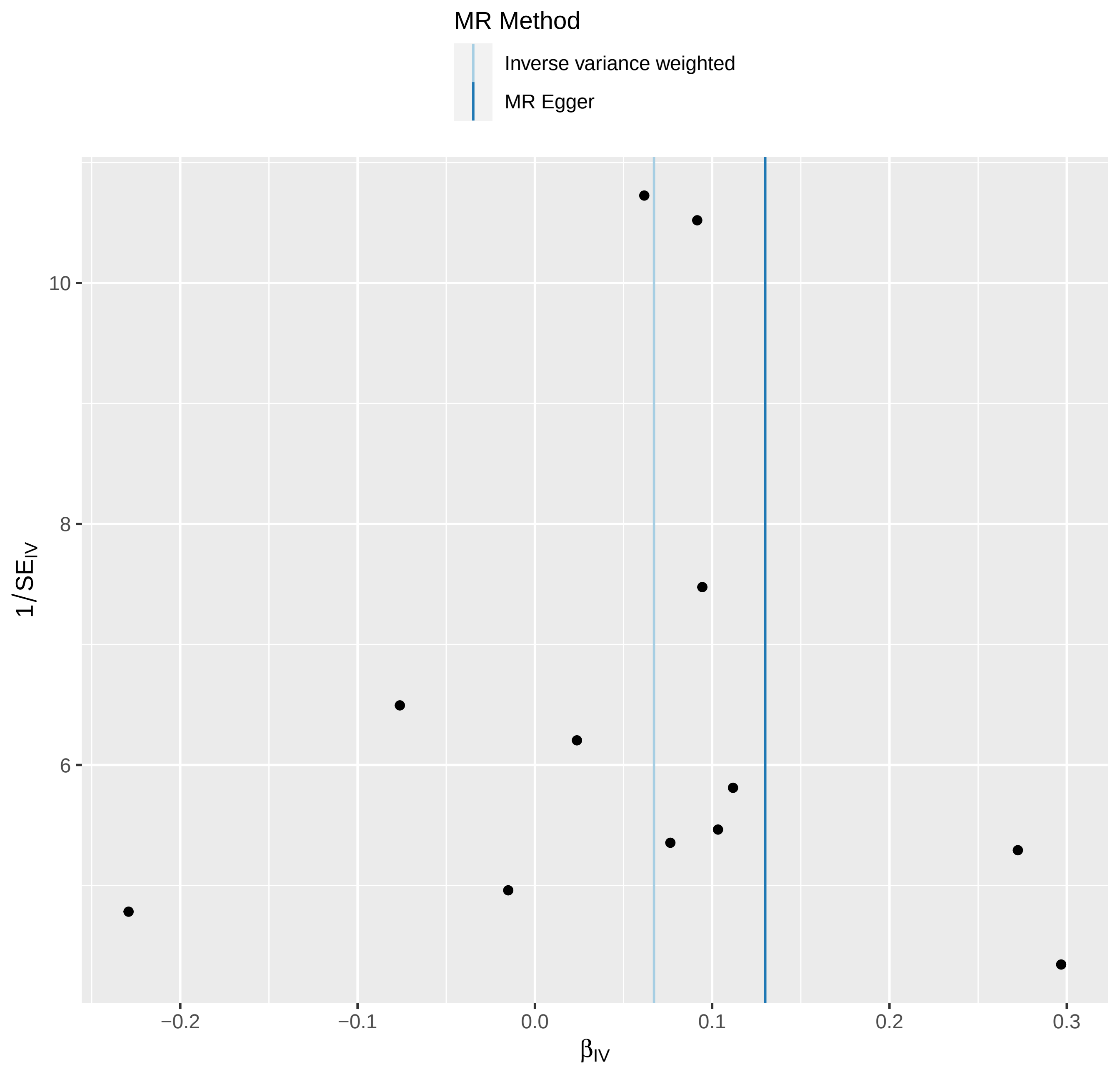

Batch 3−2036 : Selenium on Neuropilin and tolloid−like protein 2

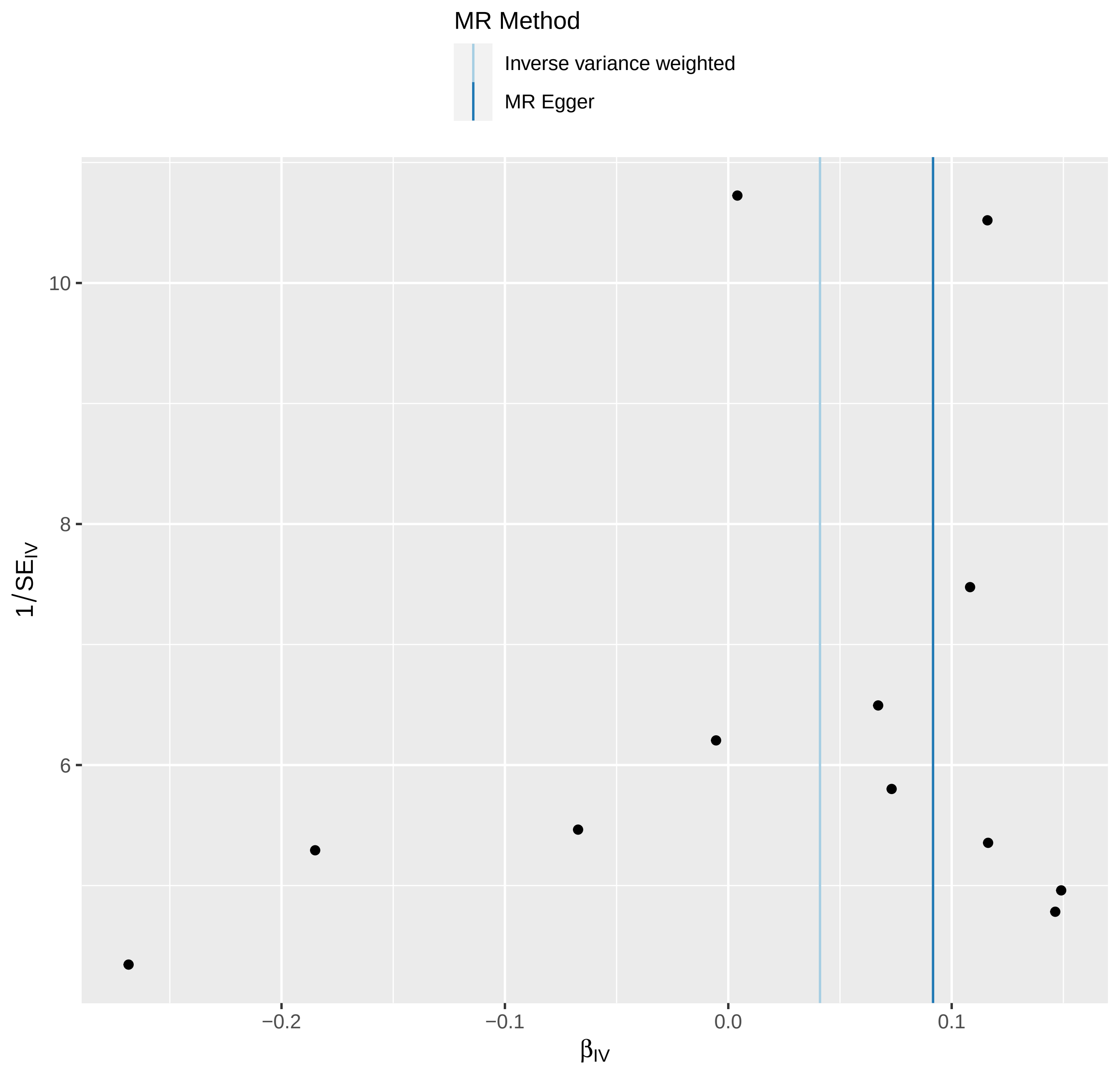

Batch 3−2037 : Selenium on E3 ubiquitin−protein ligase NEURL1

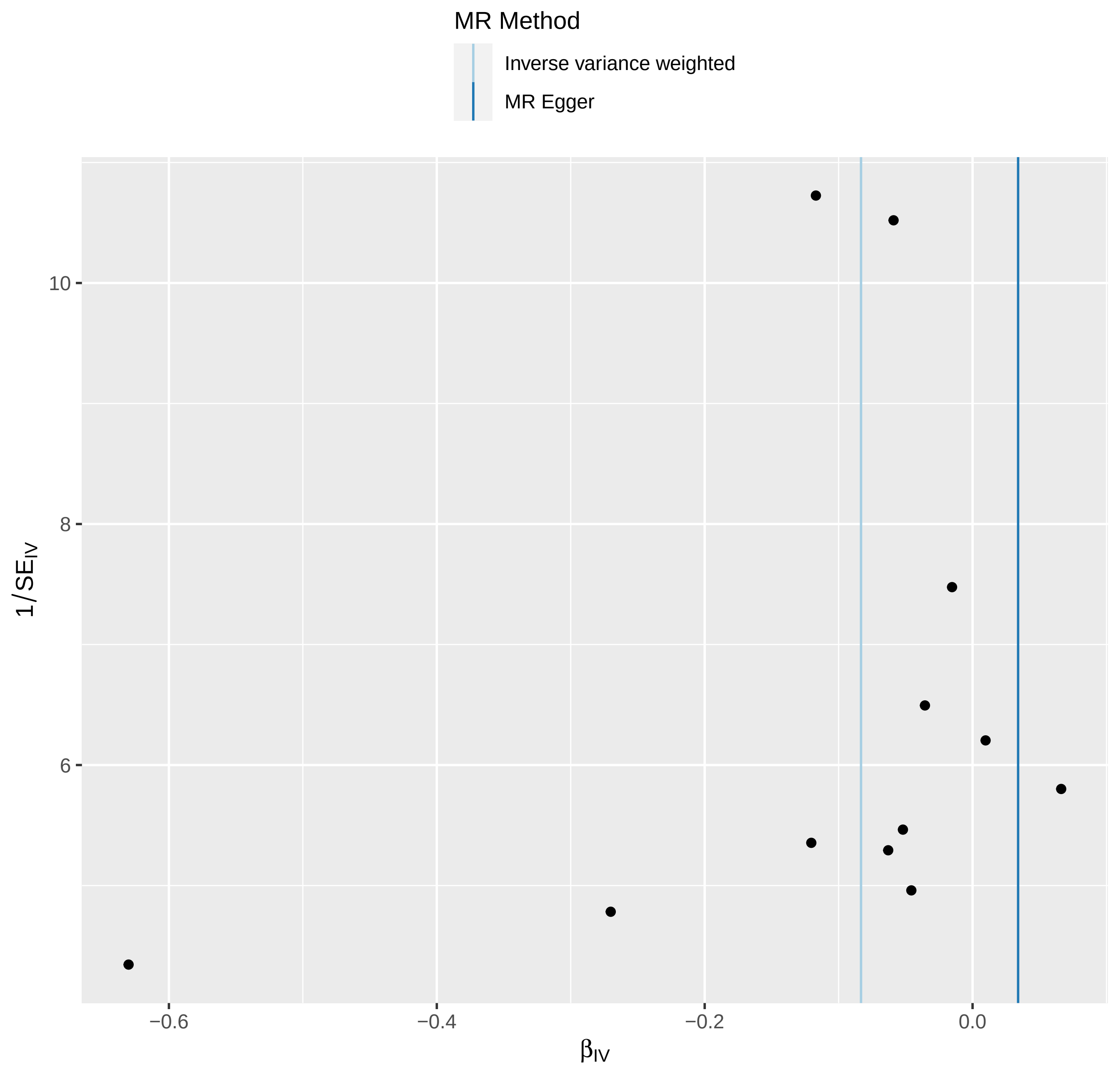

Batch 3−2038 : Selenium on Neuralized−like protein 4

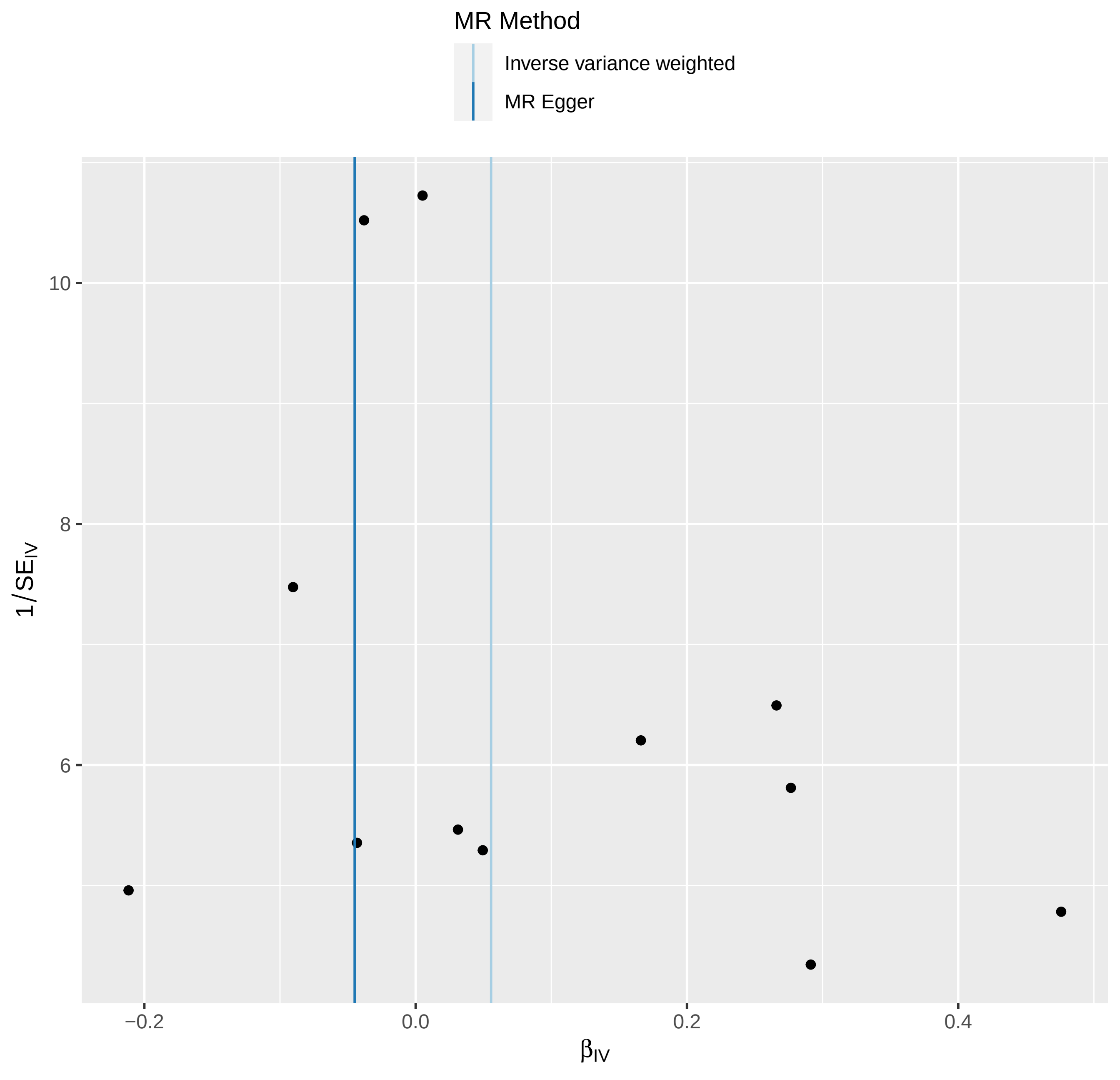

Batch 3−2039 : Selenium on Neurofascin

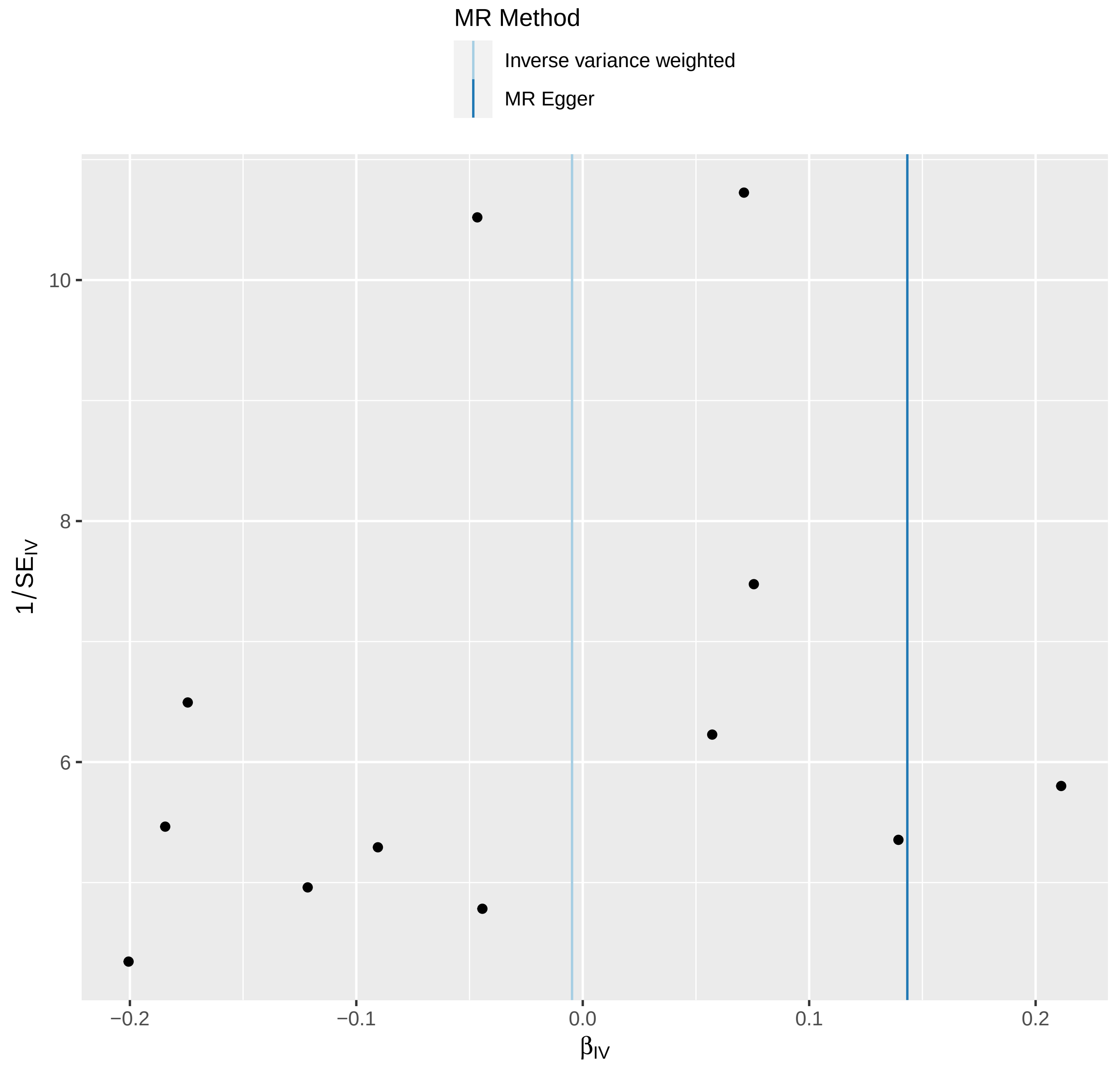

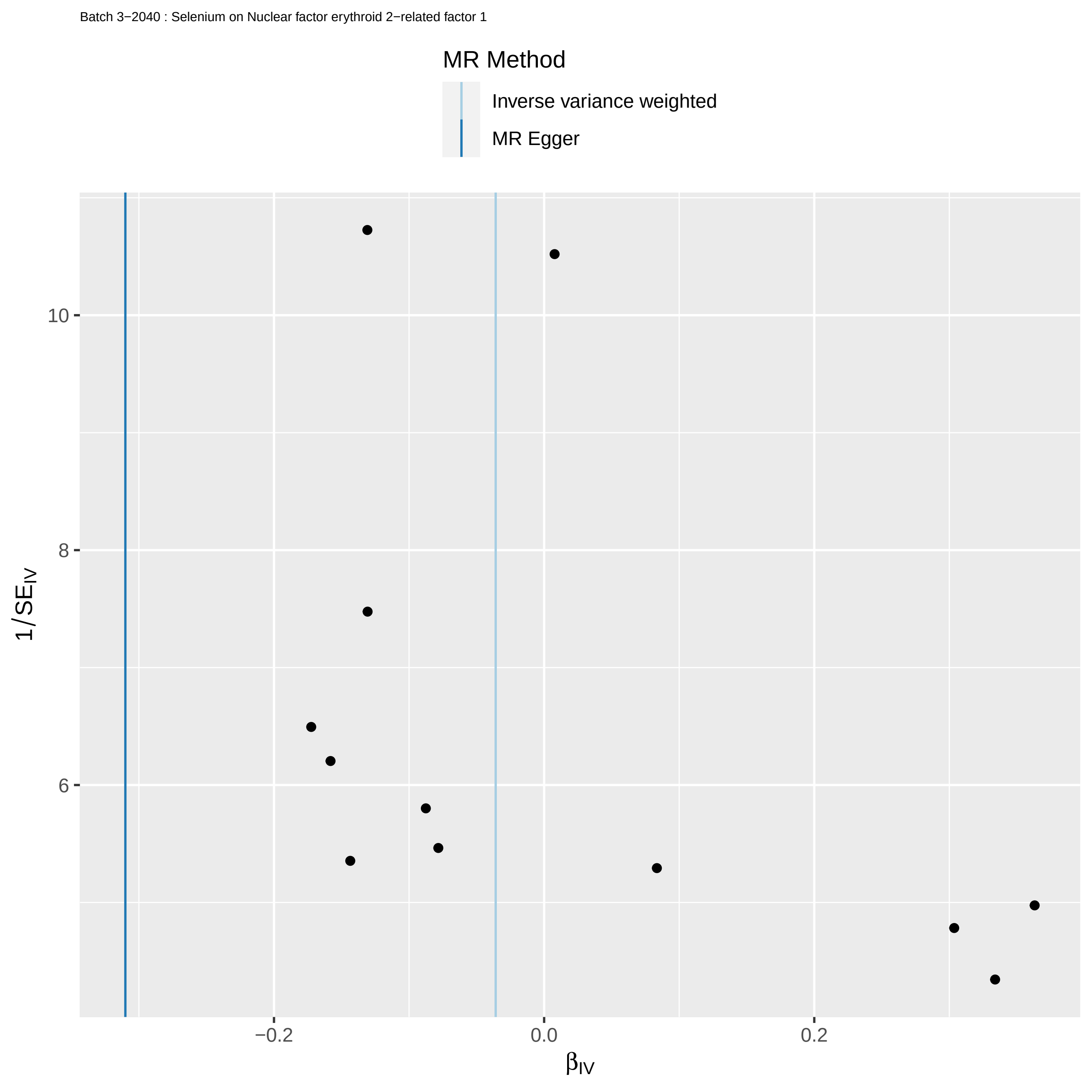
Batch 3−2040 : Selenium on Nuclear factor erythroid 2−related factor 1
MR Method
Inverse variance weighted
MR Egger
10
8
6
1/SE_IV
−0.2
0.0
0.2
β_IV

Batch 3−2041 : Selenium on NFU1 iron−sulfur cluster scaffold homolog, mitochondrial

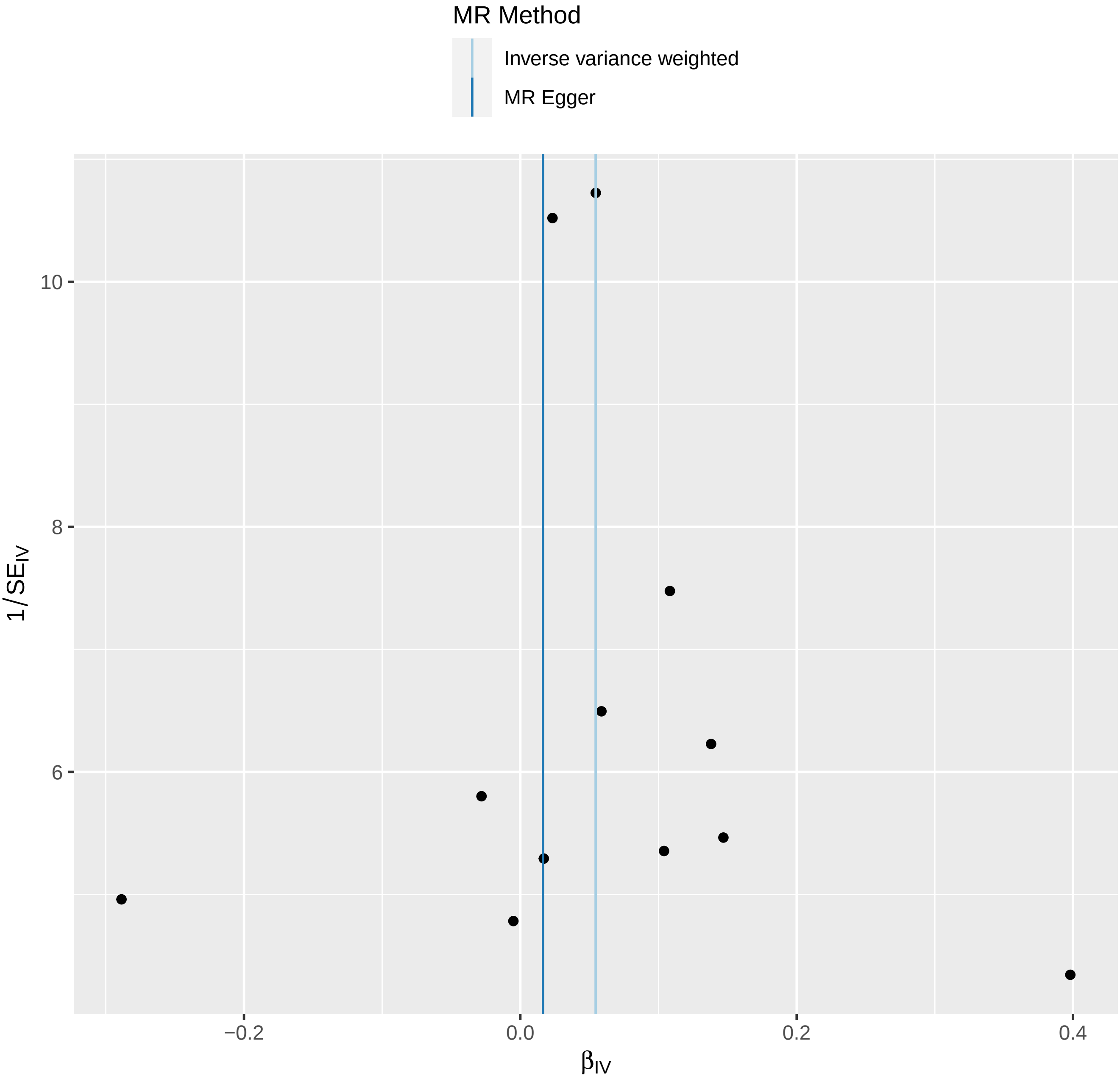

Batch 3−2042 : Selenium on beta−nerve growth factor

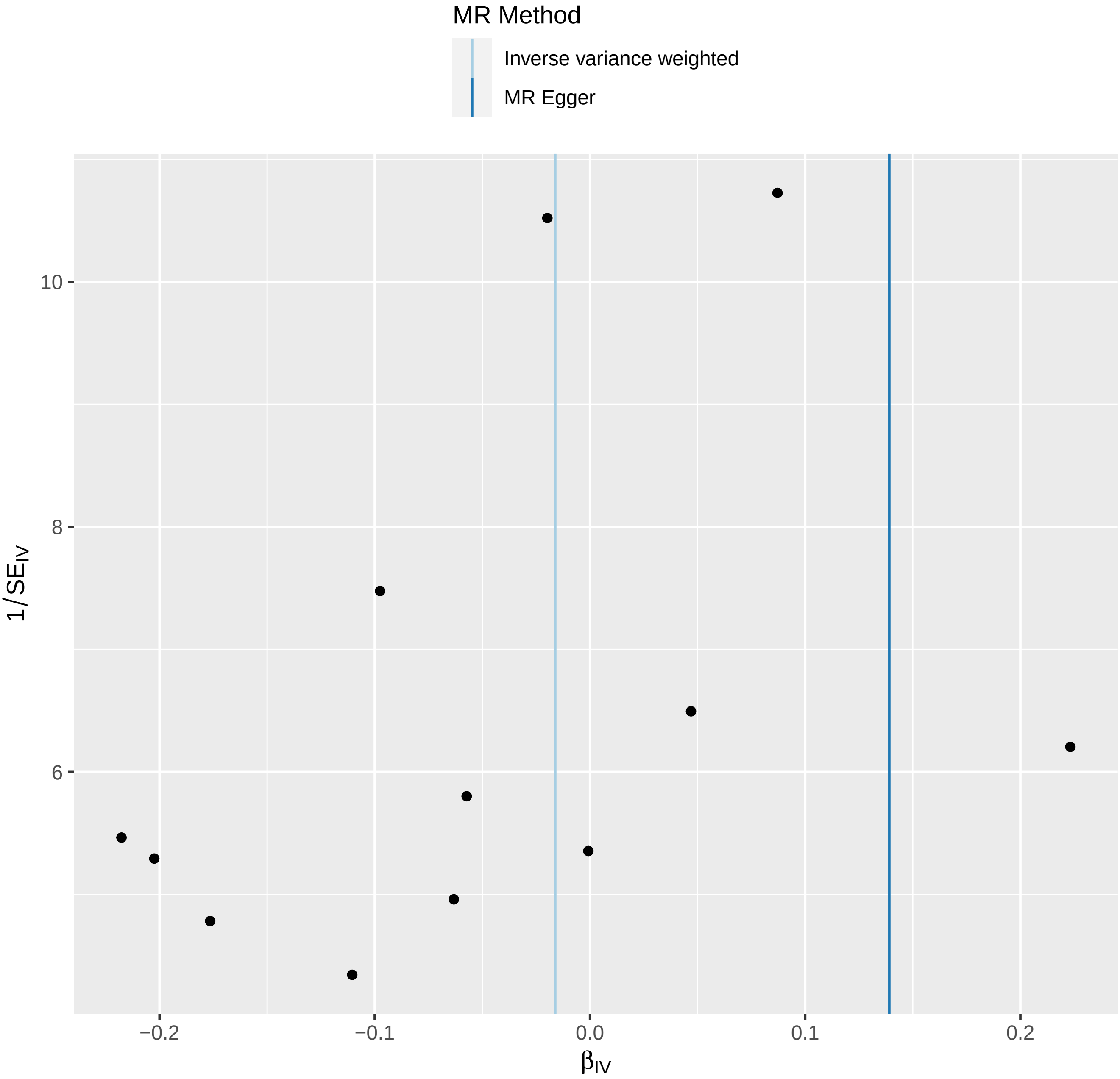

Batch 3−2043 : Selenium on Tumor necrosis factor receptor superfamily member 16

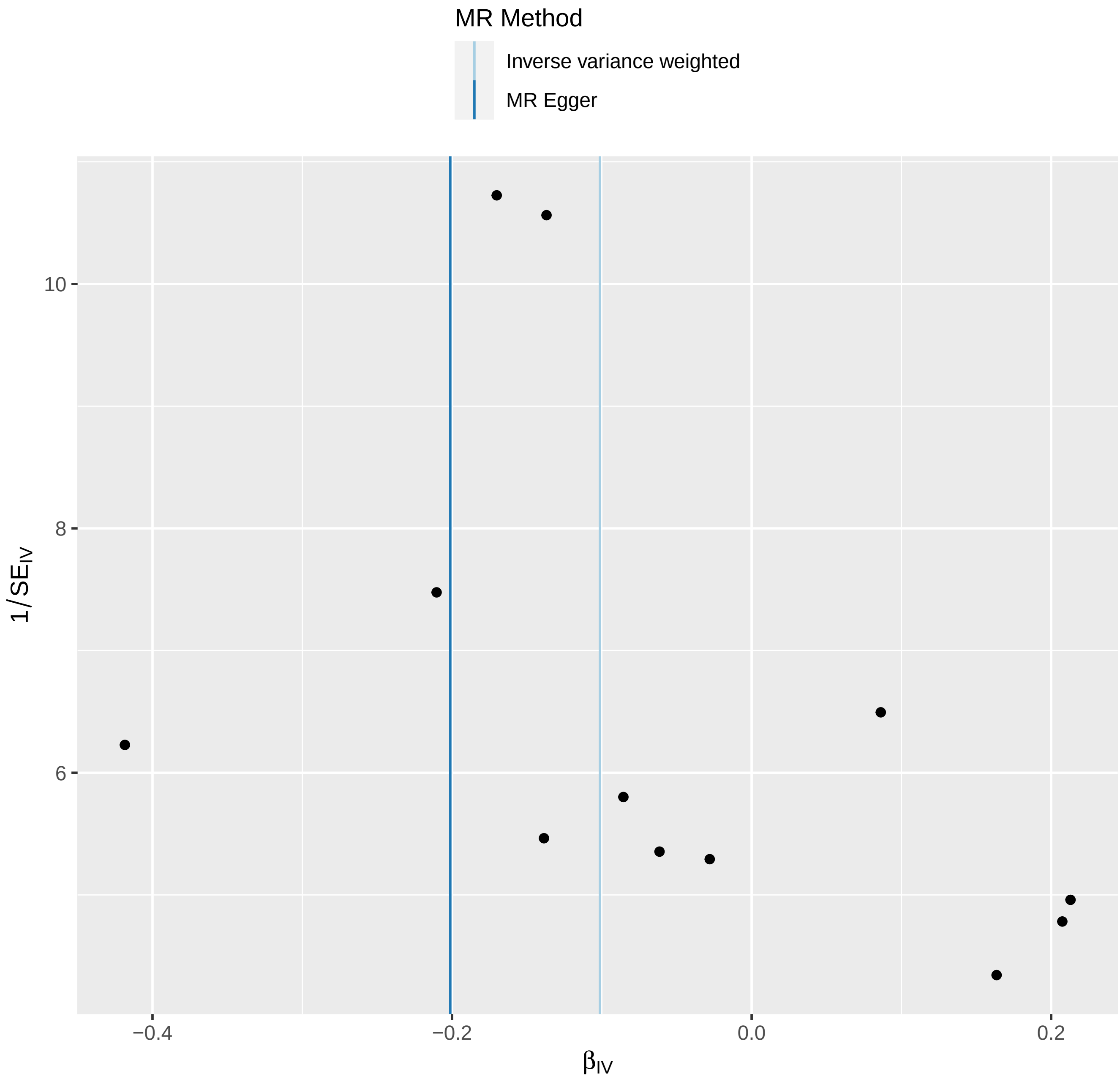

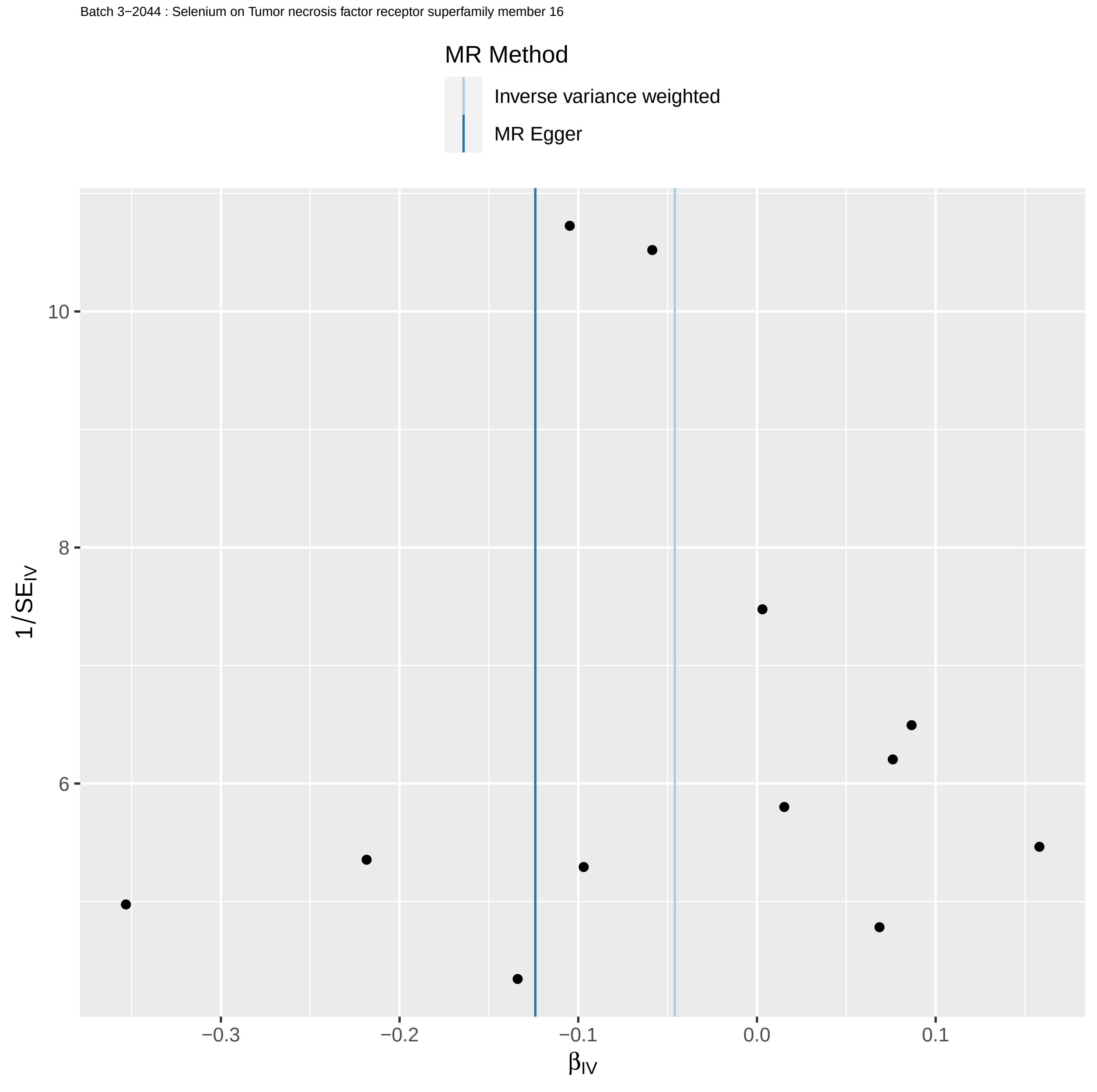

Batch 3−2044 : Selenium on Tumor necrosis factor receptor superfamily member 16
MR Method
Inverse variance weighted
MR Egger
10
8
6
1/SE_IV
−0.3
−0.2
−0.1
0.0
0.1
β_IV

Batch 3−2045 : Selenium on Neugrin

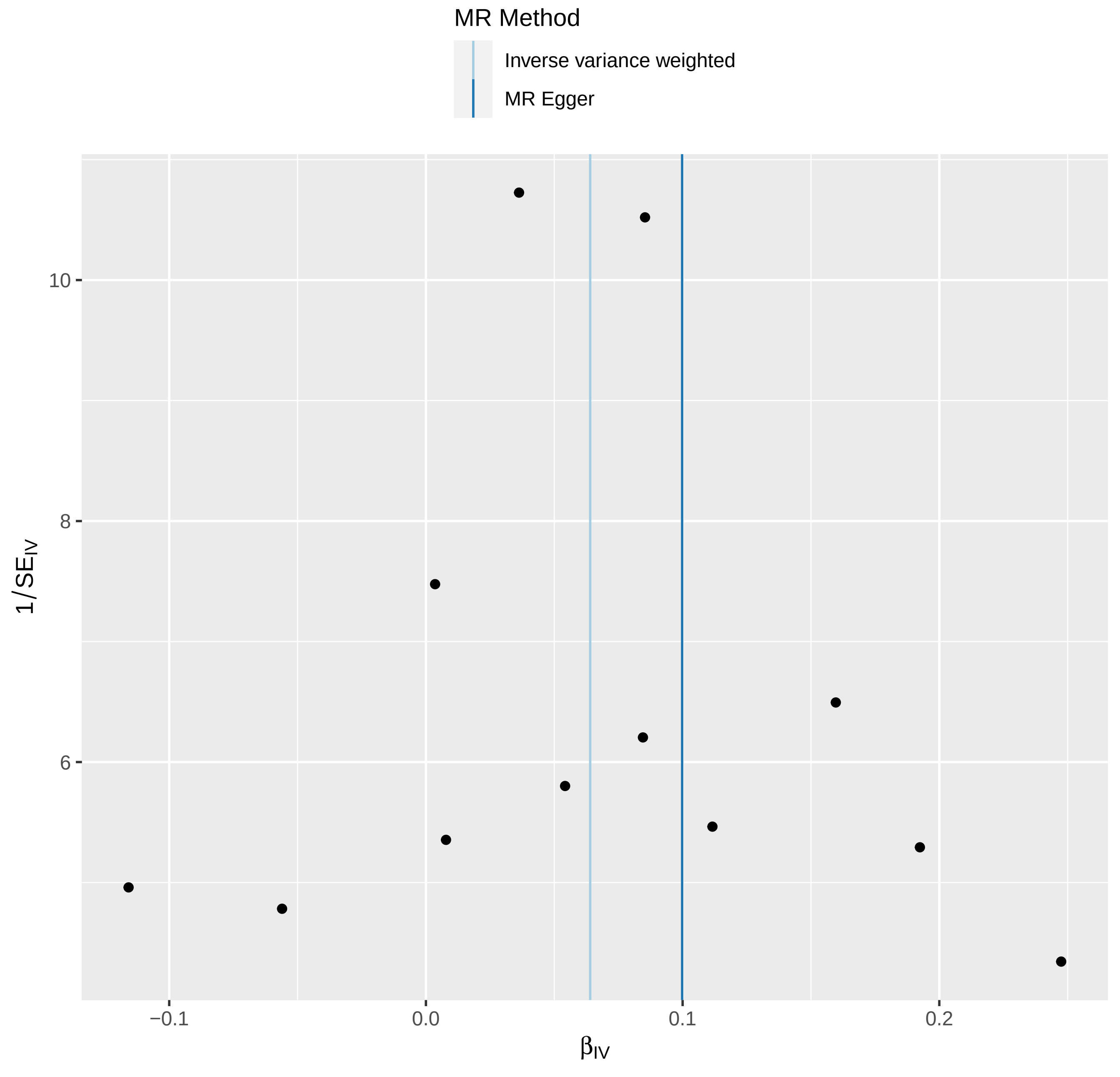

Batch 3−2046 : Selenium on Non−homologous end−joining factor 1

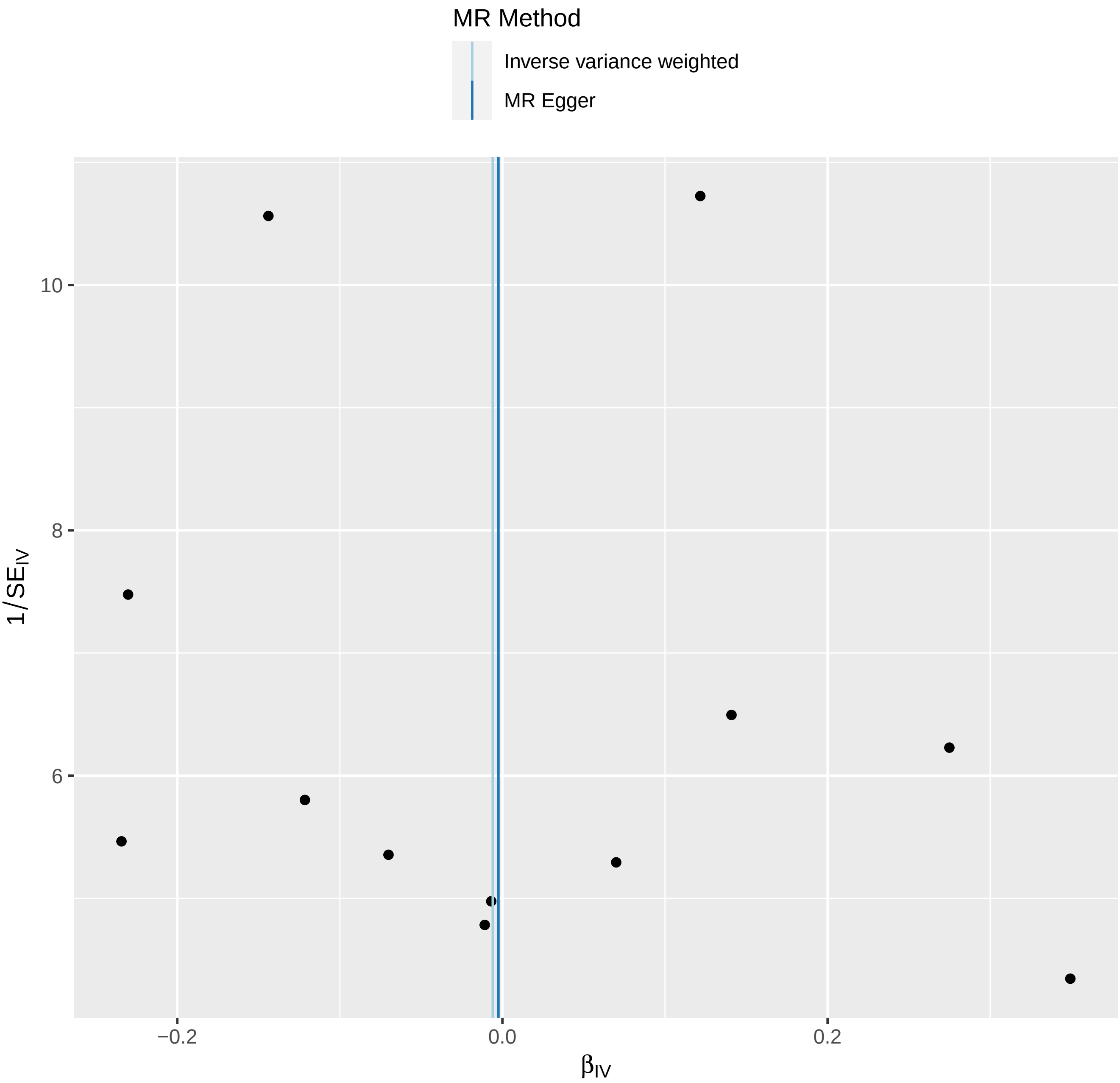

Batch 3−2047 : Selenium on NHL repeat−containing protein 3

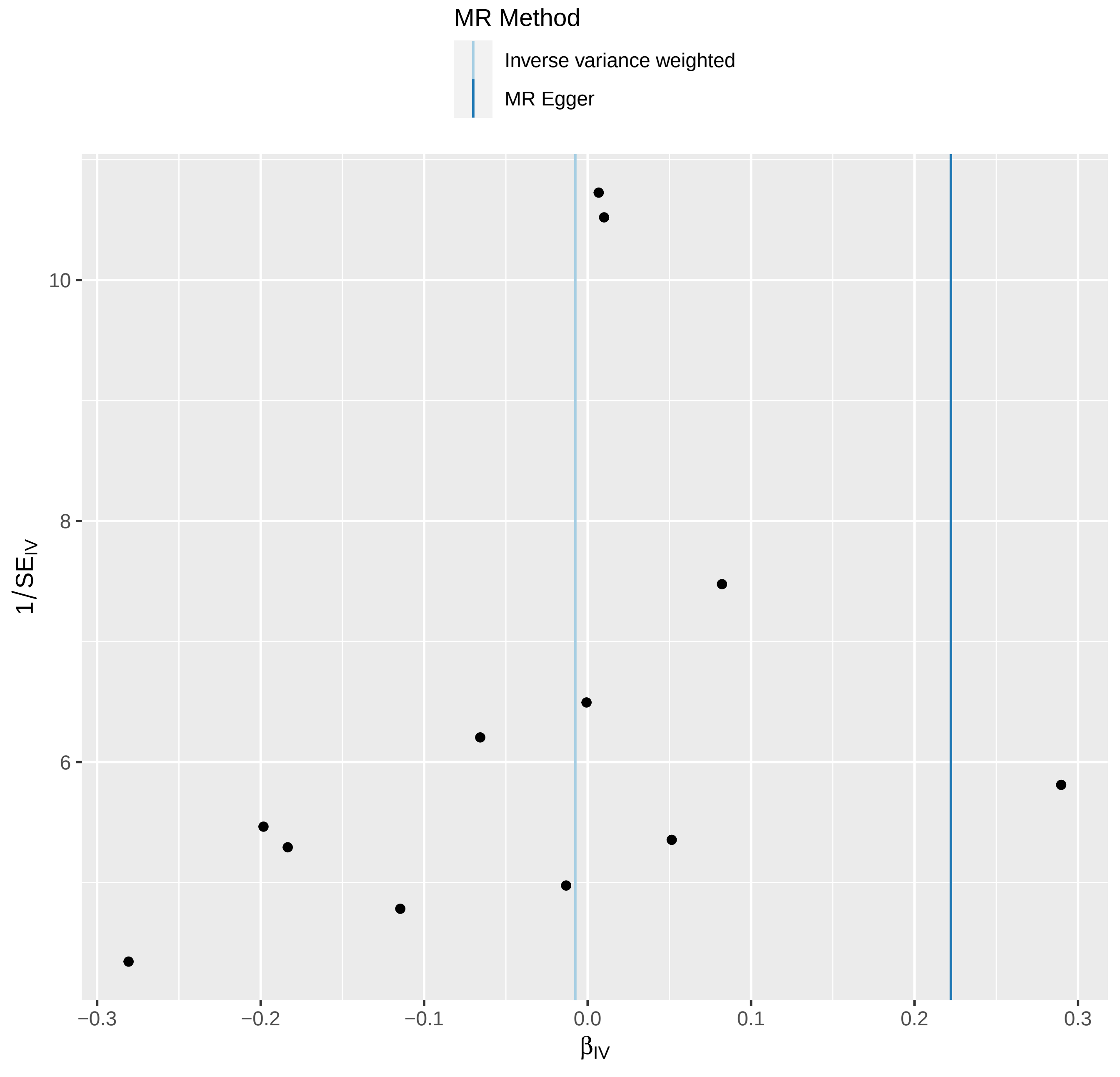

Batch 3−2048 : Selenium on NHP2−like protein 1

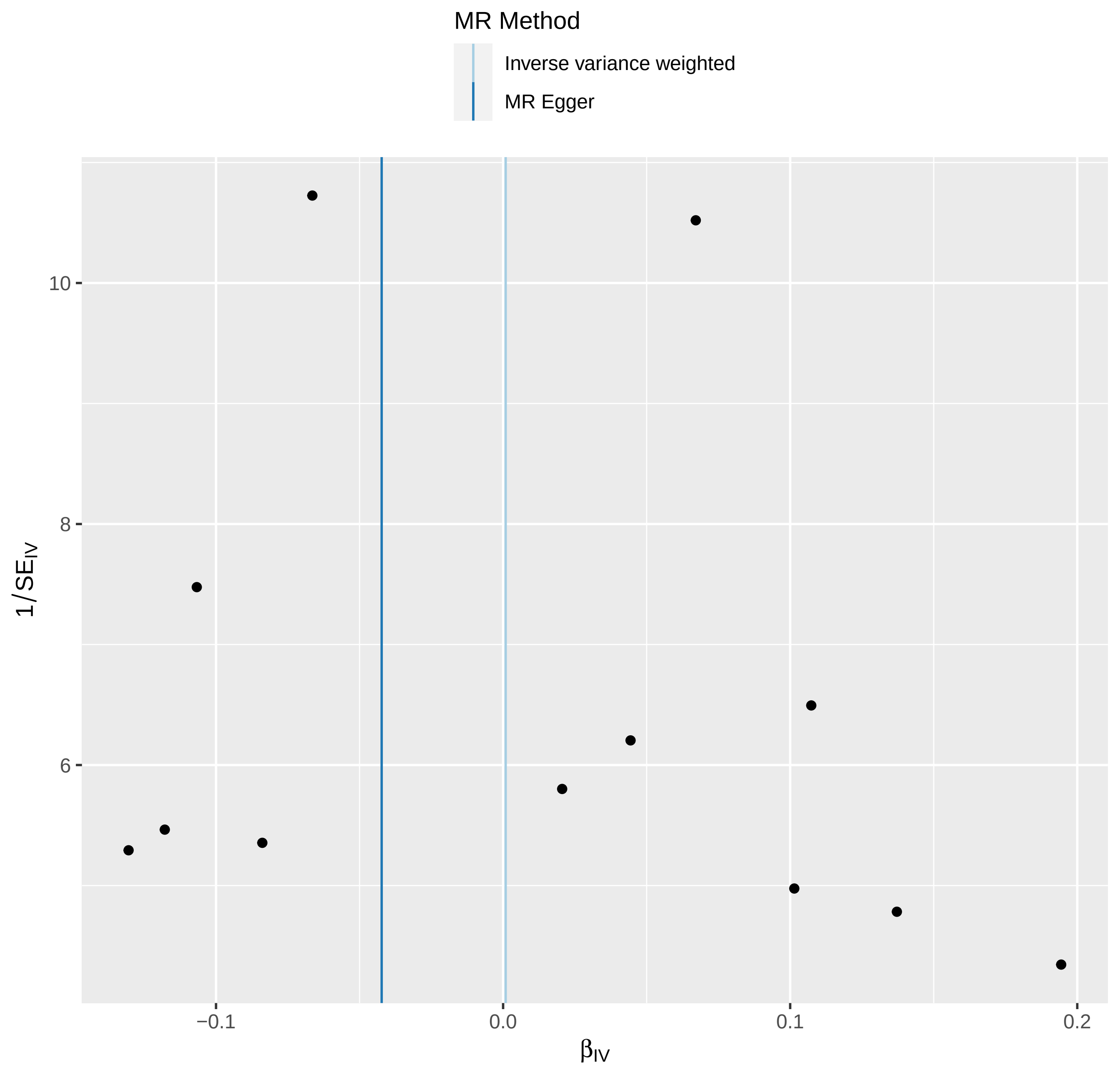

Batch 3−2049 : Selenium on Nidogen−1

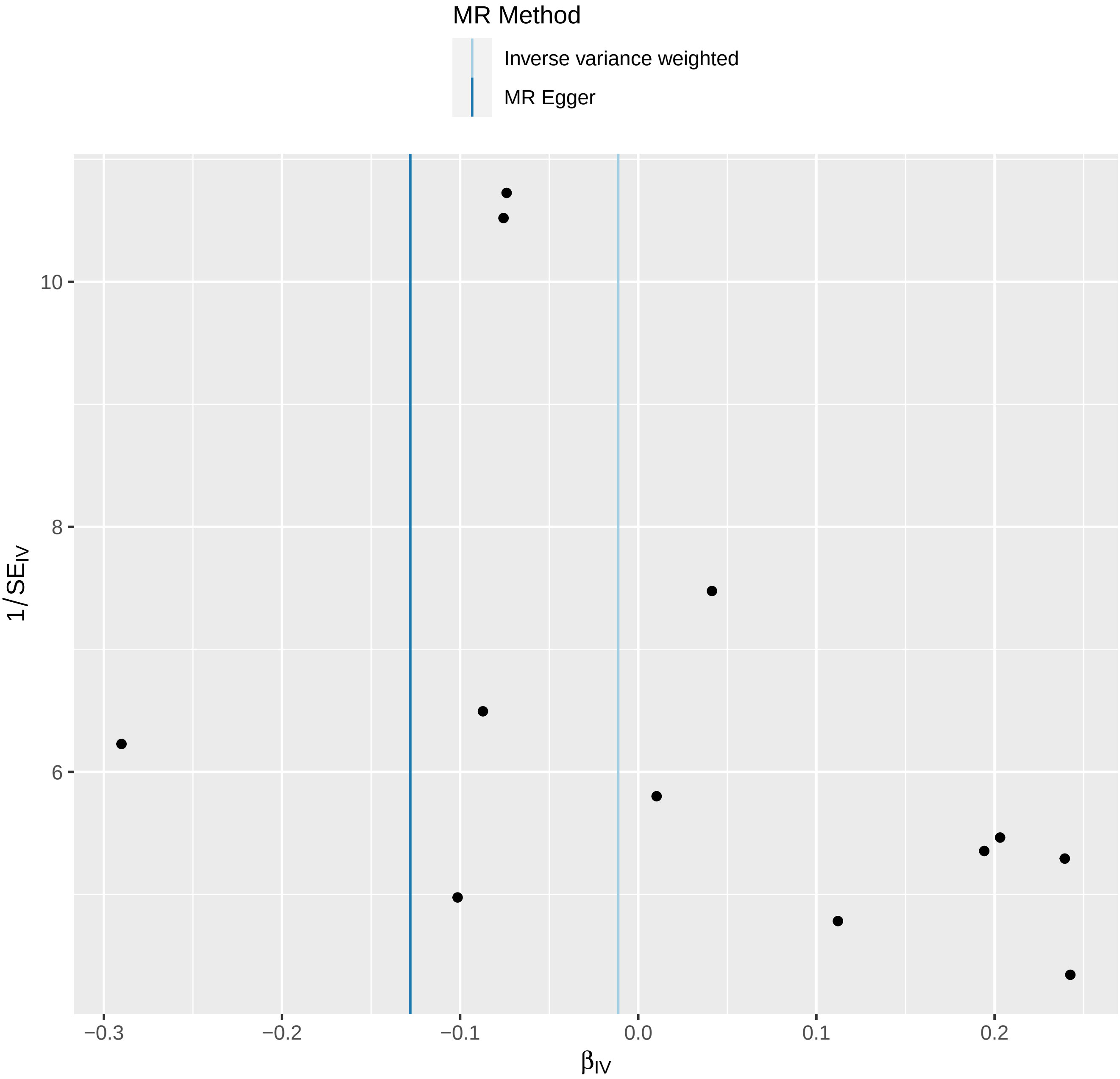

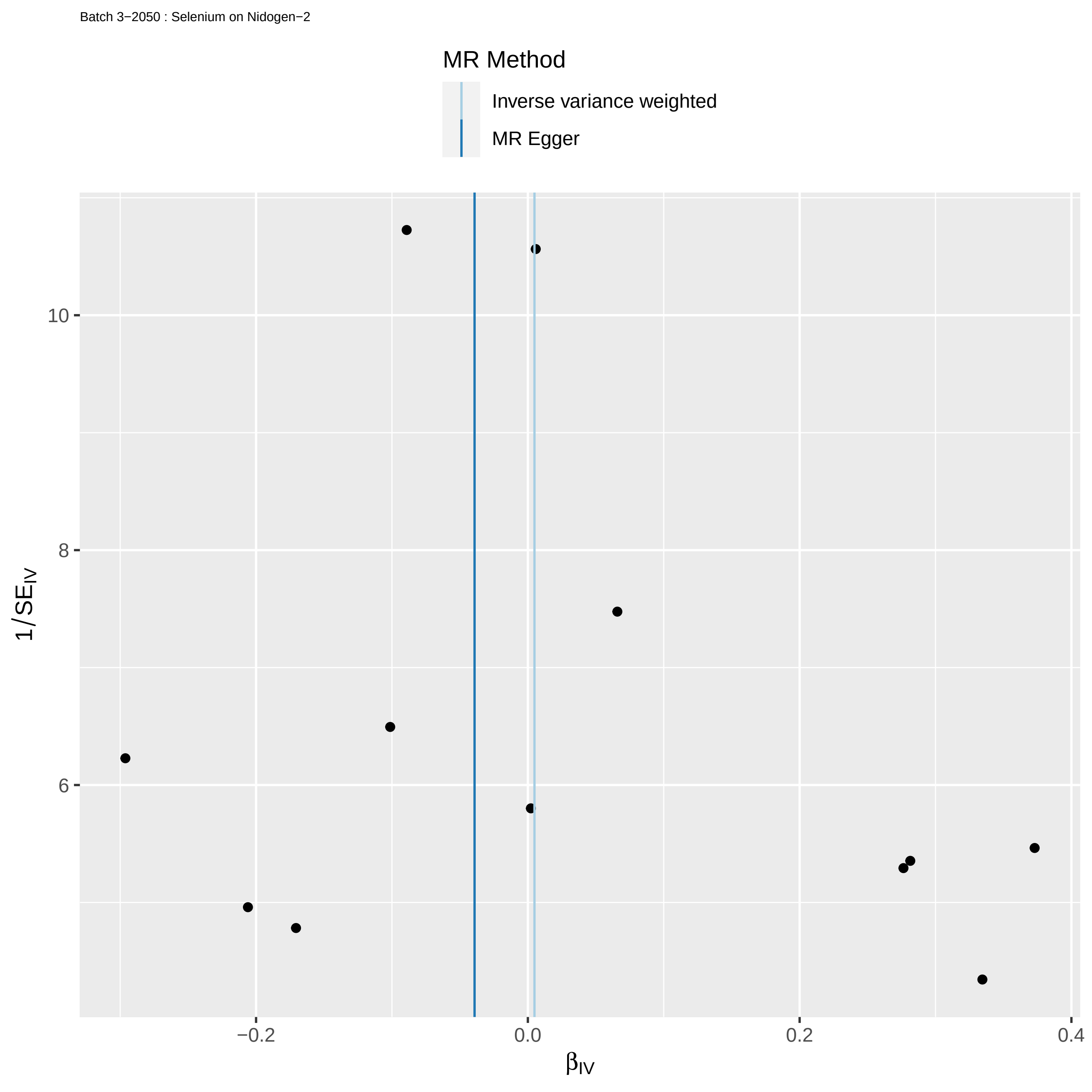
Batch 3−2050 : Selenium on Nidogen−2
MR Method
Inverse variance weighted
MR Egger
10
8
6
1/SE_IV
−0.2
0.0
0.2
0.4
β_IV

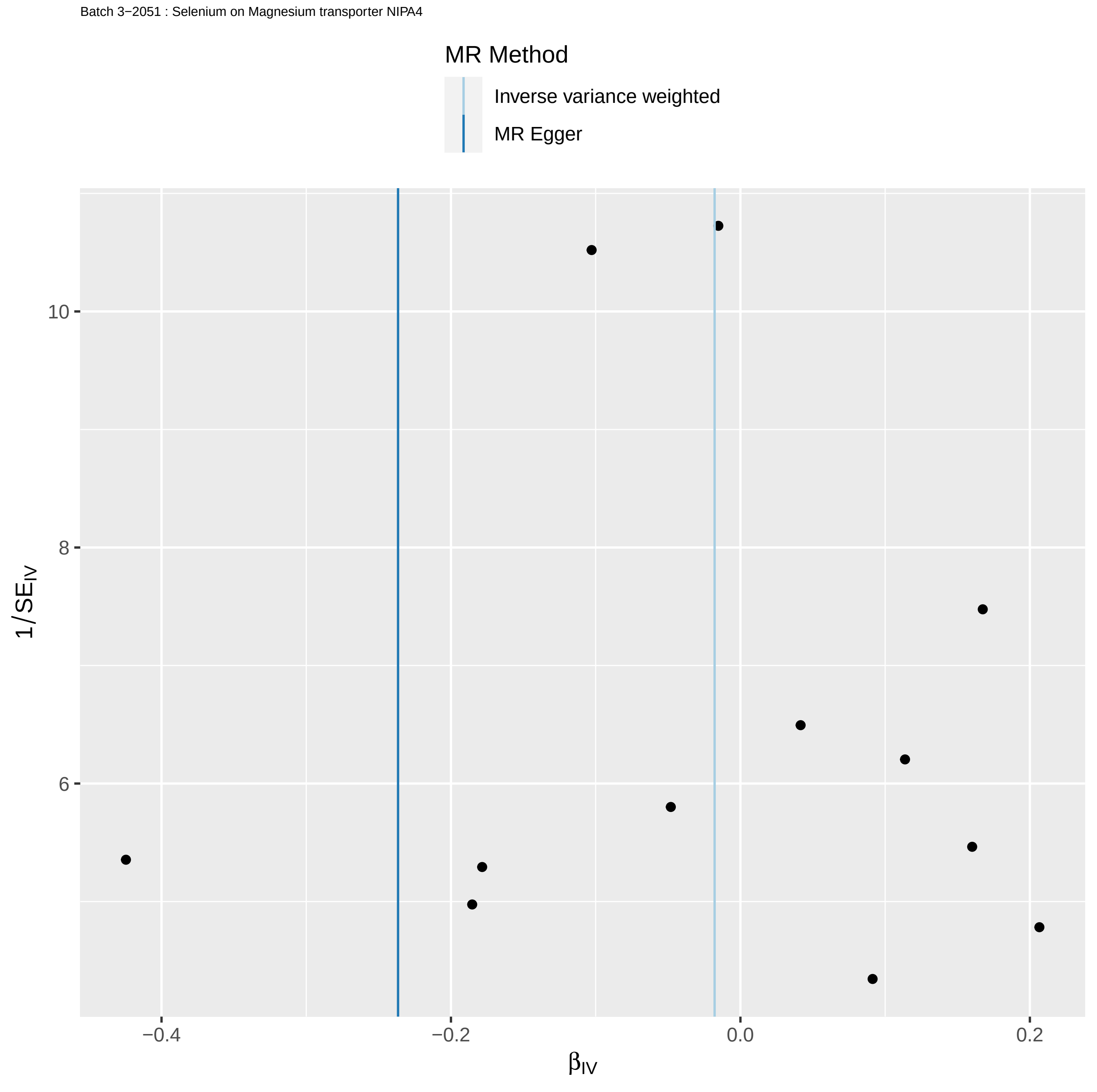
Batch 3−2051 : Selenium on Magnesium transporter NIPA4
MR Method
Inverse variance weighted
MR Egger
10
8
6
1/SE_IV
−0.4
−0.2
0.0
0.2
β_IV

Batch 3−2052 : Selenium on Nischarin

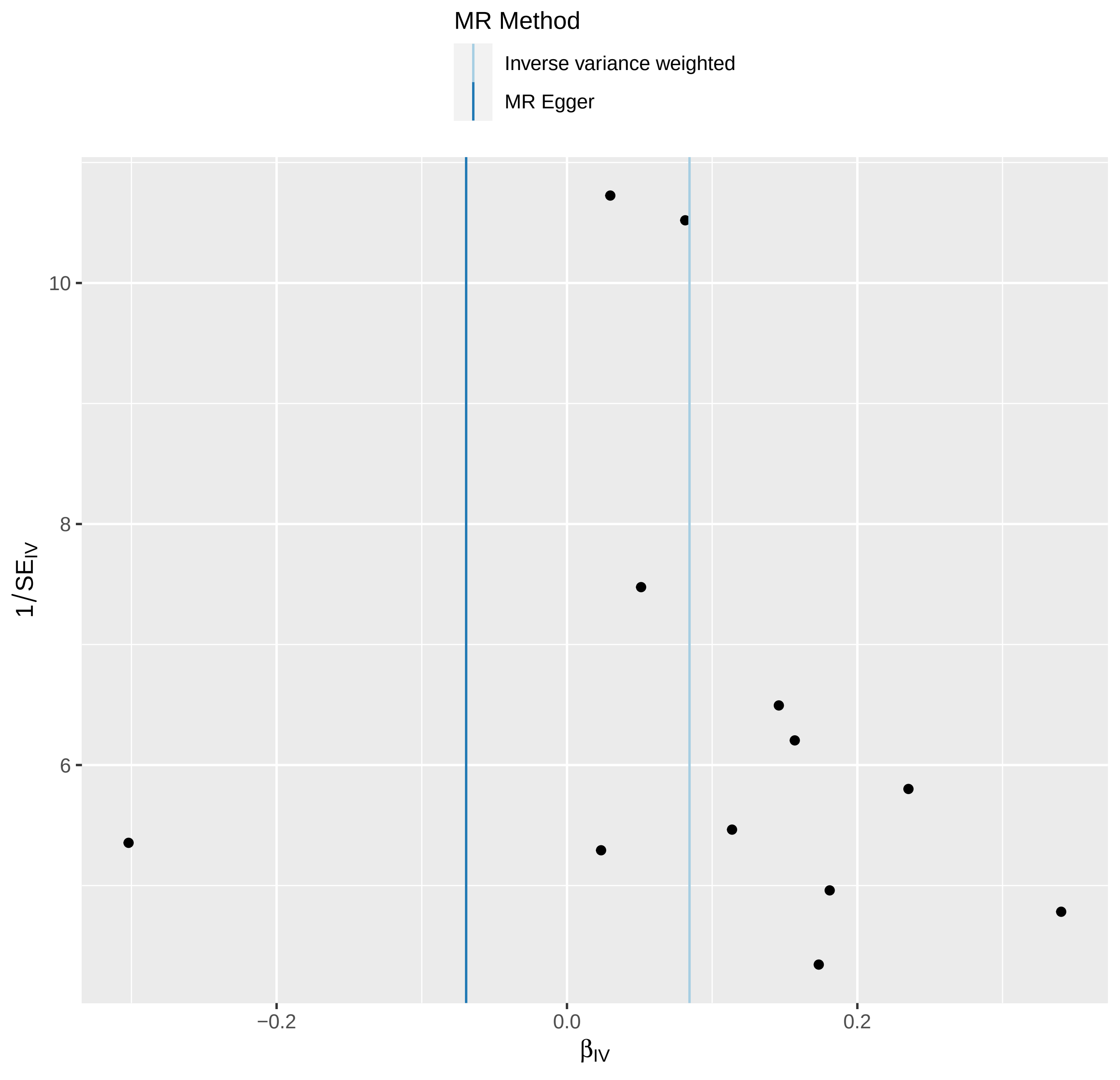

Batch 3−2053 : Selenium on Neuroligin−1

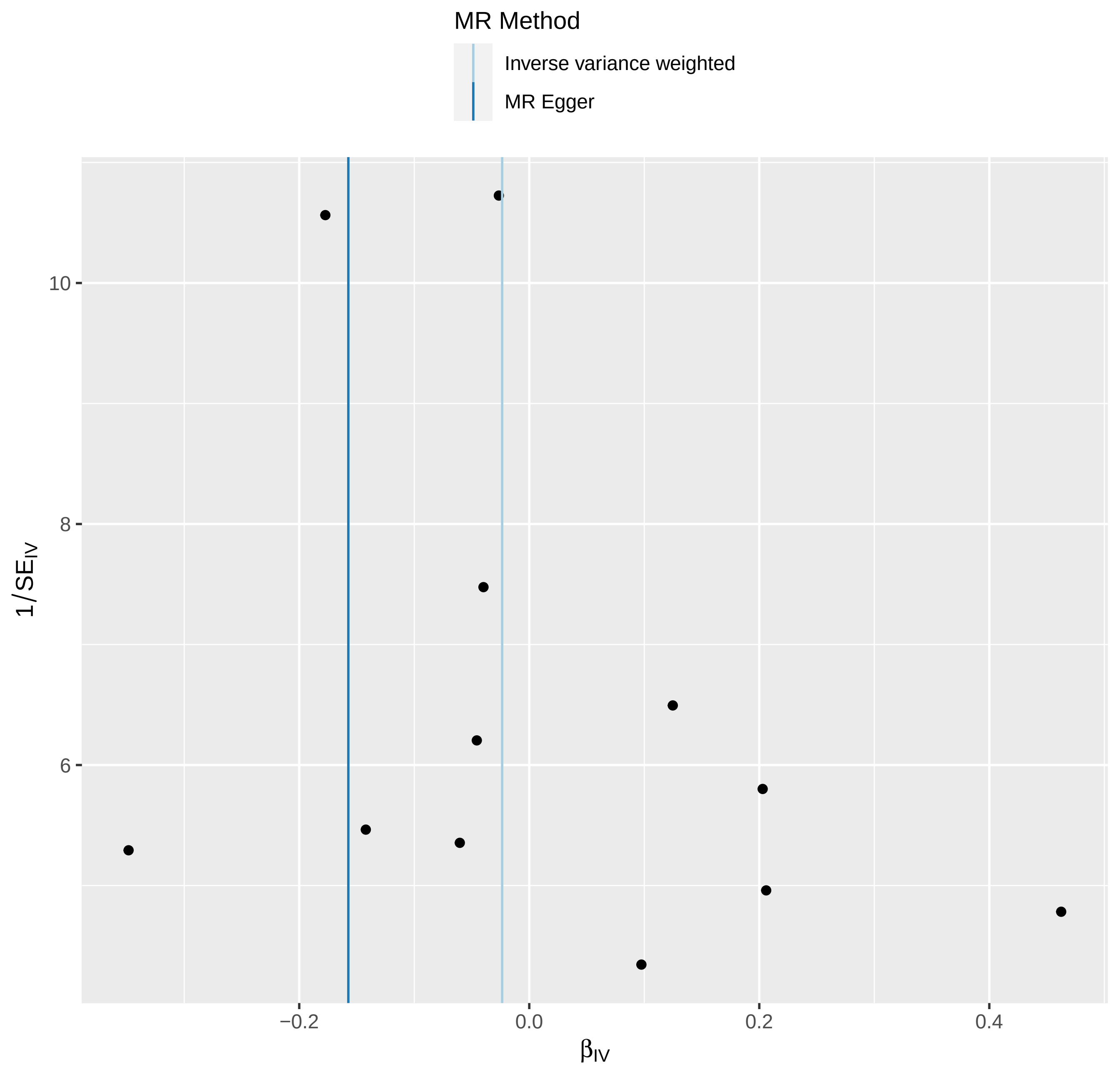

Batch 3−2054 : Selenium on Neuroligin−4, X−linked

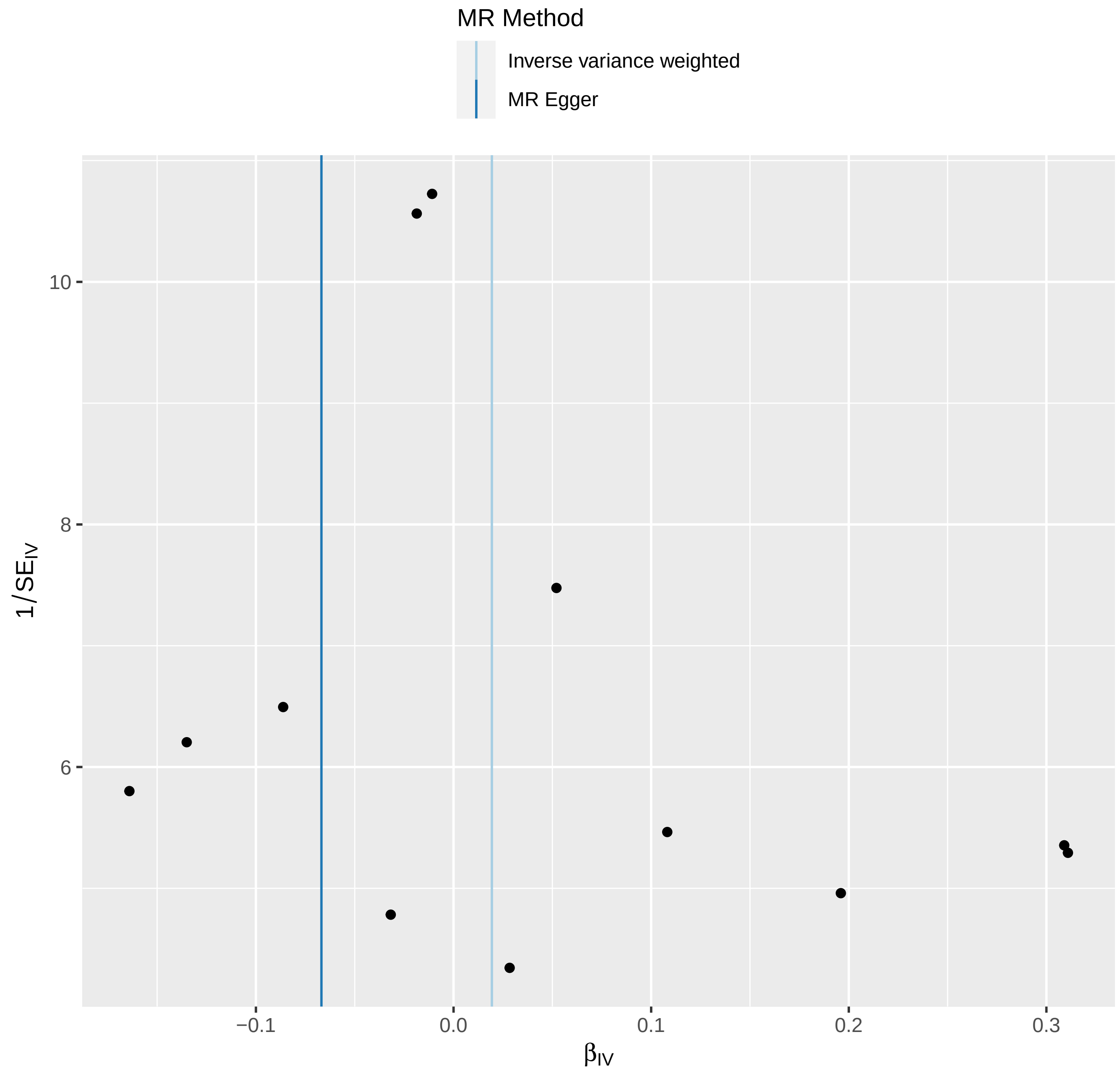

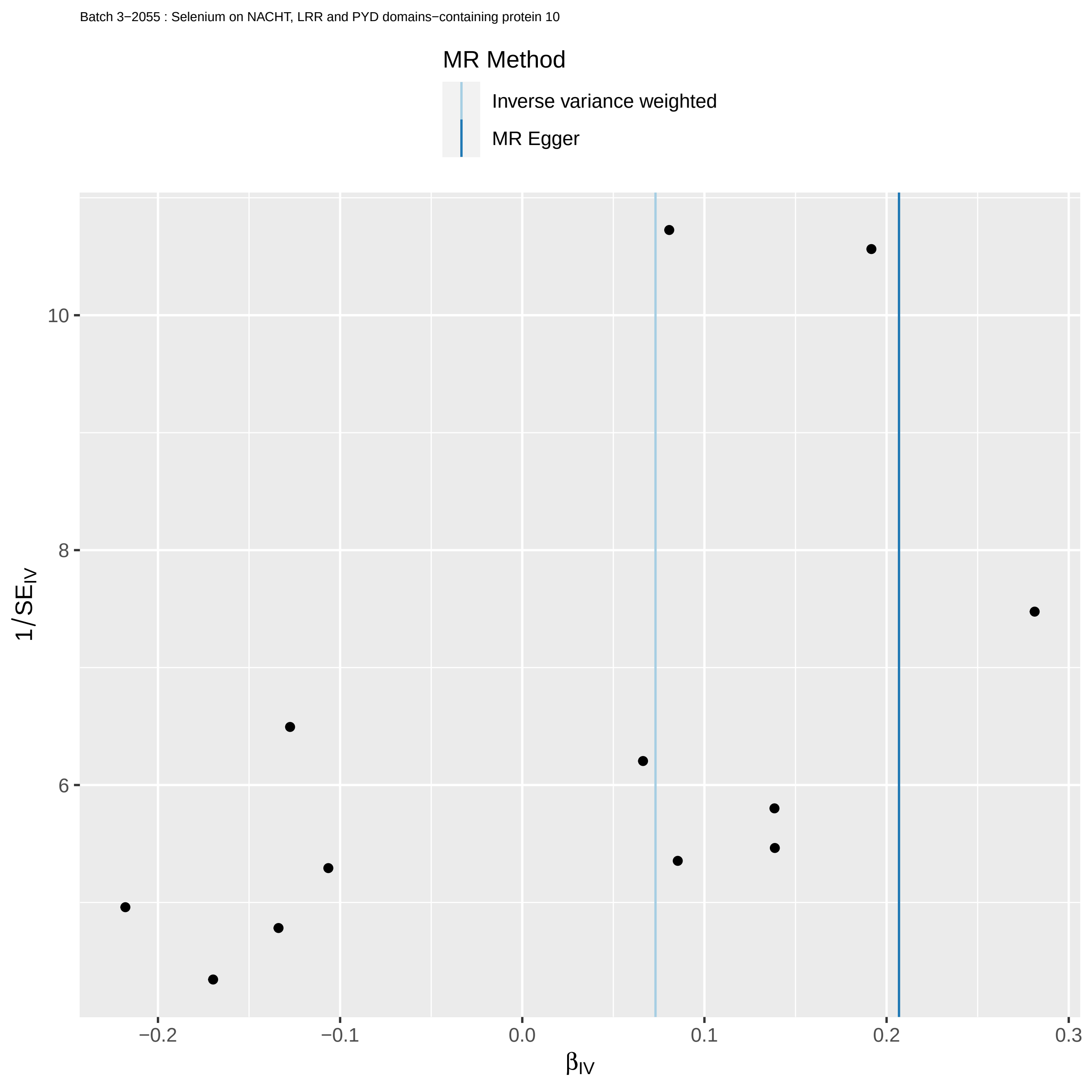
Batch 3−2055 : Selenium on NACHT, LRR and PYD domains−containing protein 10
MR Method
Inverse variance weighted
MR Egger
10
8
6
1/SE_IV
−0.2
−0.1
0.0
0.1
0.2
0.3
β_IV

Batch 3−2056 : Selenium on NACHT, LRR and PYD domains−containing protein 1

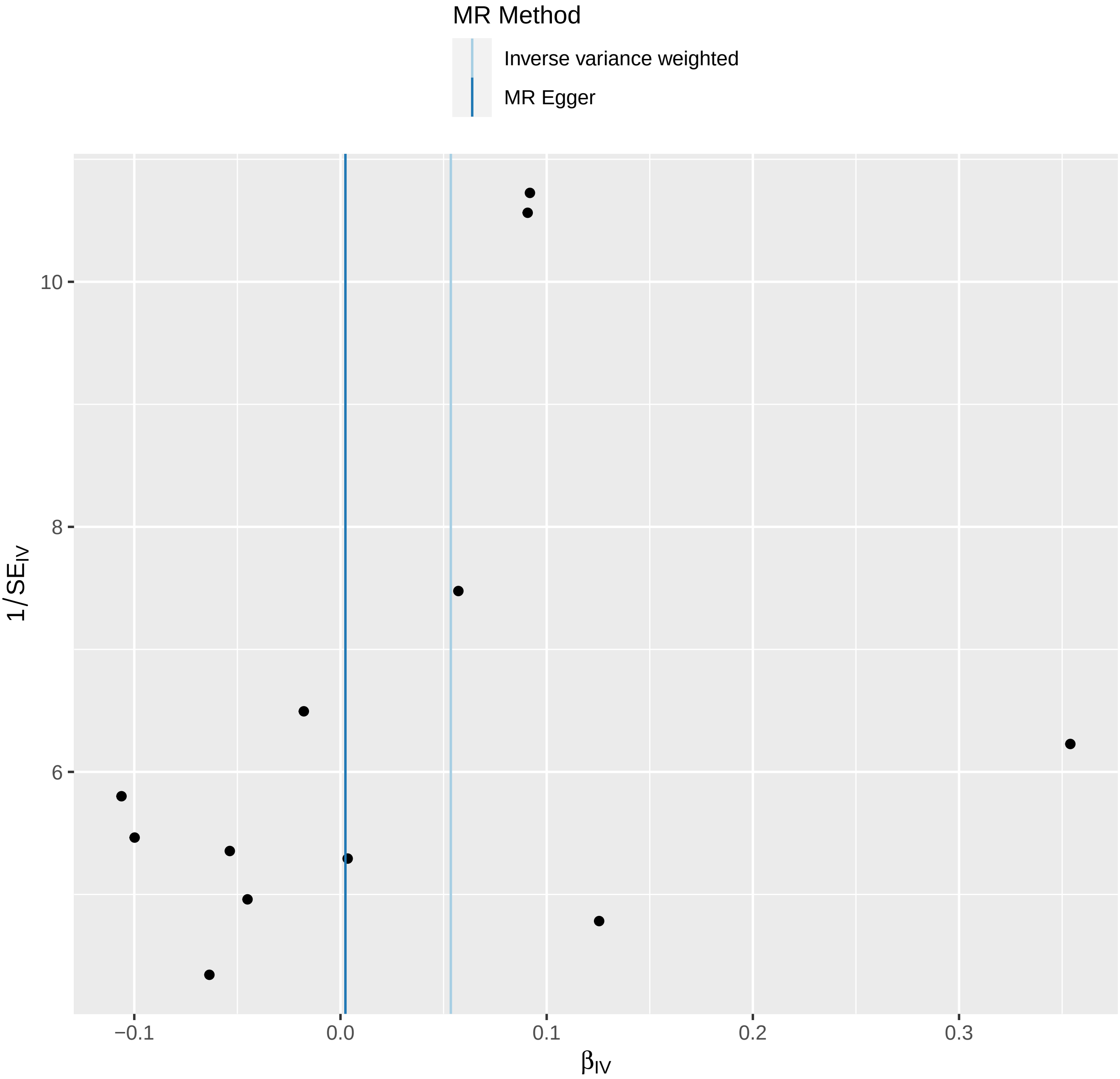

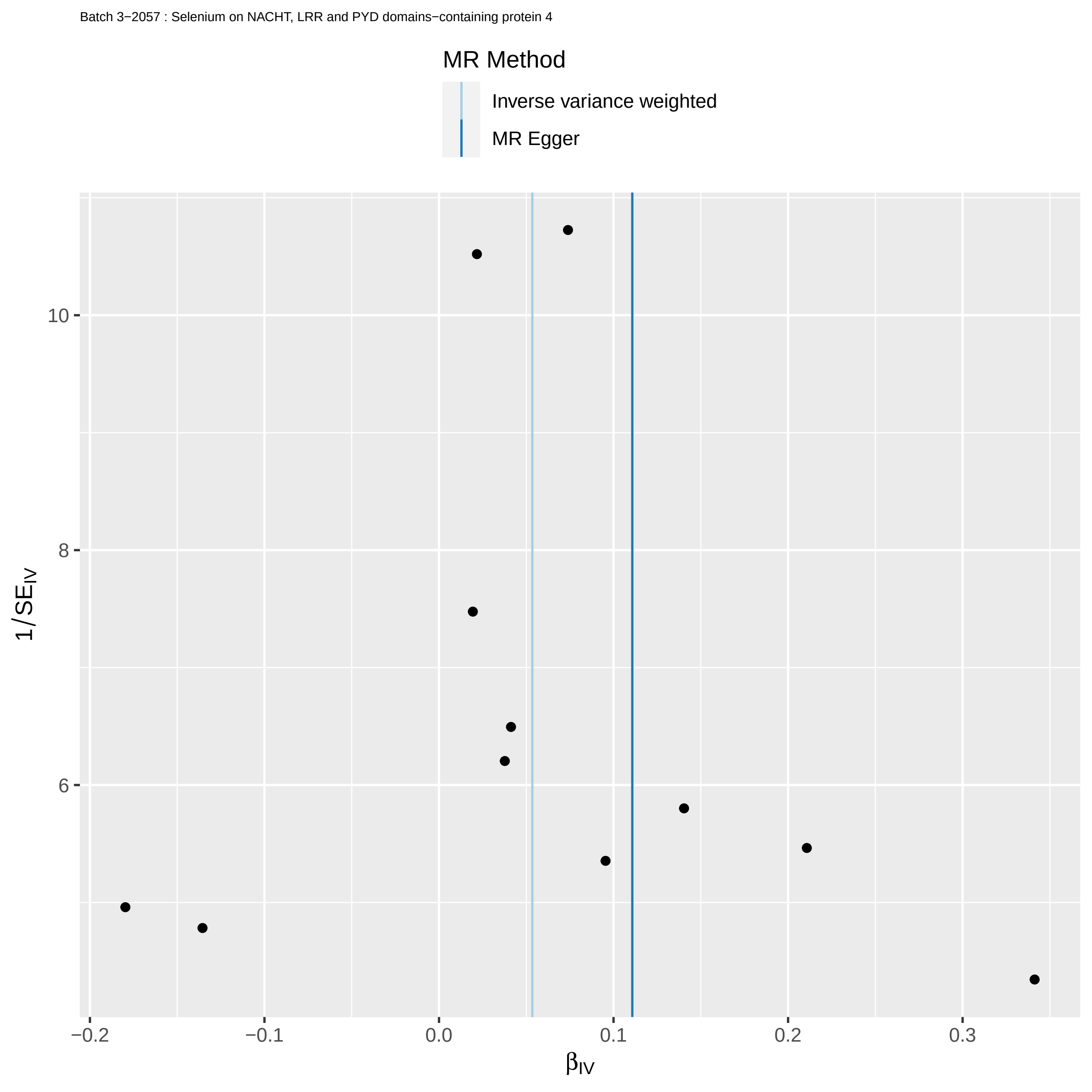
Batch 3−2057 : Selenium on NACHT, LRR and PYD domains−containing protein 4
MR Method
Inverse variance weighted
MR Egger
10
8
6
1/SE$_{IV}$
−0.2
−0.1
0.0
0.1
0.2
0.3
β$_{IV}$

Batch 3−2058 : Selenium on Neuromedin−B

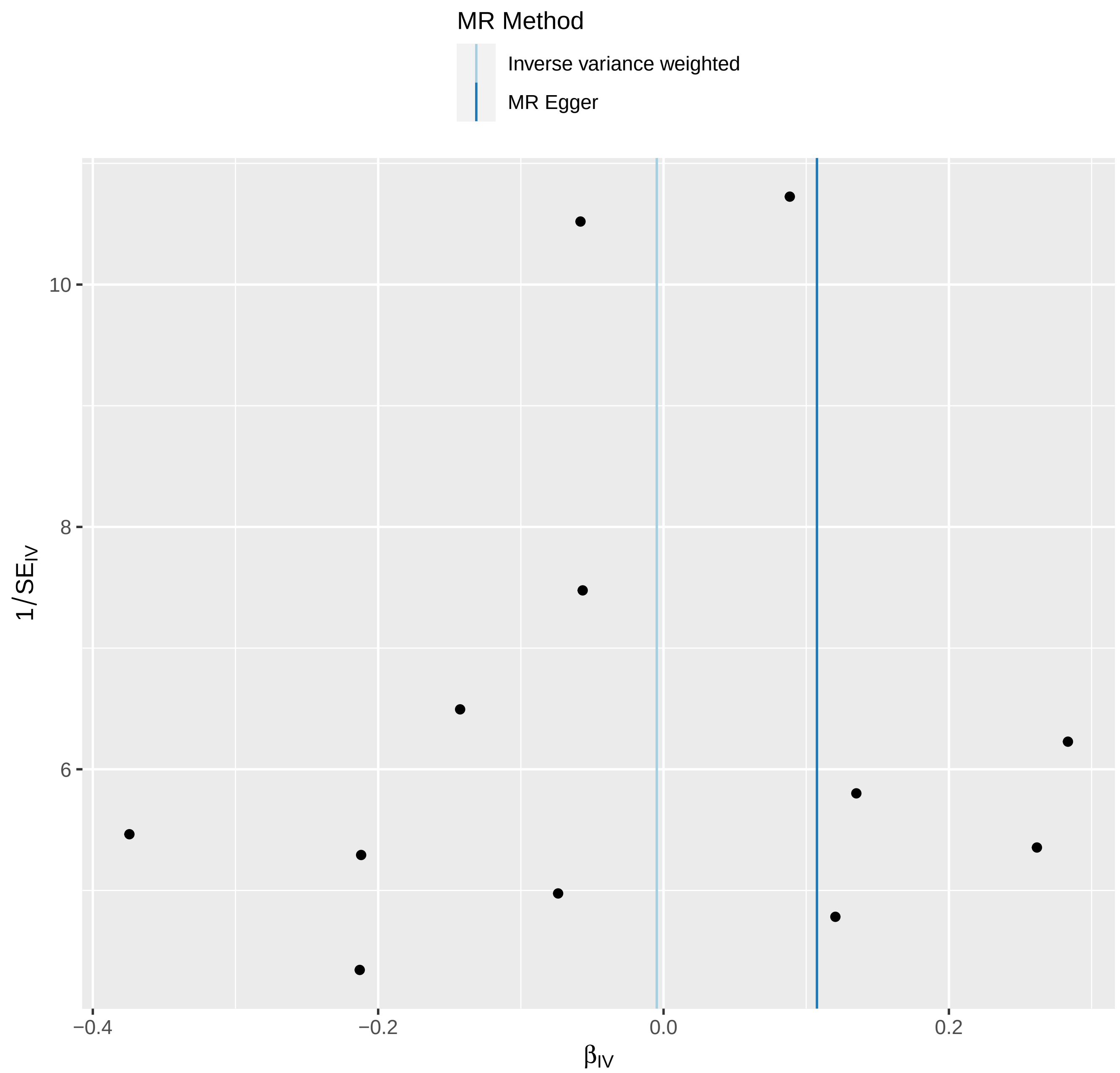

Batch 3−2059 : Selenium on Nucleoside diphosphate kinase A

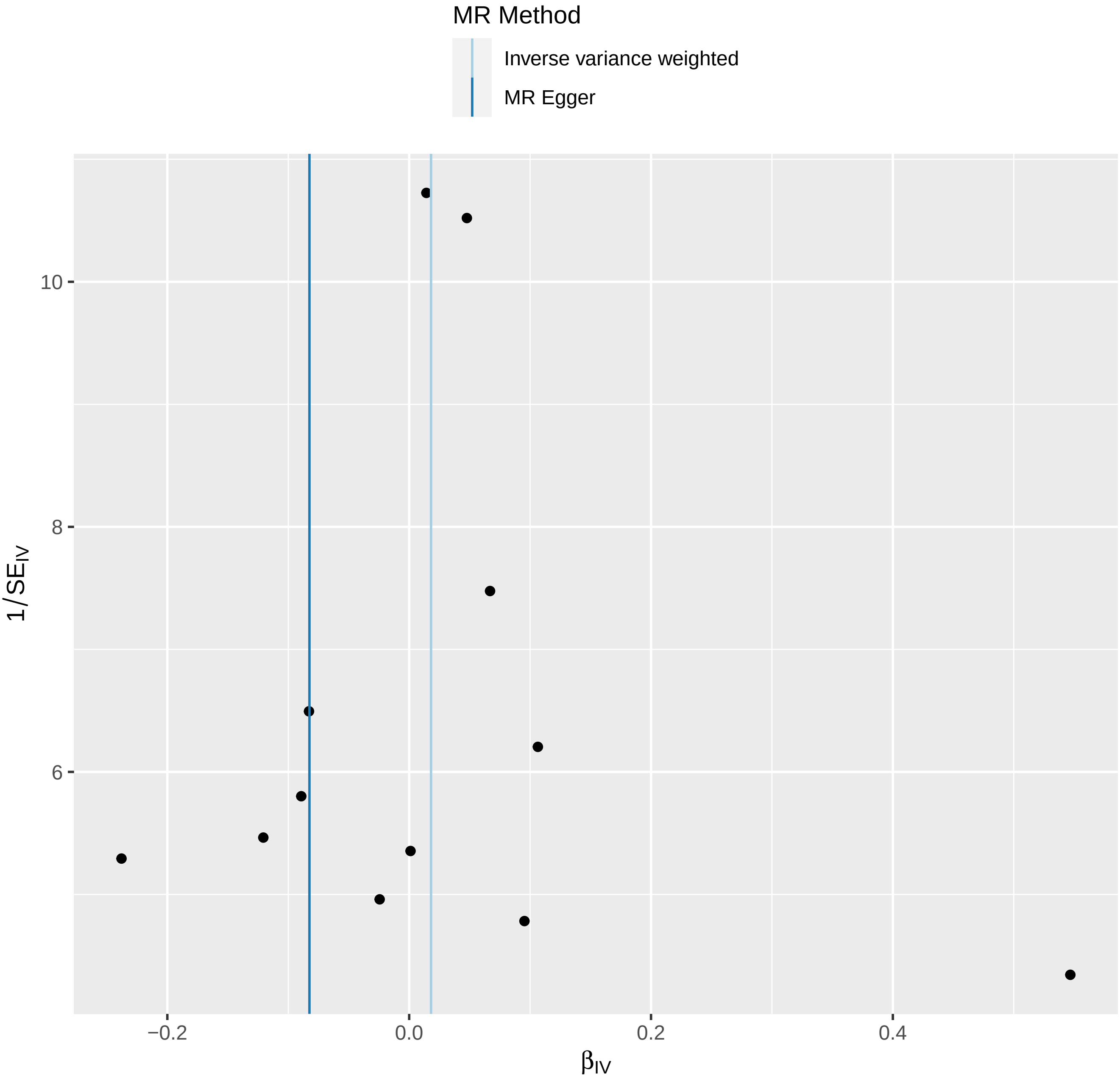

Batch 3−2060 : Selenium on Nucleoside diphosphate kinase B

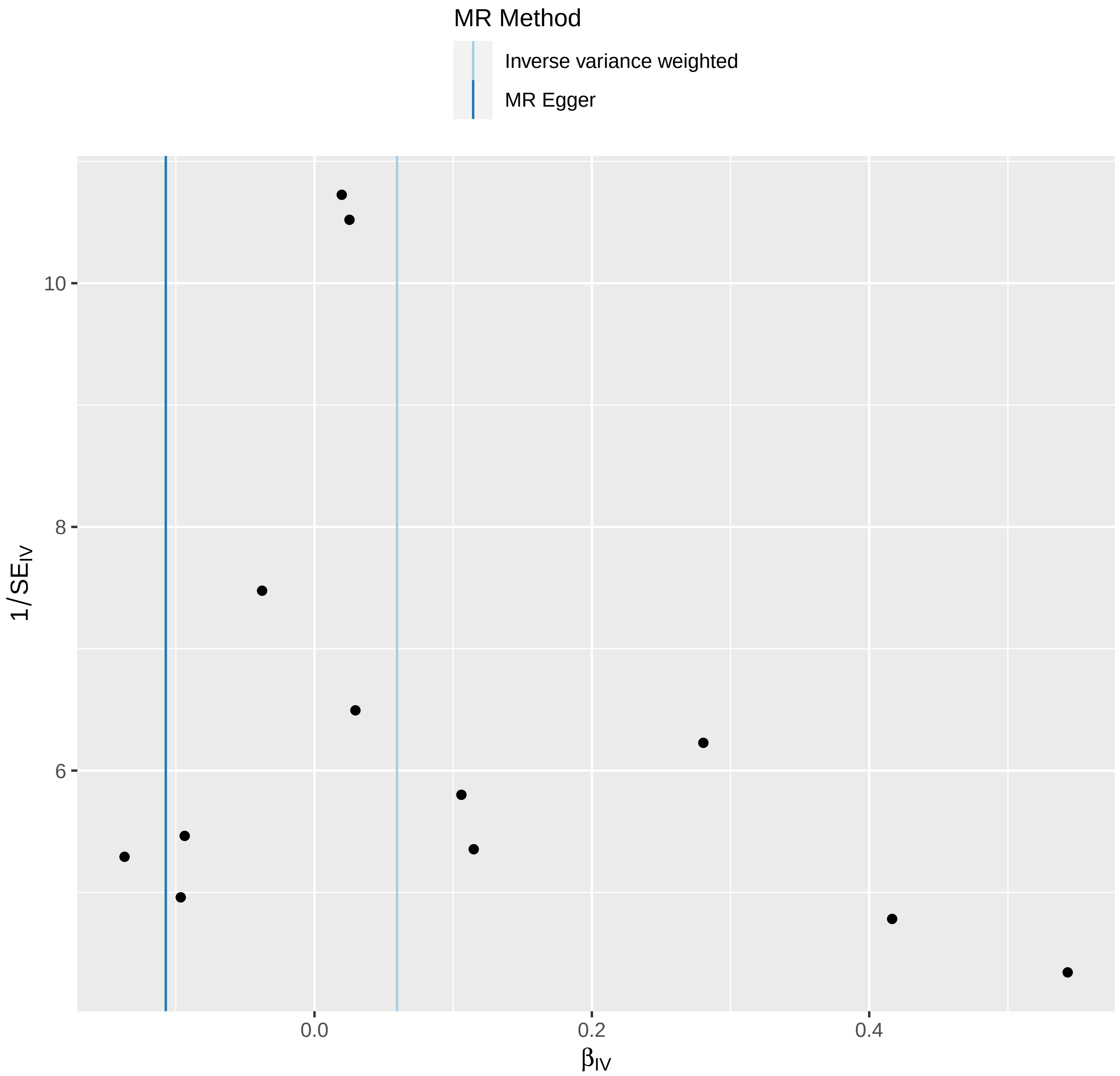

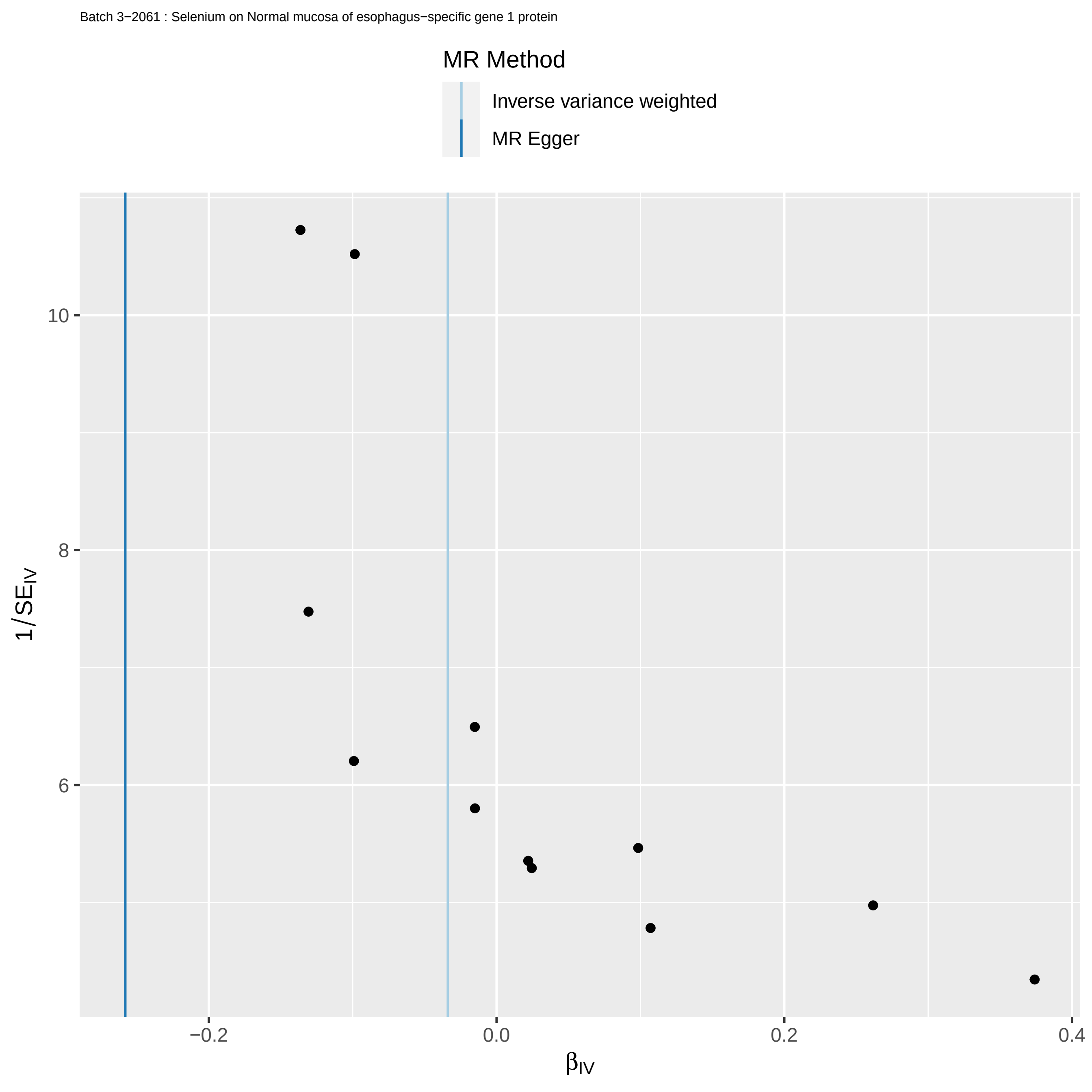

Batch 3−2061 : Selenium on Normal mucosa of esophagus−specific gene 1 protein
MR Method
Inverse variance weighted
MR Egger
10
8
6
1/SE_IV
−0.2
0.0
0.2
0.4
β_IV

Batch 3−2062 : Selenium on NmrA−like family domain−containing protein 1

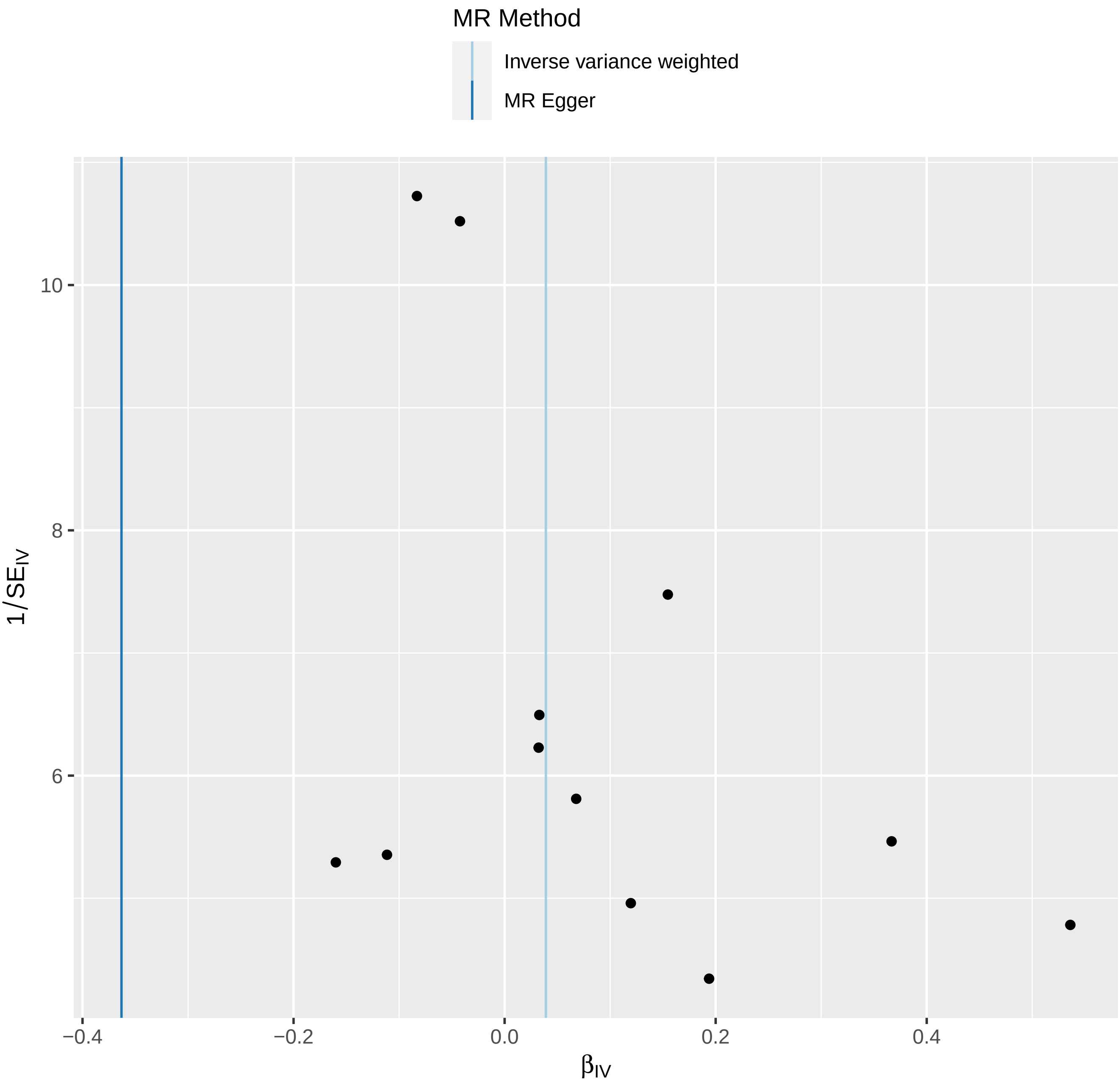

Batch 3−2063 : Selenium on Glycylpeptide N−tetradecanoyltransferase 1

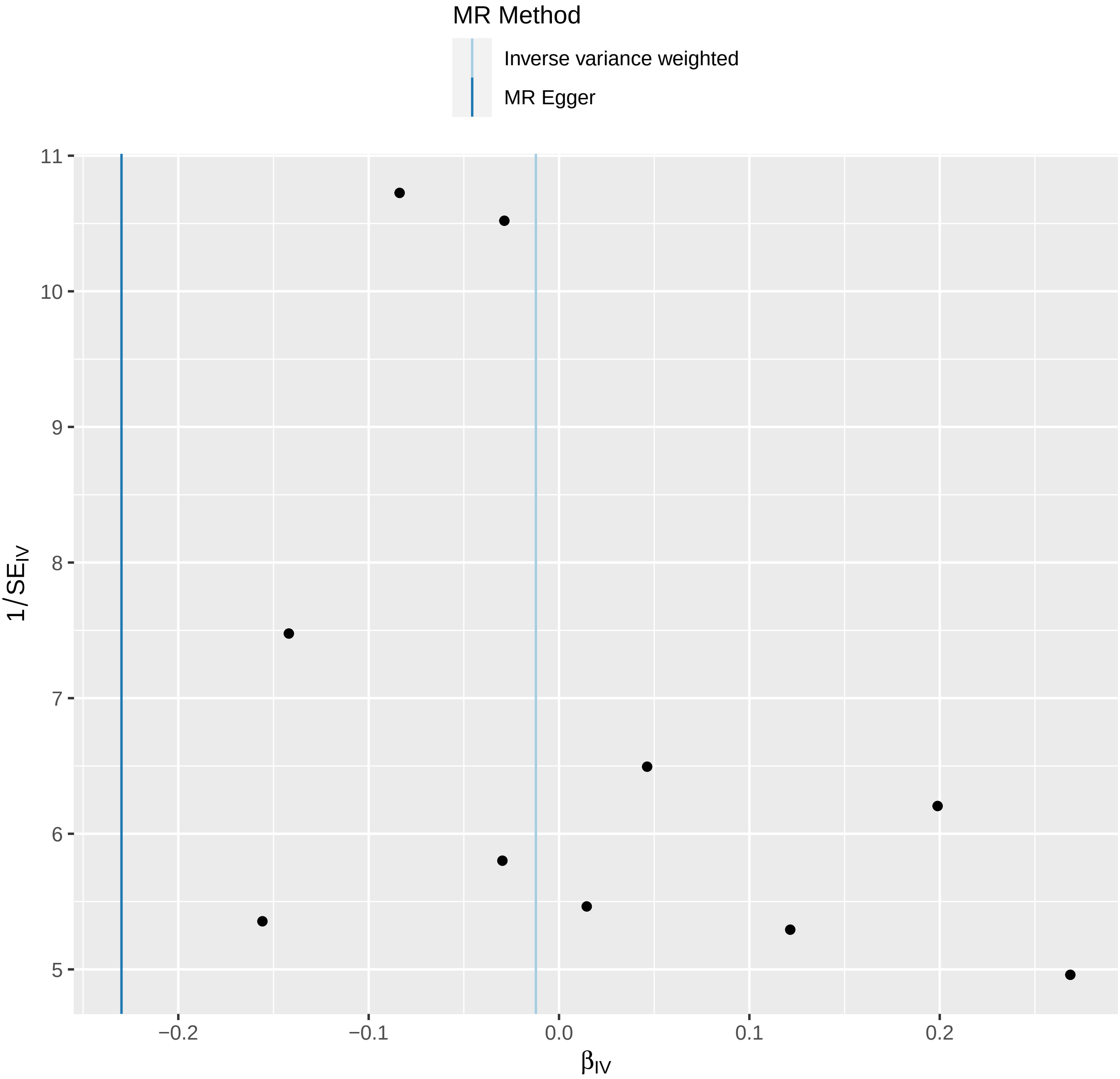

Batch 3−2064 : Selenium on Noggin

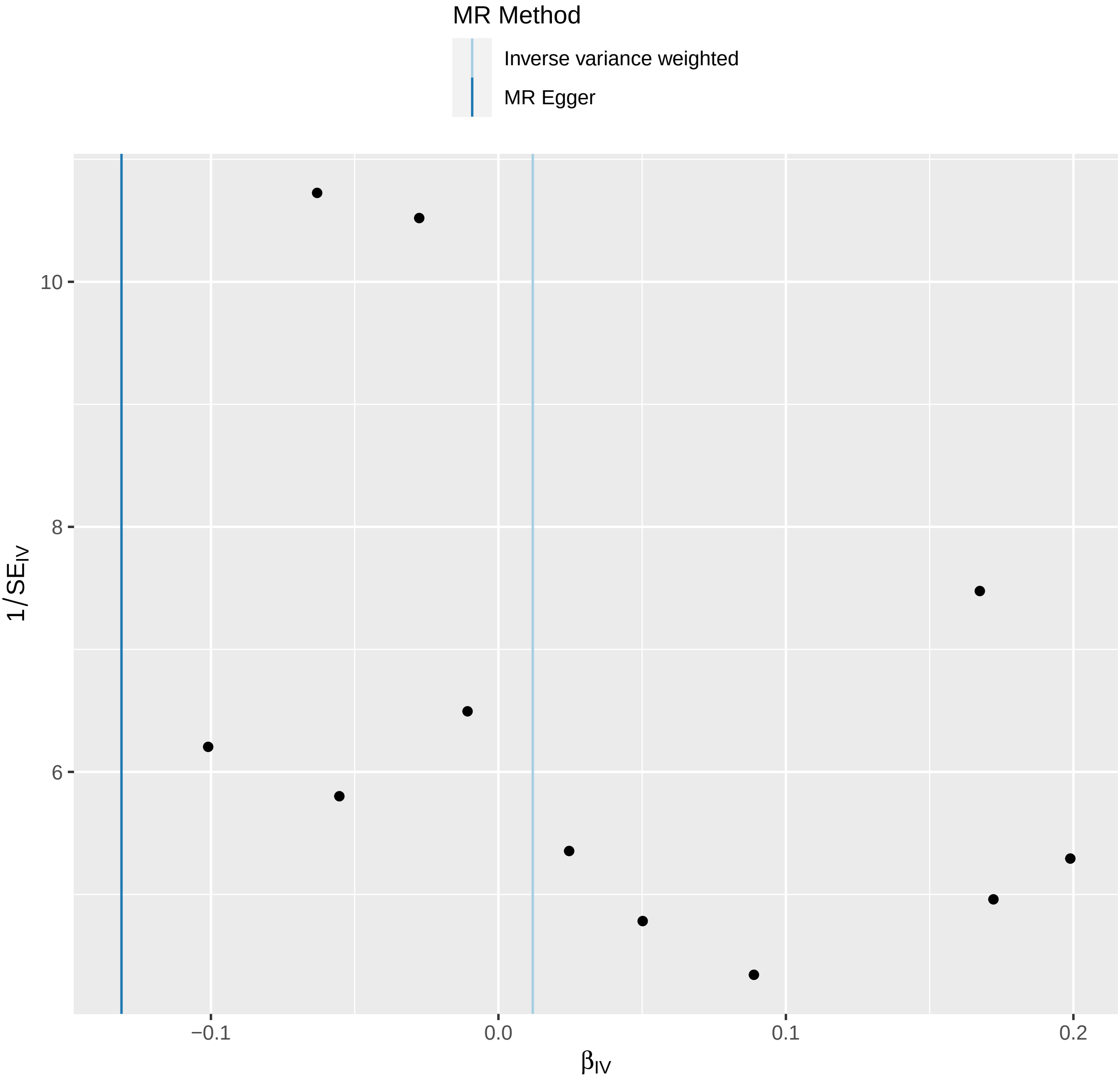

Batch 3−2065 : Selenium on NoneX

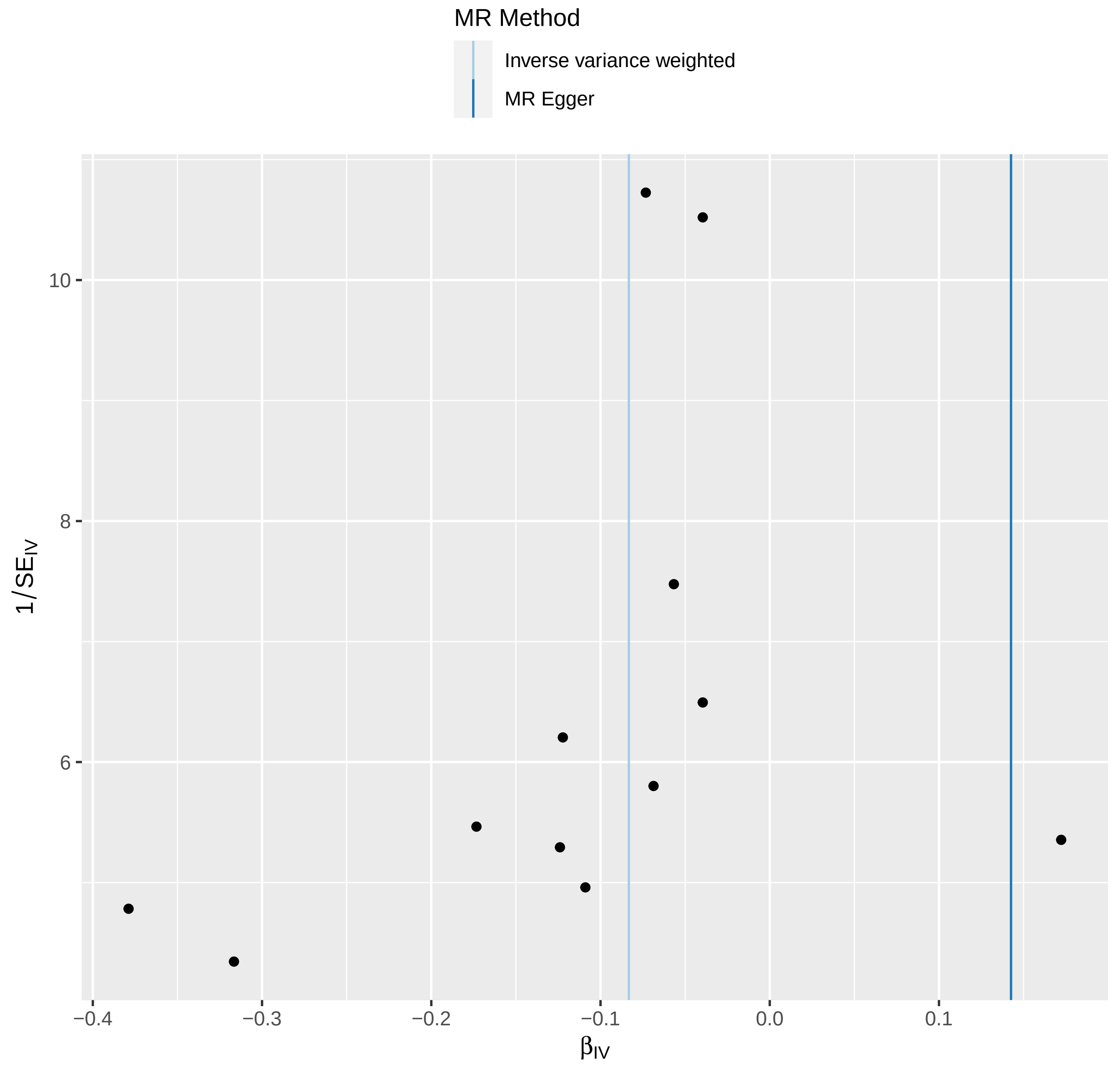

Batch 3−2066 : Selenium on NoneX

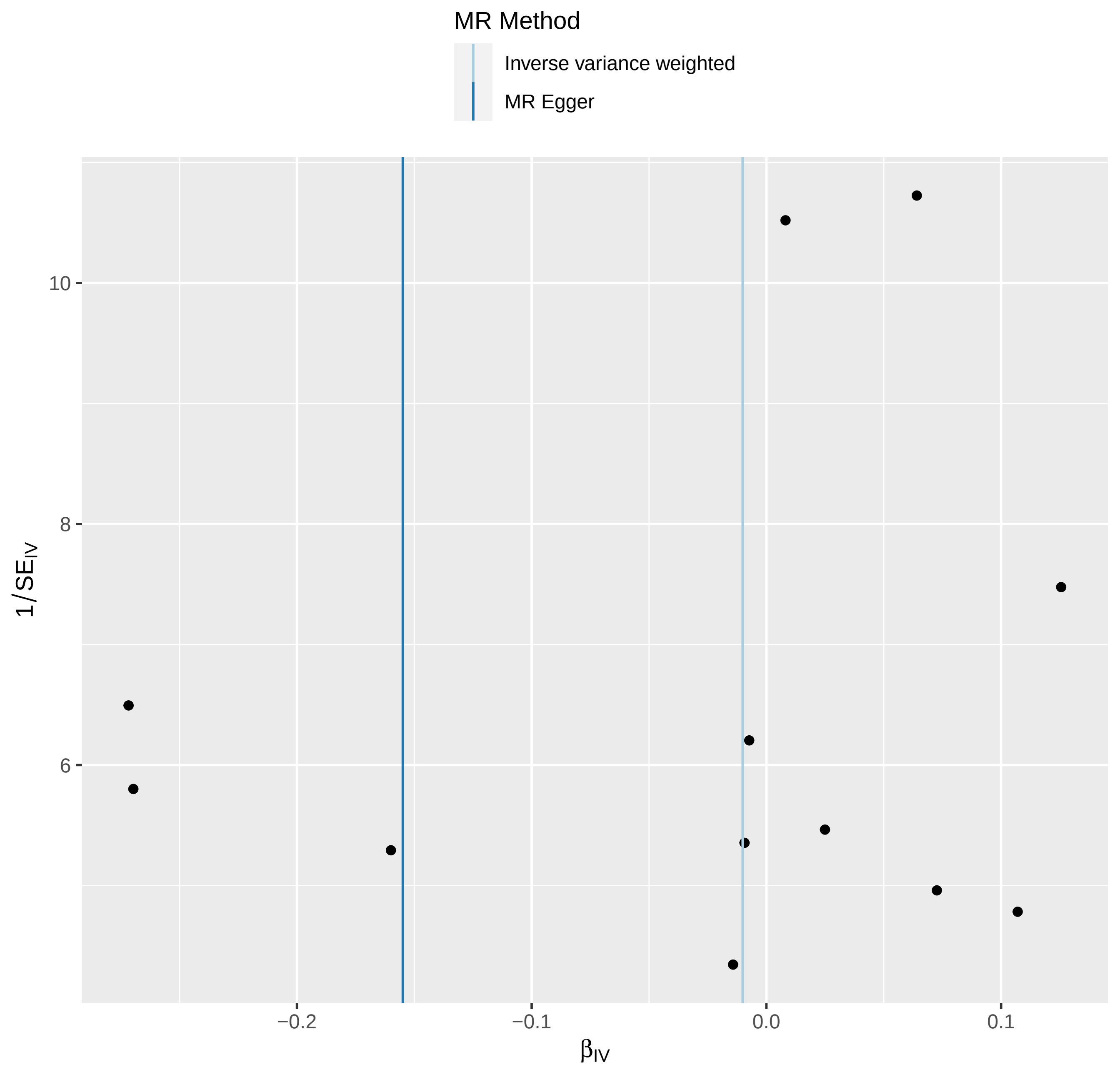

Batch 3−2067 : Selenium on Neurogenic locus notch homolog protein 1

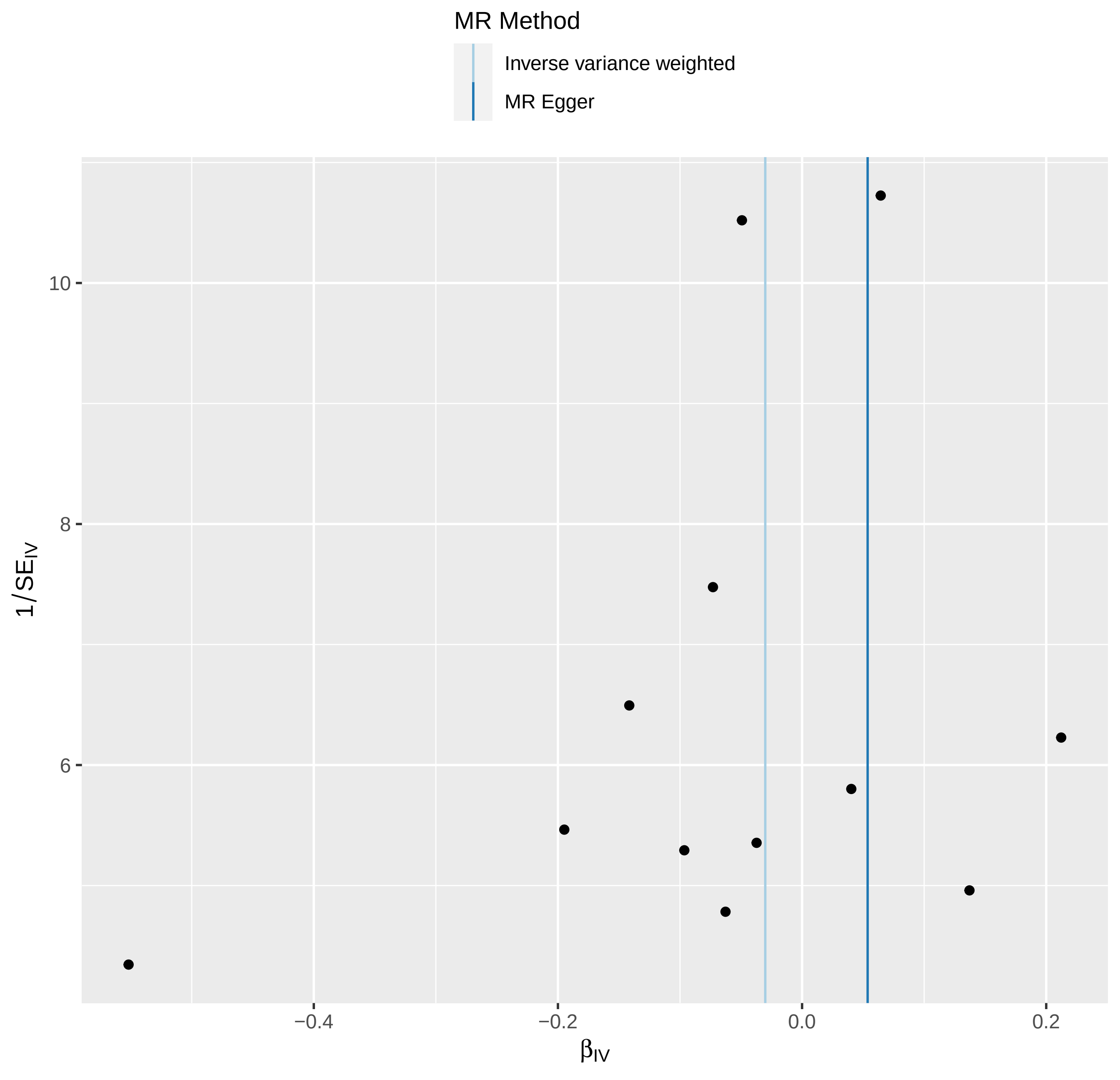

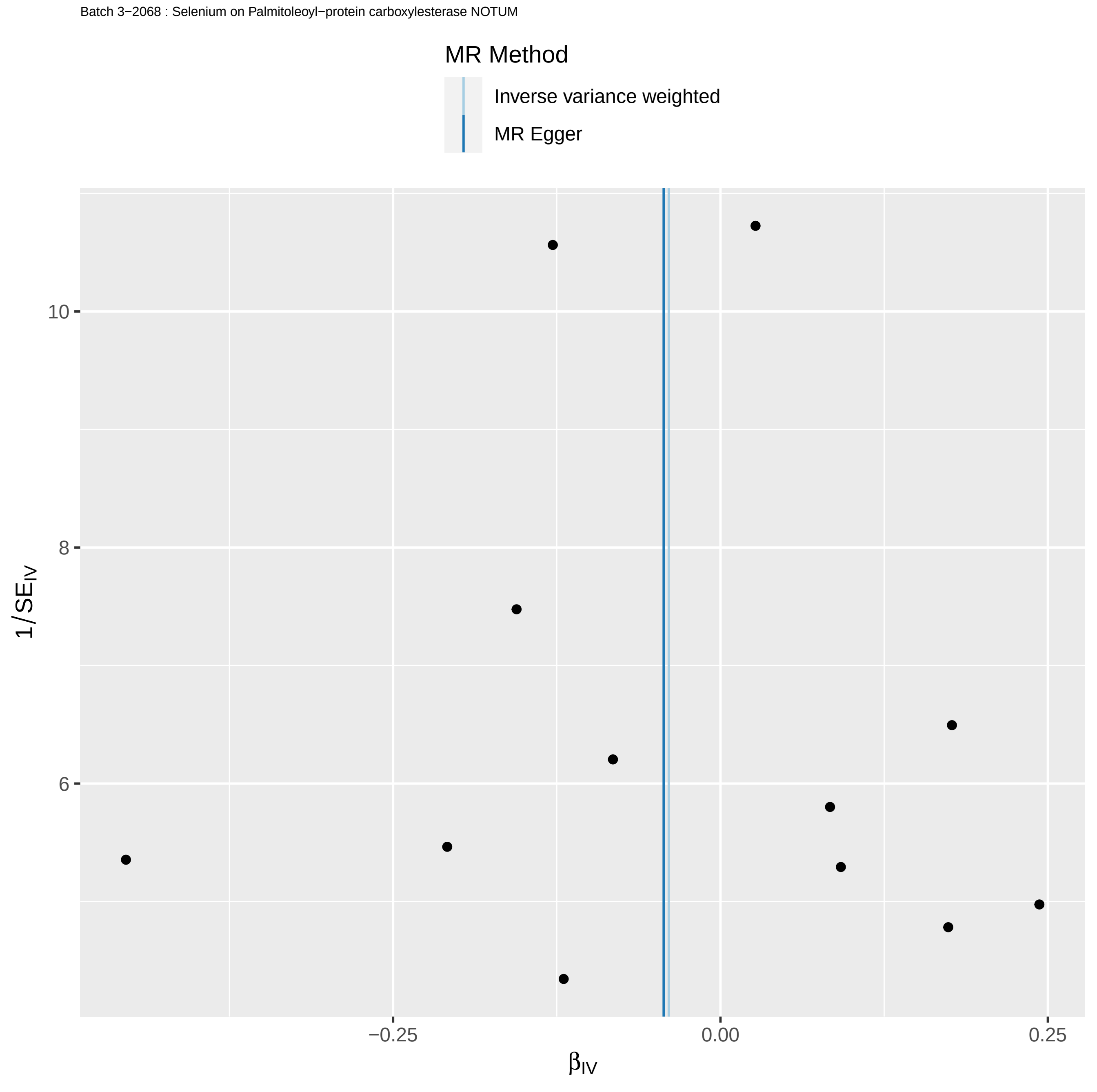
Batch 3−2068 : Selenium on Palmitoleoyl−protein carboxylesterase NOTUM
MR Method
Inverse variance weighted
MR Egger
10
8
6
1/SE$_{IV}$
−0.25
0.00
0.25
β_{IV}

Batch 3−2069 : Selenium on Protein NOV homolog

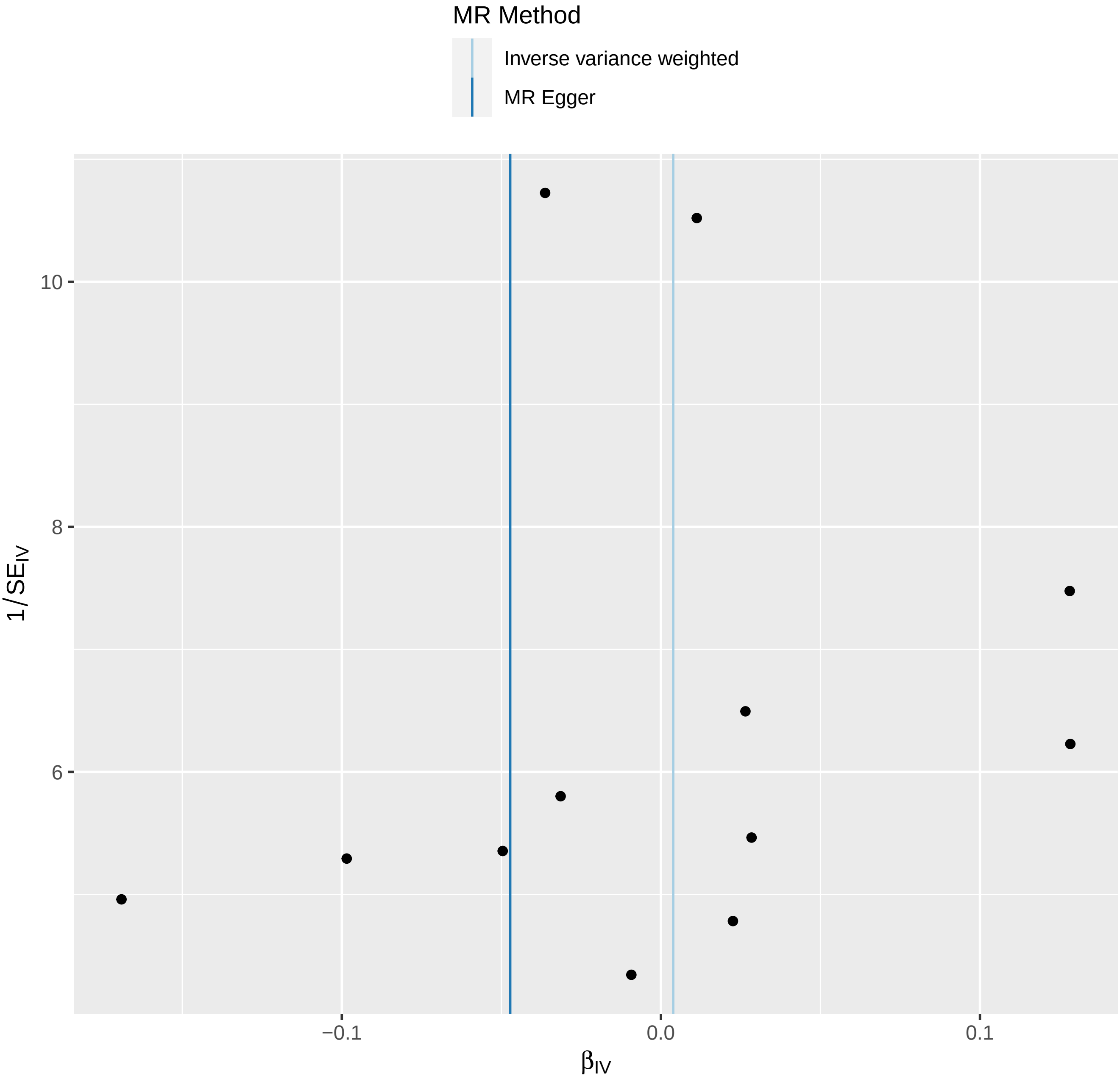

Batch 3−2070 : Selenium on RNA−binding protein Nova−1

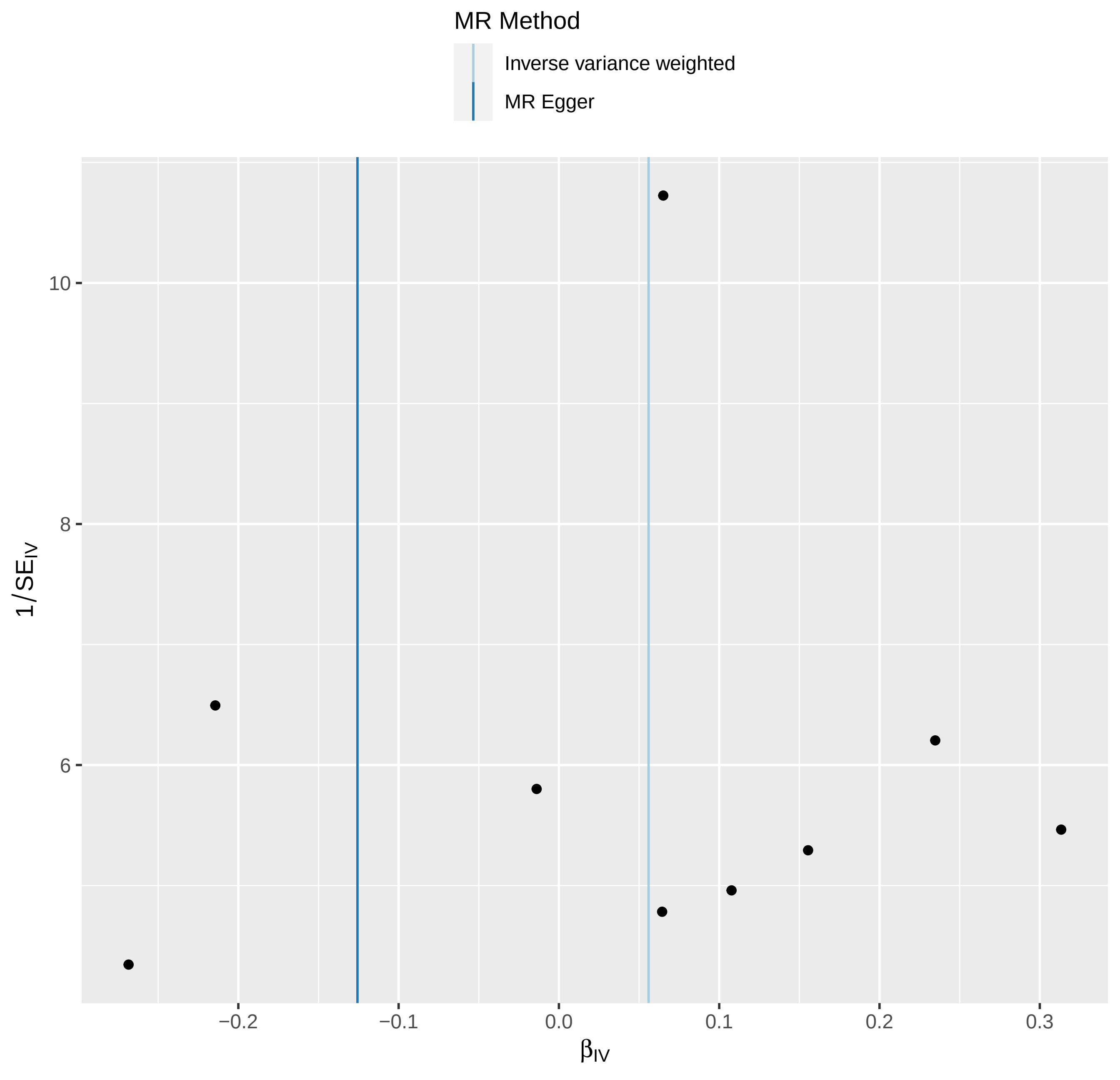

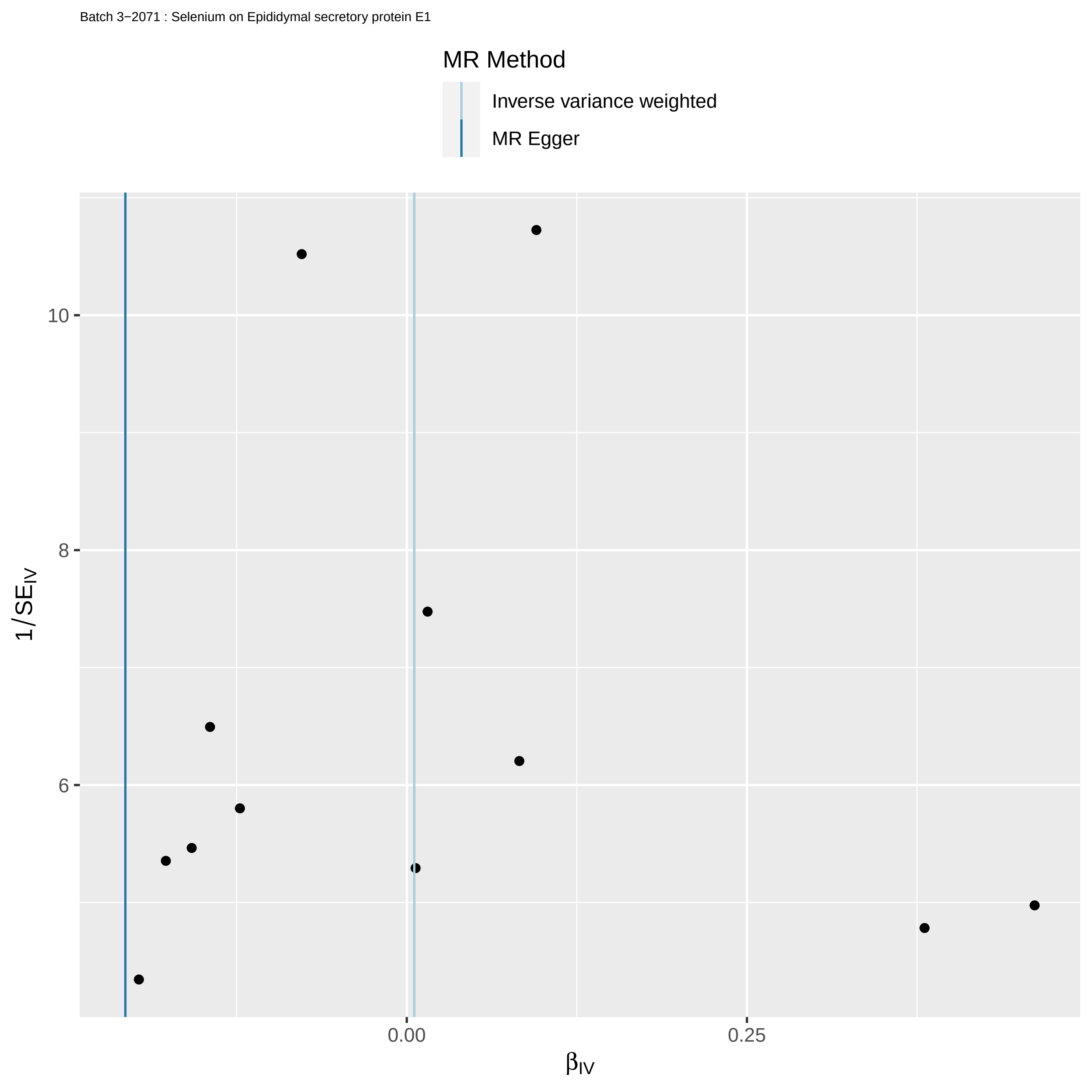

Batch 3−2071 : Selenium on Epididymal secretory protein E1
MR Method
Inverse variance weighted
MR Egger
10
8
6
1/SE_IV
0.00
0.25
β_IV

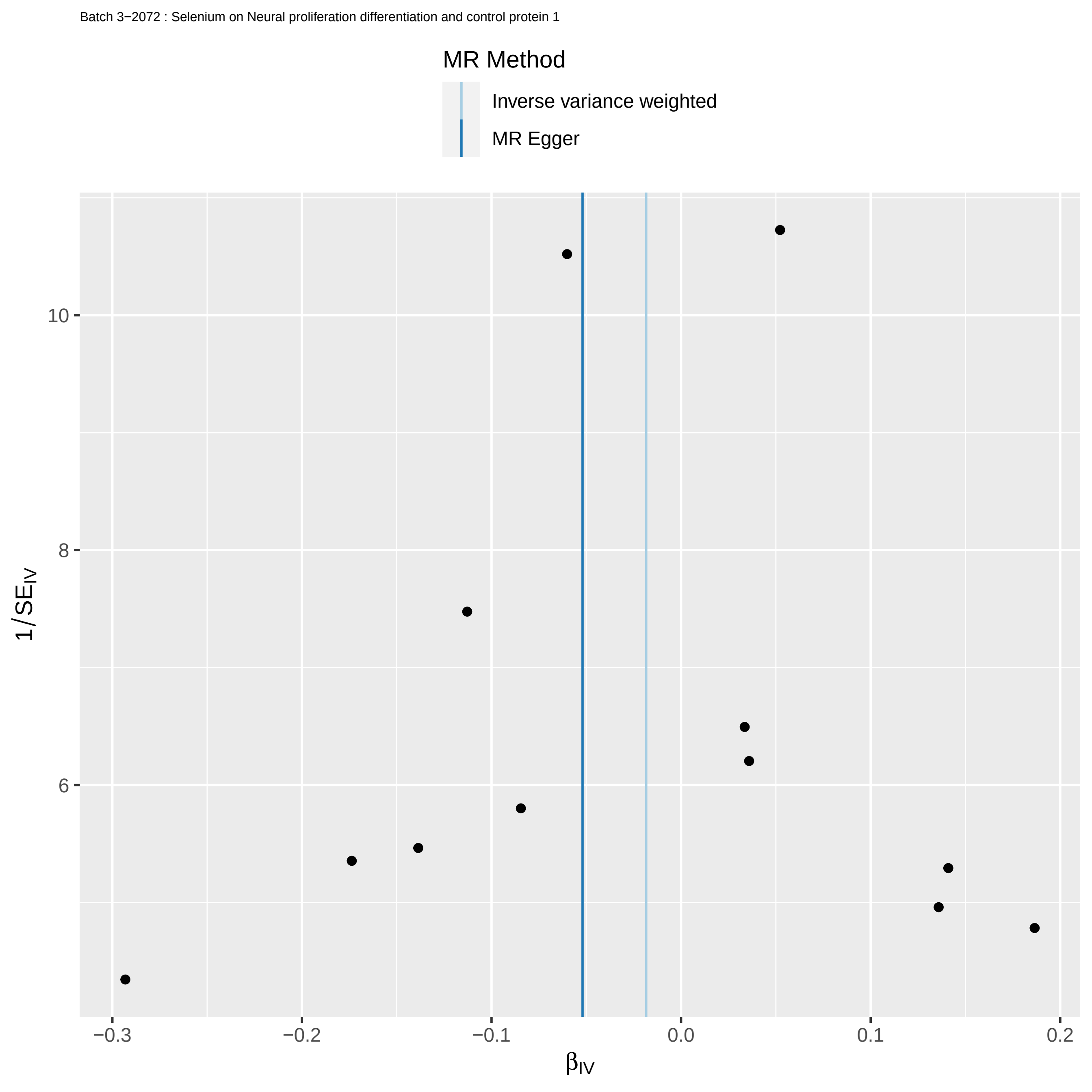
Batch 3−2072 : Selenium on Neural proliferation differentiation and control protein 1
MR Method
Inverse variance weighted
MR Egger
10
8
6
1/SE_IV
−0.3
−0.2
−0.1
0.0
0.1
0.2
β_IV

Batch 3−2073 : Selenium on Pro−FMRFamide−related neuropeptide FF

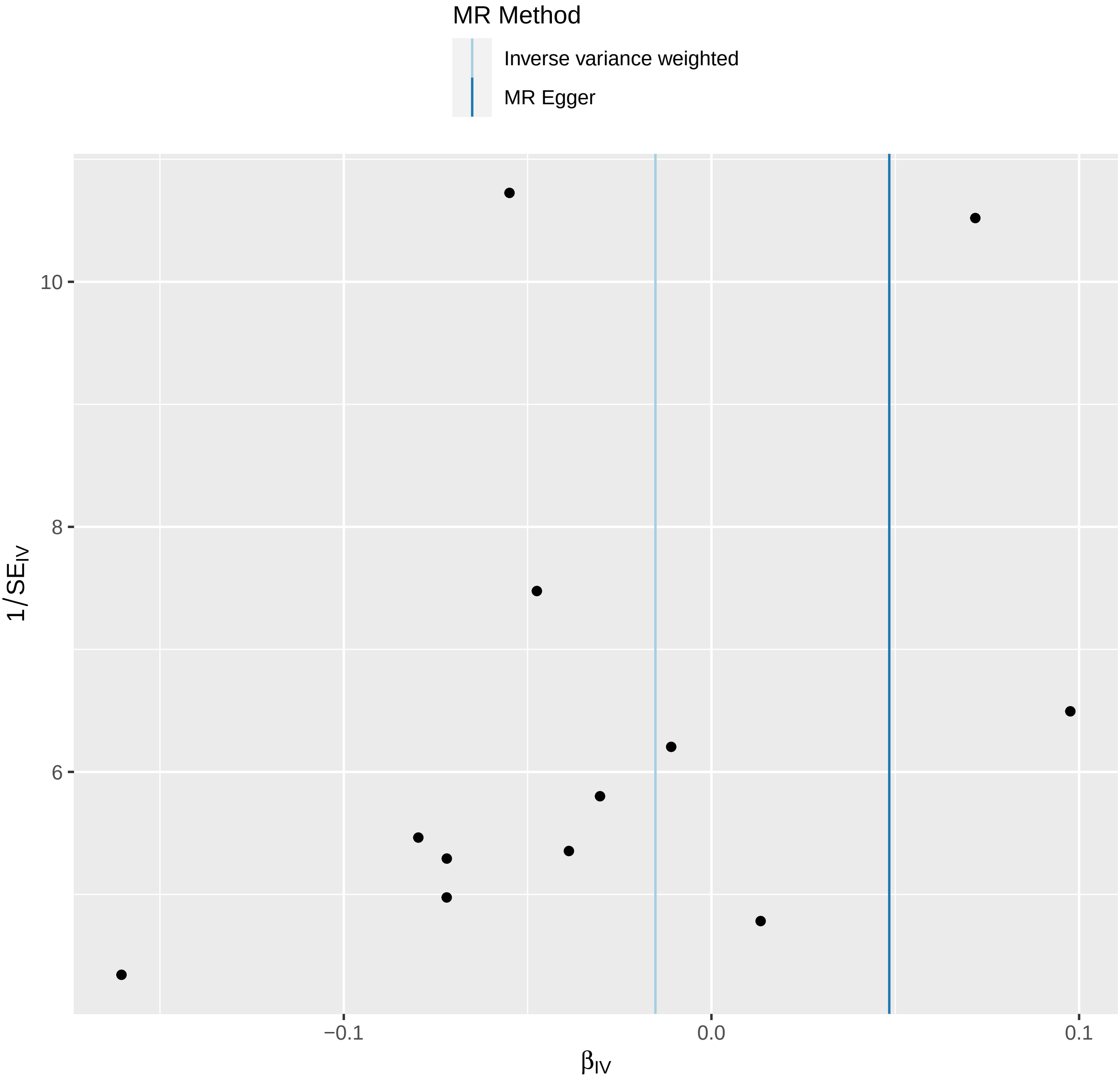

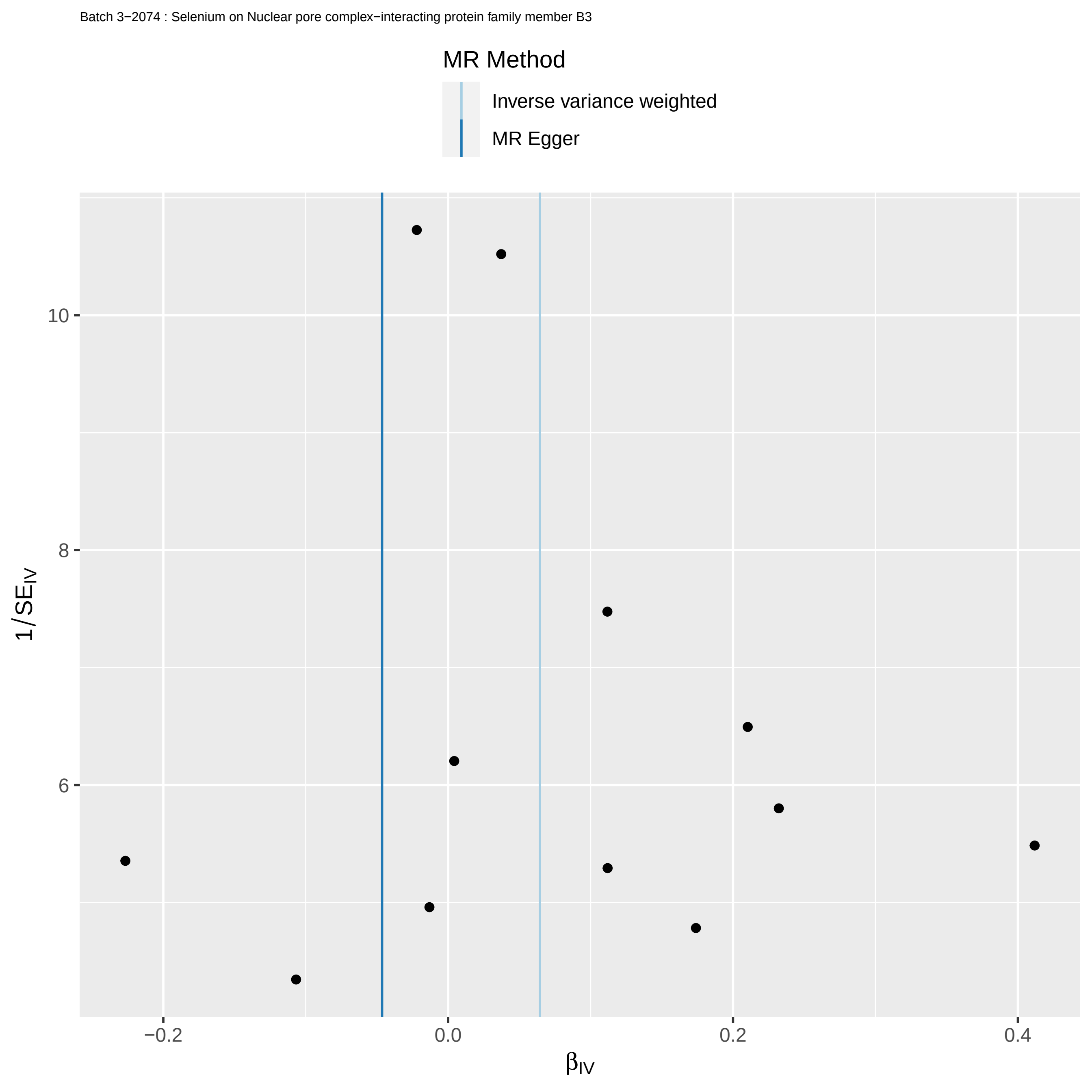

Batch 3−2074 : Selenium on Nuclear pore complex−interacting protein family member B3
MR Method
Inverse variance weighted
MR Egger
10
8
6
1/SE$_{IV}$
−0.2
0.0
0.2
0.4
β_{IV}

Batch 3−2075 : Selenium on Nuclear protein localization protein 4 homolog

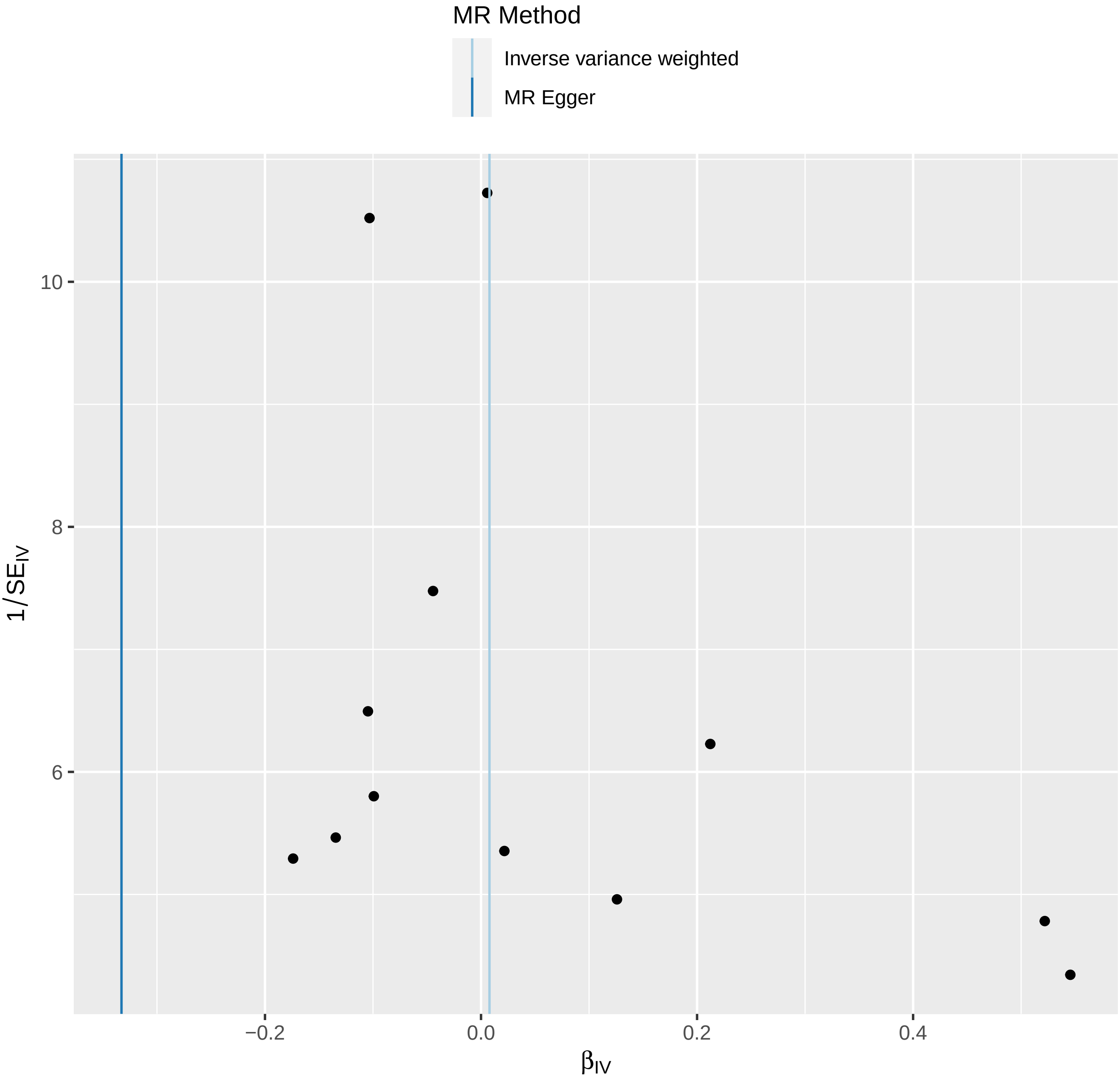

Batch 3−2076 : Selenium on Atrial natriuretic factor

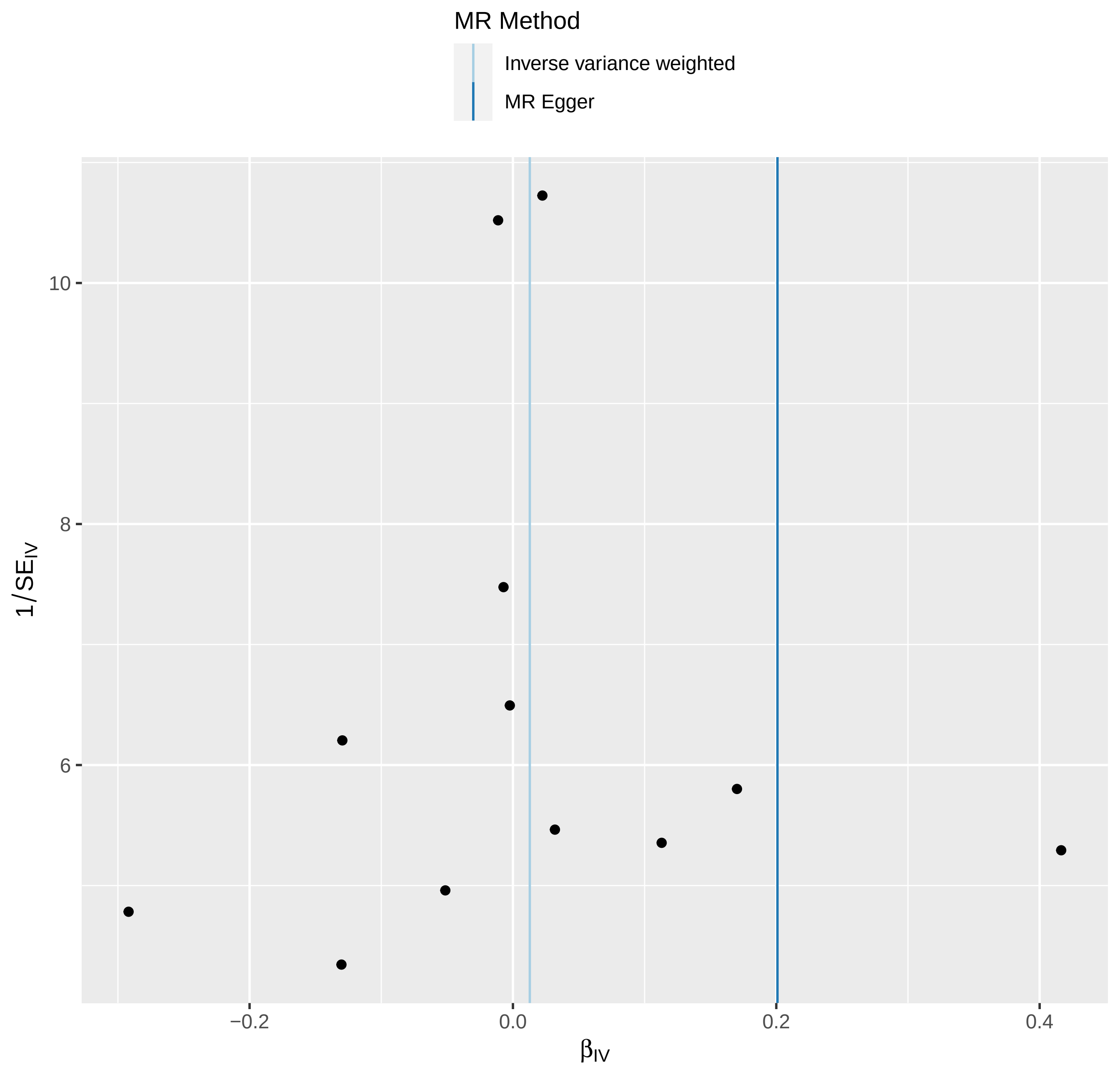

Batch 3−2077 : Selenium on Brain natriuretic peptide 32

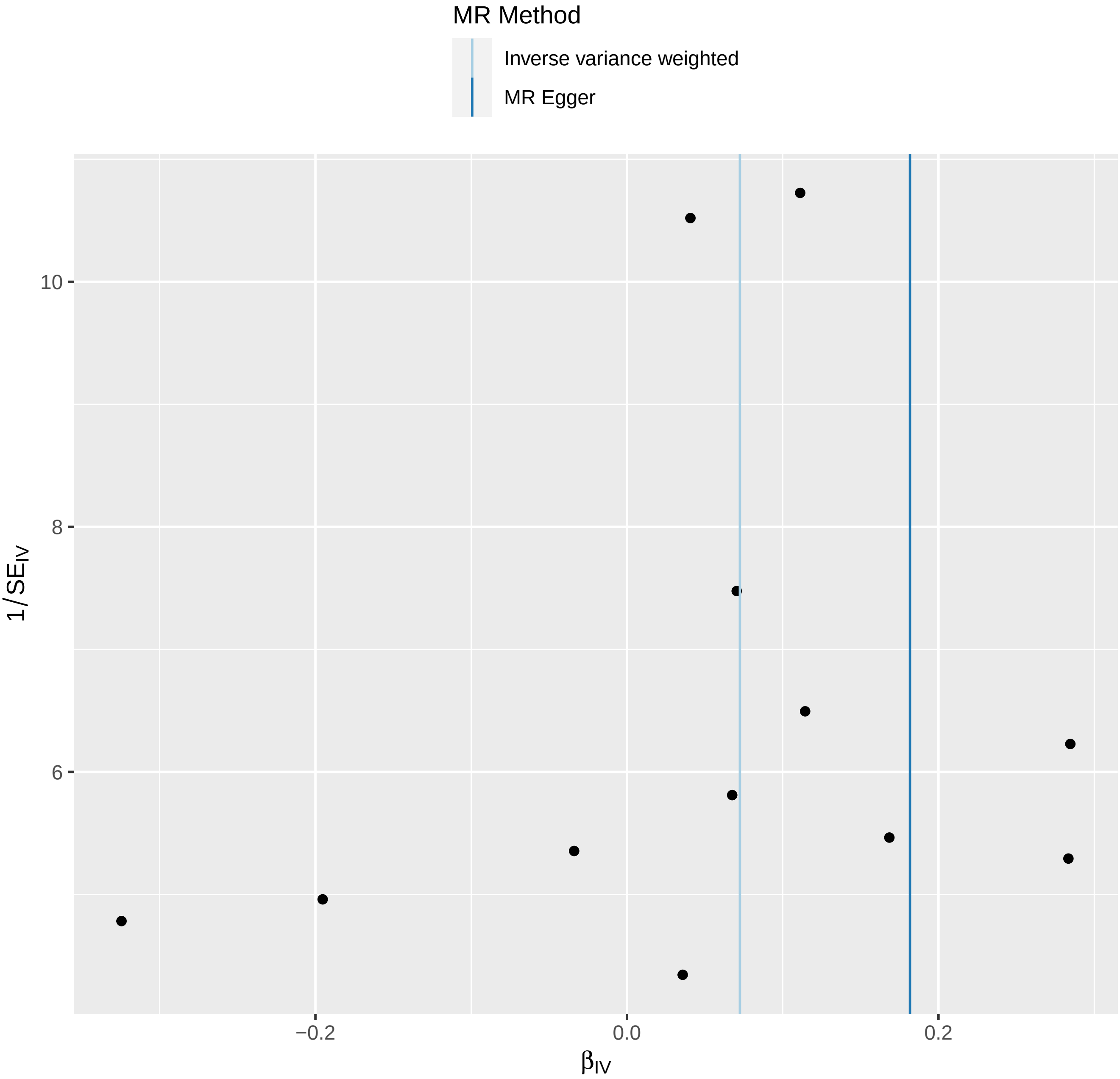

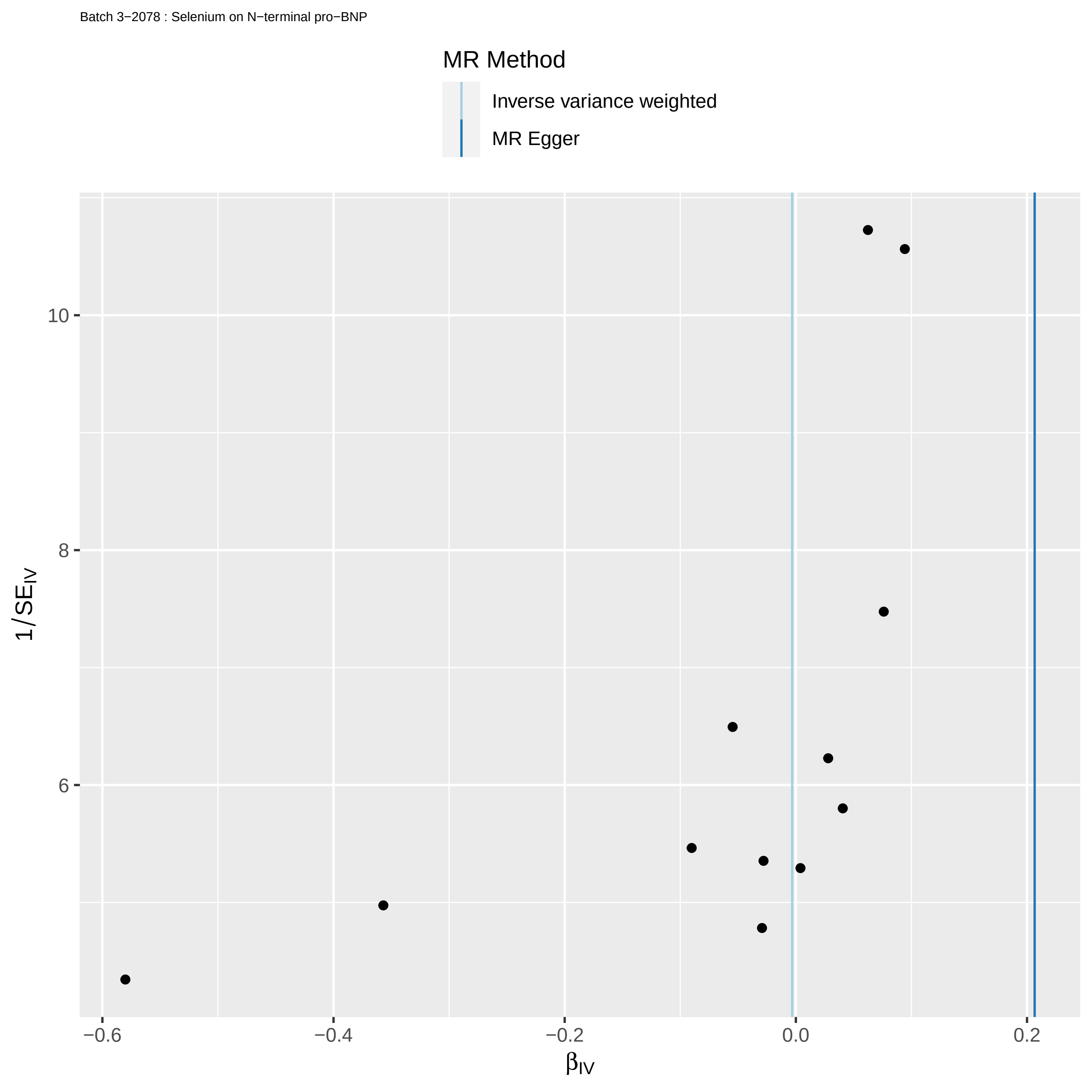
Batch 3−2078 : Selenium on N−terminal pro−BNP
MR Method
Inverse variance weighted
MR Egger
1/SE$_{IV}$
10
8
6
−0.6
−0.4
−0.2
0.0
0.2
β_{IV}

Batch 3−2079 : Selenium on Neuropeptide S

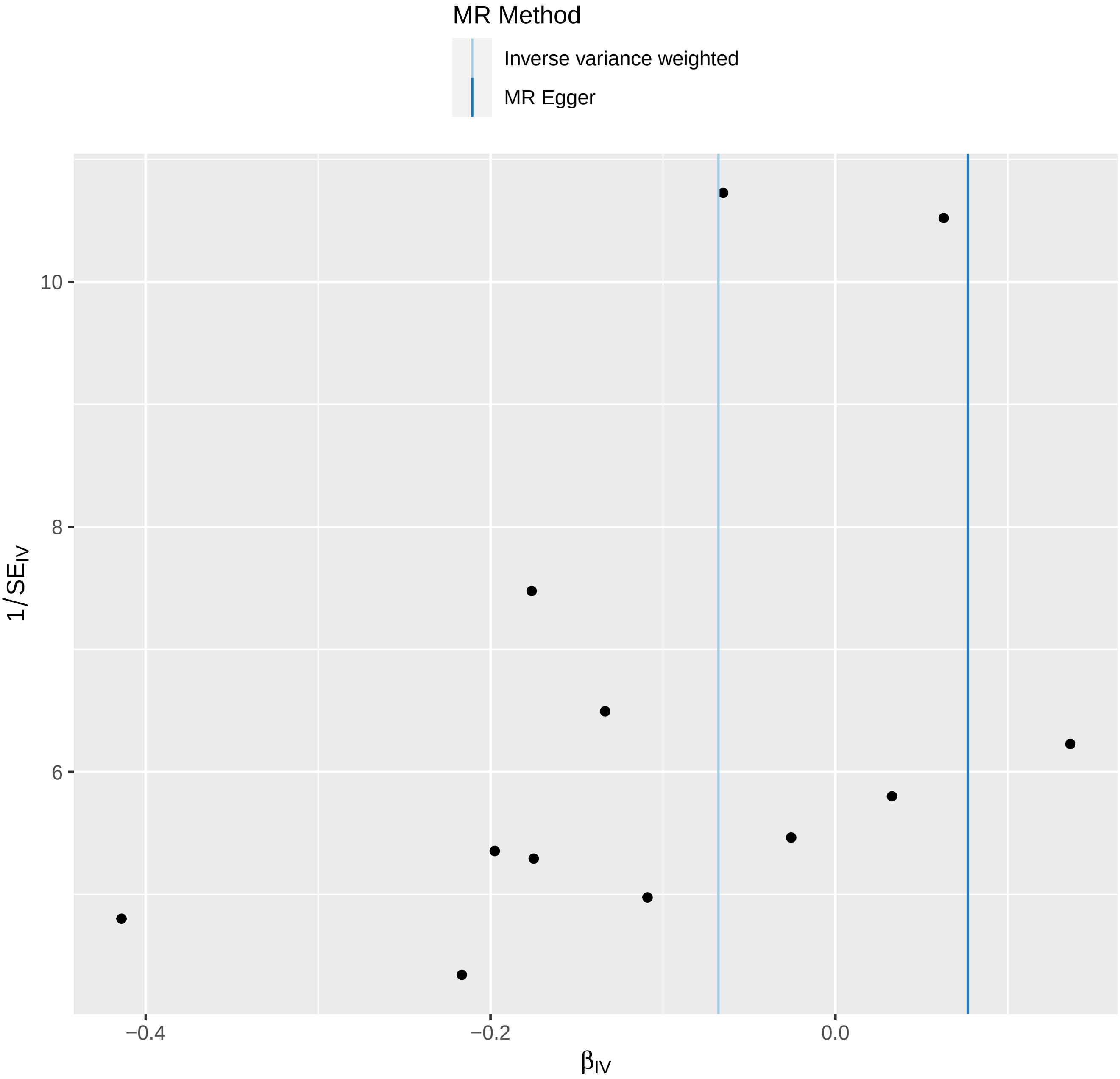

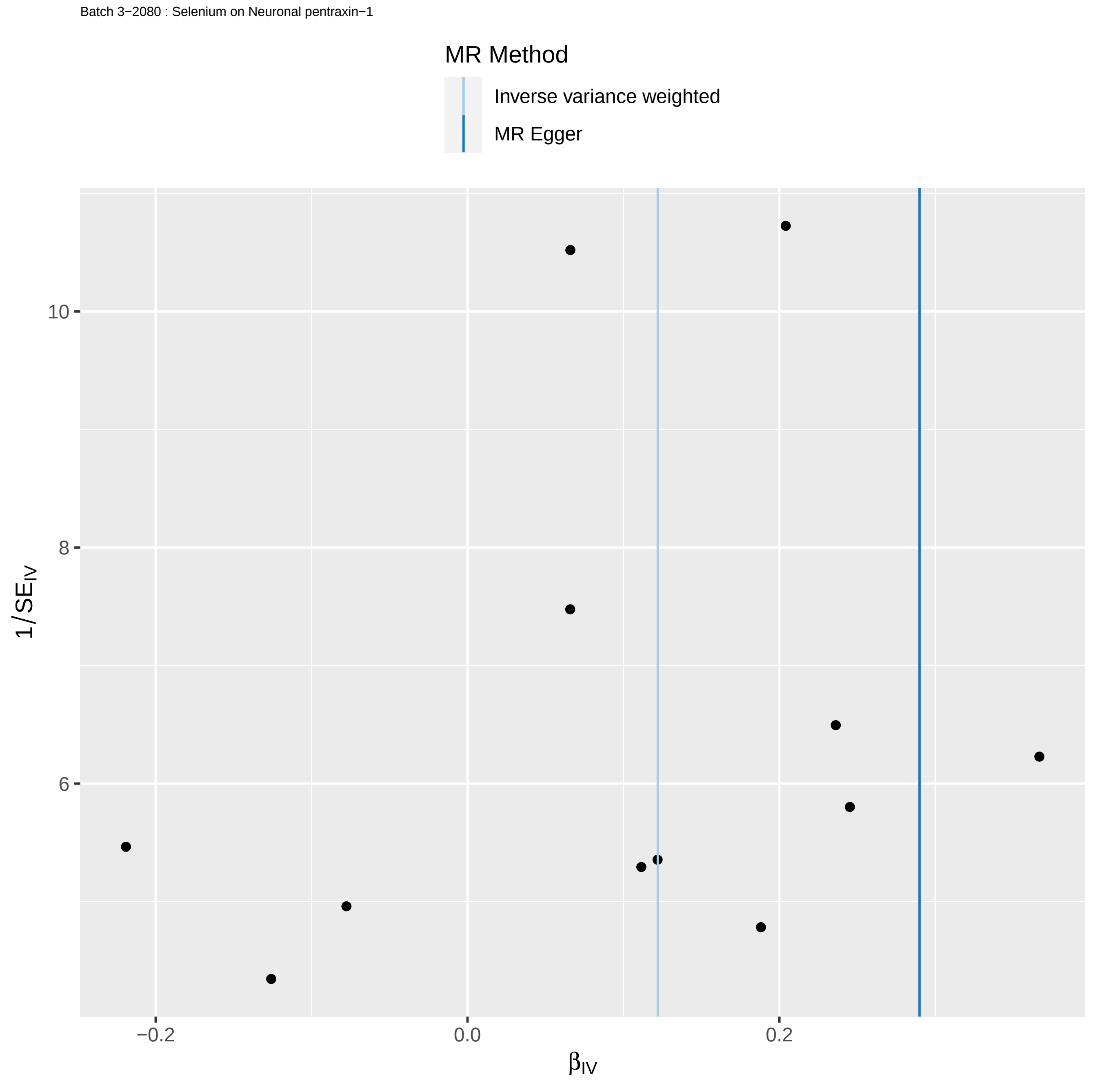

Batch 3−2080 : Selenium on Neuronal pentraxin−1
MR Method
Inverse variance weighted
MR Egger
10
8
6
1/SE_IV
−0.2
0.0
0.2
β_IV

Batch 3−2081 : Selenium on Neuronal pentraxin−2

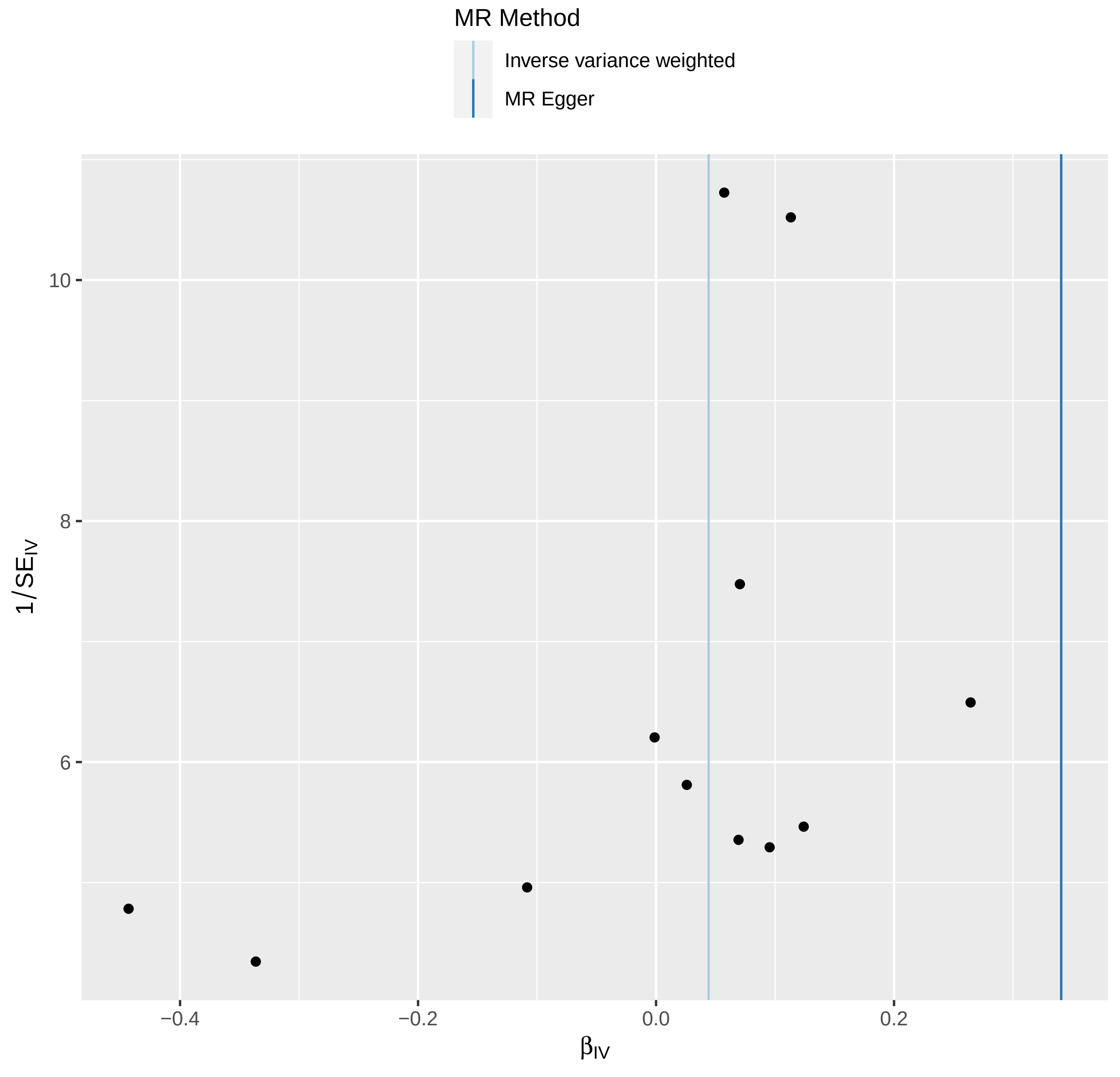

Batch 3−2082 : Selenium on Neuropeptide W

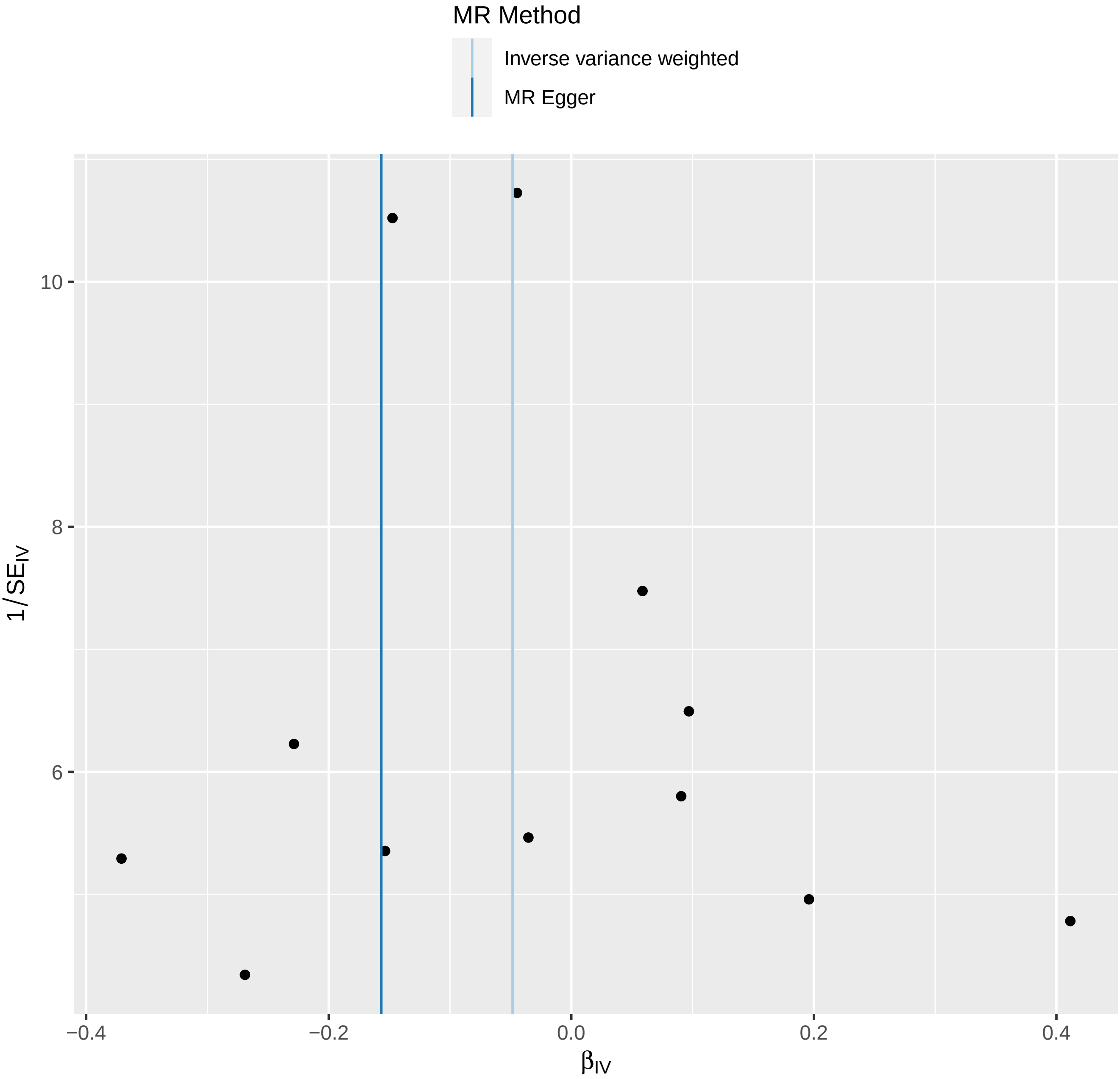

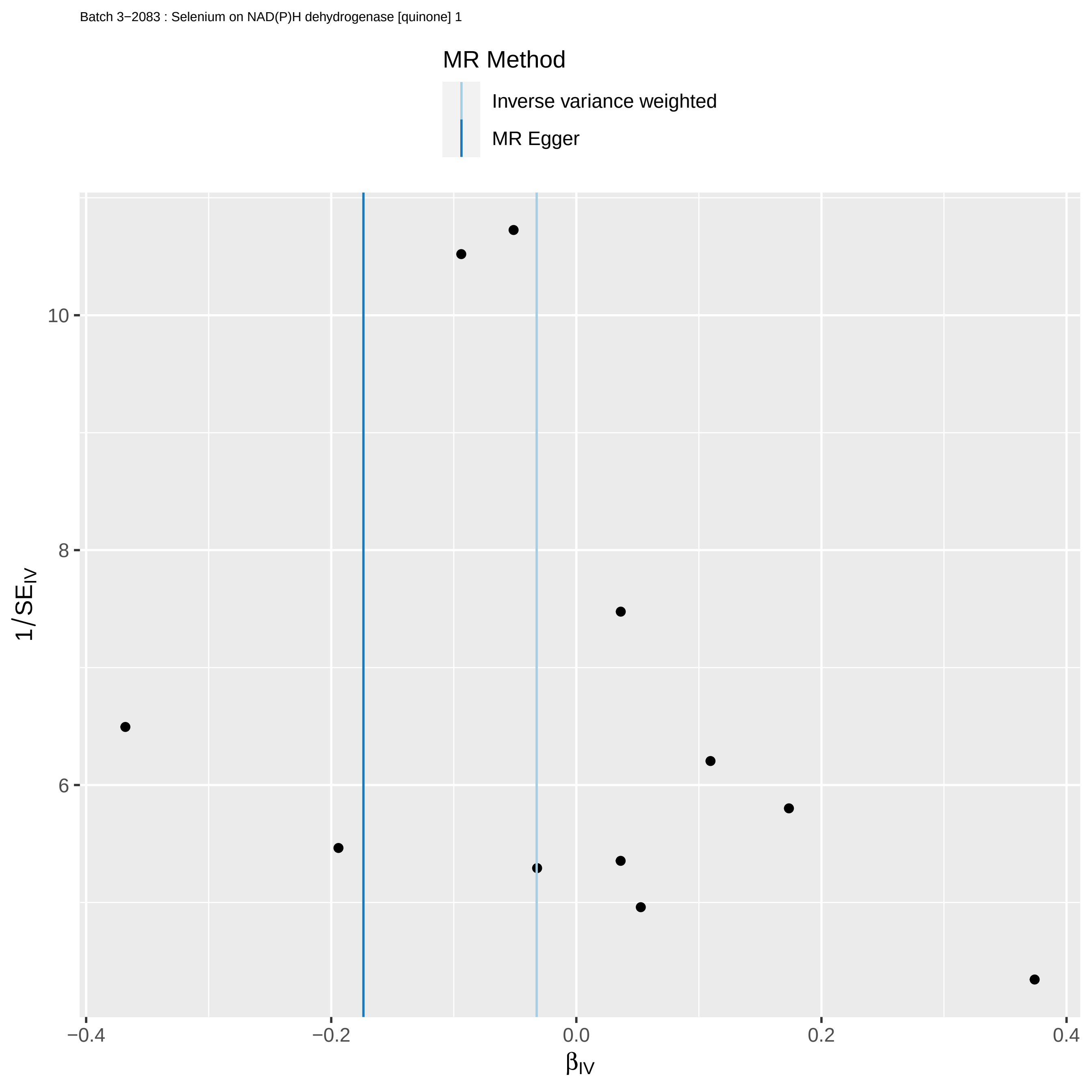
Batch 3−2083 : Selenium on NAD(P)H dehydrogenase [quinone] 1
MR Method
Inverse variance weighted
MR Egger
10
8
6
1/SE_IV
−0.4
−0.2
0.0
0.2
0.4
β_IV

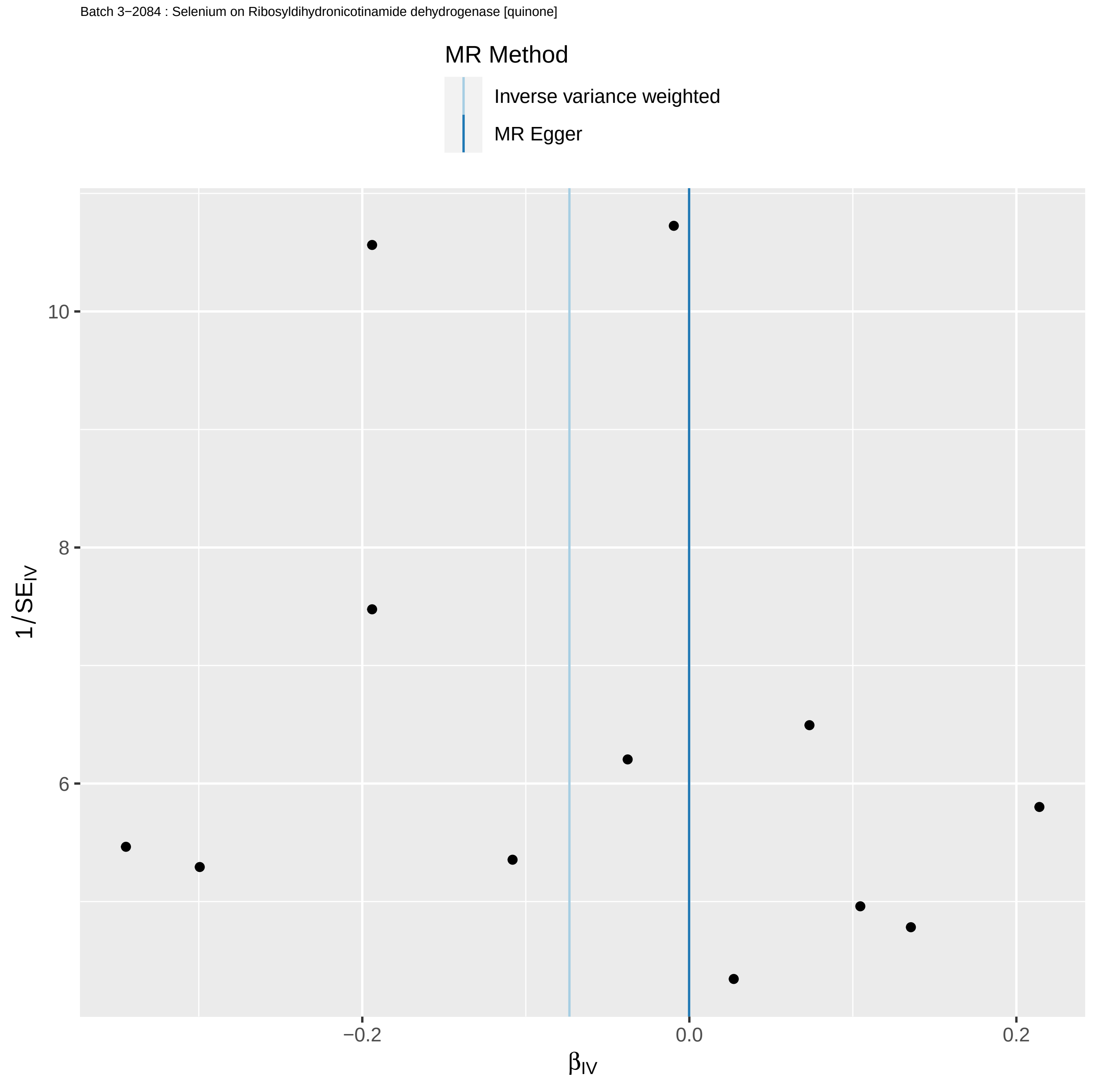
Batch 3−2084 : Selenium on Ribosyldihydronicotinamide dehydrogenase [quinone]
MR Method
Inverse variance weighted
MR Egger
10
8
6
1/SE$_{IV}$
−0.2
0.0
0.2
β$_{IV}$

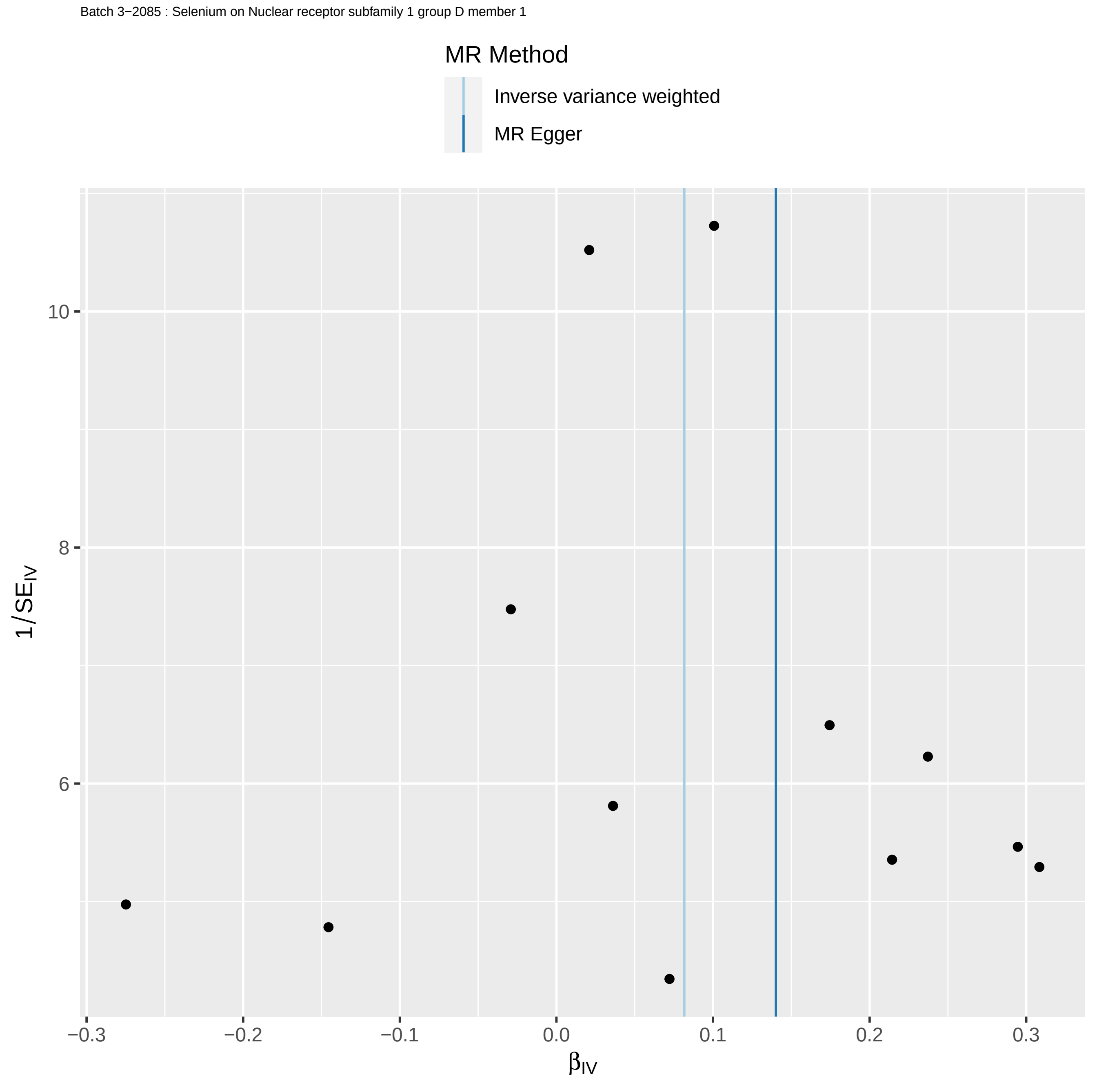

Batch 3−2085 : Selenium on Nuclear receptor subfamily 1 group D member 1
MR Method
Inverse variance weighted
MR Egger
10
8
6
1/SE$_{IV}$
−0.3
−0.2
−0.1
0.0
0.1
0.2
0.3
β$_{IV}$

Batch 3−2086 : Selenium on Nuclear receptor subfamily 1 group D member 2

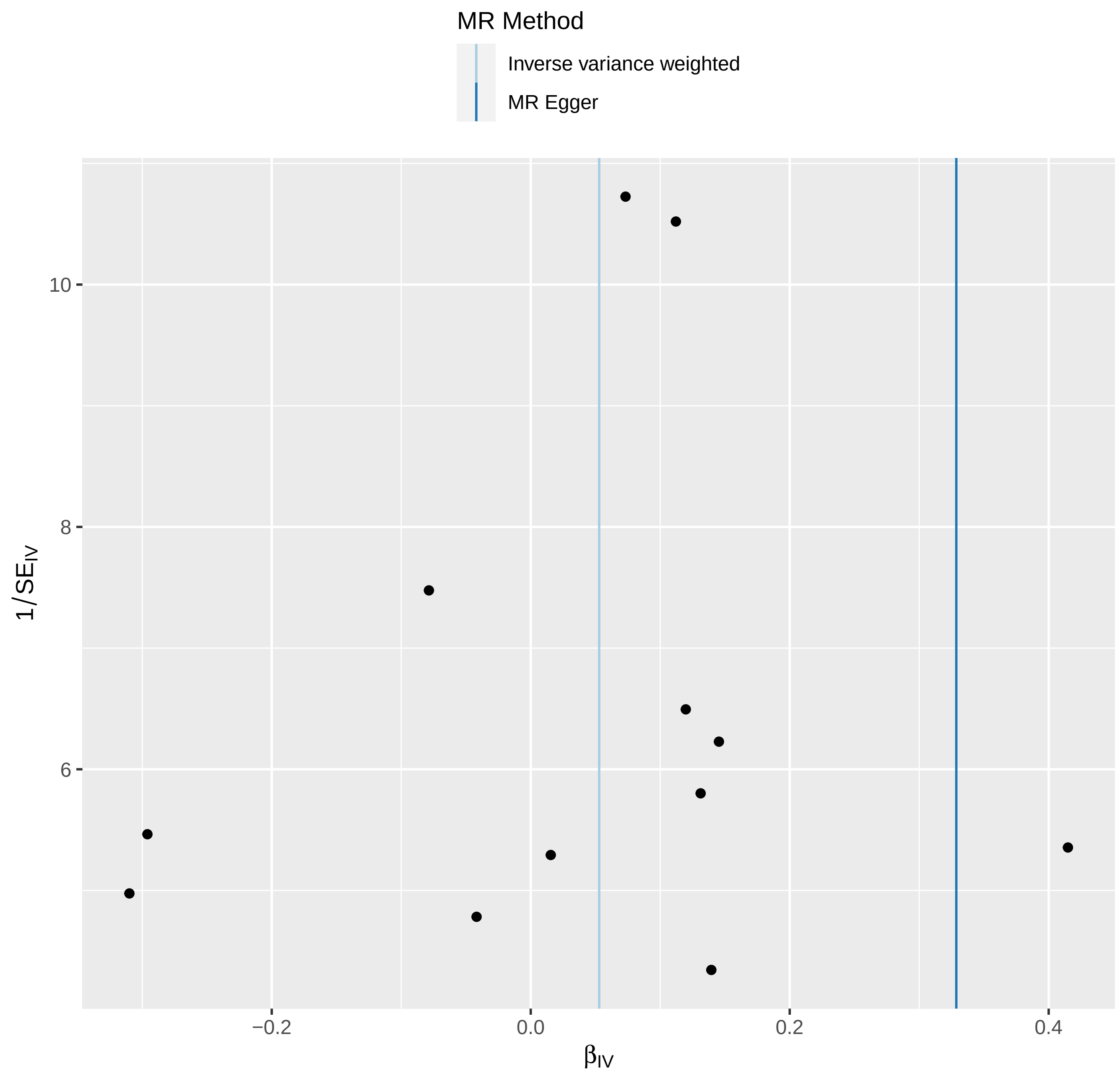

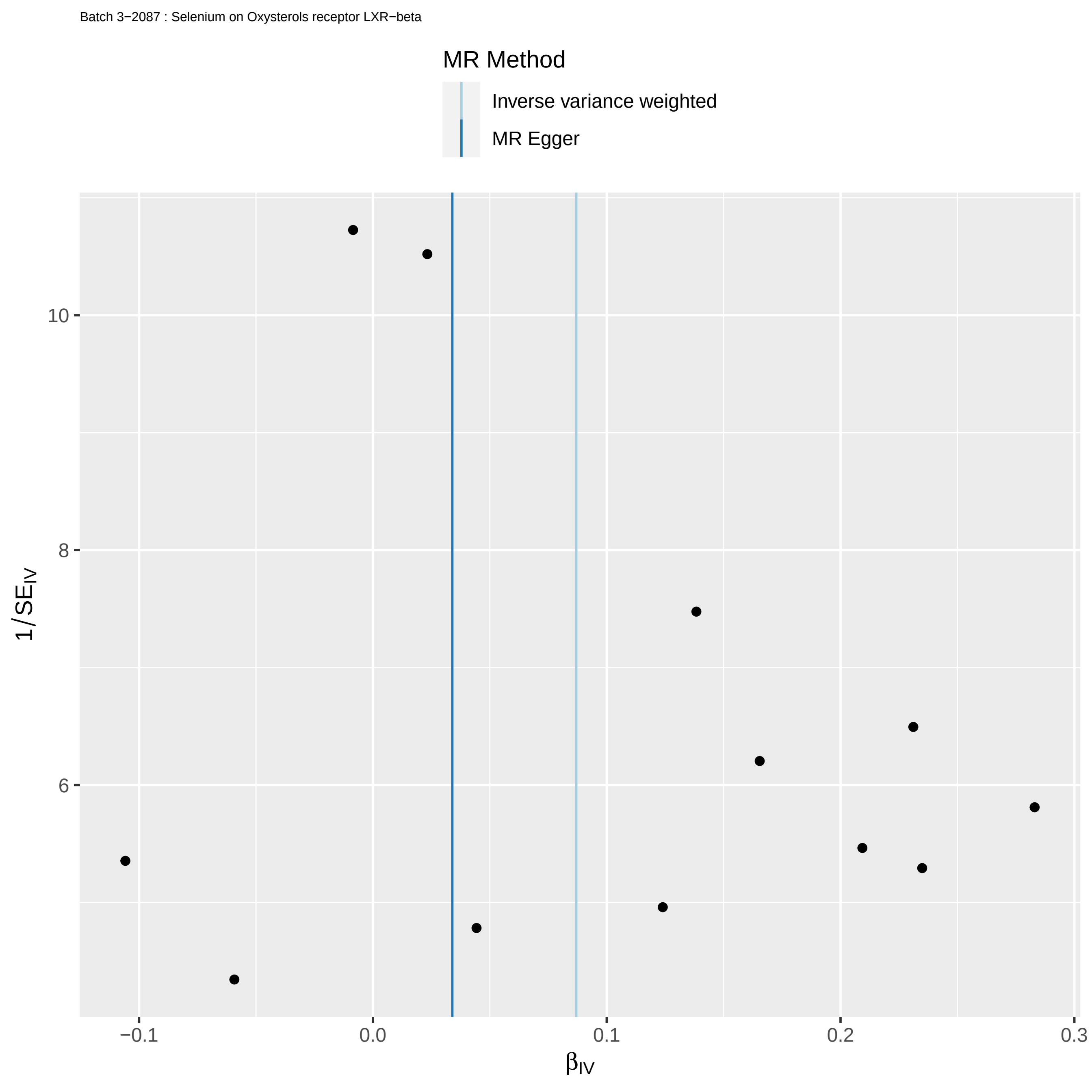

Batch 3−2087 : Selenium on Oxysterols receptor LXR−beta
MR Method
Inverse variance weighted
MR Egger
10
8
6
1/SE_IV
−0.1
0.0
0.1
0.2
0.3
β_IV

Batch 3−2088 : Selenium on Glucocorticoid receptor

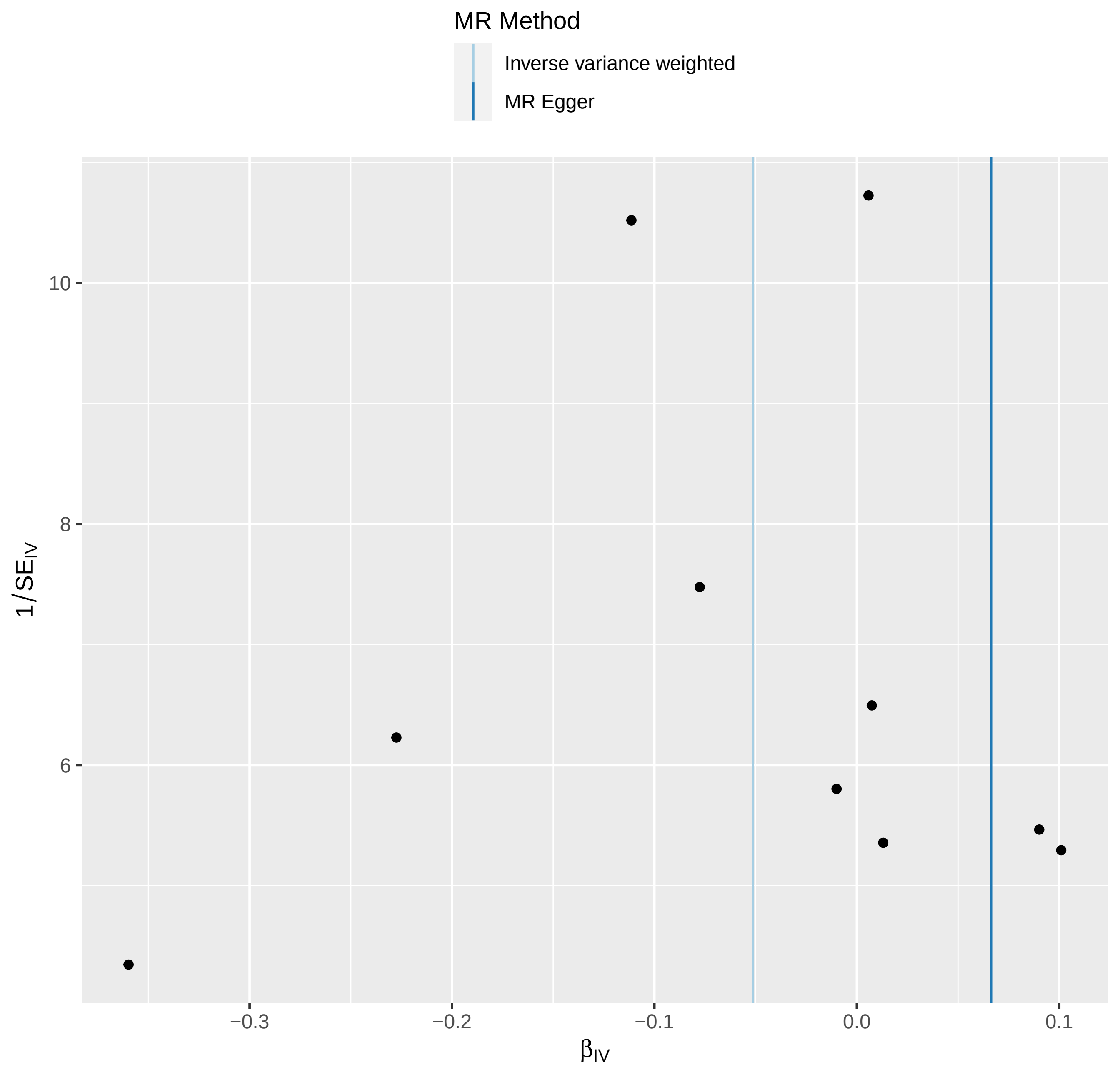

Batch 3−2089 : Selenium on Mineralocorticoid receptor

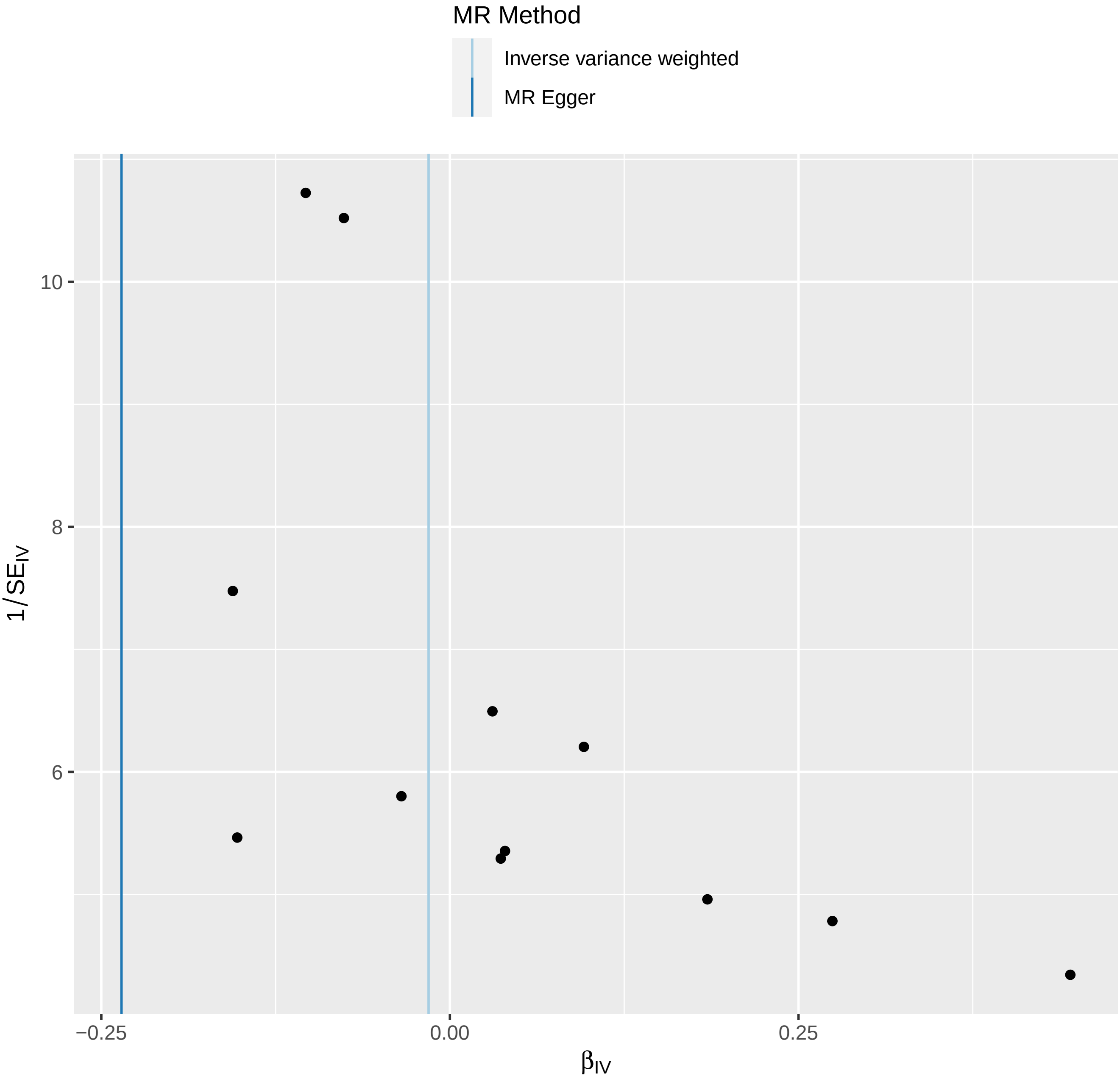

Batch 3−2090 : Selenium on Nuclear receptor subfamily 4 group A member 1

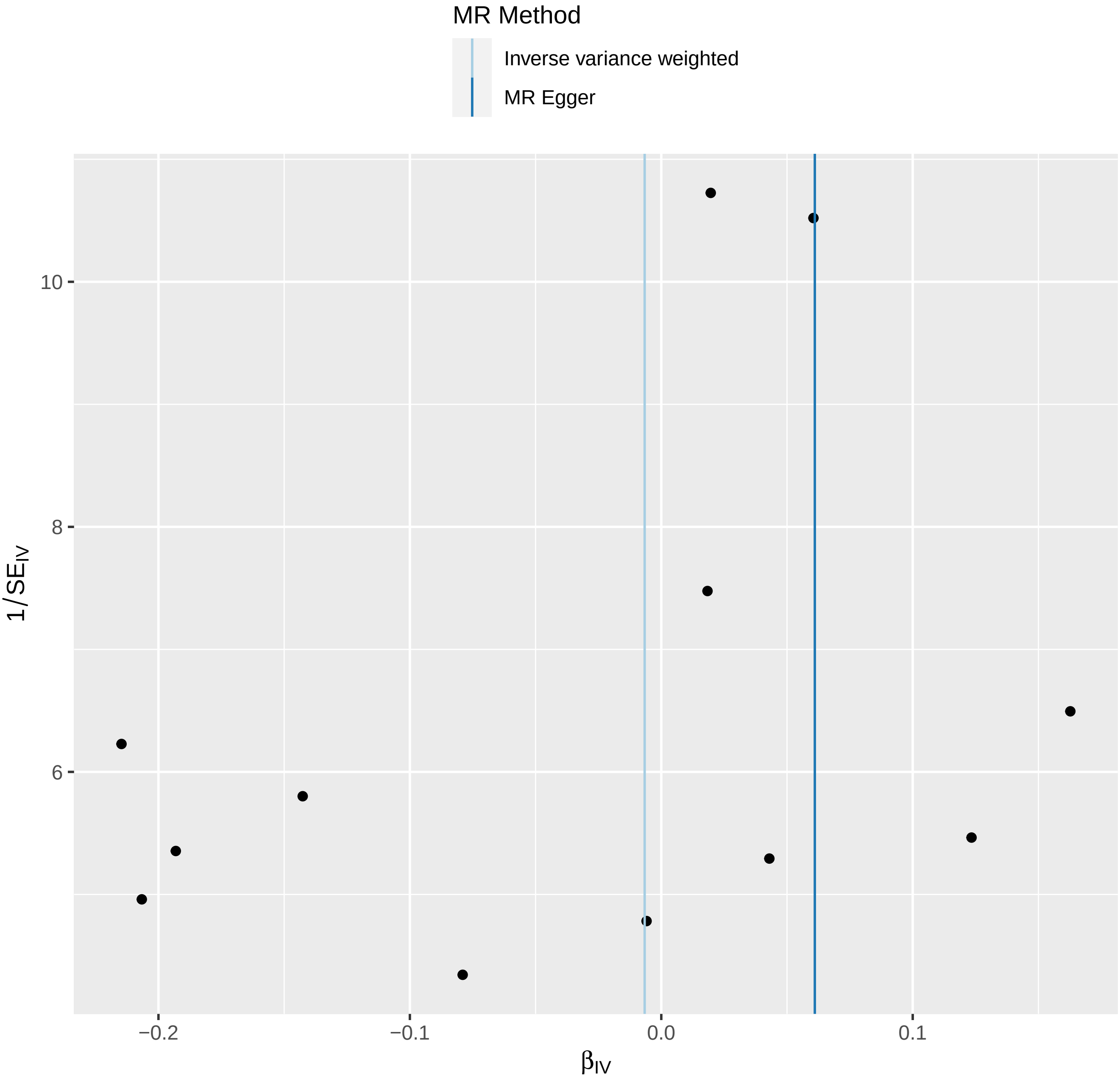

Batch 3−2091 : Selenium on Nuclear receptor subfamily 5 group A member 2

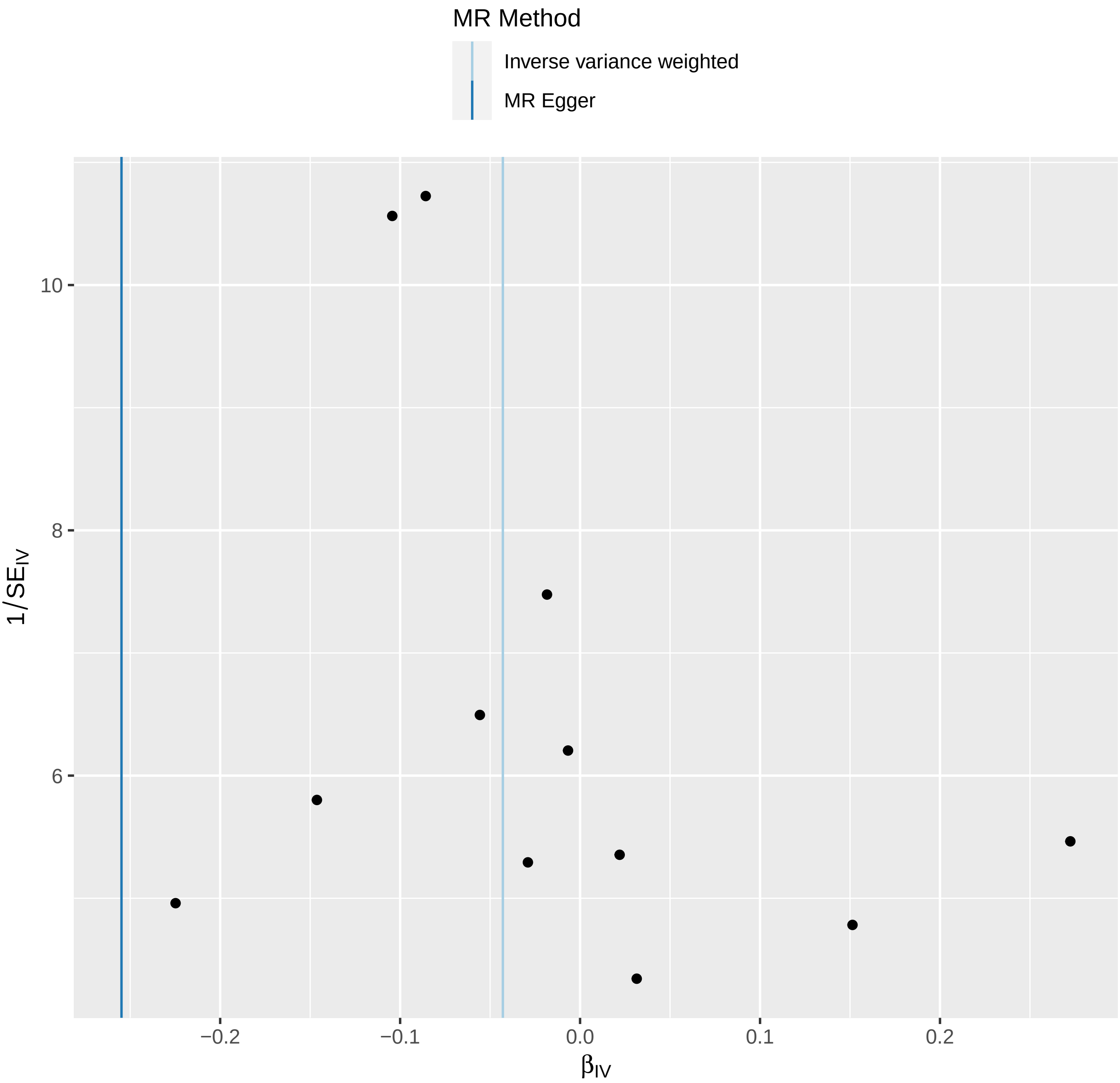

Batch 3−2092 : Selenium on Nutritionally−regulated adipose and cardiac enriched protein homolog

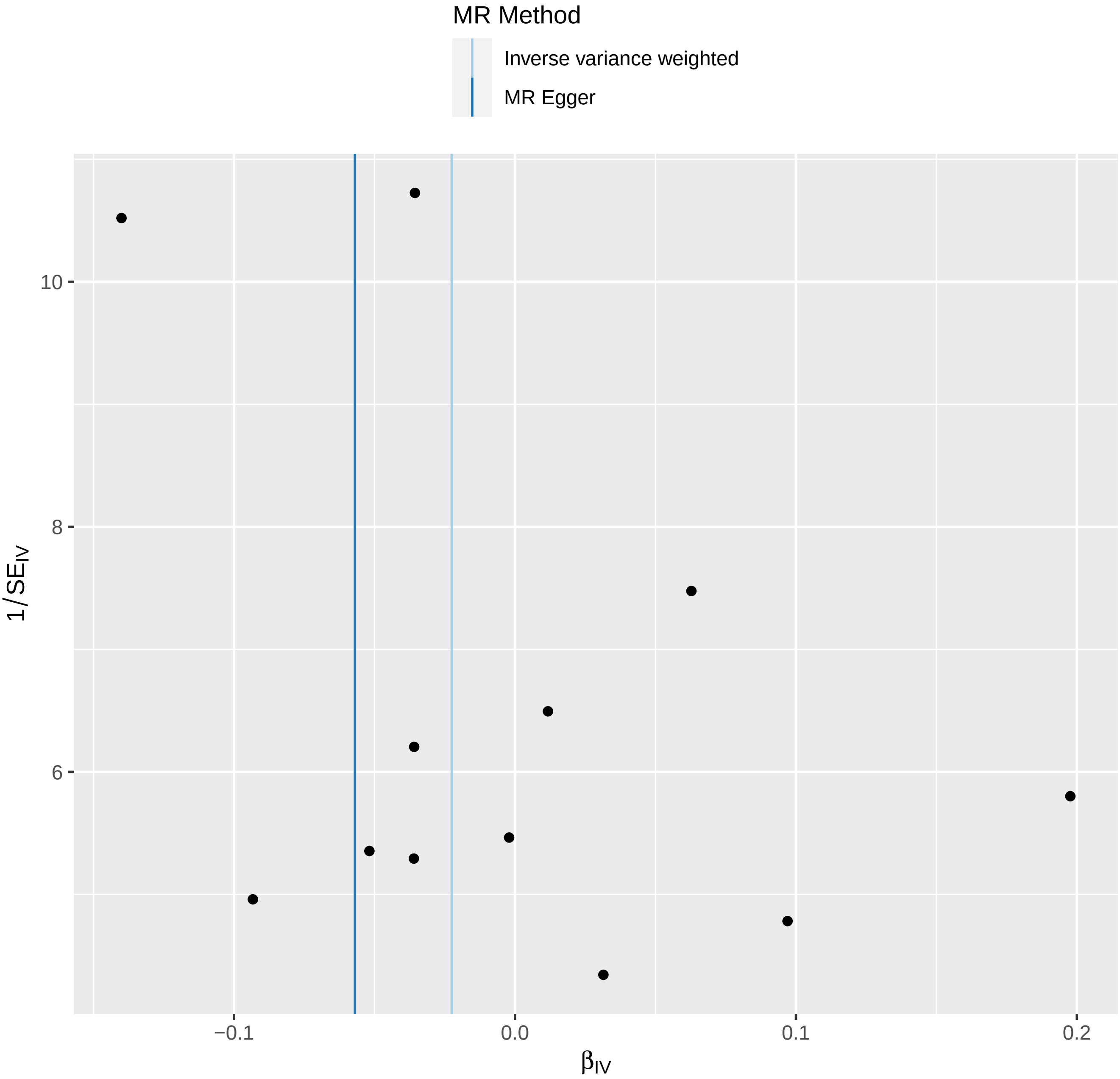

Batch 3−2093 : Selenium on Nuclear receptor−binding protein

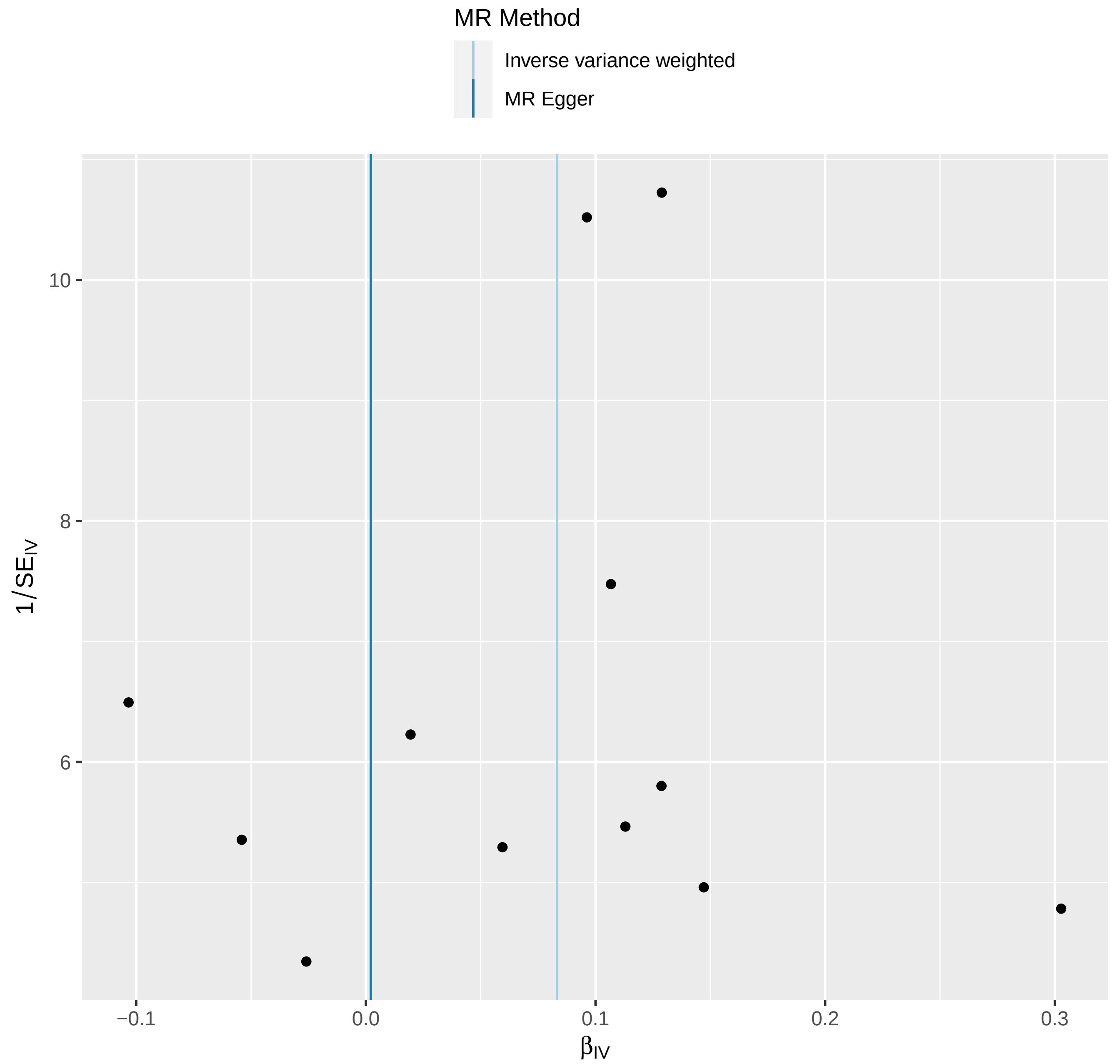

Batch 3−2094 : Selenium on Neuronal cell adhesion molecule

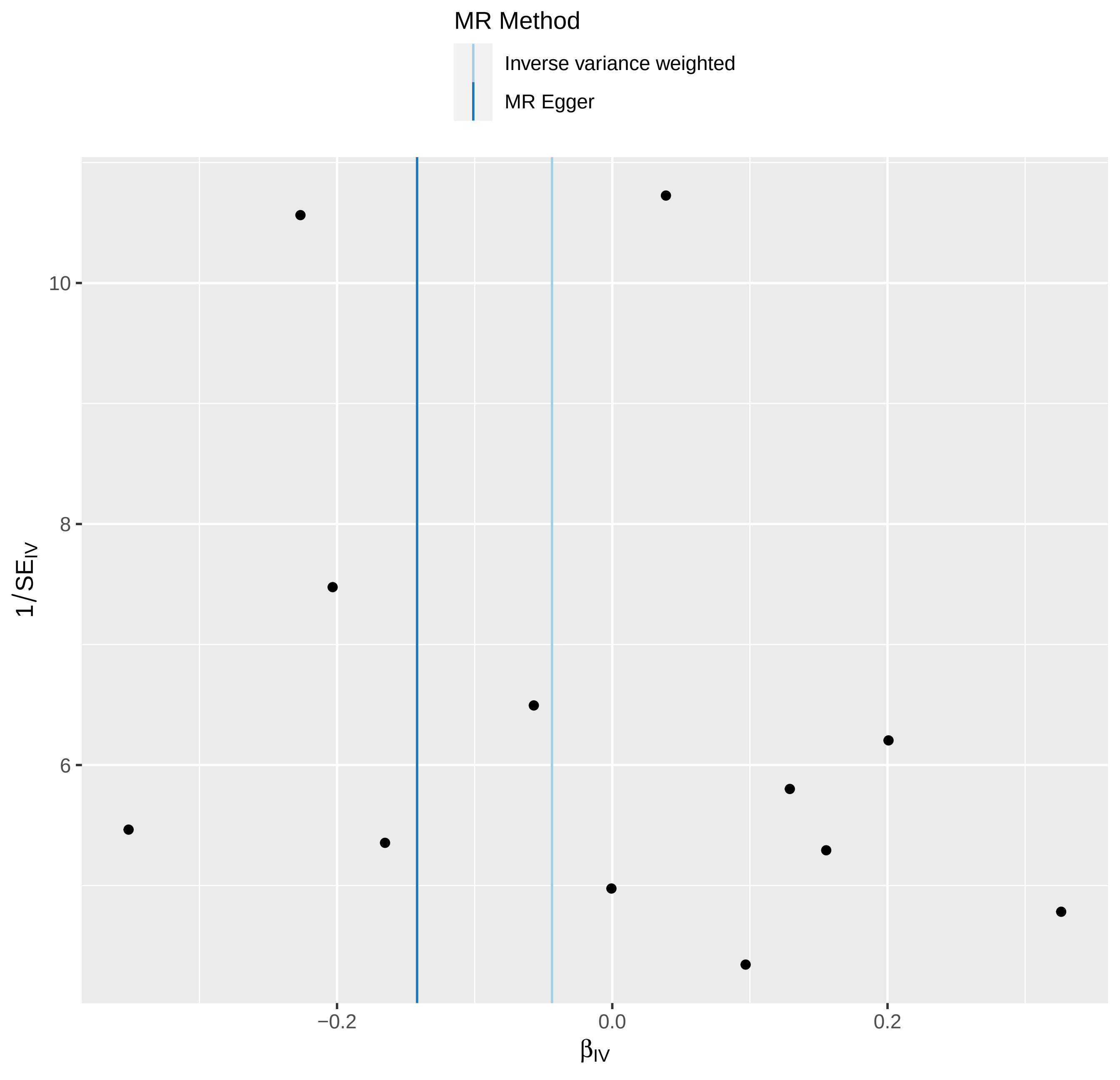

Batch 3−2095 : Selenium on Neuregulin−1

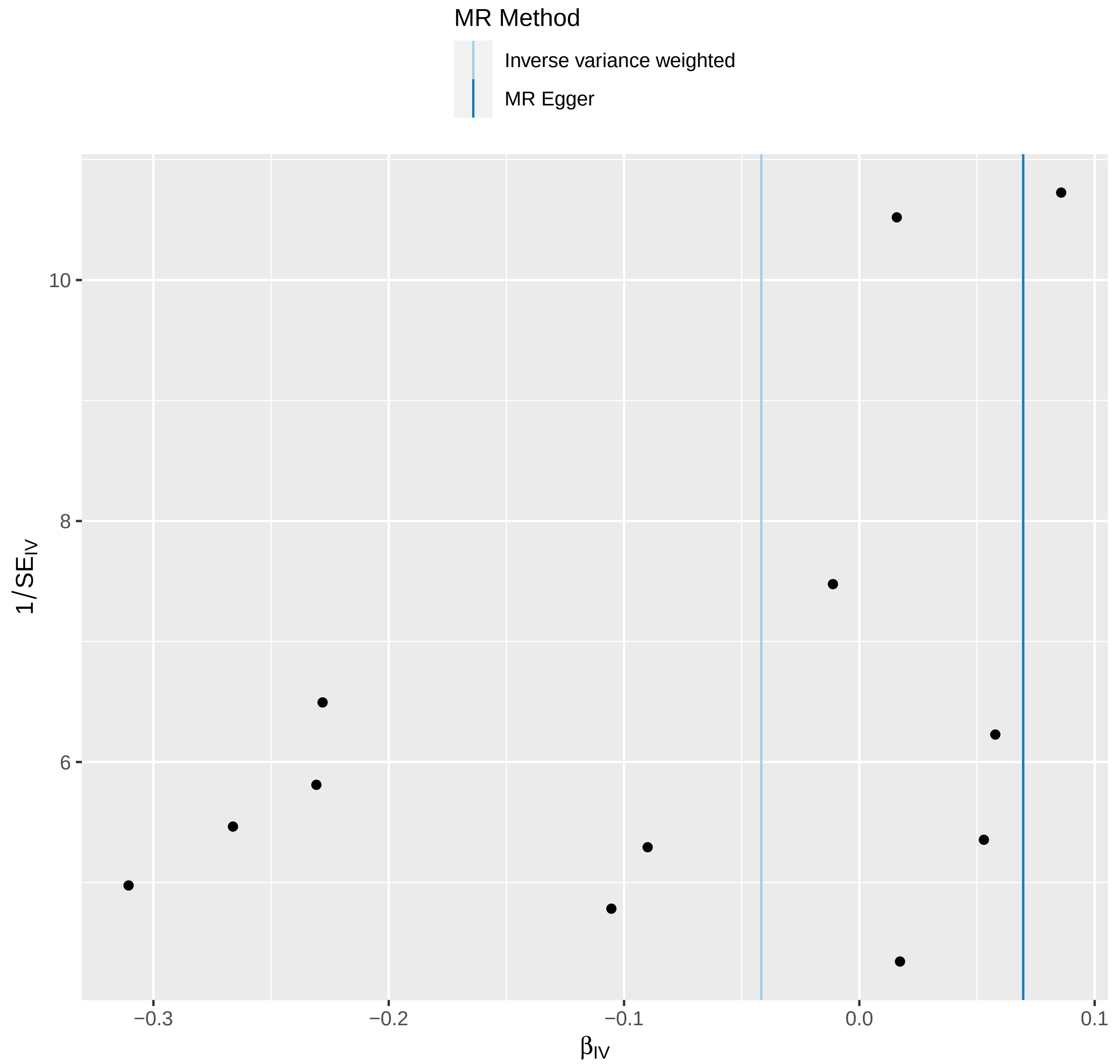

Batch 3−2096 : Selenium on Pro−neuregulin−2, membrane−bound isoform

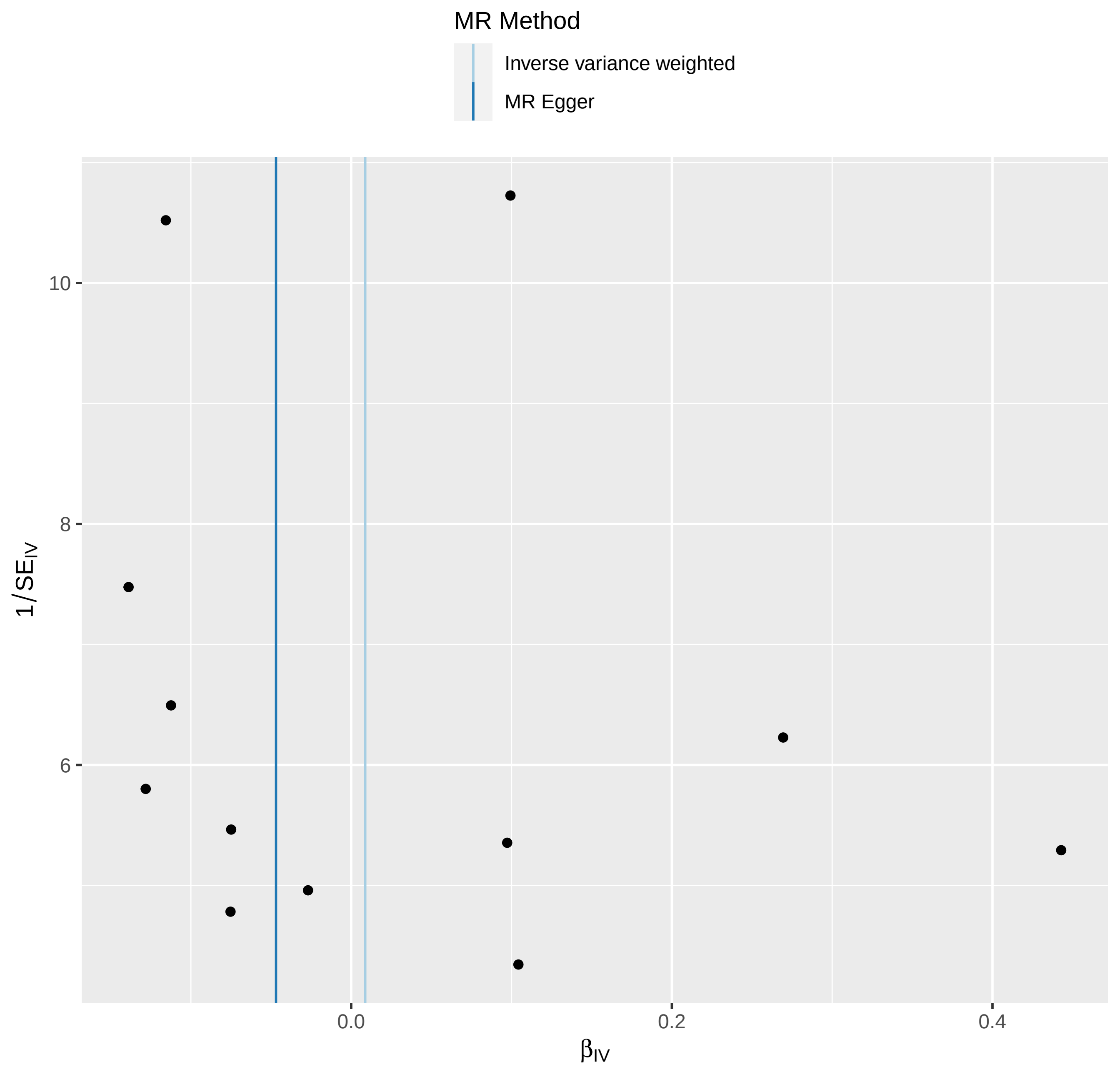

Batch 3−2097 : Selenium on Neuregulin−4

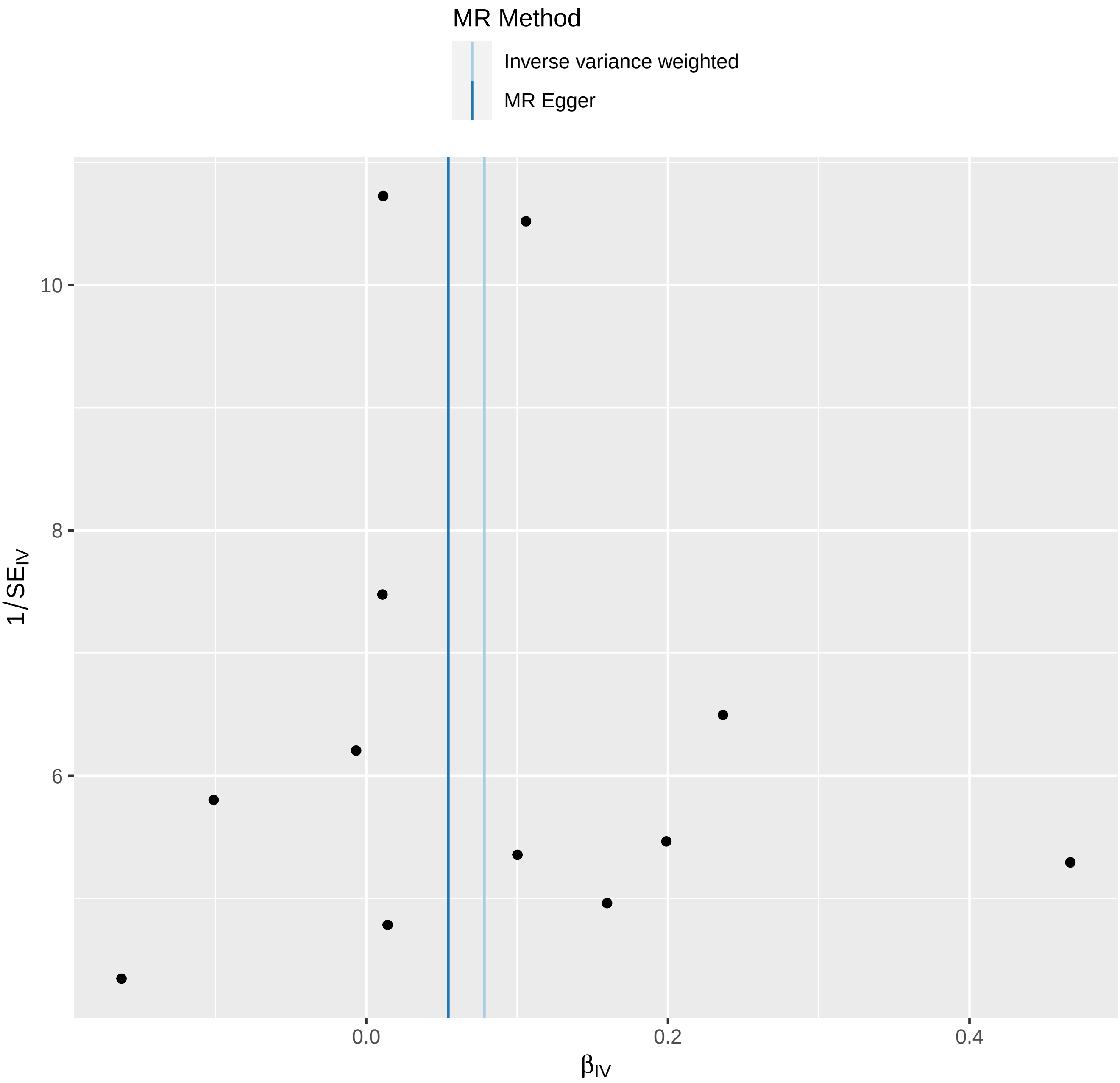

Batch 3−2098 : Selenium on Neuritin

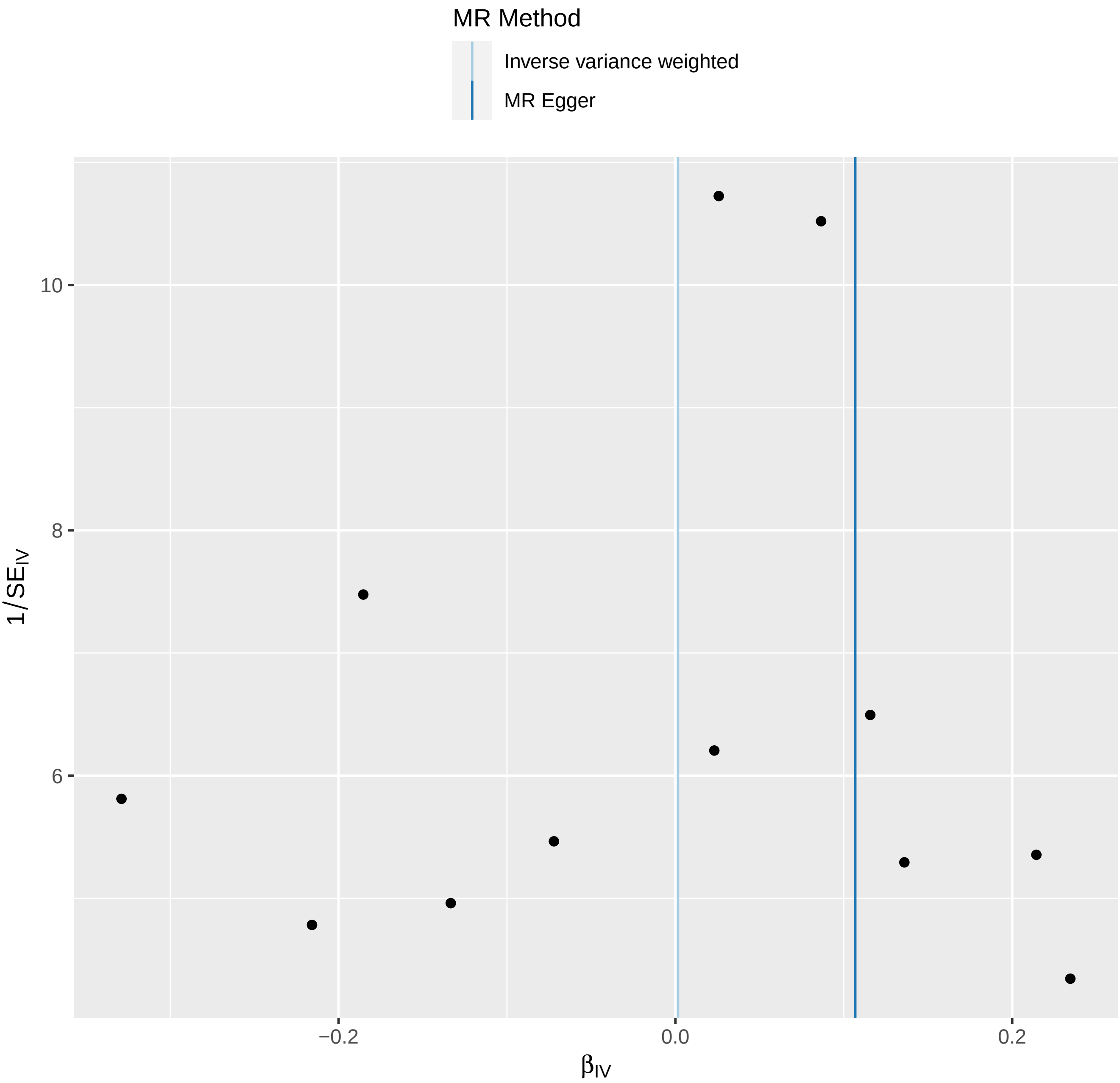

Batch 3−2099 : Selenium on Neuropilin−1

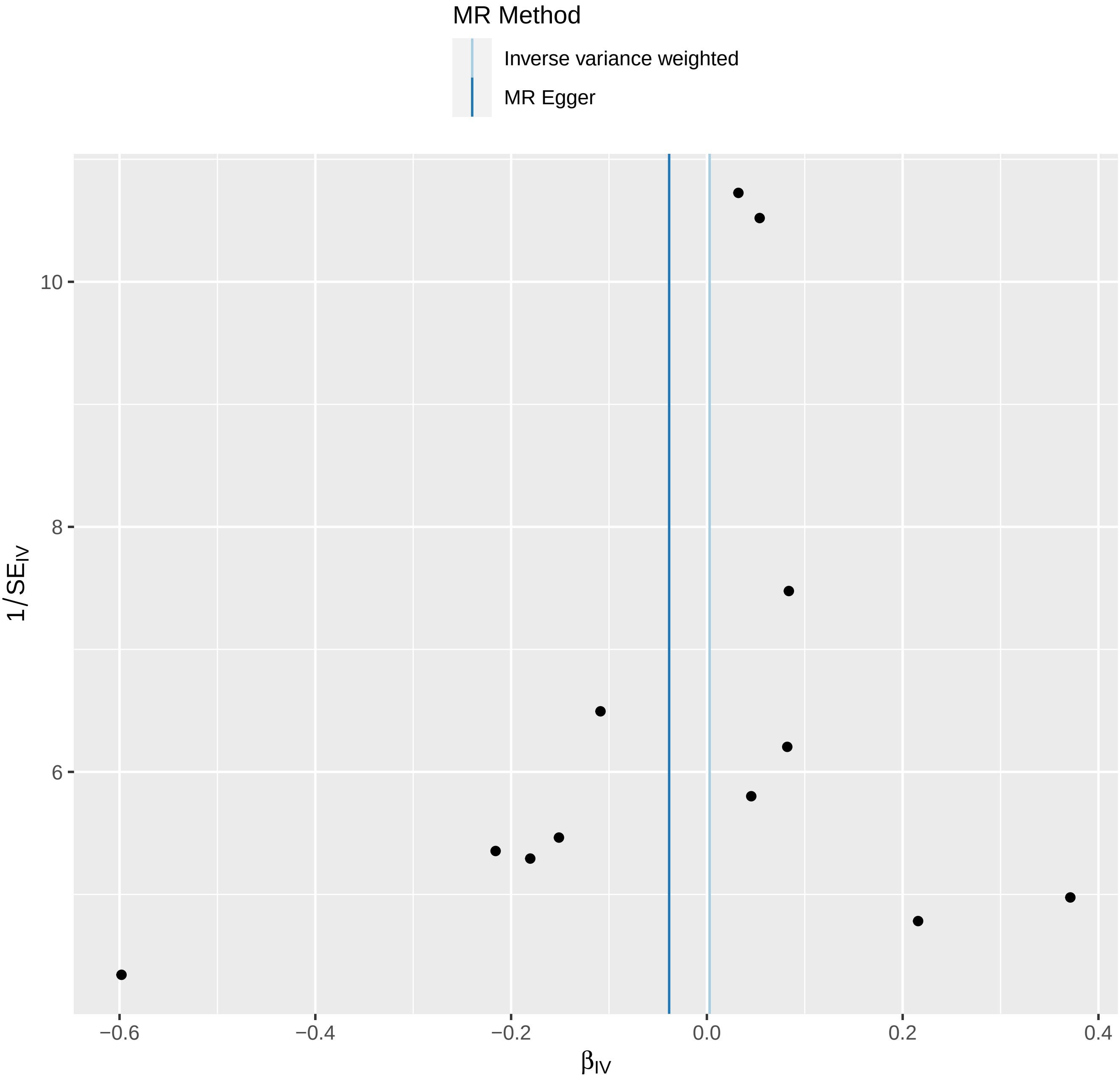

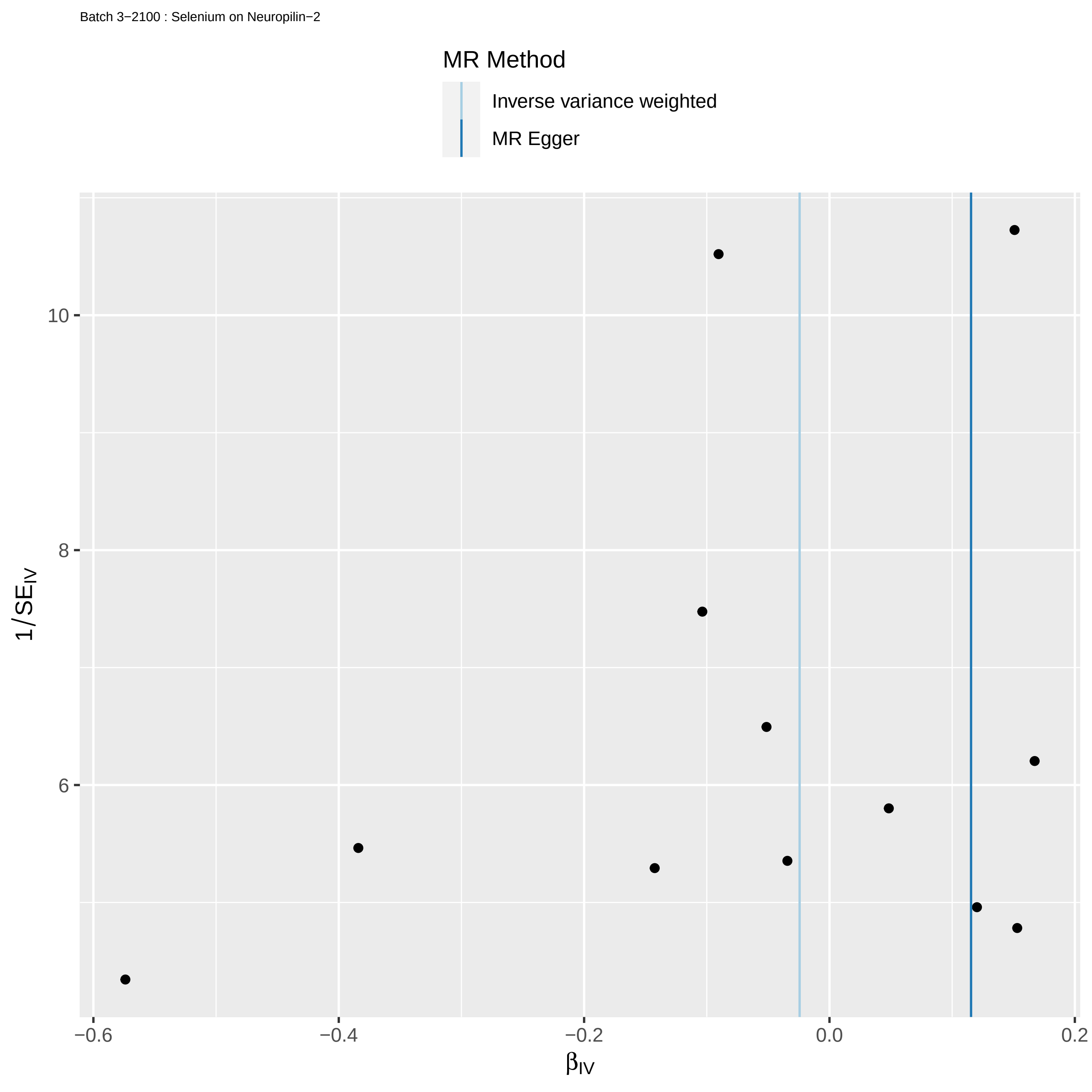
Batch 3−2100 : Selenium on Neuropilin−2
MR Method
Inverse variance weighted
MR Egger
10
8
6
1/SE$_{IV}$
−0.6
−0.4
−0.2
0.0
0.2
β$_{IV}$

Batch 3−2101 : Selenium on Neurensin−1

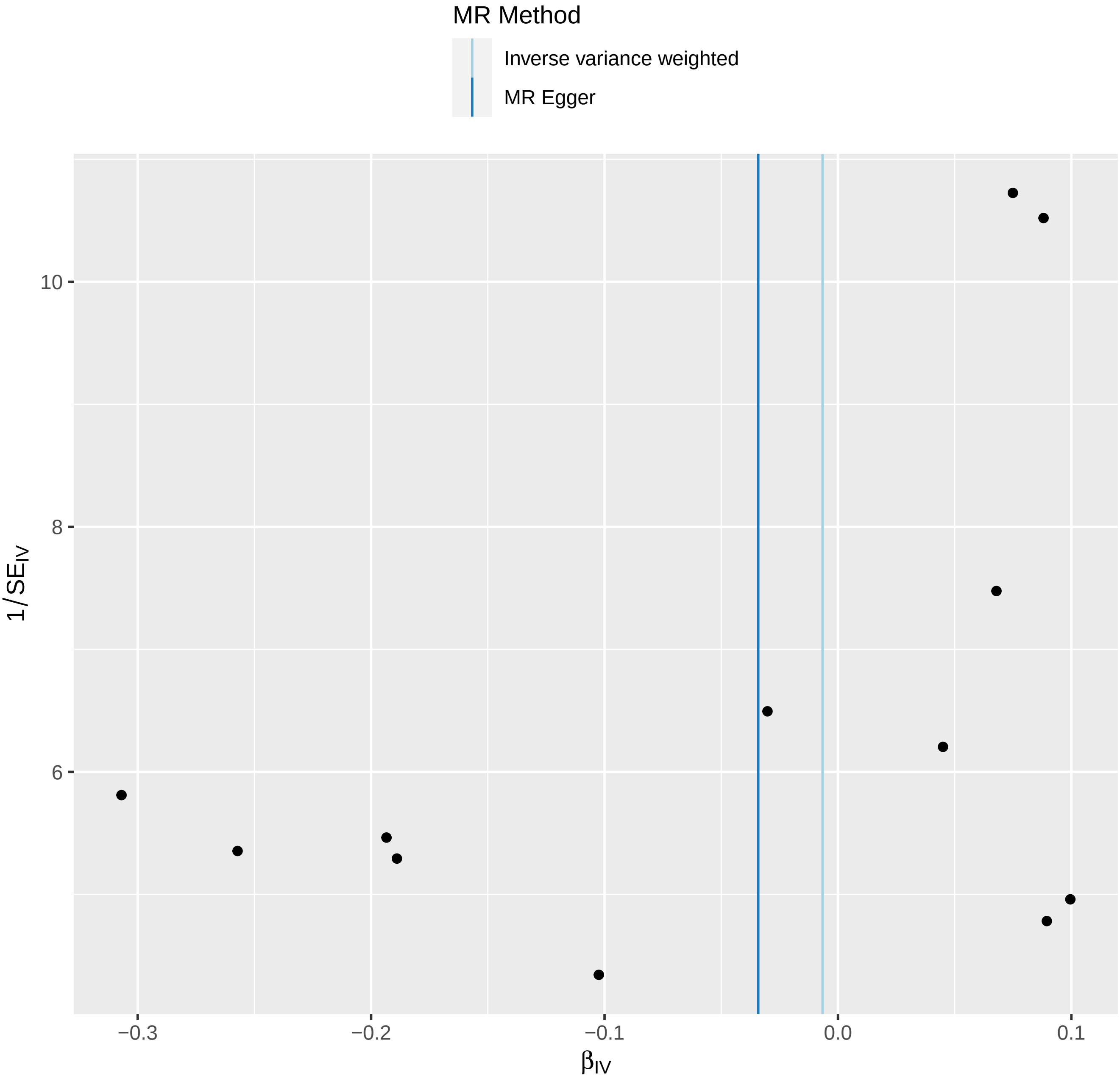

Batch 3−2102 : Selenium on Neurexin−1−beta

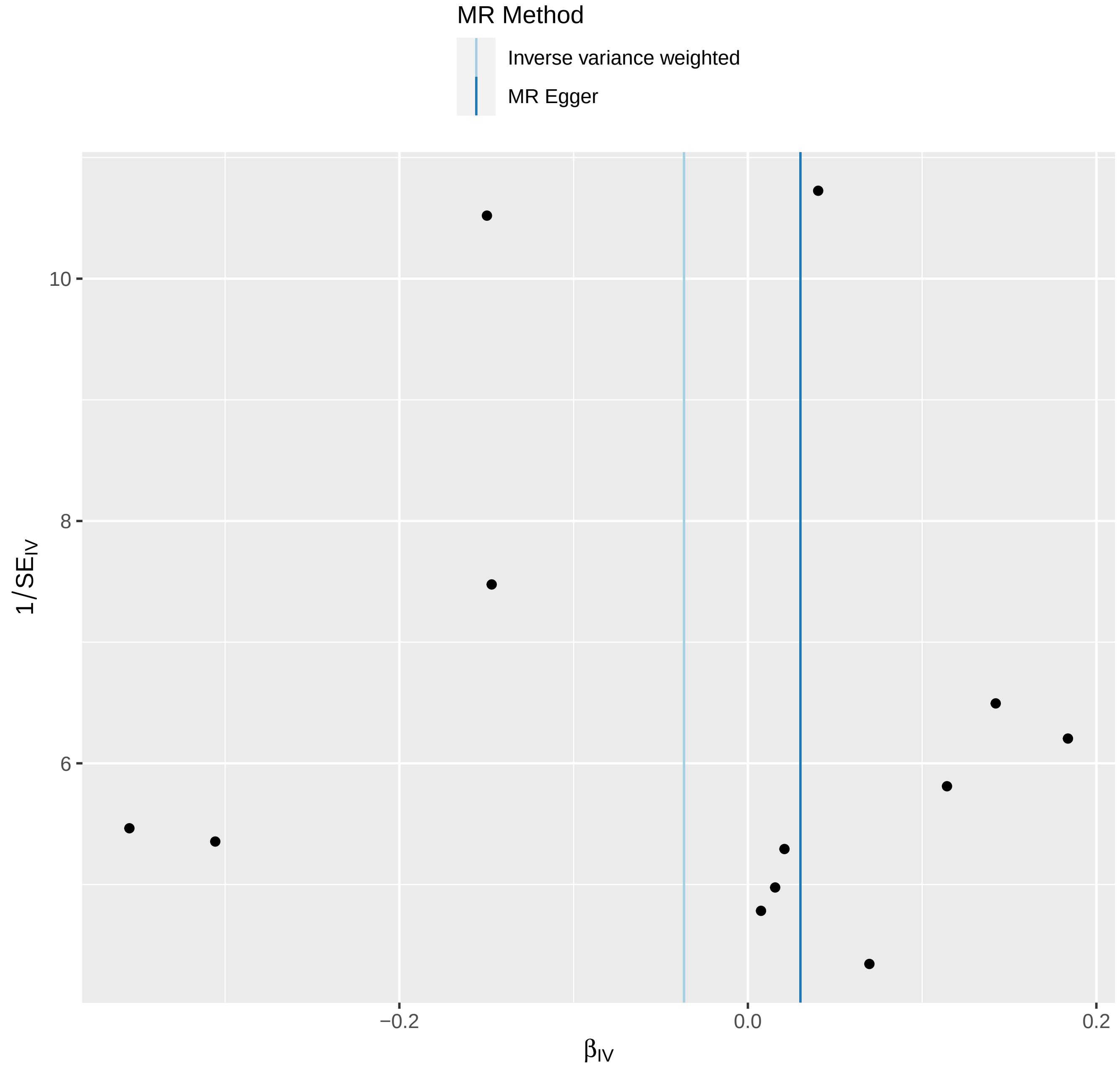

Batch 3−2103 : Selenium on Neurexin−1

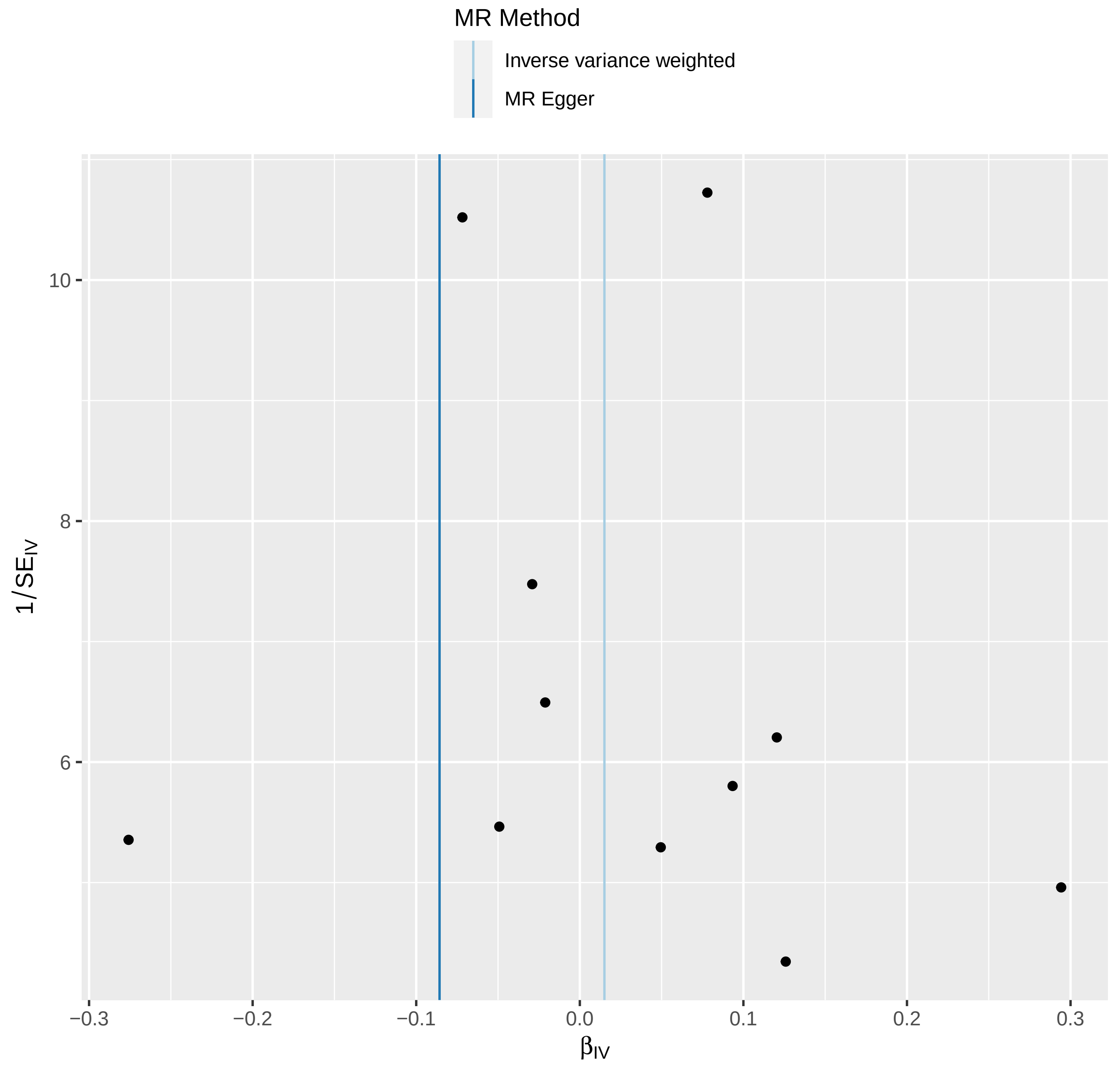

Batch 3−2104 : Selenium on Neurexin−2−beta

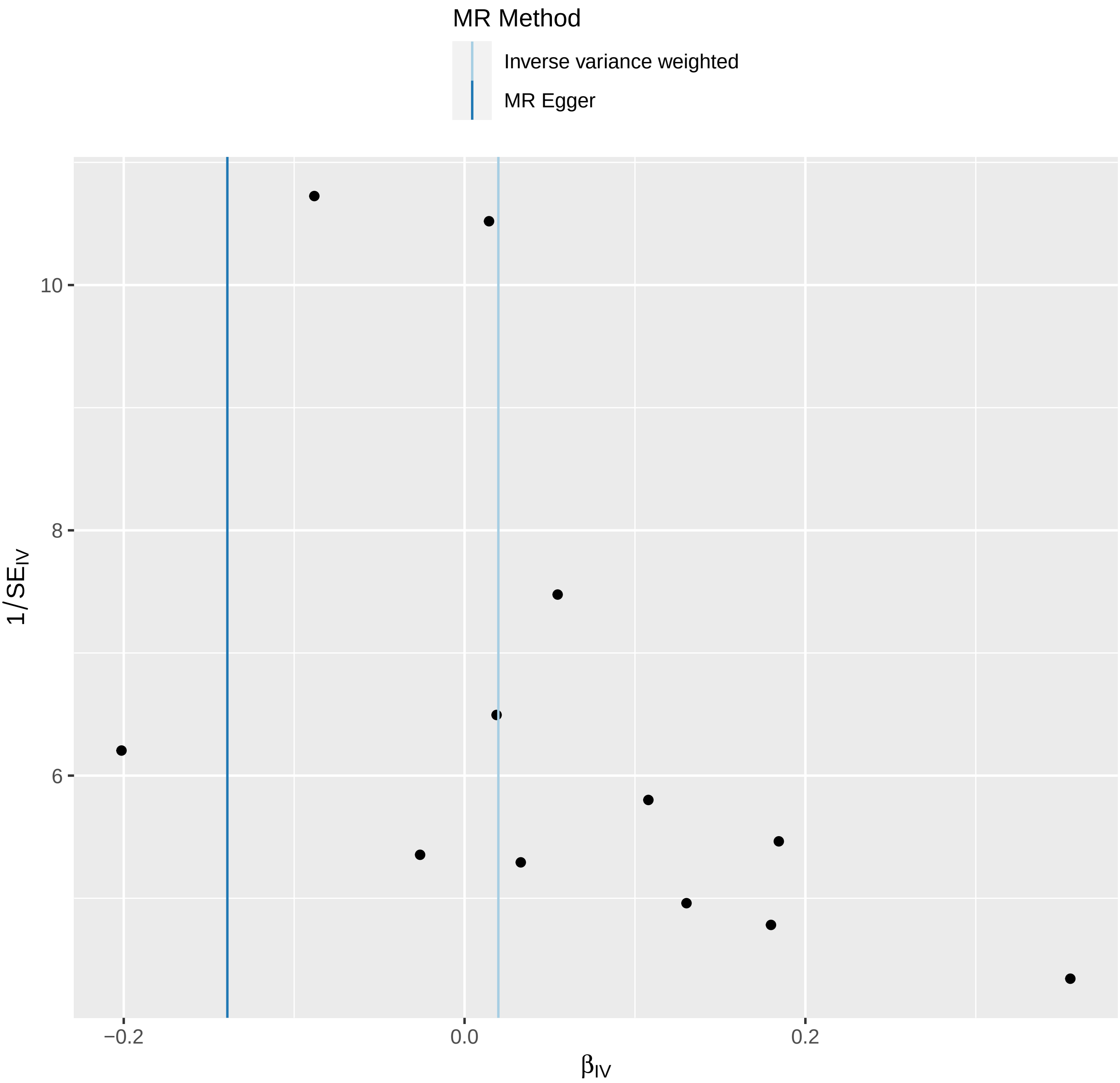

Batch 3−2105 : Selenium on Neurexin−3−beta

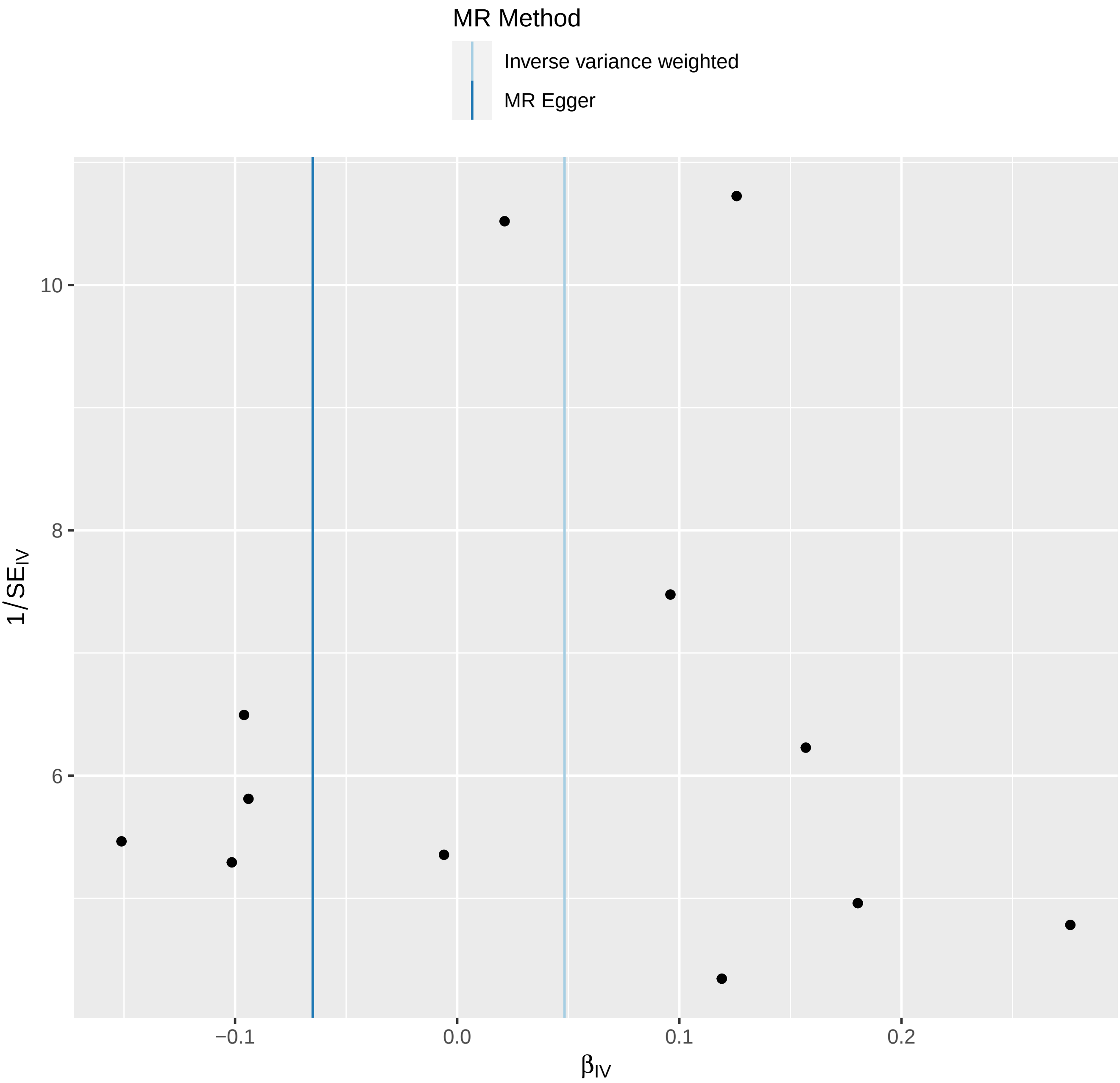

Batch 3−2106 : Selenium on Neurexin−3−beta

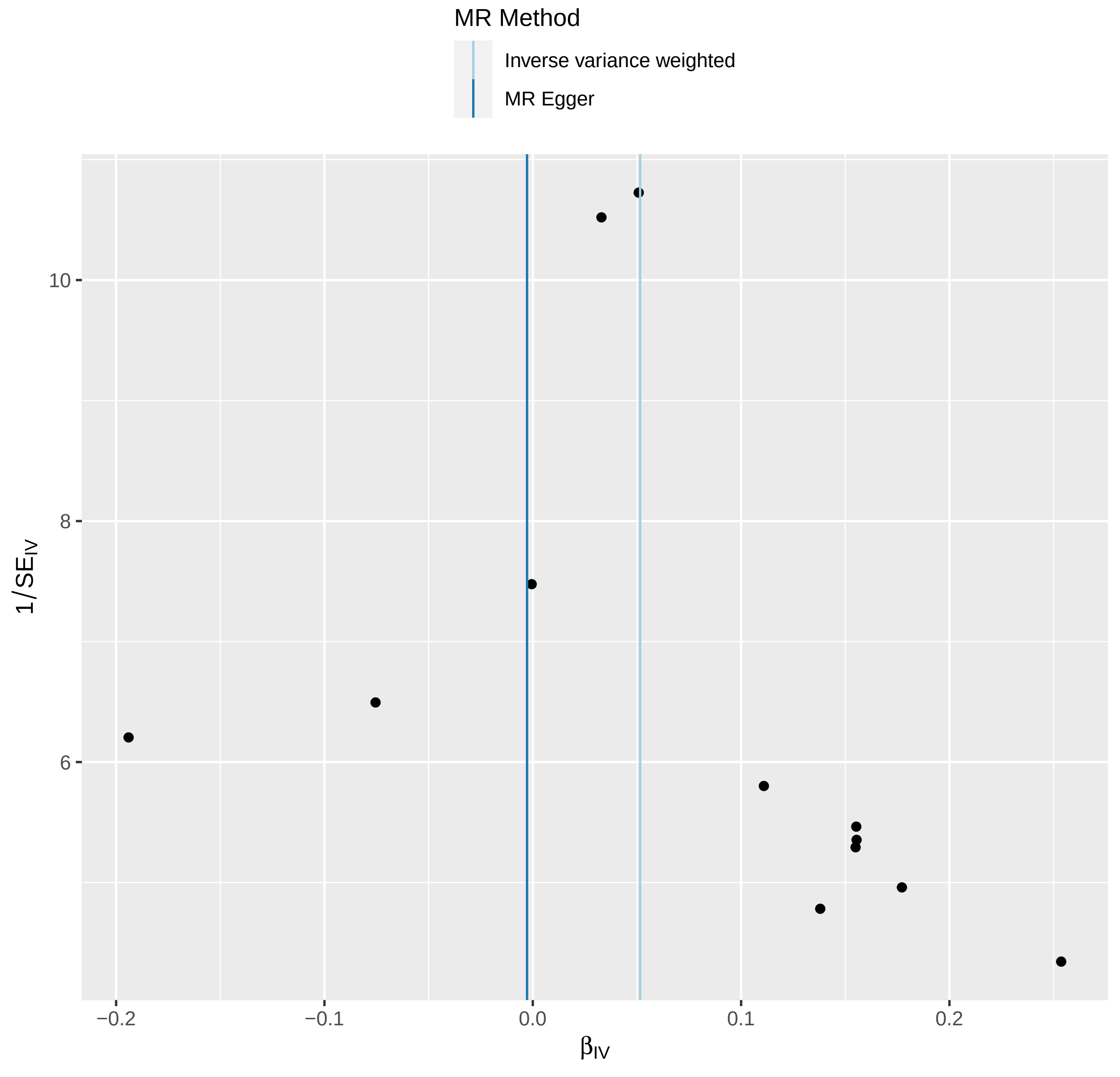

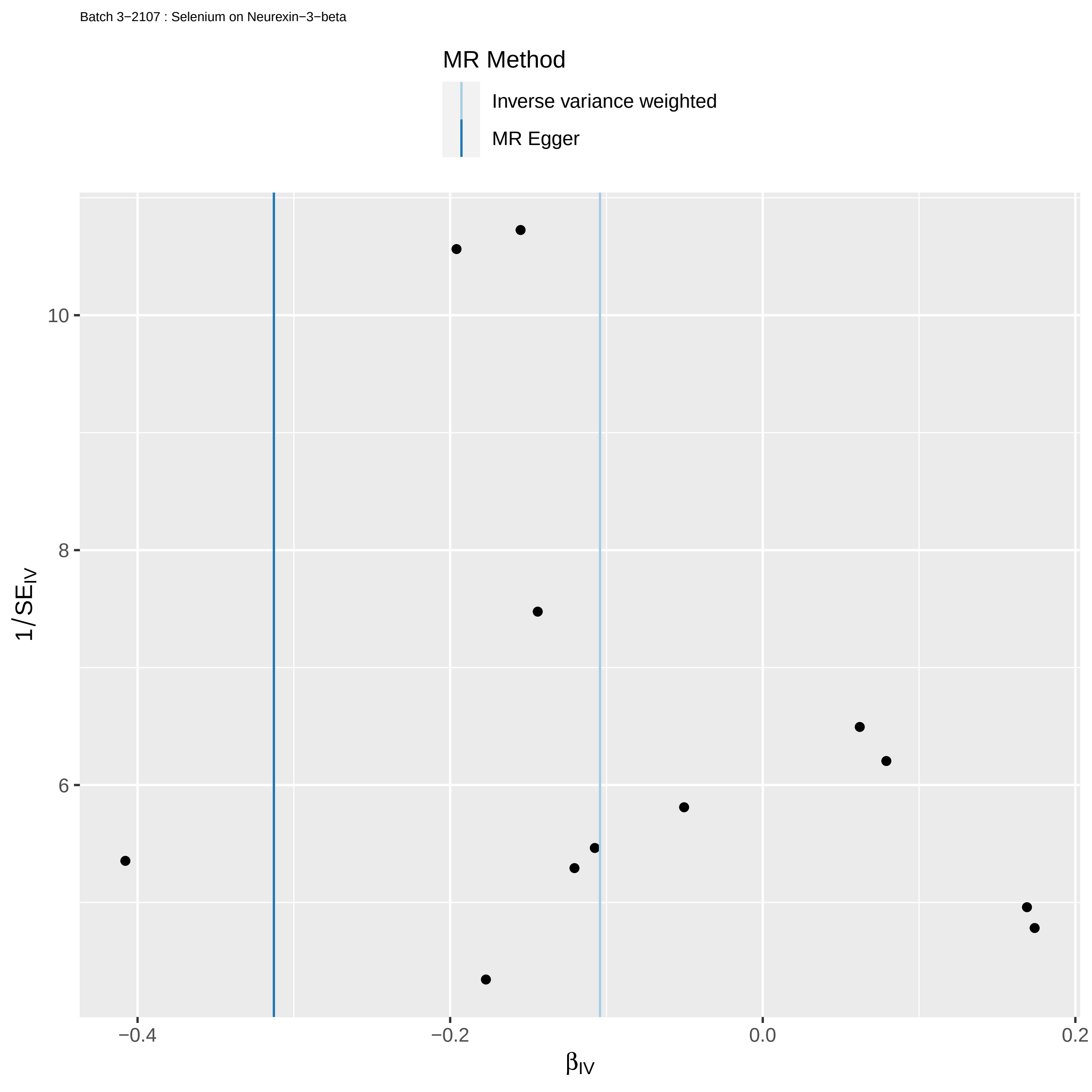
Batch 3−2107 : Selenium on Neurexin−3−beta
MR Method
Inverse variance weighted
MR Egger
10
8
6
1/SE_IV
−0.4
−0.2
0.0
0.2
β_IV

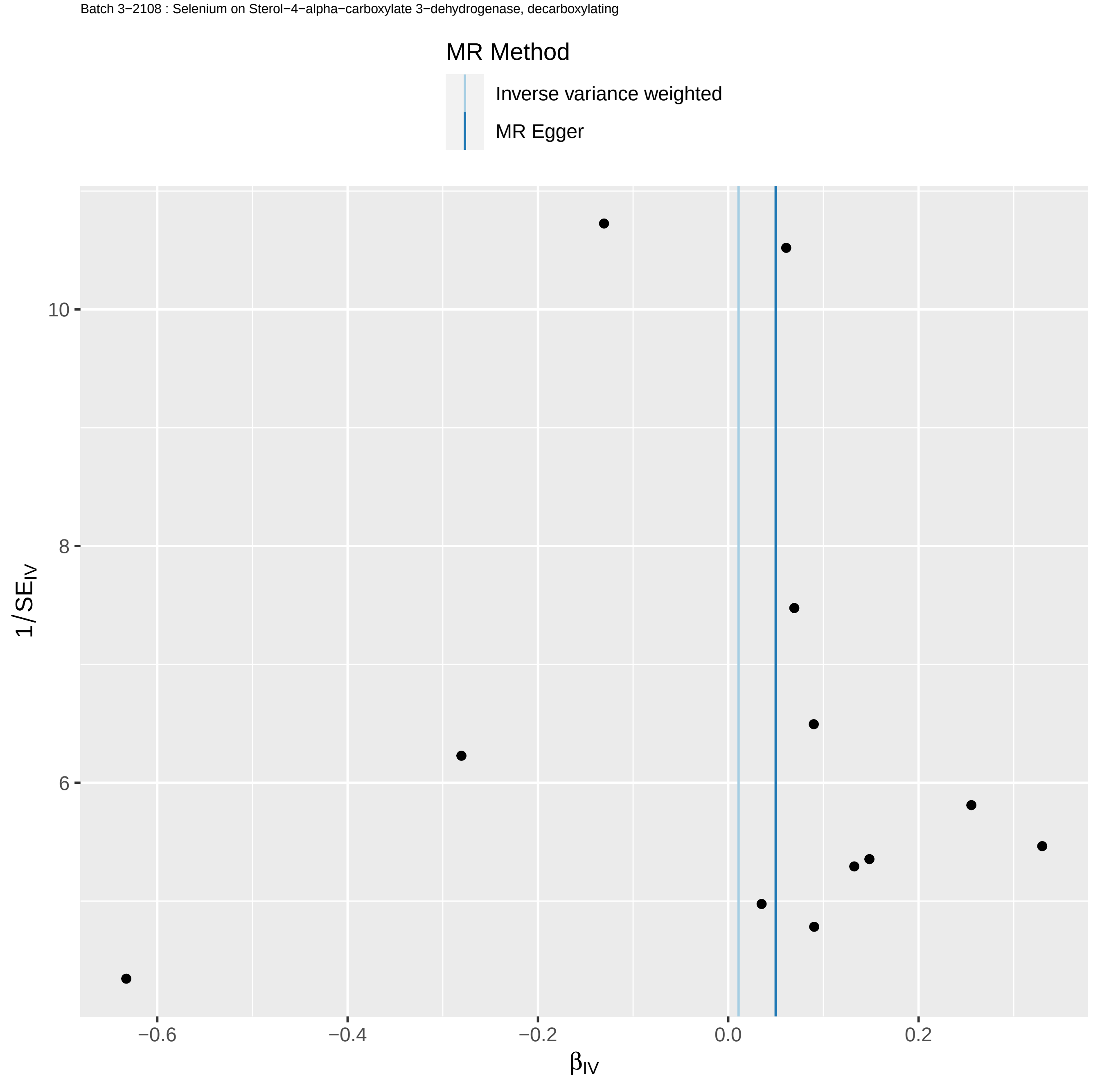

Batch 3−2108 : Selenium on Sterol−4−alpha−carboxylate 3−dehydrogenase, decarboxylating
MR Method
Inverse variance weighted
MR Egger
10
8
6
1/SE_IV
−0.6
−0.4
−0.2
0.0
0.2
β_IV

Batch 3−2109 : Selenium on Vesicle−fusing ATPase

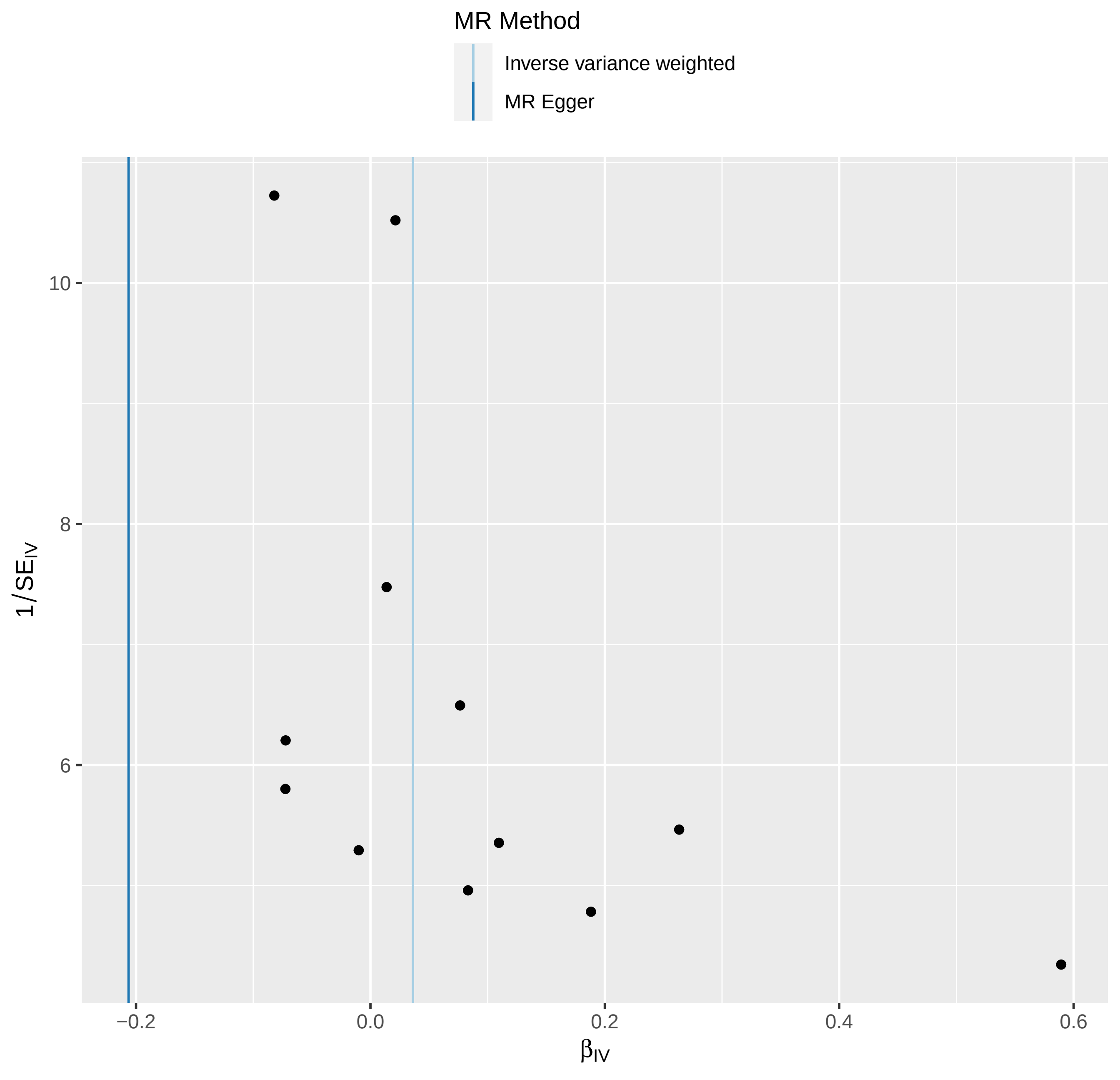

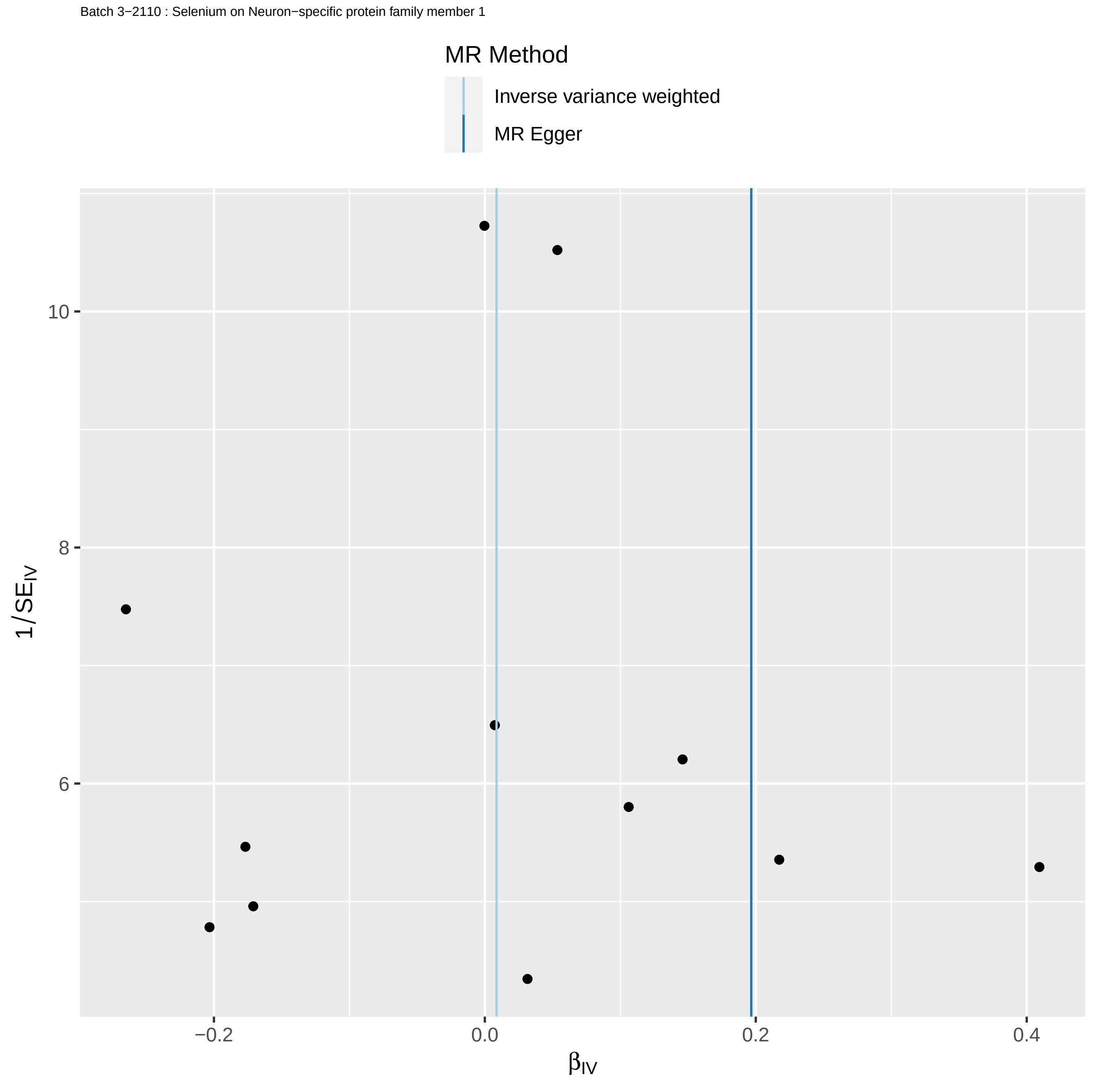
Batch 3−2110 : Selenium on Neuron−specific protein family member 1
MR Method
Inverse variance weighted
MR Egger
10
8
6
1/SE_IV
−0.2
0.0
0.2
0.4
β_IV

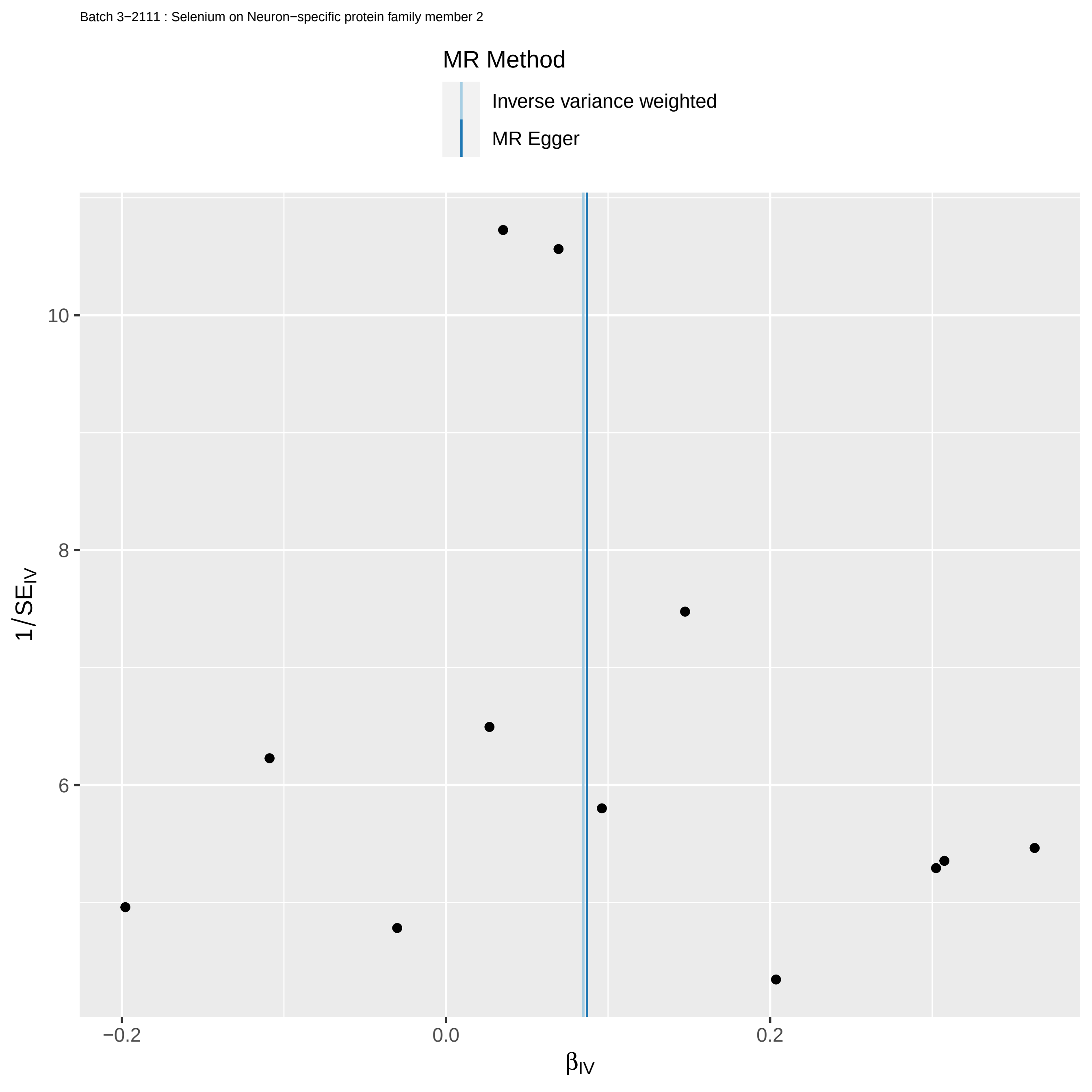

Batch 3−2111 : Selenium on Neuron−specific protein family member 2
MR Method
Inverse variance weighted
MR Egger
10
8
6
1/SE_IV
−0.2
0.0
0.2
β_IV

Batch 3−2112 : Selenium on 5'(3')−deoxyribonucleotidase, cytosolic type

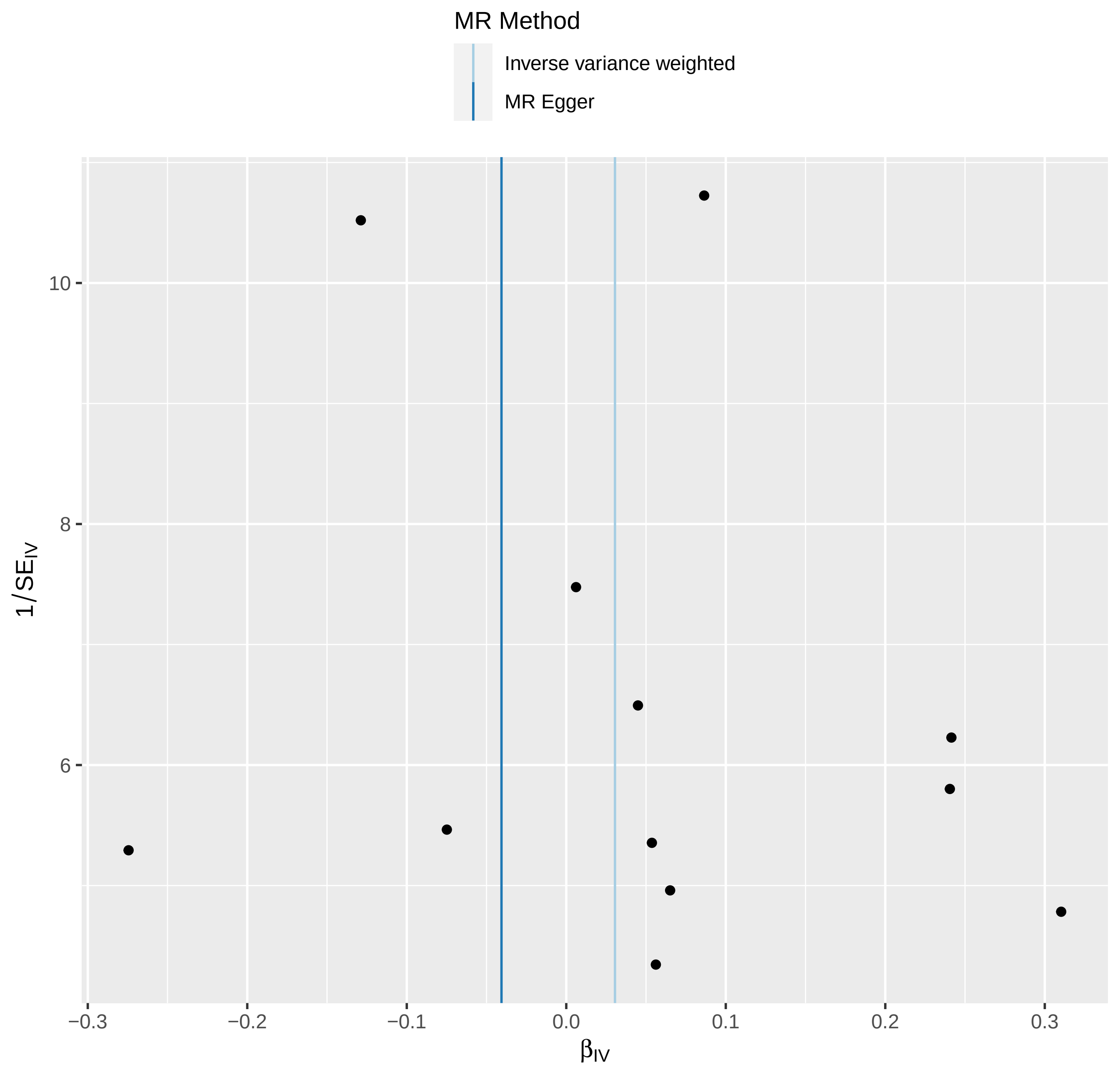

Batch 3−2113 : Selenium on 5'−Nucleotidase

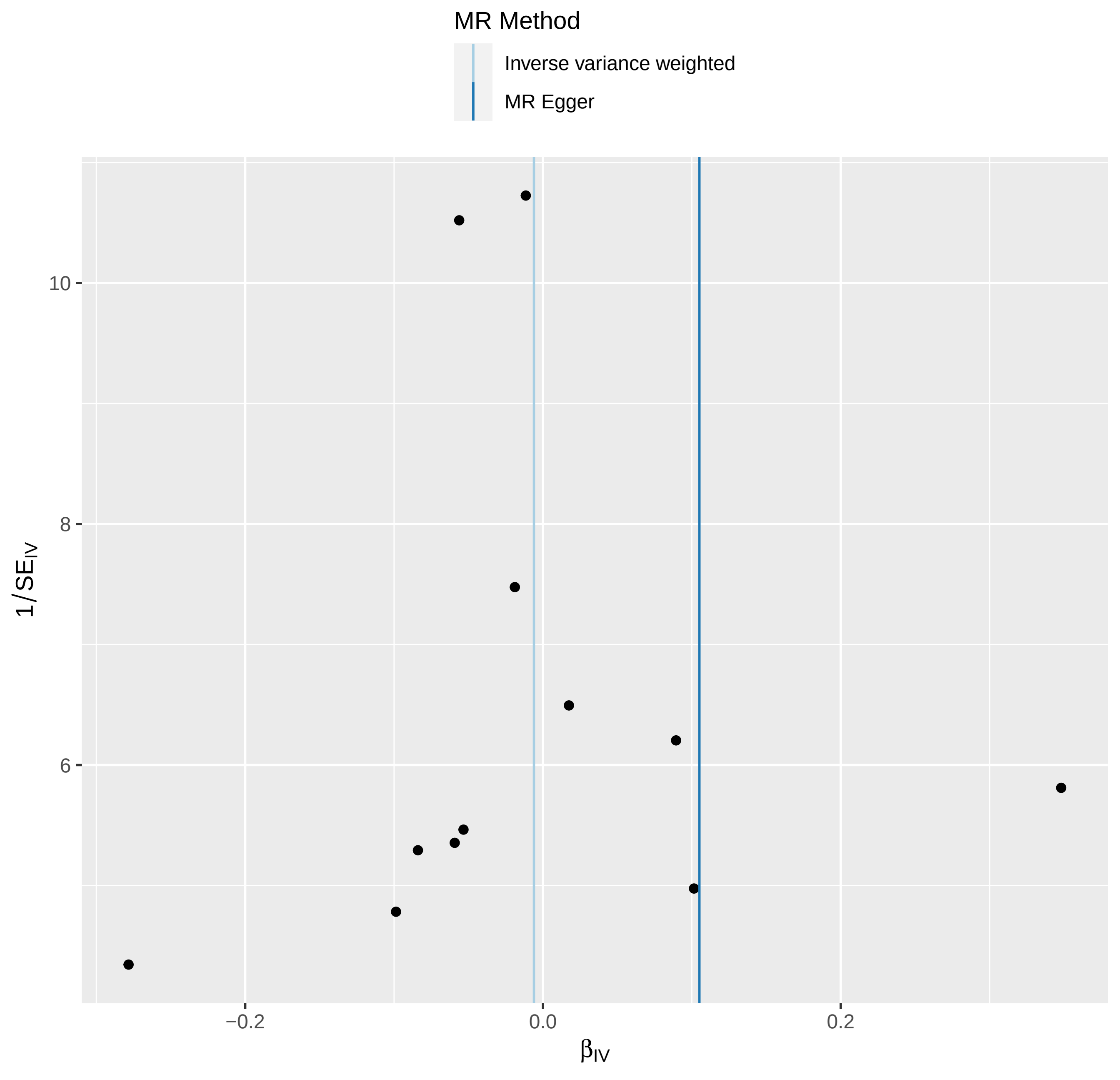

Batch 3−2114 : Selenium on Neurotrophin−3

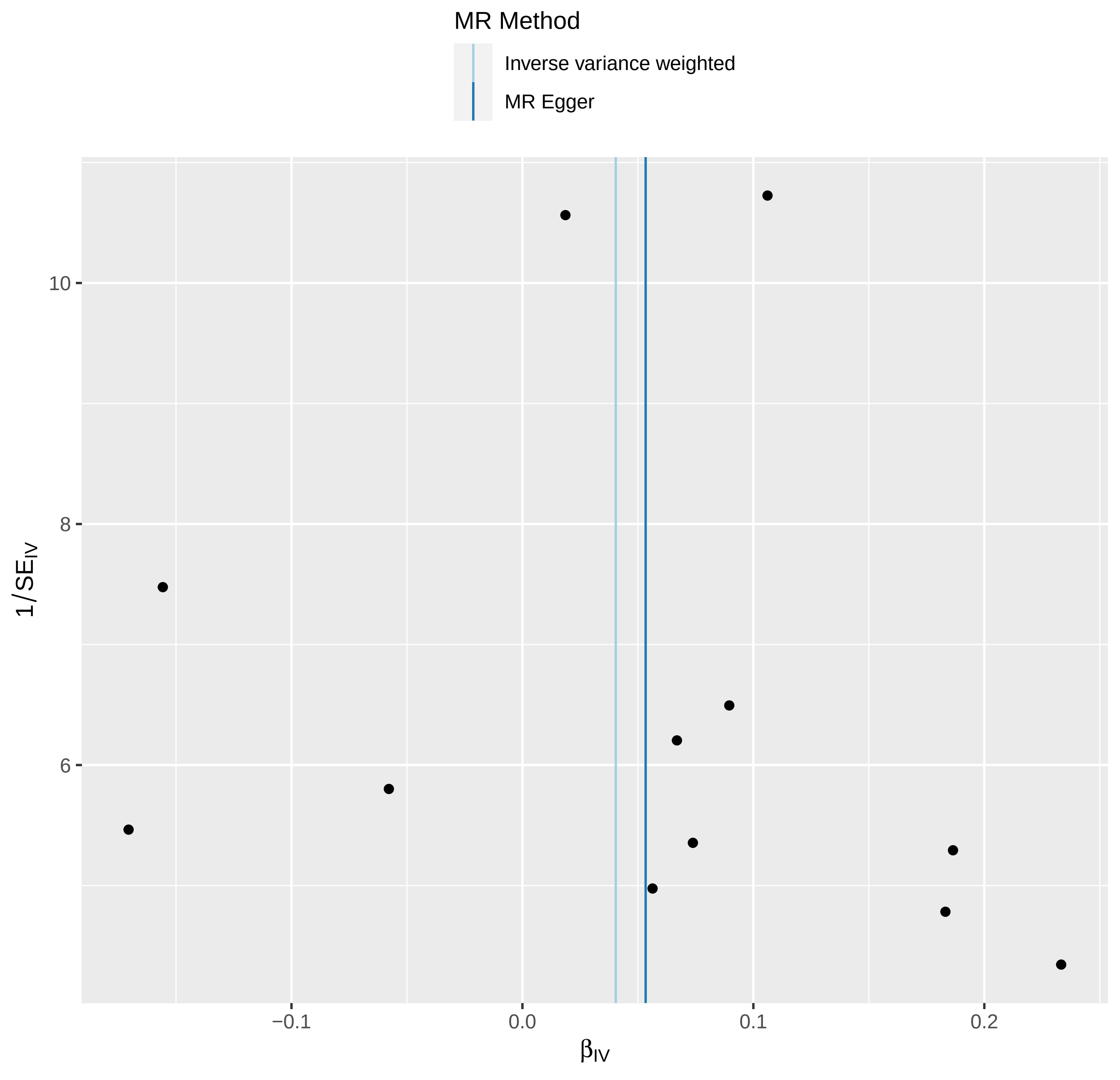

Batch 3−2115 : Selenium on Neurotrophin−4

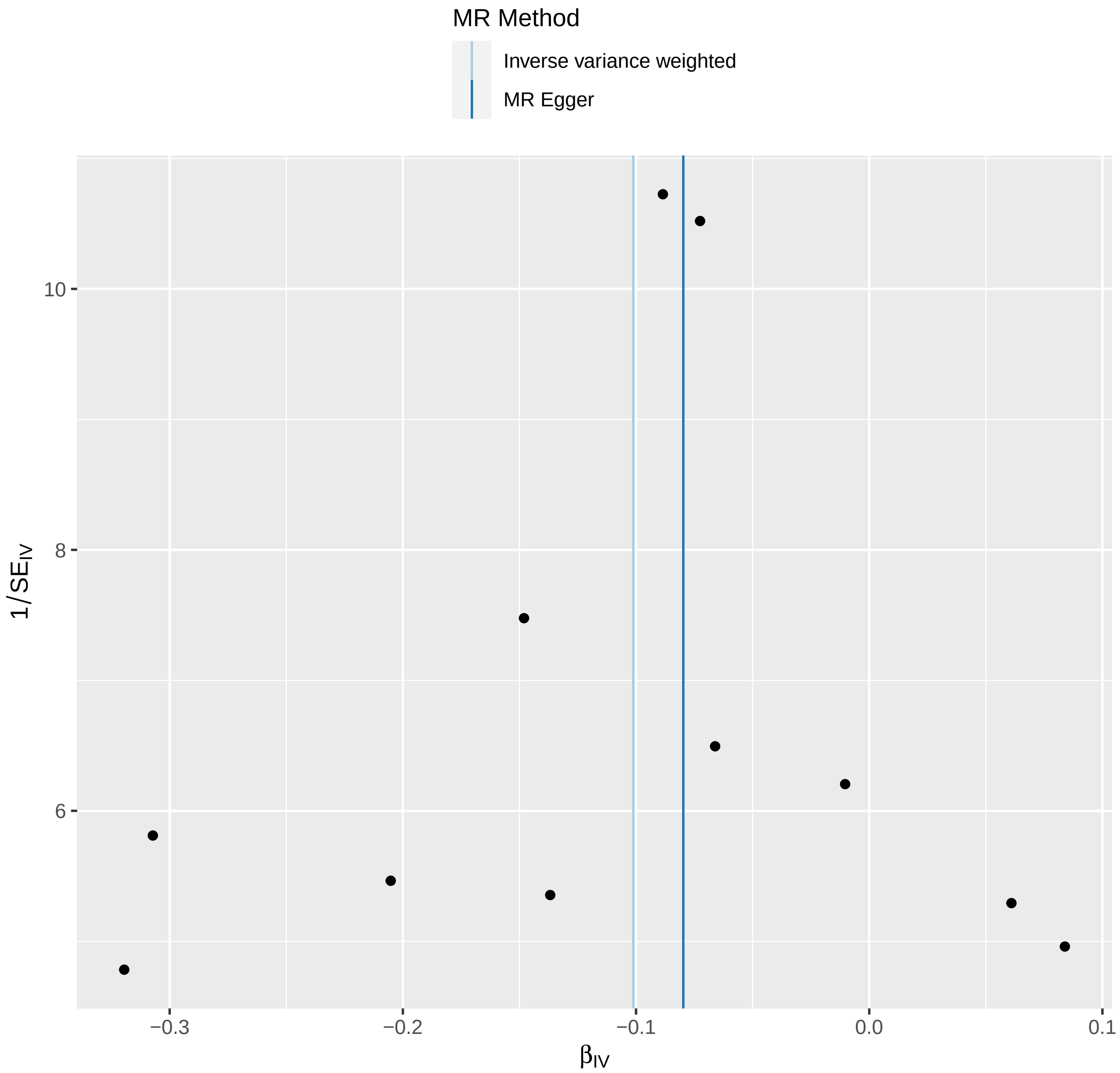

Batch 3−2116 : Selenium on N−terminal Xaa−Pro−Lys N−methyltransferase 1

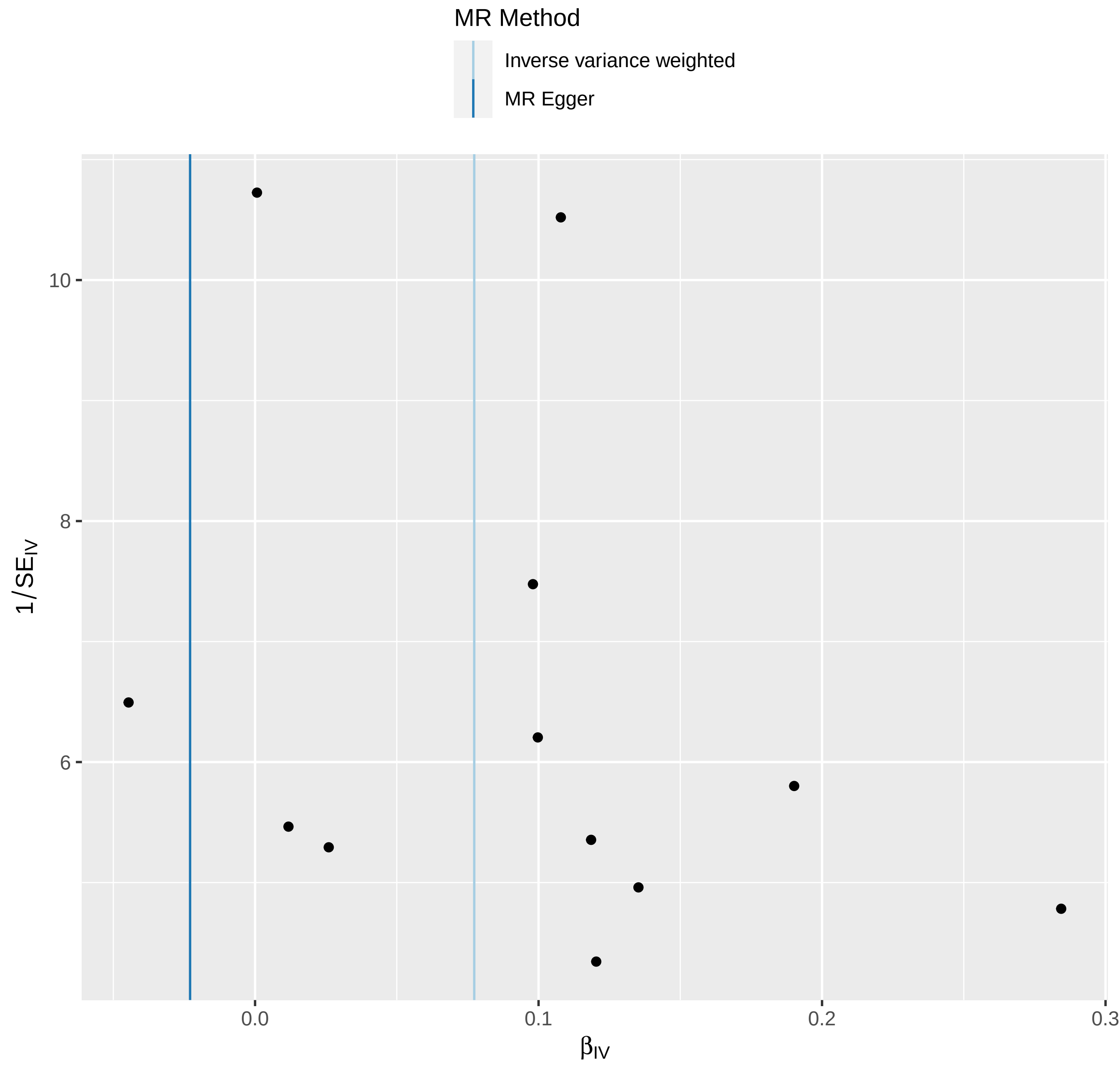

Batch 3−2117 : Selenium on Netrin−1

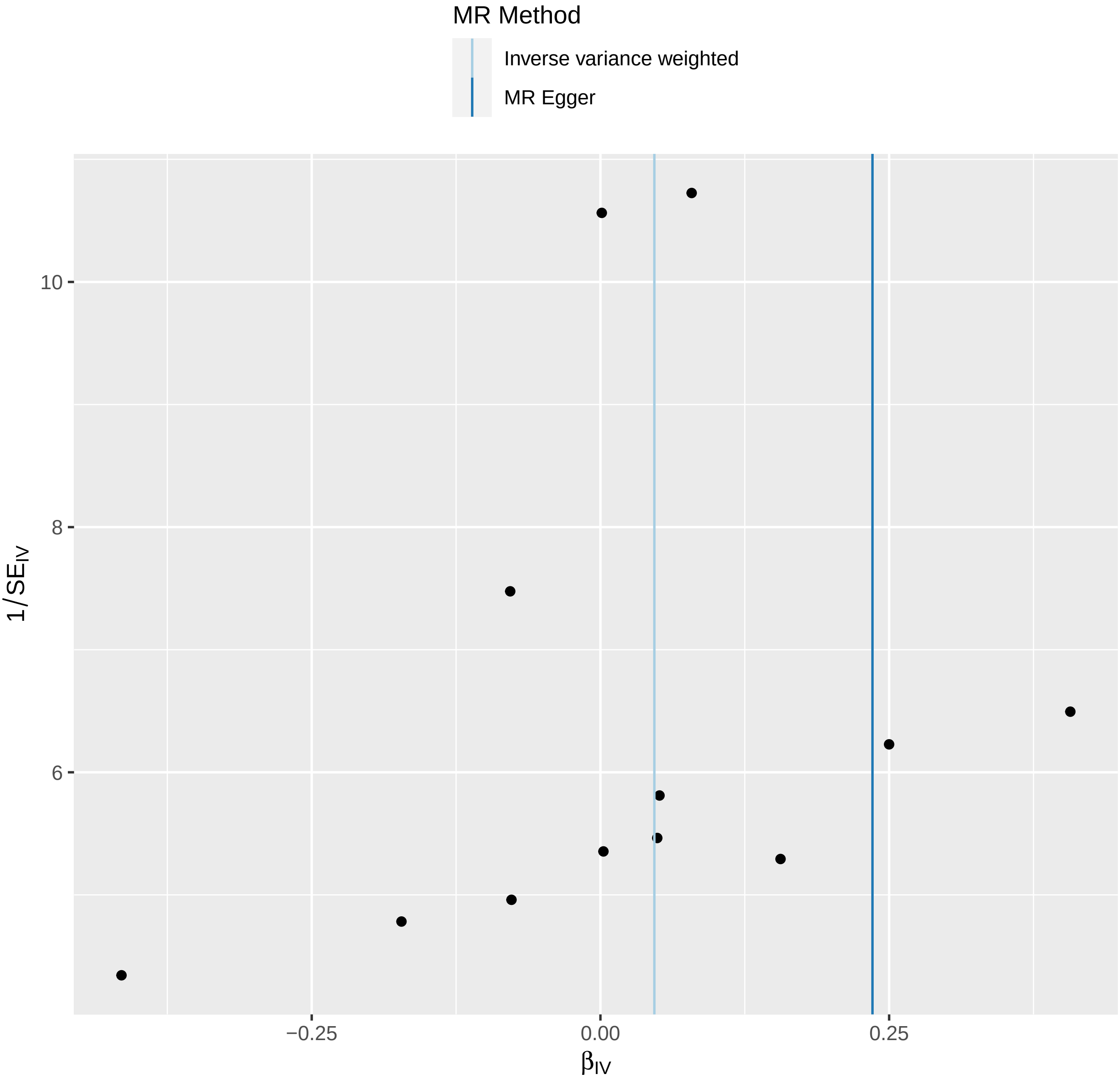

Batch 3−2118 : Selenium on Netrin−1

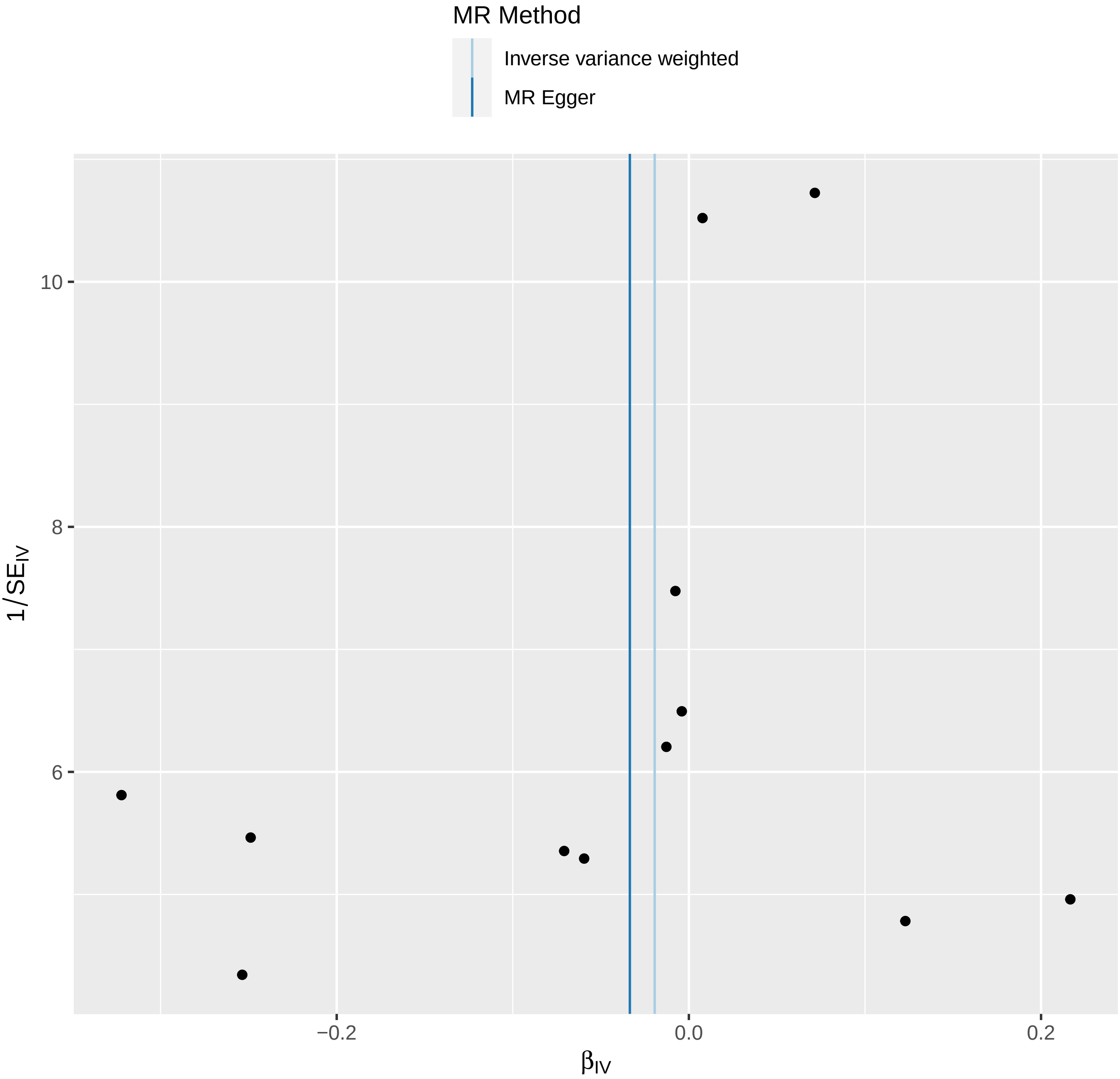

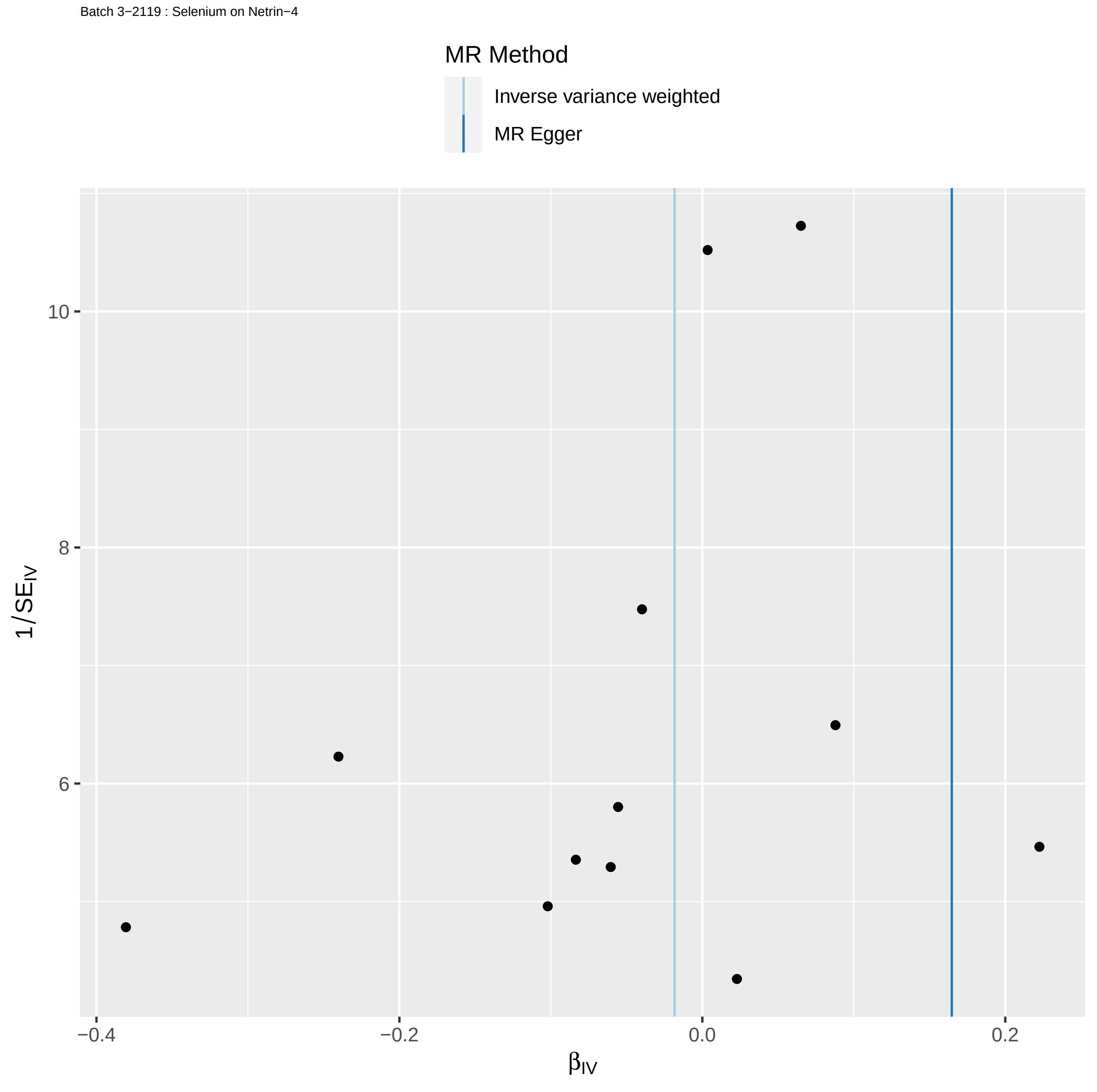
Batch 3−2119 : Selenium on Netrin−4
MR Method
Inverse variance weighted
MR Egger
10
8
6
1/SE_IV
−0.4
−0.2
0.0
0.2
β_IV

Batch 3−2120 : Selenium on Netrin−G1

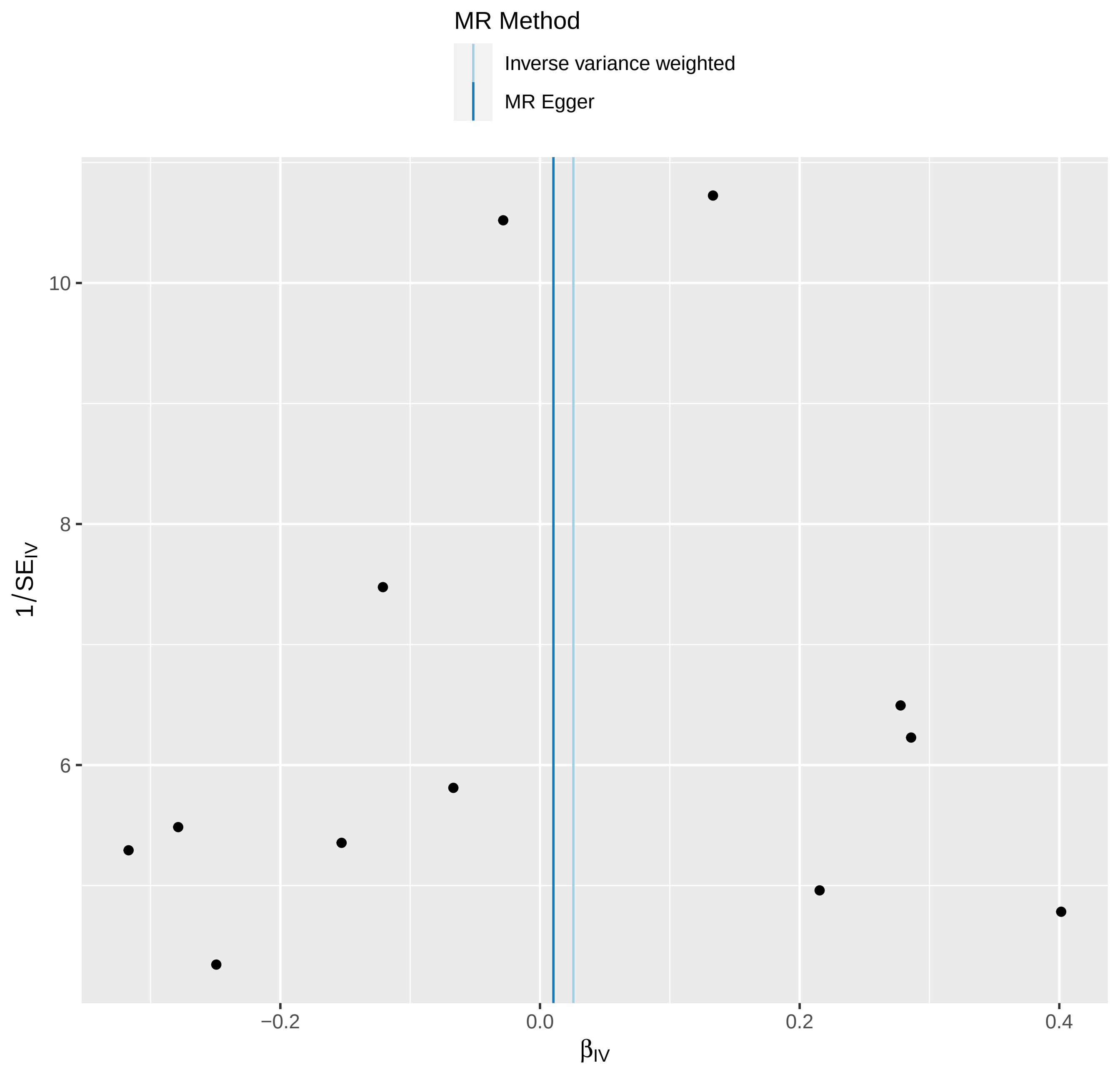

Batch 3−2121 : Selenium on High affinity nerve growth factor receptor

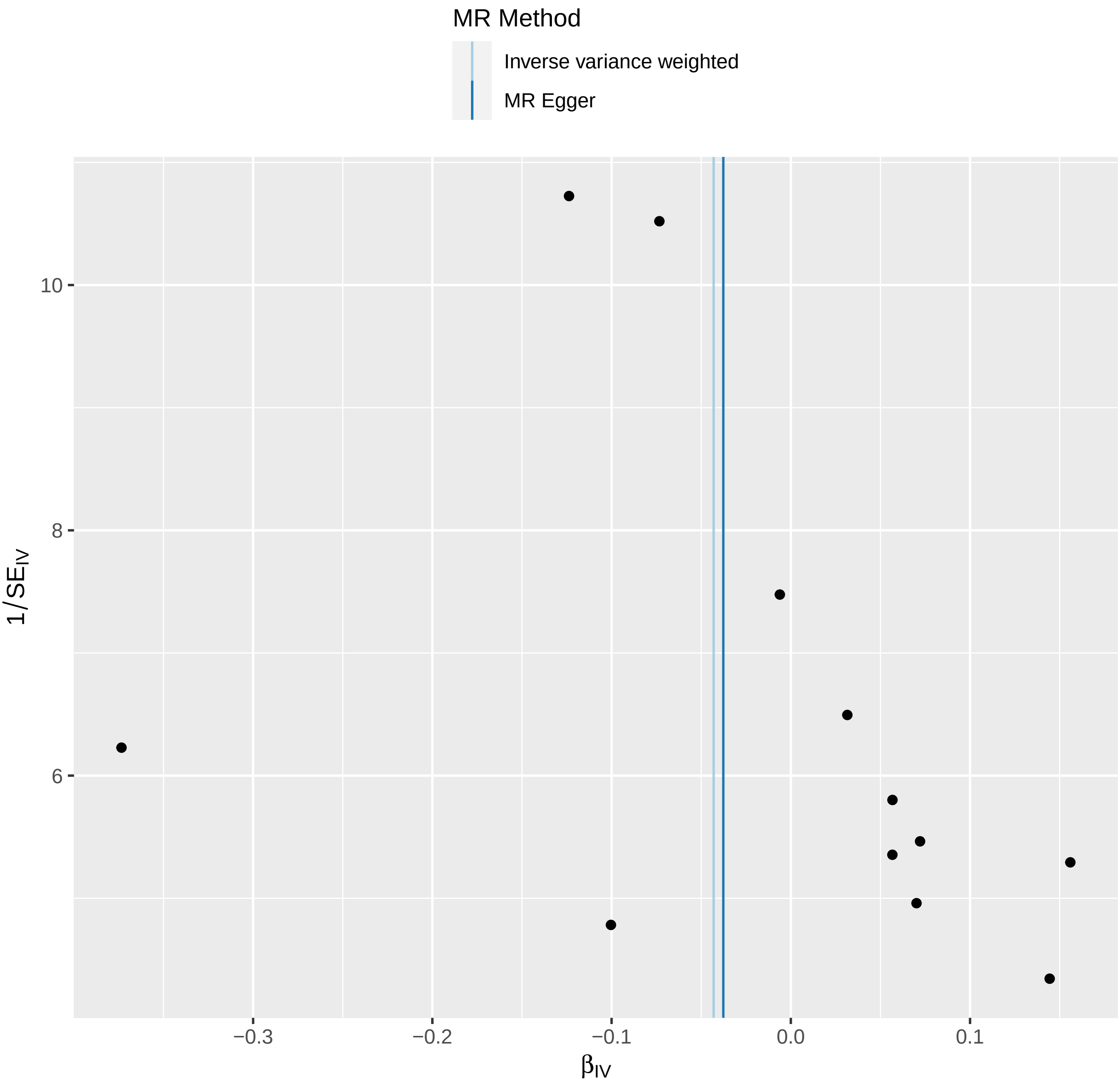

Batch 3−2122 : Selenium on BDNF/NT−3 growth factors receptor

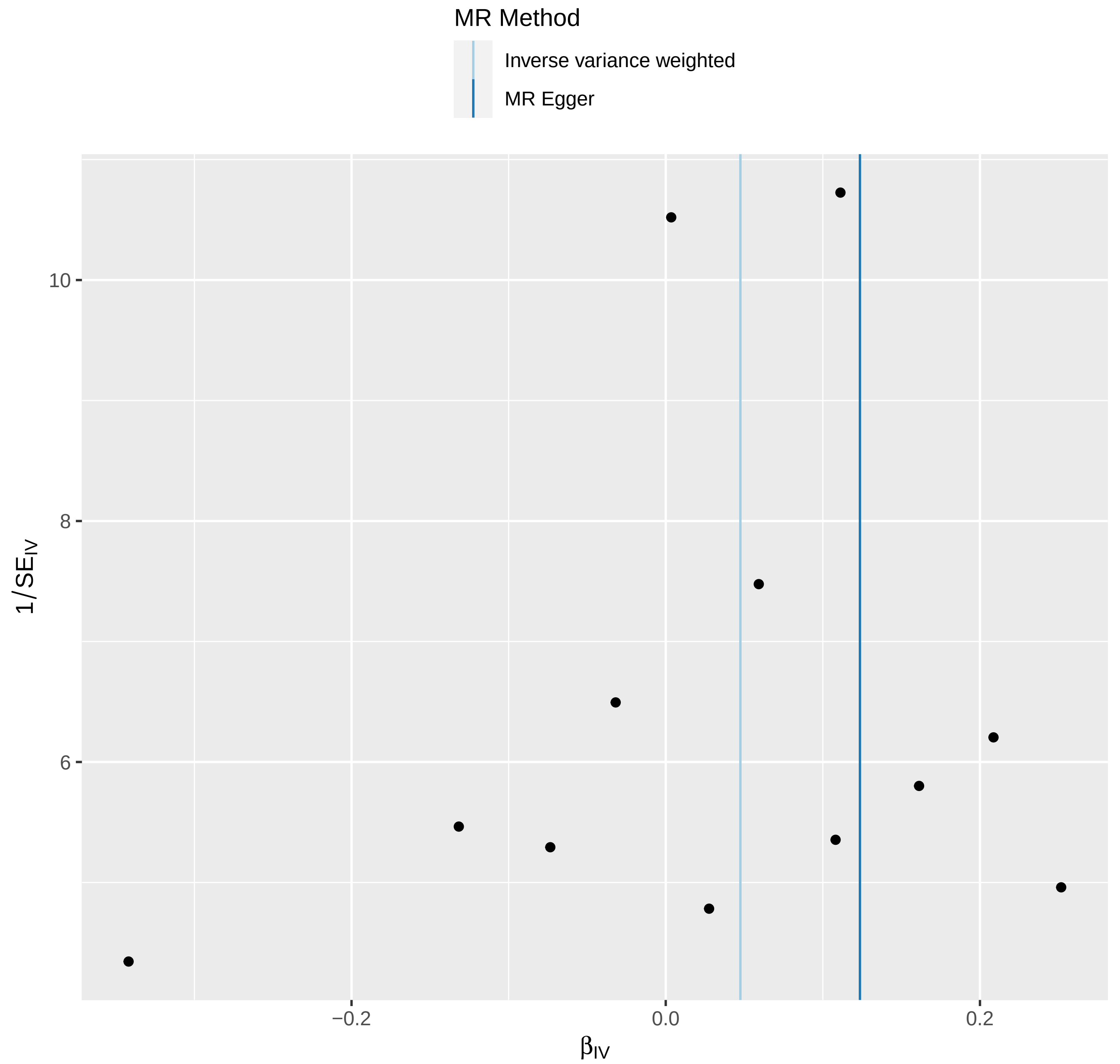

Batch 3−2123 : Selenium on NT−3 growth factor receptor

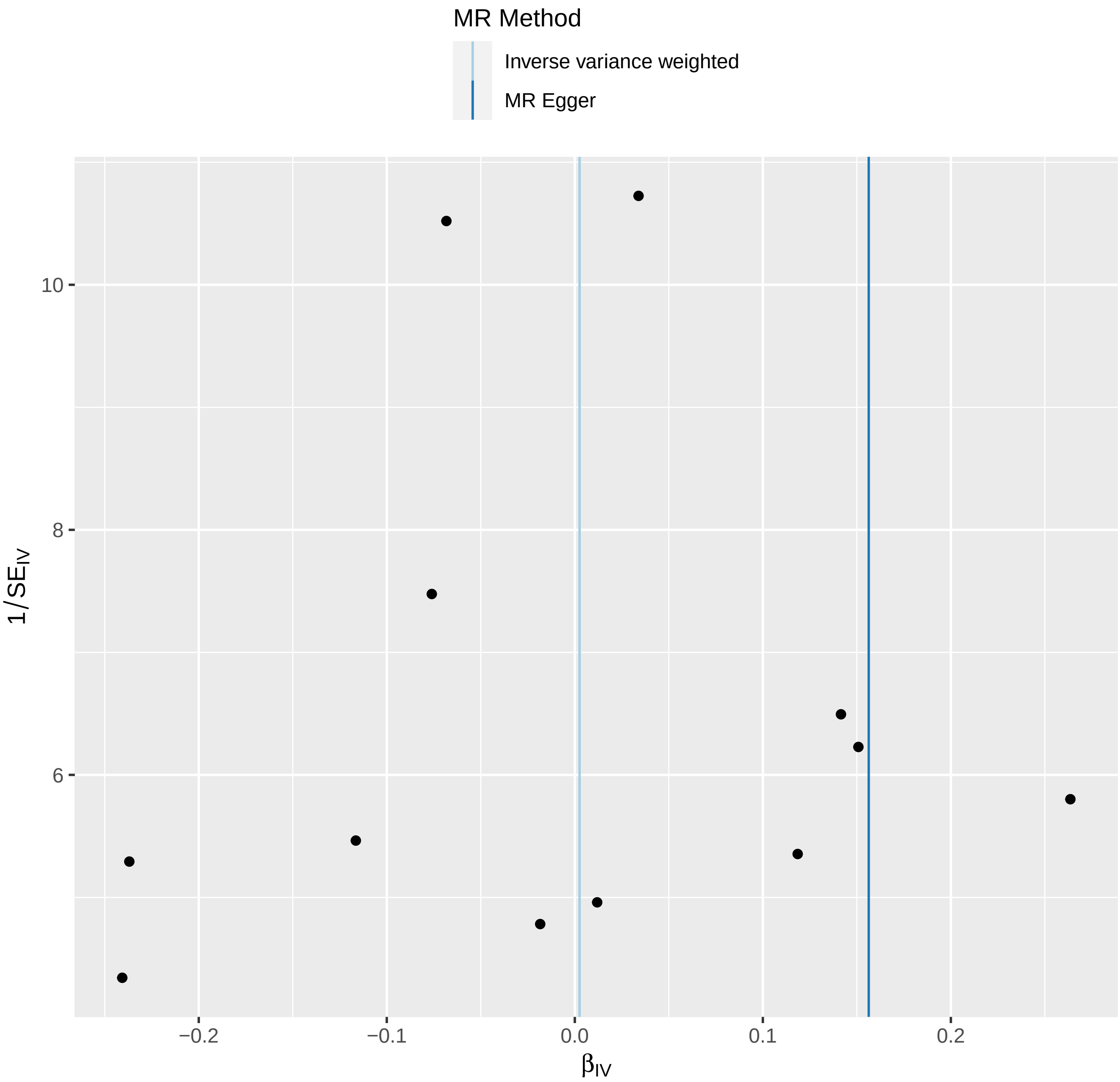

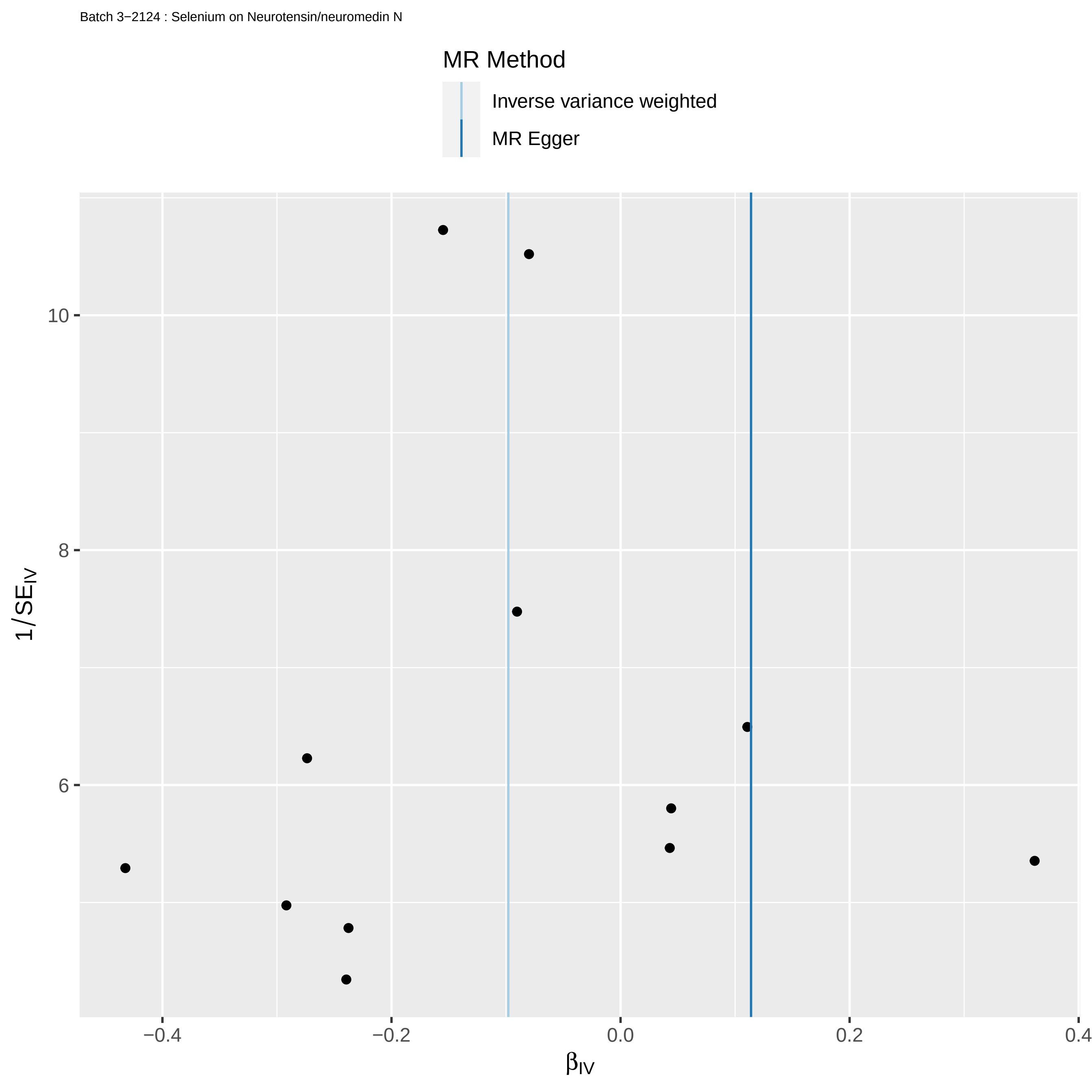
Batch 3−2124 : Selenium on Neurotensin/neuromedin N
MR Method
Inverse variance weighted
MR Egger
10
8
6
$1/SE_{IV}$
−0.4
−0.2
0.0
0.2
0.4
β_{IV}

Batch 3−2125 : Selenium on Nuclear migration protein nudC

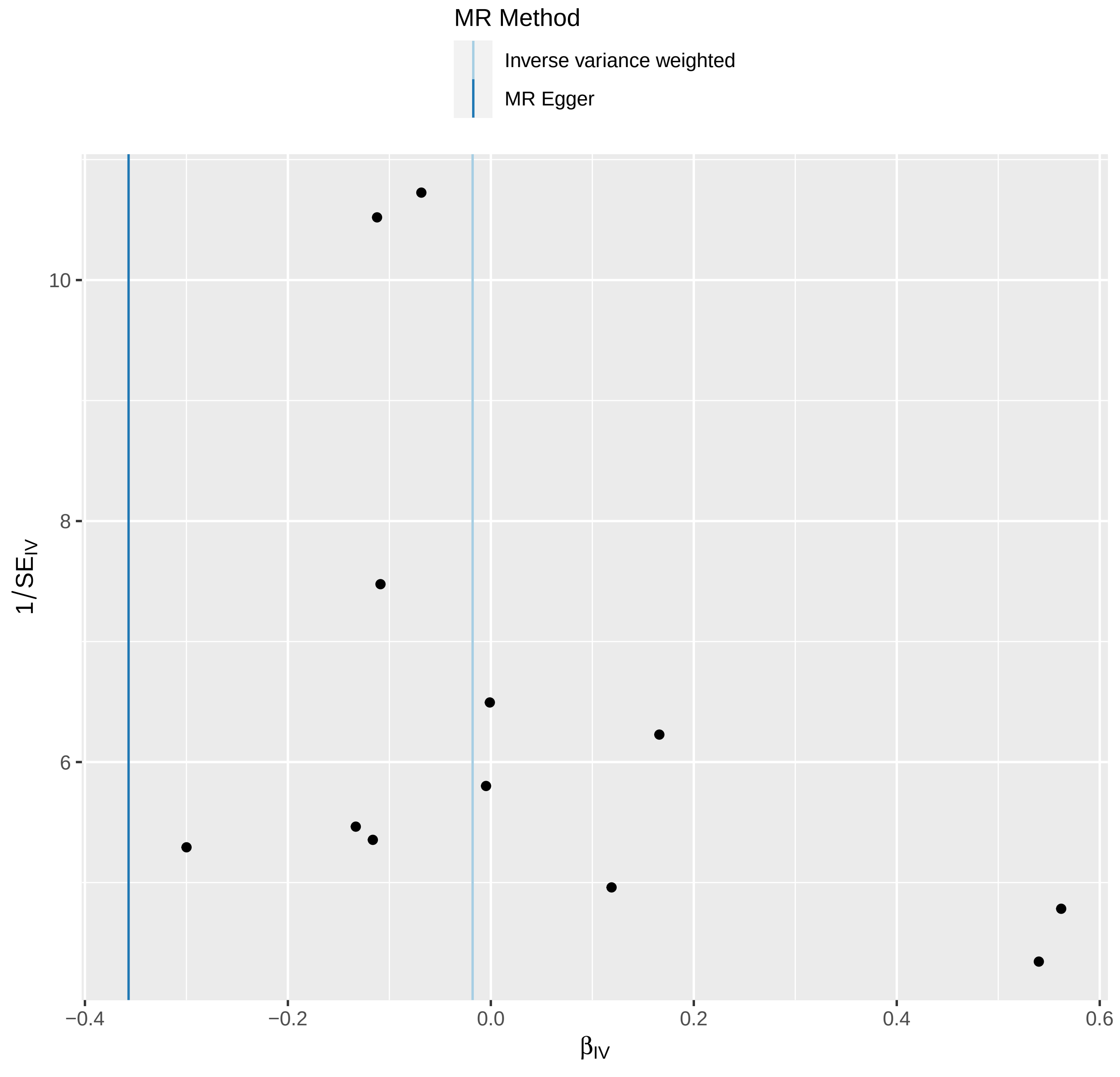

Batch 3−2126 : Selenium on Peroxisomal NADH pyrophosphatase NUDT12

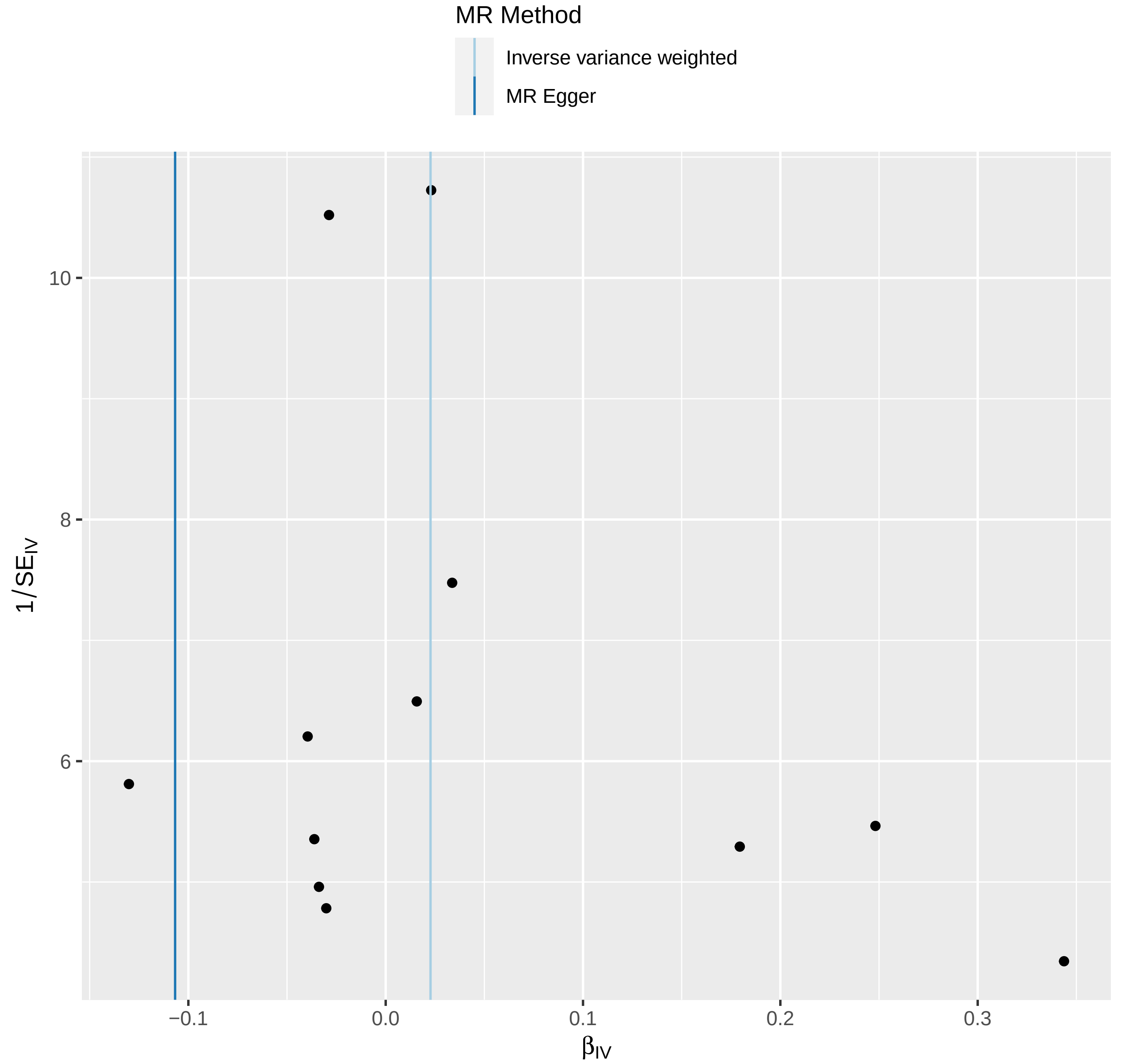

Batch 3−2127 : Selenium on Protein syndesmos

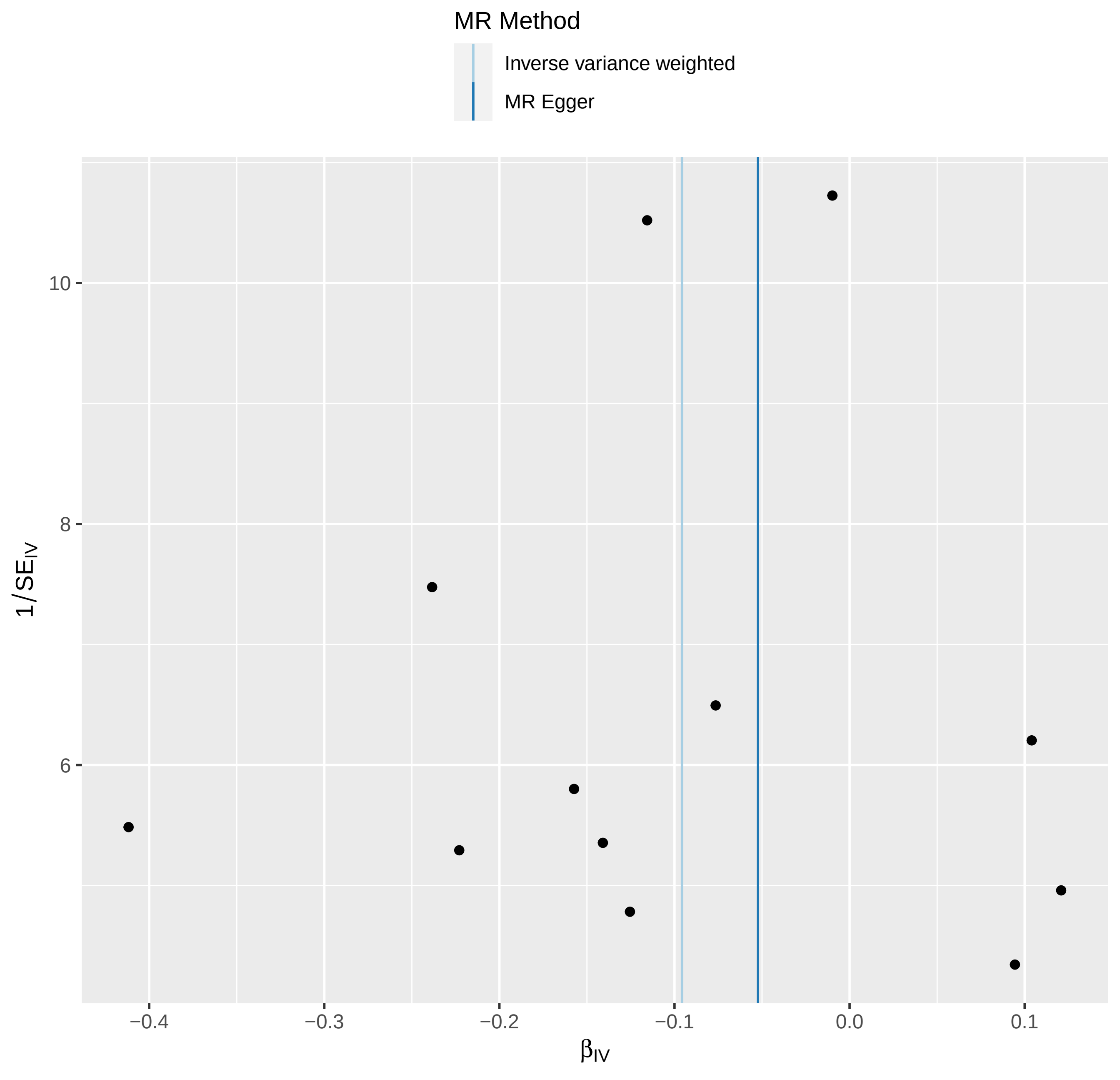

Batch 3−2128 : Selenium on Nucleoside diphosphate−linked moiety X motif 8, mitochondrial

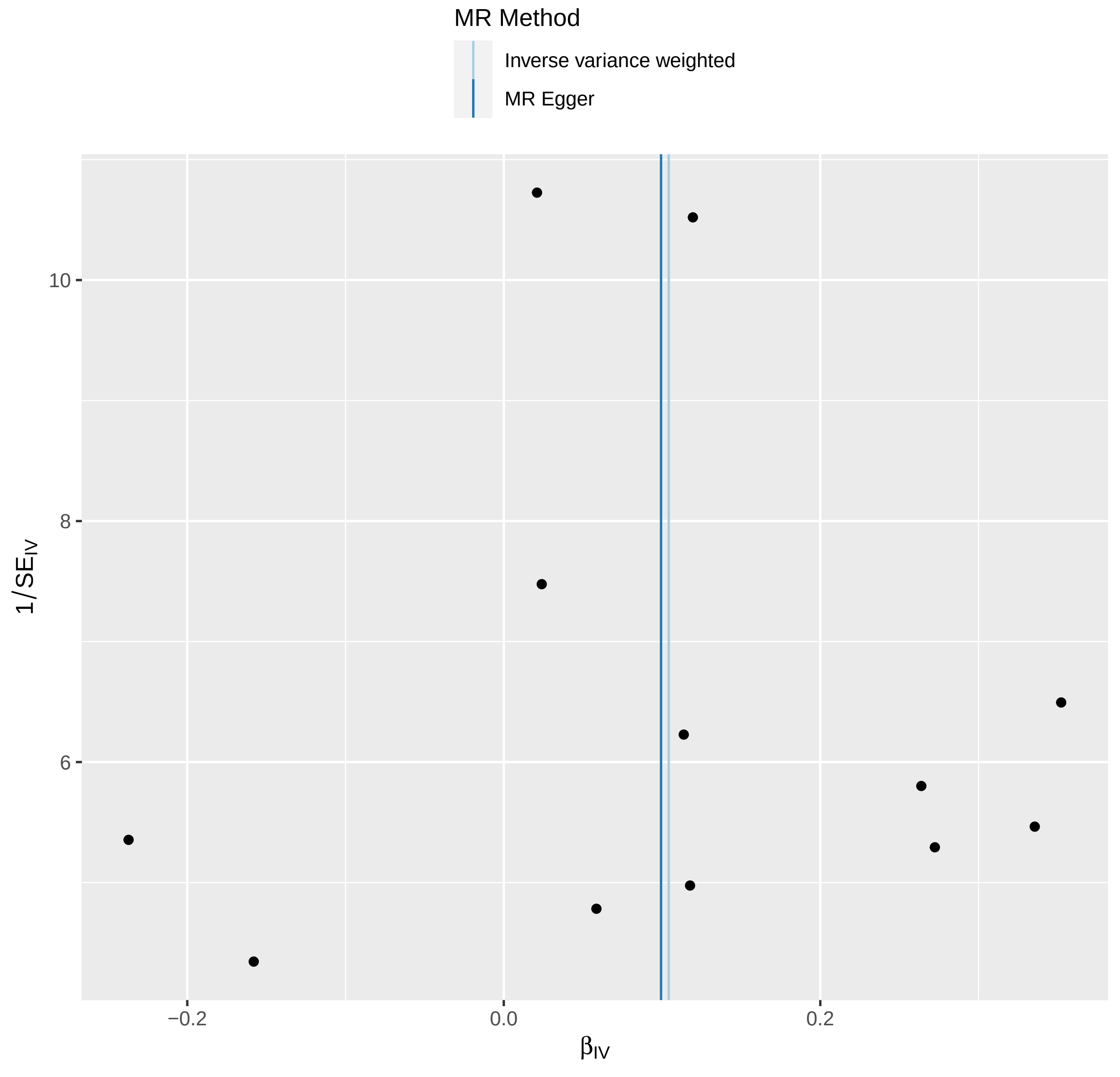

Batch 3−2129 : Selenium on ADP−ribose pyrophosphatase, mitochondrial

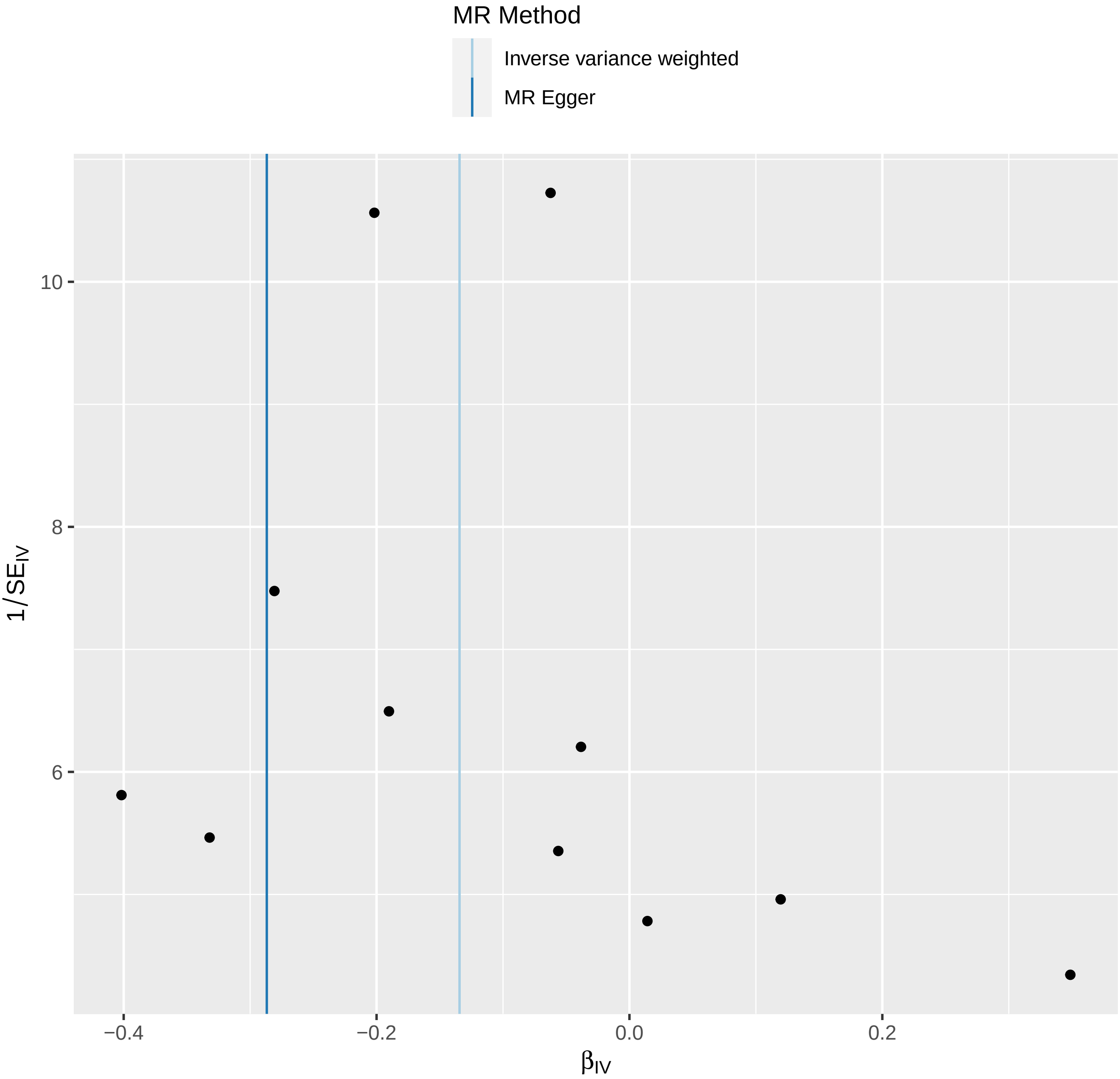

Batch 3−2130 : Selenium on Nuclear pore membrane glycoprotein 210−like

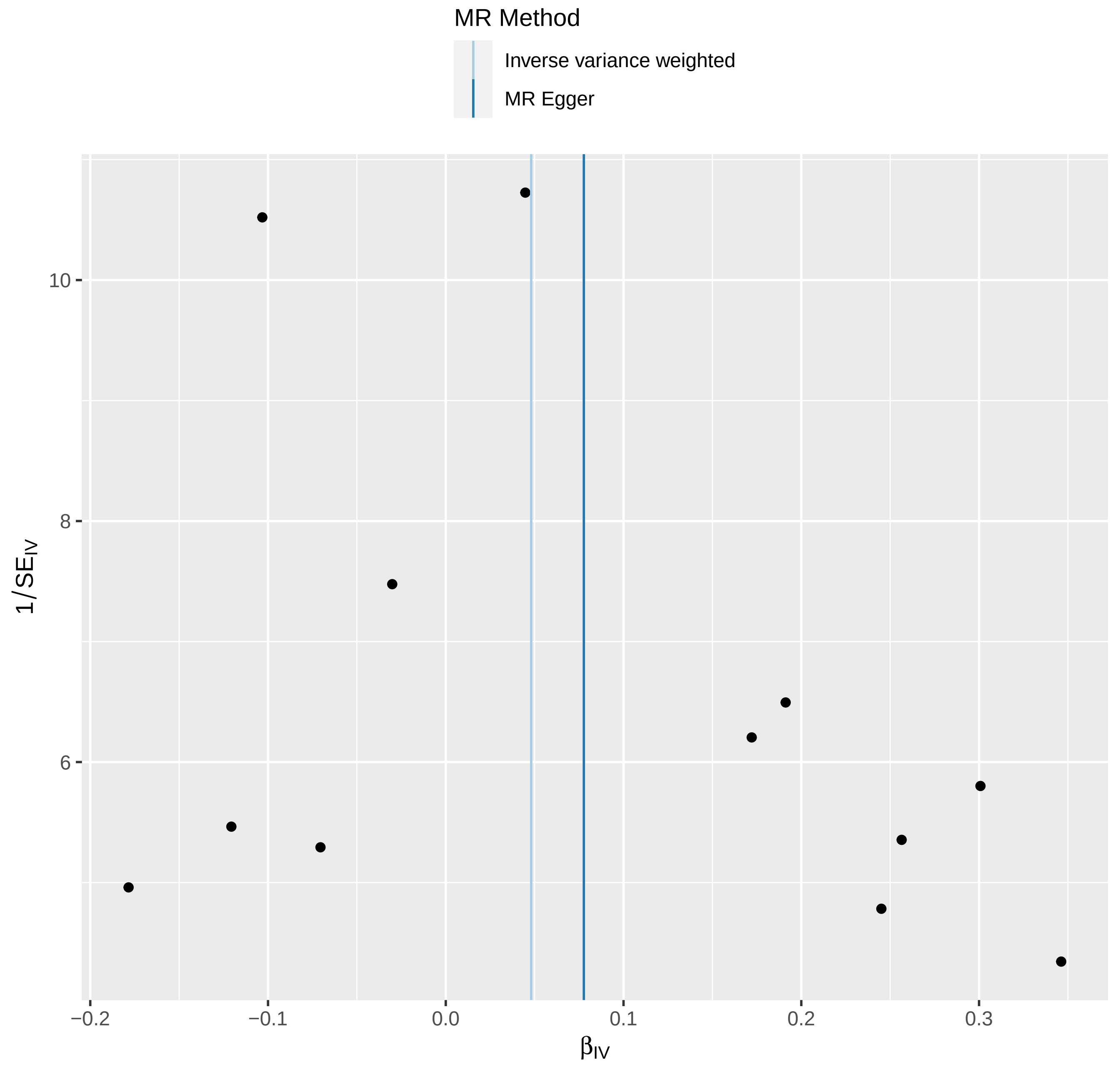

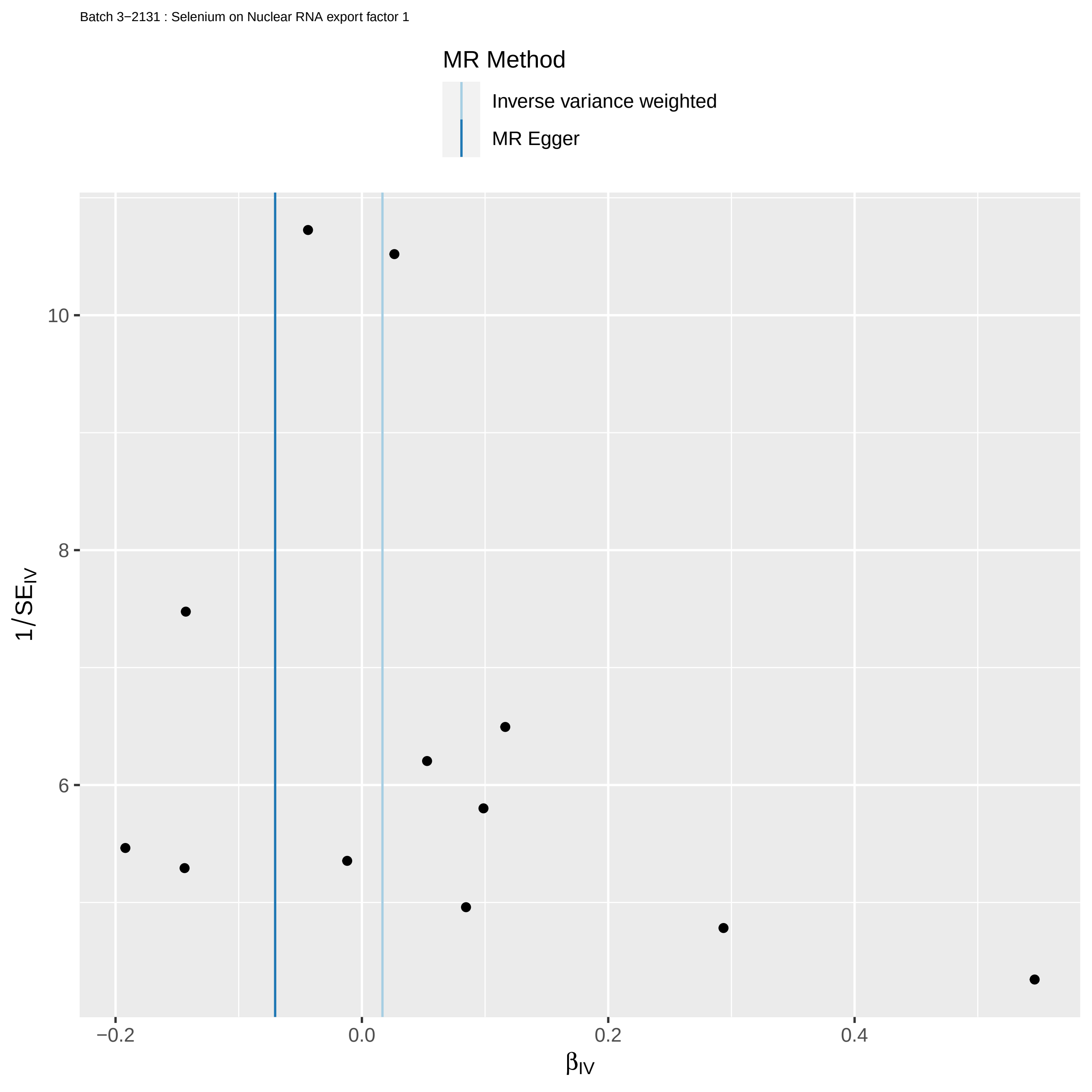

Batch 3−2131 : Selenium on Nuclear RNA export factor 1
MR Method
Inverse variance weighted
MR Egger
10
8
6
1/SE_IV
−0.2
0.0
0.2
0.4
β_IV

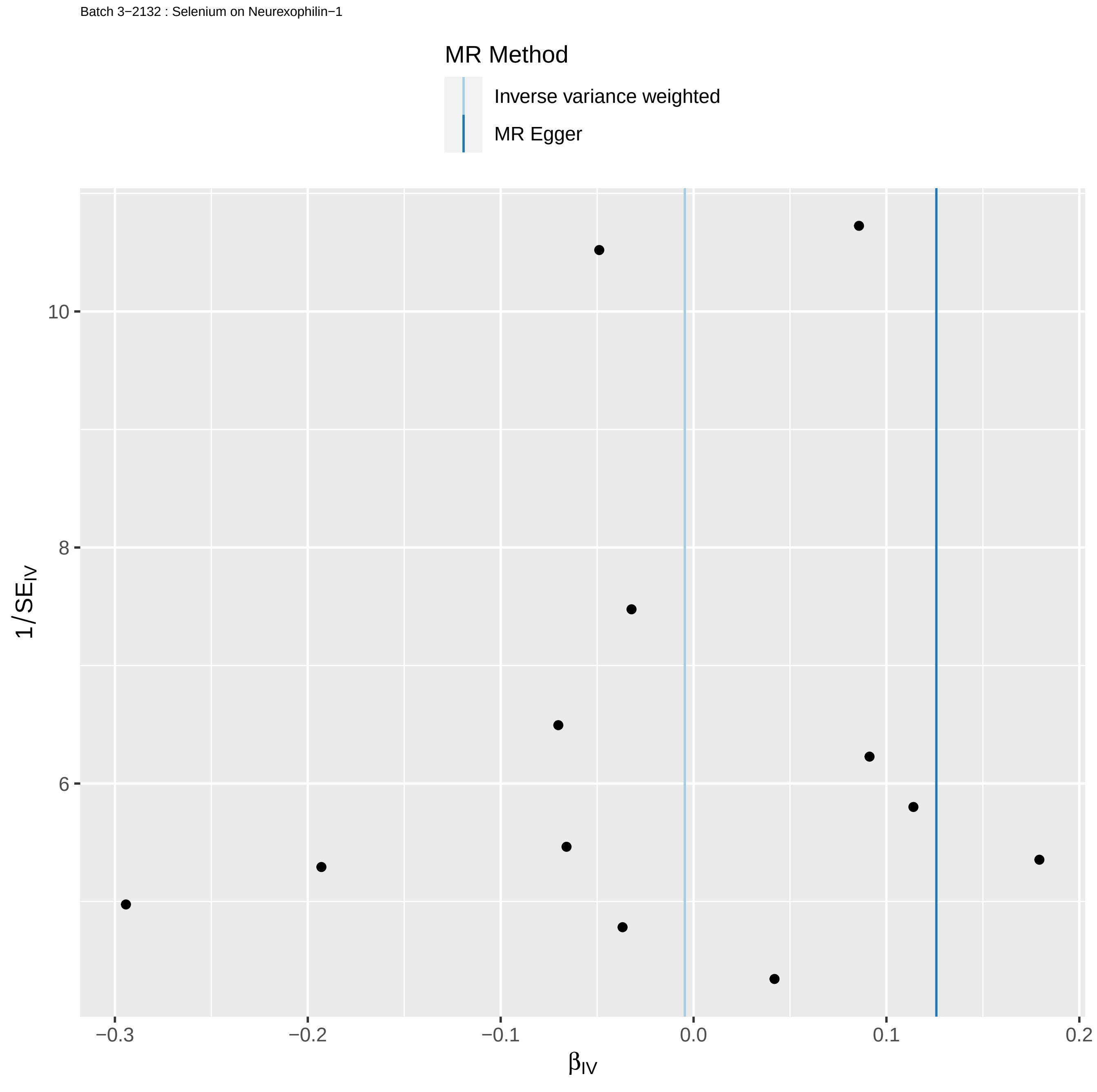

Batch 3−2132 : Selenium on Neurexophilin−1
MR Method
Inverse variance weighted
MR Egger
10
8
6
1/SE$_{IV}$
−0.3
−0.2
−0.1
0.0
0.1
0.2
β$_{IV}$

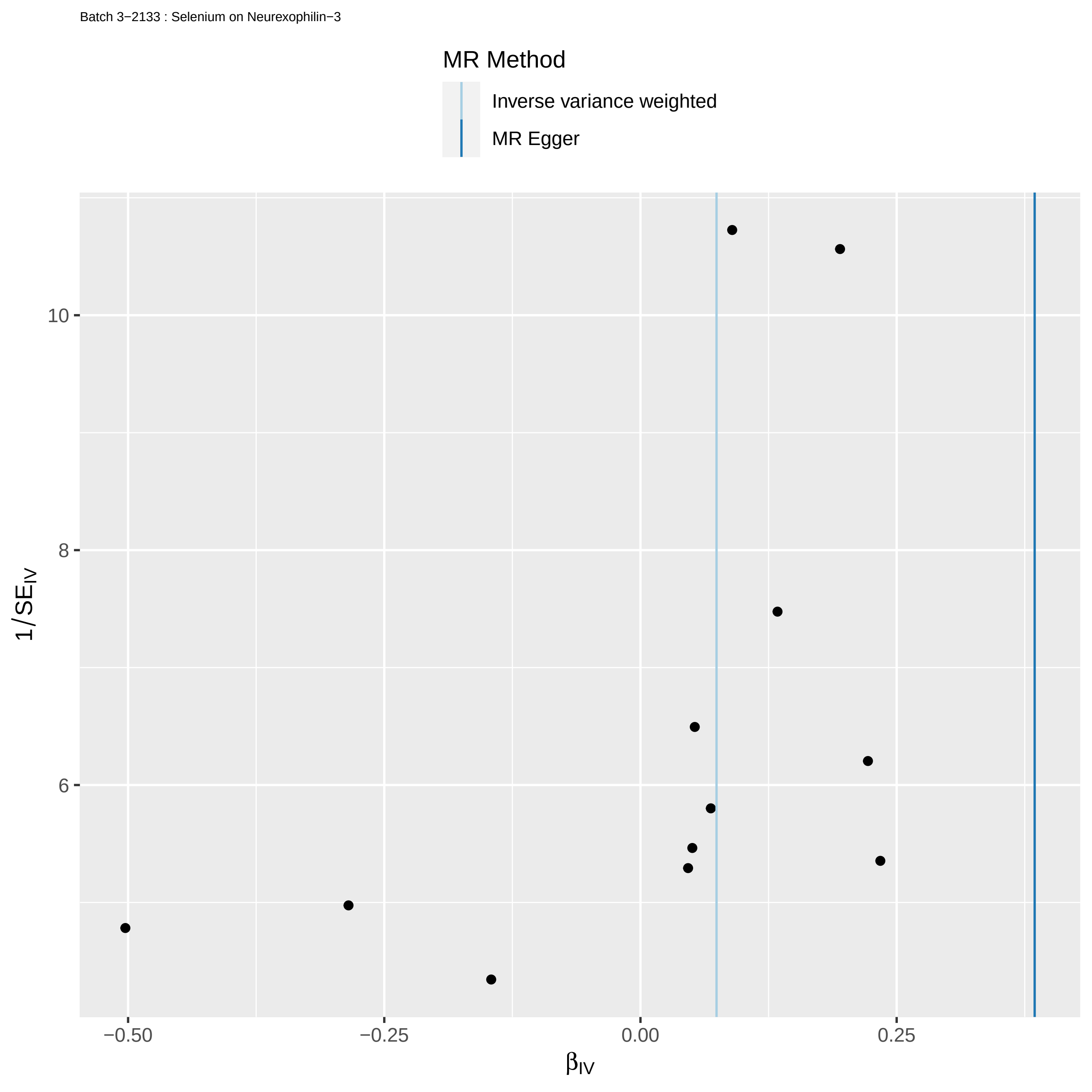

Batch 3−2133 : Selenium on Neurexophilin−3
MR Method
Inverse variance weighted
MR Egger
10
8
6
1/SE$_{IV}$
−0.50
−0.25
0.00
0.25
β$_{IV}$

Batch 3−2134 : Selenium on NTF2−related export protein 1

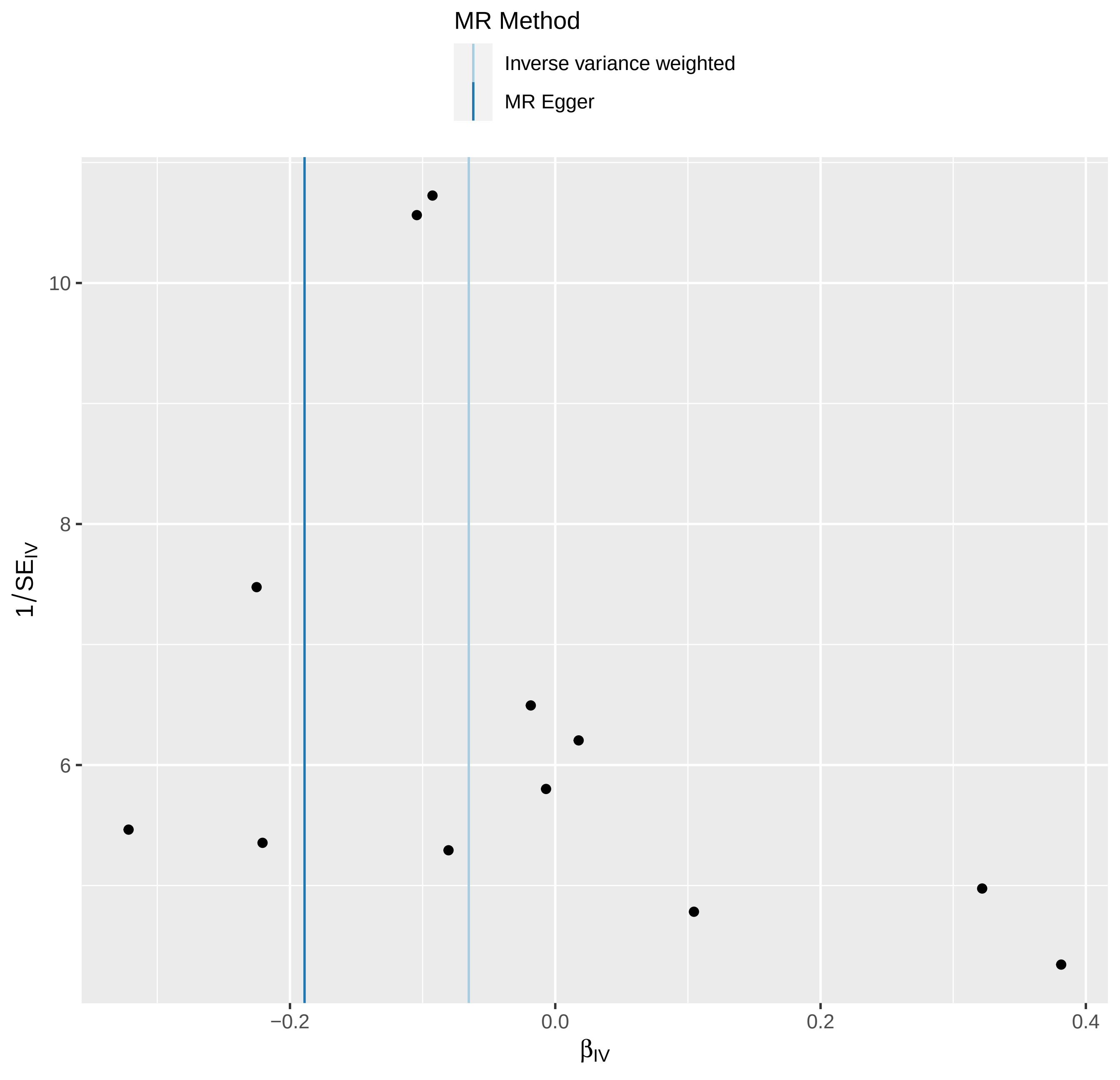

Batch 3−2135 : Selenium on Out at first protein homolog

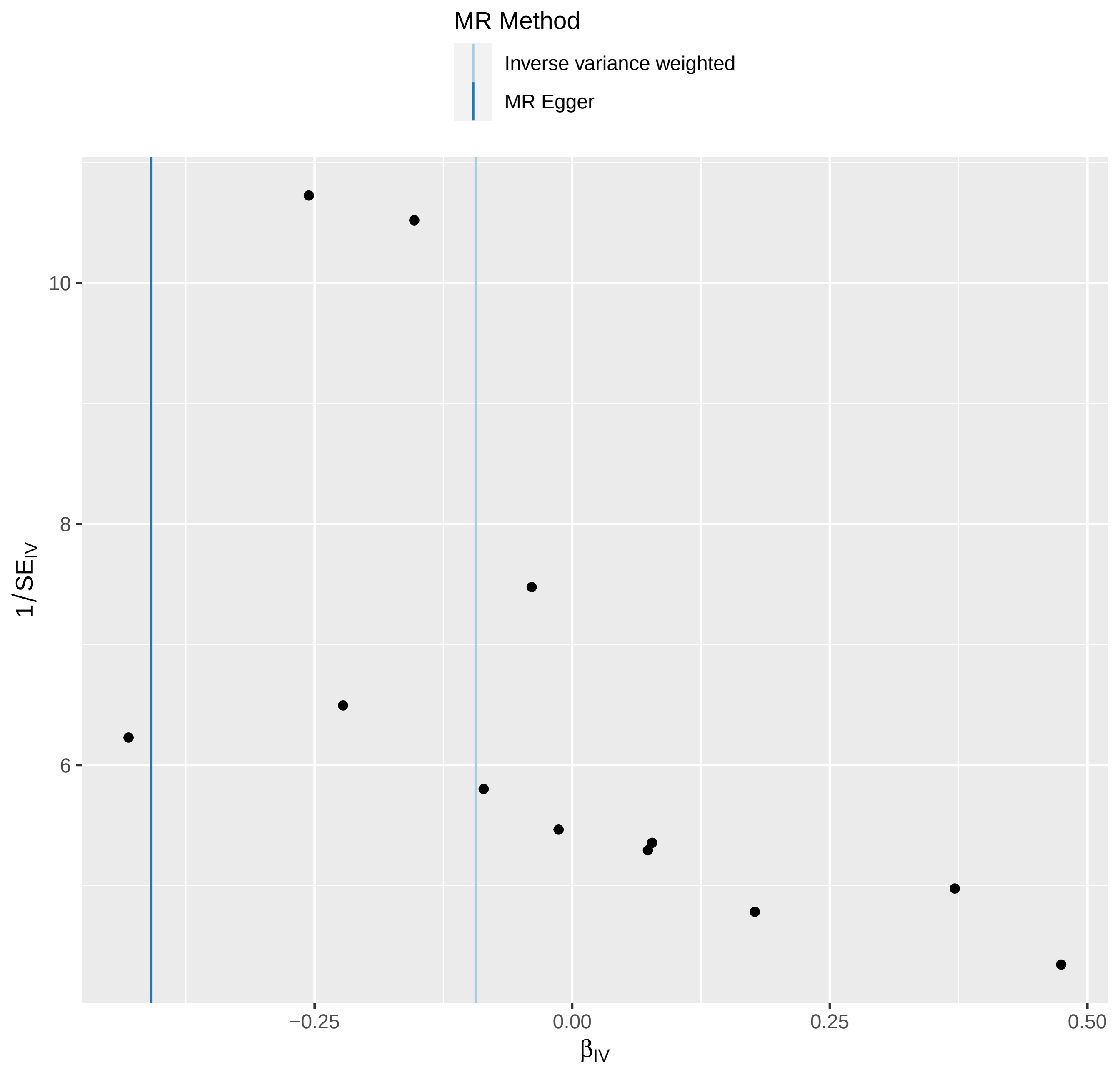

Batch 3−2136 : Selenium on 2'−5'−oligoadenylate synthase 1

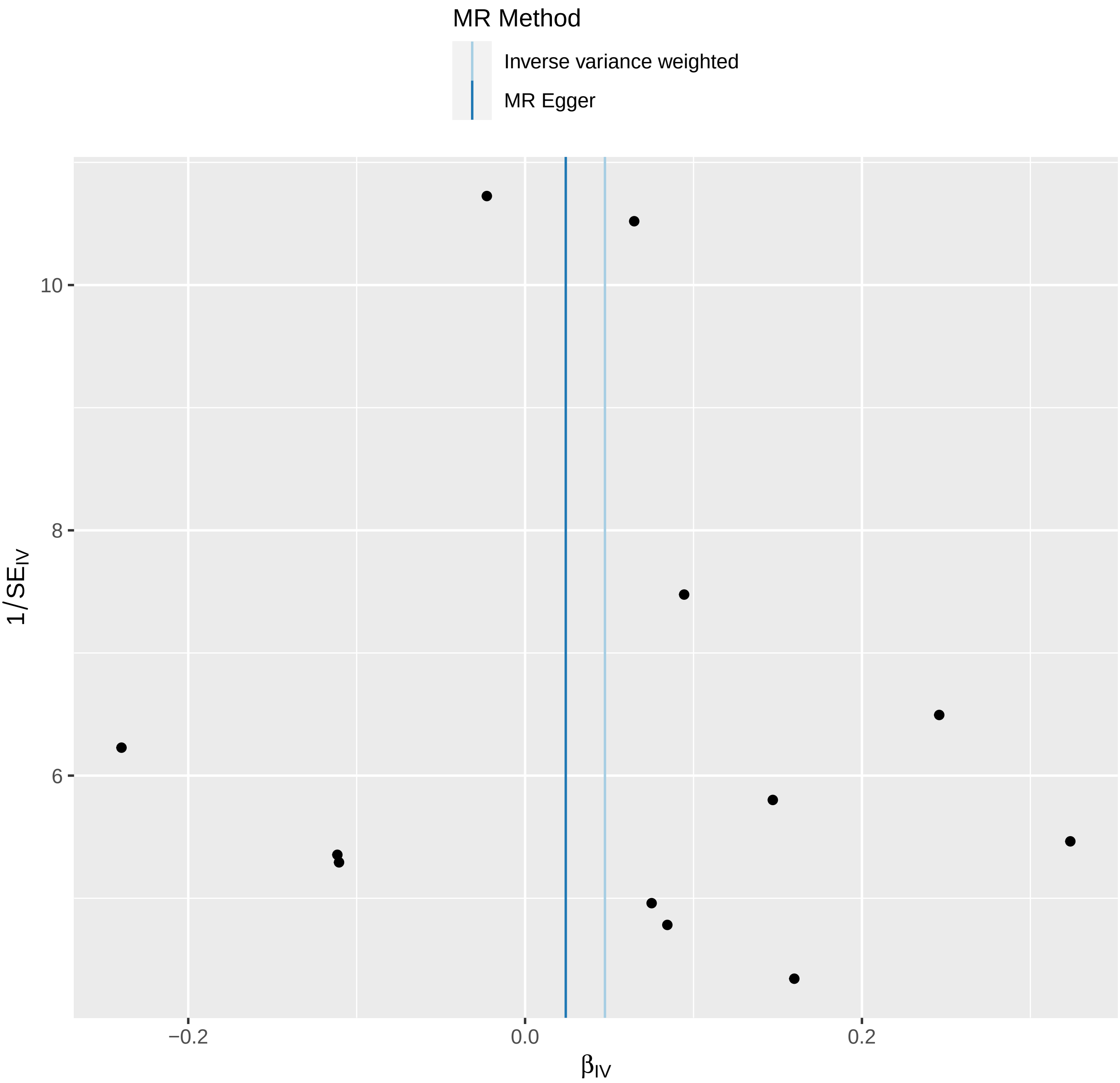

Batch 3−2137 : Selenium on Ornithine decarboxylase antizyme 1

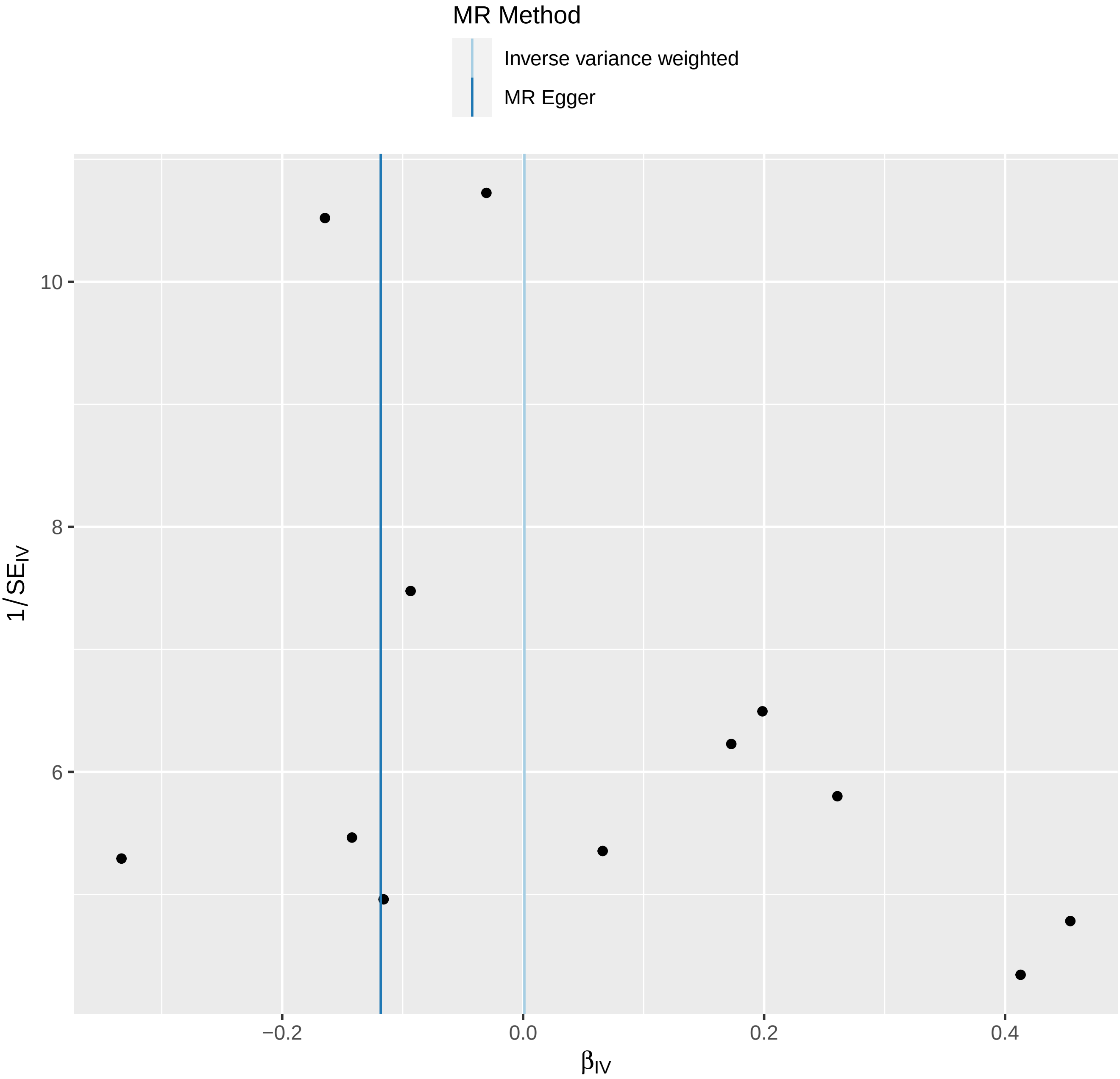

Batch 3−2138 : Selenium on Odorant−binding protein 2a

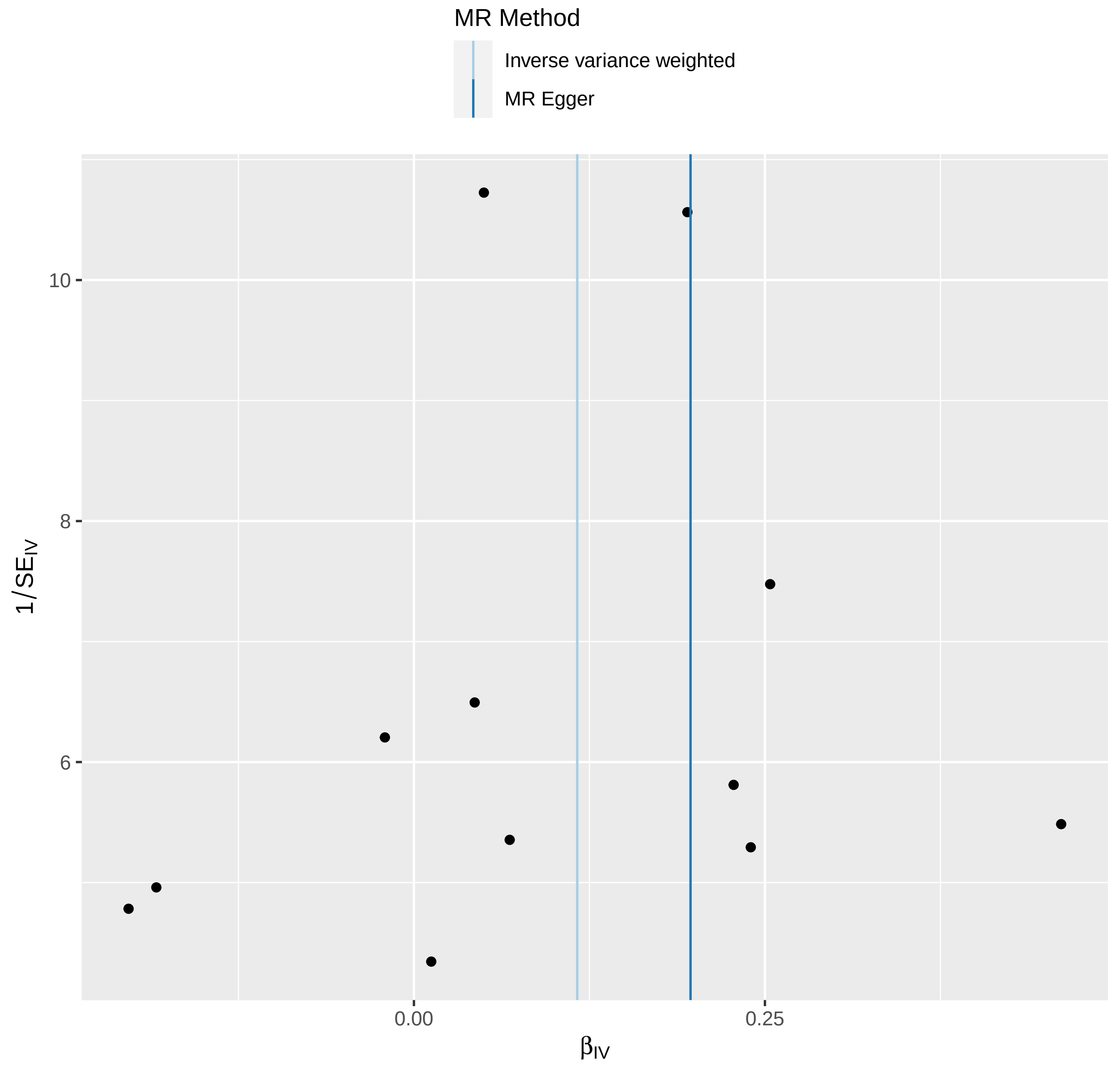

Batch 3−2139 : Selenium on Odorant−binding protein 2b

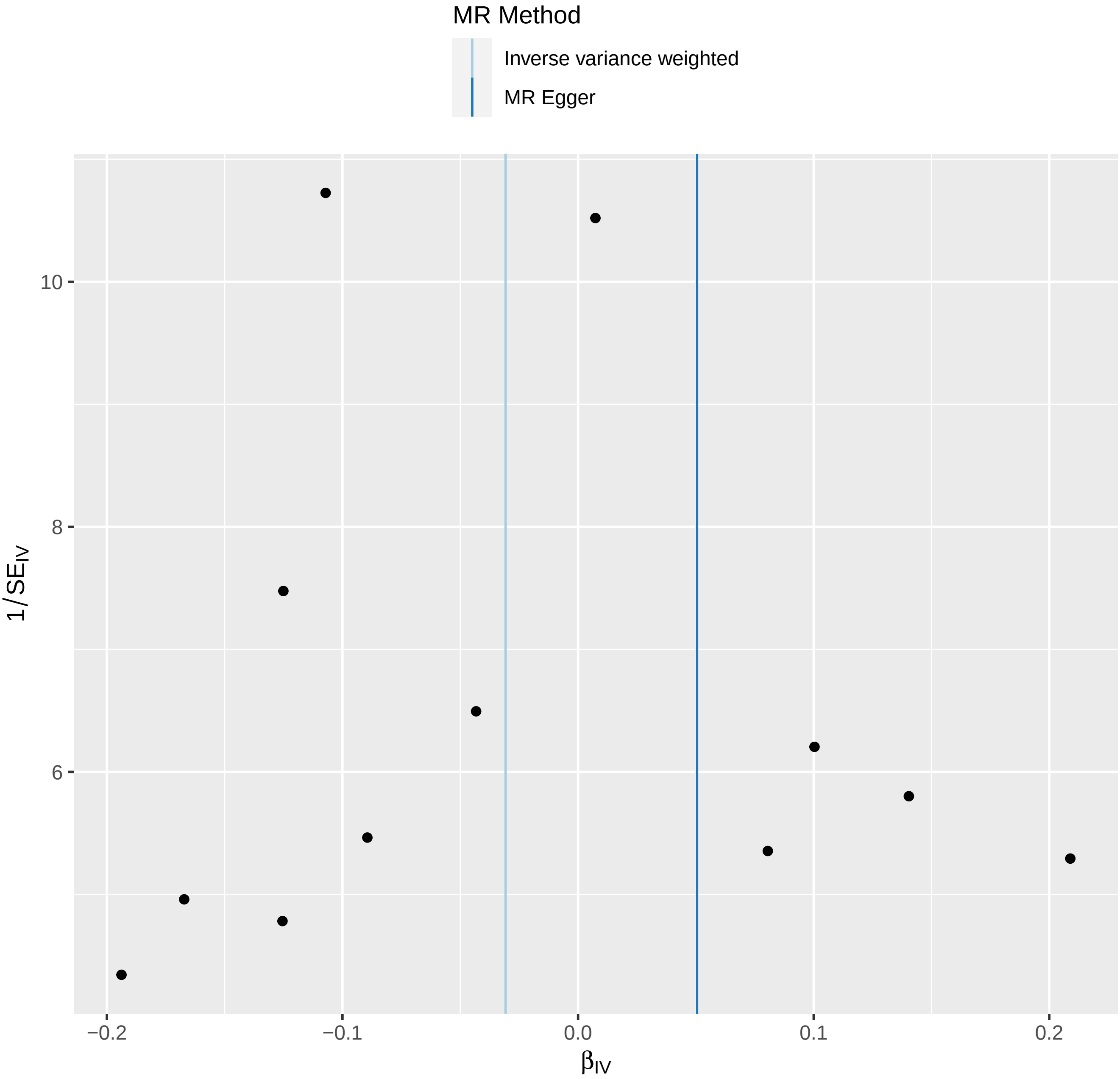

Batch 3−2140 : Selenium on OCIA domain−containing protein 1

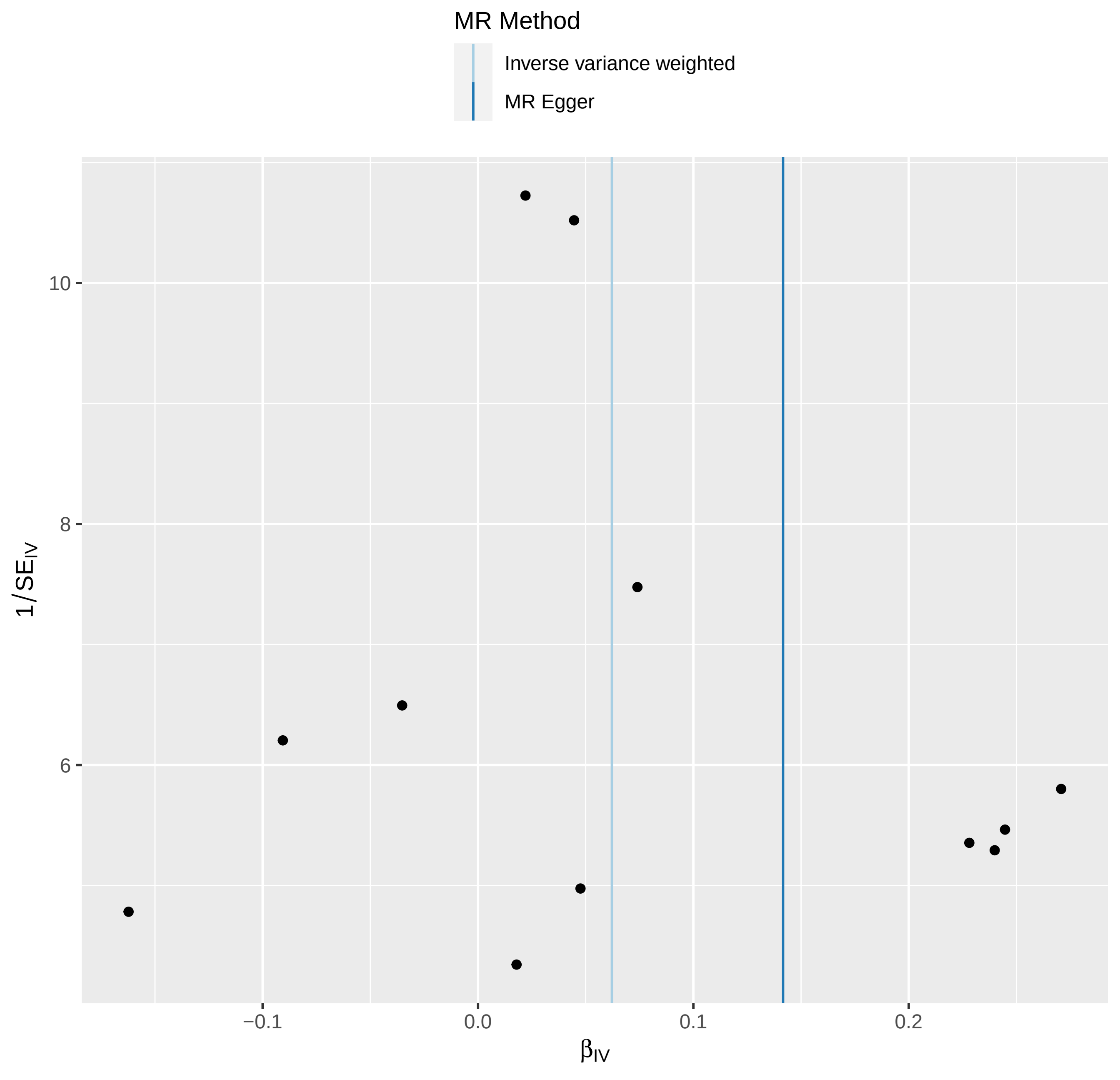

Batch 3−2141 : Selenium on Inositol polyphosphate 5−phosphatase OCRL−1

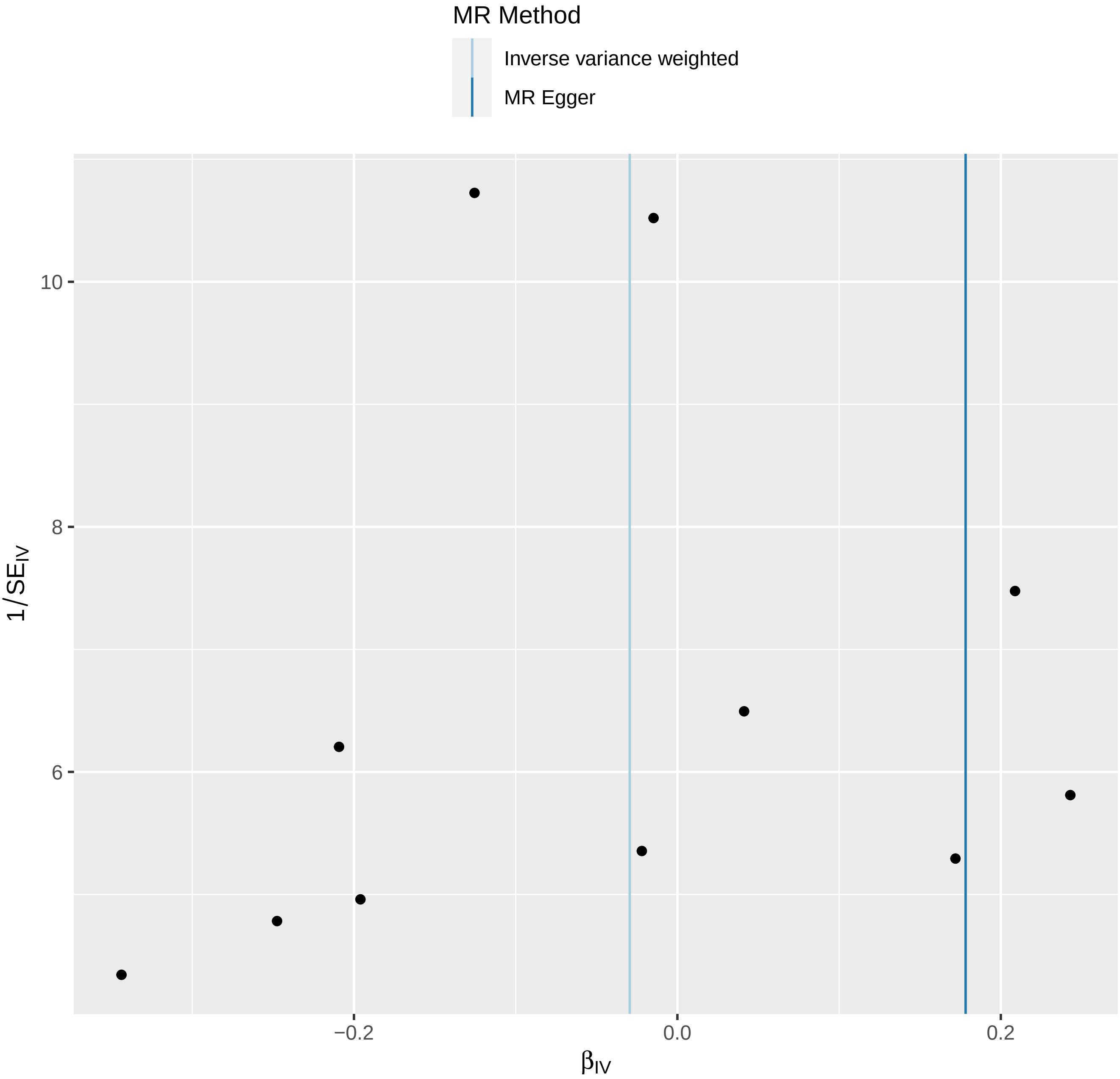

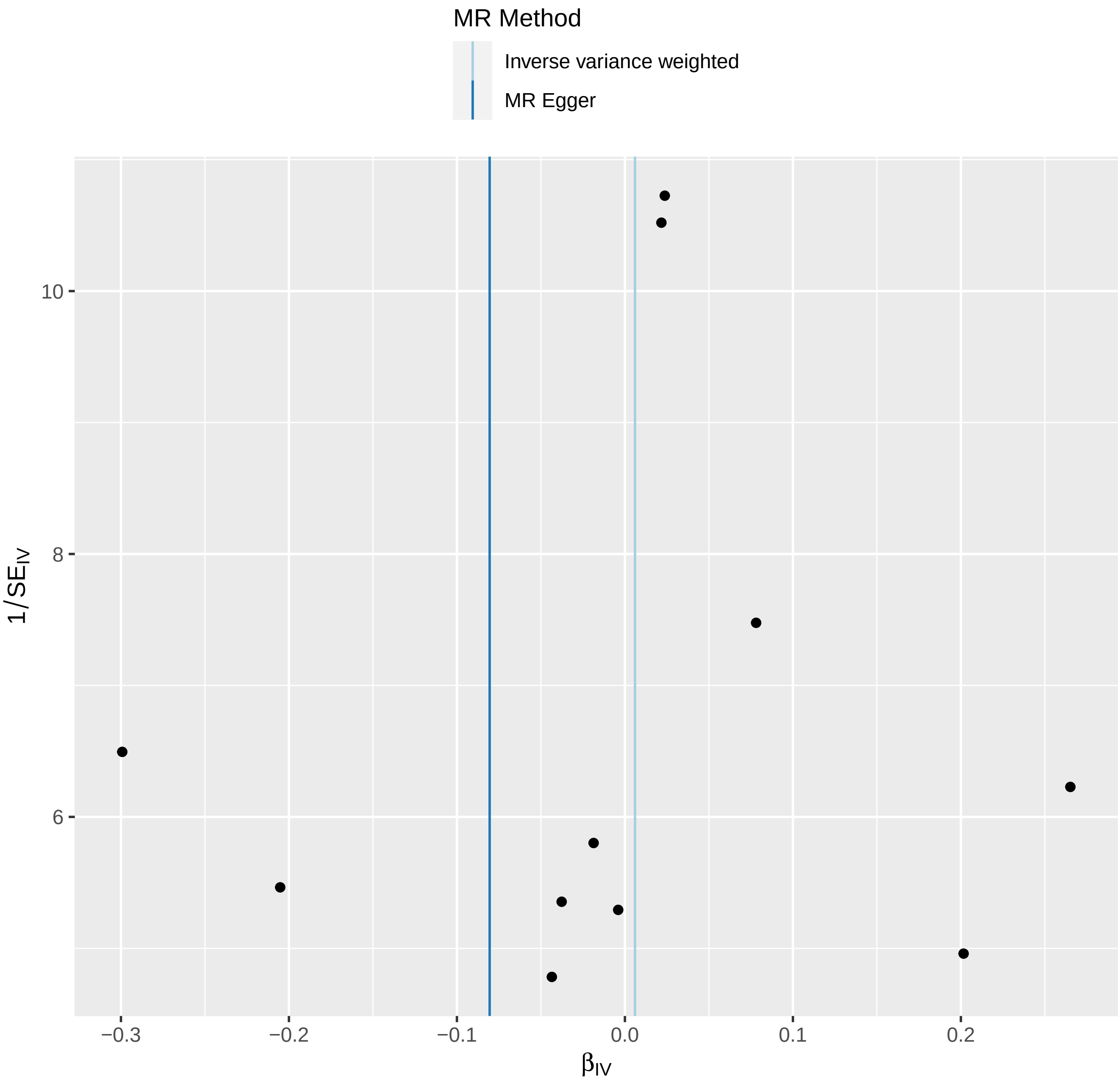
MR Method
Inverse variance weighted
MR Egger
10
8
6
1/SE$_{IV}$
−0.3
−0.2
−0.1
0.0
0.1
0.2
β$_{IV}$

Batch 3−2143 : Selenium on Obg−like ATPase 1

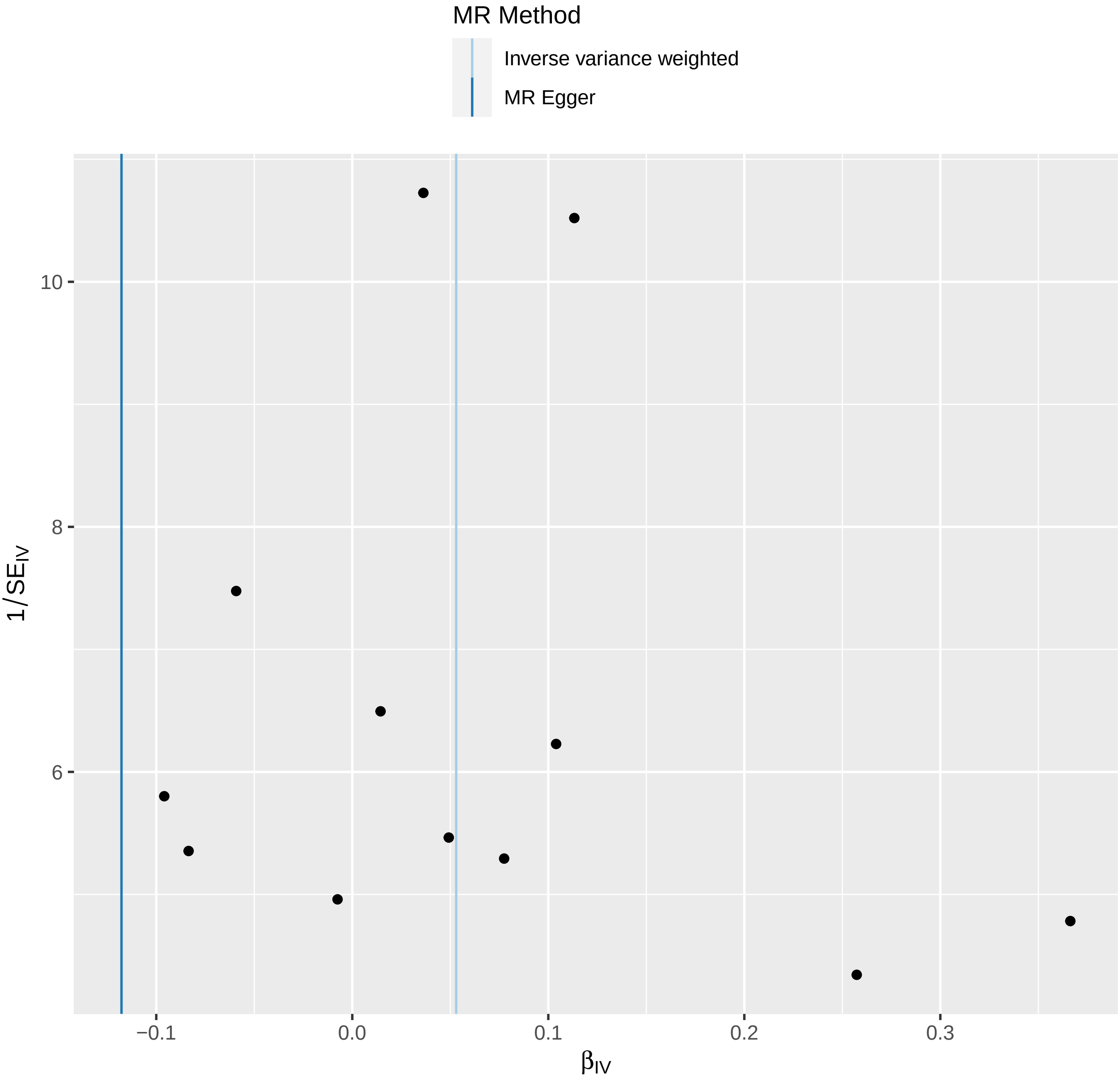

Batch 3−2144 : Selenium on Olfactomedin−like protein 3

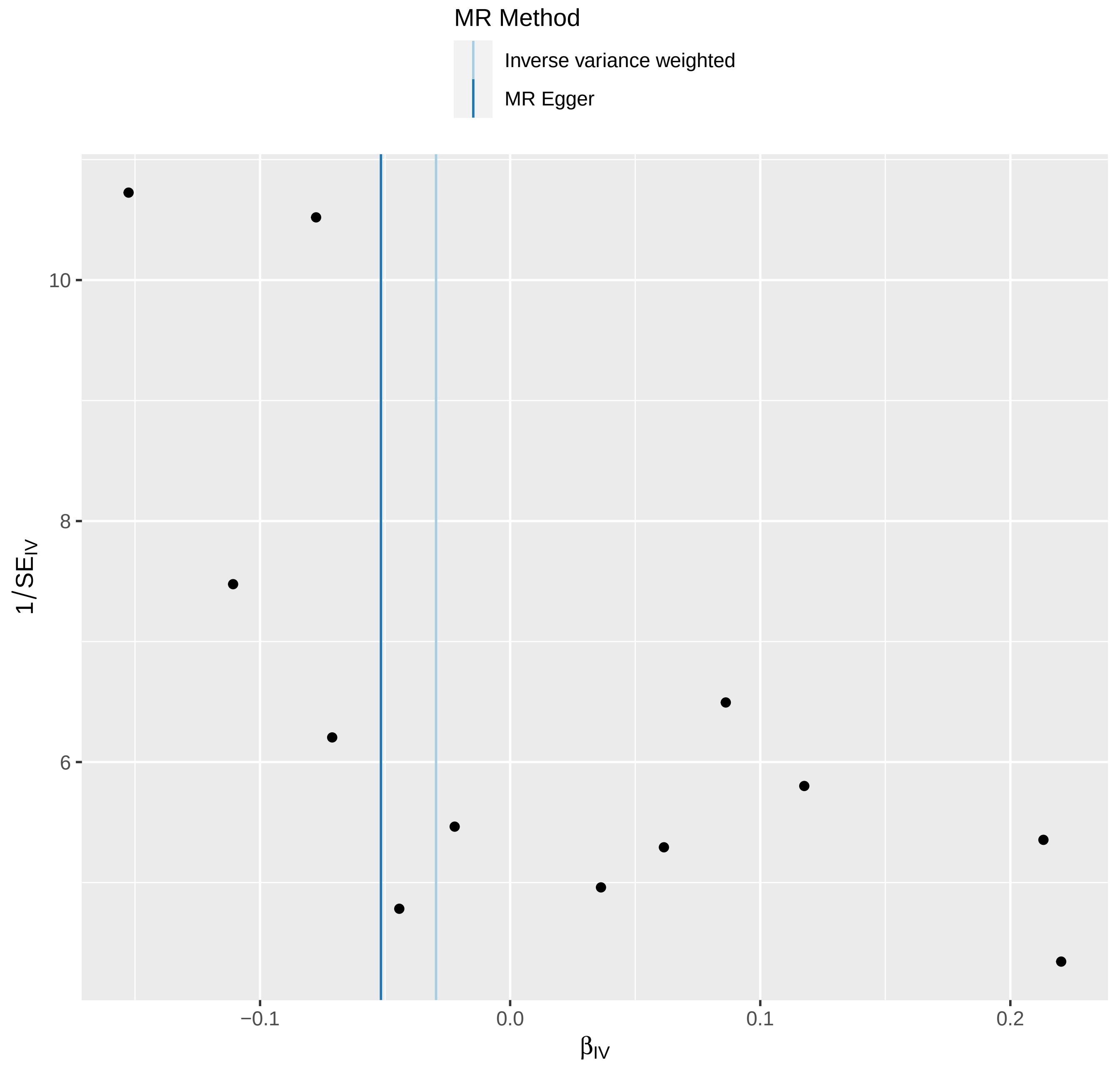

Batch 3−2145 : Selenium on Olfactomedin−like protein 3

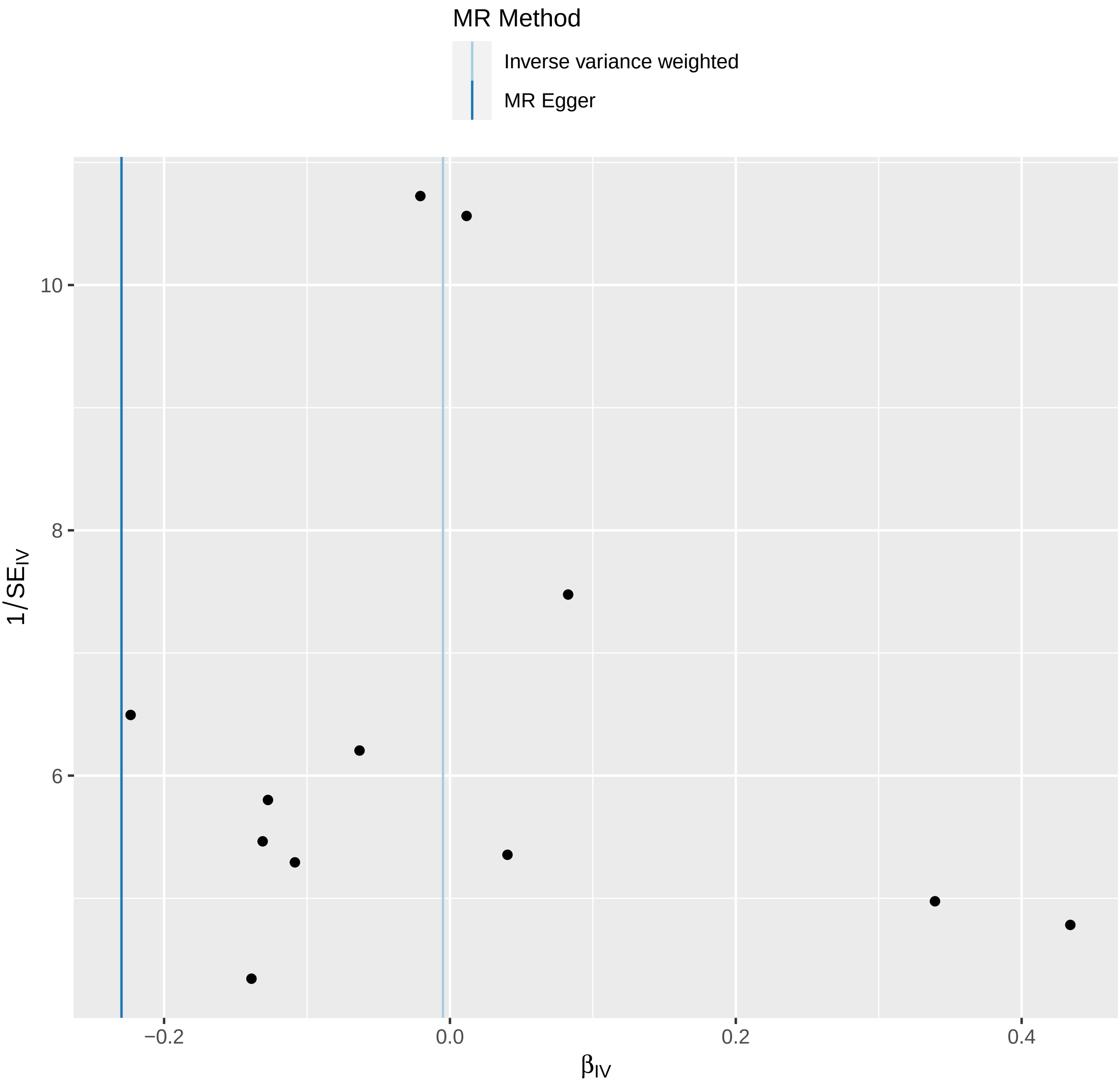

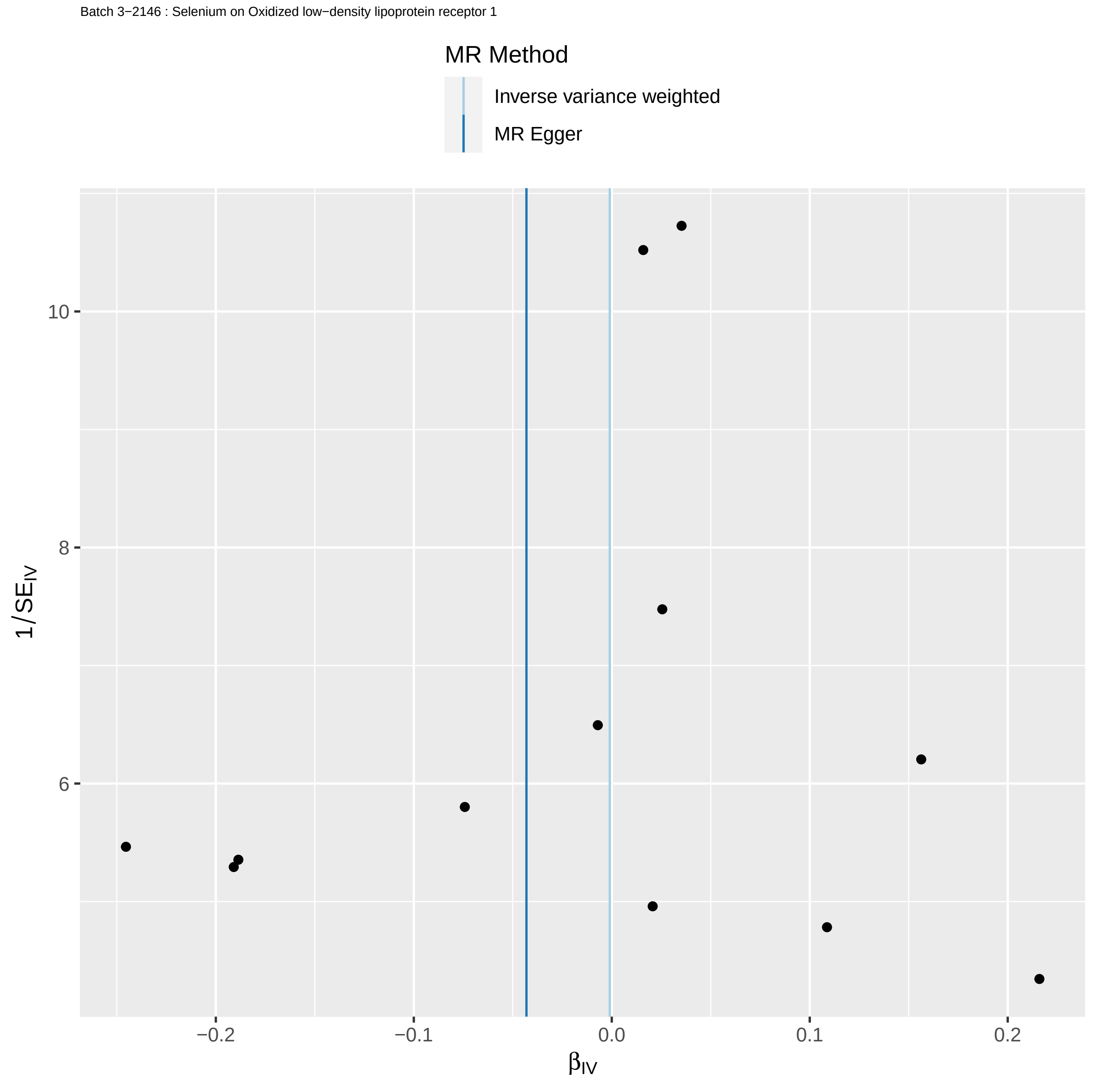
Batch 3−2146 : Selenium on Oxidized low−density lipoprotein receptor 1
MR Method
Inverse variance weighted
MR Egger
10
8
6
$1/SE_{IV}$
−0.2
−0.1
0.0
0.1
0.2
β_{IV}

Batch 3−2147 : Selenium on Osteomodulin

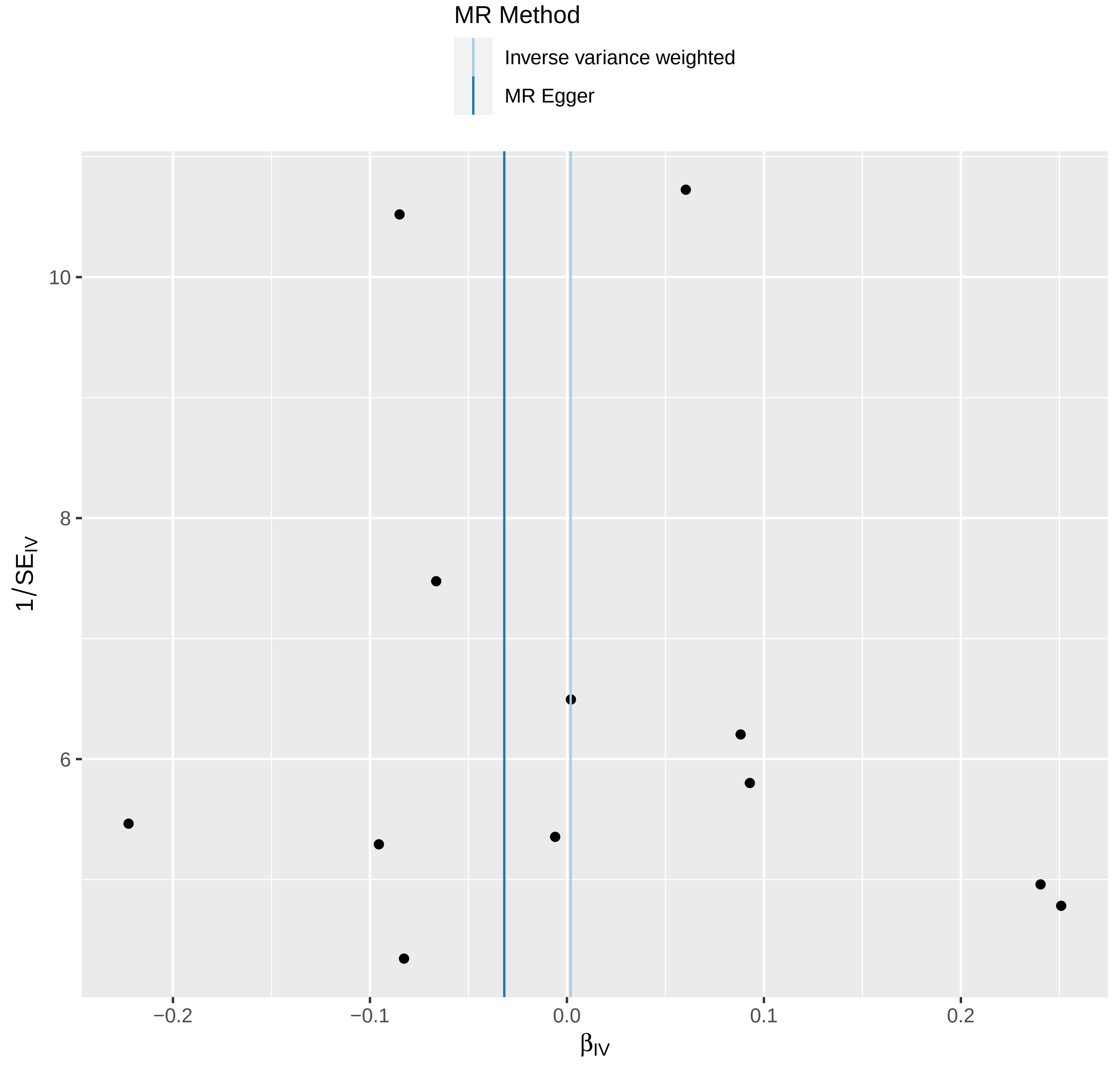

Batch 3−2148 : Selenium on Opalin

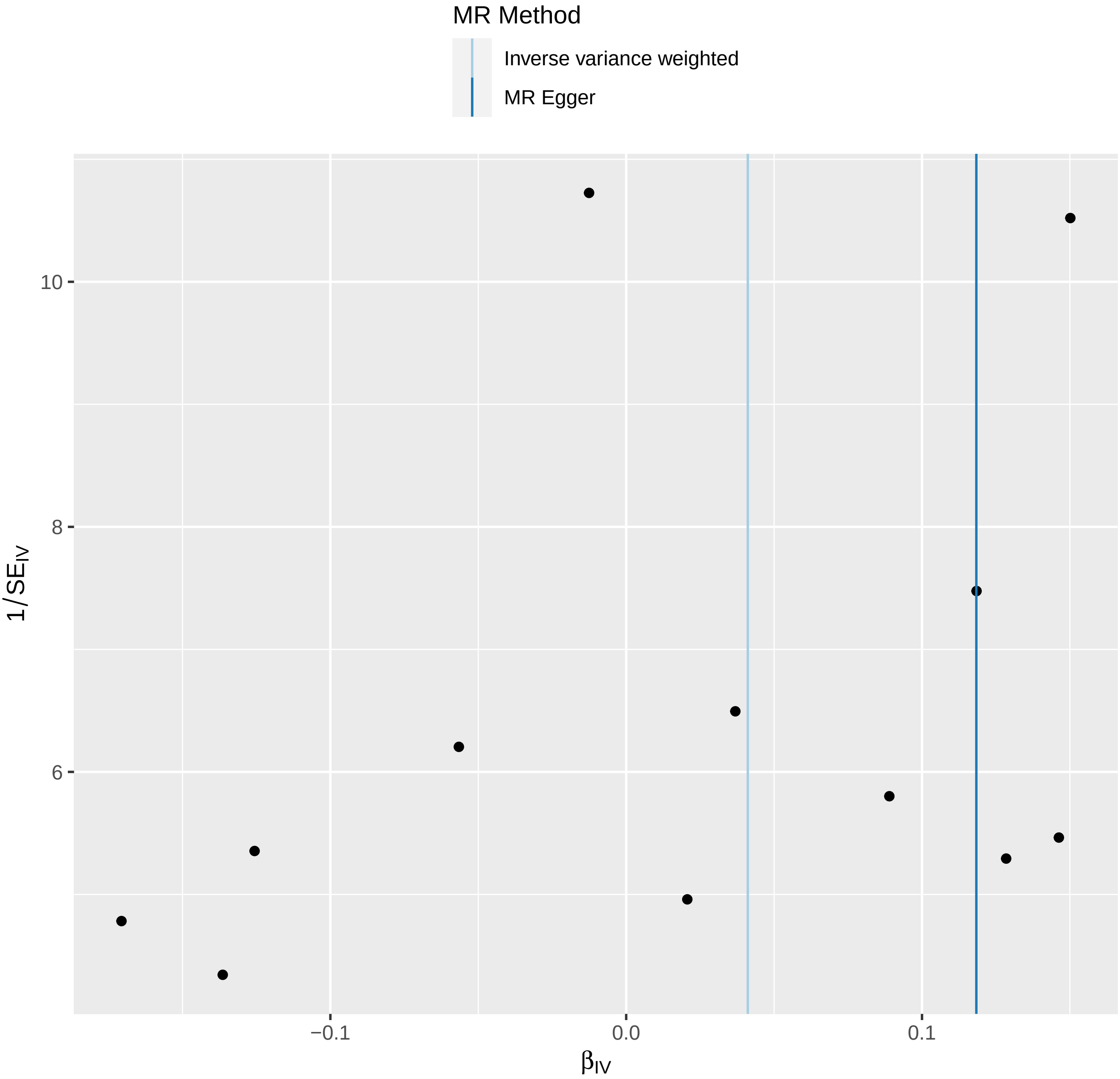

Batch 3−2149 : Selenium on Opioid−binding protein/cell adhesion molecule

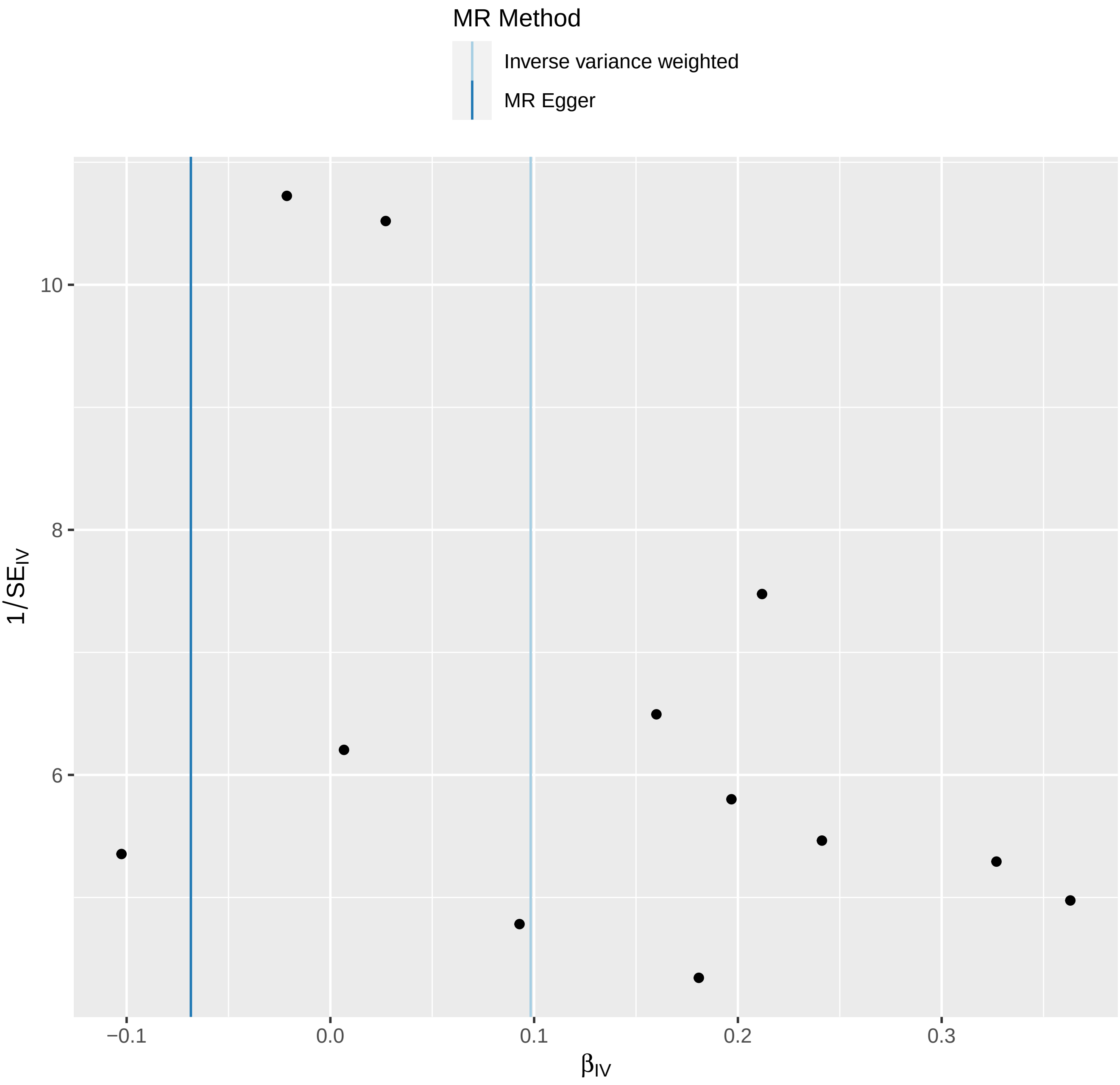

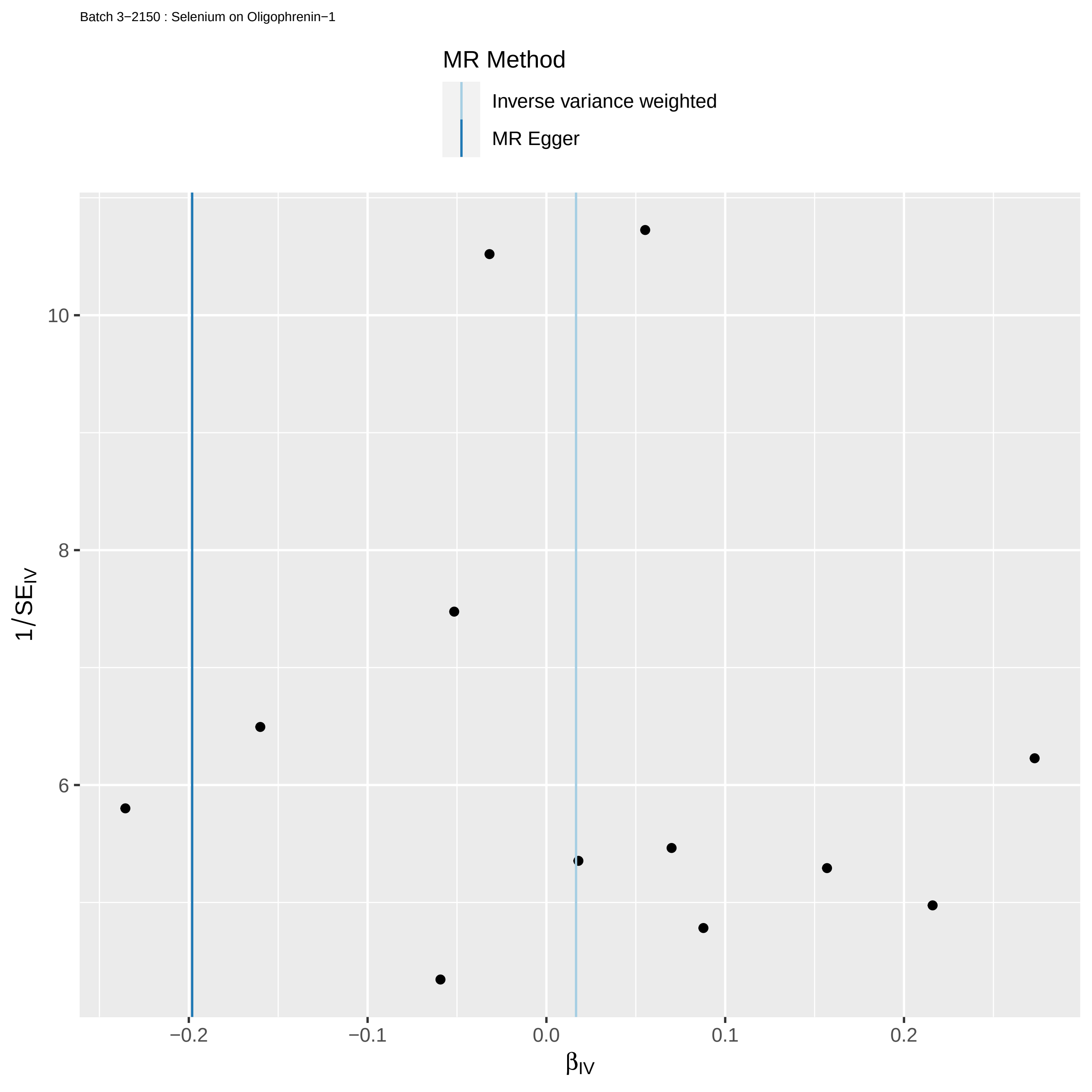

Batch 3−2150 : Selenium on Oligophrenin−1
MR Method
Inverse variance weighted
MR Egger
10
8
6
1/SE_IV
−0.2
−0.1
0.0
0.1
0.2
β_IV

Batch 3−2151 : Selenium on Origin recognition complex subunit 6

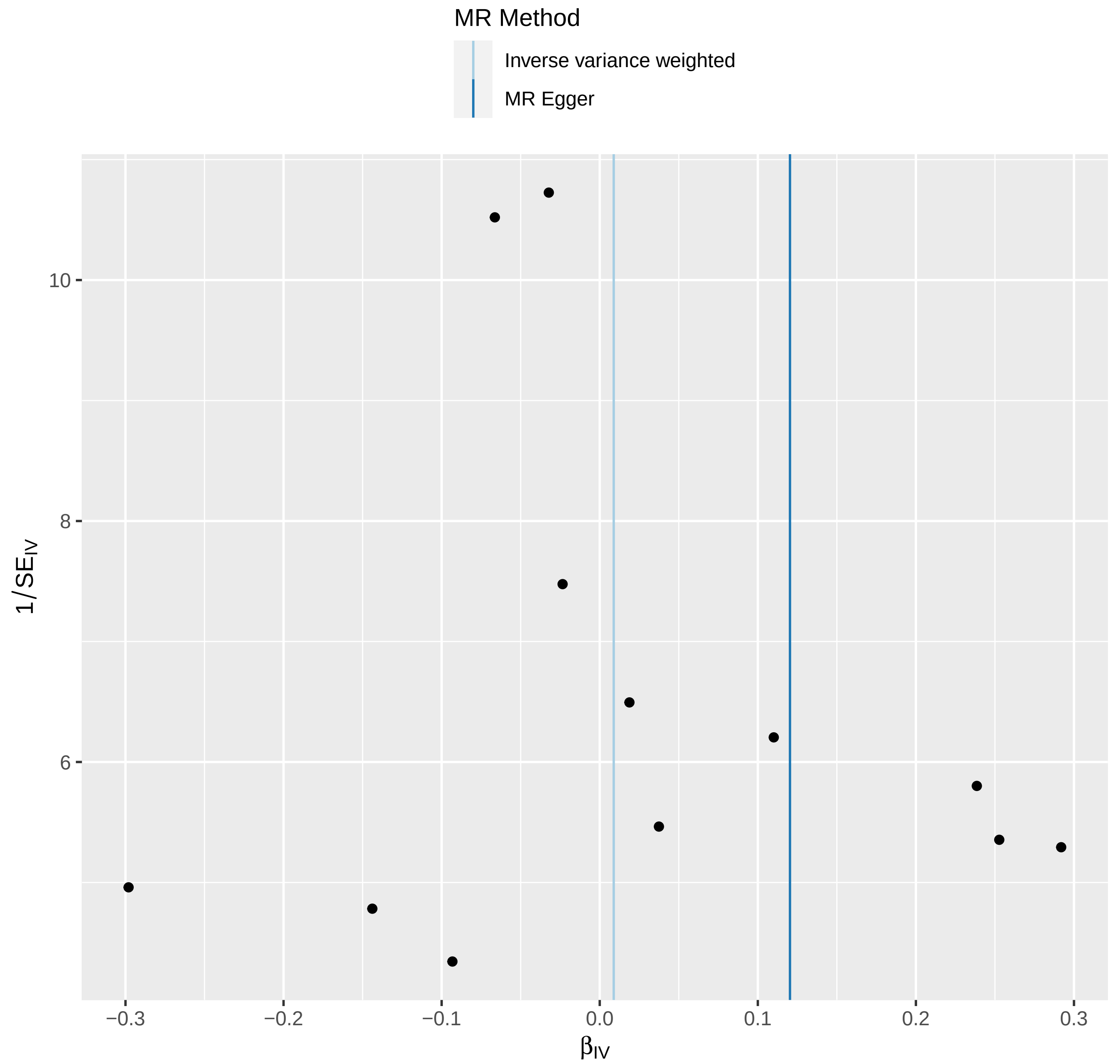

Batch 3−2152 : Selenium on Oxysterol−binding protein−related protein 11

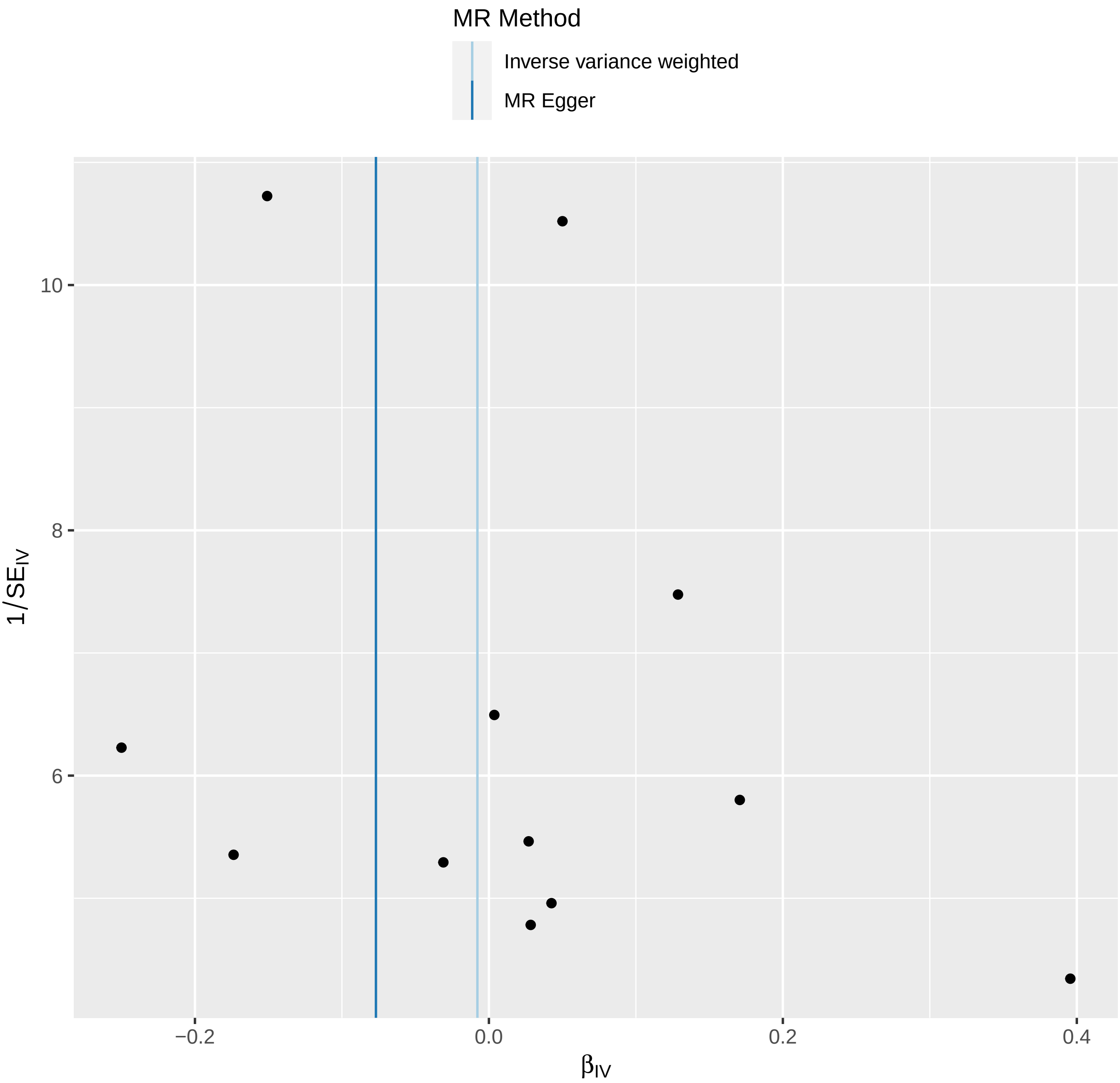

Batch 3−2153 : Selenium on Osteoclast−associated immunoglobulin−like receptor

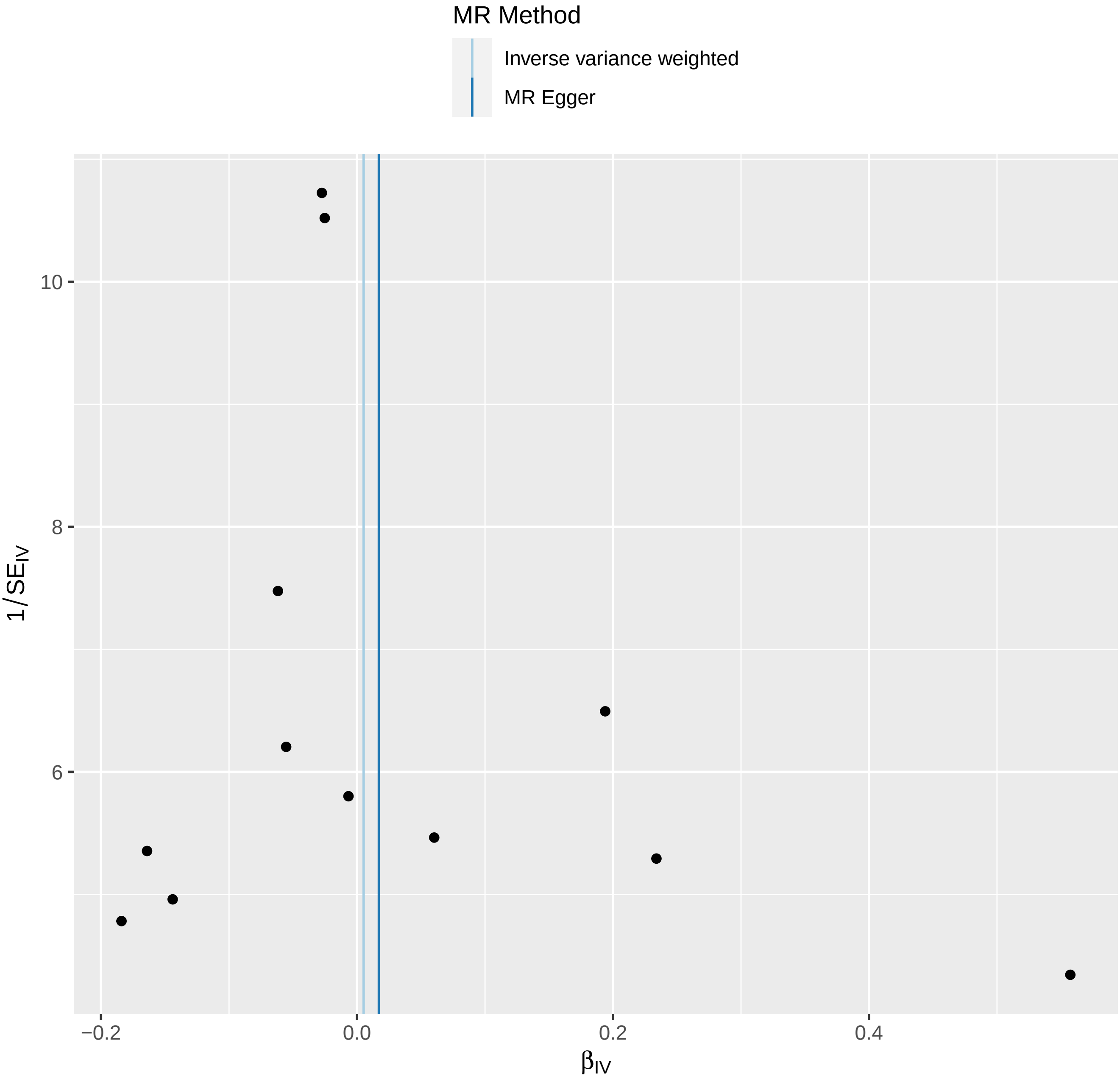

Batch 3−2154 : Selenium on Oncostatin−M

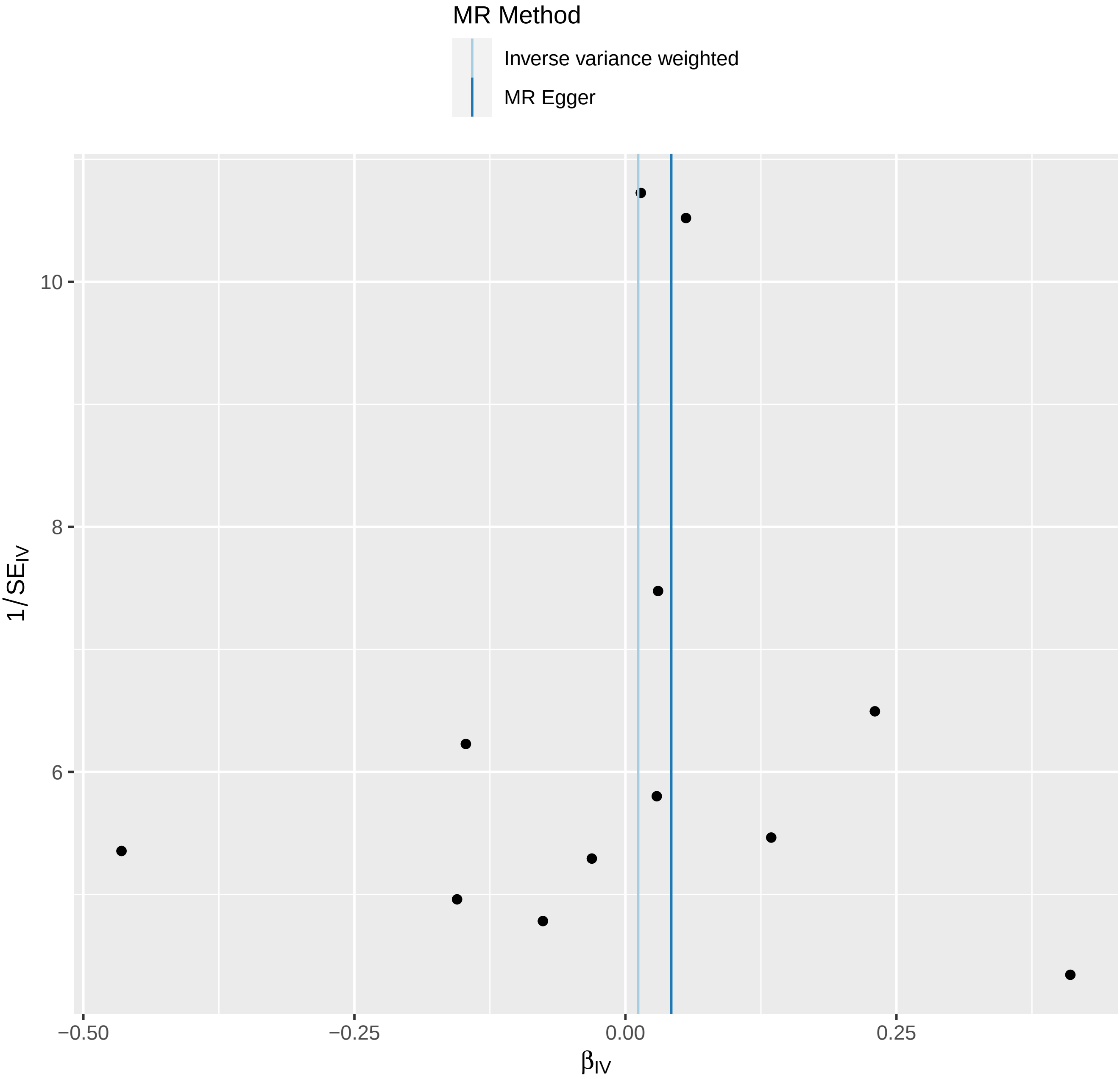

Batch 3−2155 : Selenium on Oncostatin−M

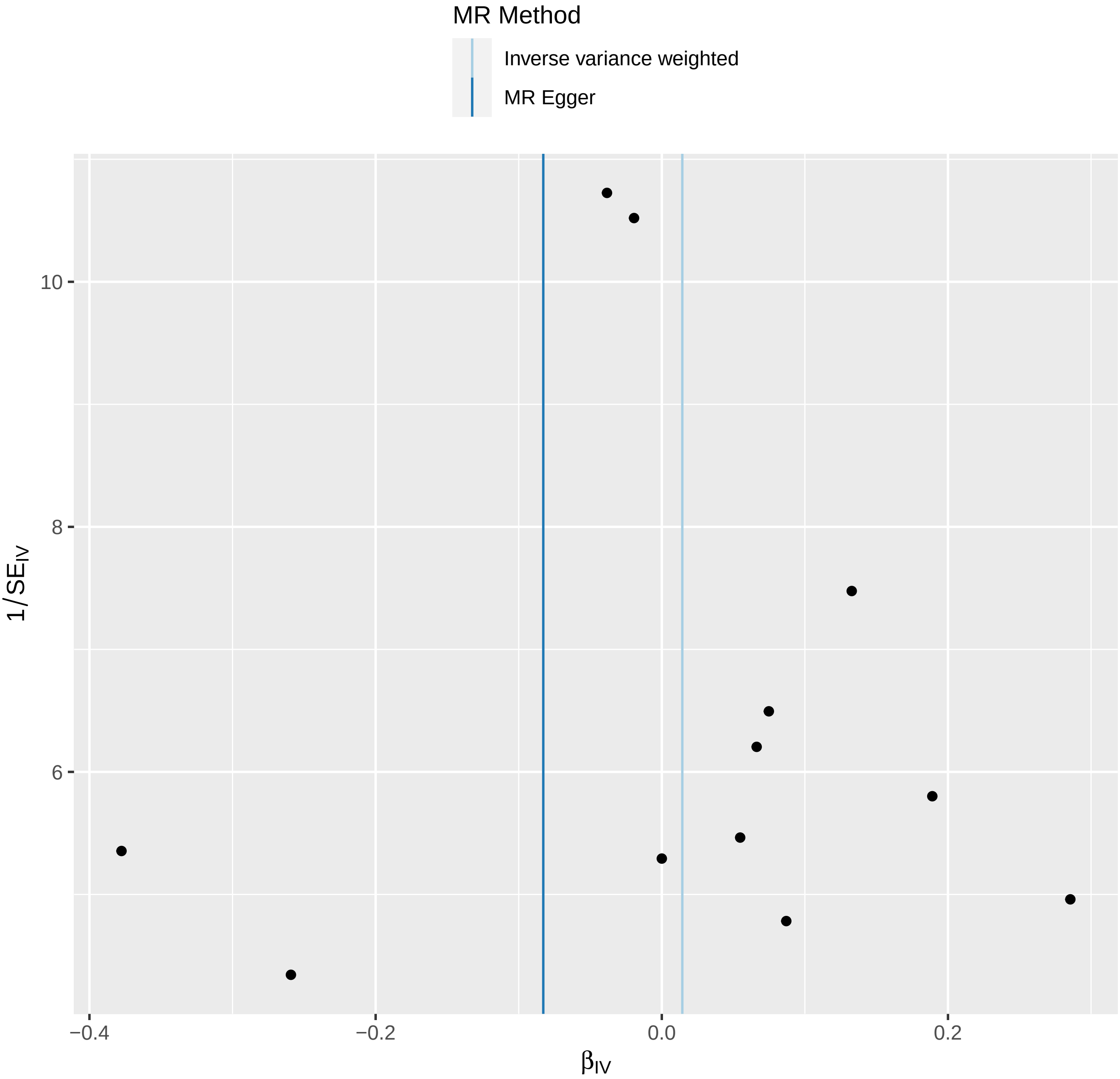

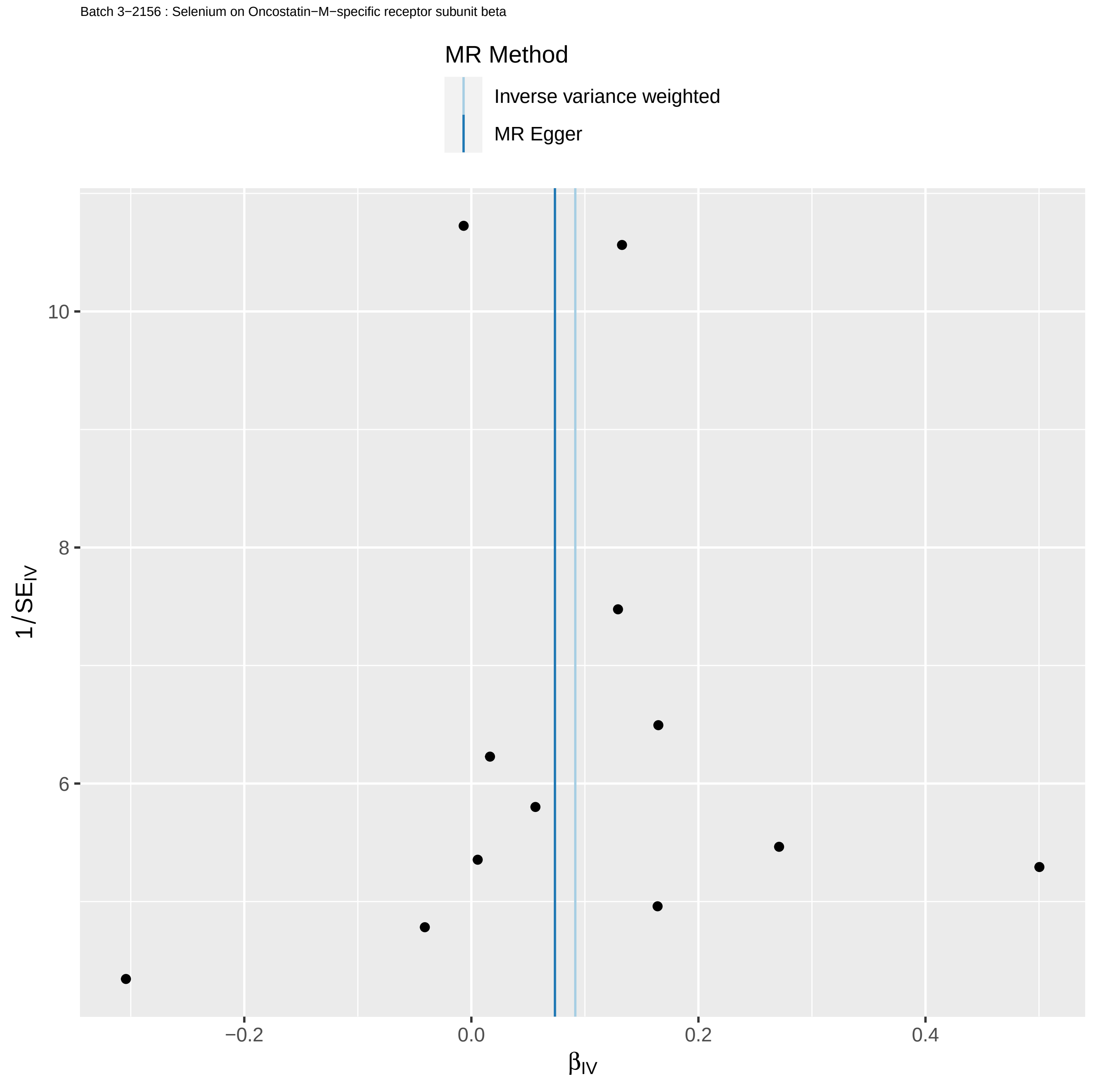
Batch 3−2156 : Selenium on Oncostatin−M−specific receptor subunit beta
MR Method
Inverse variance weighted
MR Egger
10
8
6
1/SE_IV
−0.2
0.0
0.2
0.4
β_IV

Batch 3−2157 : Selenium on Osteocrin

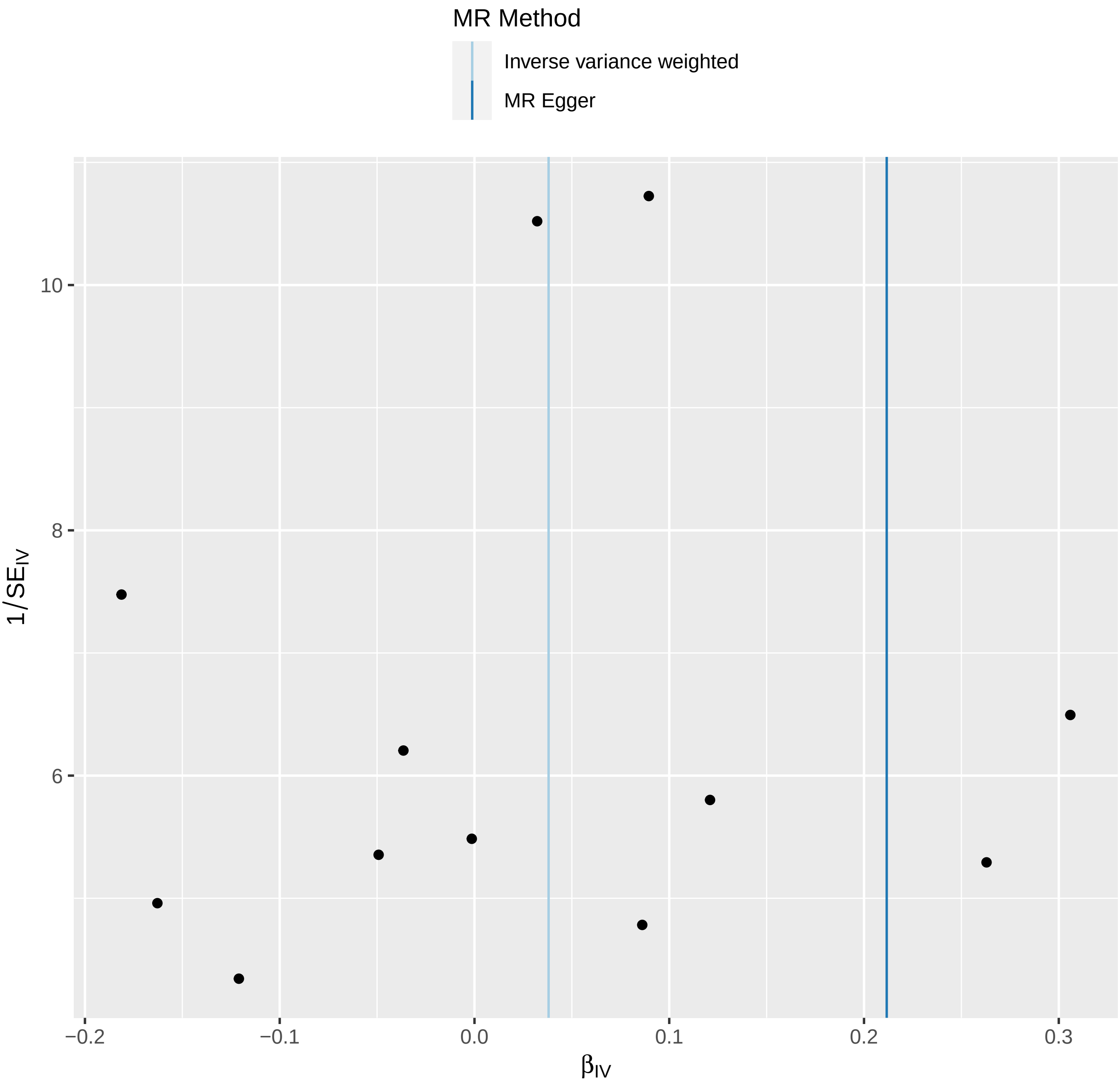

Batch 3−2158 : Selenium on Ubiquitin thioesterase OTUB2

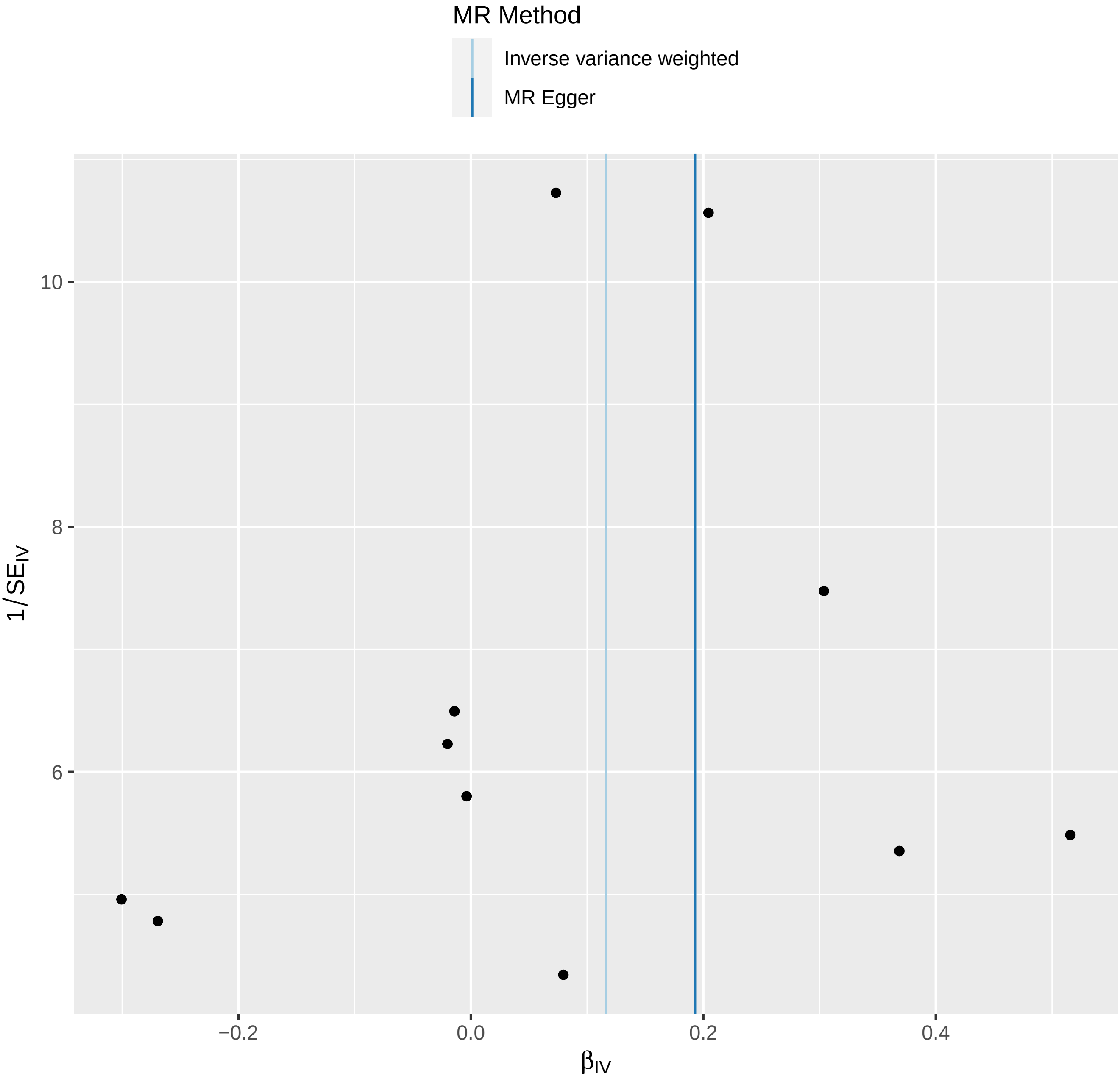

Batch 3−2159 : Selenium on Oxytocin−neurophysin 1

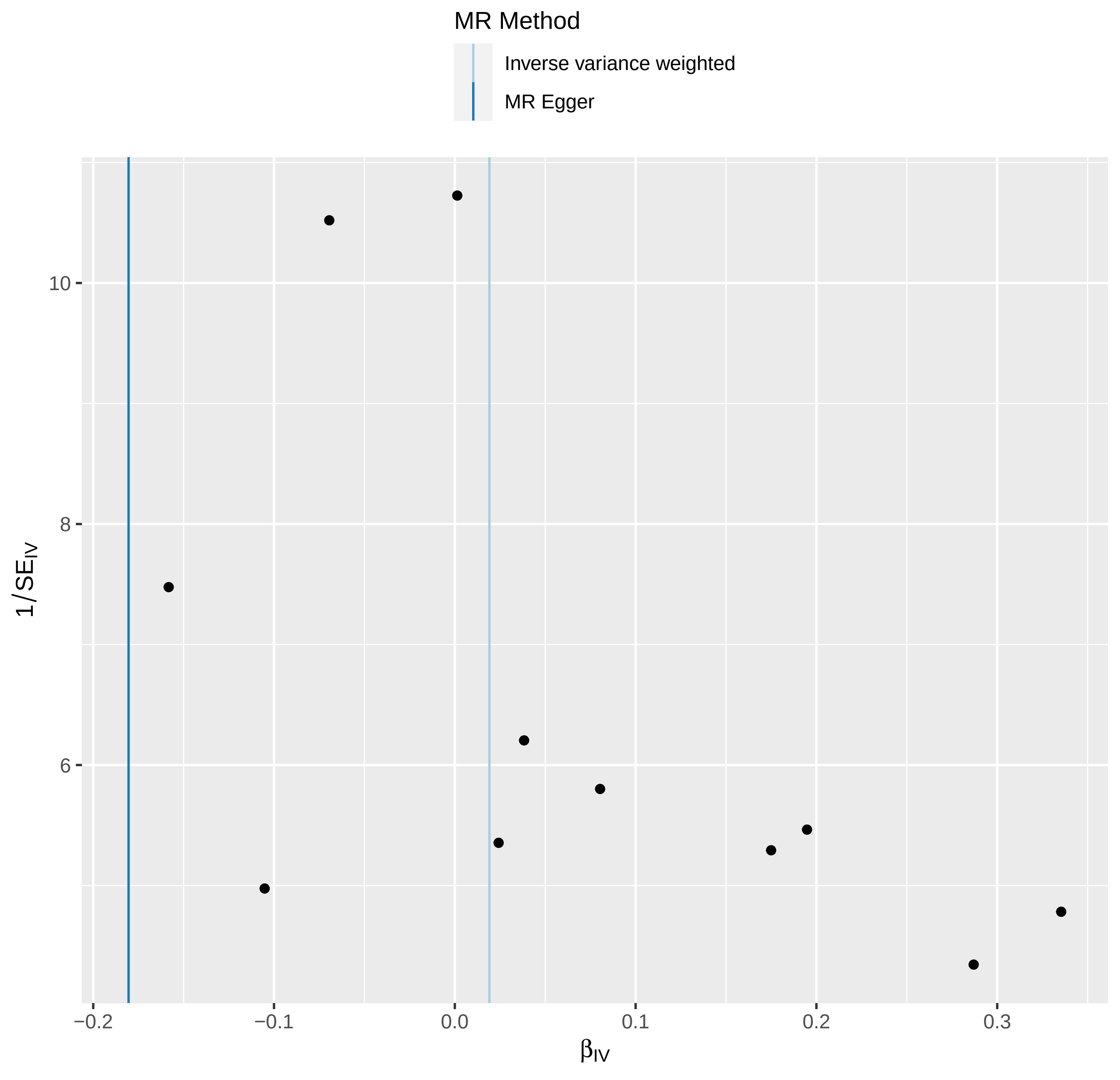

Batch 3−2160 : Selenium on P2X purinoceptor 6

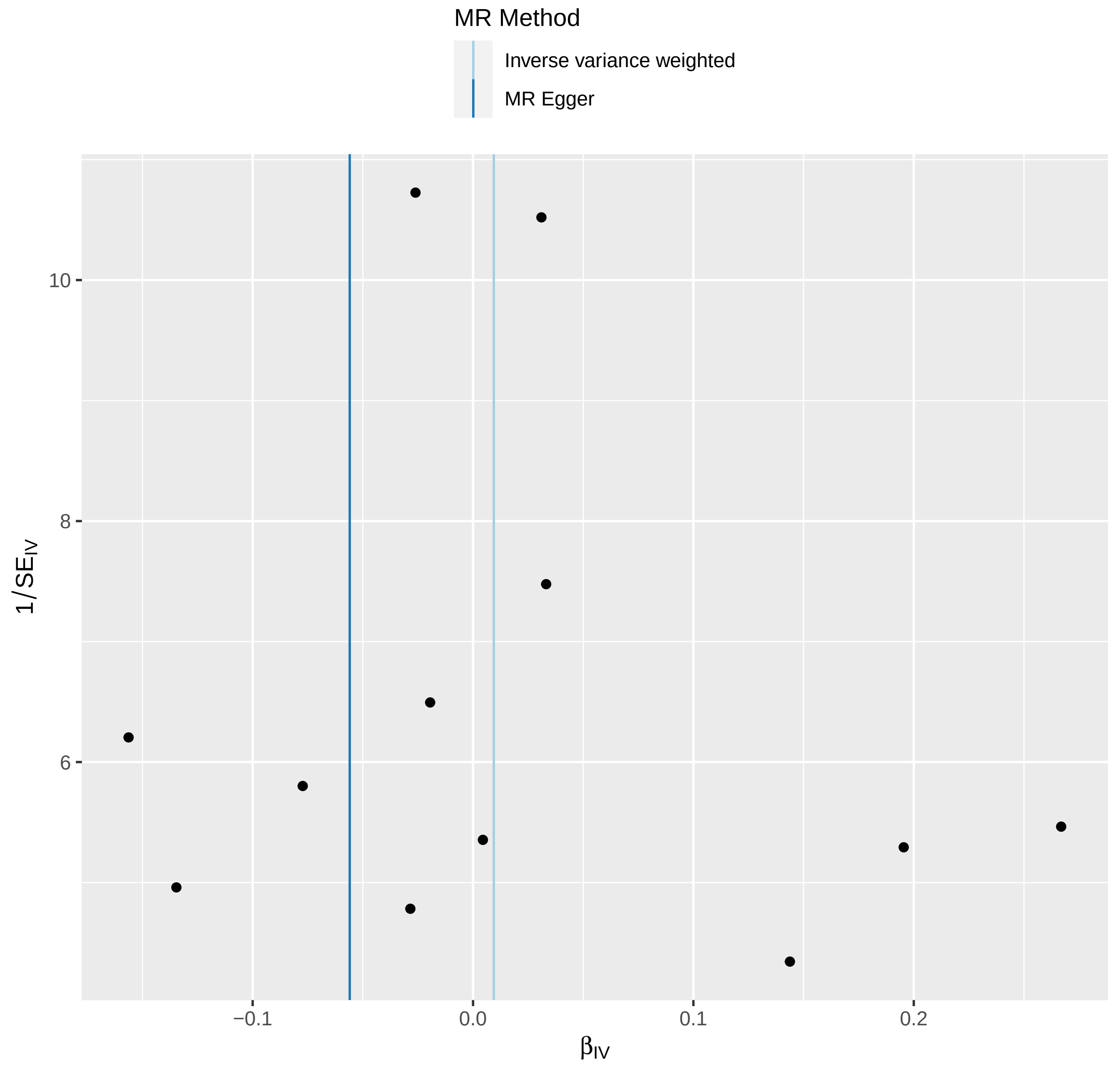

Batch 3−2161 : Selenium on Prolyl 4−hydroxylase subunit alpha−1

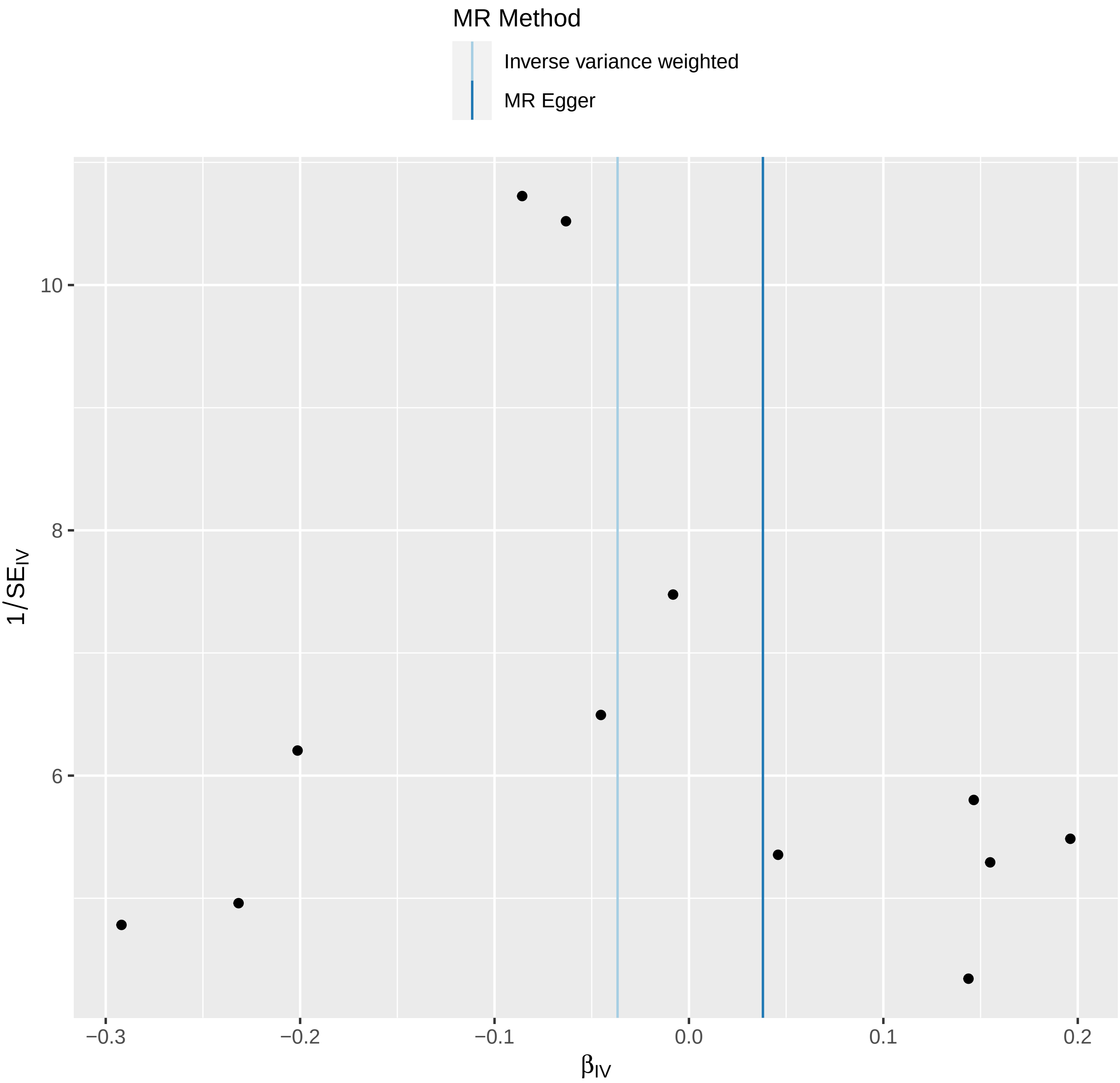

Batch 3−2162 : Selenium on Prolyl 4−hydroxylase subunit alpha−2

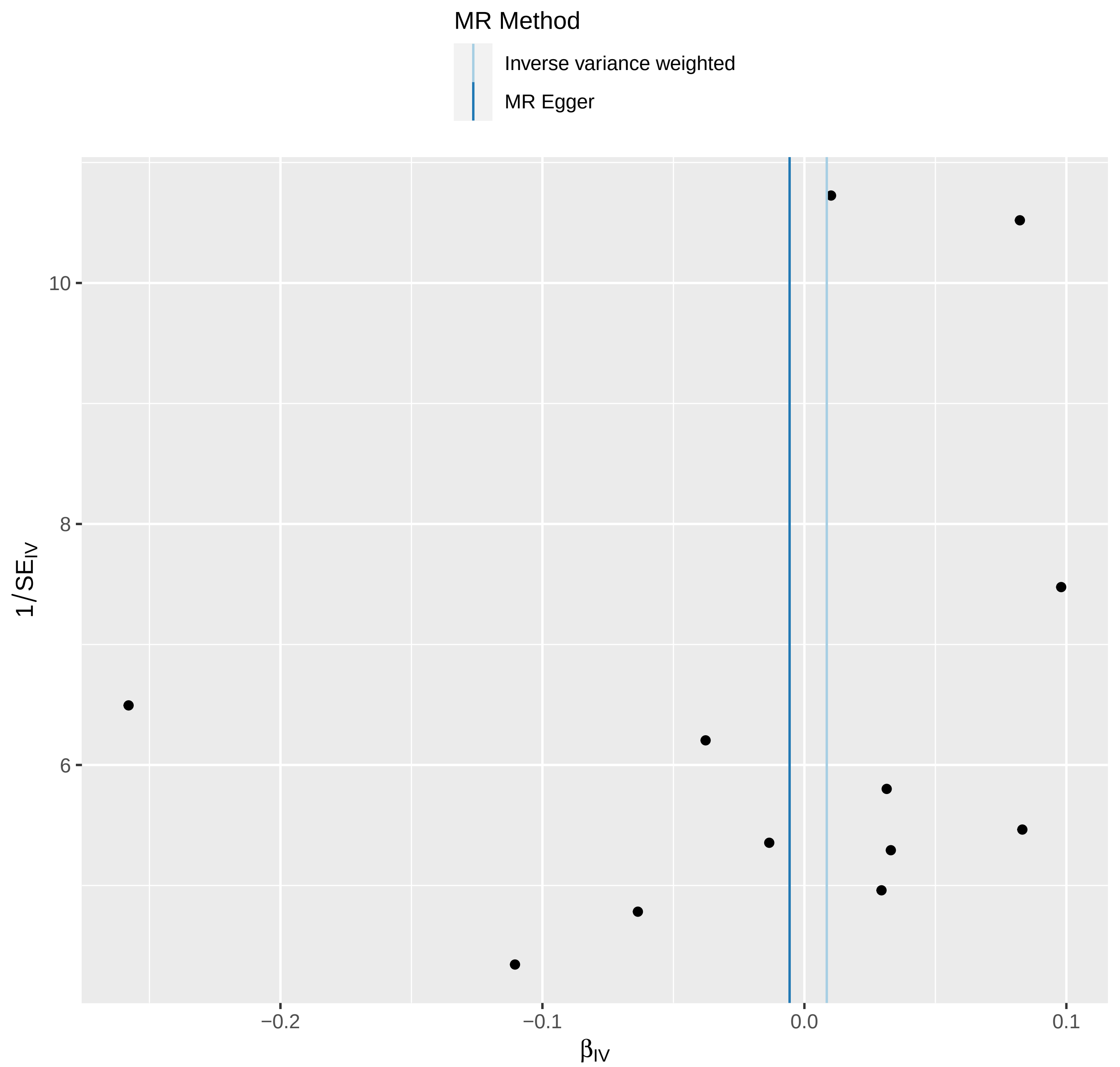

Batch 3−2163 : Selenium on Polyadenylate−binding protein 3

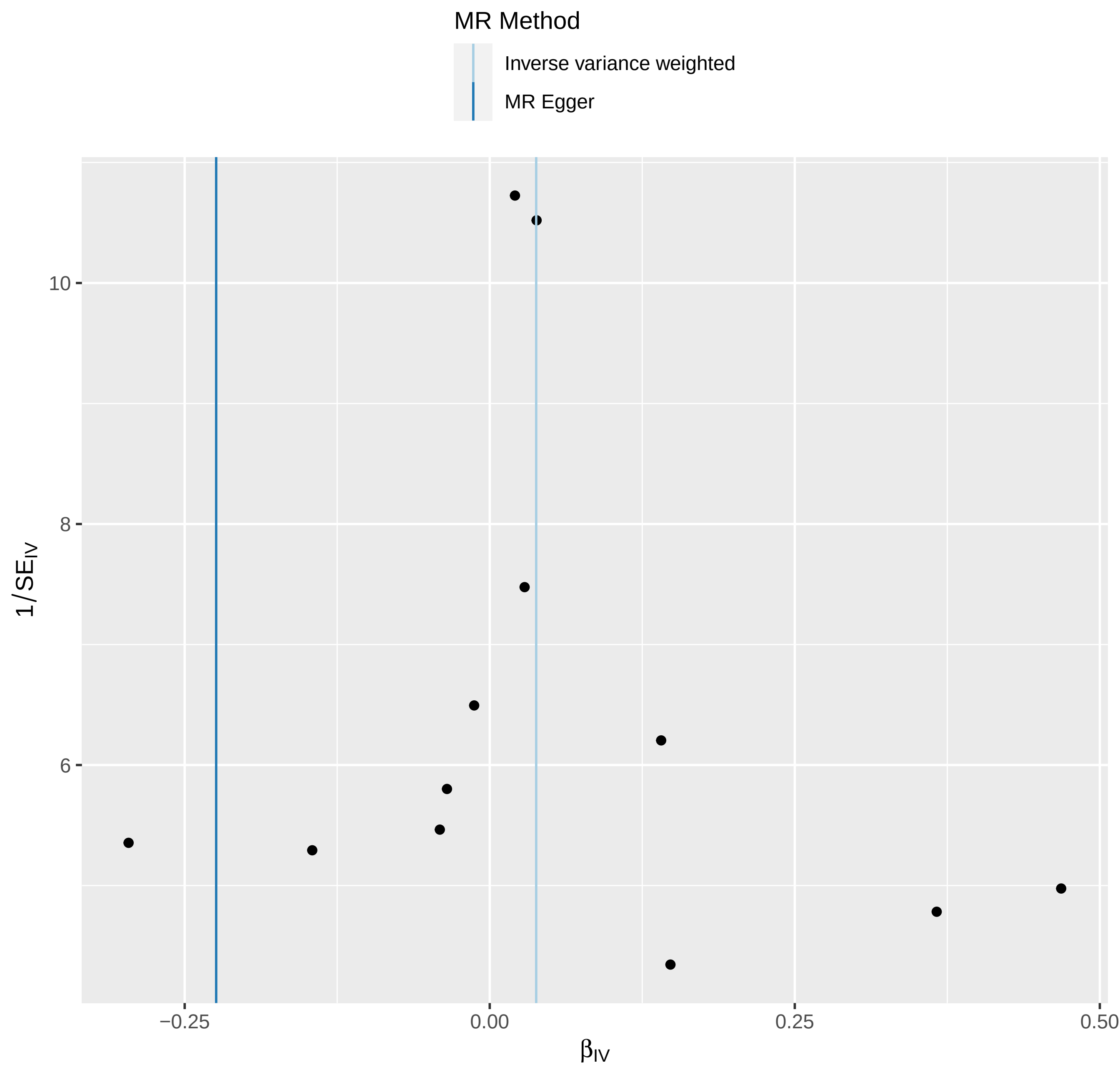

Batch 3−2164 : Selenium on Polyadenylate−binding protein 4

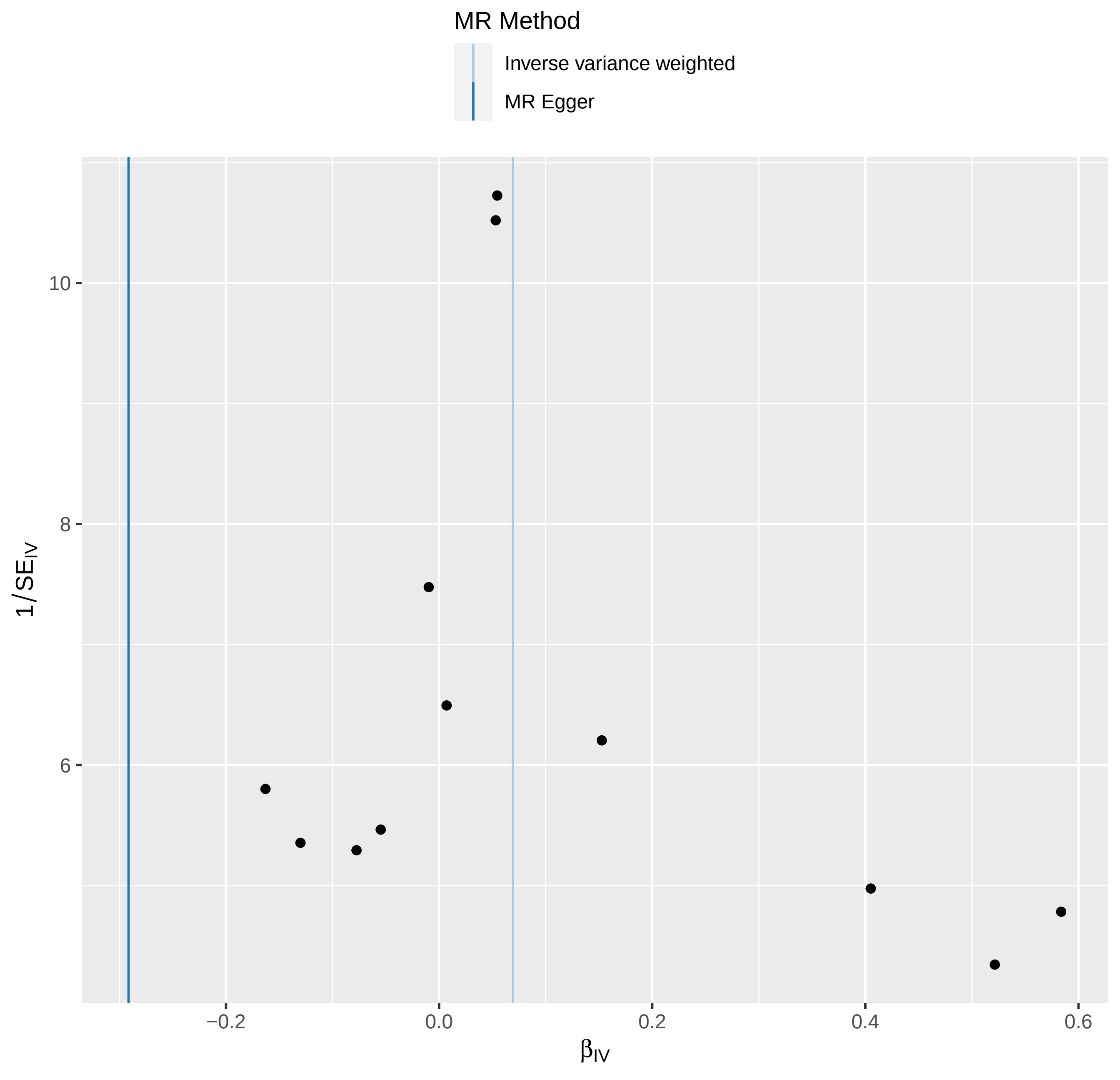

Batch 3−2165 : Selenium on Marginal zone B− and B1−cell−specific protein

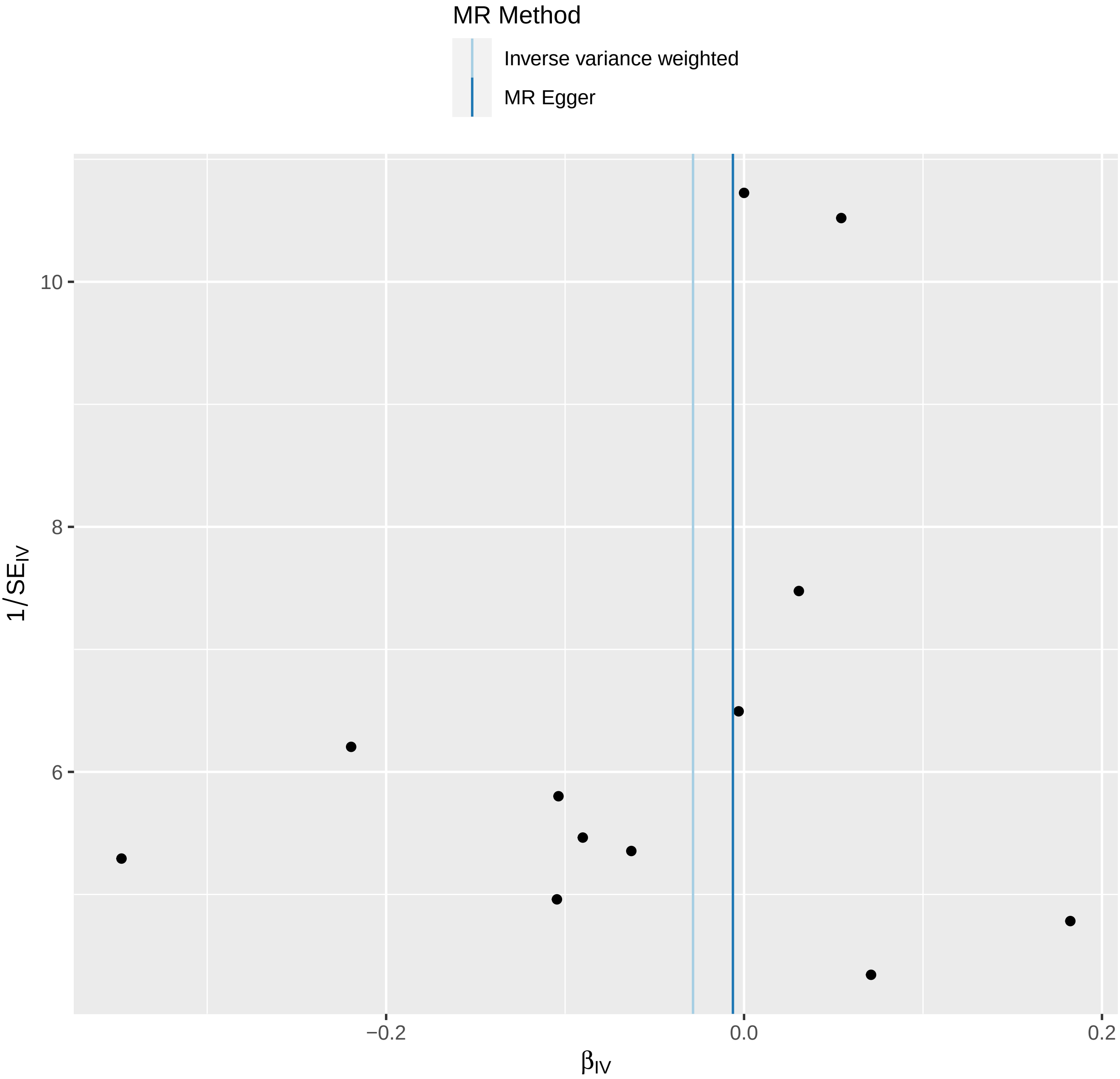

Batch 3−2166 : Selenium on Platelet−activating factor acetylhydrolase IB subunit beta

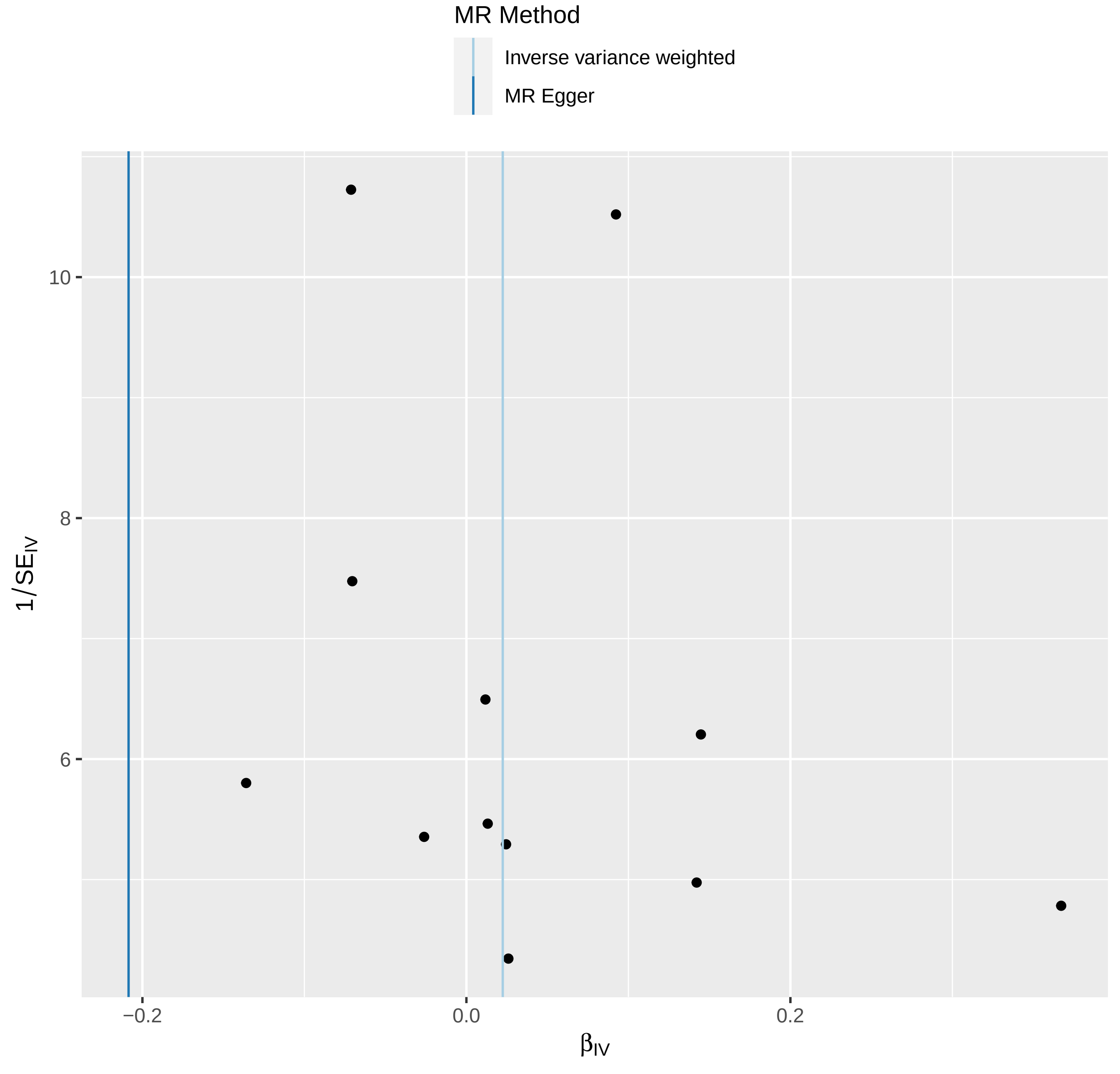

Batch 3−2167 : Selenium on Multifunctional protein ADE2

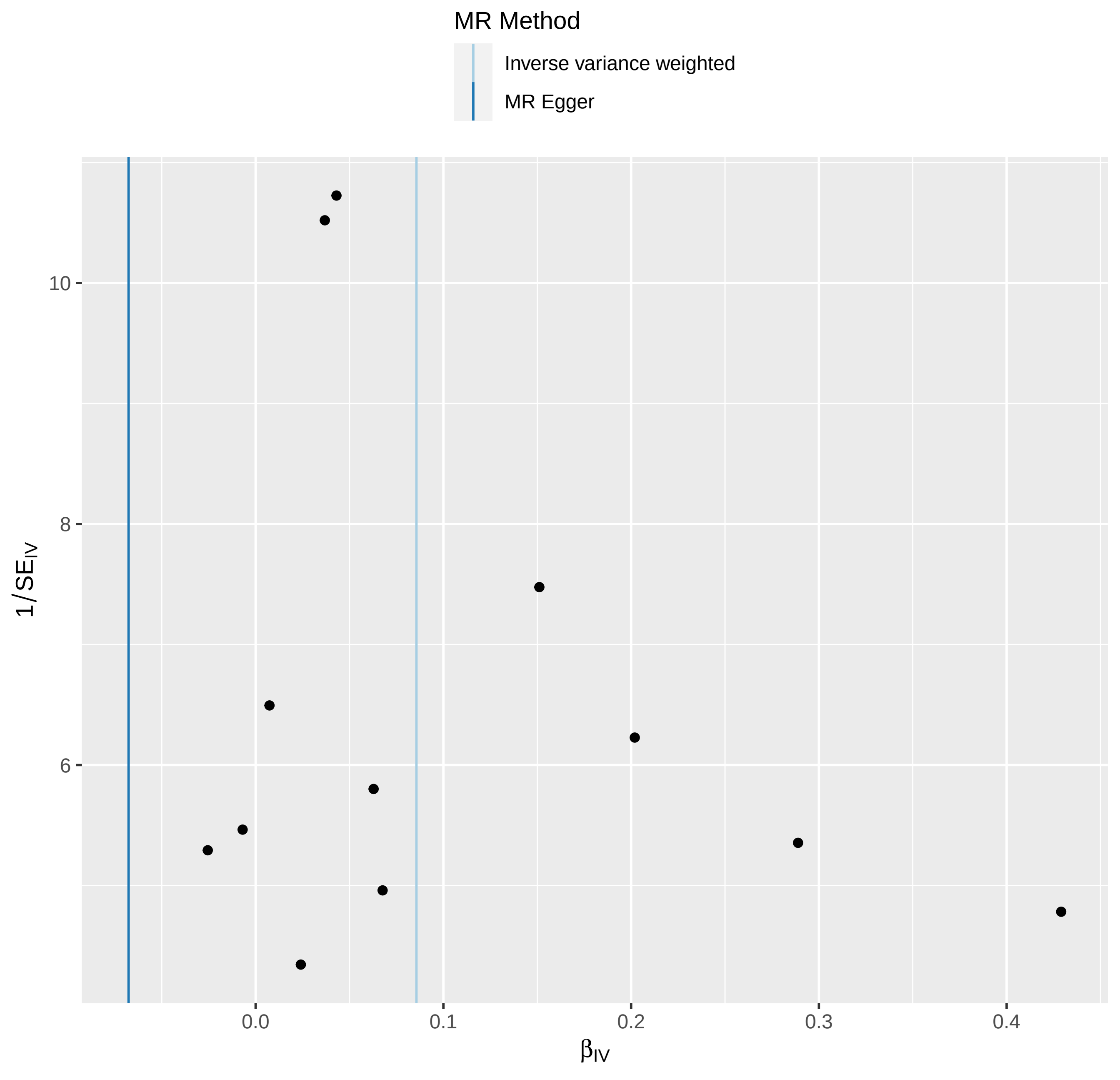

Batch 3−2168 : Selenium on Polyadenylate−binding protein−interacting protein 1

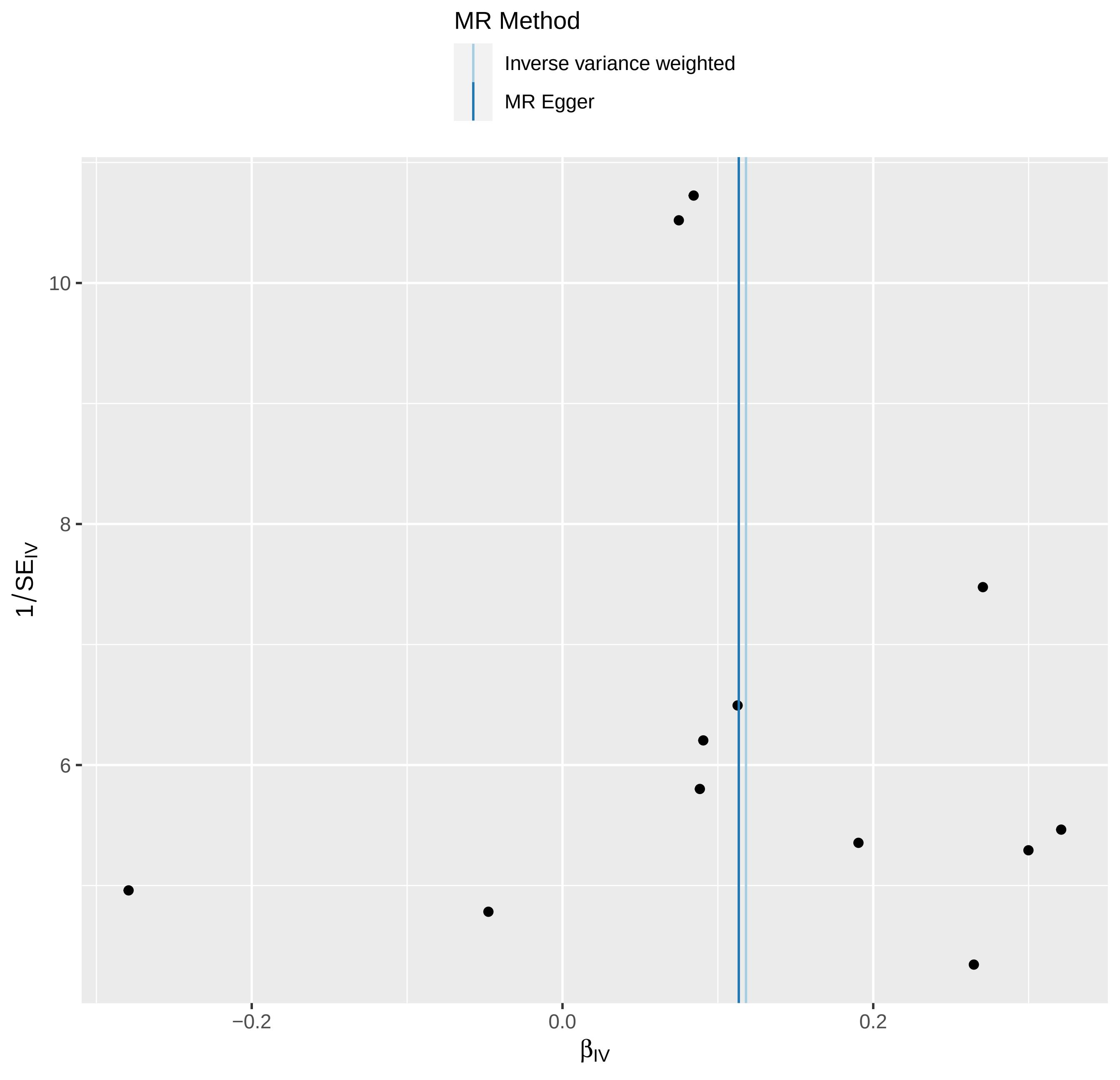

Batch 3−2169 : Selenium on Serine/threonine−protein kinase PAK 3

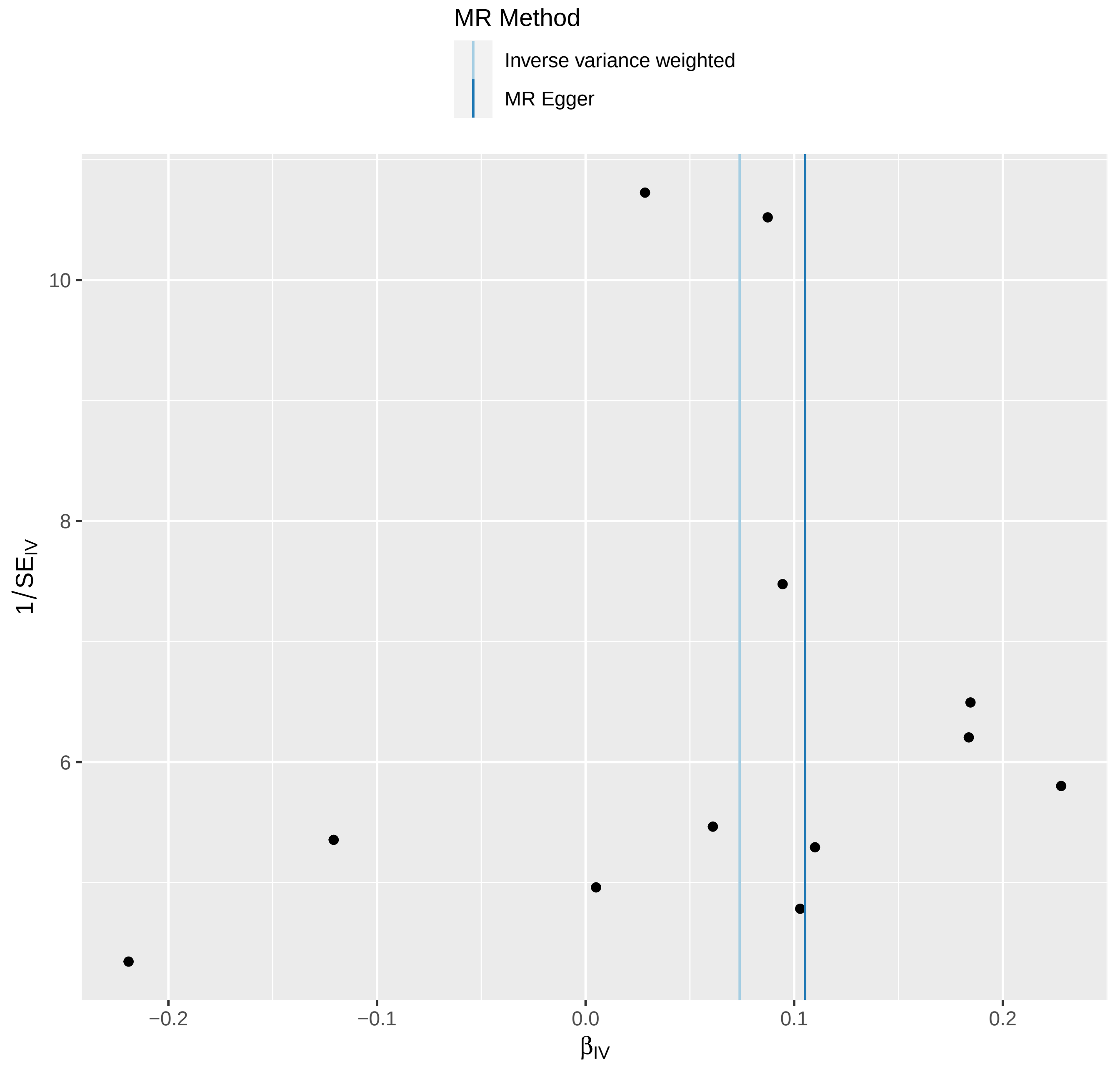

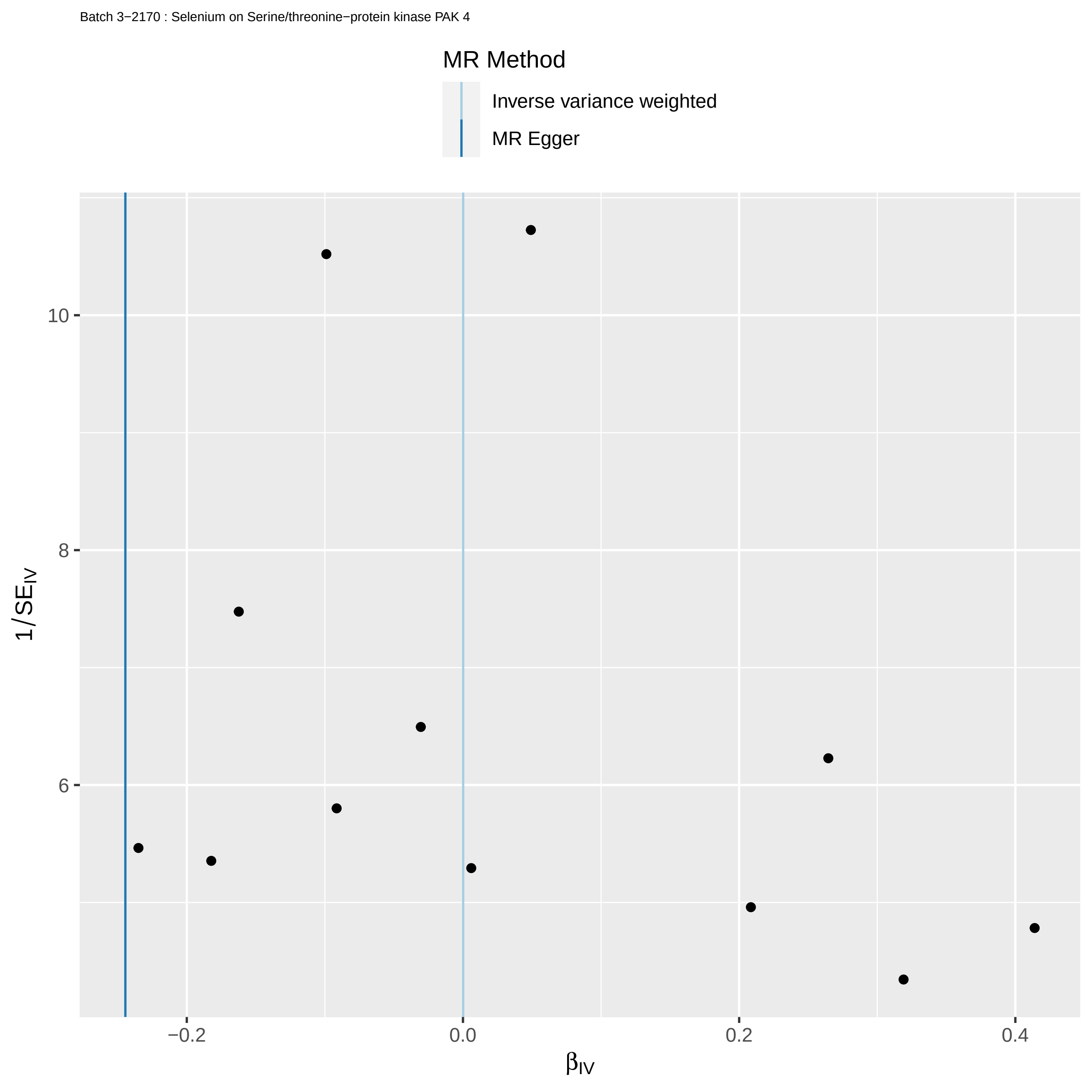
Batch 3−2170 : Selenium on Serine/threonine−protein kinase PAK 4
MR Method
Inverse variance weighted
MR Egger
10
8
6
1/SE$_{IV}$
−0.2
0.0
0.2
0.4
β$_{IV}$

Batch 3−2171 : Selenium on Serine/threonine−protein kinase PAK 7

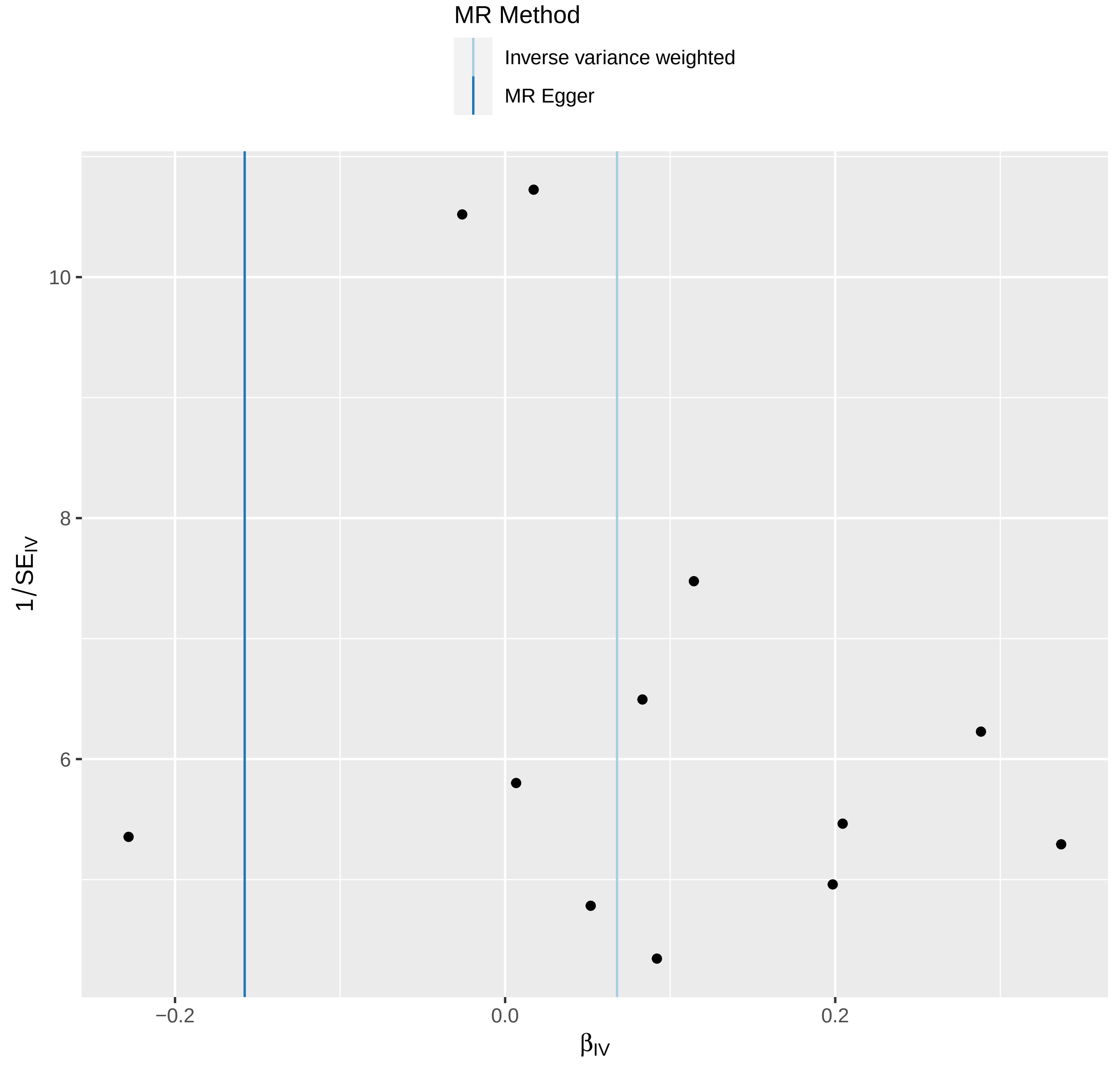

Batch 3−2172 : Selenium on Peptidyl−glycine alpha−amidating monooxygenase

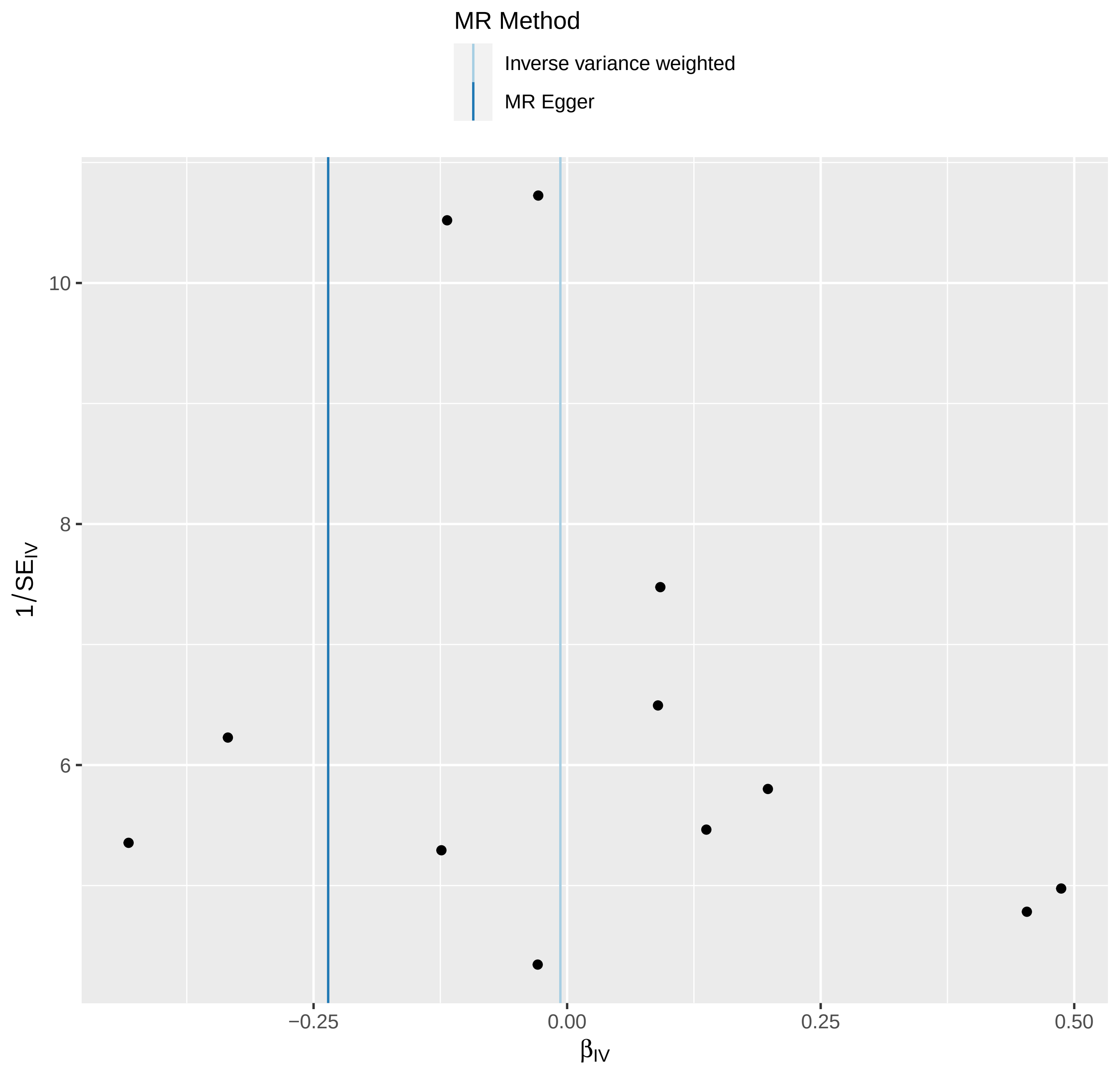

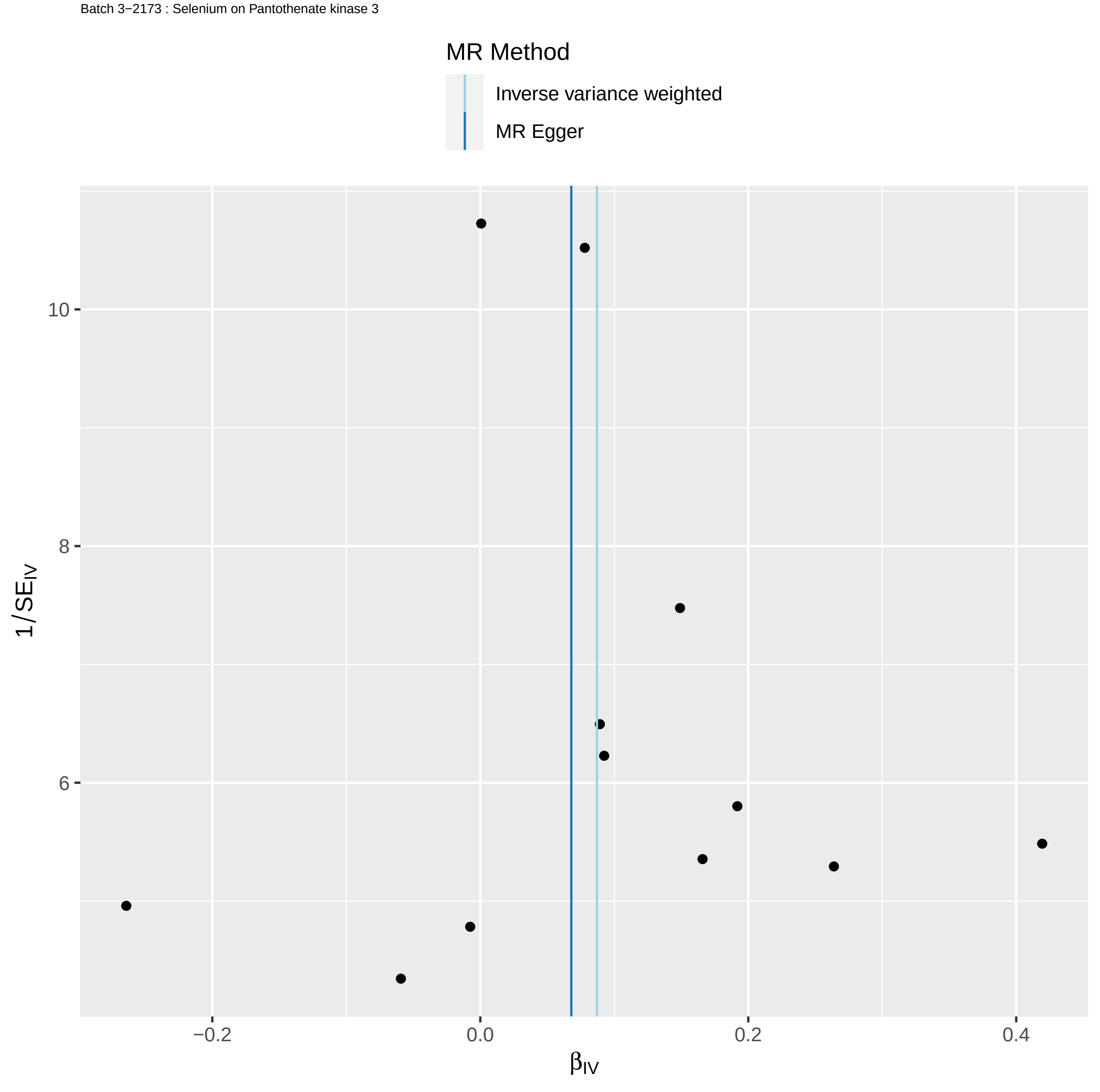
Batch 3−2173 : Selenium on Pantothenate kinase 3
MR Method
Inverse variance weighted
MR Egger
10
8
6
1/SE_IV
−0.2
0.0
0.2
0.4
β_IV

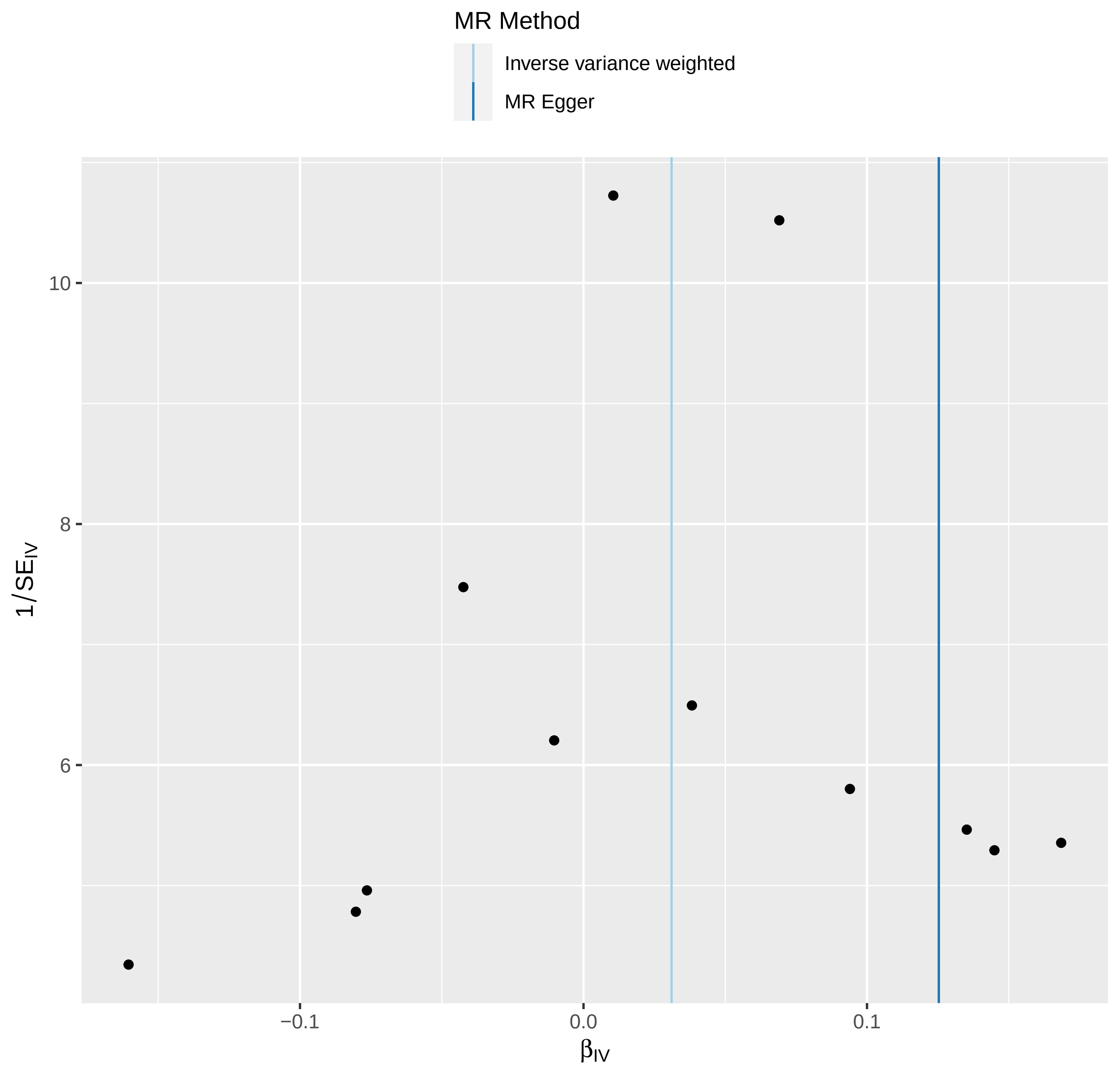
MR Method
Inverse variance weighted
MR Egger
10
8
6
1/SE$_{IV}$
−0.1
0.0
0.1
β_{IV}

Batch 3−2175 : Selenium on Poly(A) polymerase gamma

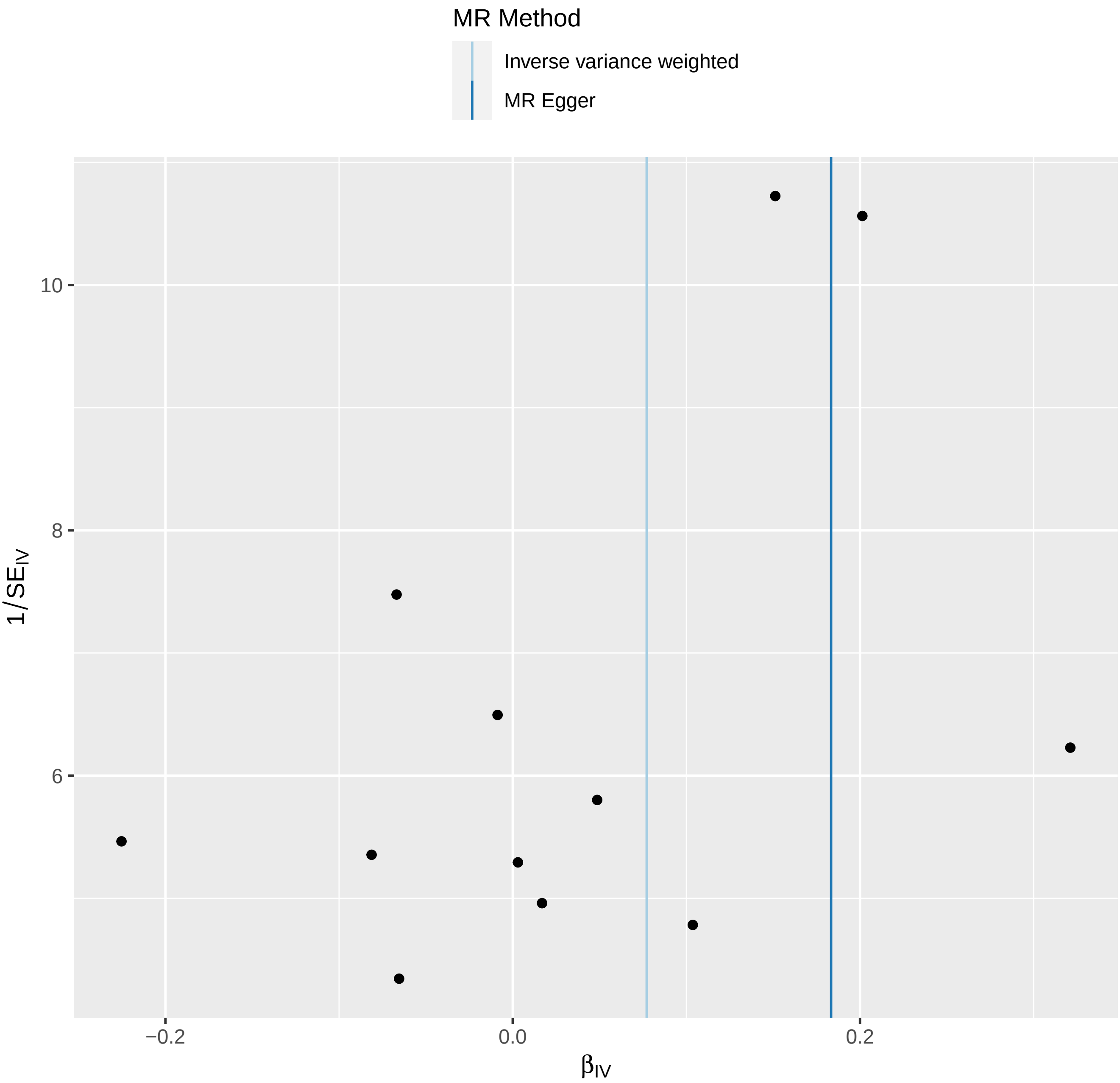

Batch 3−2176 : Selenium on Pappalysin−2

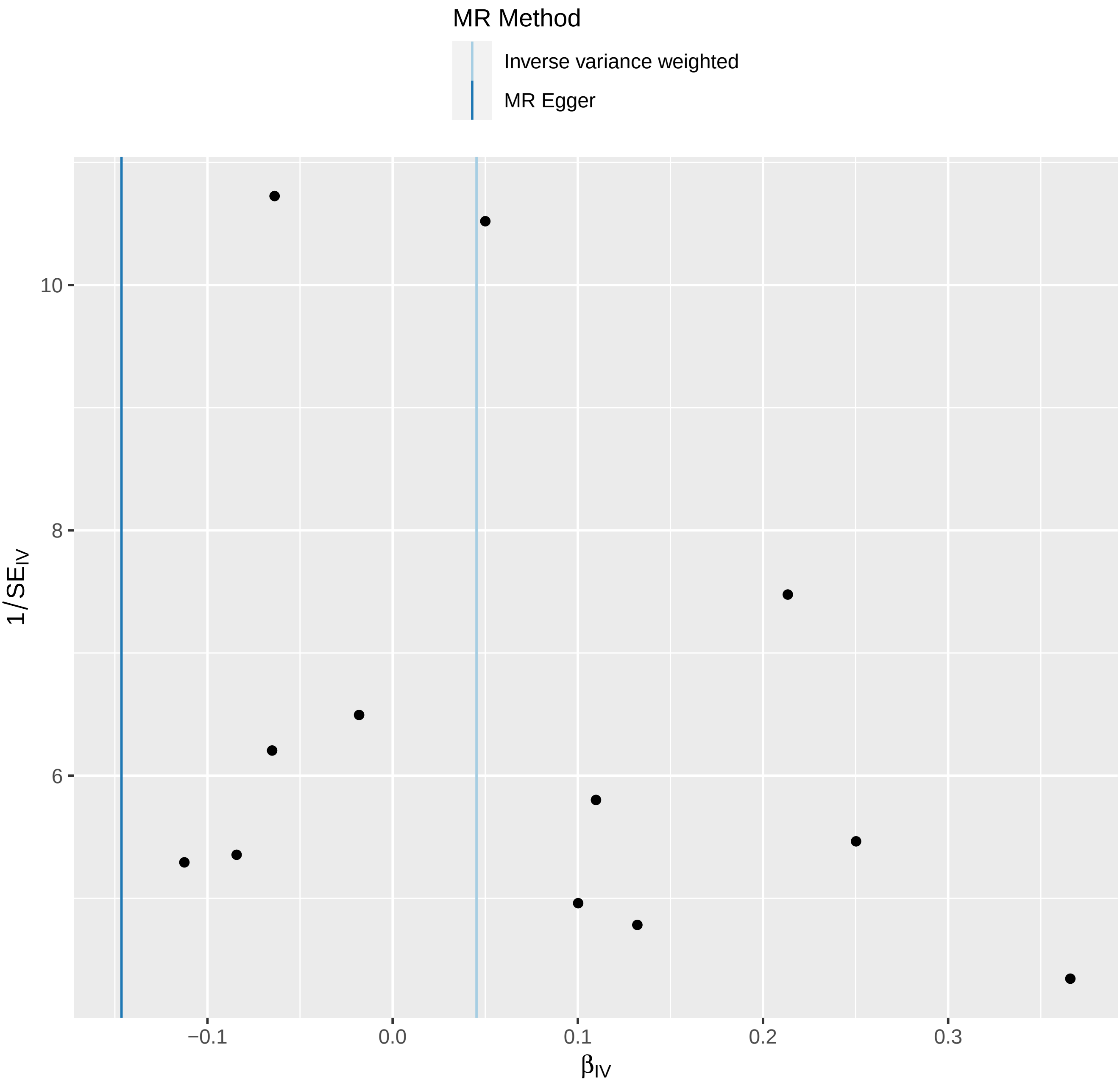

Batch 3−2177 : Selenium on Pappalysin−1

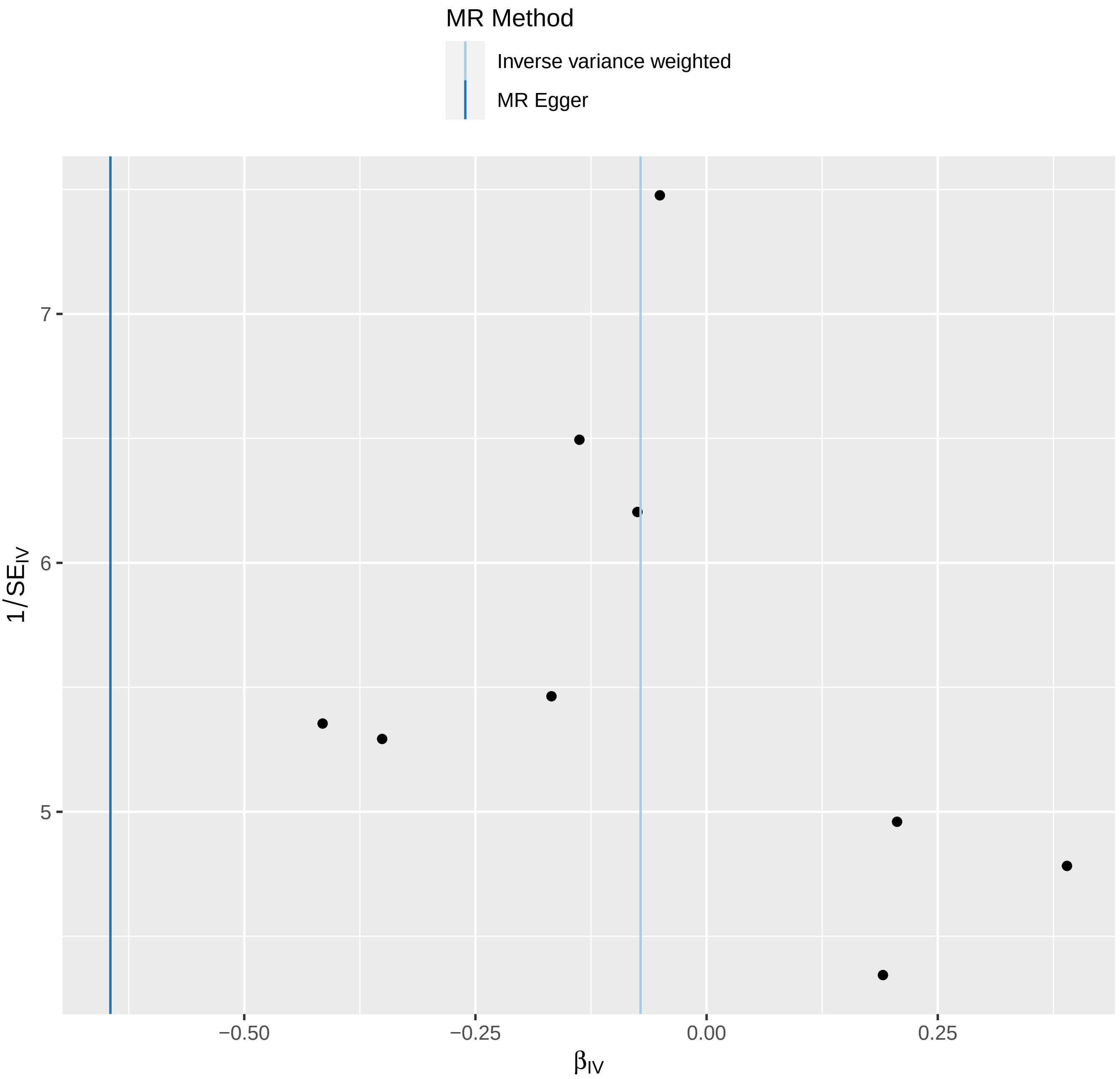

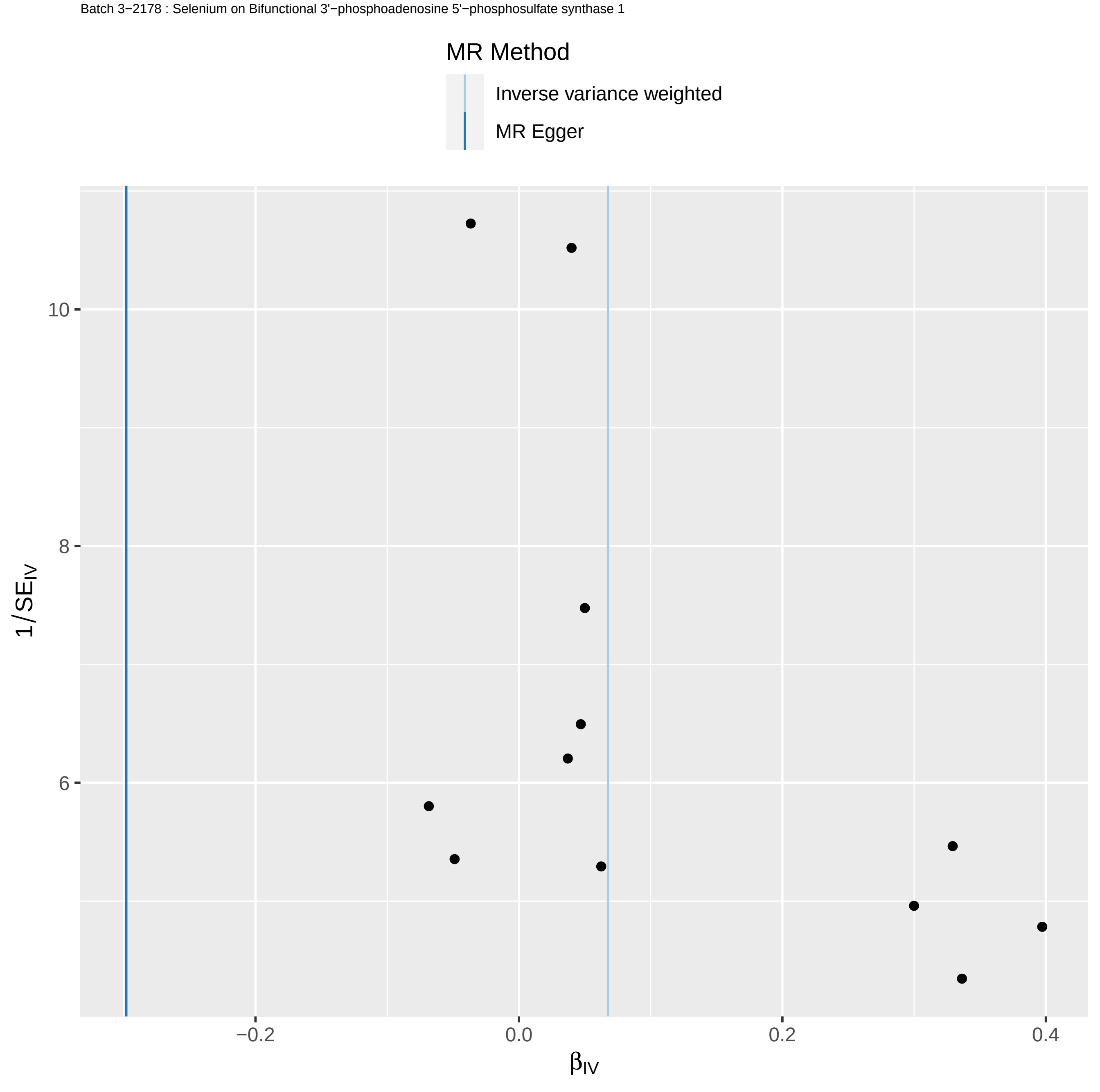
Batch 3−2178 : Selenium on Bifunctional 3'−phosphoadenosine 5'−phosphosulfate synthase 1
MR Method
Inverse variance weighted
MR Egger
10
8
6
1/SE_IV
−0.2
0.0
0.2
0.4
β_IV

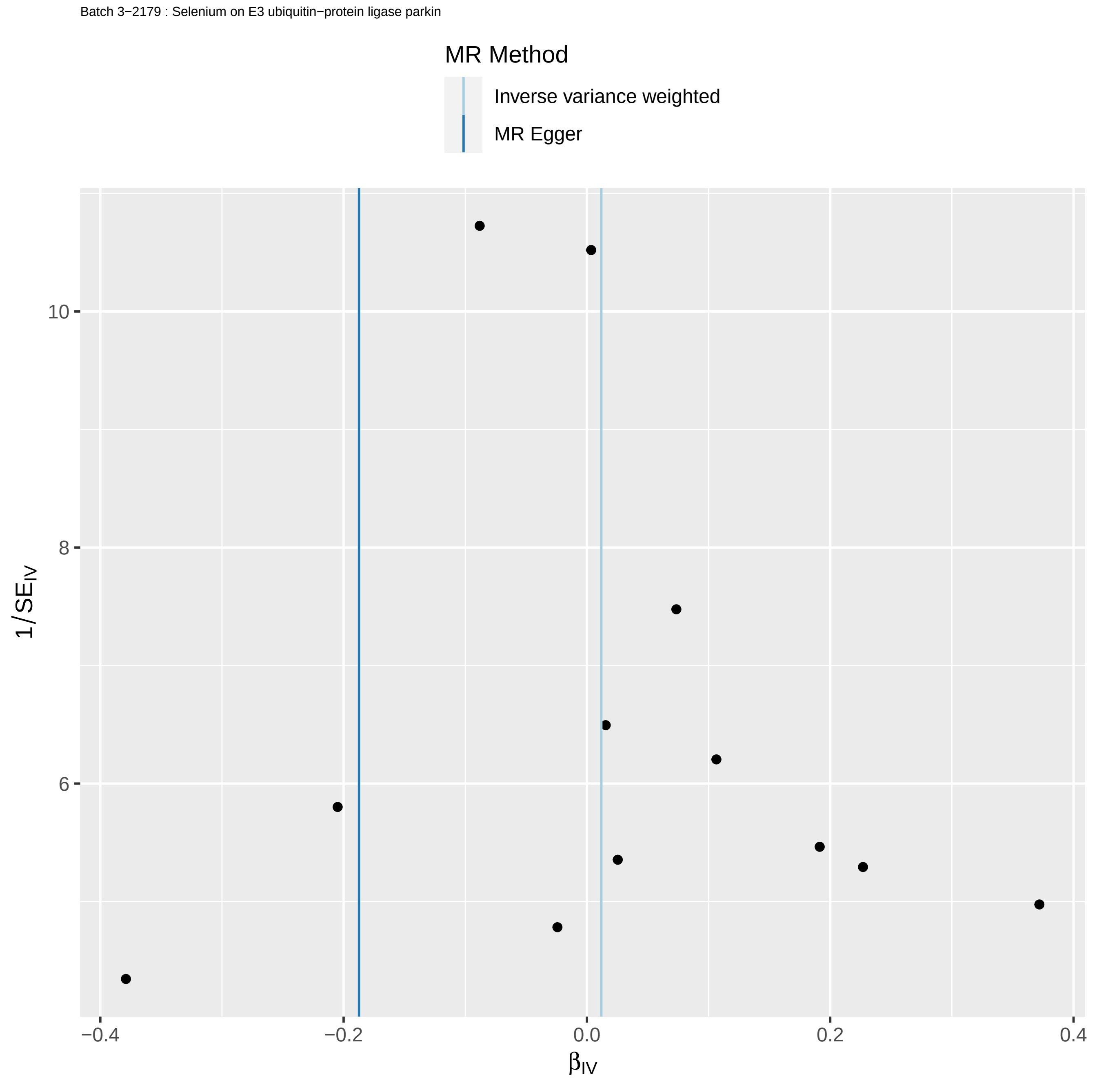

Batch 3−2179 : Selenium on E3 ubiquitin−protein ligase parkin
MR Method
Inverse variance weighted
MR Egger
10
8
6
1/SE$_{IV}$
−0.4
−0.2
0.0
0.2
0.4
β$_{IV}$

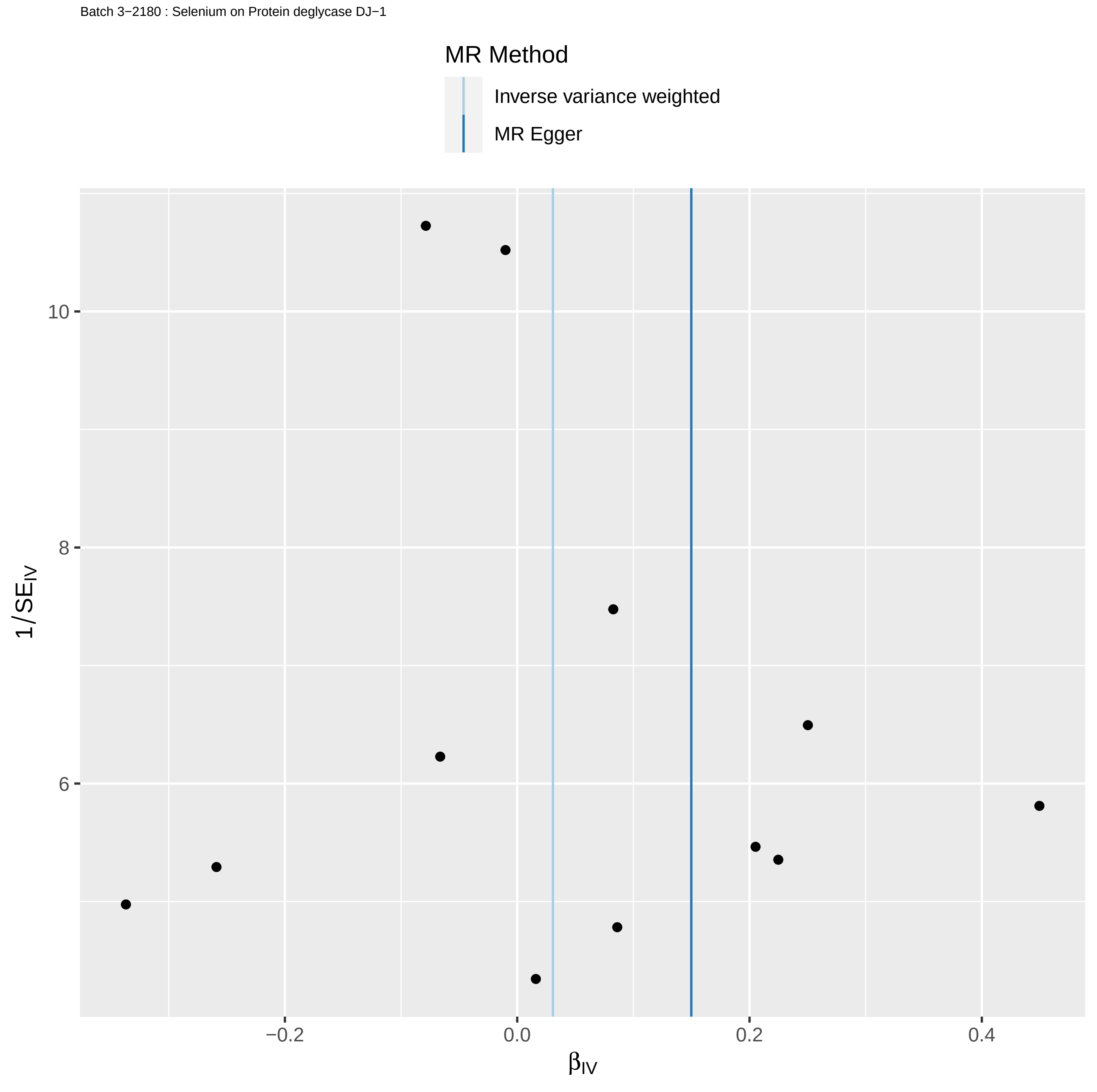
Batch 3−2180 : Selenium on Protein deglycase DJ−1
MR Method
Inverse variance weighted
MR Egger
10
8
6
−0.2
0.0
0.2
0.4
$1/SE_{IV}$
β_{IV}

Batch 3−2181 : Selenium on Protein deglycase DJ−1

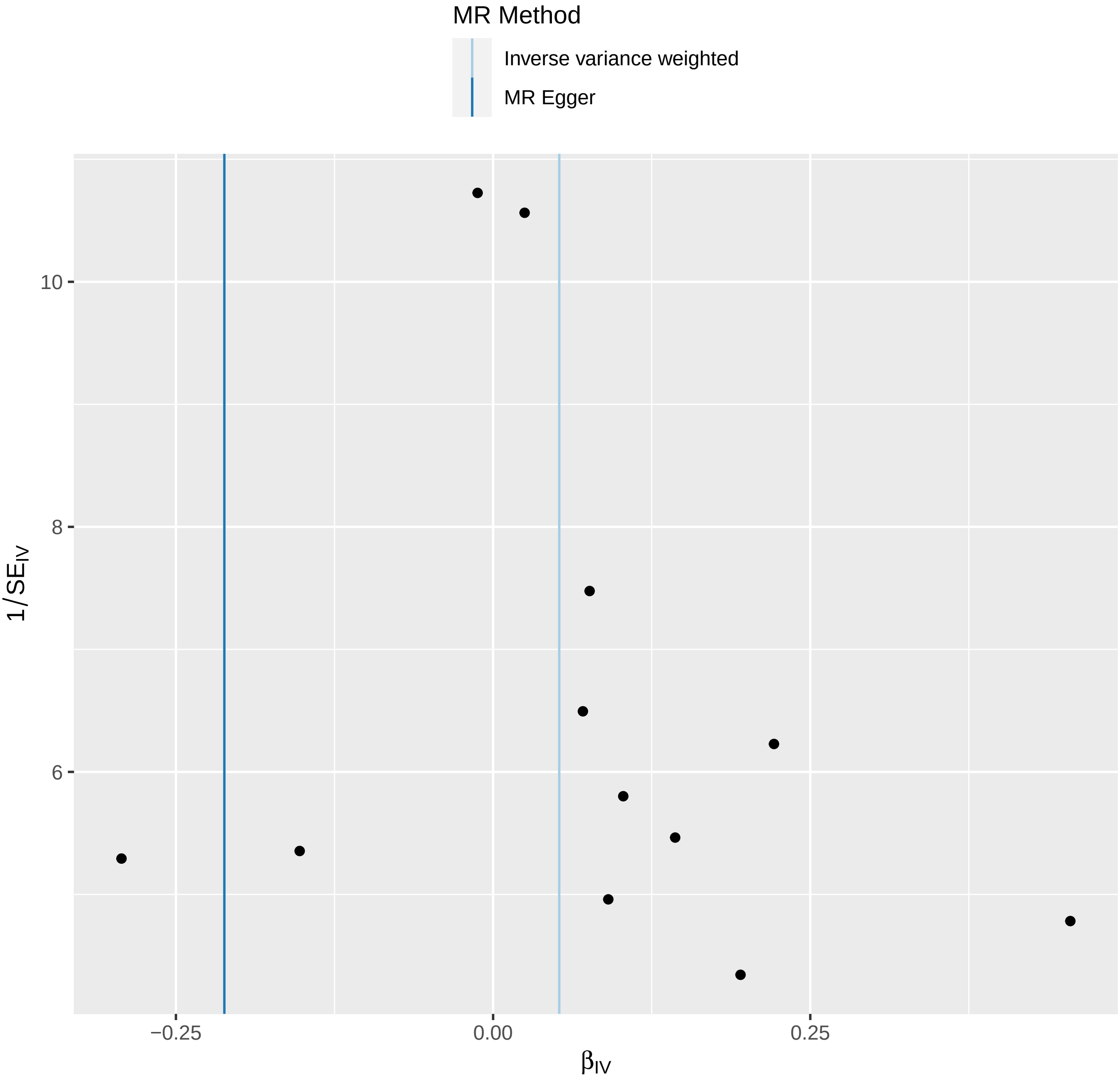

Batch 3−2182 : Selenium on Poly [ADP−ribose] polymerase 1

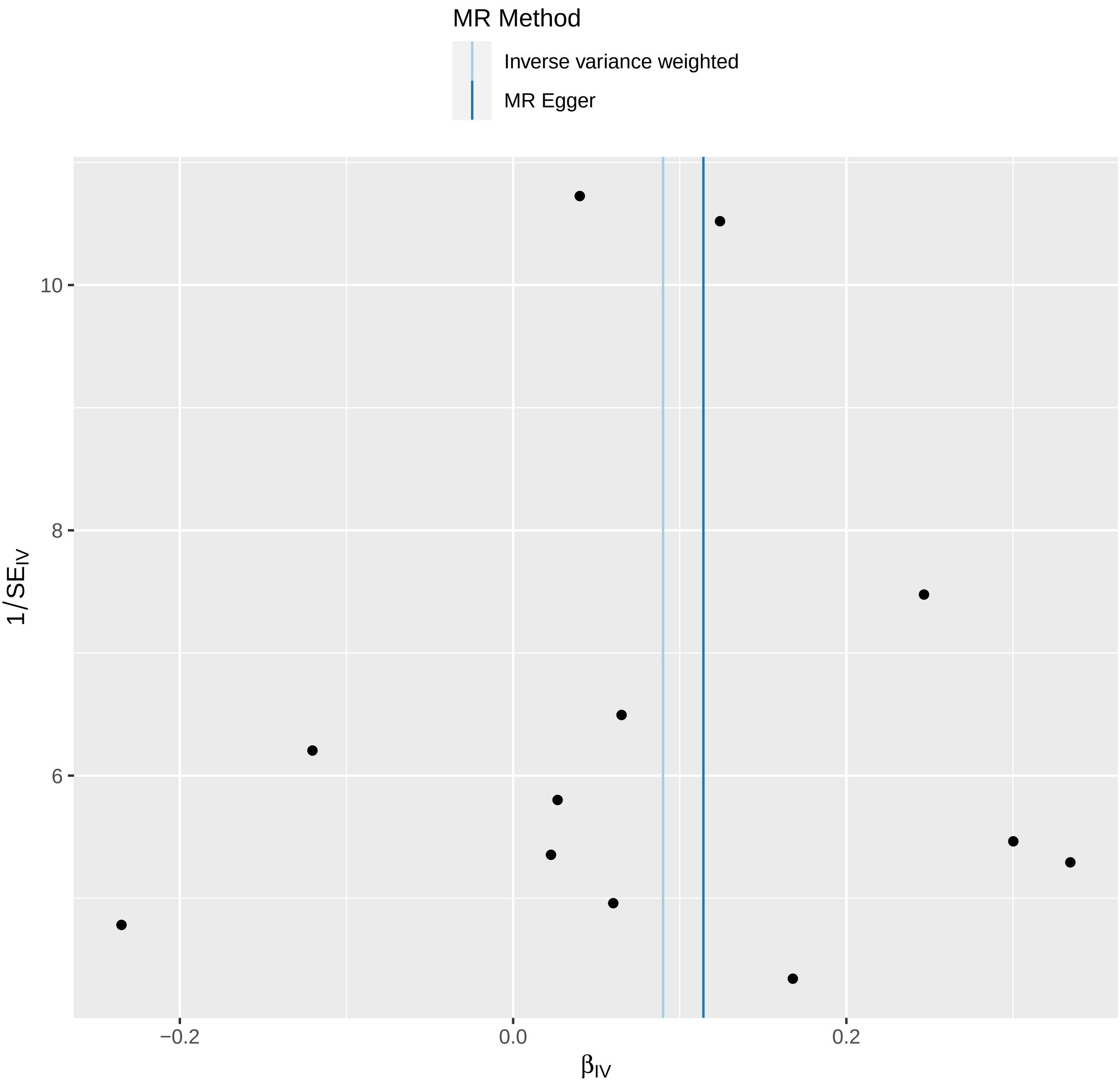

Batch 3−2183 : Selenium on Poly [ADP−ribose] polymerase 11

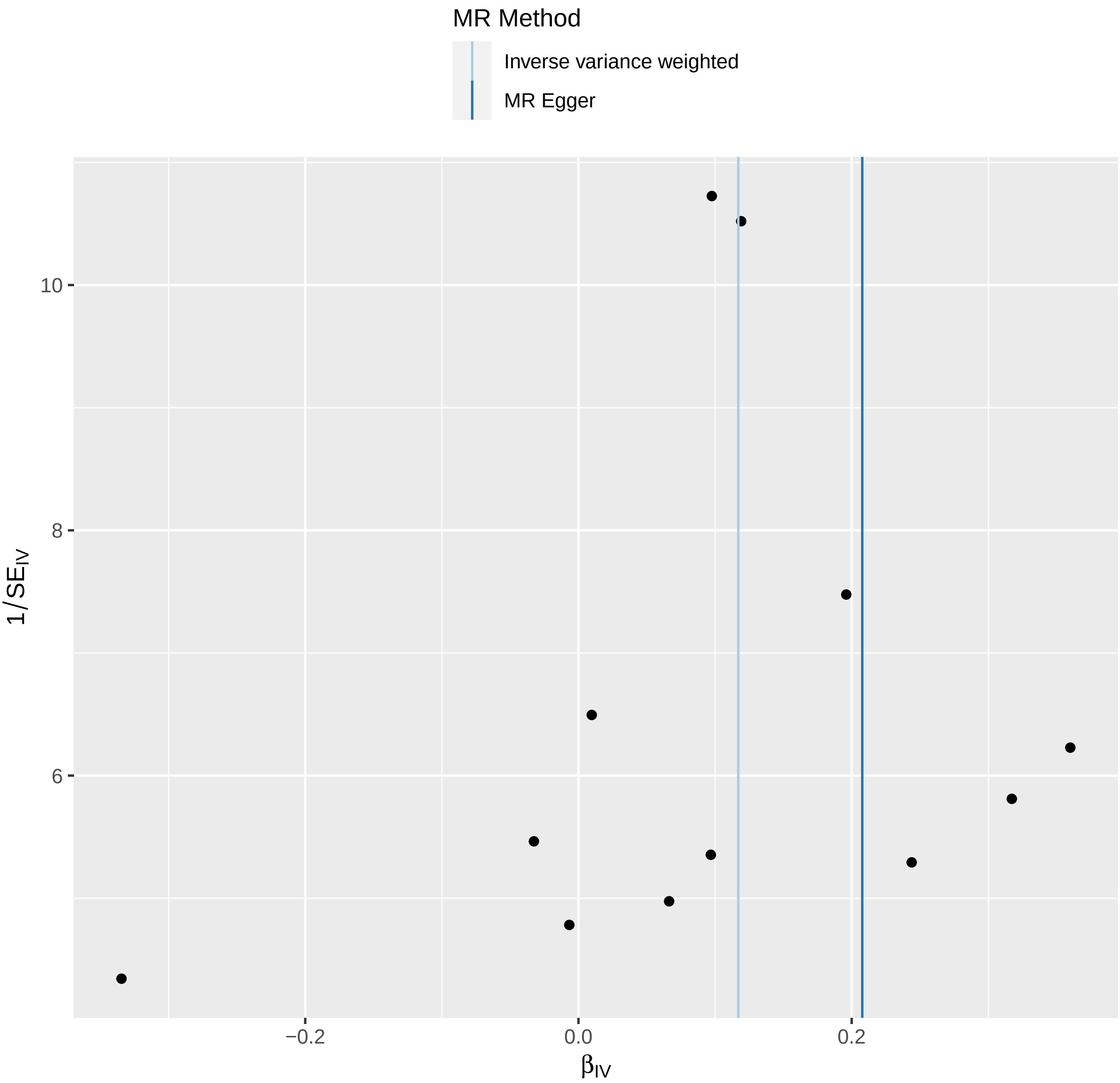

Batch 3−2184 : Selenium on Mono [ADP−ribose] polymerase PARP16

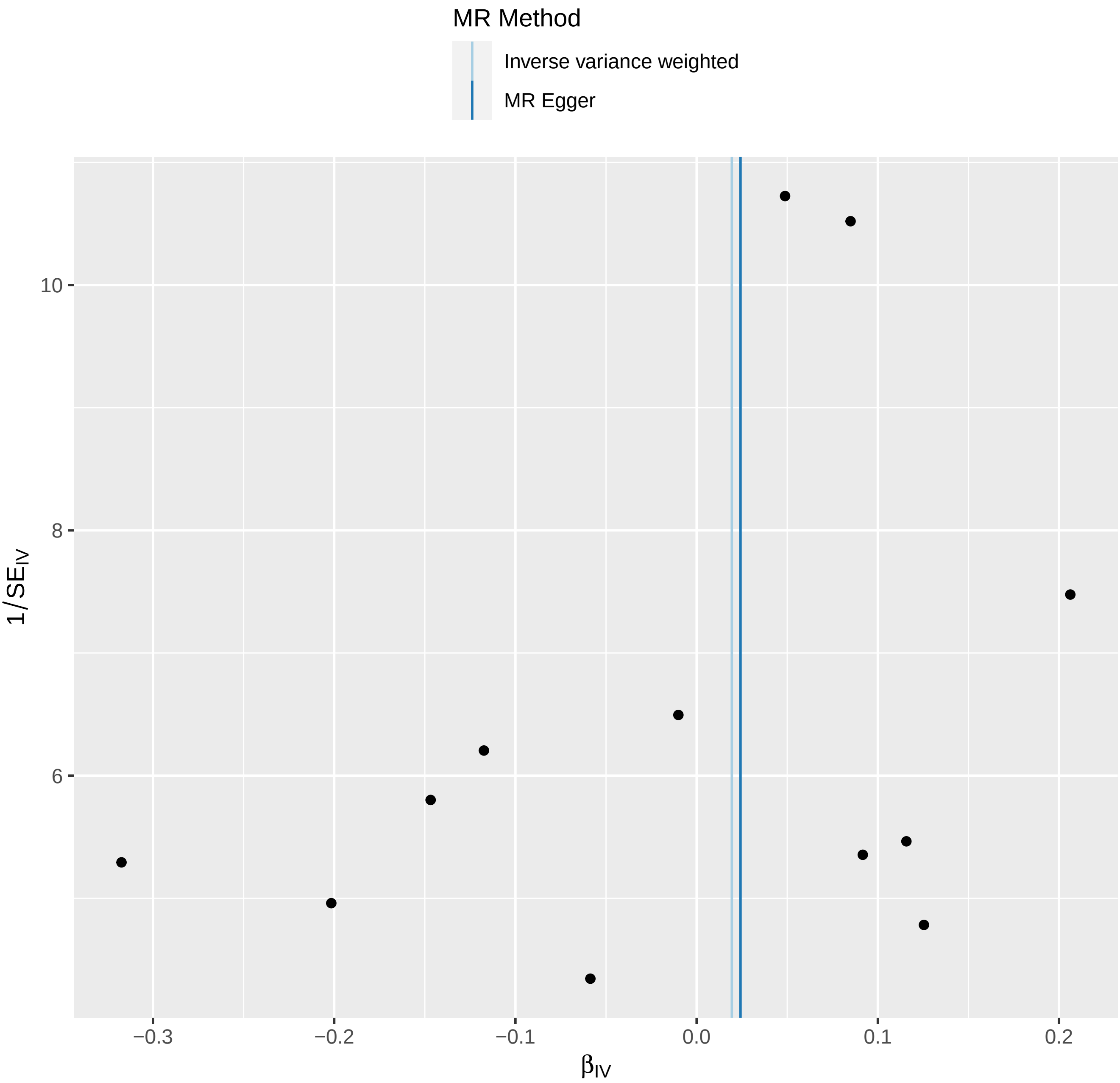

Batch 3−2185 : Selenium on Alpha−parvin

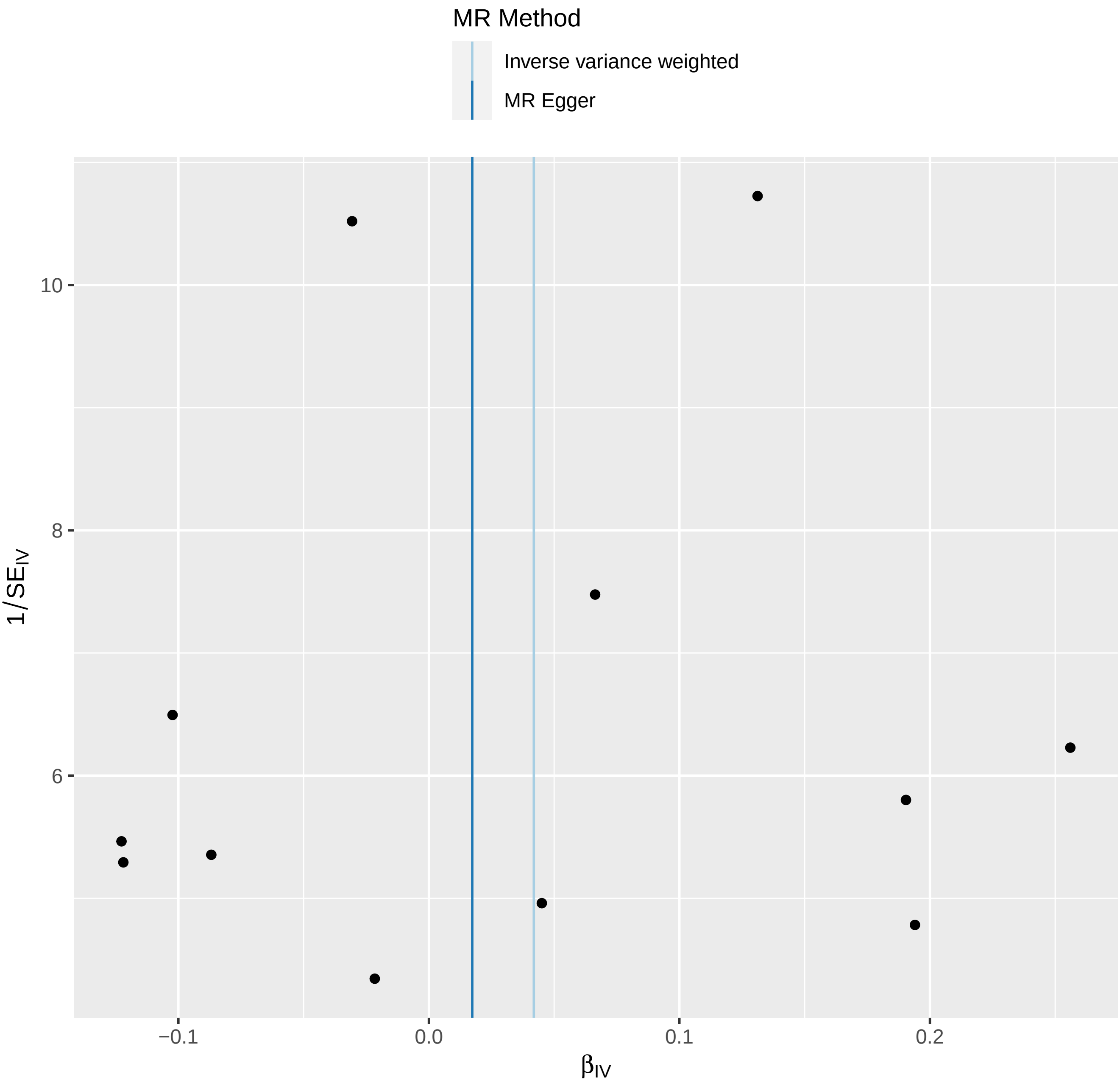

Batch 3−2186 : Selenium on Prostate and testis expressed protein 1

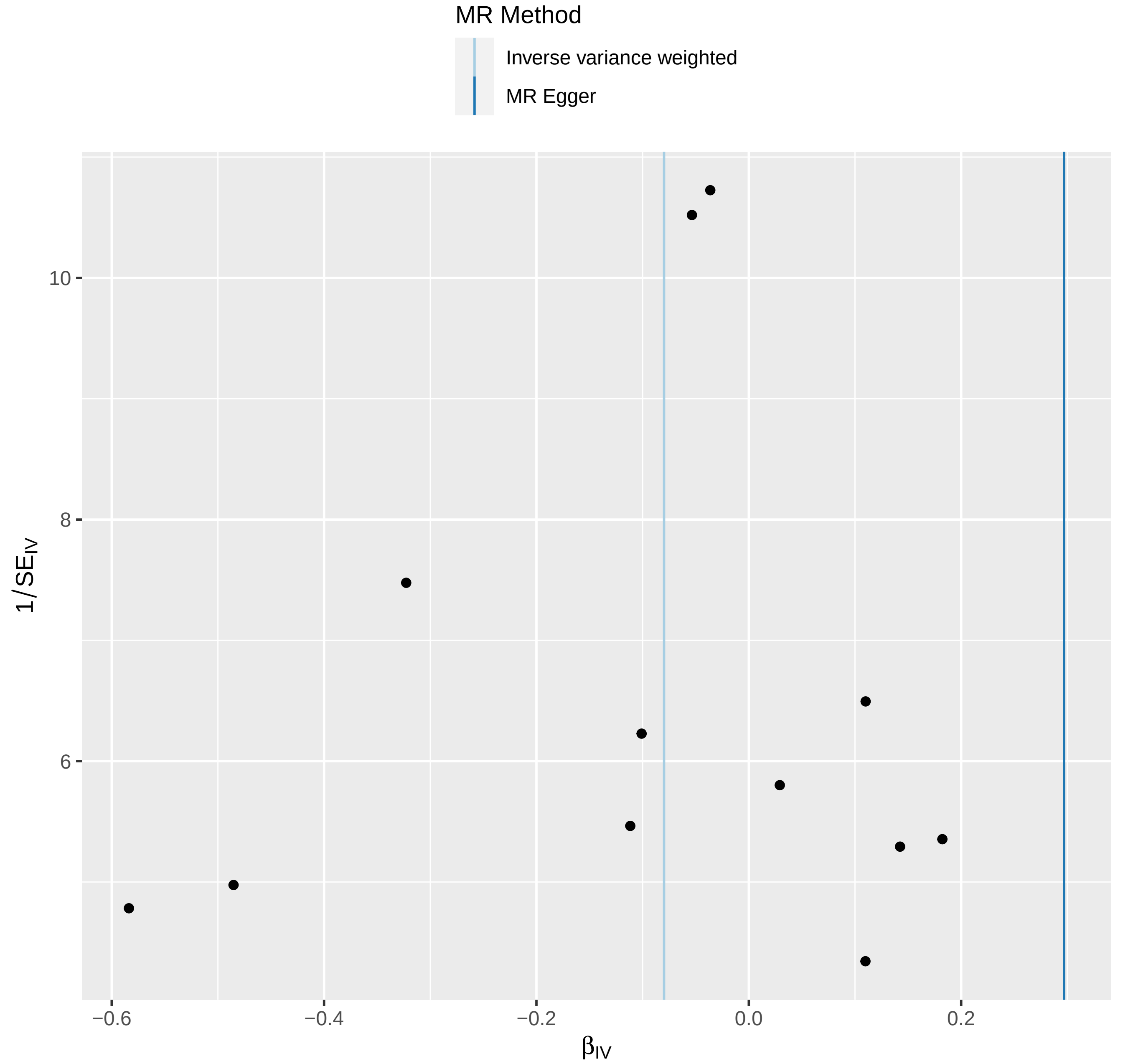

Batch 3−2187 : Selenium on Prostate and testis expressed protein 4

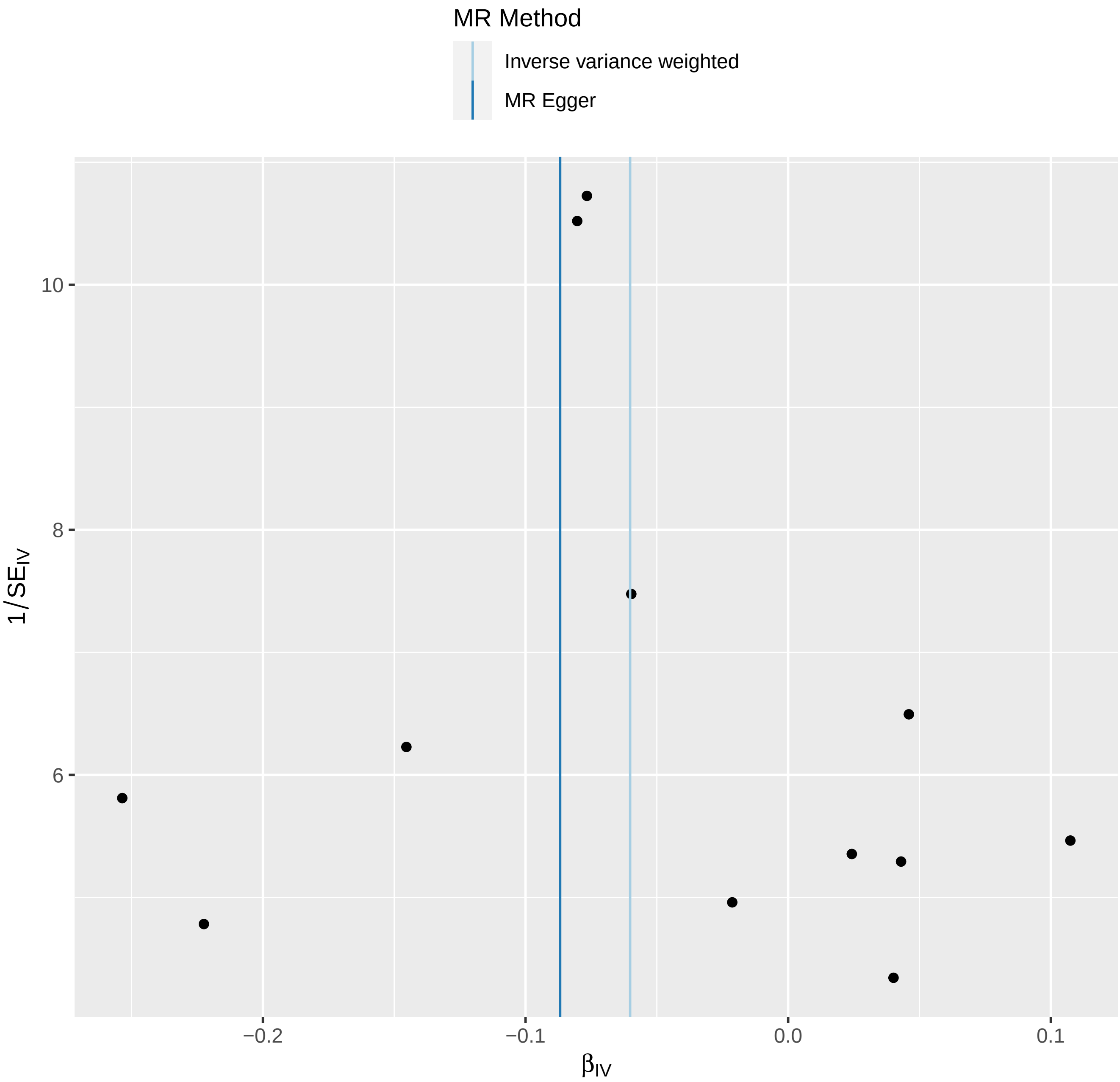

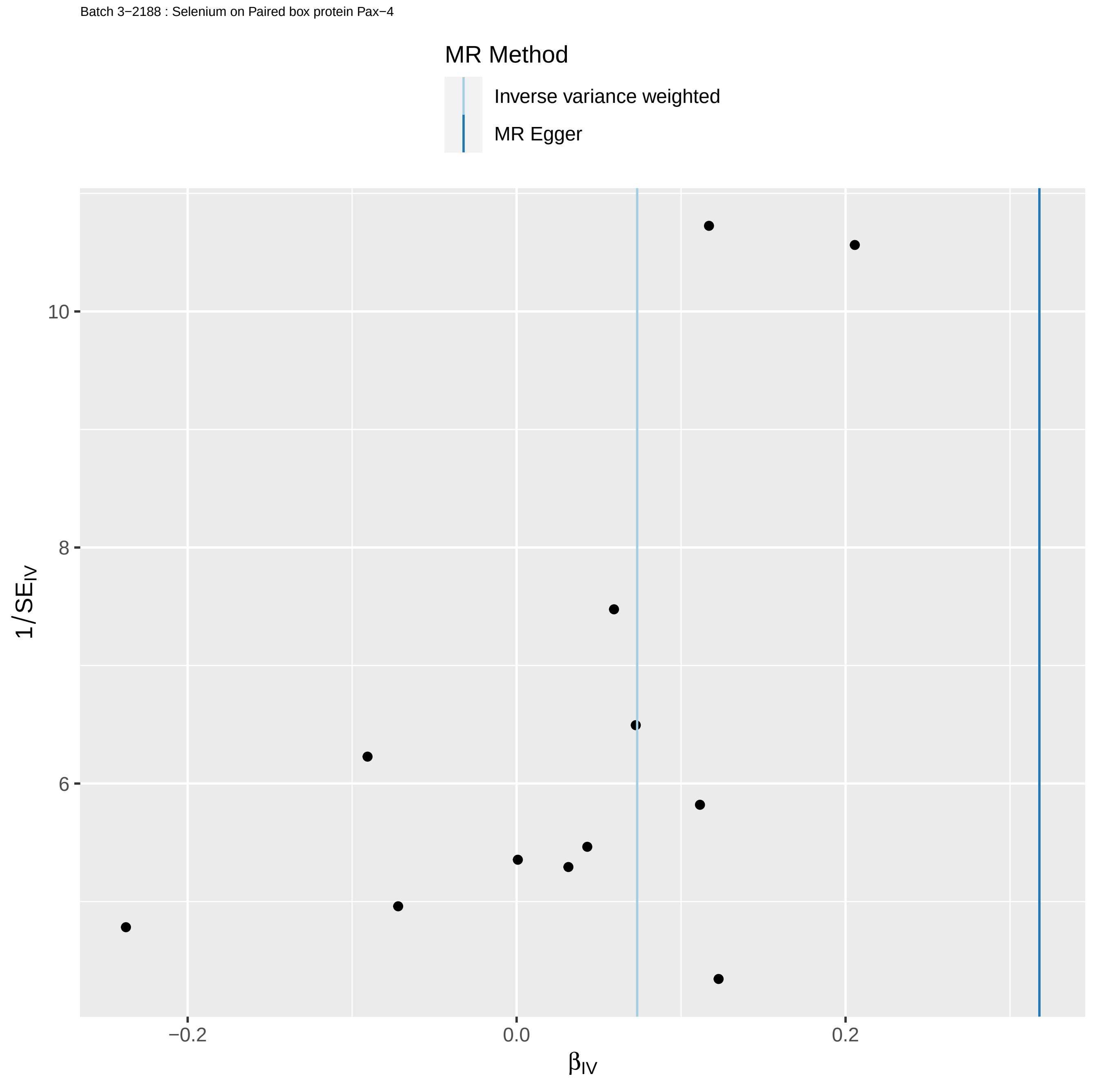

Batch 3−2188 : Selenium on Paired box protein Pax−4
MR Method
Inverse variance weighted
MR Egger
10
8
6
1/SE_IV
−0.2
0.0
0.2
β_IV

Batch 3−2189 : Selenium on PAX−interacting protein 1

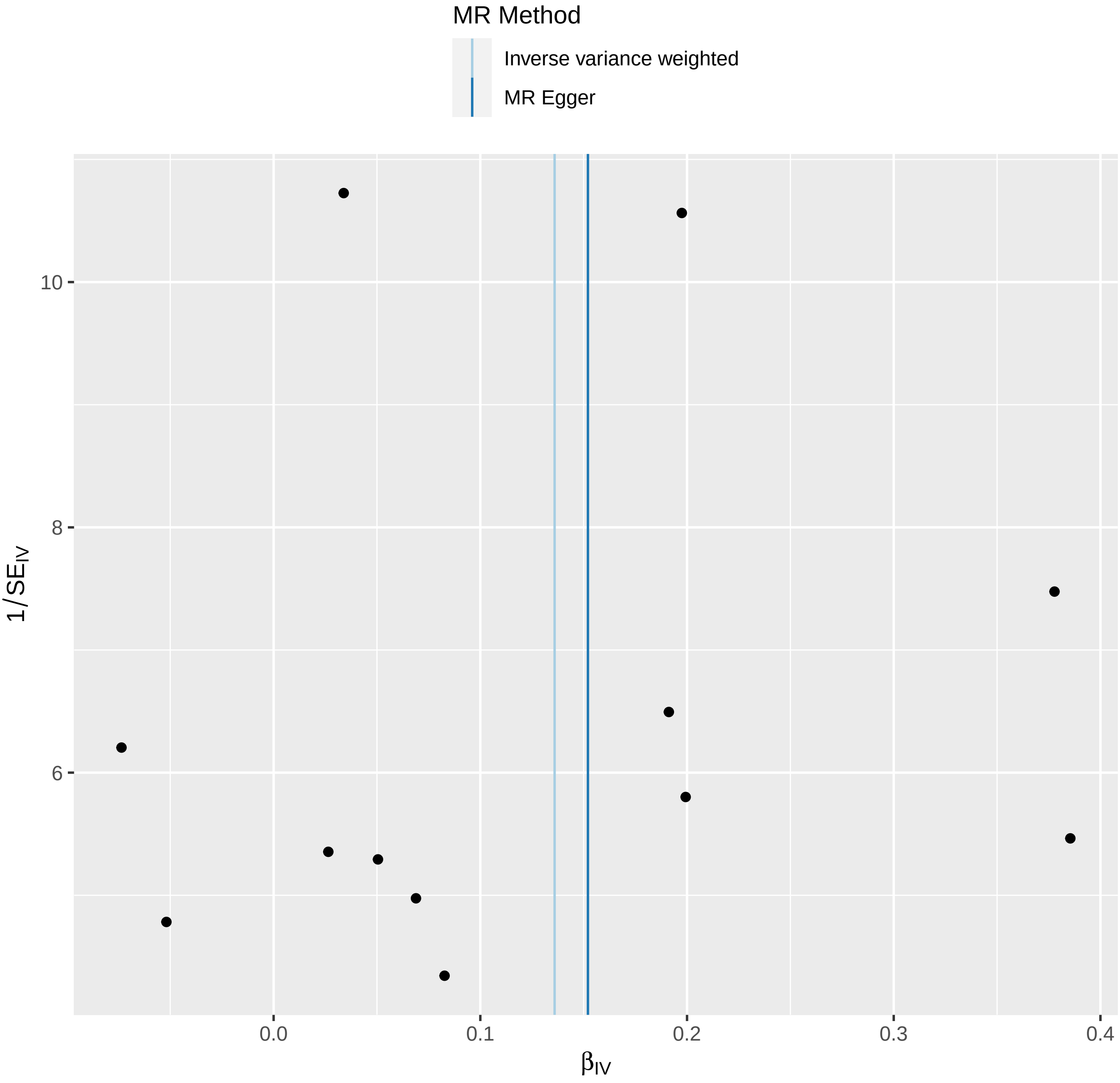

Batch 3−2190 : Selenium on Pyruvate carboxylase, mitochondrial

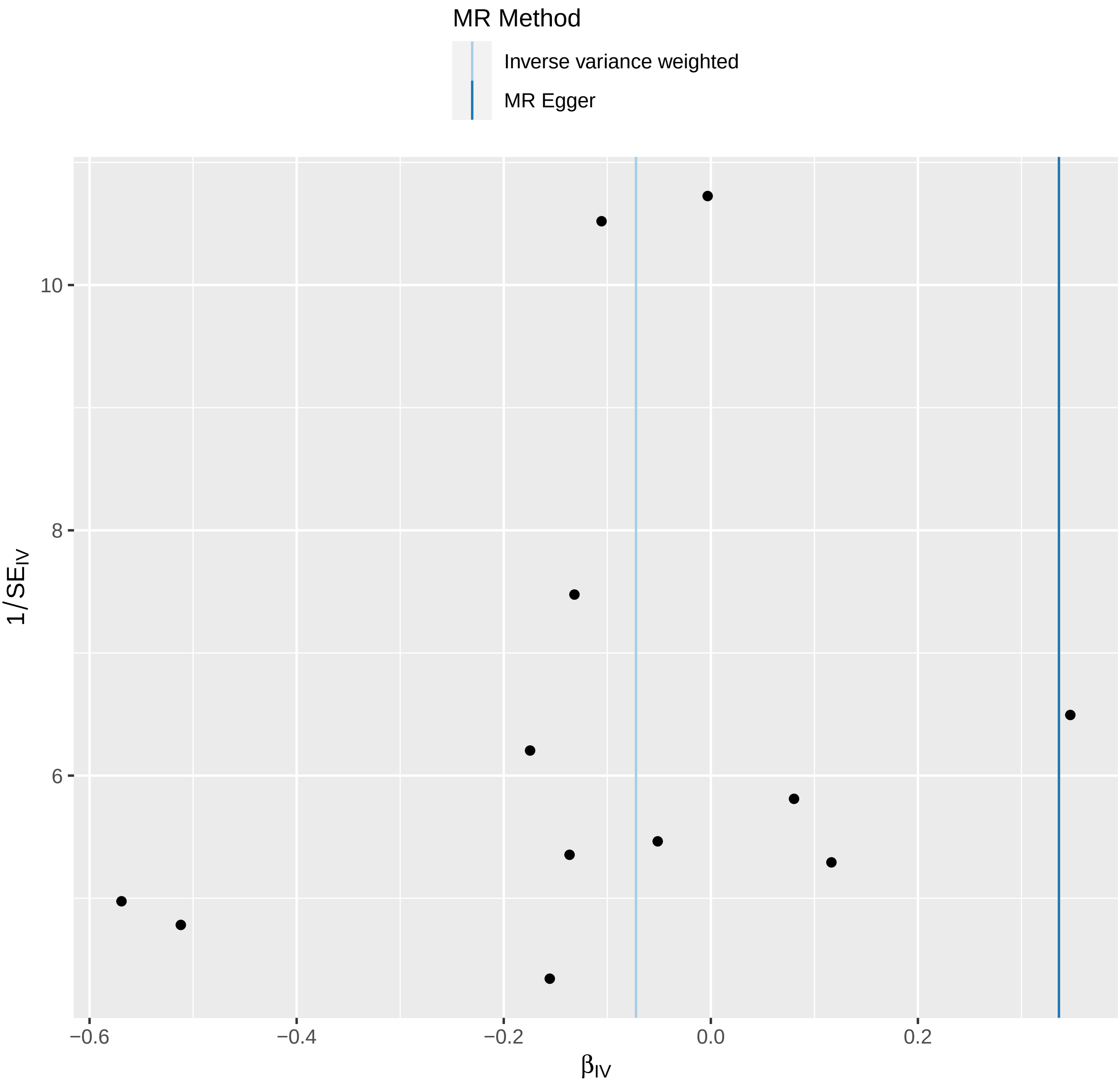

Batch 3−2191 : Selenium on Pterin−4−alpha−carbinolamine dehydratase

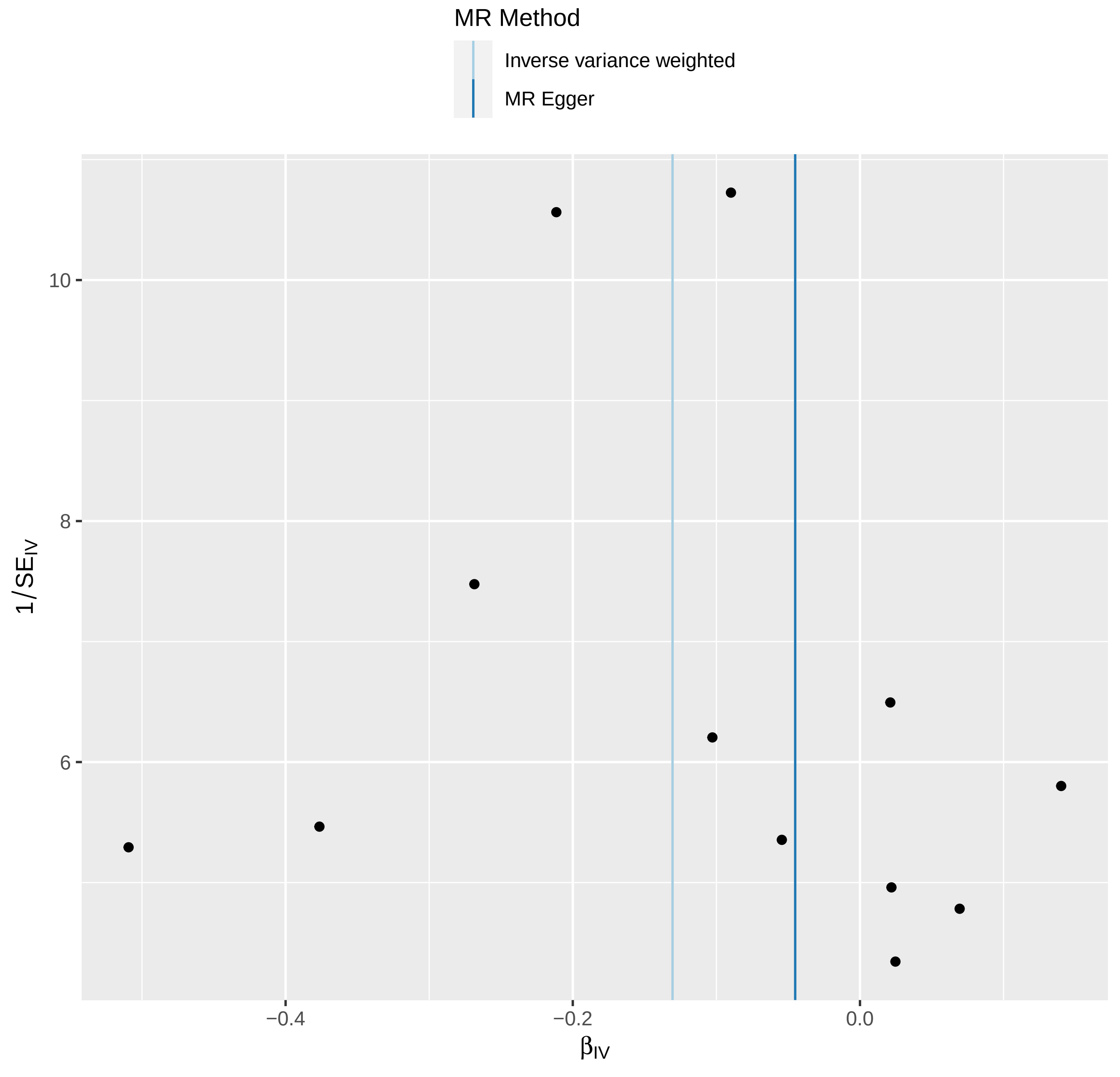

Batch 3−2192 : Selenium on Pterin−4−alpha−carbinolamine dehydratase 2

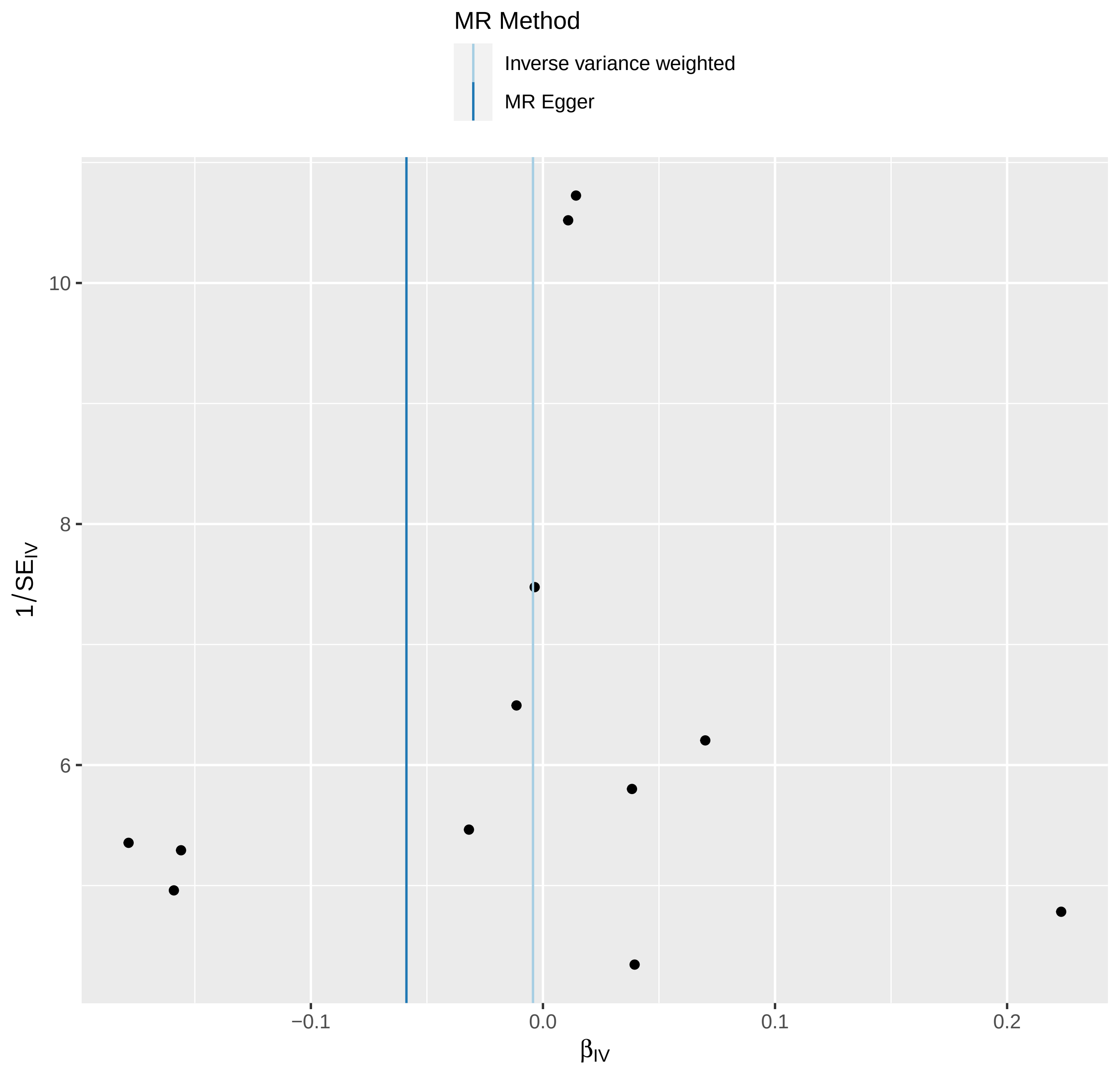

Batch 3−2193 : Selenium on Poly(rC)−binding protein 1

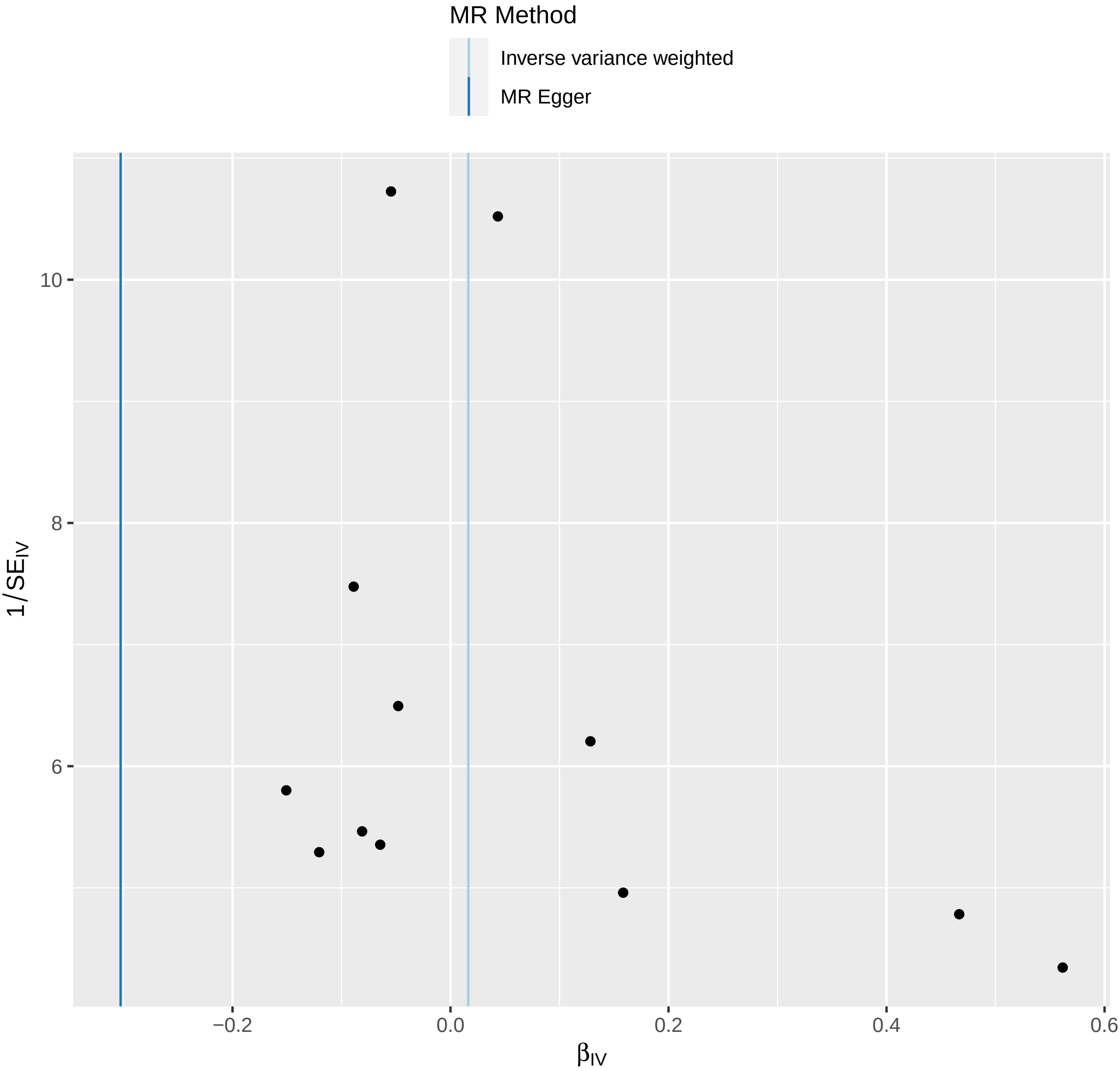

Batch 3−2194 : Selenium on Protocadherin−10

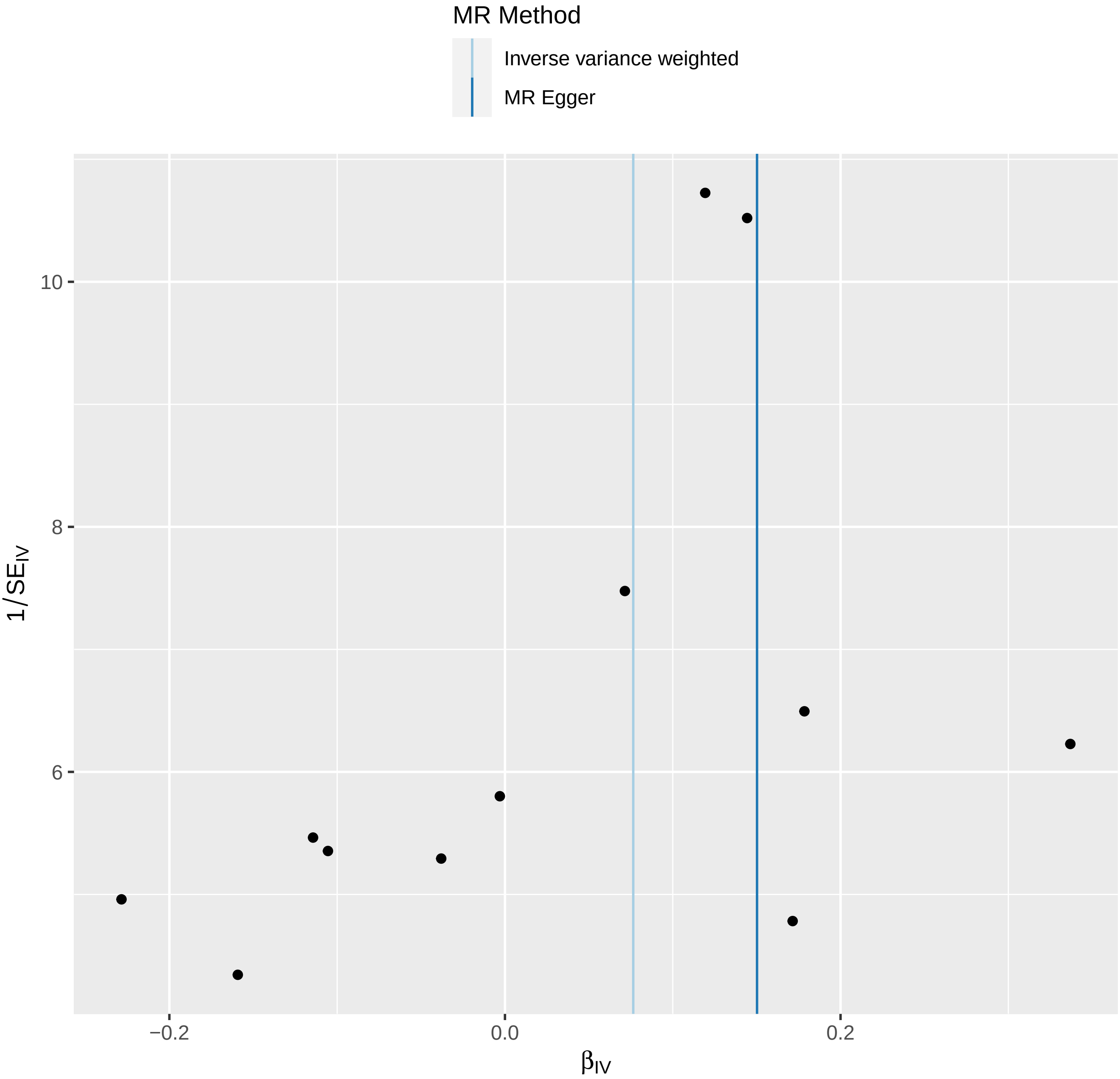

Batch 3−2195 : Selenium on Protocadherin−10

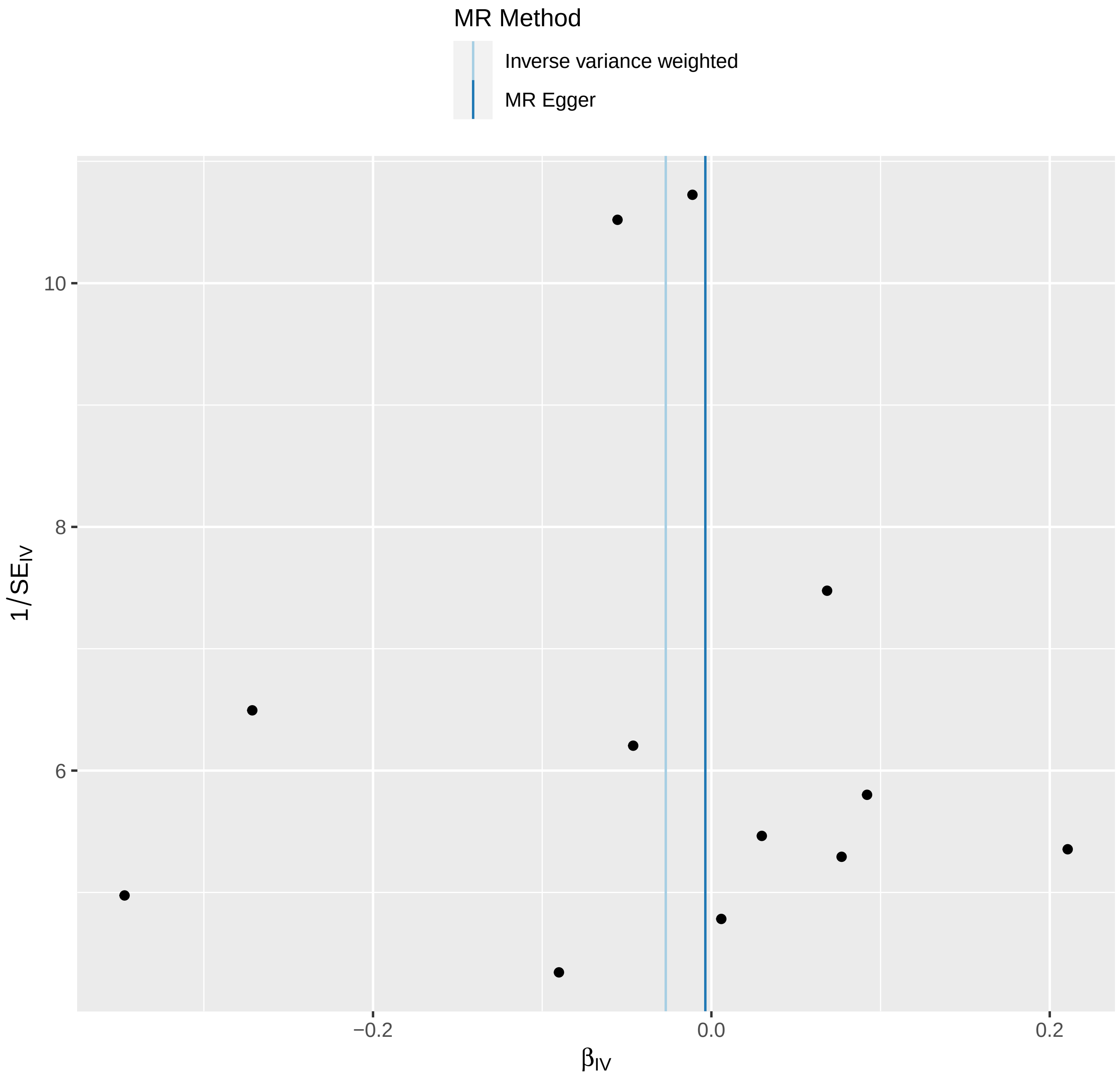

Batch 3−2196 : Selenium on Protocadherin−15

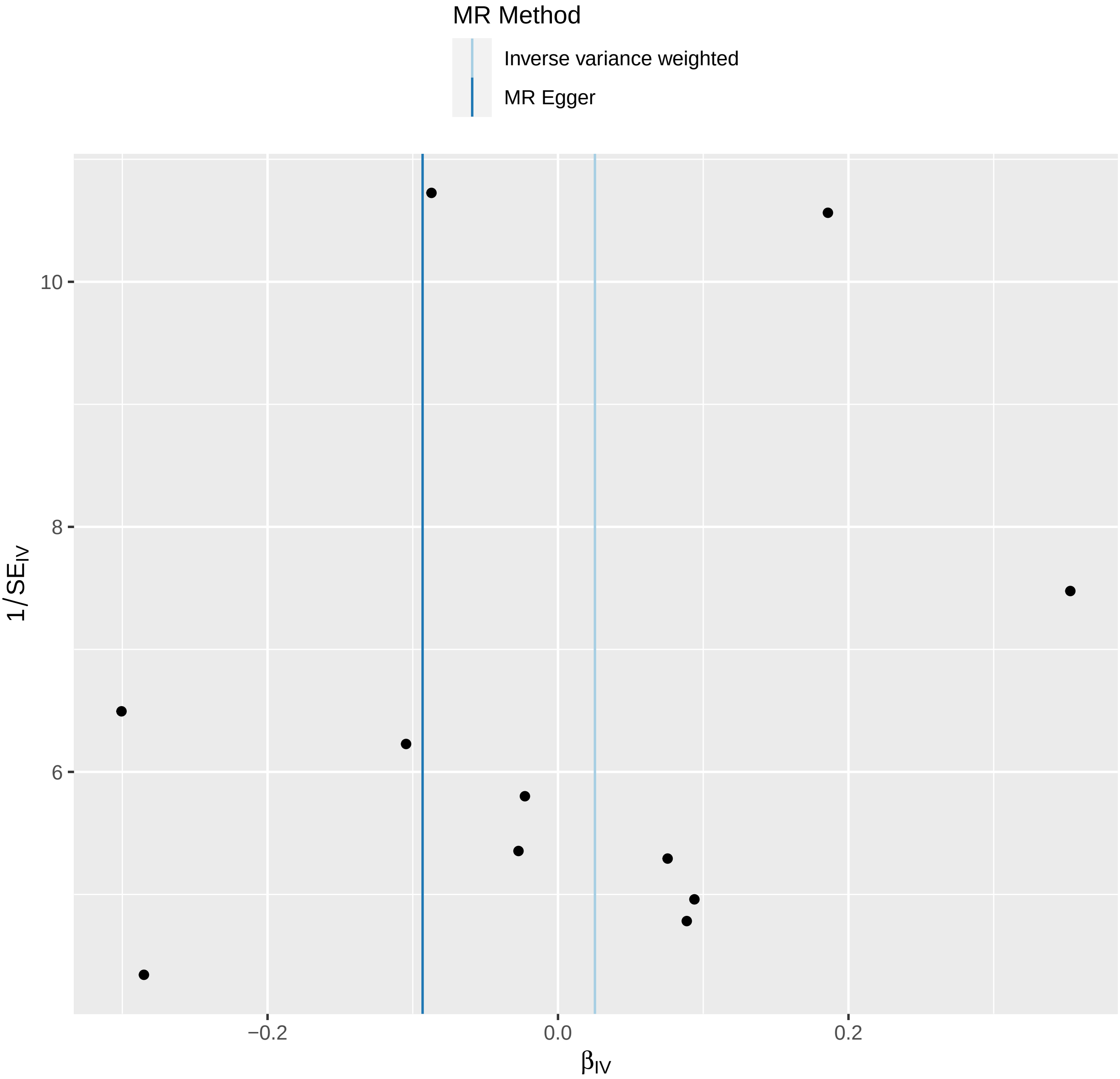

Batch 3−2197 : Selenium on Protocadherin−9

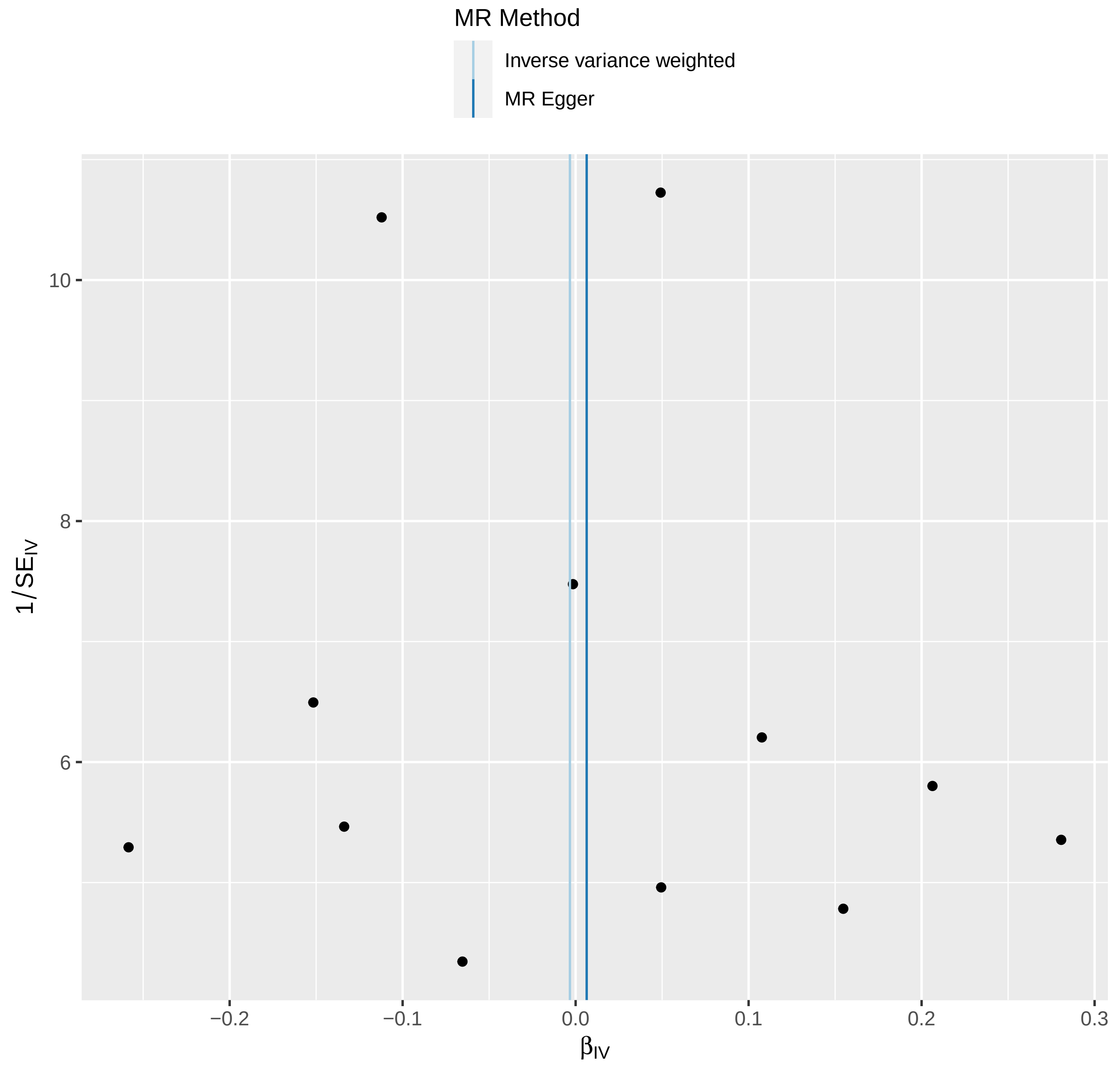

Batch 3−2198 : Selenium on Protocadherin alpha−4

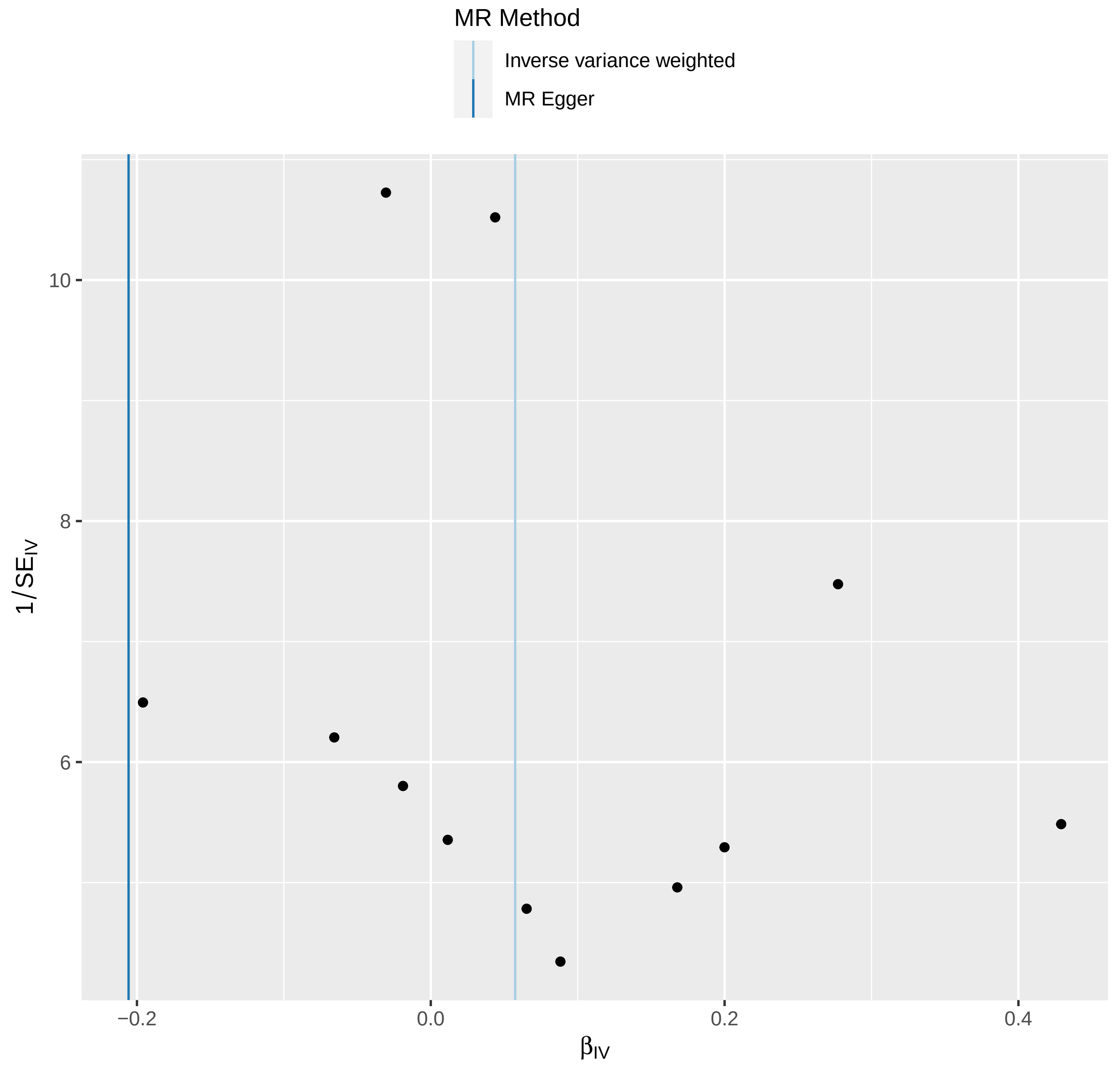

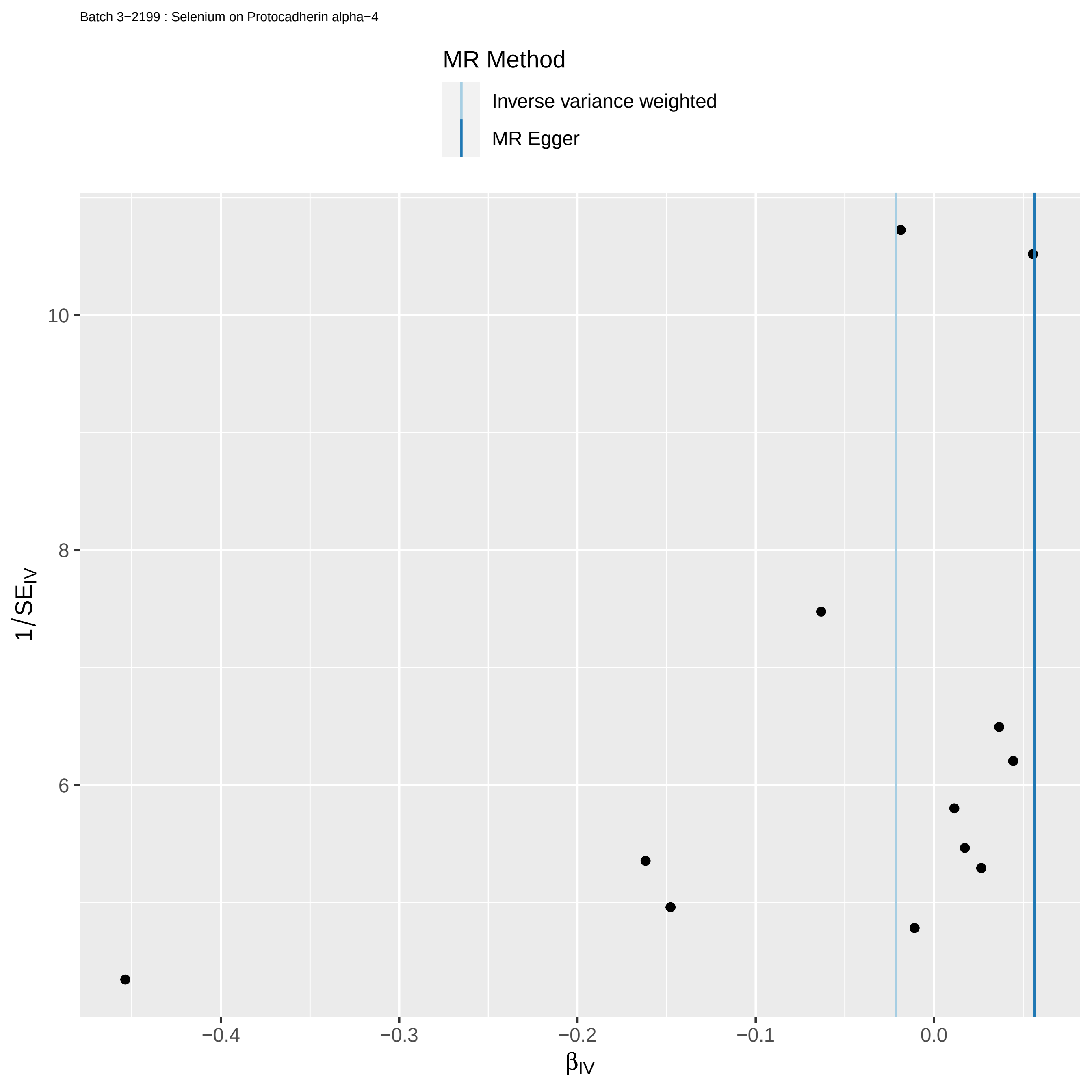
Batch 3−2199 : Selenium on Protocadherin alpha−4
MR Method
Inverse variance weighted
MR Egger
1/SE_IV
β_IV
10
8
6
−0.4
−0.3
−0.2
−0.1
0.0

Batch 3−2200 : Selenium on Protocadherin alpha−7

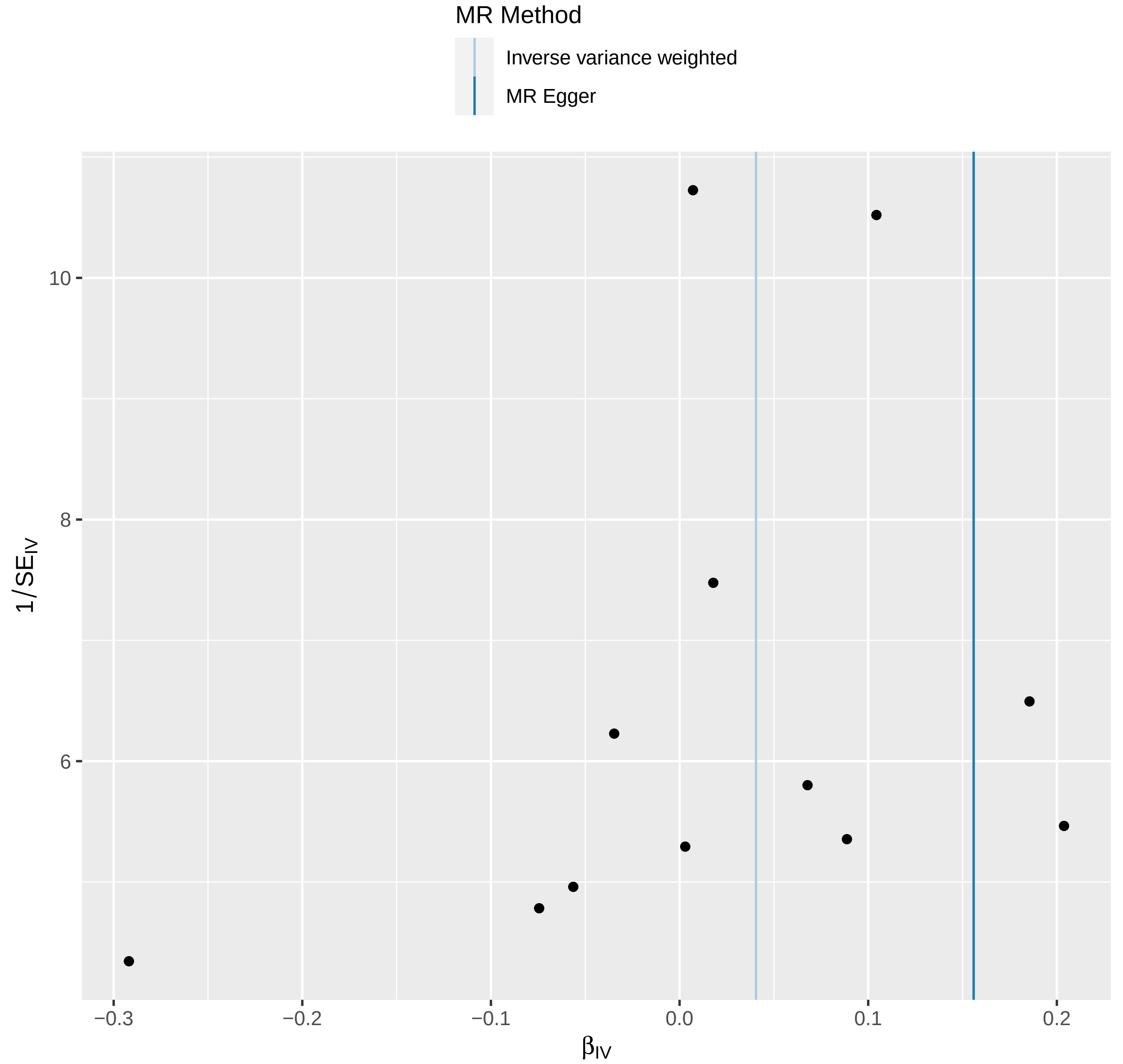

Batch 3−2201 : Selenium on Protocadherin alpha−C2

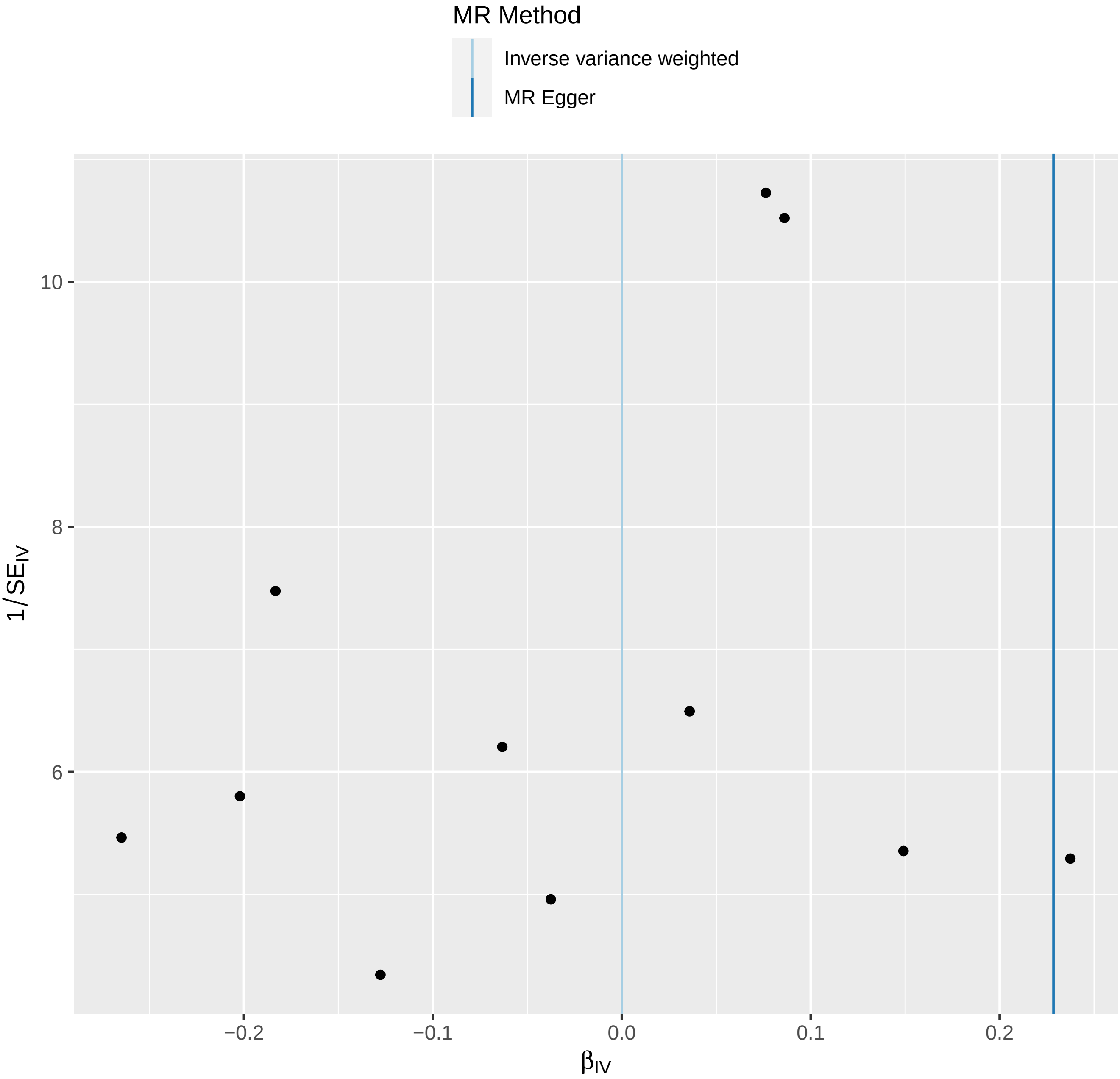

Batch 3−2202 : Selenium on Protocadherin beta−10

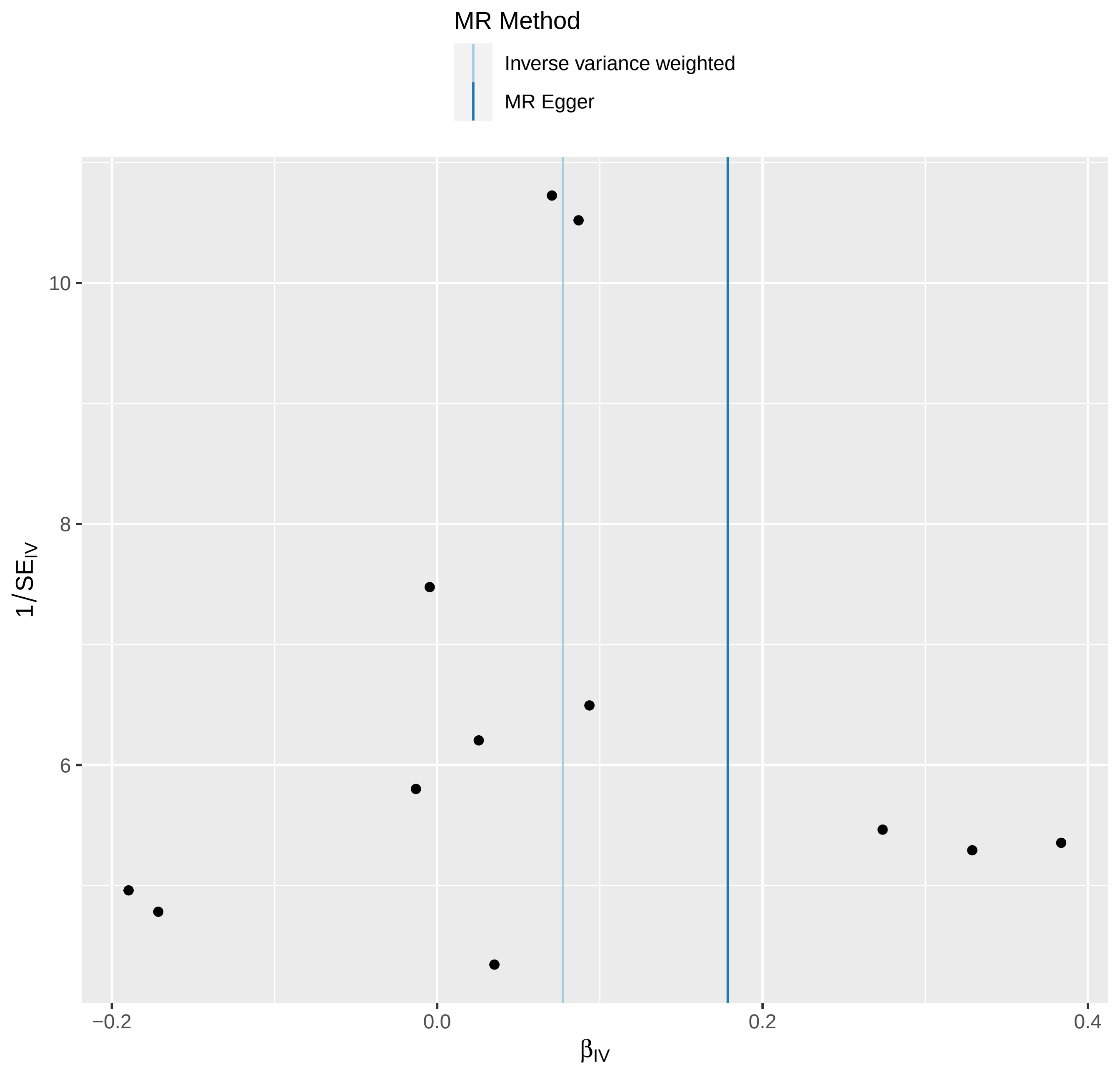

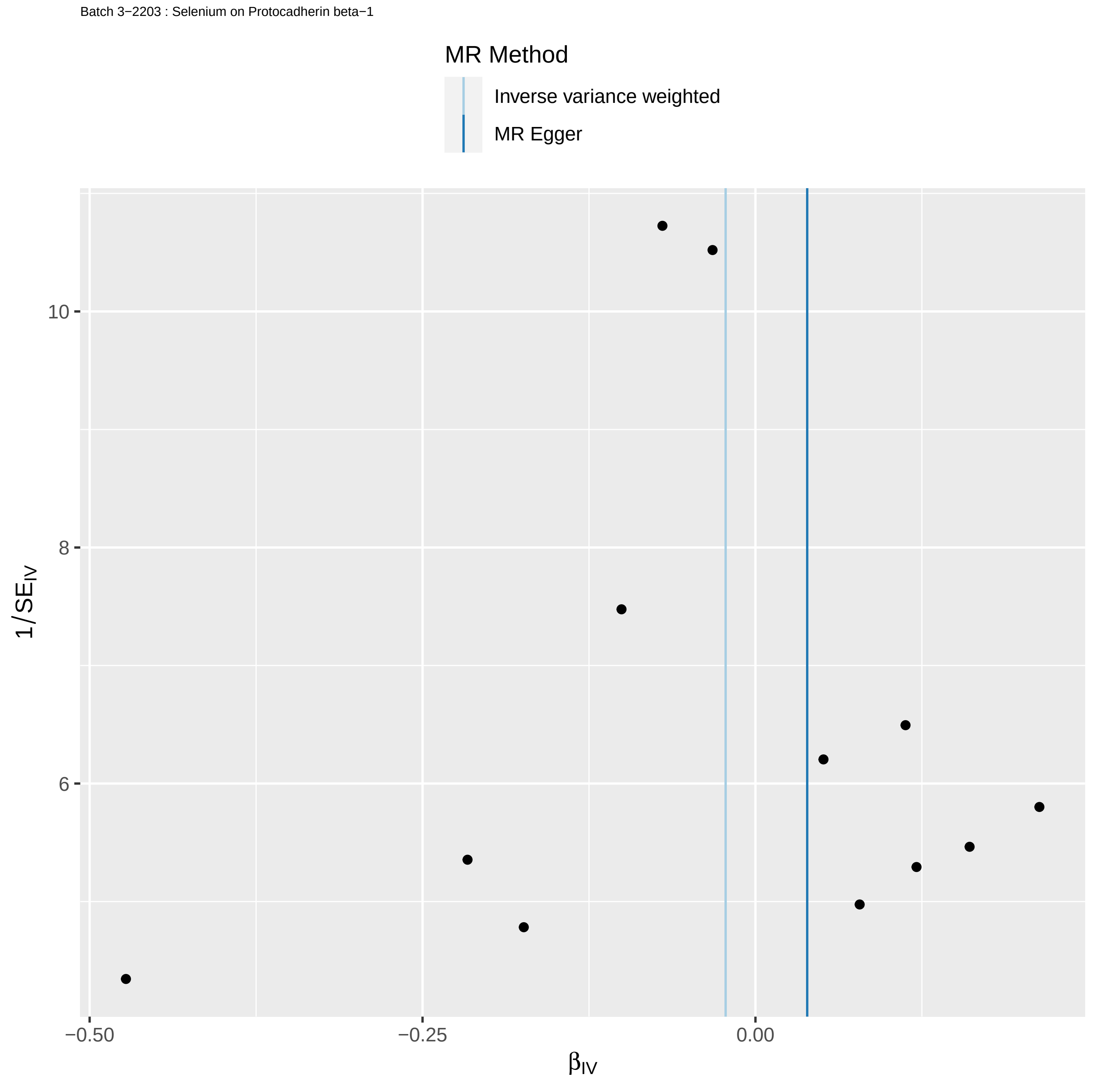
Batch 3−2203 : Selenium on Protocadherin beta−1
MR Method
Inverse variance weighted
MR Egger
10
8
6
1/SE_IV
−0.50
−0.25
0.00
β_IV

Batch 3−2204 : Selenium on Protocadherin beta−2

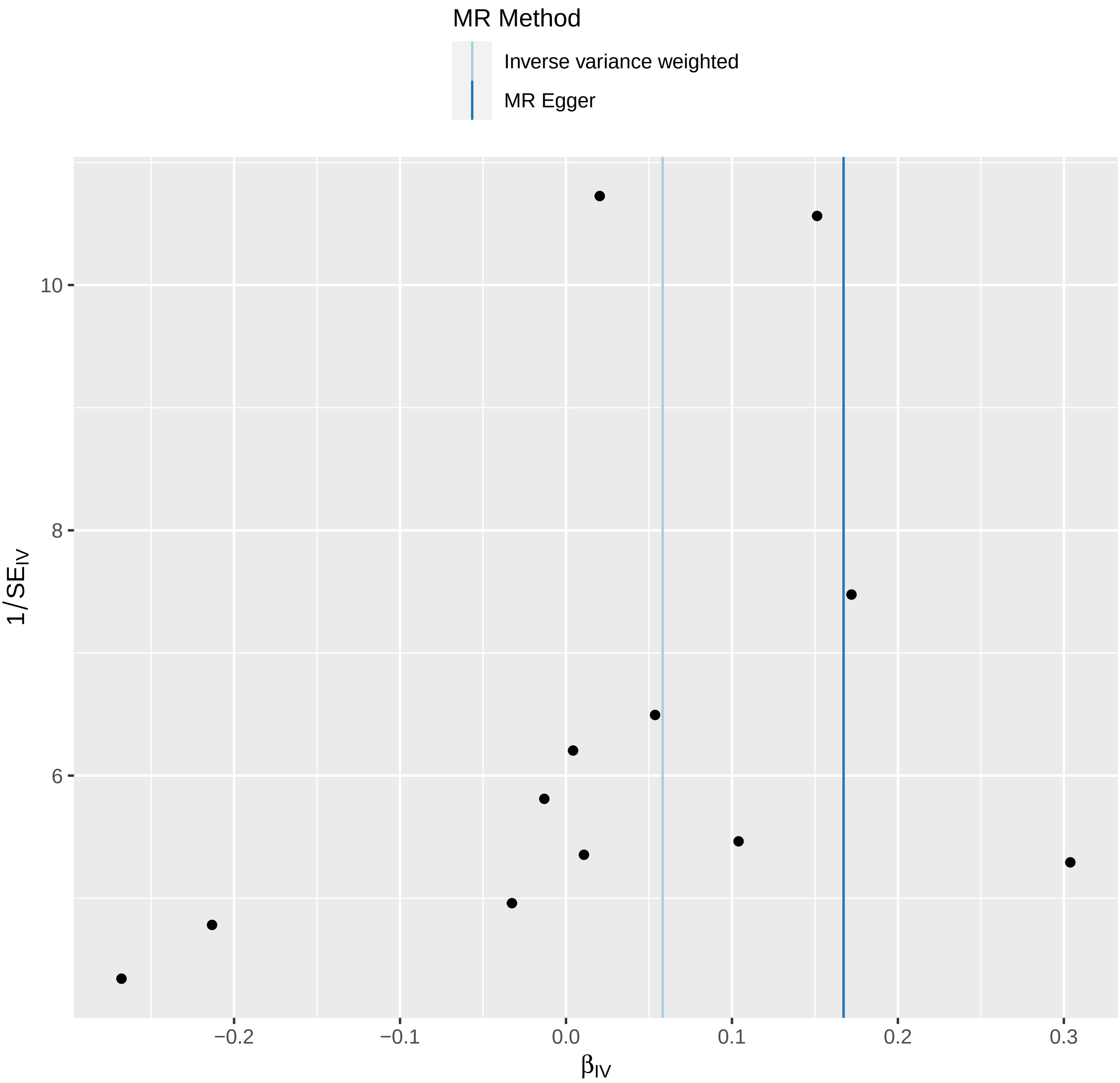

Batch 3−2205 : Selenium on Protocadherin beta−4

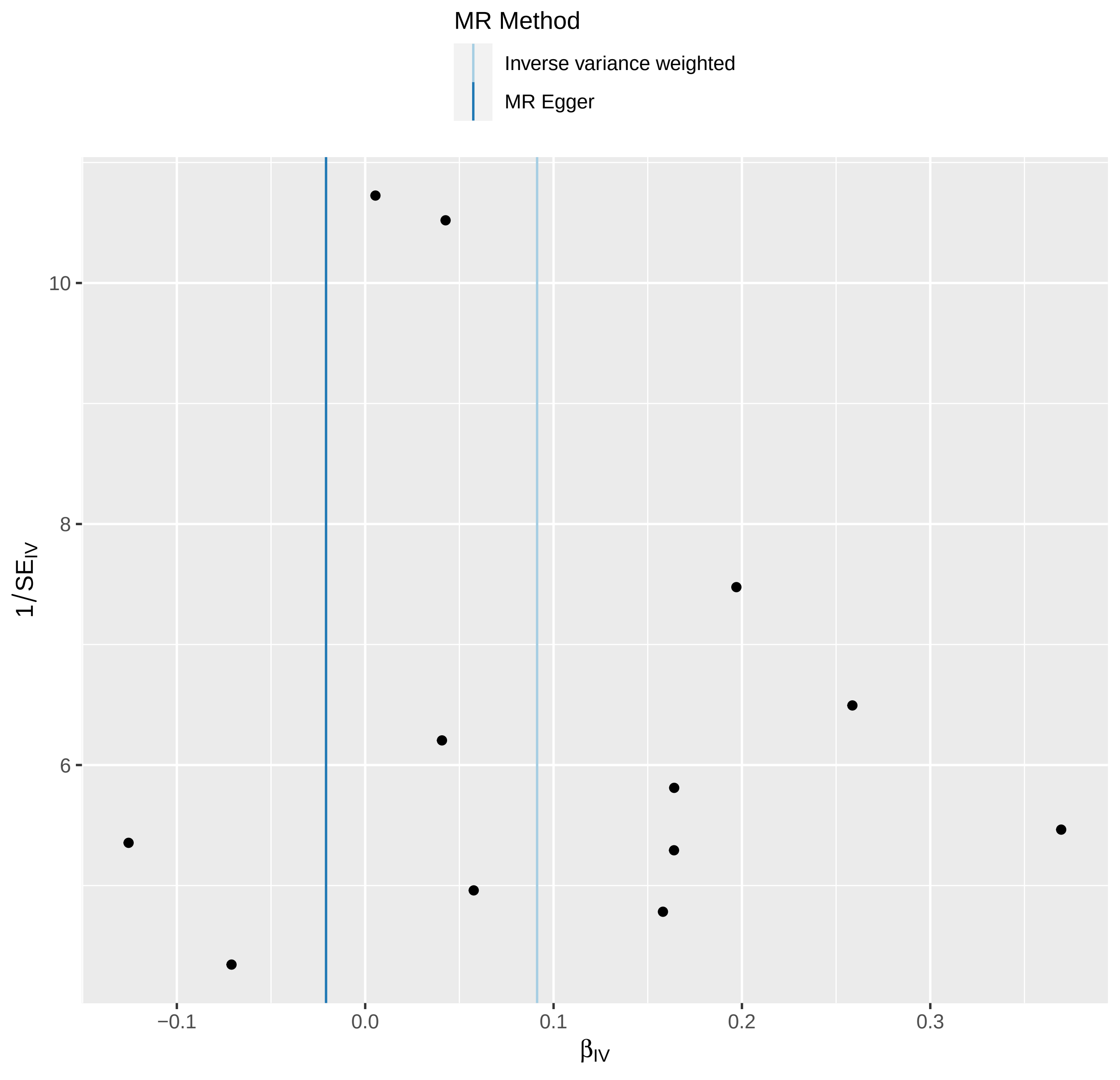

Batch 3−2206 : Selenium on Protocadherin gamma−A8

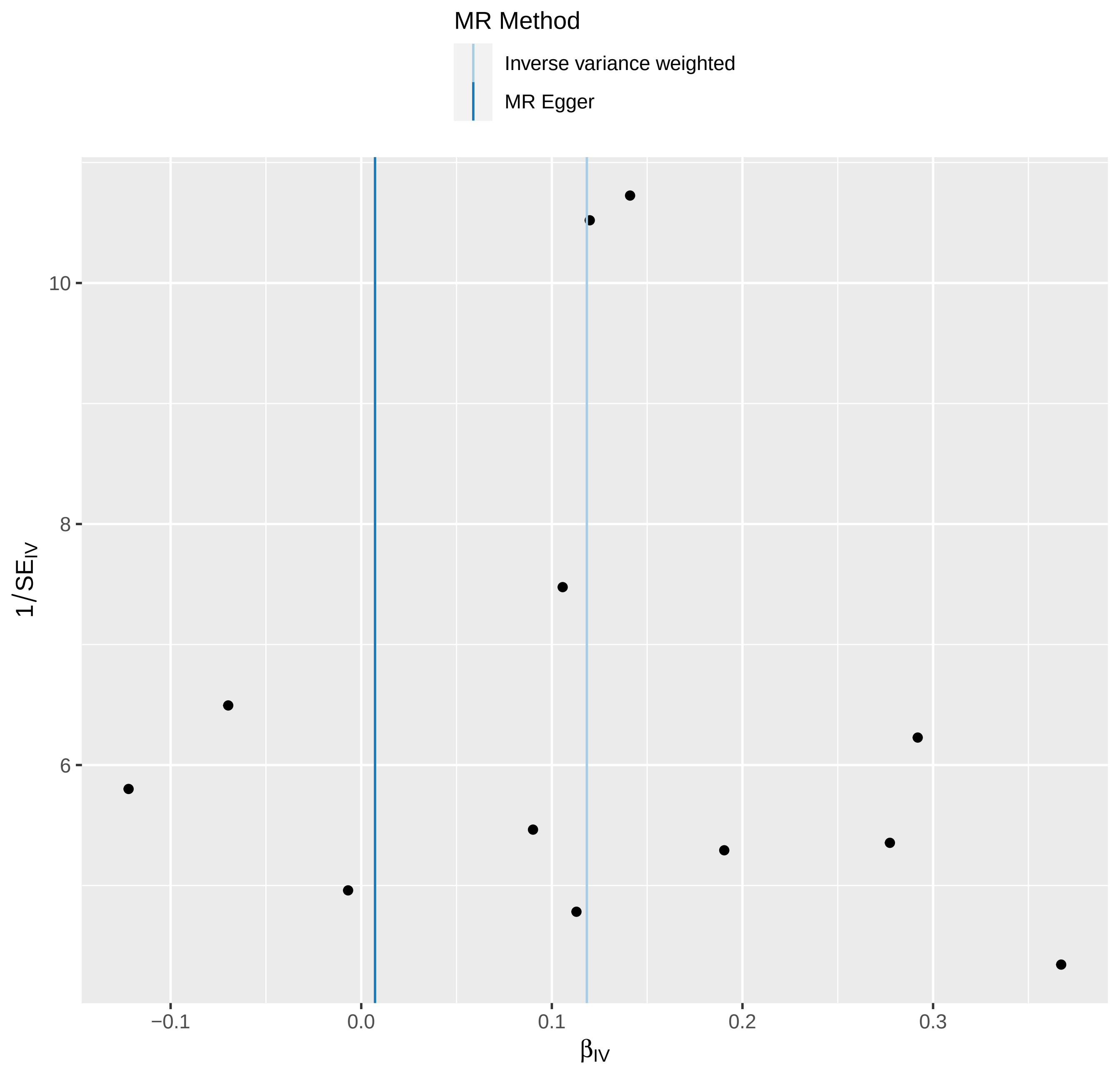

Batch 3−2207 : Selenium on Protocadherin gamma−C5

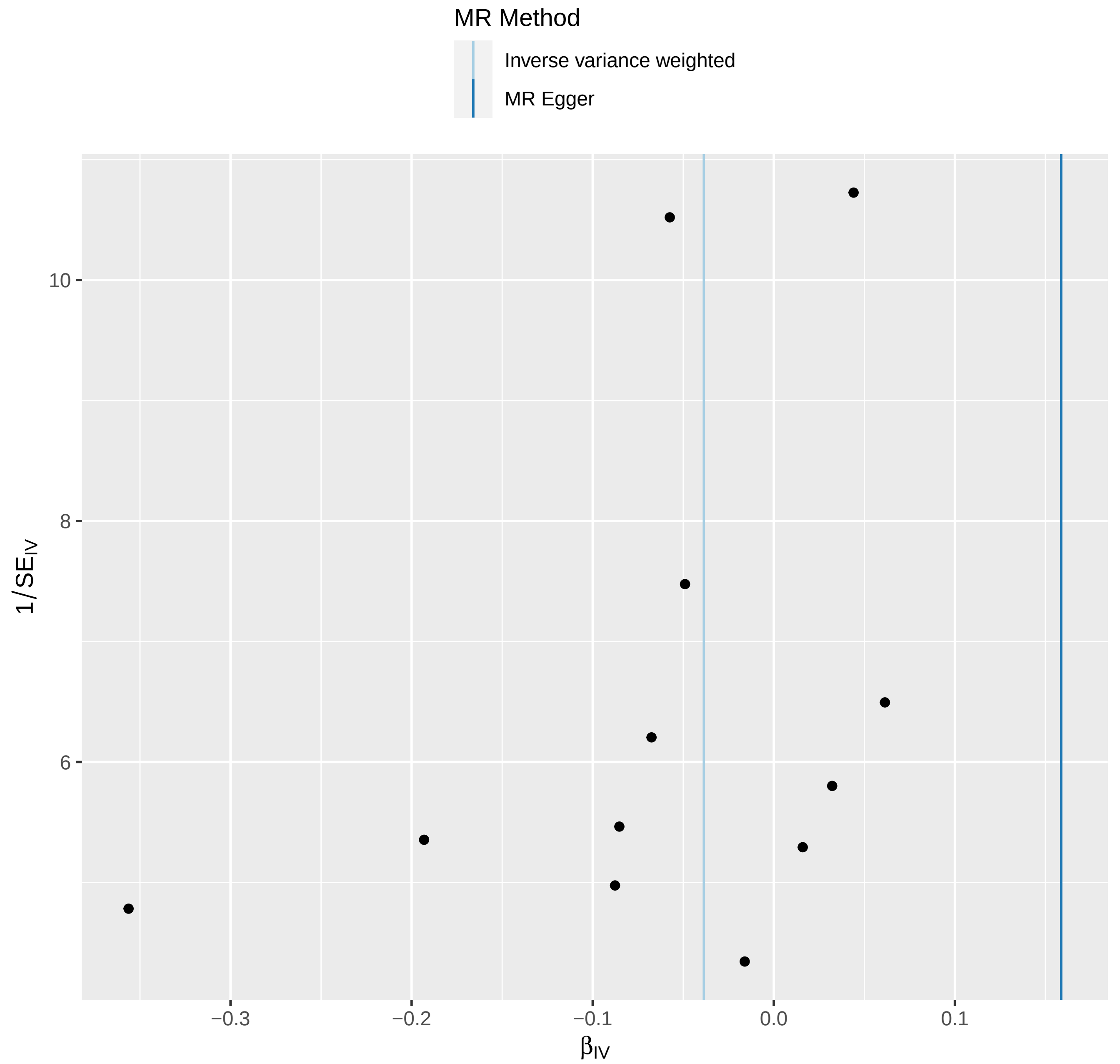

Batch 3−2208 : Selenium on Proliferating cell nuclear antigen

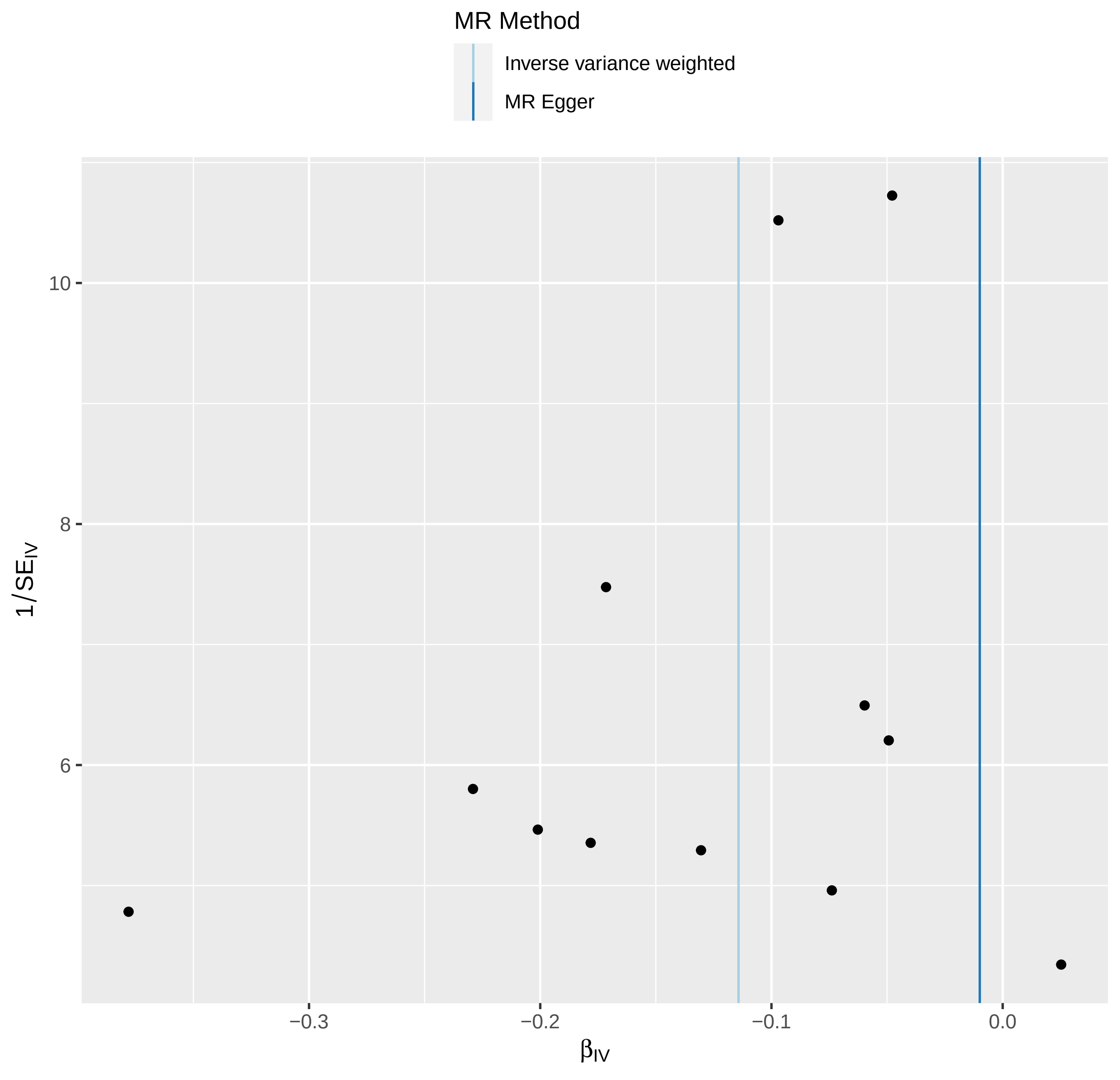

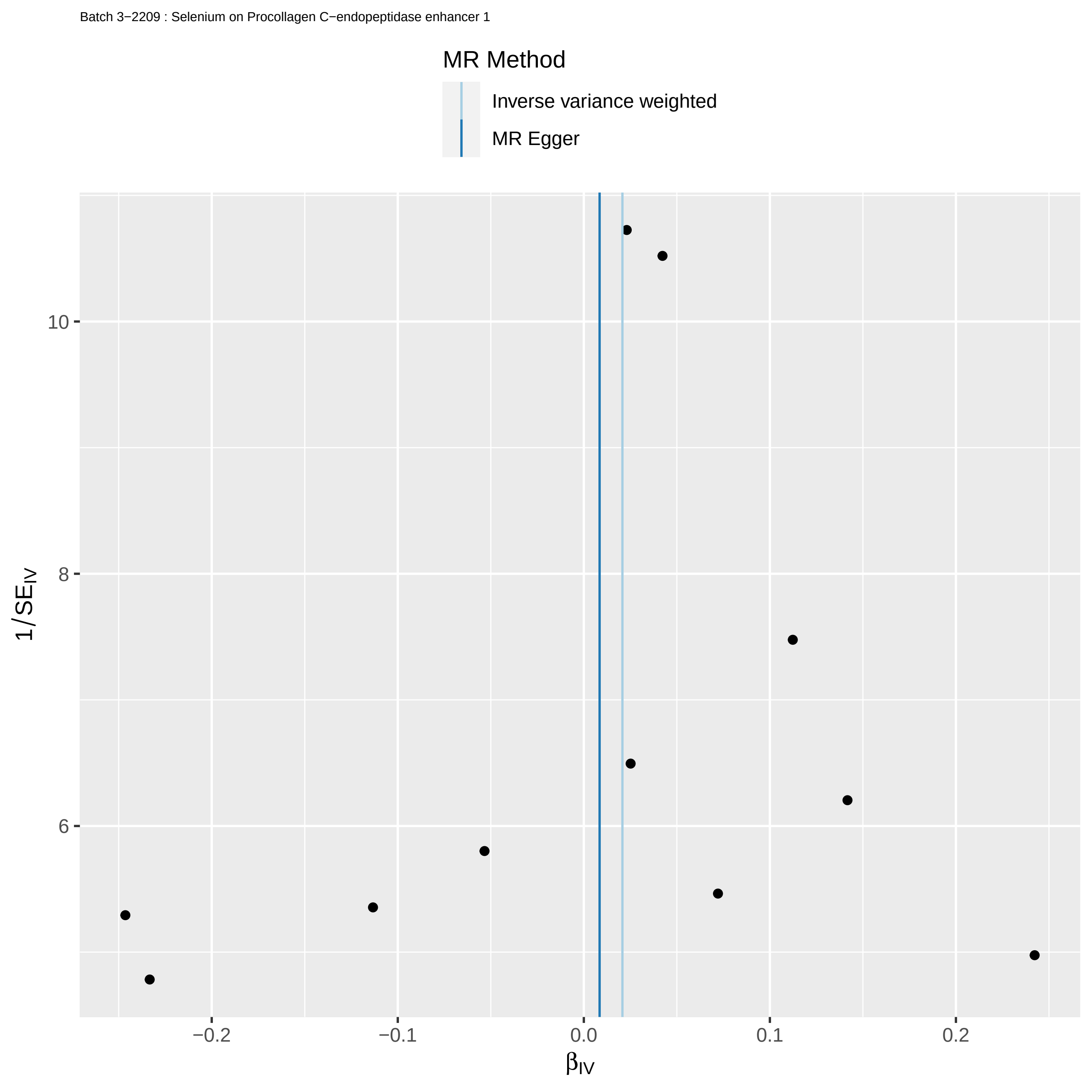

Batch 3−2209 : Selenium on Procollagen C−endopeptidase enhancer 1
MR Method
Inverse variance weighted
MR Egger
10
8
6
1/SE$_{IV}$
−0.2
−0.1
0.0
0.1
0.2
β$_{IV}$

Batch 3−2210 : Selenium on Procollagen C−endopeptidase enhancer 2

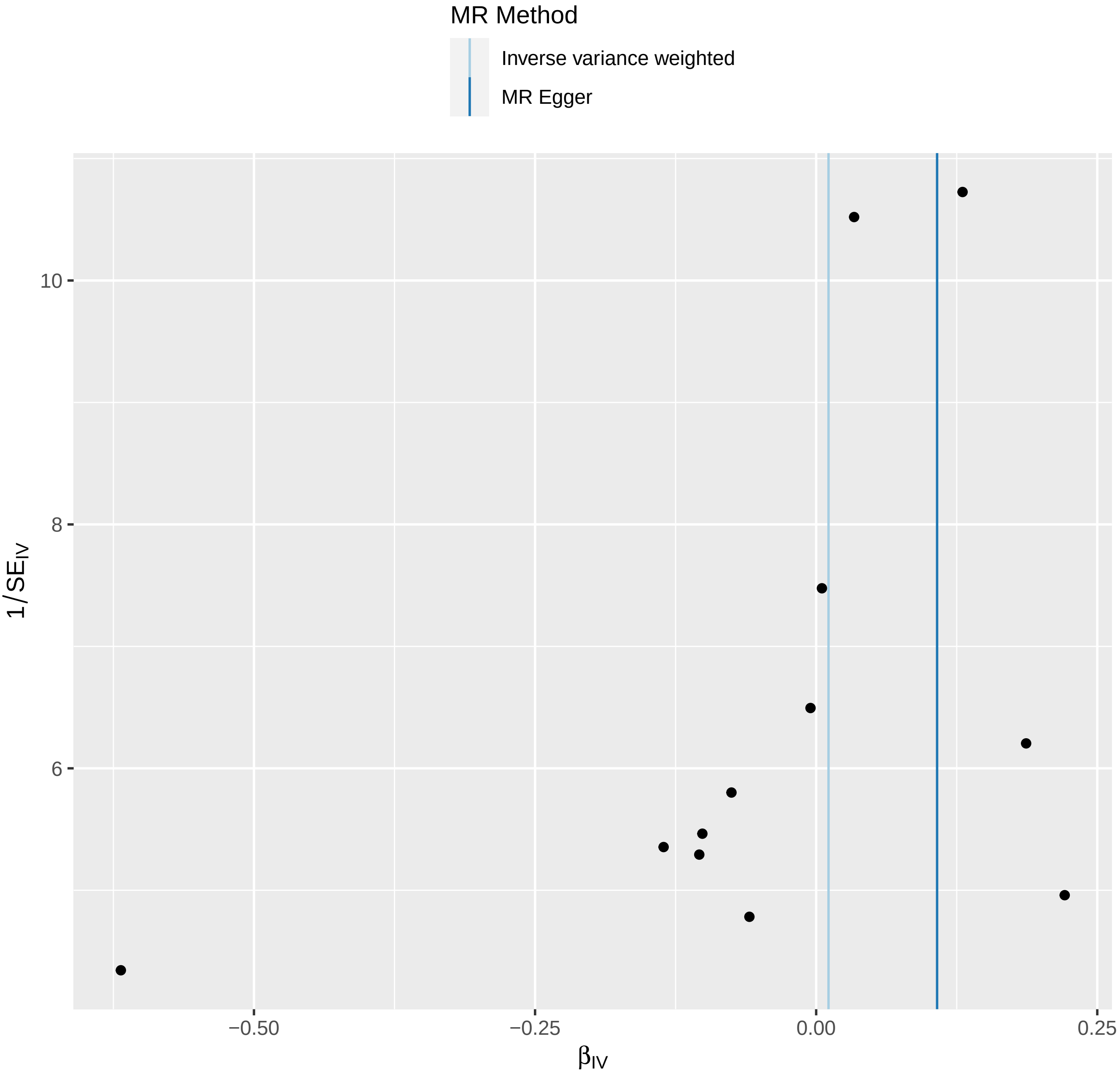

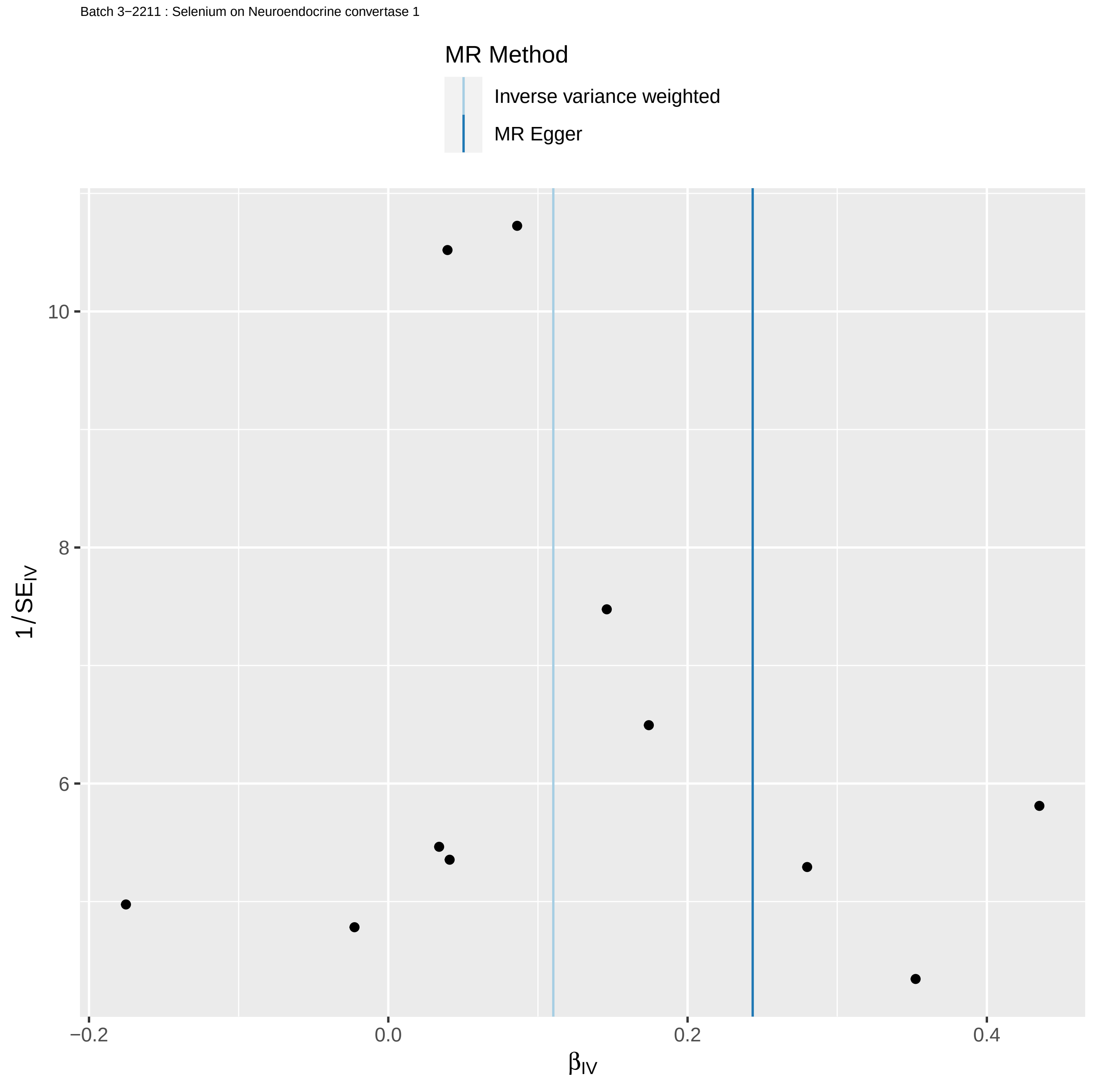
Batch 3−2211 : Selenium on Neuroendocrine convertase 1
MR Method
Inverse variance weighted
MR Egger
10
8
6
1/SE$_{IV}$
−0.2
0.0
0.2
0.4
β$_{IV}$

Batch 3−2212 : Selenium on Proprotein convertase subtilisin/kexin type 7

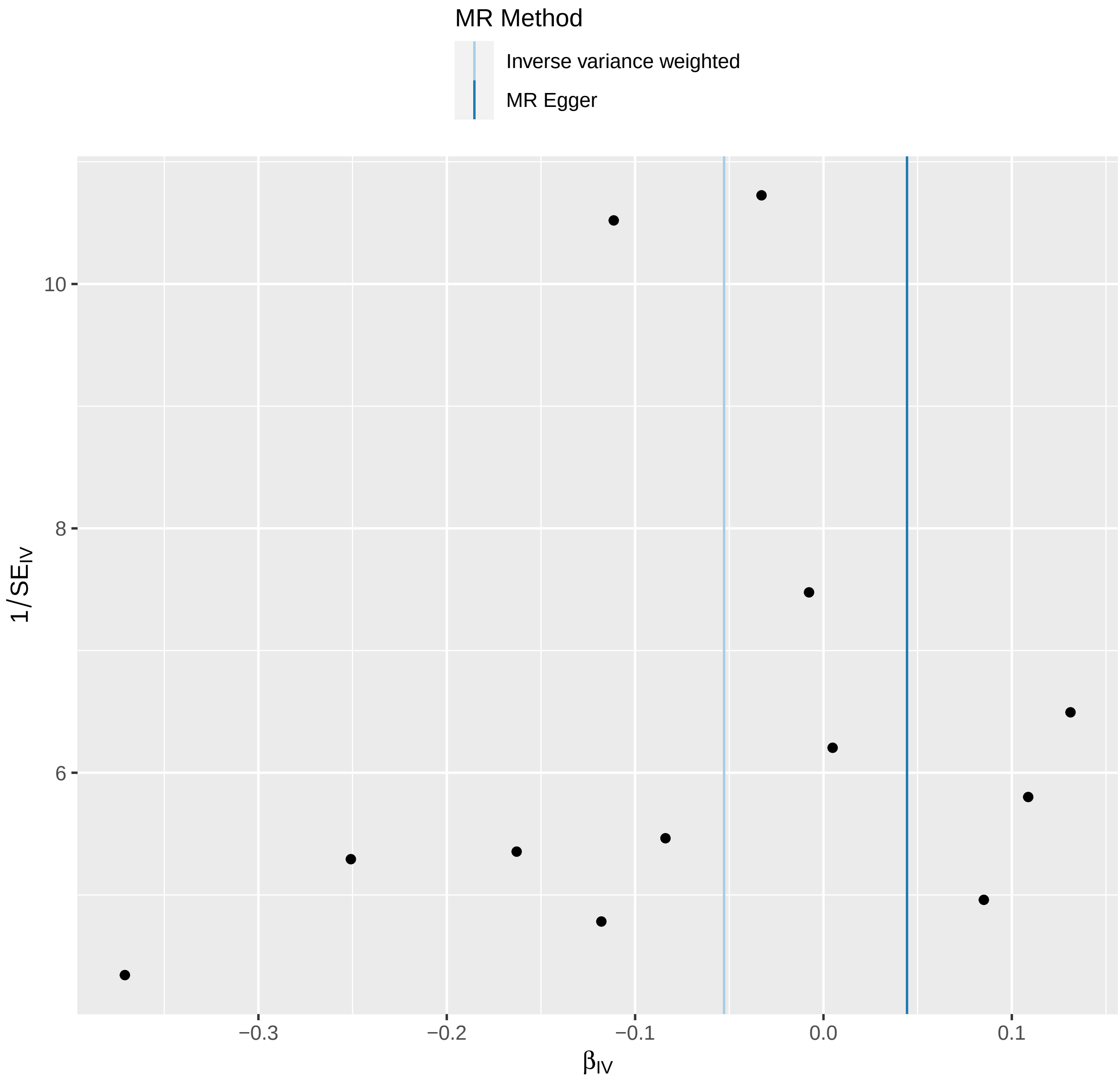

Batch 3−2213 : Selenium on Prenylcysteine oxidase−like

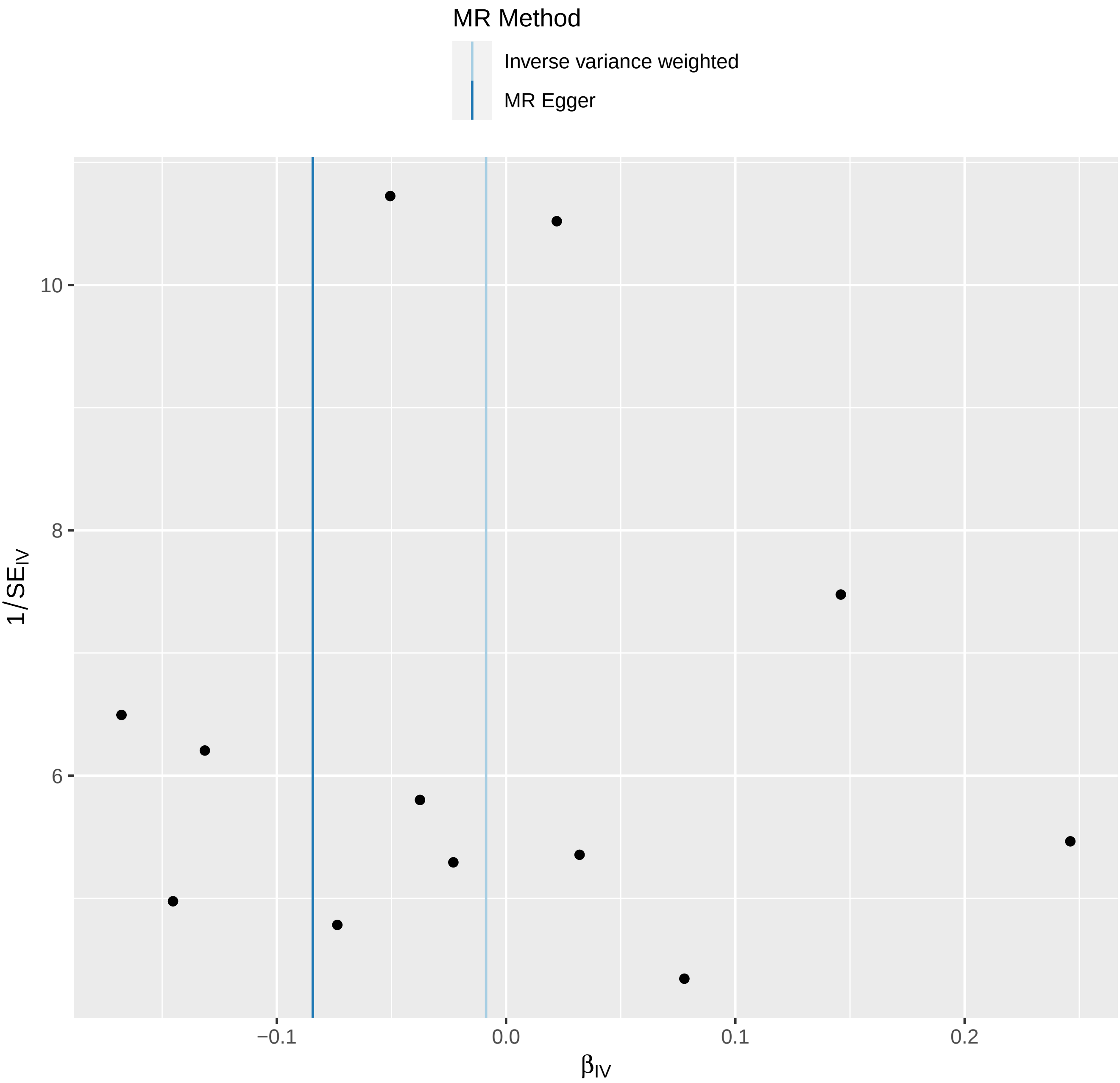

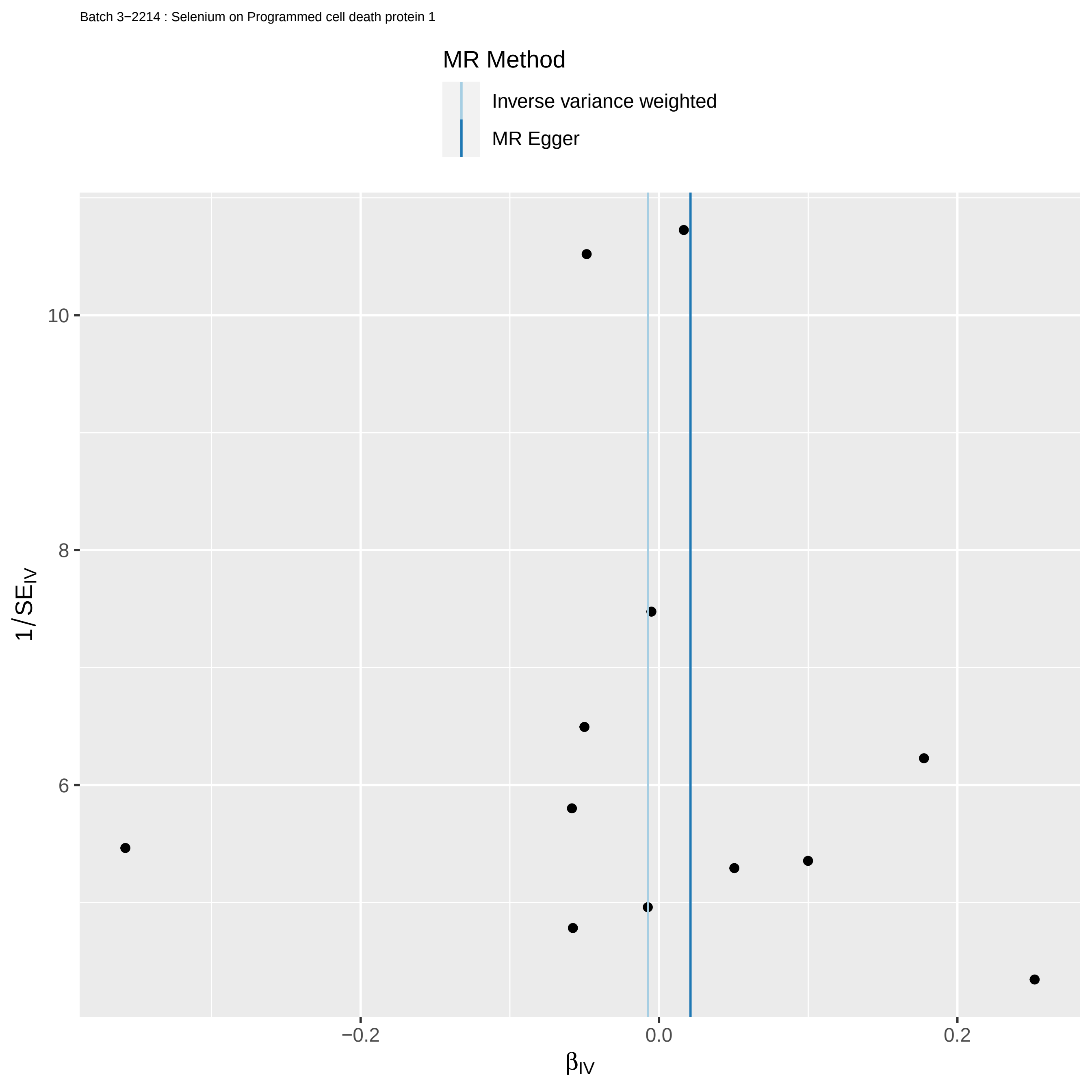
Batch 3−2214 : Selenium on Programmed cell death protein 1
MR Method
Inverse variance weighted
MR Egger
10
8
6
1/SE$_{IV}$
−0.2
0.0
0.2
β_{IV}

Batch 3−2215 : Selenium on Programmed cell death 1 ligand 2

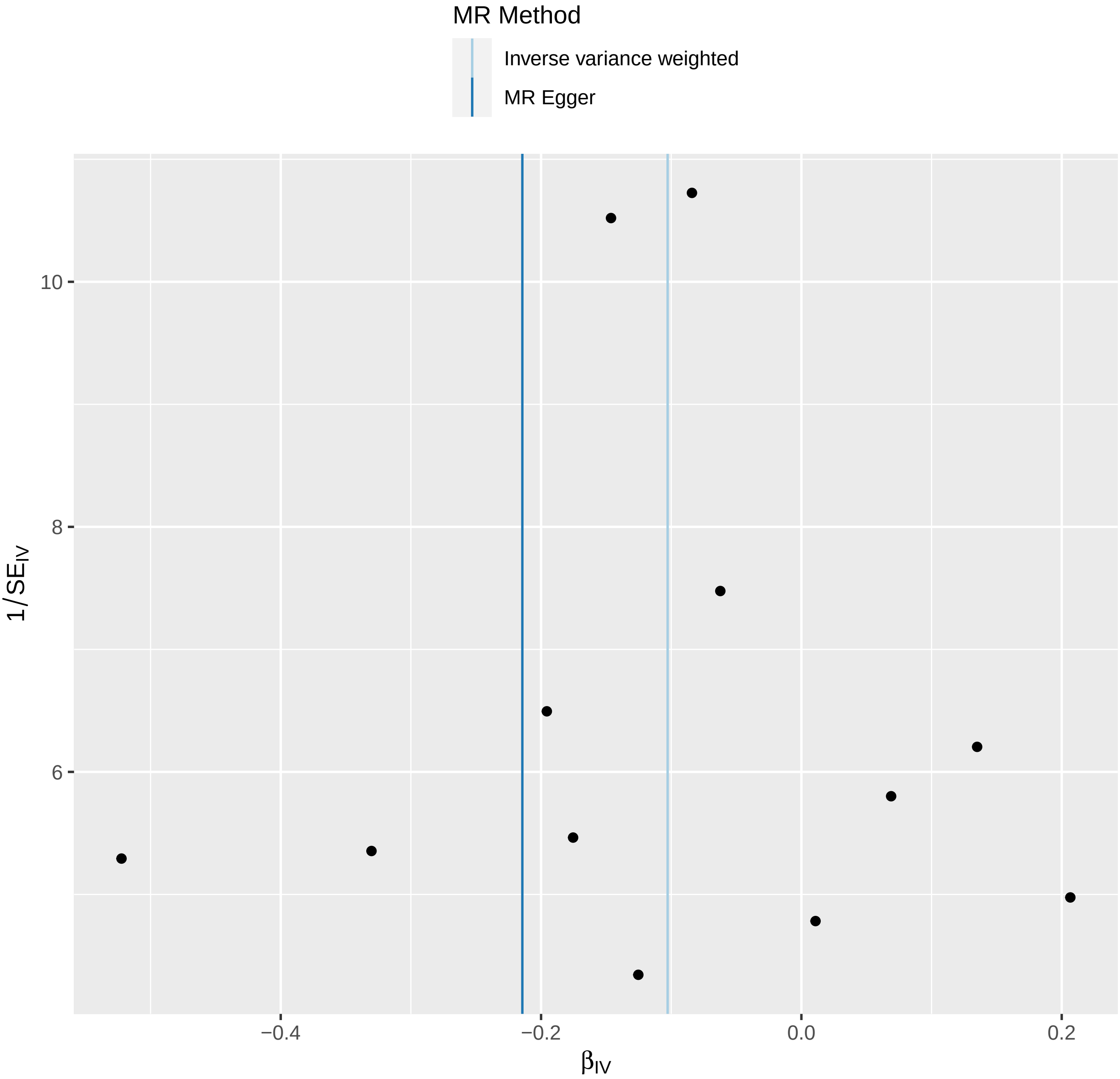

Batch 3−2216 : Selenium on Programmed cell death protein 5

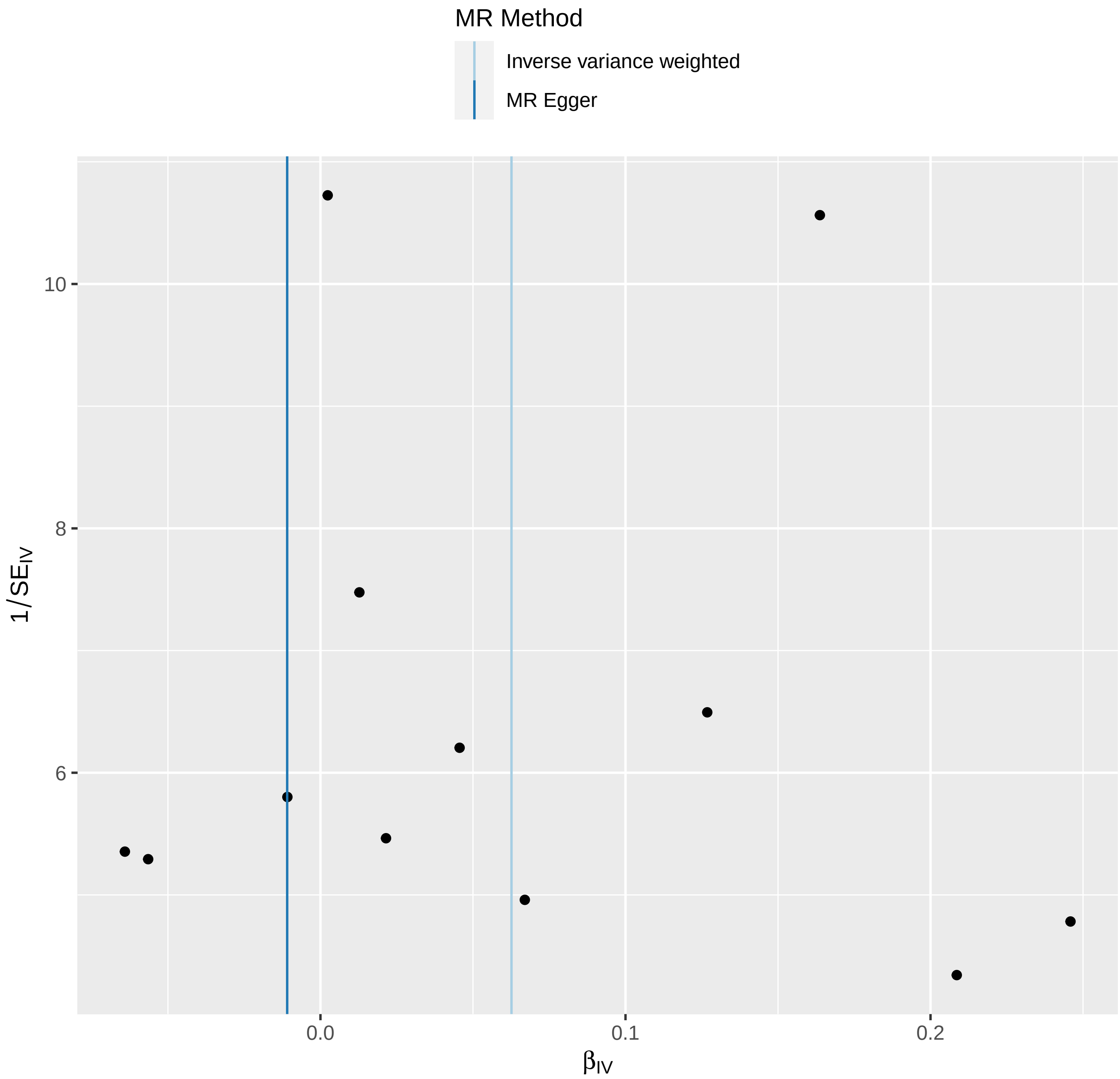

Batch 3−2217 : Selenium on Phosducin−like protein 2

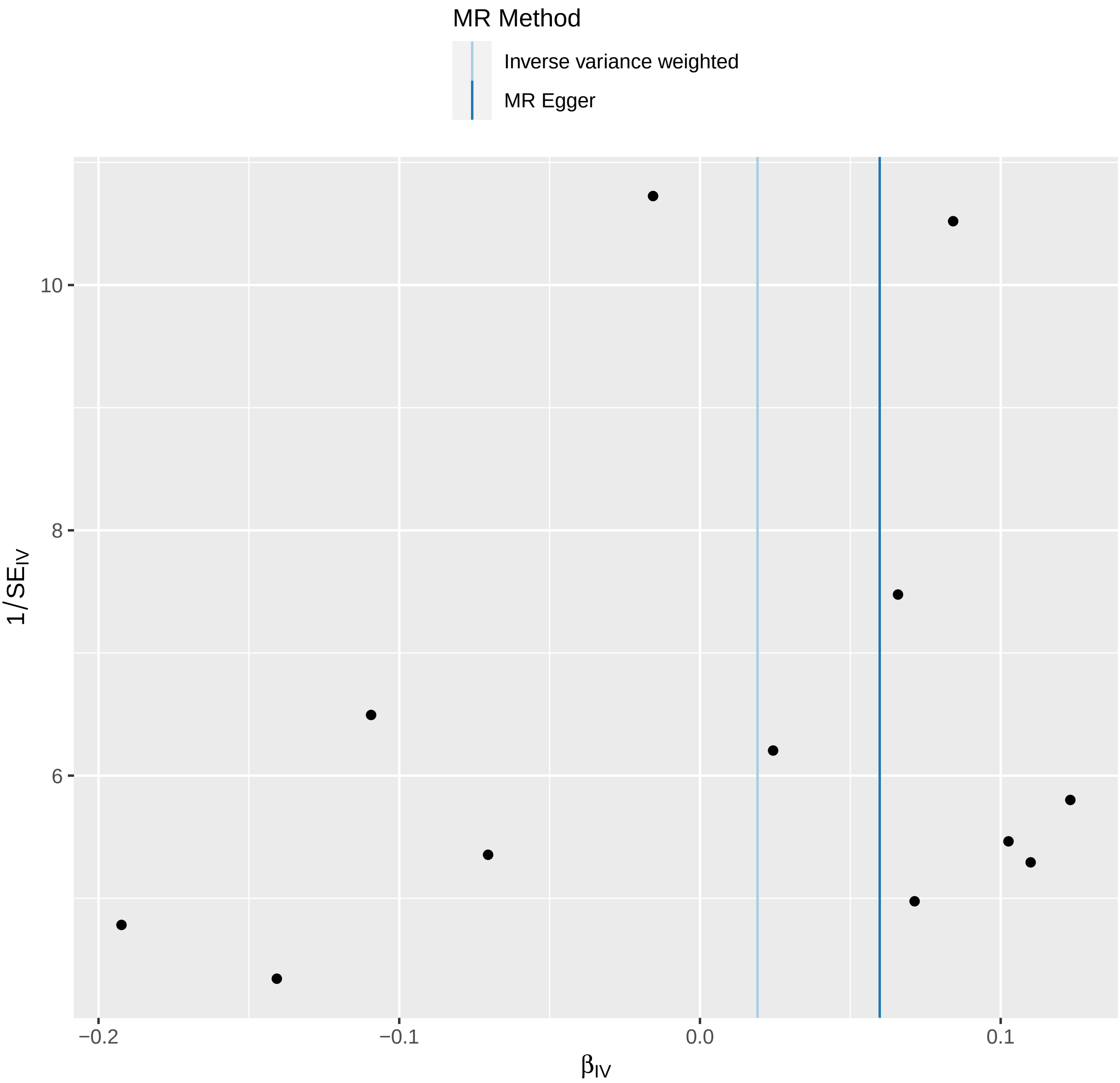

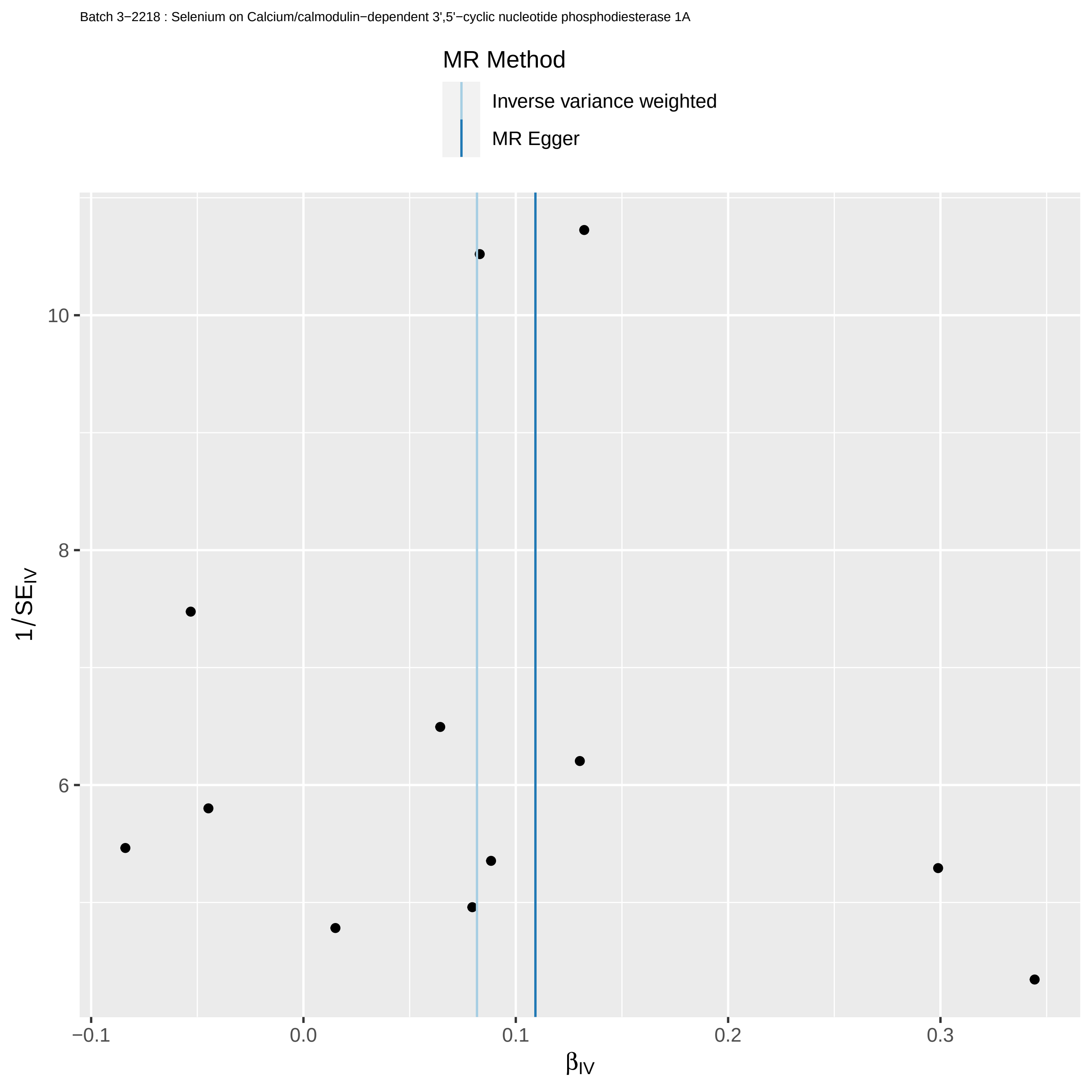

Batch 3−2218 : Selenium on Calcium/calmodulin−dependent 3',5'−cyclic nucleotide phosphodiesterase 1A
MR Method
Inverse variance weighted
MR Egger
10
8
6
1/SE_IV
−0.1
0.0
0.1
0.2
0.3
β_IV

Batch 3−2219 : Selenium on cGMP−dependent 3',5'−cyclic phosphodiesterase

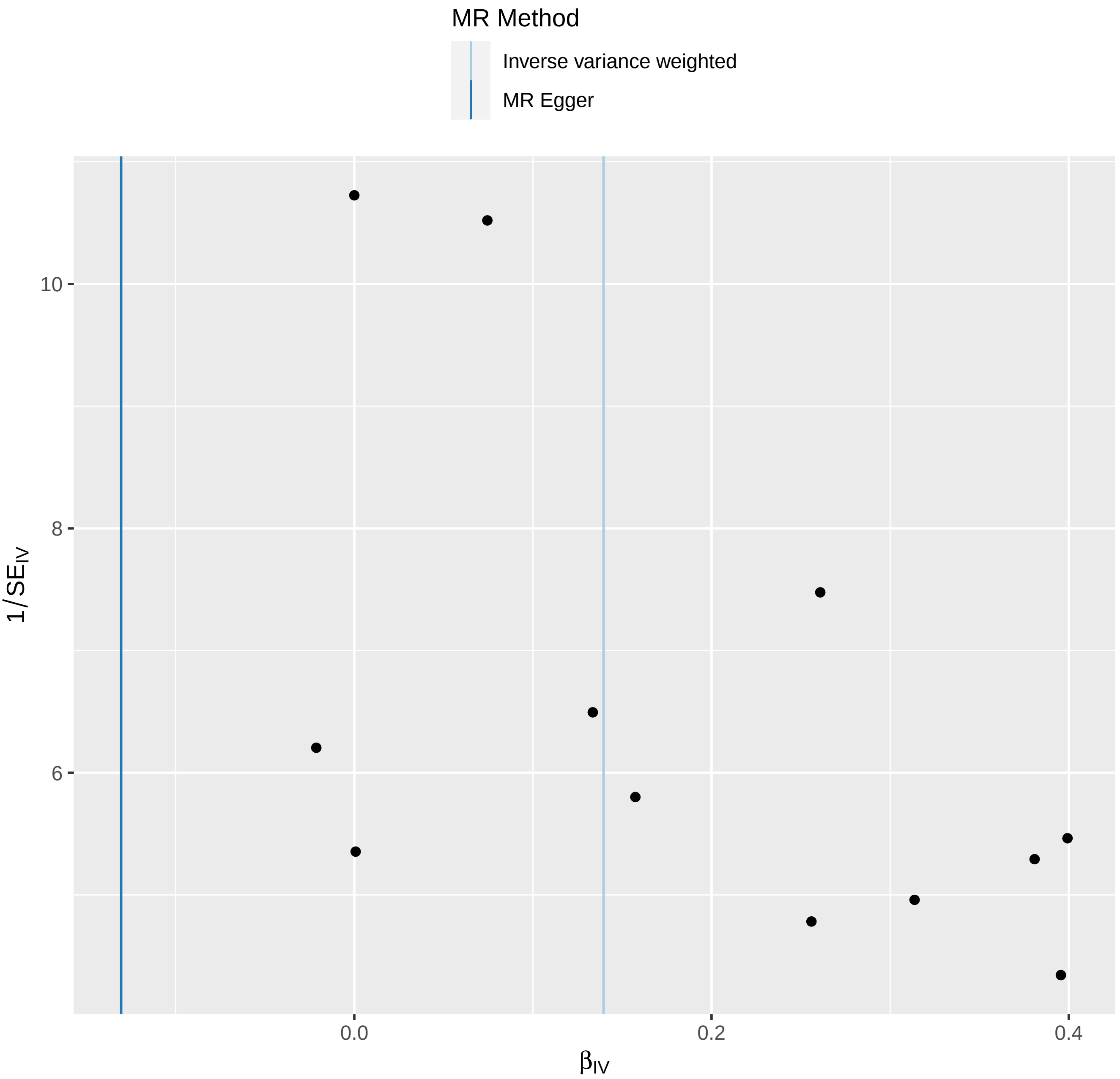

Batch 3−2220 : Selenium on cGMP−inhibited 3',5'−cyclic phosphodiesterase A

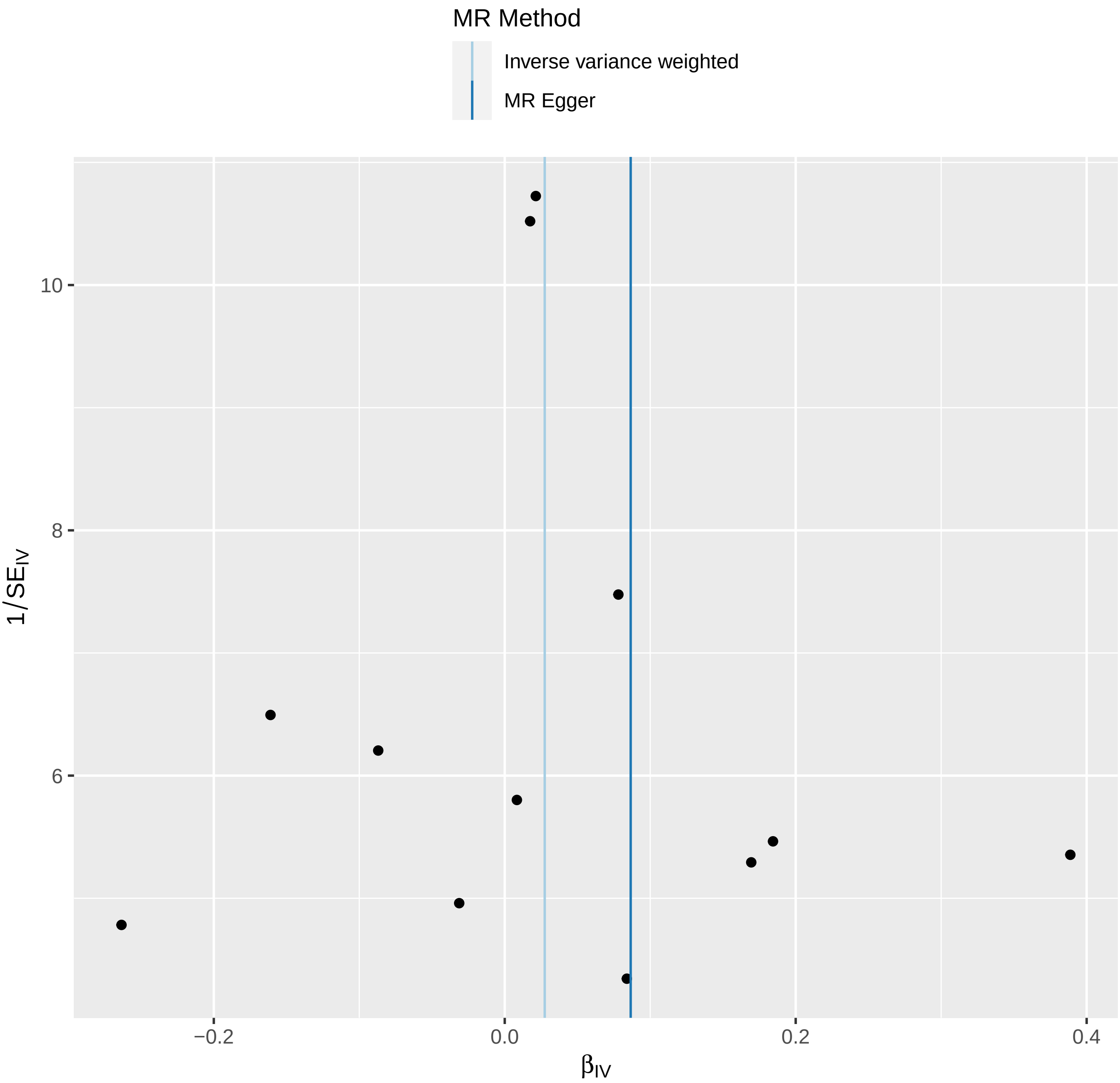

Batch 3−2221 : Selenium on cAMP−specific 3',5'−cyclic phosphodiesterase 4D

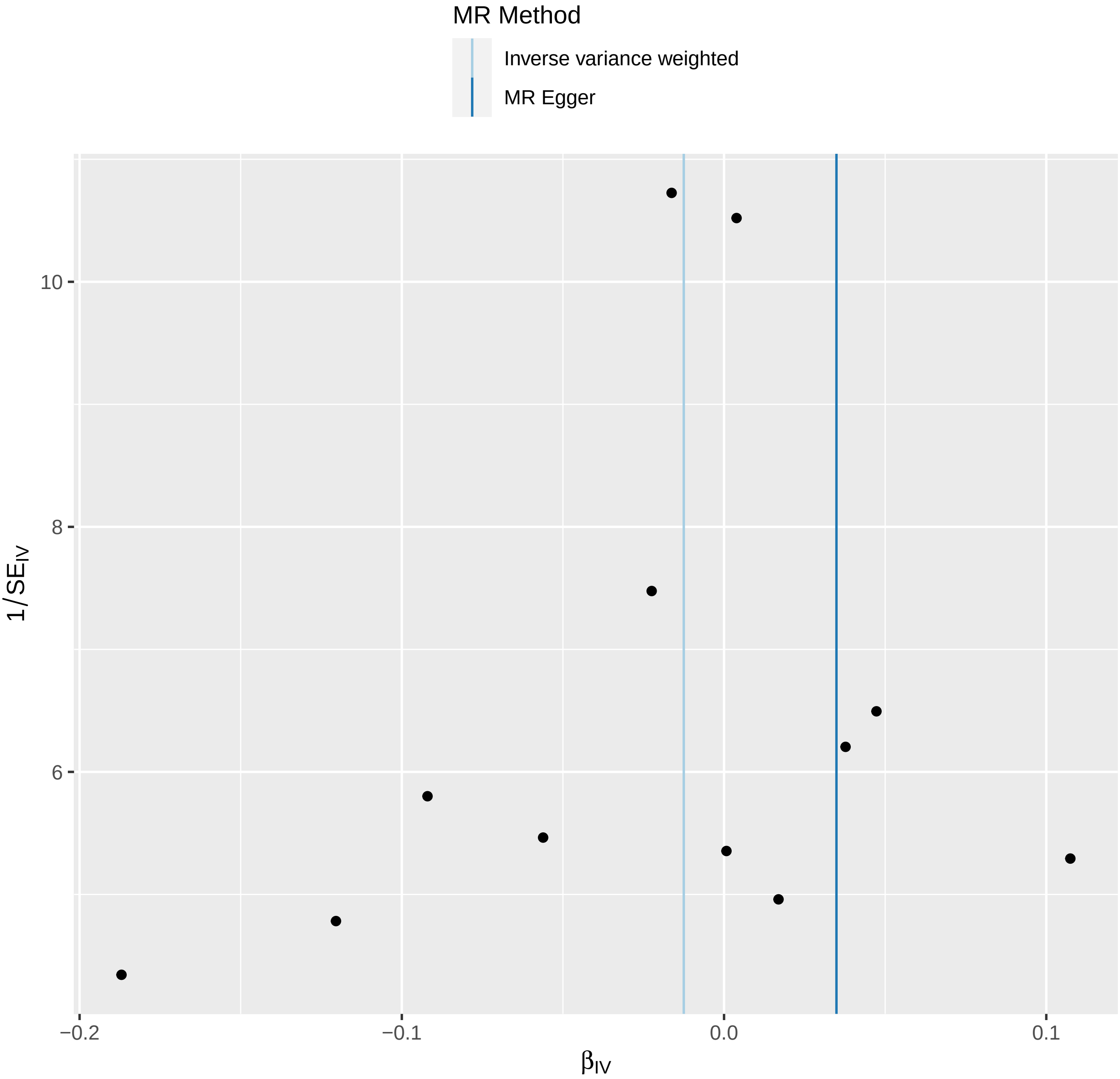

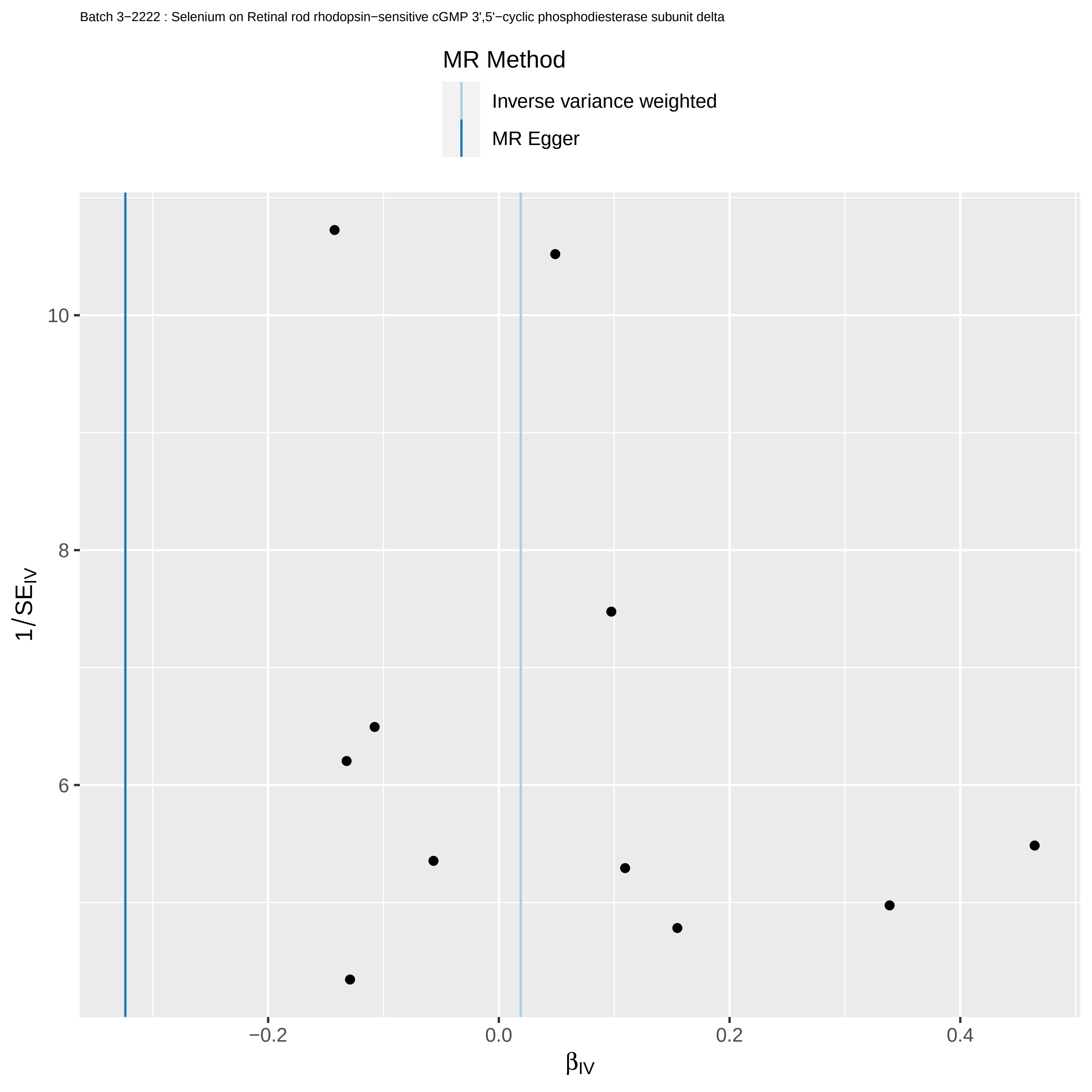

Batch 3−2222 : Selenium on Retinal rod rhodopsin−sensitive cGMP 3',5'−cyclic phosphodiesterase subunit delta
MR Method
Inverse variance weighted
MR Egger
10
8
6
1/SE_IV
−0.2
0.0
0.2
0.4
β_IV

Batch 3−2223 : Selenium on High affinity cAMP−specific 3',5'−cyclic phosphodiesterase 7A

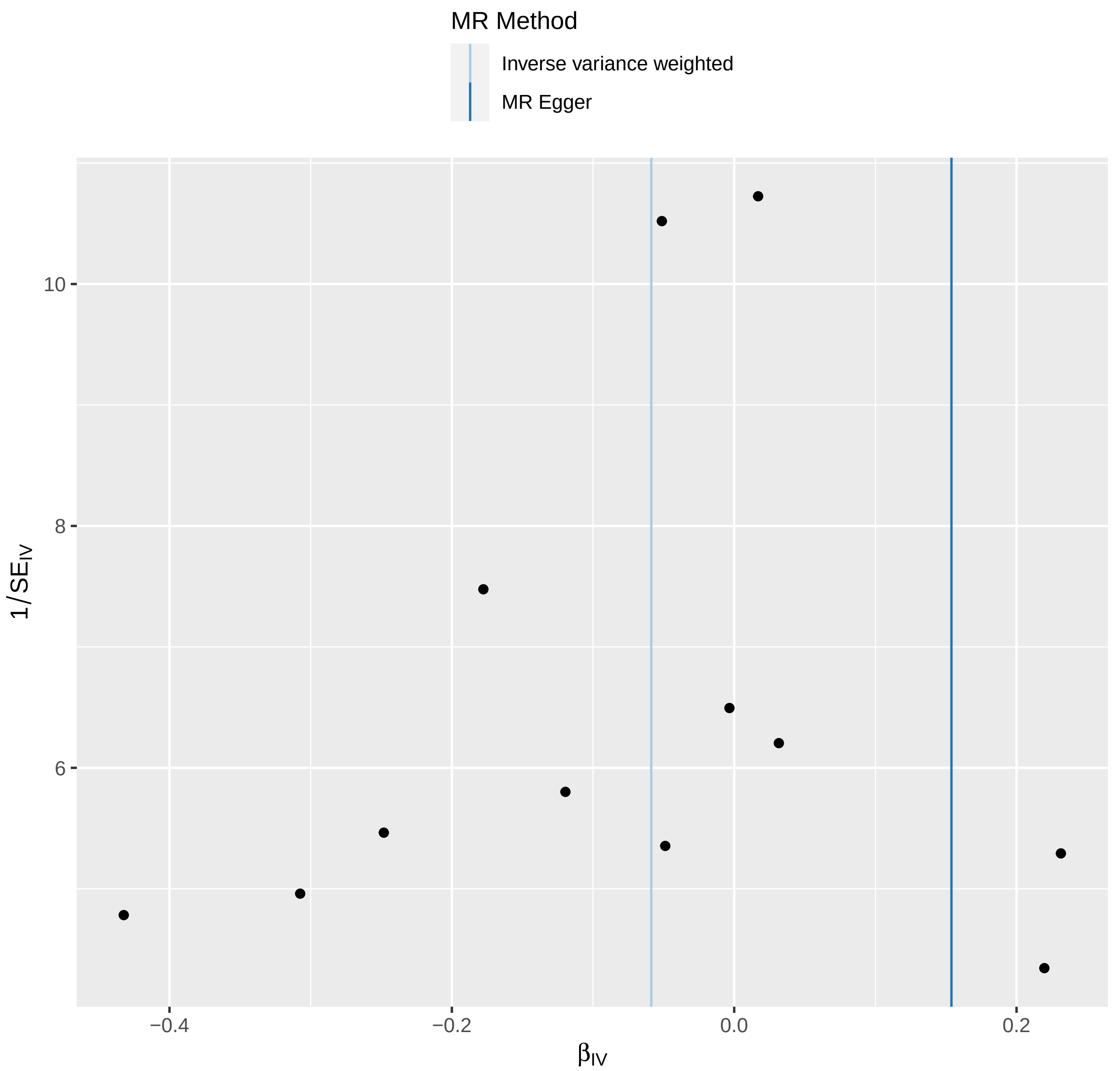

Batch 3−2224 : Selenium on High affinity cGMP−specific 3',5'−cyclic phosphodiesterase 9A

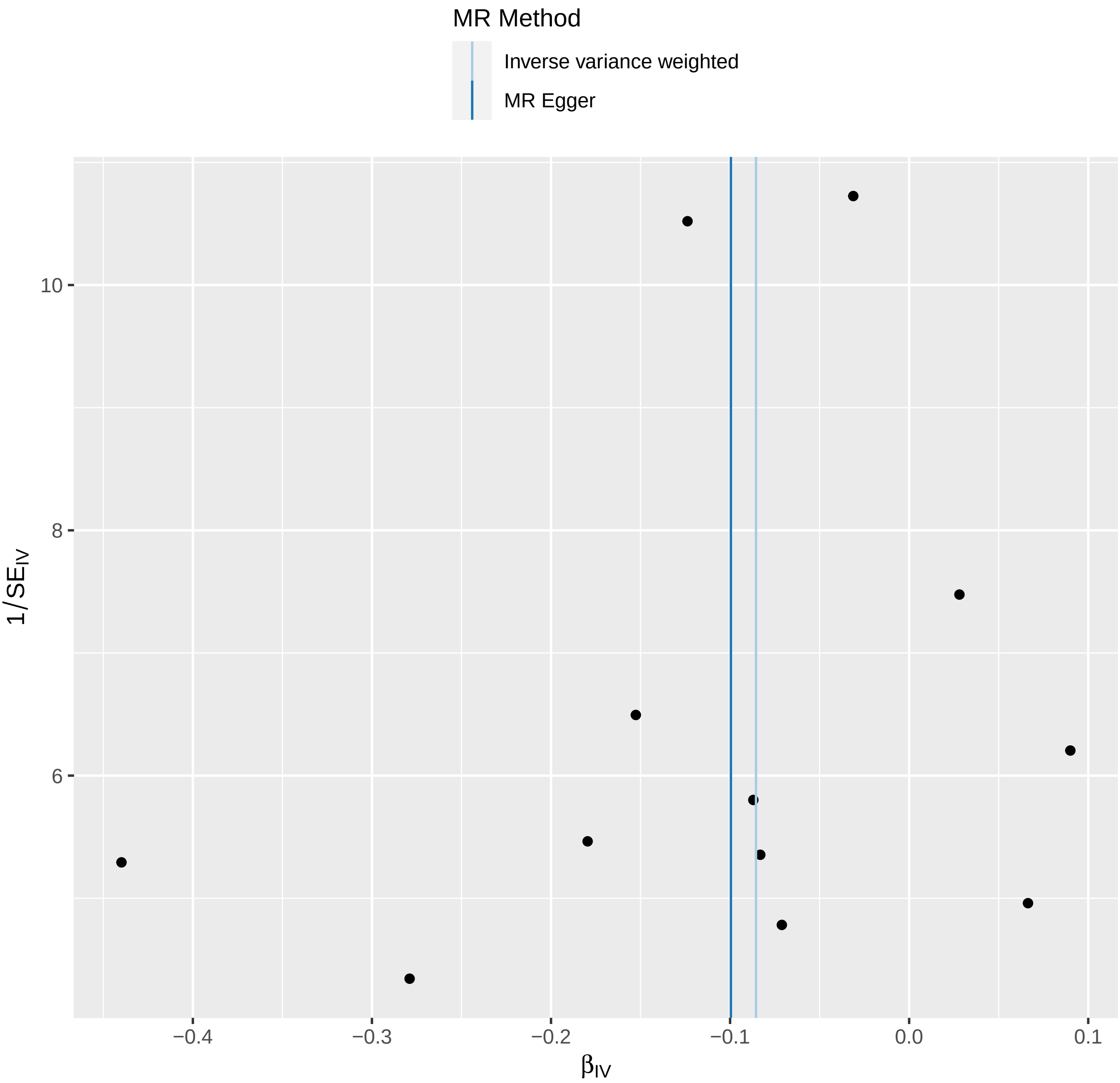

Batch 3−2225 : Selenium on Platelet−derived growth factor subunit A

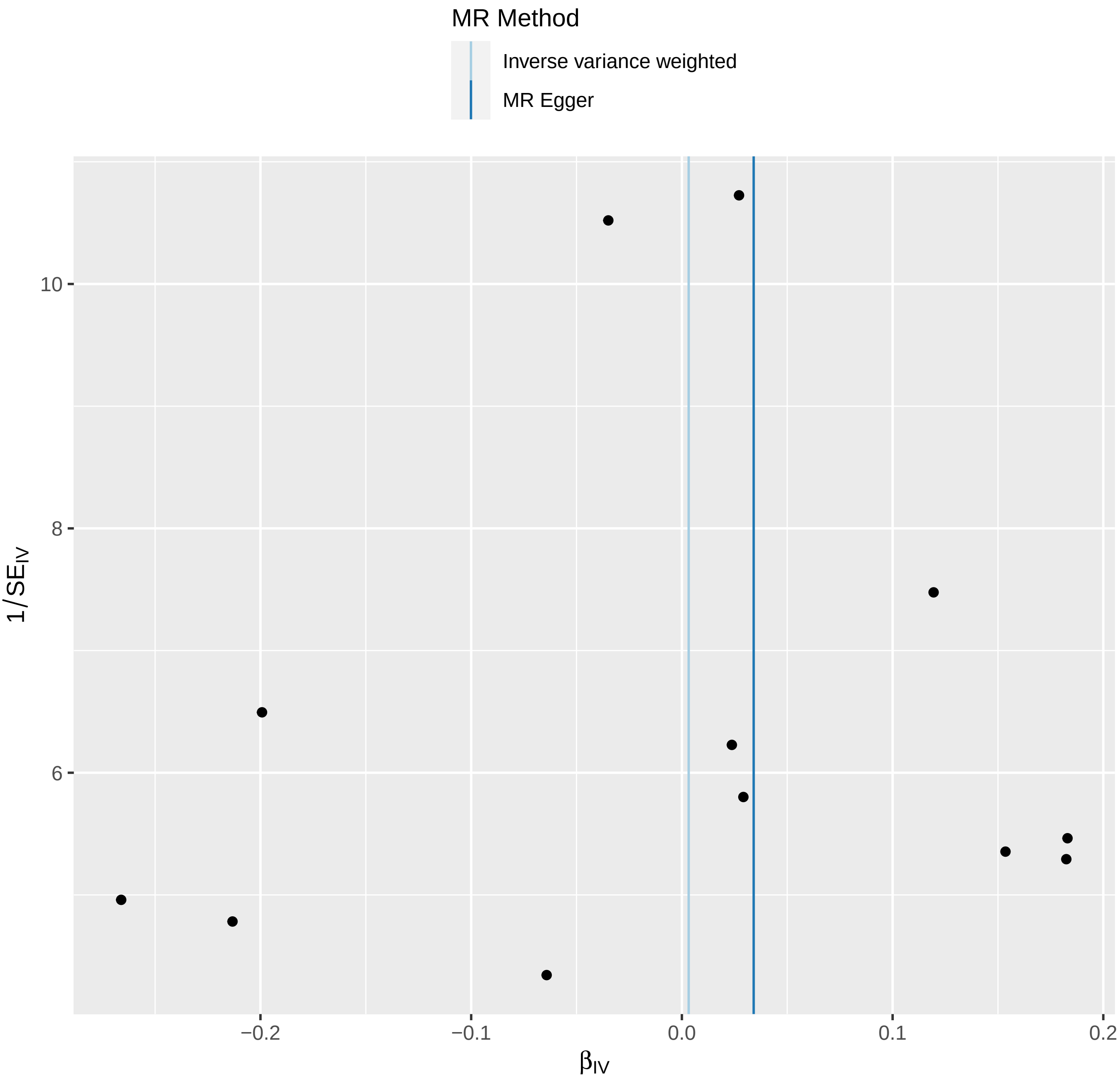

Batch 3−2226 : Selenium on Platelet−derived growth factor subunit B

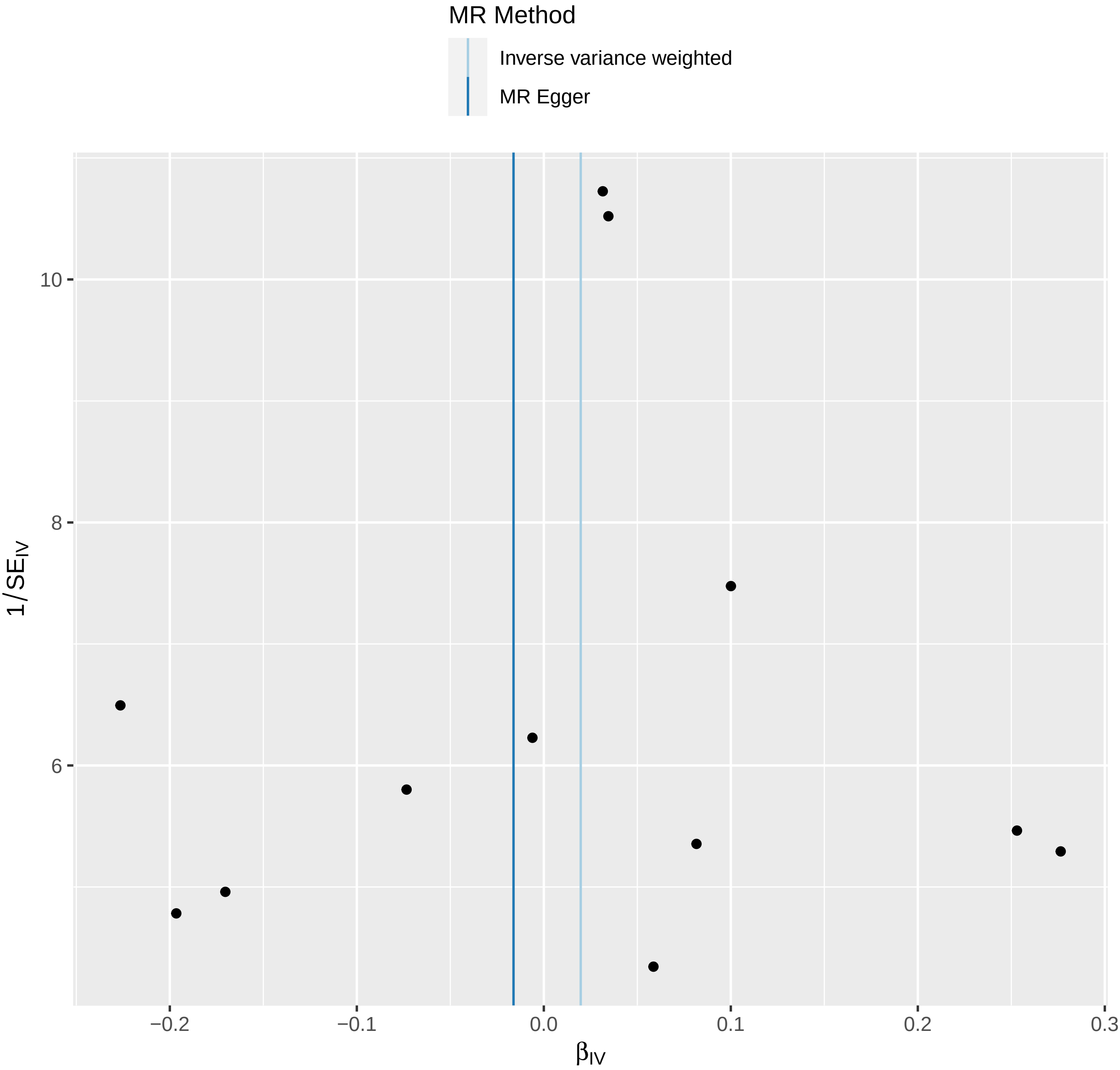

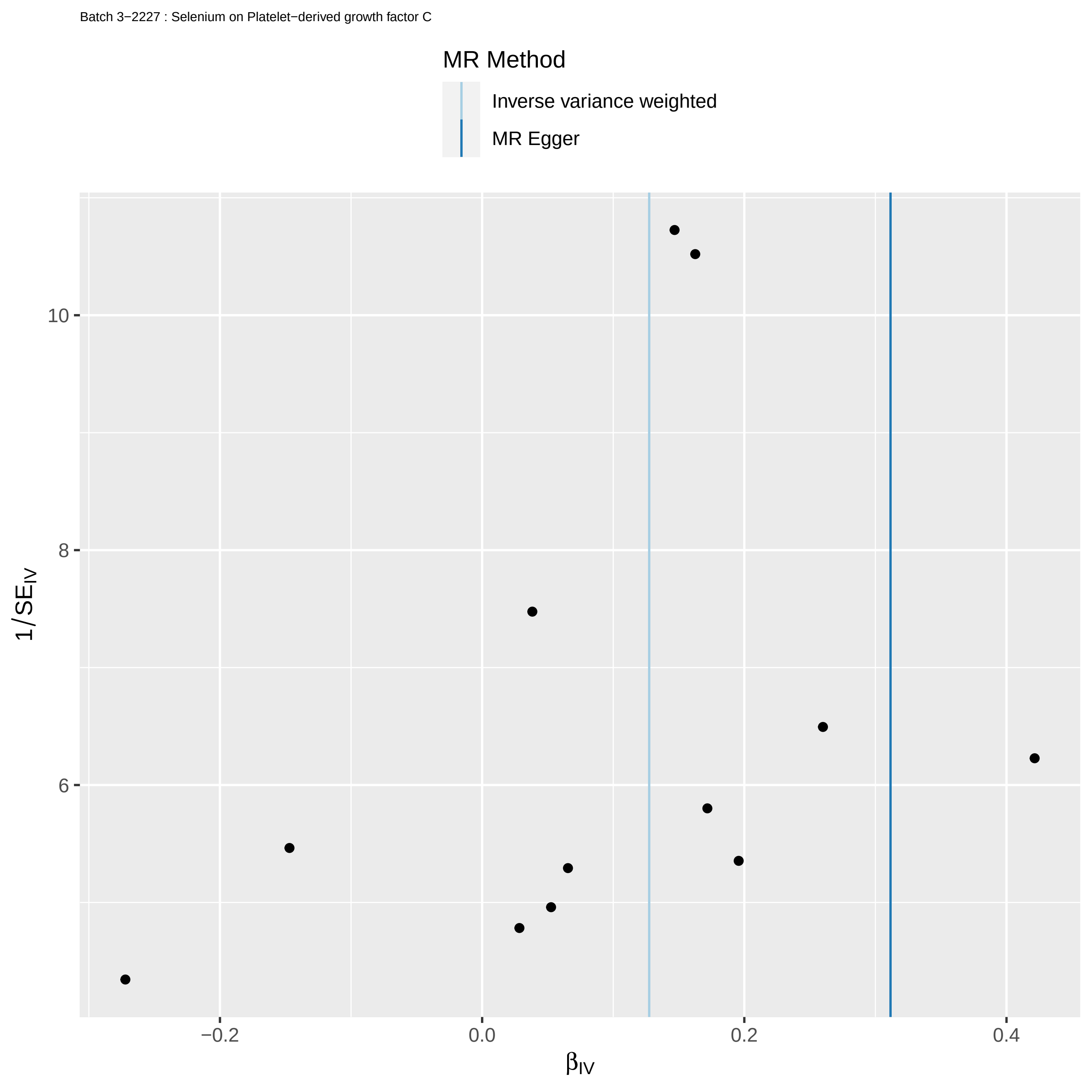
Batch 3−2227 : Selenium on Platelet−derived growth factor C
MR Method
Inverse variance weighted
MR Egger
10
8
6
−0.2
0.0
0.2
0.4
$1/SE_{IV}$
β_{IV}

Batch 3−2228 : Selenium on Platelet−derived growth factor D

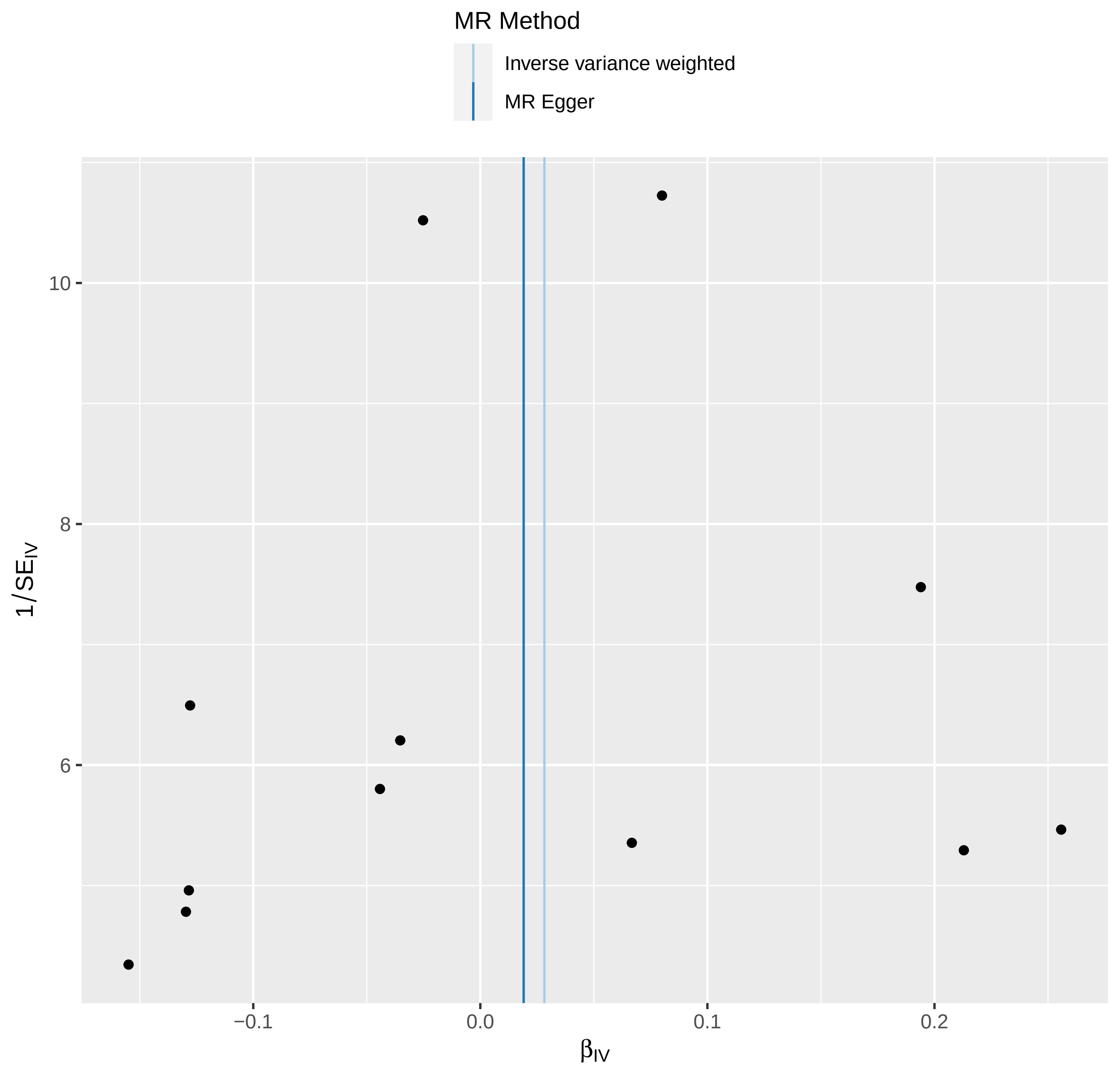

Batch 3−2229 : Selenium on Platelet−derived growth factor receptor alpha

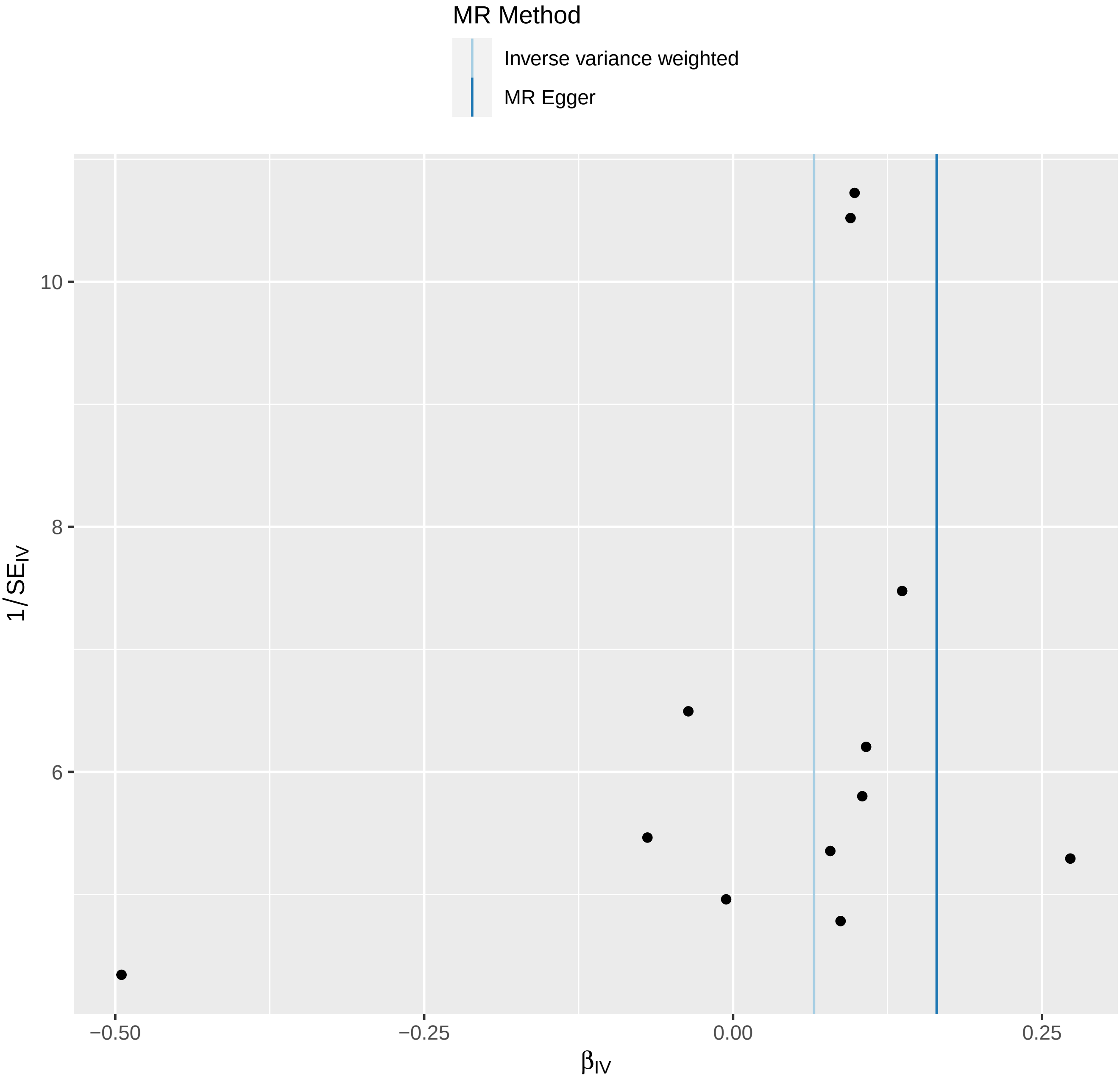

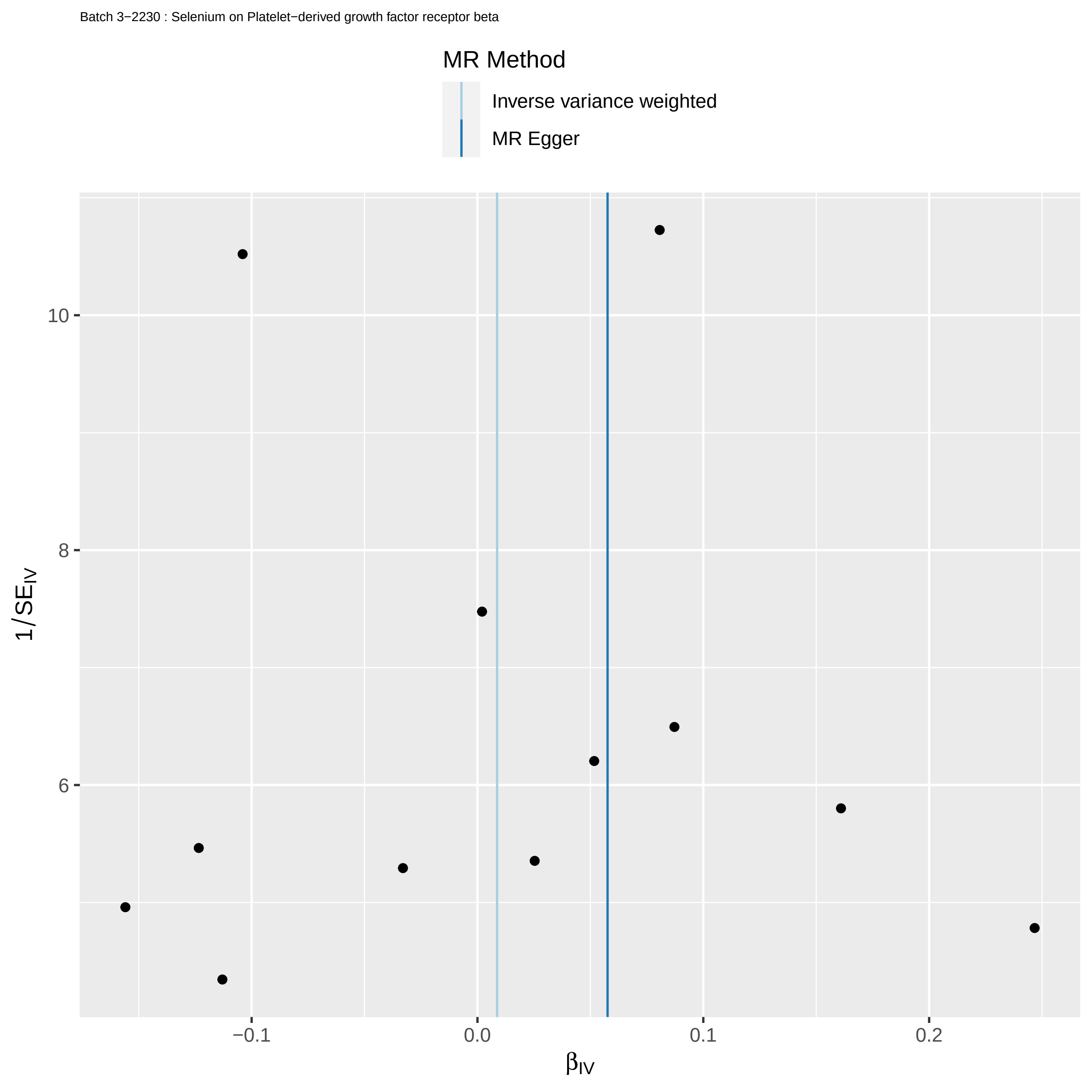
Batch 3−2230 : Selenium on Platelet−derived growth factor receptor beta
MR Method
Inverse variance weighted
MR Egger
10
8
6
−0.1
0.0
0.1
0.2
$1/SE_{IV}$
β_{IV}

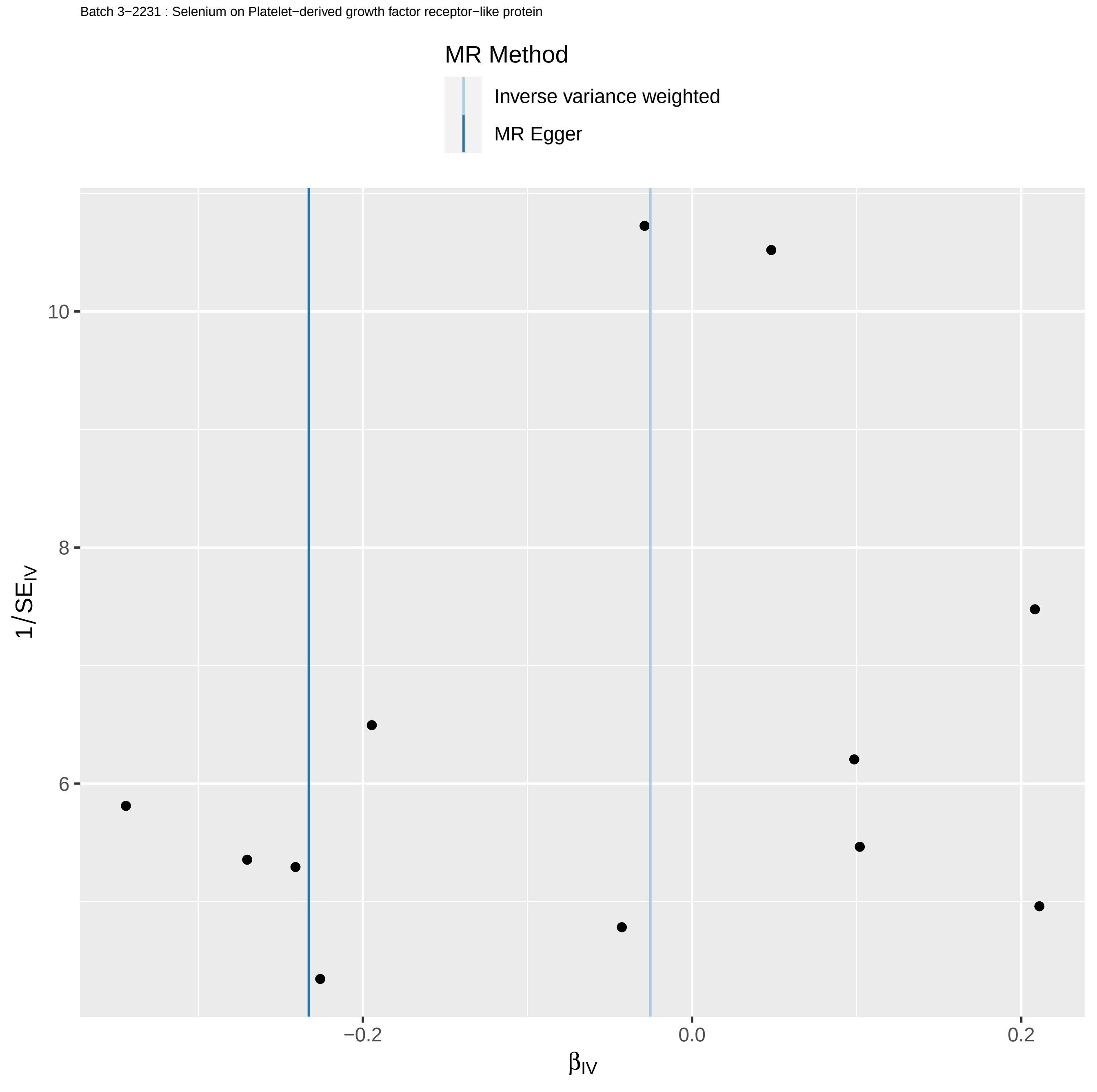
Batch 3−2231 : Selenium on Platelet−derived growth factor receptor−like protein
MR Method
Inverse variance weighted
MR Egger
10
8
6
1/SE_IV
−0.2
0.0
0.2
β_IV

Batch 3−2232 : Selenium on Protein disulfide−isomerase A3

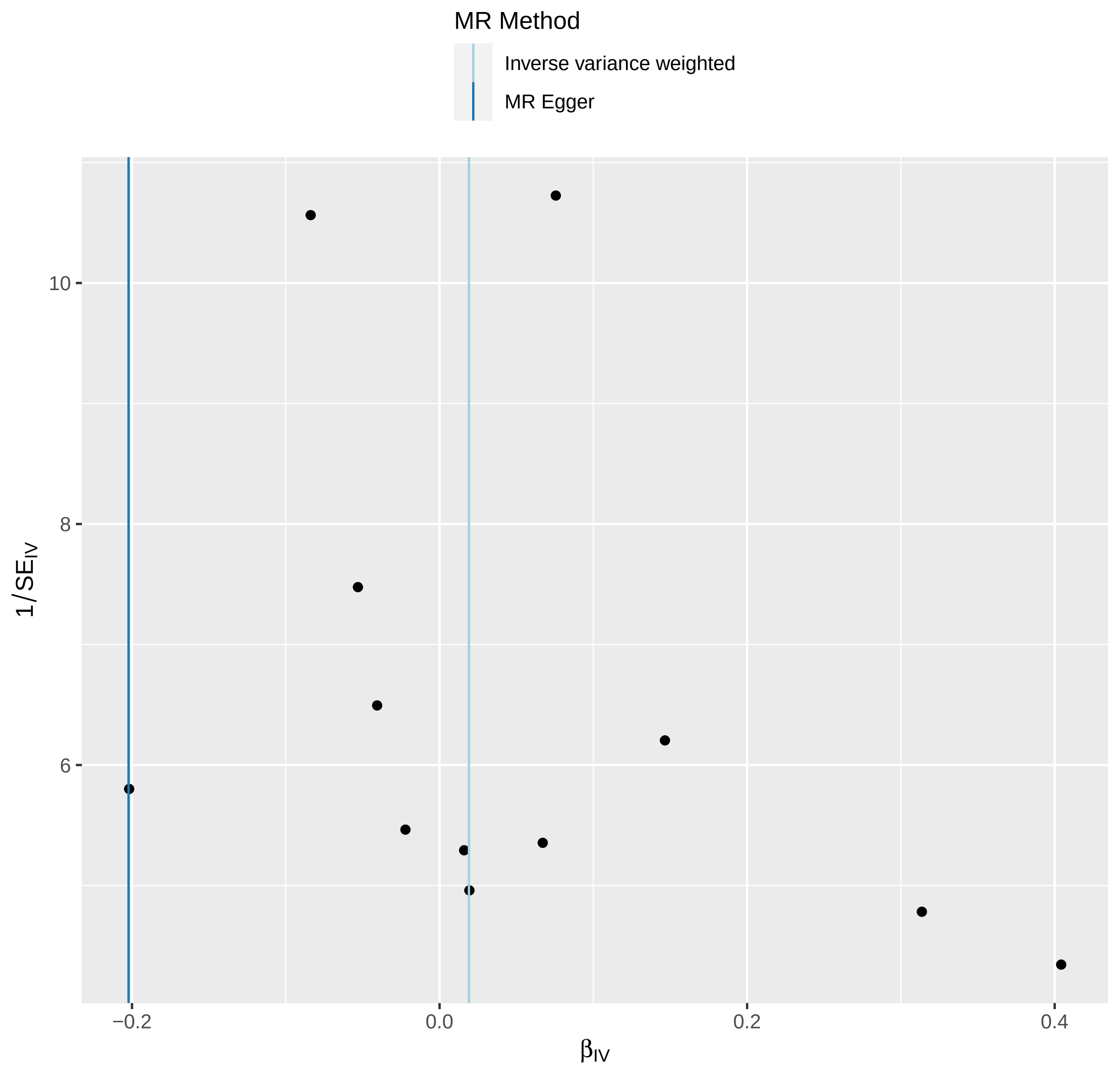

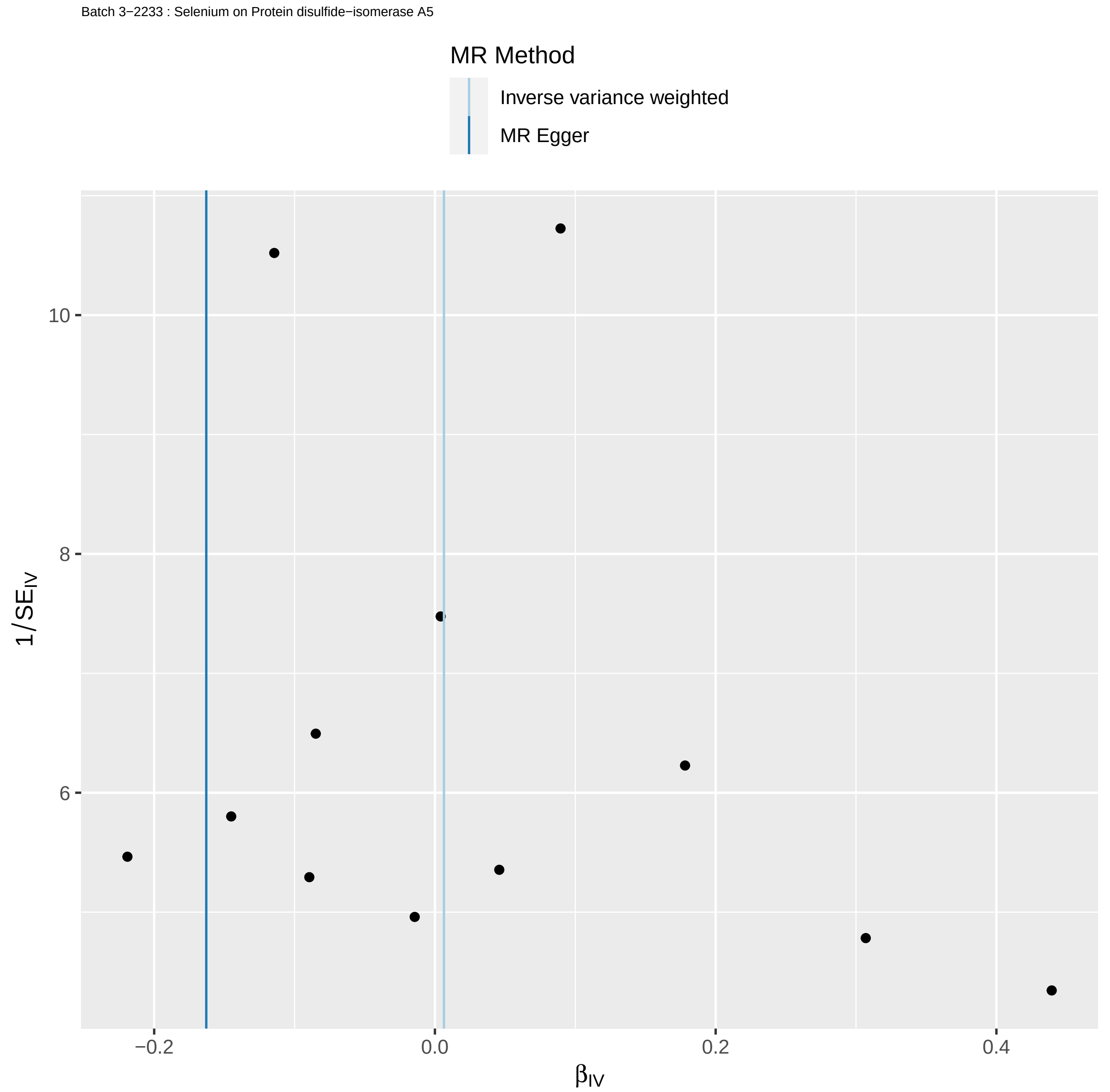

Batch 3−2233 : Selenium on Protein disulfide−isomerase A5
MR Method
Inverse variance weighted
MR Egger
10
8
6
1/SE$_{IV}$
−0.2
0.0
0.2
0.4
β_{IV}

Batch 3−2234 : Selenium on Protein disulfide−isomerase−like protein of the testis

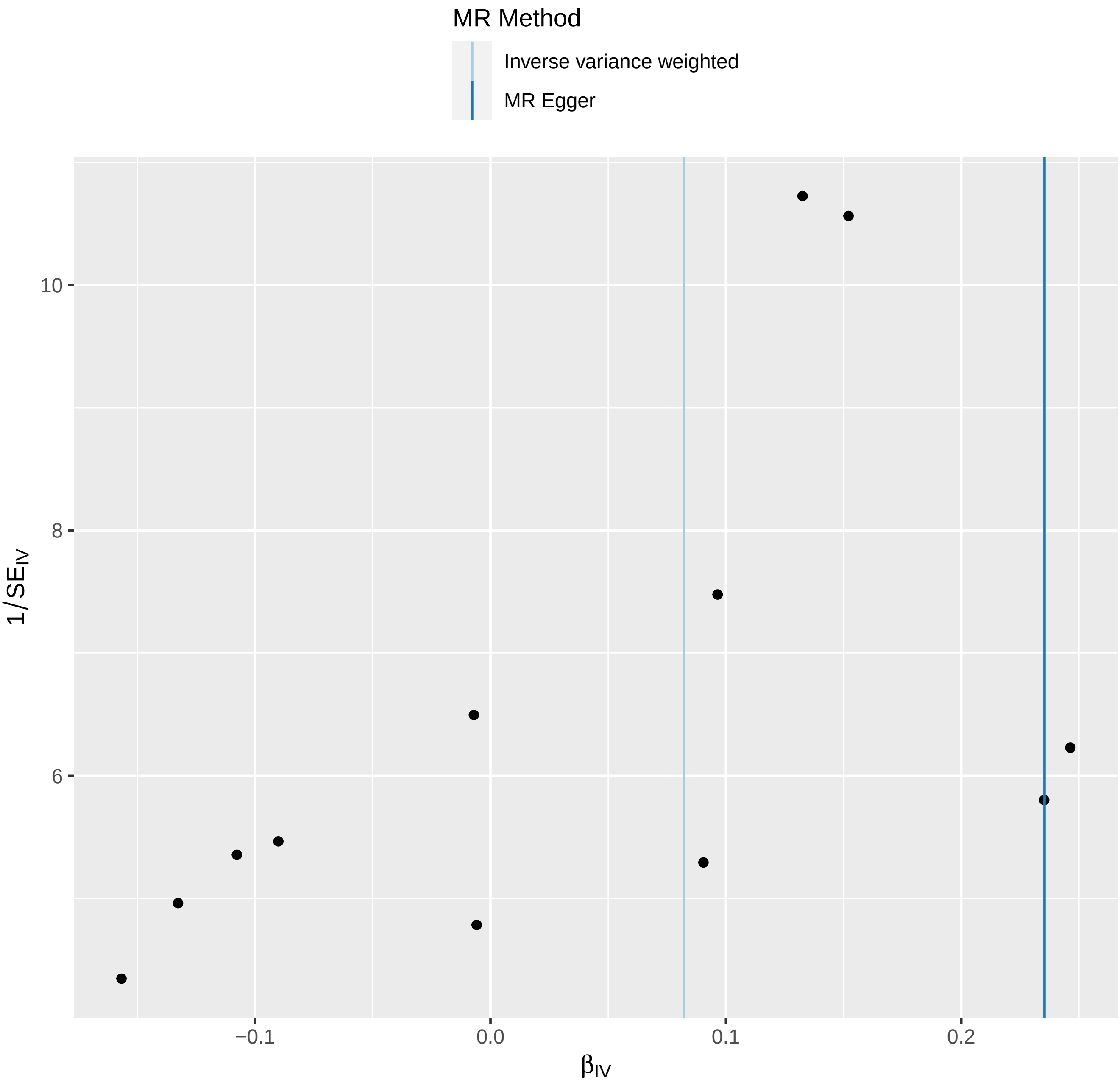

Batch 3−2235 : Selenium on [Pyruvate dehydrogenase (acetyl−transferring)] kinase isozyme 1, mitochondrial

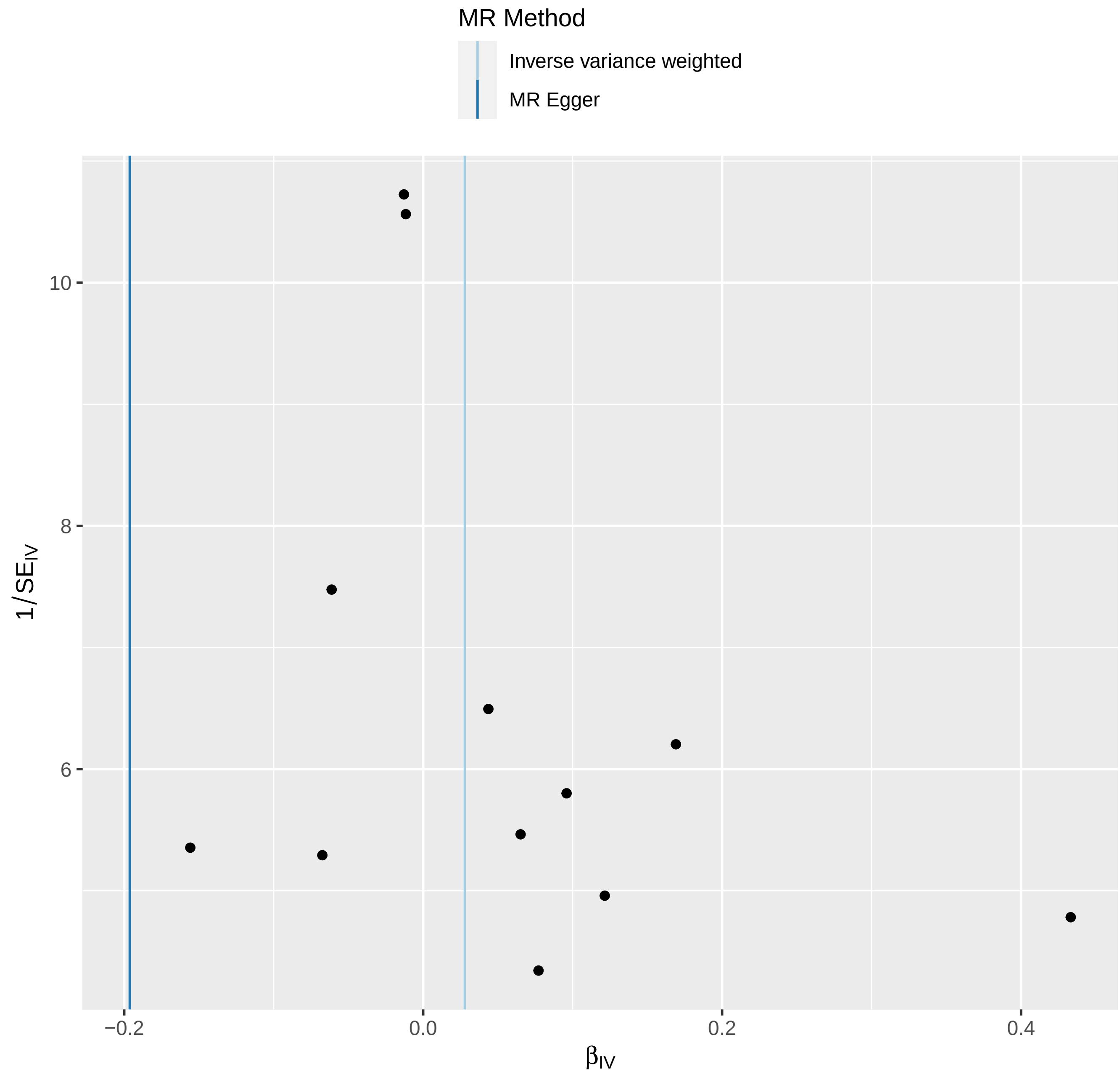

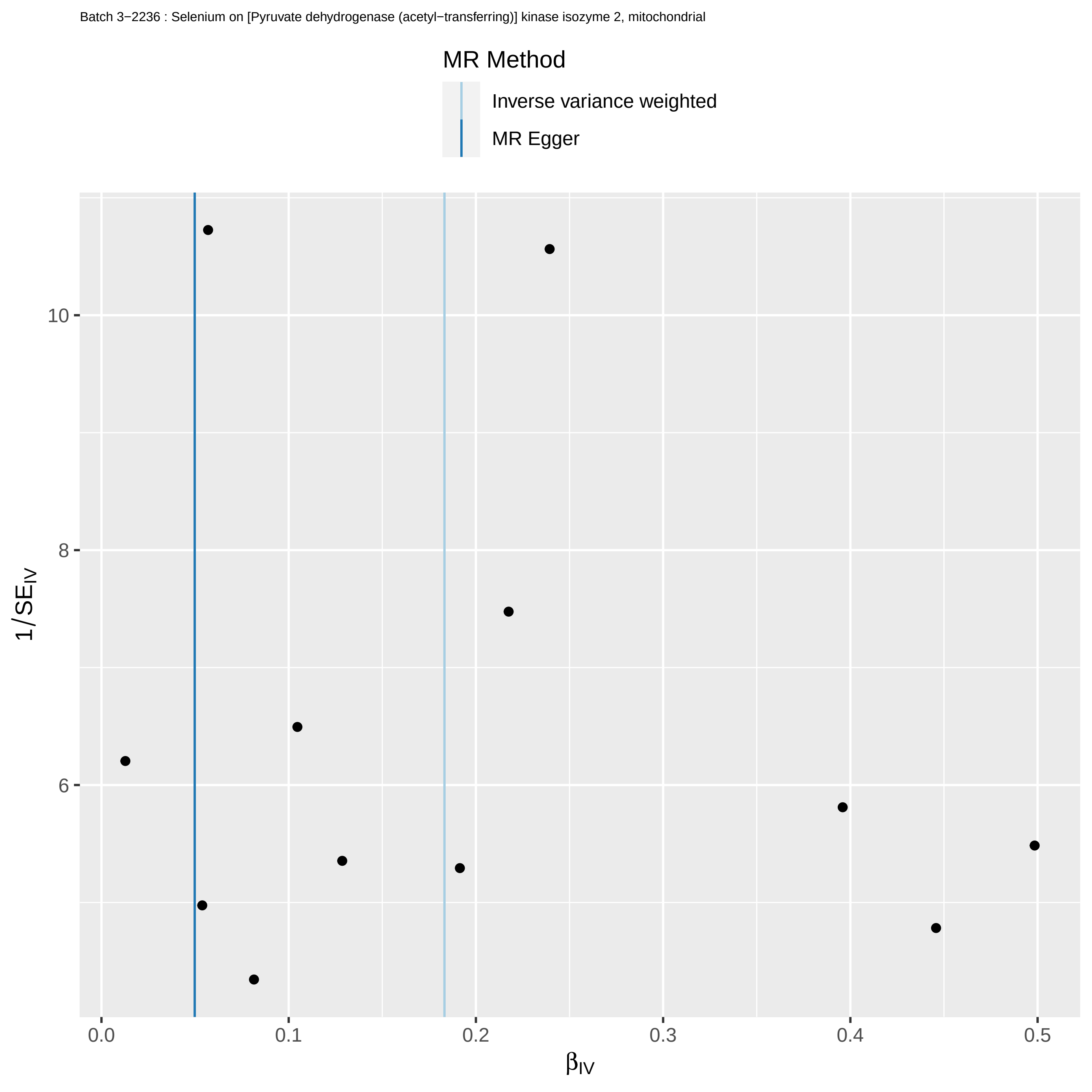
Batch 3−2236 : Selenium on [Pyruvate dehydrogenase (acetyl−transferring)] kinase isozyme 2, mitochondrial
MR Method
Inverse variance weighted
MR Egger
10
8
6
1/SE_IV
0.0
0.1
0.2
0.3
0.4
0.5
β_IV

Batch 3−2237 : Selenium on PDZ and LIM domain protein 4

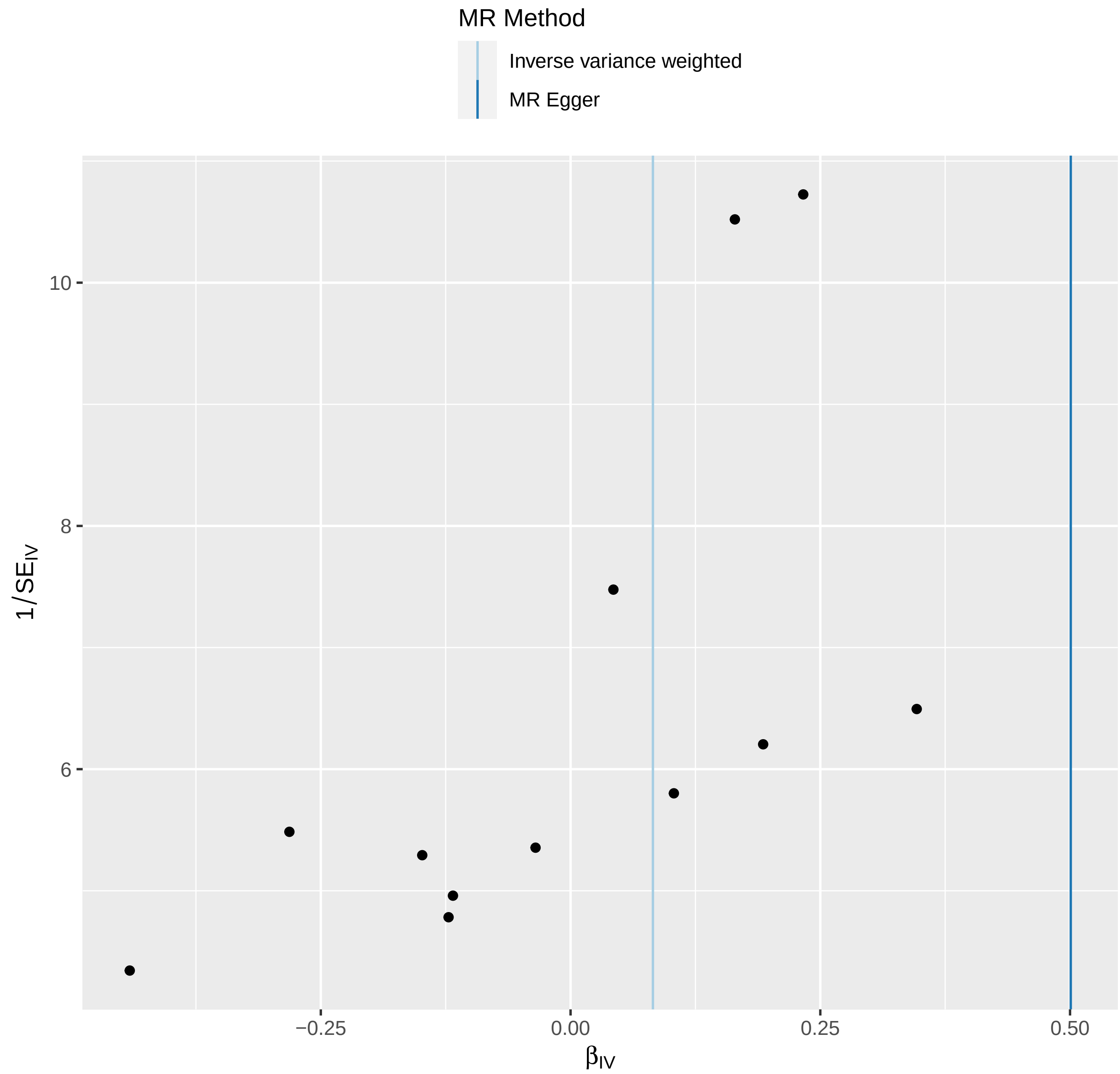

Batch 3−2238 : Selenium on p53 and DNA damage−regulated protein 1

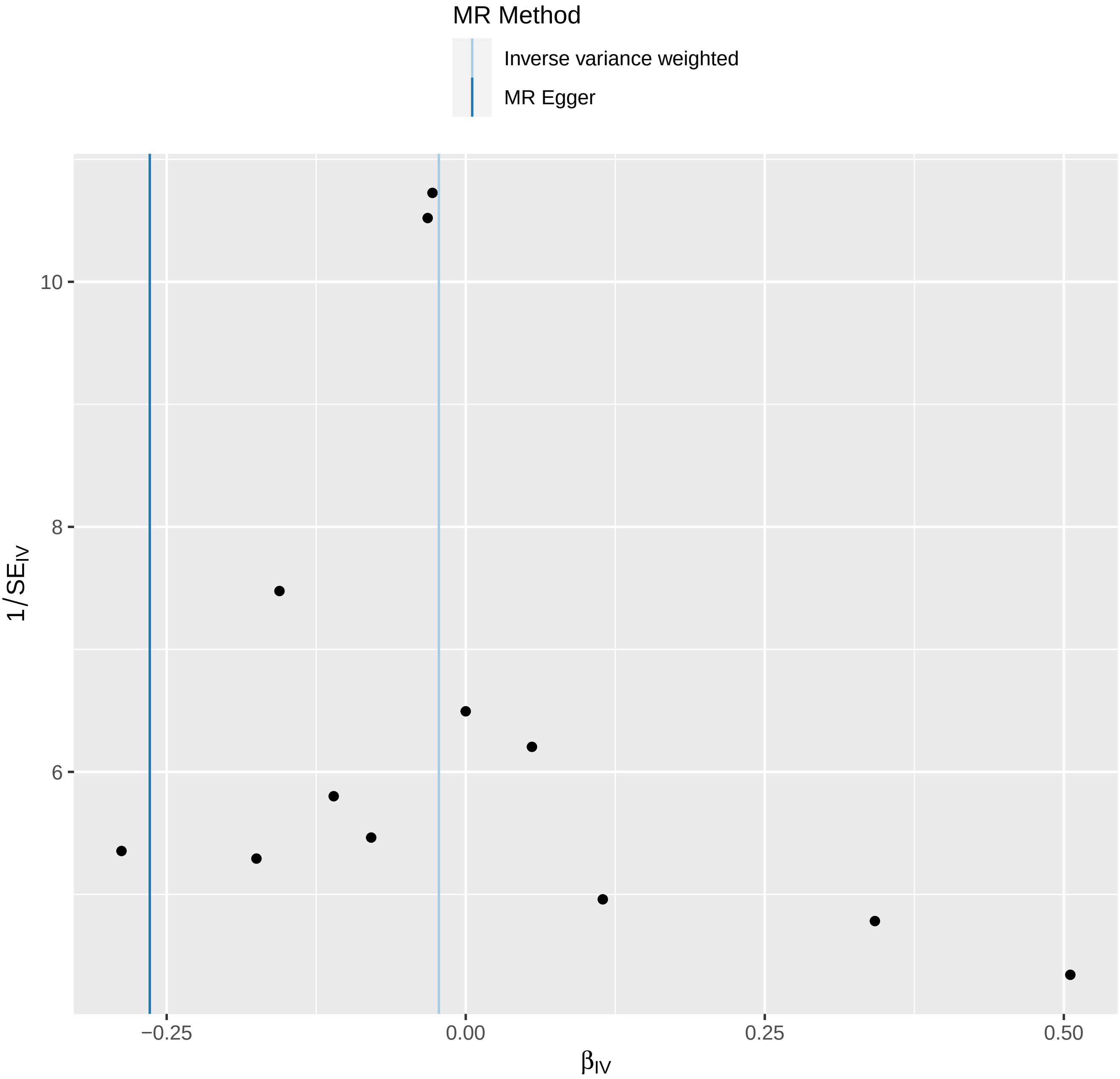

Batch 3−2239 : Selenium on Pyridoxal kinase

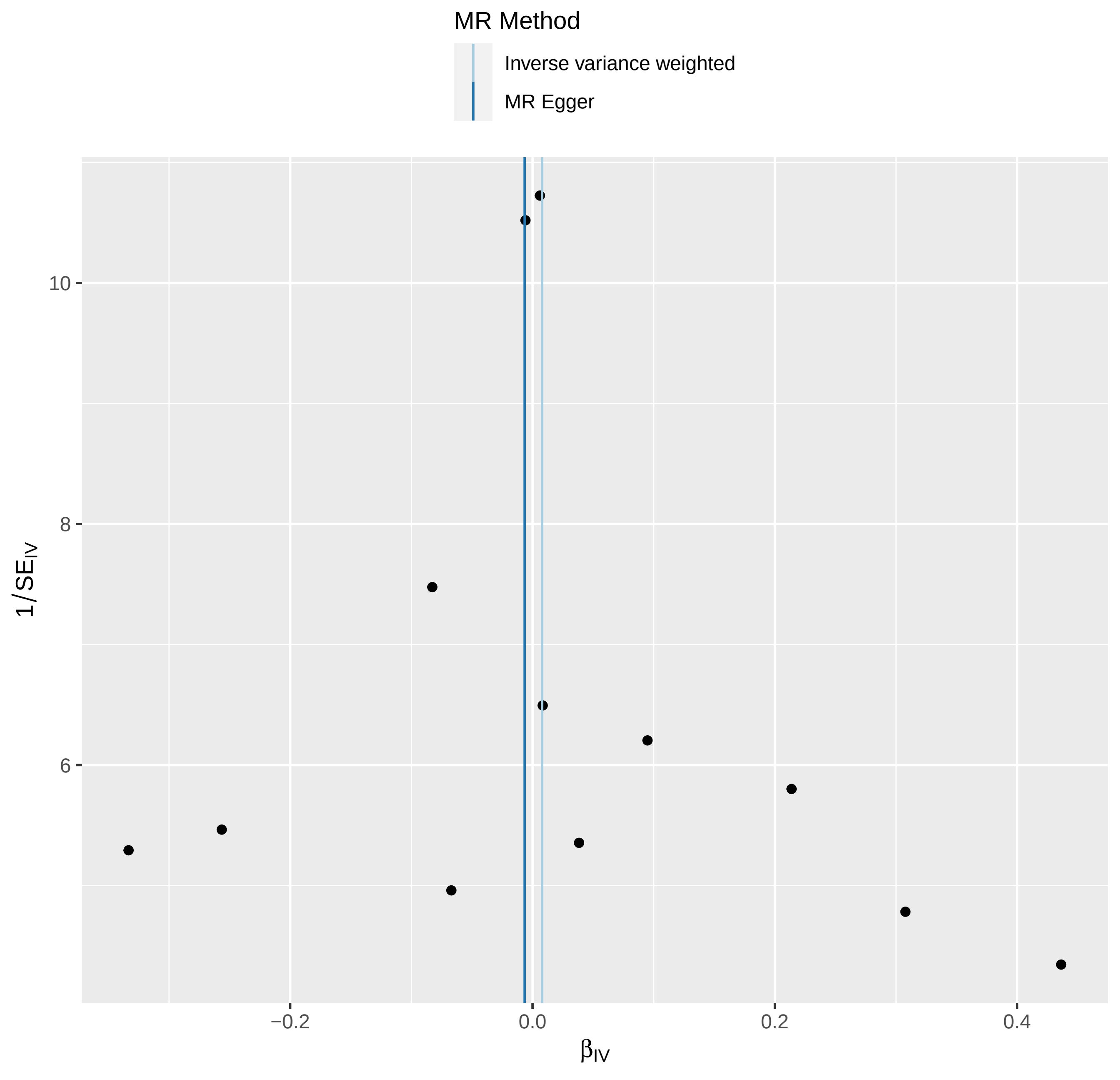

Batch 3−2240 : Selenium on Pyridoxal phosphate phosphatase

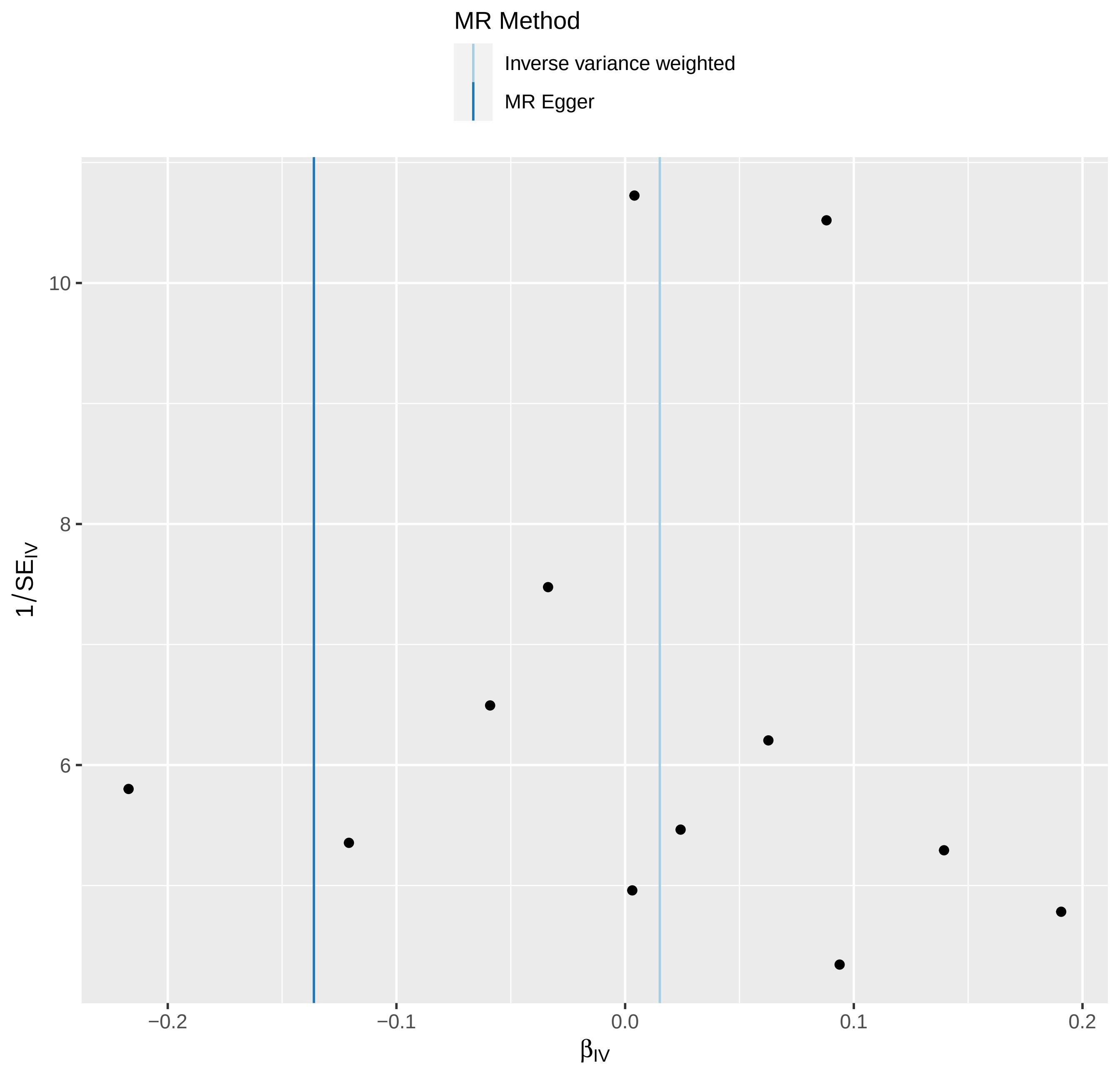

Batch 3−2241 : Selenium on PDZ domain−containing protein 11

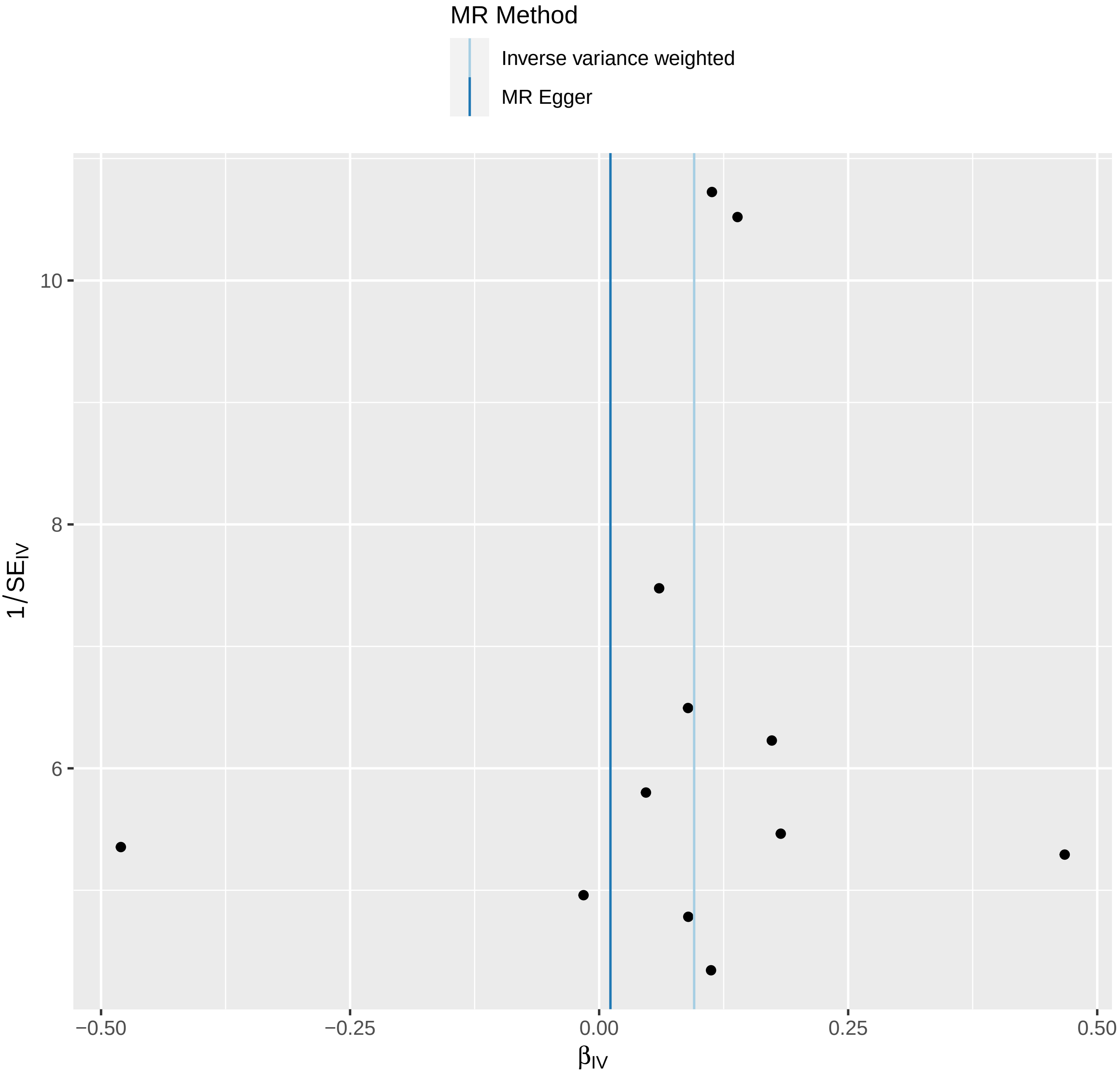

Batch 3−2242 : Selenium on PDZK1−interacting protein 1

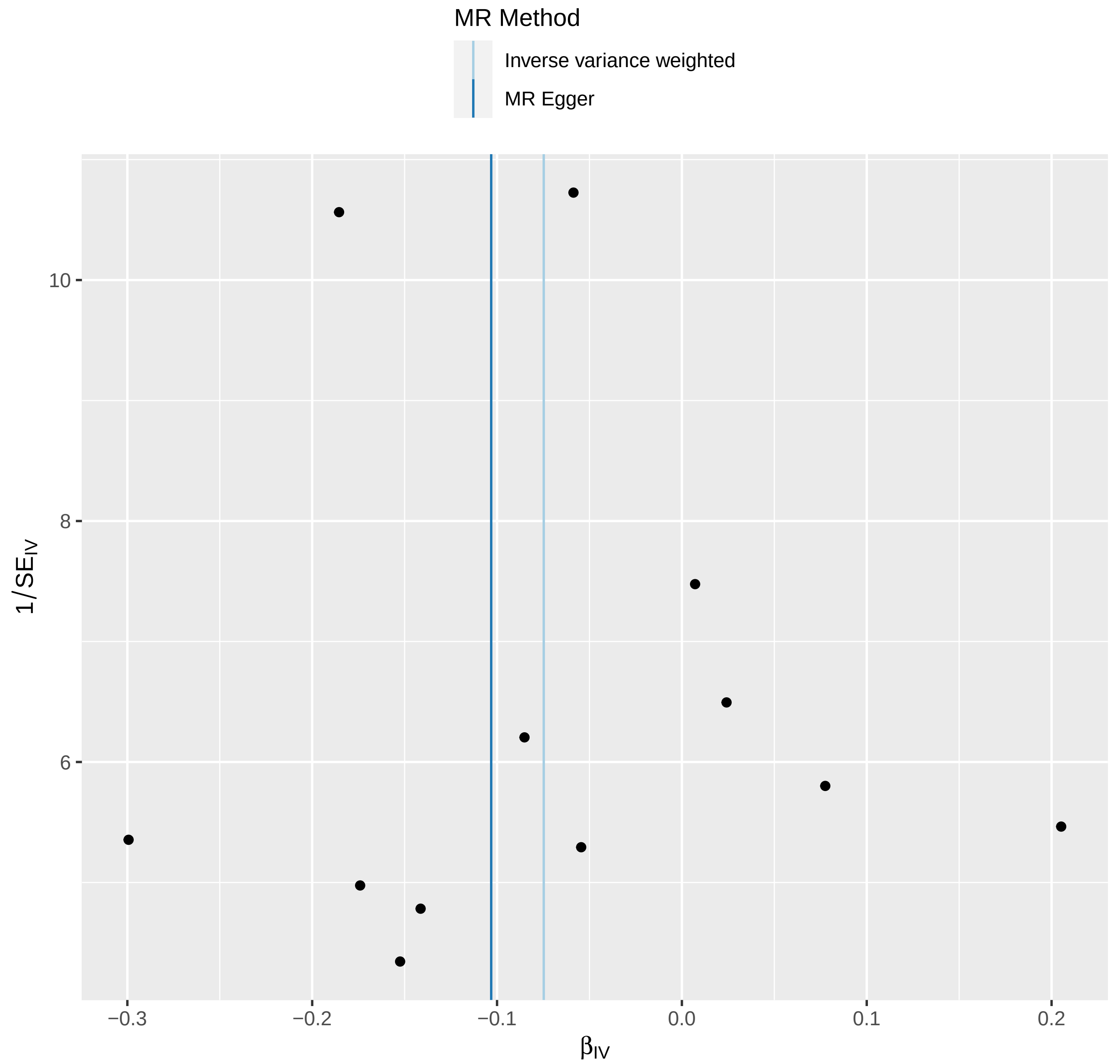

Batch 3−2243 : Selenium on Platelet endothelial aggregation receptor 1

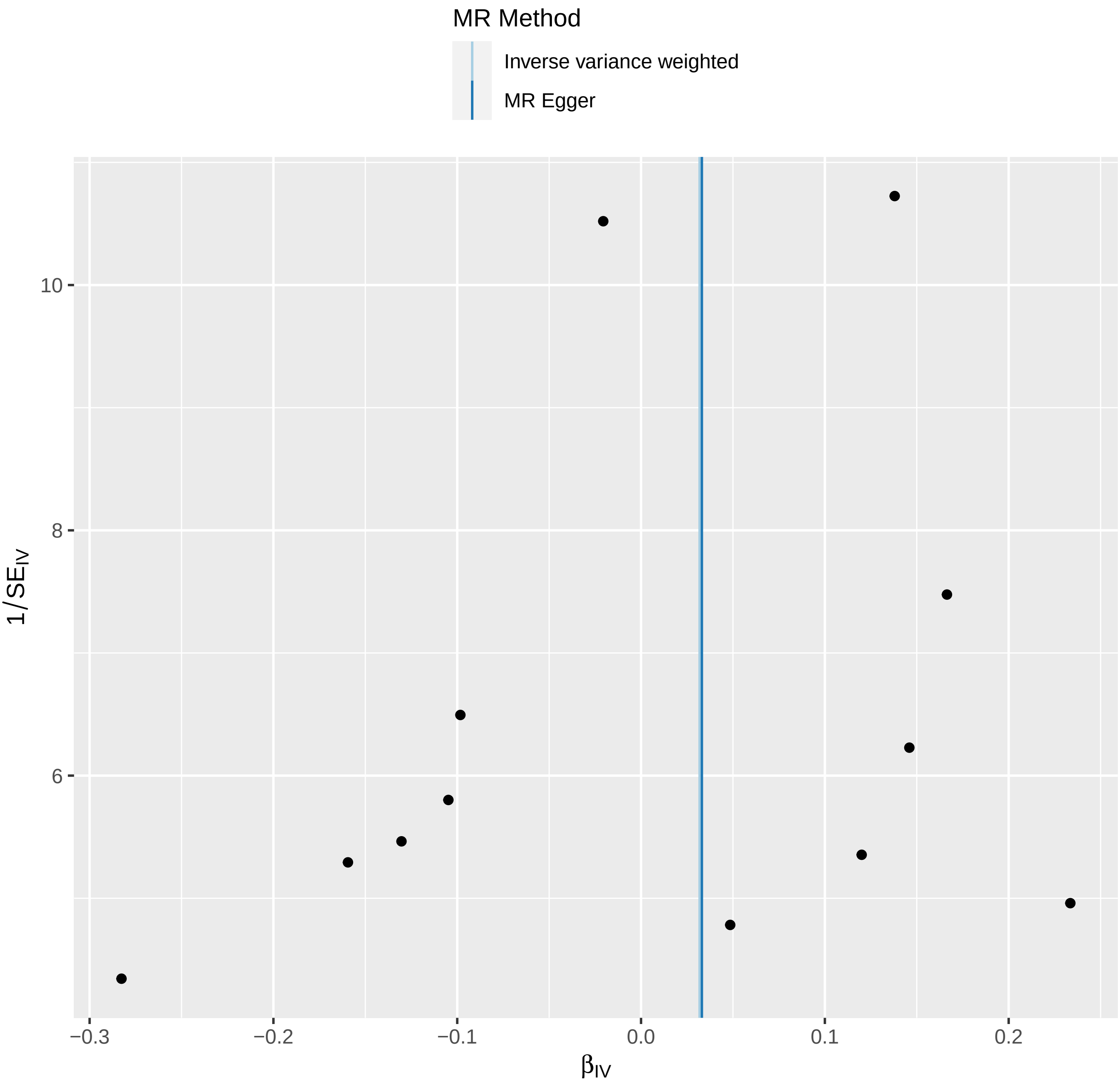

Batch 3−2244 : Selenium on Platelet endothelial aggregation receptor 1

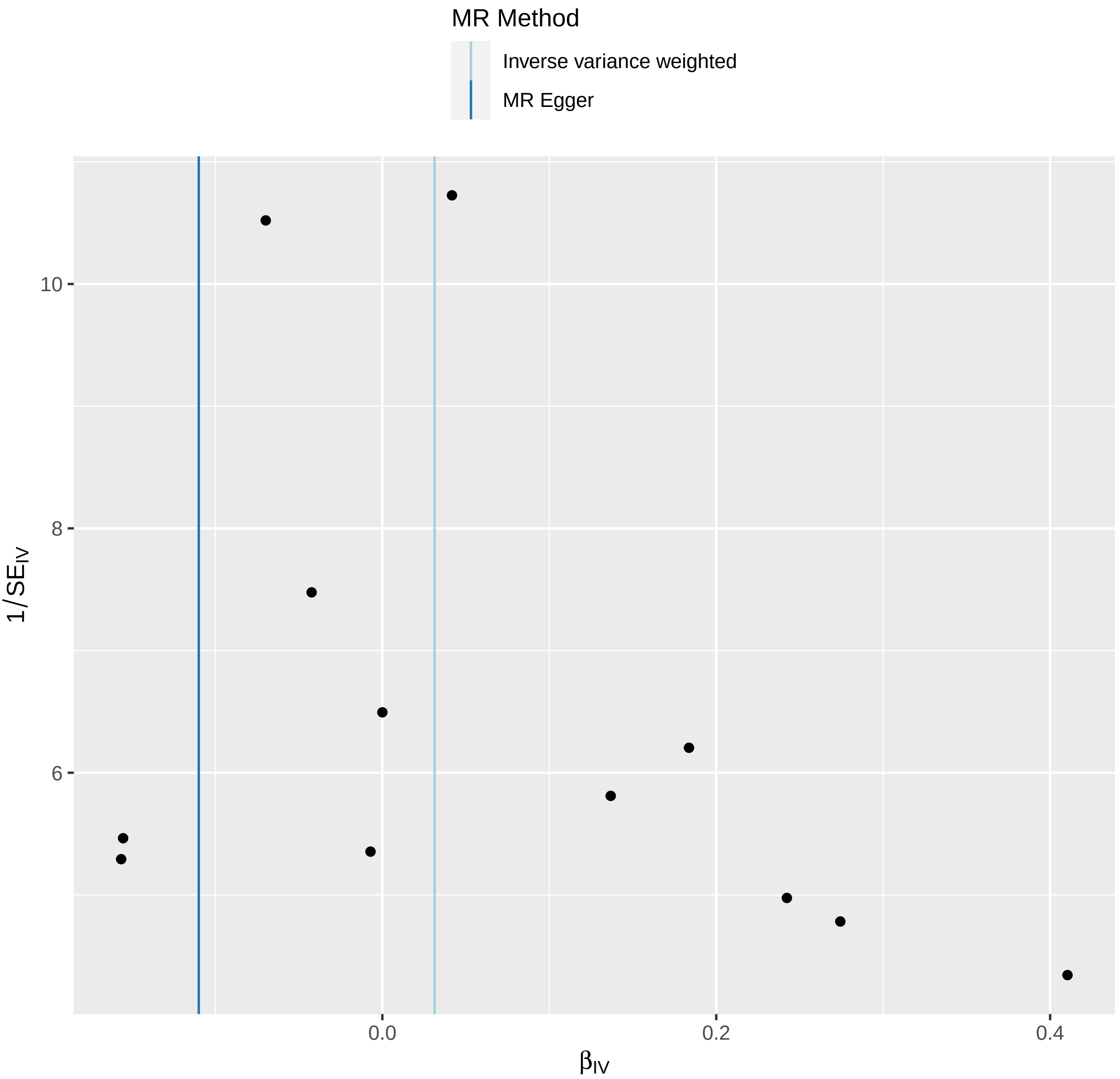

Batch 3−2245 : Selenium on Platelet endothelial cell adhesion molecule

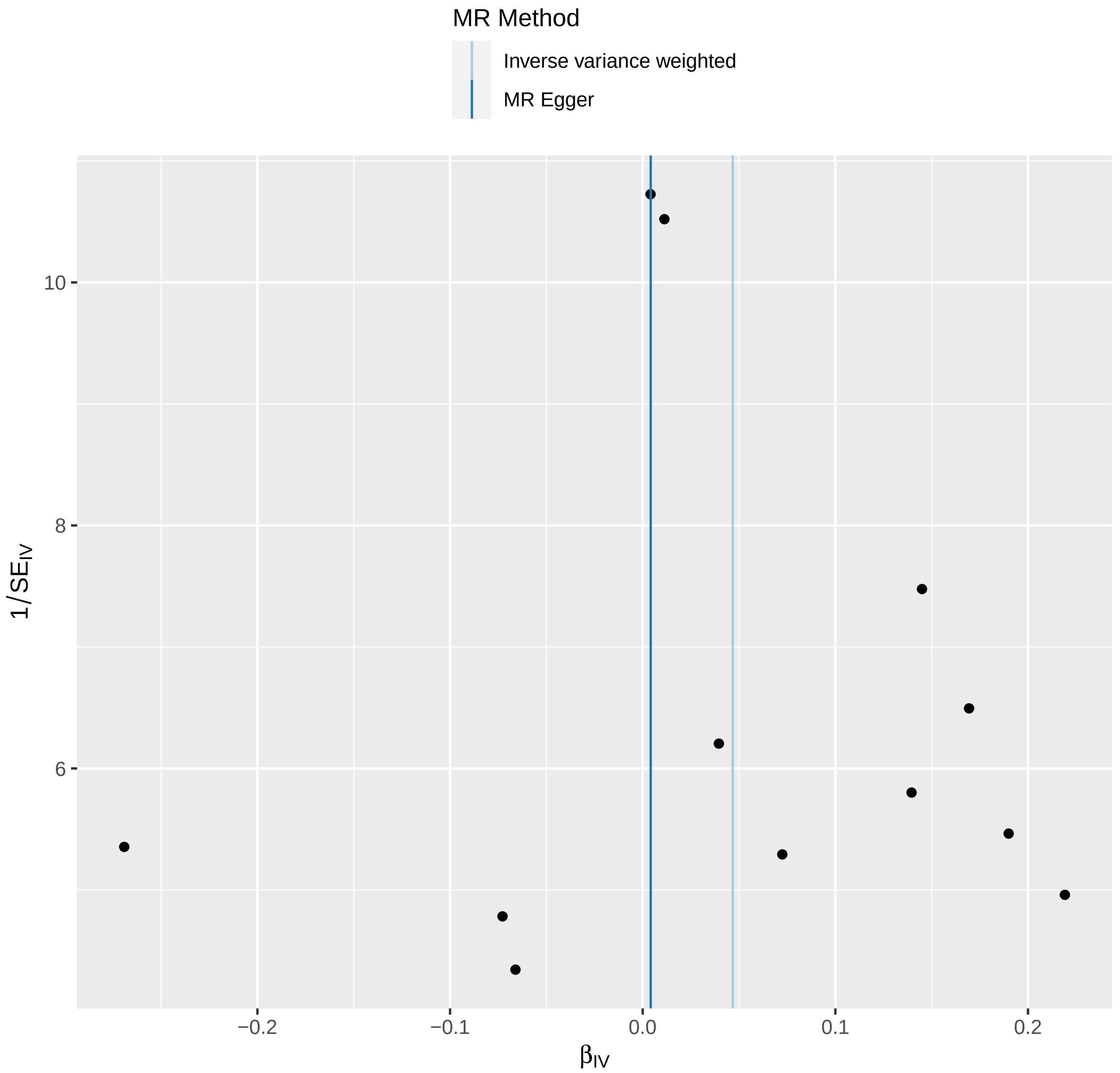

Batch 3−2246 : Selenium on E3 ubiquitin−protein ligase pellino homolog 2

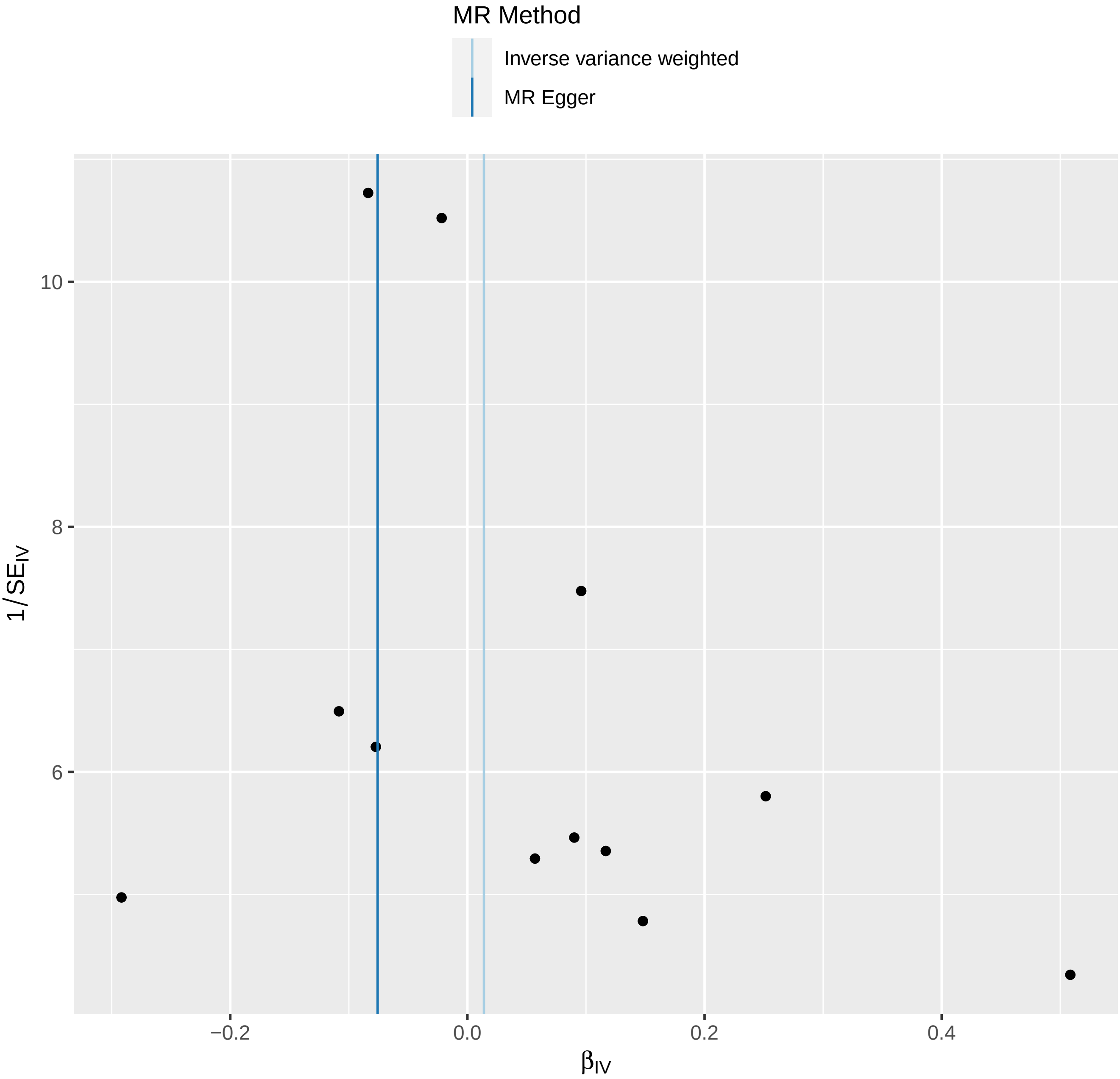

Batch 3−2247 : Selenium on Proenkephalin−A

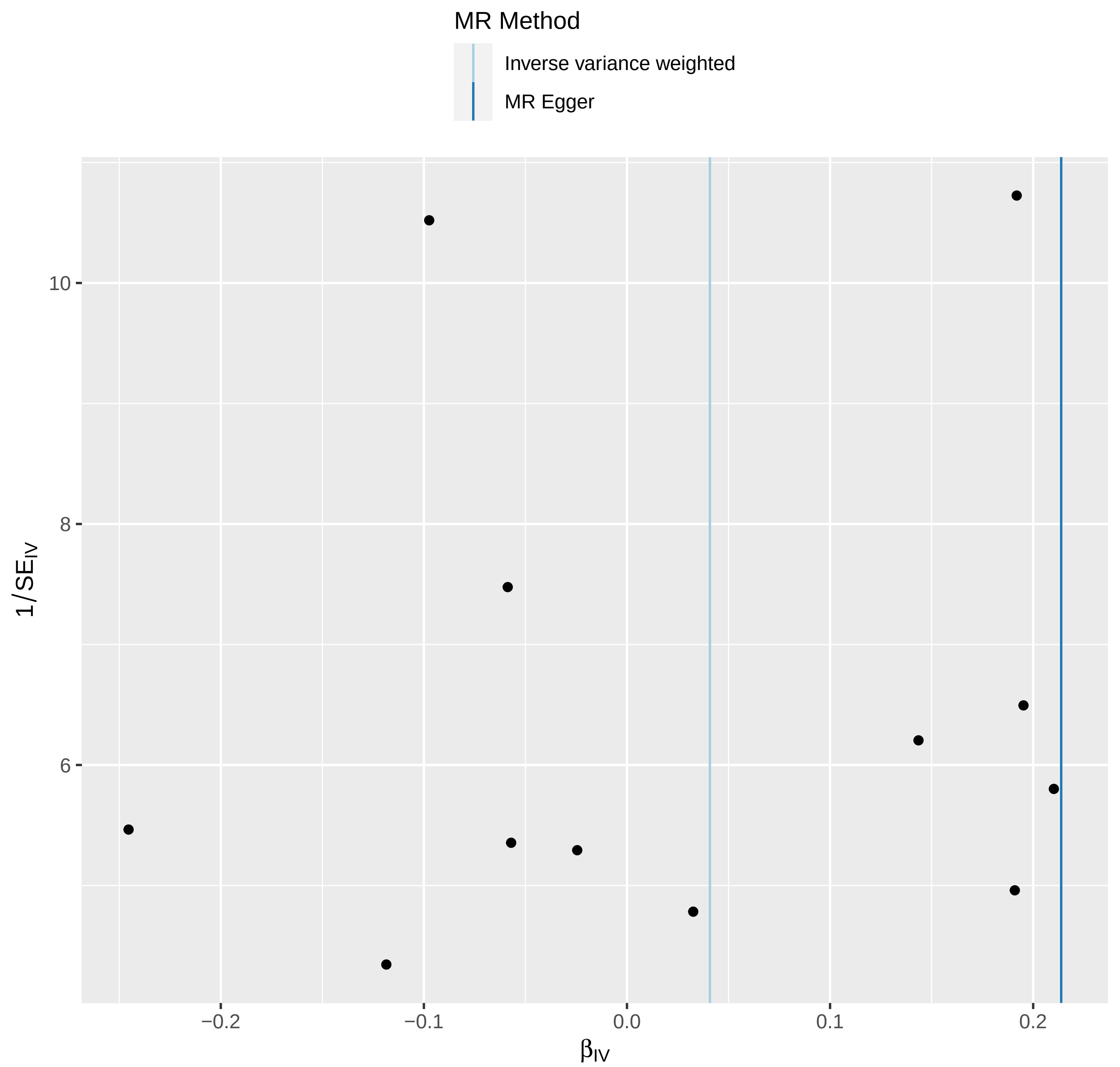

Batch 3−2248 : Selenium on Pescadillo homolog

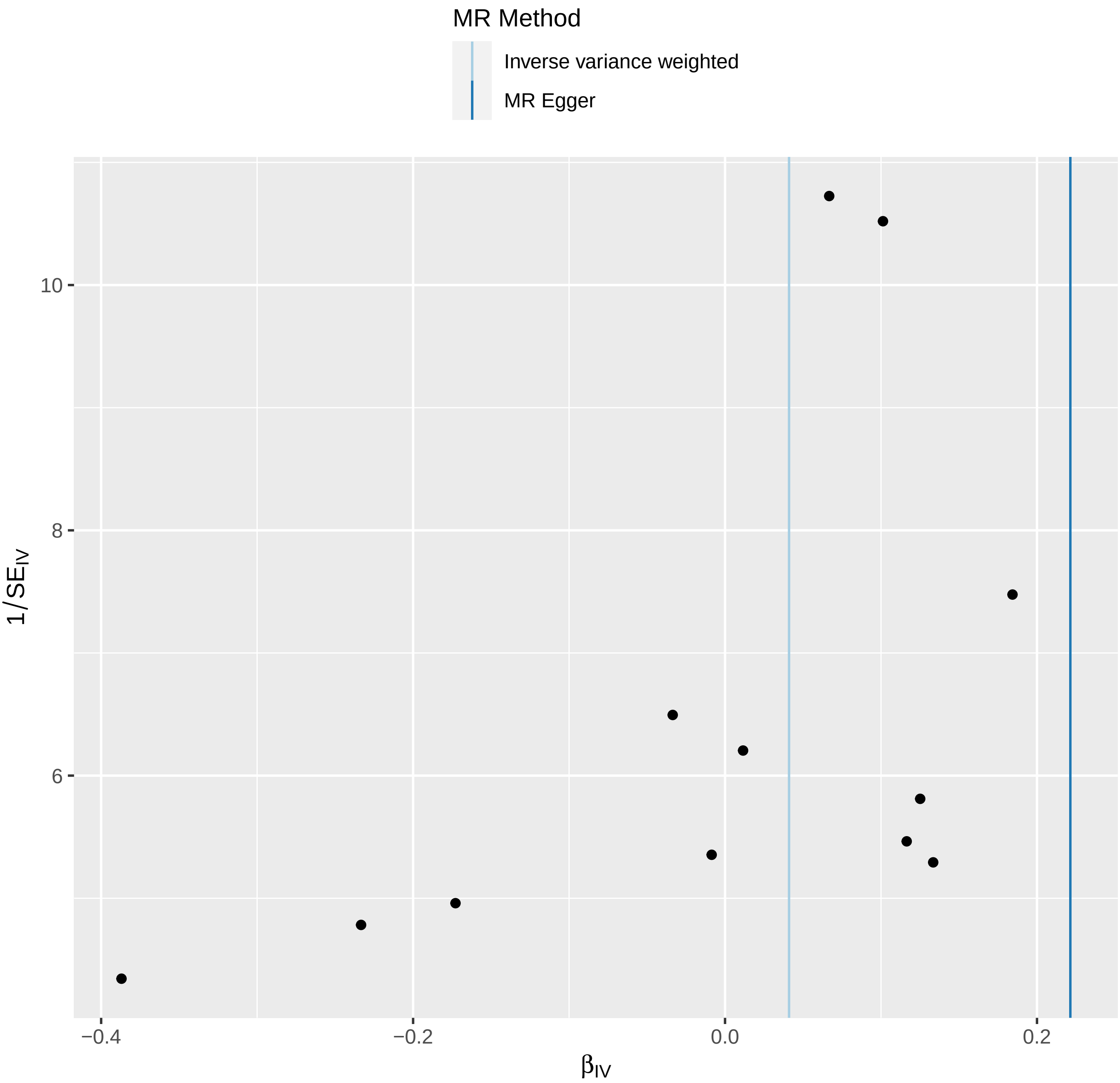

Batch 3−2249 : Selenium on Peroxisomal membrane protein PEX14

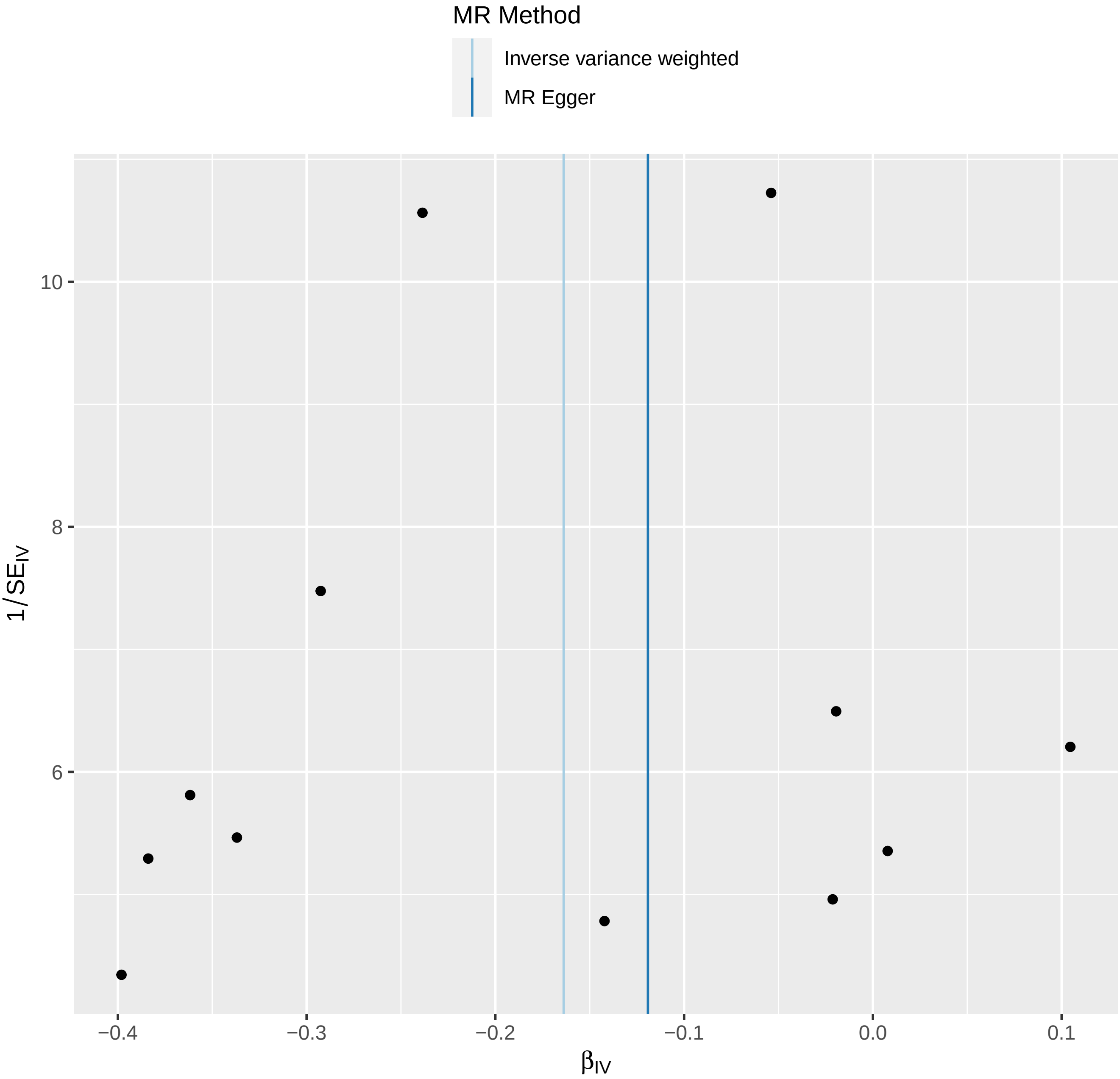

Batch 3−2250 : Selenium on Peroxisomal targeting signal 1 receptor

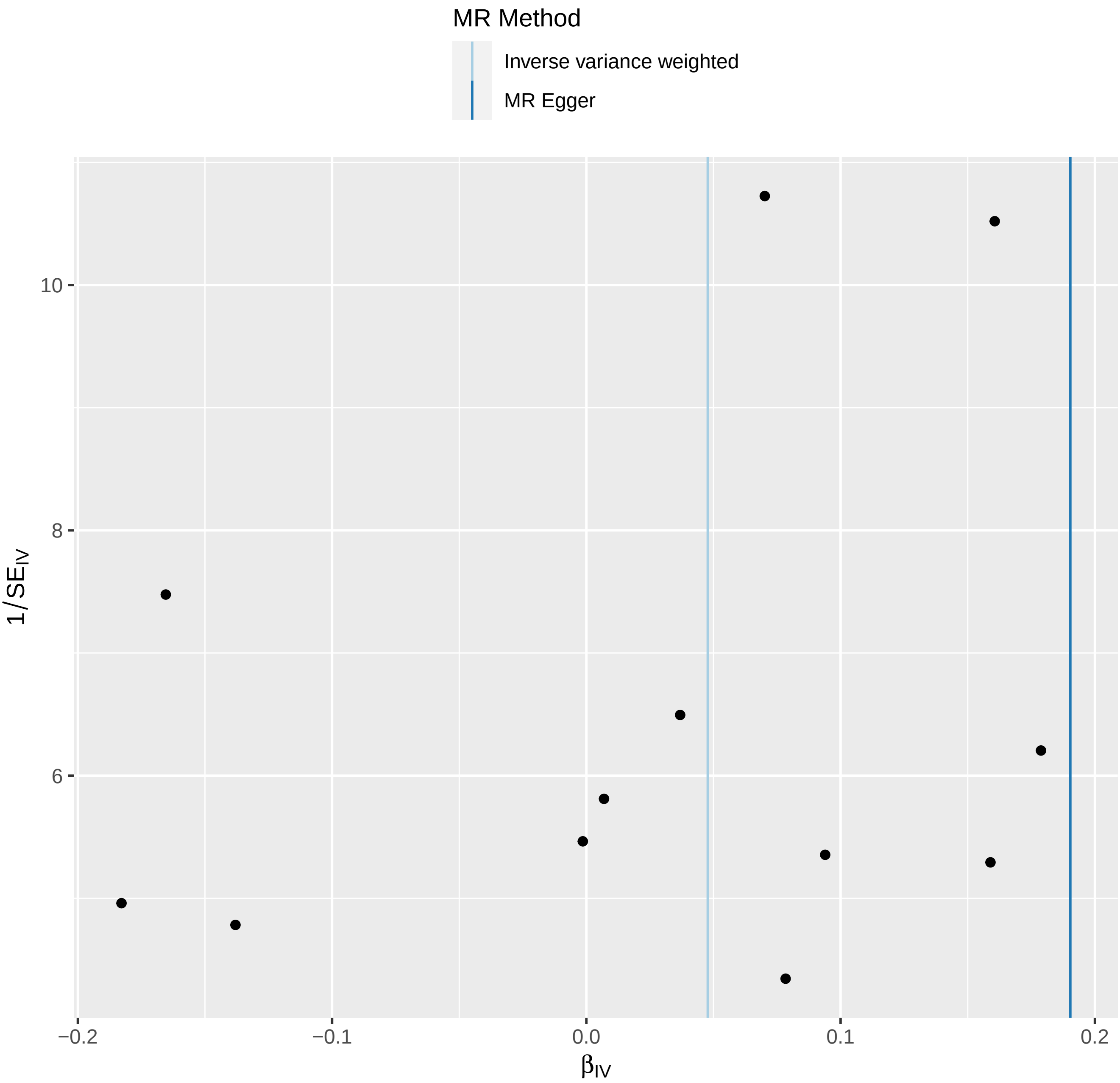

Batch 3−2251 : Selenium on Platelet factor 4 variant

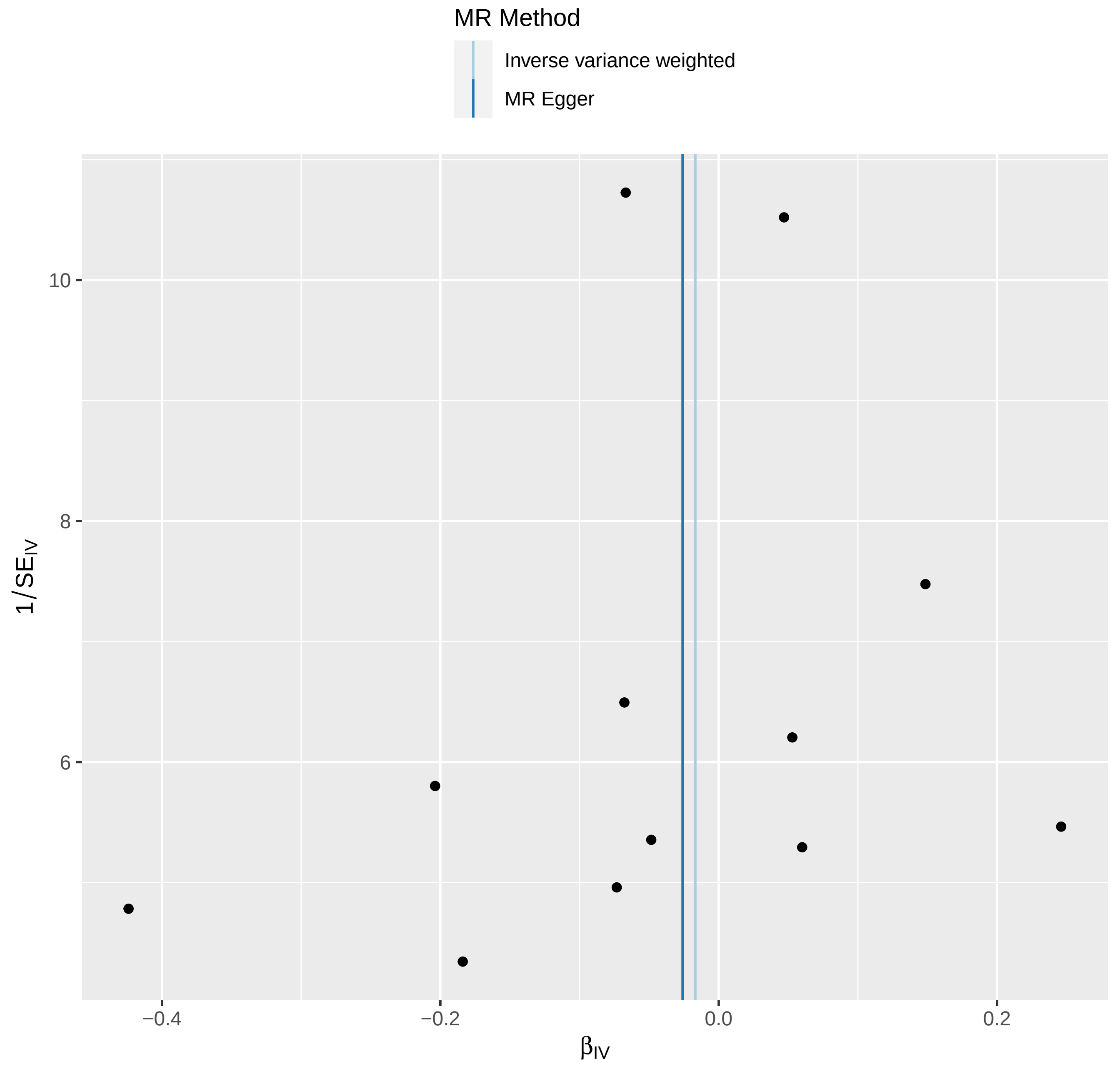

Batch 3−2252 : Selenium on Prefoldin subunit 5

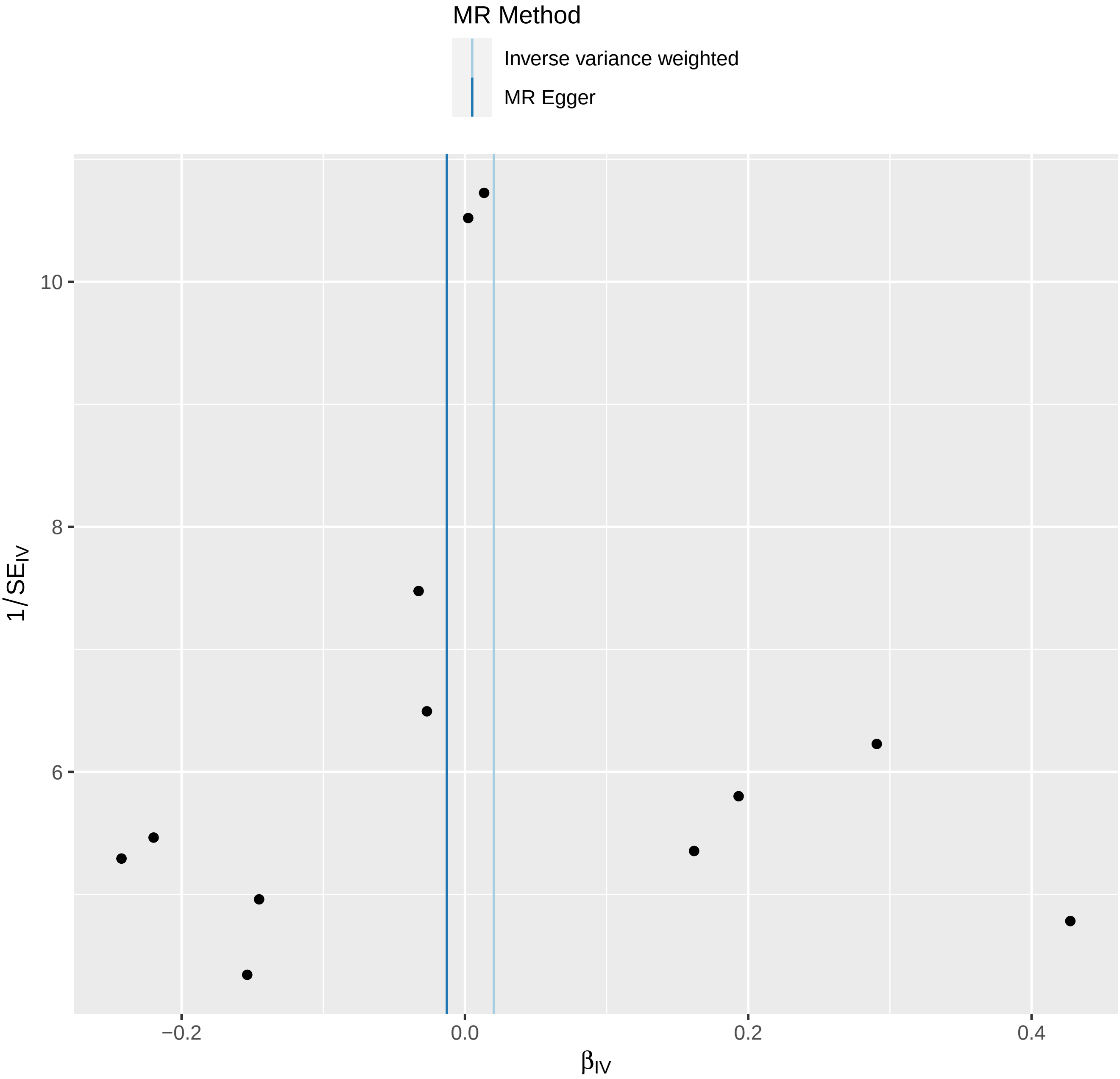

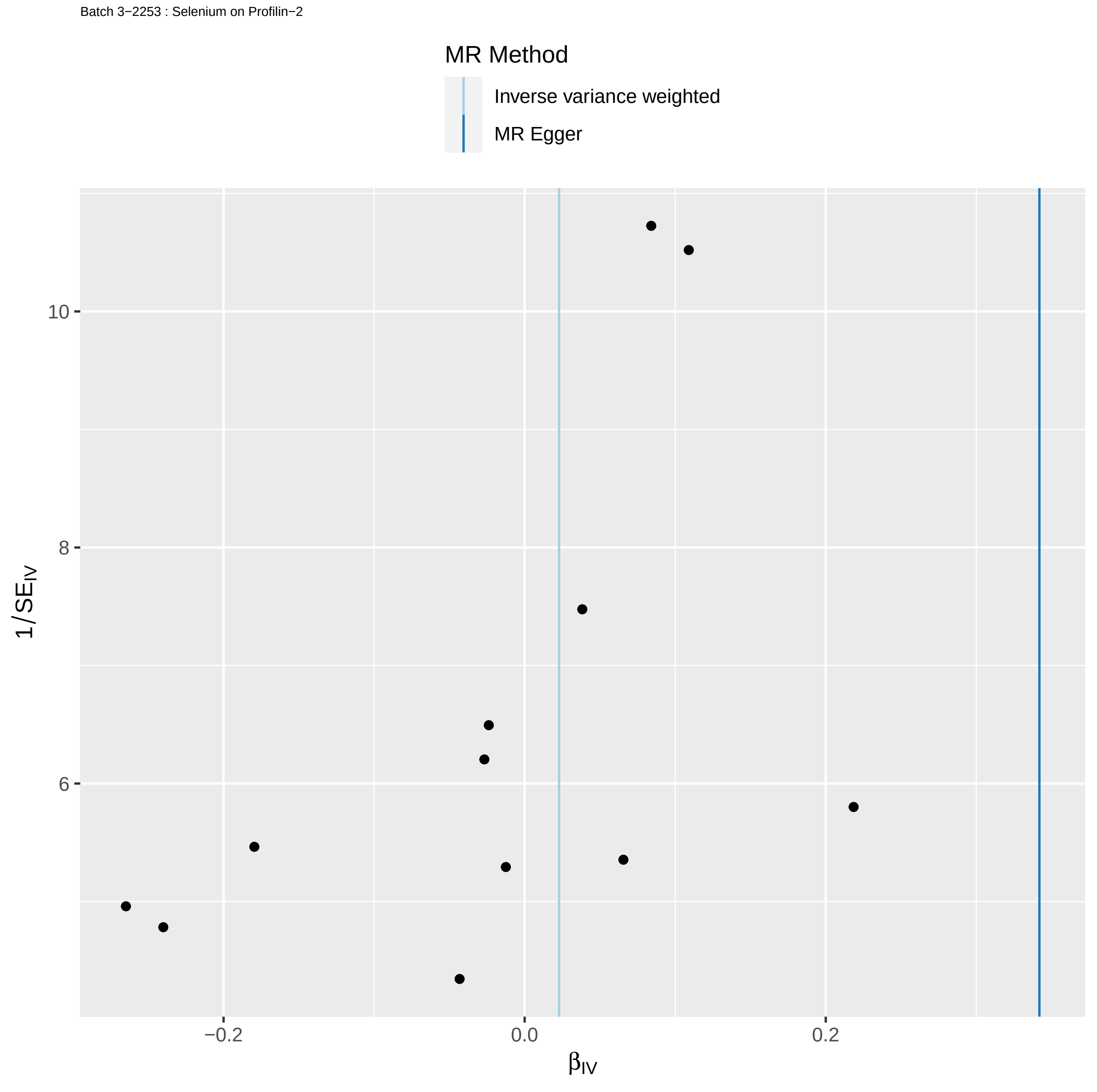

Batch 3−2253 : Selenium on Profilin−2
MR Method
Inverse variance weighted
MR Egger
10
8
6
−0.2
0.0
0.2
$1/SE_{IV}$
β_{IV}

Batch 3−2254 : Selenium on Placenta growth factor

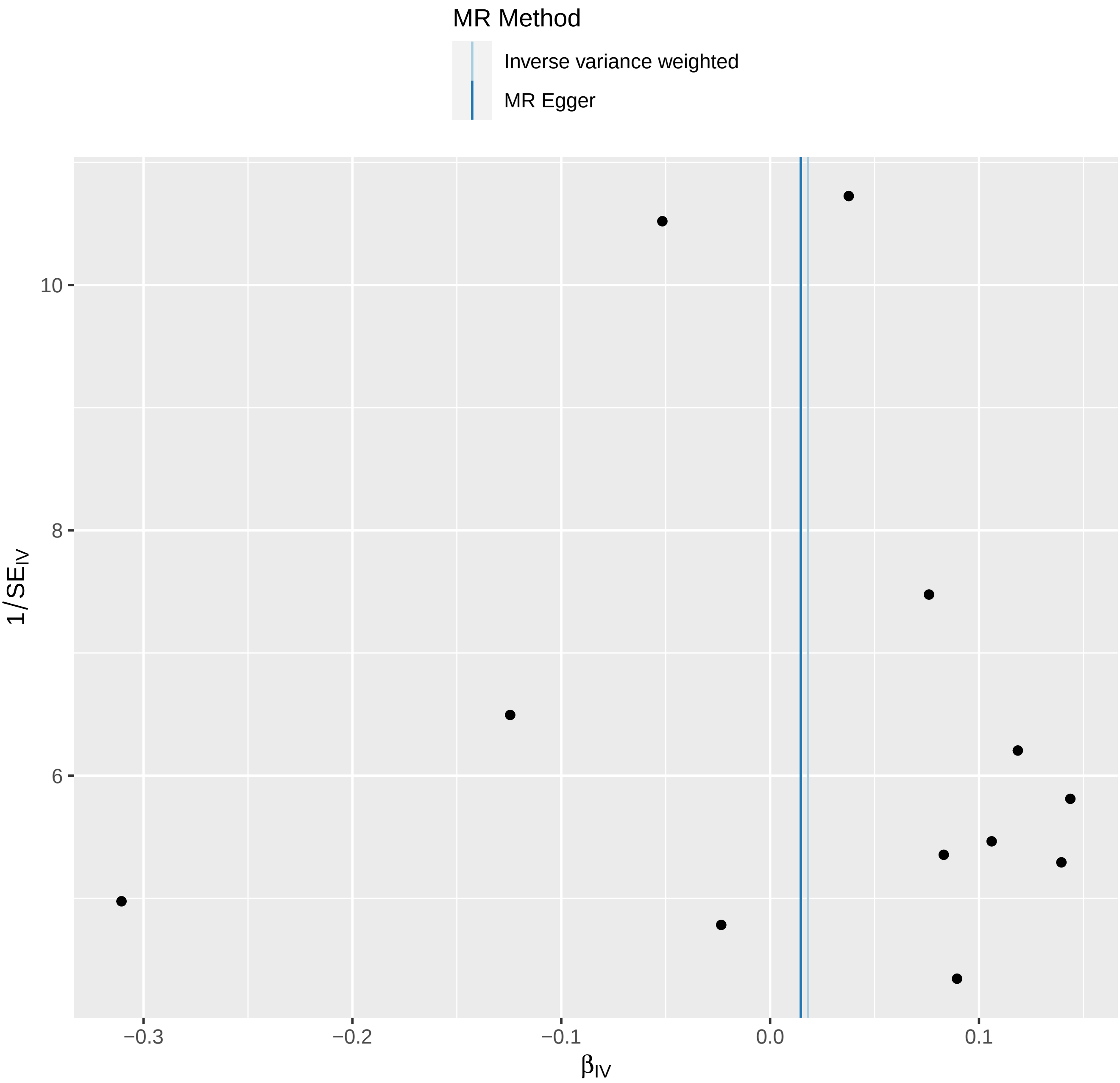

Batch 3−2255 : Selenium on Phosphoglycerate kinase 1

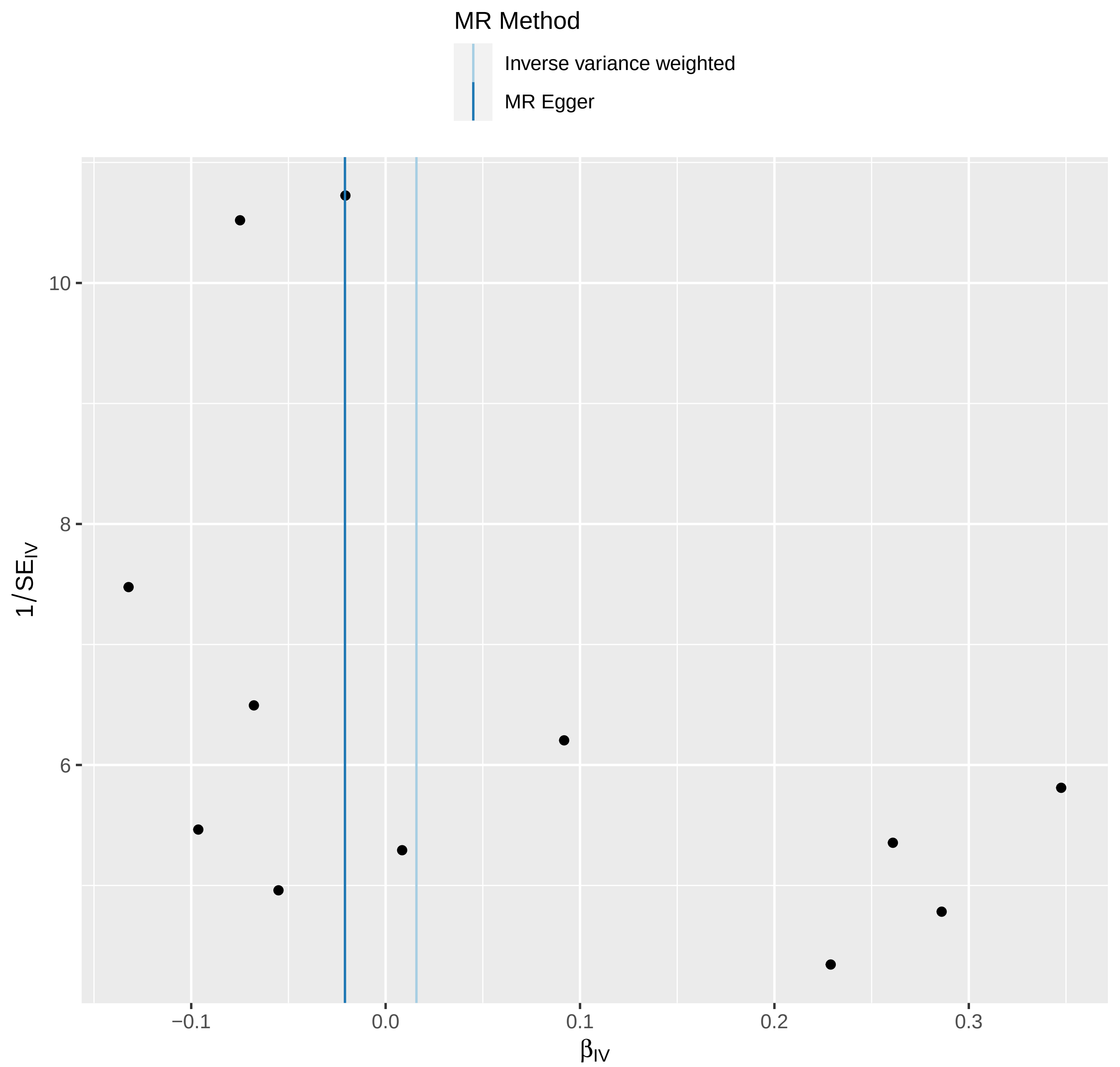

Batch 3−2256 : Selenium on Phosphoglycerate kinase 2

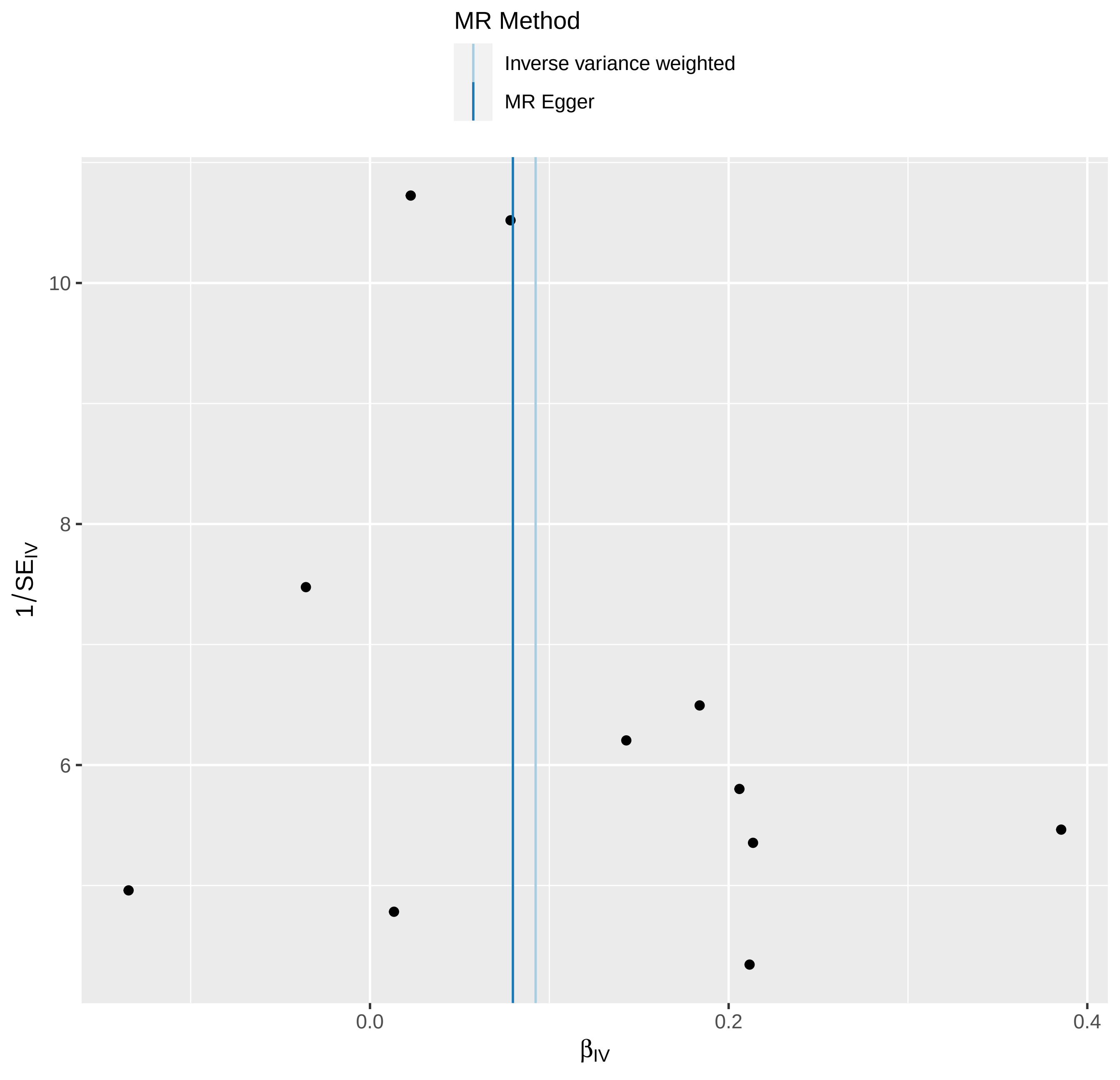

Batch 3−2257 : Selenium on Peptidoglycan recognition protein 1

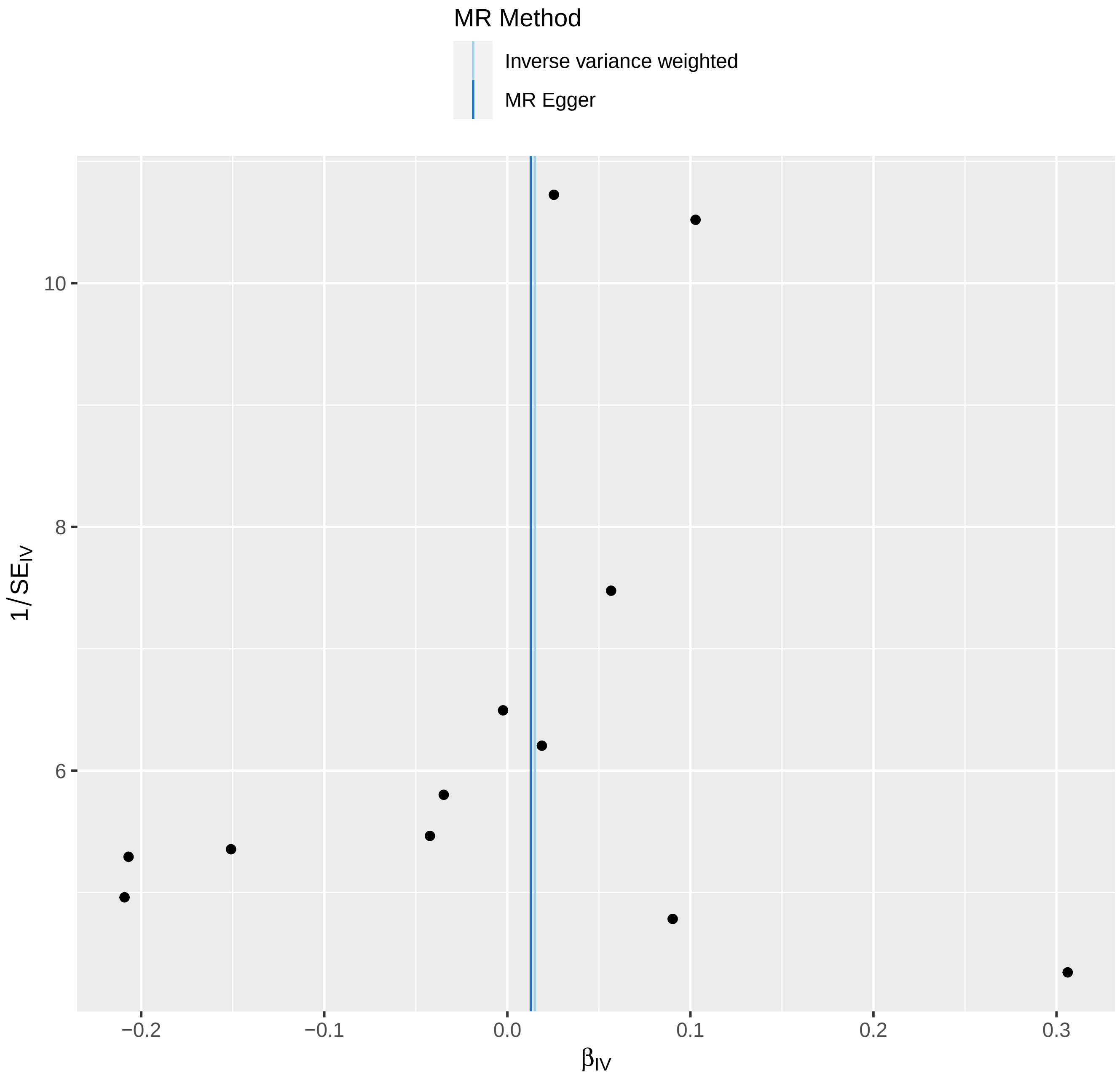

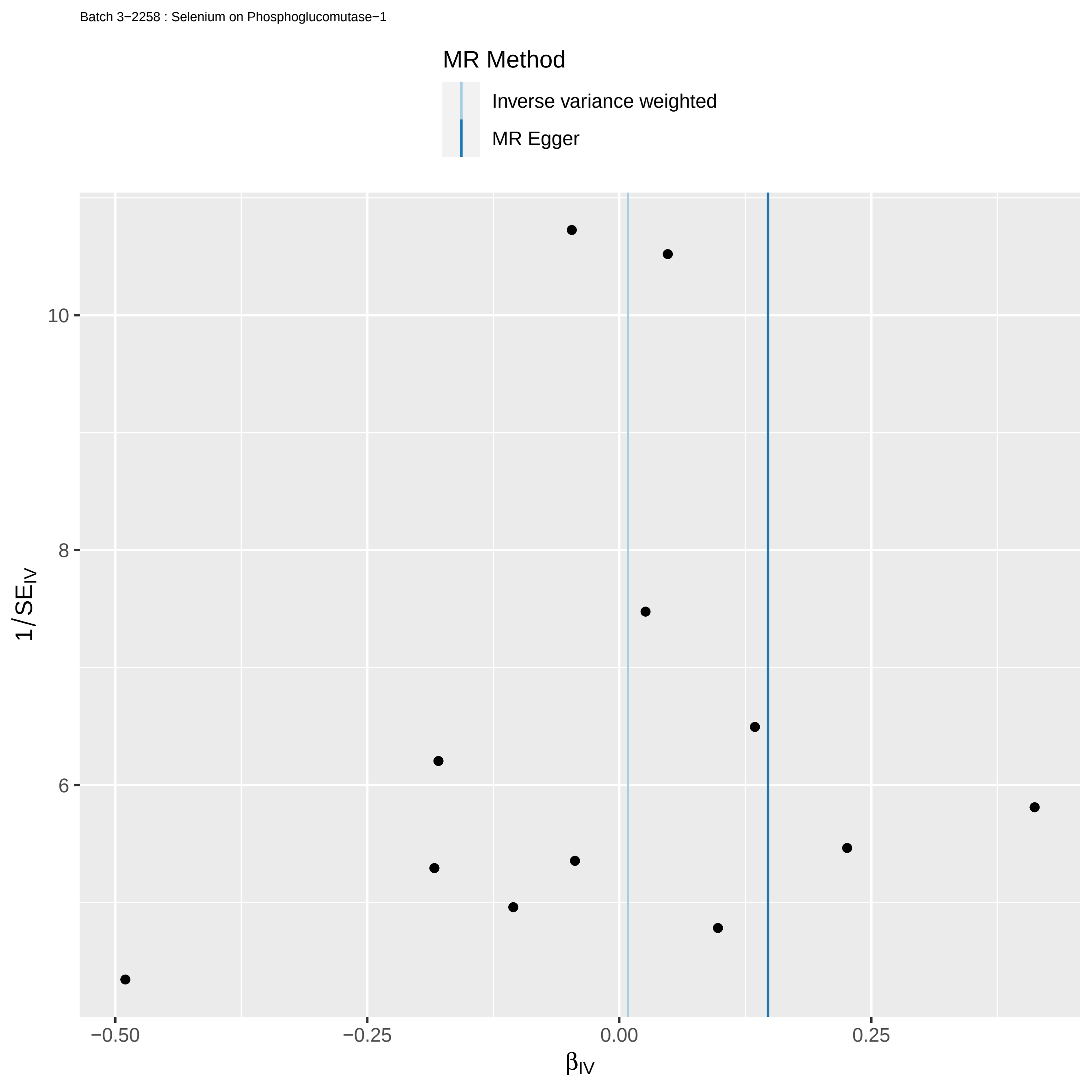
Batch 3−2258 : Selenium on Phosphoglucomutase−1
MR Method
Inverse variance weighted
MR Egger
10
8
6
$1/SE_{IV}$
−0.50
−0.25
0.00
0.25
β_{IV}

Batch 3−2259 : Selenium on Membrane−associated progesterone receptor component 1

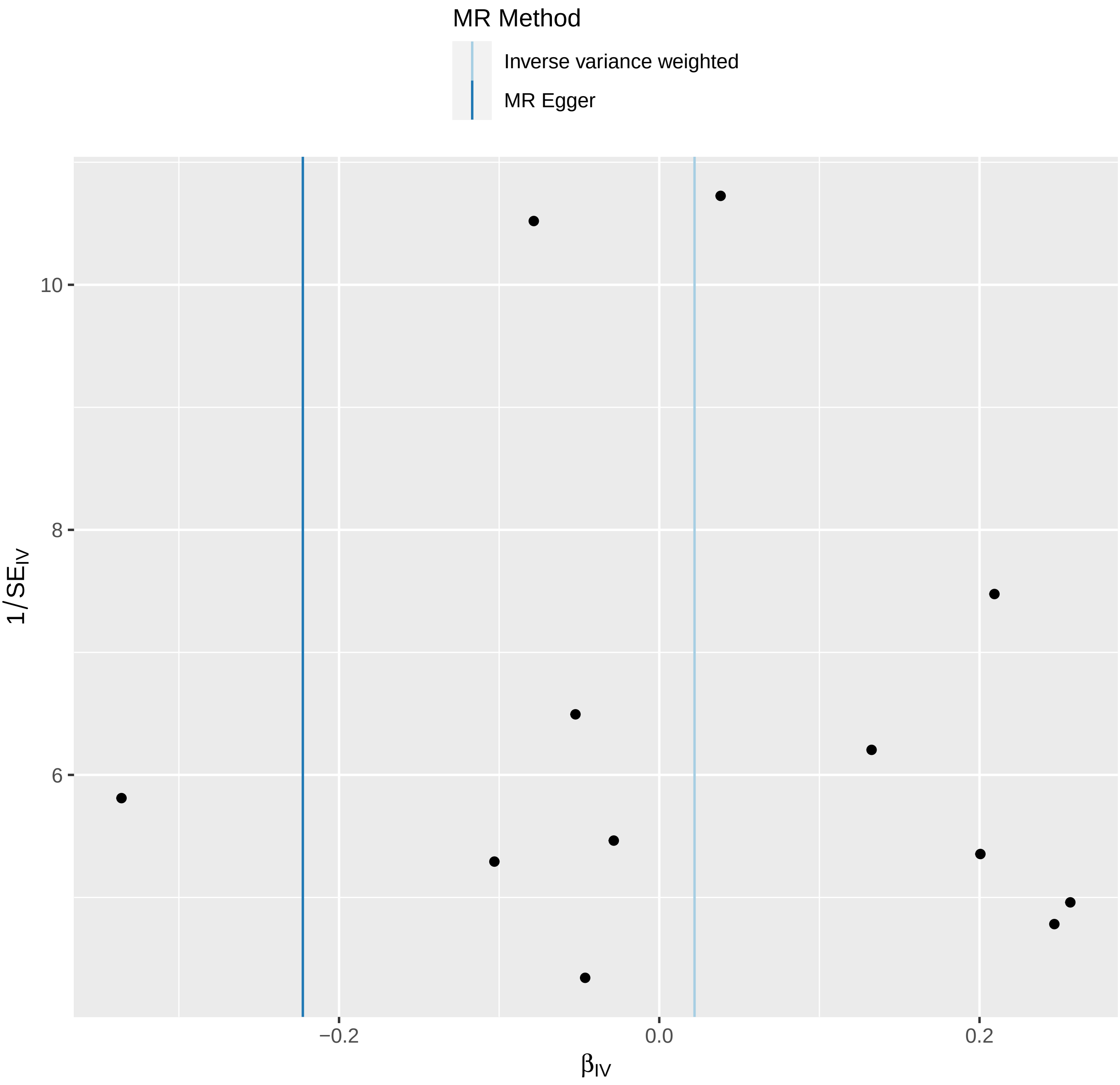

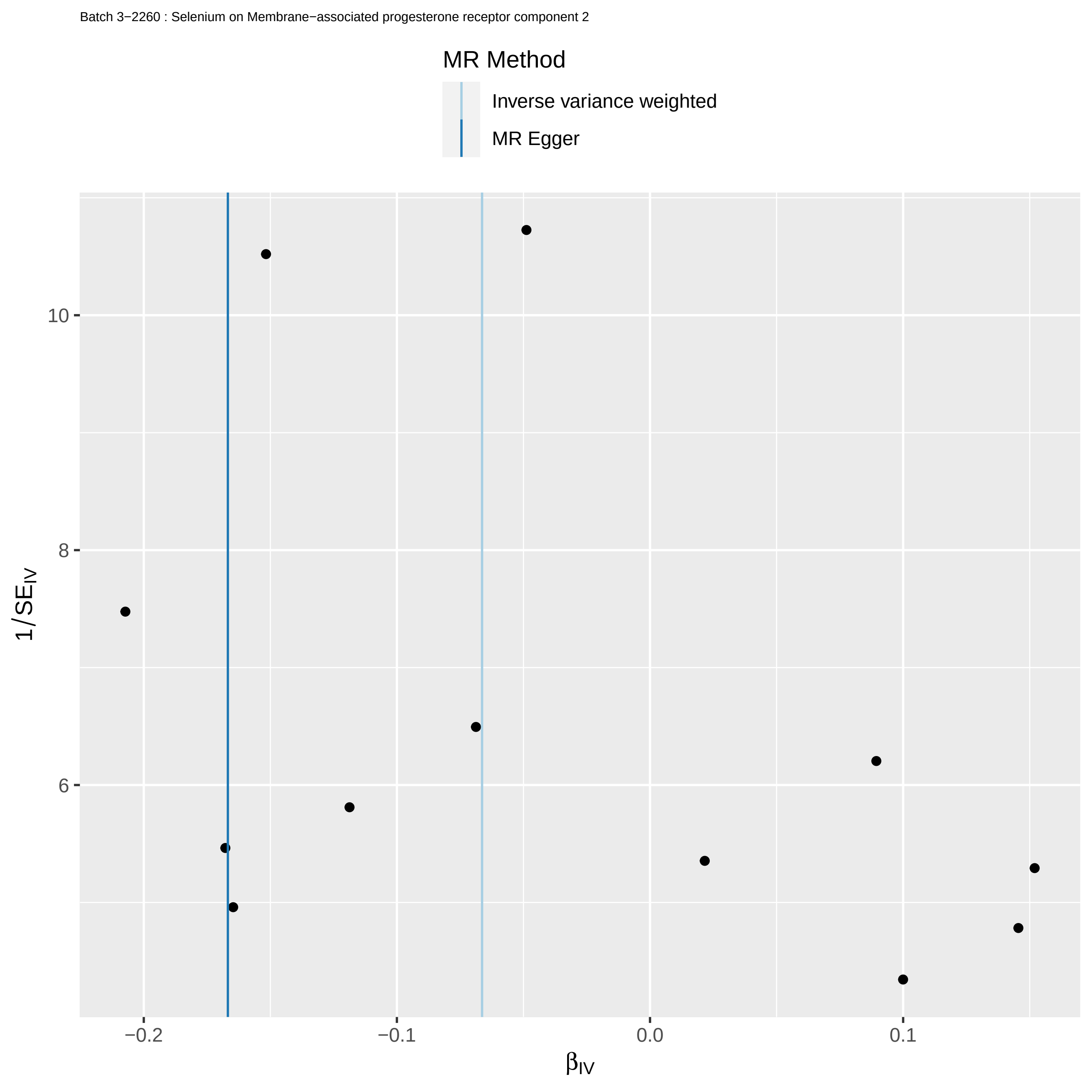

Batch 3−2260 : Selenium on Membrane−associated progesterone receptor component 2
MR Method
Inverse variance weighted
MR Egger
10
8
6
1/SE_IV
−0.2
−0.1
0.0
0.1
β_IV

Batch 3−2261 : Selenium on Membrane−associated progesterone receptor component 2

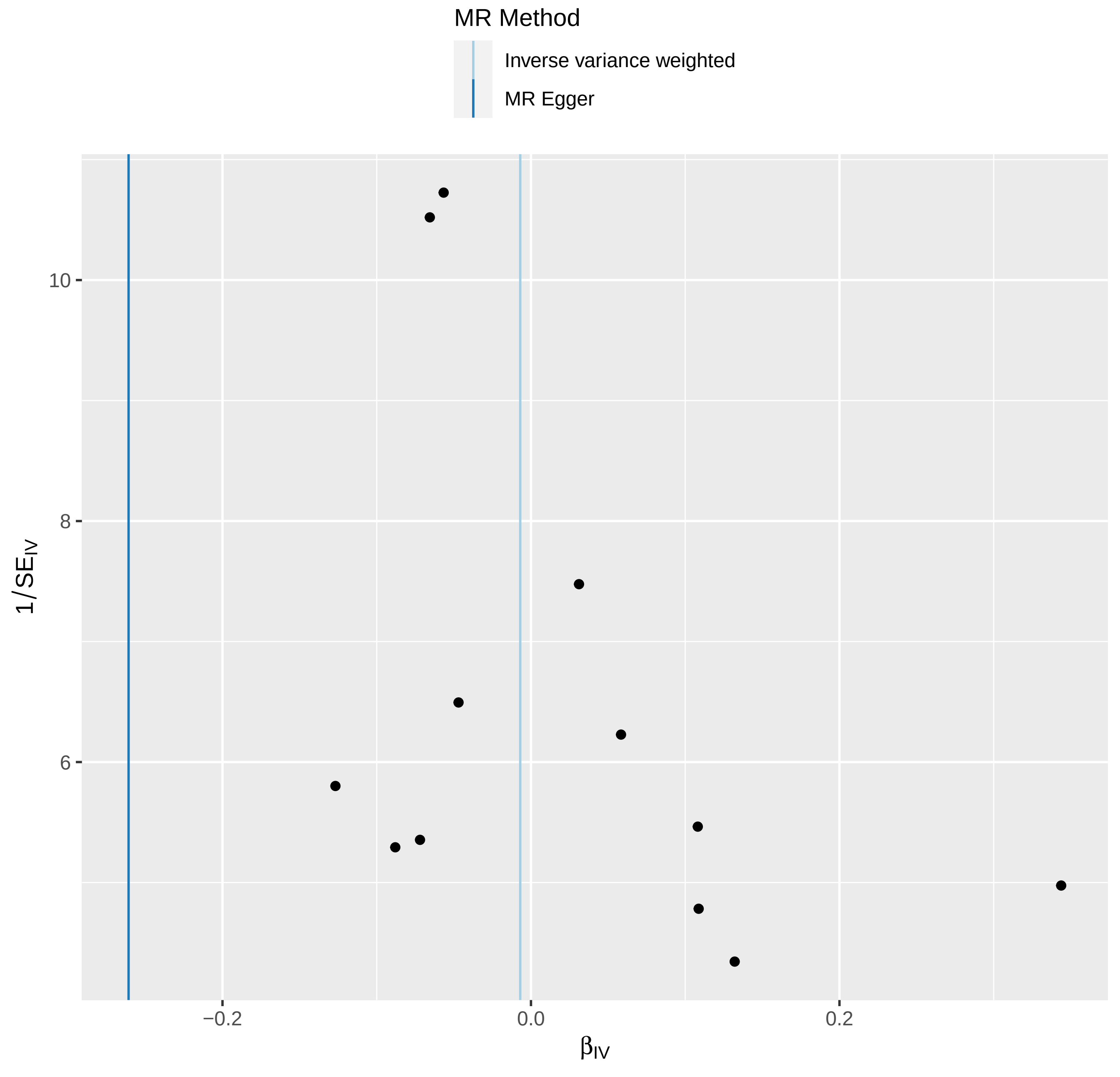

Batch 3−2262 : Selenium on Membrane−associated progesterone receptor component 2

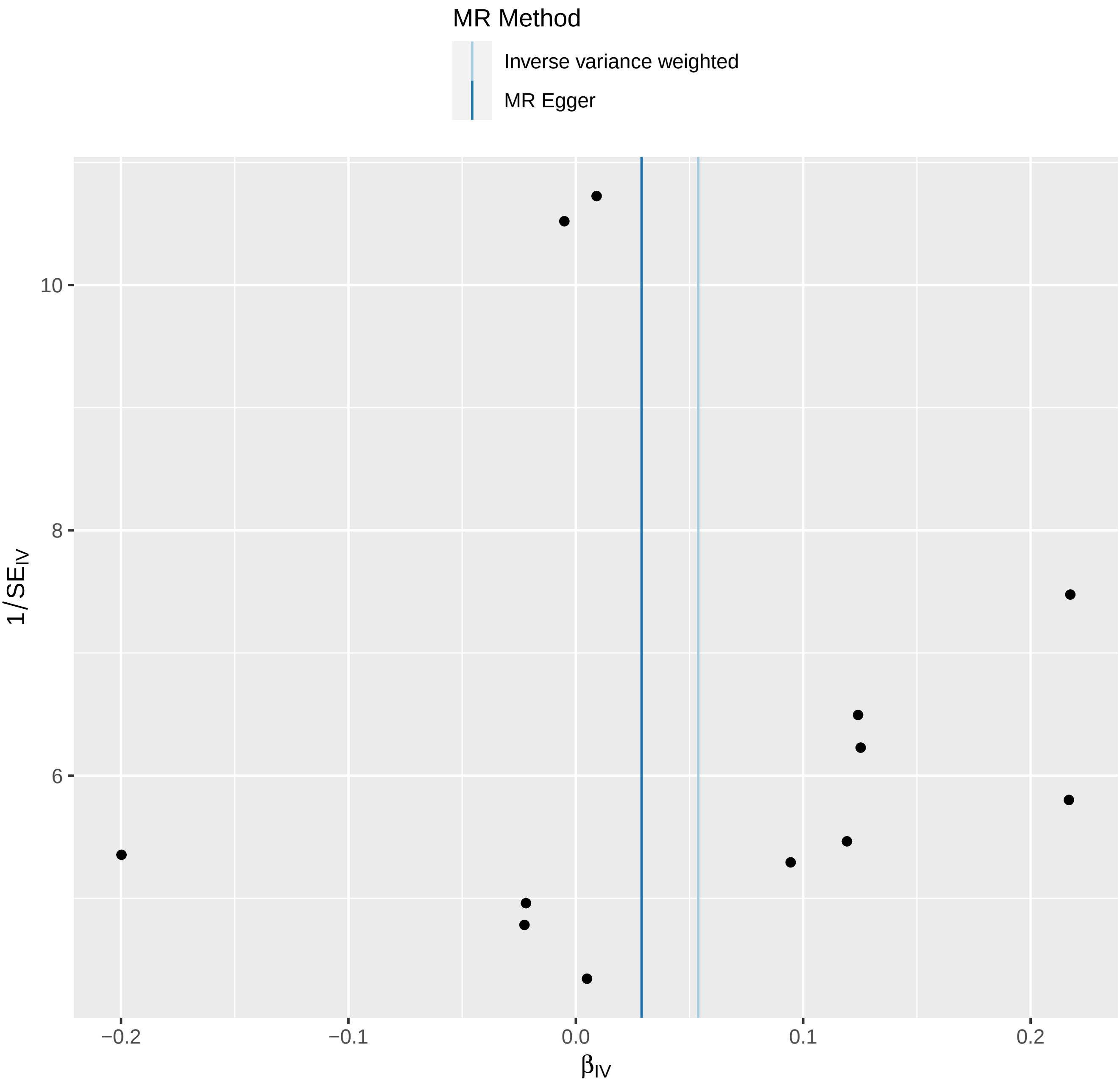

Batch 3−2263 : Selenium on PHD finger protein 3

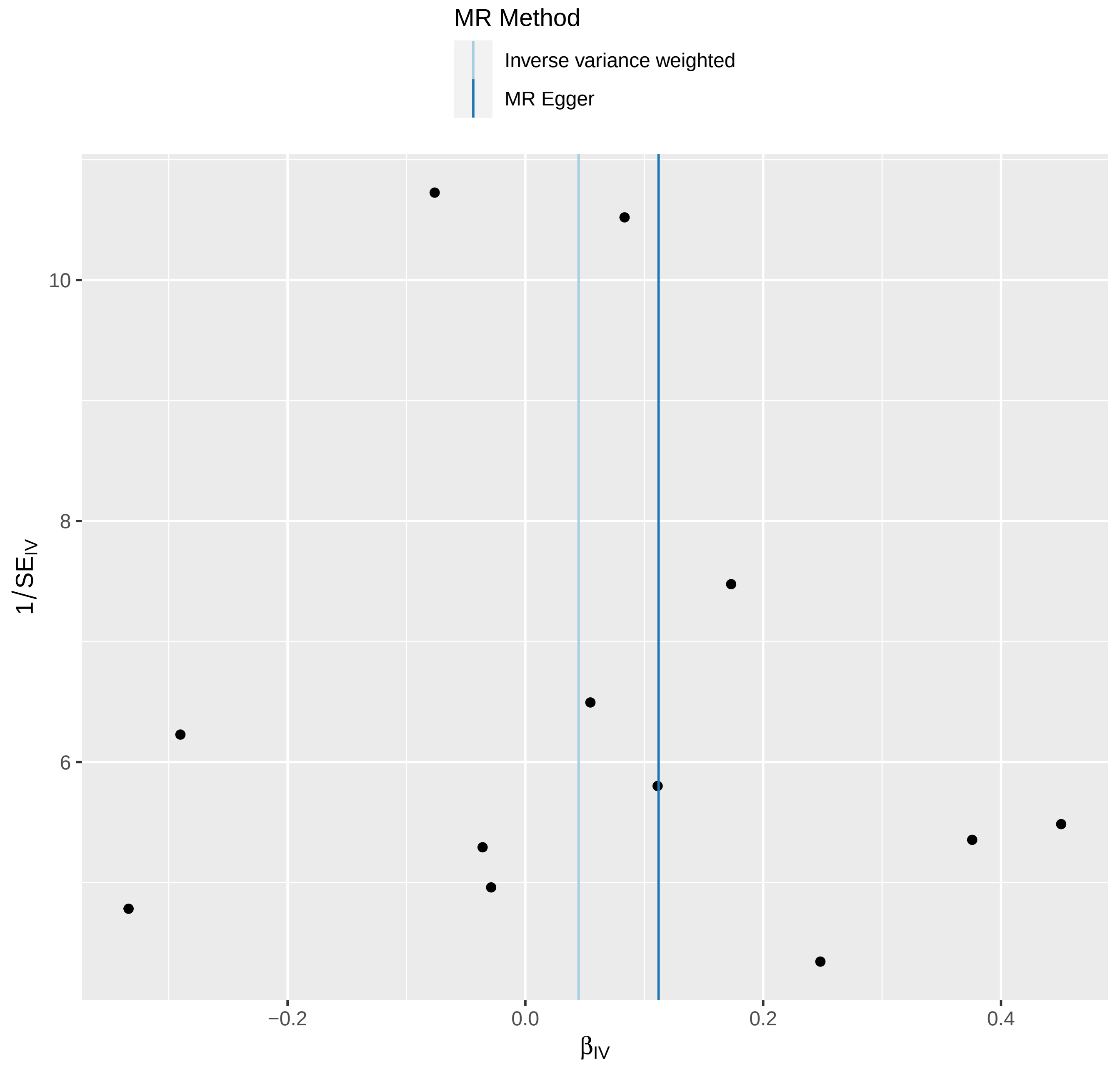

Batch 3−2264 : Selenium on Peptidase inhibitor 15

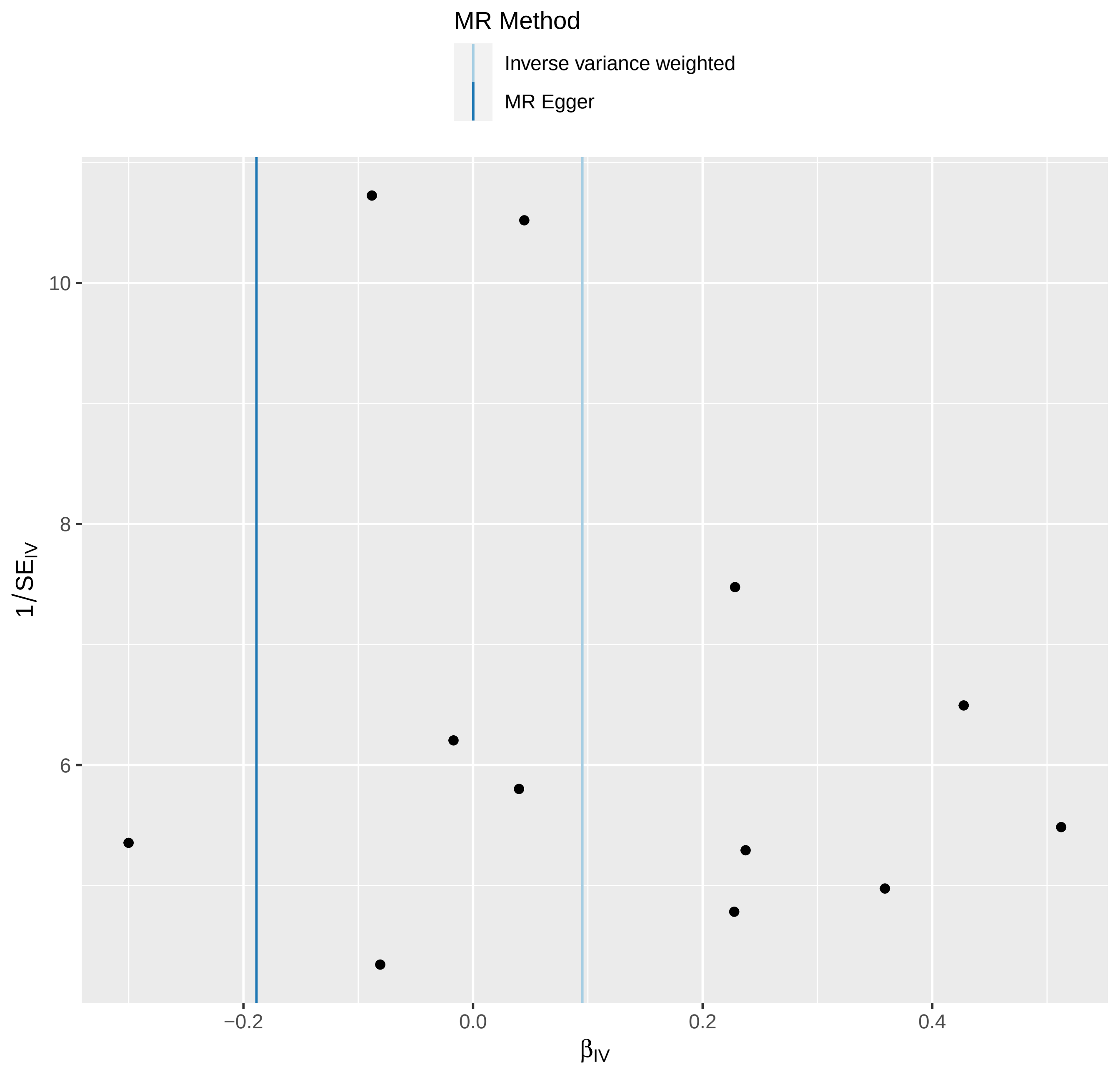

Batch 3−2265 : Selenium on Elafin

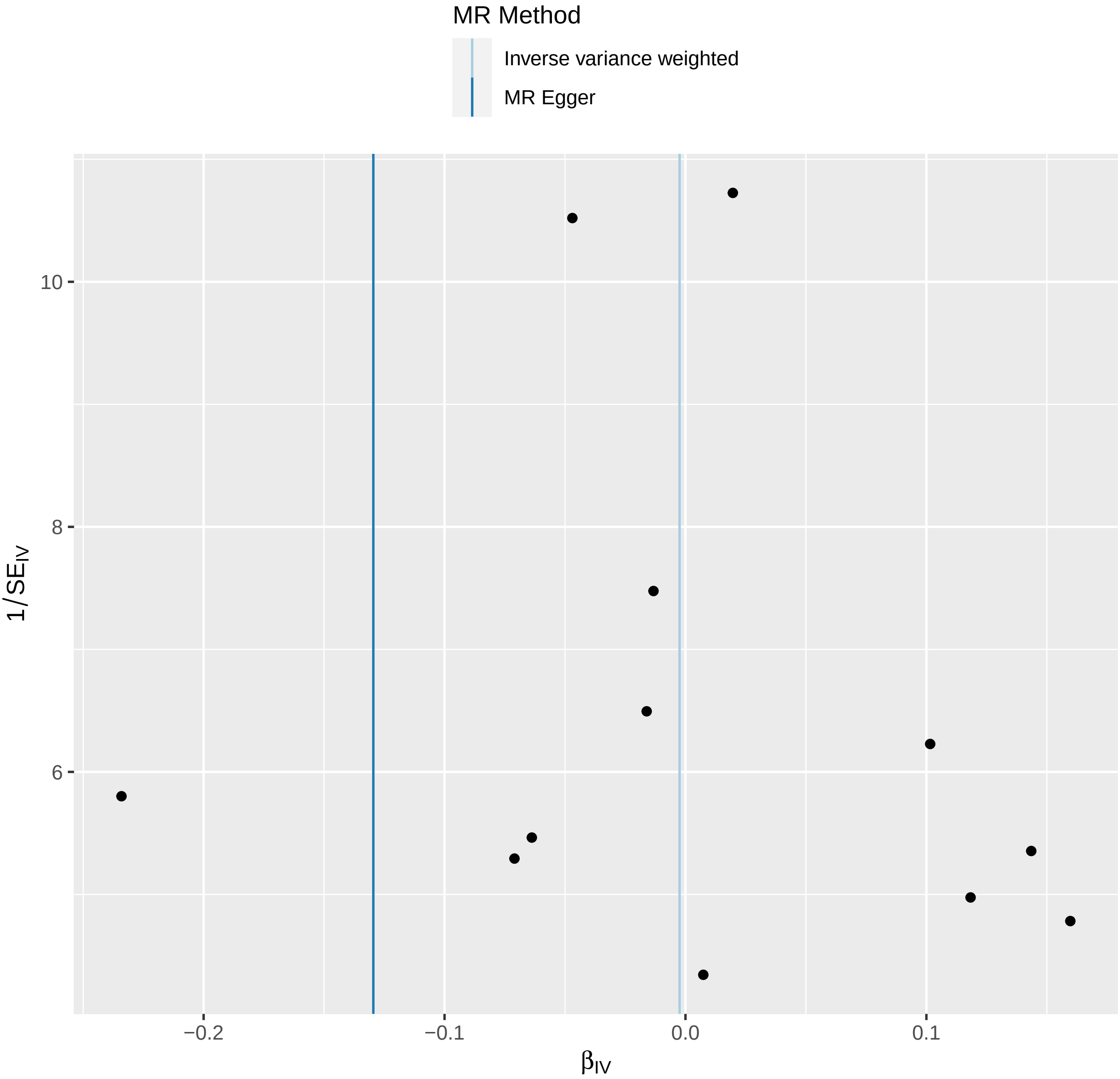

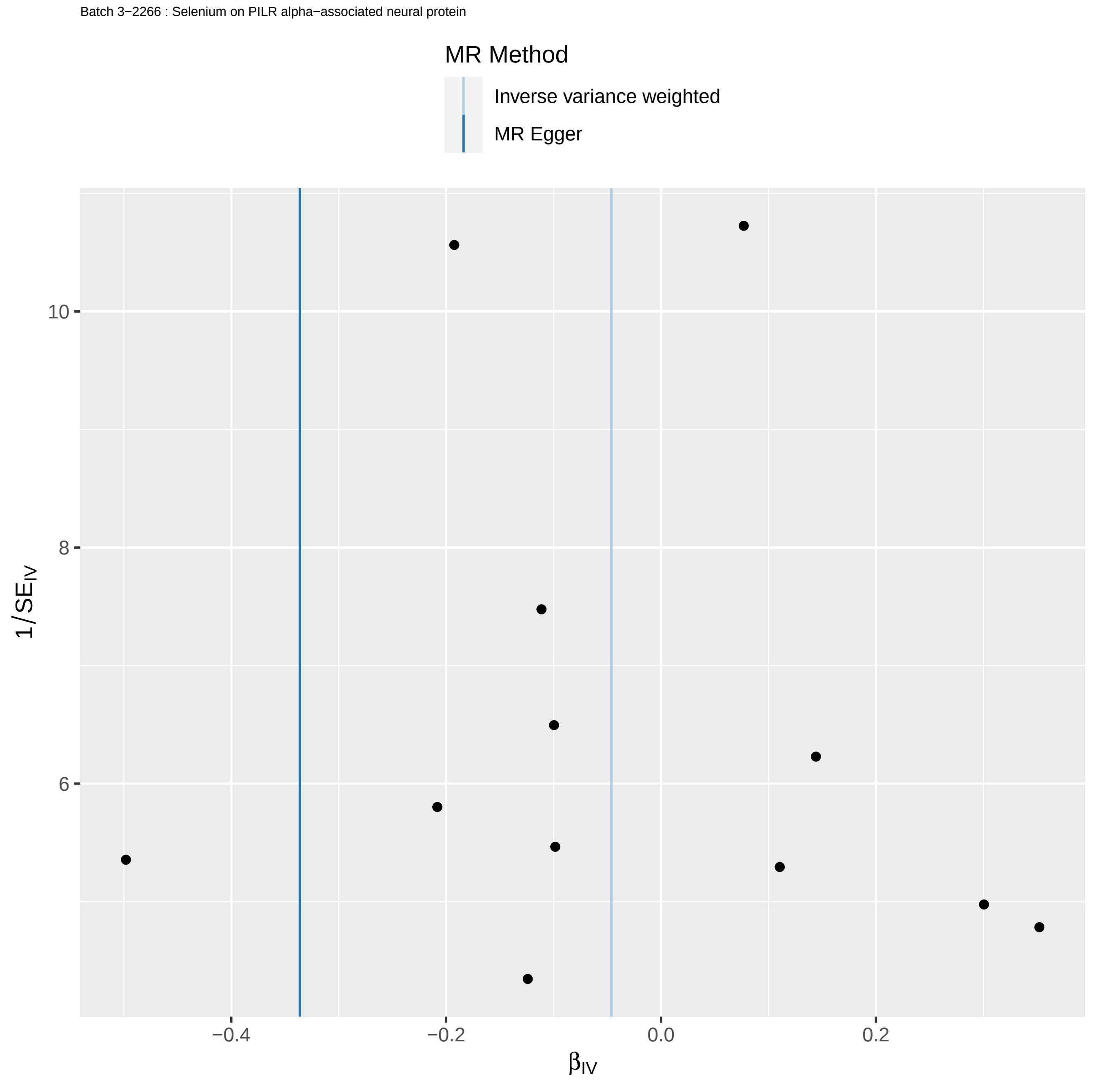

Batch 3−2266 : Selenium on PILR alpha−associated neural protein
MR Method
Inverse variance weighted
MR Egger
10
8
6
1/SE$_{IV}$
−0.4
−0.2
0.0
0.2
β$_{IV}$

Batch 3−2267 : Selenium on PILR alpha−associated neural protein

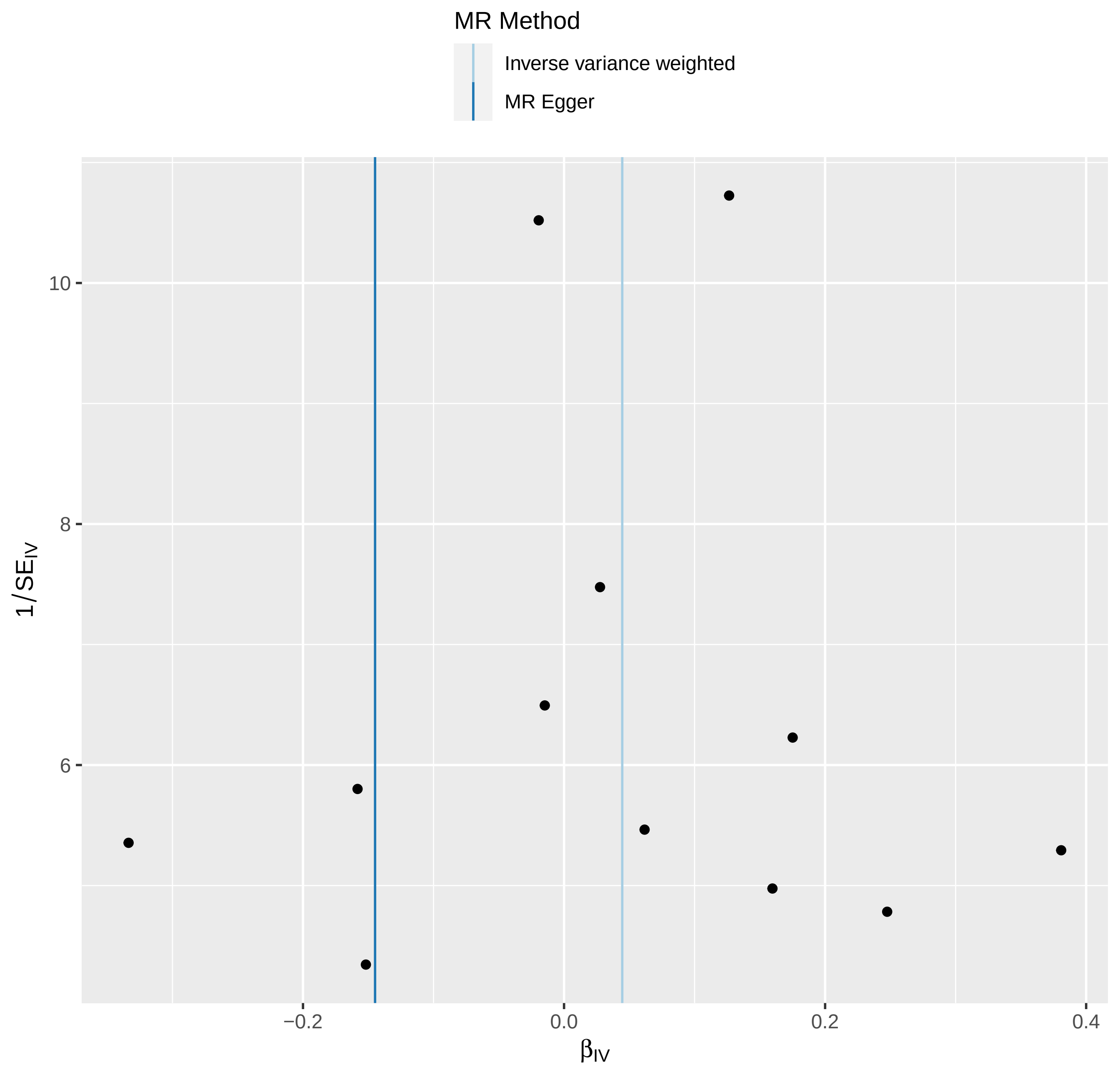

Batch 3−2268 : Selenium on Polymeric immunoglobulin receptor

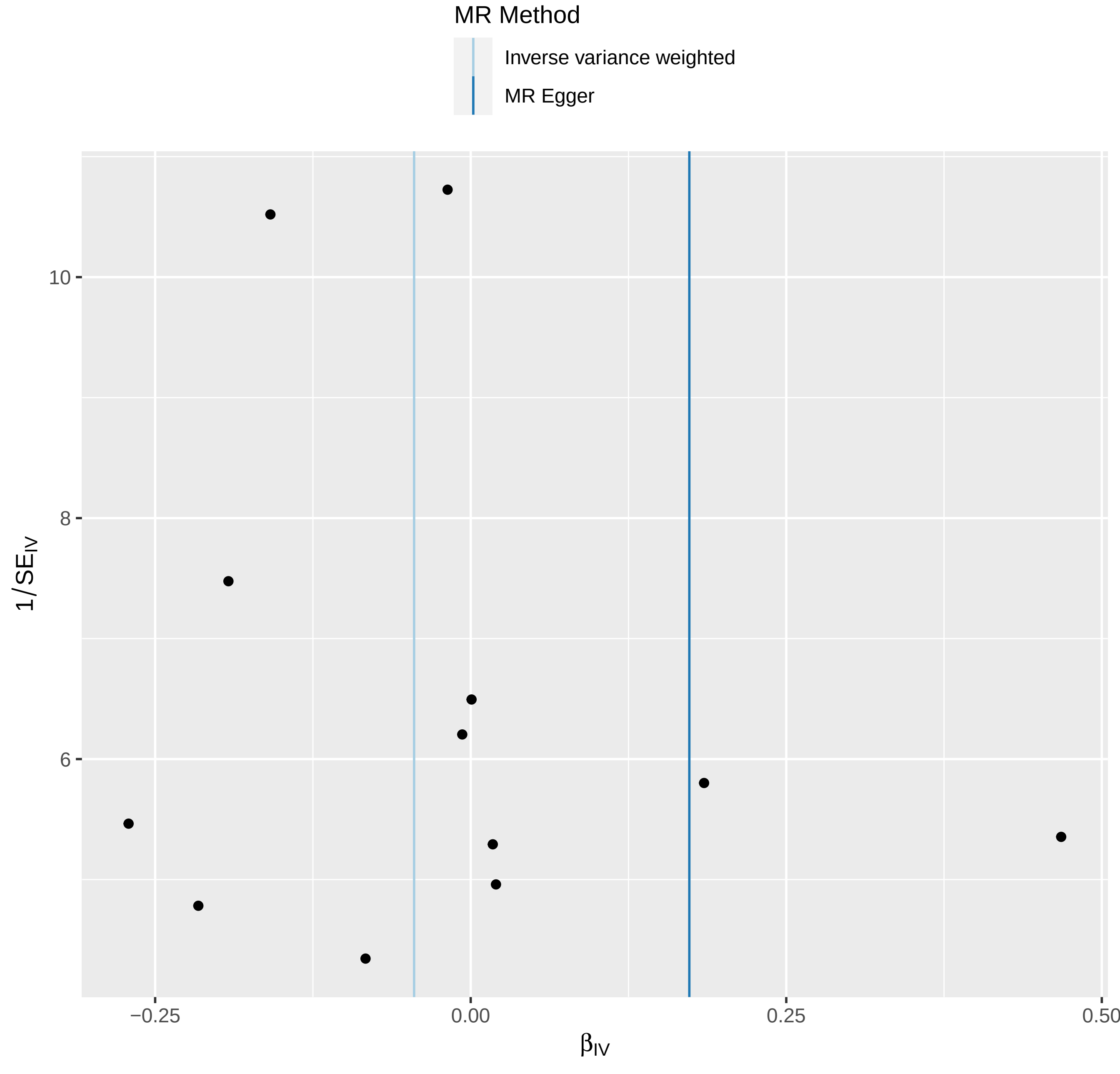

Batch 3−2269 : Selenium on Phosphatidylinositol 4−phosphate 3−kinase C2 domain−containing subunit alpha

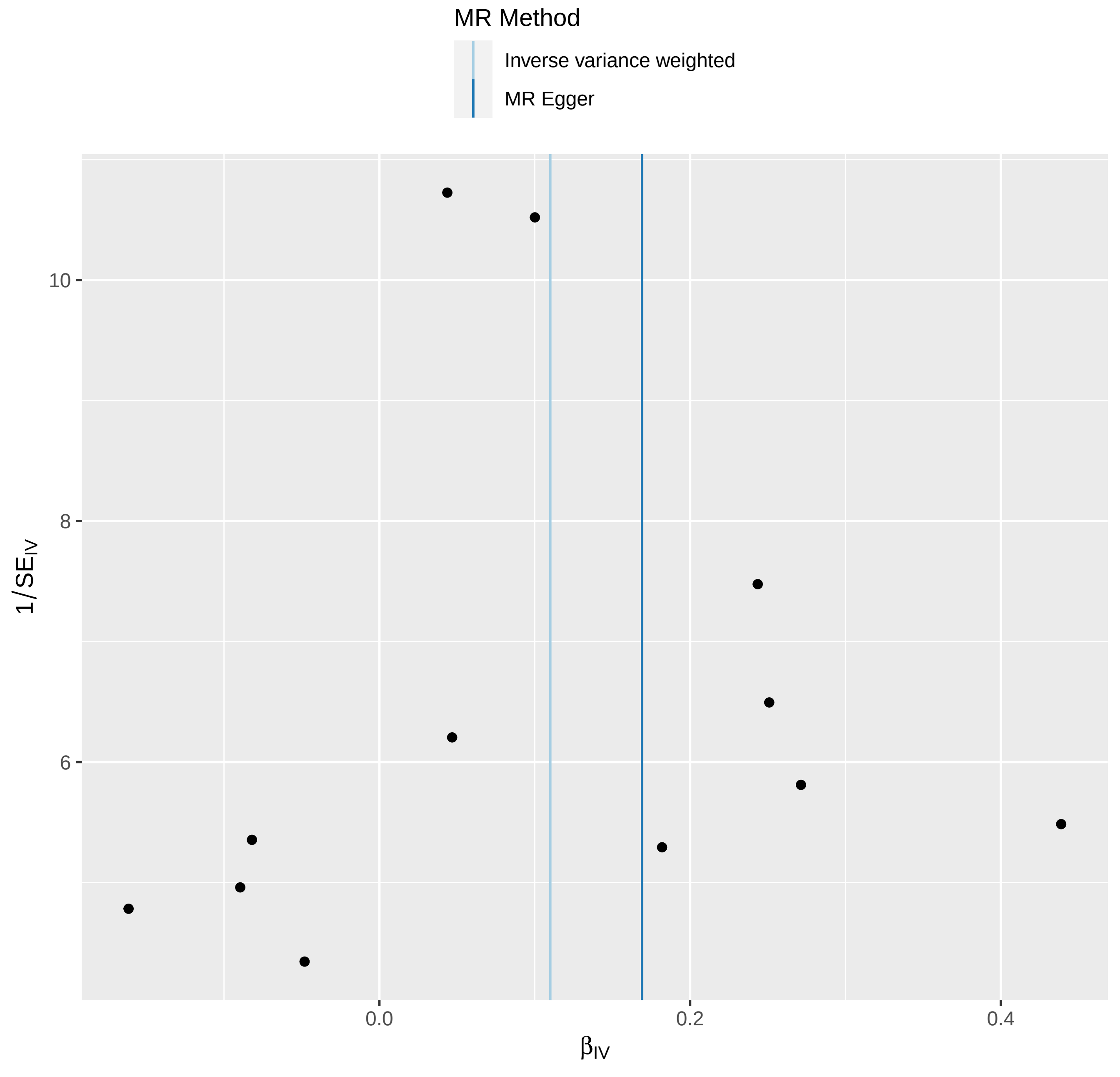

Batch 3−2270 : Selenium on PIK3CA/PIK3R1

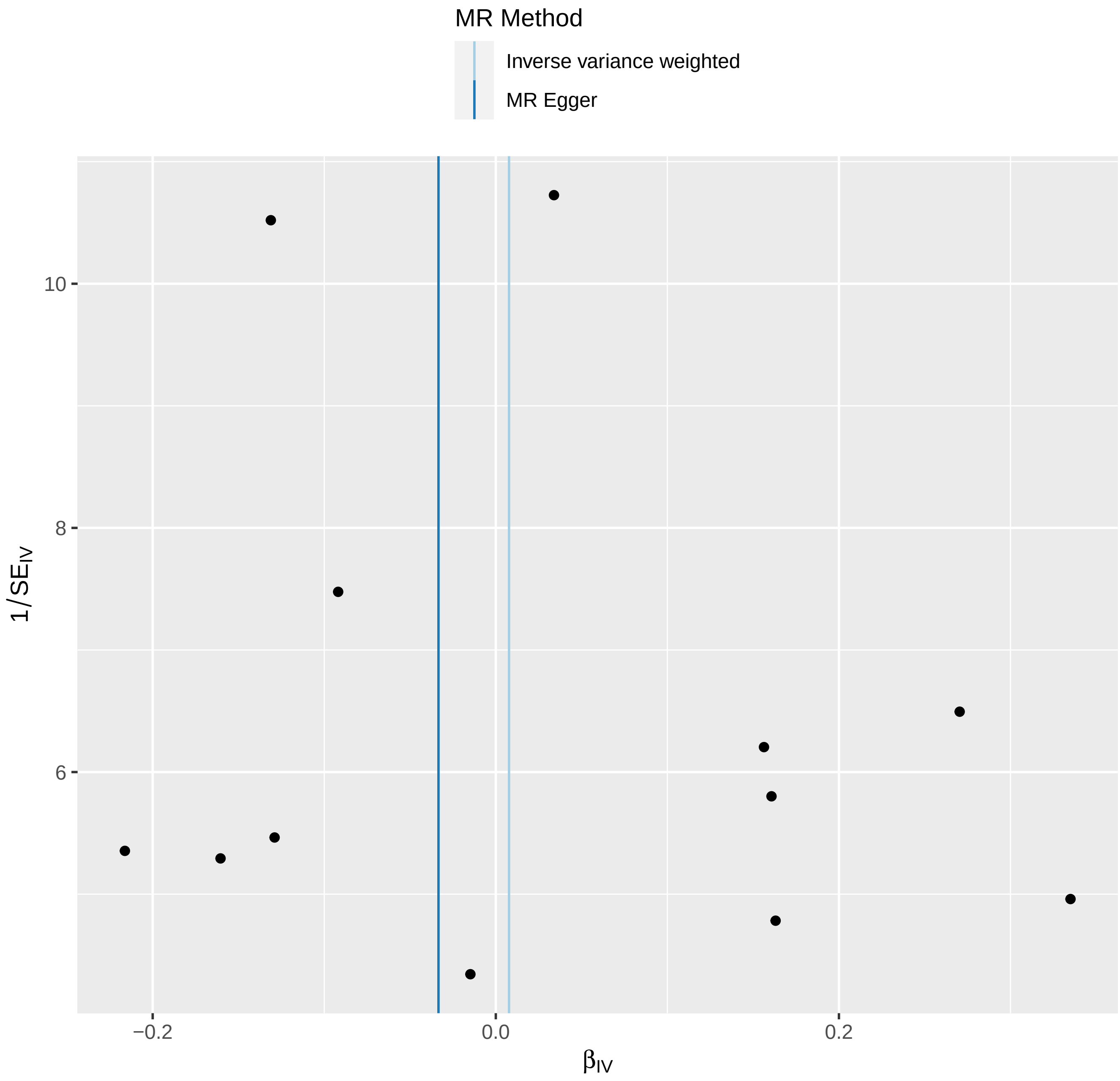

Batch 3−2271 : Selenium on Phosphatidylinositol 4,5−bisphosphate 3−kinase catalytic subunit gamma isoform

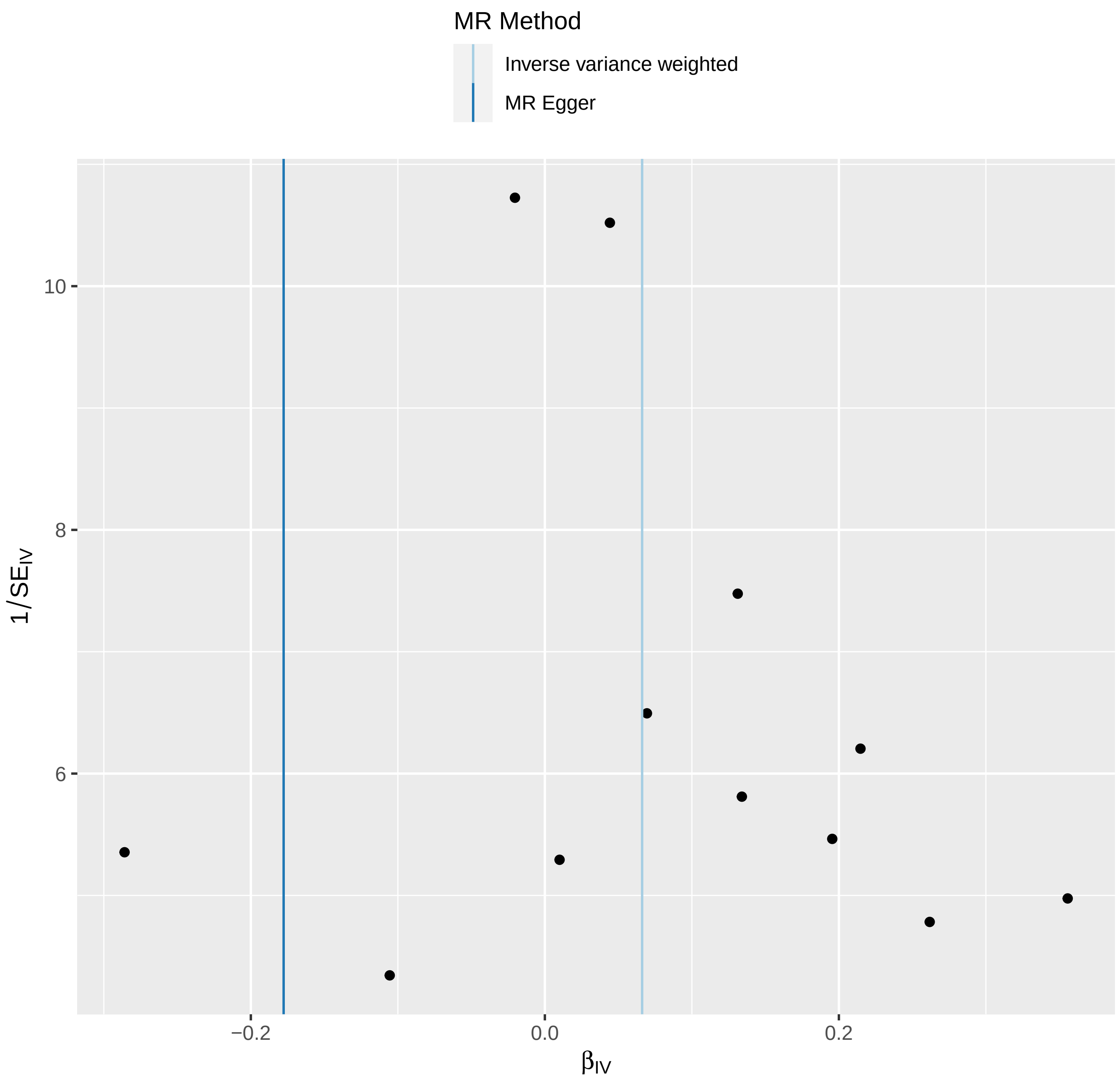

Batch 3−2272 : Selenium on Paired immunoglobulin−like type 2 receptor alpha

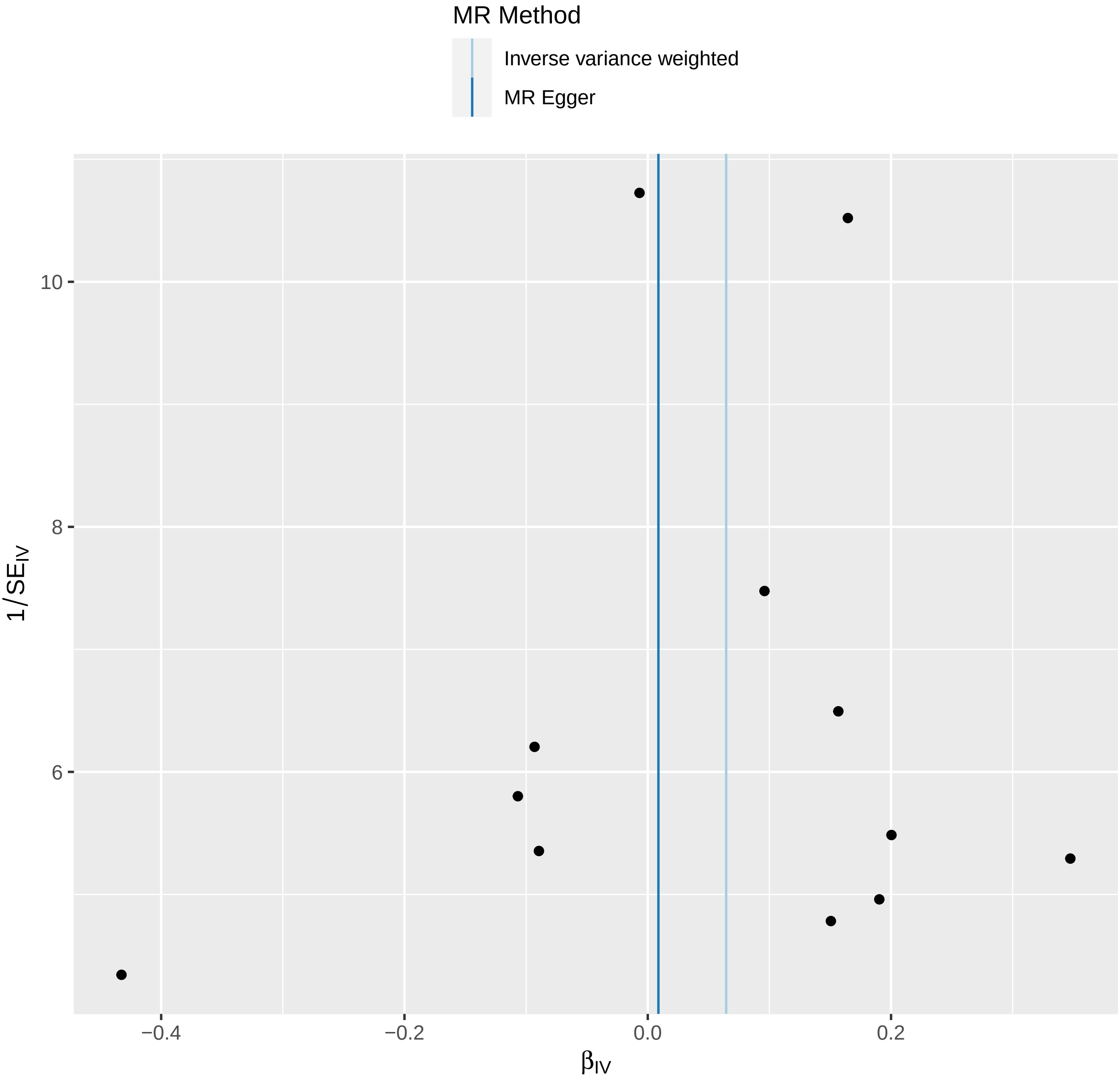

Batch 3−2273 : Selenium on Paired immunoglobulin−like type 2 receptor alpha

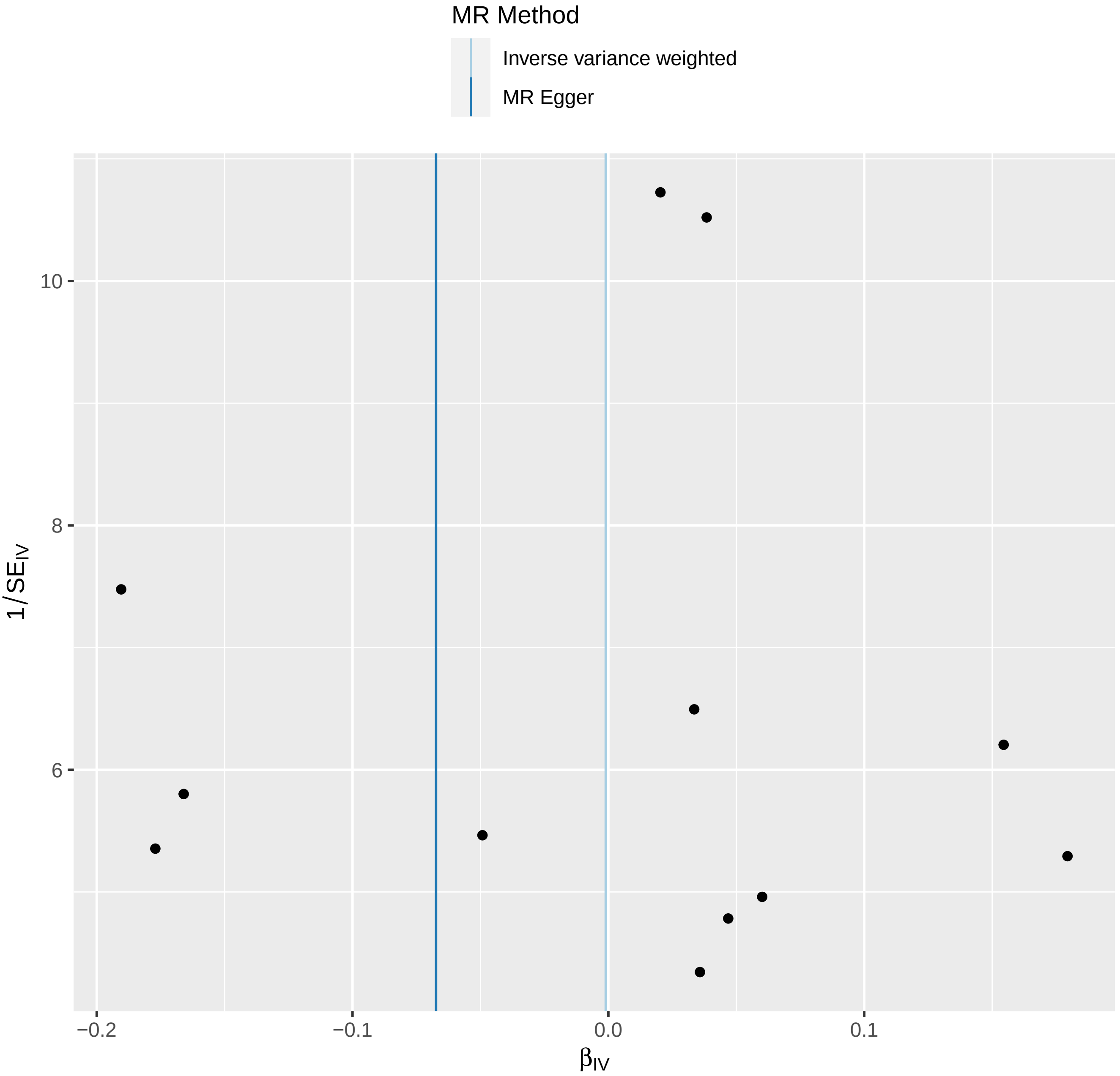

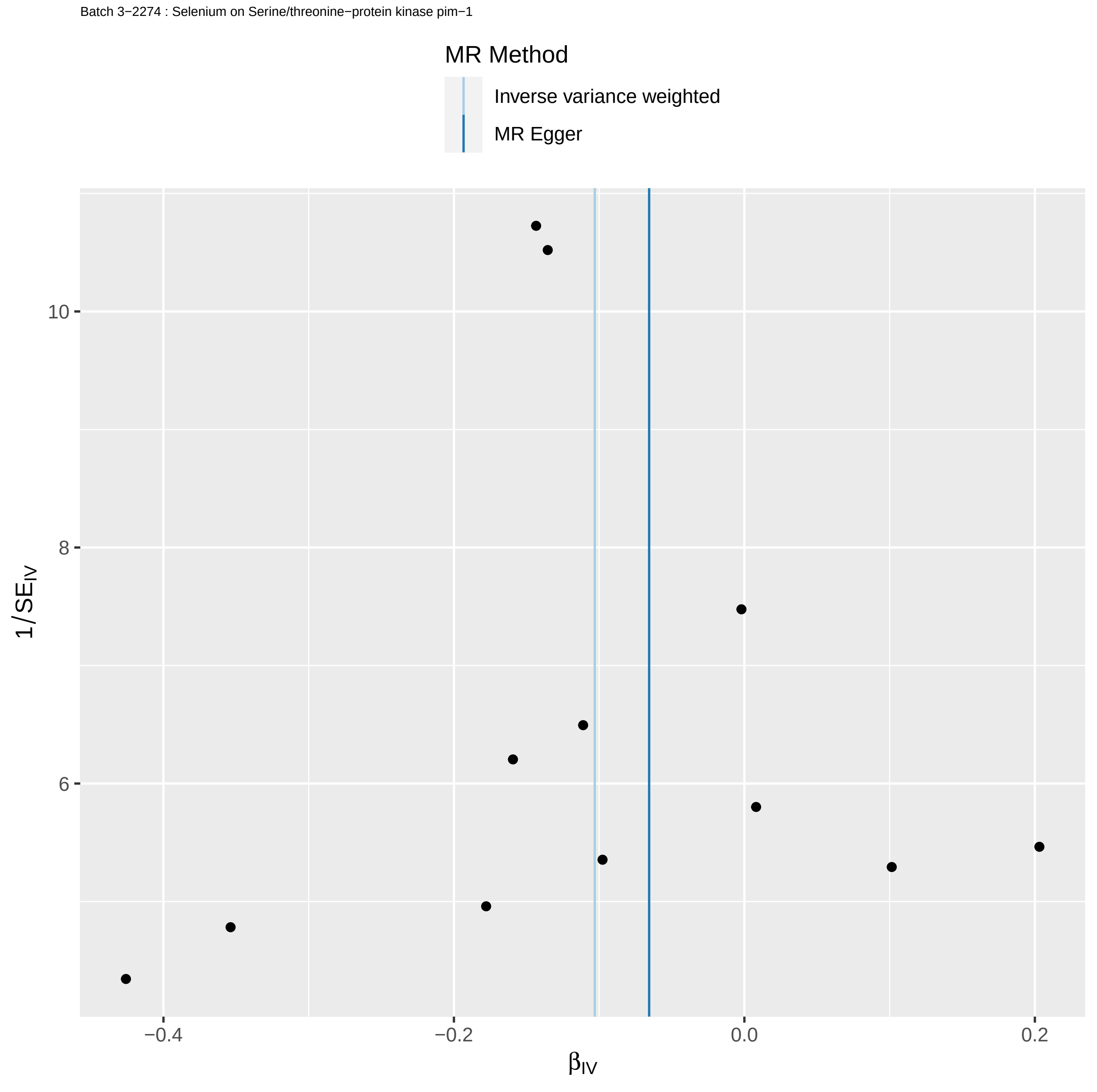

Batch 3−2274 : Selenium on Serine/threonine−protein kinase pim−1
MR Method
Inverse variance weighted
MR Egger
10
8
6
1/SE$_{IV}$
−0.4
−0.2
0.0
0.2
β$_{IV}$

Batch 3−2275 : Selenium on Peptidyl−prolyl cis−trans isomerase NIMA−interacting 4

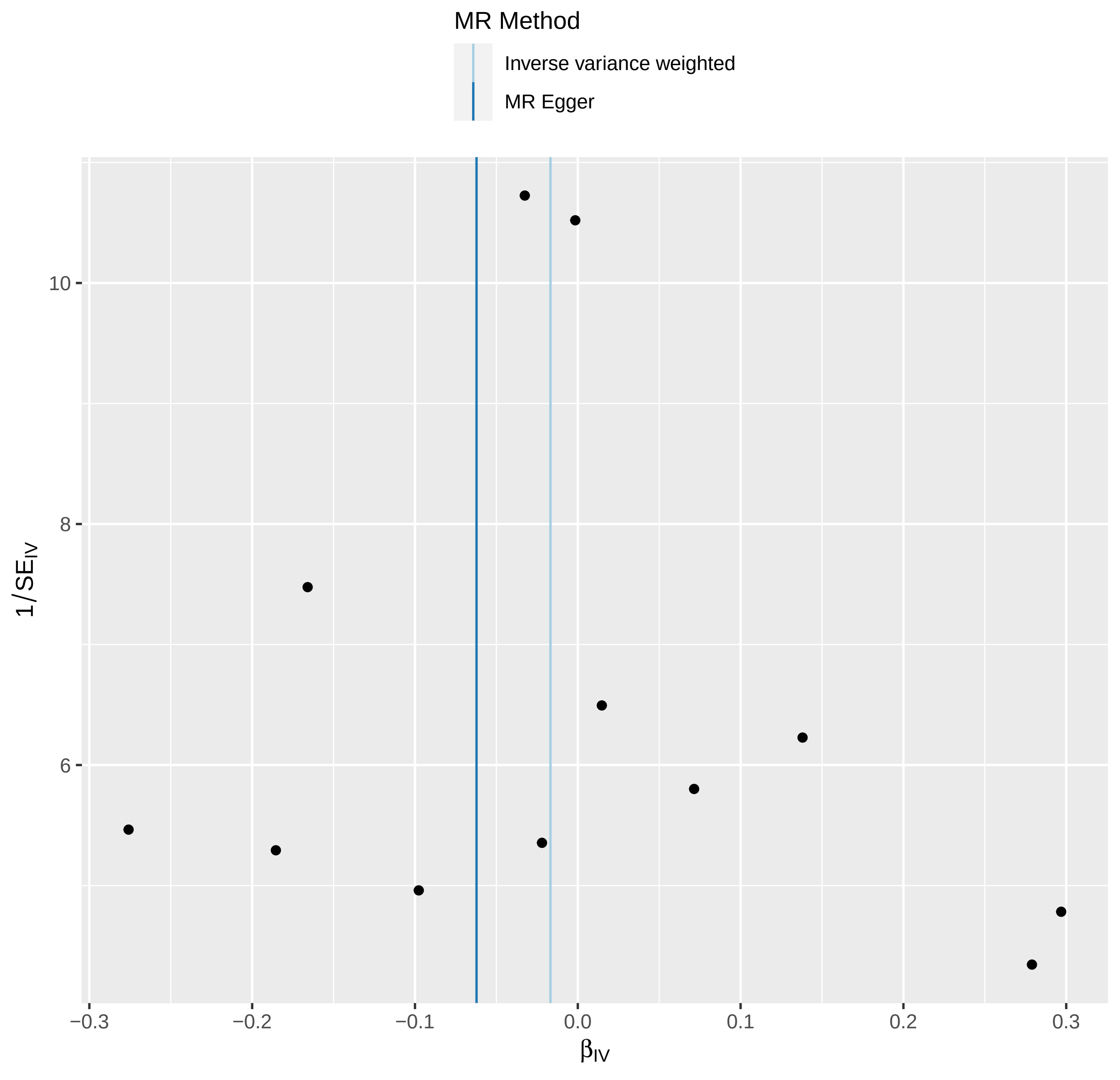

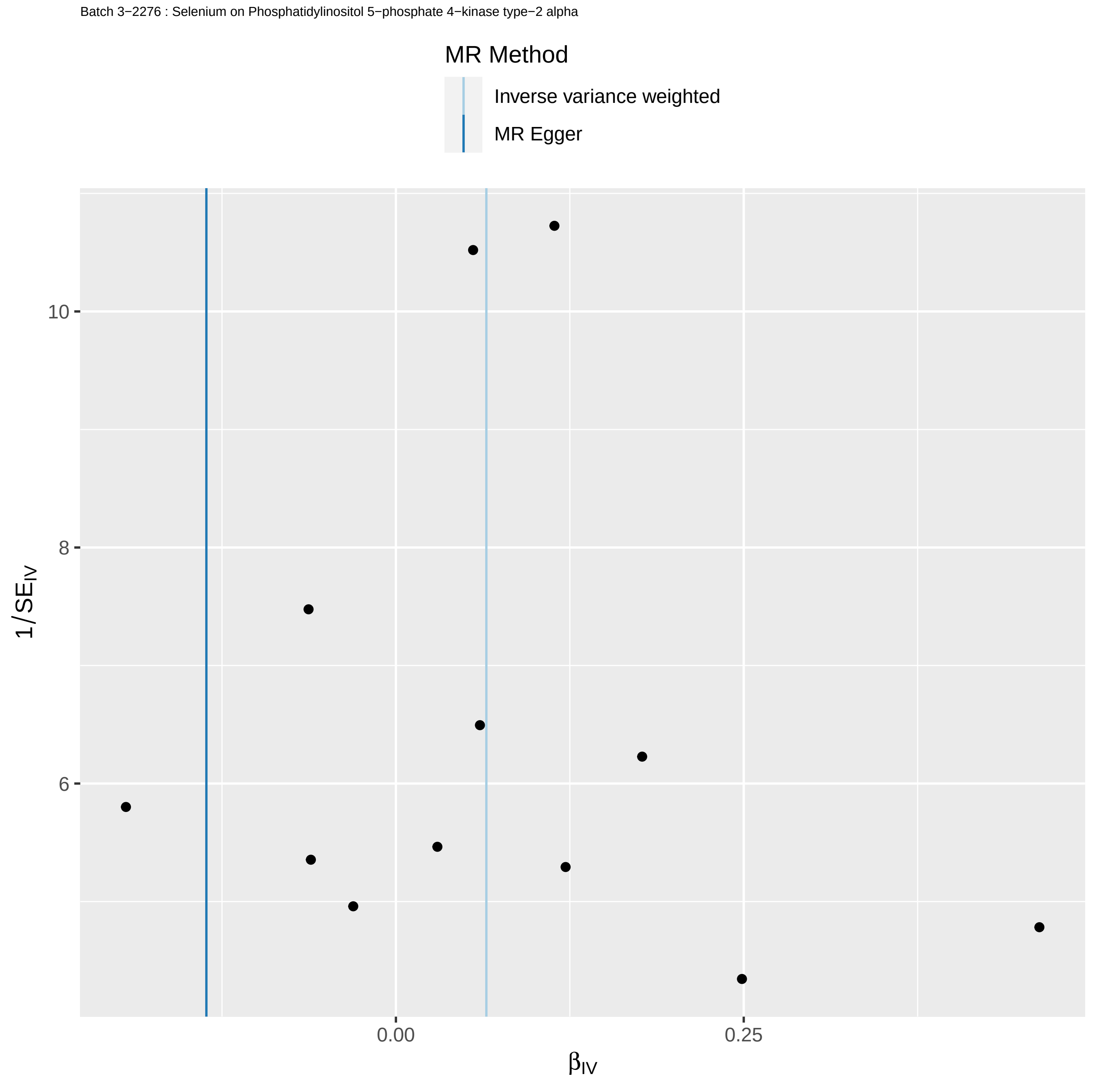
Batch 3−2276 : Selenium on Phosphatidylinositol 5−phosphate 4−kinase type−2 alpha
MR Method
Inverse variance weighted
MR Egger
10
8
6
1/SE$_{IV}$
0.00
0.25
β$_{IV}$

Batch 3−2277 : Selenium on Pirin

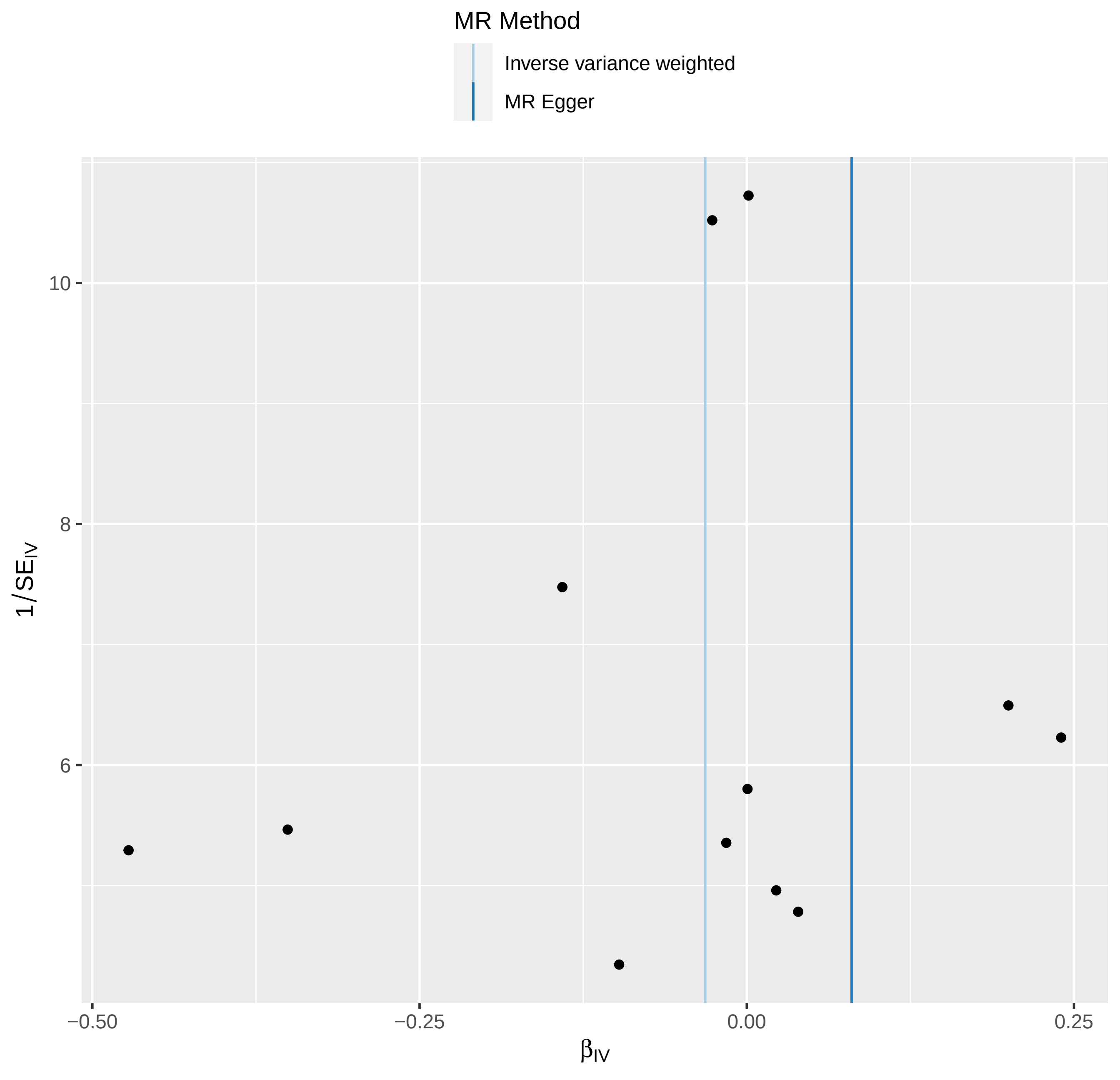

Batch 3−2278 : Selenium on Phosphatidylinositol transfer protein alpha isoform

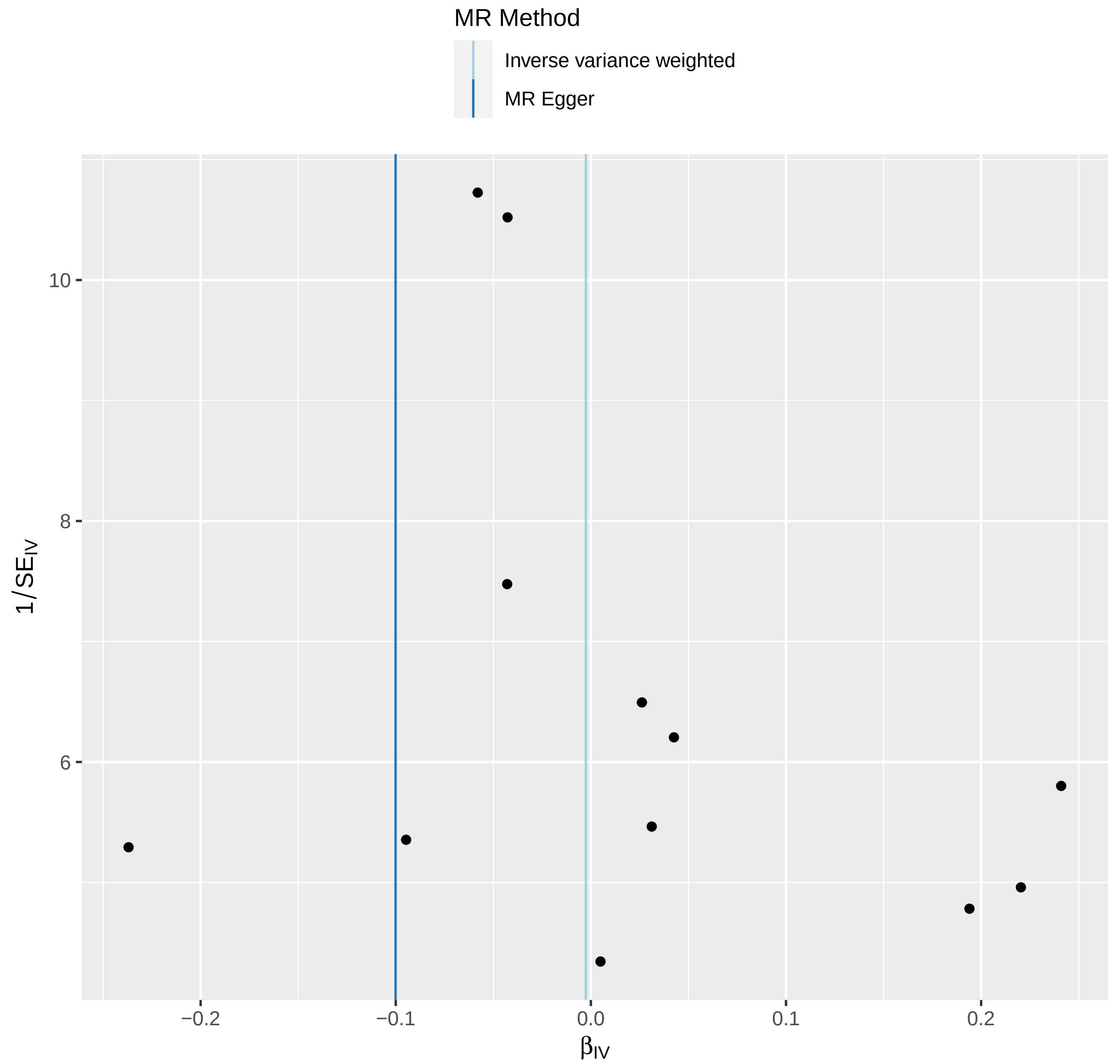

Batch 3−2279 : Selenium on Phosphatidylinositol transfer protein beta isoform

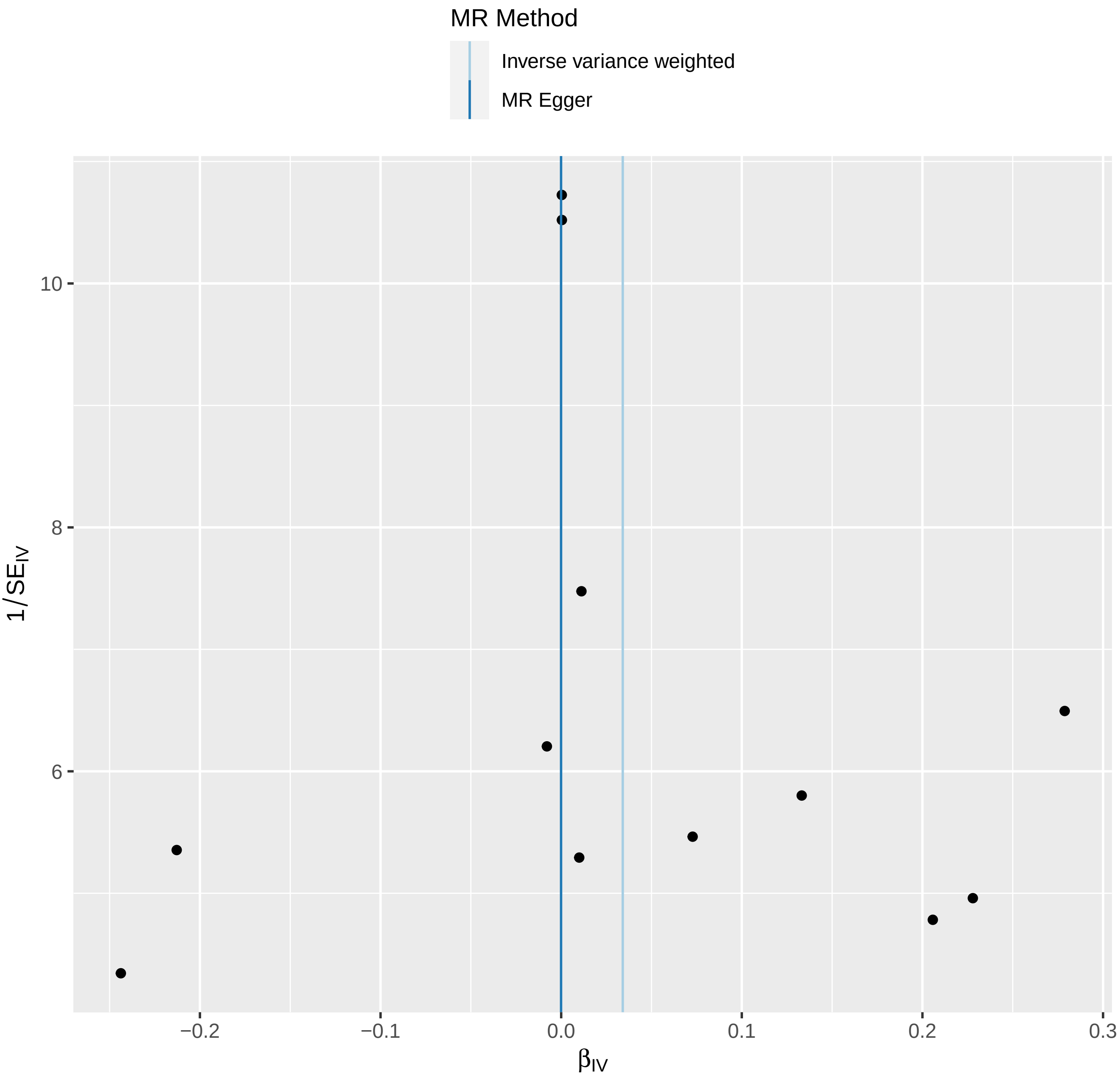

Batch 3−2280 : Selenium on Piwi−like protein 1

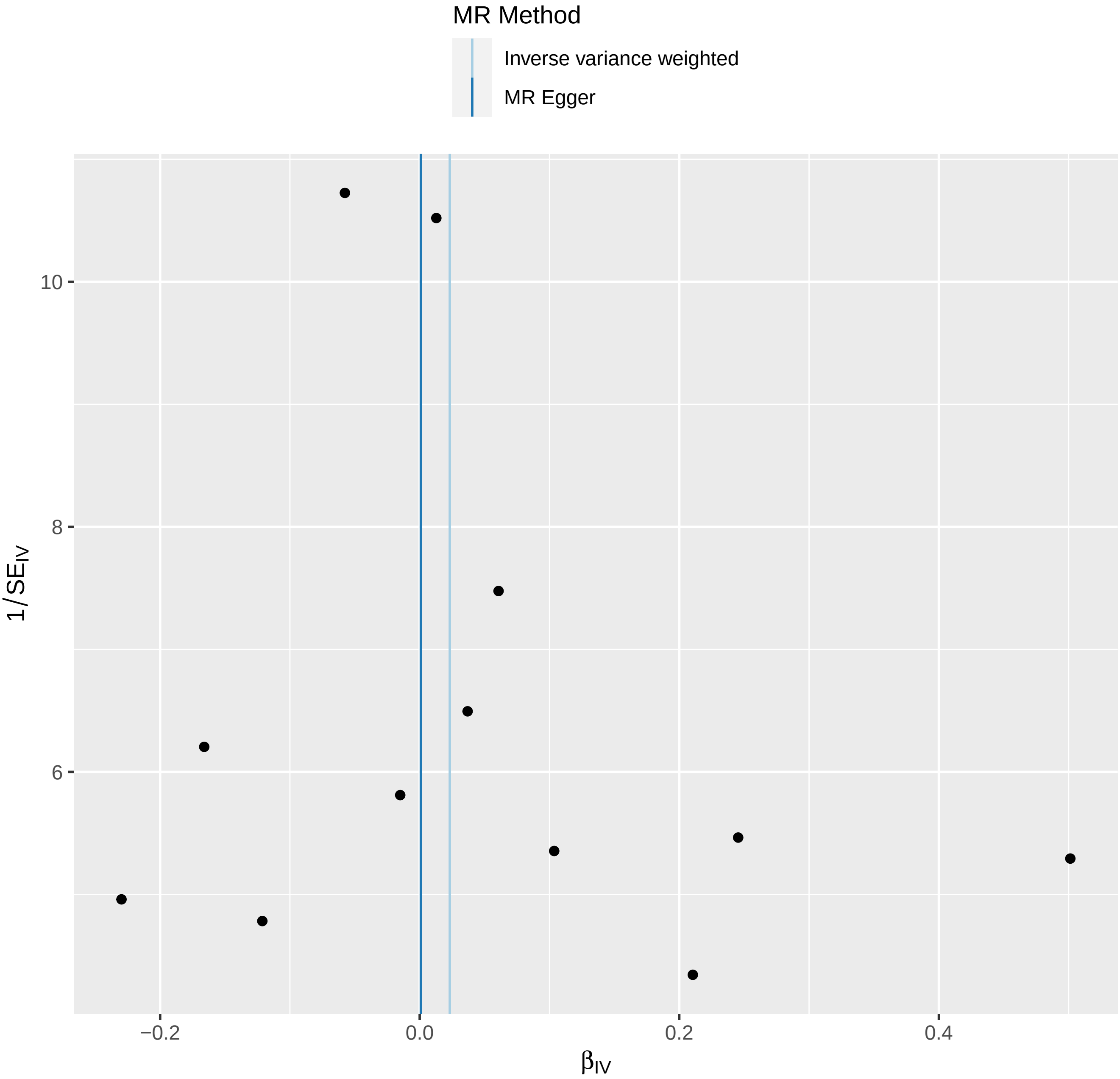

Batch 3−2281 : Selenium on Polycystin−2

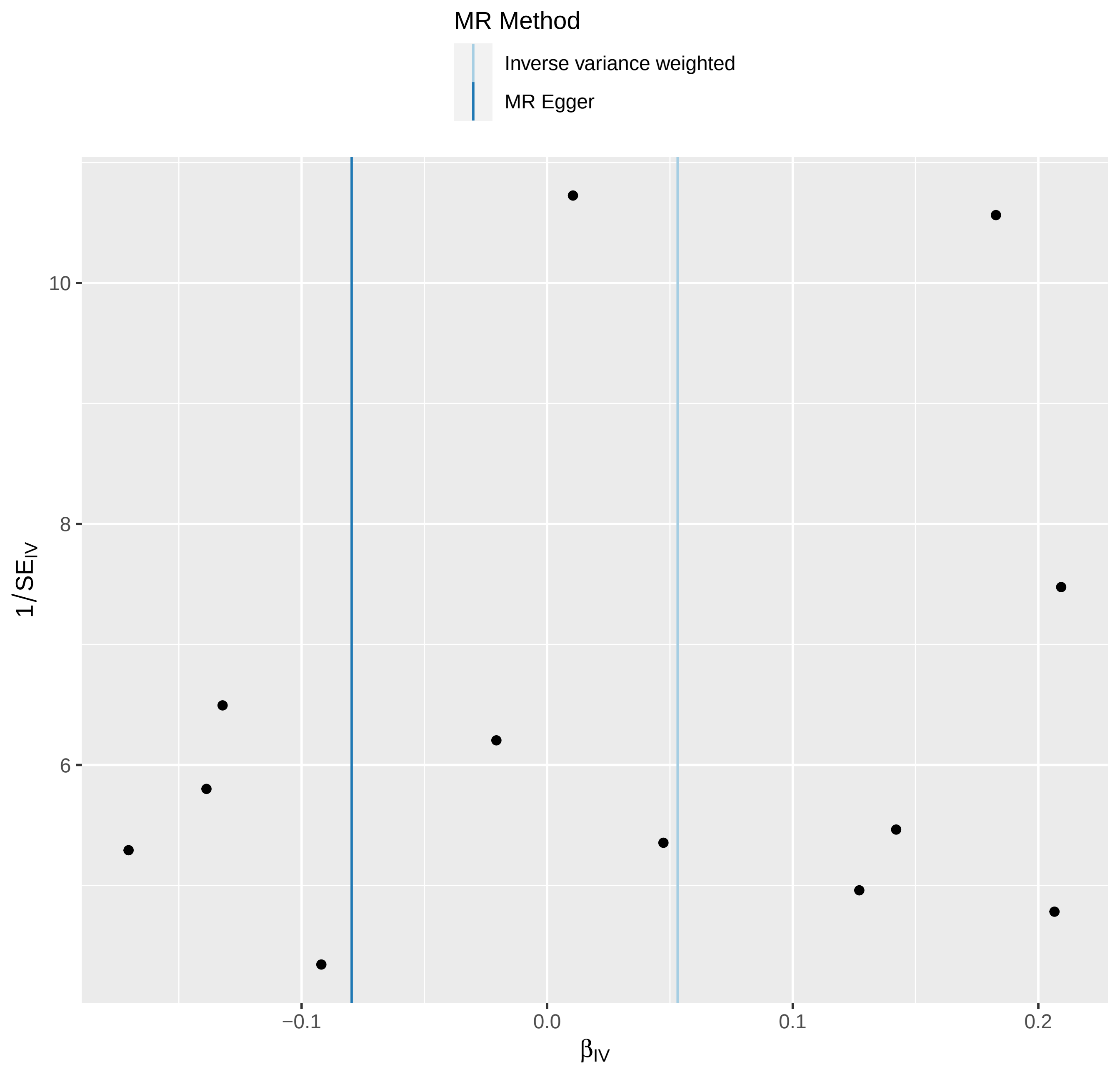

Batch 3−2282 : Selenium on Pyruvate kinase PKLR

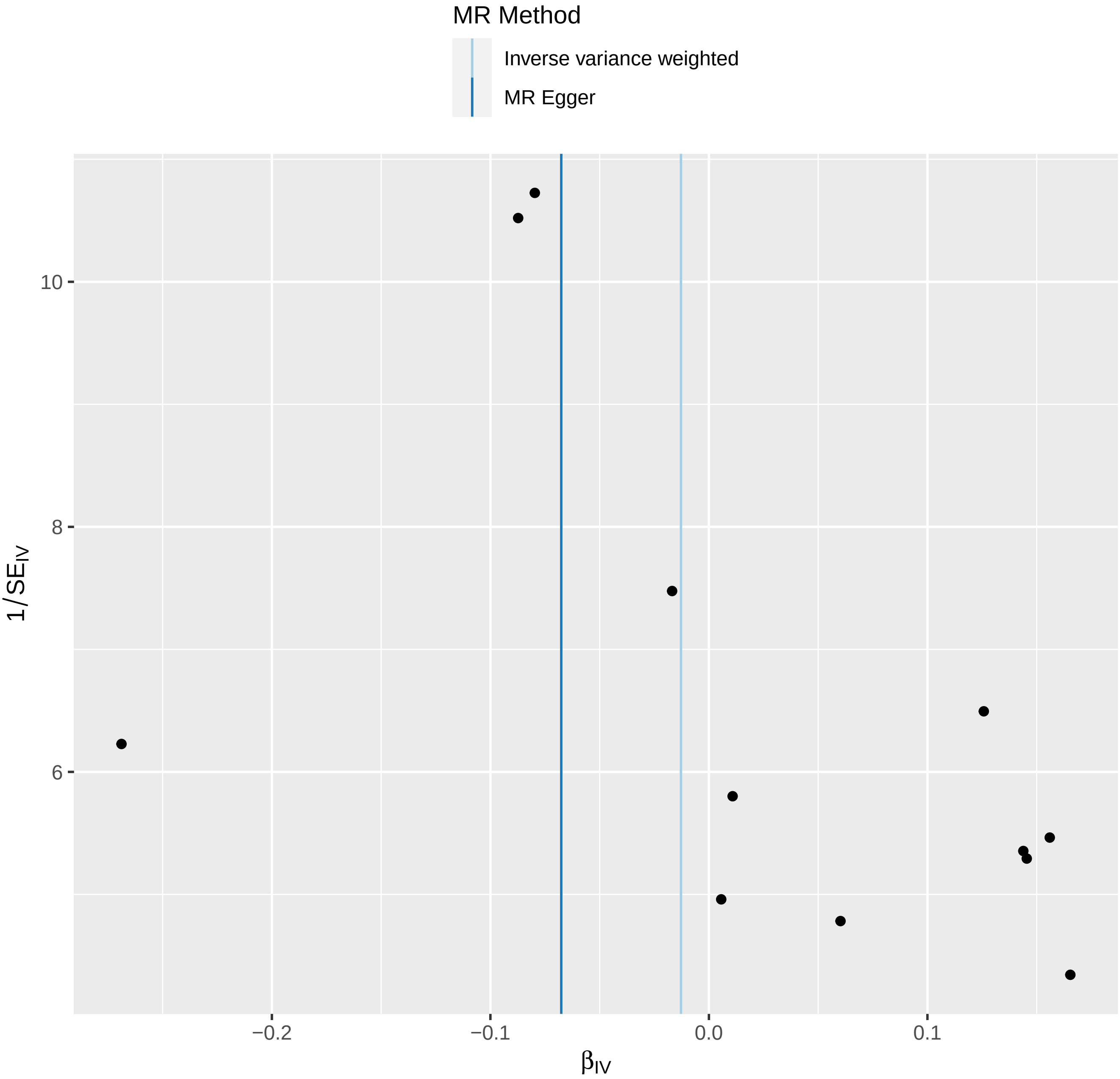

Batch 3−2283 : Selenium on Group 10 secretory phospholipase A2

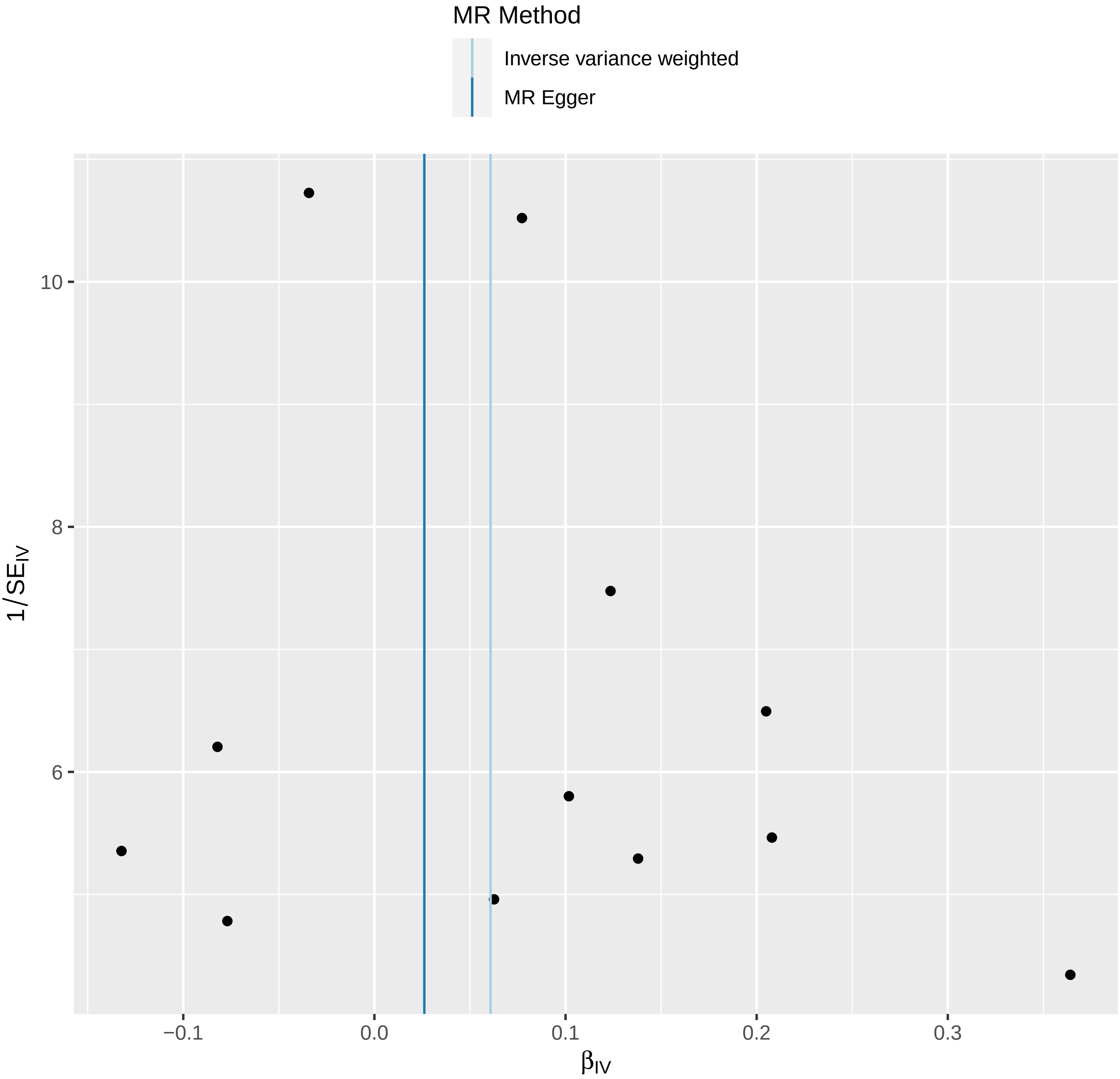

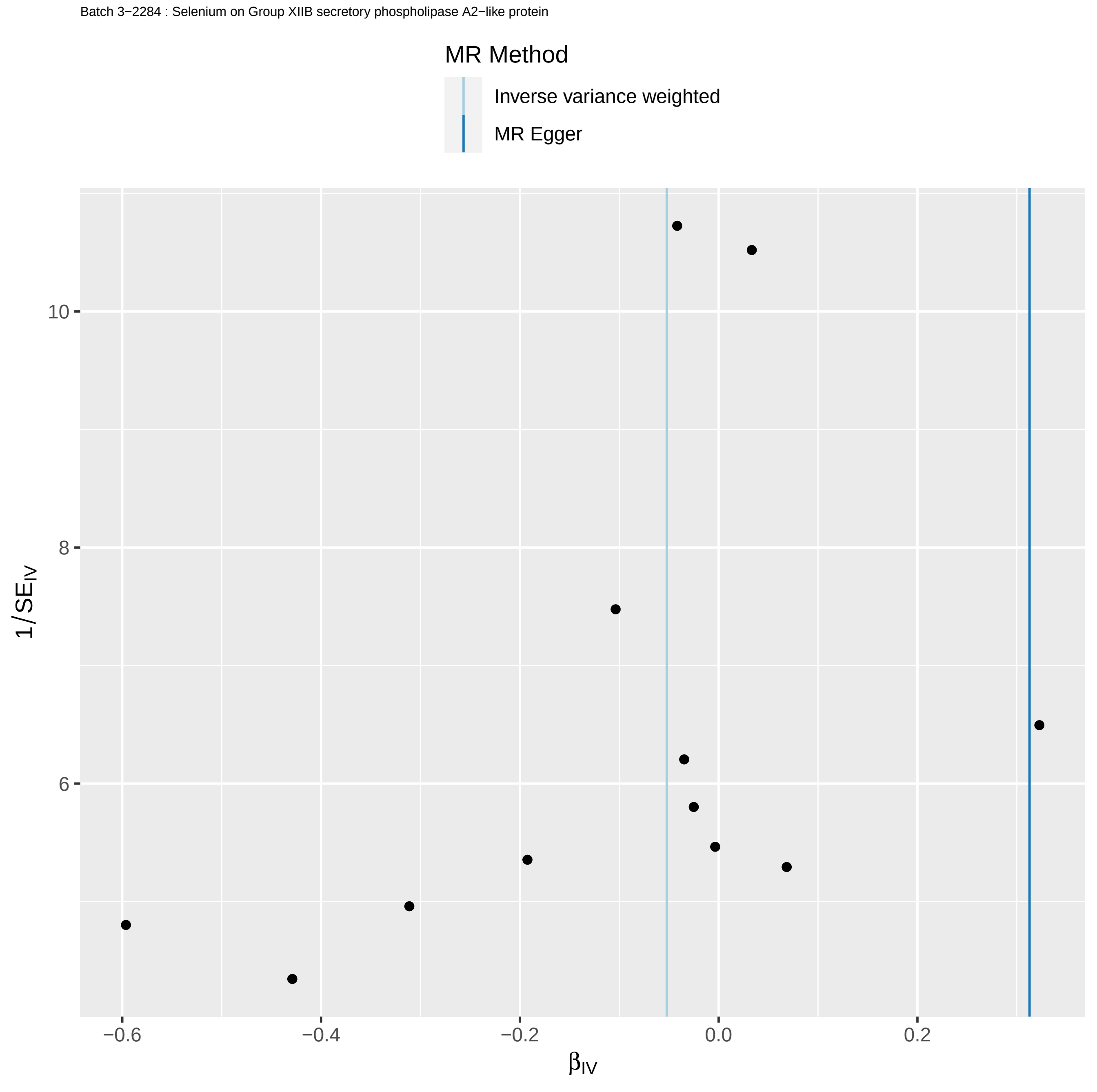

Batch 3−2284 : Selenium on Group XIIB secretory phospholipase A2−like protein
MR Method
Inverse variance weighted
MR Egger
10
8
6
1/SE$_{IV}$
−0.6
−0.4
−0.2
0.0
0.2
β$_{IV}$

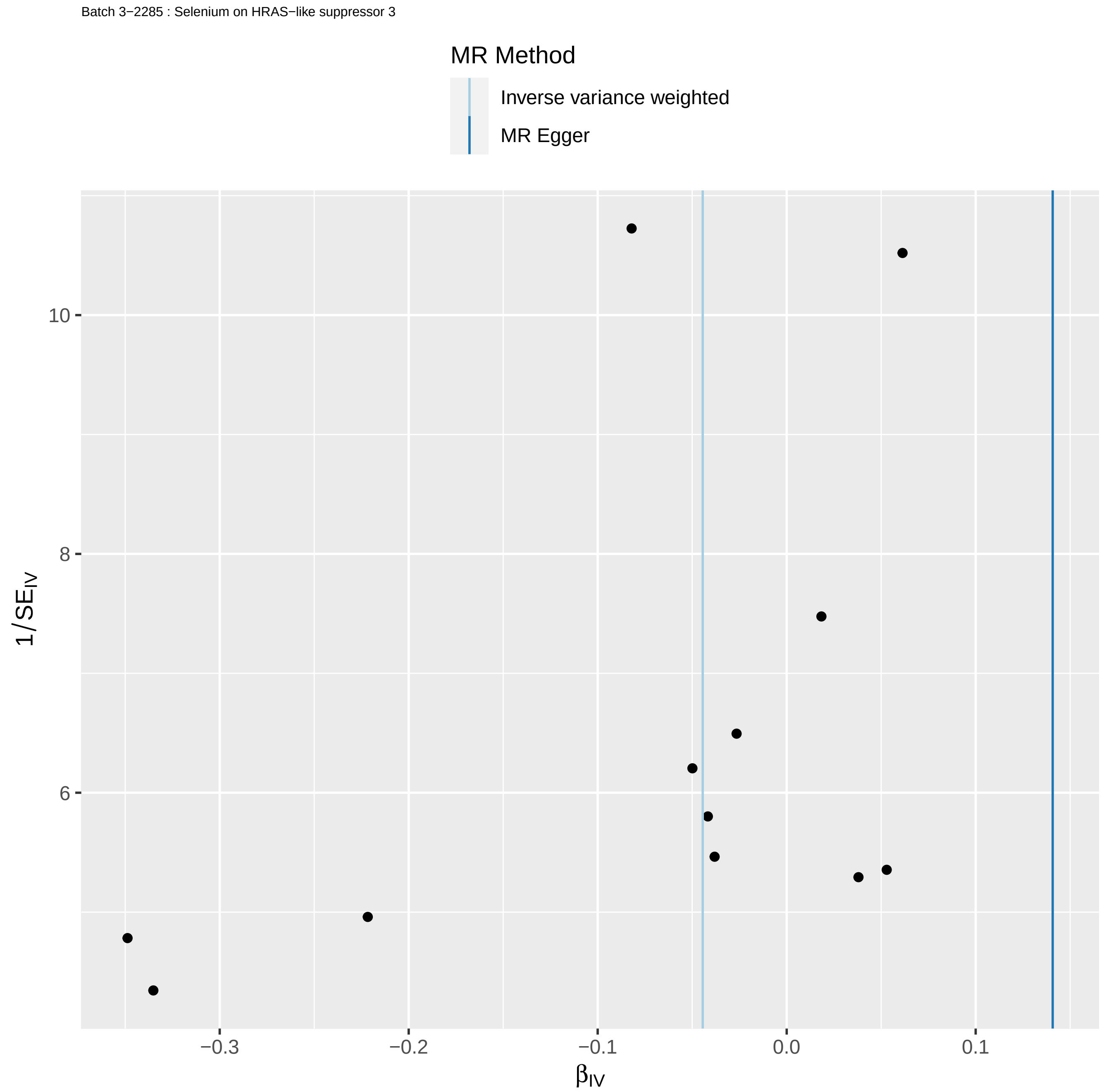

Batch 3−2285 : Selenium on HRAS−like suppressor 3
MR Method
Inverse variance weighted
MR Egger
10
8
6
1/SE$_{IV}$
−0.3
−0.2
−0.1
0.0
0.1
β$_{IV}$

Batch 3−2286 : Selenium on Phospholipase A2, membrane associated

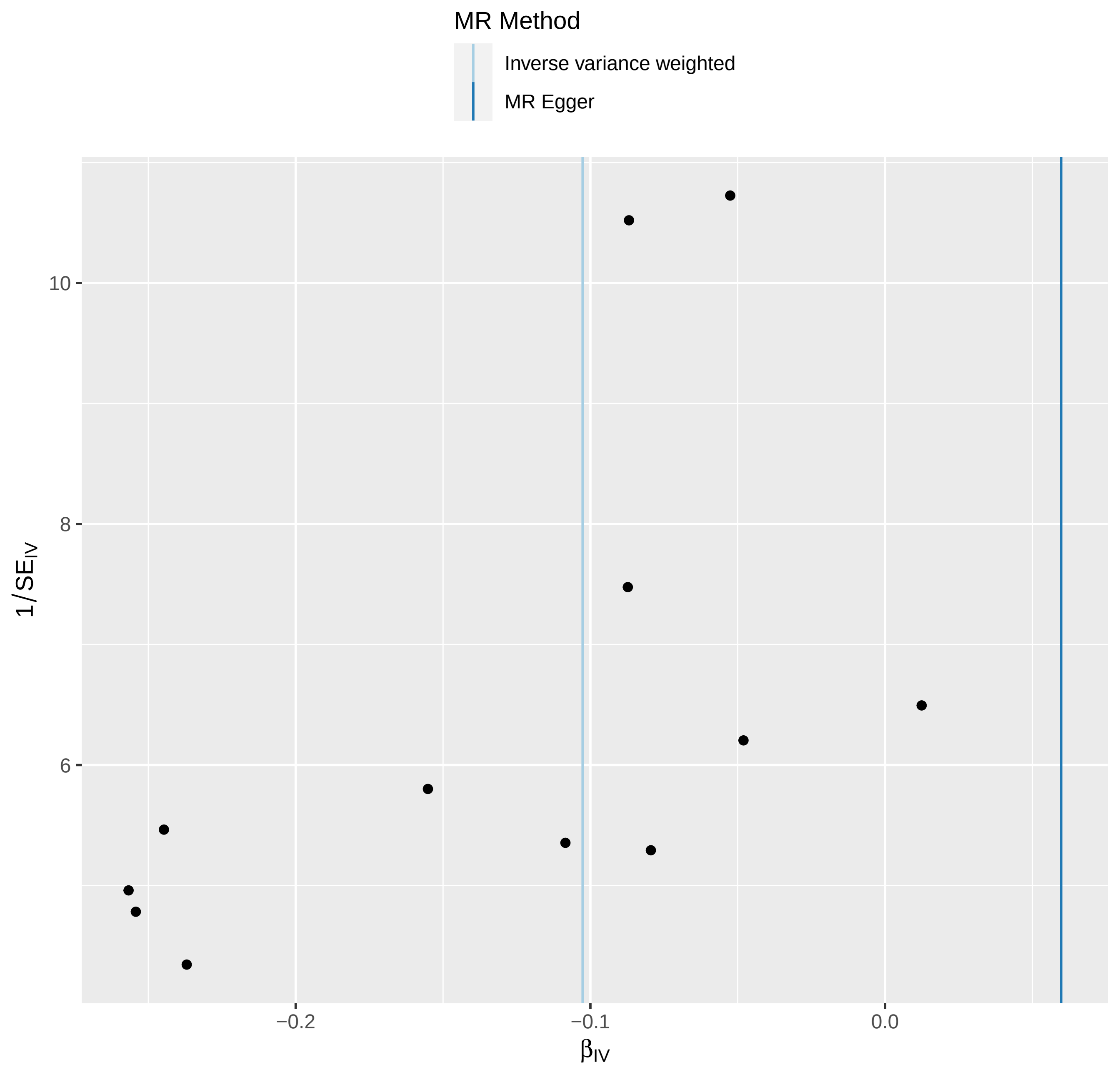

Batch 3−2287 : Selenium on Putative inactive group IIC secretory phospholipase A2

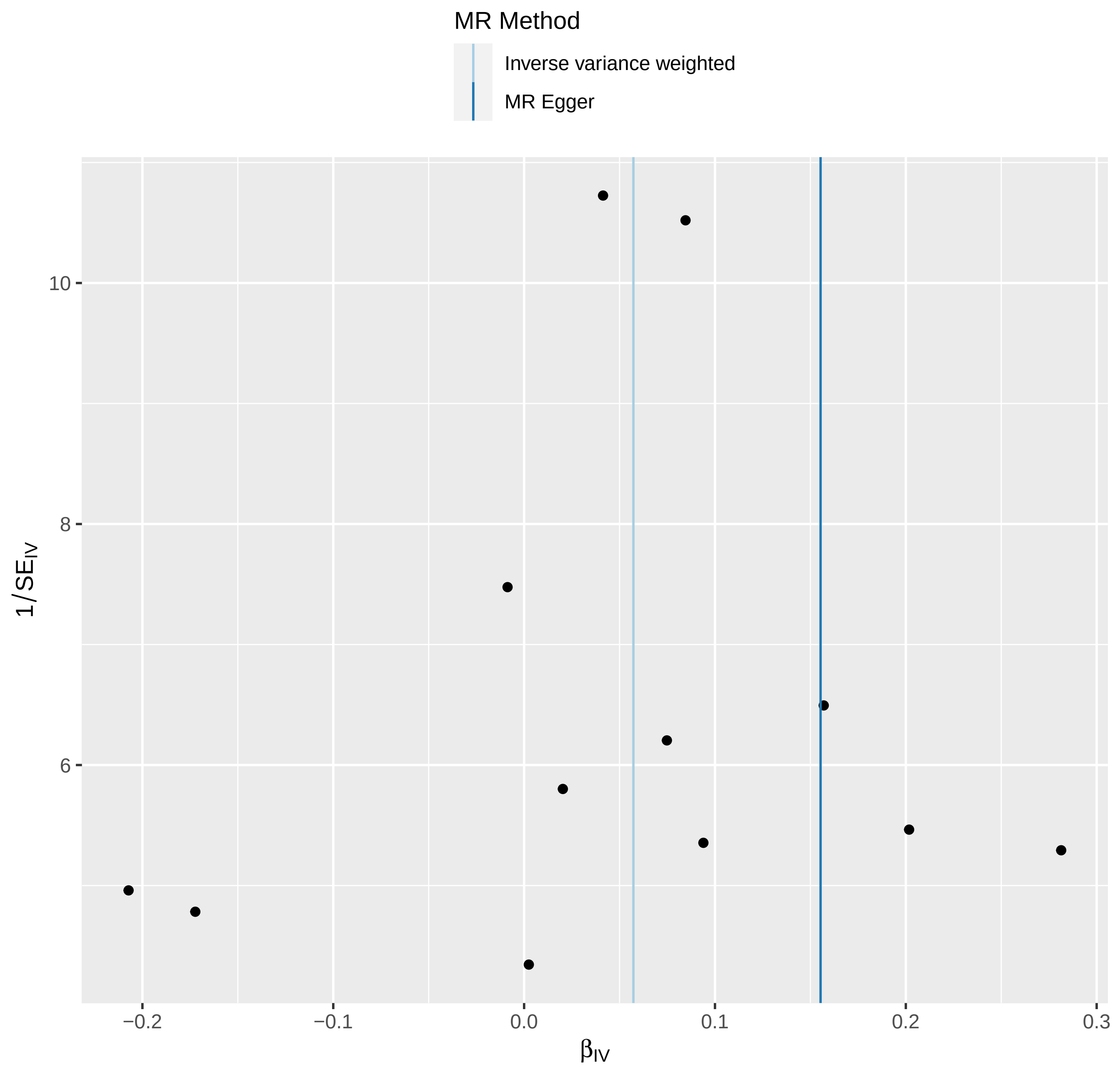

Batch 3−2288 : Selenium on Group IIE secretory phospholipase A2

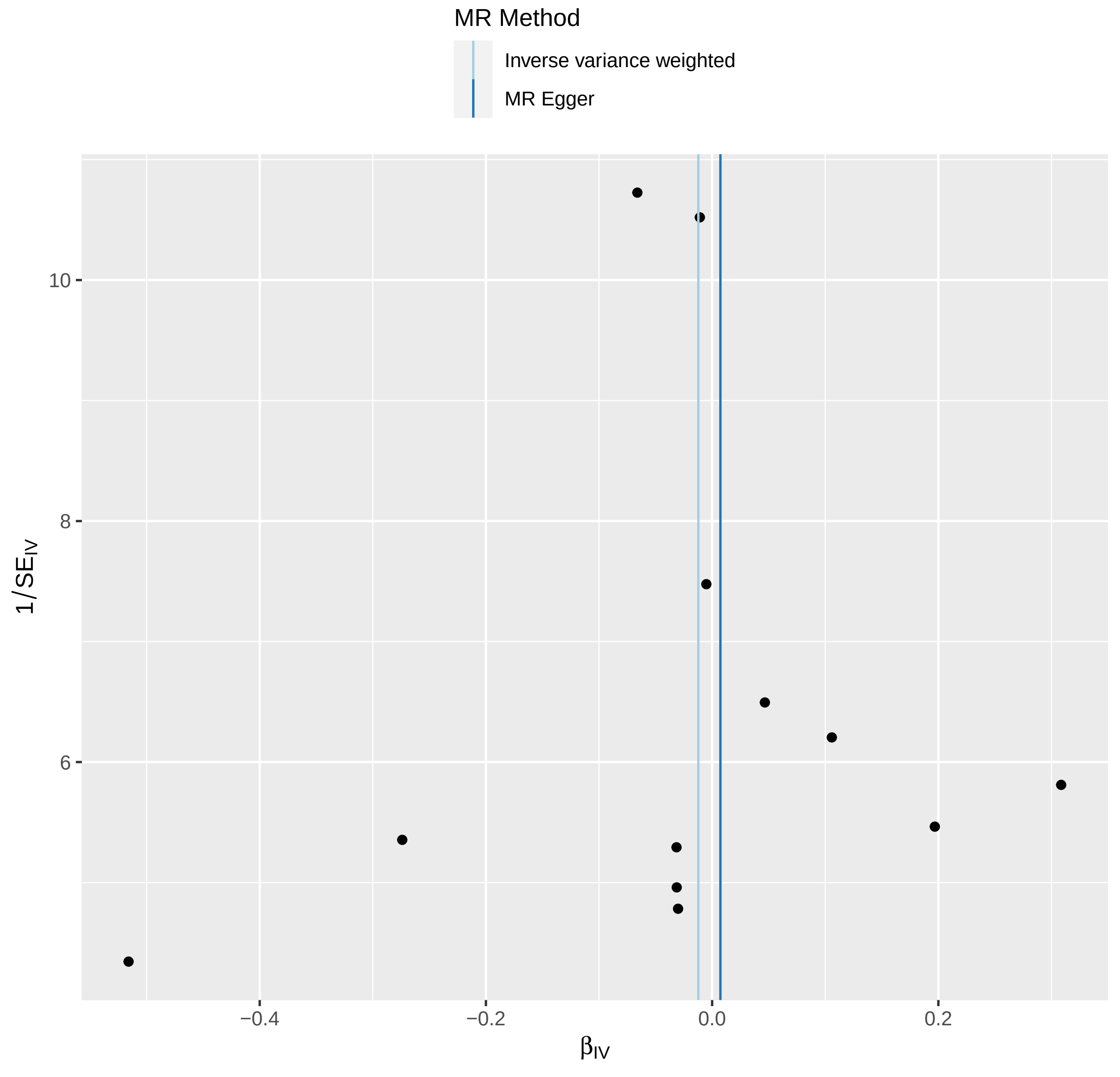

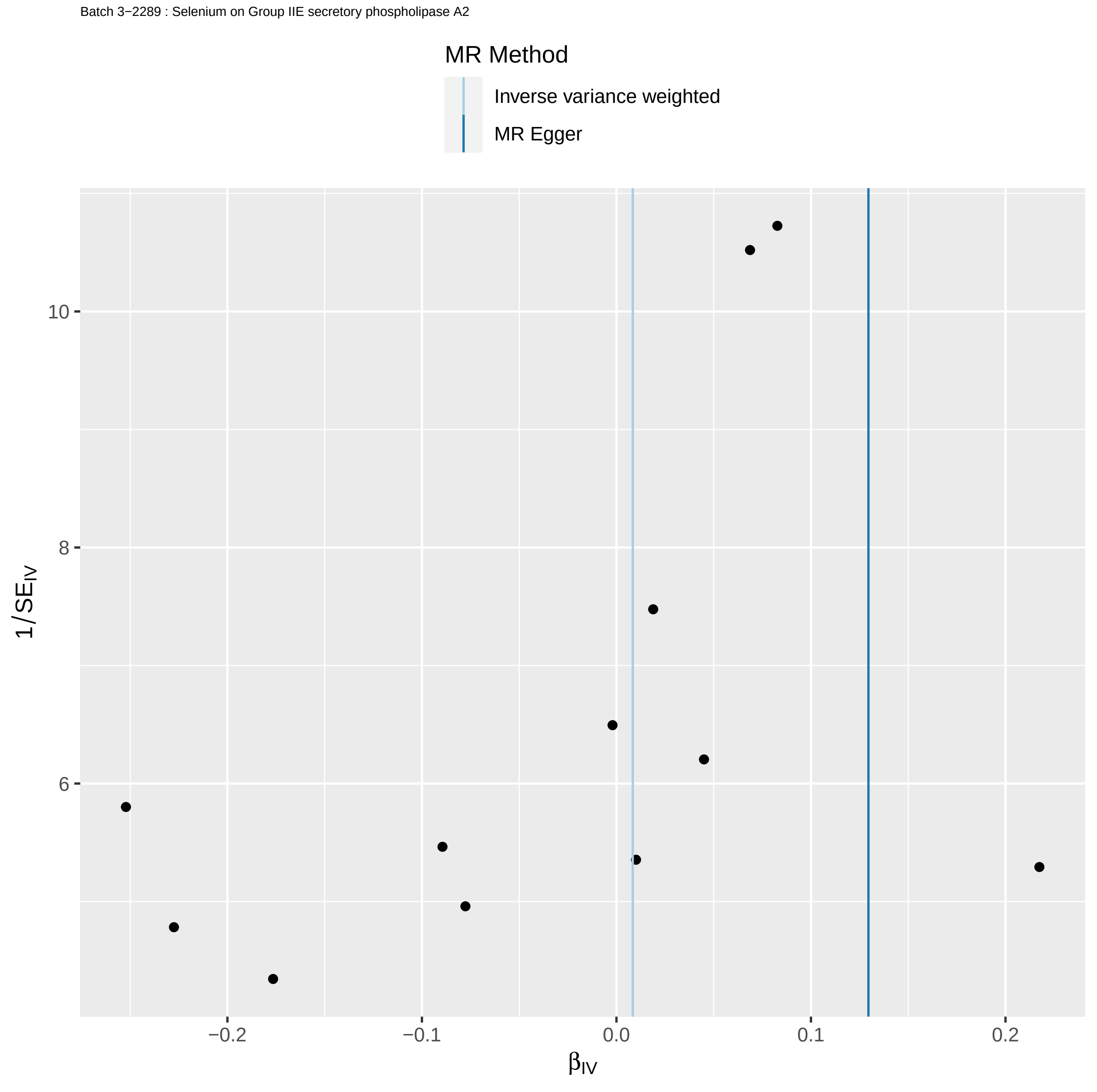

Batch 3−2289 : Selenium on Group IIE secretory phospholipase A2
MR Method
Inverse variance weighted
MR Egger
10
8
6
1/SE_IV
−0.2
−0.1
0.0
0.1
0.2
β_IV

Batch 3−2290 : Selenium on Calcium−dependent phospholipase A2

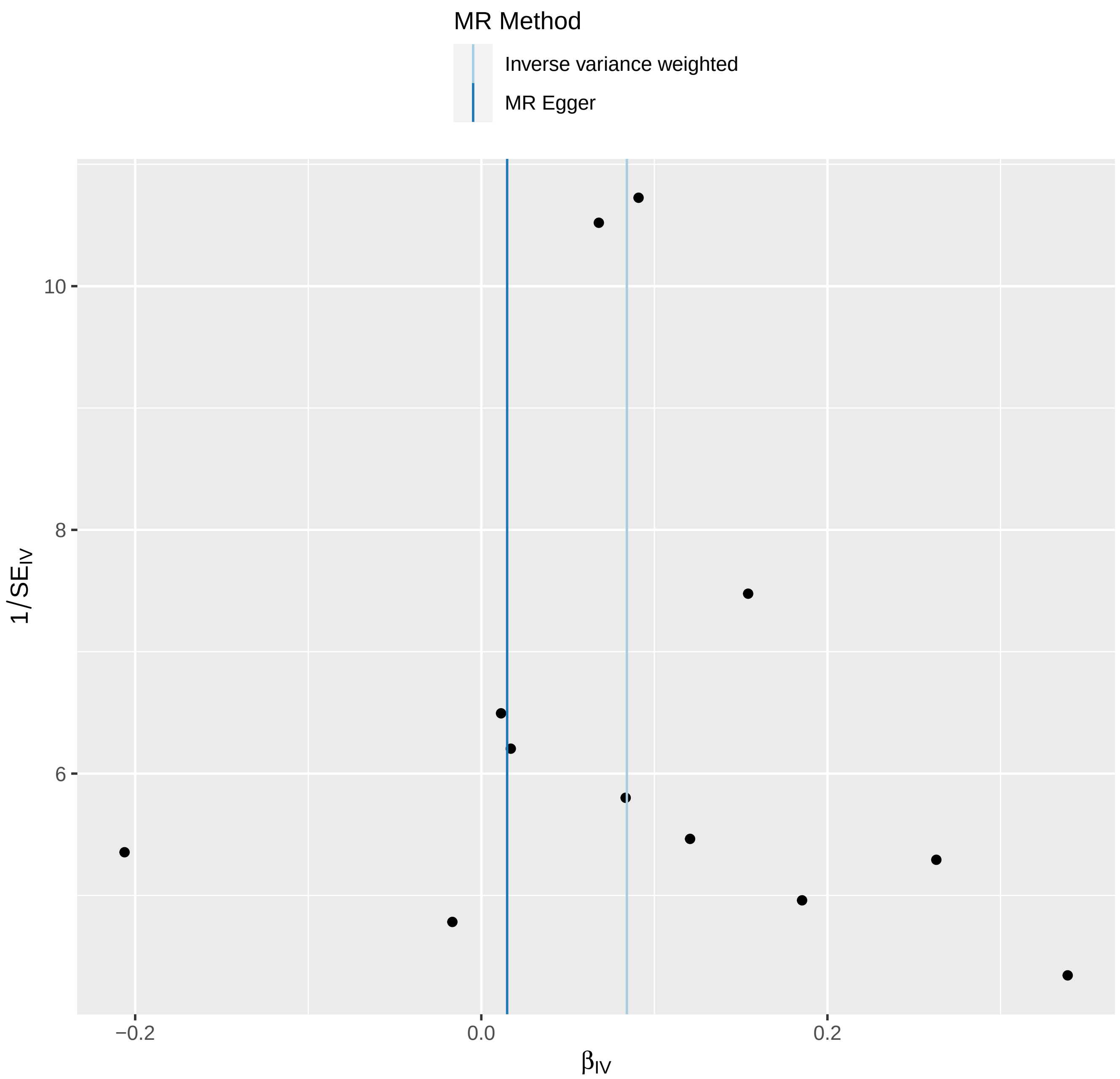

Batch 3−2291 : Selenium on Secretory phospholipase A2 receptor

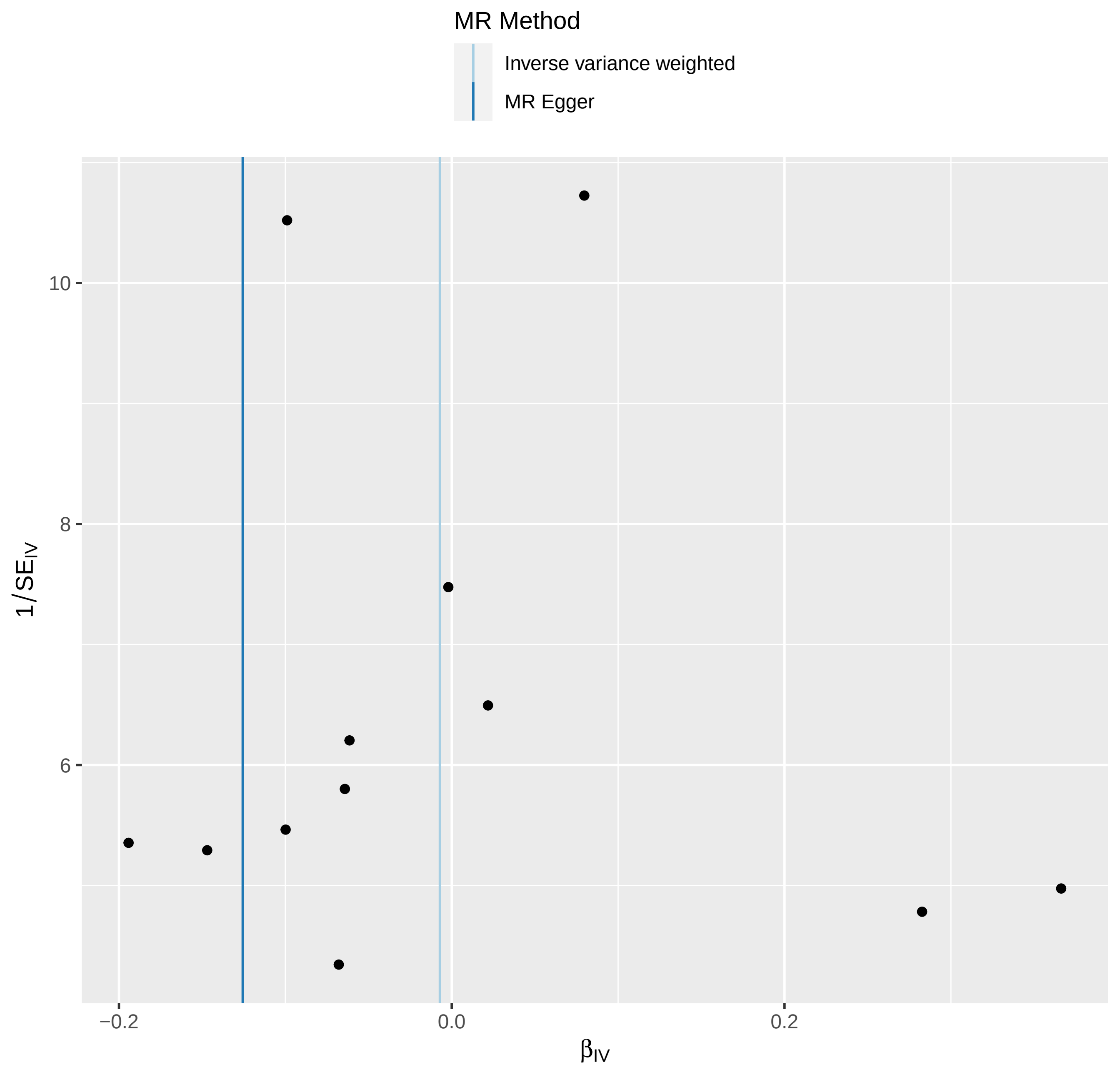

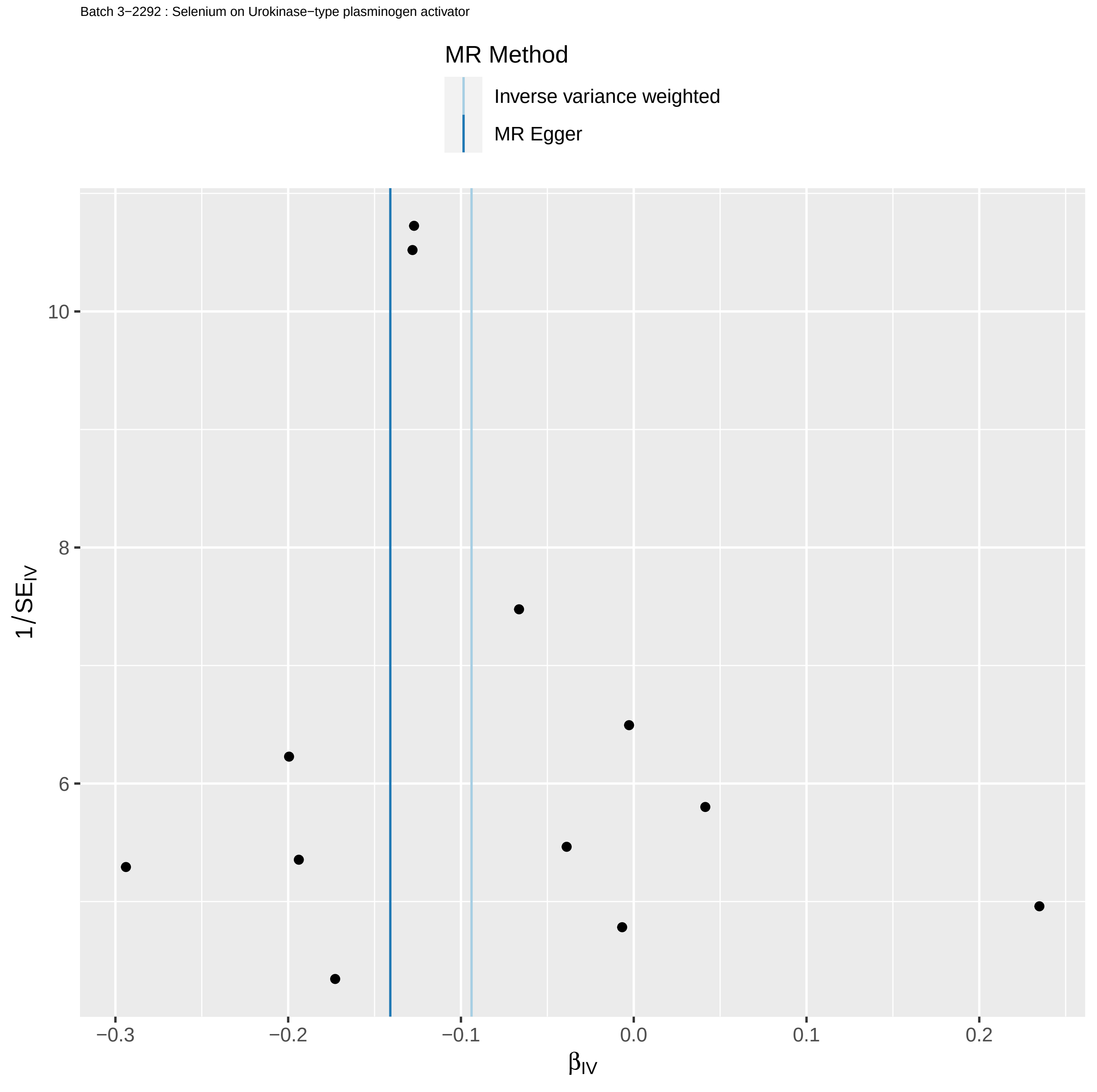
Batch 3−2292 : Selenium on Urokinase−type plasminogen activator
MR Method
Inverse variance weighted
MR Egger
10
8
6
1/SE$_{IV}$
−0.3
−0.2
−0.1
0.0
0.1
0.2
β$_{IV}$

Batch 3−2293 : Selenium on Urokinase plasminogen activator surface receptor

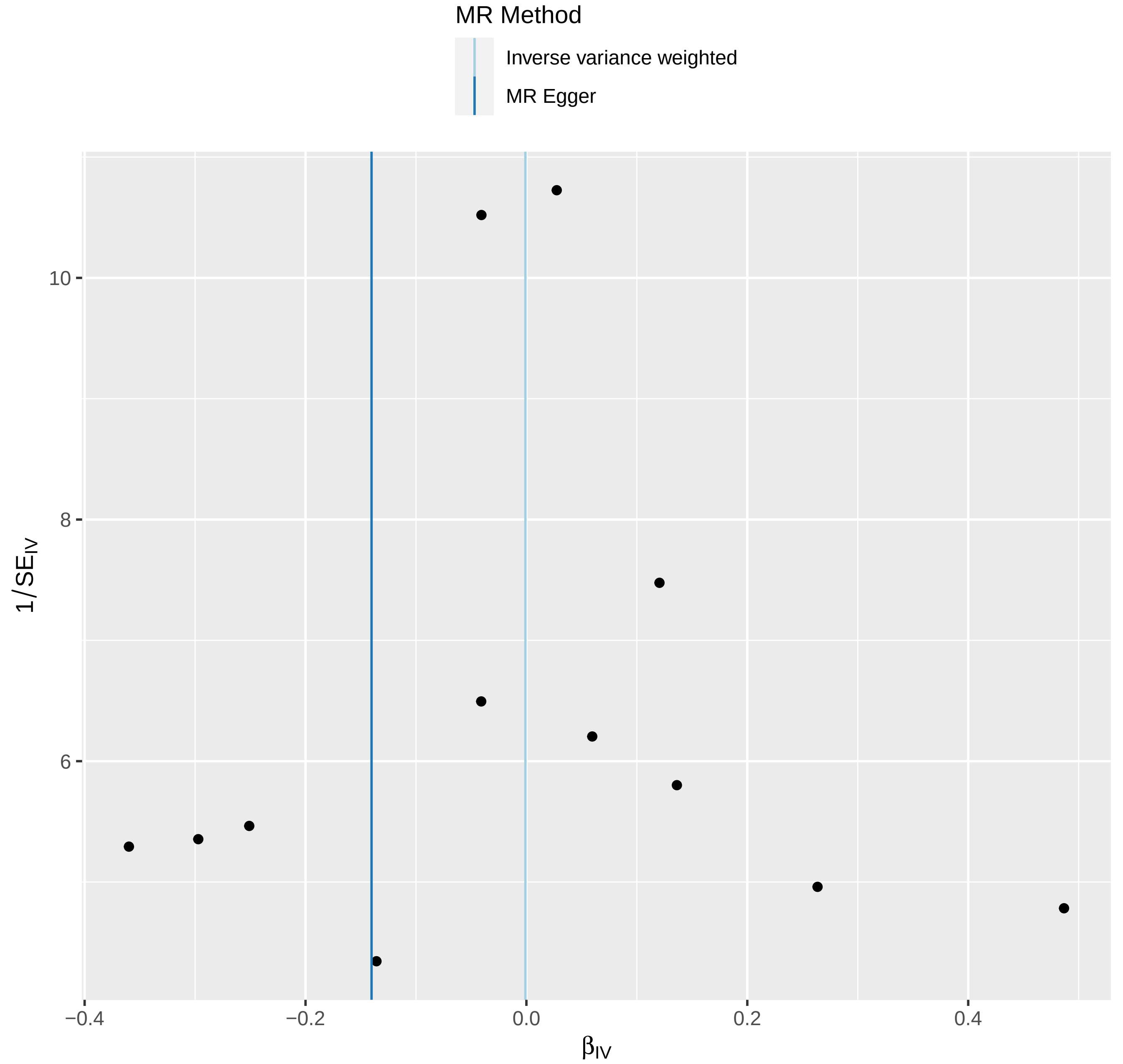

Batch 3−2294 : Selenium on Phospholipase B−like 1

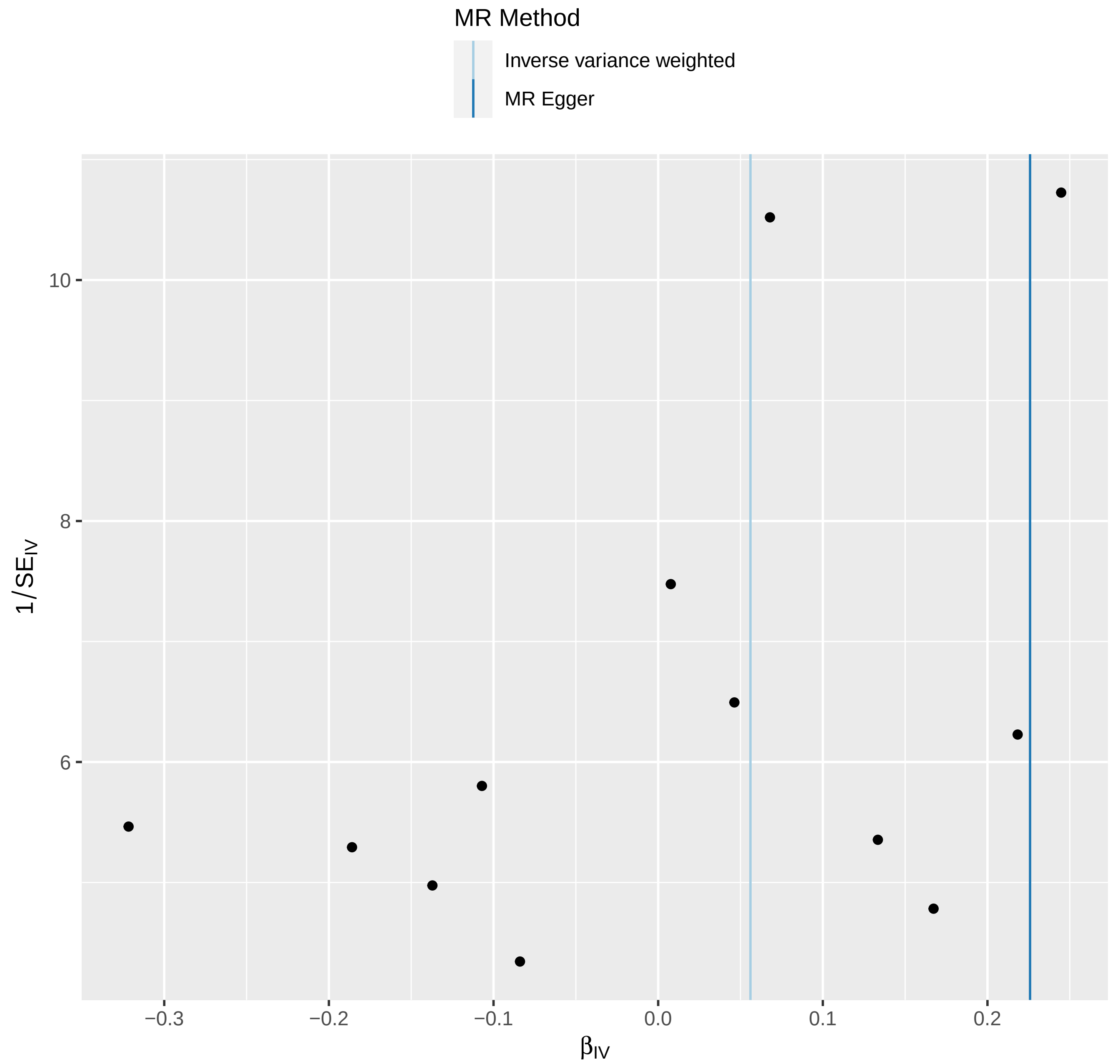

Batch 3−2295 : Selenium on Putative phospholipase B−like 2

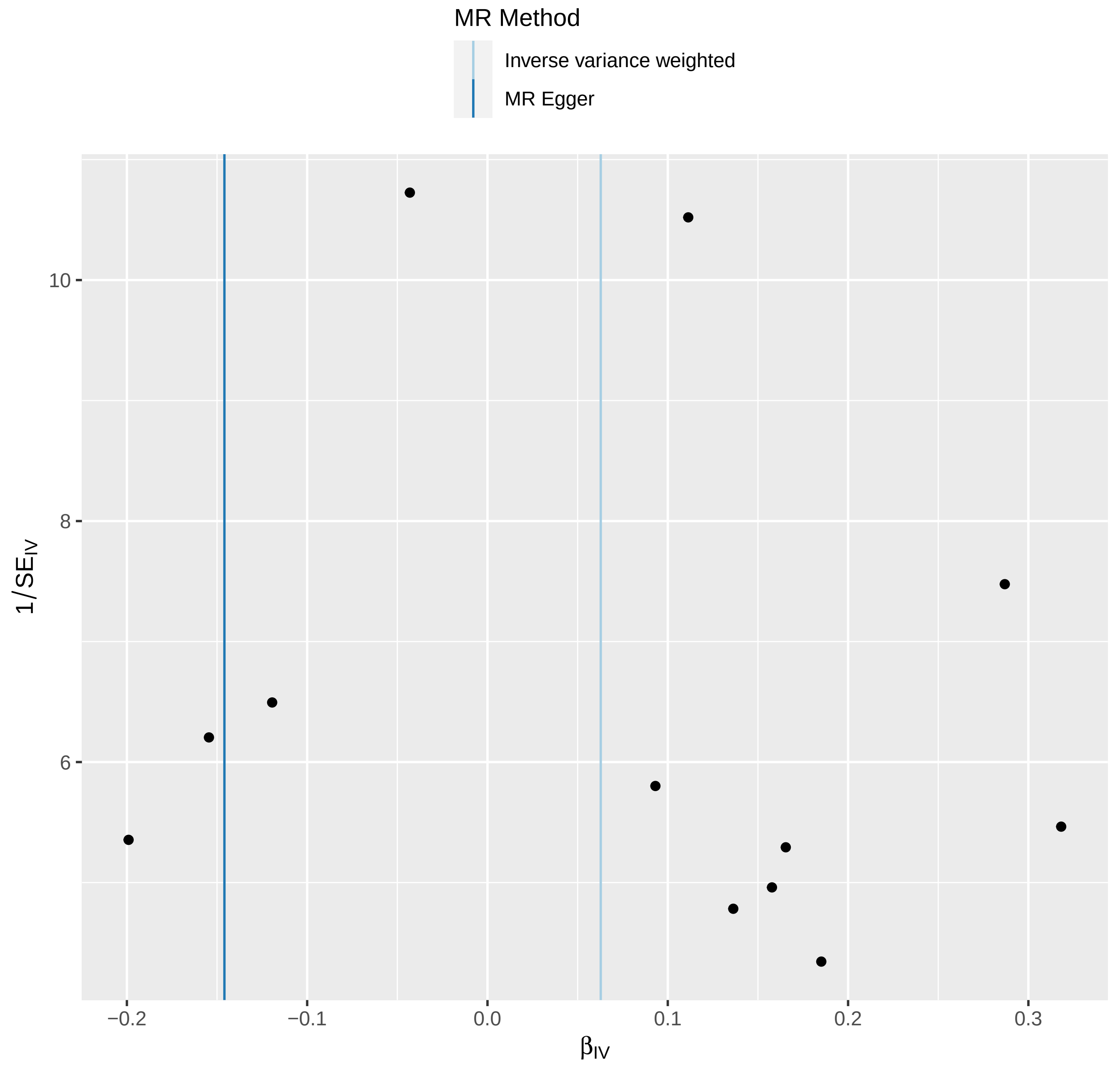

Batch 3−2296 : Selenium on 1−phosphatidylinositol 4,5−bisphosphate phosphodiesterase gamma−1

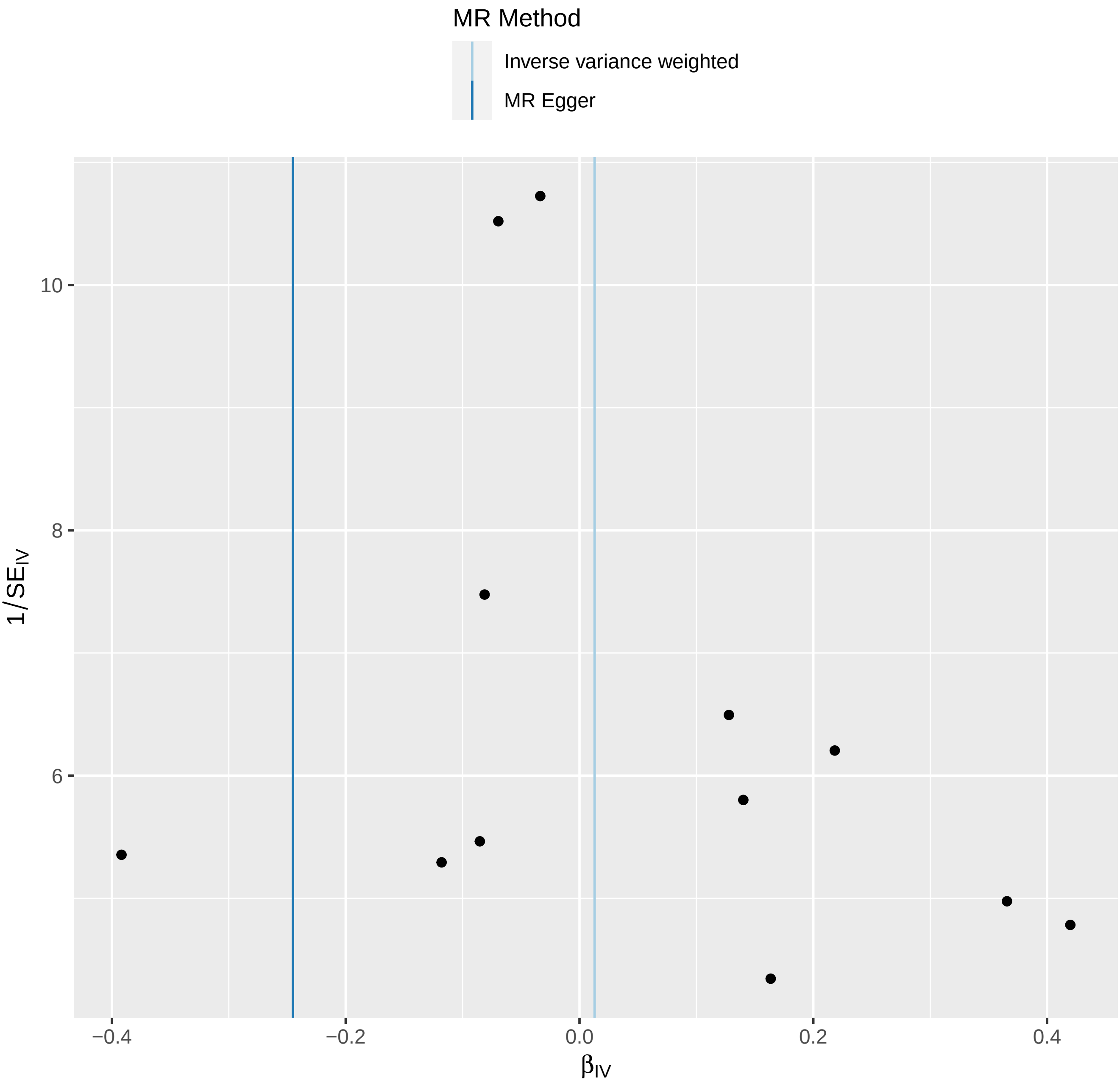

Batch 3−2297 : Selenium on Phospholipase D3

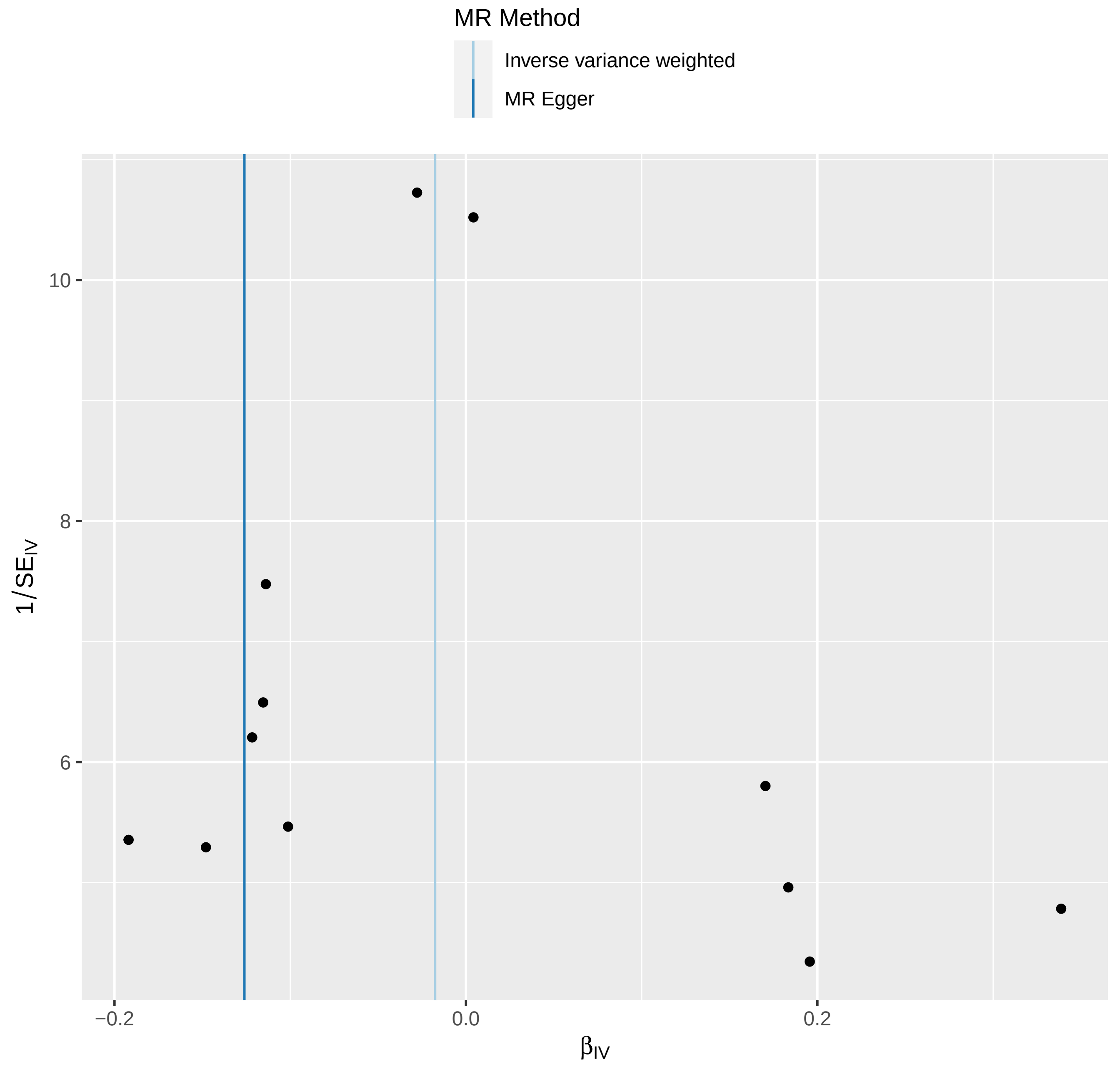

Batch 3−2298 : Selenium on Inactive phospholipase D5

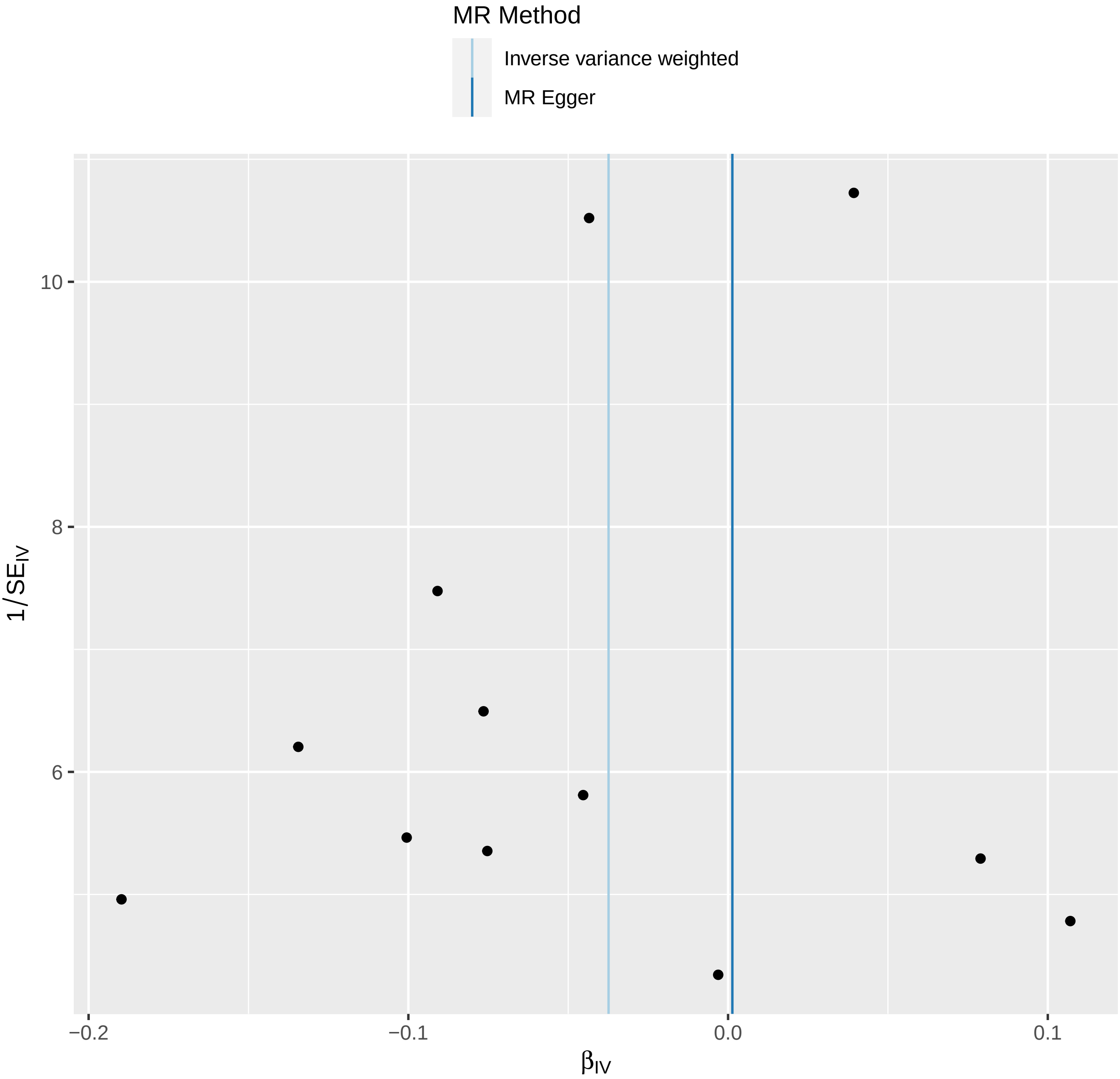

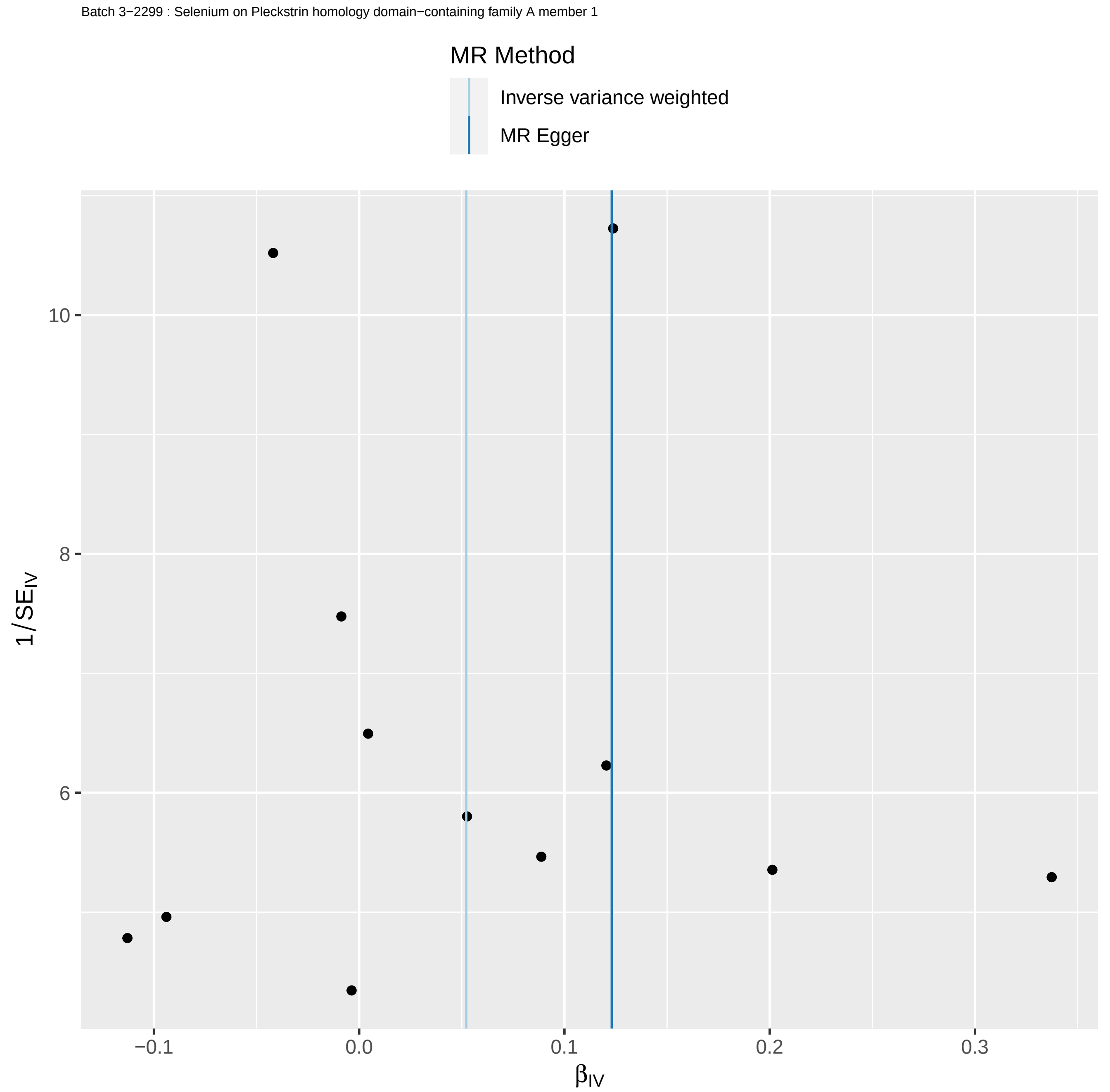
Batch 3−2299 : Selenium on Pleckstrin homology domain−containing family A member 1
MR Method
Inverse variance weighted
MR Egger
10
8
6
1/SE$_{IV}$
−0.1
0.0
0.1
0.2
0.3
β$_{IV}$

Batch 3−2300 : Selenium on Pleckstrin homology domain−containing family A member 7

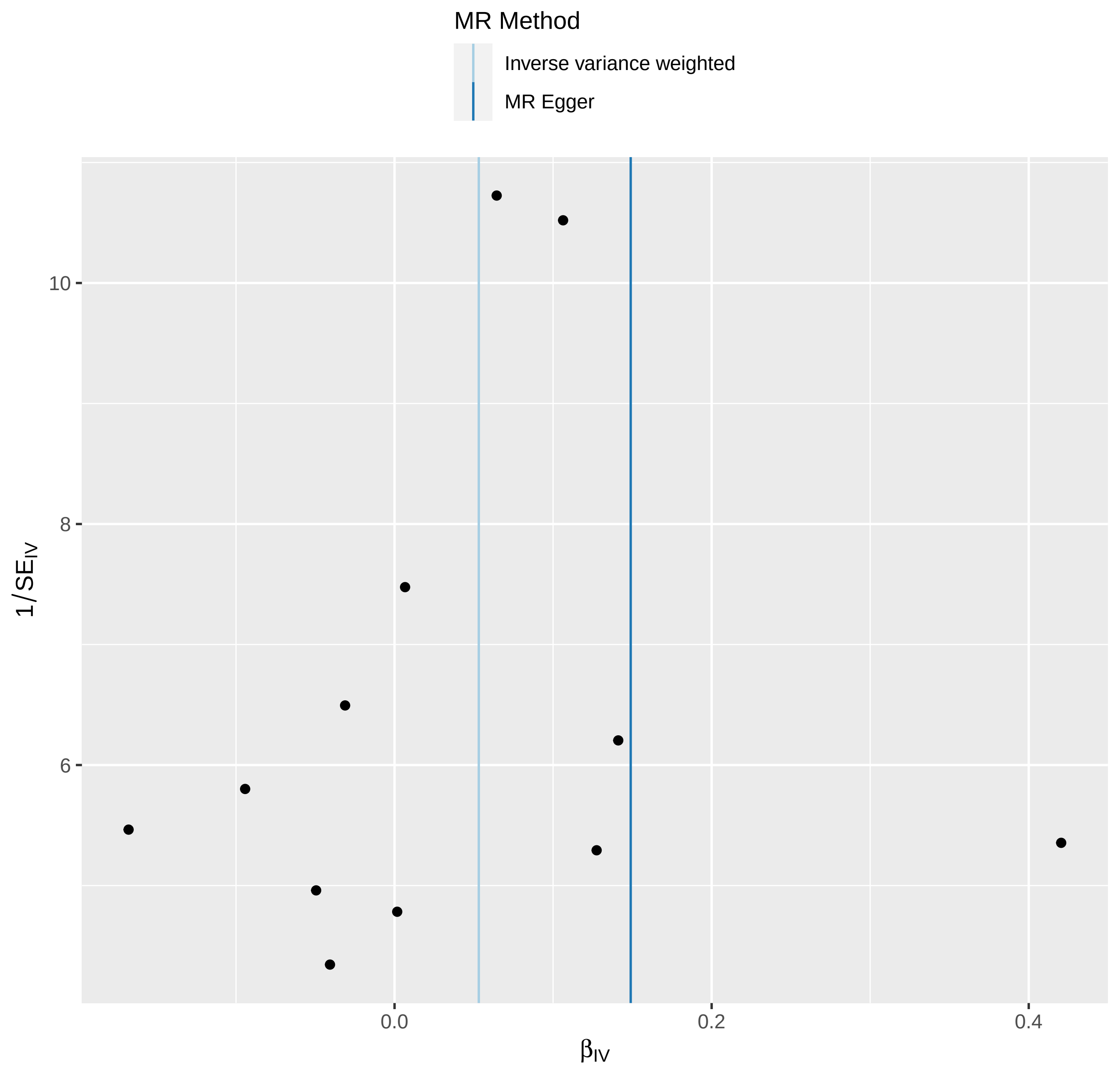

Batch 3−2301 : Selenium on Angiostatin

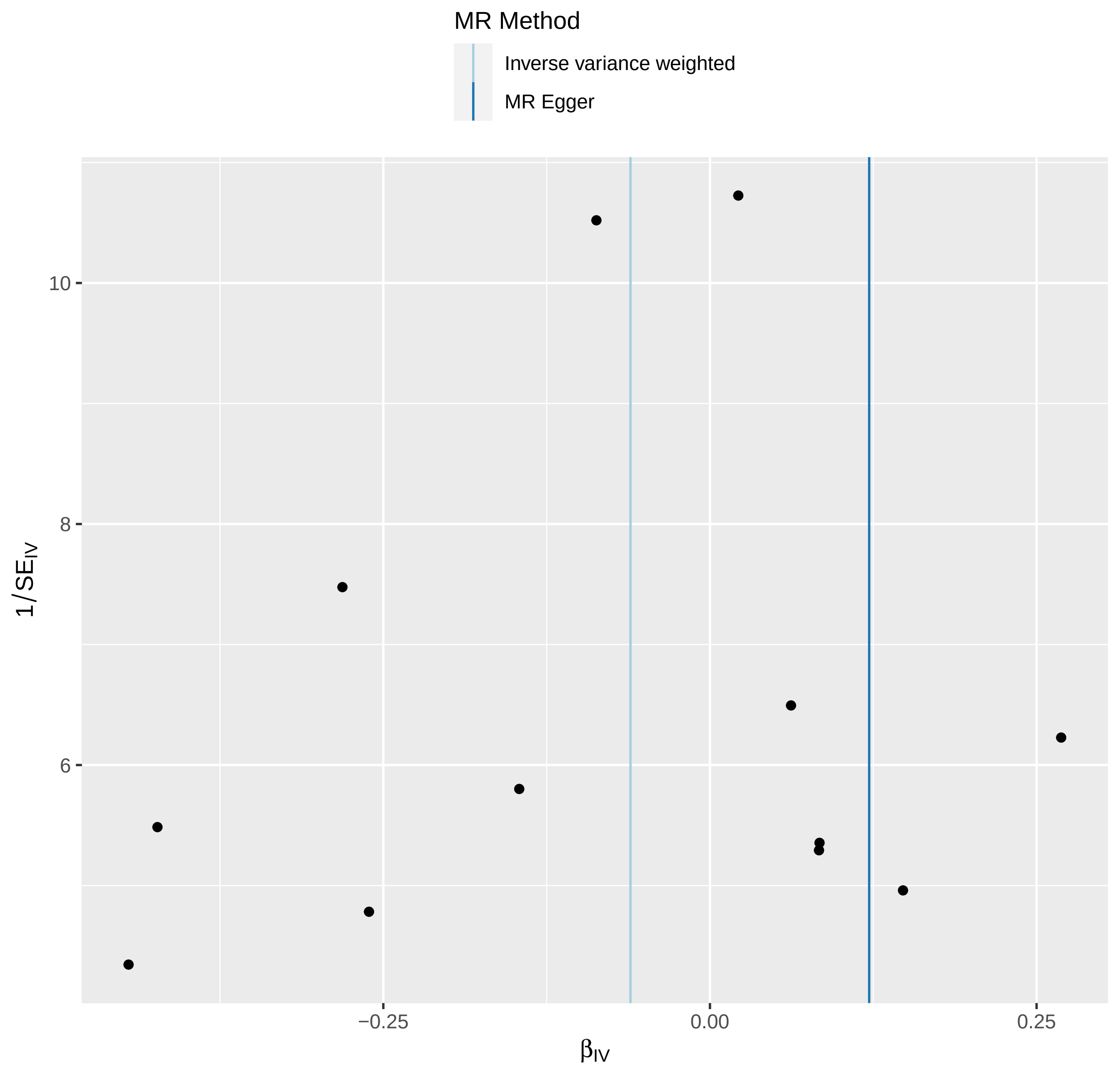

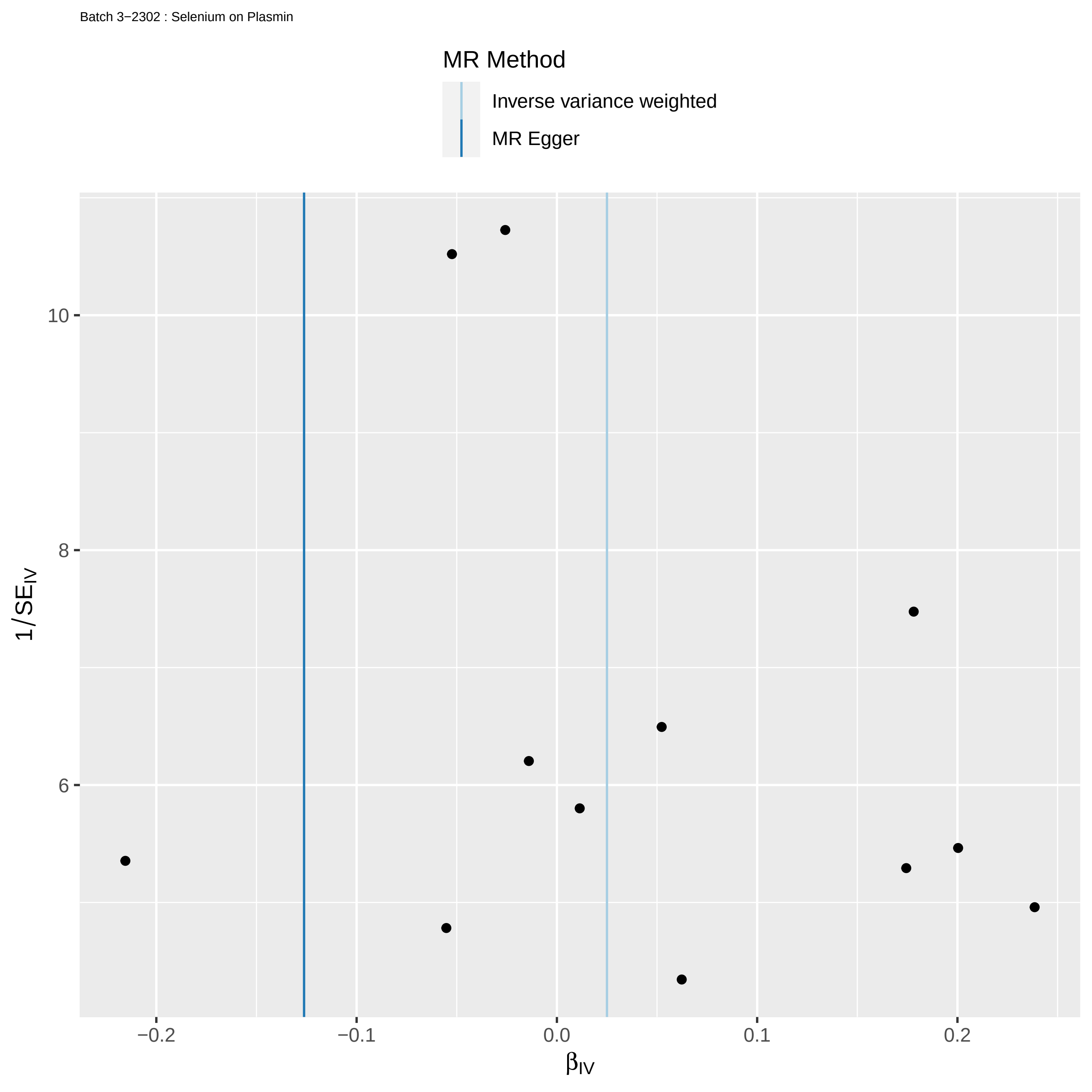

Batch 3−2302 : Selenium on Plasmin
MR Method
Inverse variance weighted
MR Egger
10
8
6
1/SE_IV
−0.2
−0.1
0.0
0.1
0.2
β_IV

Batch 3−2303 : Selenium on Serine/threonine−protein kinase PLK1

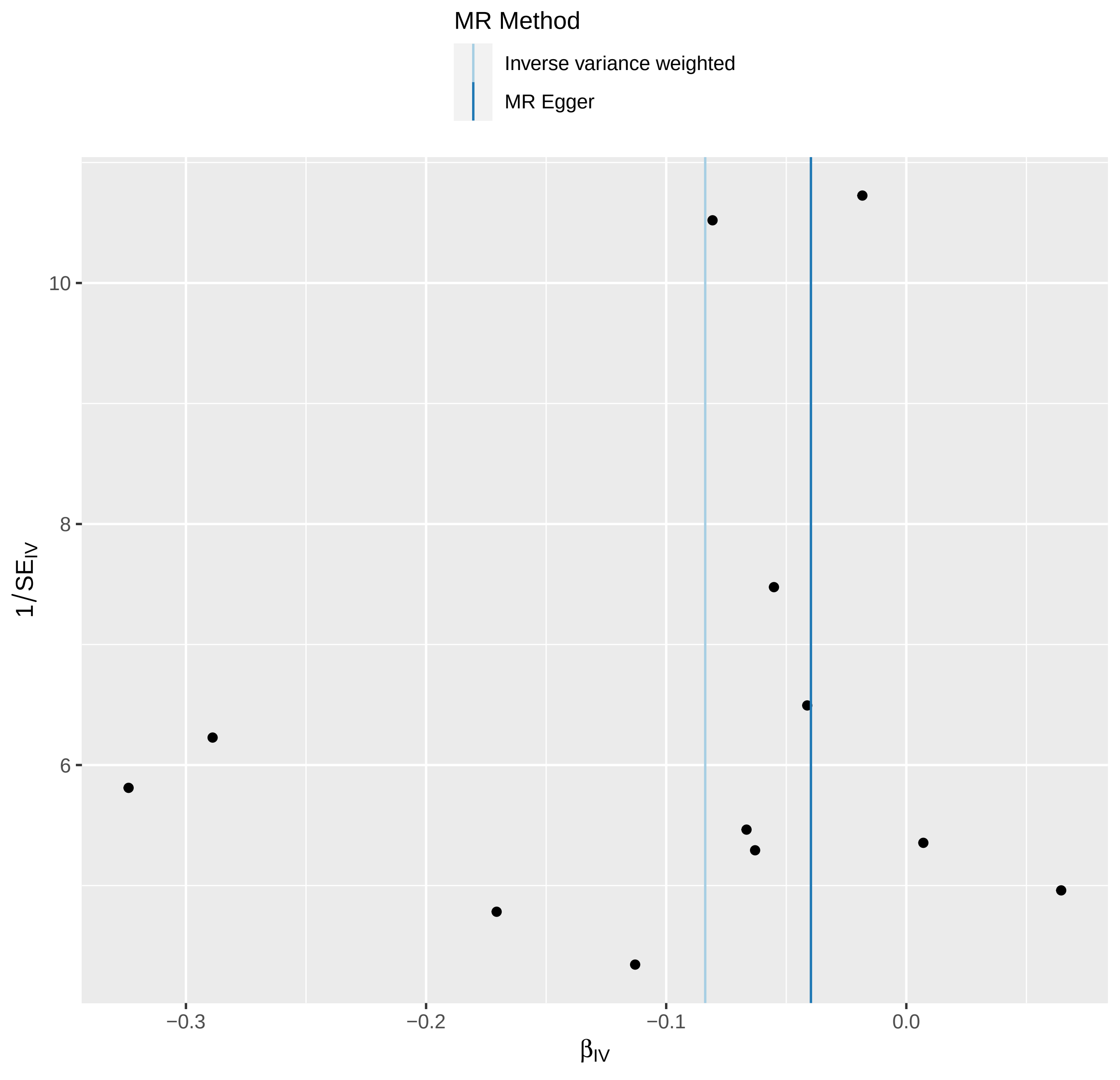

Batch 3−2304 : Selenium on Procollagen−lysine,2−oxoglutarate 5−dioxygenase 2

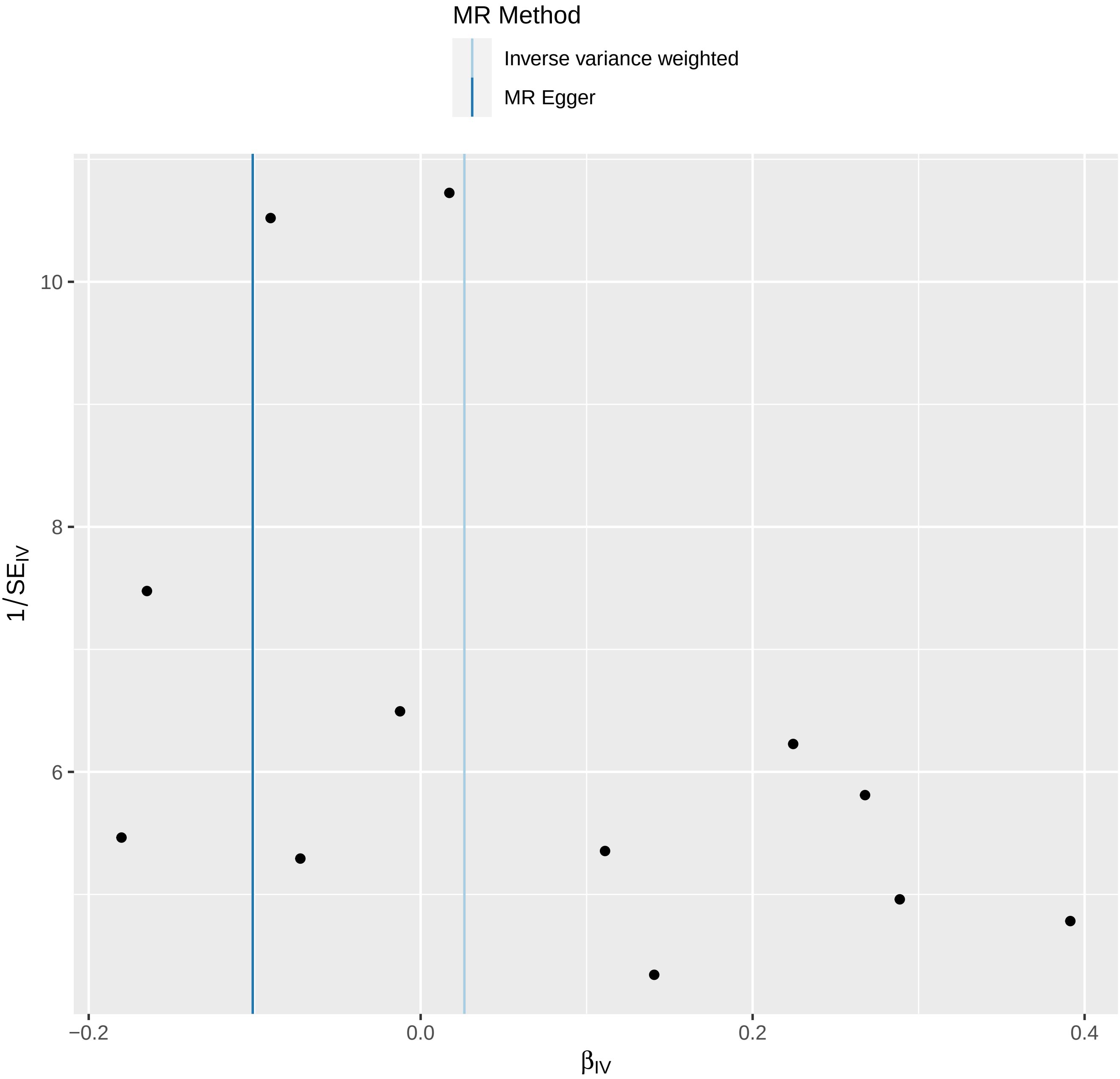

Batch 3−2305 : Selenium on Procollagen−lysine,2−oxoglutarate 5−dioxygenase 3

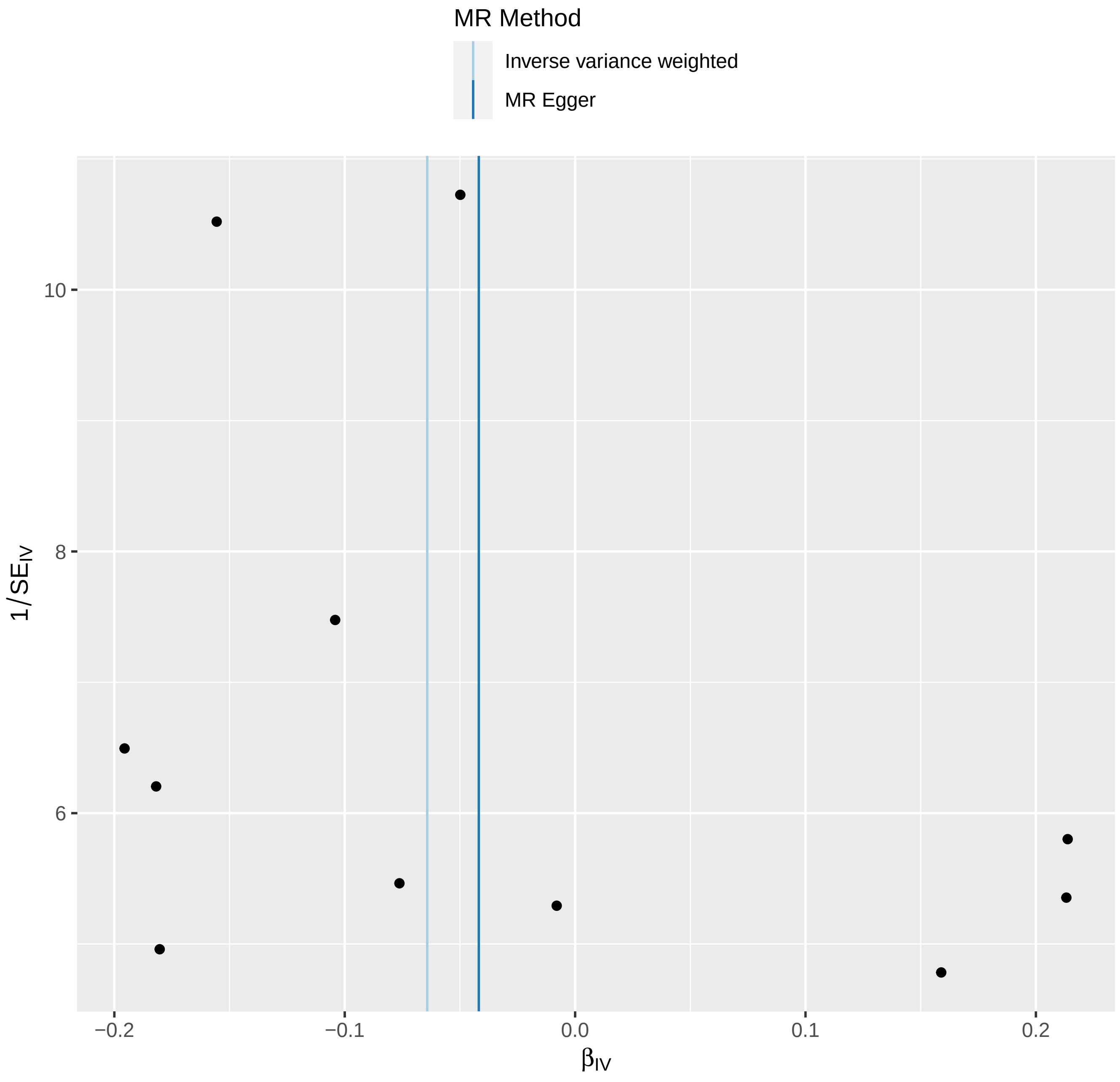

Batch 3−2306 : Selenium on Plastin−1

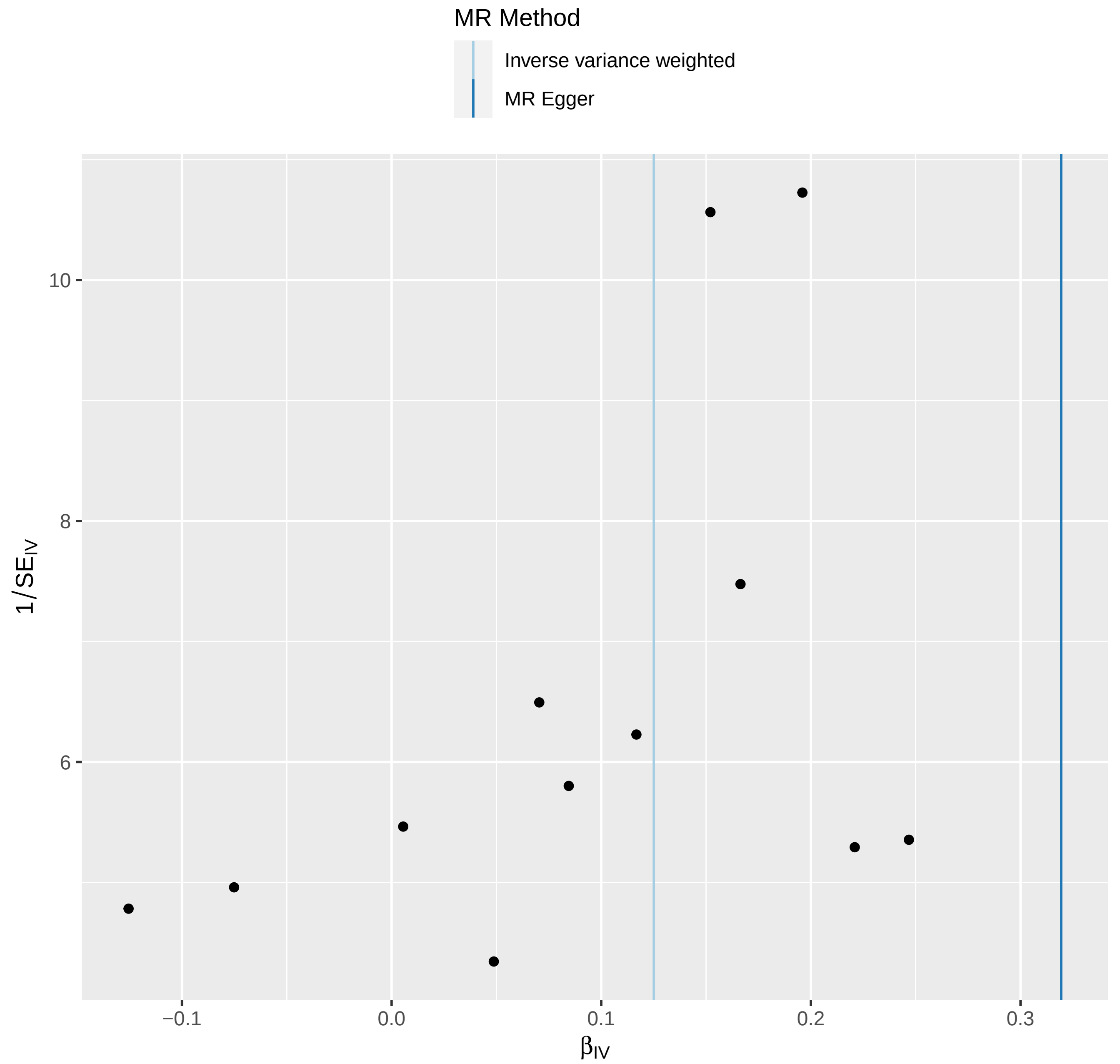

Batch 3−2307 : Selenium on Plexin−A1

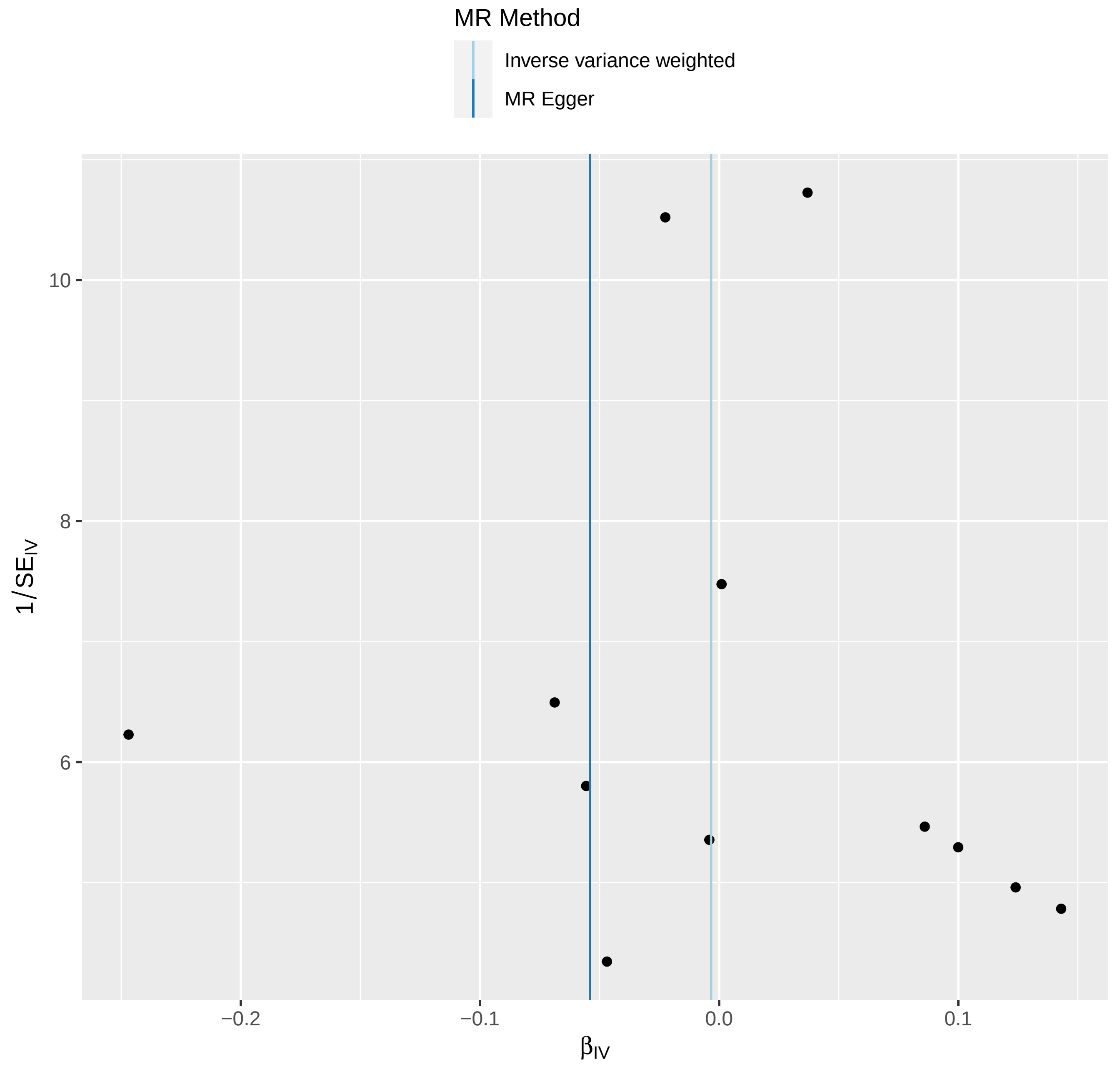

Batch 3−2308 : Selenium on Plexin−B2

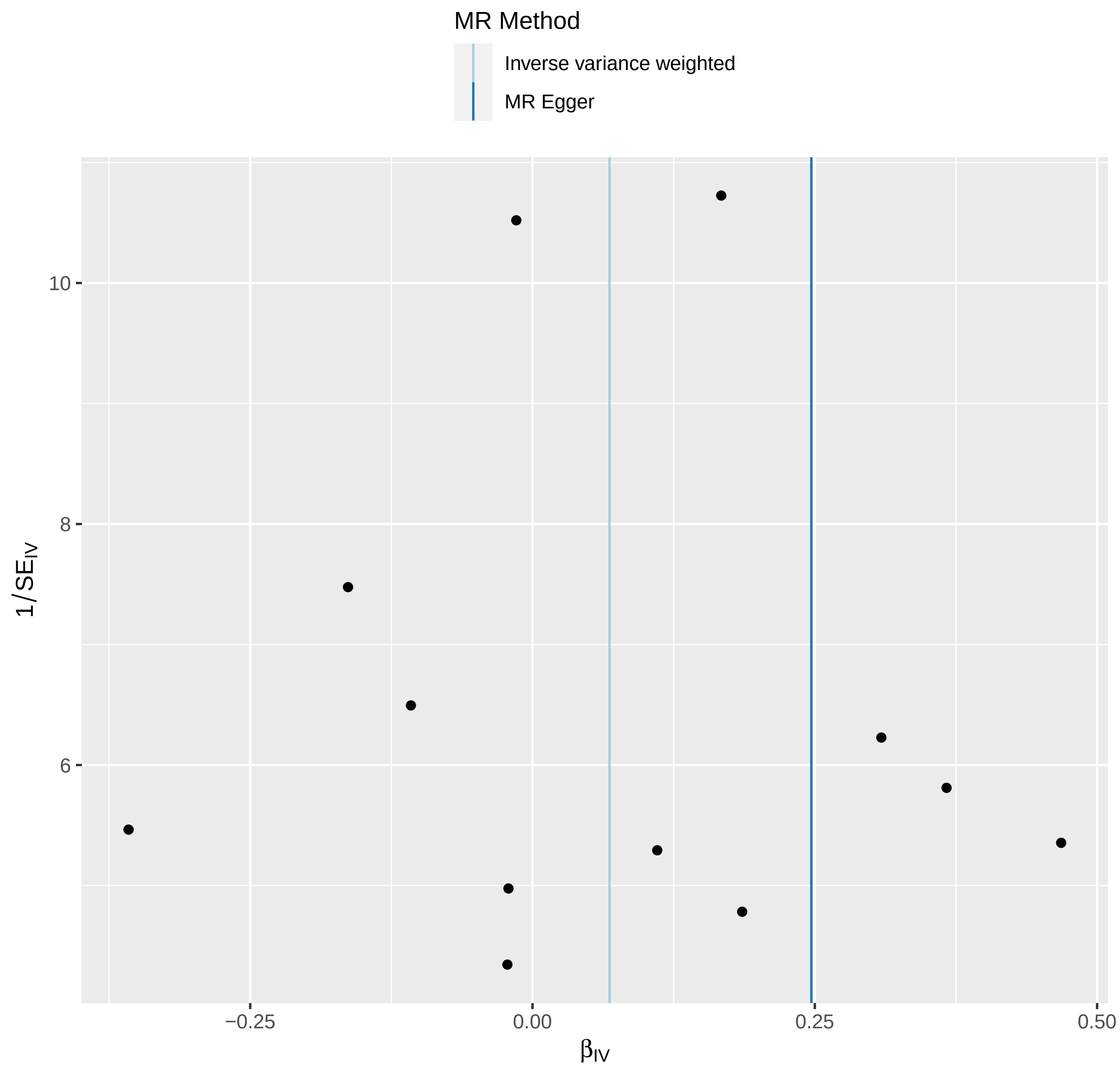

Batch 3−2309 : Selenium on Plexin−C1

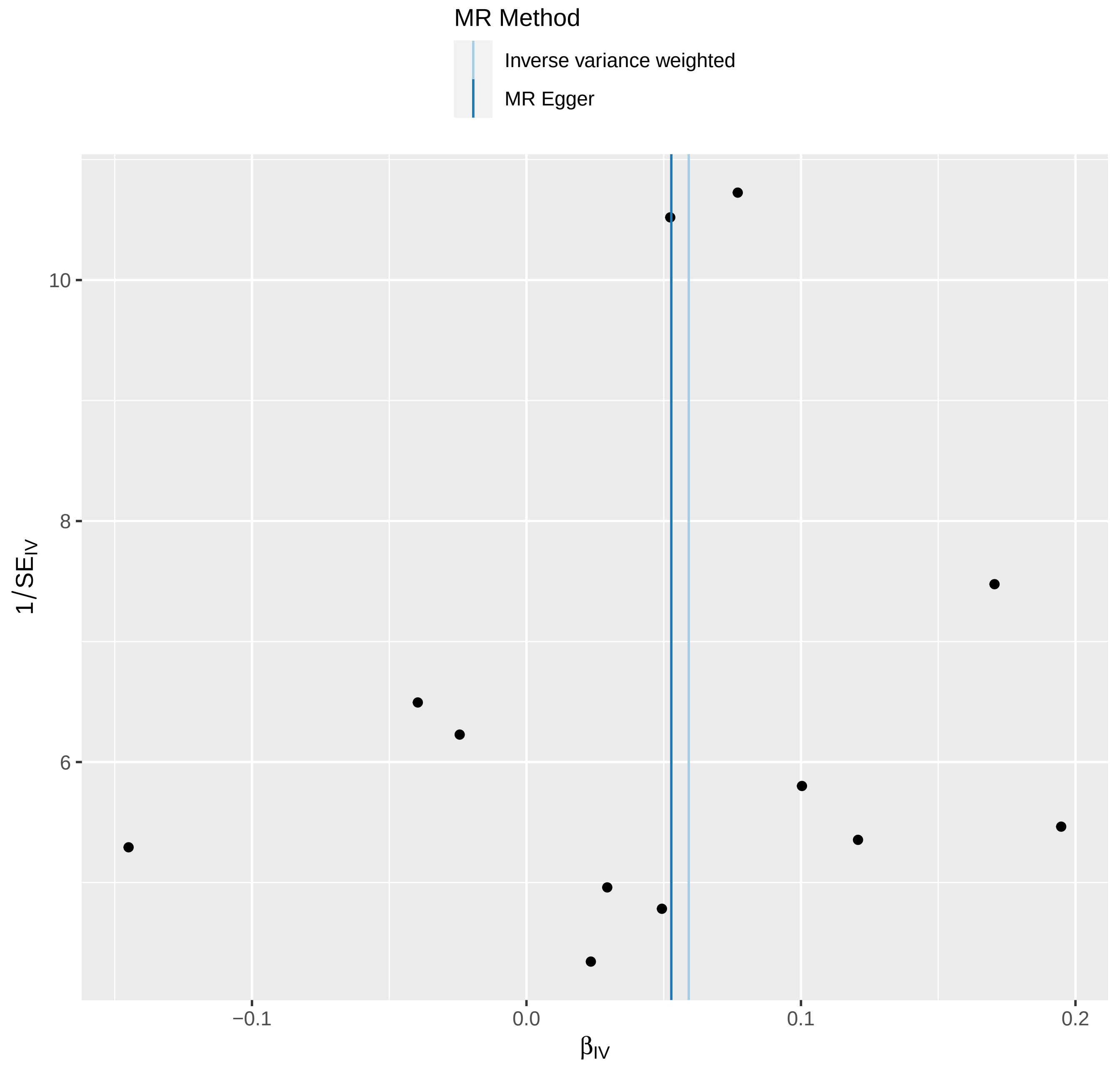

Batch 3−2310 : Selenium on Melanocyte protein PMEL

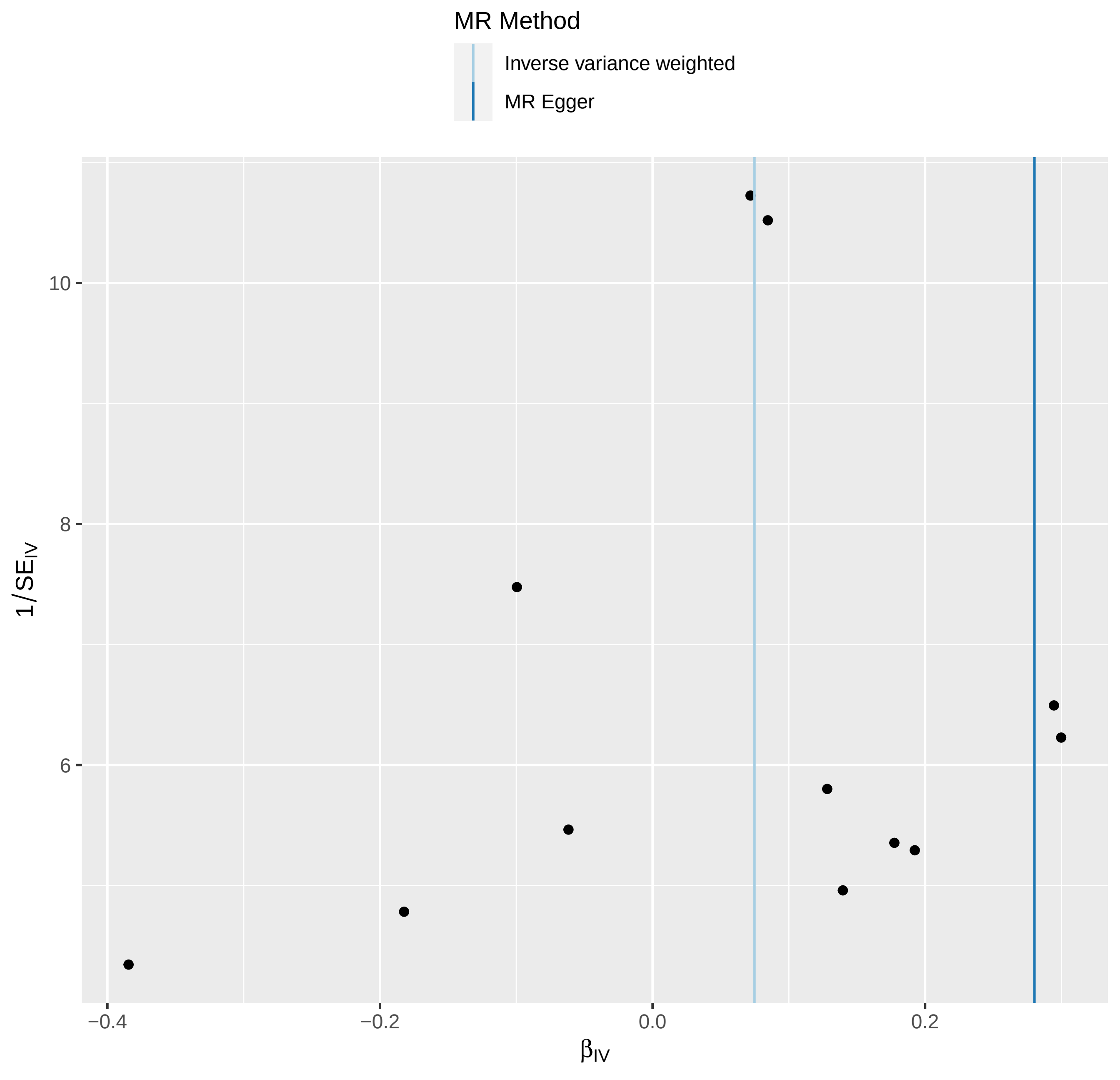

Batch 3−2311 : Selenium on Protein TMEPAI

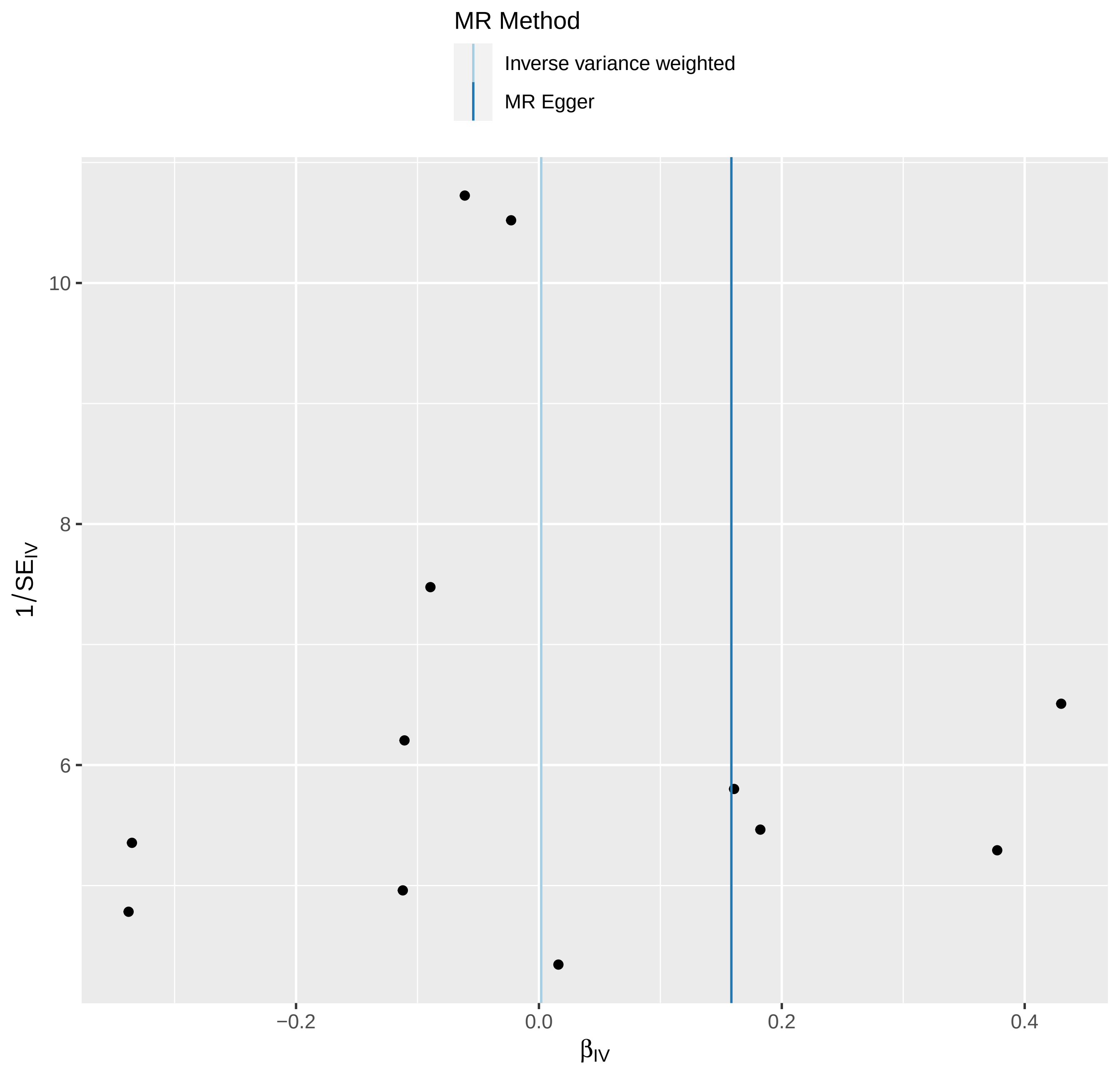

Batch 3−2312 : Selenium on Mismatch repair endonuclease PMS2

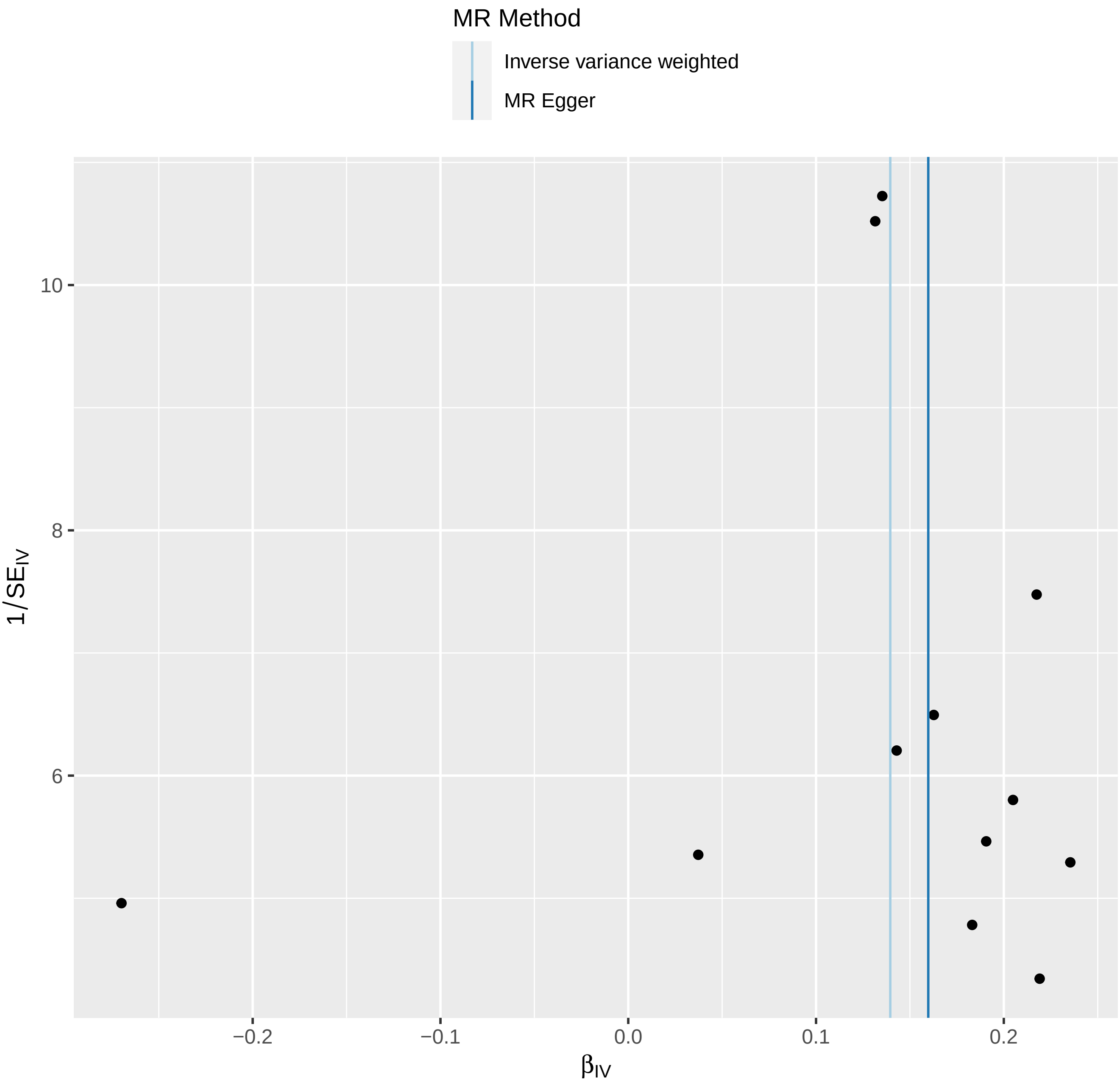

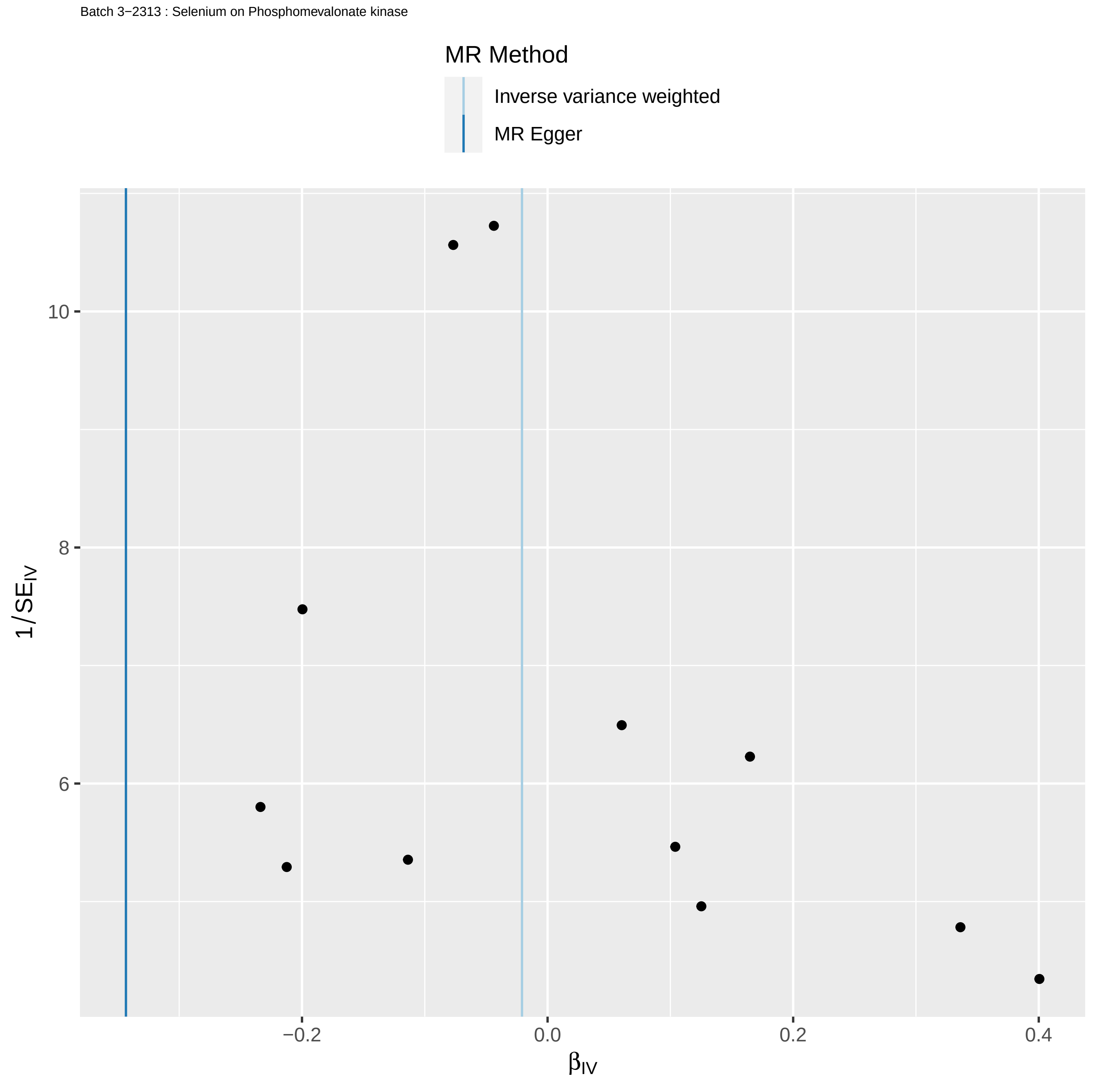
Batch 3−2313 : Selenium on Phosphomevalonate kinase
MR Method
Inverse variance weighted
MR Egger
10
8
6
1/SE_IV
−0.2
0.0
0.2
0.4
β_IV

Batch 3−2314 : Selenium on Bifunctional polynucleotide phosphatase/kinase

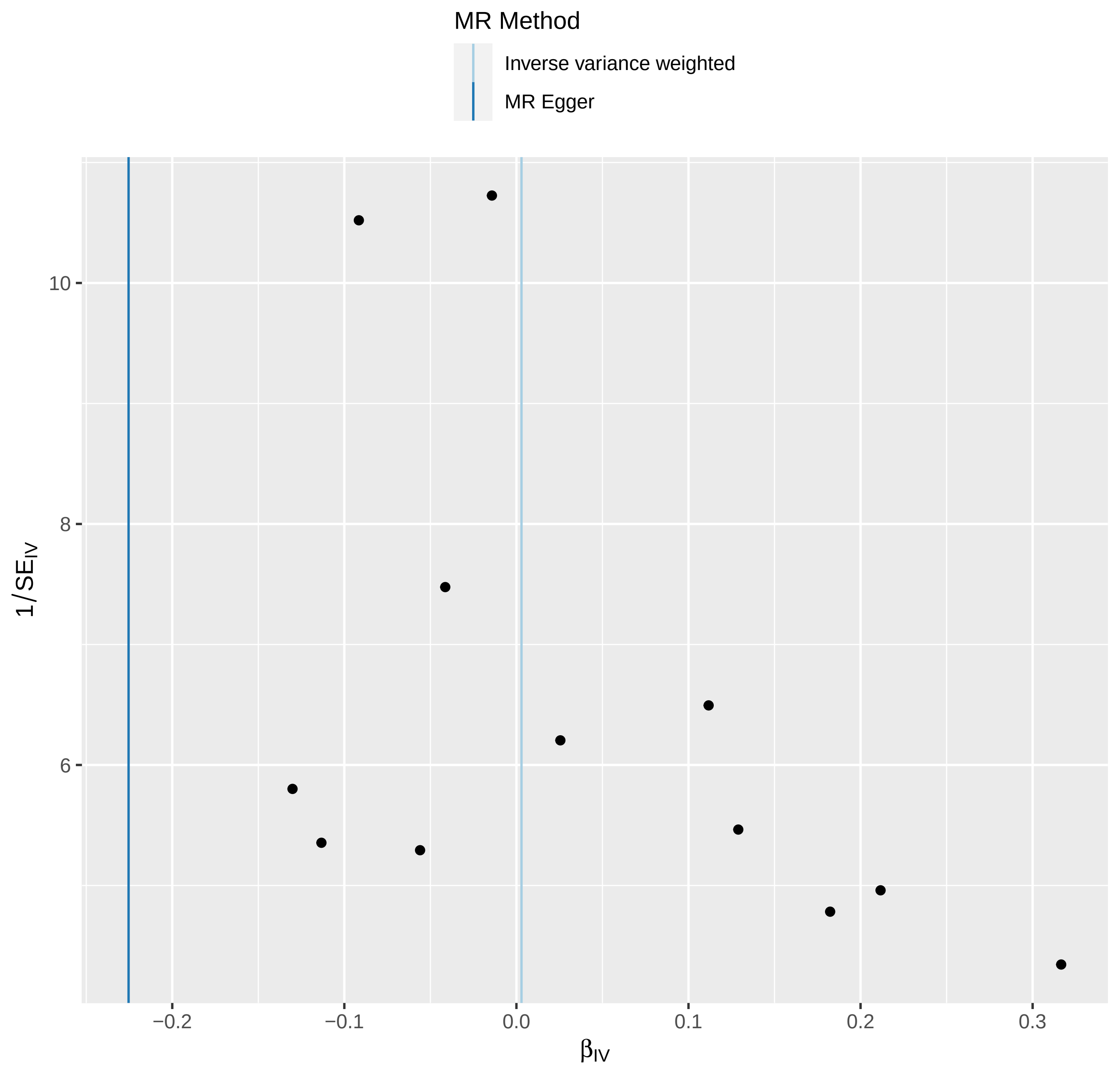

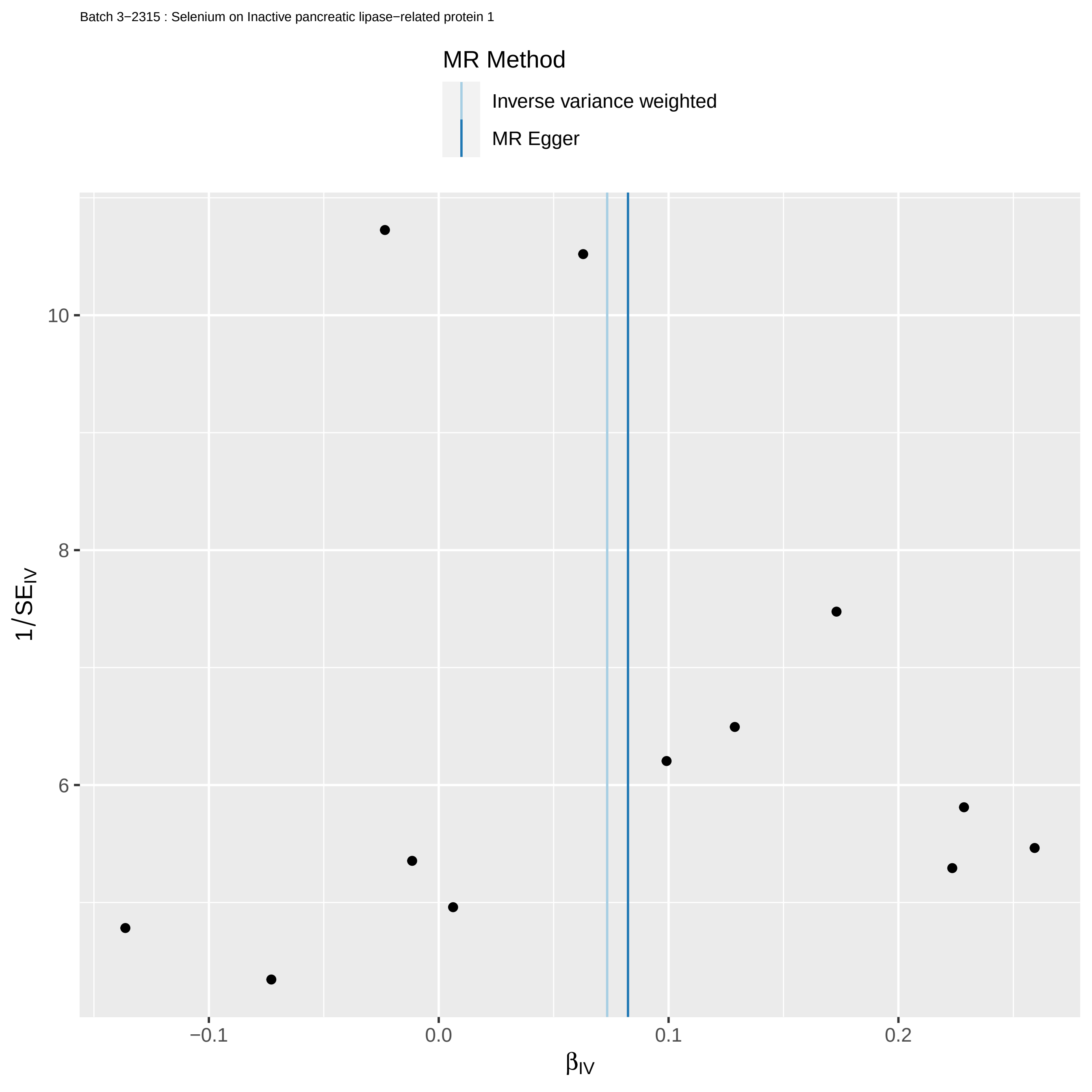

Batch 3−2315 : Selenium on Inactive pancreatic lipase−related protein 1
MR Method
Inverse variance weighted
MR Egger
10
8
6
1/SE$_{IV}$
−0.1
0.0
0.1
0.2
β$_{IV}$

Batch 3−2316 : Selenium on Purine nucleoside phosphorylase

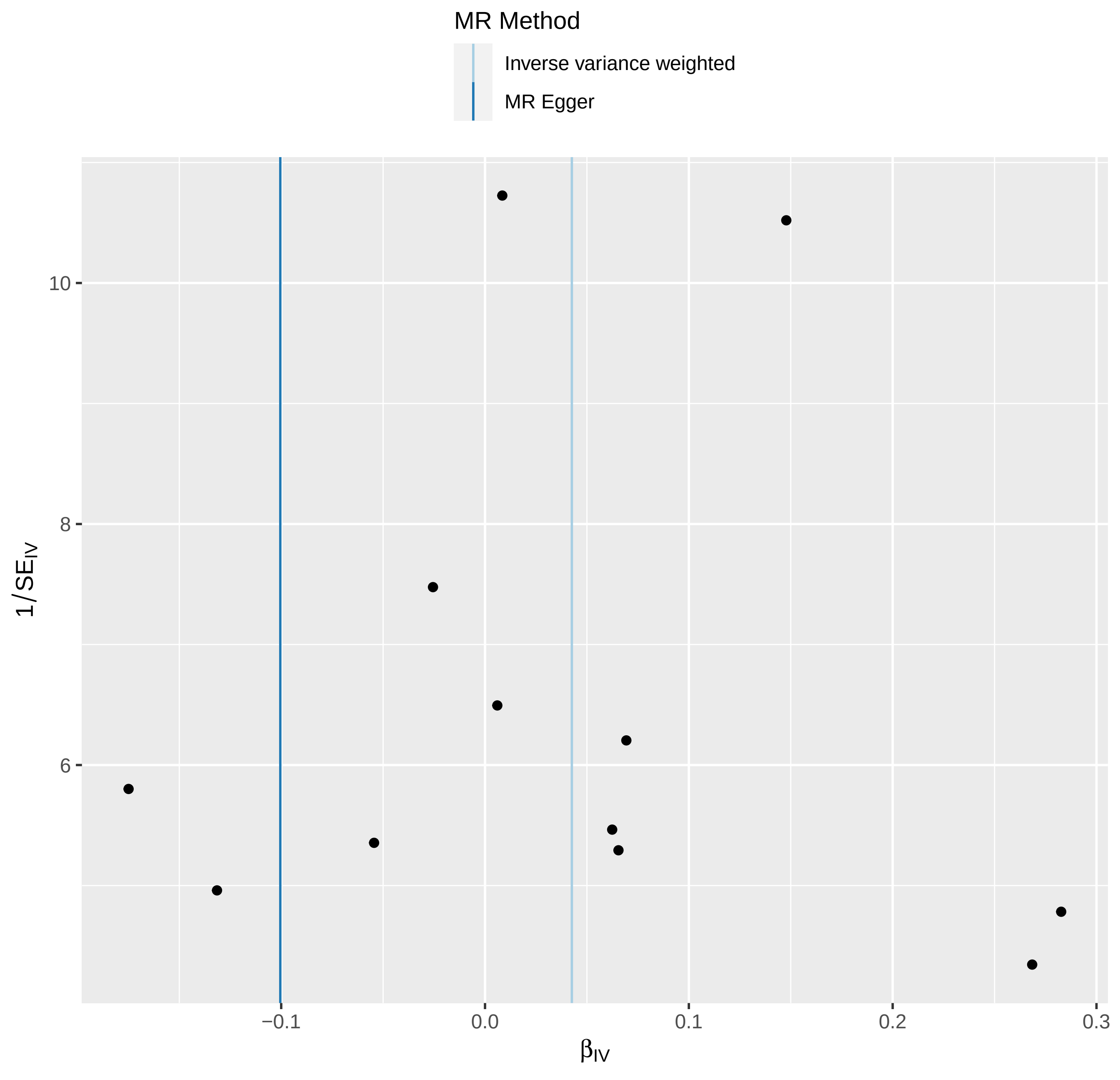

Batch 3−2317 : Selenium on Podocalyxin

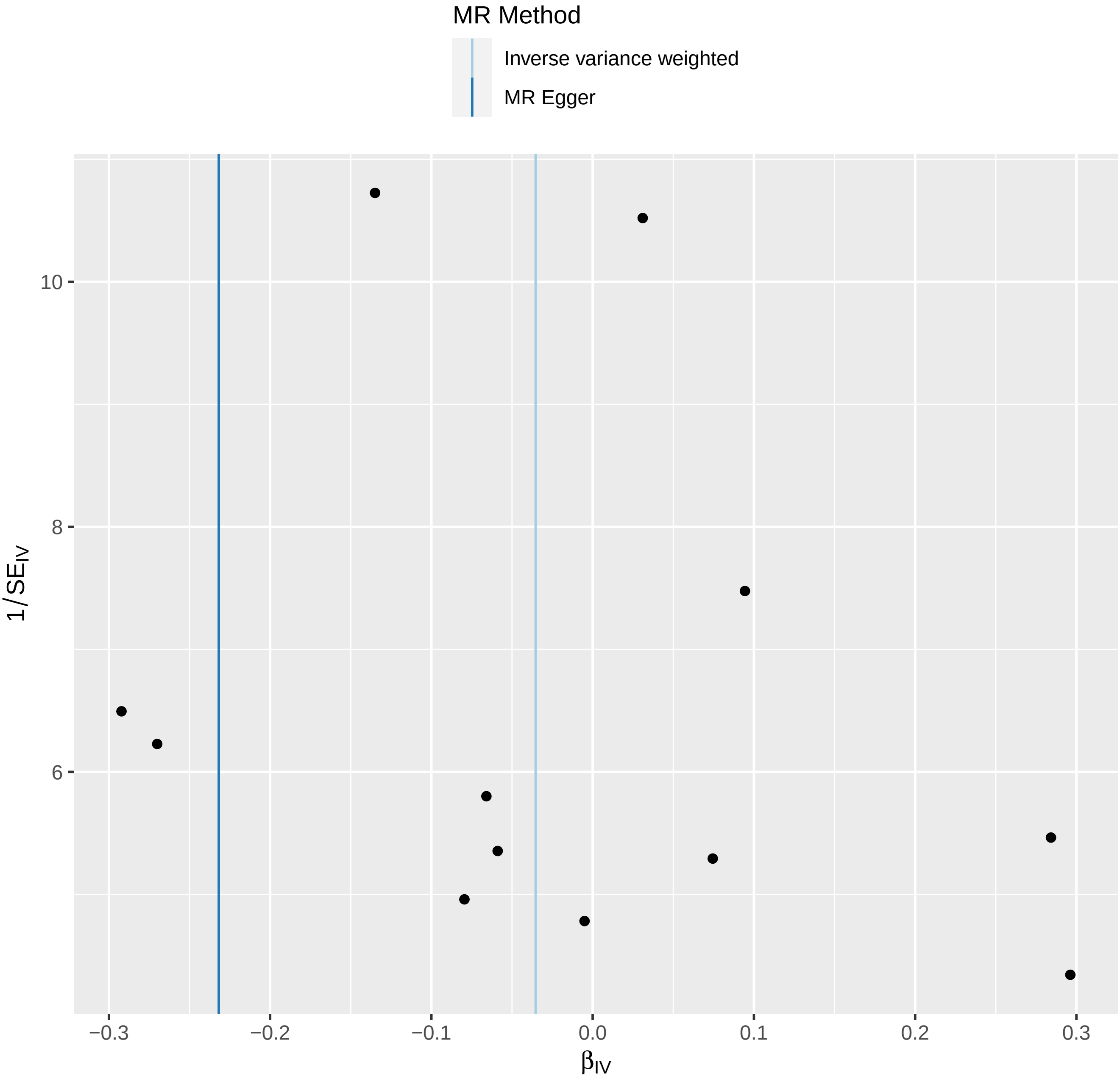

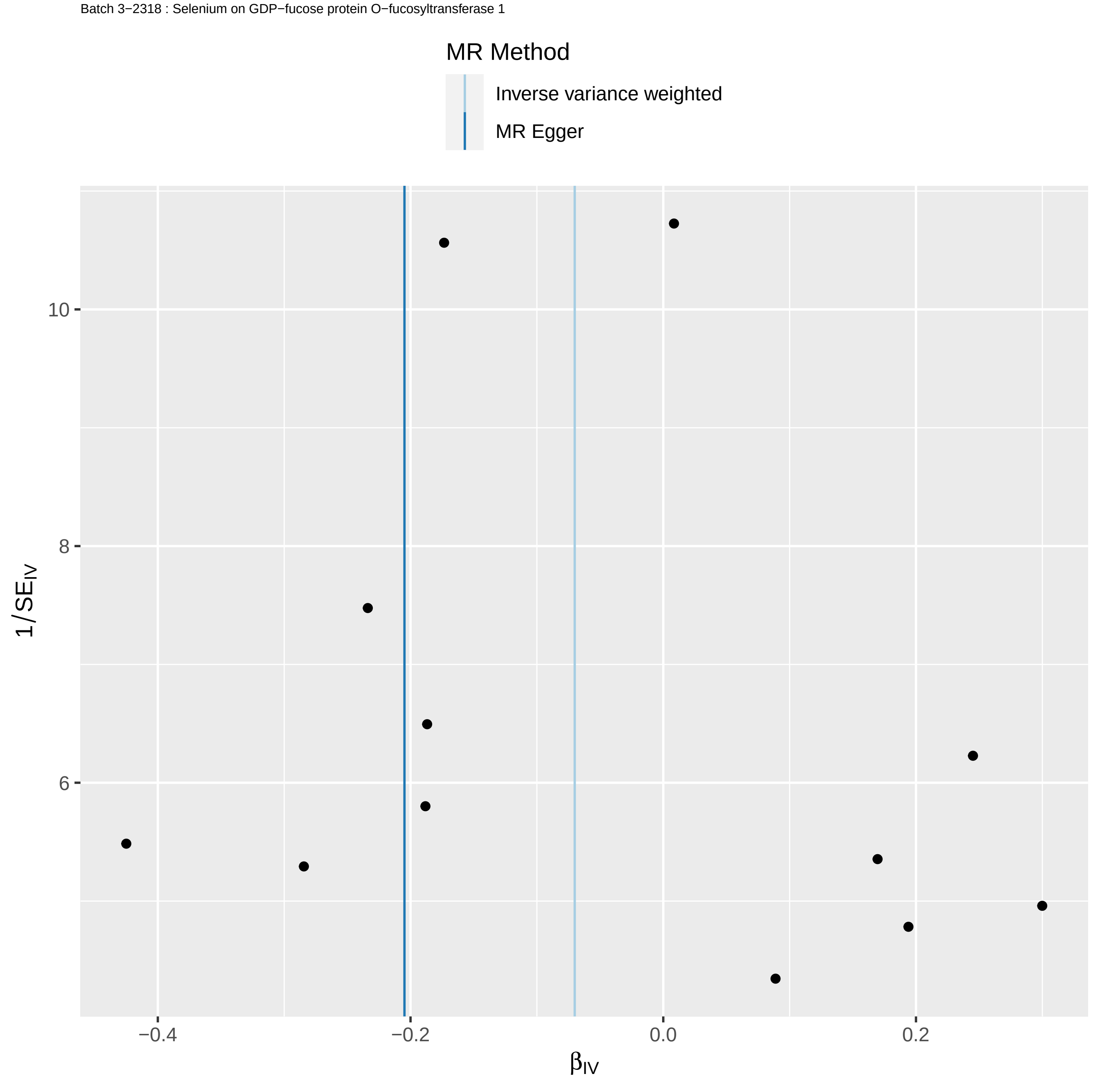
Batch 3−2318 : Selenium on GDP−fucose protein O−fucosyltransferase 1
MR Method
Inverse variance weighted
MR Egger
10
8
6
1/SE_IV
−0.4
−0.2
0.0
0.2
β_IV

Batch 3−2319 : Selenium on GDP−fucose protein O−fucosyltransferase 2

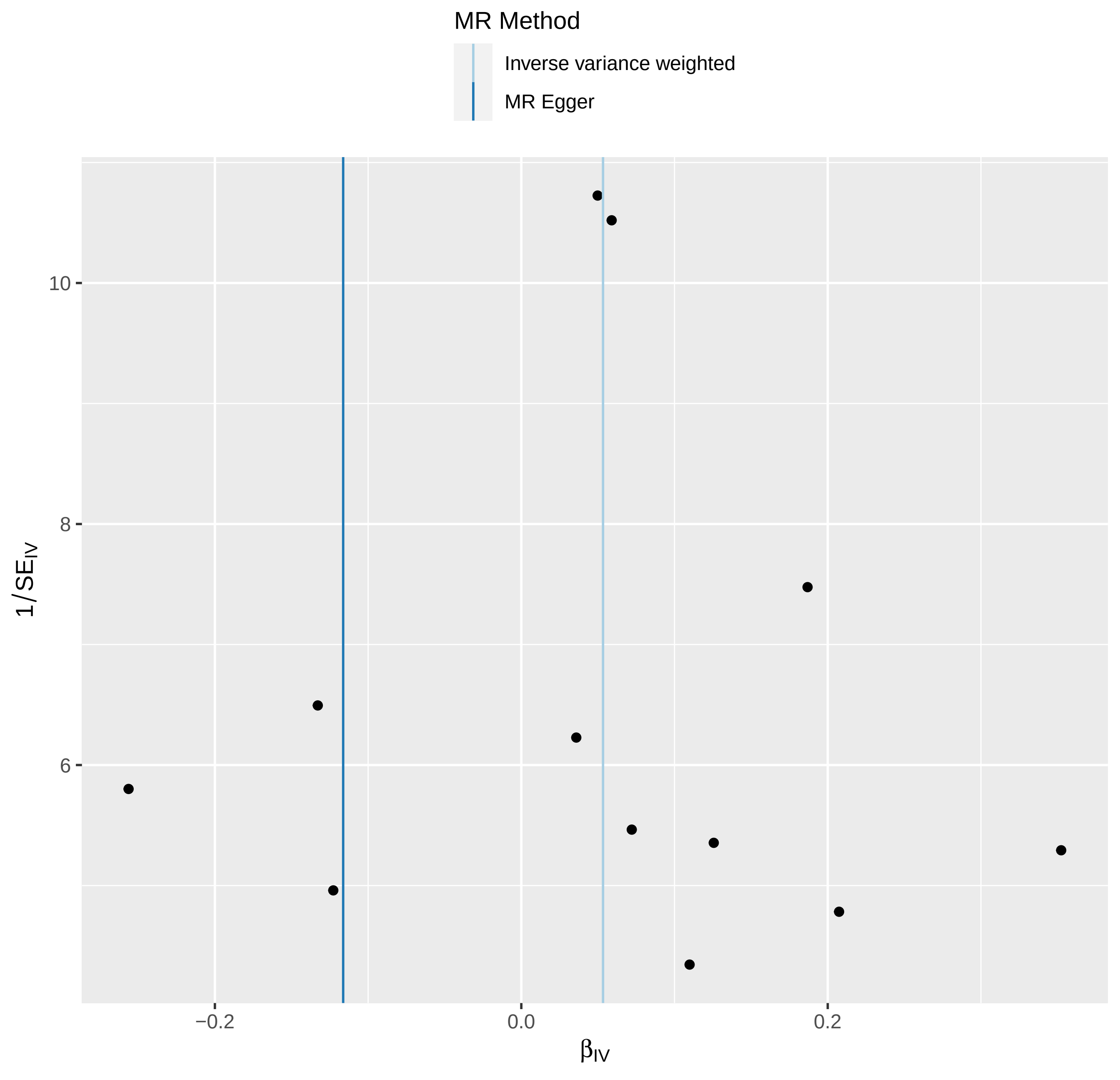

Batch 3−2320 : Selenium on Protein O−glucosyltransferase 1

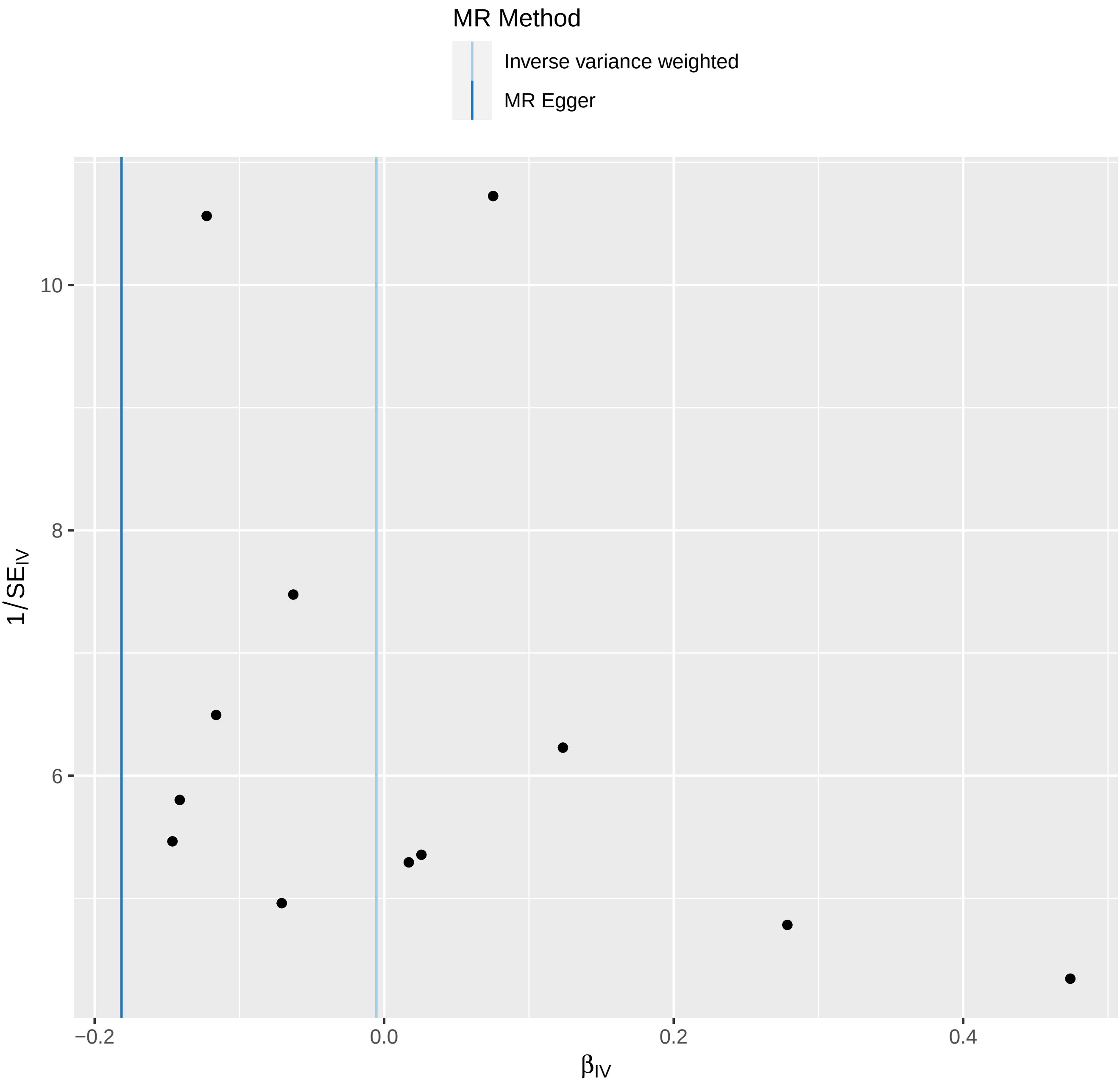

Batch 3−2321 : Selenium on DNA polymerase eta

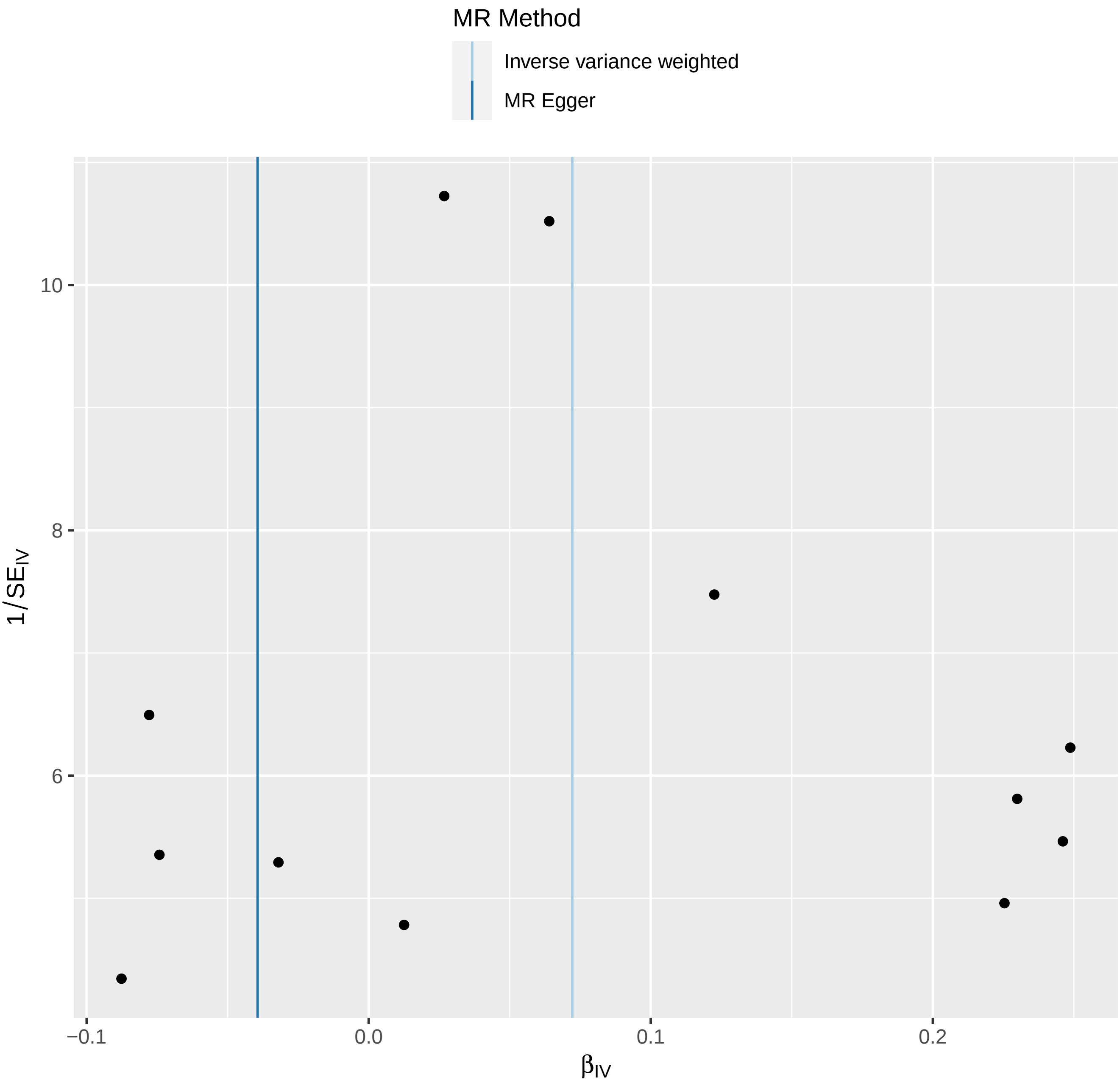

Batch 3−2322 : Selenium on DNA polymerase iota

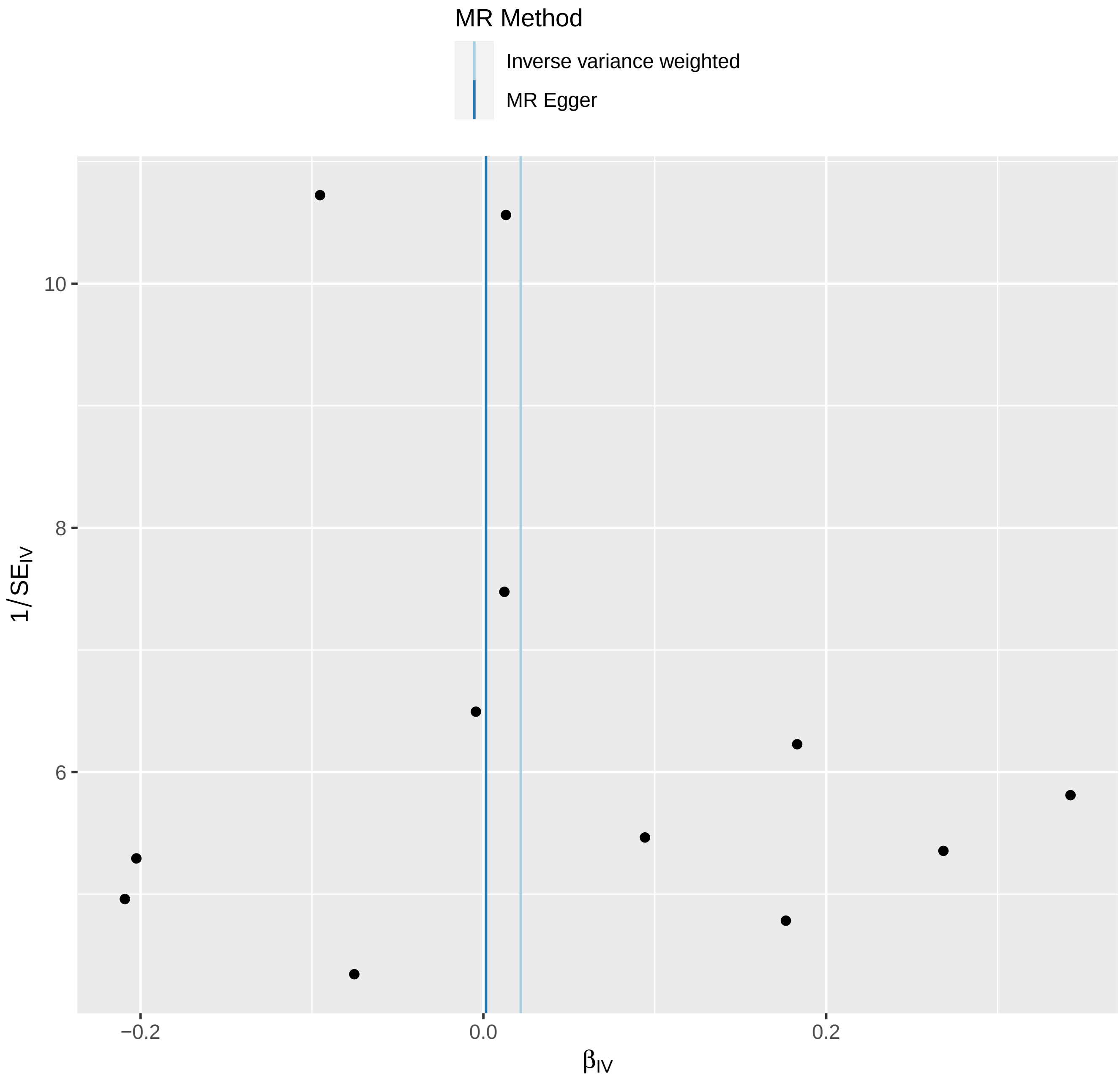

Batch 3−2323 : Selenium on DNA−directed DNA/RNA polymerase mu

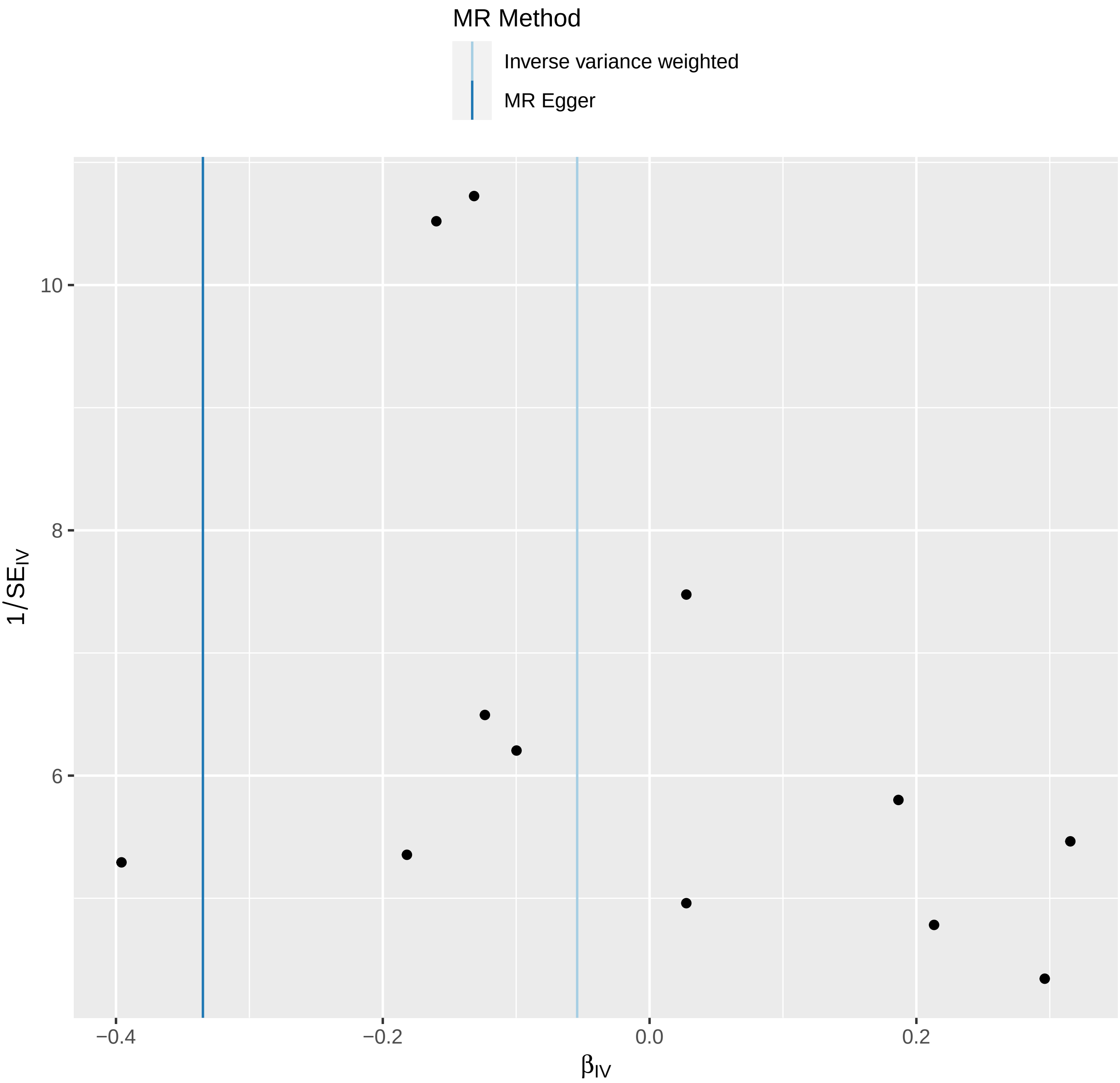

Batch 3−2324 : Selenium on DNA−directed RNA polymerases I and III subunit RPAC1

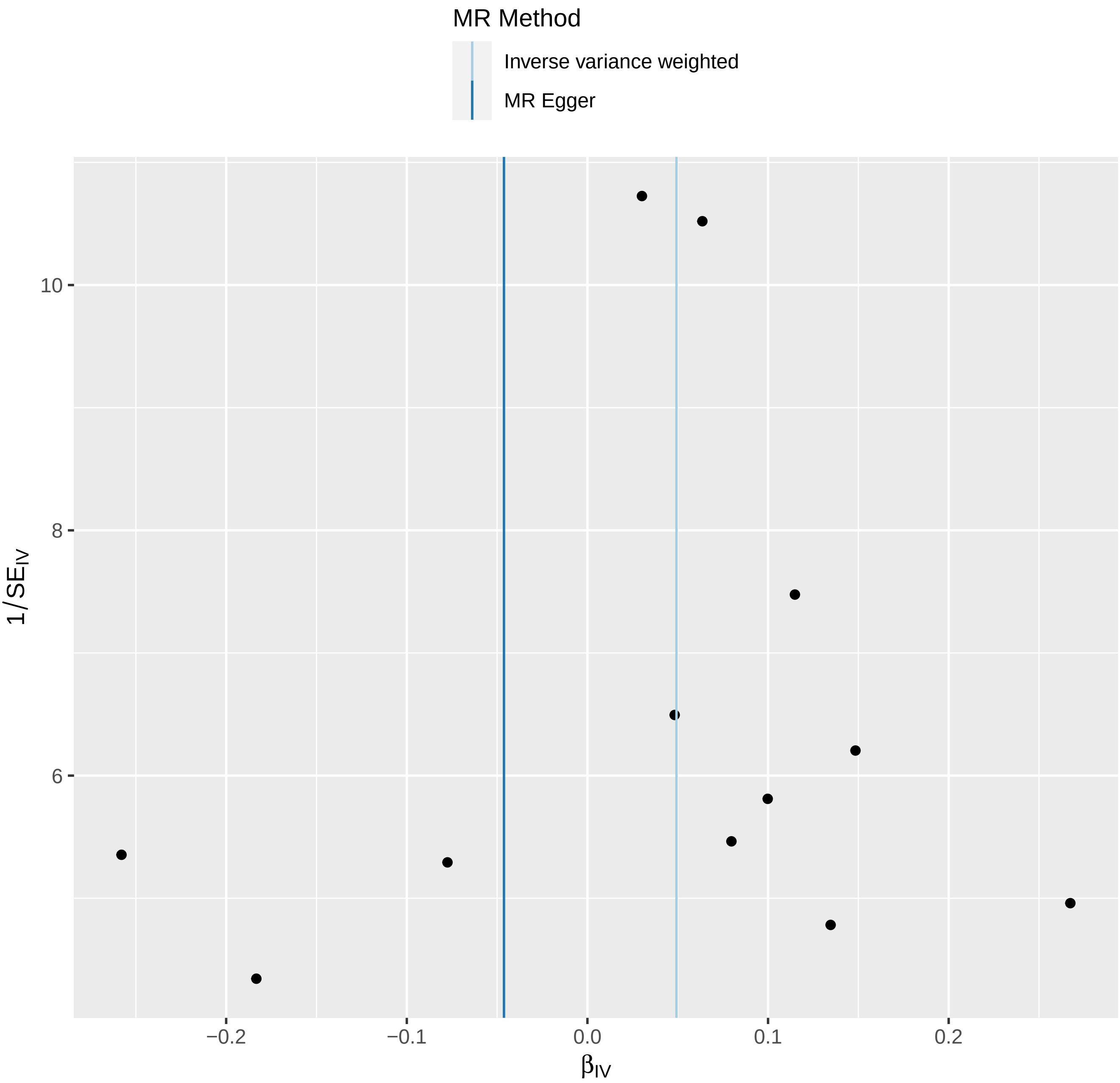

Batch 3−2325 : Selenium on Beta−endorphin

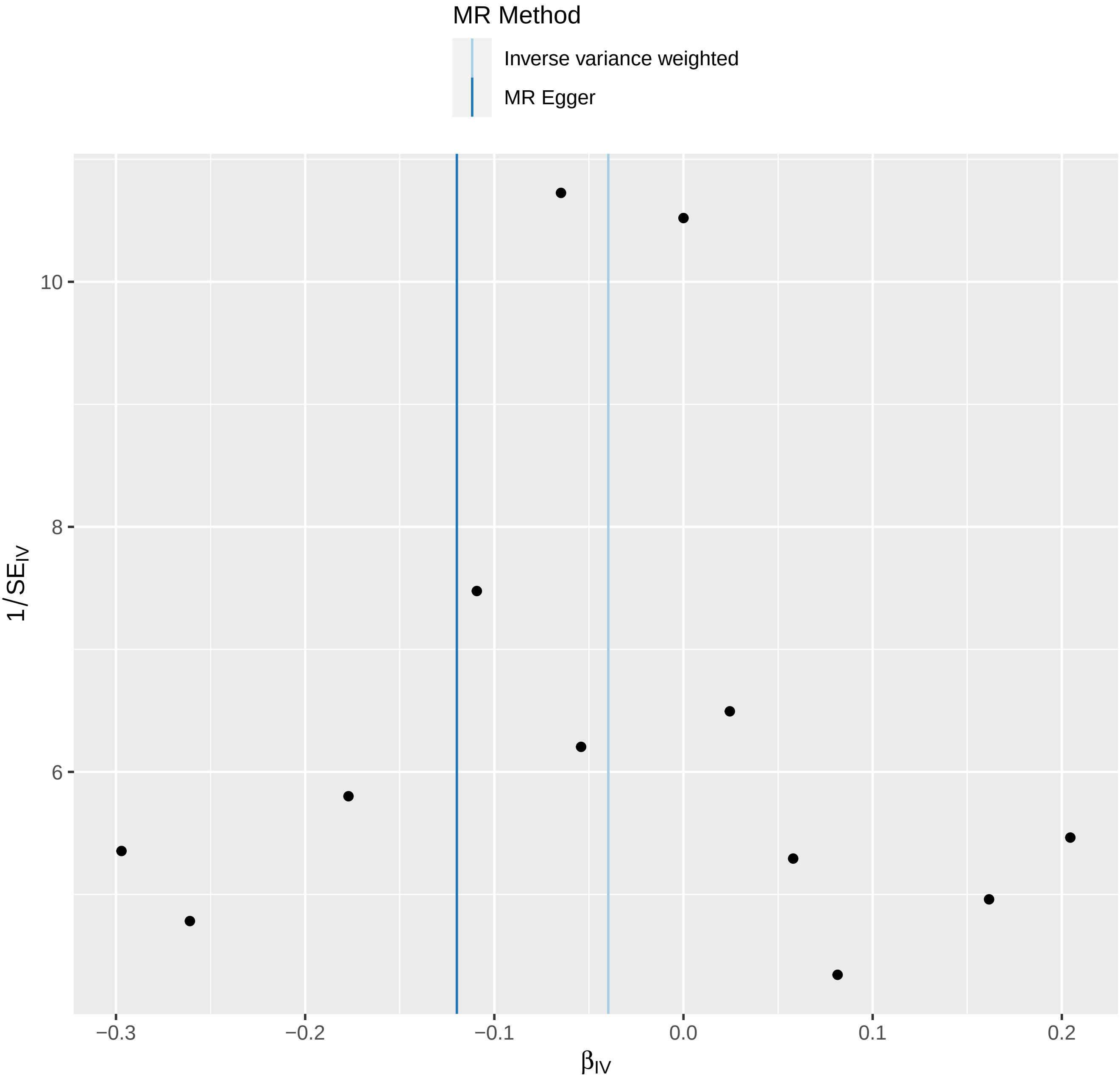

Batch 3−2326 : Selenium on Corticotropin

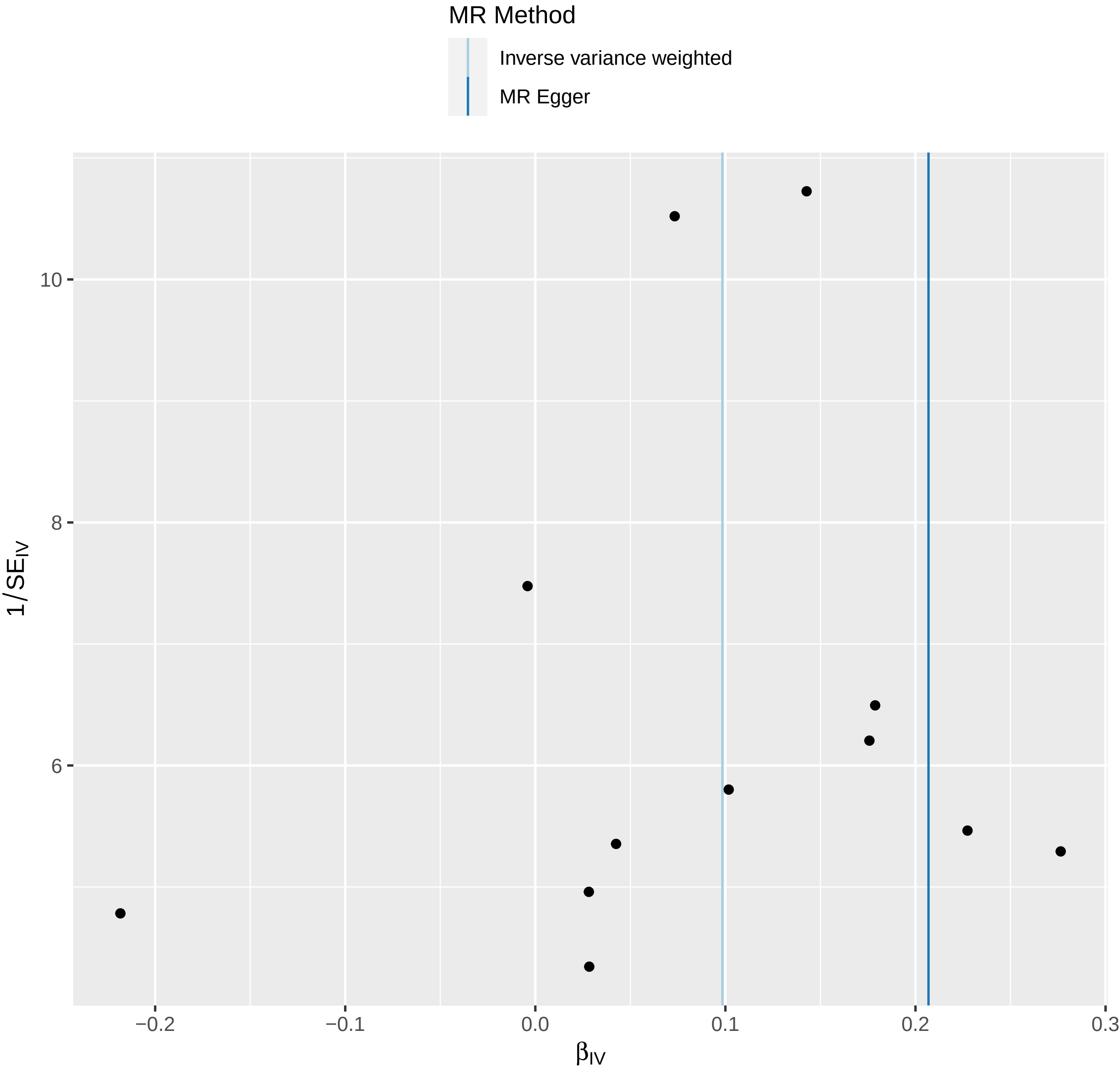

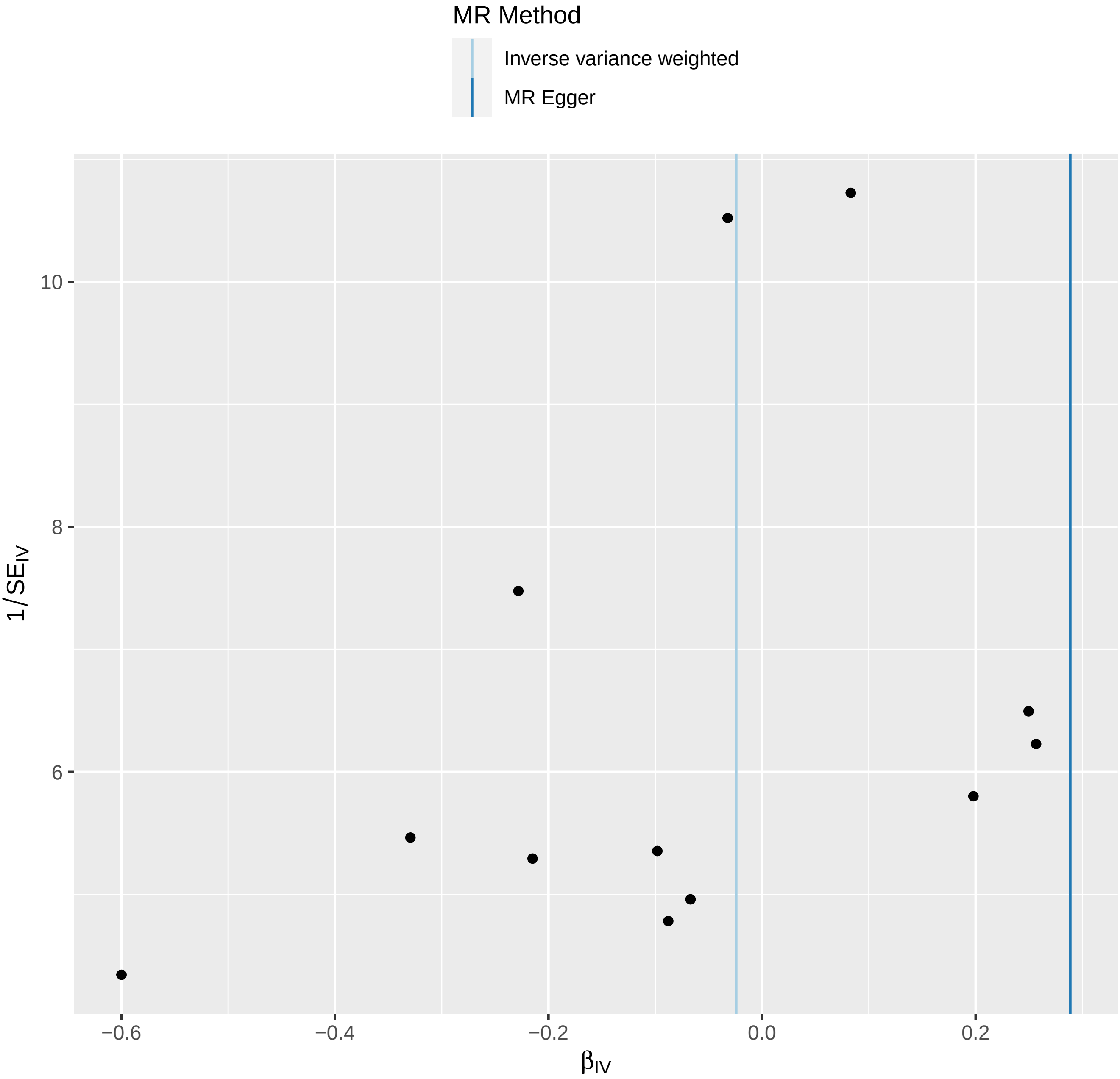
MR Method
Inverse variance weighted
MR Egger
10
8
6
1/SE$_{IV}$
−0.6
−0.4
−0.2
0.0
0.2
β$_{IV}$

Batch 3−2328 : Selenium on Protein O−linked−mannose beta−1,2−N−acetylglucosaminyltransferase 1

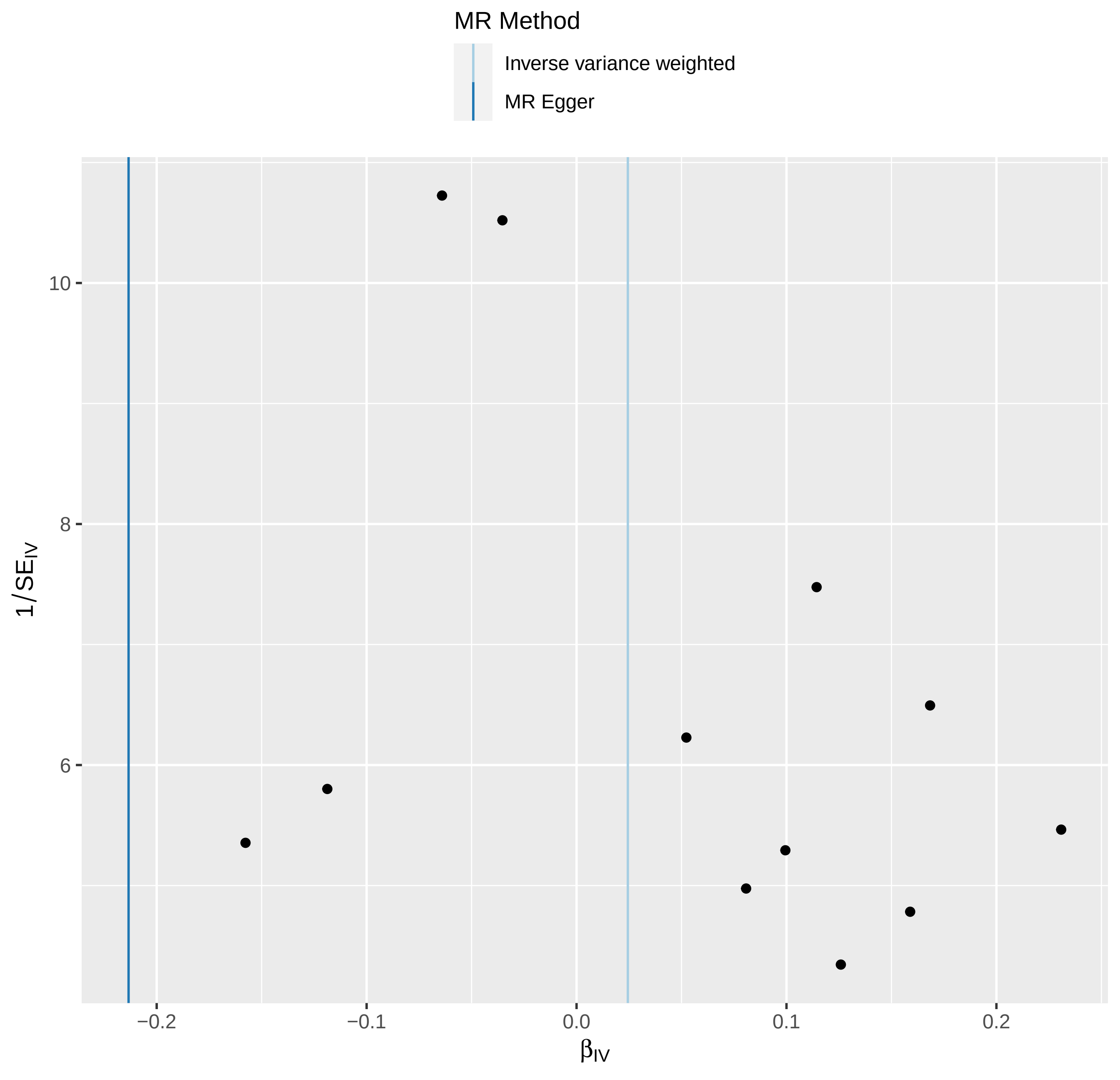

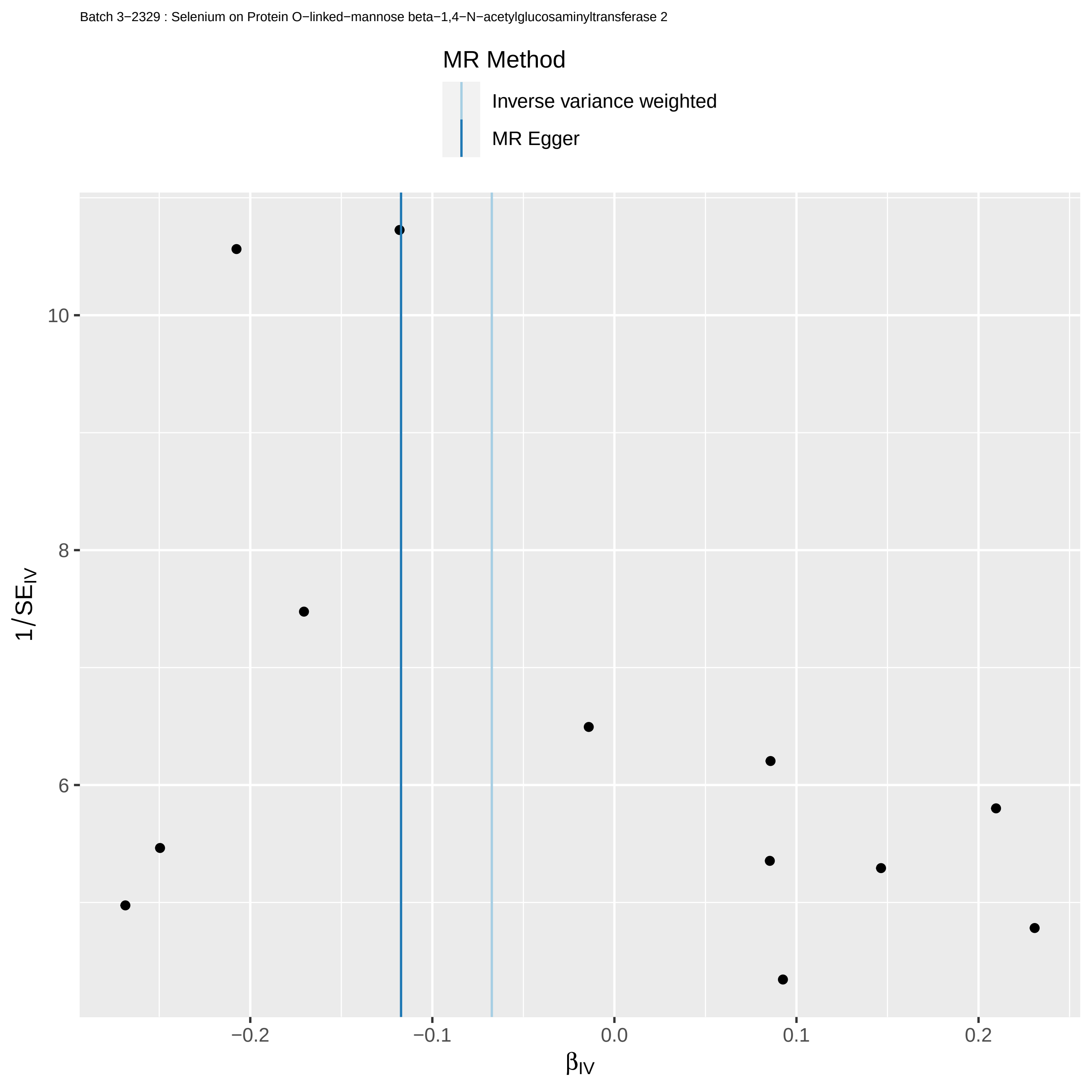
Batch 3−2329 : Selenium on Protein O−linked−mannose beta−1,4−N−acetylglucosaminyltransferase 2
MR Method
Inverse variance weighted
MR Egger
10
8
6
1/SE_IV
−0.2
−0.1
0.0
0.1
0.2
β_IV

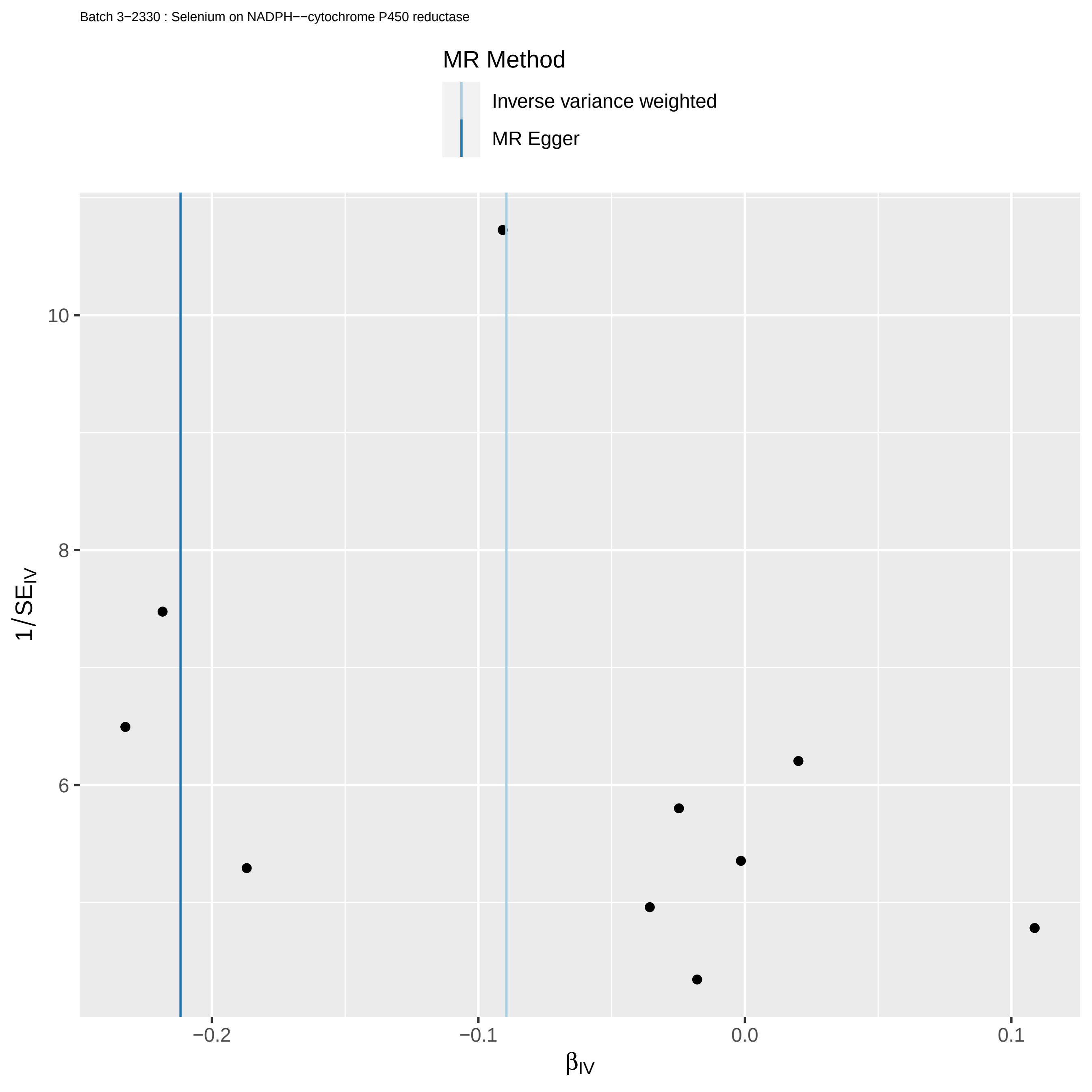
Batch 3−2330 : Selenium on NADPH−−cytochrome P450 reductase
MR Method
Inverse variance weighted
MR Egger
10
8
6
1/SE_IV
−0.2
−0.1
0.0
0.1
β_IV

Batch 3−2331 : Selenium on Periostin

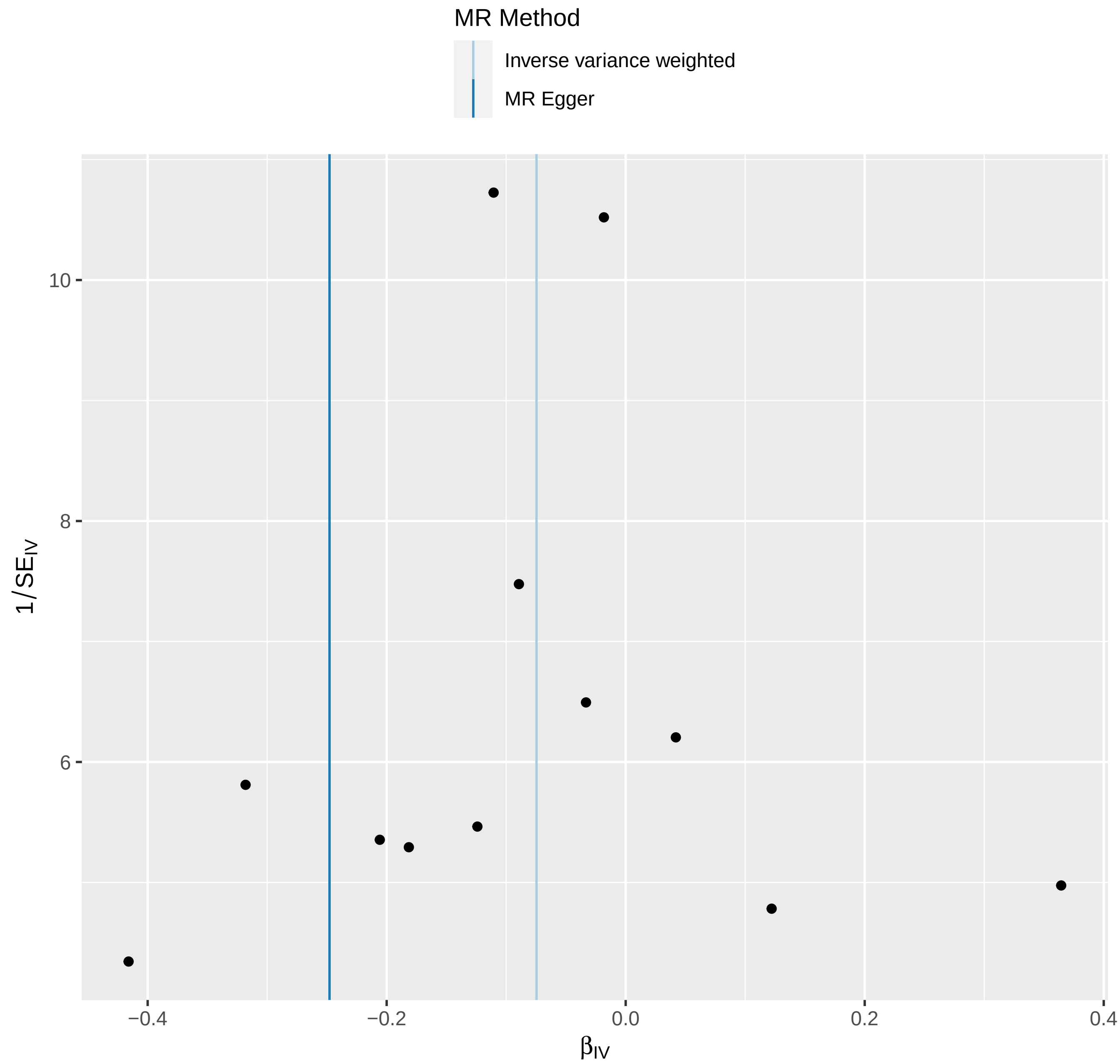

Batch 3−2332 : Selenium on Periostin

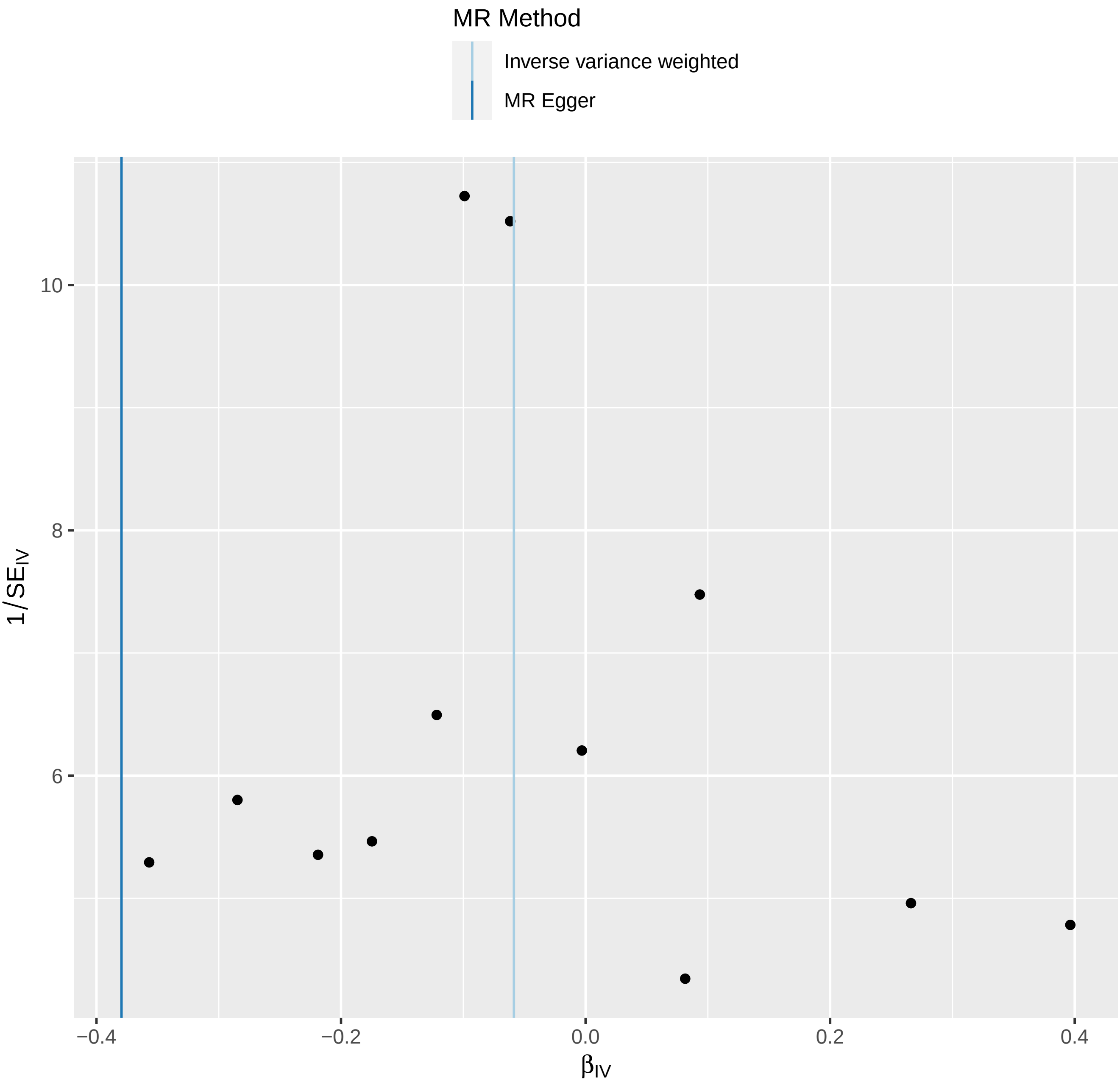

Batch 3−2333 : Selenium on Periostin

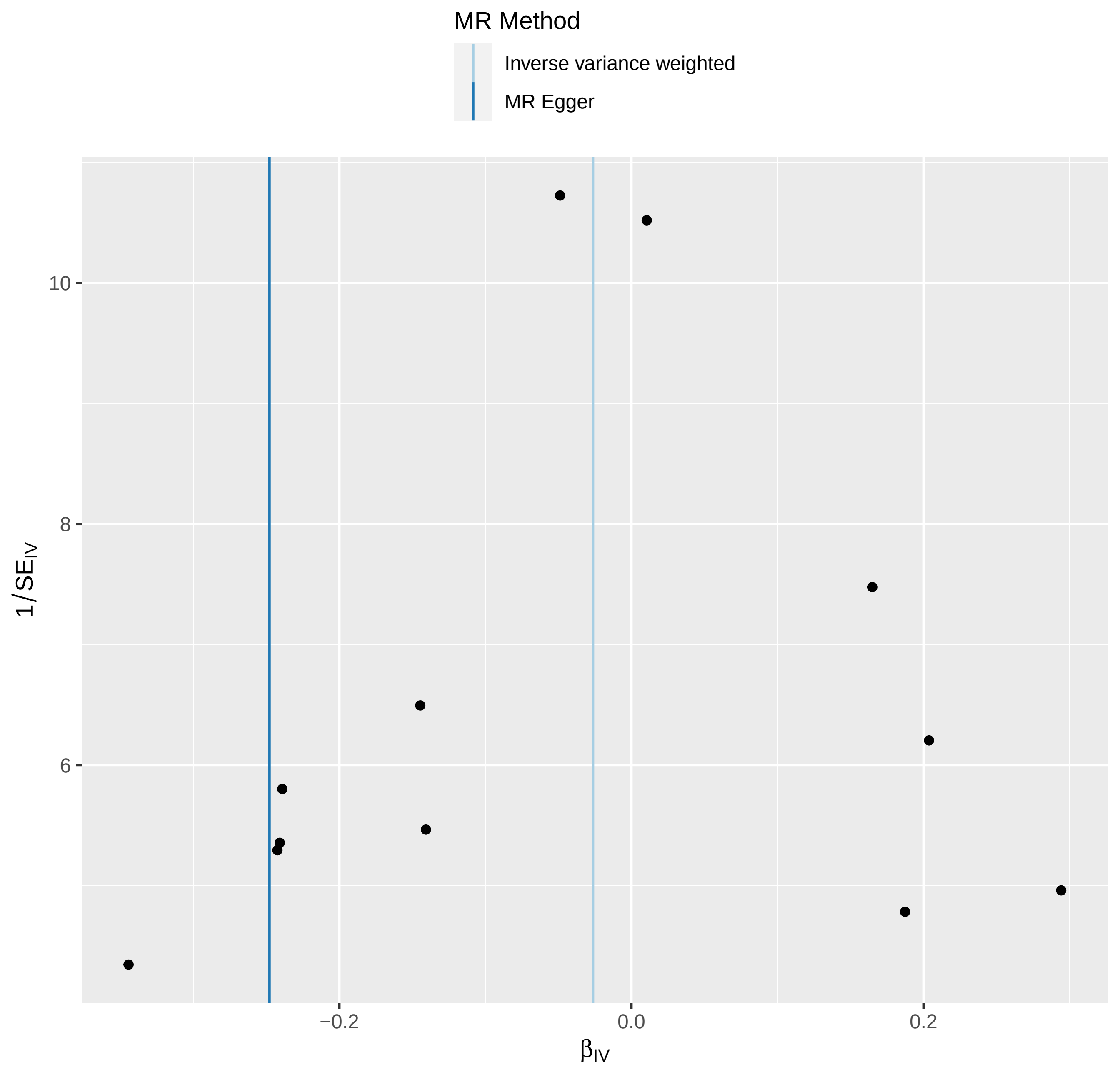

Batch 3−2334 : Selenium on POU domain, class 2, transcription factor 1

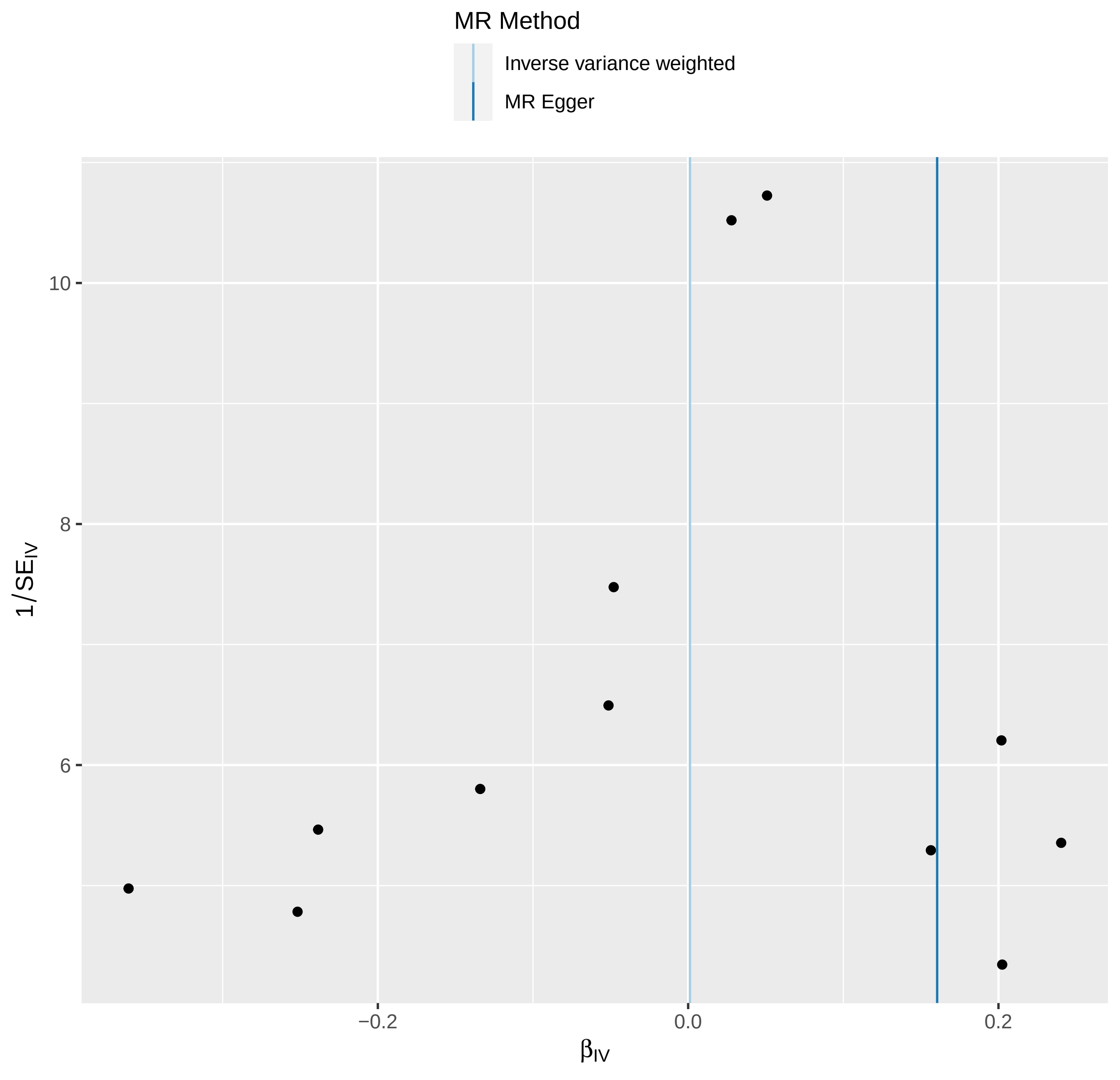

Batch 3−2335 : Selenium on Phosphatidate phosphatase PPAPDC1A

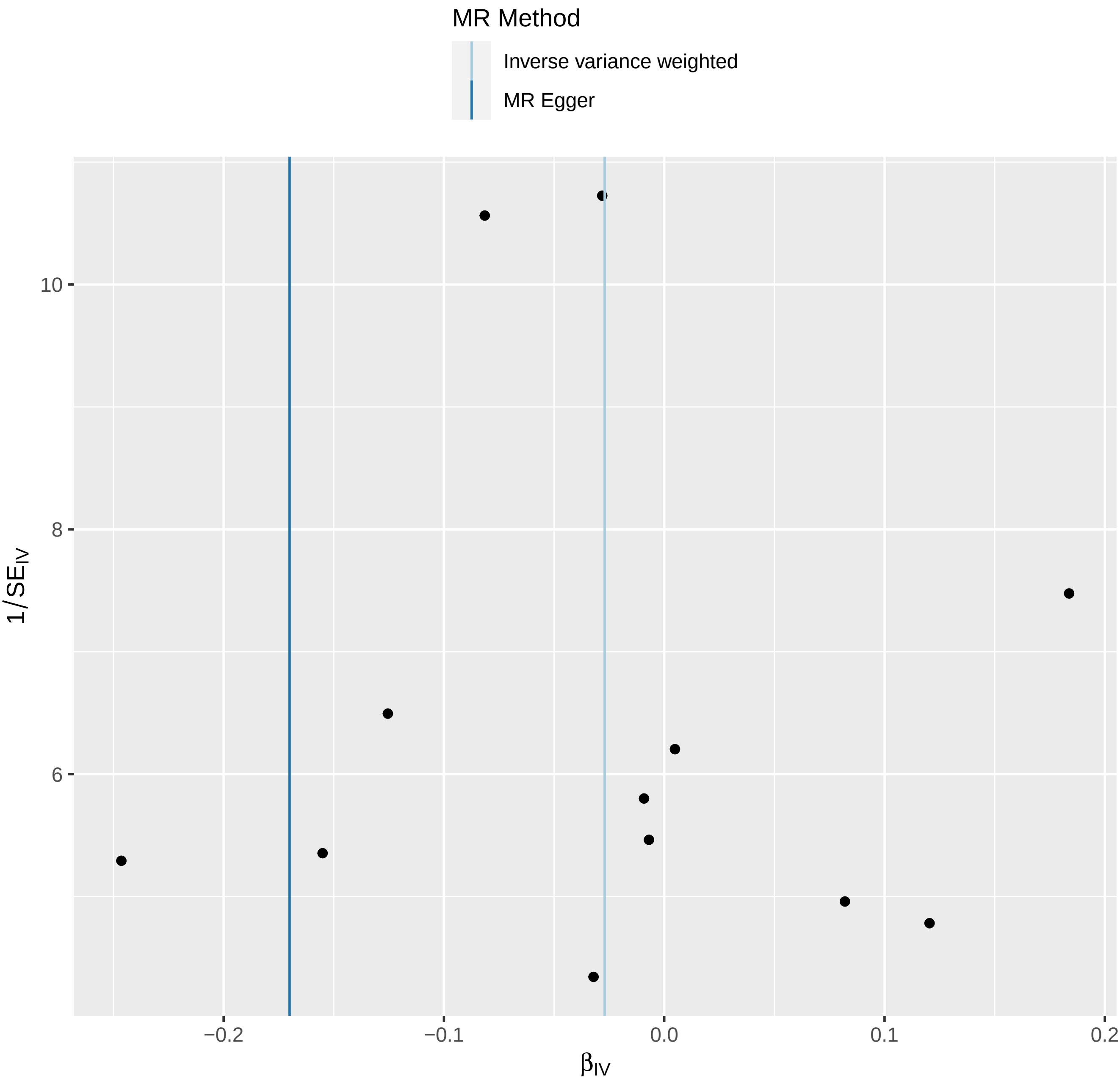

Batch 3−2336 : Selenium on Peroxisome proliferator−activated receptor alpha

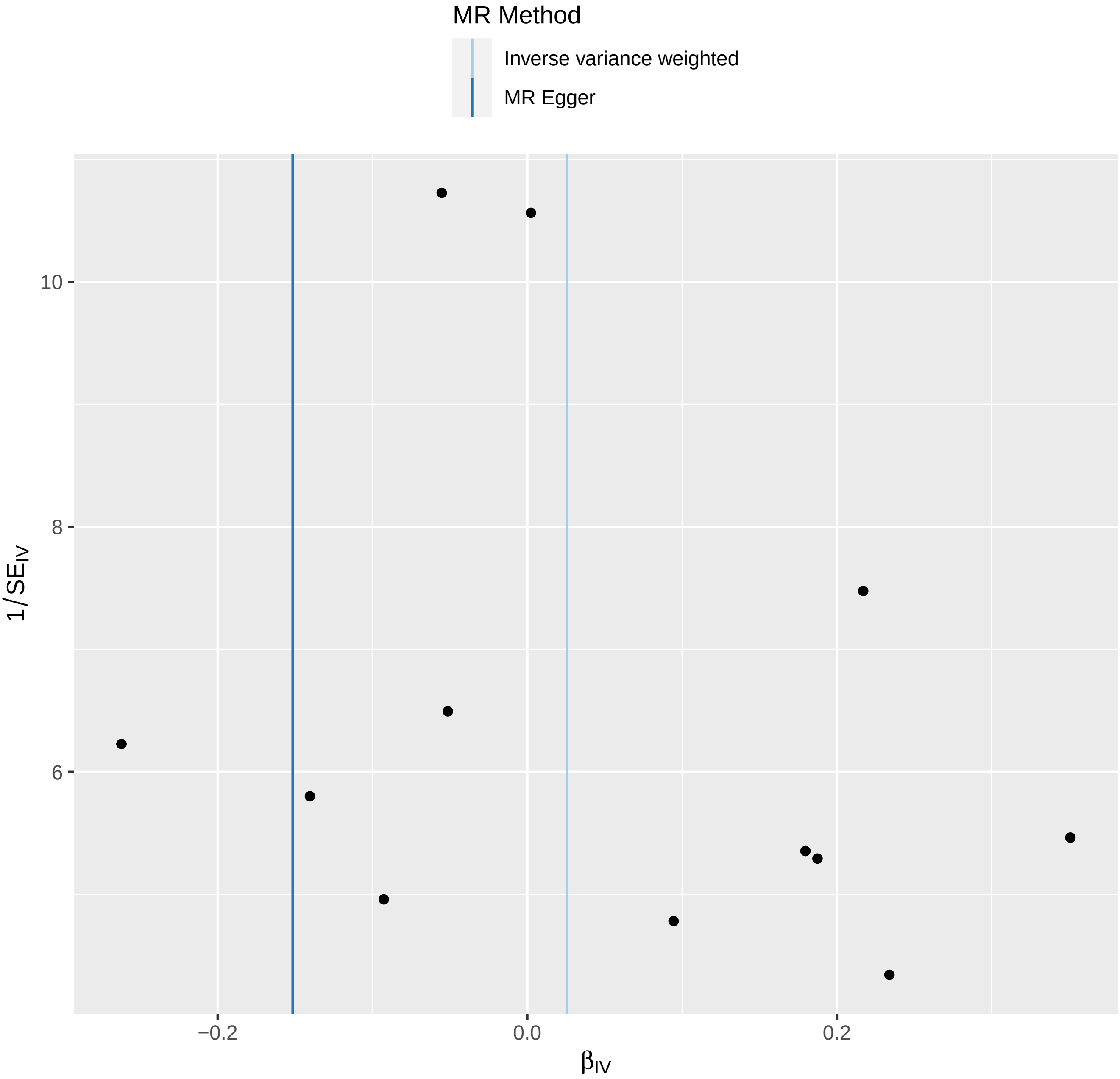

Batch 3−2337 : Selenium on Neutrophil−activating peptide 2

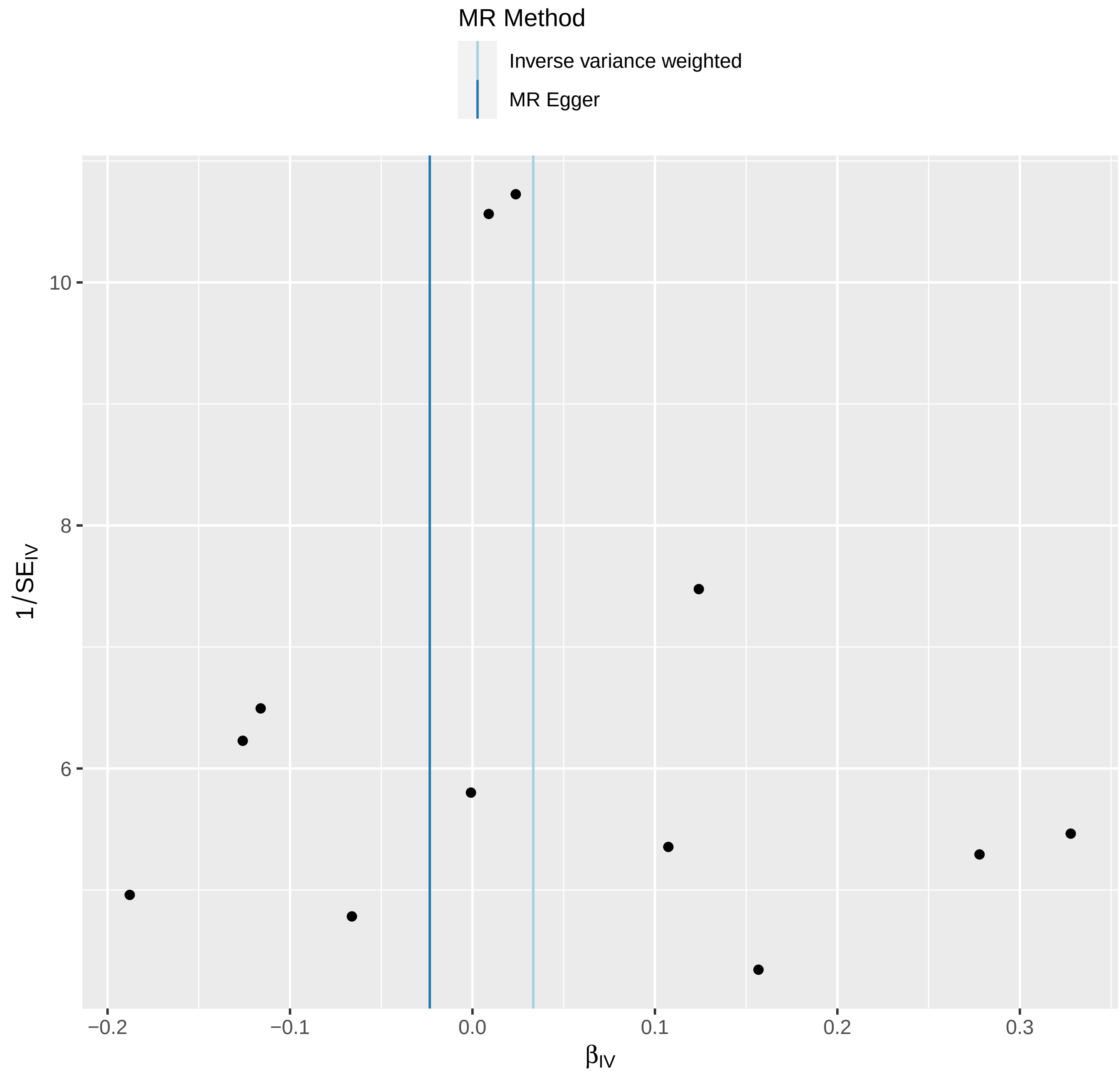

Batch 3−2338 : Selenium on Phosphopantothenoylcysteine decarboxylase

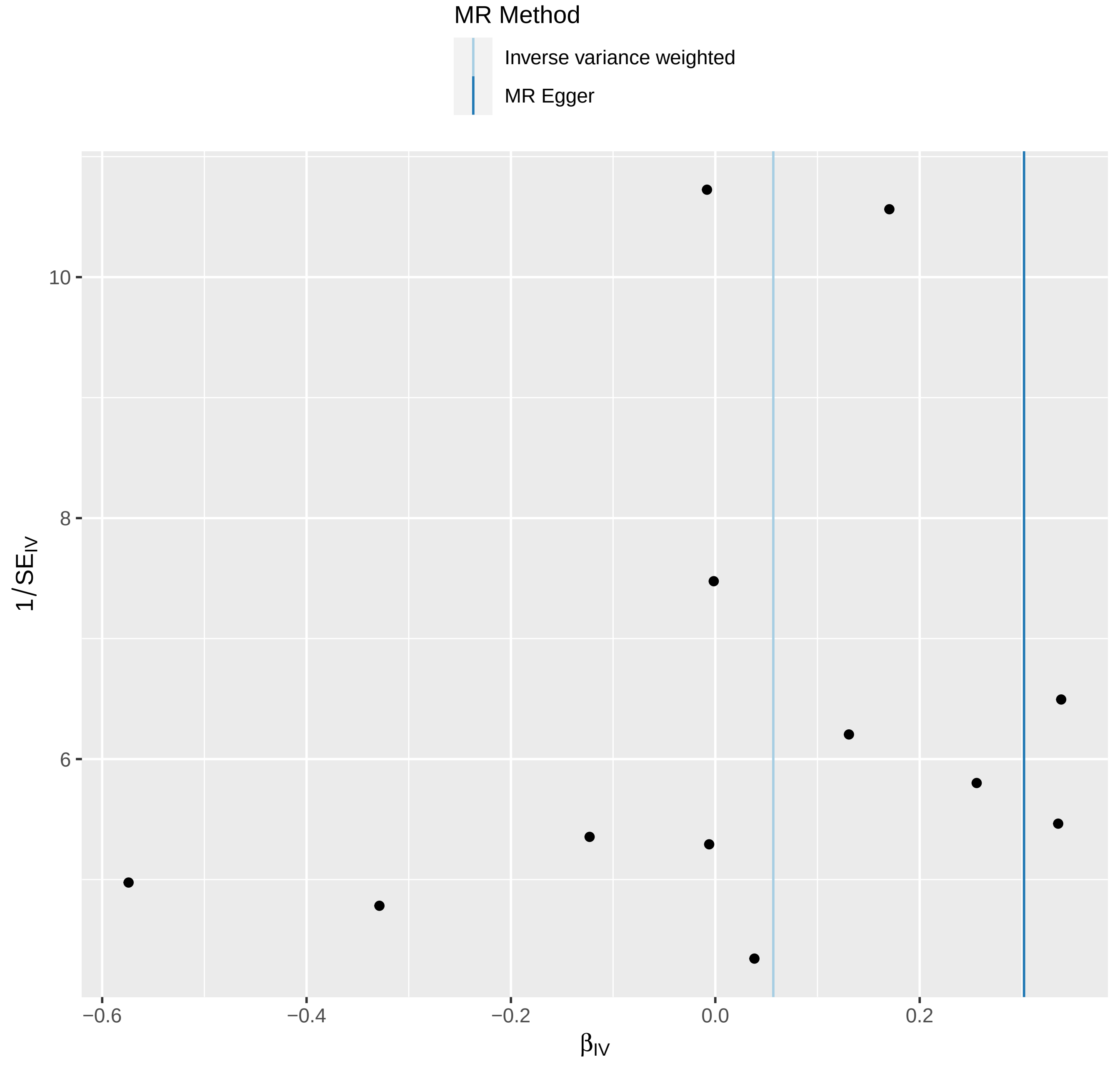

Batch 3−2339 : Selenium on Peptidyl−prolyl cis−trans isomerase A

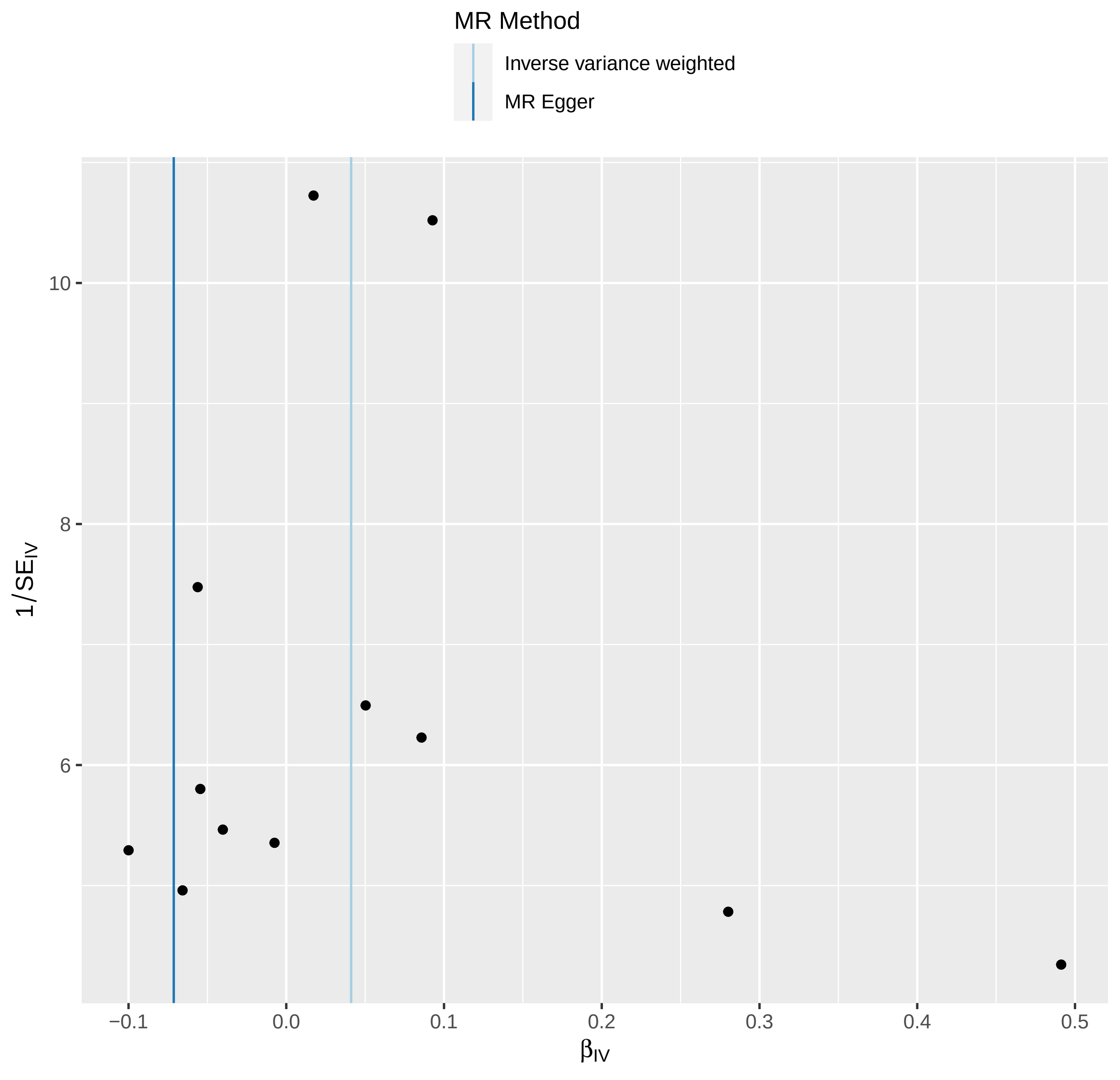

Batch 3−2340 : Selenium on Peptidyl−prolyl cis−trans isomerase B

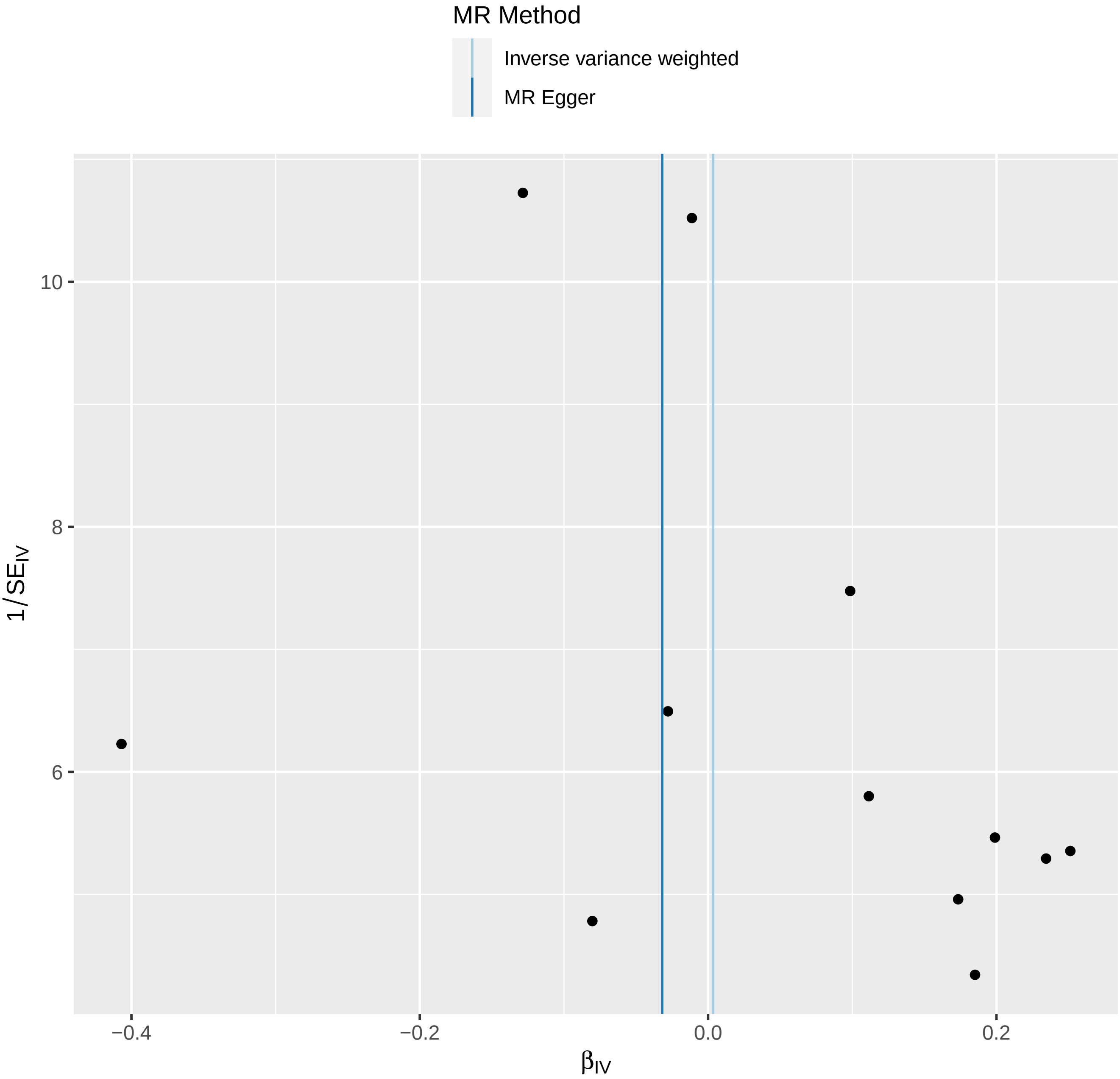

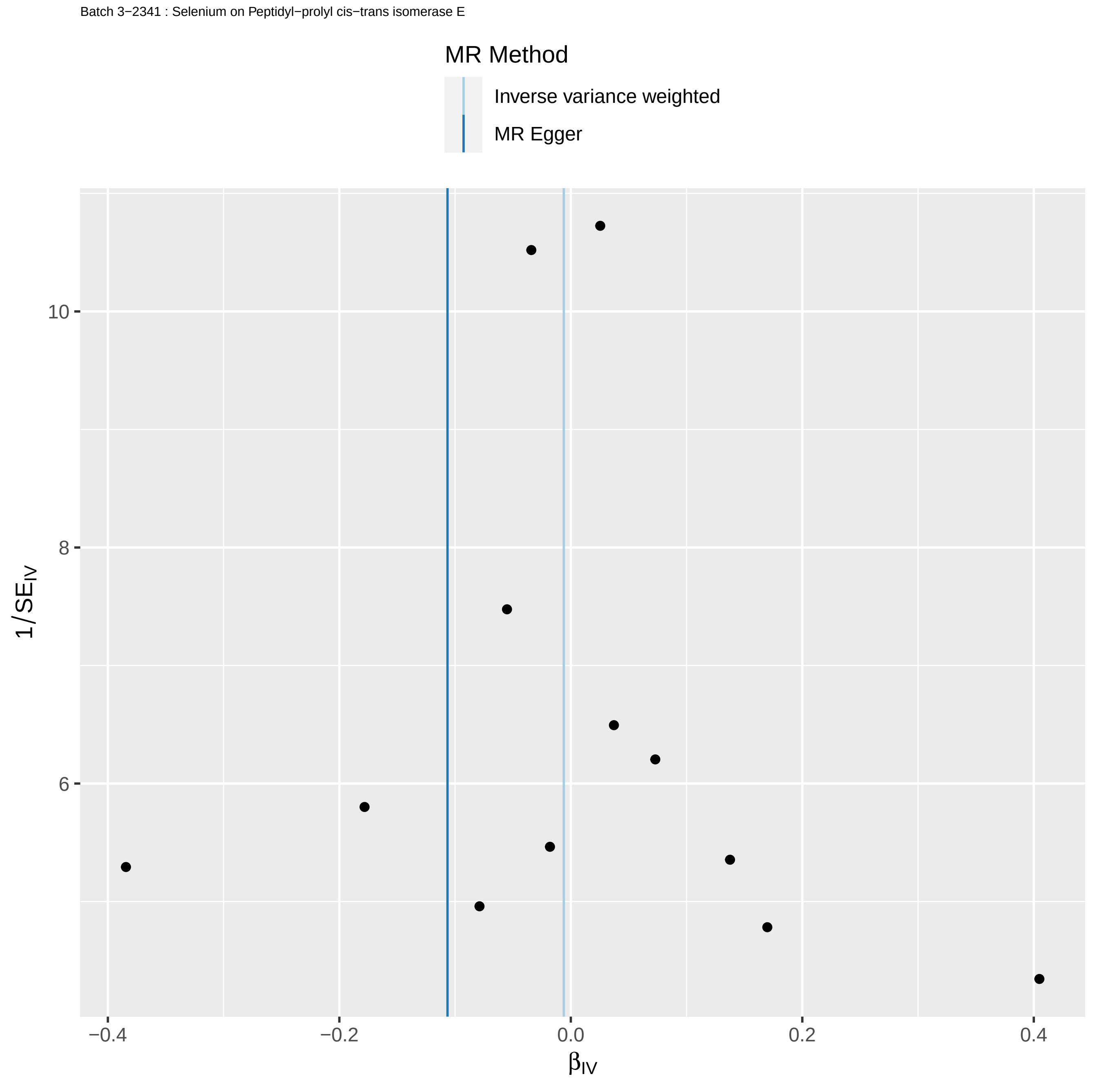

Batch 3−2341 : Selenium on Peptidyl−prolyl cis−trans isomerase E
MR Method
Inverse variance weighted
MR Egger
10
8
6
1/SE_IV
−0.4
−0.2
0.0
0.2
0.4
β_IV

Batch 3−2342 : Selenium on Peptidyl−prolyl cis−trans isomerase H

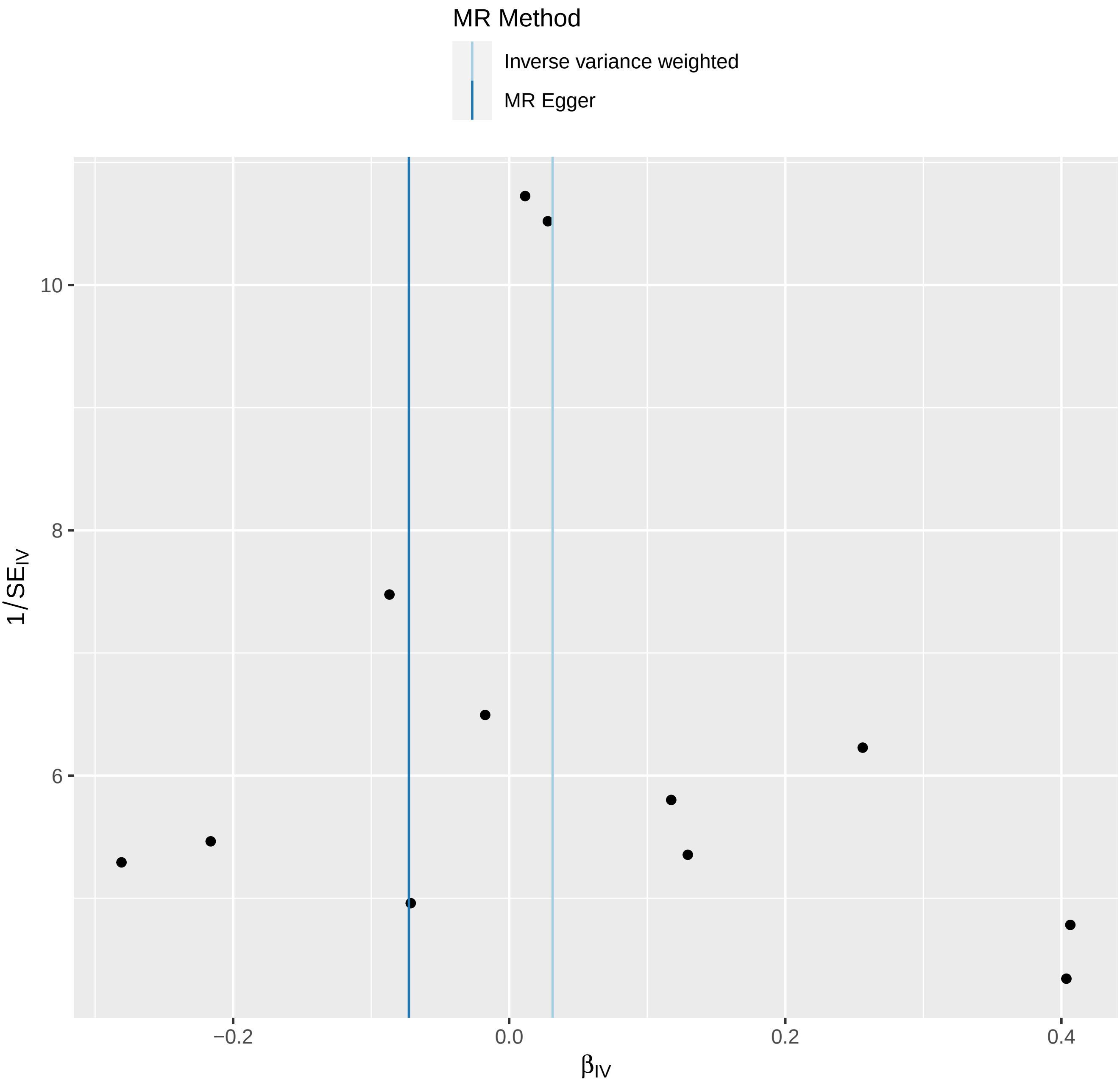

Batch 3−2343 : Selenium on Peptidyl−prolyl cis−trans isomerase−like 1

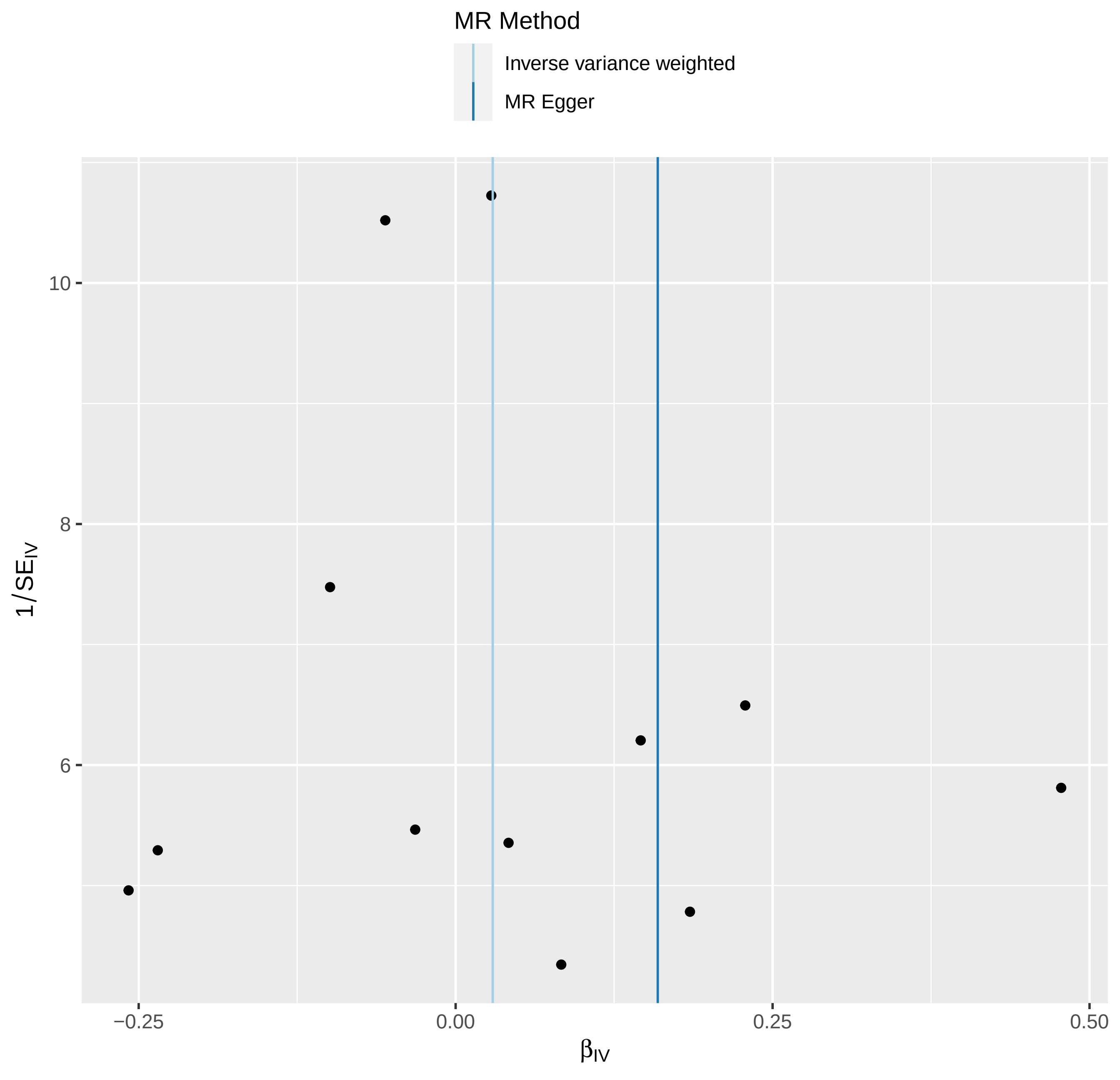

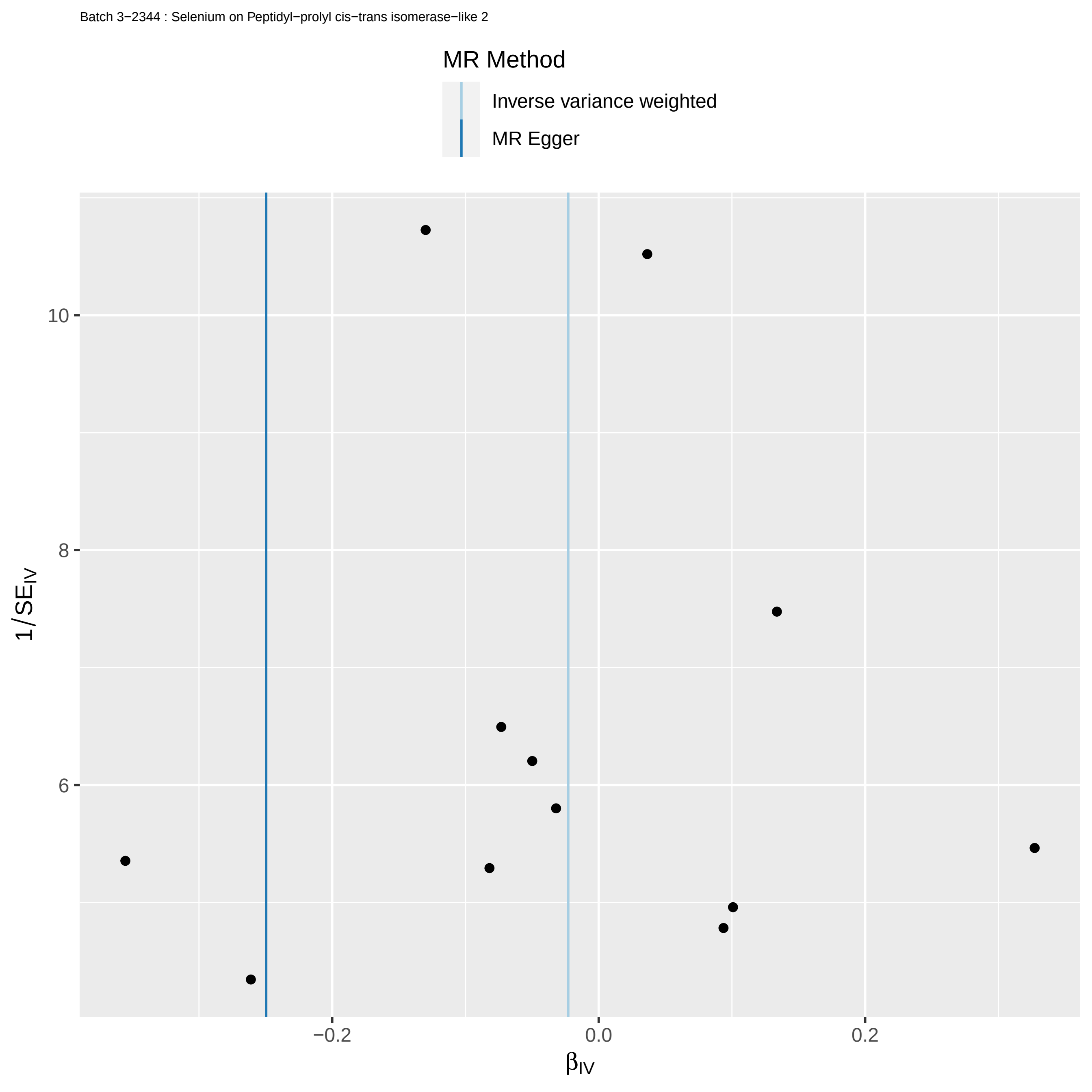
Batch 3−2344 : Selenium on Peptidyl−prolyl cis−trans isomerase−like 2
MR Method
Inverse variance weighted
MR Egger
10
8
6
1/SE_IV
−0.2
0.0
0.2
β_IV

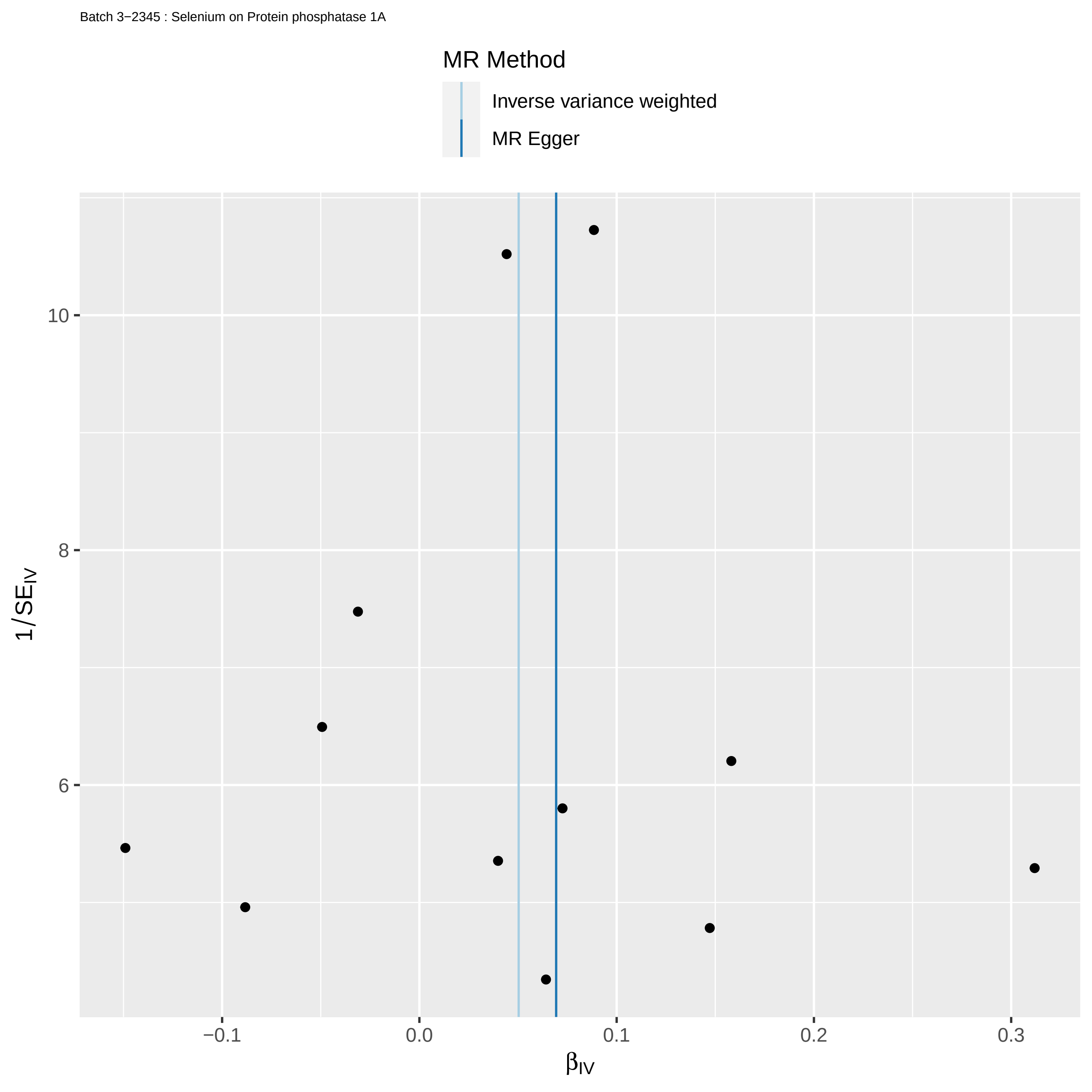

Batch 3−2345 : Selenium on Protein phosphatase 1A
MR Method
Inverse variance weighted
MR Egger
10
8
6
1/SE_IV
−0.1
0.0
0.1
0.2
0.3
β_IV

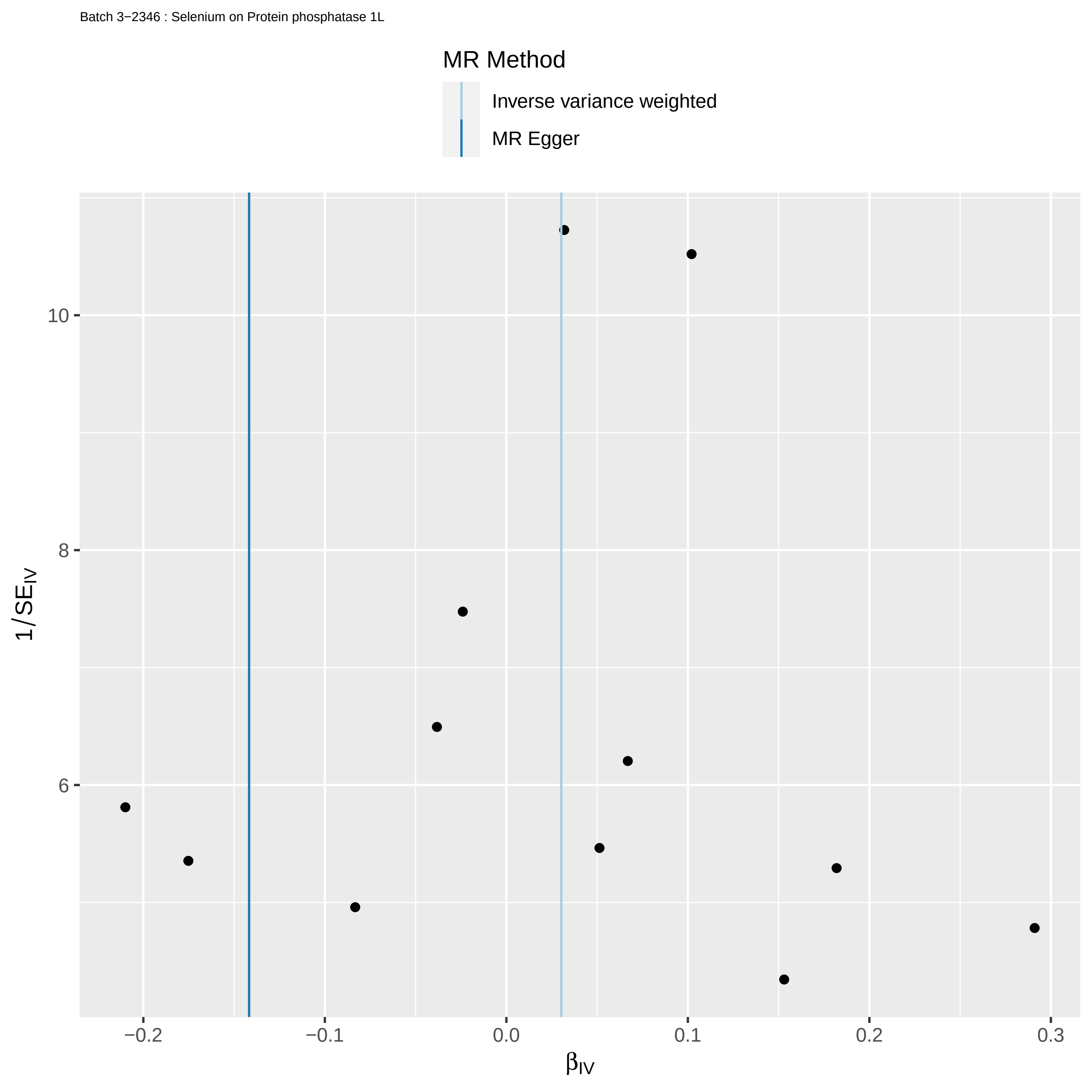

Batch 3−2346 : Selenium on Protein phosphatase 1L
MR Method
Inverse variance weighted
MR Egger
10
8
6
1/SE$_{IV}$
−0.2
−0.1
0.0
0.1
0.2
0.3
β$_{IV}$

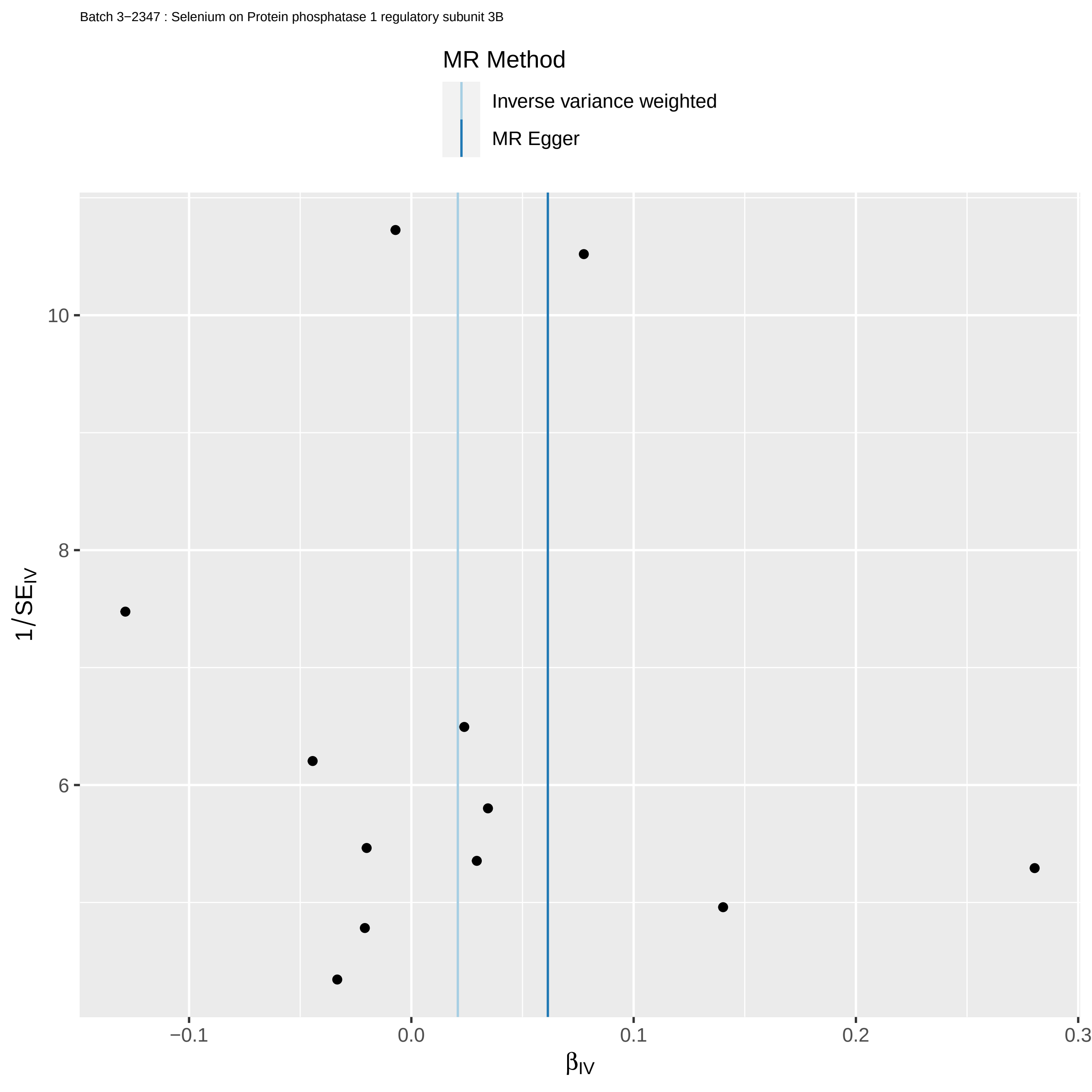
Batch 3−2347 : Selenium on Protein phosphatase 1 regulatory subunit 3B
MR Method
Inverse variance weighted
MR Egger
10
8
6
1/SE_IV
−0.1
0.0
0.1
0.2
0.3
β_IV

Batch 3−2348 : Selenium on Serine/threonine−protein phosphatase 2A 65 kDa regulatory subunit A alpha isoform

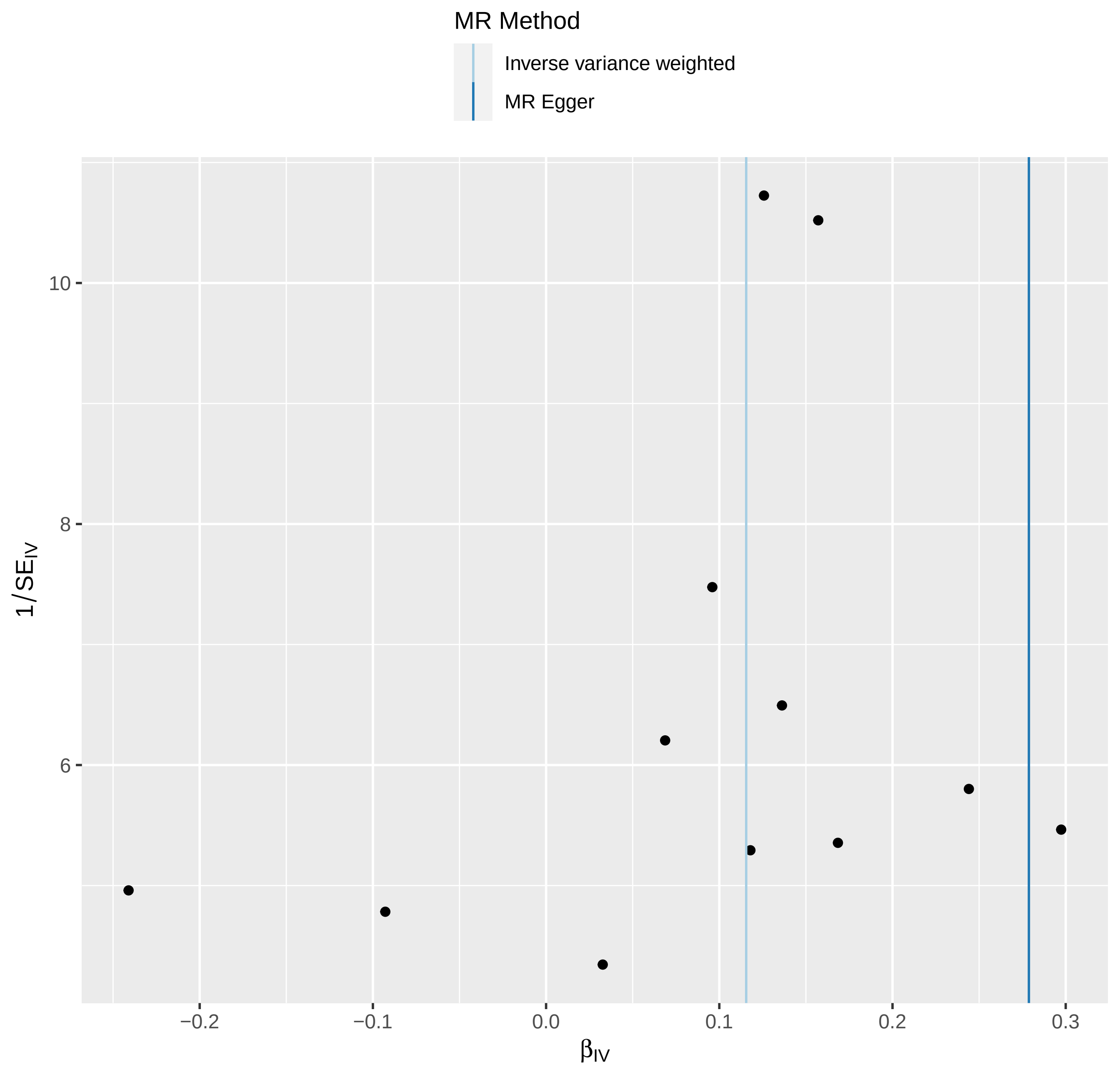

Batch 3−2349 : Selenium on Serine/threonine−protein phosphatase 2A regulatory subunit B" subunit alpha

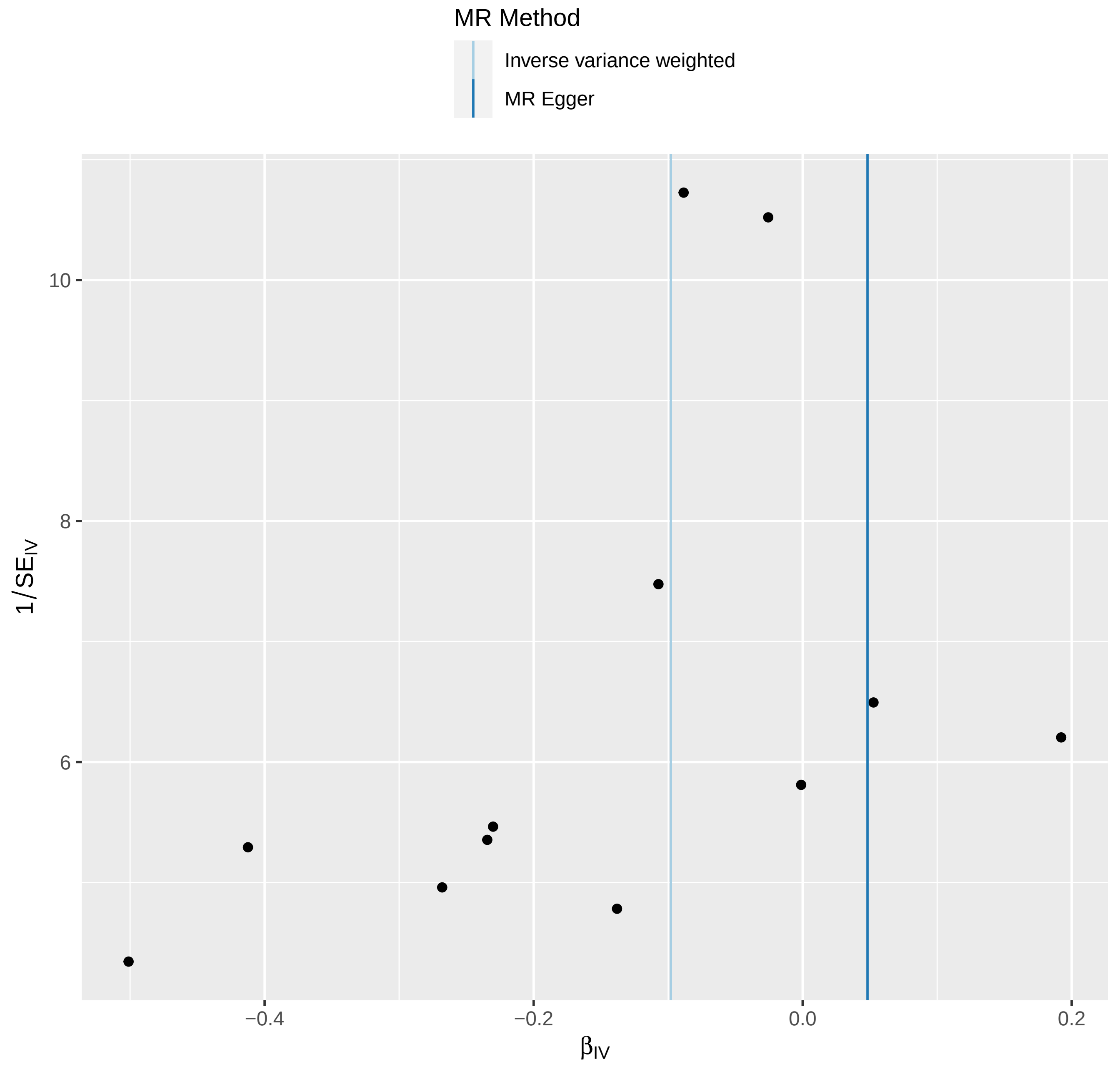

Batch 3−2350 : Selenium on Calcineurin subunit B type 1

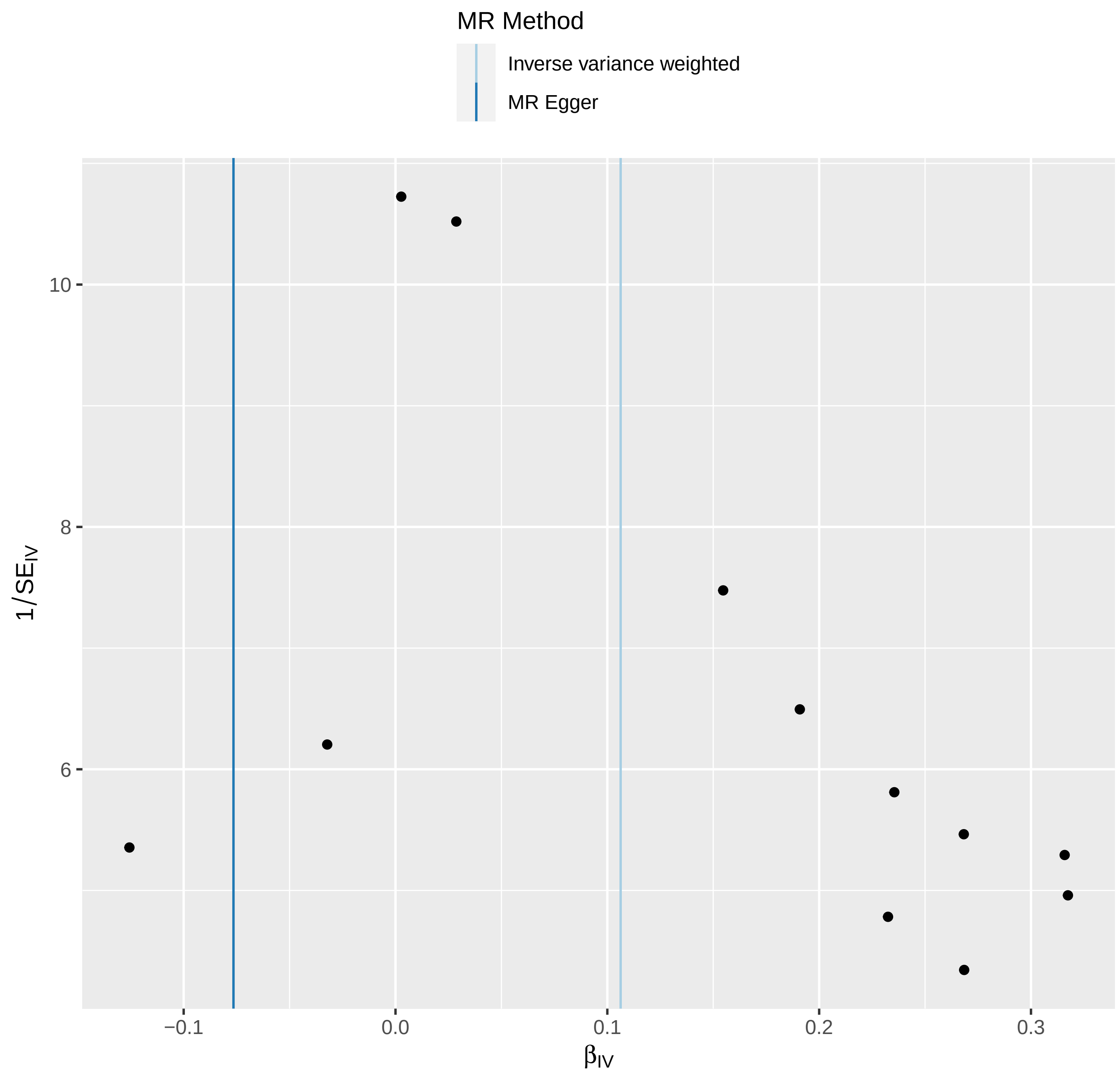

Batch 3−2351 : Selenium on Palmitoyl−protein thioesterase 1

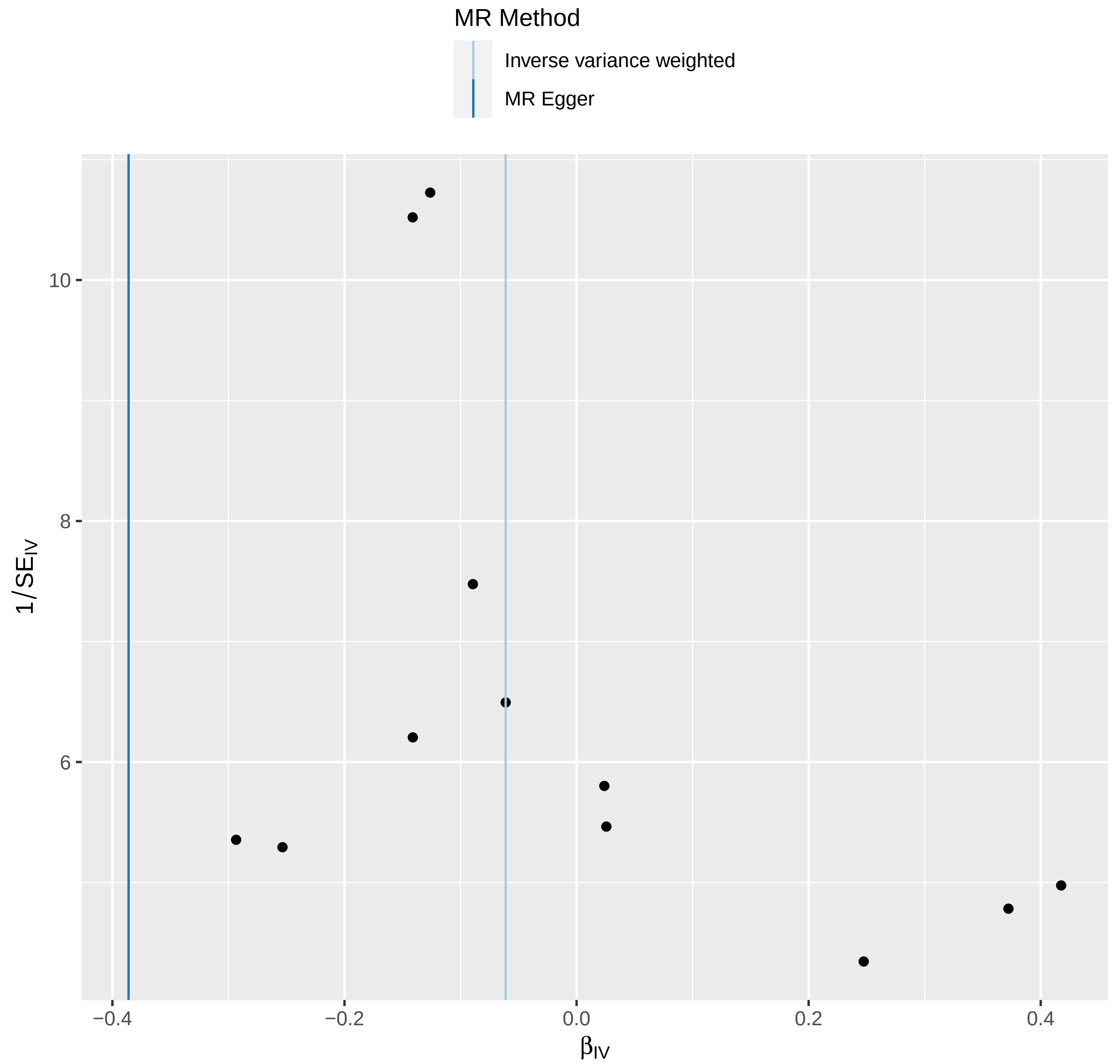

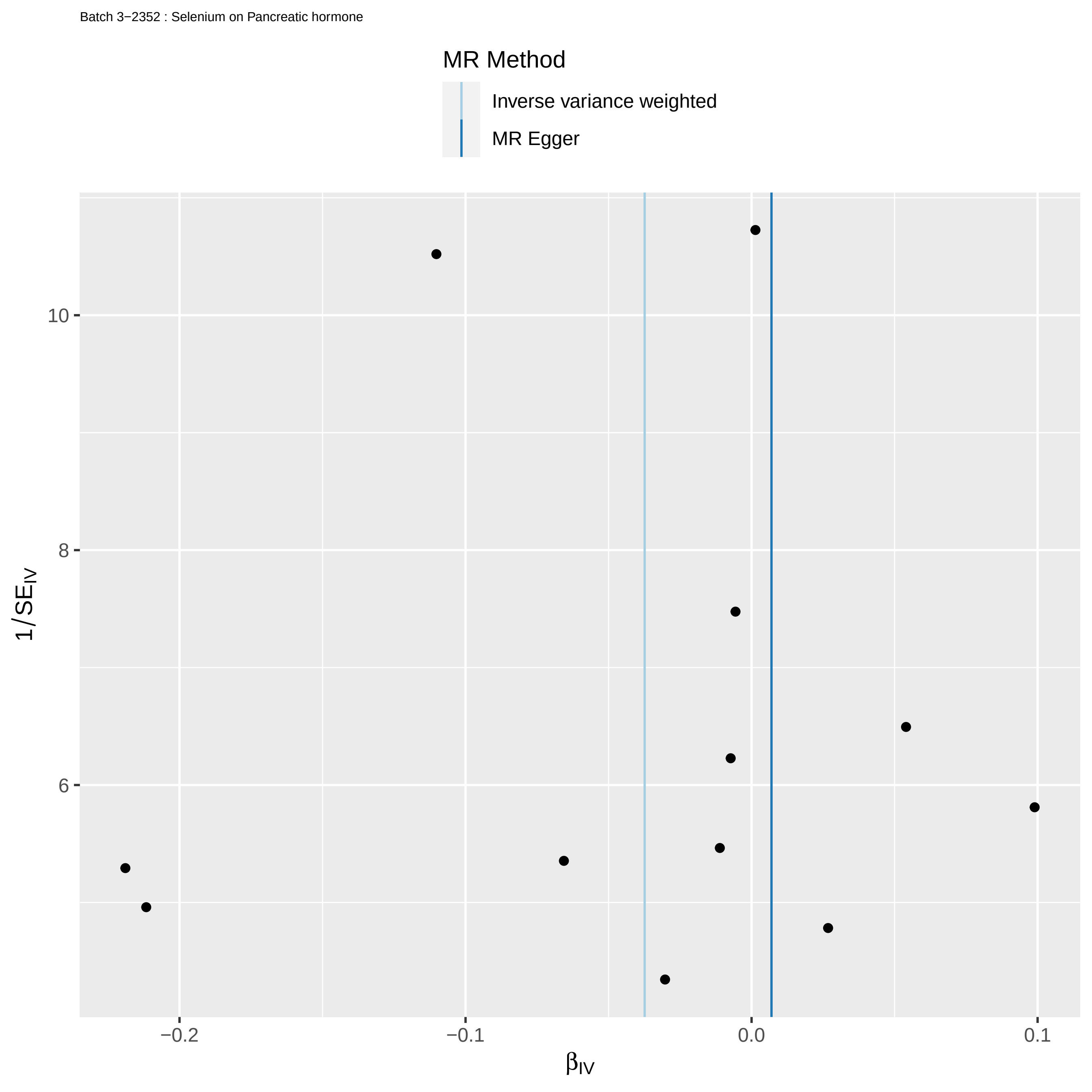
Batch 3−2352 : Selenium on Pancreatic hormone
MR Method
Inverse variance weighted
MR Egger
10
8
6
1/SE_IV
−0.2
−0.1
0.0
0.1
β_IV

Batch 3−2353 : Selenium on Putative uncharacterized protein PQLC2L

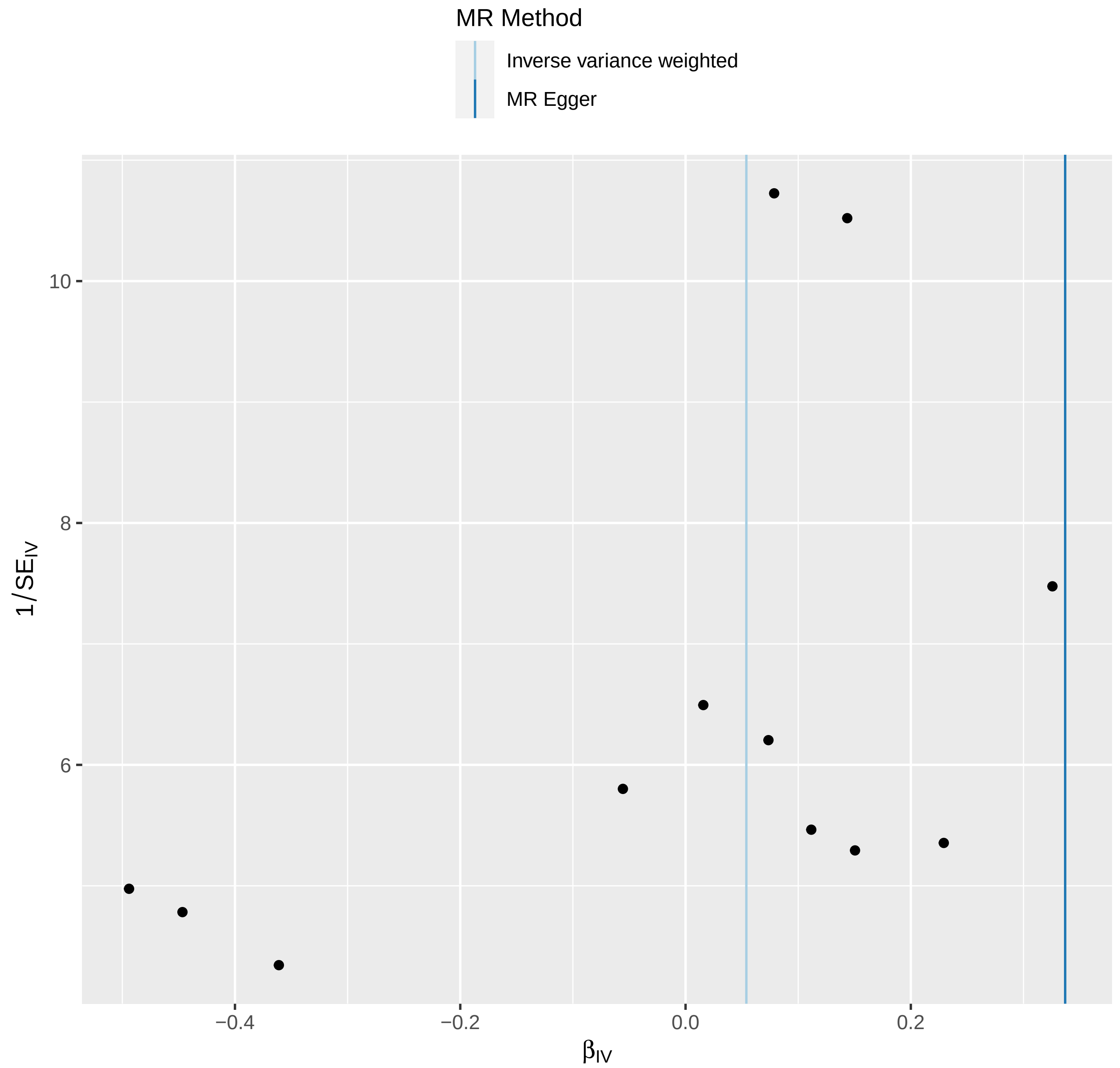

Batch 3−2354 : Selenium on Basic salivary proline−rich protein 4

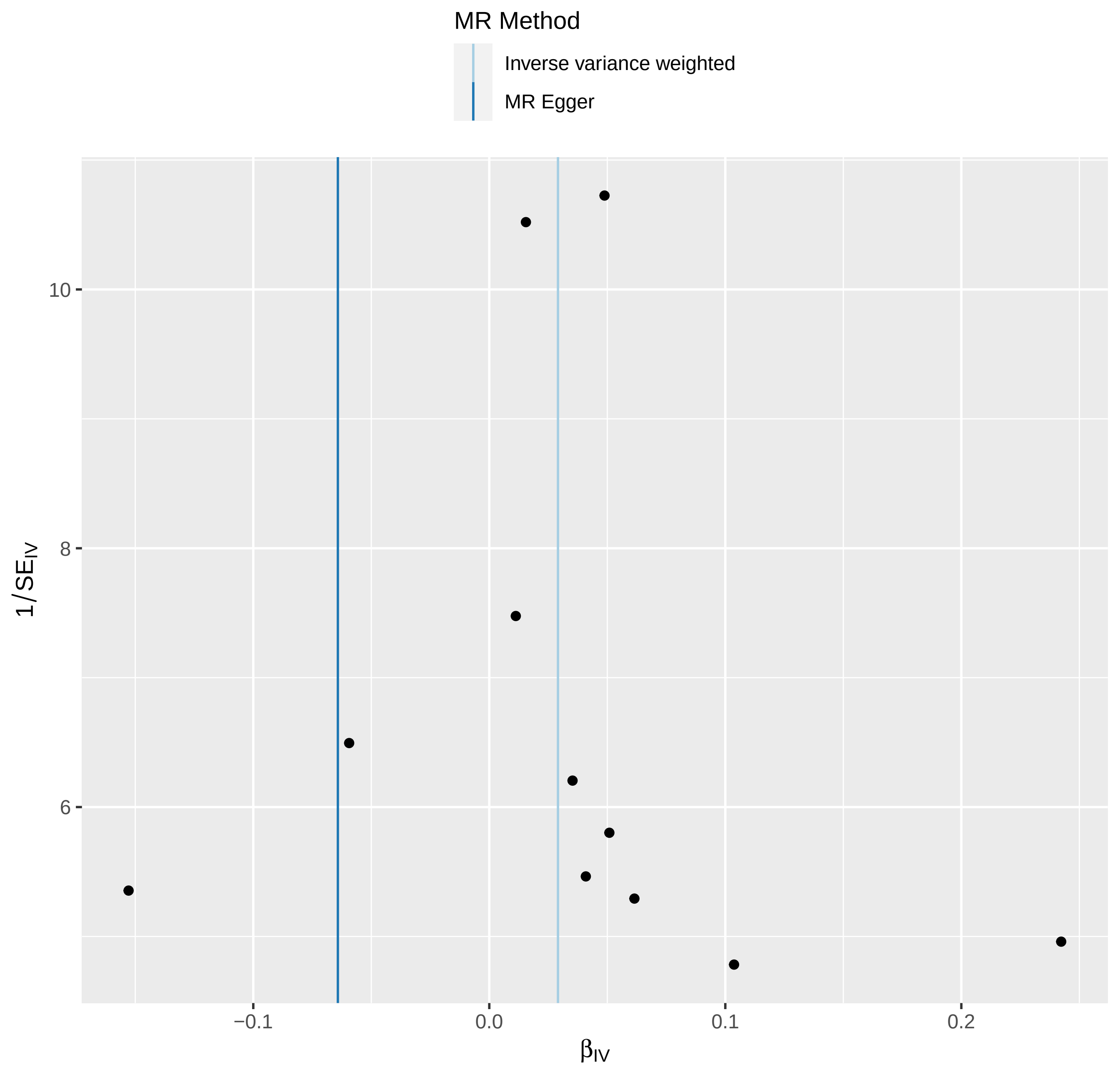

Batch 3−2355 : Selenium on Protein regulator of cytokinesis 1

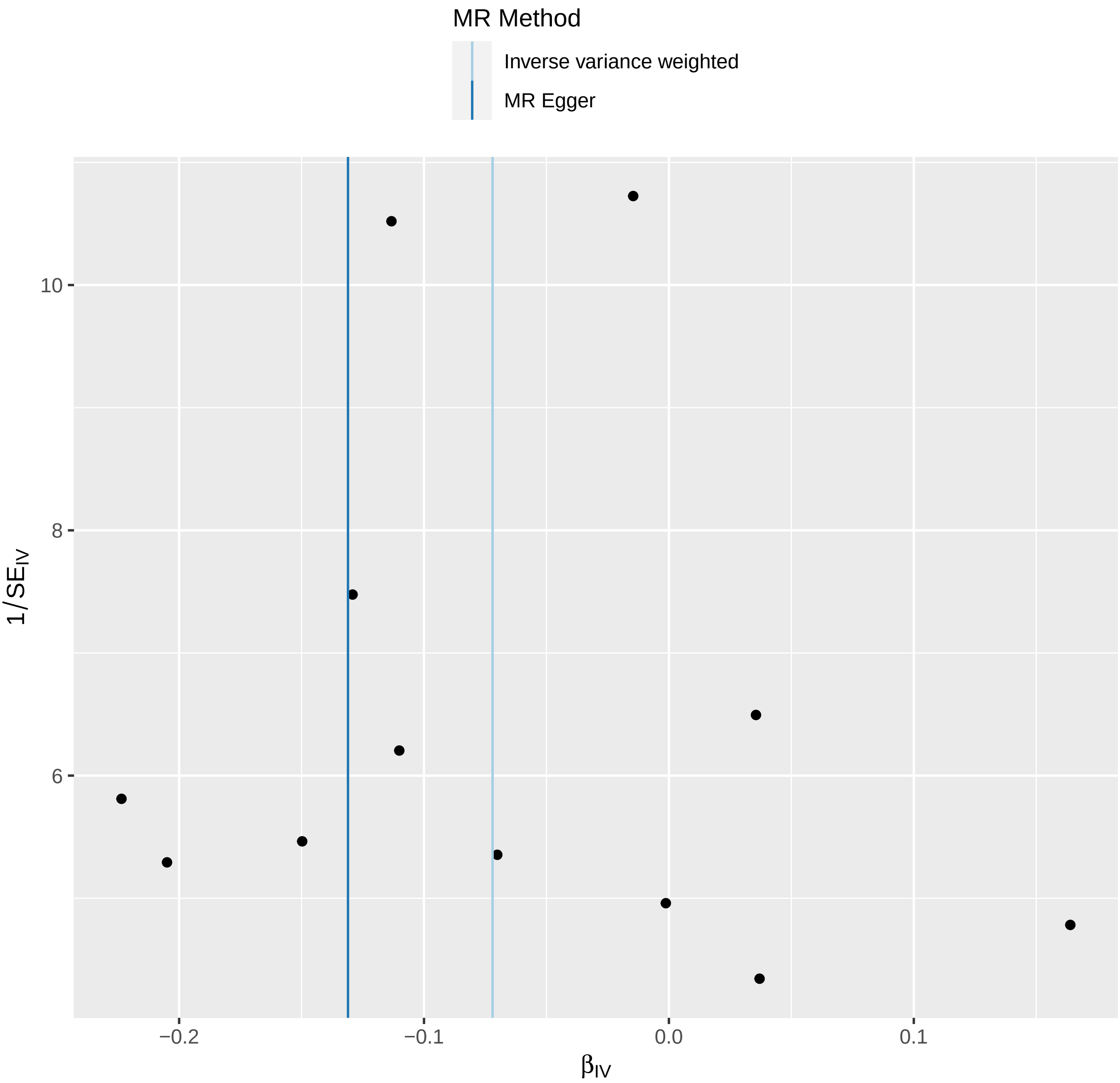

Batch 3−2356 : Selenium on Lysosomal Pro−X carboxypeptidase

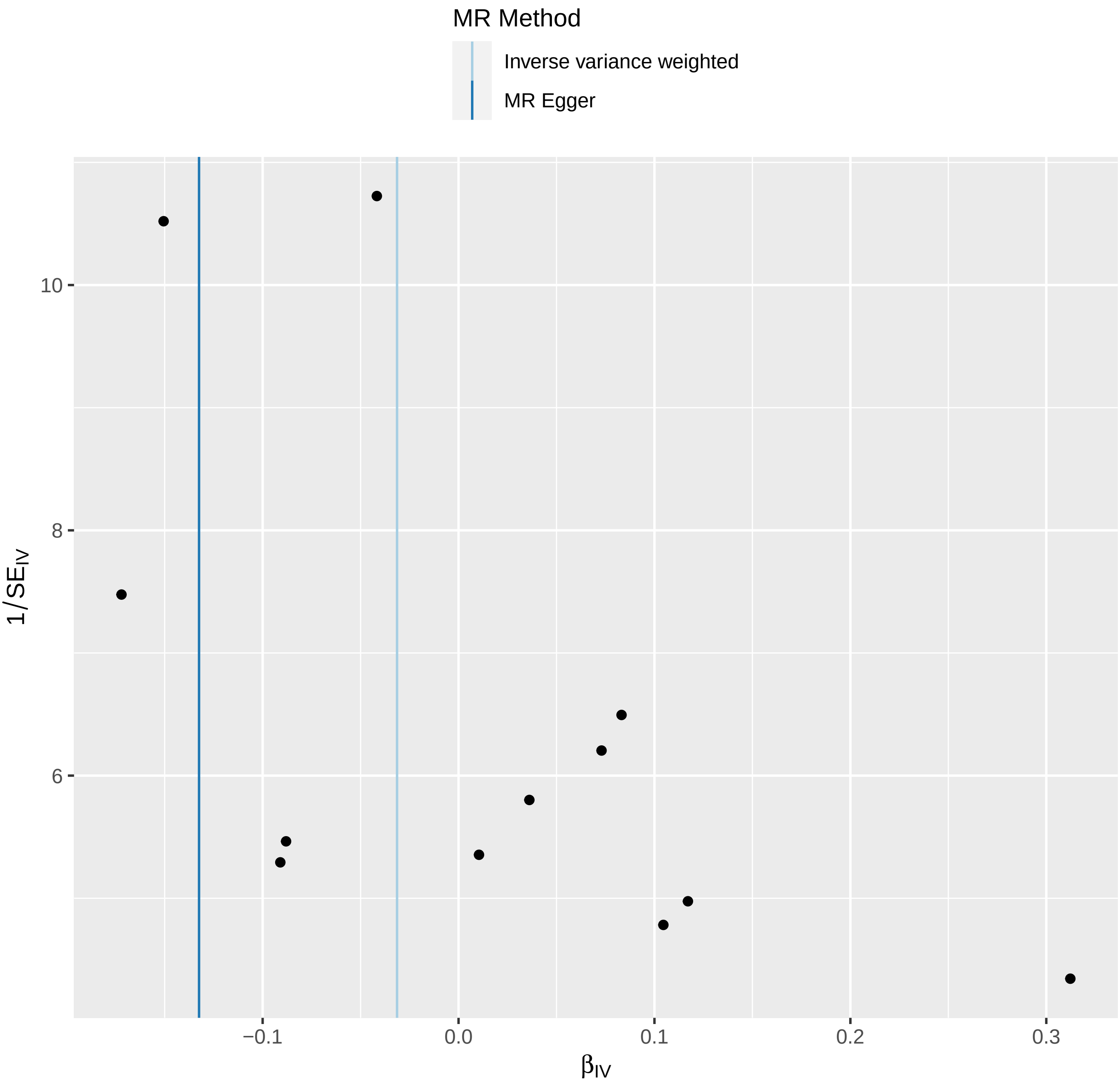

Batch 3−2357 : Selenium on PR domain zinc finger protein 1

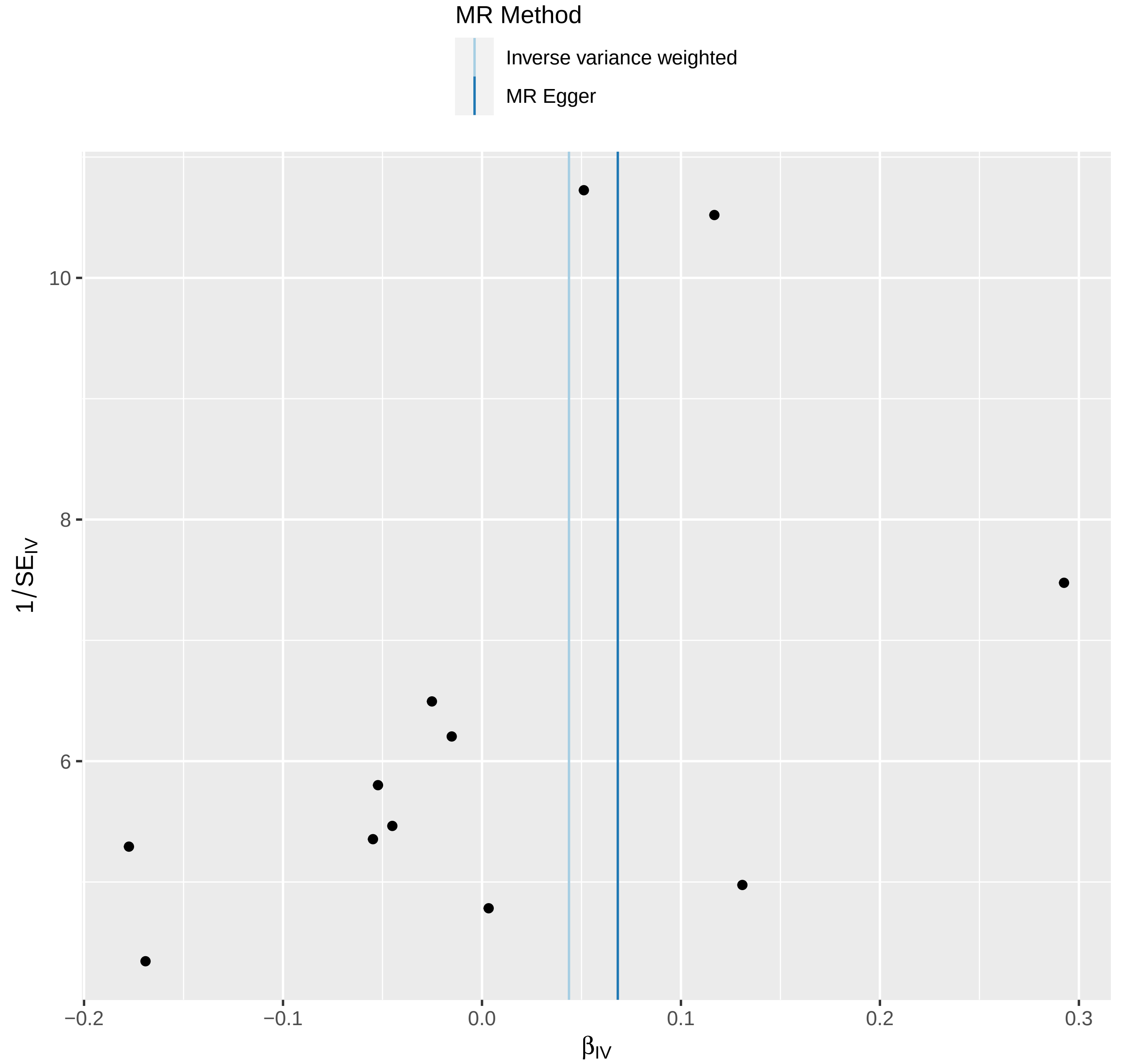

Batch 3−2358 : Selenium on PR domain zinc finger protein 4

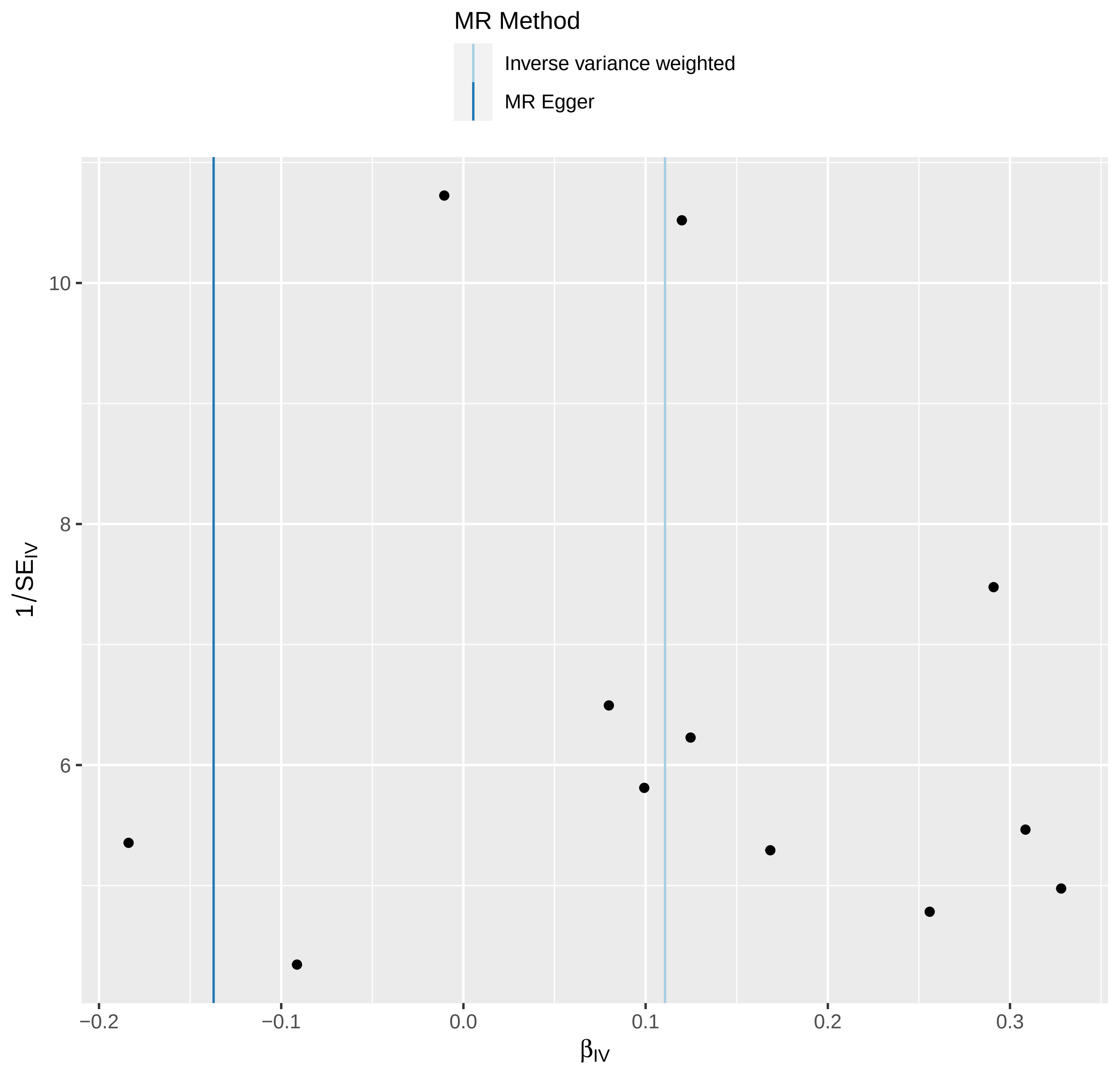

Batch 3−2359 : Selenium on Peroxiredoxin−1

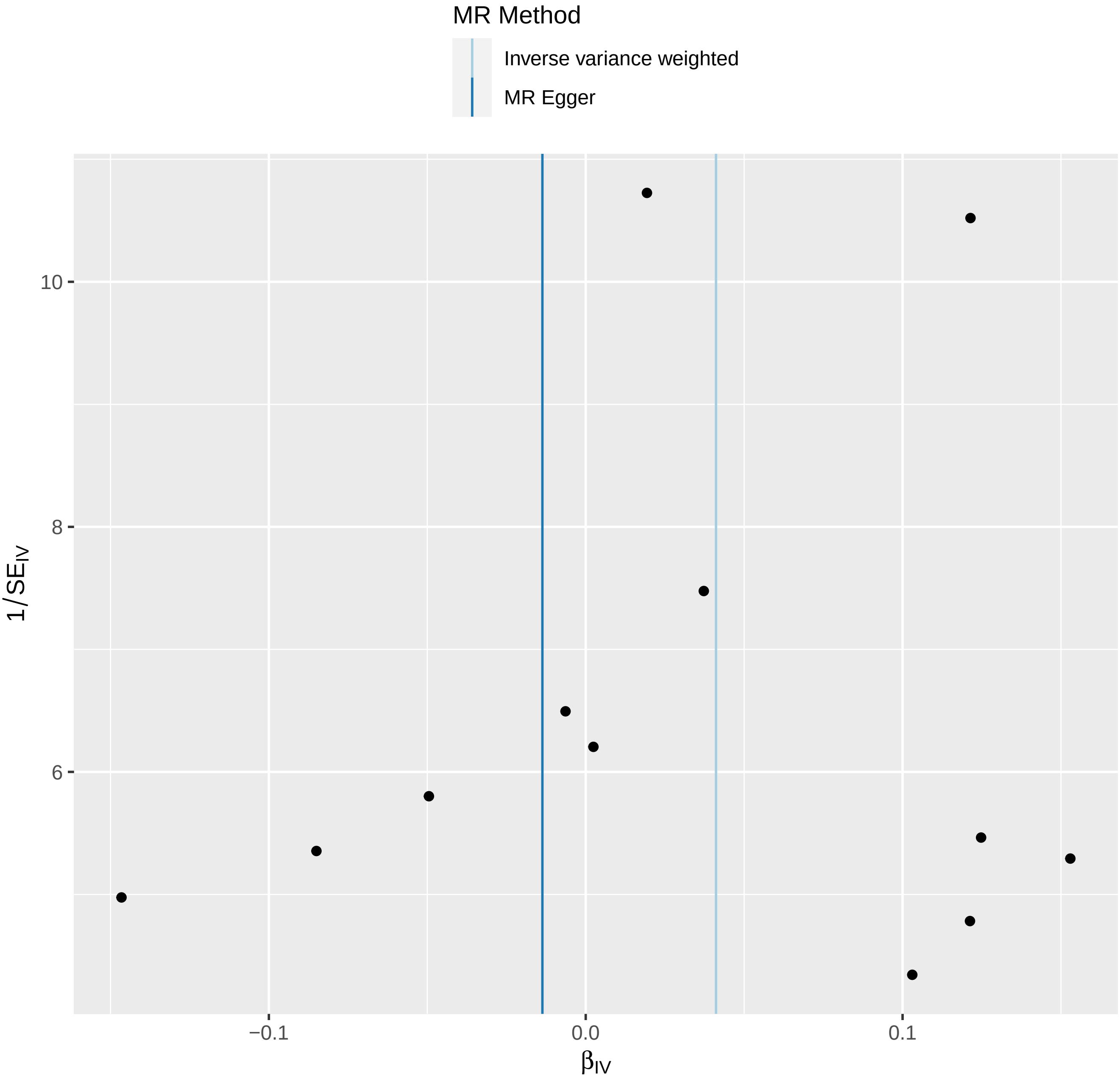

Batch 3−2360 : Selenium on Peroxiredoxin−4

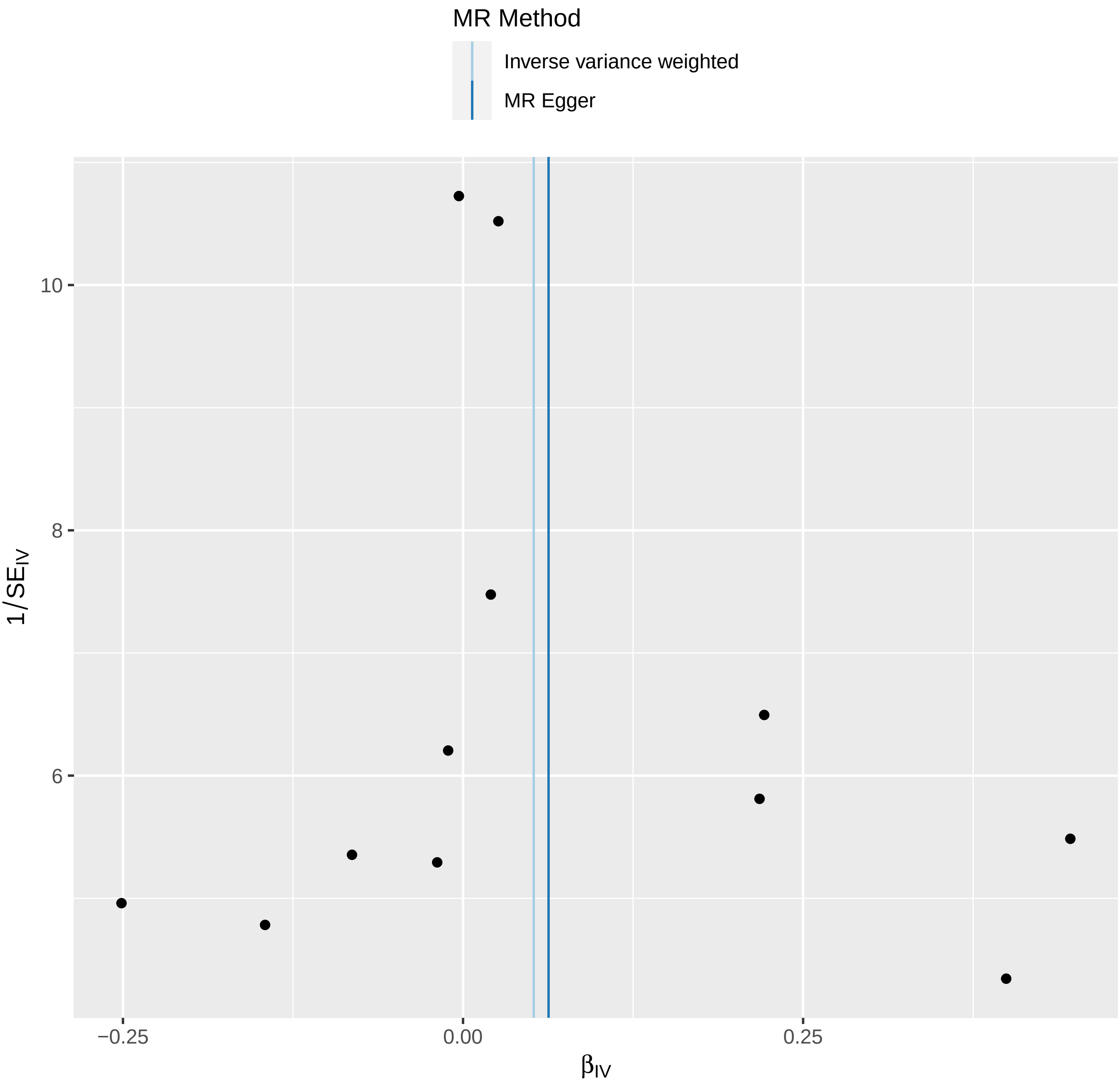

Batch 3−2361 : Selenium on Prolargin

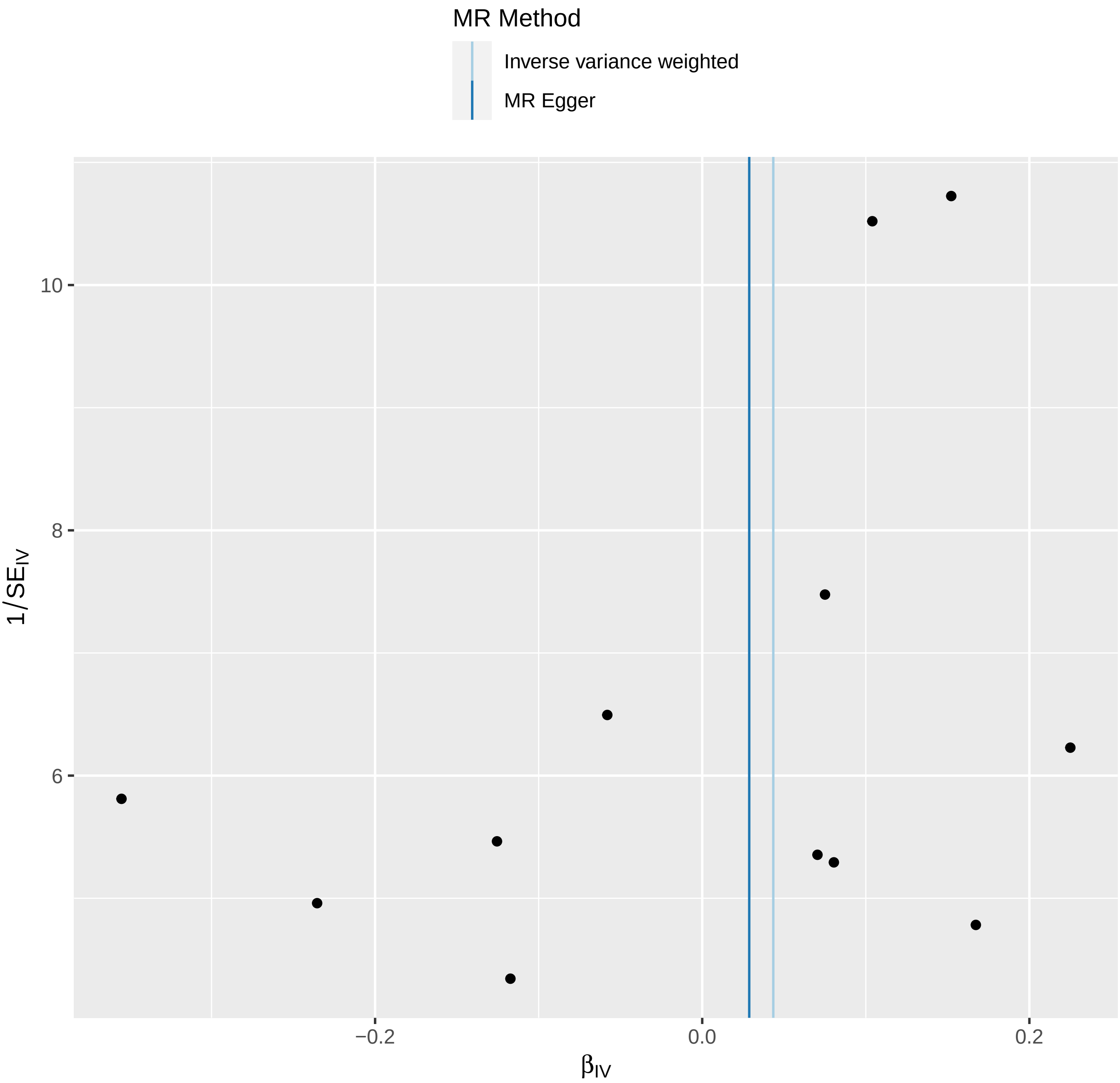

Batch 3−2362 : Selenium on Proteoglycan 3

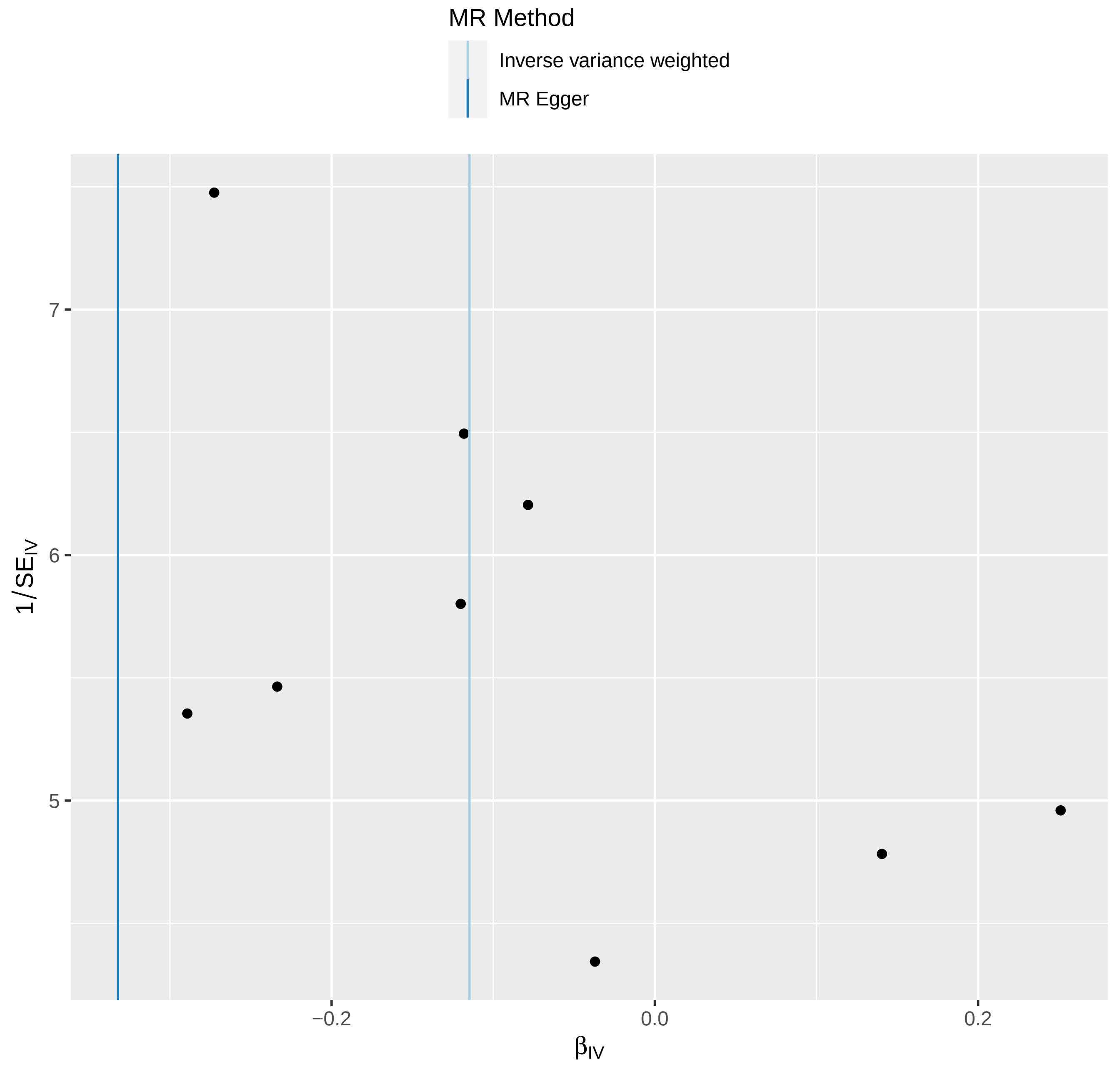

Batch 3−2363 : Selenium on Salivary acidic proline−rich phosphoprotein 1/2

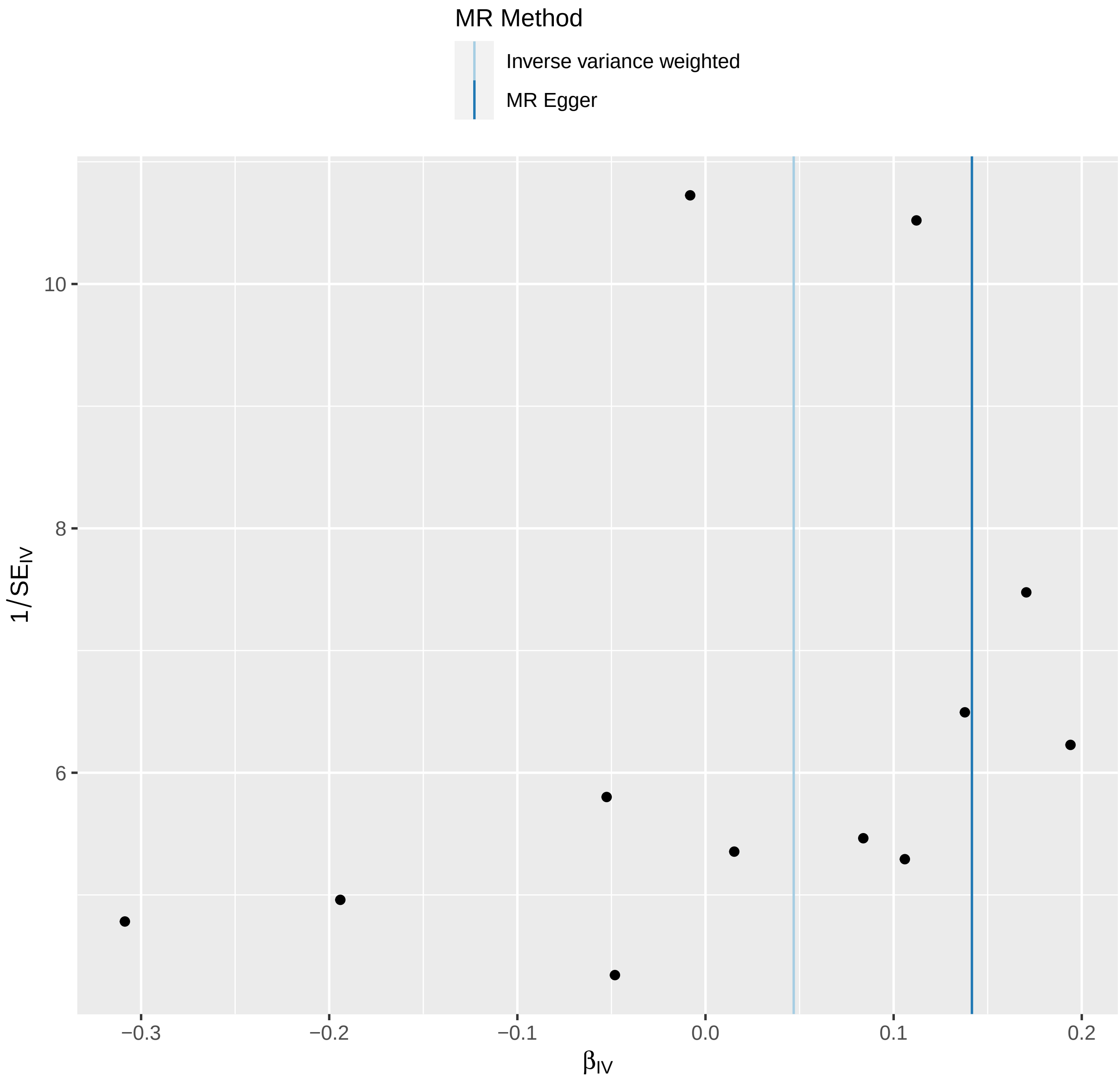

Batch 3−2364 : Selenium on DNA primase small subunit

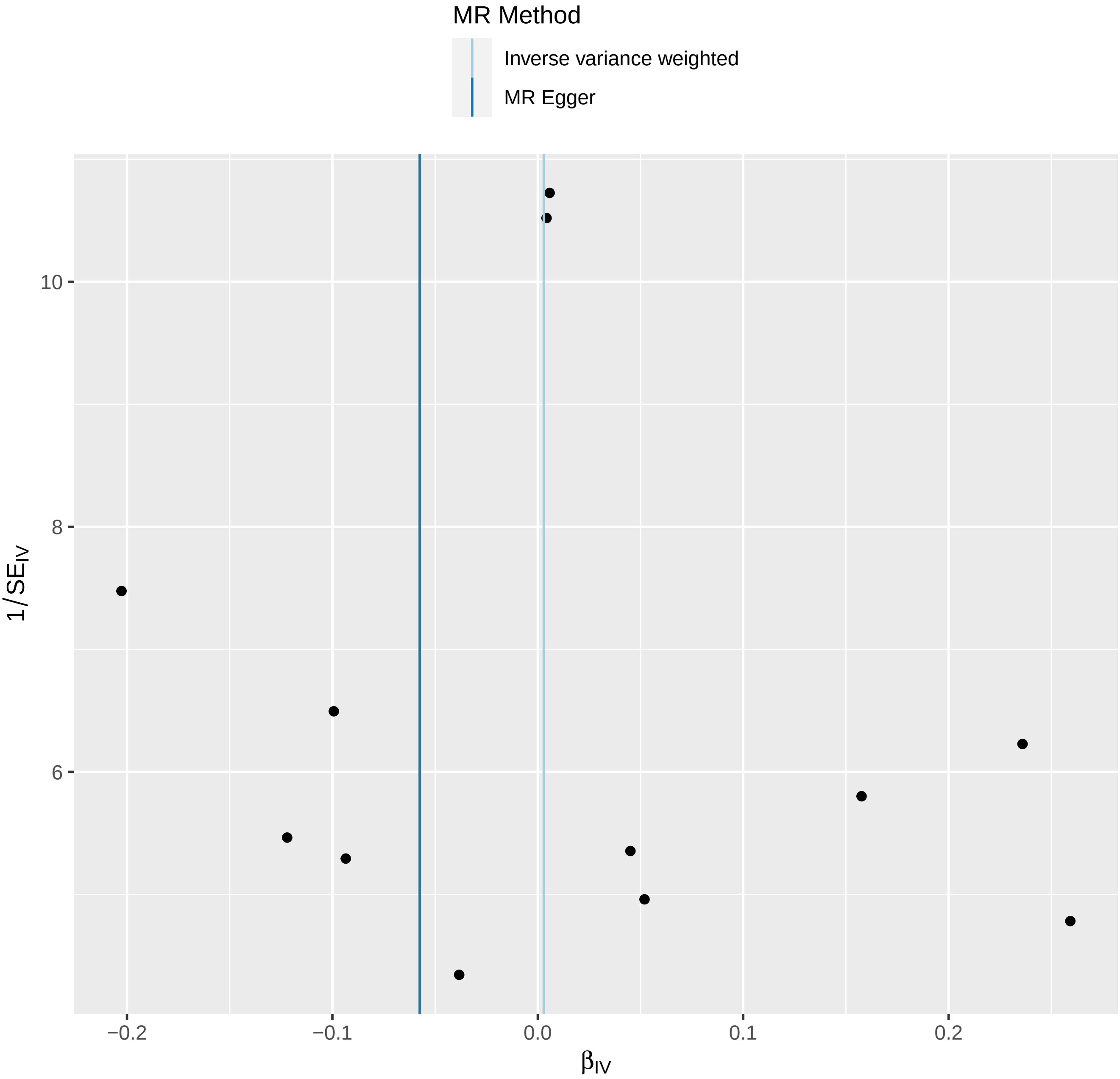

Batch 3−2365 : Selenium on AMP Kinase (alpha1beta1gamma1)

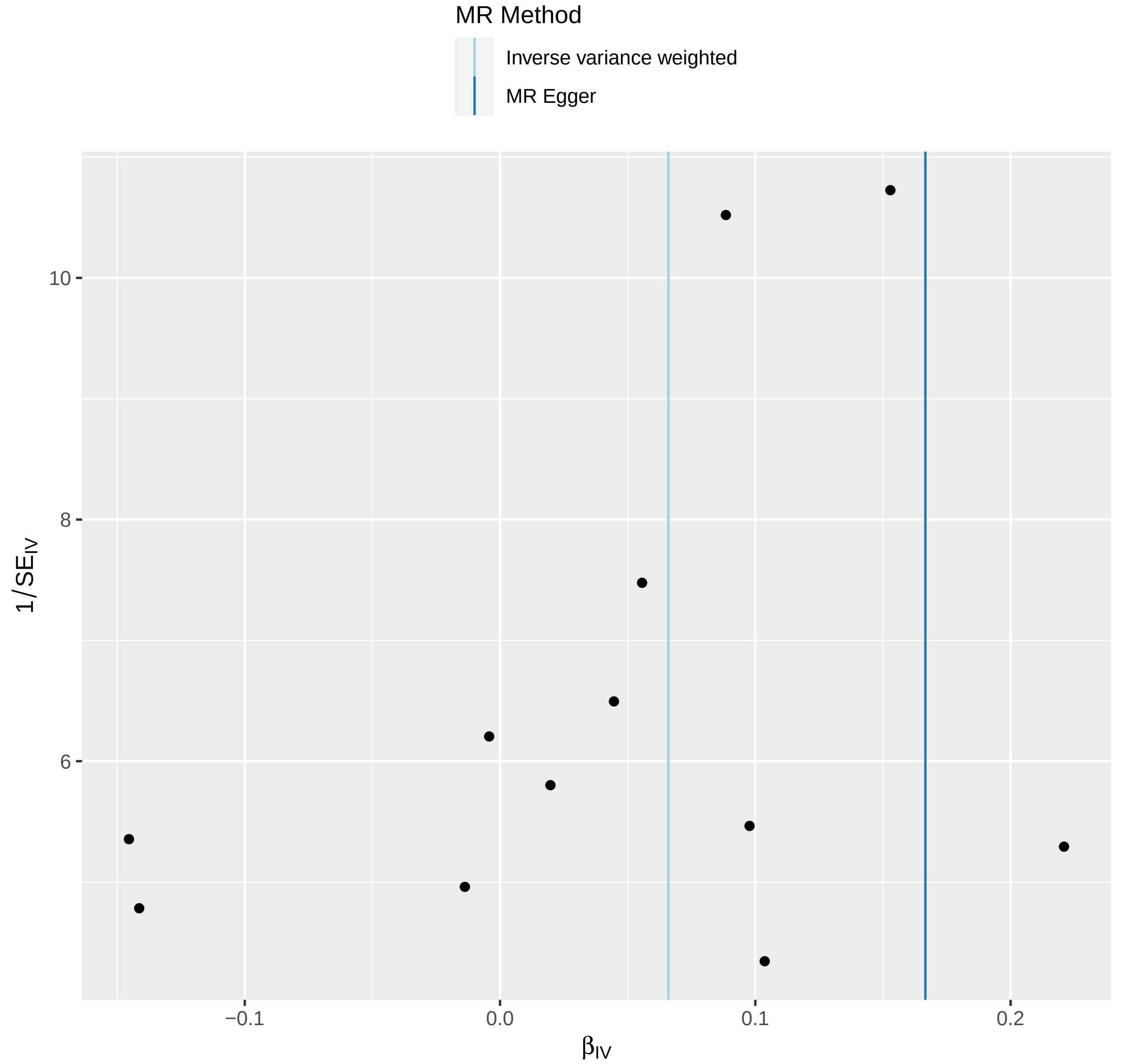

Batch 3−2366 : Selenium on AMP Kinase (alpha2beta2gamma1)

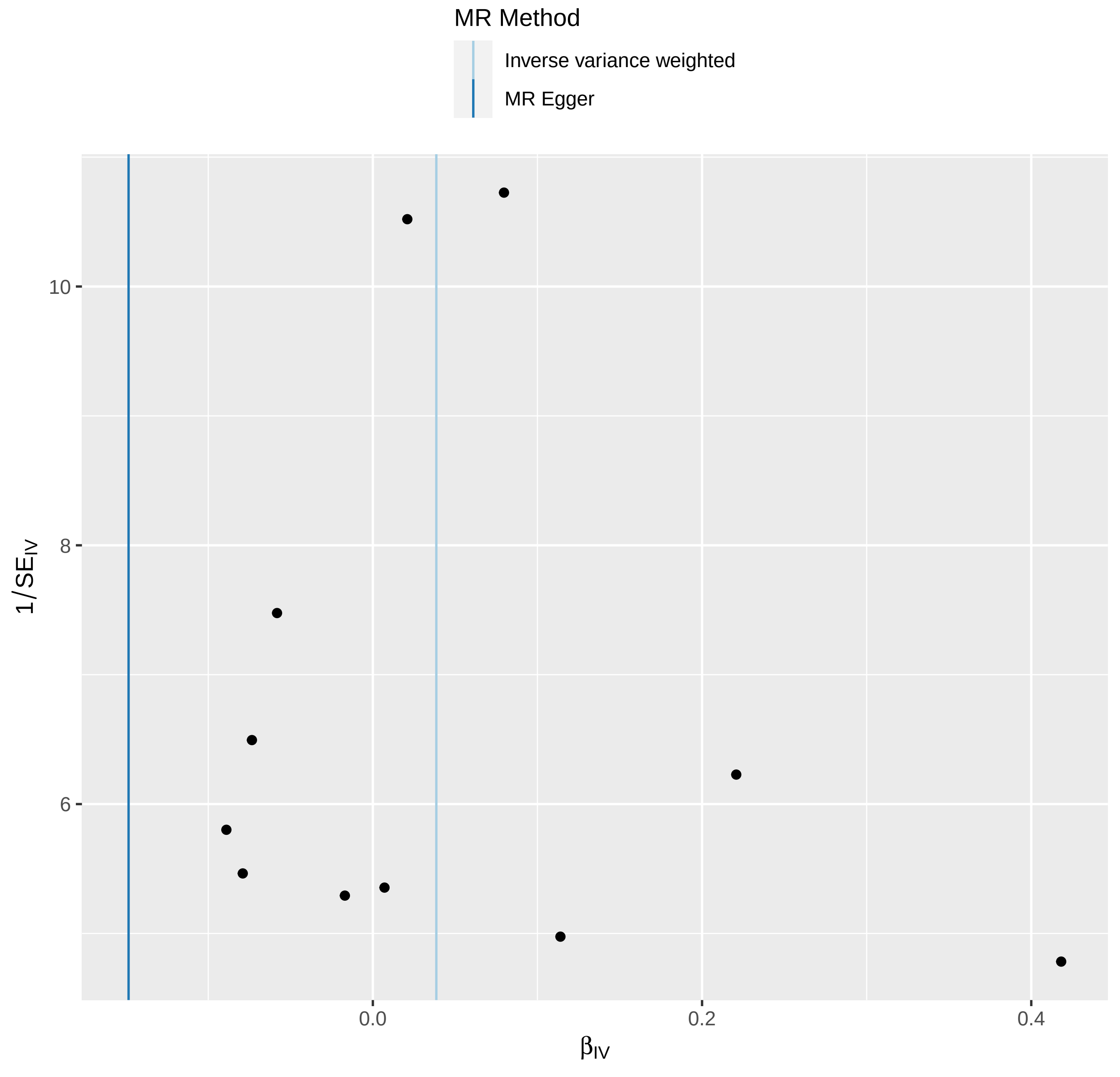

Batch 3−2367 : Selenium on cAMP−dependent protein kinase catalytic subunit alpha

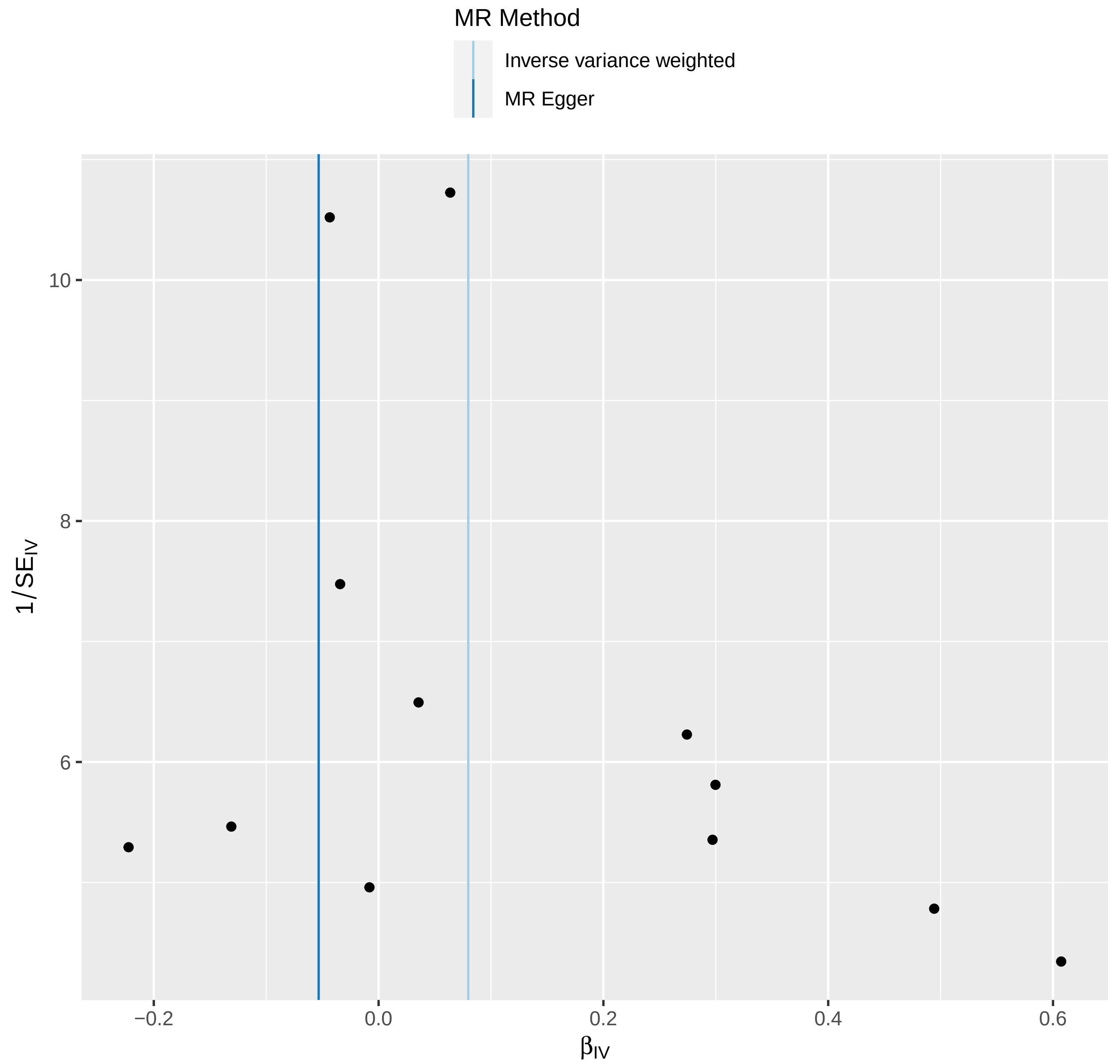

Batch 3−2368 : Selenium on cAMP−dependent protein kinase type I−beta regulatory subunit

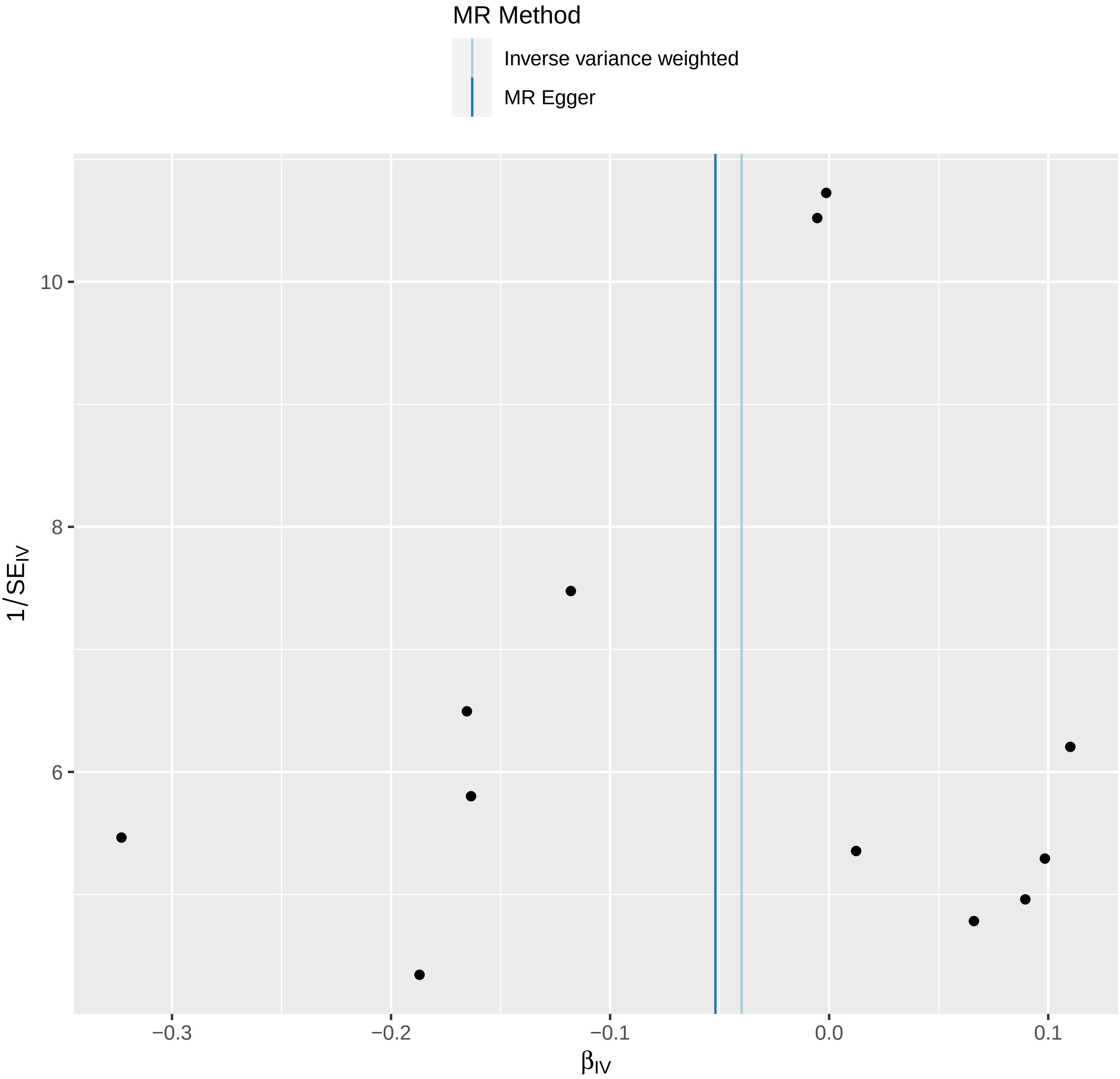

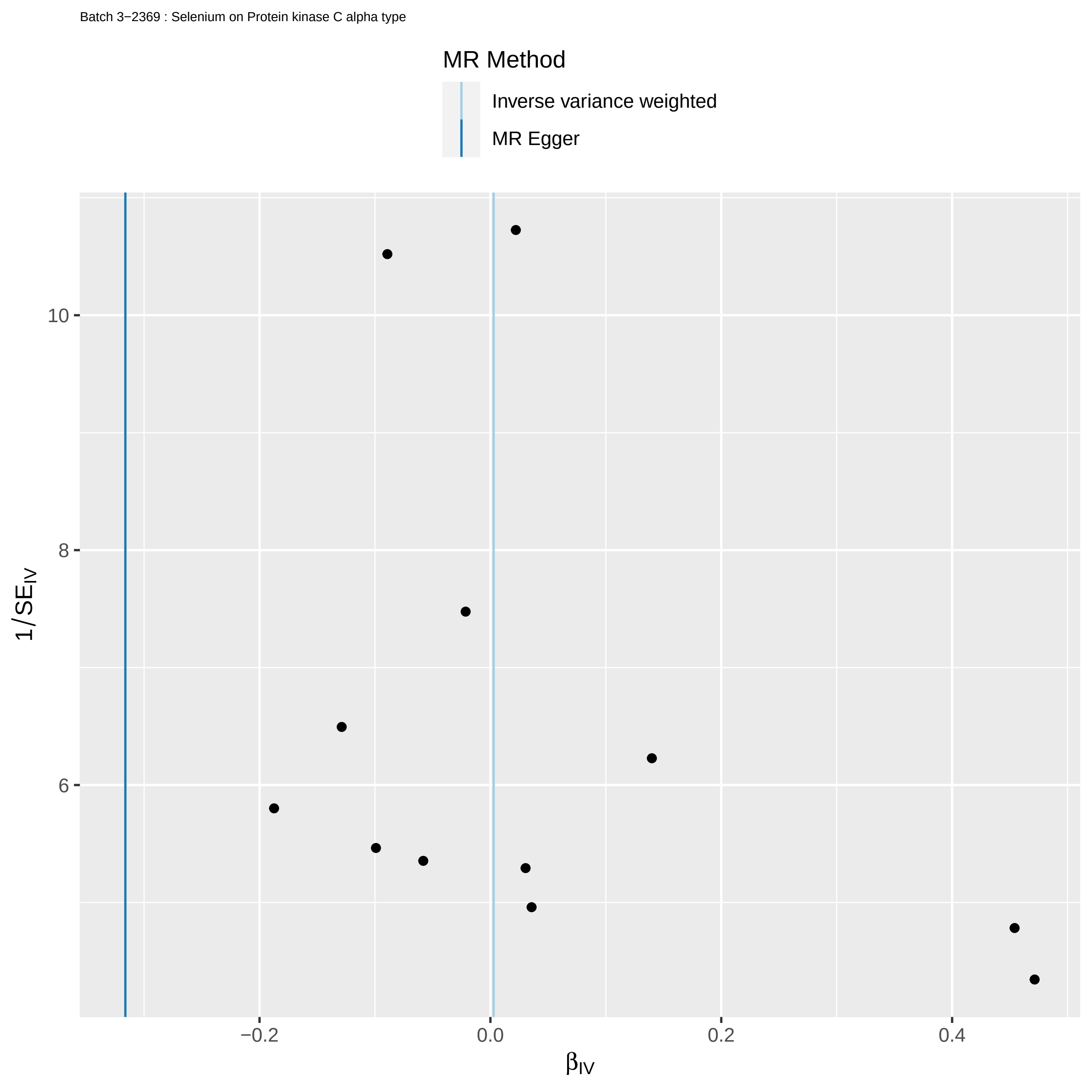
Batch 3−2369 : Selenium on Protein kinase C alpha type
MR Method
Inverse variance weighted
MR Egger
10
8
6
1/SE_IV
−0.2
0.0
0.2
0.4
β_IV

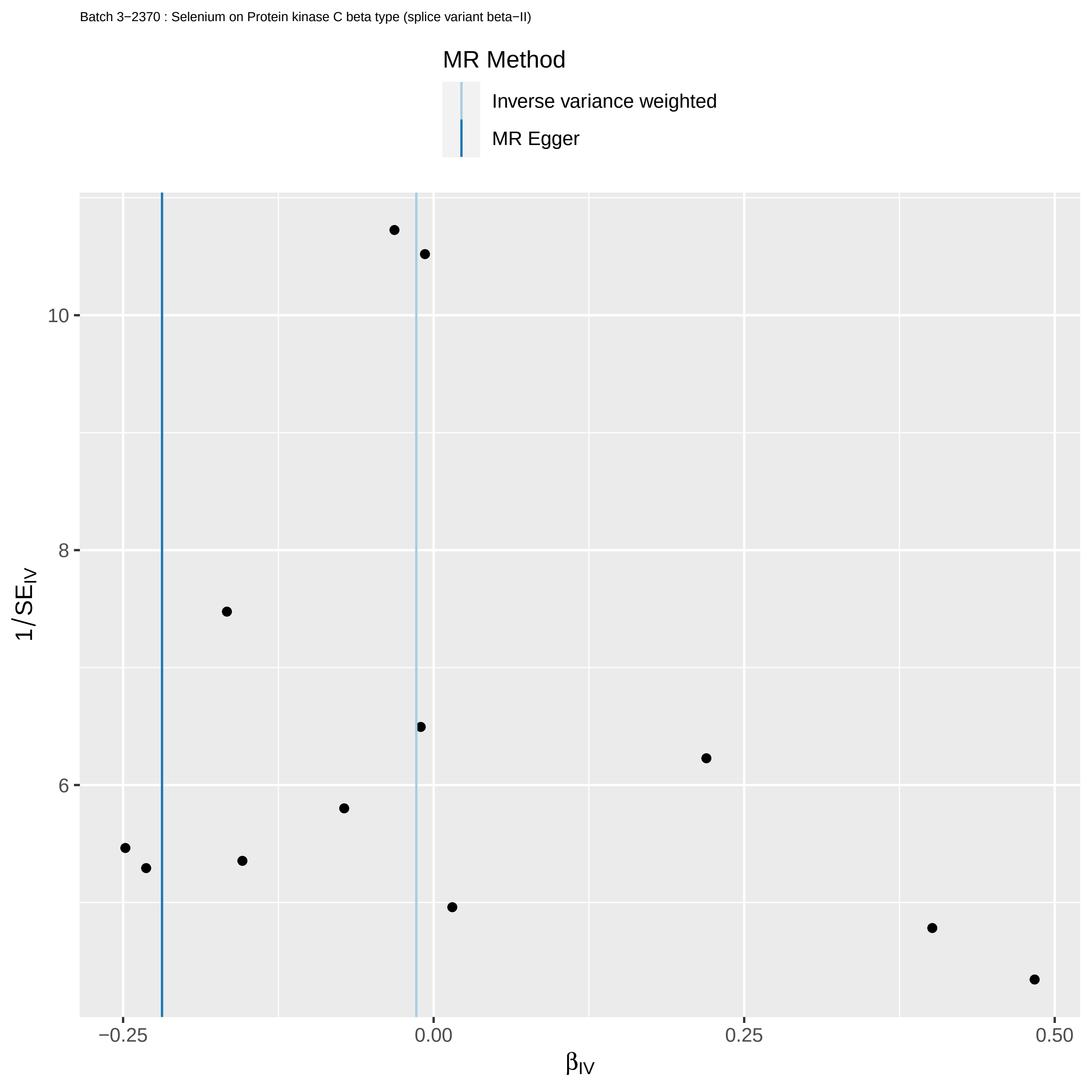
Batch 3−2370 : Selenium on Protein kinase C beta type (splice variant beta−II)
MR Method
Inverse variance weighted
MR Egger
10
8
6
1/SE_IV
−0.25
0.00
0.25
0.50
β_IV

Batch 3−2371 : Selenium on Protein kinase C iota type

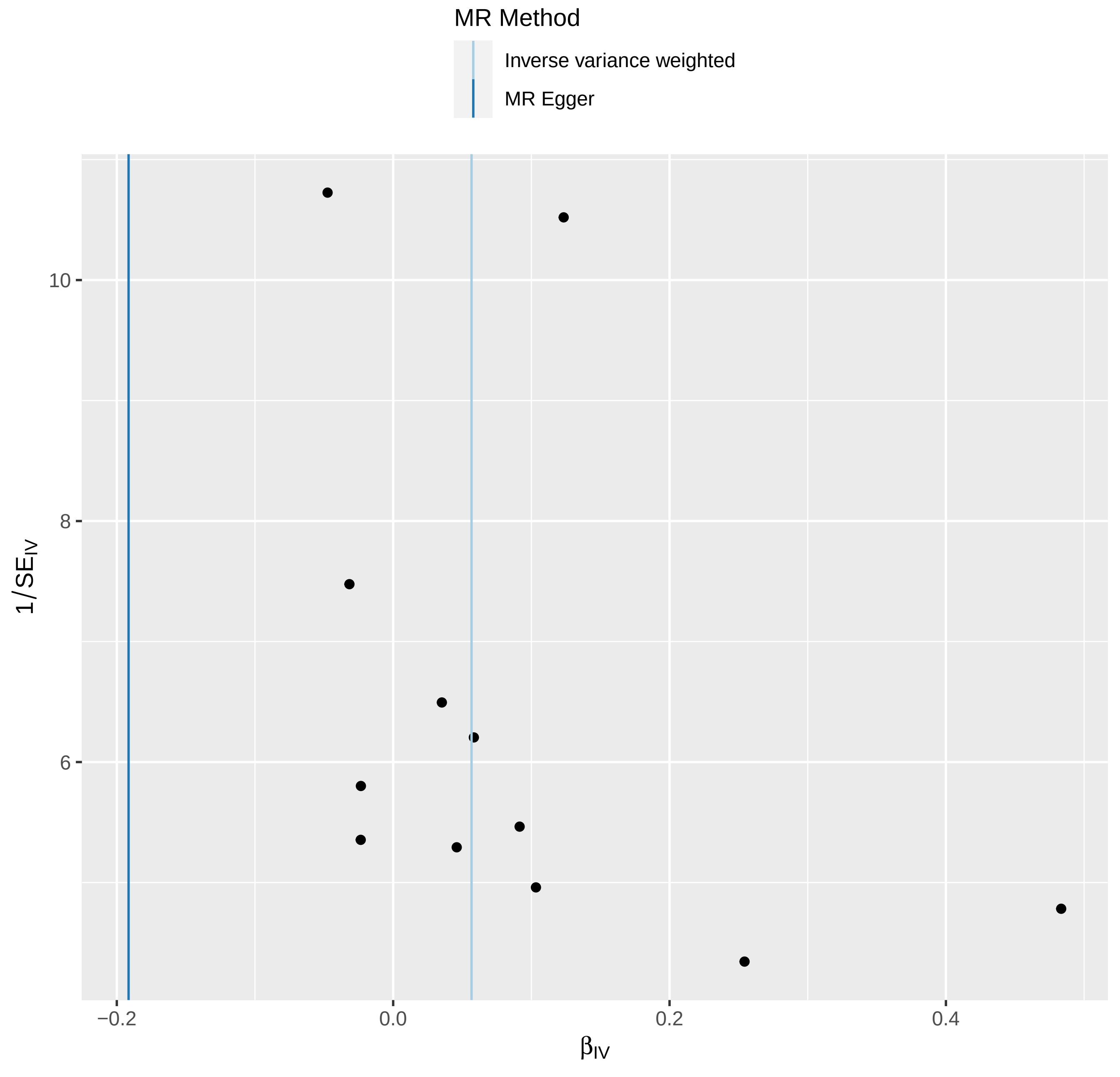

Batch 3−2372 : Selenium on Glucosidase 2 subunit beta

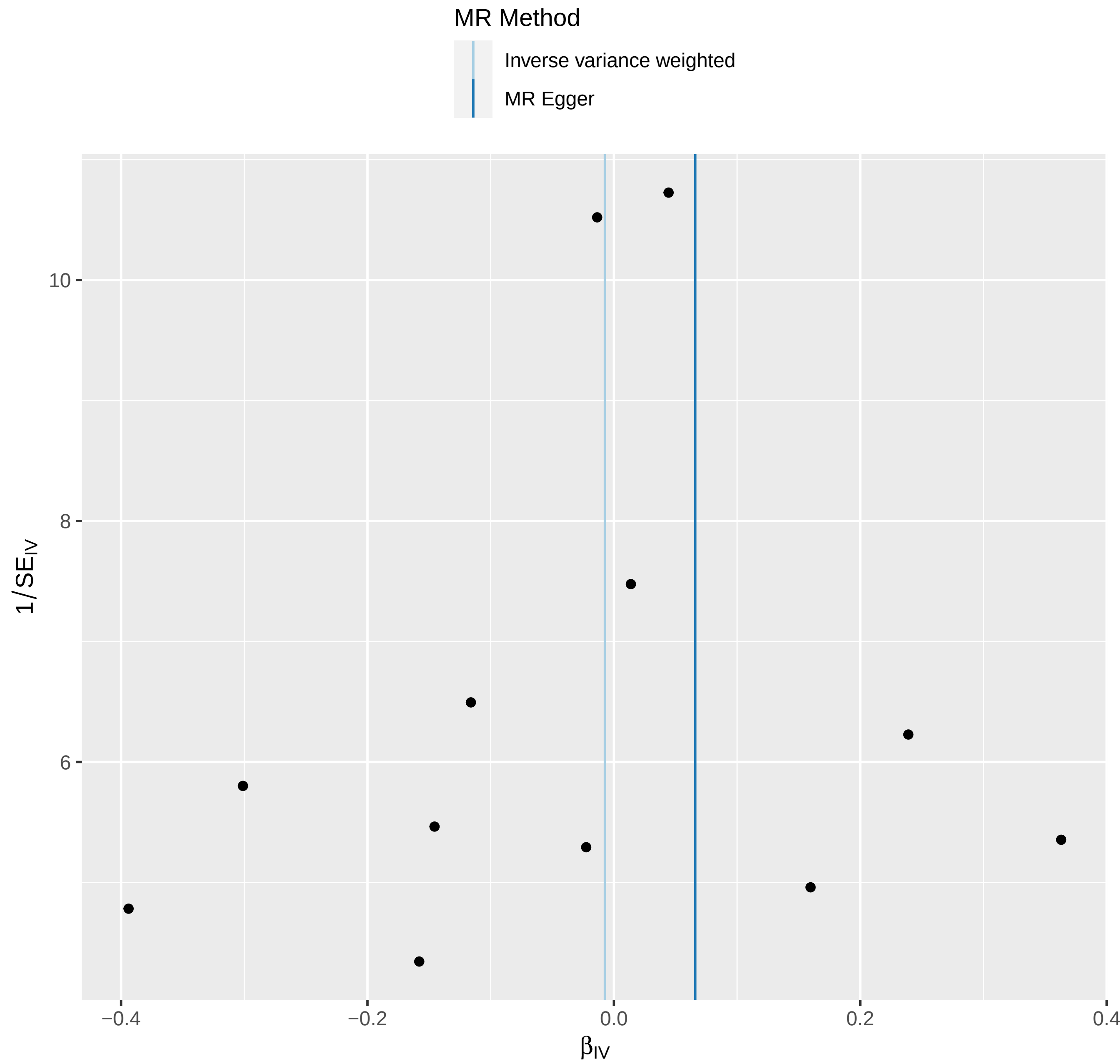

Batch 3−2373 : Selenium on Protein kinase C zeta type

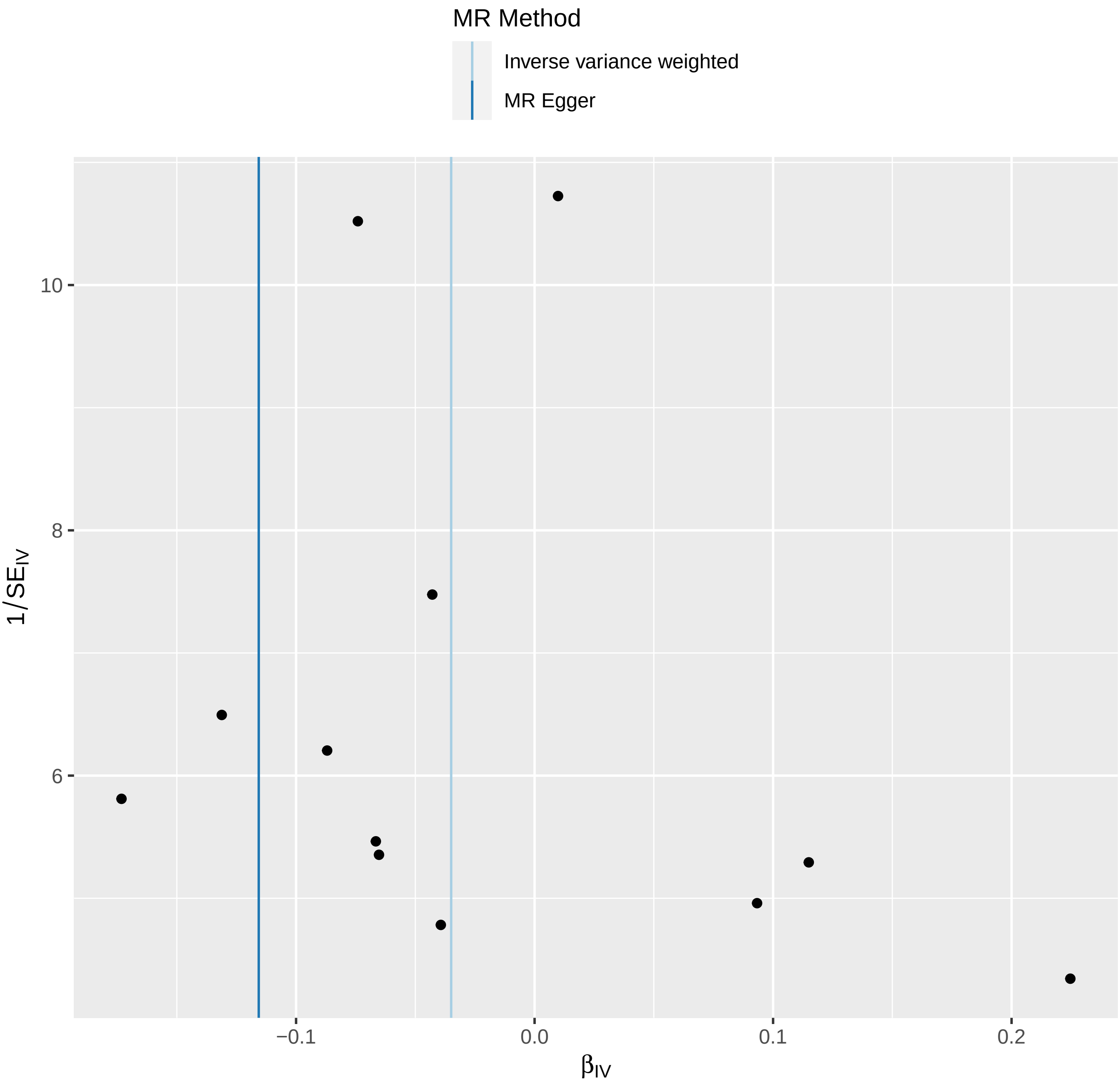

Batch 3−2374 : Selenium on cGMP−dependent protein kinase 1, beta isozyme

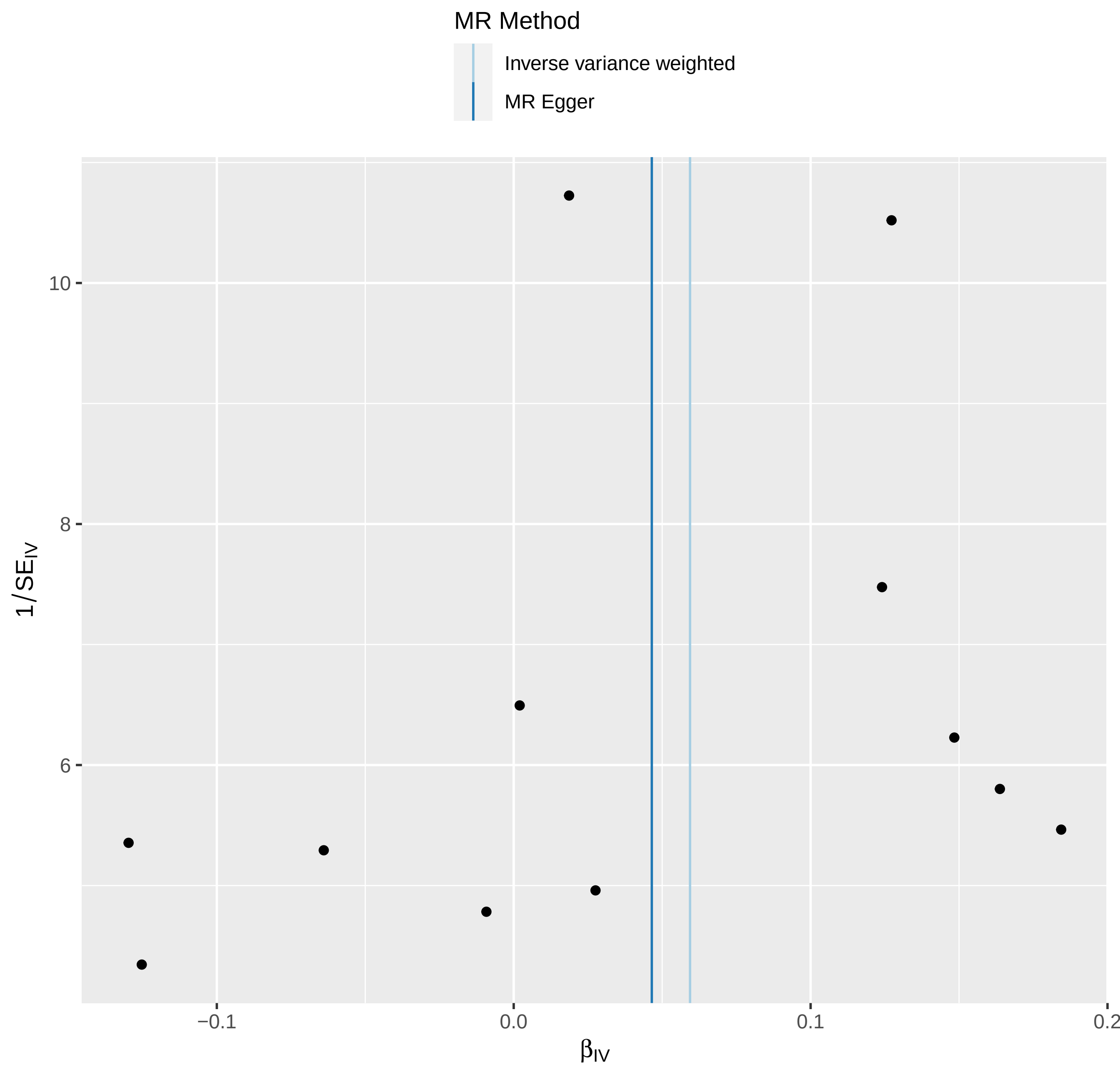

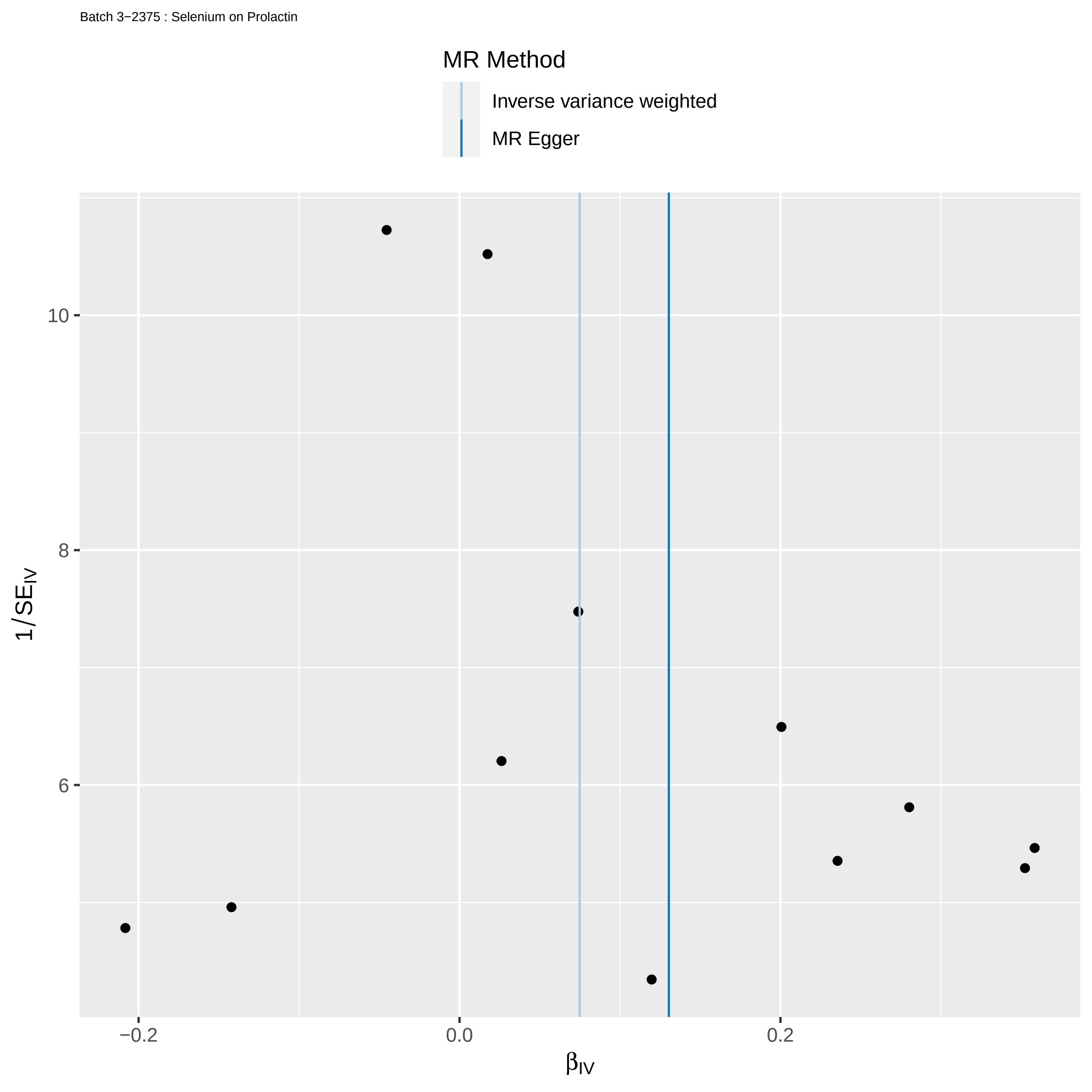
Batch 3−2375 : Selenium on Prolactin
MR Method
Inverse variance weighted
MR Egger
10
8
6
1/SE$_{IV}$
−0.2
0.0
0.2
β$_{IV}$

Batch 3−2376 : Selenium on Prolactin−releasing peptide

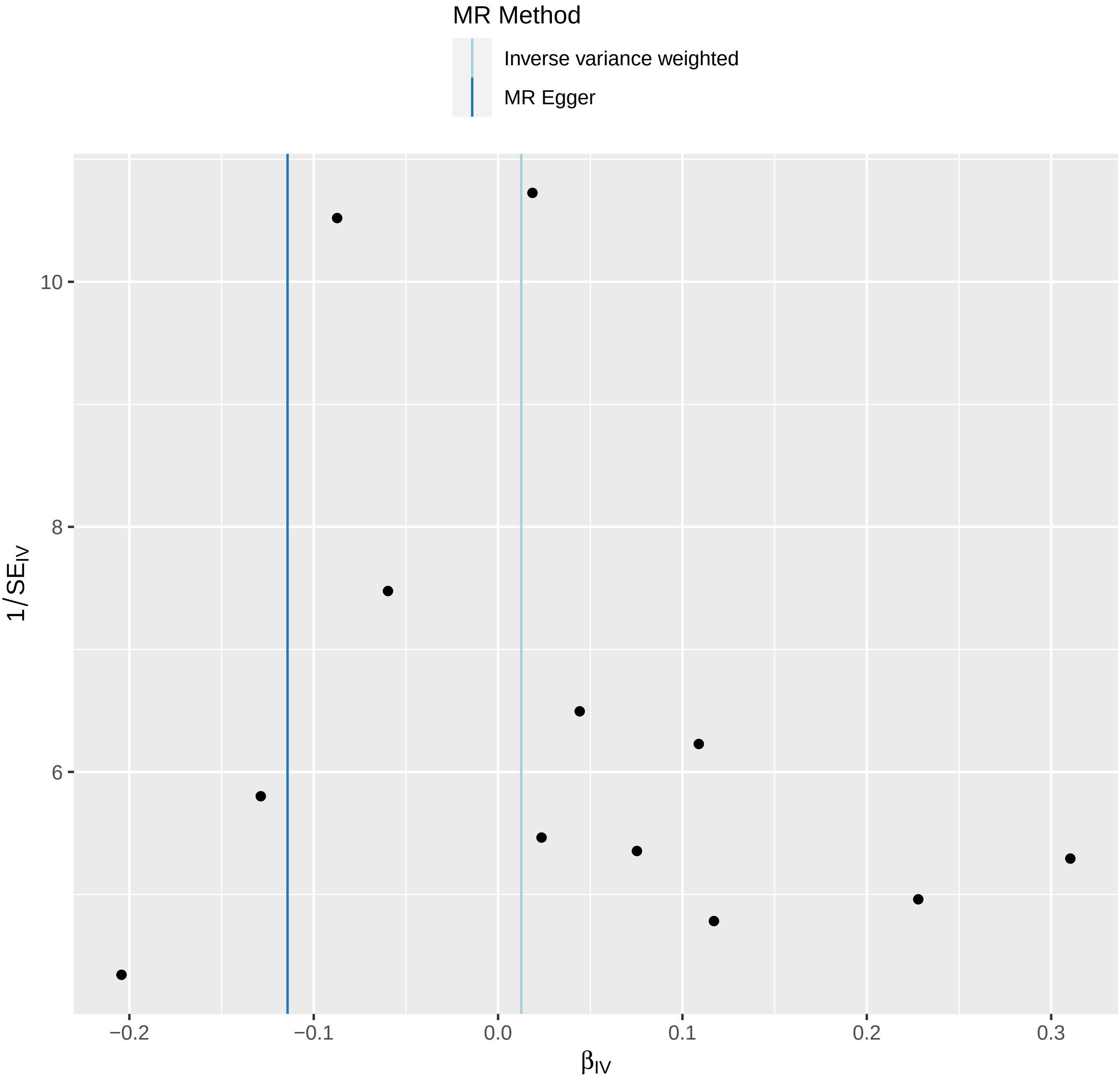

Batch 3−2377 : Selenium on Prolactin receptor

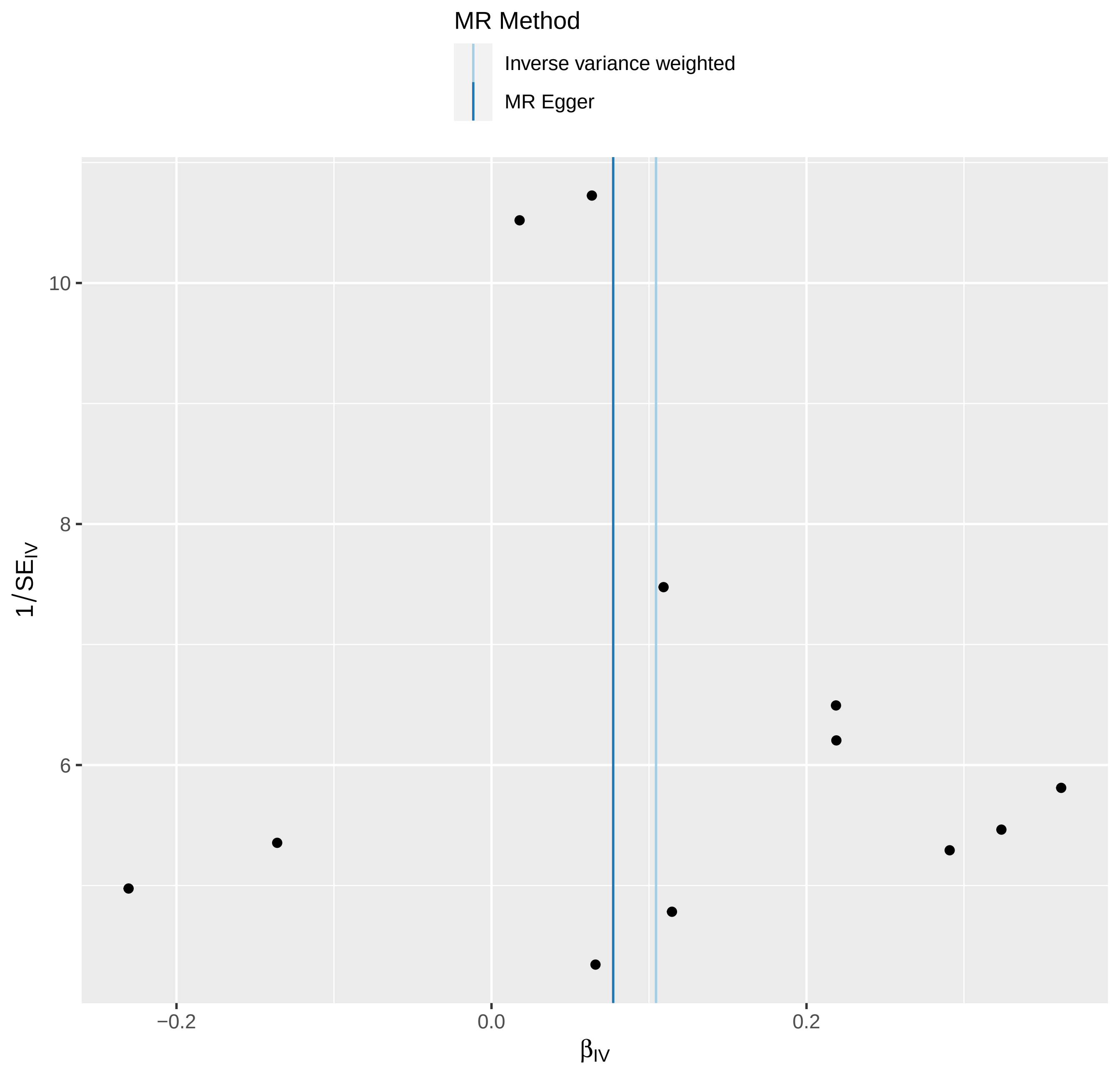

Batch 3−2378 : Selenium on Protein arginine N−methyltransferase 3

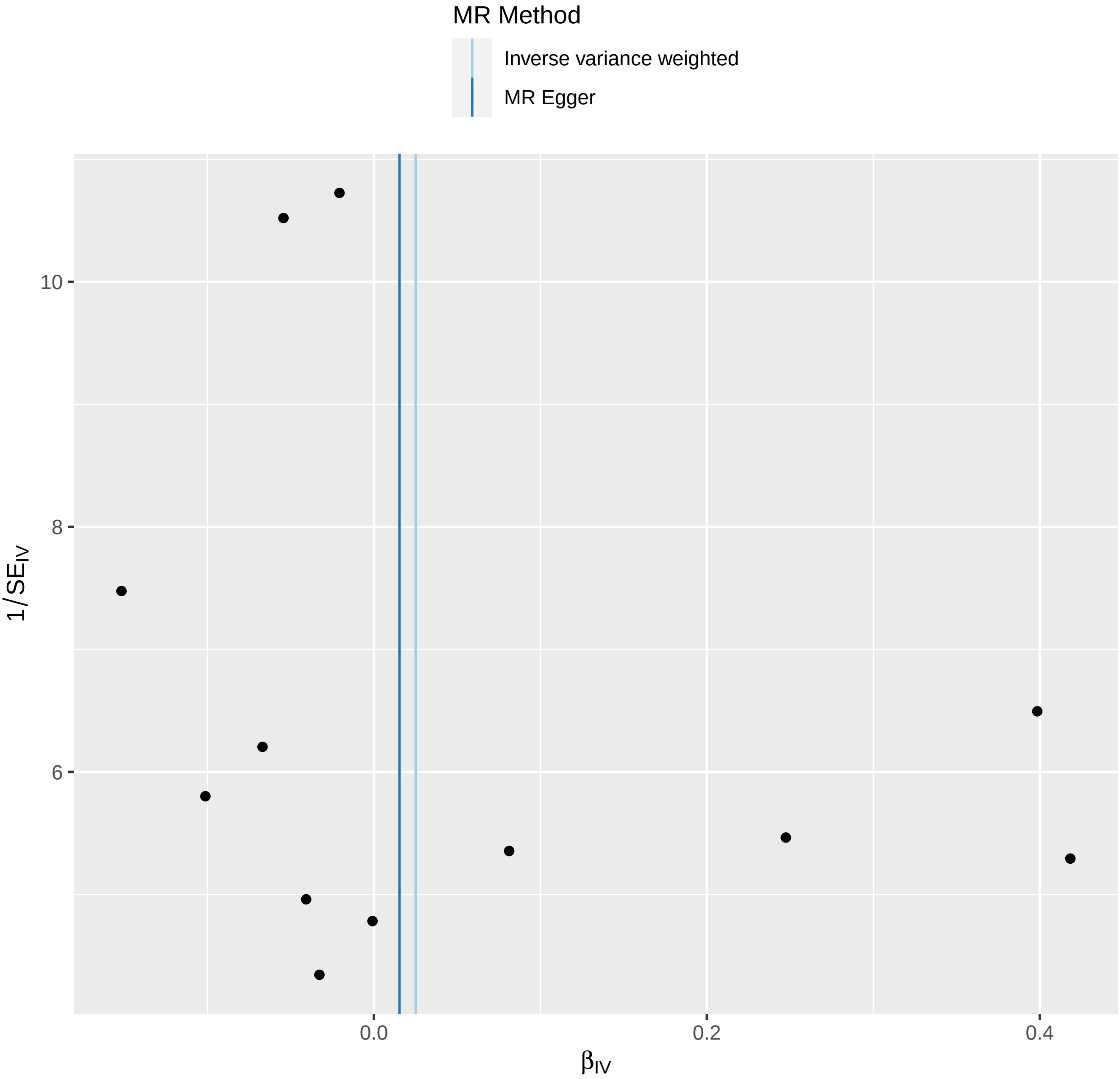

Batch 3−2379 : Selenium on Major prion protein

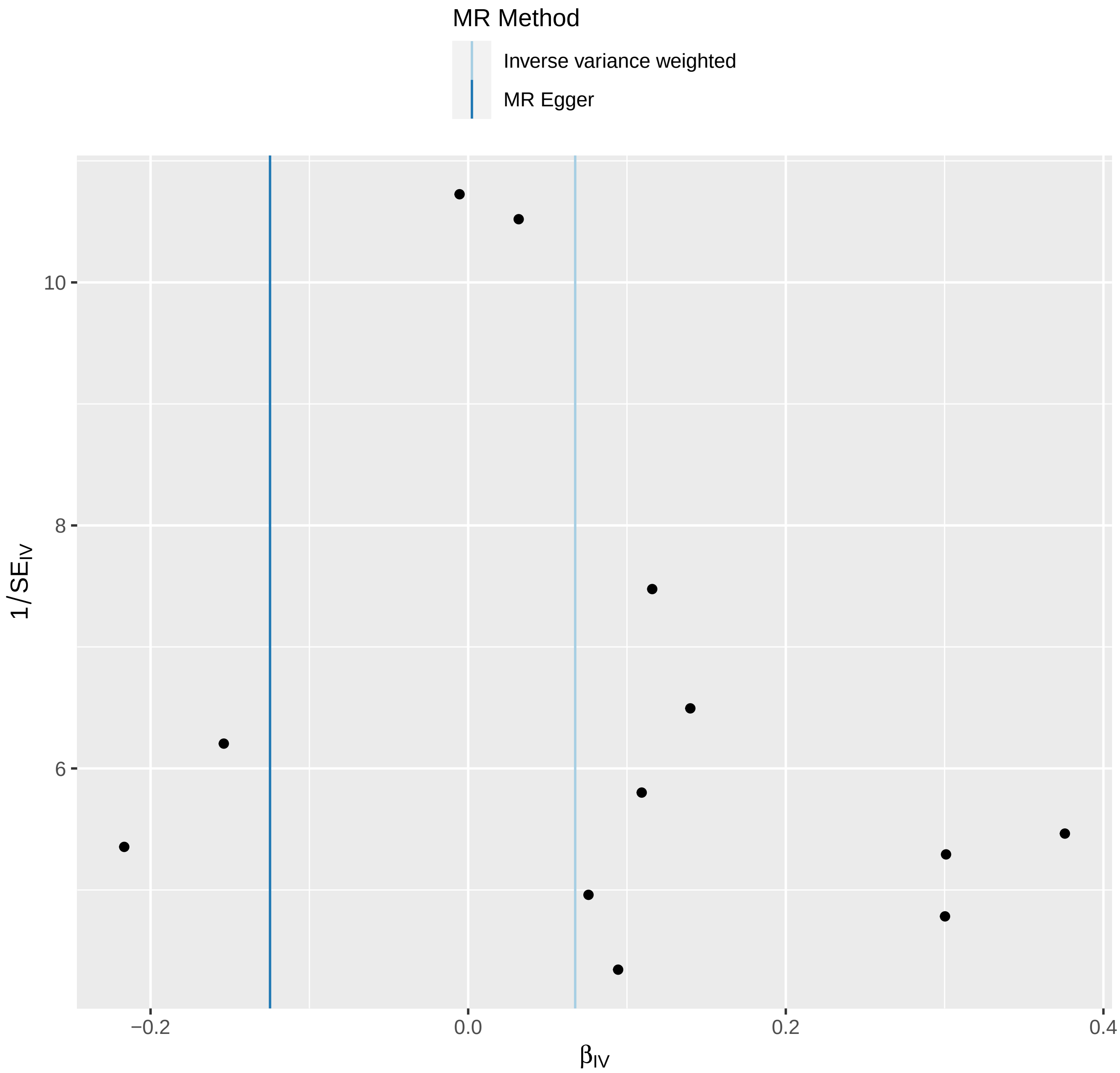

Batch 3−2380 : Selenium on Activated Protein C

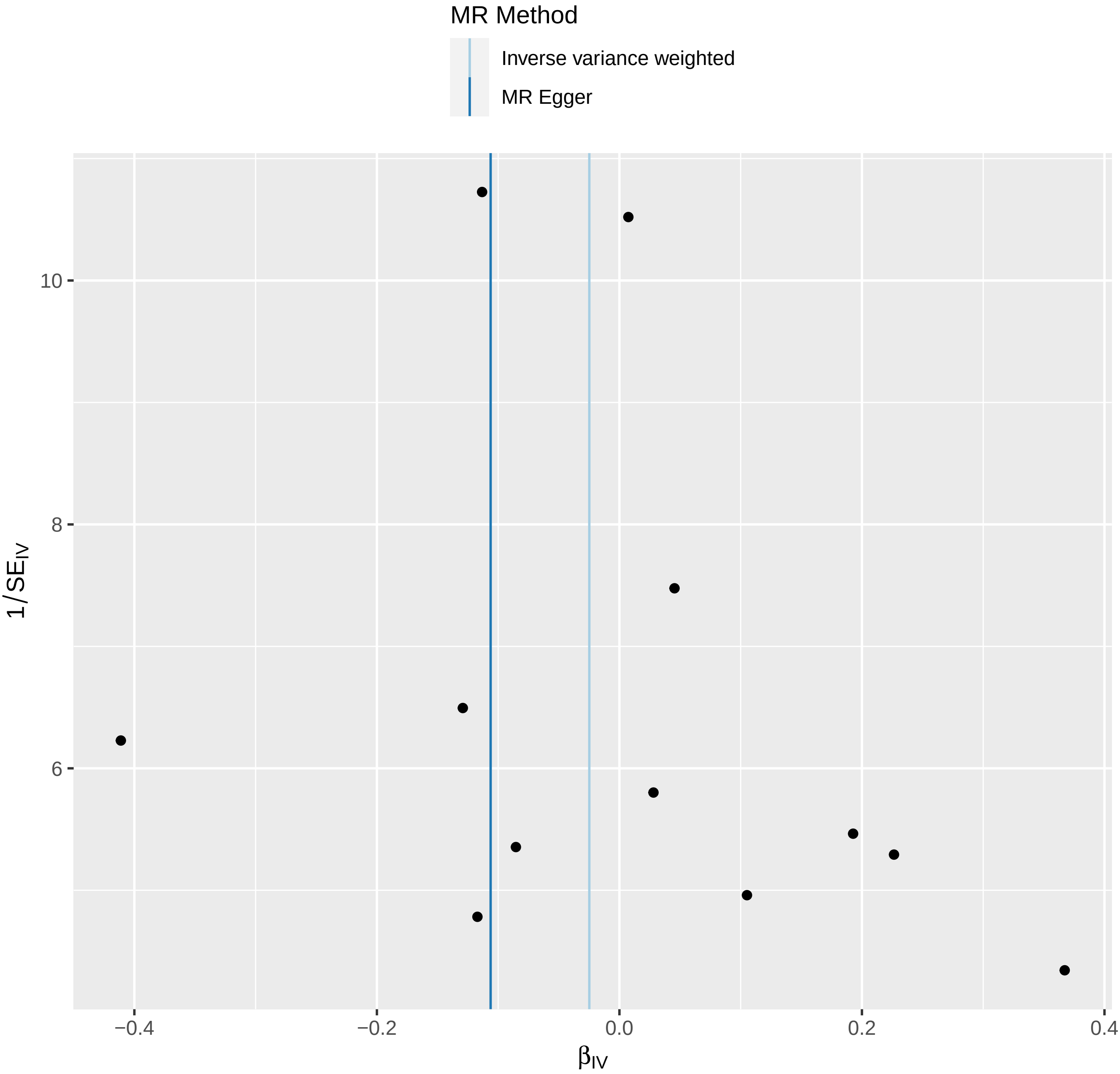

Batch 3−2381 : Selenium on Activated Protein C

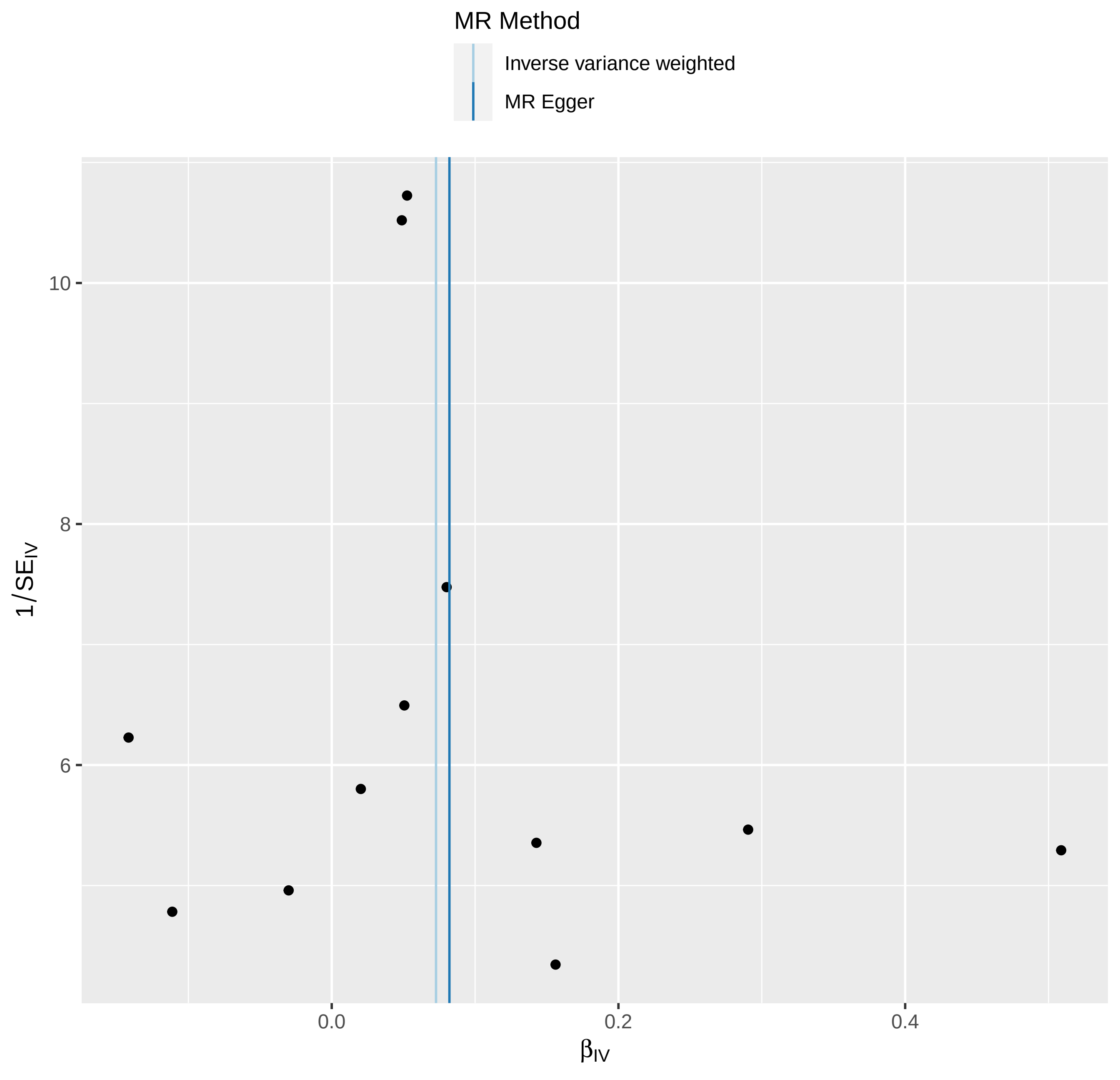

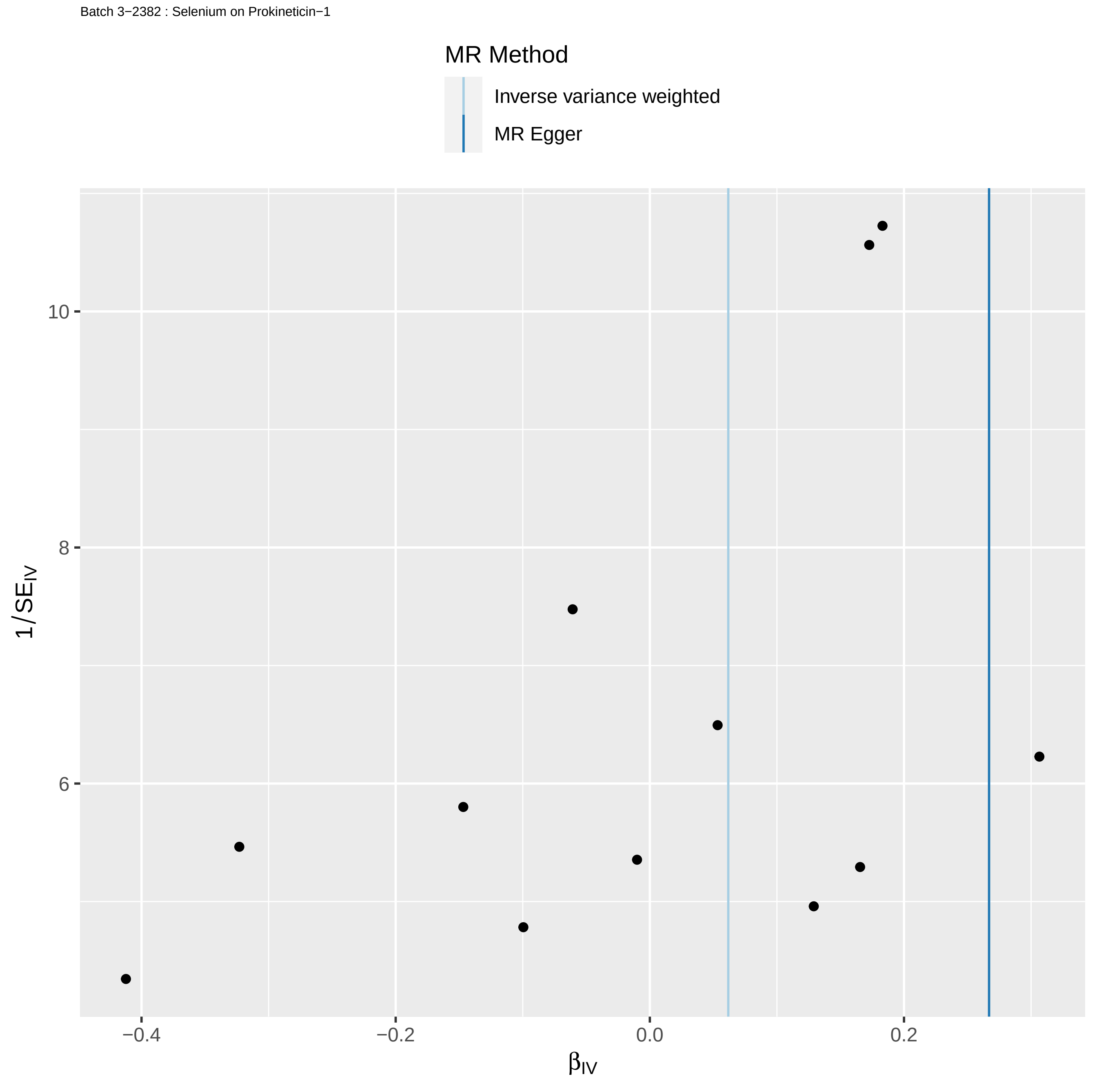

Batch 3−2382 : Selenium on Prokineticin−1
MR Method
Inverse variance weighted
MR Egger
10
8
6
1/SE$_{IV}$
−0.4
−0.2
0.0
0.2
β_{IV}

Batch 3−2383 : Selenium on Proline−rich protein 1

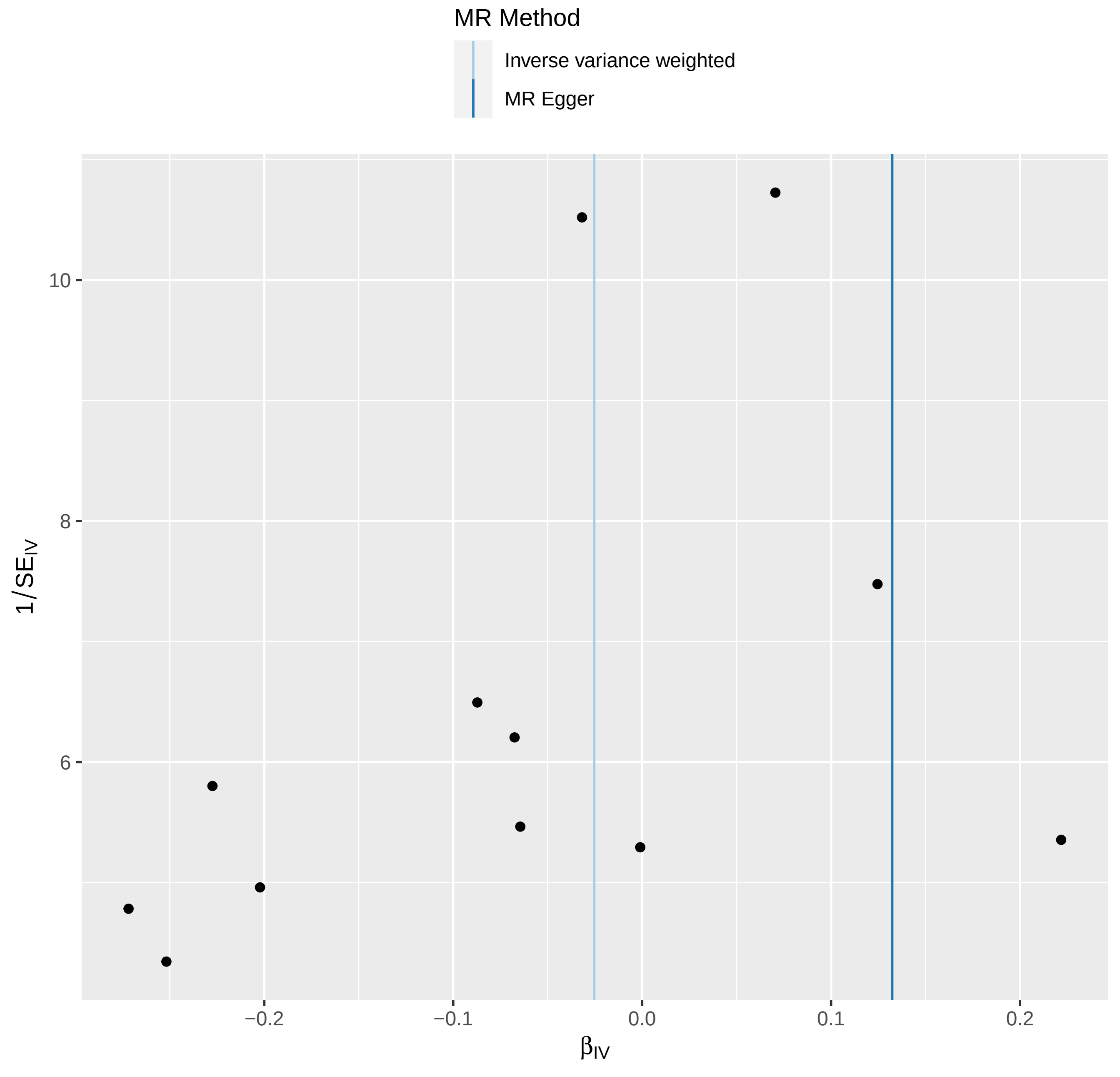

Batch 3−2384 : Selenium on Pre−mRNA−processing factor 6

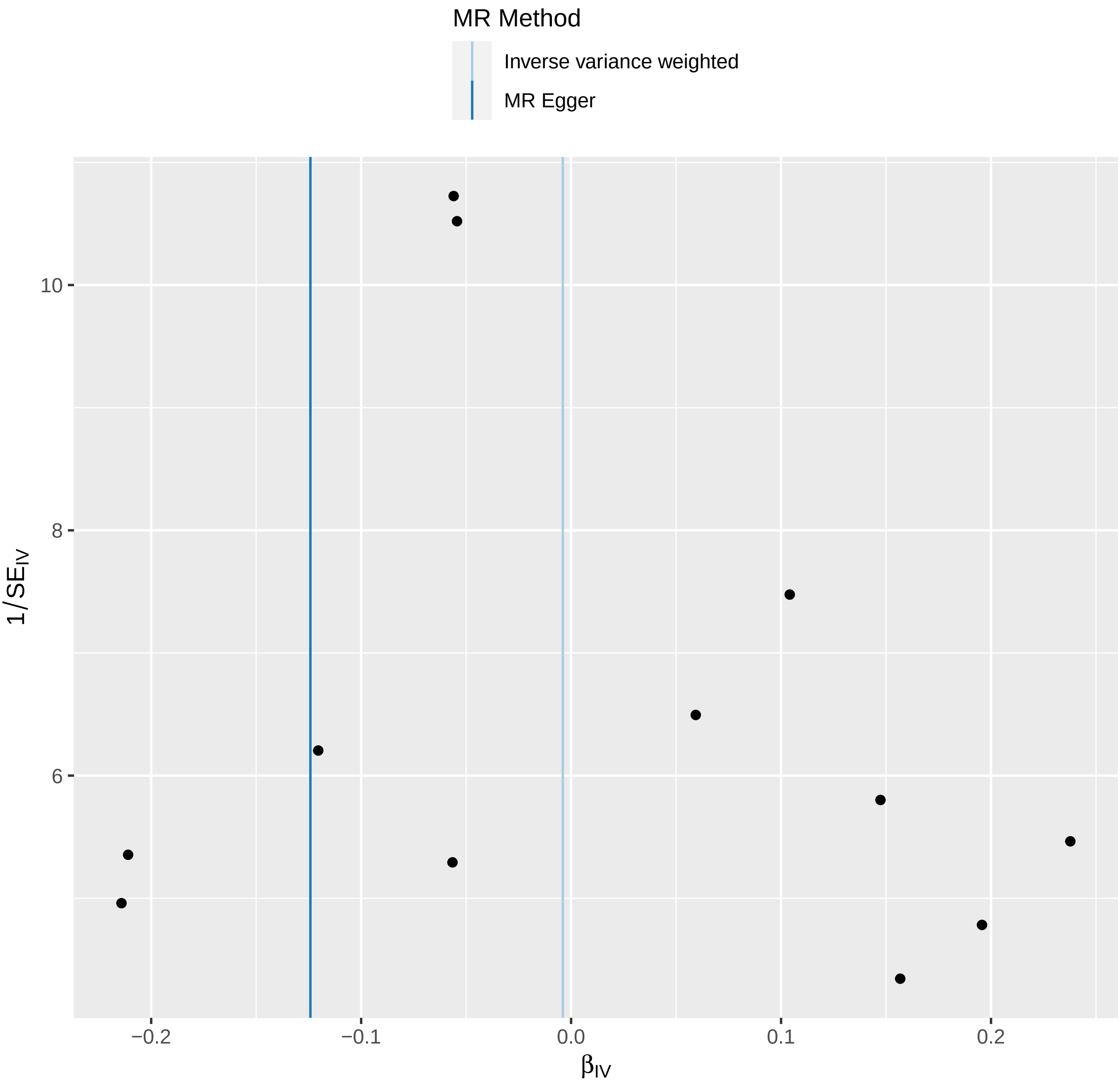

Batch 3−2385 : Selenium on Phosphoribosyl pyrophosphate synthase−associated protein 1

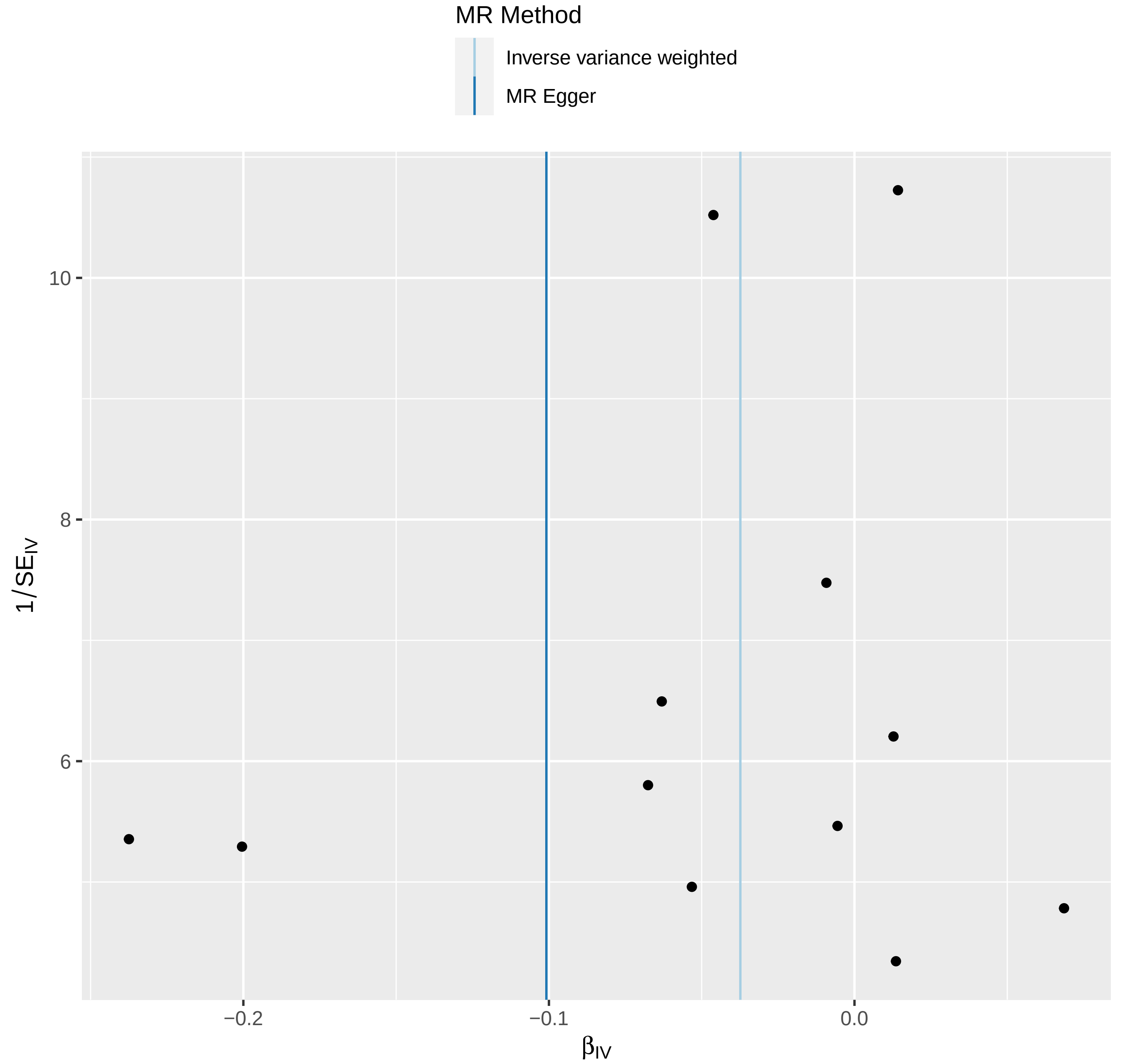

Batch 3−2386 : Selenium on Protein Largen

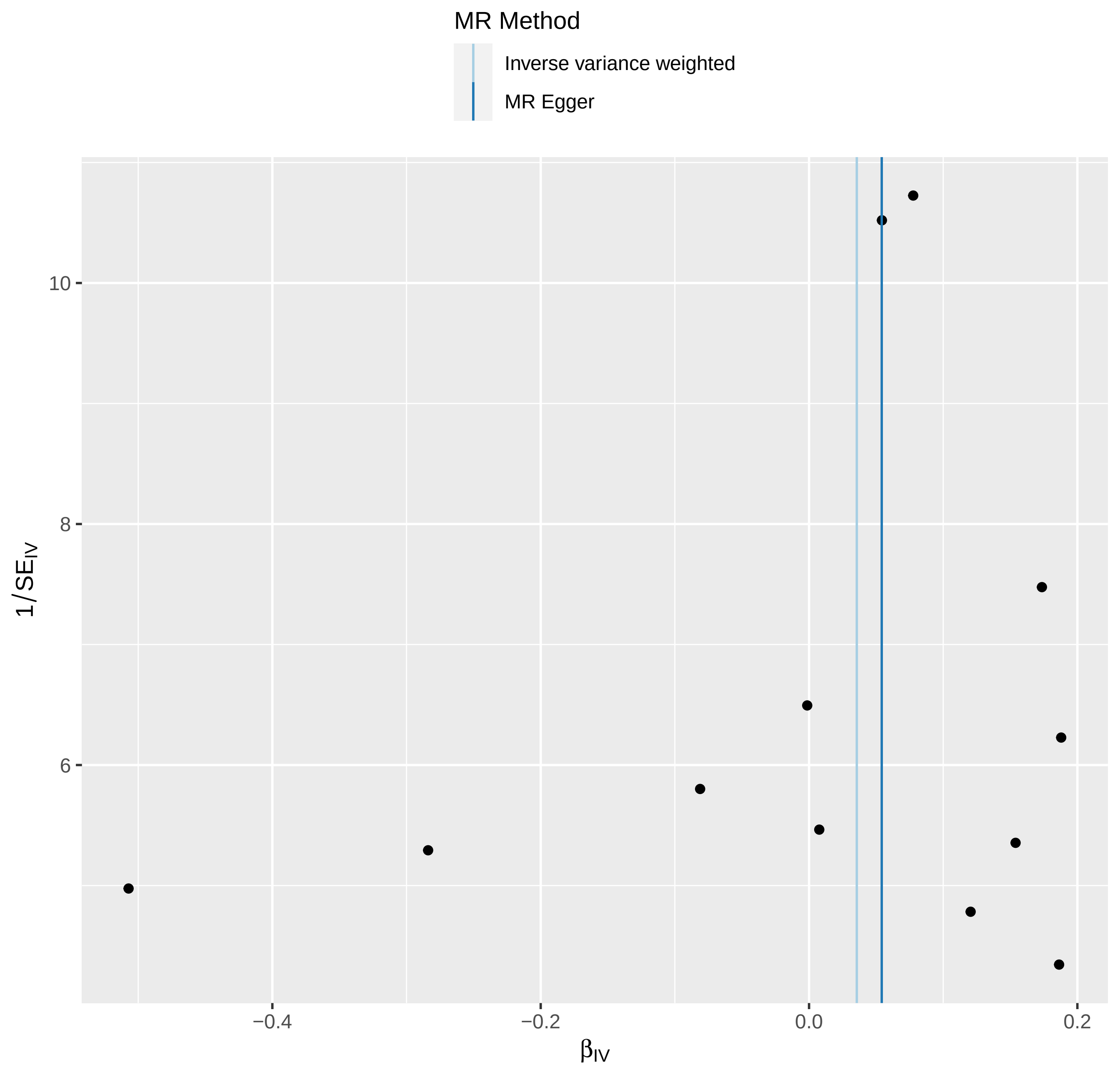

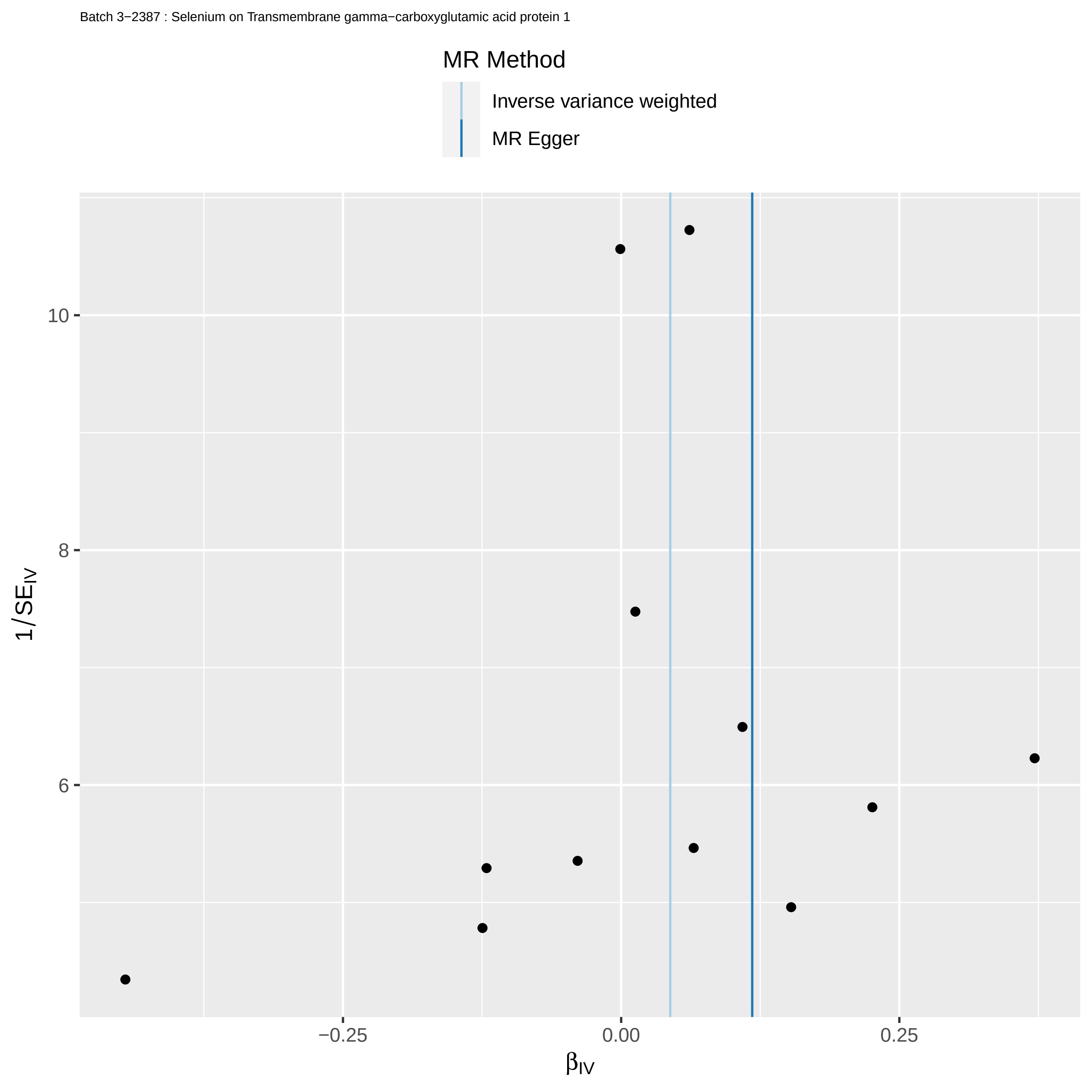
Batch 3−2387 : Selenium on Transmembrane gamma−carboxyglutamic acid protein 1
MR Method
Inverse variance weighted
MR Egger
10
8
6
1/SE_IV
−0.25
0.00
0.25
β_IV

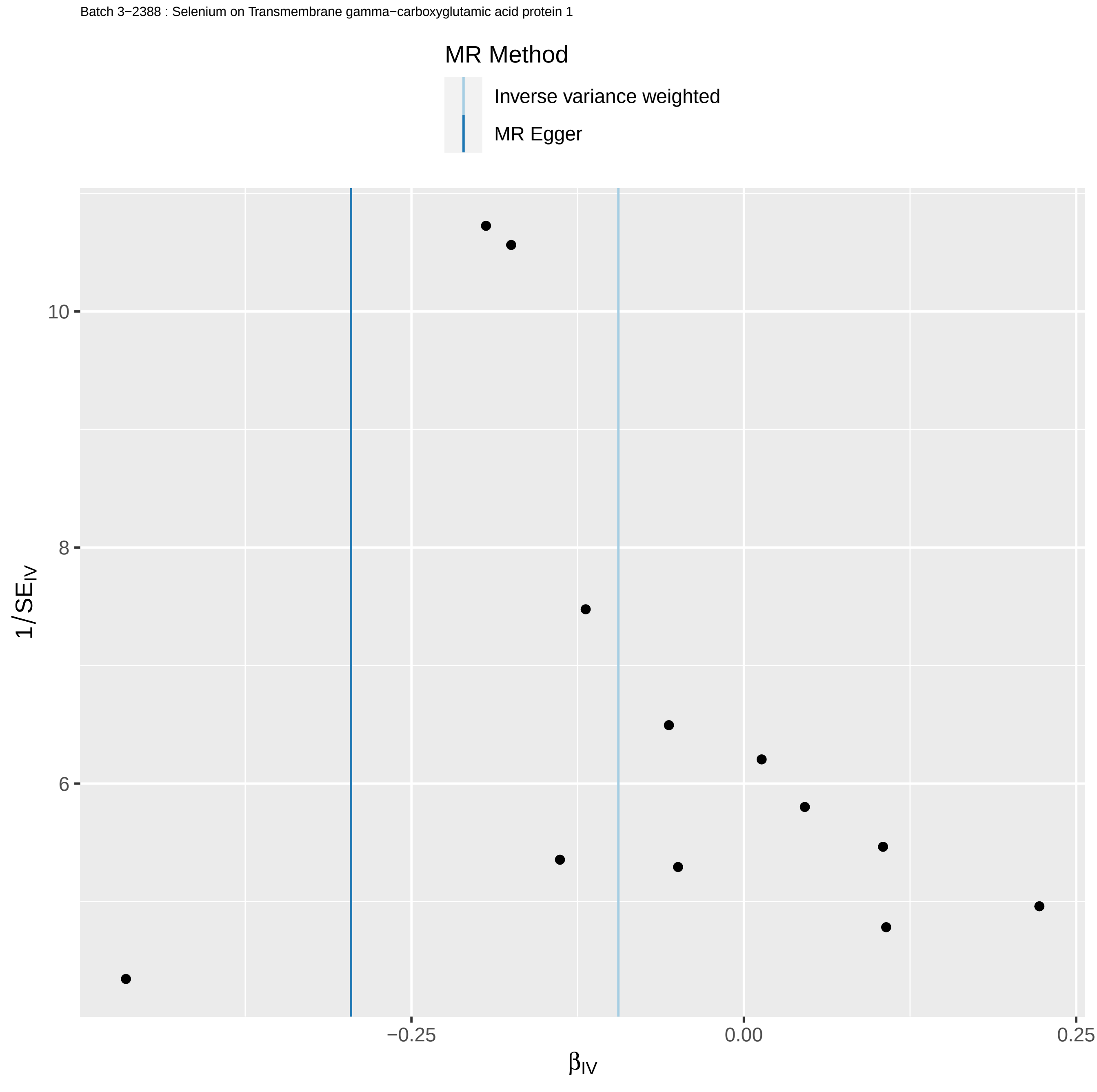
Batch 3−2388 : Selenium on Transmembrane gamma−carboxyglutamic acid protein 1
MR Method
Inverse variance weighted
MR Egger
10
8
6
1/SE_IV
−0.25
0.00
0.25
β_IV

Batch 3−2389 : Selenium on Transmembrane gamma−carboxyglutamic acid protein 4

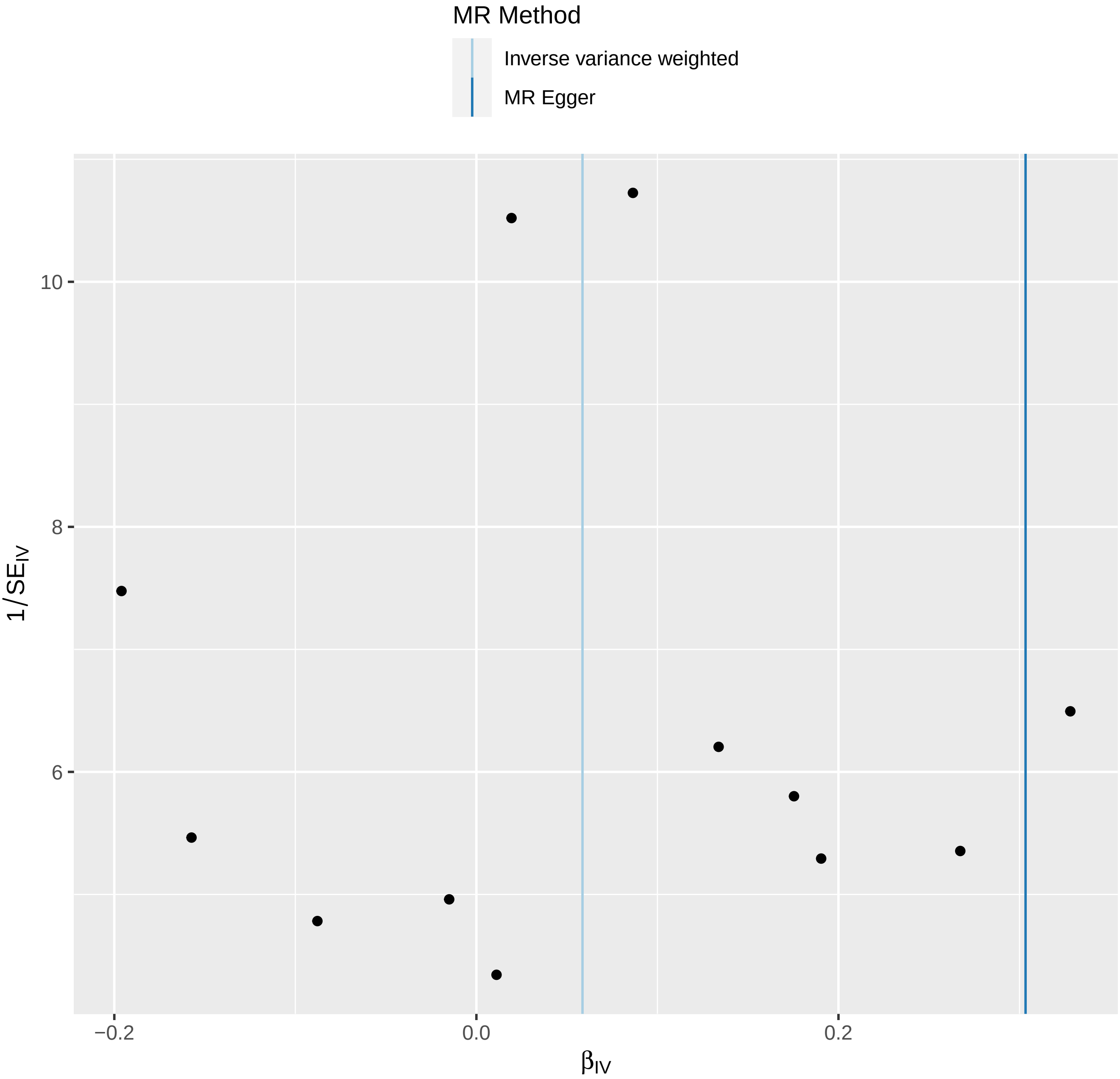

Batch 3−2390 : Selenium on Trypsin−1

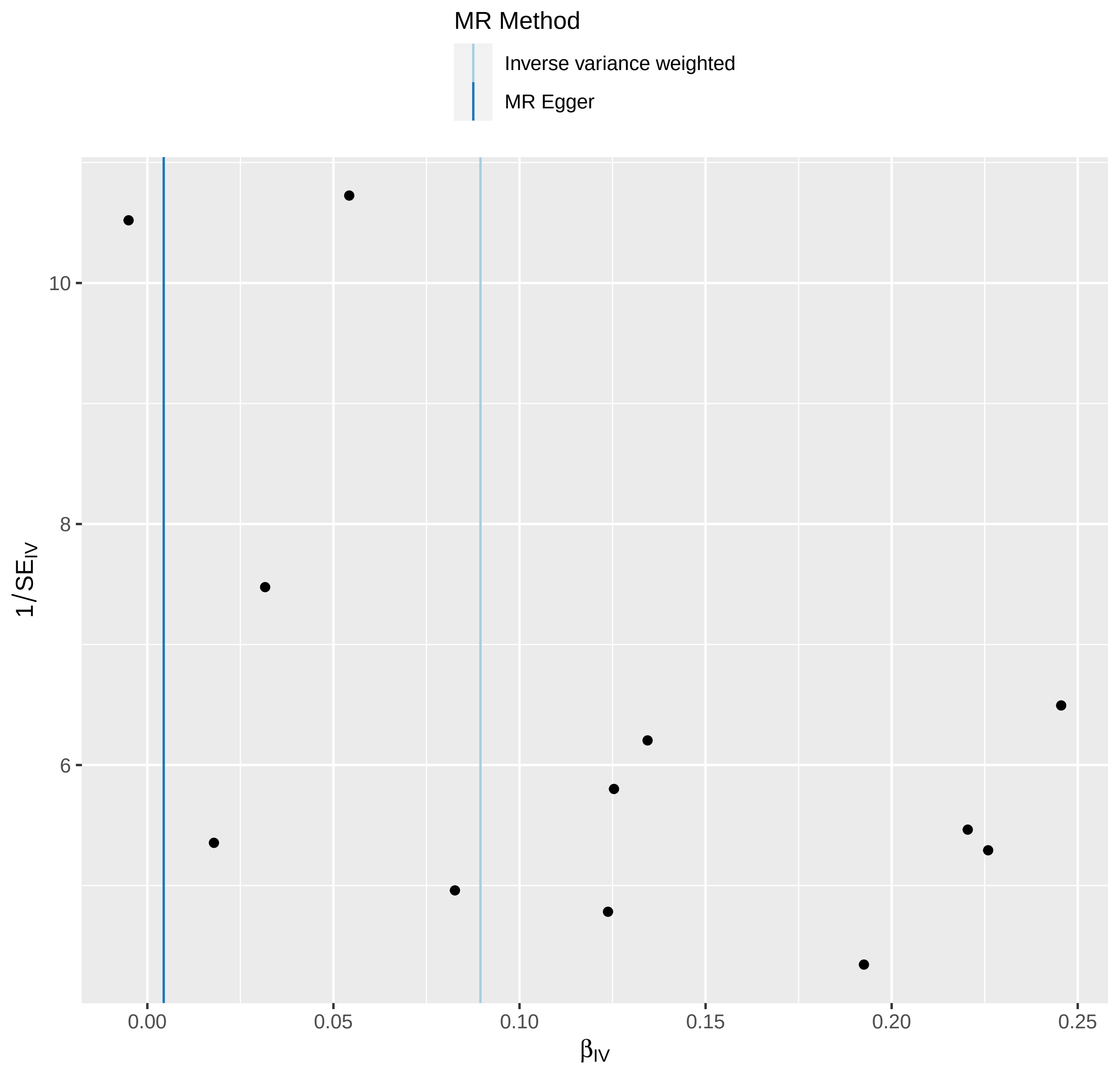

Batch 3−2391 : Selenium on Brain−specific serine protease 4

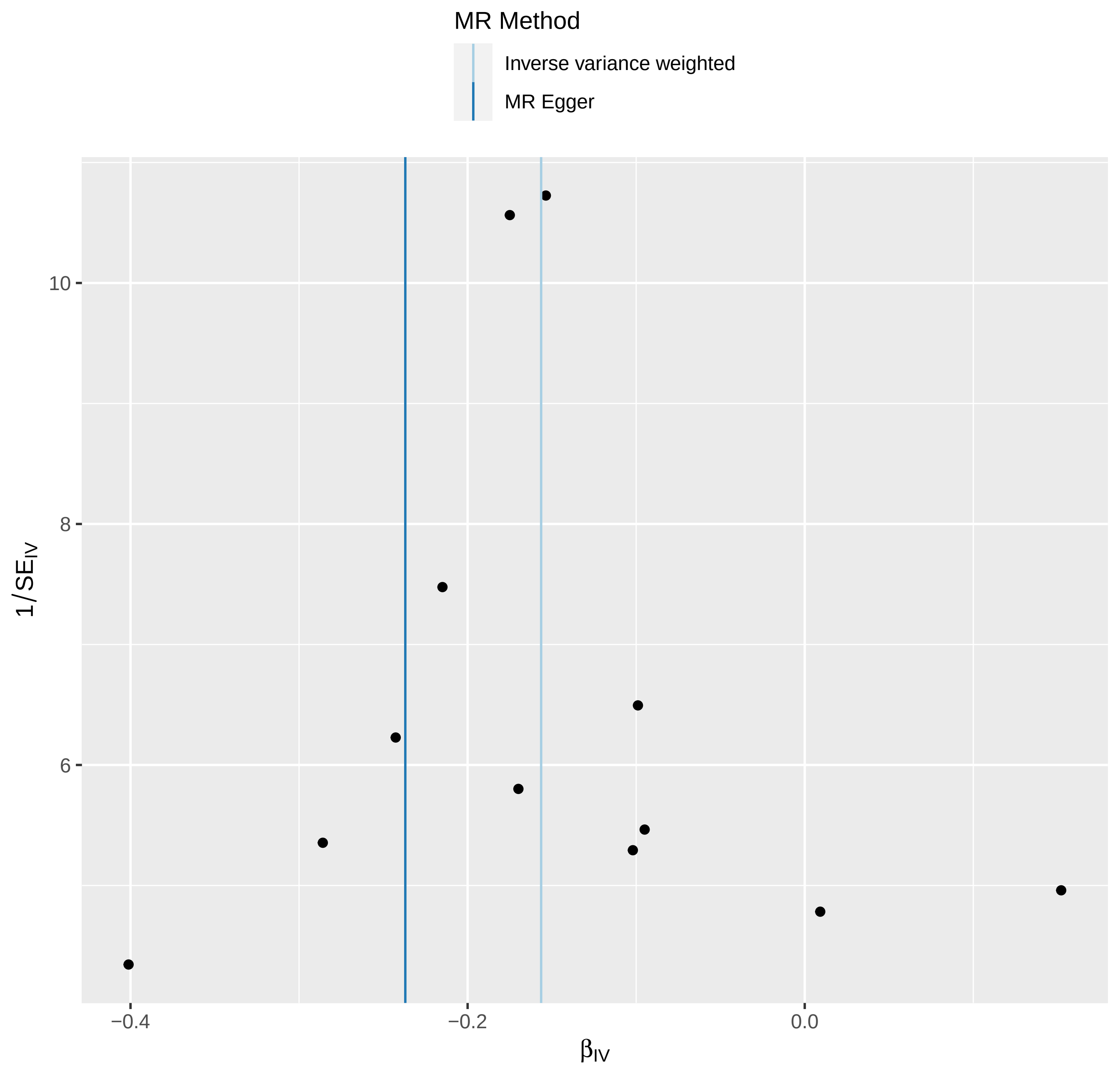

Batch 3−2392 : Selenium on Trypsin−2

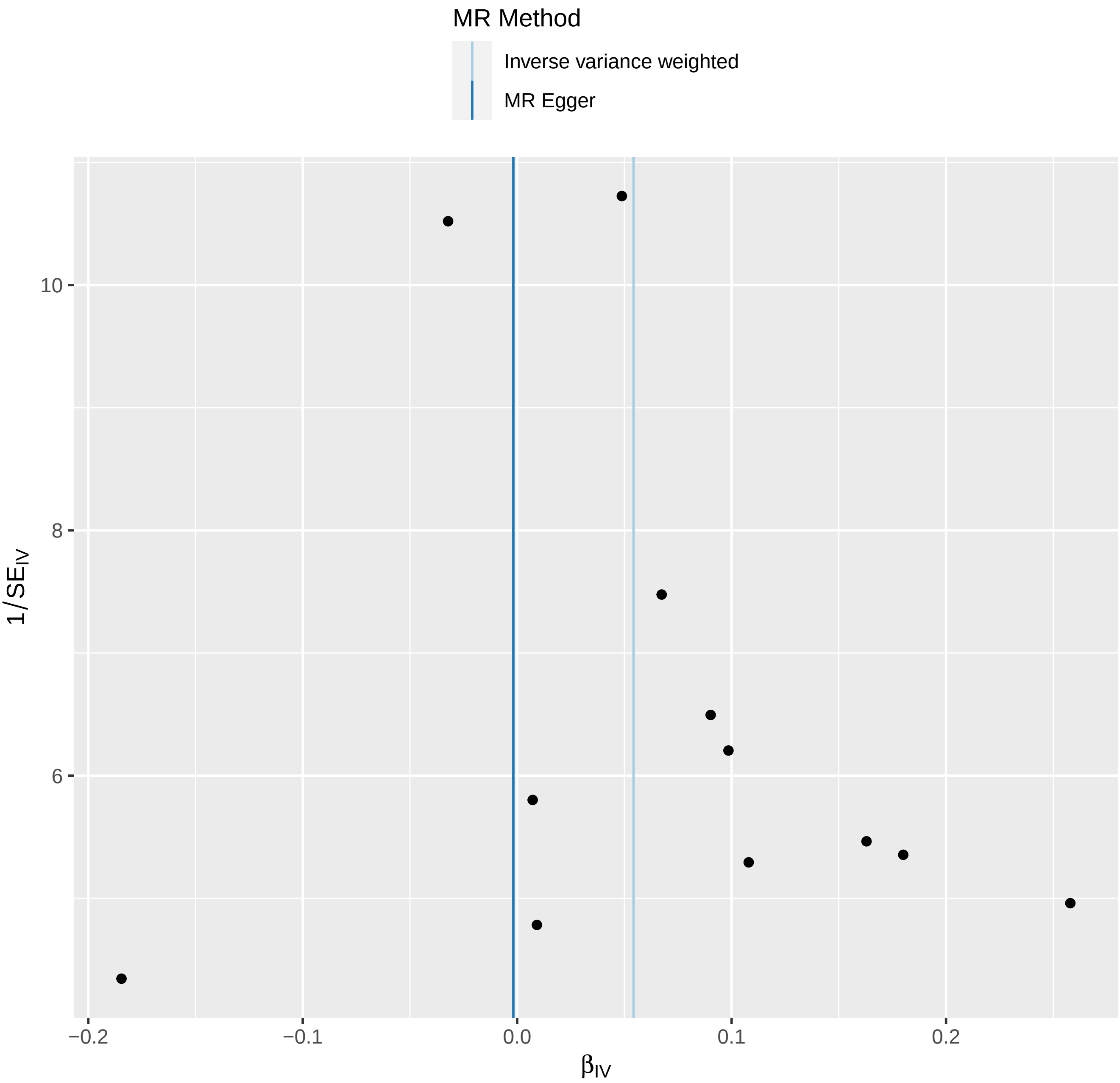

Batch 3−2393 : Selenium on Trypsin−3

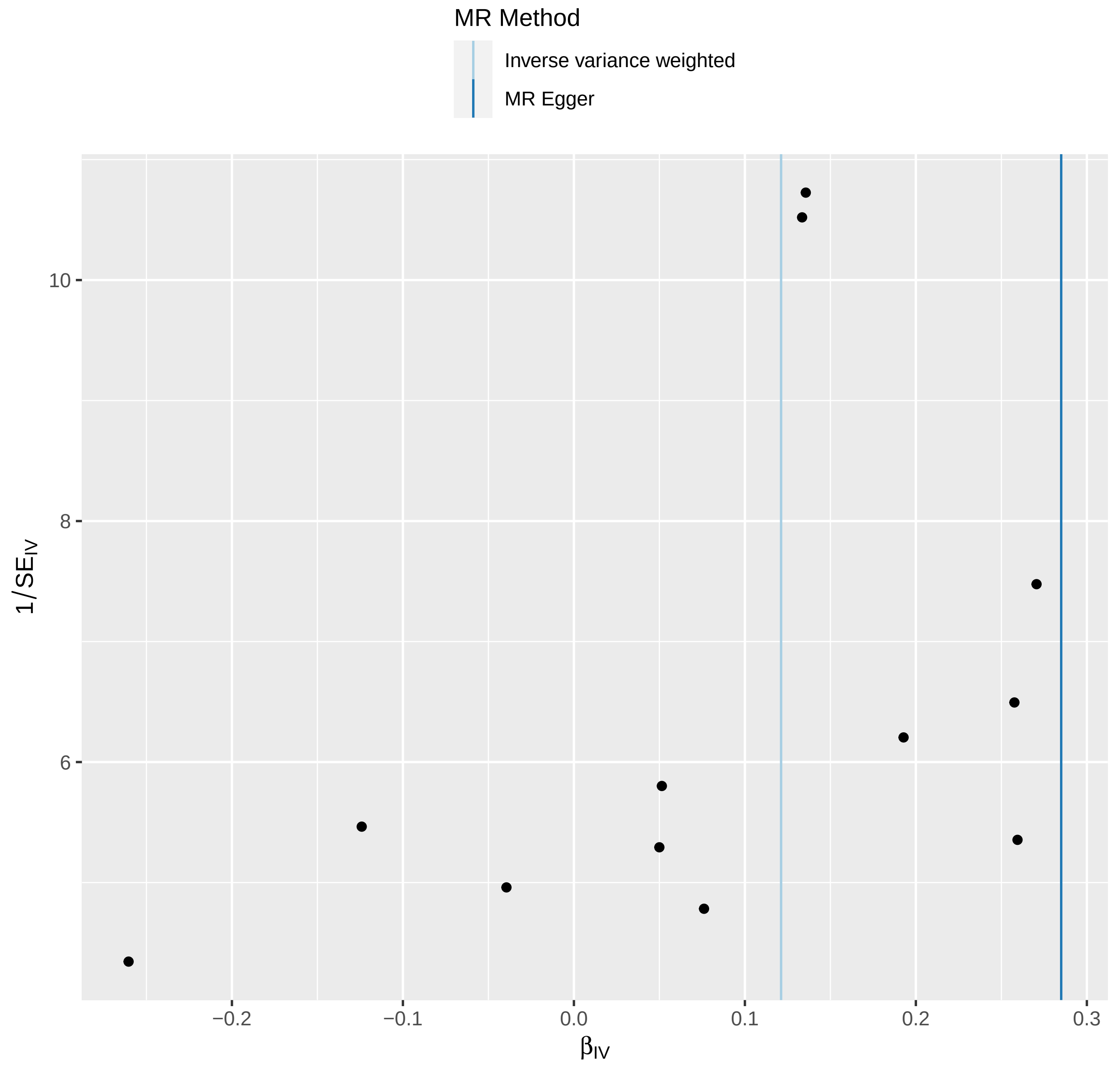

Batch 3−2394 : Selenium on Probable inactive serine protease 37

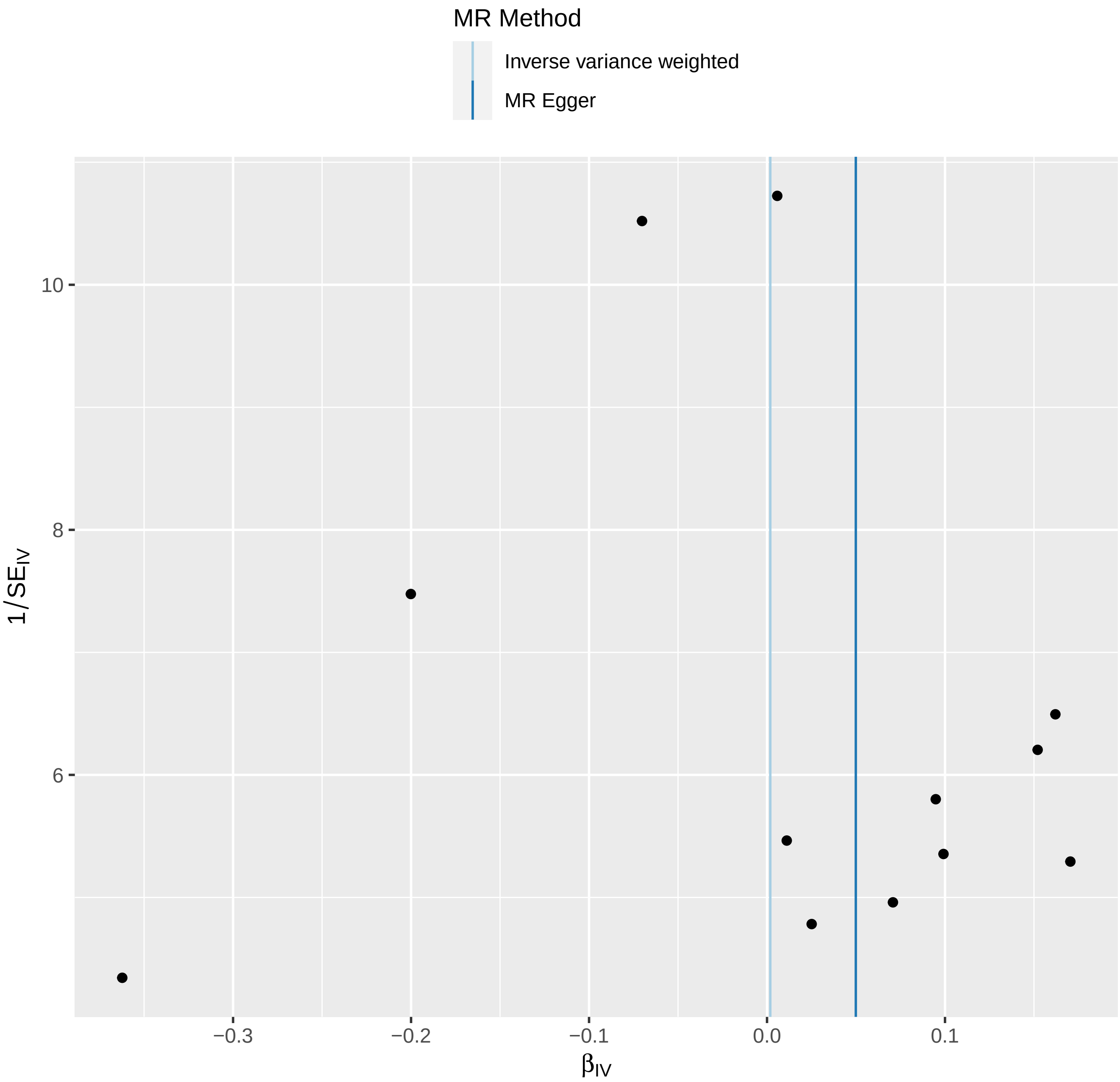

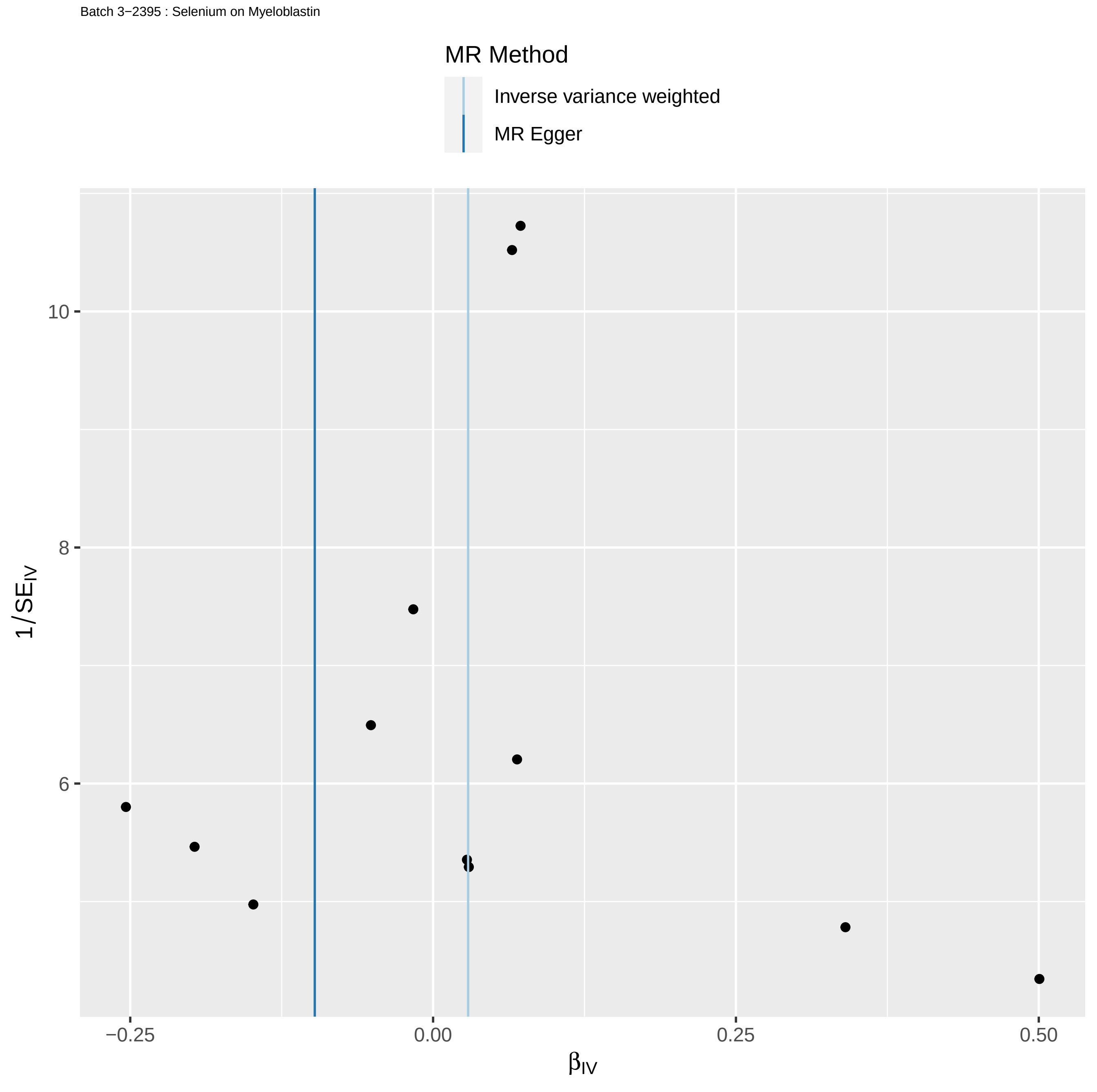
Batch 3−2395 : Selenium on Myeloblastin
MR Method
Inverse variance weighted
MR Egger
10
8
6
1/SE_IV
−0.25
0.00
0.25
0.50
β_IV

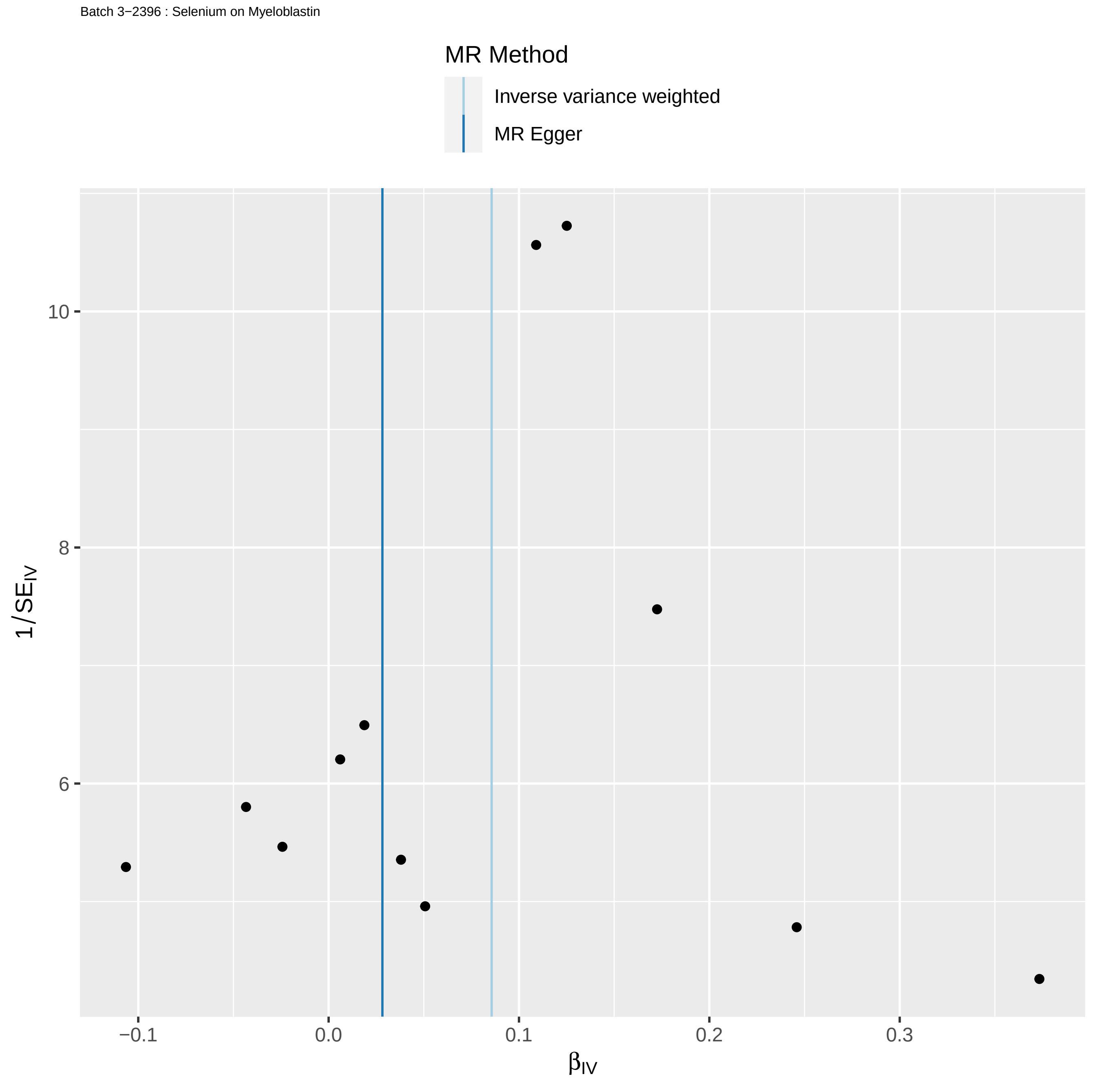
Batch 3−2396 : Selenium on Myeloblastin
MR Method
Inverse variance weighted
MR Egger
10
8
6
1/SE_IV
−0.1
0.0
0.1
0.2
0.3
β_IV

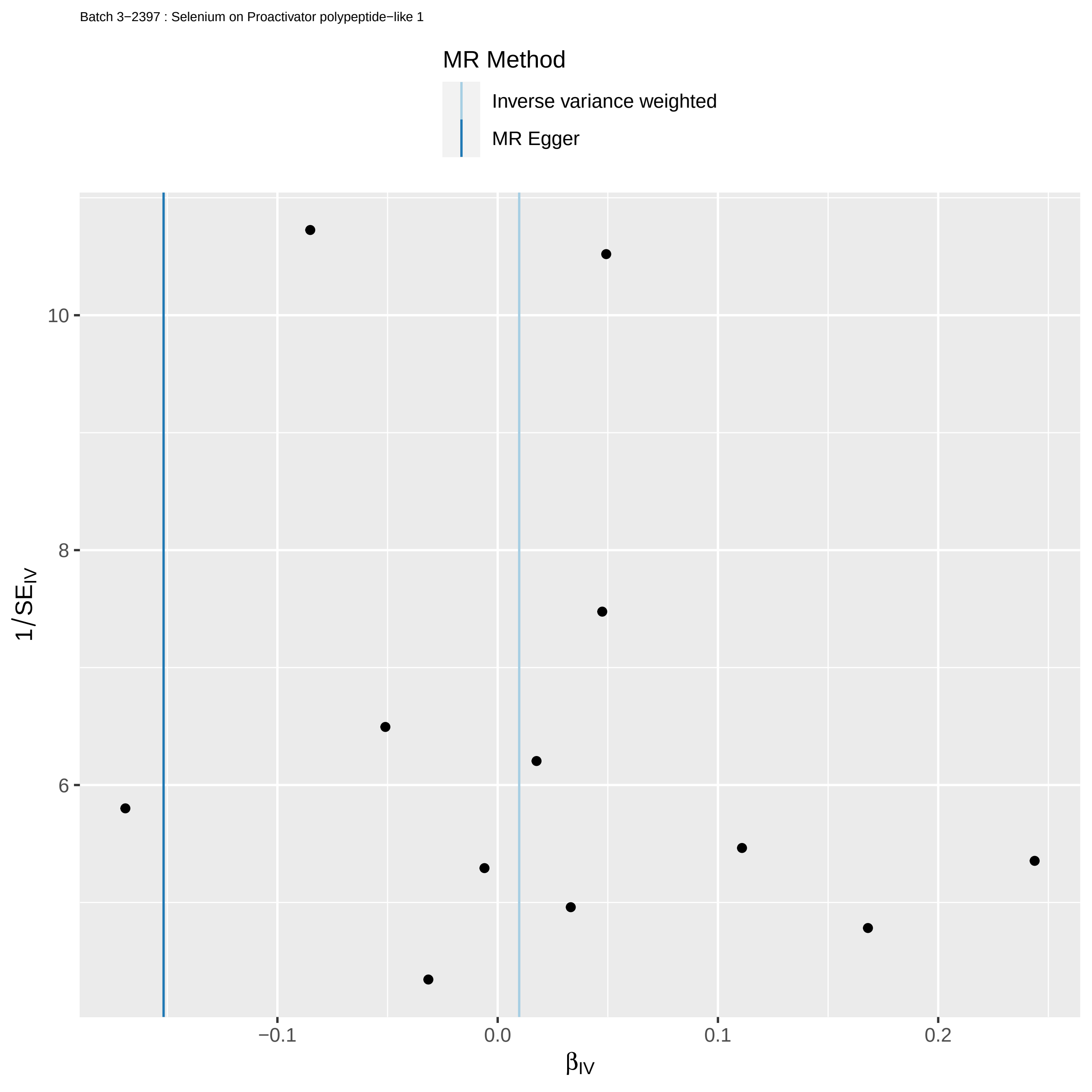
Batch 3−2397 : Selenium on Proactivator polypeptide−like 1
MR Method
Inverse variance weighted
MR Egger
10
8
6
1/SE_IV
−0.1
0.0
0.1
0.2
β_IV

Batch 3−2398 : Selenium on PH and SEC7 domain−containing protein 1

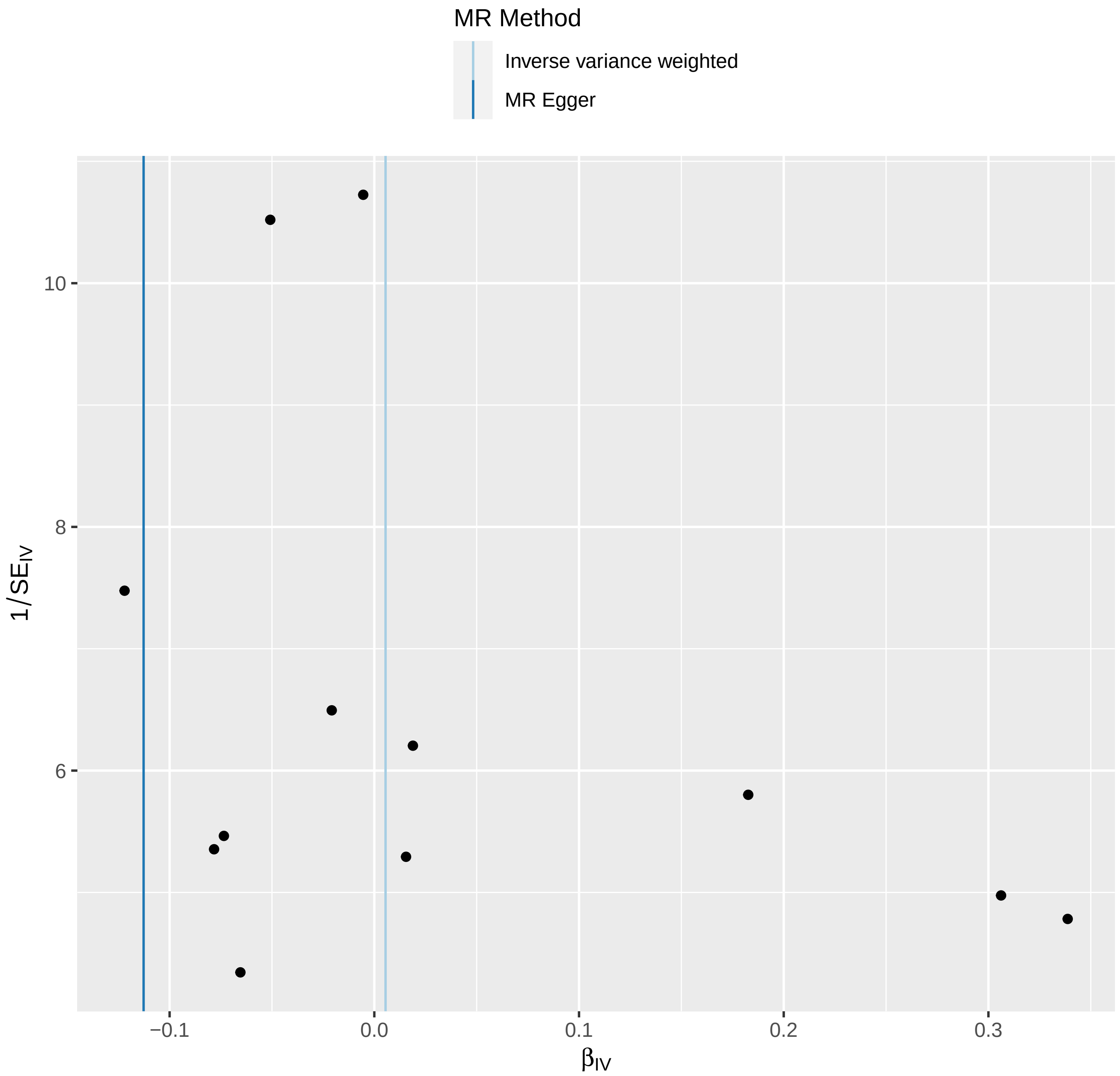

Batch 3−2399 : Selenium on PH and SEC7 domain−containing protein 2

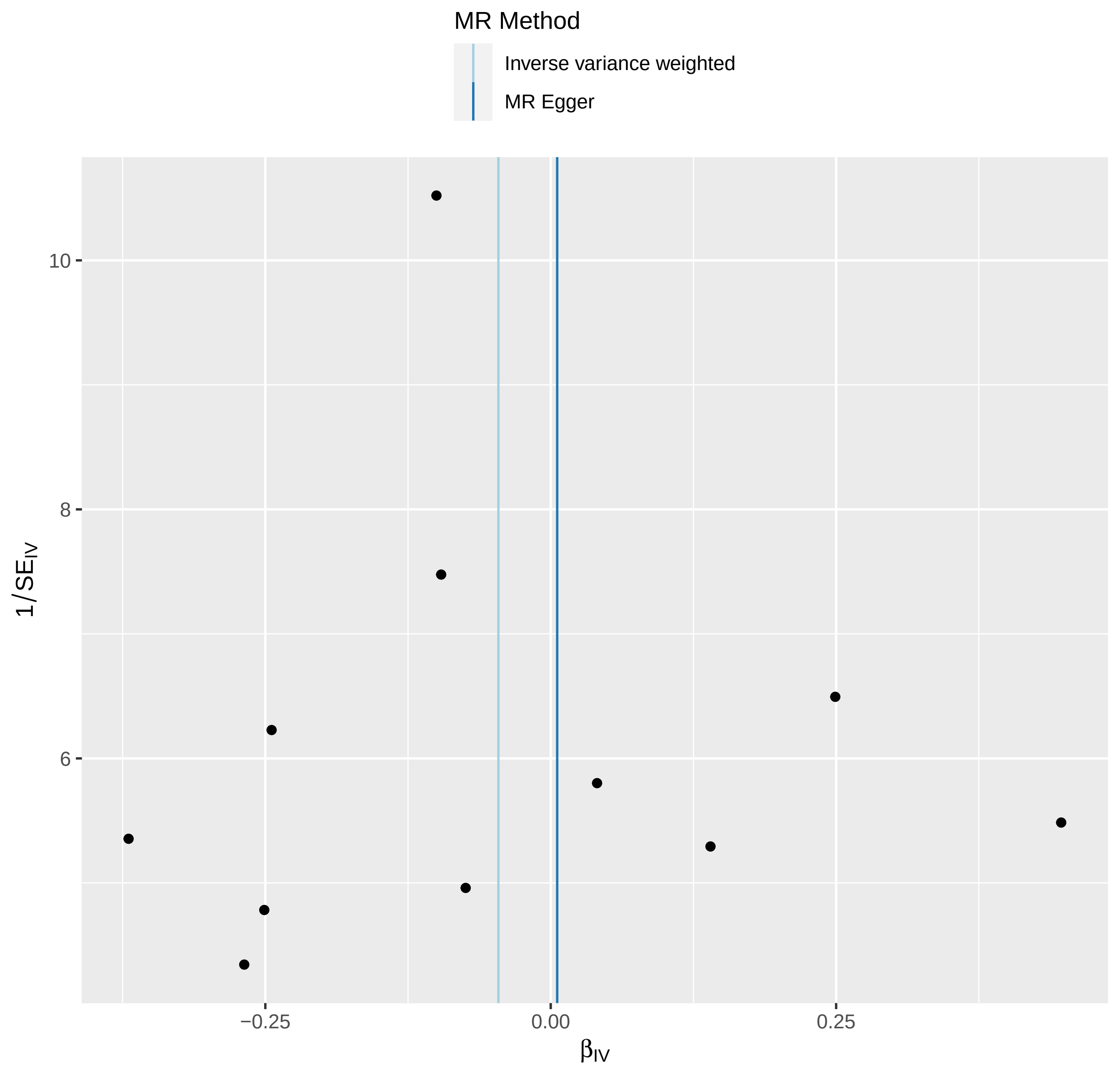

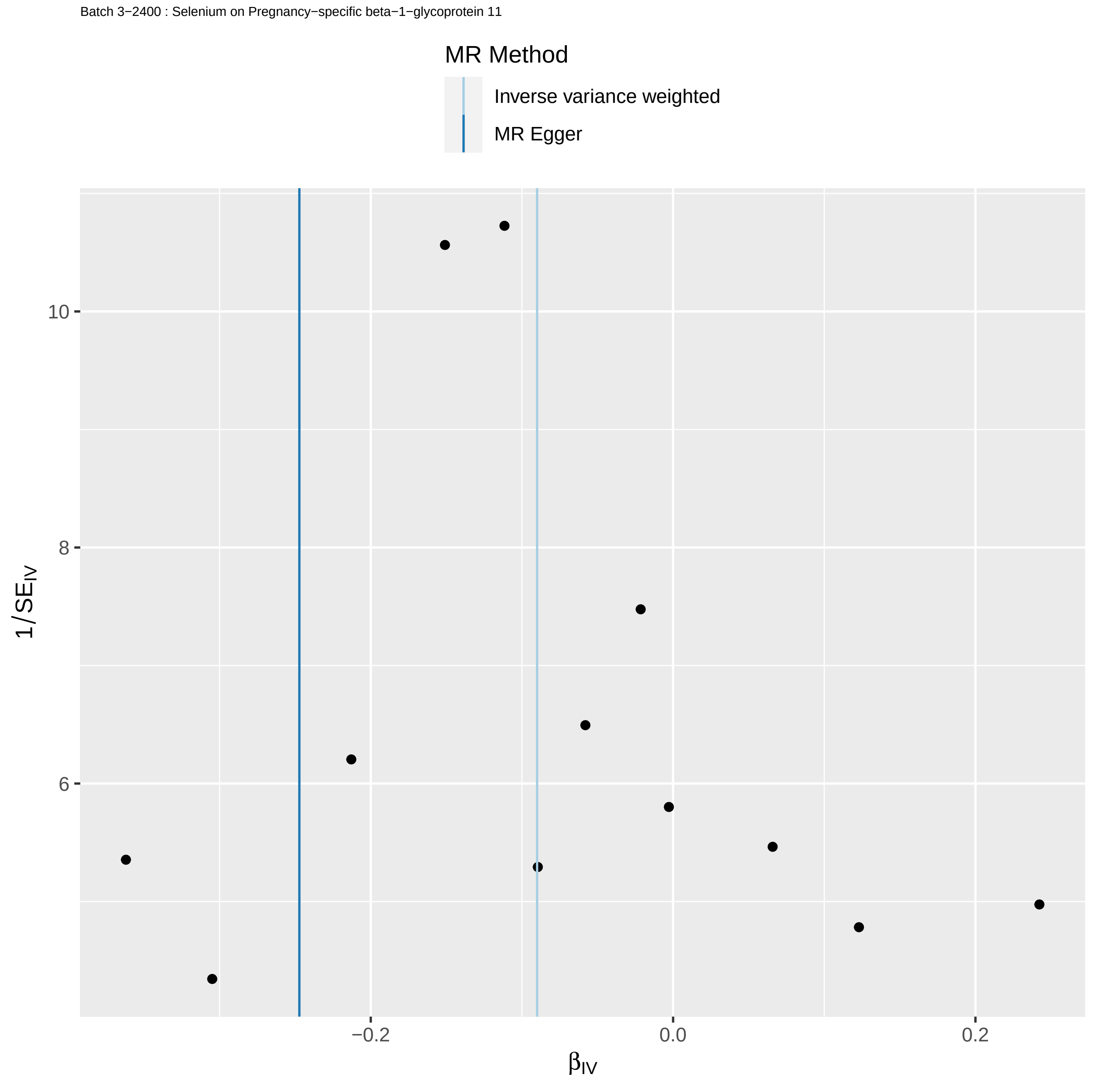
Batch 3−2400 : Selenium on Pregnancy−specific beta−1−glycoprotein 11
MR Method
Inverse variance weighted
MR Egger
10
8
6
1/SE_IV
−0.2
0.0
0.2
β_IV

Batch 3−2401 : Selenium on Pregnancy−specific beta−1−glycoprotein 2

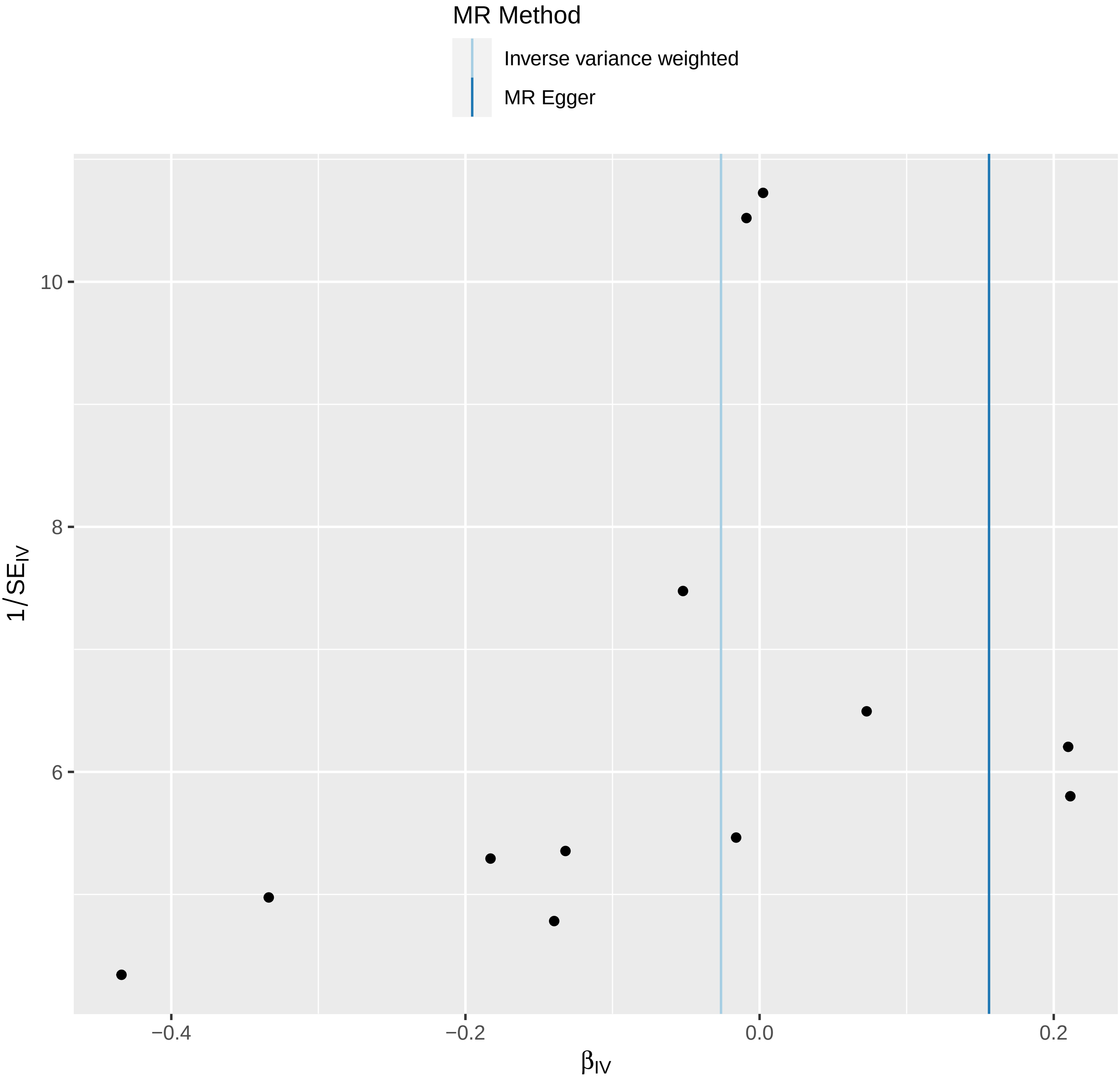

Batch 3−2402 : Selenium on Pregnancy−specific beta−1−glycoprotein 3

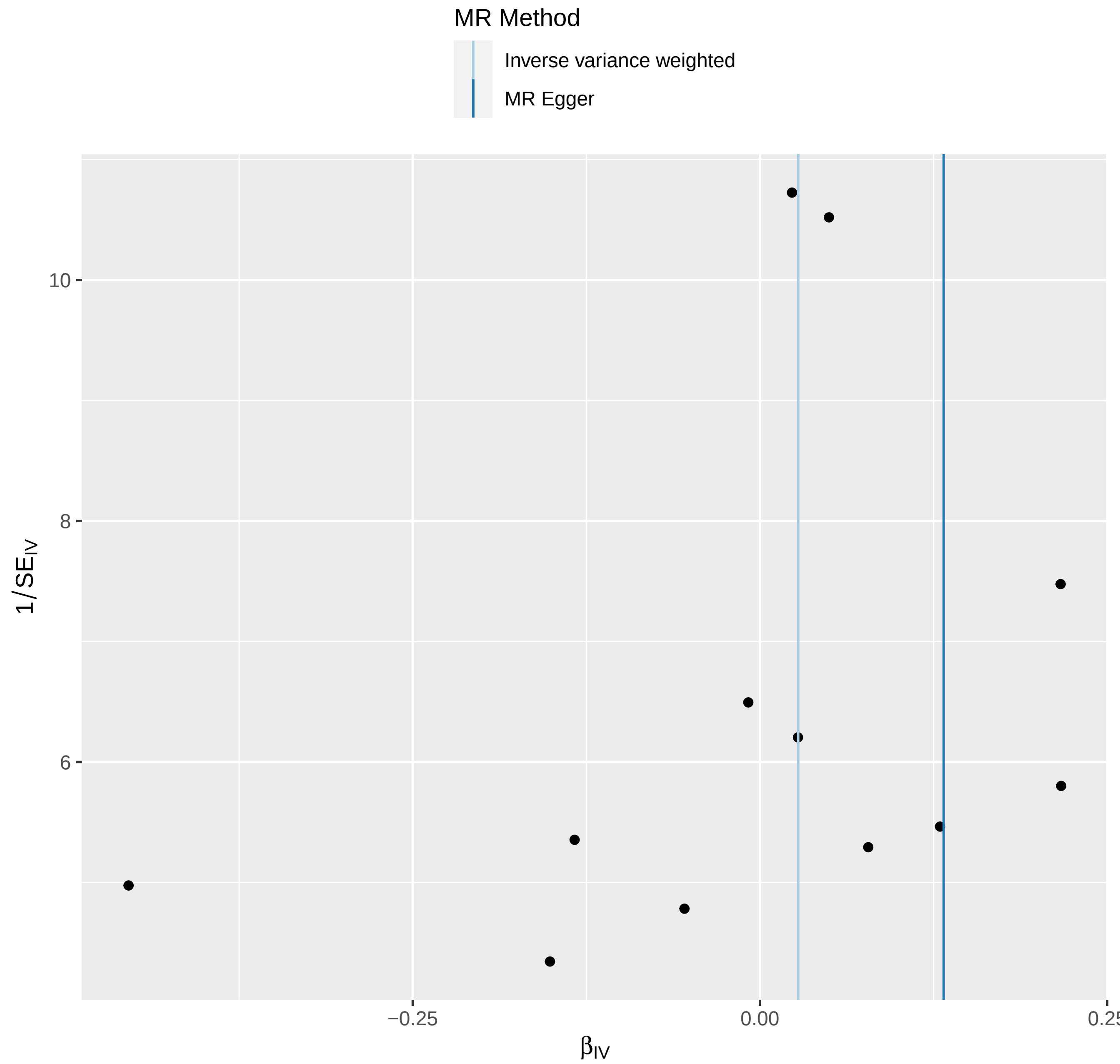

Batch 3−2403 : Selenium on Pregnancy−specific beta−1−glycoprotein 4

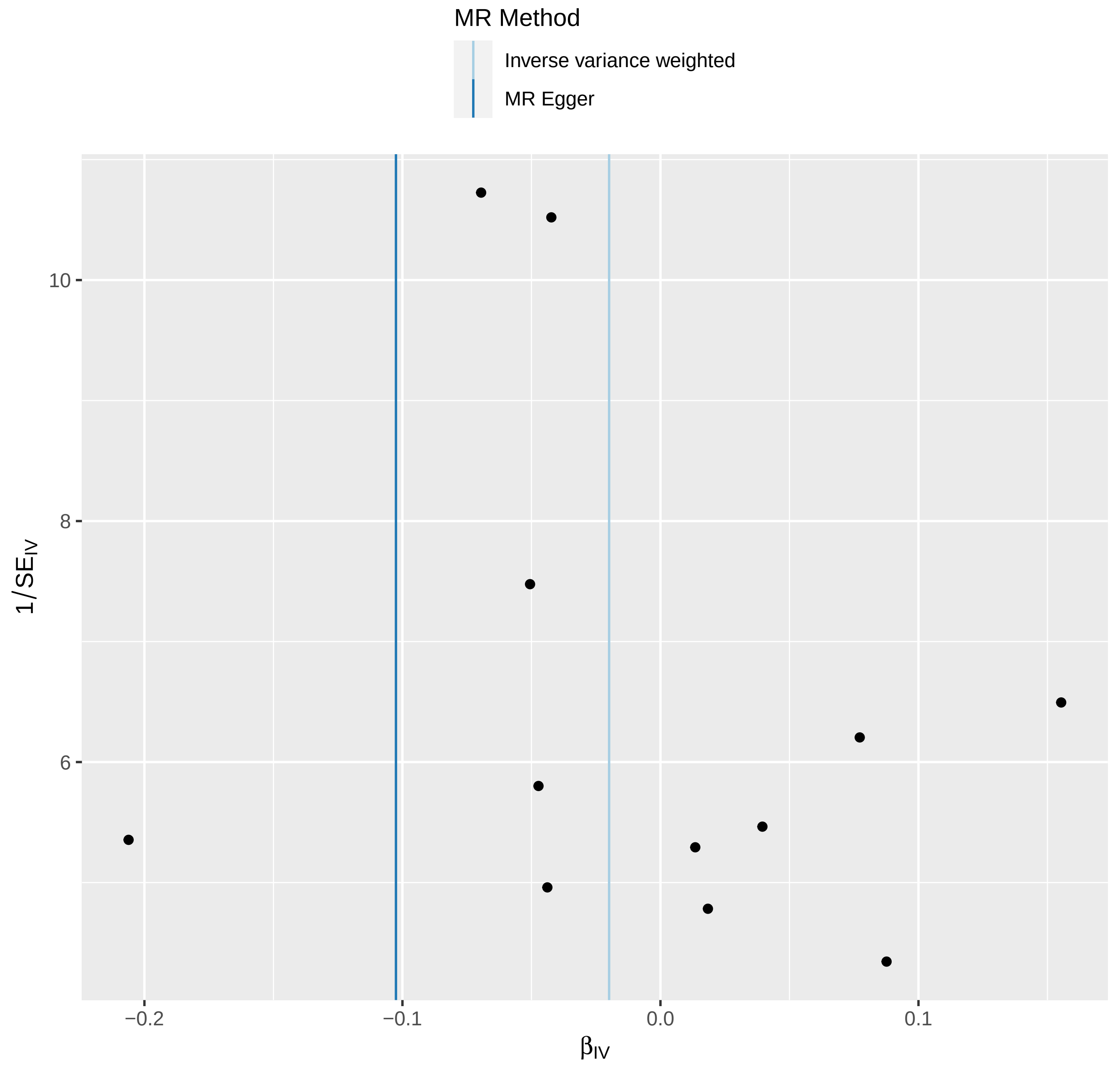

Batch 3−2404 : Selenium on Pregnancy−specific beta−1−glycoprotein 5

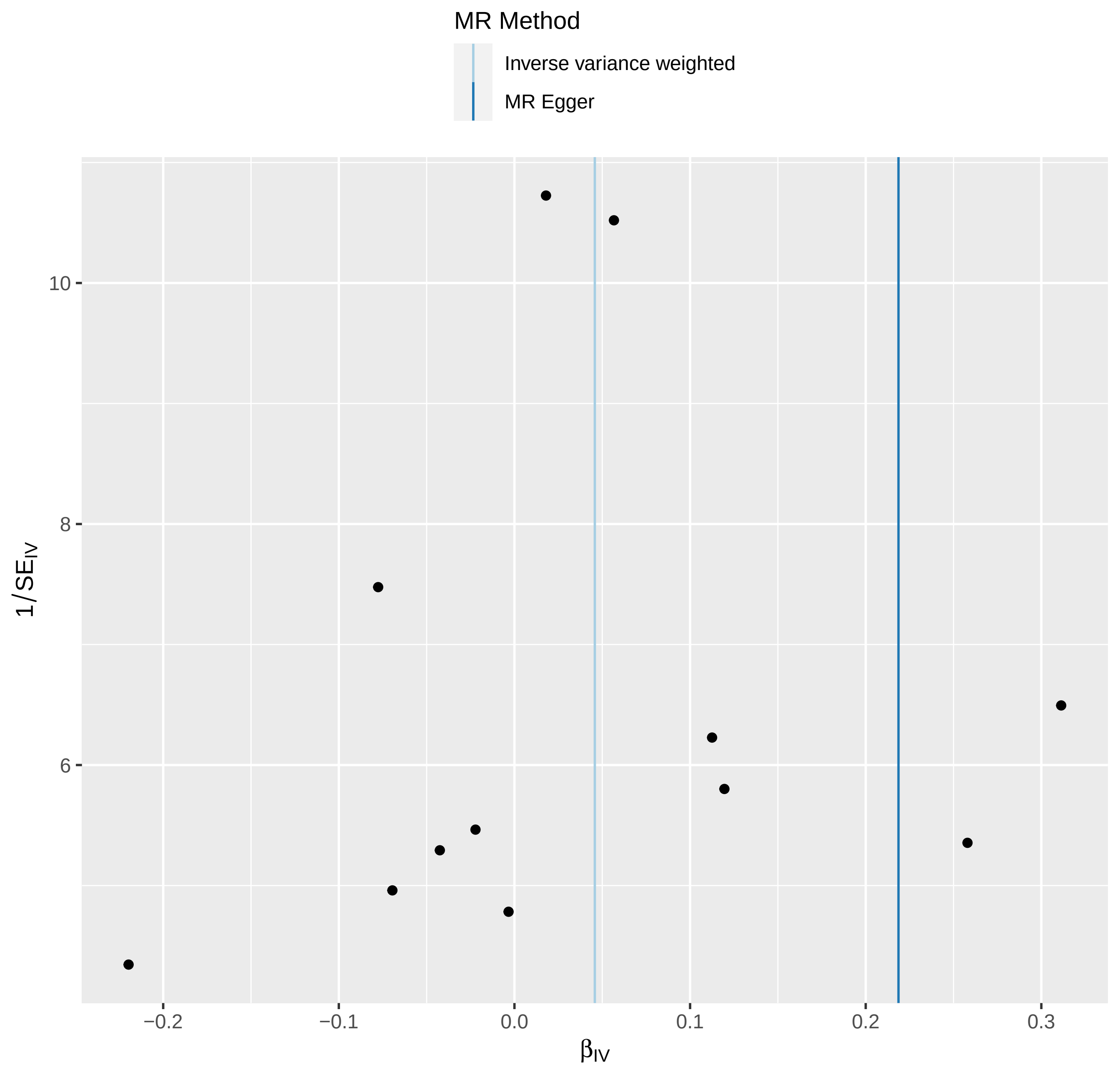

Batch 3−2405 : Selenium on Pregnancy−specific beta−1−glycoprotein 6

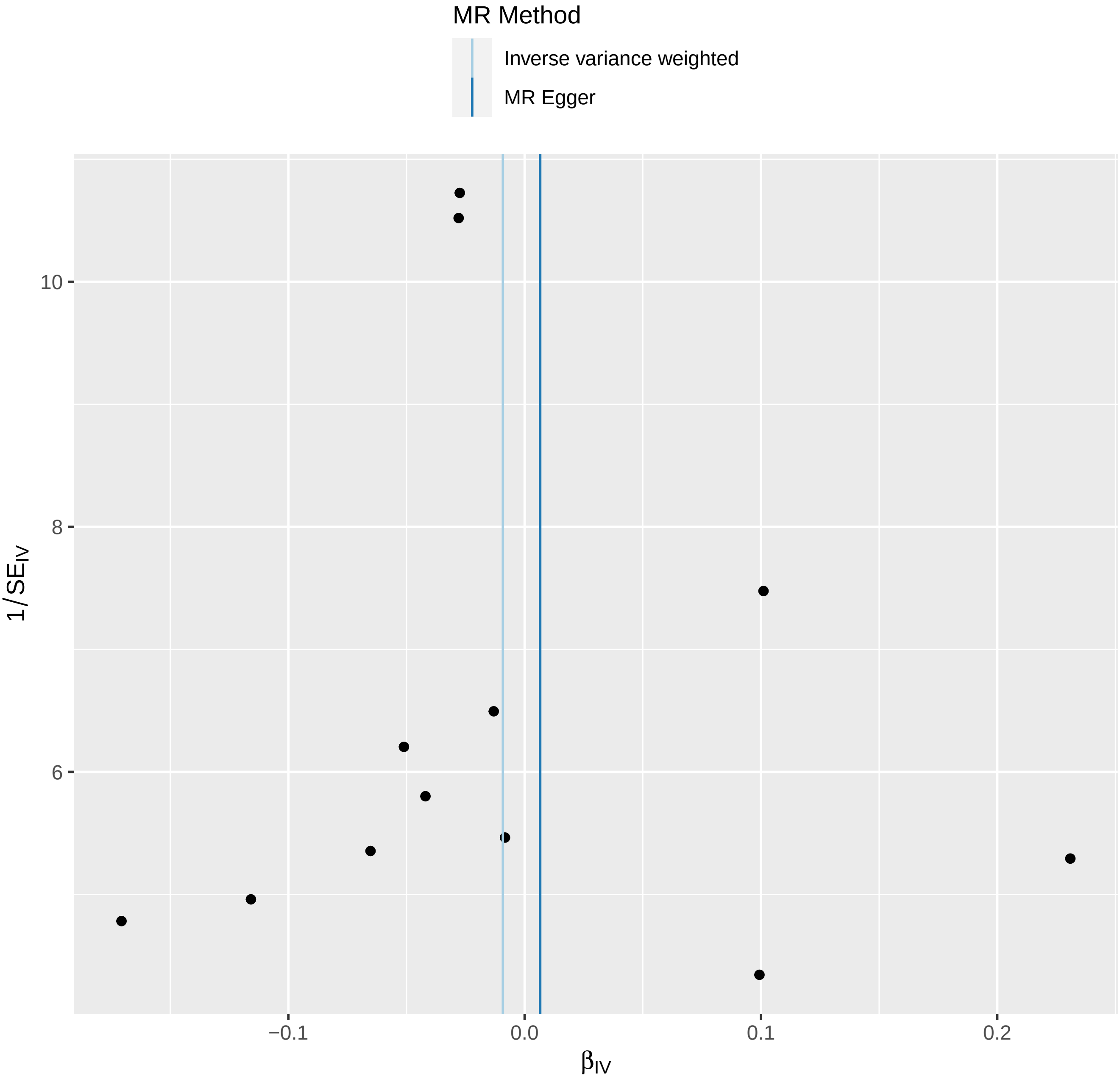

Batch 3−2406 : Selenium on Putative pregnancy−specific beta−1−glycoprotein 7

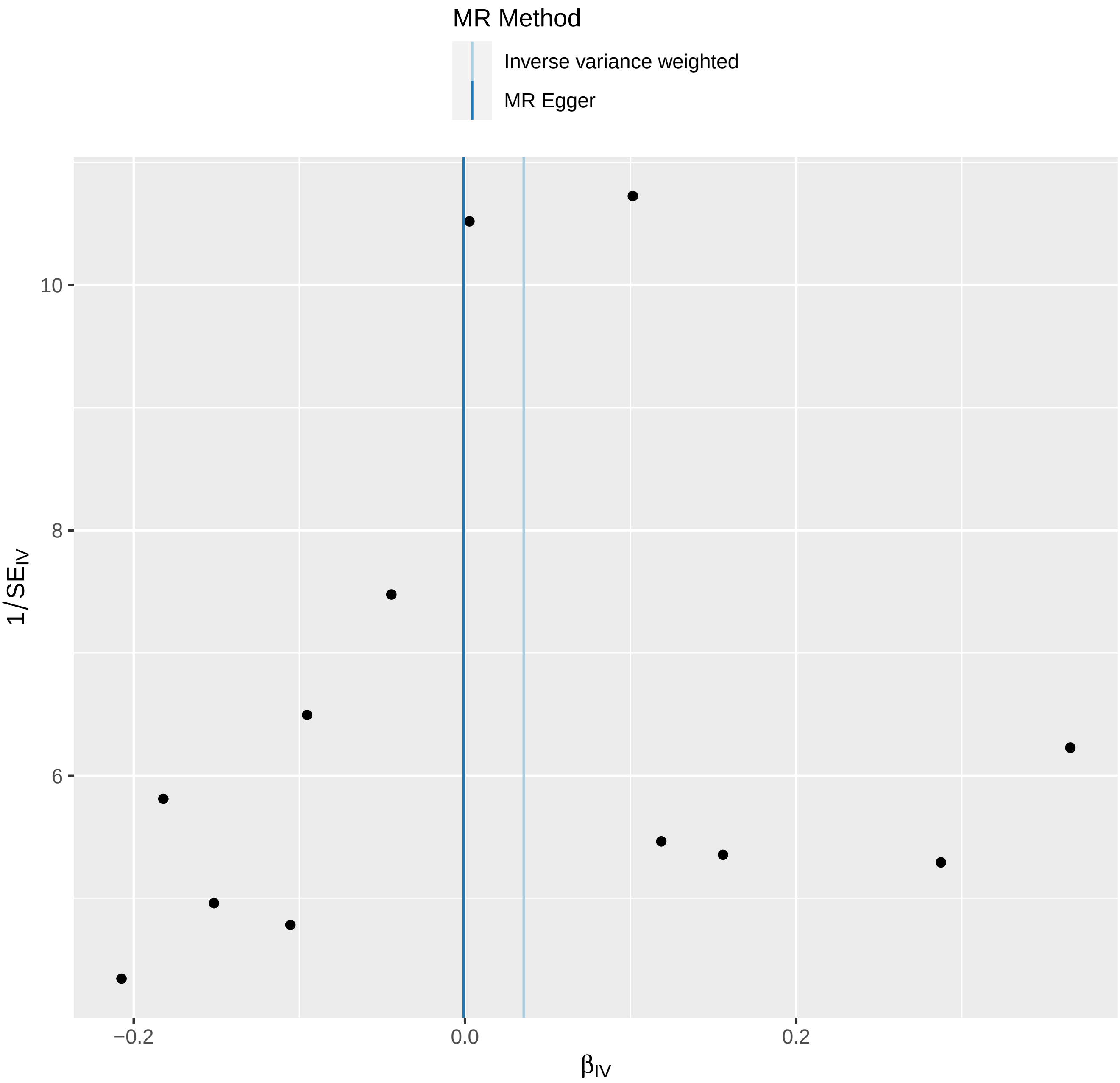

Batch 3−2407 : Selenium on Pregnancy−specific beta−1−glycoprotein 8

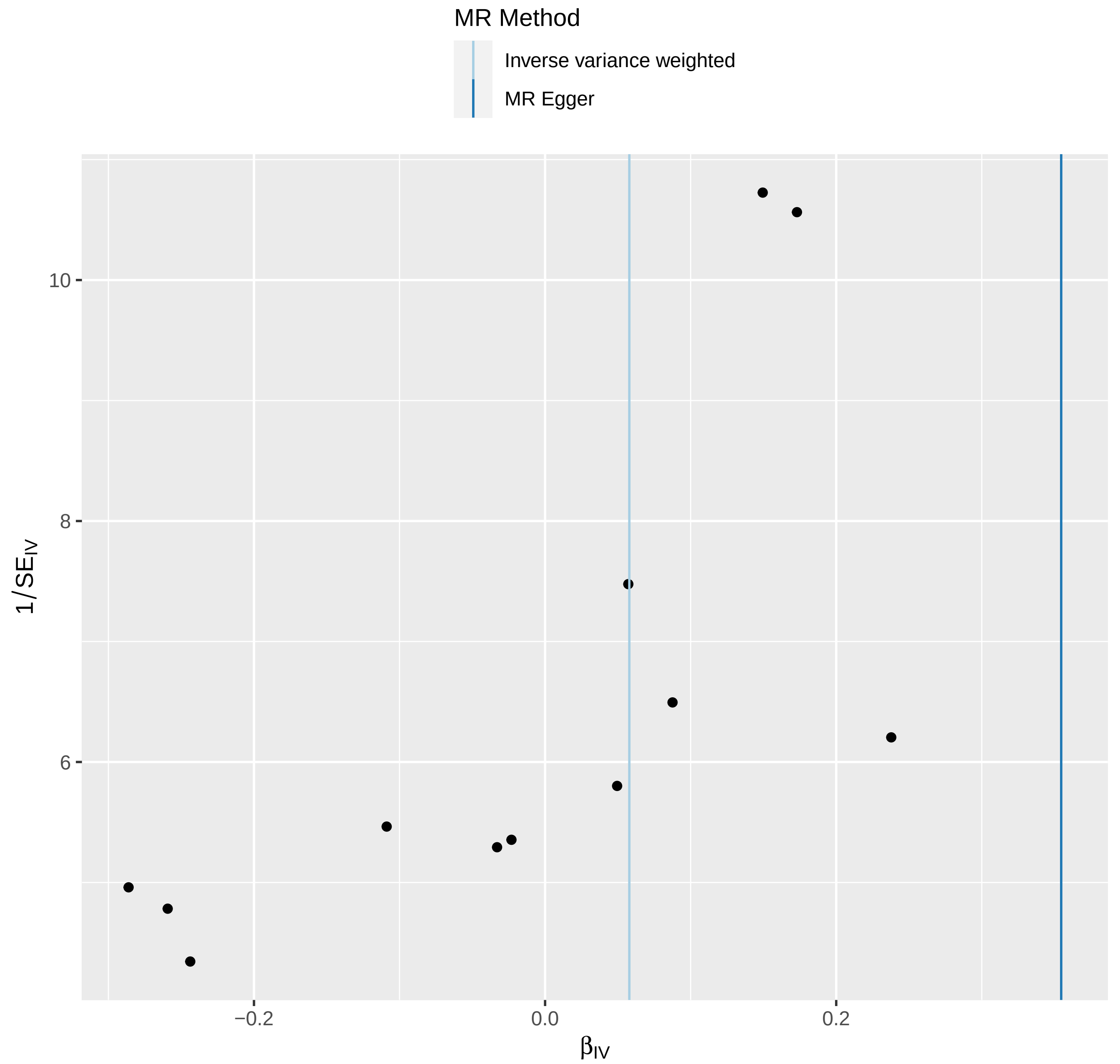

Batch 3−2408 : Selenium on Pregnancy−specific beta−1−glycoprotein 9

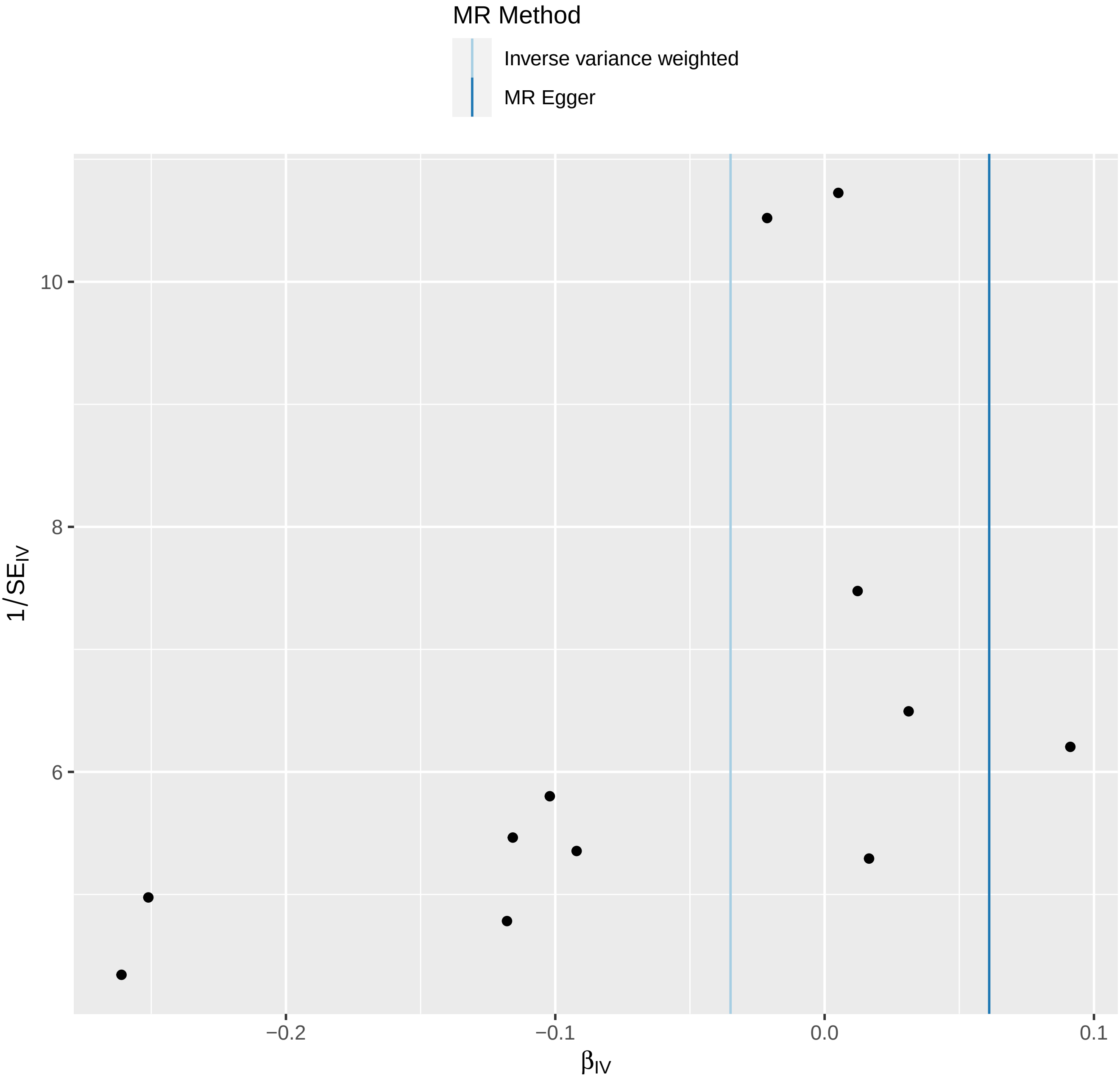

Batch 3−2409 : Selenium on Proteasome subunit alpha type−1

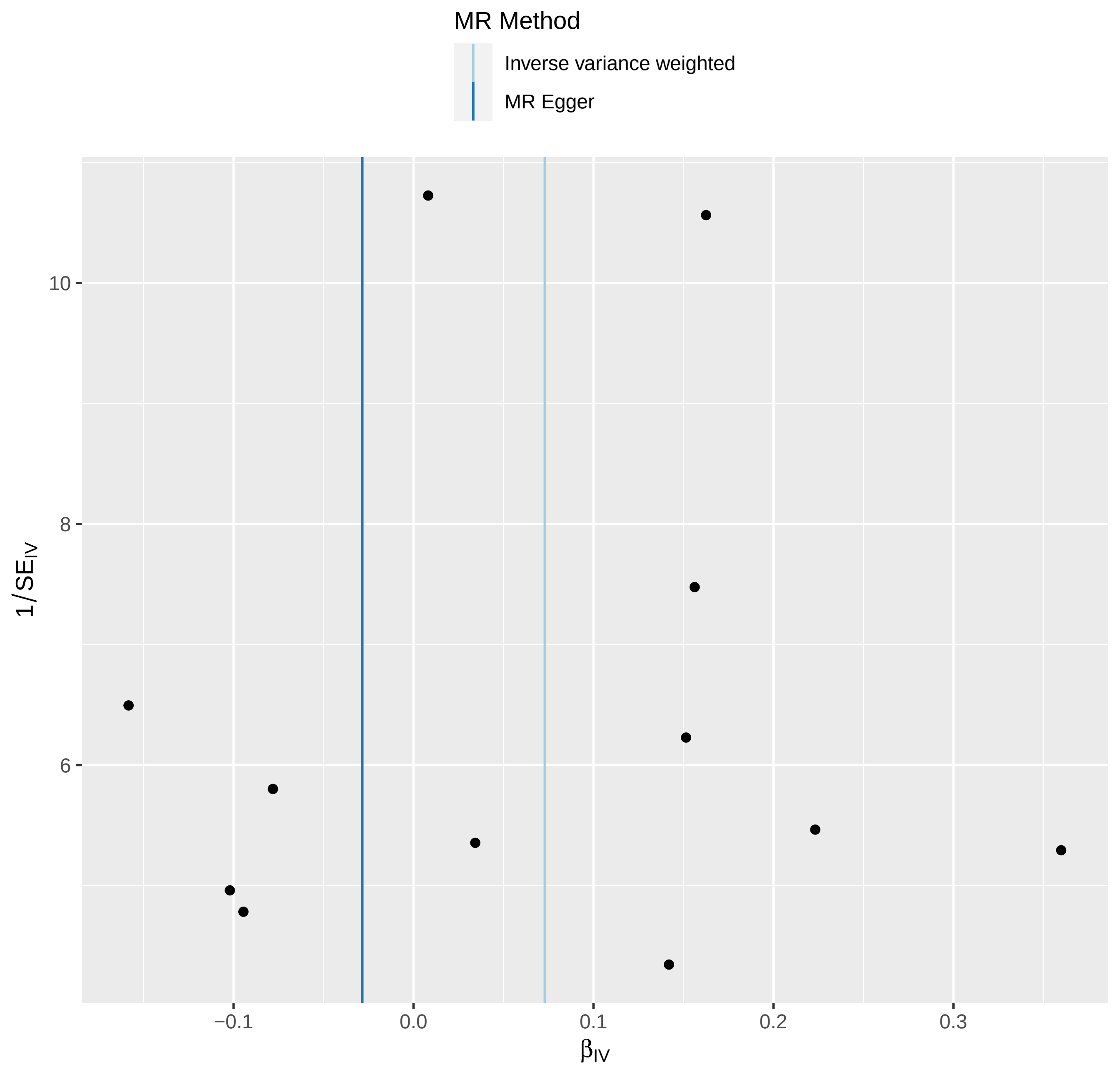

Batch 3−2410 : Selenium on Proteasome subunit alpha type−1

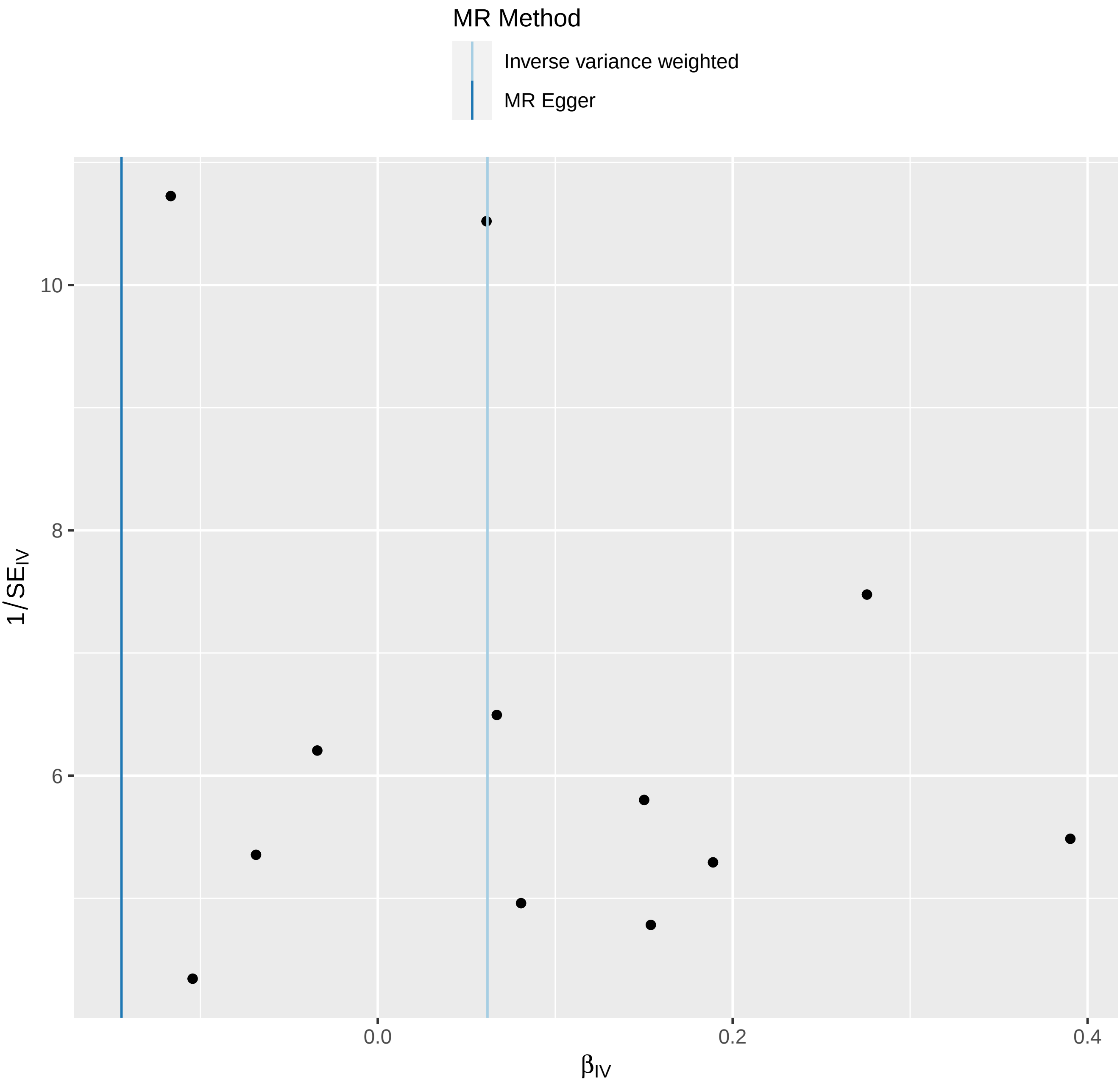

Batch 3−2411 : Selenium on Proteasome subunit alpha type−2

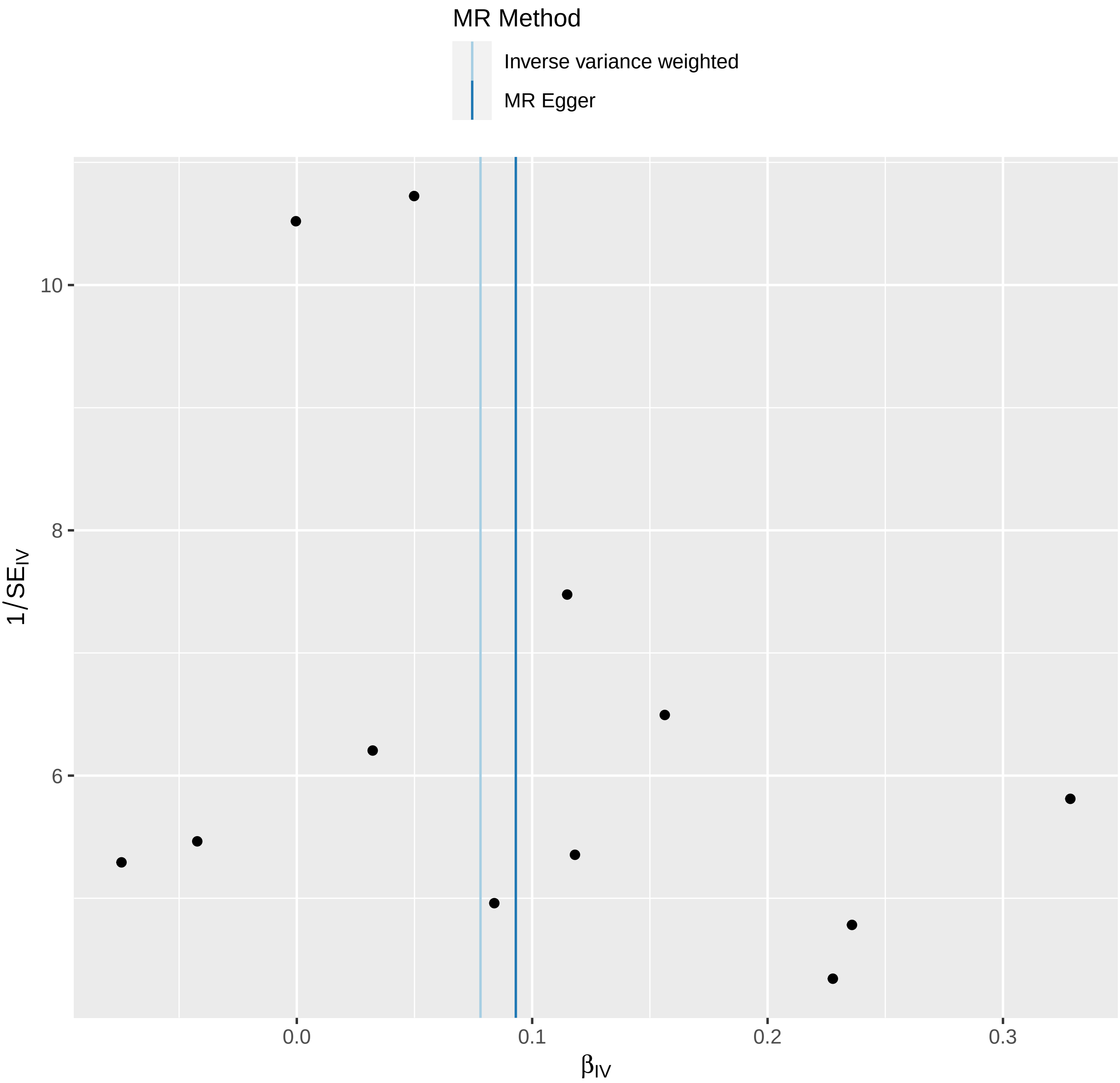

Batch 3−2412 : Selenium on Proteasome subunit alpha type−4

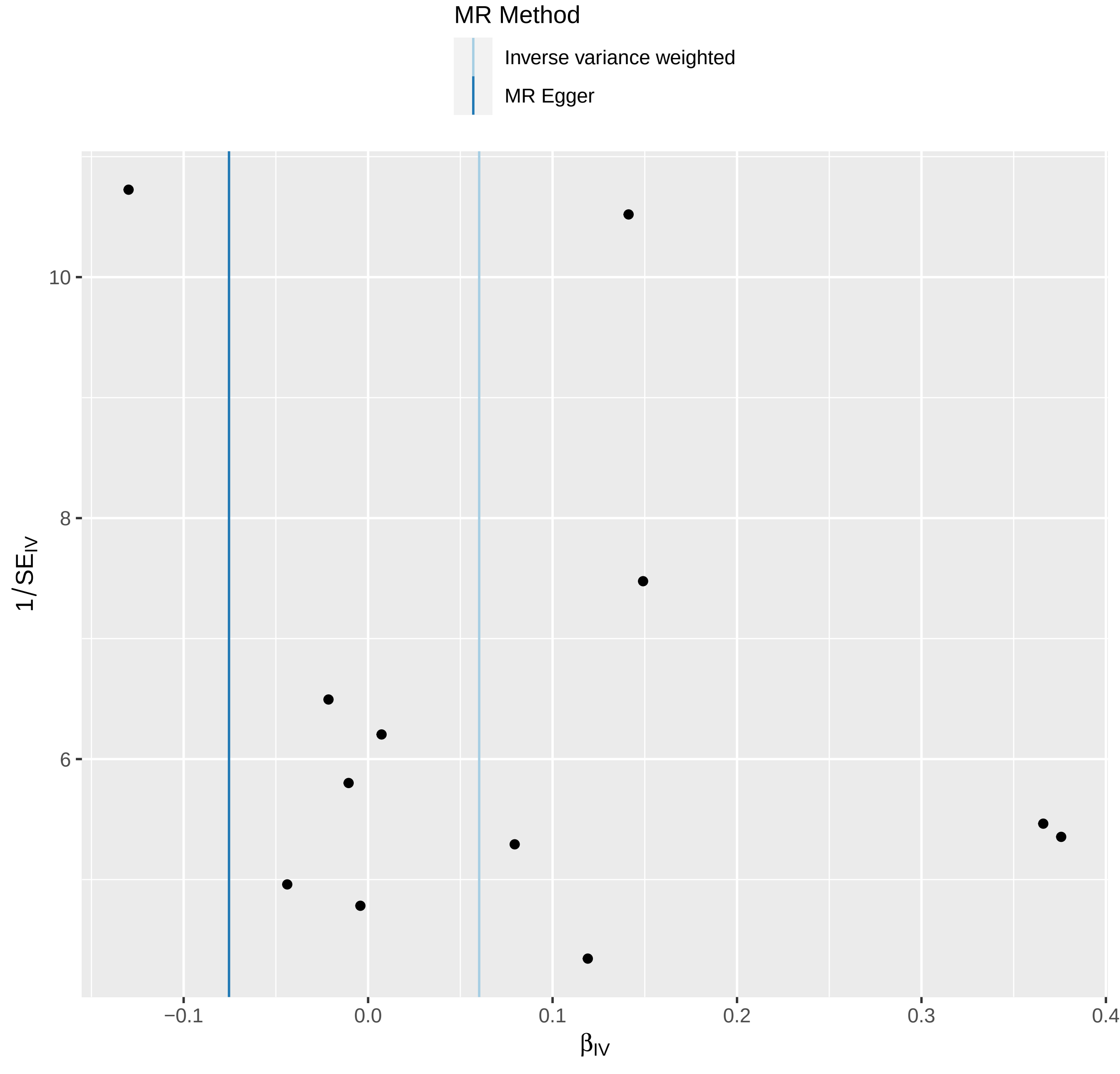

Batch 3−2413 : Selenium on Proteasome subunit alpha type−6

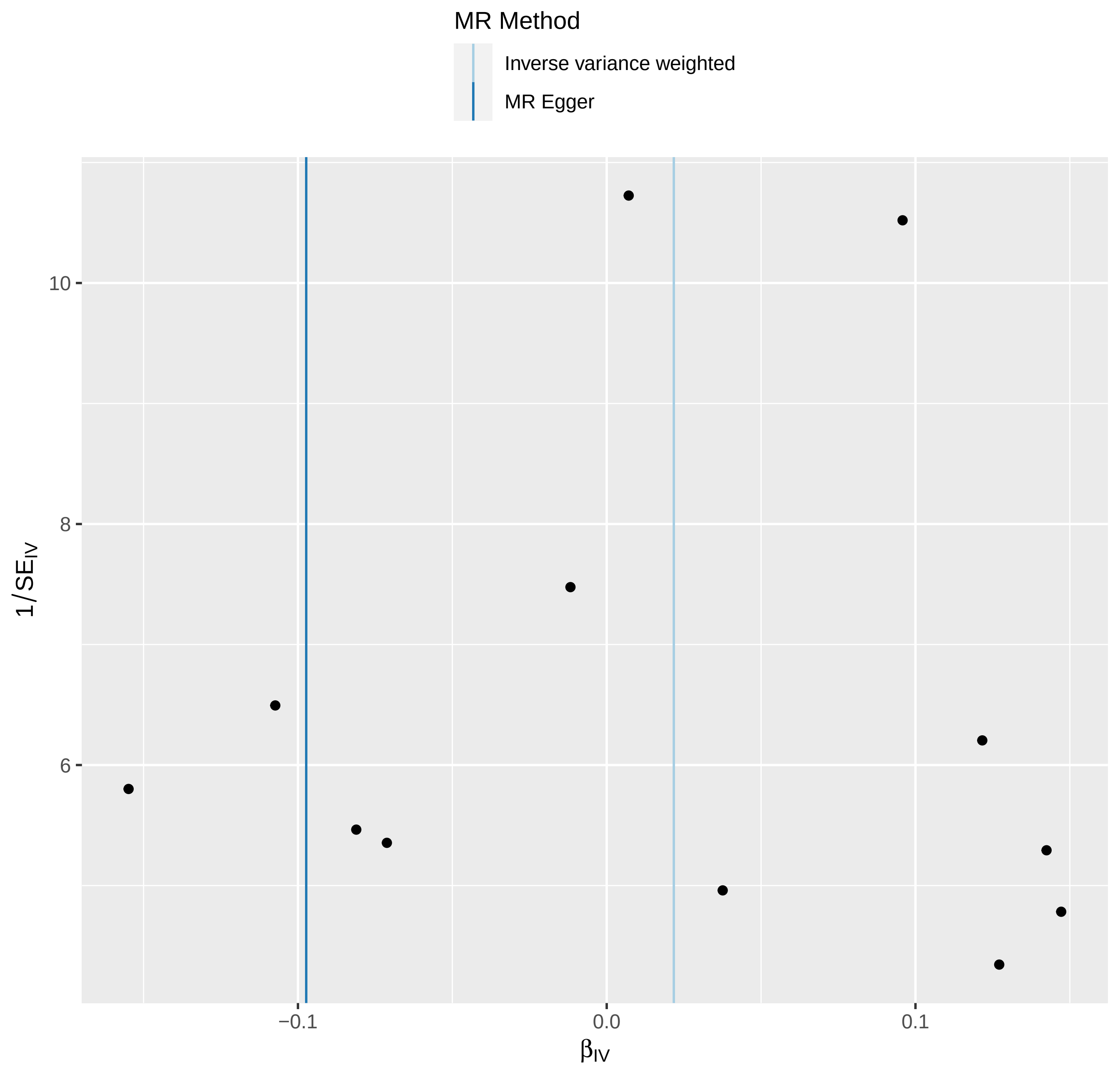

Batch 3−2414 : Selenium on Proteasome subunit beta type−5

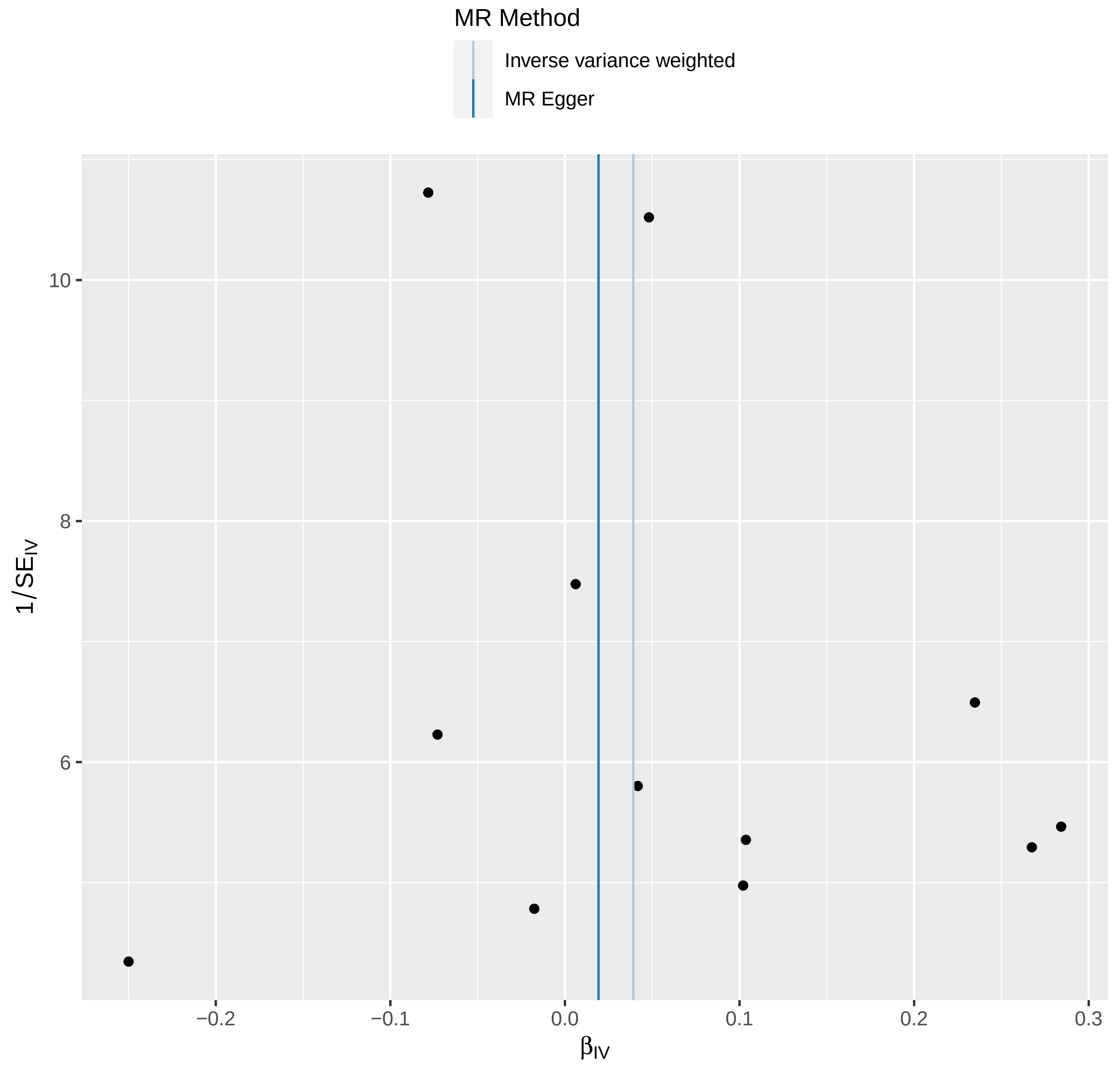

Batch 3−2415 : Selenium on 26S proteasome non−ATPase regulatory subunit 11

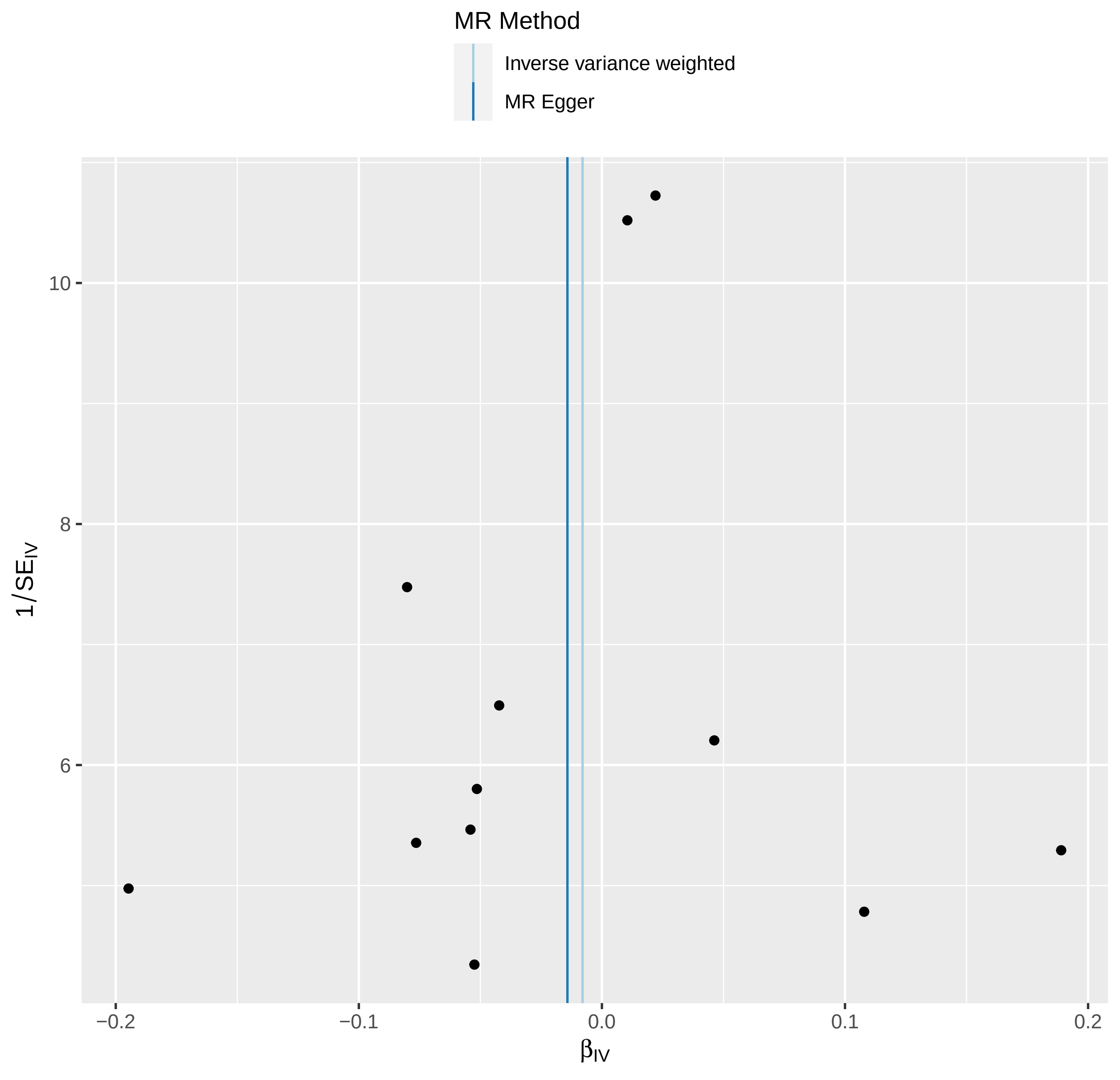

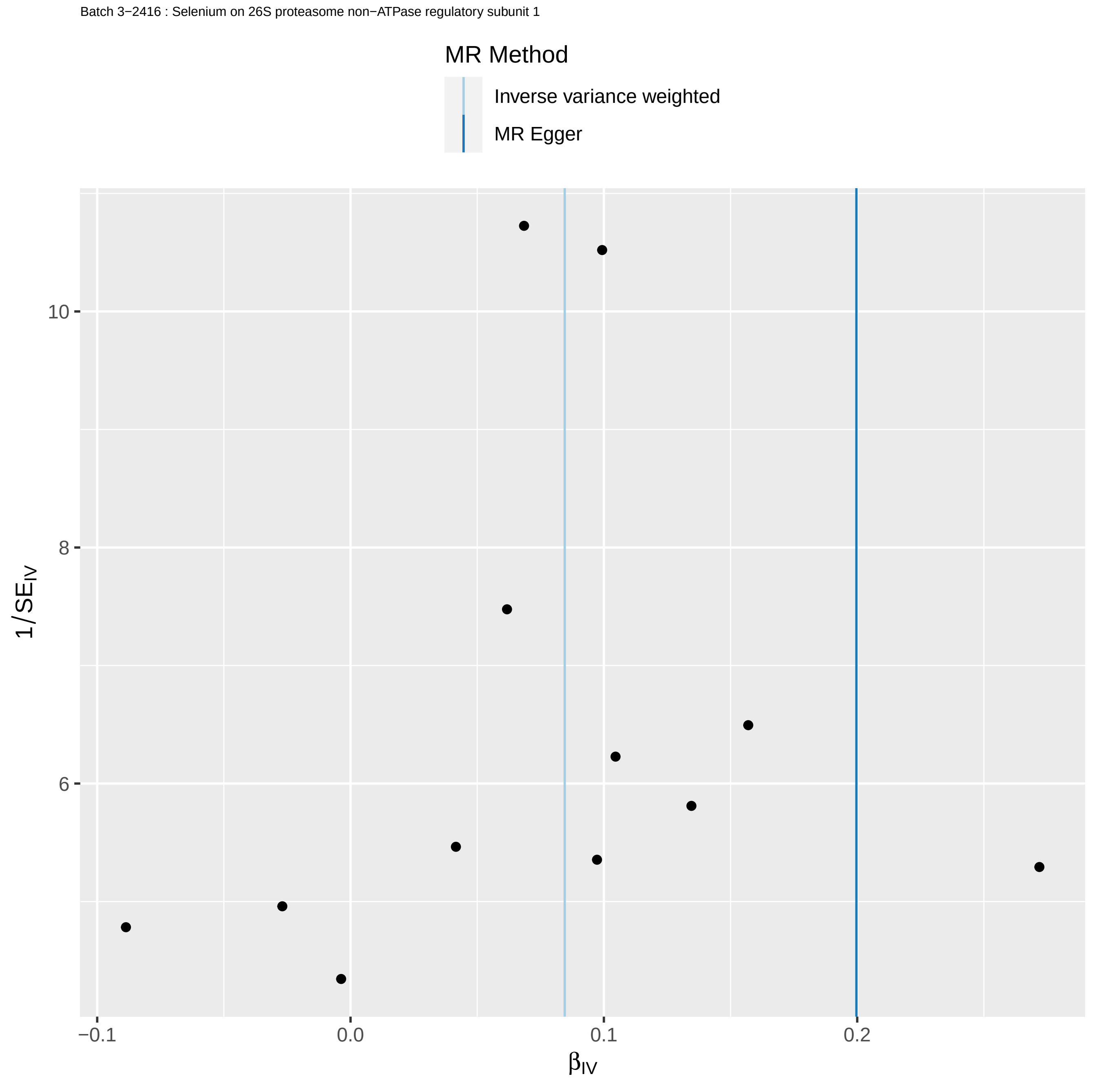

Batch 3−2416 : Selenium on 26S proteasome non−ATPase regulatory subunit 1
MR Method
Inverse variance weighted
MR Egger
10
8
6
1/SE$_{IV}$
−0.1
0.0
0.1
0.2
β$_{IV}$

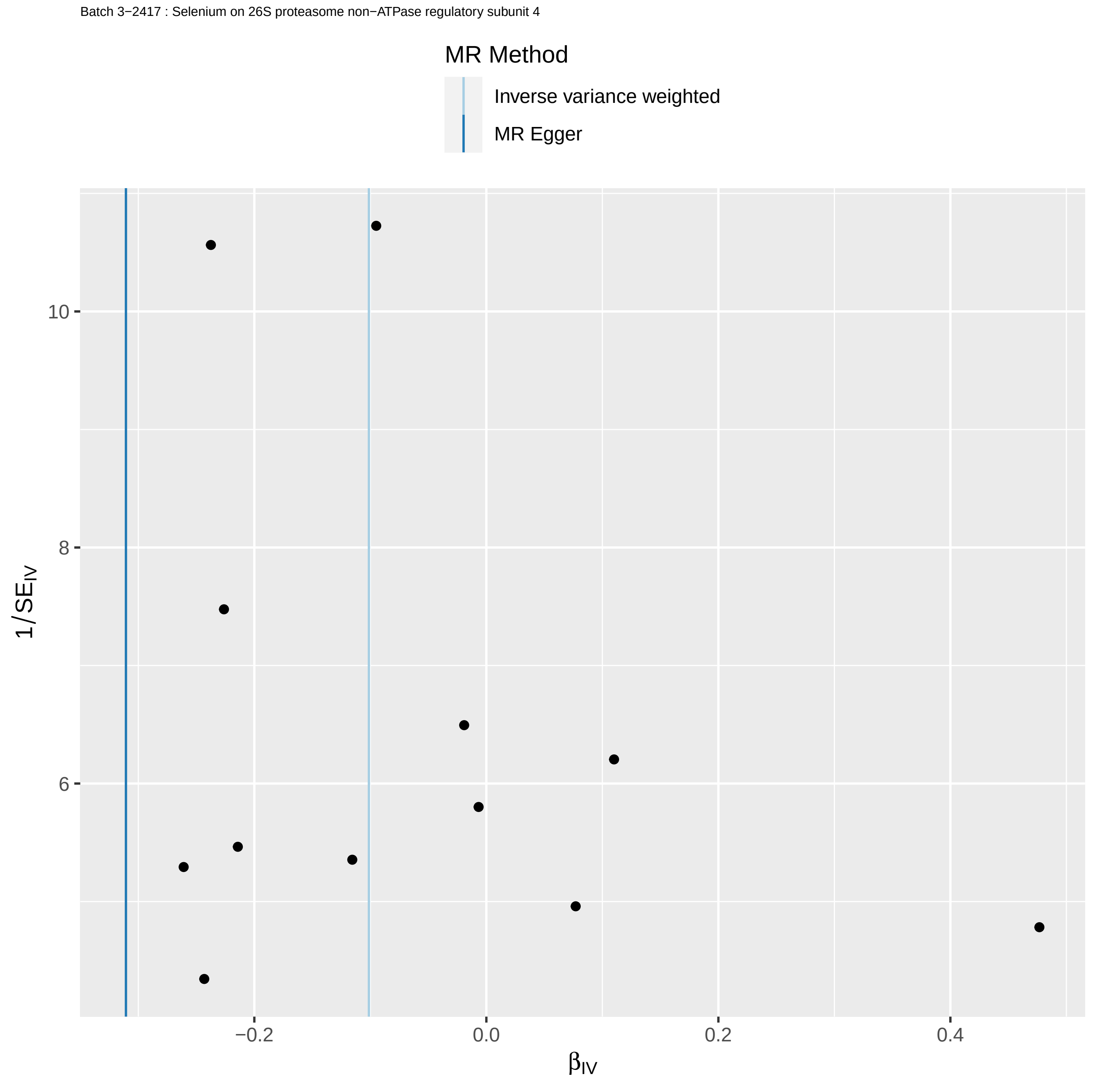

Batch 3−2417 : Selenium on 26S proteasome non−ATPase regulatory subunit 4
MR Method
Inverse variance weighted
MR Egger
10
8
6
1/SE_IV
−0.2
0.0
0.2
0.4
β_IV

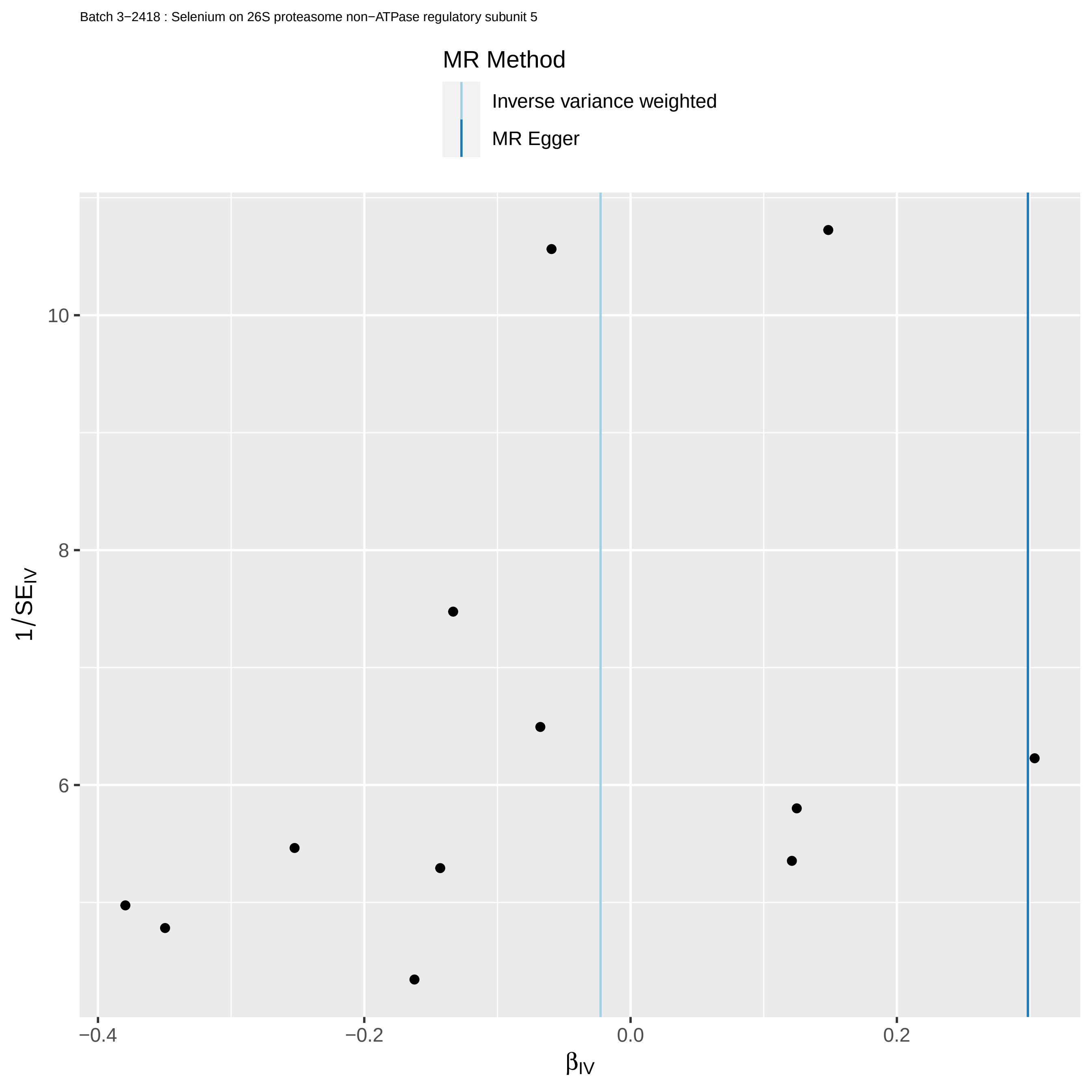
Batch 3−2418 : Selenium on 26S proteasome non−ATPase regulatory subunit 5
MR Method
Inverse variance weighted
MR Egger
10
8
6
1/SE_IV
−0.4
−0.2
0.0
0.2
β_IV

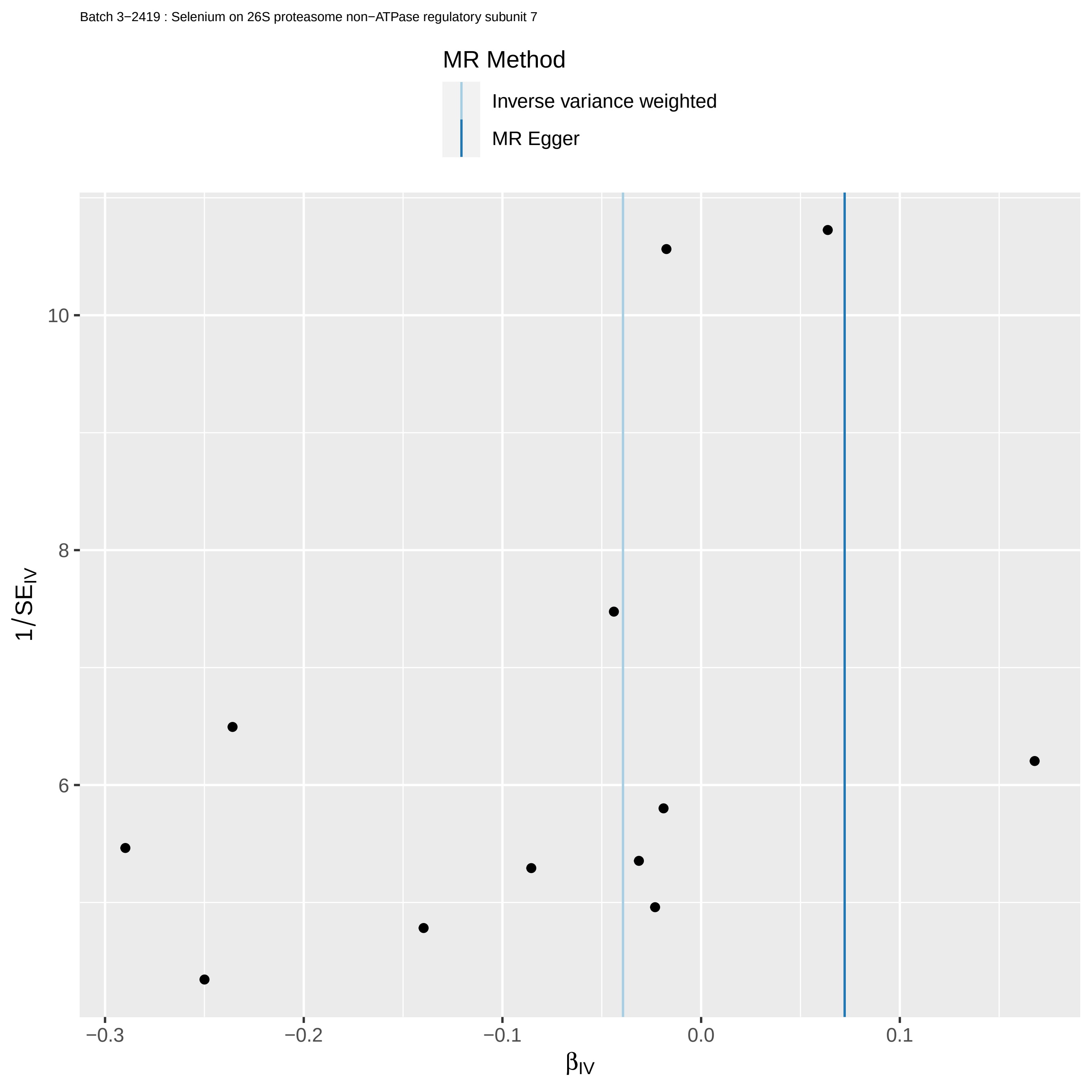
Batch 3−2419 : Selenium on 26S proteasome non−ATPase regulatory subunit 7
MR Method
Inverse variance weighted
MR Egger
10
8
6
1/SE_IV
−0.3
−0.2
−0.1
0.0
0.1
β_IV

Batch 3−2420 : Selenium on Proteasome activator complex subunit 1

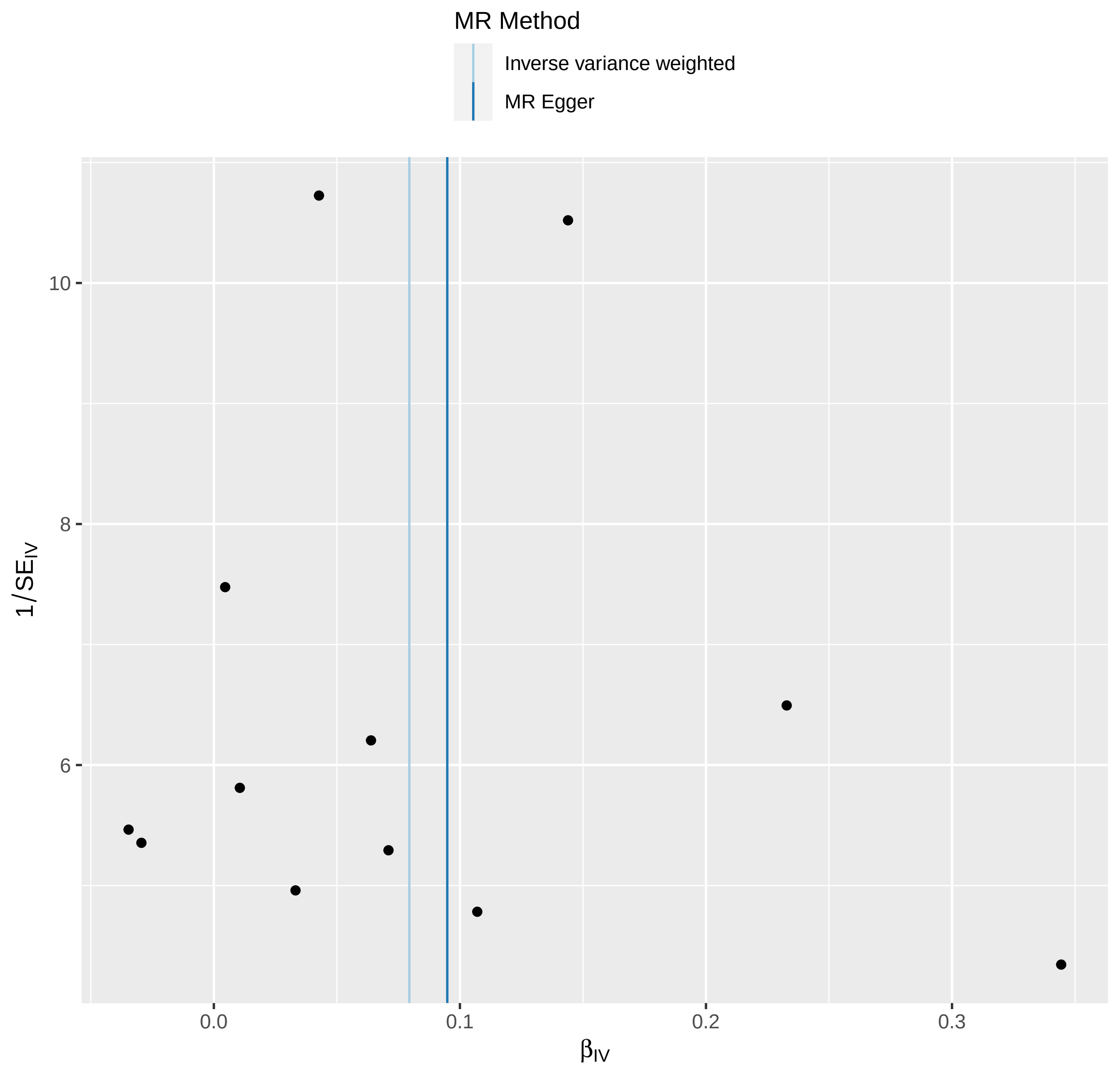

Batch 3−2421 : Selenium on Proteasome activator complex subunit 3

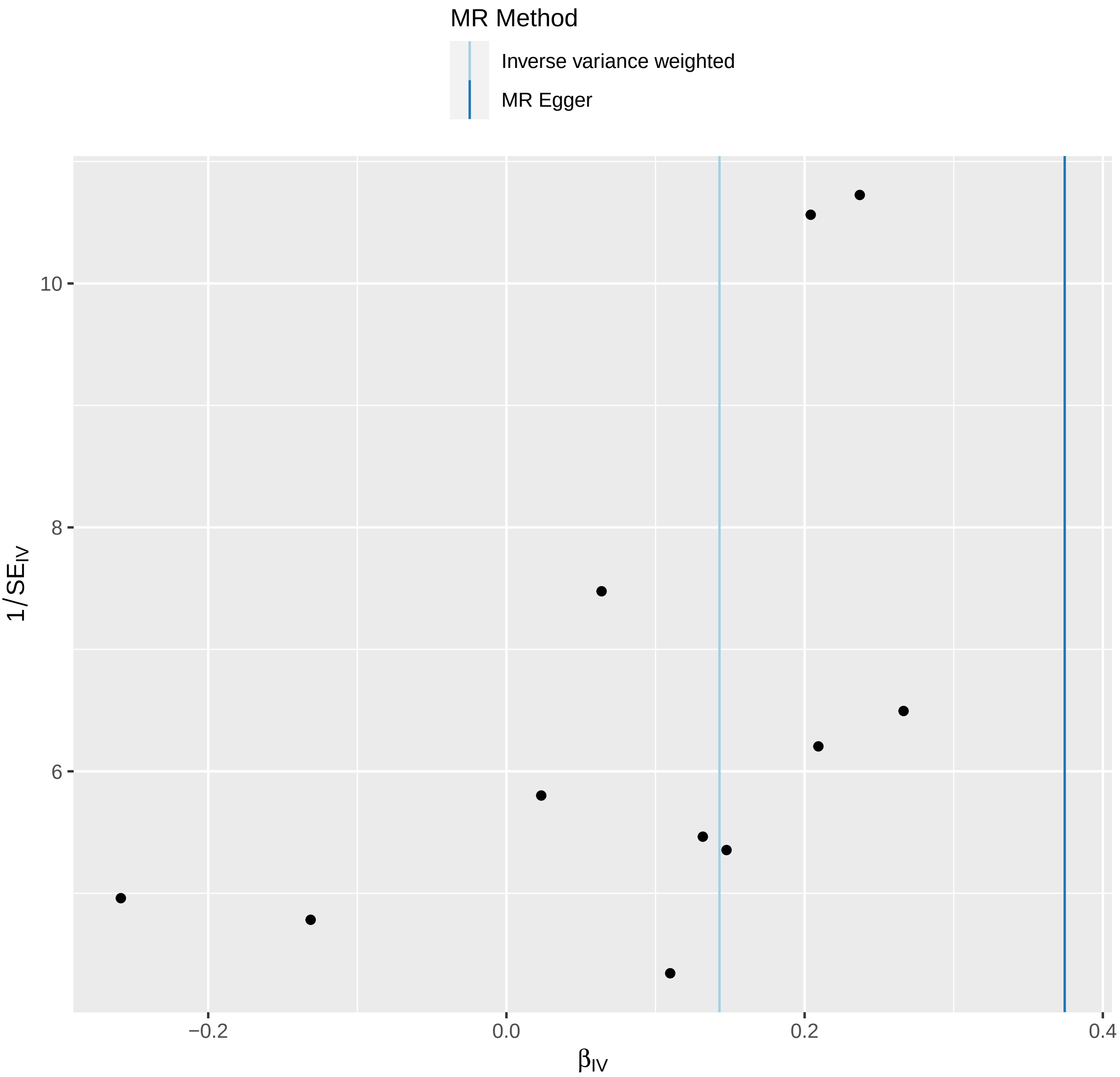

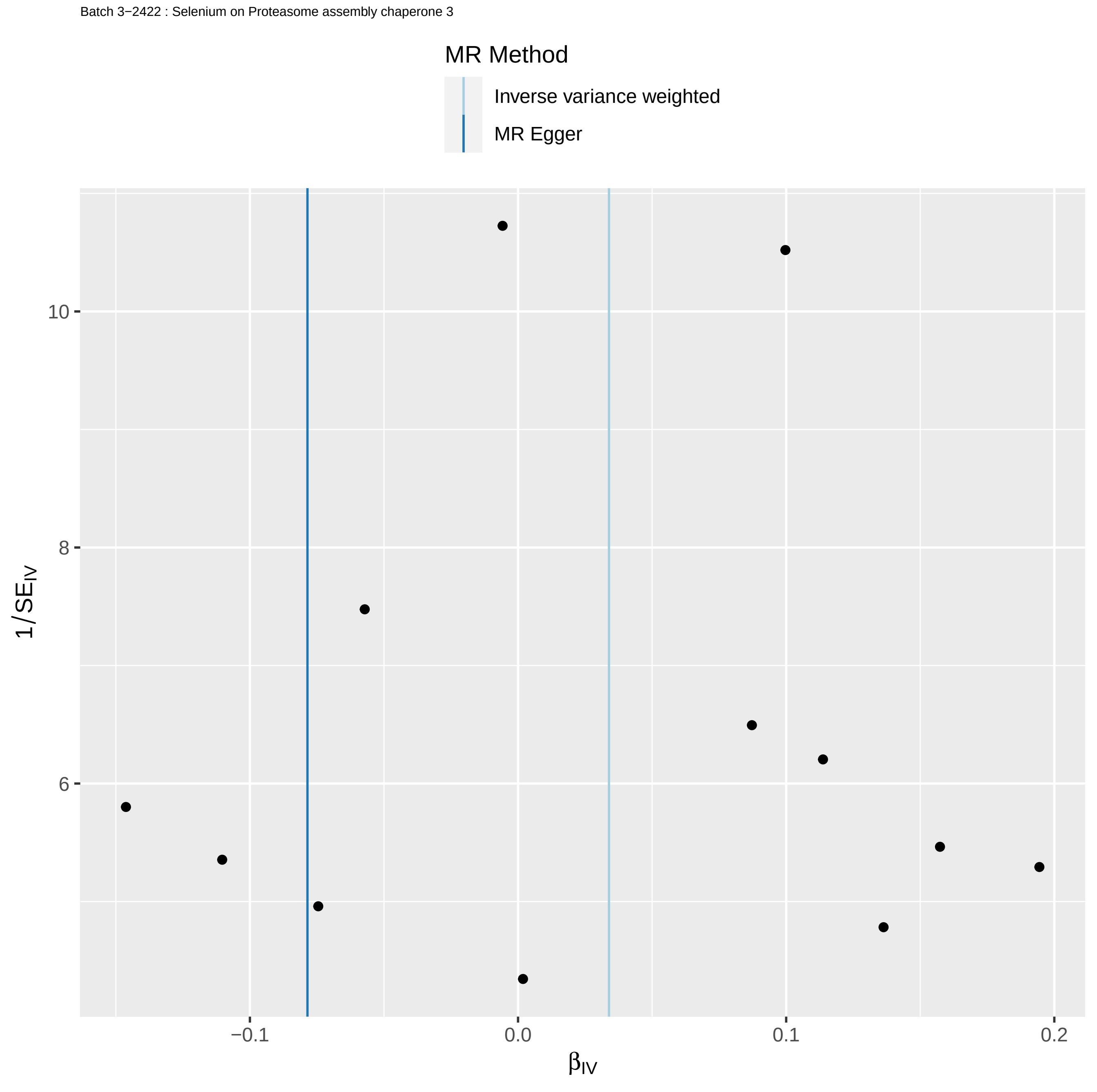
Batch 3−2422 : Selenium on Proteasome assembly chaperone 3
MR Method
Inverse variance weighted
MR Egger
10
8
6
1/SE$_{IV}$
−0.1
0.0
0.1
0.2
β_{IV}

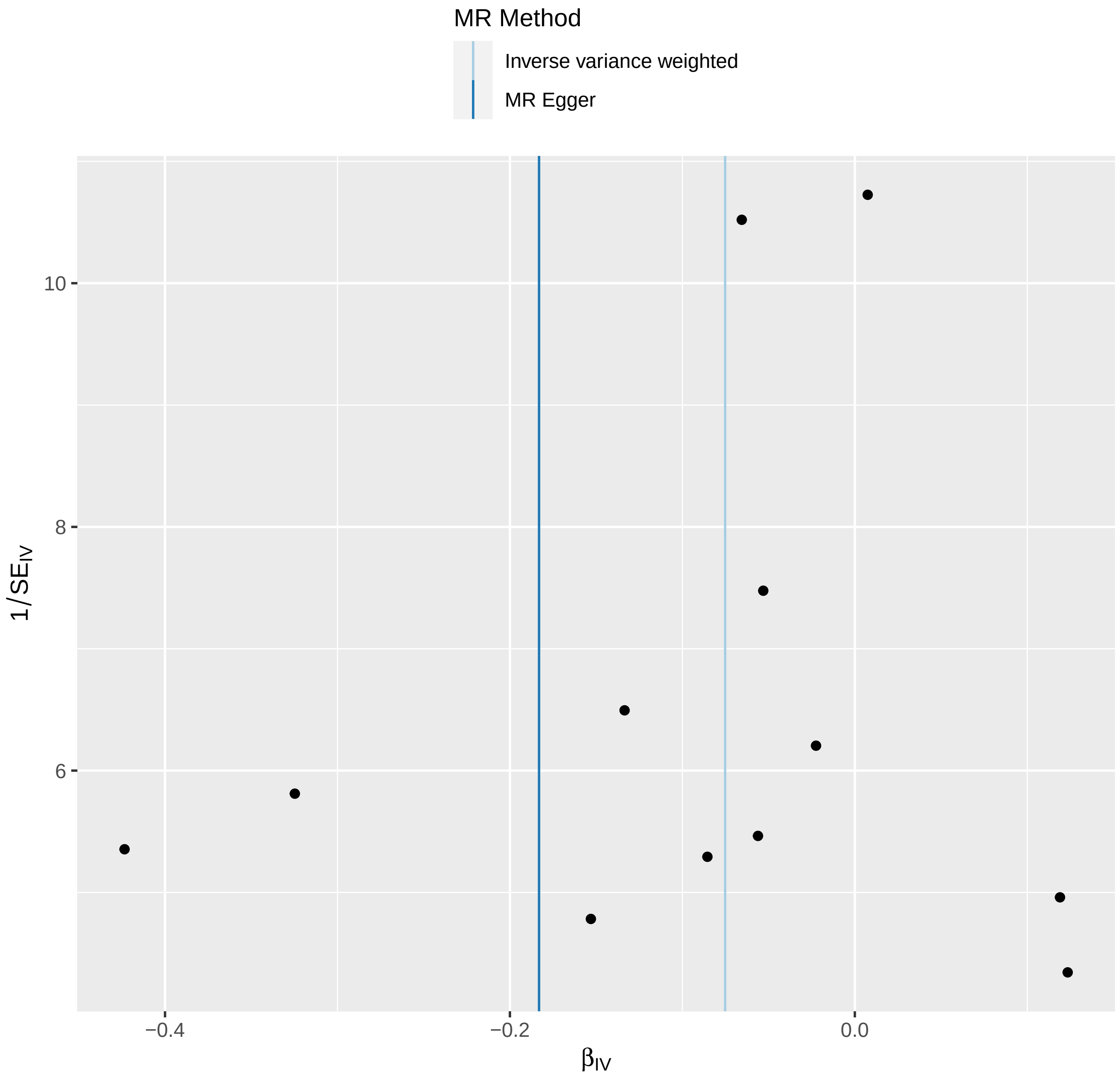
MR Method
Inverse variance weighted
MR Egger
10
8
6
1/SE$_{IV}$
−0.4
−0.2
0.0
β$_{IV}$

Batch 3−2424 : Selenium on Patched domain−containing protein 3

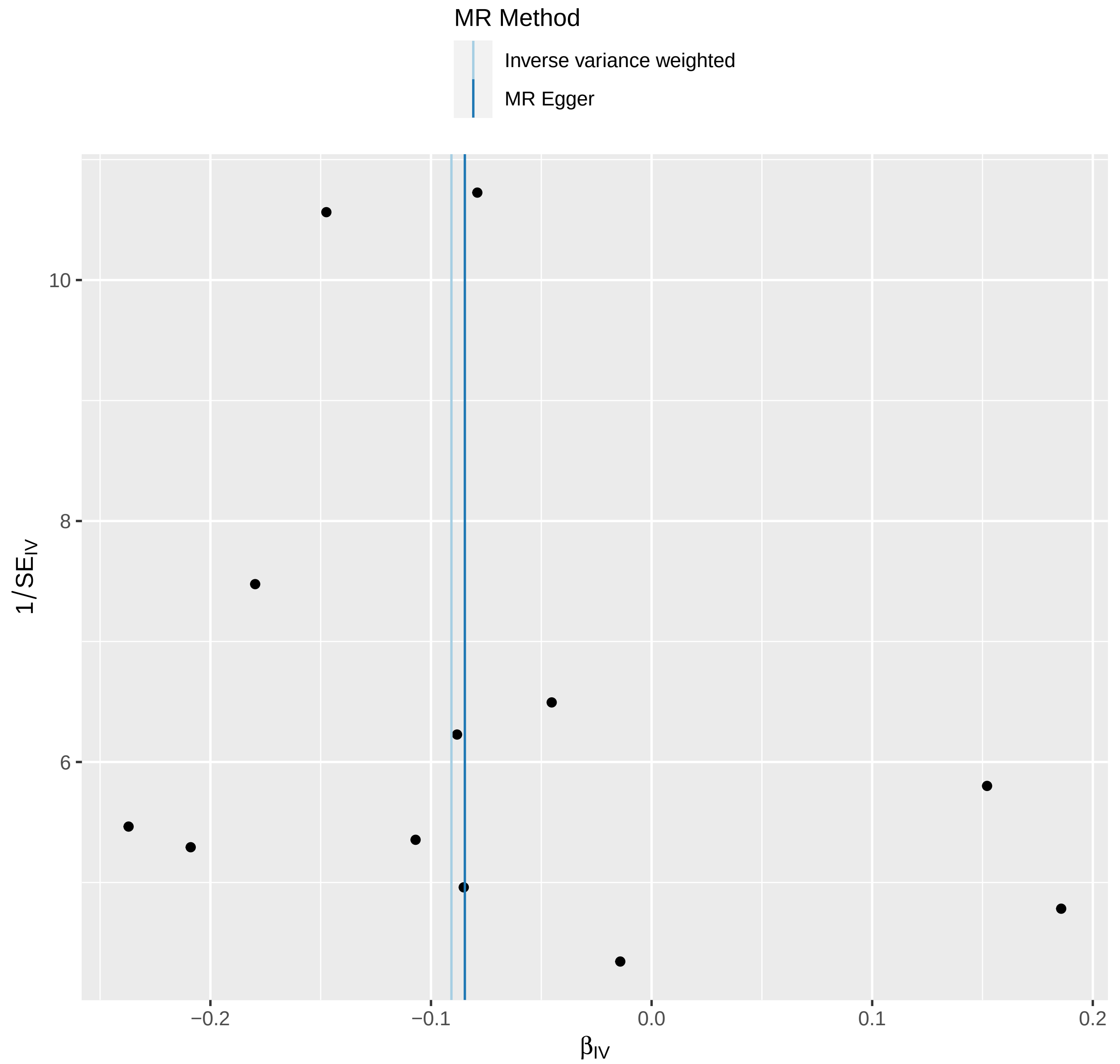

Batch 3−2425 : Selenium on Phosphatidylinositol 3,4,5−trisphosphate 3−phosphatase and dual−specificity protein phosphatase PTEN

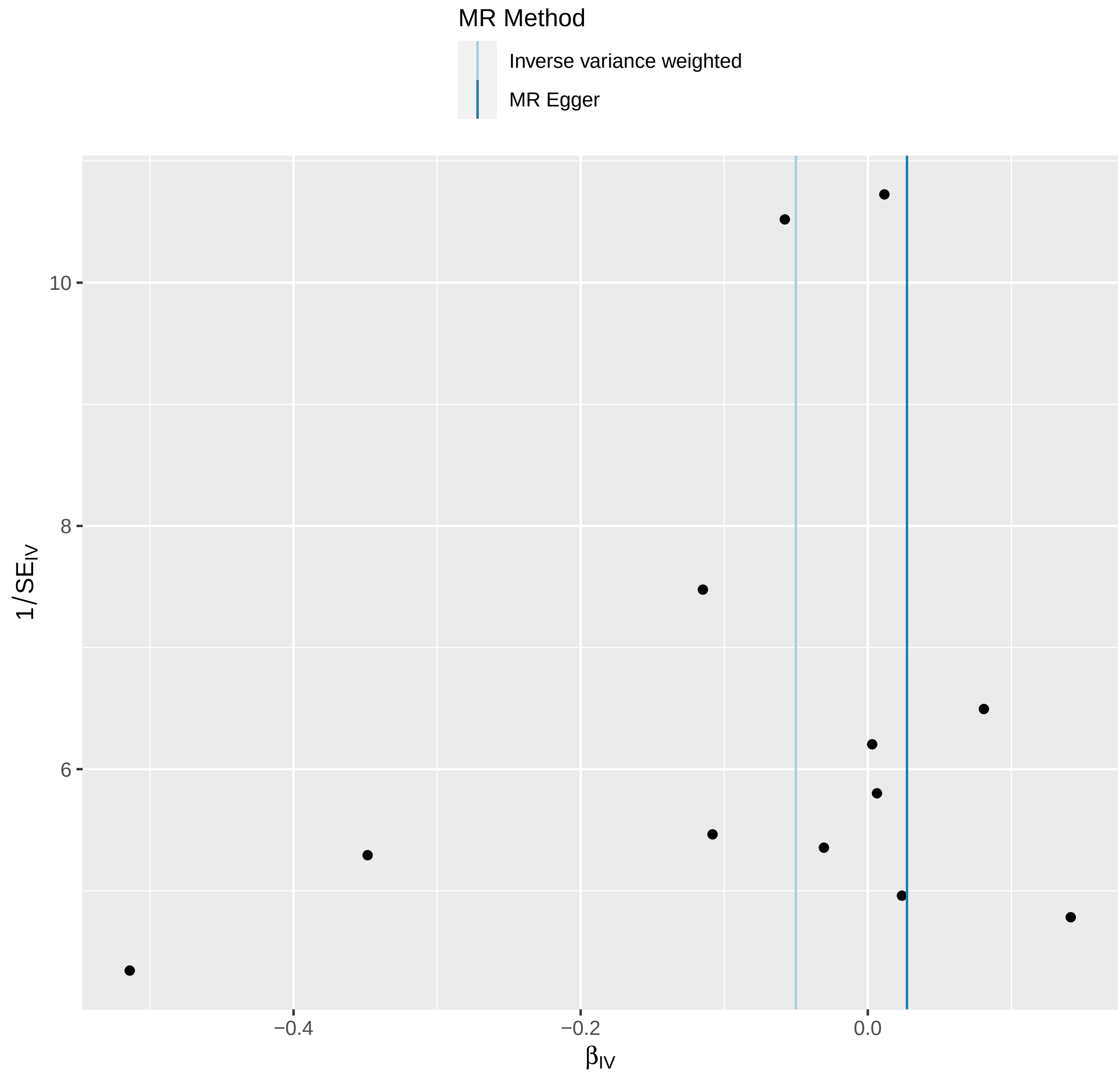

Batch 3−2426 : Selenium on Prostaglandin−H2 D−isomerase

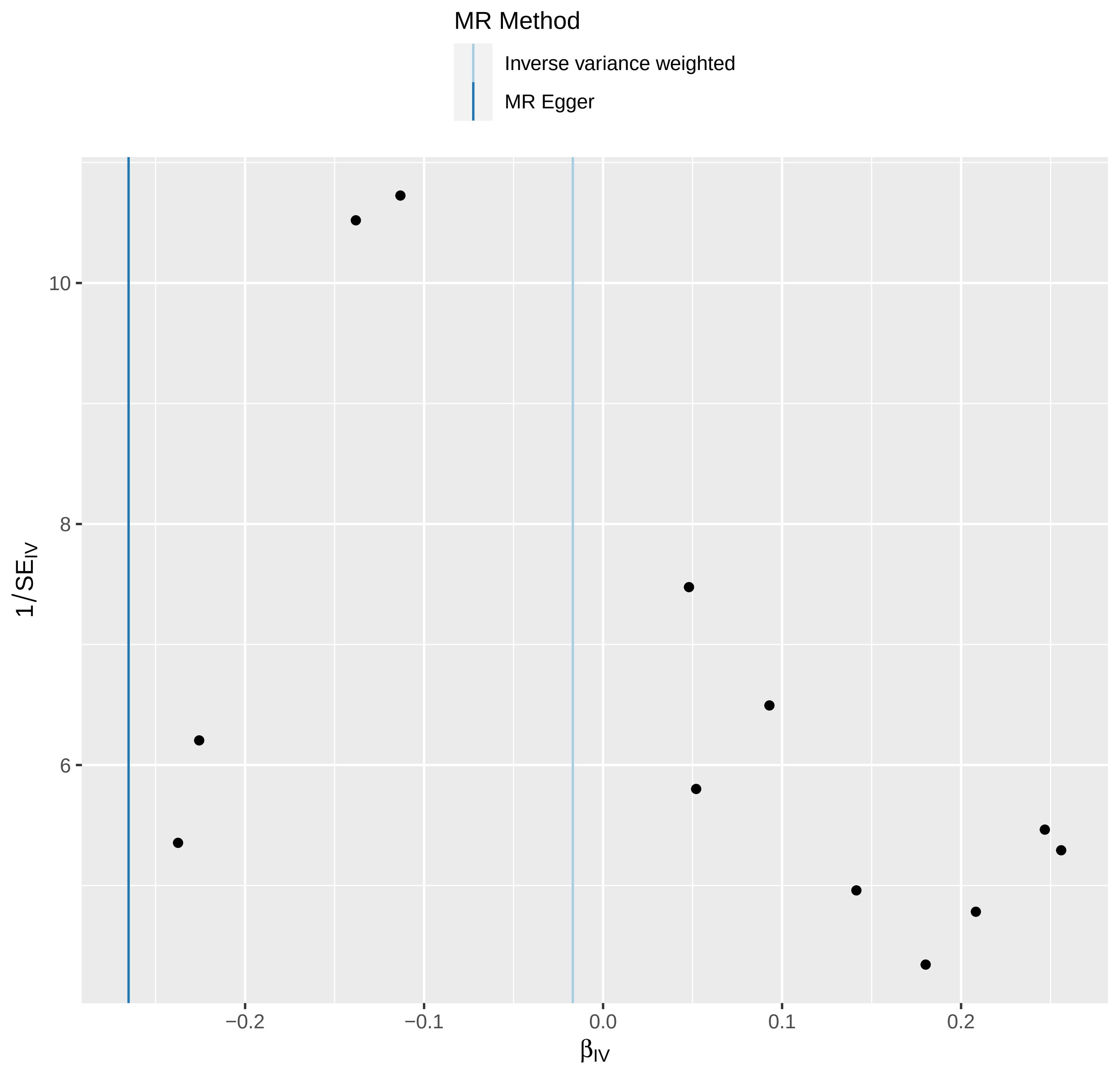

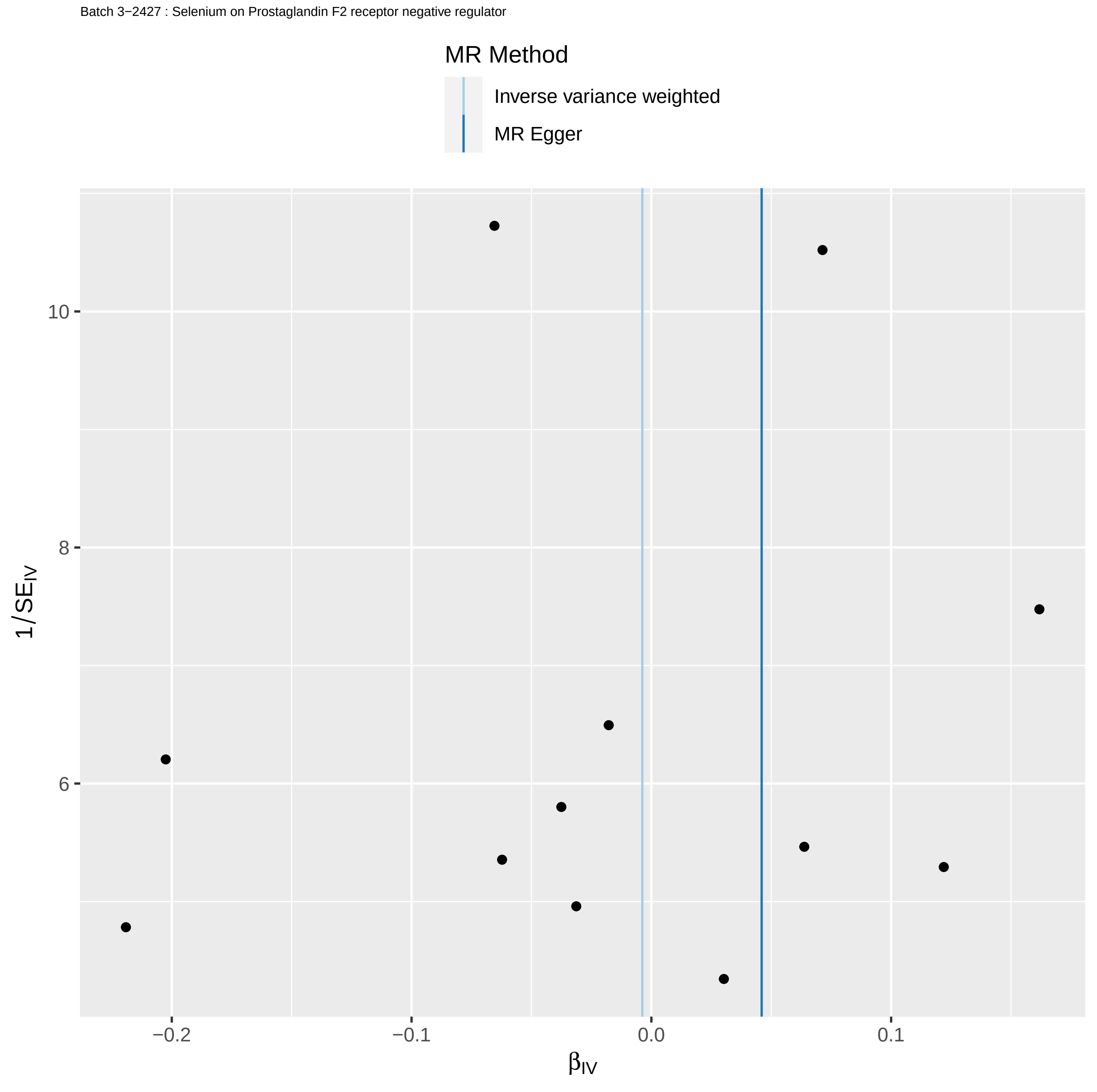
Batch 3−2427 : Selenium on Prostaglandin F2 receptor negative regulator
MR Method
Inverse variance weighted
MR Egger
10
8
6
1/SE_IV
−0.2
−0.1
0.0
0.1
β_IV

Batch 3−2428 : Selenium on Prostaglandin reductase 1

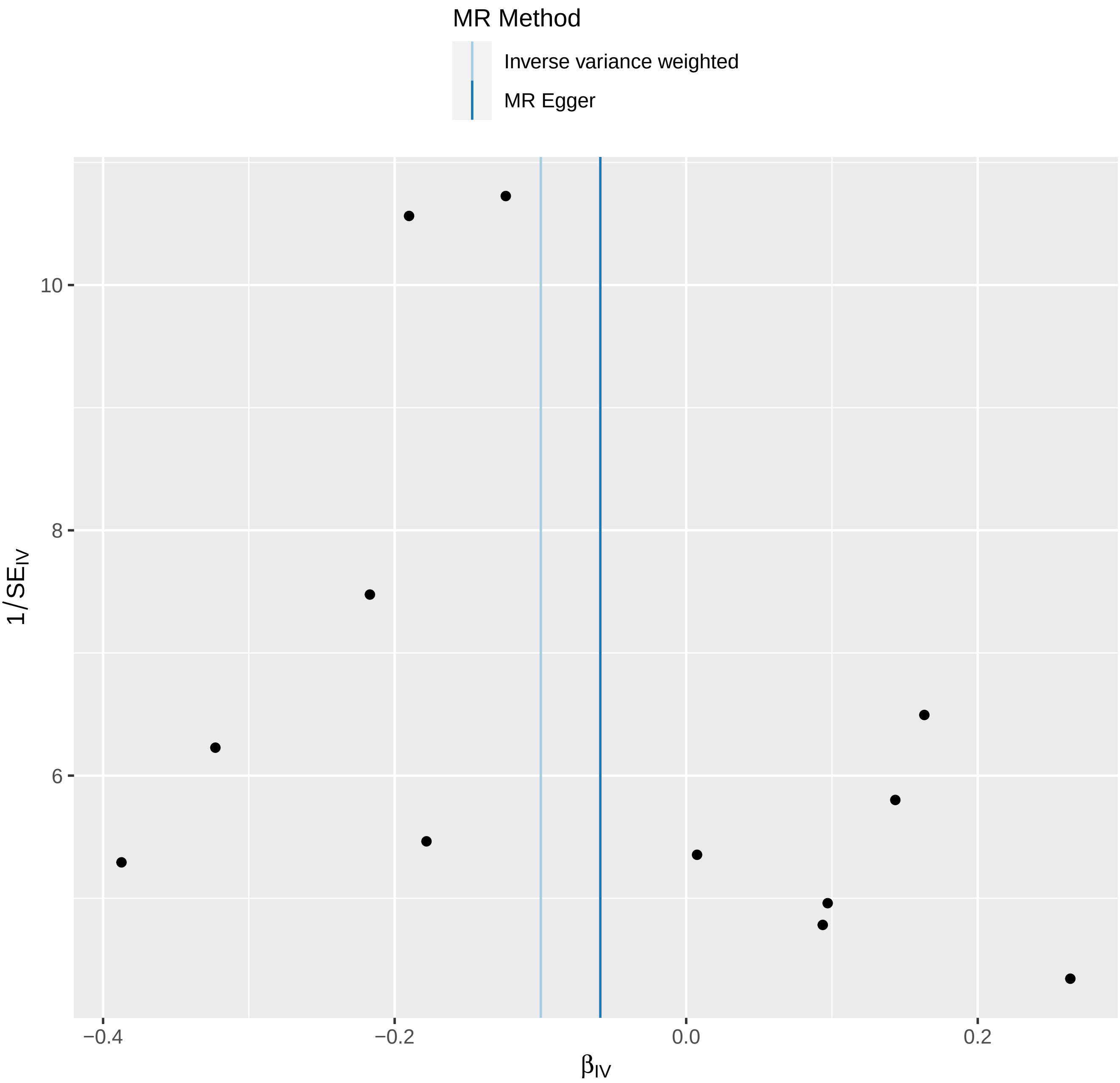

Batch 3−2429 : Selenium on Parathyroid hormone/parathyroid hormone−related peptide receptor

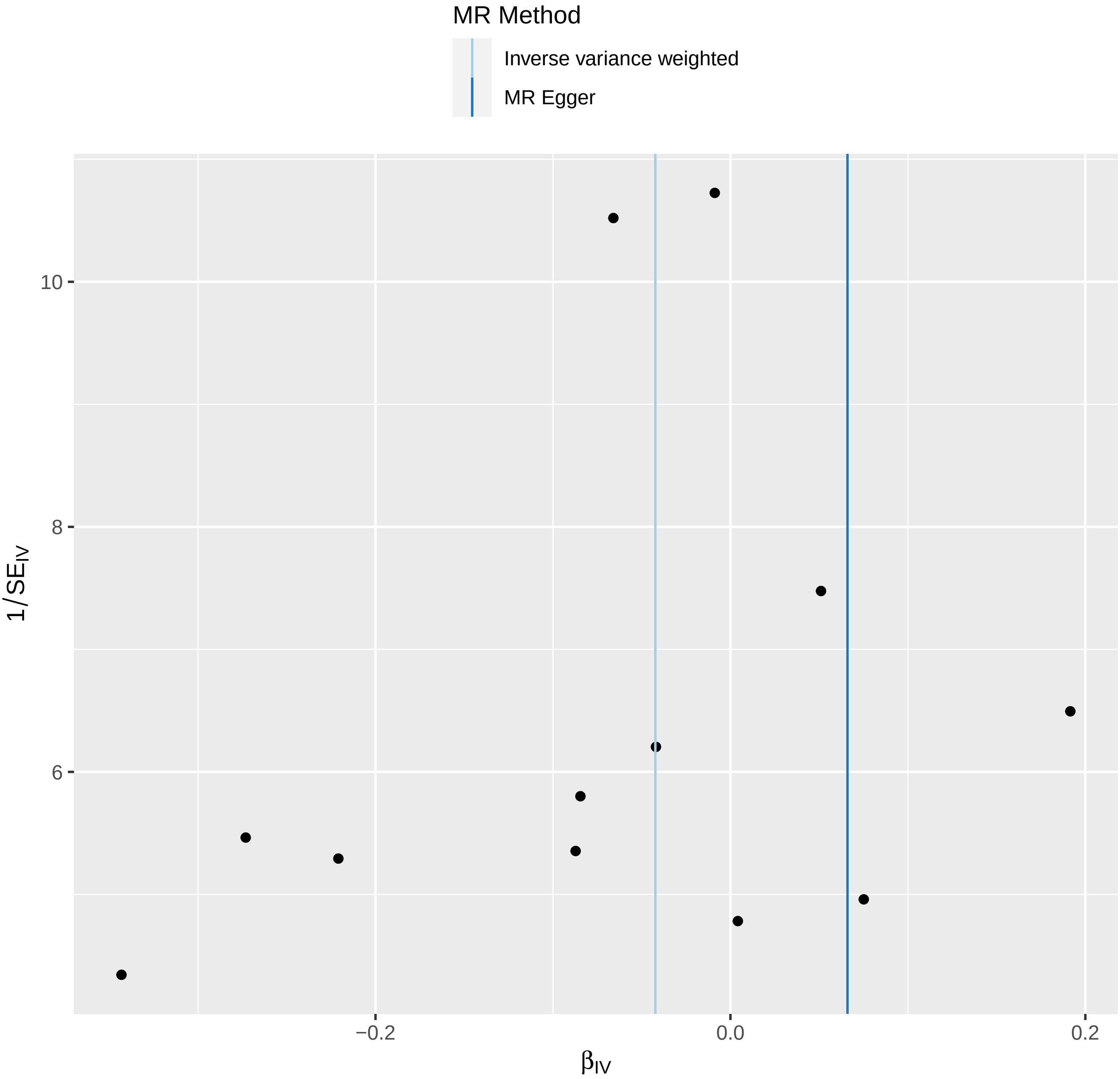

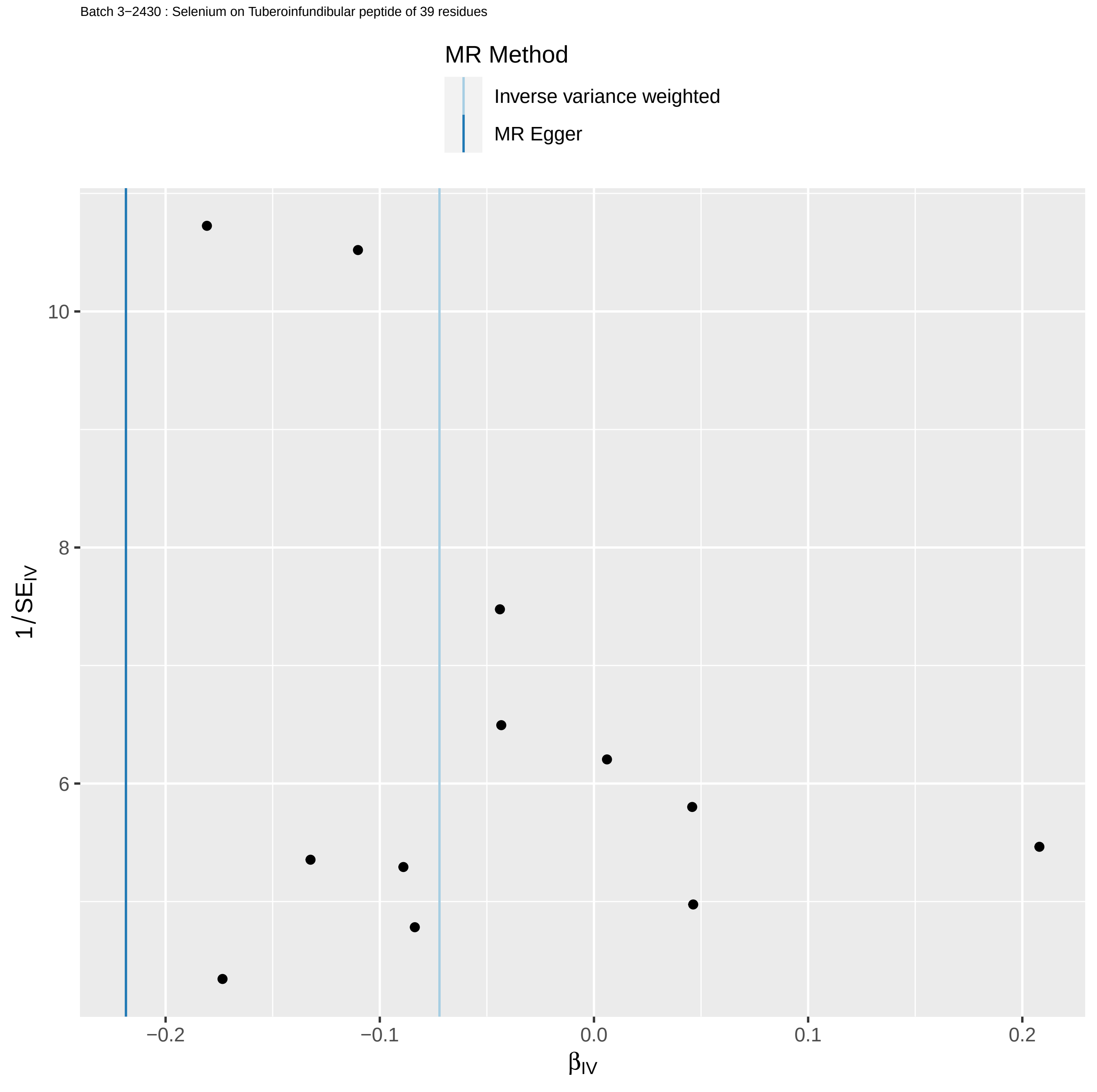

Batch 3−2430 : Selenium on Tuberoinfundibular peptide of 39 residues
MR Method
Inverse variance weighted
MR Egger
10
8
6
1/SE$_{IV}$
−0.2
−0.1
0.0
0.1
0.2
β$_{IV}$

Batch 3−2431 : Selenium on Parathyroid hormone

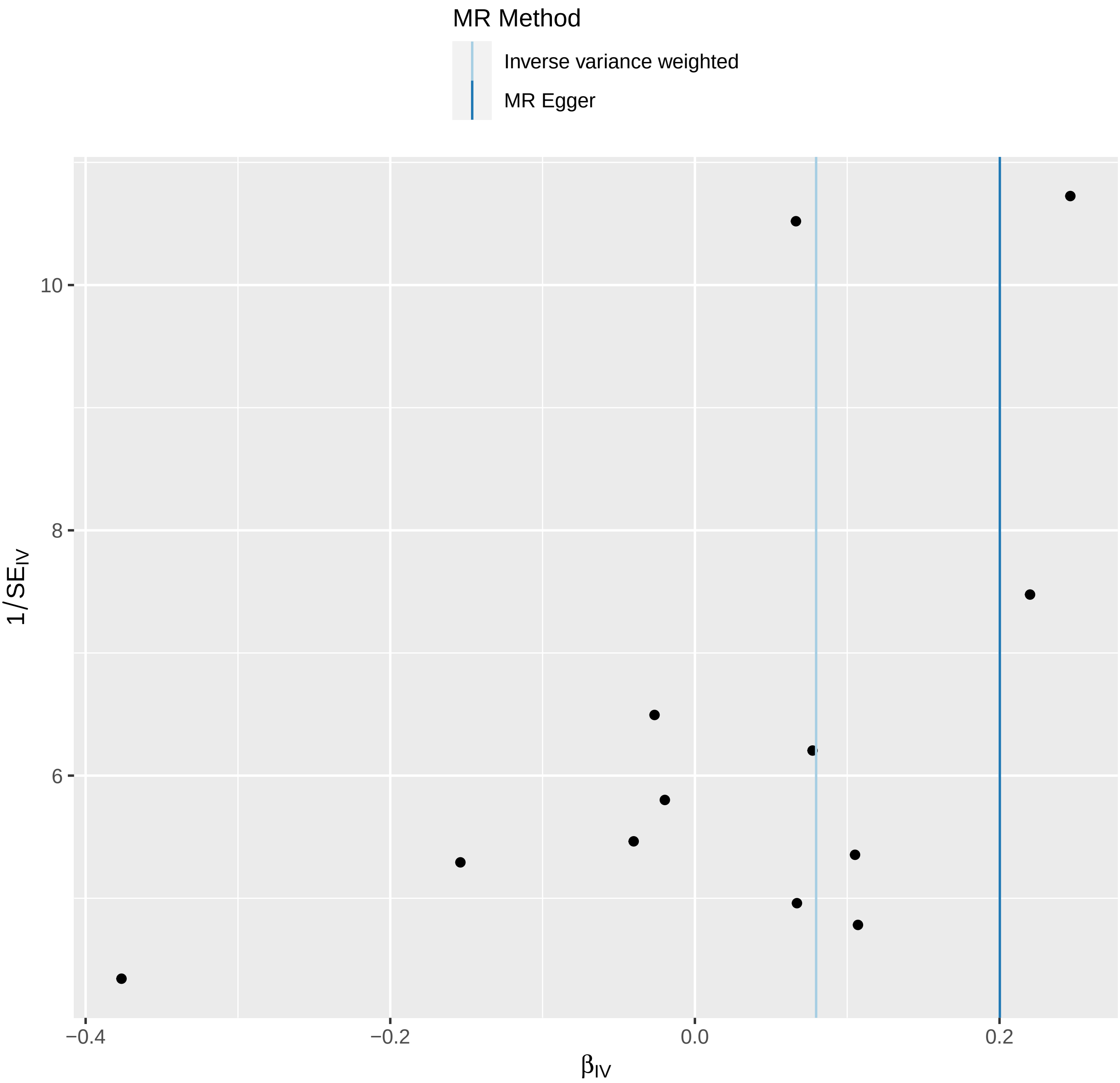

Batch 3−2432 : Selenium on Parathyroid hormone−related protein

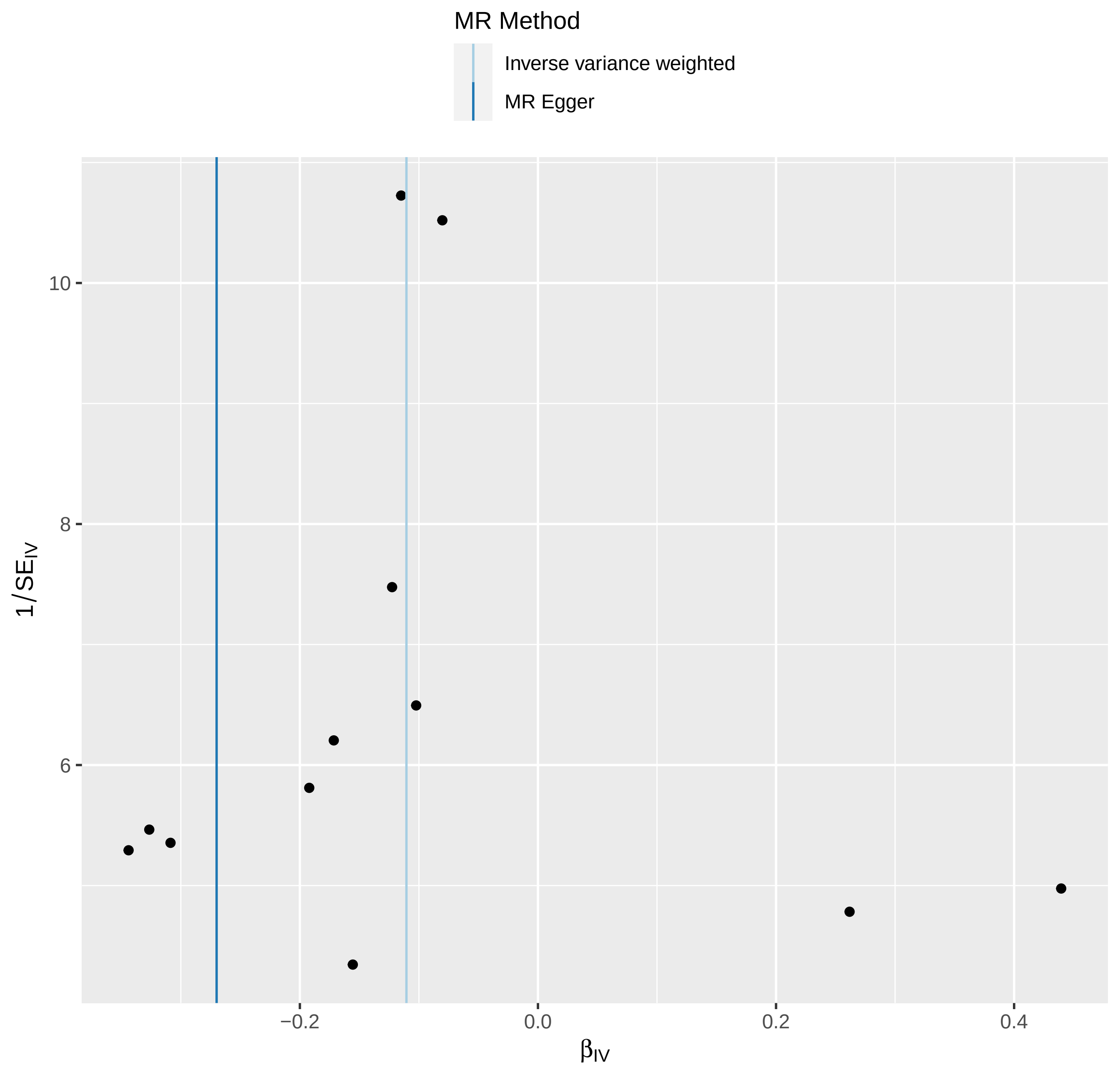

Batch 3−2433 : Selenium on Protein−tyrosine kinase 2−beta

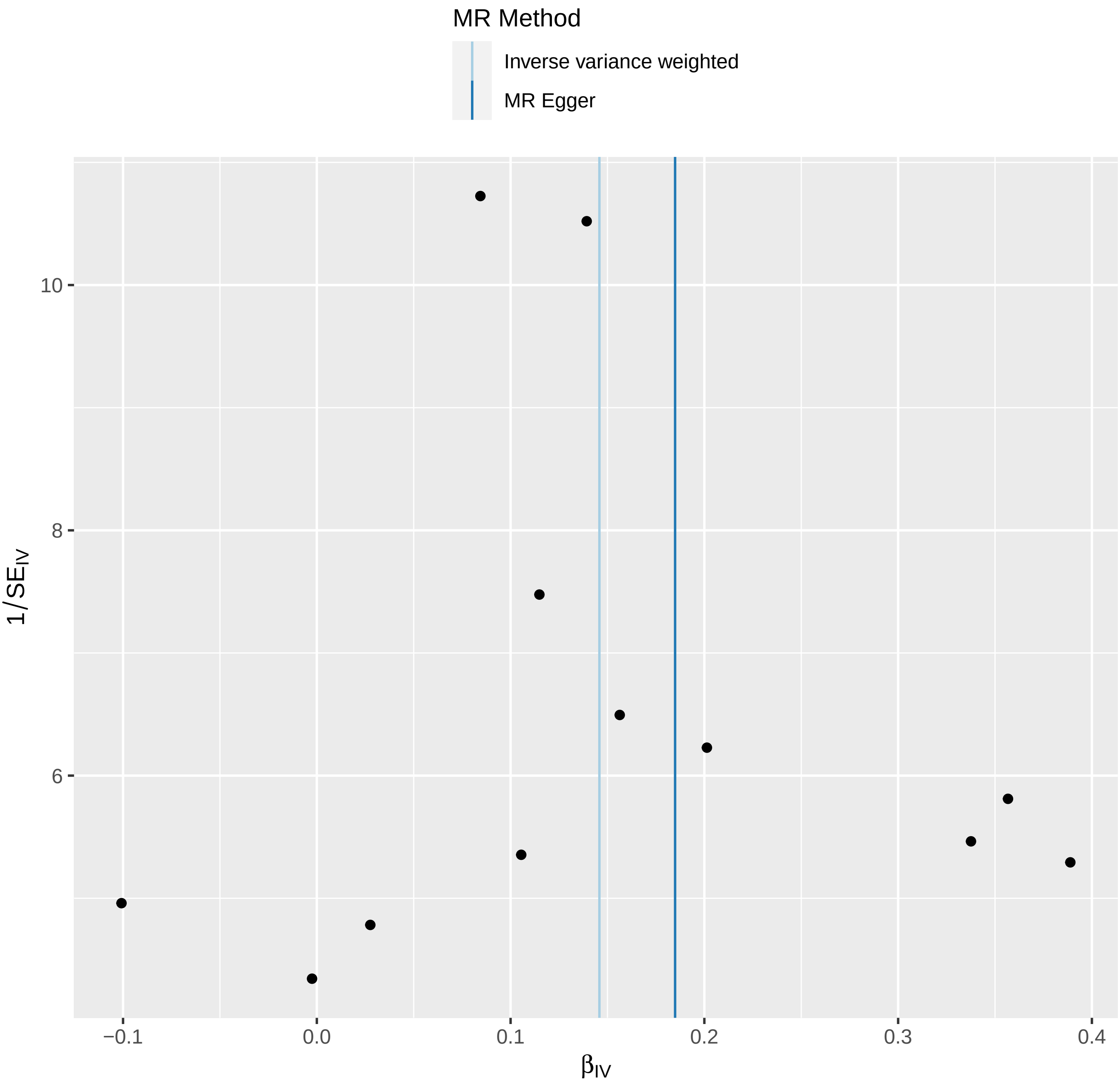

Batch 3−2434 : Selenium on Protein−tyrosine kinase 6

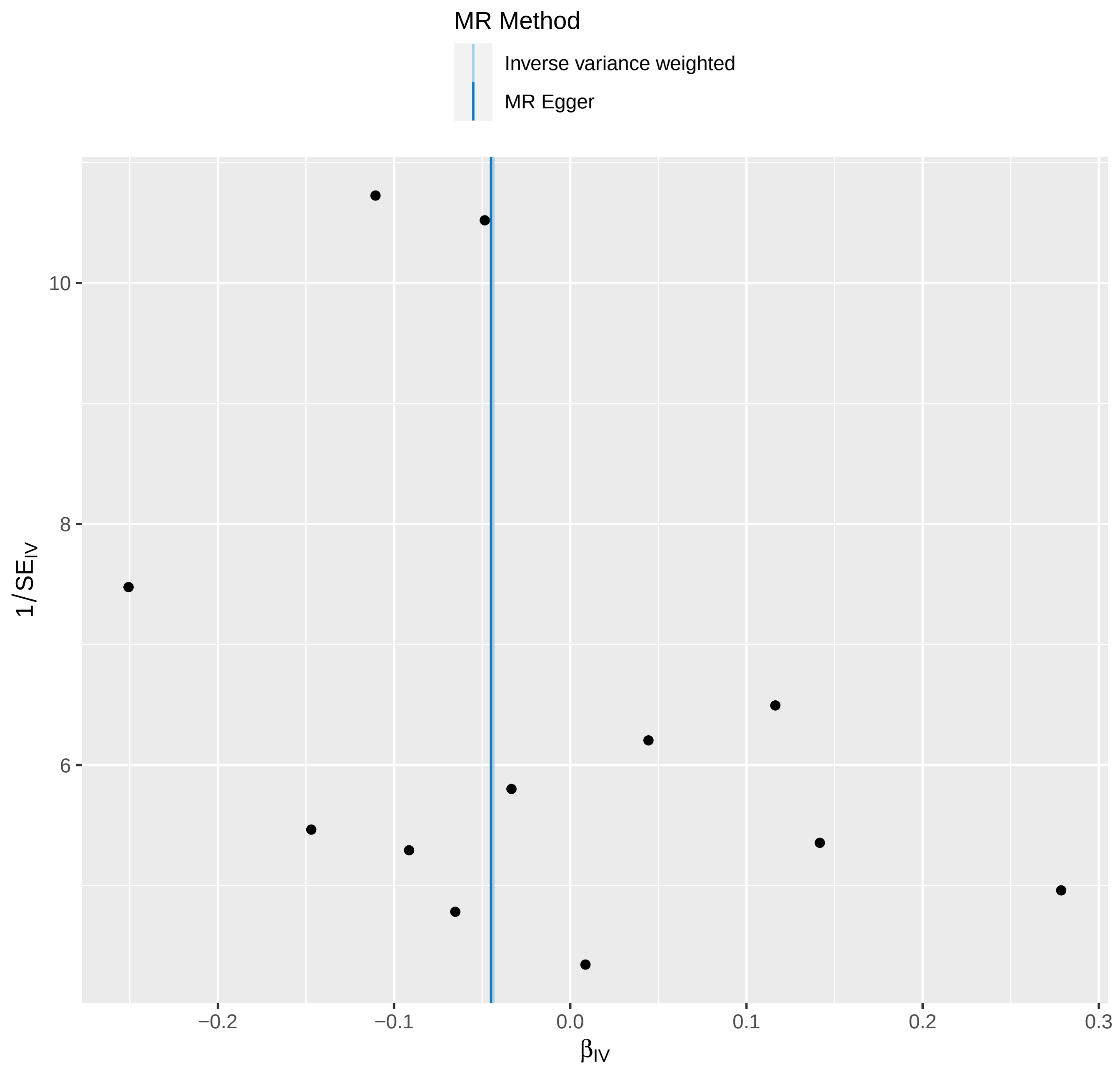

Batch 3−2435 : Selenium on Pleiotrophin

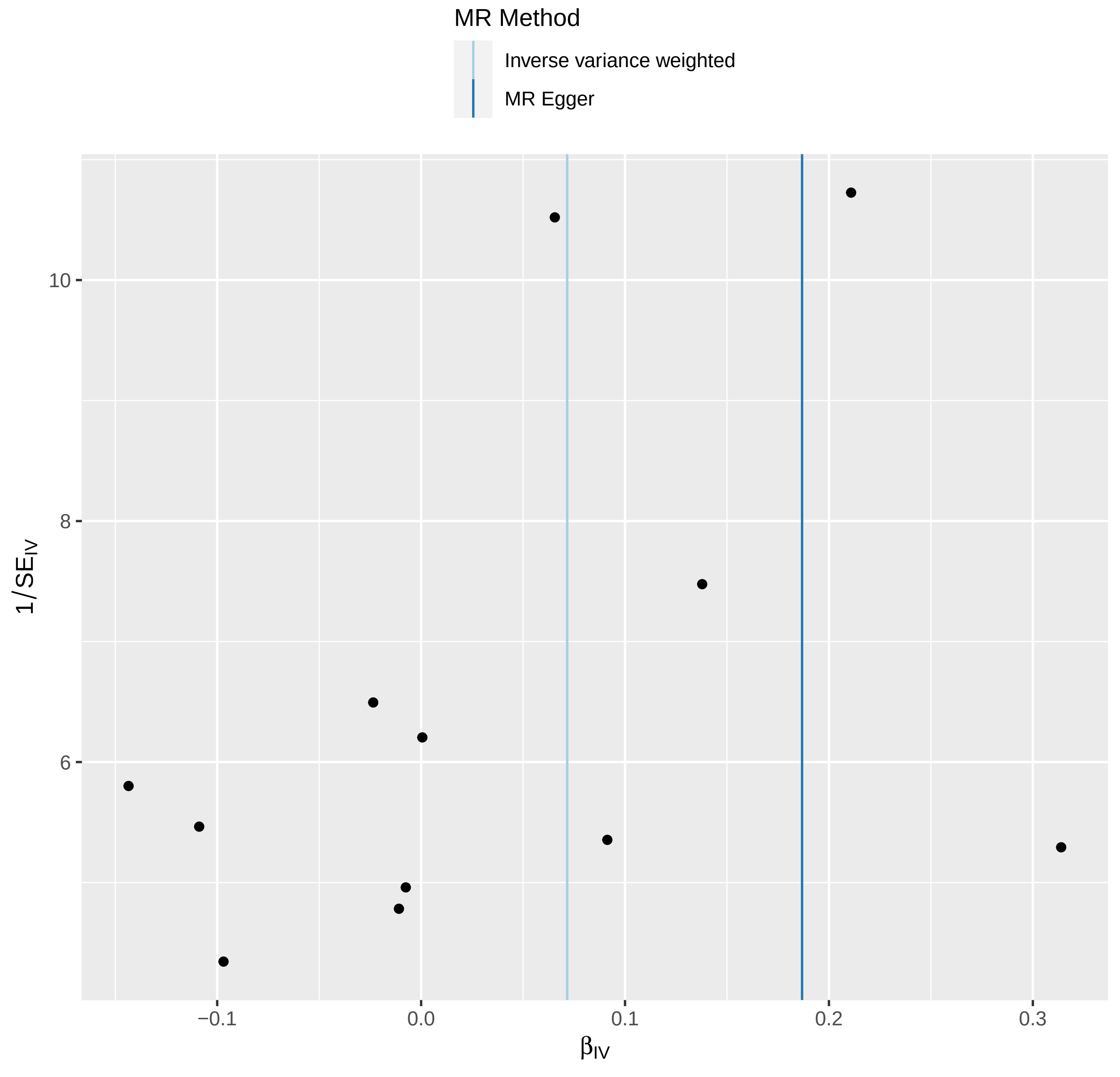

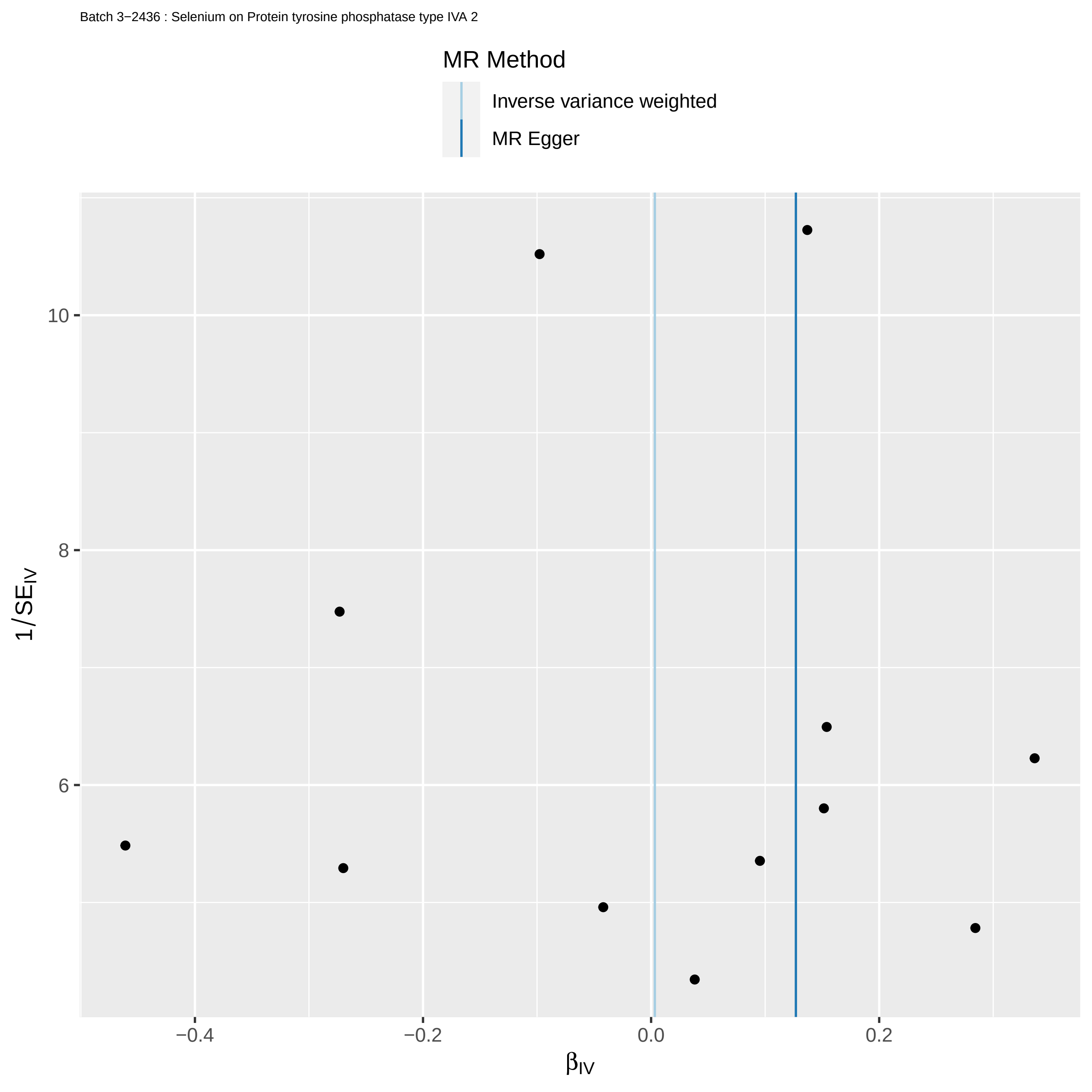
Batch 3−2436 : Selenium on Protein tyrosine phosphatase type IVA 2
MR Method
Inverse variance weighted
MR Egger
10
8
6
1/SE_IV
−0.4
−0.2
0.0
0.2
β_IV

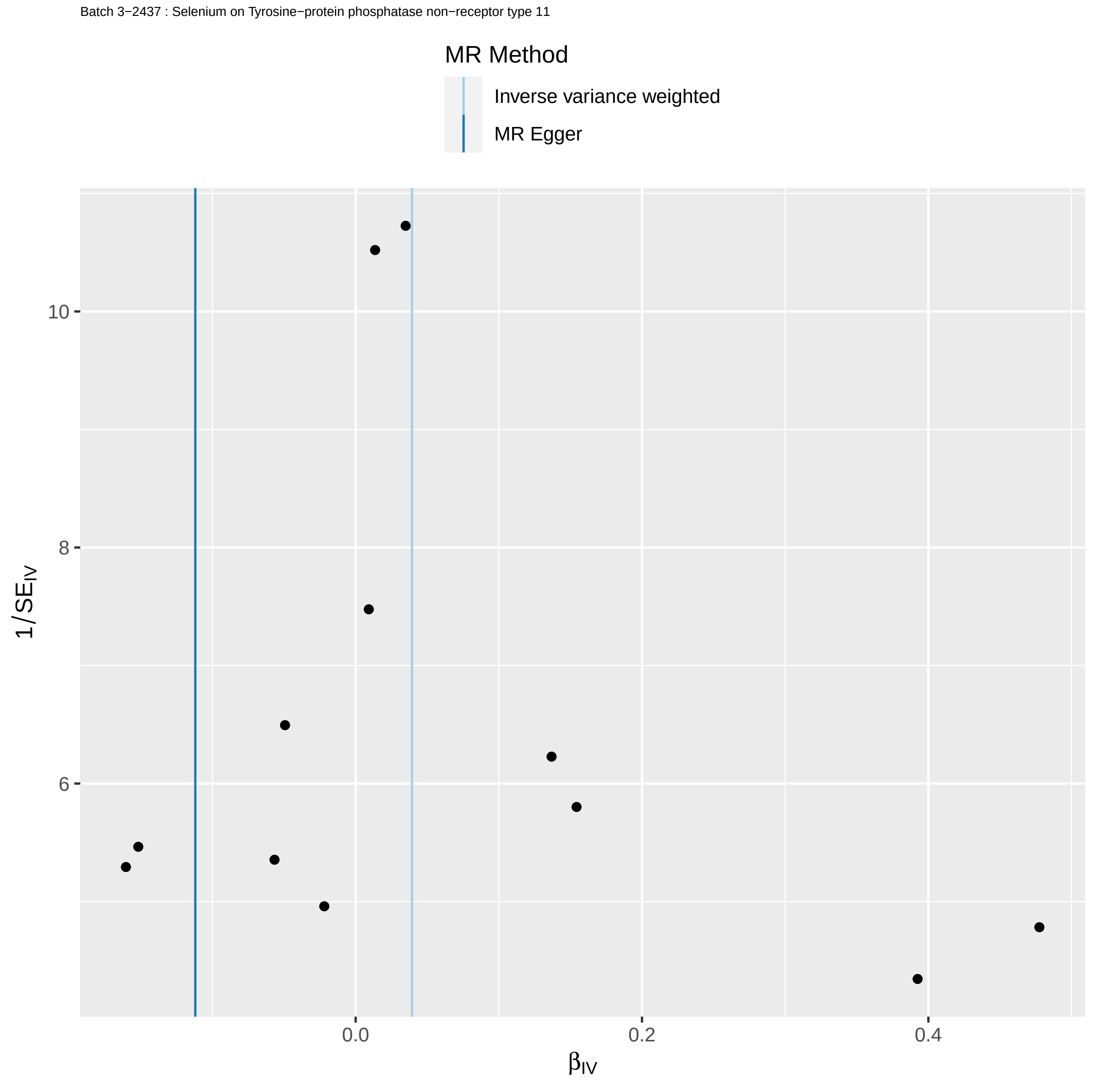
Batch 3−2437 : Selenium on Tyrosine−protein phosphatase non−receptor type 11
MR Method
Inverse variance weighted
MR Egger
10
8
6
$1/SE_{IV}$
0.0
0.2
0.4
β_{IV}

Batch 3−2438 : Selenium on Tyrosine−protein phosphatase non−receptor type 1

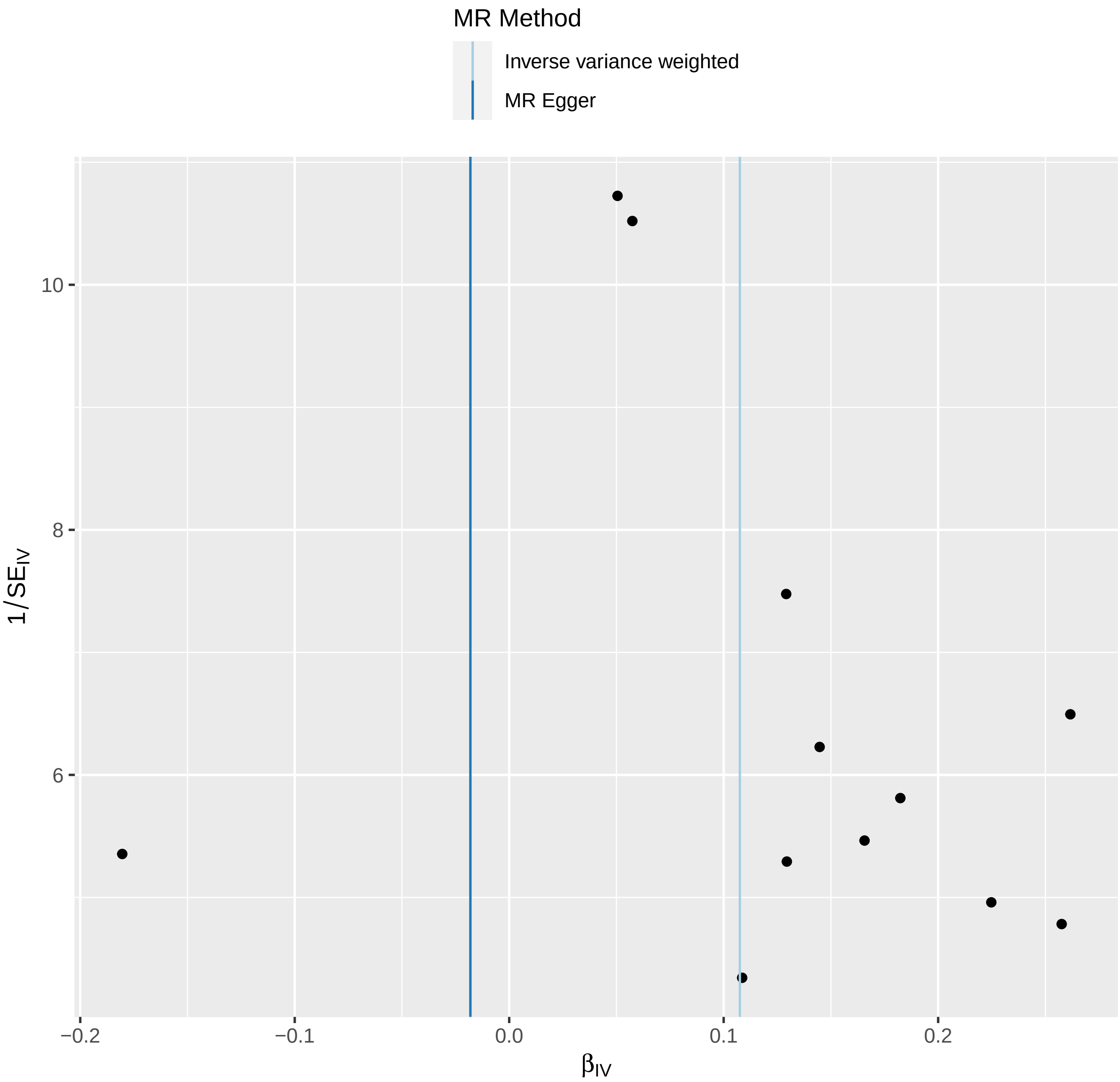

Batch 3−2439 : Selenium on Tyrosine−protein phosphatase non−receptor type 2

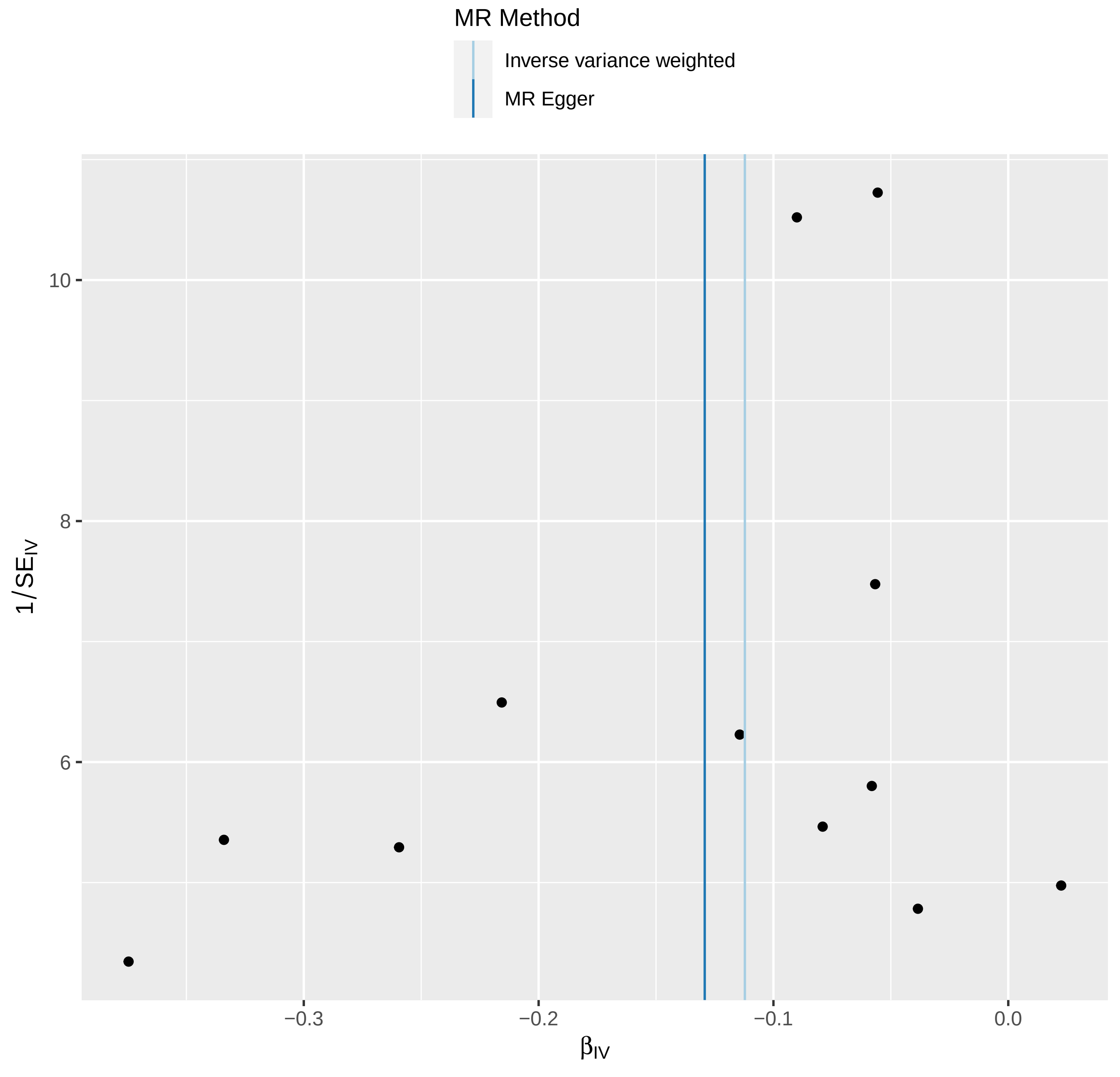

Batch 3−2440 : Selenium on Tyrosine−protein phosphatase non−receptor type 4

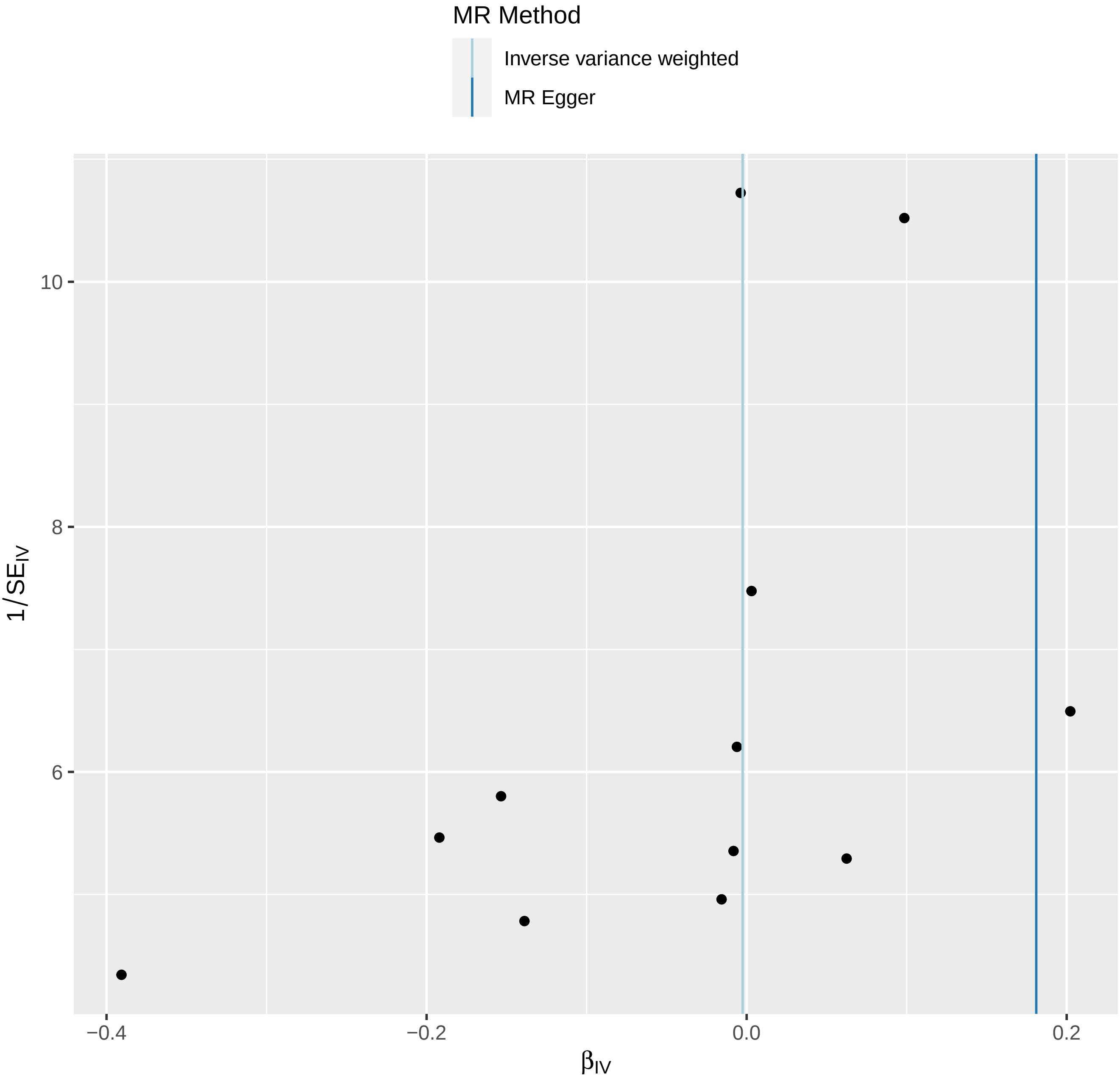

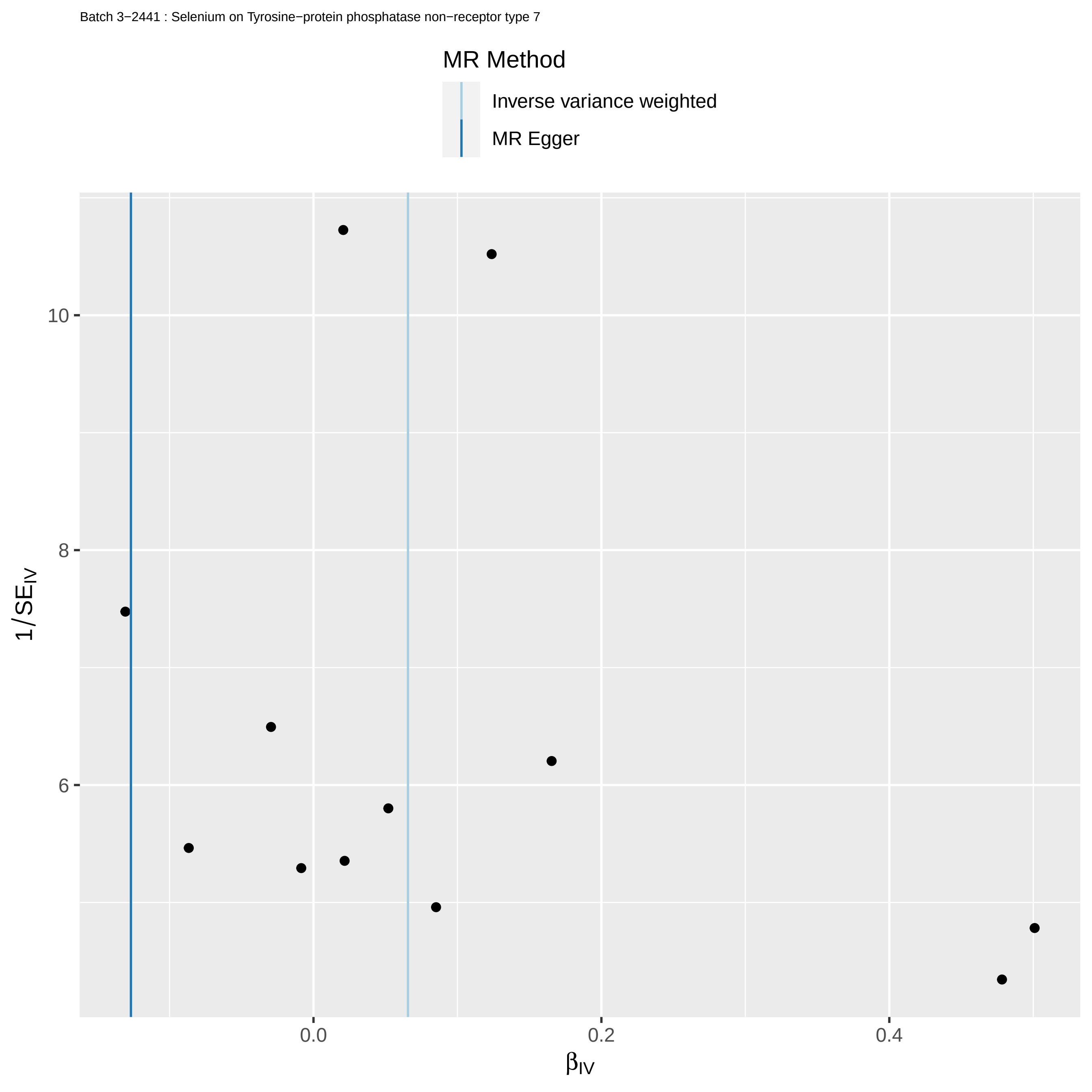

Batch 3−2441 : Selenium on Tyrosine−protein phosphatase non−receptor type 7
MR Method
Inverse variance weighted
MR Egger
10
8
6
1/SE_IV
0.0
0.2
0.4
β_IV

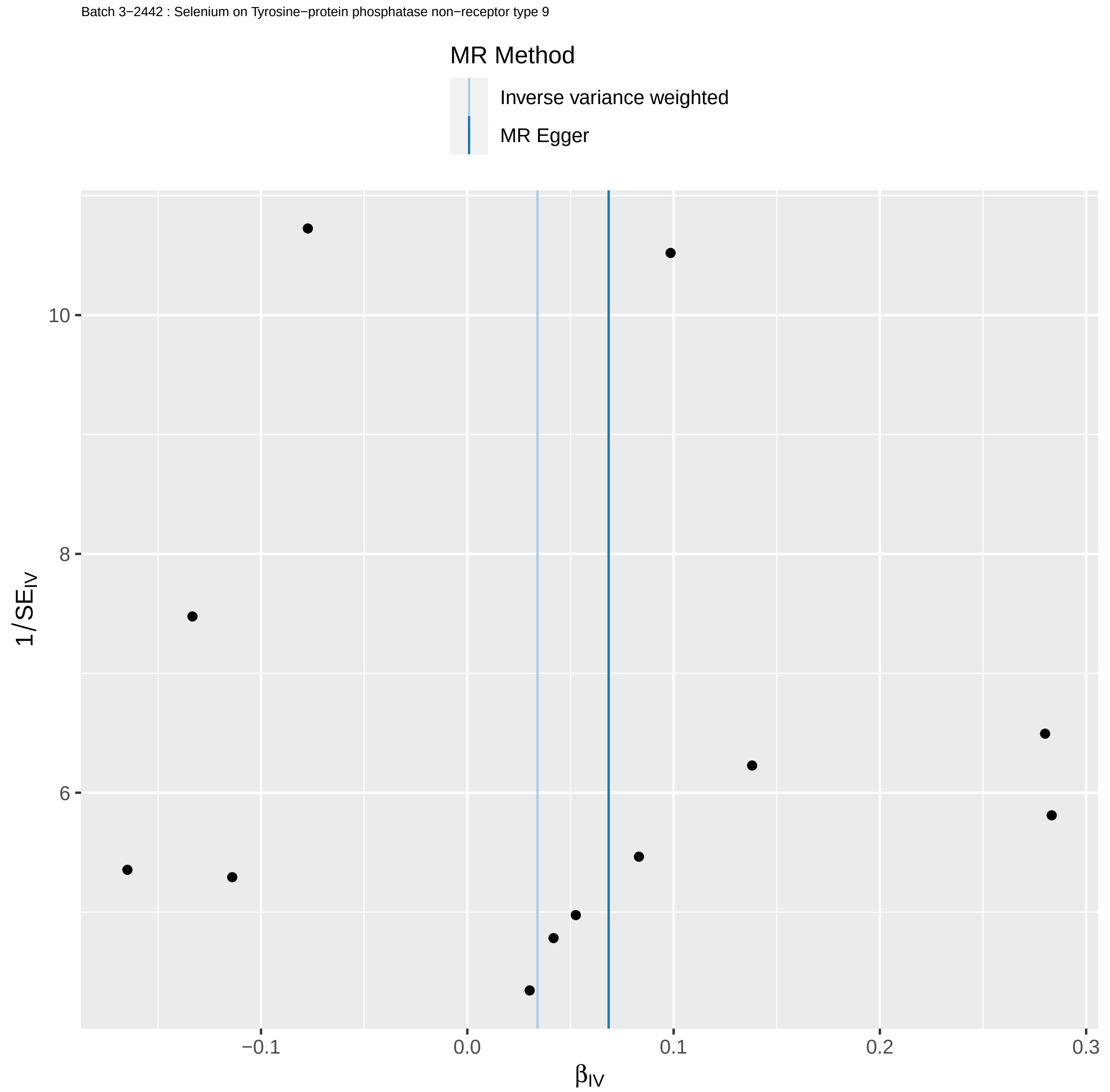

Batch 3−2442 : Selenium on Tyrosine−protein phosphatase non−receptor type 9
MR Method
Inverse variance weighted
MR Egger
10
8
6
1/SE$_{IV}$
−0.1
0.0
0.1
0.2
0.3
β$_{IV}$

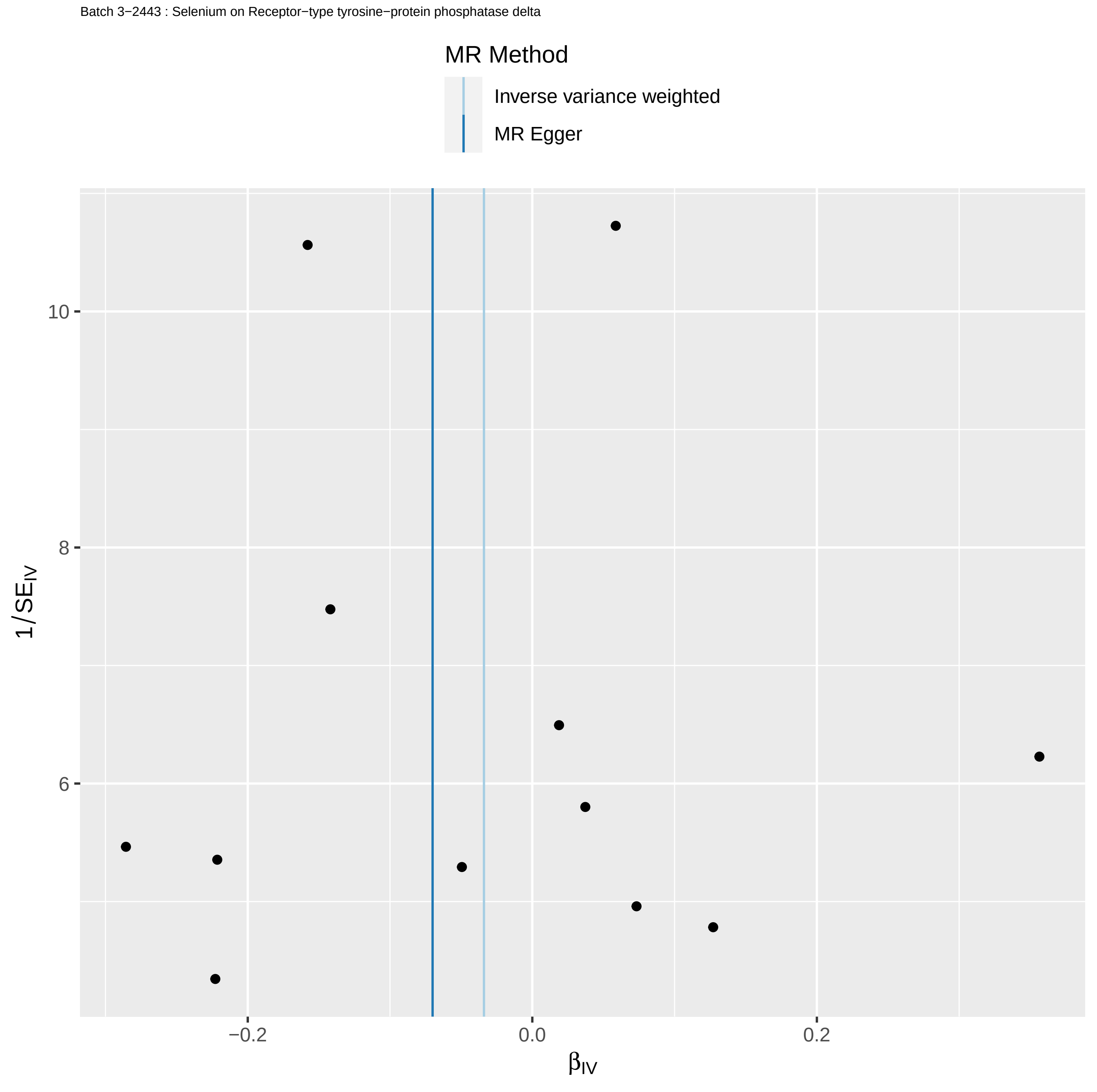
Batch 3−2443 : Selenium on Receptor−type tyrosine−protein phosphatase delta
MR Method
Inverse variance weighted
MR Egger
10
8
6
1/SE$_{IV}$
−0.2
0.0
0.2
β$_{IV}$

Batch 3−2444 : Selenium on Receptor−type tyrosine−protein phosphatase H

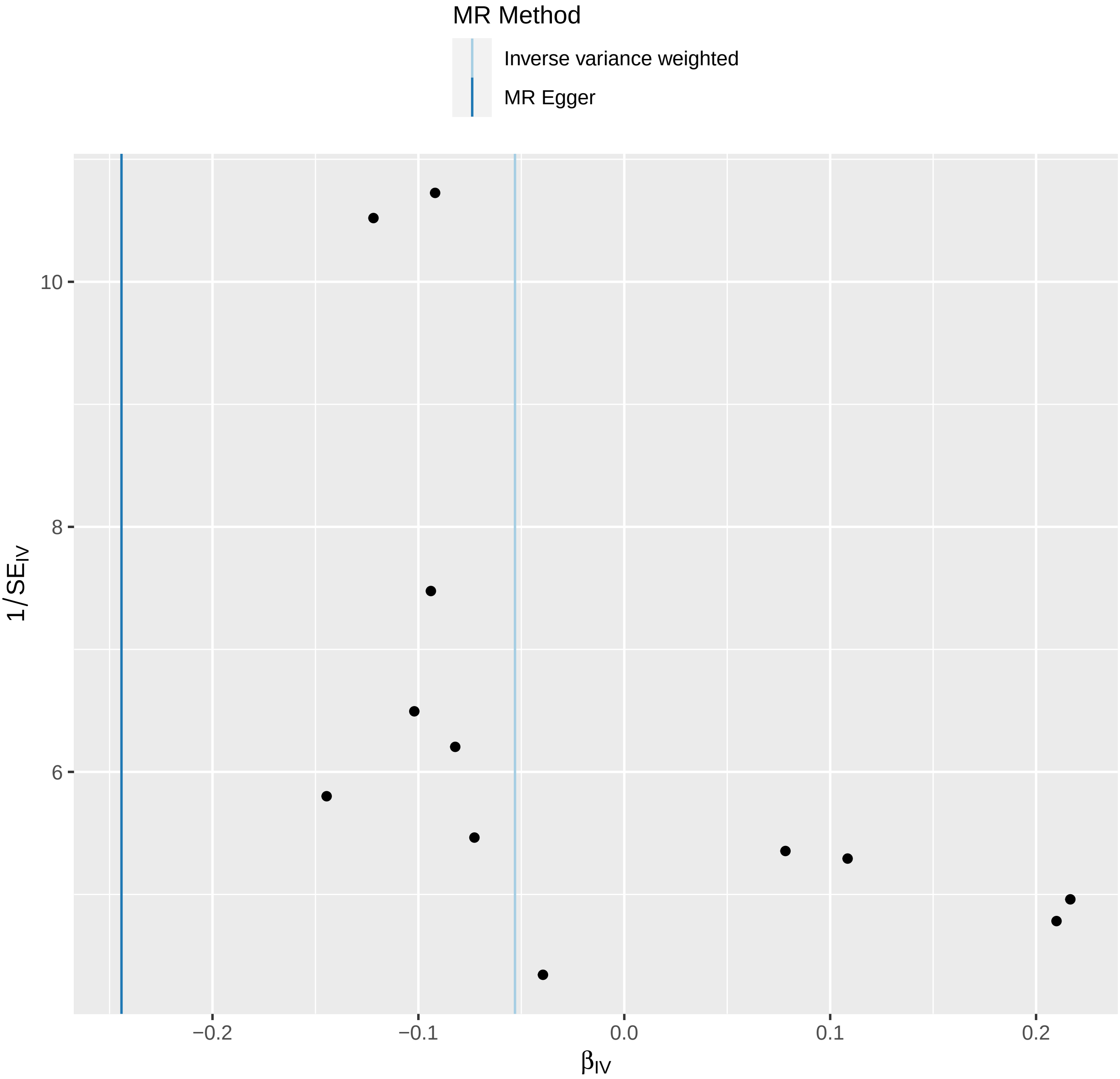

Batch 3−2445 : Selenium on Receptor−type tyrosine−protein phosphatase H

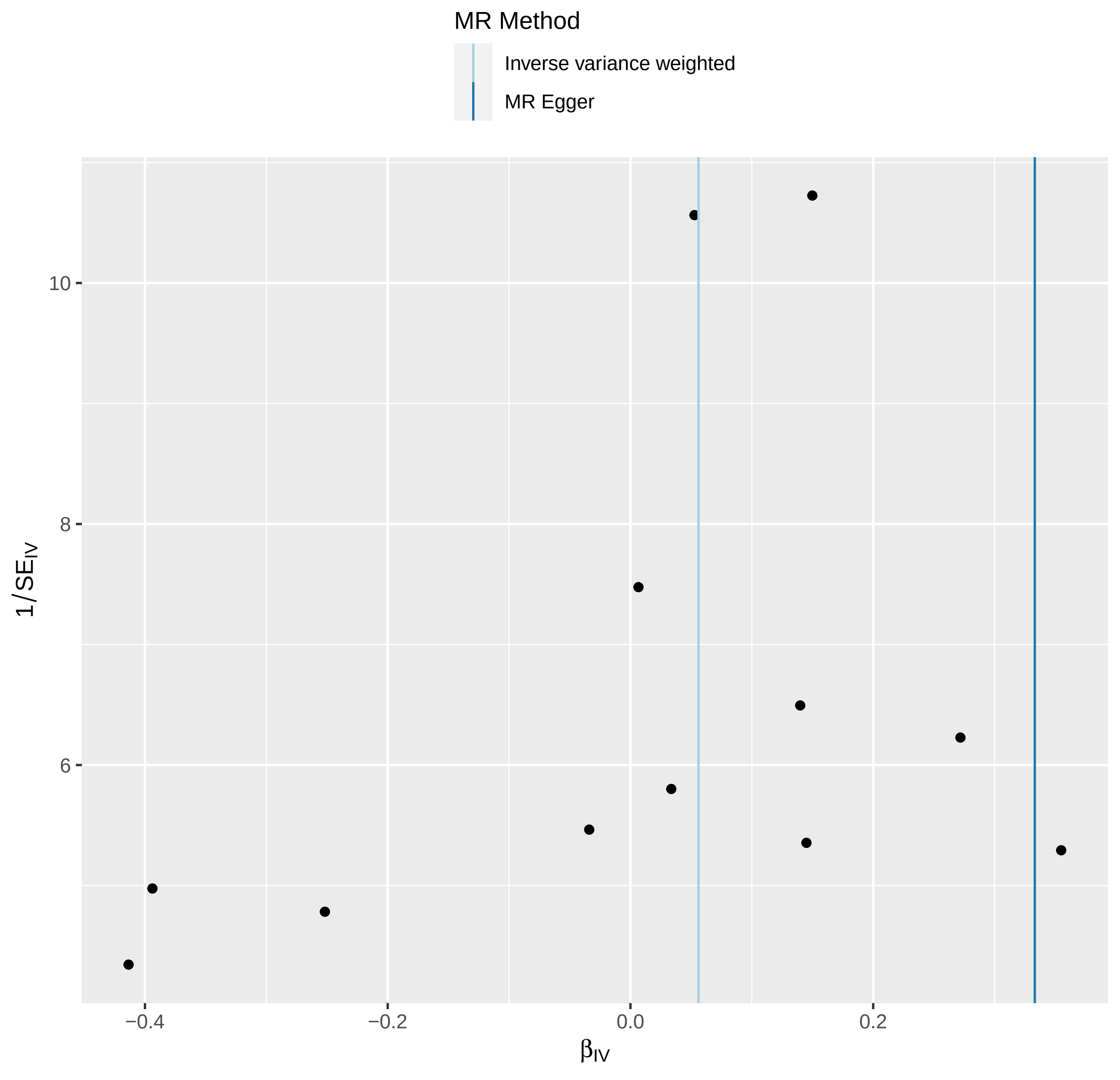

Batch 3−2446 : Selenium on Receptor−type tyrosine−protein phosphatase eta

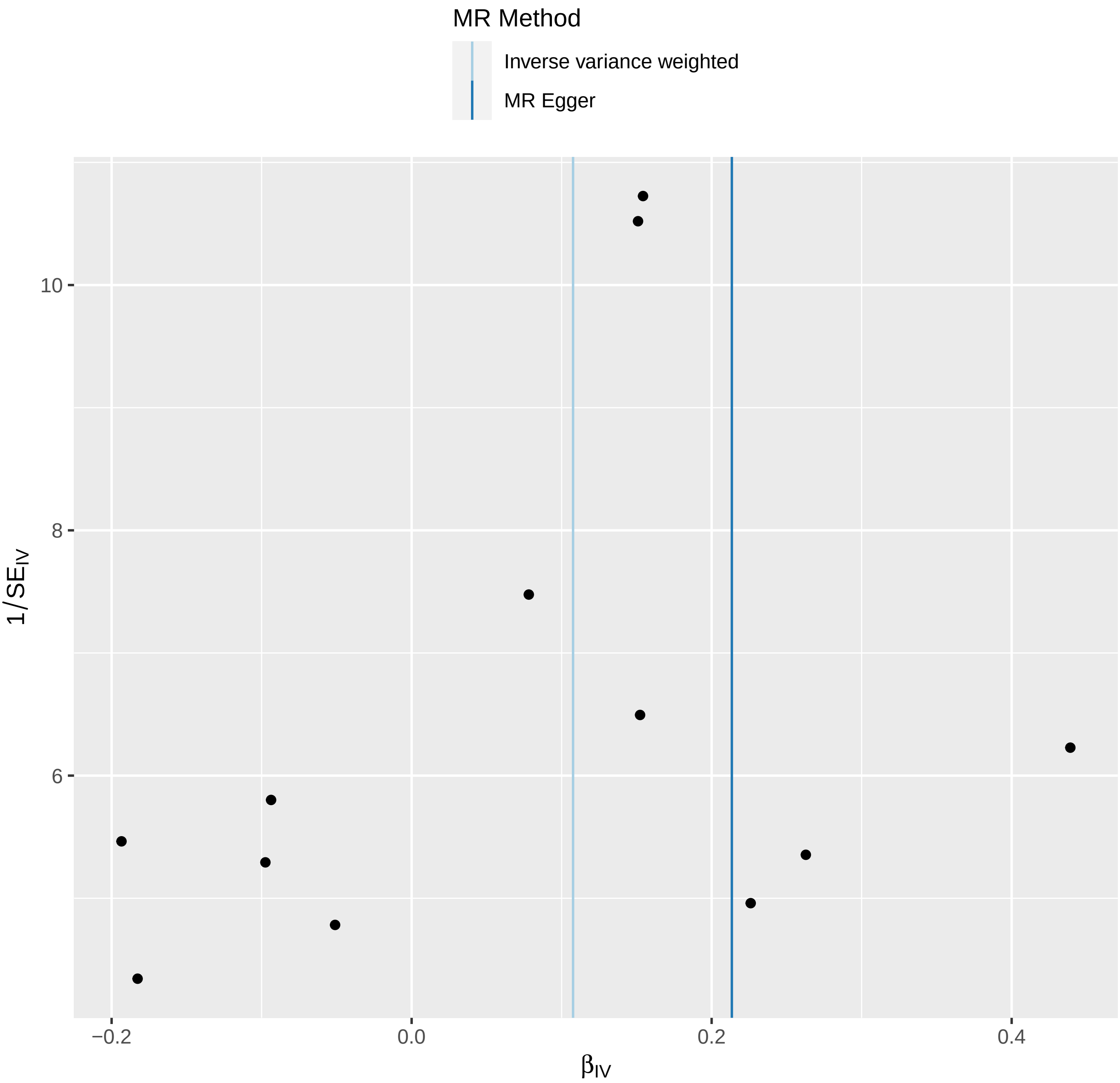

Batch 3−2447 : Selenium on Receptor−type tyrosine−protein phosphatase R

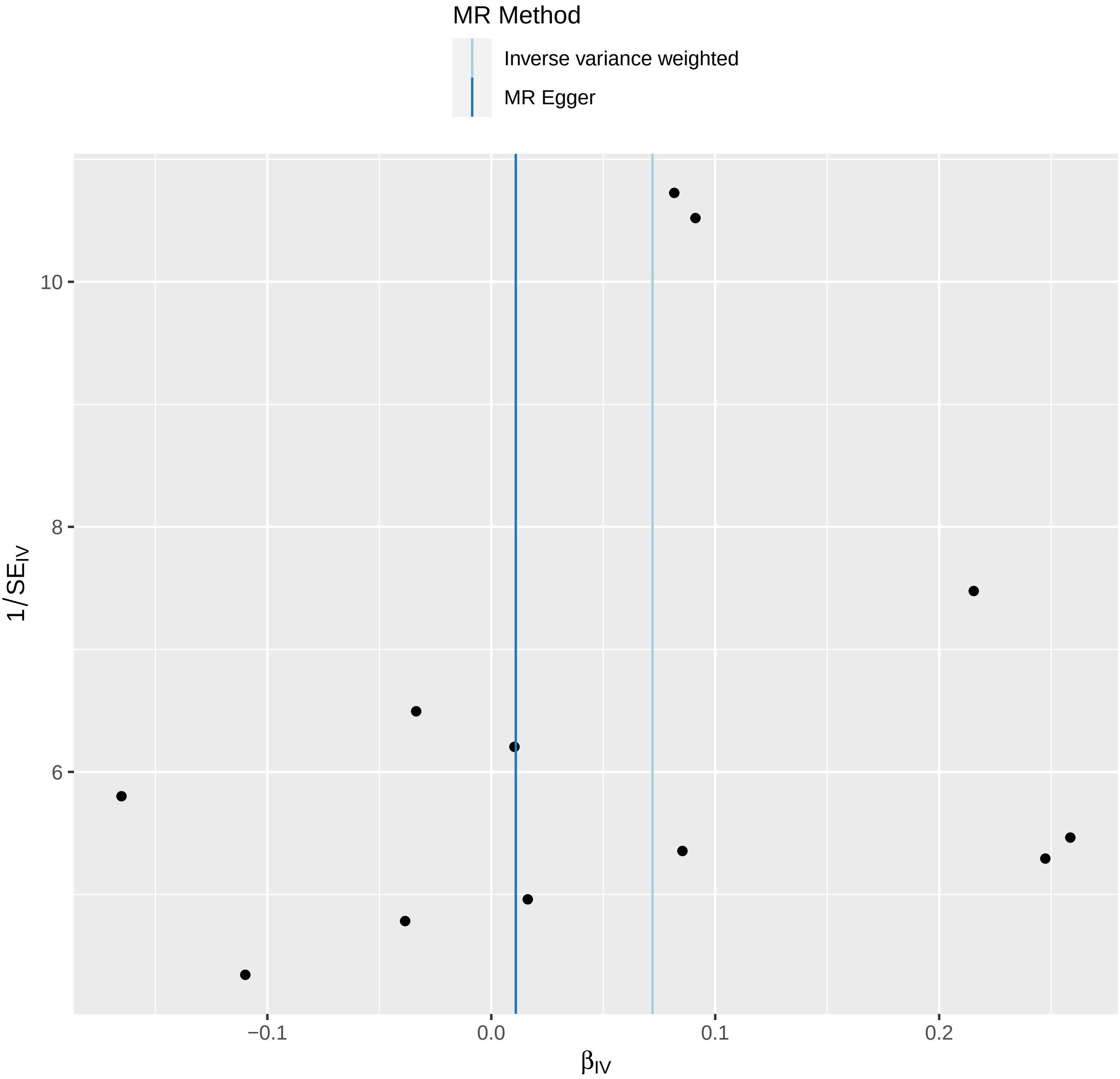

Batch 3−2448 : Selenium on Receptor−type tyrosine−protein phosphatase S

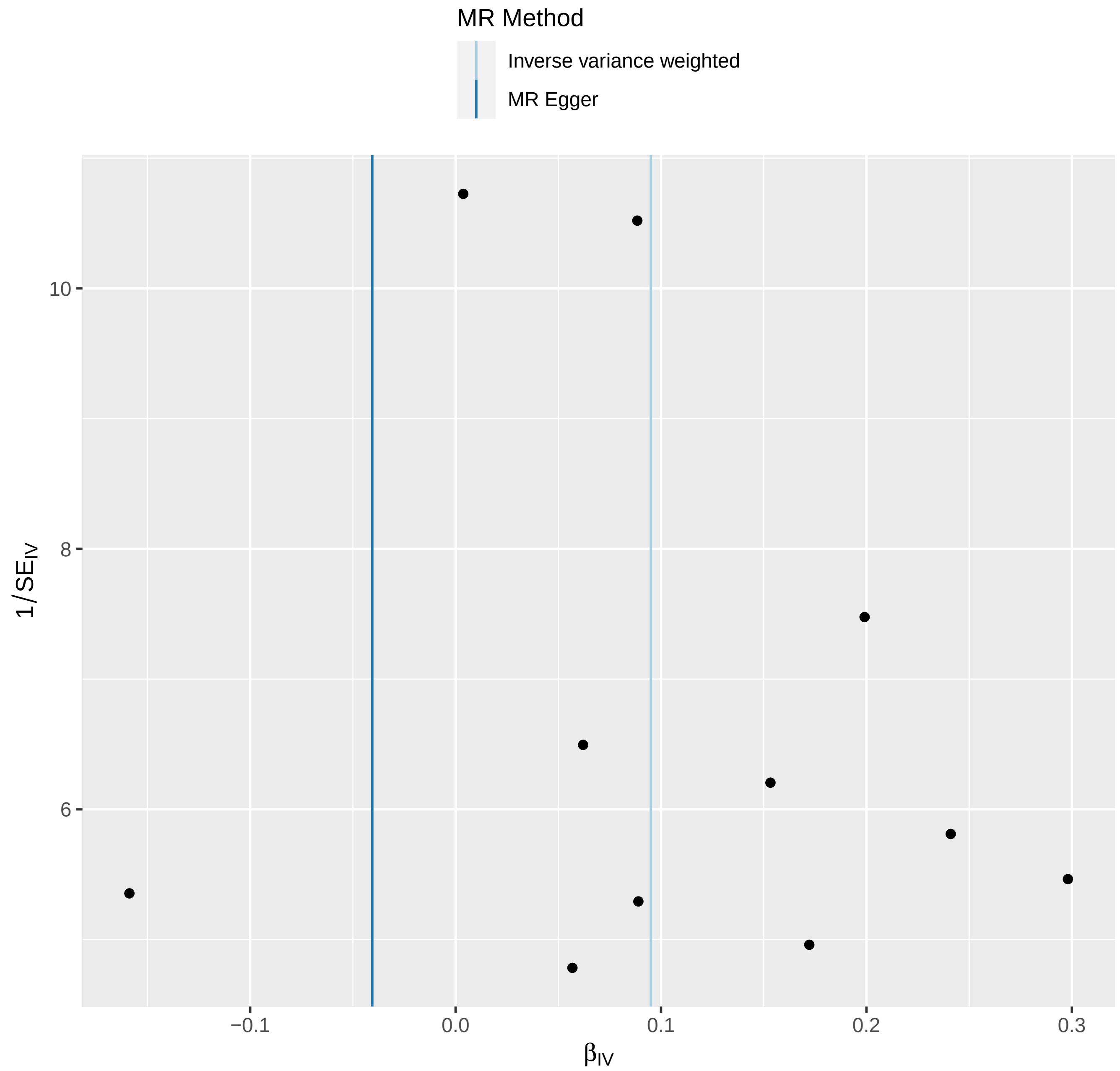

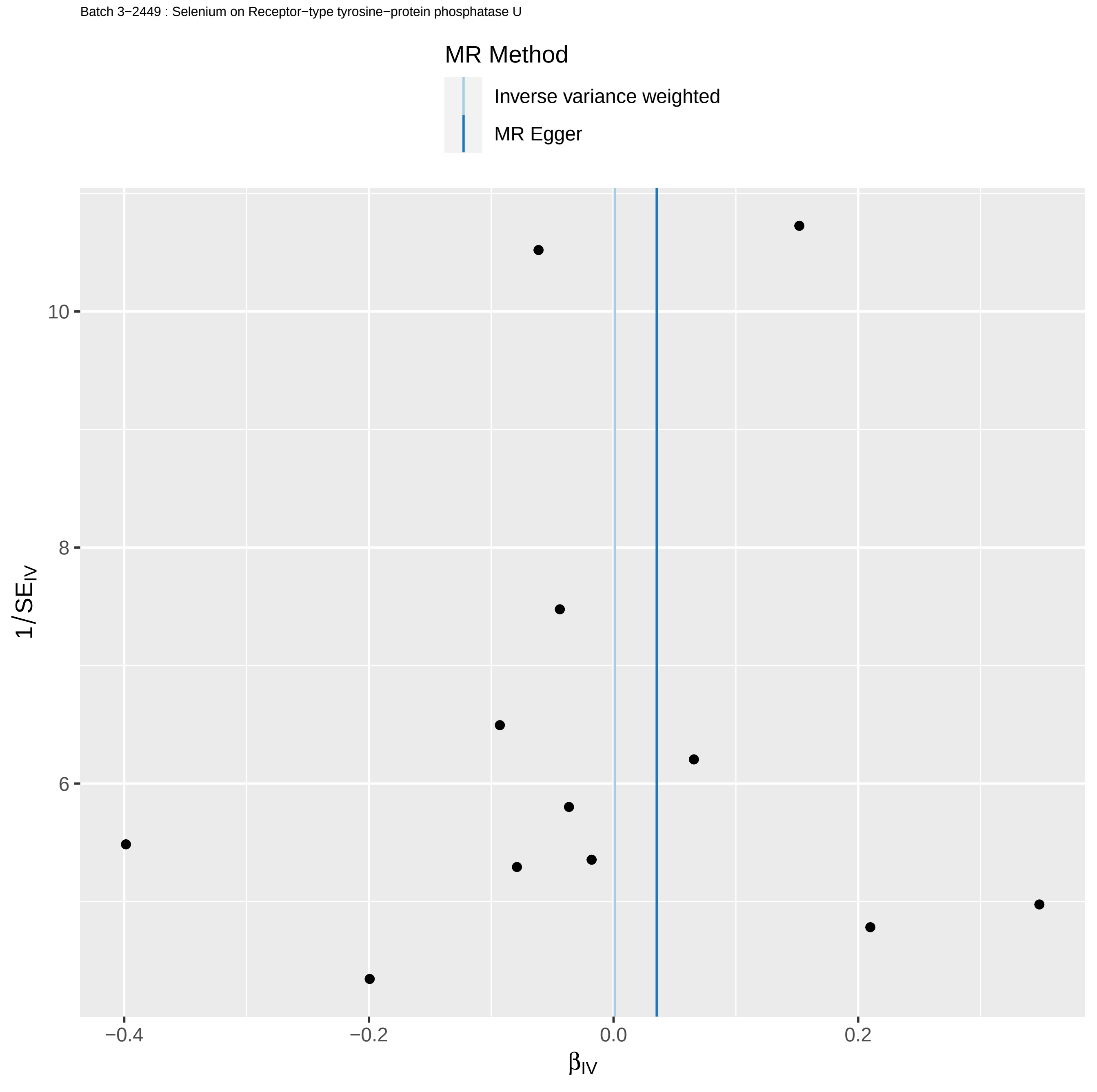
Batch 3−2449 : Selenium on Receptor−type tyrosine−protein phosphatase U
MR Method
Inverse variance weighted
MR Egger
1/SE$_{IV}$
10
8
6
−0.4
−0.2
0.0
0.2
β$_{IV}$

Batch 3−2450 : Selenium on 6−pyruvoyl tetrahydrobiopterin synthase

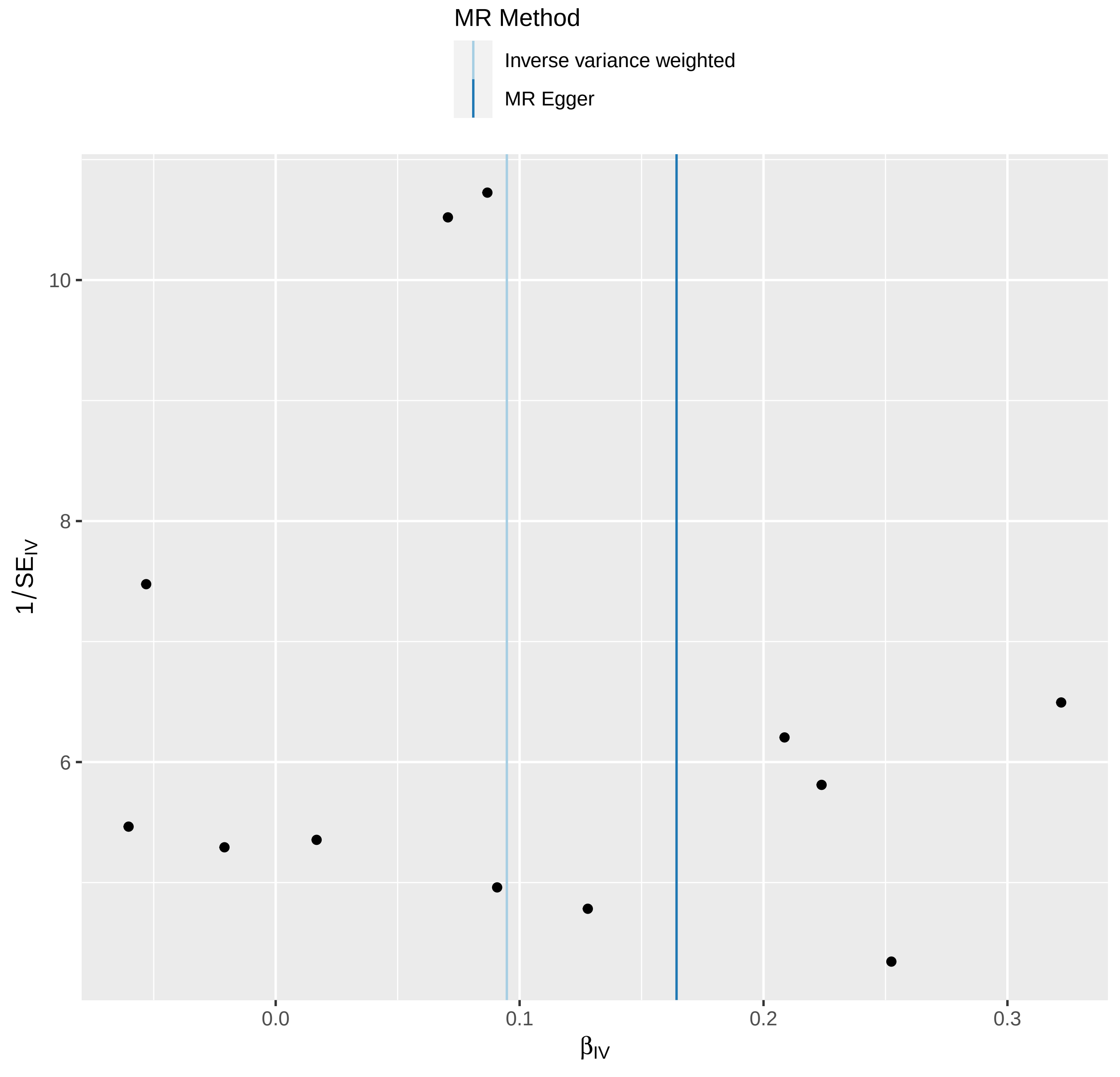

Batch 3−2451 : Selenium on Pentraxin−related protein PTX3

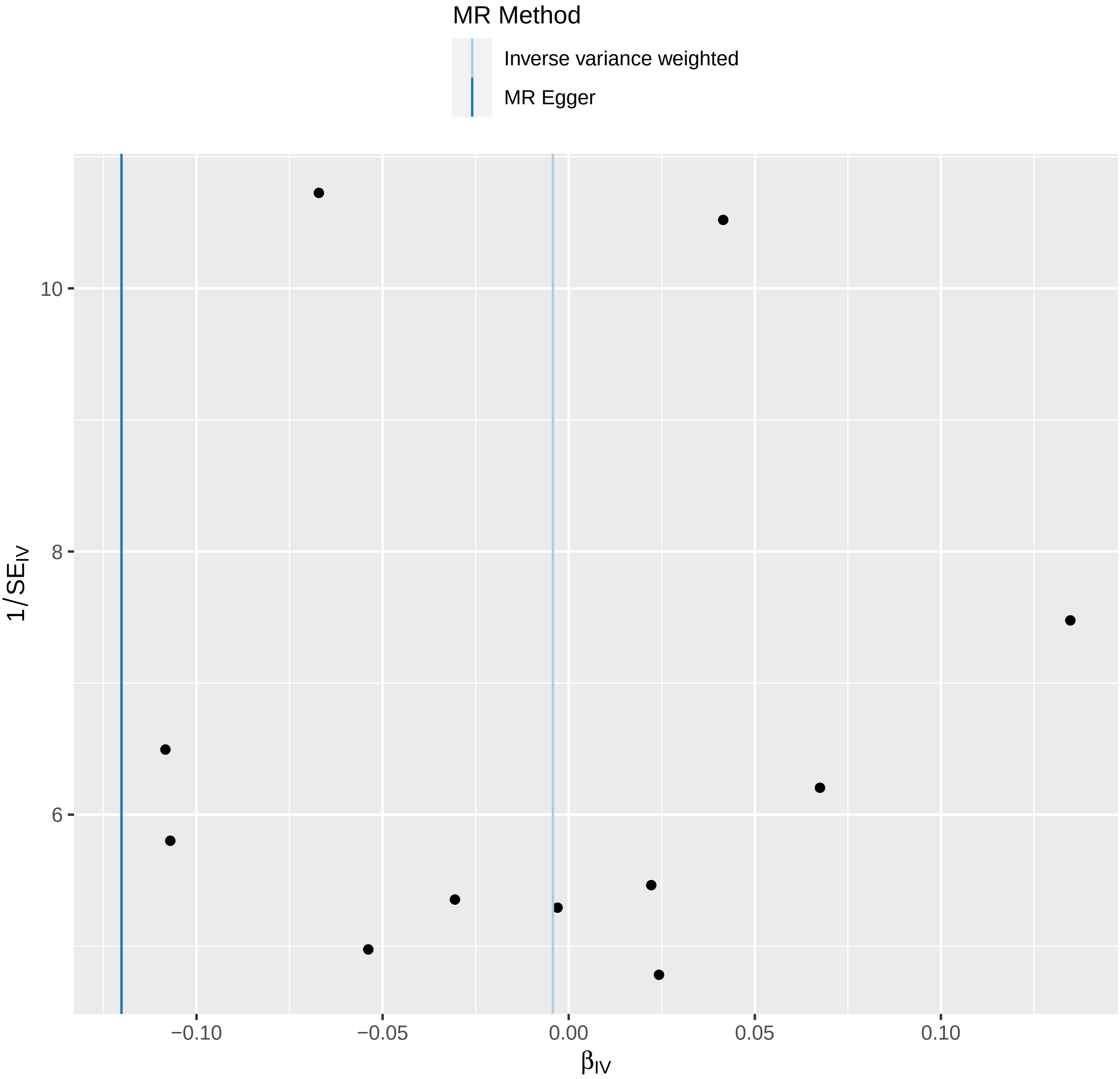

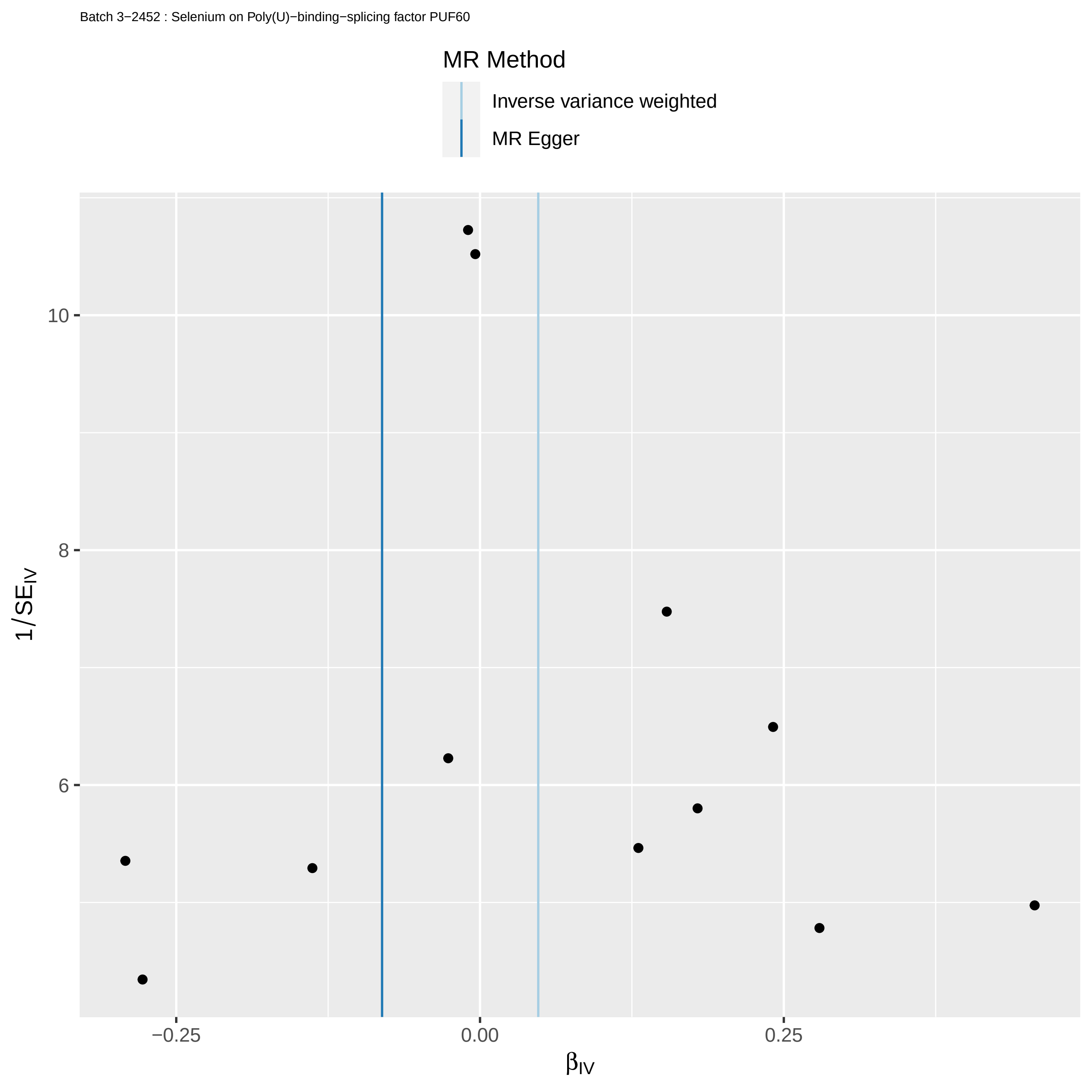
Batch 3−2452 : Selenium on Poly(U)−binding−splicing factor PUF60
MR Method
Inverse variance weighted
MR Egger
10
8
6
1/SE_IV
−0.25
0.00
0.25
β_IV

Batch 3−2453 : Selenium on Transcriptional activator protein Pur−alpha

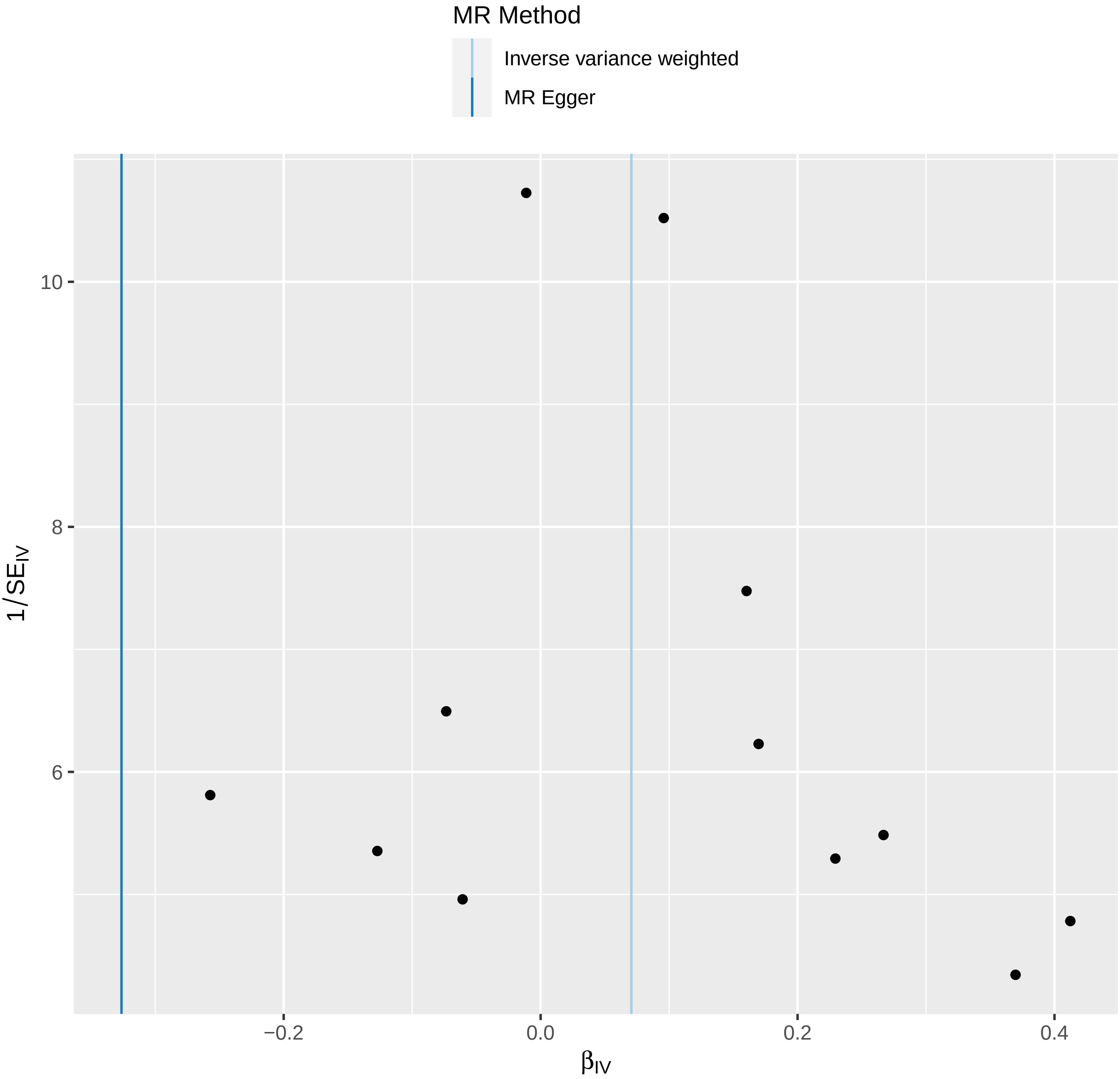

Batch 3−2454 : Selenium on tRNA pseudouridine synthase A, mitochondrial

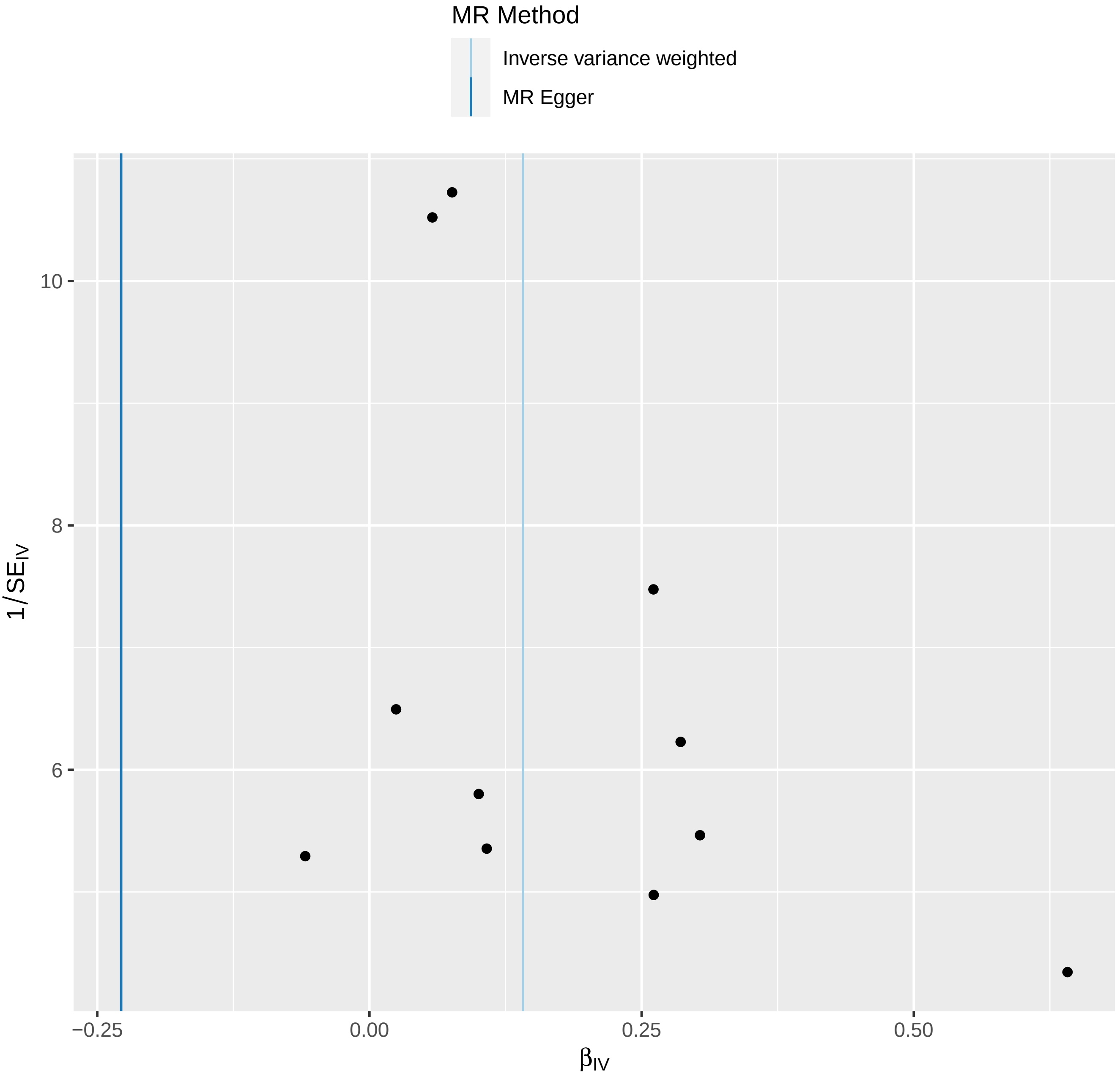

Batch 3−2455 : Selenium on Poliovirus receptor

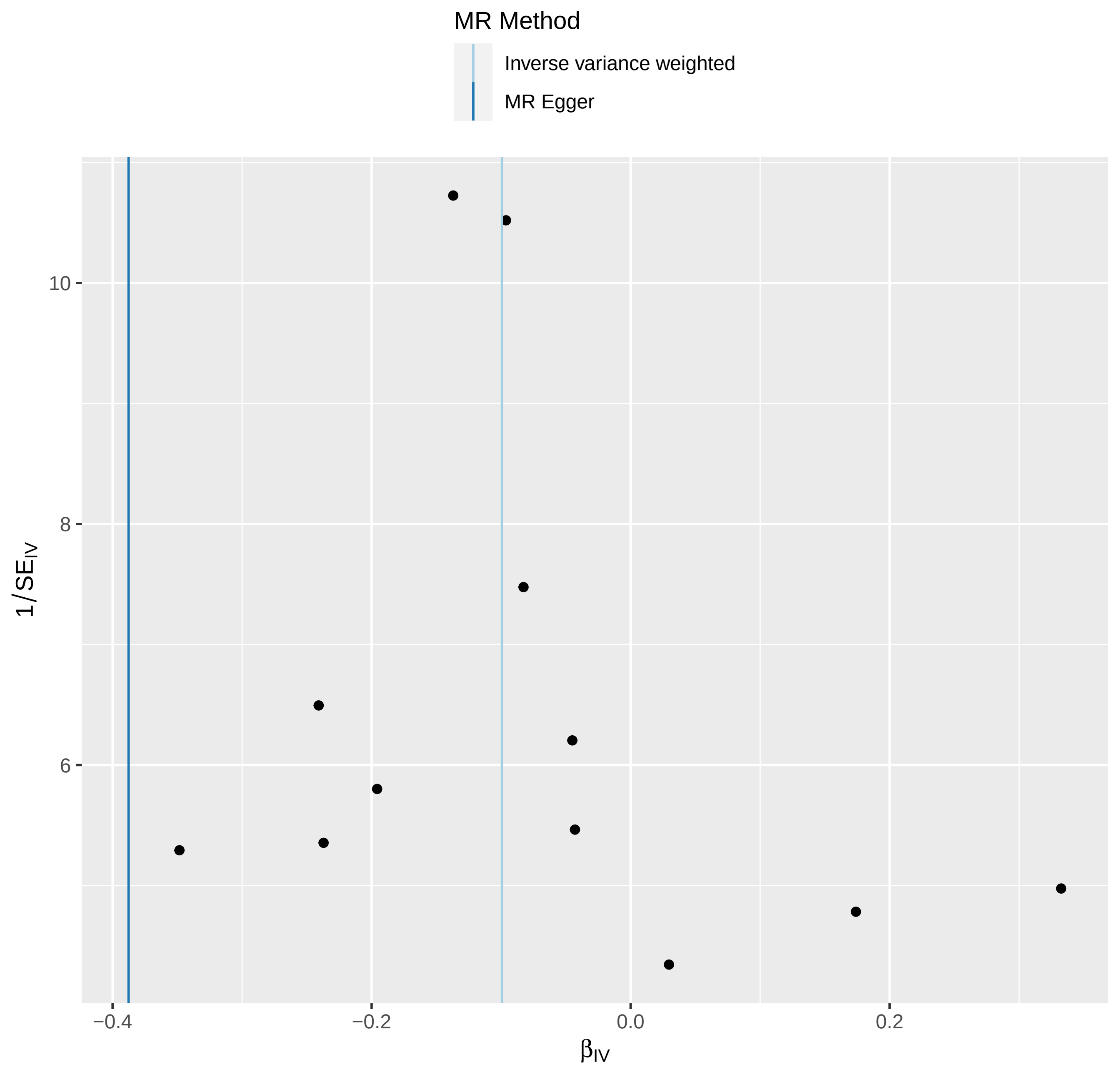

Batch 3−2456 : Selenium on Transmembrane protein PVRIG

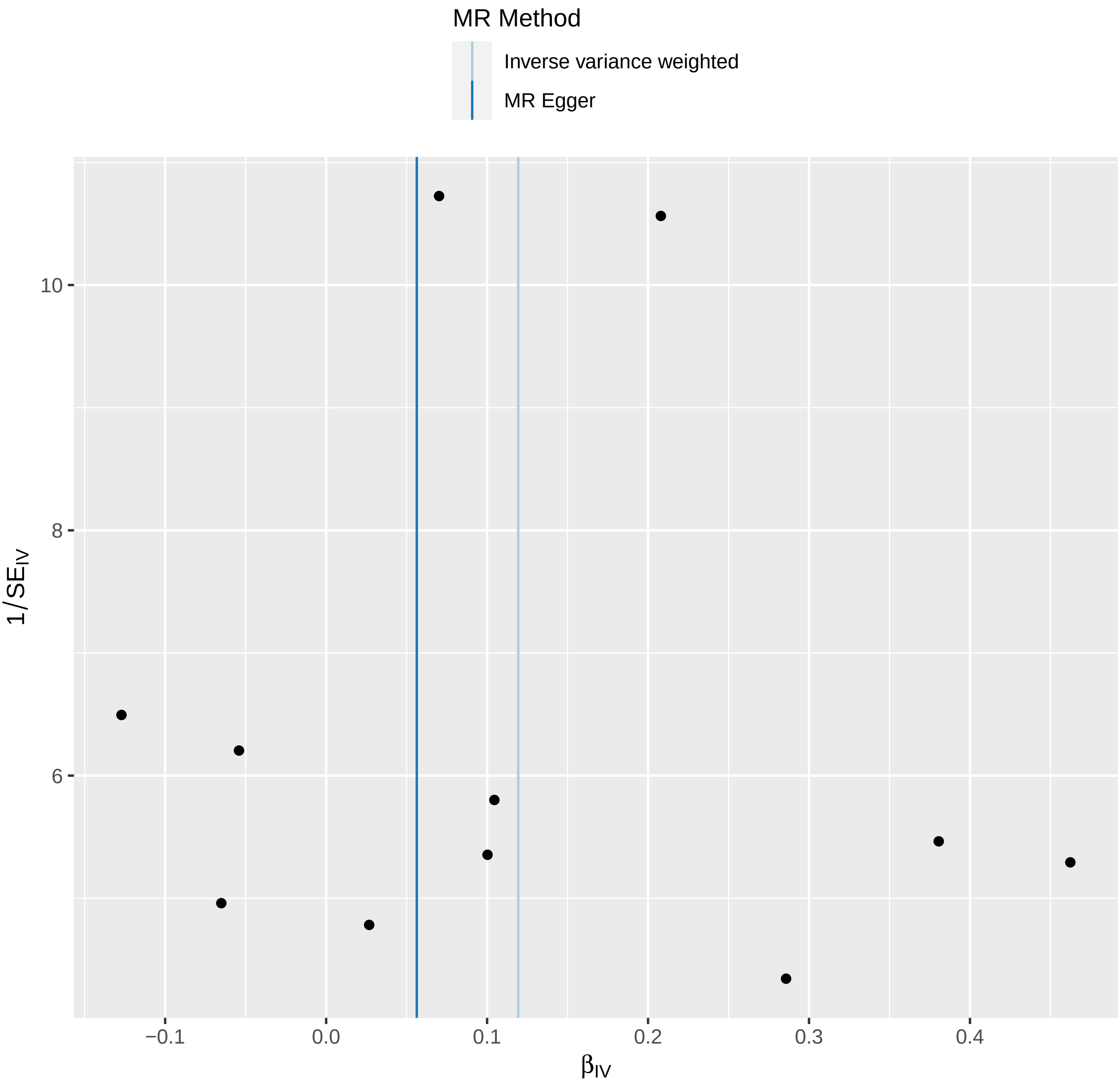

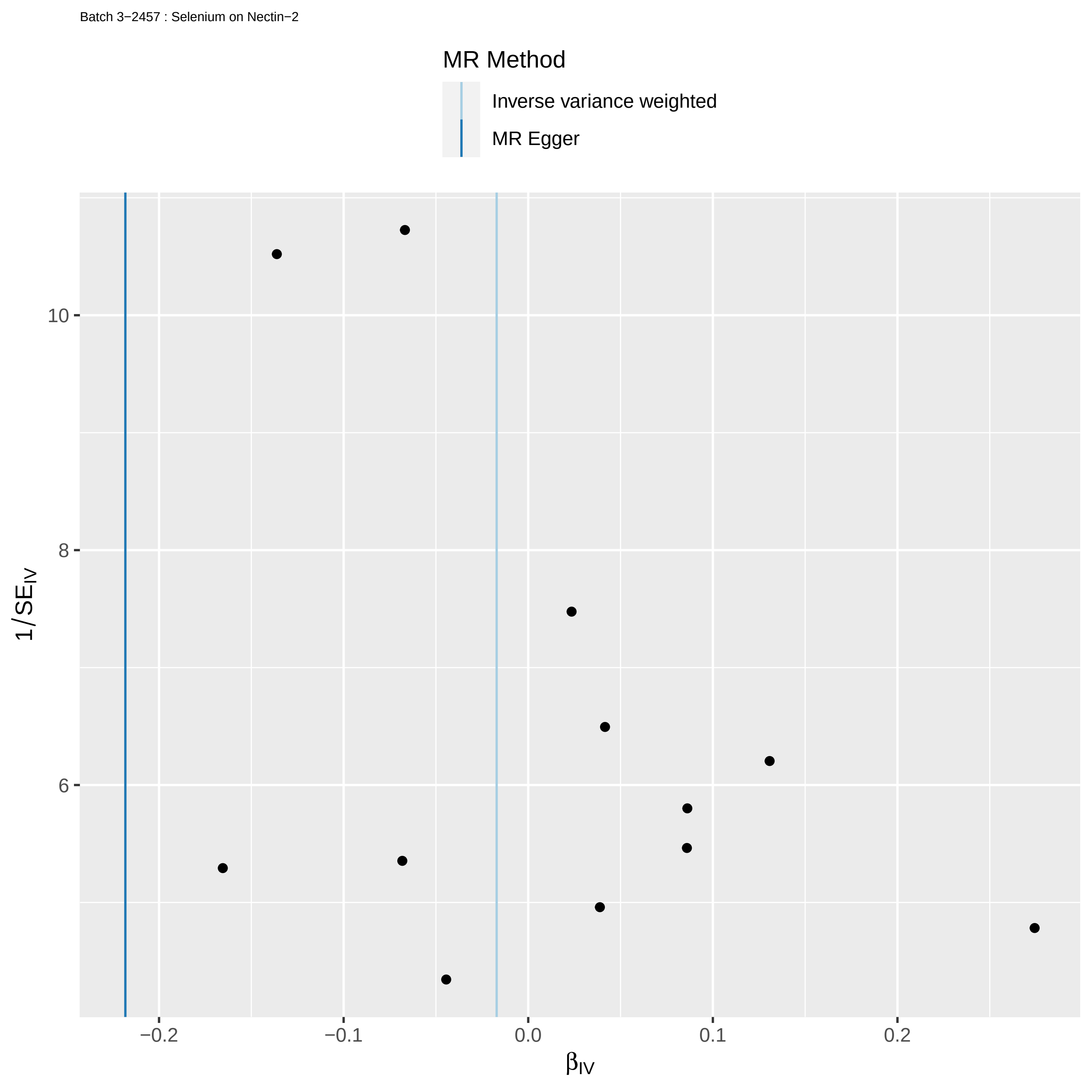
Batch 3−2457 : Selenium on Nectin−2
MR Method
Inverse variance weighted
MR Egger
10
8
6
1/SE_IV
−0.2
−0.1
0.0
0.1
0.2
β_IV

Batch 3−2458 : Selenium on Nectin−3

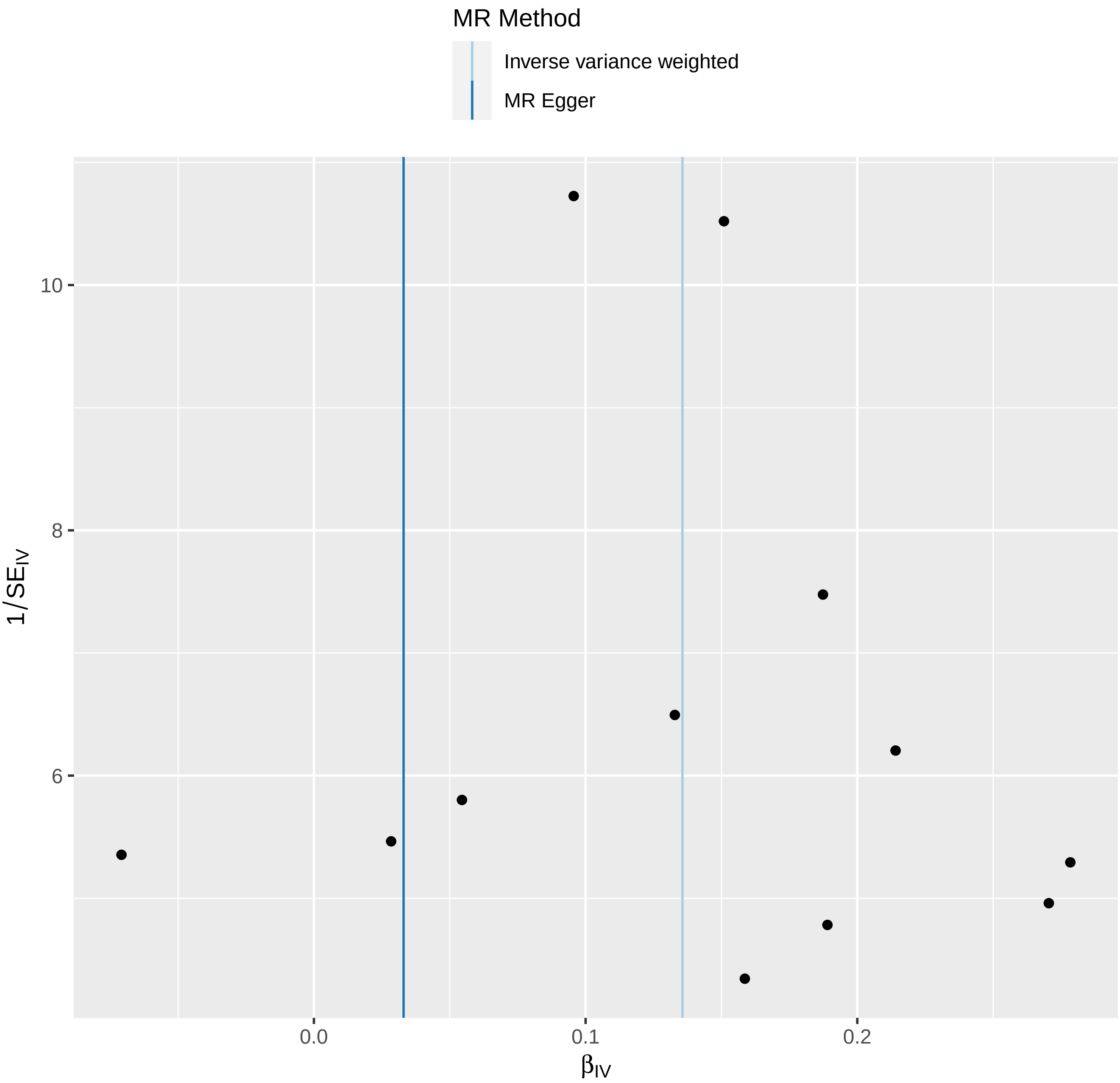

Batch 3−2459 : Selenium on Nectin−4

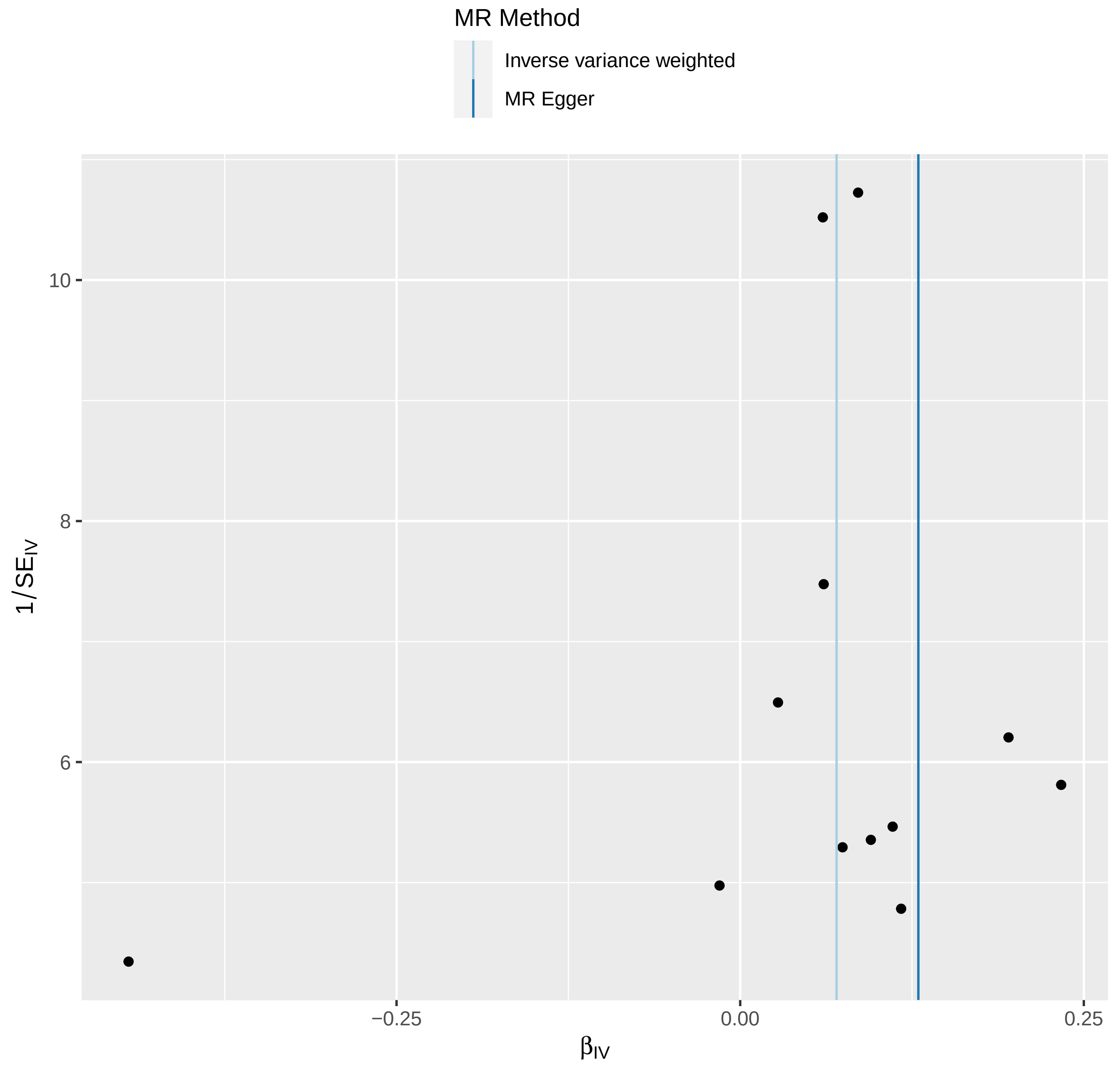

Batch 3−2460 : Selenium on Peroxidasin−like protein

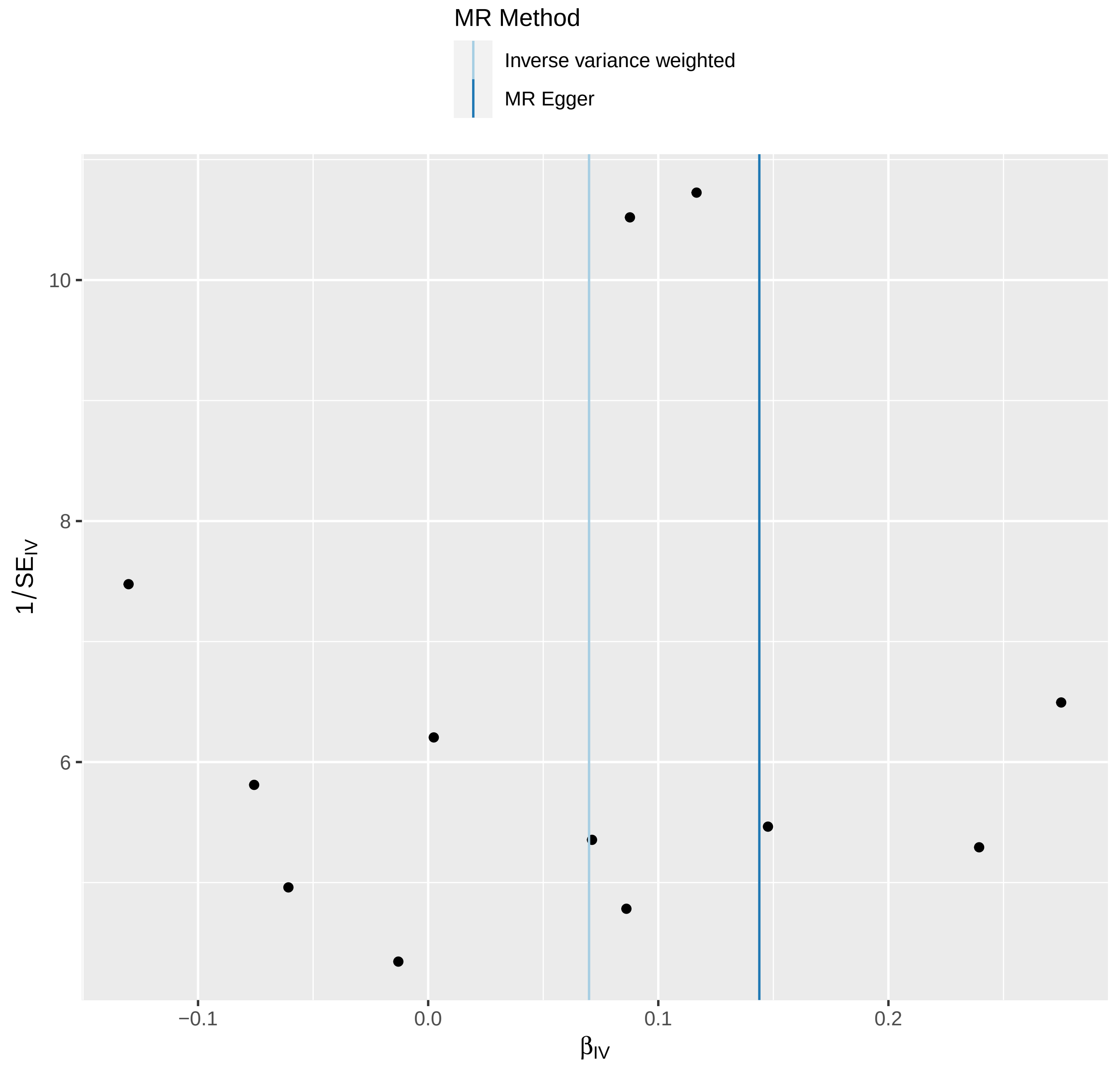

Batch 3−2461 : Selenium on Pyrin domain−containing protein 1

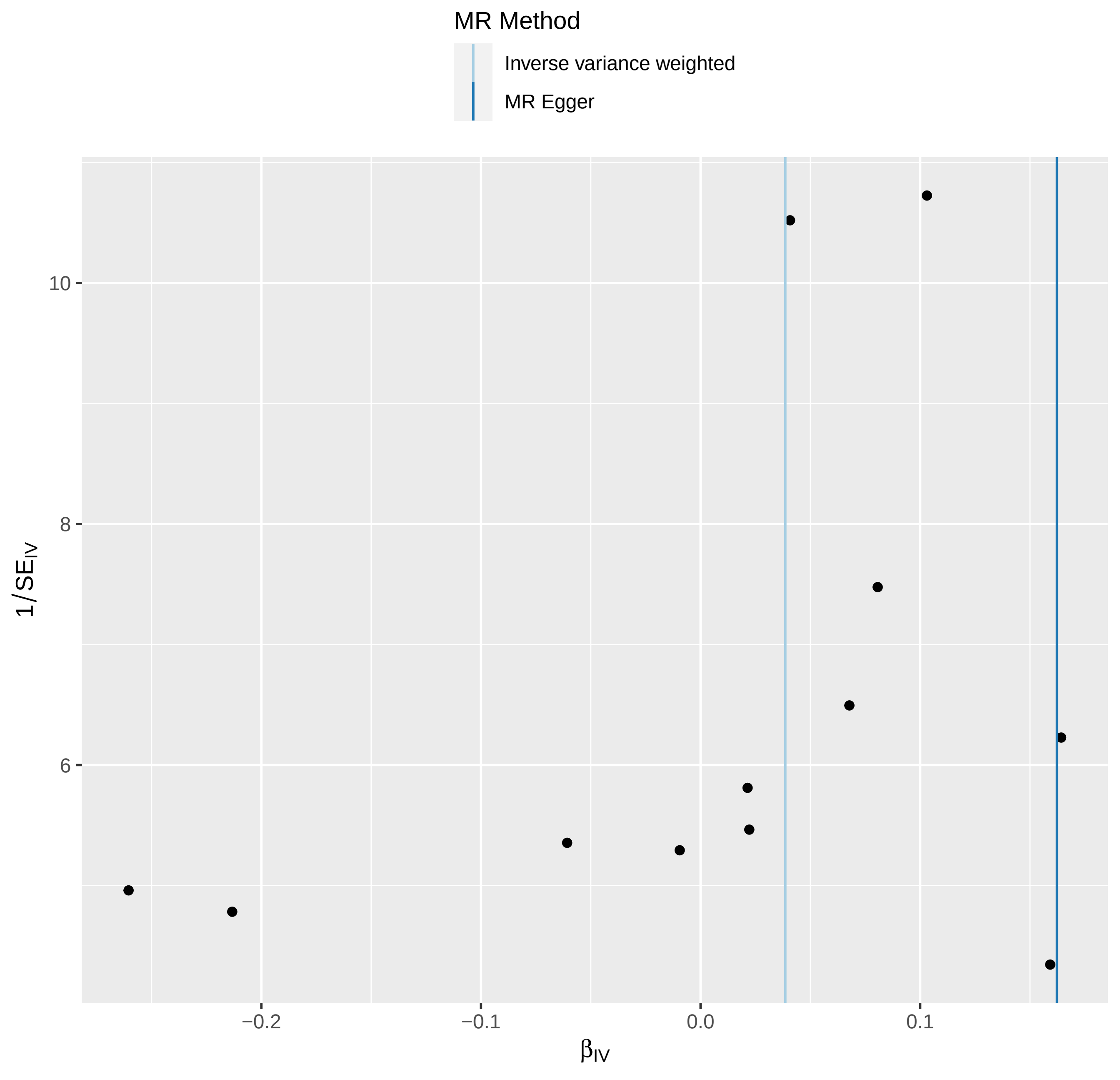

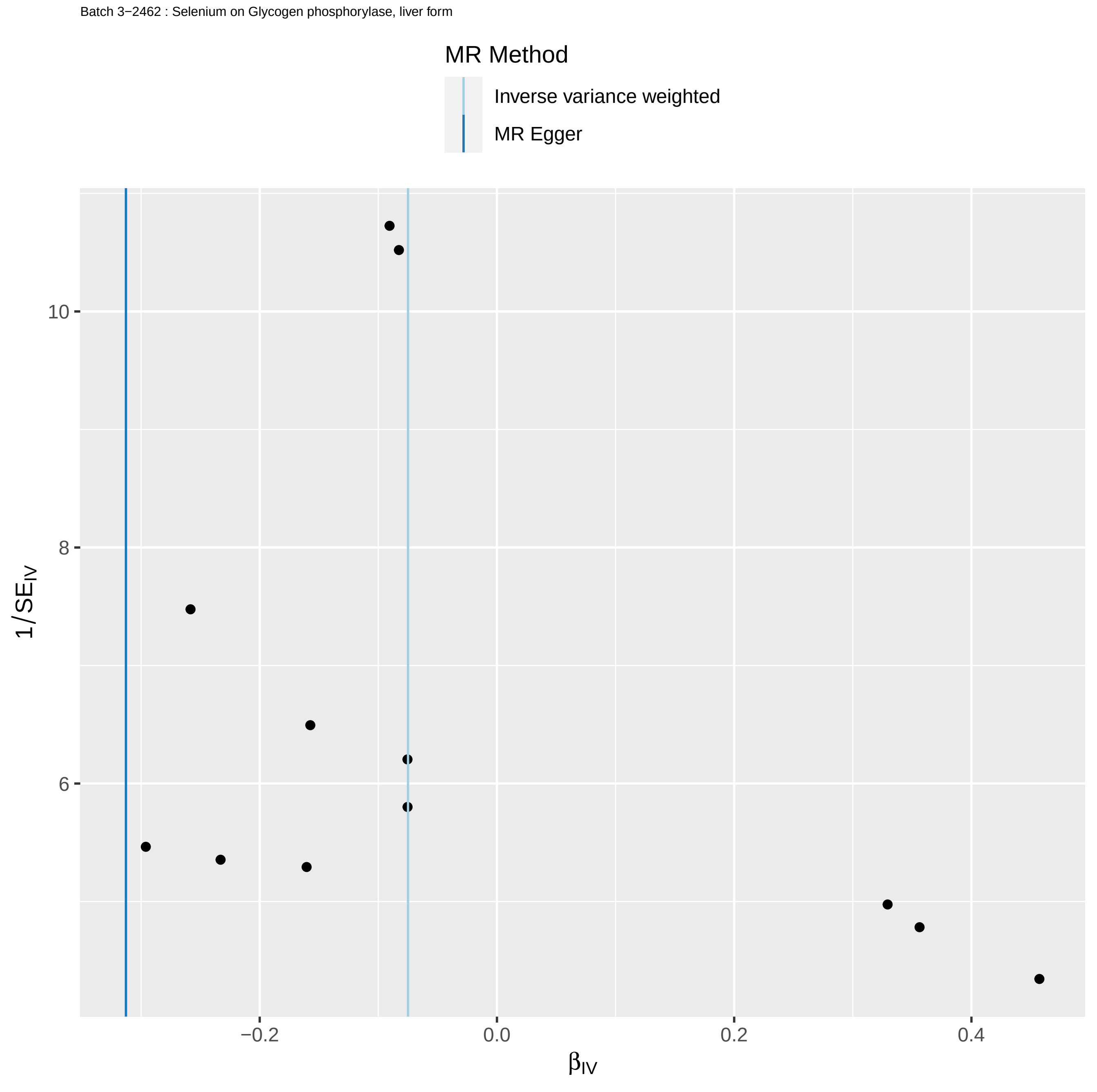

Batch 3−2462 : Selenium on Glycogen phosphorylase, liver form
MR Method
Inverse variance weighted
MR Egger
10
8
6
1/SE$_{IV}$
−0.2
0.0
0.2
0.4
β_{IV}

Batch 3−2463 : Selenium on Peptide YY

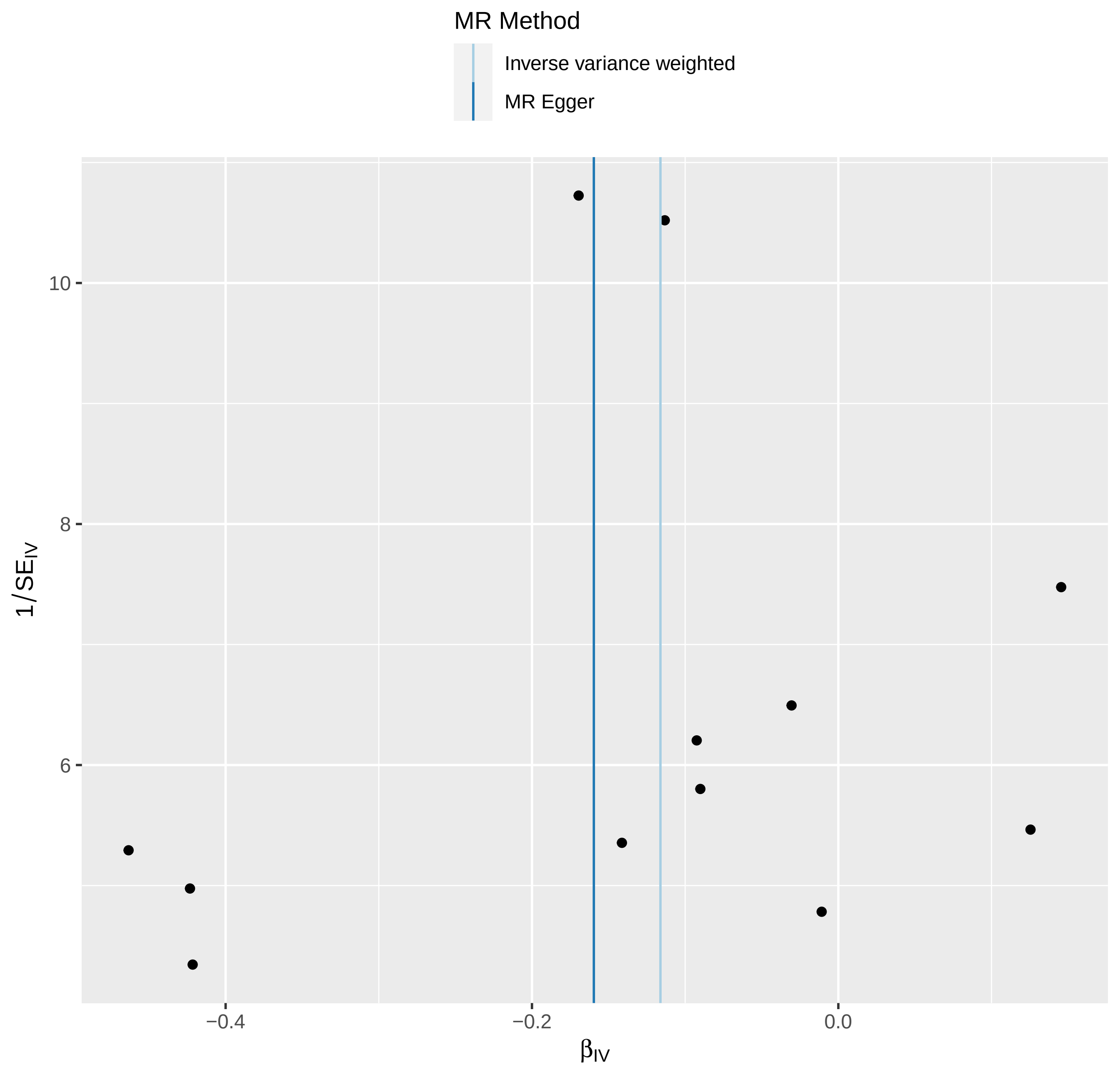

Batch 3−2464 : Selenium on Dihydropteridine reductase

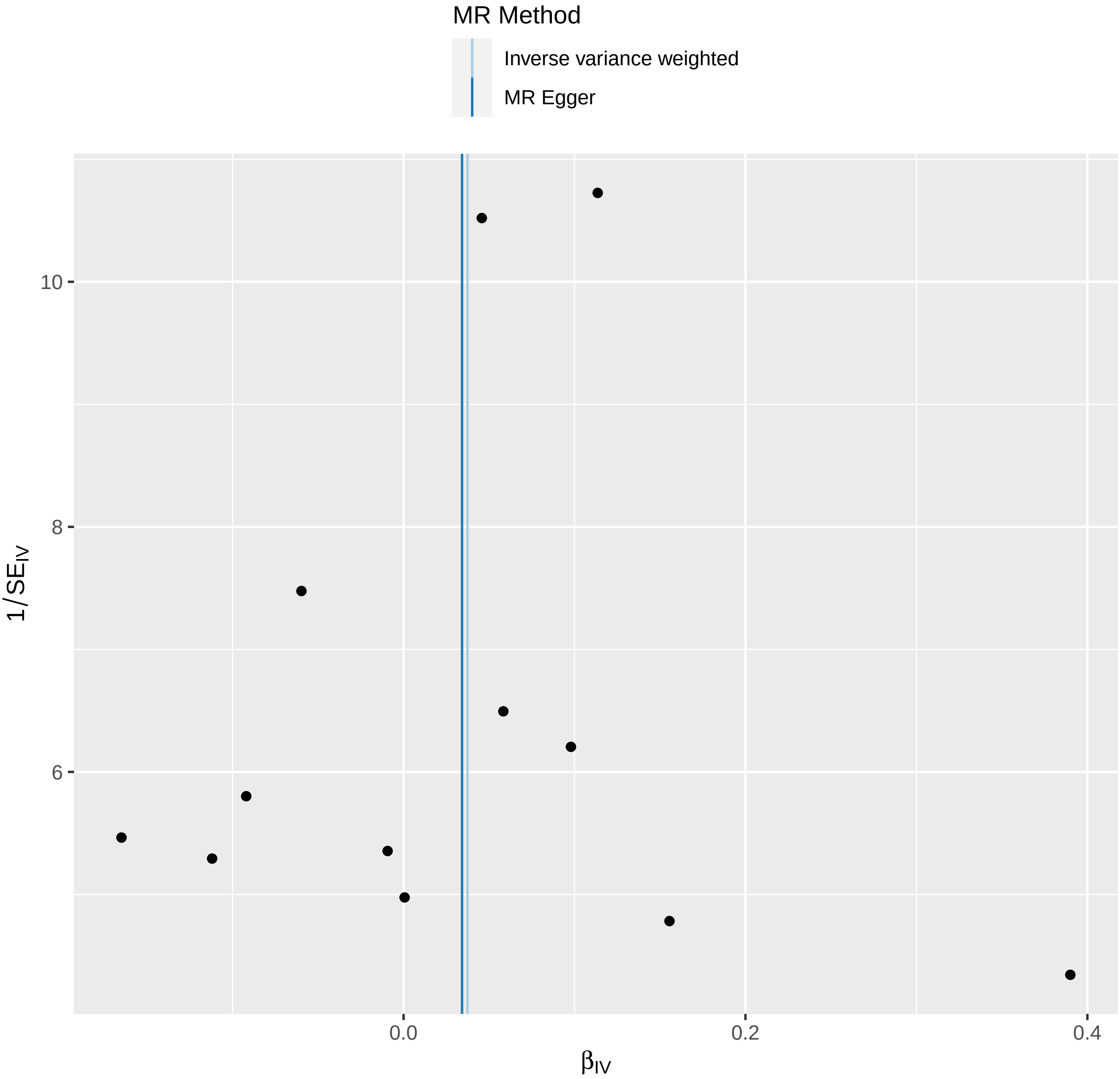

Batch 3−2465 : Selenium on Glutaminyl−peptide cyclotransferase

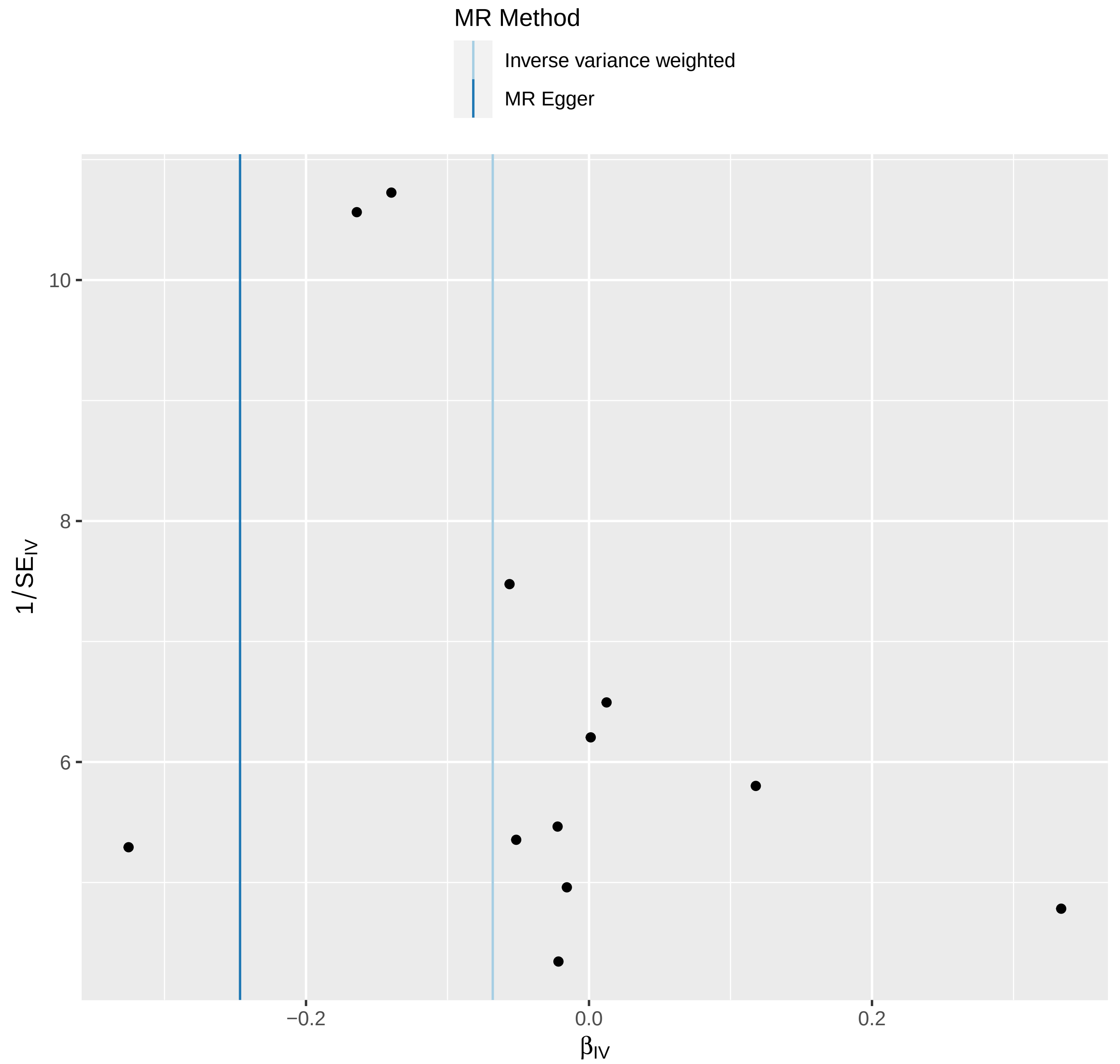

Batch 3−2466 : Selenium on Glutaminyl−peptide cyclotransferase−like protein

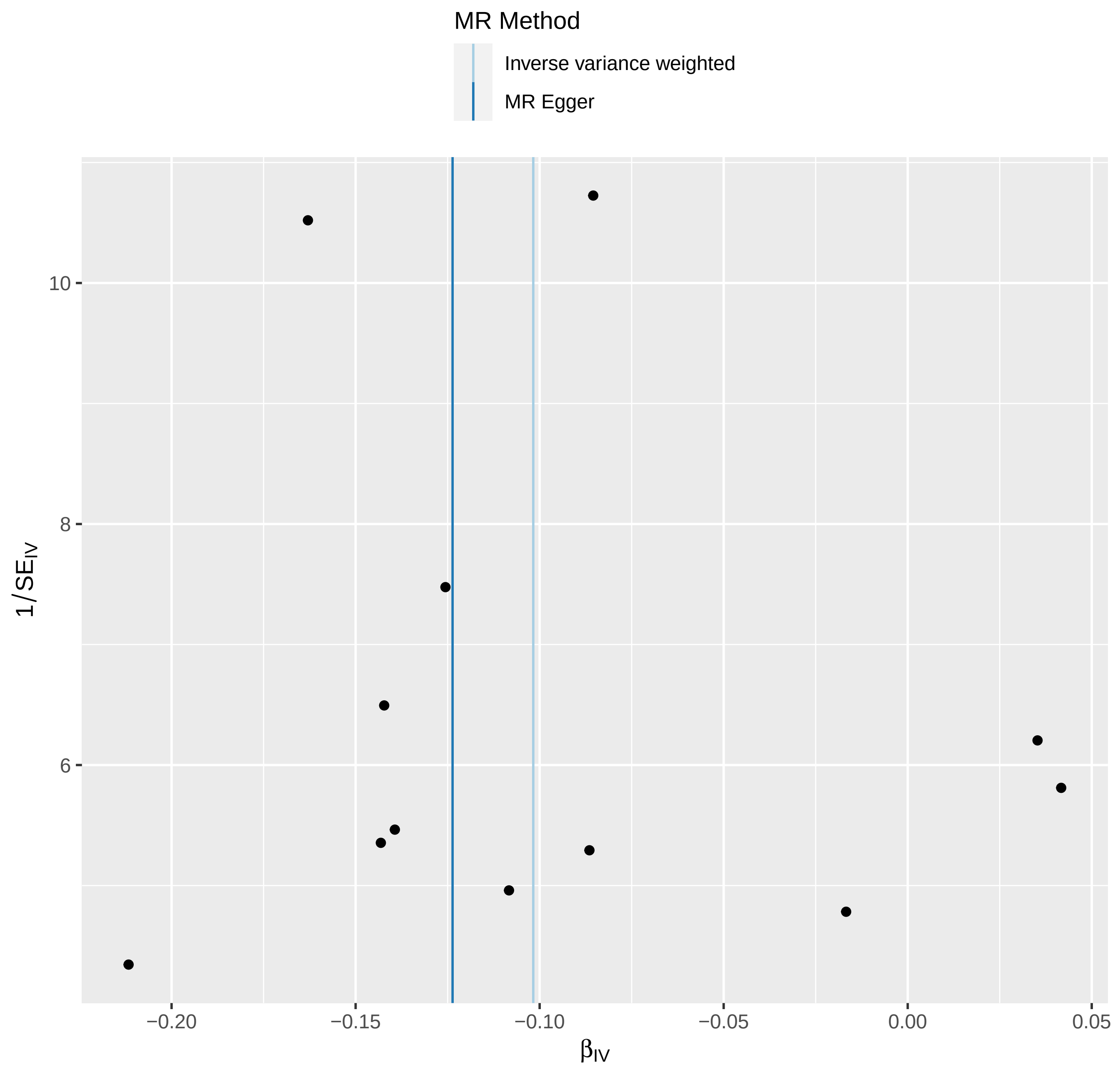

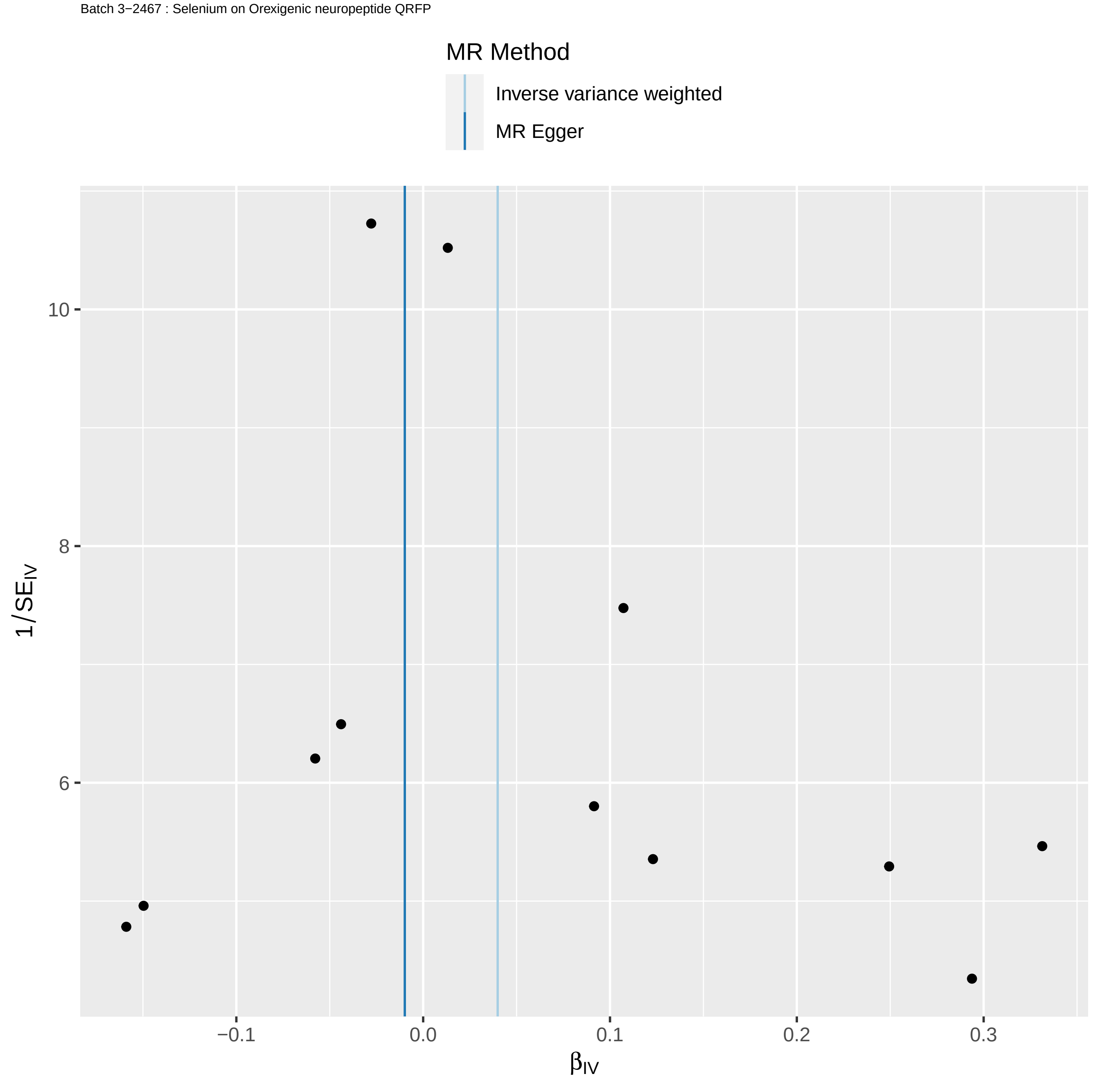
Batch 3−2467 : Selenium on Orexigenic neuropeptide QRFP
MR Method
Inverse variance weighted
MR Egger
10
8
6
1/SE$_{IV}$
−0.1
0.0
0.1
0.2
0.3
β$_{IV}$

Batch 3−2468 : Selenium on Sulfhydryl oxidase 1

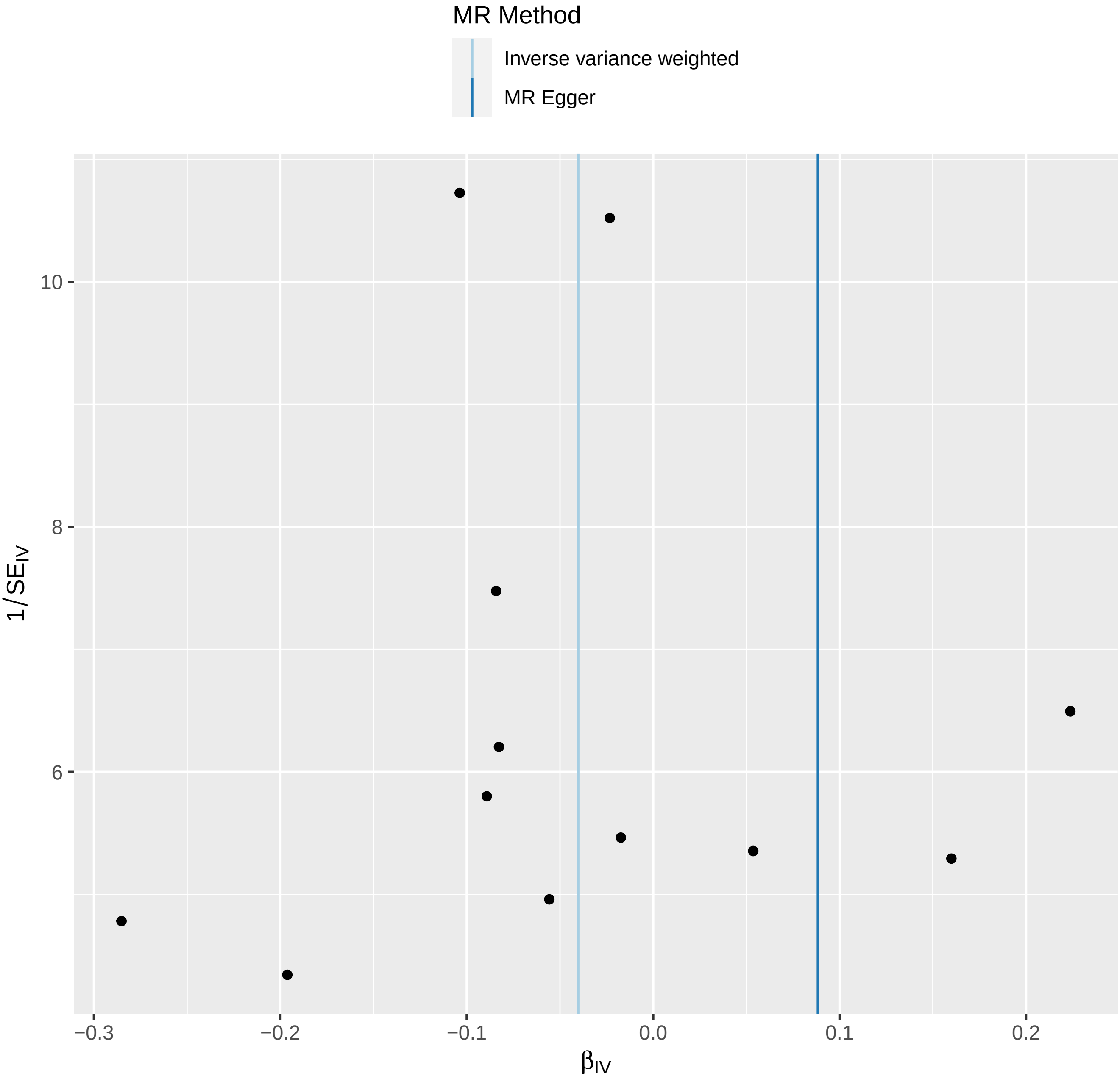

Batch 3−2469 : Selenium on Sulfhydryl oxidase 1

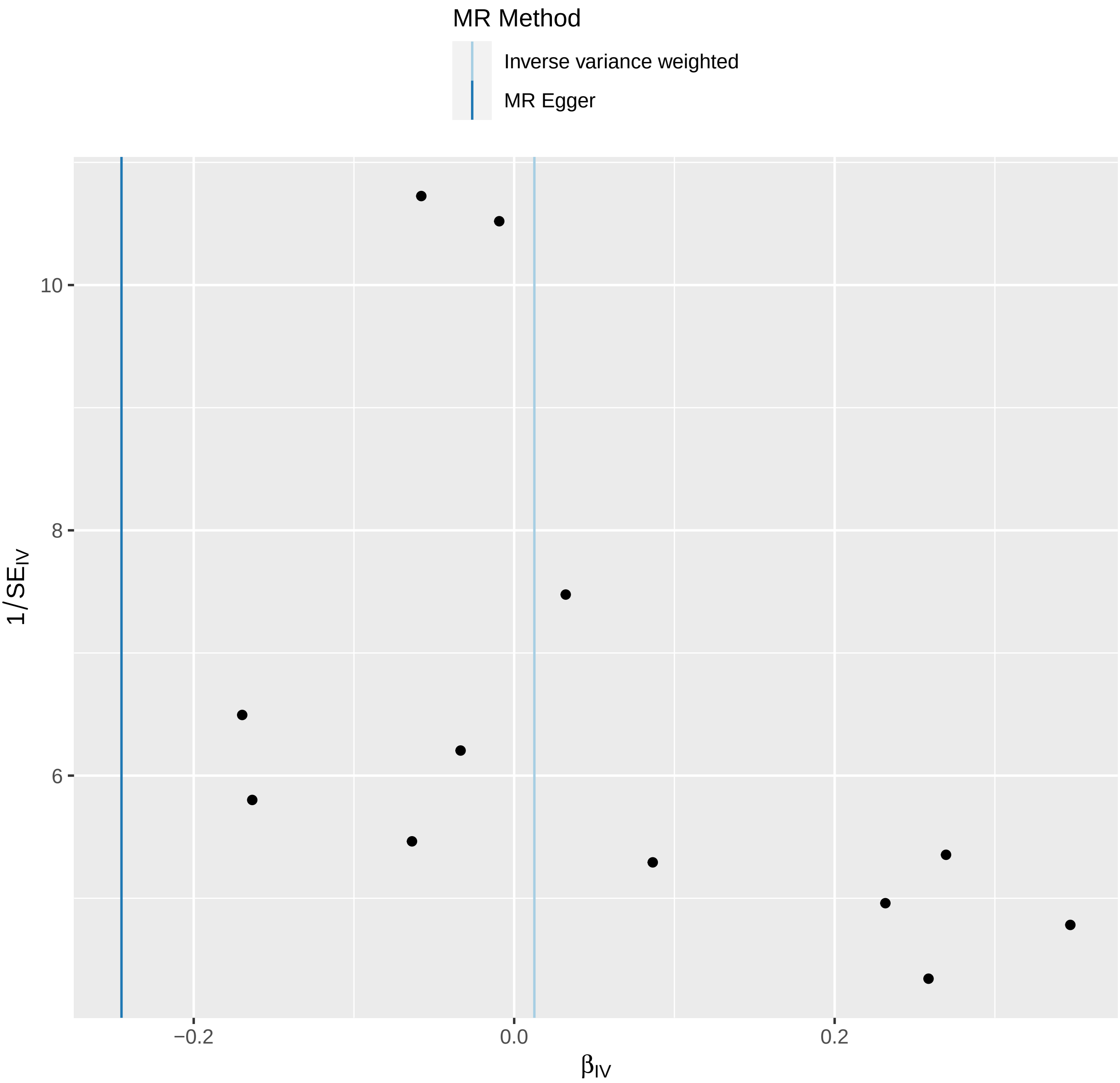

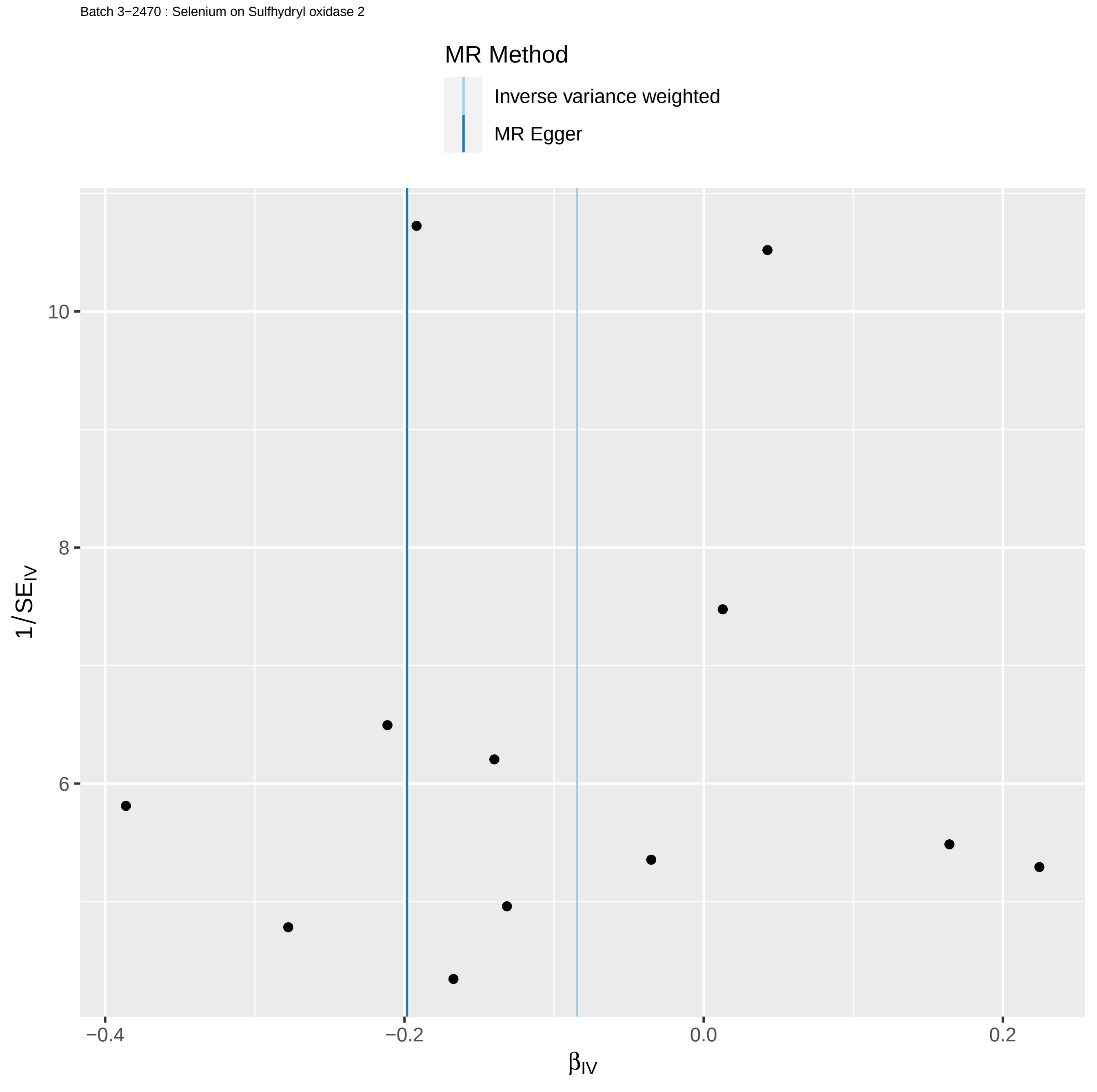
Batch 3−2470 : Selenium on Sulfhydryl oxidase 2
MR Method
Inverse variance weighted
MR Egger
10
8
6
1/SE_IV
−0.4
−0.2
0.0
0.2
β_IV

Batch 3−2471 : Selenium on Ras−related protein Rab−14

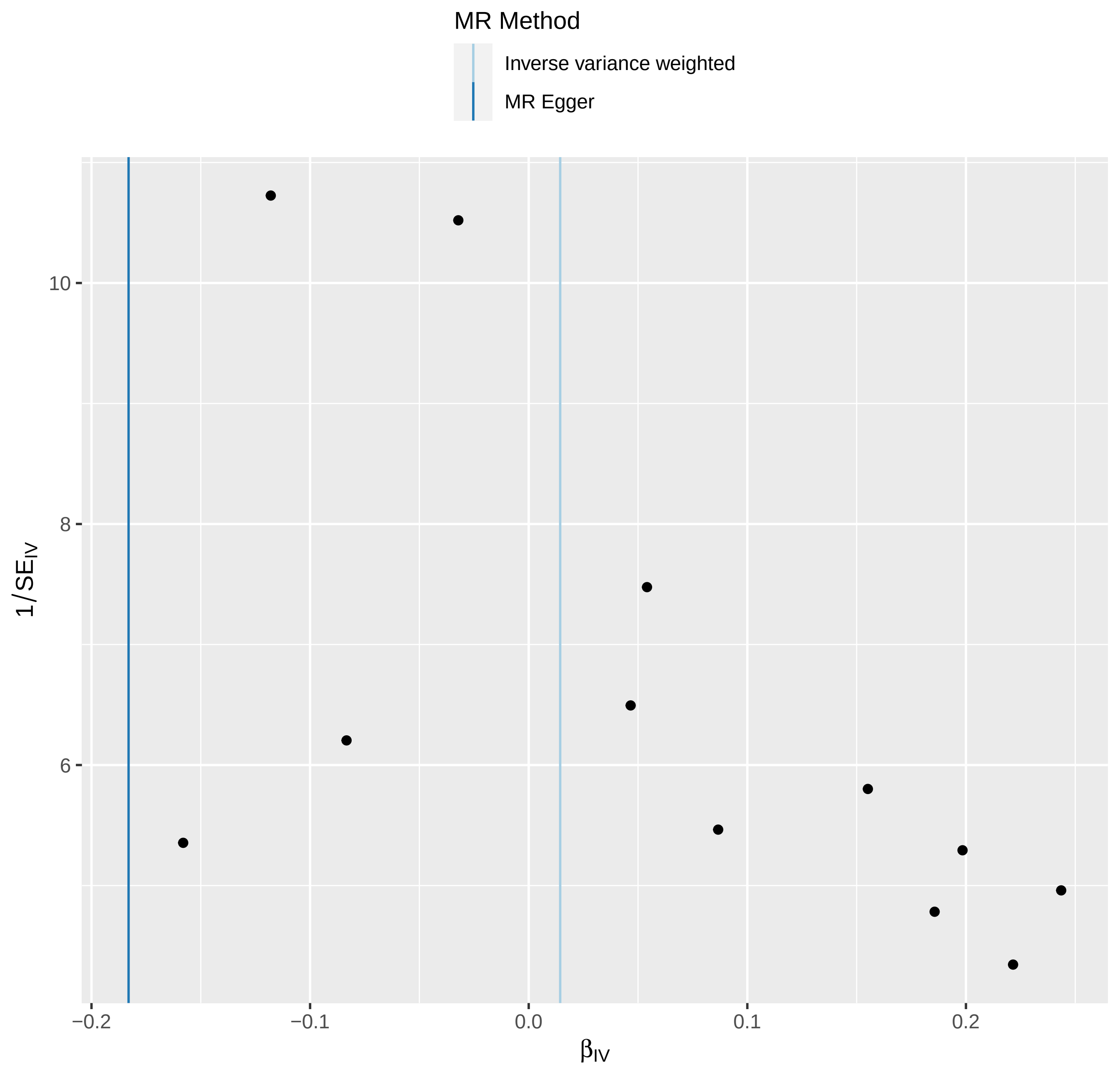

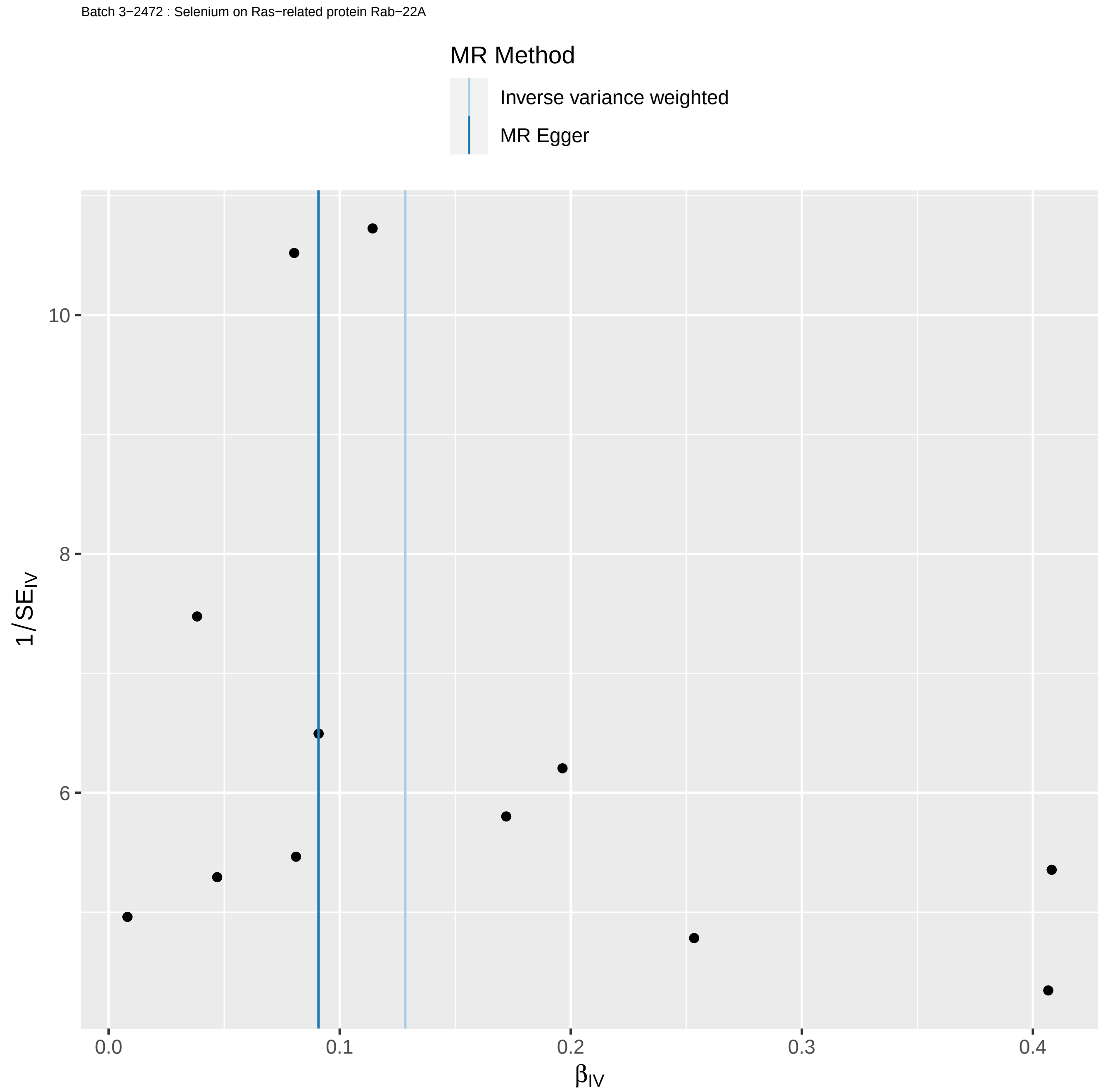

Batch 3−2472 : Selenium on Ras−related protein Rab−22A
MR Method
Inverse variance weighted
MR Egger
10
8
6
1/SE$_{IV}$
0.0
0.1
0.2
0.3
0.4
β_{IV}

Batch 3−2473 : Selenium on Ras−related protein Rab−26

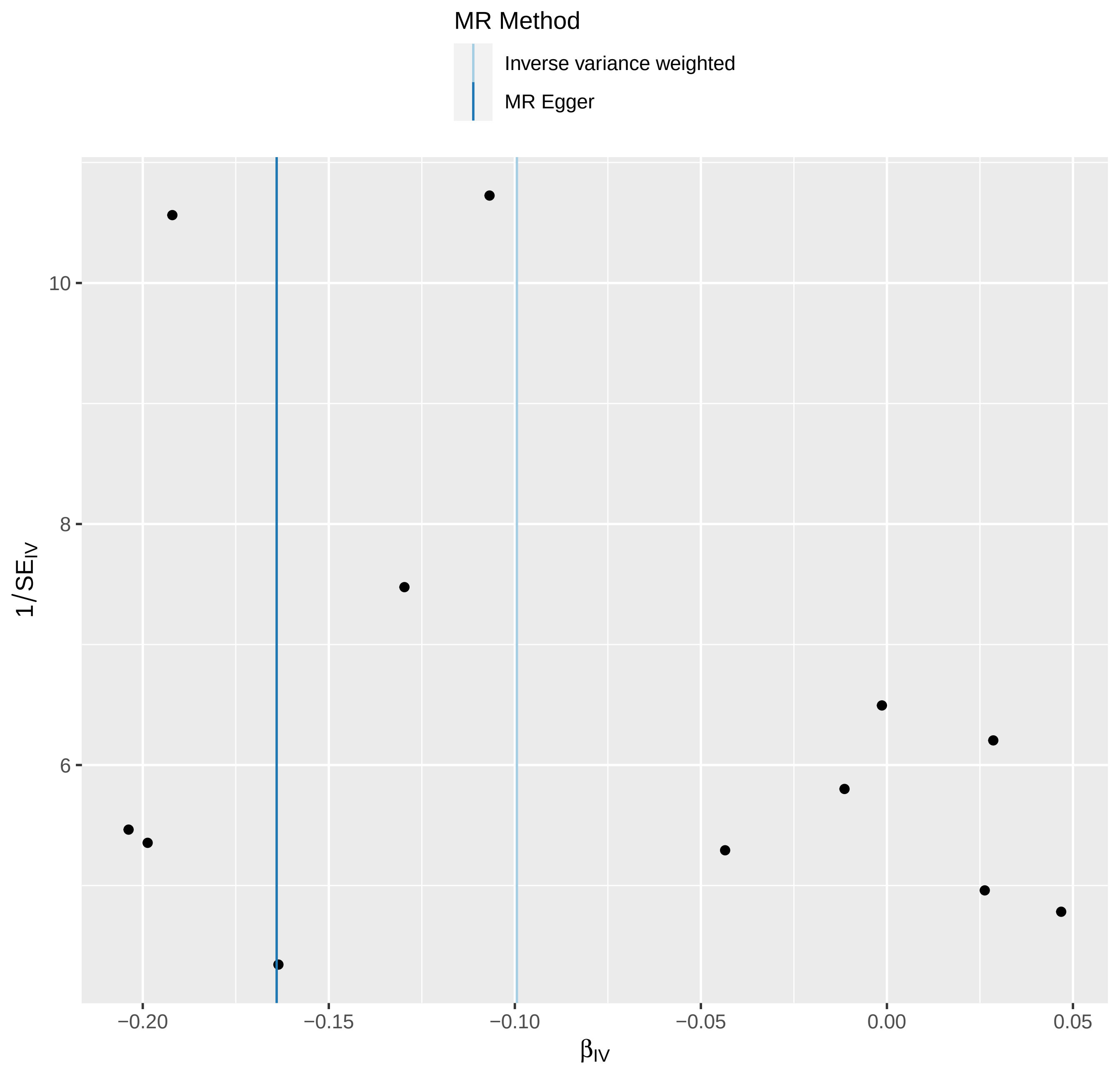

Batch 3−2474 : Selenium on Ras−related protein Rab−27A

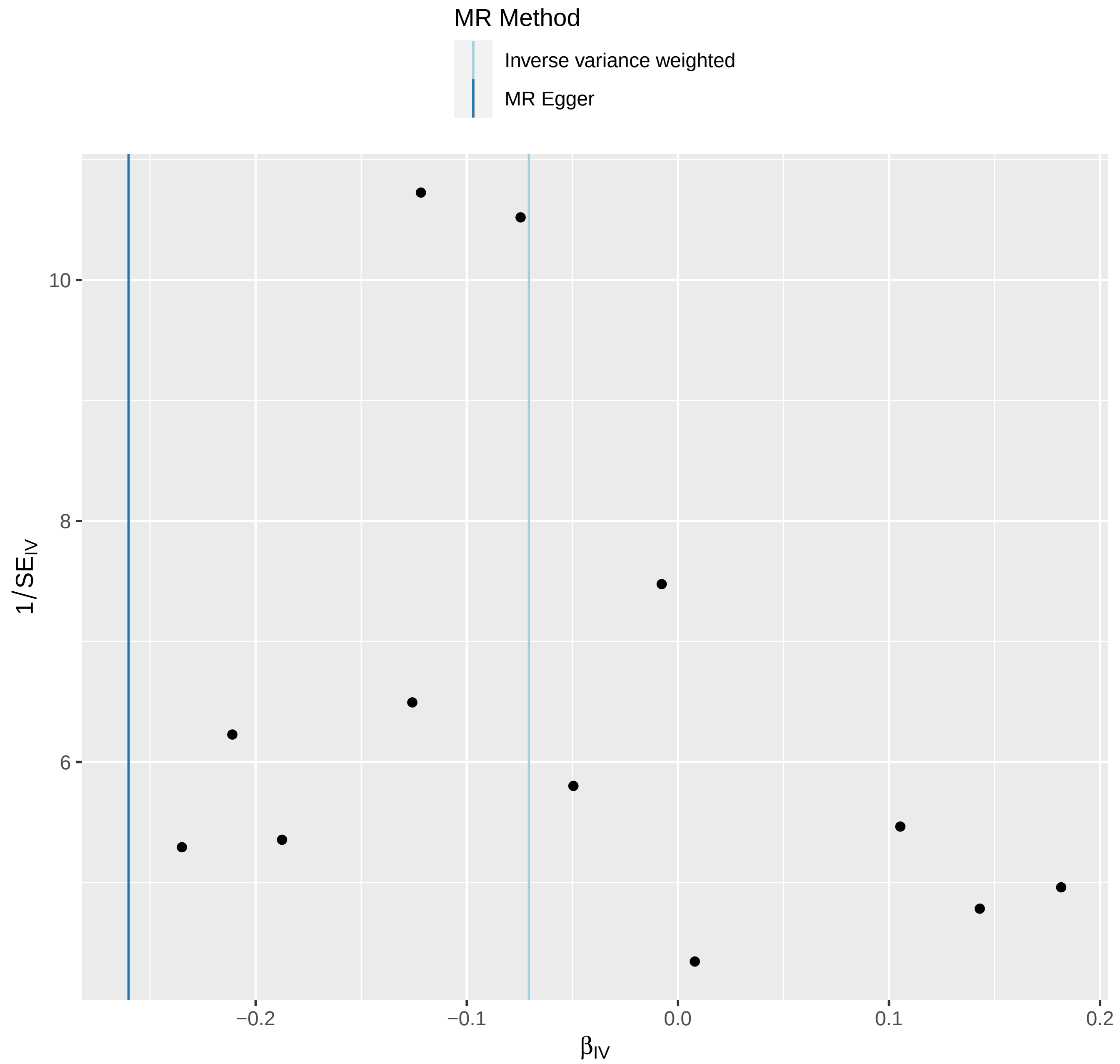

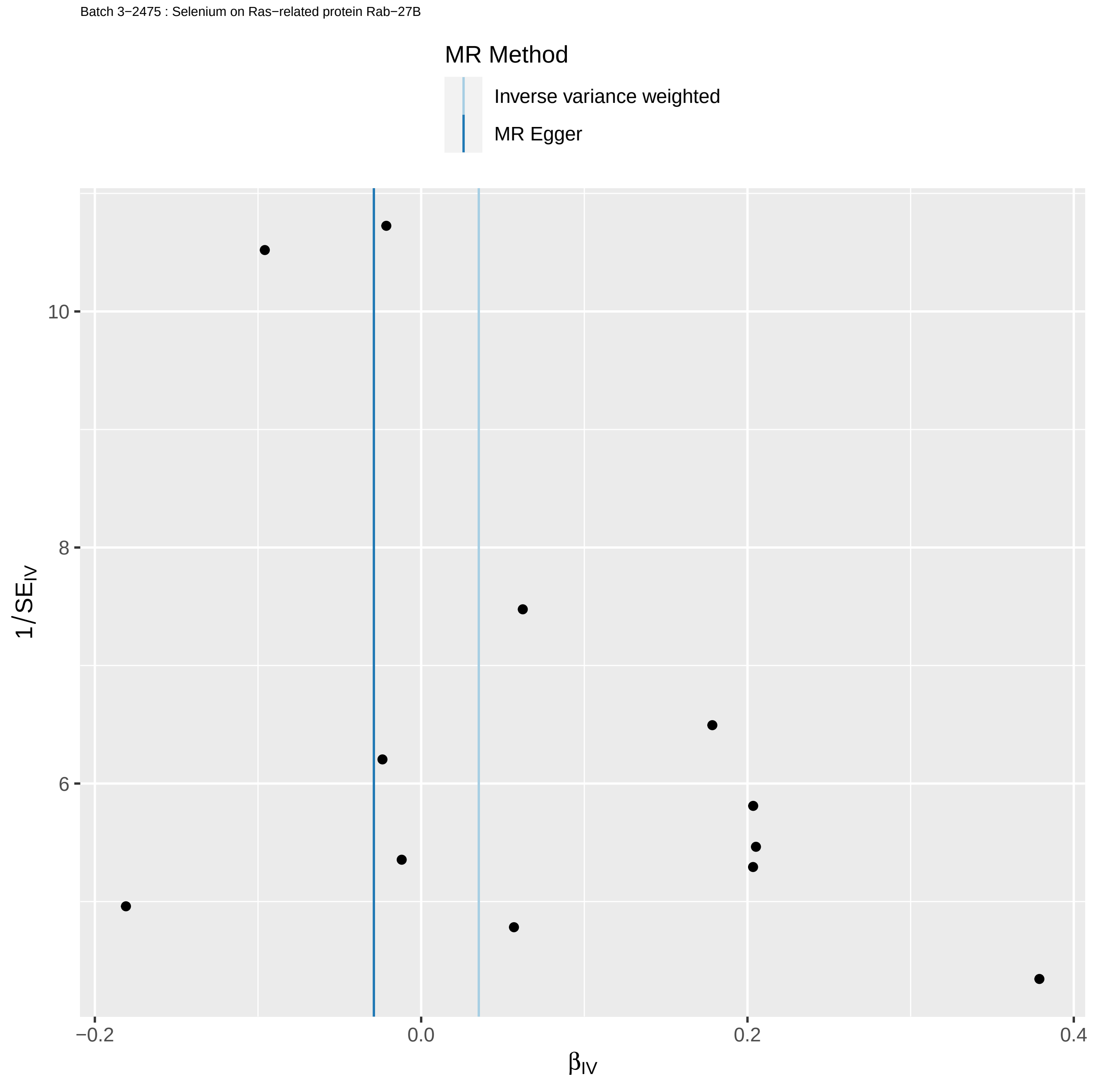

Batch 3−2475 : Selenium on Ras−related protein Rab−27B
MR Method
Inverse variance weighted
MR Egger
10
8
6
1/SE_IV
−0.2
0.0
0.2
0.4
β_IV

Batch 3−2476 : Selenium on Ras−related protein Rab−35

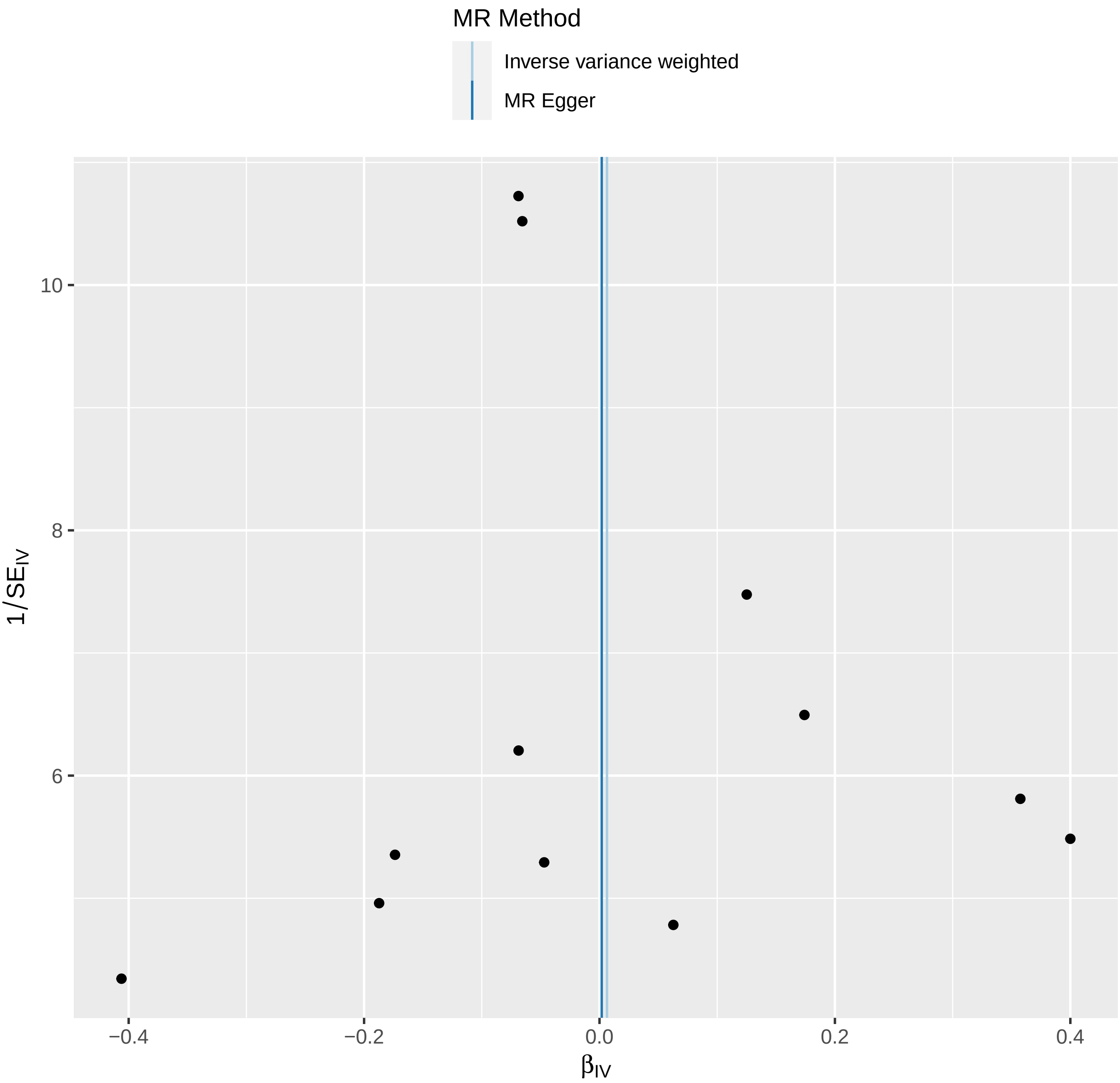

Batch 3−2477 : Selenium on Ras−related protein Rab−39B

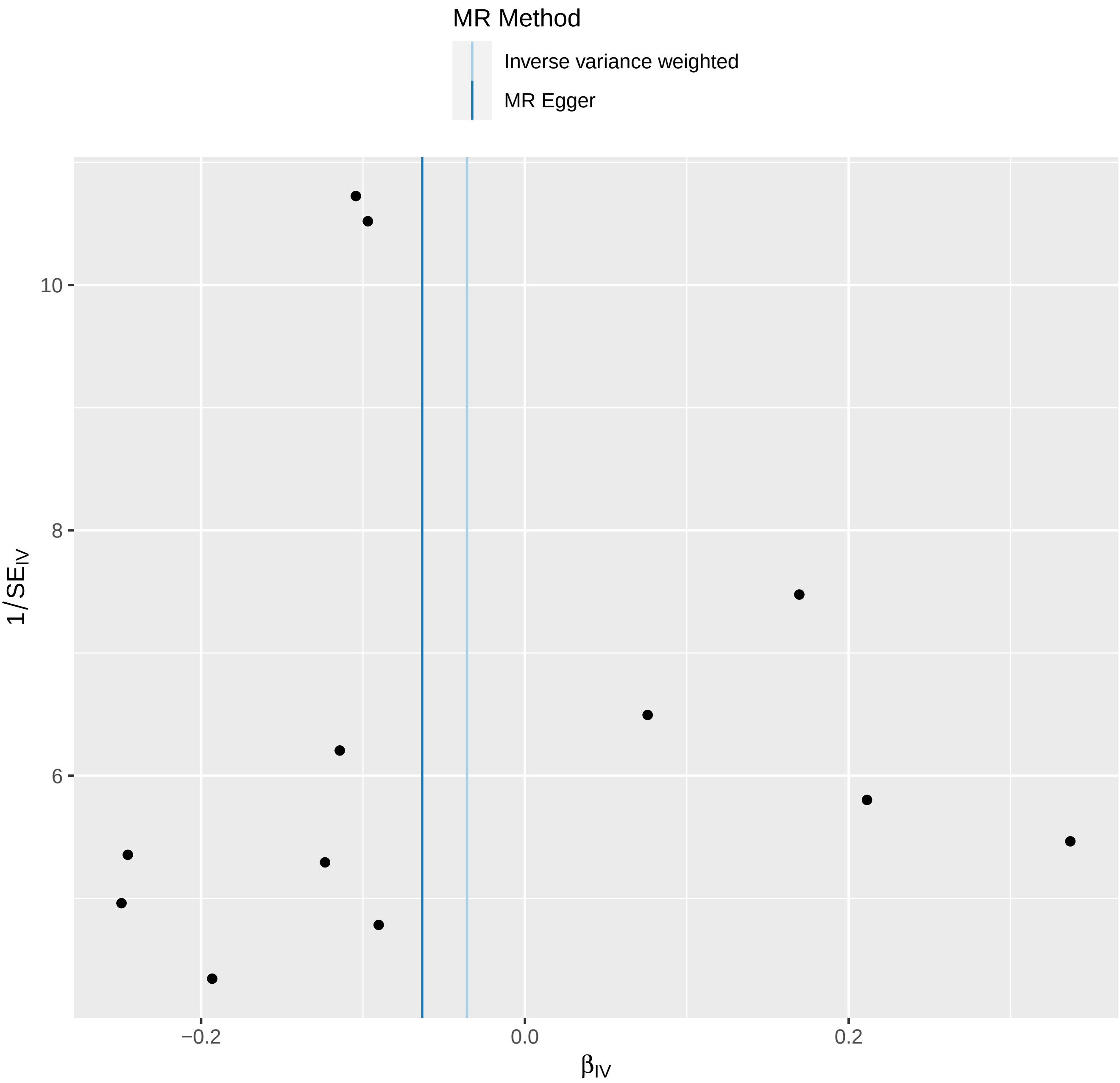

Batch 3−2478 : Selenium on Ras−related protein Rab−7b

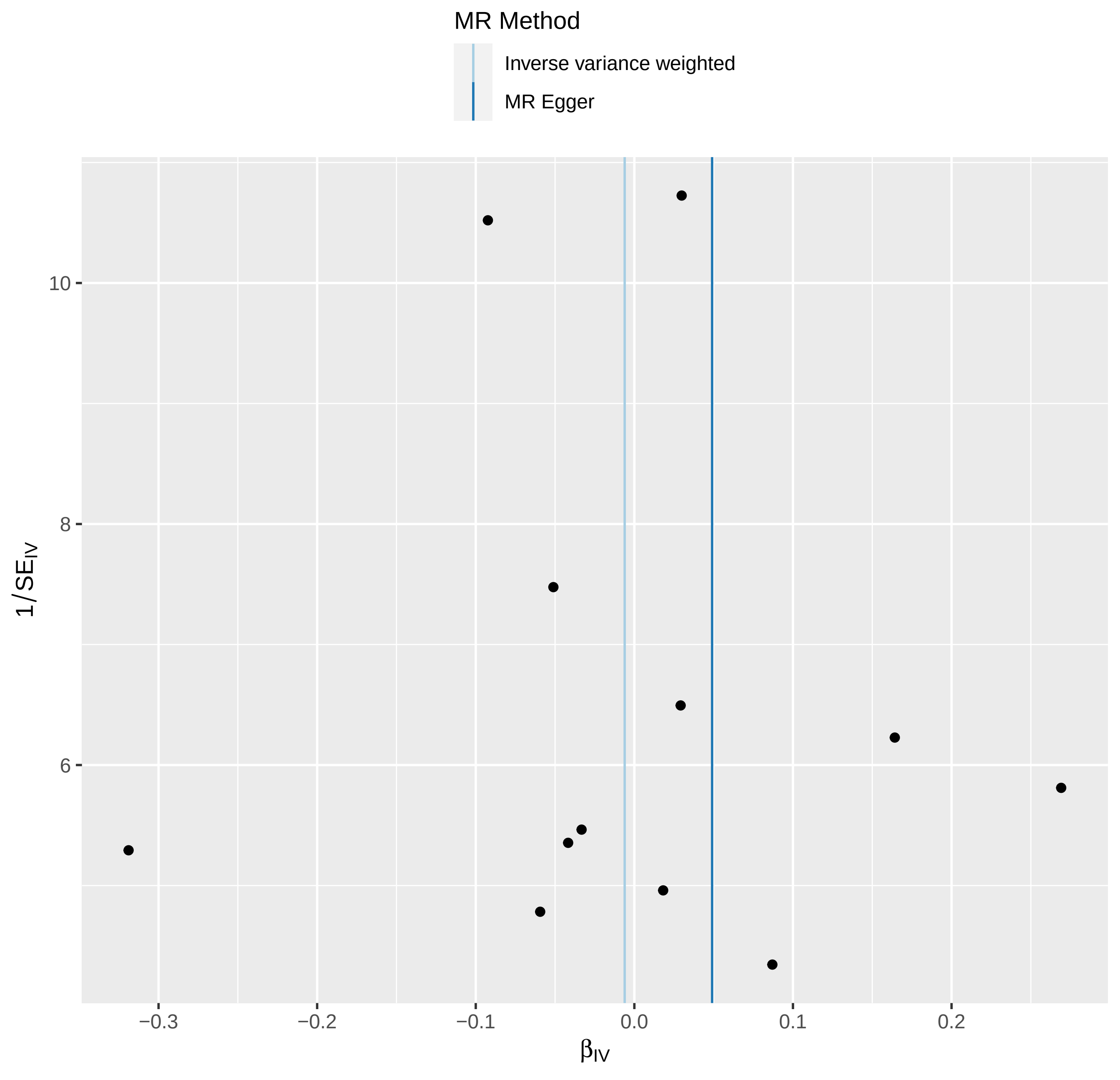

Batch 3−2479 : Selenium on Rab9 effector protein with kelch motifs

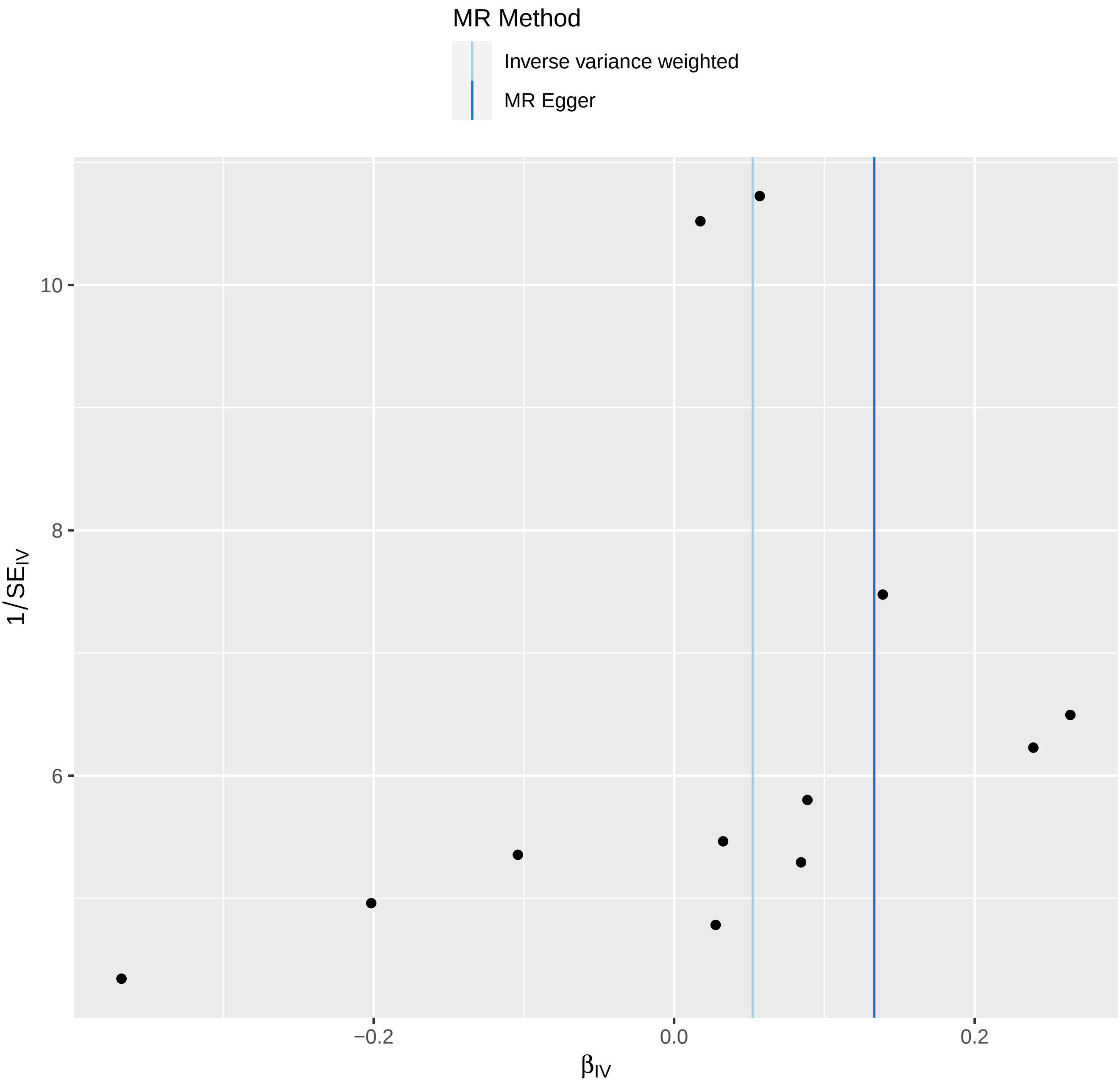

Batch 3−2480 : Selenium on Ras−related C3 botulinum toxin substrate 3

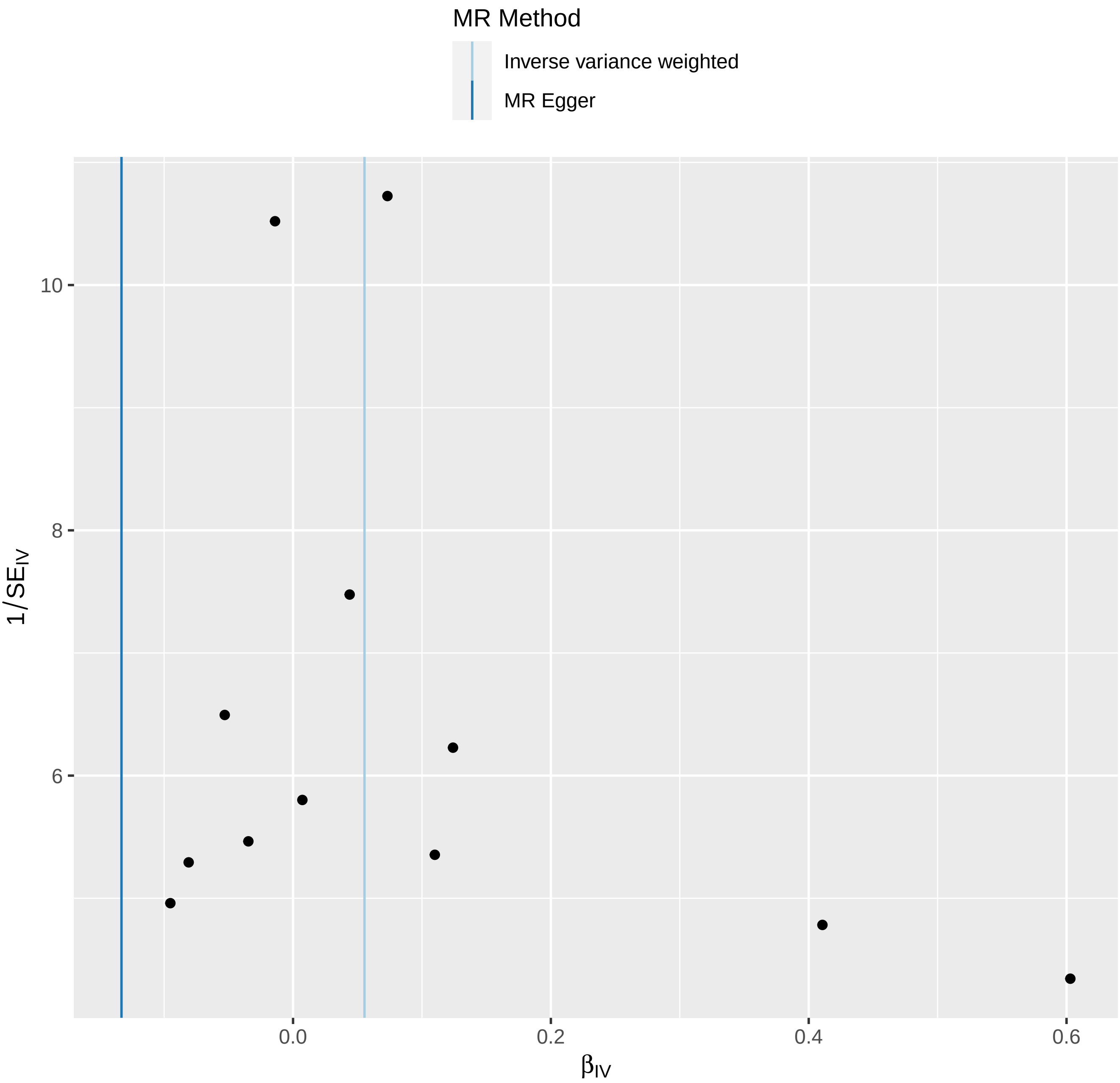

Batch 3−2481 : Selenium on Rac GTPase−activating protein 1

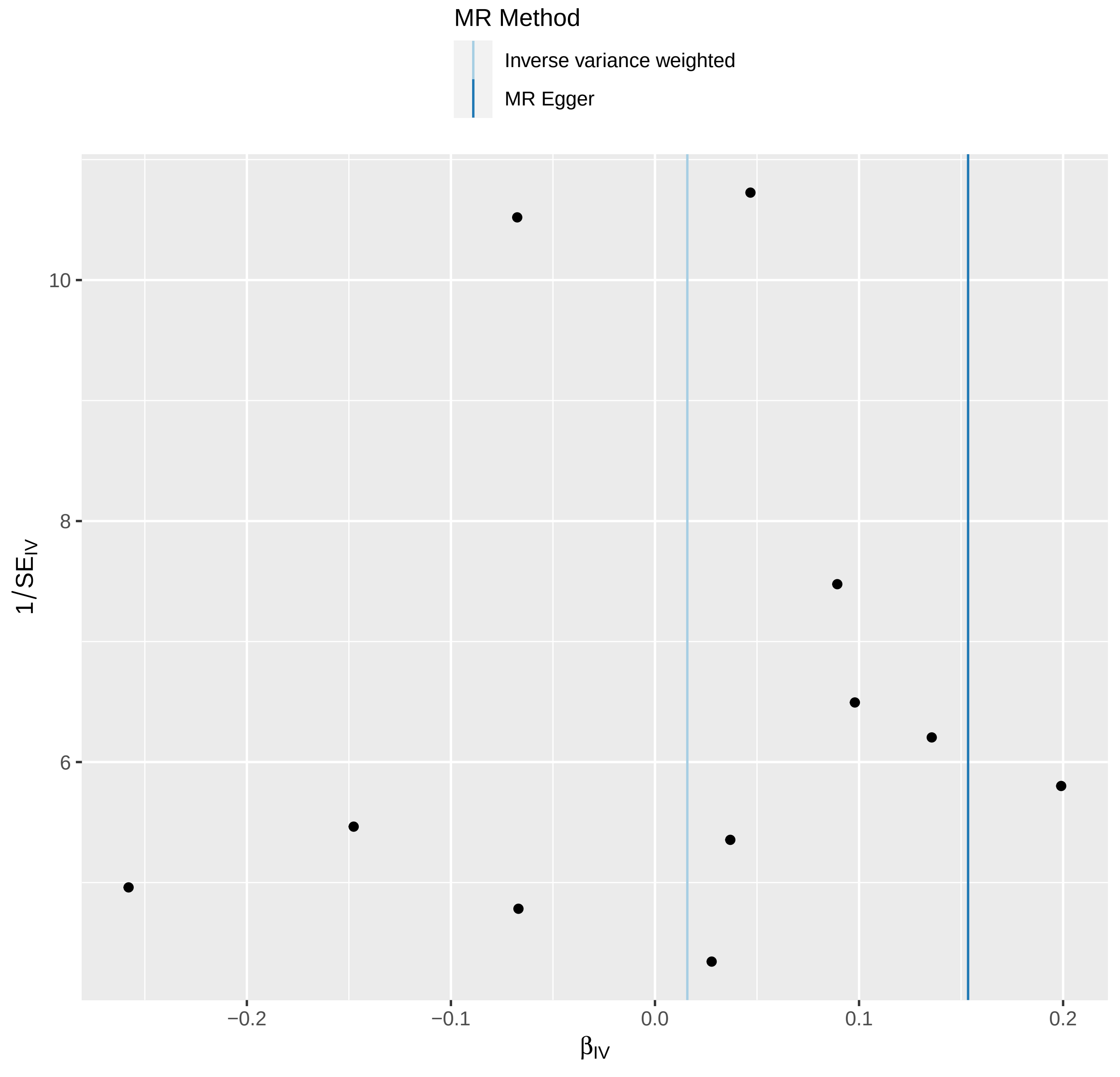

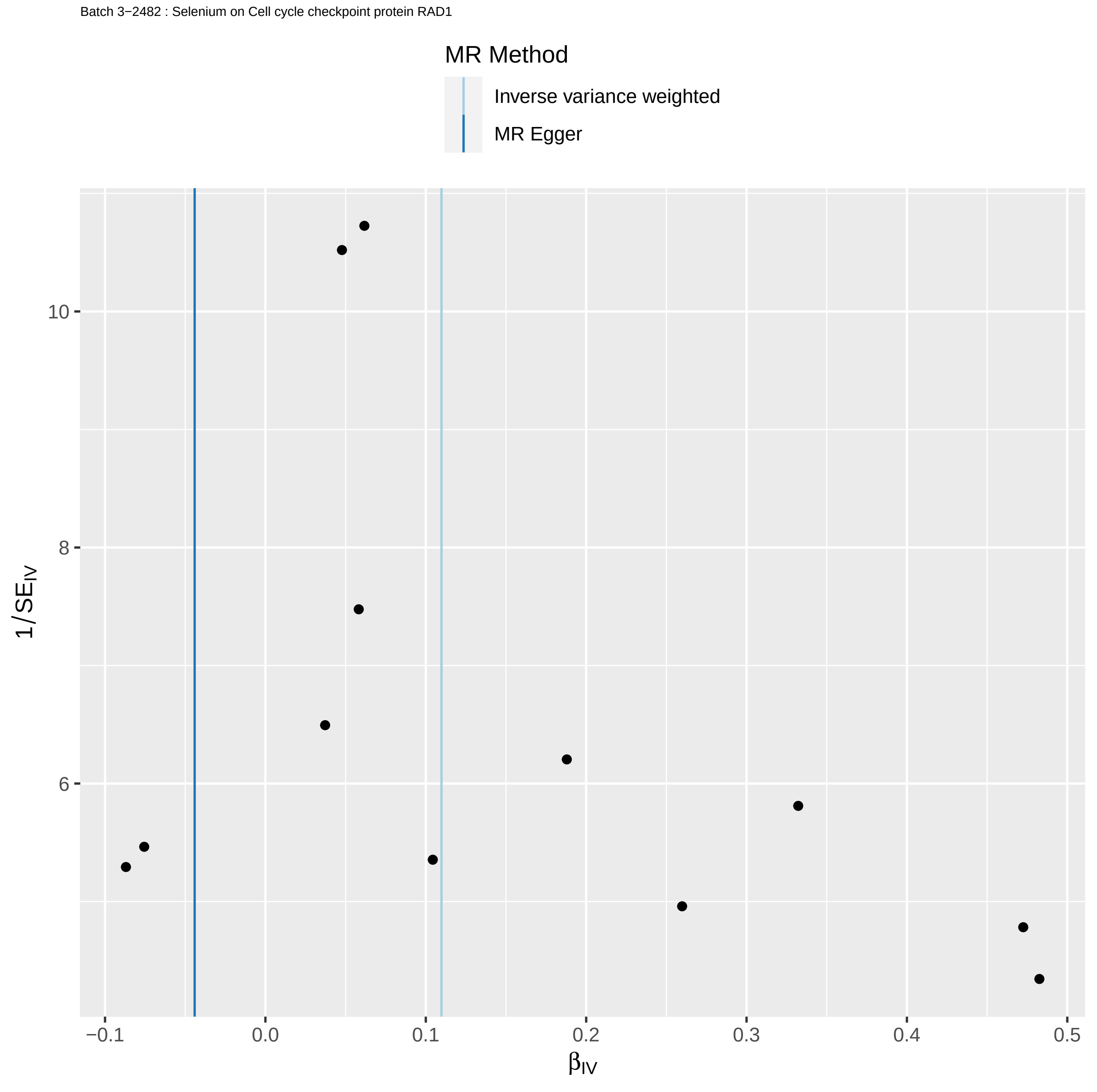
Batch 3−2482 : Selenium on Cell cycle checkpoint protein RAD1
MR Method
Inverse variance weighted
MR Egger
10
8
6
$1/SE_{IV}$
−0.1
0.0
0.1
0.2
0.3
0.4
0.5
β_{IV}

Batch 3−2483 : Selenium on UV excision repair protein RAD23 homolog A

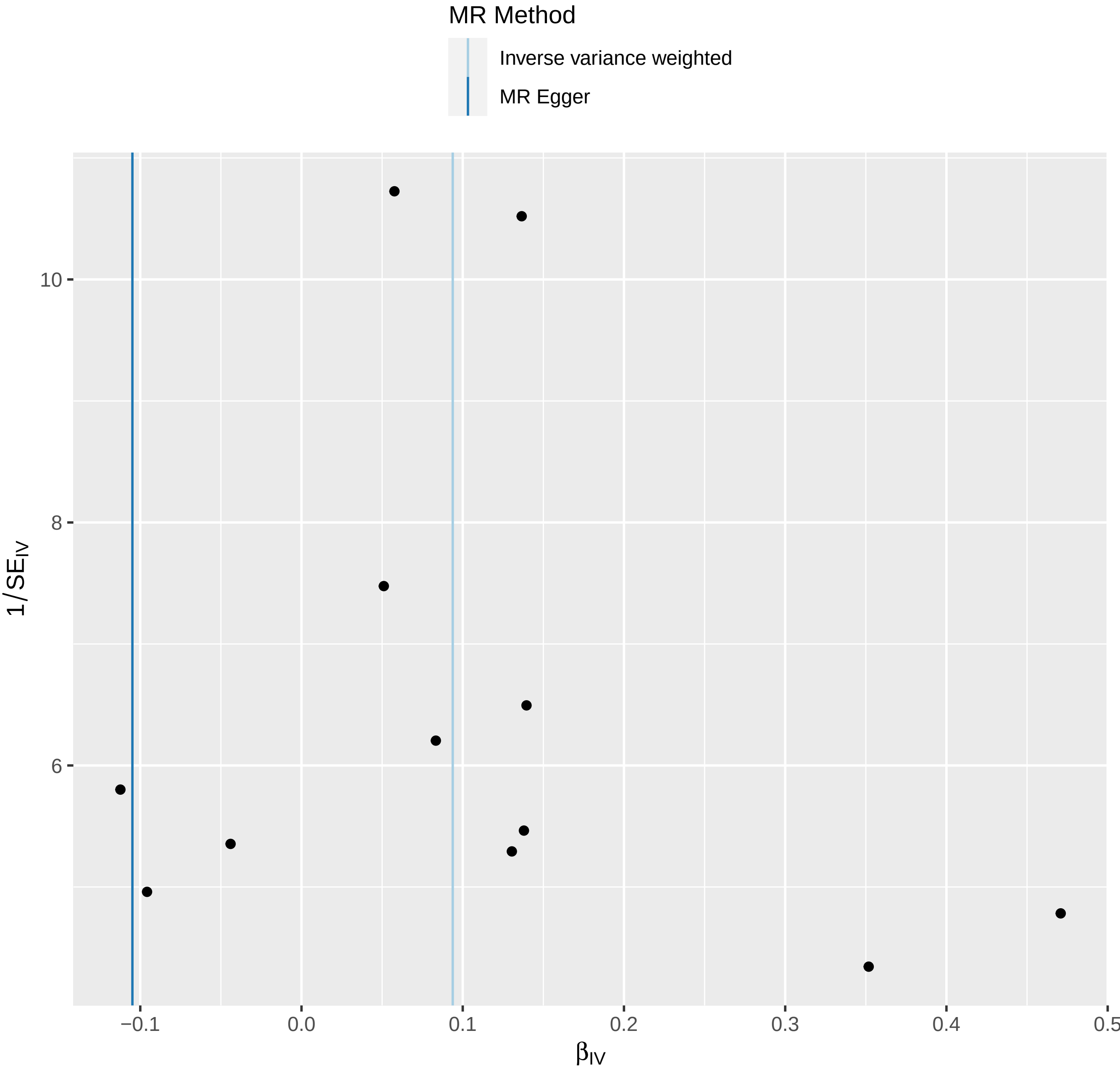

Batch 3−2484 : Selenium on UV excision repair protein RAD23 homolog B

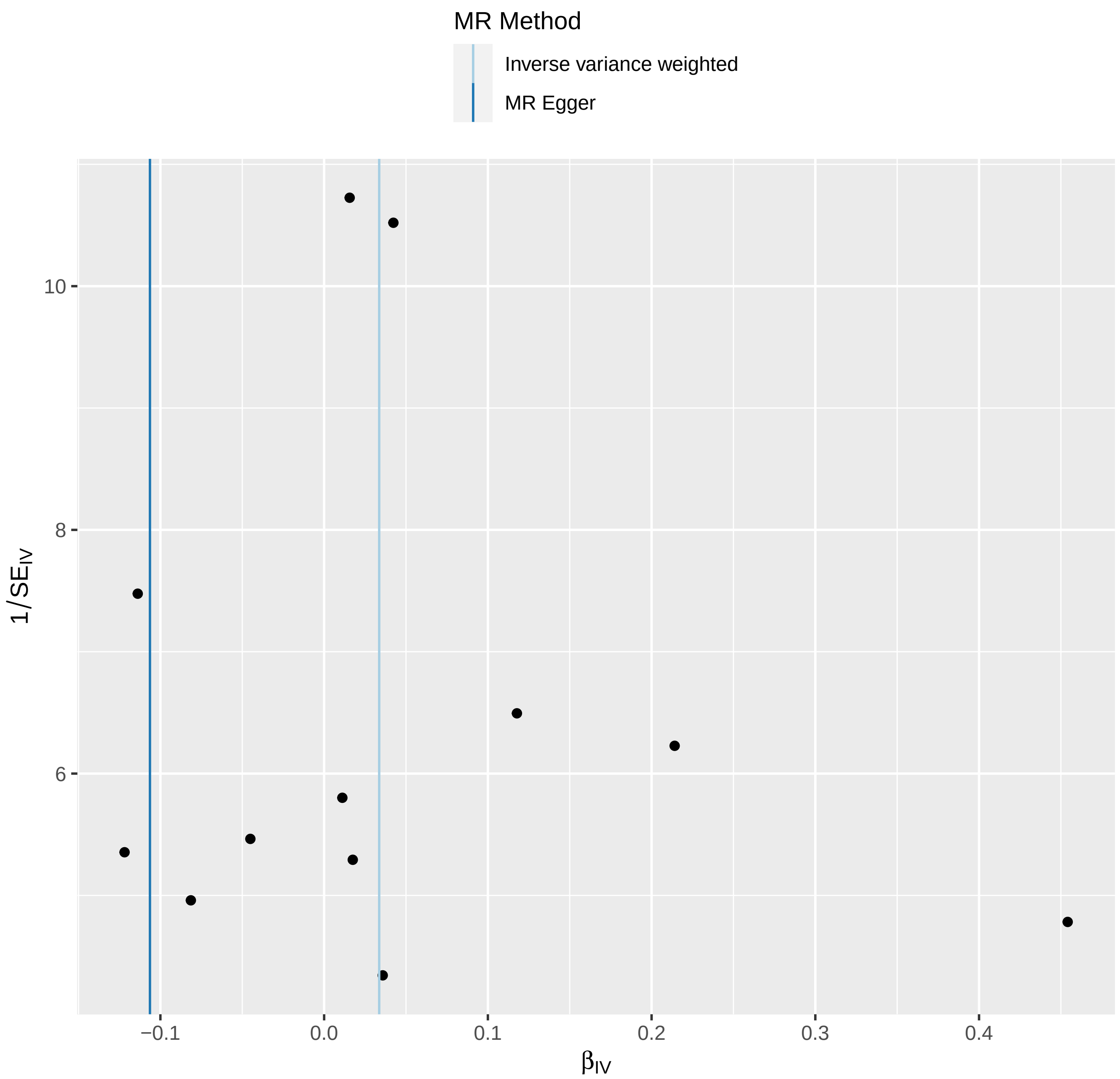

Batch 3−2485 : Selenium on DNA repair protein RAD51 homolog 1

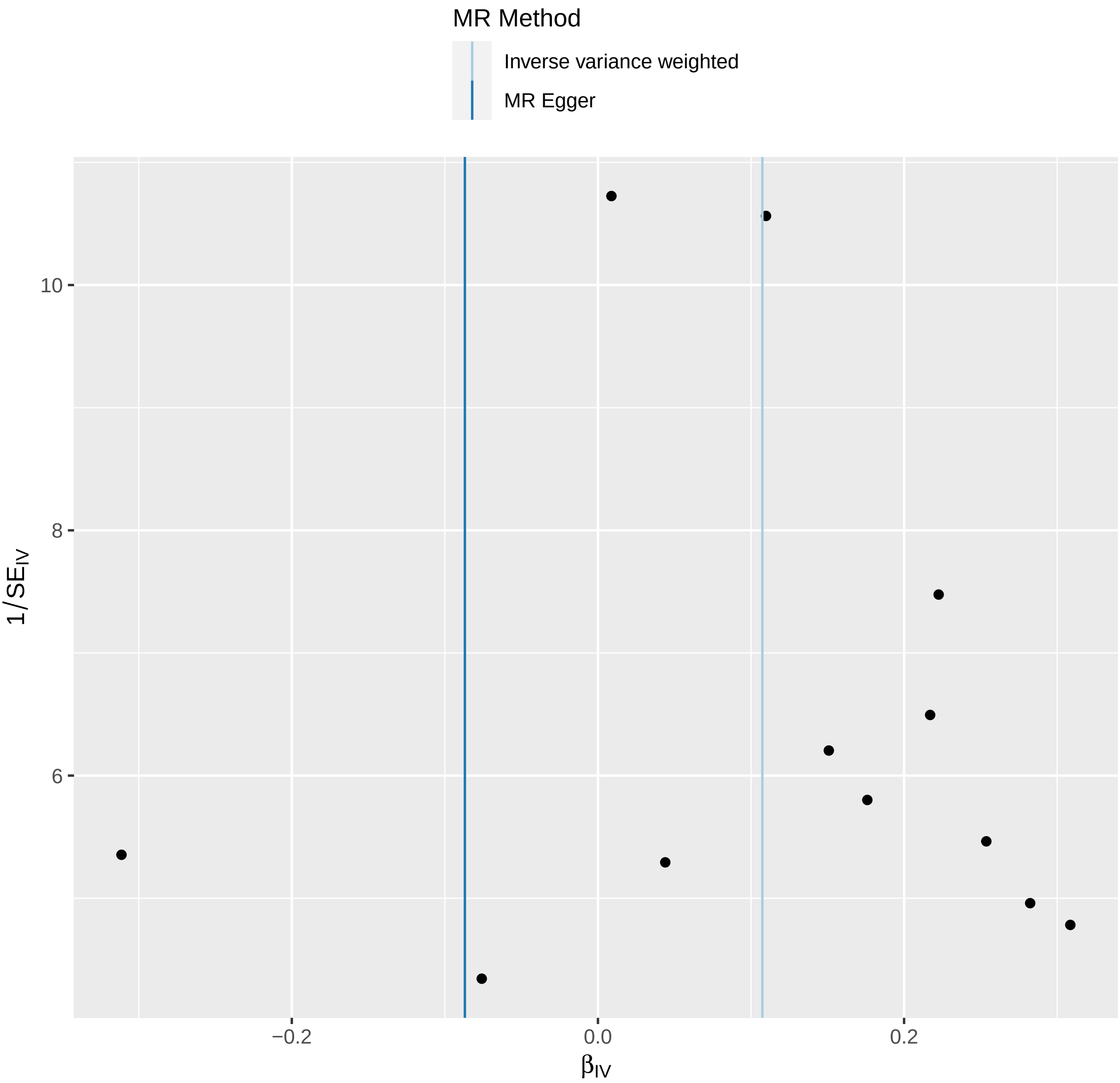

Batch 3−2486 : Selenium on DNA repair protein RAD51 homolog 4

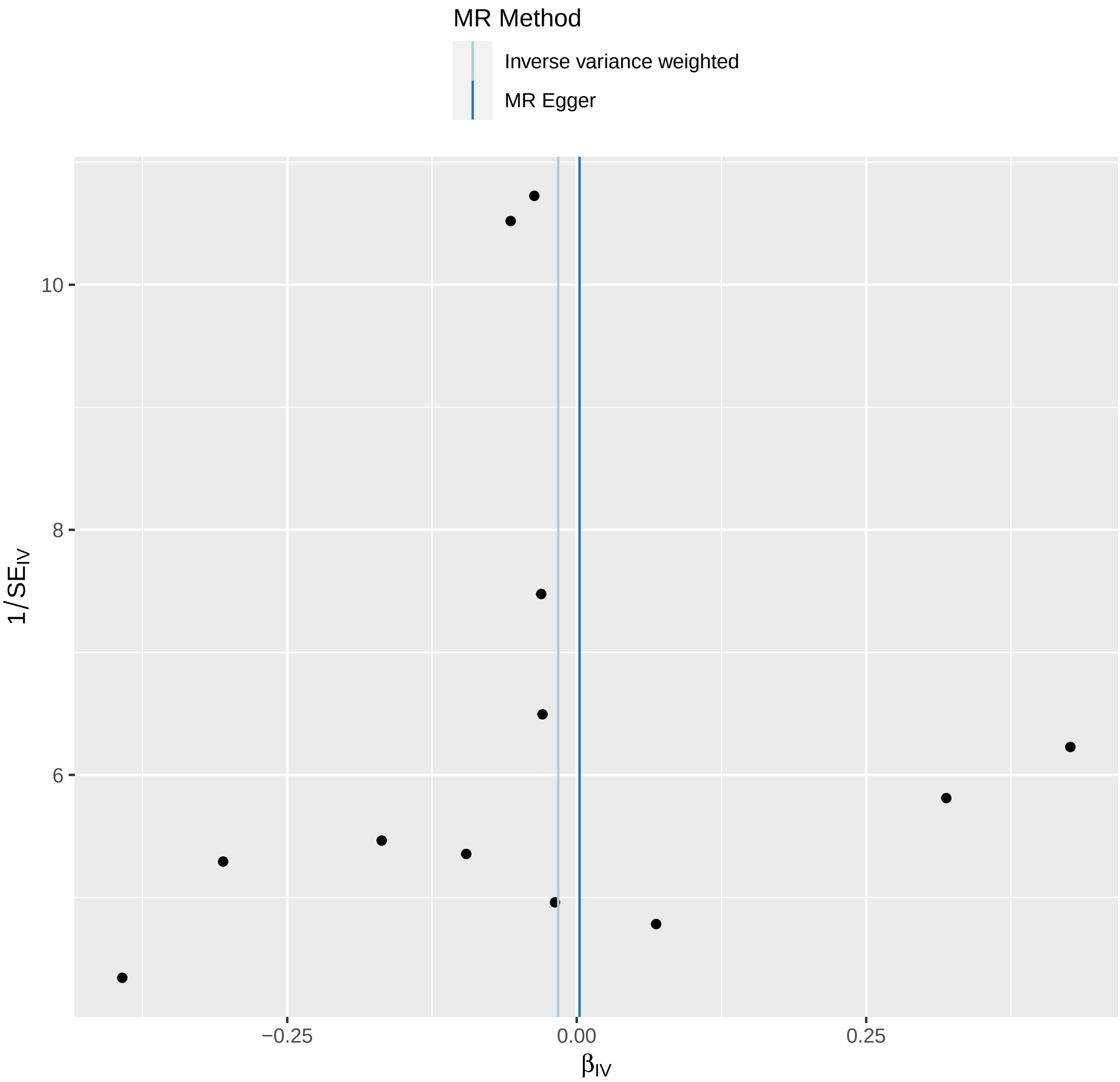

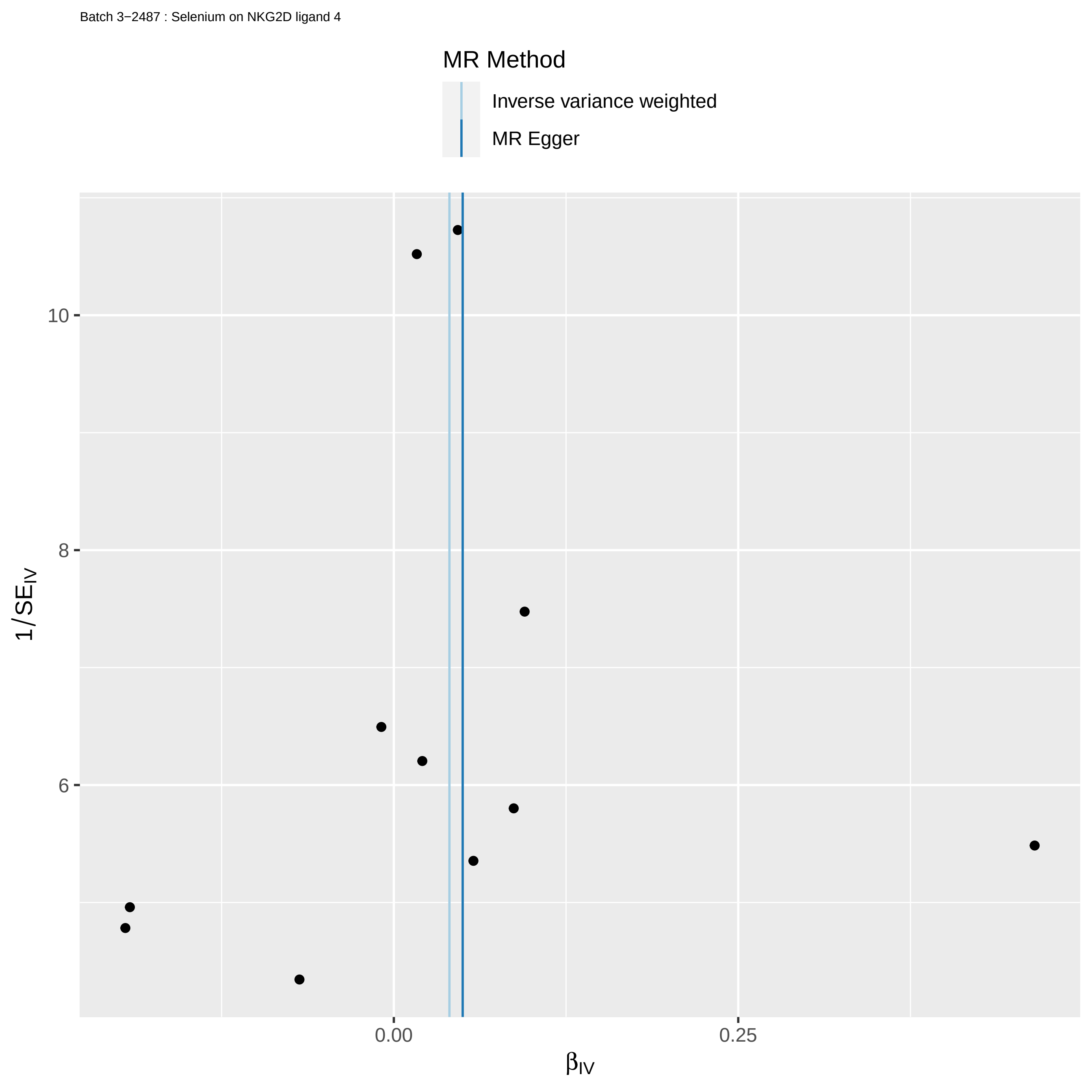

Batch 3−2487 : Selenium on NKG2D ligand 4
MR Method
Inverse variance weighted
MR Egger
10
8
6
1/SE_IV
0.00
0.25
β_IV

Batch 3−2488 : Selenium on Ran−binding protein 3

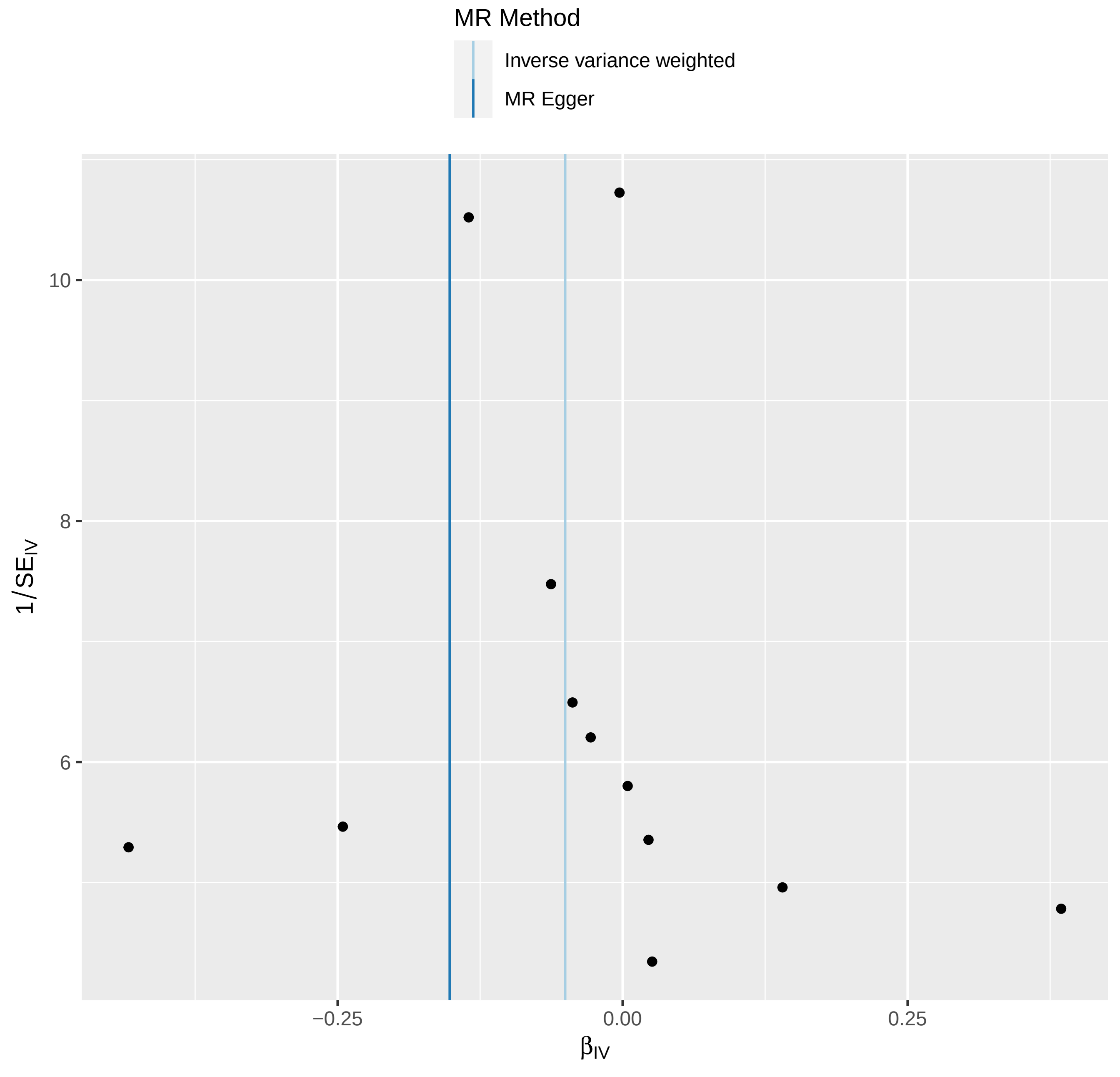

Batch 3−2489 : Selenium on Rap1 GTPase−activating protein 1

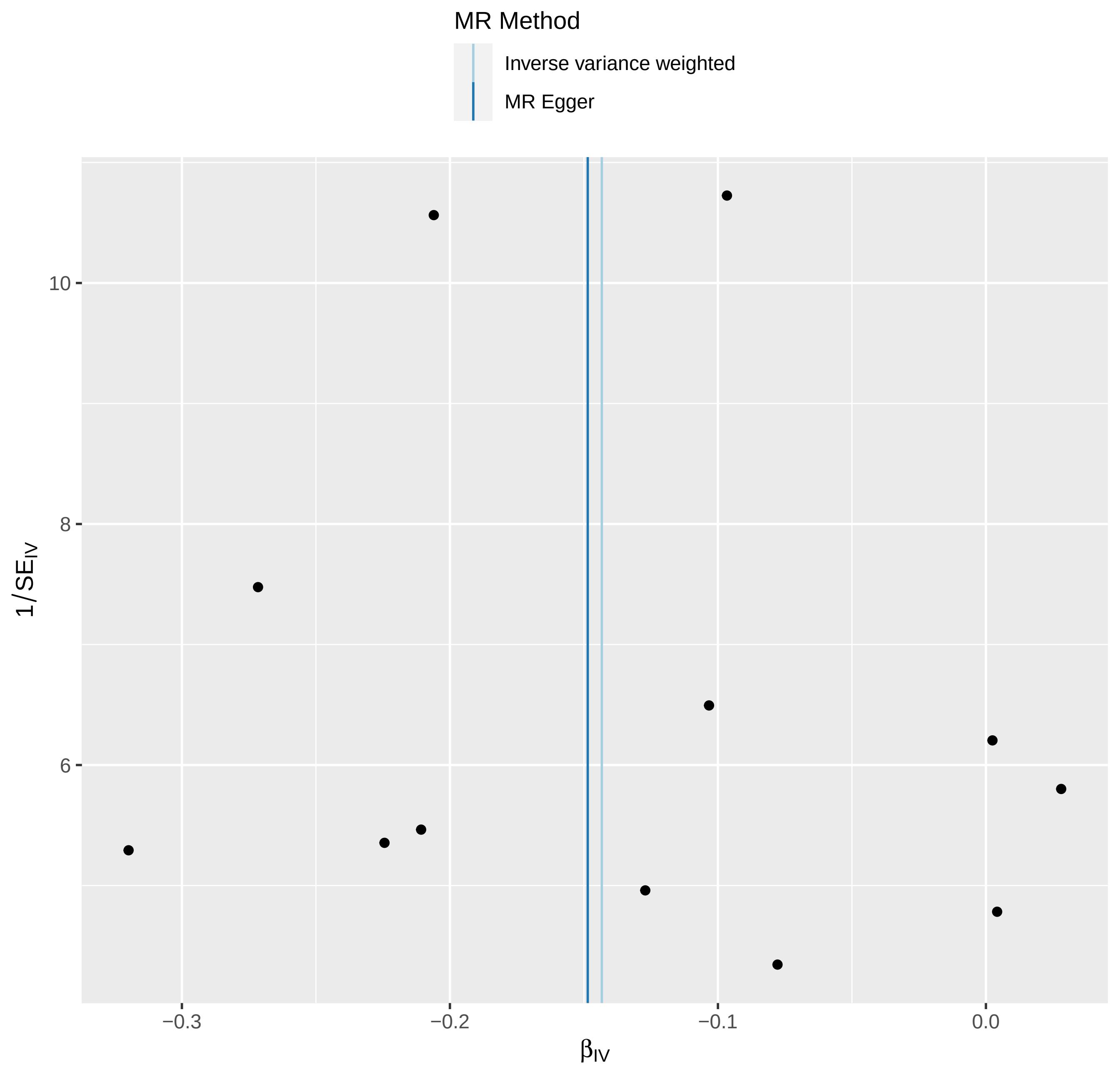

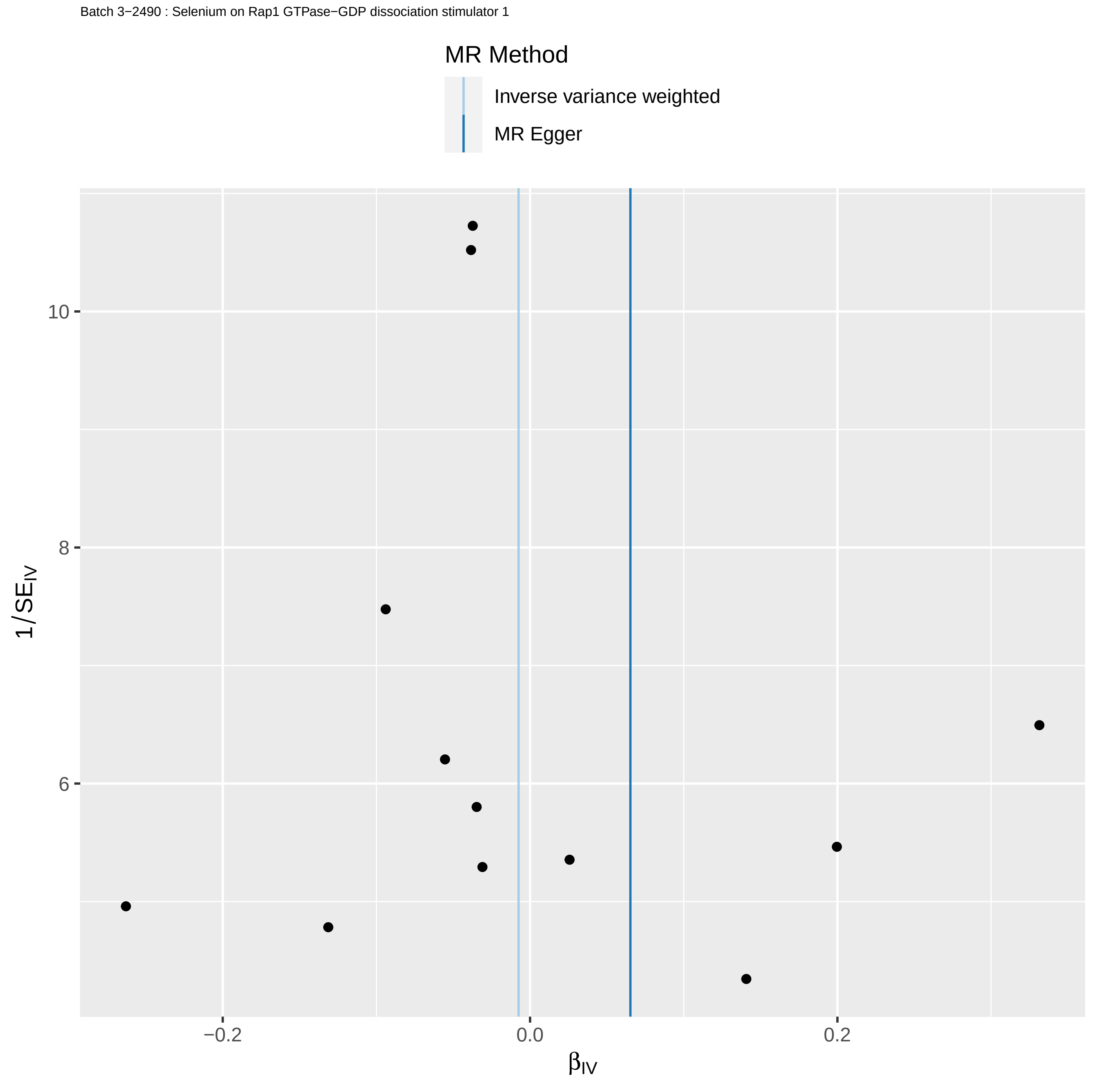

Batch 3−2490 : Selenium on Rap1 GTPase−GDP dissociation stimulator 1
MR Method
Inverse variance weighted
MR Egger
10
8
6
1/SE_IV
−0.2
0.0
0.2
β_IV

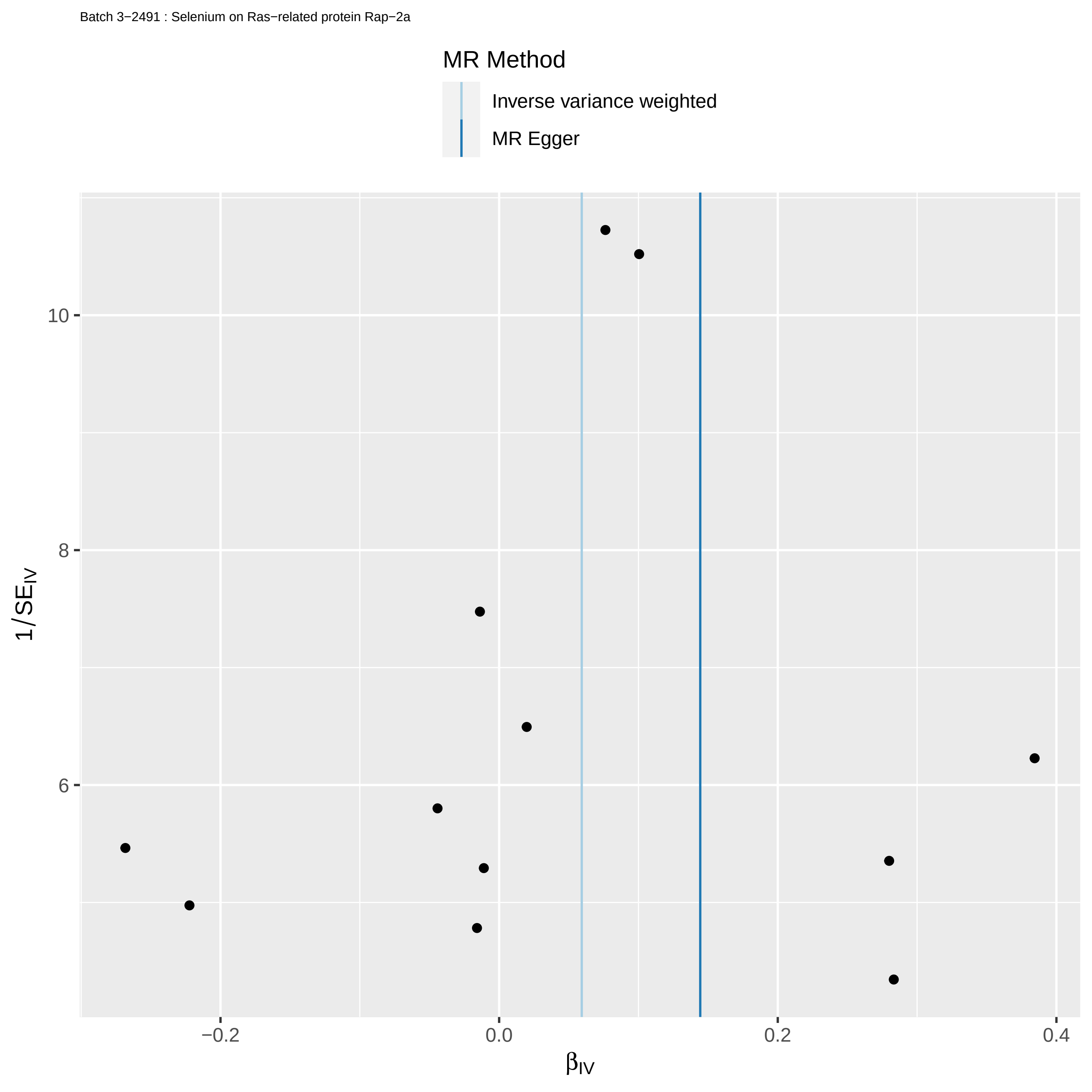

Batch 3−2491 : Selenium on Ras−related protein Rap−2a
MR Method
Inverse variance weighted
MR Egger
10
8
6
1/SE_IV
−0.2
0.0
0.2
0.4
β_IV

Batch 3−2492 : Selenium on Rap guanine nucleotide exchange factor 5

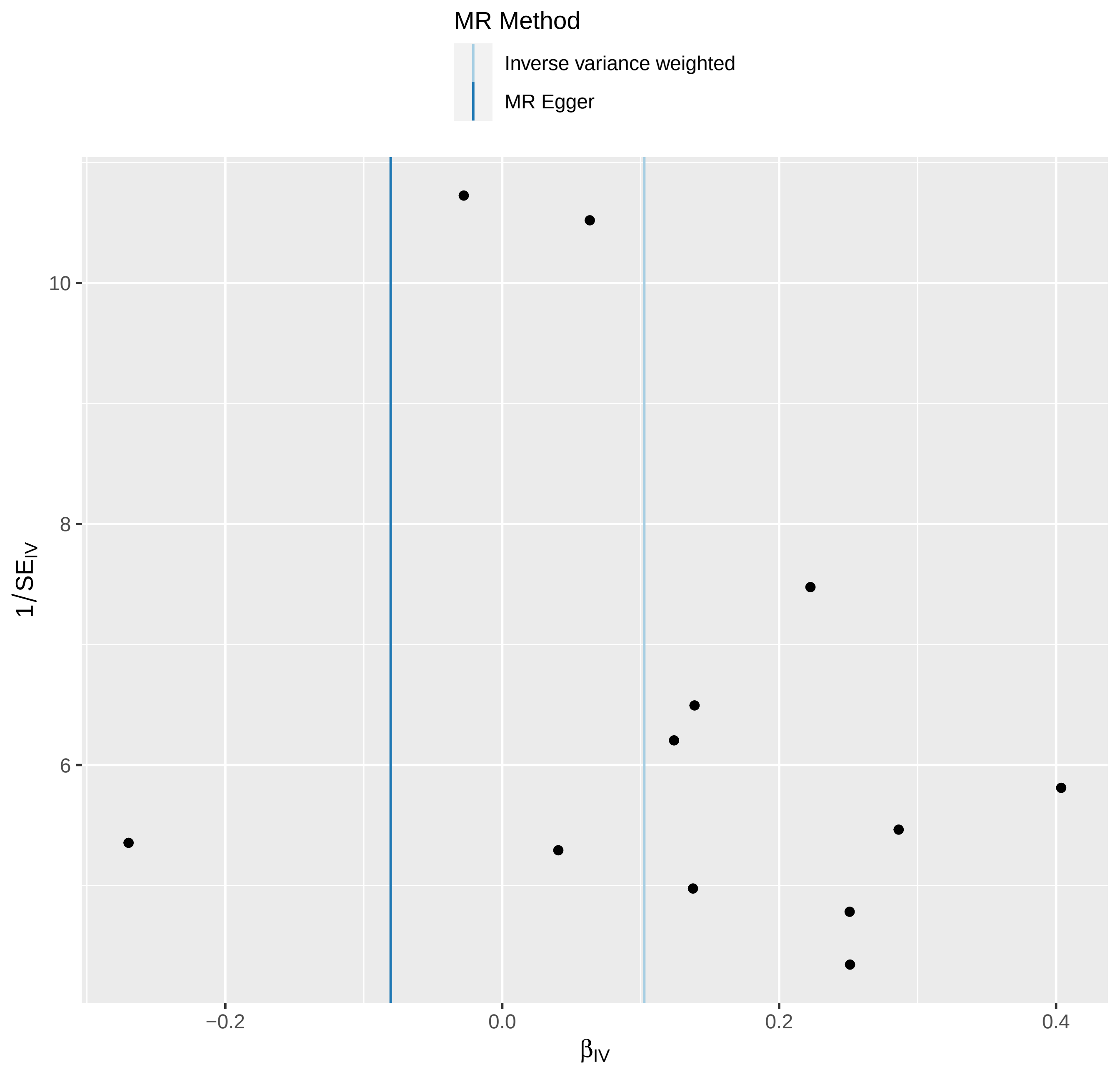

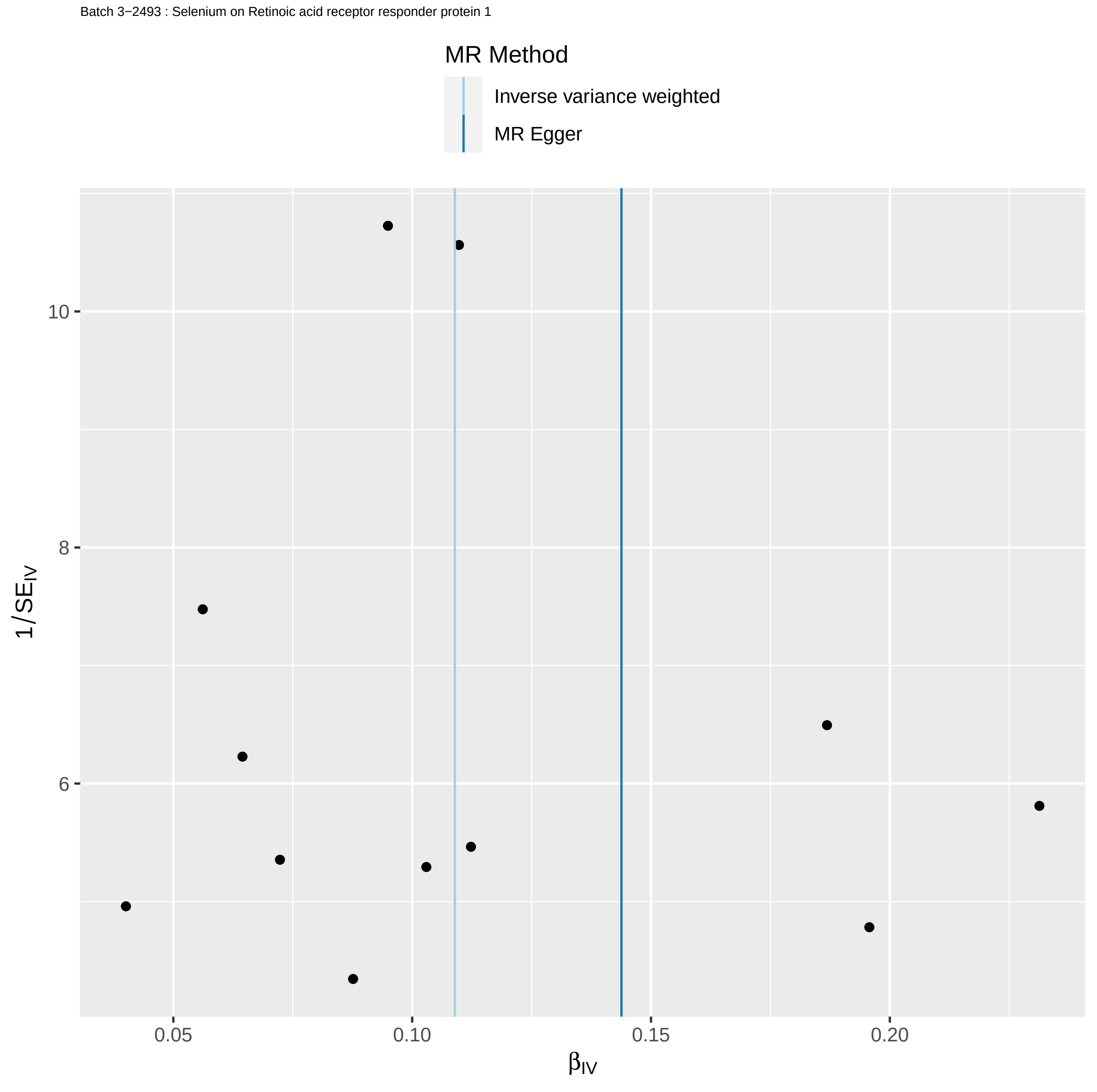

Batch 3−2493 : Selenium on Retinoic acid receptor responder protein 1
MR Method
Inverse variance weighted
MR Egger
10
8
6
1/SE$_{IV}$
0.05
0.10
0.15
0.20
β$_{IV}$

Batch 3−2494 : Selenium on Retinoic acid receptor responder protein 3

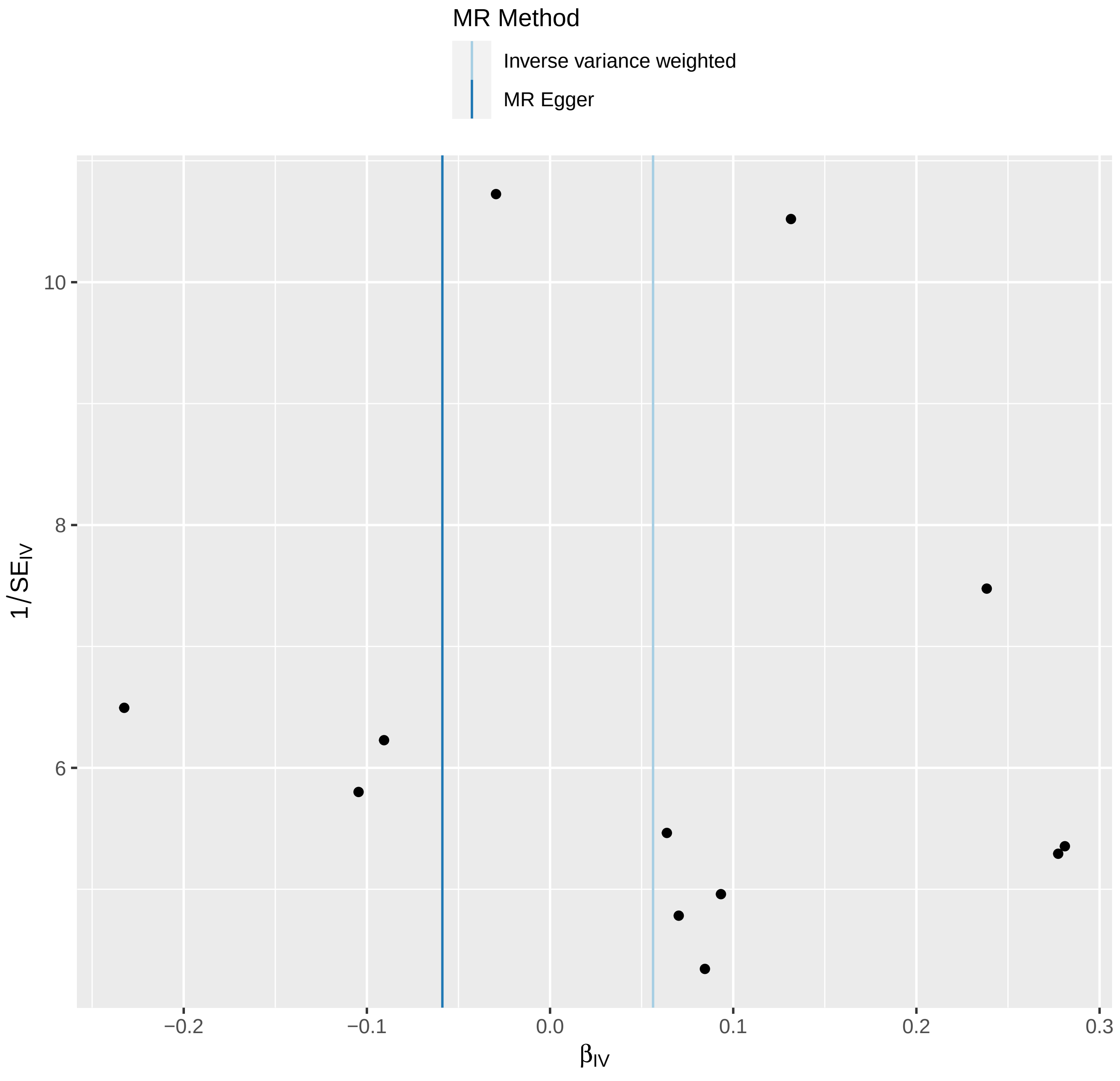

Batch 3−2495 : Selenium on Ras GTPase−activating protein 1

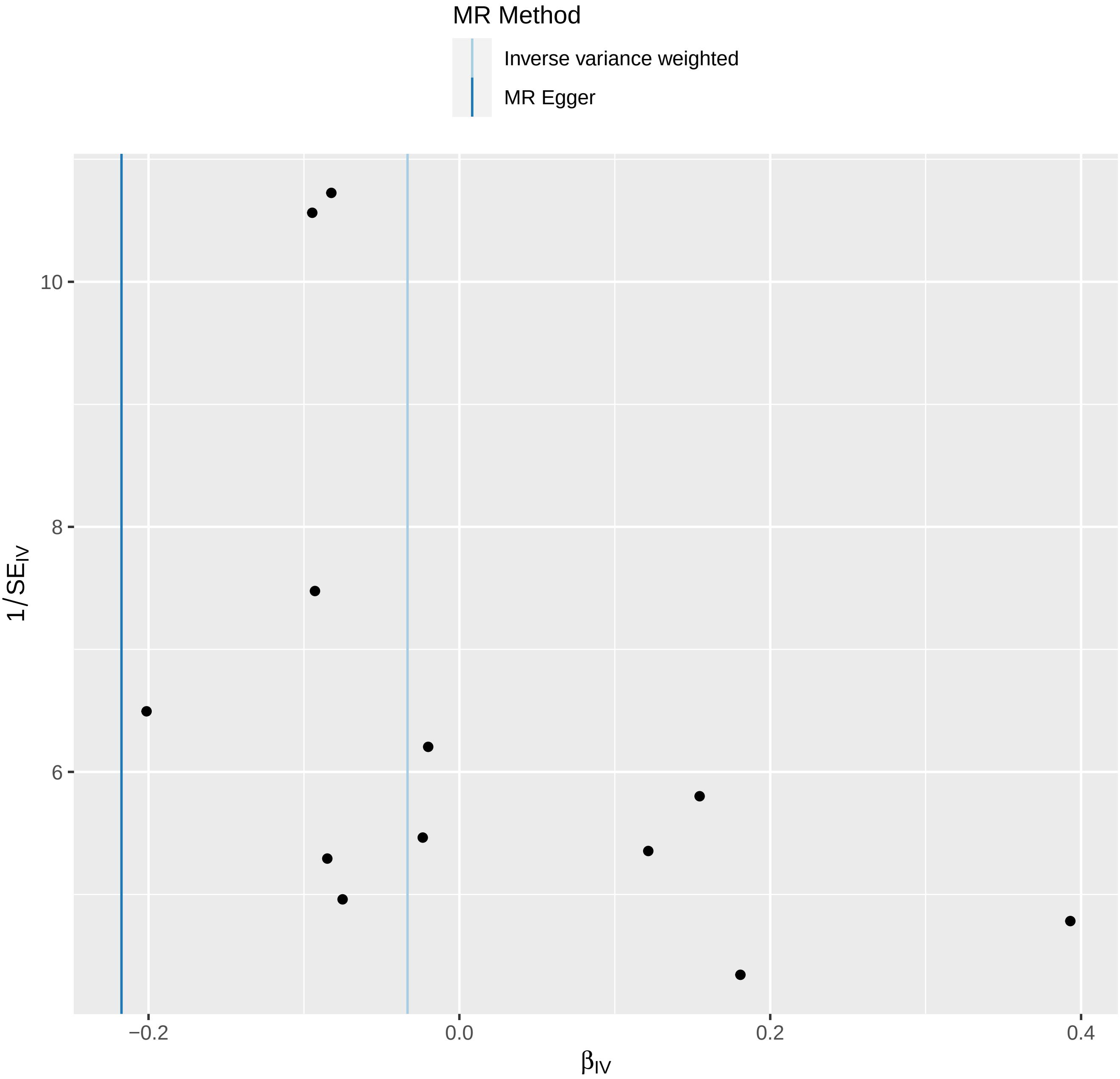

Batch 3−2496 : Selenium on Retinoblastoma−associated protein

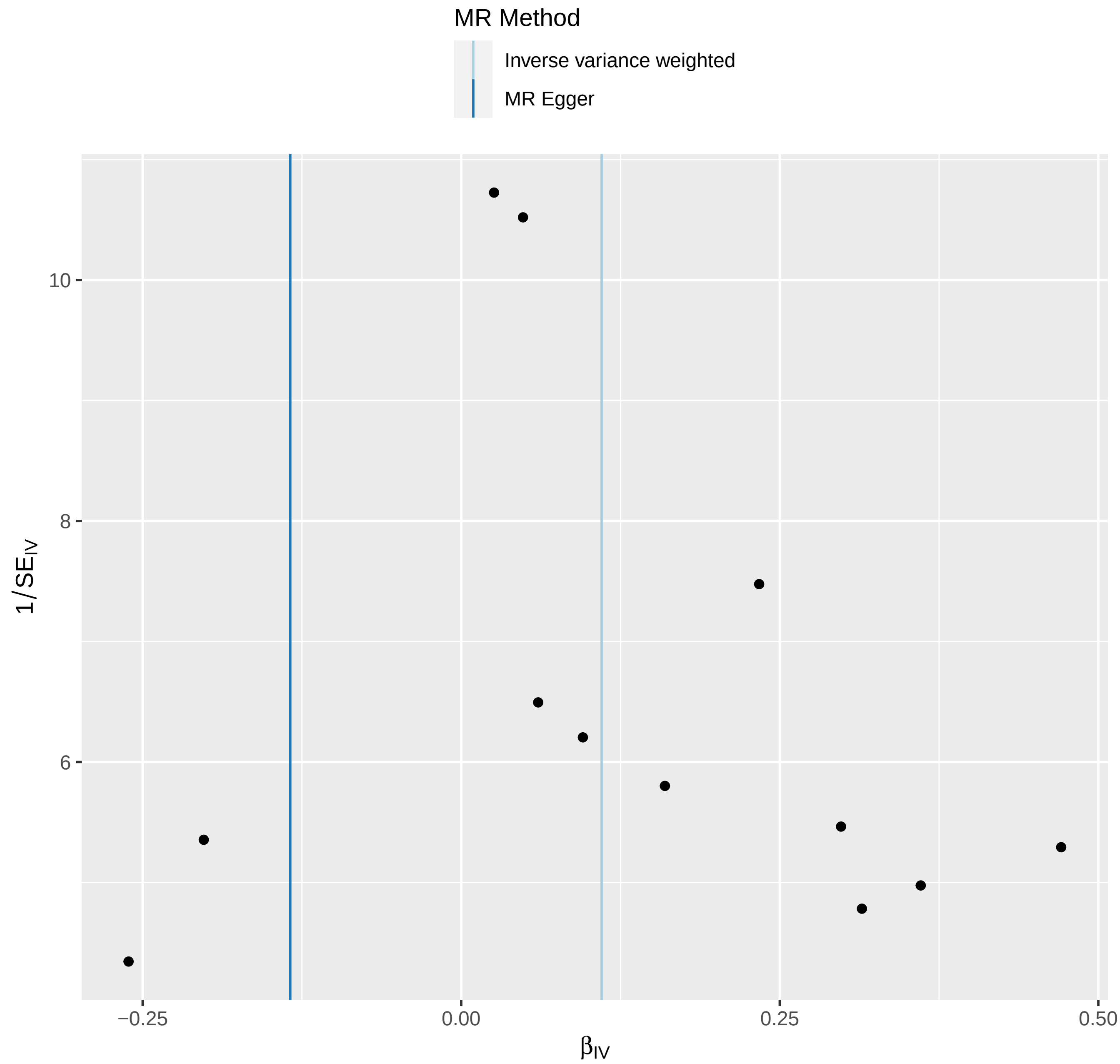

Batch 3−2497 : Selenium on Retinoblastoma−binding protein 5

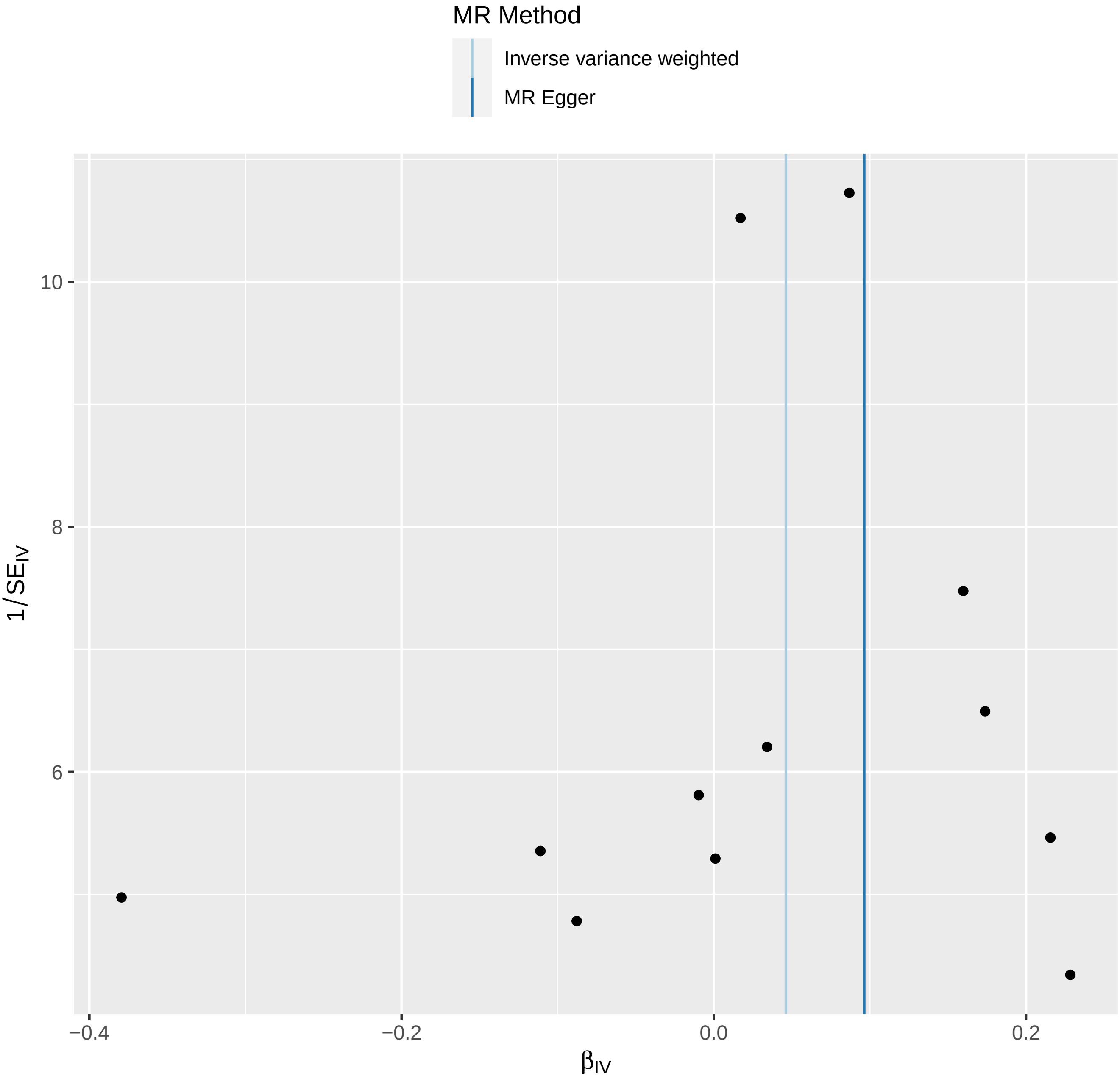

Batch 3−2498 : Selenium on E3 ubiquitin−protein ligase RBBP6

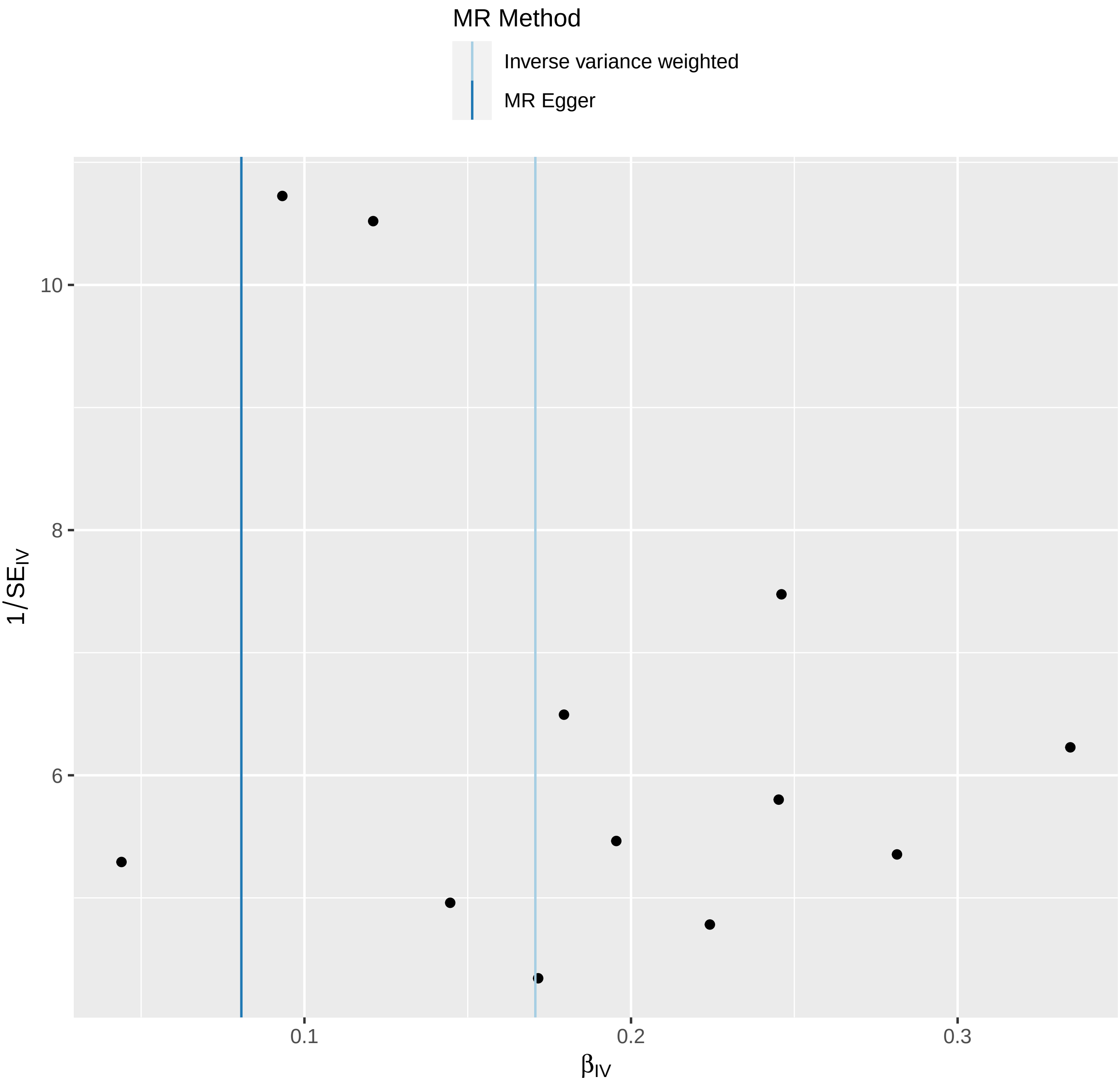

Batch 3−2499 : Selenium on RNA binding protein fox−1 homolog 2

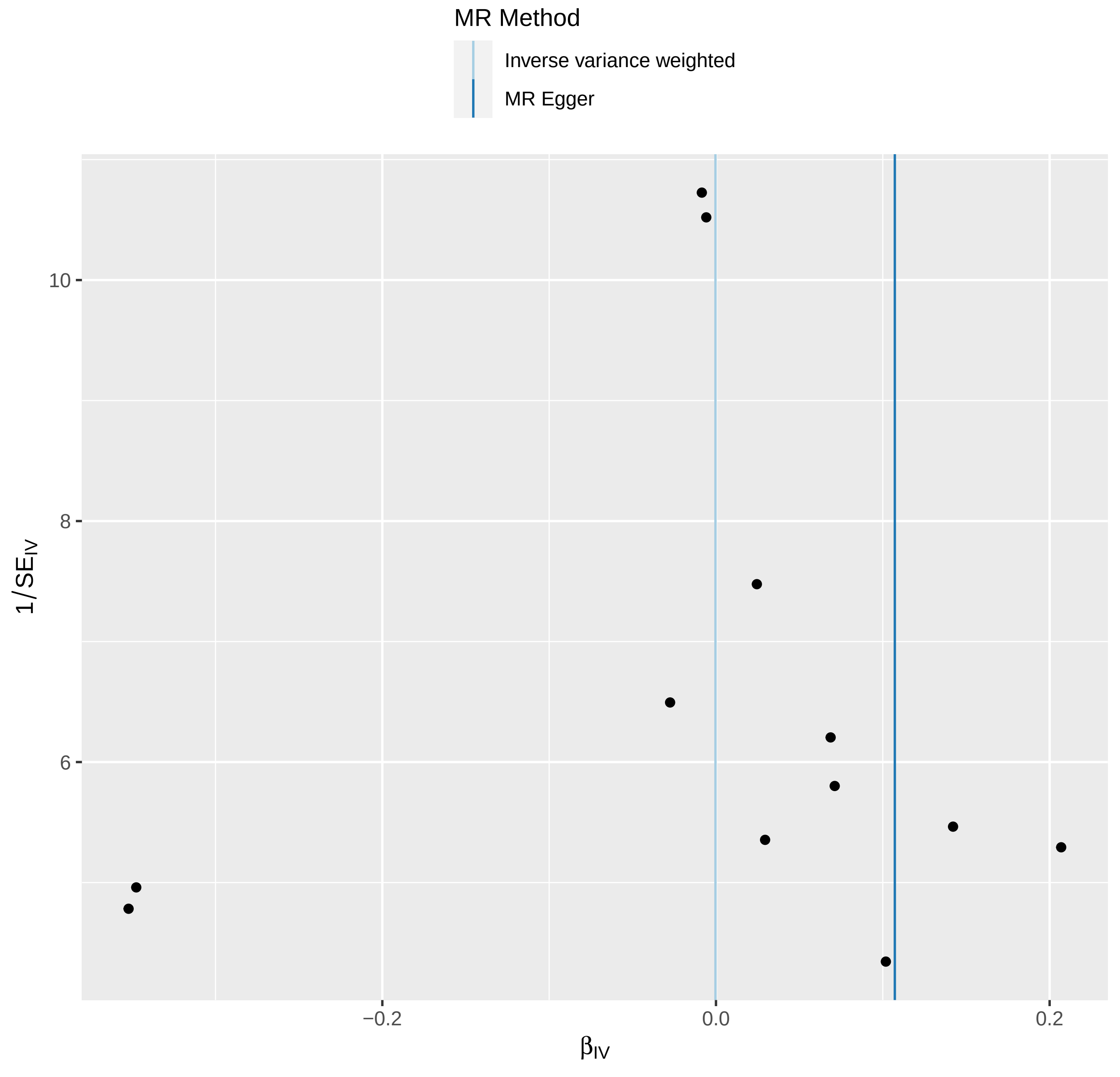

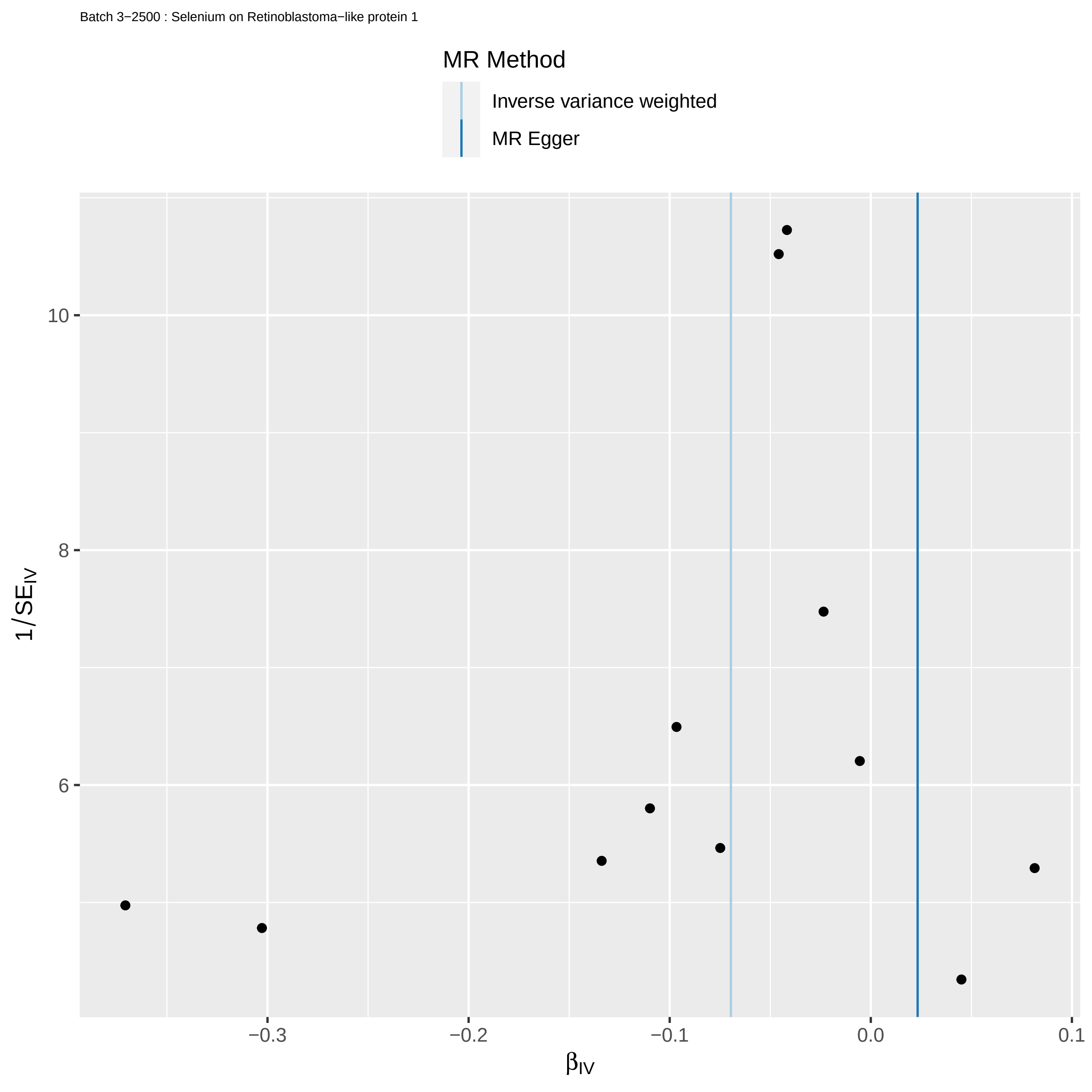
Batch 3−2500 : Selenium on Retinoblastoma−like protein 1
MR Method
Inverse variance weighted
MR Egger
10
8
6
1/SE_IV
−0.3
−0.2
−0.1
0.0
0.1
β_IV

Batch 3−2501 : Selenium on Probable RNA−binding protein 19

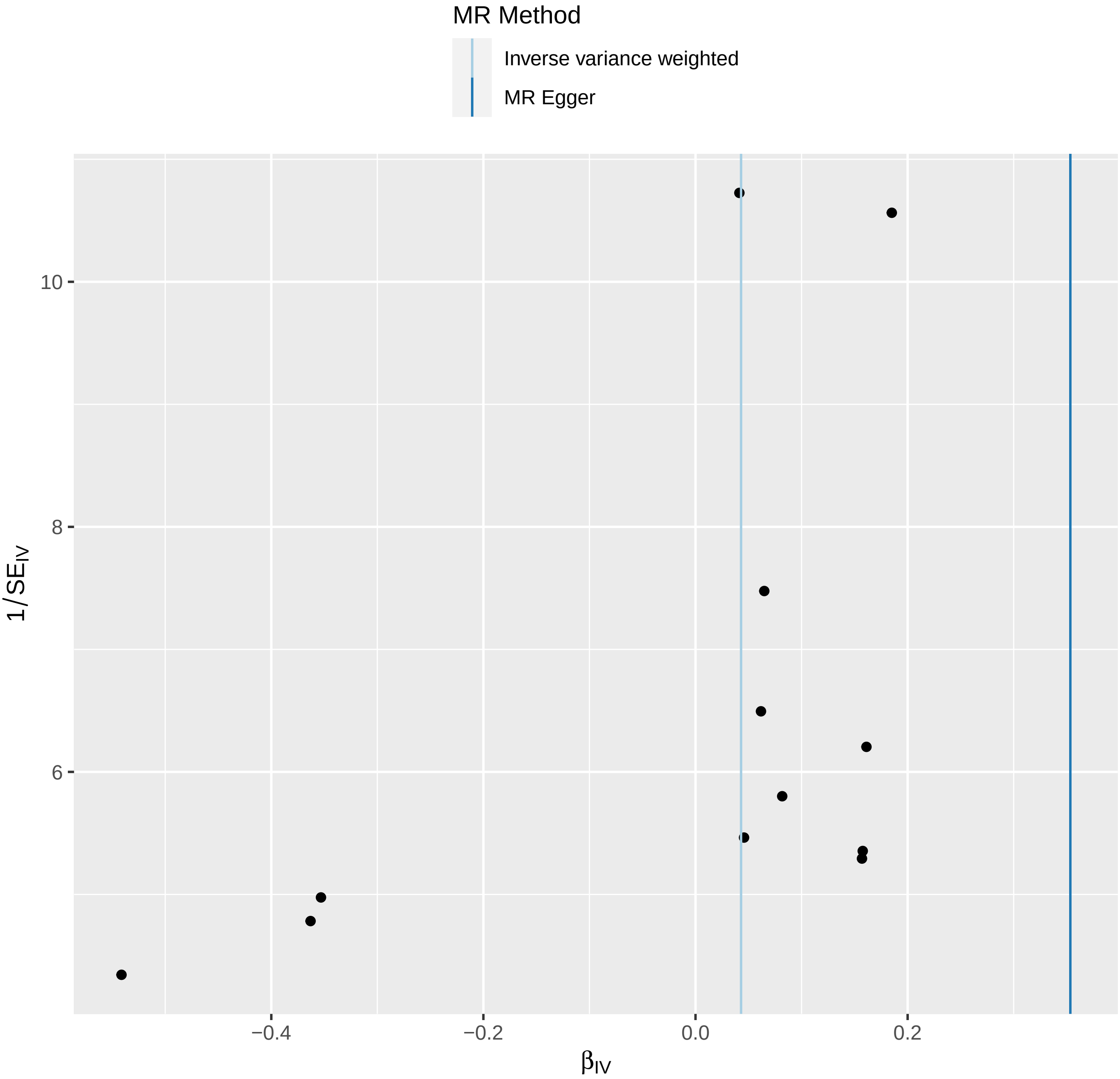

Batch 3−2502 : Selenium on Probable RNA−binding protein 23

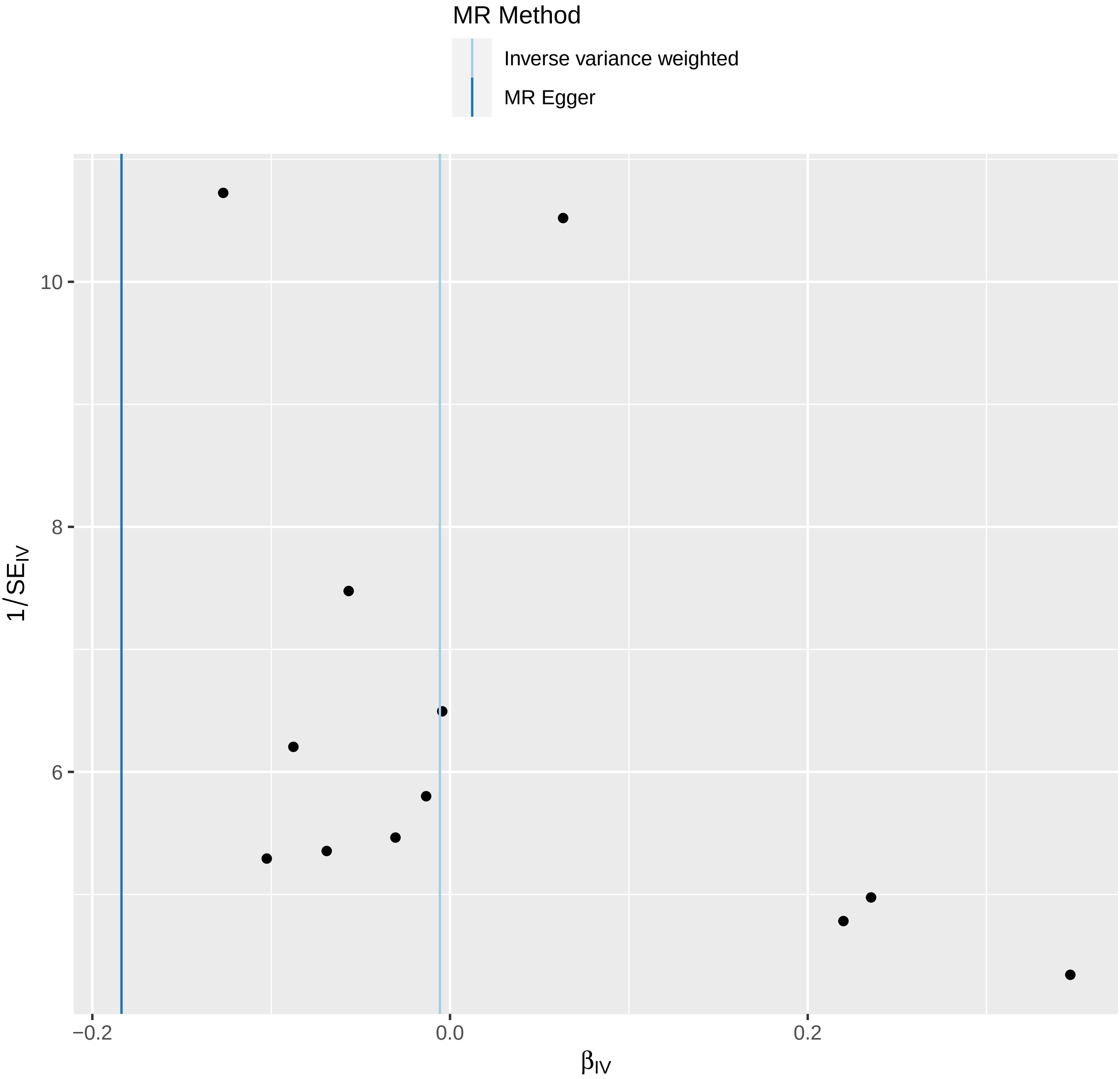

Batch 3−2503 : Selenium on RNA−binding protein 24

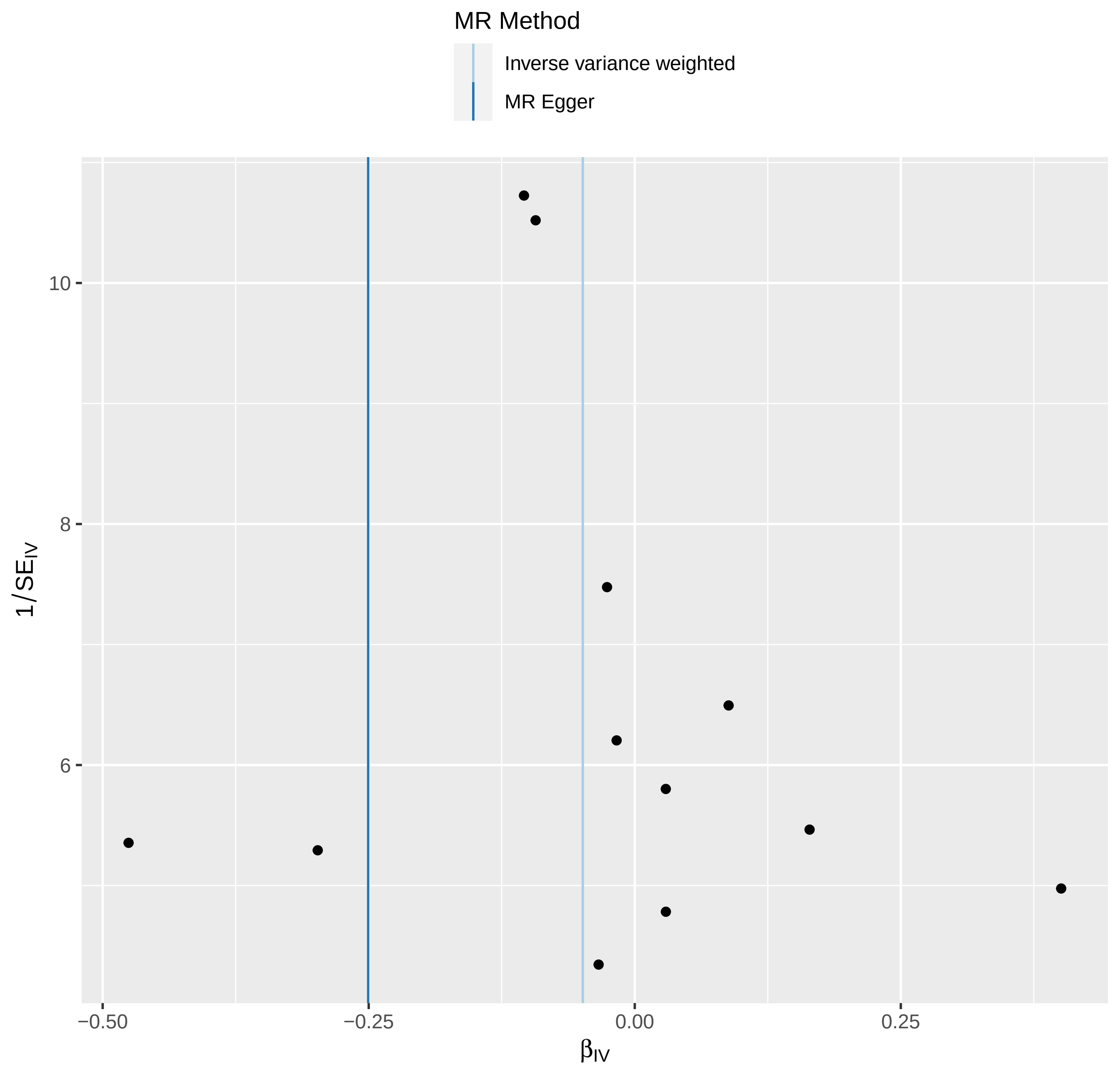

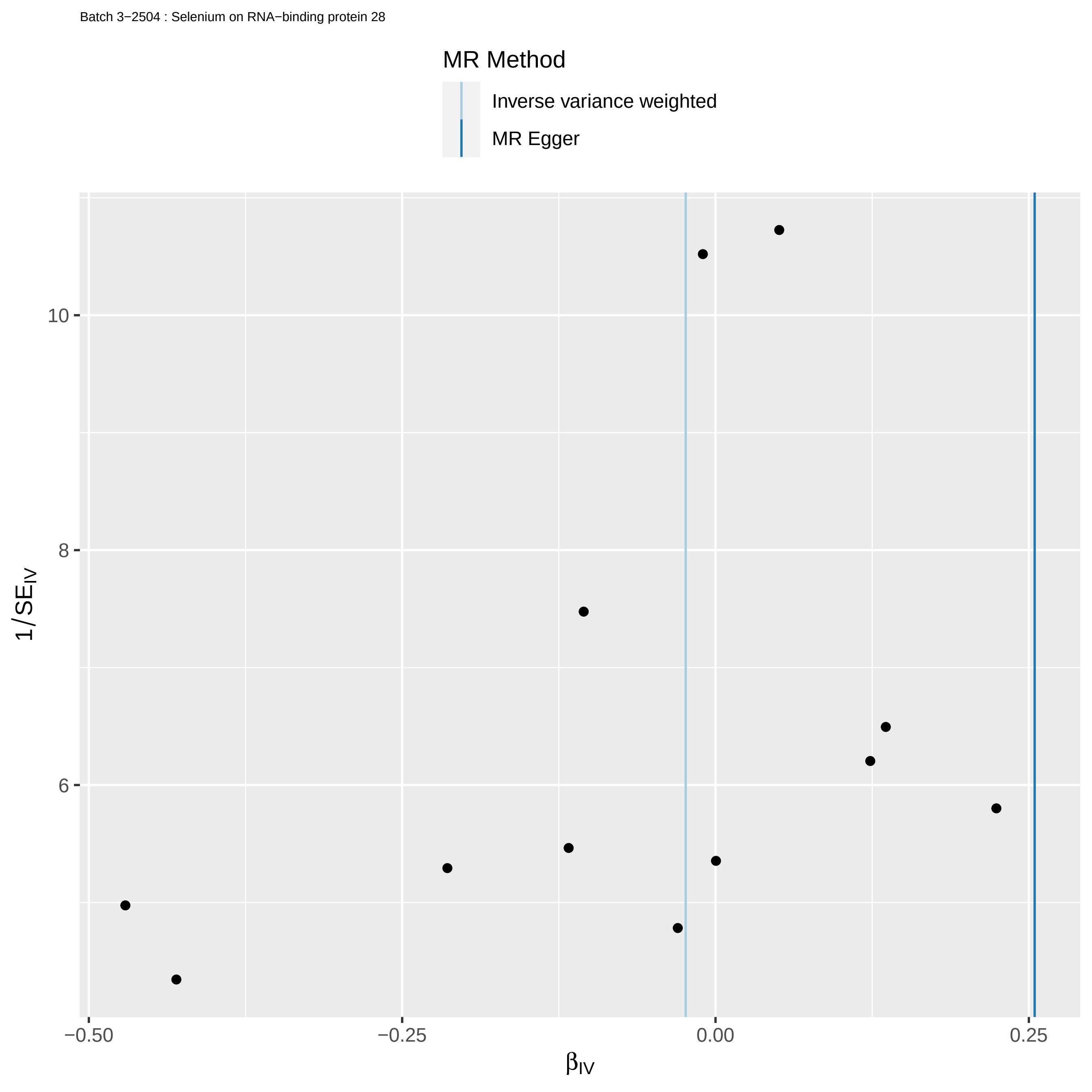
Batch 3−2504 : Selenium on RNA−binding protein 28
MR Method
Inverse variance weighted
MR Egger
10
8
6
1/SE$_{IV}$
−0.50
−0.25
0.00
0.25
β$_{IV}$

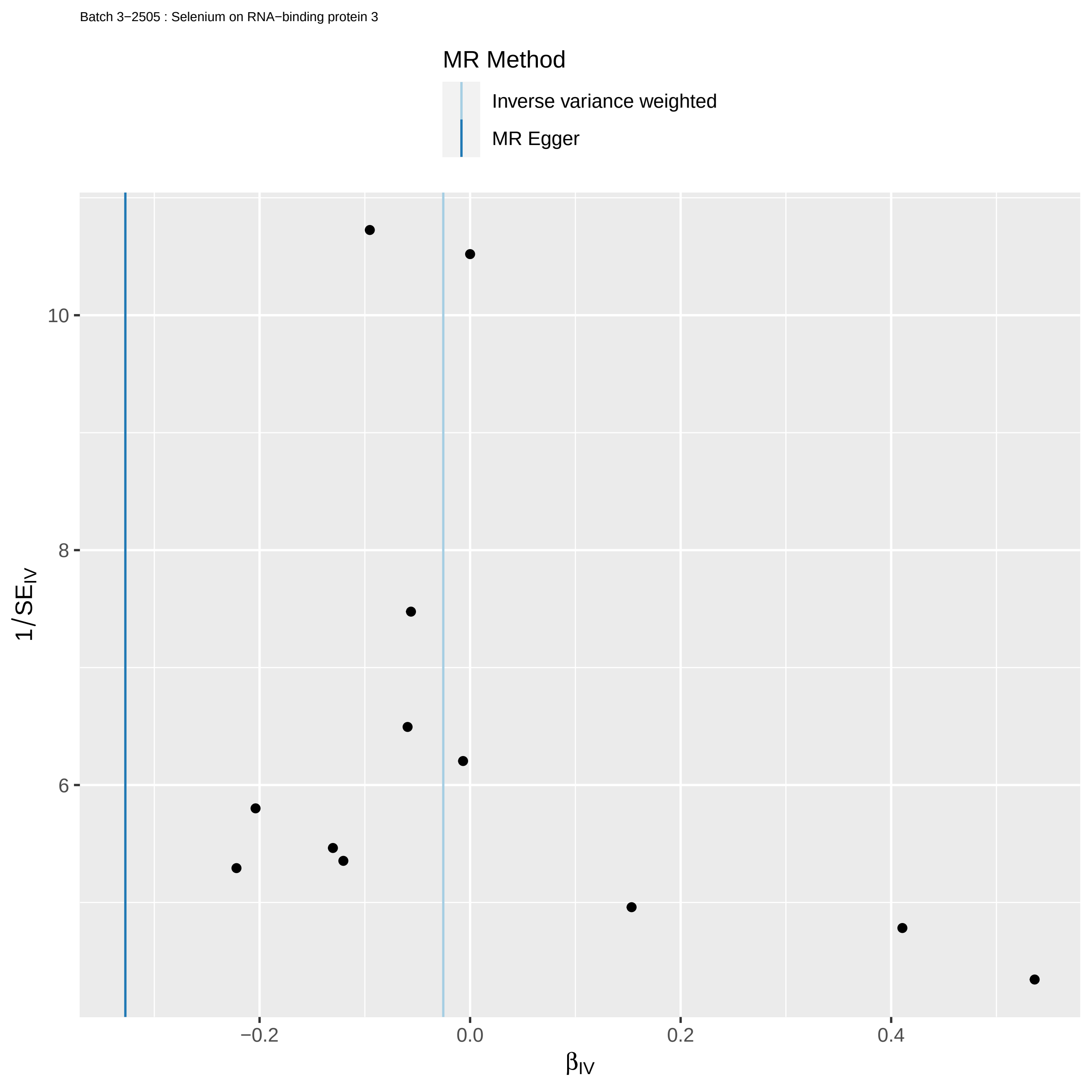

Batch 3−2505 : Selenium on RNA−binding protein 3
MR Method
Inverse variance weighted
MR Egger
10
8
6
1/SE$_{IV}$
−0.2
0.0
0.2
0.4
β$_{IV}$

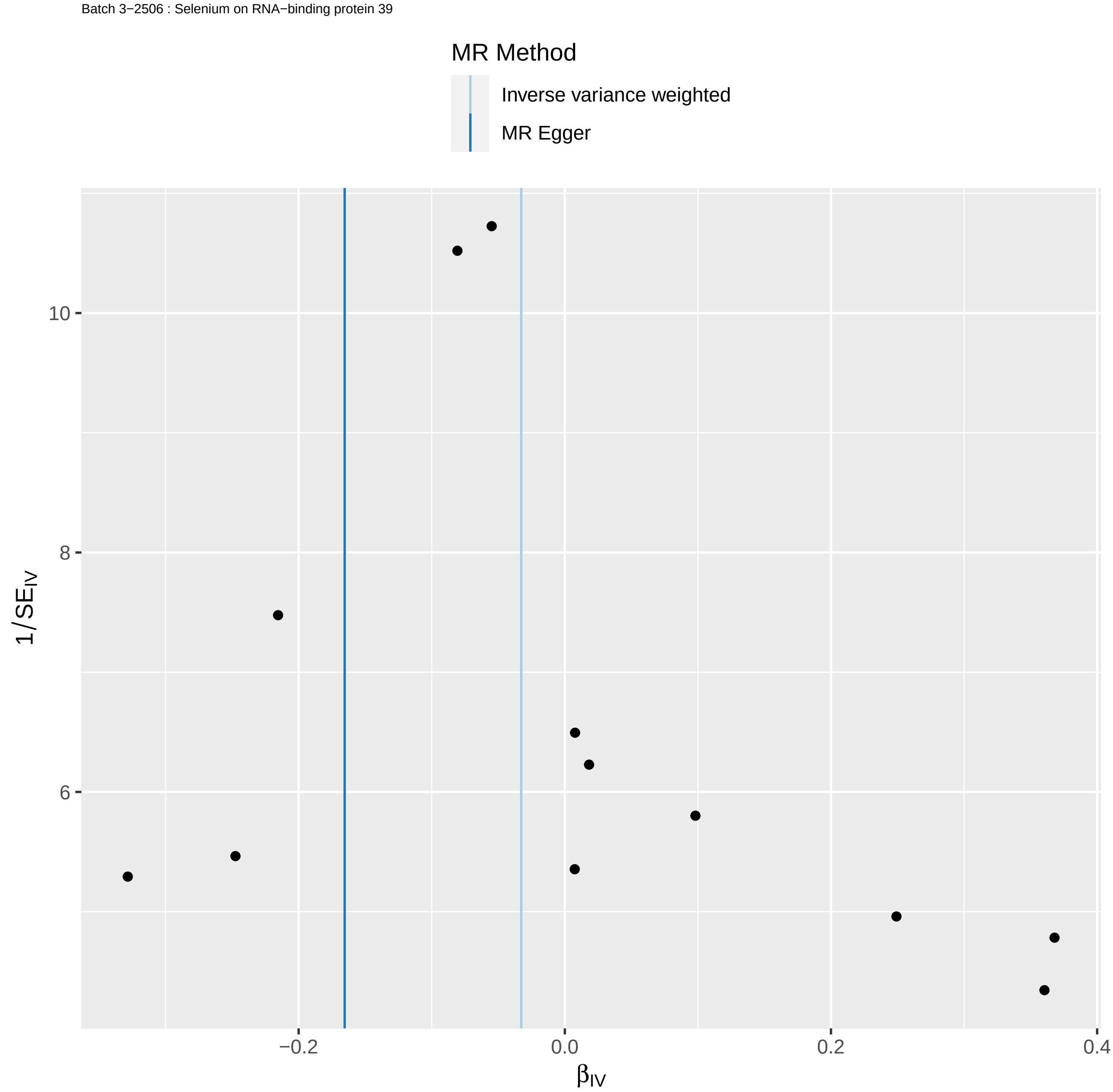
Batch 3−2506 : Selenium on RNA−binding protein 39
MR Method
Inverse variance weighted
MR Egger
10
8
6
1/SE_IV
−0.2
0.0
0.2
0.4
β_IV

Batch 3−2507 : Selenium on Retinol−binding protein 4

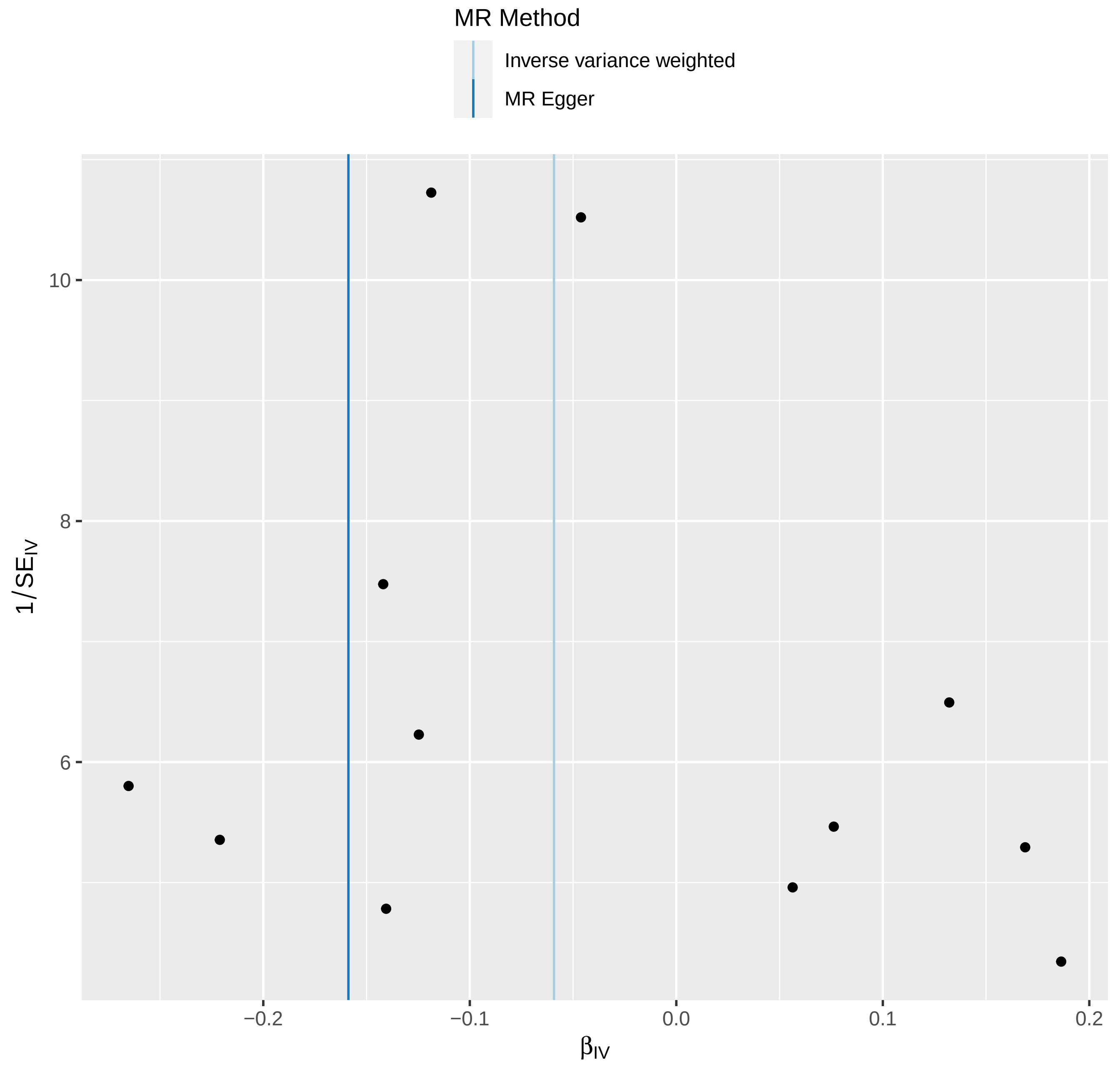

Batch 3−2508 : Selenium on Retinoid−binding protein 7

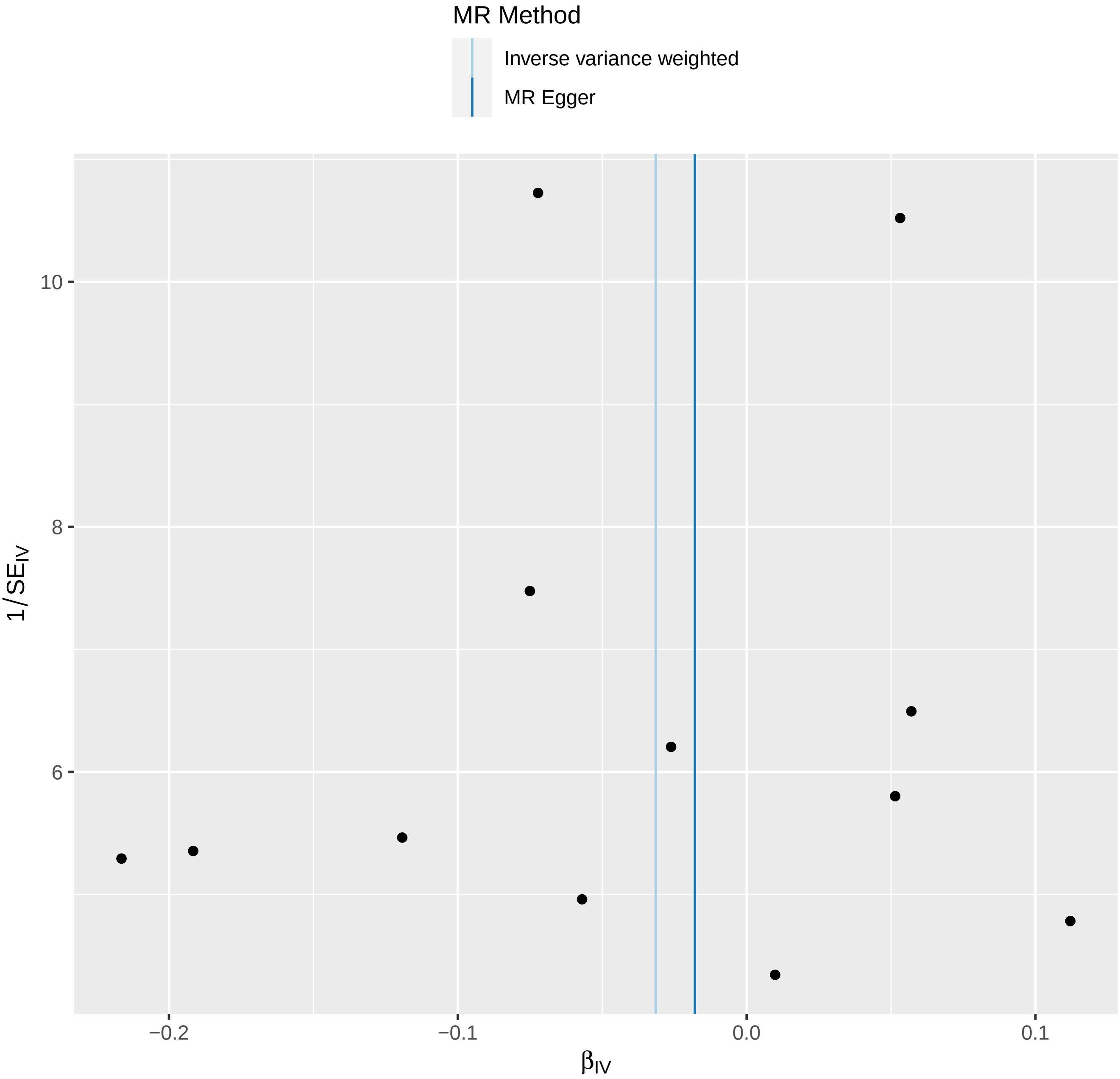

Batch 3−2509 : Selenium on Calcipressin−1

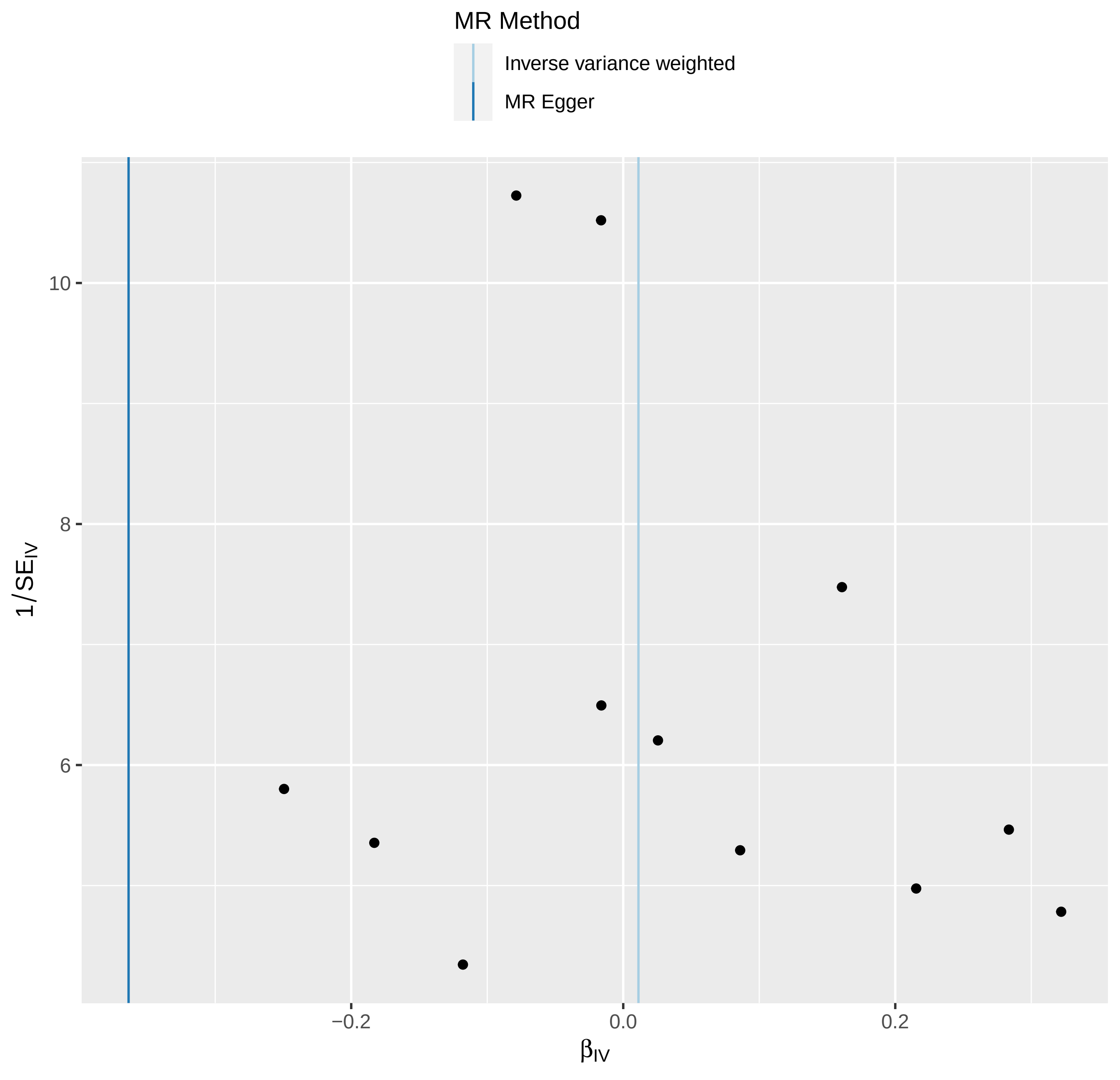

Batch 3−2510 : Selenium on Reticulocalbin−2

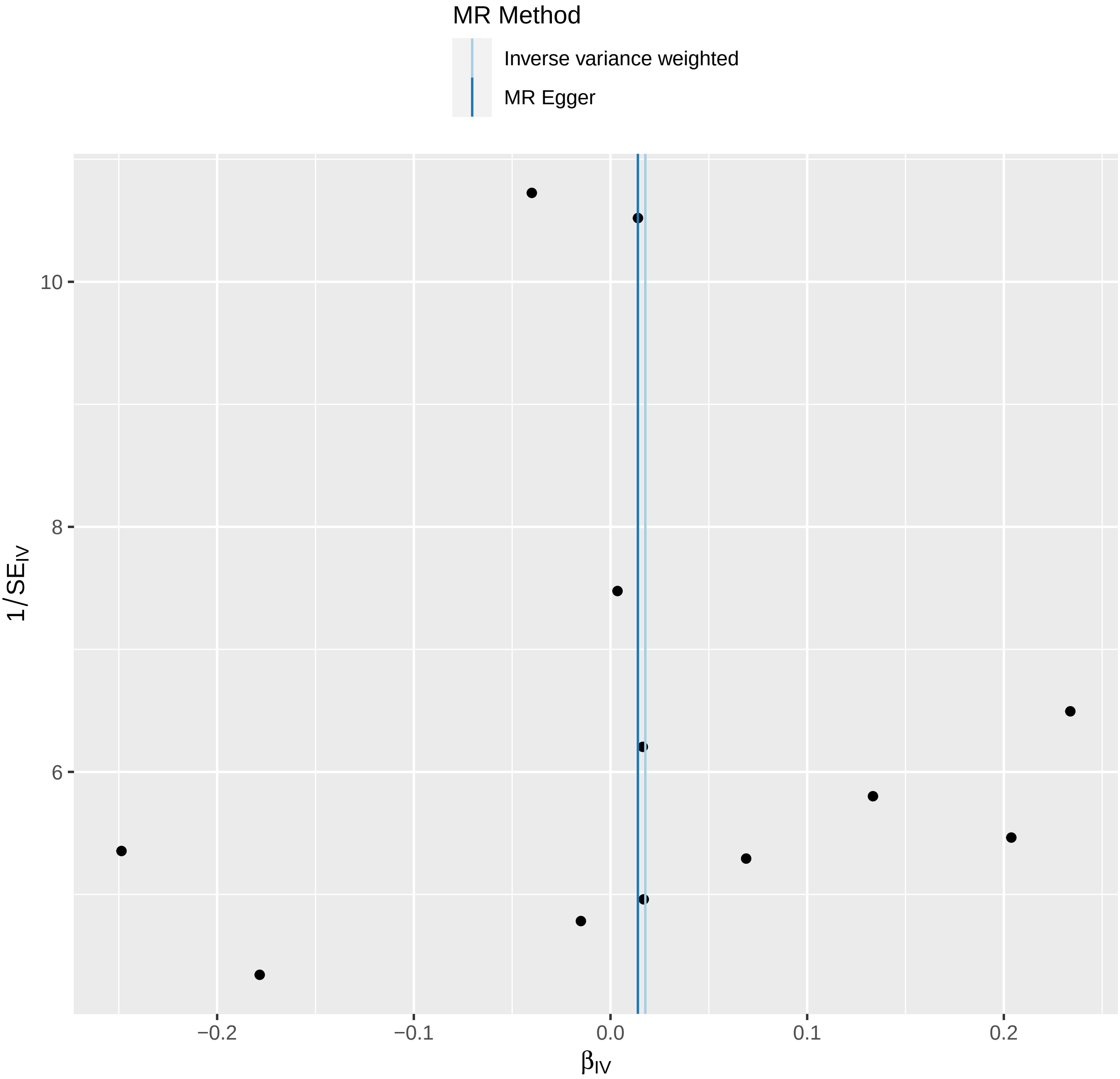

Batch 3−2511 : Selenium on Recoverin

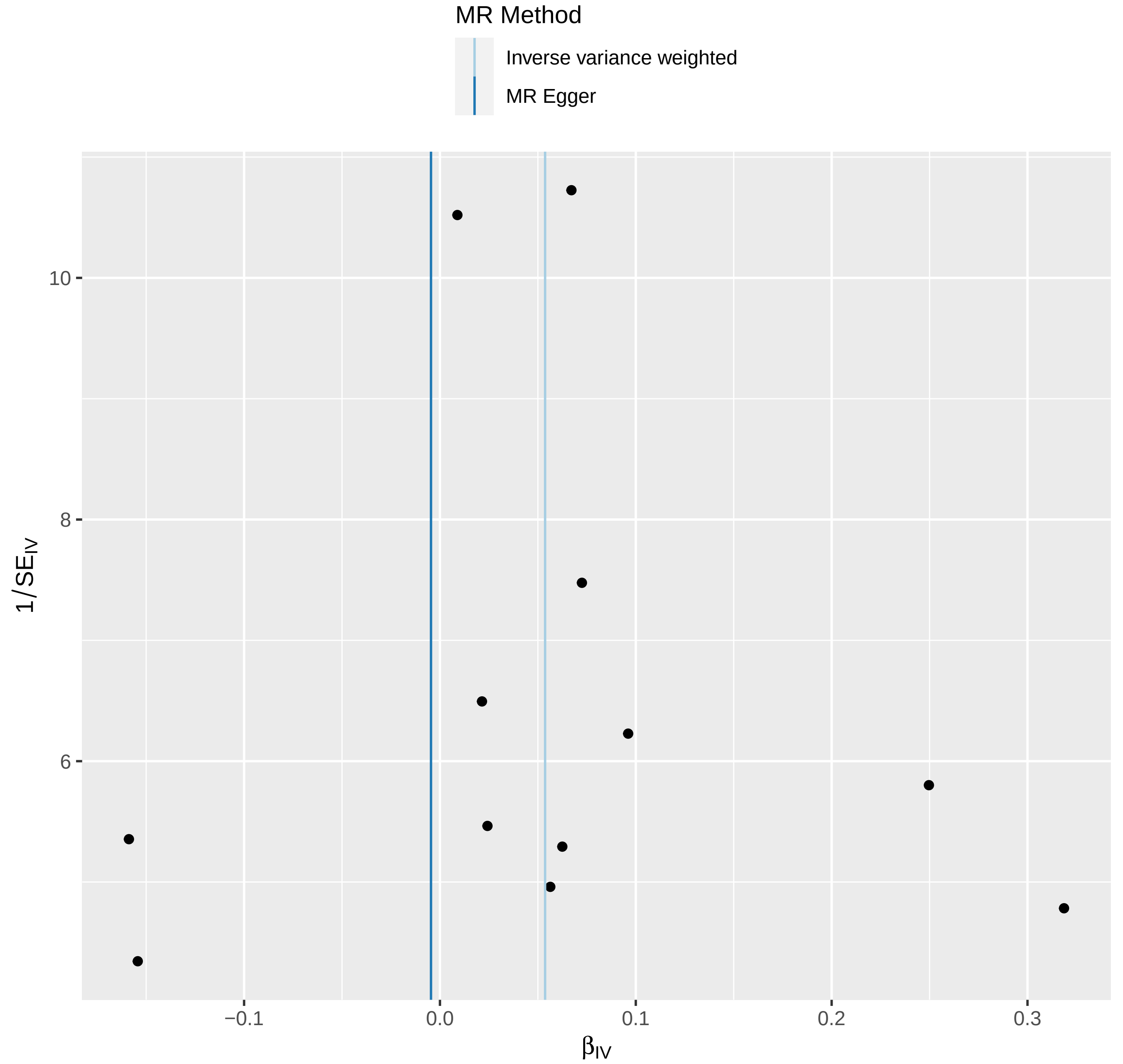

Batch 3−2512 : Selenium on Retinol dehydrogenase 16

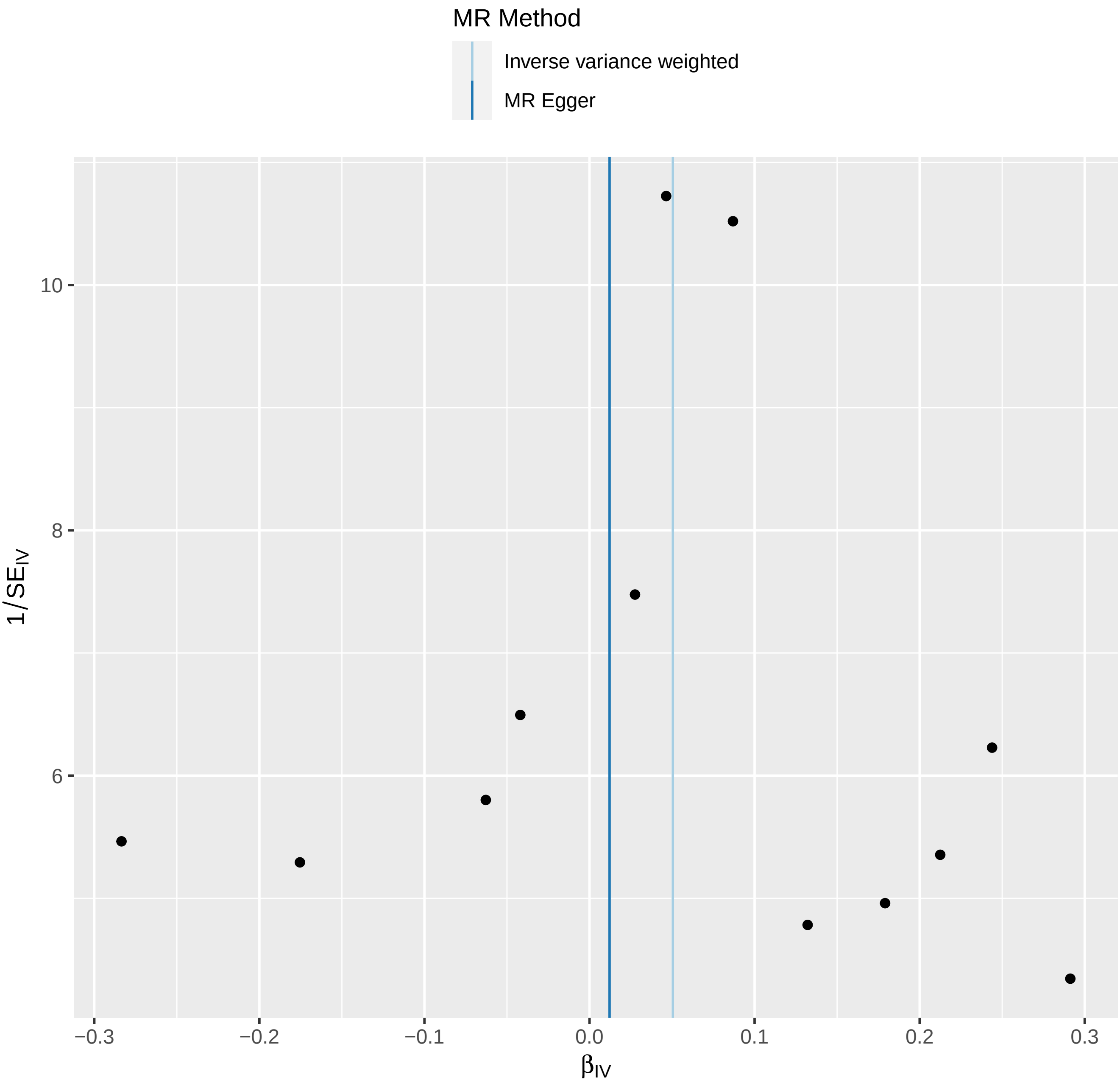

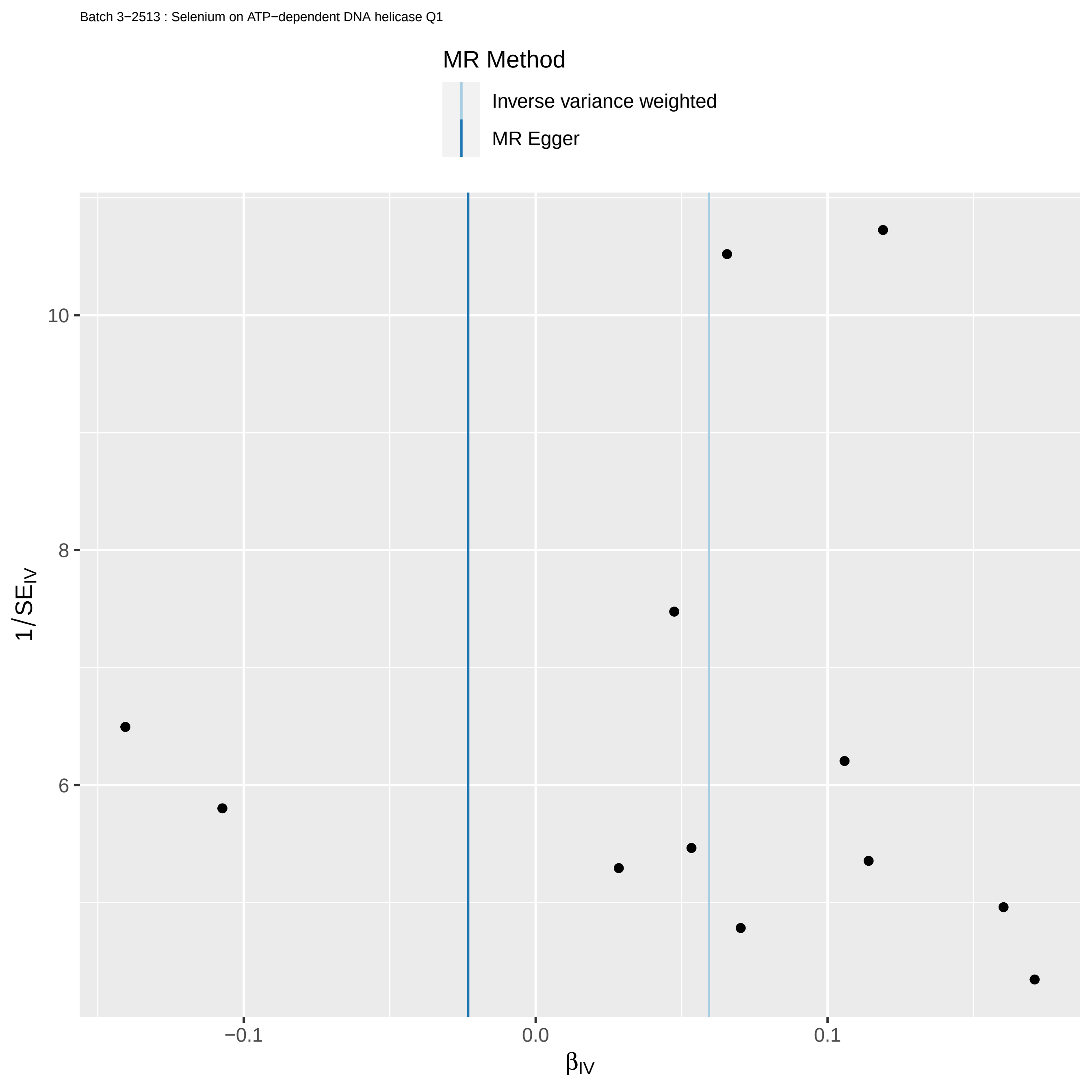
Batch 3−2513 : Selenium on ATP−dependent DNA helicase Q1
MR Method
Inverse variance weighted
MR Egger
10
8
6
$1/SE_{IV}$
−0.1
0.0
0.1
β_{IV}

Batch 3−2514 : Selenium on Regenerating islet−derived protein 3−alpha

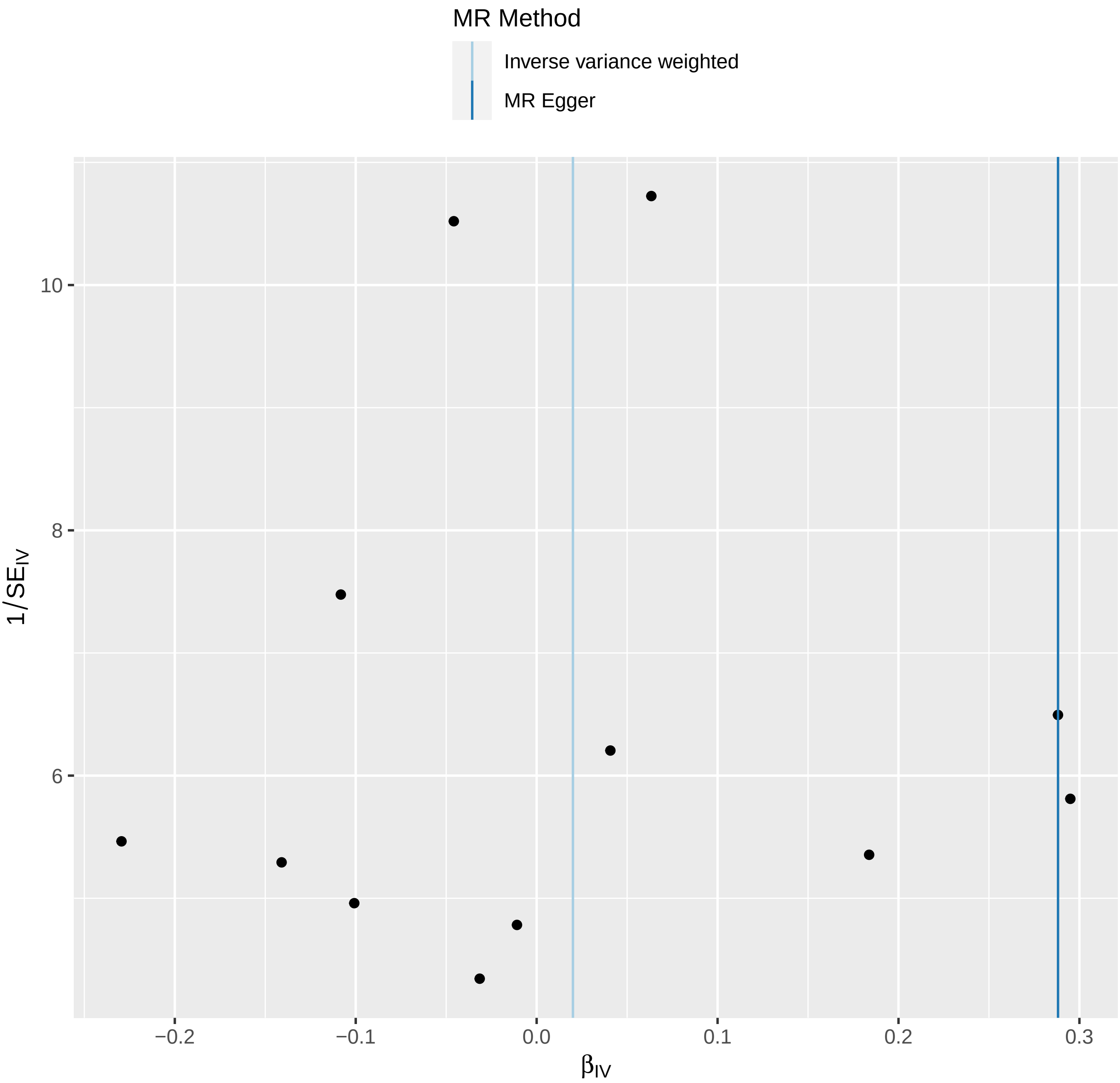

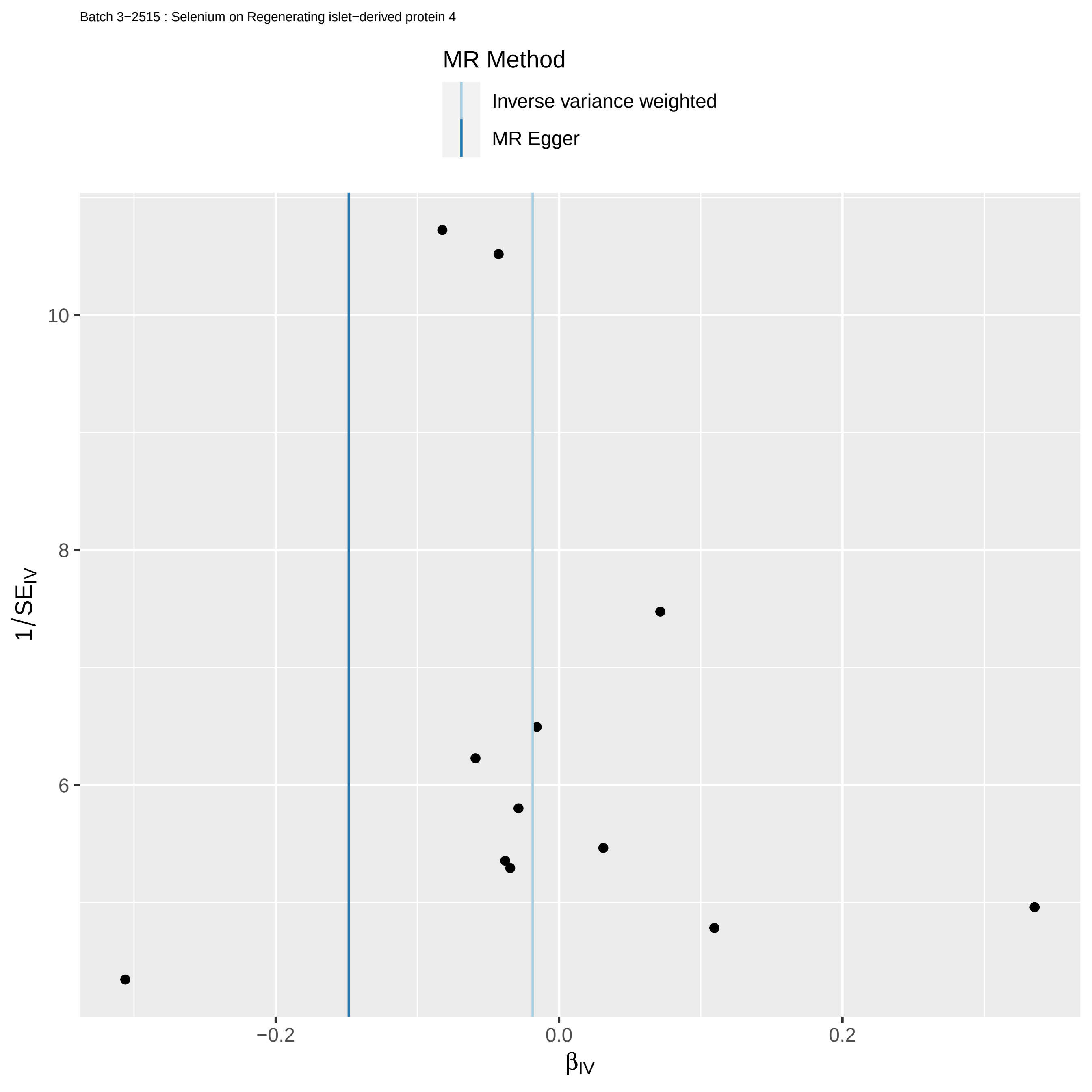

Batch 3−2515 : Selenium on Regenerating islet−derived protein 4
MR Method
Inverse variance weighted
MR Egger
10
8
6
1/SE$_{IV}$
−0.2
0.0
0.2
β$_{IV}$

Batch 3−2516 : Selenium on Transcription factor RelB

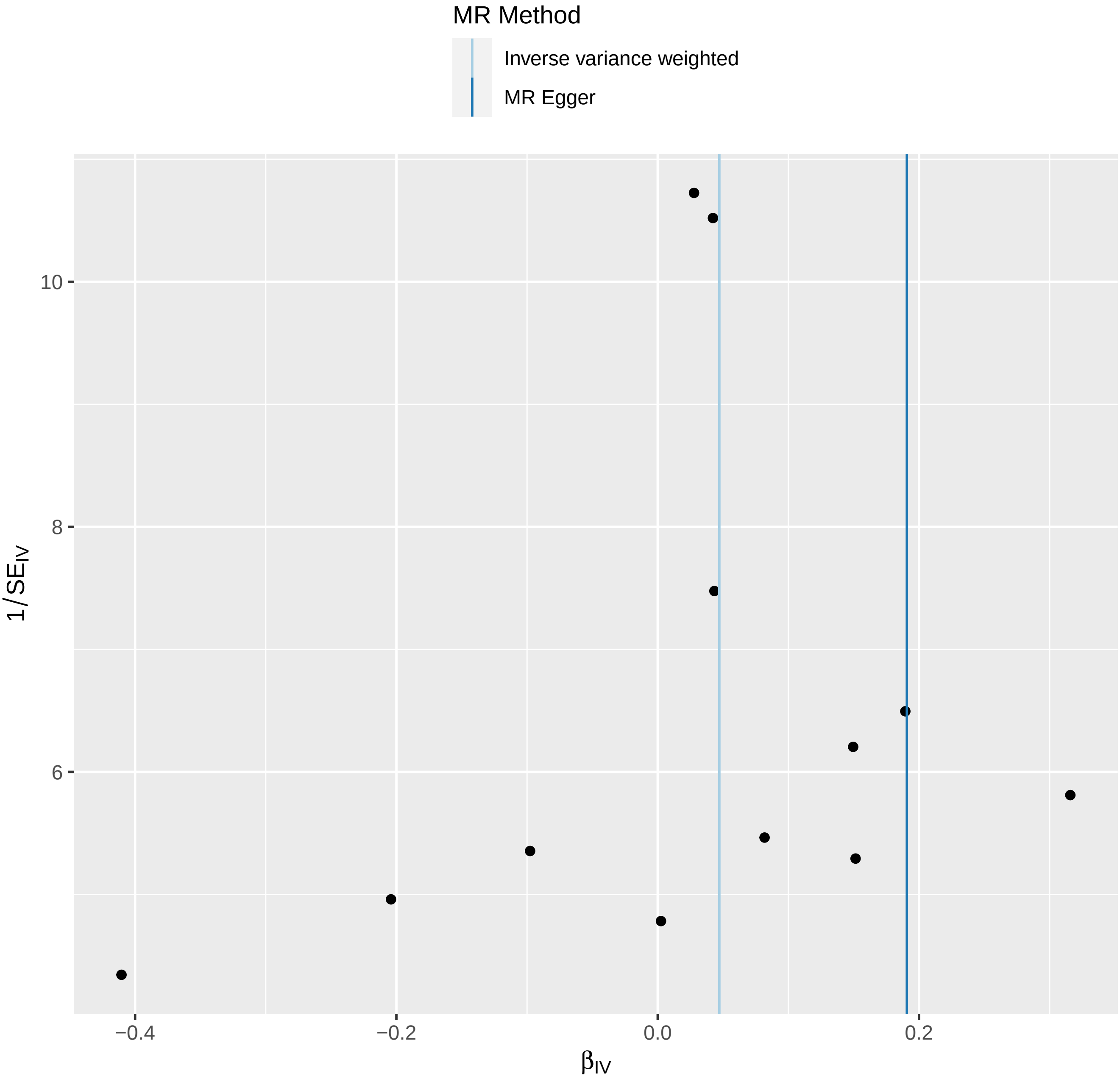

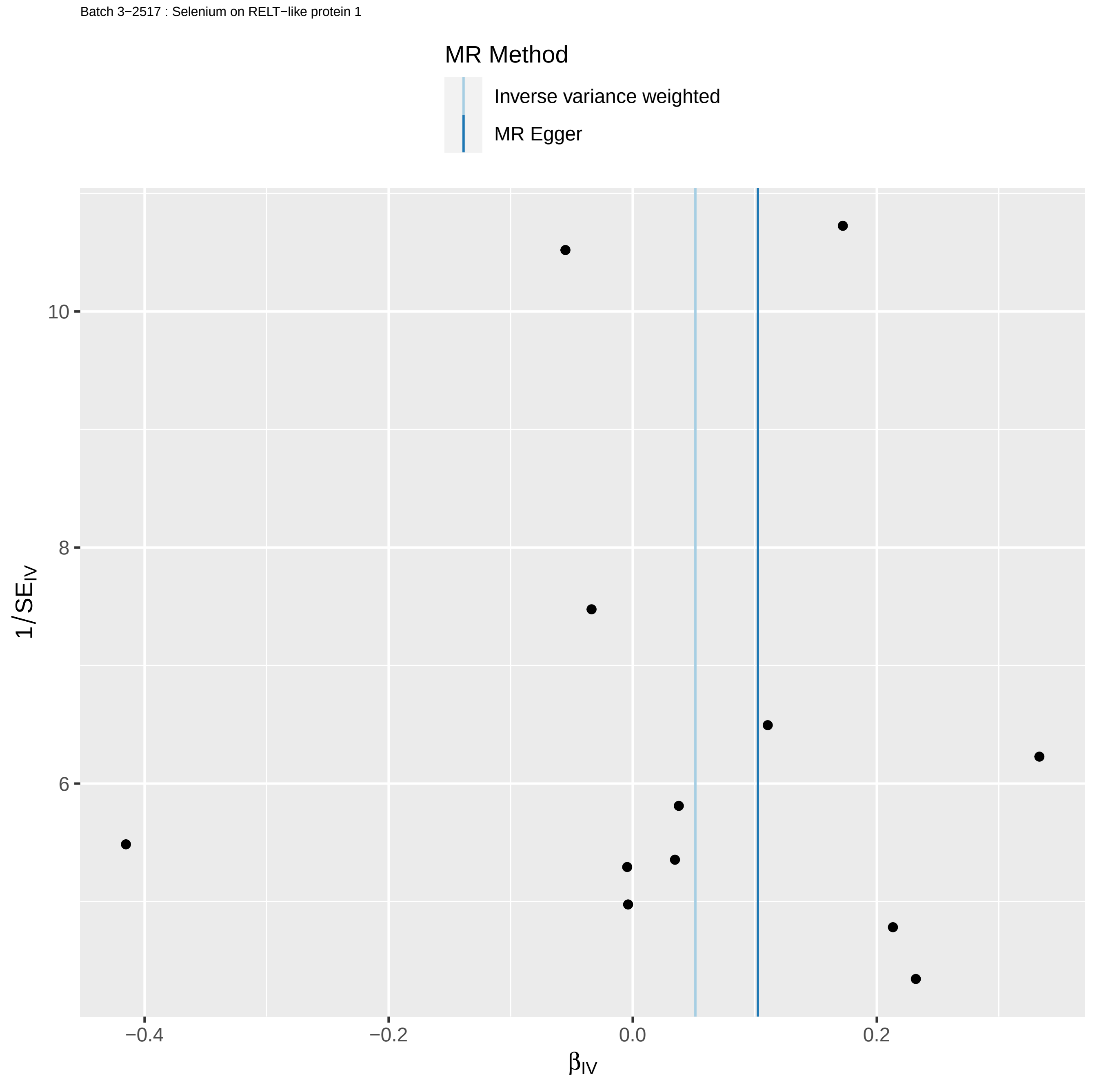

Batch 3−2517 : Selenium on RELT−like protein 1
MR Method
Inverse variance weighted
MR Egger
10
8
6
1/SE_IV
−0.4
−0.2
0.0
0.2
β_IV

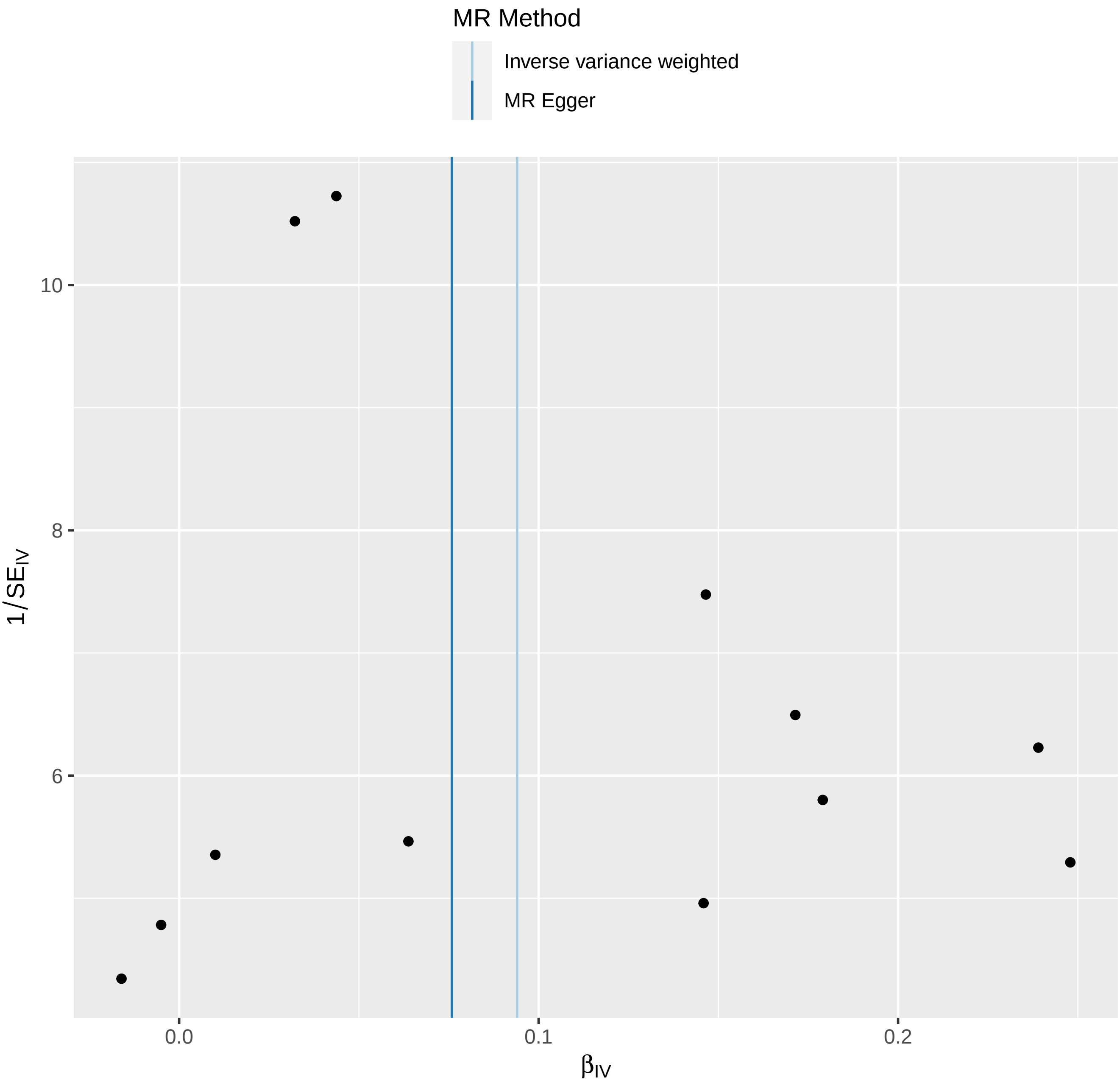
MR Method
Inverse variance weighted
MR Egger
10
8
6
1/SE_IV
0.0
0.1
0.2
β_IV

Batch 3−2519 : Selenium on Tumor necrosis factor receptor superfamily member 19L

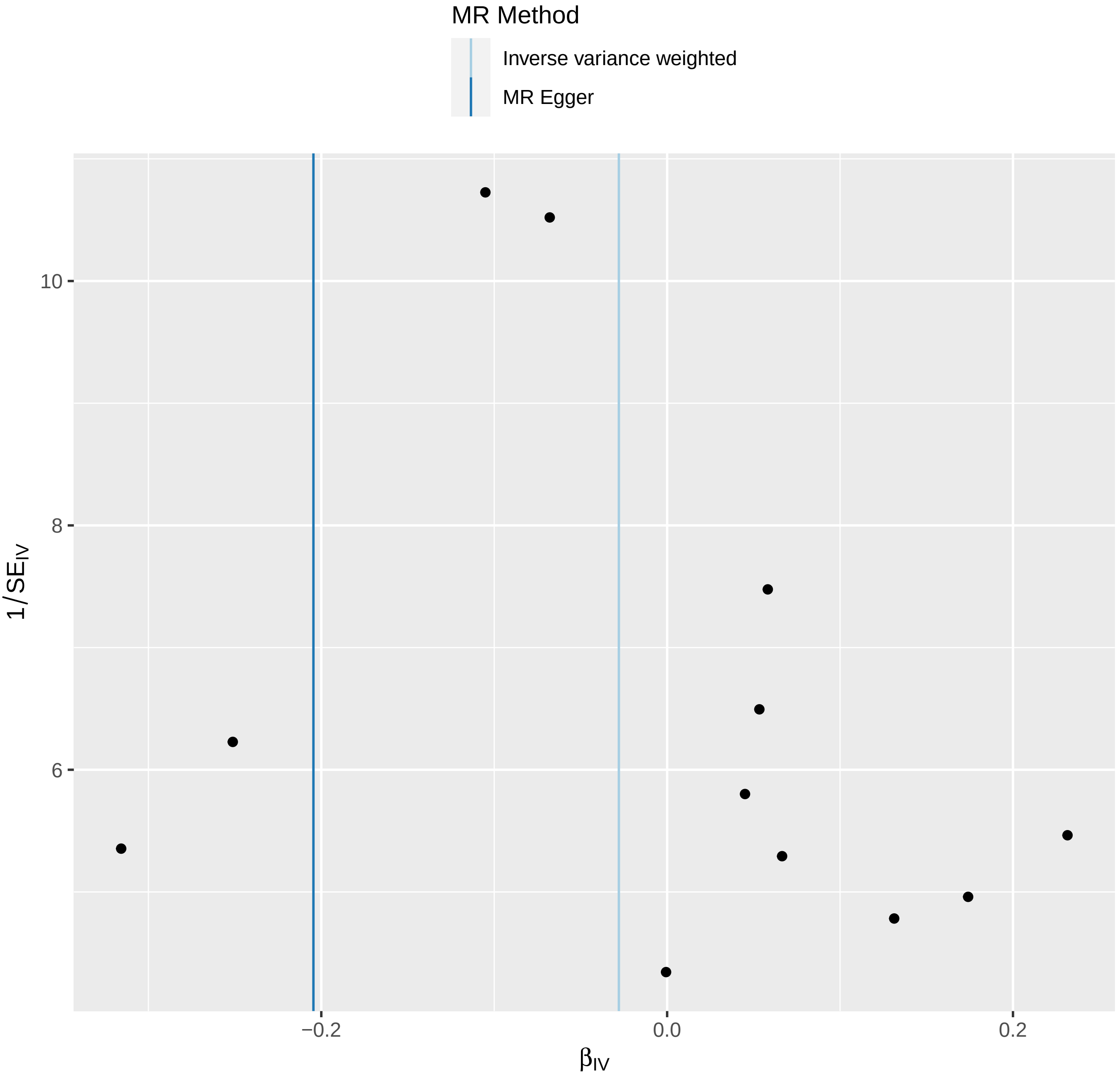

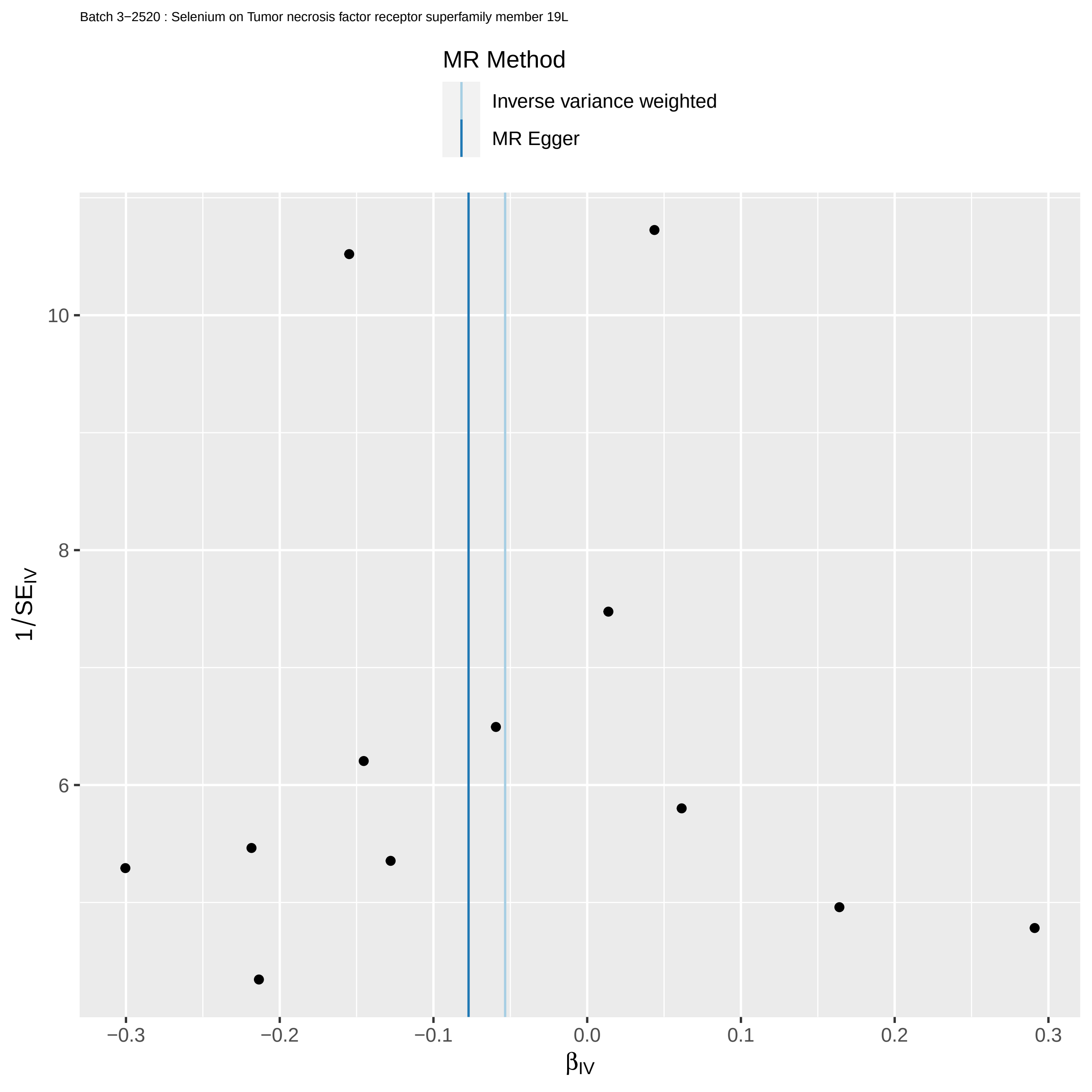
Batch 3−2520 : Selenium on Tumor necrosis factor receptor superfamily member 19L
MR Method
Inverse variance weighted
MR Egger
10
8
6
1/SE$_{IV}$
−0.3
−0.2
−0.1
0.0
0.1
0.2
0.3
β$_{IV}$

Batch 3−2521 : Selenium on Renin

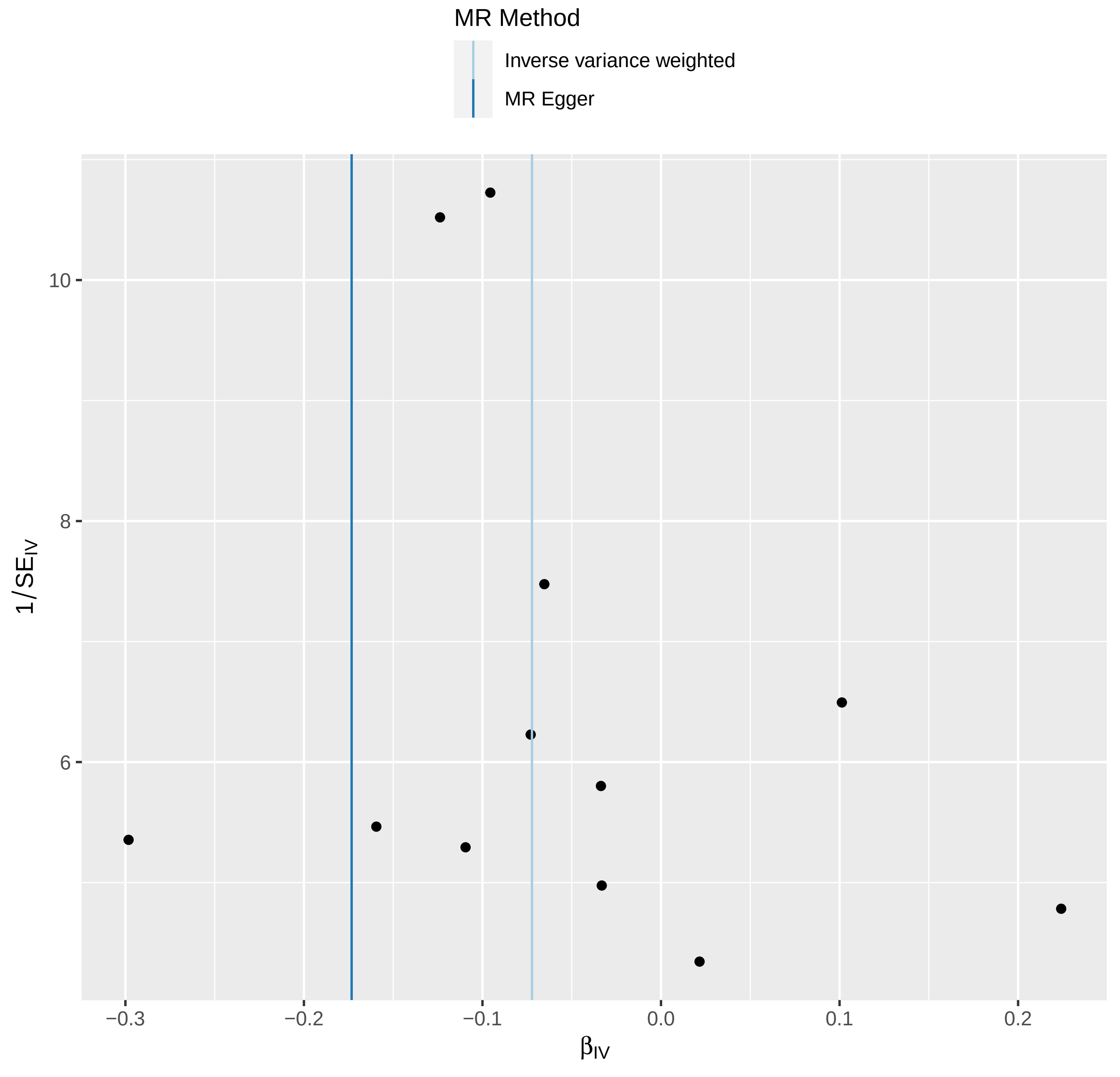

Batch 3−2522 : Selenium on Replication initiator 1

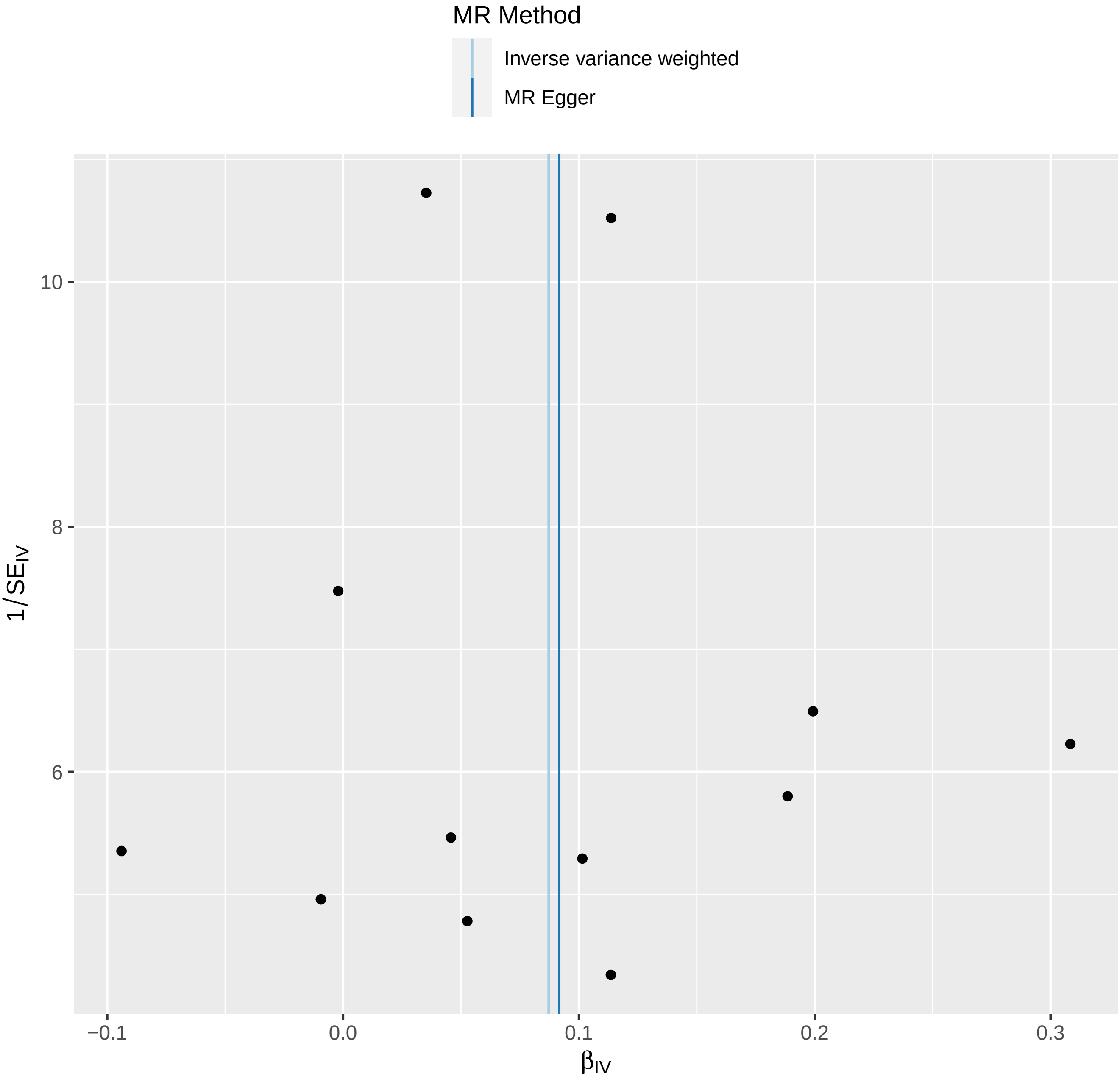

Batch 3−2523 : Selenium on Proto−oncogene tyrosine−protein kinase receptor Ret

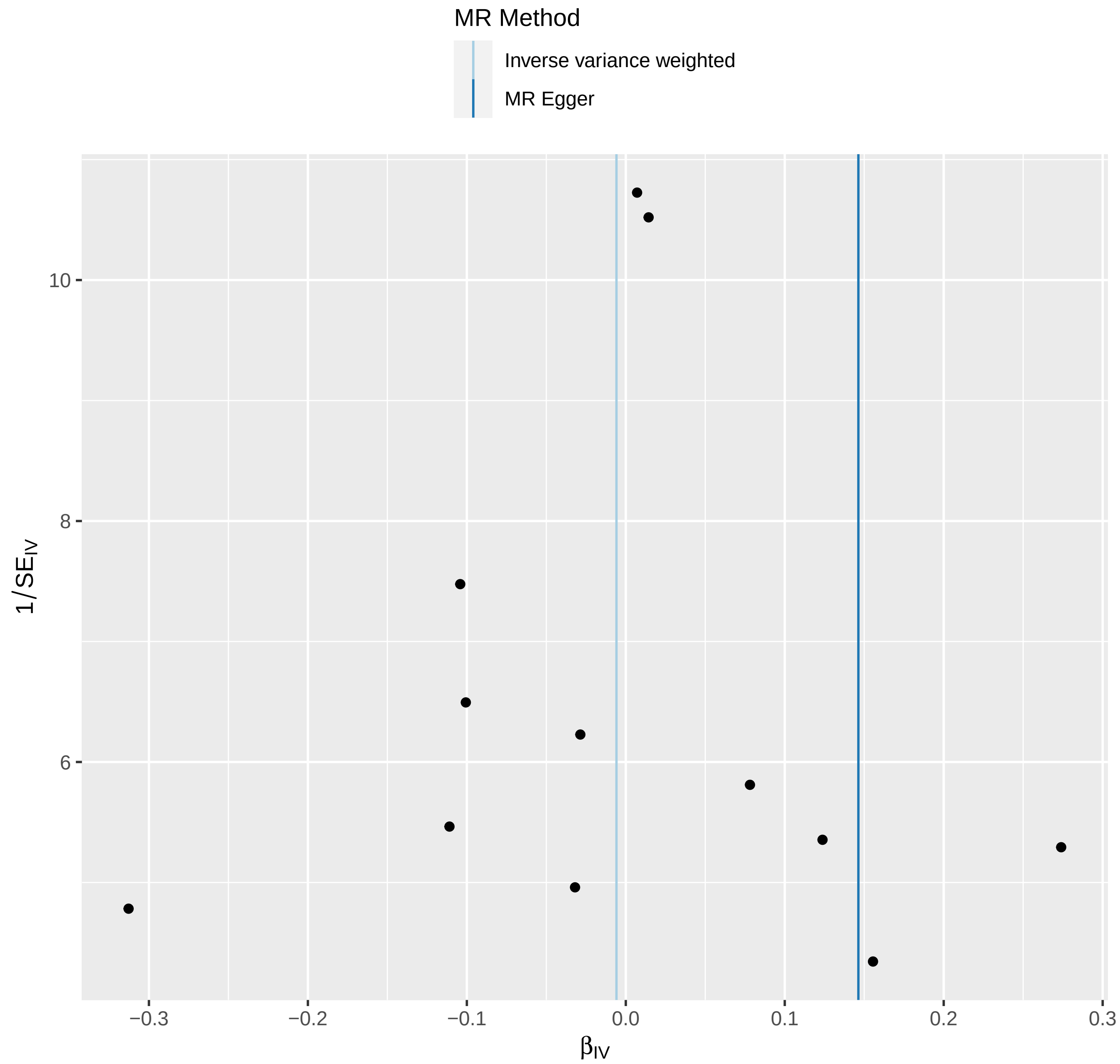

Batch 3−2524 : Selenium on Resistin

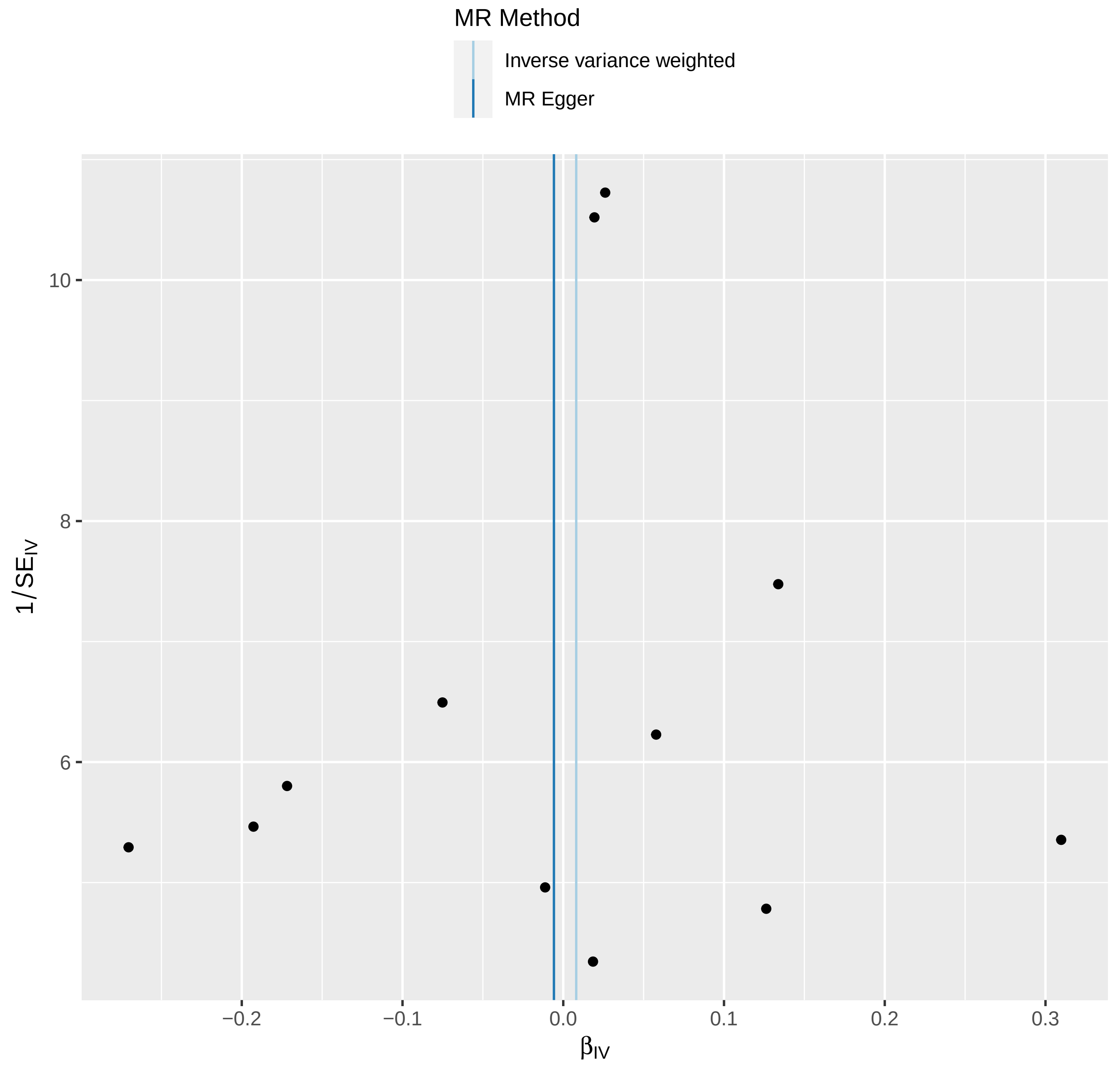

Batch 3−2525 : Selenium on All−trans−retinol 13,14−reductase

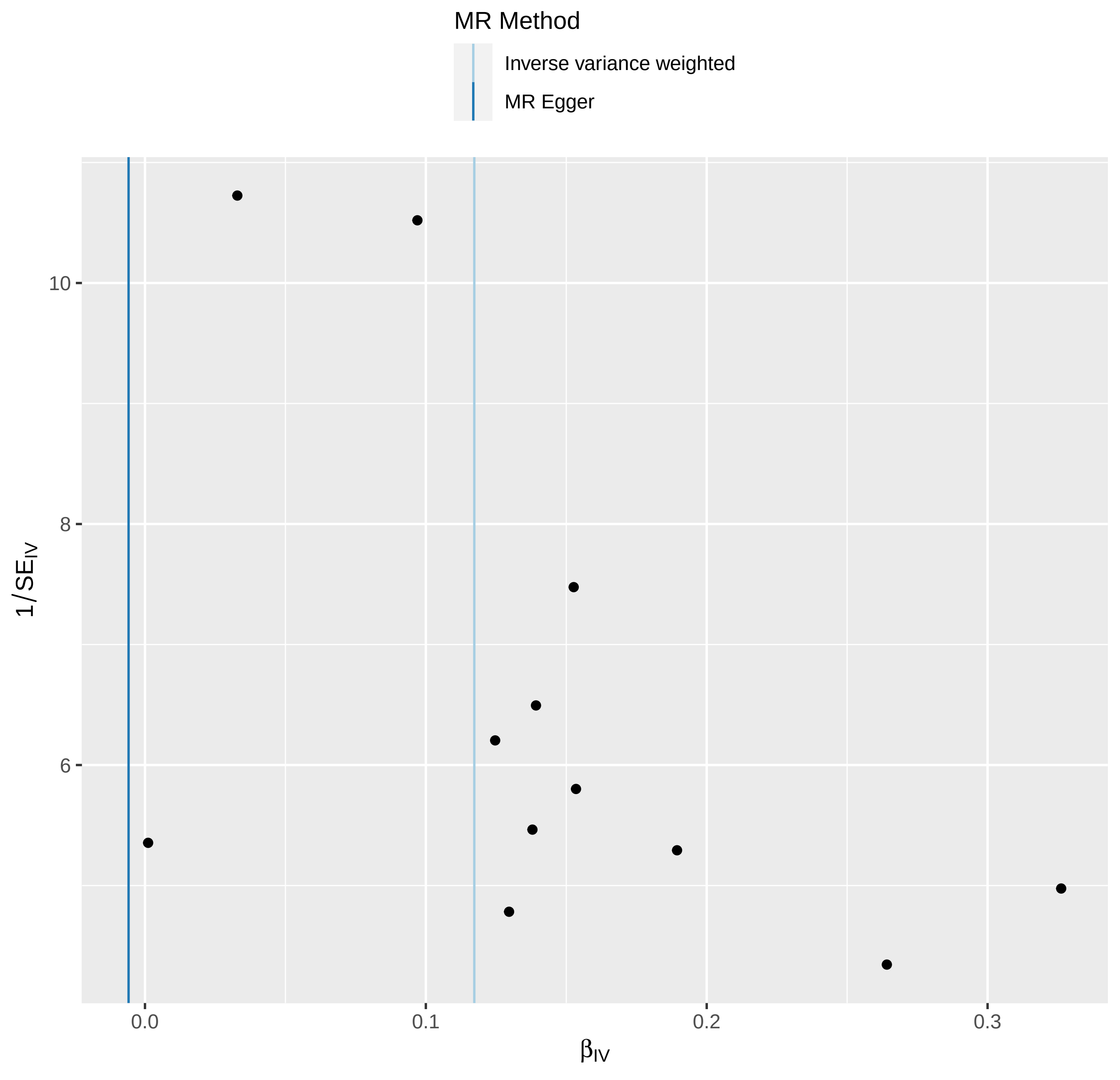

Batch 3−2526 : Selenium on Oligoribonuclease, mitochondrial

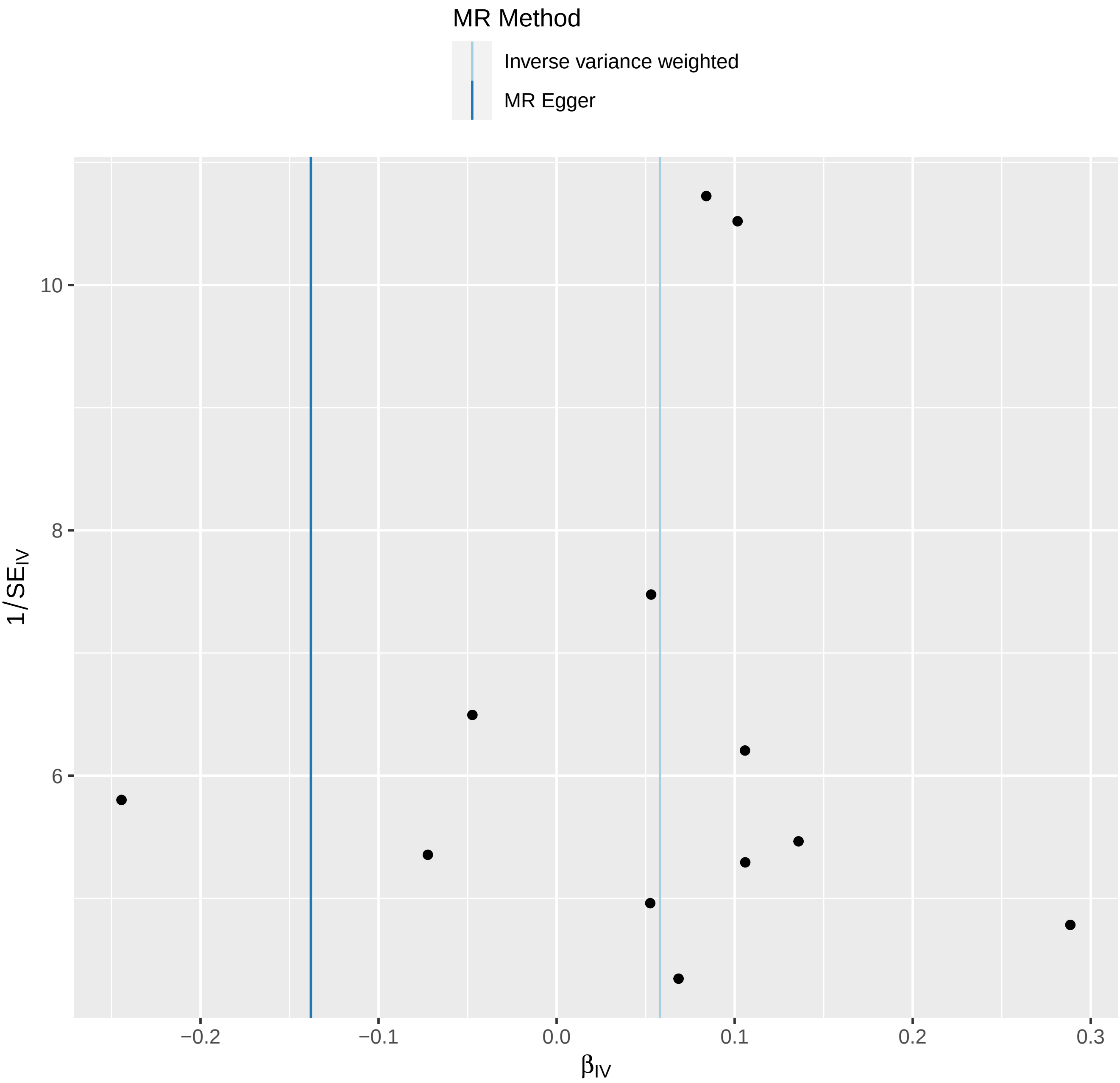

Batch 3−2527 : Selenium on Rieske domain−containing protein

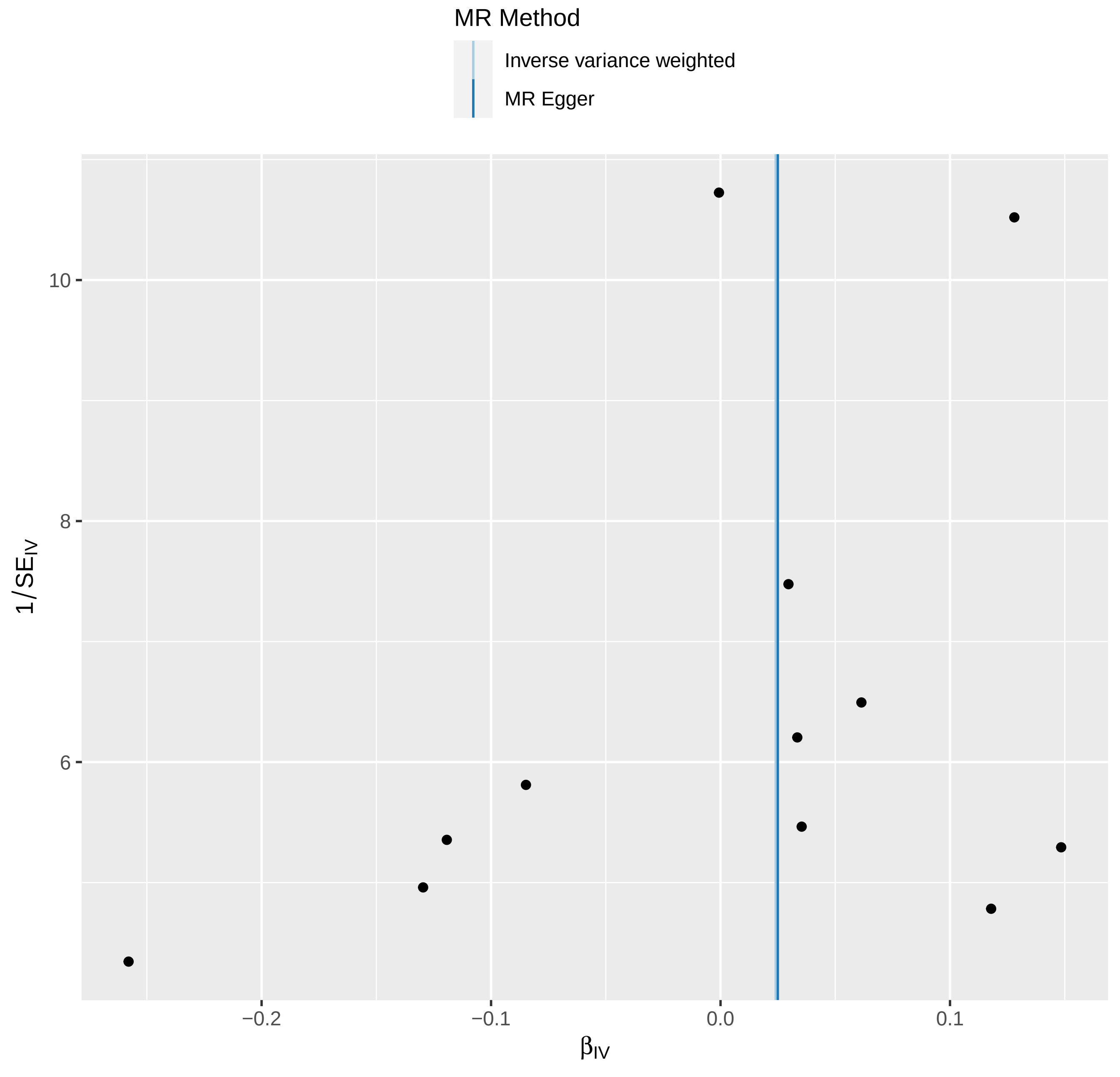

Batch 3−2528 : Selenium on E3 ubiquitin−protein ligase rififylin

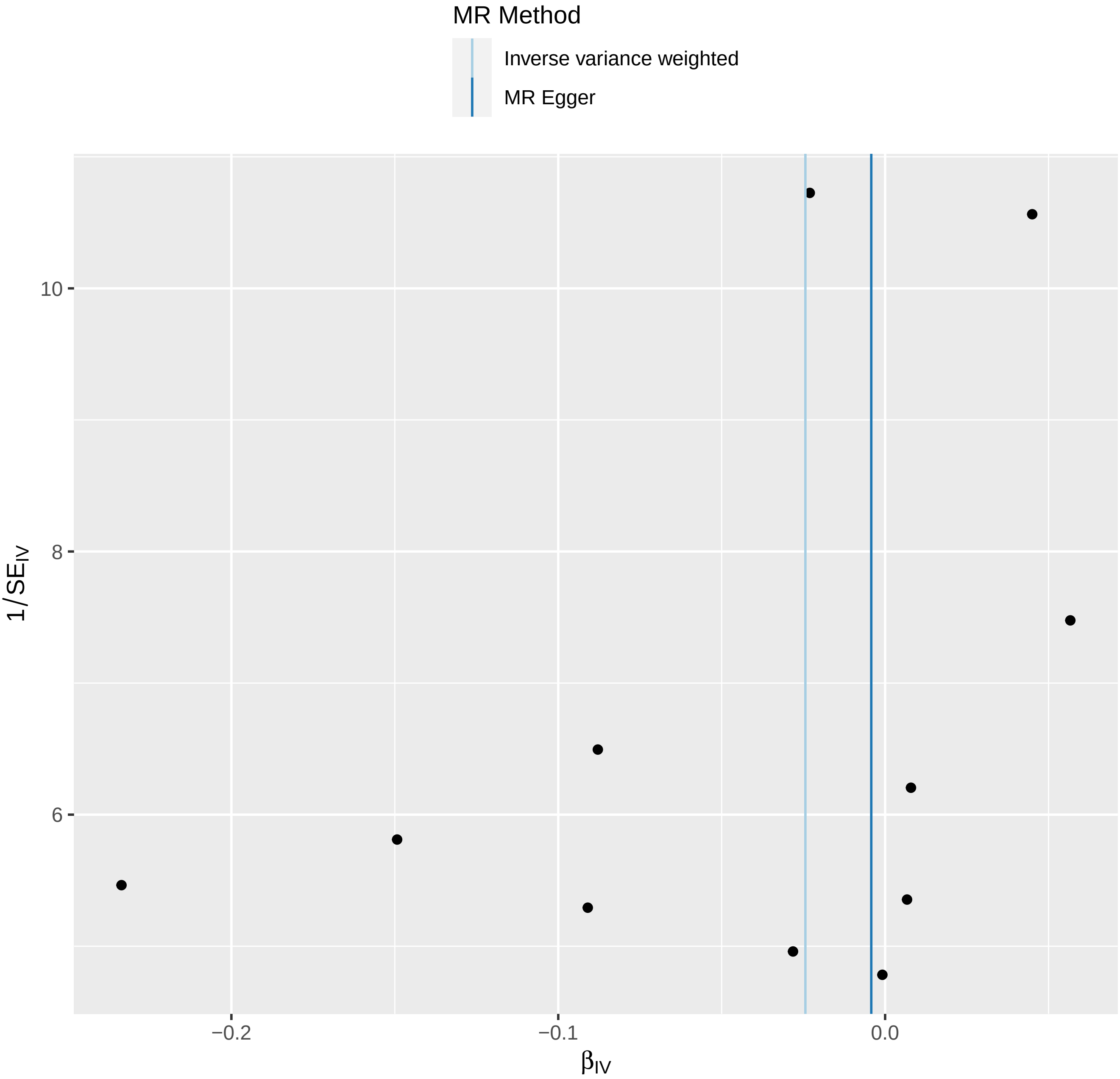

Batch 3−2529 : Selenium on Riboflavin kinase

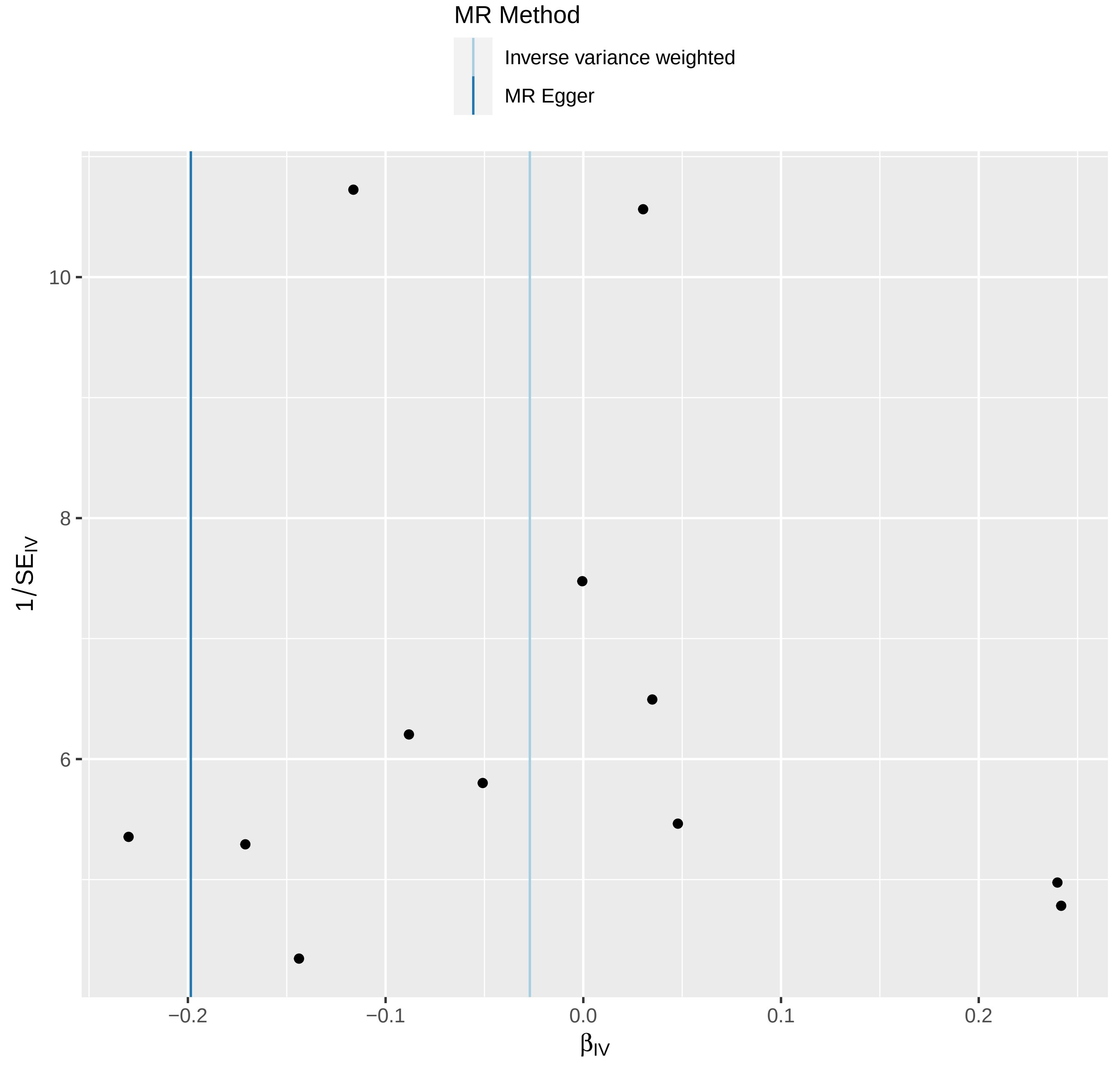

Batch 3−2530 : Selenium on Beta−1,3−N−acetylglucosaminyltransferase radical fringe

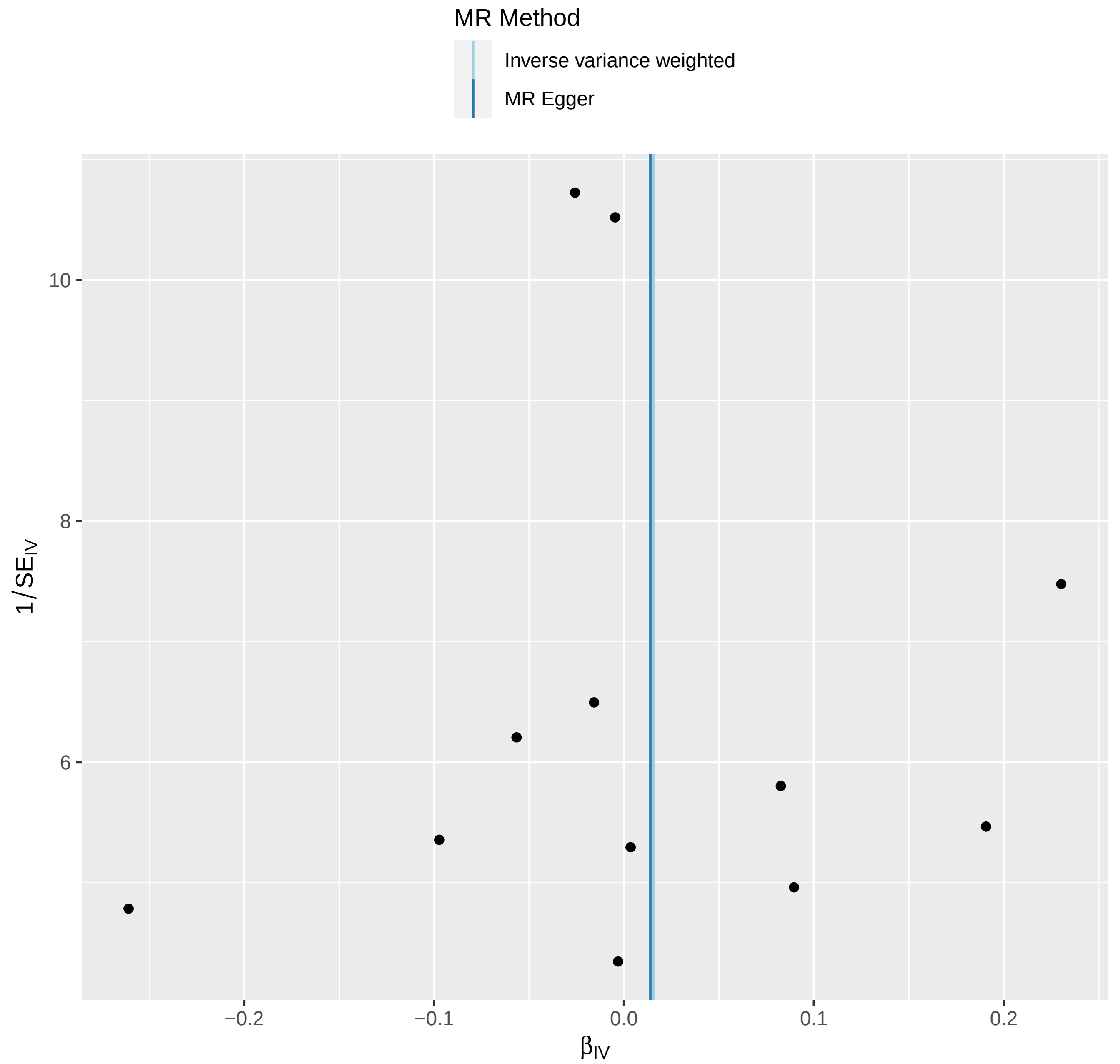

Batch 3−2531 : Selenium on Regulatory factor X−associated protein

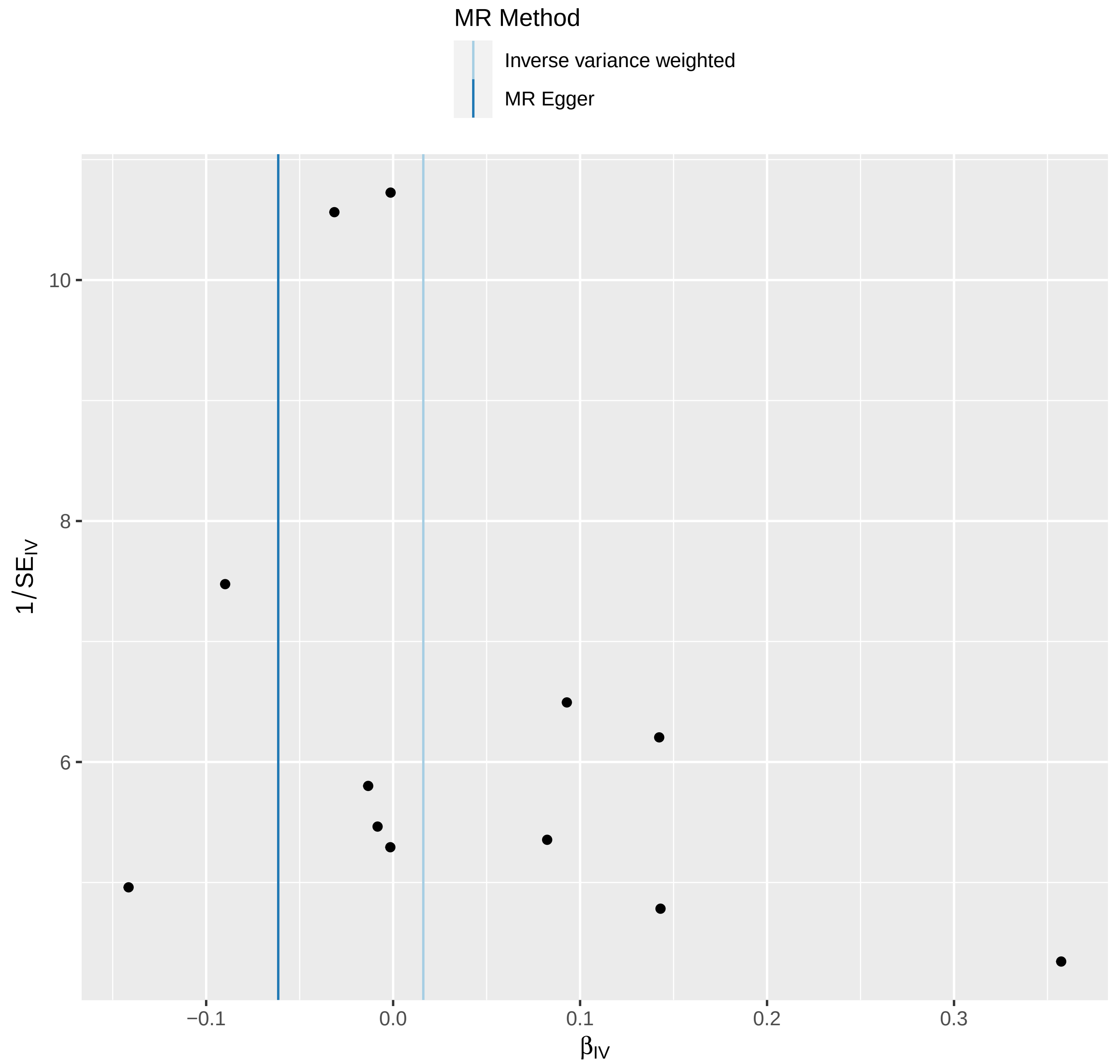

Batch 3−2532 : Selenium on Repulsive guidance molecule A

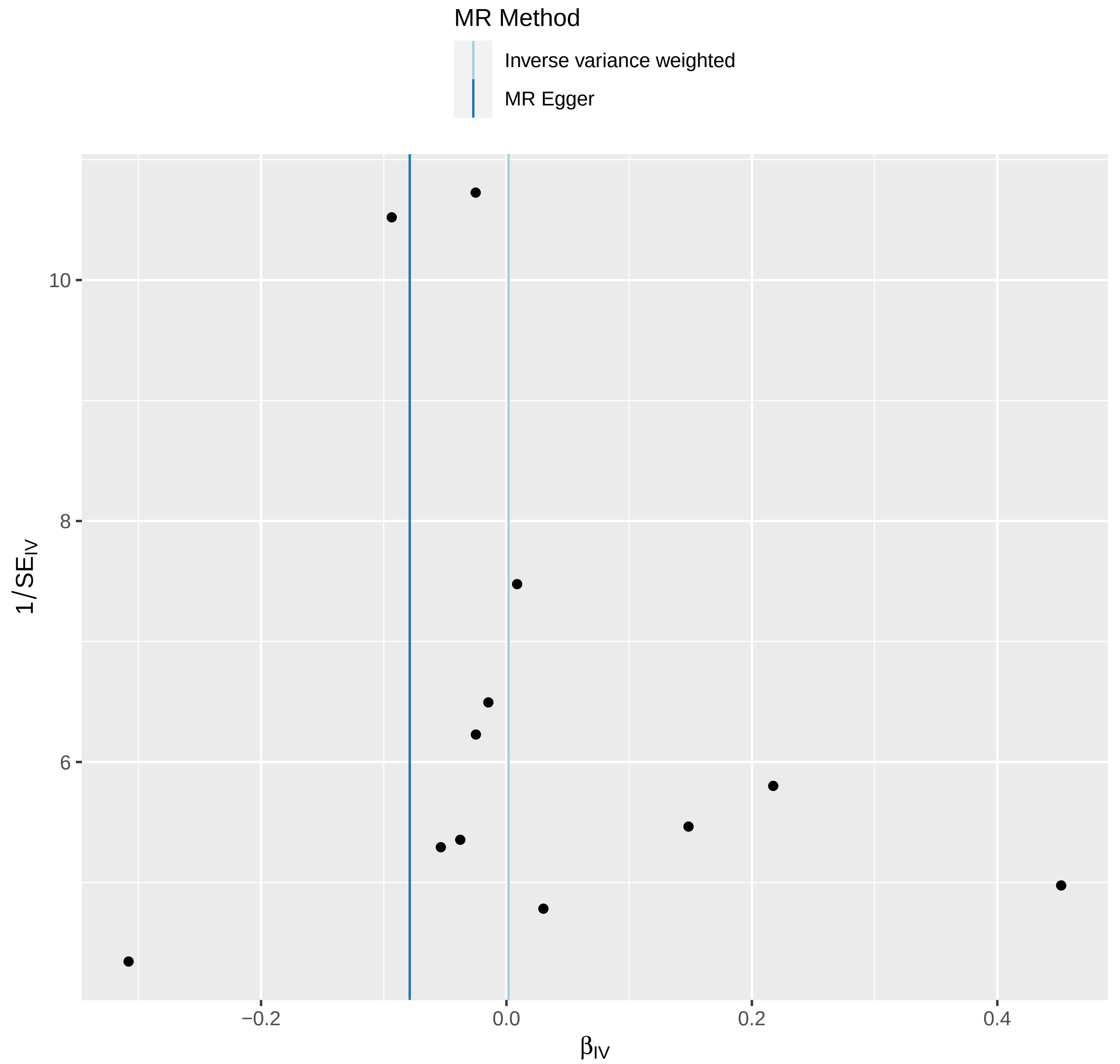

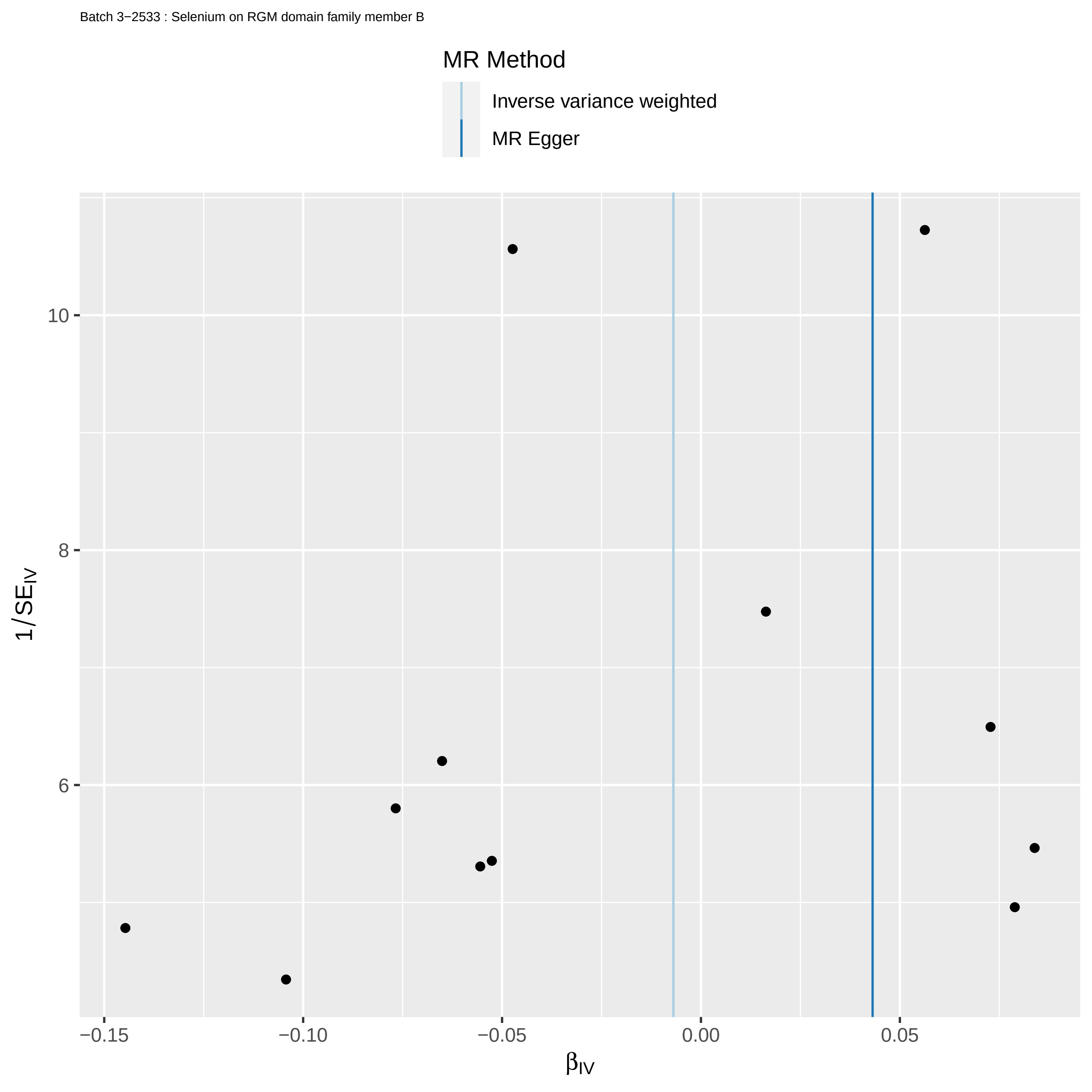

Batch 3−2533 : Selenium on RGM domain family member B
MR Method
Inverse variance weighted
MR Egger
10
8
6
1/SE_IV
−0.15
−0.10
−0.05
0.00
0.05
β_IV

Batch 3−2534 : Selenium on Regulator of G−protein signaling 10

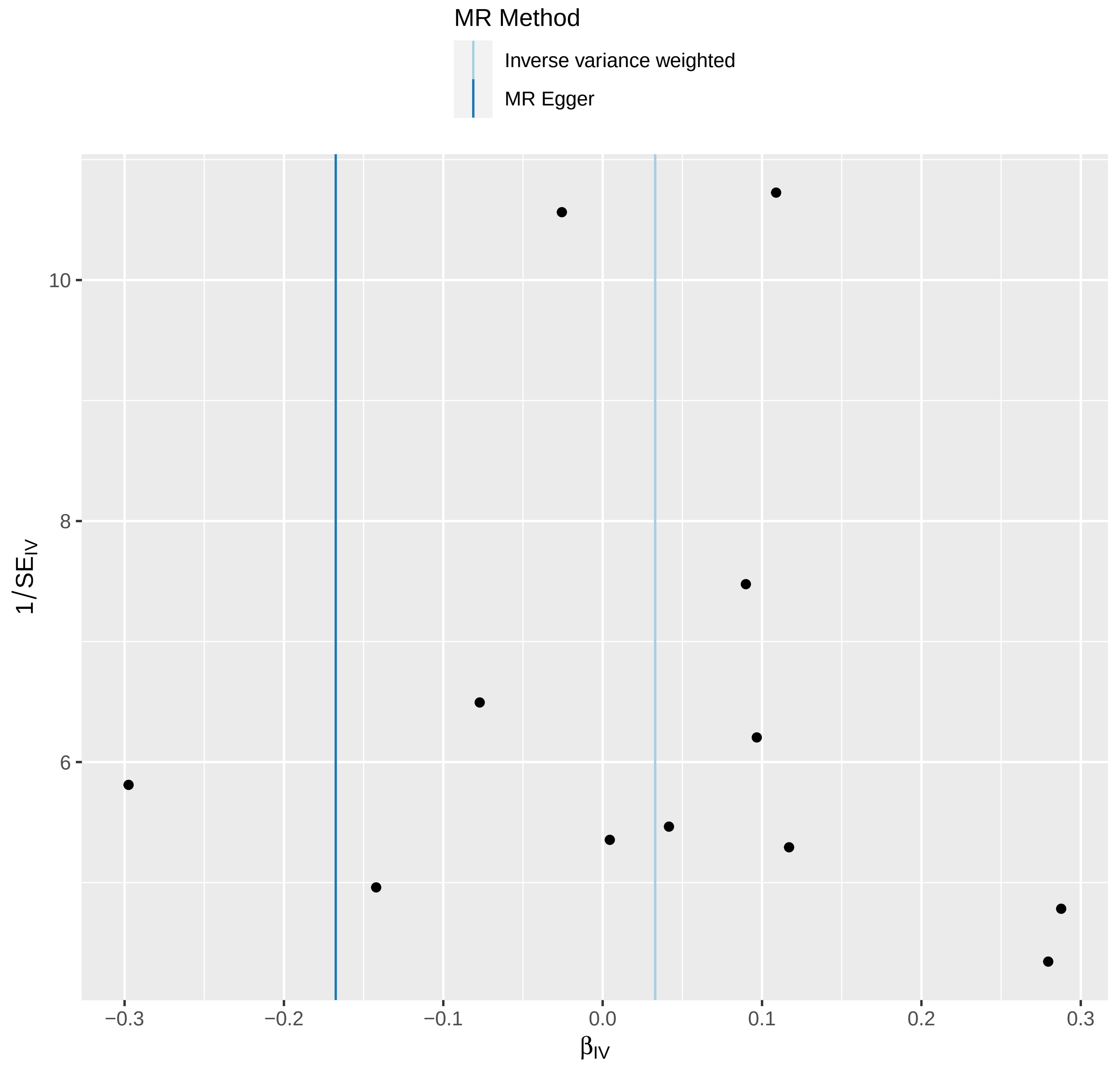

Batch 3−2535 : Selenium on Regulator of G−protein signaling 18

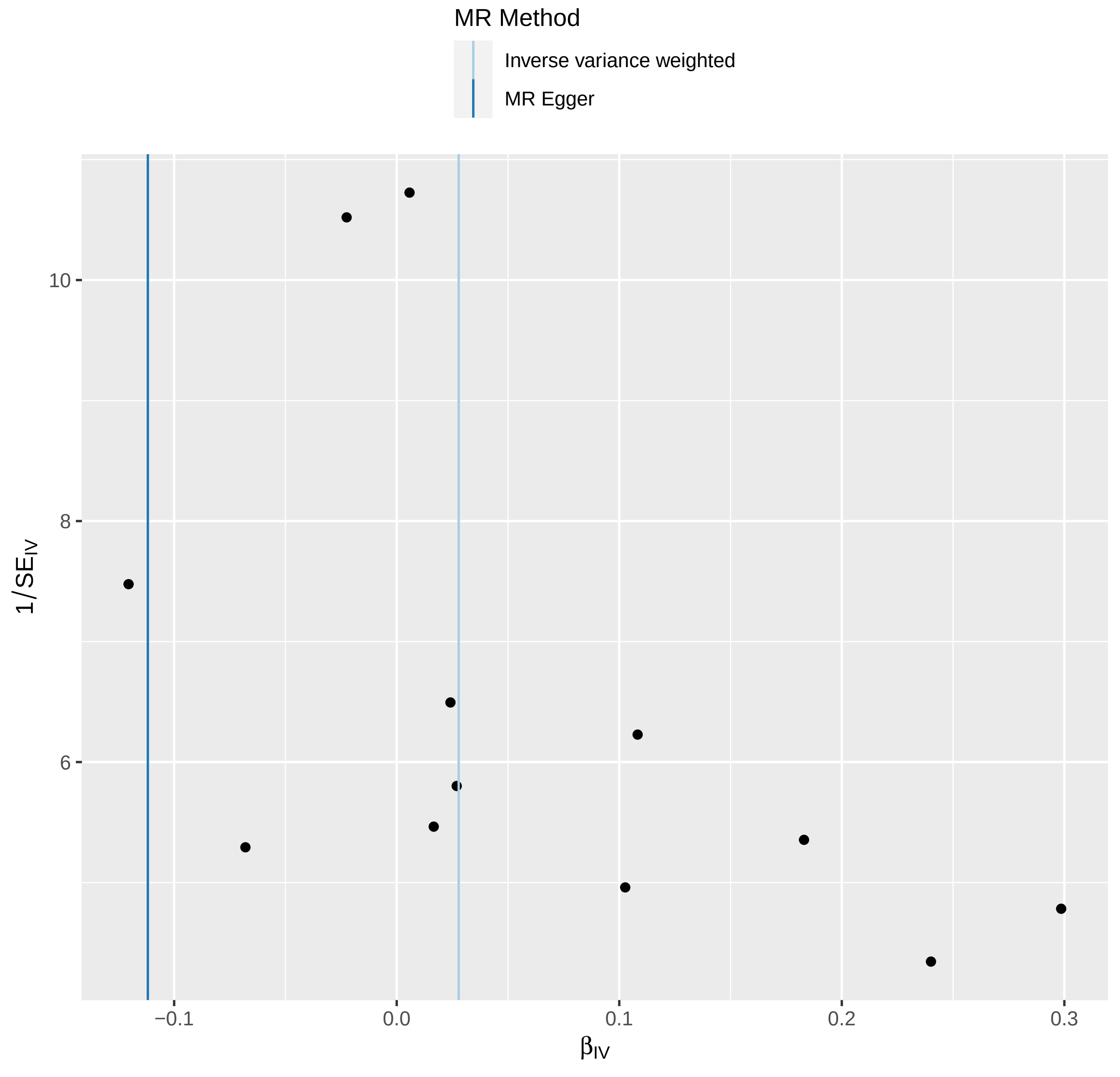

Batch 3−2536 : Selenium on Regulator of G−protein signaling 19

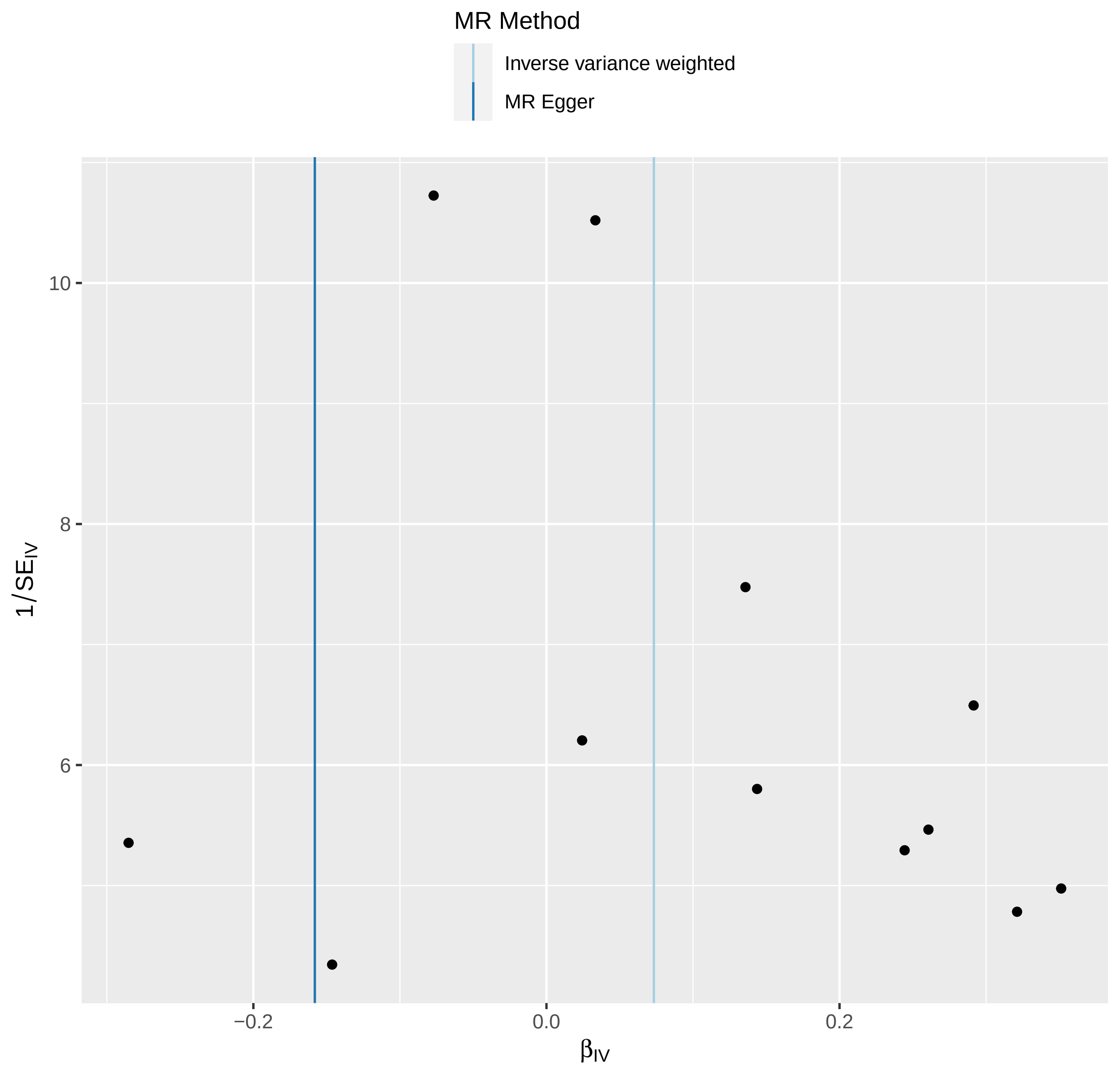

Batch 3−2537 : Selenium on Regulator of G−protein signaling 3

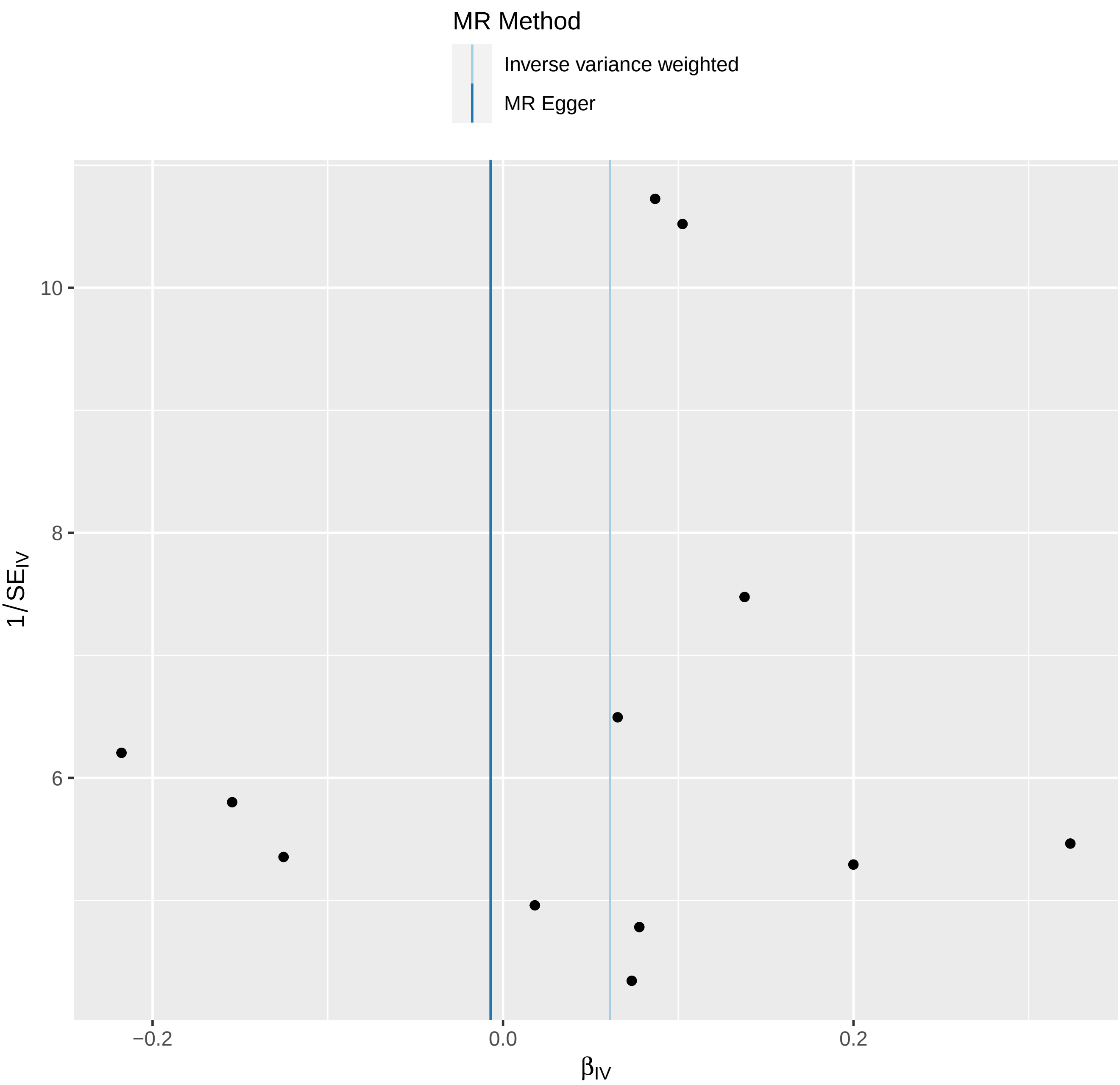

Batch 3−2538 : Selenium on Regulator of G−protein signaling 8

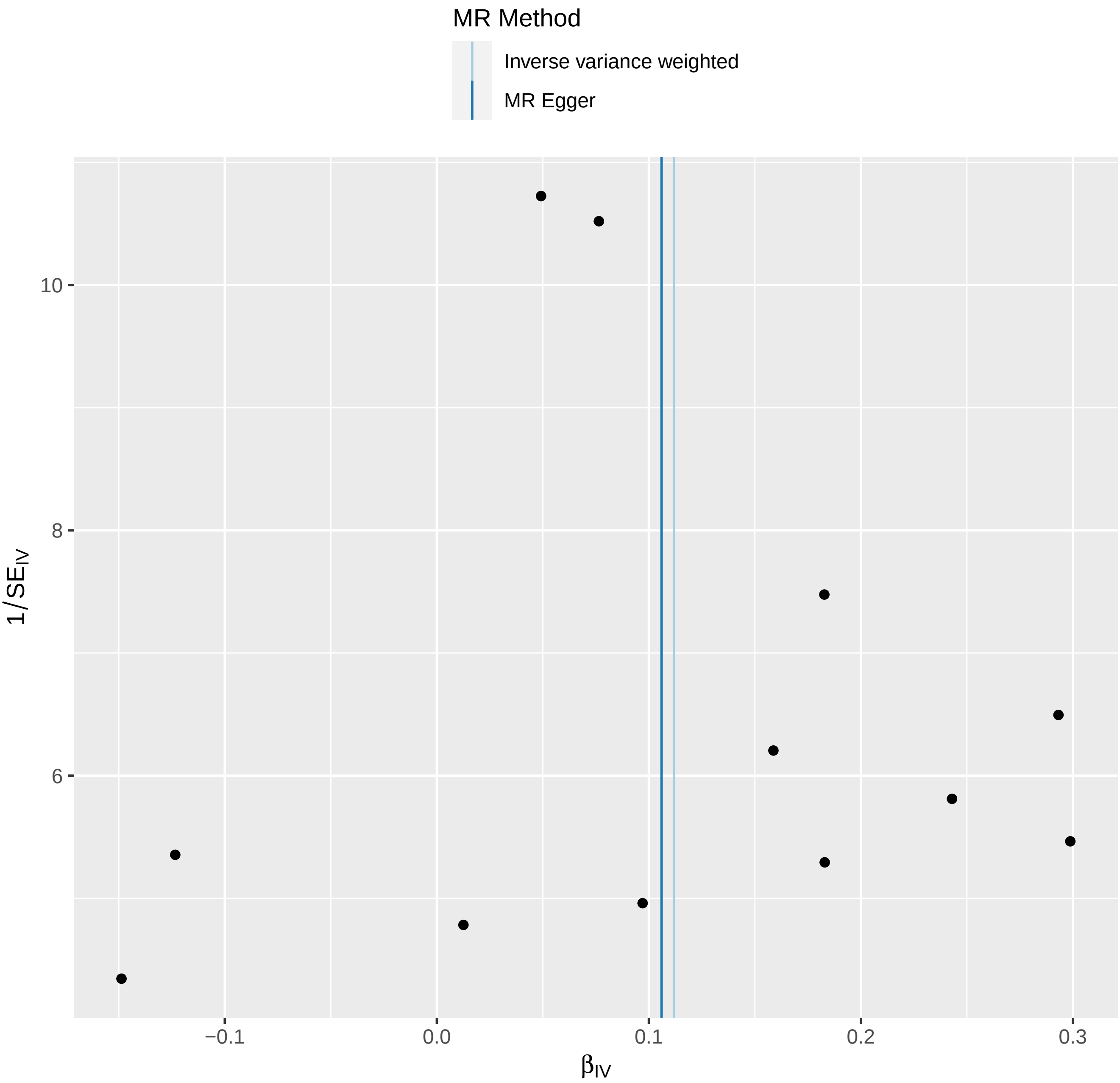

Batch 3−2539 : Selenium on Transforming protein RhoA

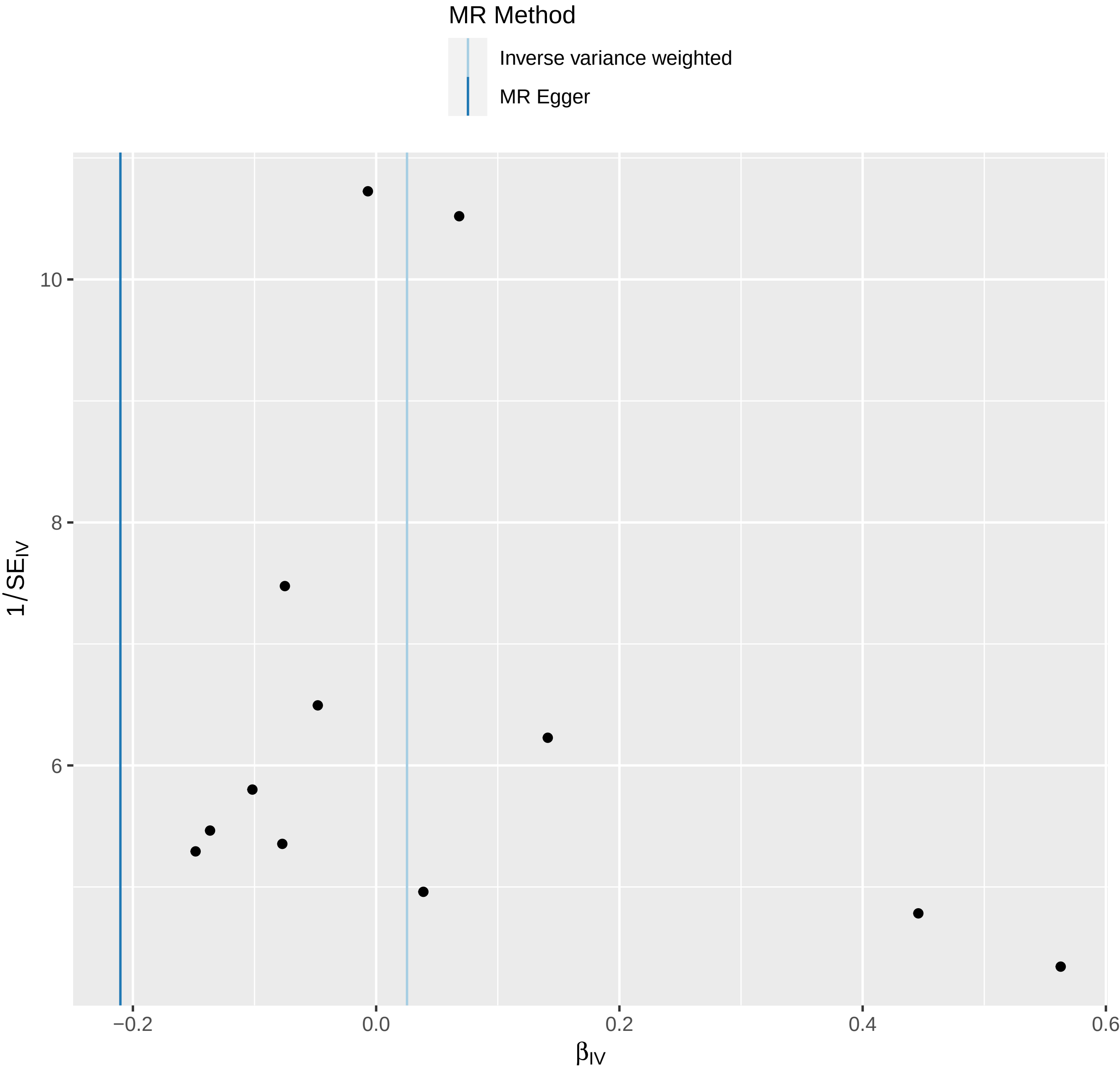

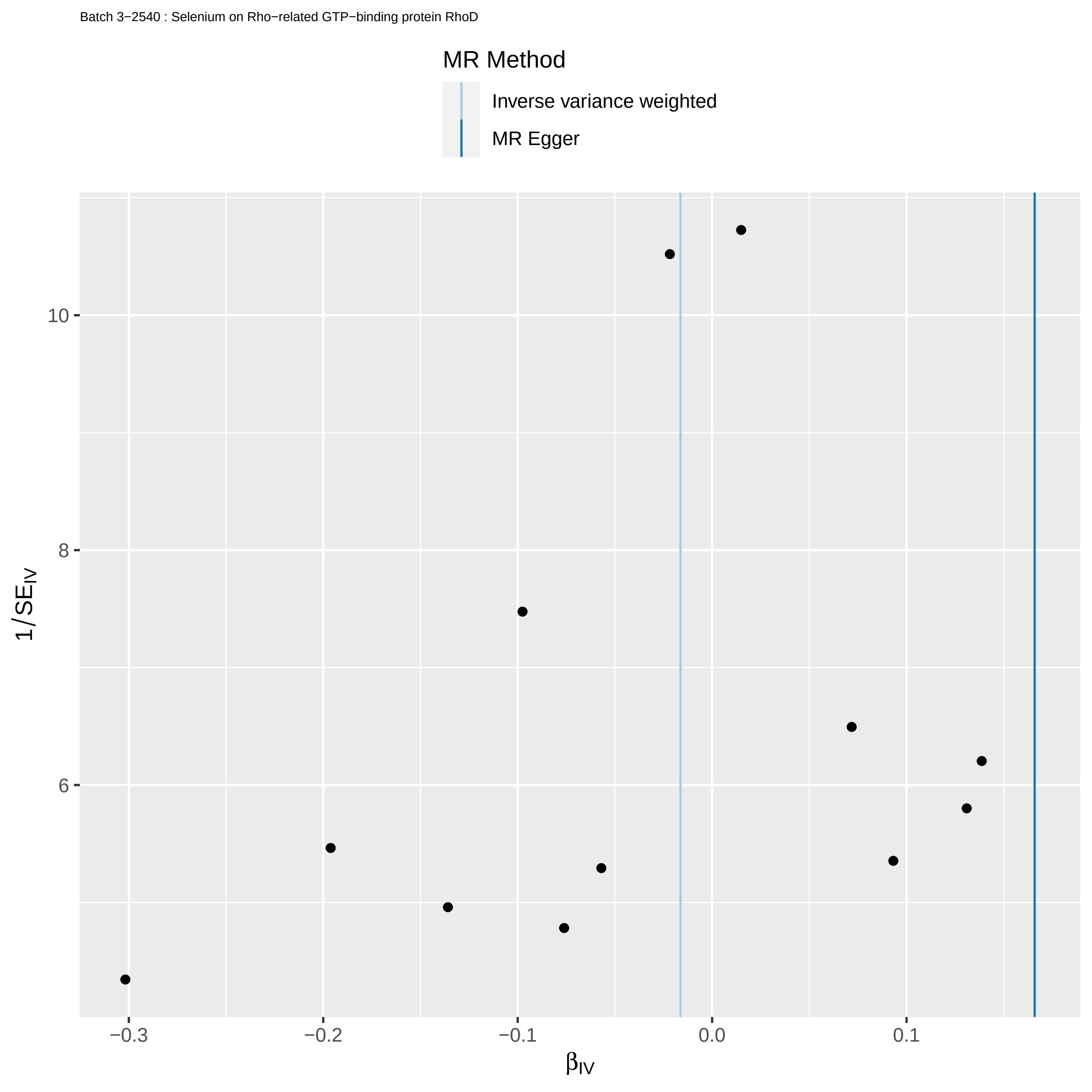

Batch 3−2540 : Selenium on Rho−related GTP−binding protein RhoD
MR Method
Inverse variance weighted
MR Egger
10
8
6
1/SE$_{IV}$
−0.3
−0.2
−0.1
0.0
0.1
β$_{IV}$

Batch 3−2541 : Selenium on Rho−related GTP−binding protein RhoG

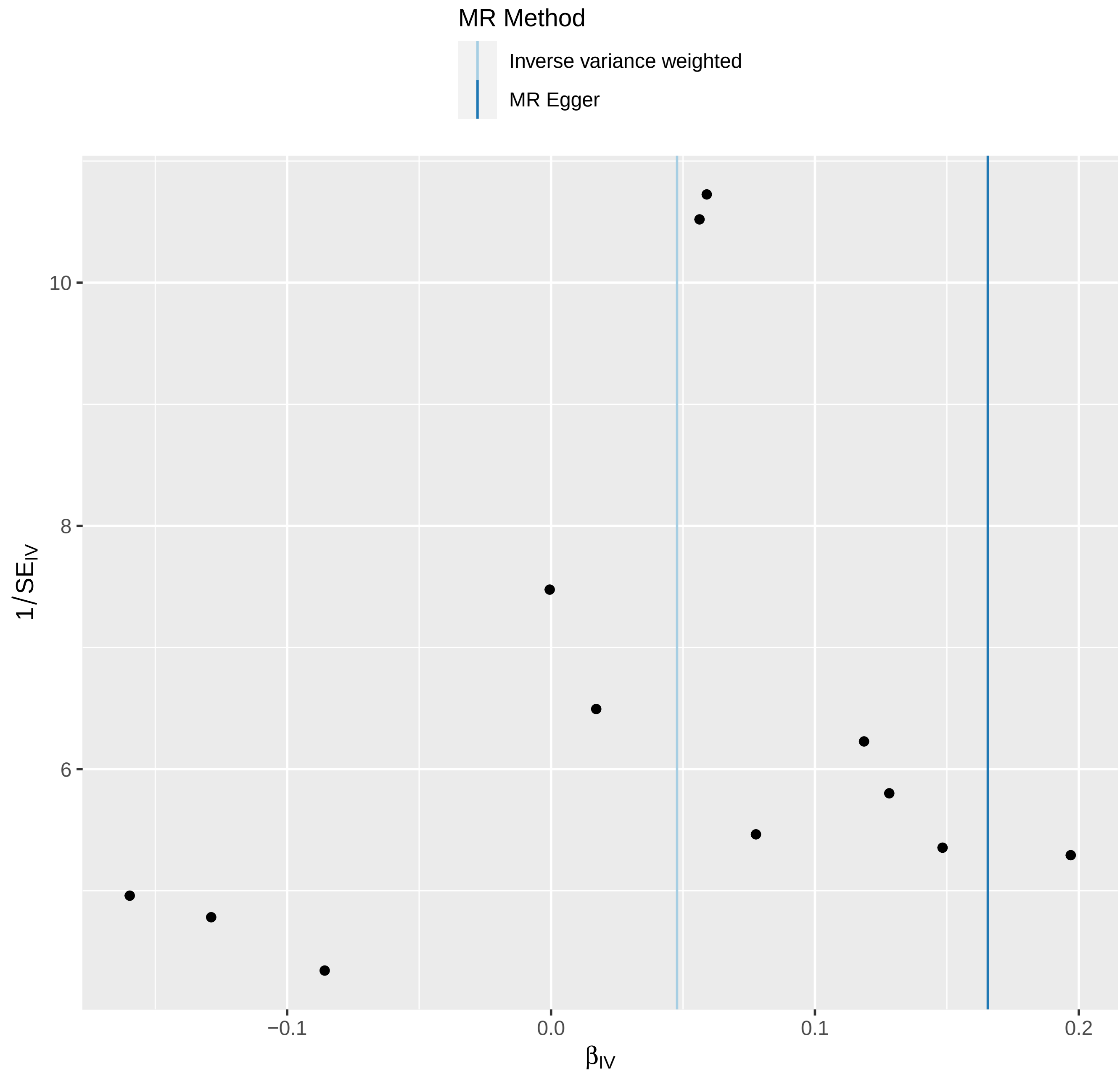

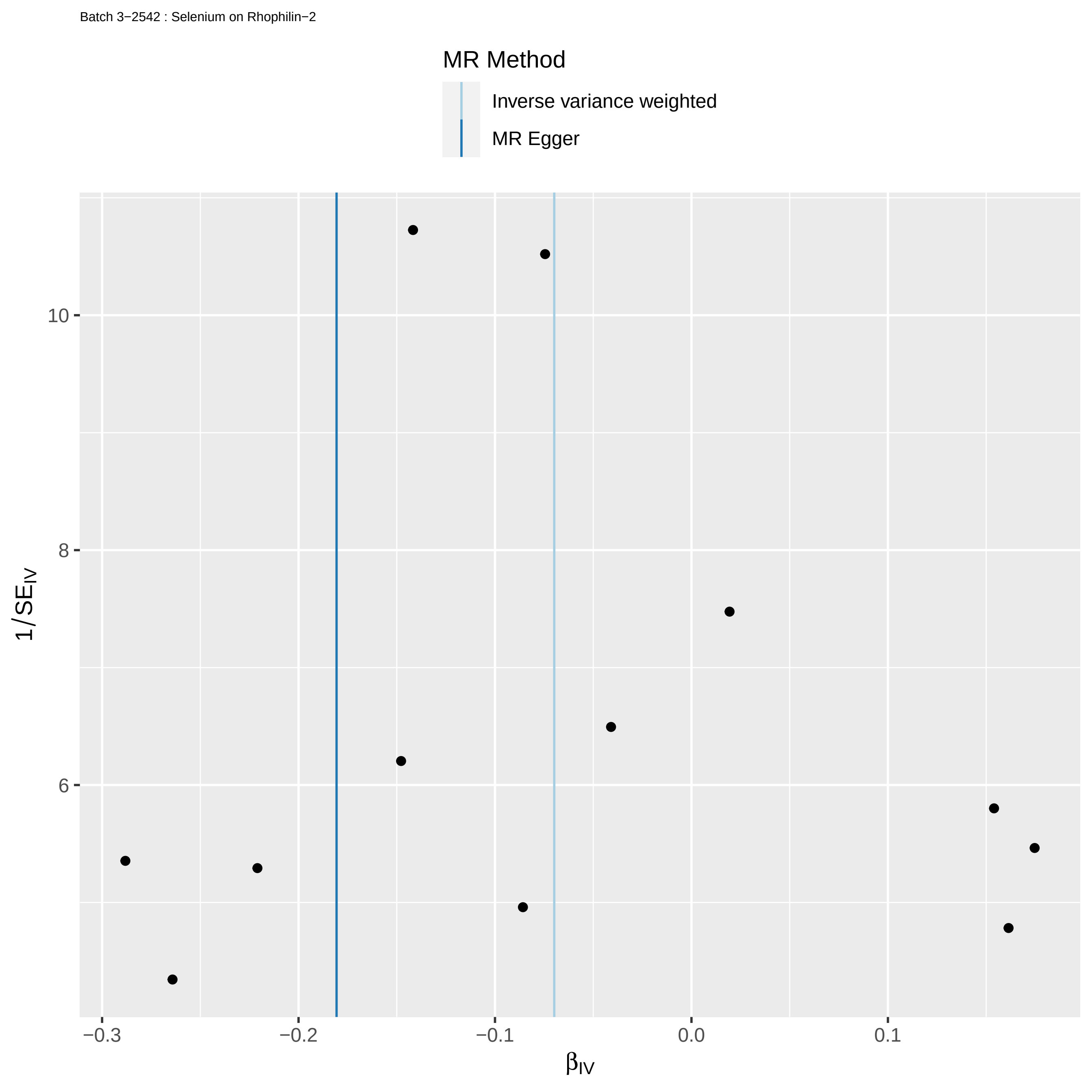

Batch 3−2542 : Selenium on Rhophilin−2
MR Method
Inverse variance weighted
MR Egger
10
8
6
1/SE_IV
−0.3
−0.2
−0.1
0.0
0.1
β_IV

Batch 3−2543 : Selenium on Protein RIC−3

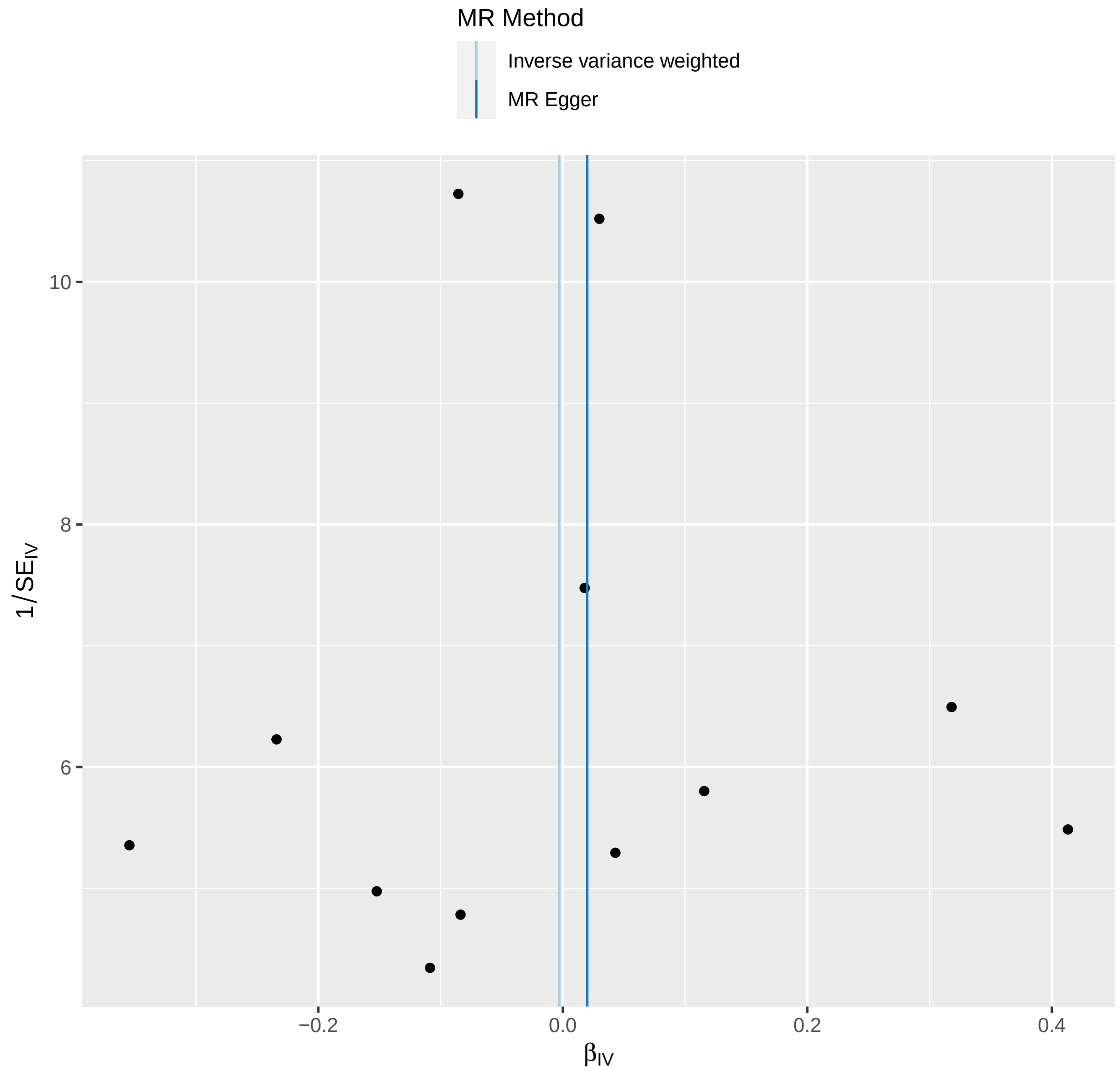

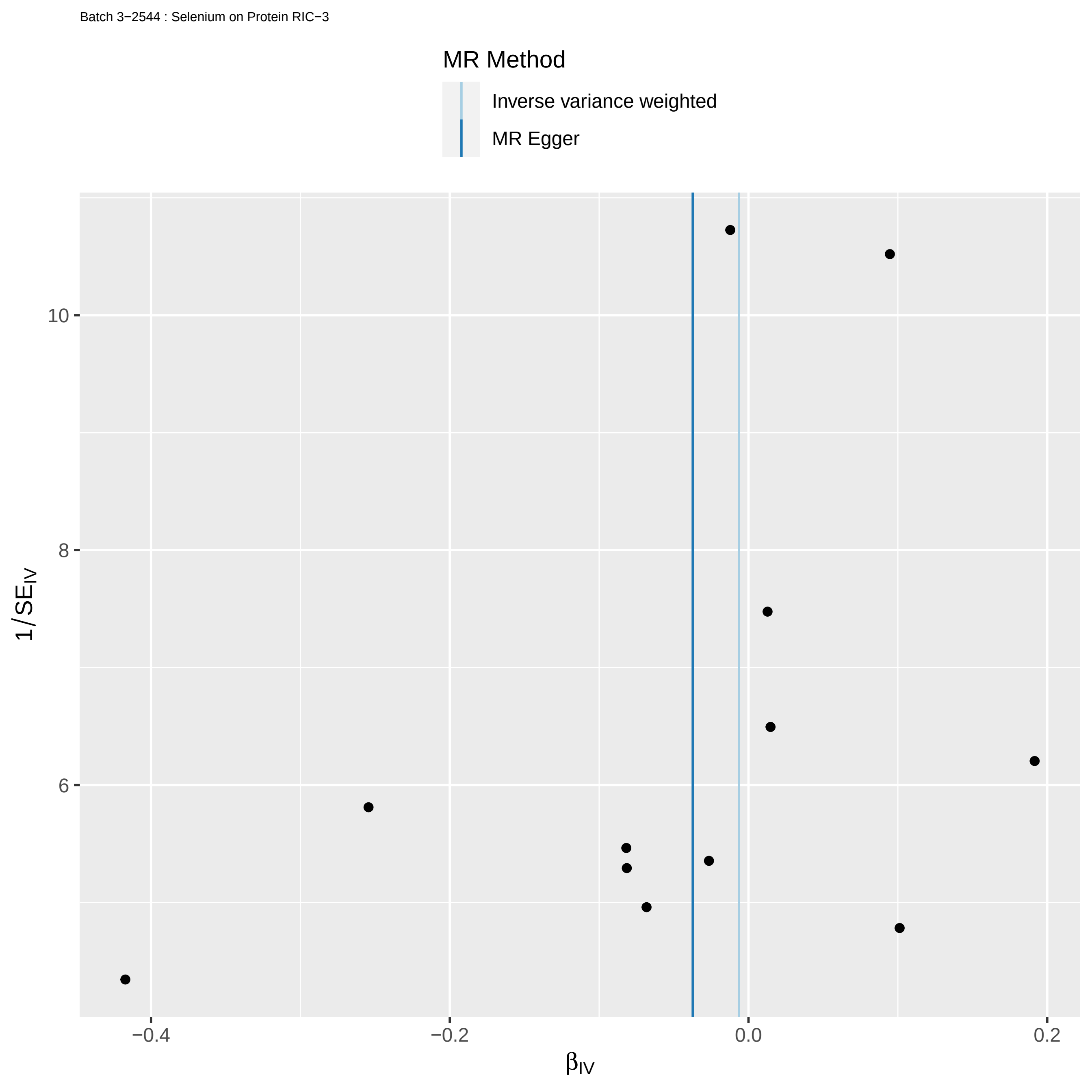
Batch 3−2544 : Selenium on Protein RIC−3
MR Method
Inverse variance weighted
MR Egger
10
8
6
1/SE_IV
−0.4
−0.2
0.0
0.2
β_IV

Batch 3−2545 : Selenium on Receptor−interacting serine/threonine−protein kinase 2

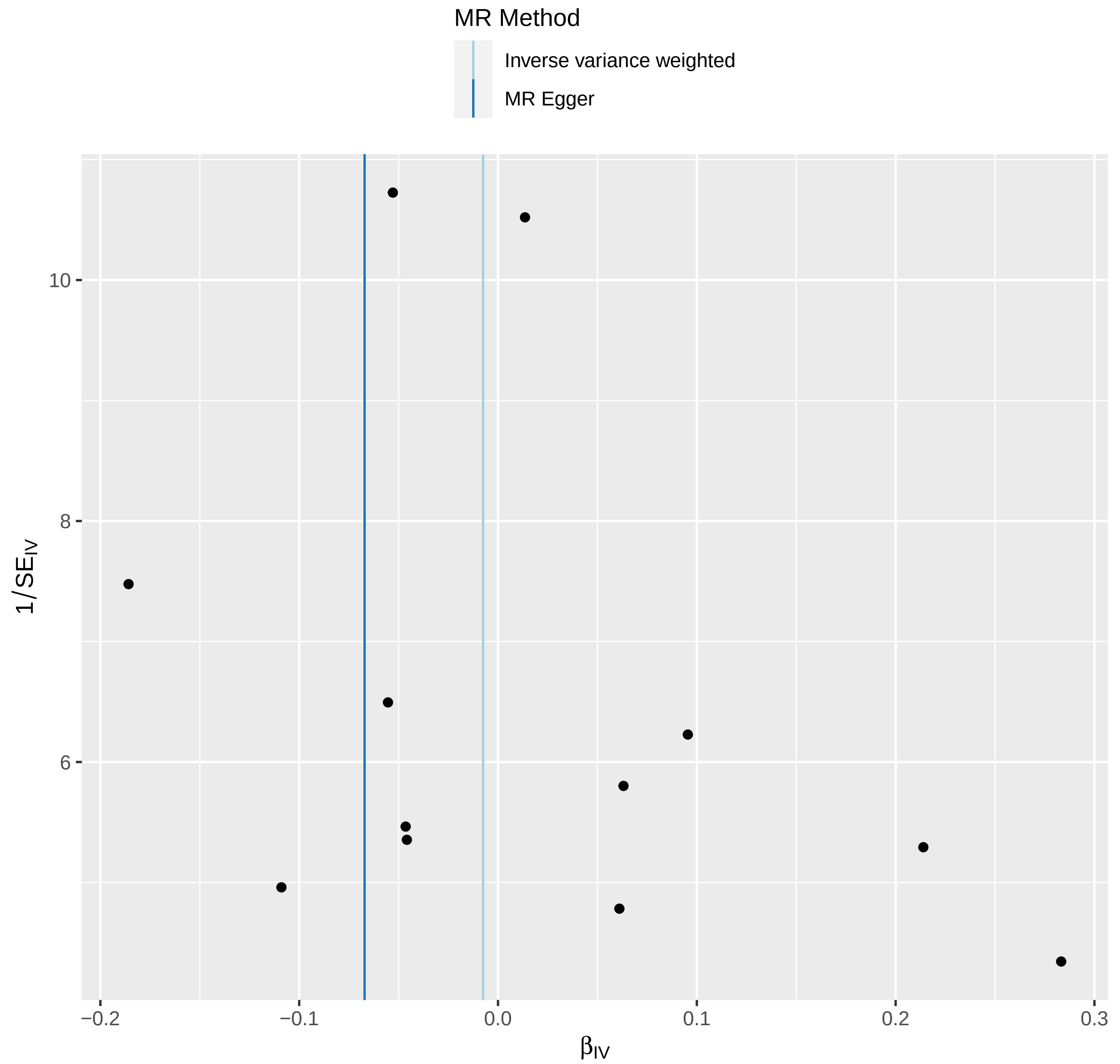

Batch 3−2546 : Selenium on Receptor−interacting serine/threonine−protein kinase 2

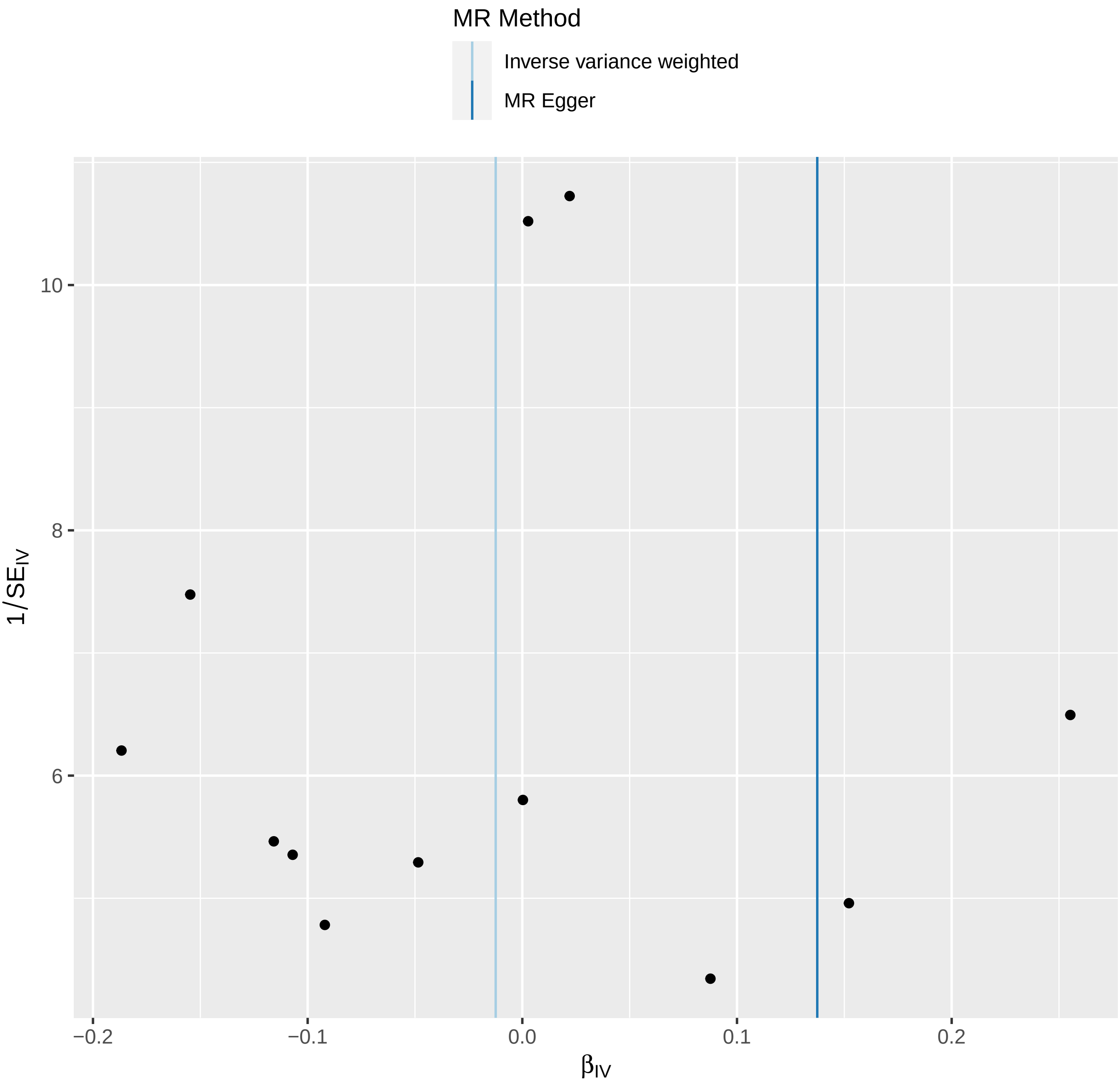

Batch 3−2547 : Selenium on Protein ripply1

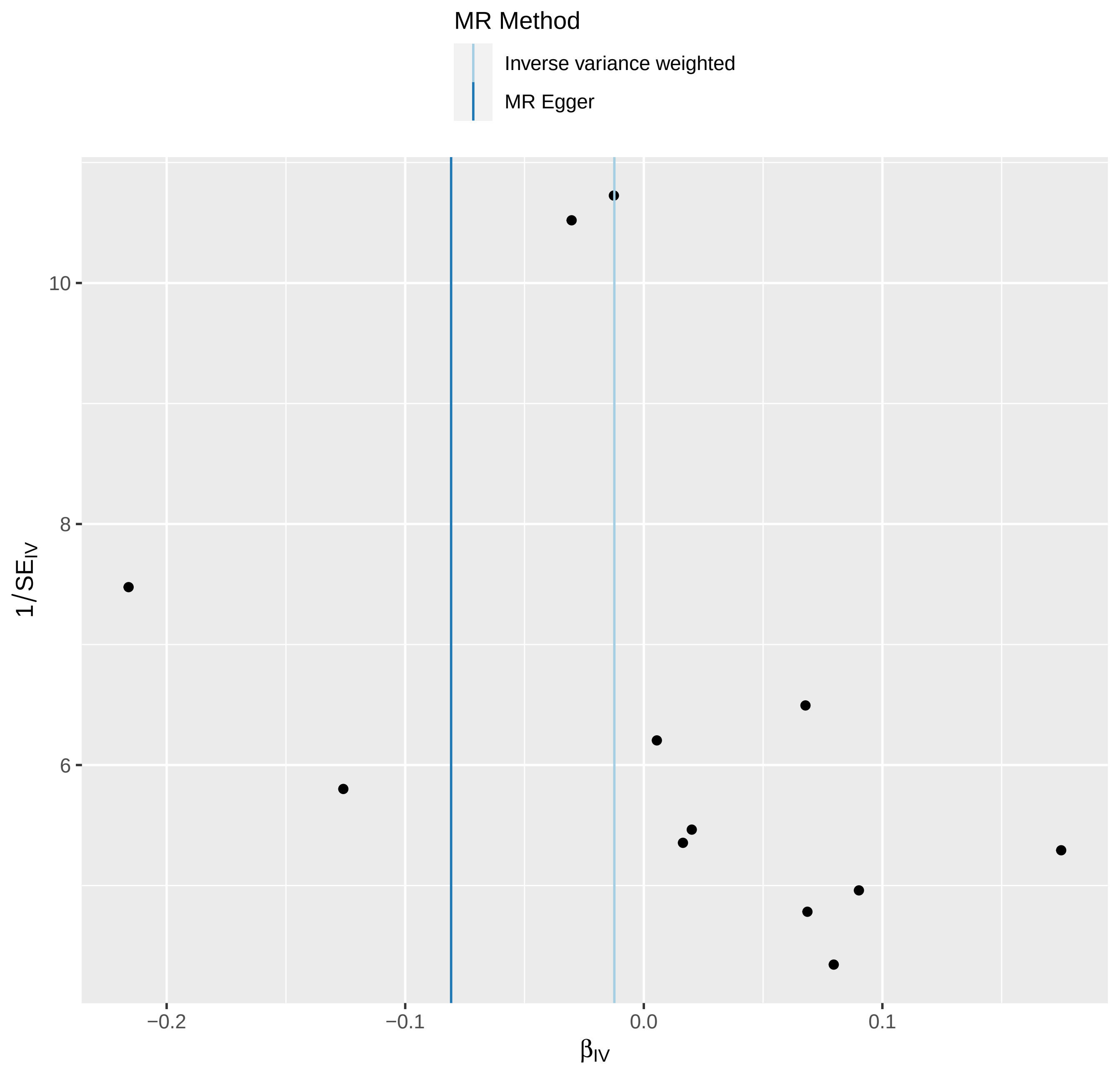

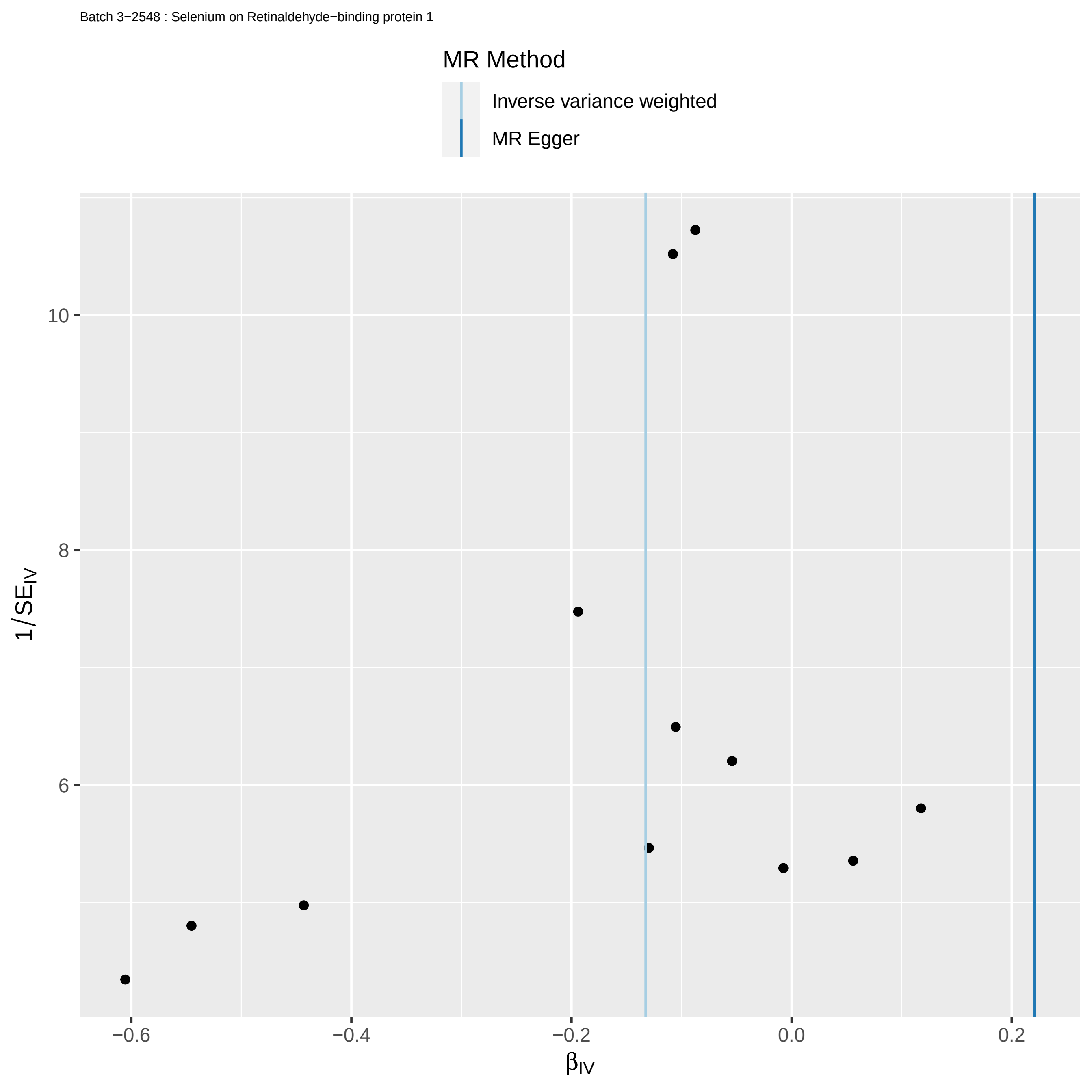

Batch 3−2548 : Selenium on Retinaldehyde−binding protein 1
MR Method
Inverse variance weighted
MR Egger
10
8
6
1/SE$_{IV}$
−0.6
−0.4
−0.2
0.0
0.2
β$_{IV}$

Batch 3−2549 : Selenium on Regulator of microtubule dynamics protein 1

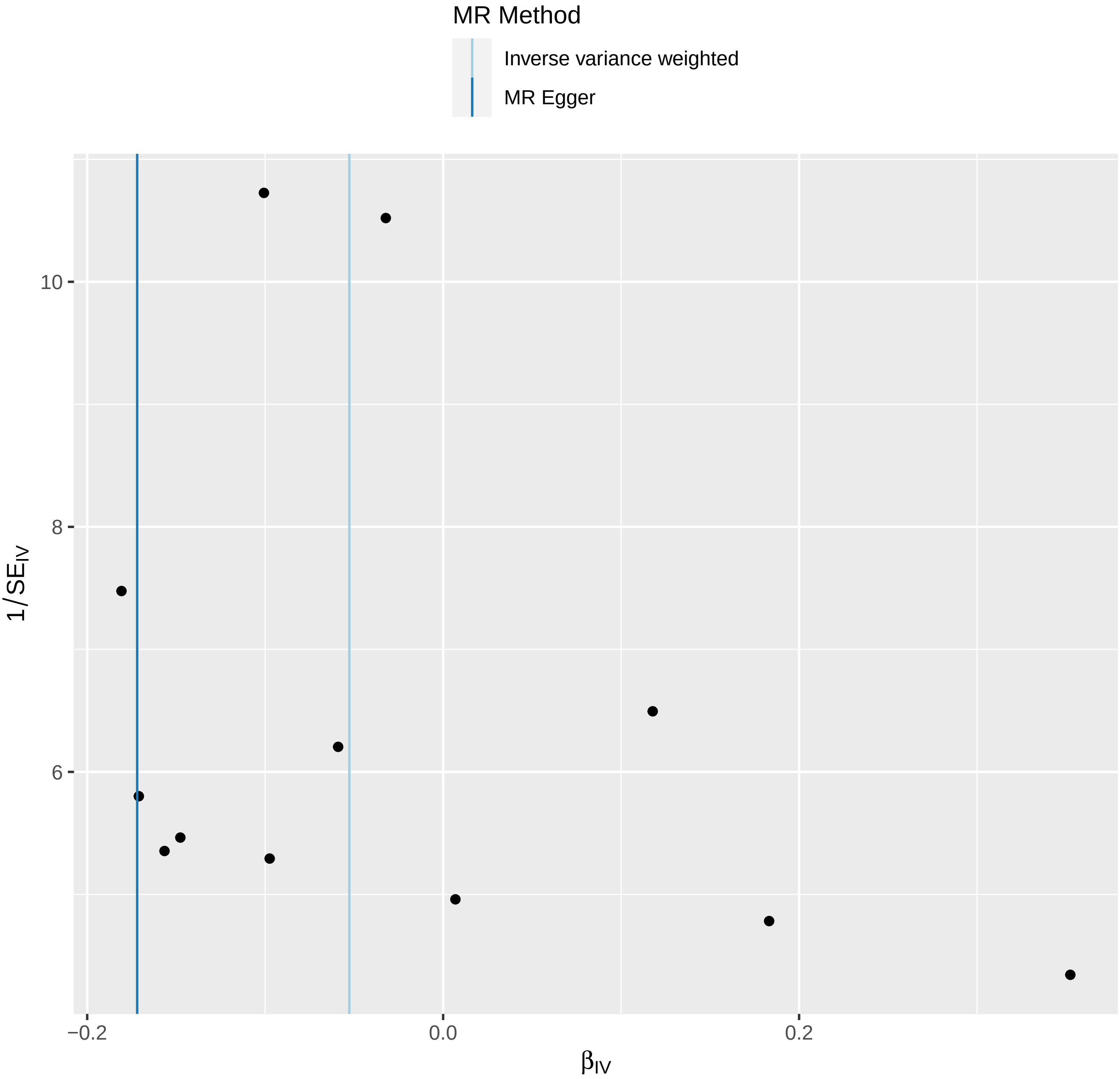

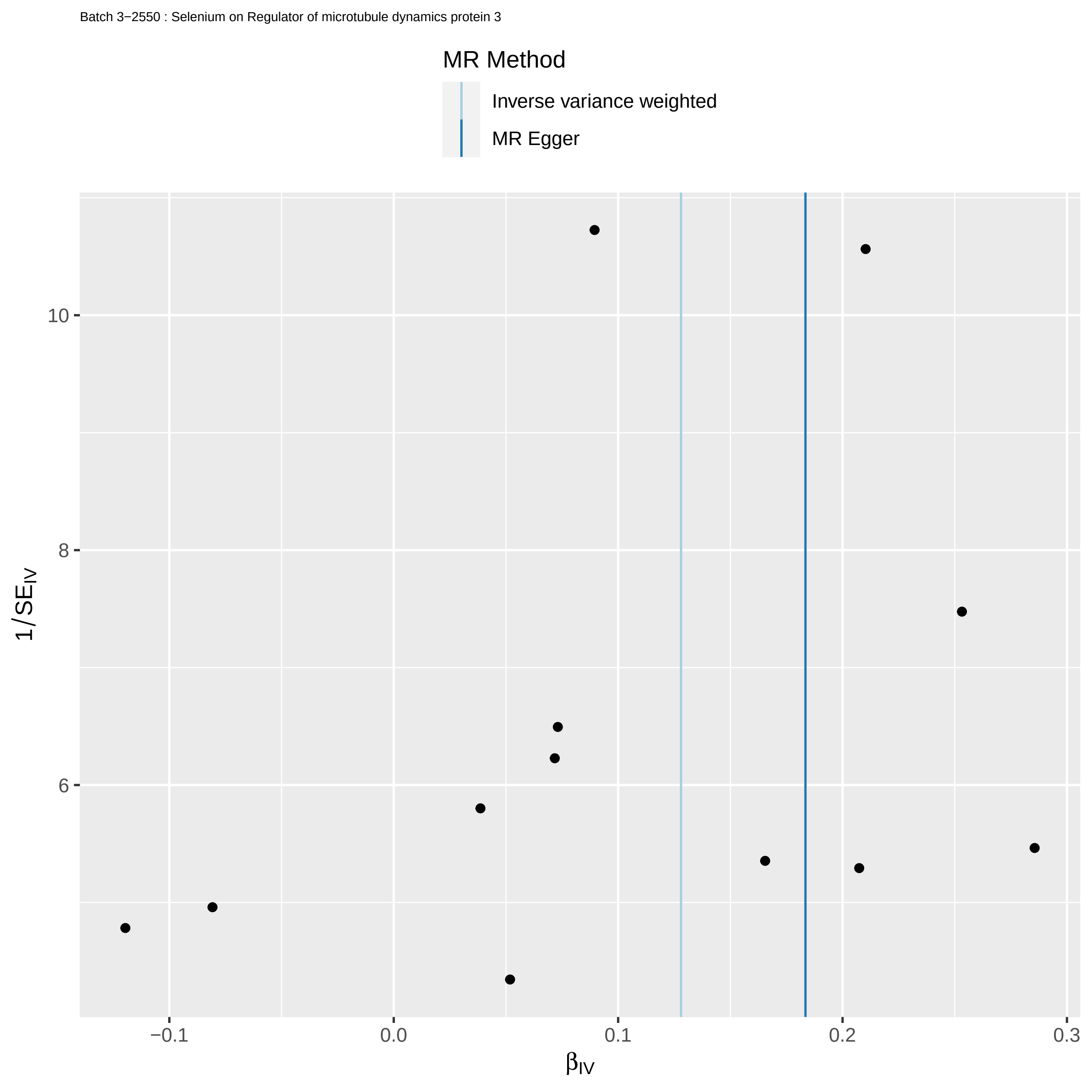

Batch 3−2550 : Selenium on Regulator of microtubule dynamics protein 3
MR Method
Inverse variance weighted
MR Egger
10
8
6
1/SE_IV
−0.1
0.0
0.1
0.2
0.3
β_IV

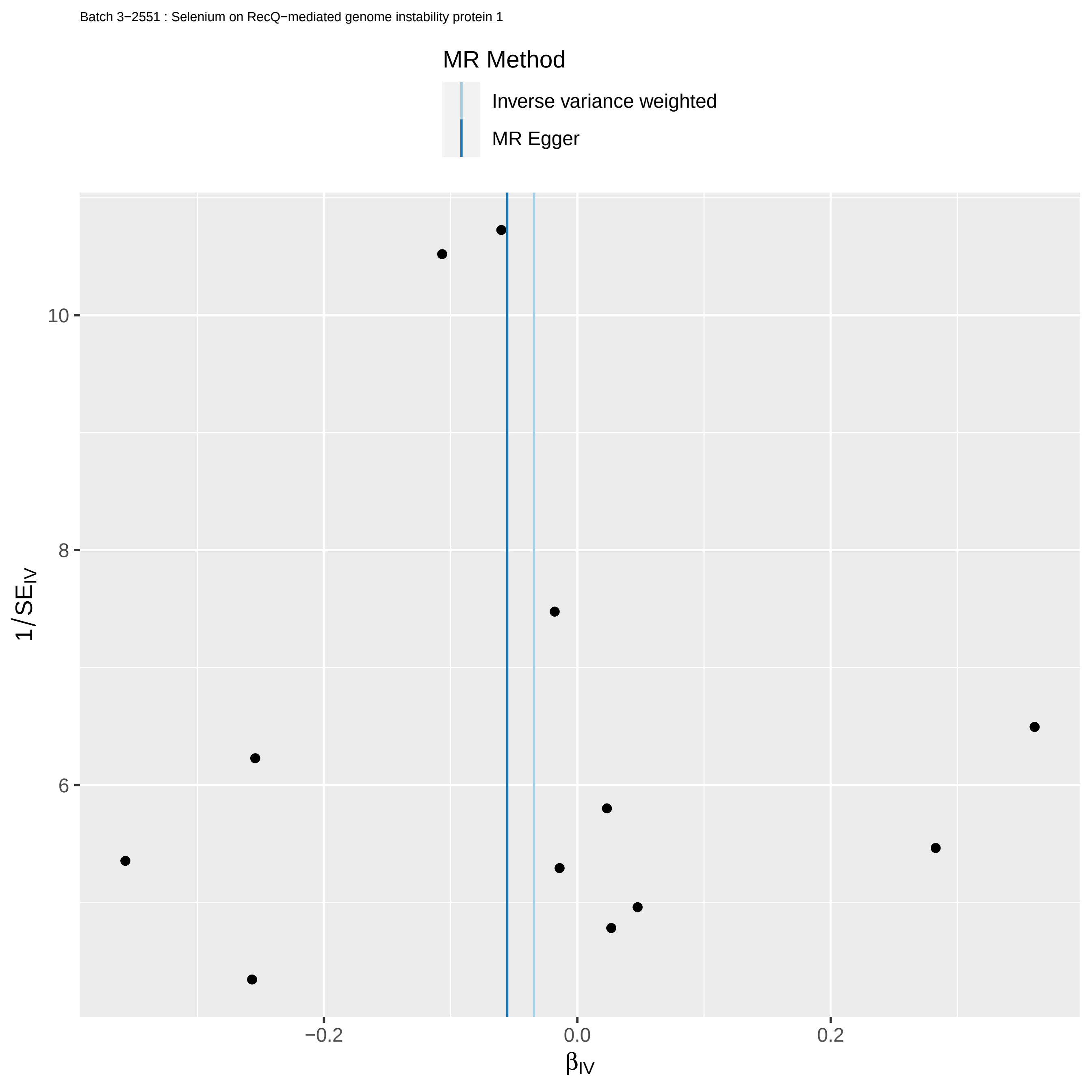

Batch 3−2551 : Selenium on RecQ−mediated genome instability protein 1
MR Method
Inverse variance weighted
MR Egger
10
8
6
1/SE_IV
−0.2
0.0
0.2
β_IV

Batch 3−2552 : Selenium on Inactive ribonuclease−like protein 10

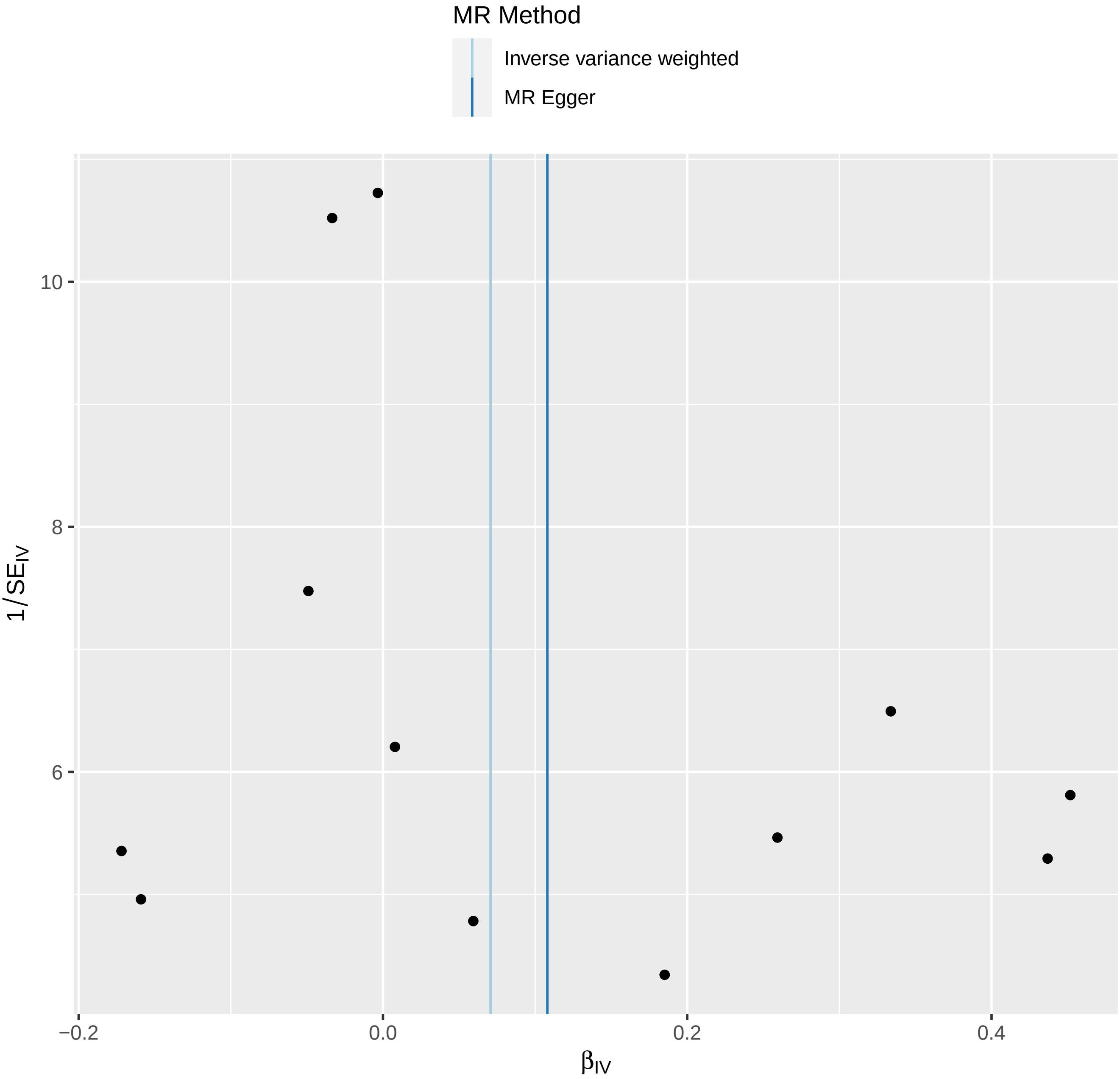

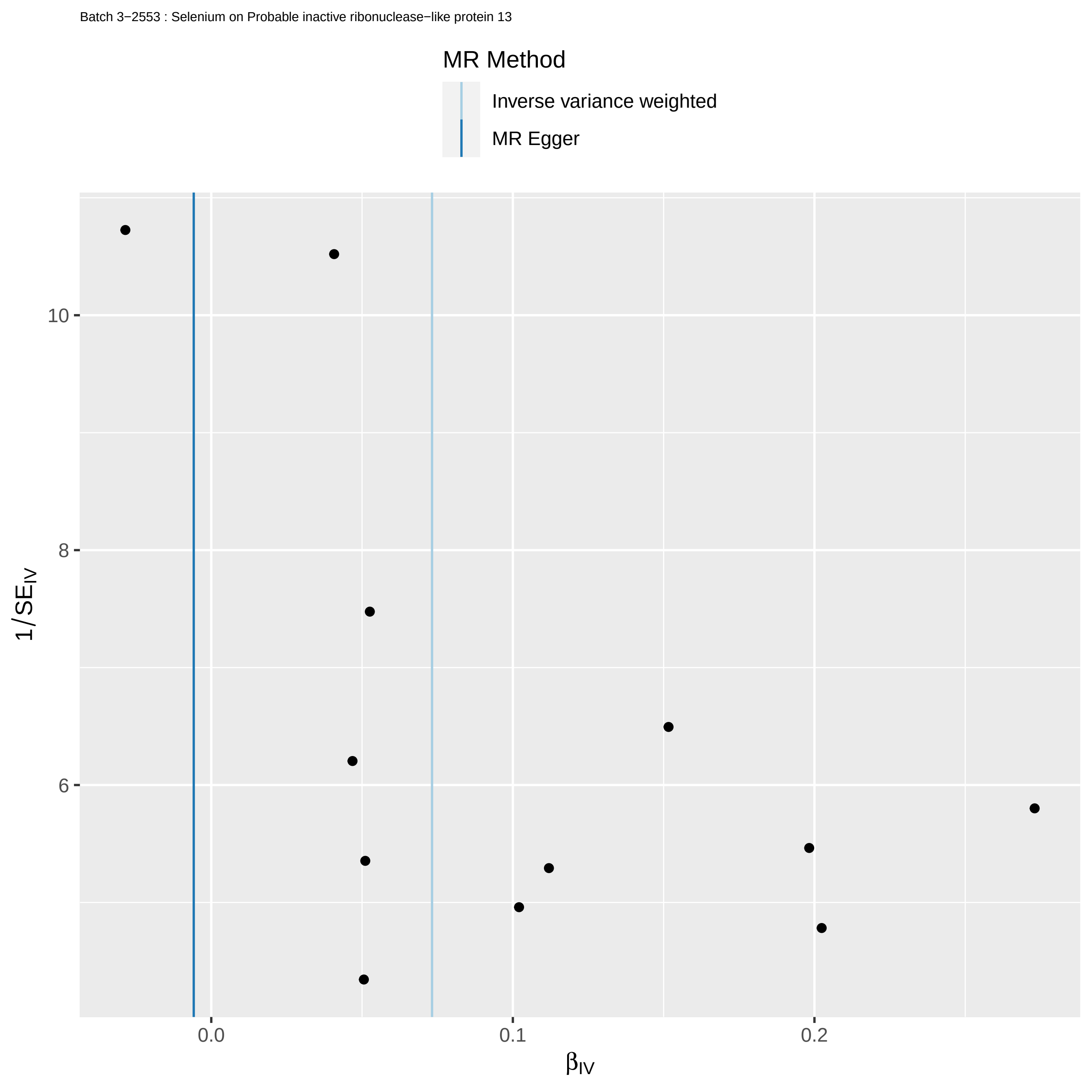
Batch 3−2553 : Selenium on Probable inactive ribonuclease−like protein 13
MR Method
Inverse variance weighted
MR Egger
10
8
6
0.0
0.1
0.2
1/SE_IV
β_IV

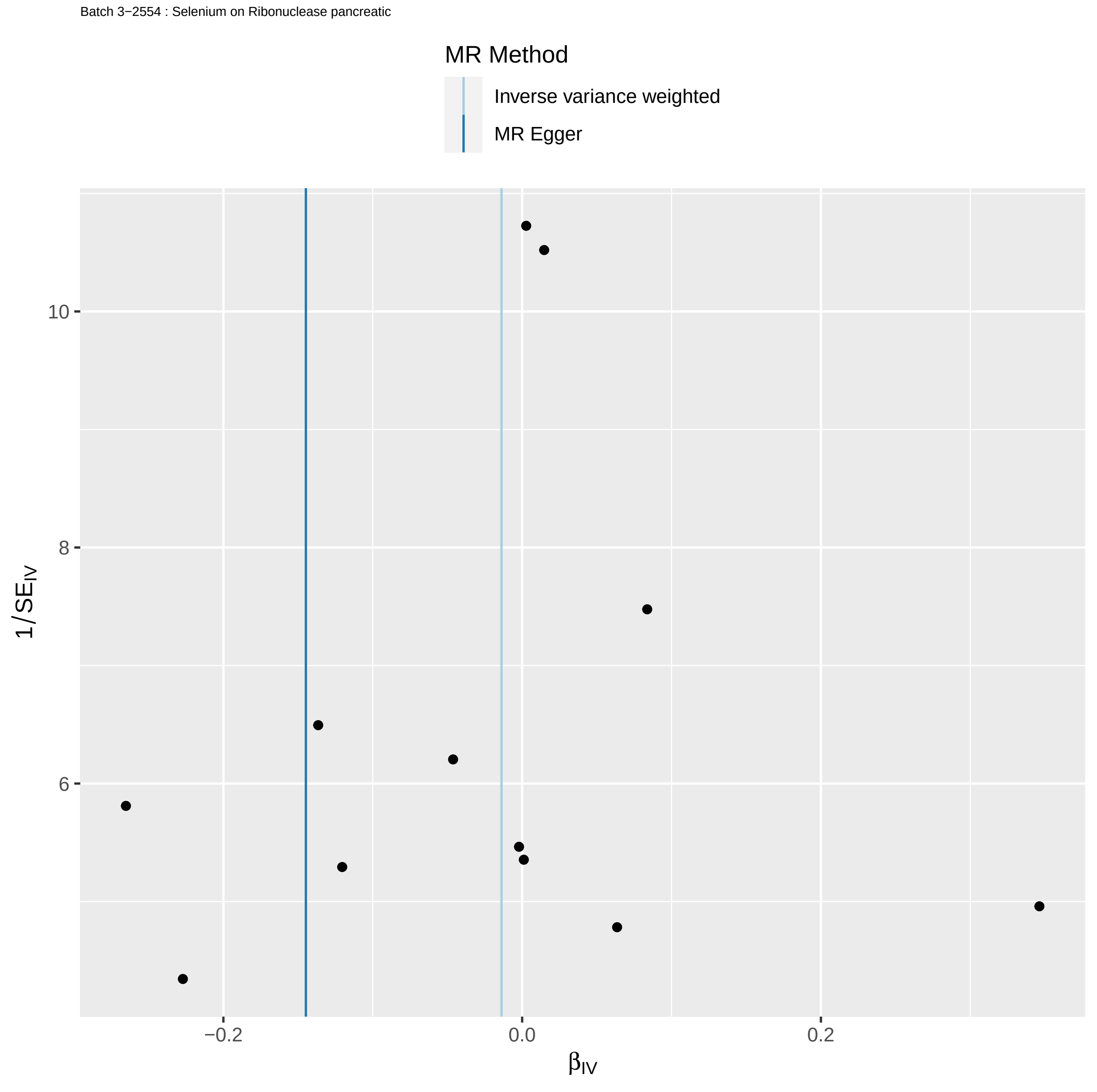
Batch 3−2554 : Selenium on Ribonuclease pancreatic
MR Method
Inverse variance weighted
MR Egger
10
8
6
1/SE$_{IV}$
−0.2
0.0
0.2
β$_{IV}$

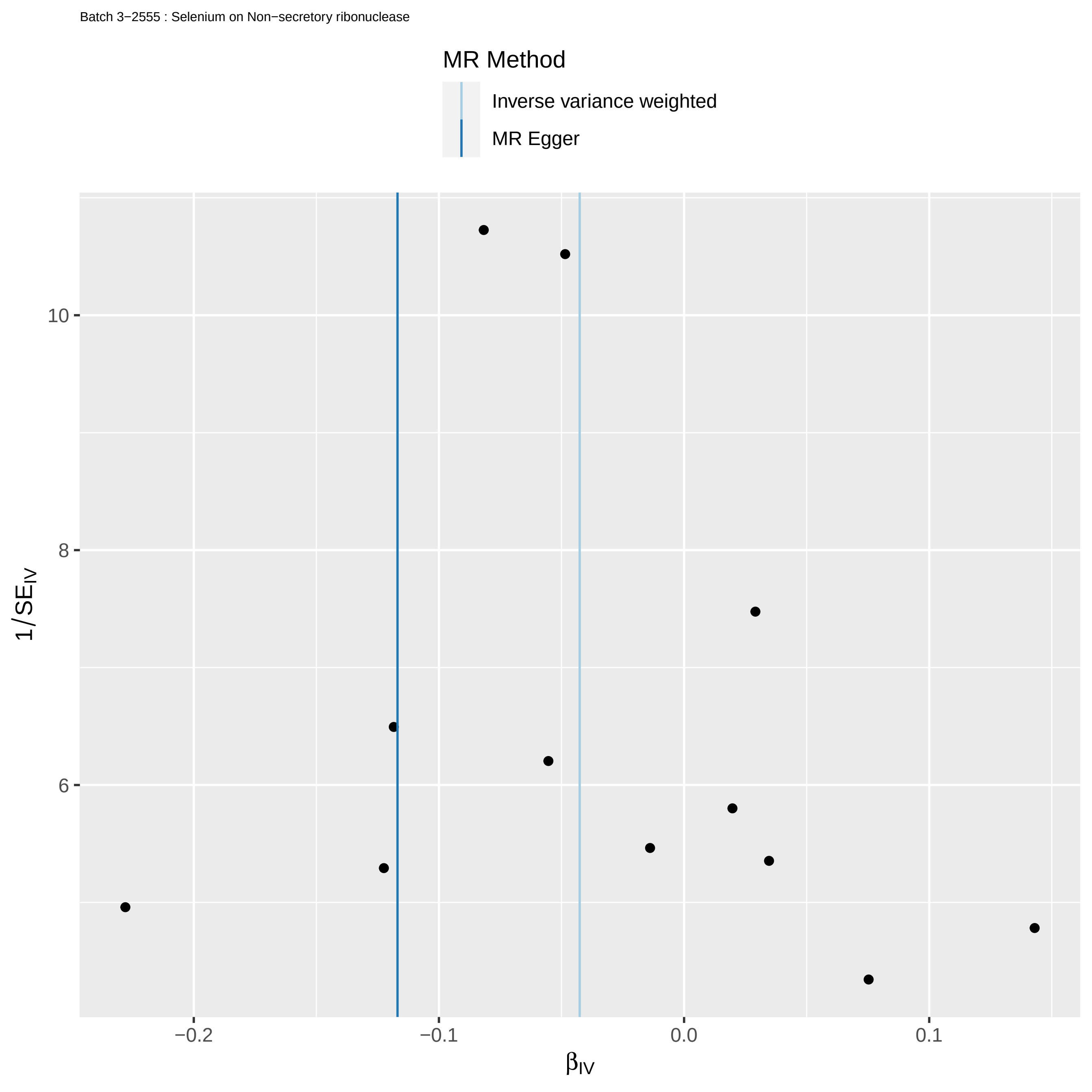

Batch 3−2555 : Selenium on Non−secretory ribonuclease
MR Method
Inverse variance weighted
MR Egger
10
8
6
1/SE_IV
−0.2
−0.1
0.0
0.1
β_IV

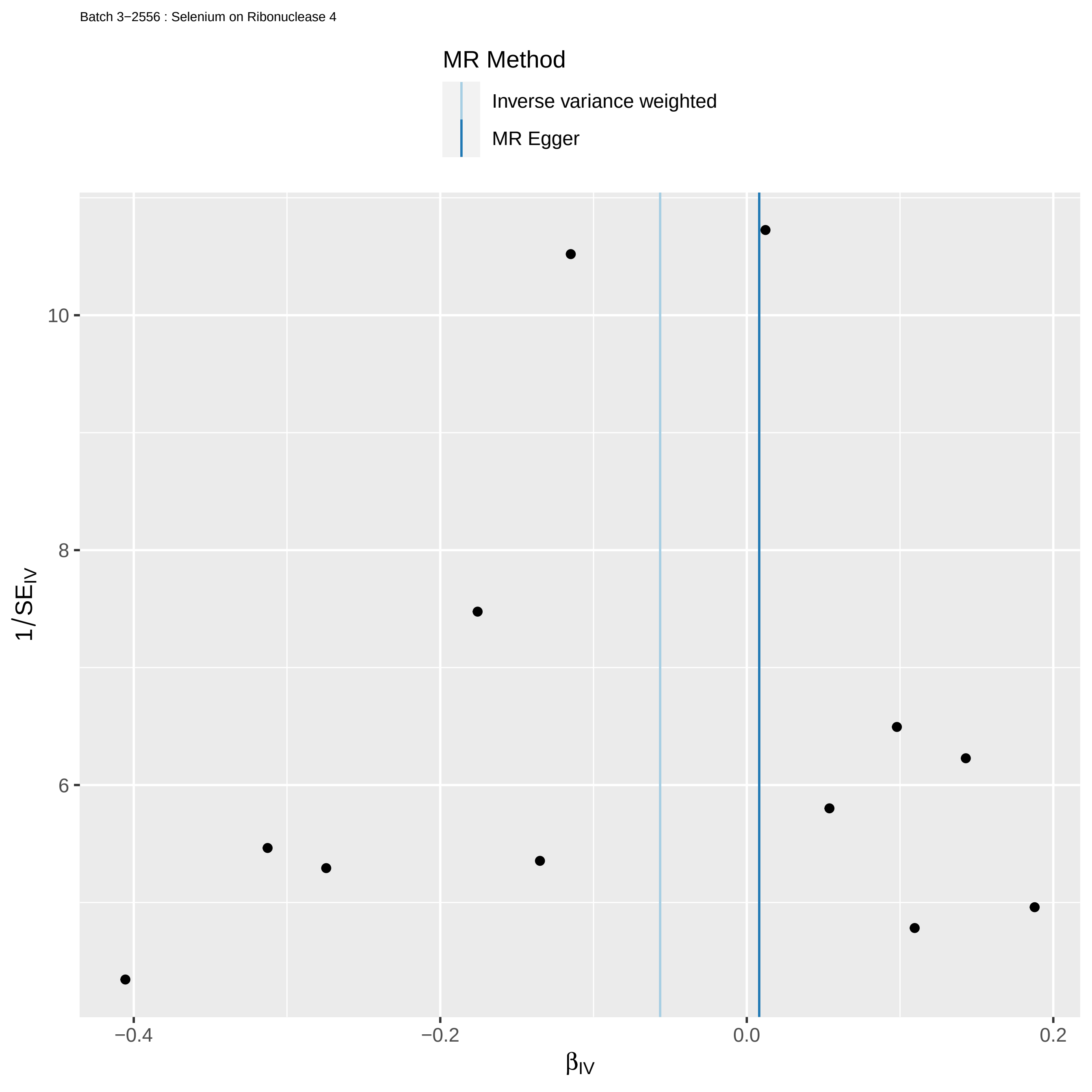
Batch 3−2556 : Selenium on Ribonuclease 4
MR Method
Inverse variance weighted
MR Egger
10
8
6
1/SE_IV
−0.4
−0.2
0.0
0.2
β_IV

Batch 3−2557 : Selenium on Ribonuclease K6

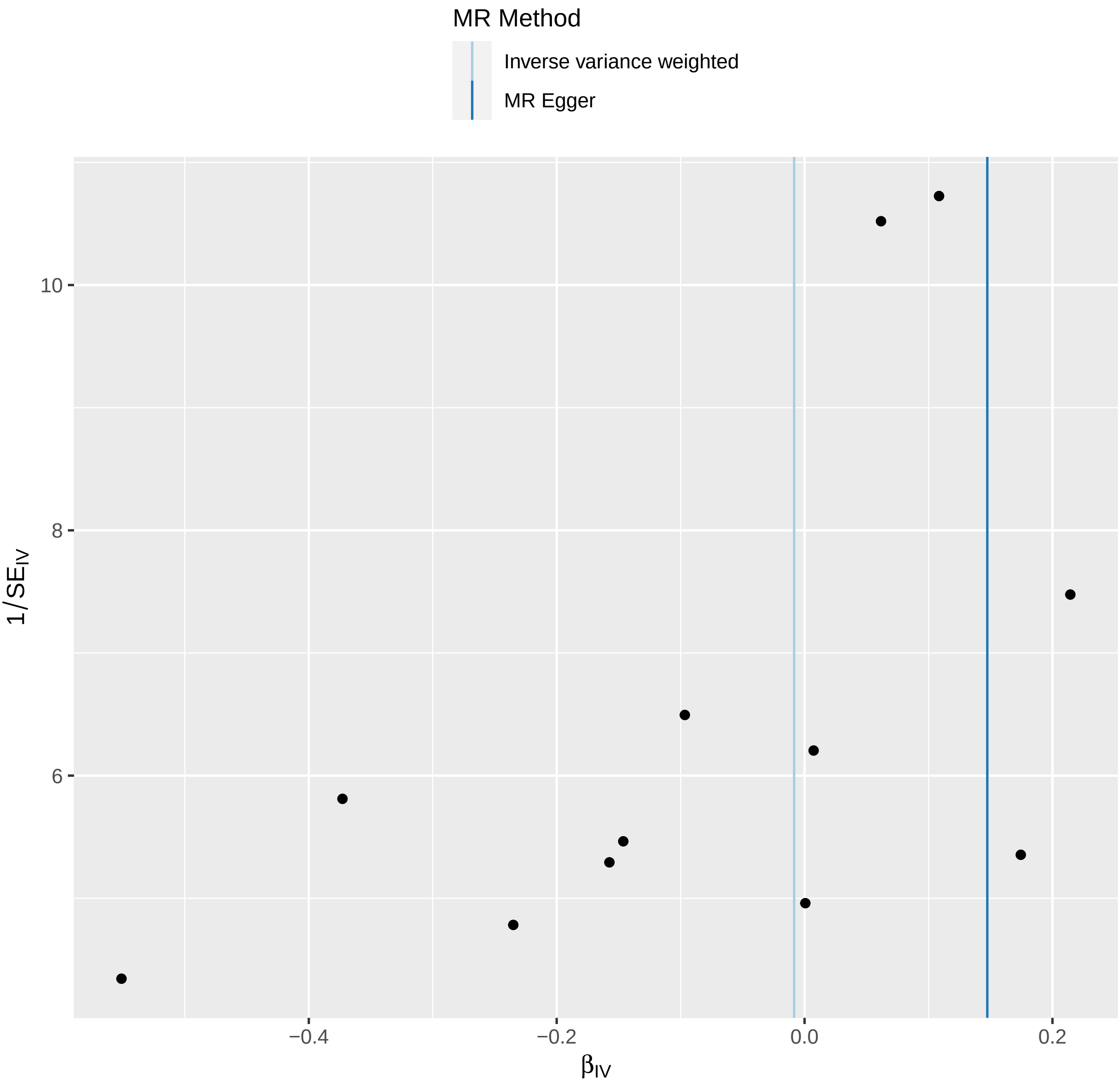

Batch 3−2558 : Selenium on Ribonuclease H1

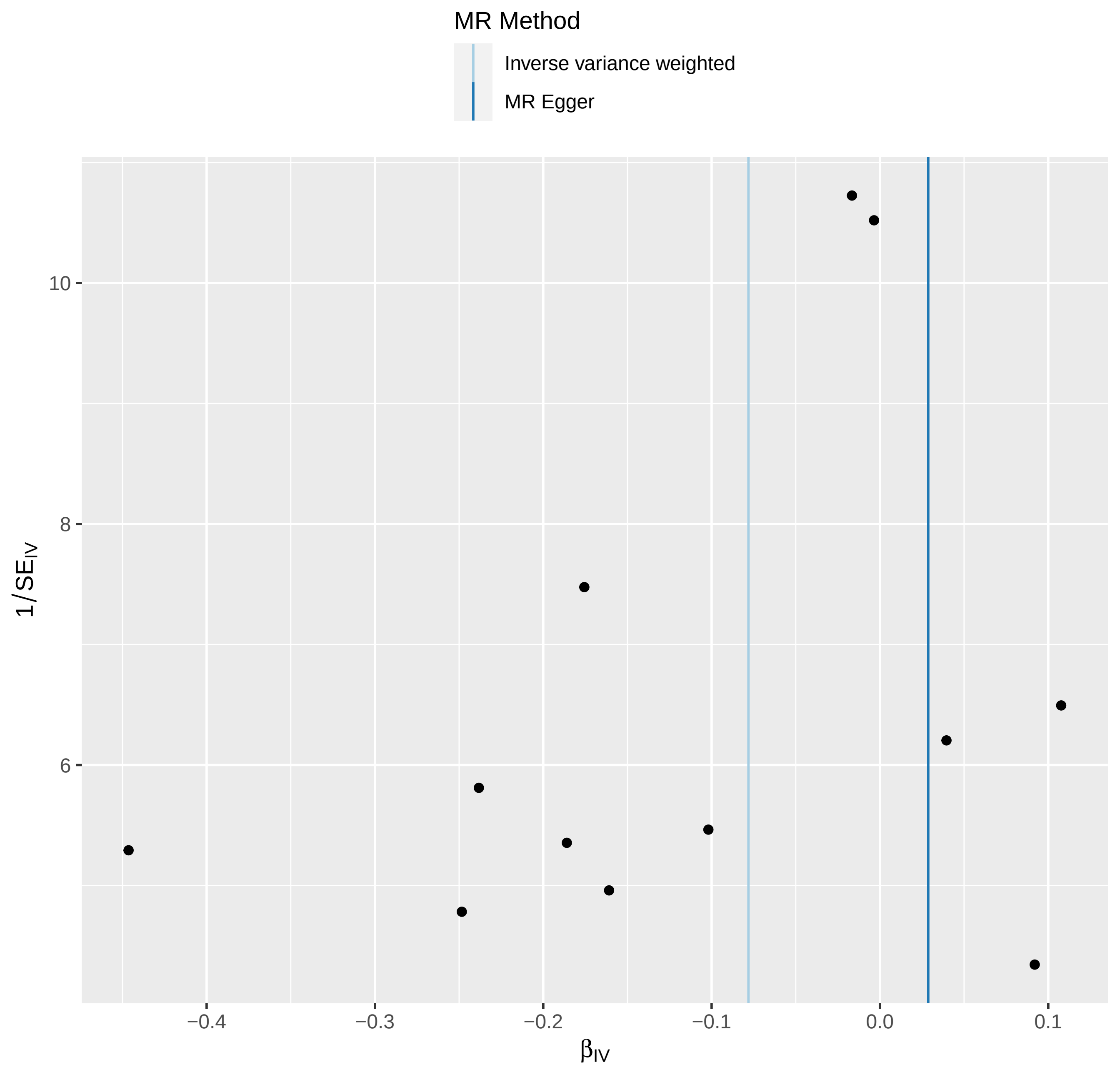

Batch 3−2559 : Selenium on Rho−related GTP−binding protein Rho6

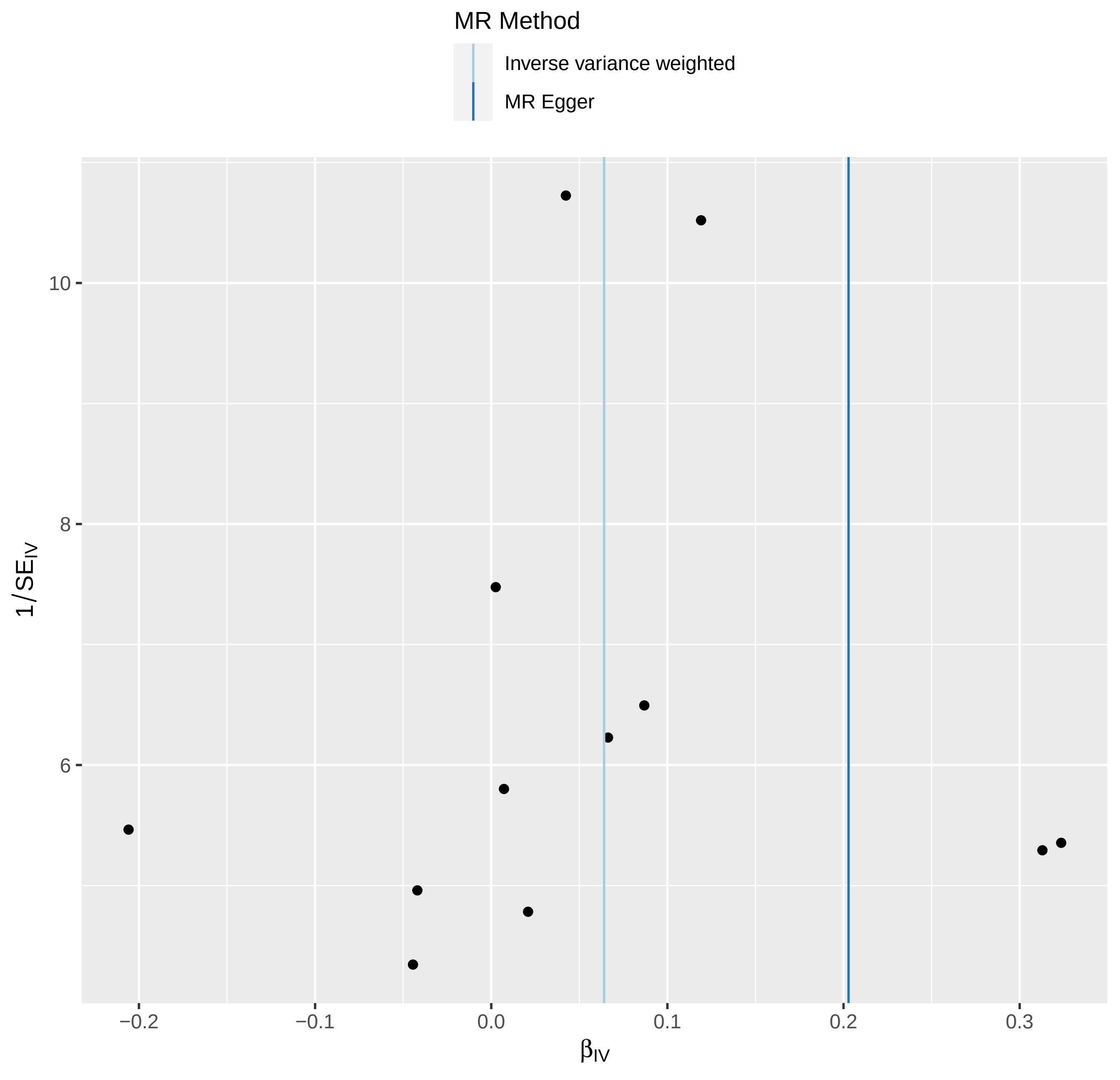

Batch 3−2560 : Selenium on E3 ubiquitin−protein ligase RNF114

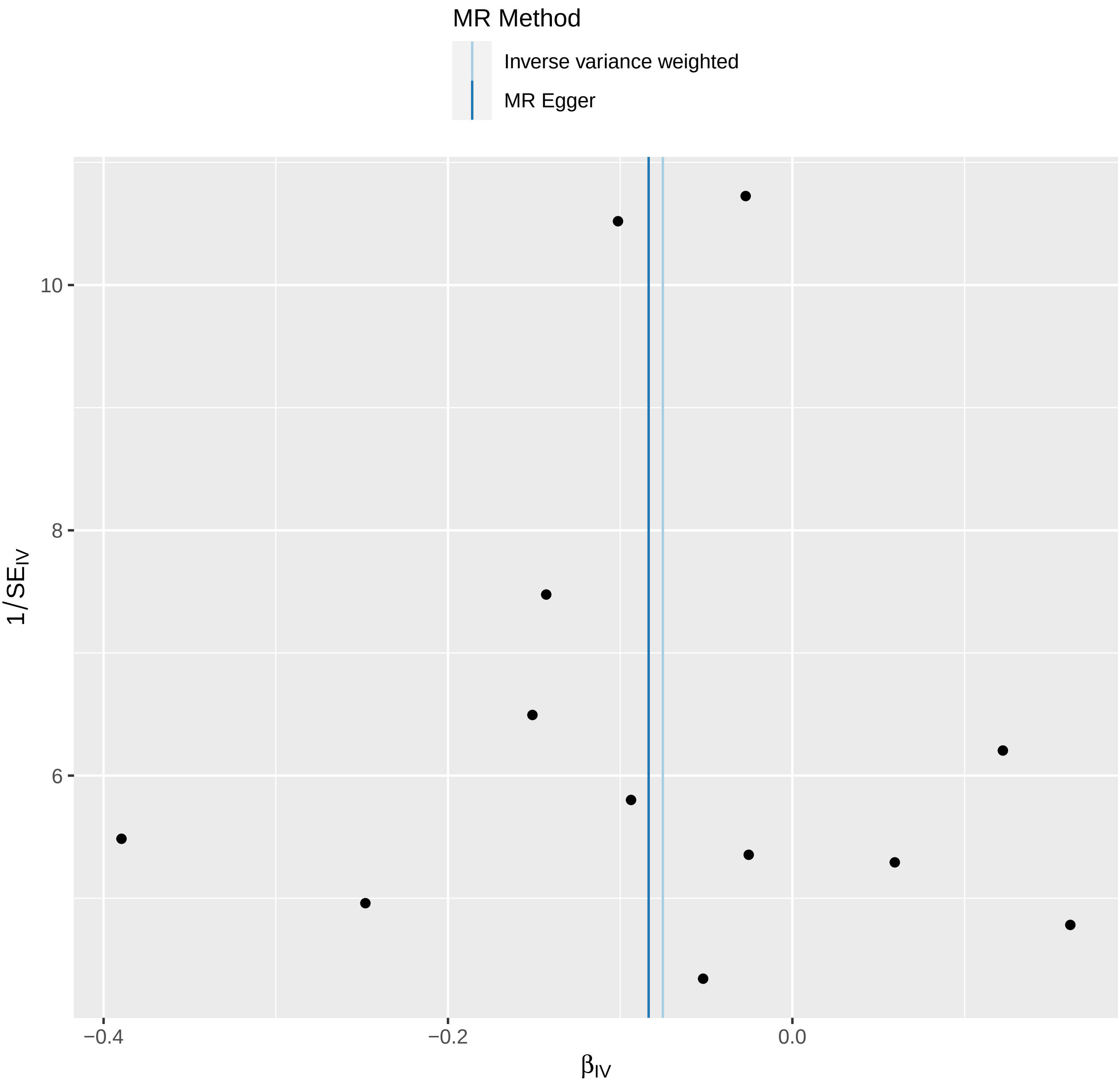

Batch 3−2561 : Selenium on RING finger protein 122

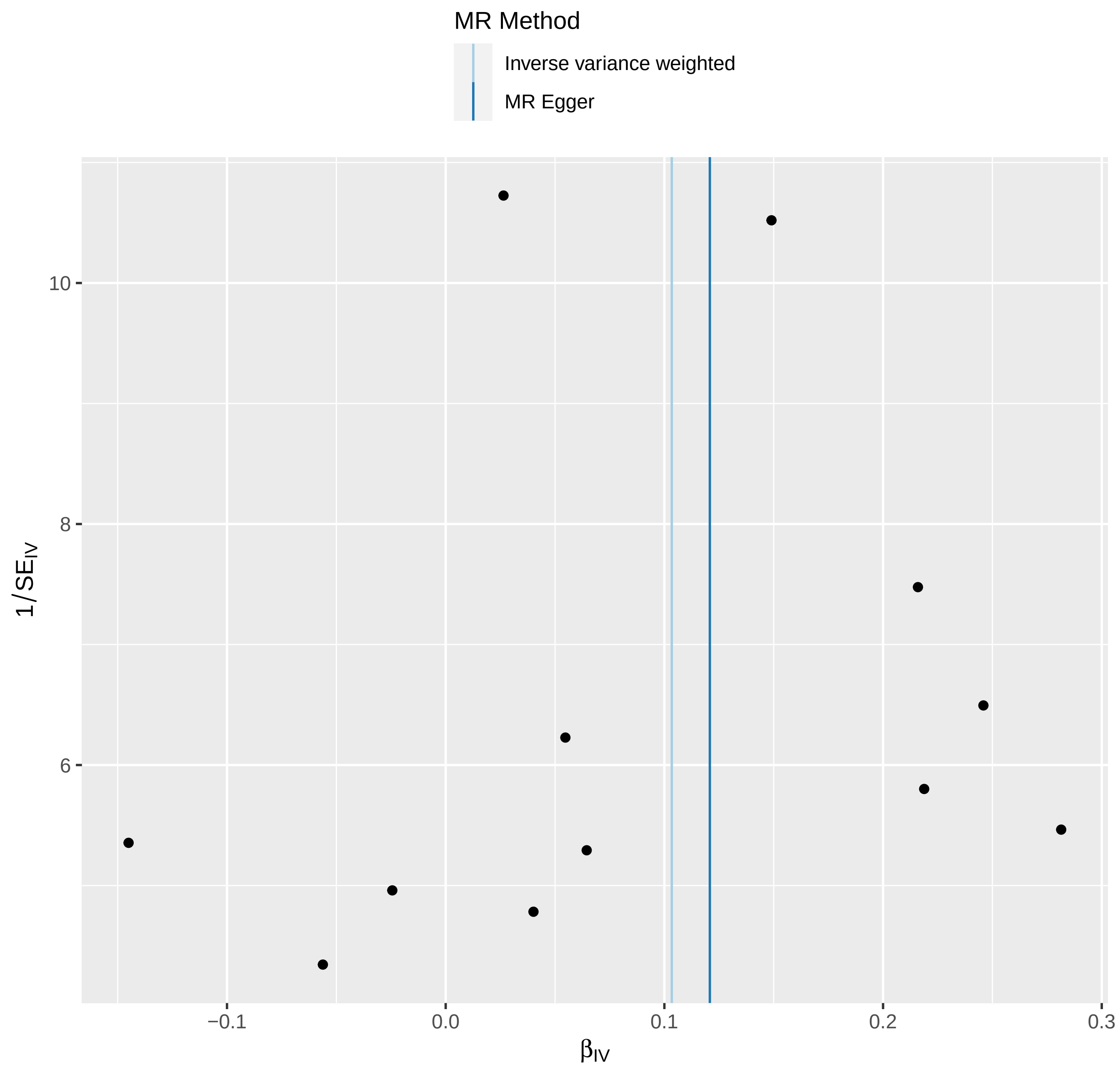

Batch 3−2562 : Selenium on E3 ubiquitin−protein ligase RNF128

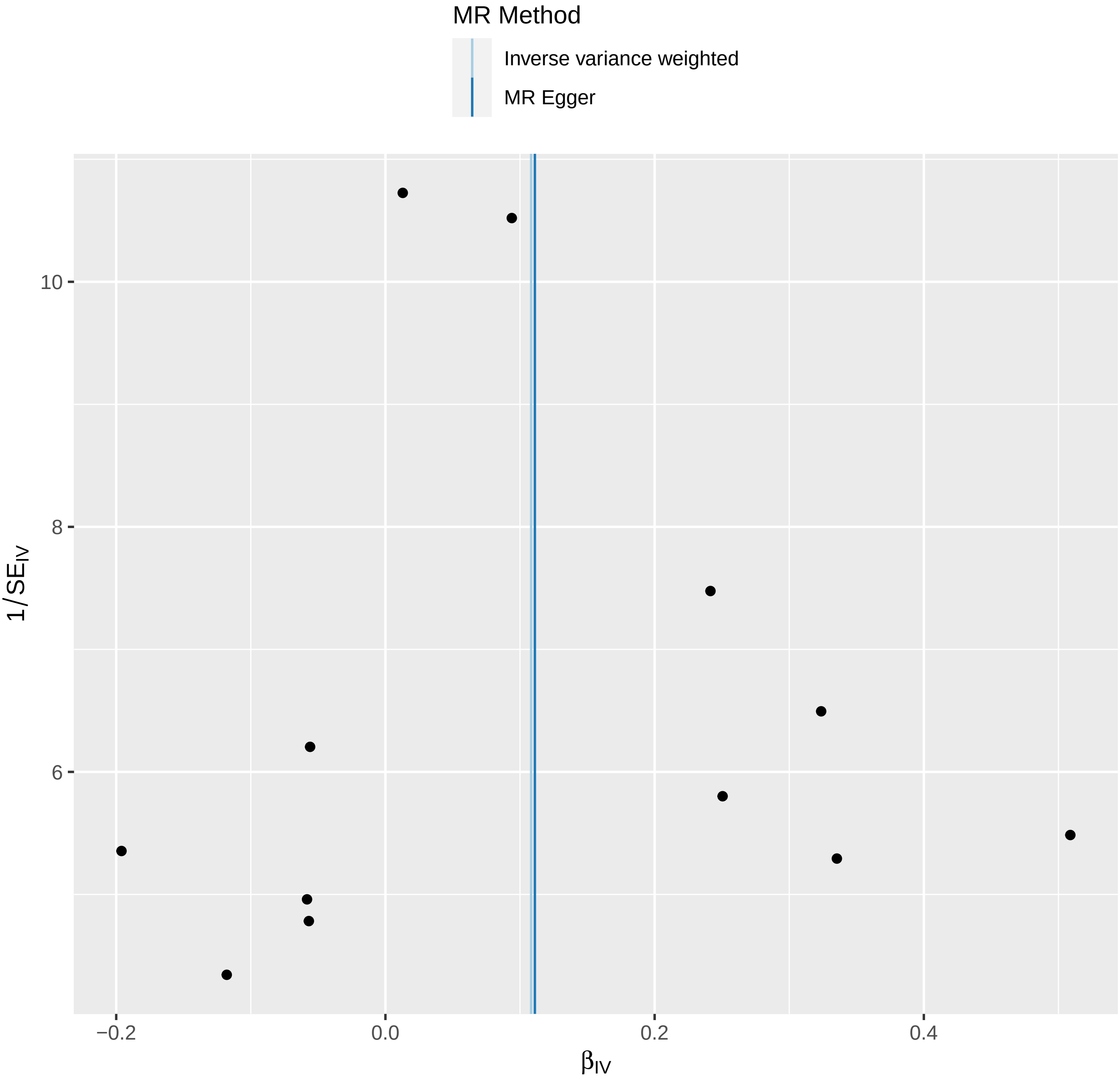

Batch 3−2563 : Selenium on E3 ubiquitin−protein ligase RNF13

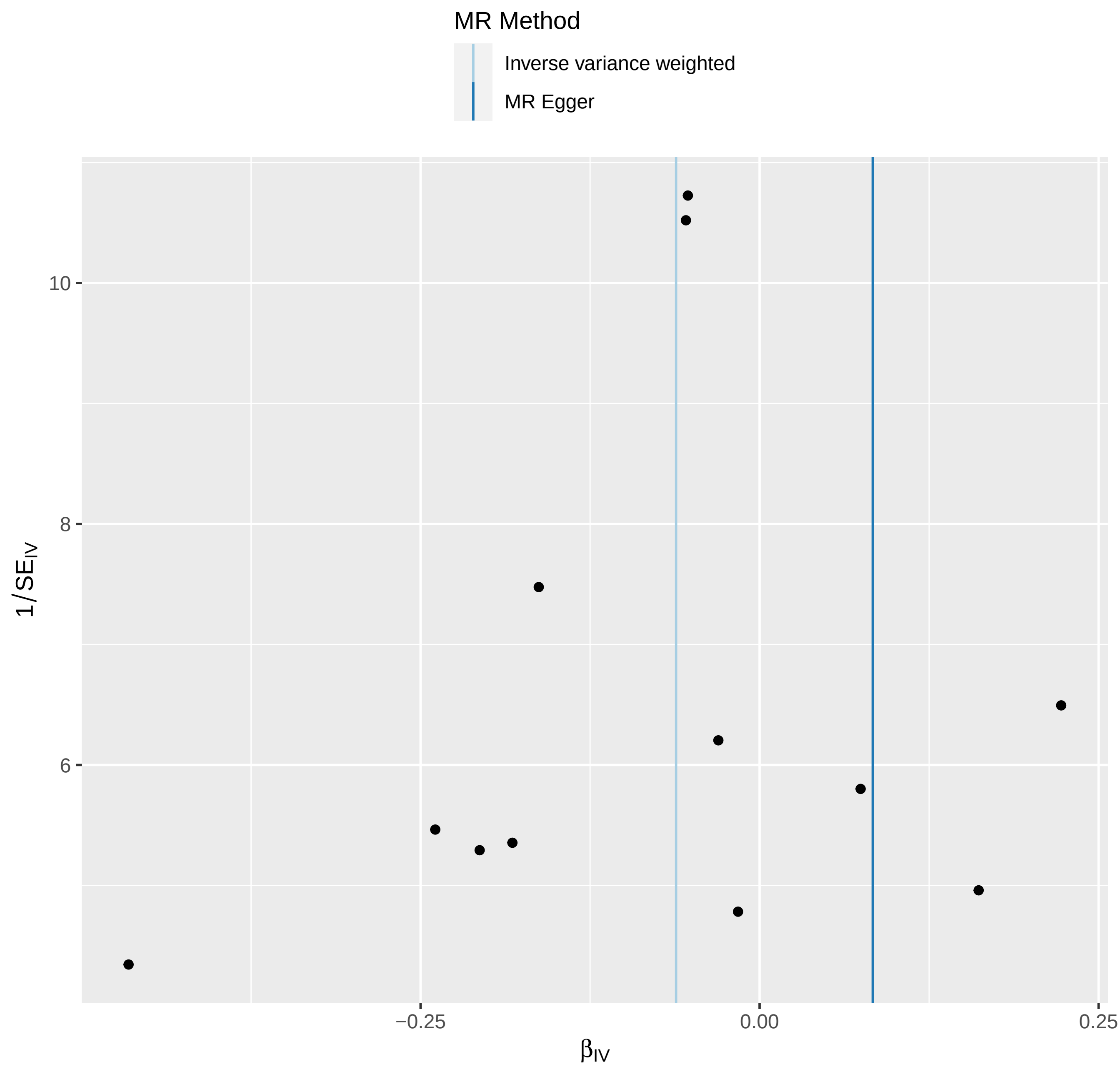

Batch 3−2564 : Selenium on RING finger protein 148

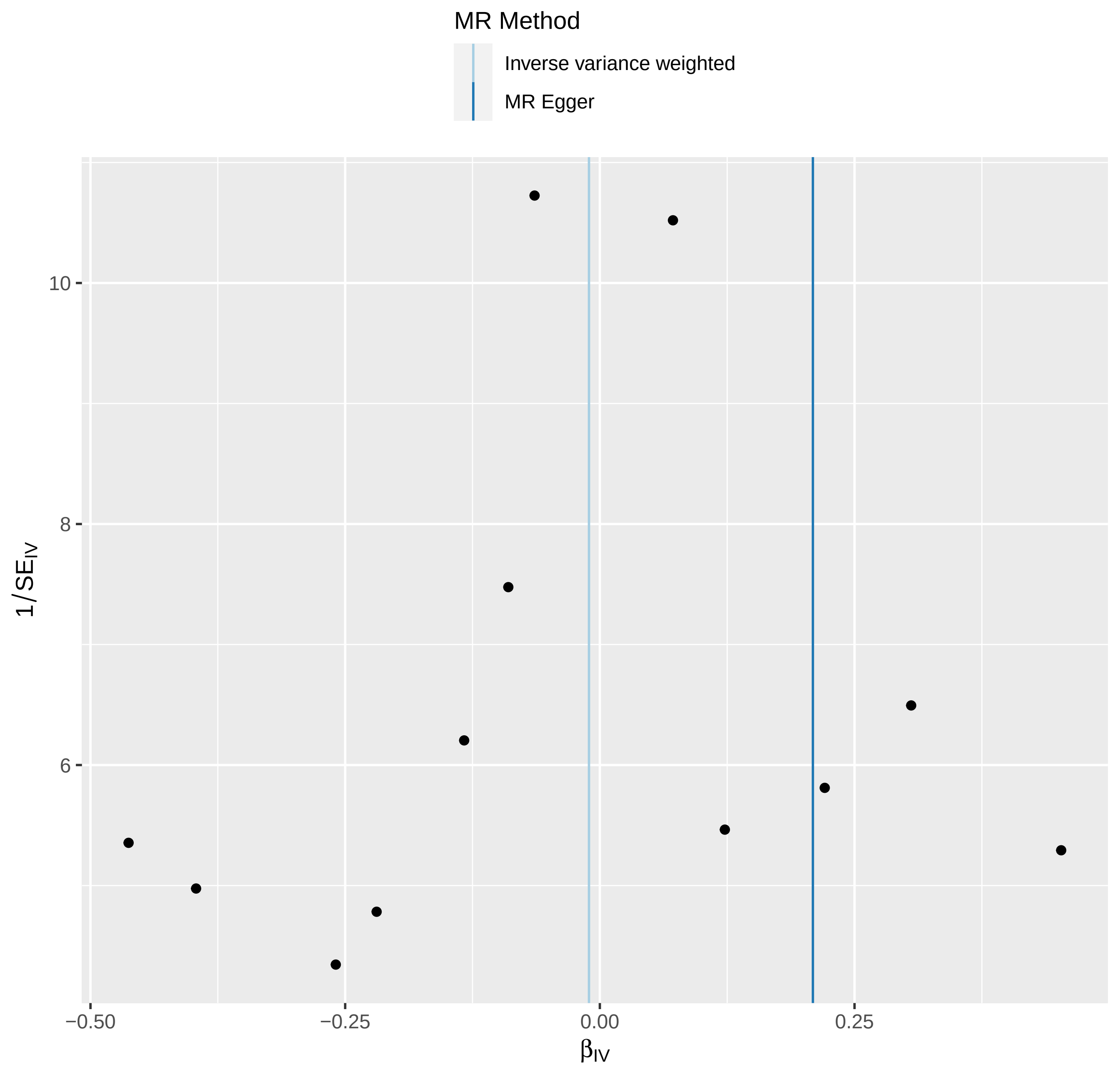

Batch 3−2565 : Selenium on RING finger protein 148

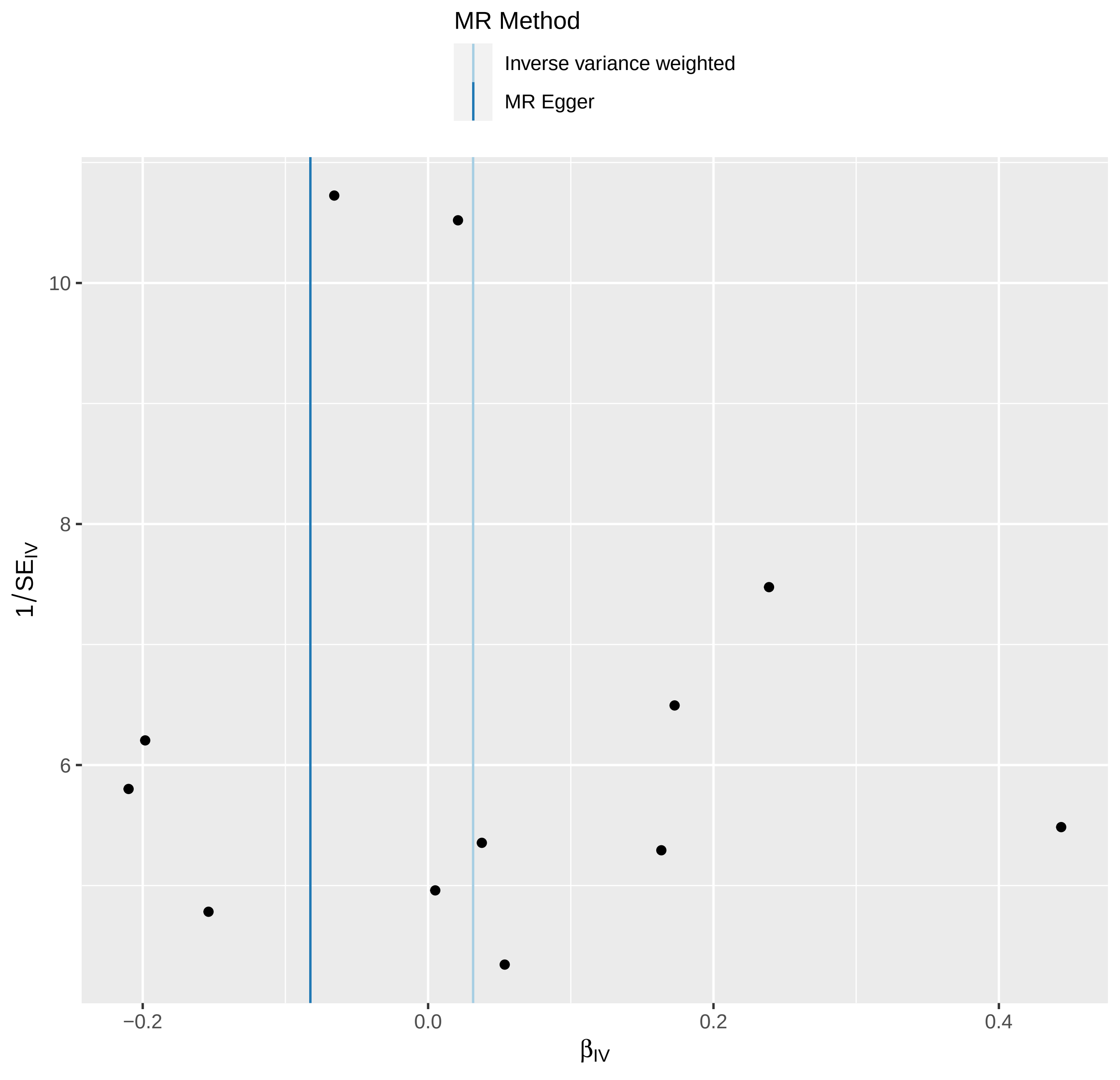

Batch 3−2566 : Selenium on E3 ubiquitin−protein ligase RNF149

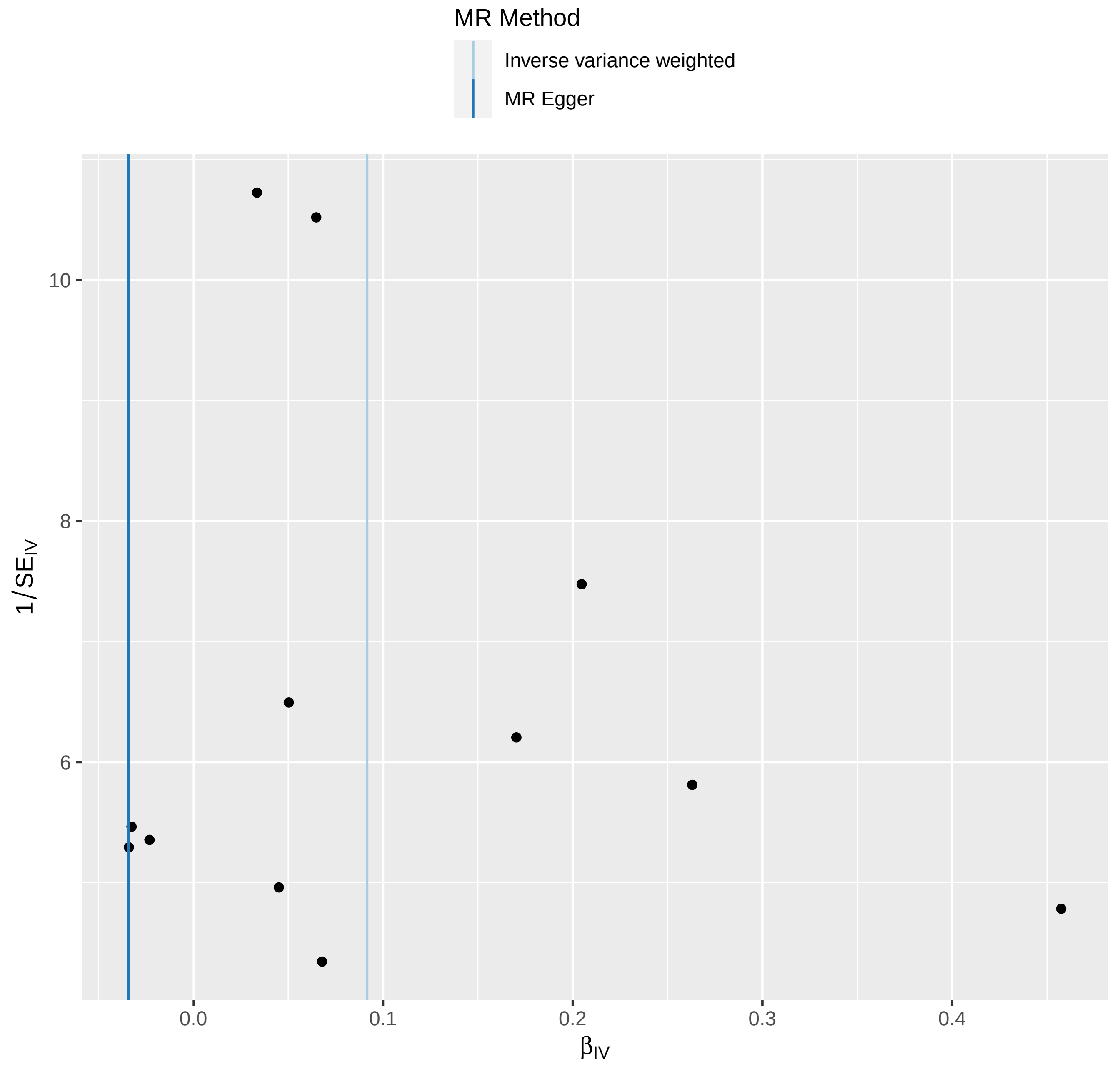

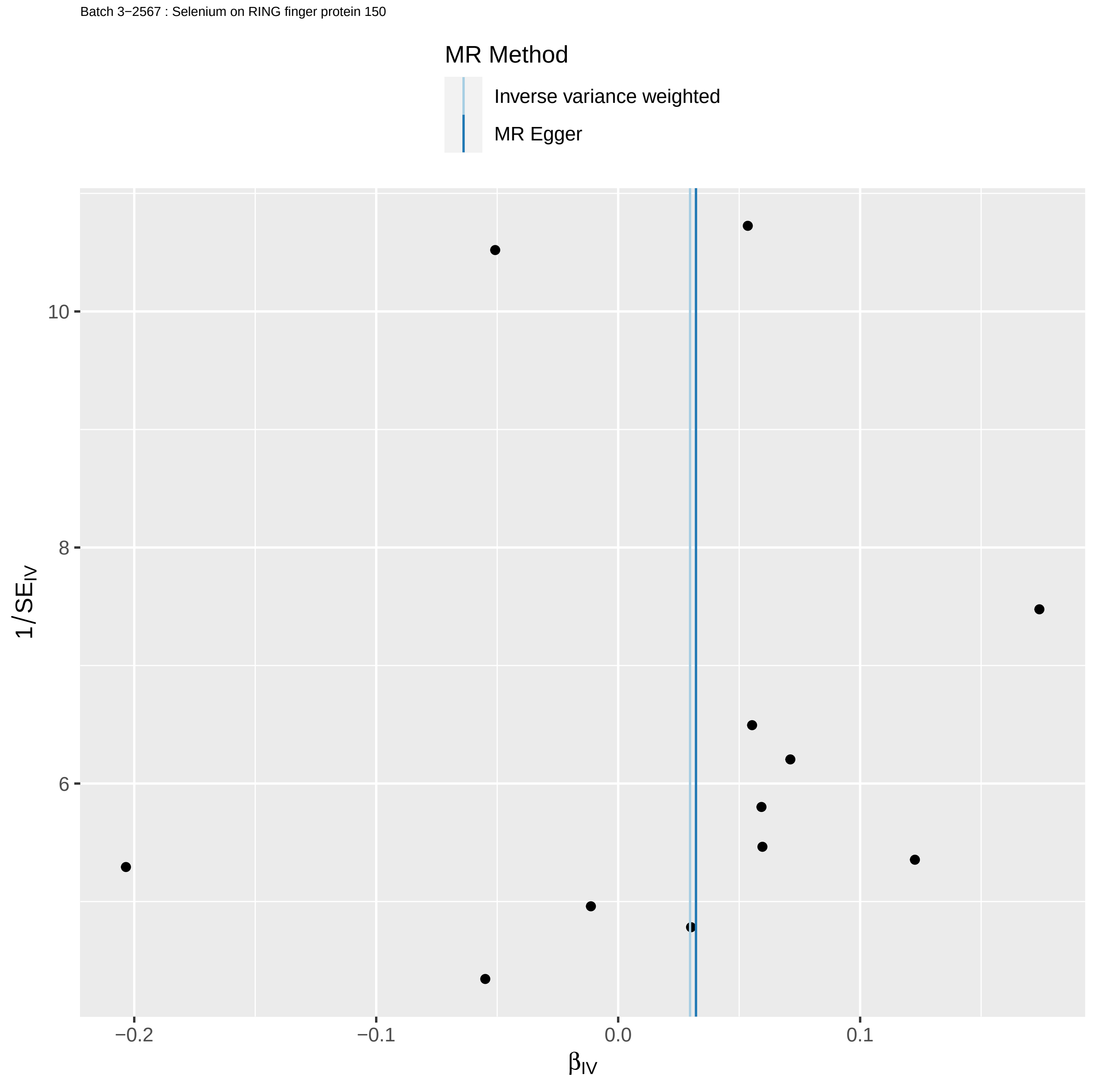
Batch 3−2567 : Selenium on RING finger protein 150
MR Method
Inverse variance weighted
MR Egger
10
8
6
1/SE_IV
−0.2
−0.1
0.0
0.1
β_IV

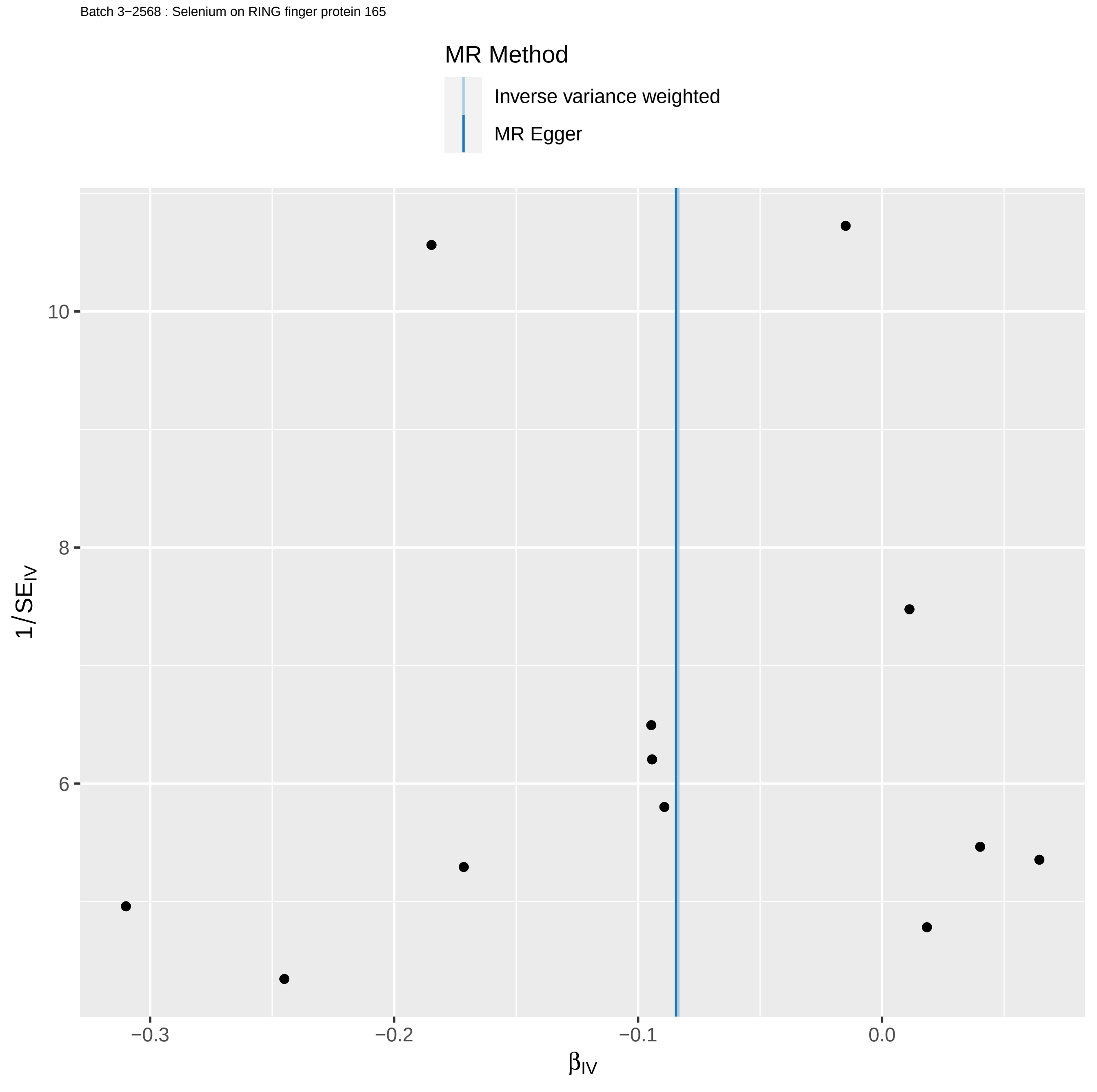

Batch 3−2568 : Selenium on RING finger protein 165
MR Method
Inverse variance weighted
MR Egger
10
8
6
1/SE_IV
−0.3
−0.2
−0.1
0.0
β_IV

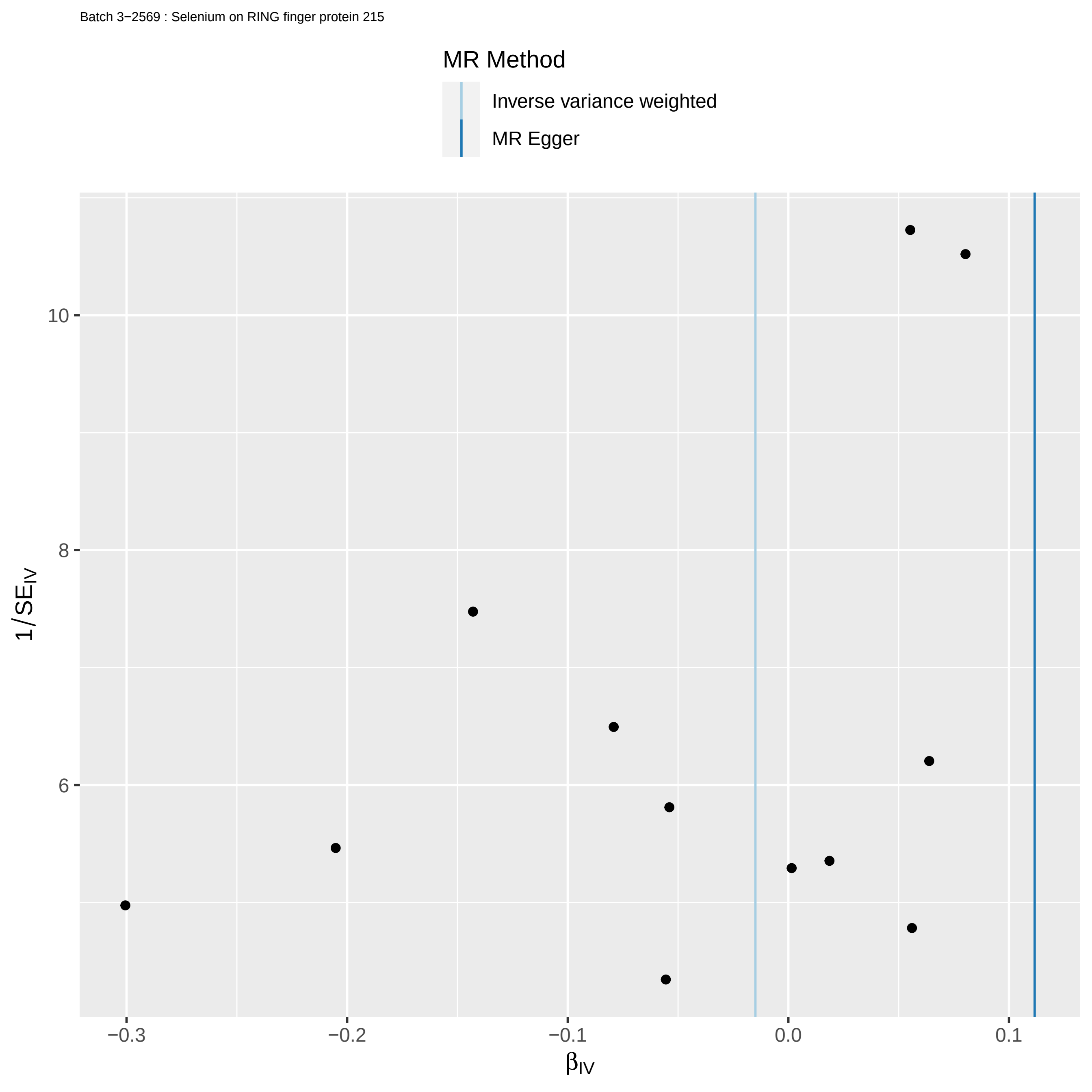

Batch 3−2569 : Selenium on RING finger protein 215
MR Method
Inverse variance weighted
MR Egger
10
8
6
1/SE$_{IV}$
−0.3
−0.2
−0.1
0.0
0.1
β$_{IV}$

Batch 3−2570 : Selenium on RING finger protein 219

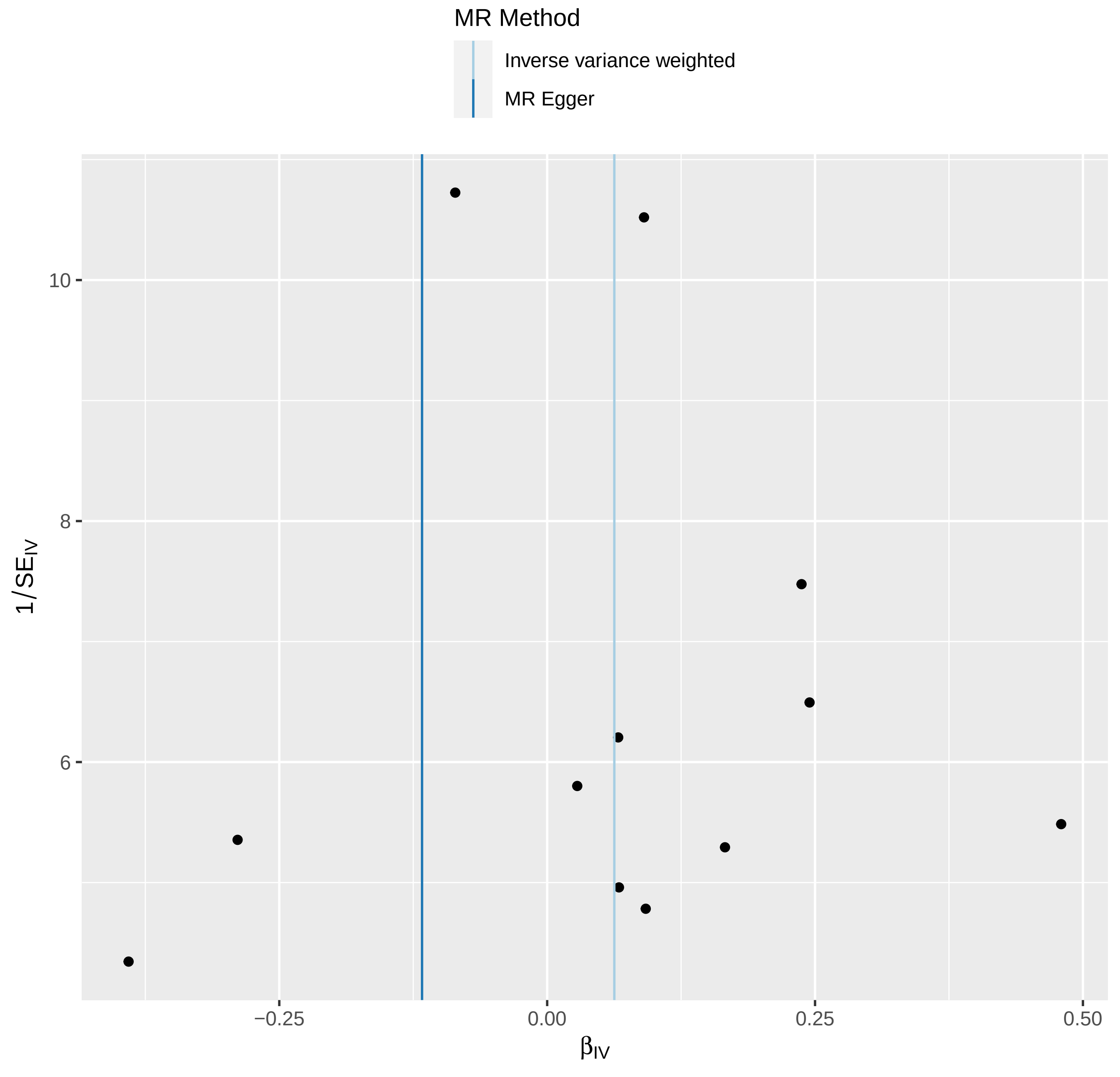

Batch 3−2571 : Selenium on E3 ubiquitin−protein ligase RNF34

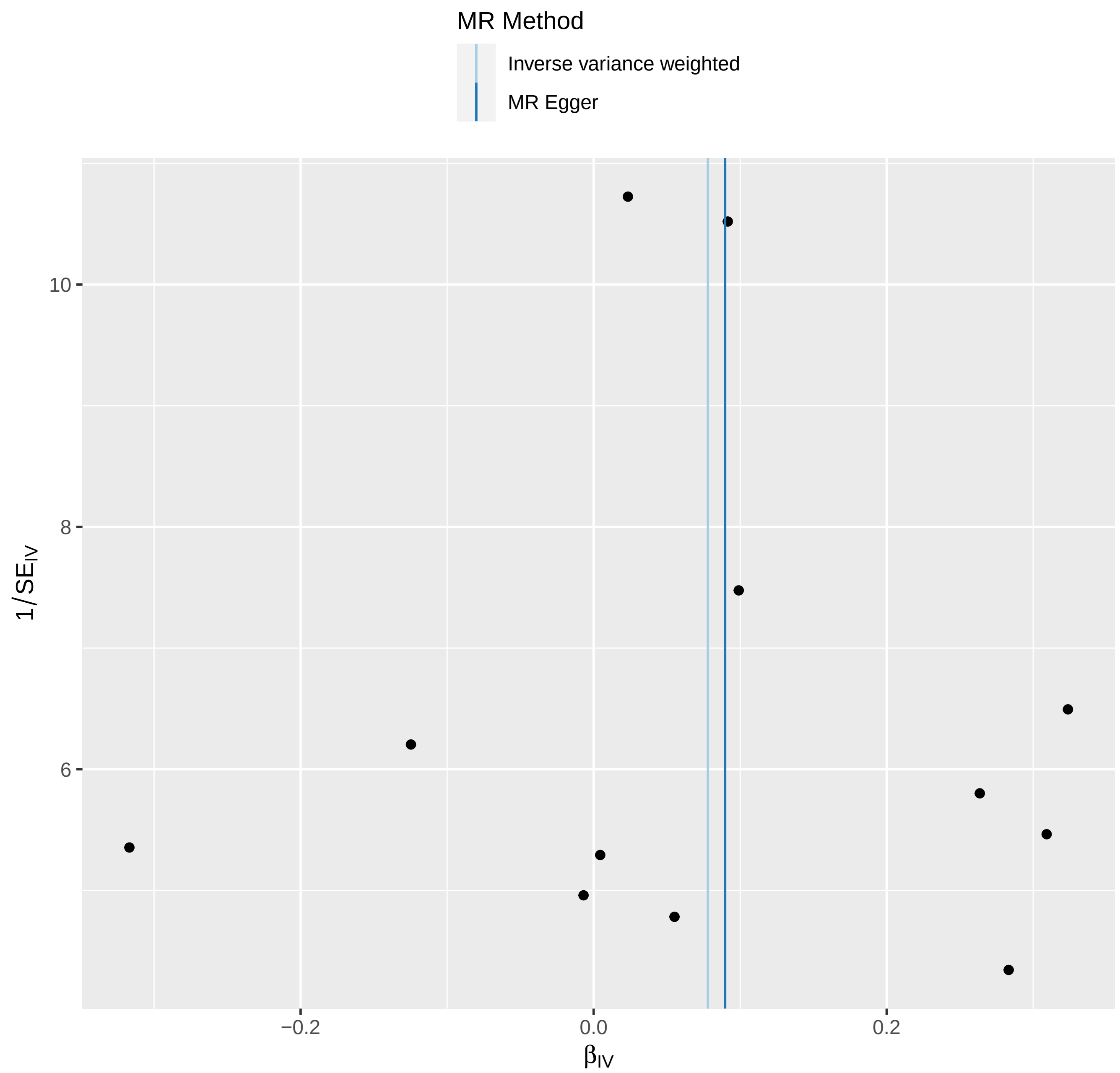

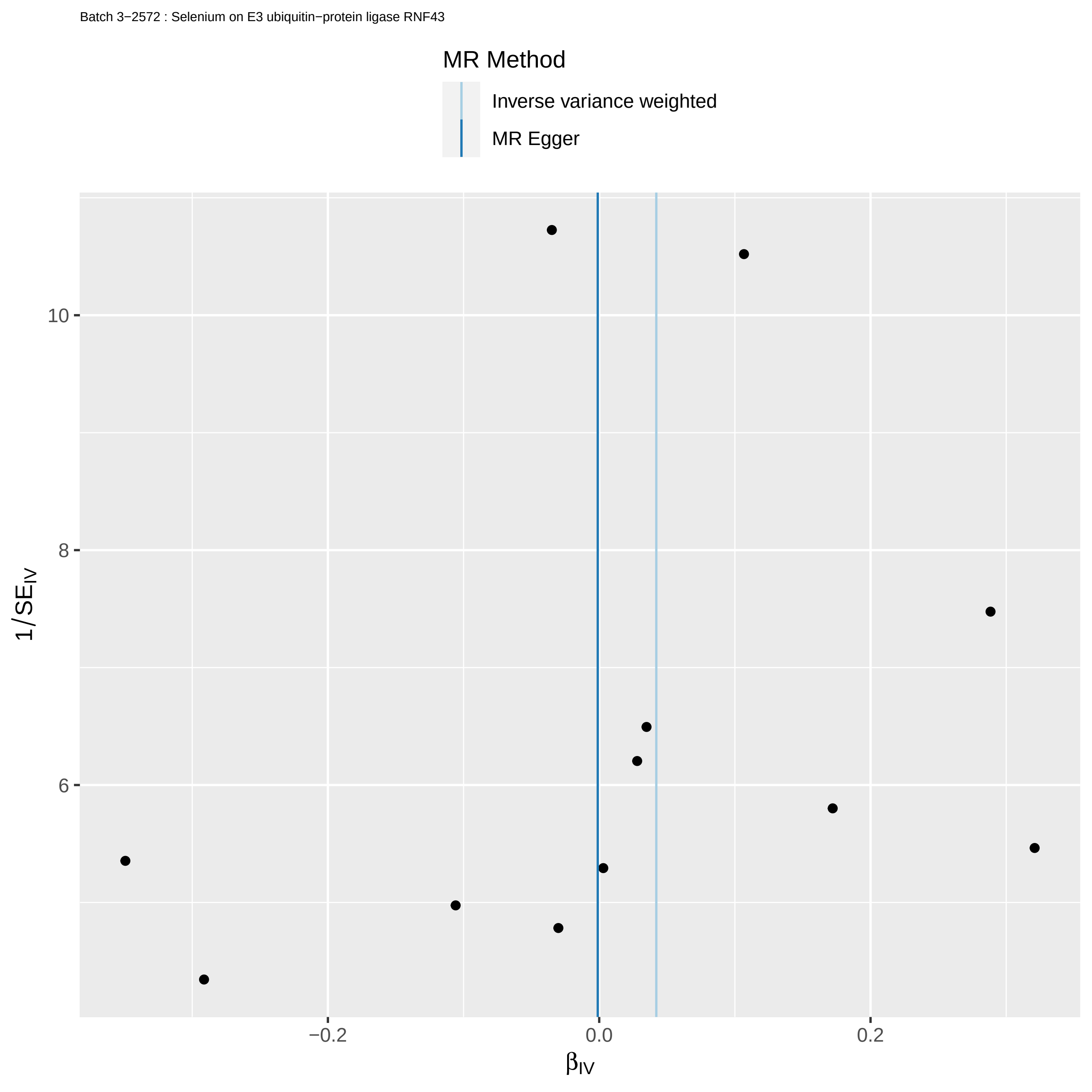
Batch 3−2572 : Selenium on E3 ubiquitin−protein ligase RNF43
MR Method
Inverse variance weighted
MR Egger
10
8
6
1/SE_IV
−0.2
0.0
0.2
β_IV

Batch 3−2573 : Selenium on E3 ubiquitin−protein ligase RNF8

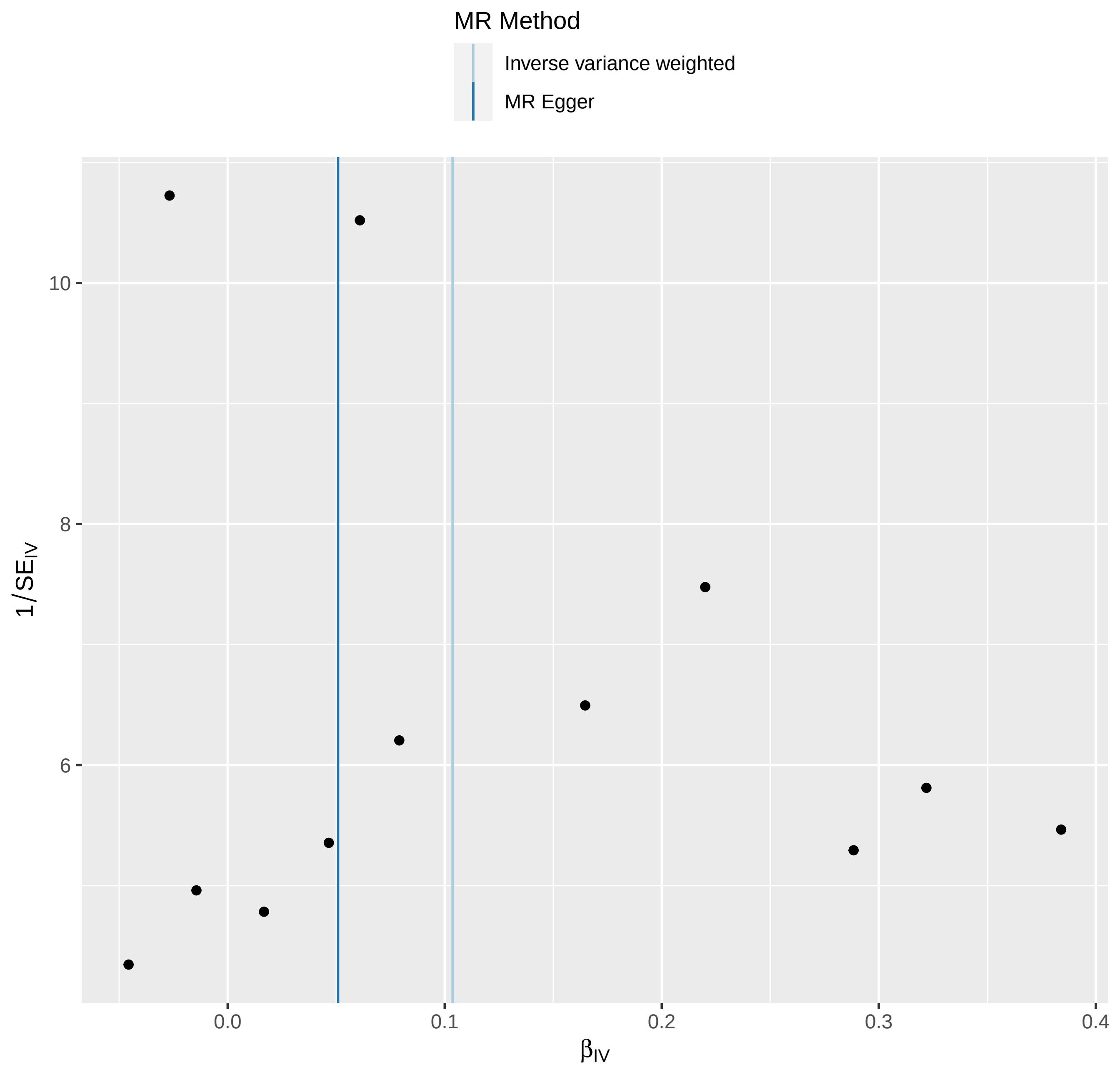

Batch 3−2574 : Selenium on mRNA−capping enzyme

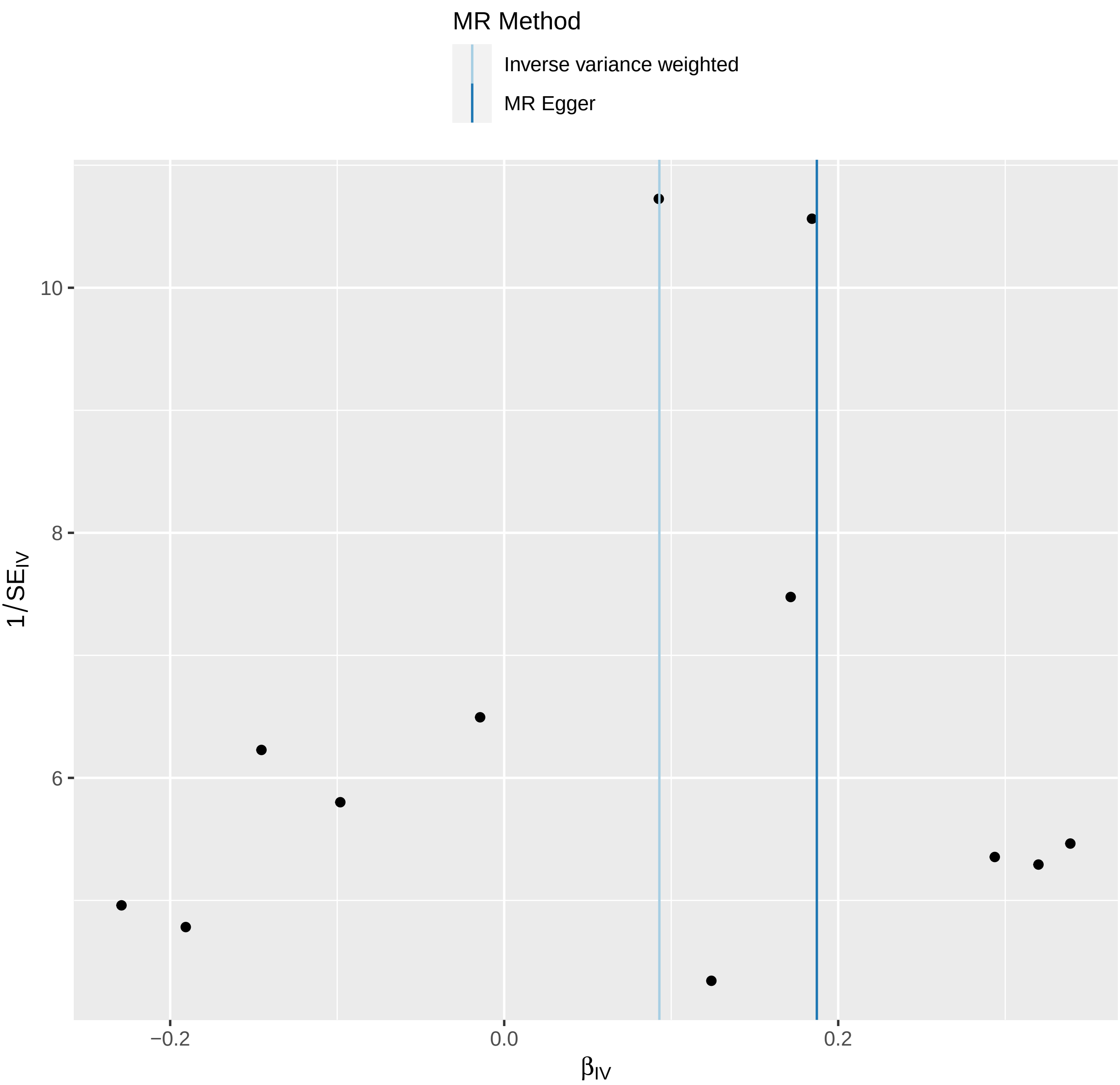

Batch 3−2575 : Selenium on rRNA methyltransferase 3, mitochondrial

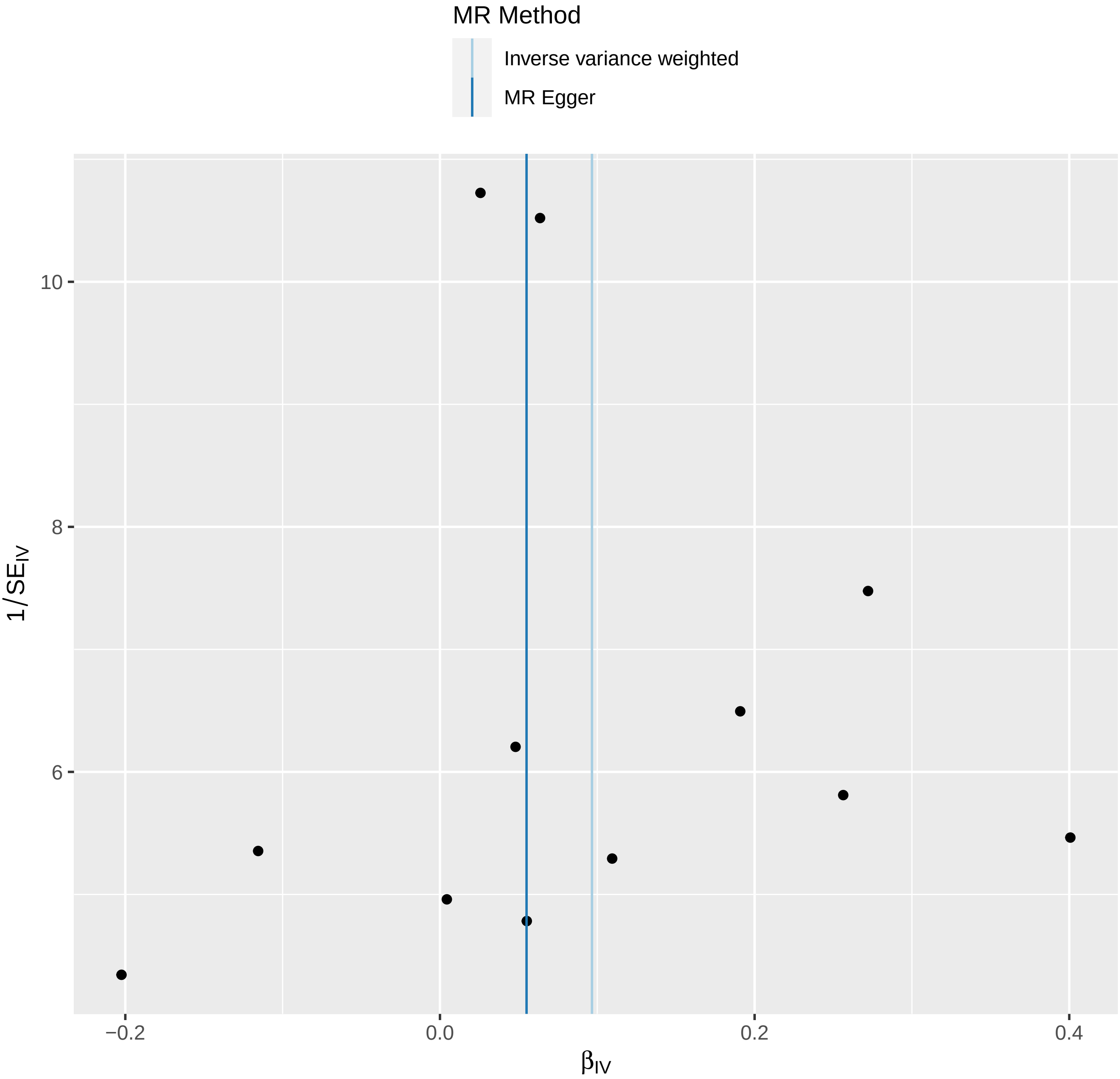

Batch 3−2576 : Selenium on RNA−binding protein 40

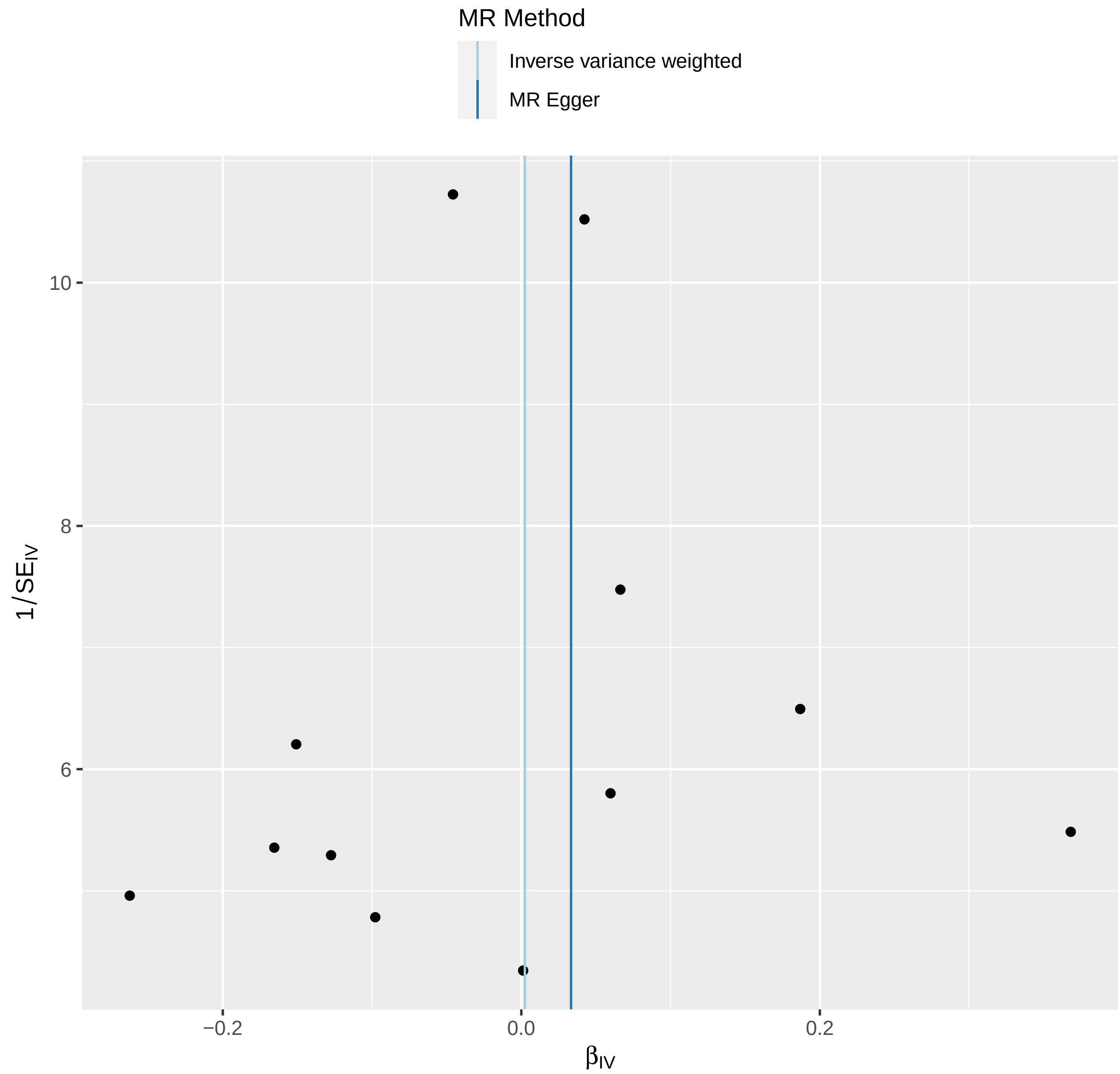

Batch 3−2577 : Selenium on Roundabout homolog 1

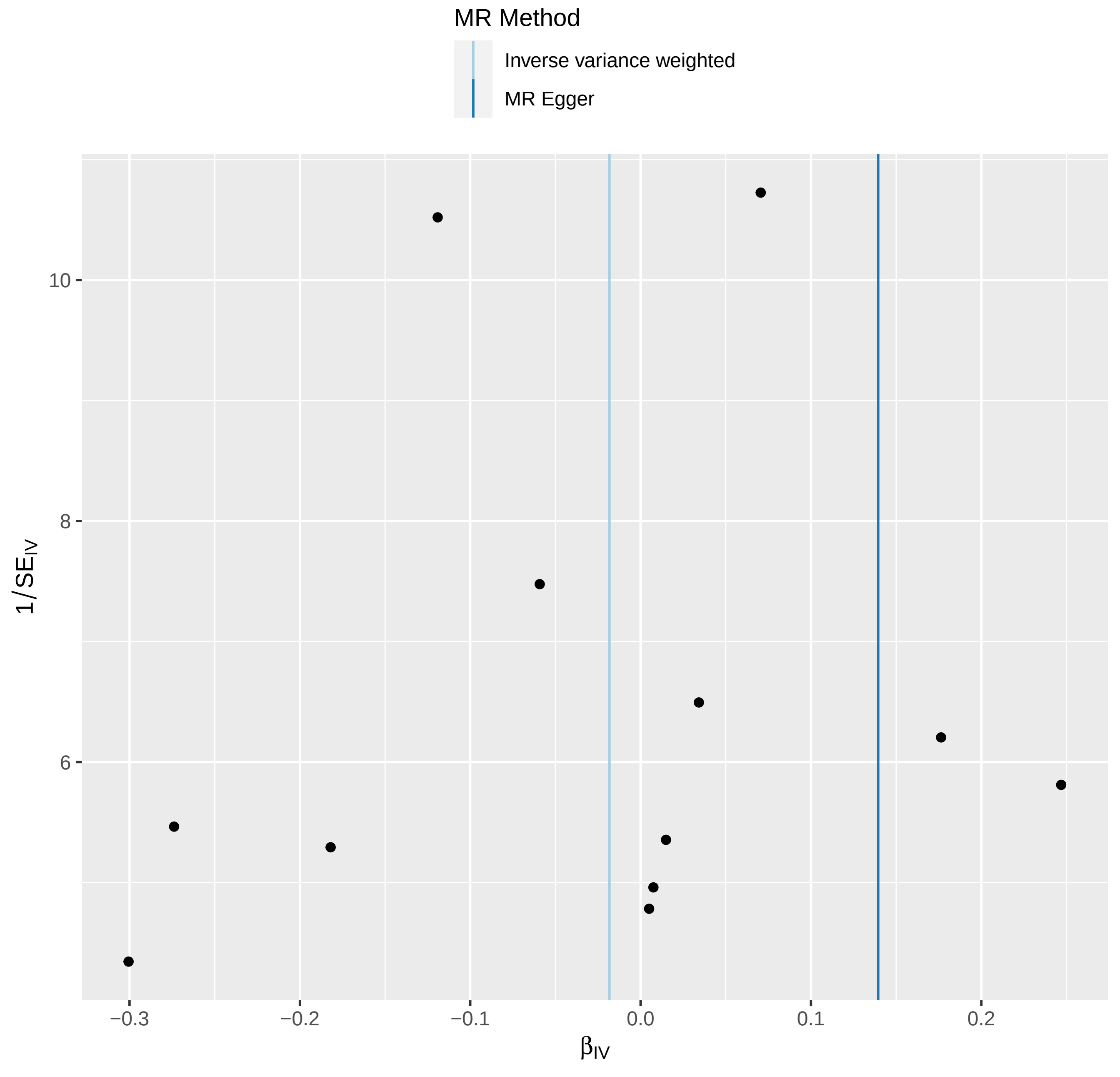

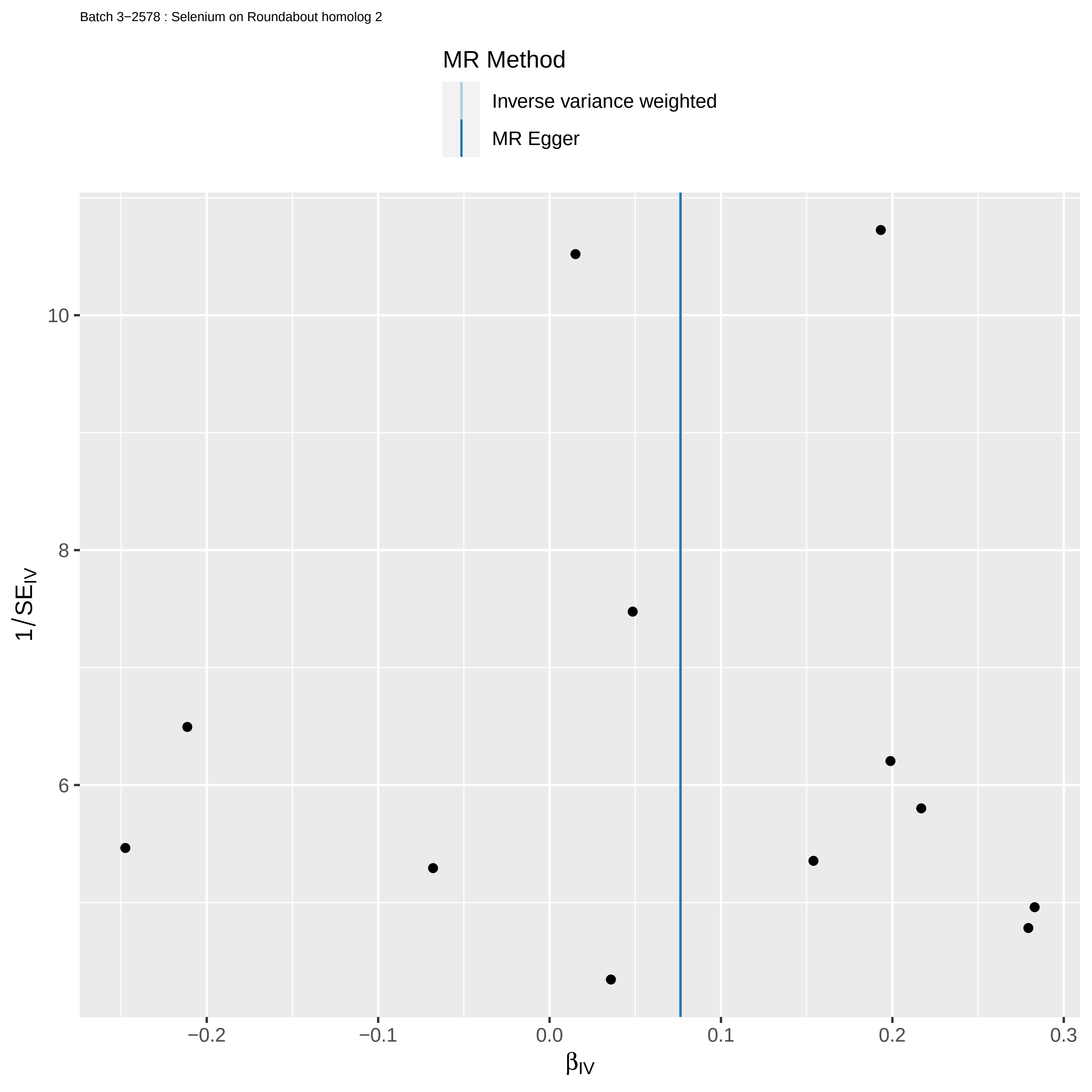

Batch 3−2578 : Selenium on Roundabout homolog 2
MR Method
Inverse variance weighted
MR Egger
10
8
6
1/SE_IV
−0.2
−0.1
0.0
0.1
0.2
0.3
β_IV

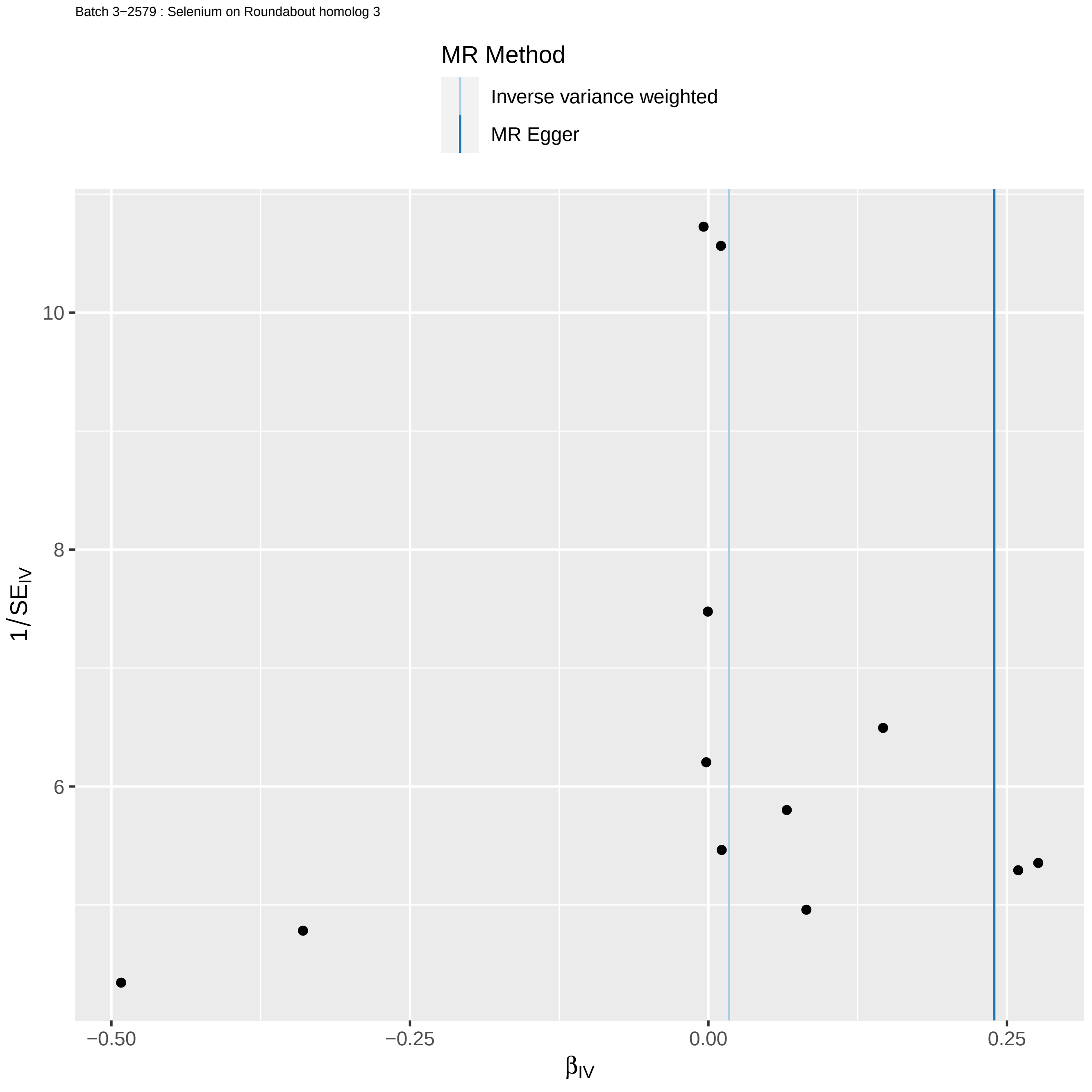

Batch 3−2579 : Selenium on Roundabout homolog 3
MR Method
Inverse variance weighted
MR Egger
10
8
6
−0.50
−0.25
0.00
0.25
$1/SE_{IV}$
β_{IV}

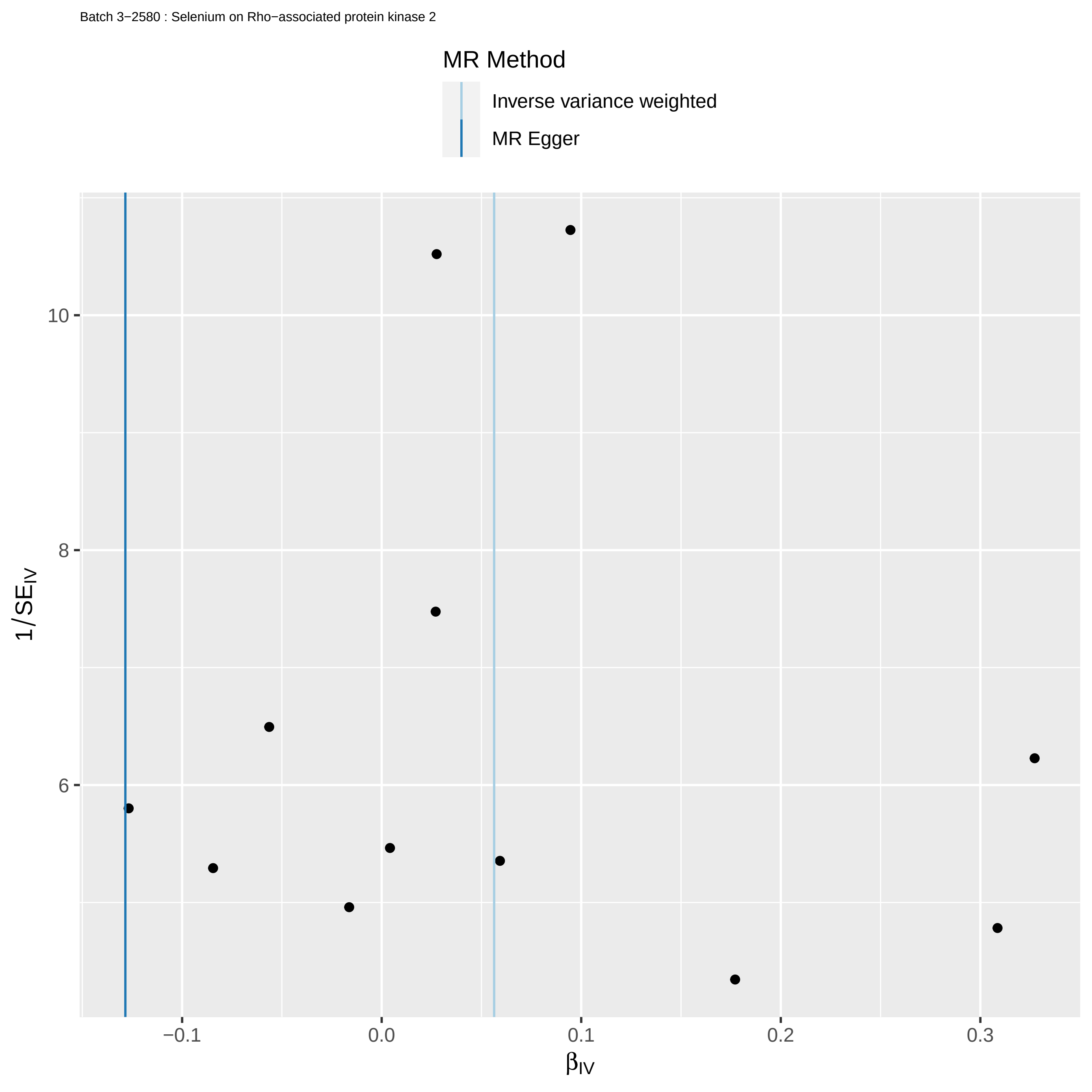

Batch 3−2580 : Selenium on Rho−associated protein kinase 2
MR Method
Inverse variance weighted
MR Egger
10
8
6
1/SE$_{IV}$
−0.1
0.0
0.1
0.2
0.3
β_{IV}

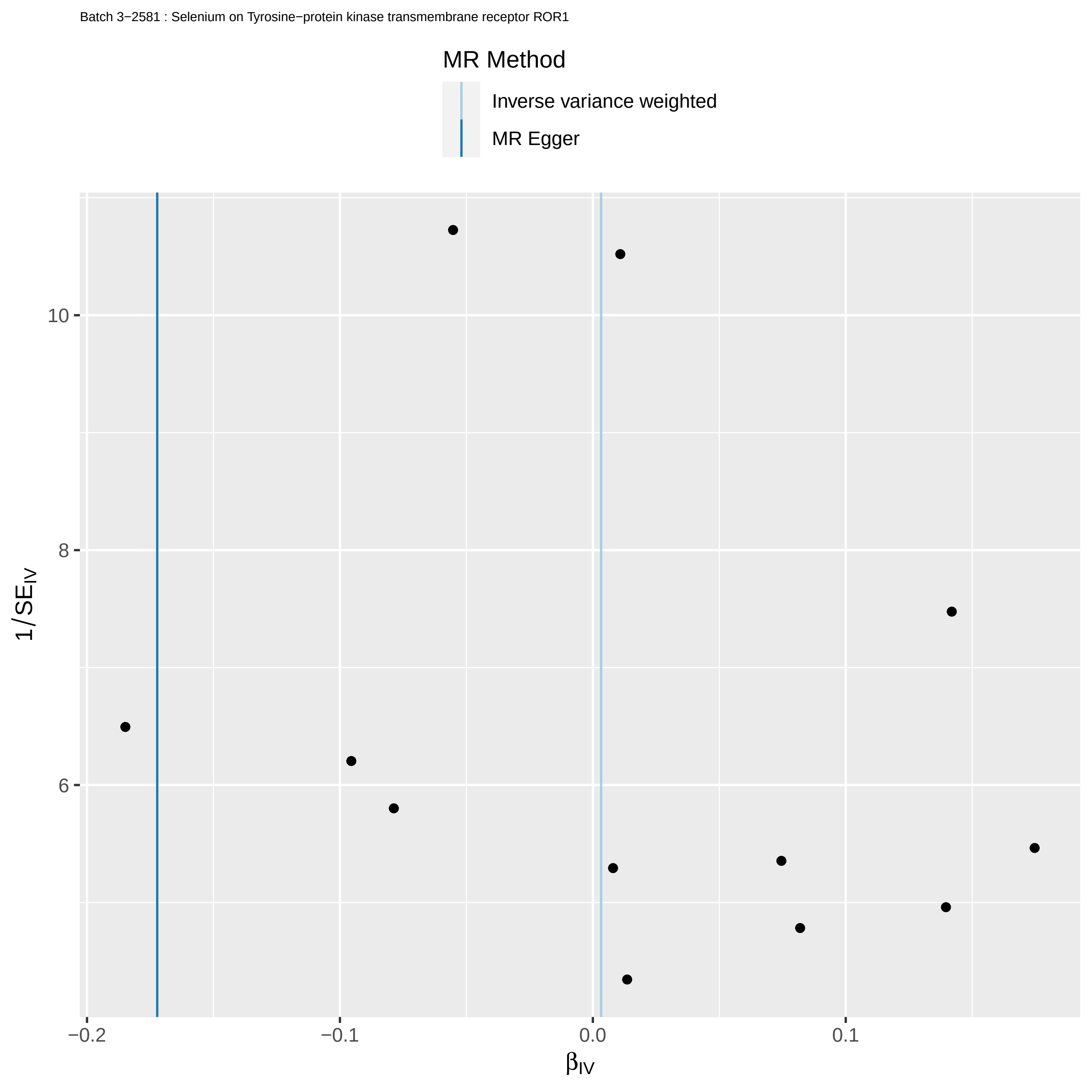

Batch 3−2581 : Selenium on Tyrosine−protein kinase transmembrane receptor ROR1
MR Method
Inverse variance weighted
MR Egger
10
8
6
1/SE_IV
−0.2
−0.1
0.0
0.1
β_IV

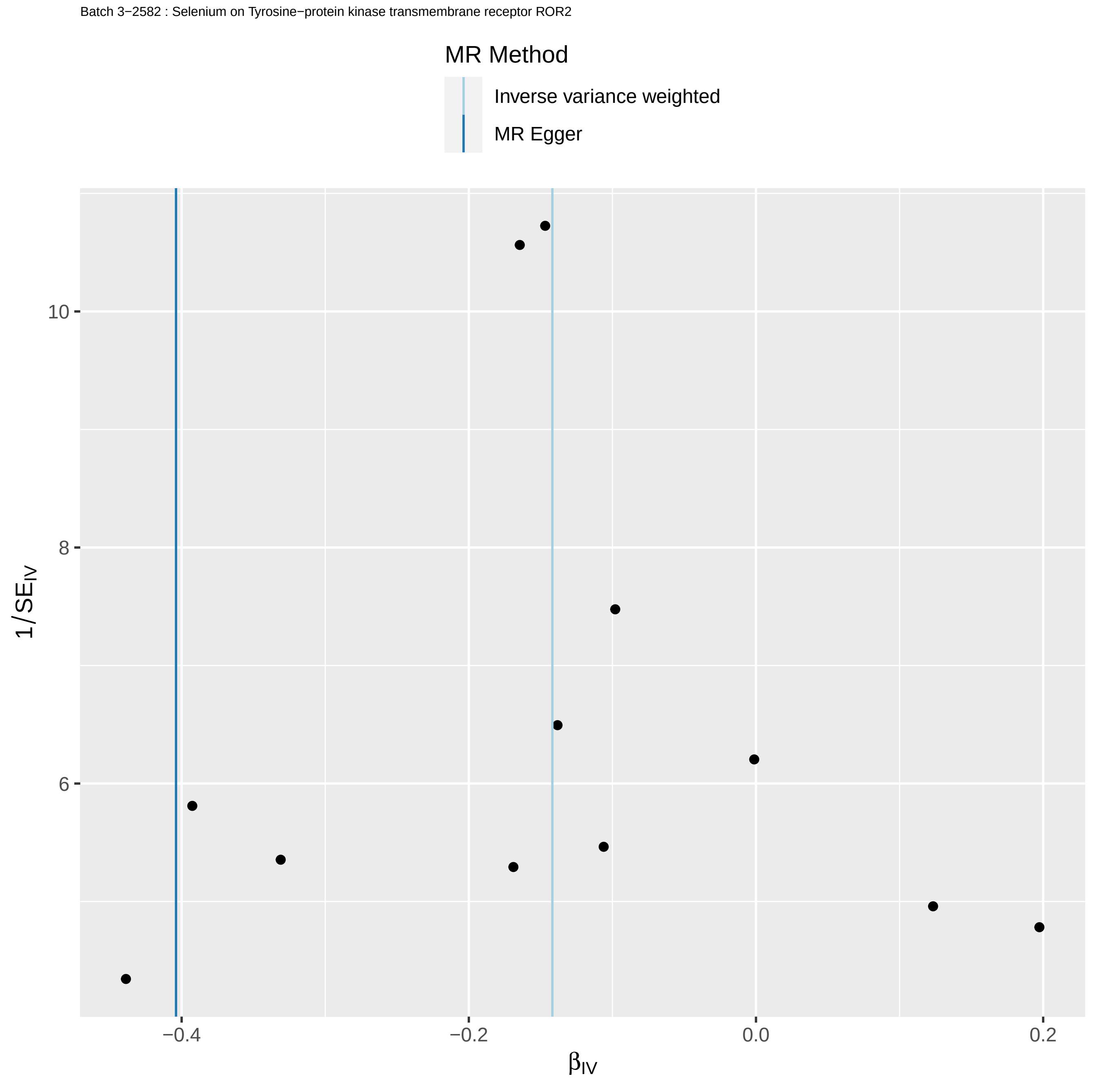
Batch 3−2582 : Selenium on Tyrosine−protein kinase transmembrane receptor ROR2
MR Method
Inverse variance weighted
MR Egger
10
8
6
1/SE$_{IV}$
−0.4
−0.2
0.0
0.2
β_{IV}

Batch 3−2583 : Selenium on Nuclear receptor ROR−beta

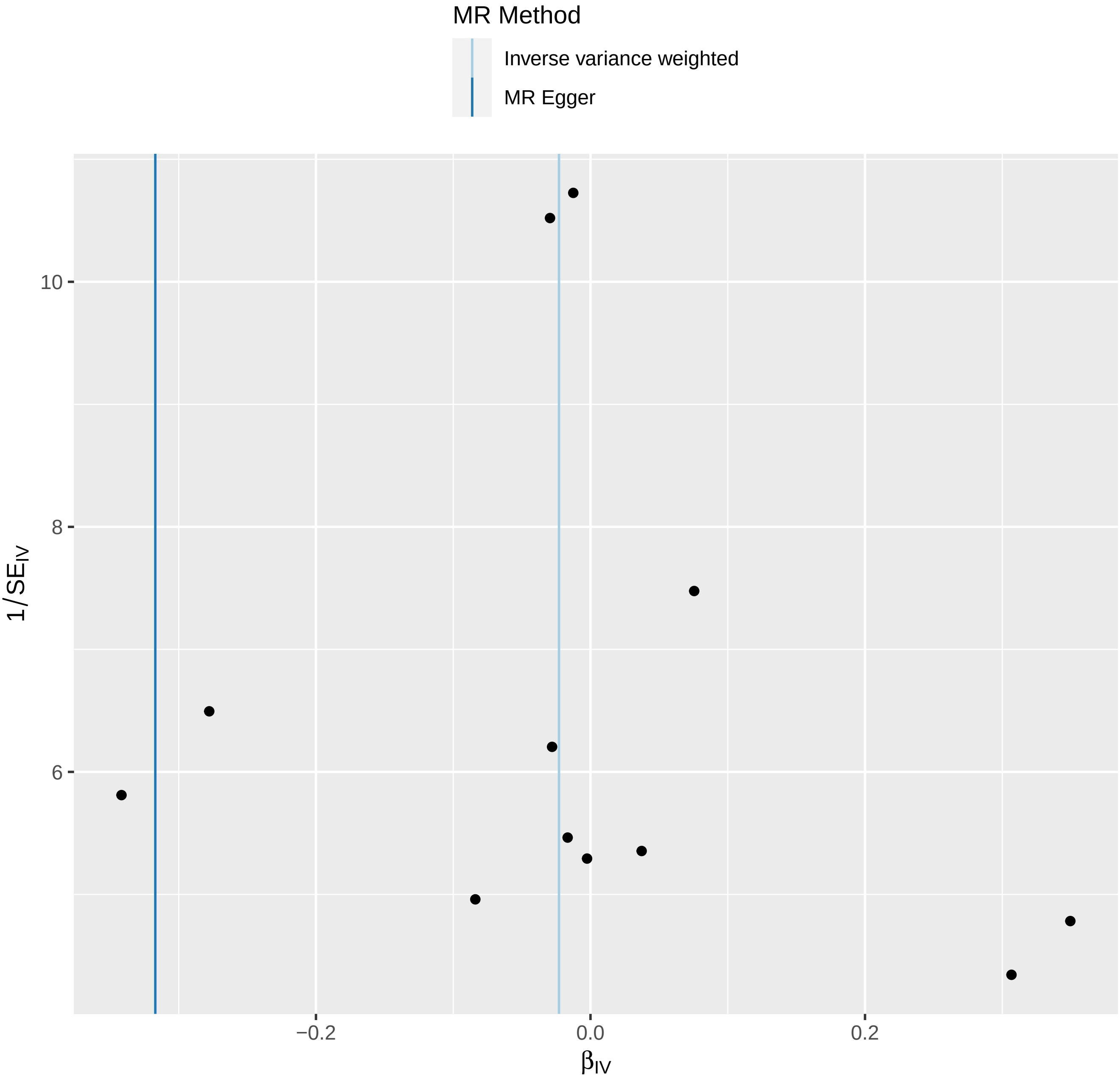

Batch 3−2584 : Selenium on Ribulose−phosphate 3−epimerase

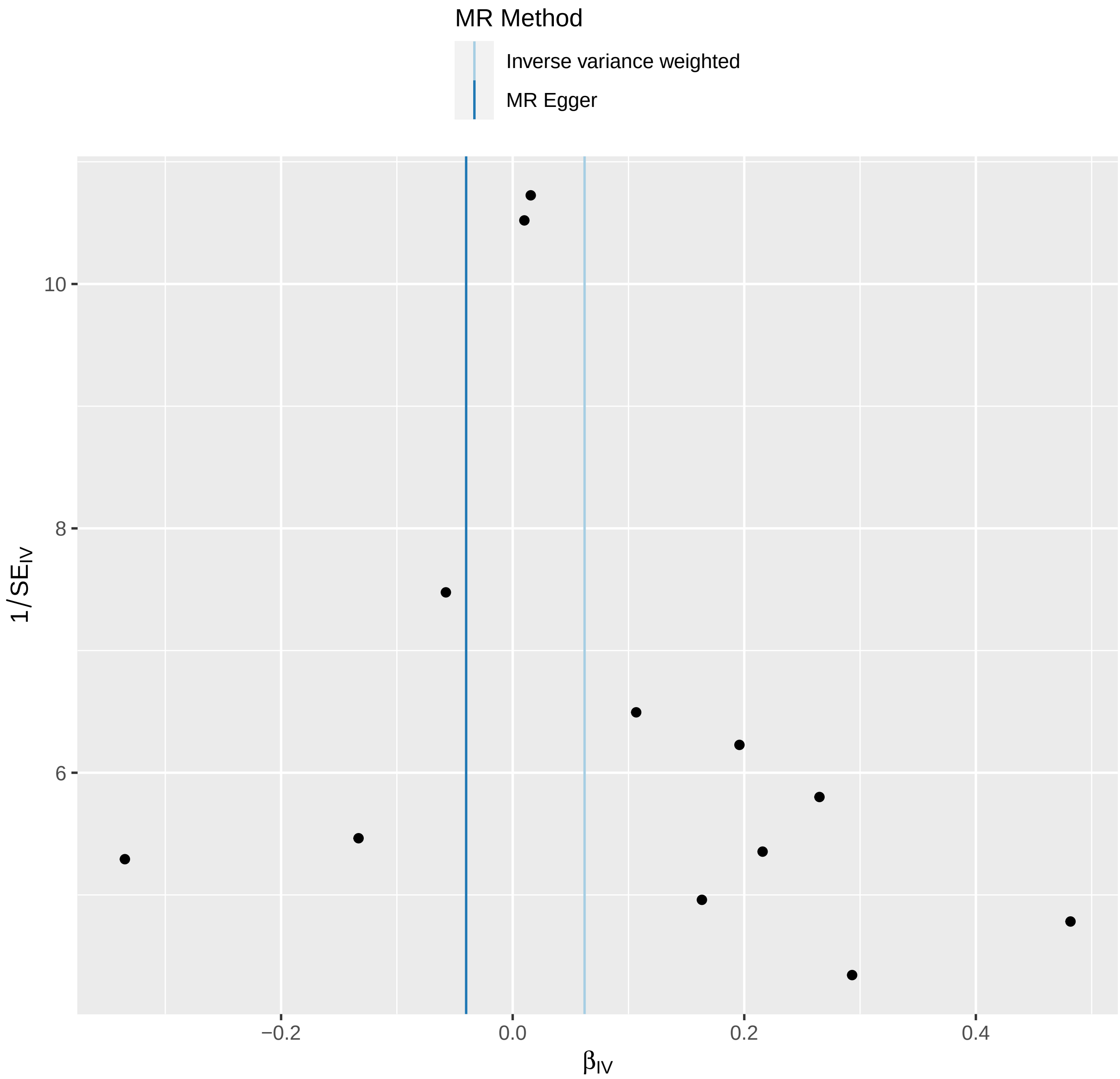

Batch 3−2585 : Selenium on Ribose−5−phosphate isomerase

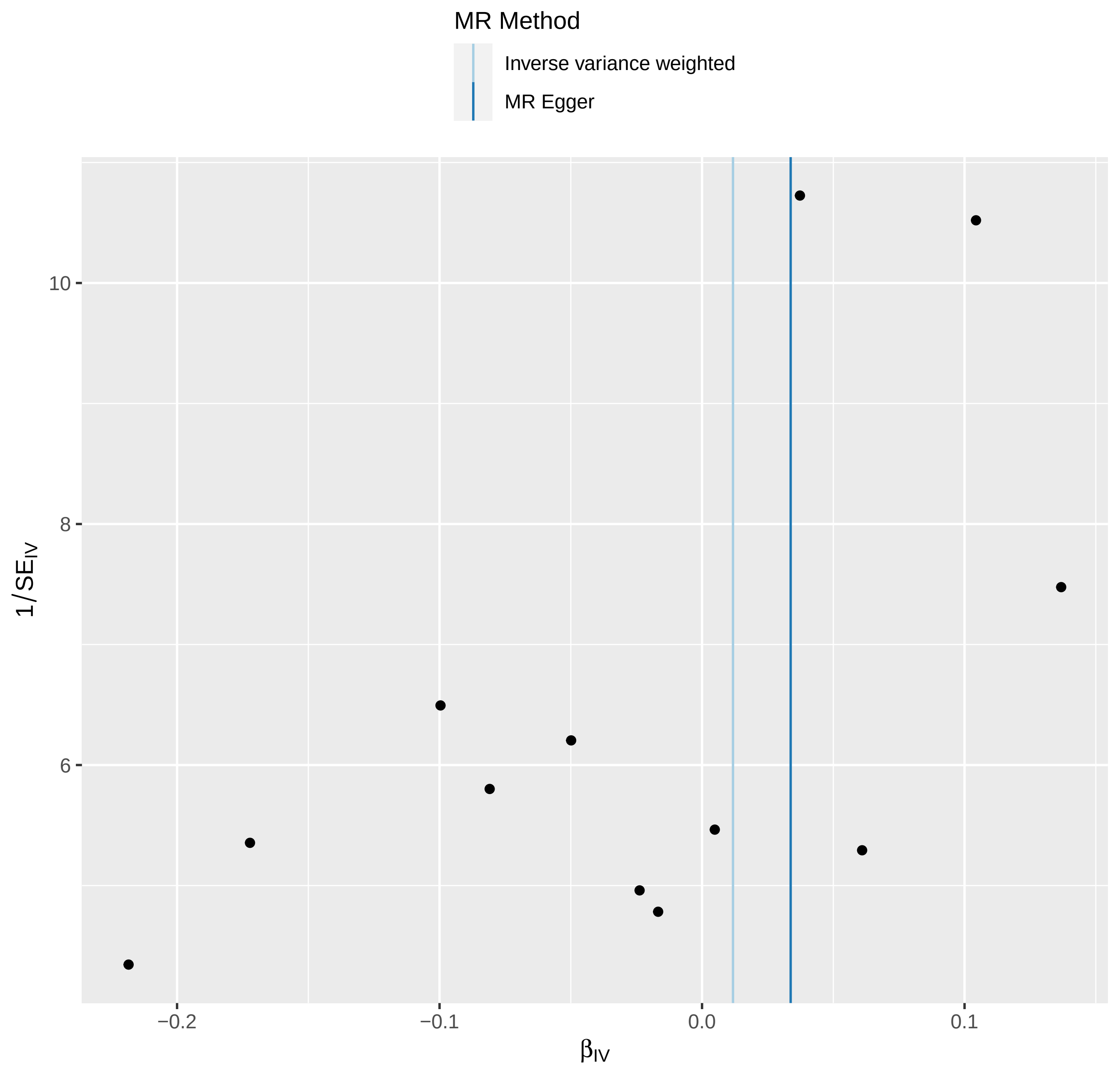

Batch 3−2586 : Selenium on 60S ribosomal protein L30

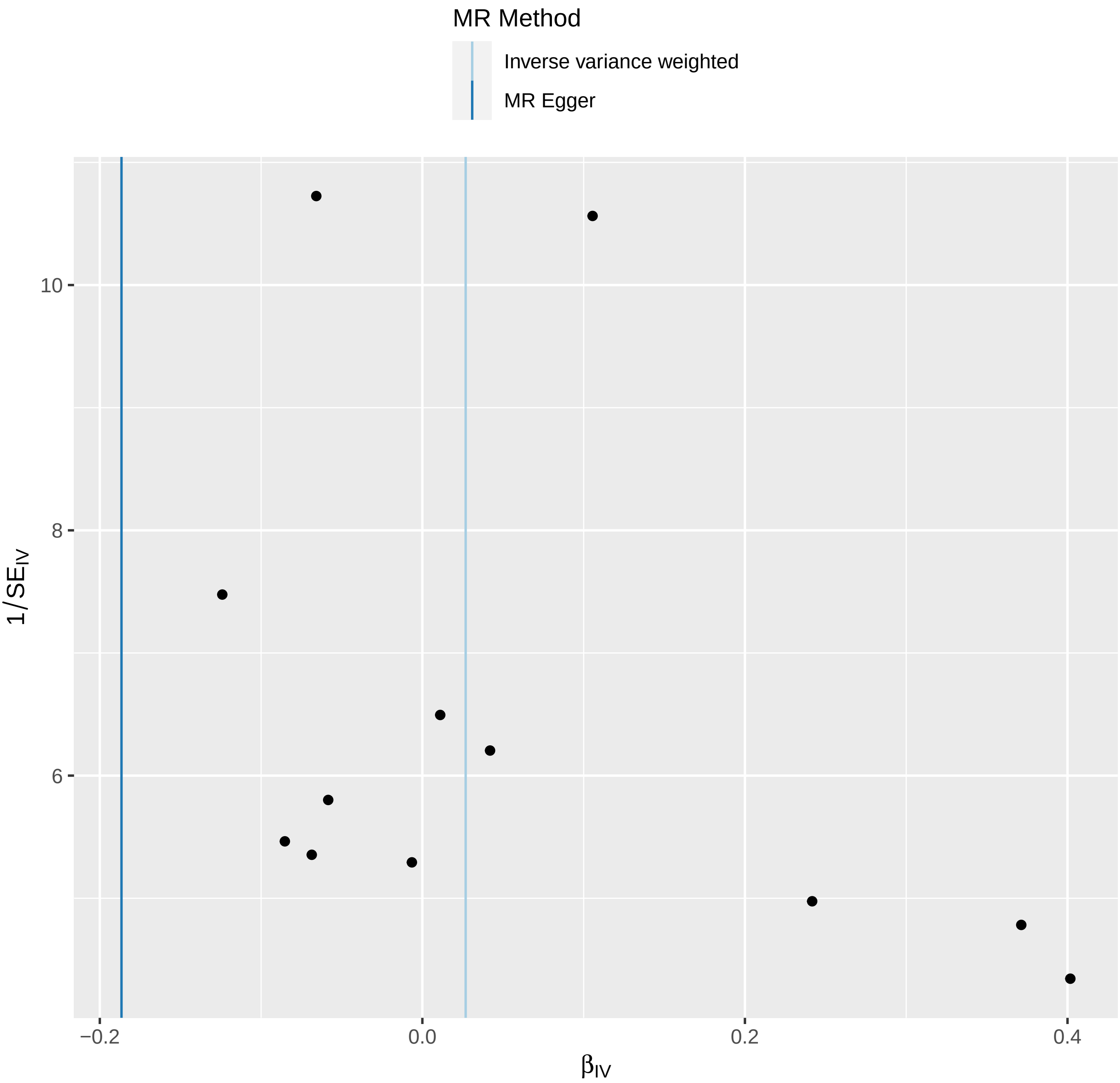

Batch 3−2587 : Selenium on 60S acidic ribosomal protein P2

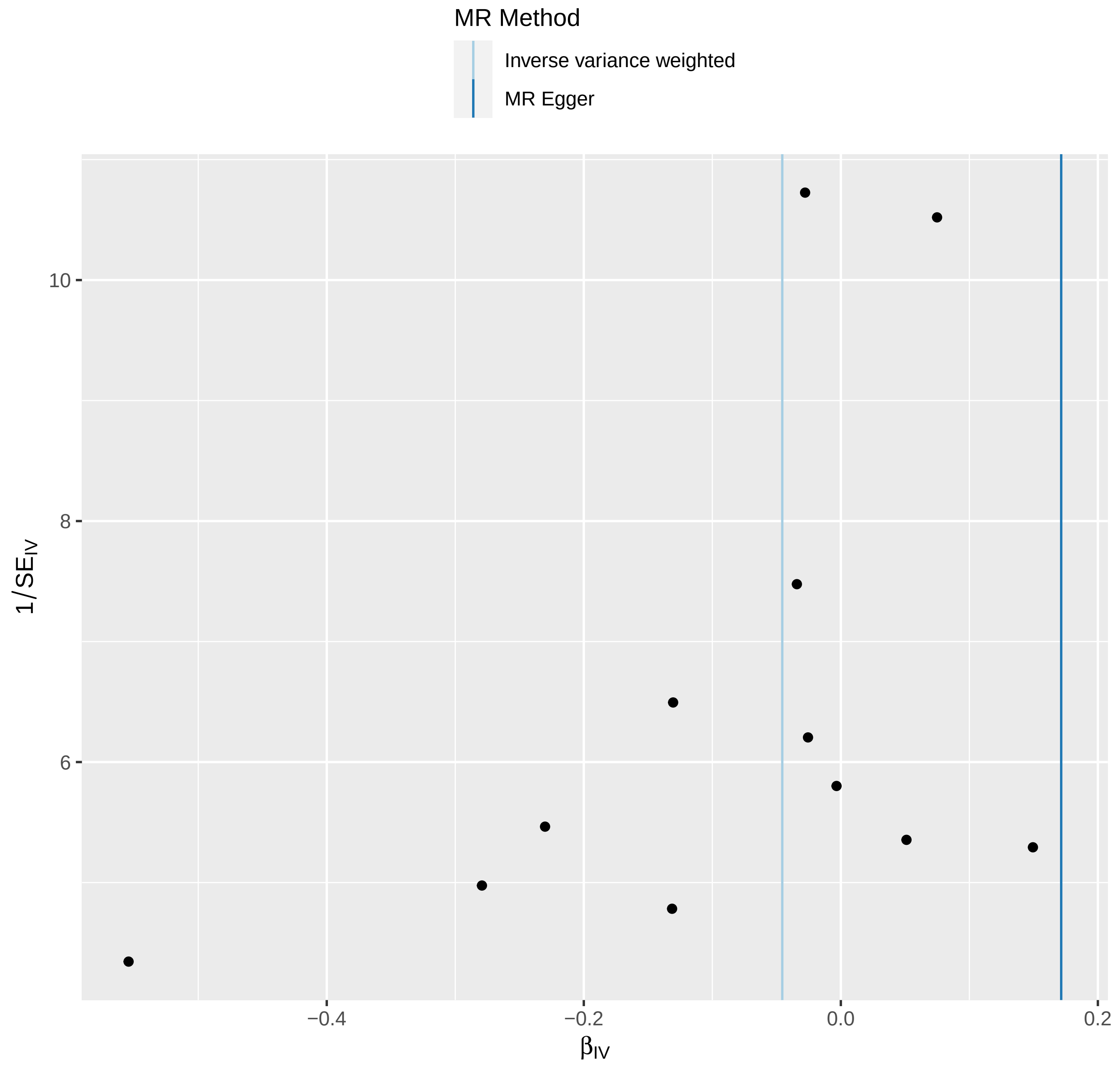

Batch 3−2588 : Selenium on Dolichyl−diphosphooligosaccharide−−protein glycosyltransferase subunit 1

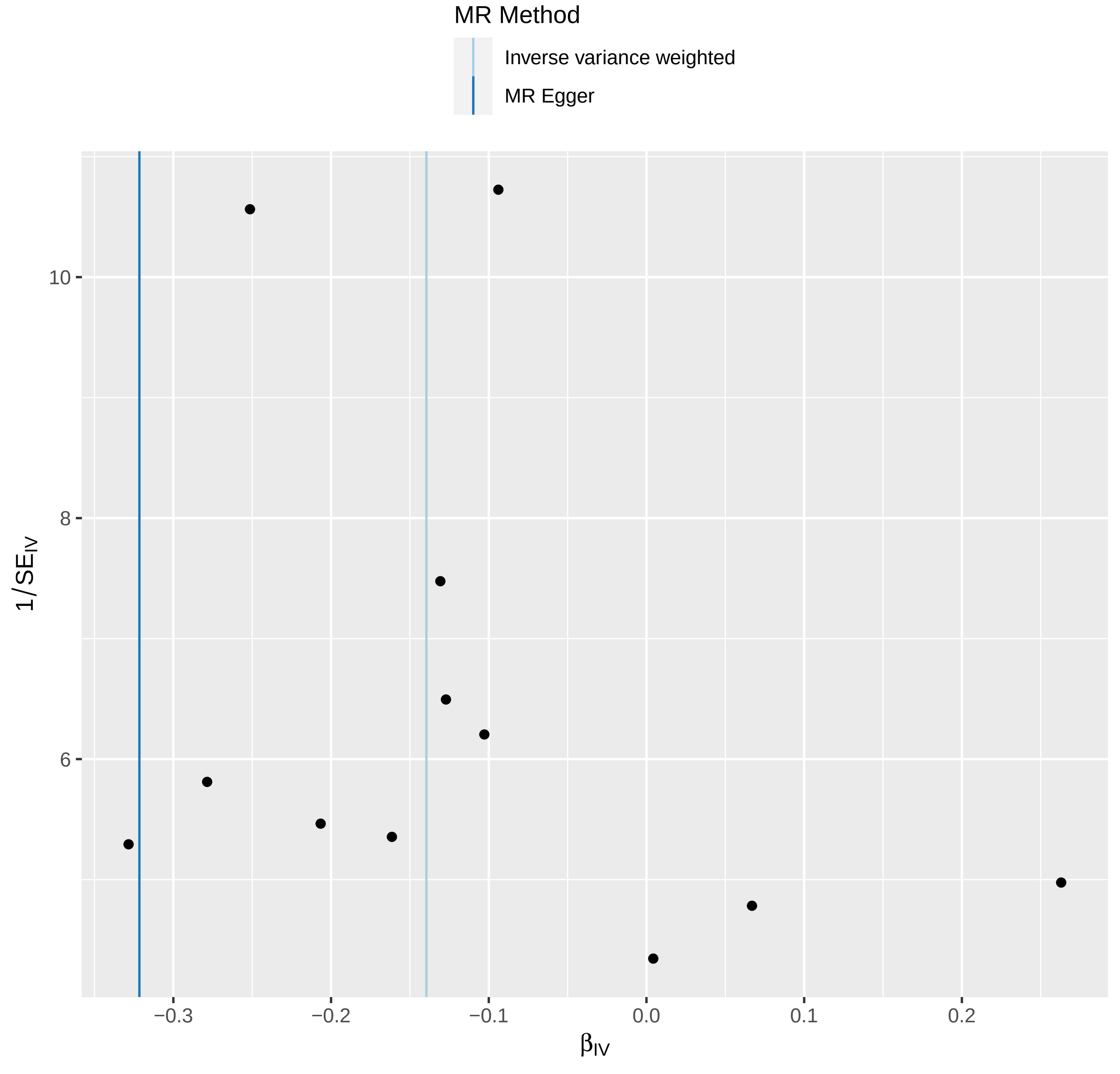

Batch 3−2589 : Selenium on Dolichyl−diphosphooligosaccharide−−protein glycosyltransferase subunit 1

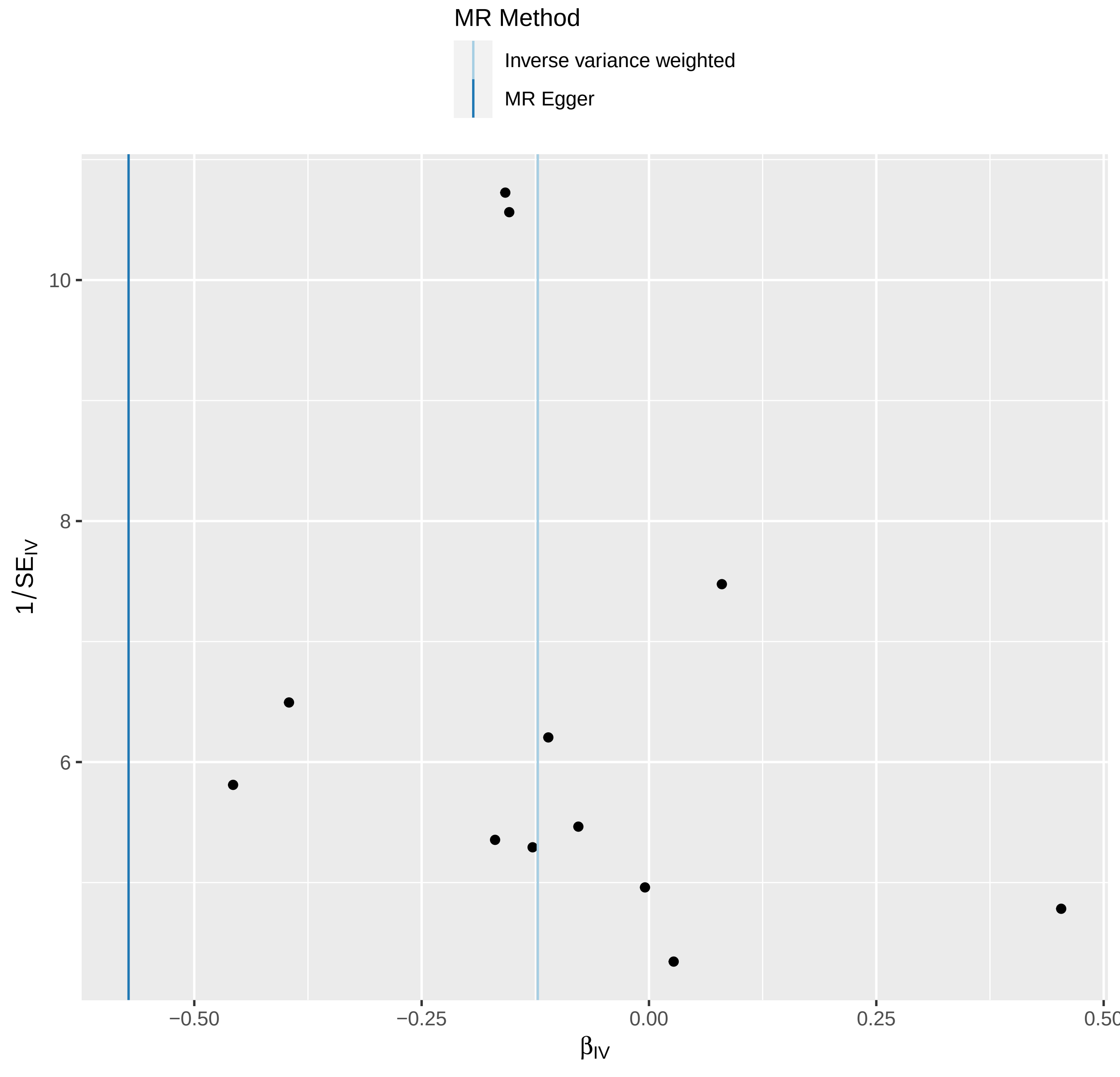

Batch 3−2590 : Selenium on Regulation of nuclear pre−mRNA domain−containing protein 1A

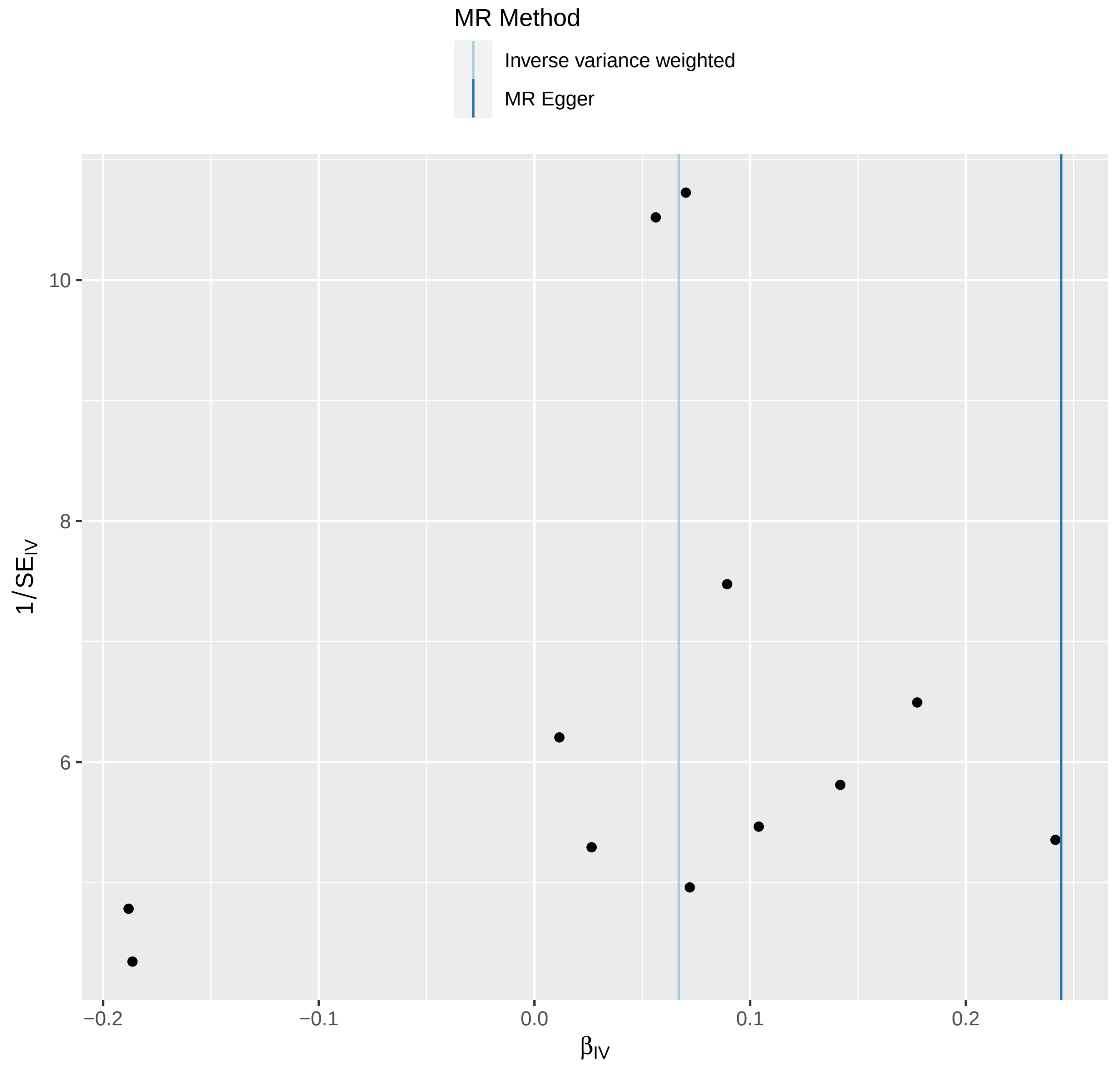

Batch 3−2591 : Selenium on Ubiquitin+1, truncated mutation for UbB

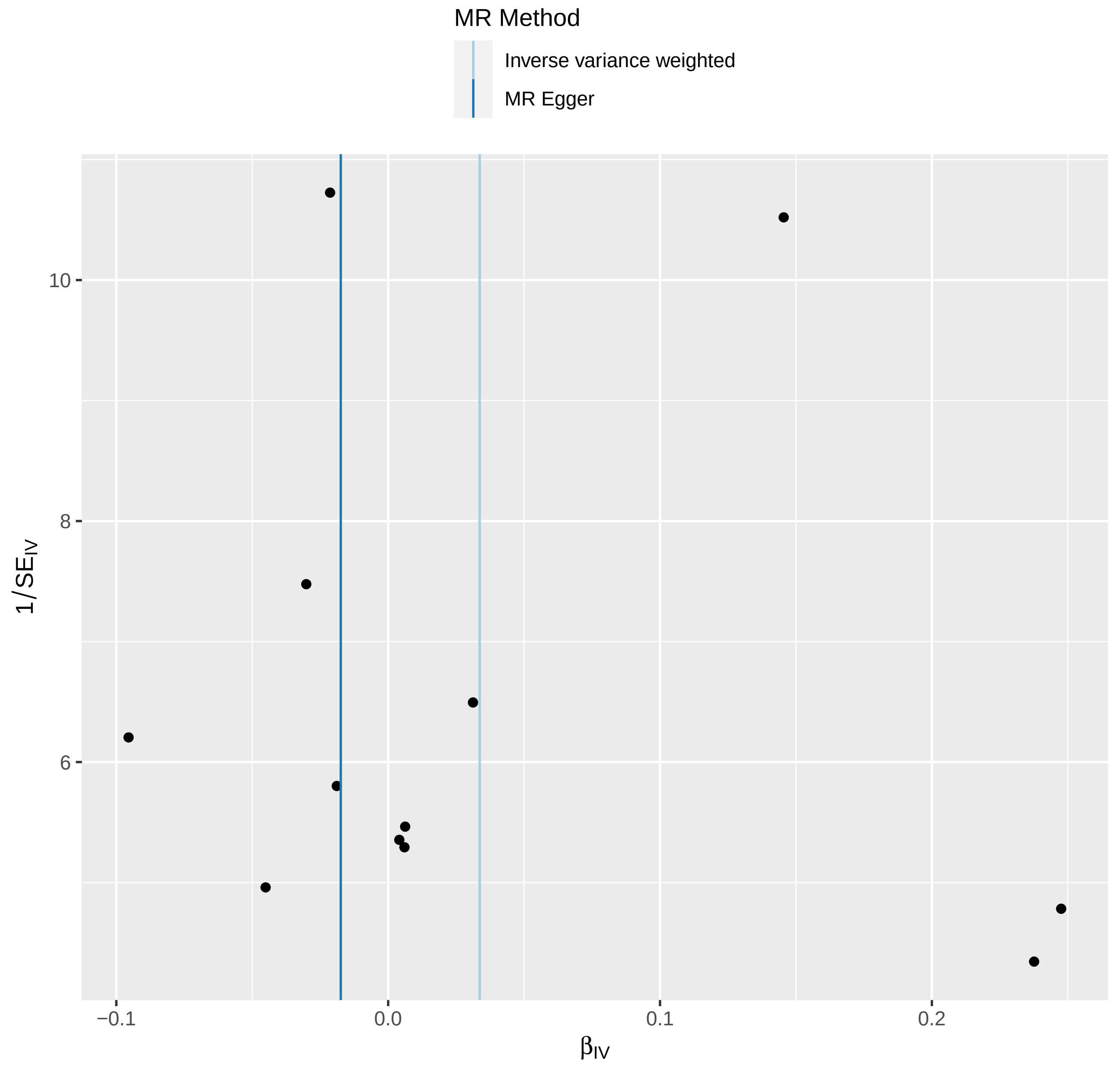

Batch 3−2592 : Selenium on Ubiquitin

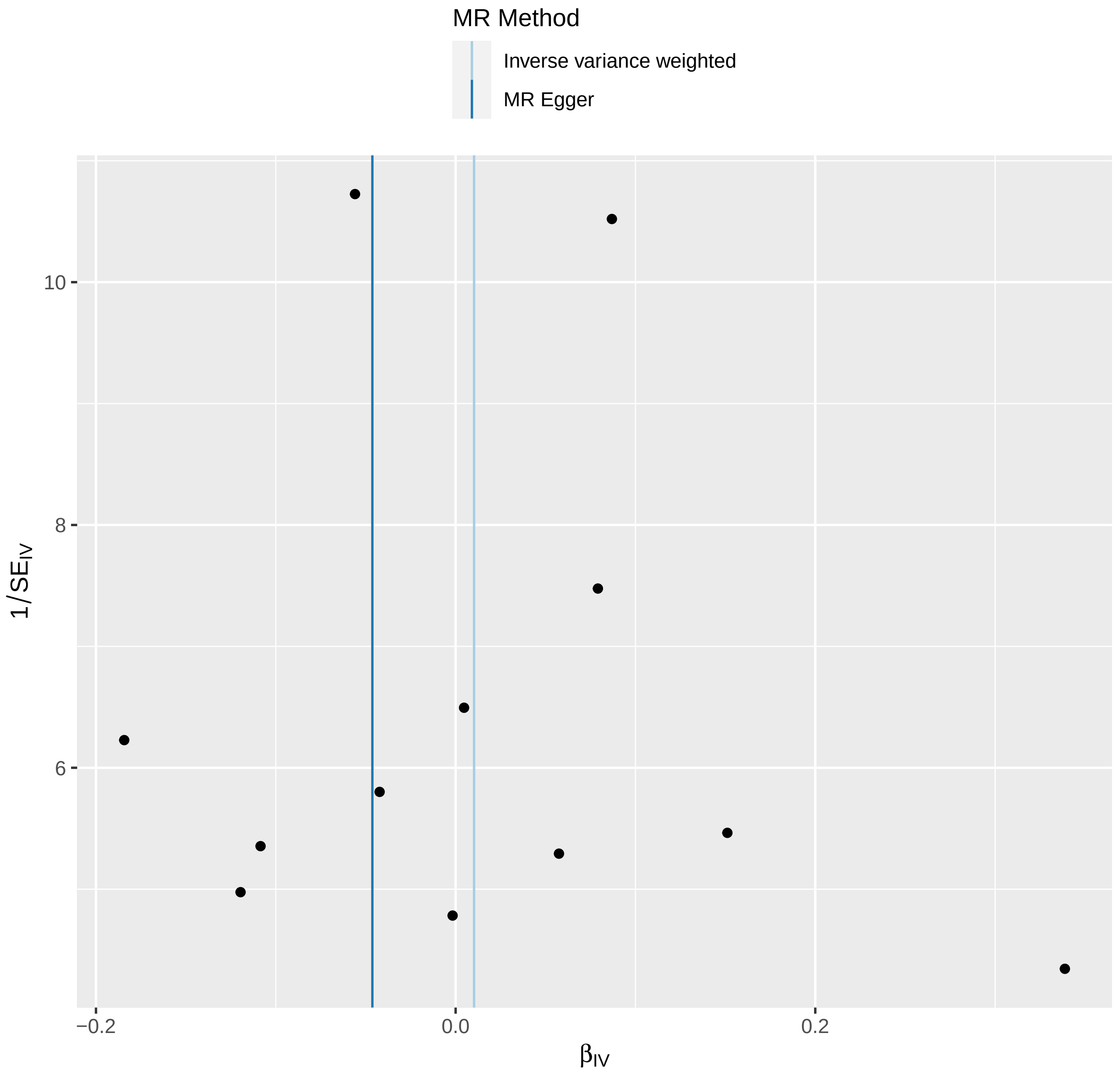

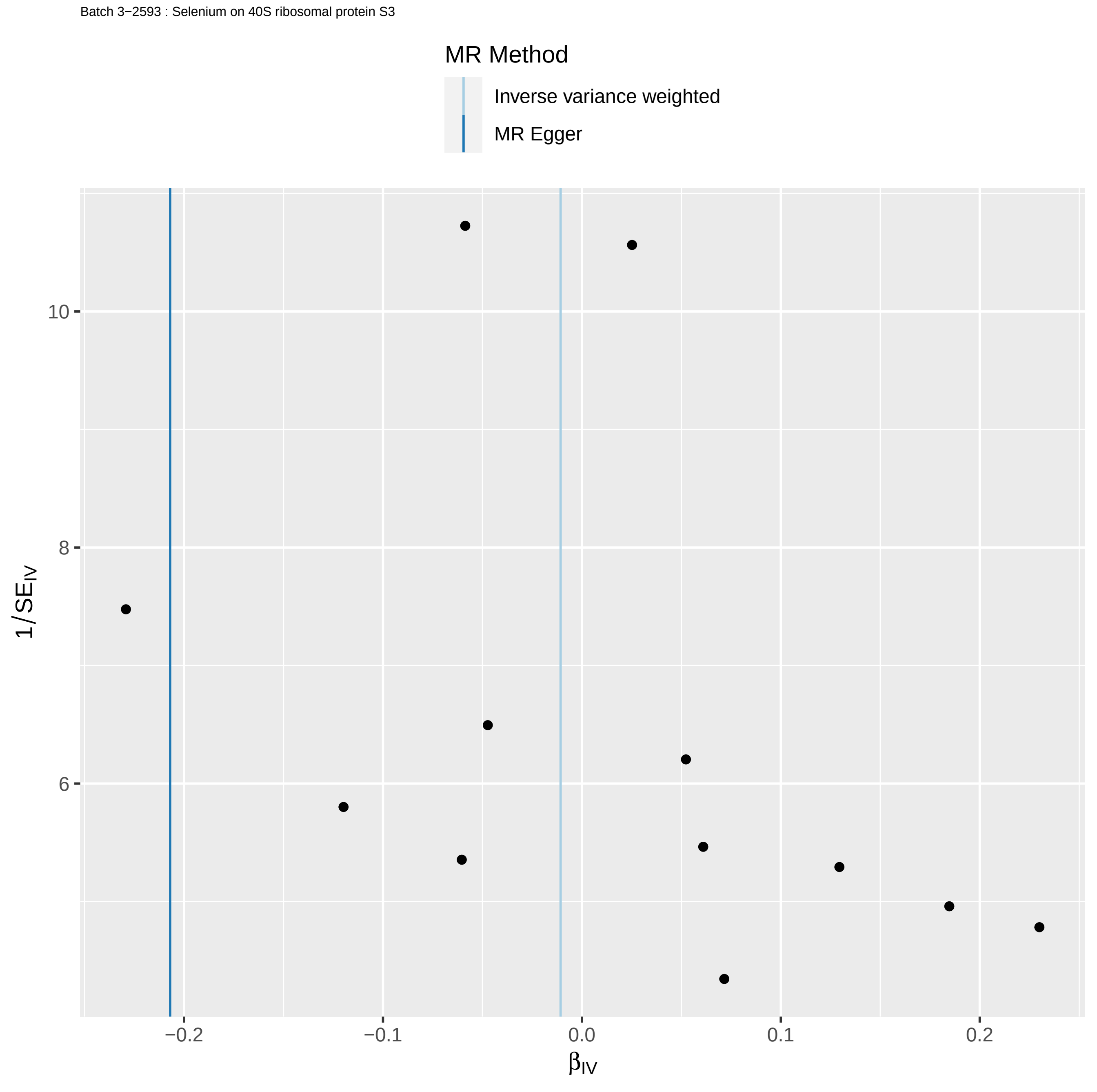

Batch 3−2593 : Selenium on 40S ribosomal protein S3
MR Method
Inverse variance weighted
MR Egger
10
8
6
1/SE$_{IV}$
−0.2
−0.1
0.0
0.1
0.2
β$_{IV}$

Batch 3−2594 : Selenium on 40S ribosomal protein S3a

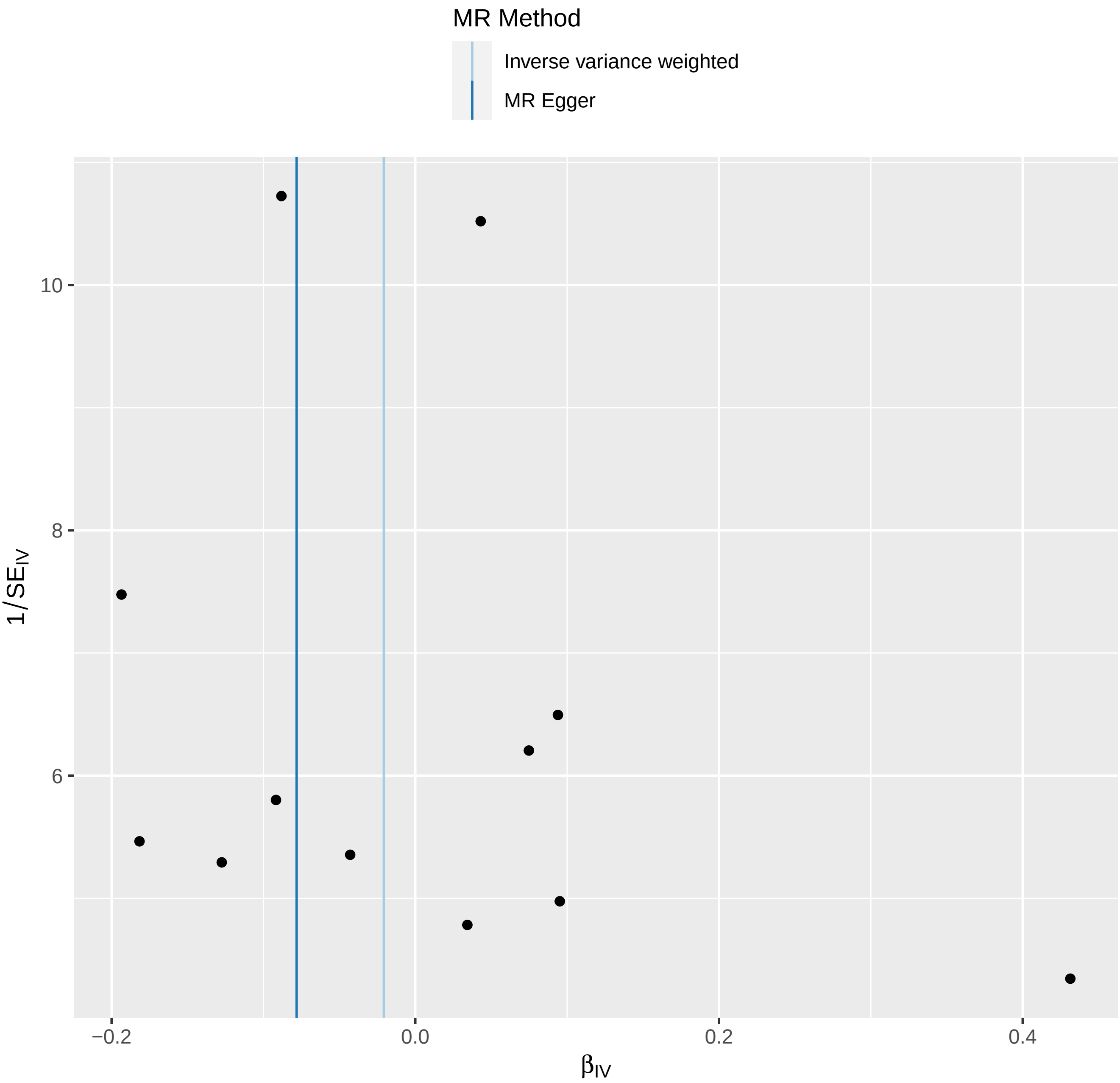

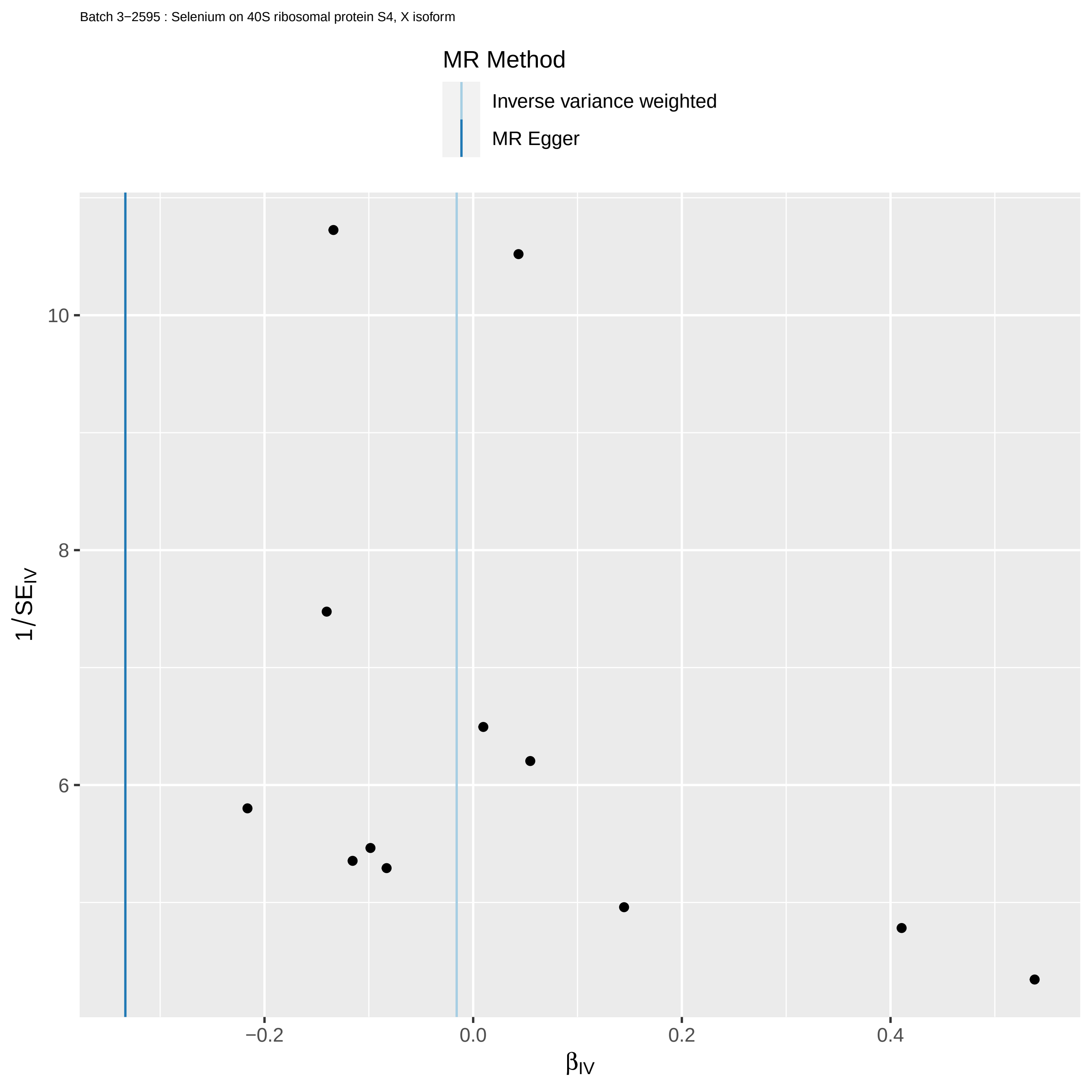

Batch 3−2595 : Selenium on 40S ribosomal protein S4, X isoform
MR Method
Inverse variance weighted
MR Egger
10
8
6
1/SE$_{IV}$
−0.2
0.0
0.2
0.4
β$_{IV}$

Batch 3−2596 : Selenium on Ribosomal protein S6 kinase alpha−6

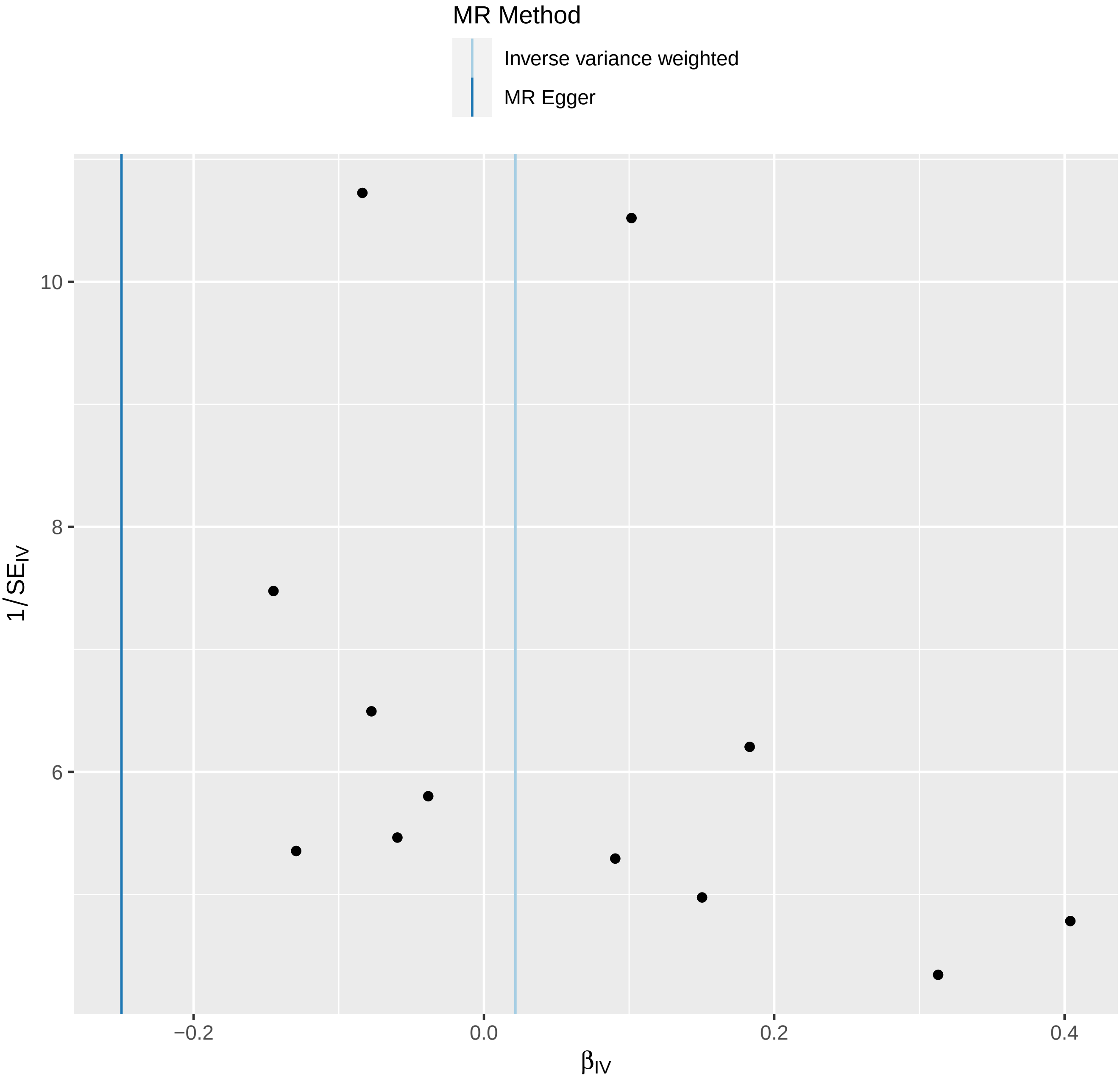

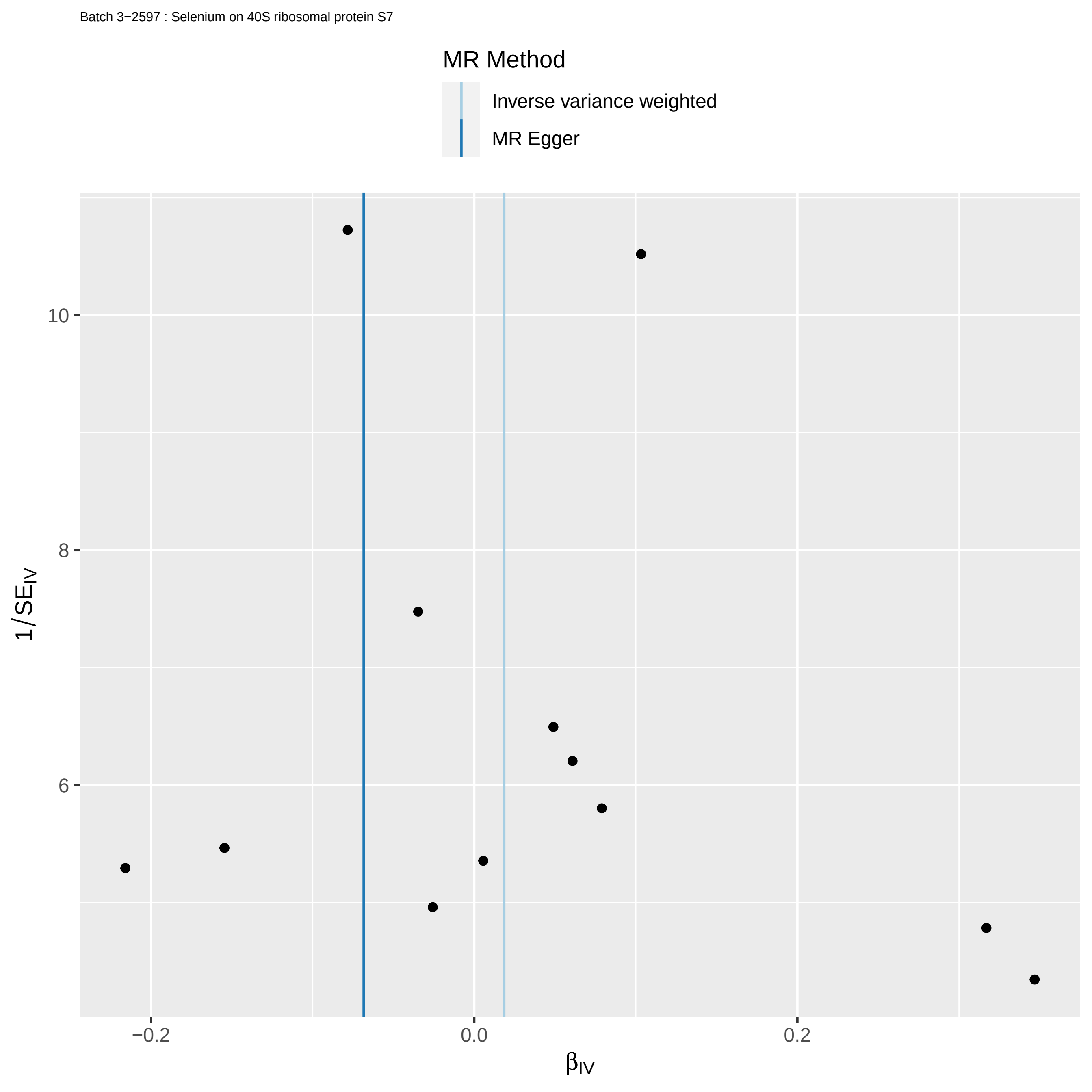
Batch 3−2597 : Selenium on 40S ribosomal protein S7
MR Method
Inverse variance weighted
MR Egger
10
8
6
1/SE_IV
−0.2
0.0
0.2
β_IV

Batch 3−2598 : Selenium on 40S ribosomal protein SA

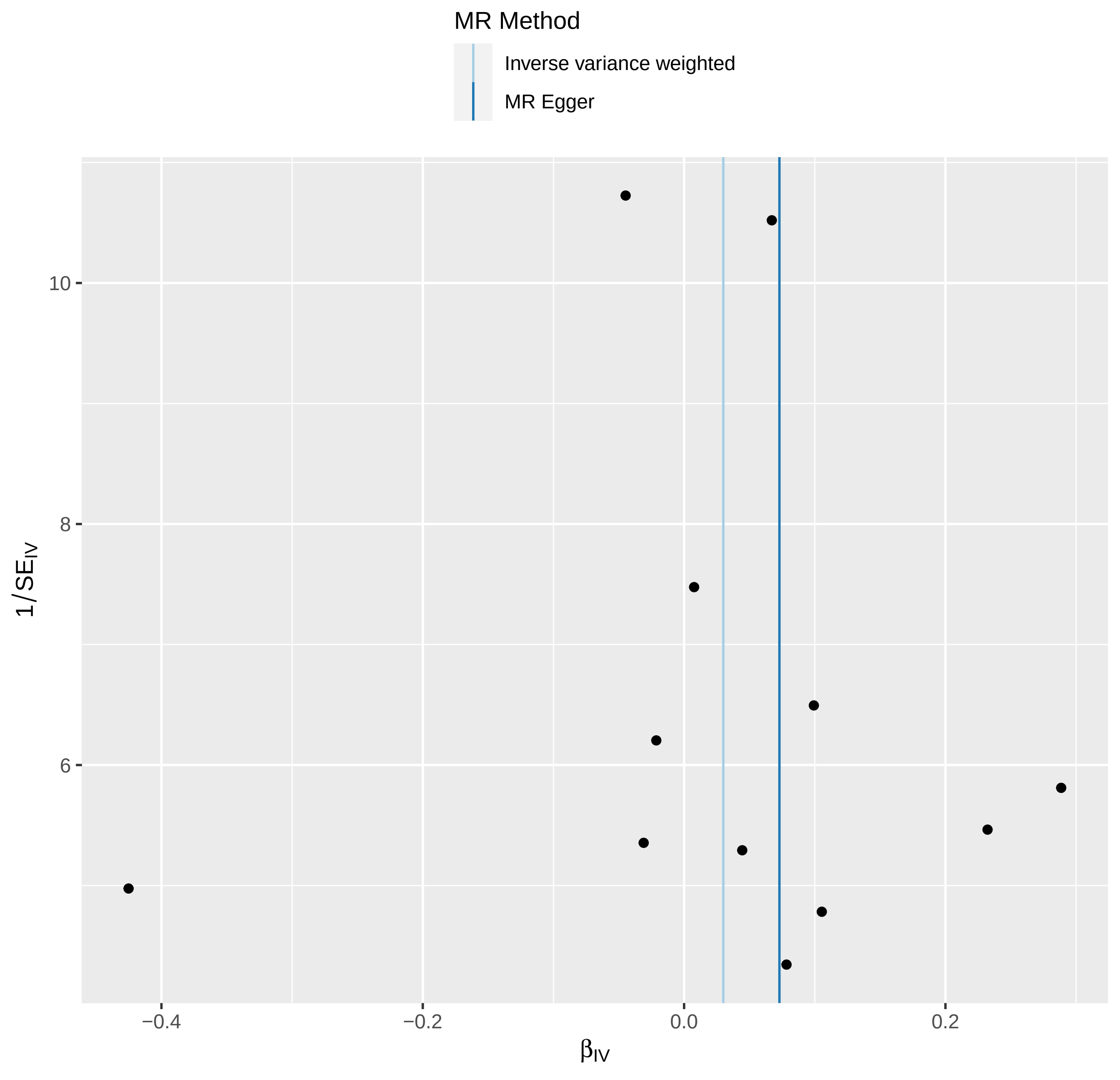

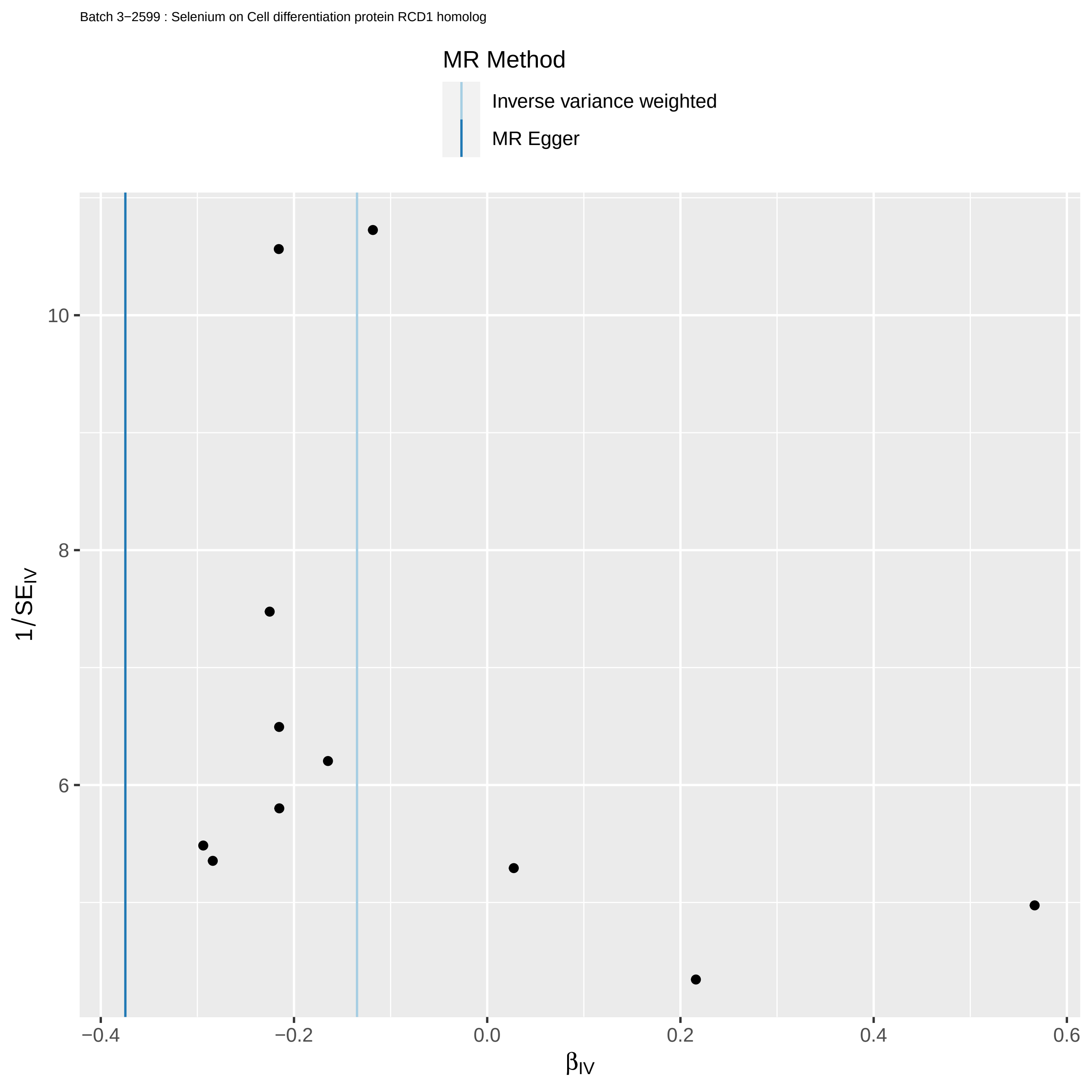
Batch 3−2599 : Selenium on Cell differentiation protein RCD1 homolog
MR Method
Inverse variance weighted
MR Egger
10
8
6
1/SE_IV
−0.4
−0.2
0.0
0.2
0.4
0.6
β_IV

Batch 3−2600 : Selenium on Ribosome−binding protein 1

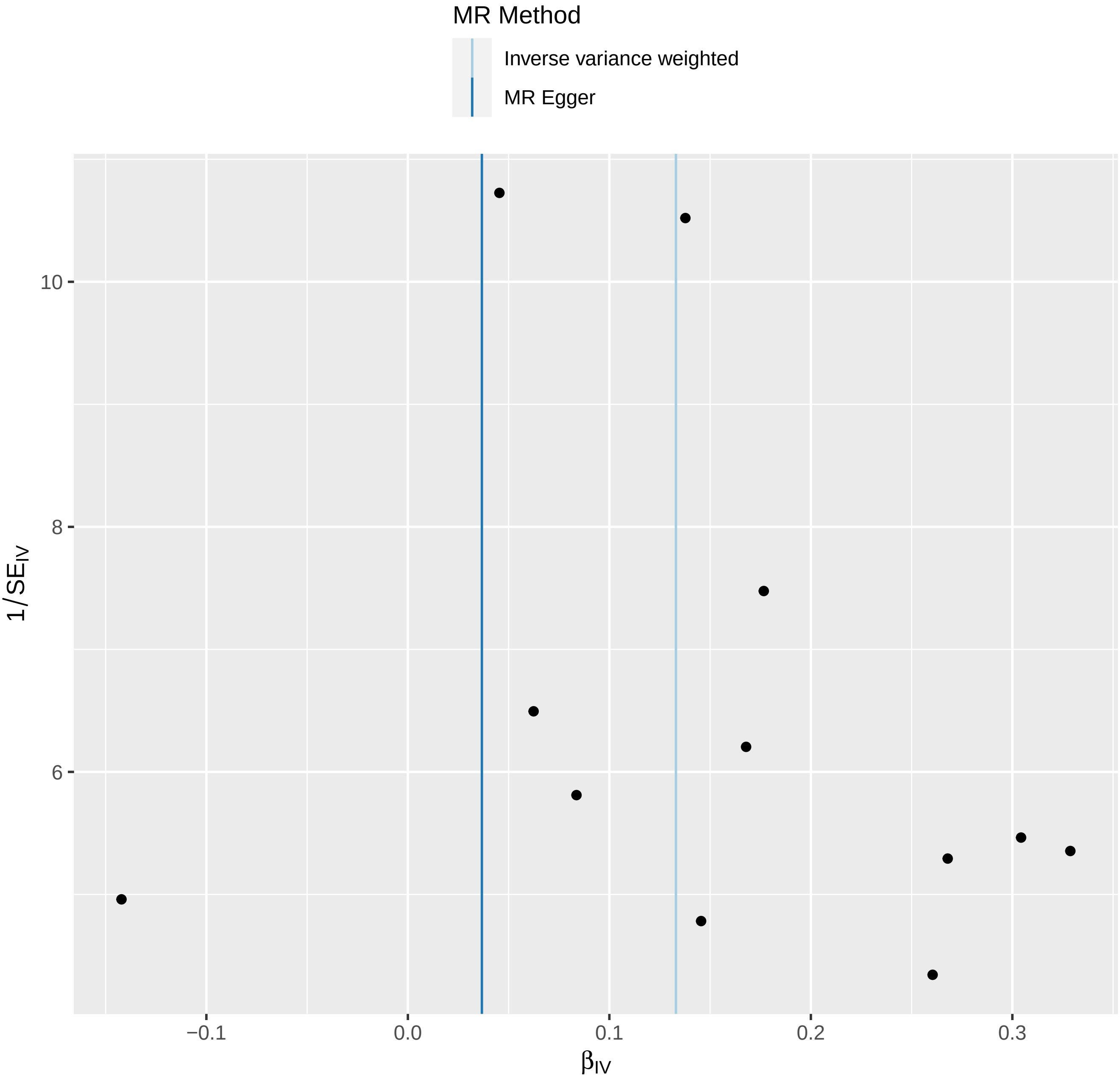

Batch 3−2601 : Selenium on Ribonucleoside−diphosphate reductase large subunit

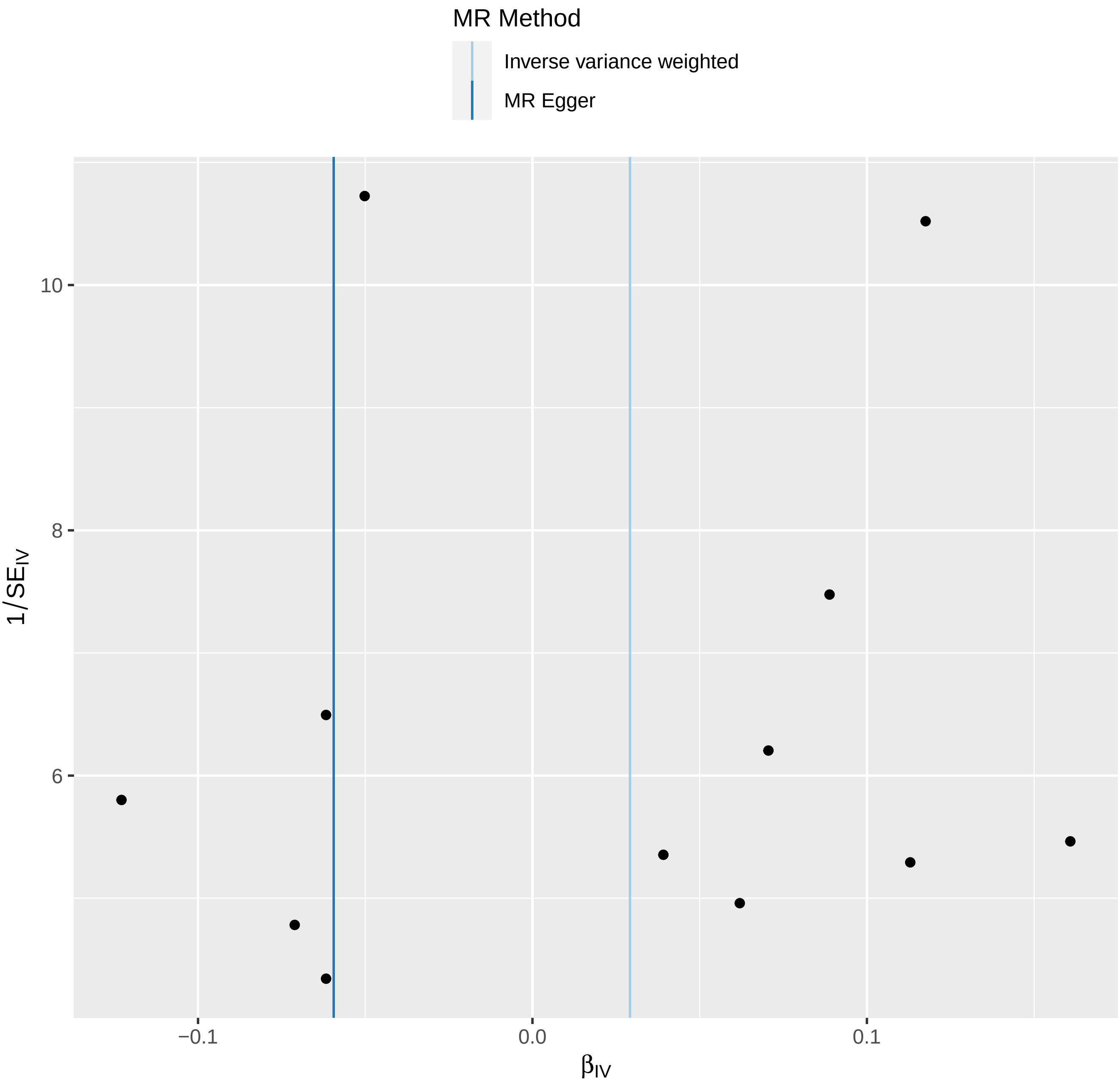

Batch 3−2602 : Selenium on Ribonucleoside−diphosphate reductase subunit M2 B

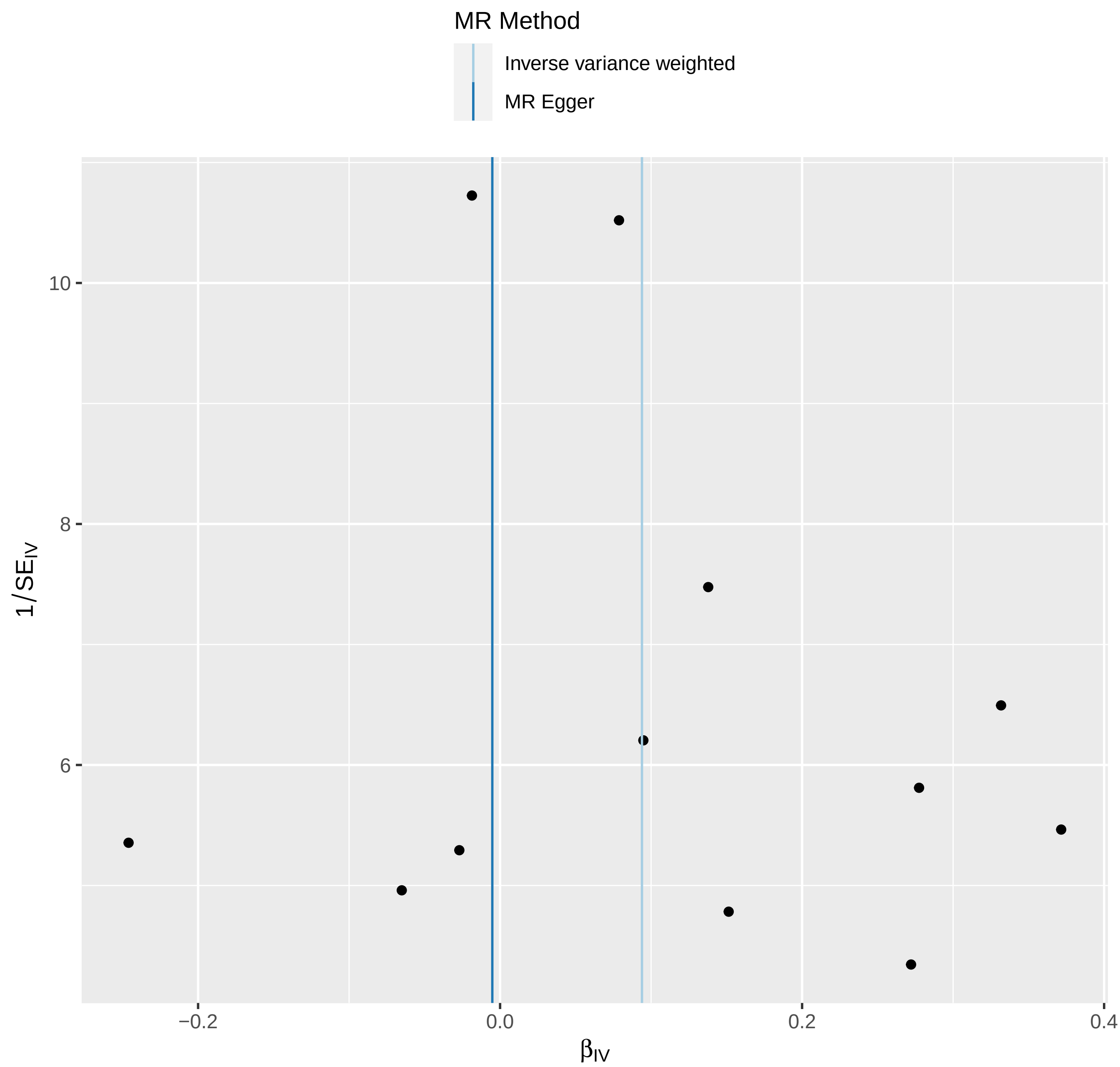

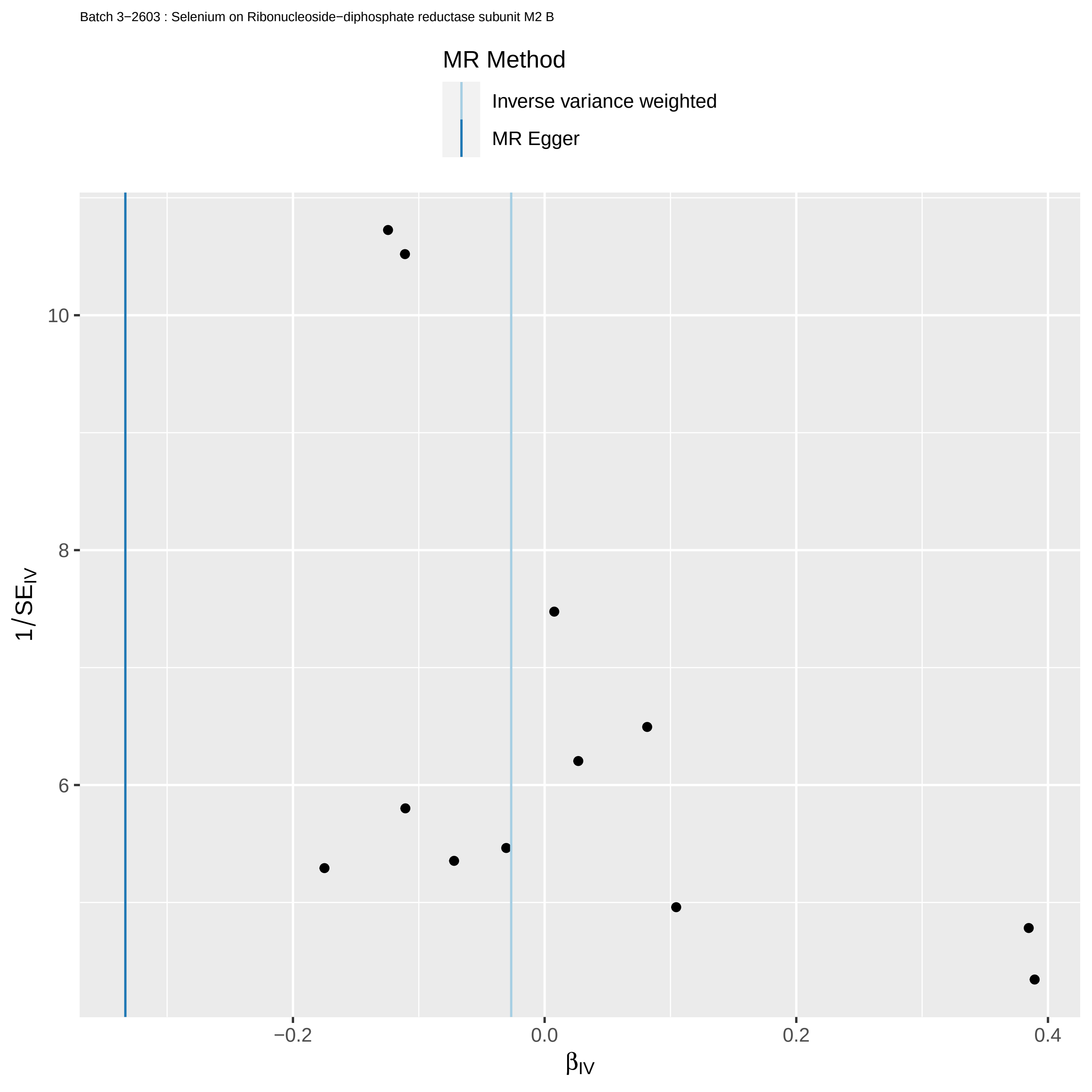

Batch 3−2603 : Selenium on Ribonucleoside−diphosphate reductase subunit M2 B
MR Method
Inverse variance weighted
MR Egger
10
8
6
$1/SE_{IV}$
−0.2
0.0
0.2
0.4
β_{IV}

Batch 3−2604 : Selenium on Retinoschisin

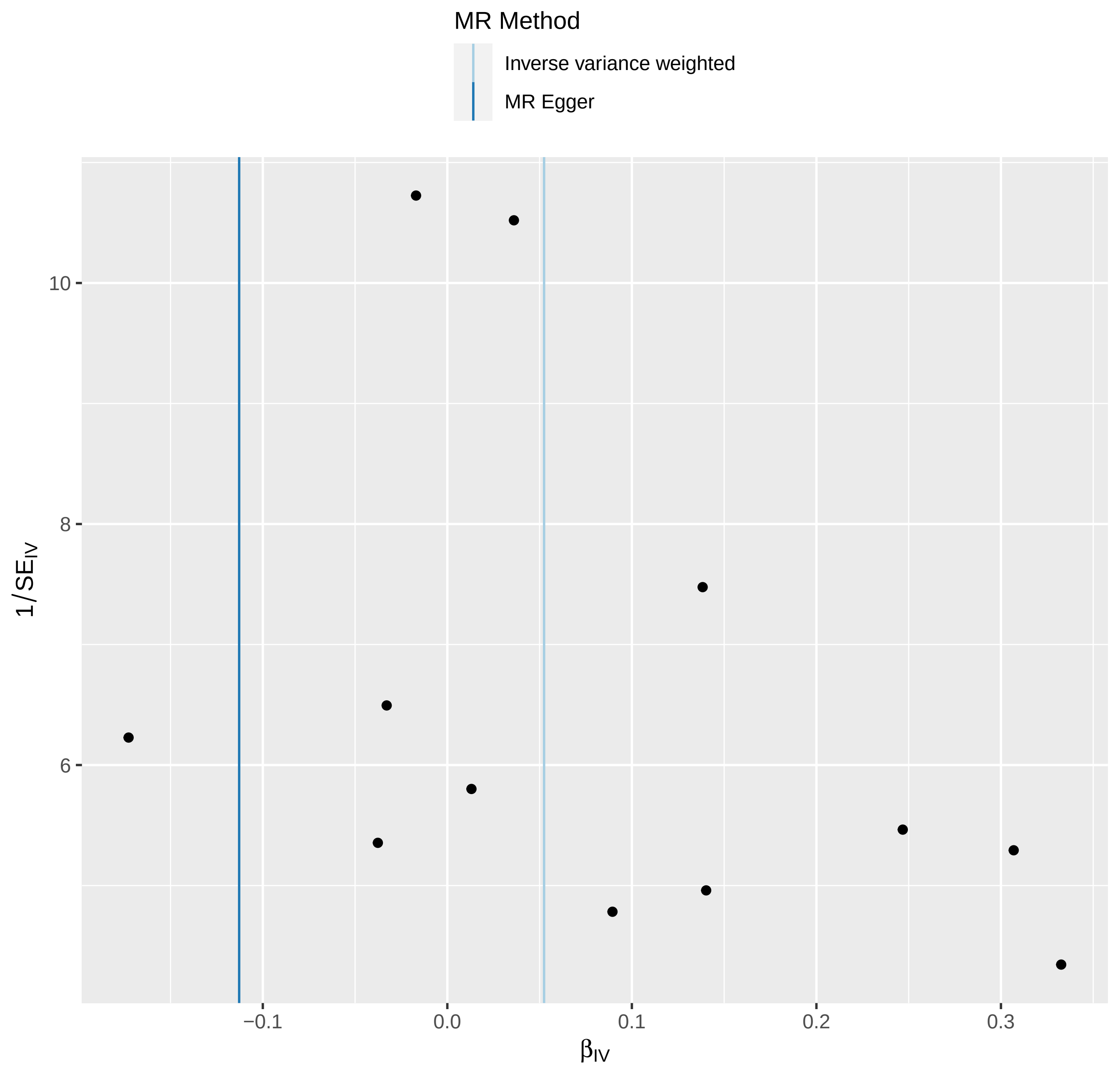

Batch 3−2605 : Selenium on R−spondin−3

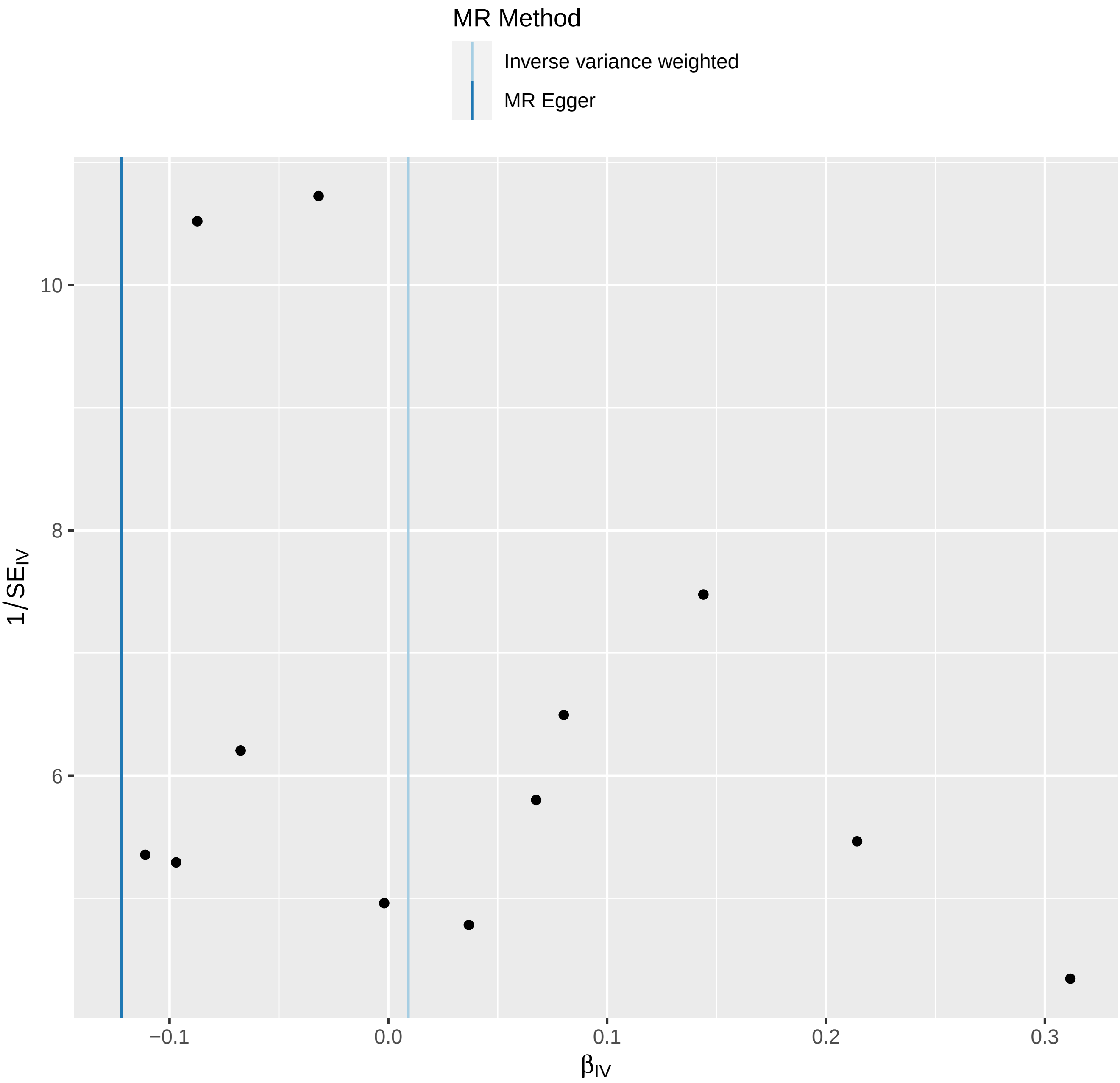

Batch 3−2606 : Selenium on R−spondin−4

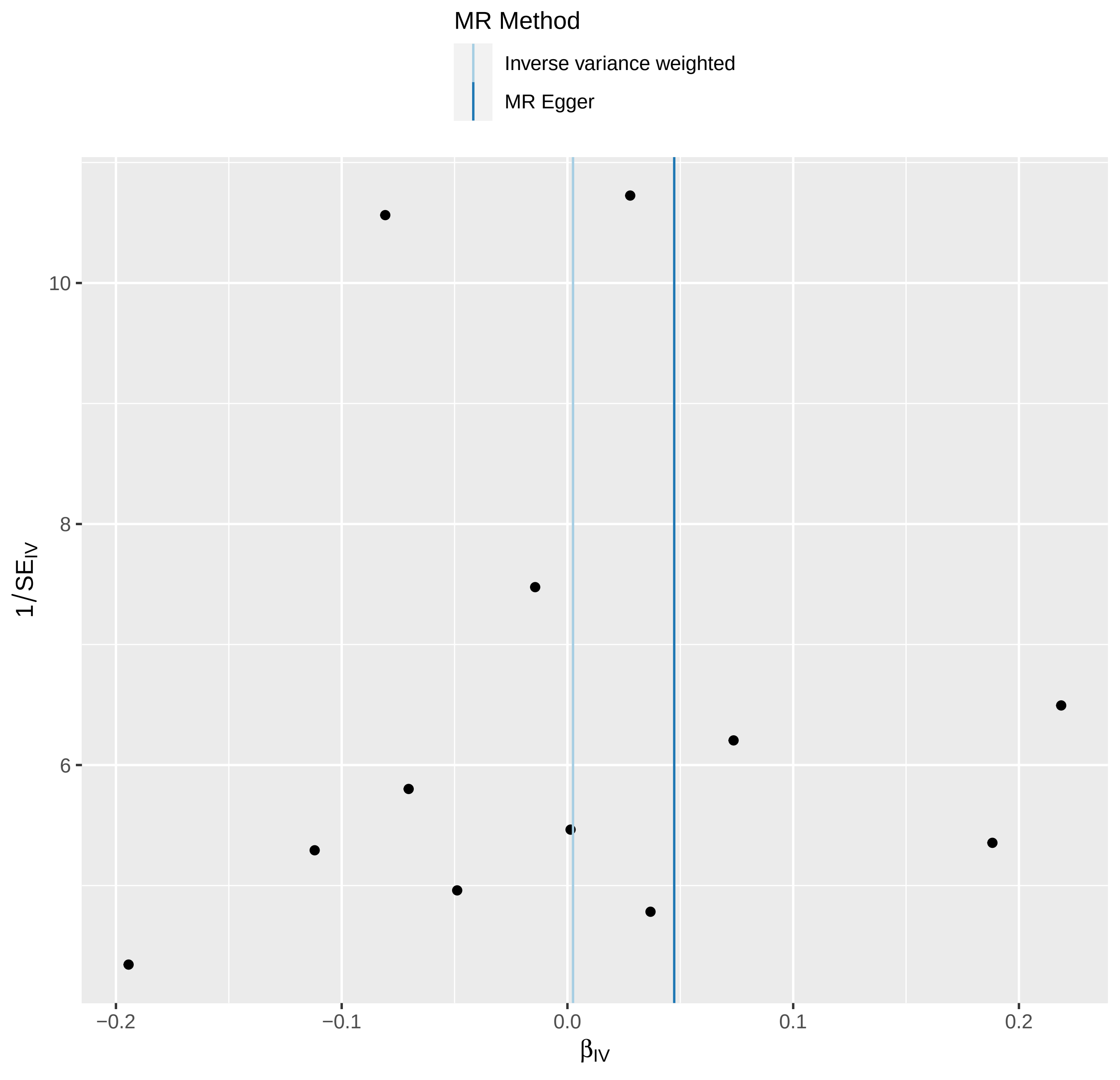

Batch 3−2607 : Selenium on Arginine/serine−rich protein 1

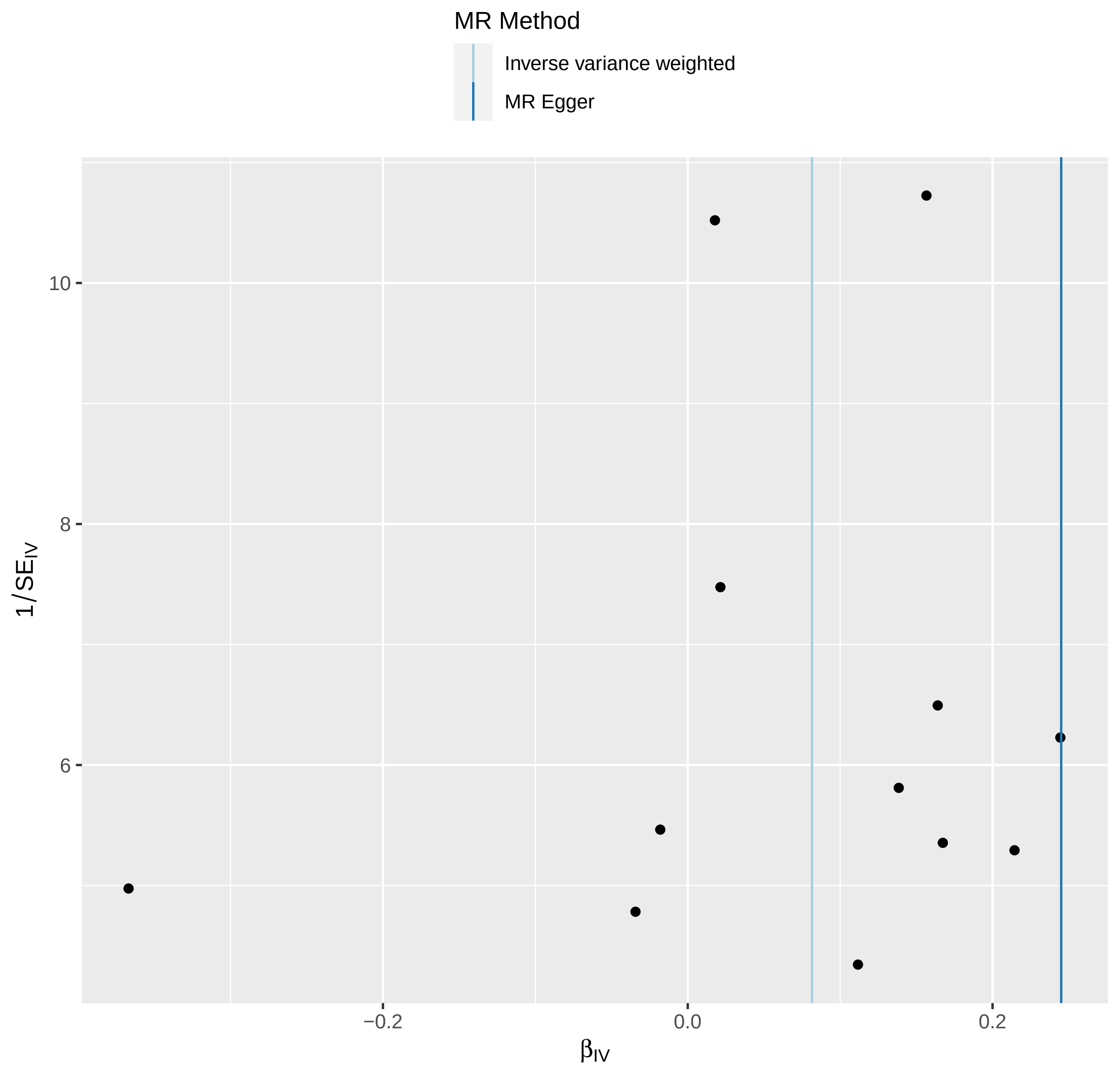

Batch 3−2608 : Selenium on Retbindin

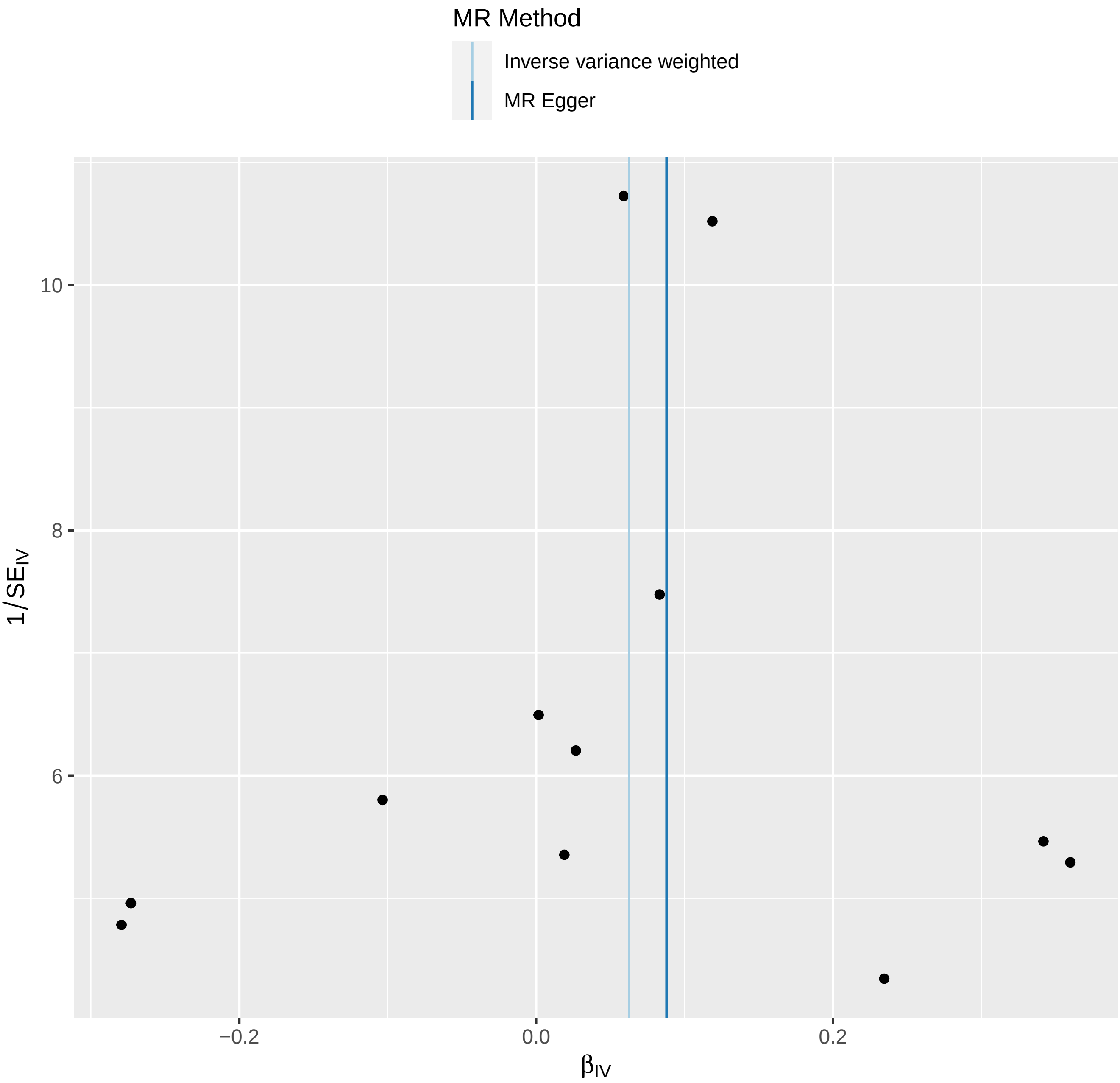

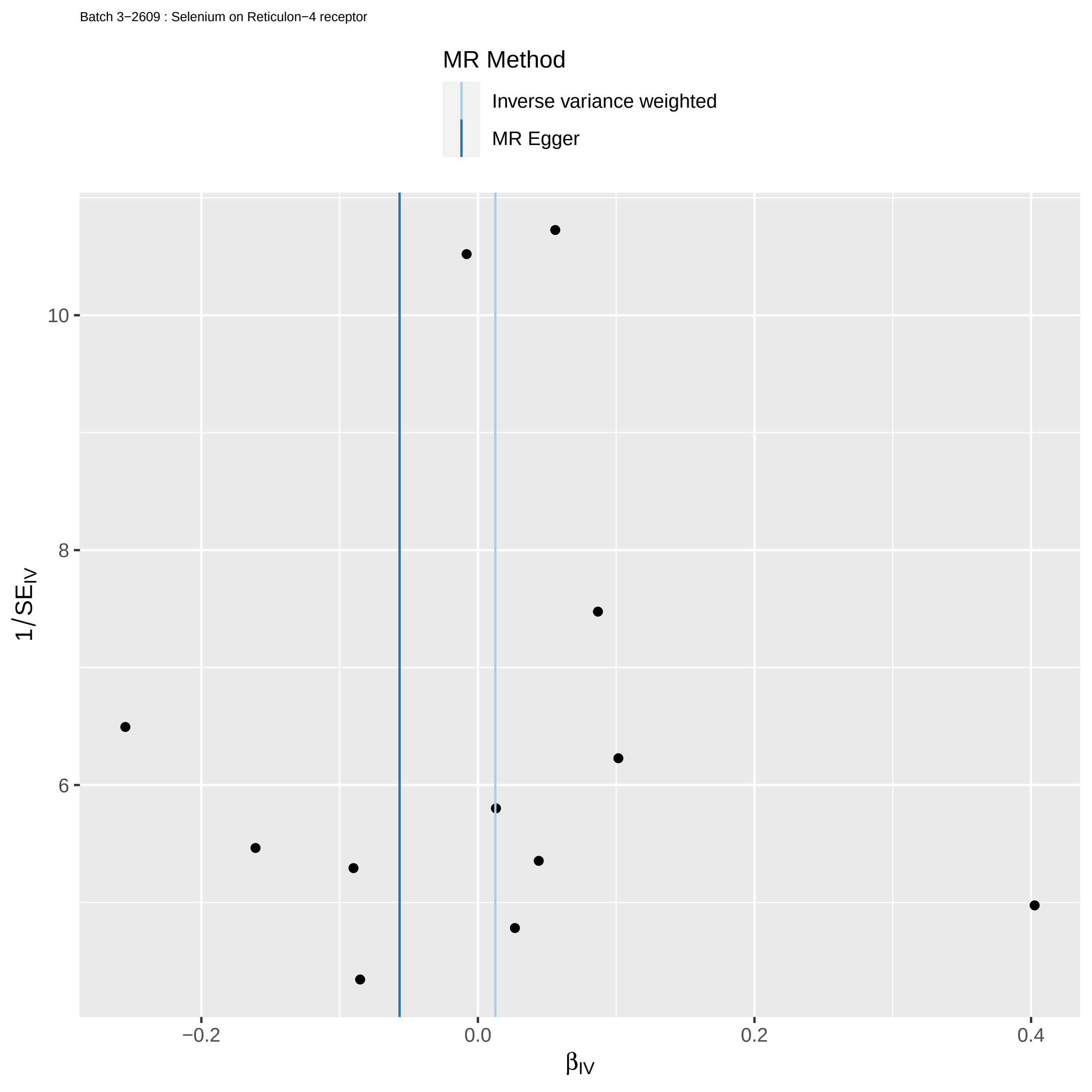

Batch 3−2609 : Selenium on Reticulon−4 receptor
MR Method
Inverse variance weighted
MR Egger
10
8
6
1/SE_IV
−0.2
0.0
0.2
0.4
β_IV

Batch 3−2610 : Selenium on RUN and FYVE domain−containing protein 1

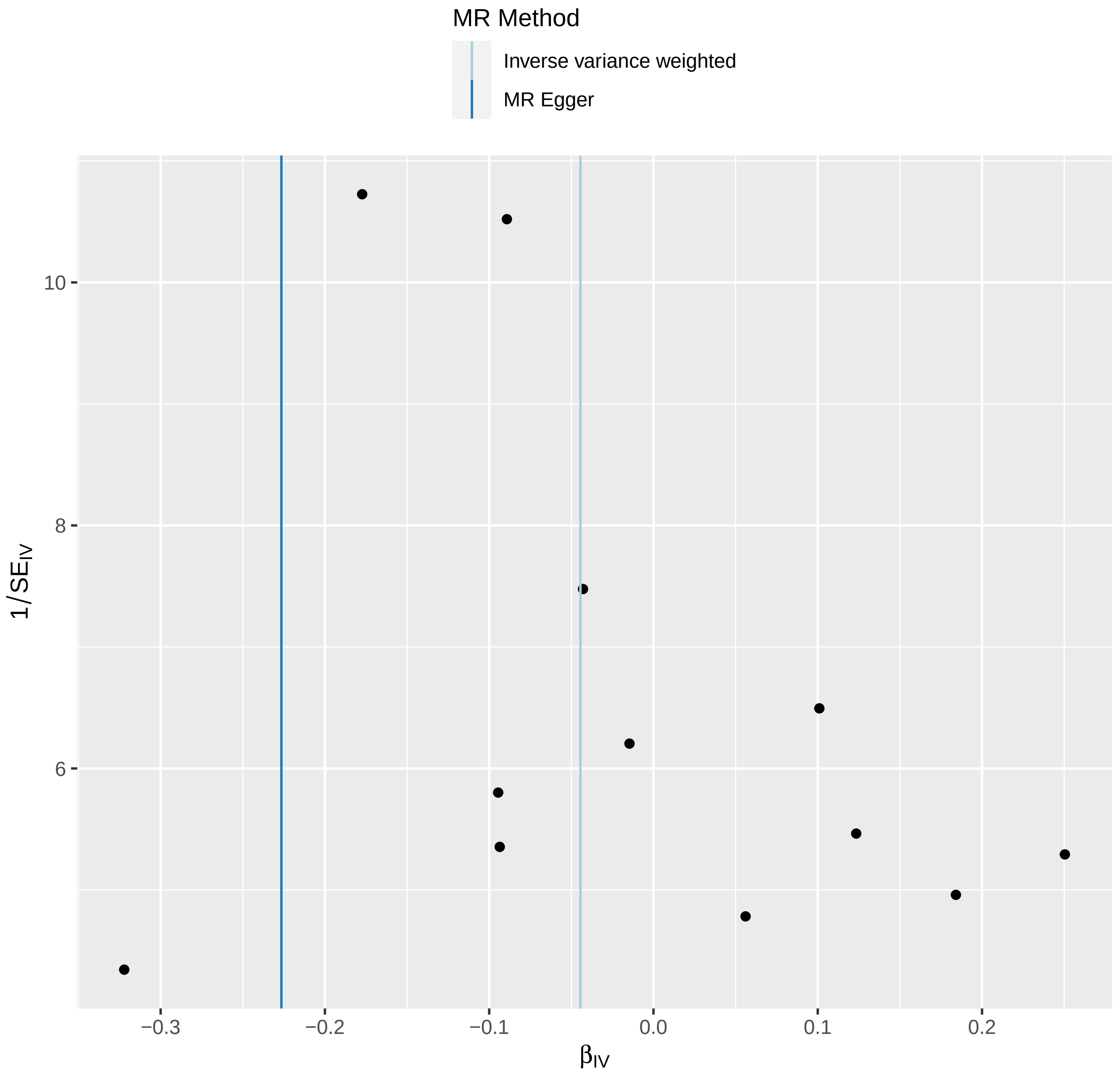

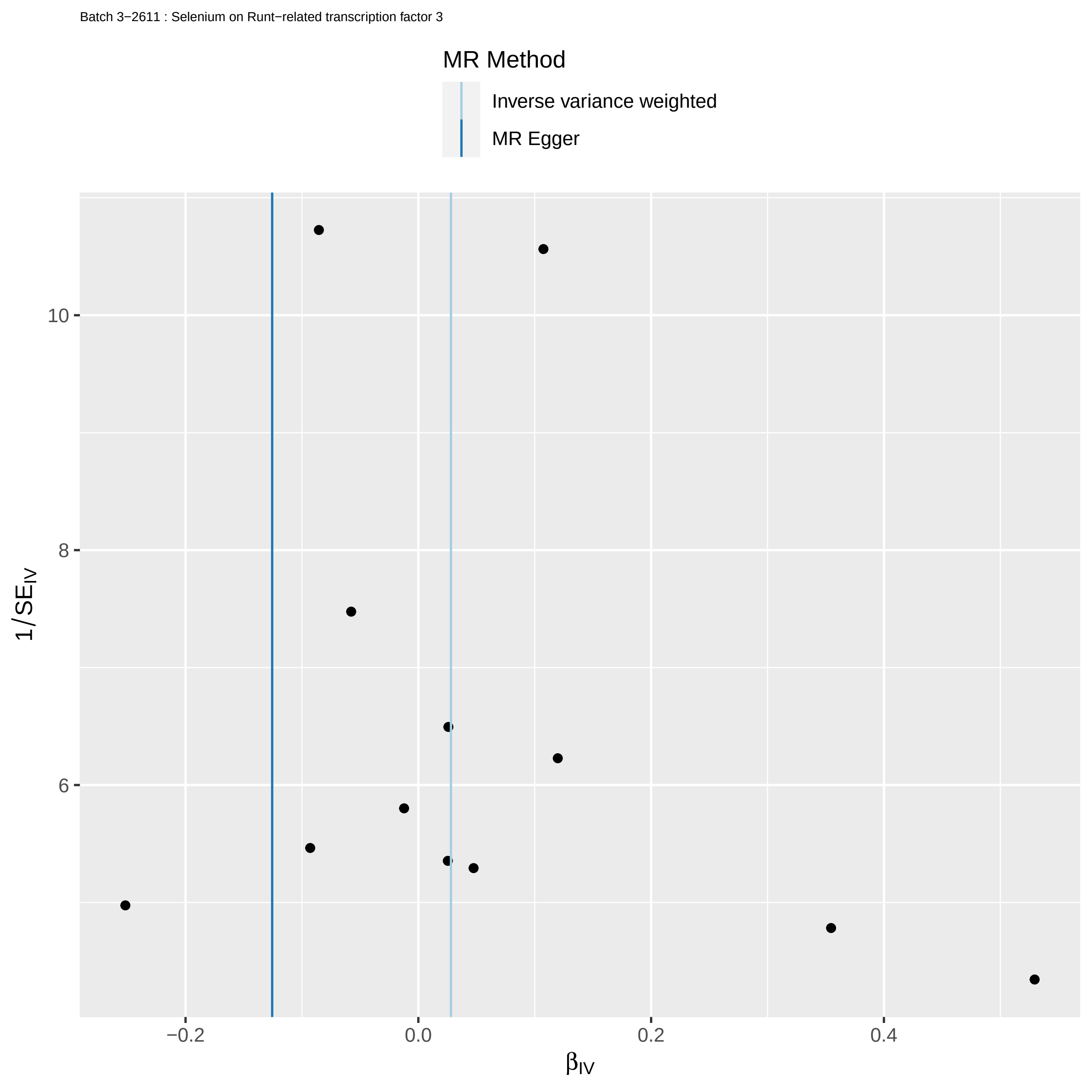

Batch 3−2611 : Selenium on Runt−related transcription factor 3
MR Method
Inverse variance weighted
MR Egger
10
8
6
1/SE$_{IV}$
−0.2
0.0
0.2
0.4
β$_{IV}$

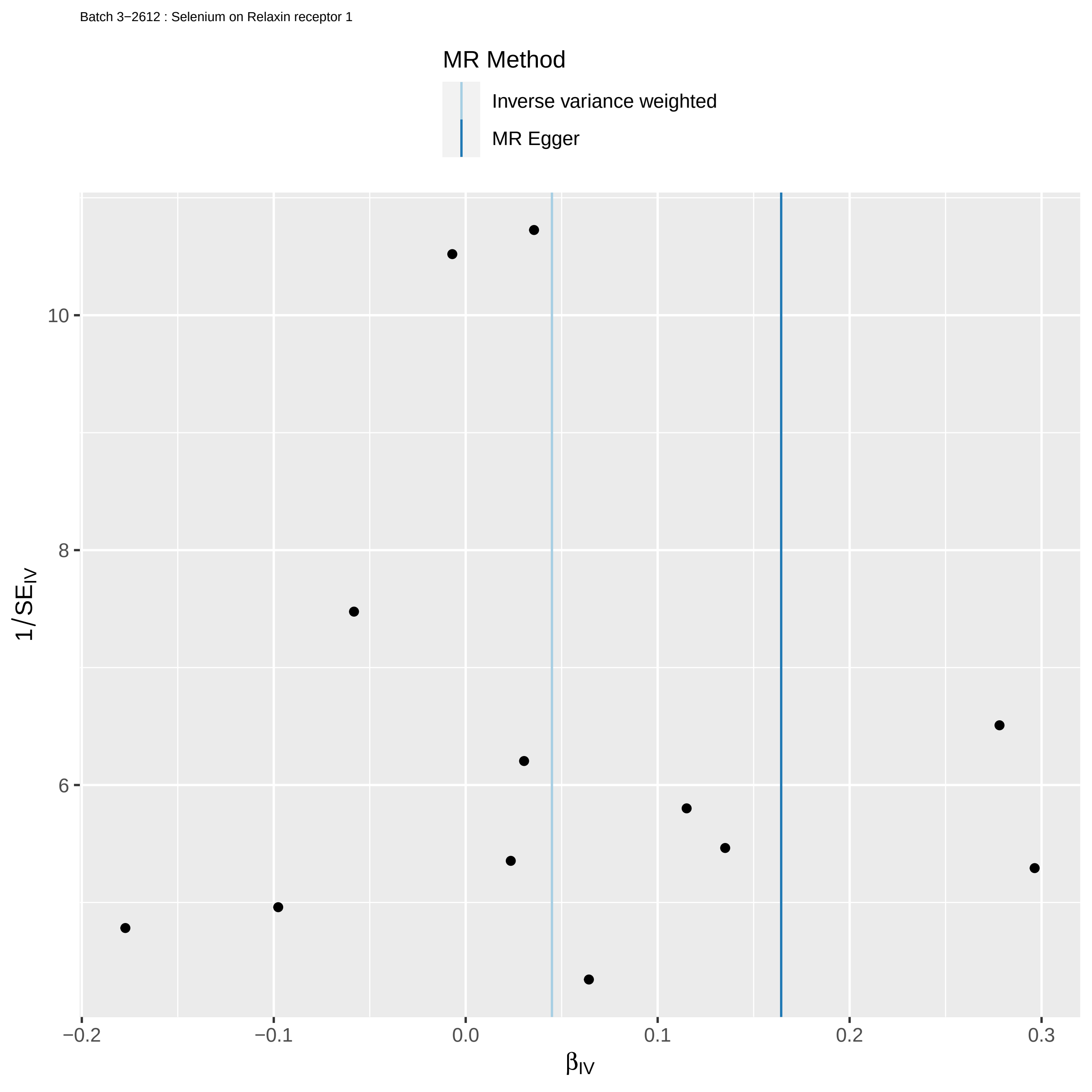
Batch 3−2612 : Selenium on Relaxin receptor 1
MR Method
Inverse variance weighted
MR Egger
10
8
6
1/SE_IV
−0.2
−0.1
0.0
0.1
0.2
0.3
β_IV

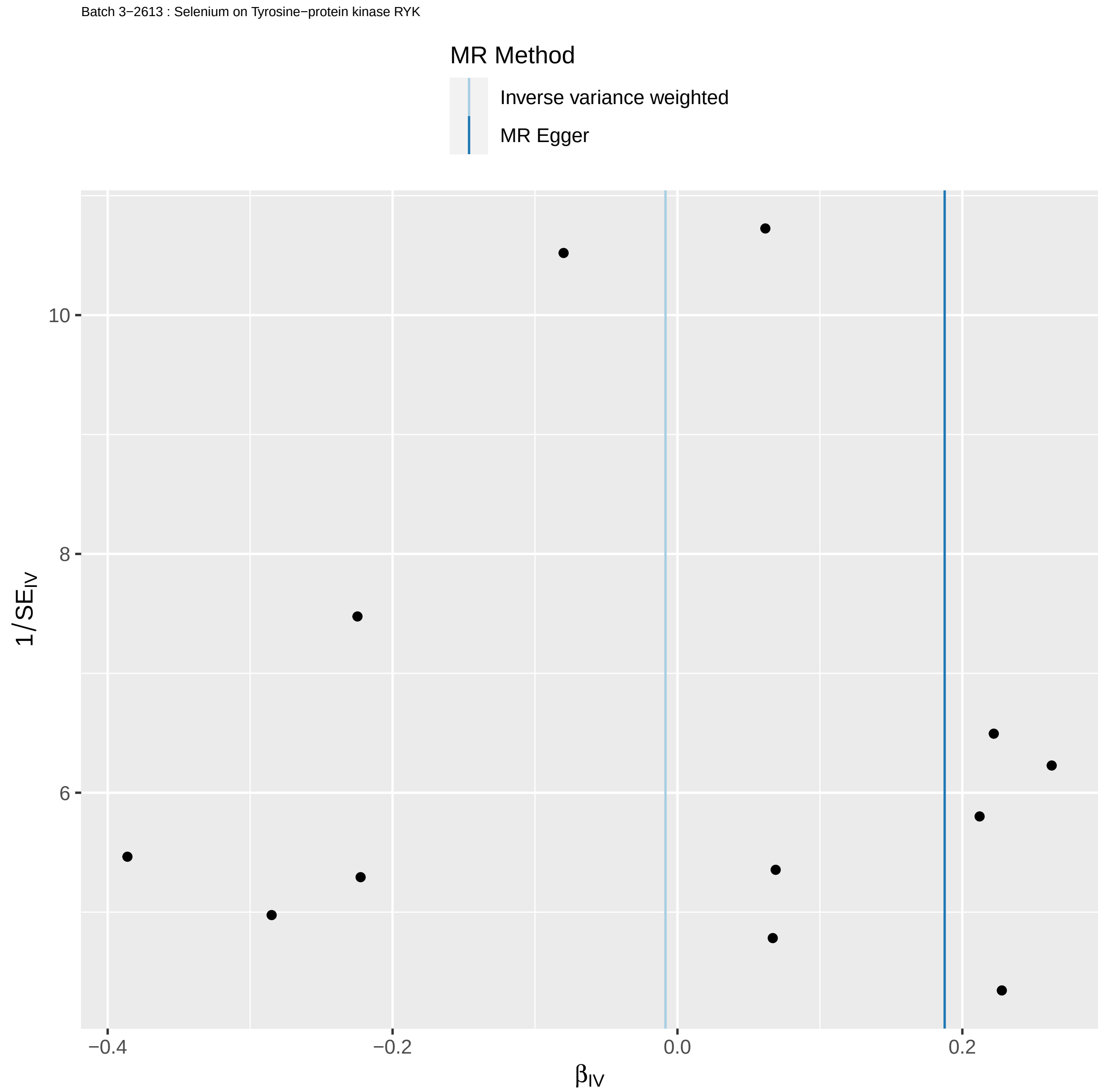
Batch 3−2613 : Selenium on Tyrosine−protein kinase RYK
MR Method
Inverse variance weighted
MR Egger
10
8
6
1/SE$_{IV}$
−0.4
−0.2
0.0
0.2
β$_{IV}$

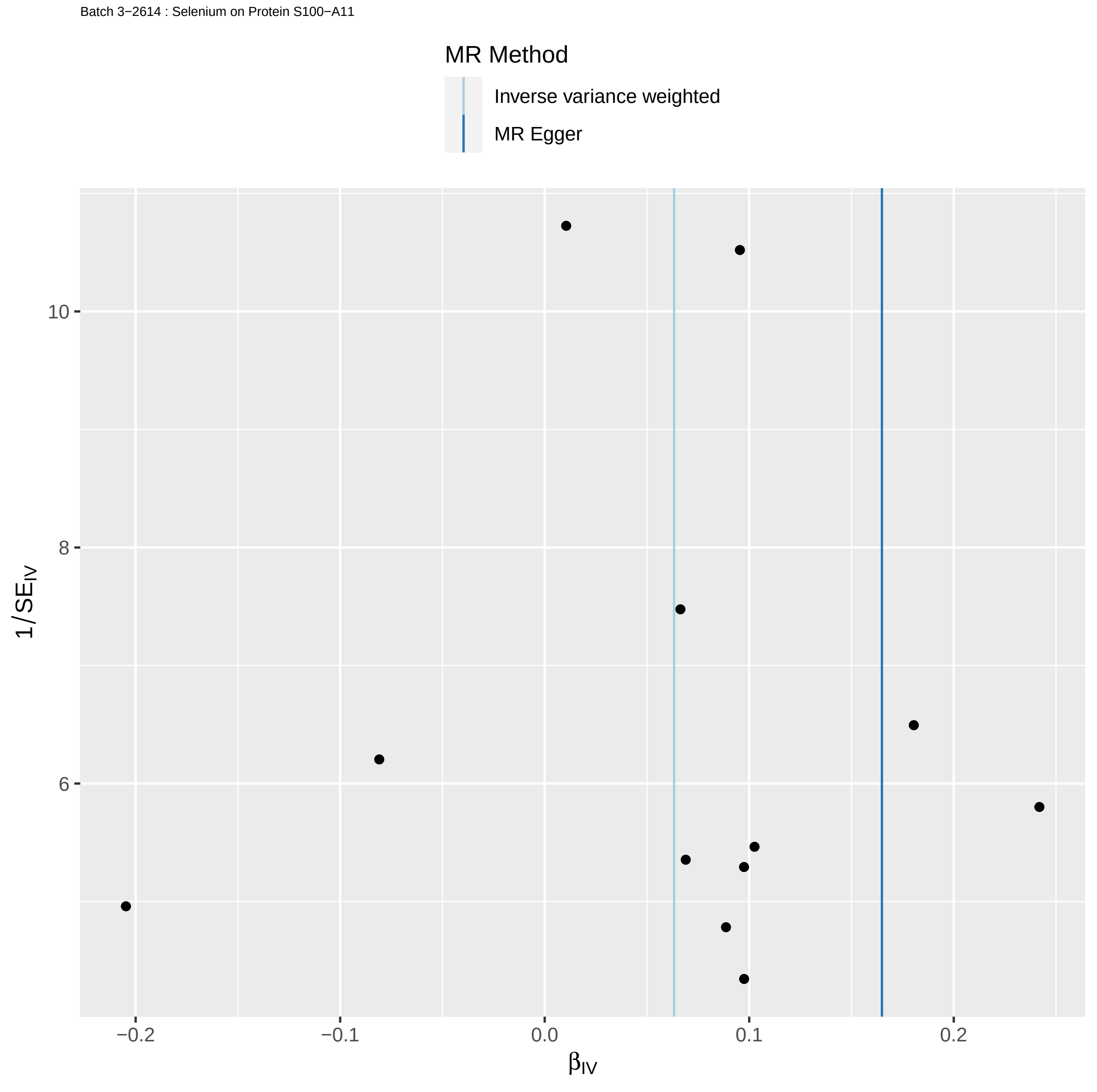
Batch 3−2614 : Selenium on Protein S100−A11
MR Method
Inverse variance weighted
MR Egger
10
8
6
1/SE_IV
−0.2
−0.1
0.0
0.1
0.2
β_IV

Batch 3−2615 : Selenium on Protein S100−A12

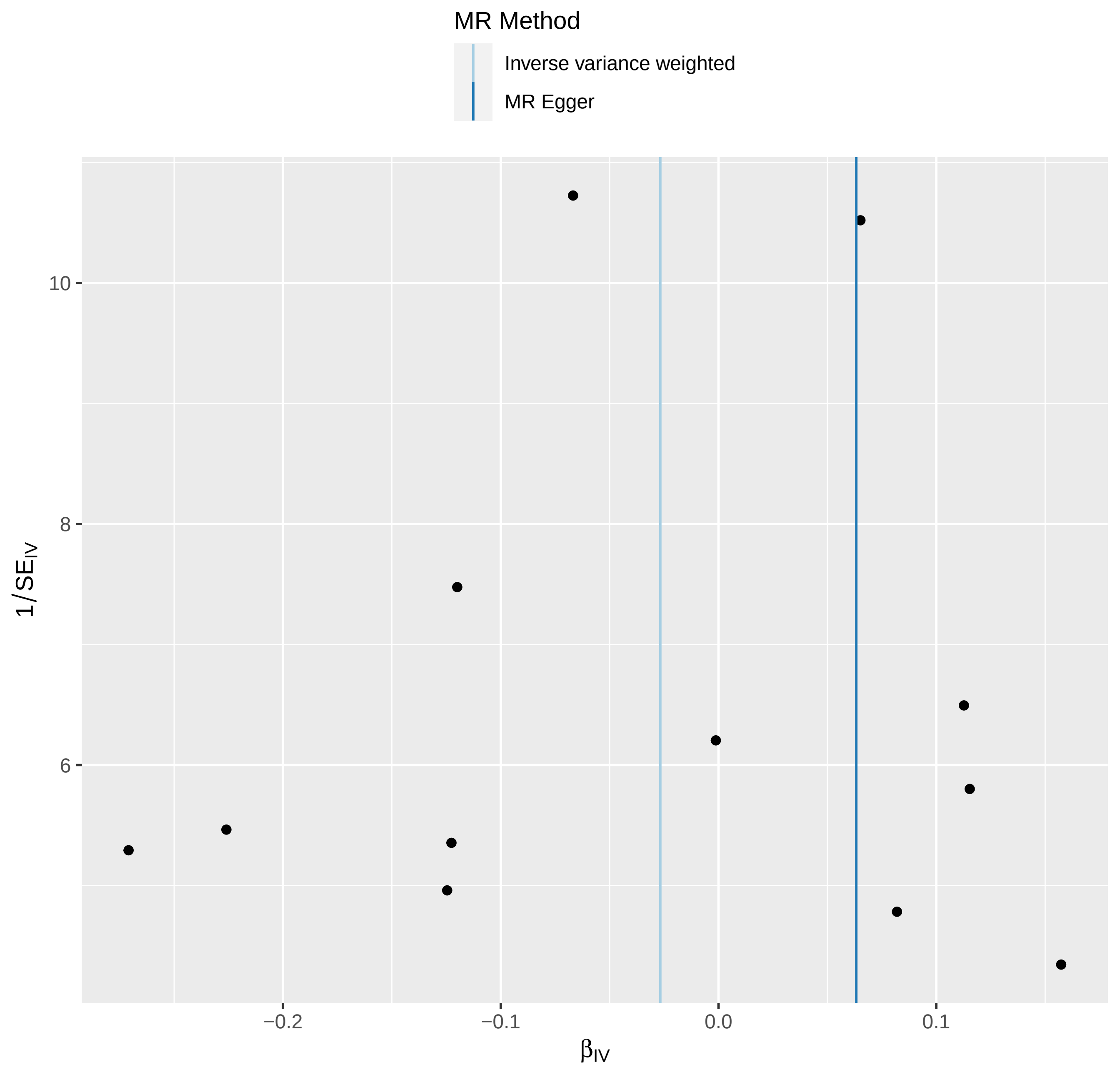

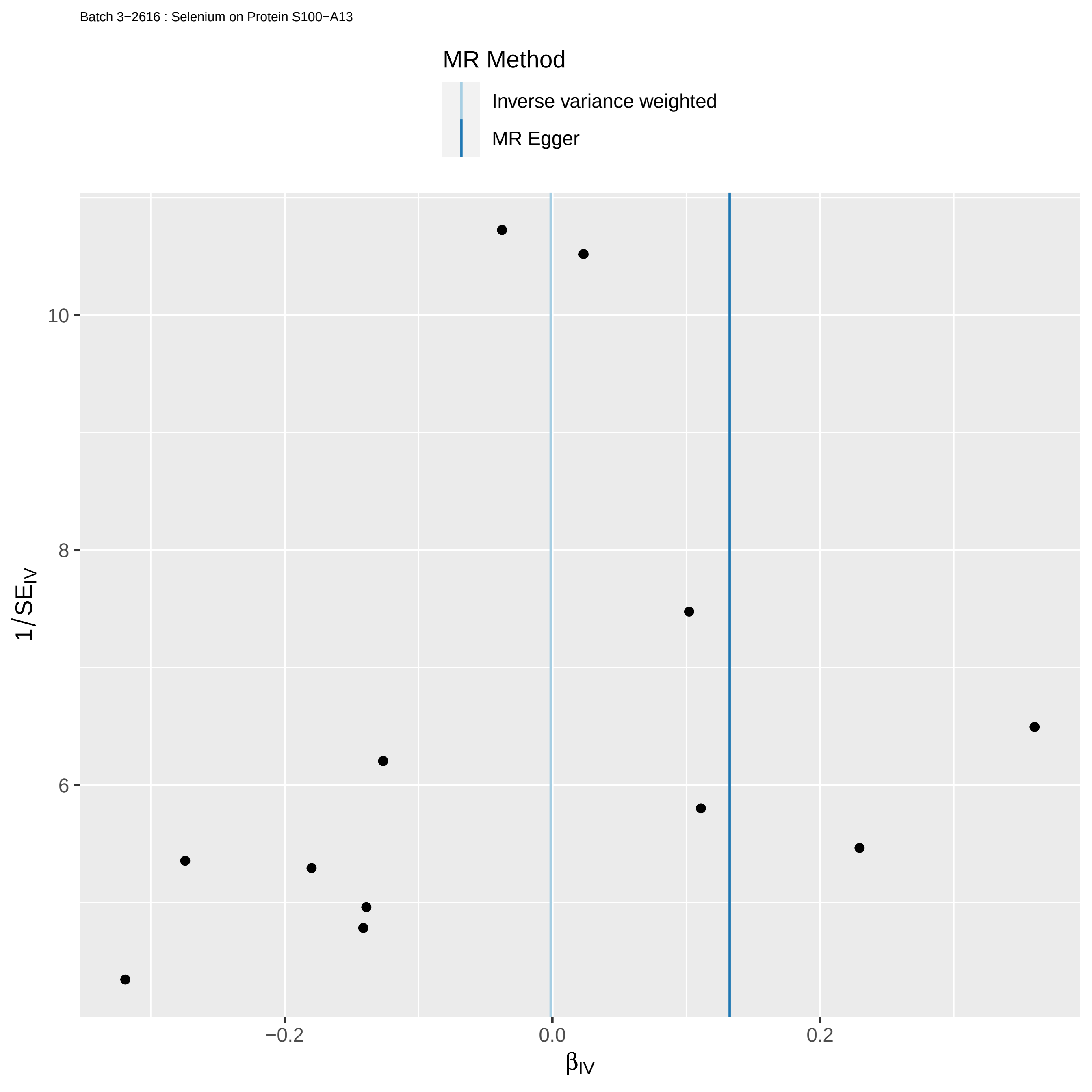

Batch 3−2616 : Selenium on Protein S100−A13
MR Method
Inverse variance weighted
MR Egger
10
8
6
1/SE$_{IV}$
−0.2
0.0
0.2
β$_{IV}$

Batch 3−2617 : Selenium on Protein S100−A2

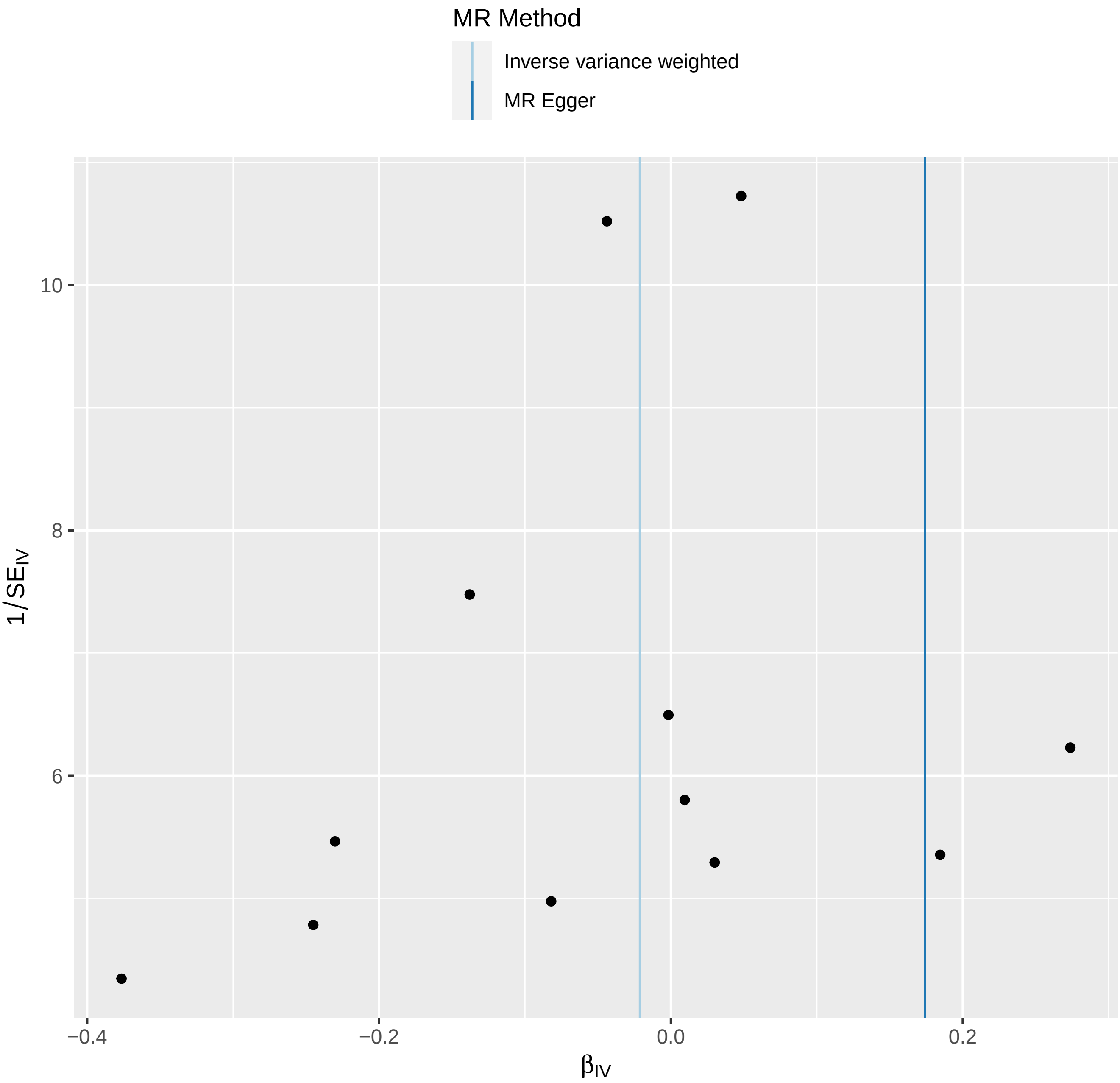

Batch 3−2618 : Selenium on Protein S100−A4

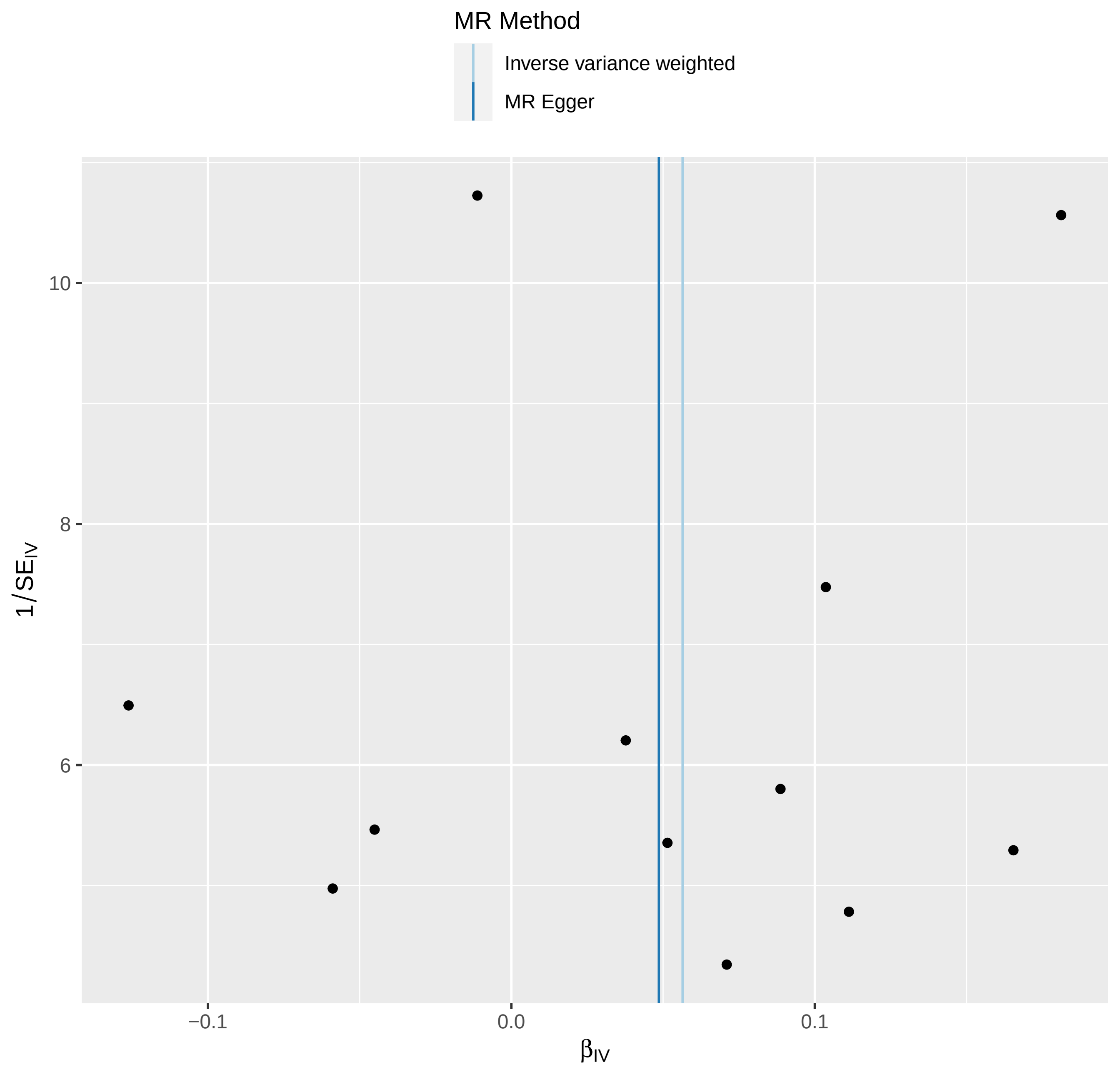

Batch 3−2619 : Selenium on Protein S100−A5

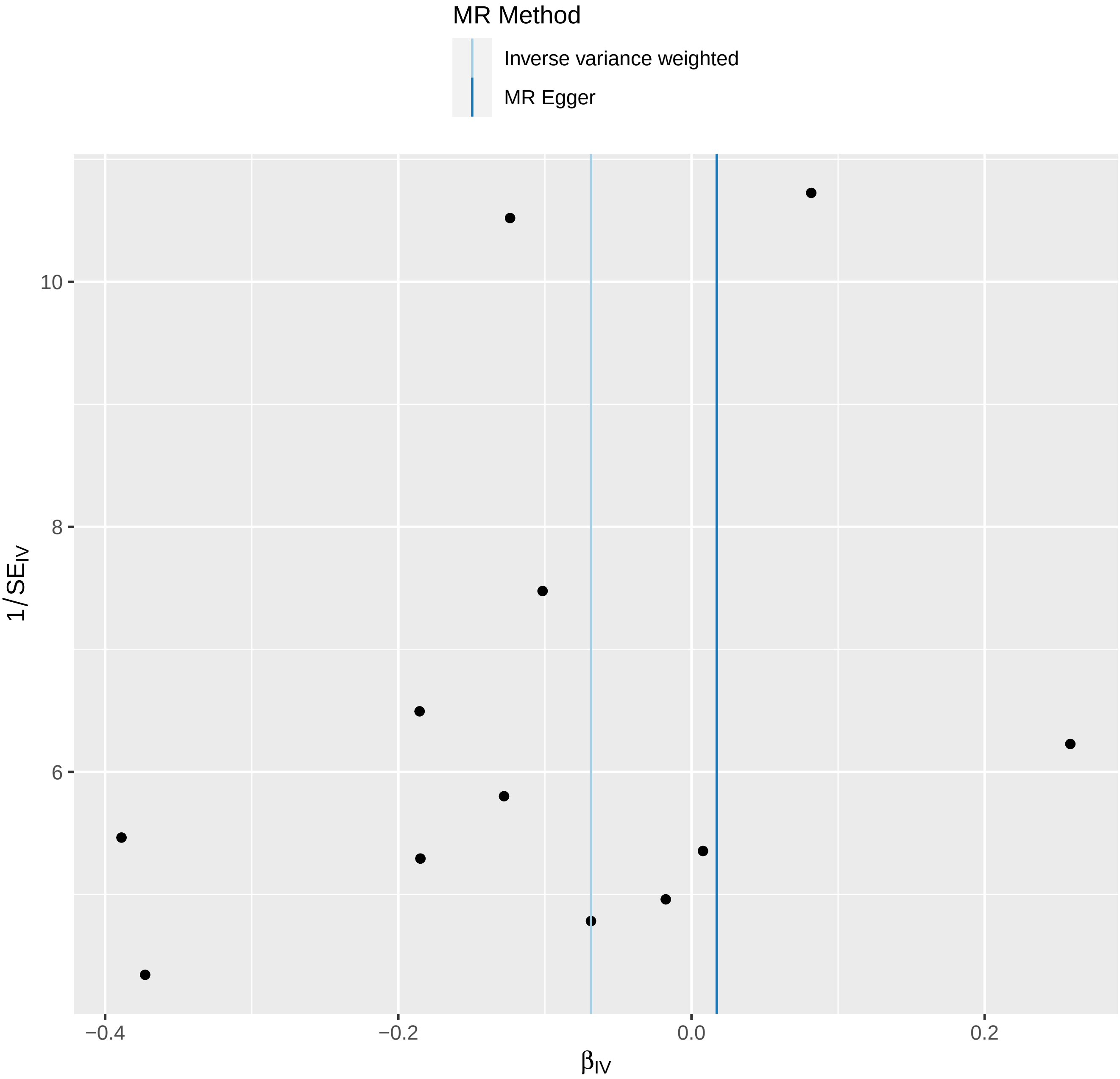

Batch 3−2620 : Selenium on Protein S100−A6

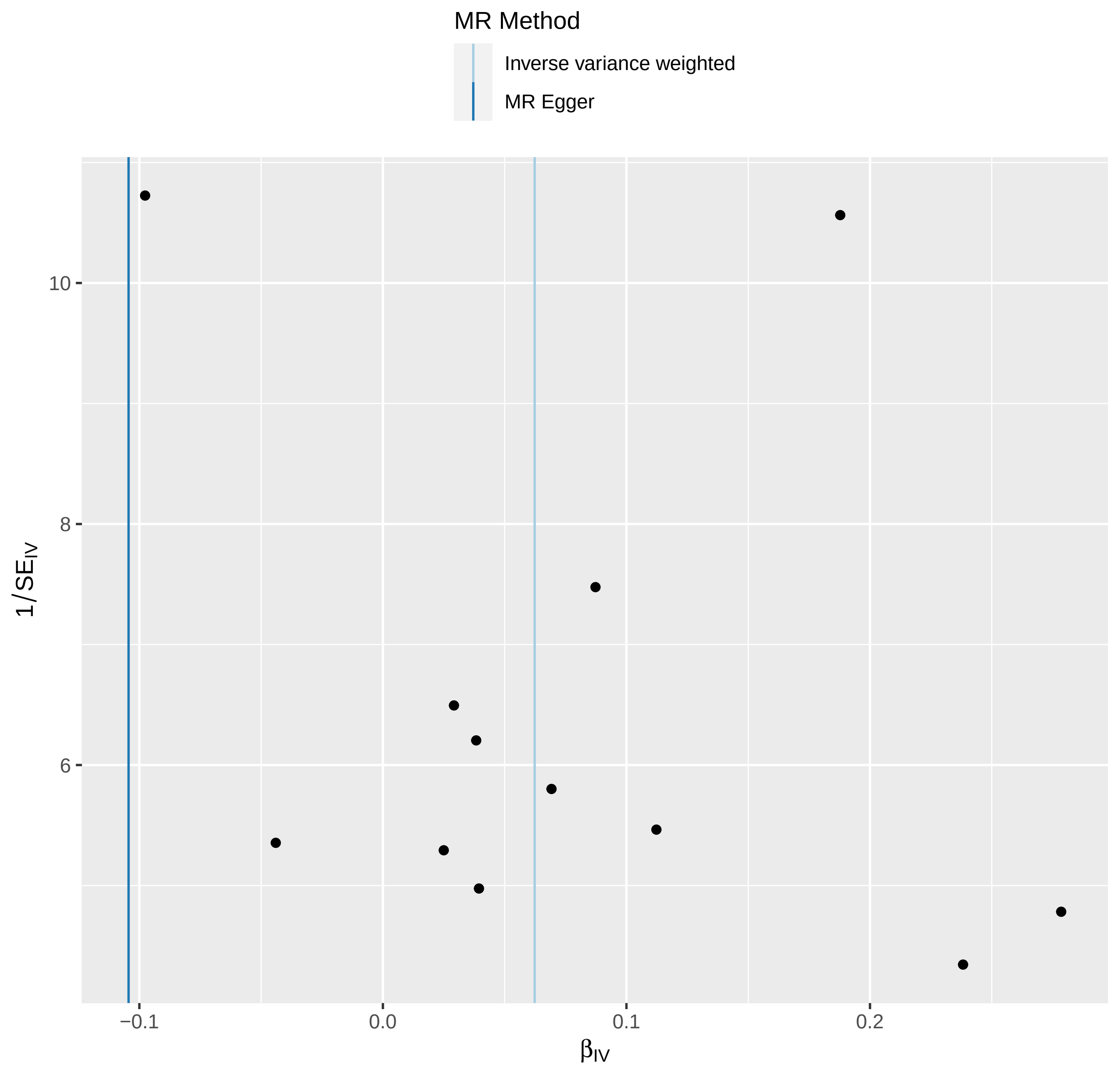

Batch 3−2621 : Selenium on Protein S100−A7

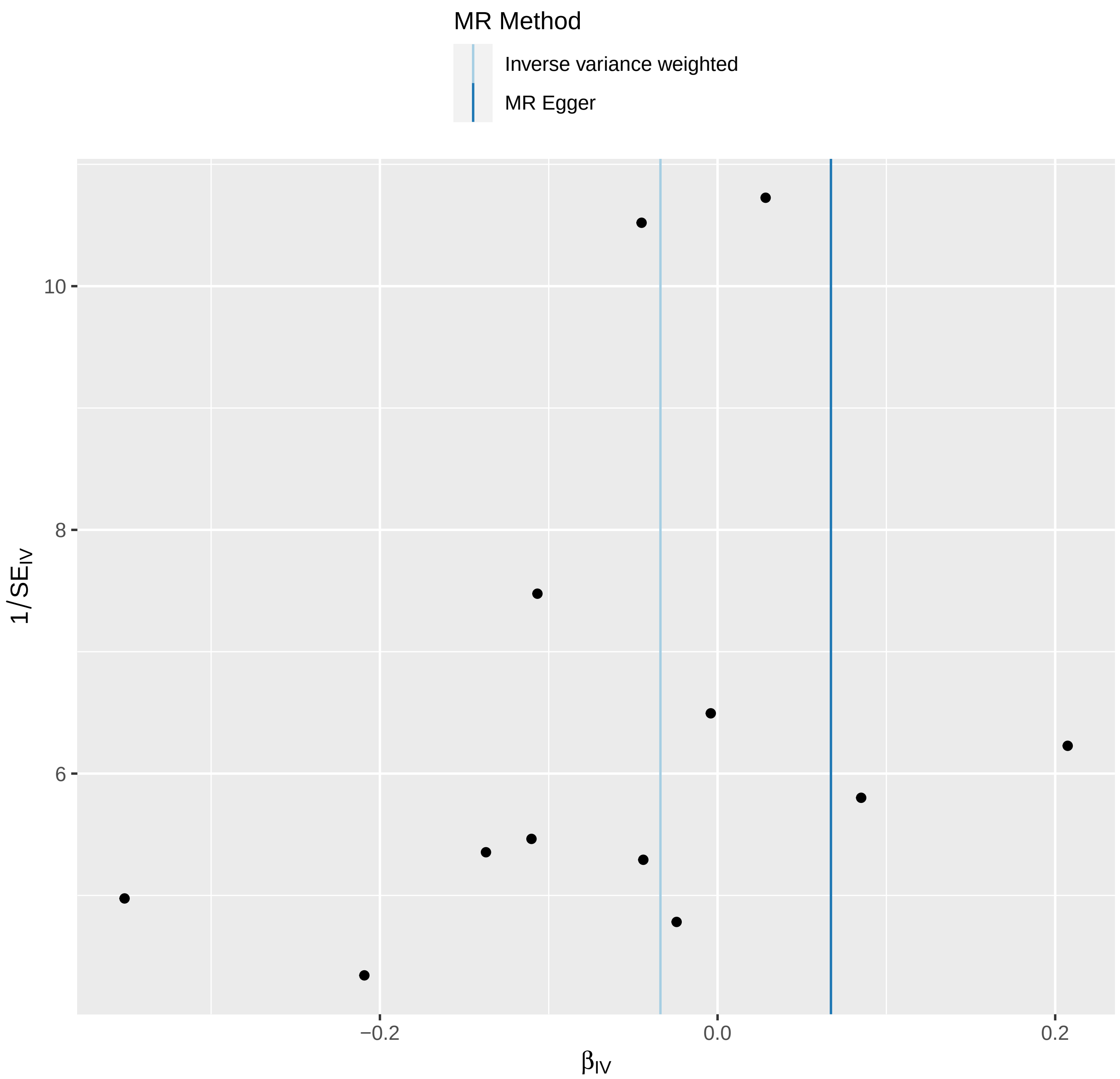

Batch 3−2622 : Selenium on Protein S100−A9

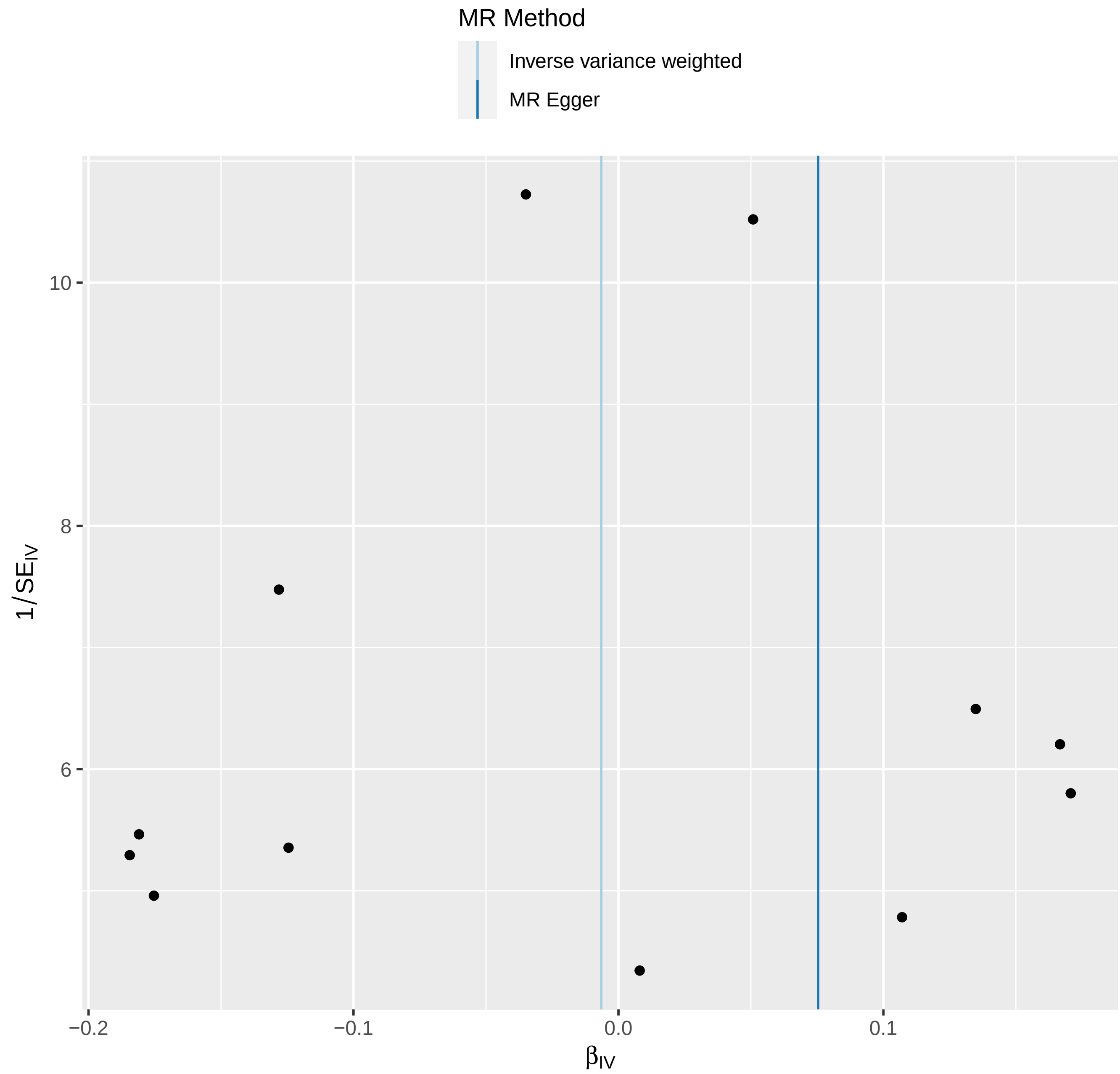

Batch 3−2623 : Selenium on Serum amyloid A−1 protein

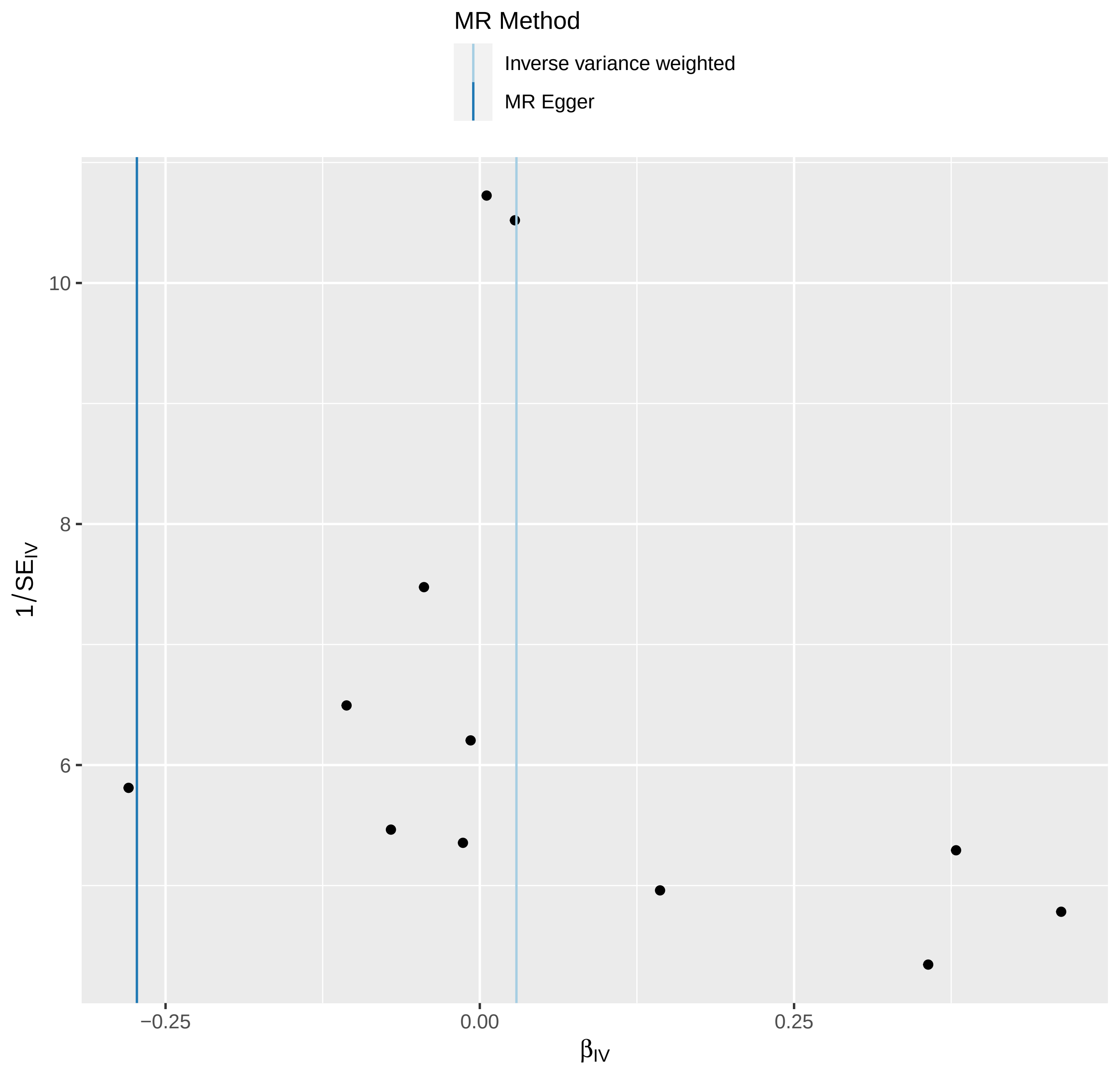

Batch 3−2624 : Selenium on Deoxynucleoside triphosphate triphosphohydrolase SAMHD1

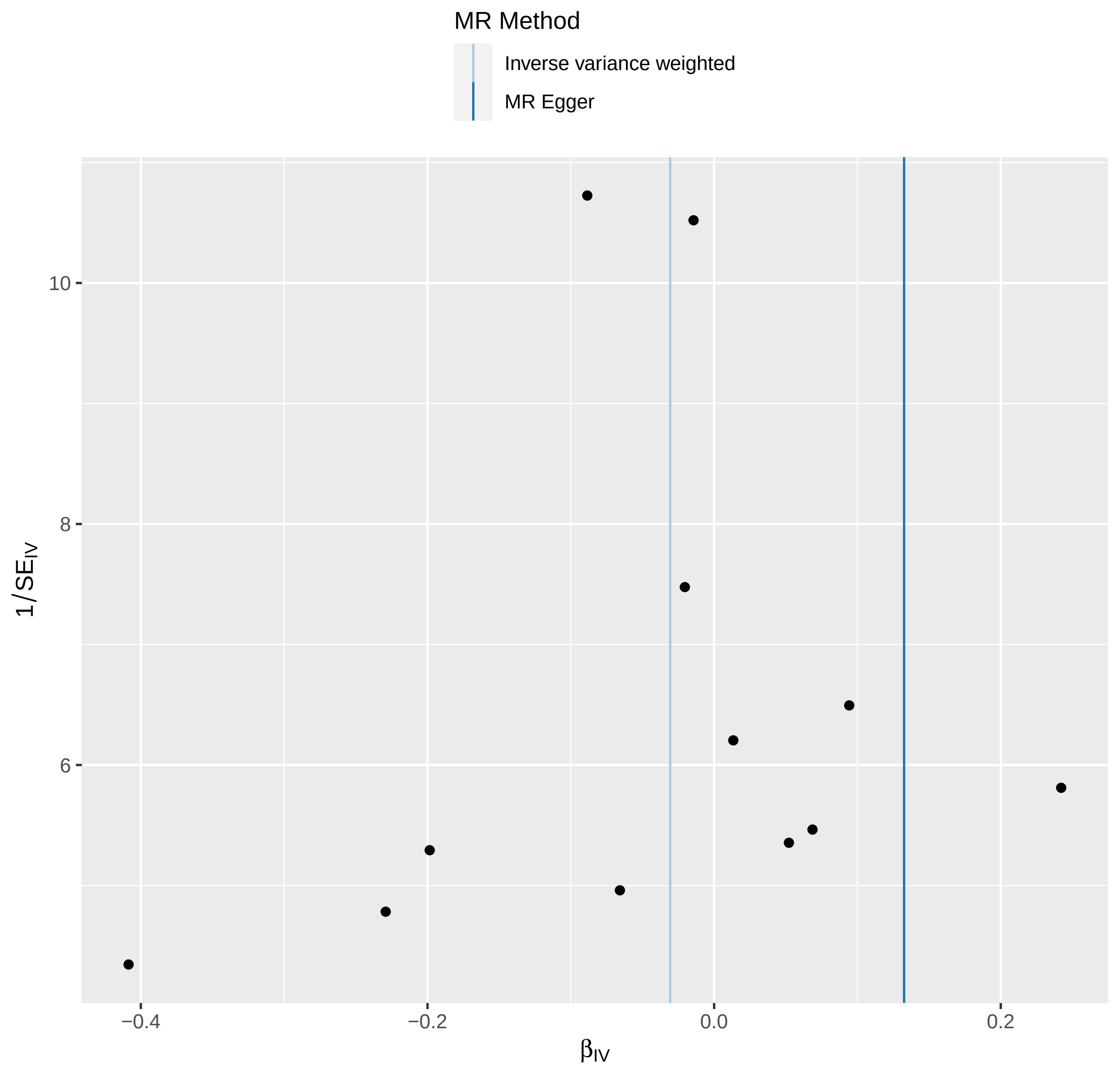

Batch 3−2625 : Selenium on Histone deacetylase complex subunit SAP18

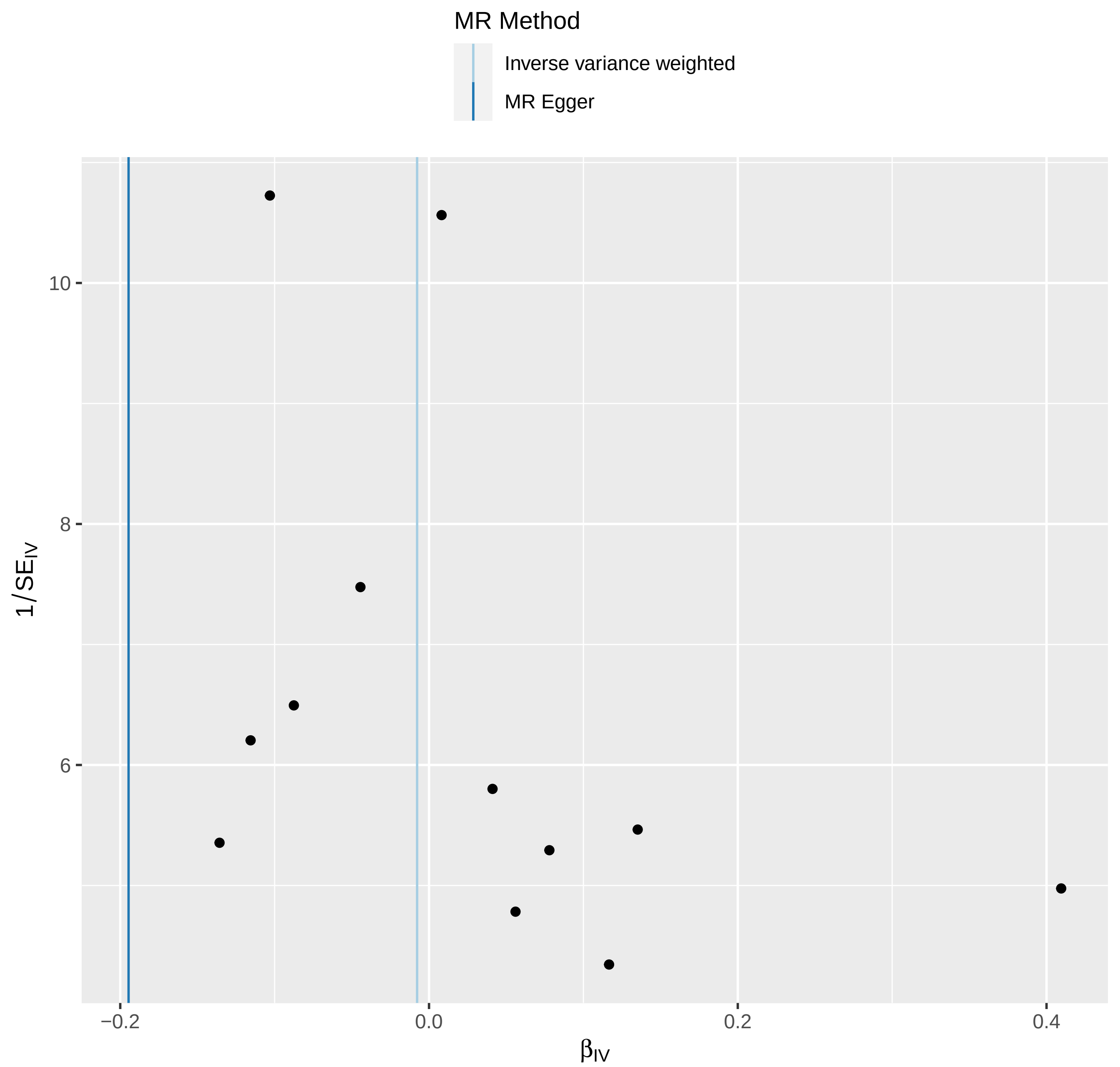

Batch 3−2626 : Selenium on Histone deacetylase complex subunit SAP30

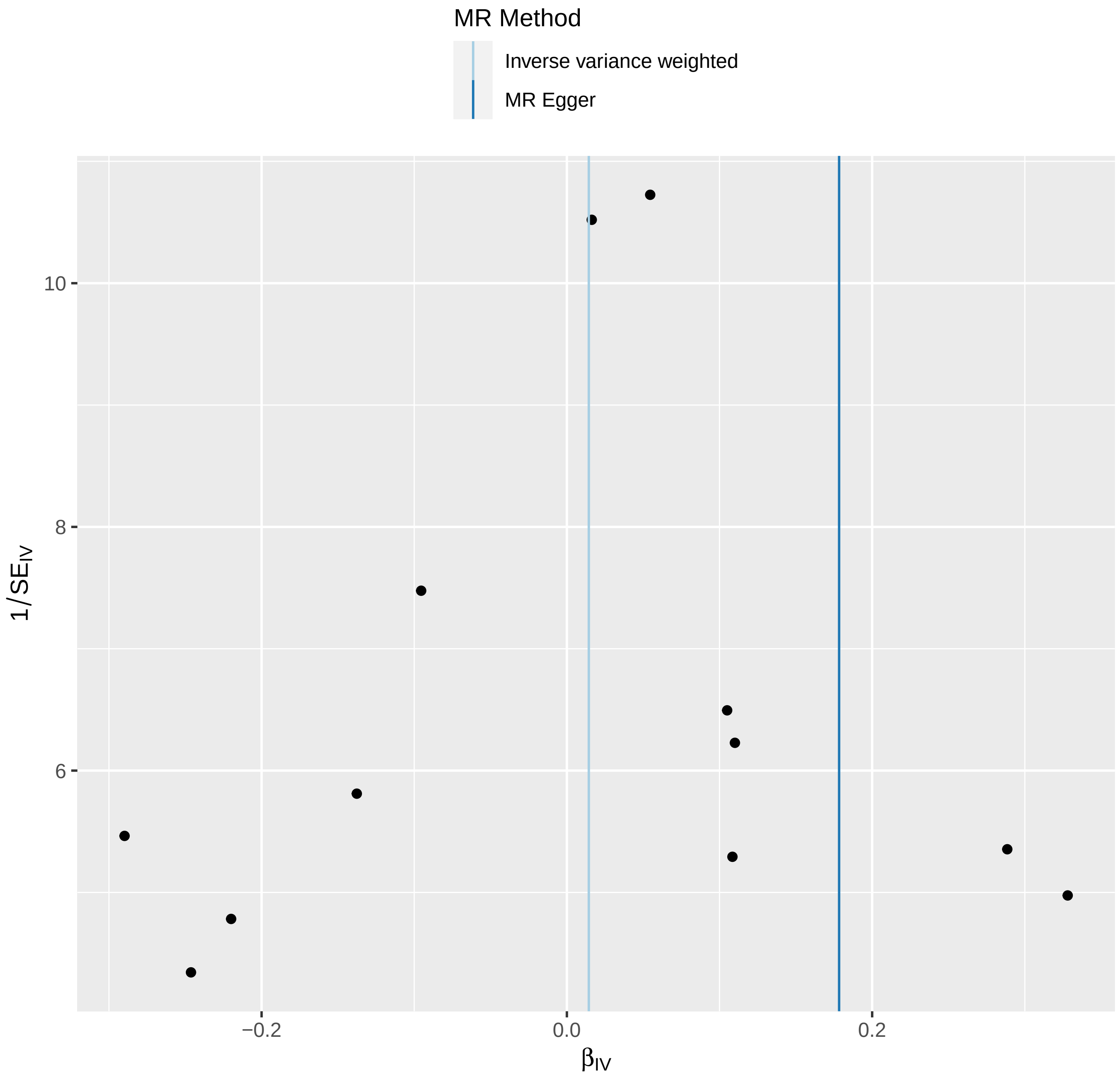

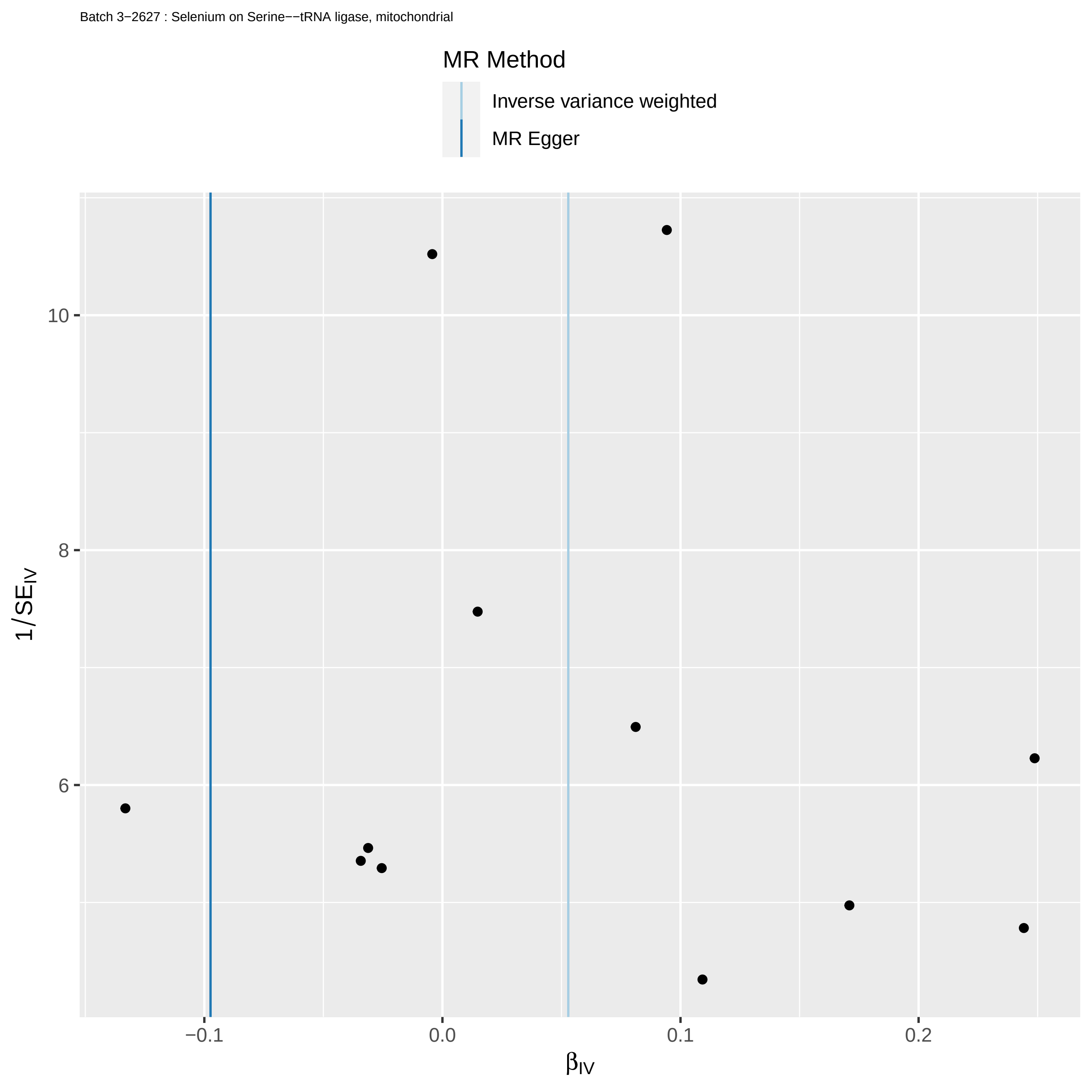
Batch 3−2627 : Selenium on Serine−−tRNA ligase, mitochondrial
MR Method
Inverse variance weighted
MR Egger
10
8
6
1/SE_IV
−0.1
0.0
0.1
0.2
β_IV

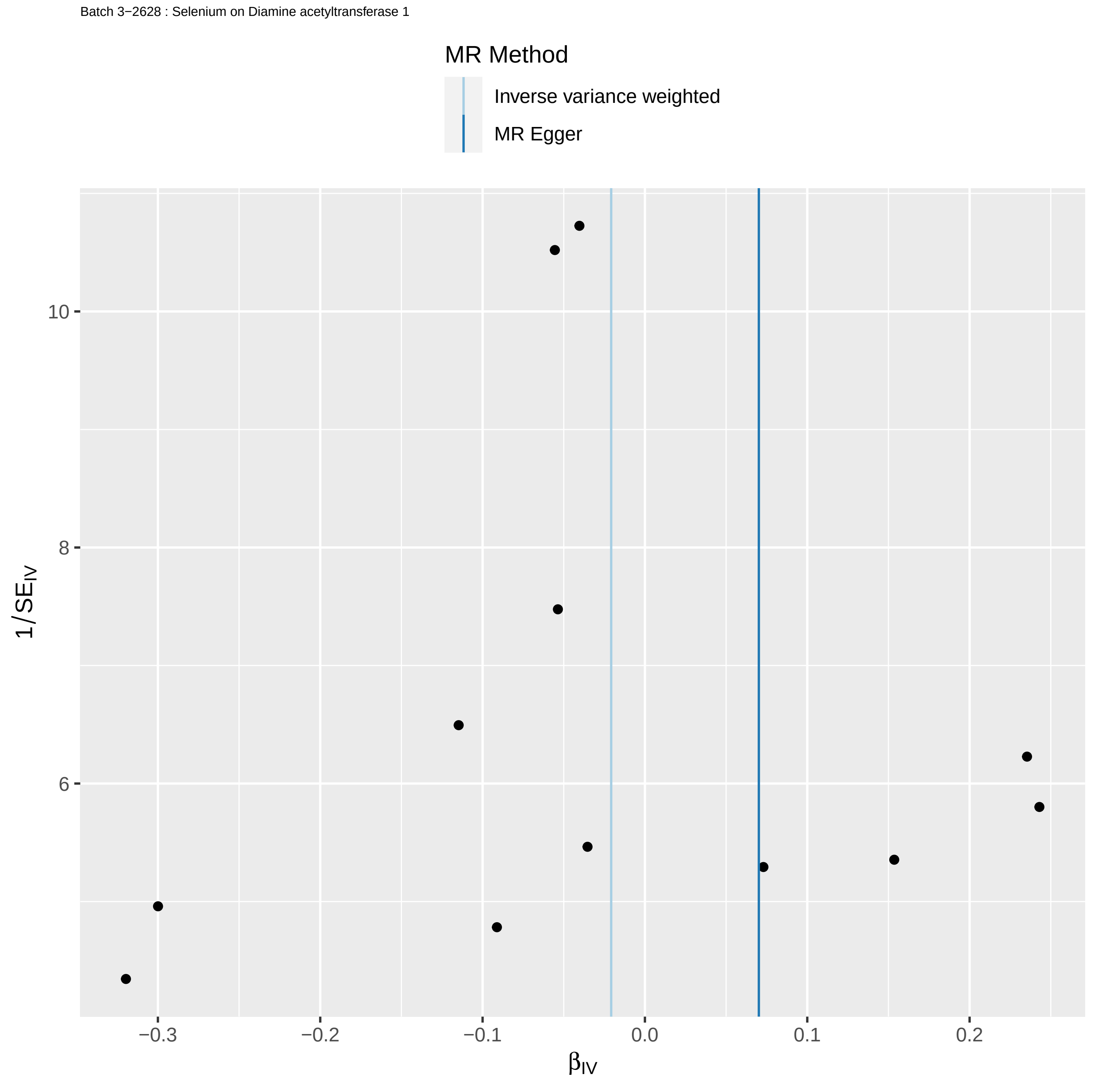

Batch 3−2628 : Selenium on Diamine acetyltransferase 1
MR Method
Inverse variance weighted
MR Egger
10
8
6
1/SE_IV
−0.3
−0.2
−0.1
0.0
0.1
0.2
β_IV

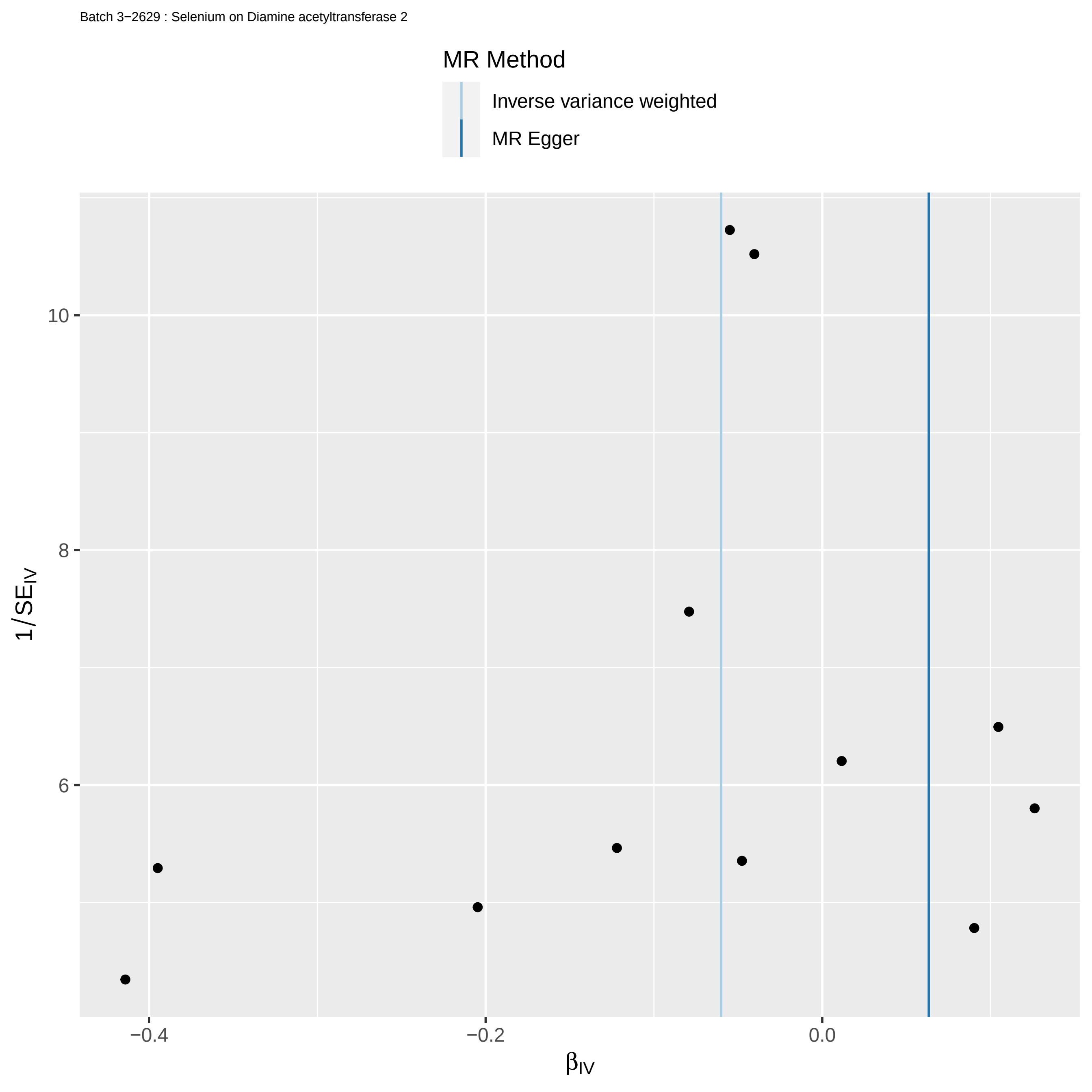

Batch 3−2629 : Selenium on Diamine acetyltransferase 2
MR Method
Inverse variance weighted
MR Egger
10
8
6
$1/SE_{IV}$
−0.4
−0.2
0.0
β_{IV}

Batch 3−2630 : Selenium on DNA−binding protein SATB1

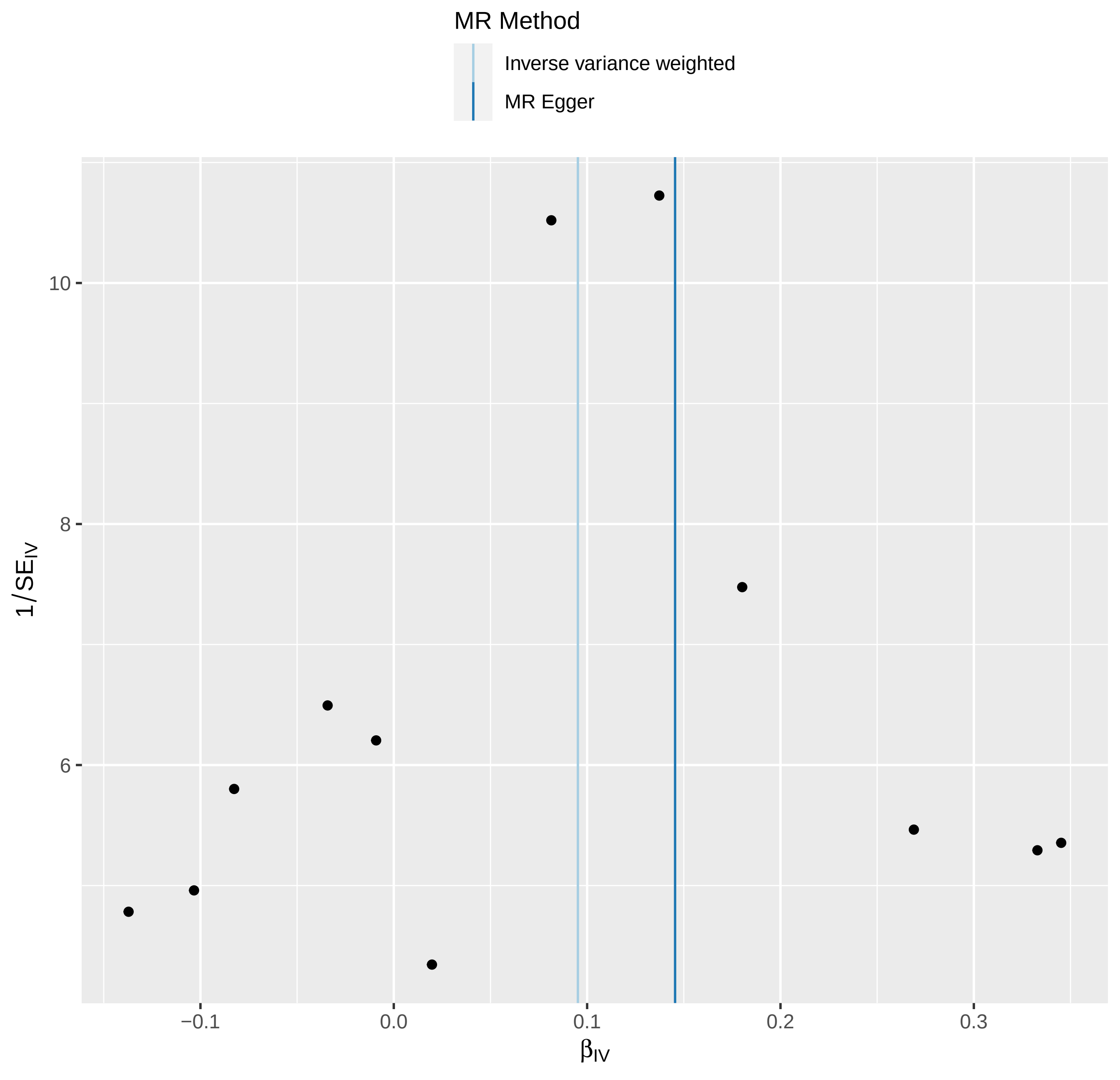

Batch 3−2631 : Selenium on DNA−binding protein SATB2

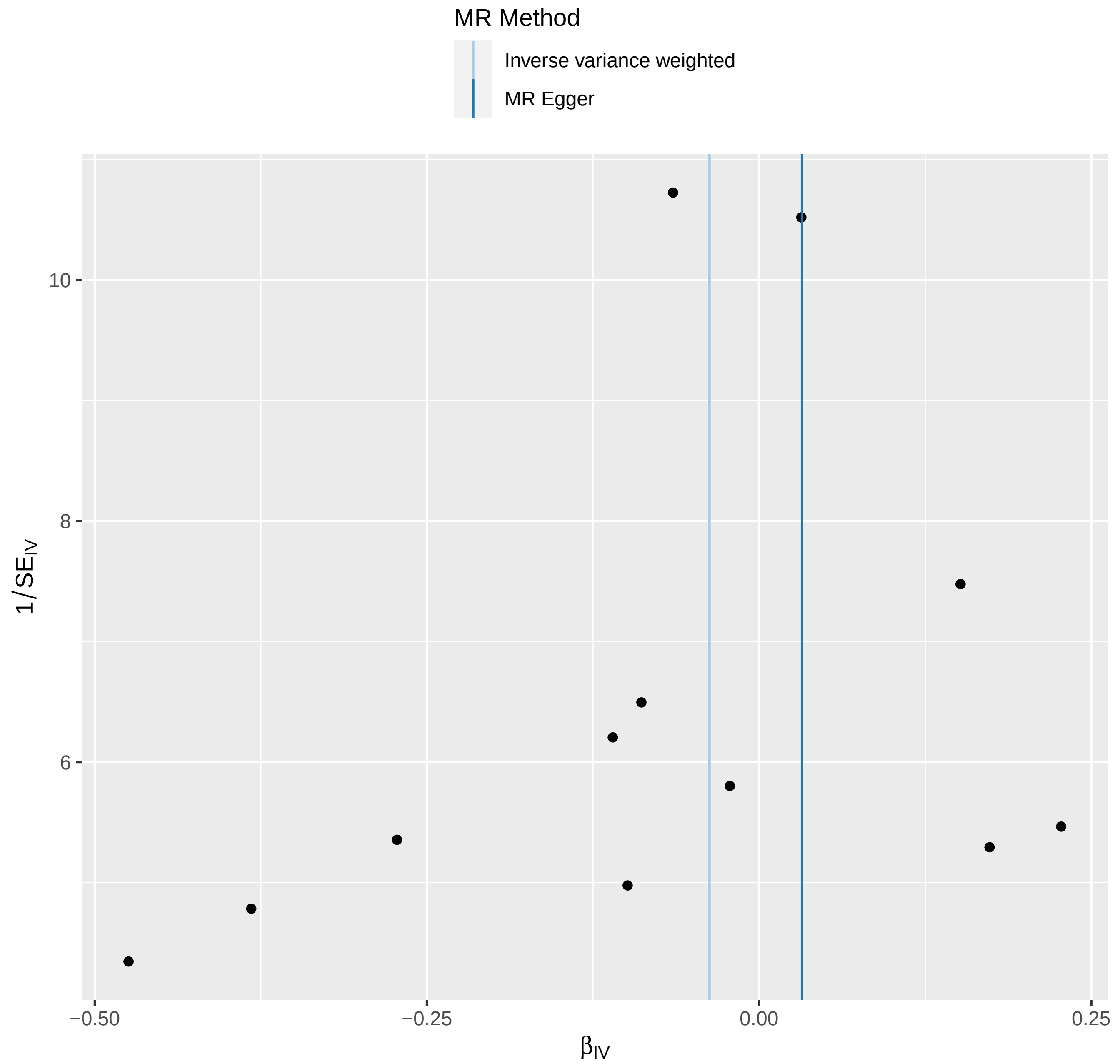

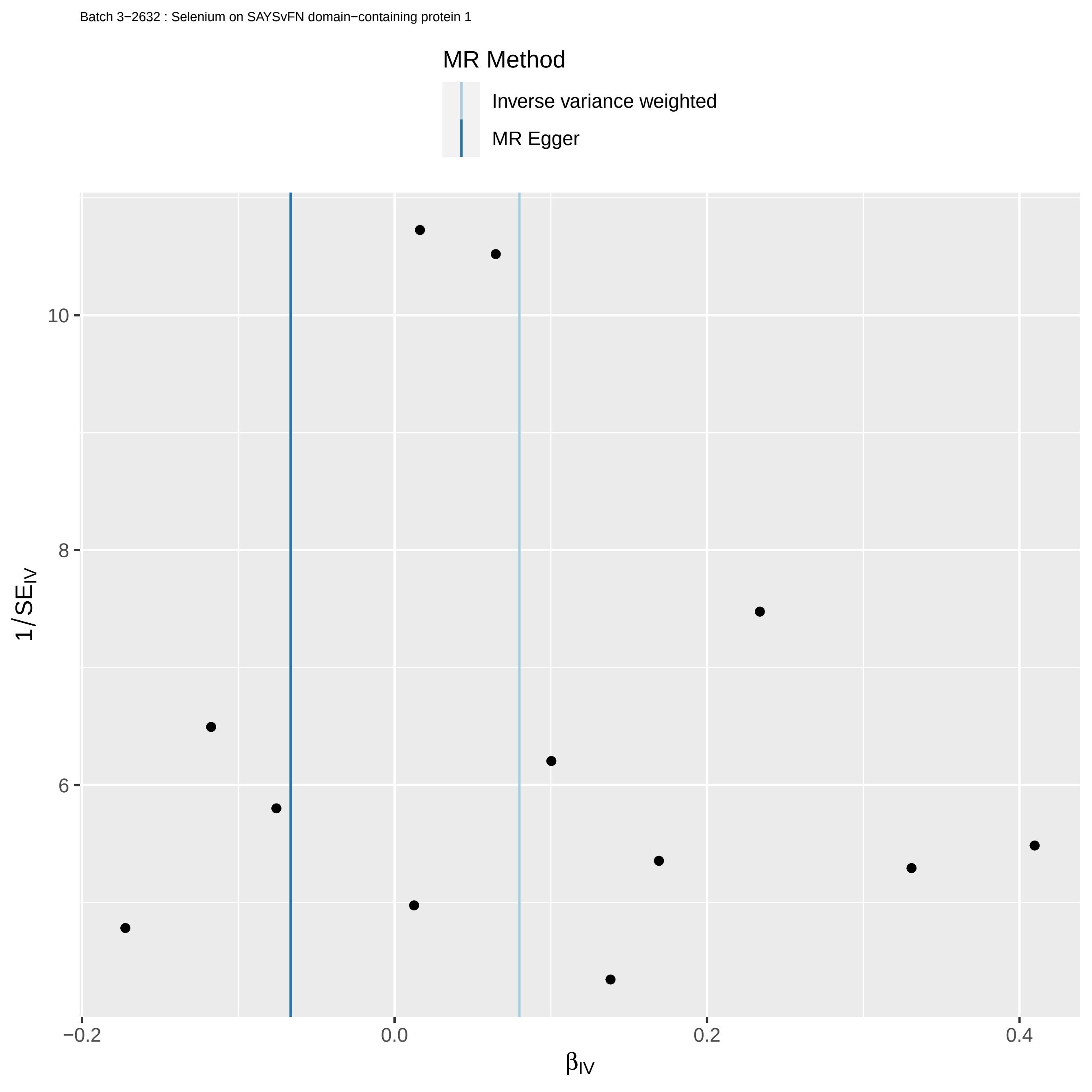
Batch 3−2632 : Selenium on SAYSvFN domain−containing protein 1
MR Method
Inverse variance weighted
MR Egger
10
8
6
1/SE_IV
−0.2
0.0
0.2
0.4
β_IV

Batch 3−2633 : Selenium on Suprabasin

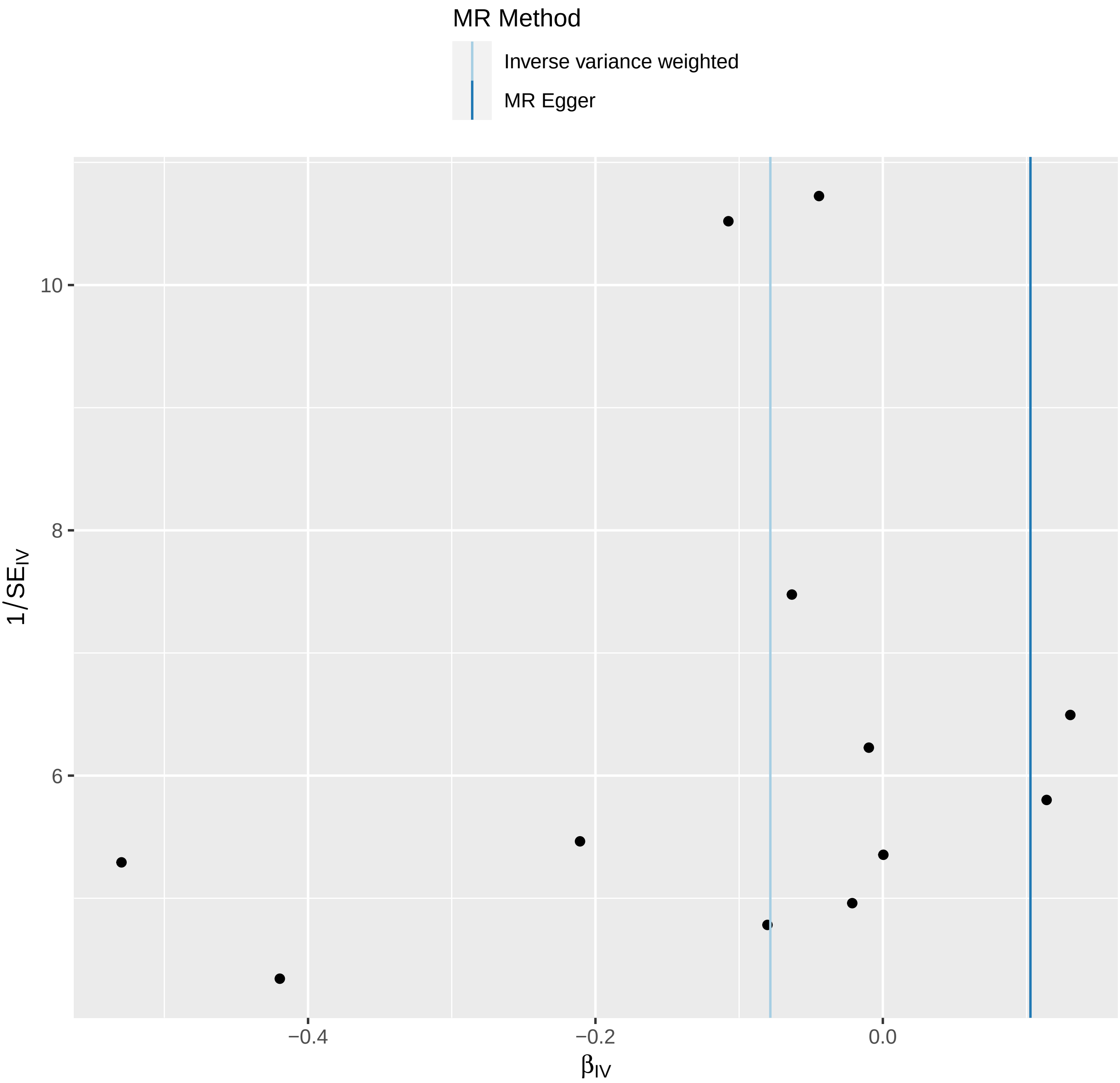

Batch 3−2634 : Selenium on Secretory carrier−associated membrane protein 5

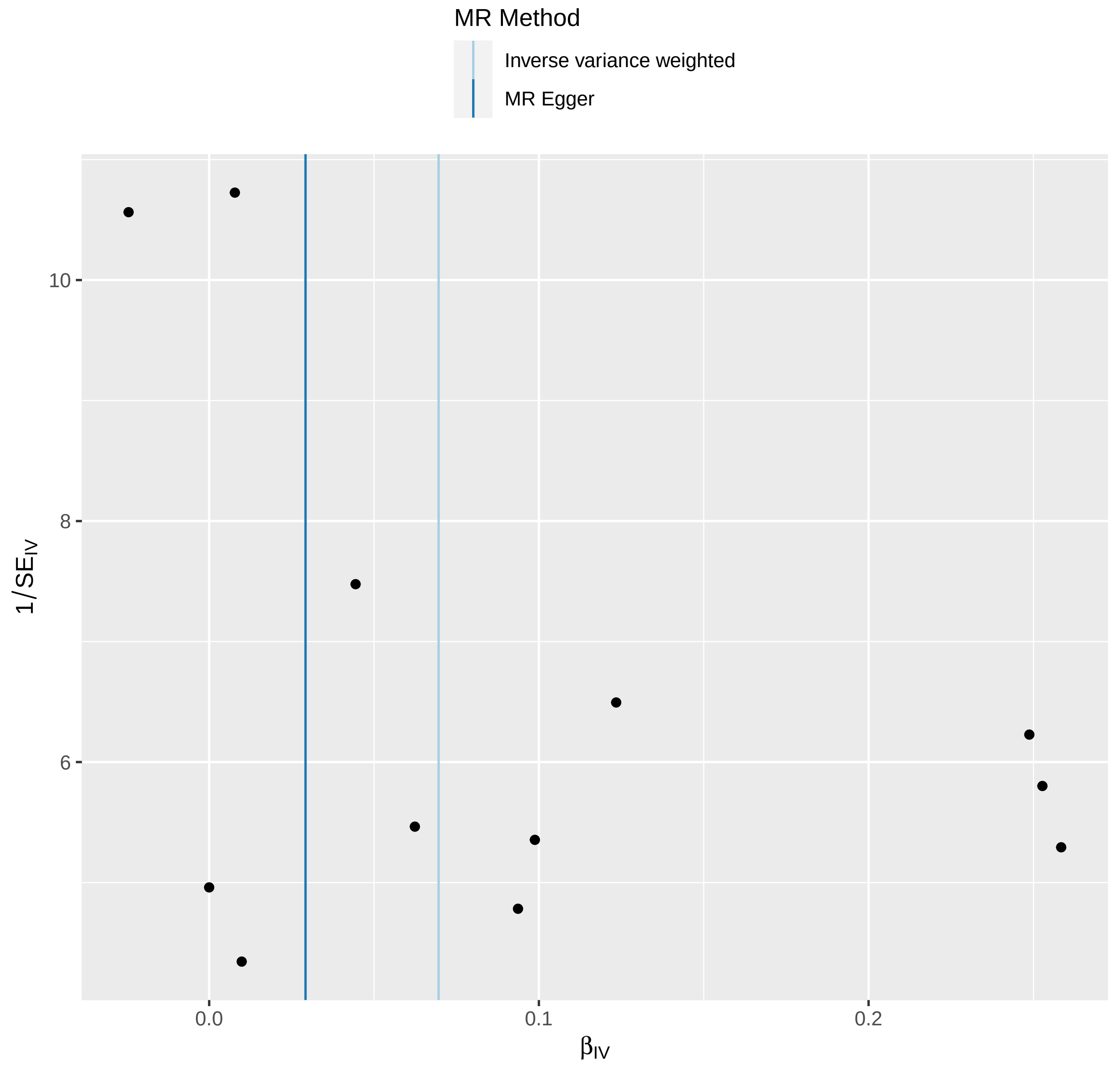

Batch 3−2635 : Selenium on Scavenger receptor class A member 3

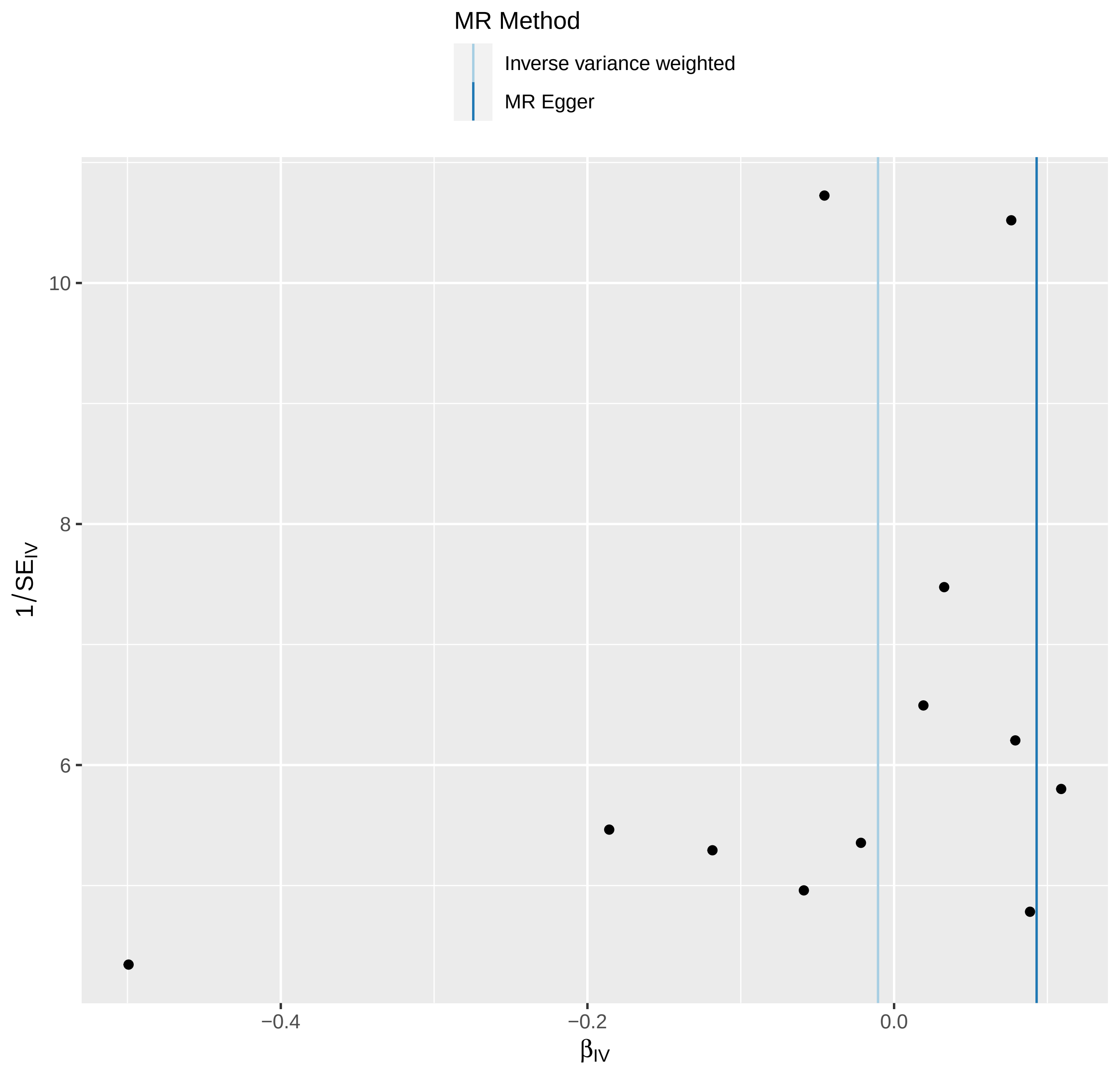

Batch 3−2636 : Selenium on Scavenger receptor class A member 3

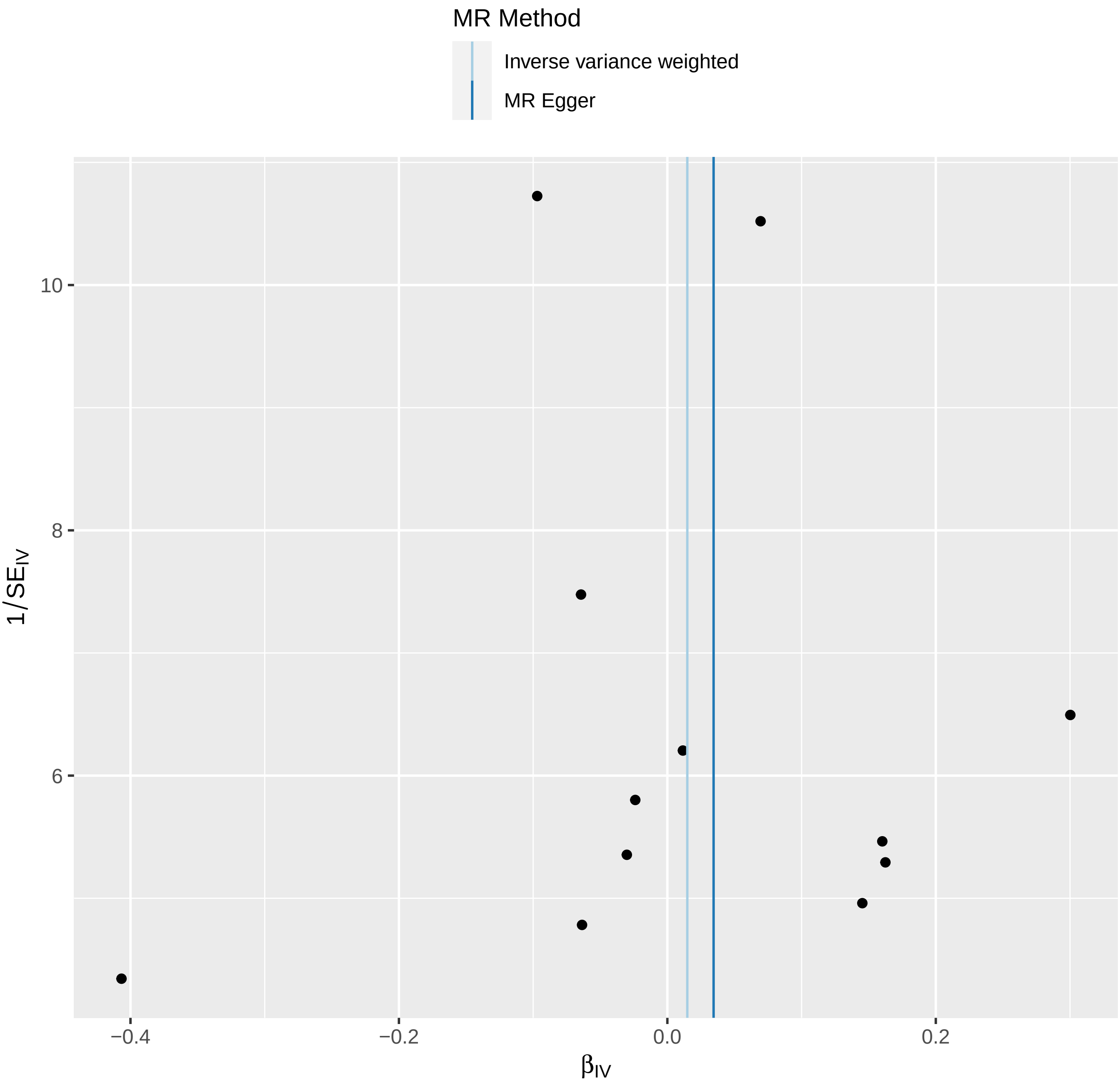

Batch 3−2637 : Selenium on Scavenger receptor class A member 3

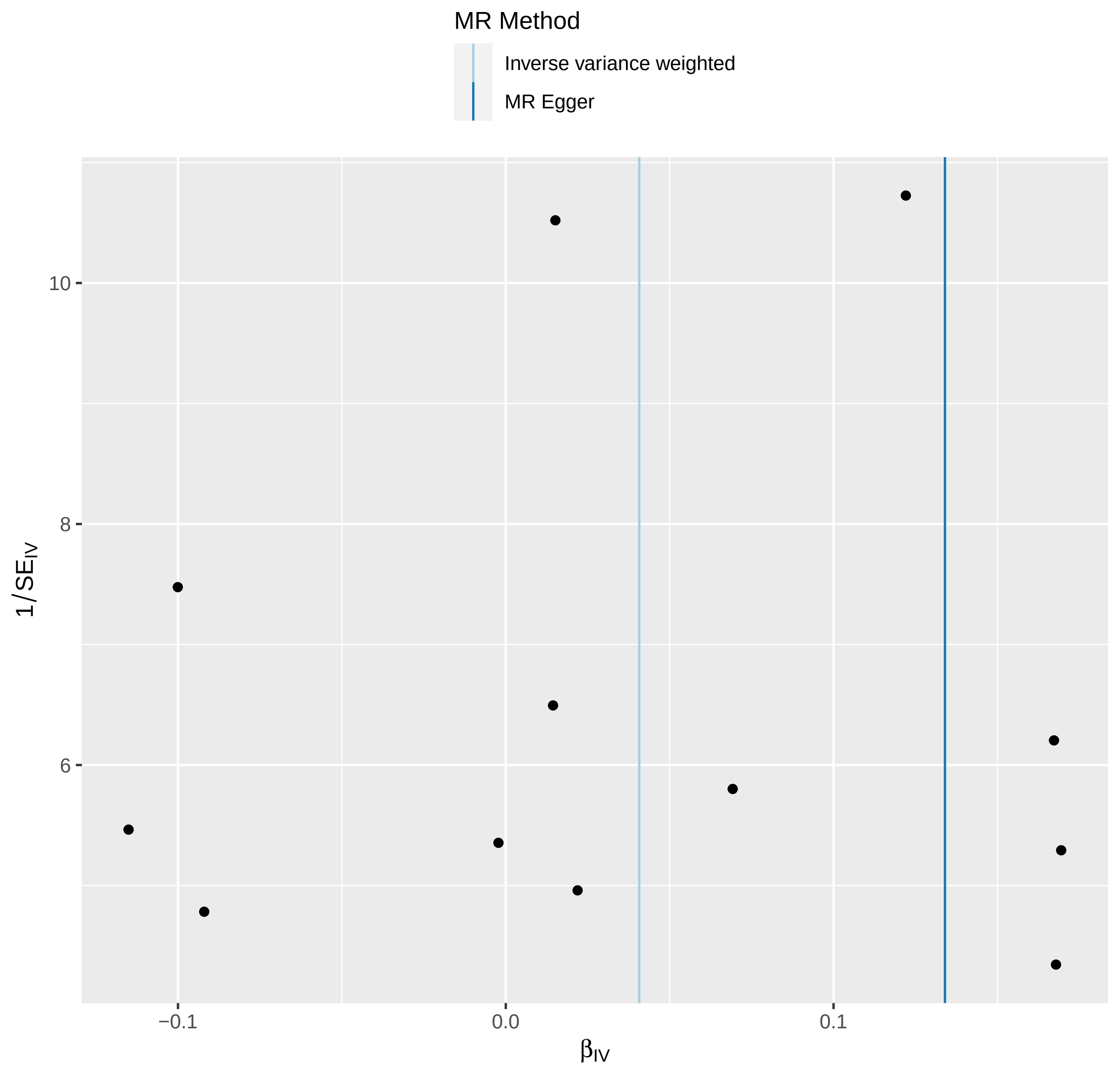

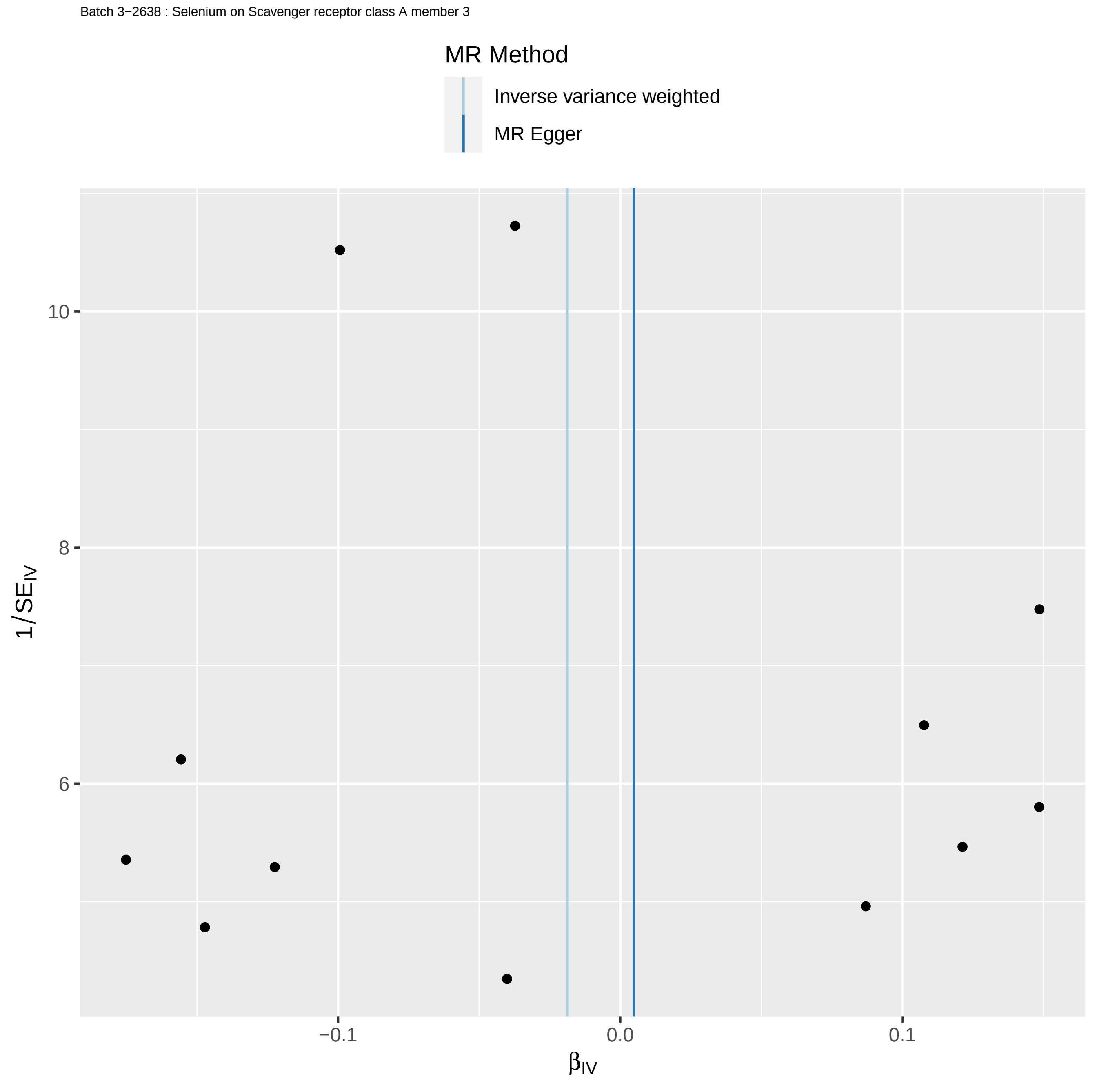

Batch 3−2638 : Selenium on Scavenger receptor class A member 3
MR Method
Inverse variance weighted
MR Egger
10
8
6
1/SE_IV
−0.1
0.0
0.1
β_IV

Batch 3−2639 : Selenium on Scavenger receptor class A member 5

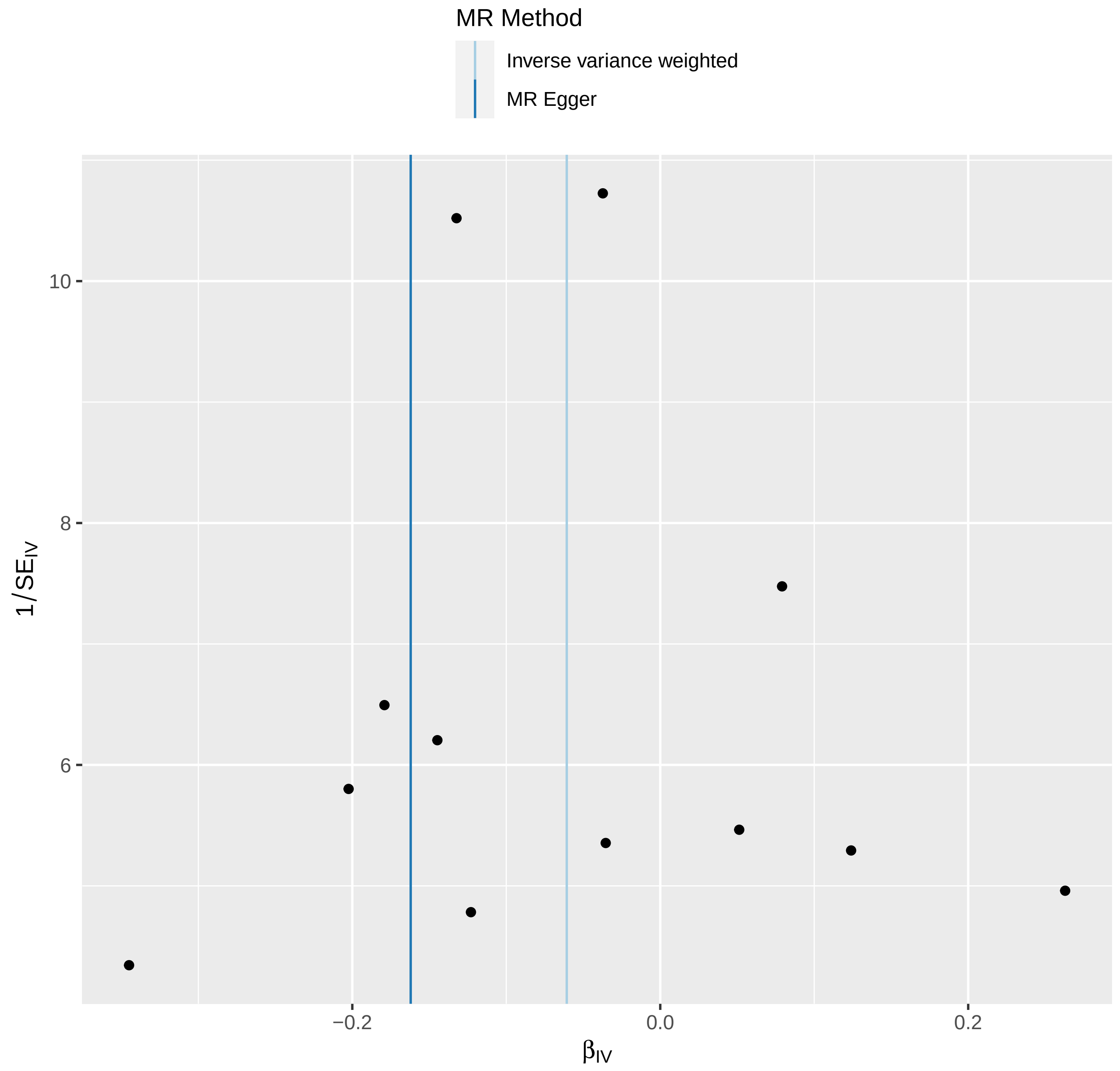

Batch 3−2640 : Selenium on Lysosome membrane protein 2

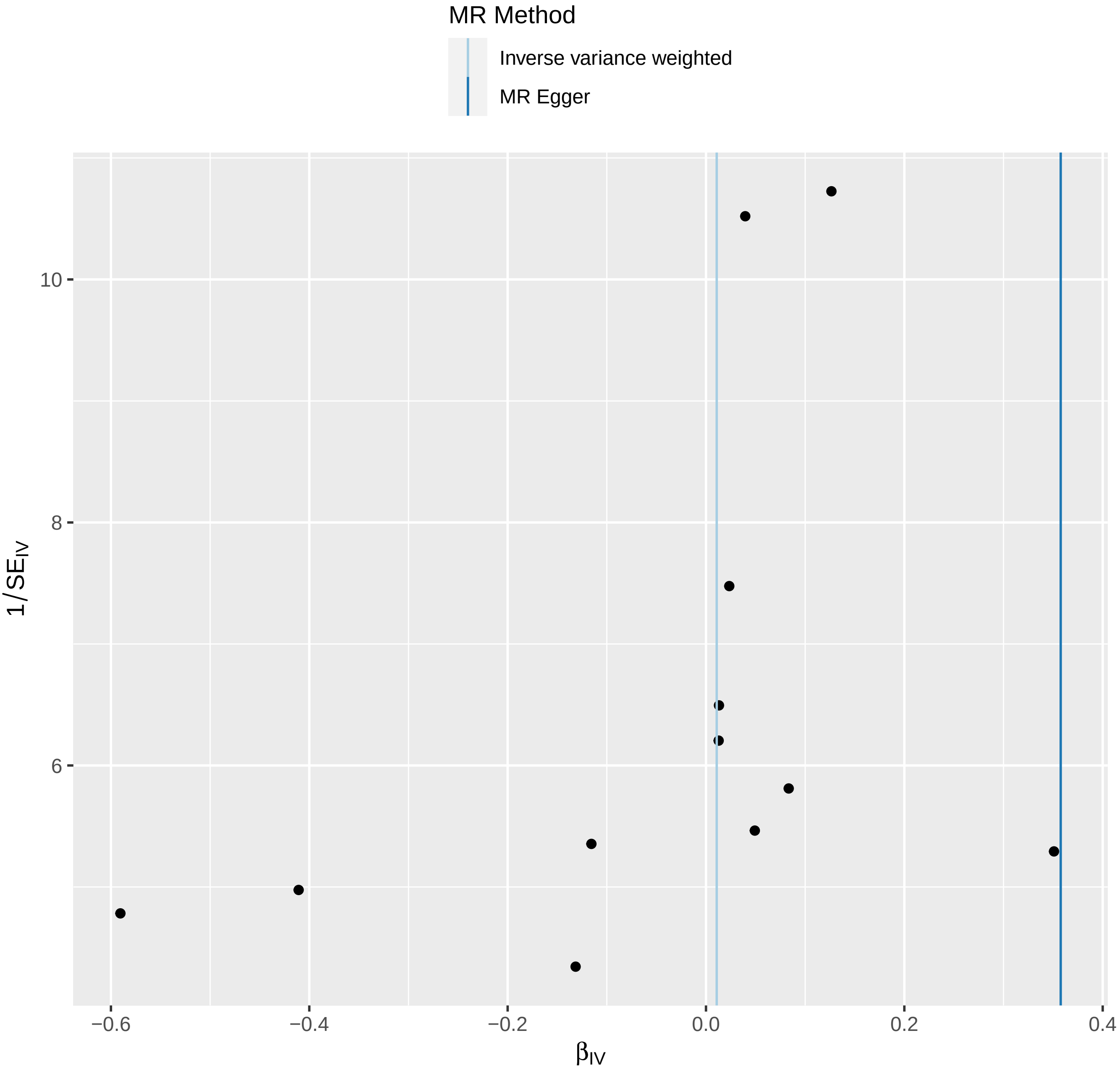

Batch 3−2641 : Selenium on Scavenger receptor class F member 2

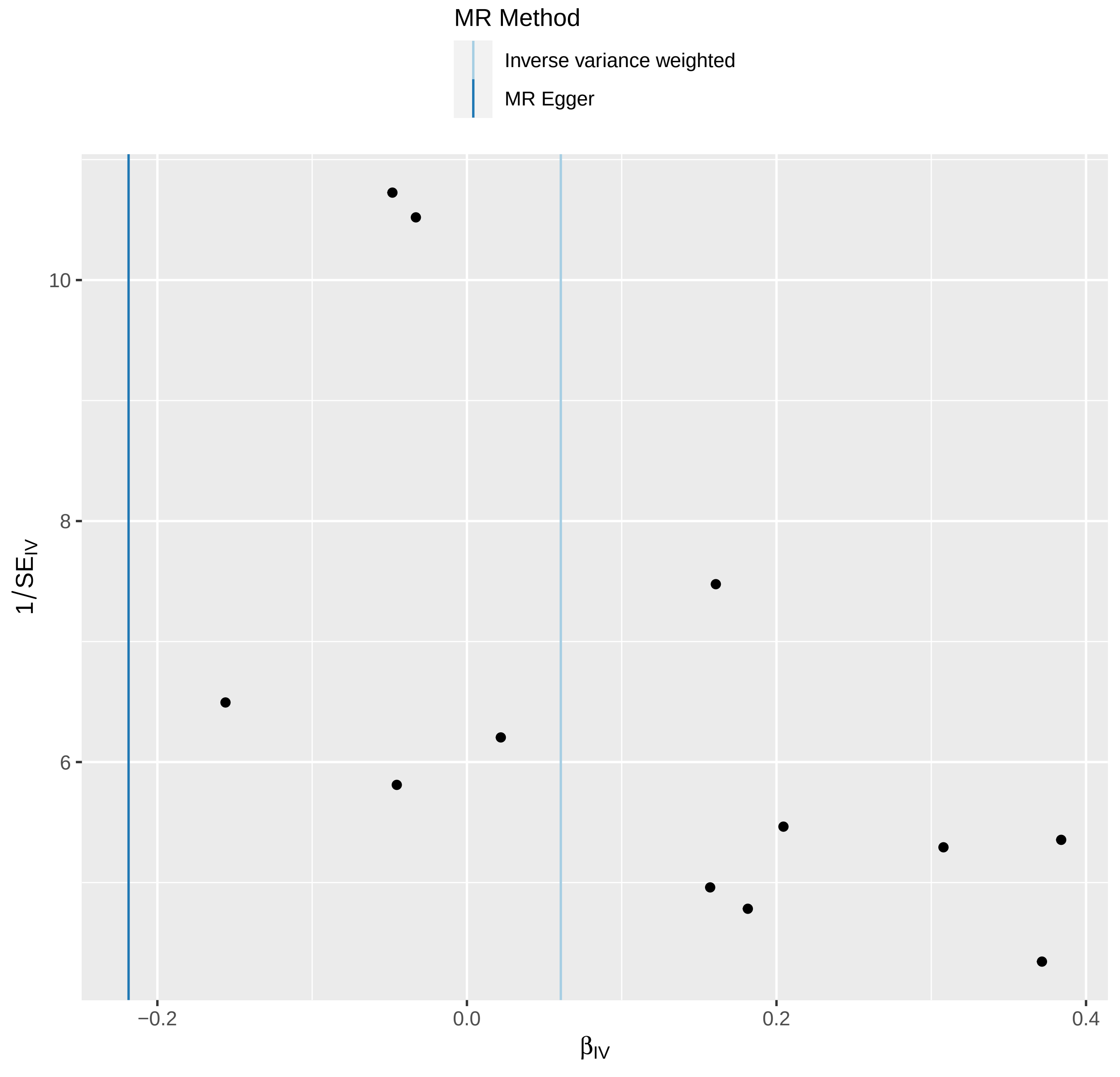

Batch 3−2642 : Selenium on Scavenger receptor class F member 2

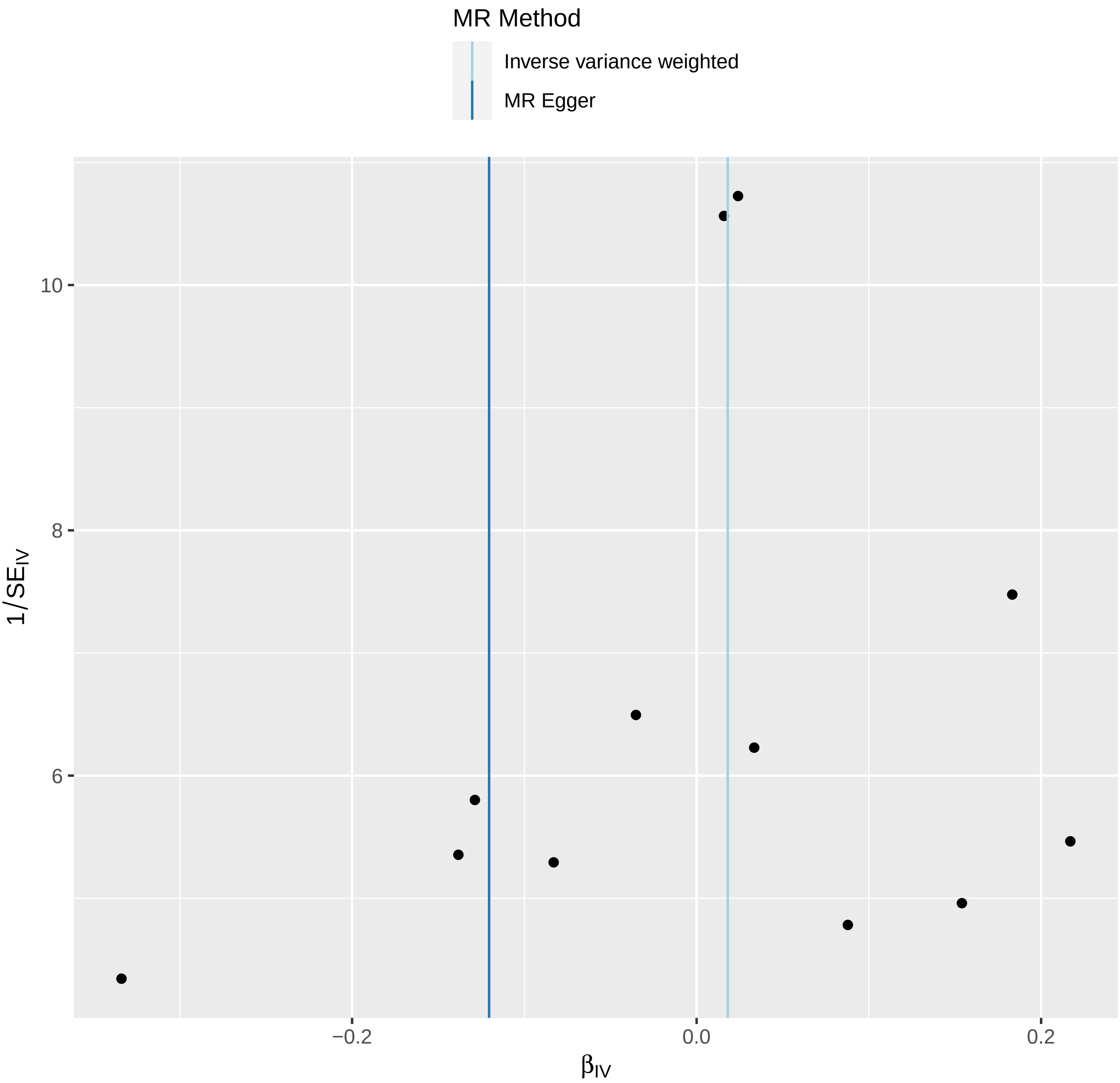

Batch 3−2643 : Selenium on Scavenger receptor class F member 2

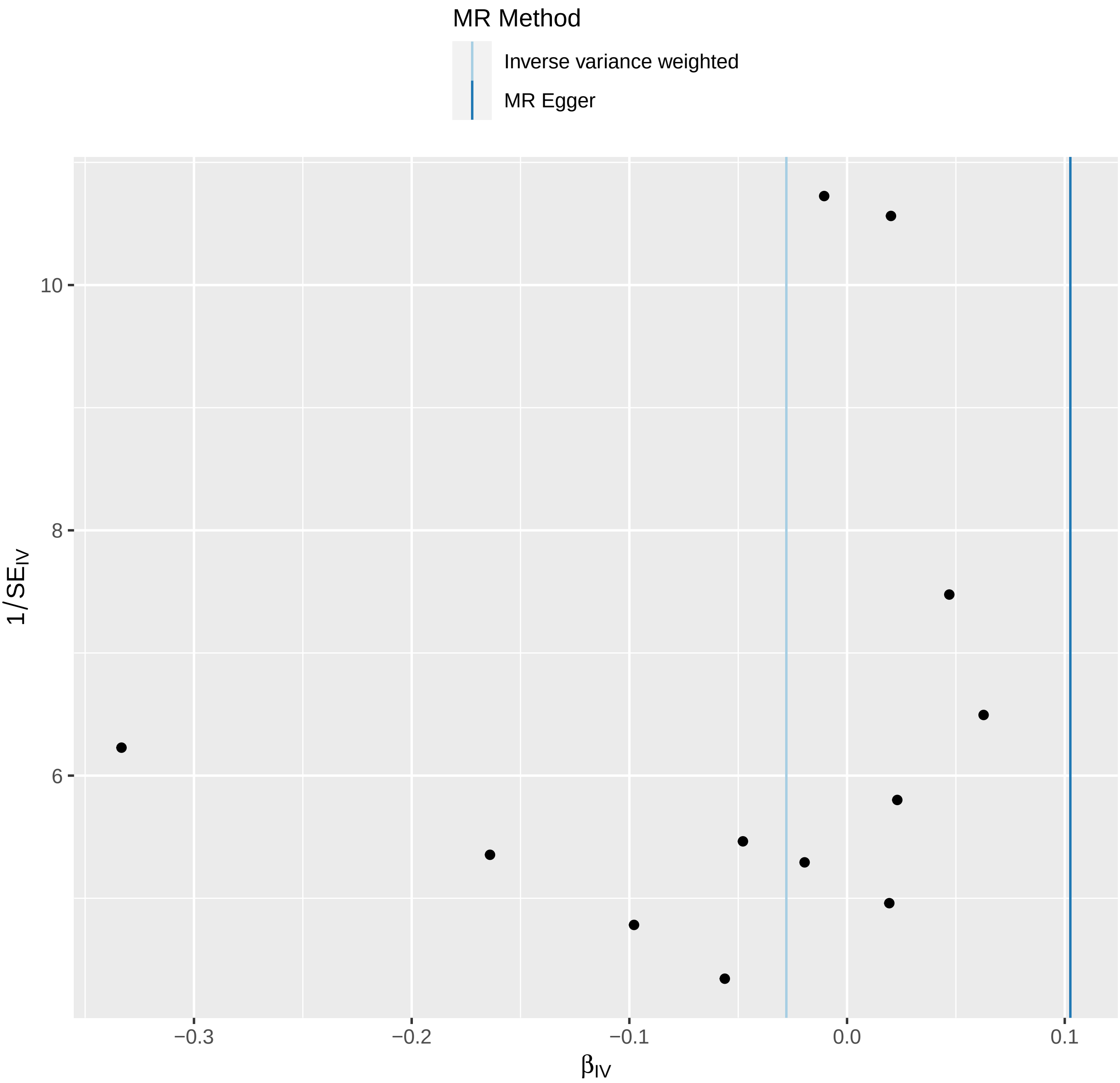

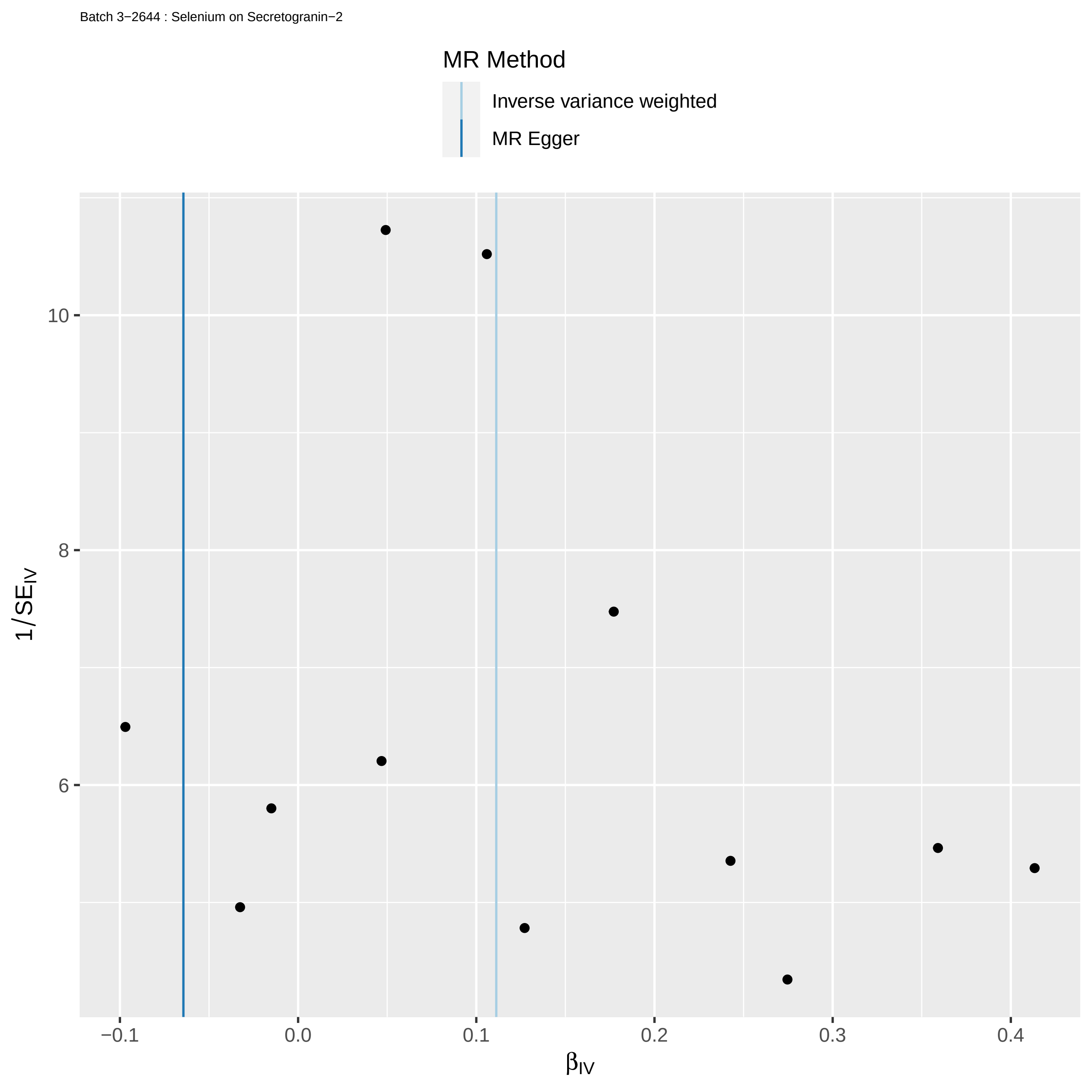

Batch 3−2644 : Selenium on Secretogranin−2
MR Method
Inverse variance weighted
MR Egger
10
8
6
1/SE_IV
−0.1
0.0
0.1
0.2
0.3
0.4
β_IV

Batch 3−2645 : Selenium on Secretogranin−3

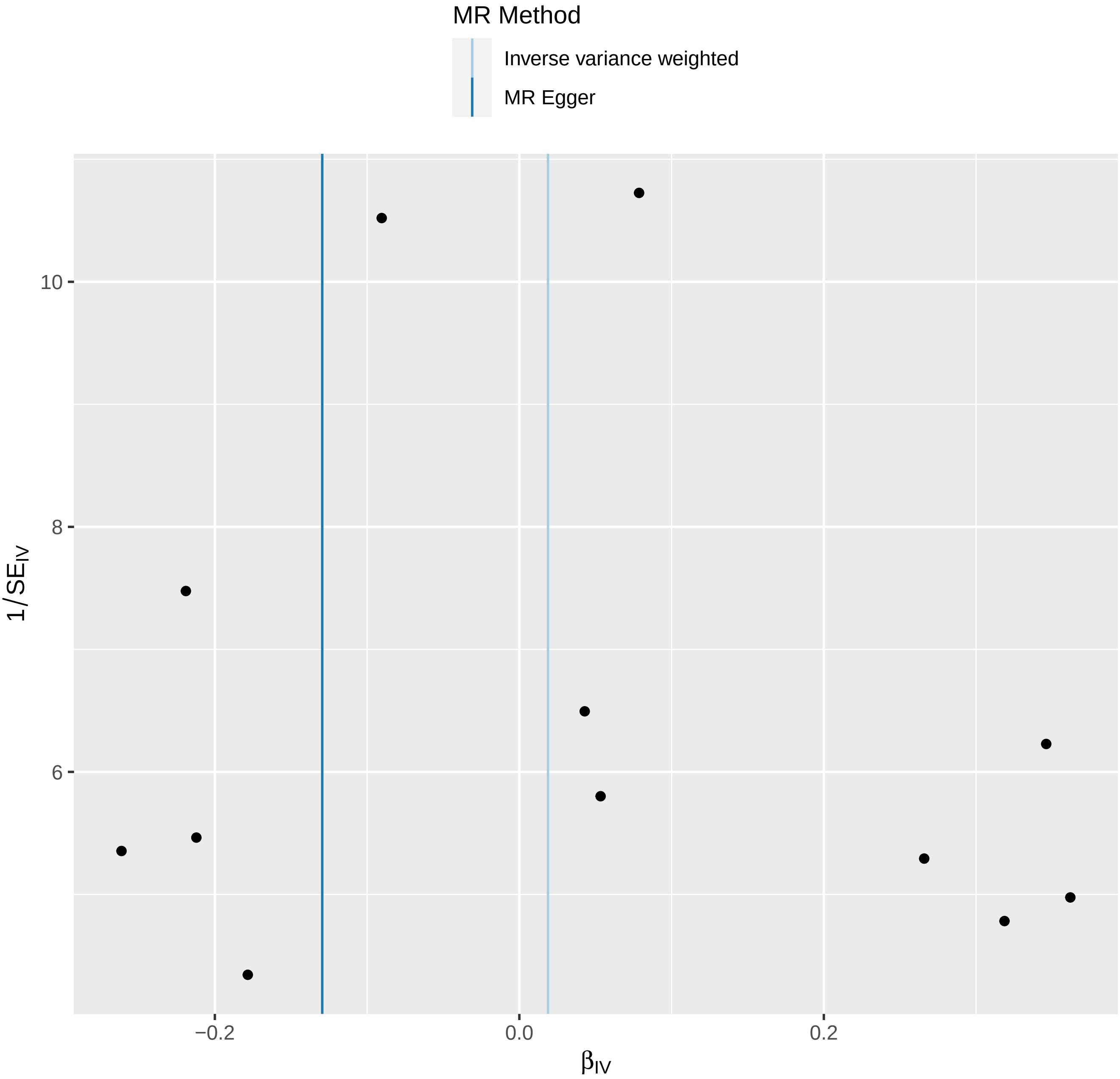

Batch 3−2646 : Selenium on Secretoglobin family 1C member 1

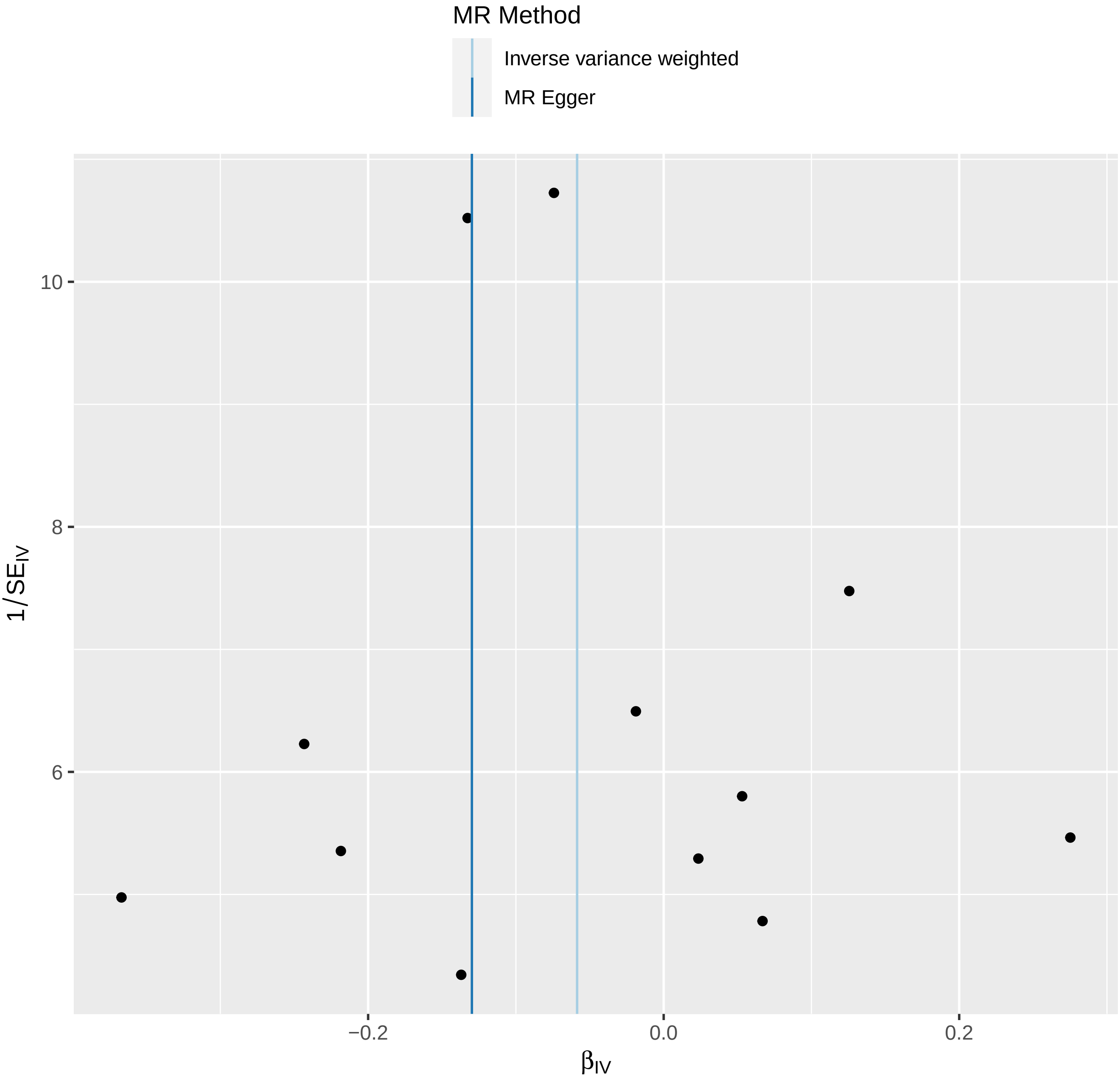

Batch 3−2647 : Selenium on Secretoglobin family 1D member 2

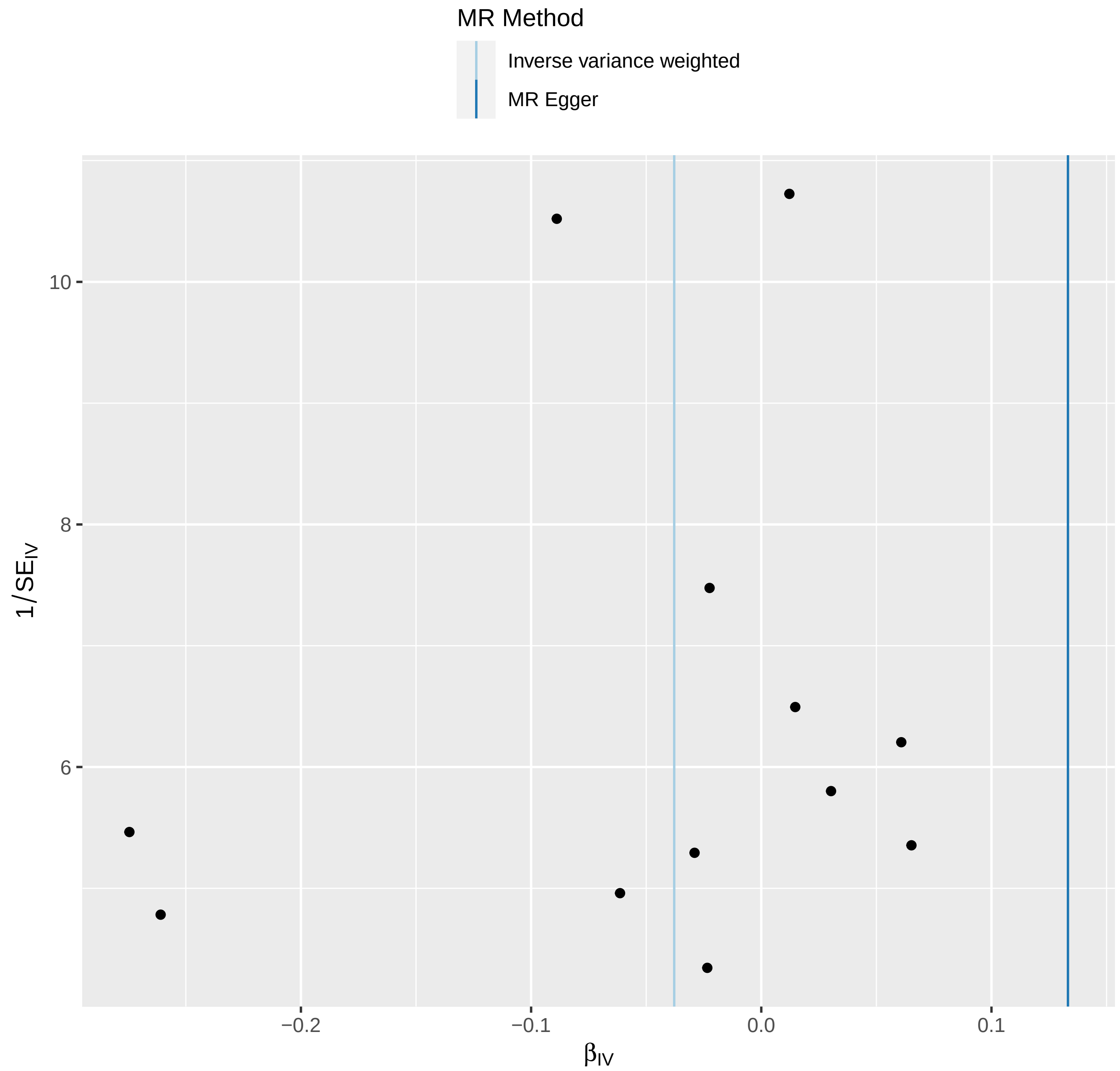

Batch 3−2648 : Selenium on Mammaglobin−B

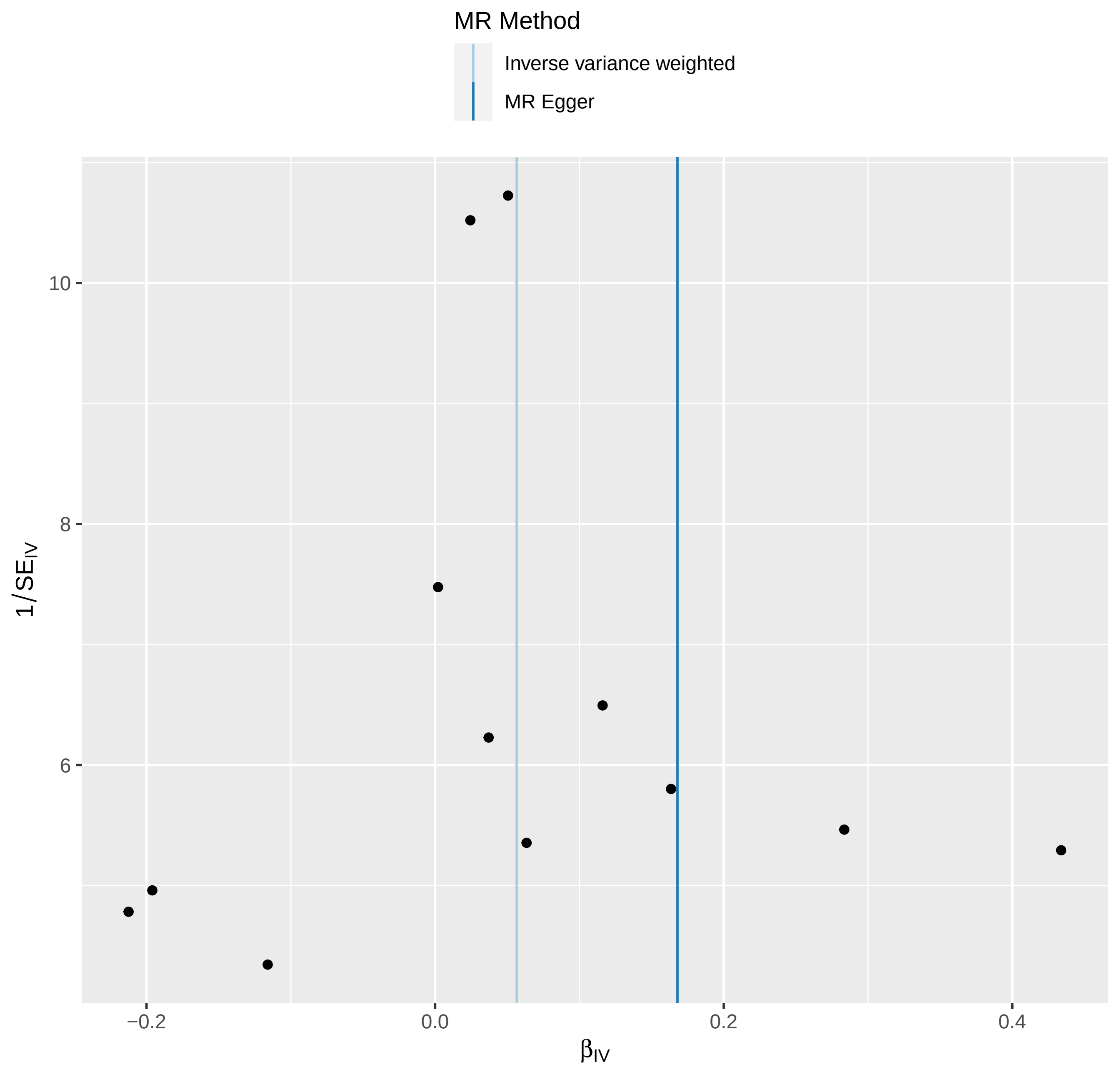

Batch 3−2649 : Selenium on Secretagogin

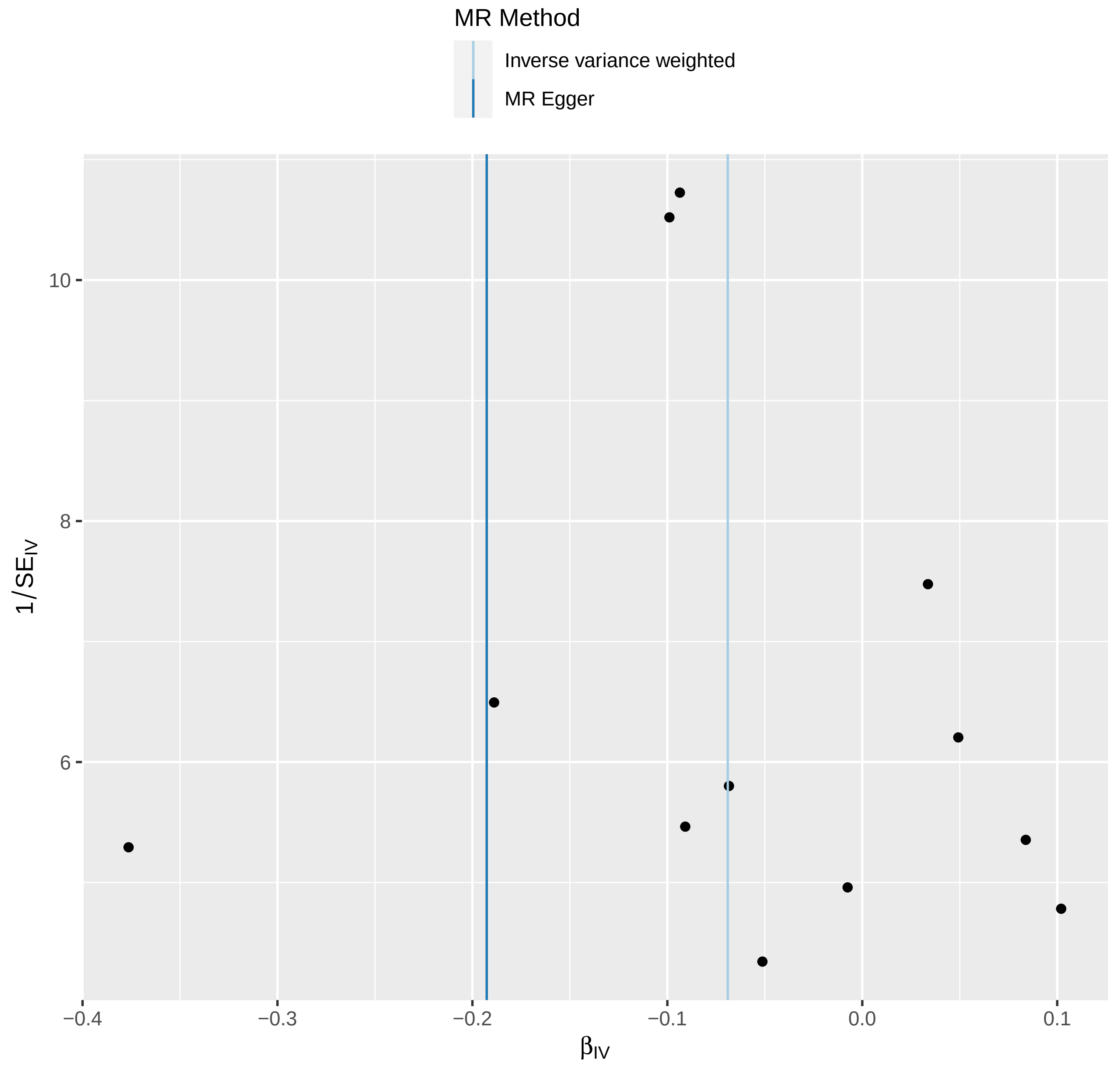

Batch 3−2650 : Selenium on Adseverin

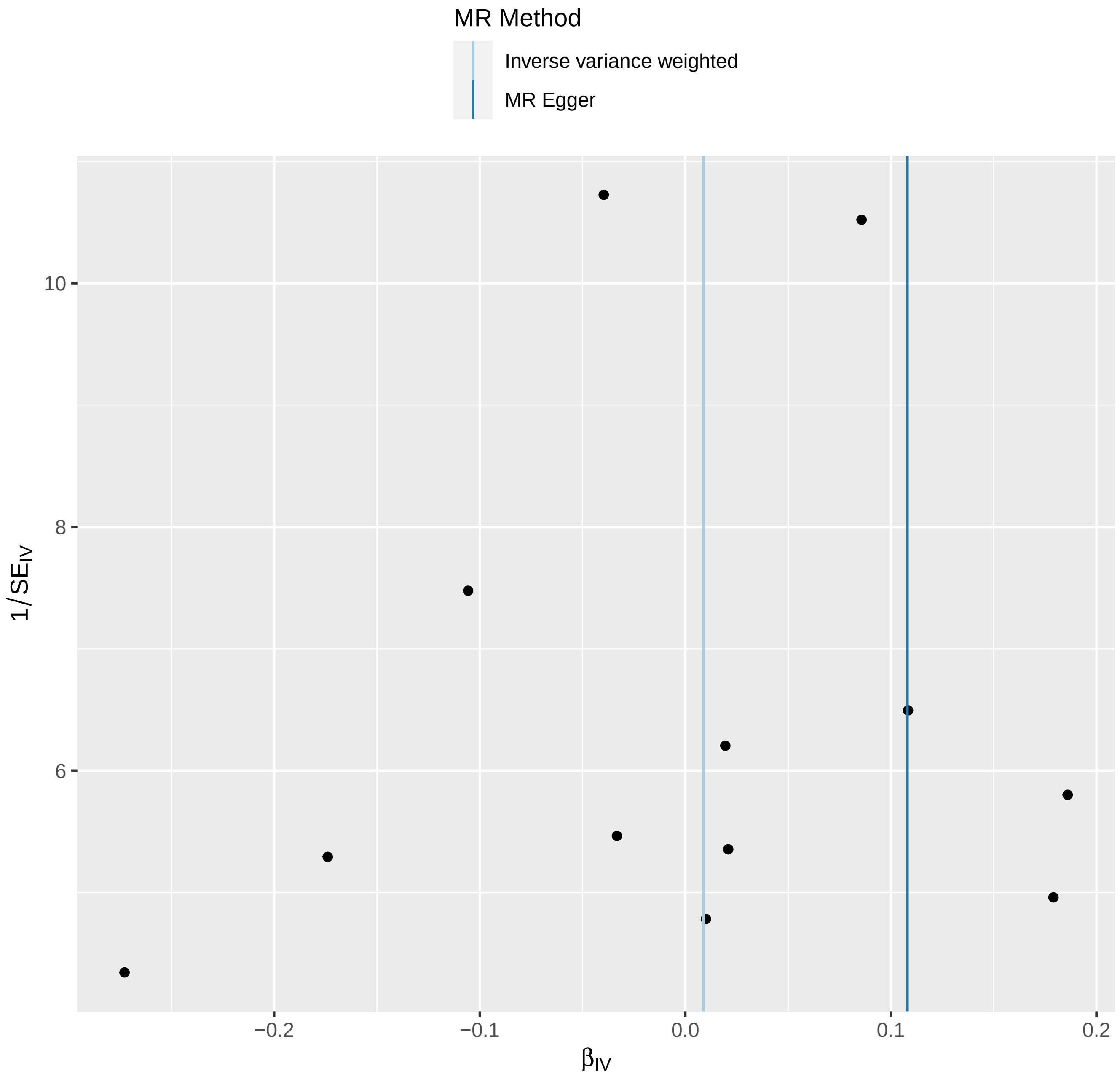

Batch 3−2651 : Selenium on Polycomb protein SCMH1

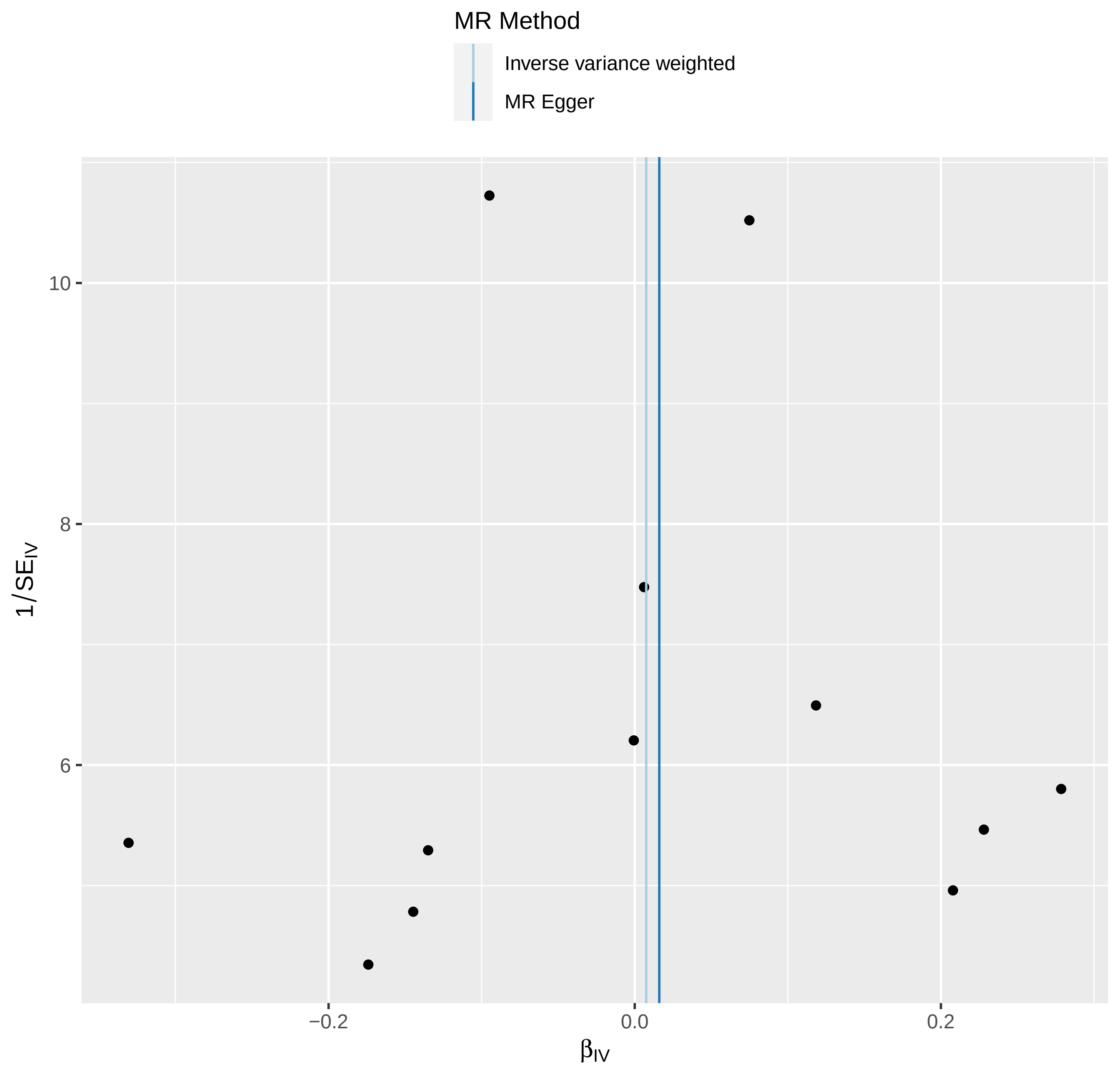

Batch 3−2652 : Selenium on Sodium channel subunit beta−2

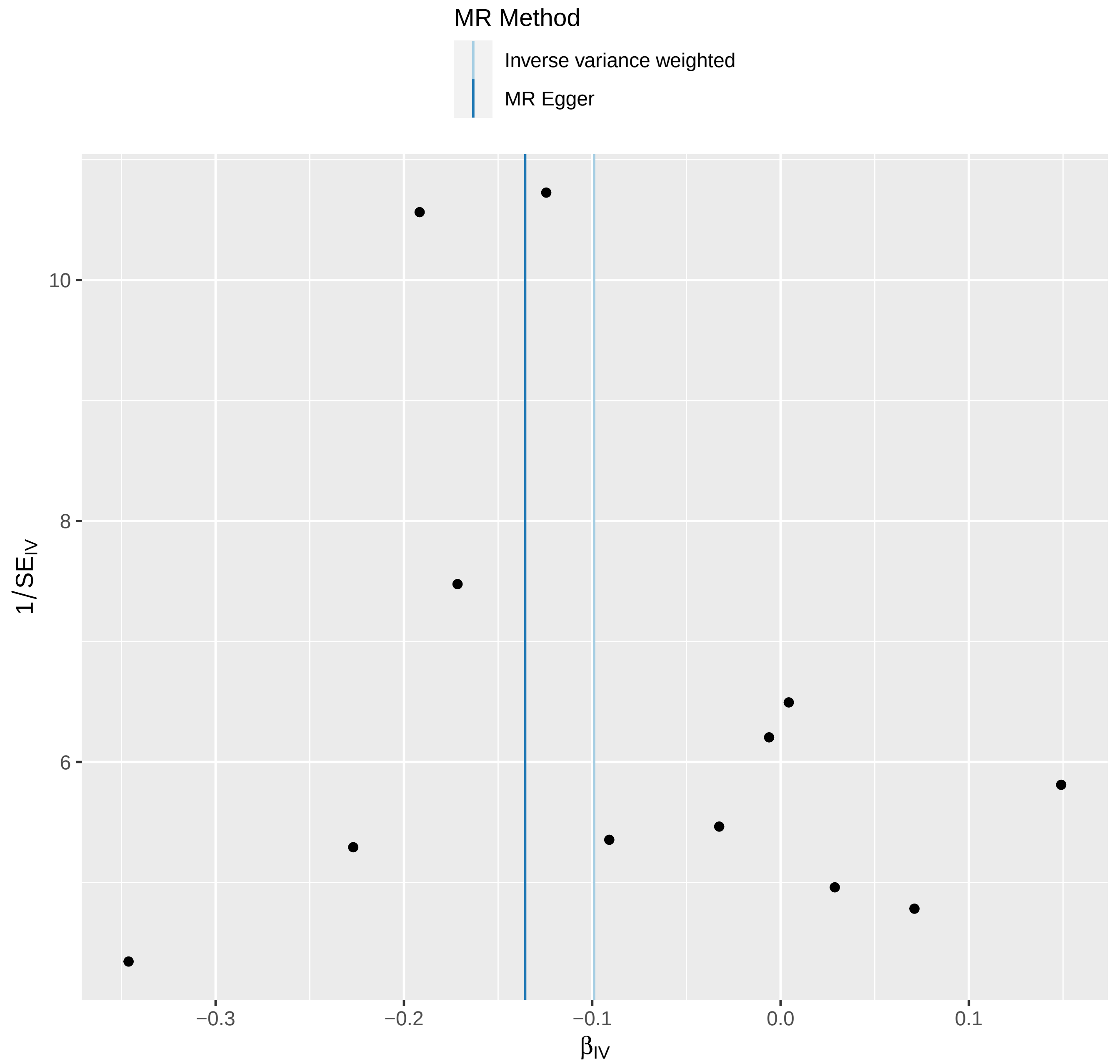

Batch 3−2653 : Selenium on Protein SCO1 homolog, mitochondrial

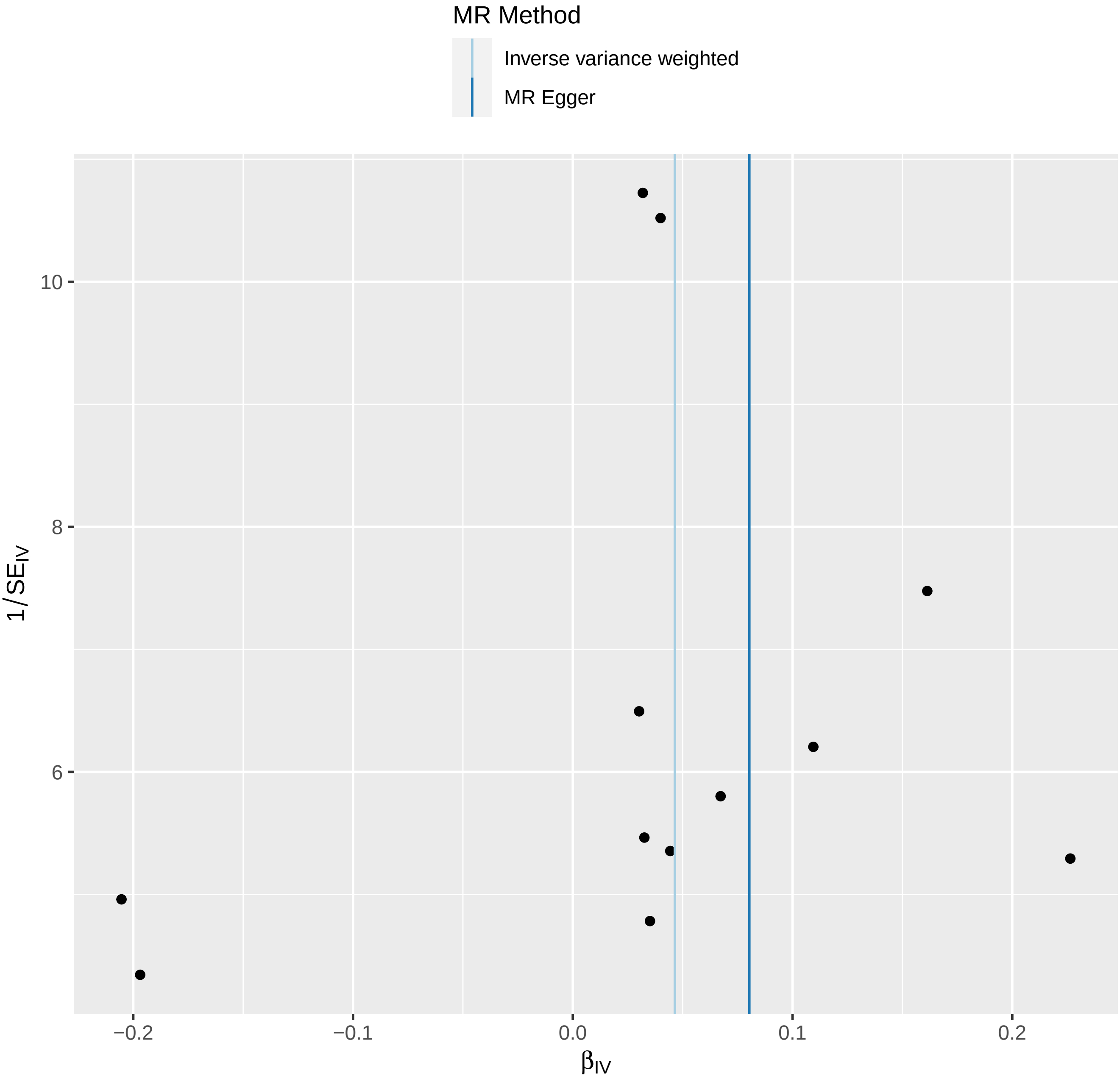

Batch 3−2654 : Selenium on SCP2 sterol−binding domain−containing protein 1

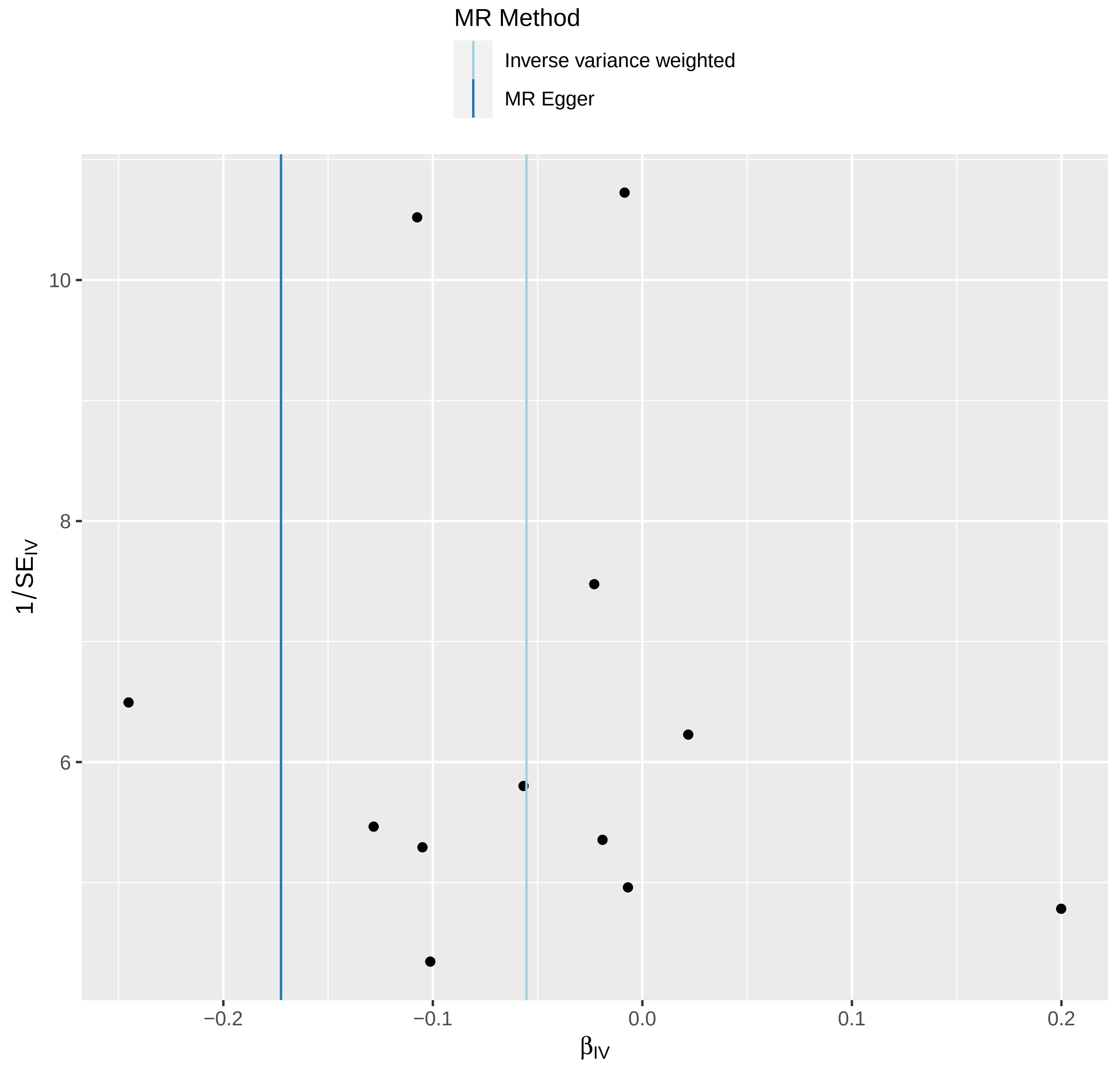

Batch 3−2655 : Selenium on Signal peptide, CUB and EGF−like domain−containing protein 1

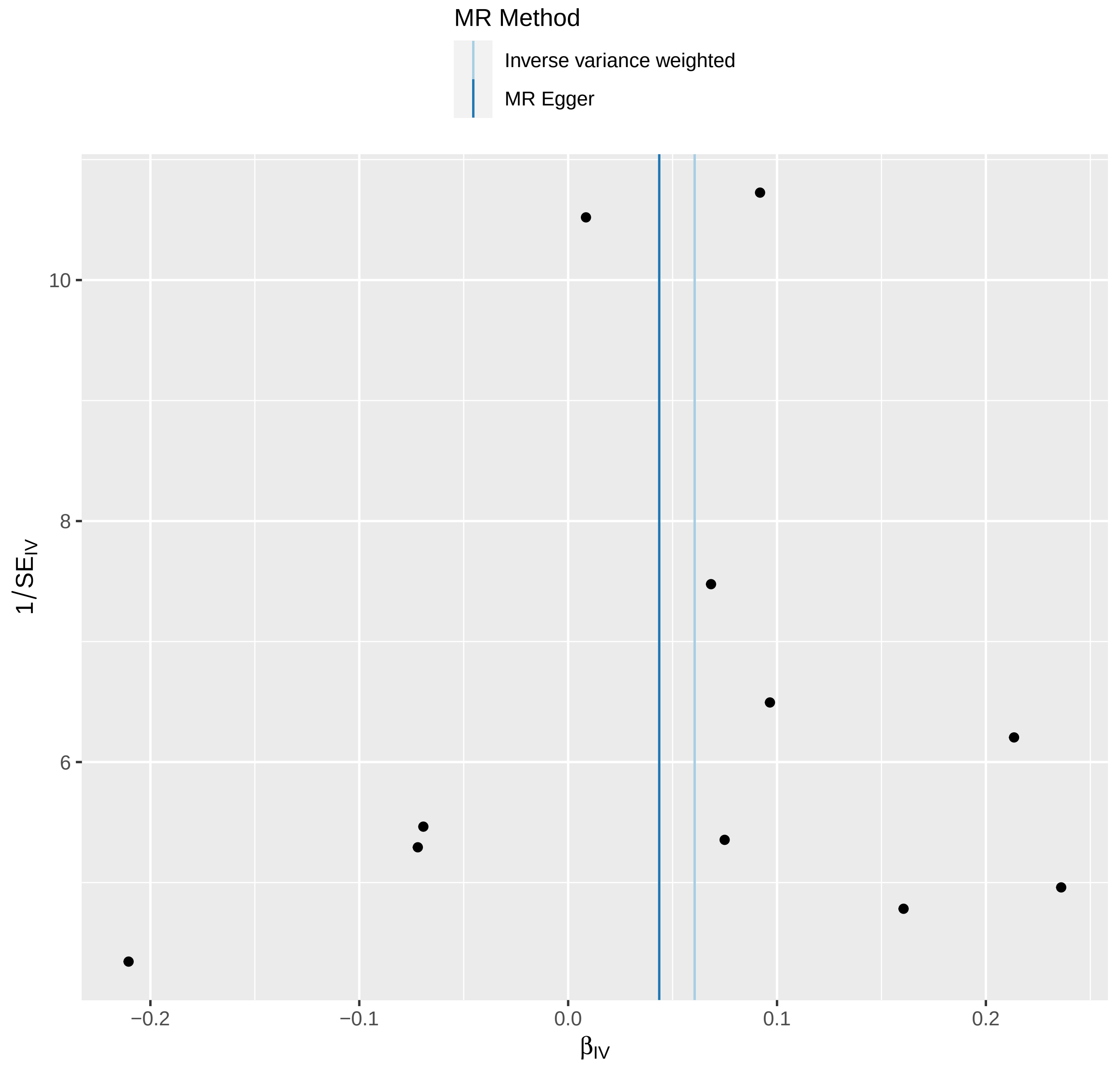

Batch 3−2656 : Selenium on Stromal cell−derived factor 2−like protein 1

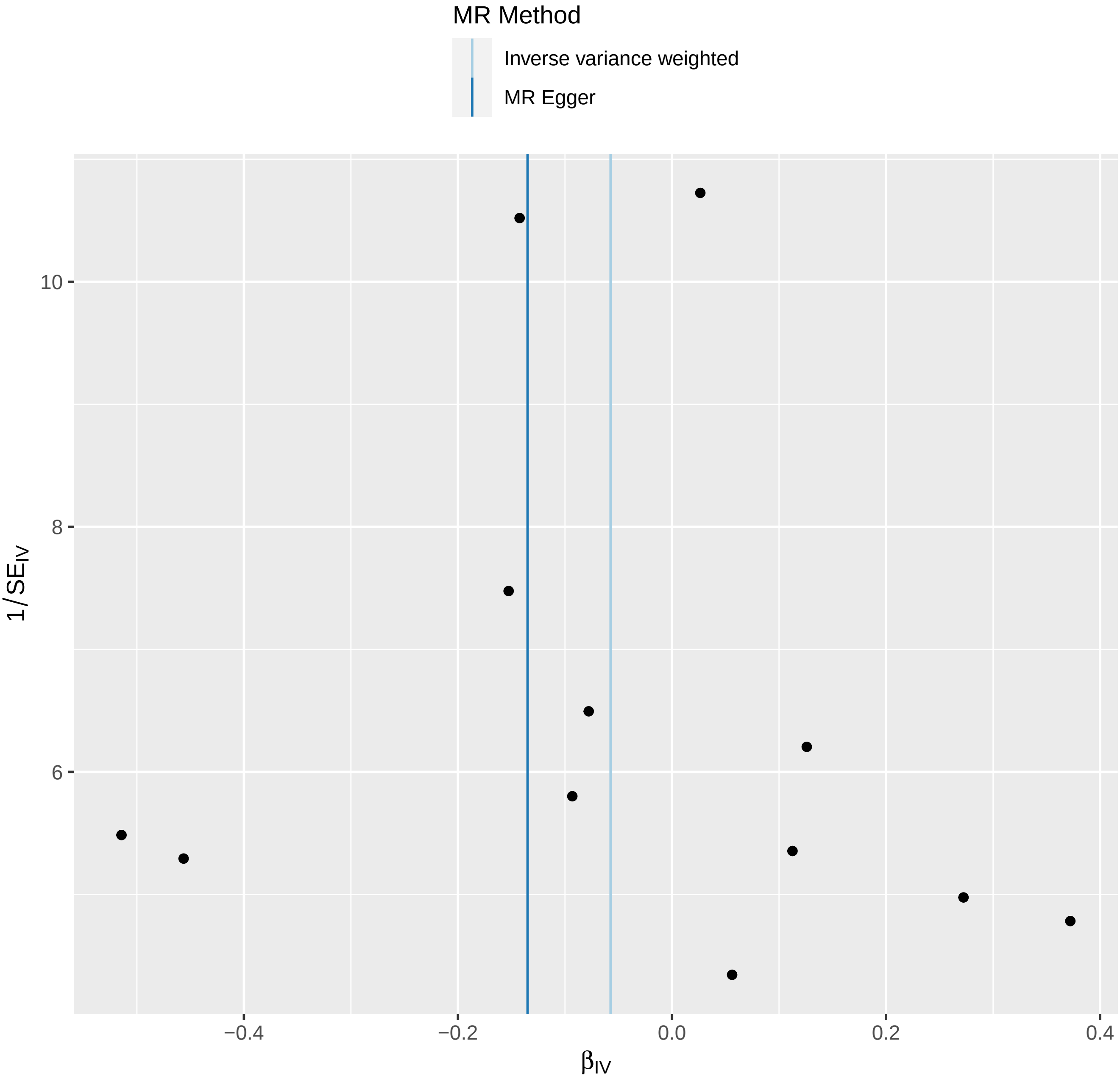

Batch 3−2657 : Selenium on Succinate dehydrogenase assembly factor 2, mitochondrial

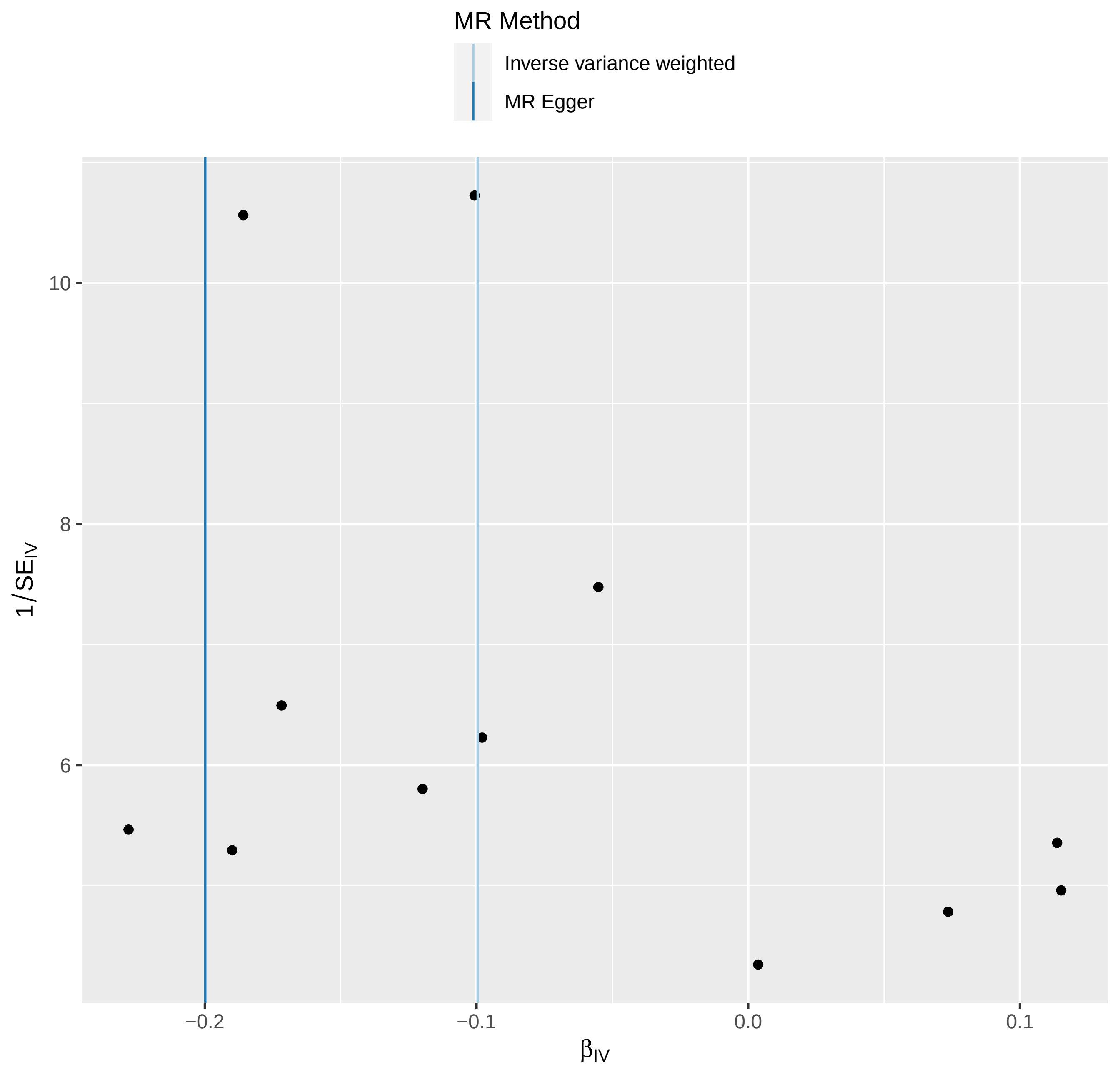

Batch 3−2658 : Selenium on Signal peptidase complex catalytic subunit SEC11C

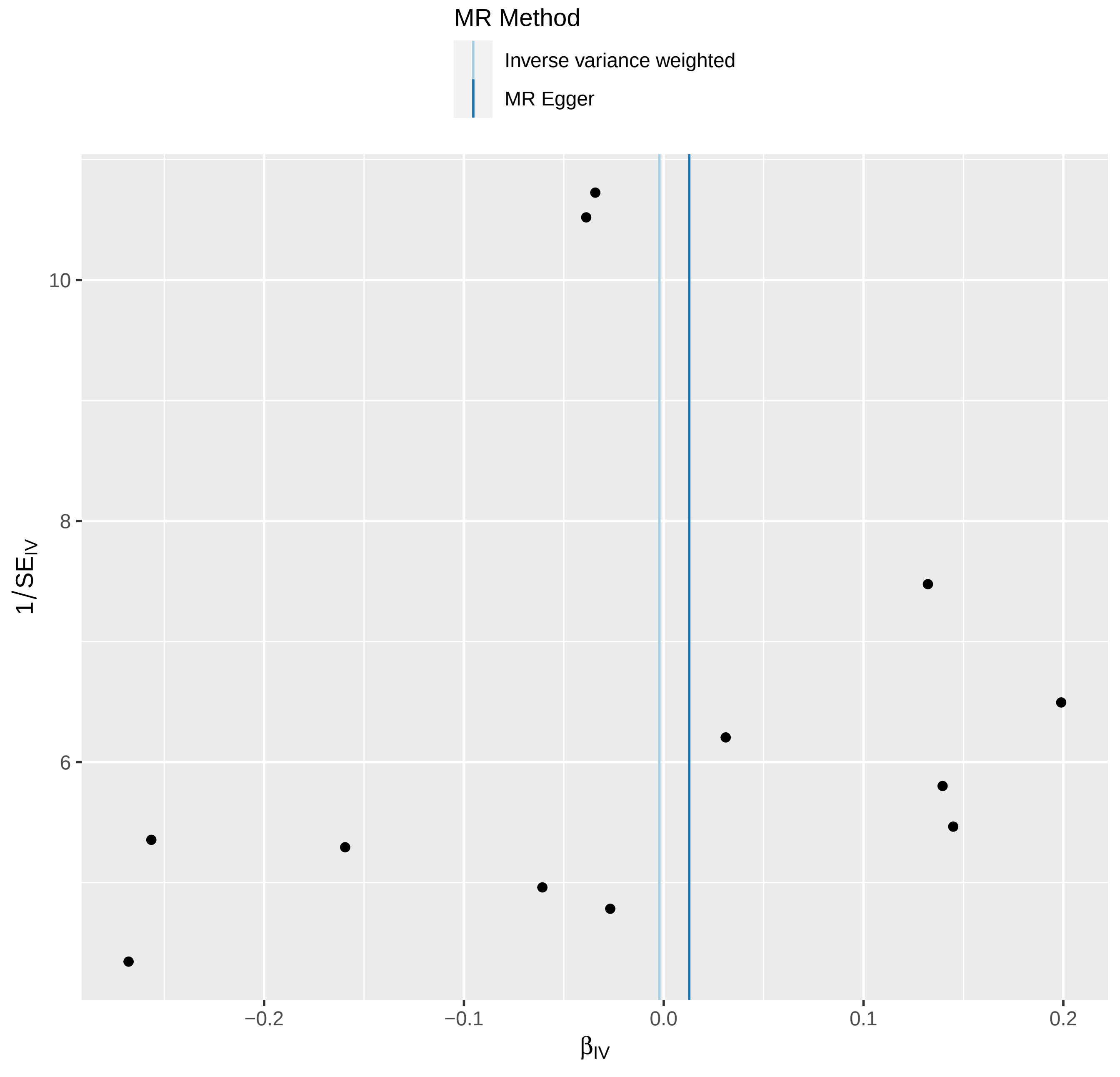

Batch 3−2659 : Selenium on Protein SEC13 homolog

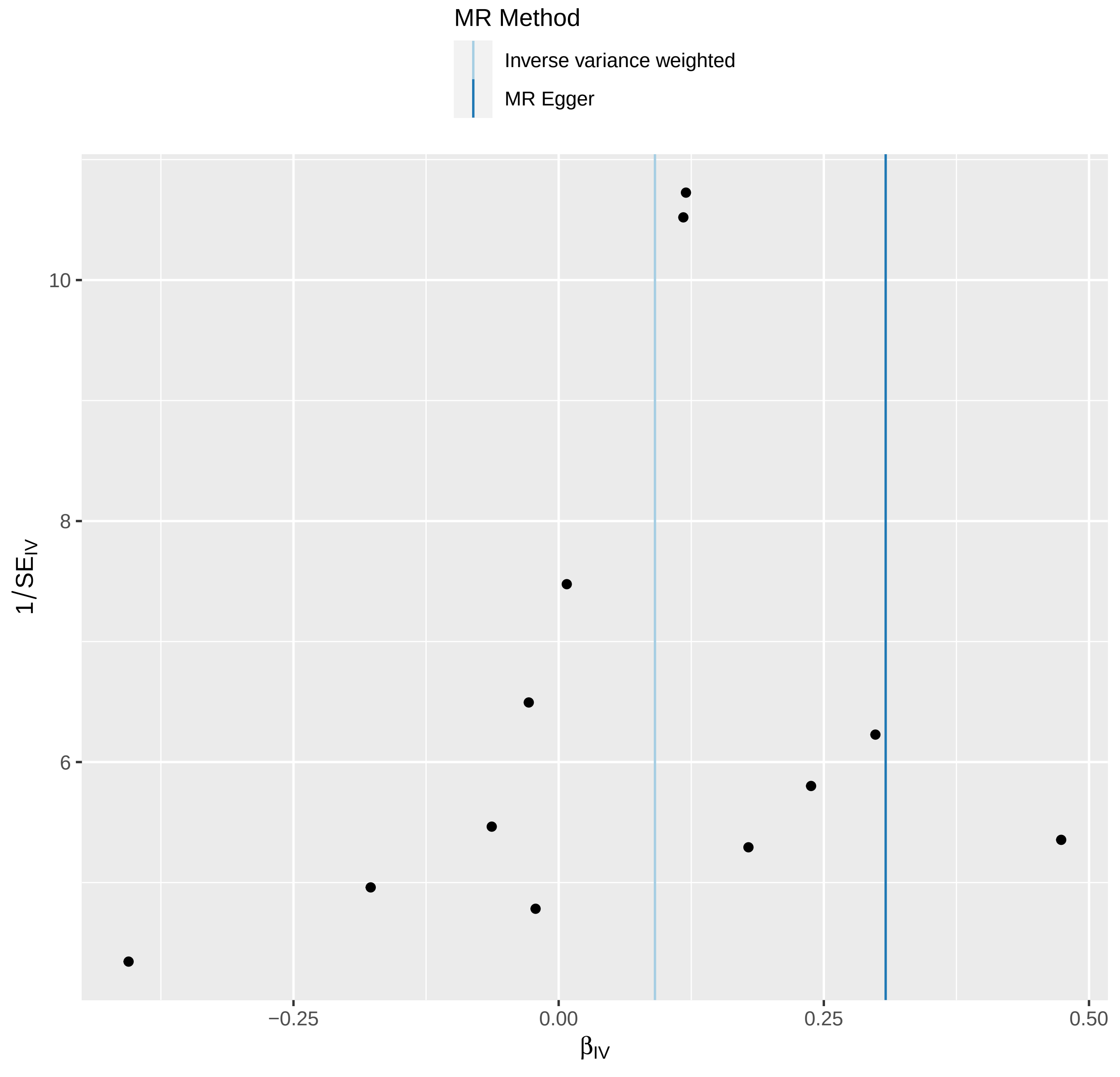

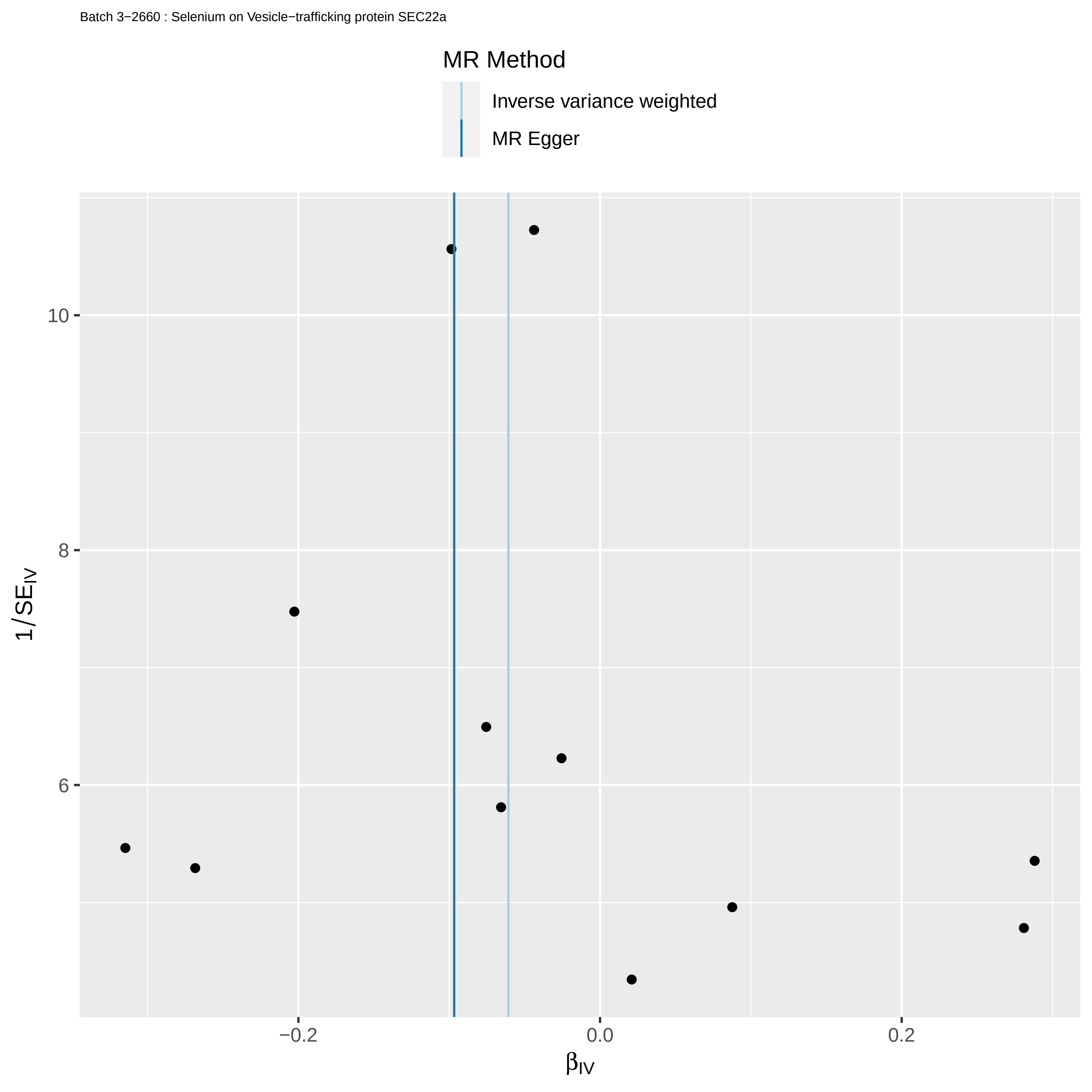

Batch 3−2660 : Selenium on Vesicle−trafficking protein SEC22a
MR Method
Inverse variance weighted
MR Egger
10
8
6
1/SE$_{IV}$
−0.2
0.0
0.2
β_{IV}

Batch 3−2661 : Selenium on Protein transport protein Sec61 subunit beta

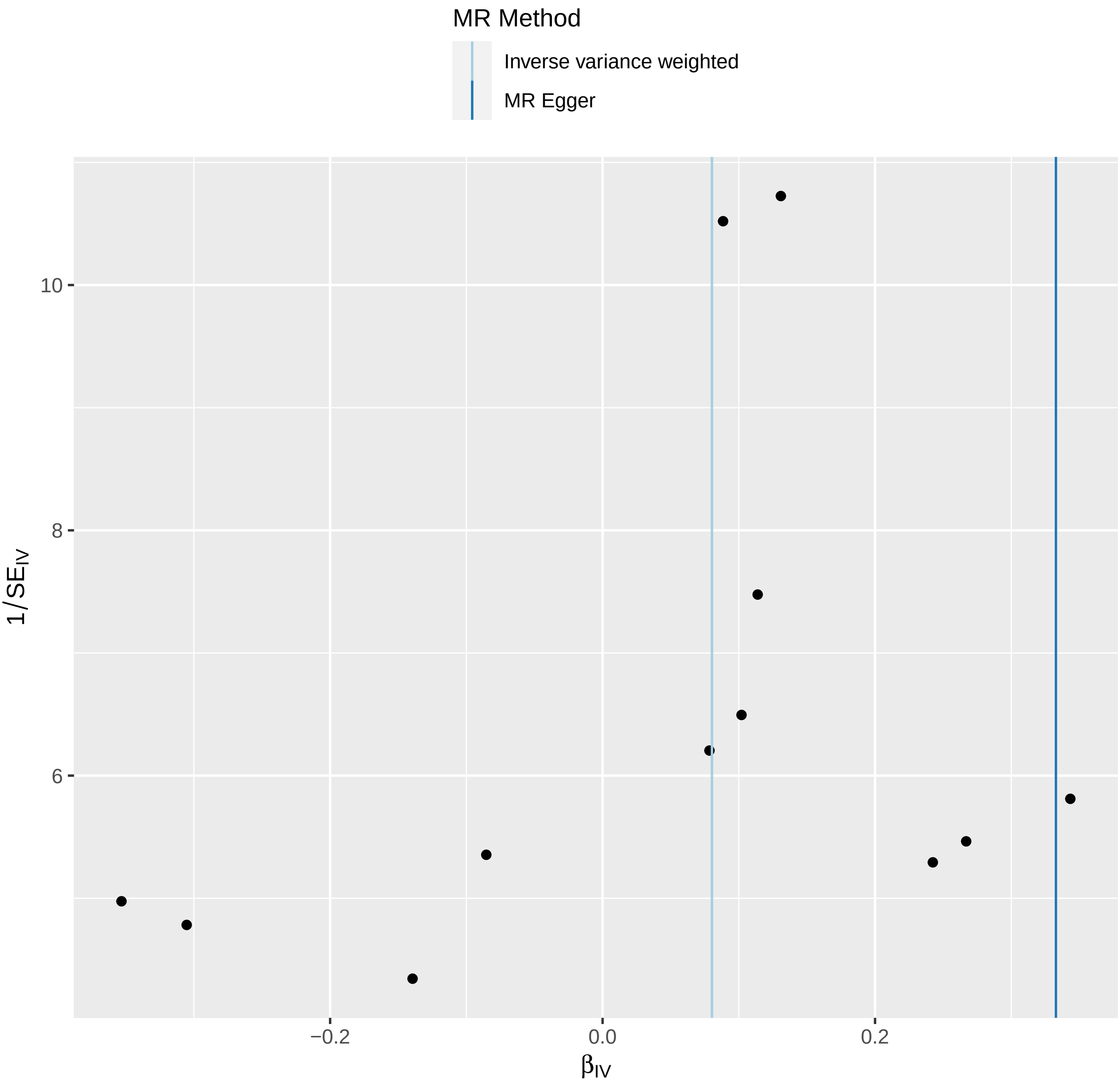

Batch 3−2662 : Selenium on Protein transport protein Sec61 subunit gamma

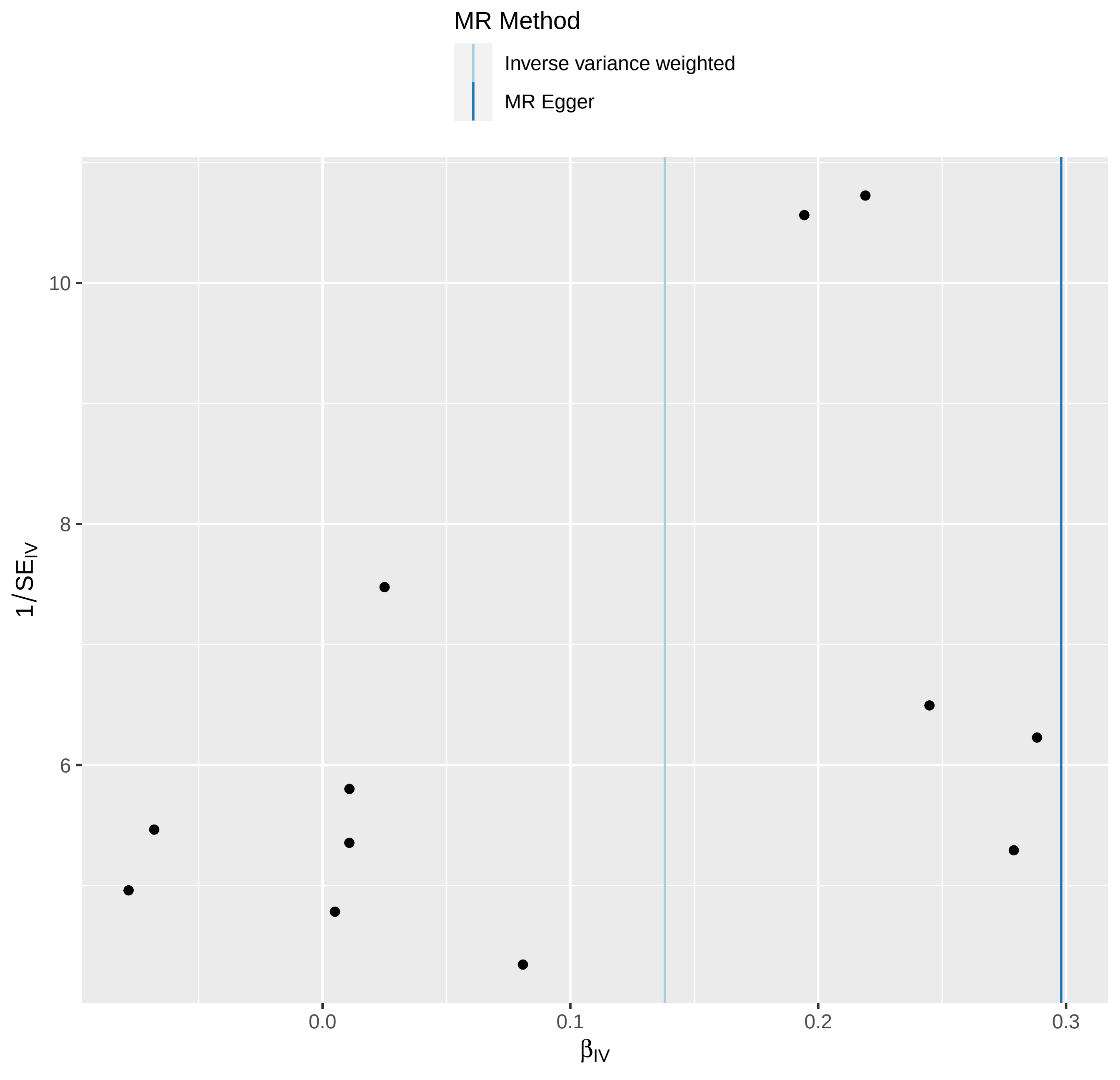

Batch 3−2663 : Selenium on Secreted and transmembrane protein 1

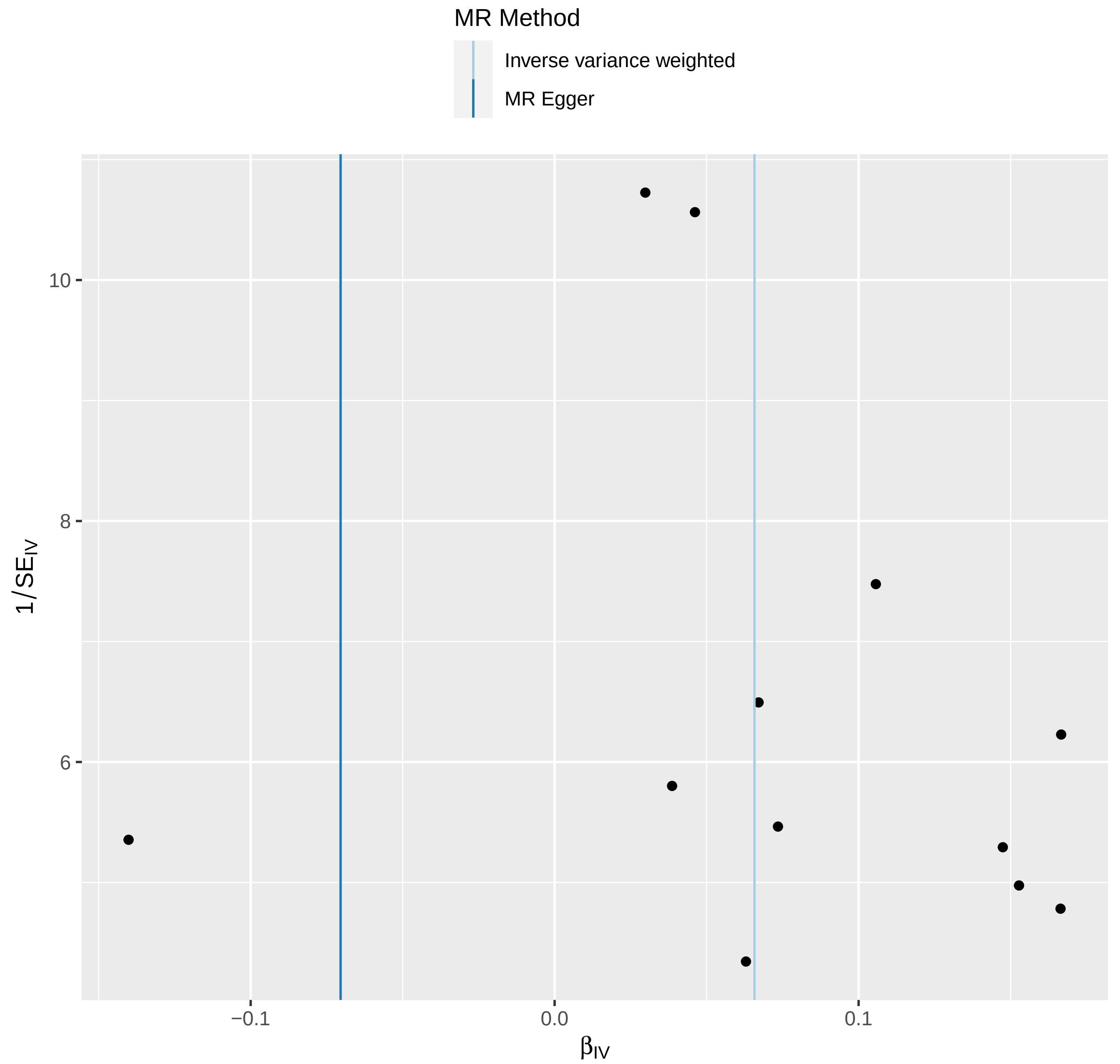

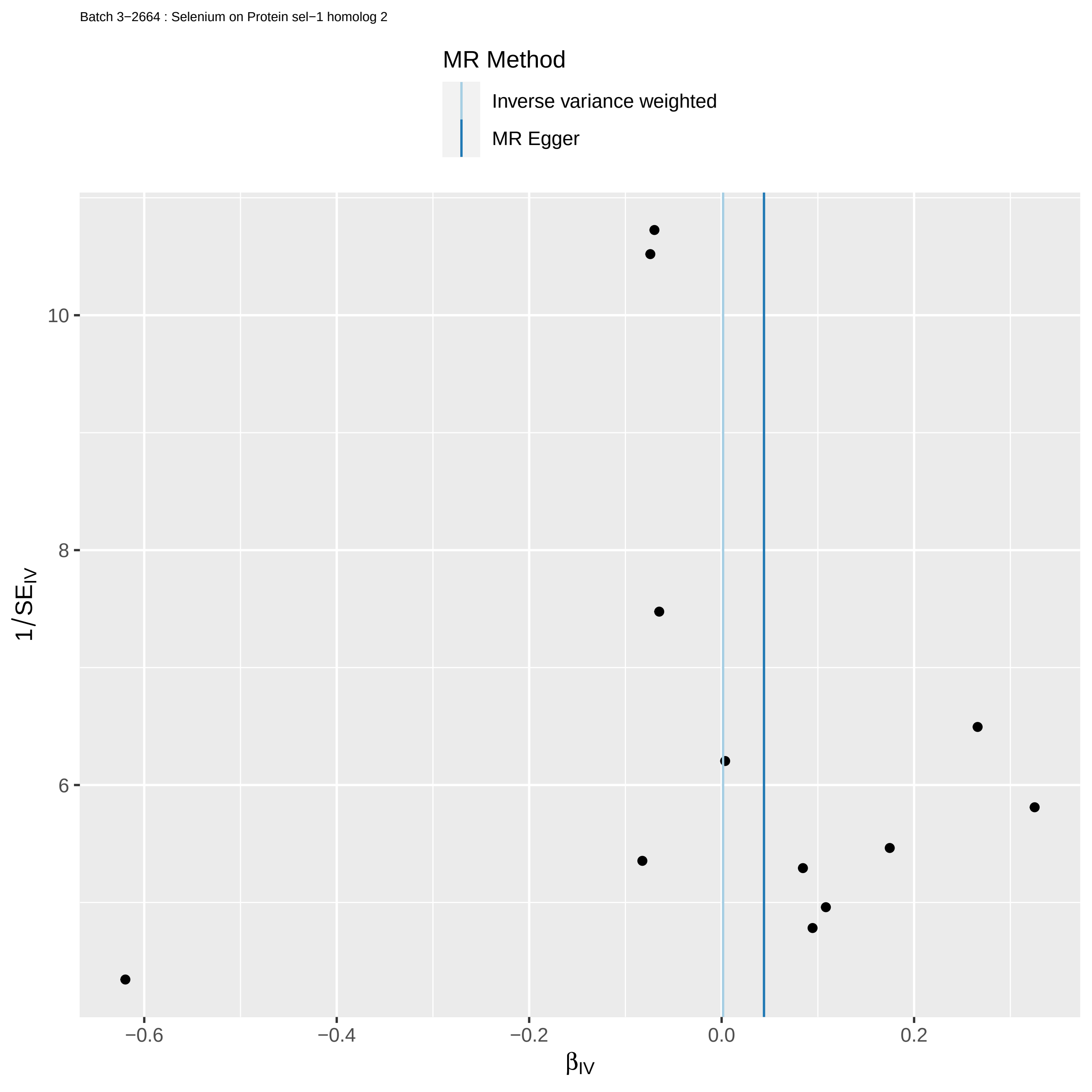
Batch 3−2664 : Selenium on Protein sel−1 homolog 2
MR Method
Inverse variance weighted
MR Egger
10
8
6
1/SE$_{IV}$
−0.6
−0.4
−0.2
0.0
0.2
β_{IV}

Batch 3−2665 : Selenium on E−selectin

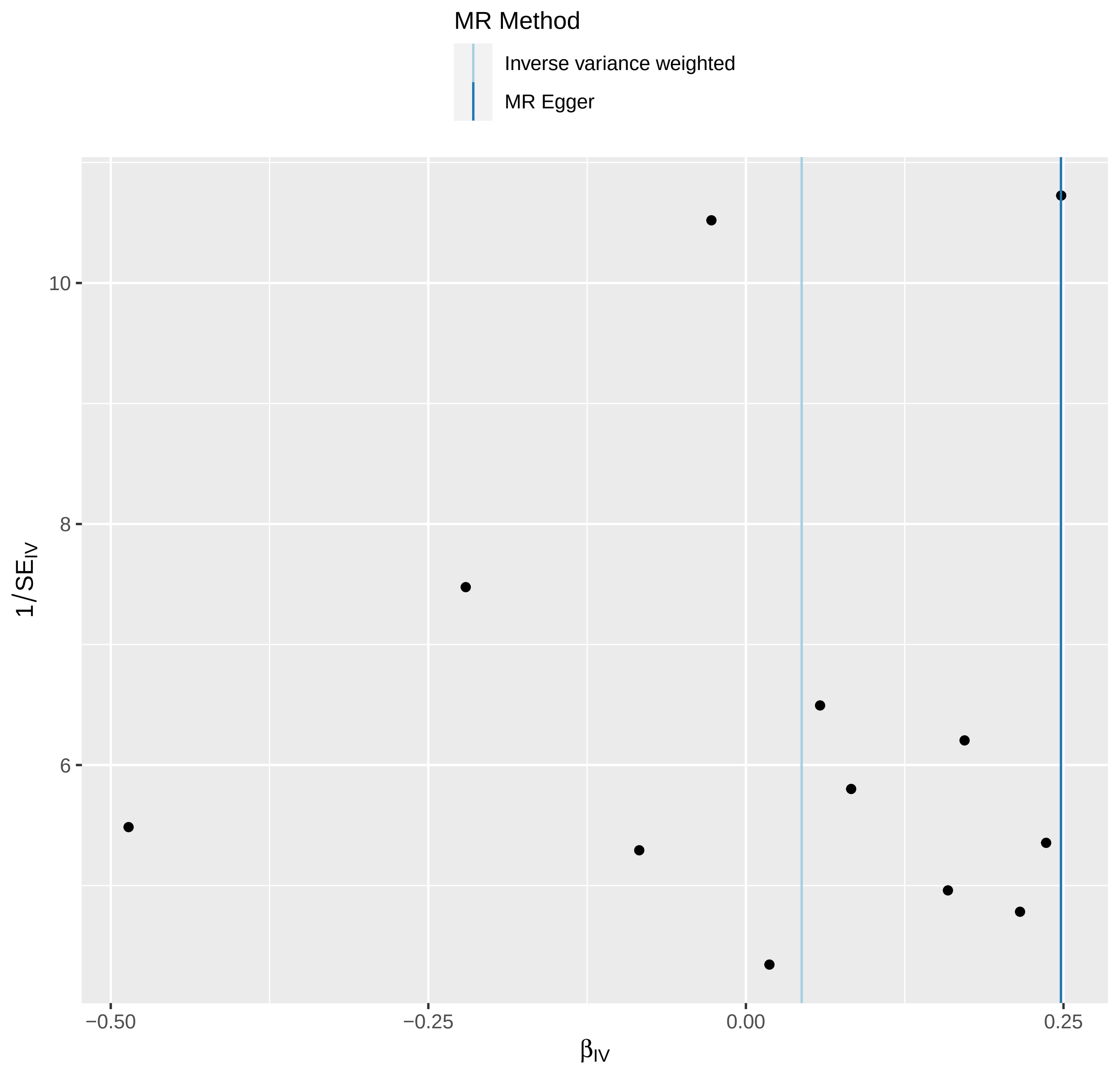

Batch 3−2666 : Selenium on L−Selectin

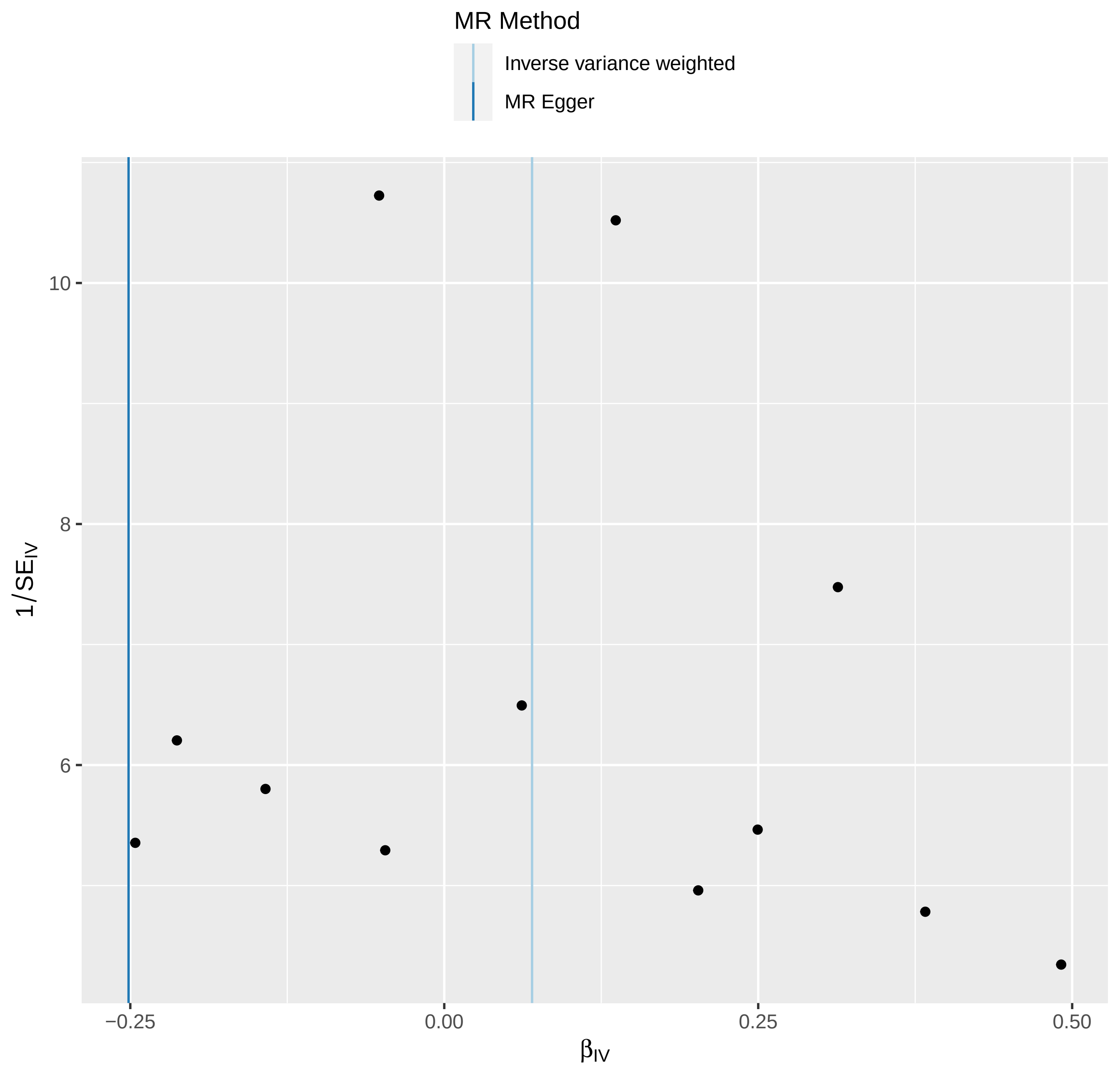

Batch 3−2667 : Selenium on P−selectin

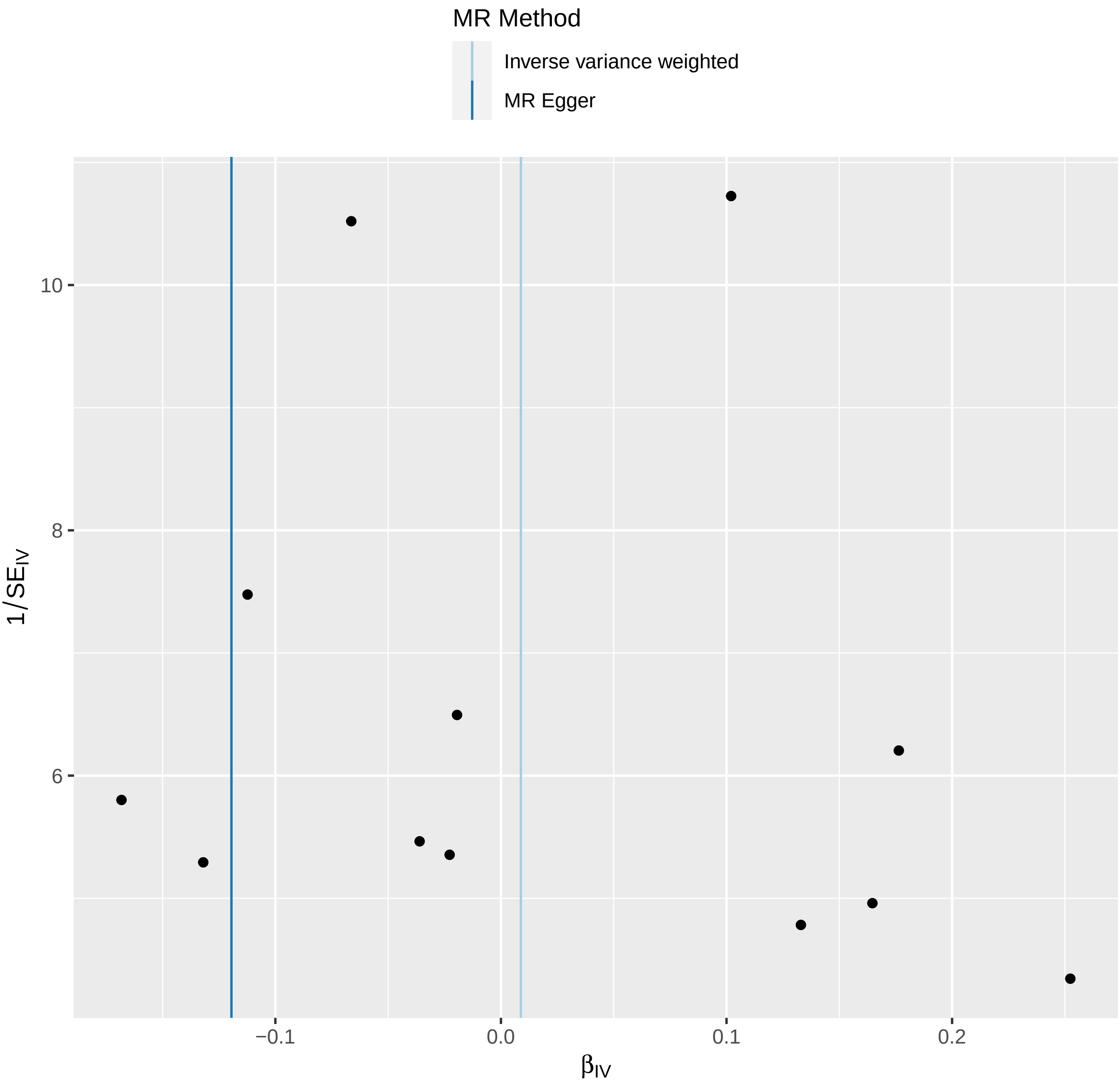

Batch 3−2668 : Selenium on P−selectin glycoprotein ligand 1

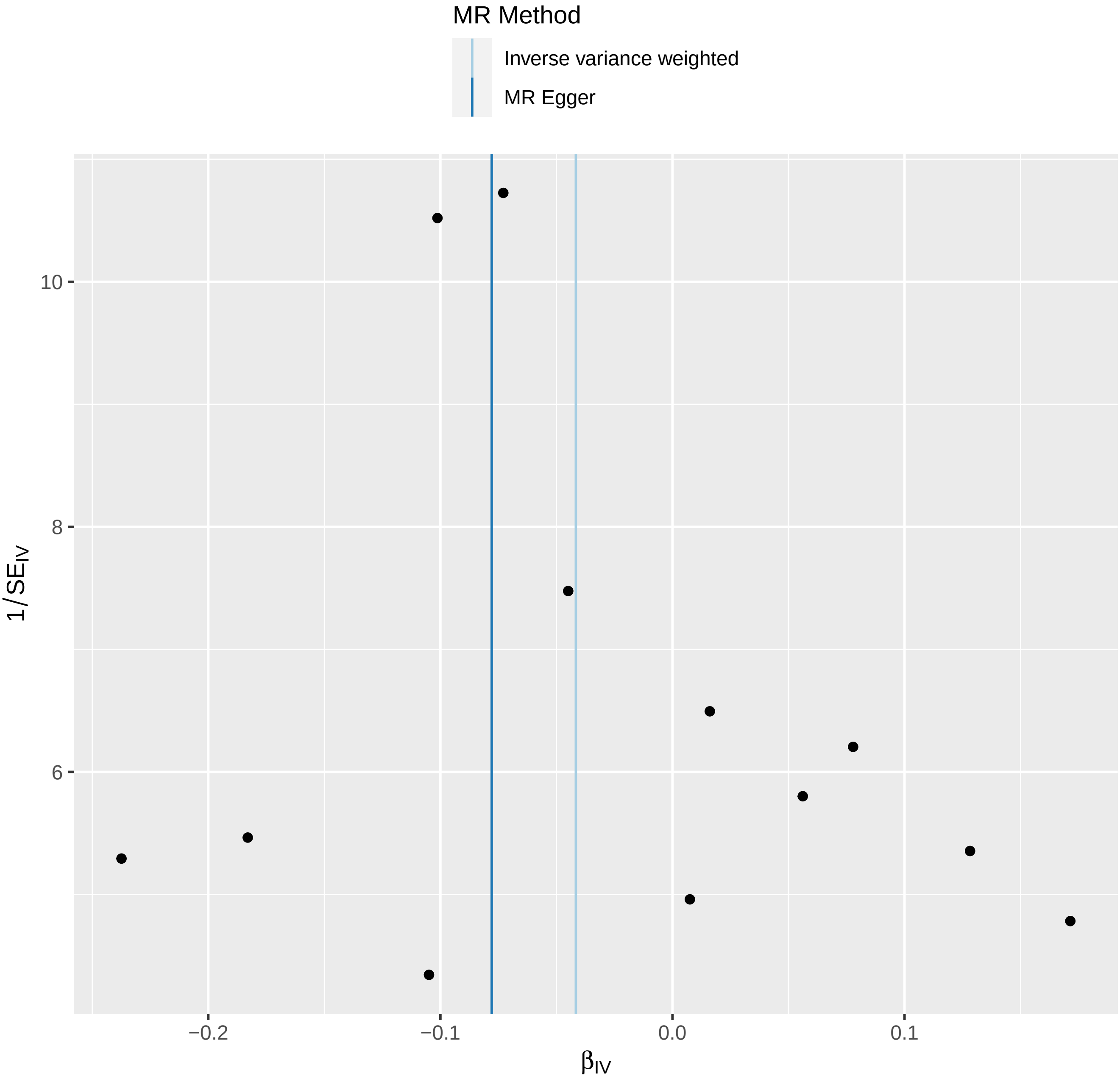

Batch 3−2669 : Selenium on Semaphorin−3A

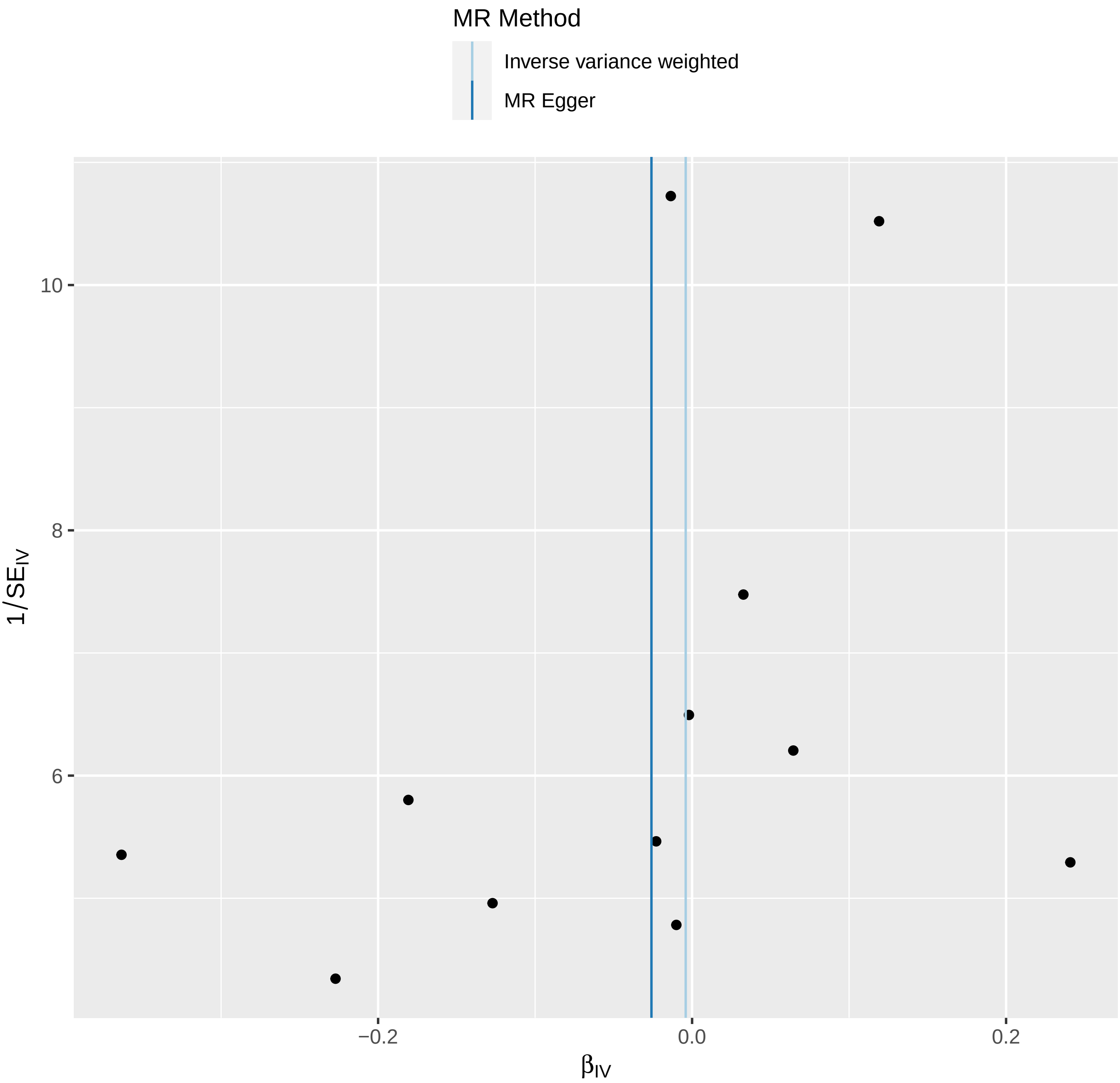

Batch 3−2670 : Selenium on Semaphorin−3B

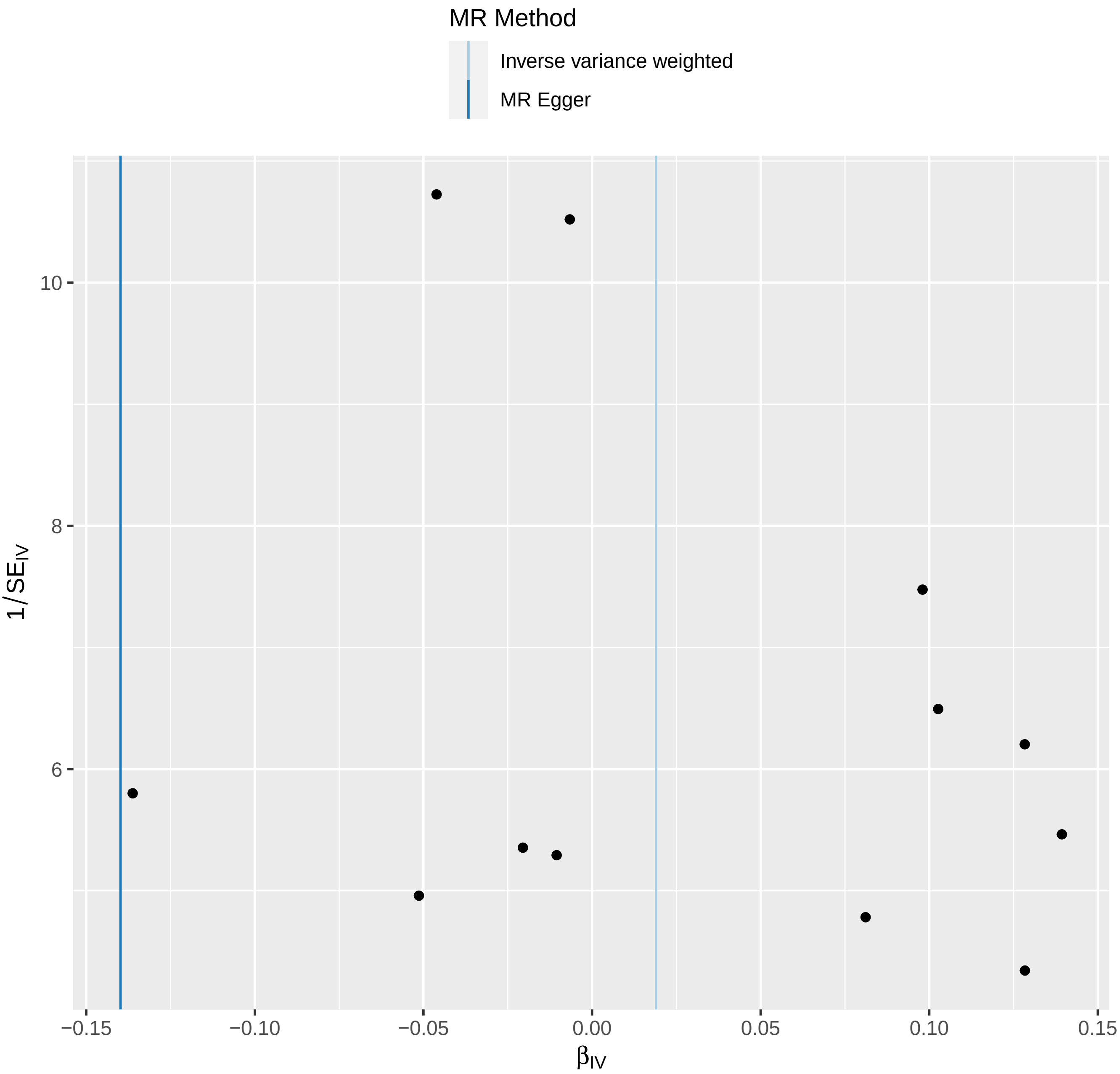

Batch 3−2671 : Selenium on Semaphorin−3C

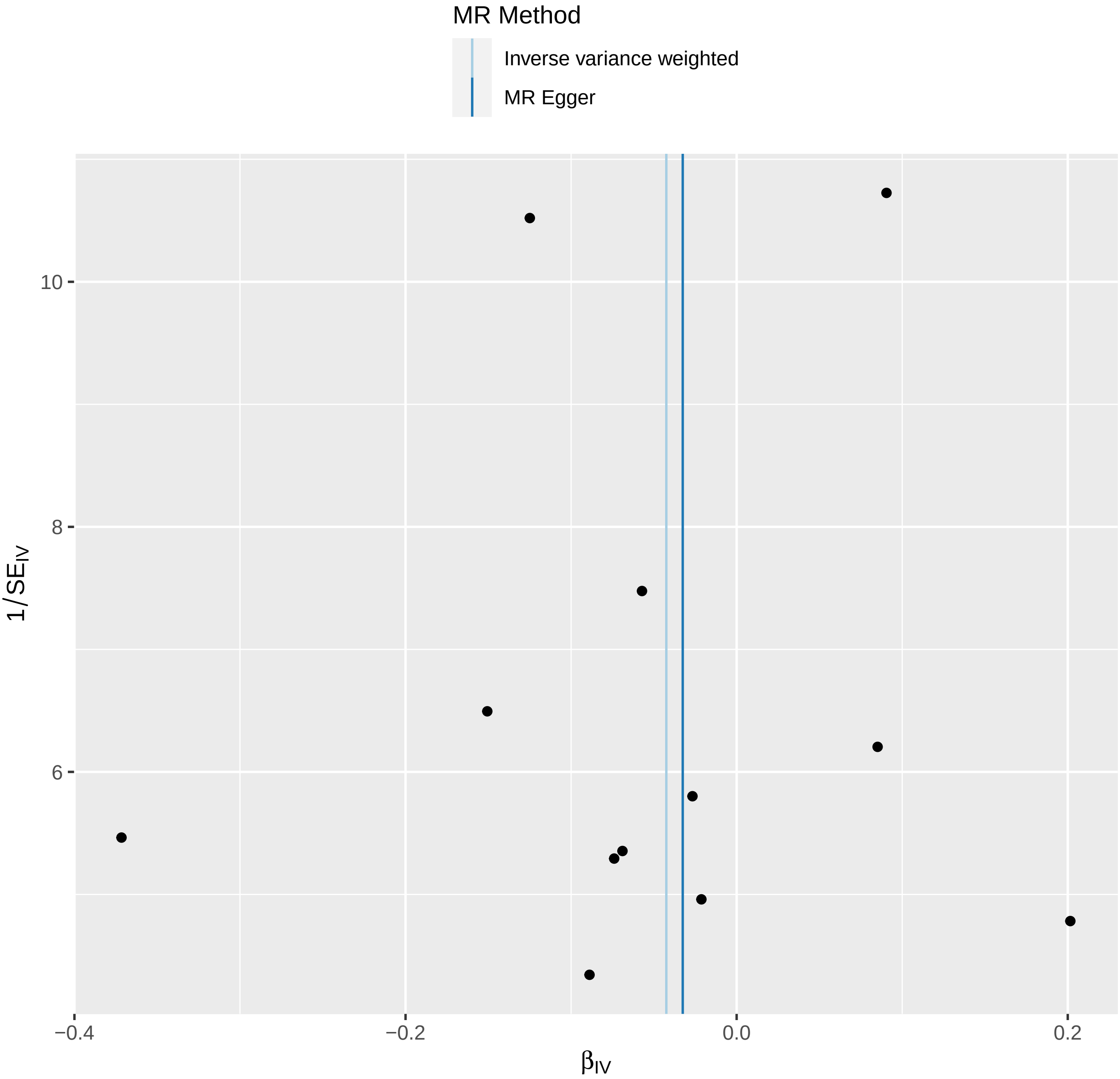

Batch 3−2672 : Selenium on Semaphorin−3E

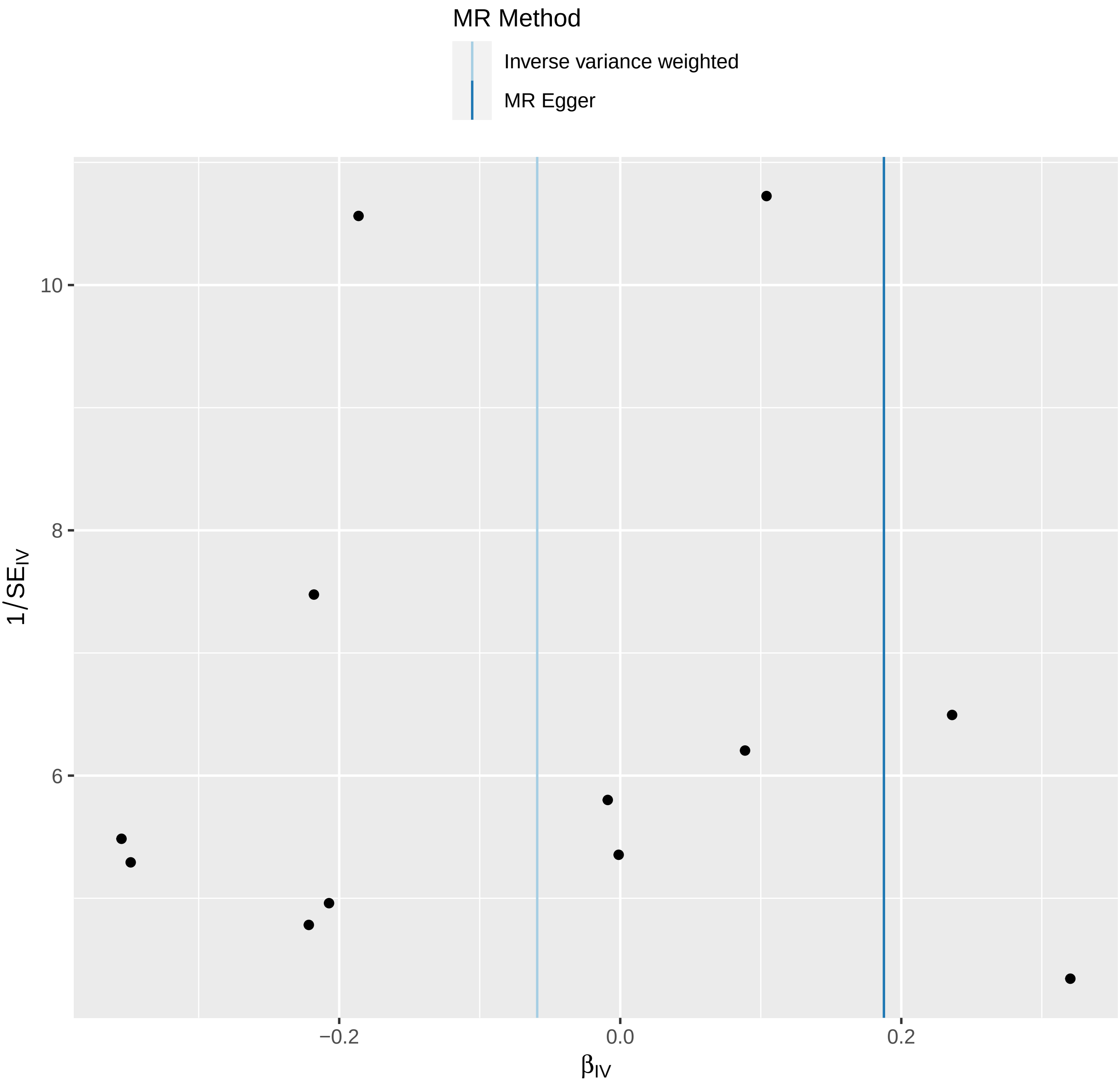

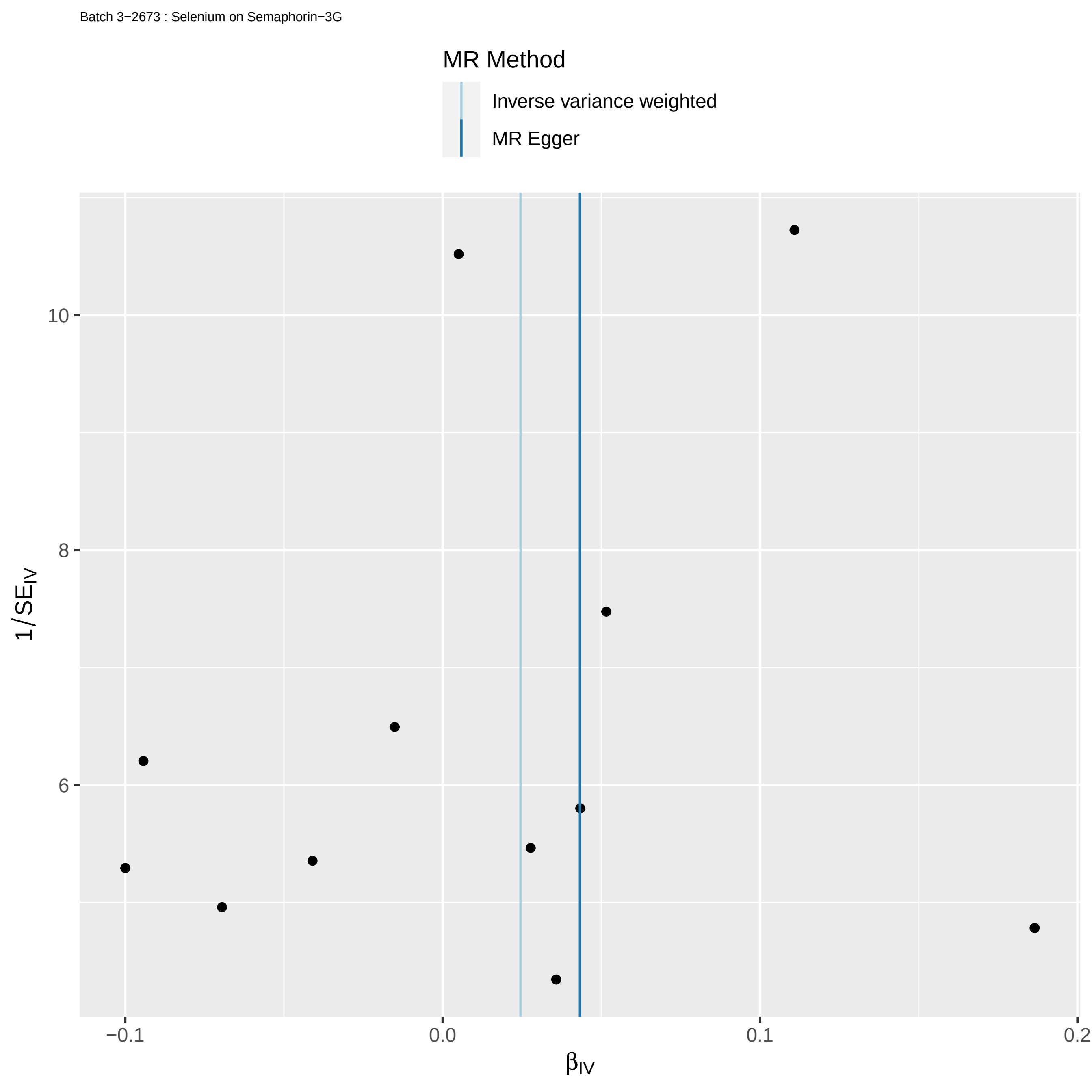

Batch 3−2673 : Selenium on Semaphorin−3G
MR Method
Inverse variance weighted
MR Egger
10
8
6
1/SE$_{IV}$
−0.1
0.0
0.1
0.2
β$_{IV}$

Batch 3−2674 : Selenium on Semaphorin−4C

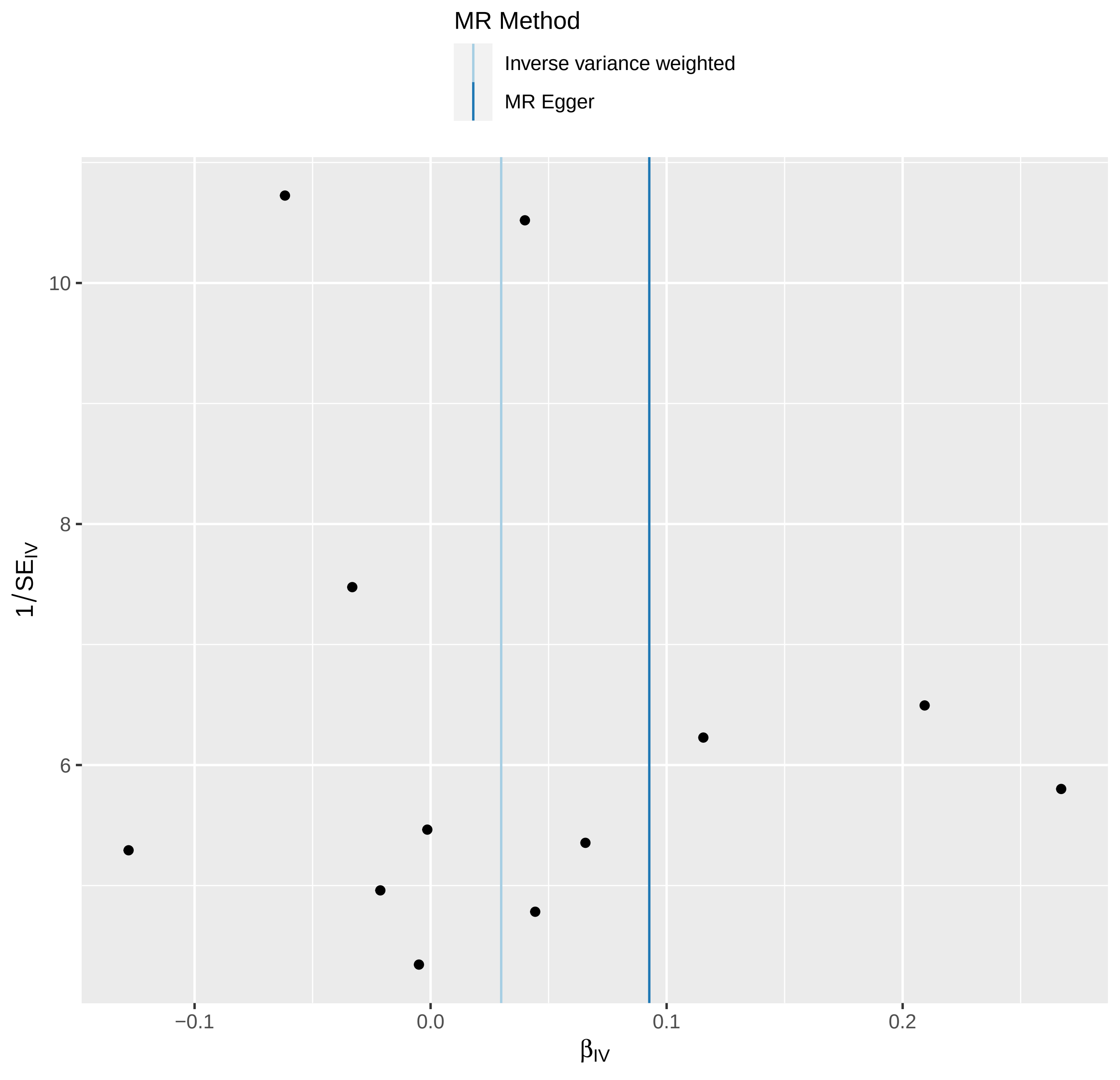

Batch 3−2675 : Selenium on Semaphorin−4D

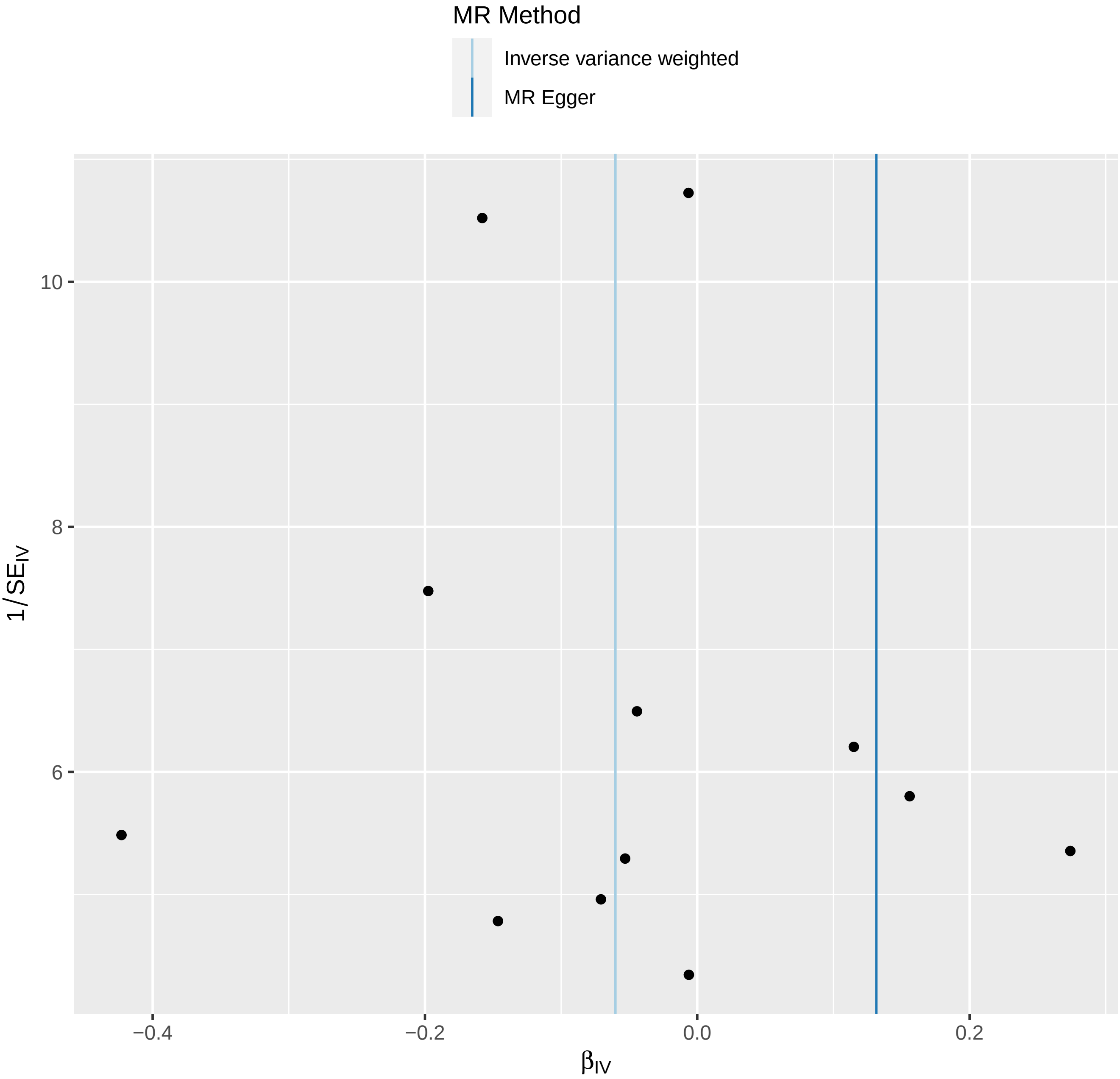

Batch 3−2676 : Selenium on Semaphorin−5A

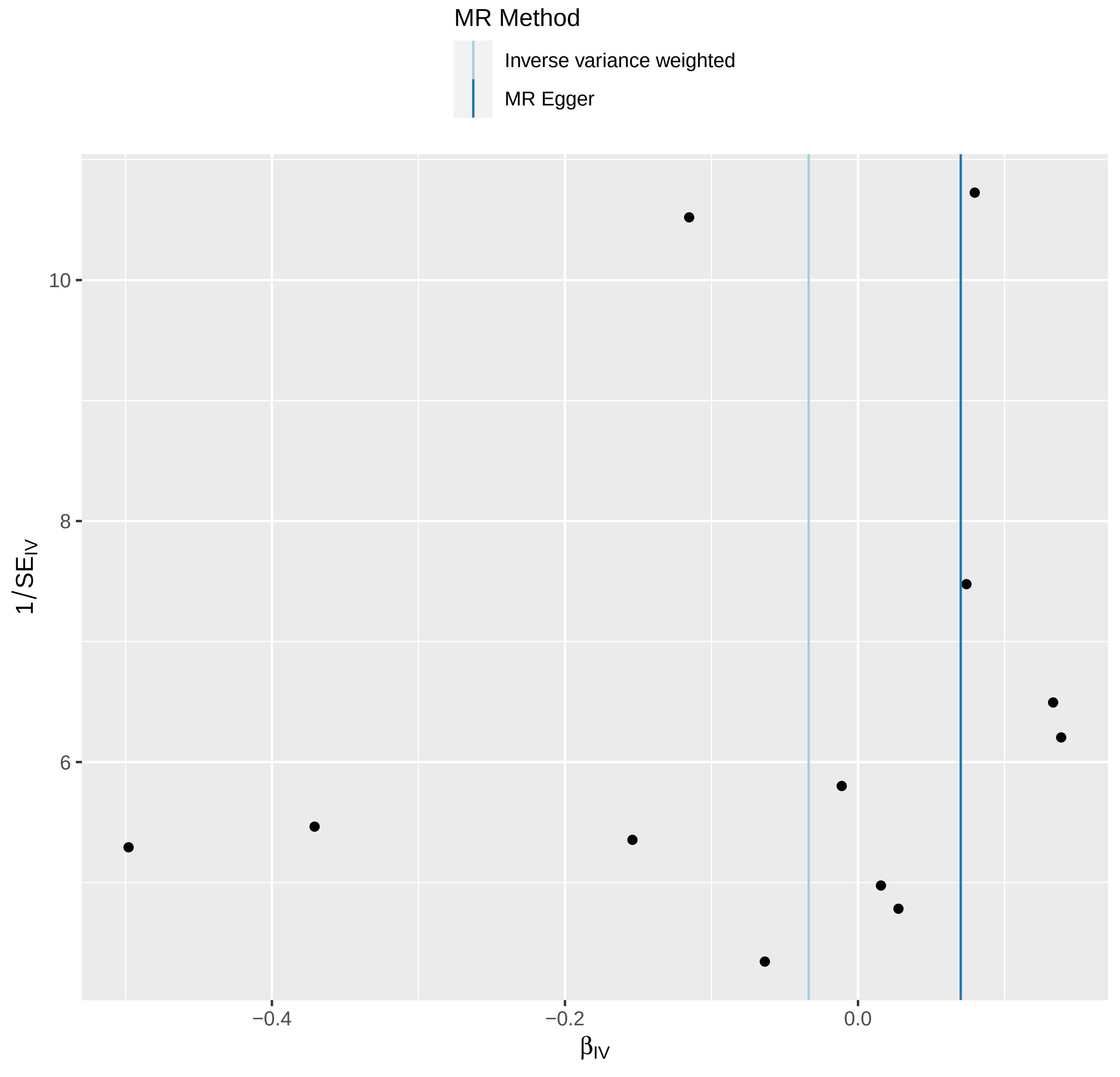

Batch 3−2677 : Selenium on Semaphorin−6A

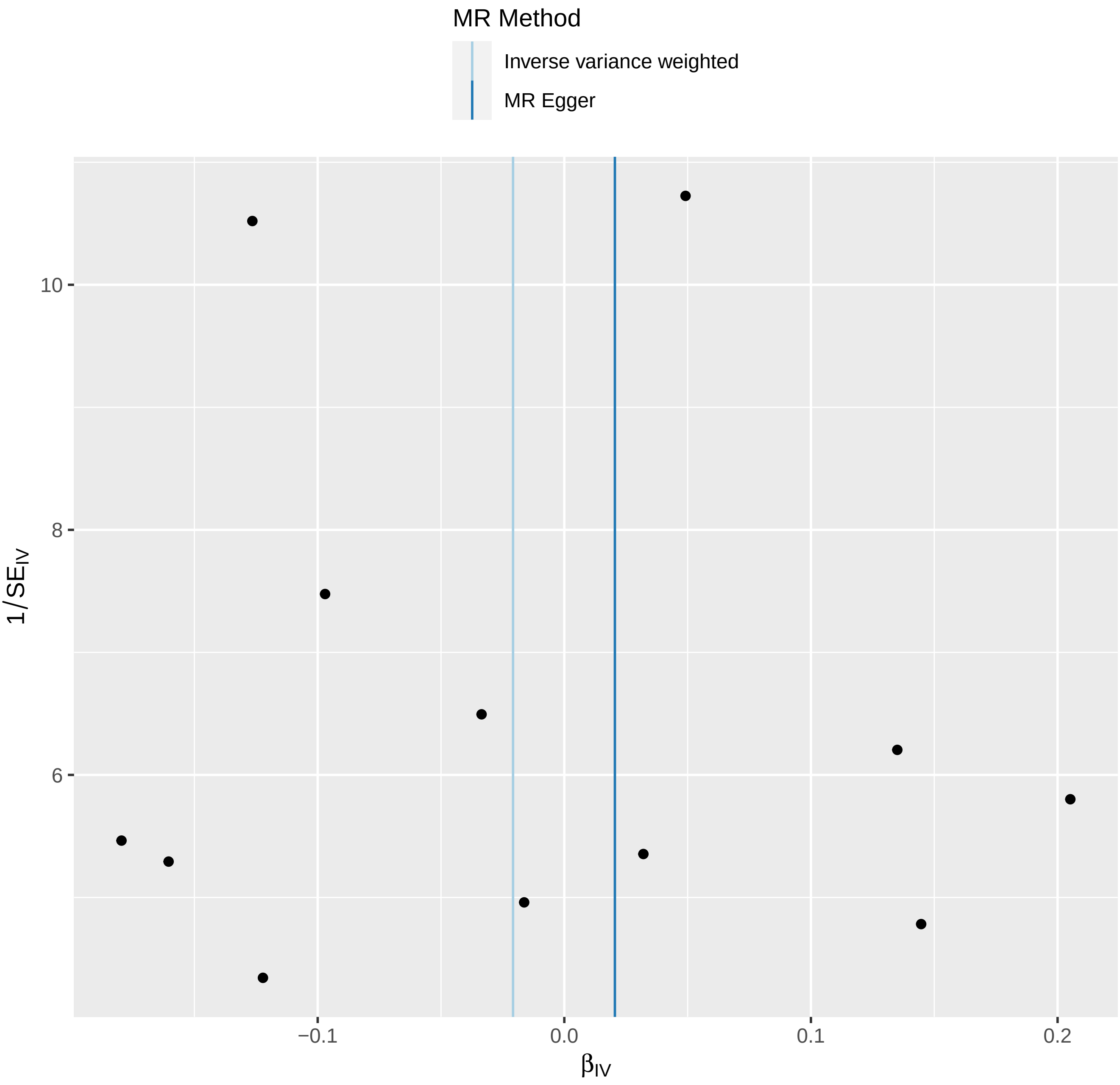

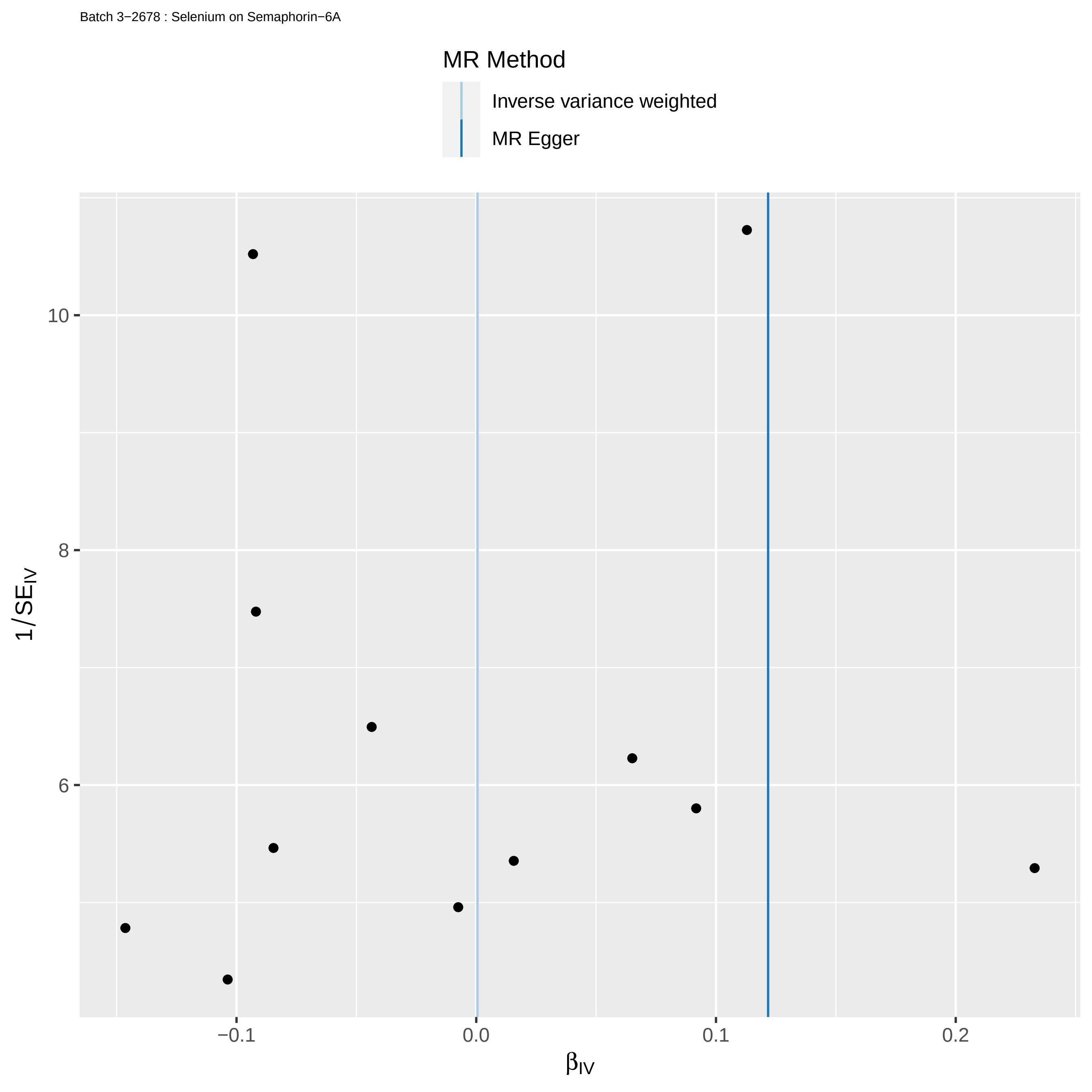
Batch 3−2678 : Selenium on Semaphorin−6A
MR Method
Inverse variance weighted
MR Egger
10
8
6
1/SE_IV
−0.1
0.0
0.1
0.2
β_IV

Batch 3−2679 : Selenium on Semaphorin−6C

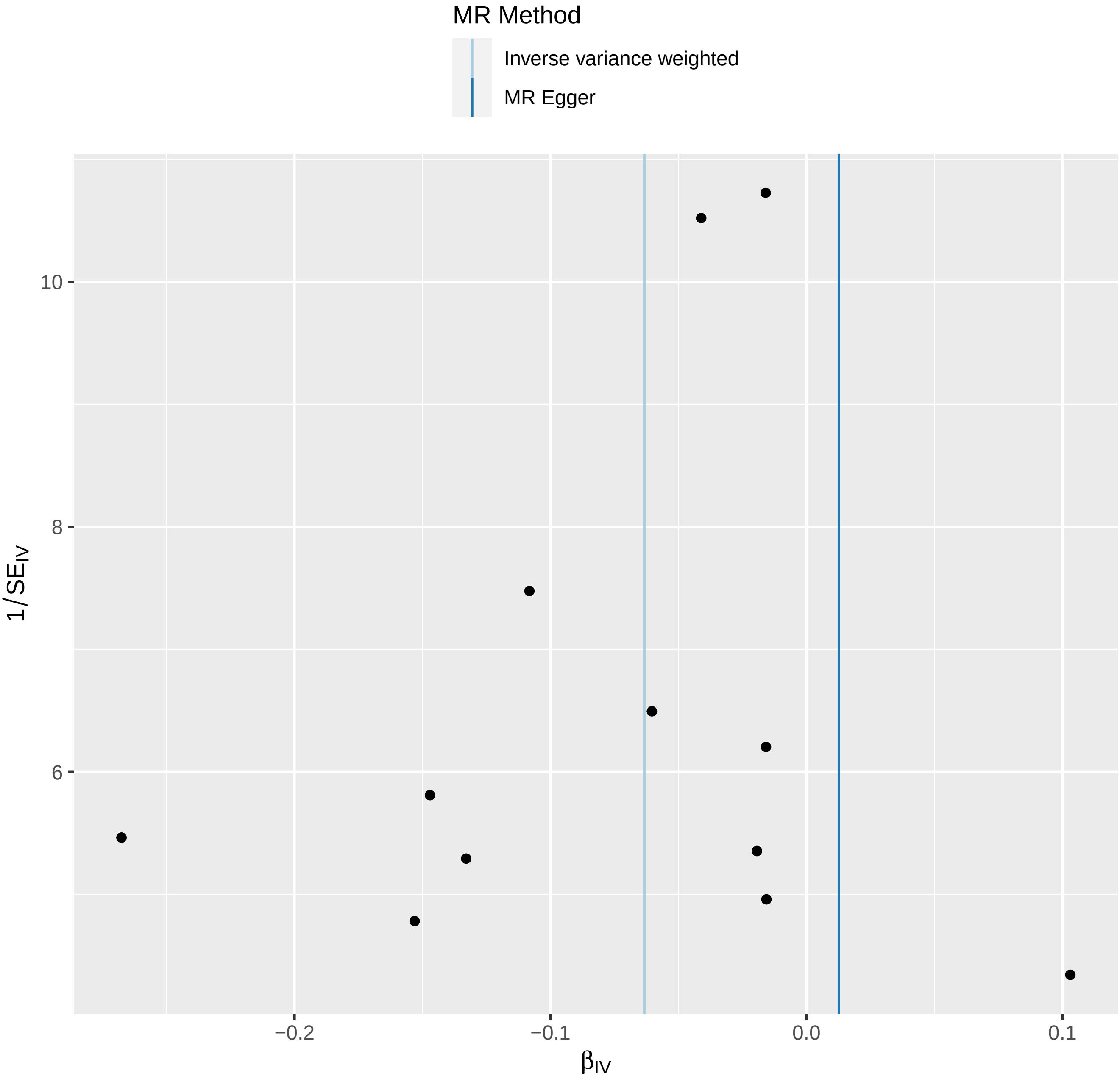

Batch 3−2680 : Selenium on Semaphorin−7A

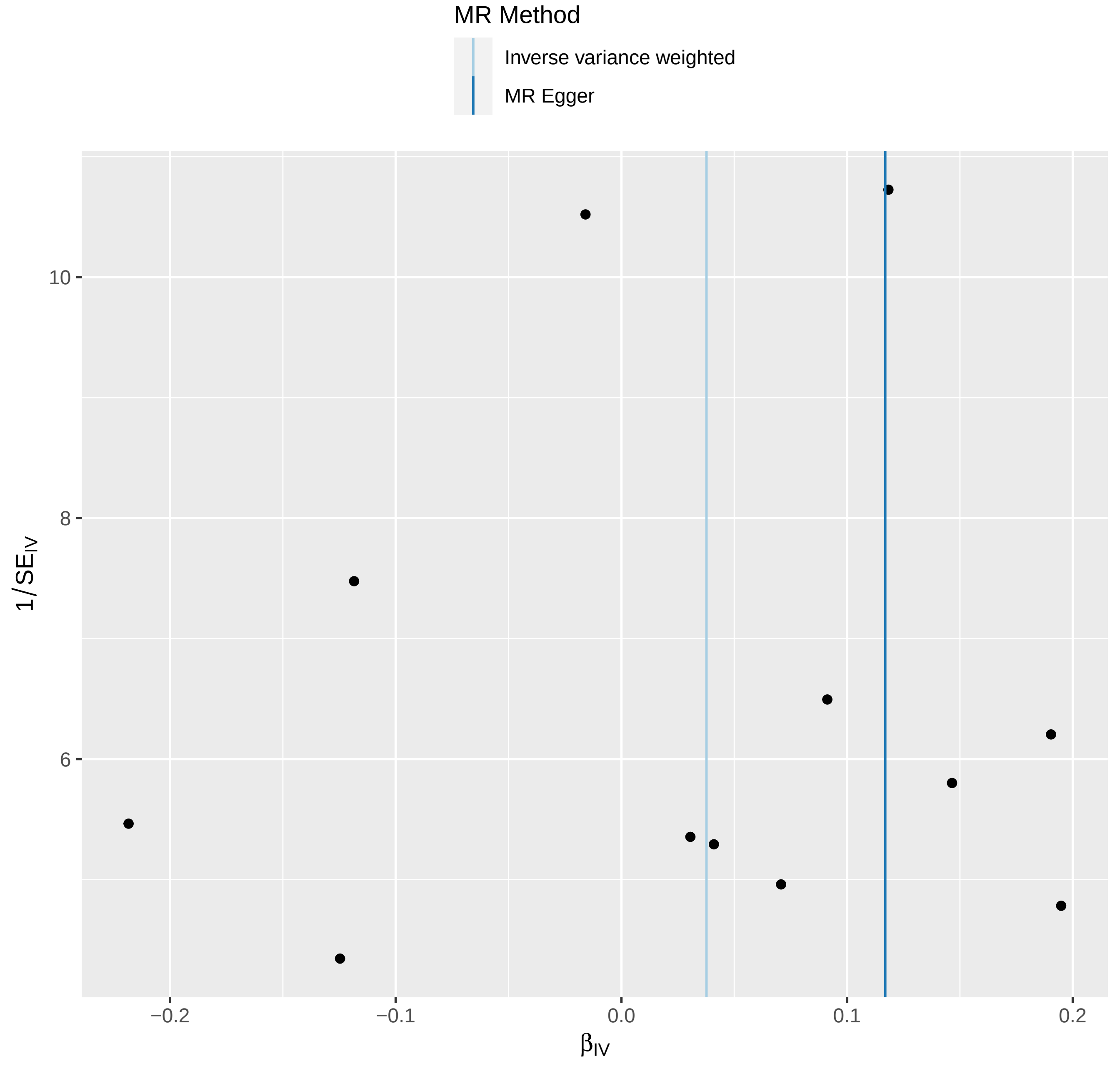

Batch 3−2681 : Selenium on Semenogelin−1

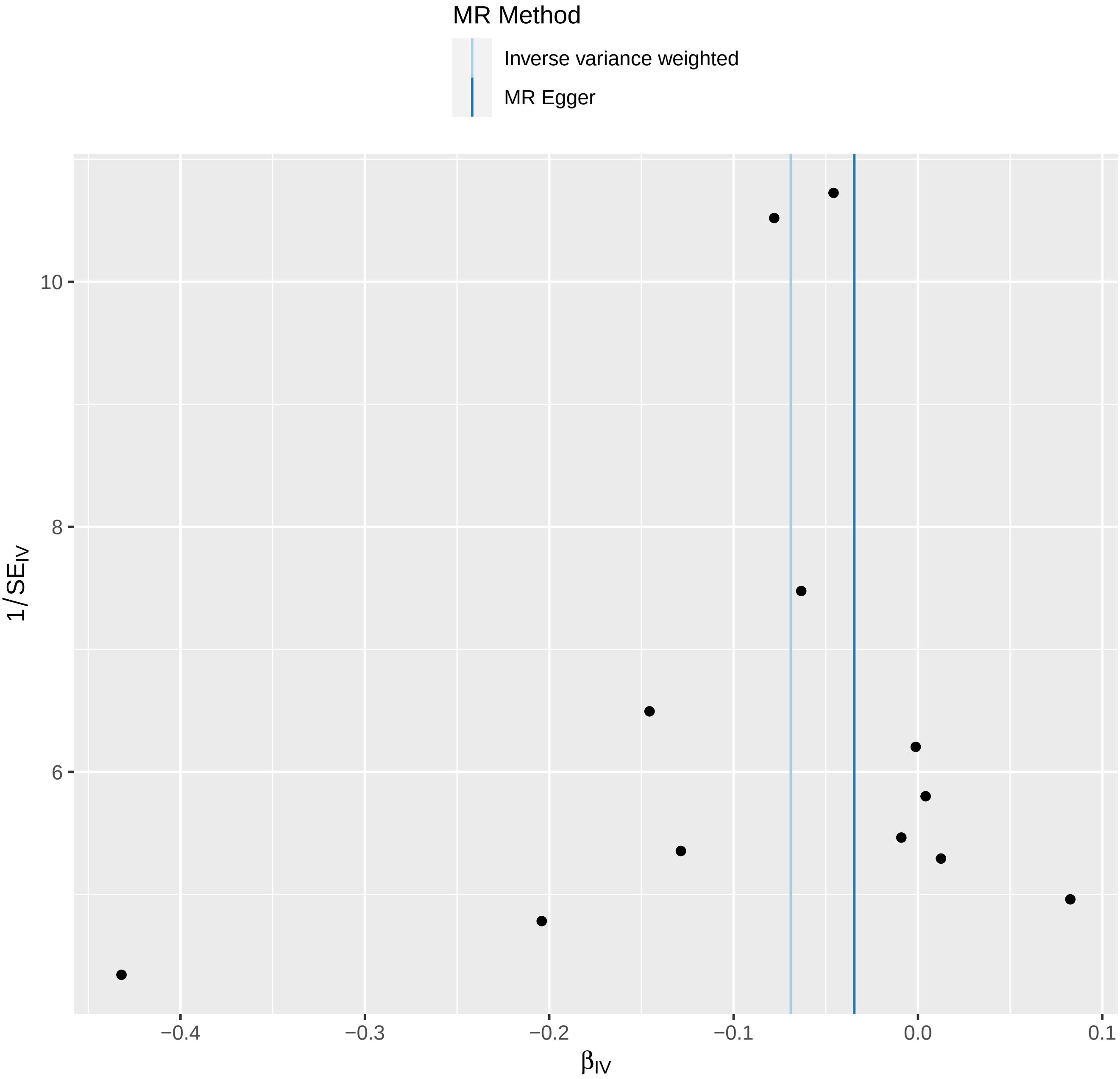

Batch 3−2682 : Selenium on Semenogelin−2

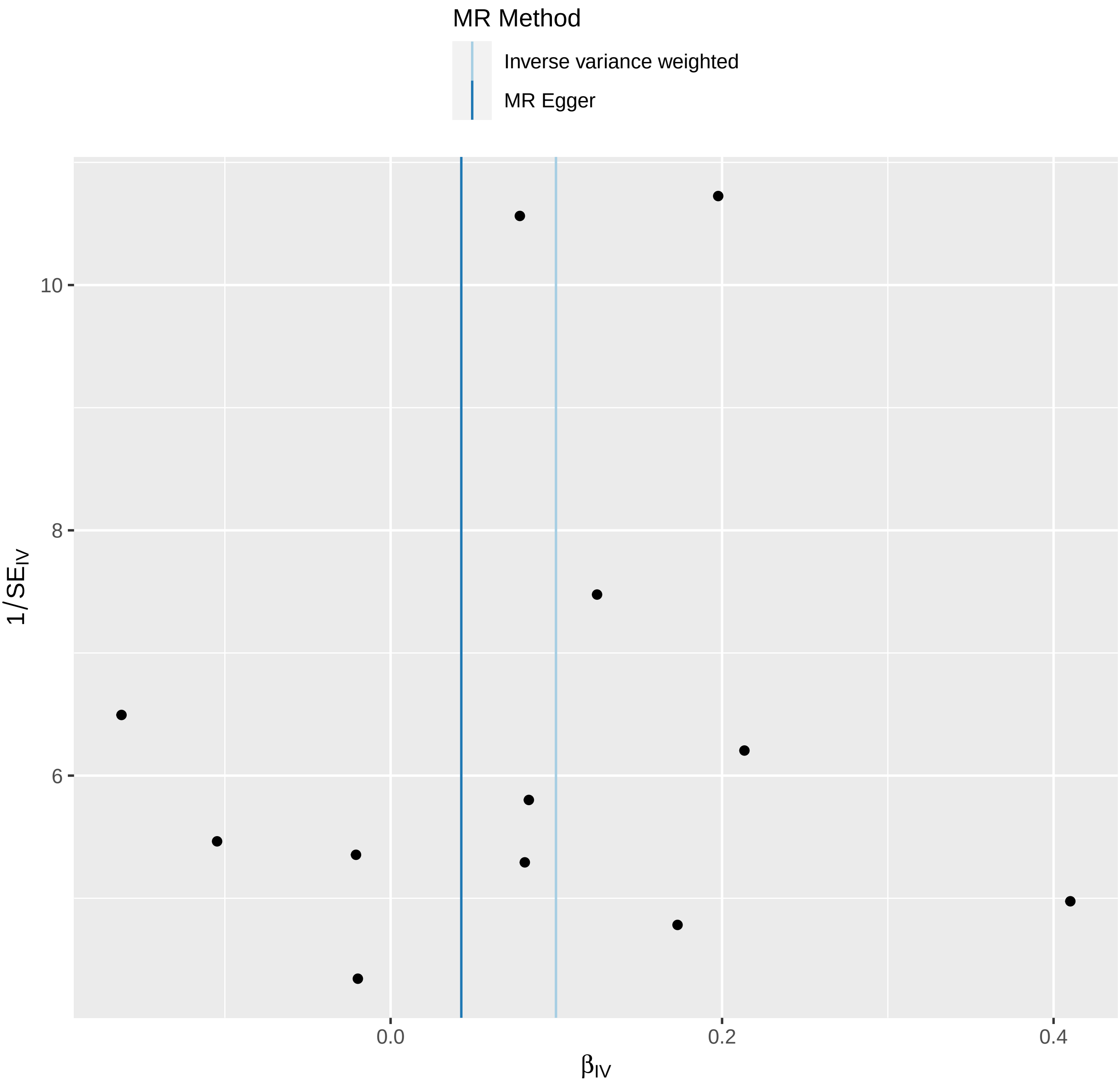

Batch 3−2683 : Selenium on Sentrin−specific protease 7

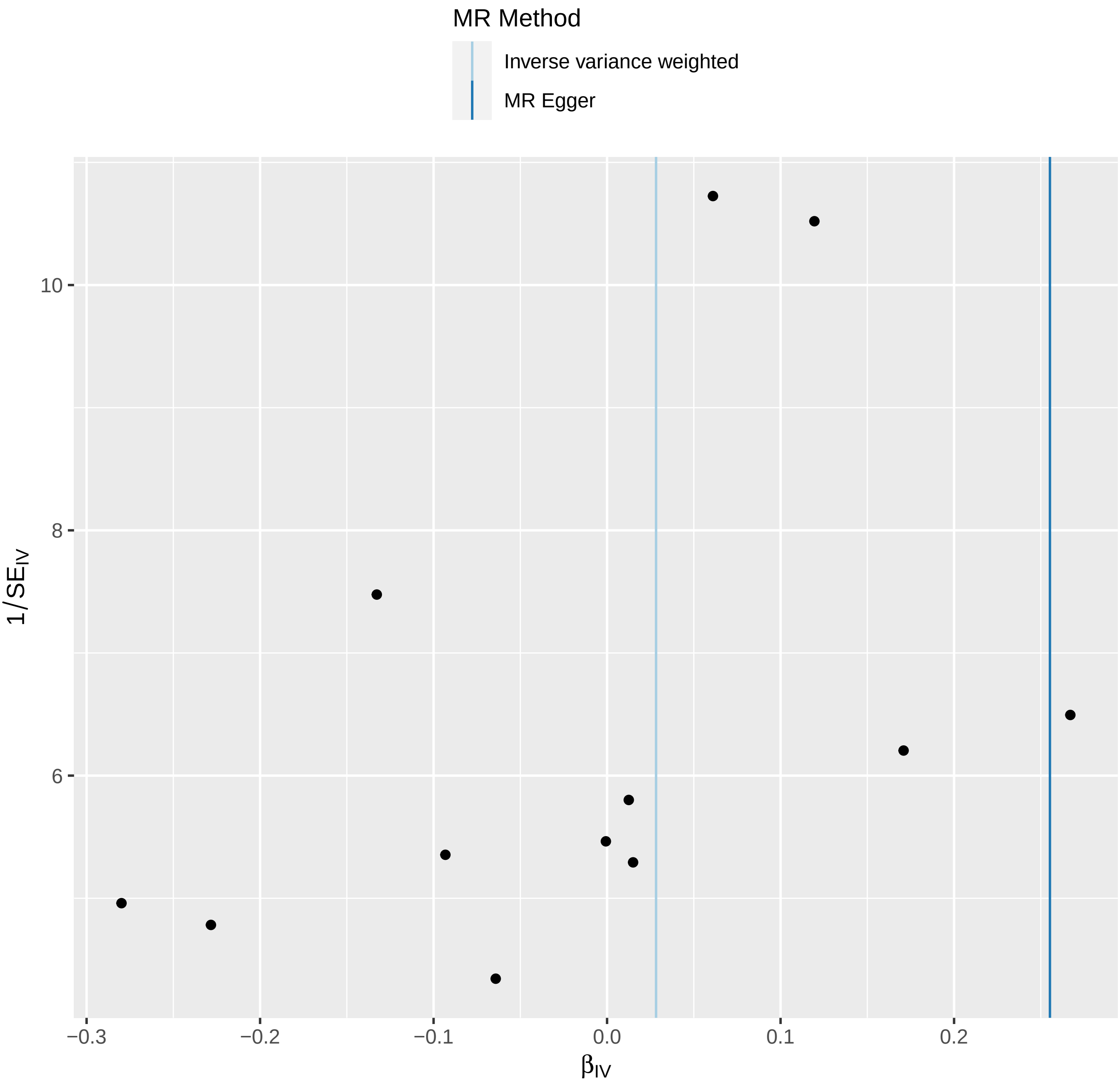

Batch 3−2684 : Selenium on Selenide, water dikinase 1

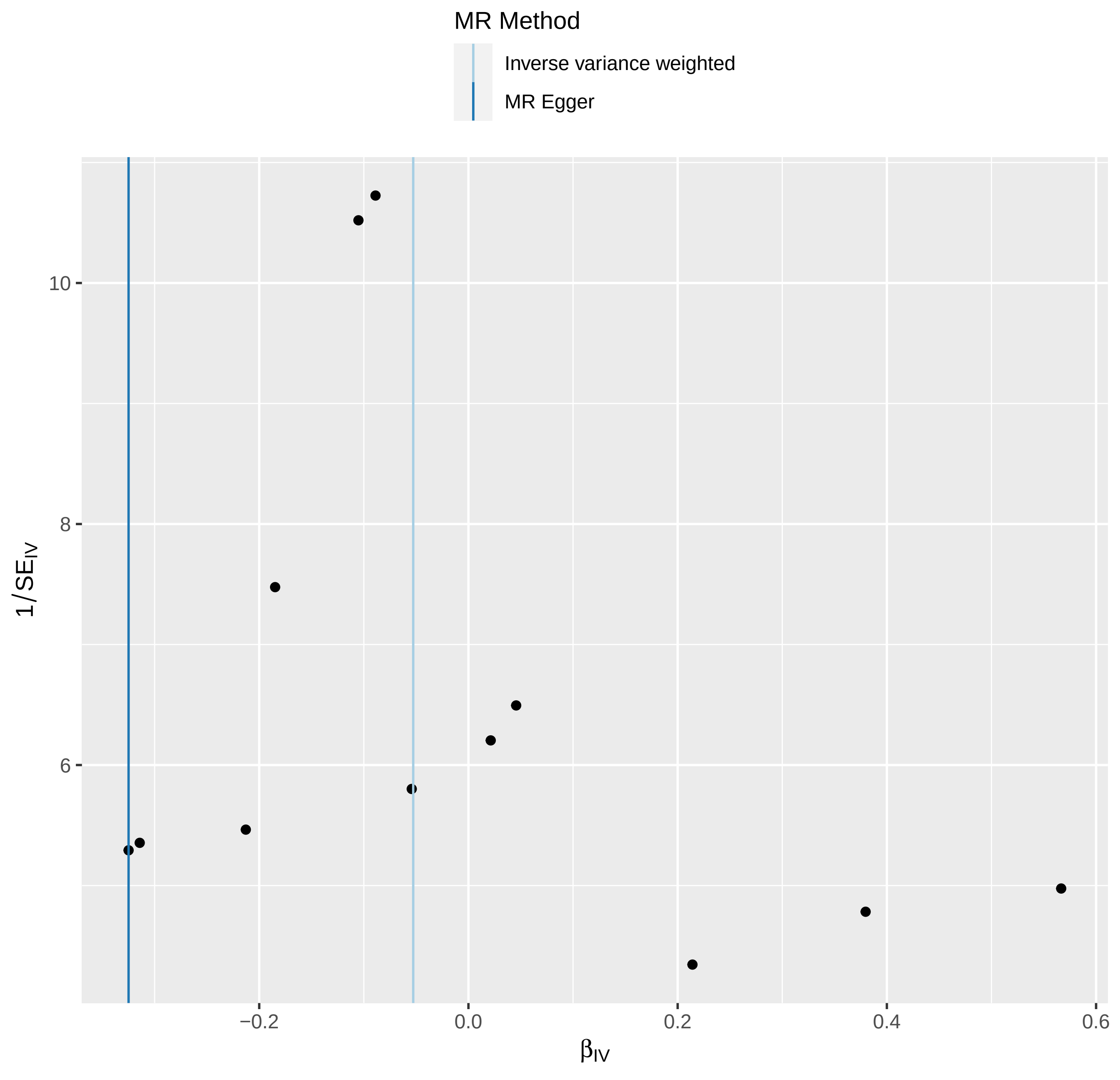

Batch 3−2685 : Selenium on Septin−10

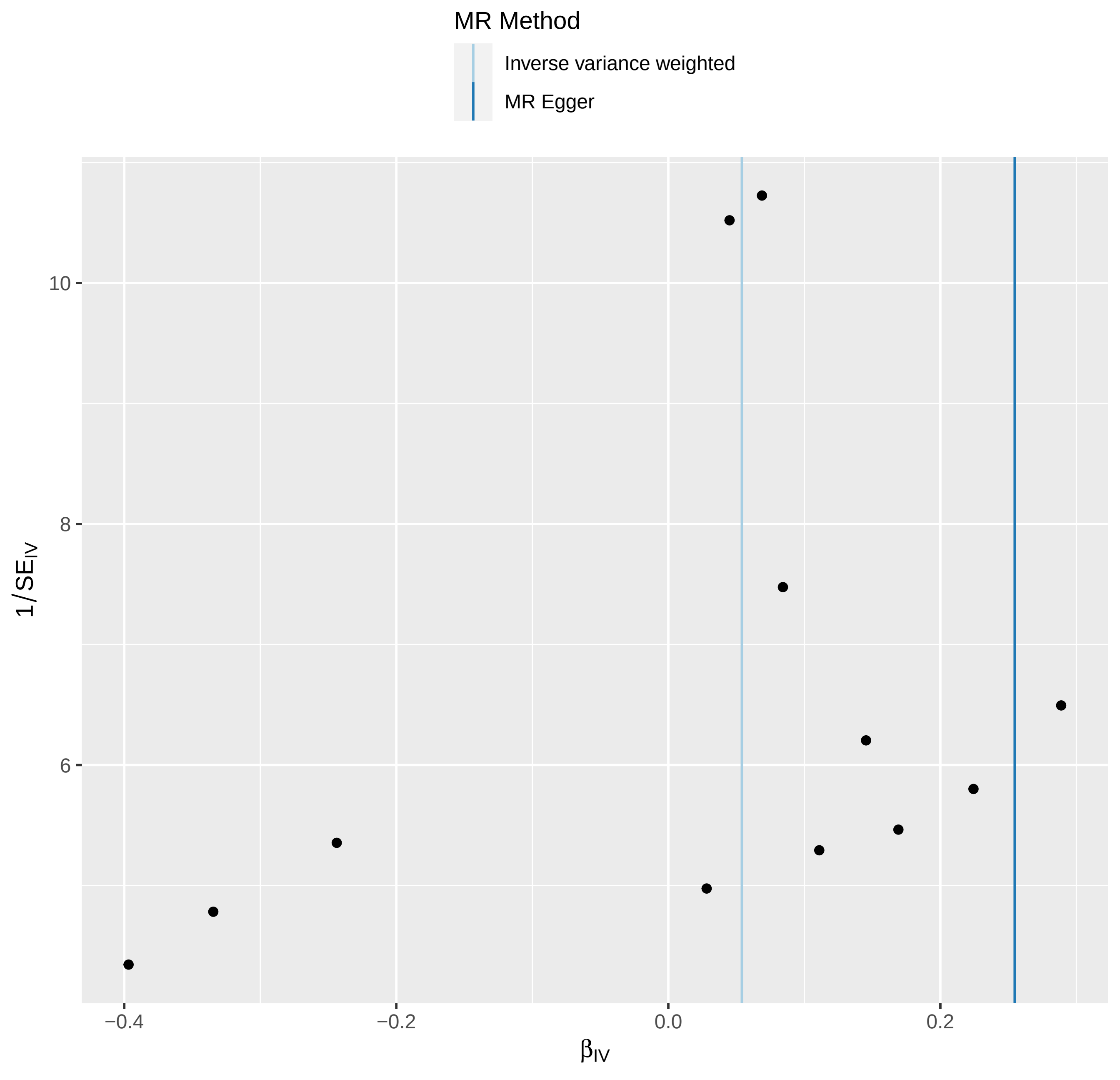

Batch 3−2686 : Selenium on Protein SERAC1

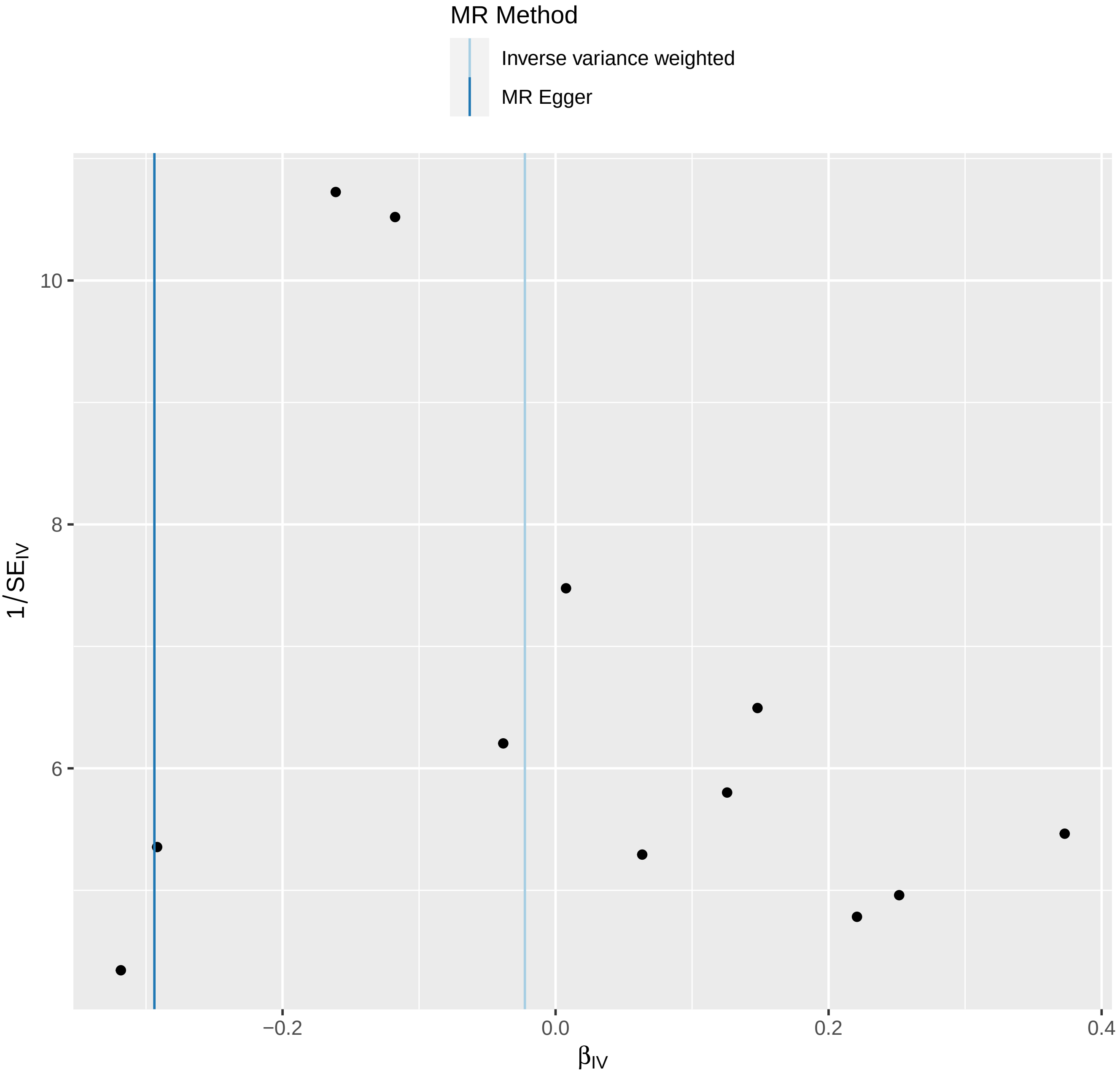

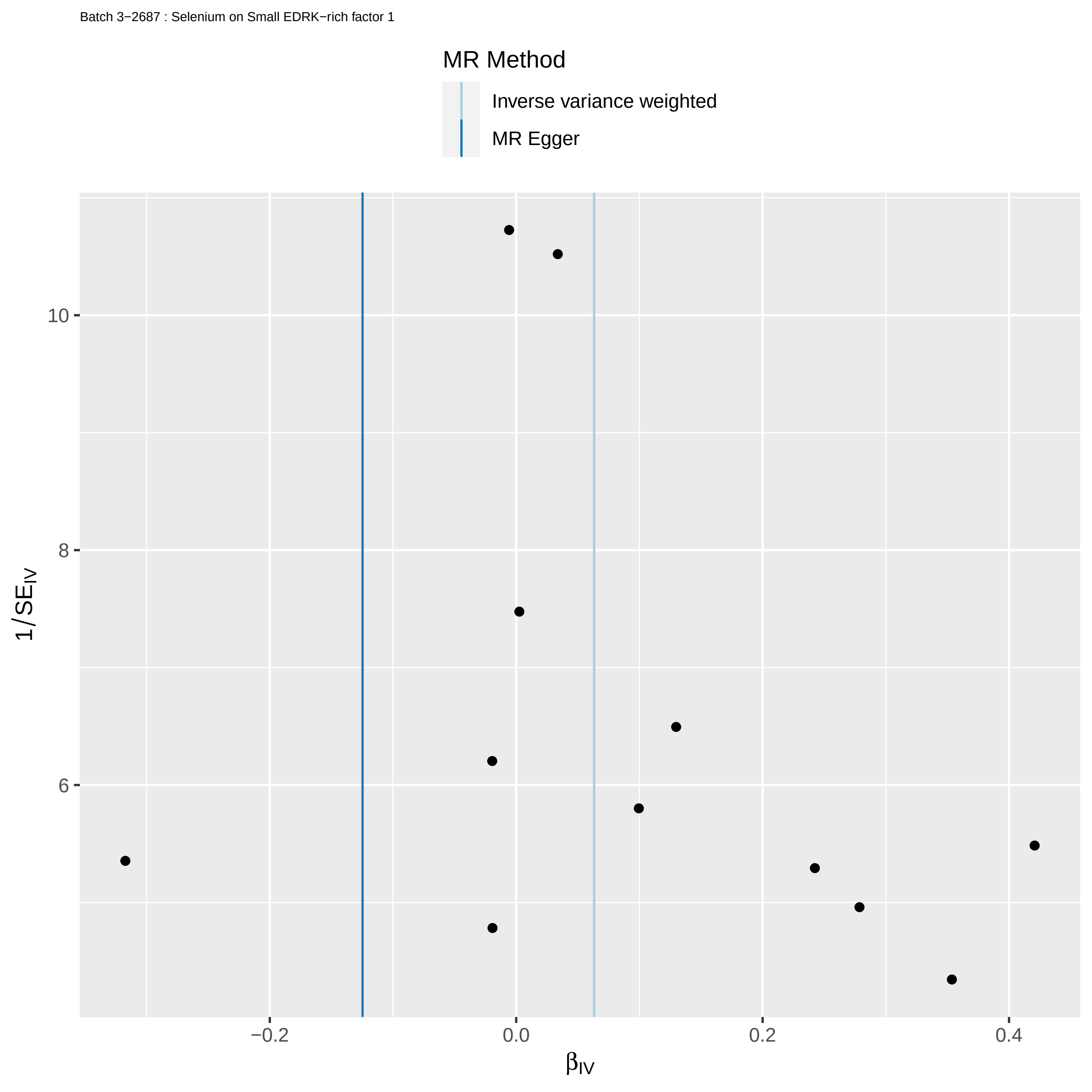

Batch 3−2687 : Selenium on Small EDRK−rich factor 1
MR Method
Inverse variance weighted
MR Egger
10
8
6
1/SE$_{IV}$
−0.2
0.0
0.2
0.4
β$_{IV}$

Batch 3−2688 : Selenium on Protein Z−dependent protease inhibitor

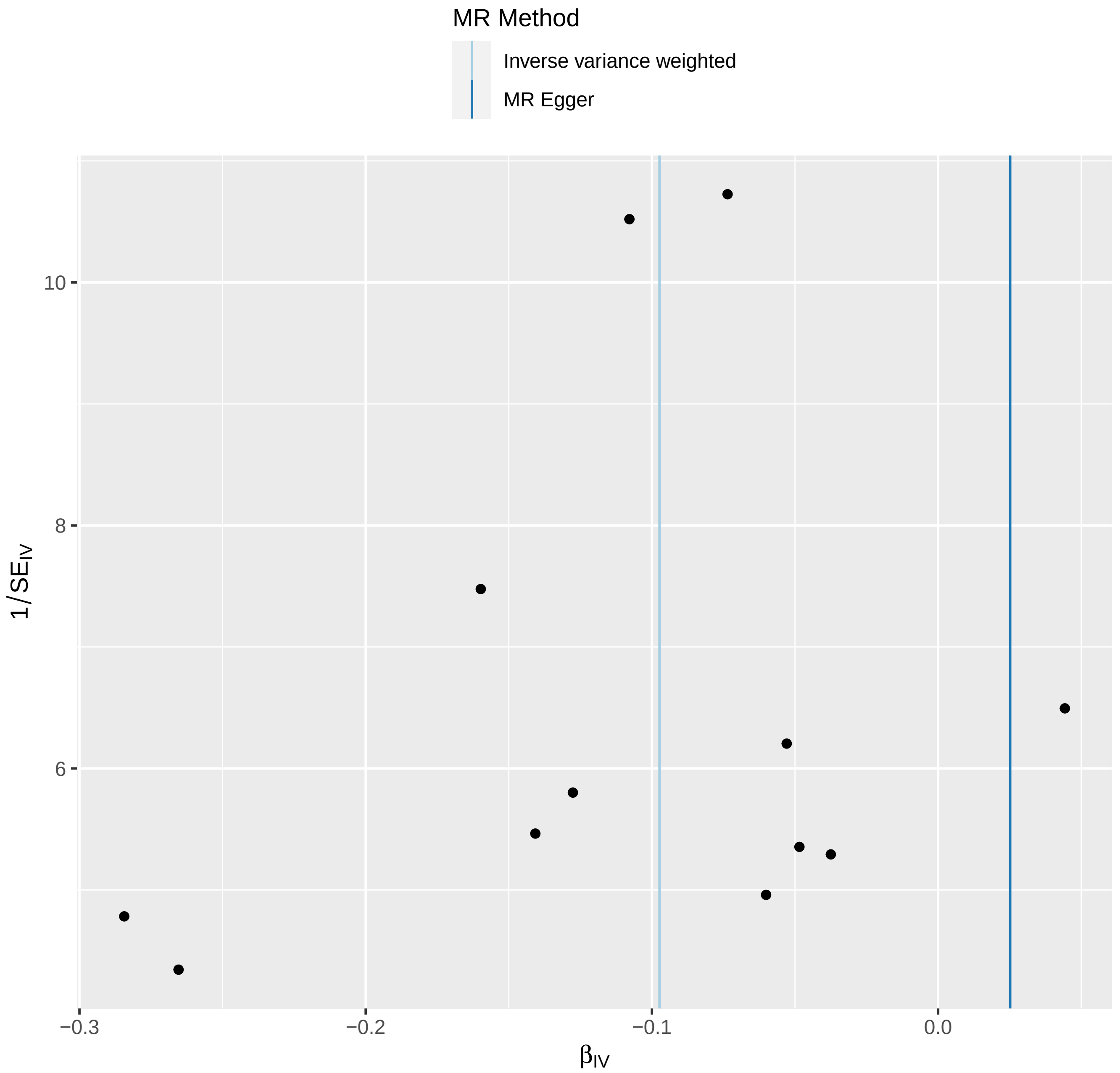

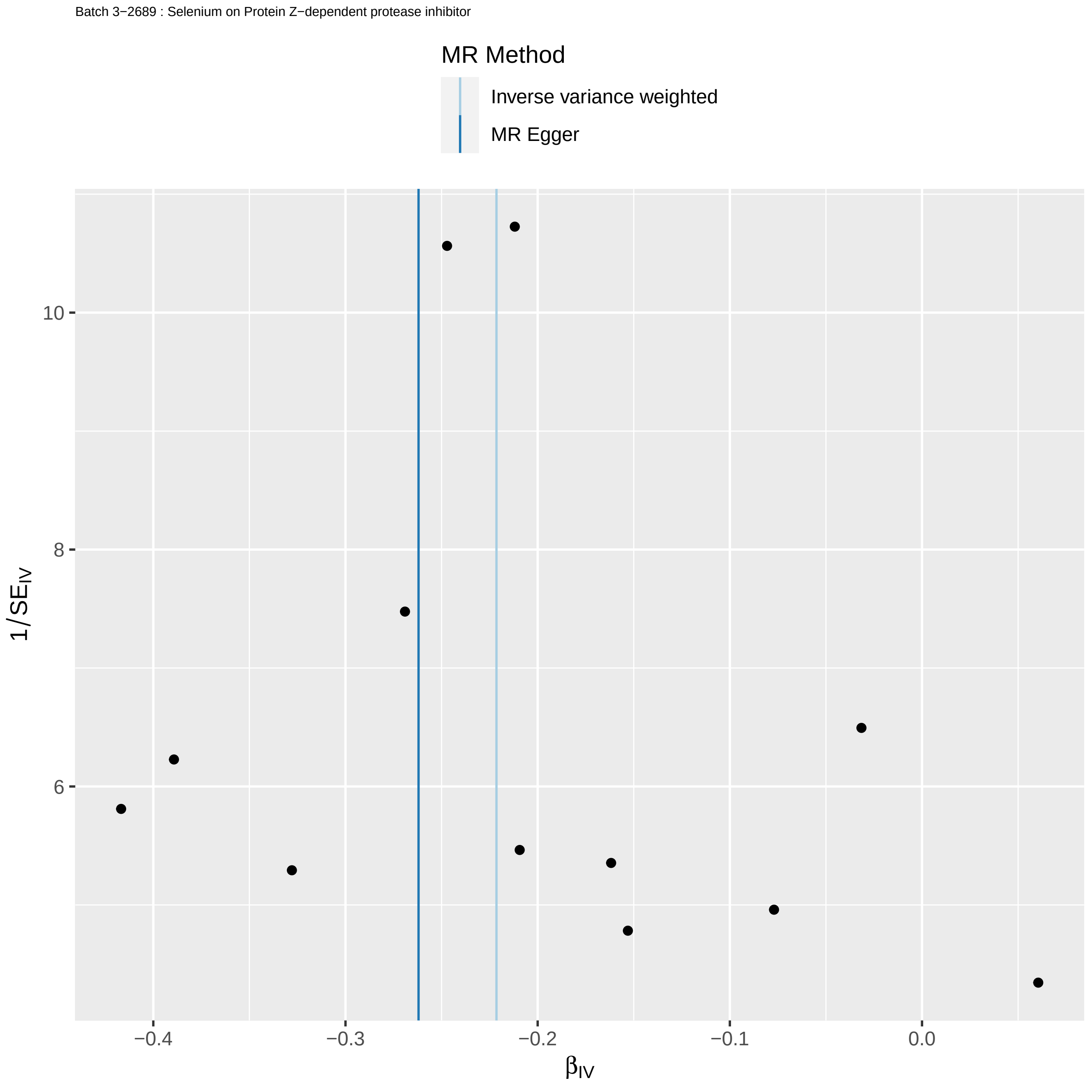
Batch 3−2689 : Selenium on Protein Z−dependent protease inhibitor
MR Method
Inverse variance weighted
MR Egger
10
8
6
1/SE$_{IV}$
−0.4
−0.3
−0.2
−0.1
0.0
β$_{IV}$

Batch 3−2690 : Selenium on Serpin A12

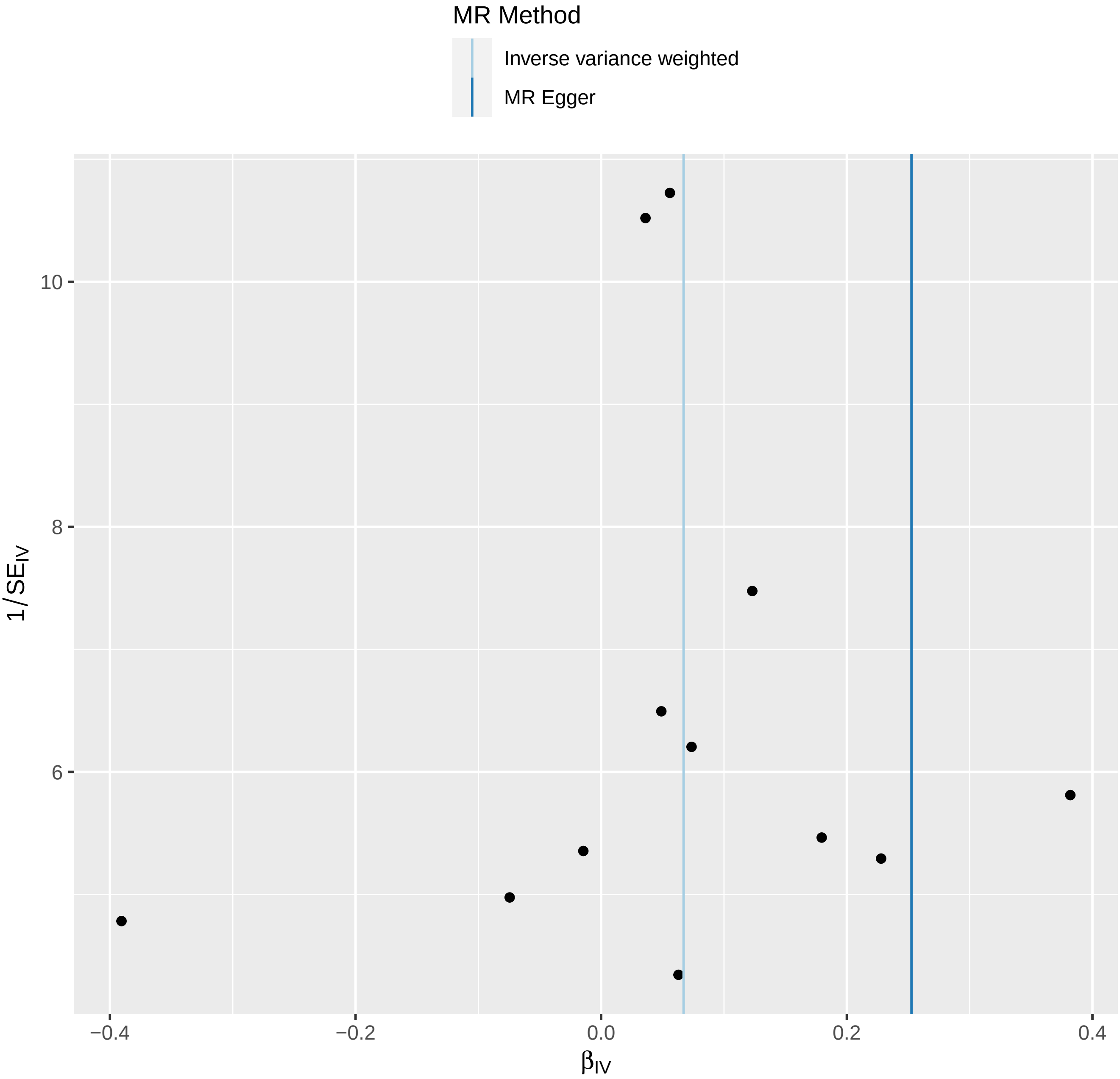

Batch 3−2691 : Selenium on Kallistatin

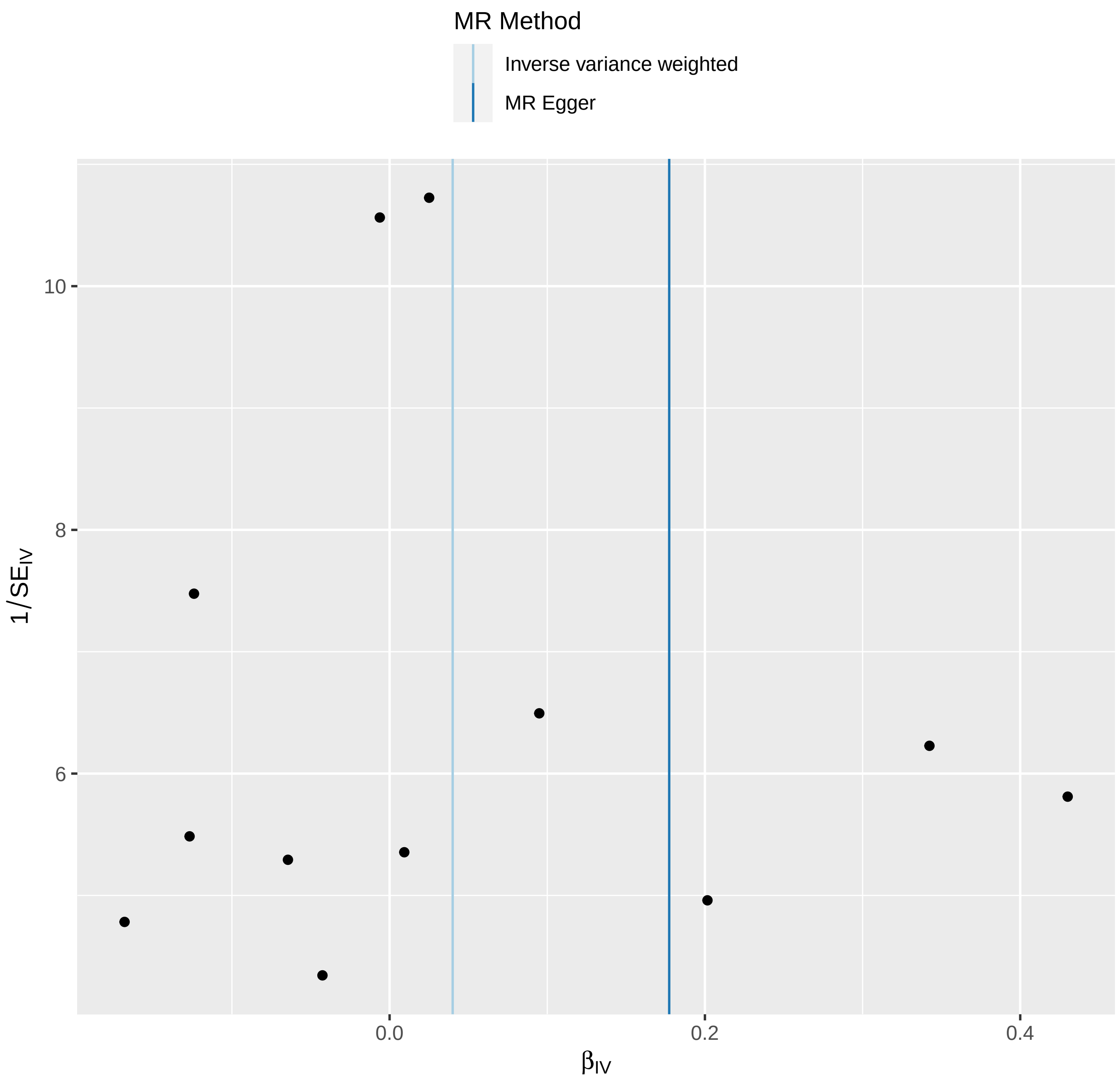

Batch 3−2692 : Selenium on Kallistatin

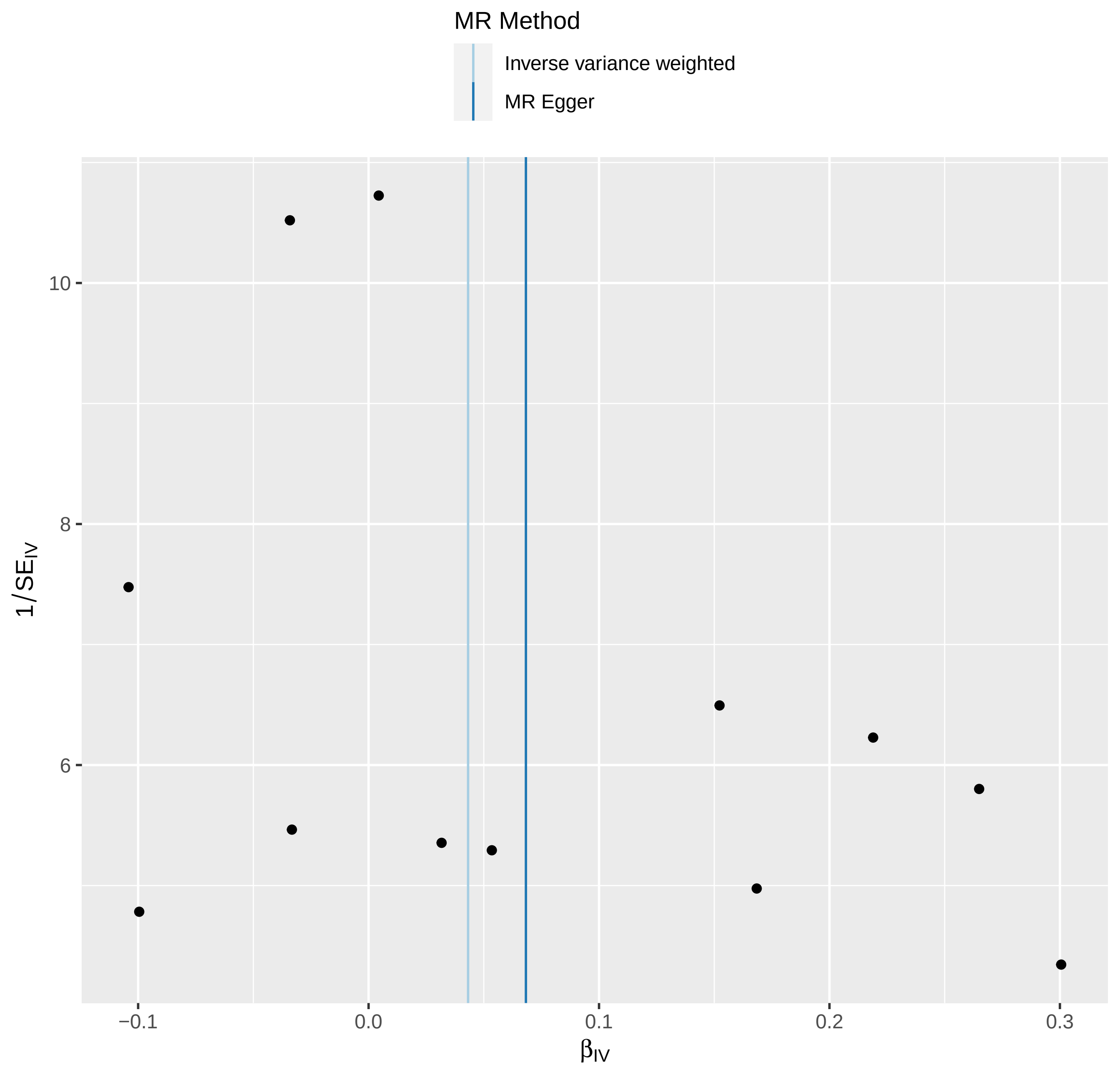

Batch 3−2693 : Selenium on Corticosteroid−binding globulin

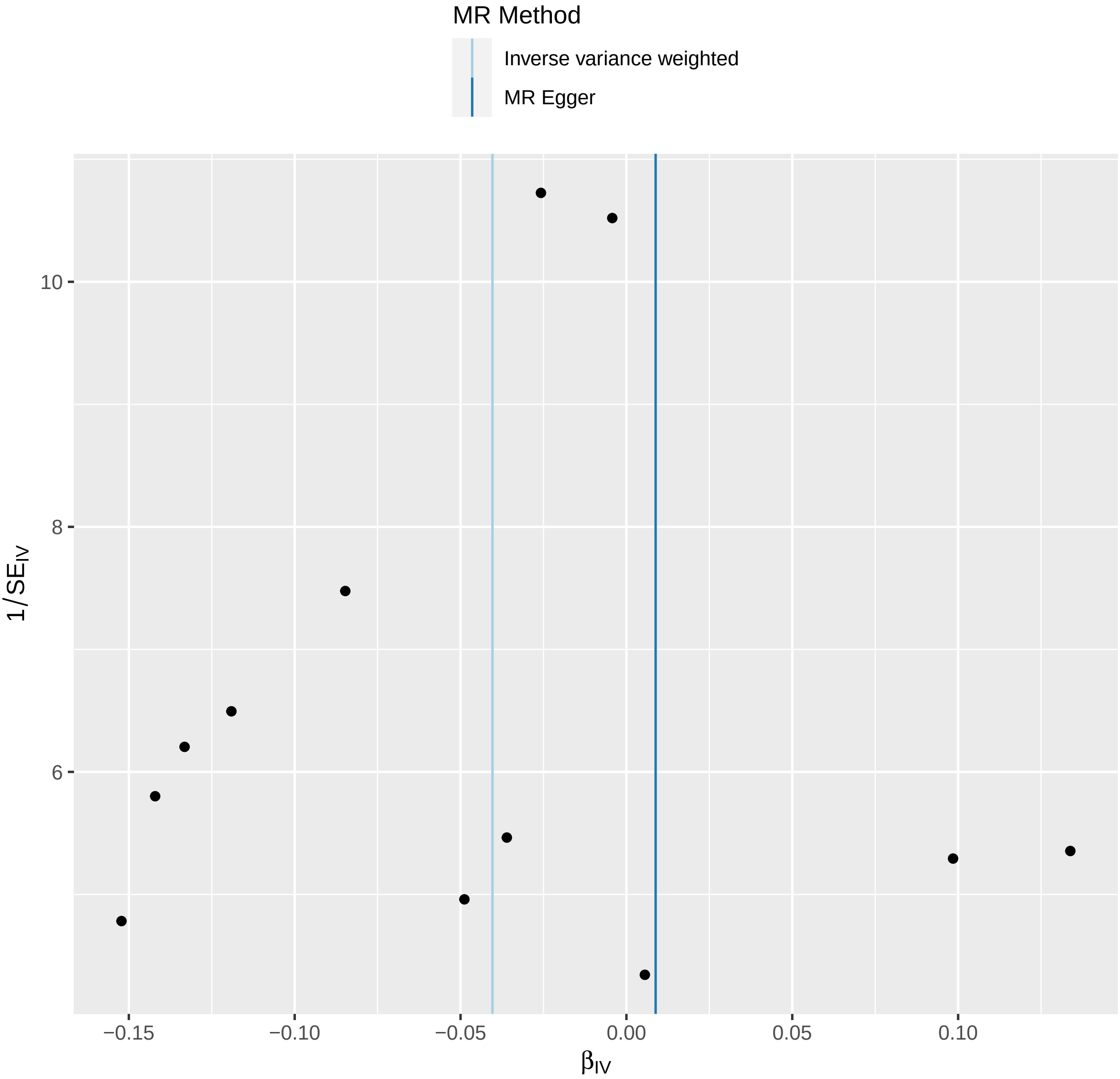

Batch 3−2694 : Selenium on Serpin A9

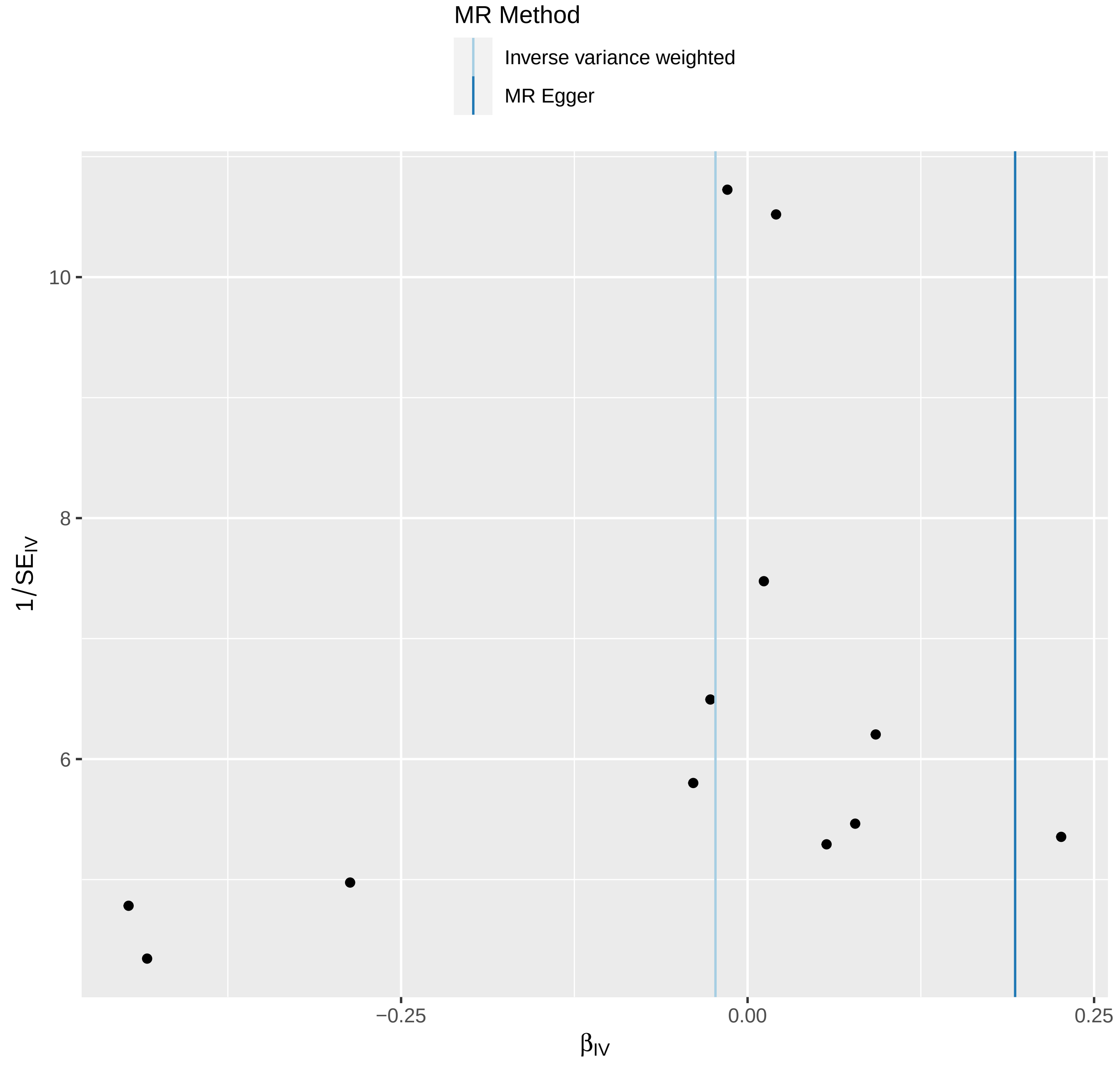

Batch 3−2695 : Selenium on Antithrombin−III

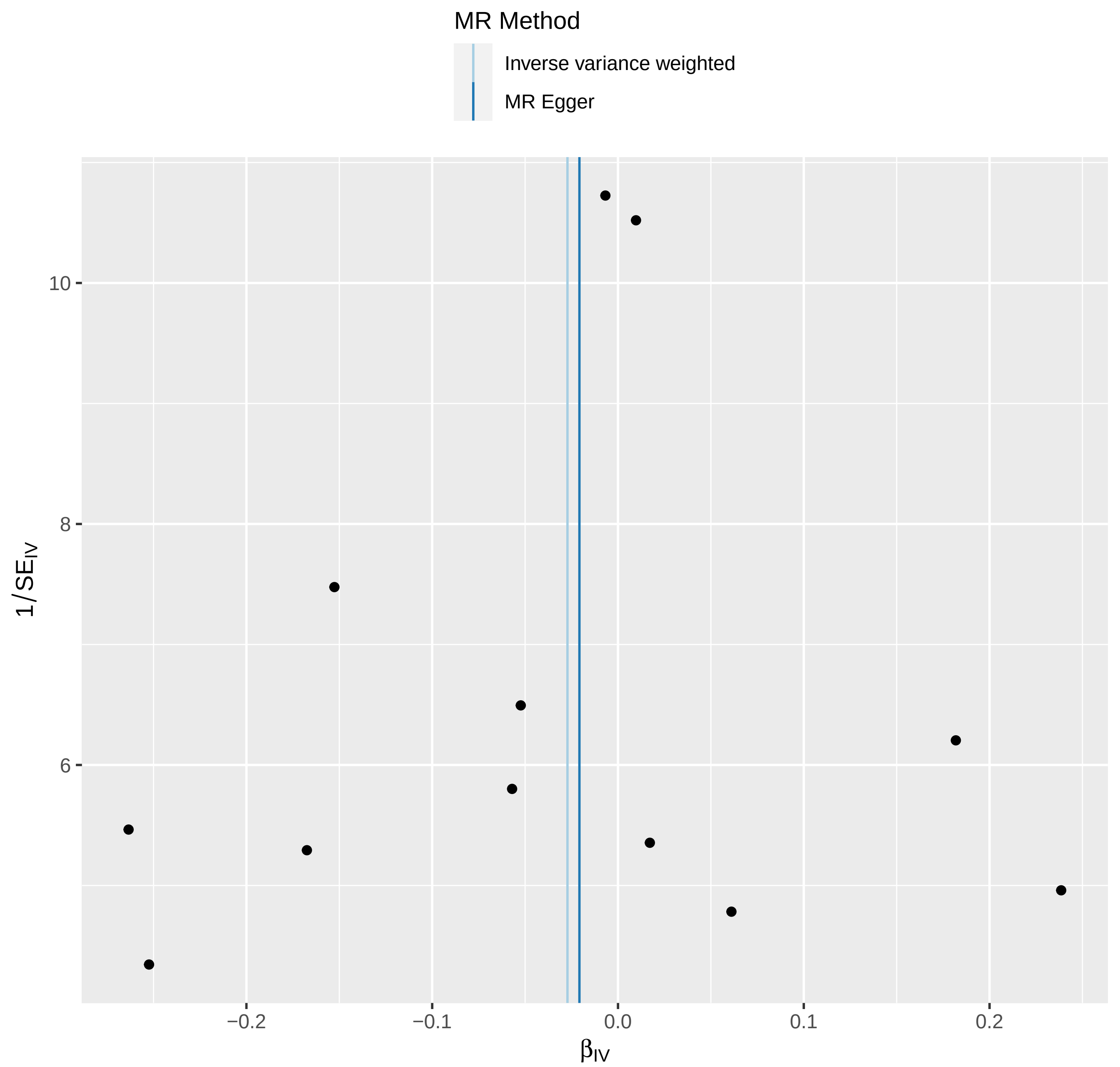

Batch 3−2696 : Selenium on Plasminogen activator inhibitor 1

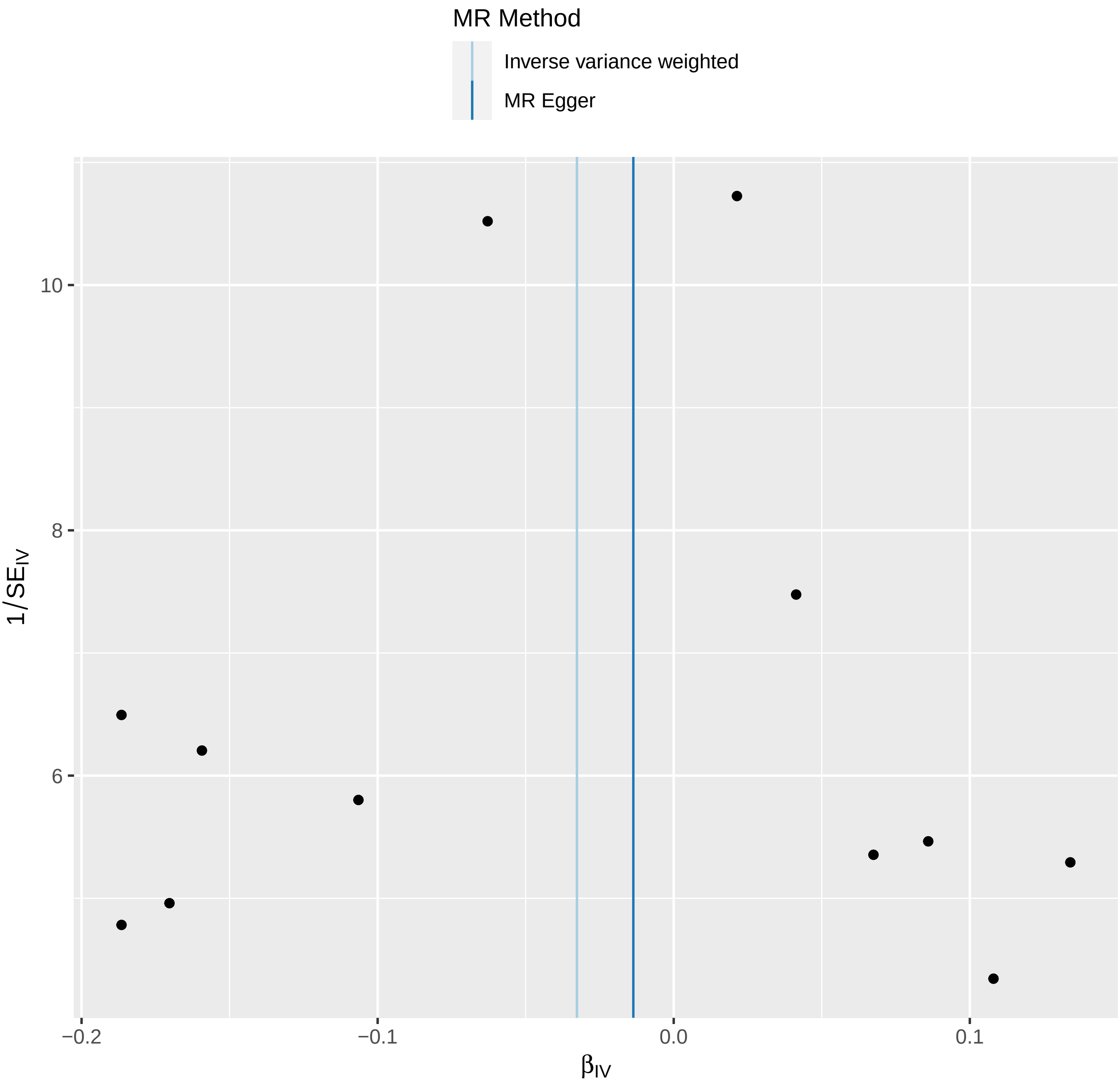

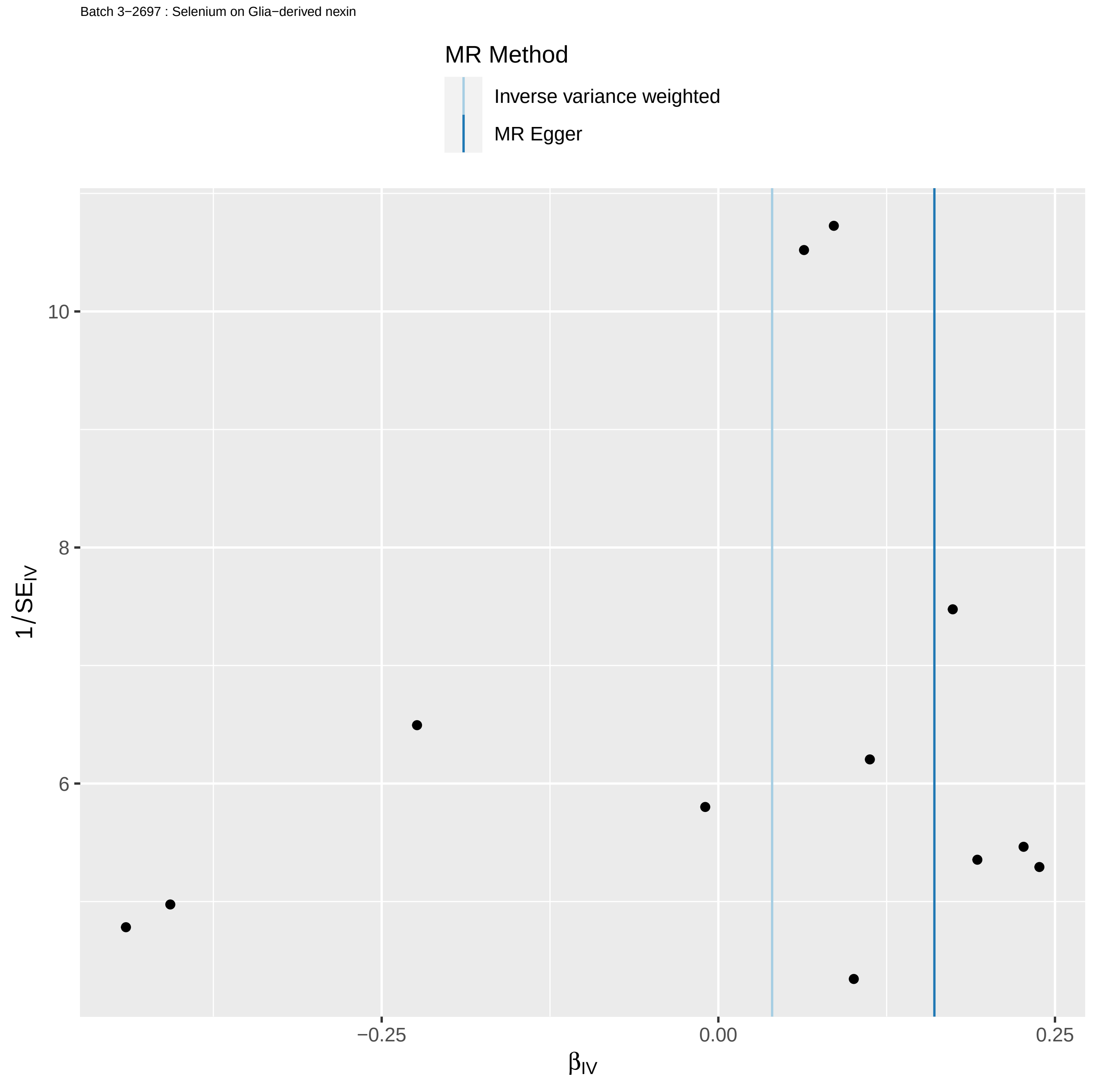

Batch 3−2697 : Selenium on Glia−derived nexin
MR Method
Inverse variance weighted
MR Egger
10
8
6
1/SE_IV
−0.25
0.00
0.25
β_IV

Batch 3−2698 : Selenium on Pigment epithelium−derived factor

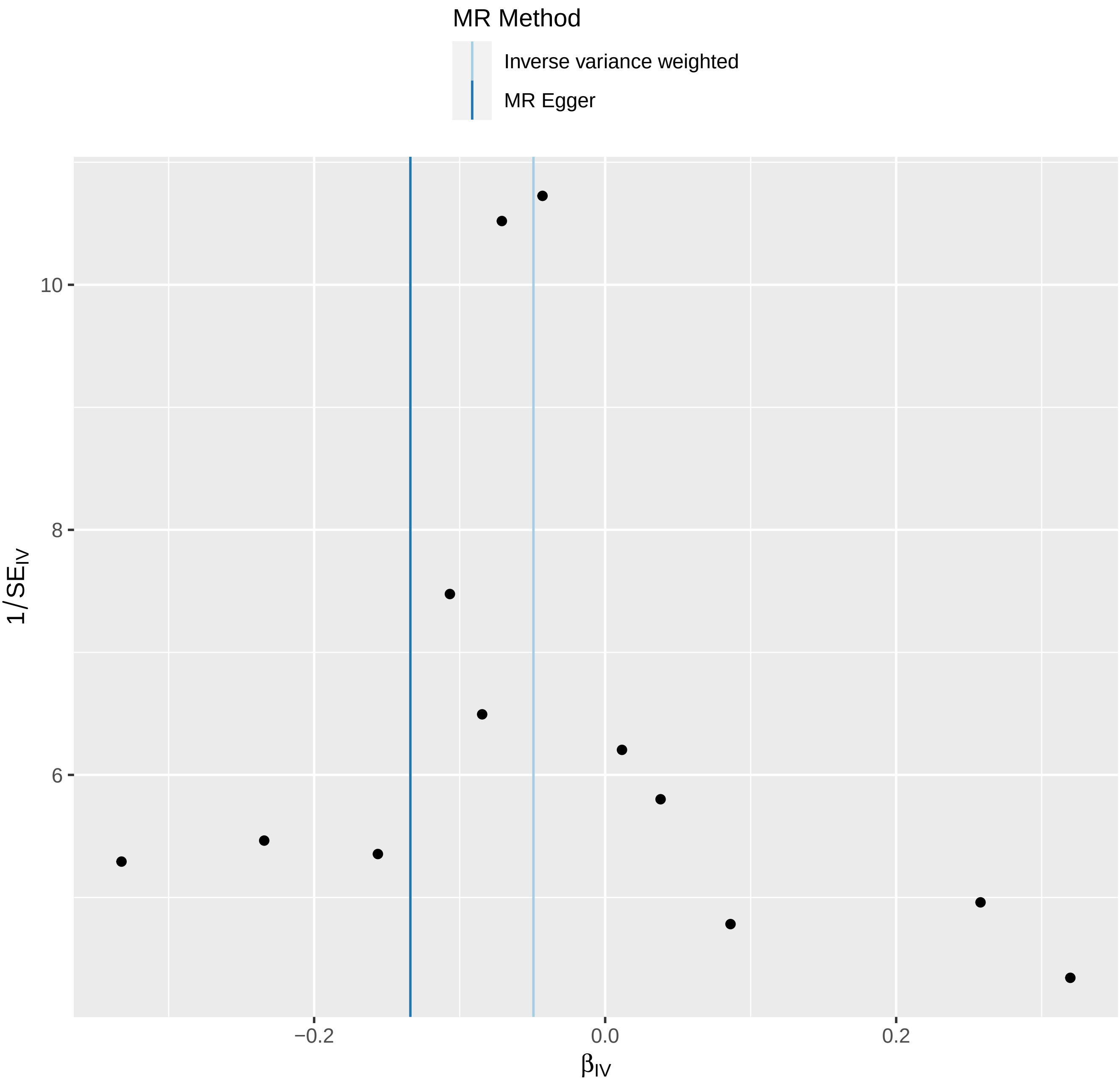

Batch 3−2699 : Selenium on Pigment epithelium−derived factor

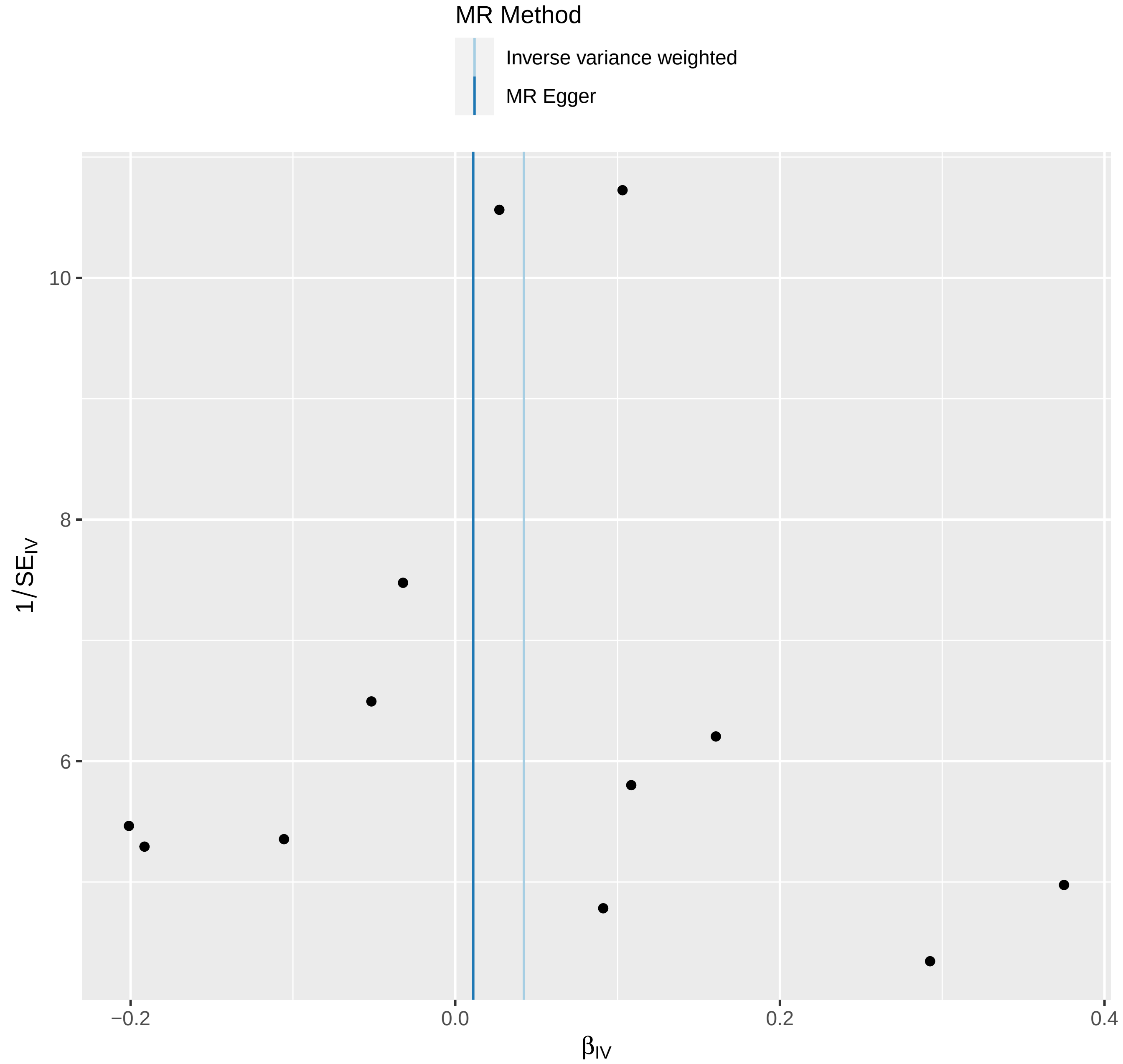

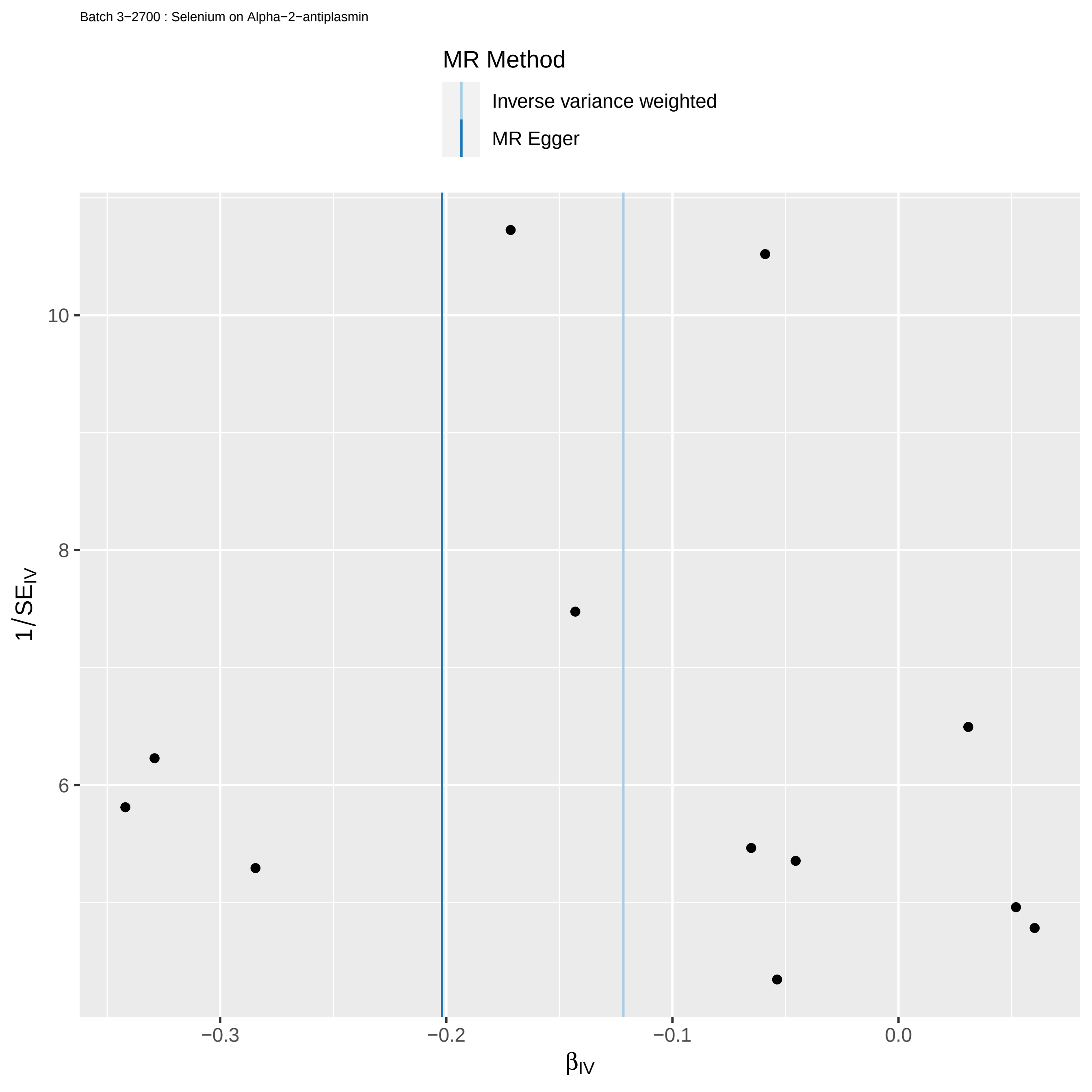

Batch 3−2700 : Selenium on Alpha−2−antiplasmin
MR Method
Inverse variance weighted
MR Egger
10
8
6
1/SE_IV
−0.3
−0.2
−0.1
0.0
β_IV

Batch 3−2701 : Selenium on Plasma protease C1 inhibitor

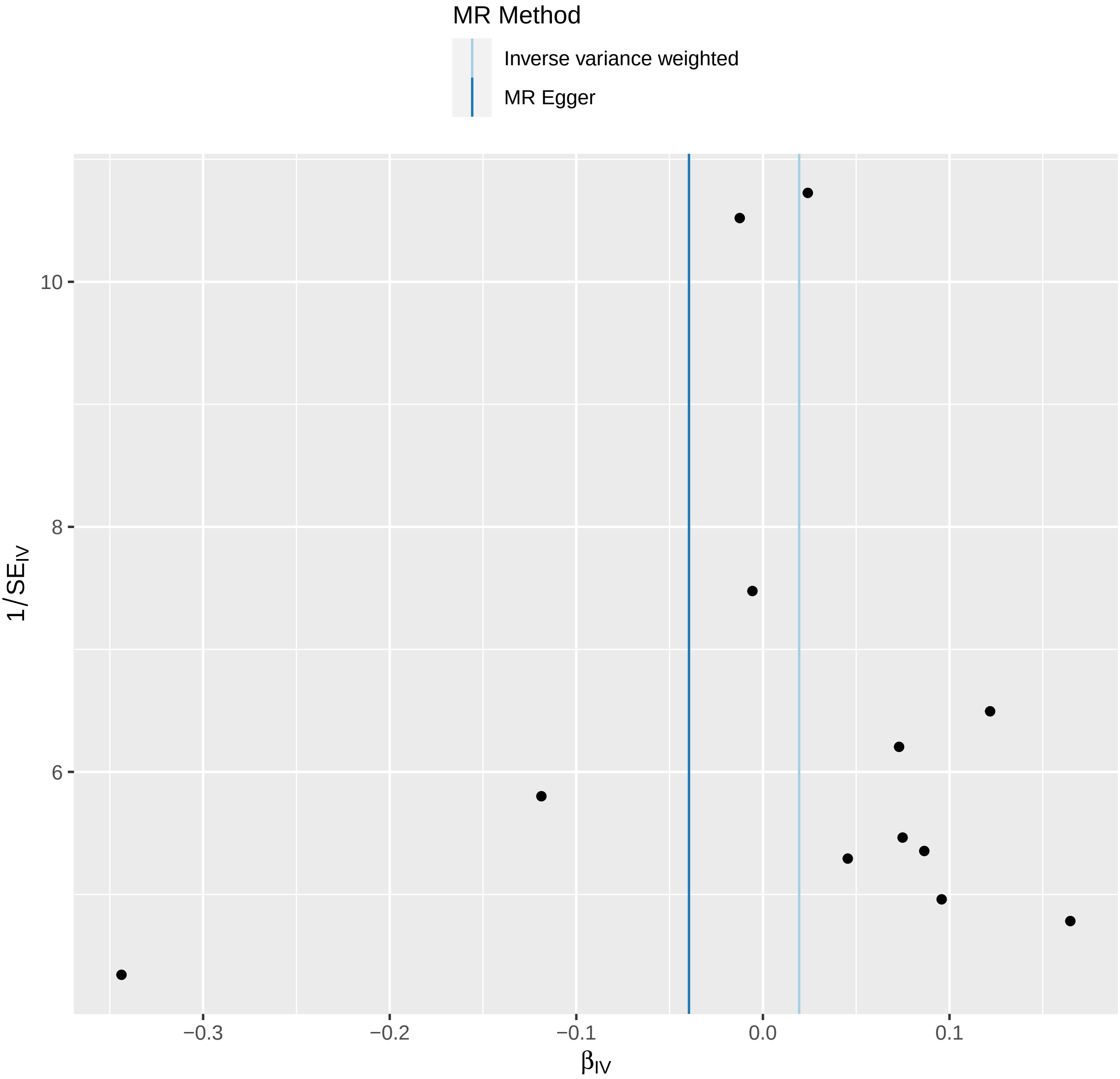

Batch 3−2702 : Selenium on Serpin I2

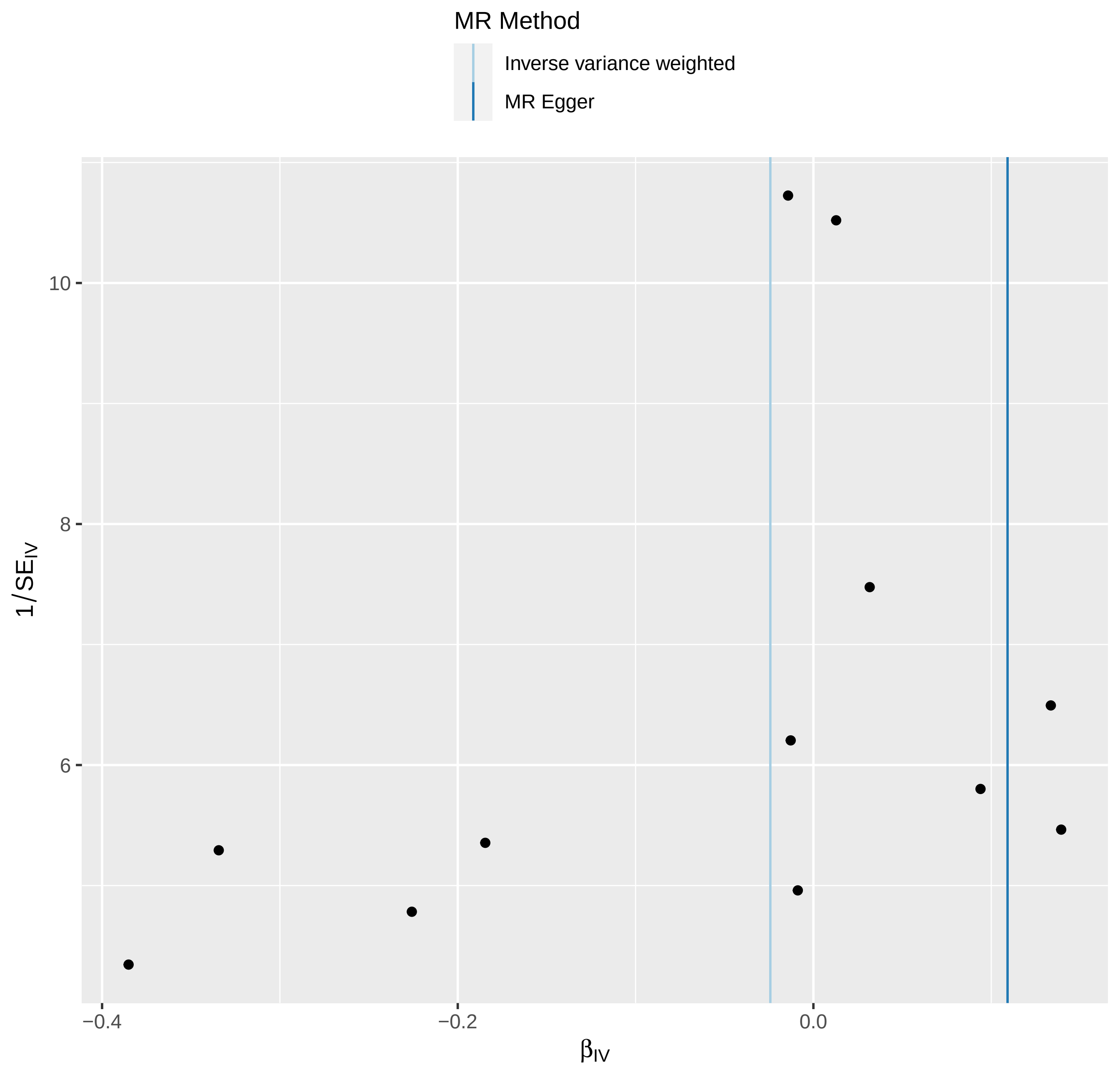

Batch 3−2703 : Selenium on Histone−lysine N−methyltransferase SETD2

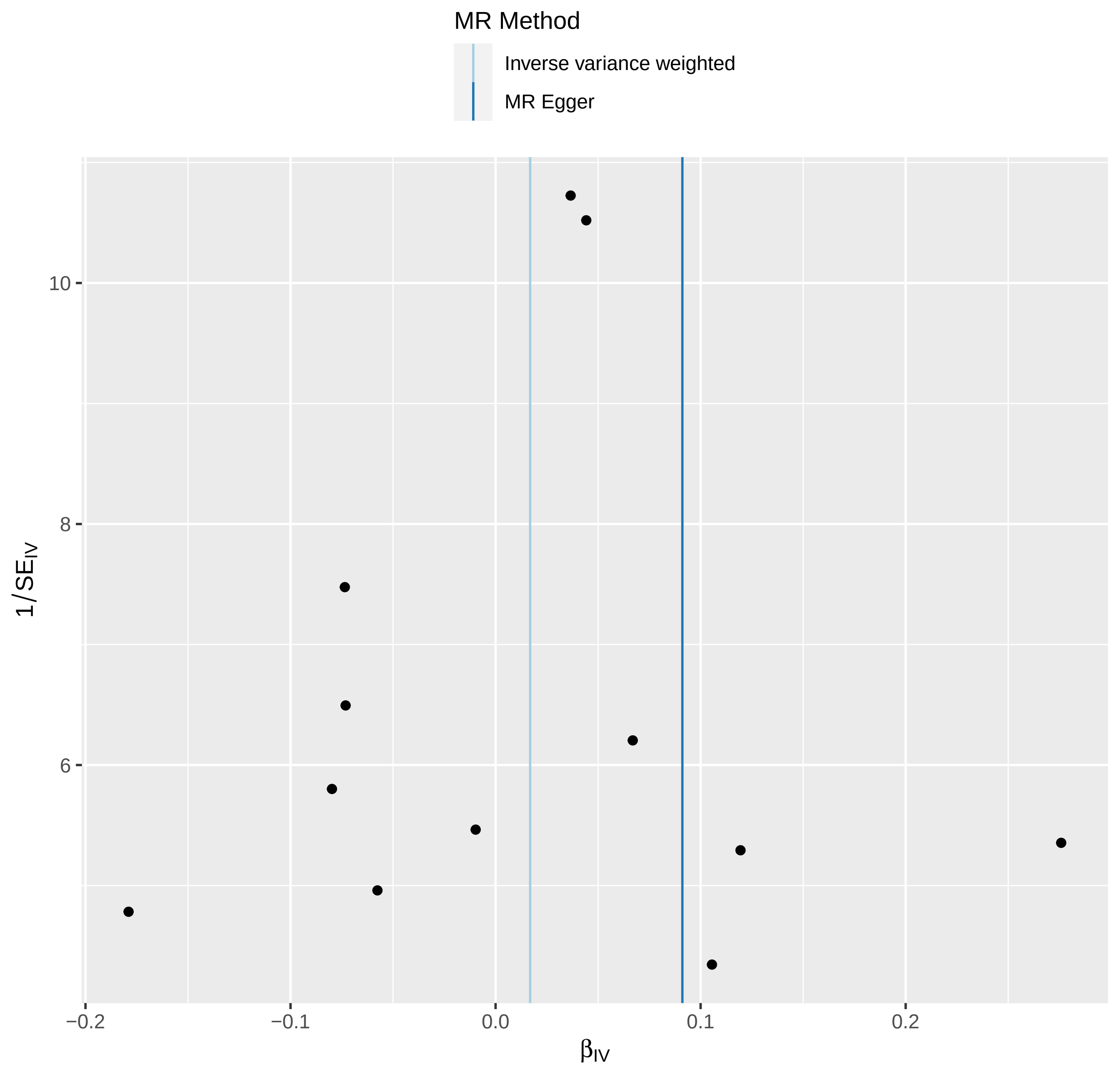

Batch 3−2704 : Selenium on Histone−lysine N−methyltransferase SETMAR

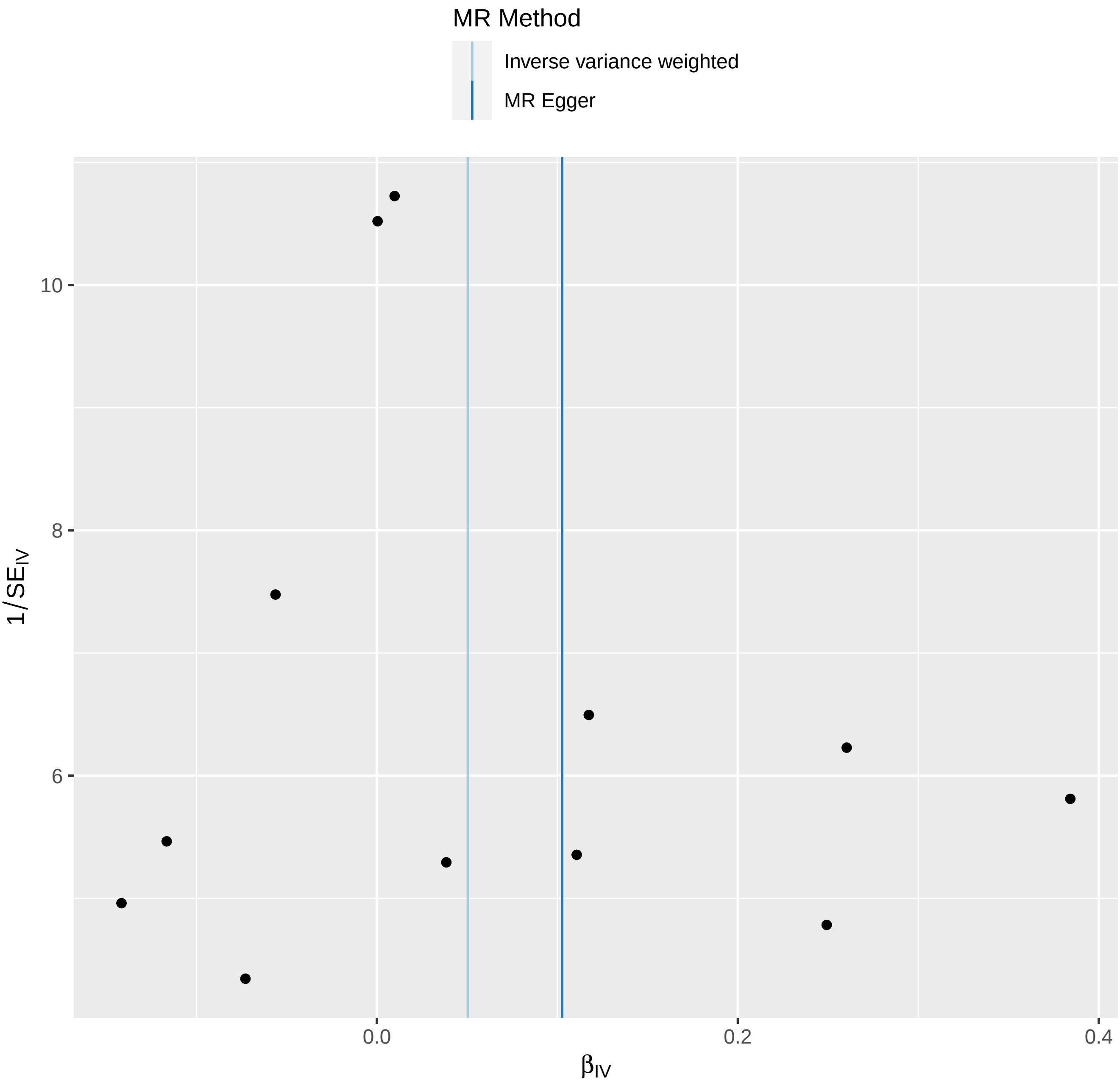

Batch 3−2705 : Selenium on Seizure 6−like protein 2

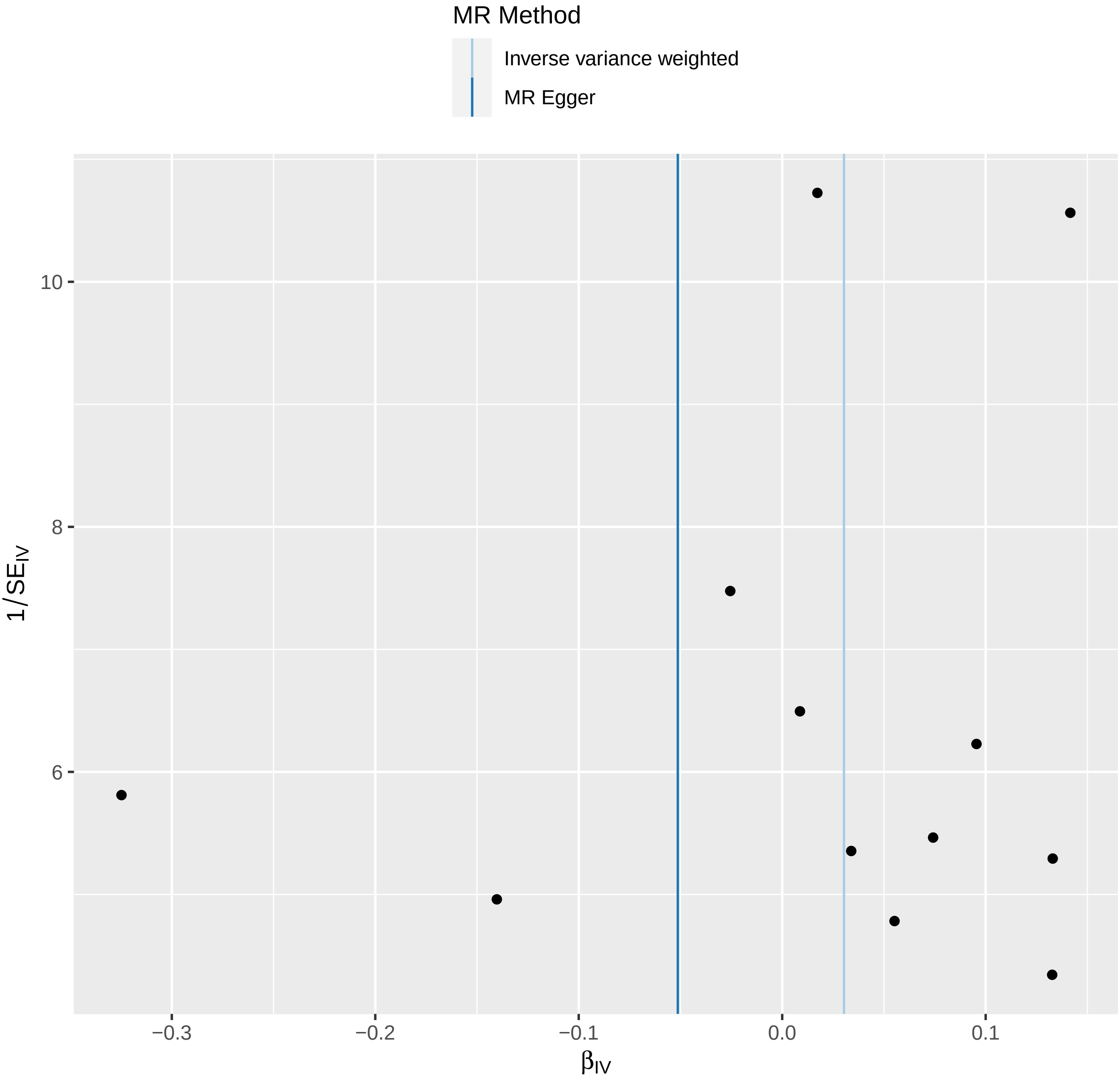

Batch 3−2706 : Selenium on Splicing factor 1

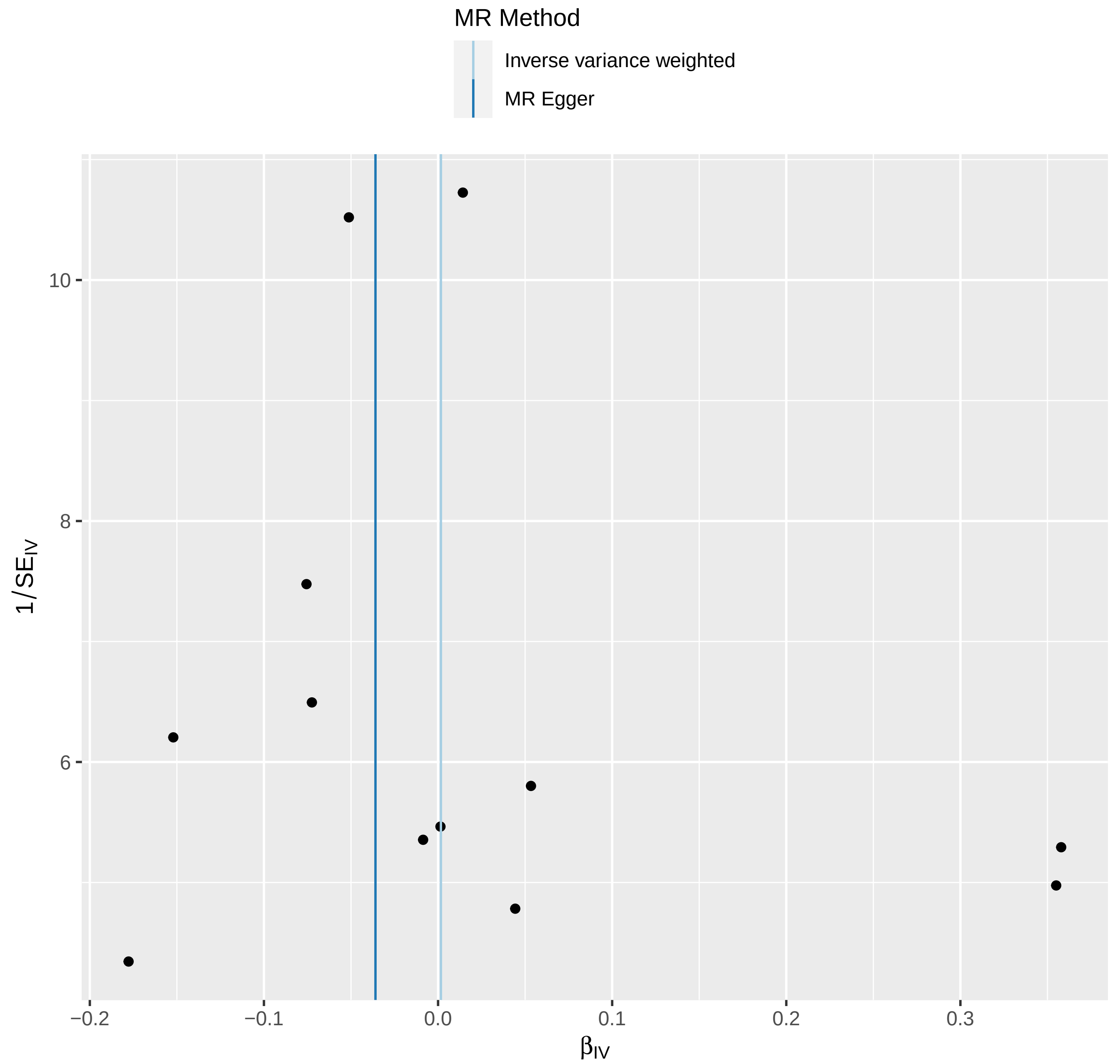

Batch 3−2707 : Selenium on Splicing factor 3B subunit 4

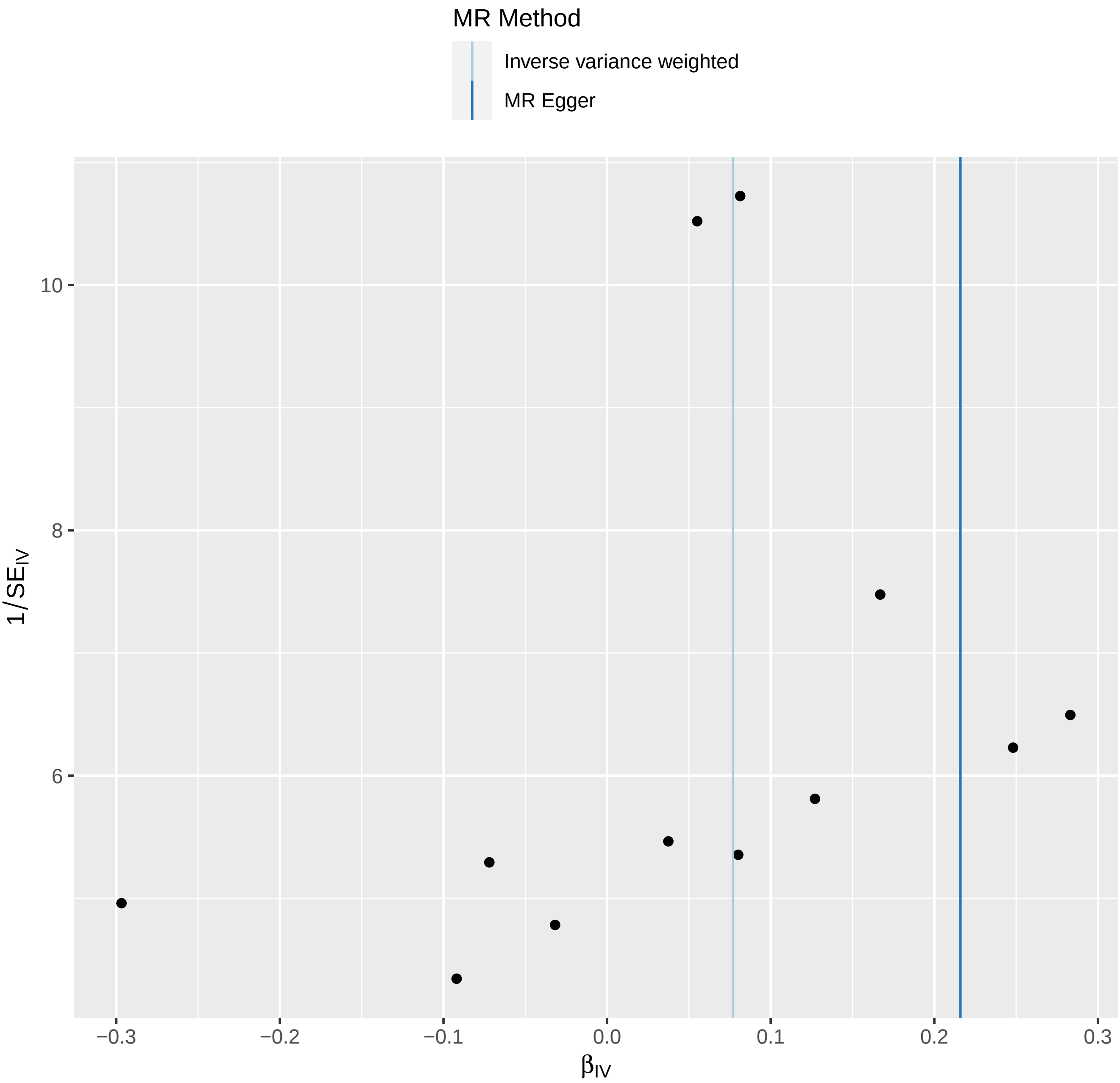

Batch 3−2708 : Selenium on 14−3−3 protein sigma

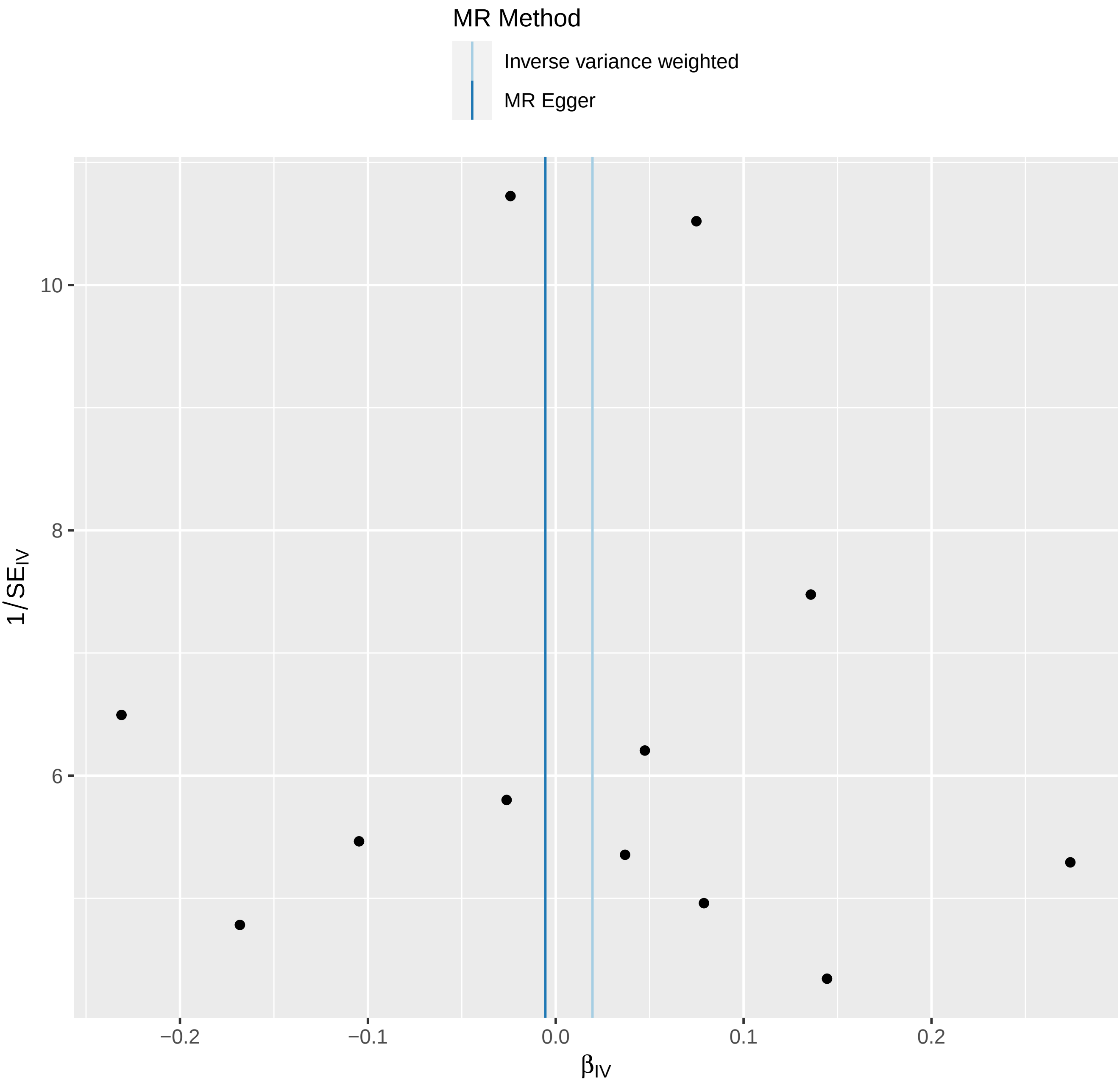

Batch 3−2709 : Selenium on Secreted frizzled−related protein 1

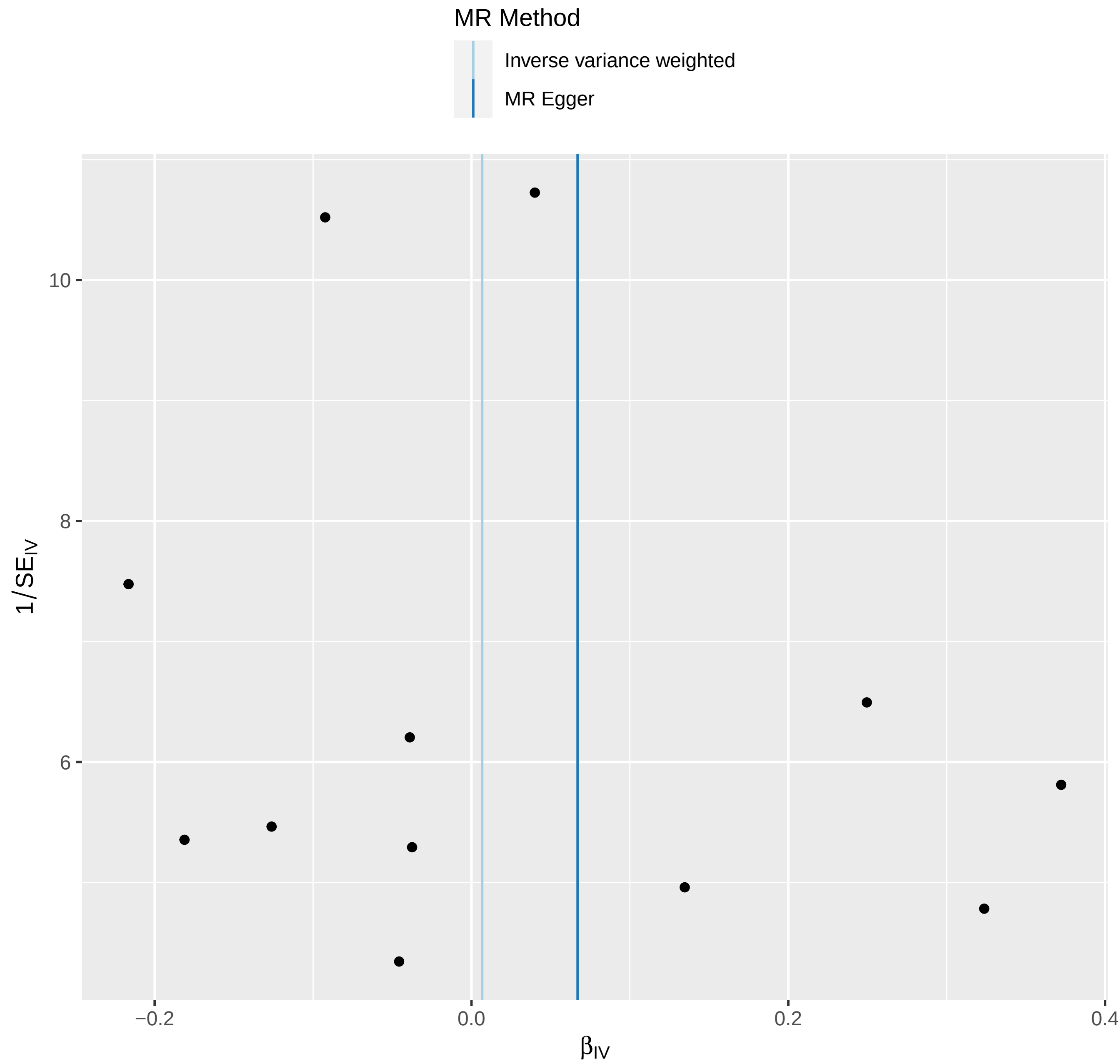

Batch 3−2710 : Selenium on Secreted frizzled−related protein 2

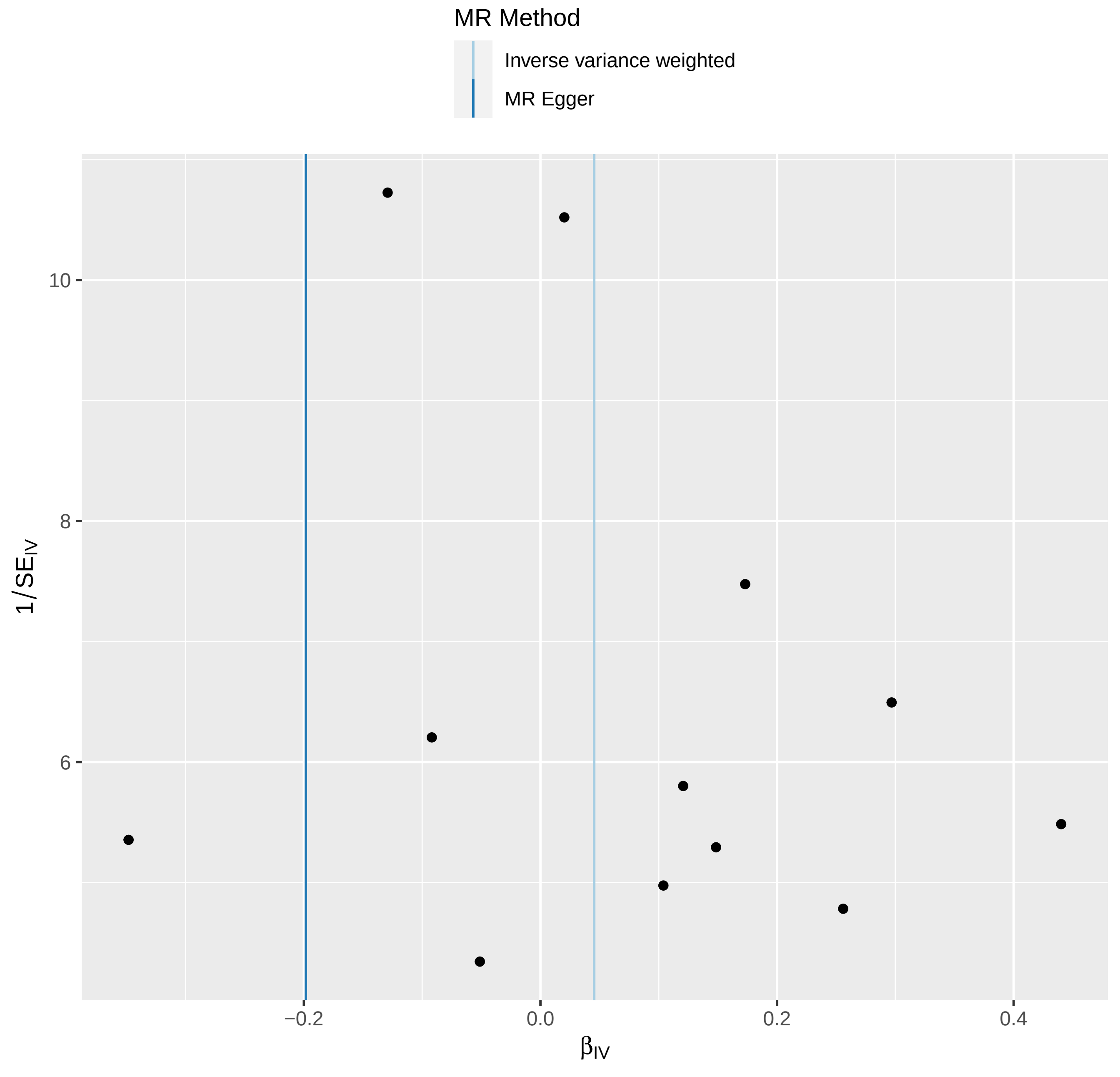

Batch 3−2711 : Selenium on Pulmonary surfactant−associated protein C

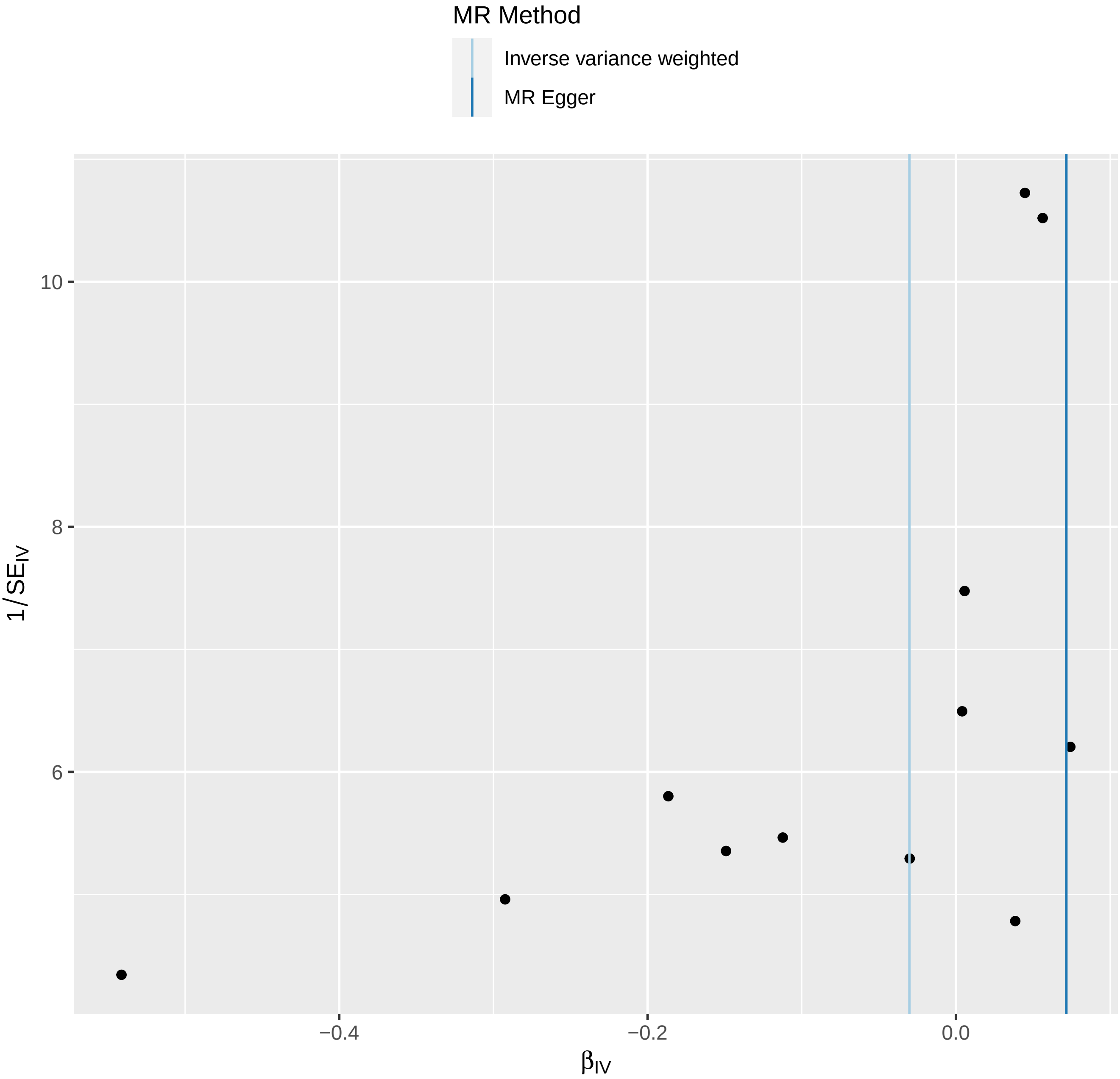

Batch 3−2712 : Selenium on Pulmonary surfactant−associated protein D

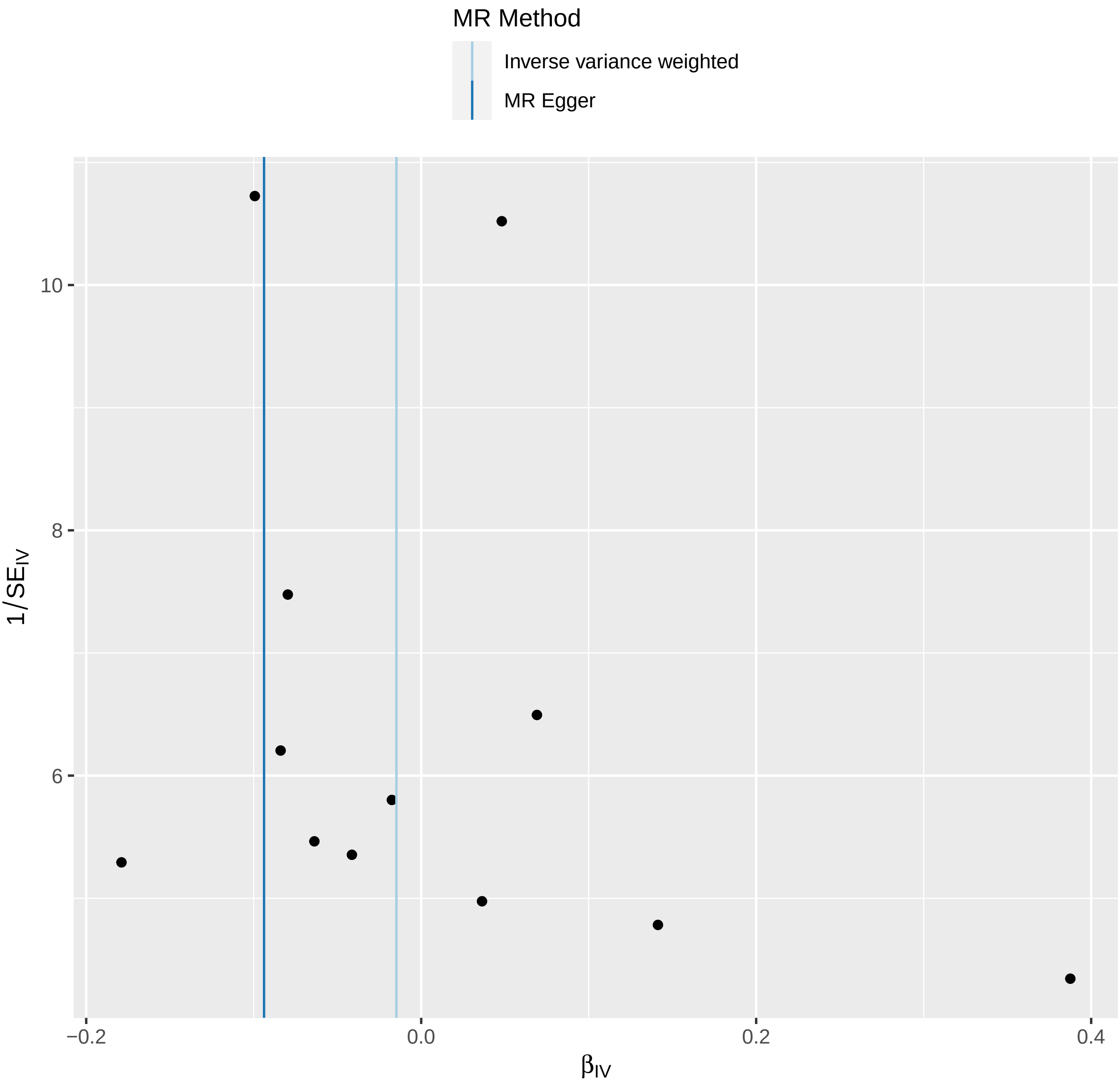

Batch 3−2713 : Selenium on Beta−sarcoglycan

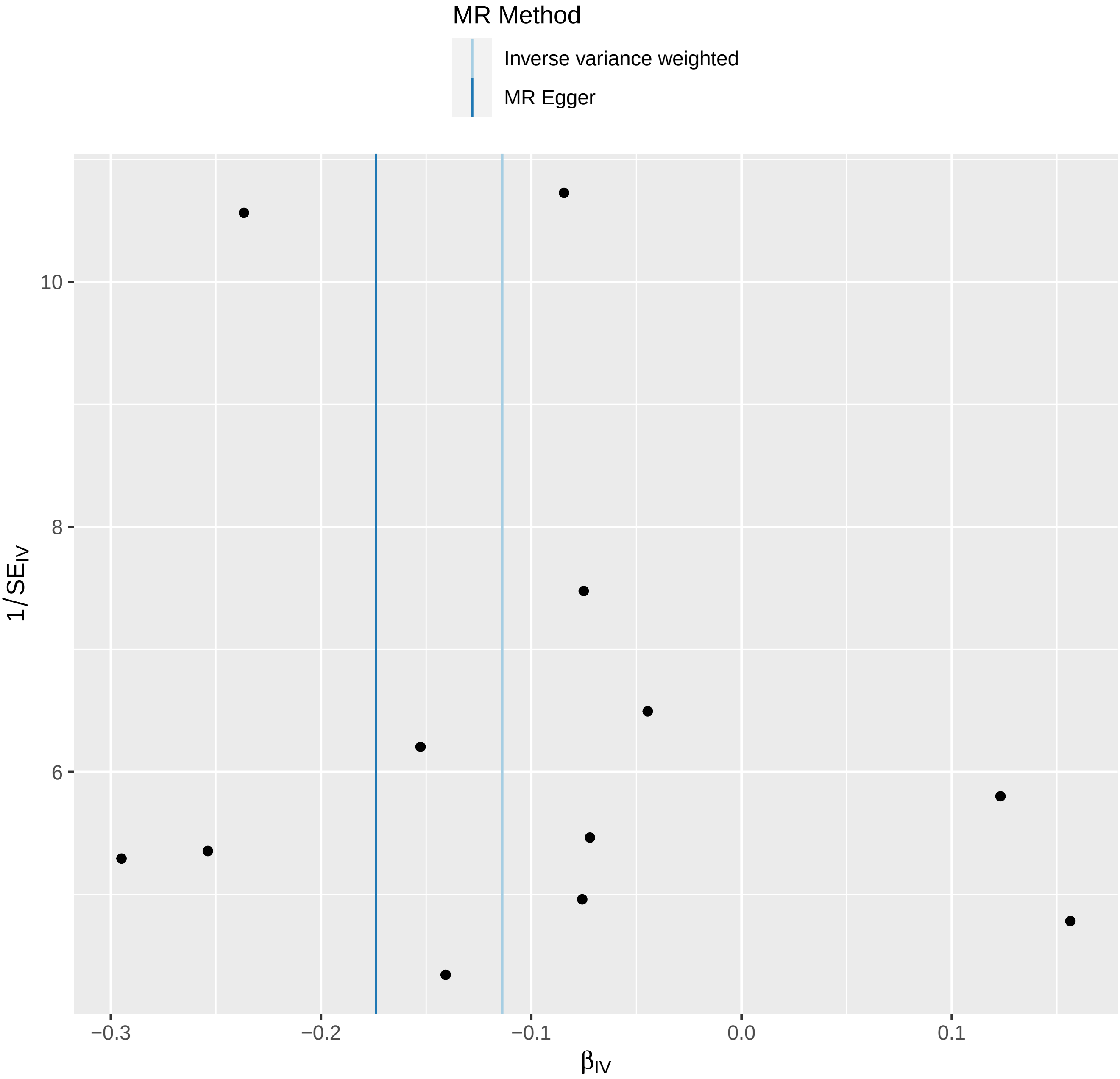

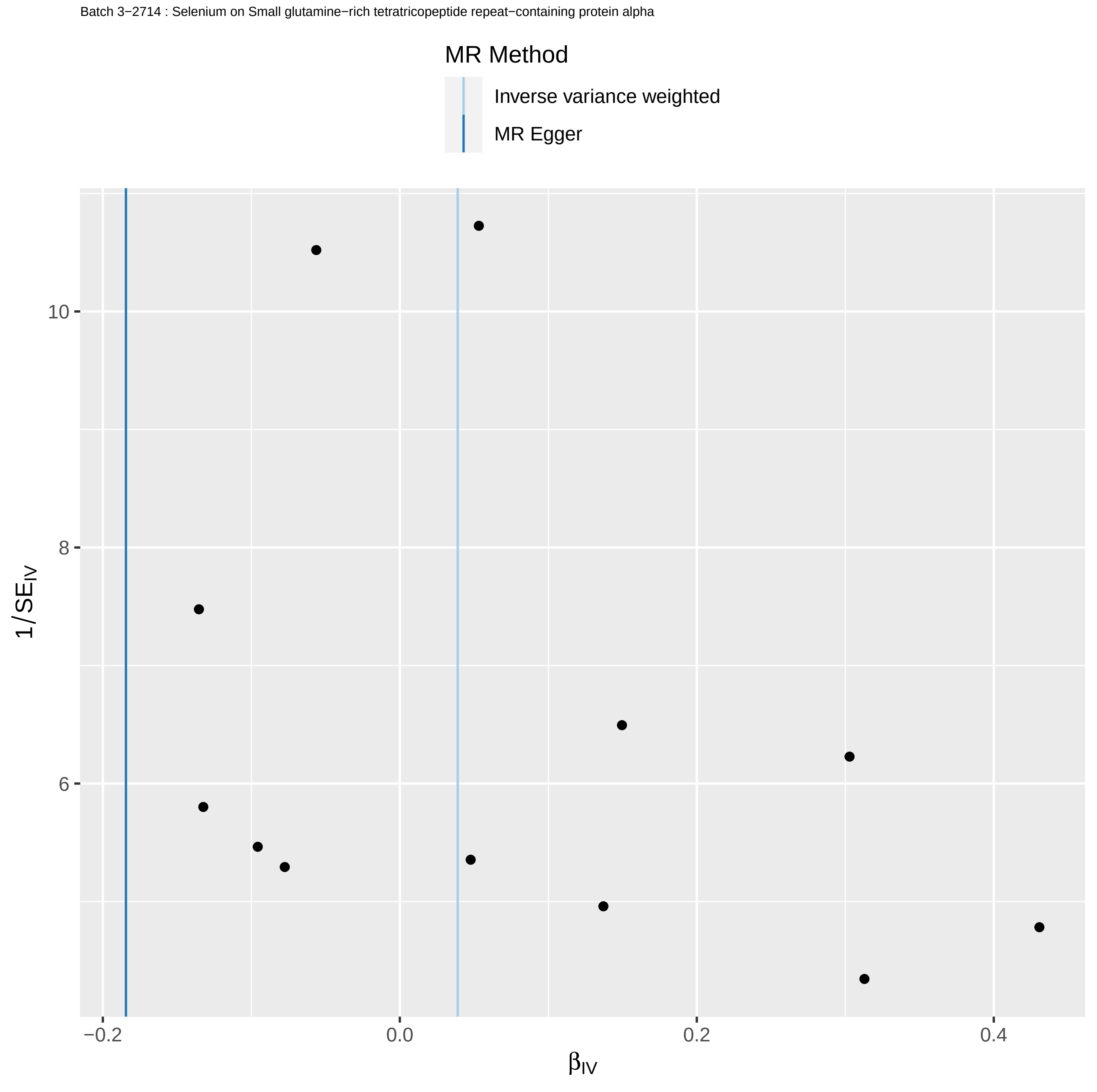
Batch 3−2714 : Selenium on Small glutamine−rich tetratricopeptide repeat−containing protein alpha
MR Method
Inverse variance weighted
MR Egger
10
8
6
1/SE_IV
−0.2
0.0
0.2
0.4
β_IV

Batch 3−2715 : Selenium on SH2 domain−containing protein 1A

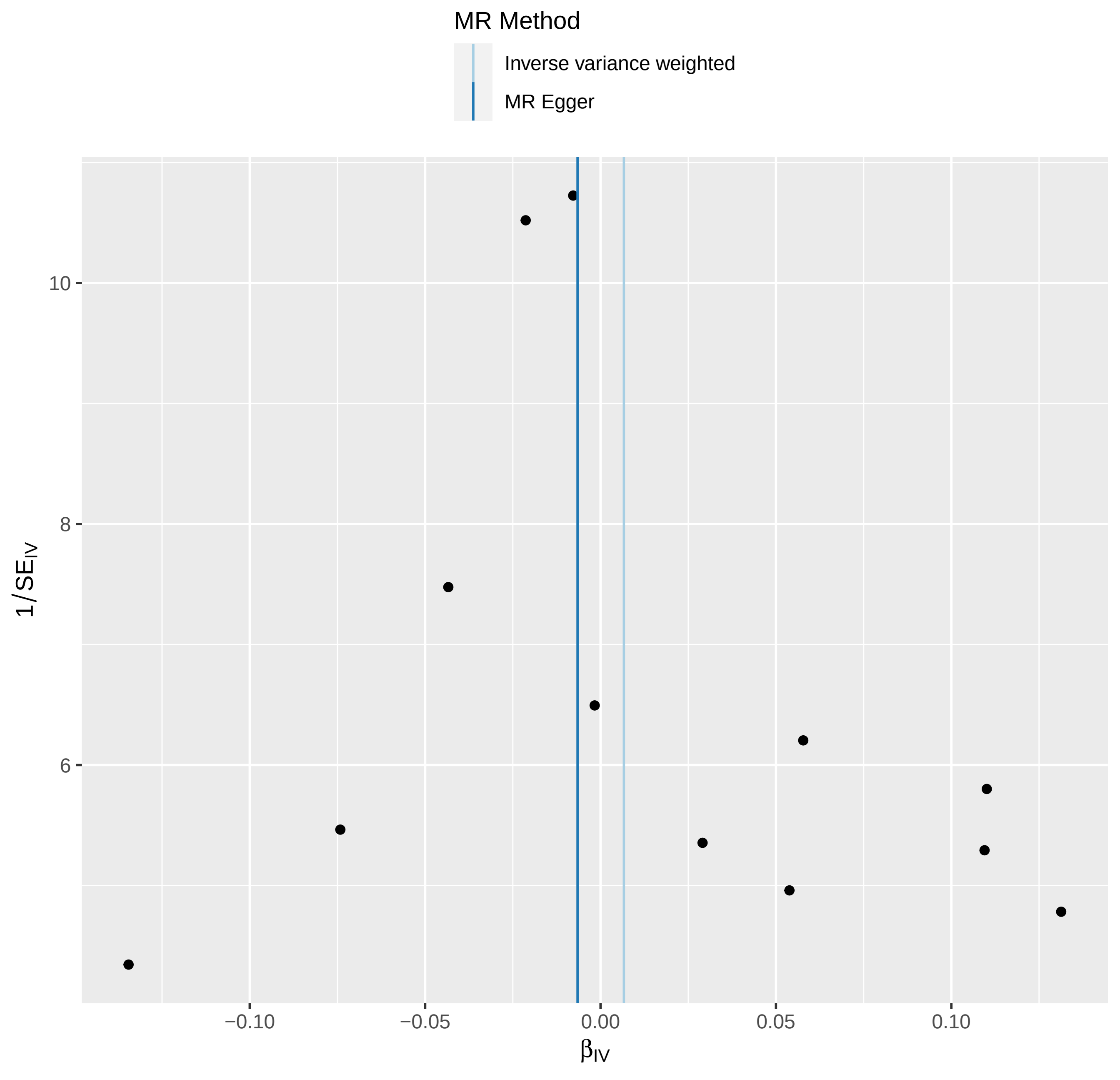

Batch 3−2716 : Selenium on SH3 domain−binding protein 2

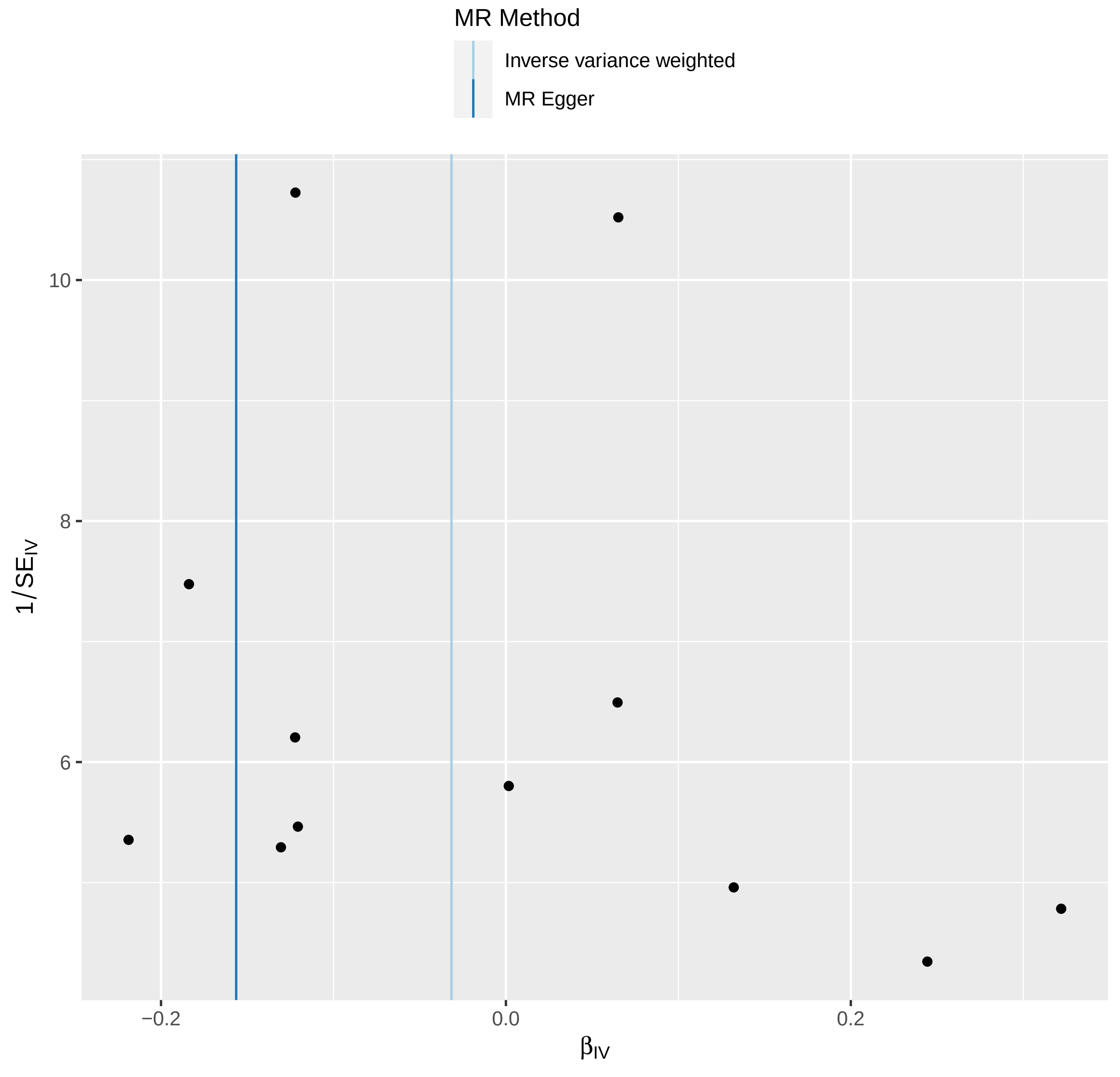

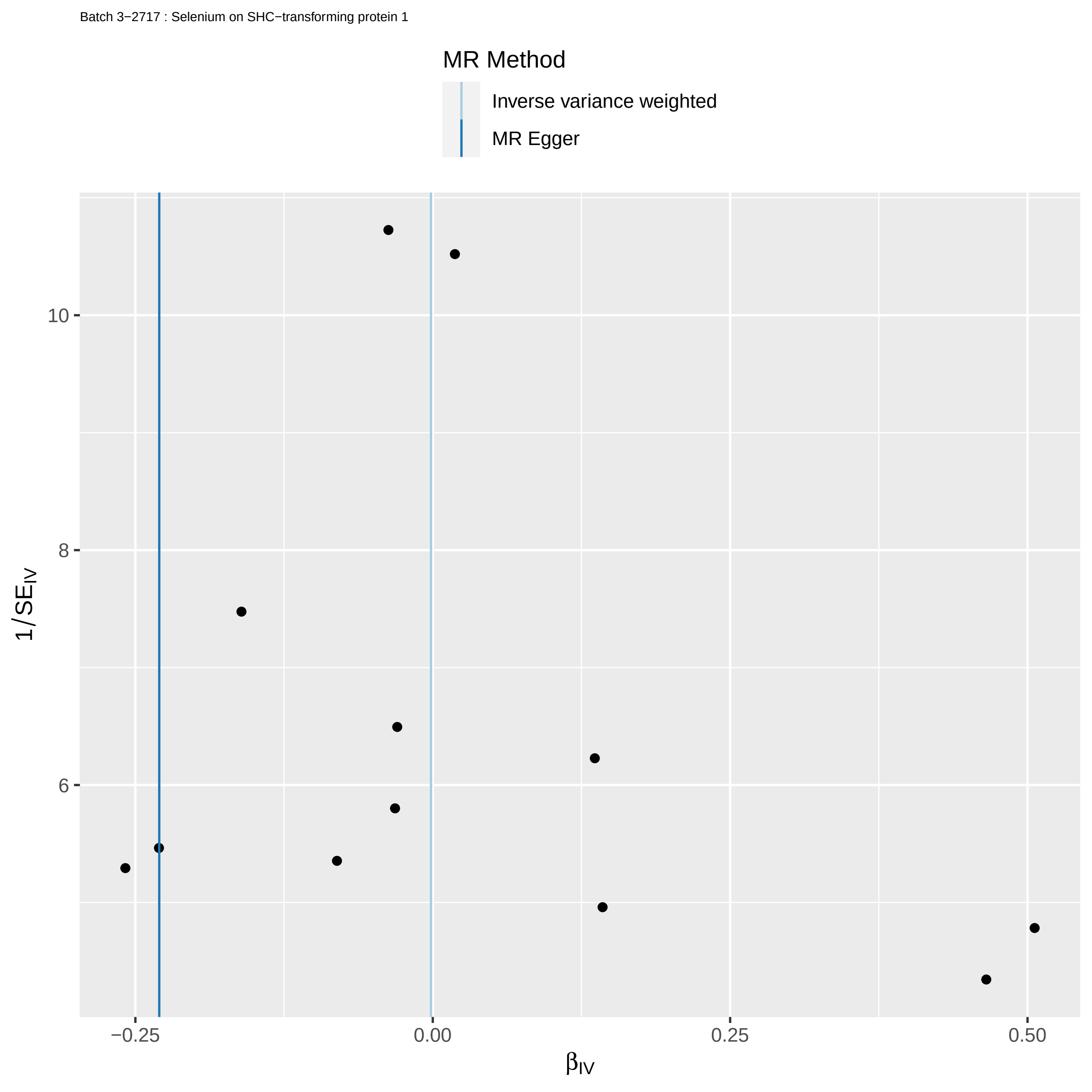
Batch 3−2717 : Selenium on SHC−transforming protein 1
MR Method
Inverse variance weighted
MR Egger
10
8
6
1/SE$_{IV}$
−0.25
0.00
0.25
0.50
β$_{IV}$

Batch 3−2718 : Selenium on SHC−transforming protein 2

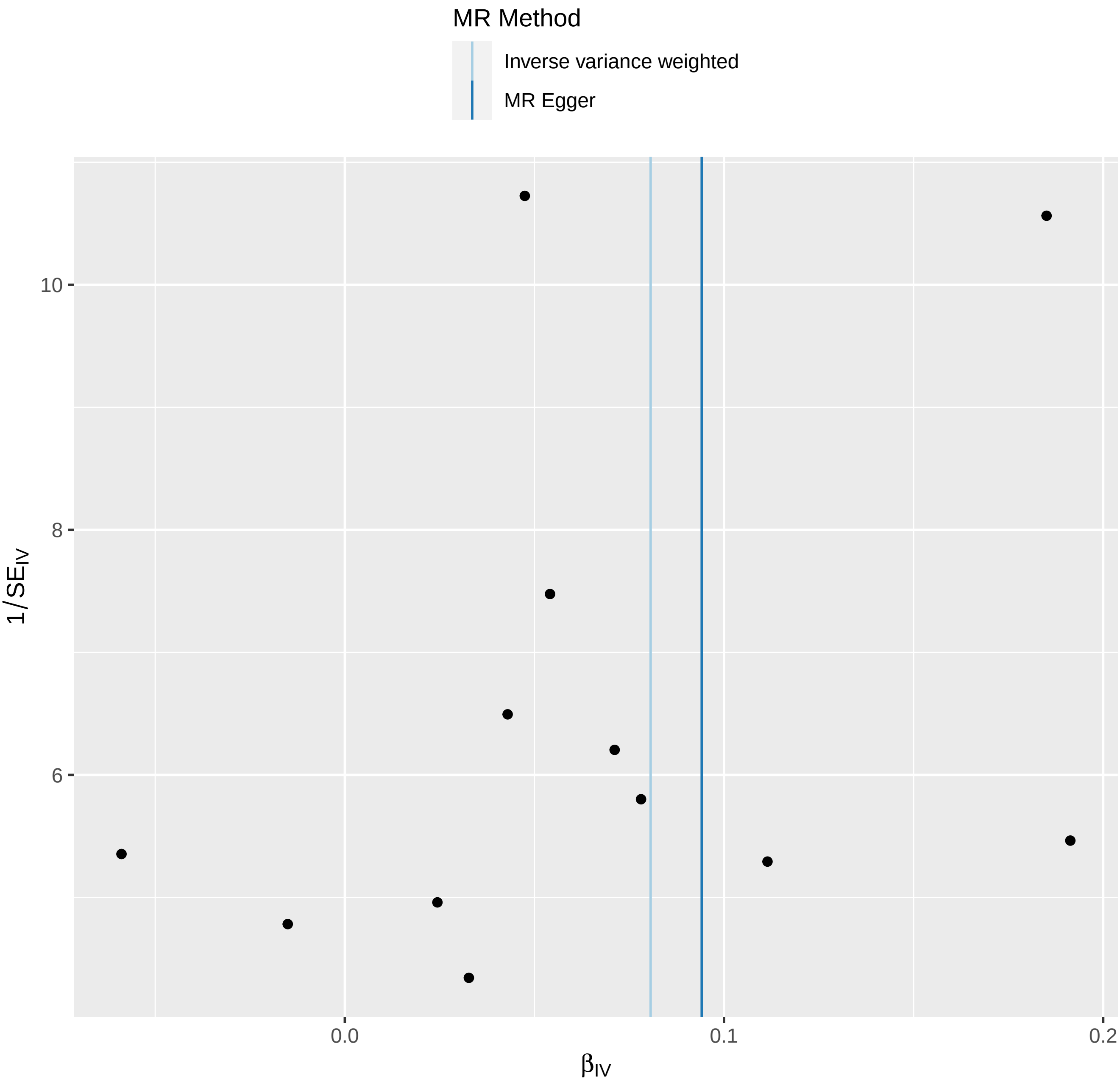

Batch 3−2719 : Selenium on SHC−transforming protein 4

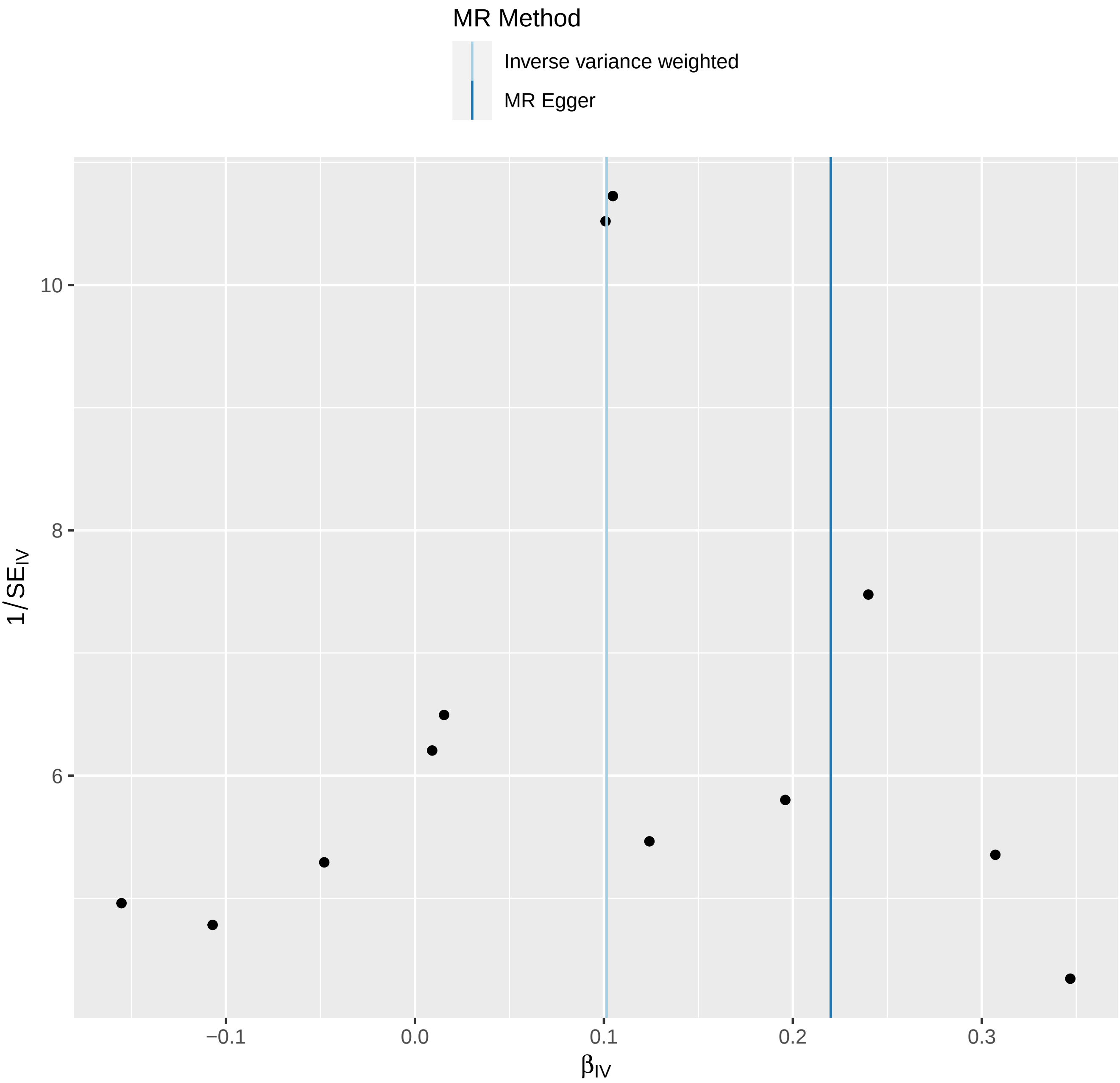

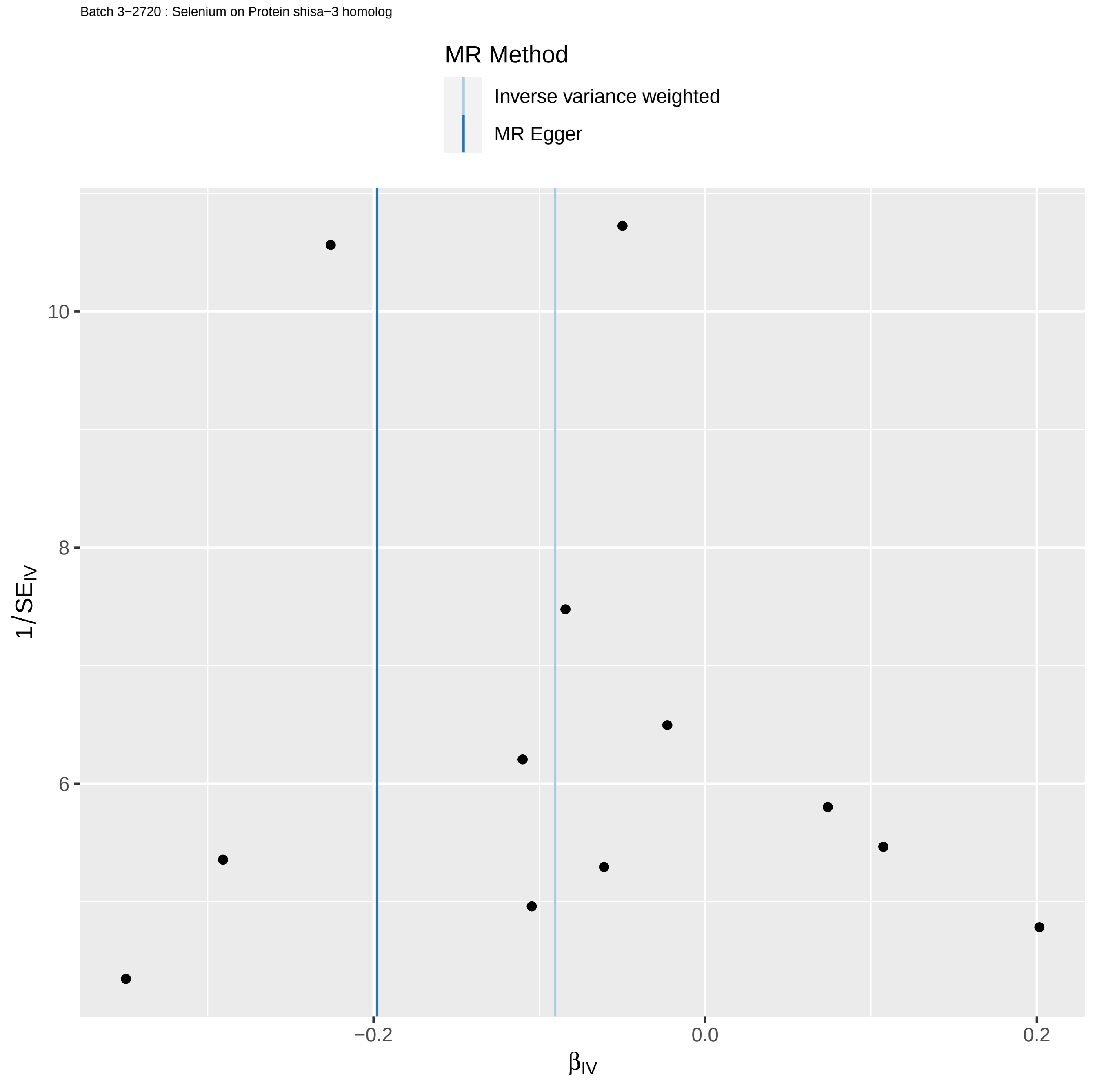

Batch 3−2720 : Selenium on Protein shisa−3 homolog
MR Method
Inverse variance weighted
MR Egger
10
8
6
1/SE_IV
−0.2
0.0
0.2
β_IV

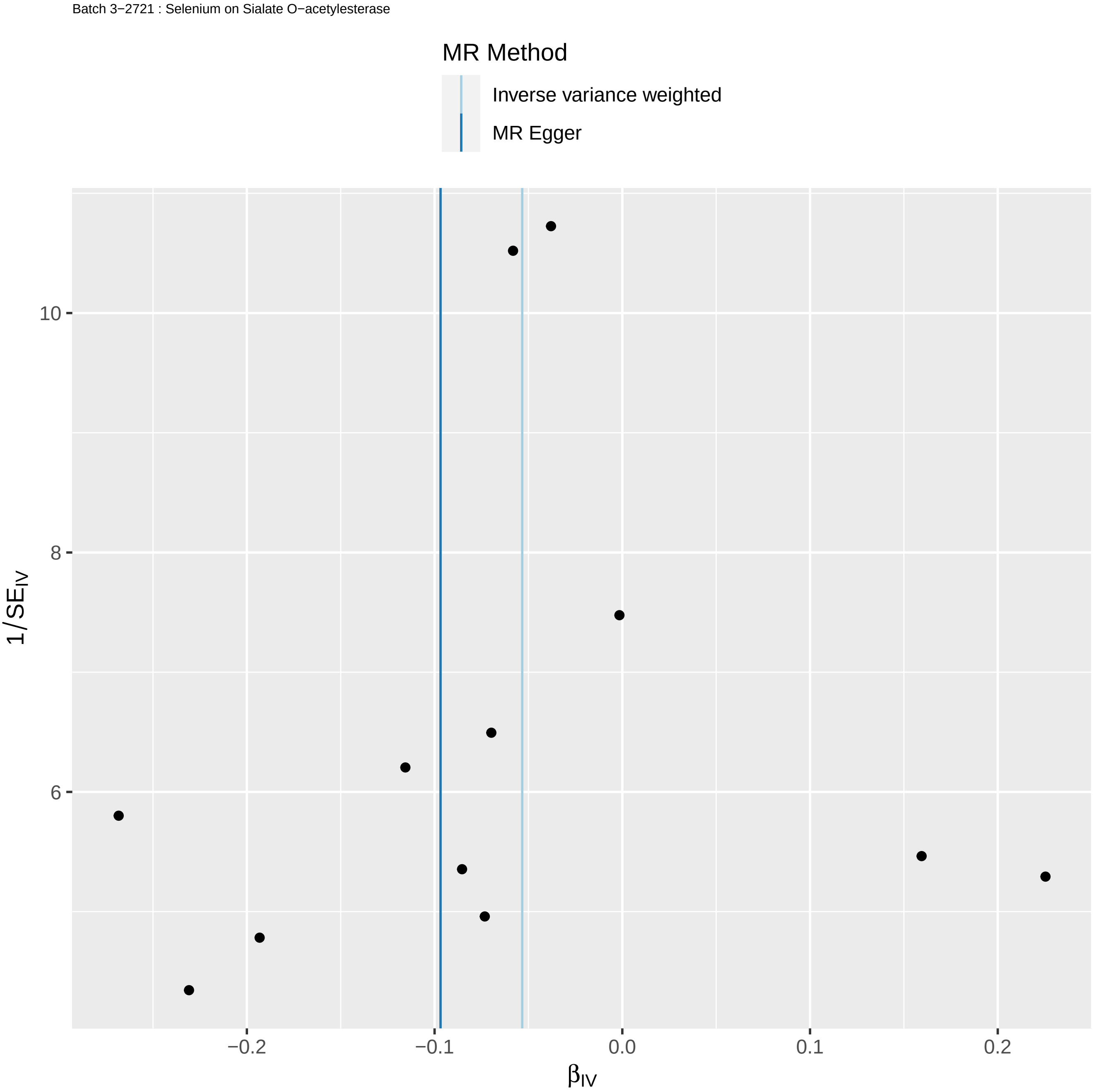
Batch 3−2721 : Selenium on Sialate O−acetylesterase
MR Method
Inverse variance weighted
MR Egger
10
8
6
1/SE$_{IV}$
−0.2
−0.1
0.0
0.1
0.2
β$_{IV}$

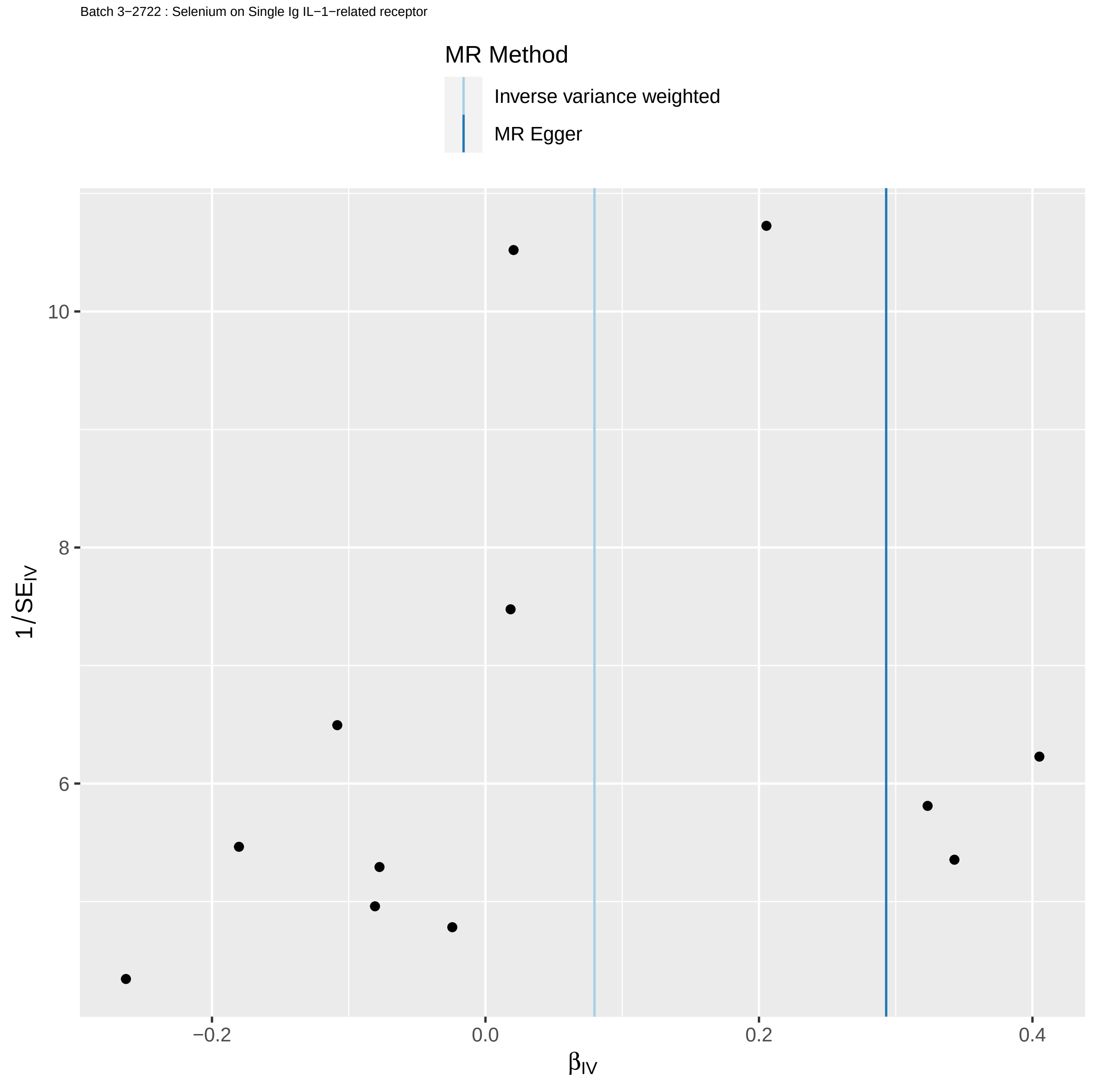

Batch 3−2722 : Selenium on Single Ig IL−1−related receptor
MR Method
Inverse variance weighted
MR Egger
10
8
6
1/SE_IV
−0.2
0.0
0.2
0.4
β_IV

Batch 3−2723 : Selenium on Sialic acid−binding Ig−like lectin 10

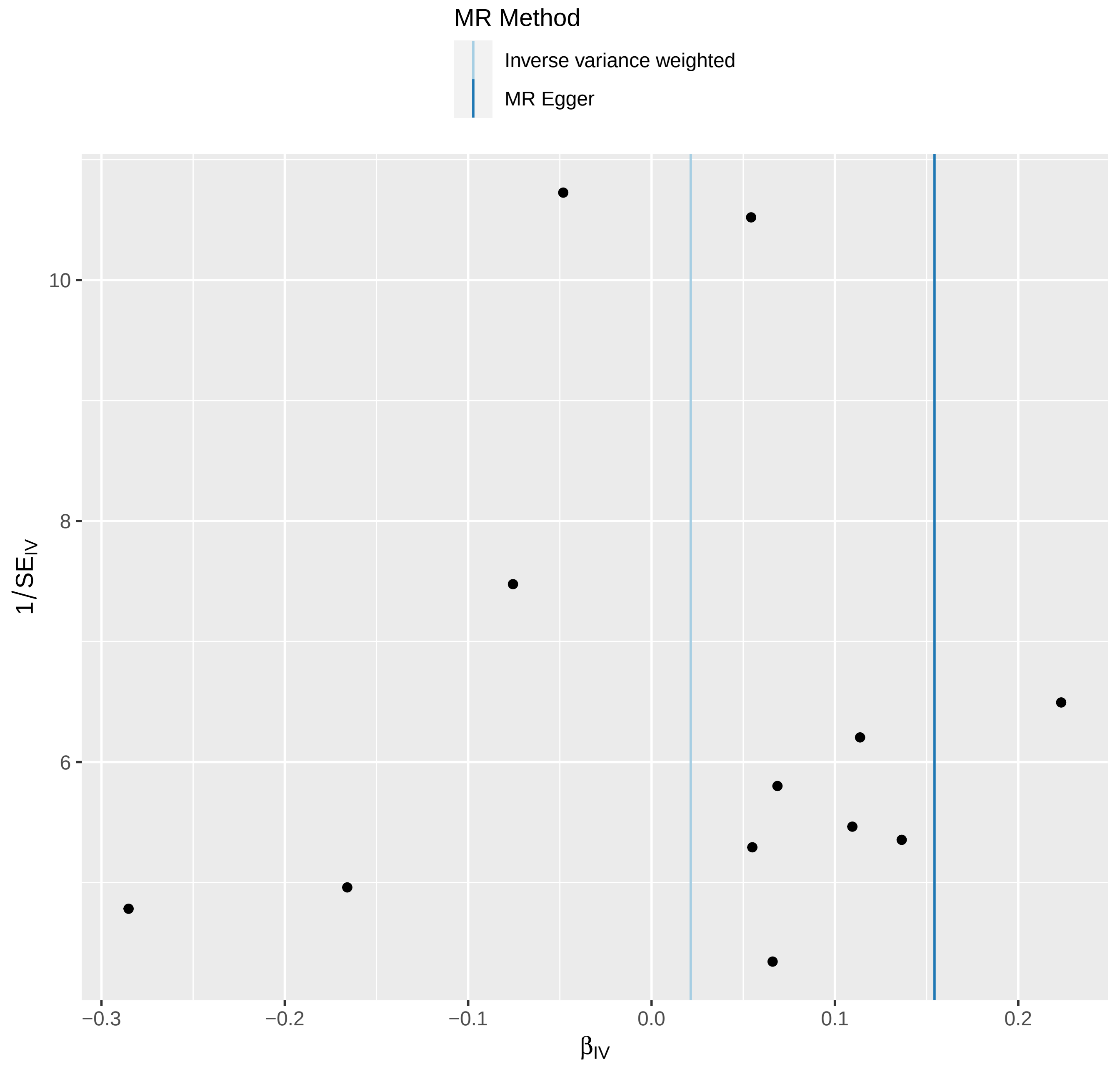

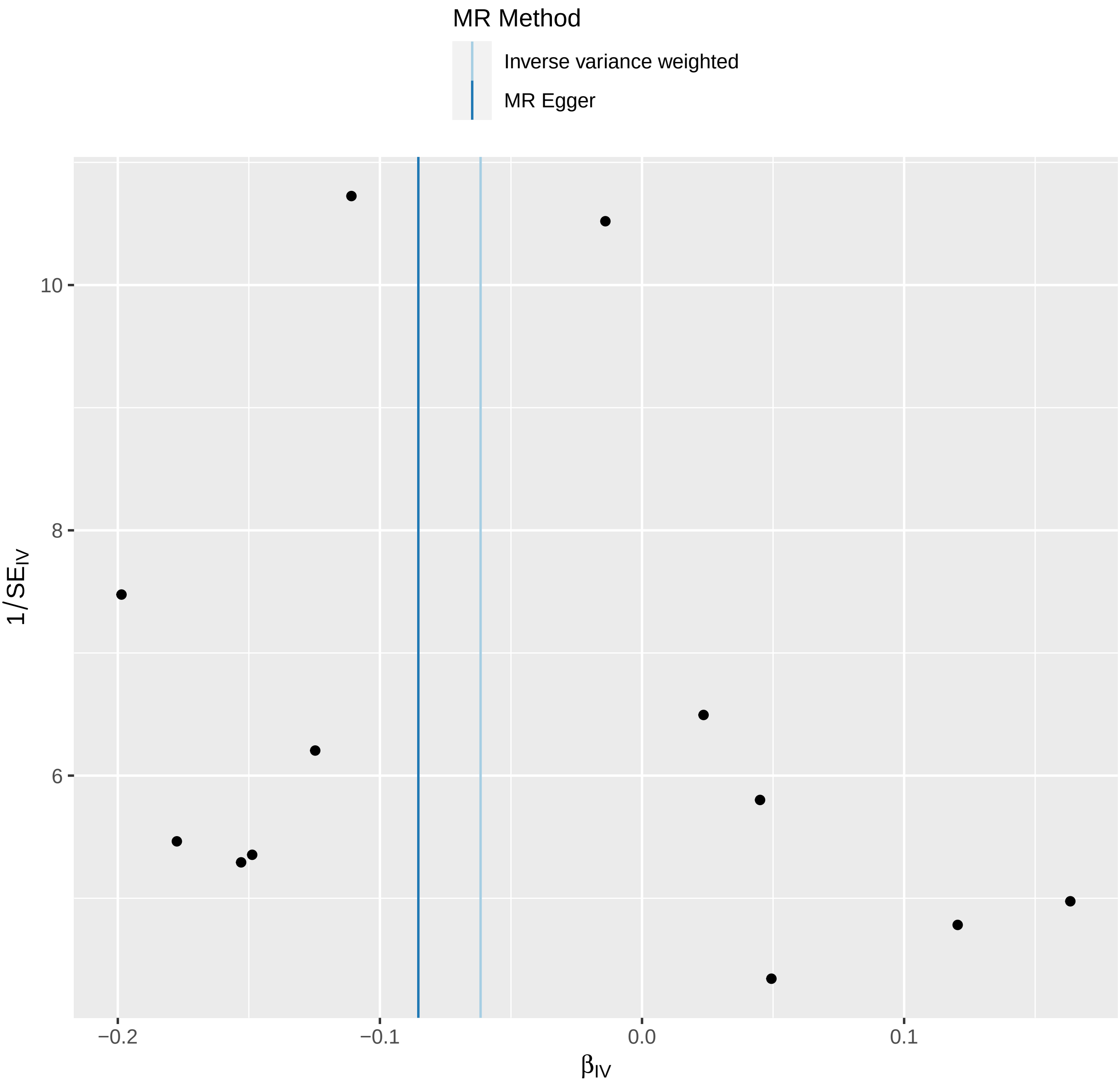
MR Method
Inverse variance weighted
MR Egger
10
8
6
1/SE$_{IV}$
−0.2
−0.1
0.0
0.1
β_{IV}

Batch 3−2725 : Selenium on Sialic acid−binding Ig−like lectin 12

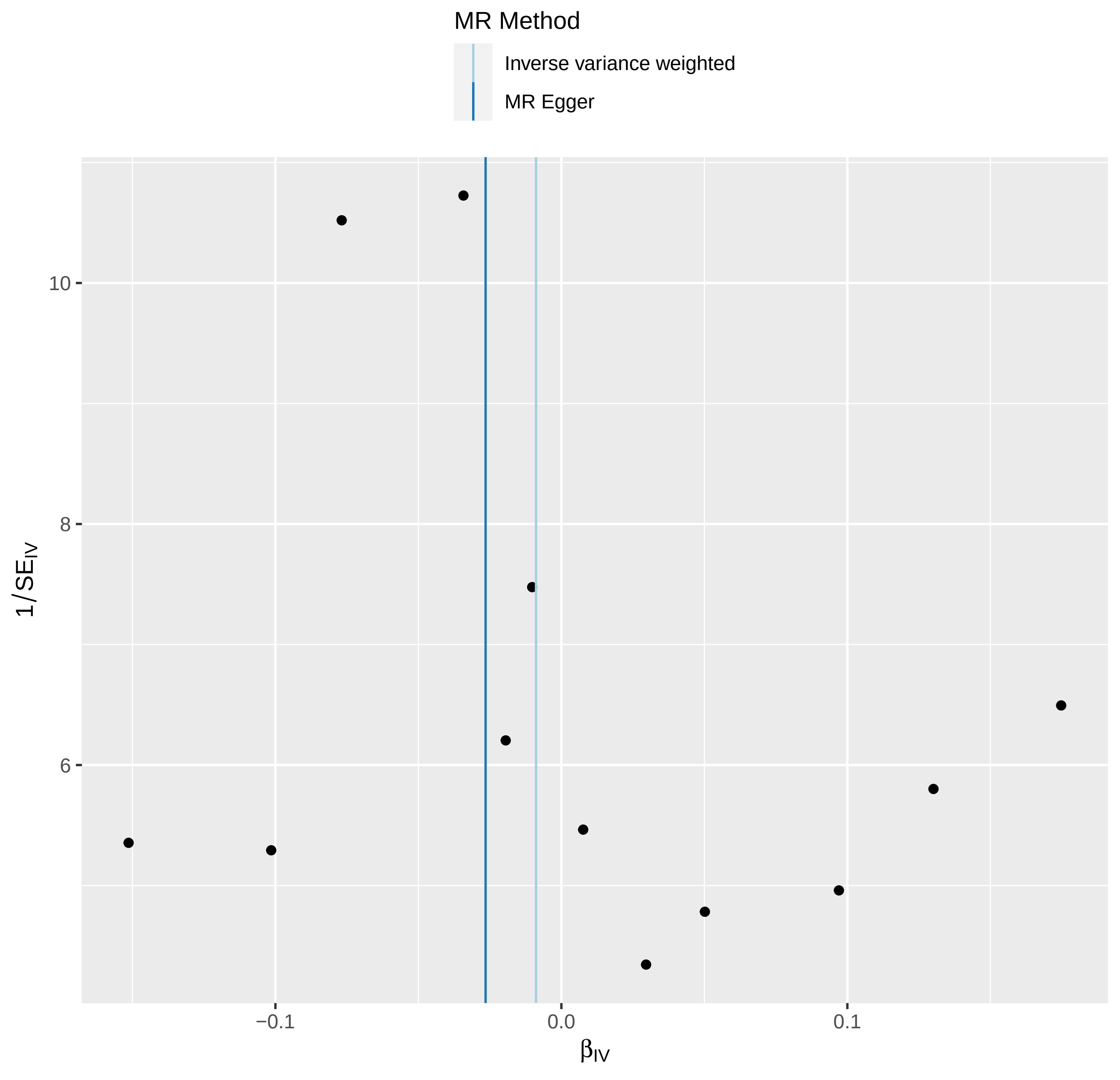

Batch 3−2726 : Selenium on Sialic acid−binding Ig−like lectin 12

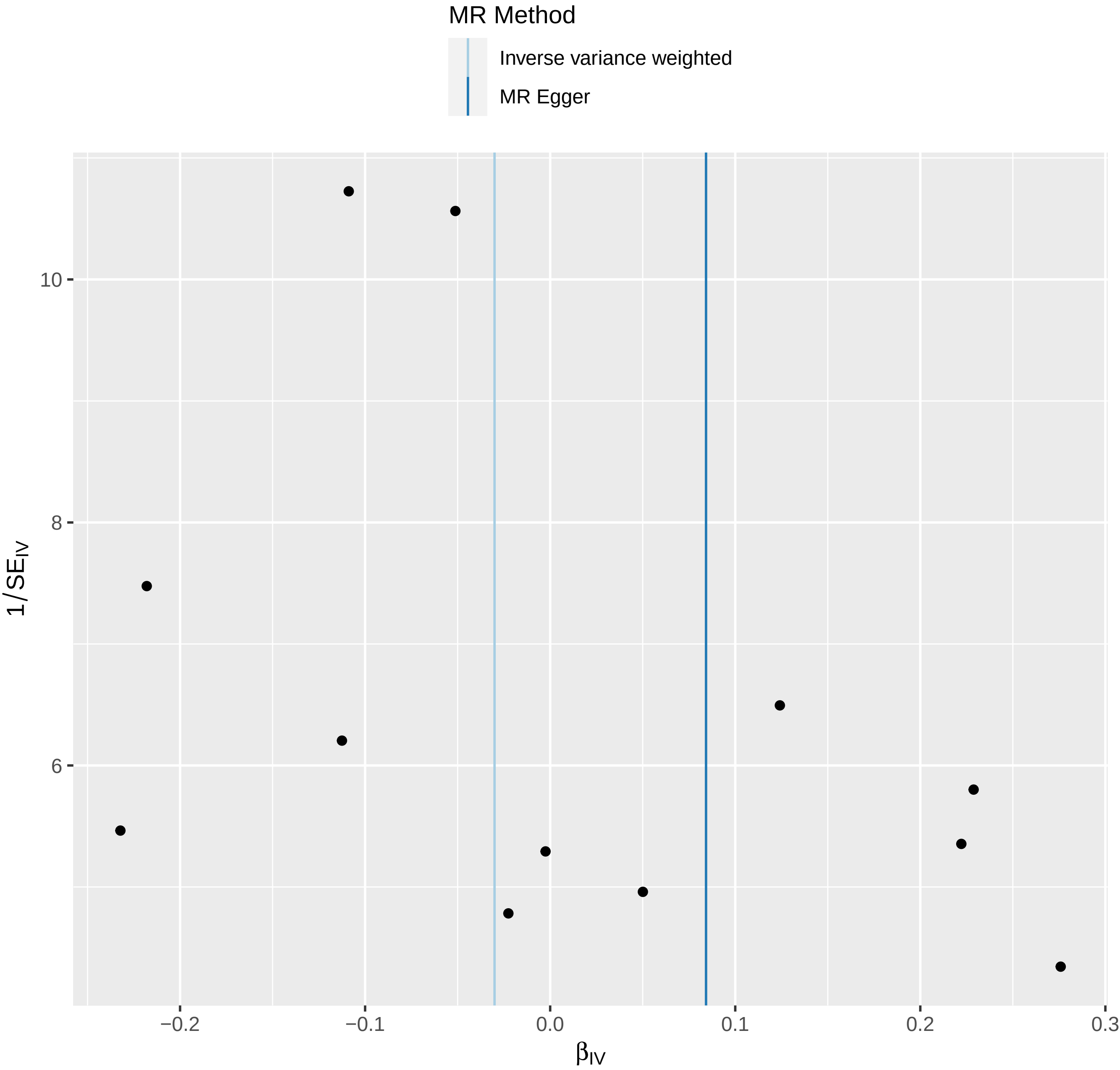

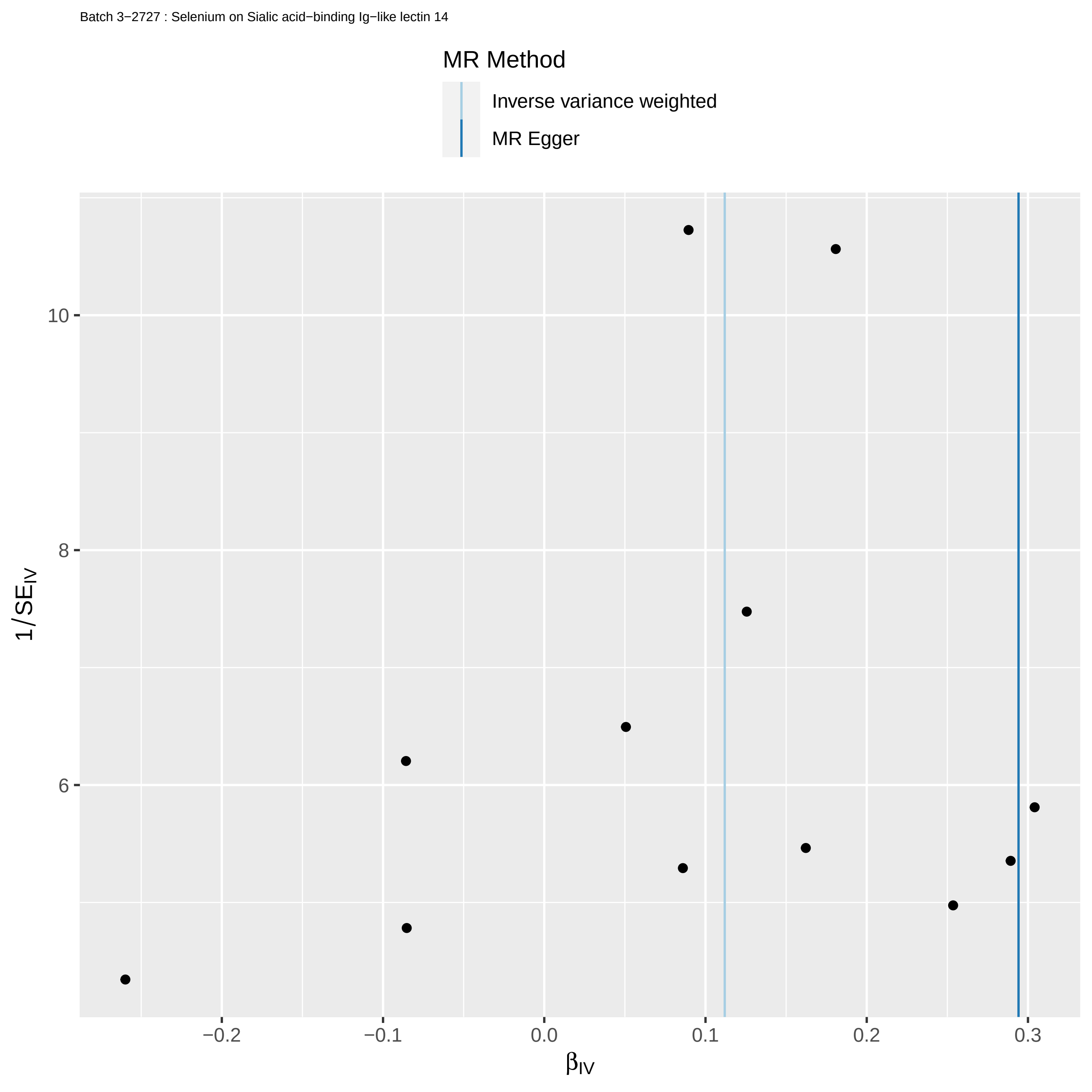

Batch 3−2727 : Selenium on Sialic acid−binding Ig−like lectin 14
MR Method
Inverse variance weighted
MR Egger
10
8
6
1/SE_IV
−0.2
−0.1
0.0
0.1
0.2
0.3
β_IV

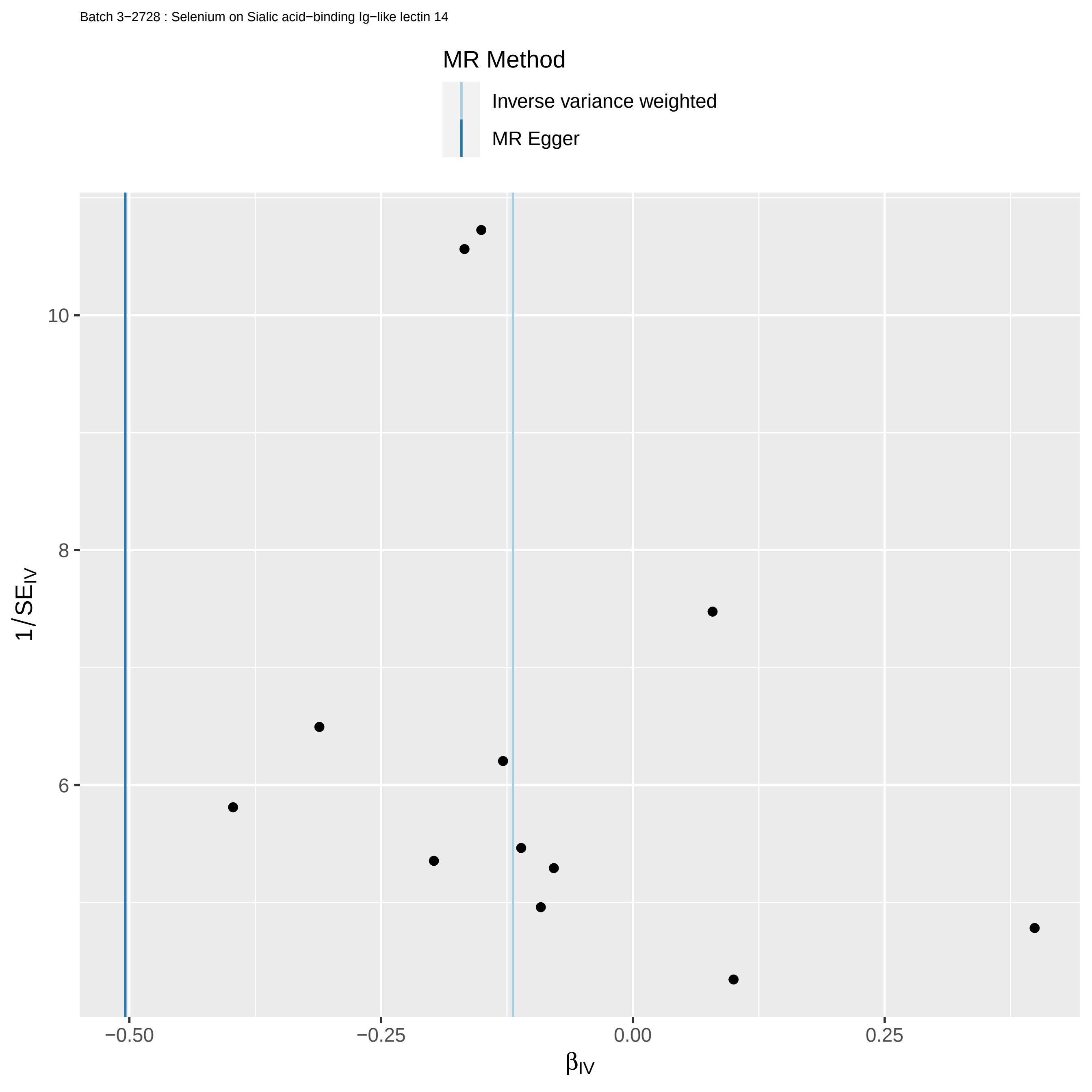

Batch 3−2728 : Selenium on Sialic acid−binding Ig−like lectin 14
MR Method
Inverse variance weighted
MR Egger
10
8
6
1/SE$_{IV}$
−0.50
−0.25
0.00
0.25
β$_{IV}$

Batch 3−2729 : Selenium on Sialic acid−binding Ig−like lectin 7

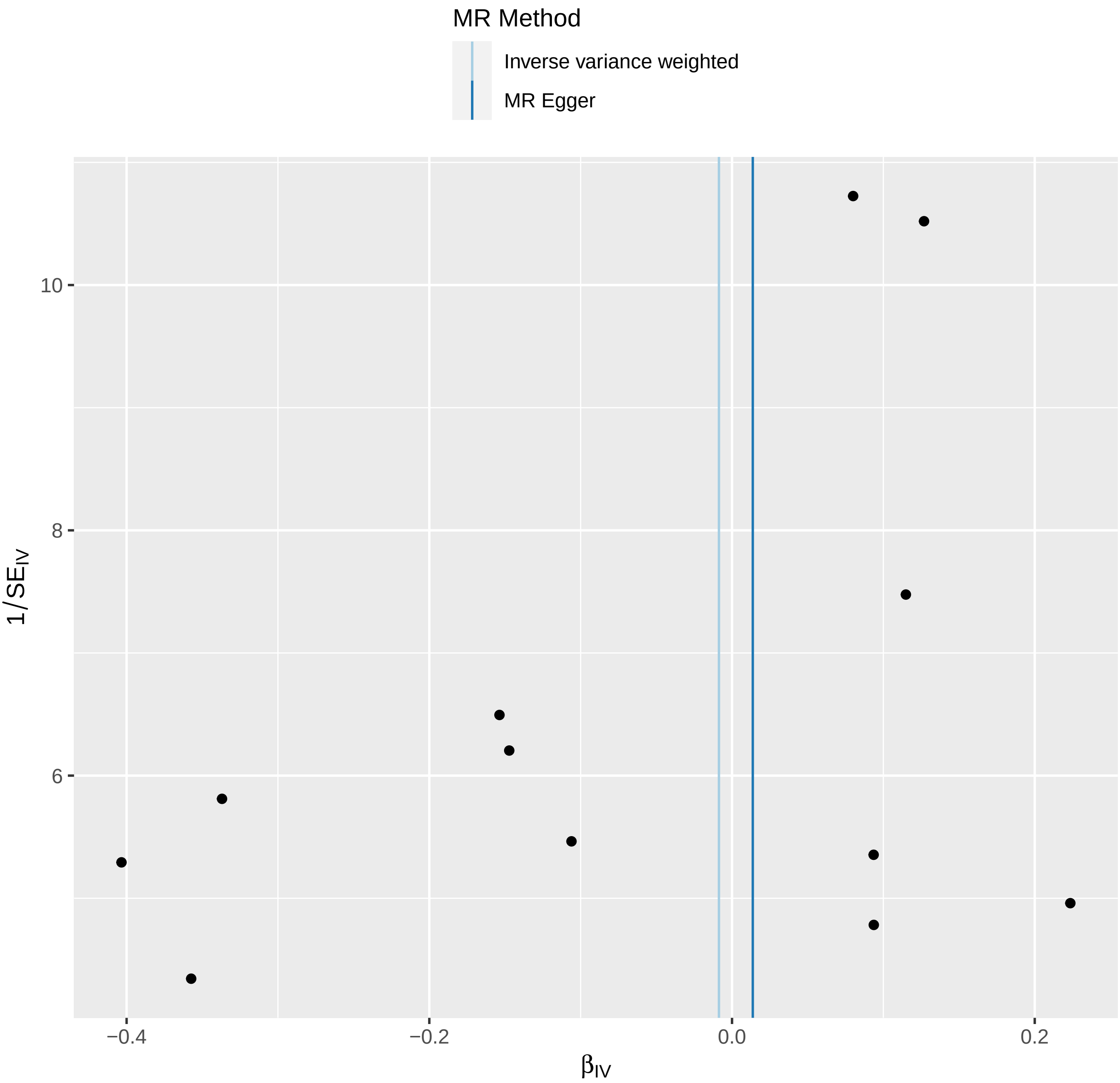

Batch 3−2730 : Selenium on Sialic acid−binding Ig−like lectin 8

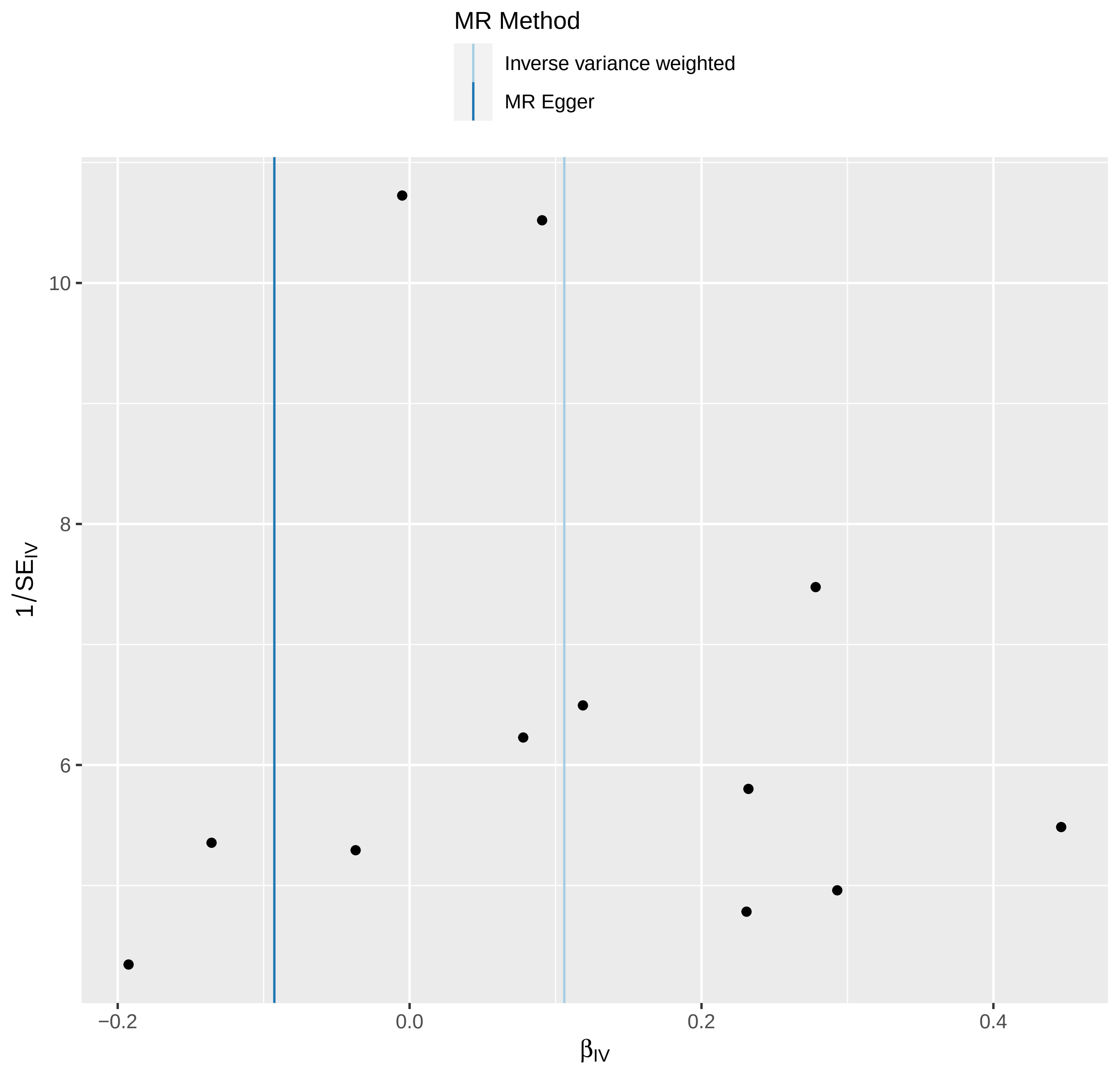

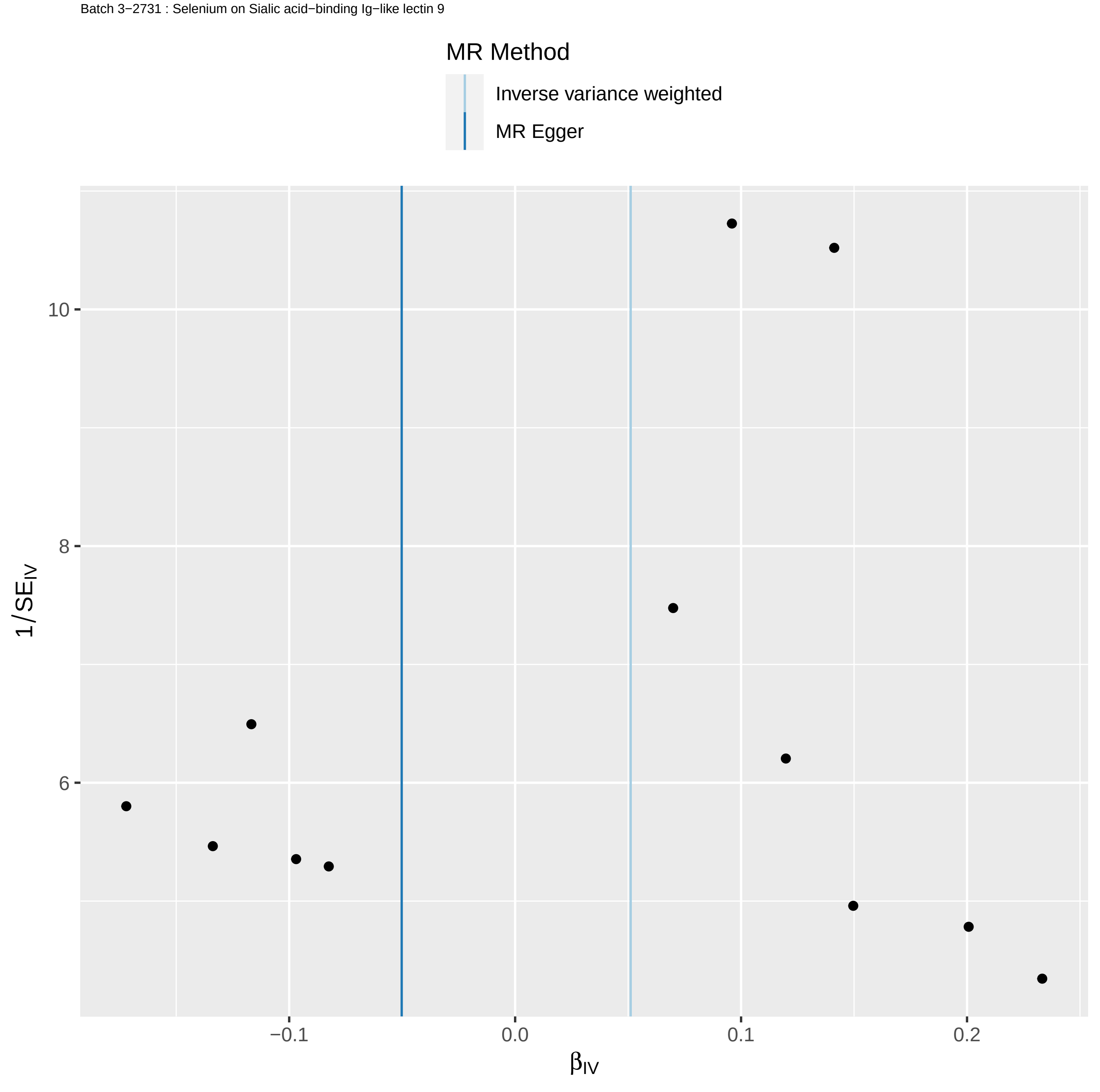

Batch 3−2731 : Selenium on Sialic acid−binding Ig−like lectin 9
MR Method
Inverse variance weighted
MR Egger
10
8
6
1/SE_IV
−0.1
0.0
0.1
0.2
β_IV

Batch 3−2732 : Selenium on Tyrosine−protein phosphatase non−receptor type substrate 1

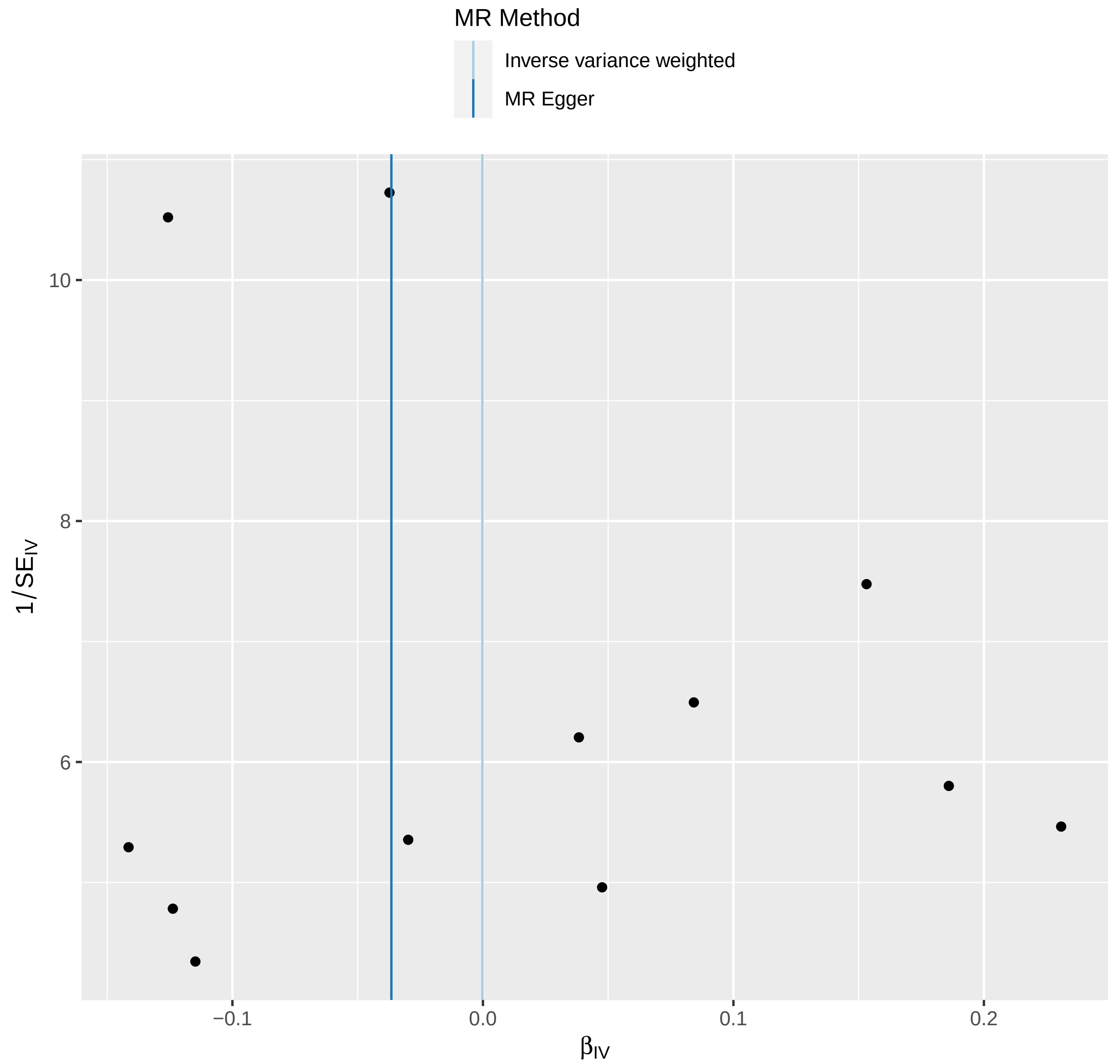

Batch 3−2733 : Selenium on Signal−regulatory protein beta−1

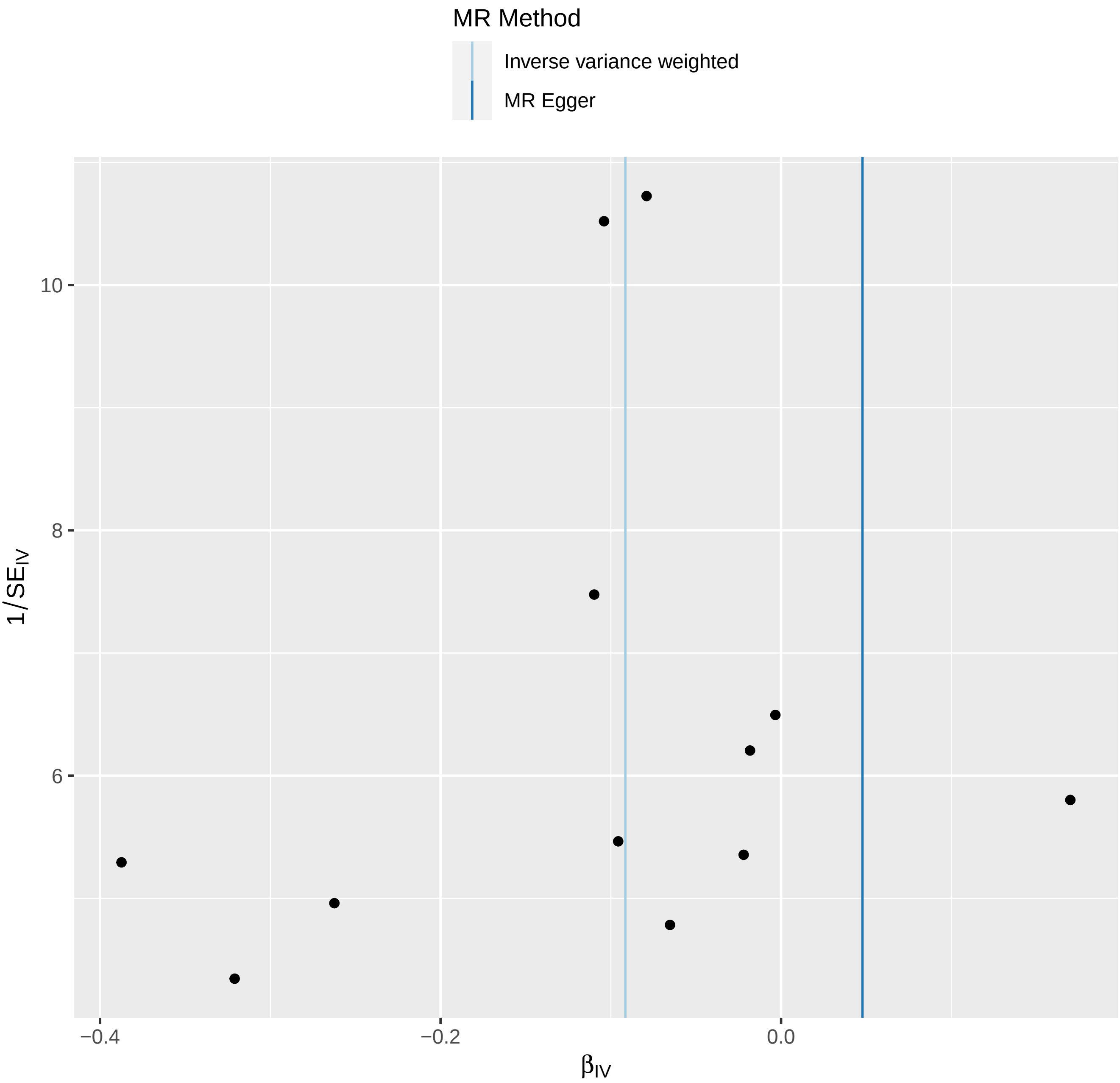

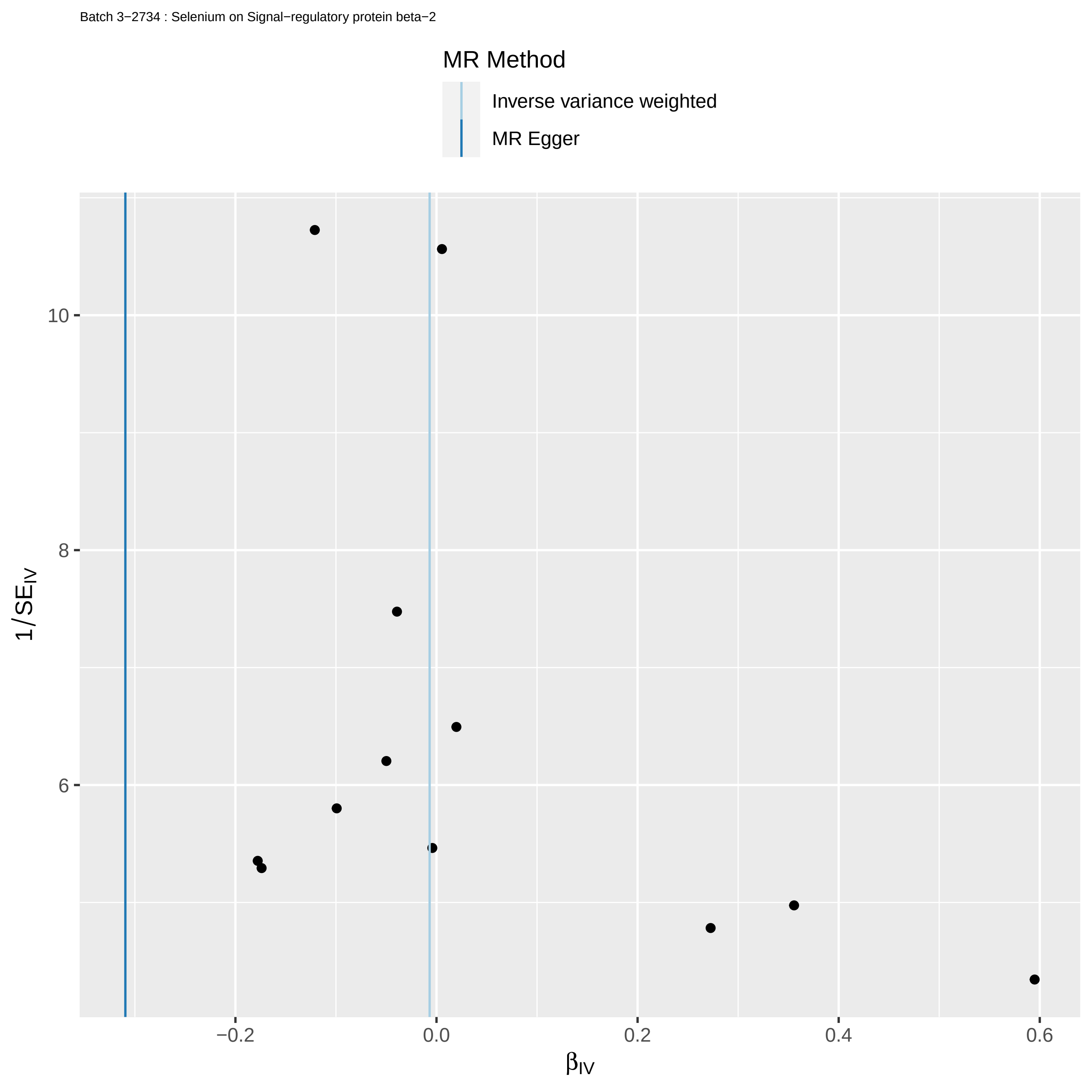

Batch 3−2734 : Selenium on Signal−regulatory protein beta−2
MR Method
Inverse variance weighted
MR Egger
10
8
6
1/SE_IV
−0.2
0.0
0.2
0.4
0.6
β_IV

Batch 3−2735 : Selenium on Signal−regulatory protein gamma

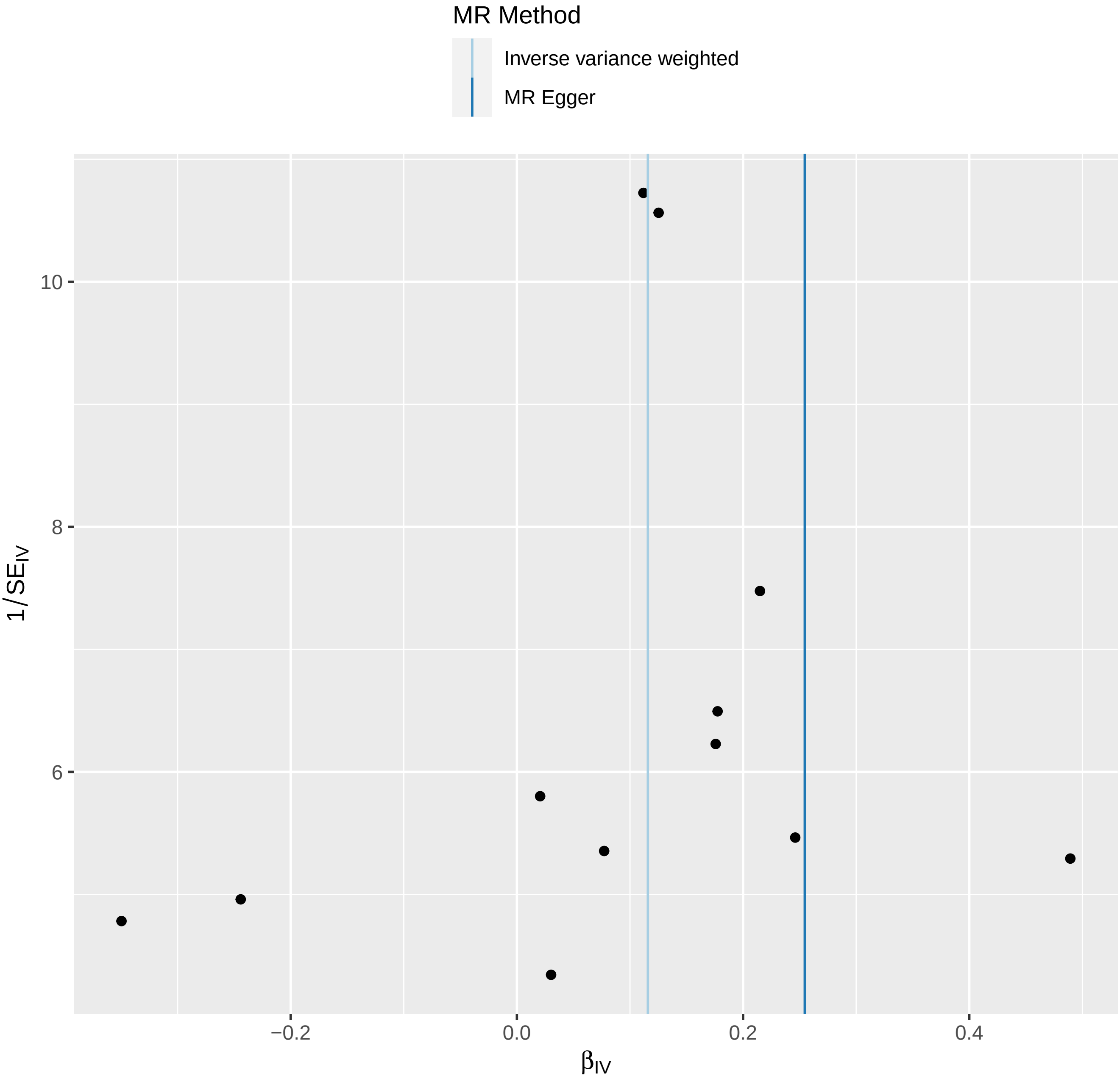

Batch 3−2736 : Selenium on NAD−dependent protein deacetylase sirtuin−2

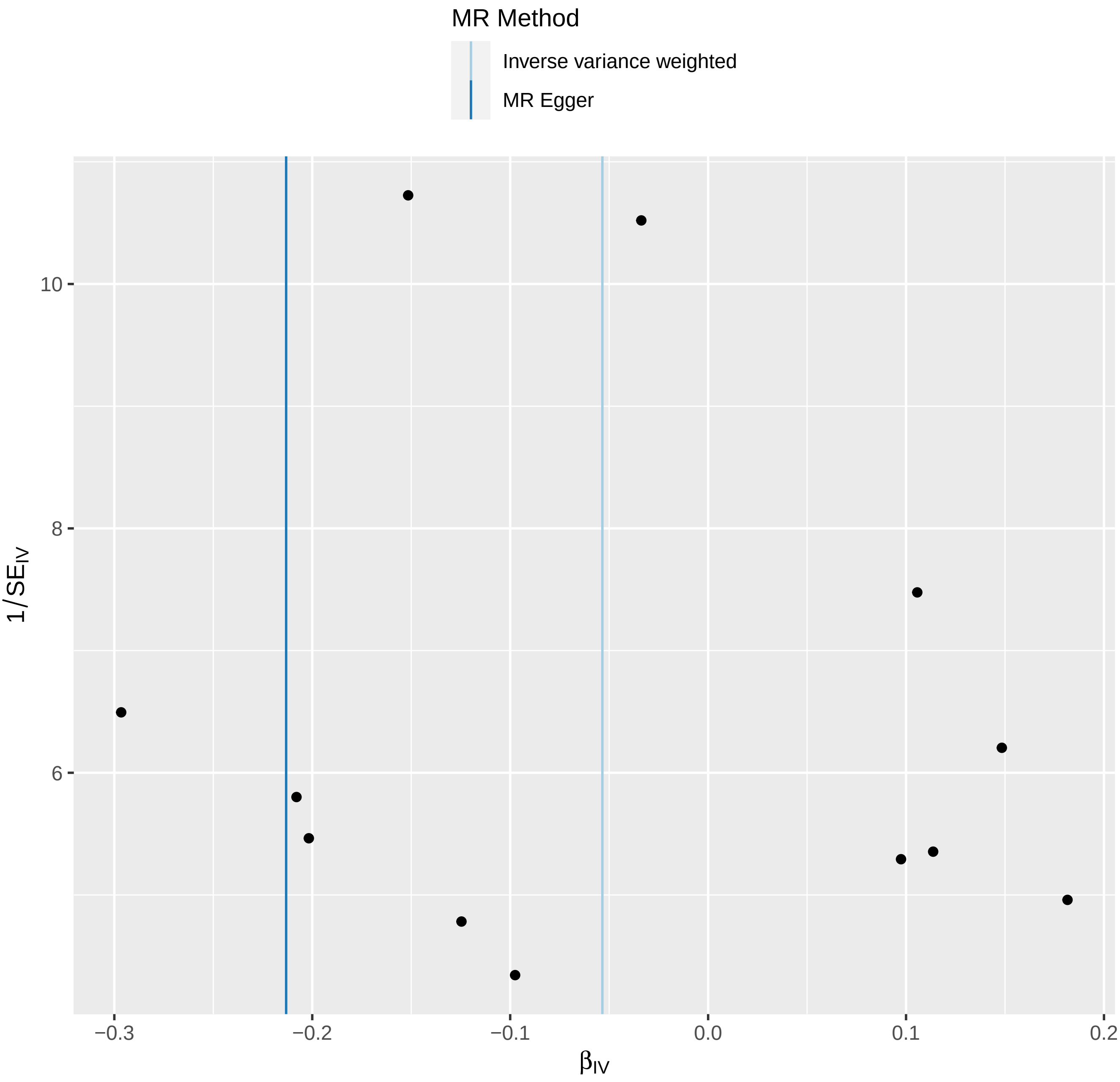

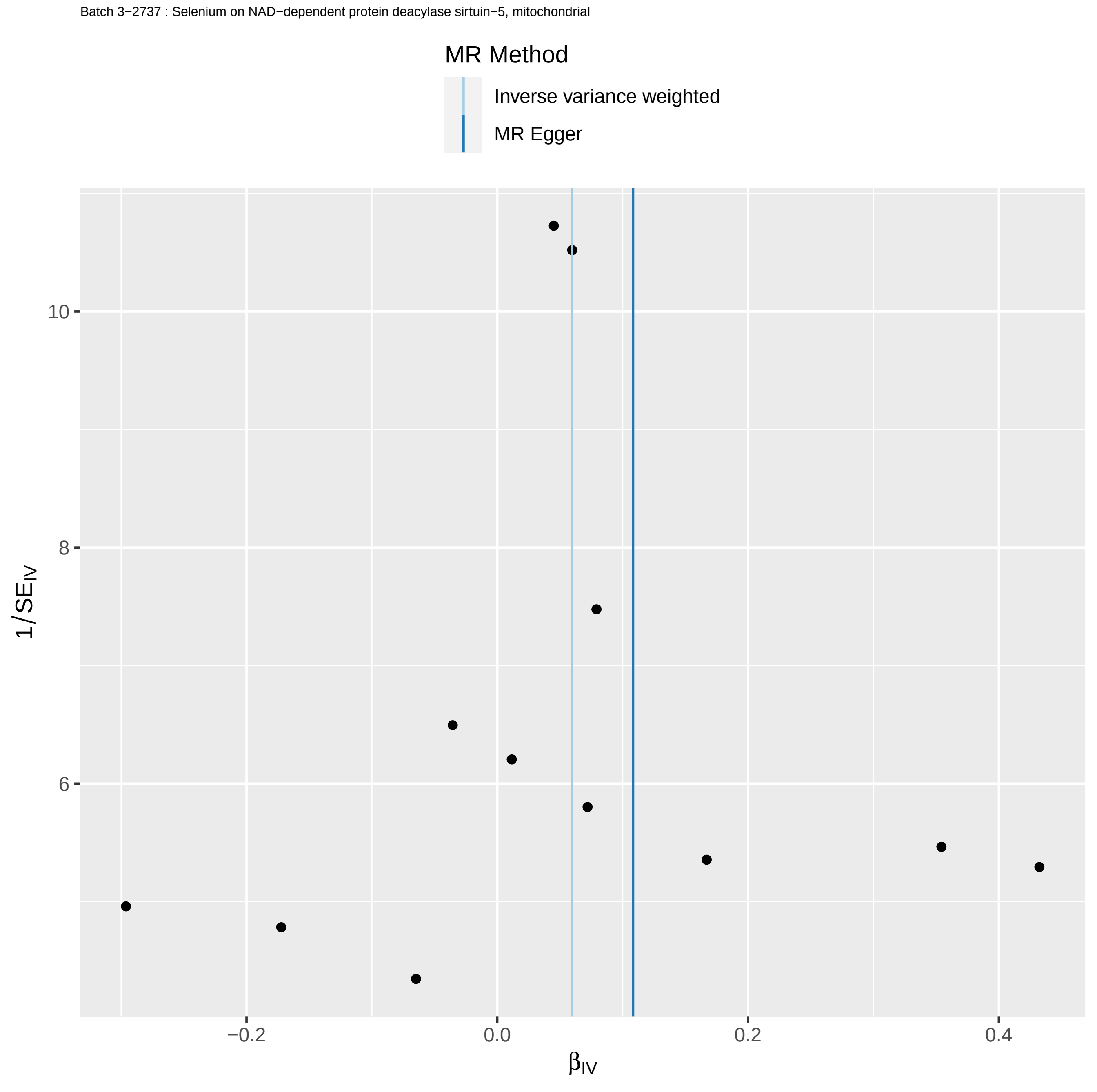
Batch 3−2737 : Selenium on NAD−dependent protein deacylase sirtuin−5, mitochondrial
MR Method
Inverse variance weighted
MR Egger
10
8
6
1/SE_IV
−0.2
0.0
0.2
0.4
β_IV

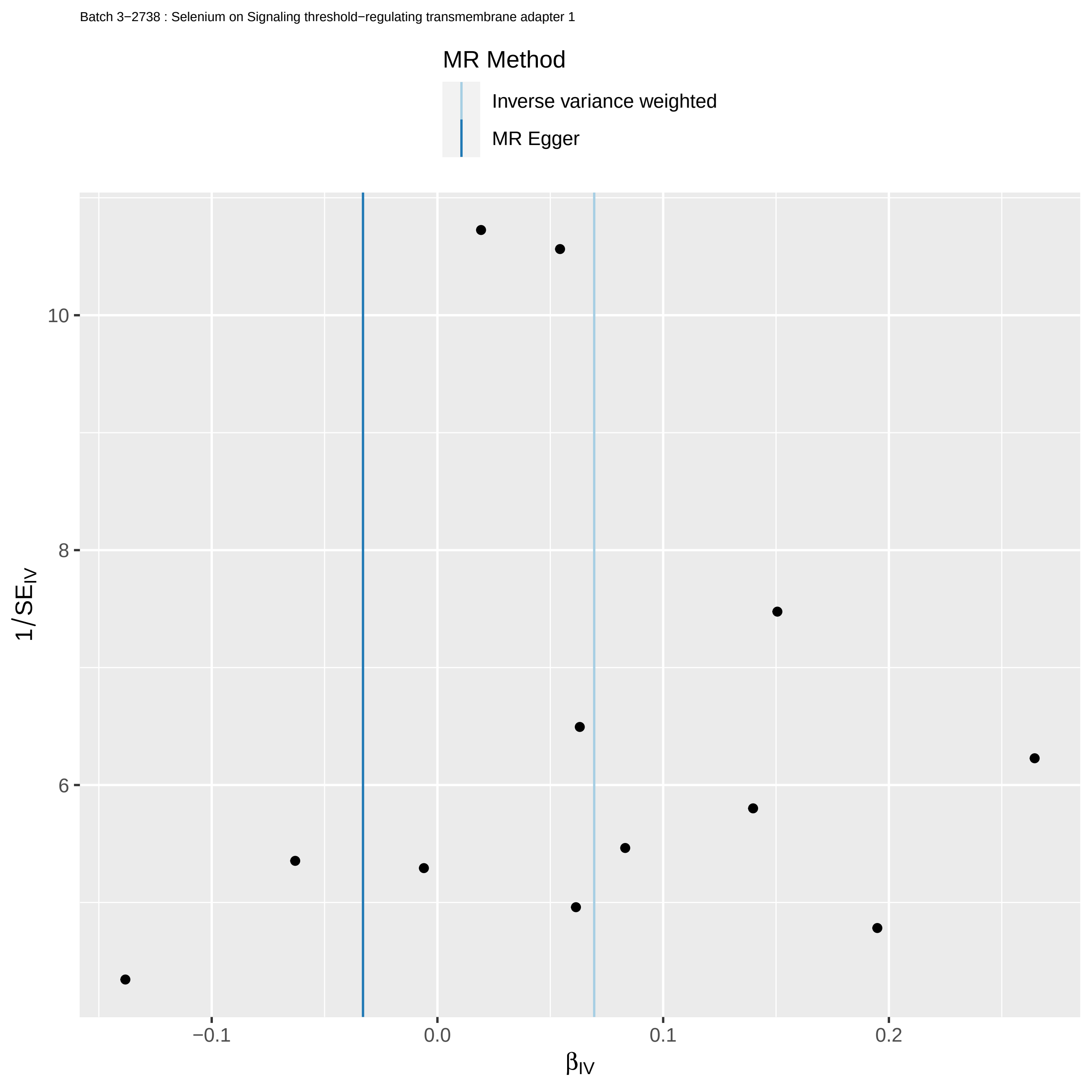

Batch 3−2738 : Selenium on Signaling threshold−regulating transmembrane adapter 1
MR Method
Inverse variance weighted
MR Egger
10
8
6
$1/SE_{IV}$
−0.1
0.0
0.1
0.2
β_{IV}

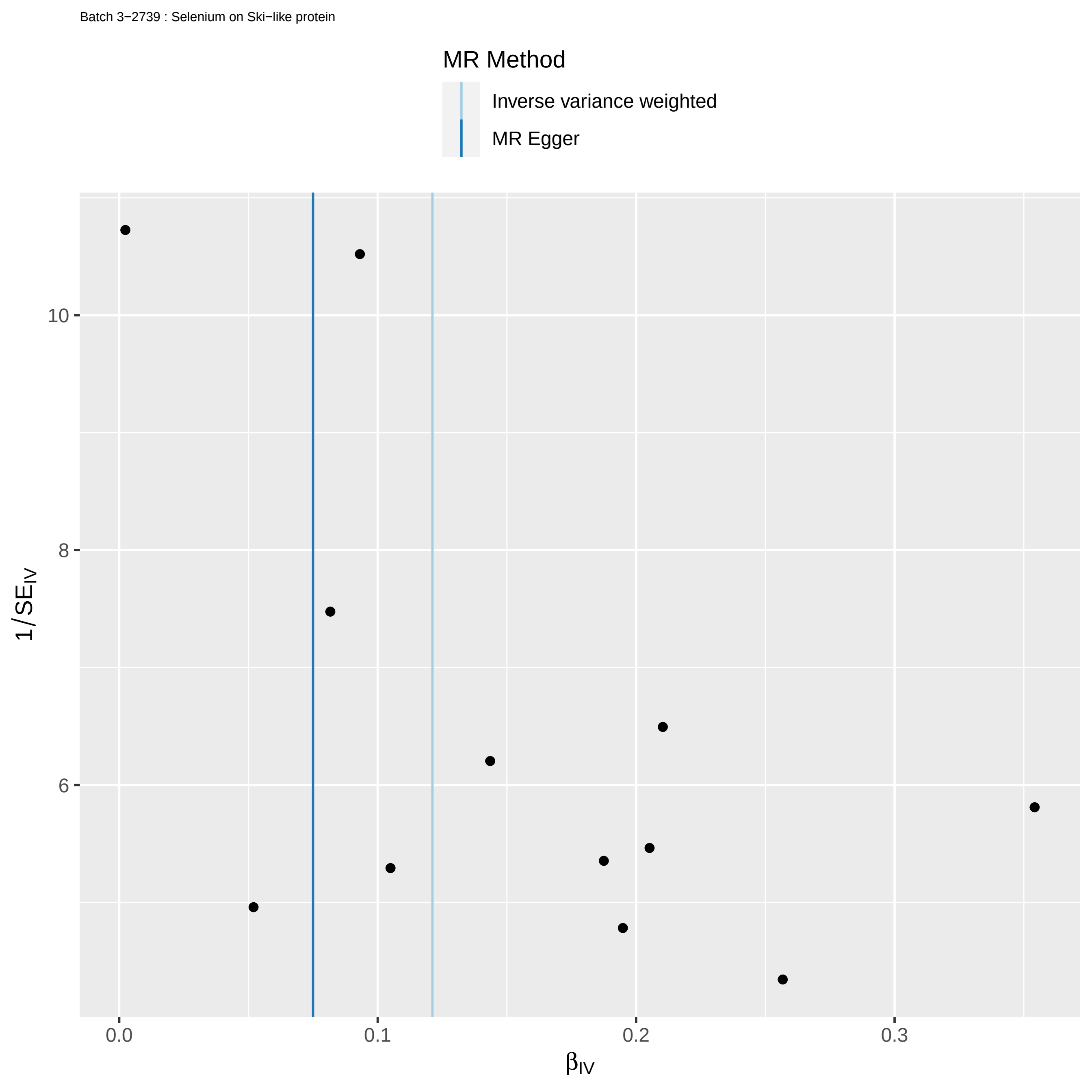
Batch 3−2739 : Selenium on Ski−like protein
MR Method
Inverse variance weighted
MR Egger
10
8
6
1/SE$_{IV}$
0.0
0.1
0.2
0.3
β$_{IV}$

Batch 3−2740 : Selenium on S−phase kinase−associated protein 1

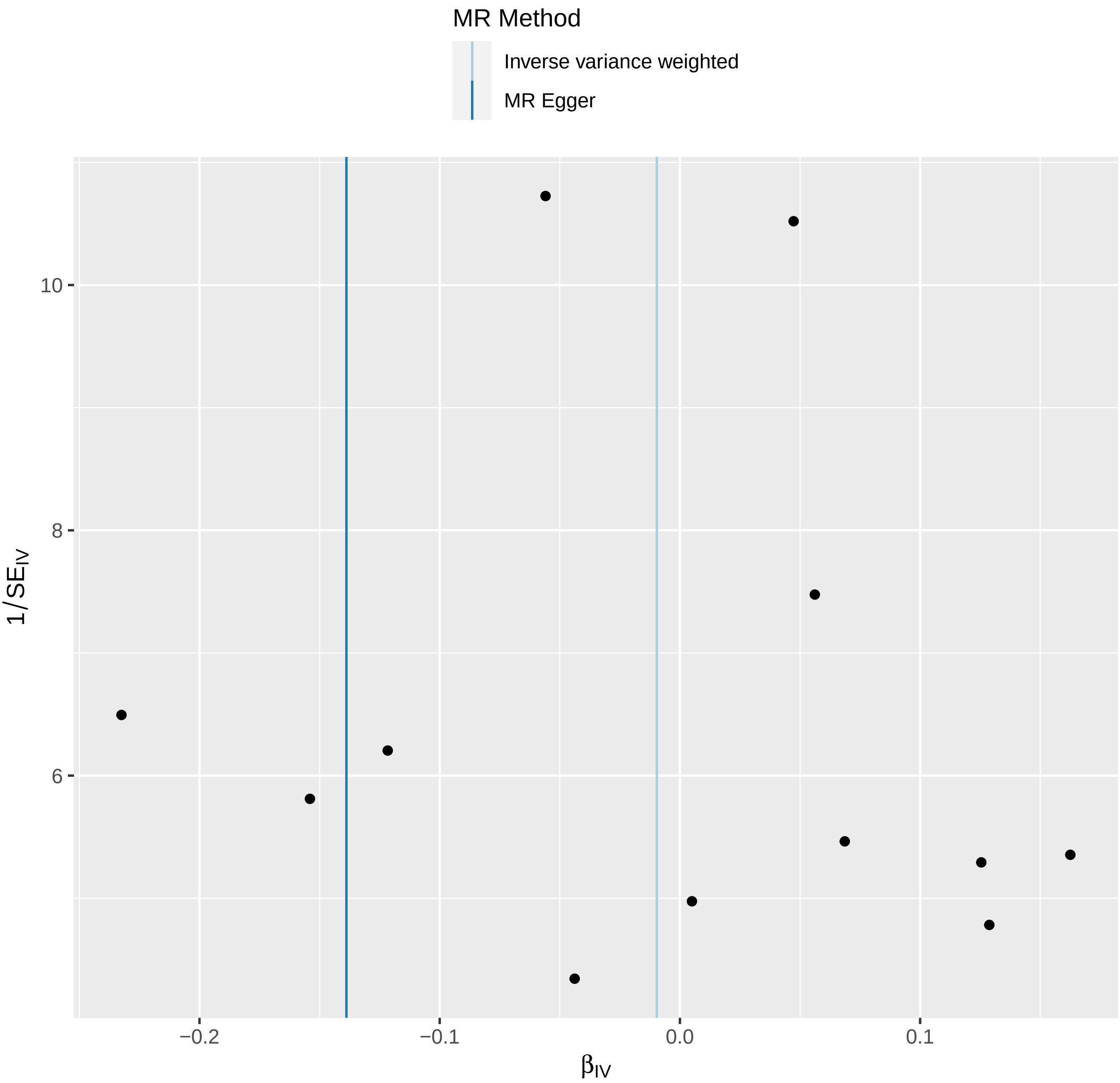

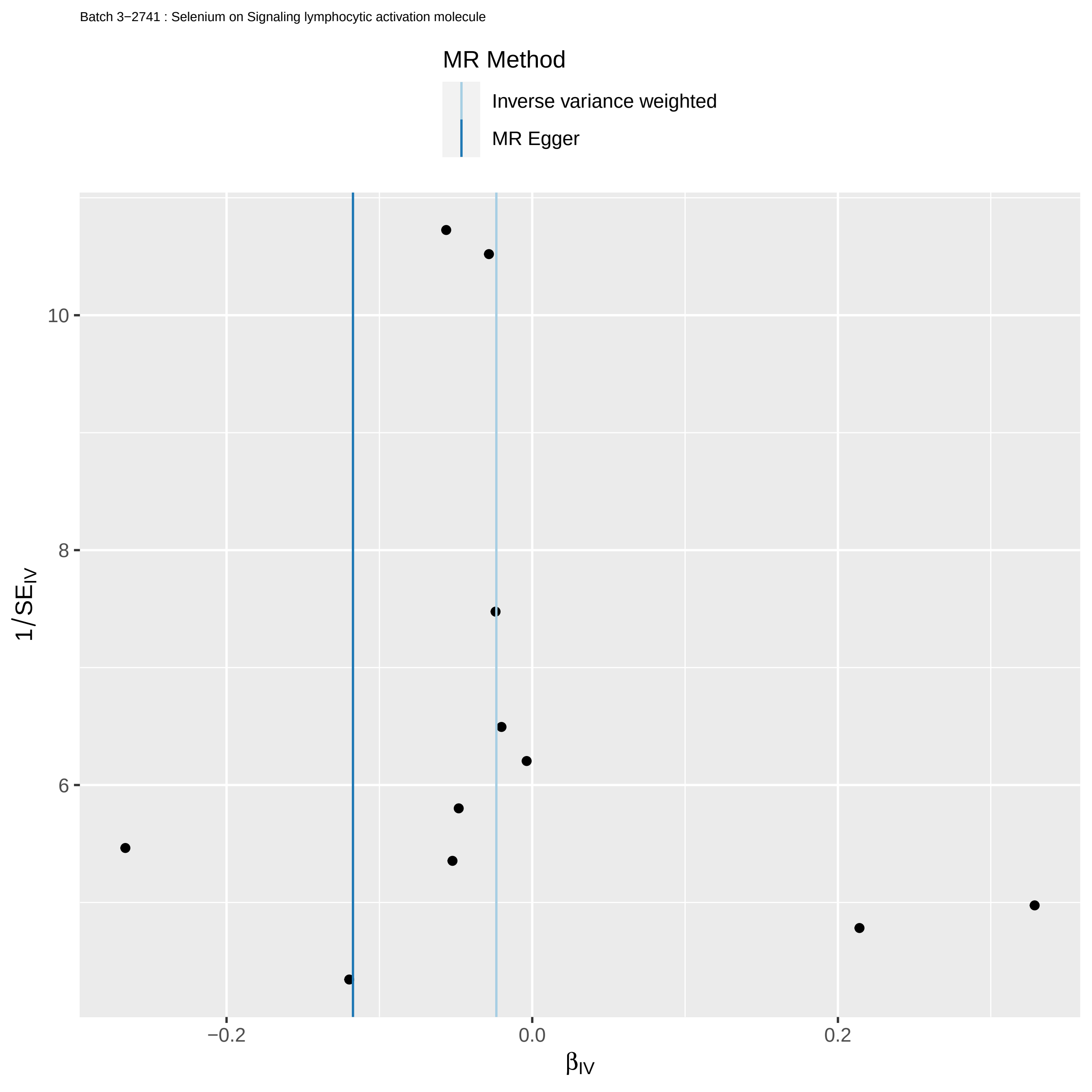

Batch 3−2741 : Selenium on Signaling lymphocytic activation molecule
MR Method
Inverse variance weighted
MR Egger
10
8
6
1/SE$_{IV}$
−0.2
0.0
0.2
β$_{IV}$

Batch 3−2742 : Selenium on SLAM family member 6

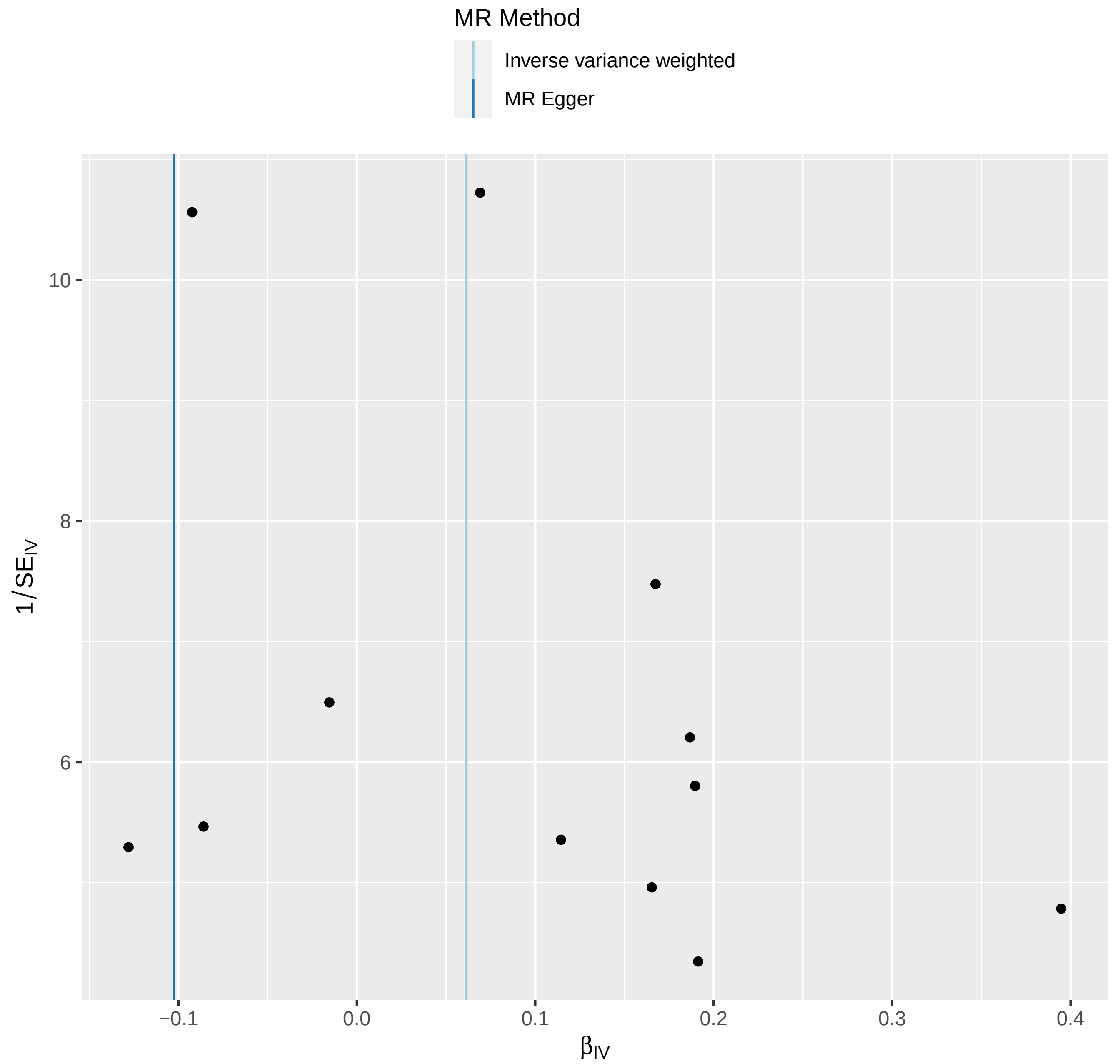

Batch 3−2743 : Selenium on SLAM family member 7

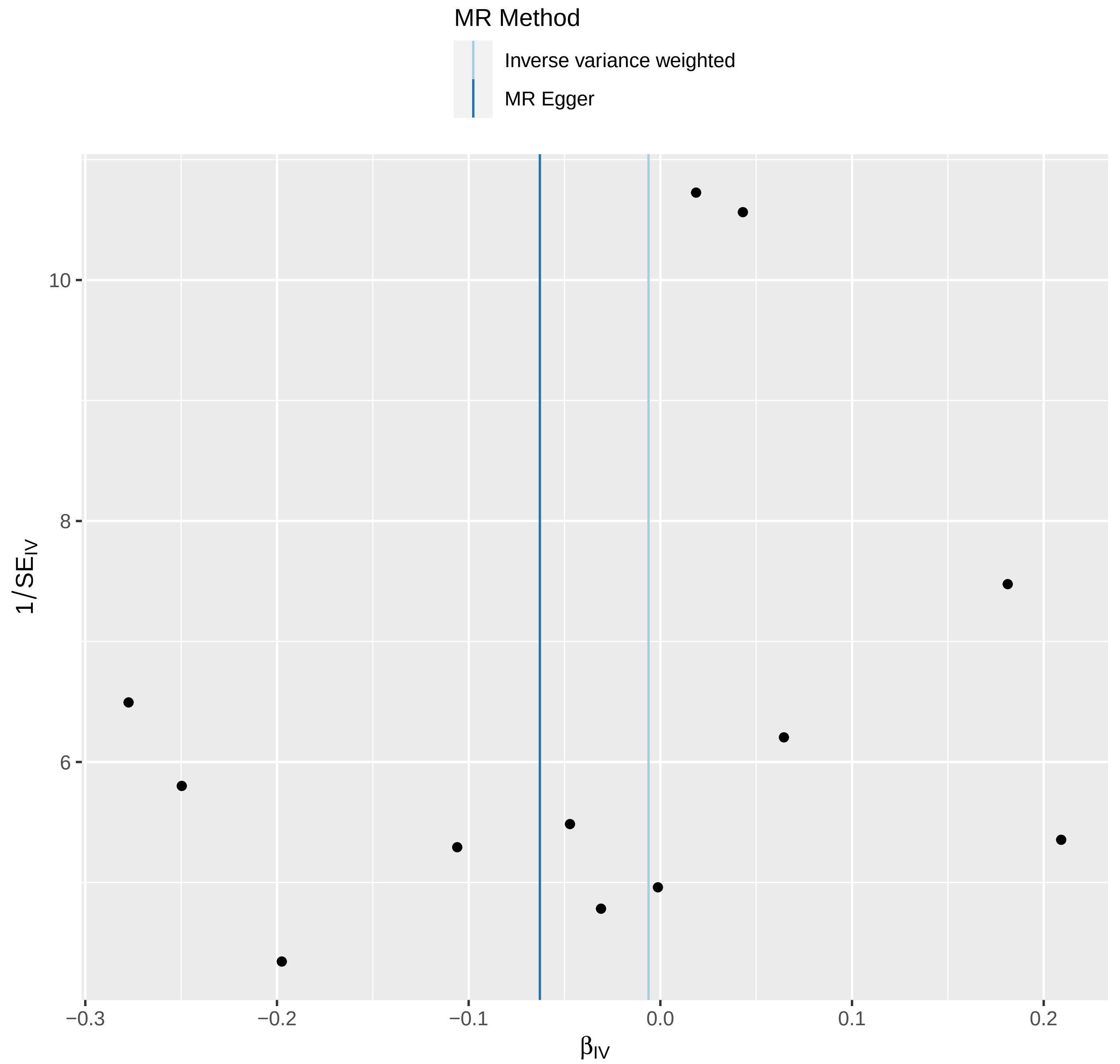

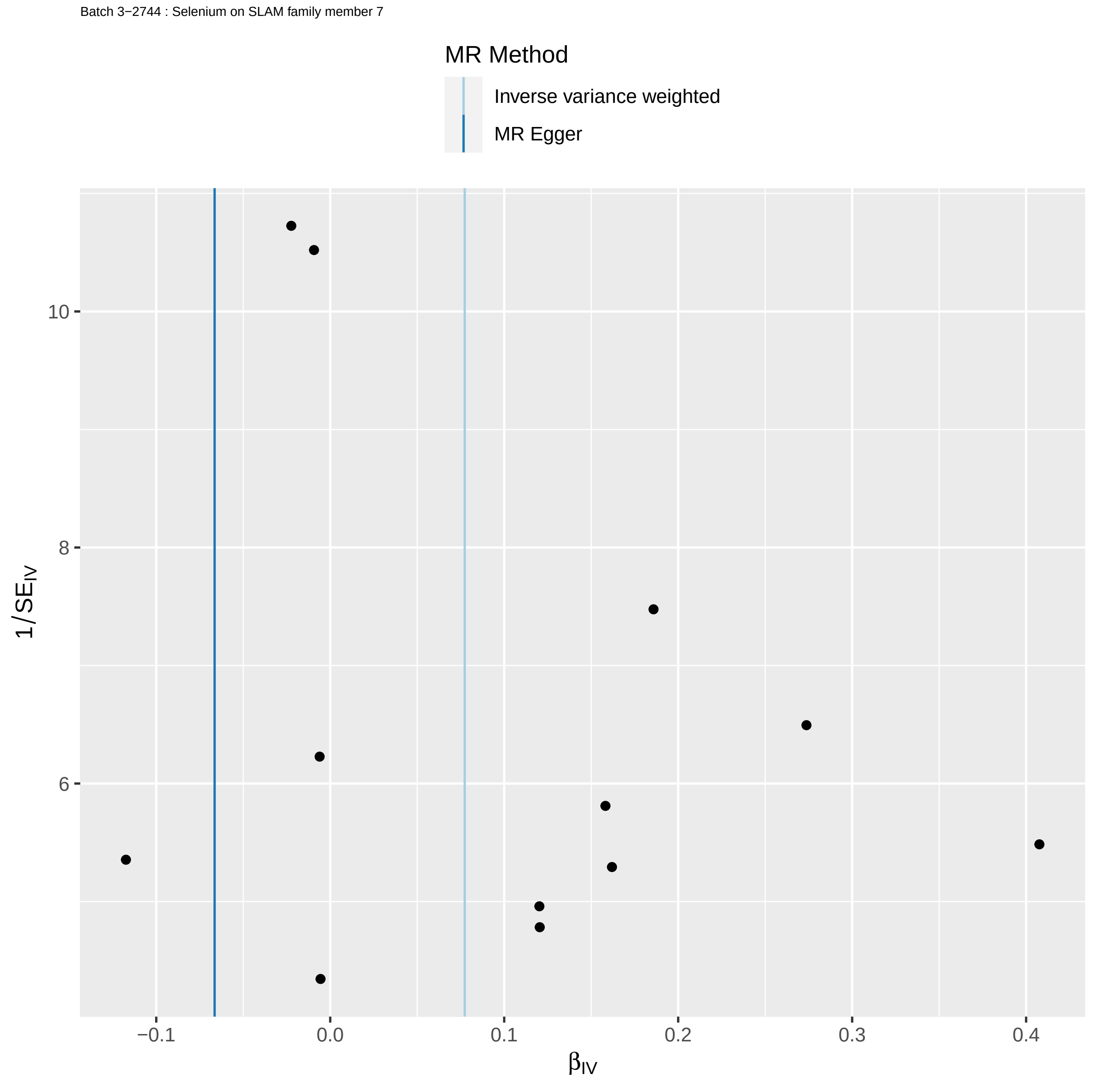

Batch 3−2744 : Selenium on SLAM family member 7
MR Method
Inverse variance weighted
MR Egger
10
8
6
1/SE$_{IV}$
−0.1
0.0
0.1
0.2
0.3
0.4
β$_{IV}$

Batch 3−2745 : Selenium on SLAM family member 8

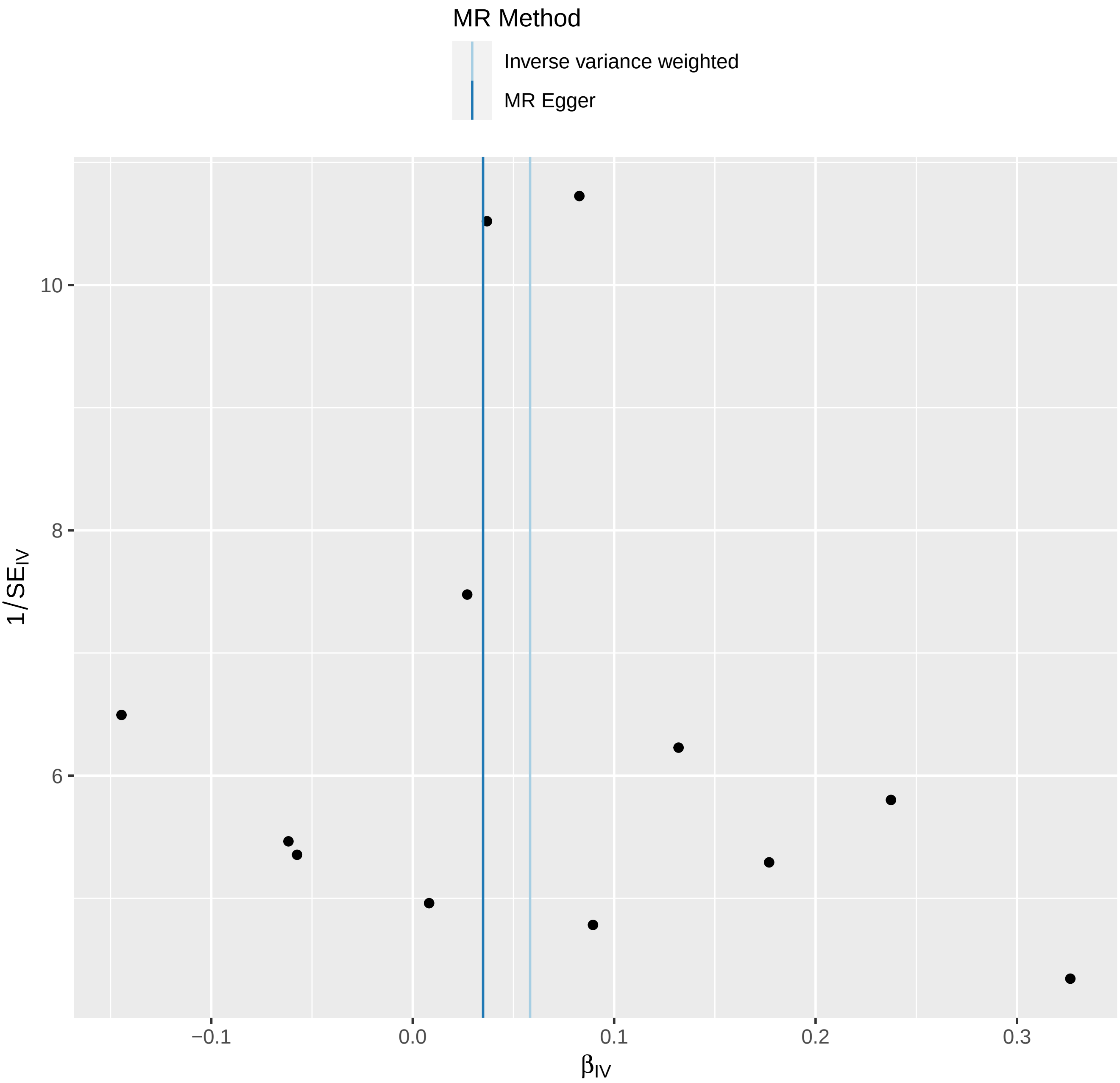

Batch 3−2746 : Selenium on Urea transporter 1

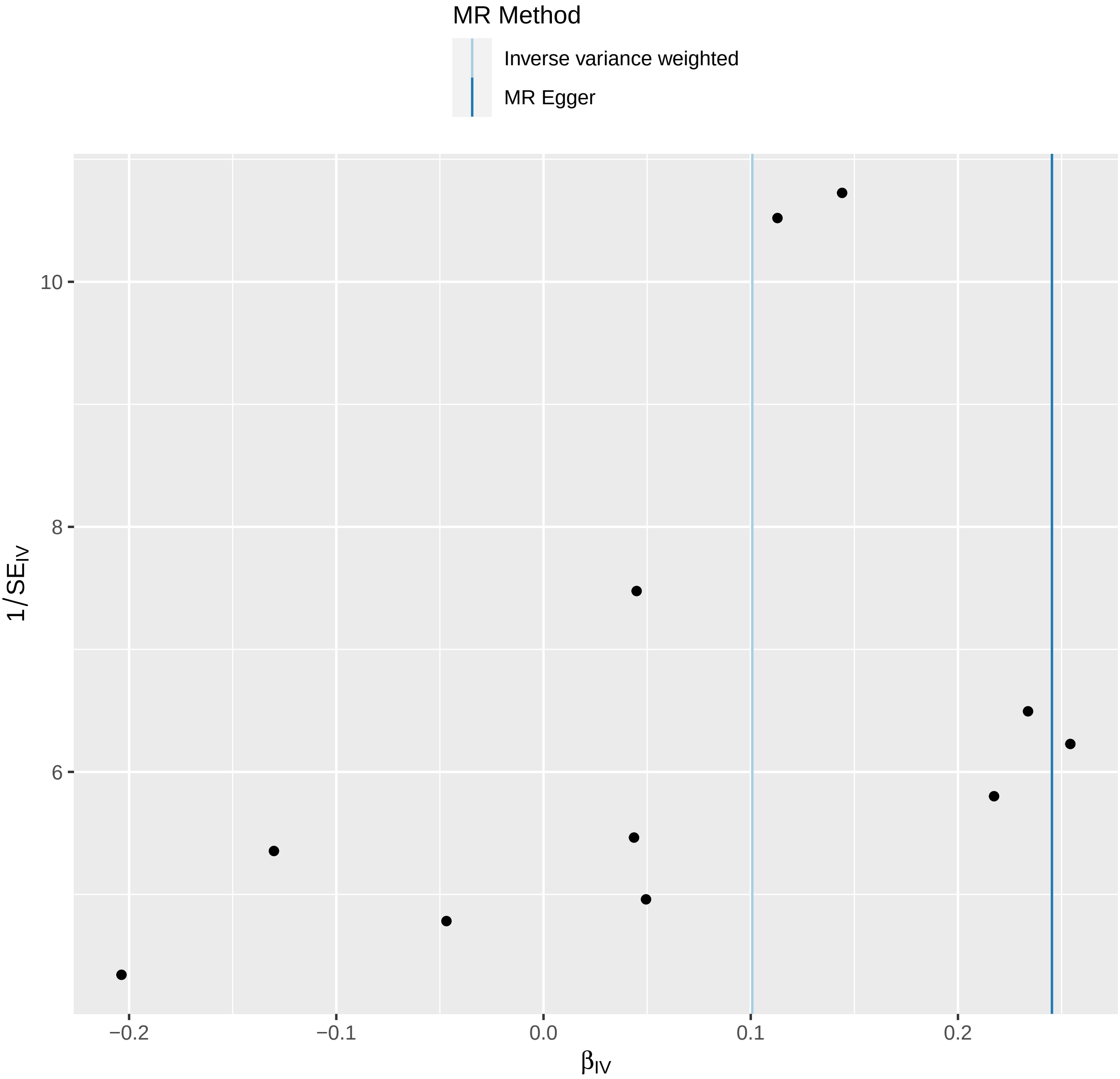

Batch 3−2747 : Selenium on Urea transporter 2

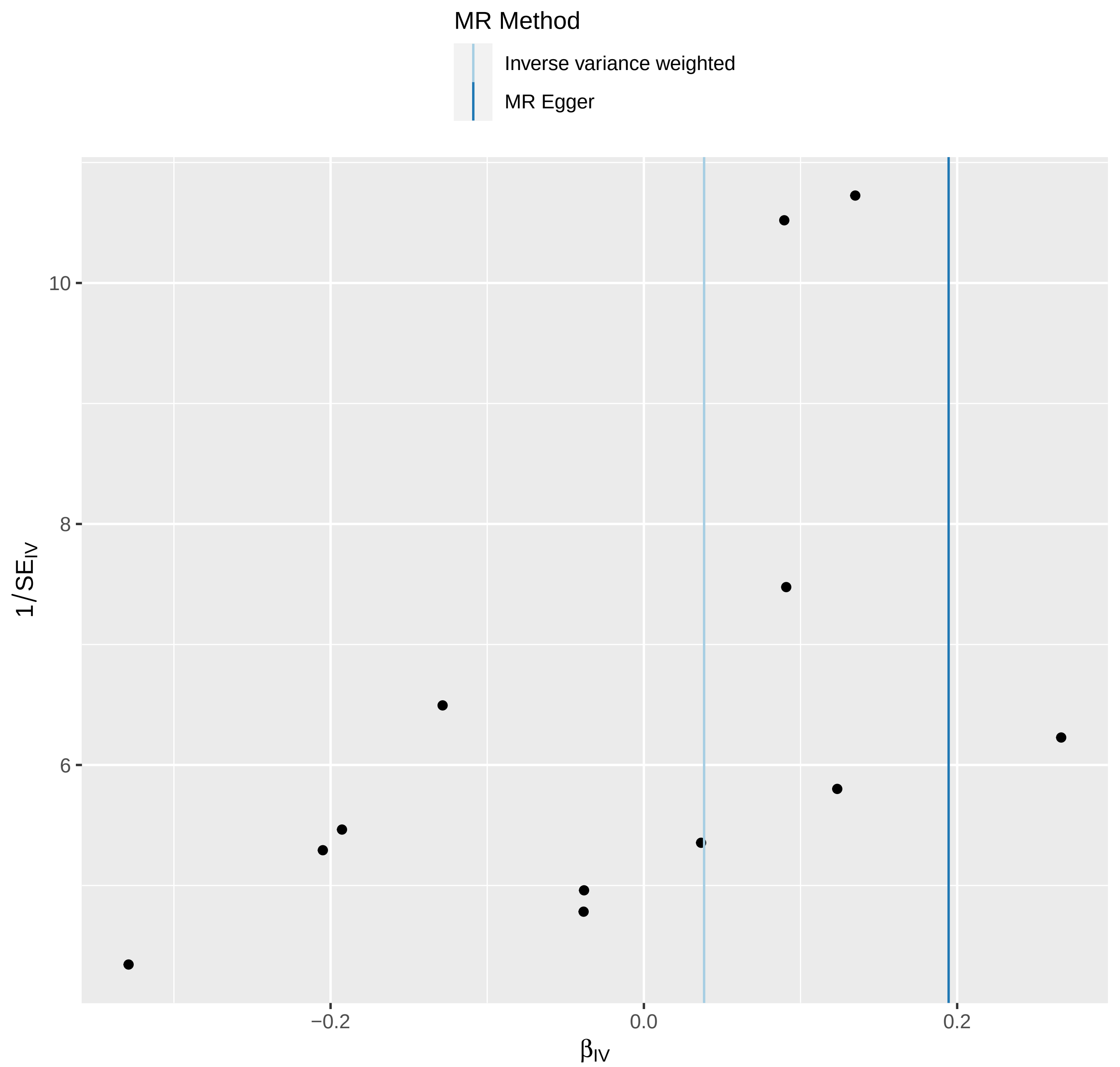

Batch 3−2748 : Selenium on Solute carrier family 22 member 16

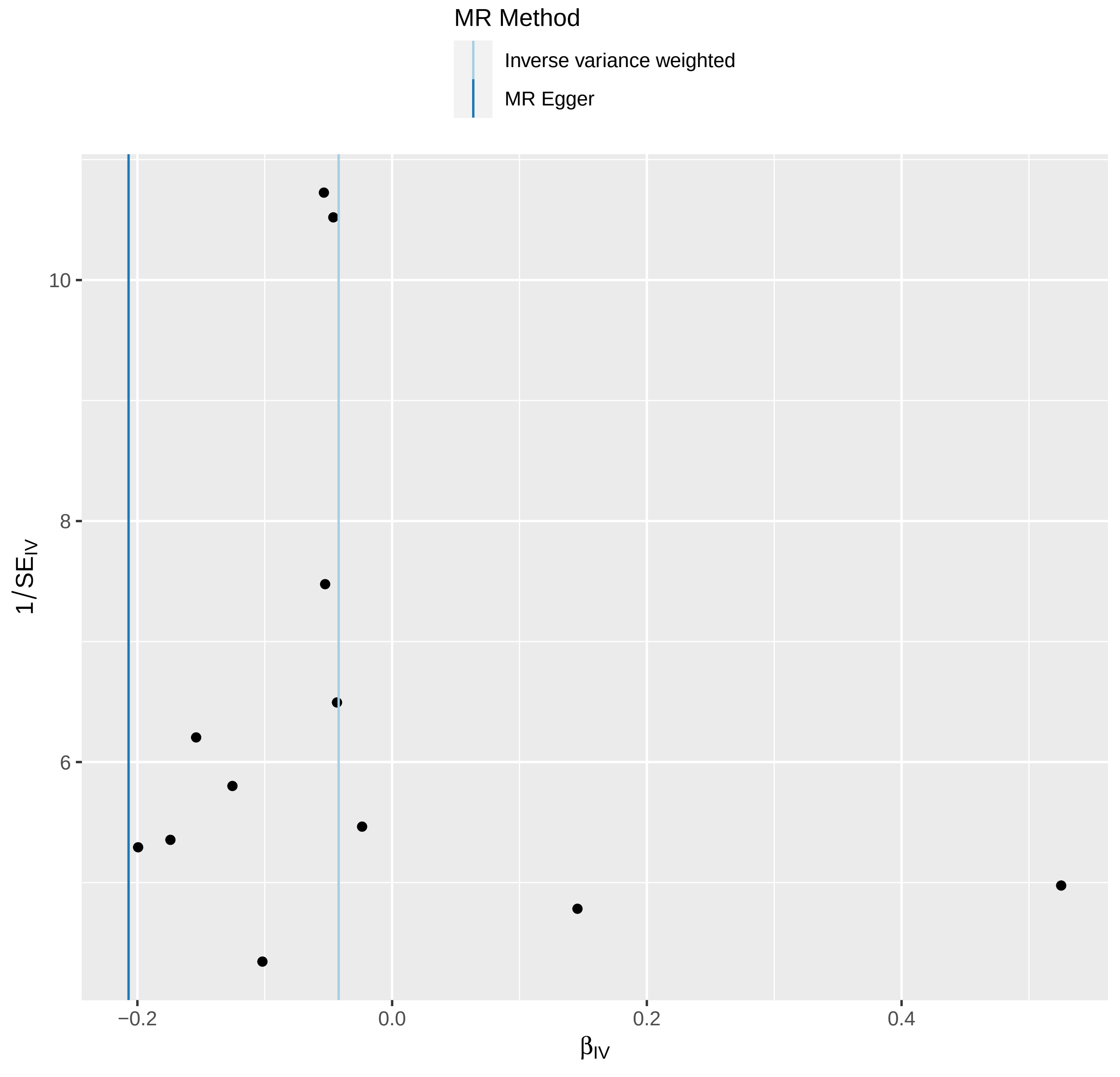

Batch 3−2749 : Selenium on Mitochondrial glutamate carrier 2

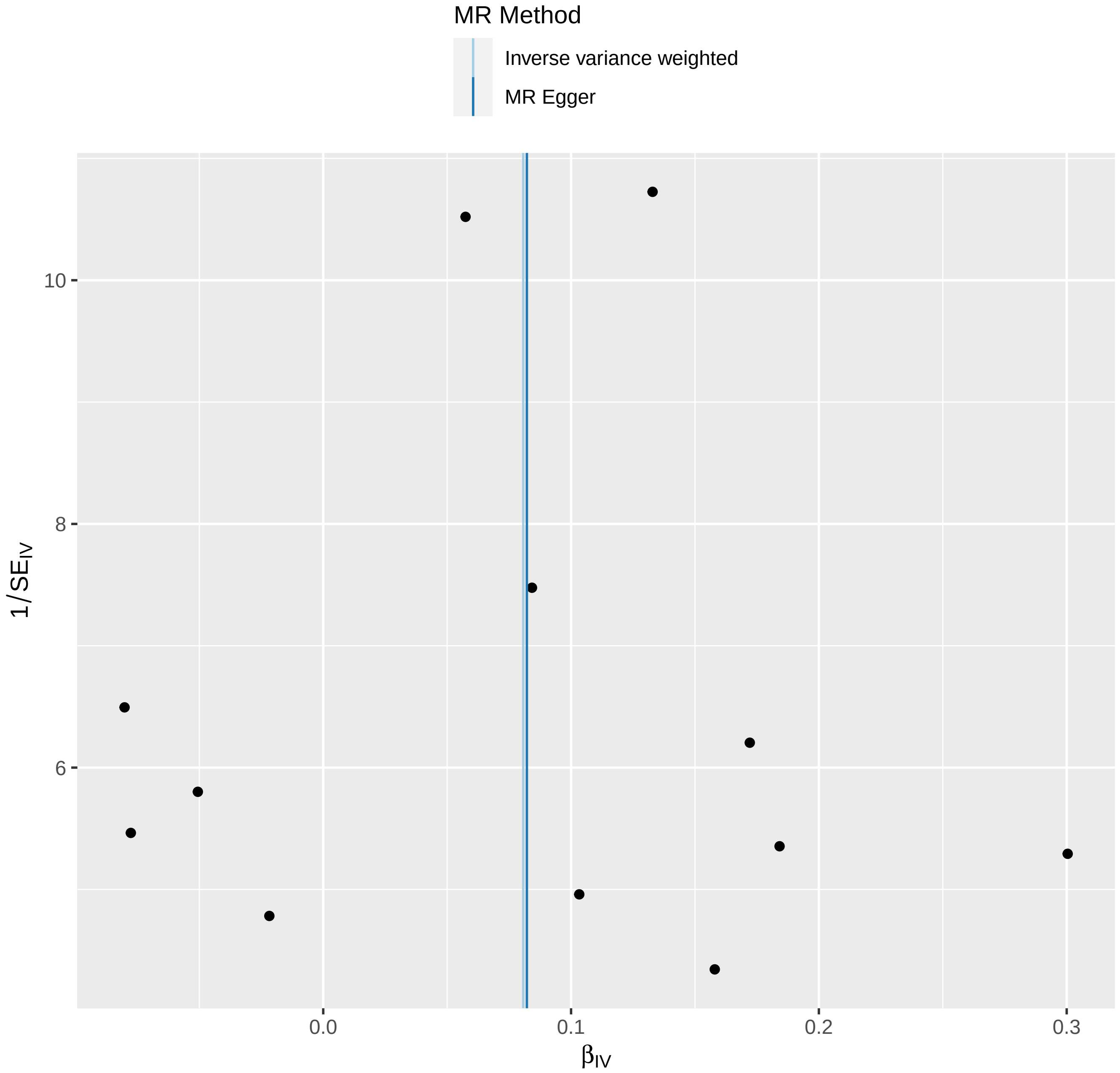

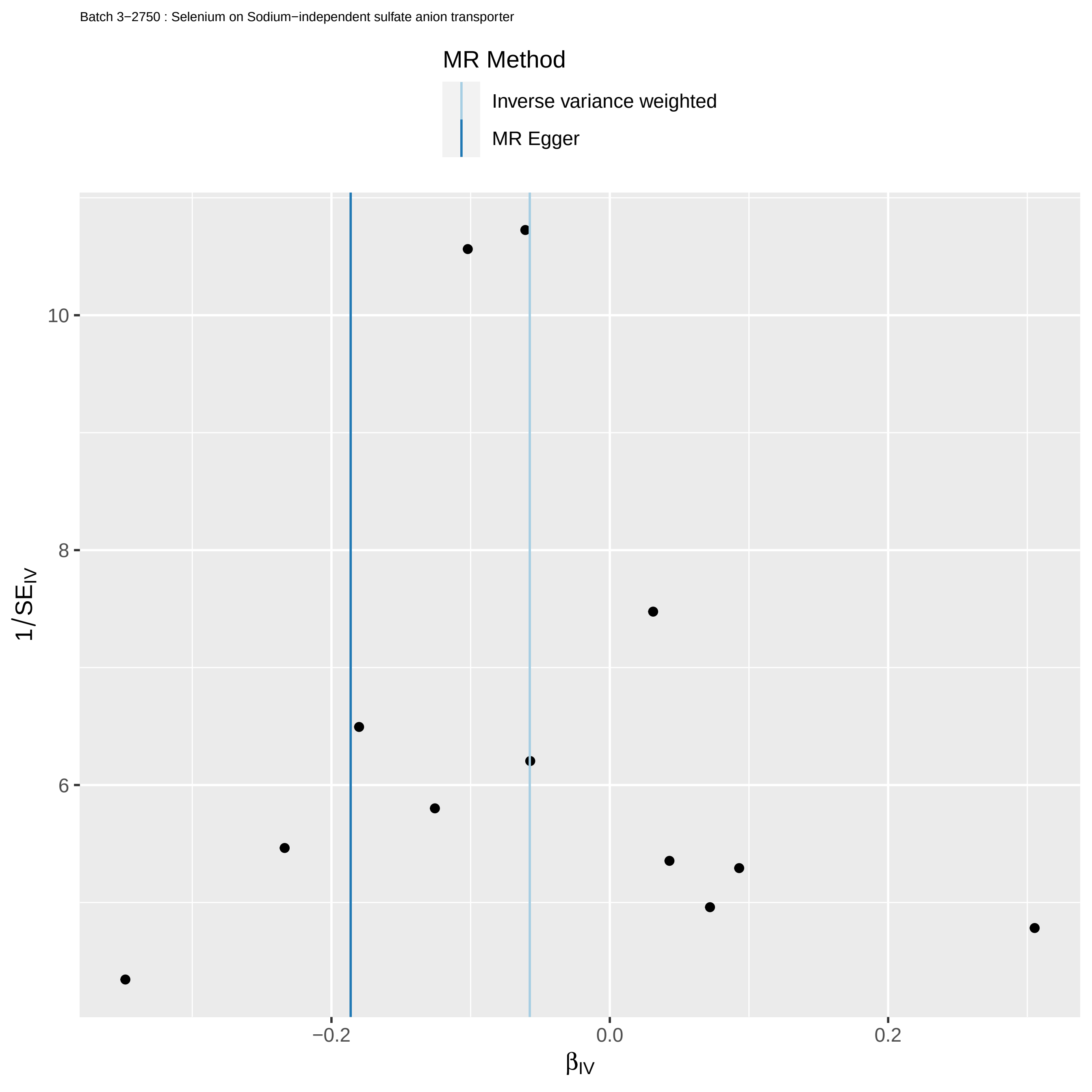

Batch 3−2750 : Selenium on Sodium−independent sulfate anion transporter
MR Method
Inverse variance weighted
MR Egger
10
8
6
1/SE$_{IV}$
−0.2
0.0
0.2
β$_{IV}$

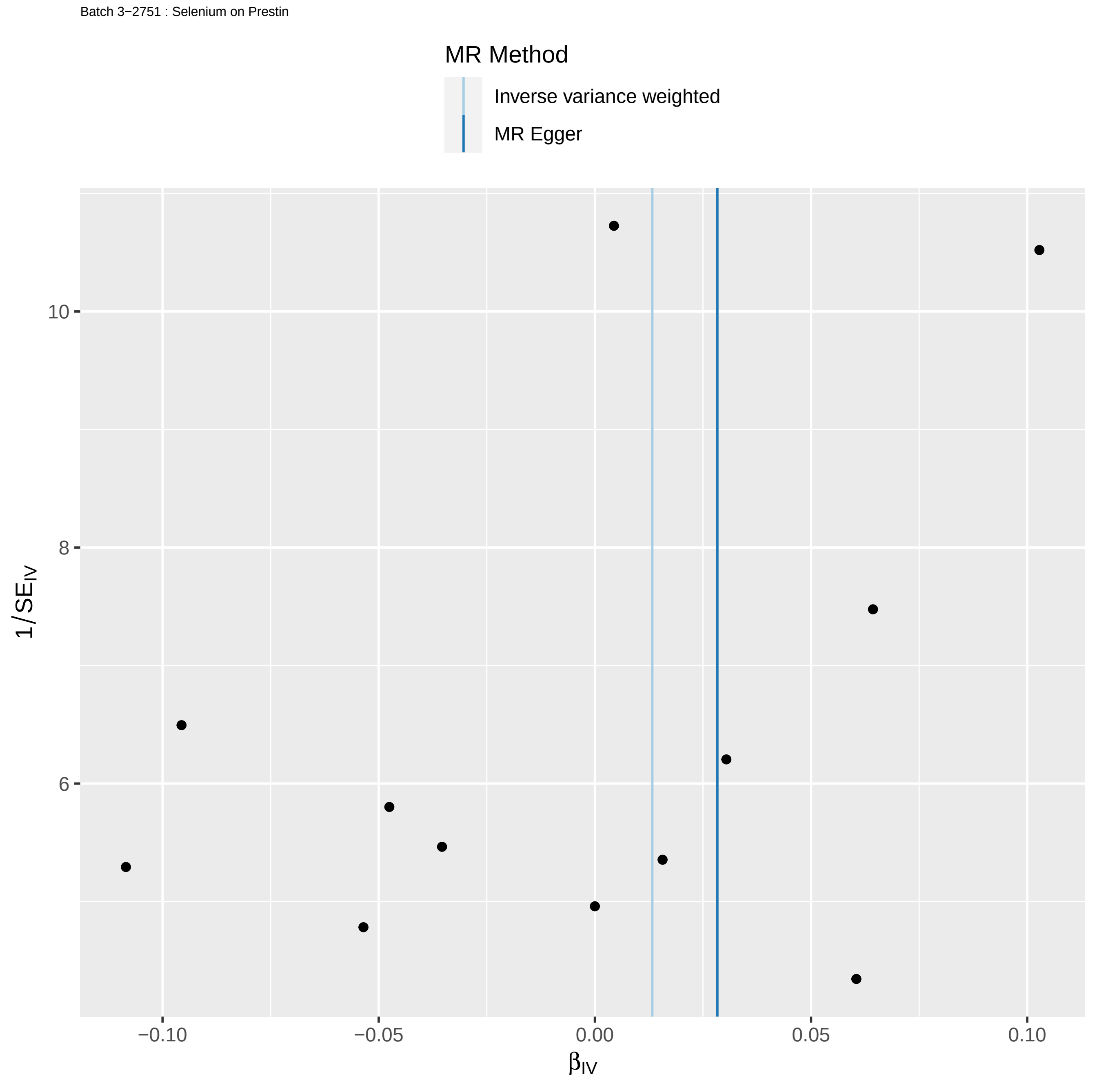
Batch 3−2751 : Selenium on Prestin
MR Method
Inverse variance weighted
MR Egger
10
8
6
1/SE_IV
−0.10
−0.05
0.00
0.05
0.10
β_IV

Batch 3−2752 : Selenium on Very long−chain acyl−CoA synthetase

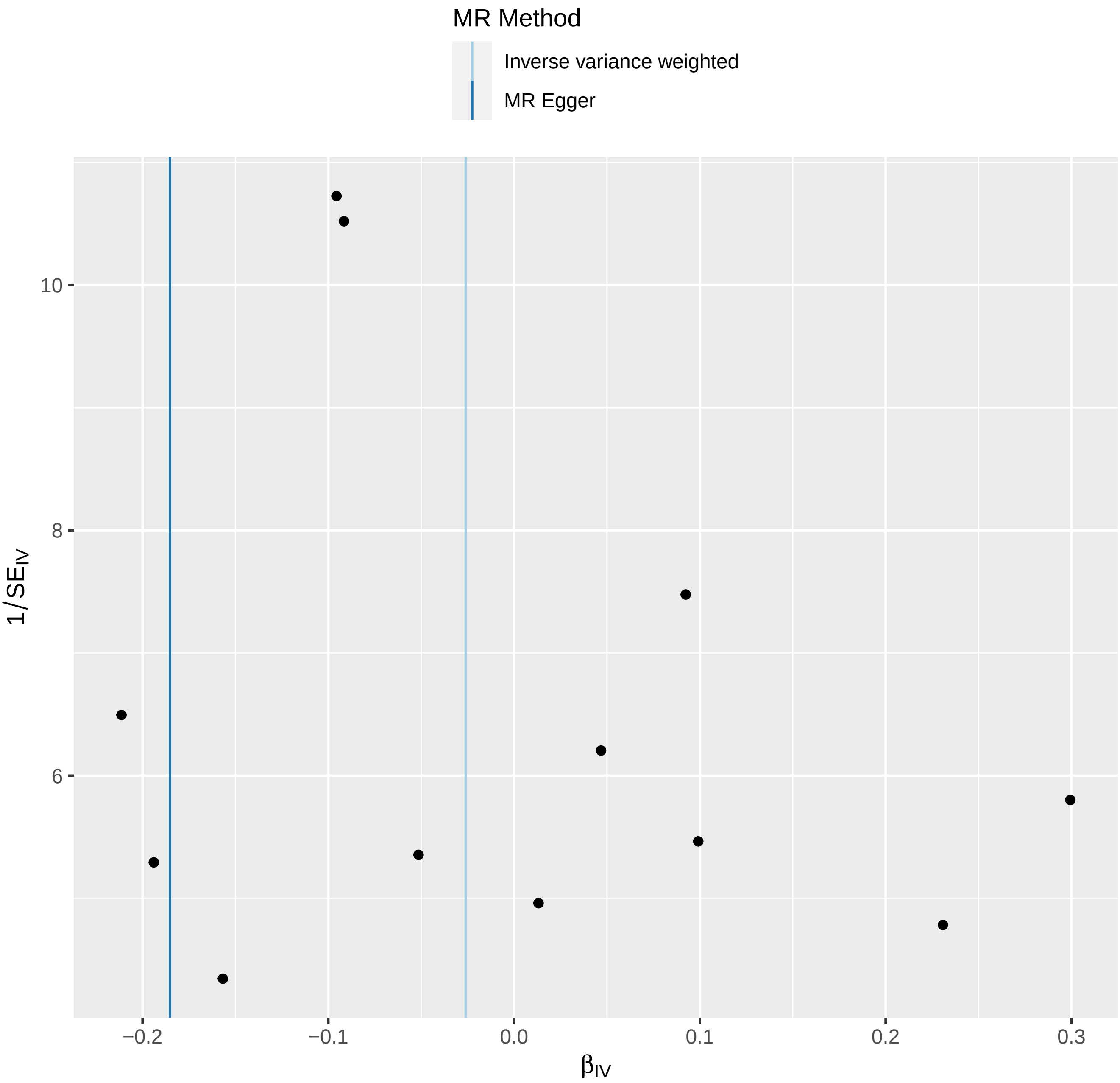

Batch 3−2753 : Selenium on Zinc transporter 3

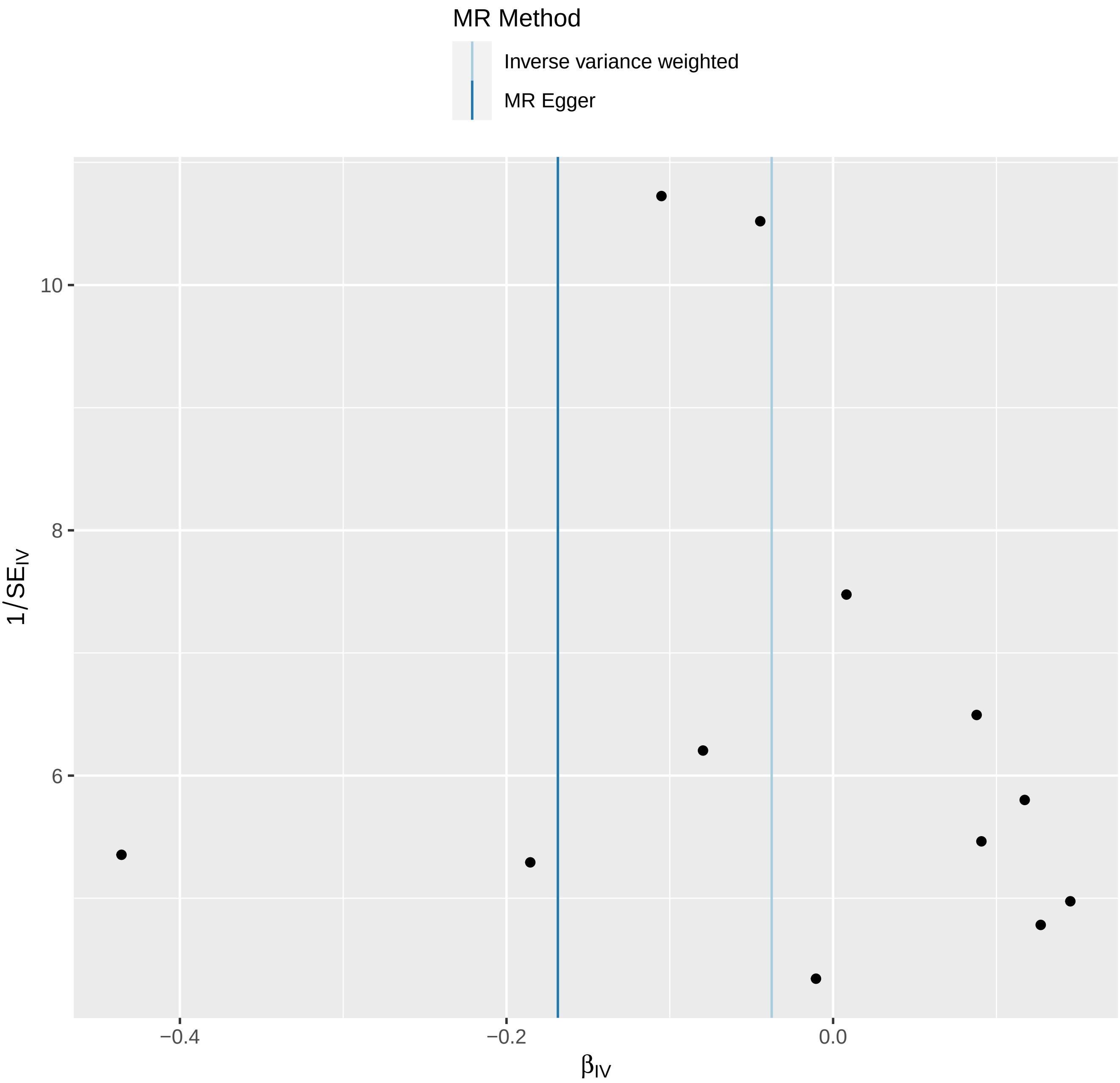

Batch 3−2754 : Selenium on Zinc transporter 5

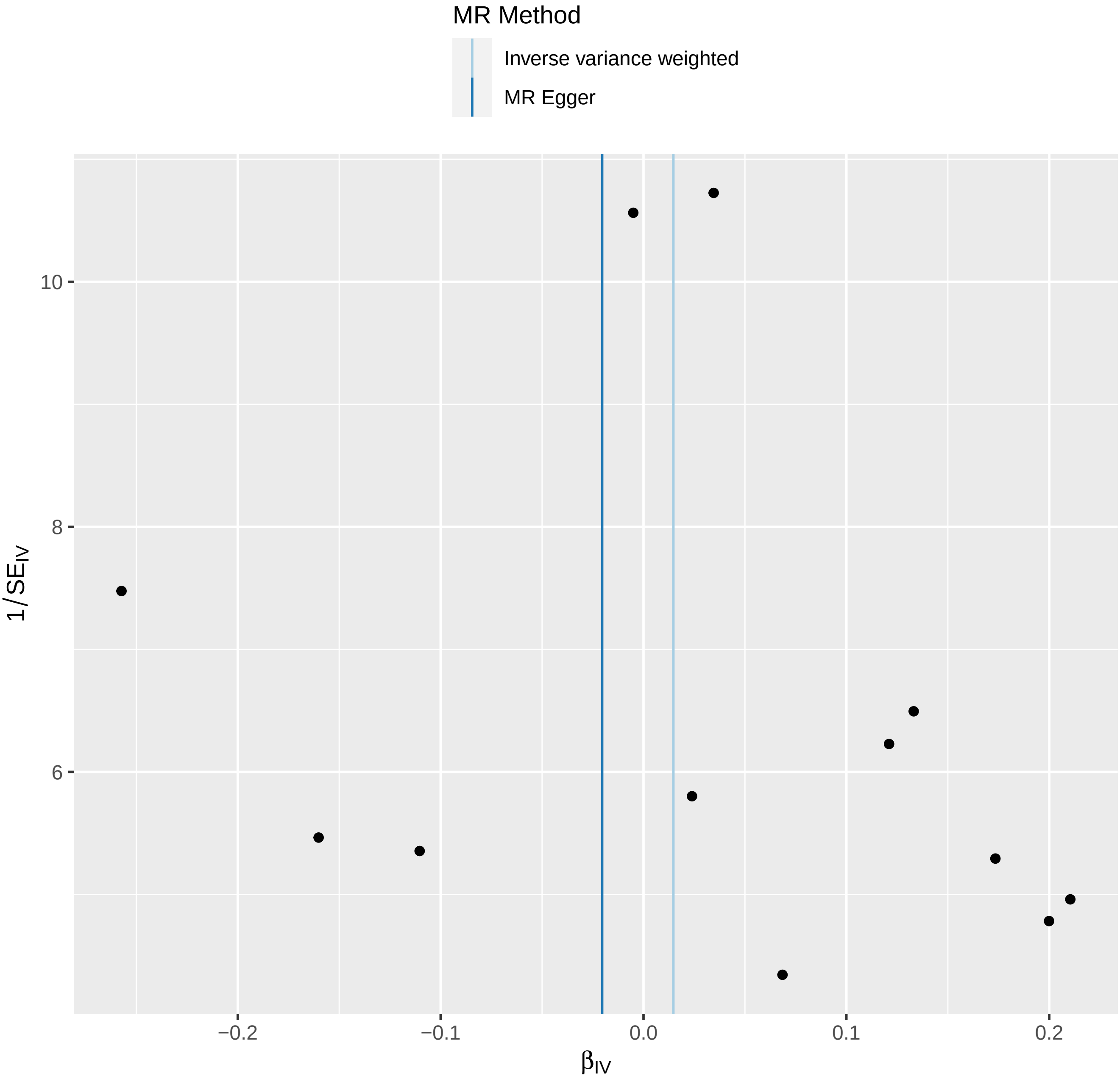

Batch 3−2755 : Selenium on Adenosine 3'−phospho 5'−phosphosulfate transporter 2

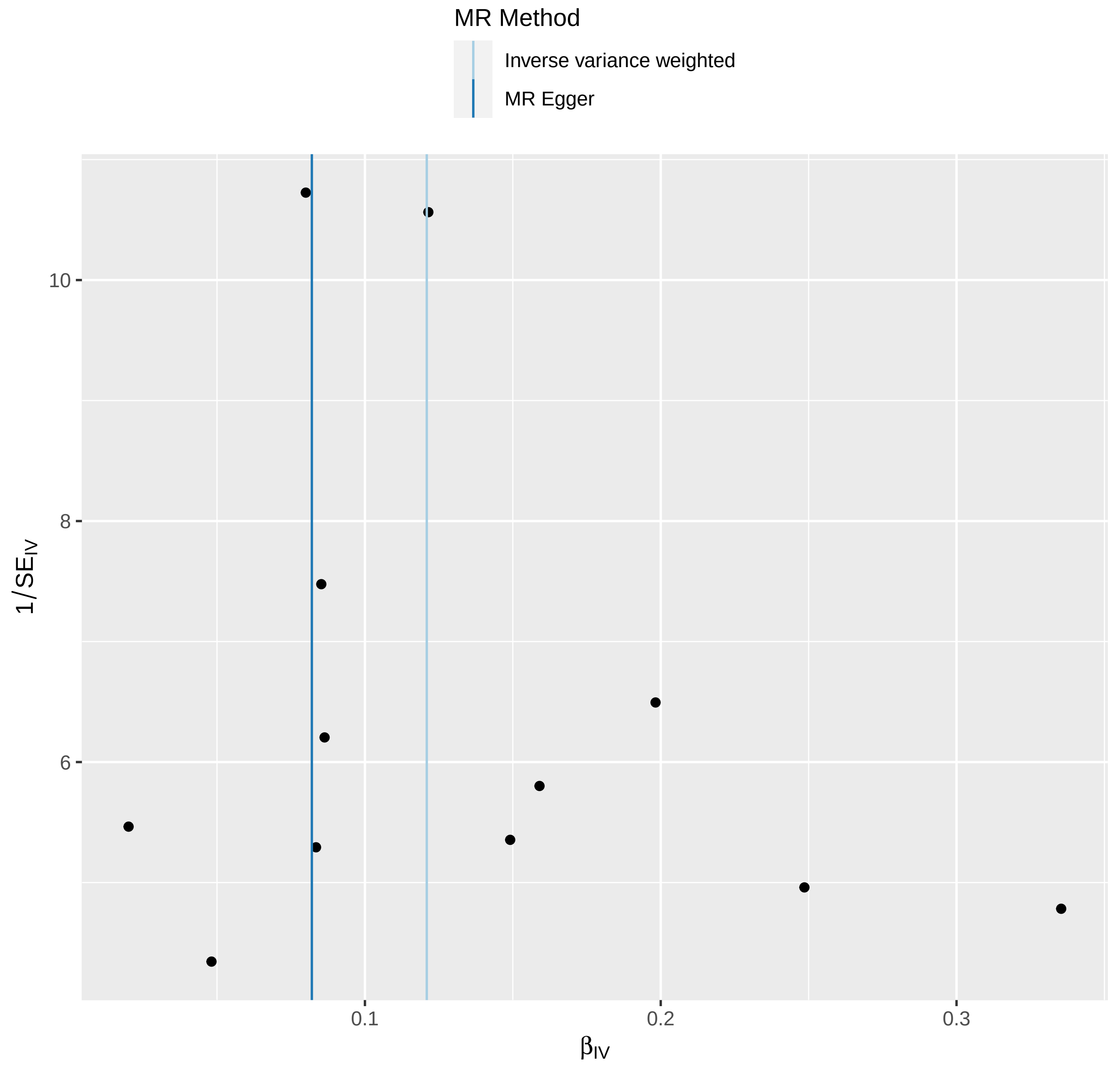

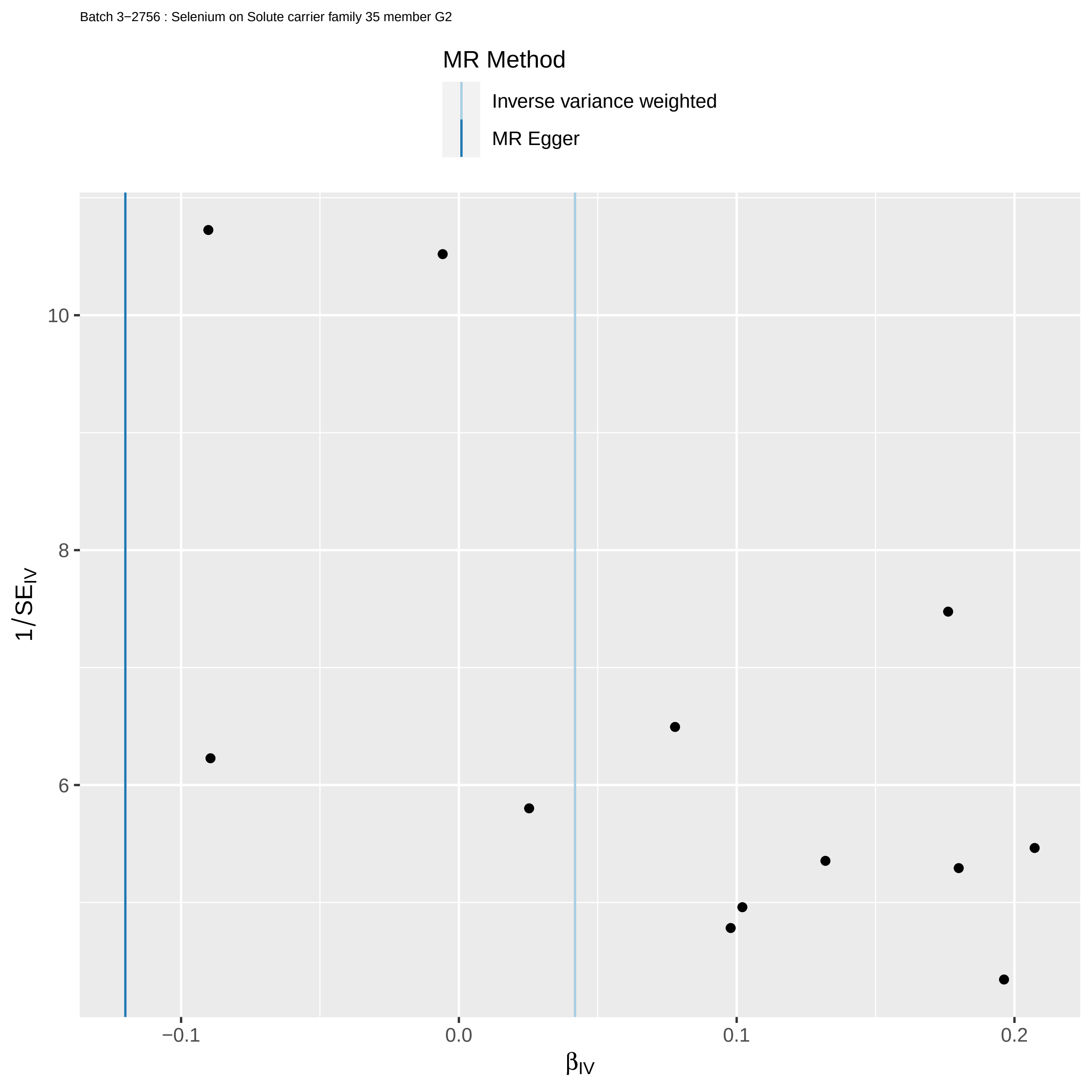
Batch 3−2756 : Selenium on Solute carrier family 35 member G2
MR Method
Inverse variance weighted
MR Egger
10
8
6
1/SE$_{IV}$
−0.1
0.0
0.1
0.2
β$_{IV}$

Batch 3−2757 : Selenium on Solute carrier family 41 member 2

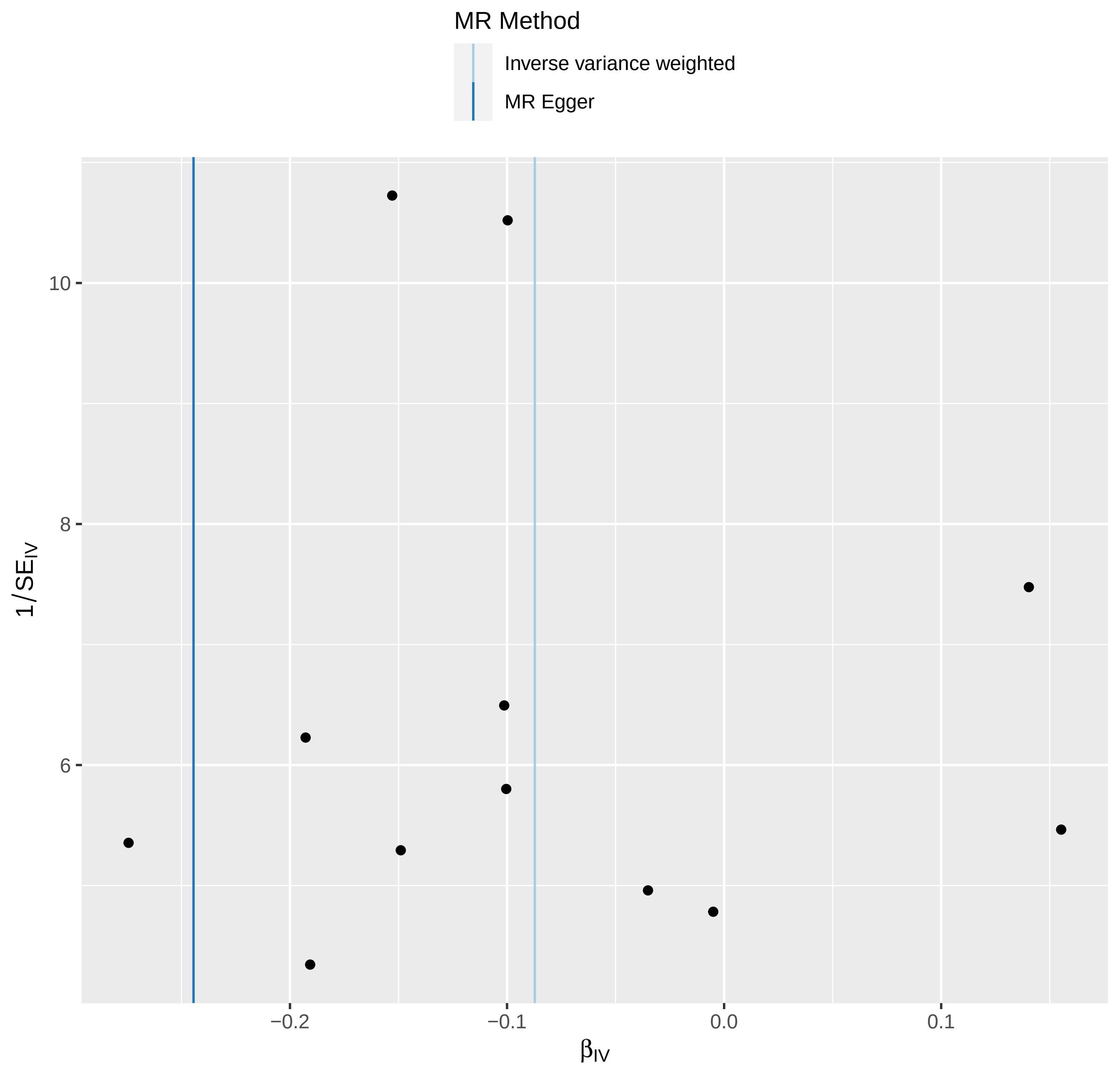

Batch 3−2758 : Selenium on Electroneutral sodium bicarbonate exchanger 1

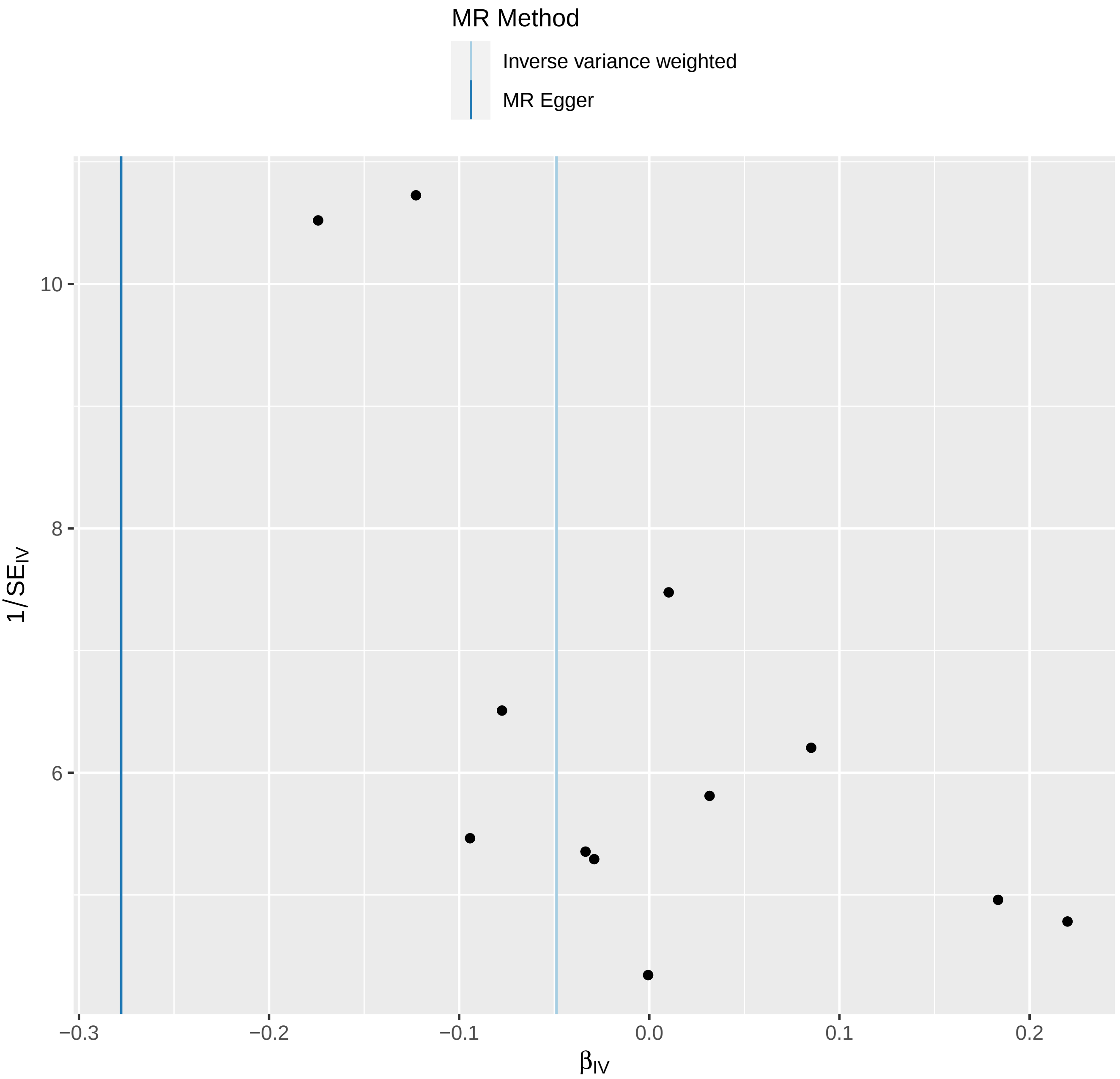

Batch 3−2759 : Selenium on Sodium/iodide cotransporter

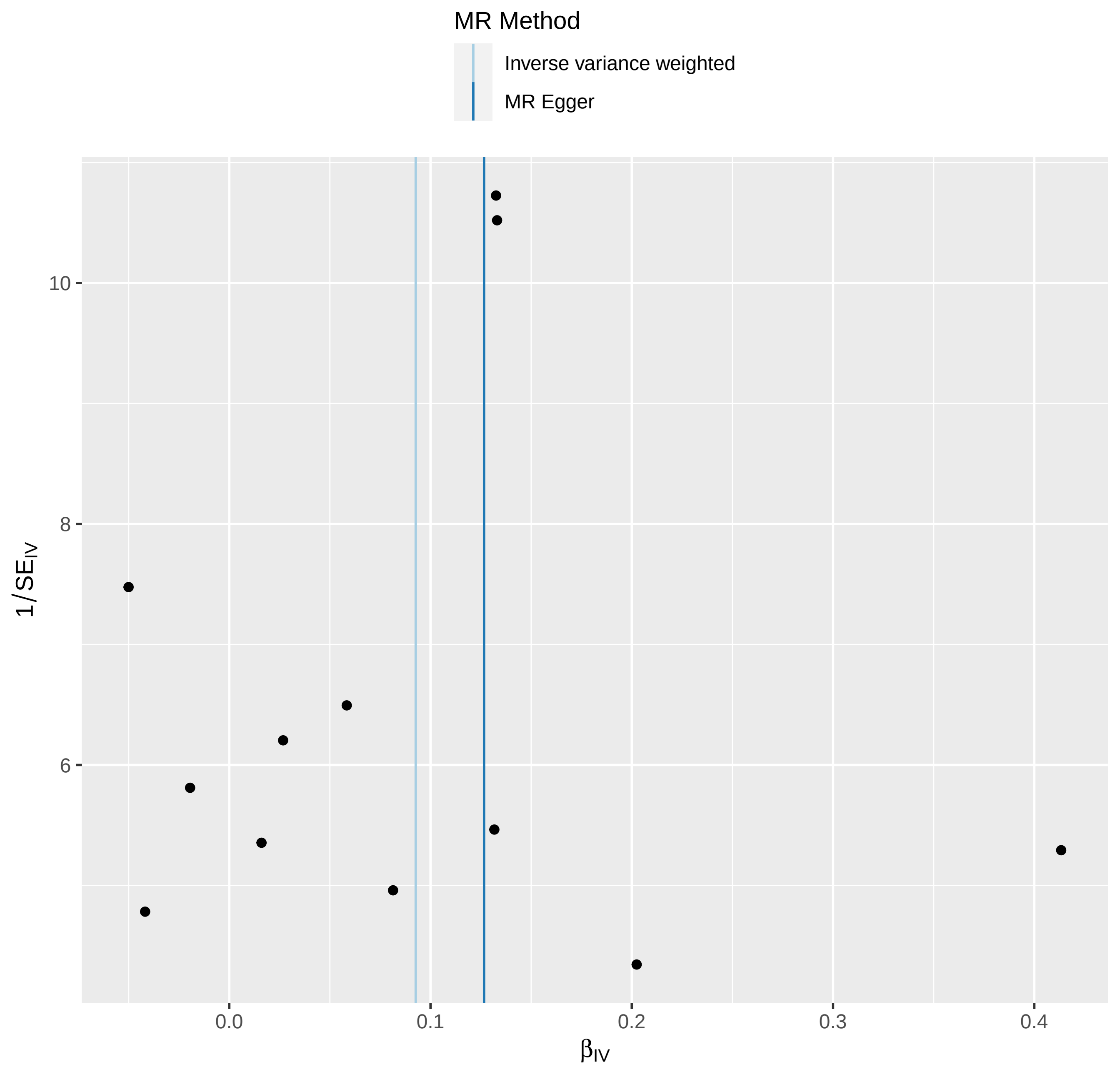

Batch 3−2760 : Selenium on Sodium−coupled monocarboxylate transporter 1

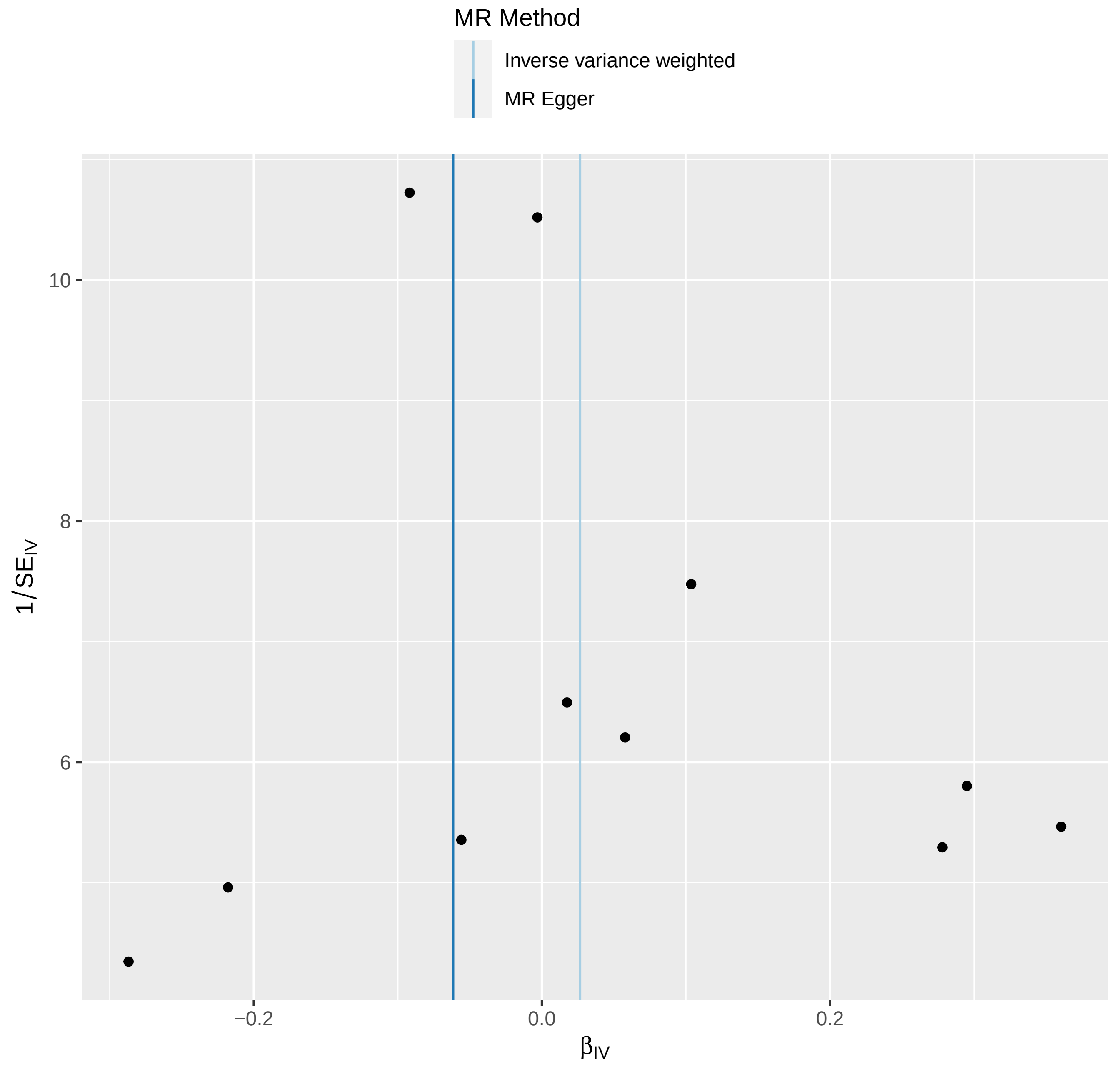

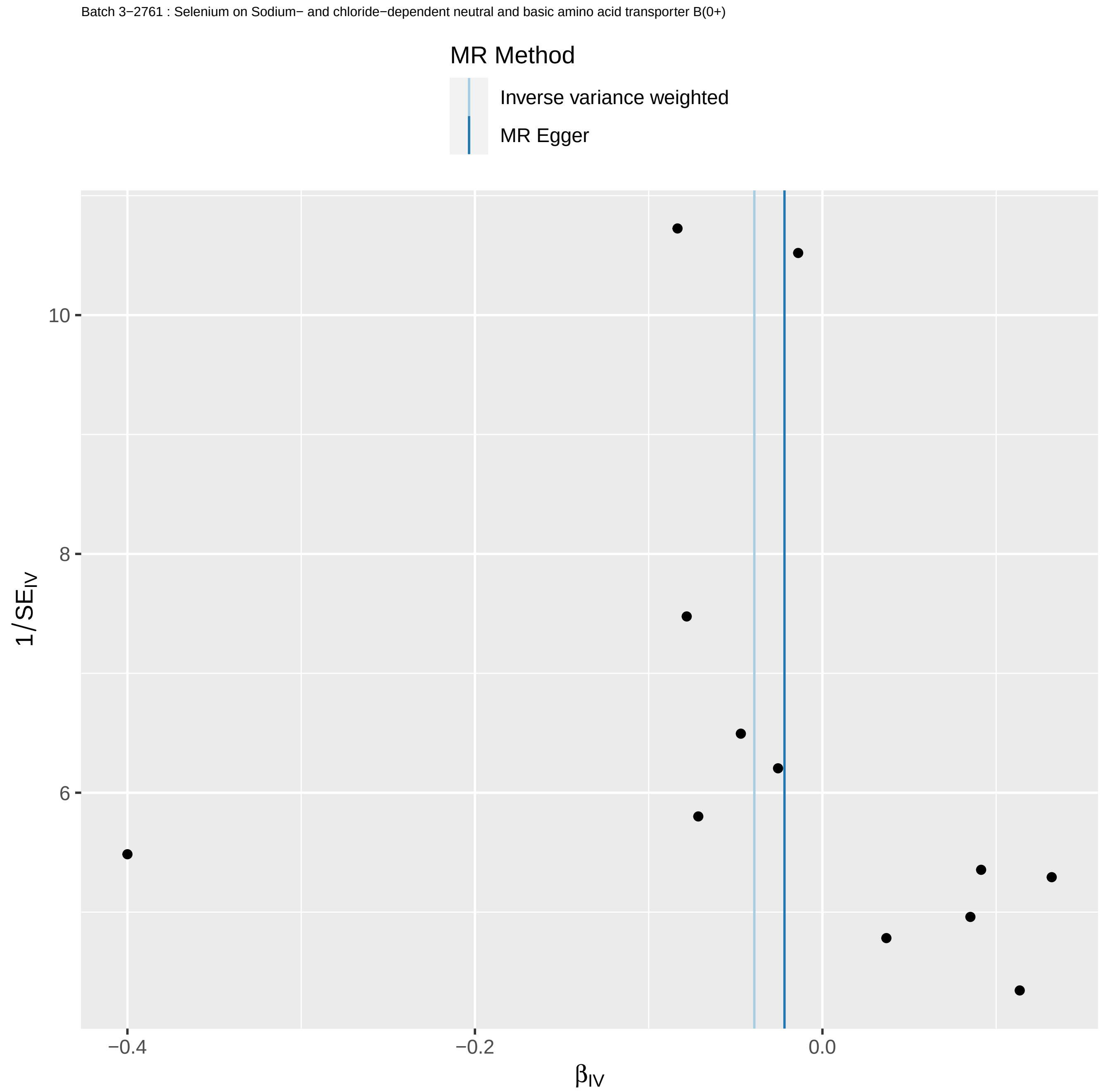
Batch 3−2761 : Selenium on Sodium− and chloride−dependent neutral and basic amino acid transporter B(0+)
MR Method
Inverse variance weighted
MR Egger
10
8
6
1/SE_IV
−0.4
−0.2
0.0
β_IV

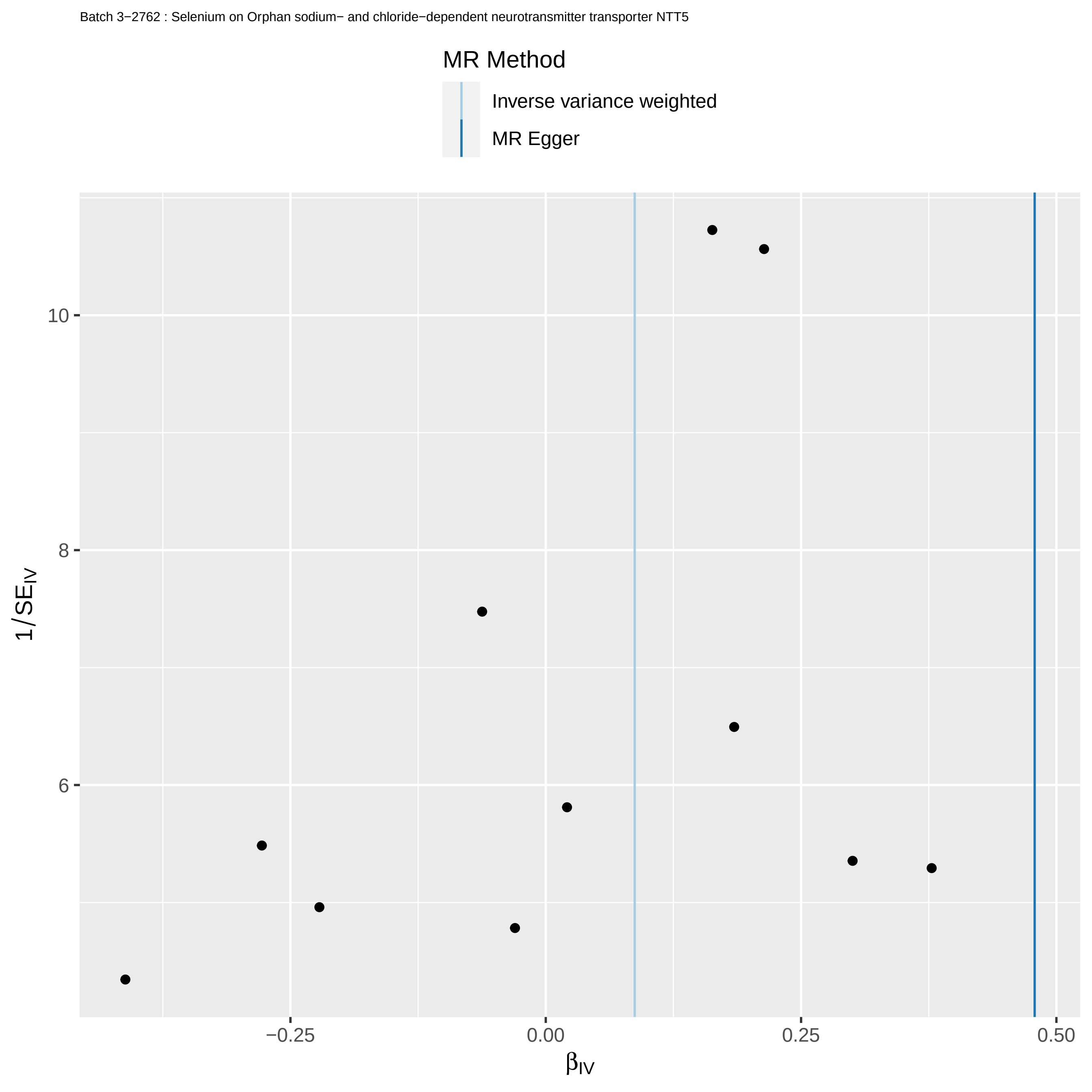

Batch 3−2762 : Selenium on Orphan sodium− and chloride−dependent neurotransmitter transporter NTT5
MR Method
Inverse variance weighted
MR Egger
10
8
6
1/SE$_{IV}$
−0.25
0.00
0.25
0.50
β$_{IV}$

Batch 3−2763 : Selenium on Sodium− and chloride−dependent glycine transporter 1

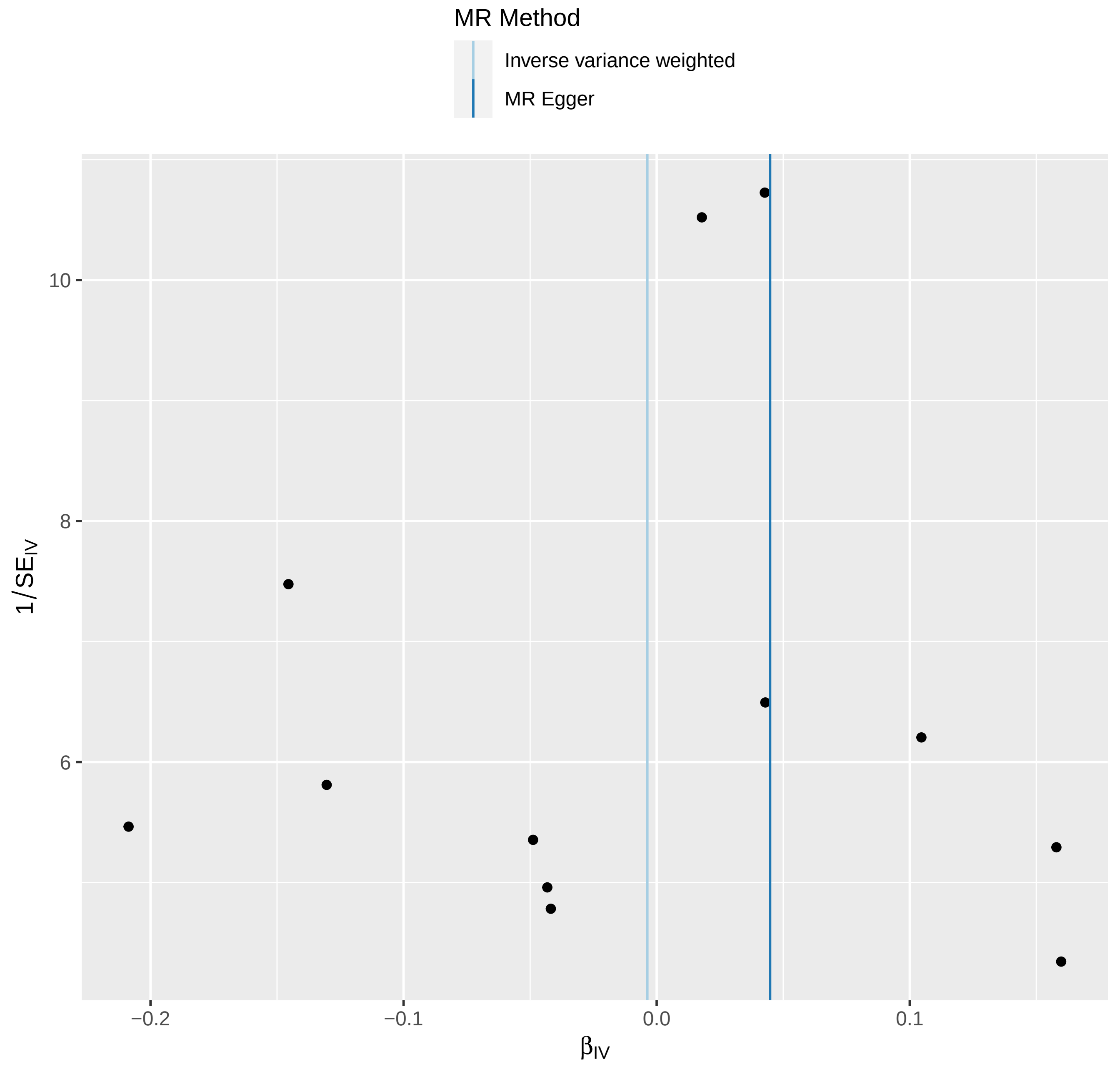

Batch 3−2764 : Selenium on Mitochondrial sodium/hydrogen exchanger 9B2

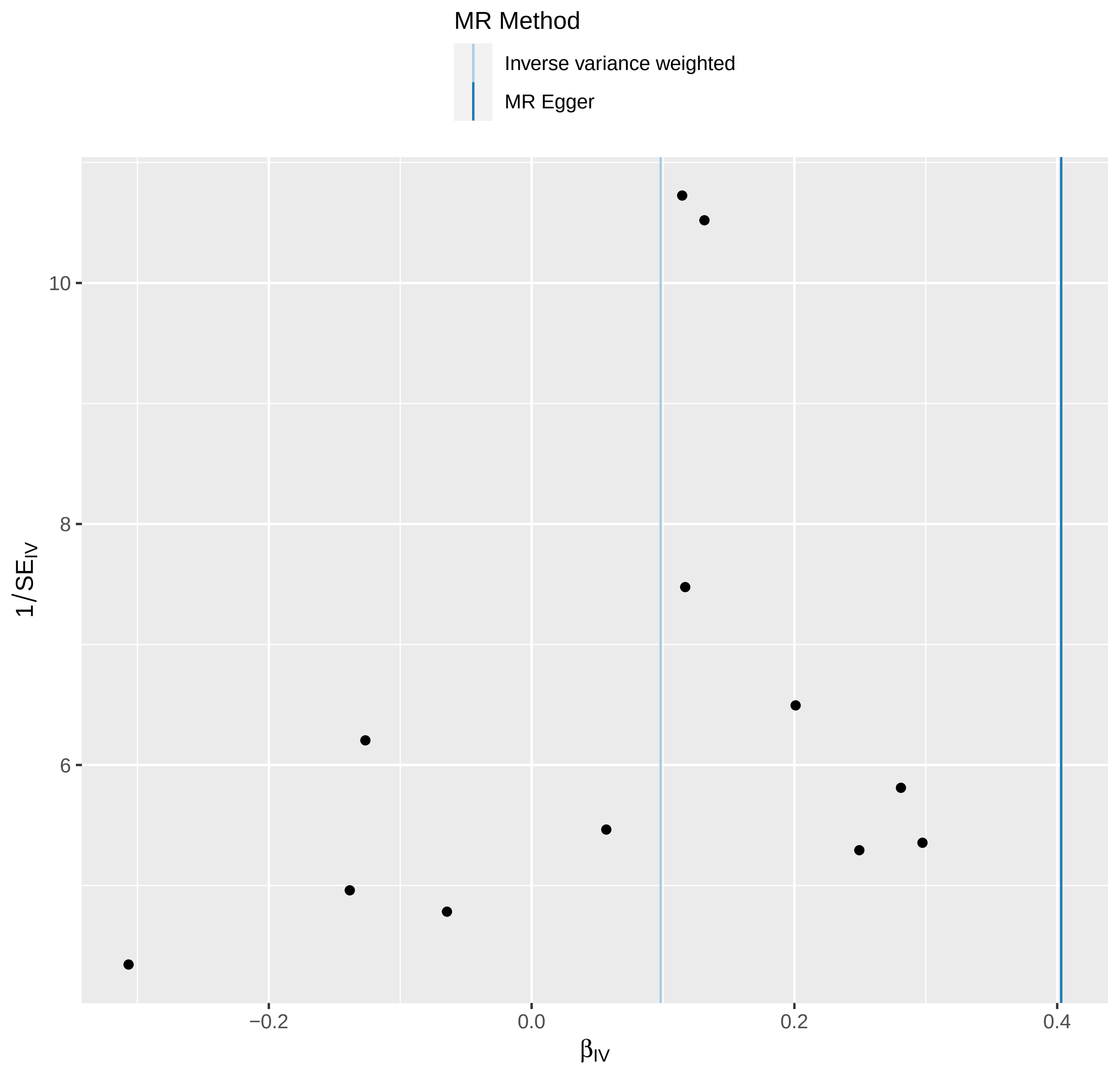

Batch 3−2765 : Selenium on Solute carrier organic anion transporter family member 5A1

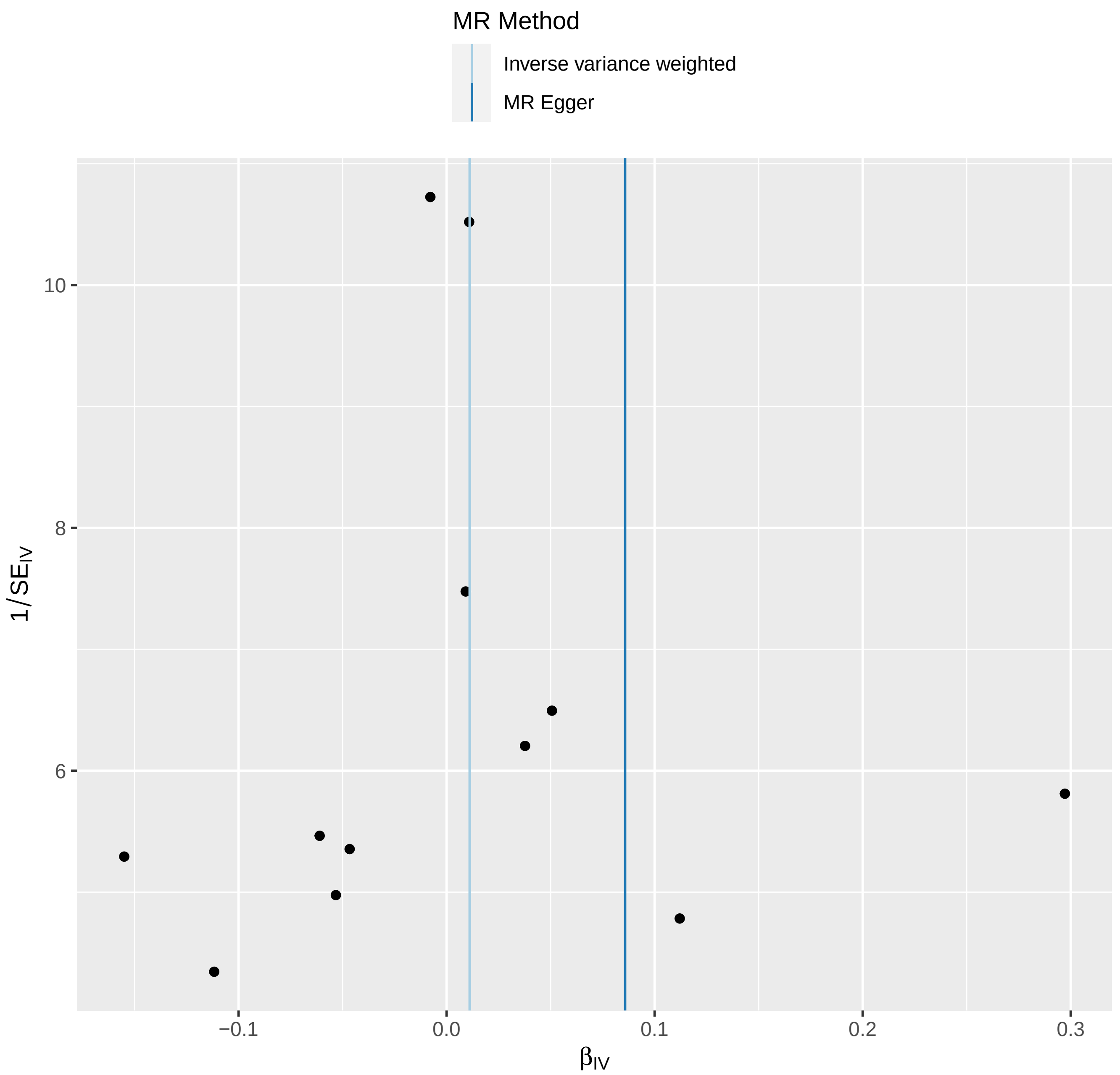

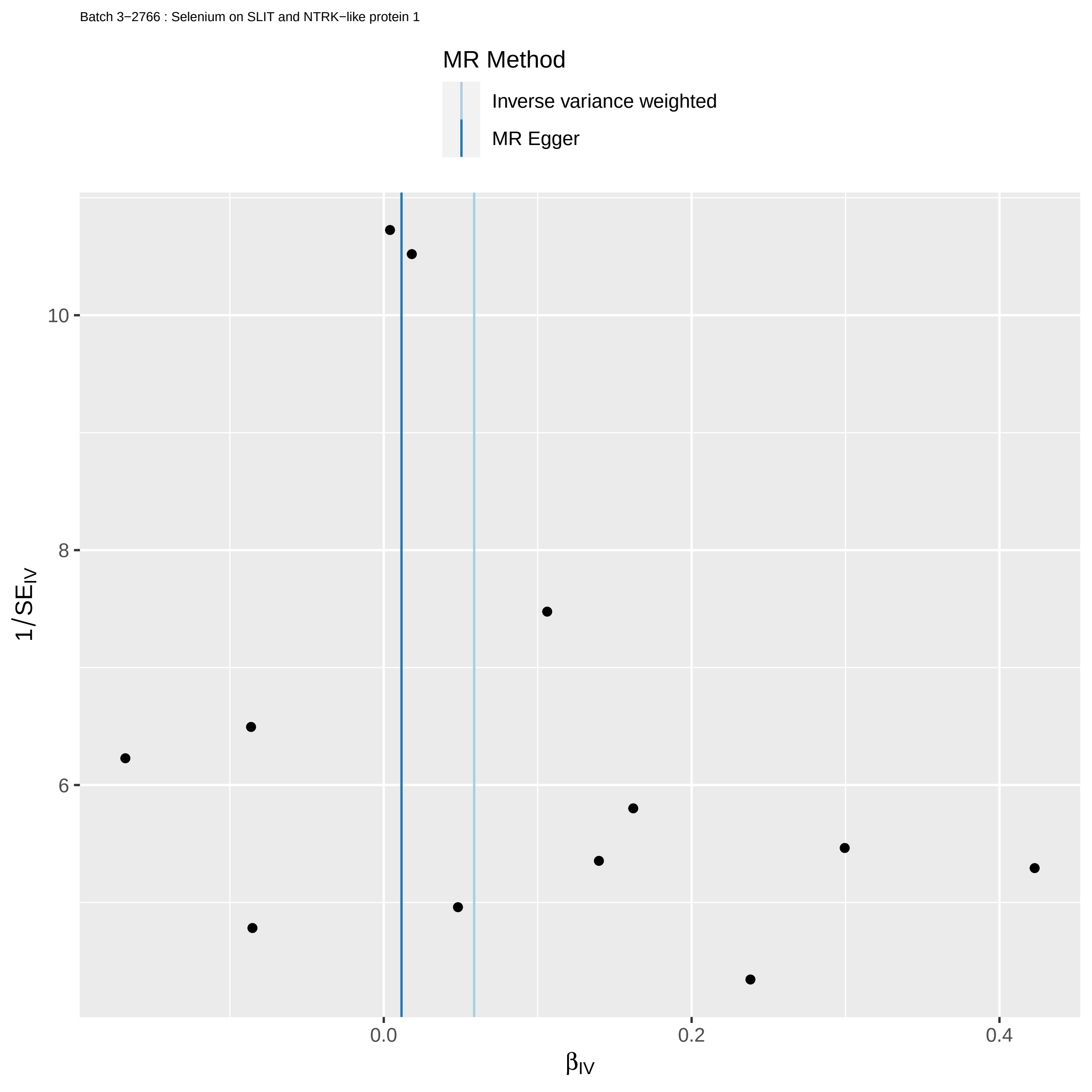

Batch 3−2766 : Selenium on SLIT and NTRK−like protein 1
MR Method
Inverse variance weighted
MR Egger
10
8
6
1/SE$_{IV}$
0.0
0.2
0.4
β$_{IV}$

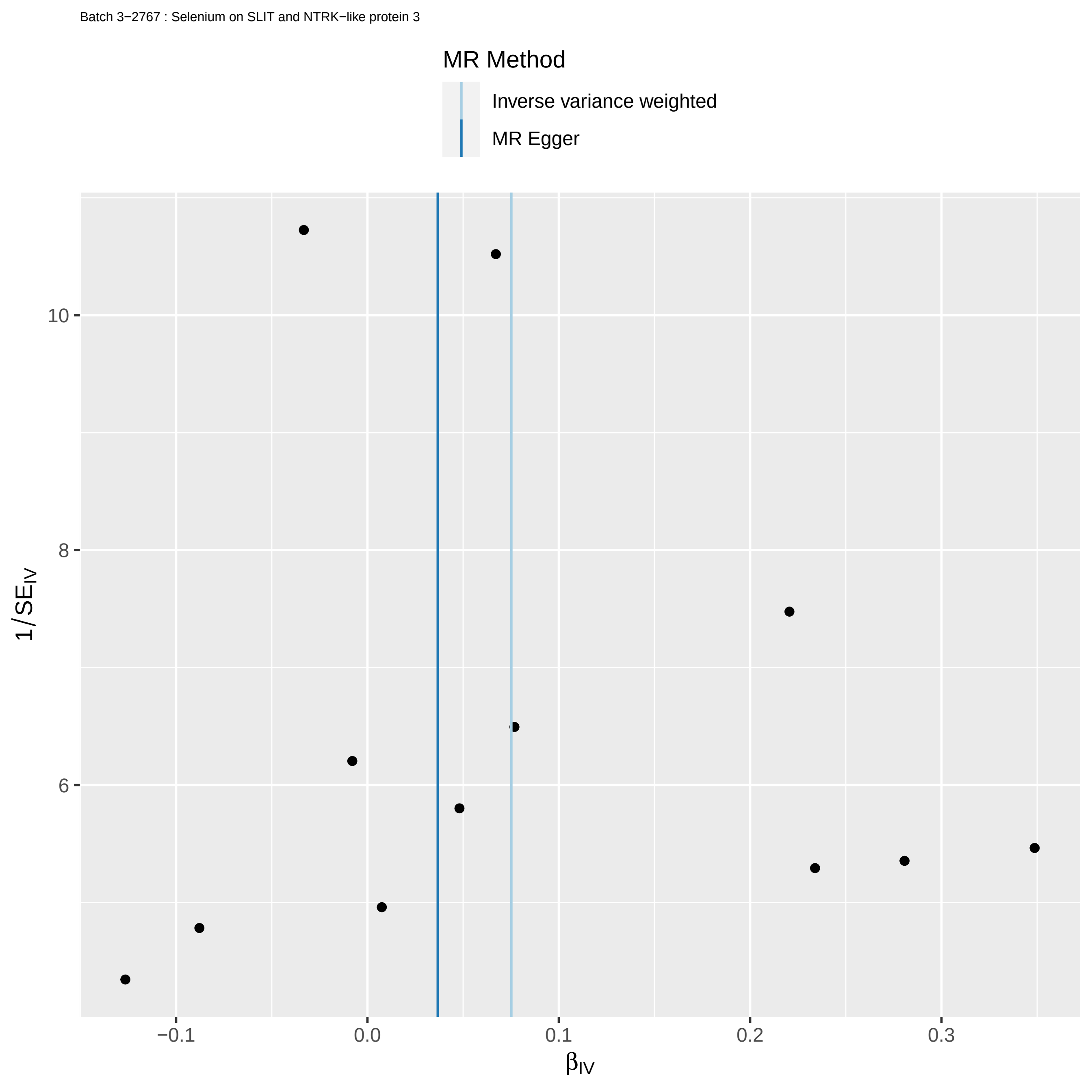
Batch 3−2767 : Selenium on SLIT and NTRK−like protein 3
MR Method
Inverse variance weighted
MR Egger
10
8
6
1/SE_IV
−0.1
0.0
0.1
0.2
0.3
β_IV

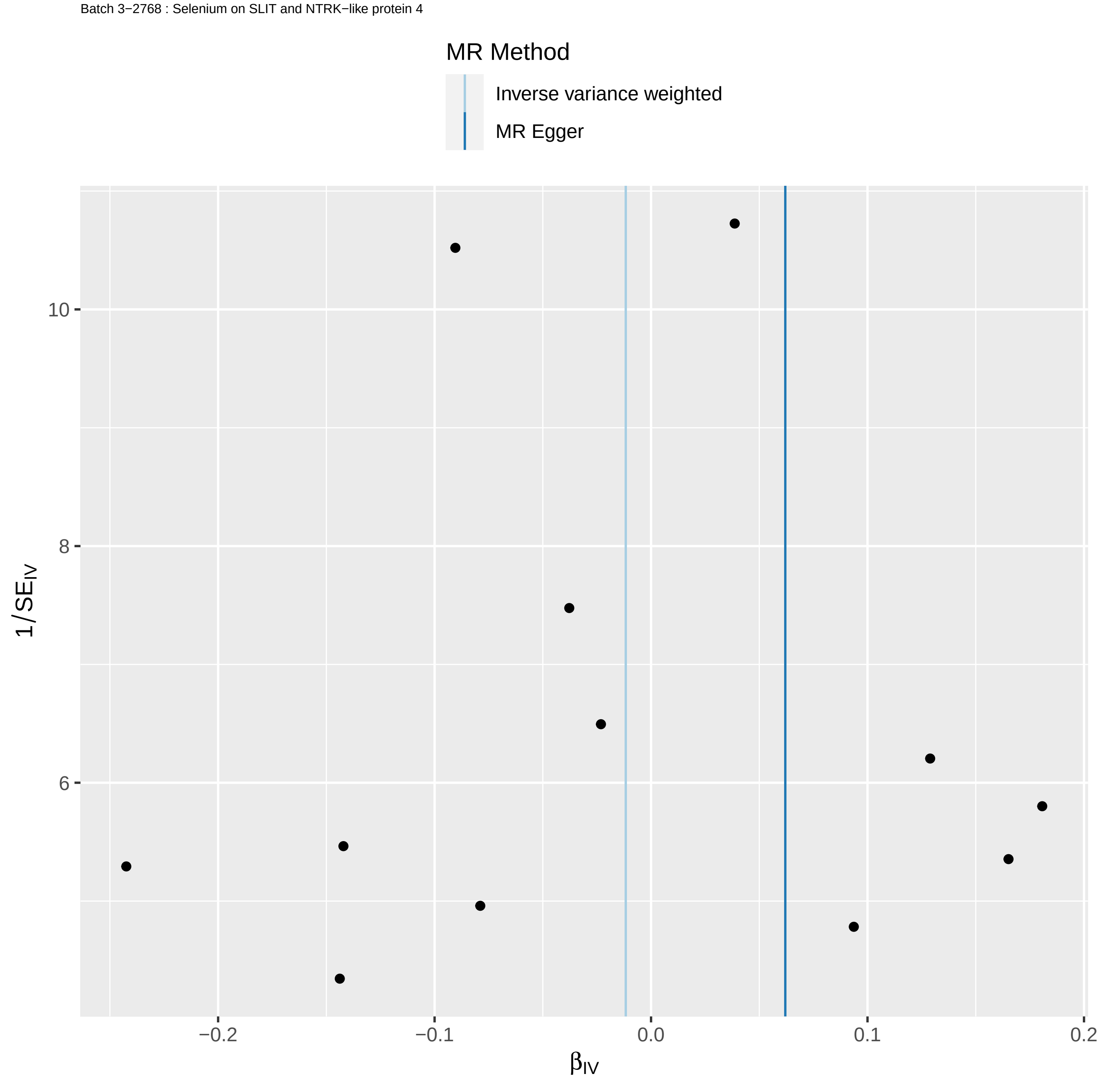

Batch 3−2768 : Selenium on SLIT and NTRK−like protein 4
MR Method
Inverse variance weighted
MR Egger
10
8
6
1/SE_IV
−0.2
−0.1
0.0
0.1
0.2
β_IV

Batch 3−2769 : Selenium on SLIT and NTRK−like protein 5

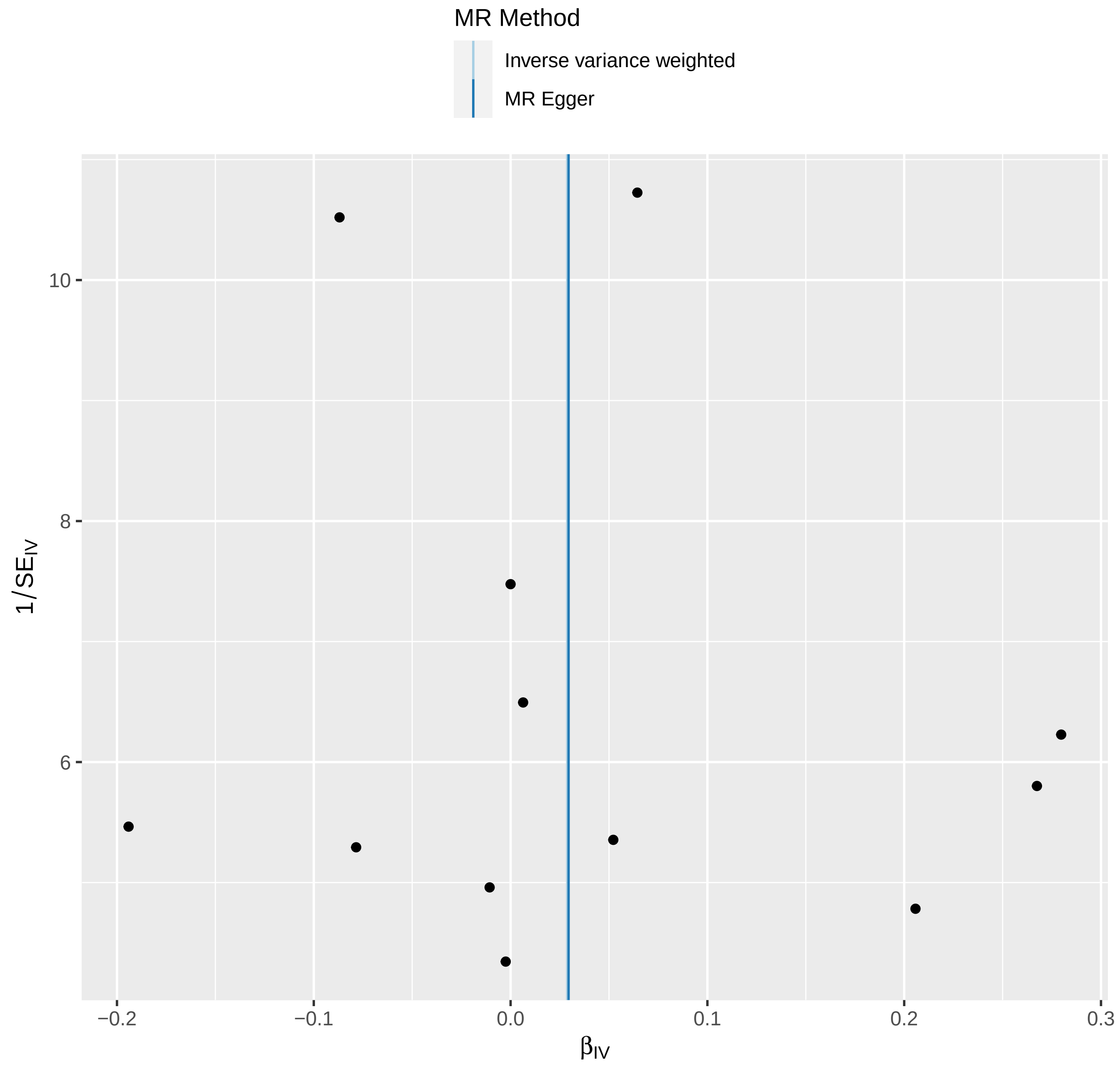

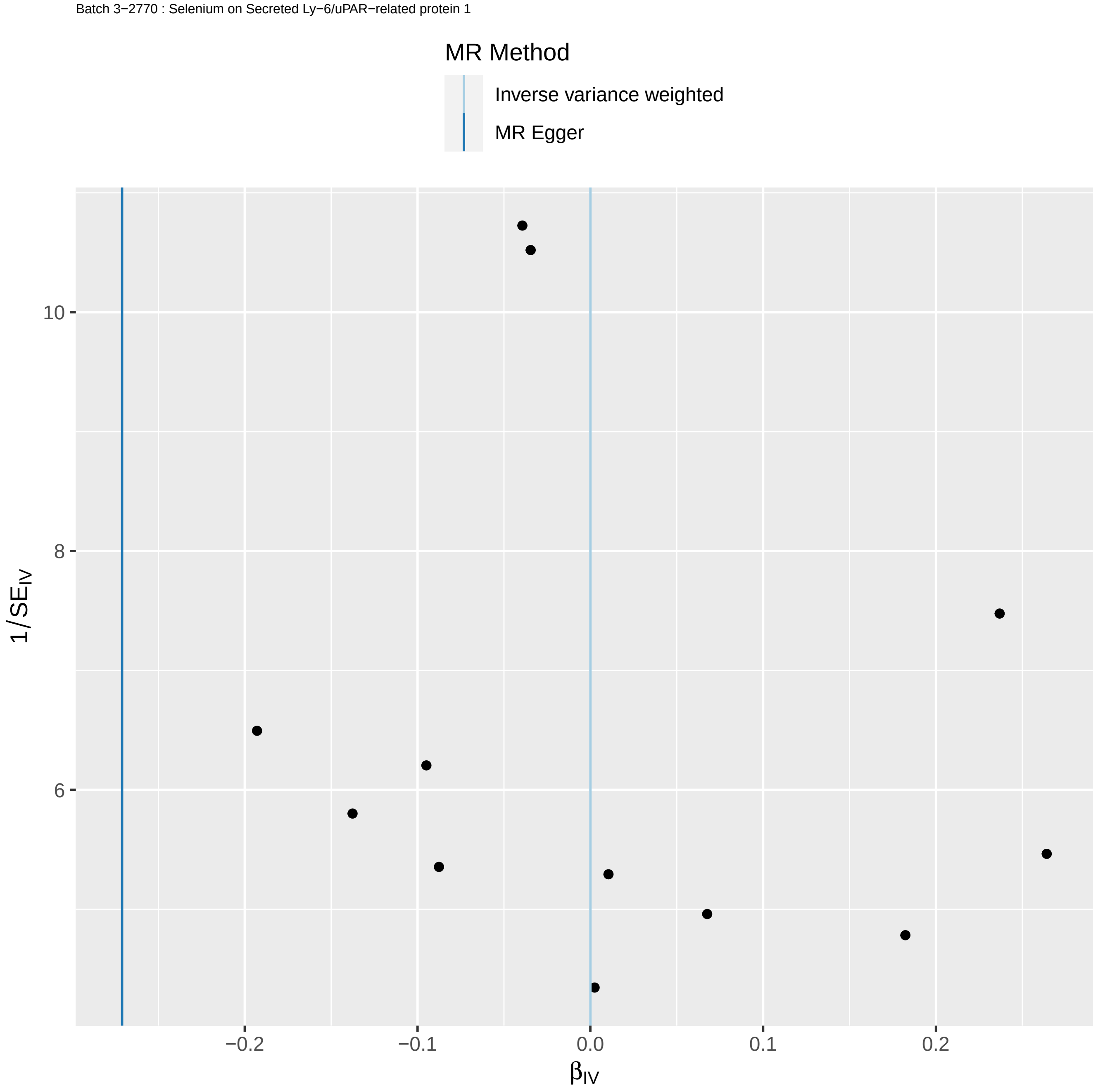

Batch 3−2770 : Selenium on Secreted Ly−6/uPAR−related protein 1
MR Method
Inverse variance weighted
MR Egger
10
8
6
1/SE$_{IV}$
−0.2
−0.1
0.0
0.1
0.2
β$_{IV}$

Batch 3−2771 : Selenium on Mothers against decapentaplegic homolog 2

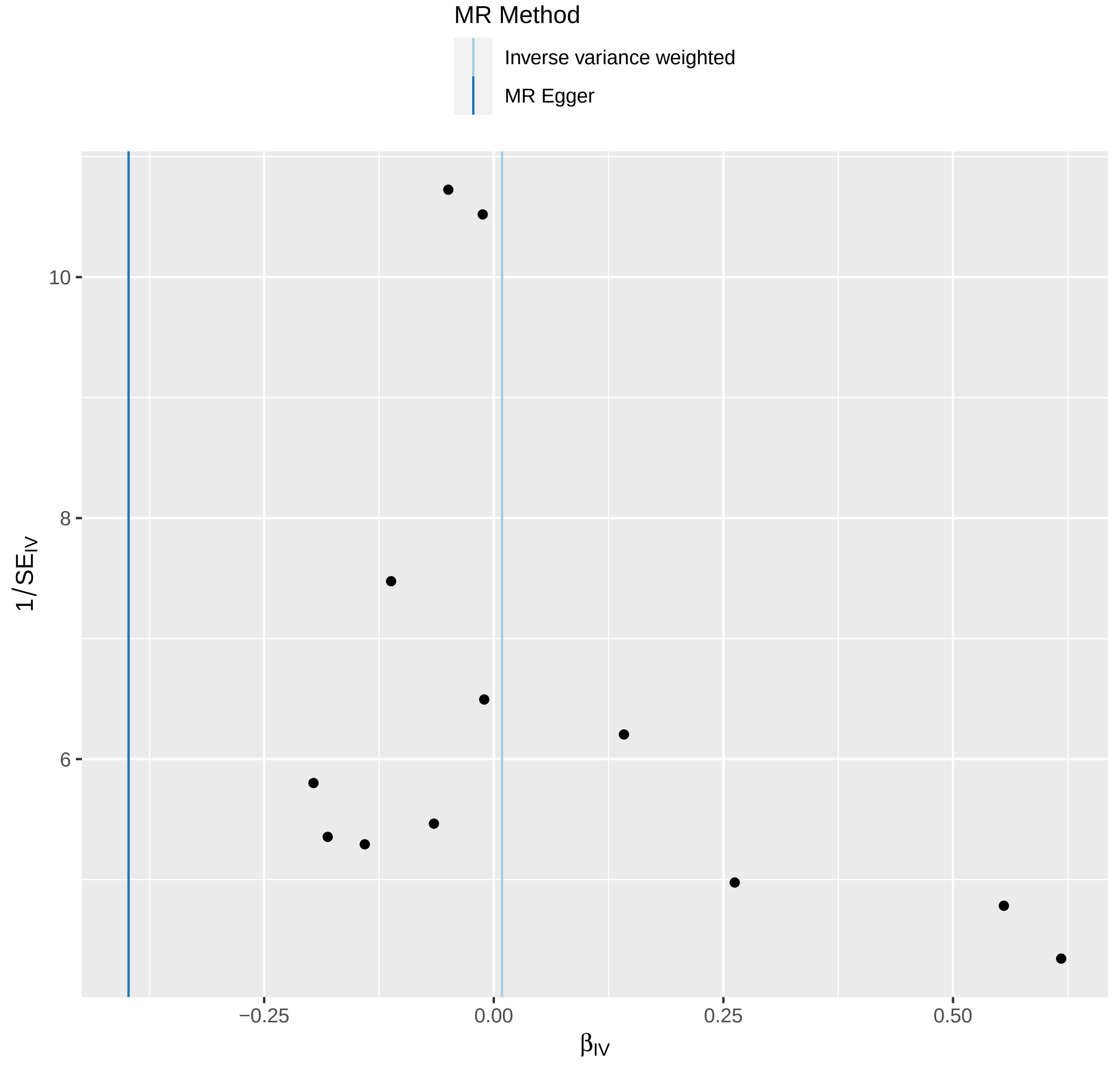

Batch 3−2772 : Selenium on Mothers against decapentaplegic homolog 4

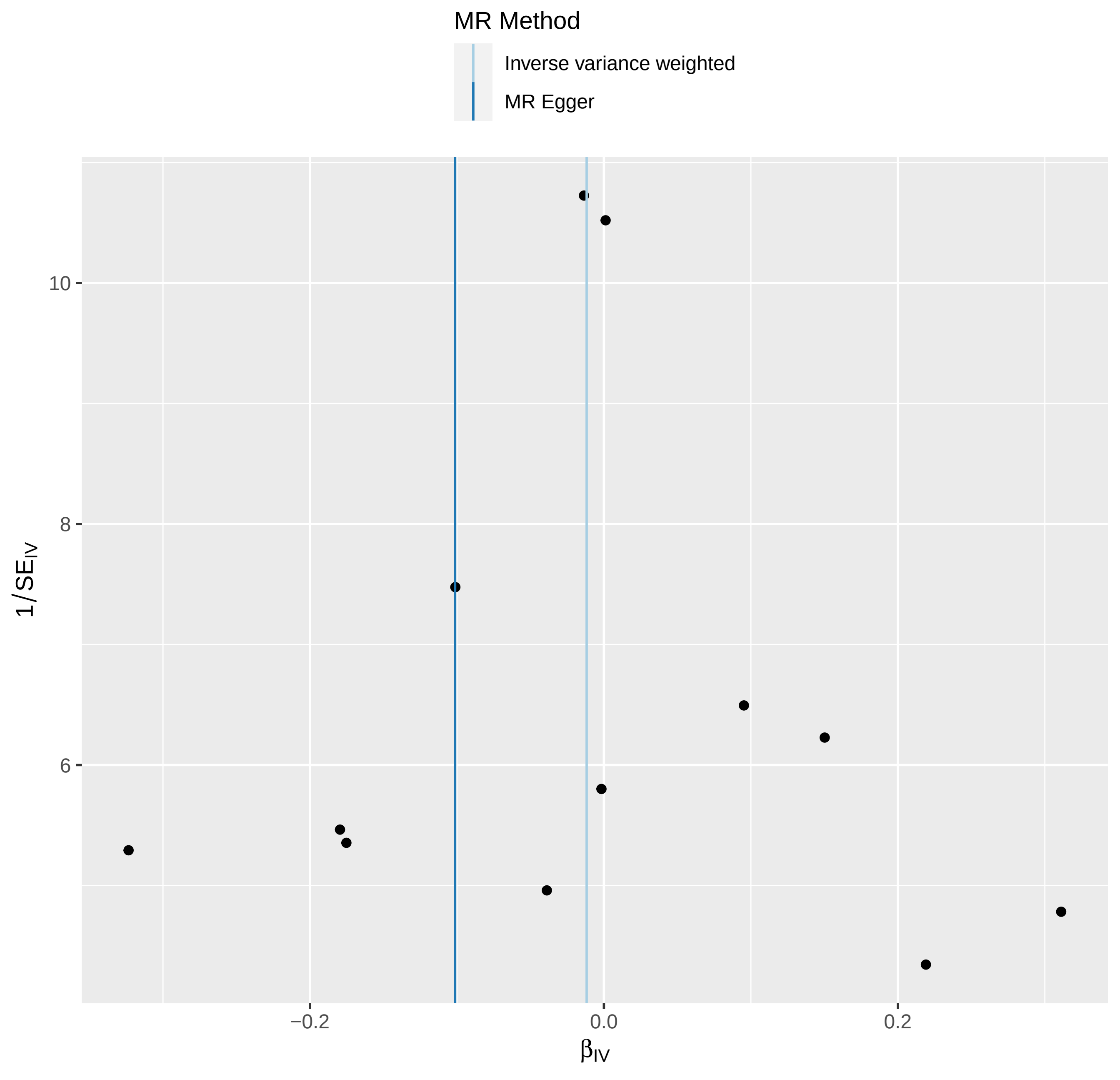

Batch 3−2773 : Selenium on Stromal membrane−associated protein 1

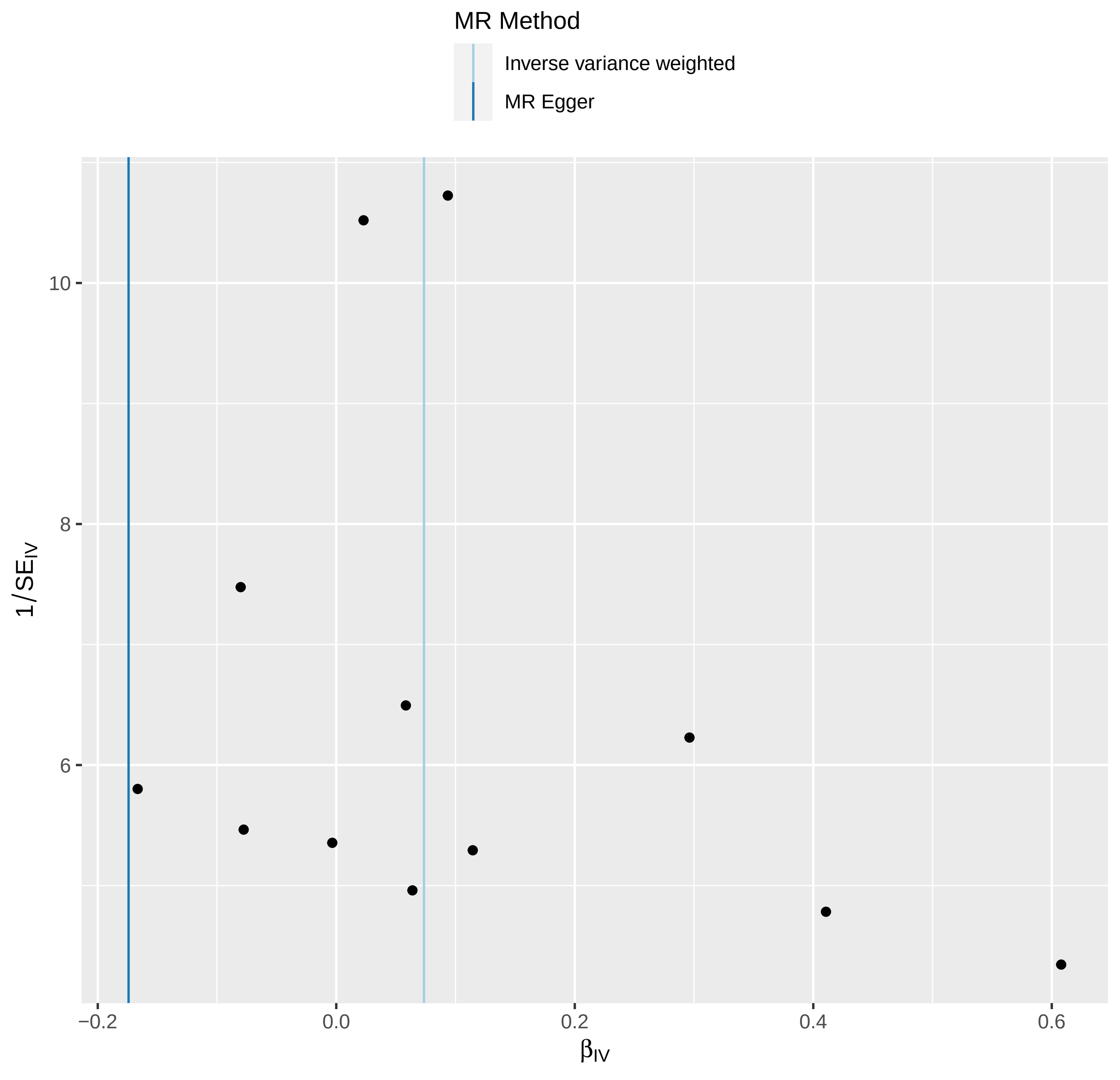

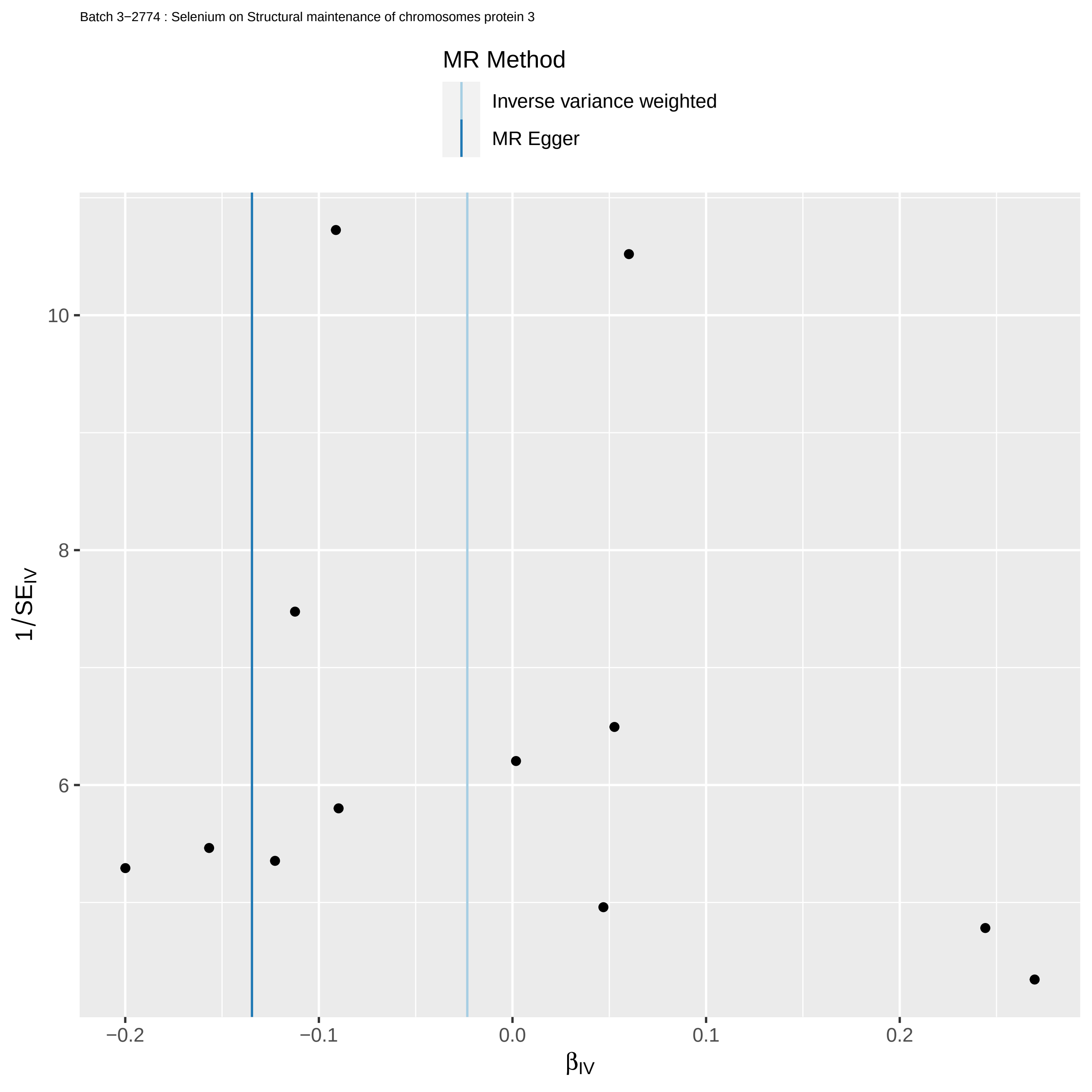

Batch 3−2774 : Selenium on Structural maintenance of chromosomes protein 3
MR Method
Inverse variance weighted
MR Egger
10
8
6
1/SE_IV
−0.2
−0.1
0.0
0.1
0.2
β_IV

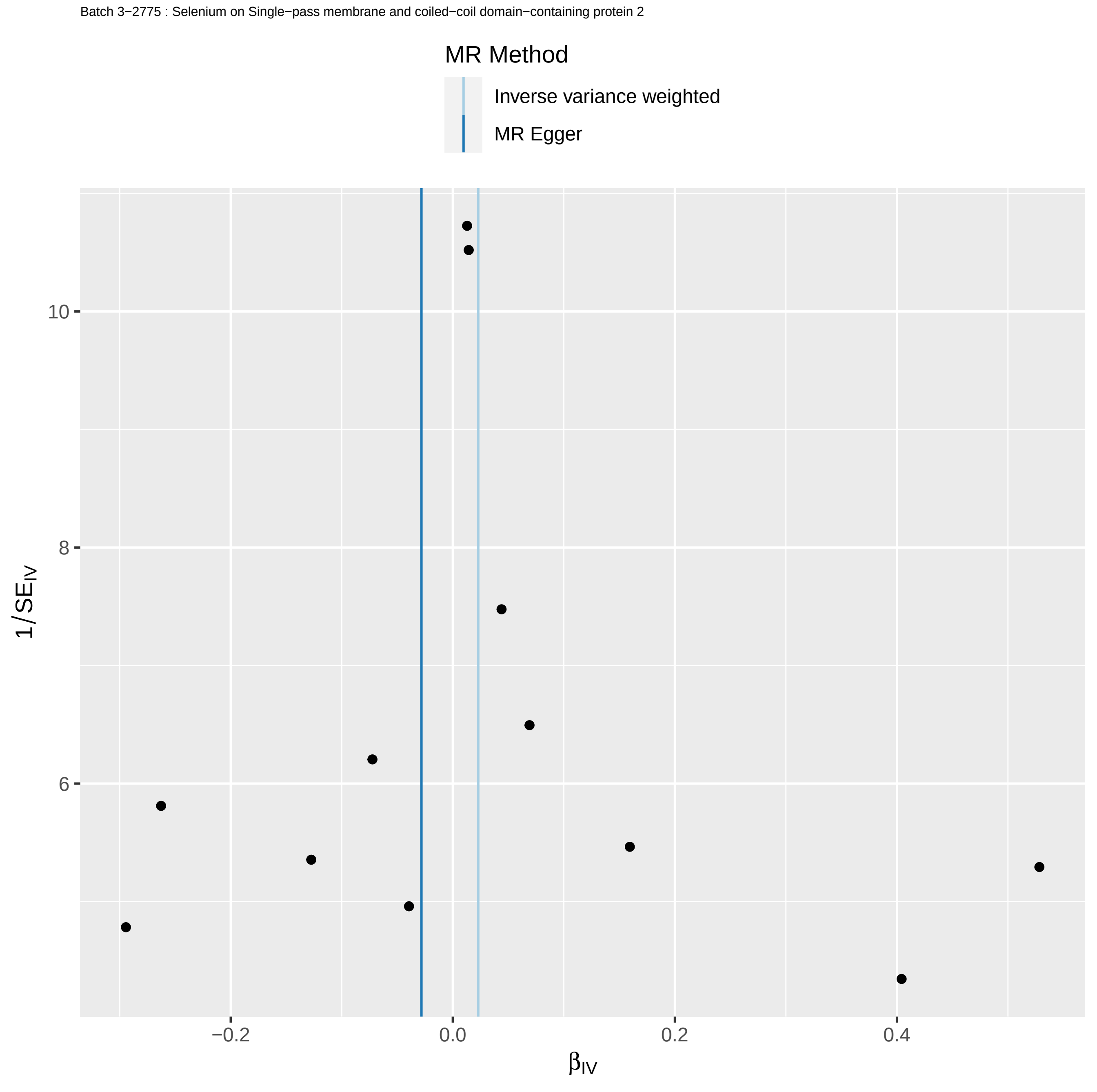

Batch 3−2775 : Selenium on Single−pass membrane and coiled−coil domain−containing protein 2
MR Method
Inverse variance weighted
MR Egger
10
8
6
1/SE_IV
−0.2
0.0
0.2
0.4
β_IV

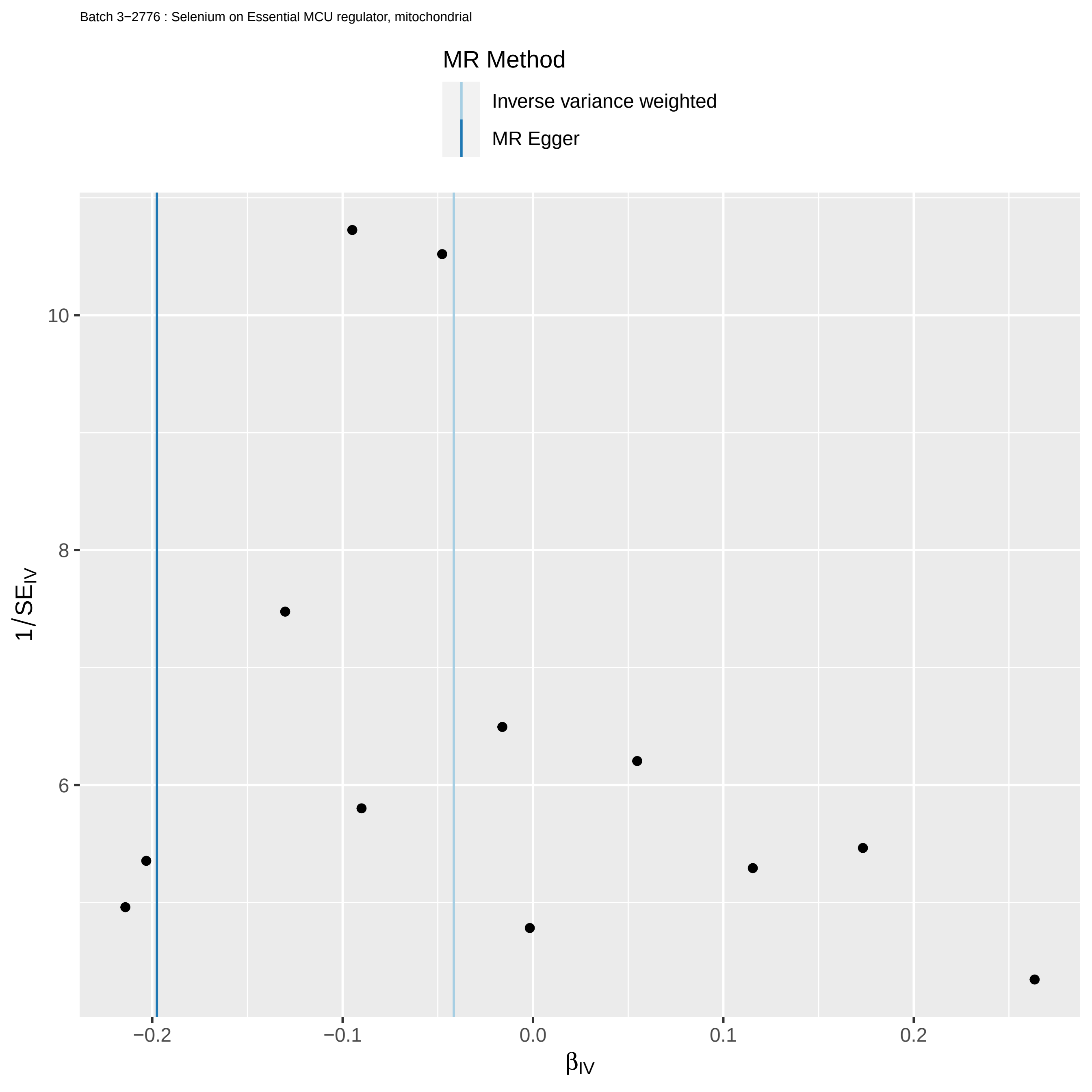
Batch 3−2776 : Selenium on Essential MCU regulator, mitochondrial
MR Method
Inverse variance weighted
MR Egger
10
8
6
1/SE_IV
−0.2
−0.1
0.0
0.1
0.2
β_IV

Batch 3−2777 : Selenium on Small integral membrane protein 10

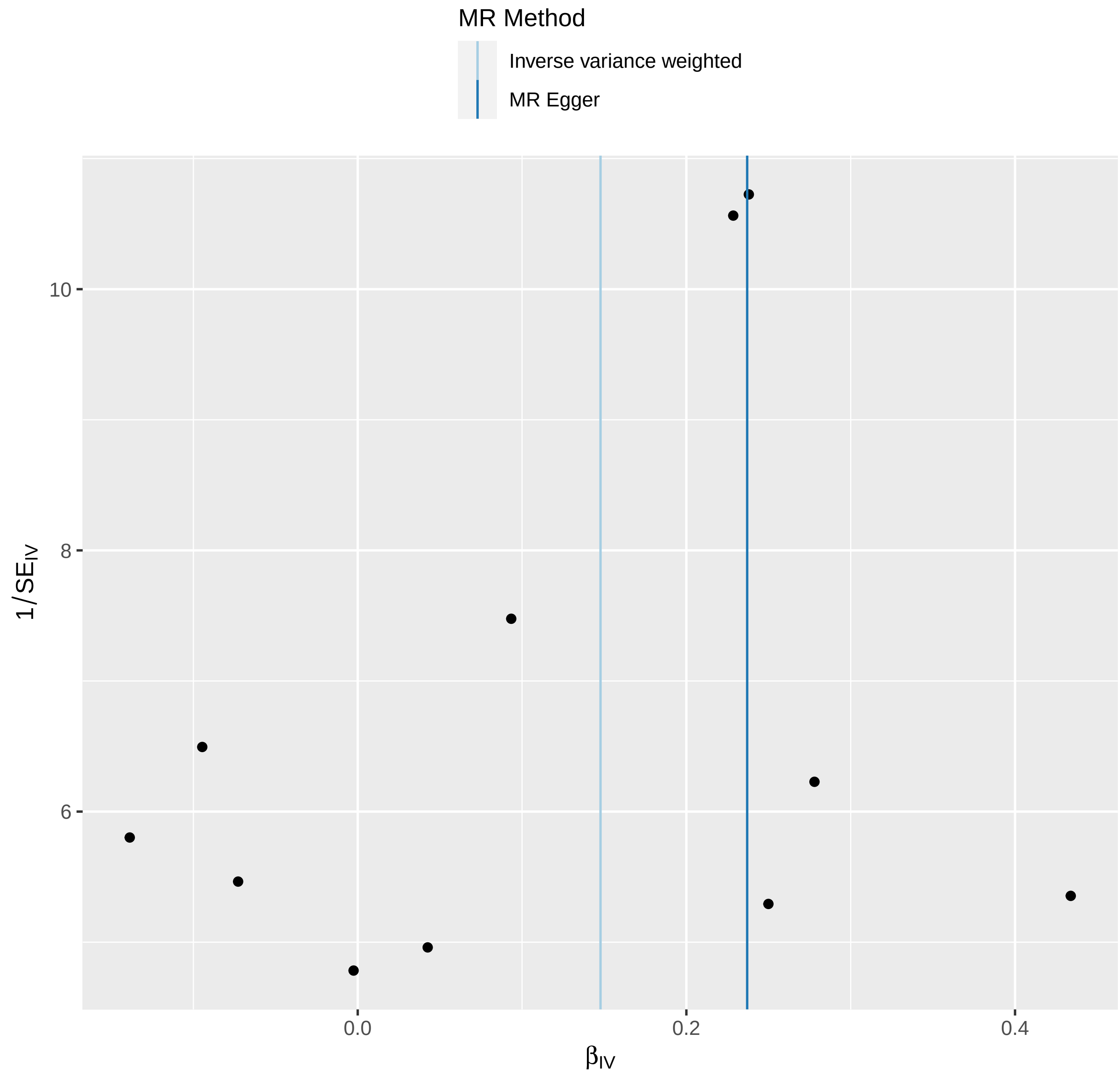

Batch 3−2778 : Selenium on Small integral membrane protein 9

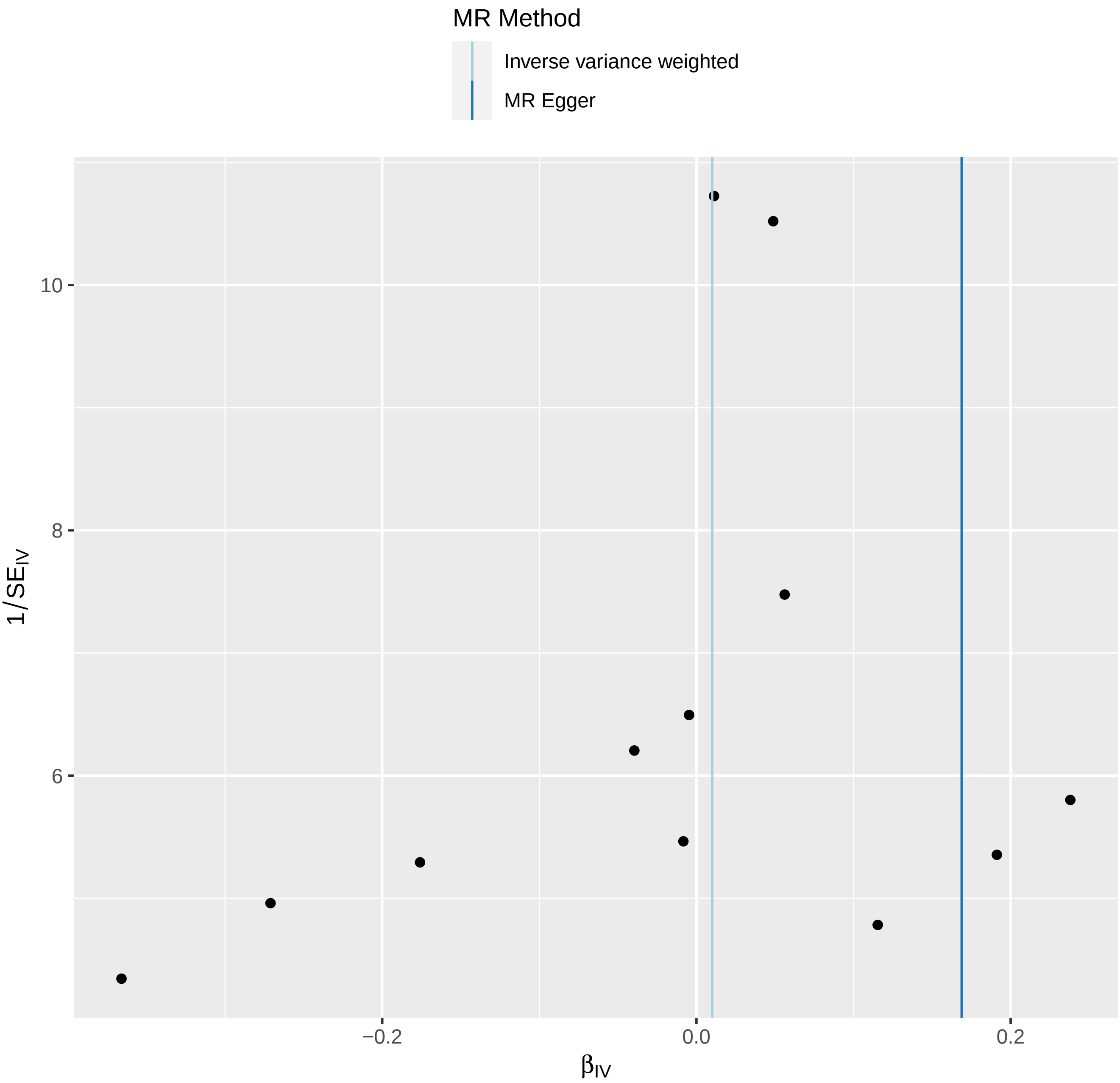

Batch 3−2779 : Selenium on SPARC−related modular calcium−binding protein 1

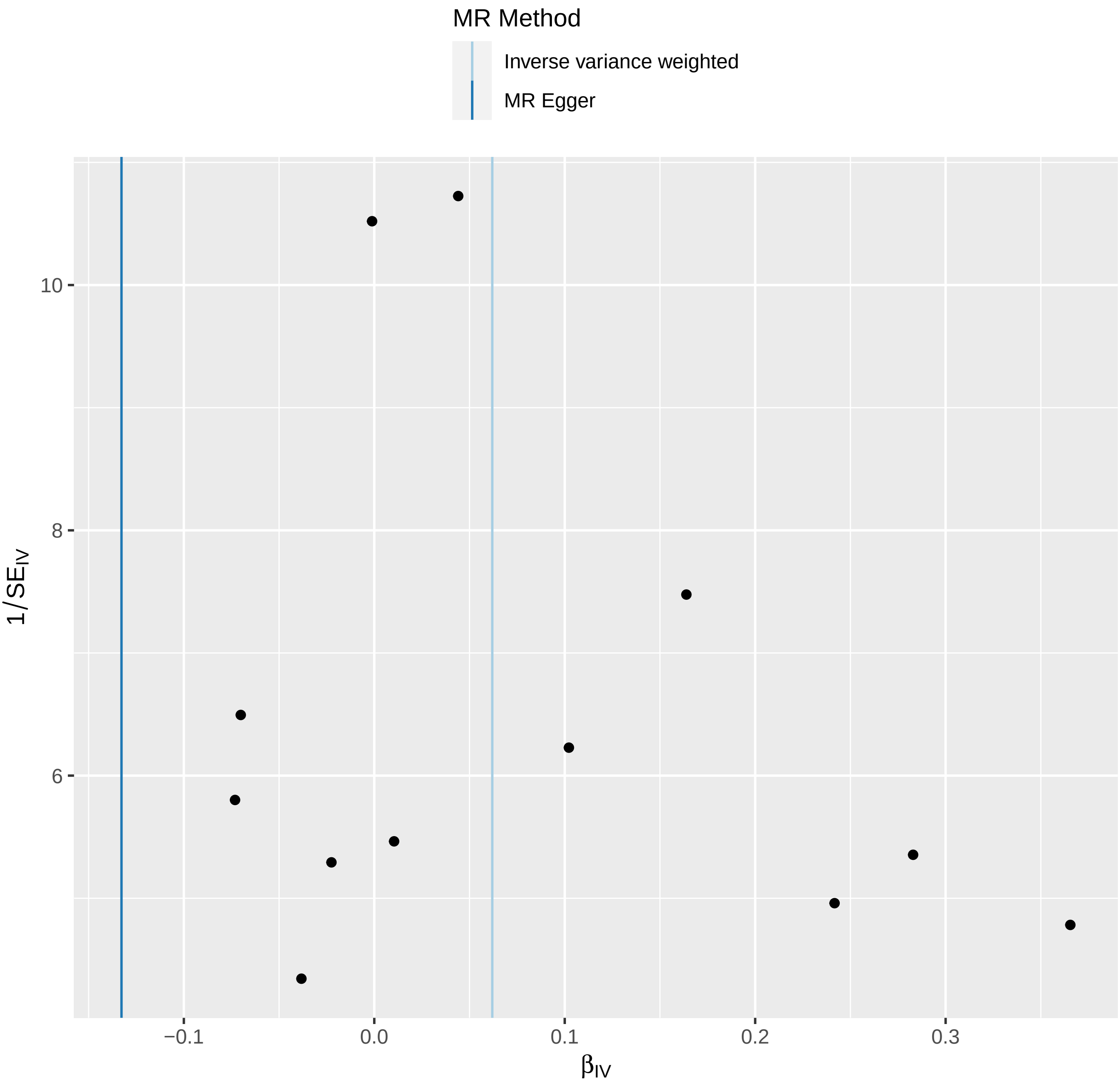

Batch 3−2780 : Selenium on Acid sphingomyelinase−like phosphodiesterase 3a

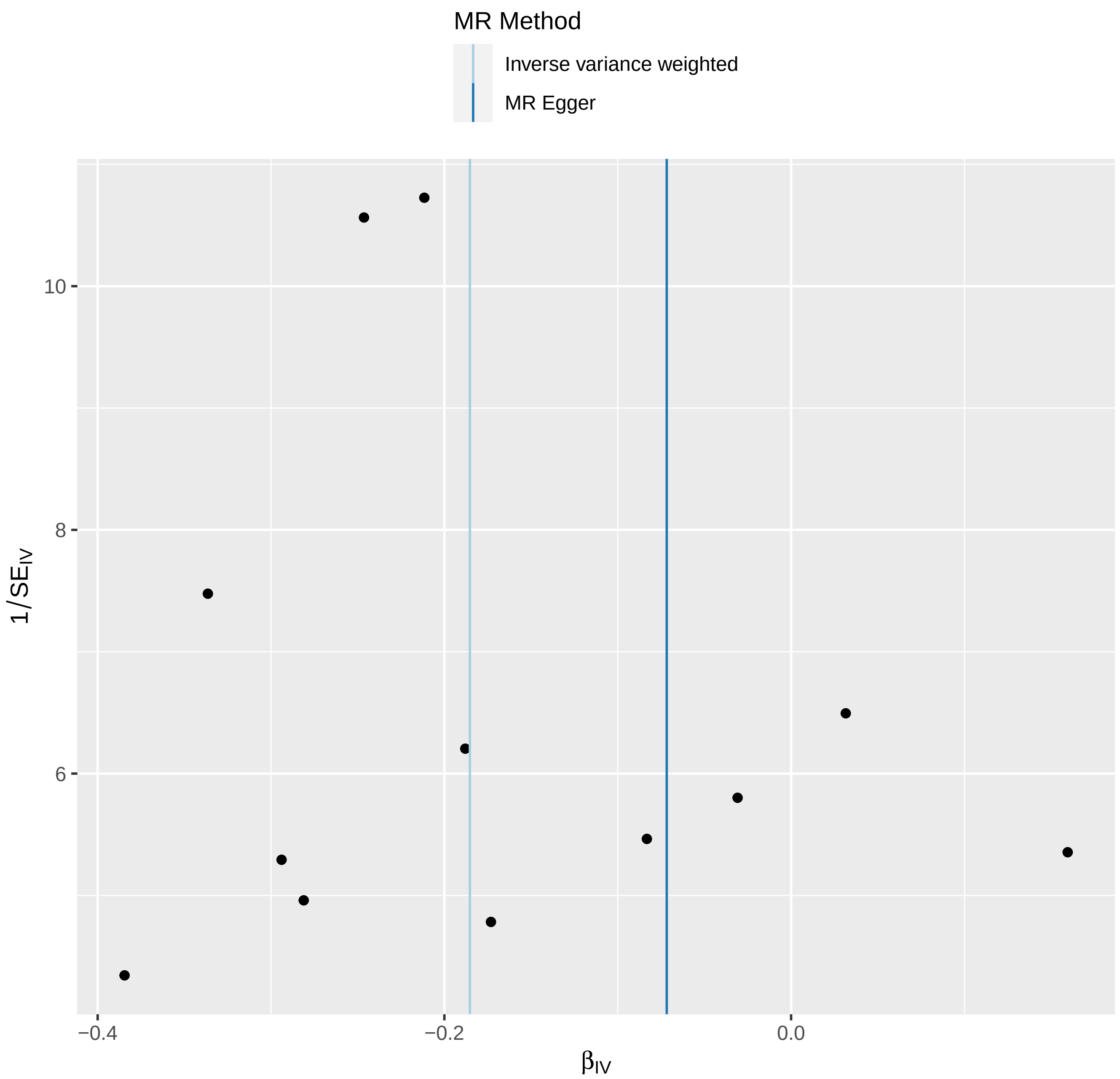

Batch 3−2781 : Selenium on Acid sphingomyelinase−like phosphodiesterase 3a

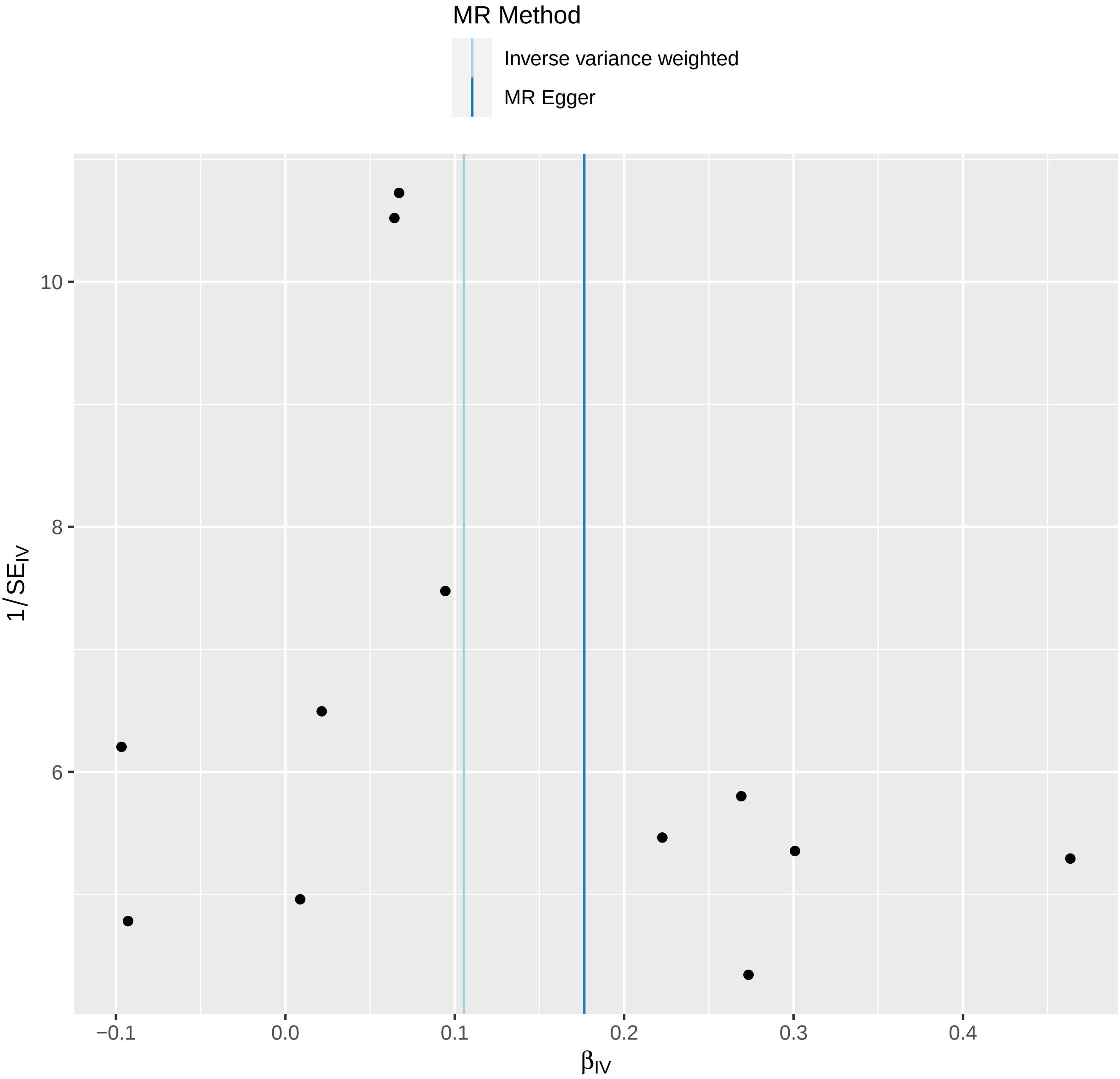

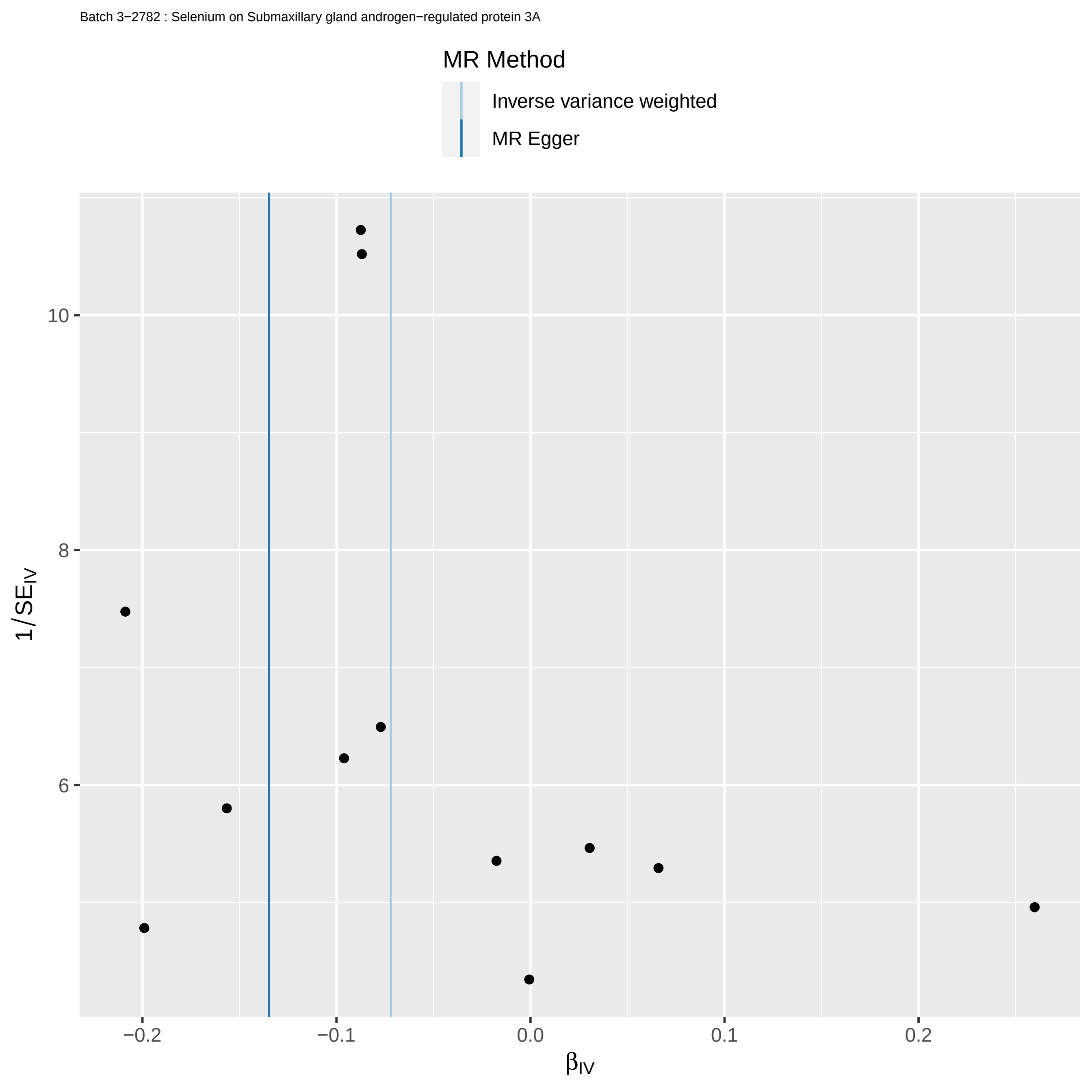

Batch 3−2782 : Selenium on Submaxillary gland androgen−regulated protein 3A
MR Method
Inverse variance weighted
MR Egger
10
8
6
$1/SE_{IV}$
−0.2
−0.1
0.0
0.1
0.2
β_{IV}

Batch 3−2783 : Selenium on Submaxillary gland androgen−regulated protein 3B

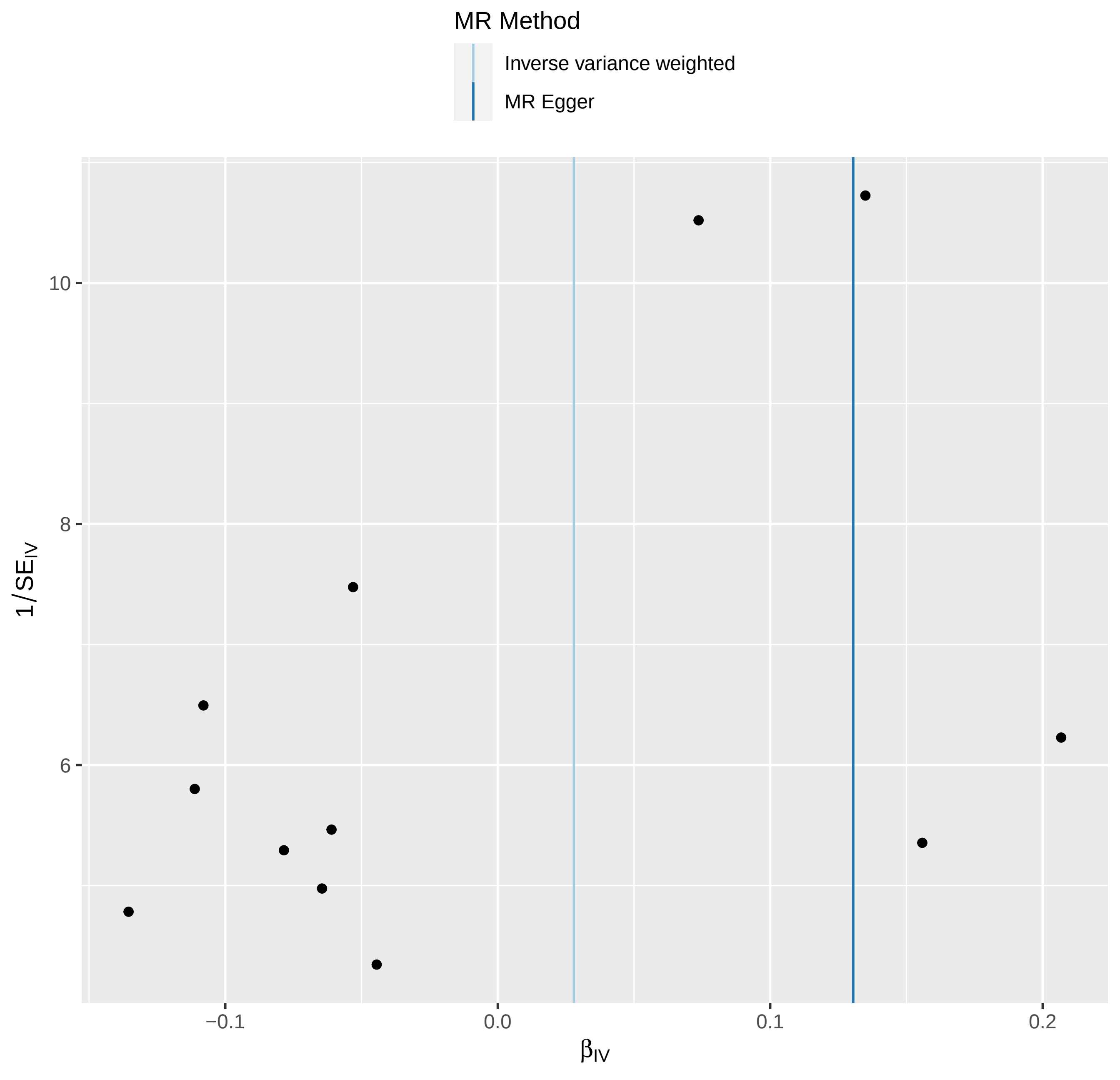

Batch 3−2784 : Selenium on N−lysine methyltransferase SMYD2

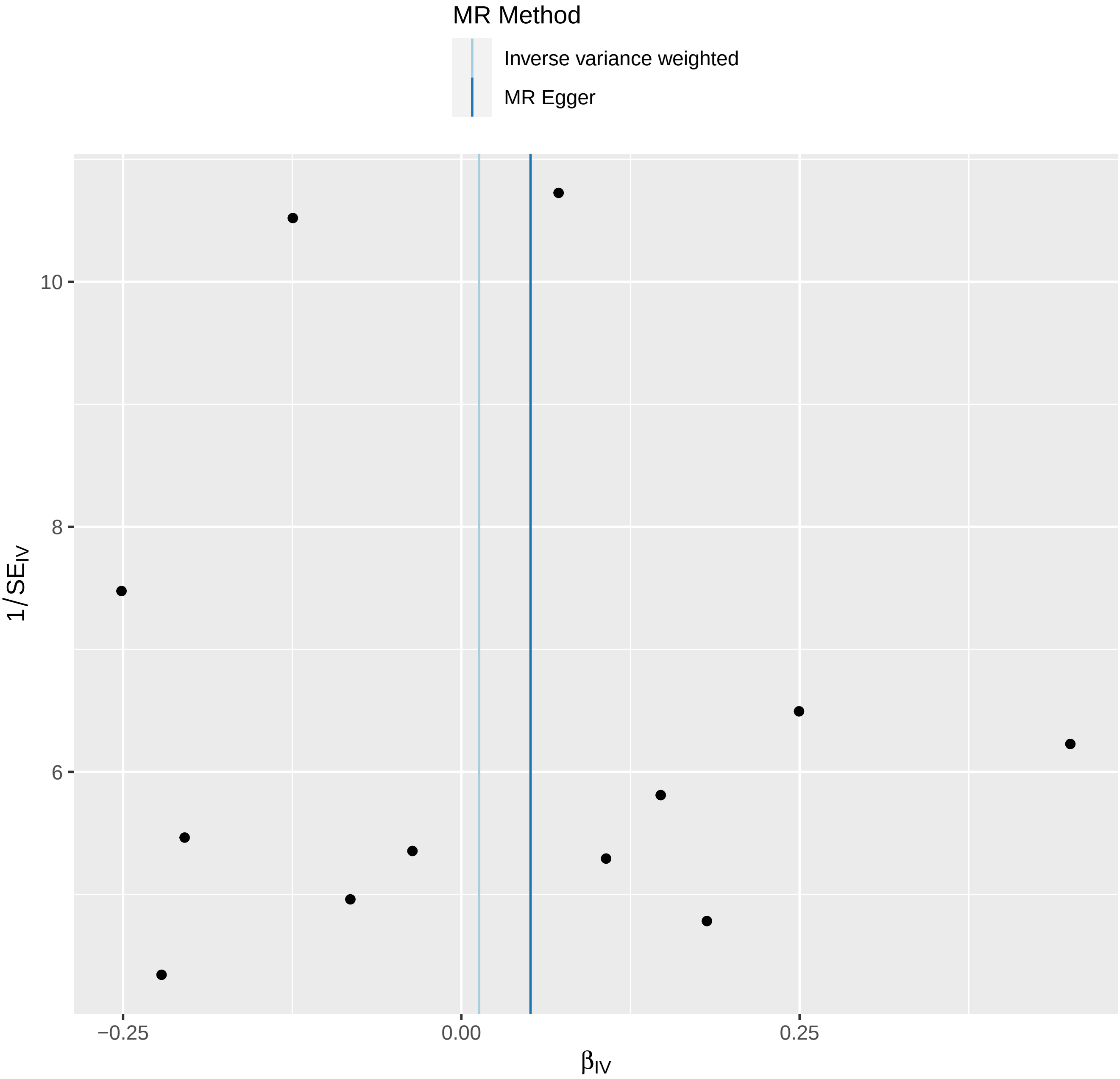

Batch 3−2785 : Selenium on Zinc finger protein SNAI2

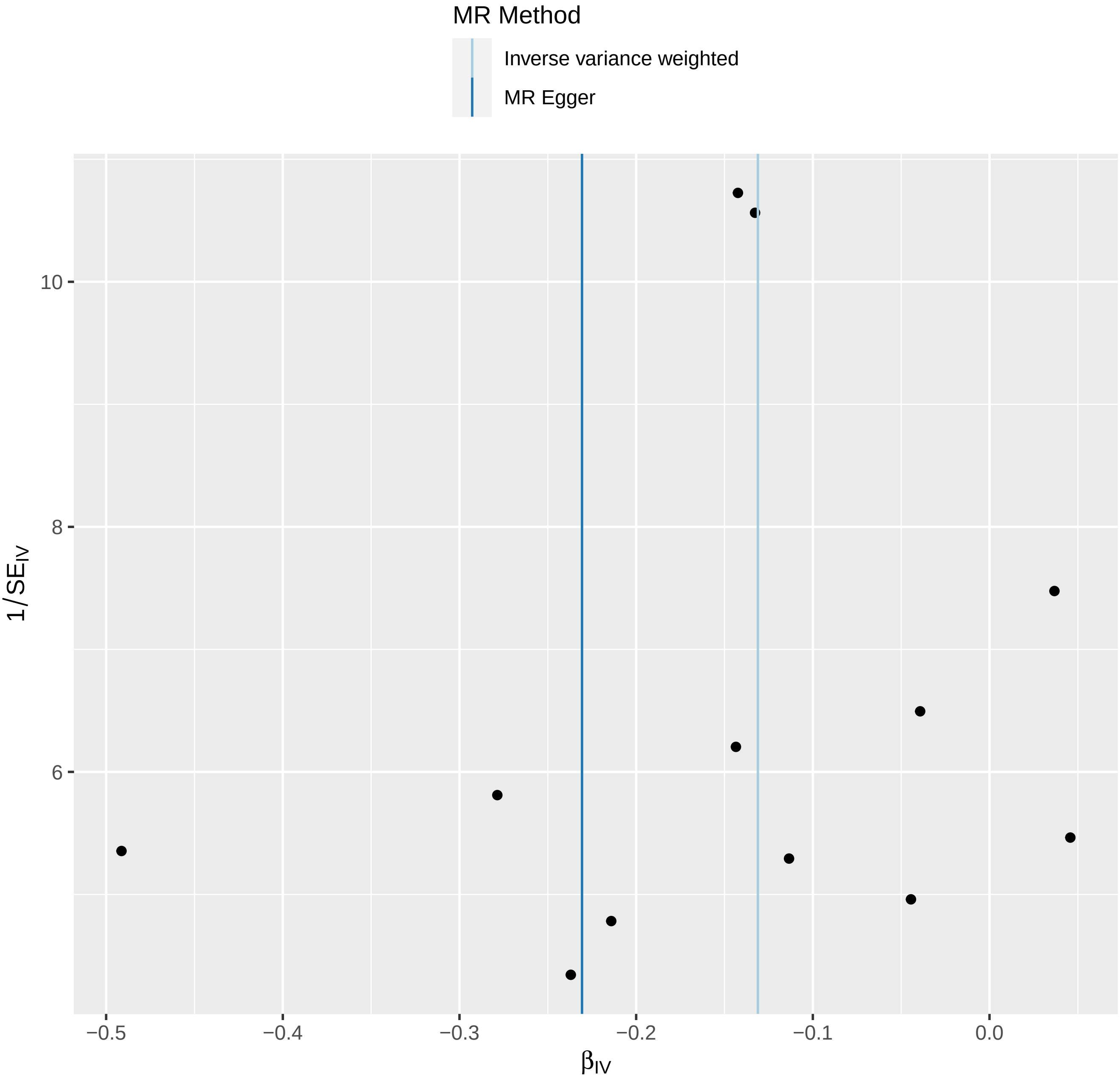

Batch 3−2786 : Selenium on Synaptosomal−associated protein 25

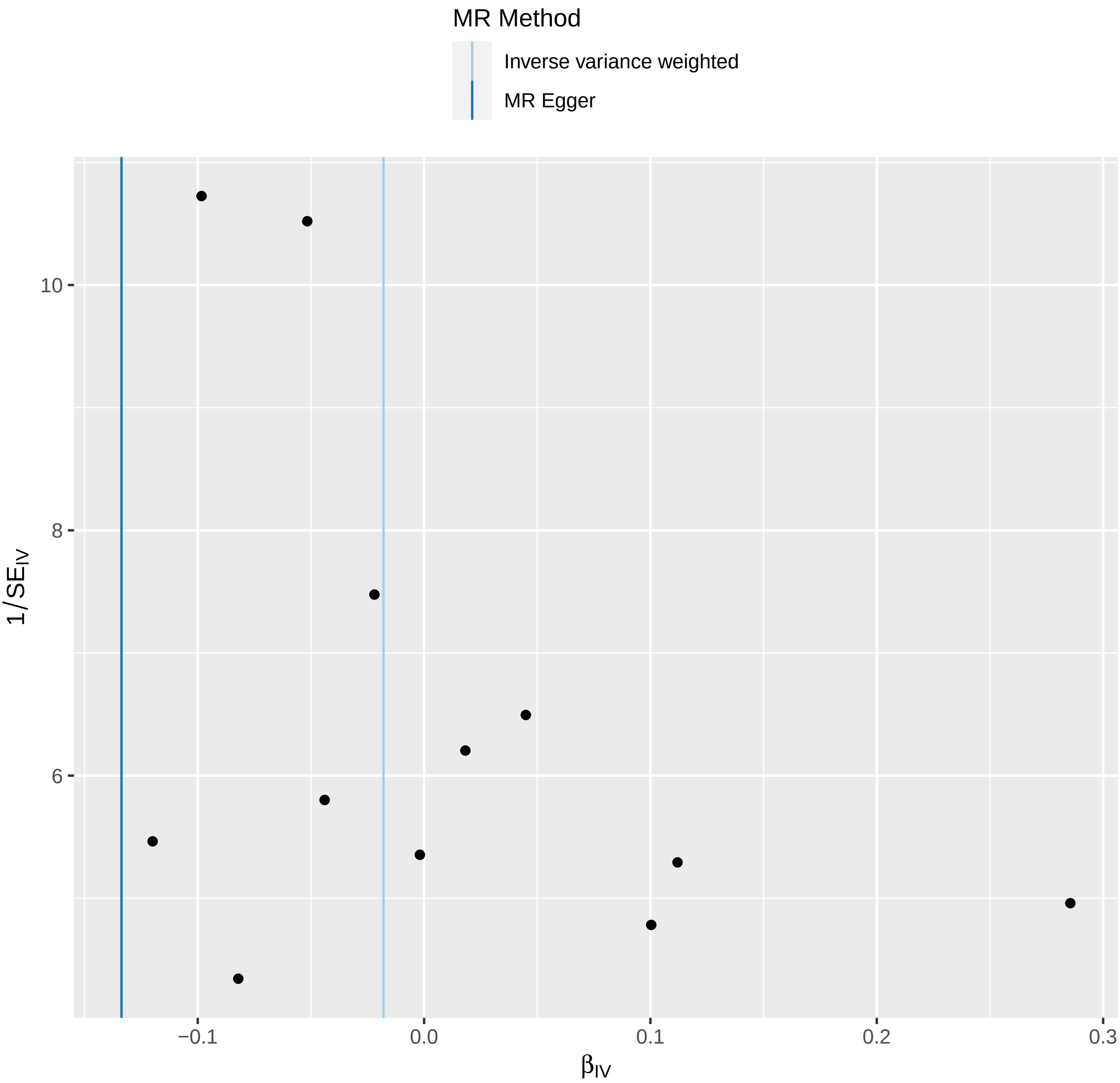

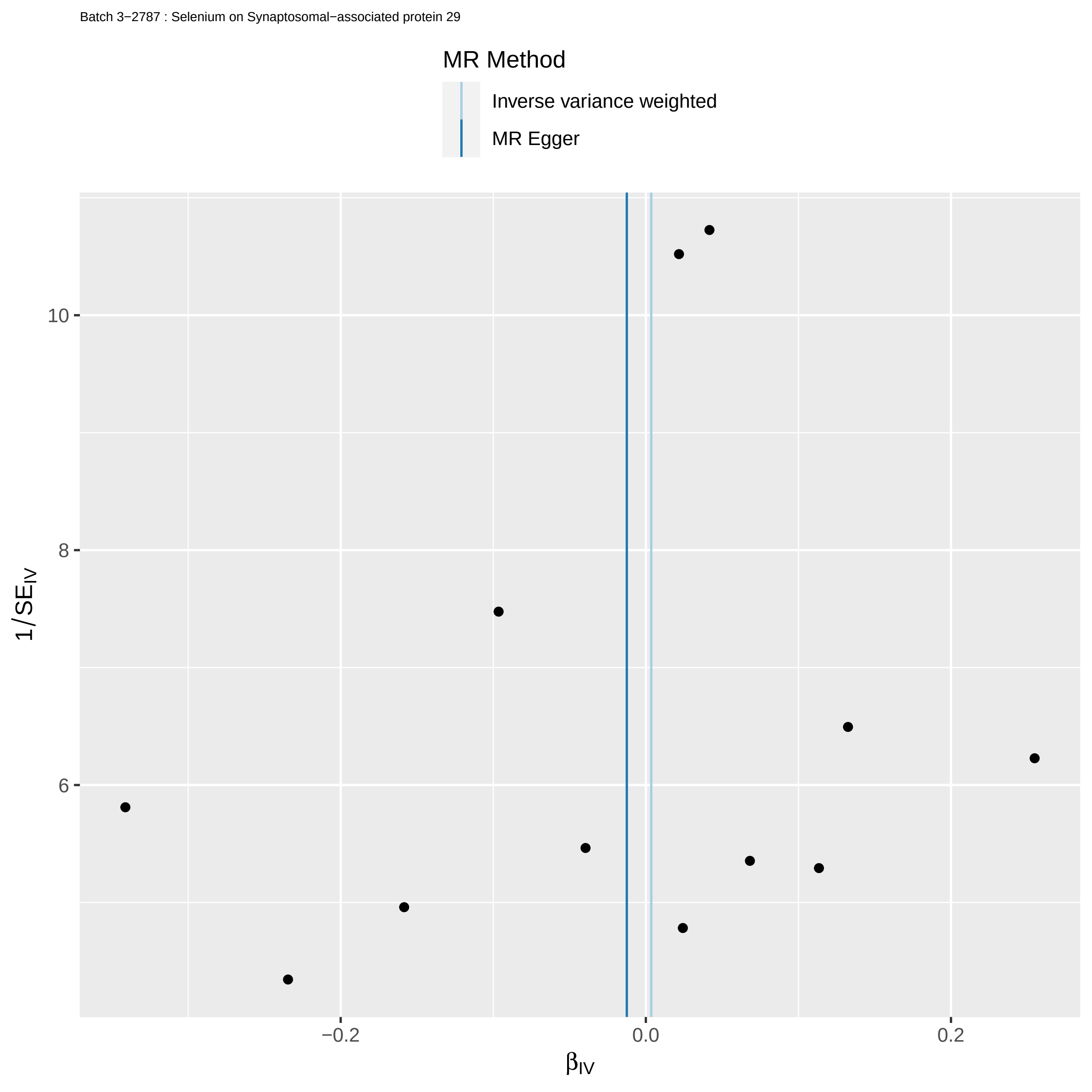
Batch 3−2787 : Selenium on Synaptosomal−associated protein 29
MR Method
Inverse variance weighted
MR Egger
10
8
6
1/SE_IV
−0.2
0.0
0.2
β_IV

Batch 3−2788 : Selenium on SNARE−associated protein Snapin

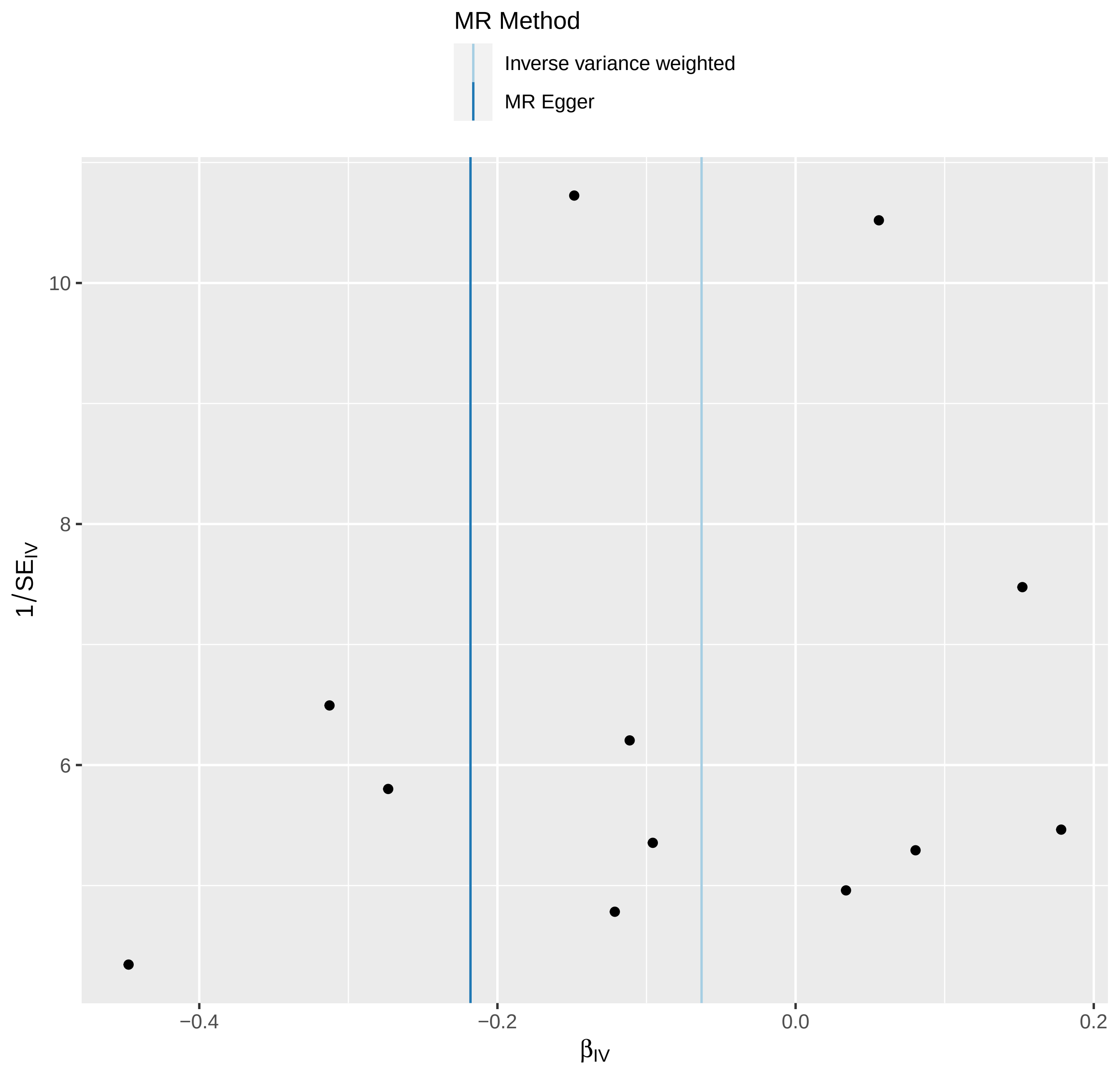

Batch 3−2789 : Selenium on Alpha−synuclein

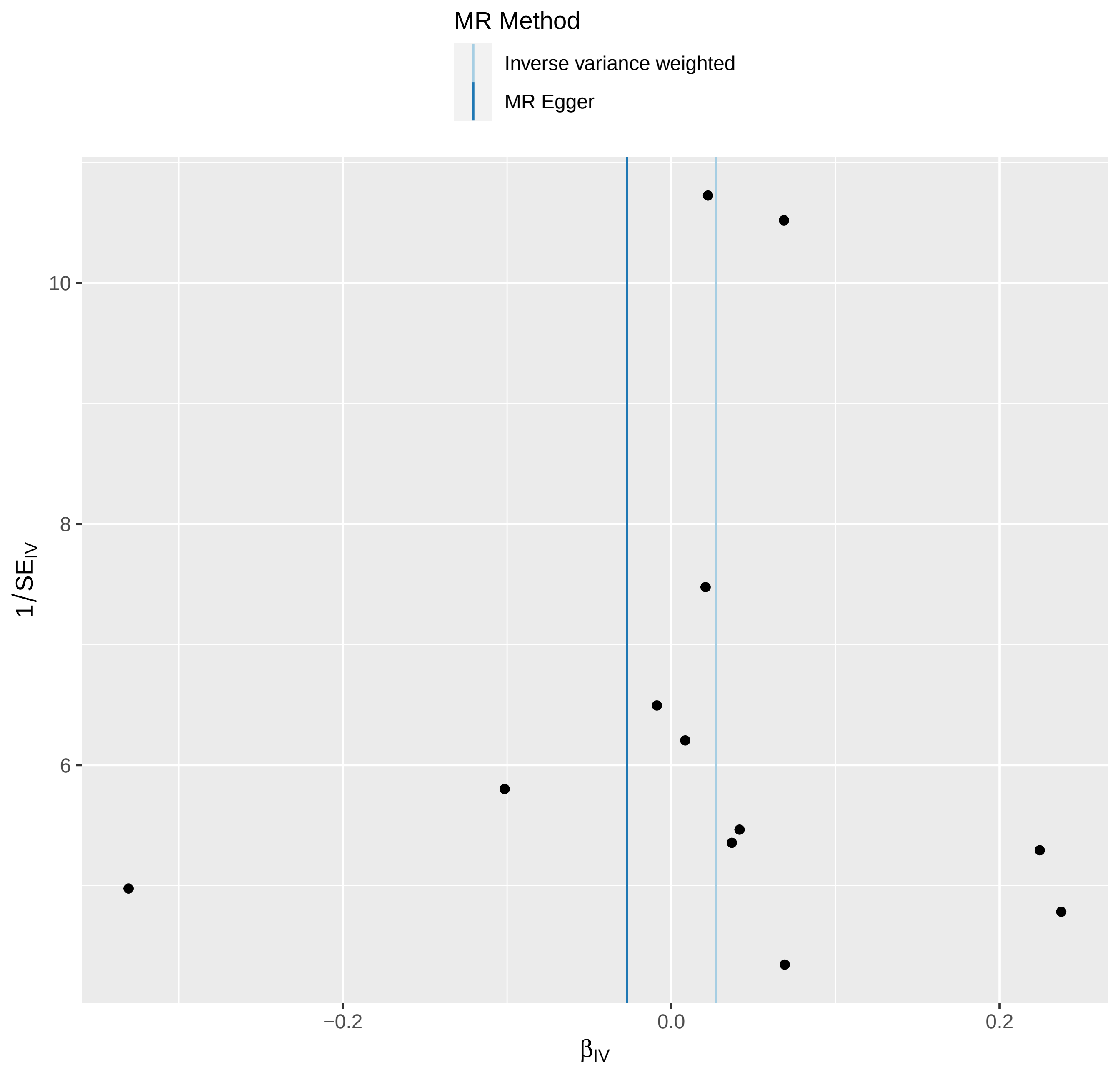

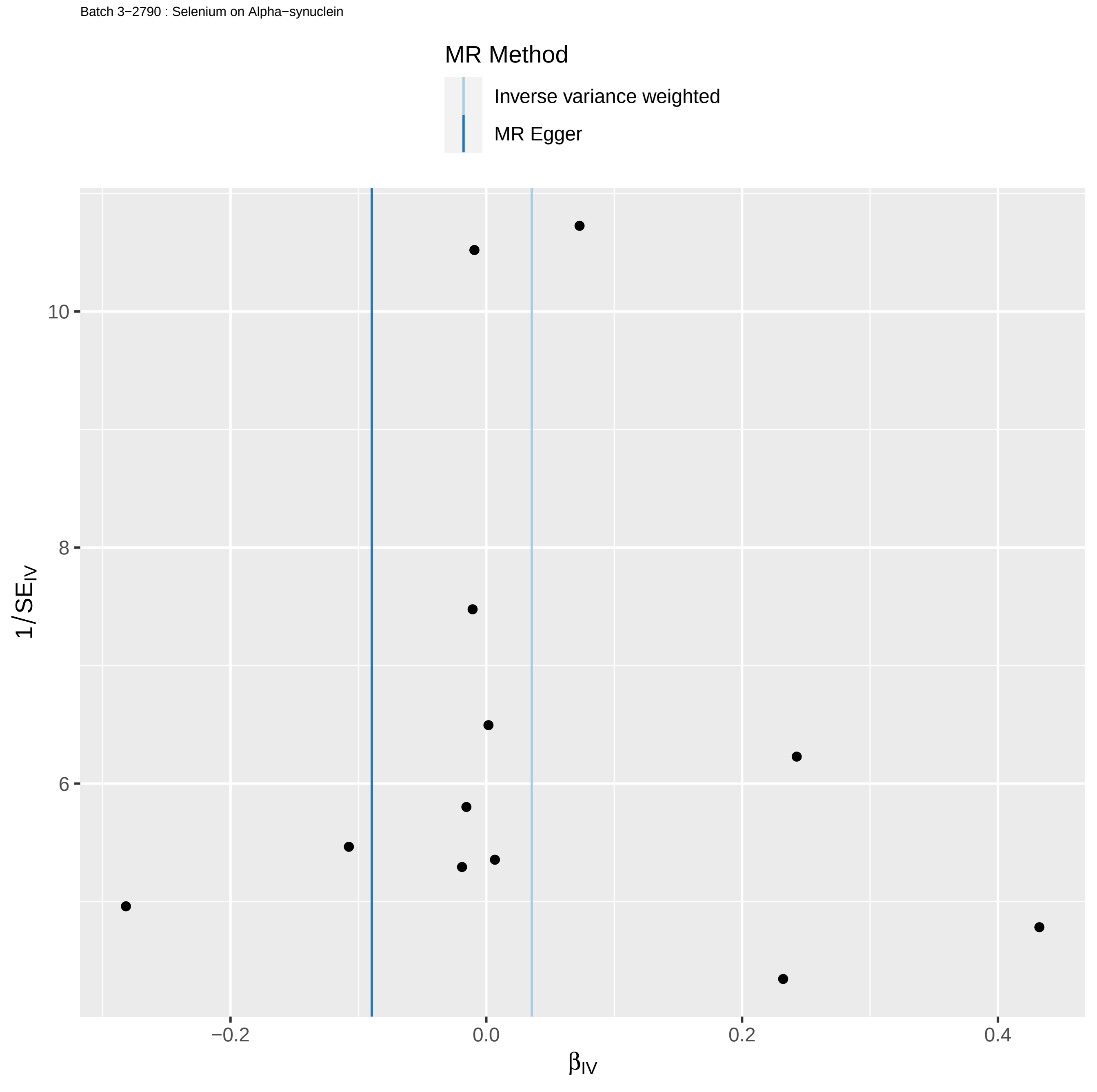

Batch 3−2790 : Selenium on Alpha−synuclein
MR Method
Inverse variance weighted
MR Egger
10
8
6
$1/SE_{IV}$
−0.2
0.0
0.2
0.4
β_{IV}

Batch 3−2791 : Selenium on U1 small nuclear ribonucleoprotein A

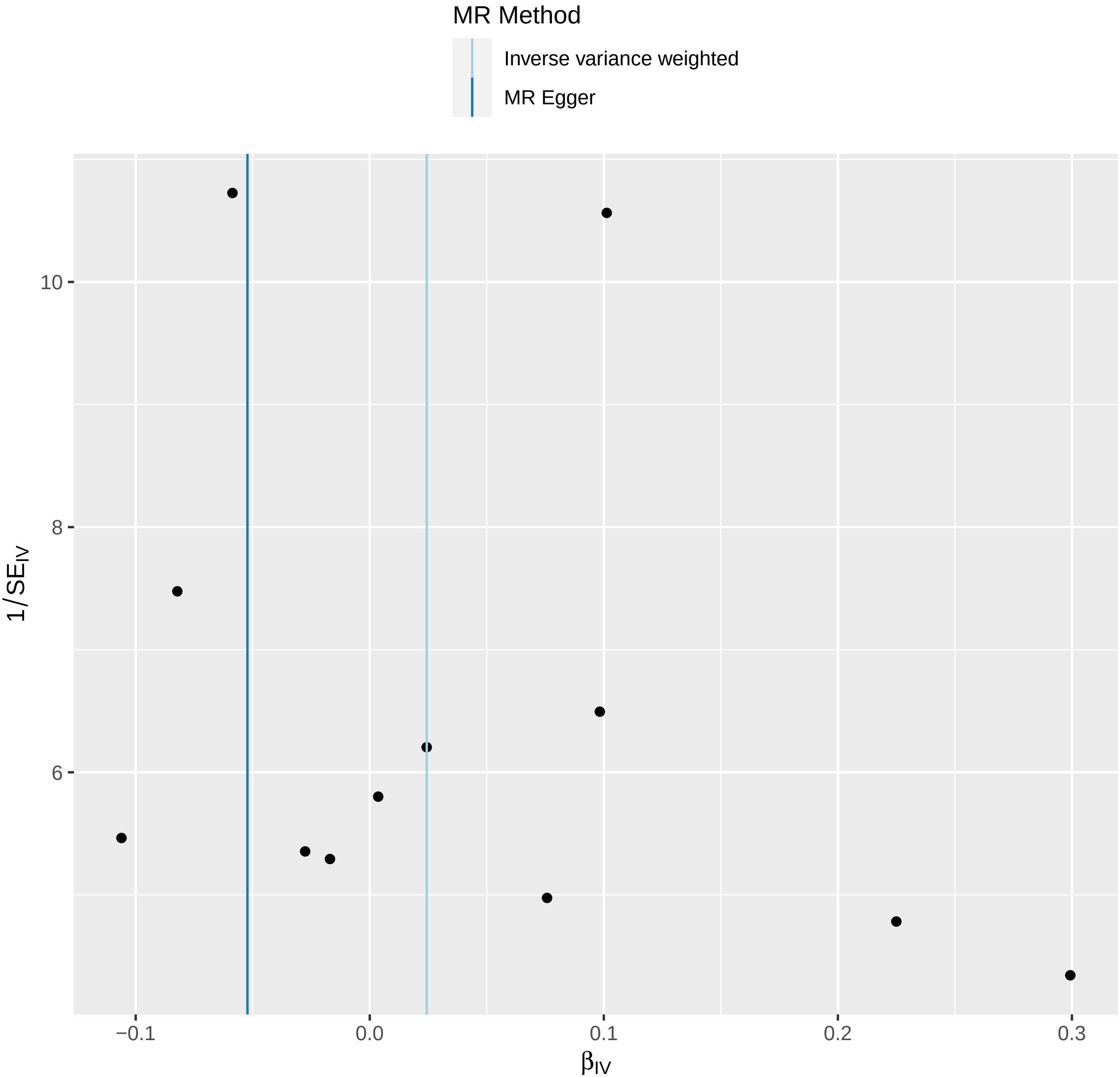

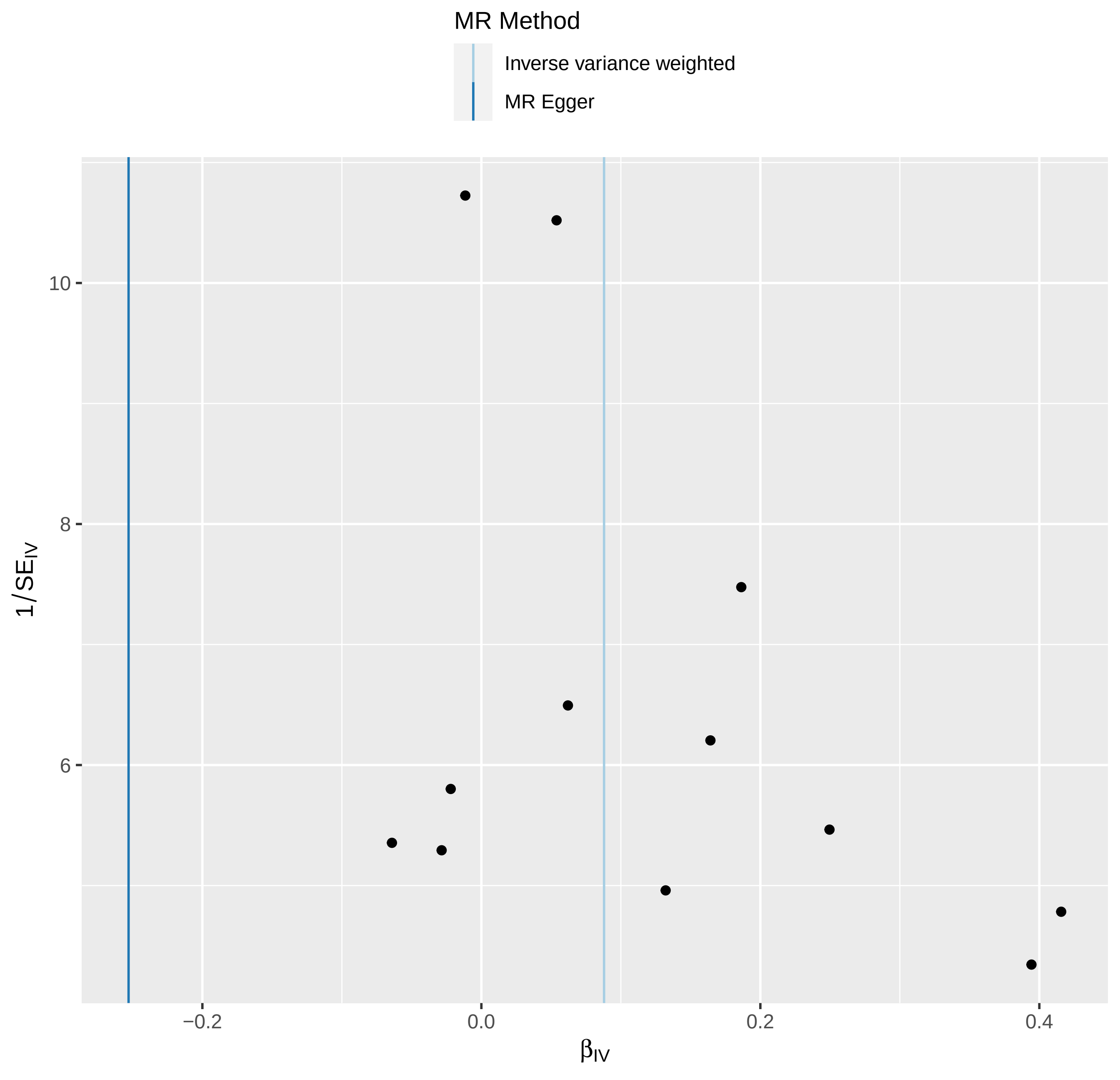
MR Method
Inverse variance weighted
MR Egger
10
8
6
1/SE$_{IV}$
−0.2
0.0
0.2
0.4
β$_{IV}$

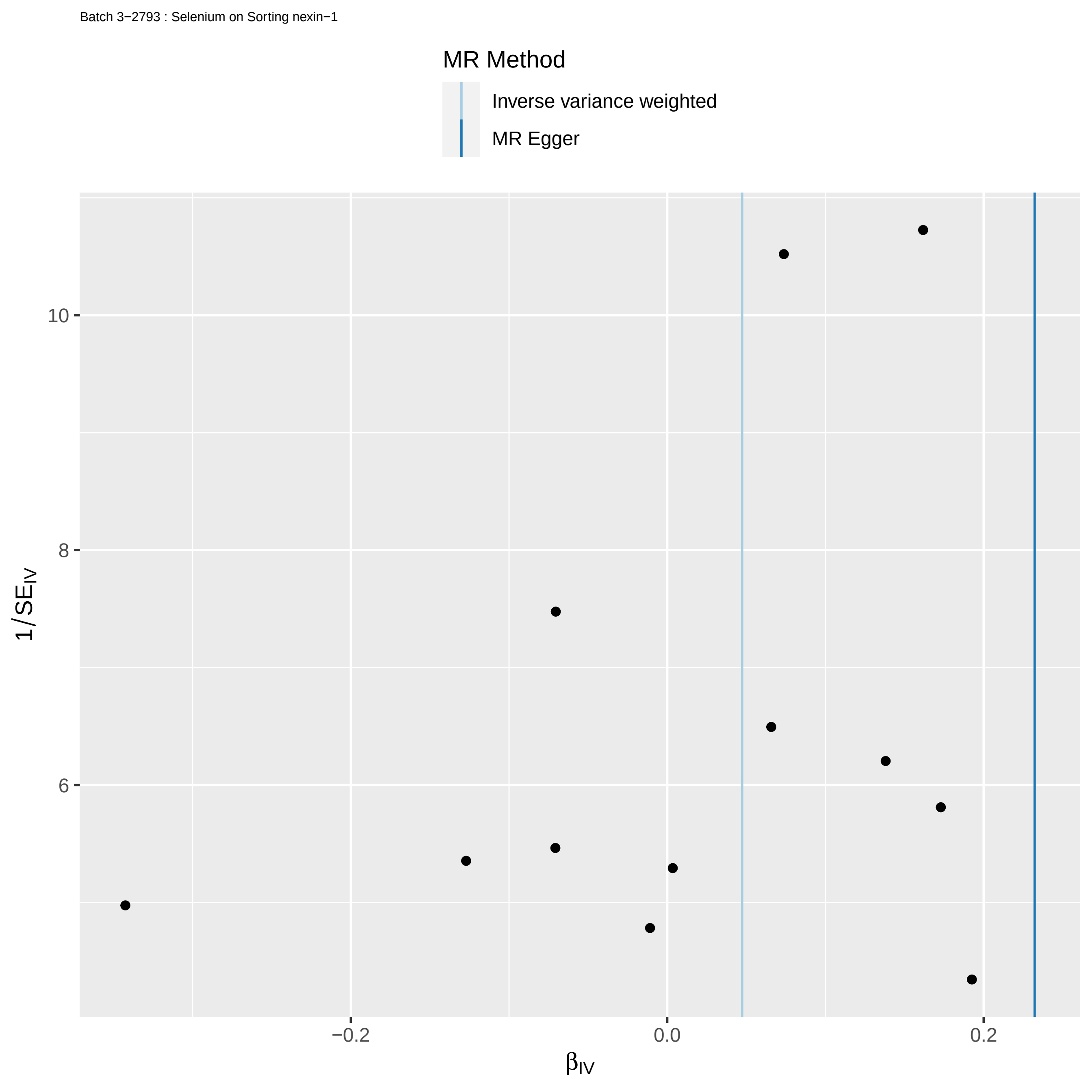

Batch 3−2793 : Selenium on Sorting nexin−1
MR Method
Inverse variance weighted
MR Egger
10
8
6
1/SE_IV
−0.2
0.0
0.2
β_IV

Batch 3−2794 : Selenium on Sorting nexin−4

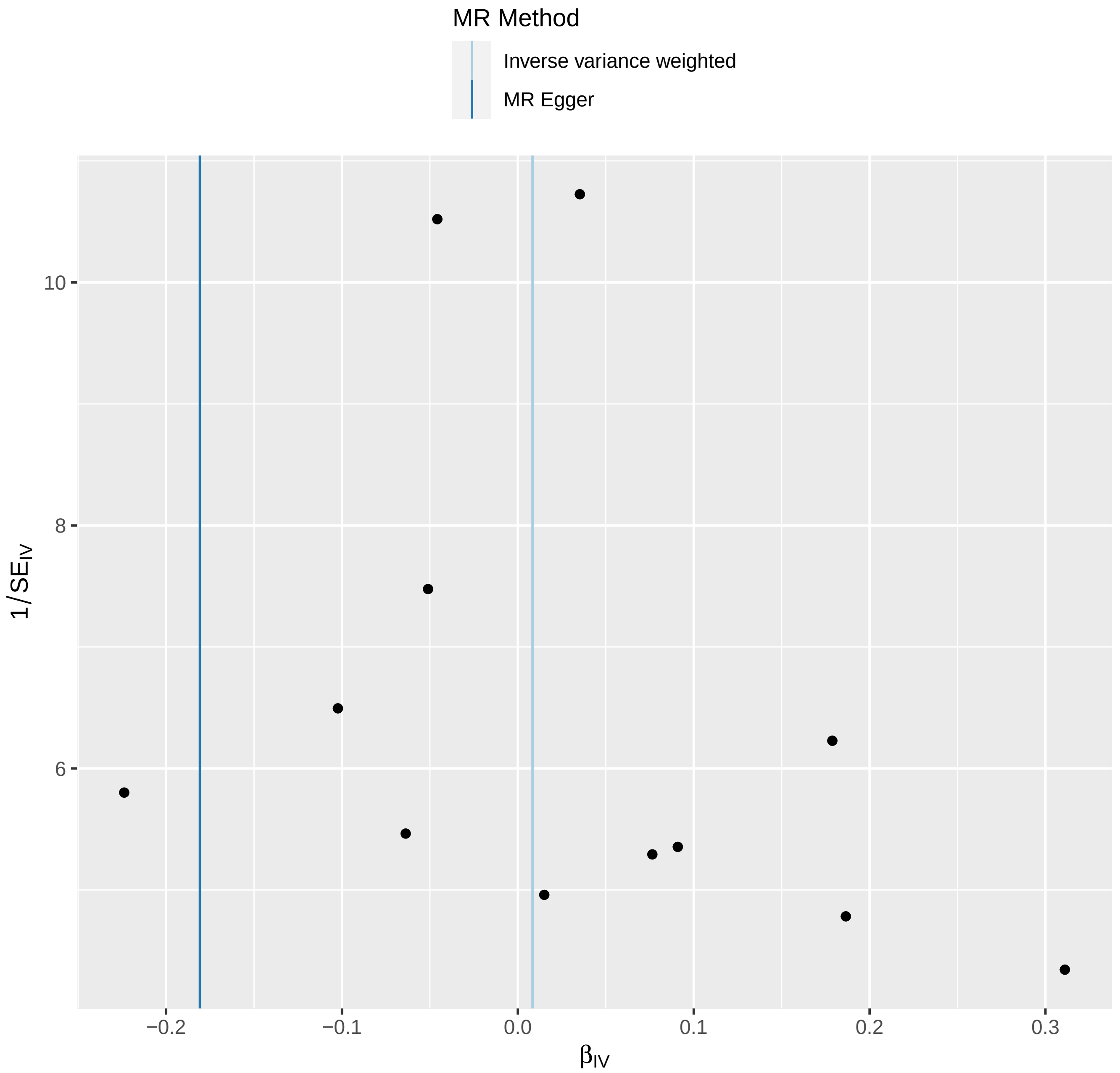

Batch 3−2795 : Selenium on Sorting nexin−7

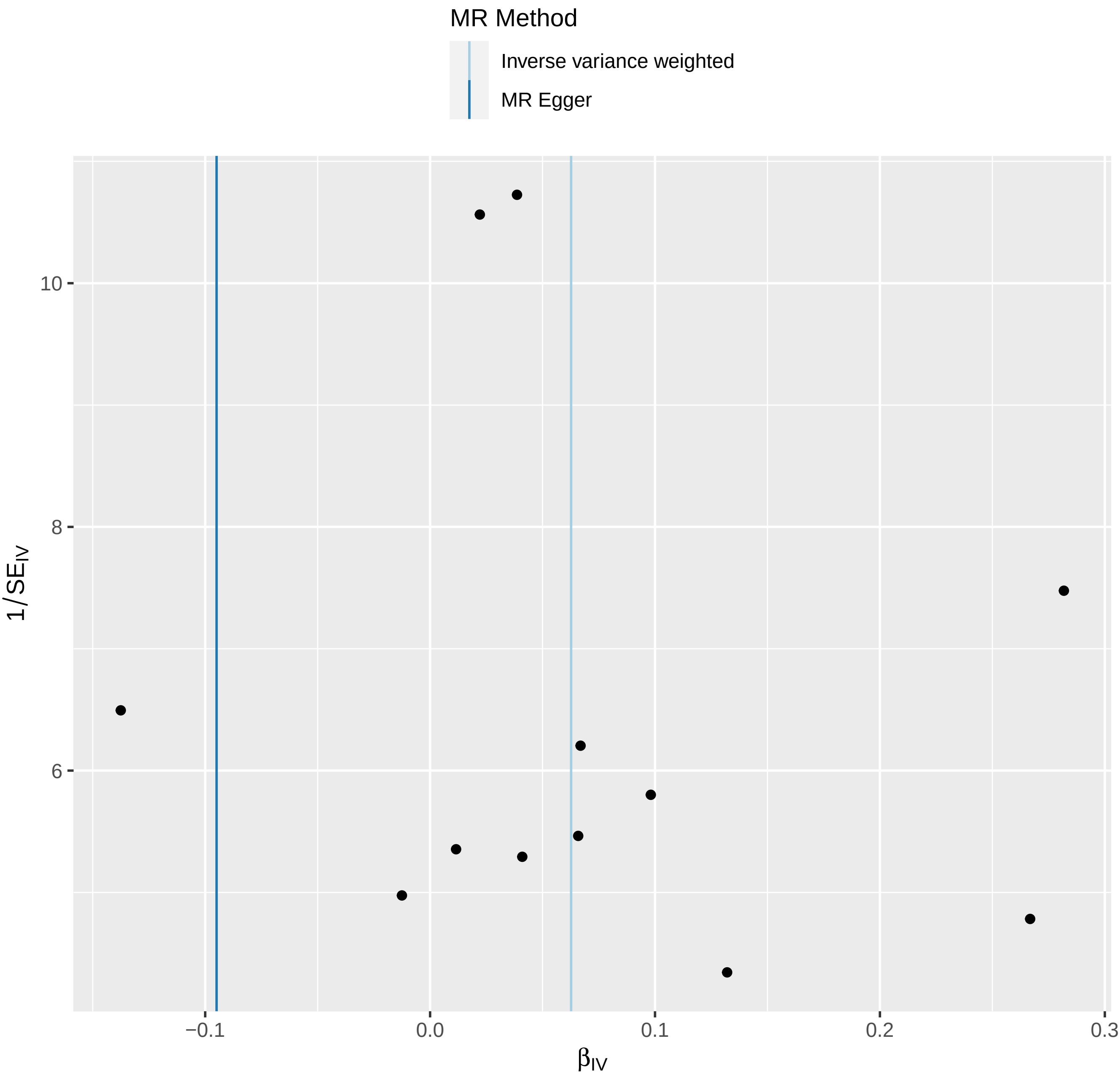

Batch 3−2796 : Selenium on Sorting nexin−8

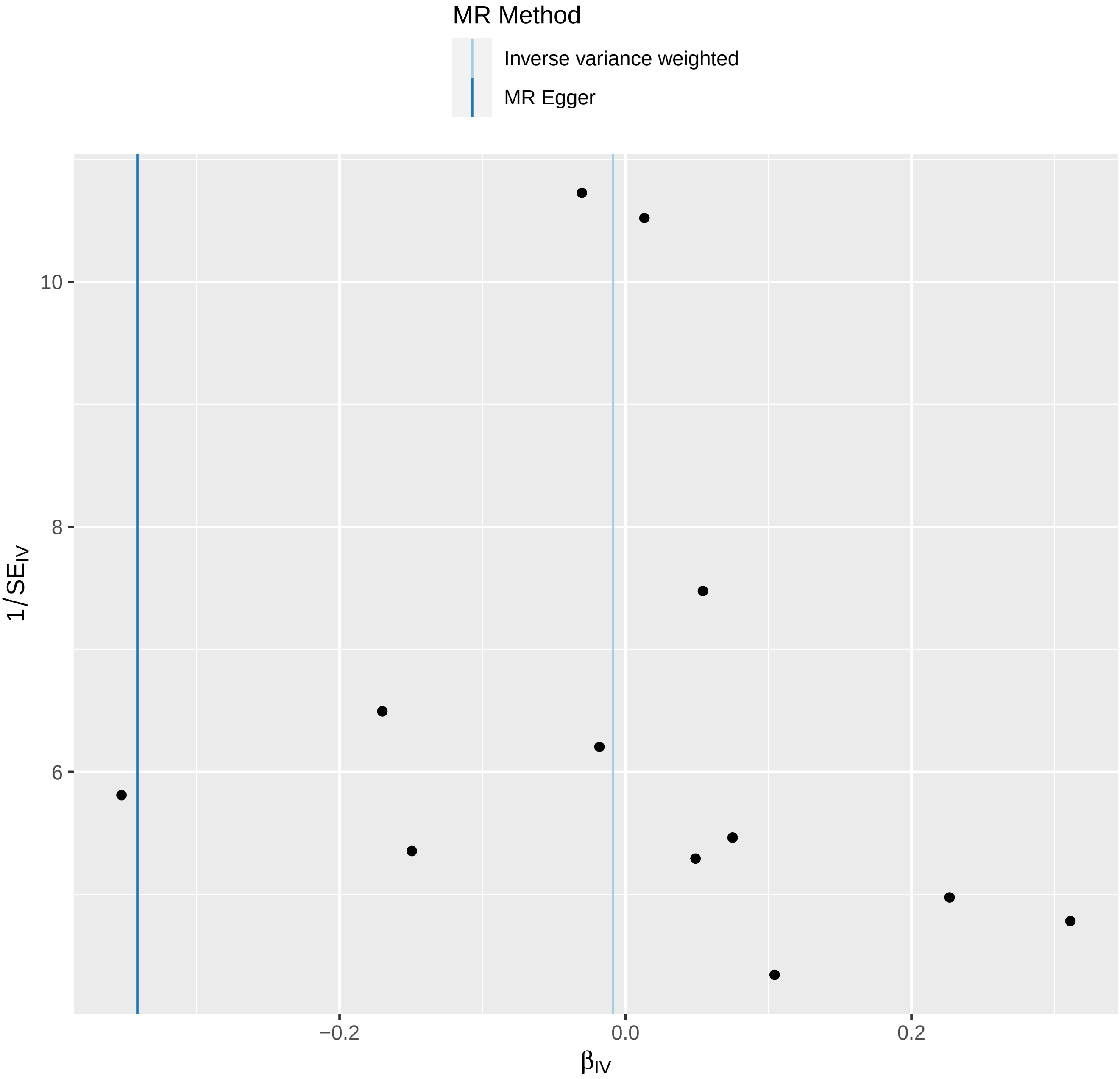

Batch 3−2797 : Selenium on Suppressor of cytokine signaling 3

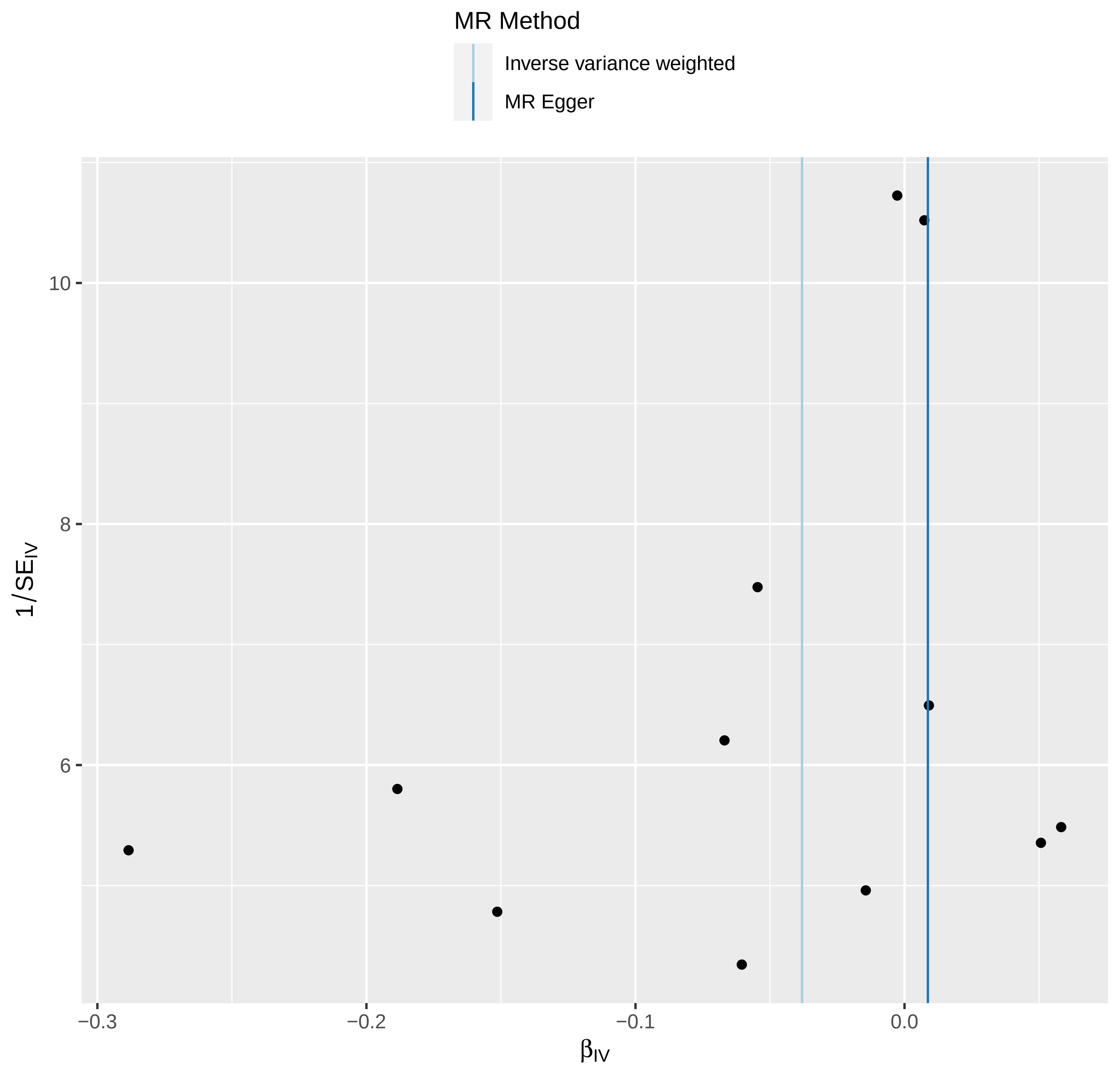

Batch 3−2798 : Selenium on Suppressor of cytokine signaling 7

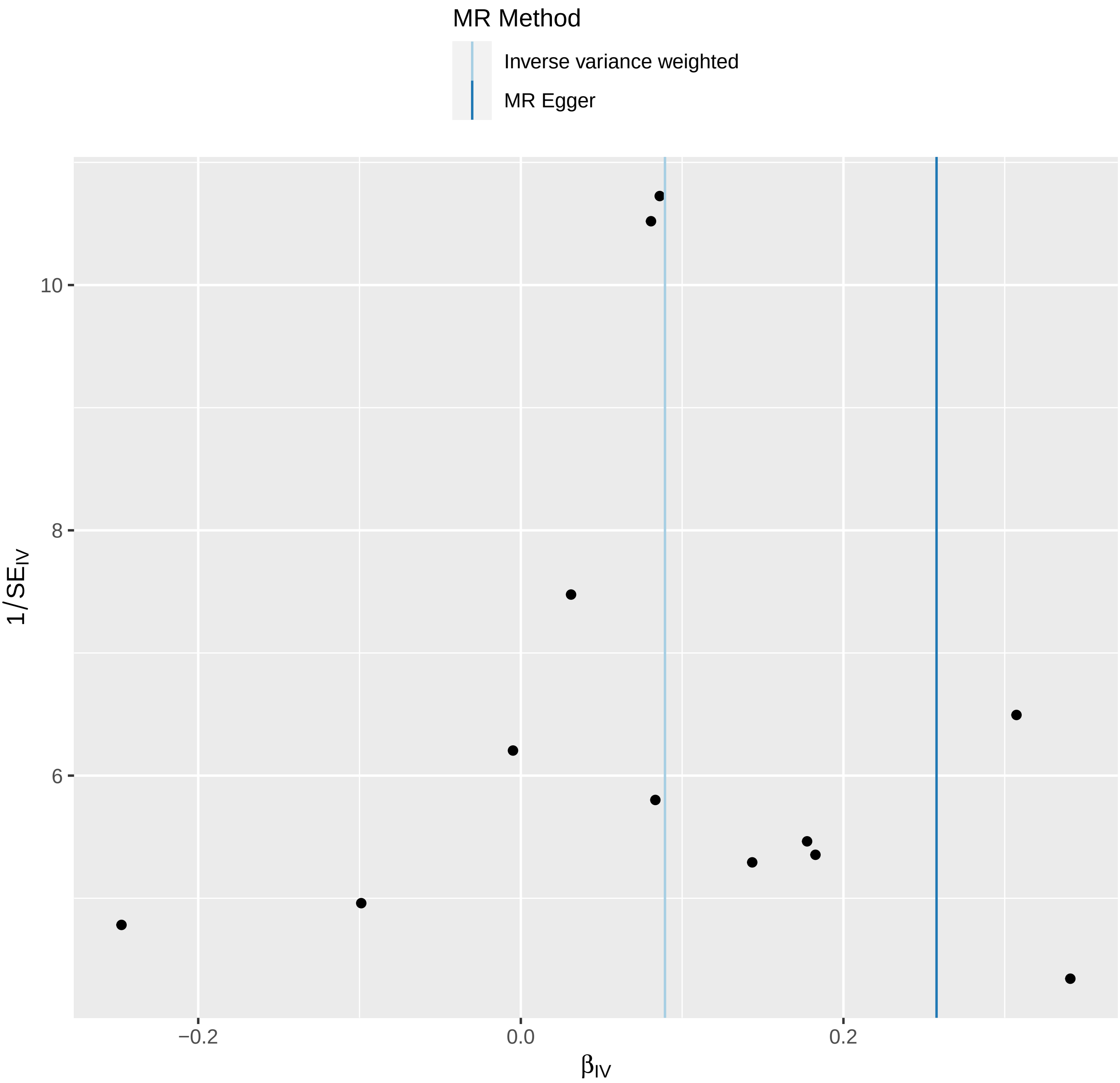

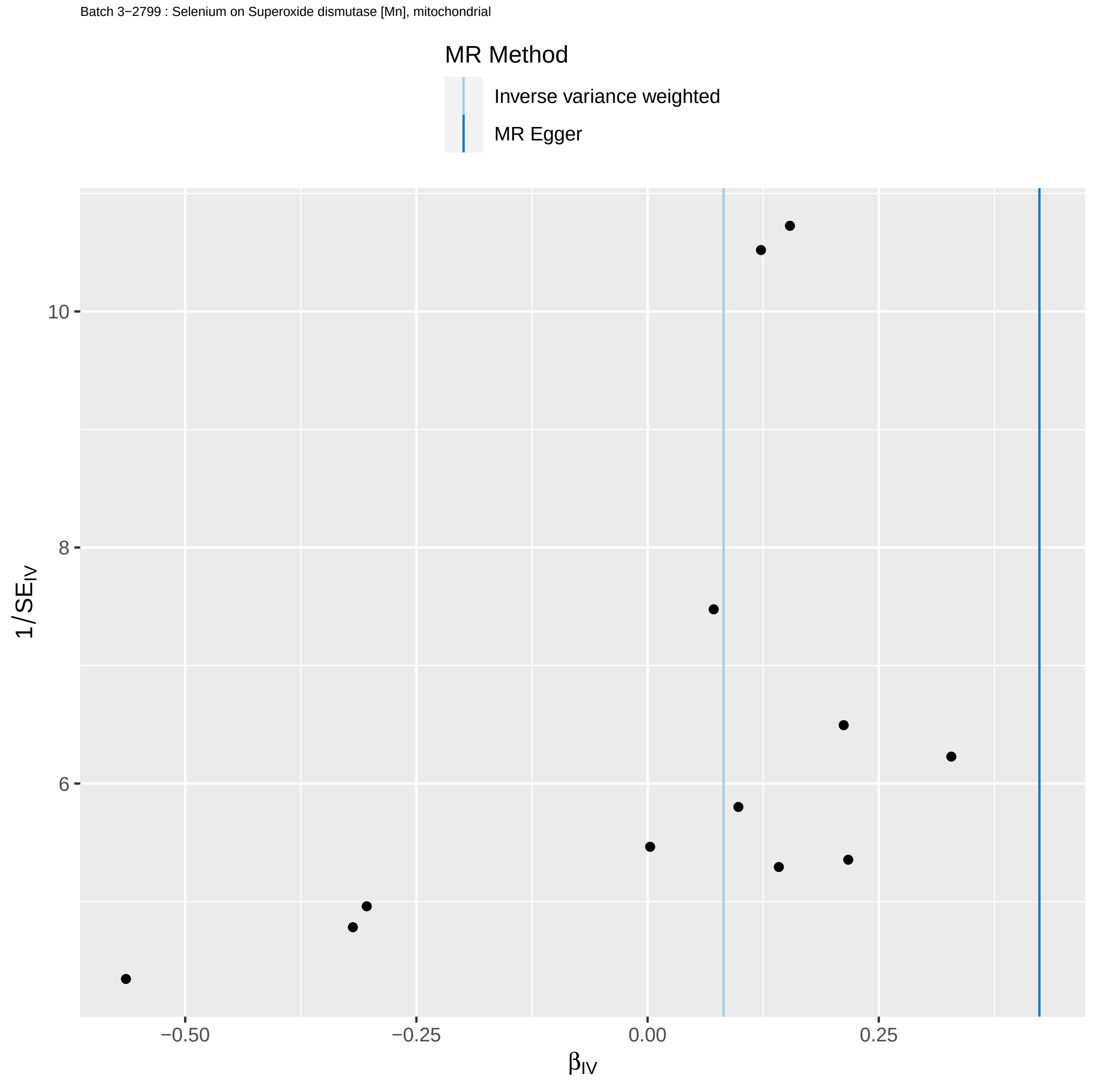
Batch 3−2799 : Selenium on Superoxide dismutase [Mn], mitochondrial
MR Method
Inverse variance weighted
MR Egger
10
8
6
1/SE_IV
−0.50
−0.25
0.00
0.25
β_IV

Batch 3−2800 : Selenium on Extracellular superoxide dismutase [Cu−Zn]

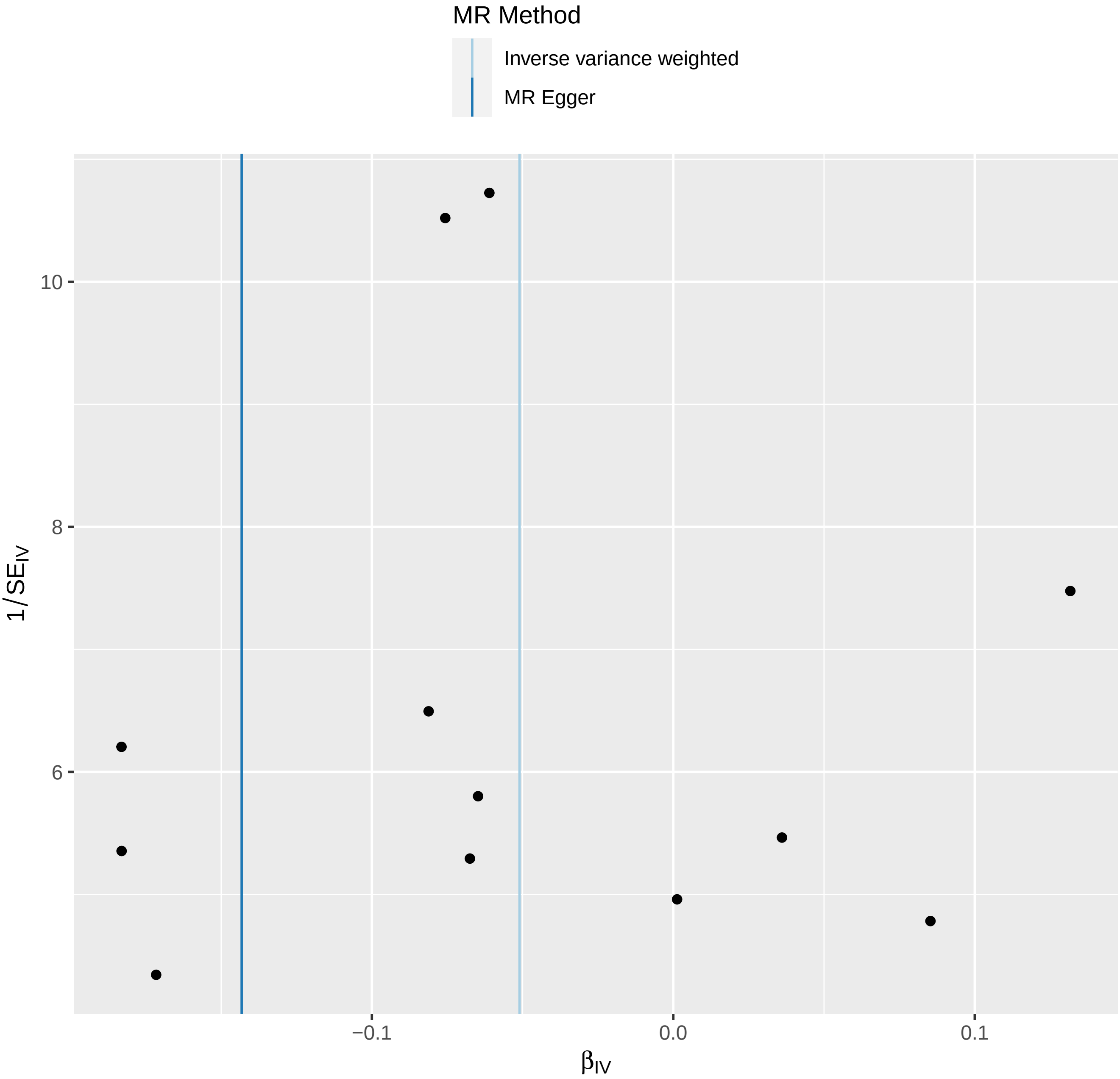

Batch 3−2801 : Selenium on Vinexin b

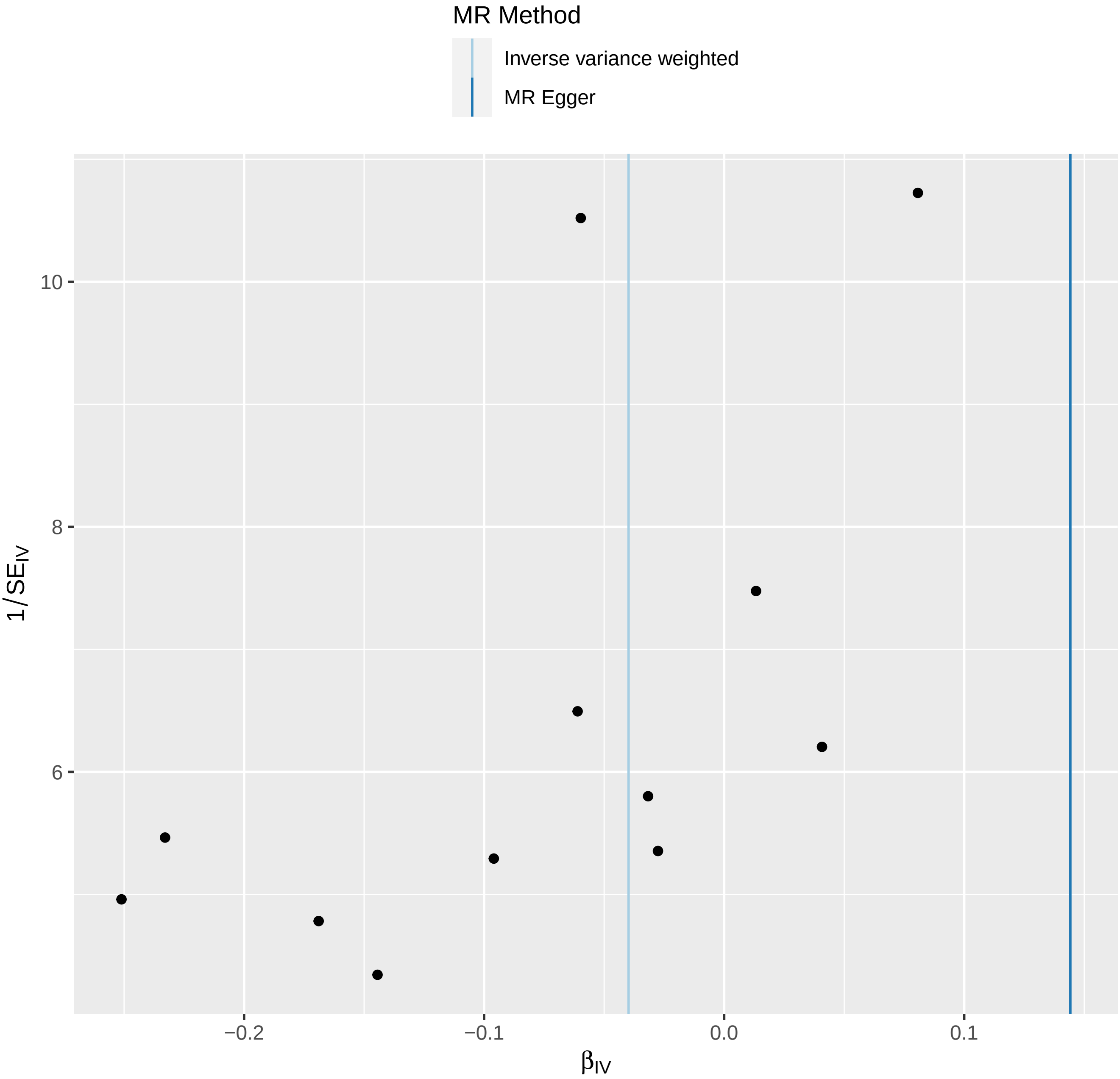

Batch 3−2802 : Selenium on Sortilin

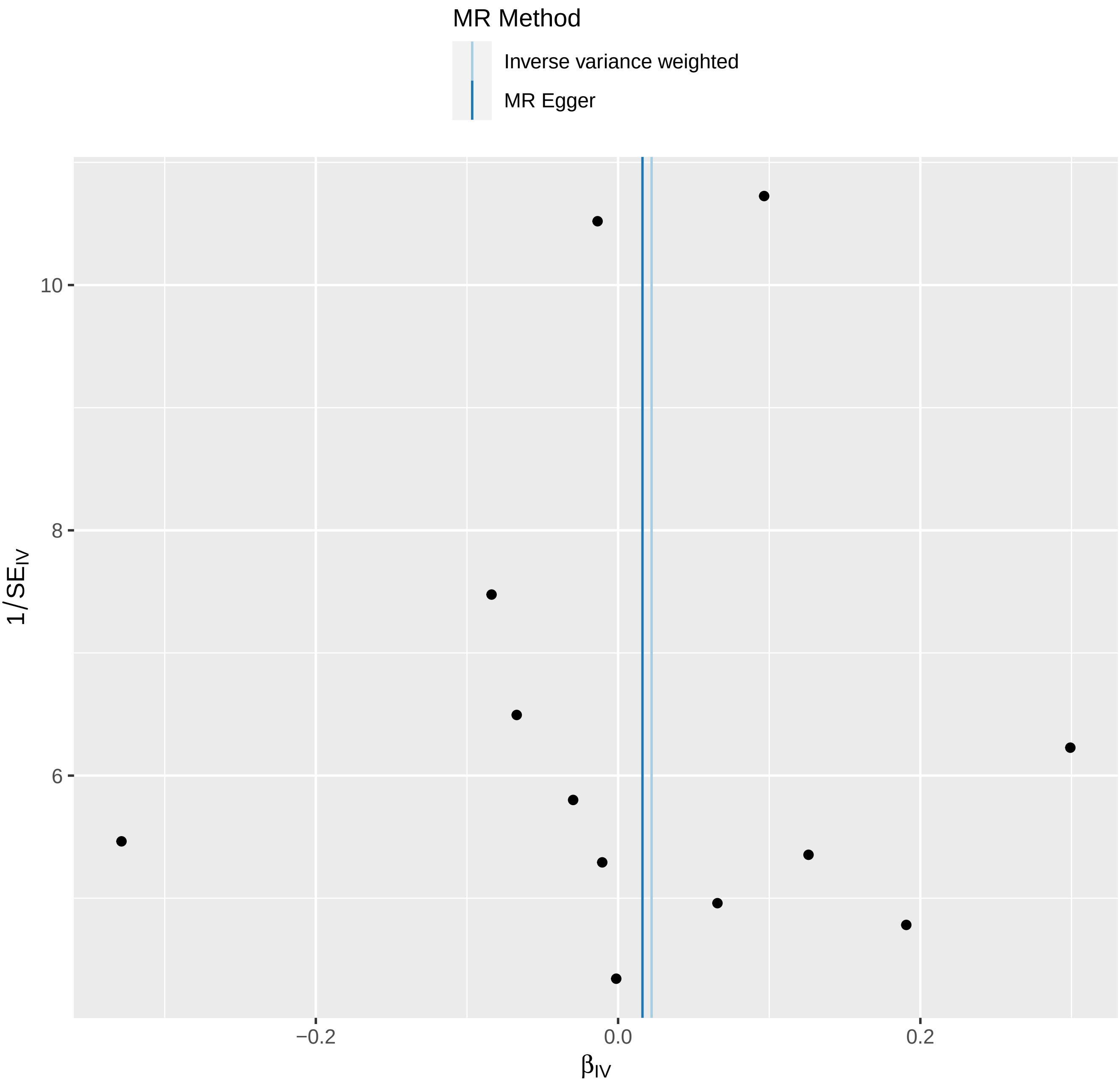

Batch 3−2803 : Selenium on Sclerostin

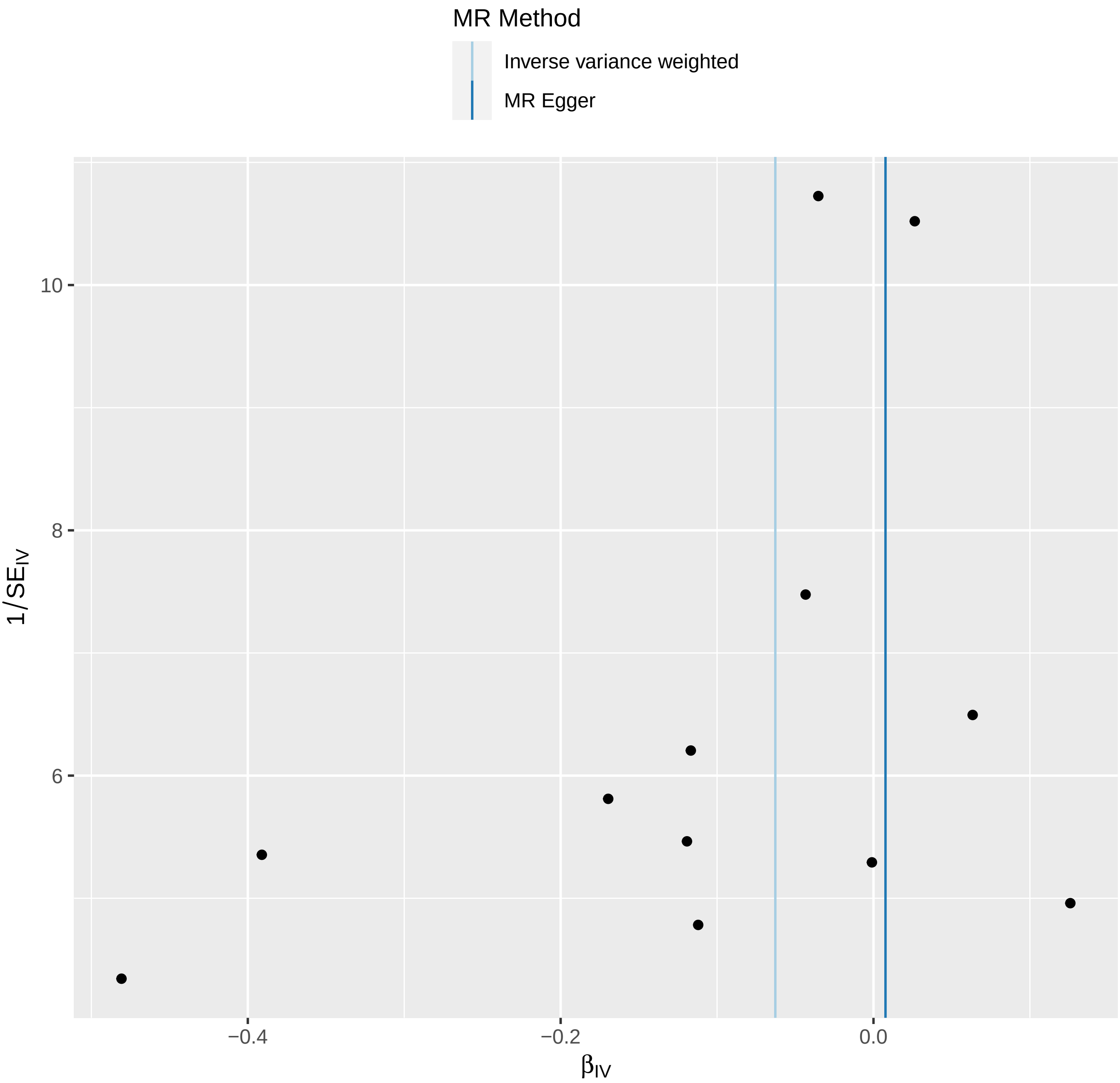

Batch 3−2804 : Selenium on Sperm acrosome membrane−associated protein 3

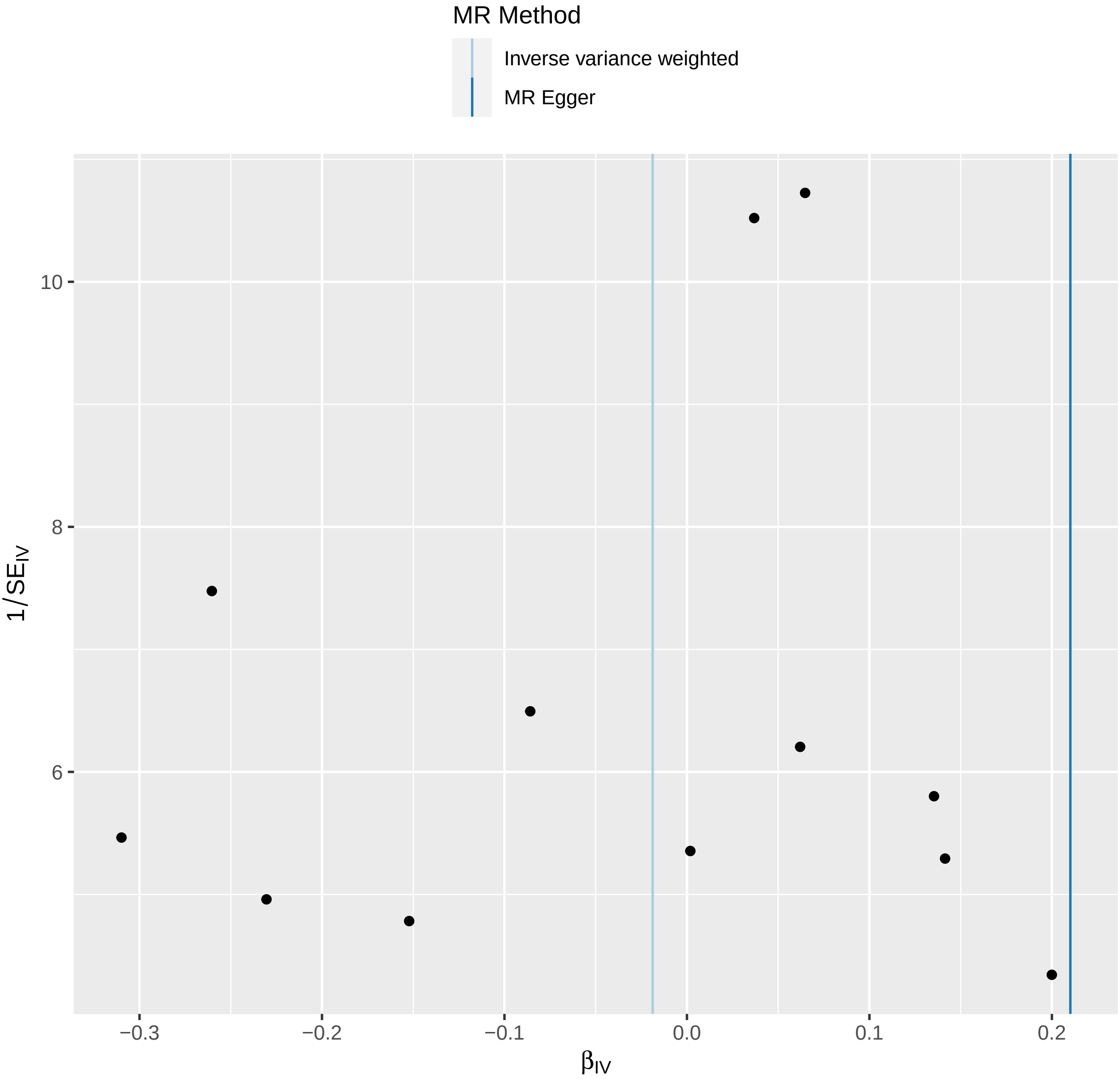

Batch 3−2805 : Selenium on Protein SPACA7

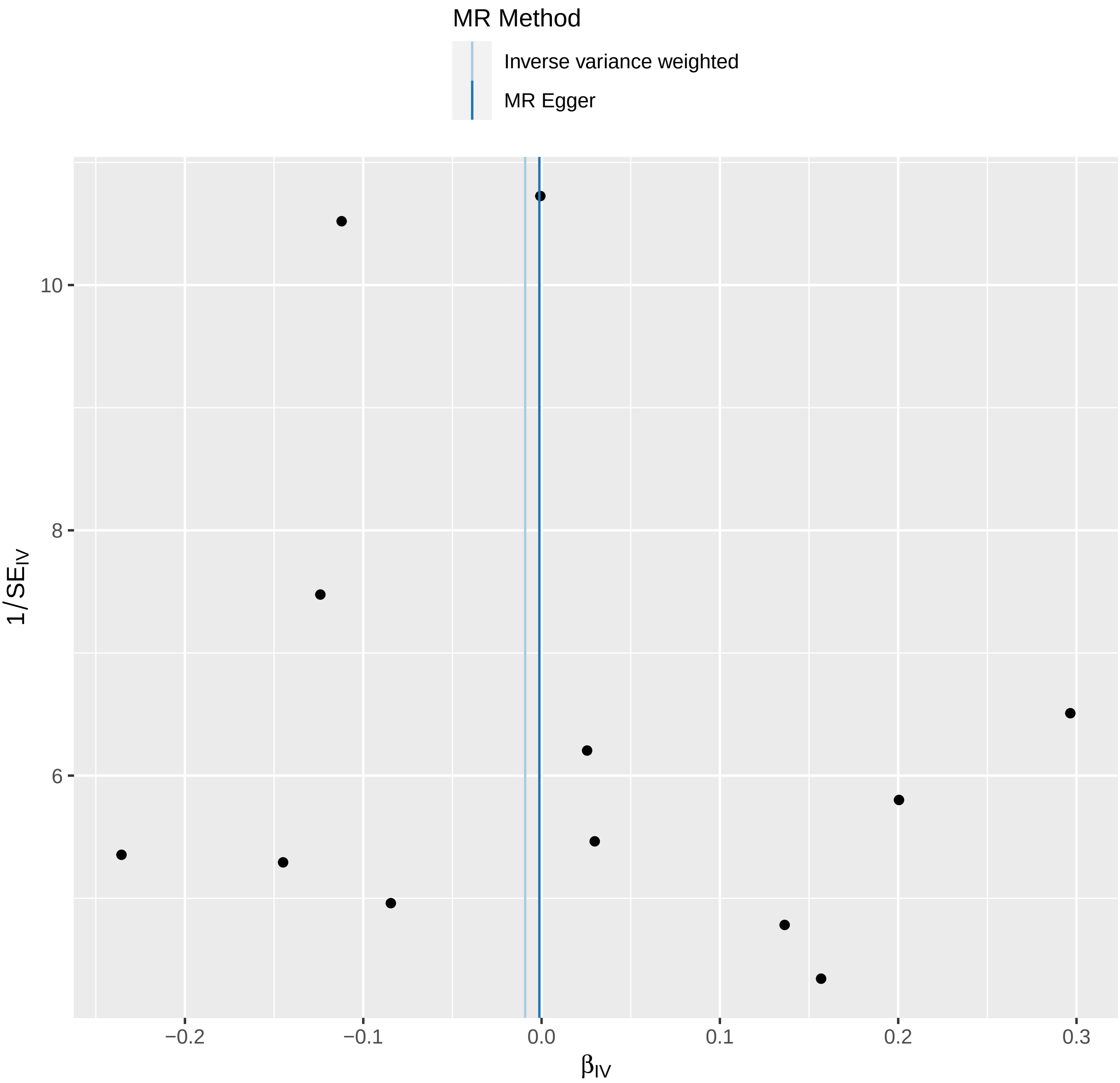

Batch 3−2806 : Selenium on Sperm−associated antigen 11A

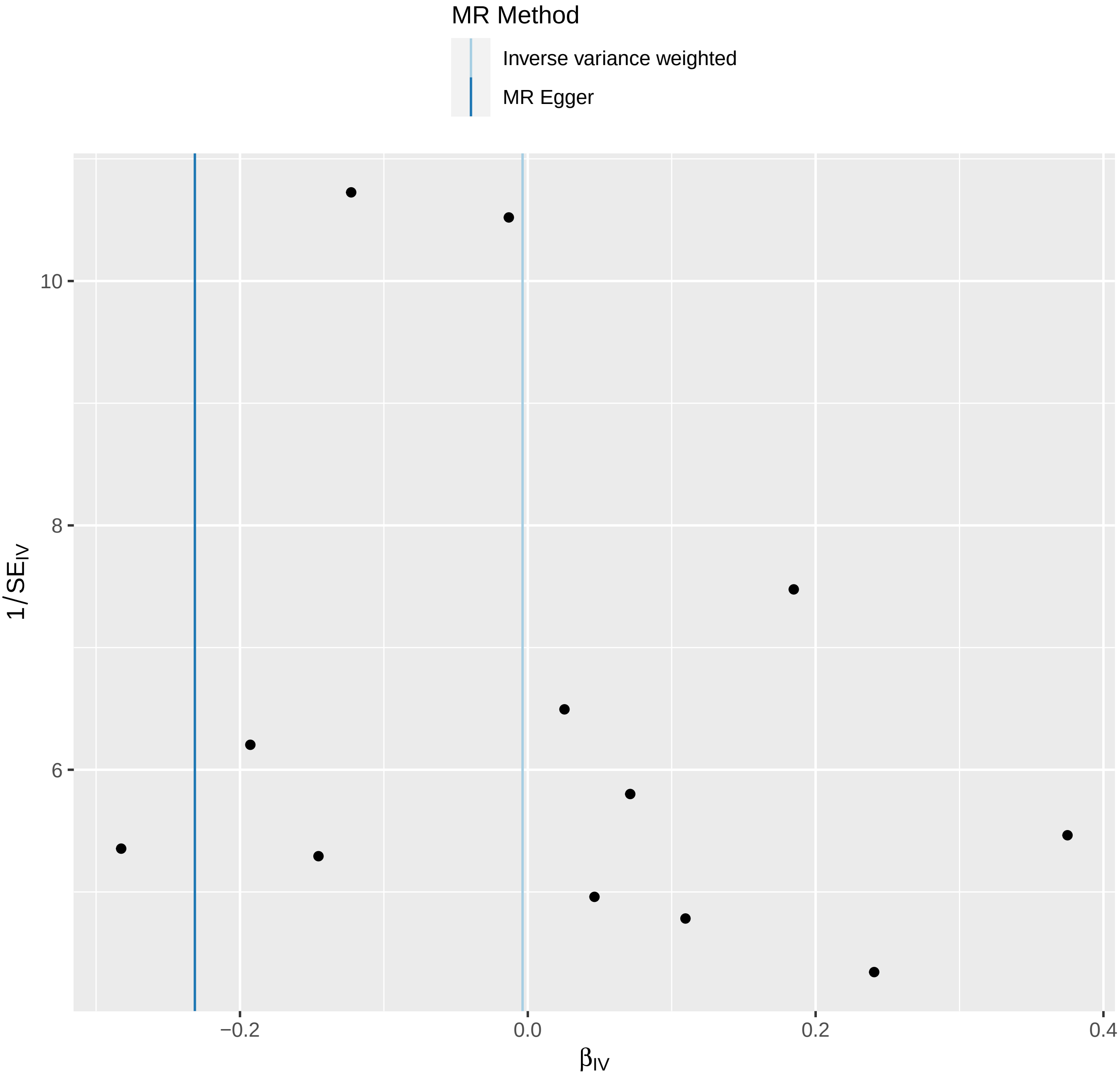

Batch 3−2807 : Selenium on Sperm−associated antigen 11B

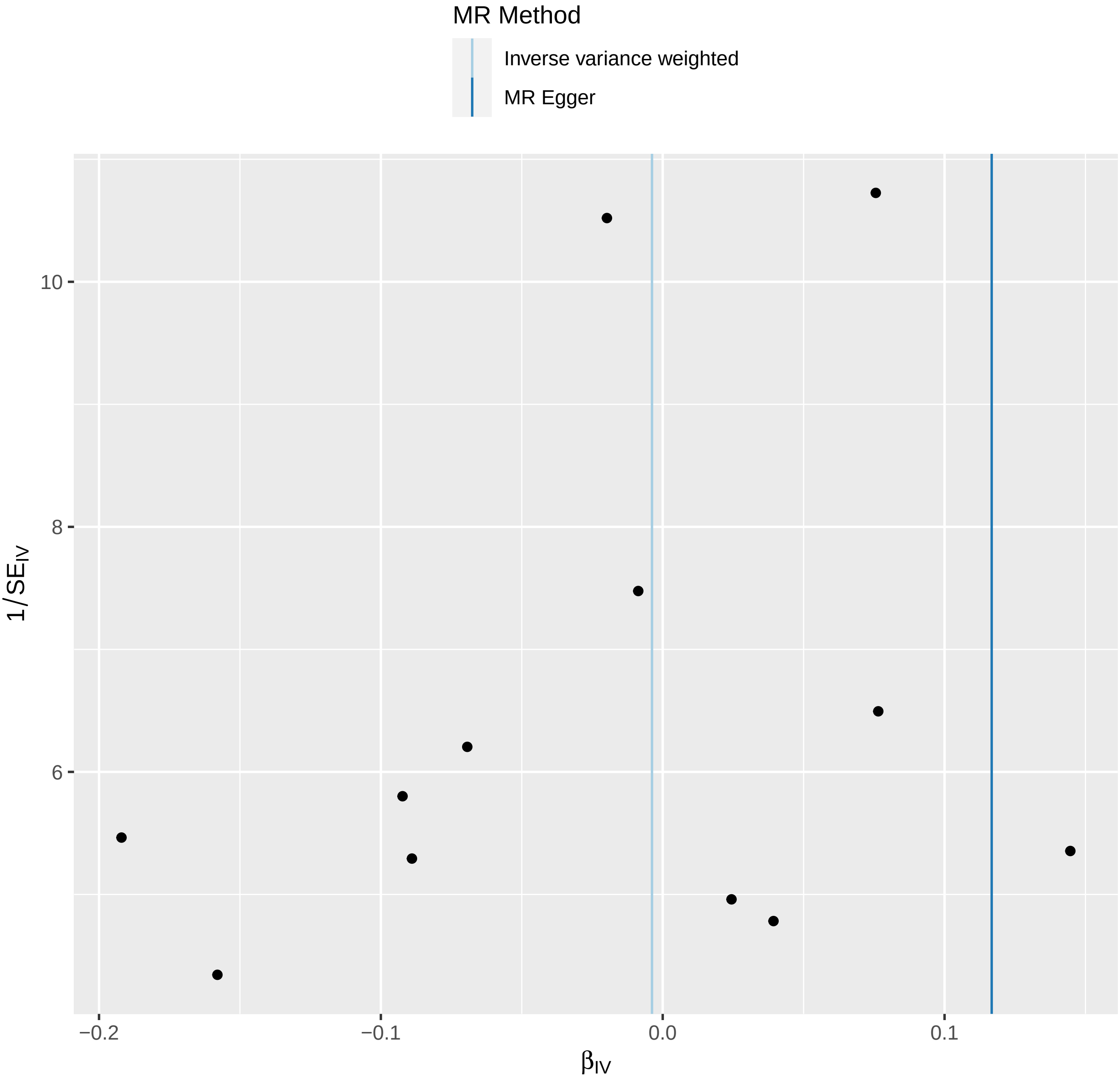

Batch 3−2808 : Selenium on SPARC

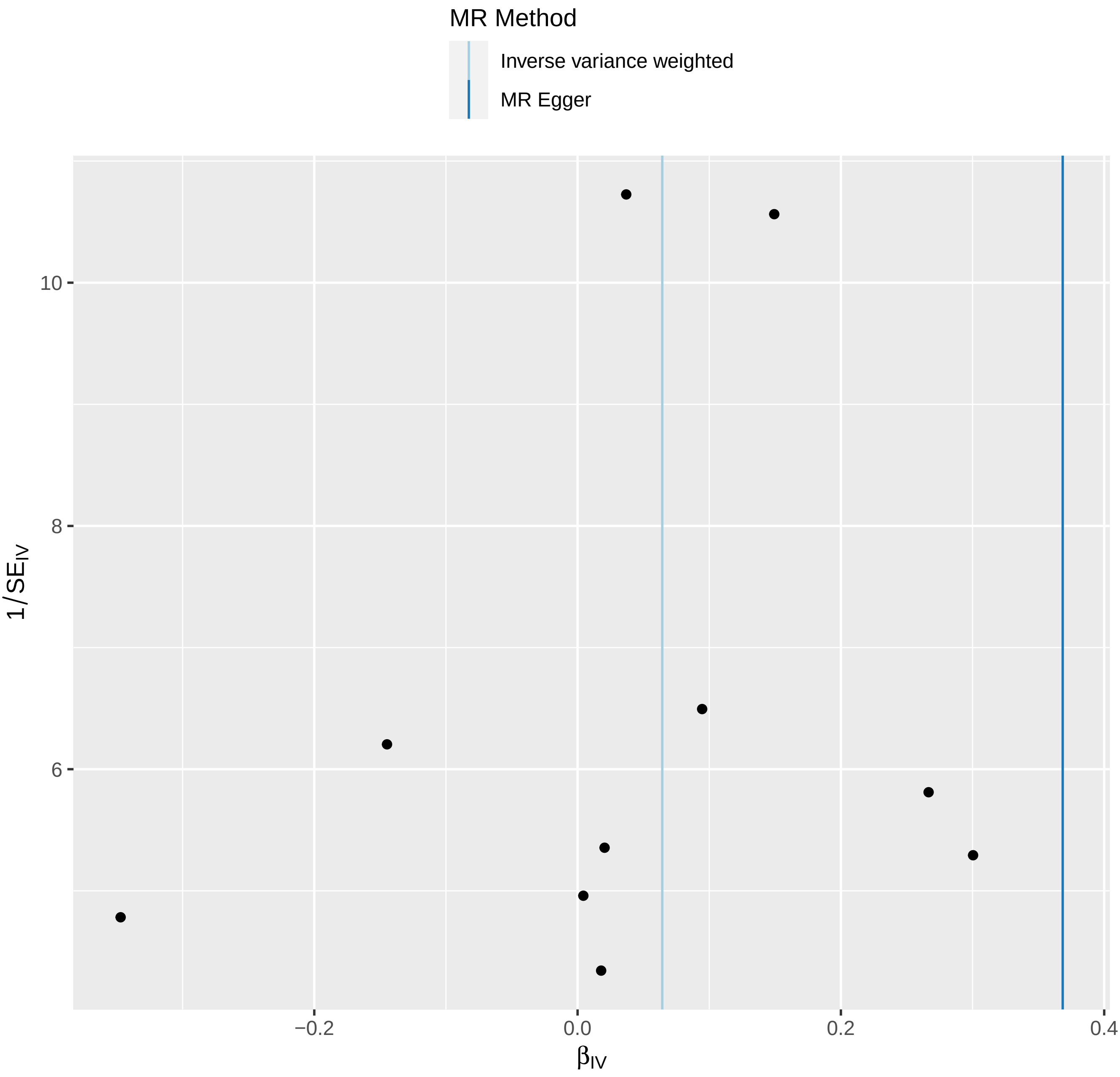

Batch 3−2809 : Selenium on SPARC

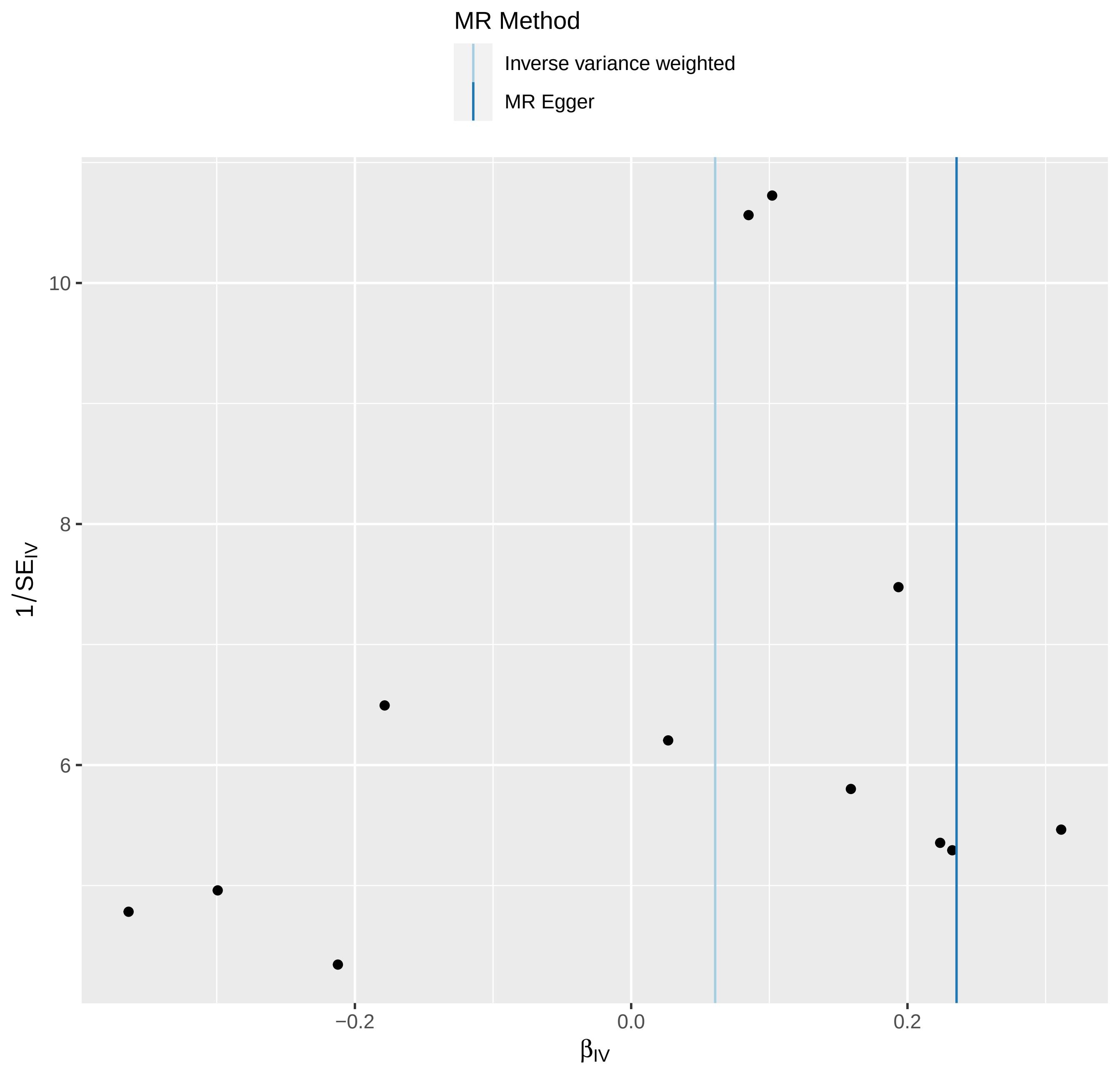

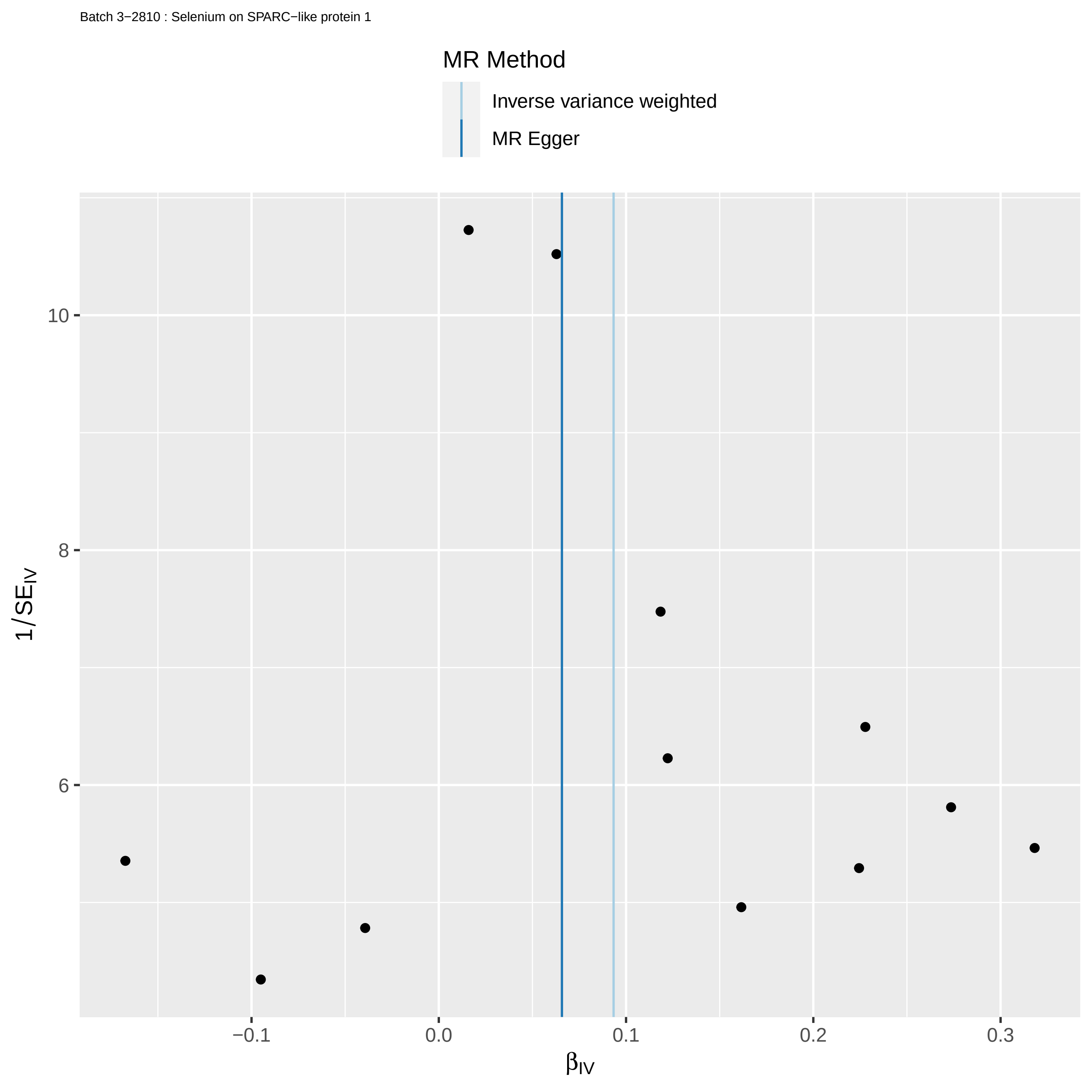
Batch 3−2810 : Selenium on SPARC−like protein 1
MR Method
Inverse variance weighted
MR Egger
10
8
6
1/SE_IV
−0.1
0.0
0.1
0.2
0.3
β_IV

Batch 3−2811 : Selenium on SPARC−like protein 1

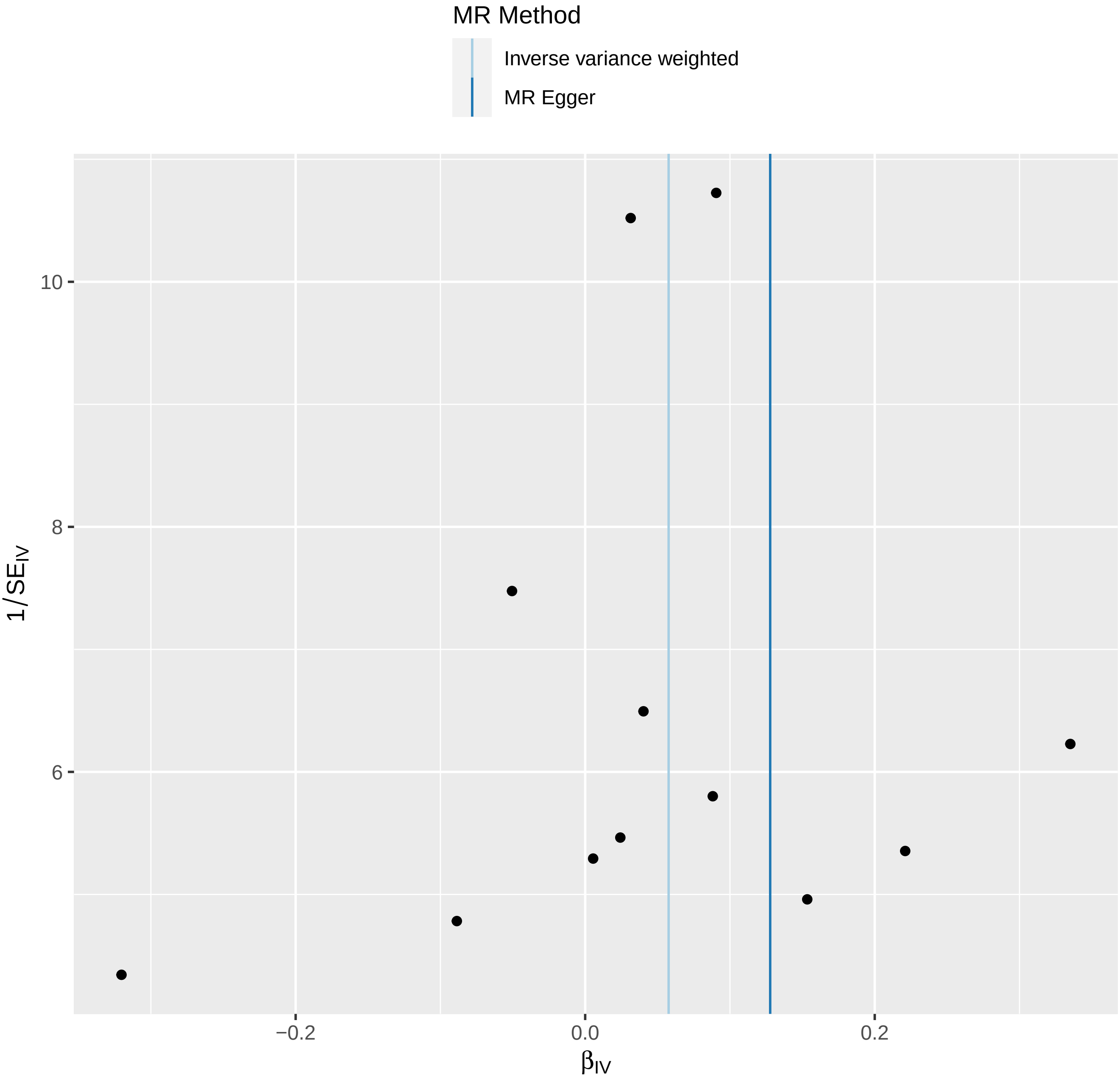

Batch 3−2812 : Selenium on Spastin

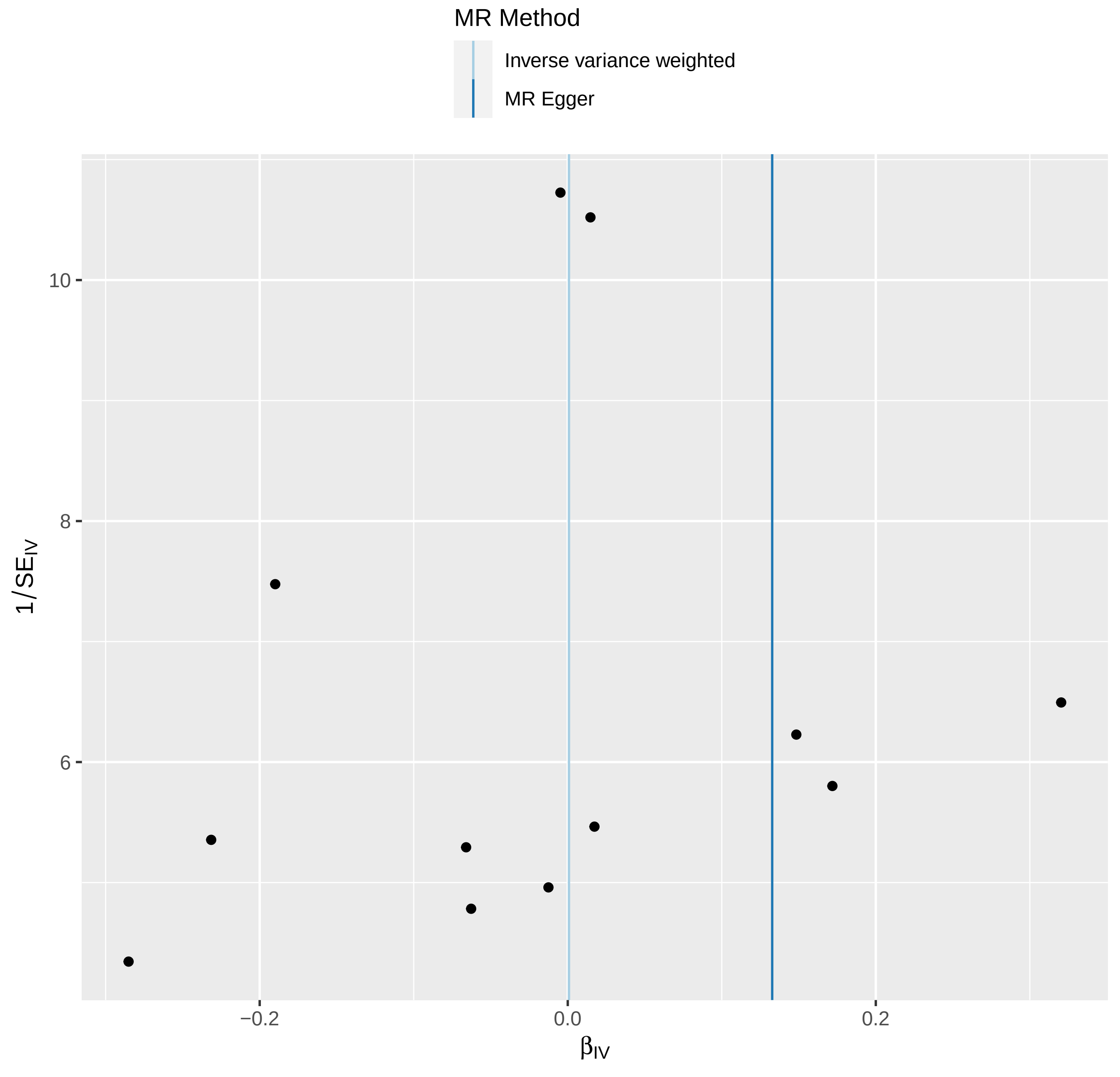

Batch 3−2813 : Selenium on Spermatogenesis−associated protein 20

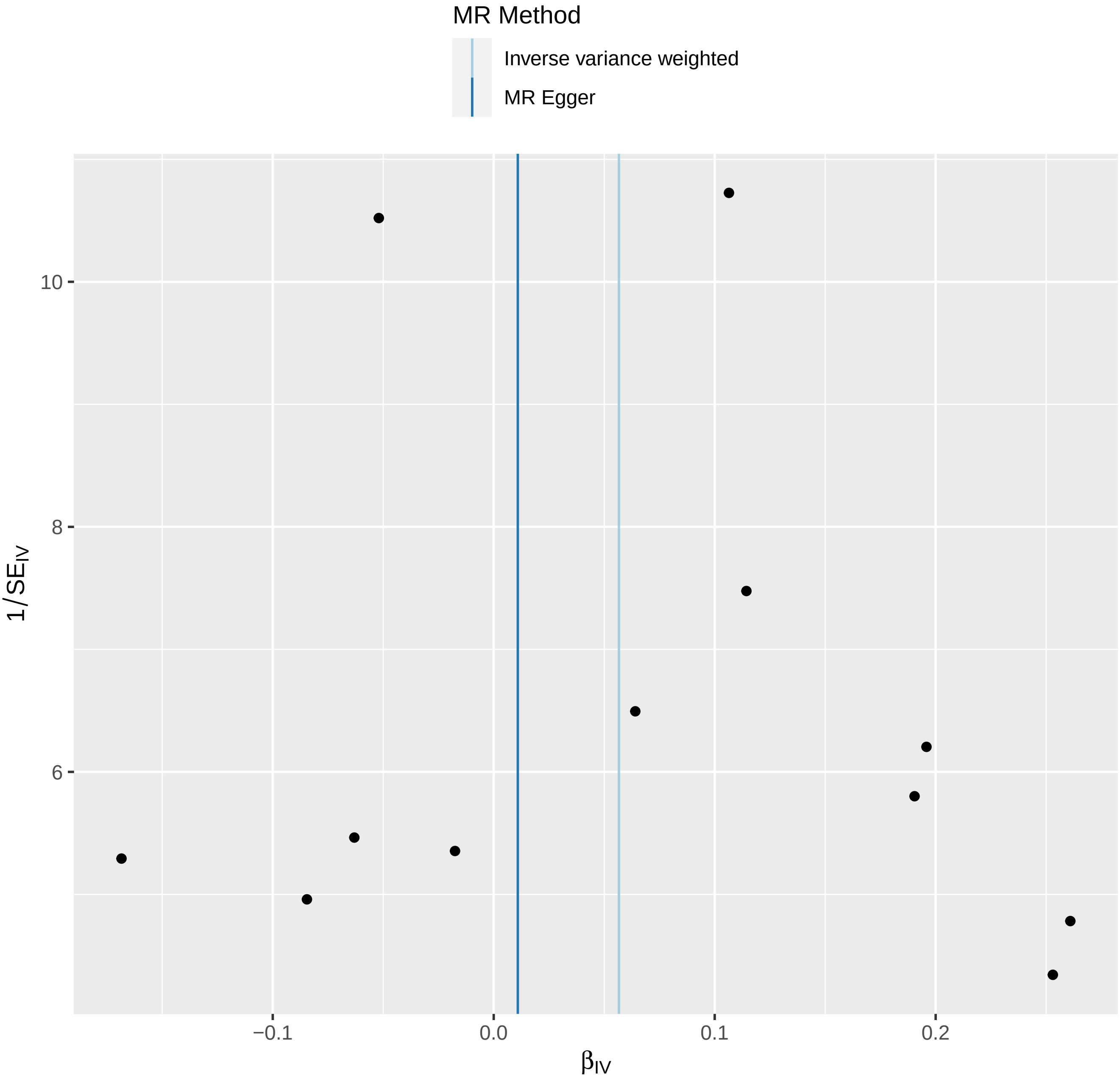

Batch 3−2814 : Selenium on Spermatogenesis−associated protein 9

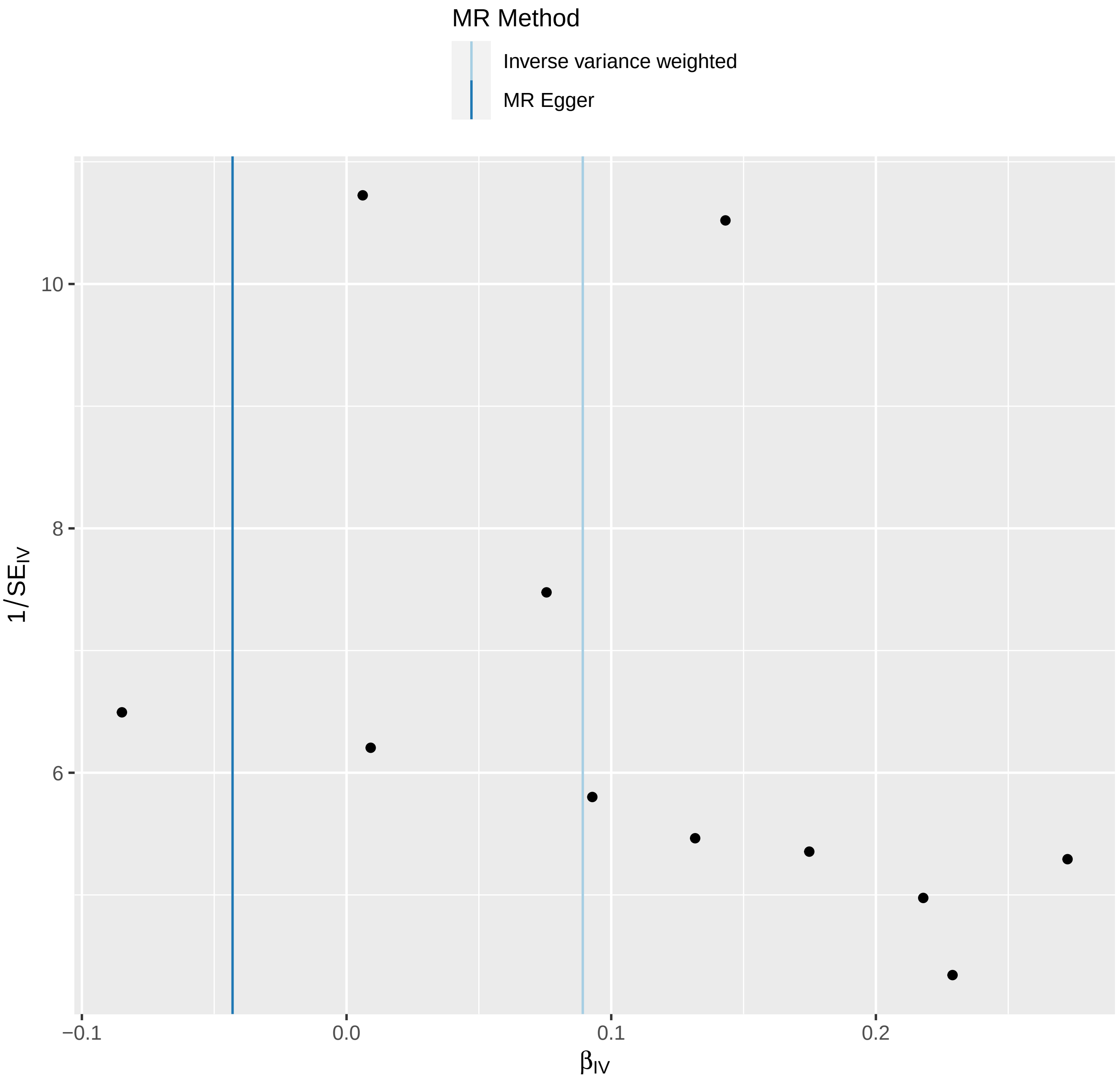

Batch 3−2815 : Selenium on Maspardin

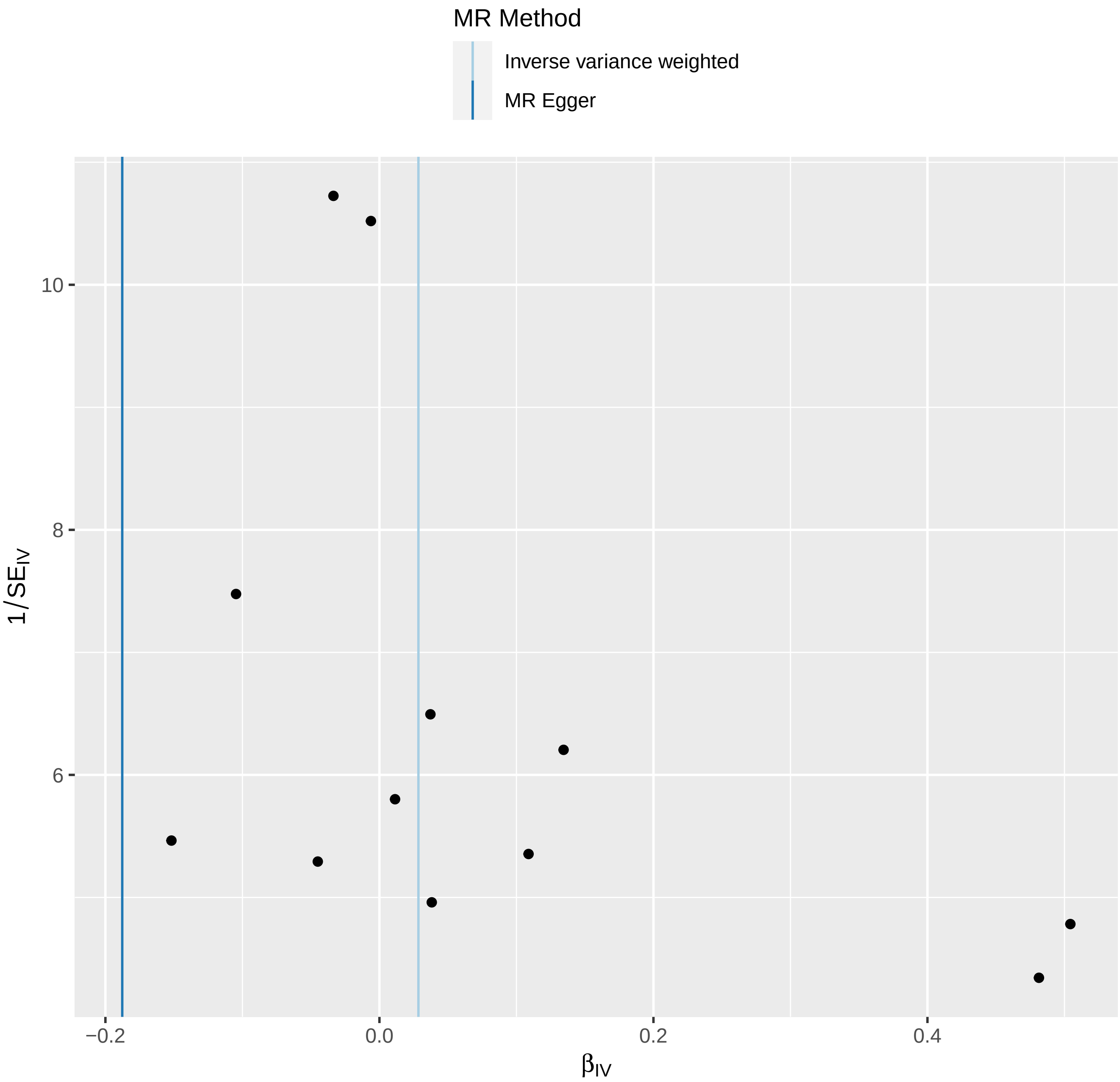

Batch 3−2816 : Selenium on Sphingosine kinase 2

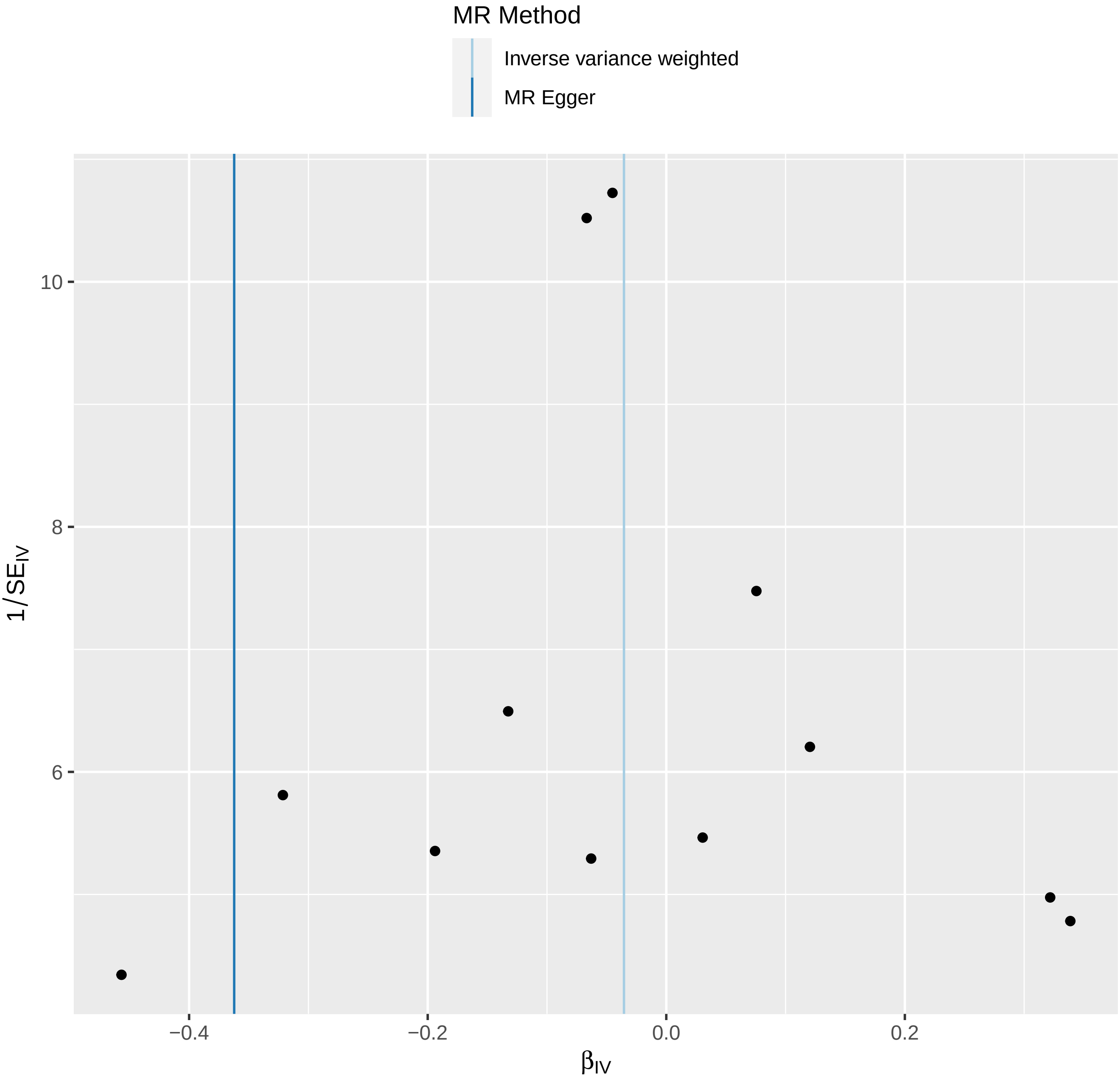

Batch 3−2817 : Selenium on Serine protease inhibitor Kazal−type 13

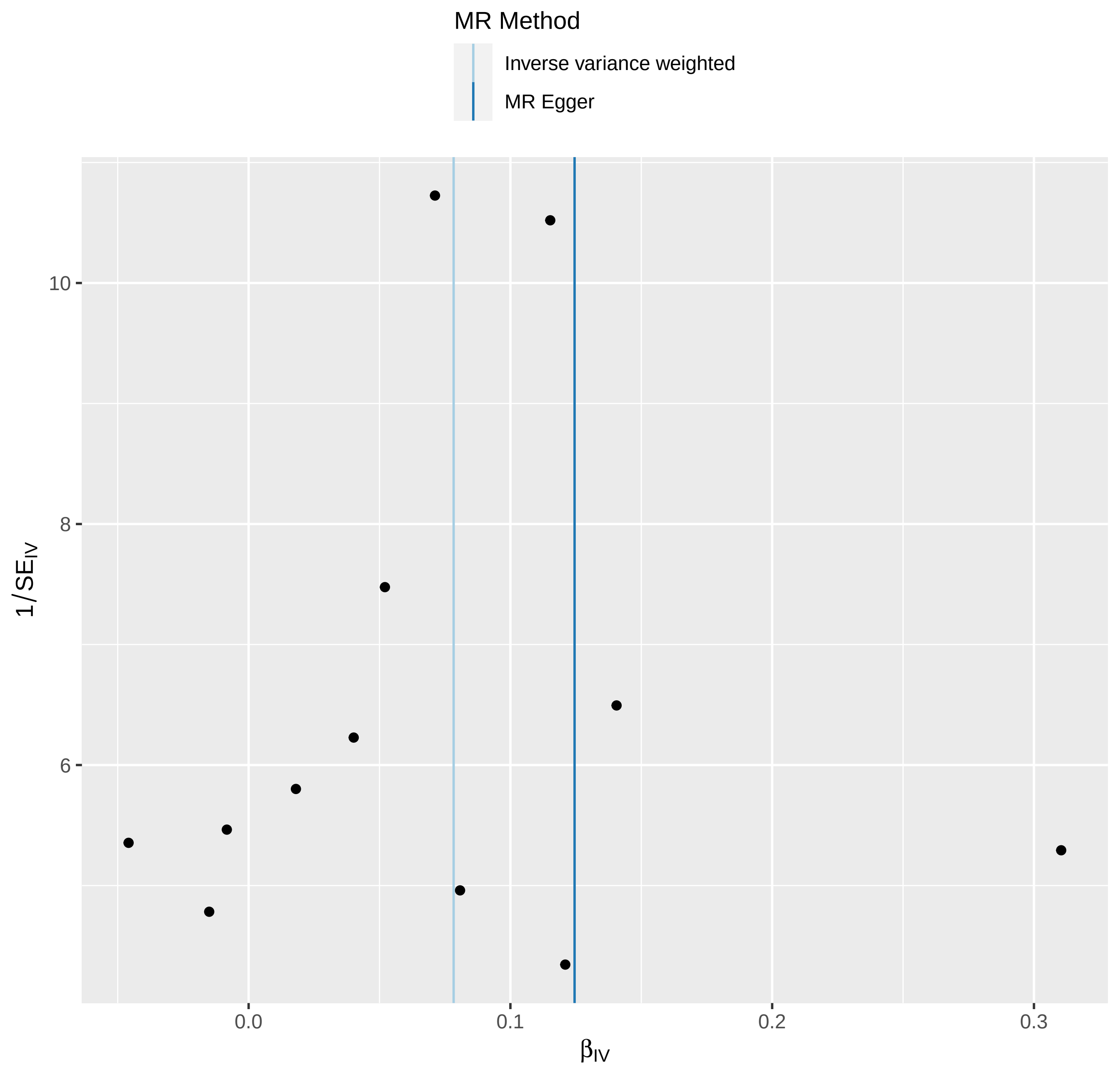

Batch 3−2818 : Selenium on Serine protease inhibitor Kazal−type 2

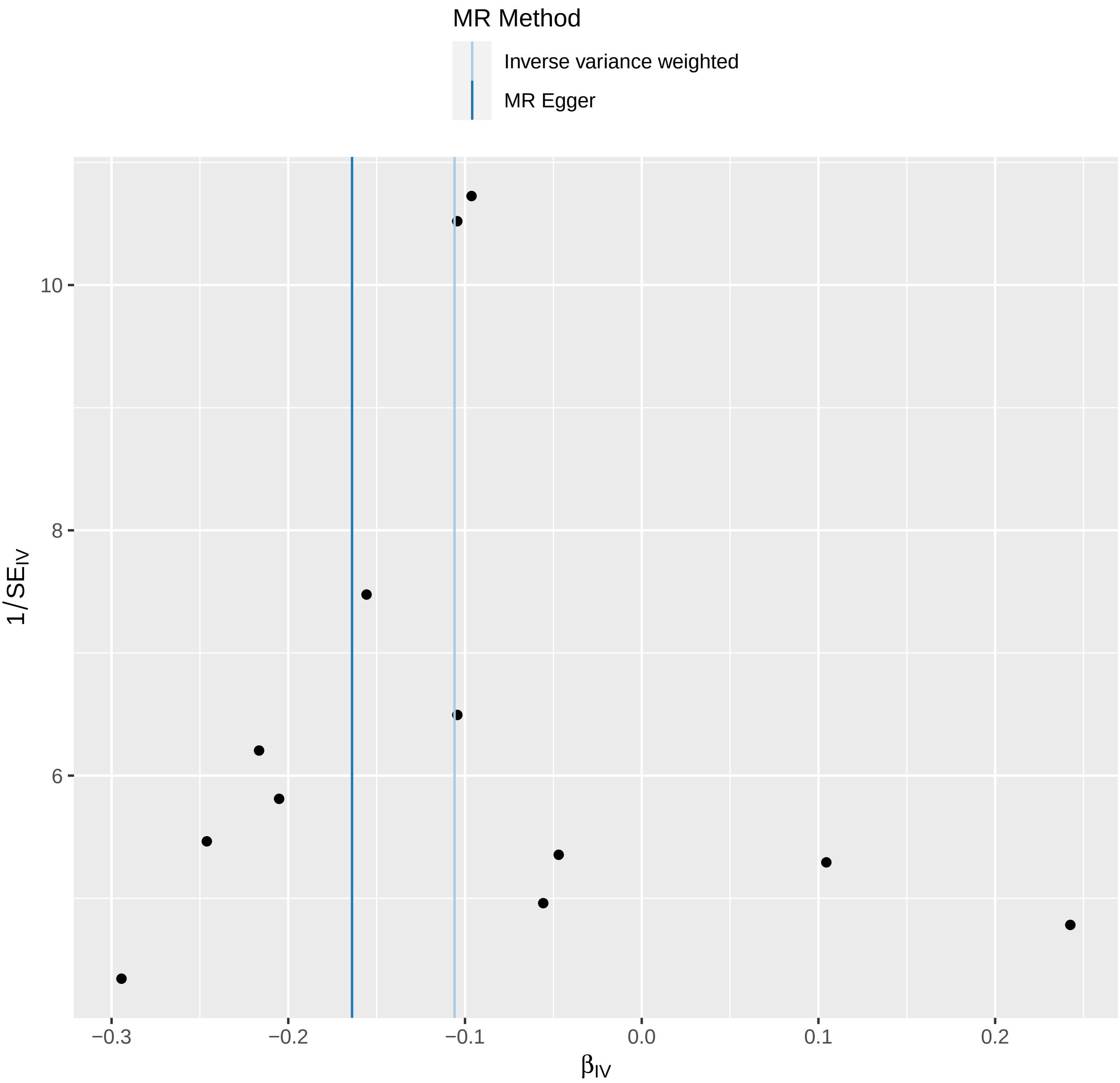

Batch 3−2819 : Selenium on Serine protease inhibitor Kazal−type 5

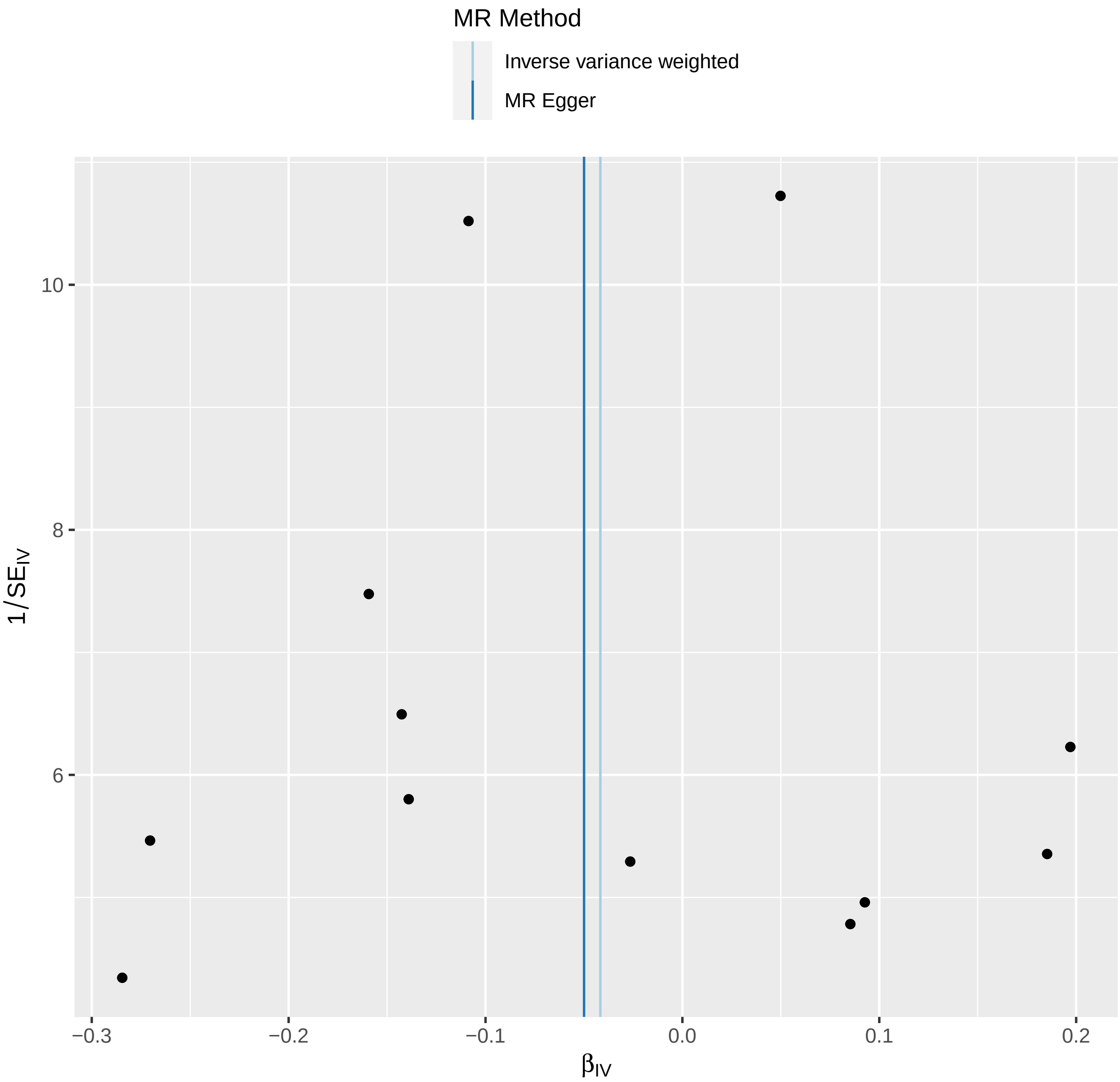

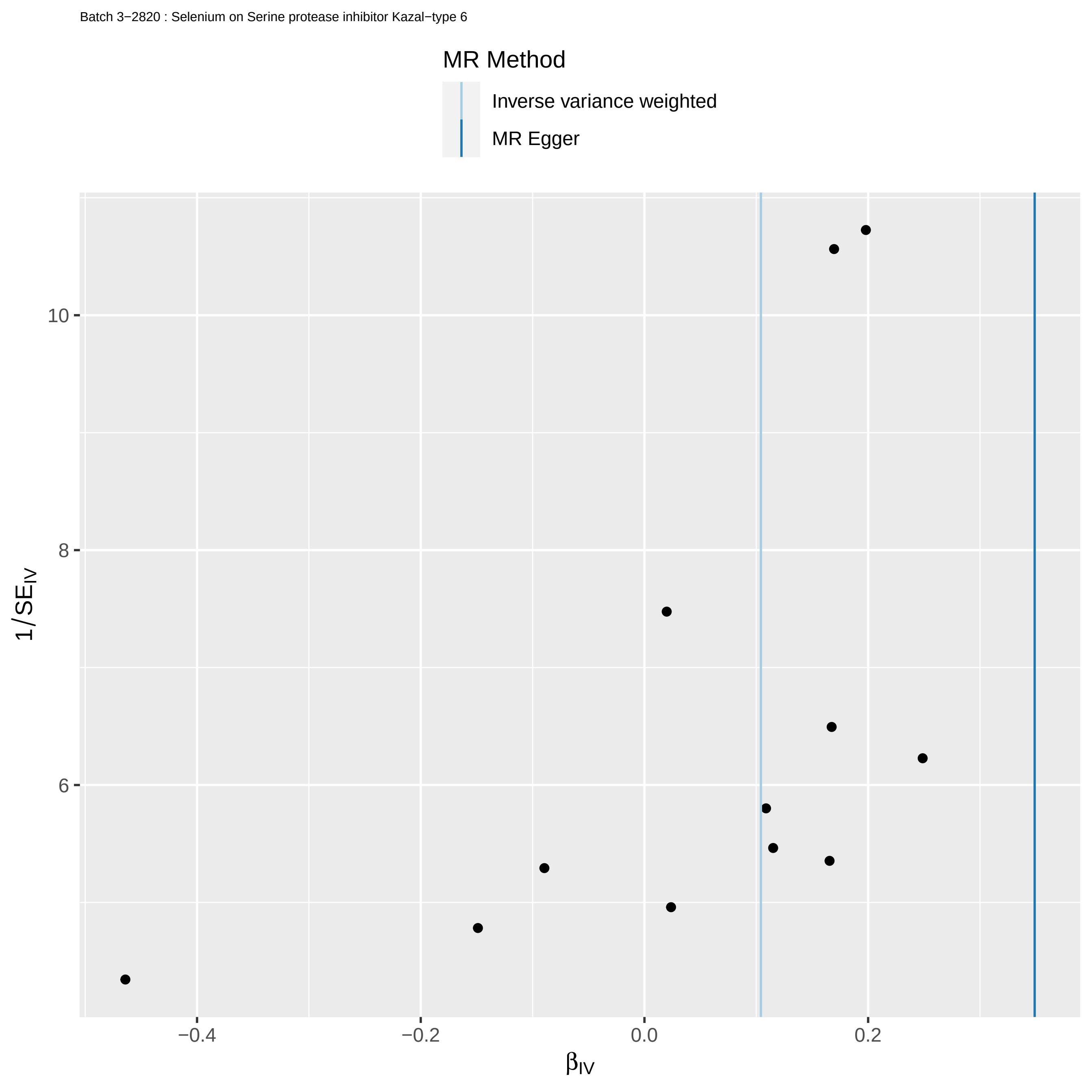
Batch 3−2820 : Selenium on Serine protease inhibitor Kazal−type 6
MR Method
Inverse variance weighted
MR Egger
10
8
6
1/SE$_{IV}$
−0.4
−0.2
0.0
0.2
β$_{IV}$

Batch 3−2821 : Selenium on Serine protease inhibitor Kazal−type 8

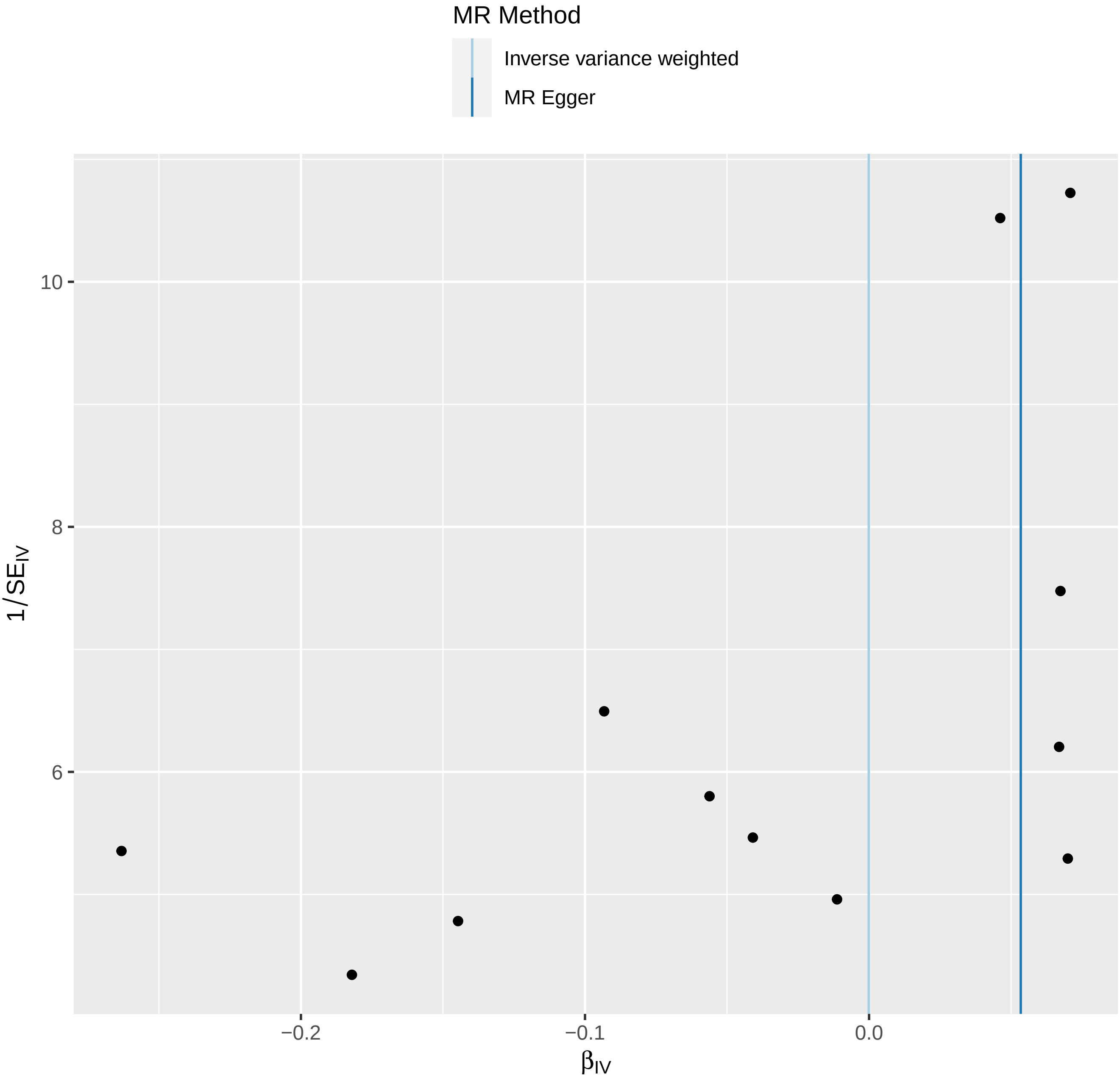

Batch 3−2822 : Selenium on Serine protease inhibitor Kazal−type 9

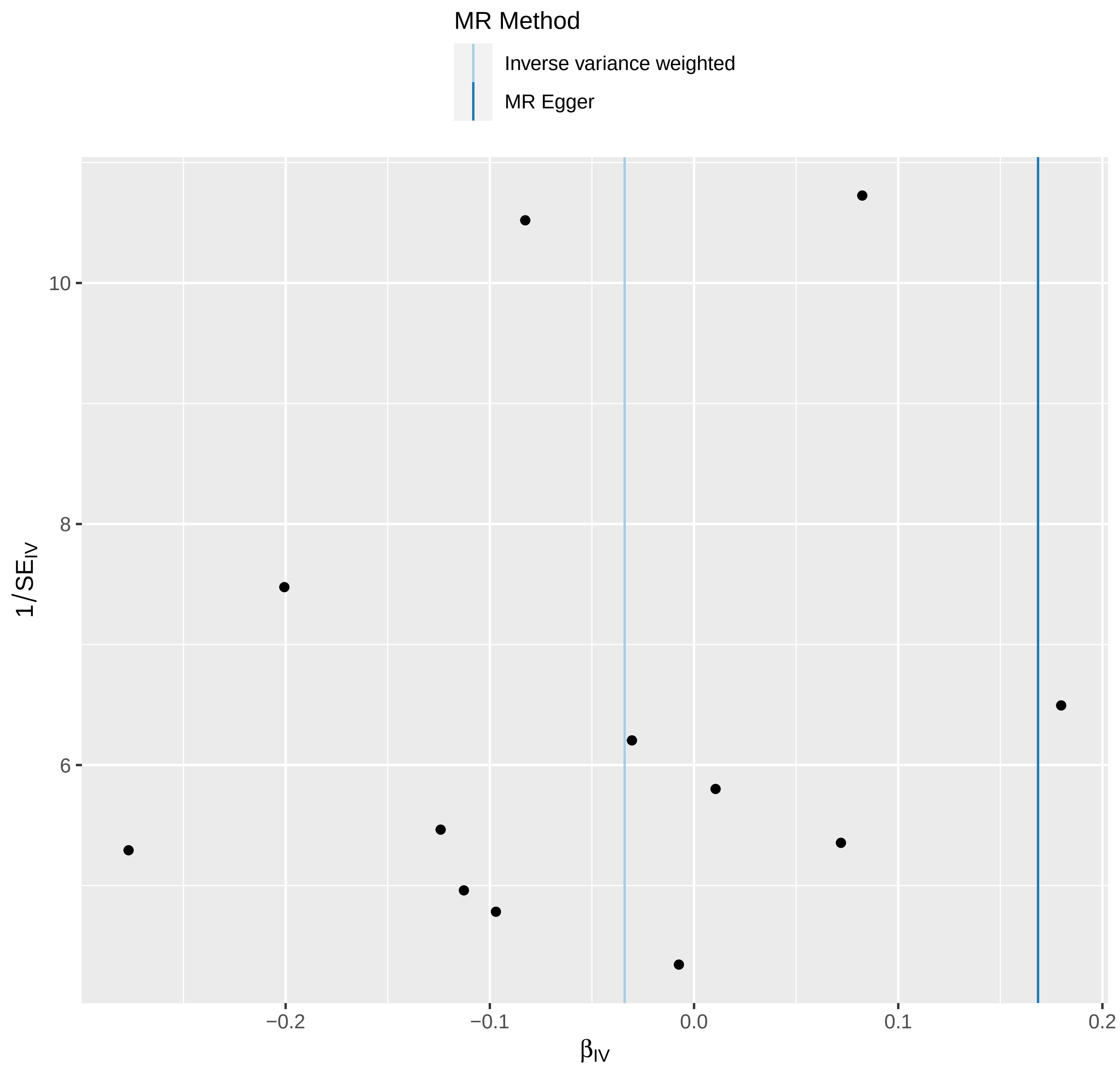

Batch 3−2823 : Selenium on Kunitz−type protease inhibitor 1

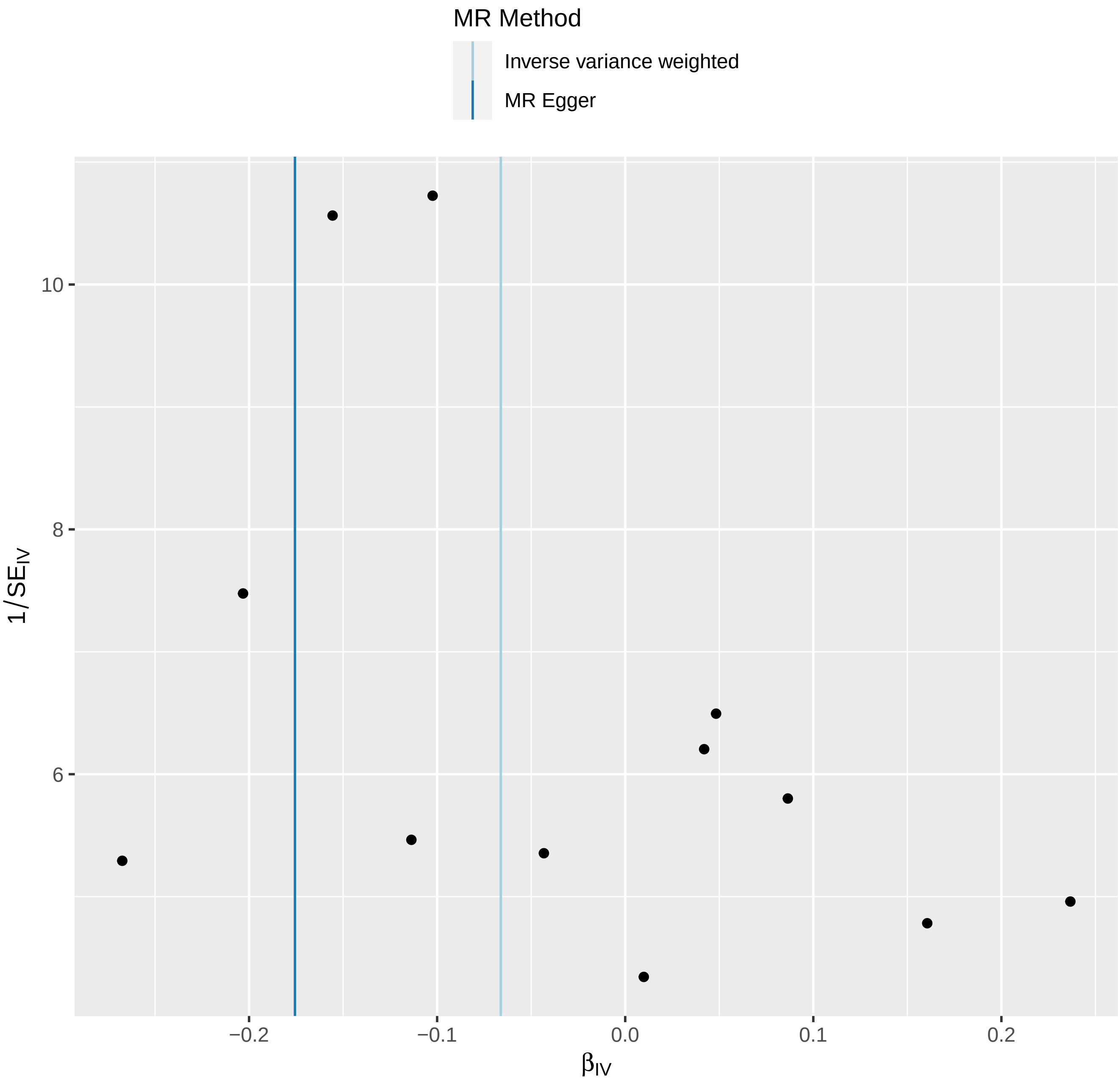

Batch 3−2824 : Selenium on Kunitz−type protease inhibitor 2

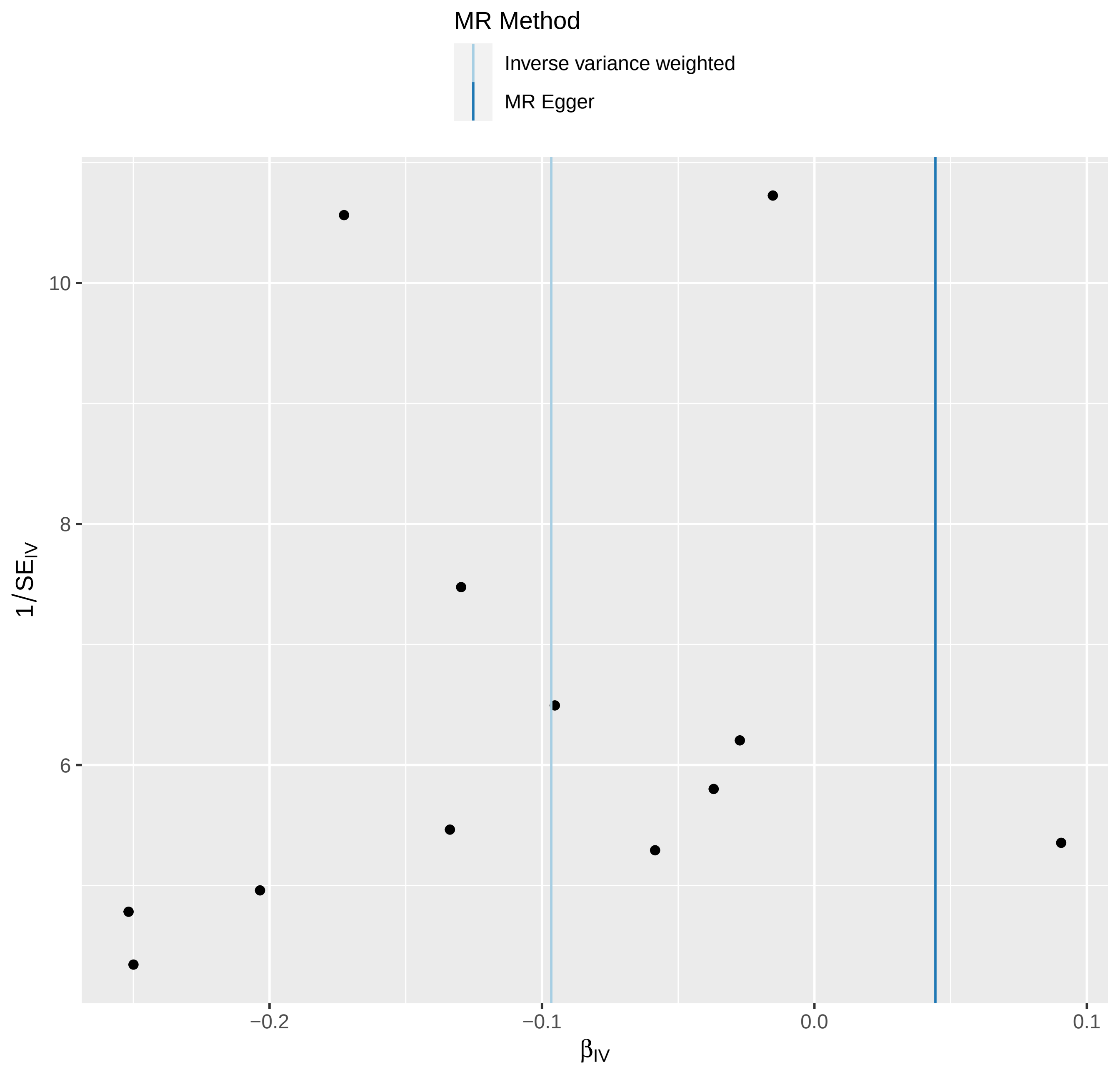

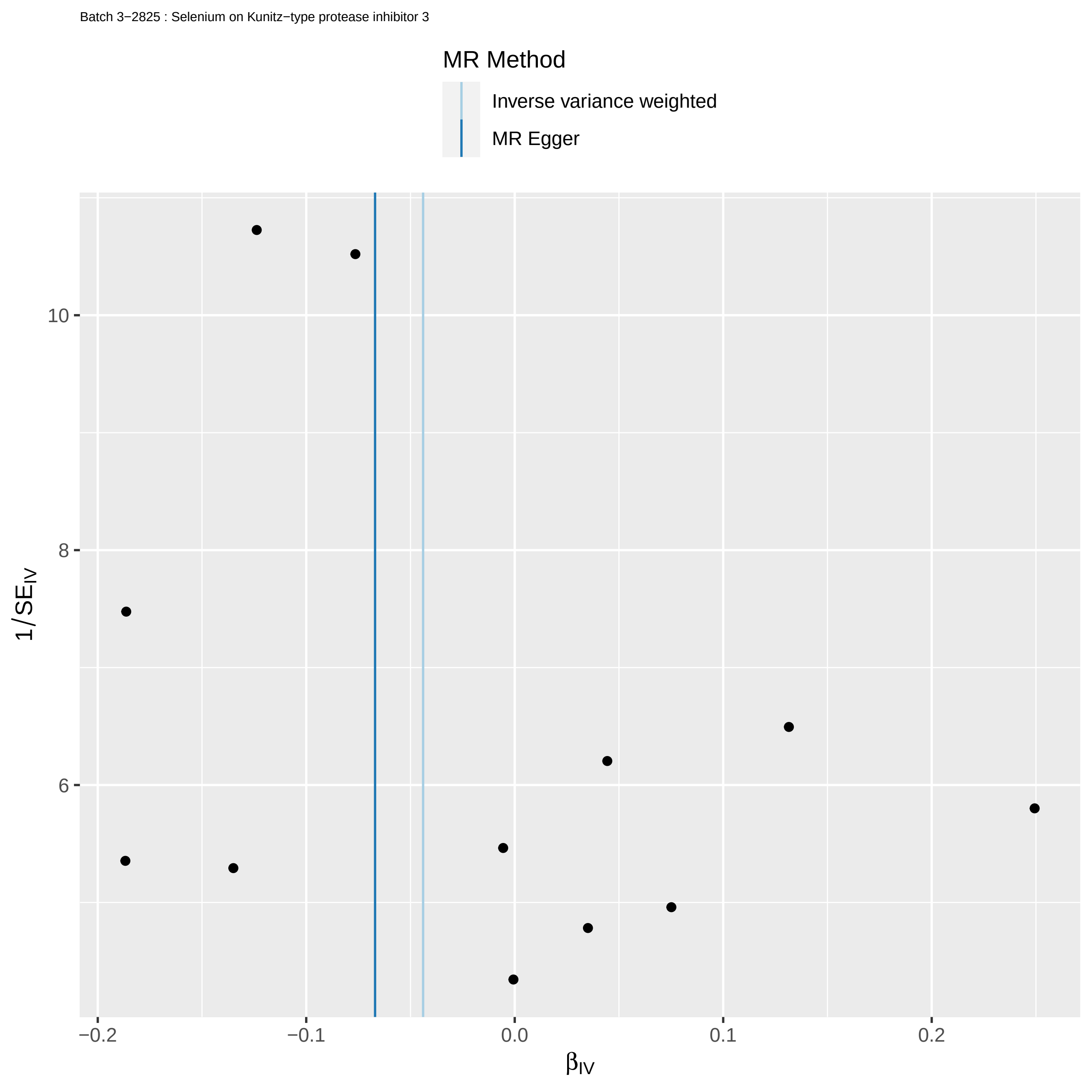
Batch 3−2825 : Selenium on Kunitz−type protease inhibitor 3
MR Method
Inverse variance weighted
MR Egger
10
8
6
1/SE_IV
−0.2
−0.1
0.0
0.1
0.2
β_IV

Batch 3−2826 : Selenium on Testican−1

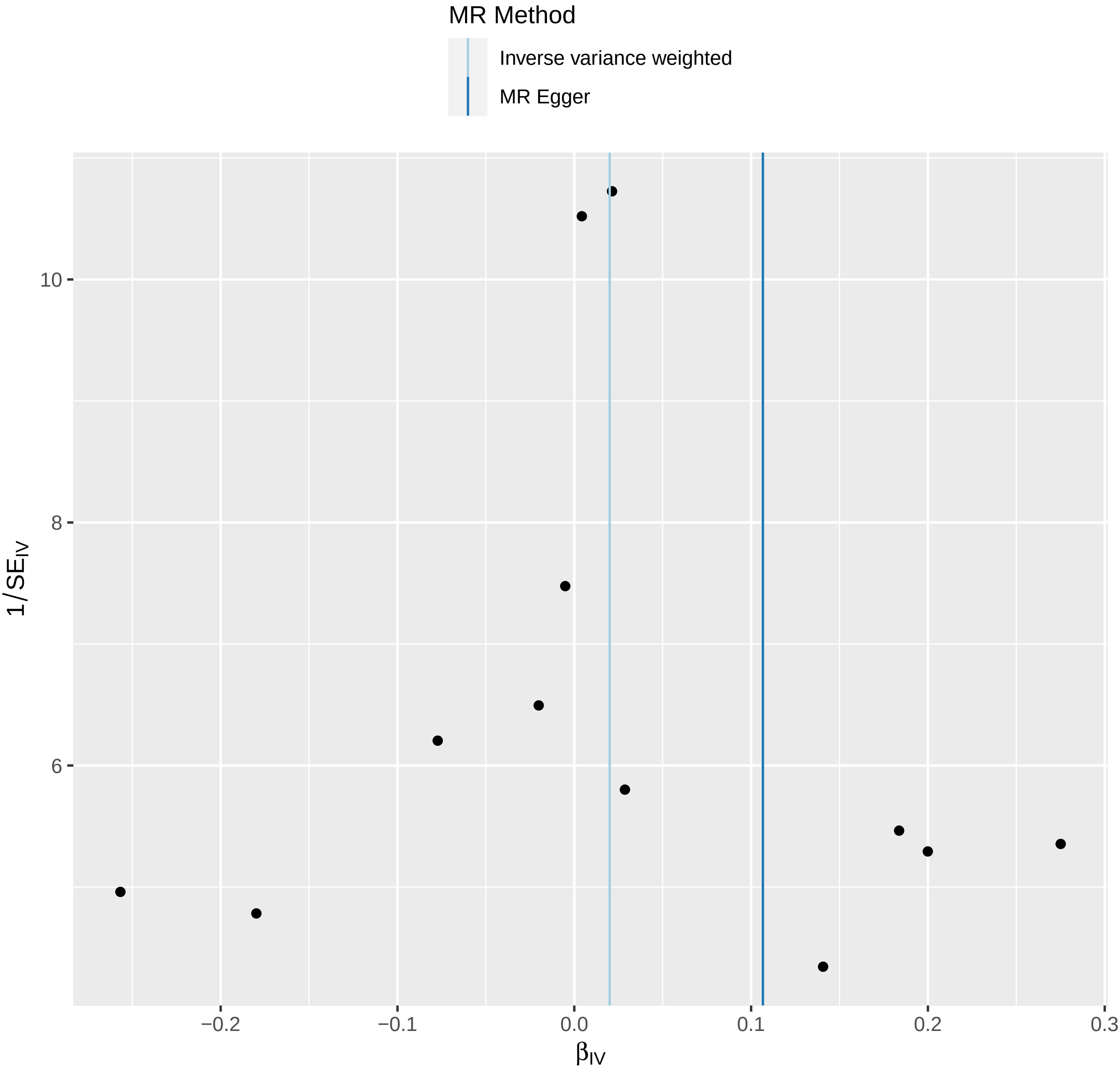

Batch 3−2827 : Selenium on Testican−2

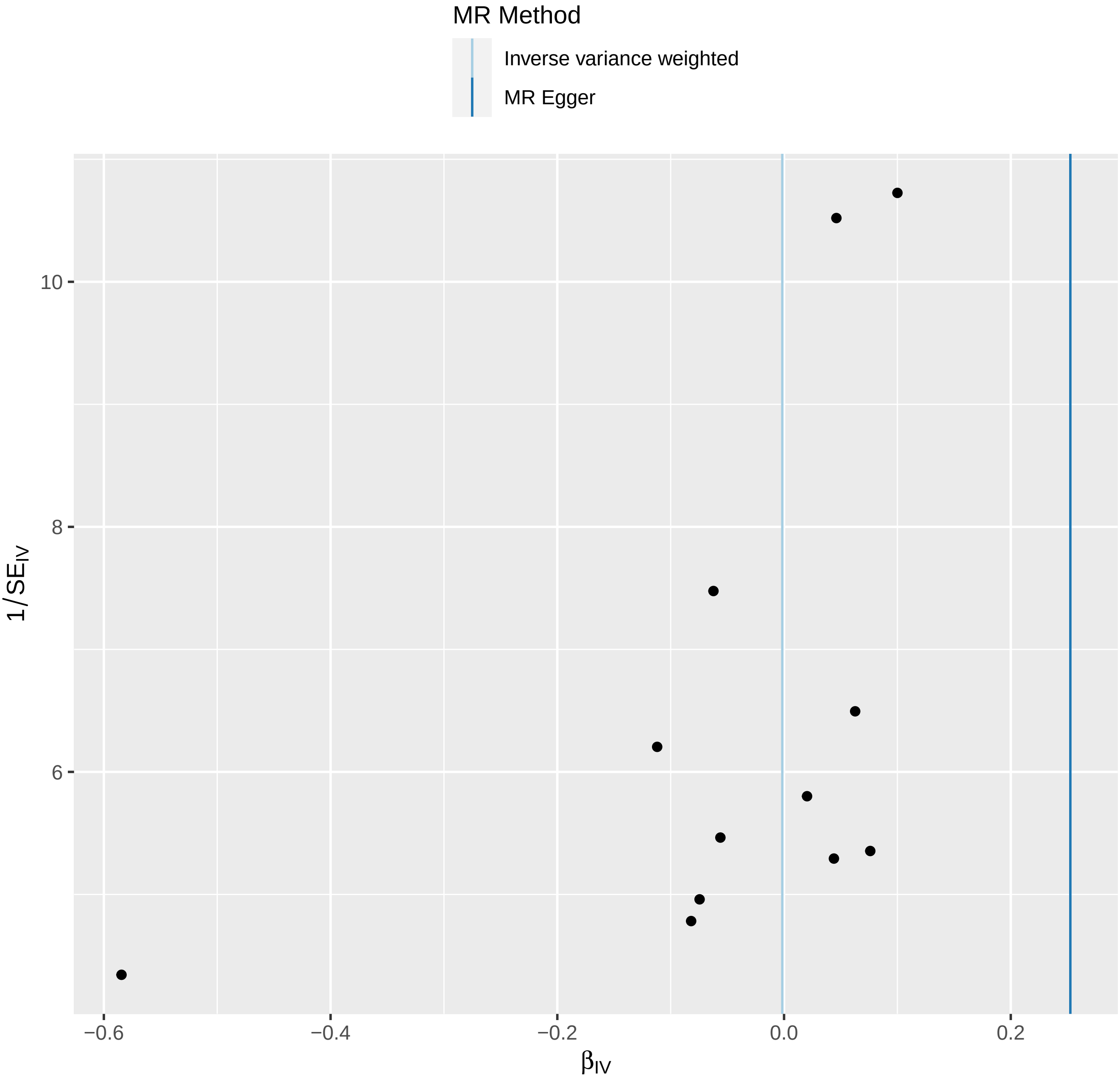

Batch 3−2828 : Selenium on Testican−3

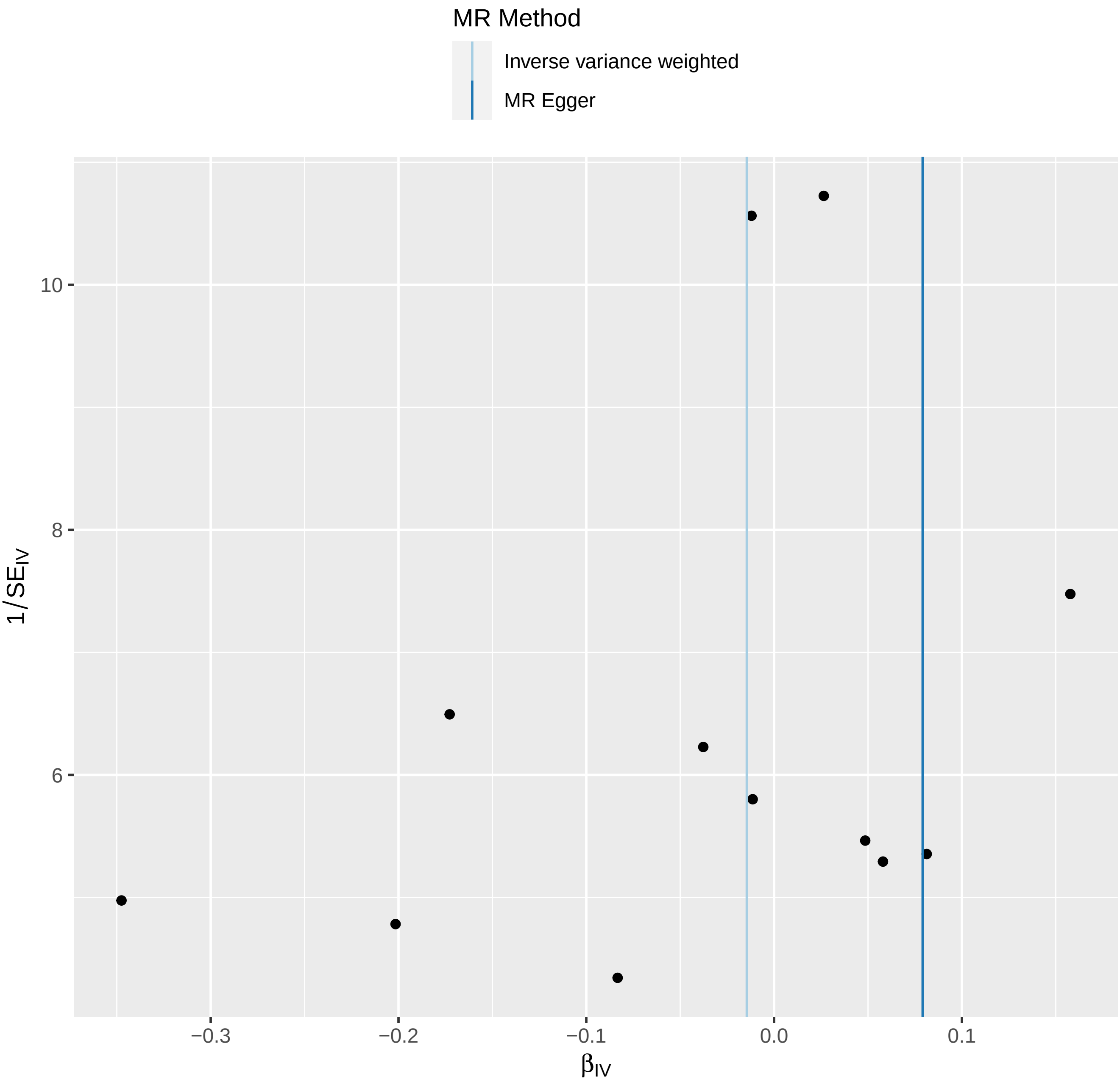

Batch 3−2829 : Selenium on Spondin−1

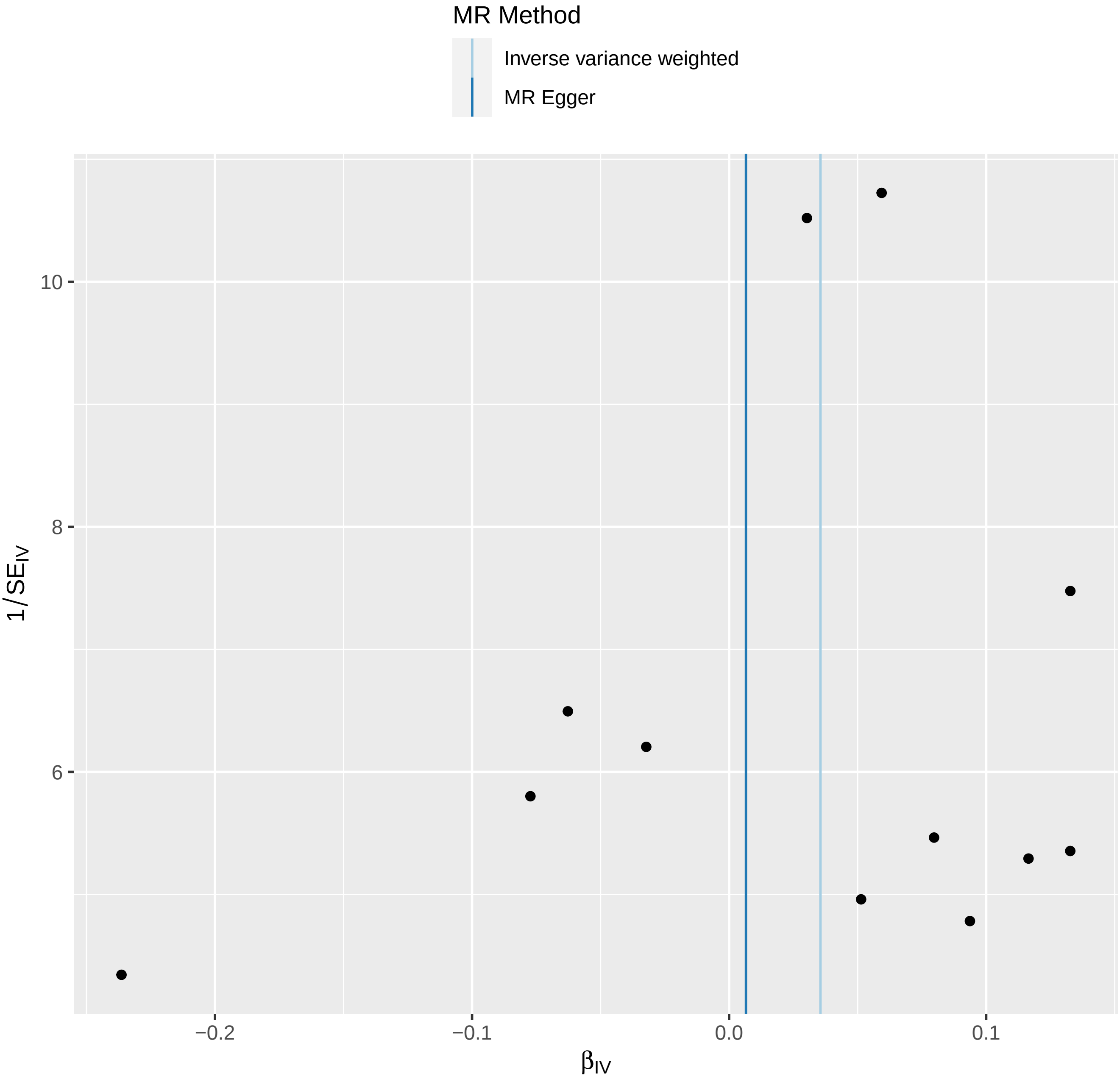

Batch 3−2830 : Selenium on Speckle−type POZ protein

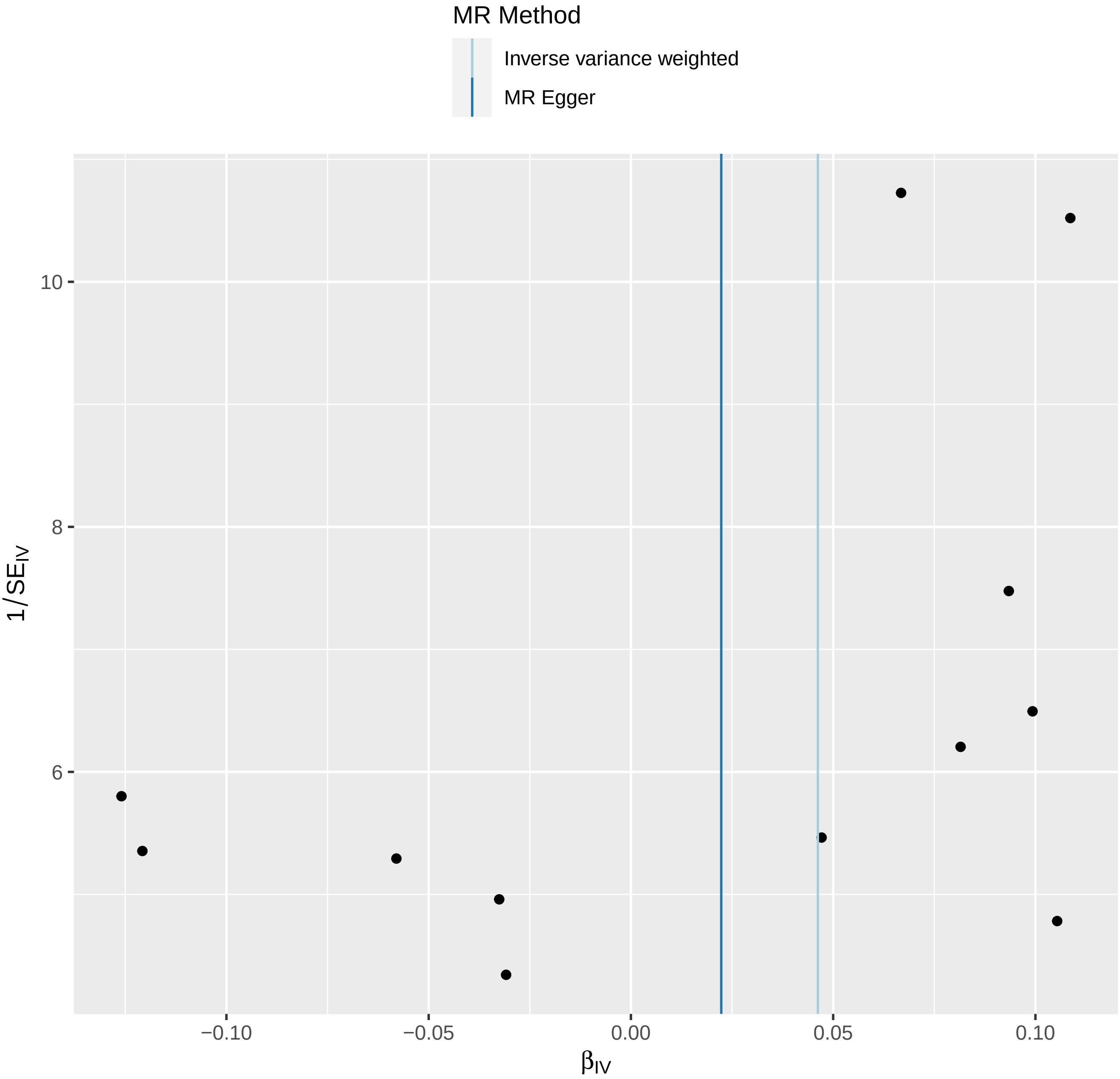

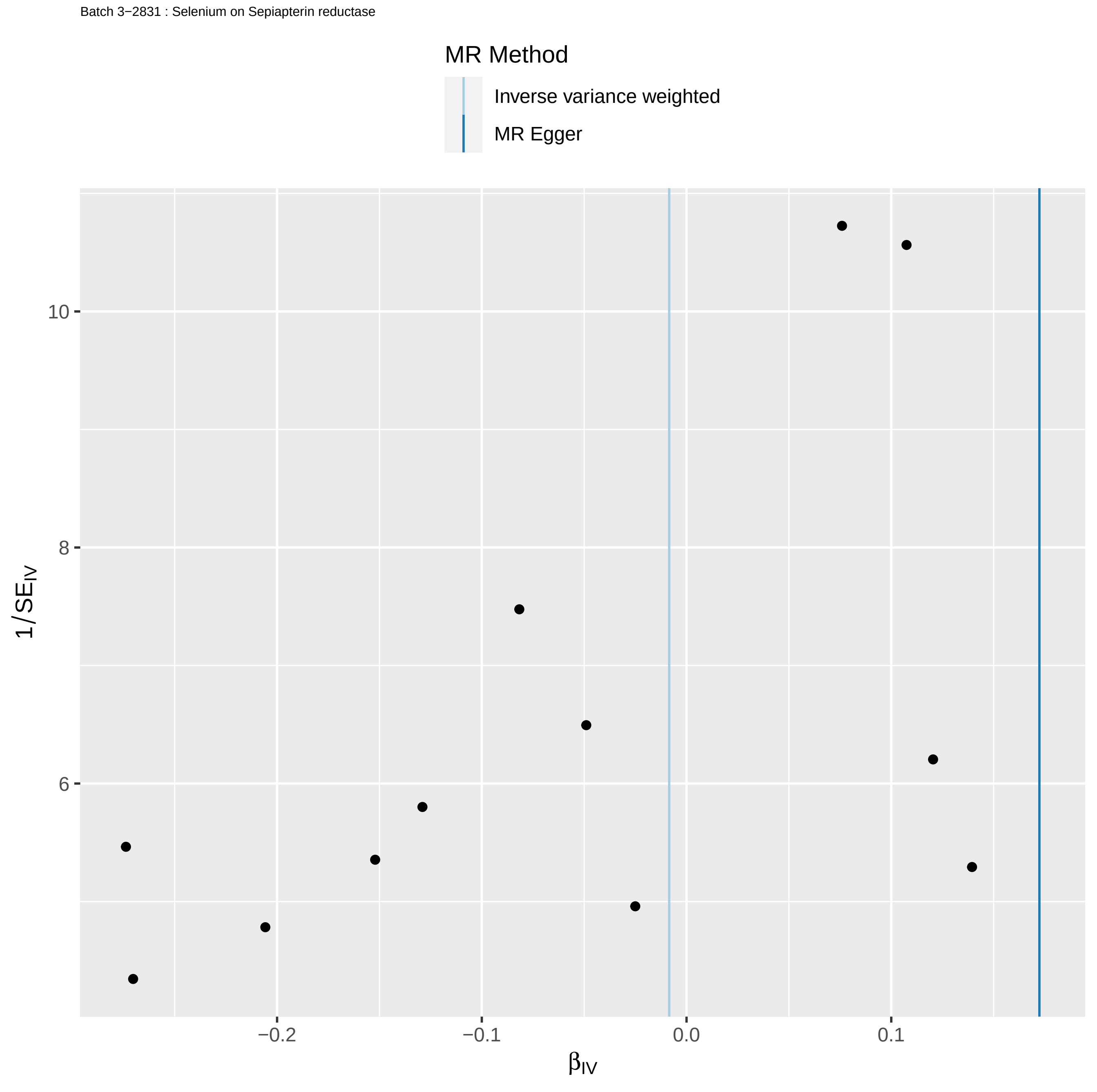

Batch 3−2831 : Selenium on Sepiapterin reductase
MR Method
Inverse variance weighted
MR Egger
10
8
6
1/SE_IV
−0.2
−0.1
0.0
0.1
β_IV

Batch 3−2832 : Selenium on Sprouty−related, EVH1 domain−containing protein 1

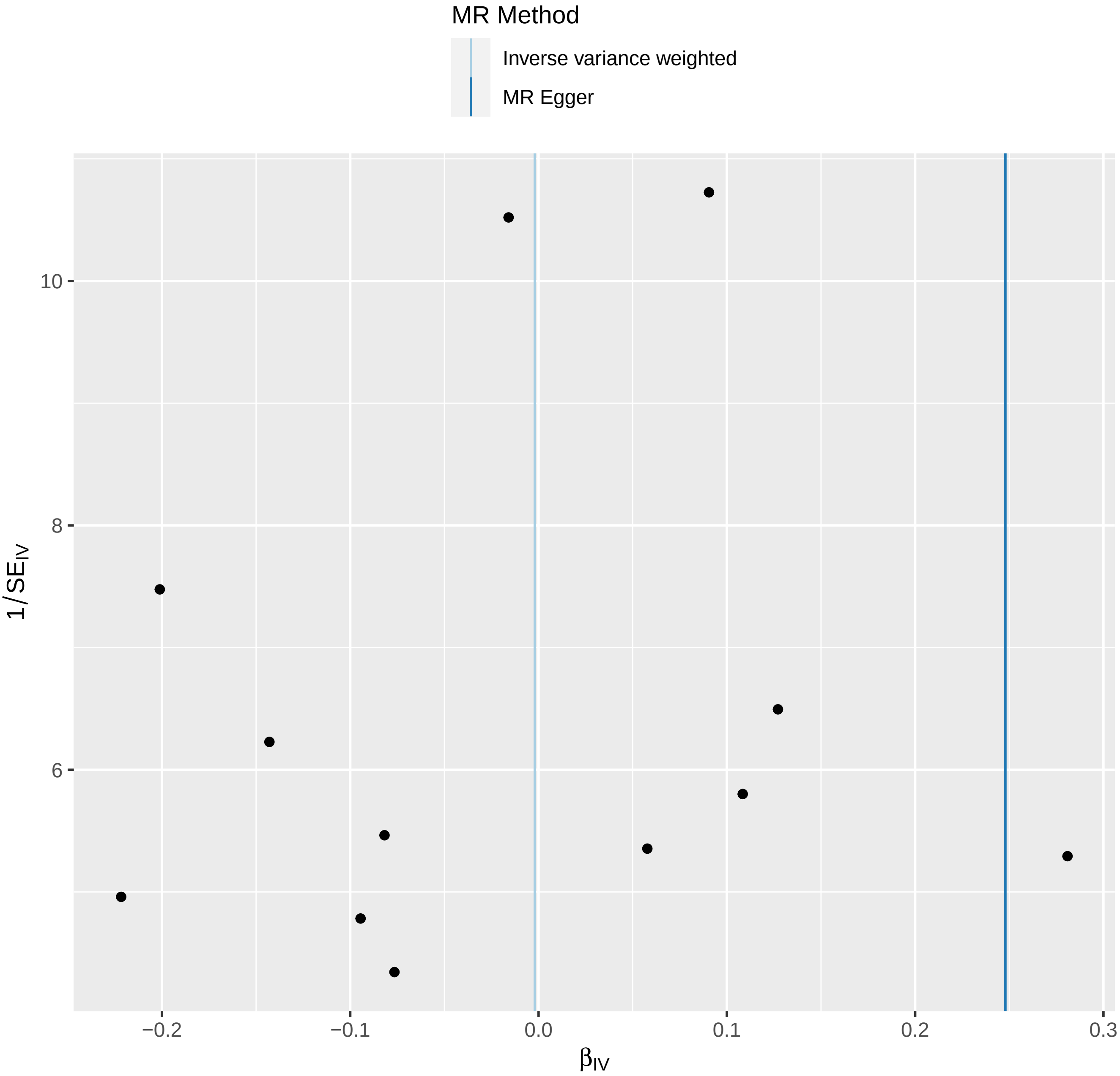

Batch 3−2833 : Selenium on Protein sprouty homolog 1

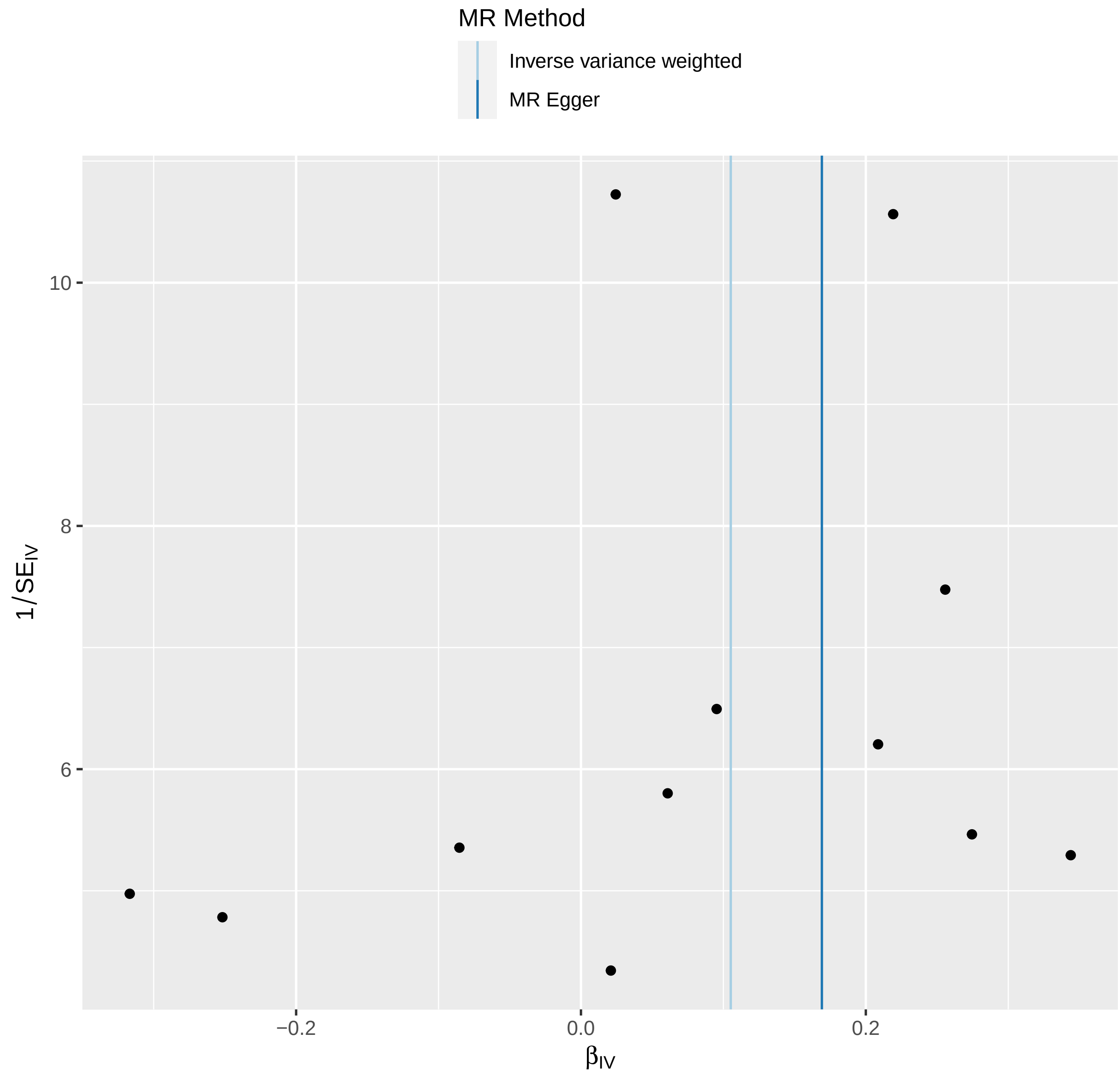

Batch 3−2834 : Selenium on SPRY domain−containing SOCS box protein 1

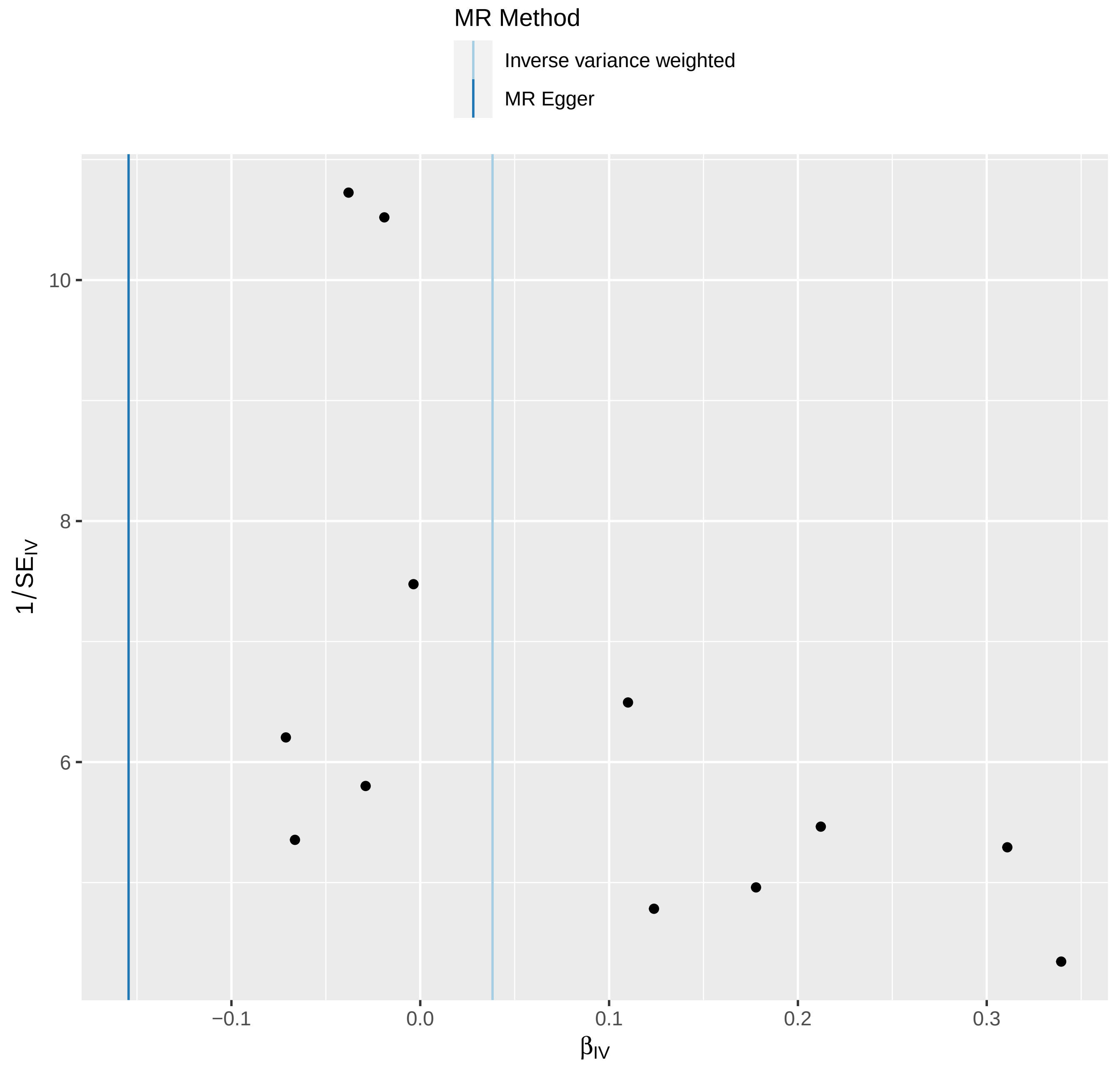

Batch 3−2835 : Selenium on Spectrin alpha chain, non−erythrocytic 1

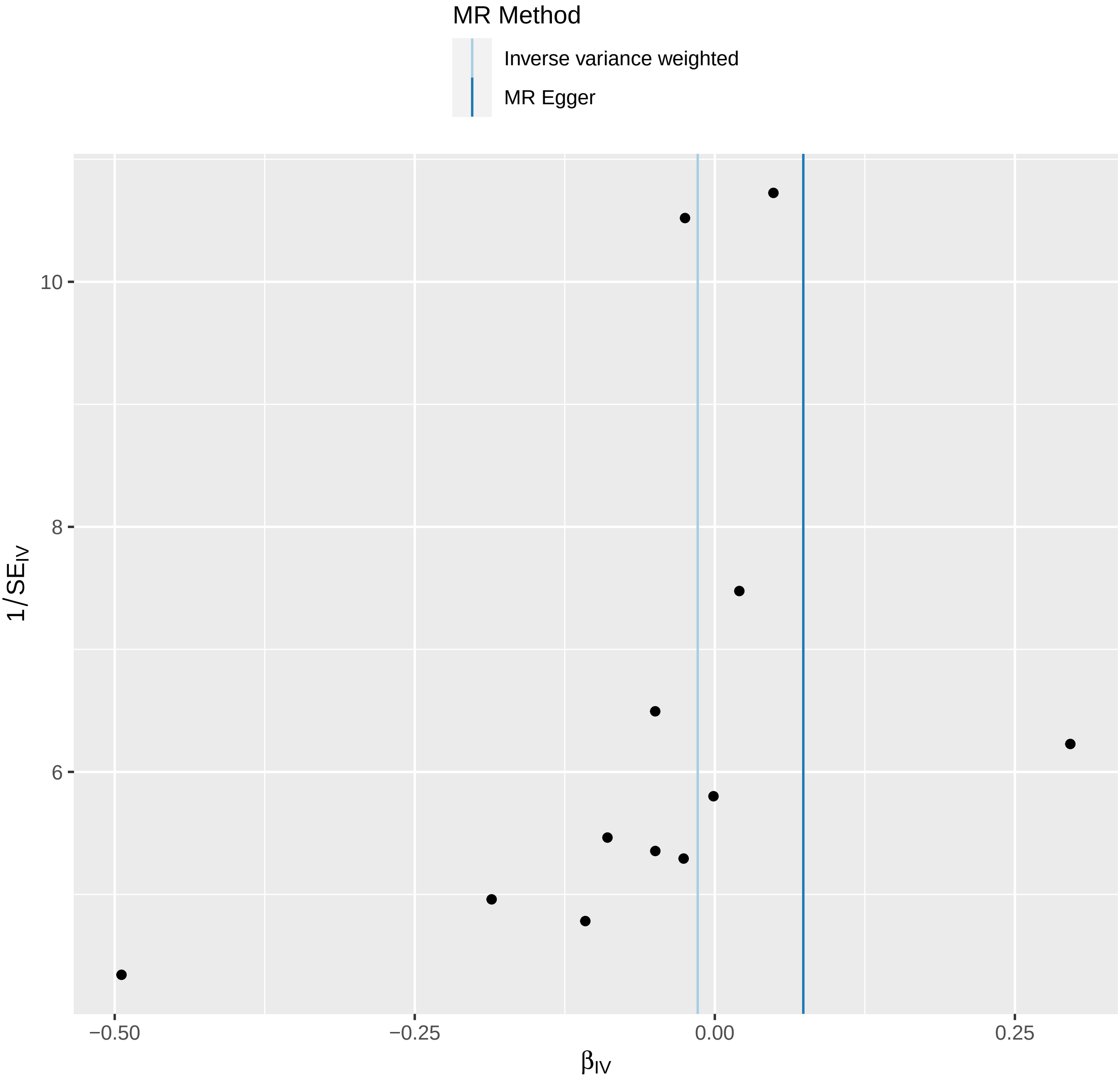

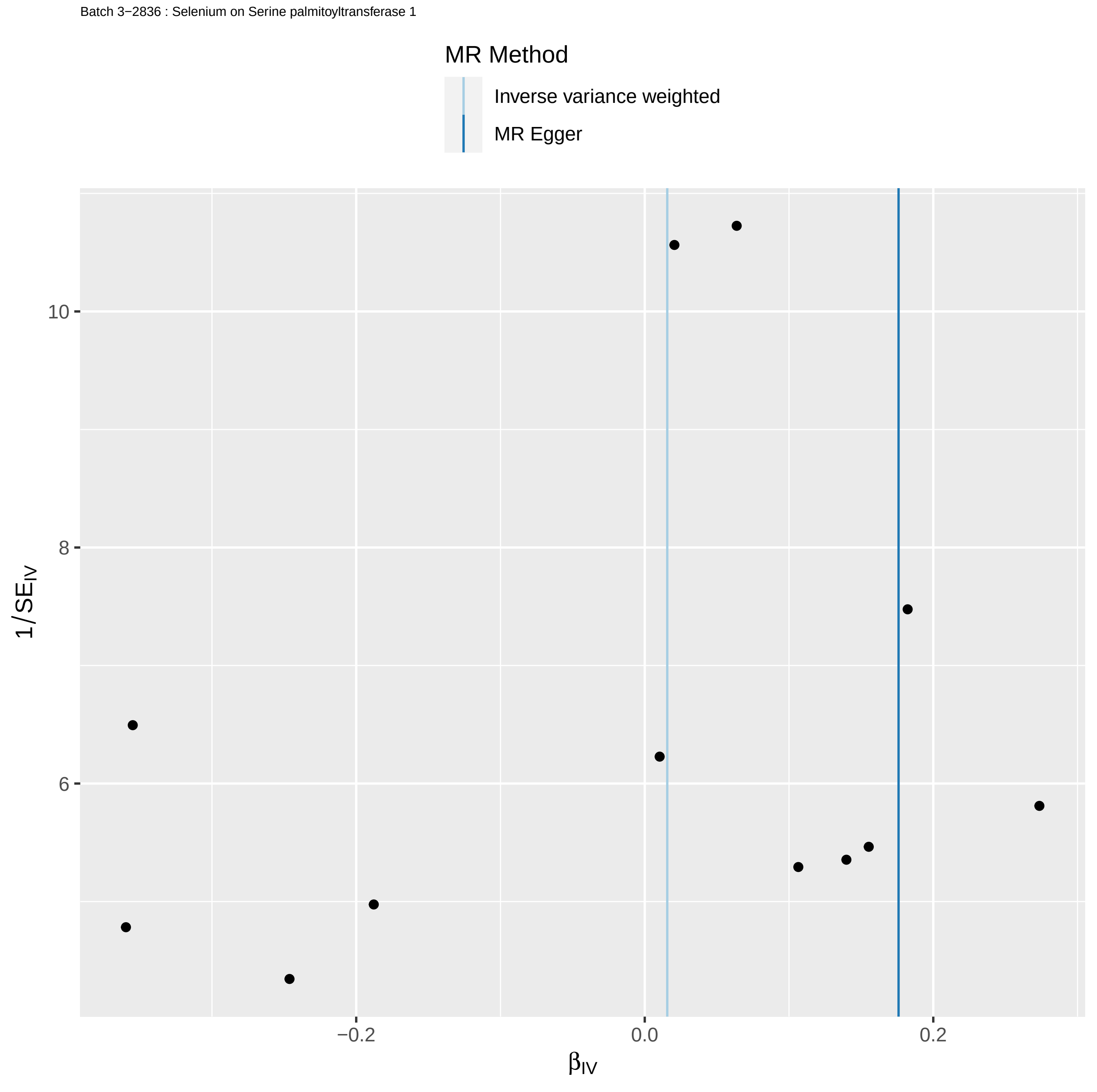

Batch 3−2836 : Selenium on Serine palmitoyltransferase 1
MR Method
Inverse variance weighted
MR Egger
10
8
6
1/SE_IV
−0.2
0.0
0.2
β_IV

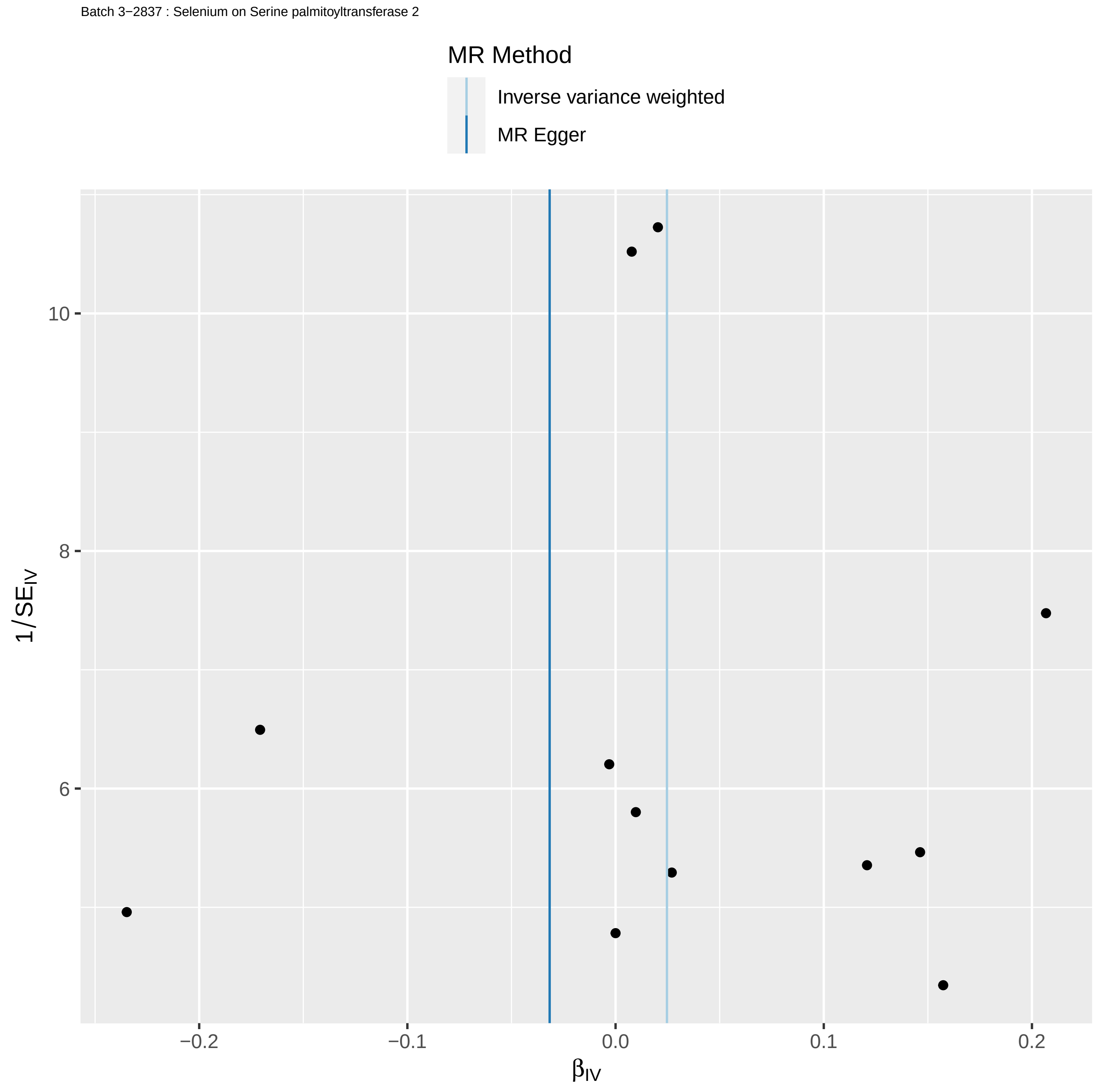

Batch 3−2837 : Selenium on Serine palmitoyltransferase 2
MR Method
Inverse variance weighted
MR Egger
10
8
6
1/SE_IV
−0.2
−0.1
0.0
0.1
0.2
β_IV

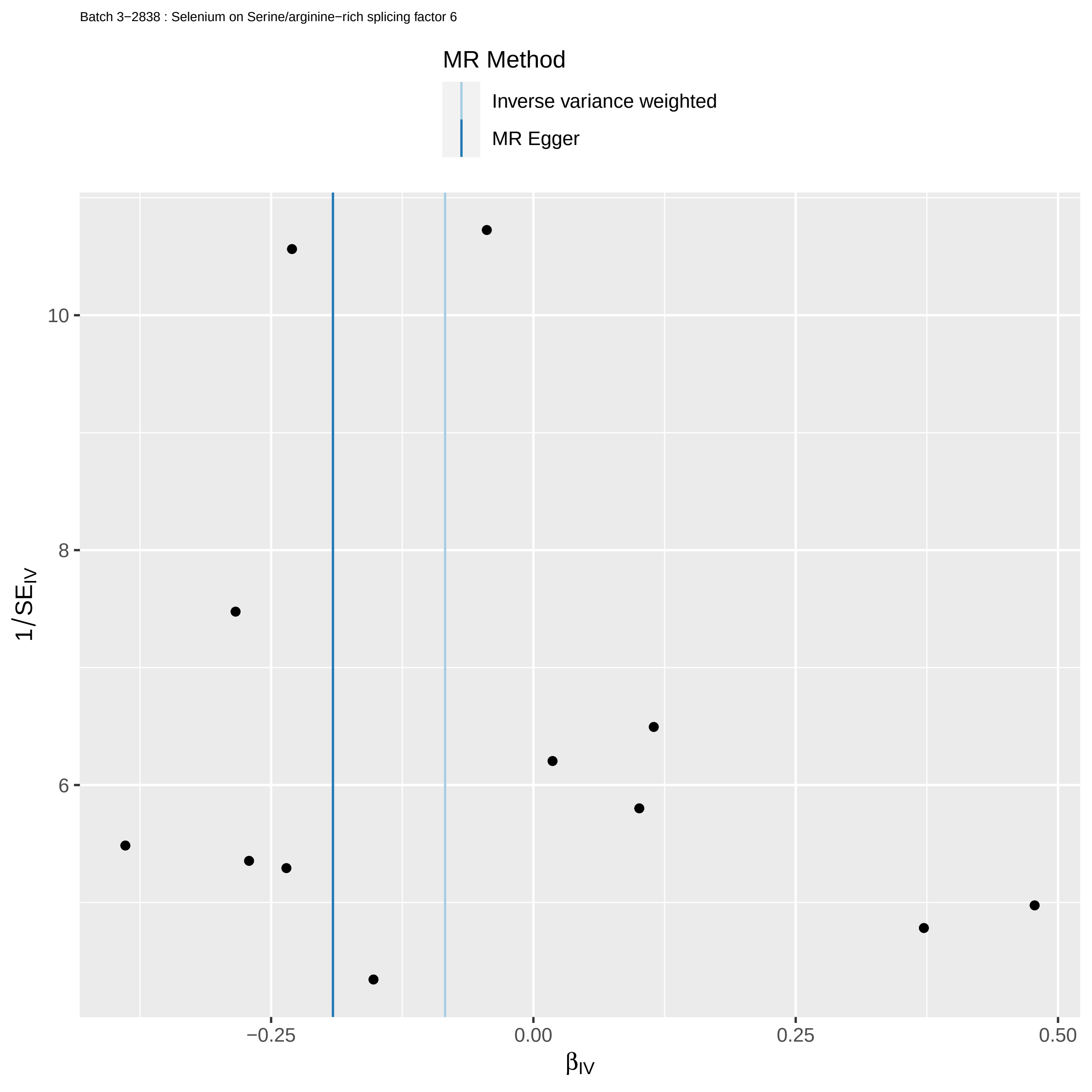
Batch 3−2838 : Selenium on Serine/arginine−rich splicing factor 6
MR Method
Inverse variance weighted
MR Egger
10
8
6
1/SE$_{IV}$
−0.25
0.00
0.25
0.50
β$_{IV}$

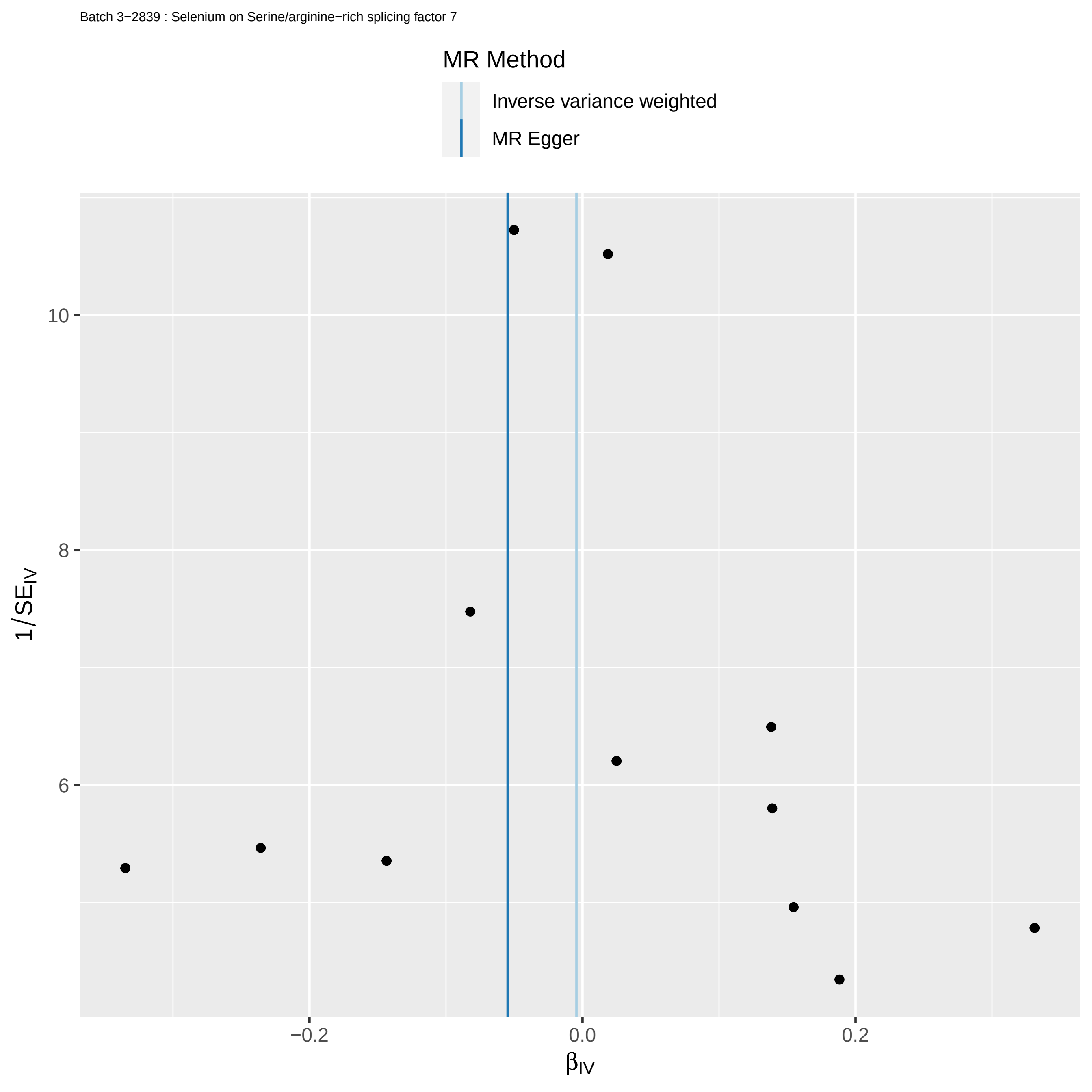
Batch 3−2839 : Selenium on Serine/arginine−rich splicing factor 7
MR Method
Inverse variance weighted
MR Egger
10
8
6
1/SE_IV
−0.2
0.0
0.2
β_IV

Batch 3−2840 : Selenium on Sulfiredoxin−1

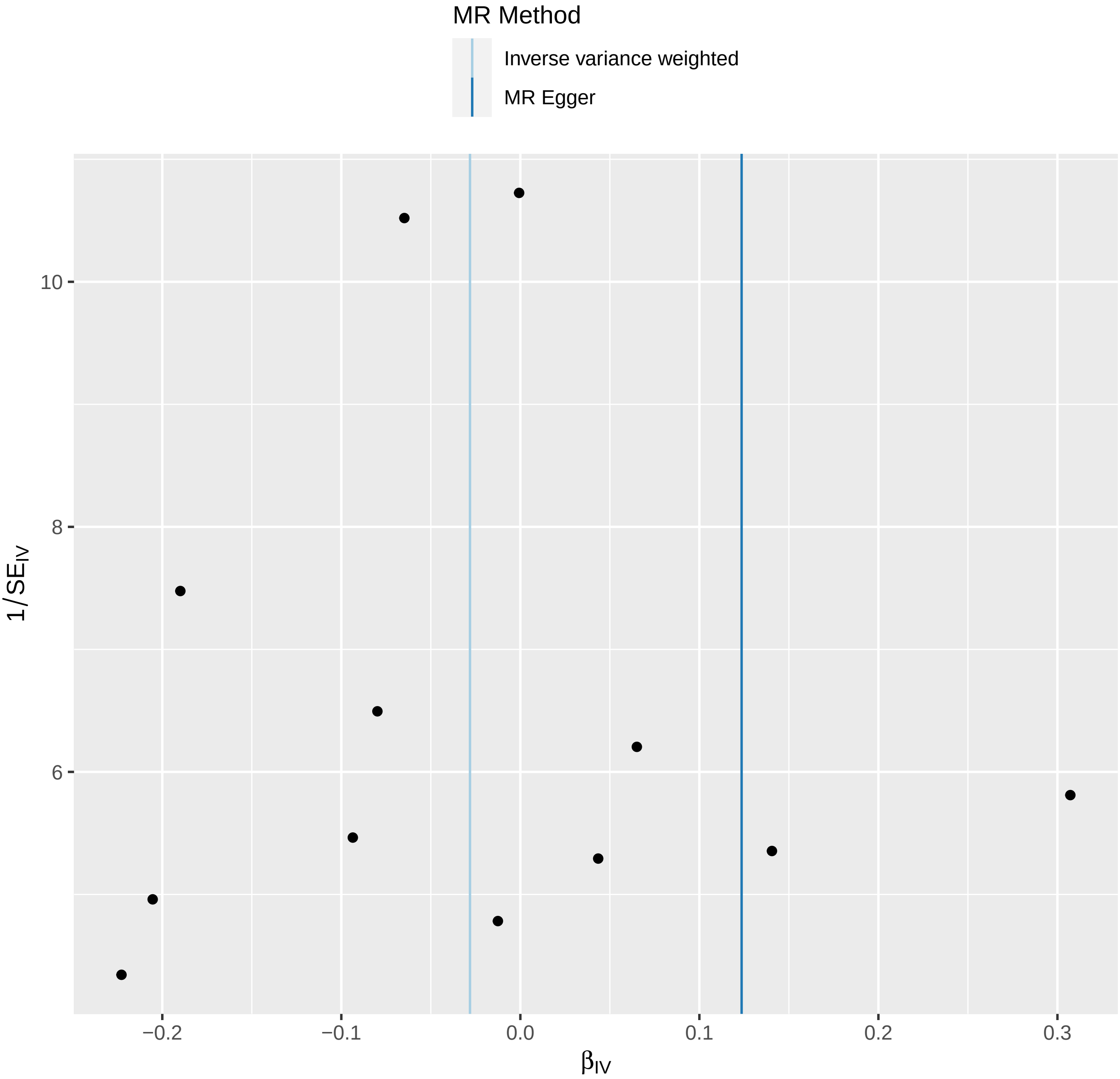

Batch 3−2841 : Selenium on Lupus La protein

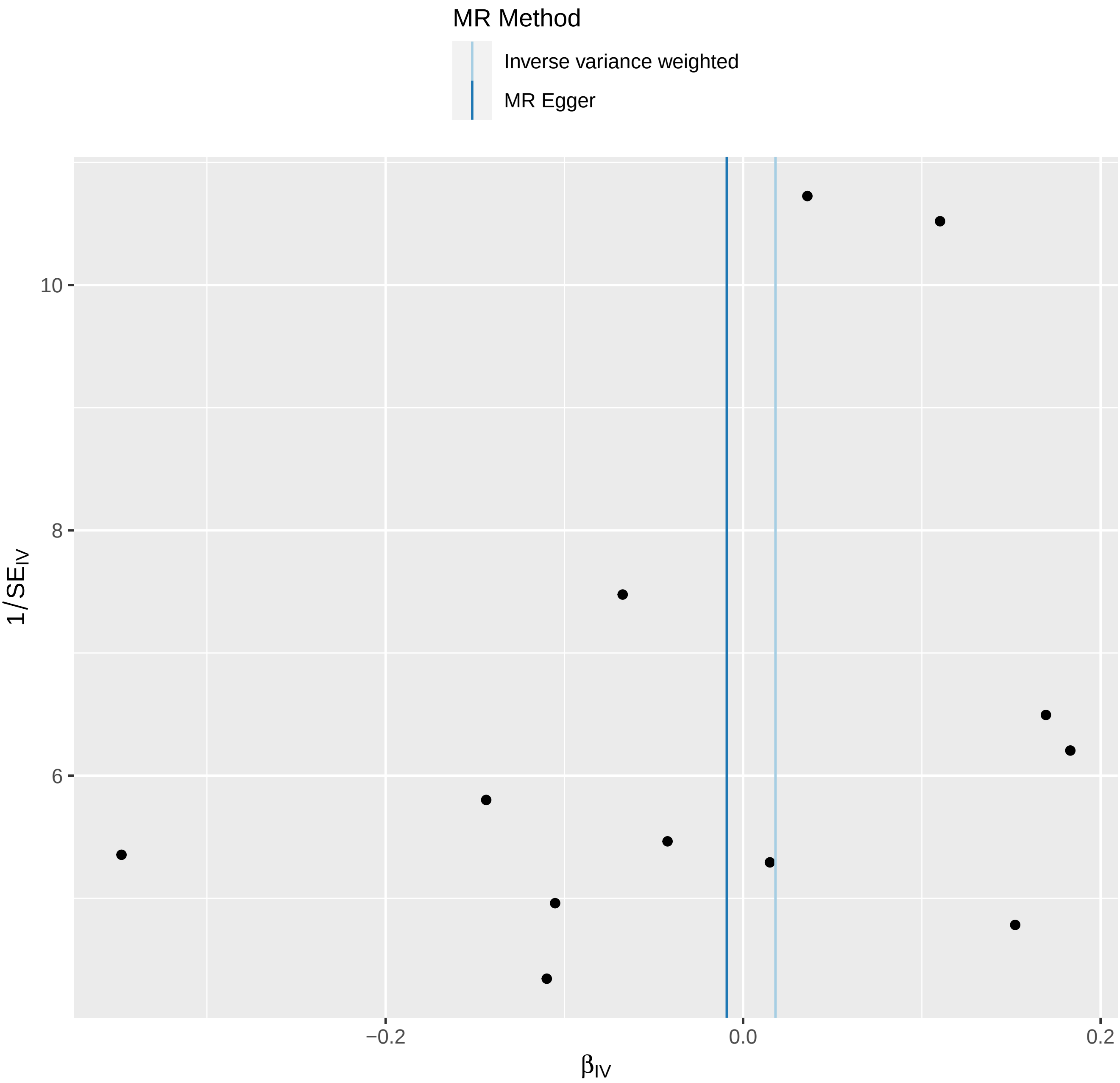

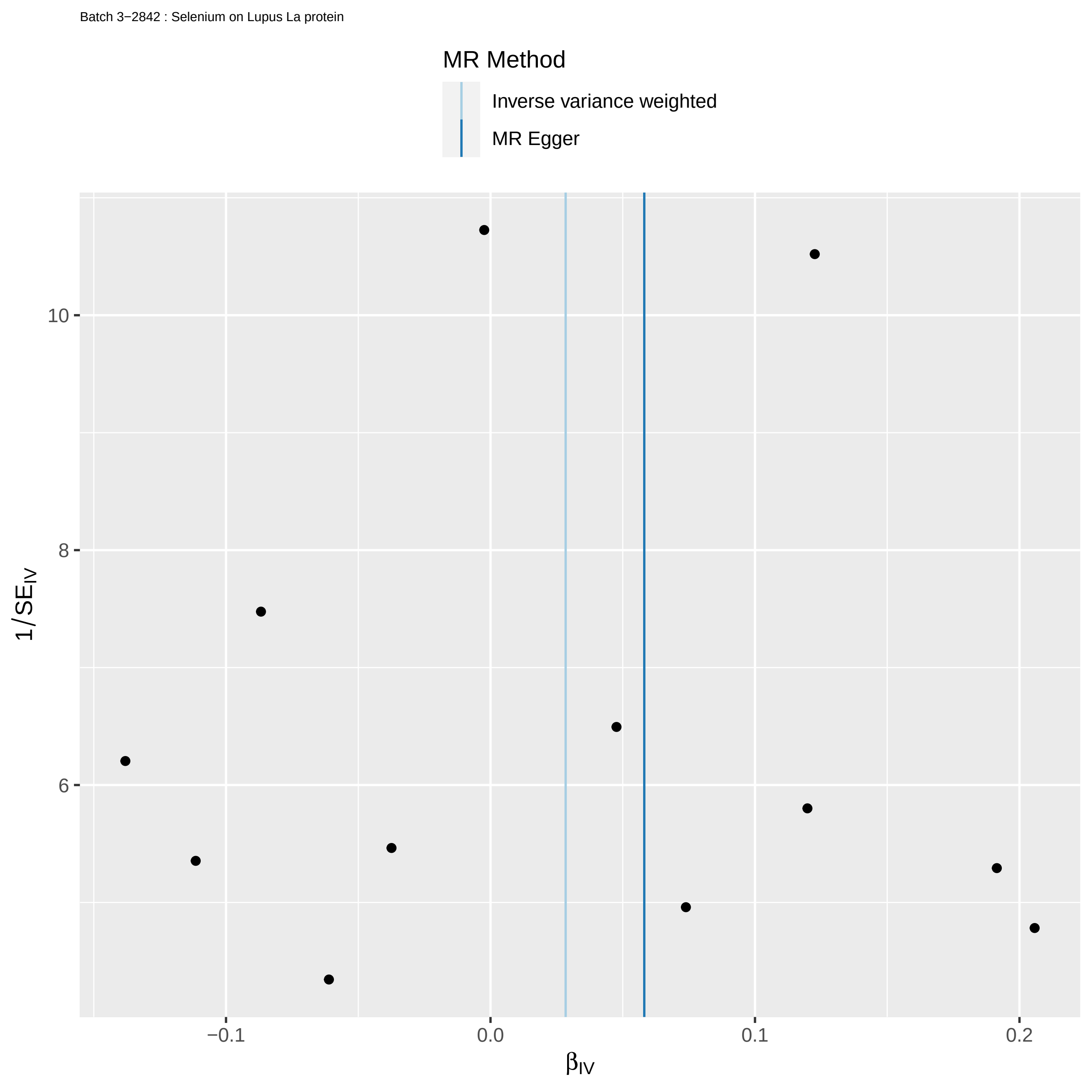

Batch 3−2842 : Selenium on Lupus La protein
MR Method
Inverse variance weighted
MR Egger
10
8
6
1/SE$_{IV}$
−0.1
0.0
0.1
0.2
β$_{IV}$

Batch 3−2843 : Selenium on Serine−rich single−pass membrane protein 1

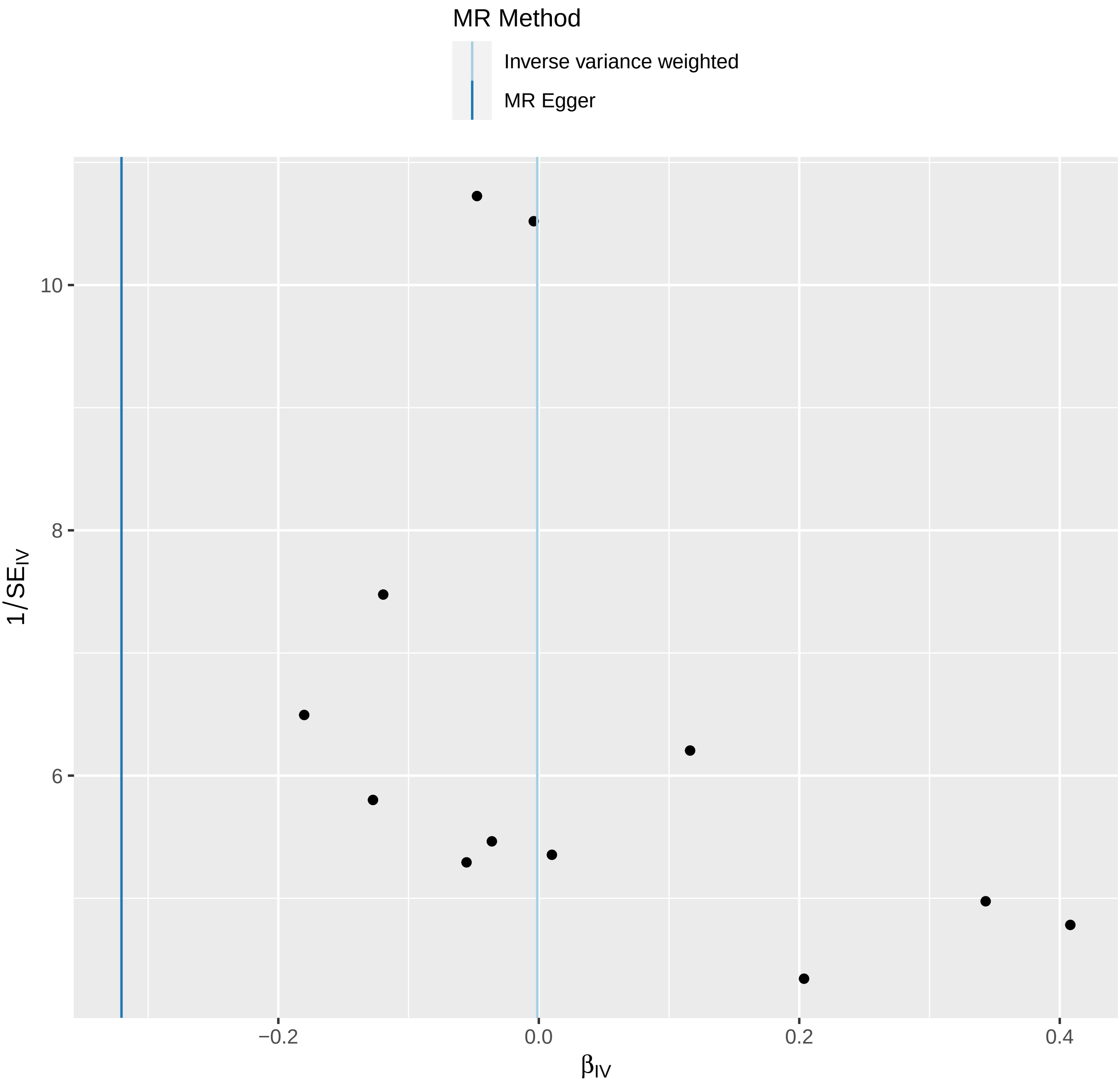

Batch 3−2844 : Selenium on Translocon−associated protein subunit alpha

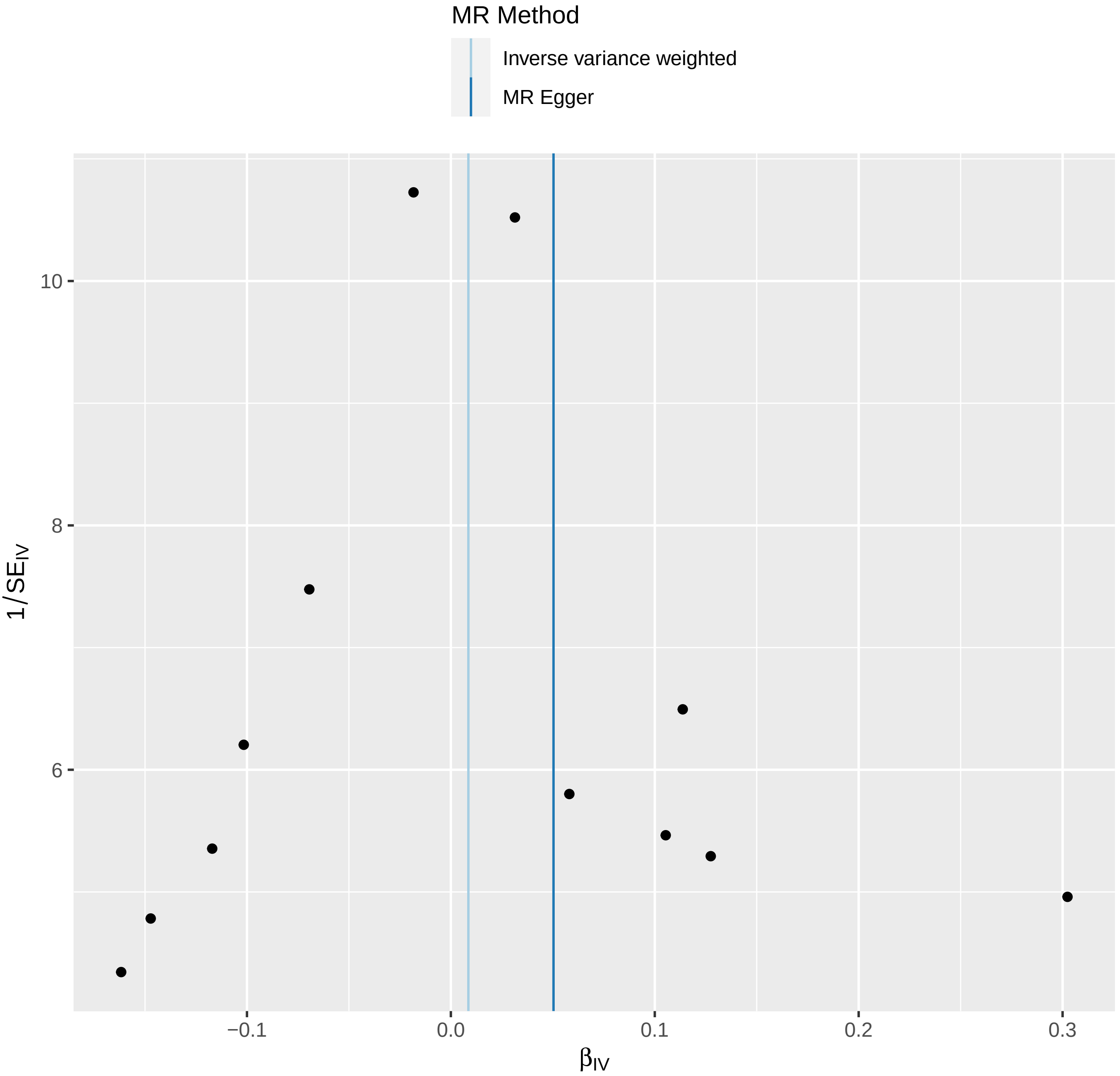

Batch 3−2845 : Selenium on FACT complex subunit SSRP1

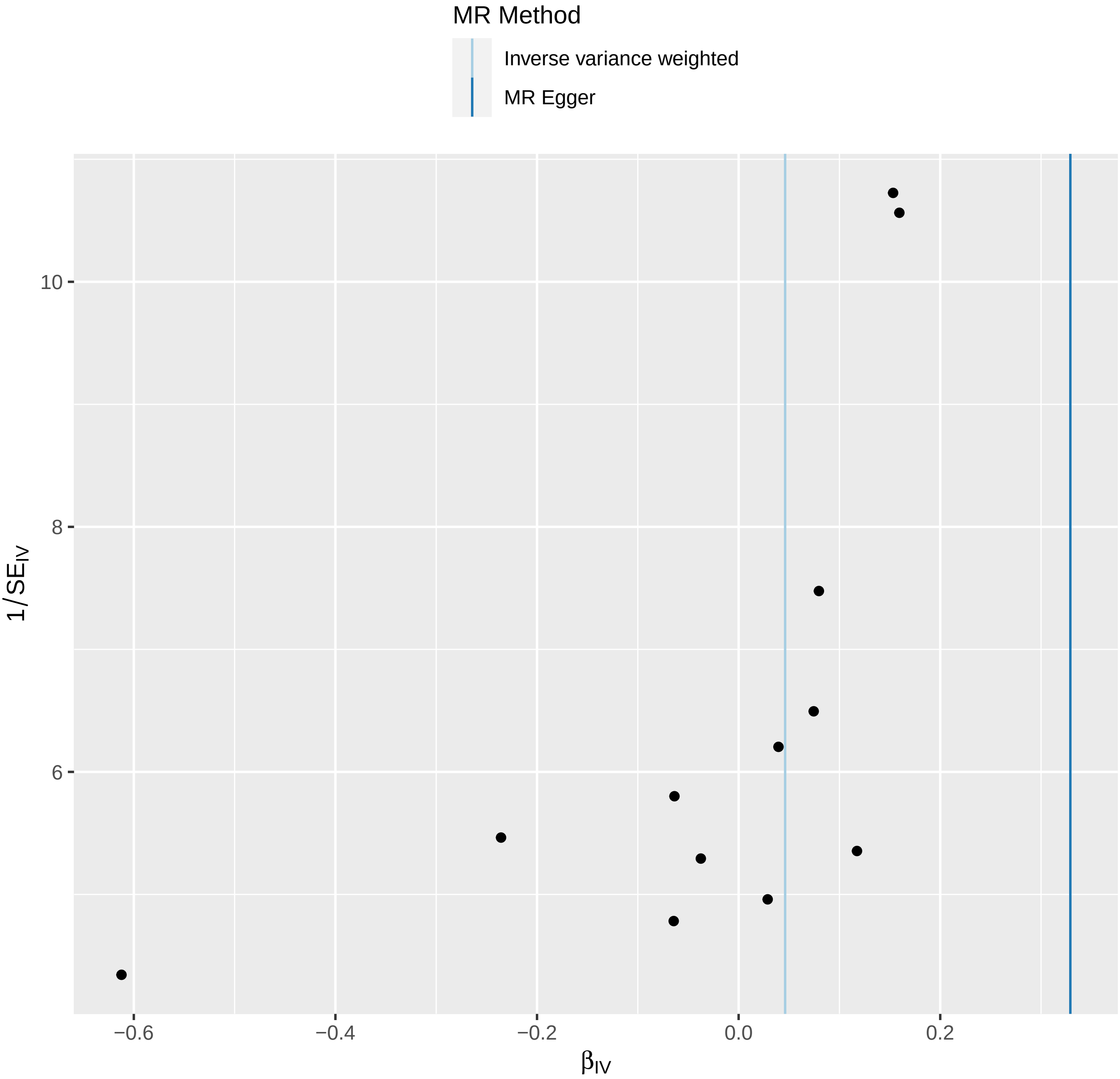

Batch 3−2846 : Selenium on Somatostatin−28

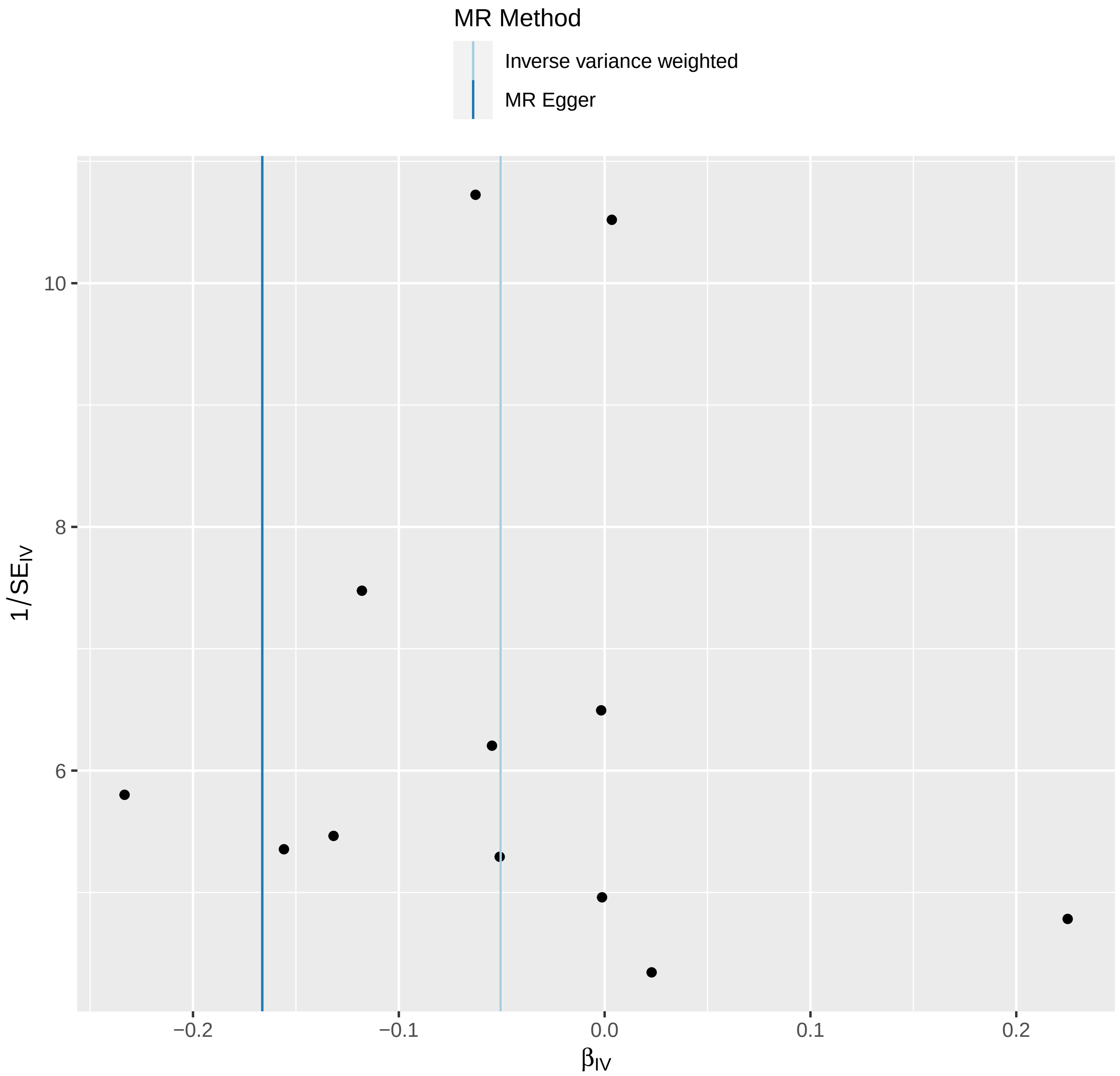

Batch 3−2847 : Selenium on RNA polymerase II subunit A C−terminal domain phosphatase SSU72

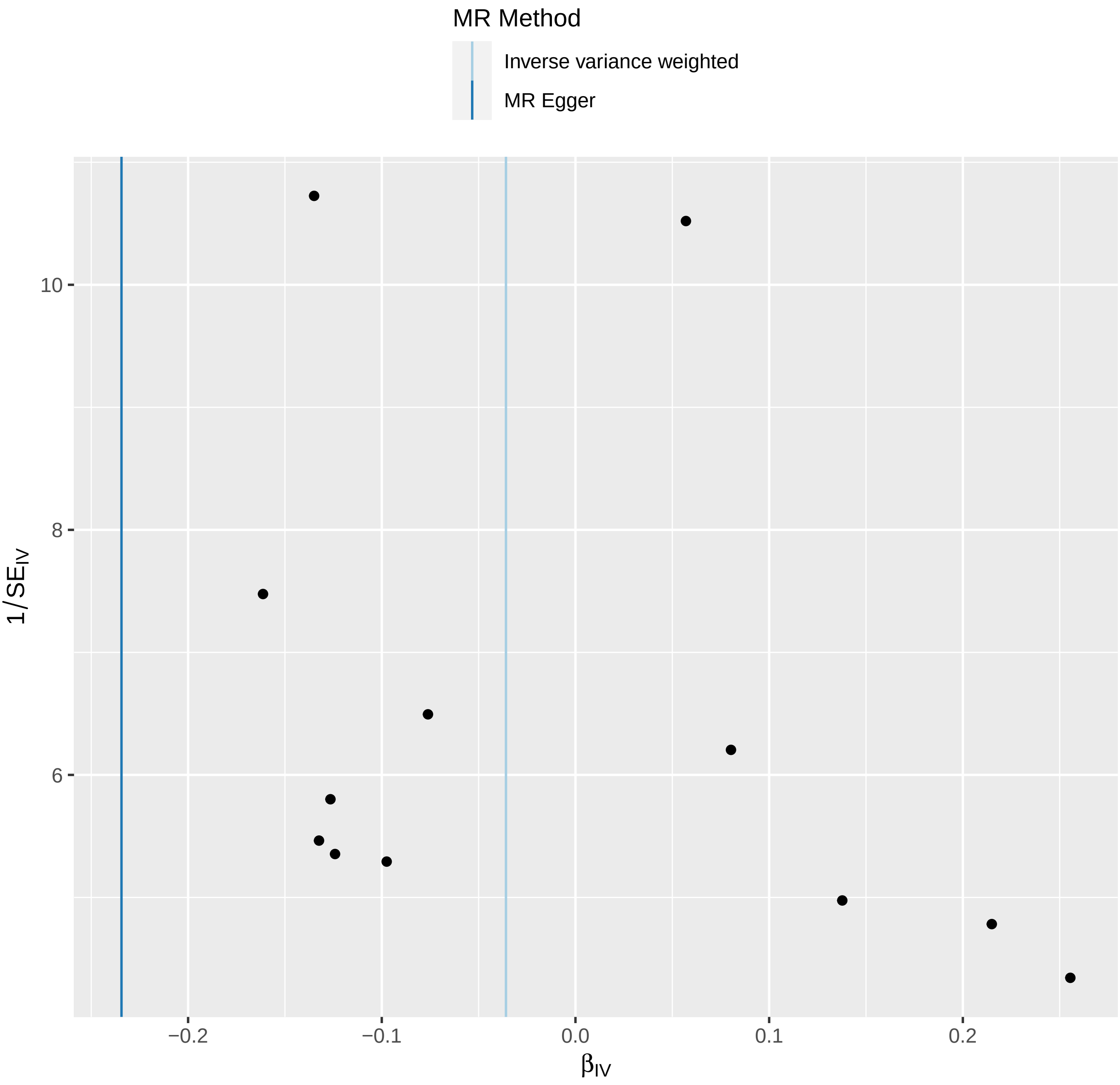

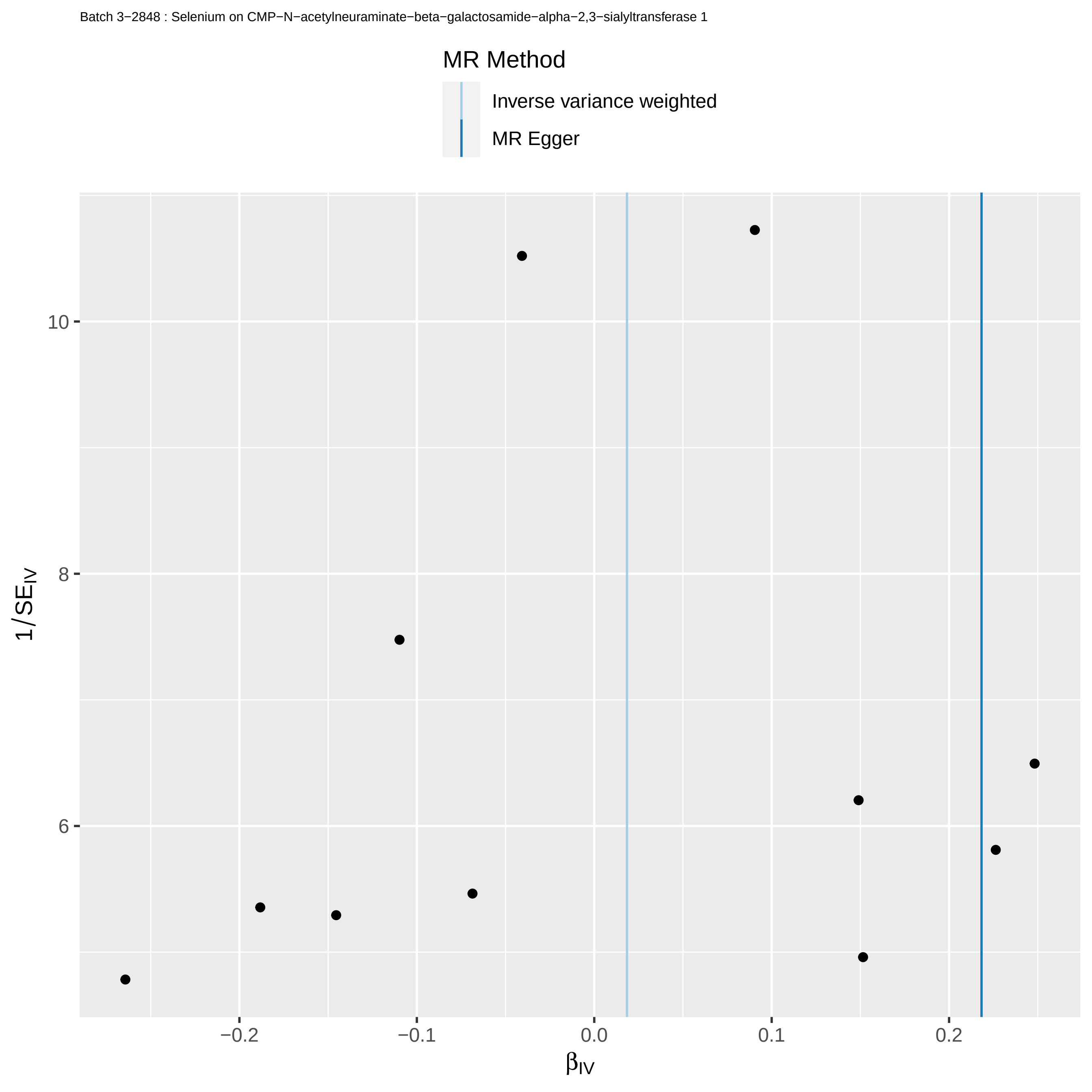

Batch 3−2848 : Selenium on CMP−N−acetylneuraminate−beta−galactosamide−alpha−2,3−sialyltransferase 1
MR Method
Inverse variance weighted
MR Egger
10
8
6
−0.2
−0.1
0.0
0.1
0.2
$1/SE_{IV}$
β_{IV}

Batch 3−2849 : Selenium on CMP−N−acetylneuraminate−beta−galactosamide−alpha−2,3−sialyltransferase 2

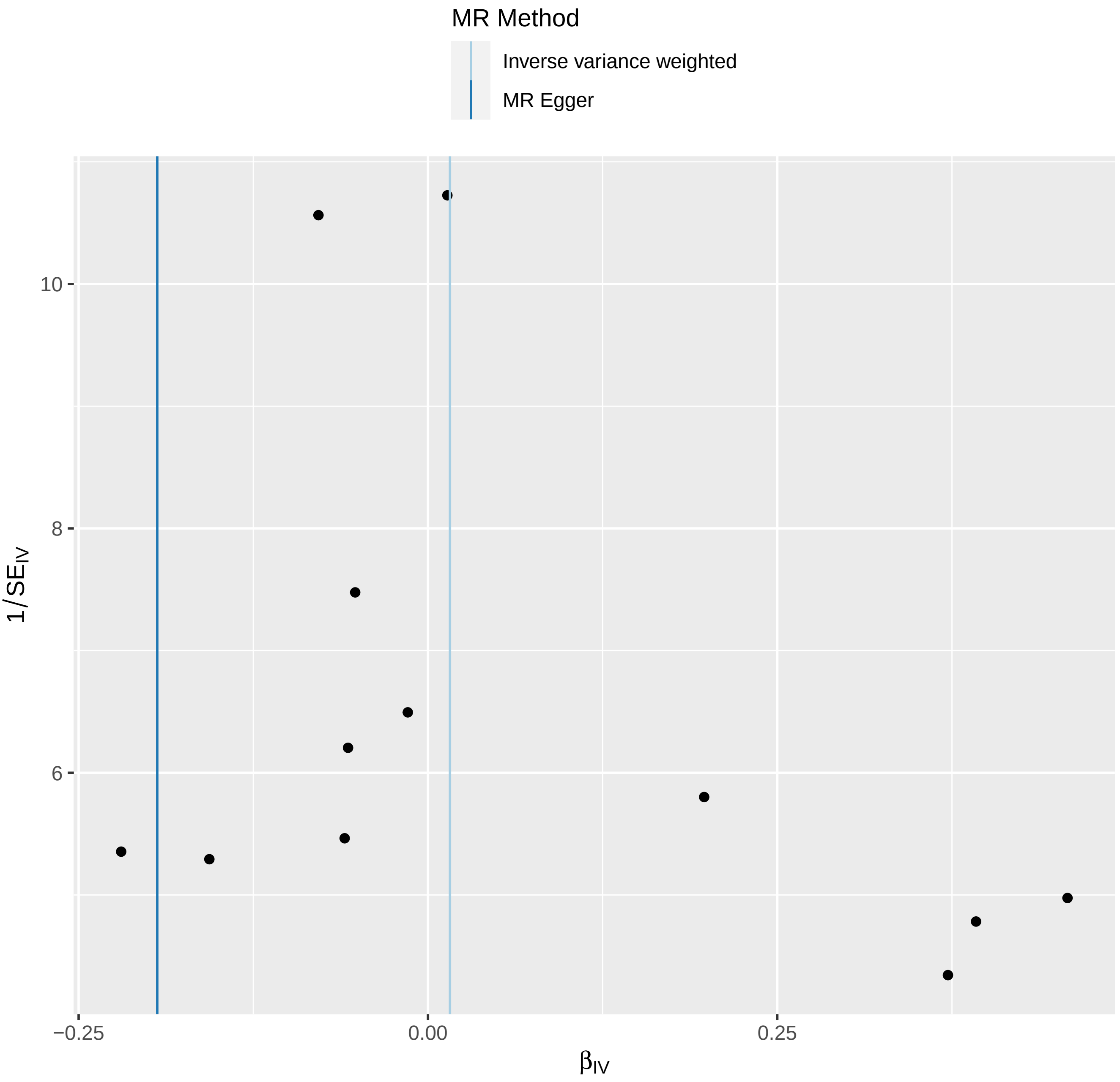

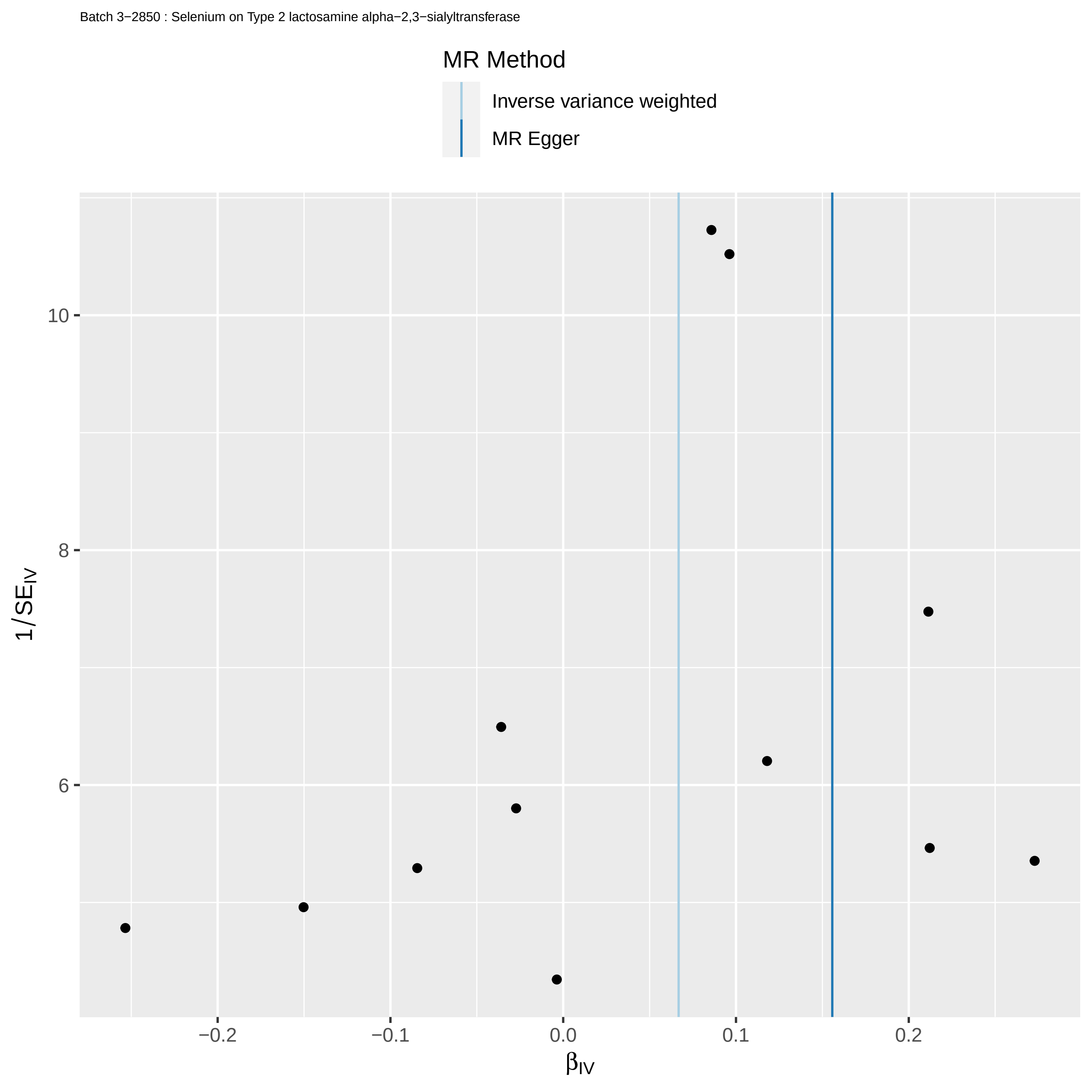
Batch 3−2850 : Selenium on Type 2 lactosamine alpha−2,3−sialyltransferase
MR Method
Inverse variance weighted
MR Egger
10
8
6
1/SE_IV
−0.2
−0.1
0.0
0.1
0.2
β_IV

Batch 3−2851 : Selenium on Beta−galactoside alpha−2,6−sialyltransferase 1

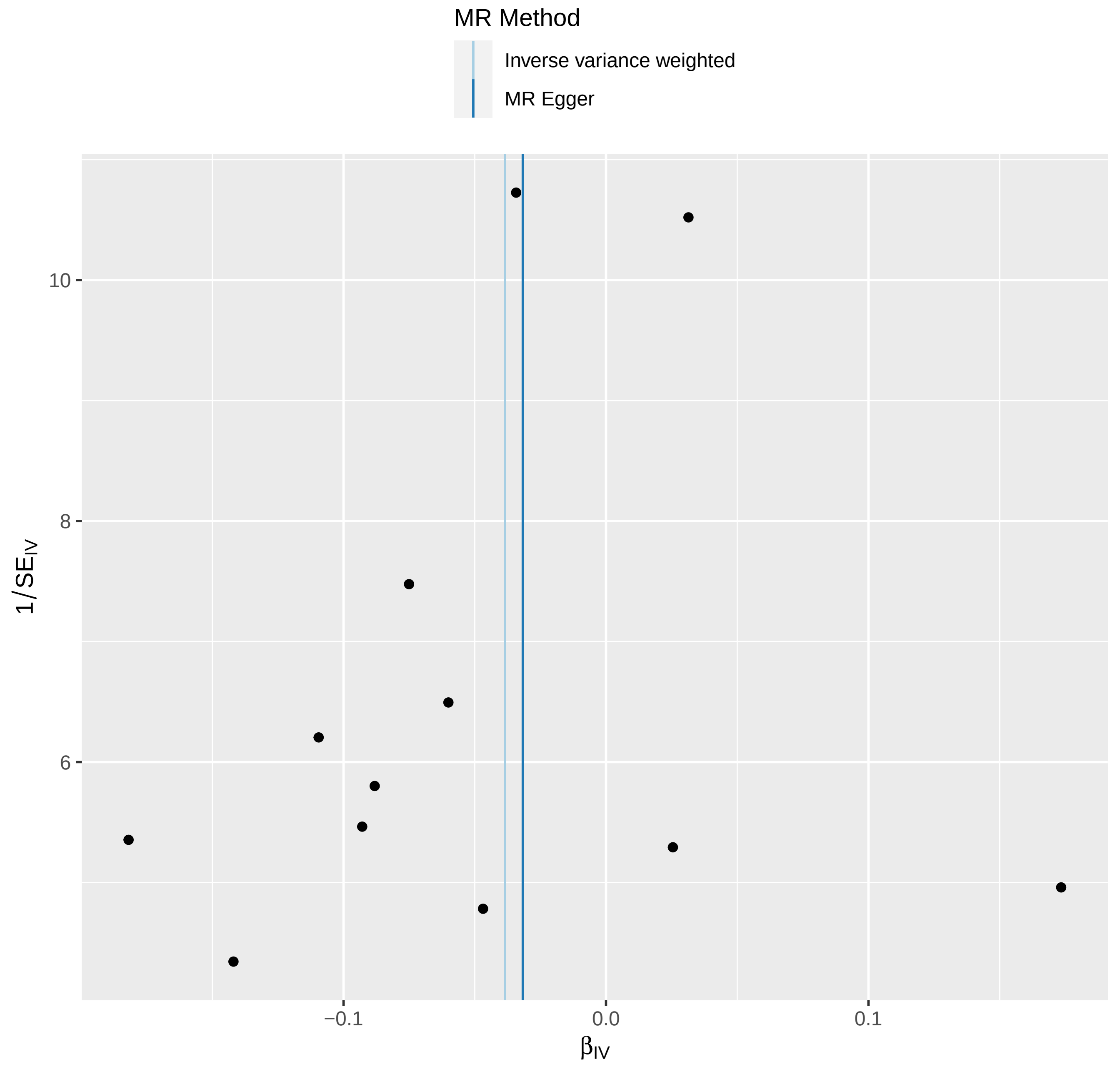

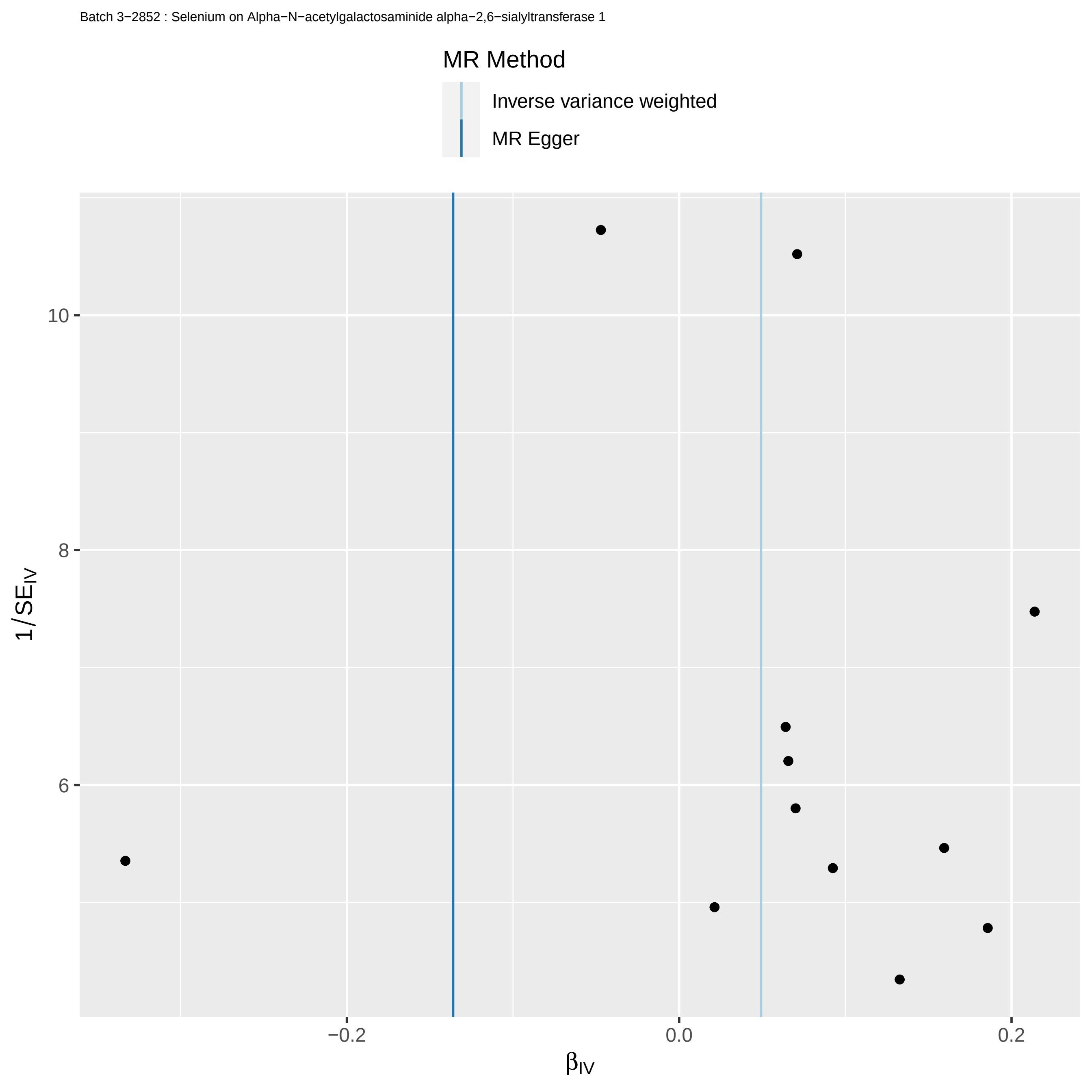

Batch 3−2852 : Selenium on Alpha−N−acetylgalactosaminide alpha−2,6−sialyltransferase 1
MR Method
Inverse variance weighted
MR Egger
10
8
6
1/SE$_{IV}$
−0.2
0.0
0.2
β$_{IV}$

Batch 3−2853 : Selenium on Alpha−N−acetylgalactosaminide alpha−2,6−sialyltransferase 2

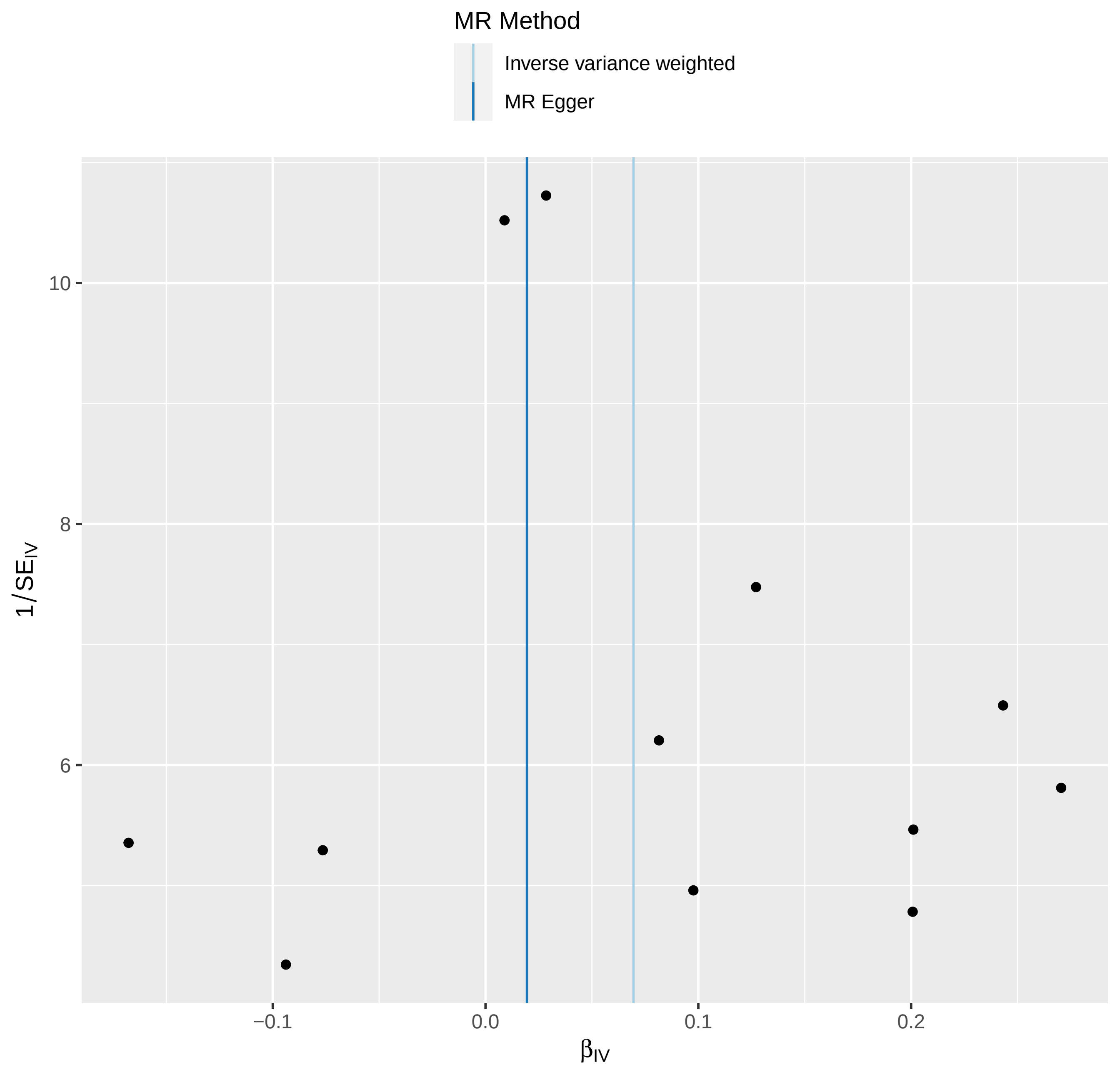

Batch 3−2854 : Selenium on Alpha−N−acetylgalactosaminide alpha−2,6−sialyltransferase 3

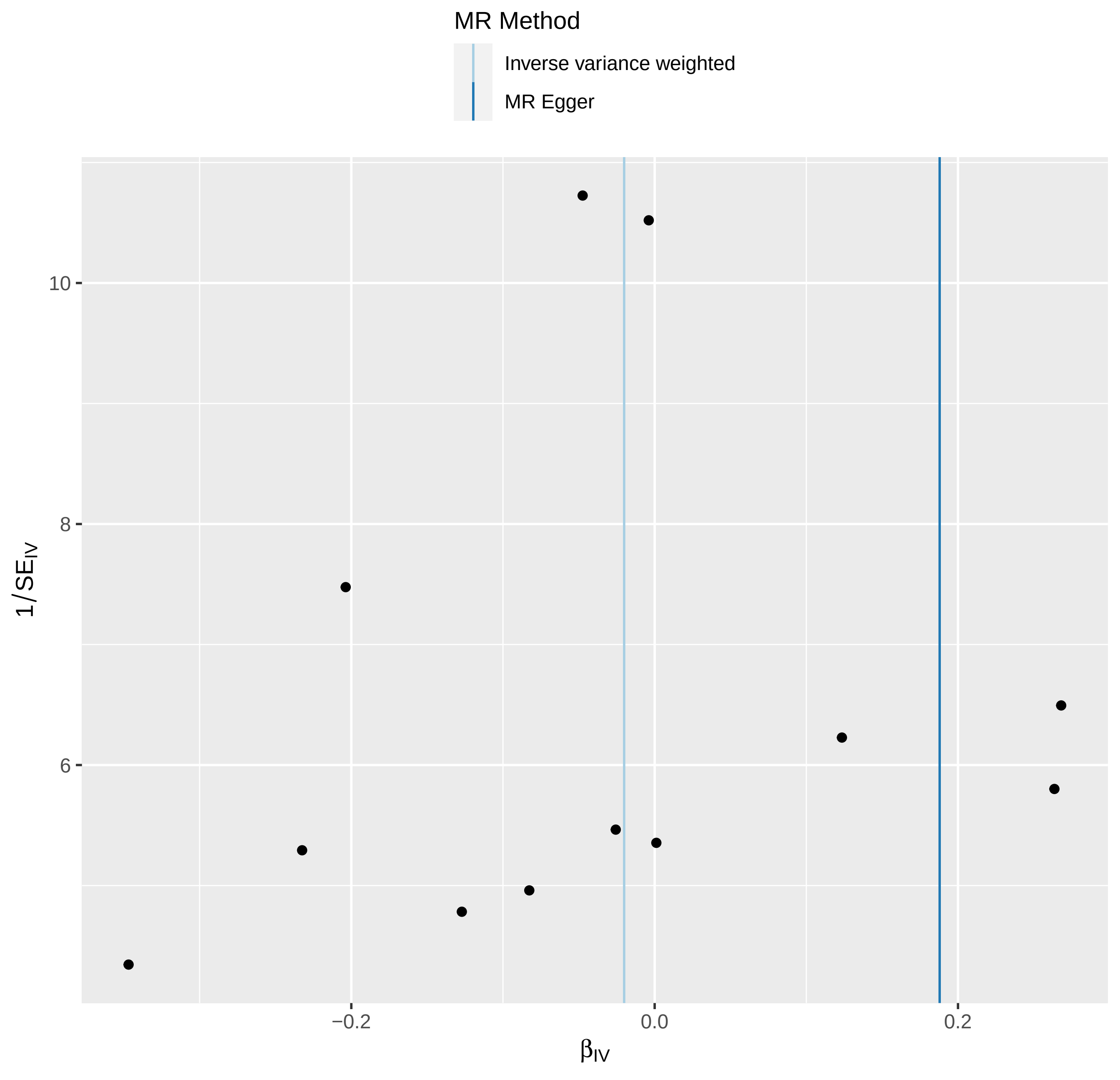

Batch 3−2855 : Selenium on Alpha−N−acetylgalactosaminide alpha−2,6−sialyltransferase 3

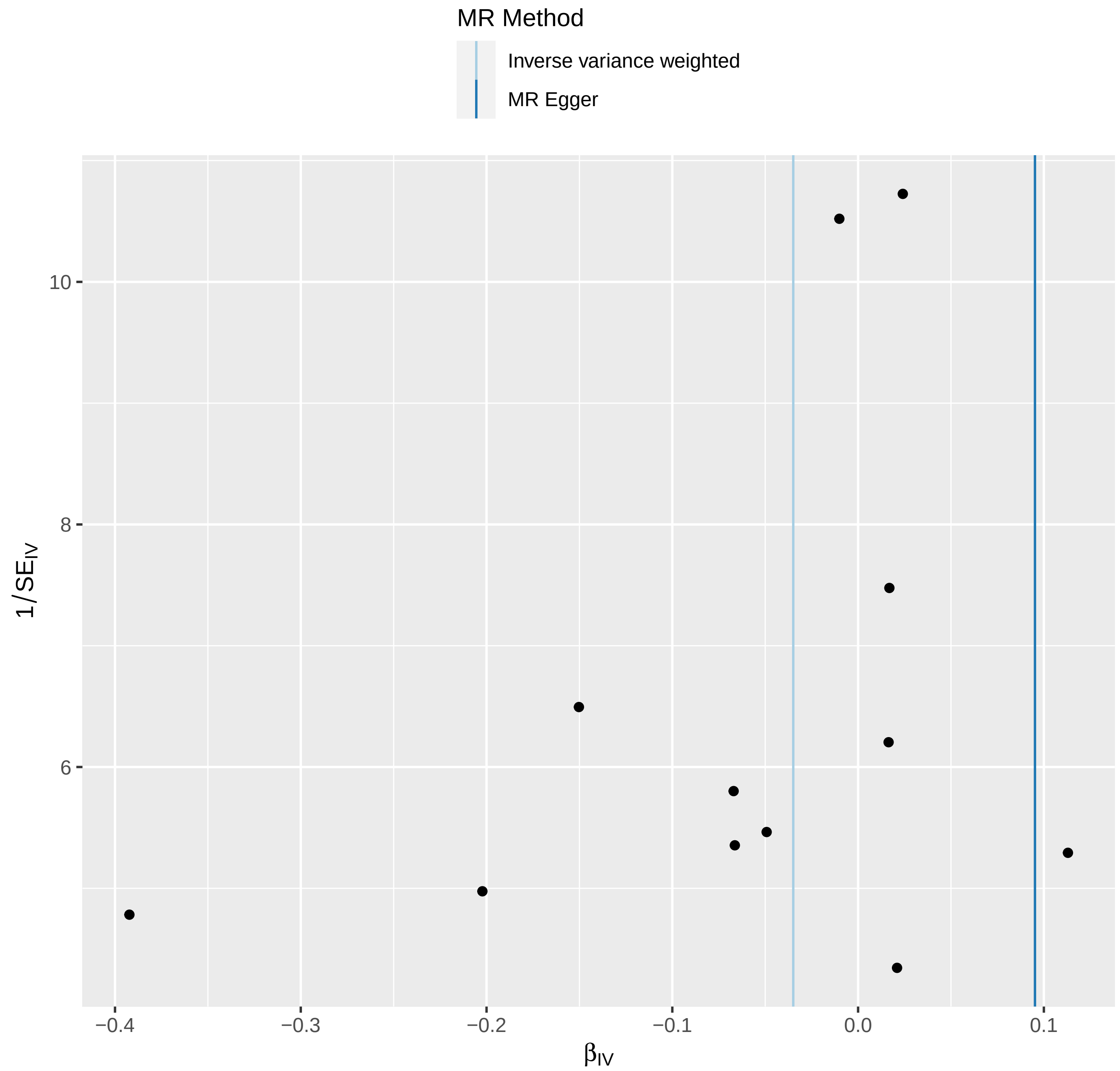

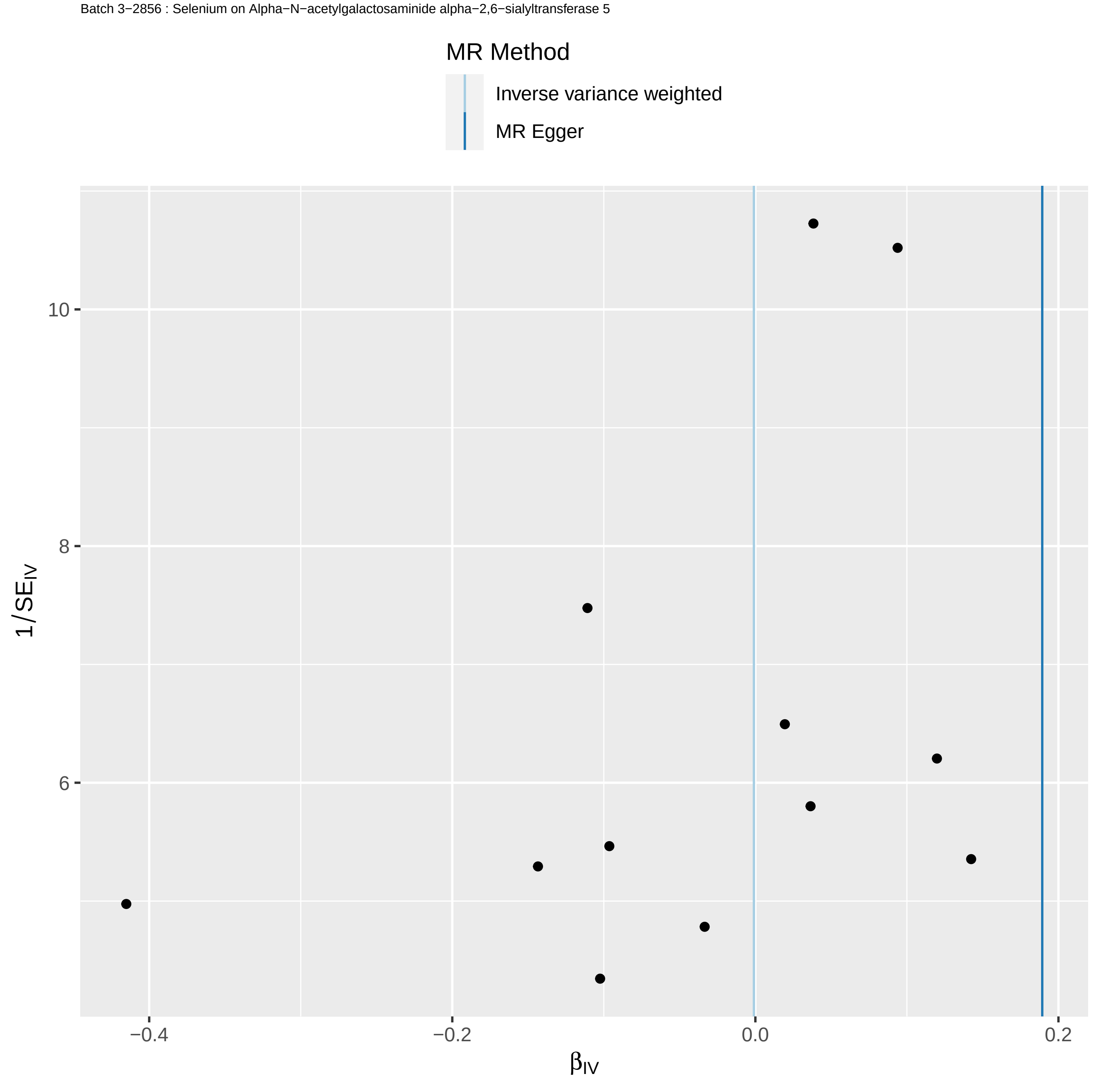

Batch 3−2856 : Selenium on Alpha−N−acetylgalactosaminide alpha−2,6−sialyltransferase 5
MR Method
Inverse variance weighted
MR Egger
10
8
6
1/SE_IV
−0.4
−0.2
0.0
0.2
β_IV

Batch 3−2857 : Selenium on Alpha−N−acetylgalactosaminide alpha−2,6−sialyltransferase 6

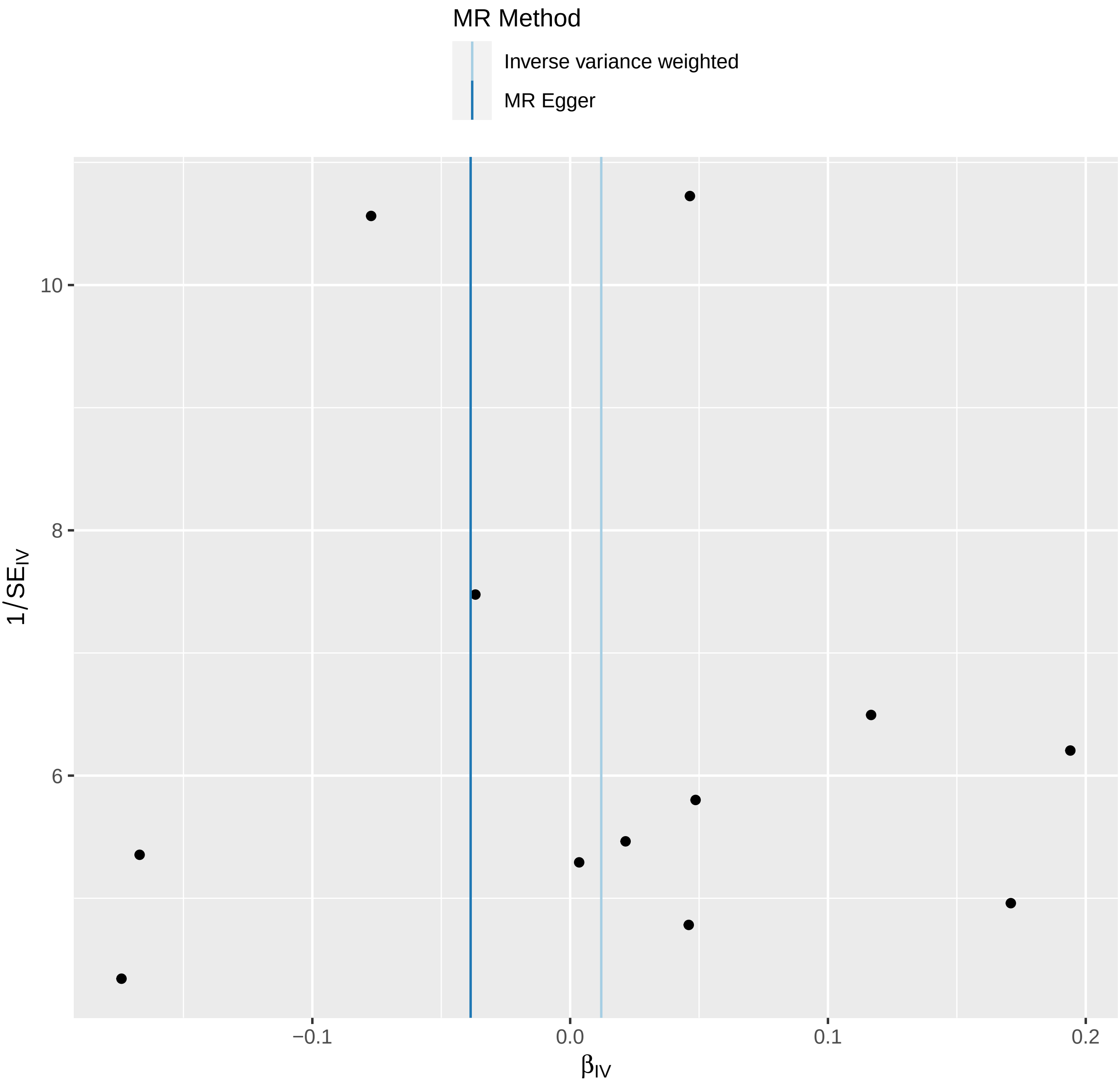

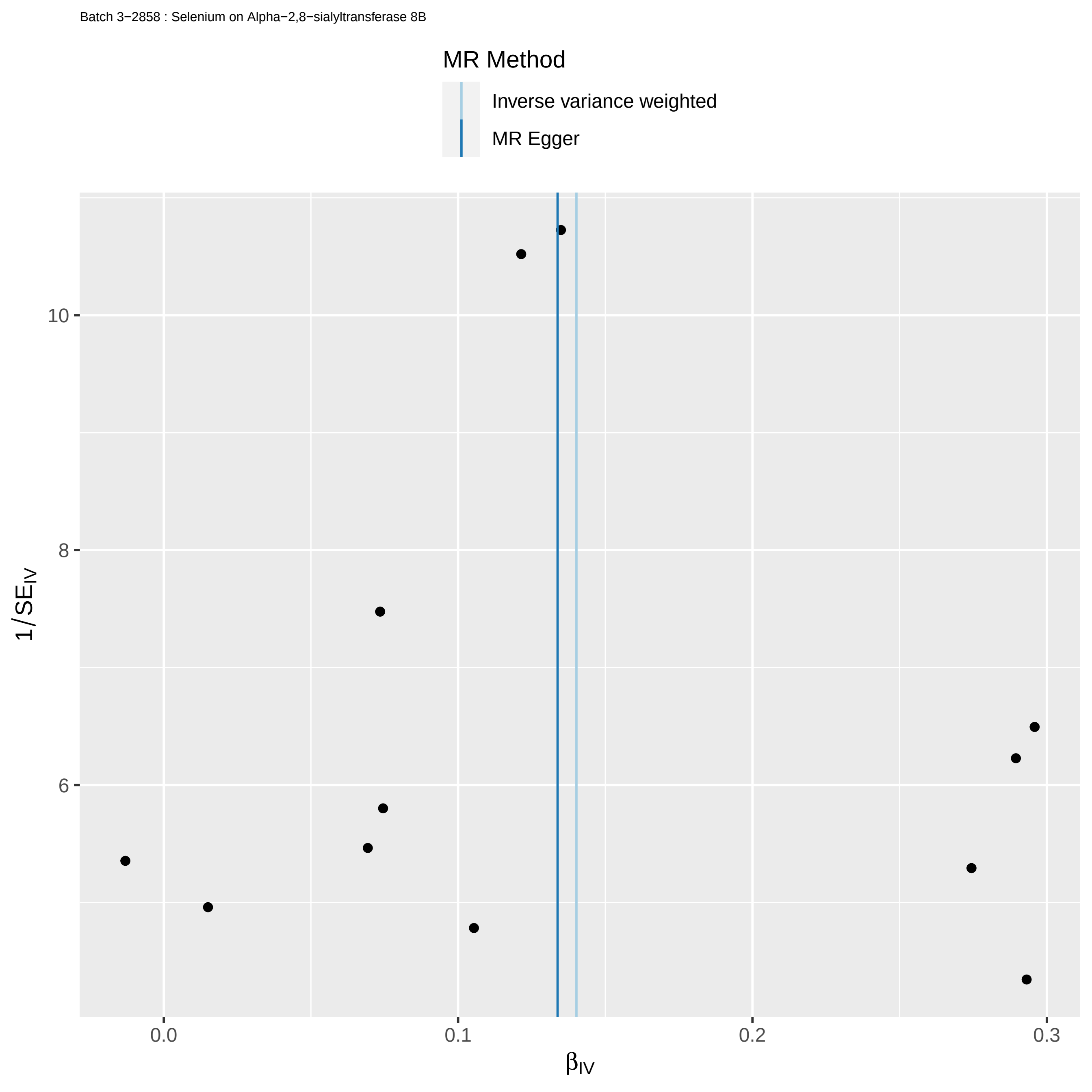
Batch 3−2858 : Selenium on Alpha−2,8−sialyltransferase 8B
MR Method
Inverse variance weighted
MR Egger
10
8
6
1/SE_IV
0.0
0.1
0.2
0.3
β_IV

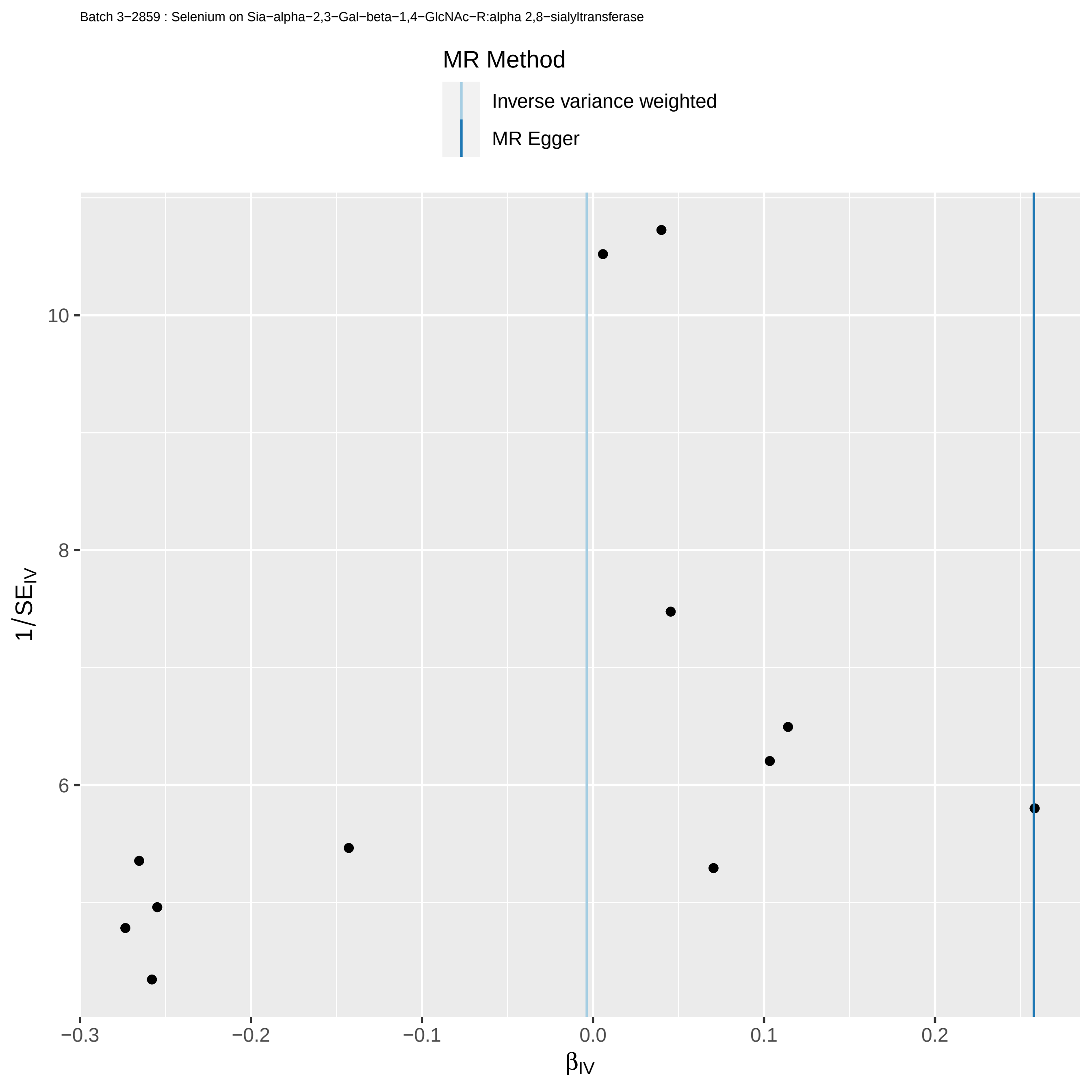

Batch 3−2859 : Selenium on Sia−alpha−2,3−Gal−beta−1,4−GlcNAc−R:alpha 2,8−sialyltransferase
MR Method
Inverse variance weighted
MR Egger
10
8
6
1/SE_IV
−0.3
−0.2
−0.1
0.0
0.1
0.2
β_IV

Batch 3−2860 : Selenium on CMP−N−acetylneuraminate−poly−alpha−2,8−sialyltransferase

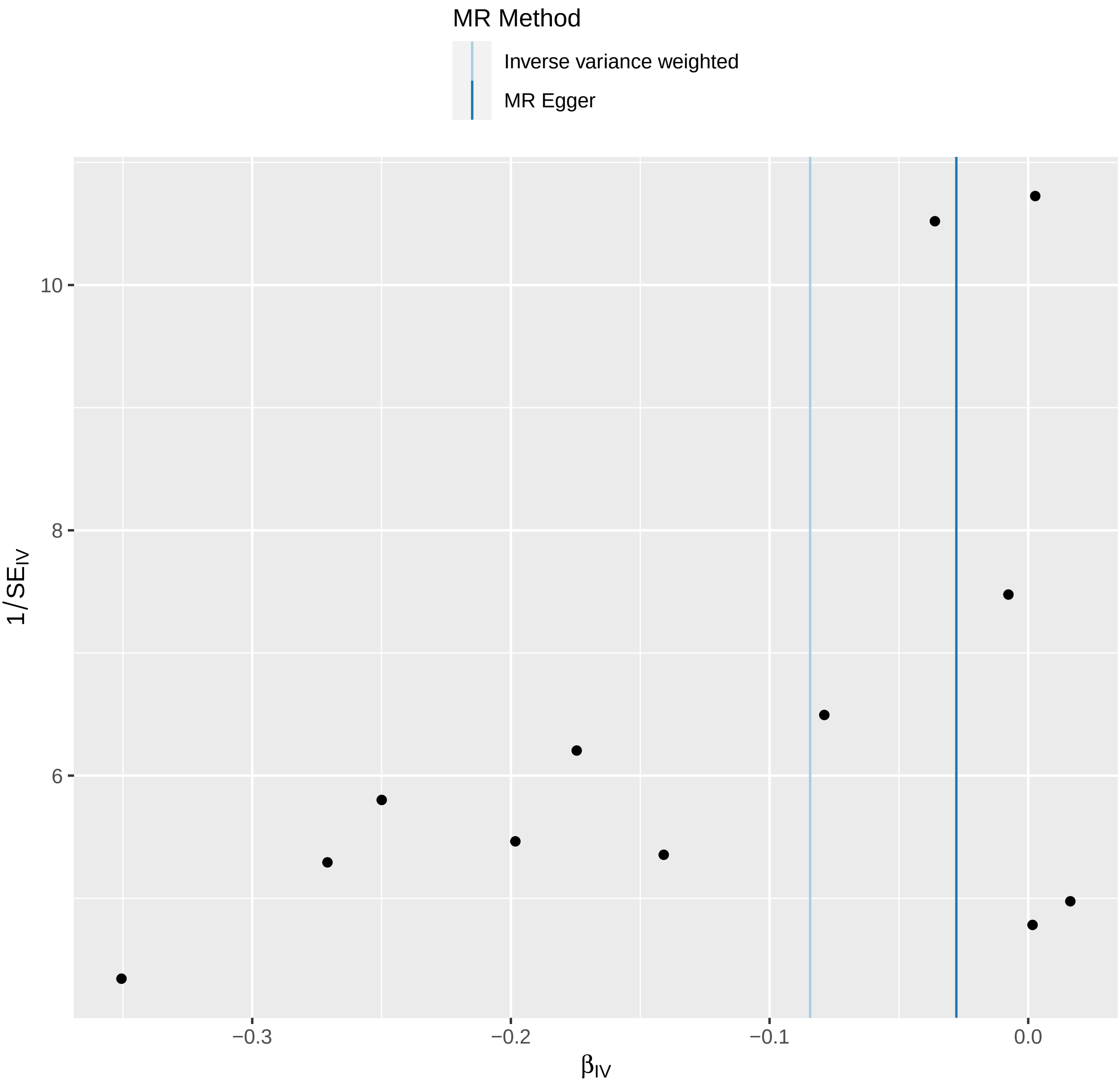

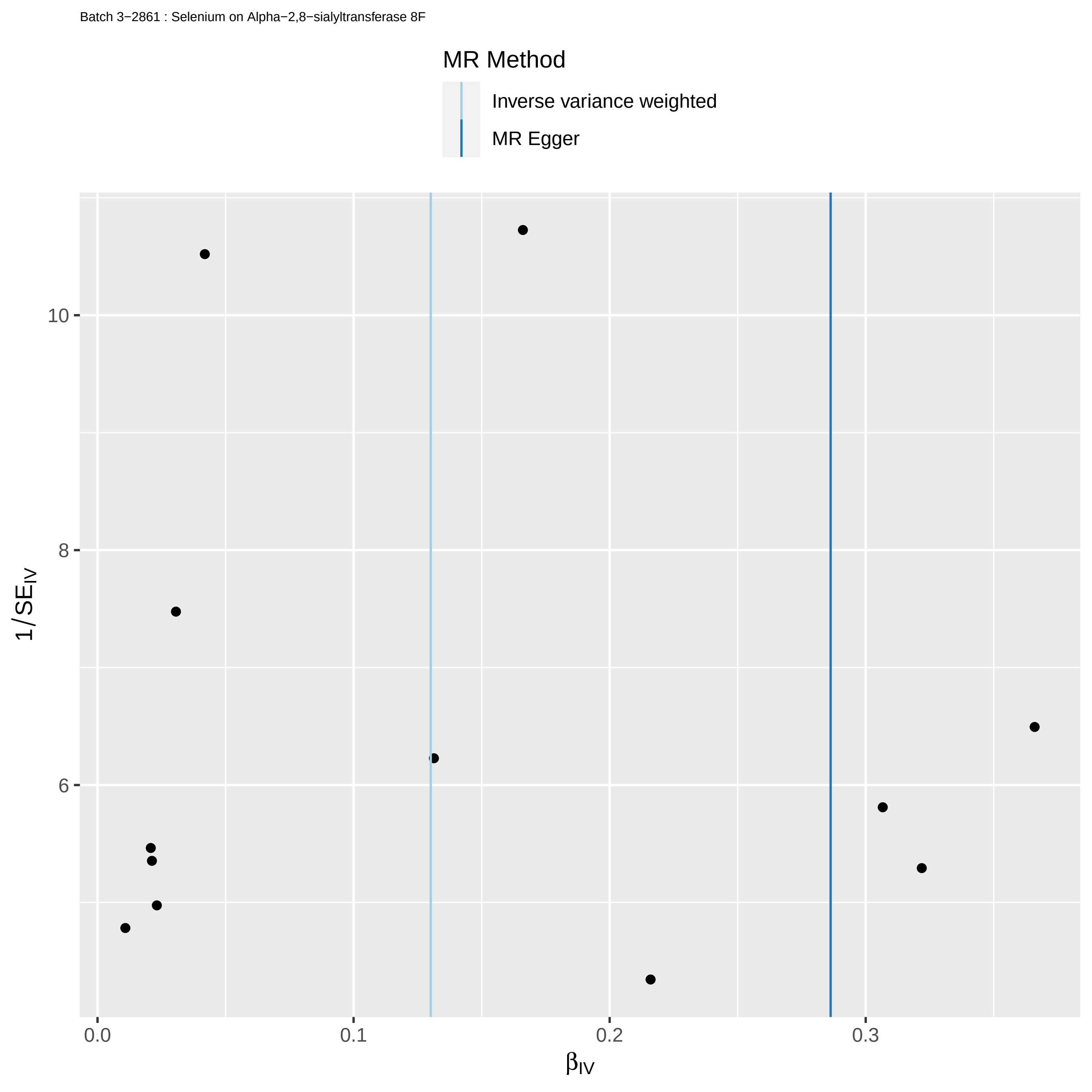
Batch 3−2861 : Selenium on Alpha−2,8−sialyltransferase 8F
MR Method
Inverse variance weighted
MR Egger
10
8
6
1/SE$_{IV}$
0.0
0.1
0.2
0.3
β$_{IV}$

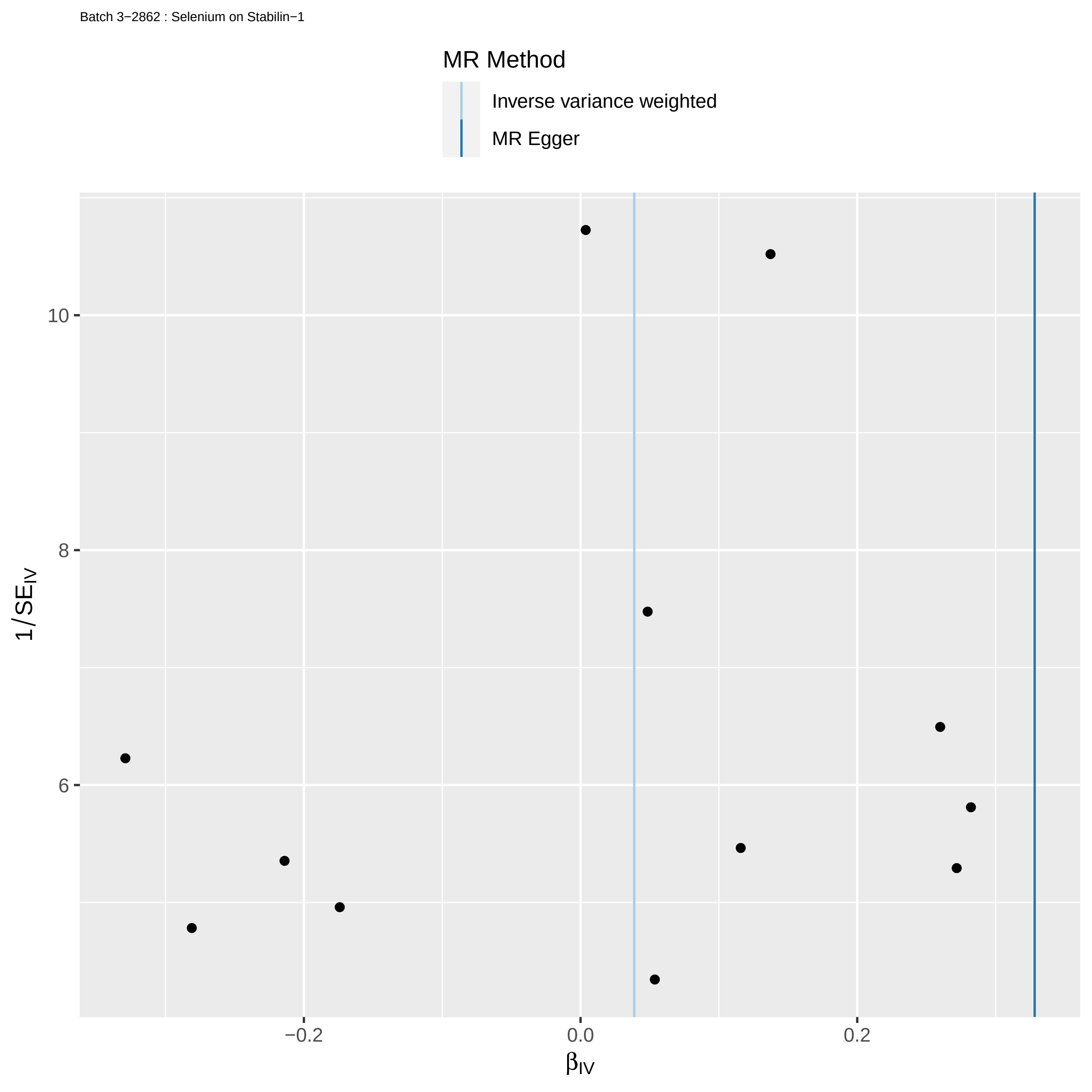
Batch 3−2862 : Selenium on Stabilin−1
MR Method
Inverse variance weighted
MR Egger
10
8
6
1/SE_IV
−0.2
0.0
0.2
β_IV

Batch 3−2863 : Selenium on Stabilin−2

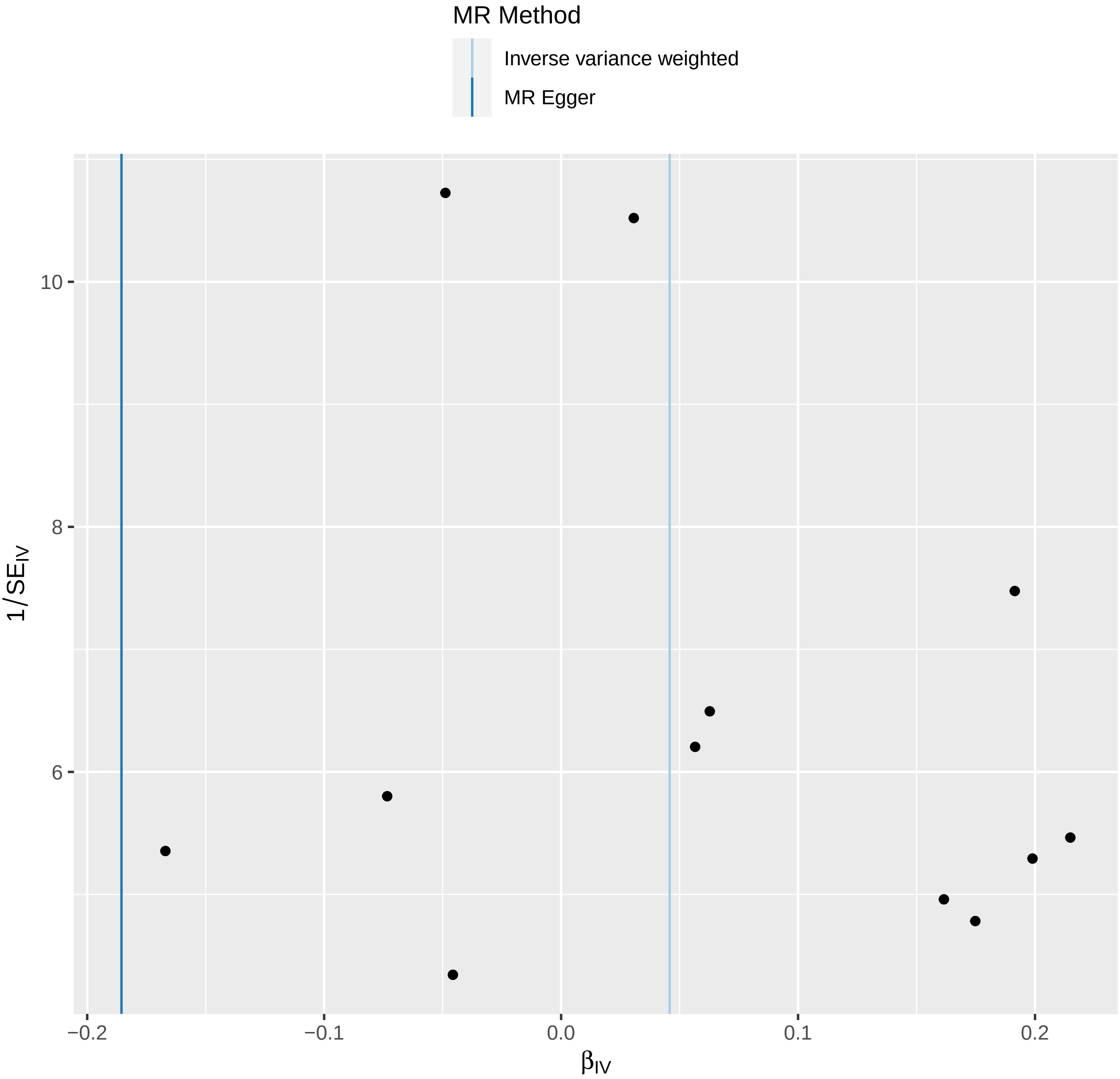

Batch 3−2864 : Selenium on AMSH−like protease

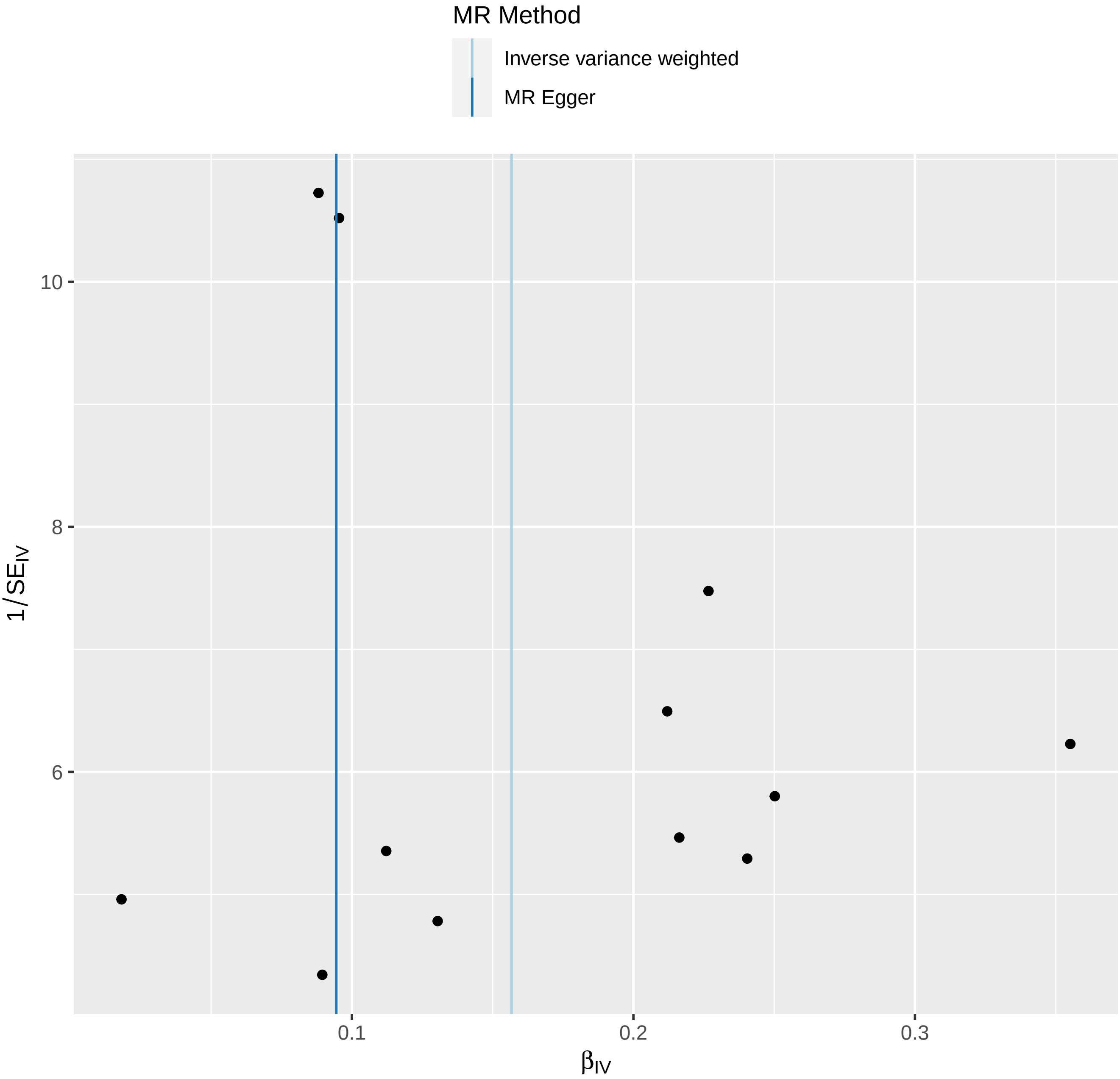

Batch 3−2865 : Selenium on Signal−transducing adaptor protein 1

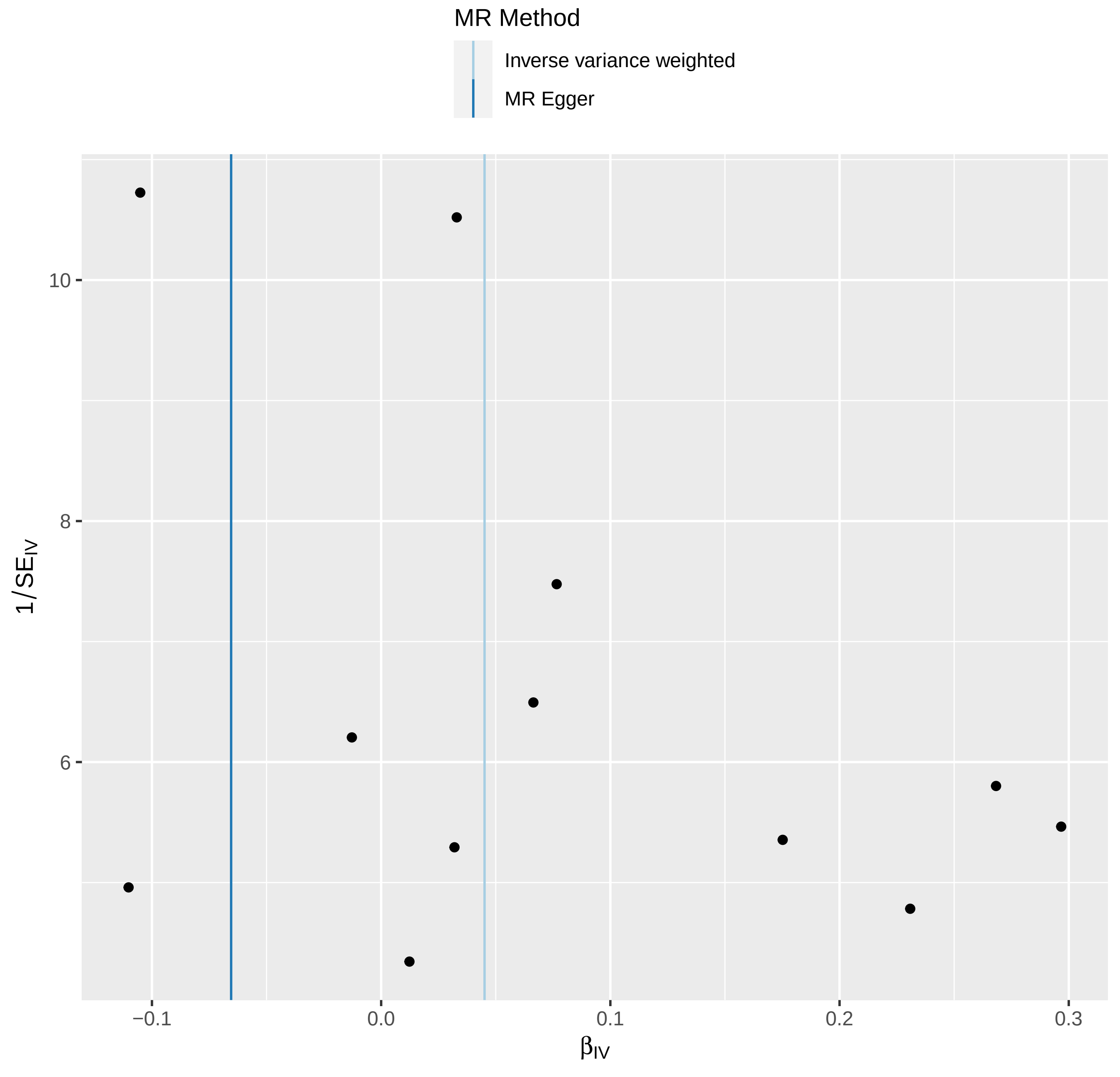

Batch 3−2866 : Selenium on Steroidogenic acute regulatory protein, mitochondrial

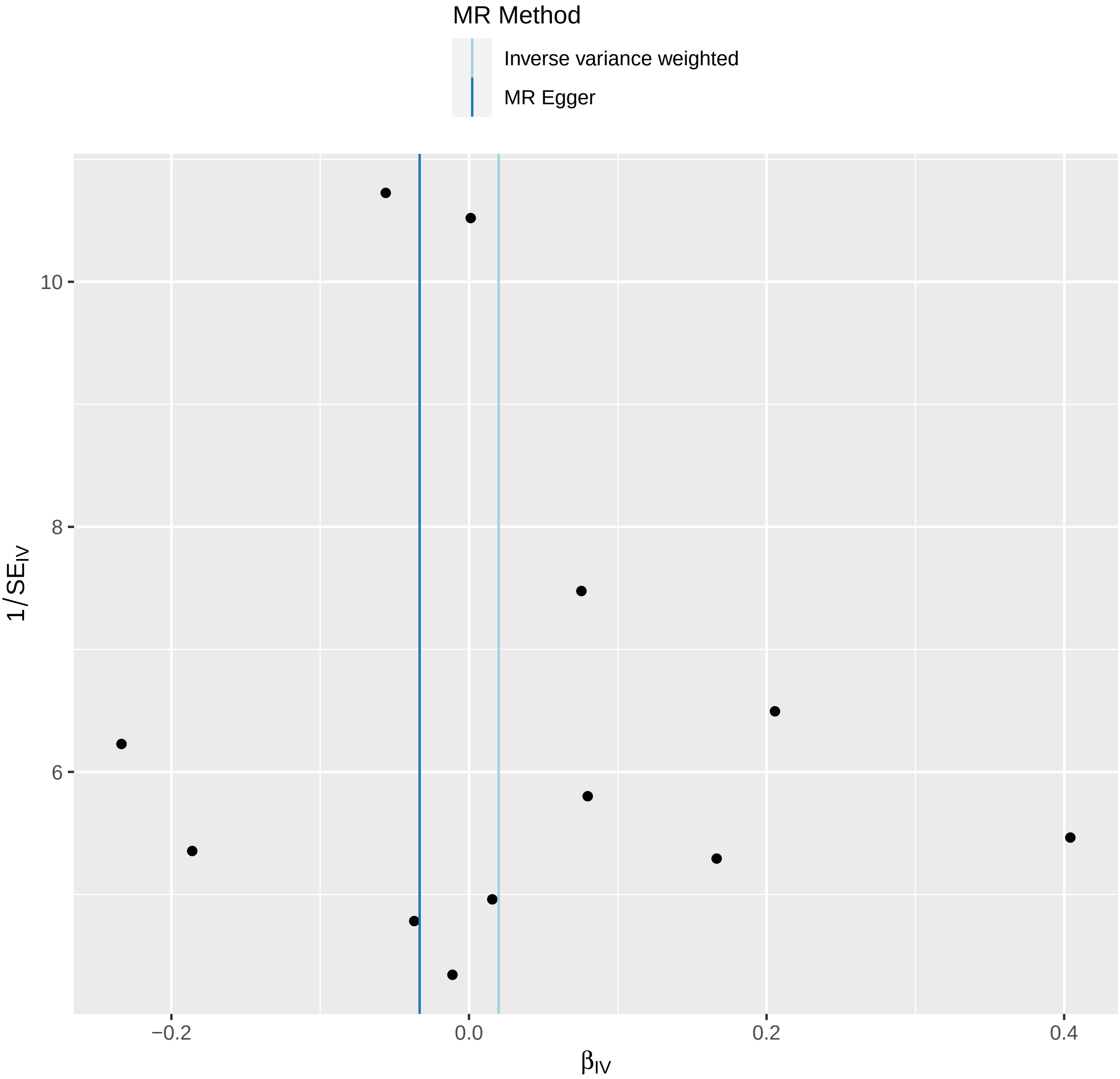

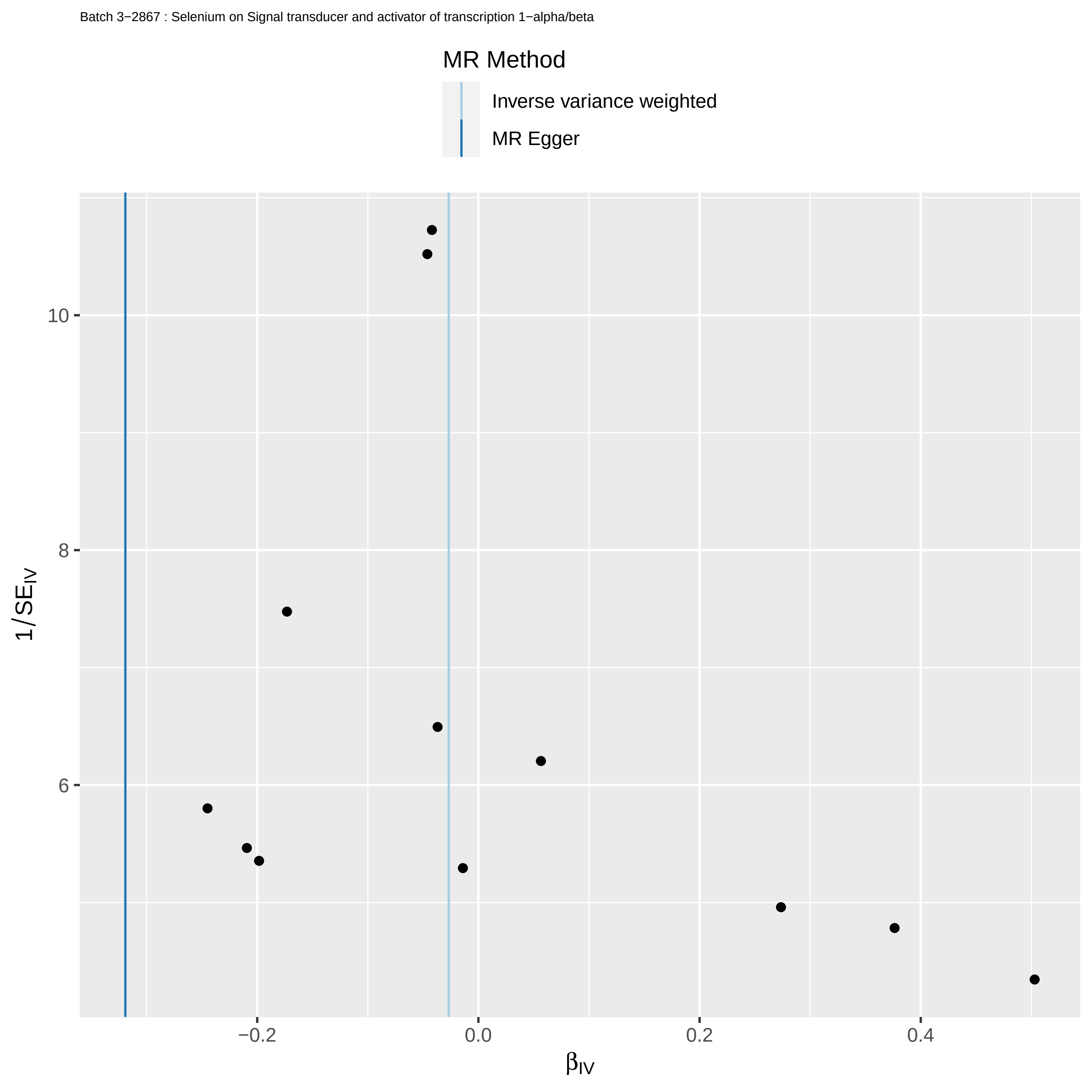

Batch 3−2867 : Selenium on Signal transducer and activator of transcription 1−alpha/beta
MR Method
Inverse variance weighted
MR Egger
10
8
6
1/SE_IV
−0.2
0.0
0.2
0.4
β_IV

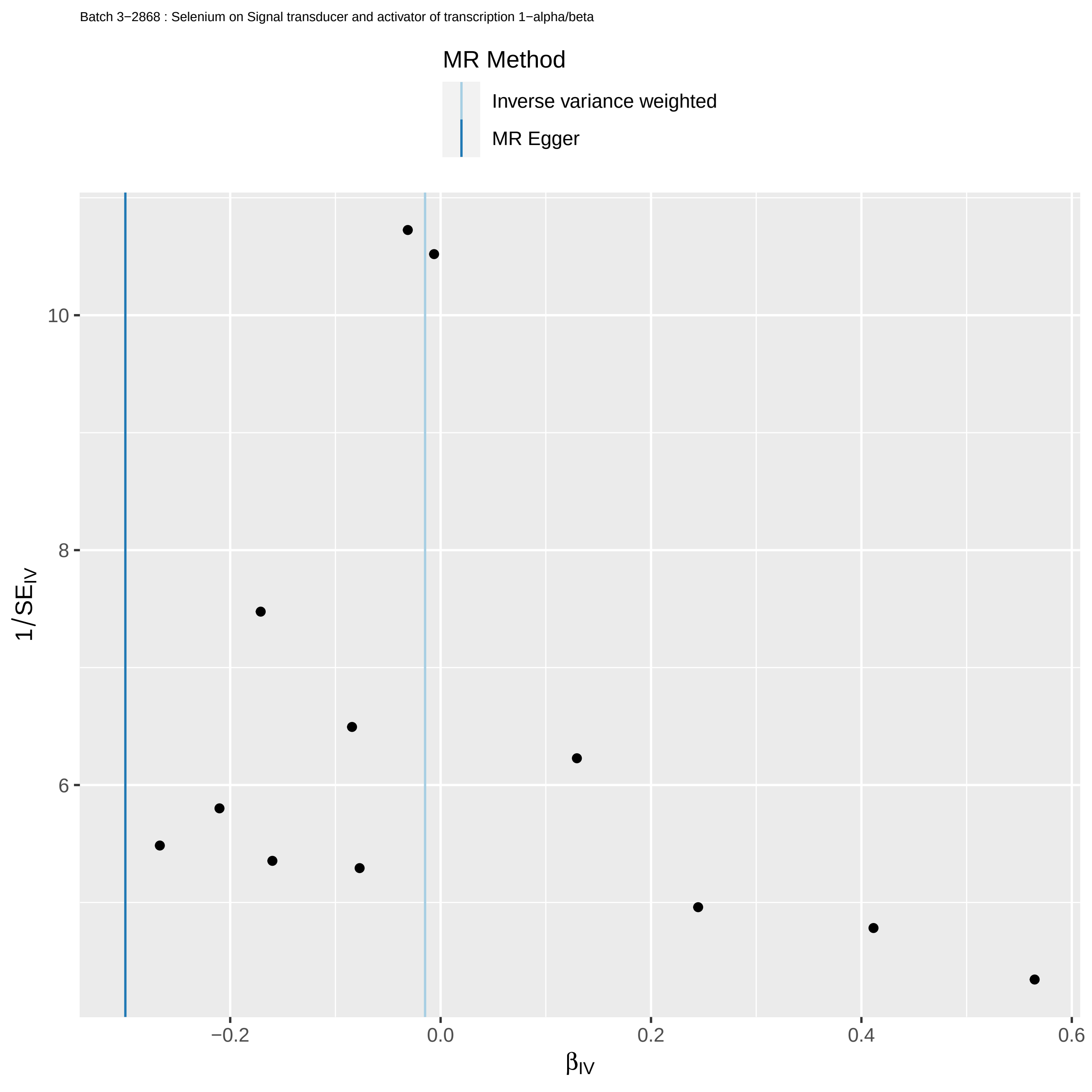

Batch 3−2868 : Selenium on Signal transducer and activator of transcription 1−alpha/beta
MR Method
Inverse variance weighted
MR Egger
10
8
6
1/SE_IV
−0.2
0.0
0.2
0.4
0.6
β_IV

Batch 3−2869 : Selenium on Signal transducer and activator of transcription 3

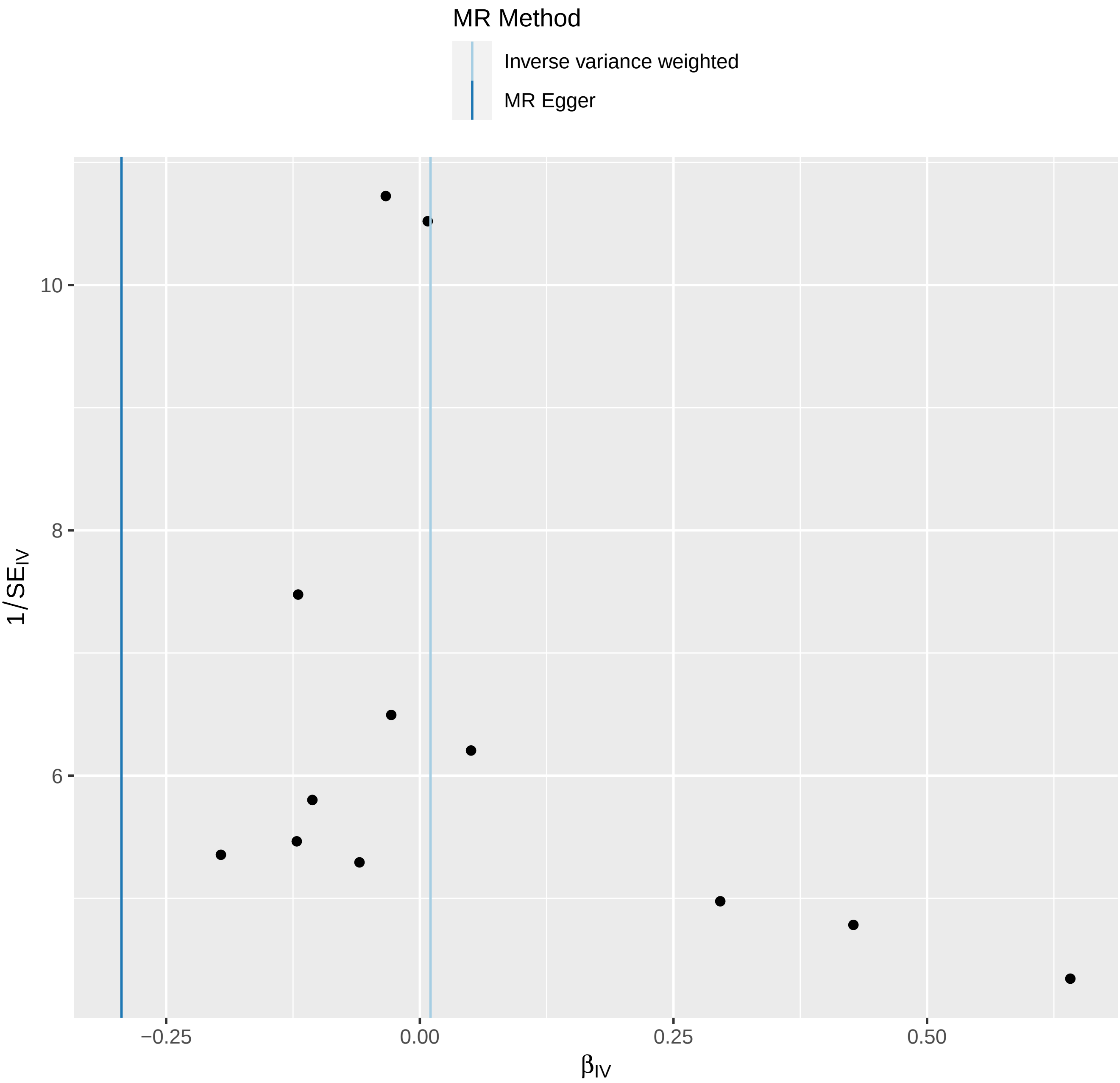

Batch 3−2870 : Selenium on Signal transducer and activator of transcription 6

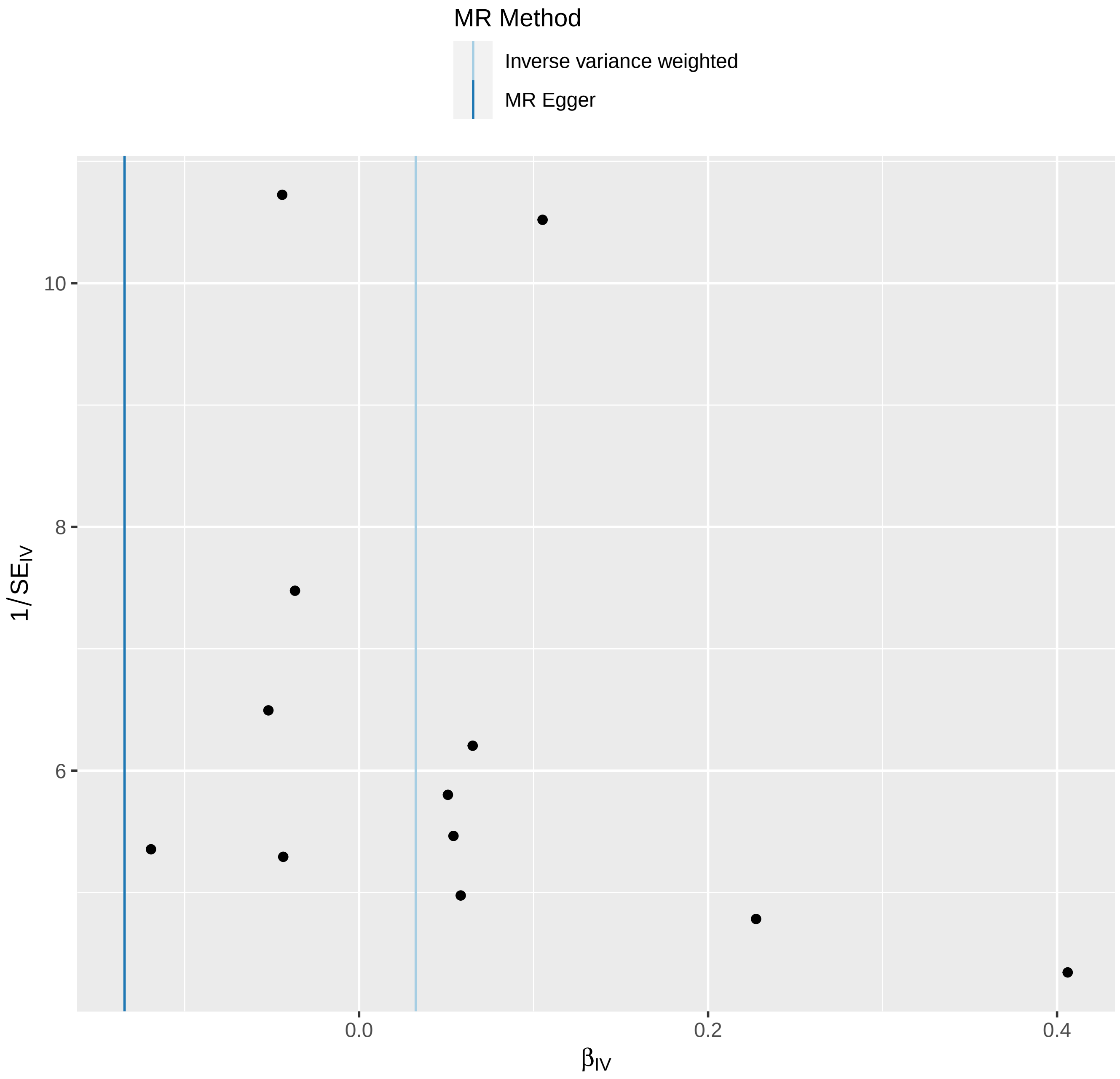

Batch 3−2871 : Selenium on Double−stranded RNA−binding protein Staufen homolog 1

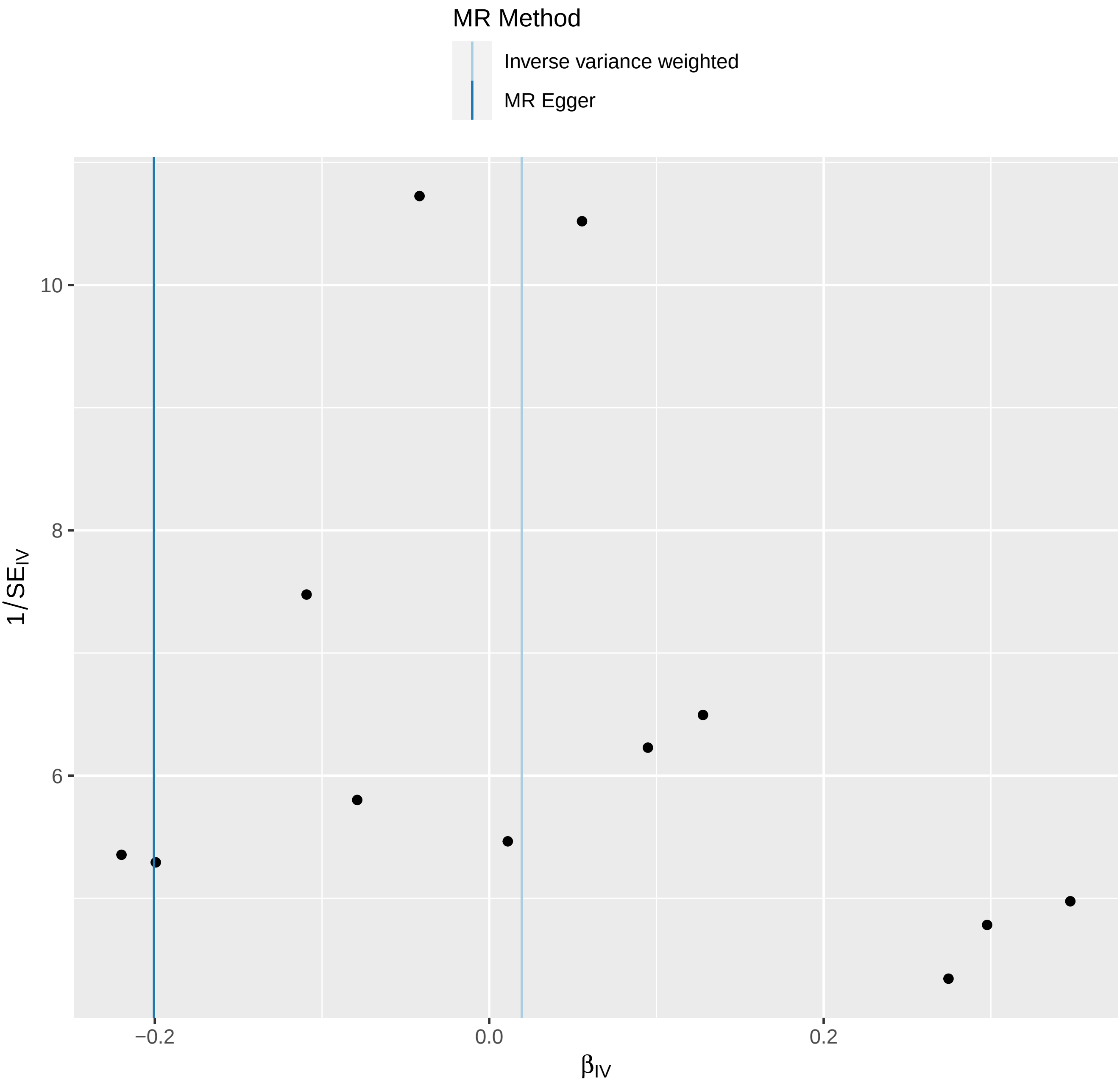

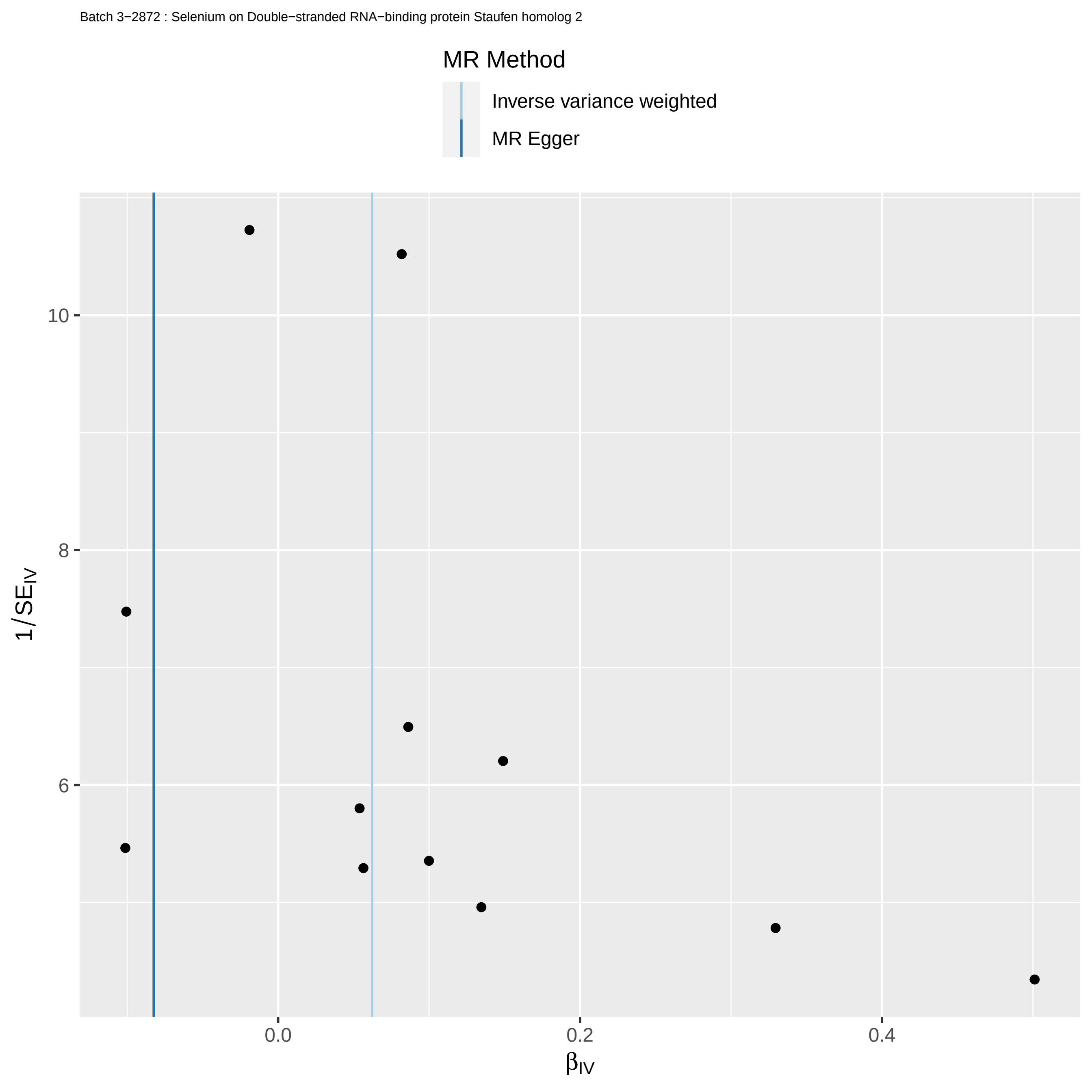
Batch 3−2872 : Selenium on Double−stranded RNA−binding protein Staufen homolog 2
MR Method
Inverse variance weighted
MR Egger
10
8
6
1/SE$_{IV}$
0.0
0.2
0.4
β_{IV}

Batch 3−2873 : Selenium on Stanniocalcin−1

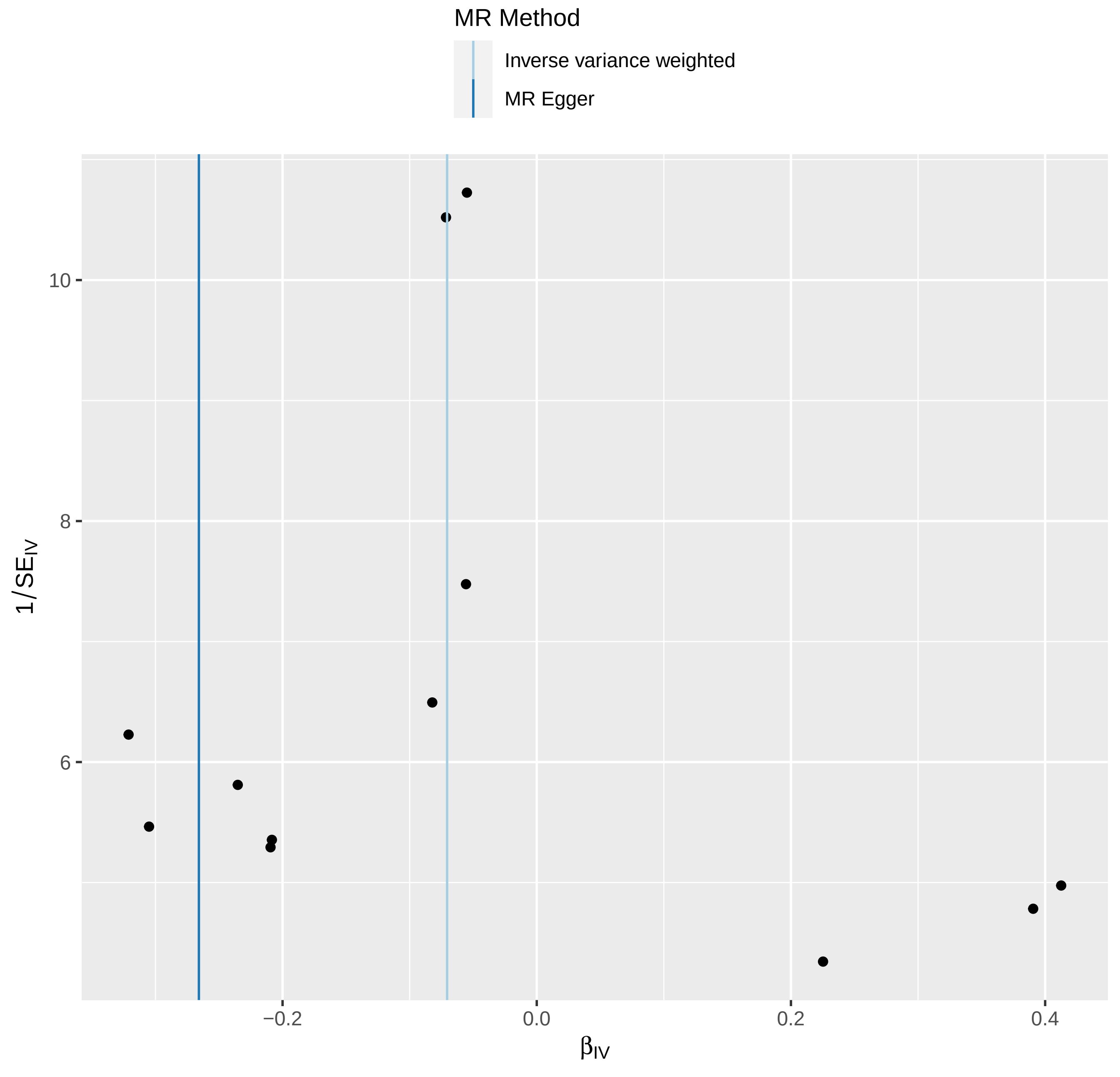

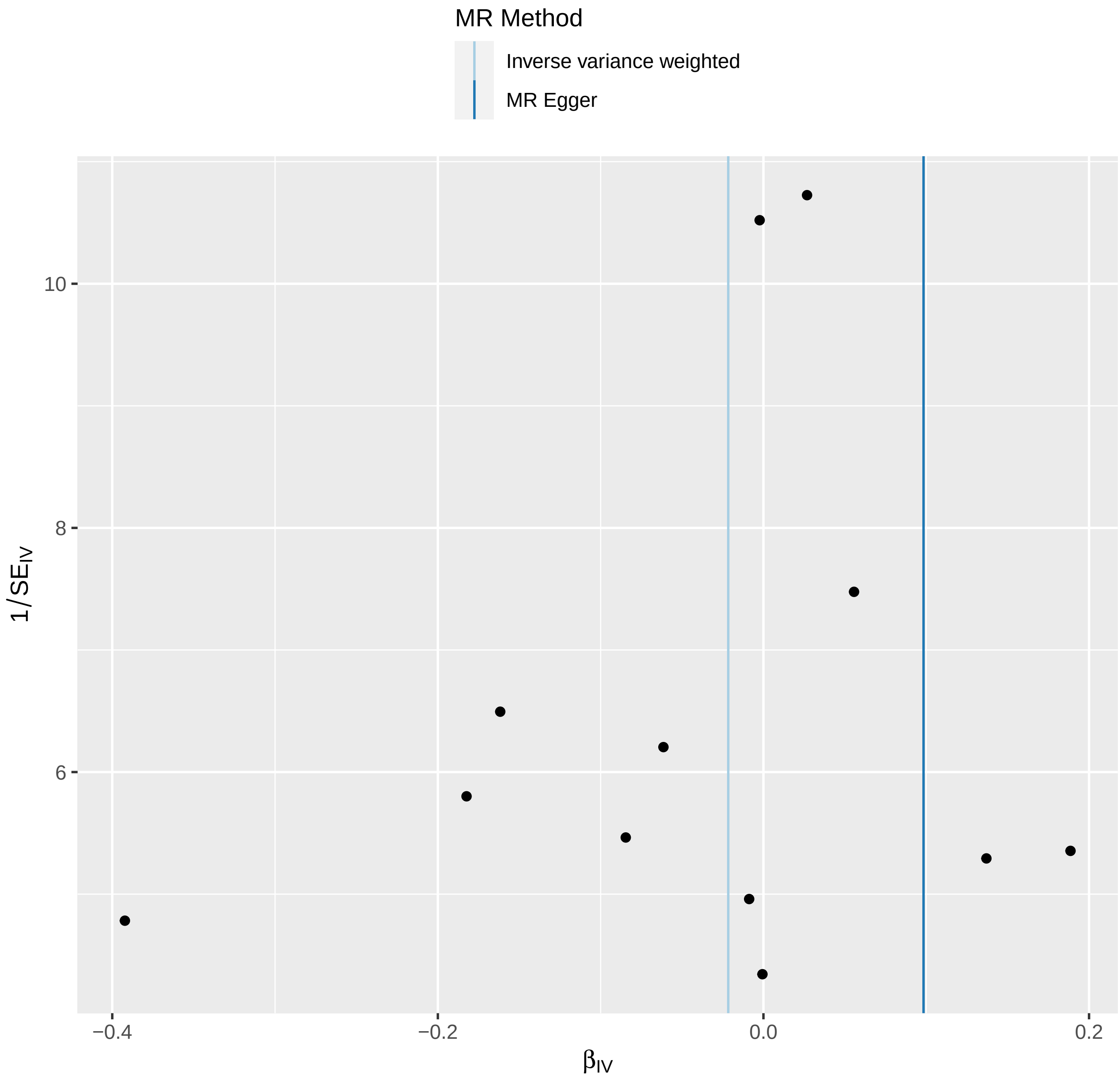
MR Method
Inverse variance weighted
MR Egger
10
8
6
1/SE_IV
−0.4
−0.2
0.0
0.2
β_IV

Batch 3−2875 : Selenium on Stromal interaction molecule 1

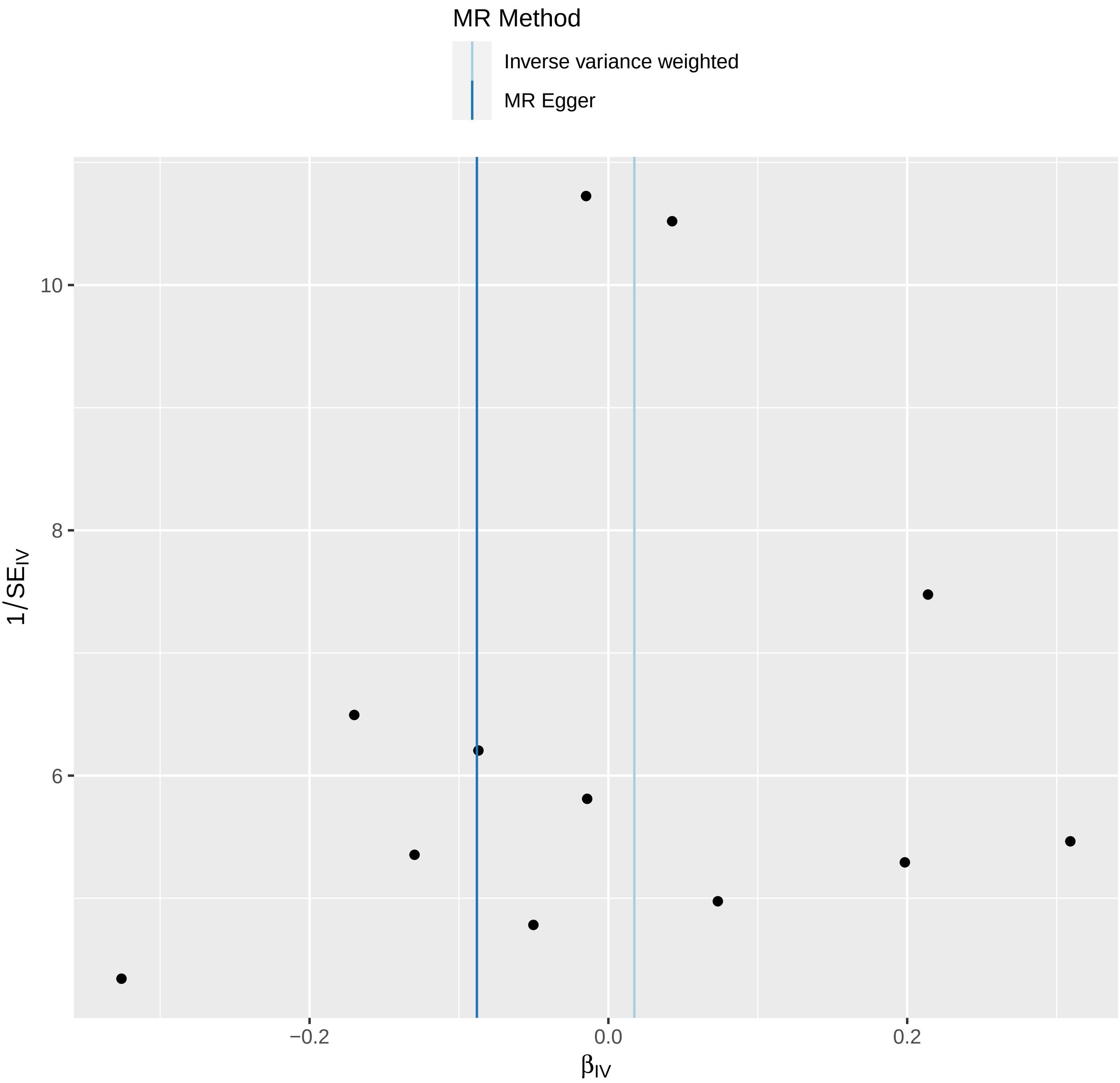

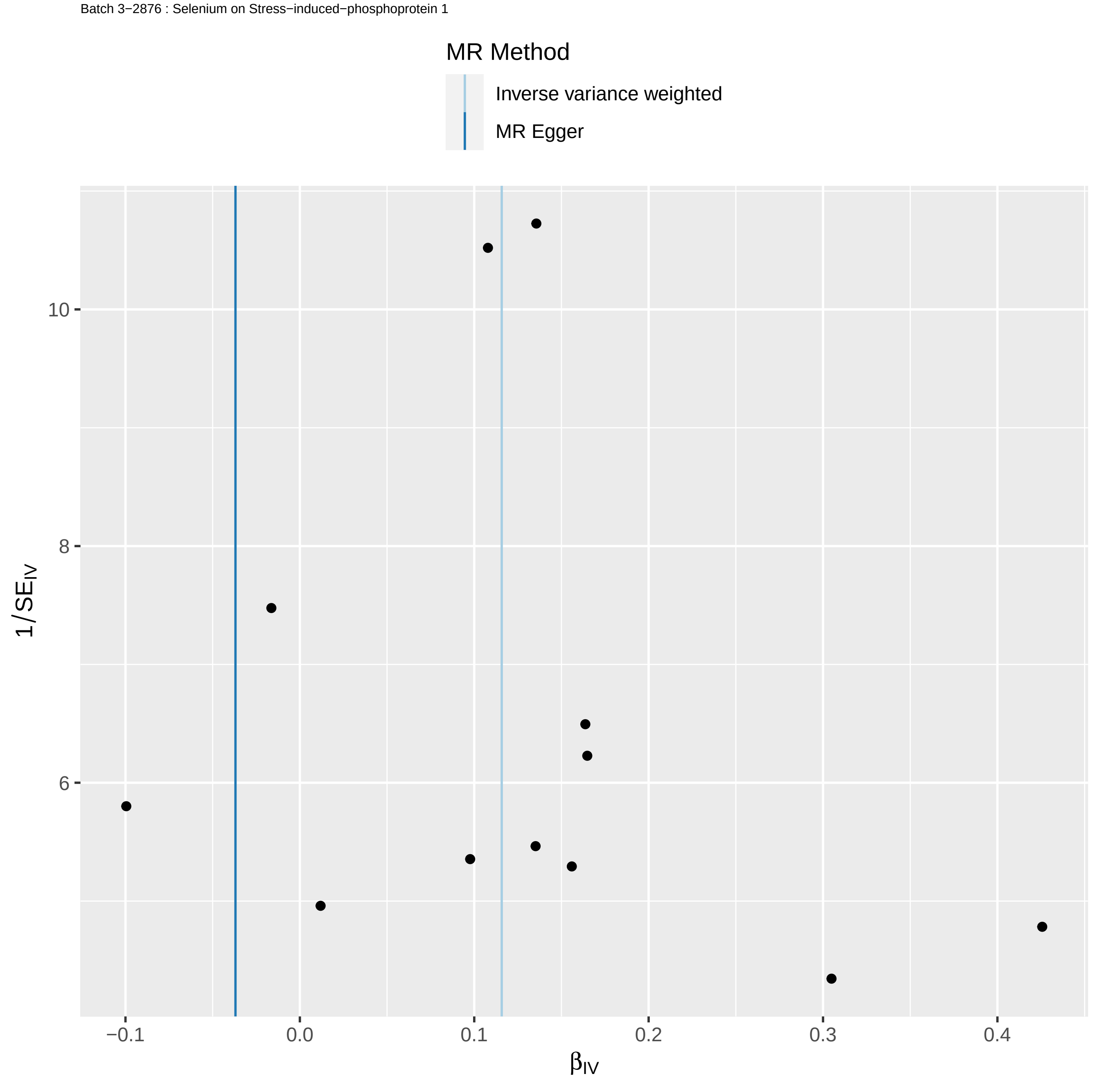

Batch 3−2876 : Selenium on Stress−induced−phosphoprotein 1
MR Method
Inverse variance weighted
MR Egger
10
8
6
1/SE_IV
−0.1
0.0
0.1
0.2
0.3
0.4
β_IV

Batch 3−2877 : Selenium on Serine/threonine−protein kinase 16

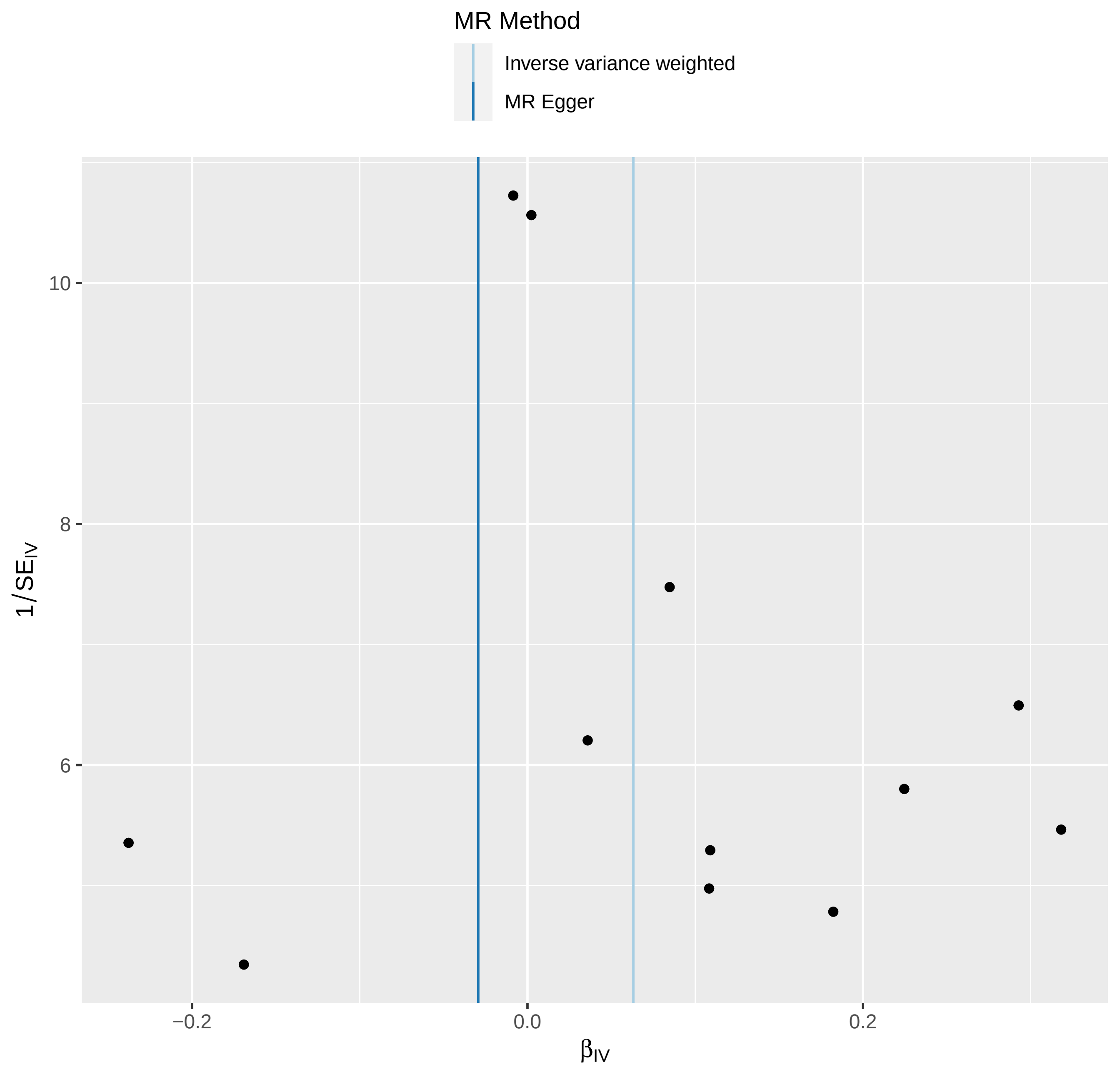

Batch 3−2878 : Selenium on Serine/threonine−protein kinase 17B

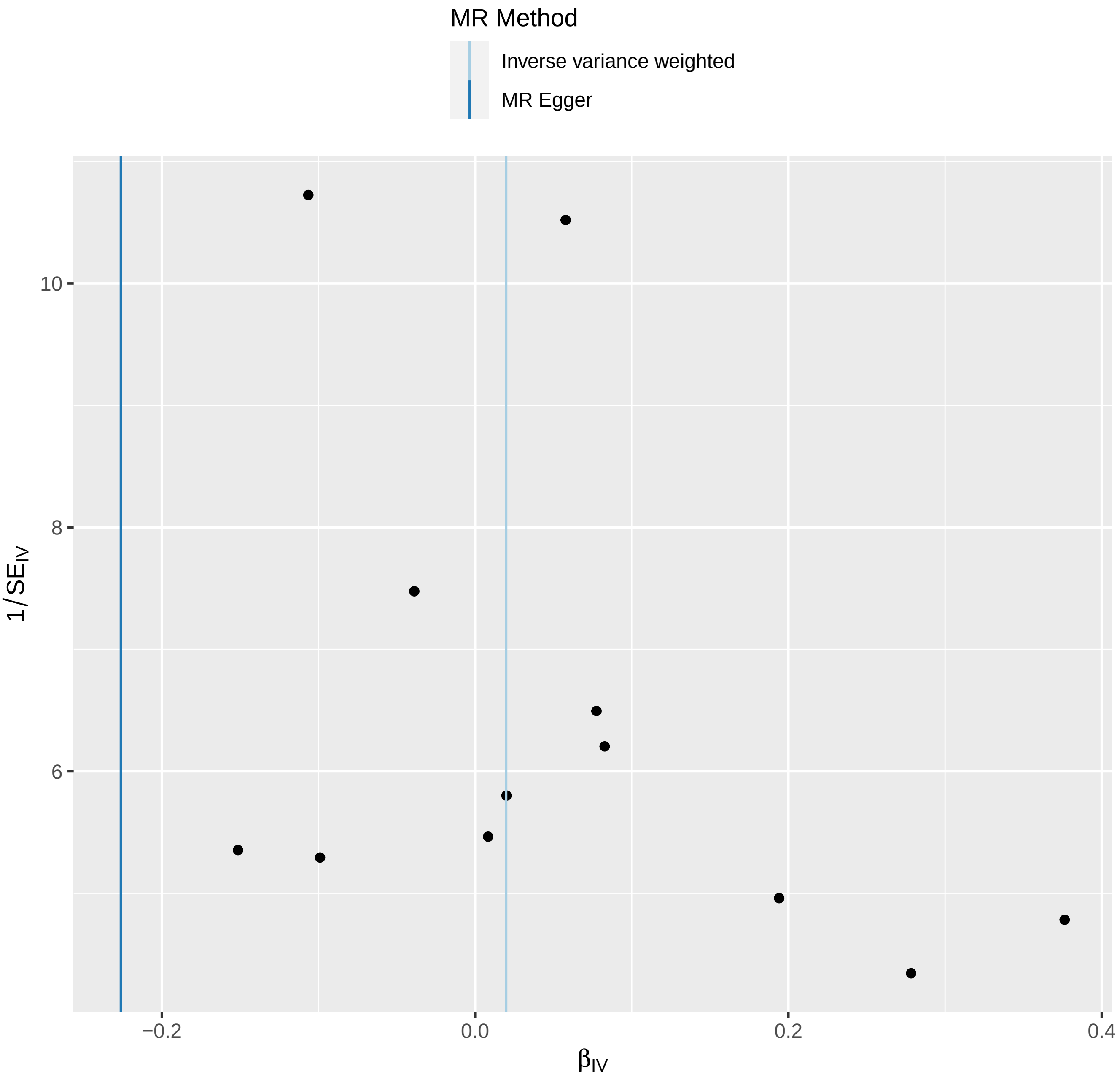

Batch 3−2879 : Selenium on Stathmin−2

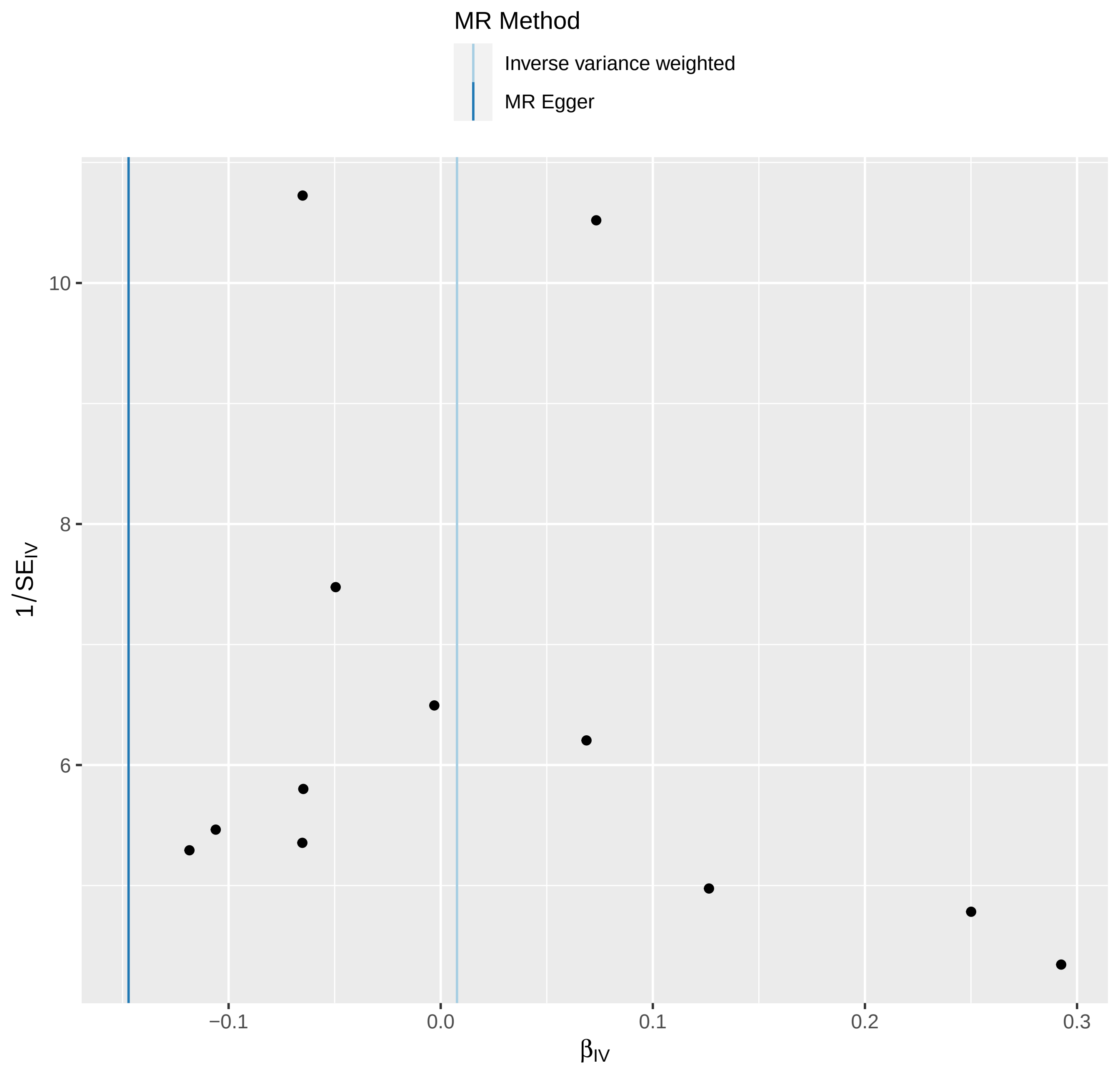

Batch 3−2880 : Selenium on Stathmin−3

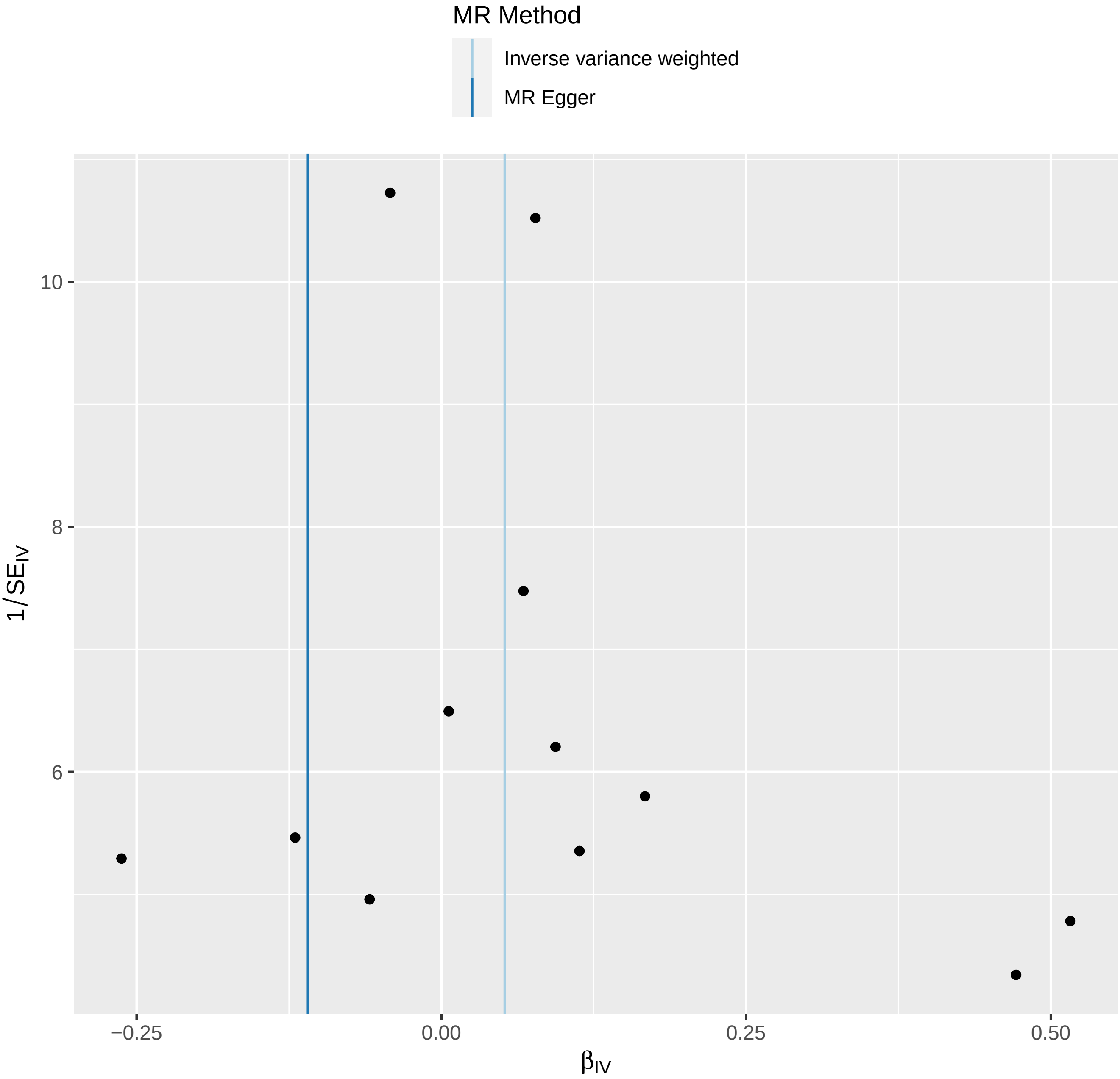

Batch 3−2881 : Selenium on Stathmin−4

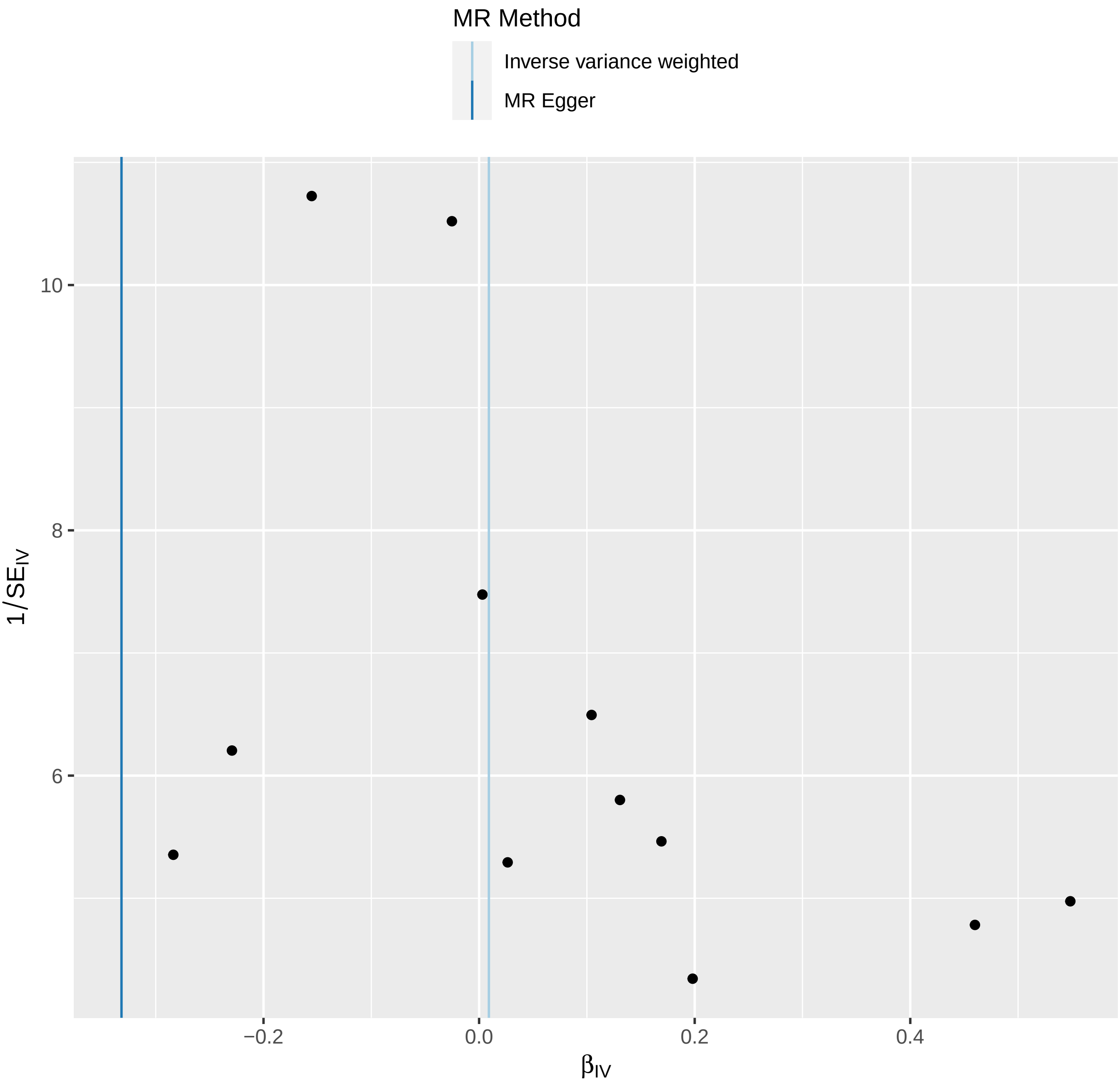

Batch 3−2882 : Selenium on Erythrocyte band 7 integral membrane protein

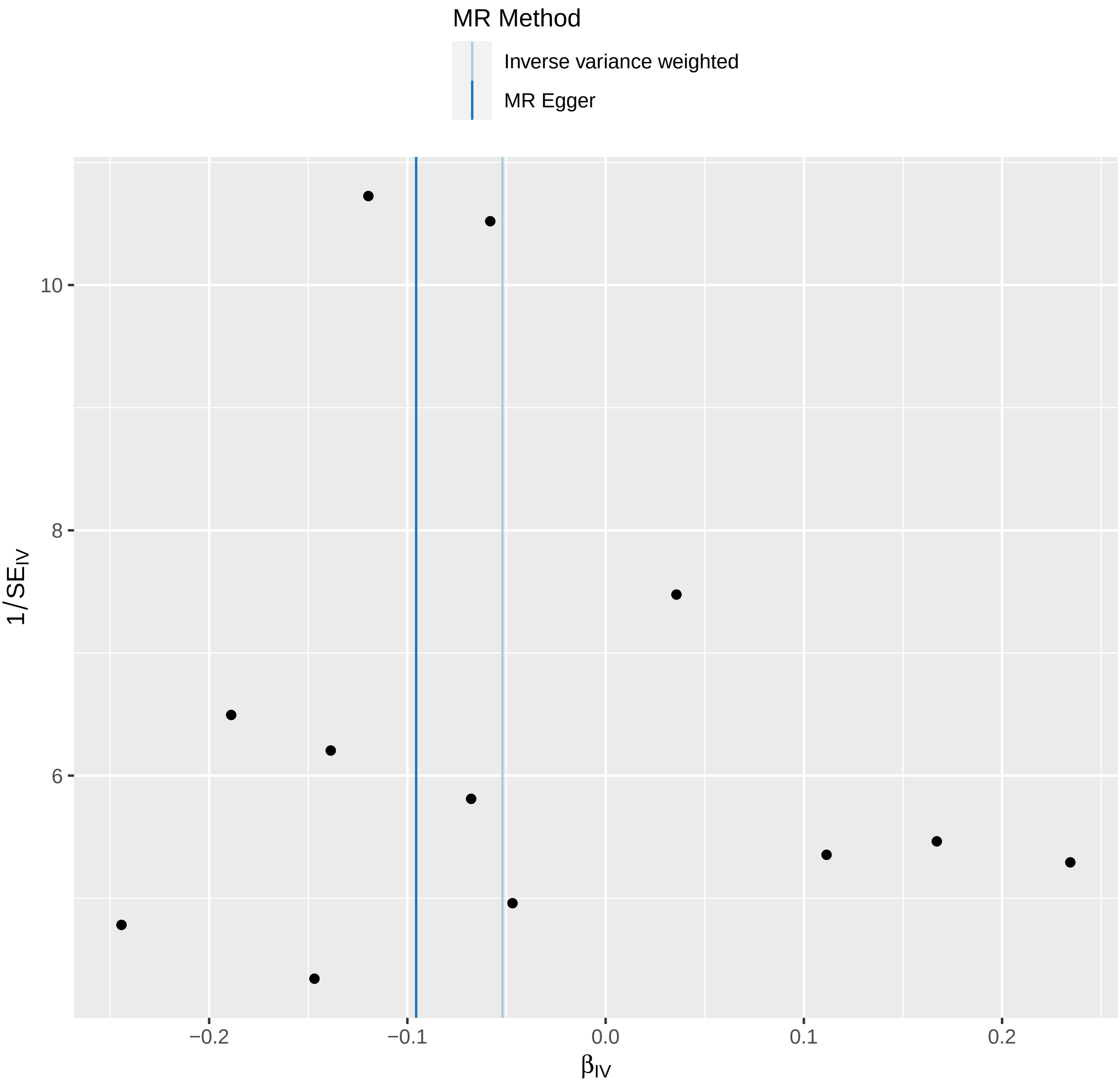

Batch 3−2883 : Selenium on Syntaxin−10

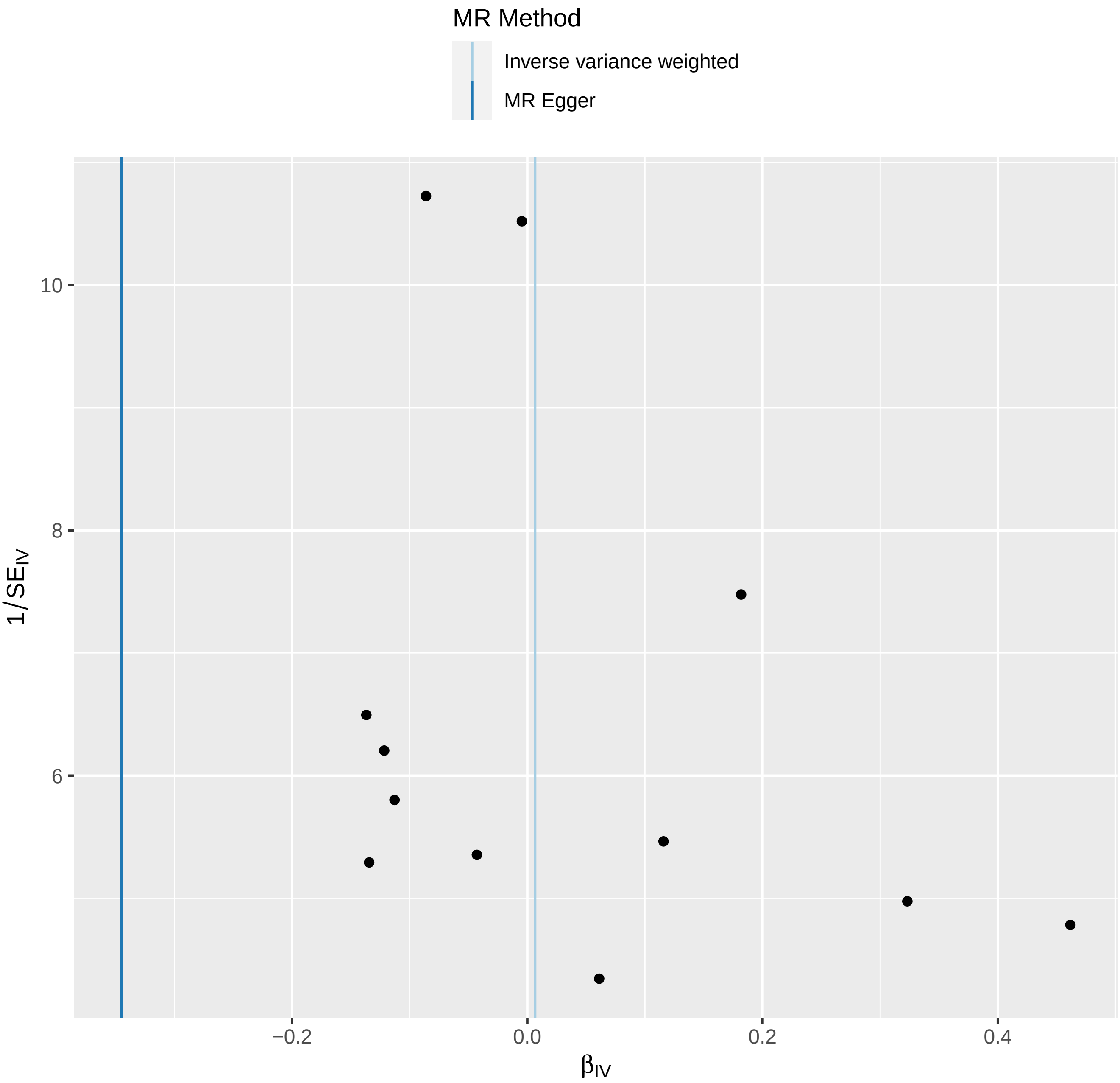

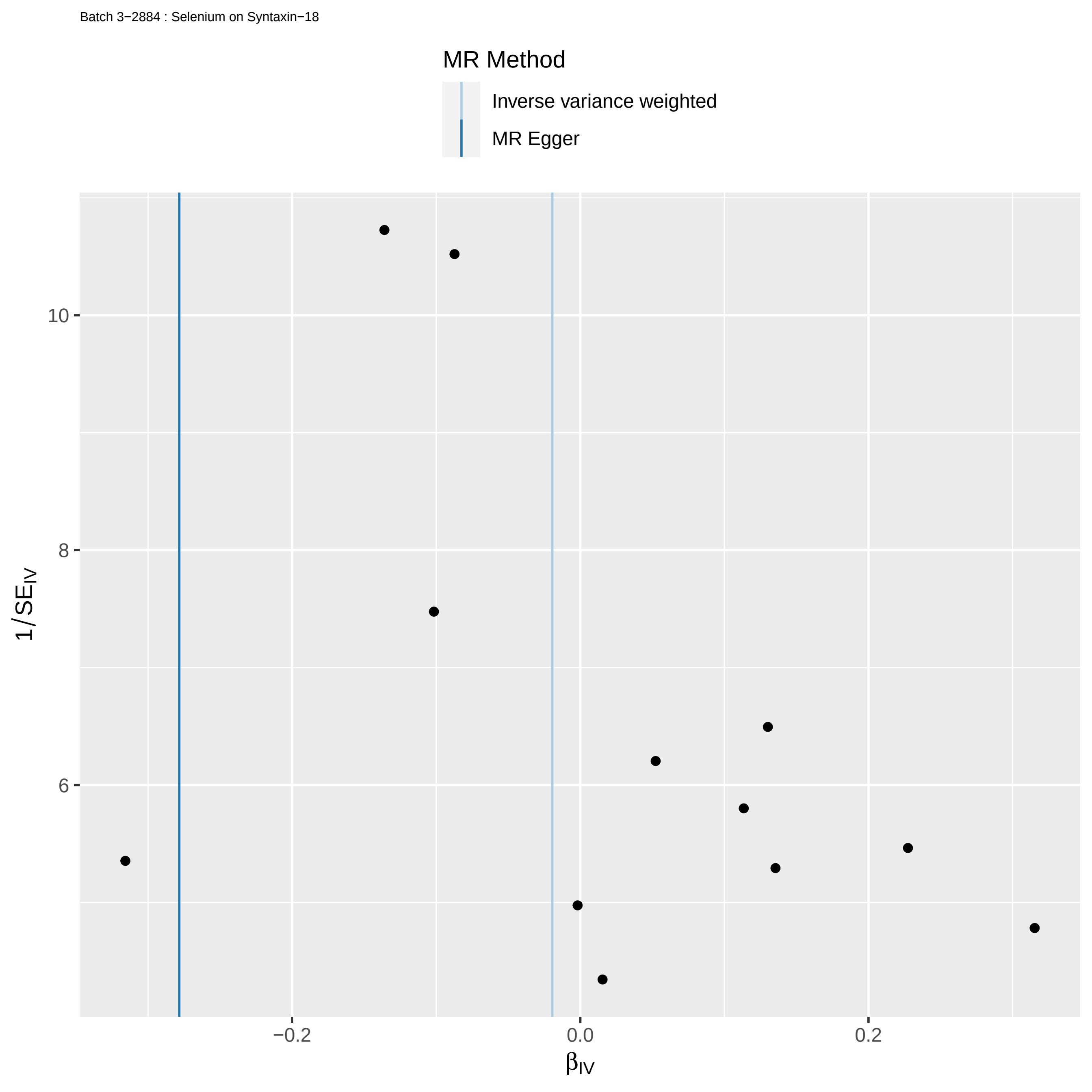

Batch 3−2884 : Selenium on Syntaxin−18
MR Method
Inverse variance weighted
MR Egger
10
8
6
1/SE_IV
−0.2
0.0
0.2
β_IV

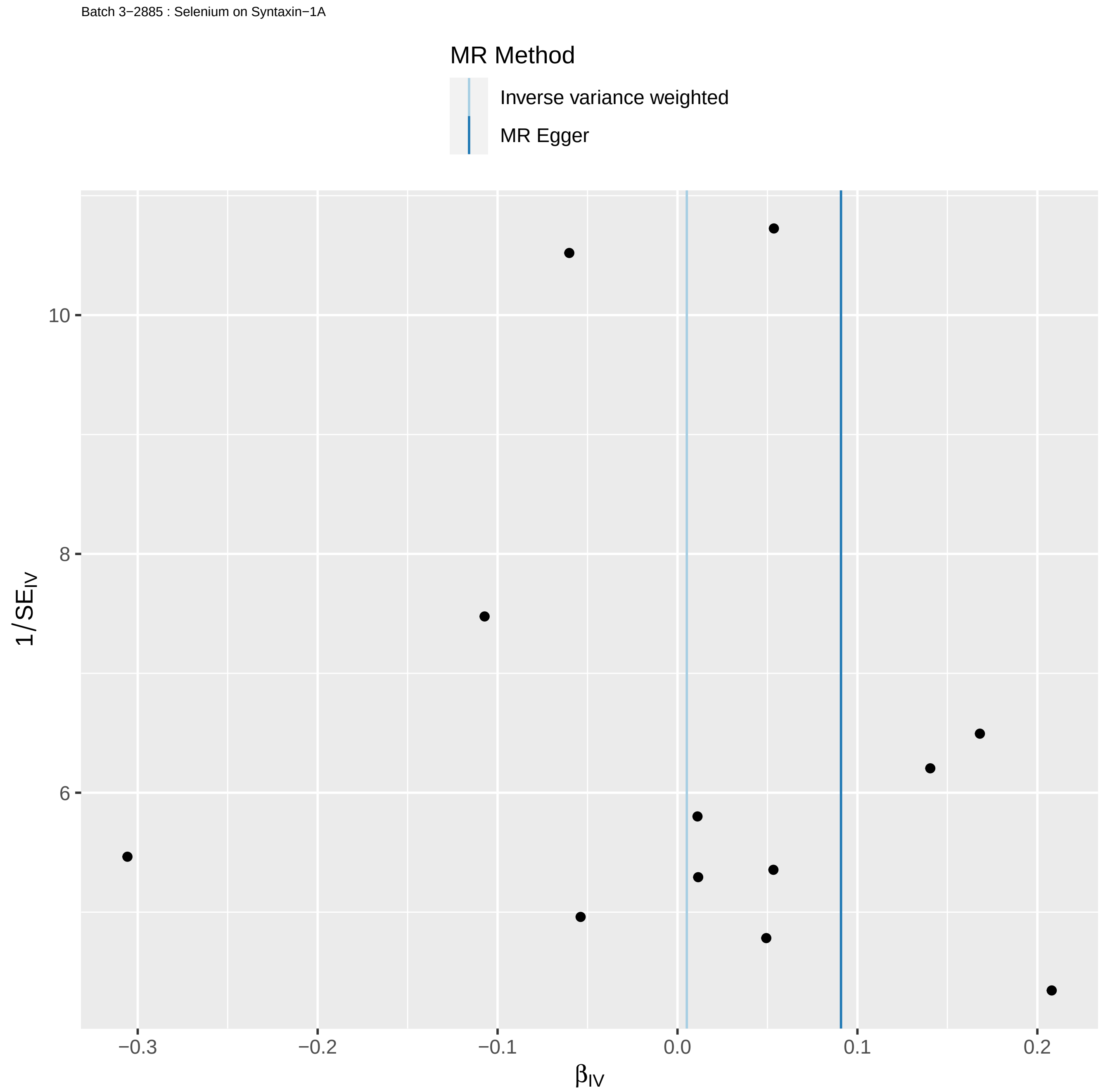
Batch 3−2885 : Selenium on Syntaxin−1A
MR Method
Inverse variance weighted
MR Egger
10
8
6
1/SE_IV
−0.3
−0.2
−0.1
0.0
0.1
0.2
β_IV

Batch 3−2886 : Selenium on Syntaxin−1B

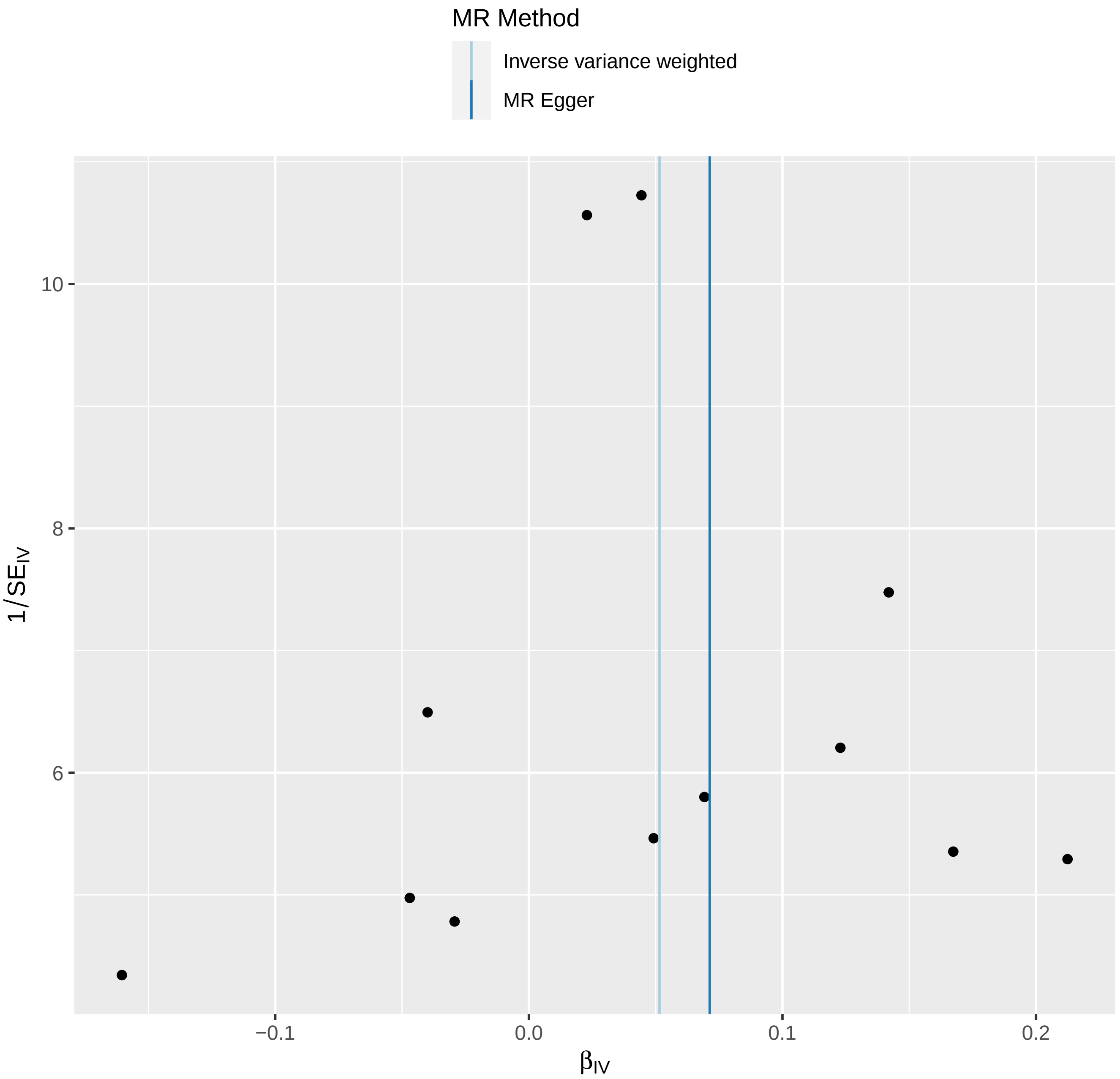

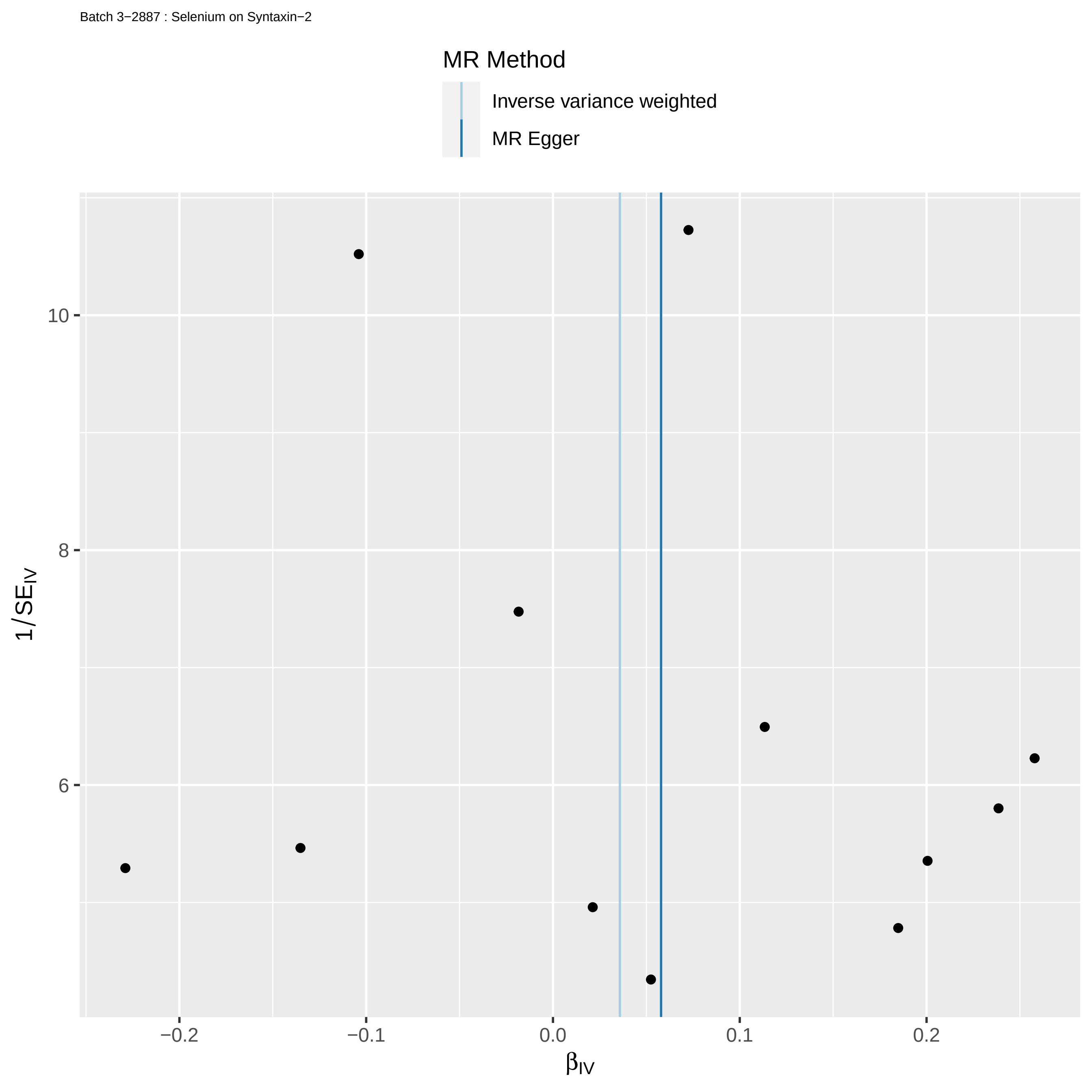

Batch 3−2887 : Selenium on Syntaxin−2
MR Method
Inverse variance weighted
MR Egger
10
8
6
1/SE_IV
−0.2
−0.1
0.0
0.1
0.2
β_IV

Batch 3−2888 : Selenium on Syntaxin−6

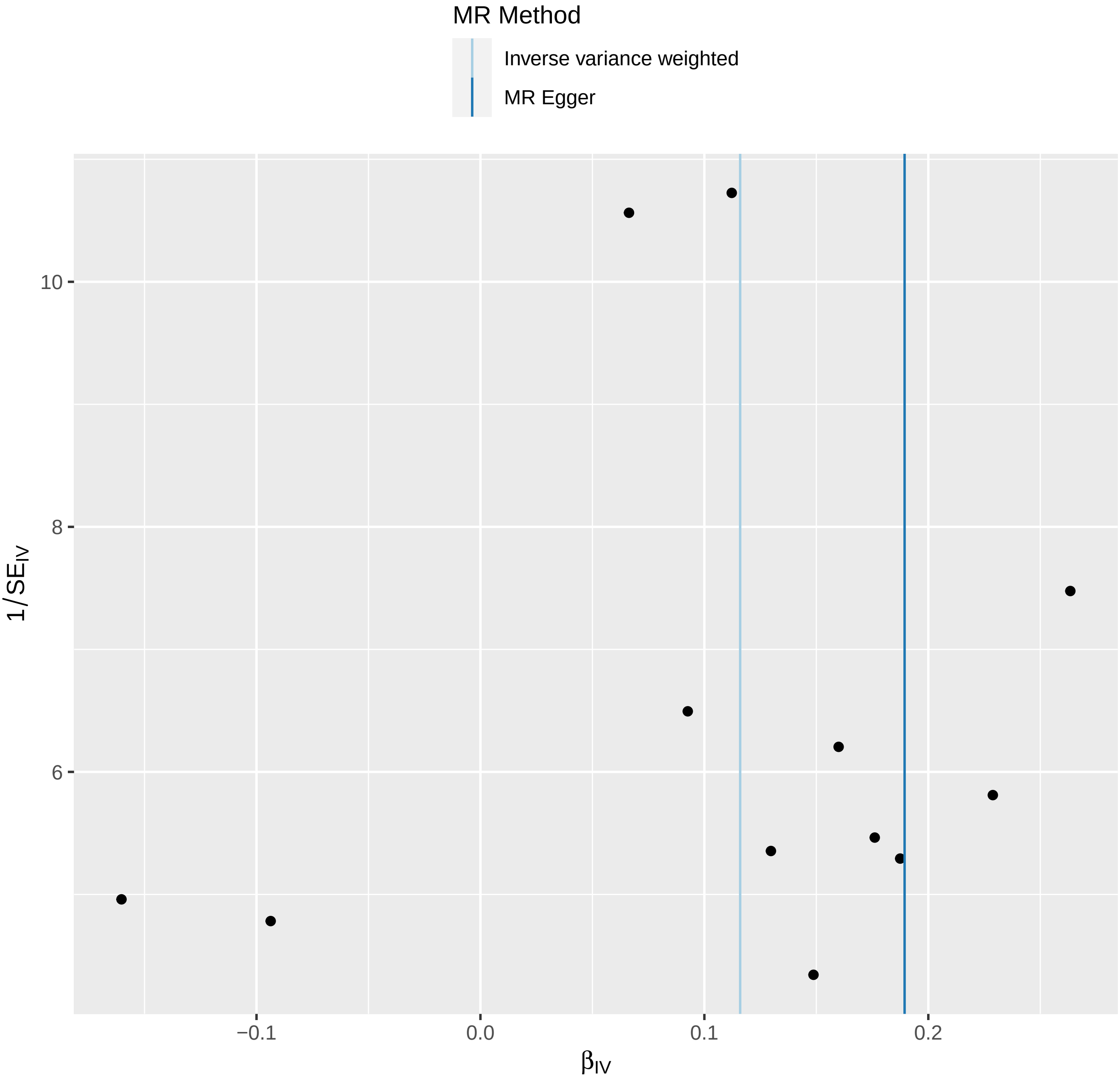

Batch 3−2889 : Selenium on Syntaxin−7

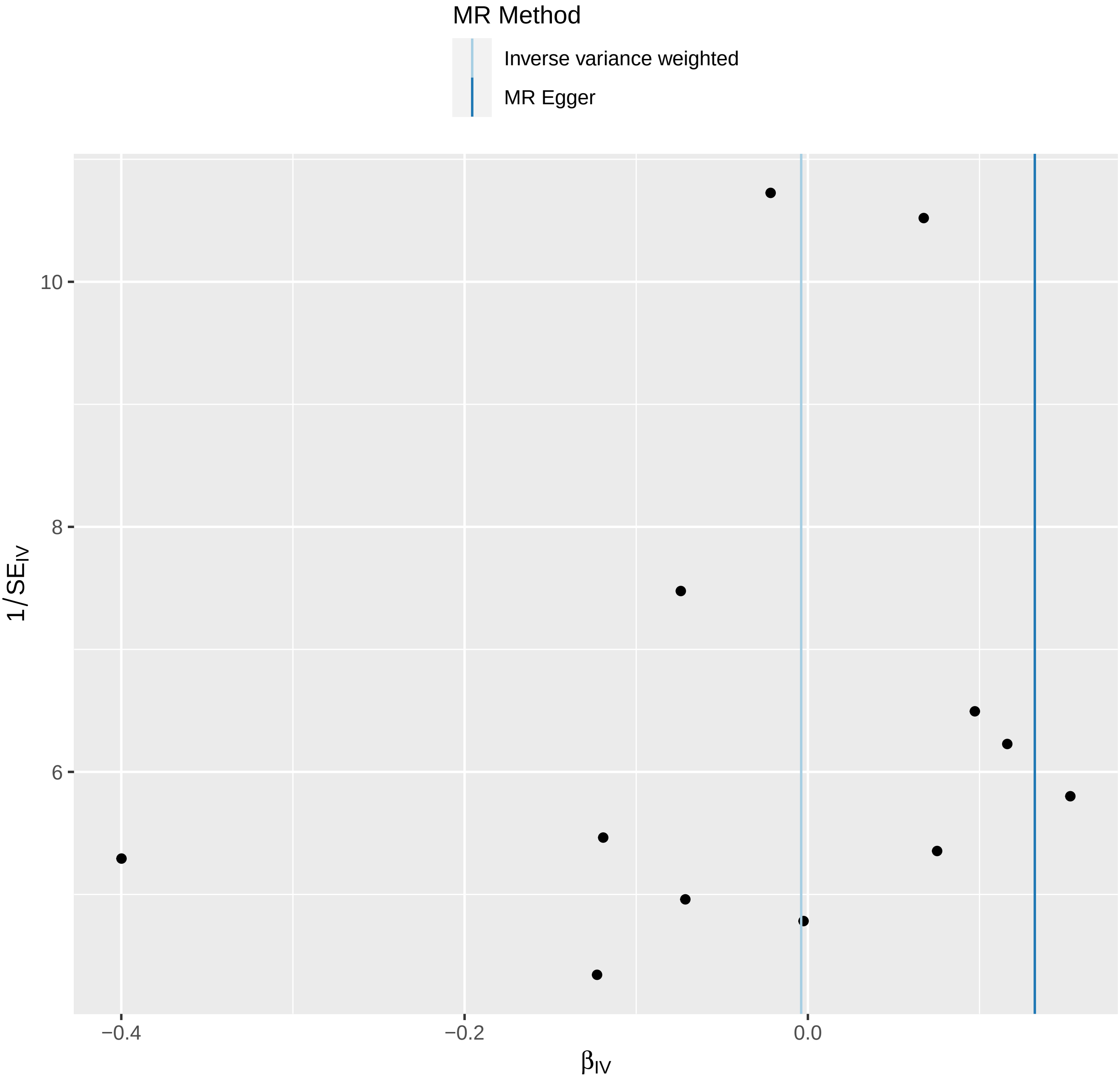

Batch 3−2890 : Selenium on Syntaxin−8

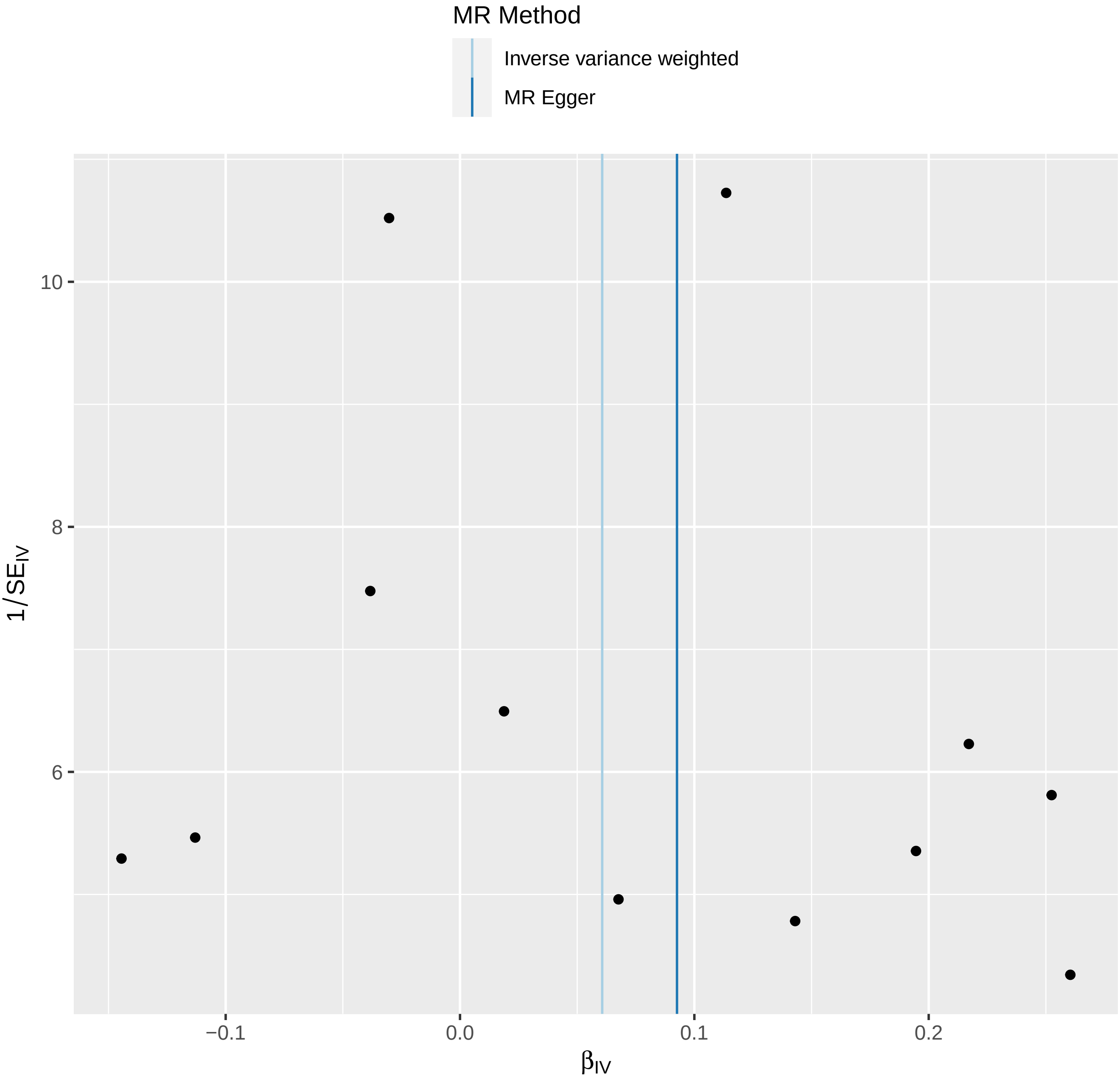

Batch 3−2891 : Selenium on Extracellular sulfatase Sulf−2

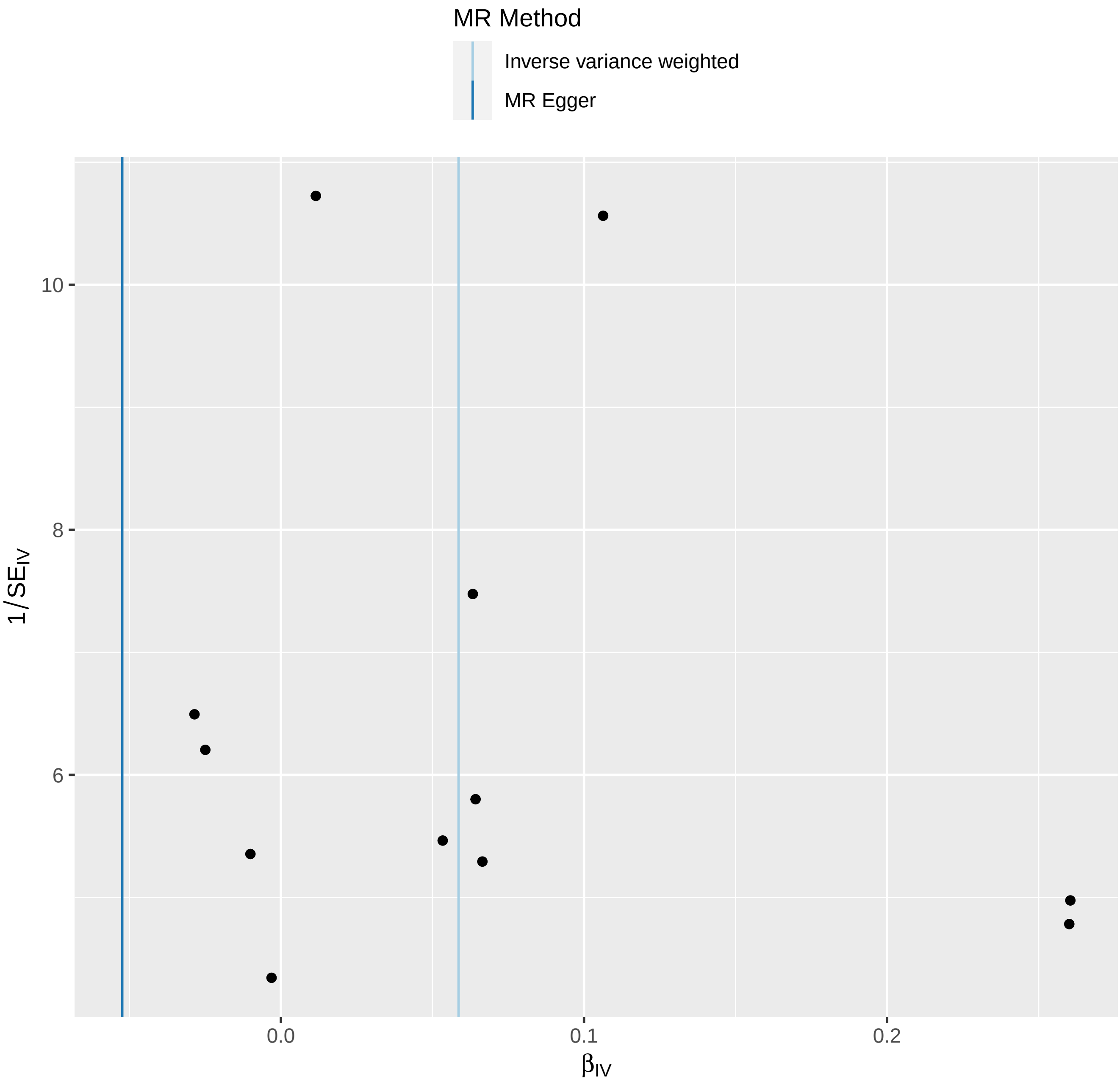

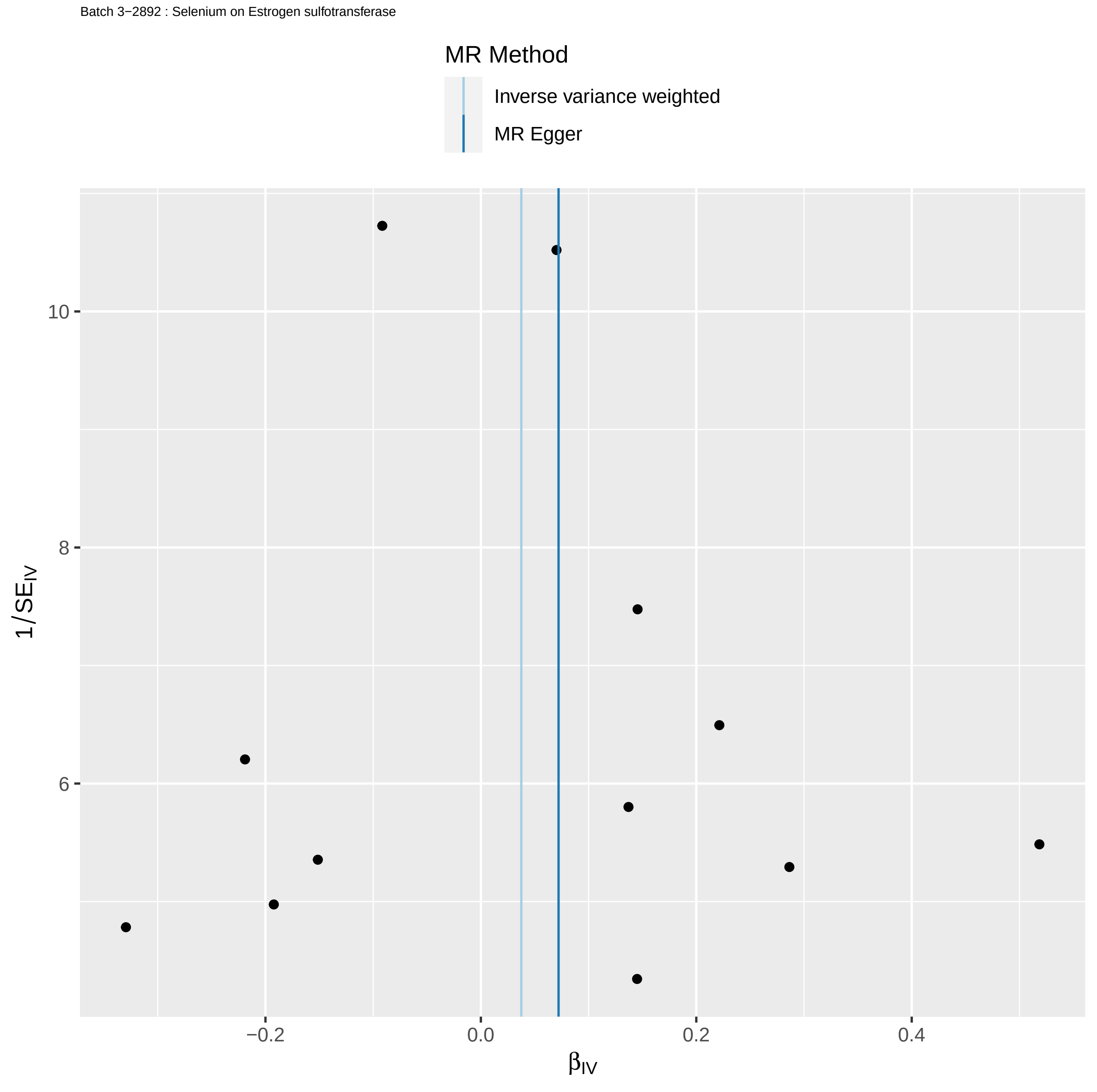

Batch 3−2892 : Selenium on Estrogen sulfotransferase
MR Method
Inverse variance weighted
MR Egger
10
8
6
1/SE_IV
−0.2
0.0
0.2
0.4
β_IV

Batch 3−2893 : Selenium on Bile salt sulfotransferase

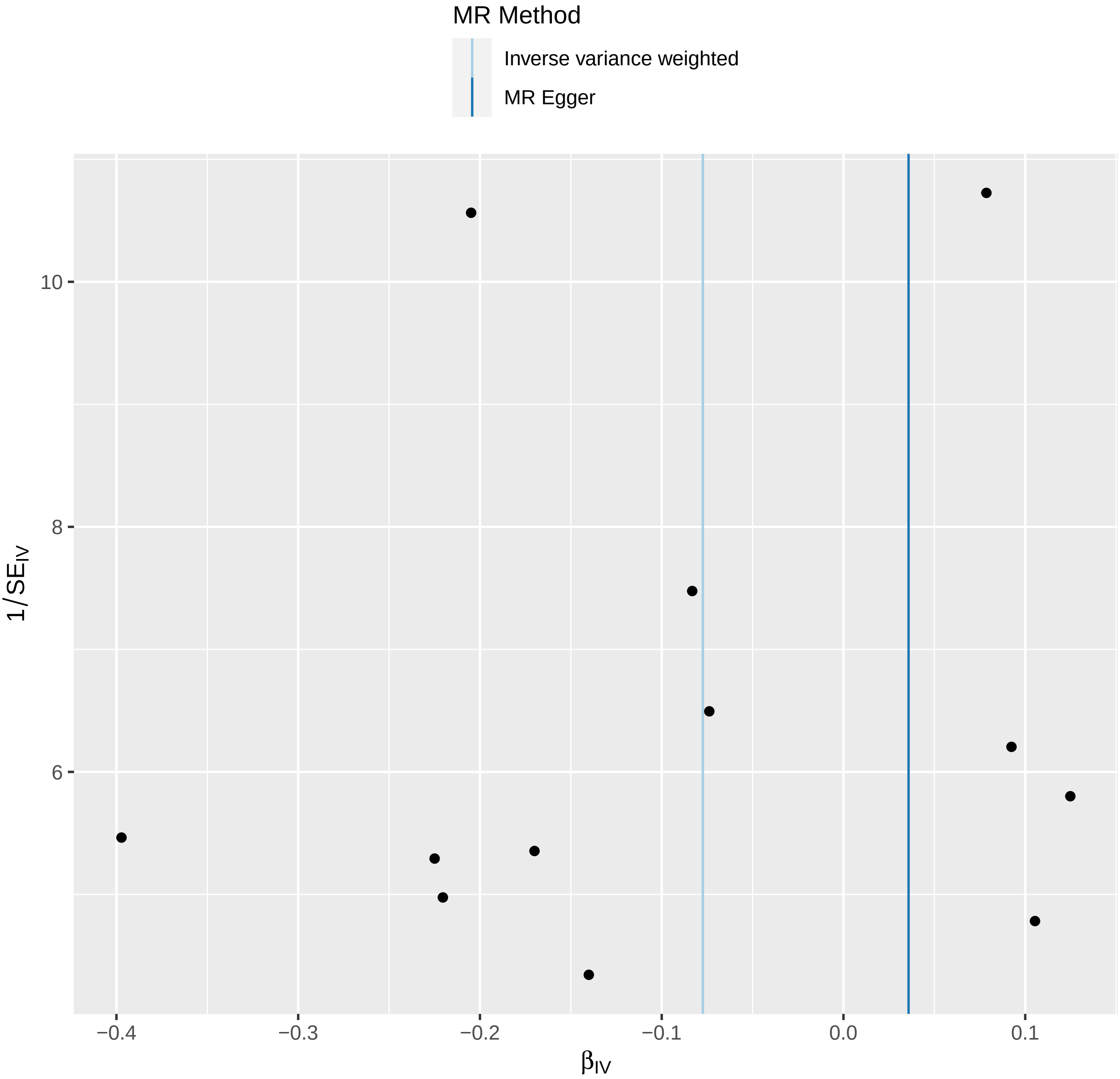

Batch 3−2894 : Selenium on Sulfotransferase 4A1

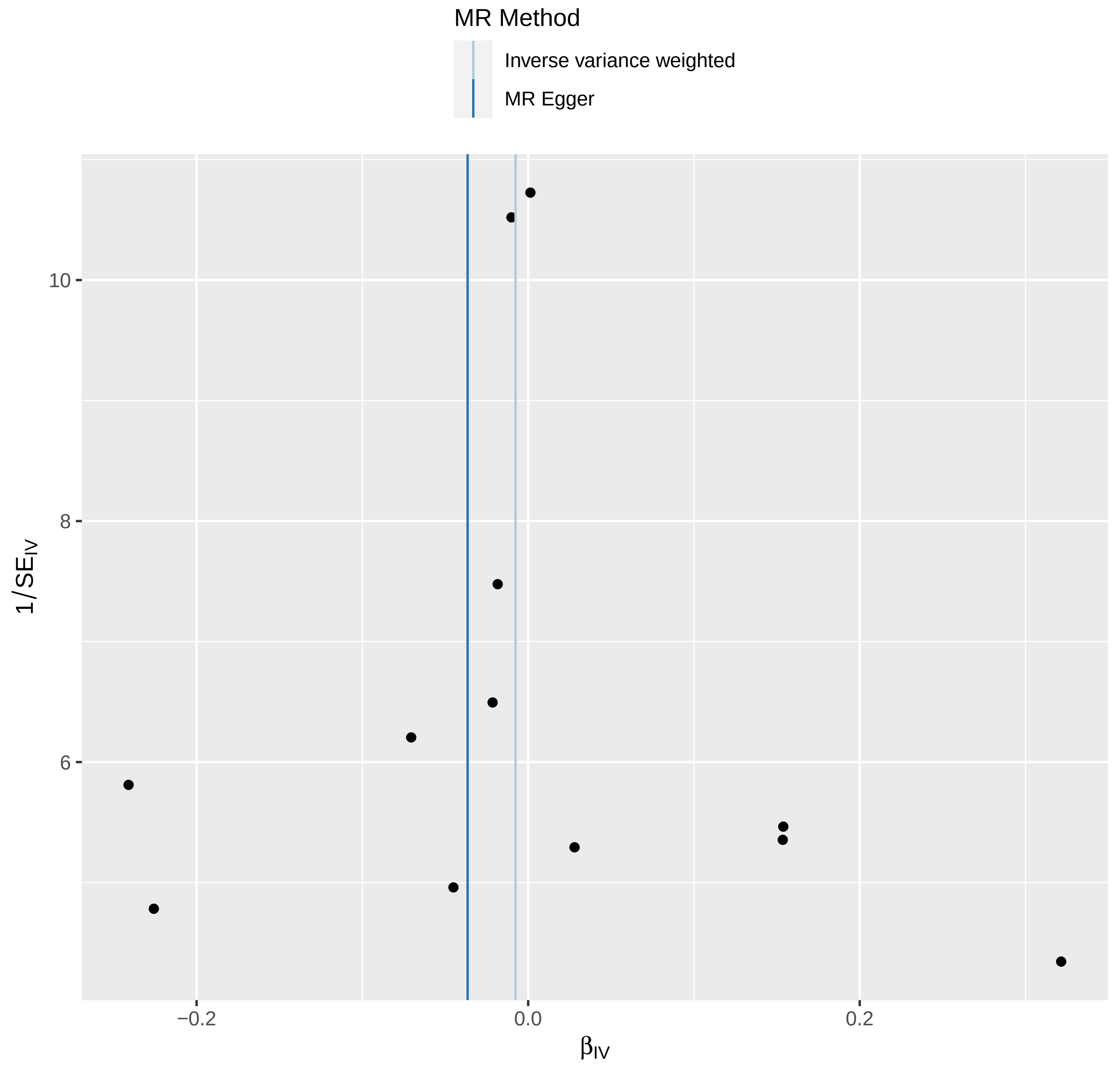

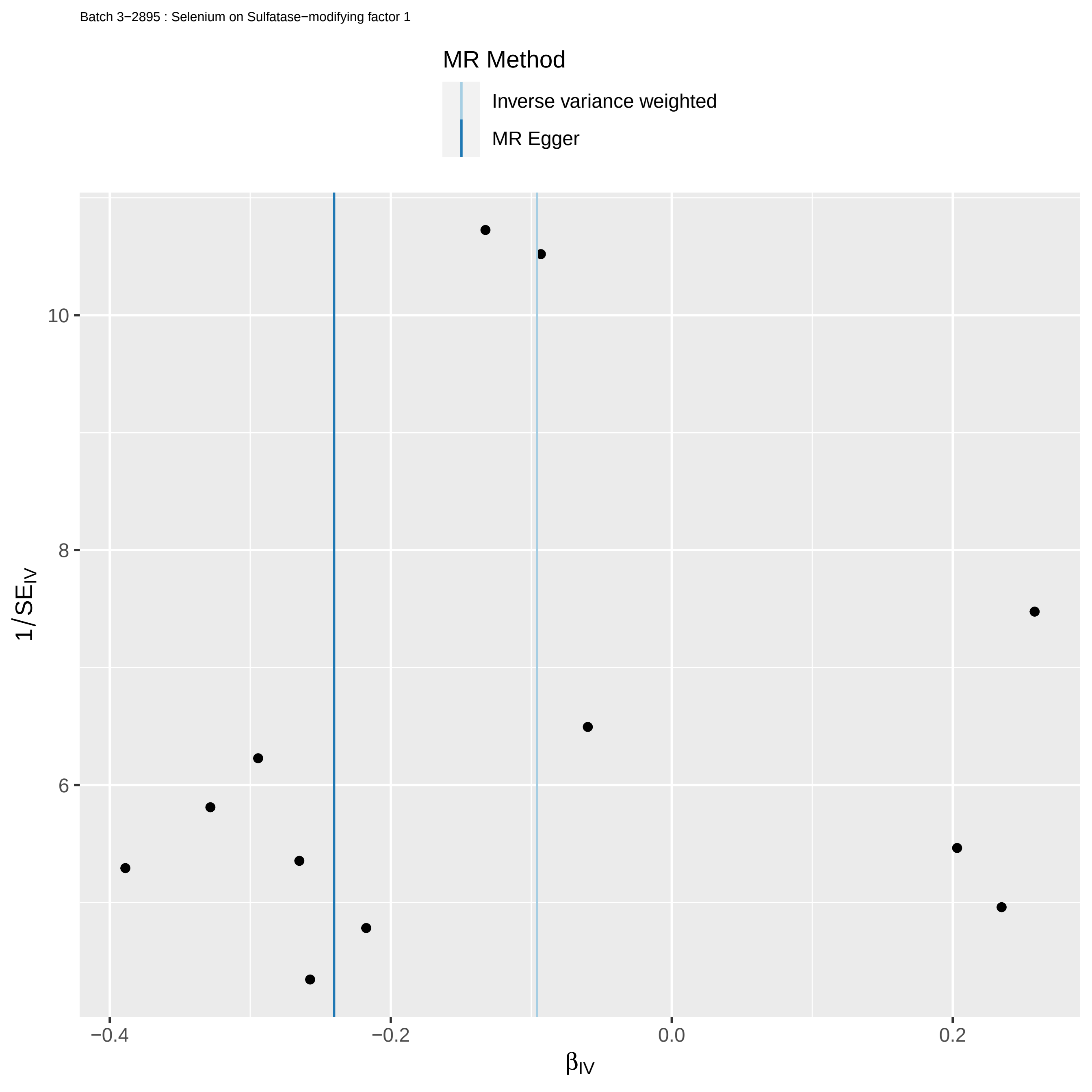

Batch 3−2895 : Selenium on Sulfatase−modifying factor 1
MR Method
Inverse variance weighted
MR Egger
10
8
6
1/SE_IV
−0.4
−0.2
0.0
0.2
β_IV

Batch 3−2896 : Selenium on Small ubiquitin−related modifier 3

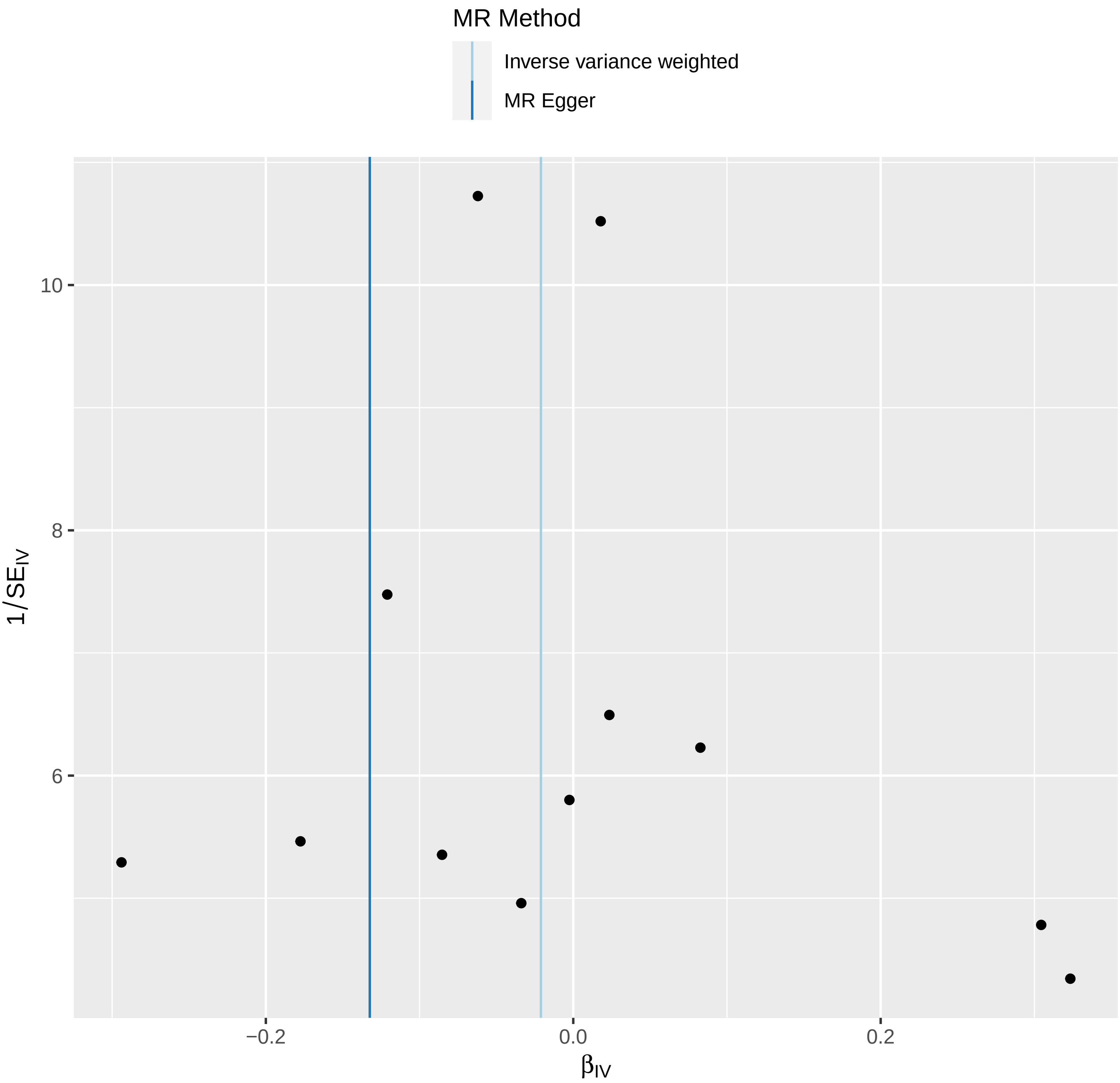

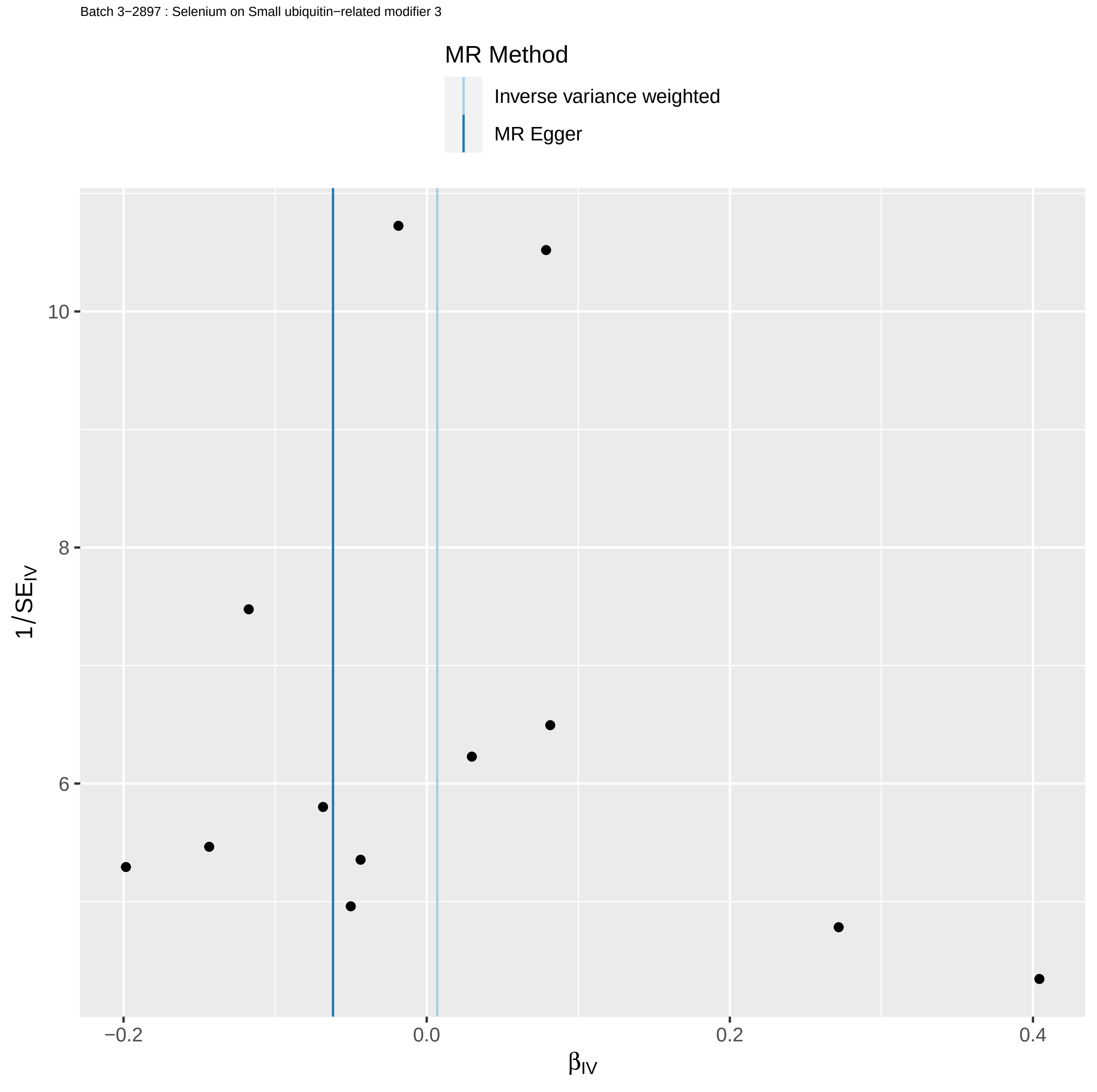

Batch 3−2897 : Selenium on Small ubiquitin−related modifier 3
MR Method
Inverse variance weighted
MR Egger
10
8
6
1/SE_IV
−0.2
0.0
0.2
0.4
β_IV

Batch 3−2898 : Selenium on SUN domain−containing protein 3

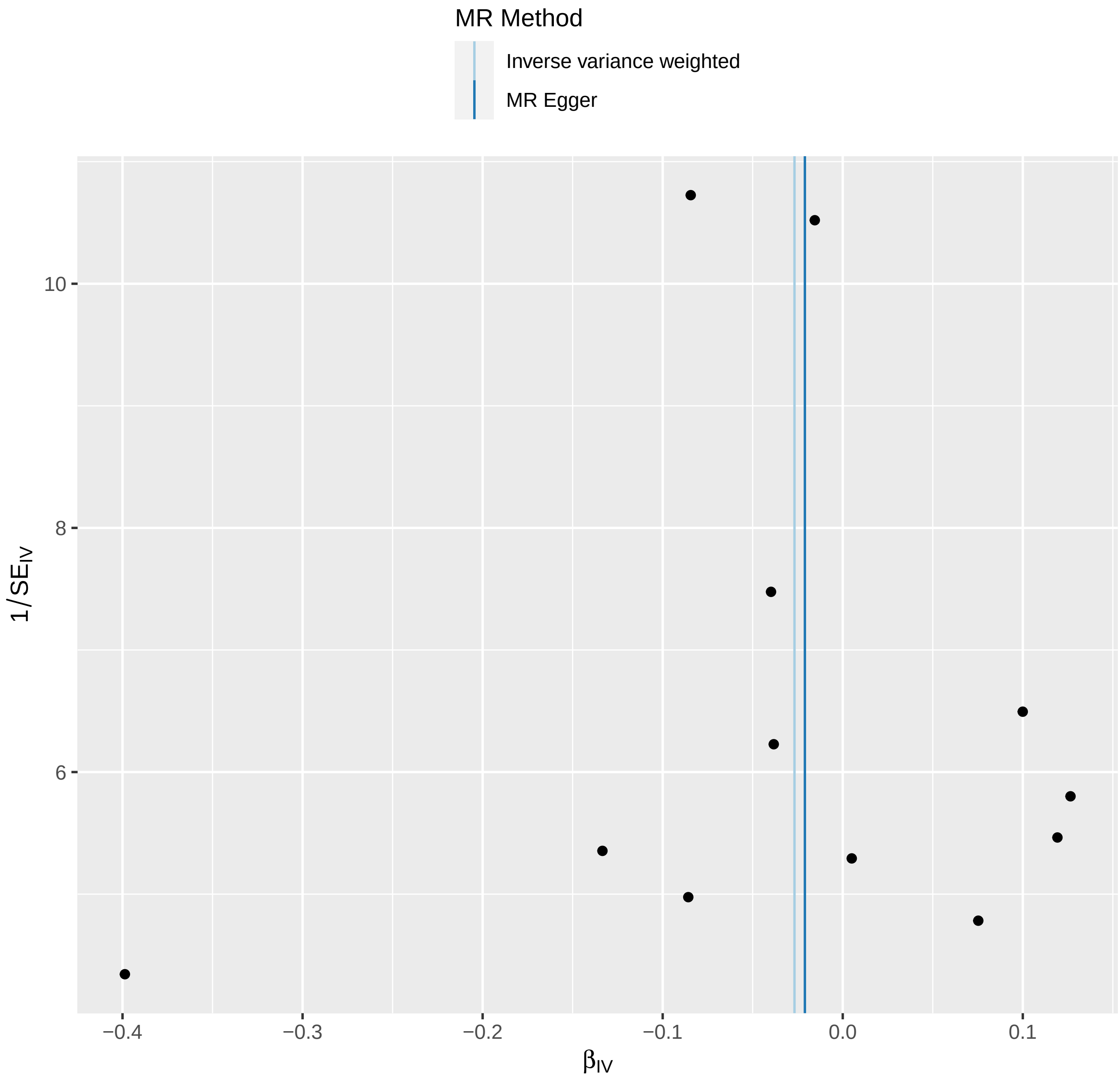

Batch 3−2899 : Selenium on SUN domain−containing protein 5

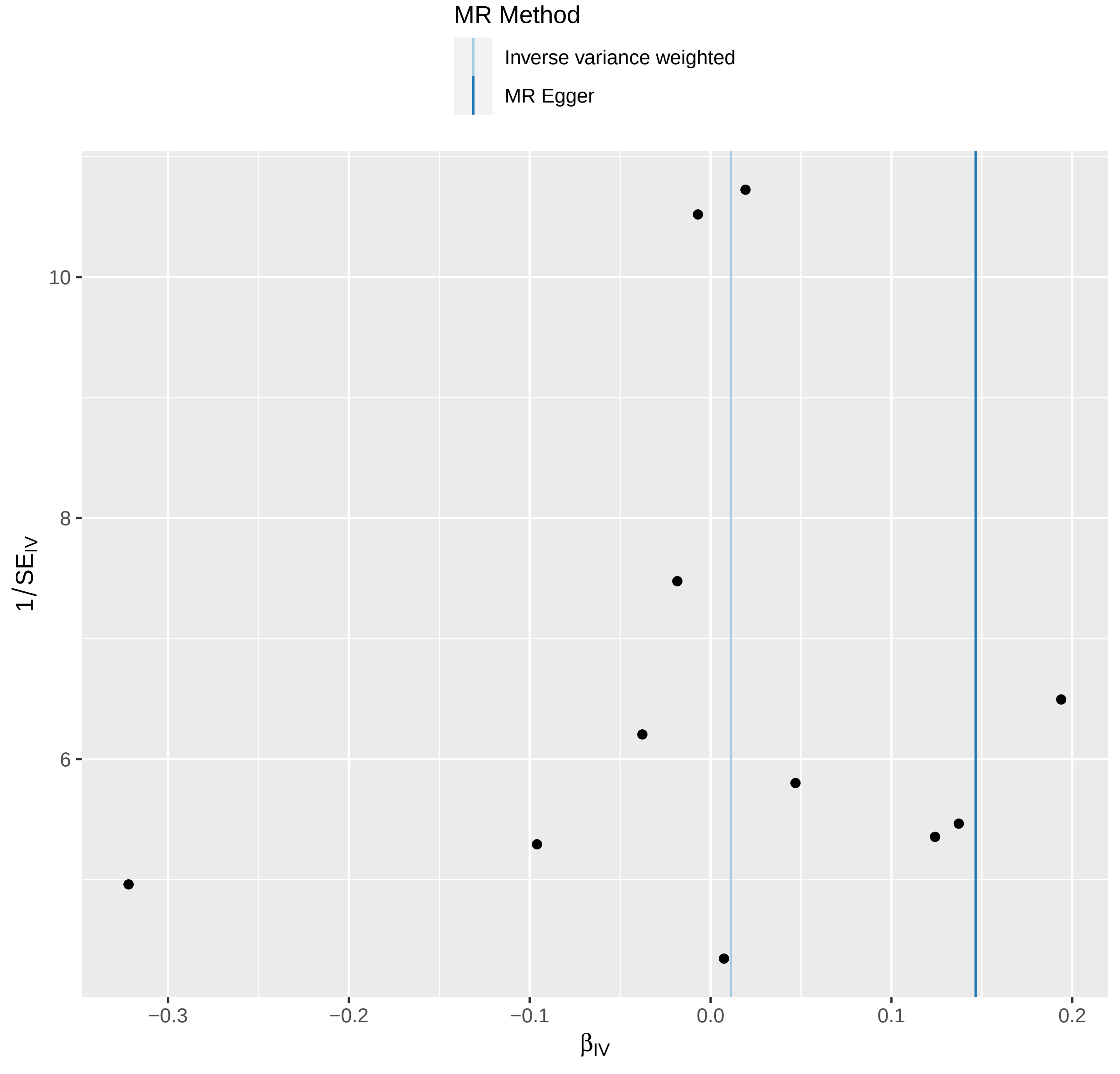

Batch 3−2900 : Selenium on Surfeit locus protein 1

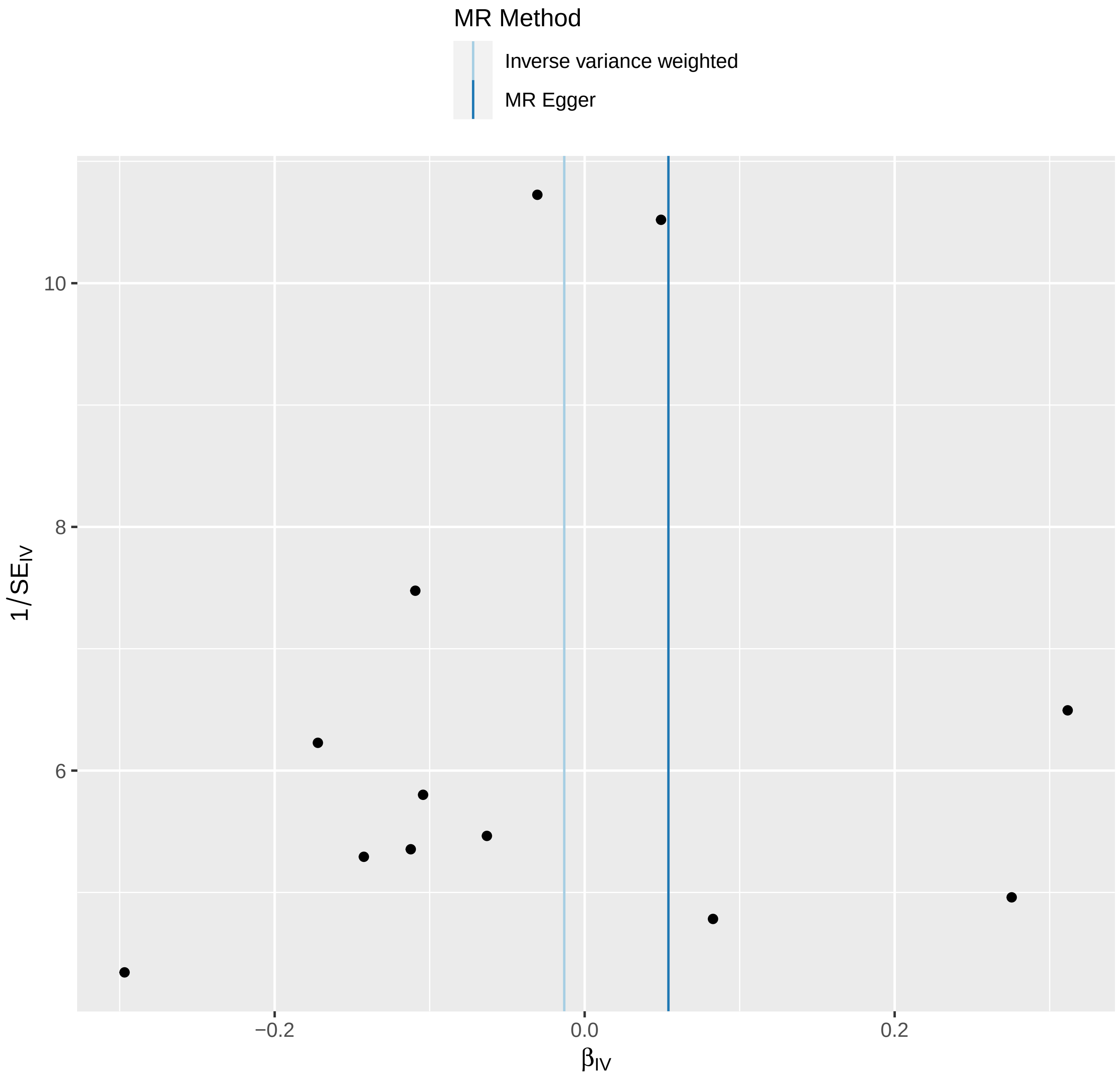

Batch 3−2901 : Selenium on Sushi domain−containing protein 1

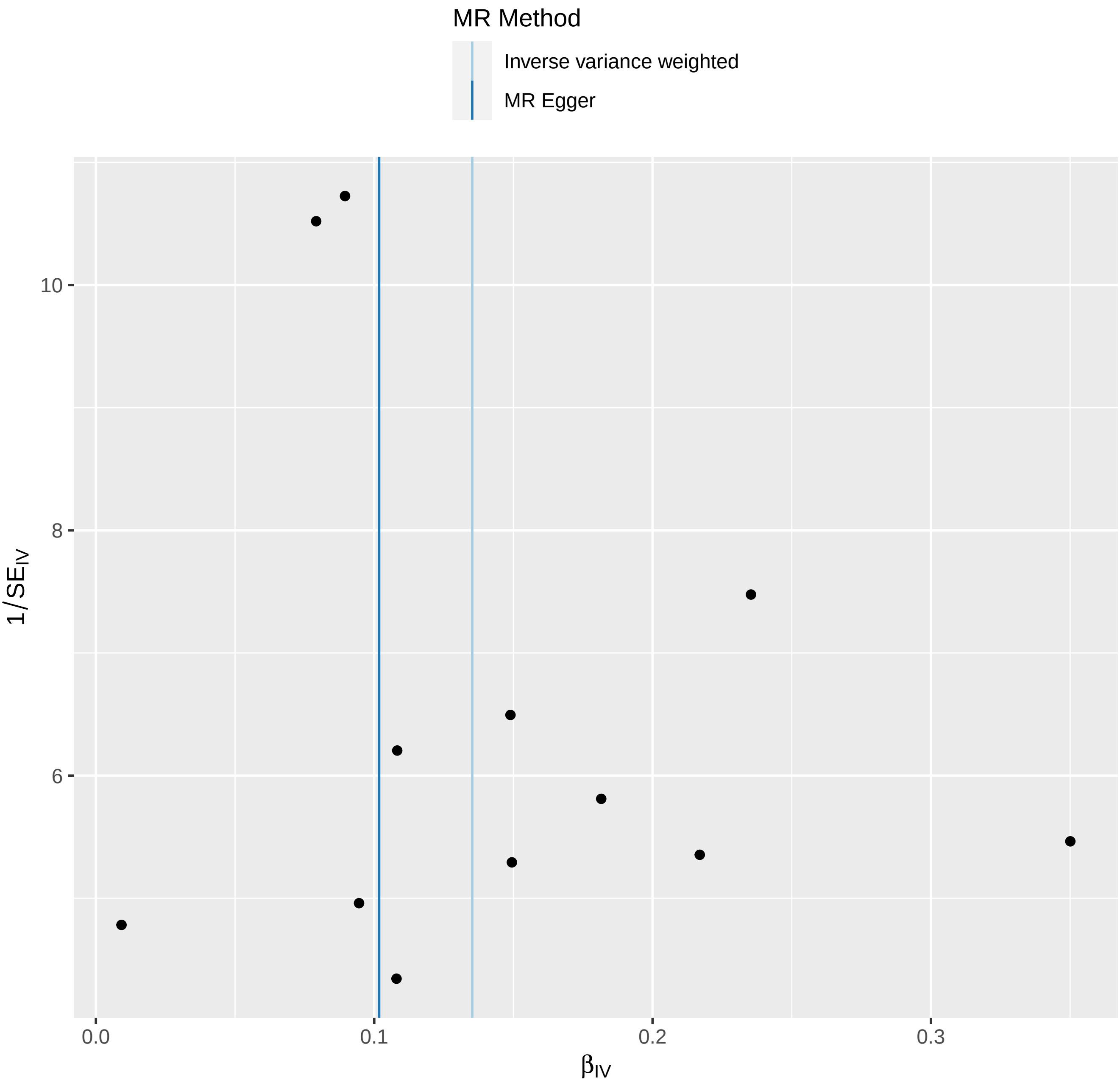

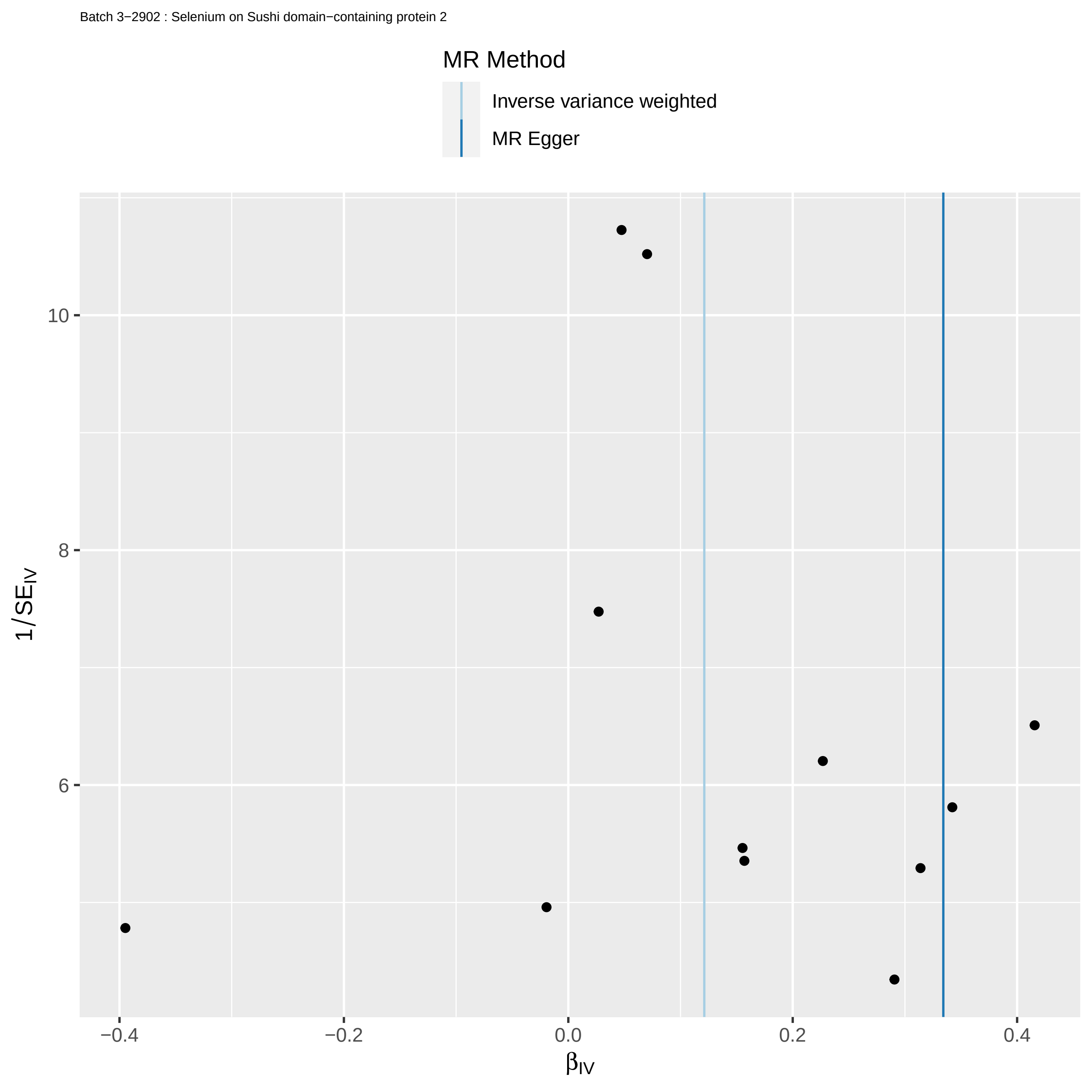
Batch 3−2902 : Selenium on Sushi domain−containing protein 2
MR Method
Inverse variance weighted
MR Egger
10
8
6
−0.4
−0.2
0.0
0.2
0.4
1/SE_IV
β_IV

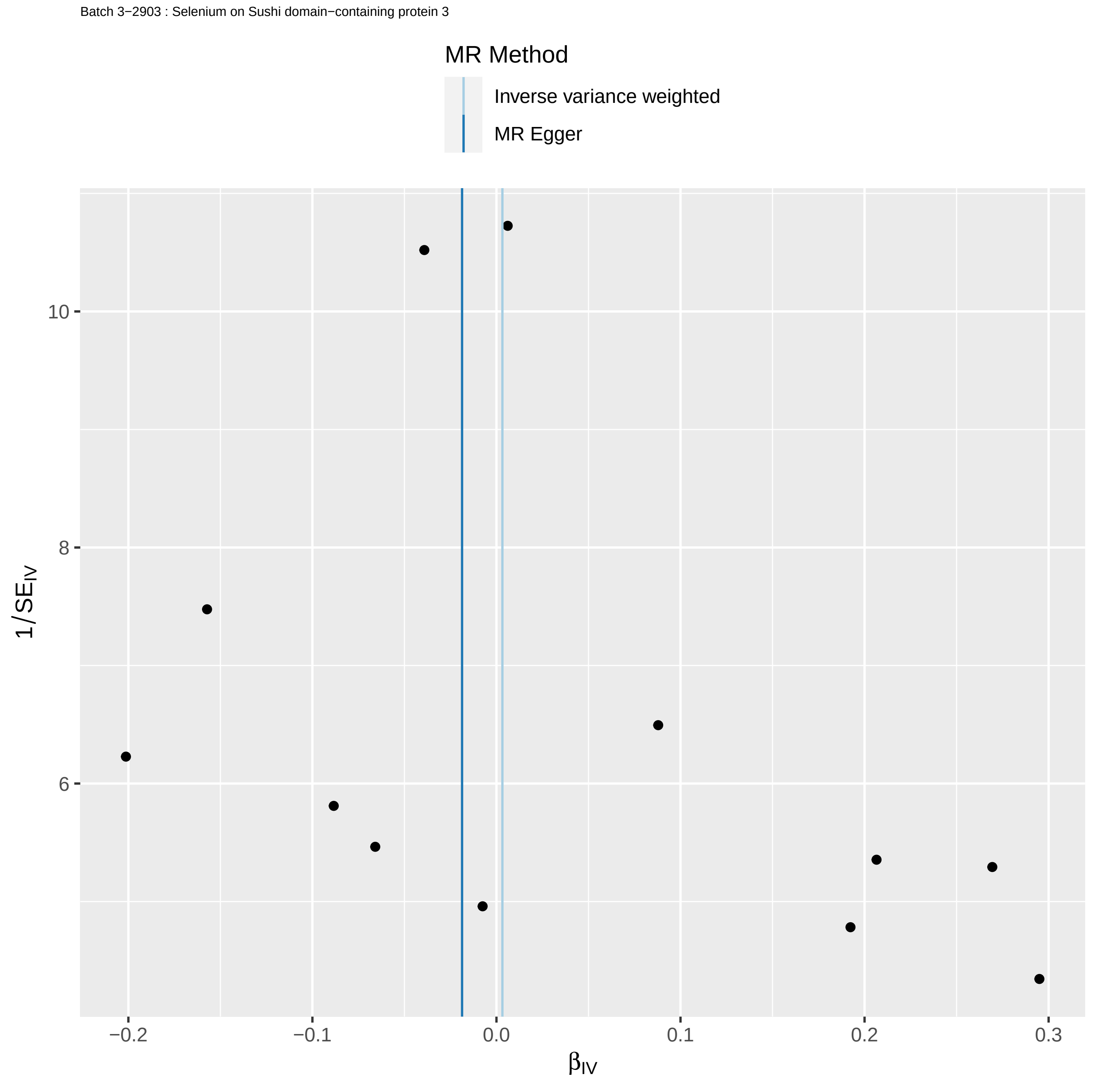

Batch 3−2903 : Selenium on Sushi domain−containing protein 3
MR Method
Inverse variance weighted
MR Egger
10
8
6
1/SE$_{IV}$
−0.2
−0.1
0.0
0.1
0.2
0.3
β$_{IV}$

Batch 3−2904 : Selenium on Histone−lysine N−methyltransferase SUV420H2

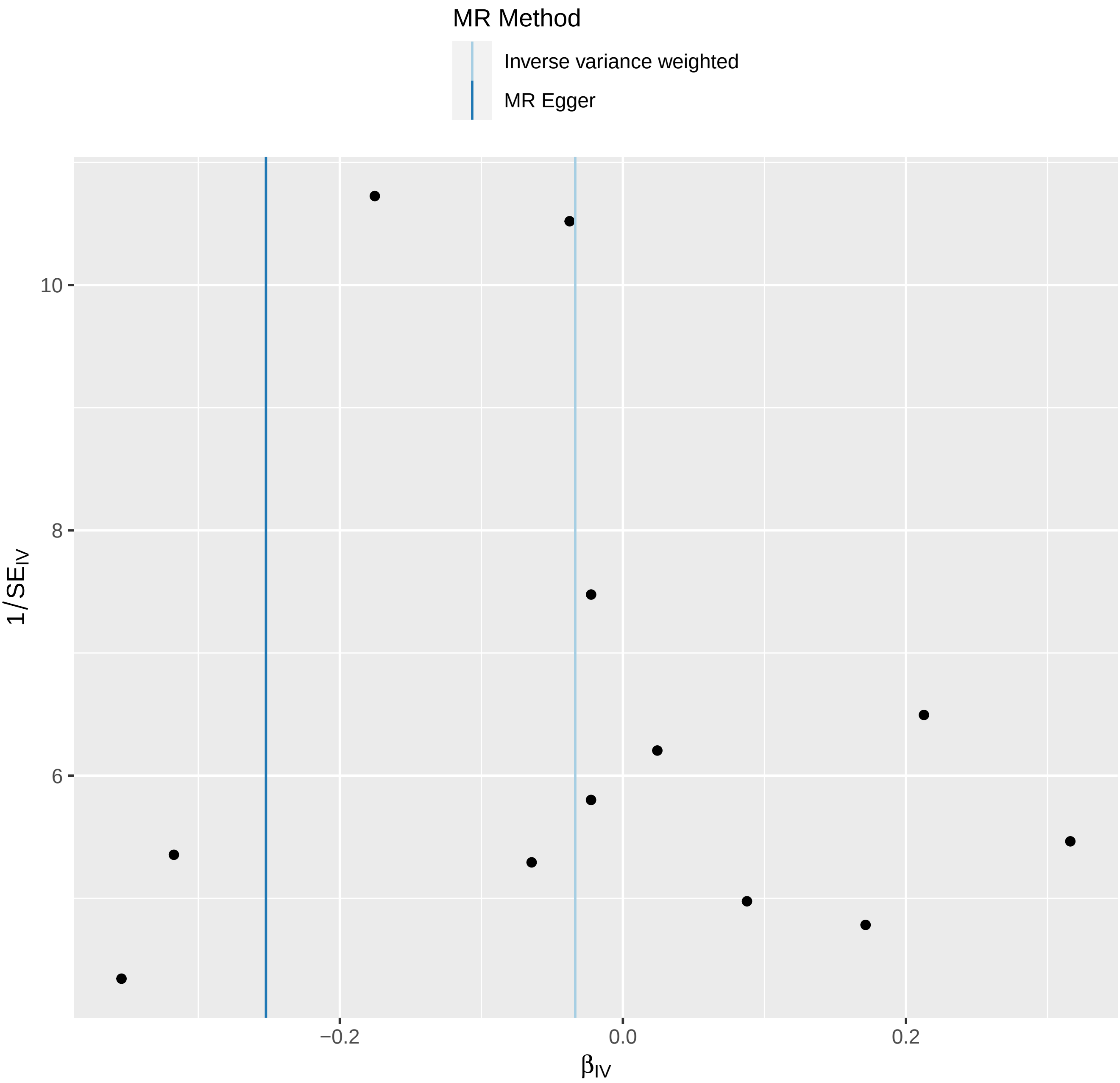

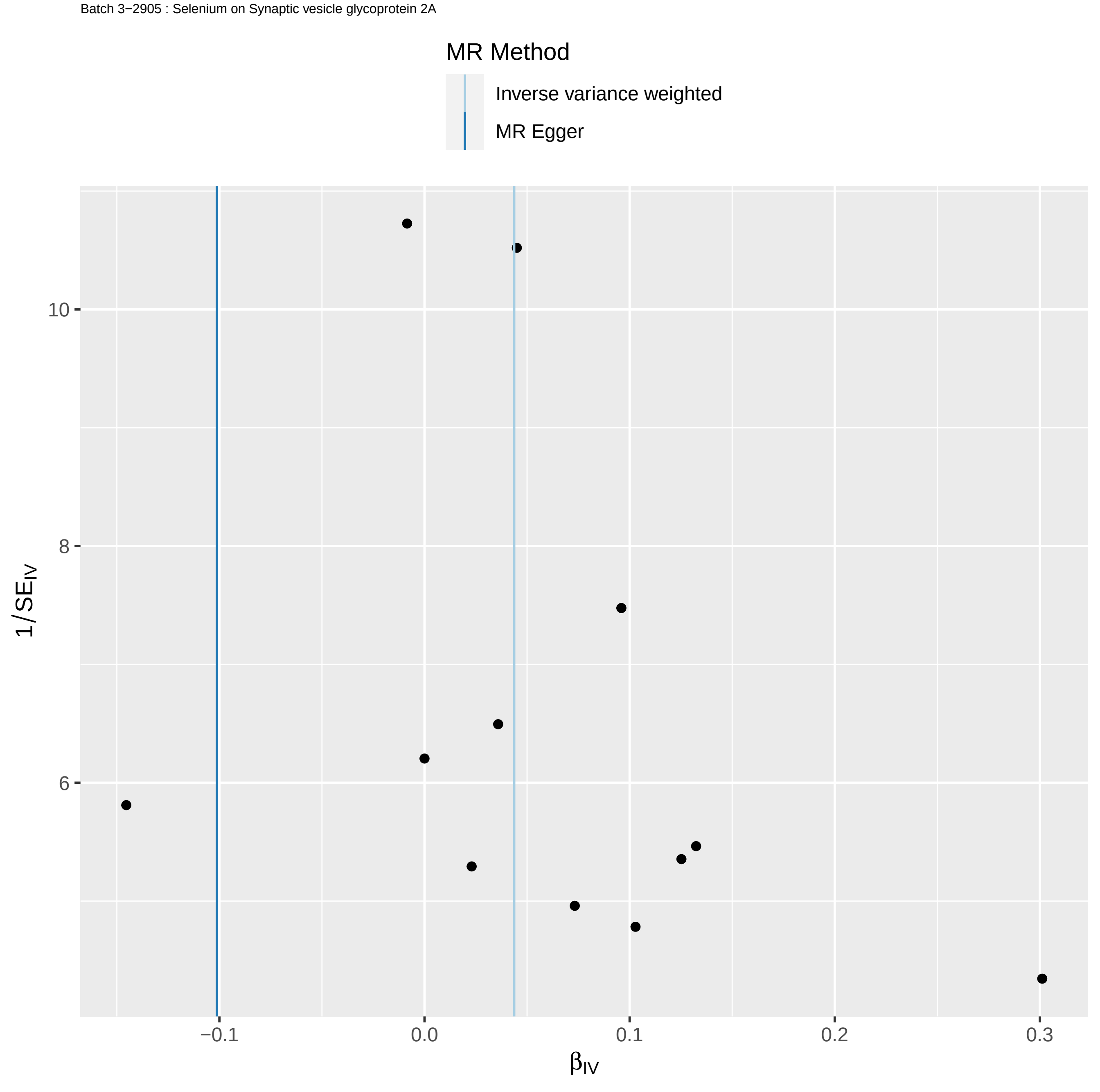
Batch 3−2905 : Selenium on Synaptic vesicle glycoprotein 2A
MR Method
Inverse variance weighted
MR Egger
10
8
6
1/SE_IV
−0.1
0.0
0.1
0.2
0.3
β_IV

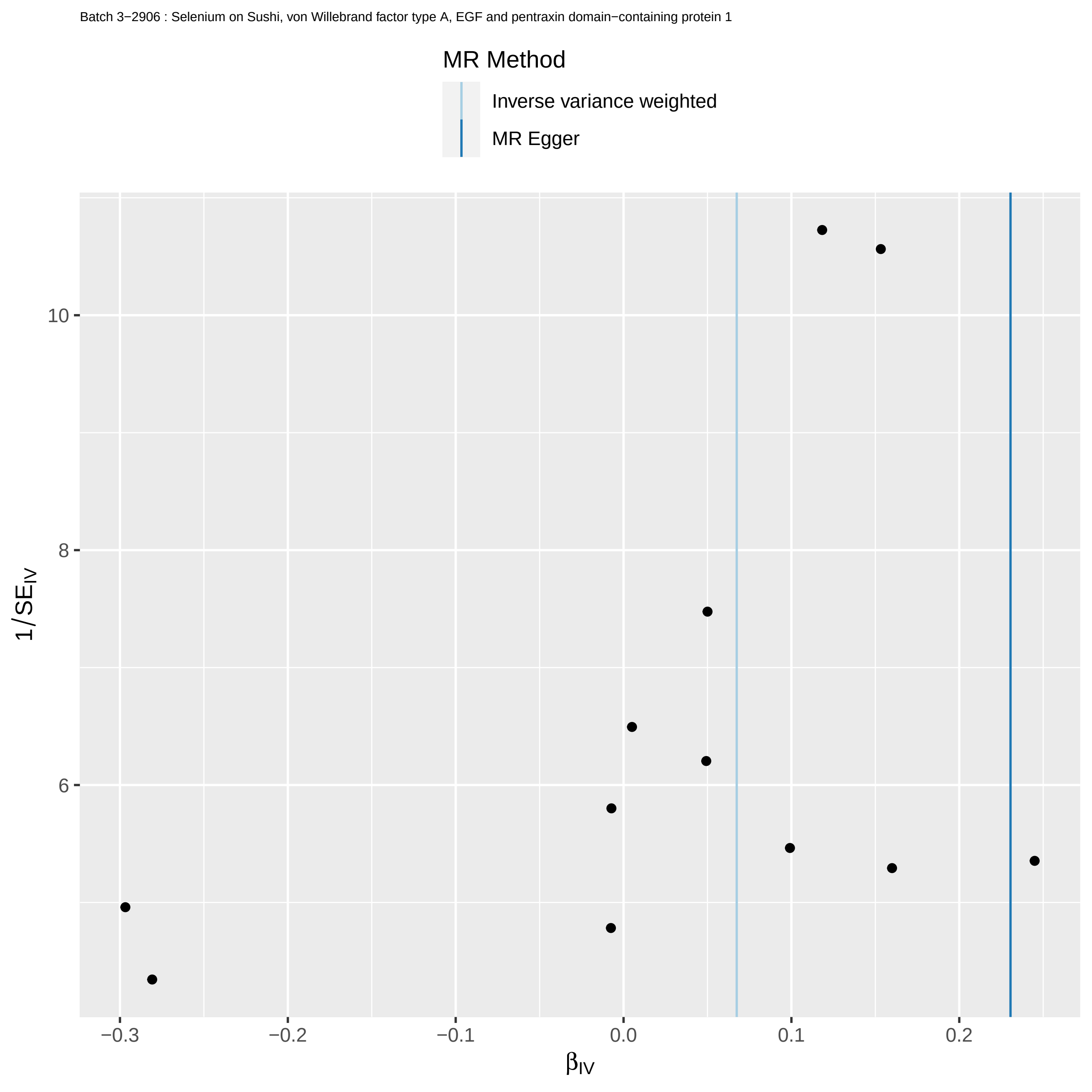

Batch 3−2906 : Selenium on Sushi, von Willebrand factor type A, EGF and pentraxin domain−containing protein 1
MR Method
Inverse variance weighted
MR Egger
10
8
6
1/SE_IV
−0.3
−0.2
−0.1
0.0
0.1
0.2
β_IV

Batch 3−2907 : Selenium on Sushi, von Willebrand factor type A, EGF and pentraxin domain−containing protein 1

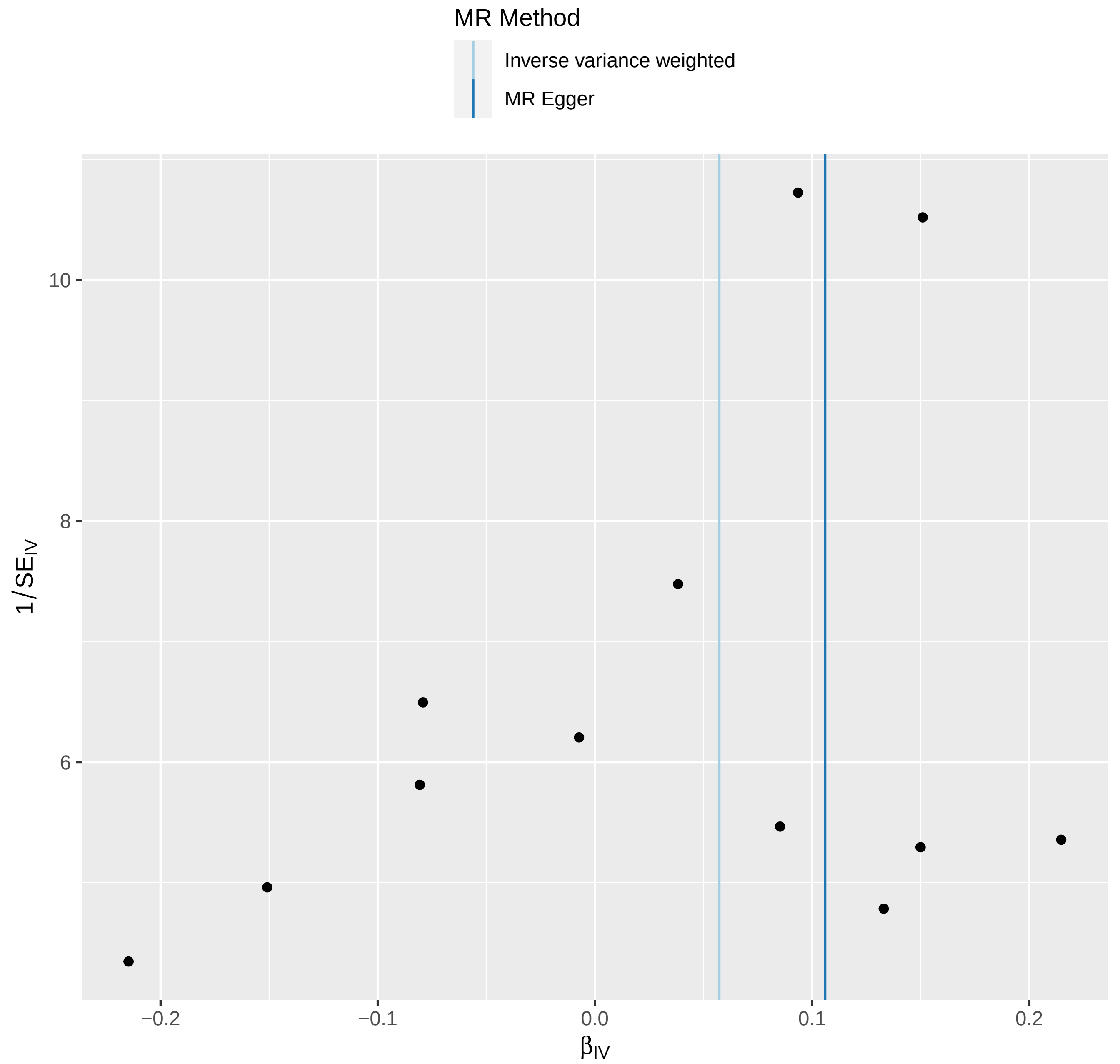

Batch 3−2908 : Selenium on Switch−associated protein 70

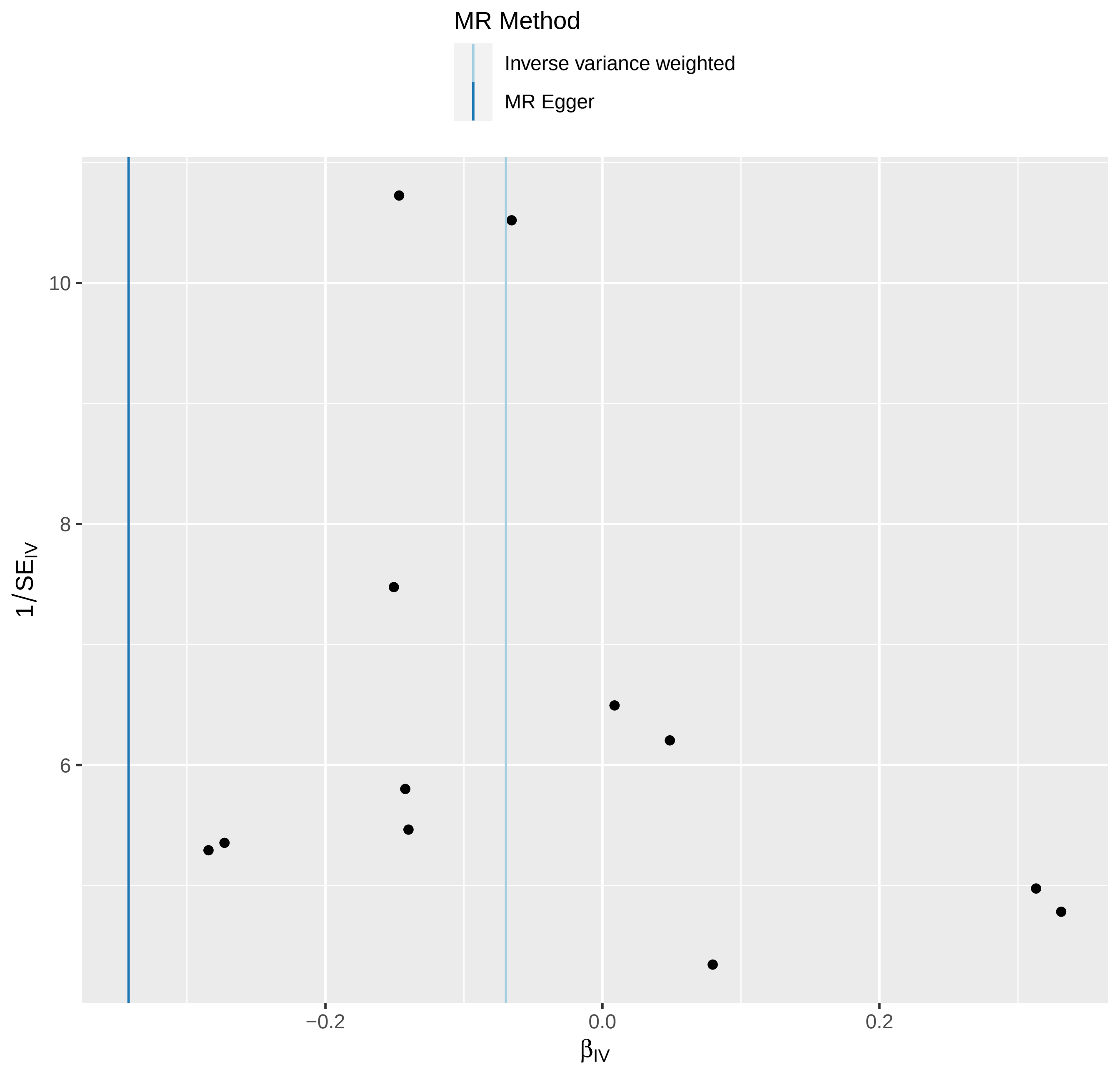

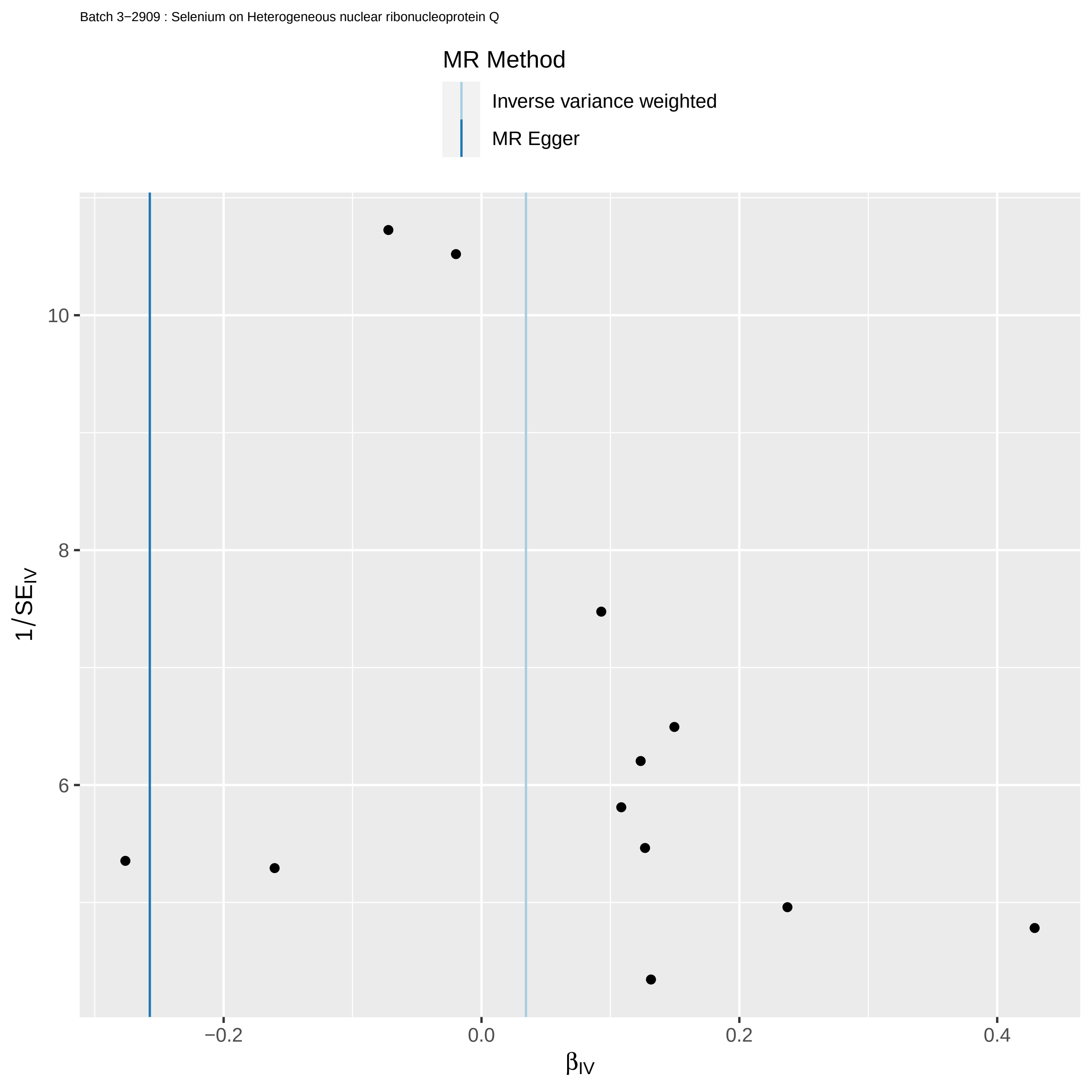

Batch 3−2909 : Selenium on Heterogeneous nuclear ribonucleoprotein Q
MR Method
Inverse variance weighted
MR Egger
10
8
6
$1/SE_{IV}$
−0.2
0.0
0.2
0.4
β_{IV}

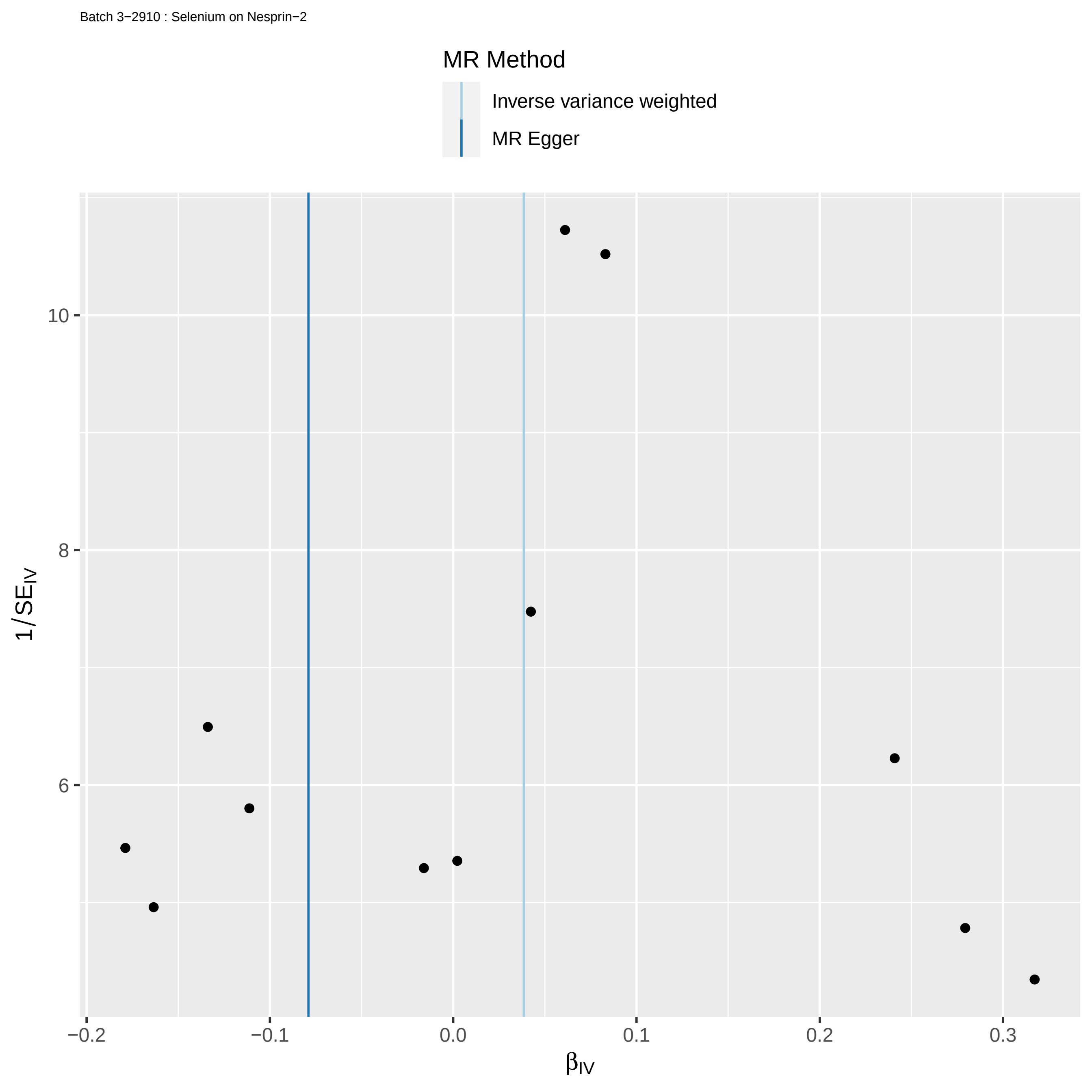

Batch 3−2910 : Selenium on Nesprin−2
MR Method
Inverse variance weighted
MR Egger
10
8
6
1/SE_IV
−0.2
−0.1
0.0
0.1
0.2
0.3
β_IV

Batch 3−2911 : Selenium on Synaptotagmin−11

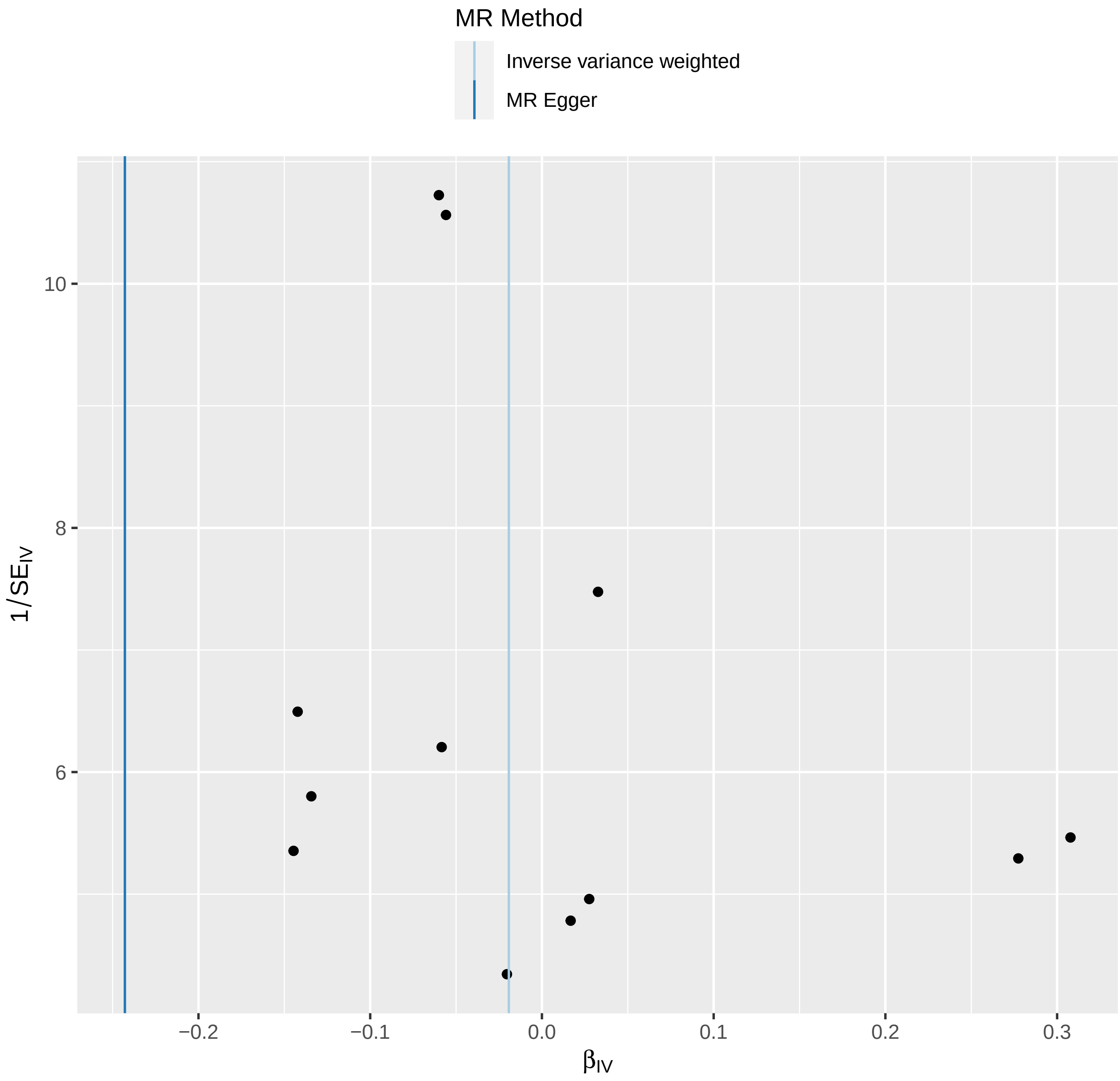

Batch 3−2912 : Selenium on Synaptotagmin−17

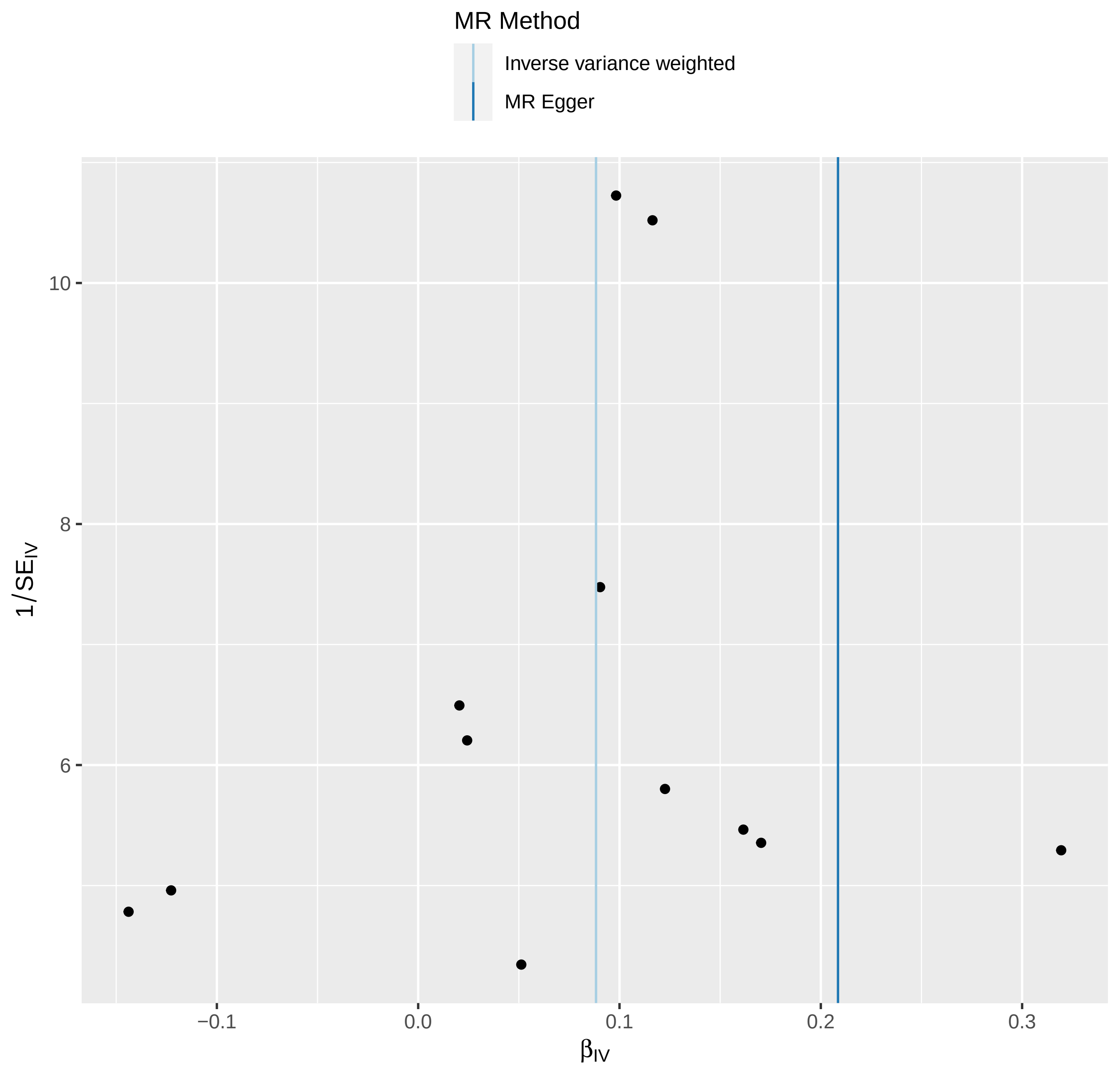

Batch 3−2913 : Selenium on Synaptotagmin−2

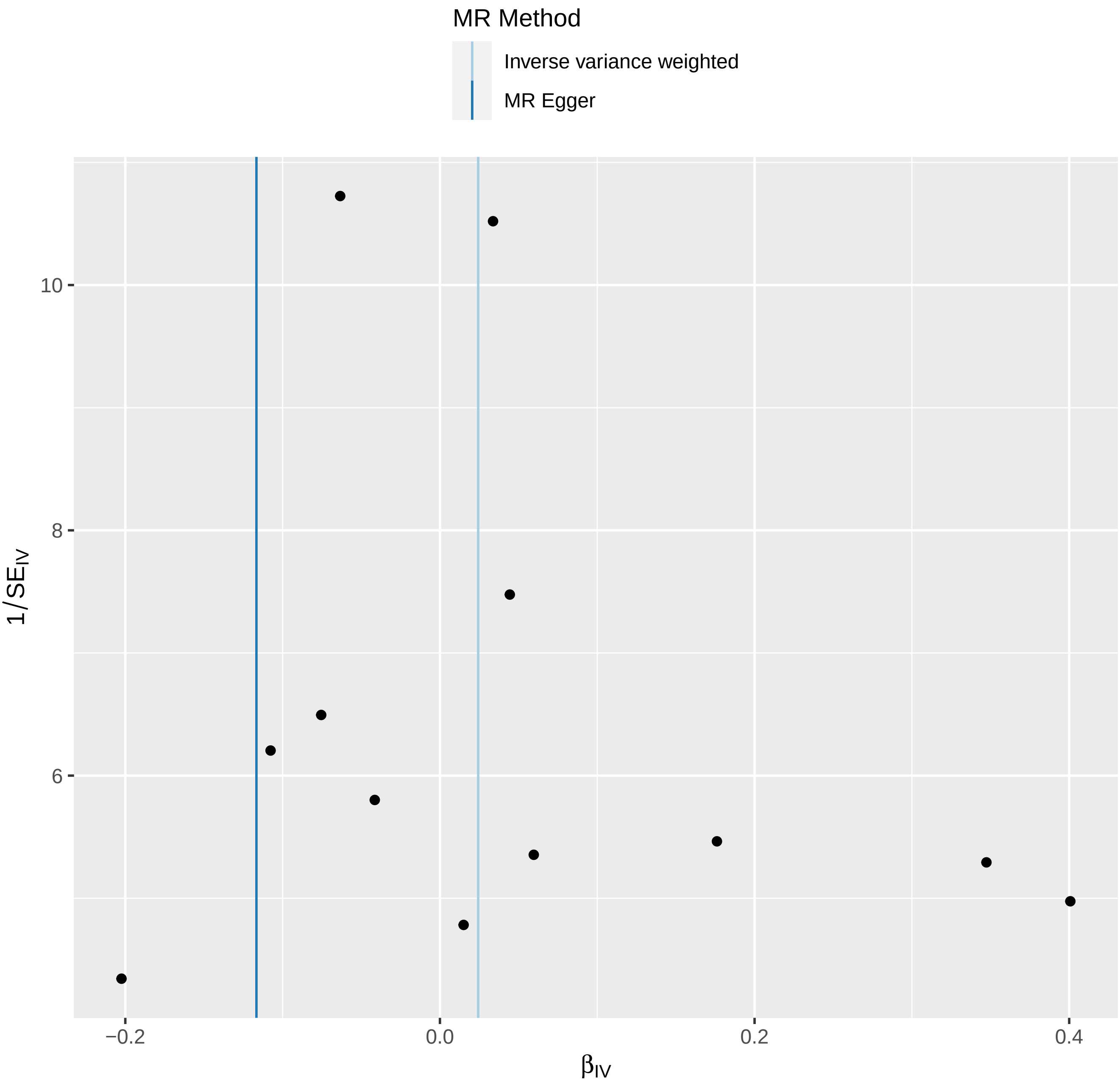

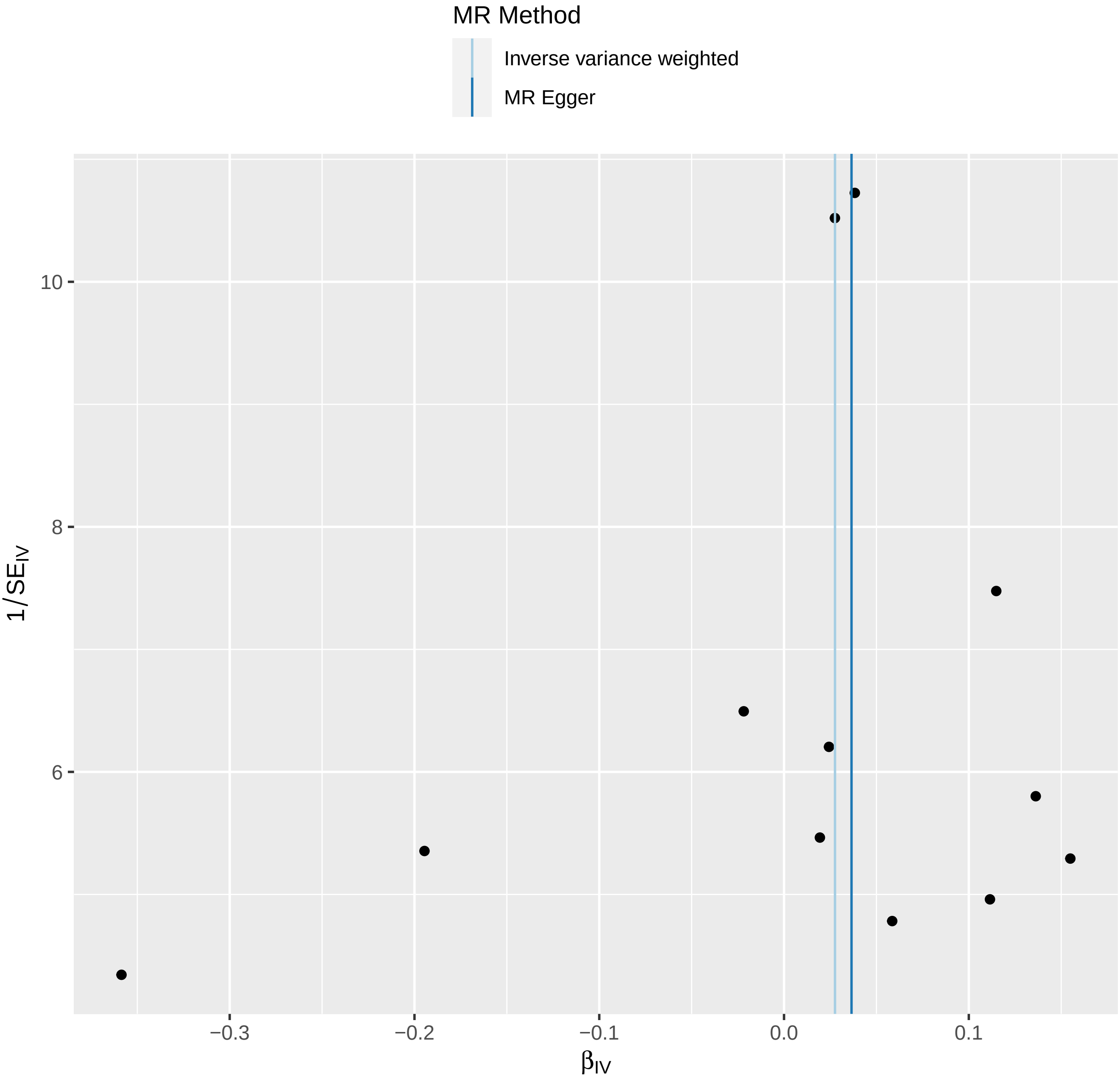
MR Method
Inverse variance weighted
MR Egger
10
8
6
1/SE_IV
−0.3
−0.2
−0.1
0.0
0.1
β_IV

Batch 3−2915 : Selenium on Synaptotagmin−5

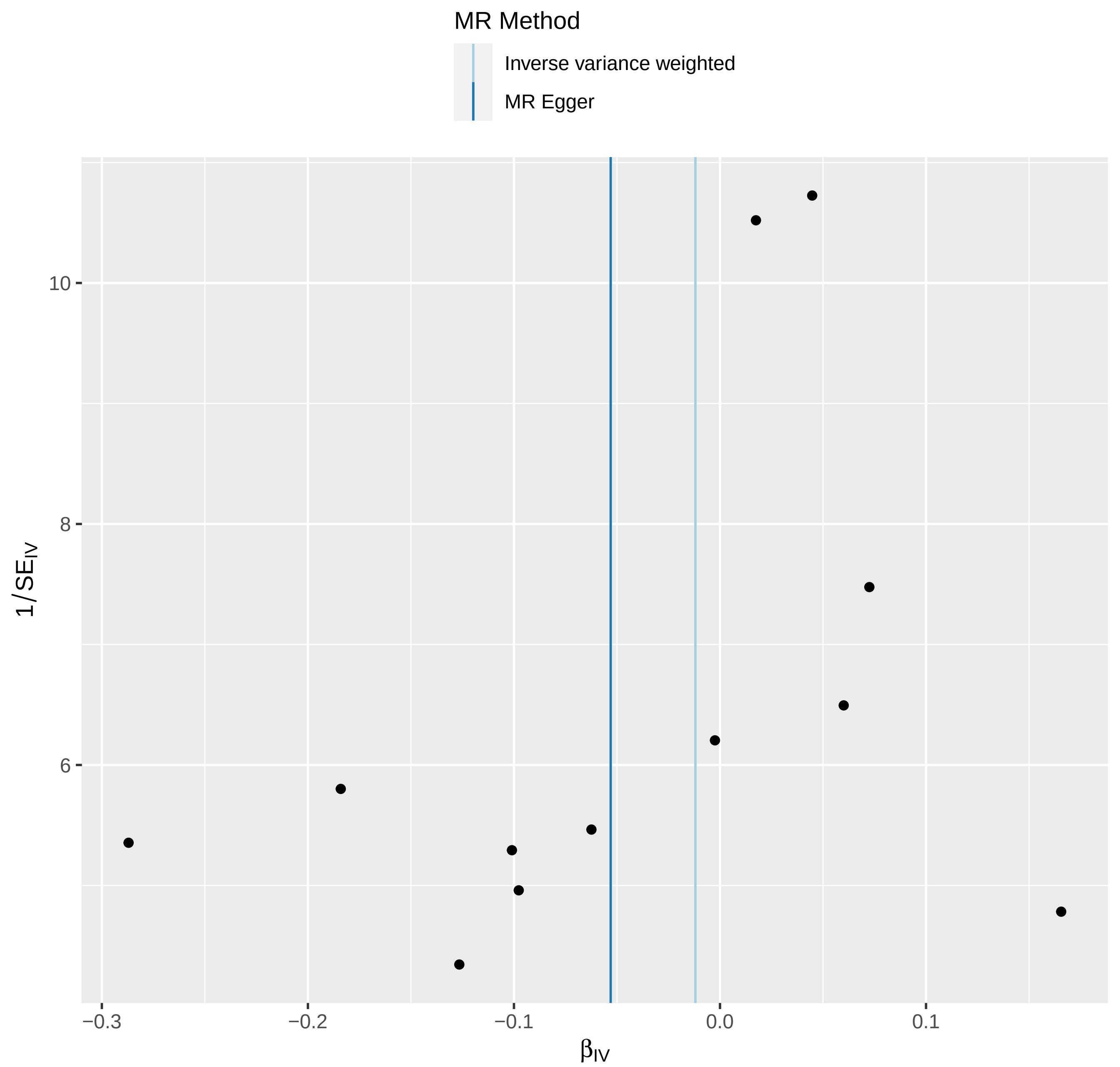

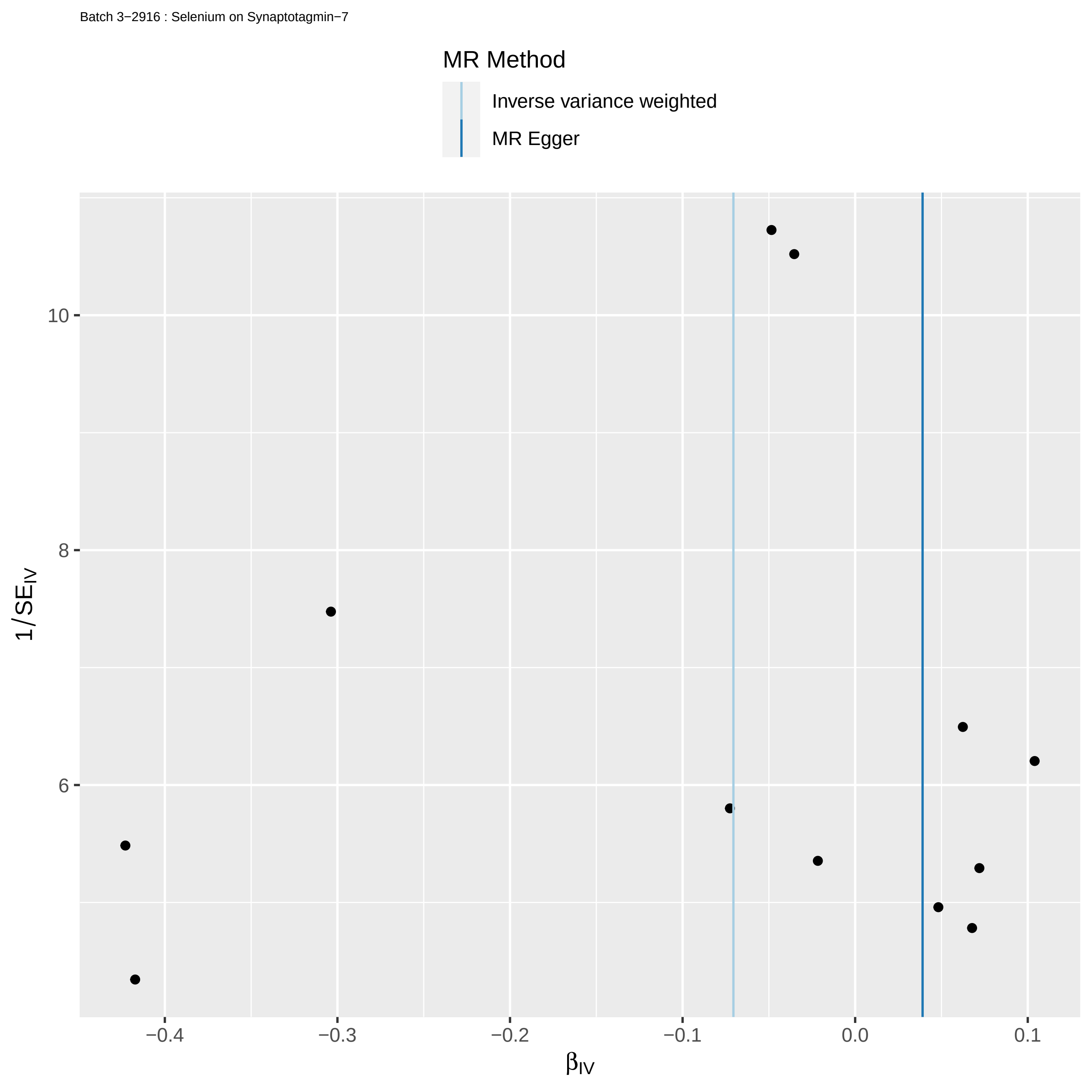

Batch 3−2916 : Selenium on Synaptotagmin−7
MR Method
Inverse variance weighted
MR Egger
1/SE_IV
10
8
6
β_IV
−0.4
−0.3
−0.2
−0.1
0.0
0.1

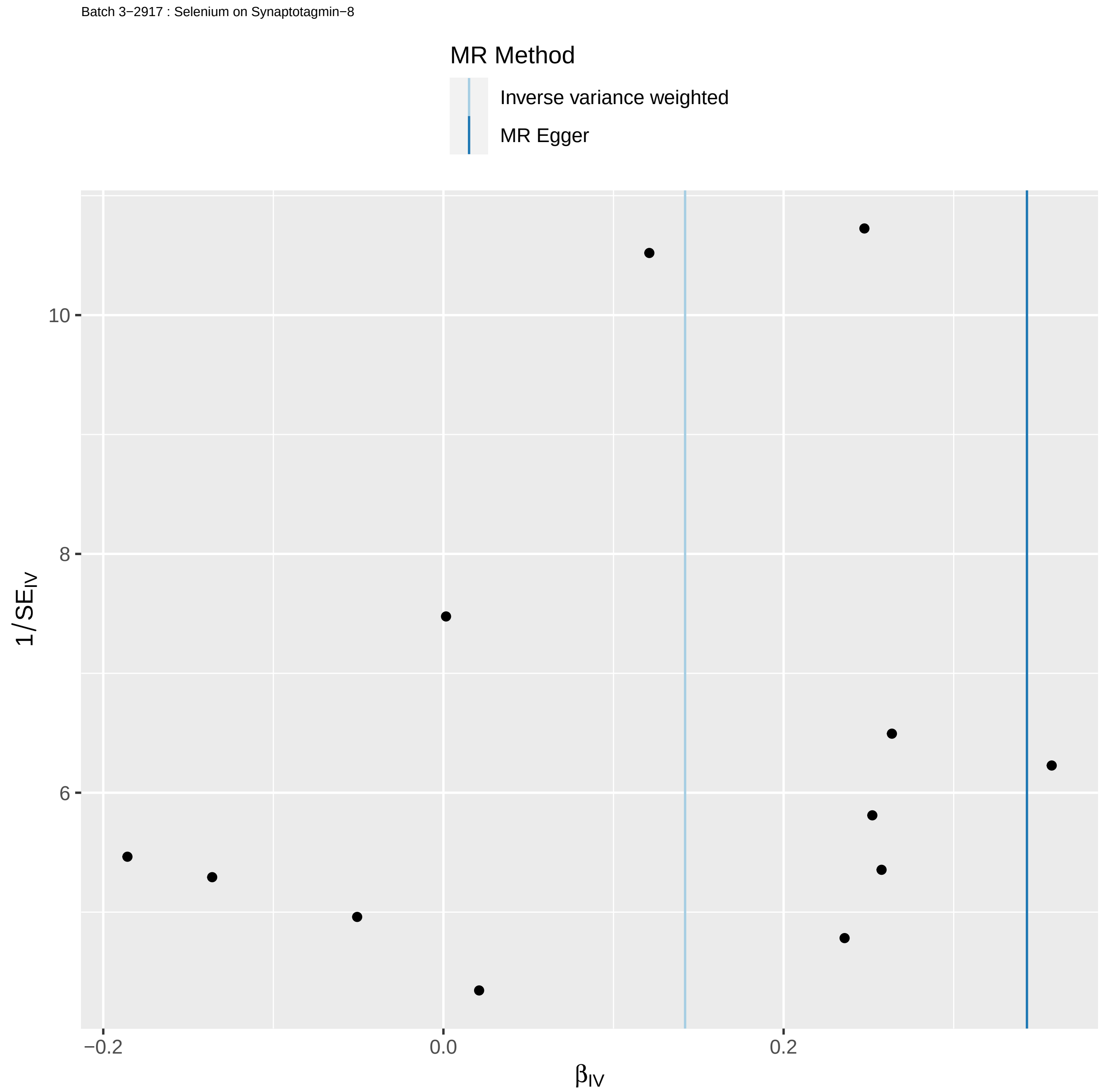

Batch 3−2917 : Selenium on Synaptotagmin−8
MR Method
Inverse variance weighted
MR Egger
10
8
6
−0.2
0.0
0.2
1/SE$_{IV}$
β$_{IV}$

Batch 3−2918 : Selenium on Synaptotagmin−like protein 1

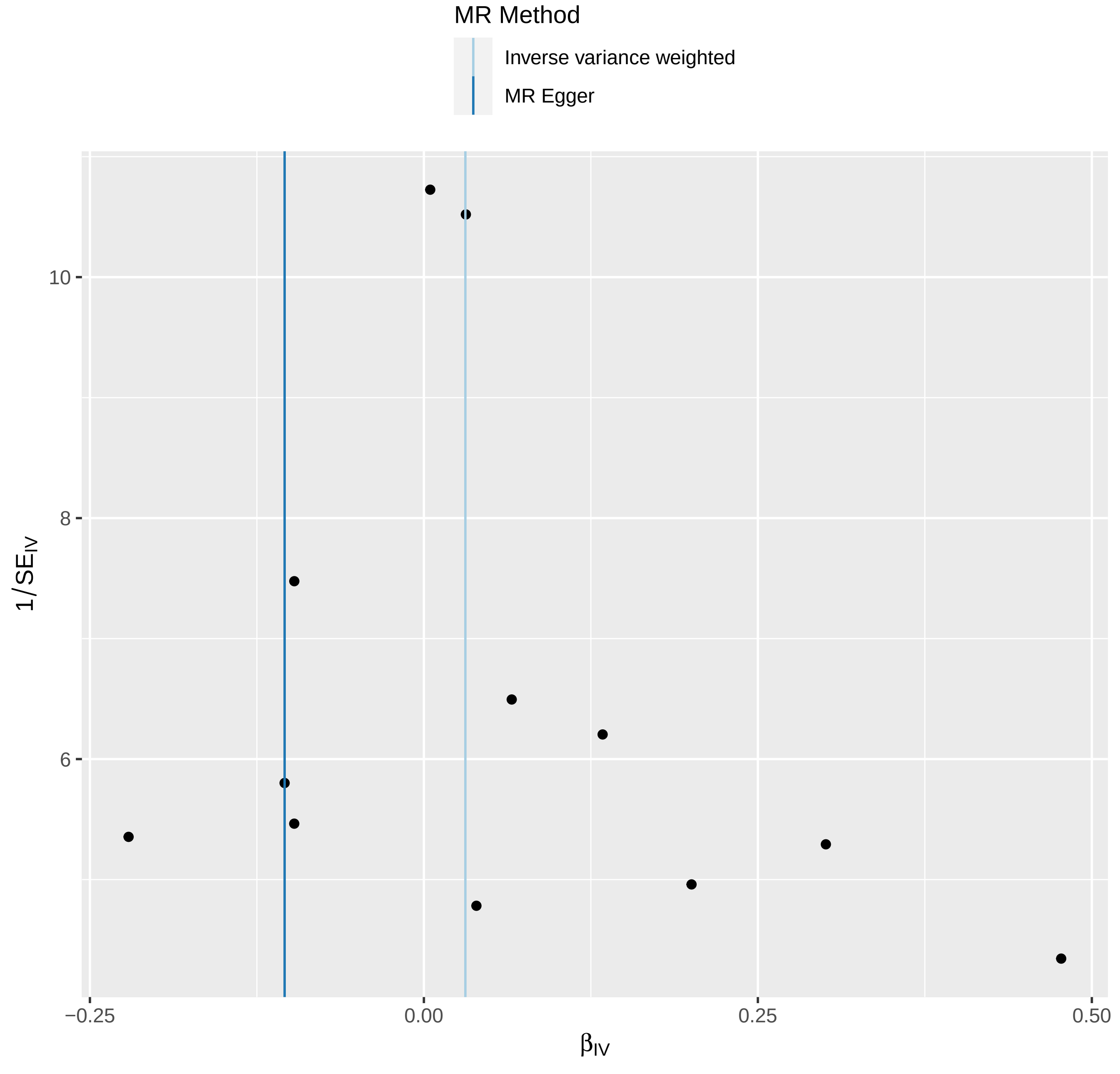

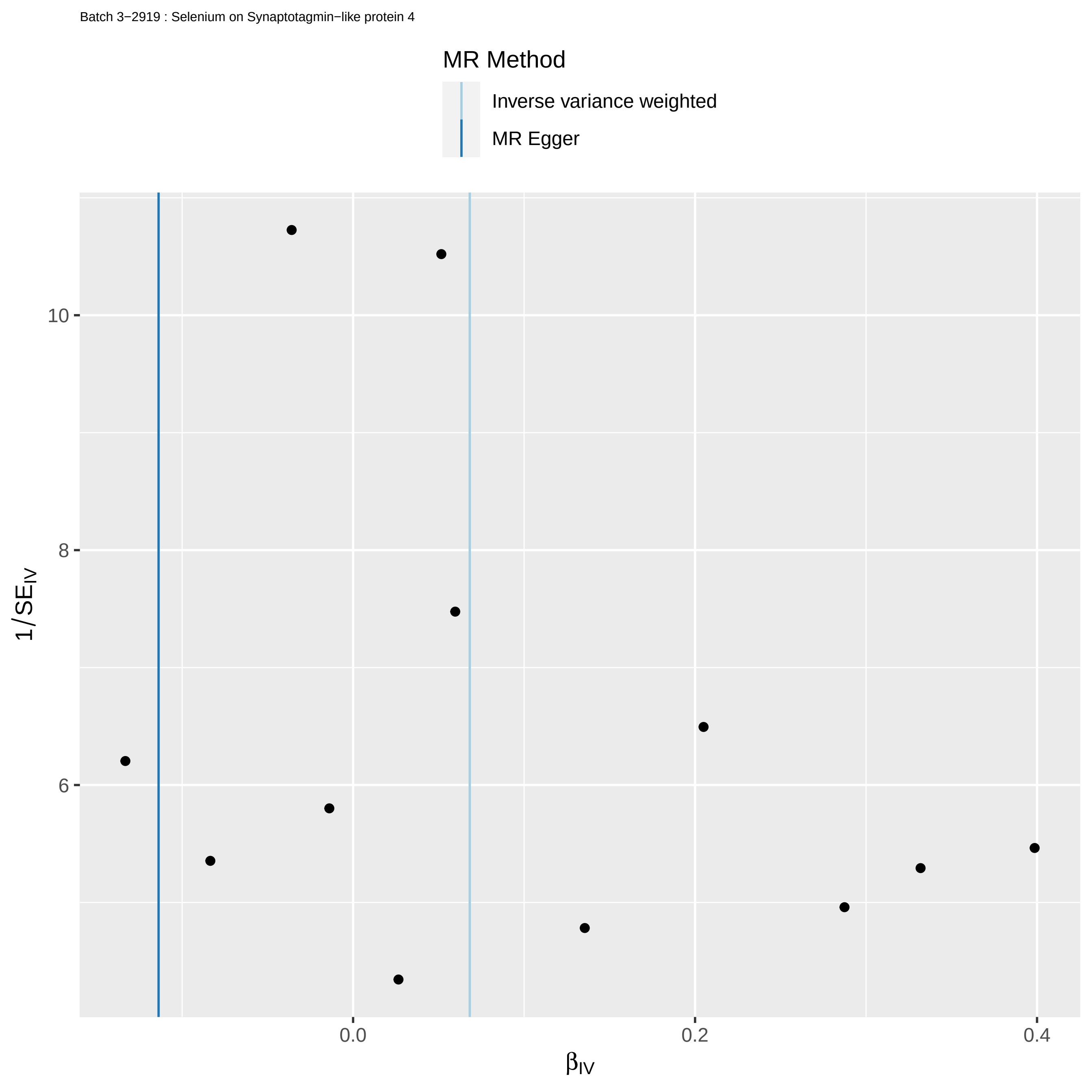

Batch 3−2919 : Selenium on Synaptotagmin−like protein 4
MR Method
Inverse variance weighted
MR Egger
10
8
6
1/SE_IV
0.0
0.2
0.4
β_IV

Batch 3−2920 : Selenium on Protachykinin−1

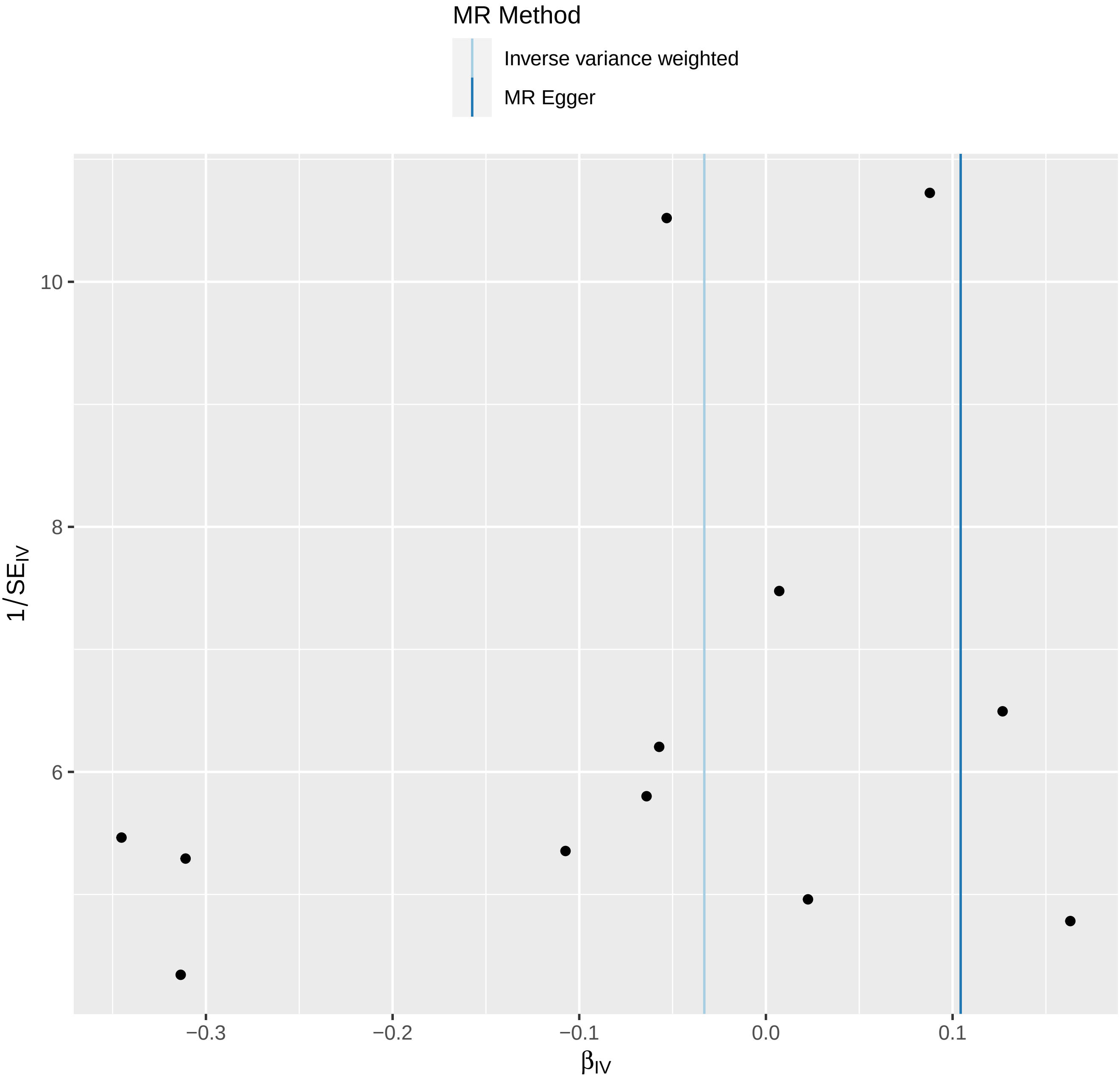

Batch 3−2921 : Selenium on Tachykinin−3

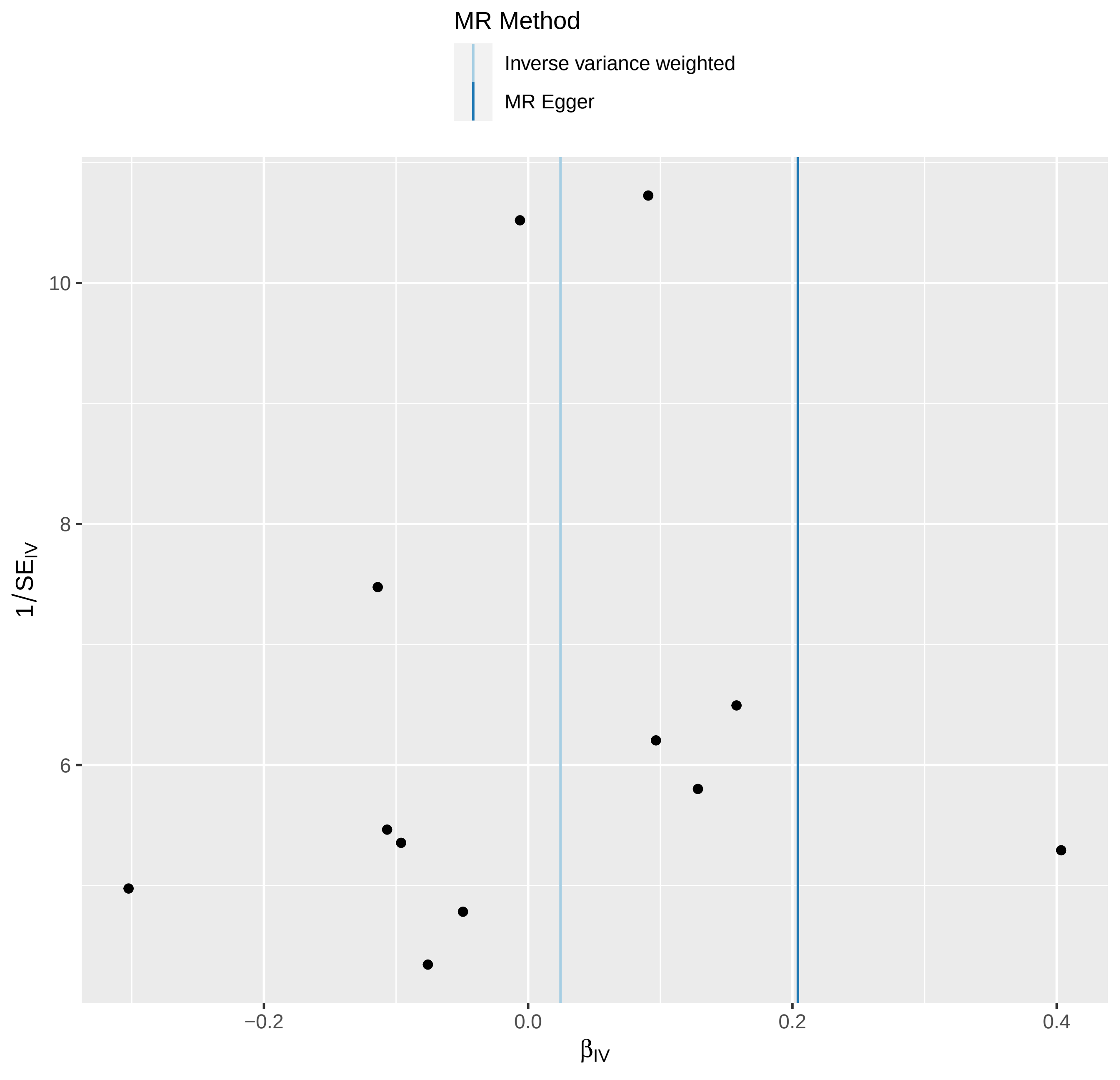

Batch 3−2922 : Selenium on Tachykinin−4

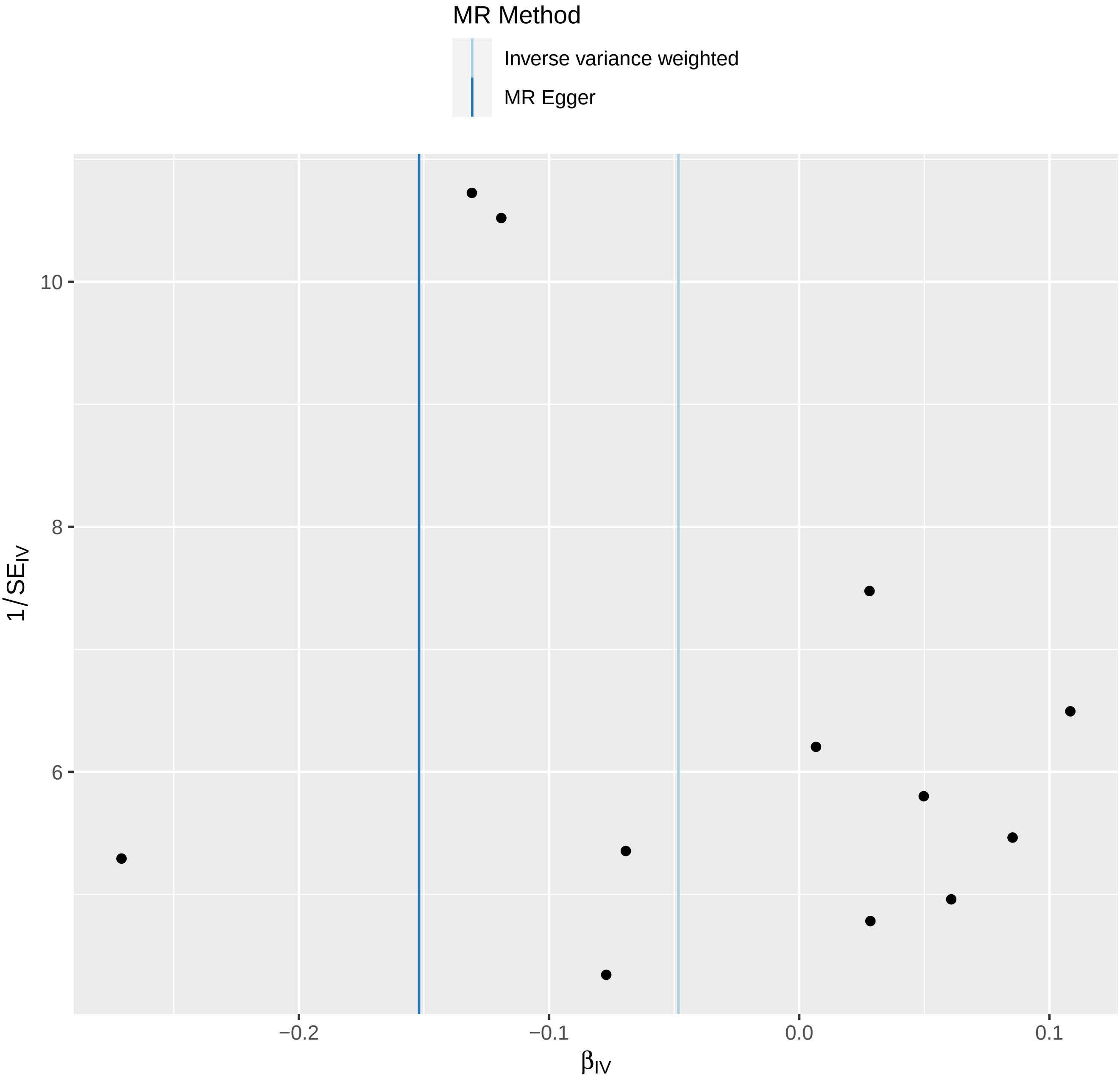

Batch 3−2923 : Selenium on Tumor−associated calcium signal transducer 2

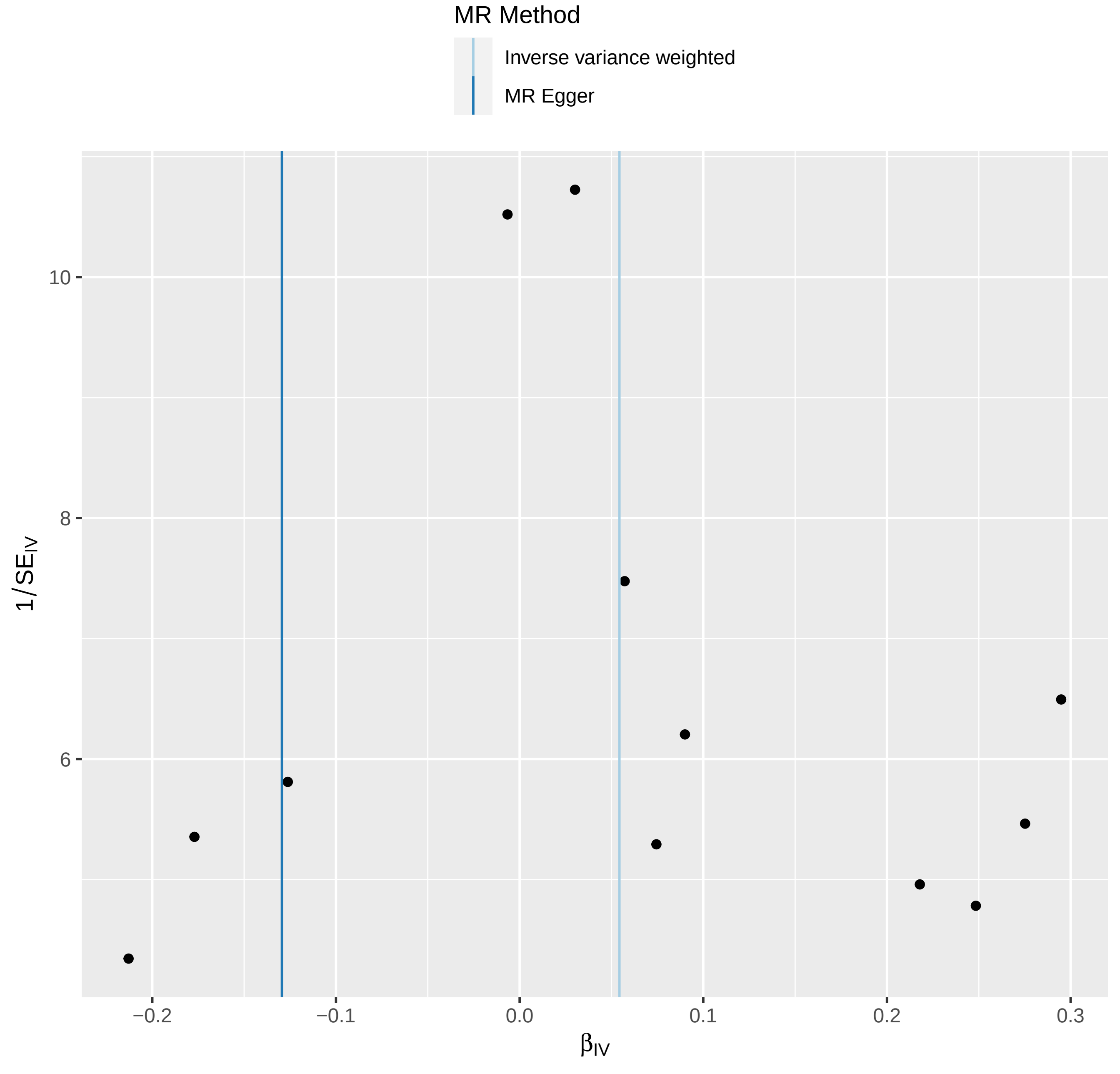

Batch 3−2924 : Selenium on Tumor−associated calcium signal transducer 2

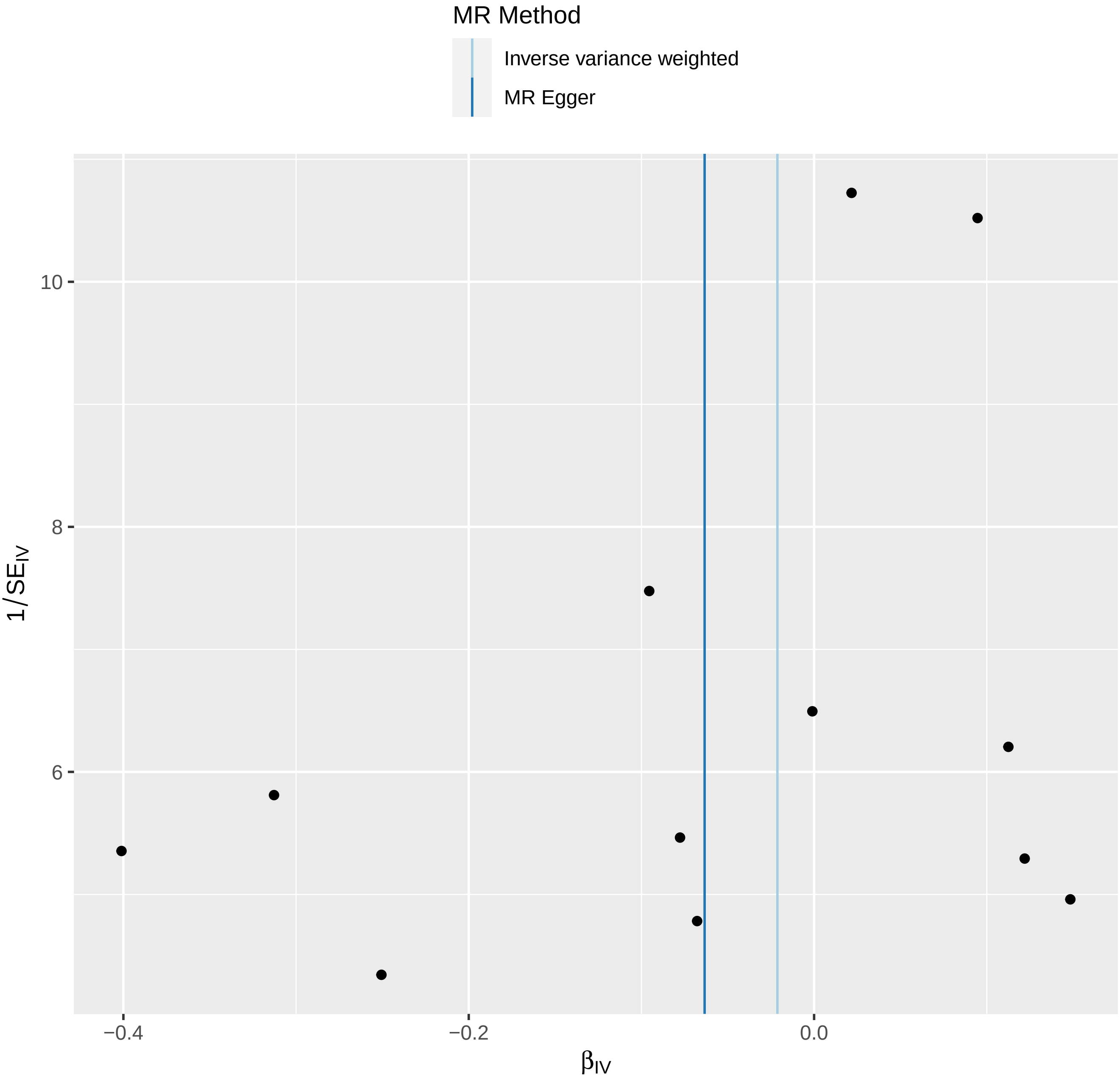

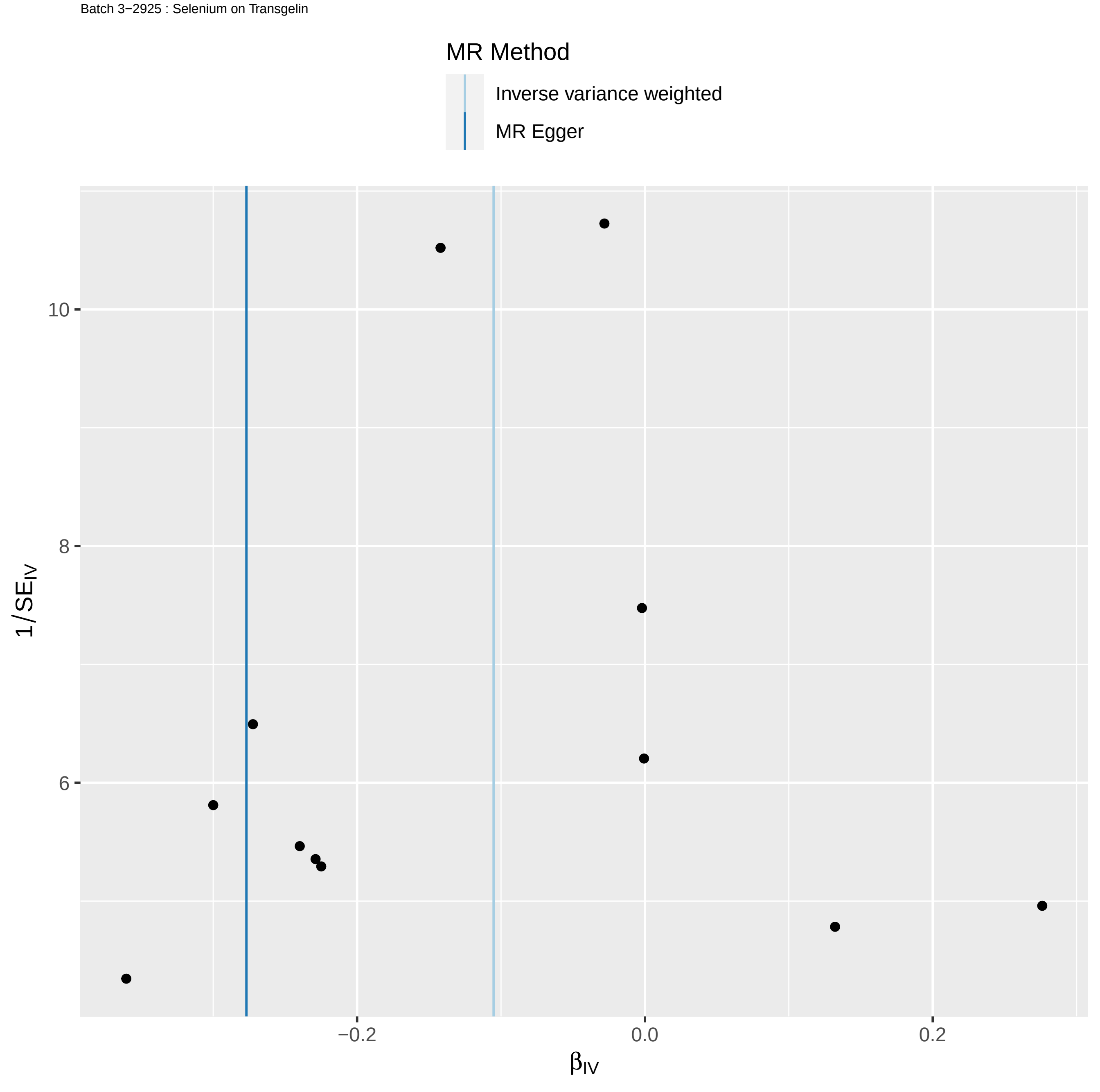

Batch 3−2925 : Selenium on Transgelin
MR Method
Inverse variance weighted
MR Egger
10
8
6
1/SE_IV
−0.2
0.0
0.2
β_IV

Batch 3−2926 : Selenium on Tapasin

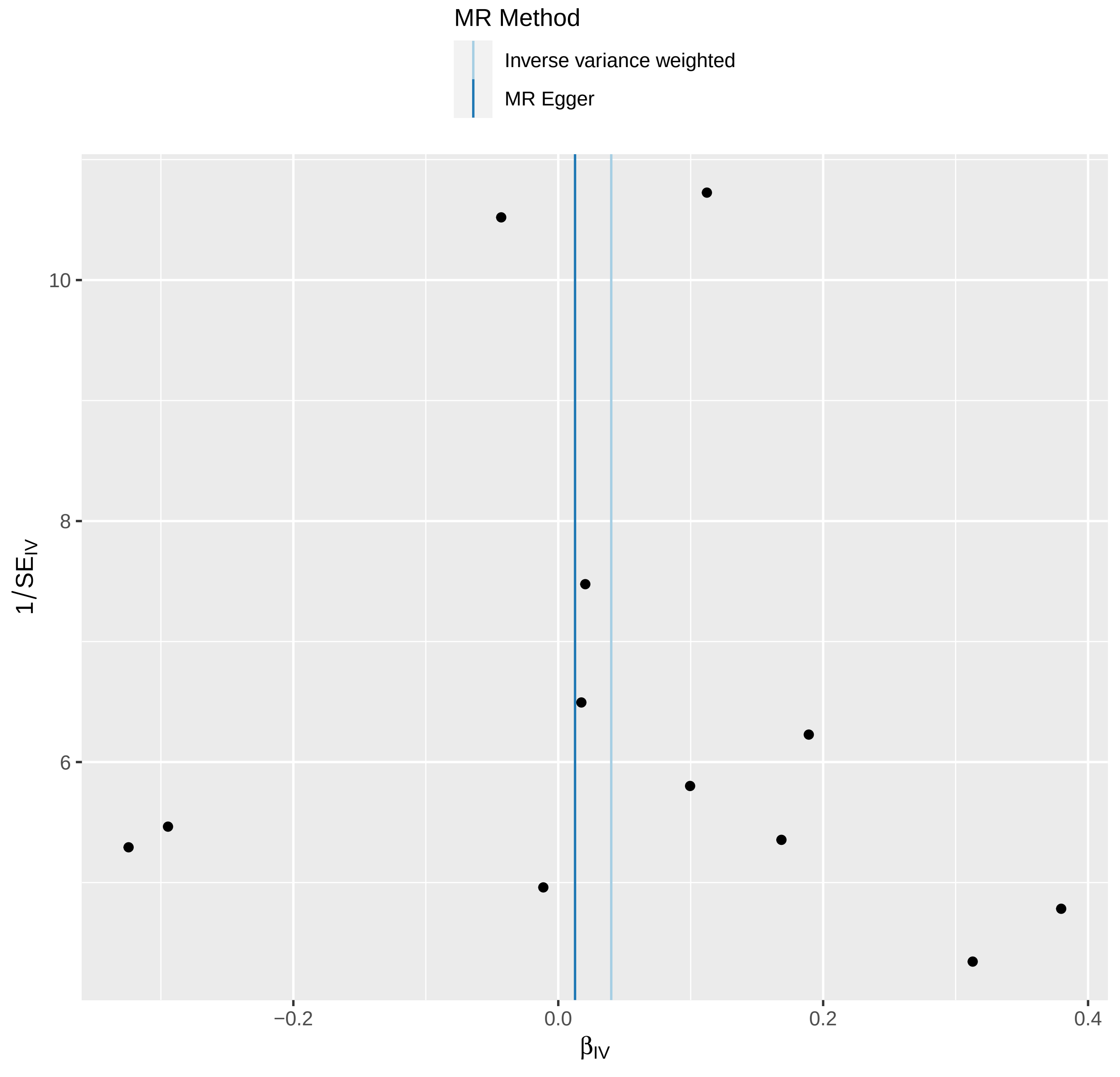

Batch 3−2927 : Selenium on Tapasin−related protein

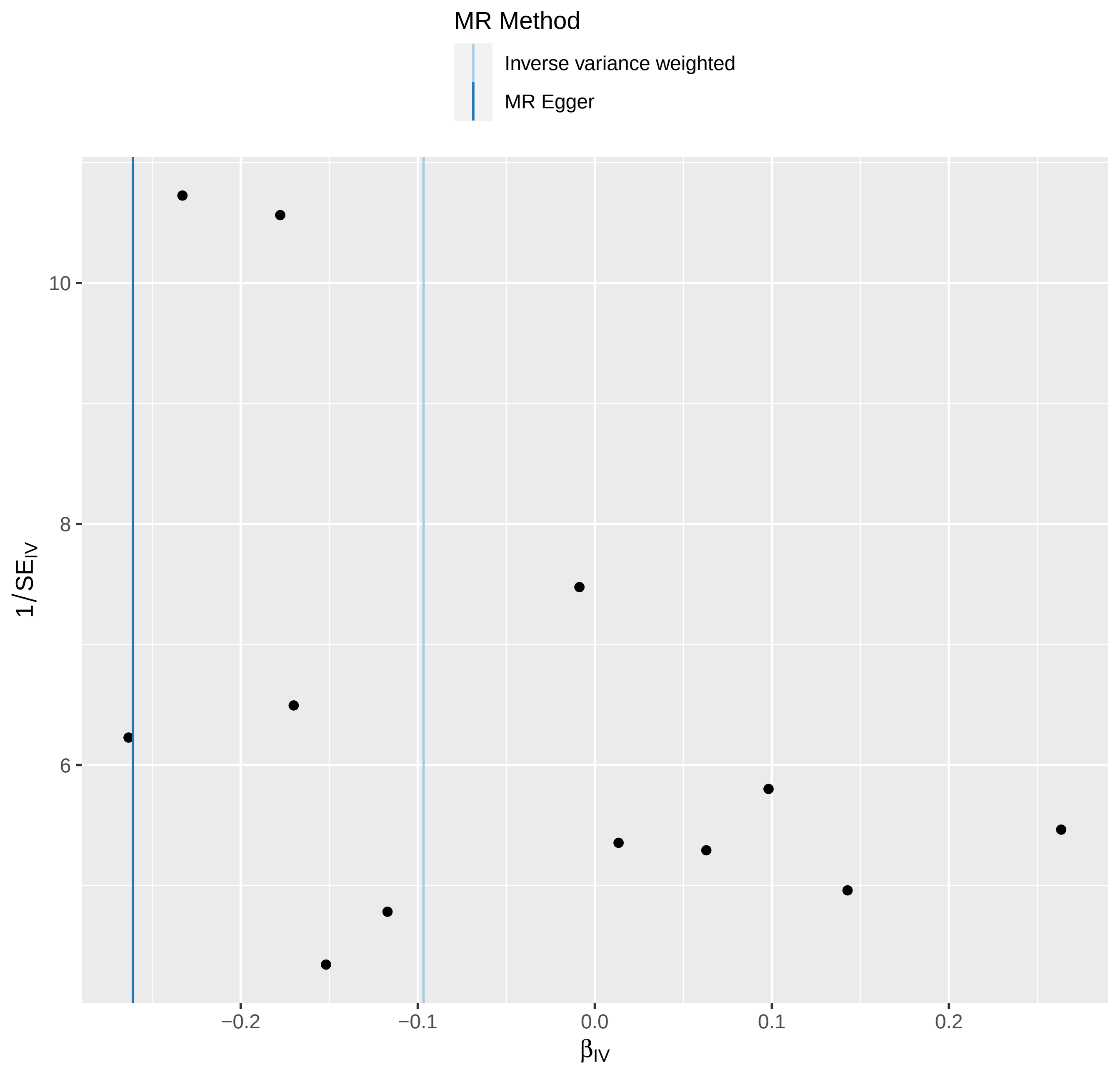

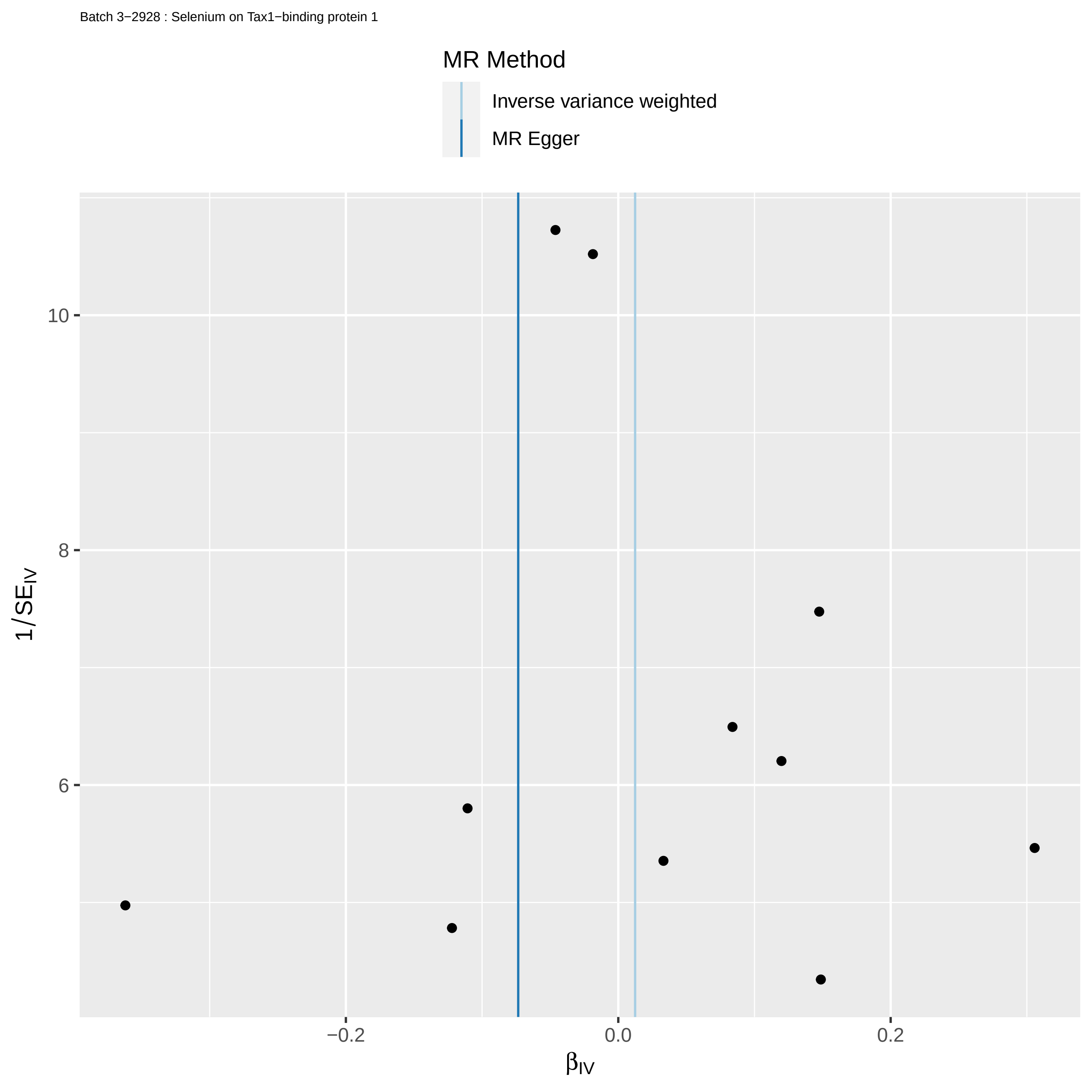
Batch 3−2928 : Selenium on Tax1−binding protein 1
MR Method
Inverse variance weighted
MR Egger
10
8
6
1/SE$_{IV}$
−0.2
0.0
0.2
β$_{IV}$

Batch 3−2929 : Selenium on Tax1−binding protein 3

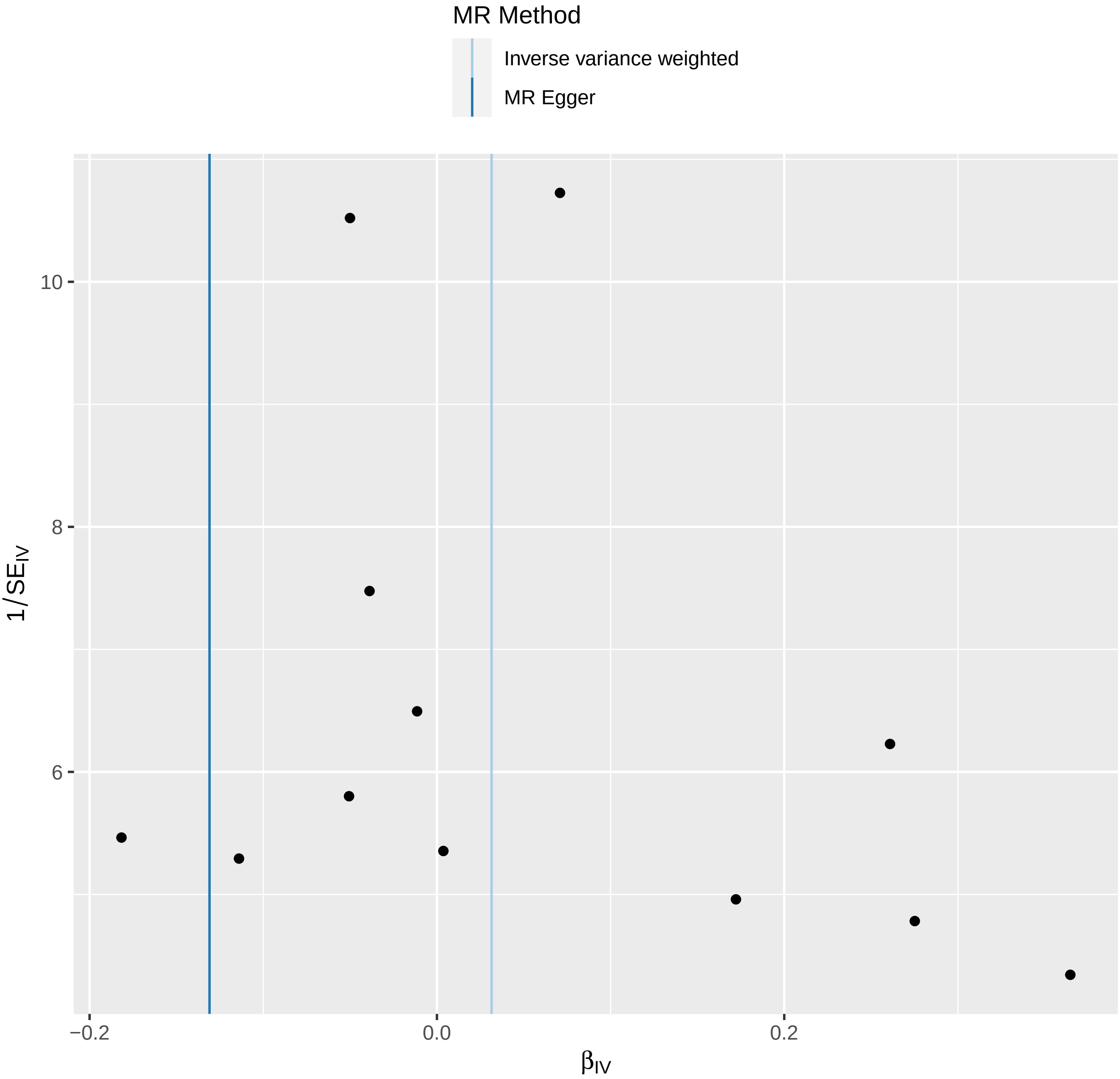

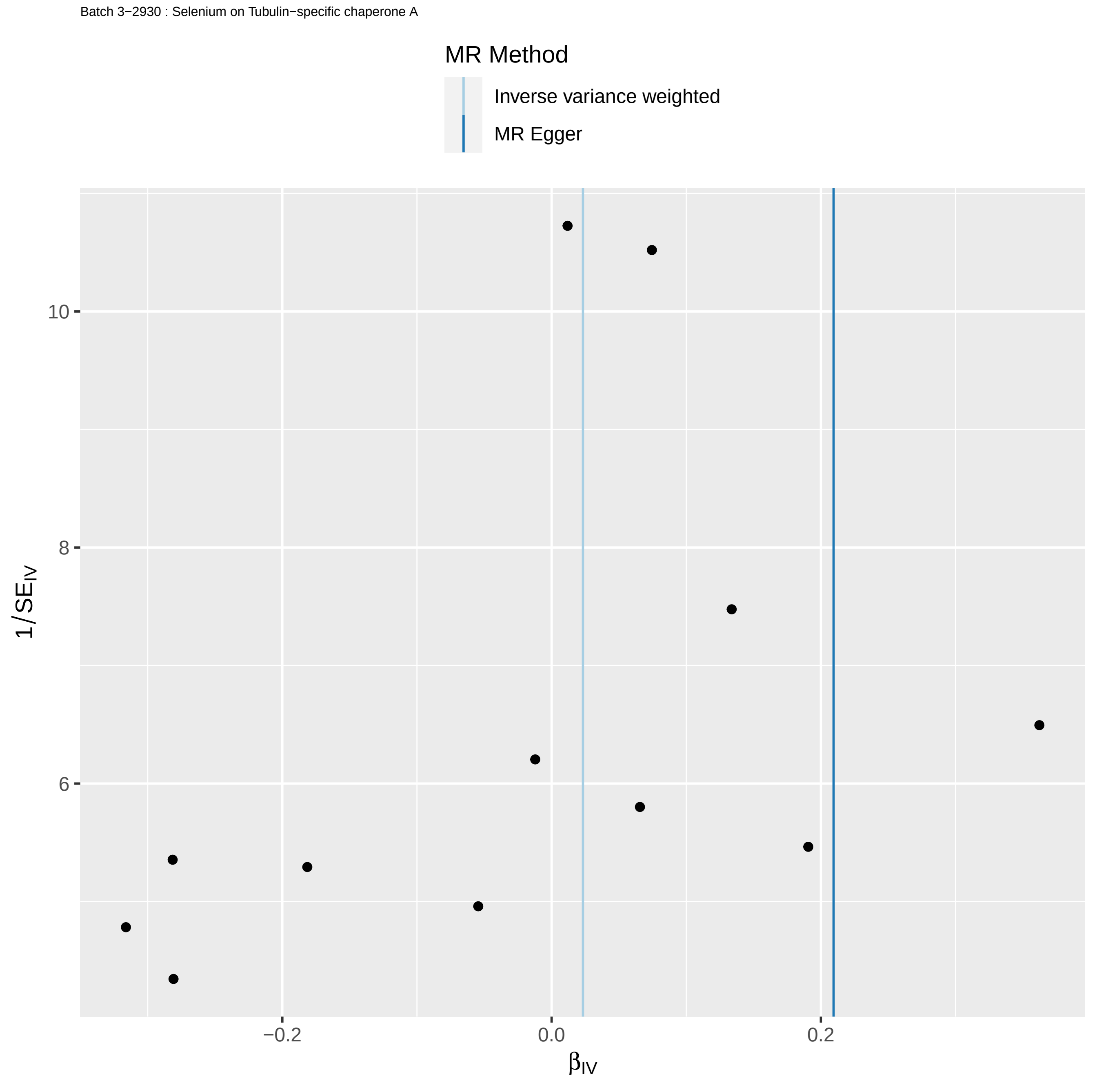
Batch 3−2930 : Selenium on Tubulin−specific chaperone A
MR Method
Inverse variance weighted
MR Egger
10
8
6
1/SE_IV
−0.2
0.0
0.2
β_IV

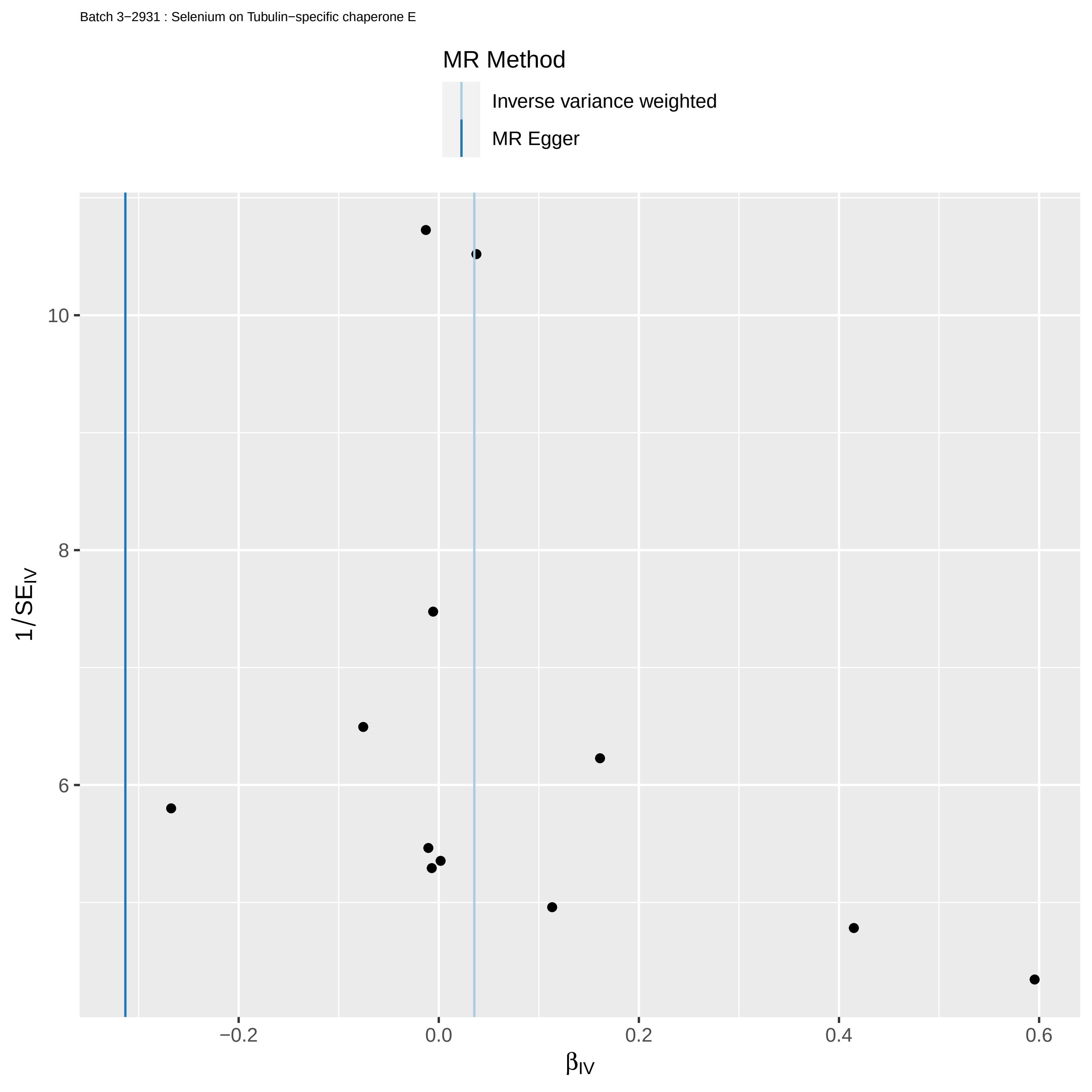

Batch 3−2931 : Selenium on Tubulin−specific chaperone E
MR Method
Inverse variance weighted
MR Egger
10
8
6
1/SE_IV
−0.2
0.0
0.2
0.4
0.6
β_IV

Batch 3−2932 : Selenium on Serine/threonine−protein kinase TBK1

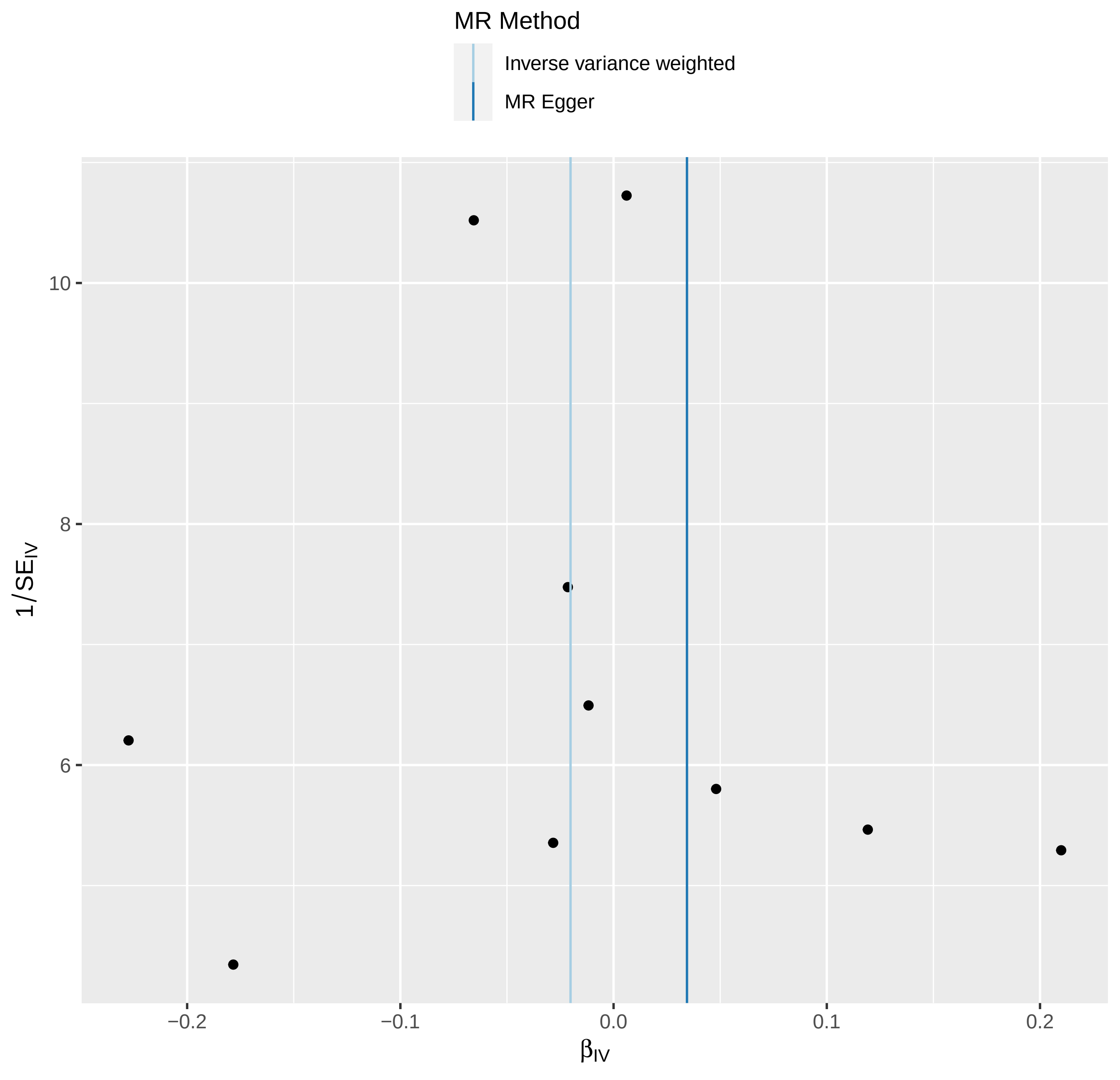

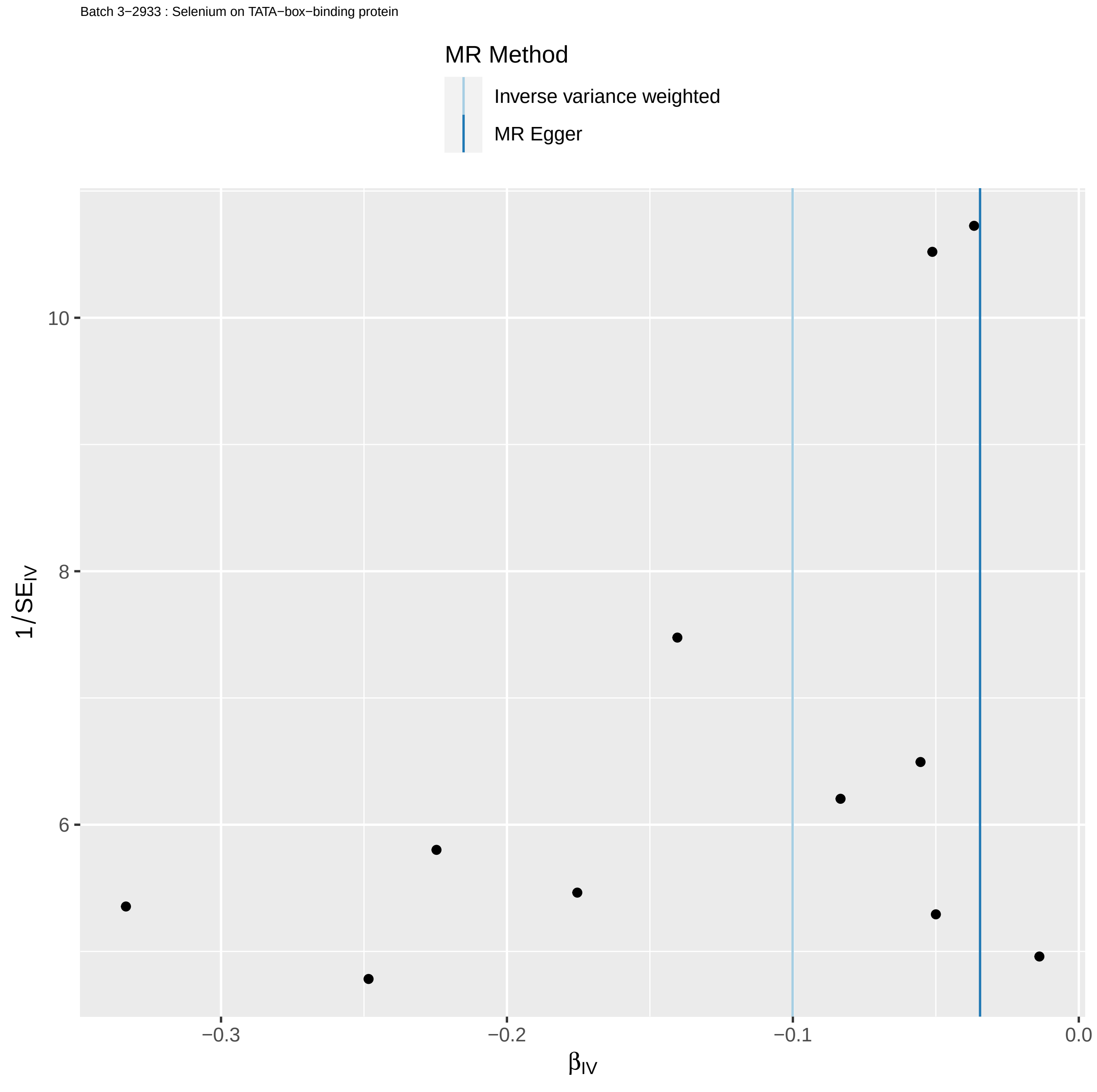
Batch 3−2933 : Selenium on TATA−box−binding protein
MR Method
Inverse variance weighted
MR Egger
10
8
6
$1/SE_{IV}$
−0.3
−0.2
−0.1
0.0
β_{IV}

Batch 3−2934 : Selenium on T−box transcription factor TBX22

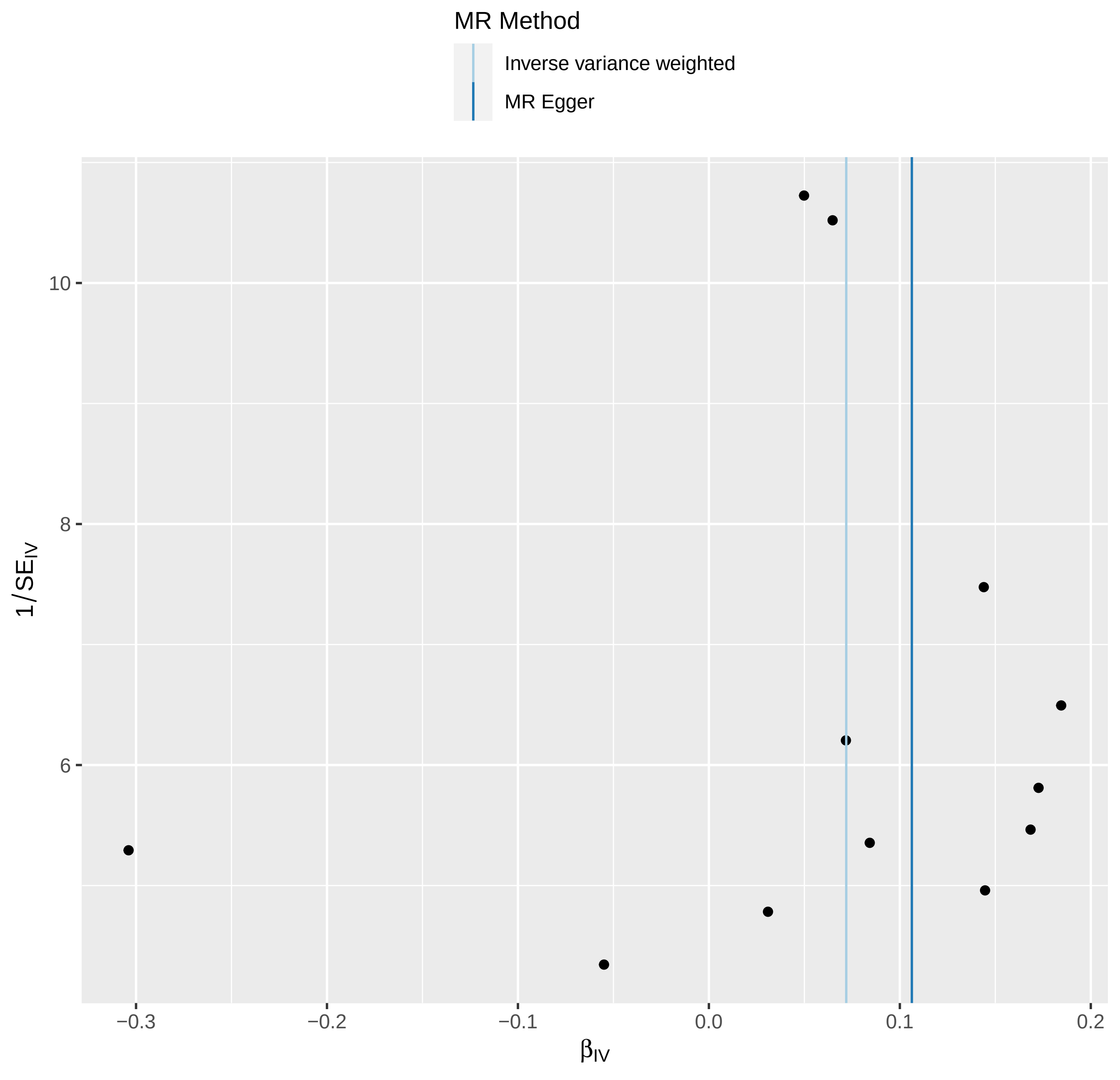

Batch 3−2935 : Selenium on T−box transcription factor TBX3

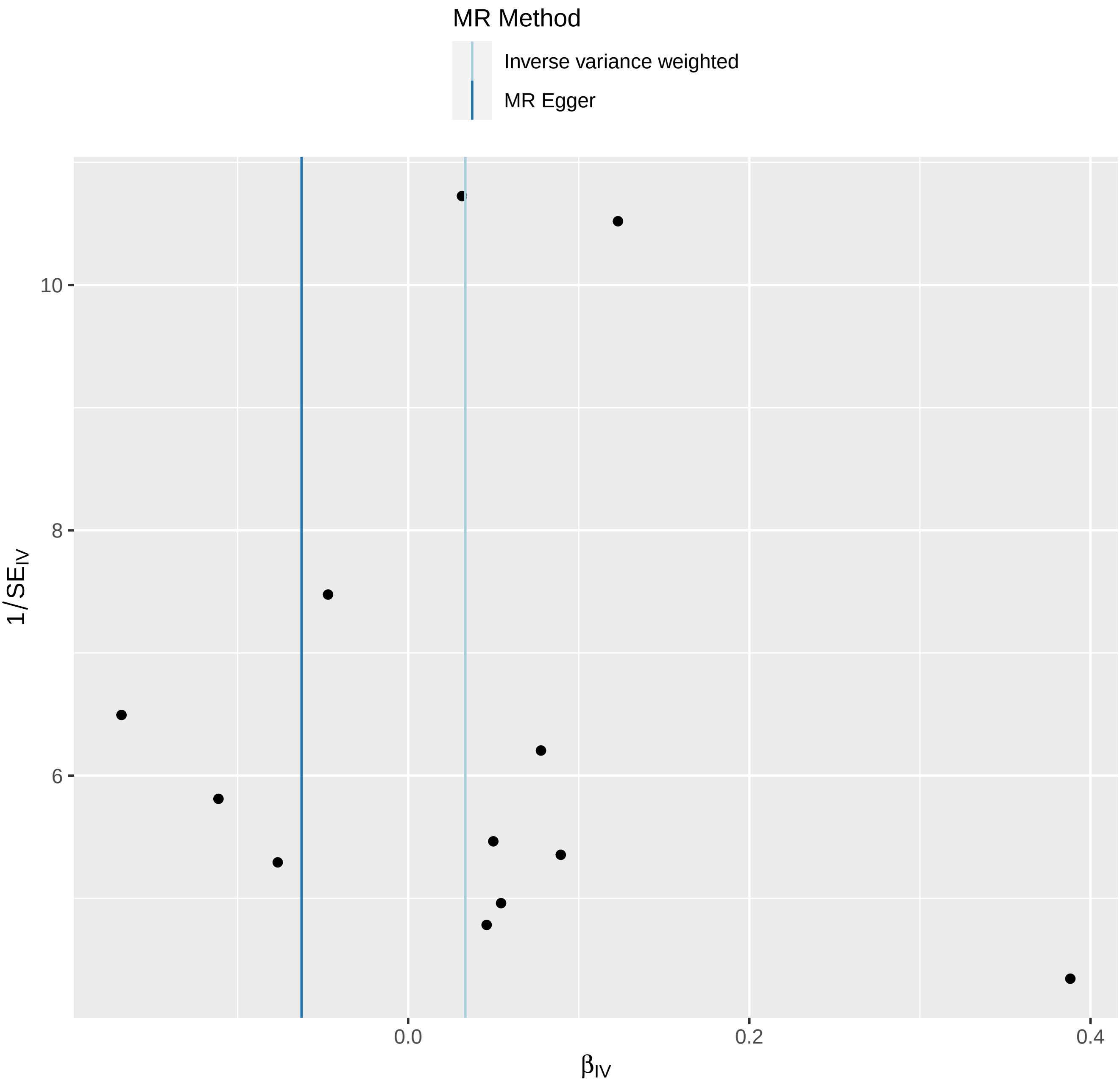

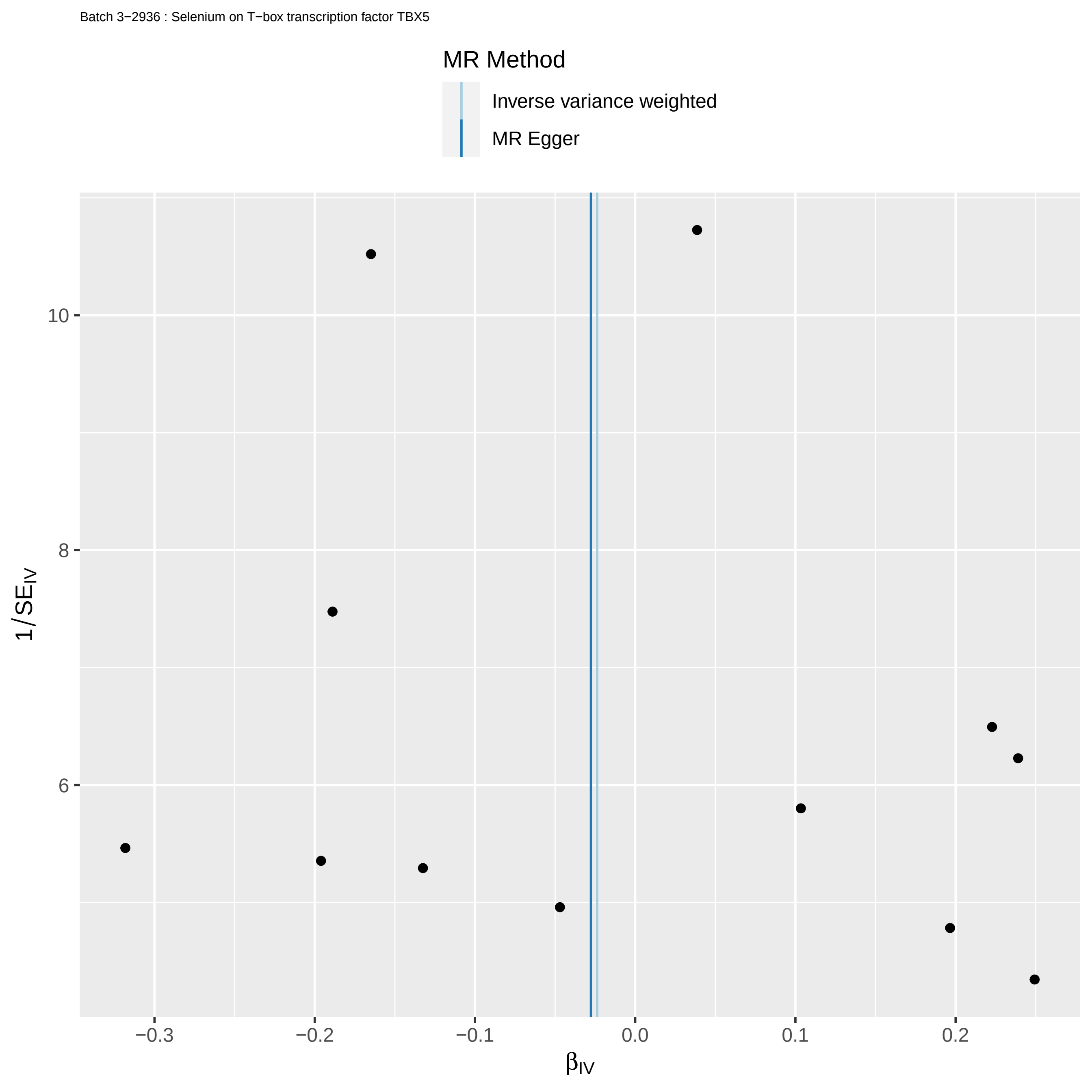

Batch 3−2936 : Selenium on T−box transcription factor TBX5
MR Method
Inverse variance weighted
MR Egger
10
8
6
1/SE_IV
−0.3
−0.2
−0.1
0.0
0.1
0.2
β_IV

Batch 3−2937 : Selenium on Thromboxane−A synthase

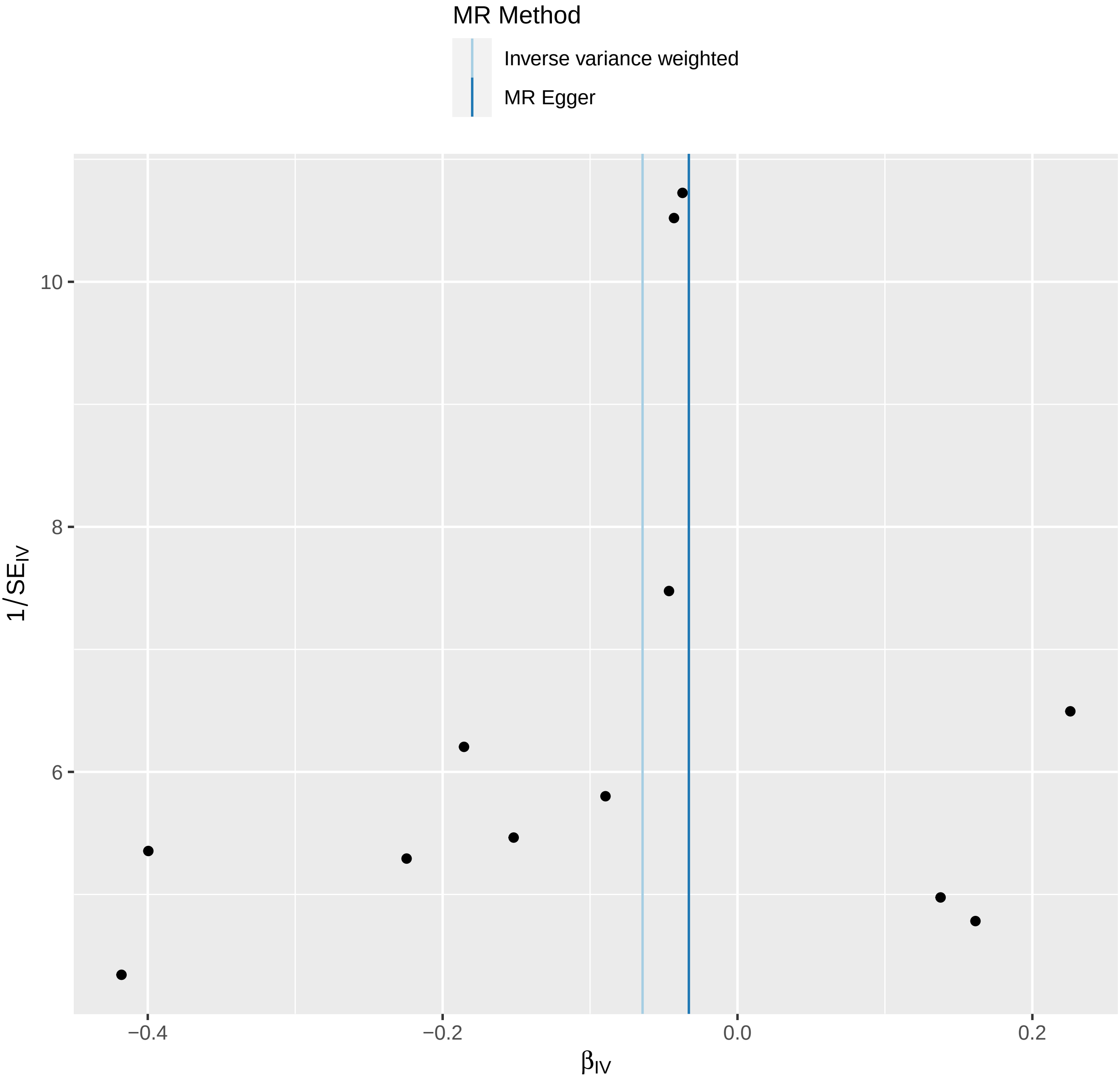

Batch 3−2938 : Selenium on Transcobalamin−1

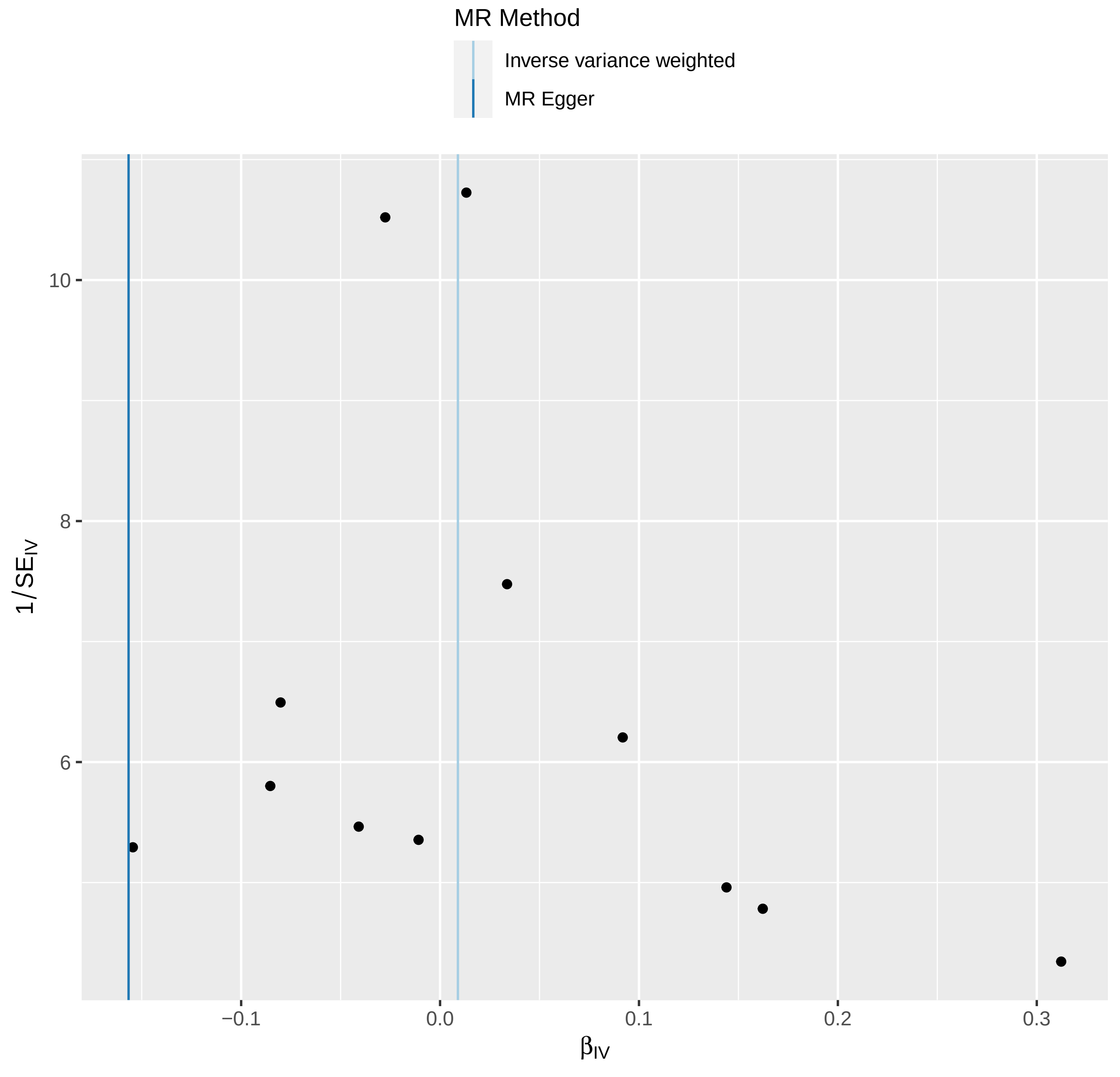

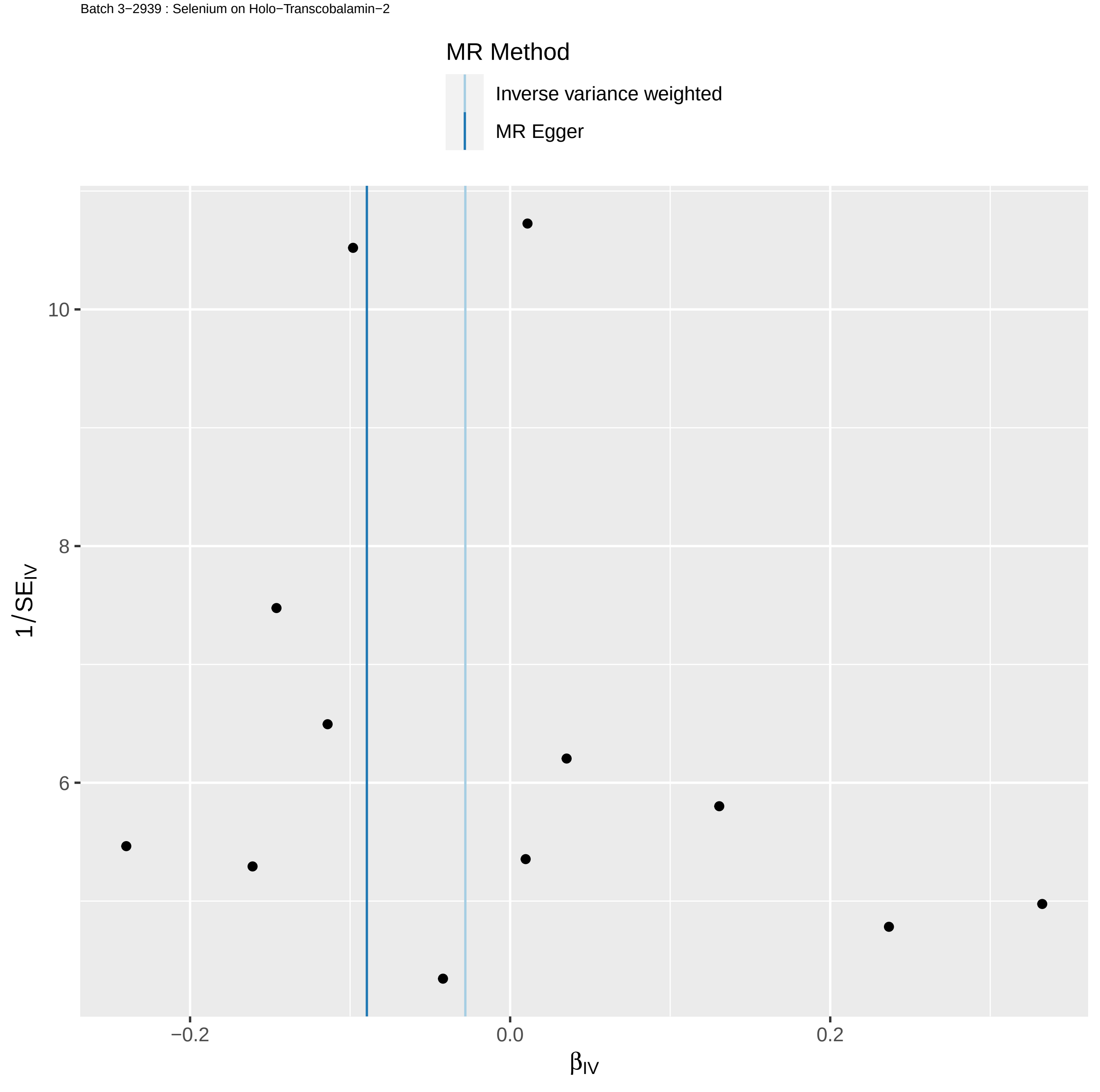

Batch 3−2939 : Selenium on Holo−Transcobalamin−2
MR Method
Inverse variance weighted
MR Egger
10
8
6
1/SE_IV
−0.2
0.0
0.2
β_IV

Batch 3−2940 : Selenium on Teratocarcinoma−derived growth factor 1

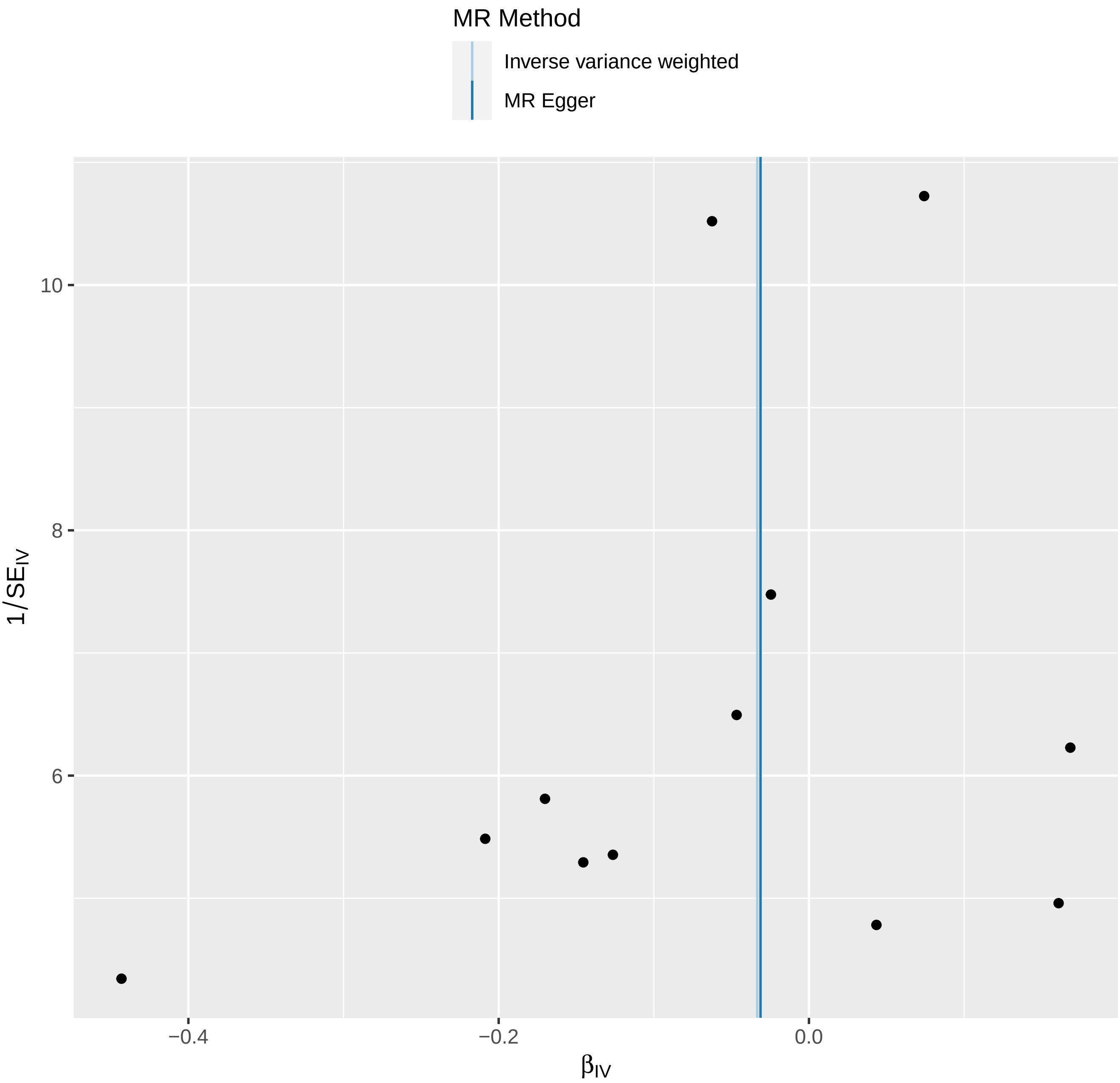

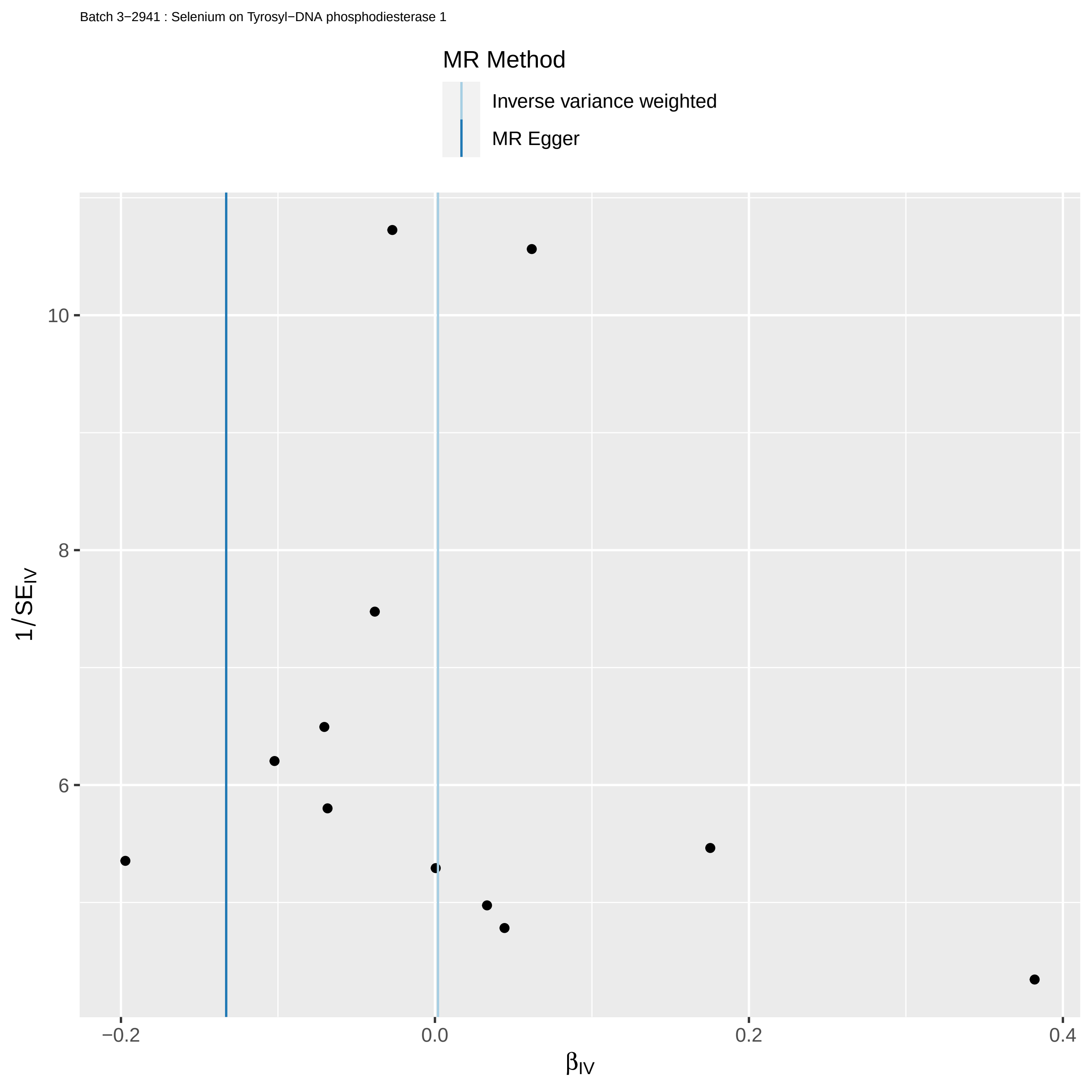

Batch 3−2941 : Selenium on Tyrosyl−DNA phosphodiesterase 1
MR Method
Inverse variance weighted
MR Egger
10
8
6
1/SE_IV
−0.2
0.0
0.2
0.4
β_IV

Batch 3−2942 : Selenium on Transcriptional enhancer factor TEF−5

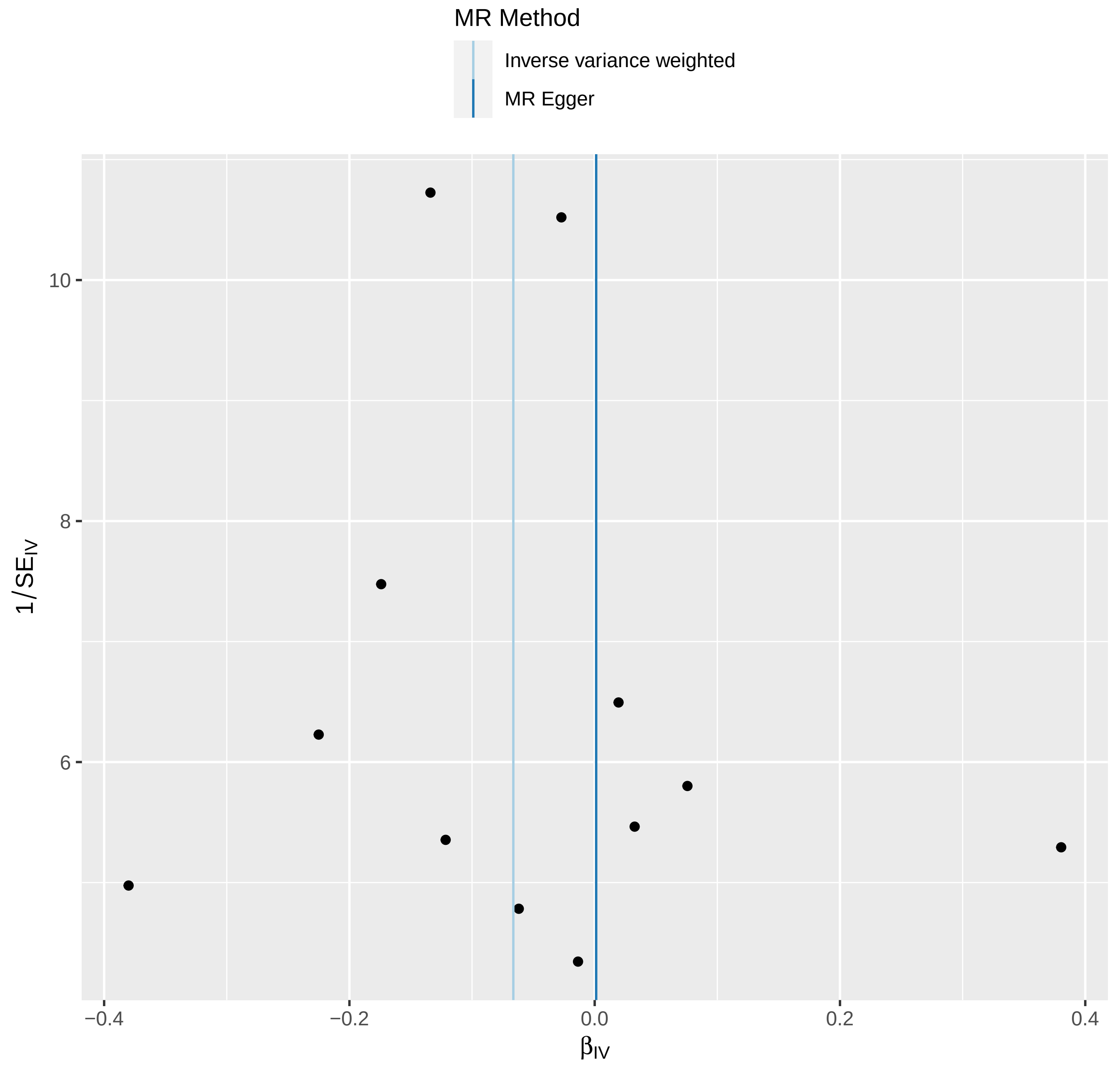

Batch 3−2943 : Selenium on Transcriptional enhancer factor TEF−3

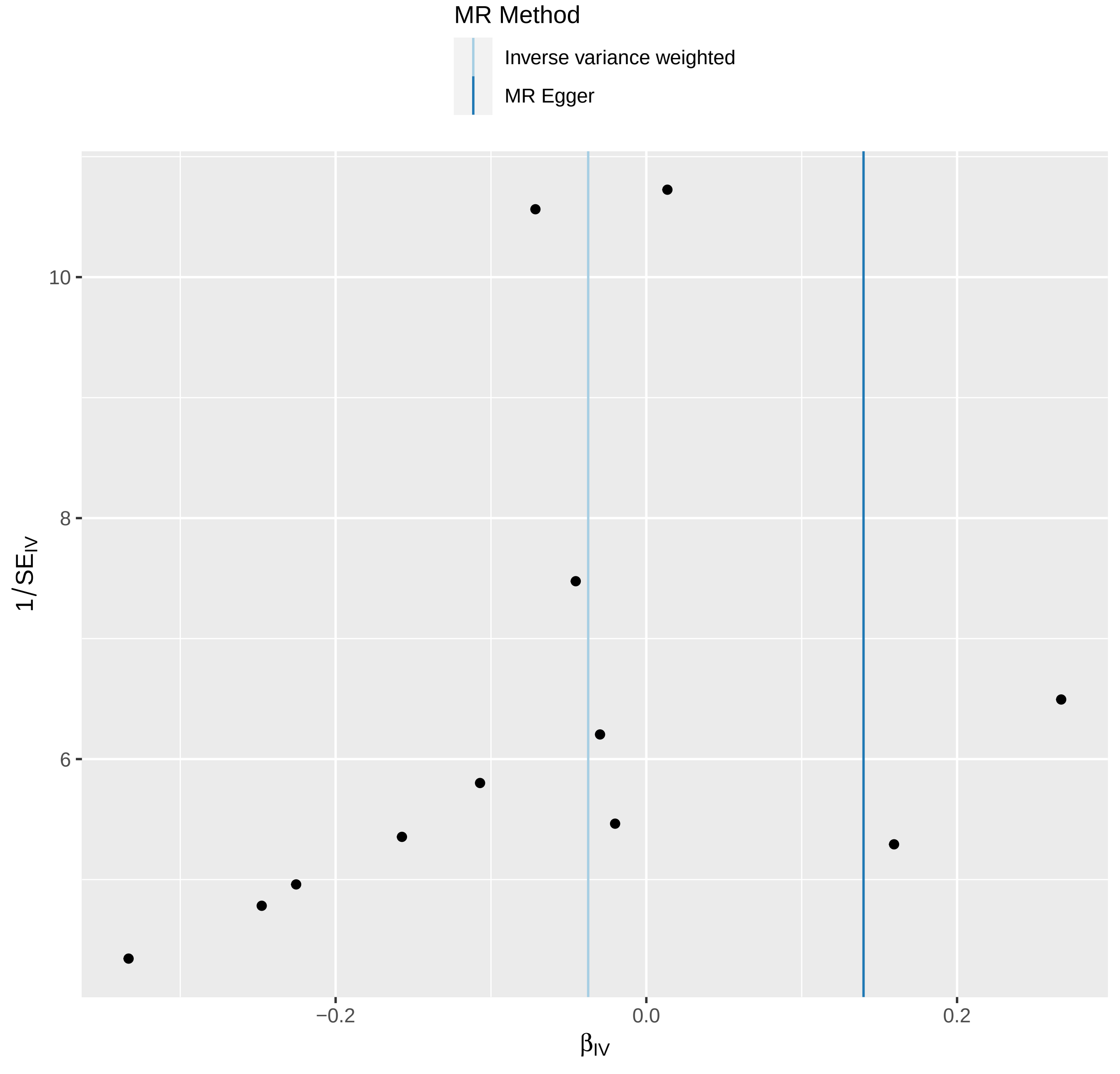

Batch 3−2944 : Selenium on Angiopoietin−1 receptor, soluble

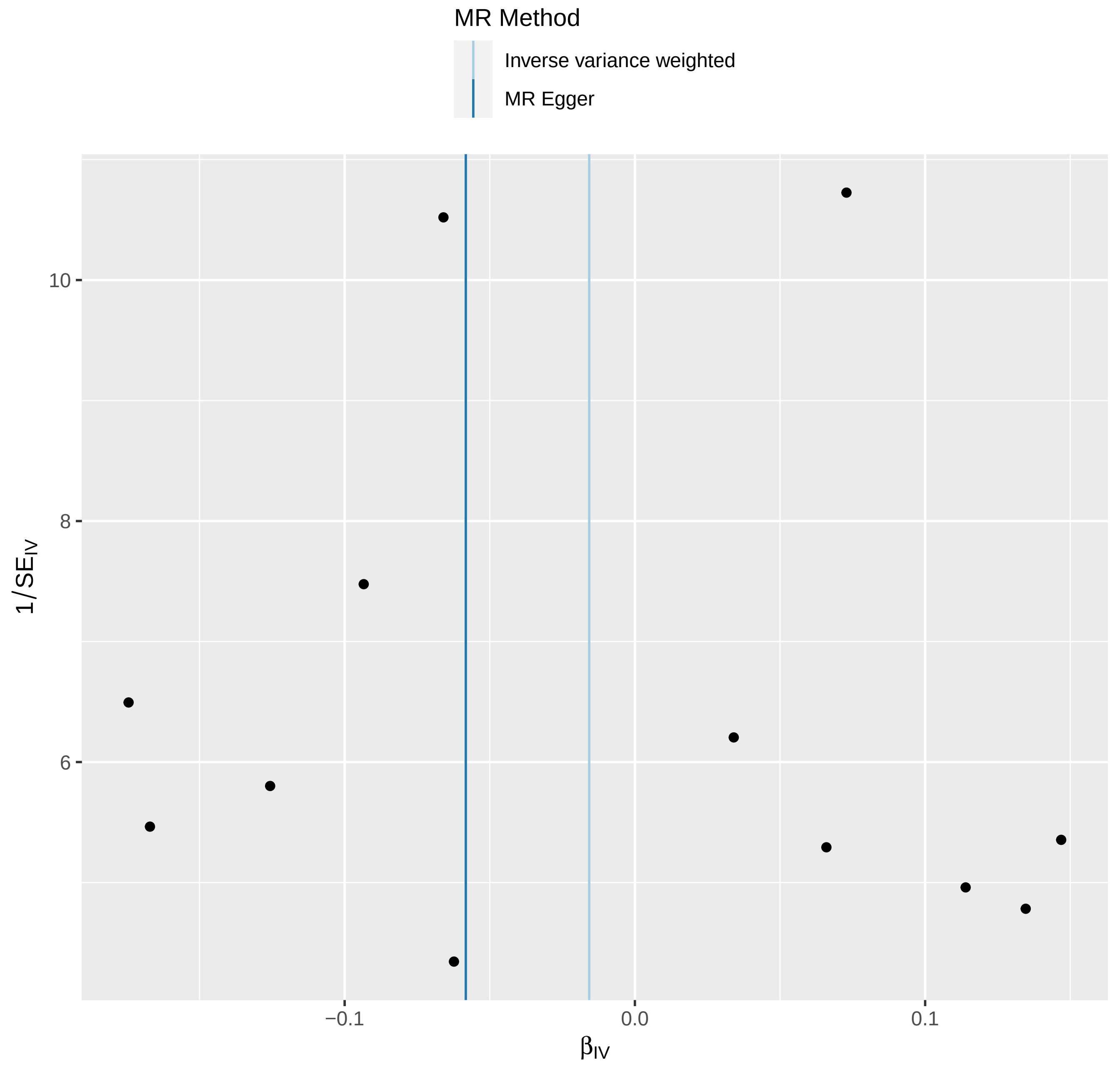

Batch 3−2945 : Selenium on Teneurin−3

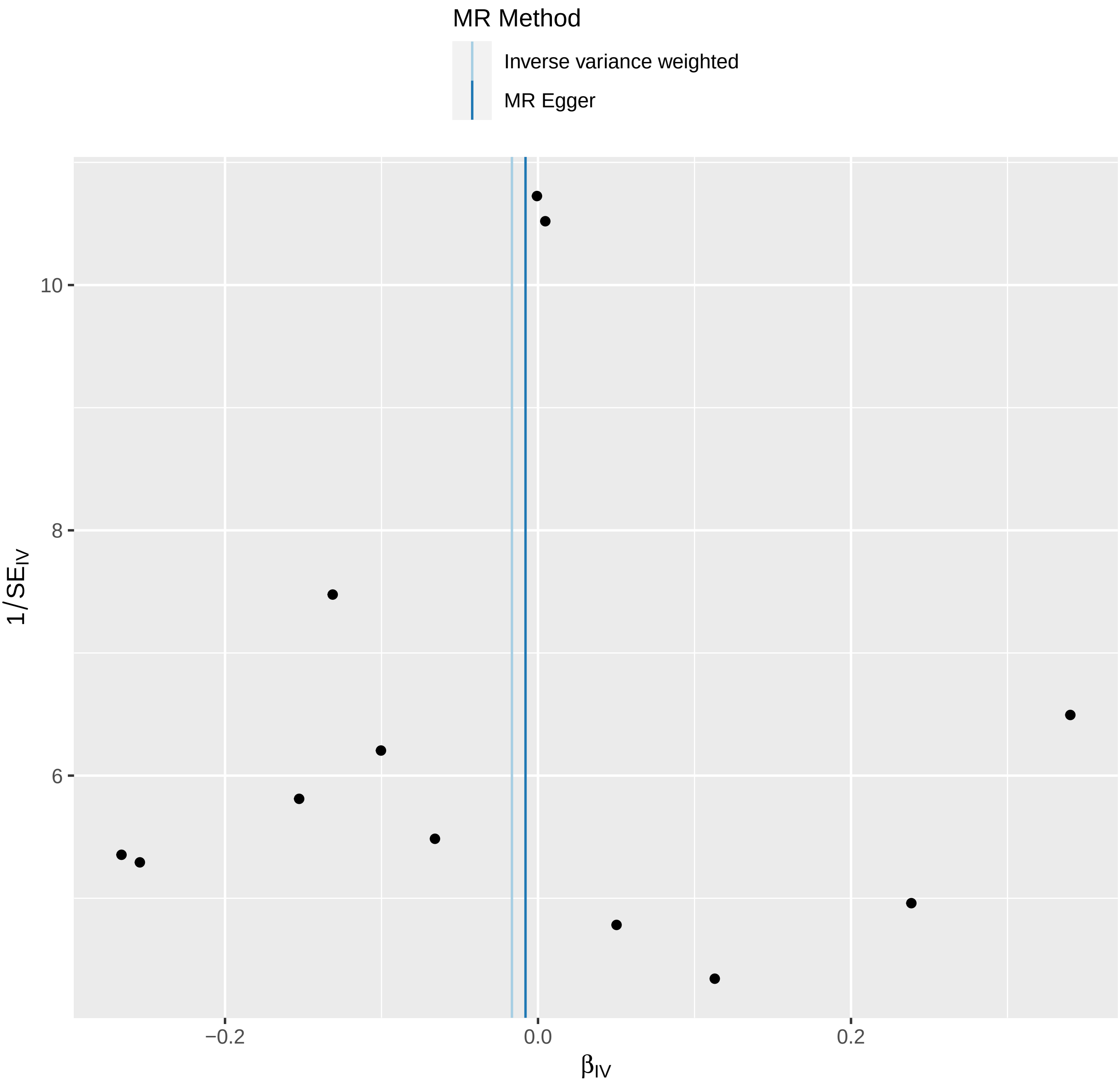

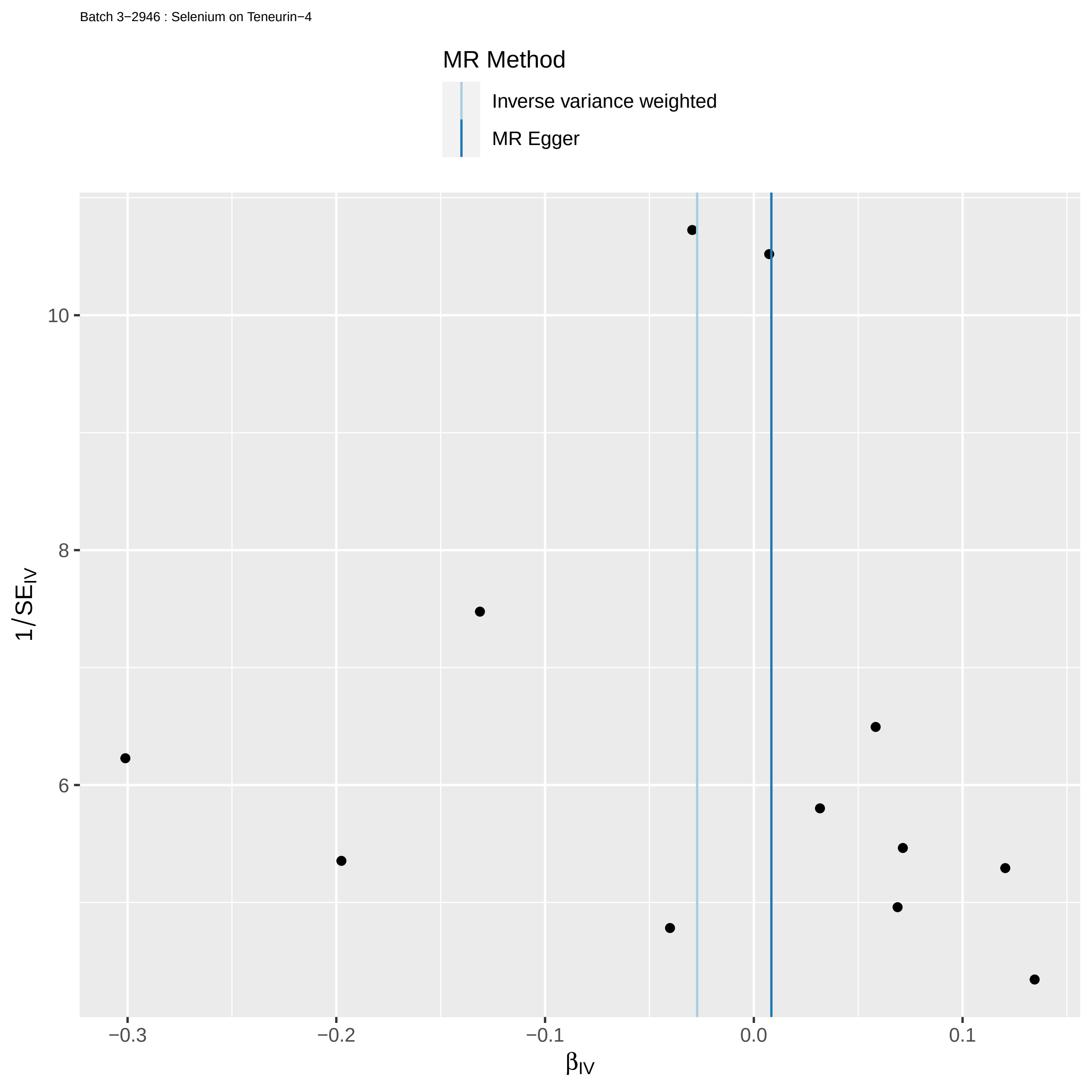

Batch 3−2946 : Selenium on Teneurin−4
MR Method
Inverse variance weighted
MR Egger
10
8
6
1/SE_IV
−0.3
−0.2
−0.1
0.0
0.1
β_IV

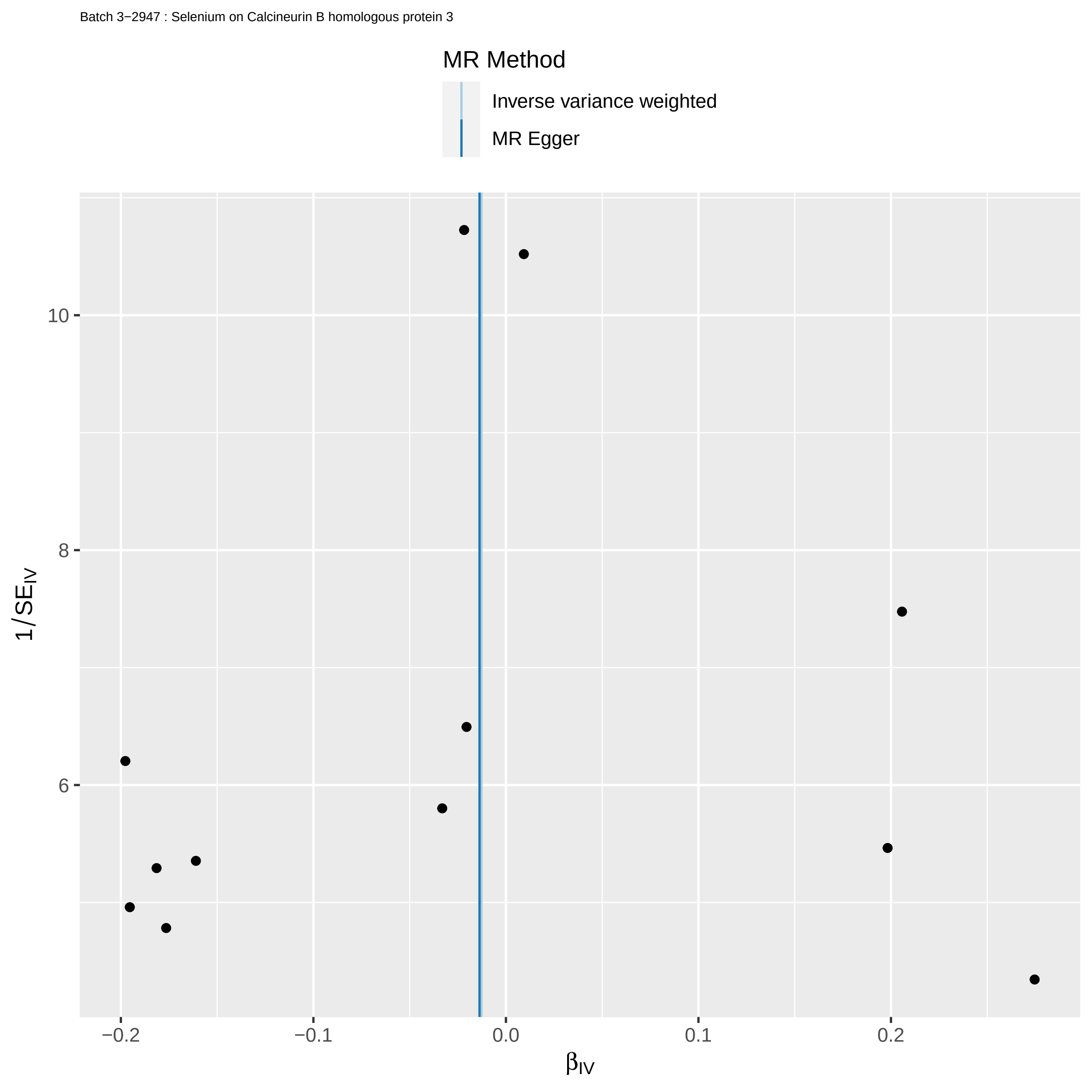

Batch 3−2947 : Selenium on Calcineurin B homologous protein 3
MR Method
Inverse variance weighted
MR Egger
10
8
6
$1/SE_{IV}$
−0.2
−0.1
0.0
0.1
0.2
β_{IV}

Batch 3−2948 : Selenium on Testis−expressed sequence 29 protein

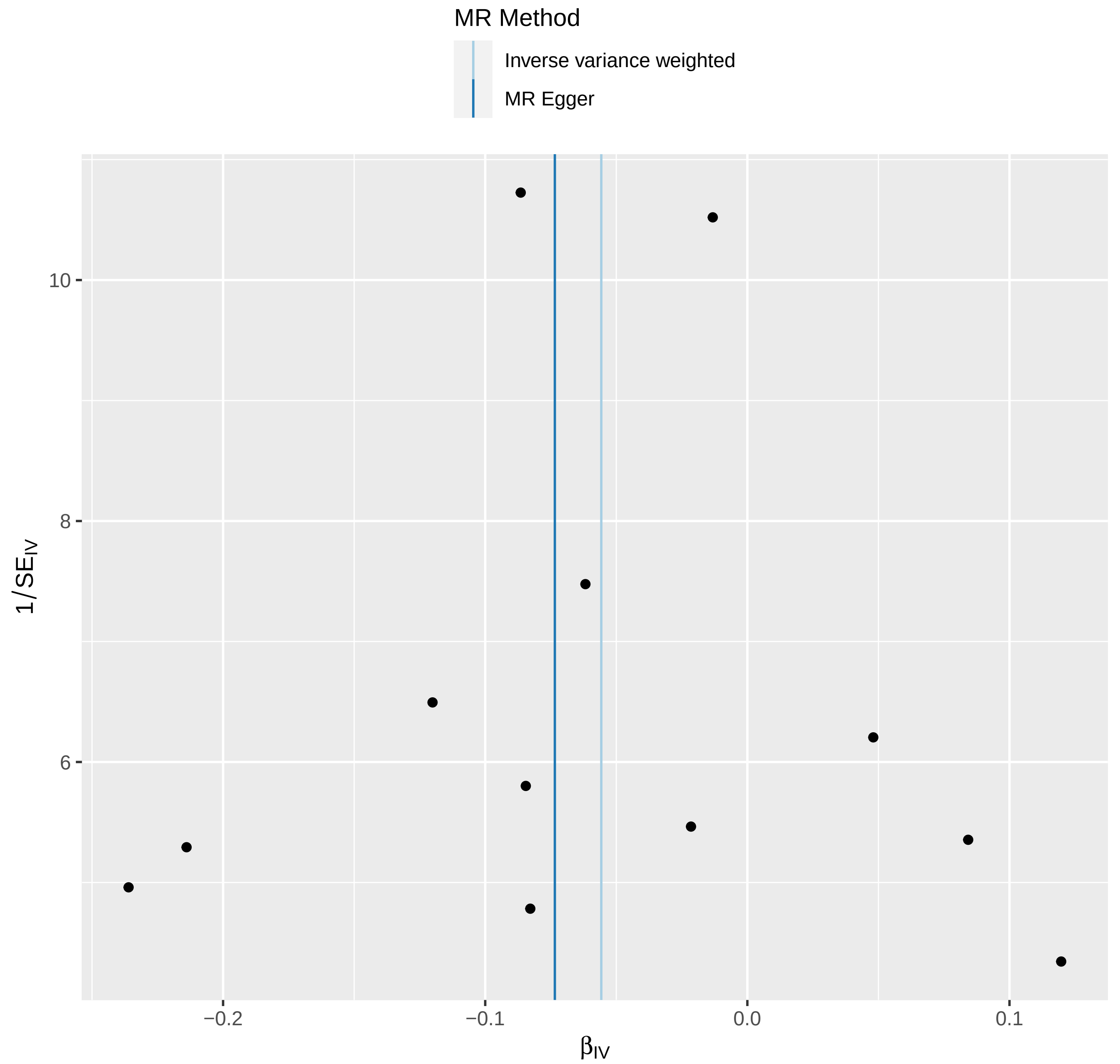

Batch 3−2949 : Selenium on Testis−expressed sequence 29 protein

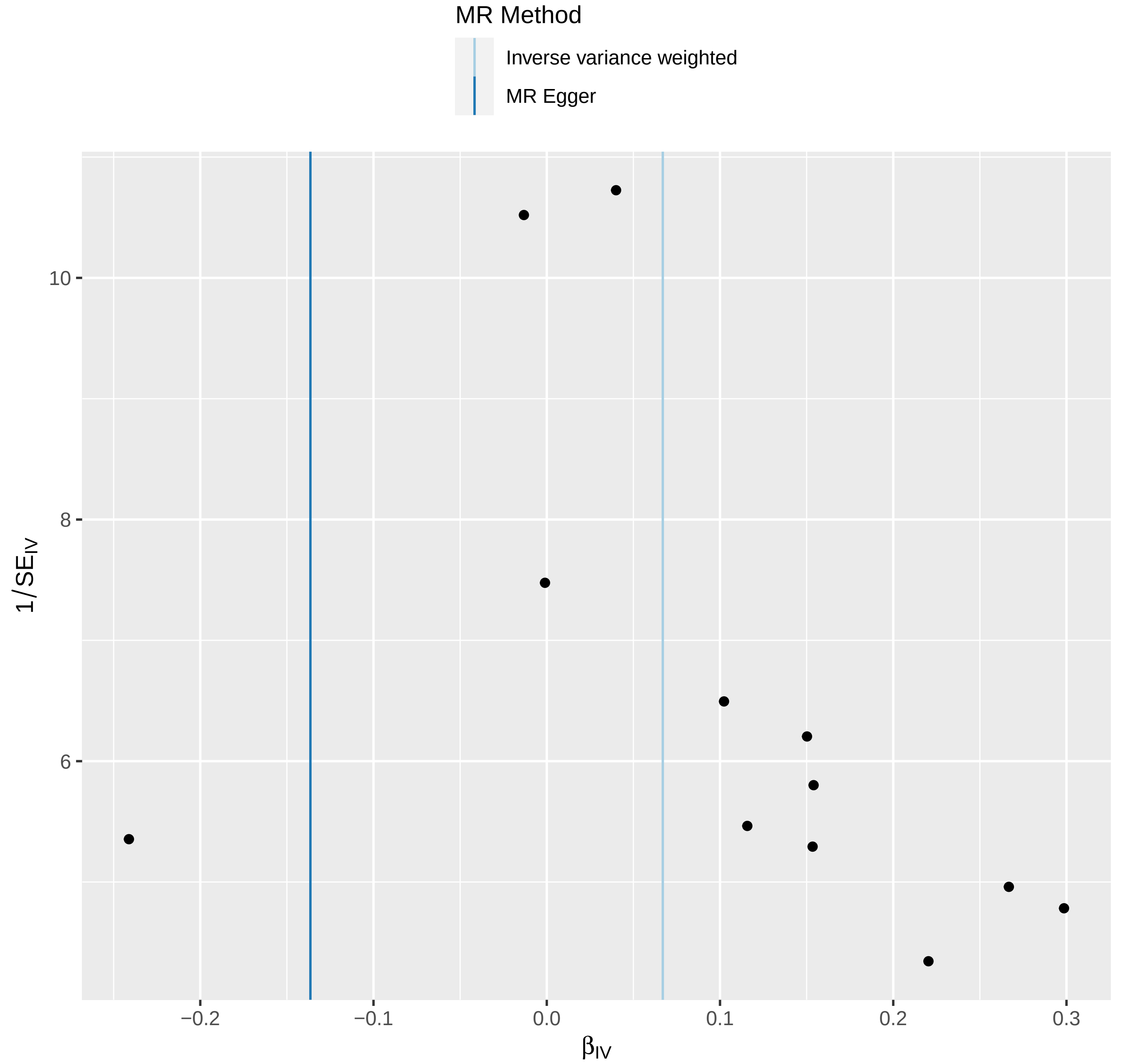

Batch 3−2950 : Selenium on Serotransferrin

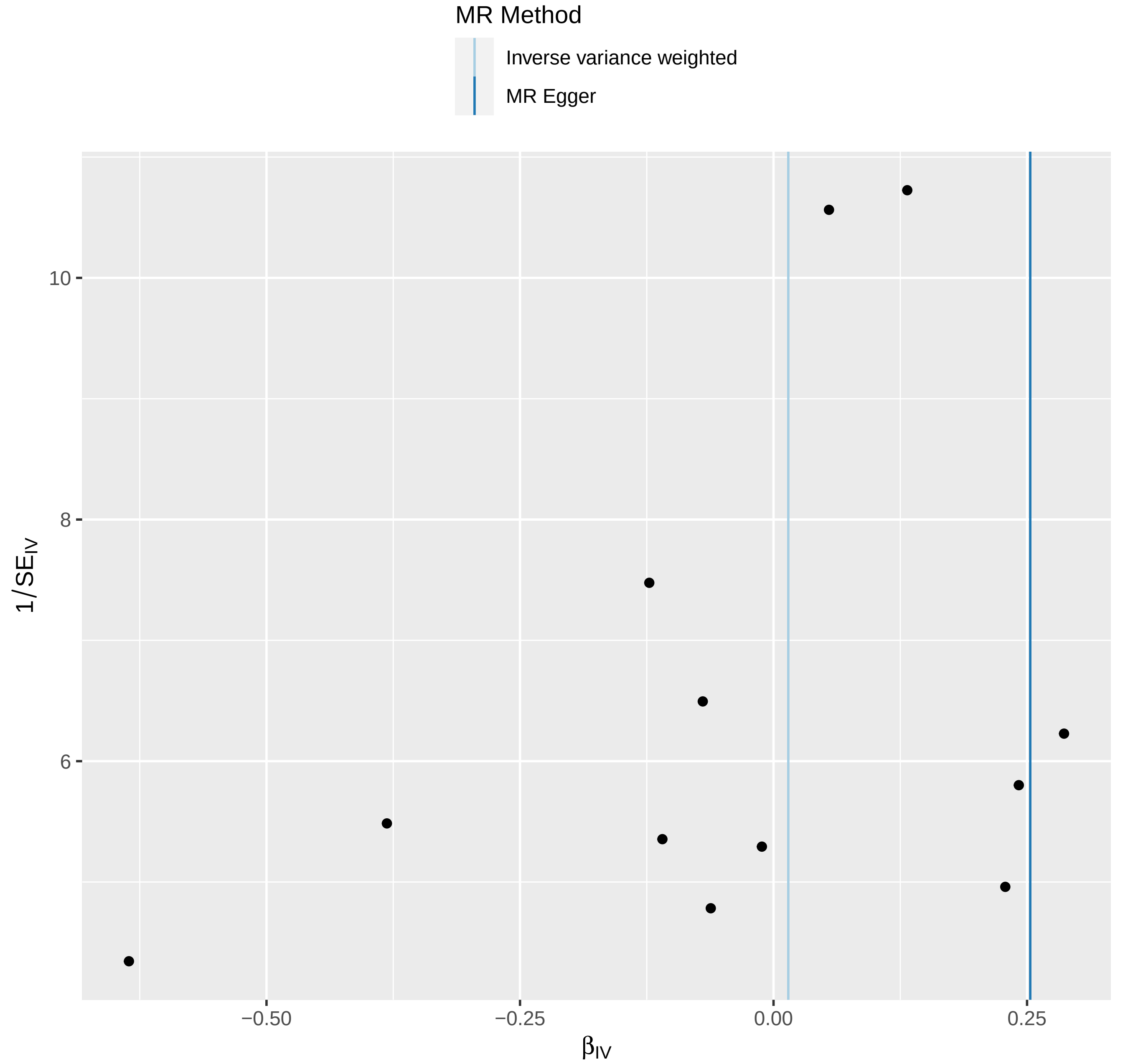

Batch 3−2951 : Selenium on Trefoil factor 1

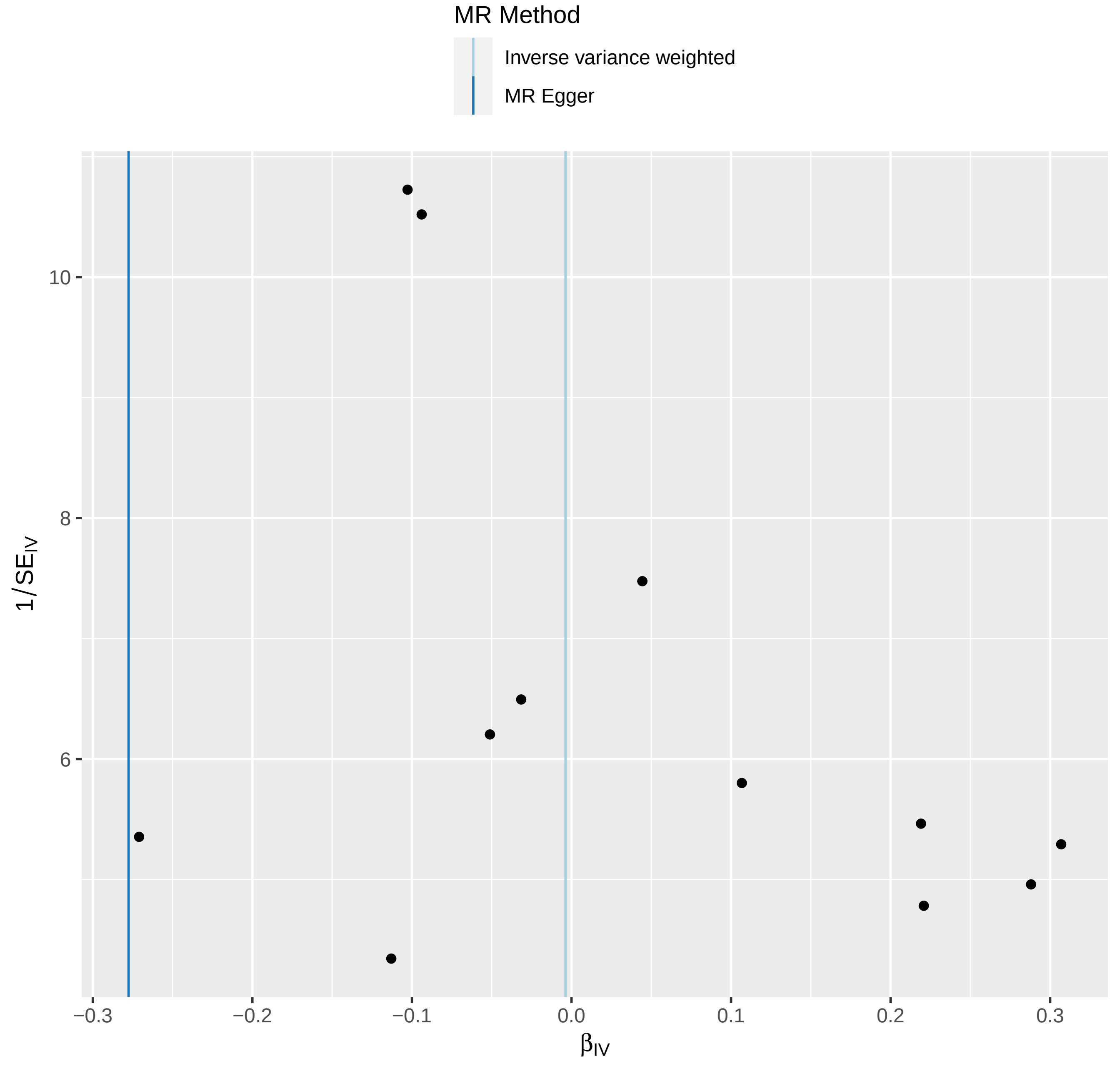

Batch 3−2952 : Selenium on Trefoil factor 2

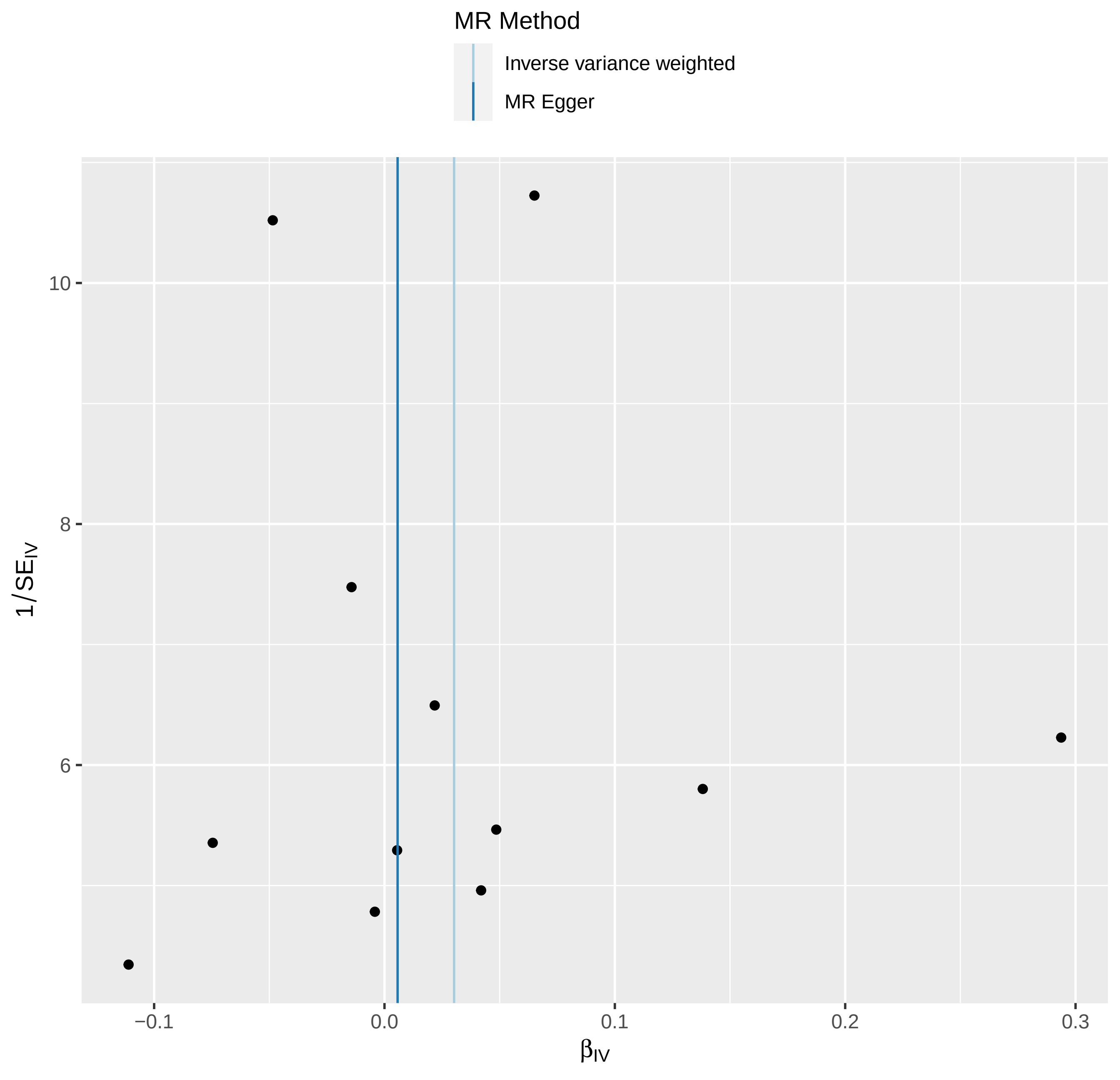

Batch 3−2953 : Selenium on Trefoil factor 3

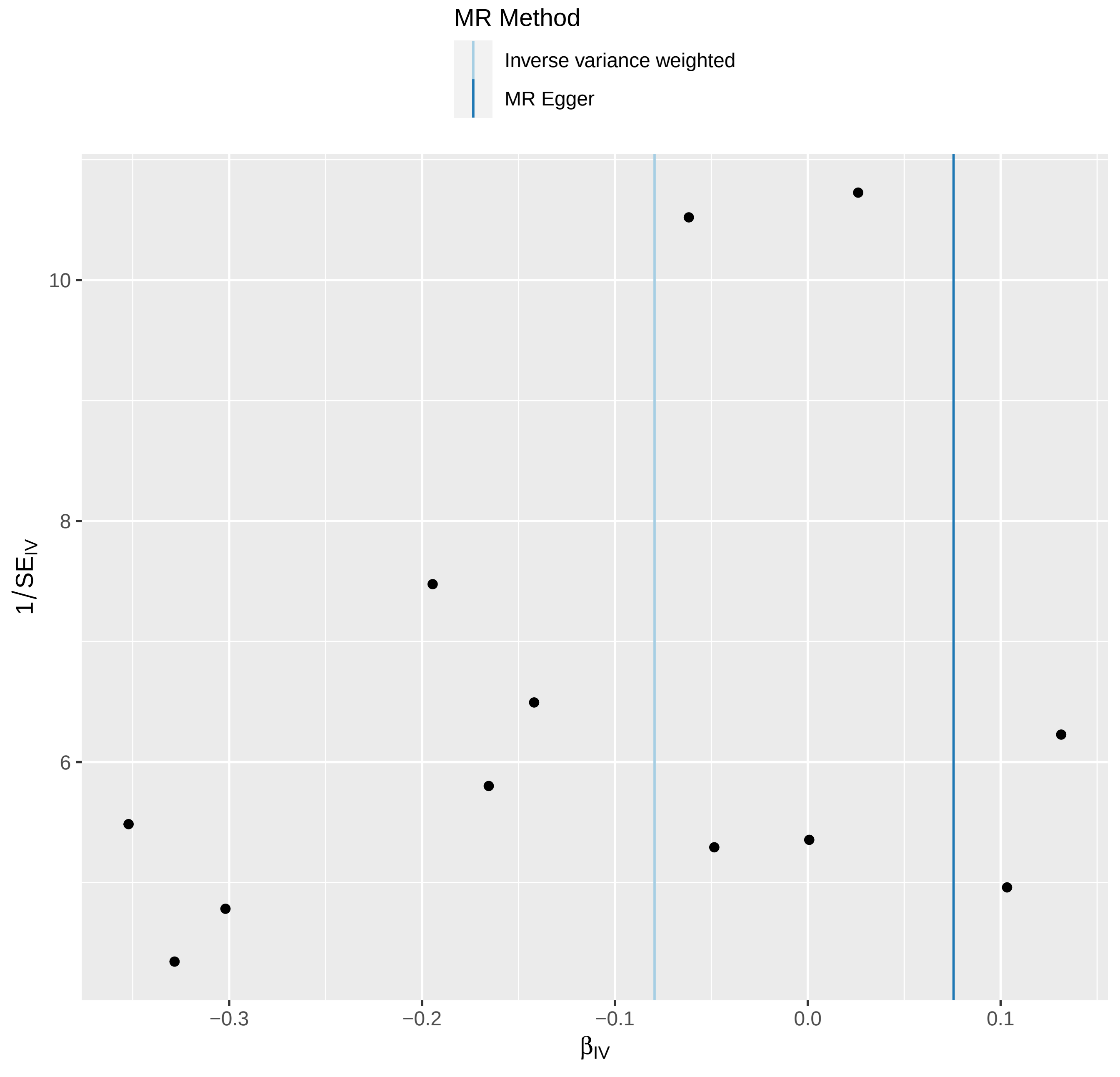

Batch 3−2954 : Selenium on Trefoil factor 3

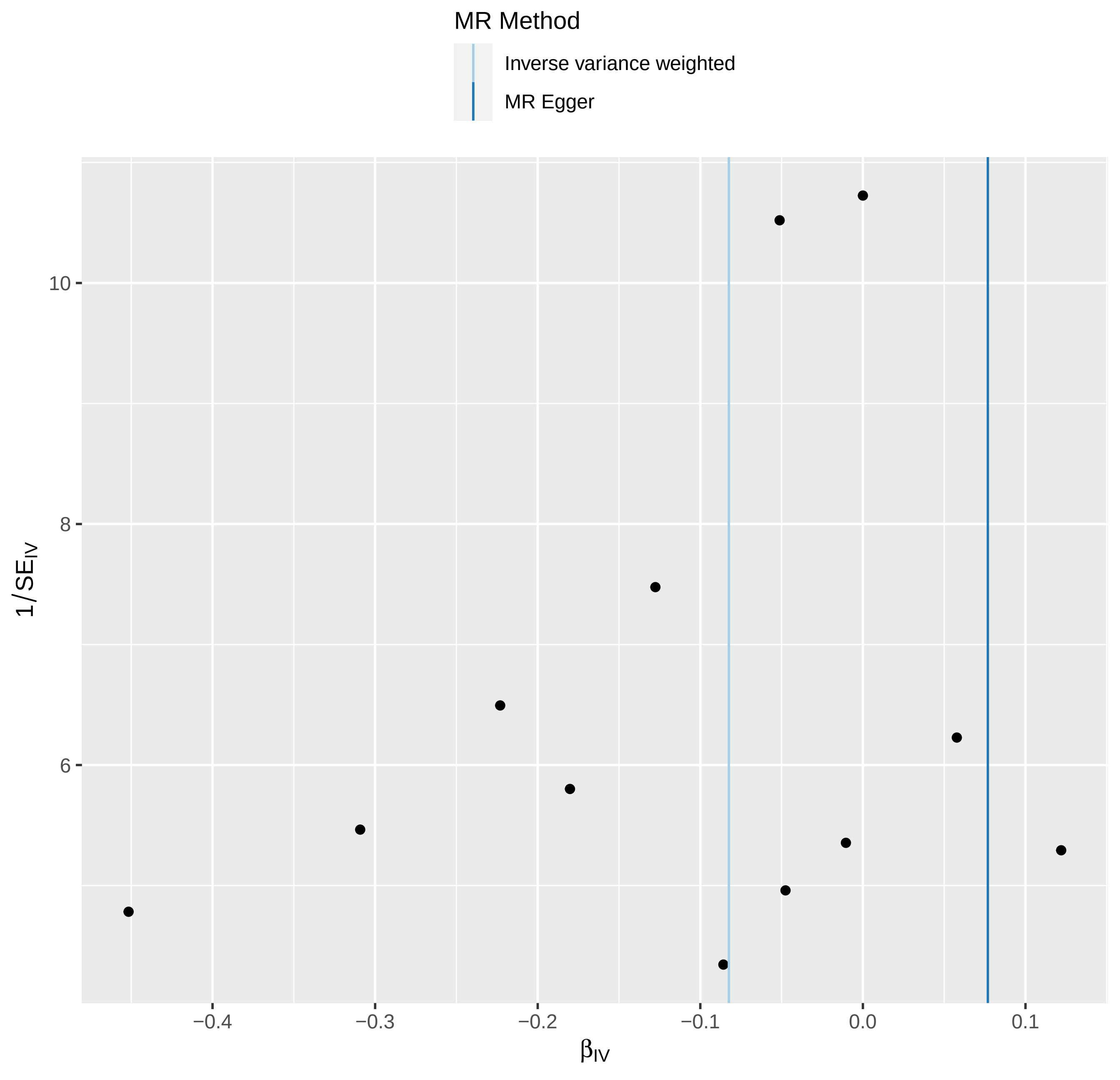

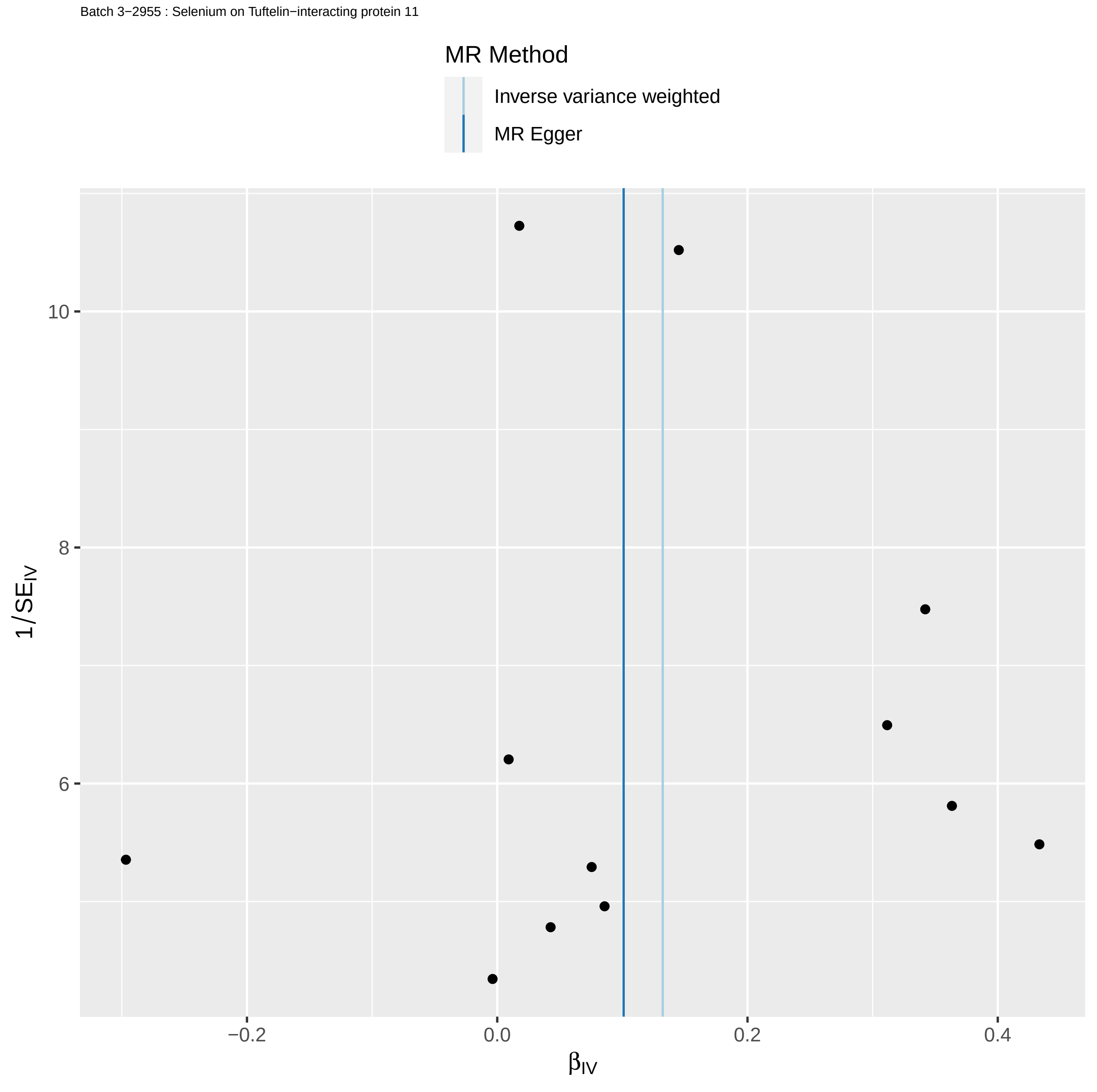

Batch 3−2955 : Selenium on Tuftelin−interacting protein 11
MR Method
Inverse variance weighted
MR Egger
10
8
6
1/SE$_{IV}$
−0.2
0.0
0.2
0.4
β$_{IV}$

Batch 3−2956 : Selenium on Tissue factor pathway inhibitor 2

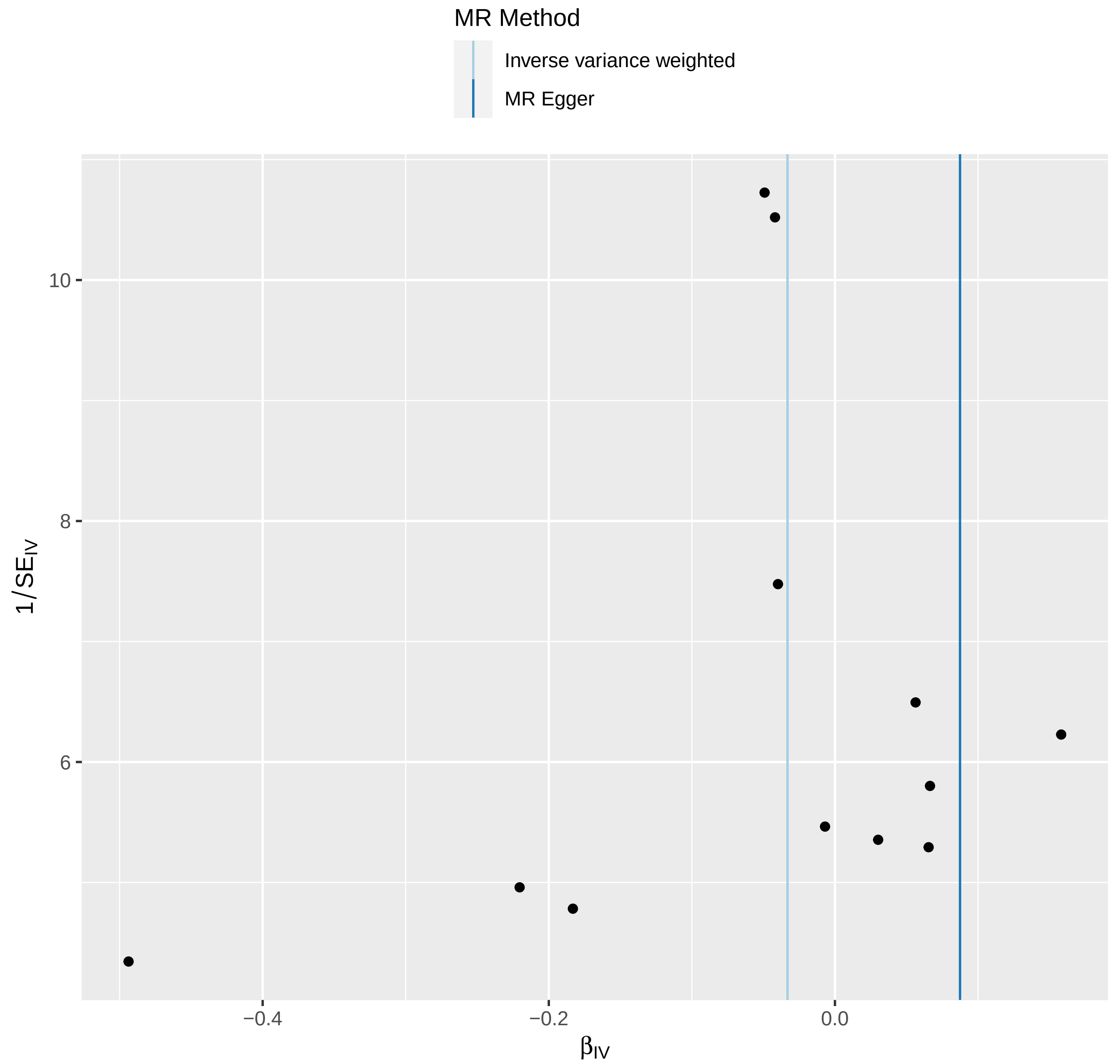

Batch 3−2957 : Selenium on Tissue factor pathway inhibitor

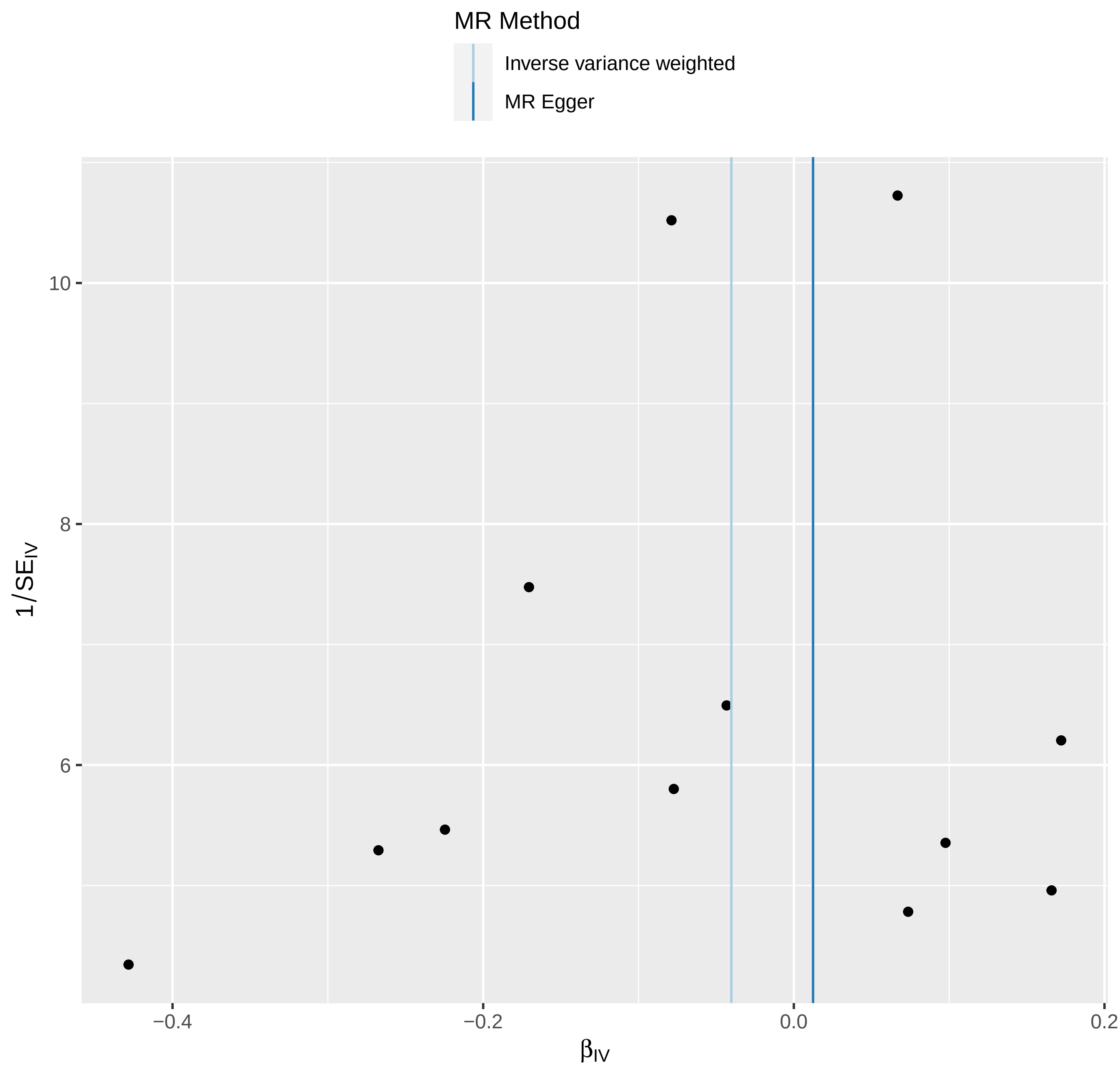

Batch 3−2958 : Selenium on Transferrin receptor protein 1

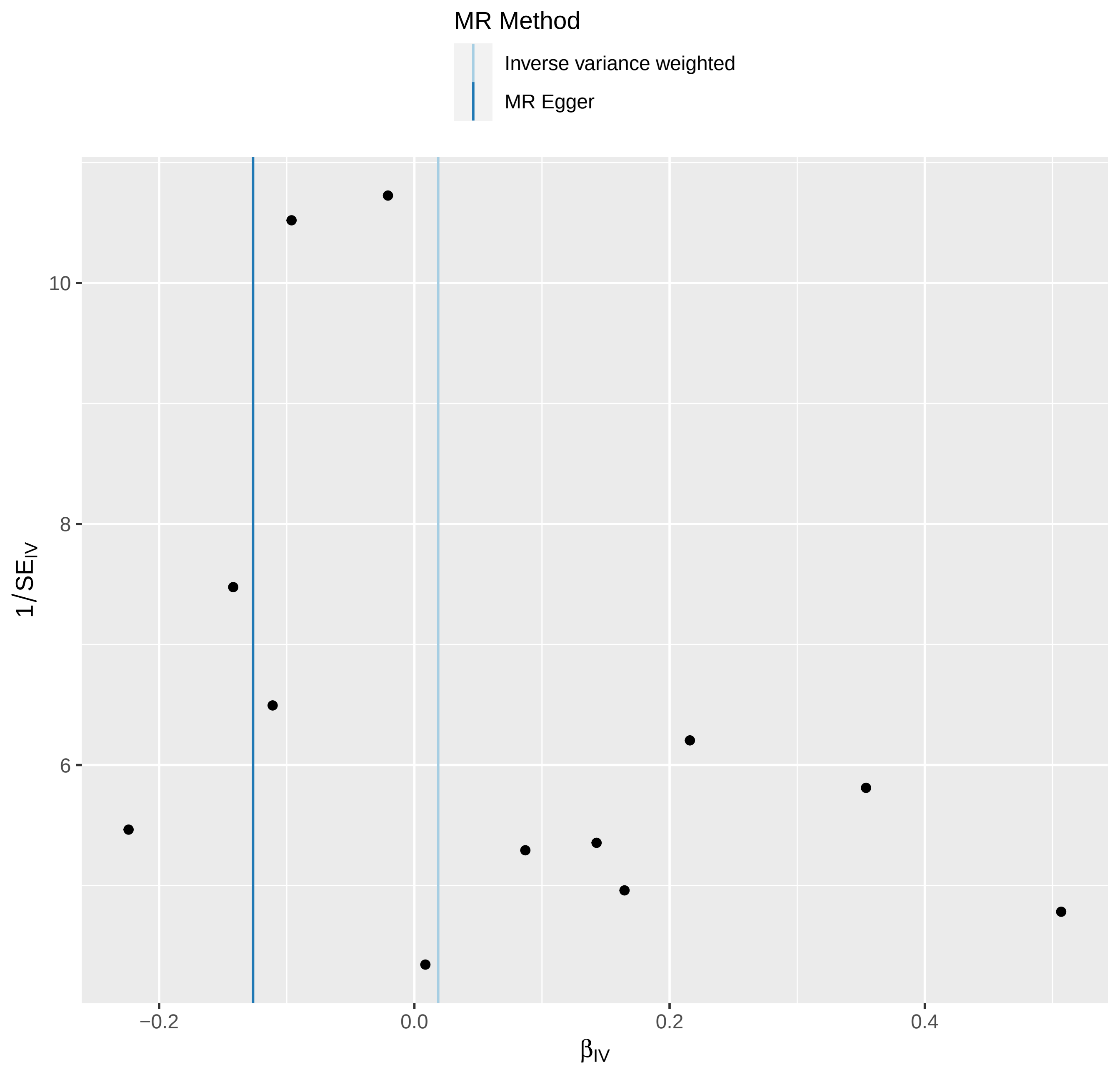

Batch 3−2959 : Selenium on Transferrin receptor protein 1

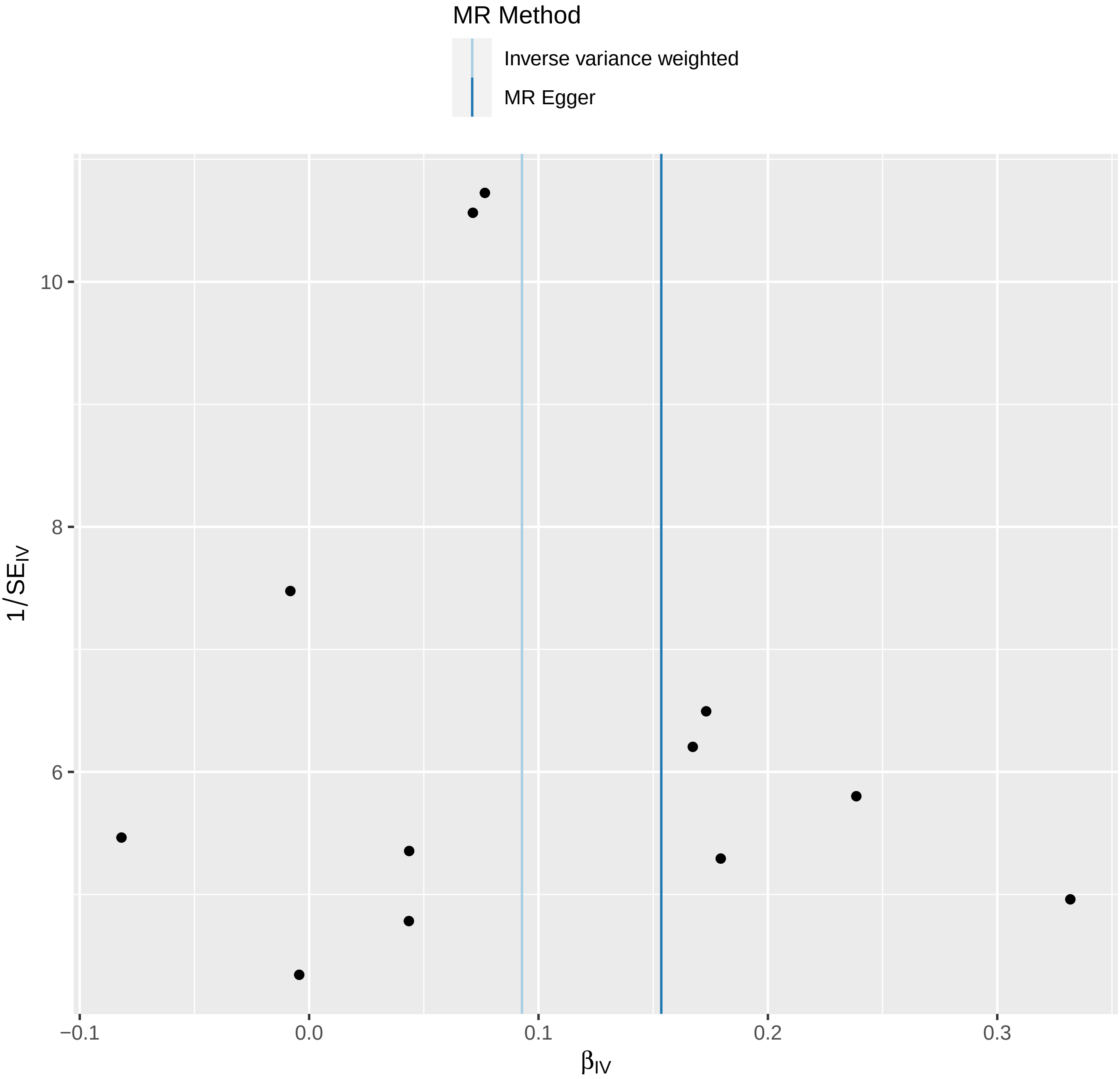

Batch 3−2960 : Selenium on Thyroglobulin

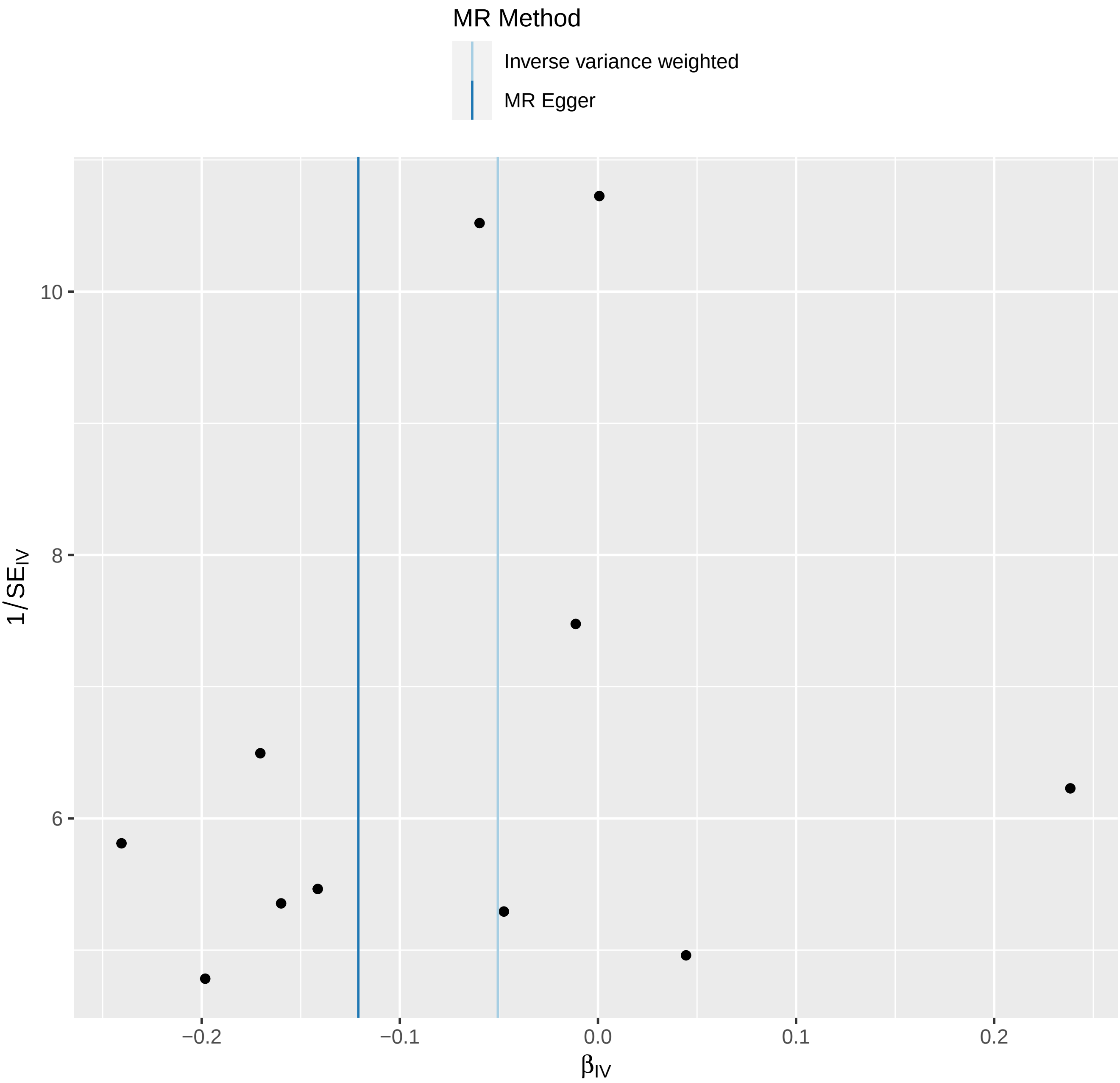

Batch 3−2961 : Selenium on Transforming growth factor alpha

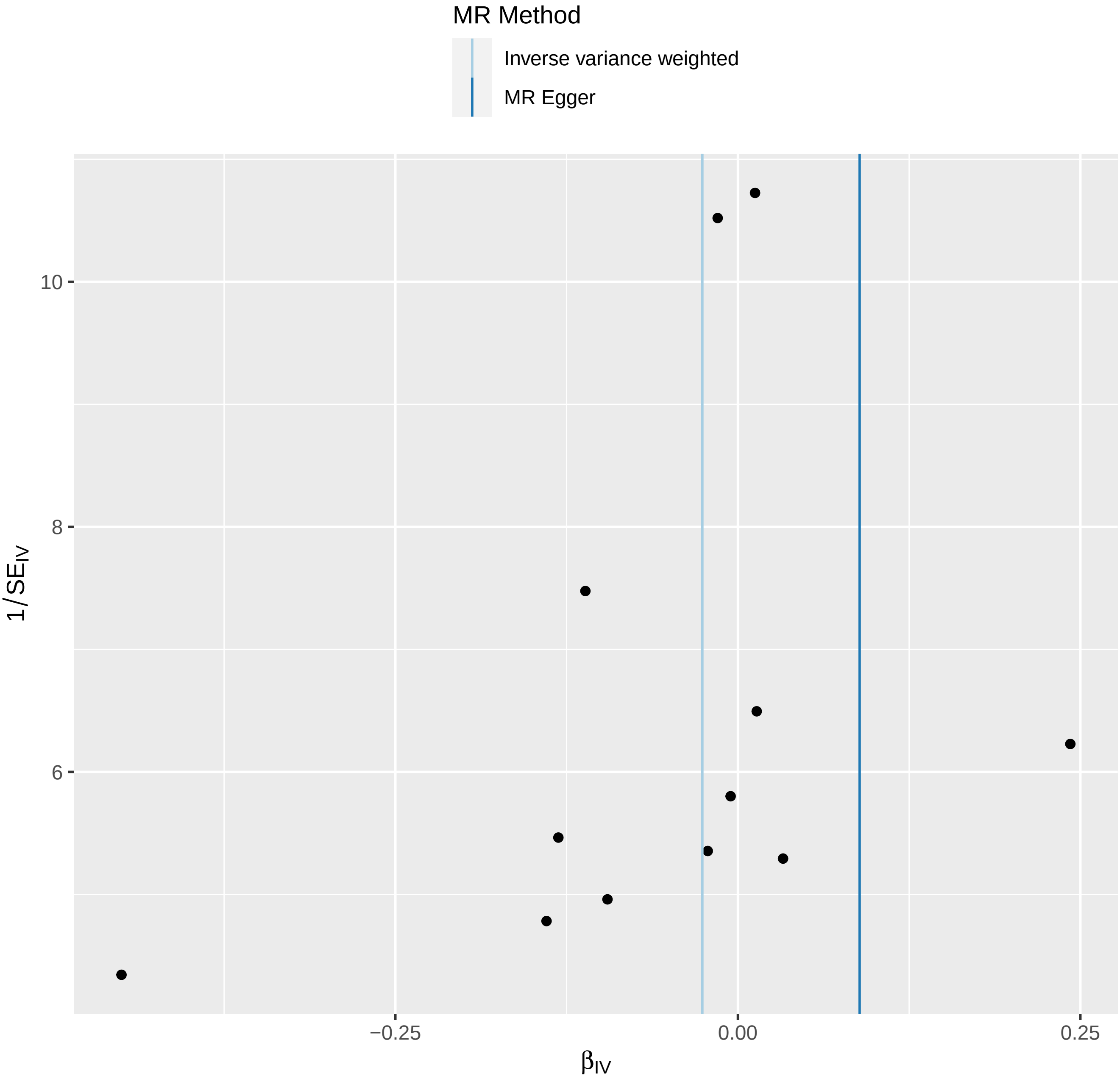

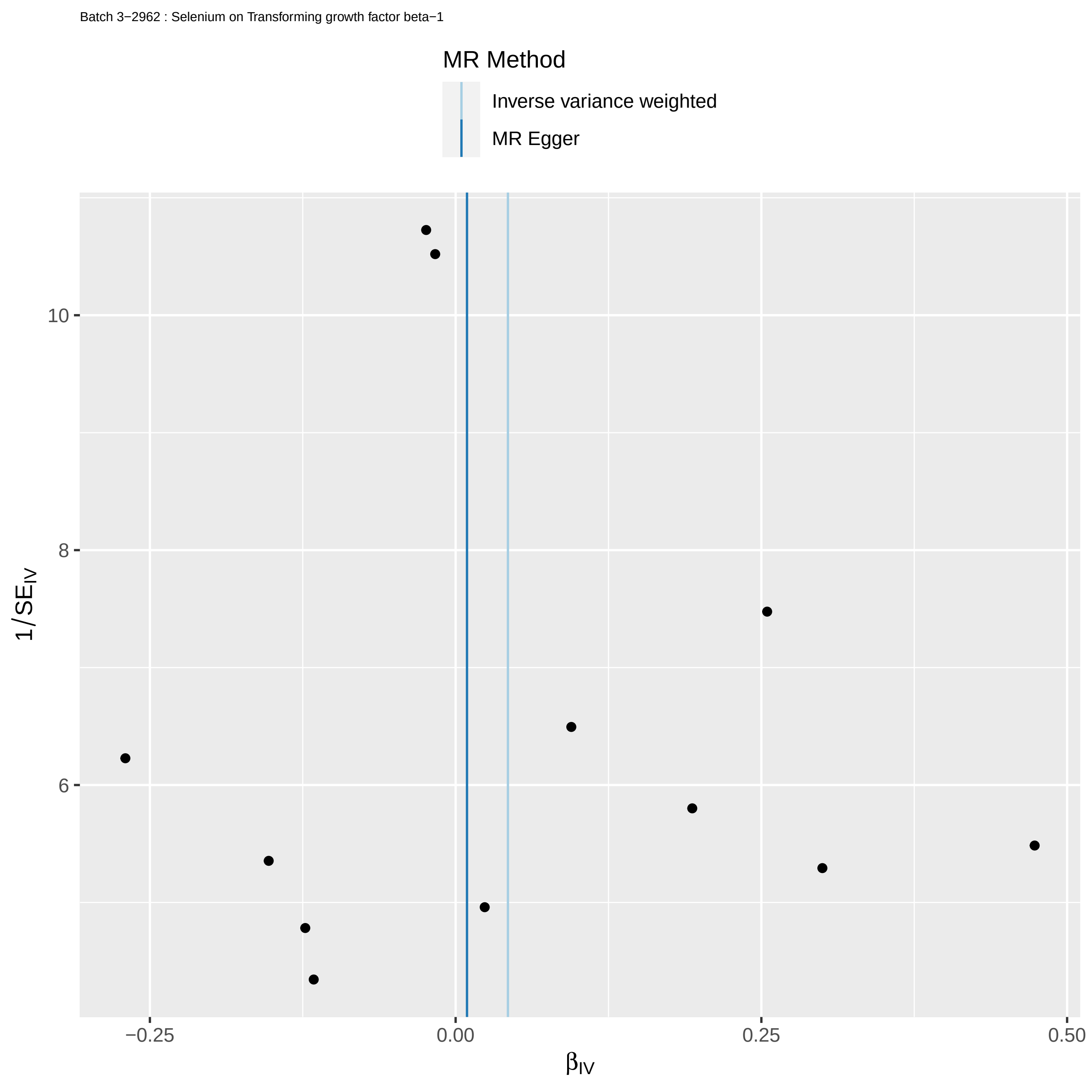
Batch 3−2962 : Selenium on Transforming growth factor beta−1
MR Method
Inverse variance weighted
MR Egger
10
8
6
1/SE$_{IV}$
−0.25
0.00
0.25
0.50
β$_{IV}$

Batch 3−2963 : Selenium on Transforming growth factor beta−1−induced transcript 1 protein

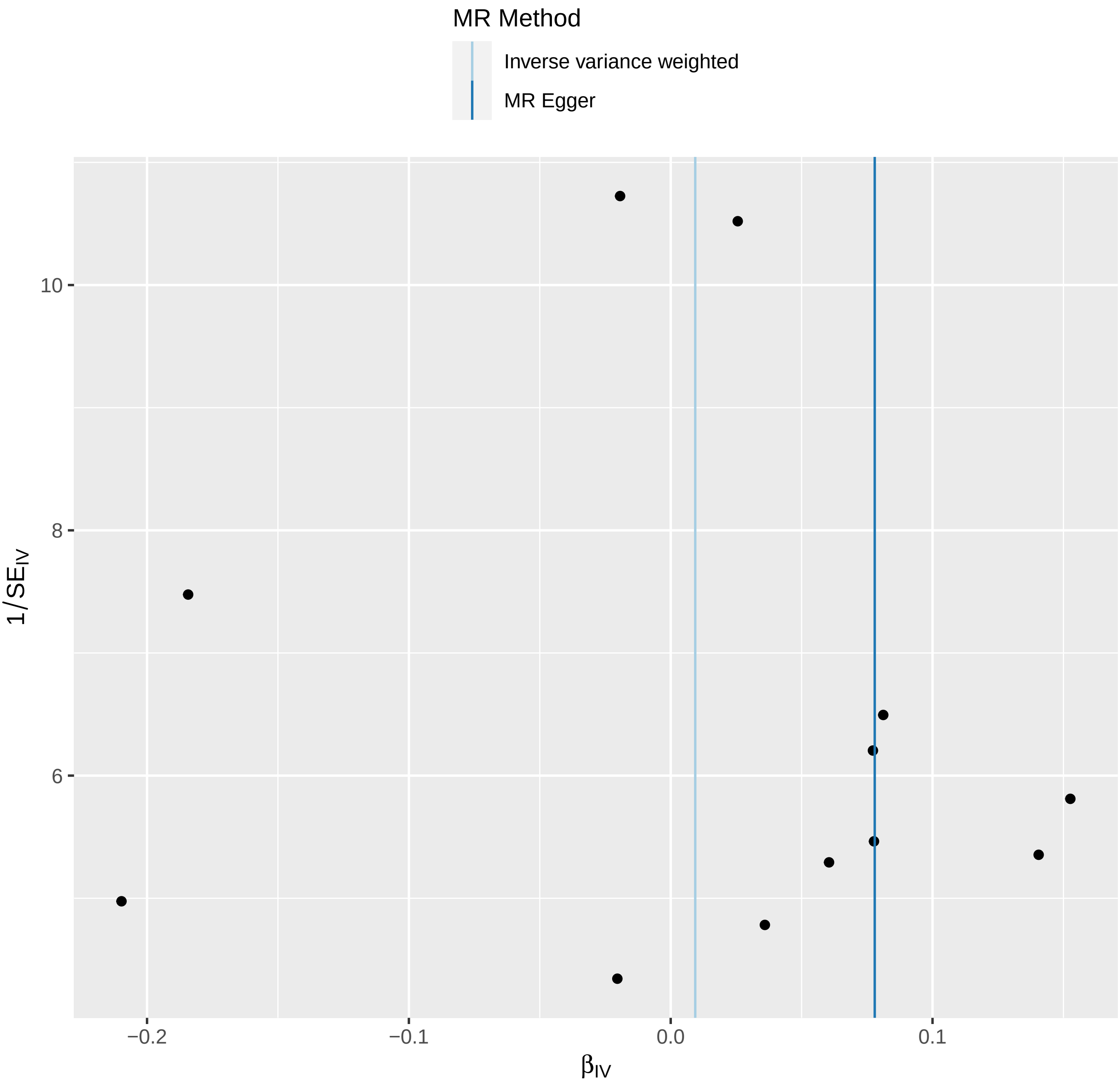

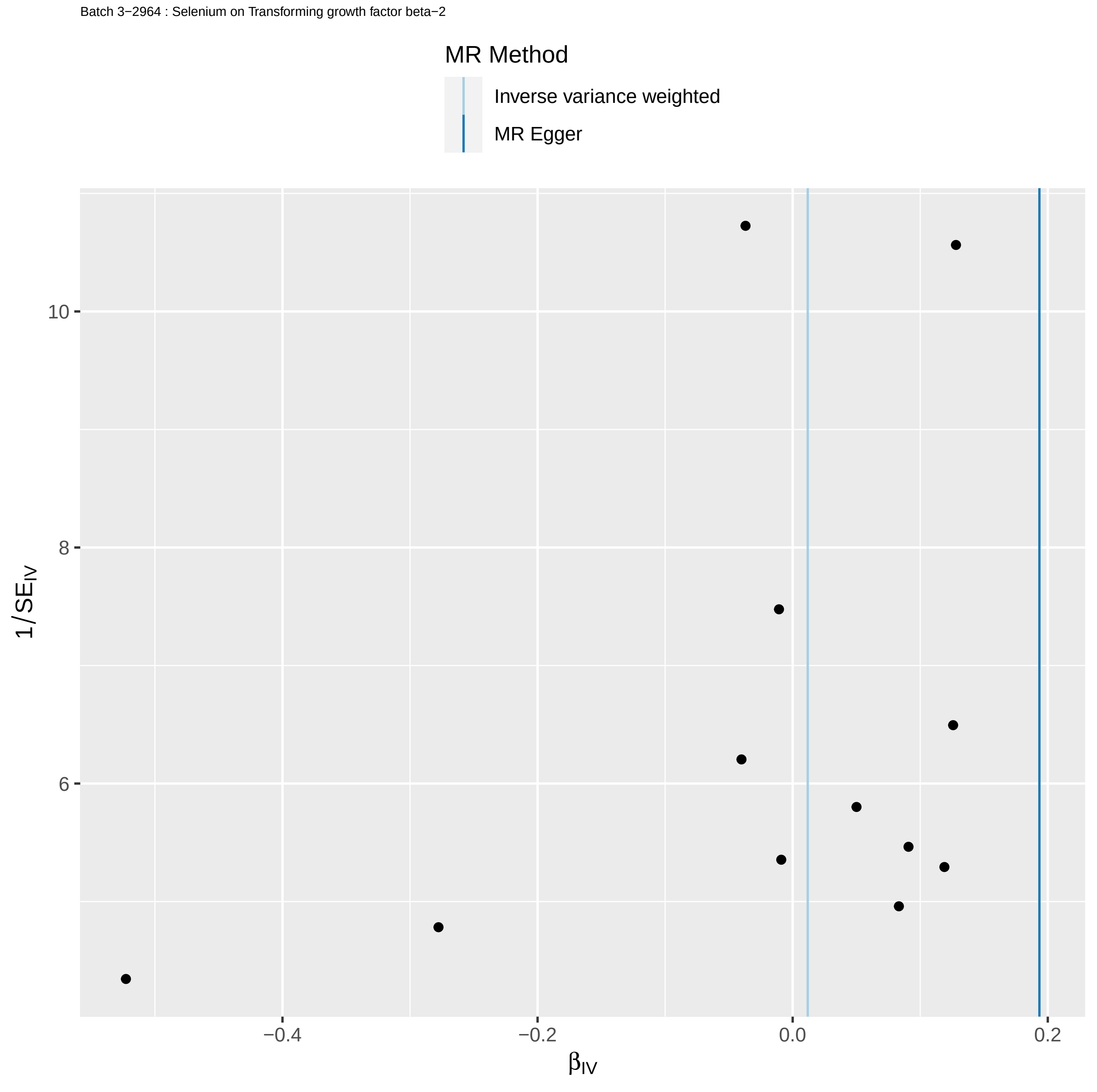

Batch 3−2964 : Selenium on Transforming growth factor beta−2
MR Method
Inverse variance weighted
MR Egger
10
8
6
1/SE_IV
−0.4
−0.2
0.0
0.2
β_IV

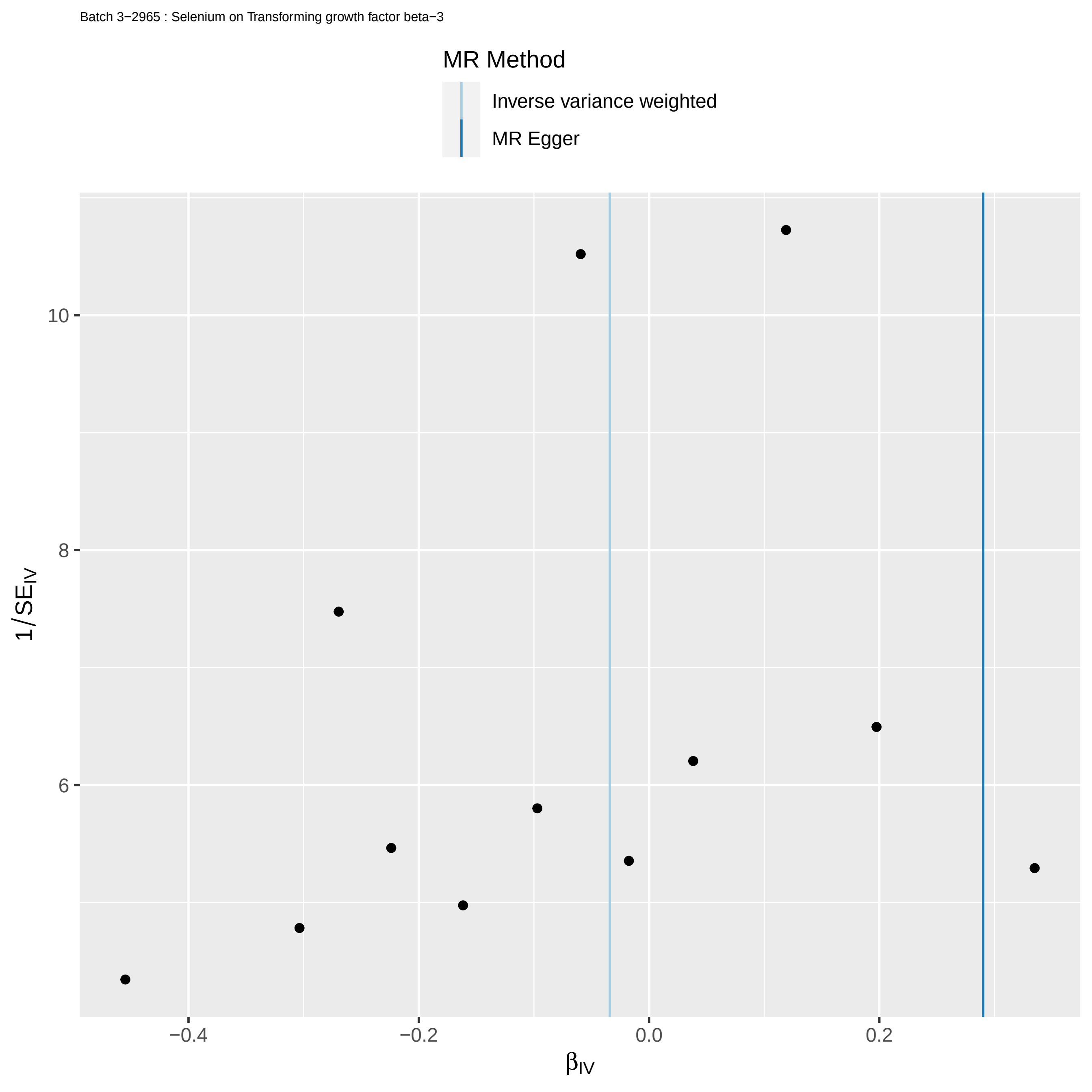

Batch 3−2965 : Selenium on Transforming growth factor beta−3
MR Method
Inverse variance weighted
MR Egger
10
8
6
1/SE_IV
−0.4
−0.2
0.0
0.2
β_IV

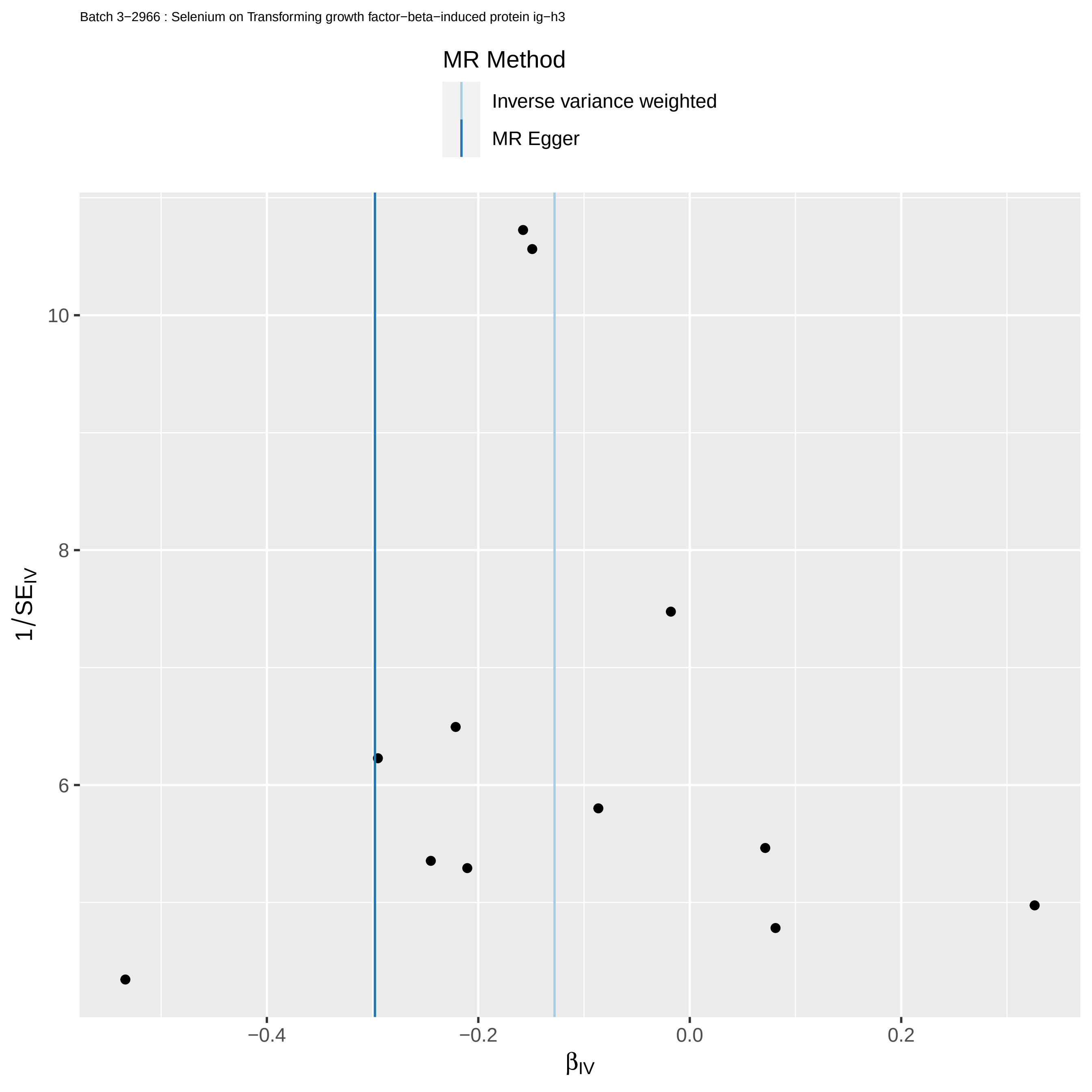
Batch 3−2966 : Selenium on Transforming growth factor−beta−induced protein ig−h3
MR Method
Inverse variance weighted
MR Egger
10
8
6
1/SE_IV
−0.4
−0.2
0.0
0.2
β_IV

Batch 3−2967 : Selenium on TGF−beta receptor type−2

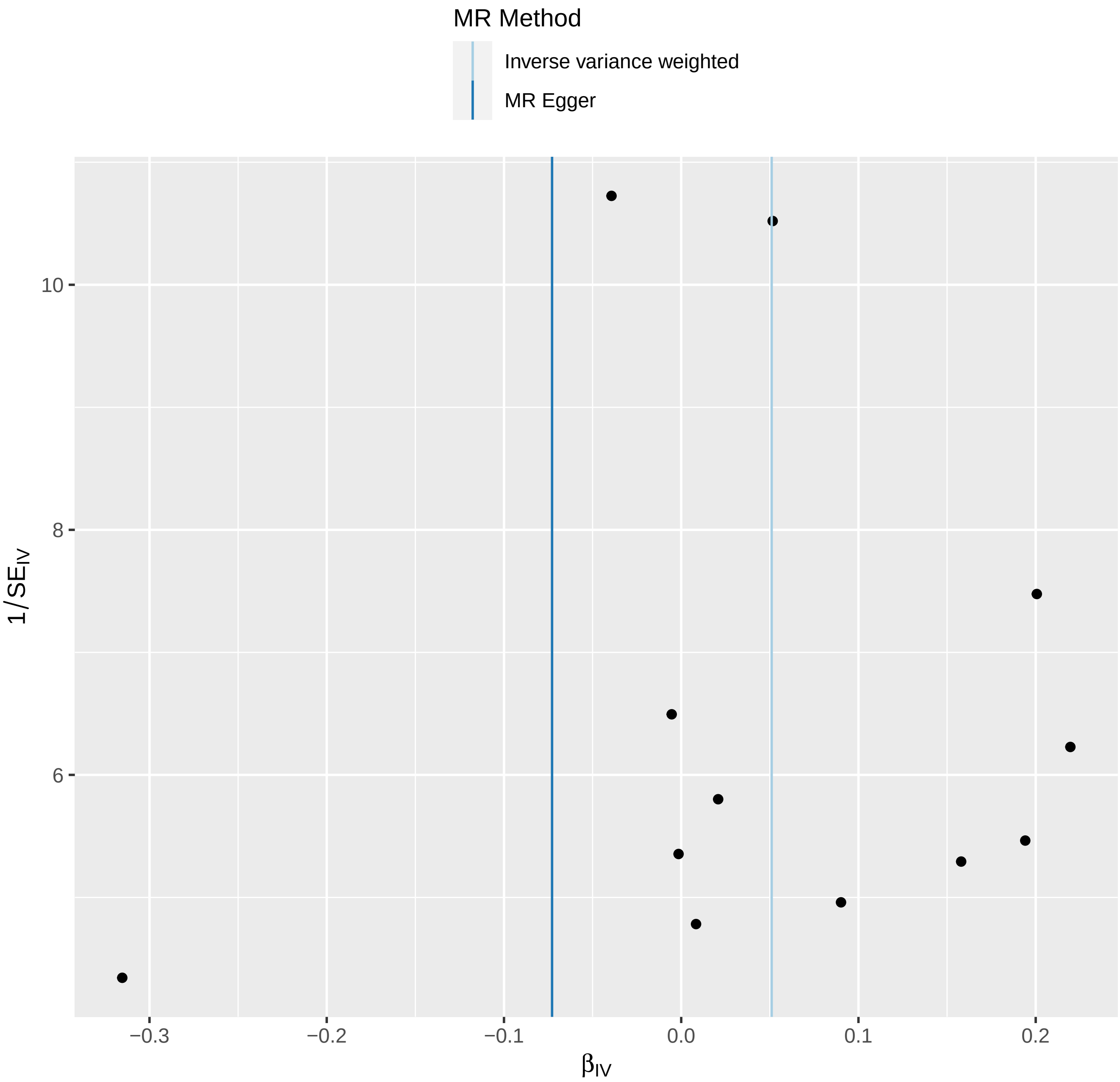

Batch 3−2968 : Selenium on Transforming growth factor beta receptor type 3

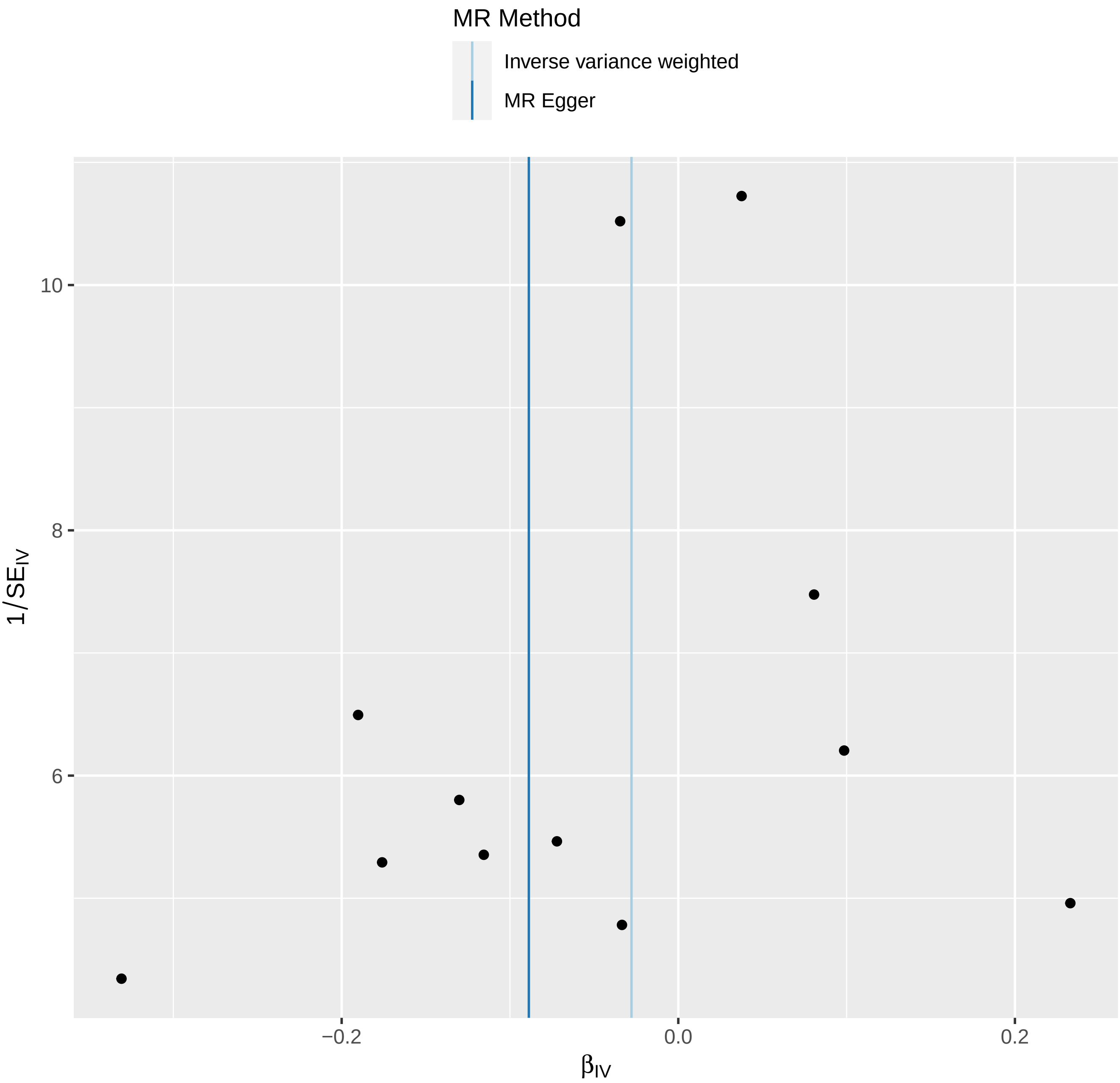

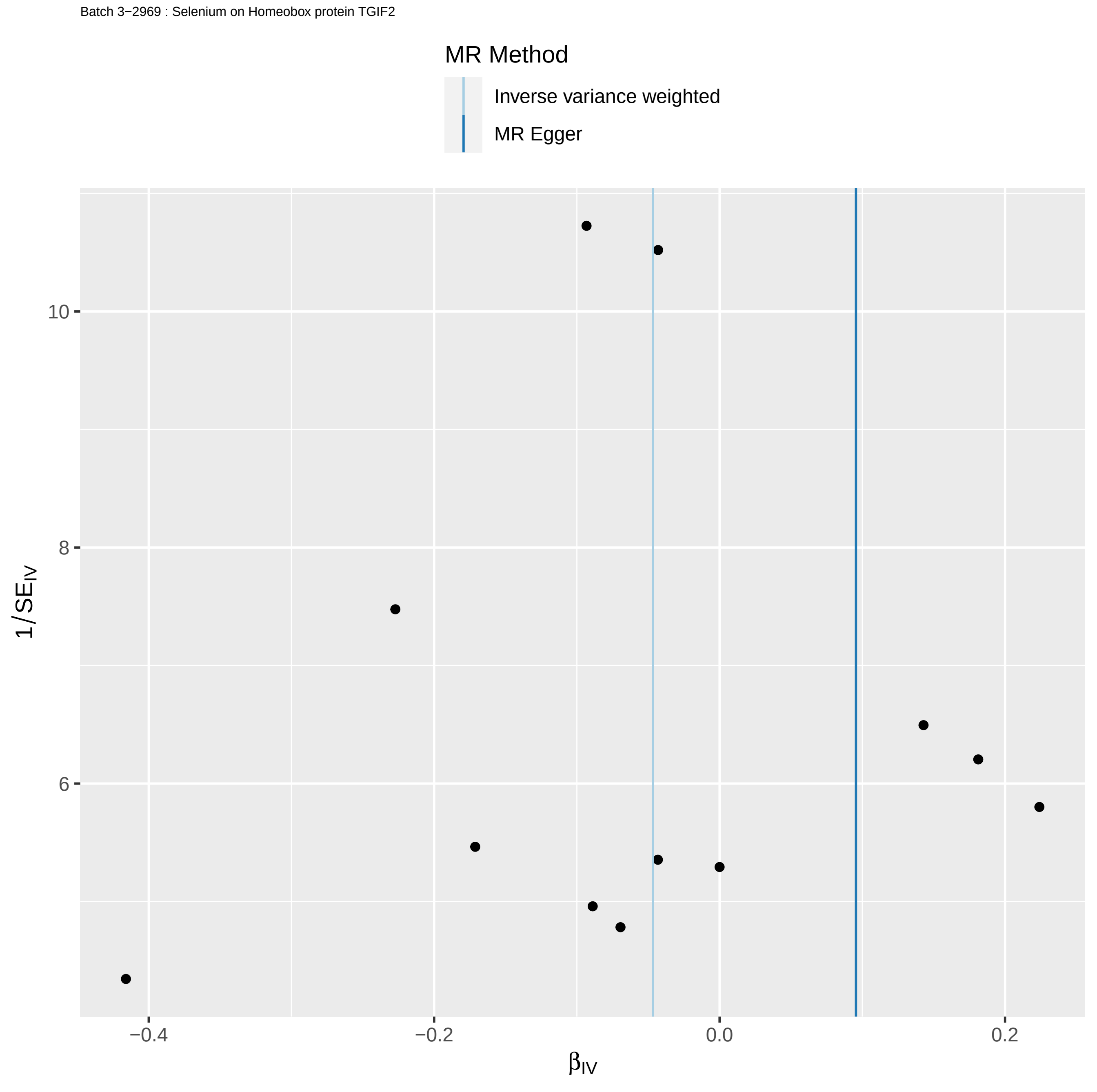
Batch 3−2969 : Selenium on Homeobox protein TGIF2
MR Method
Inverse variance weighted
MR Egger
10
8
6
$1/SE_{IV}$
−0.4
−0.2
0.0
0.2
β_{IV}

Batch 3−2970 : Selenium on Thrombospondin−2

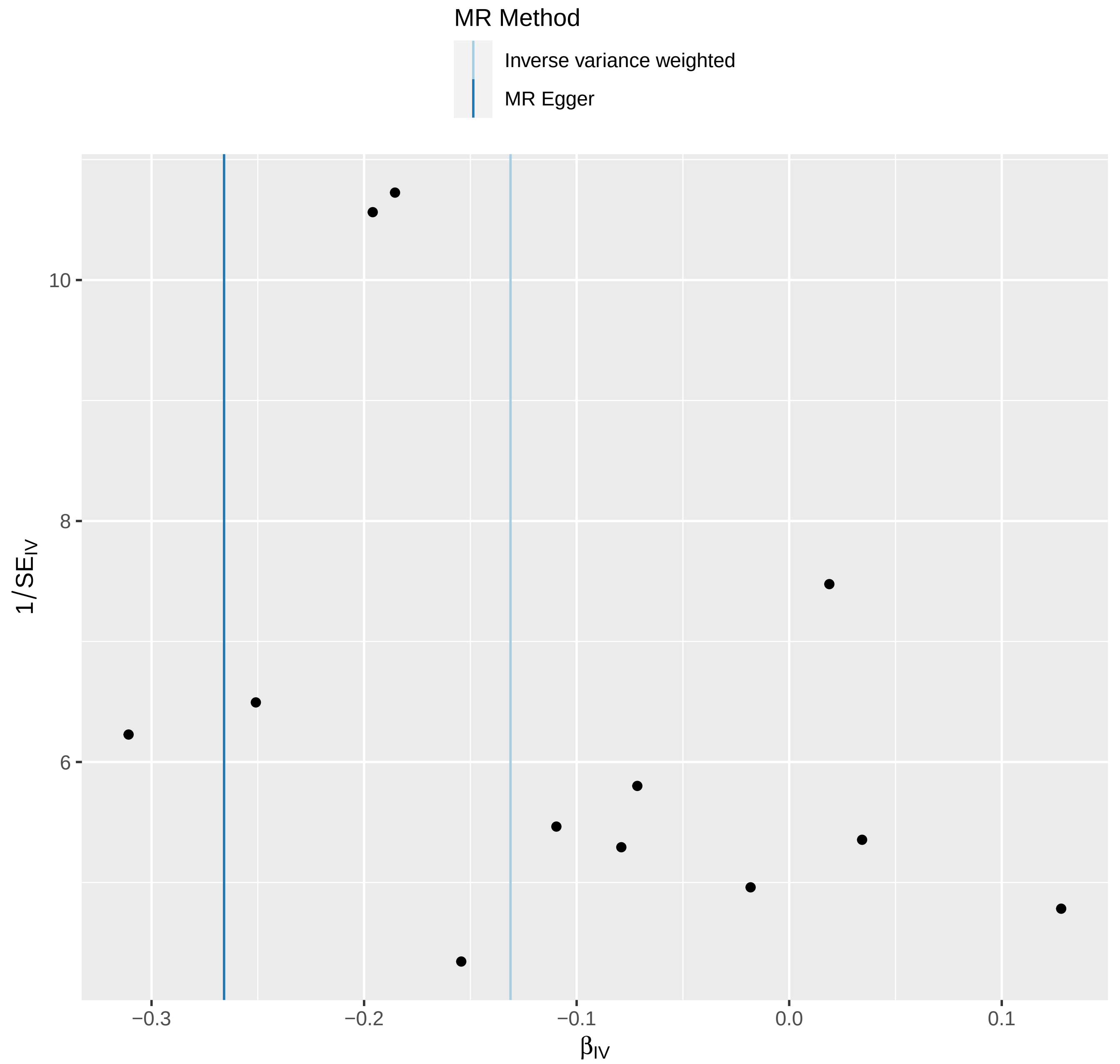

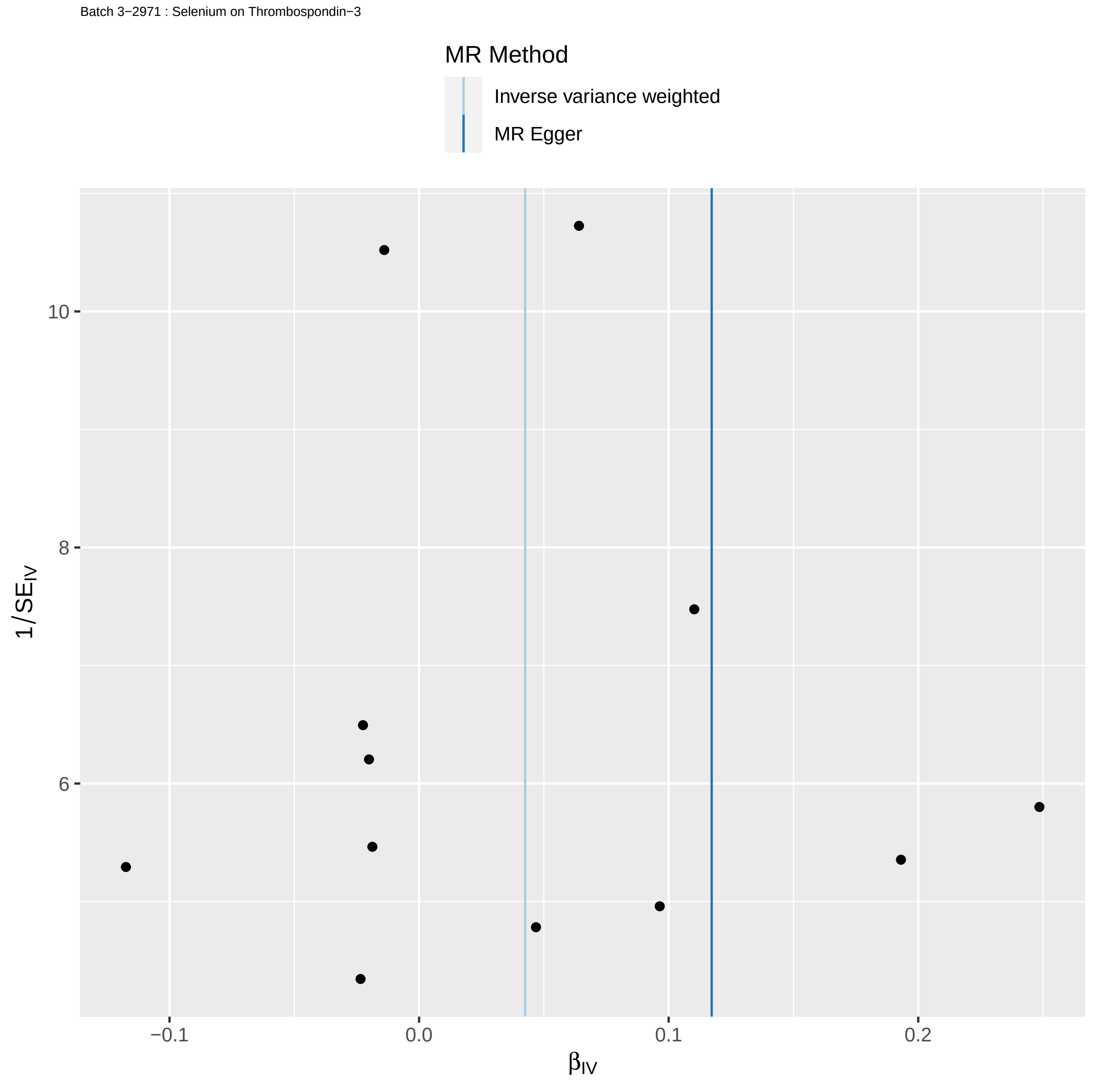

Batch 3−2971 : Selenium on Thrombospondin−3
MR Method
Inverse variance weighted
MR Egger
10
8
6
1/SE$_{IV}$
−0.1
0.0
0.1
0.2
β$_{IV}$

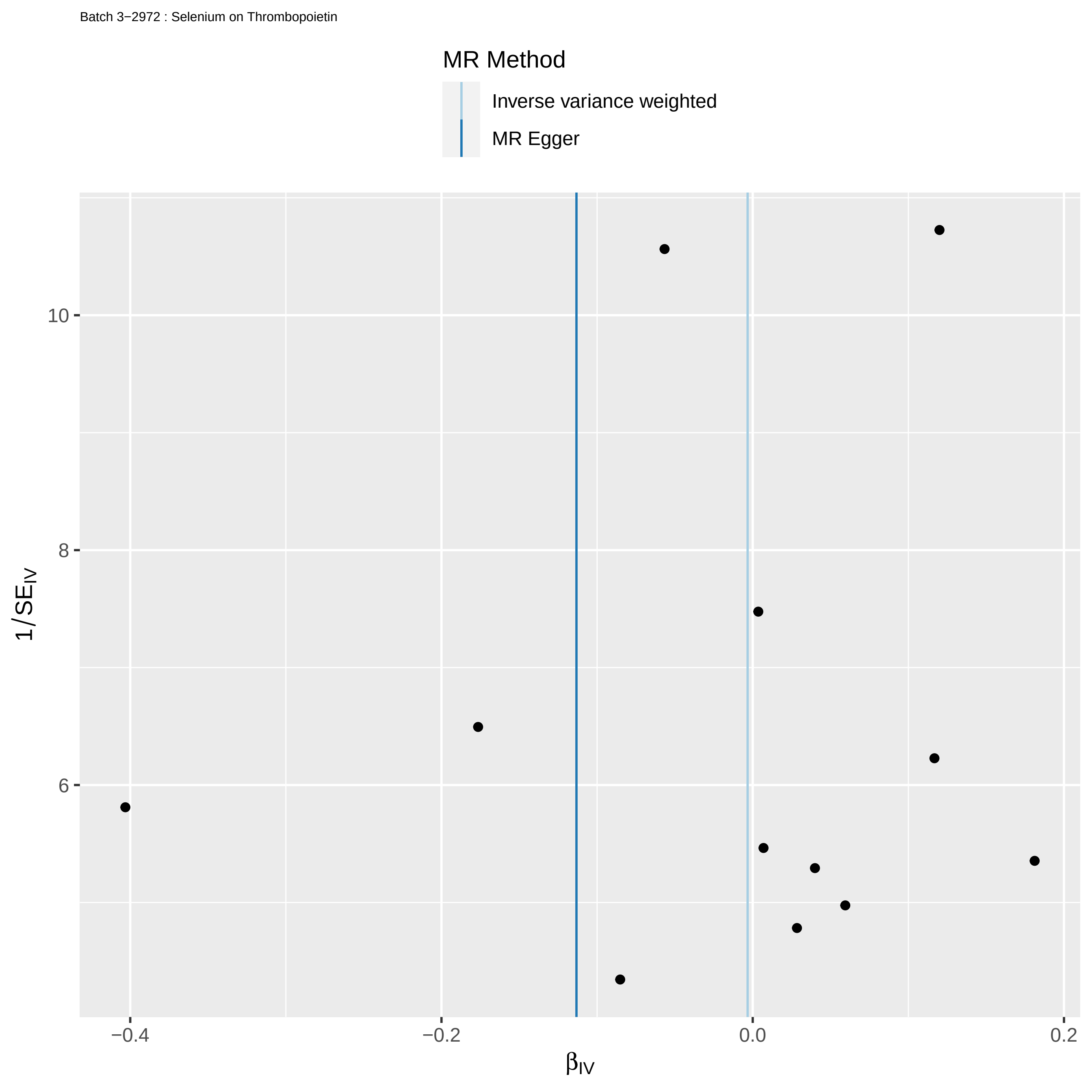

Batch 3−2972 : Selenium on Thrombopoietin
MR Method
Inverse variance weighted
MR Egger
10
8
6
−0.4
−0.2
0.0
0.2
$1/SE_{IV}$
β_{IV}

Batch 3−2973 : Selenium on Thrombopoietin

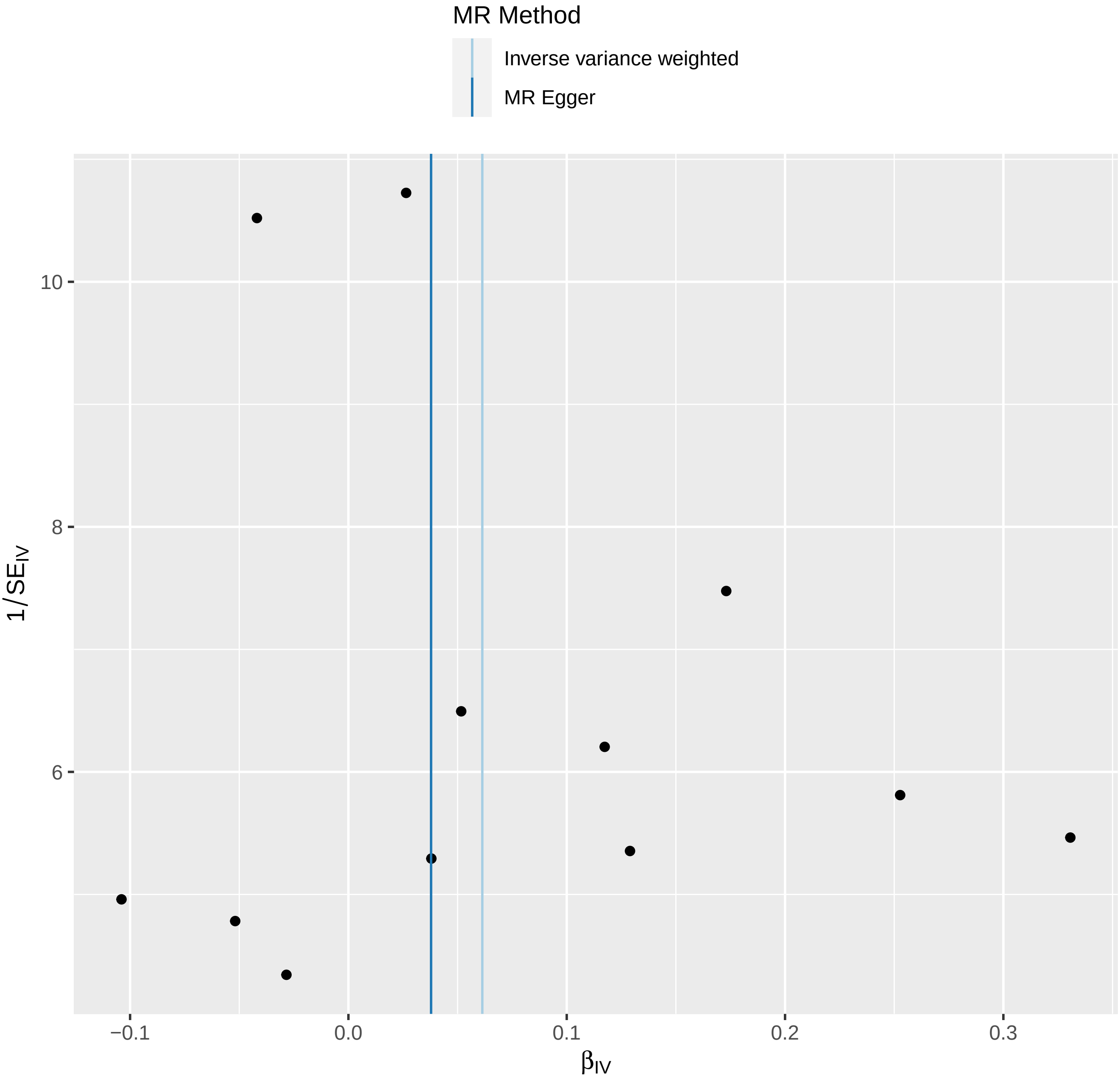

Batch 3−2974 : Selenium on Thyroid hormone receptor alpha

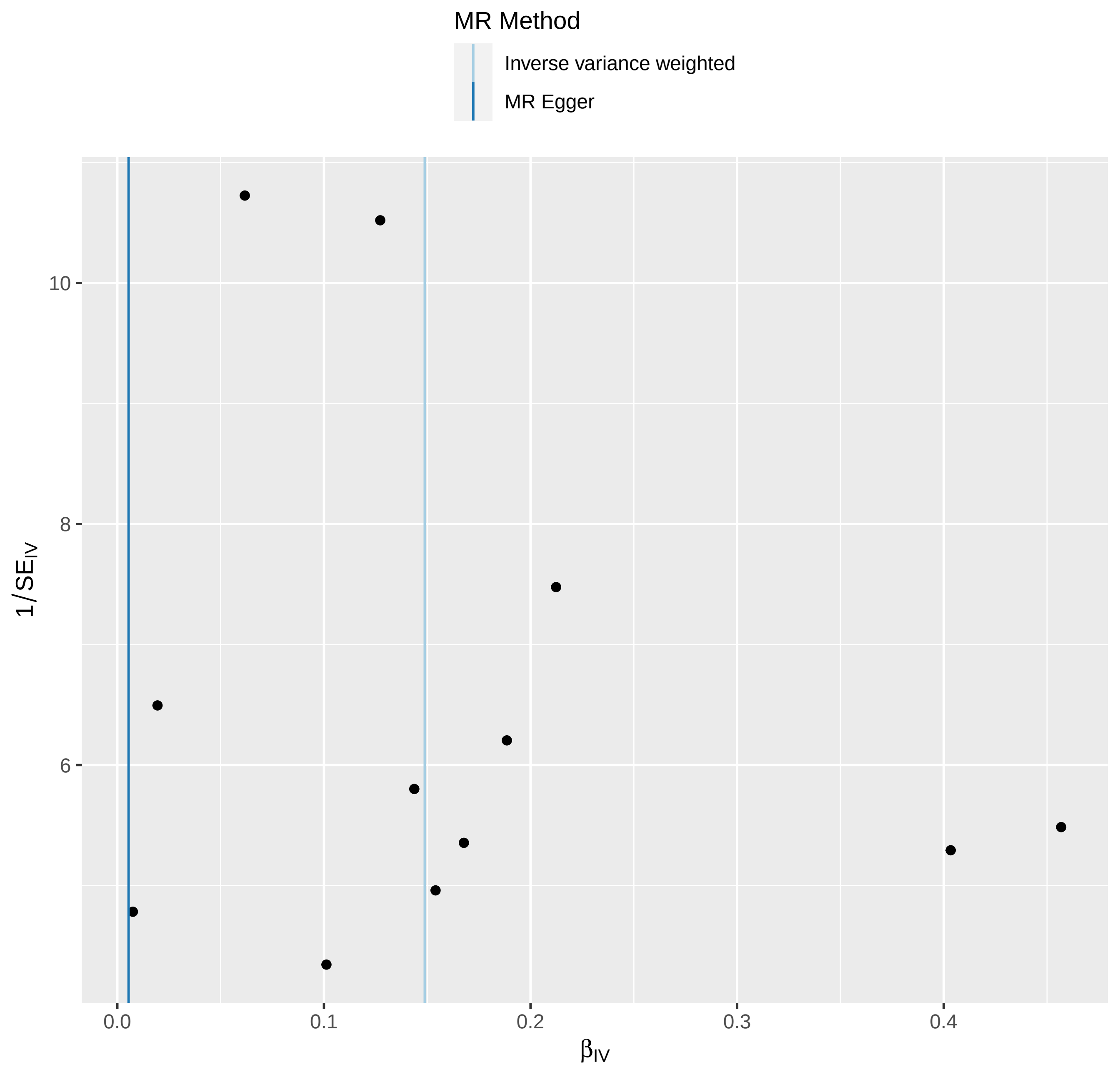

Batch 3−2975 : Selenium on Thrombospondin type−1 domain−containing protein 1

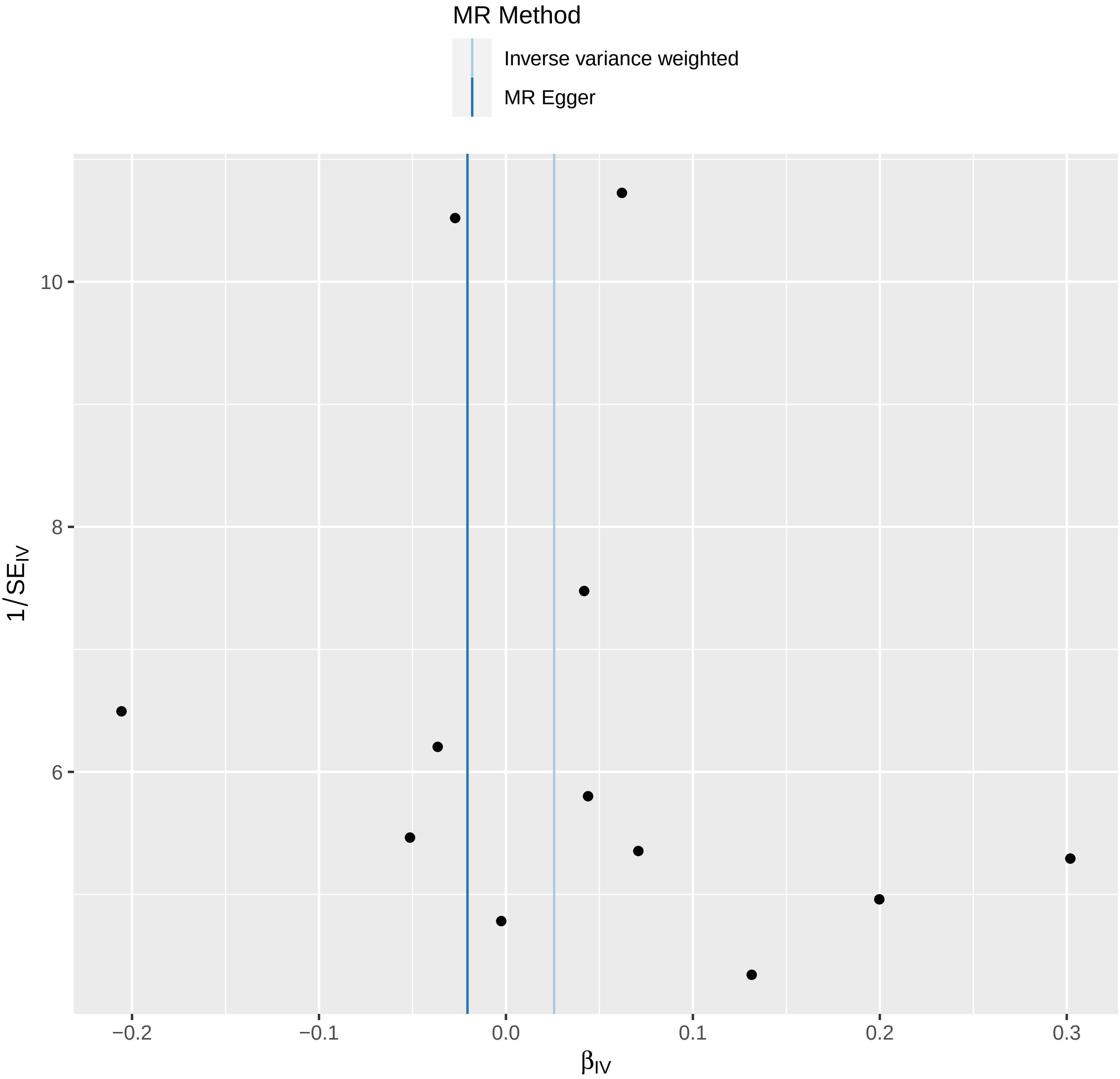

Batch 3−2976 : Selenium on Thrombospondin type−1 domain−containing protein 7A

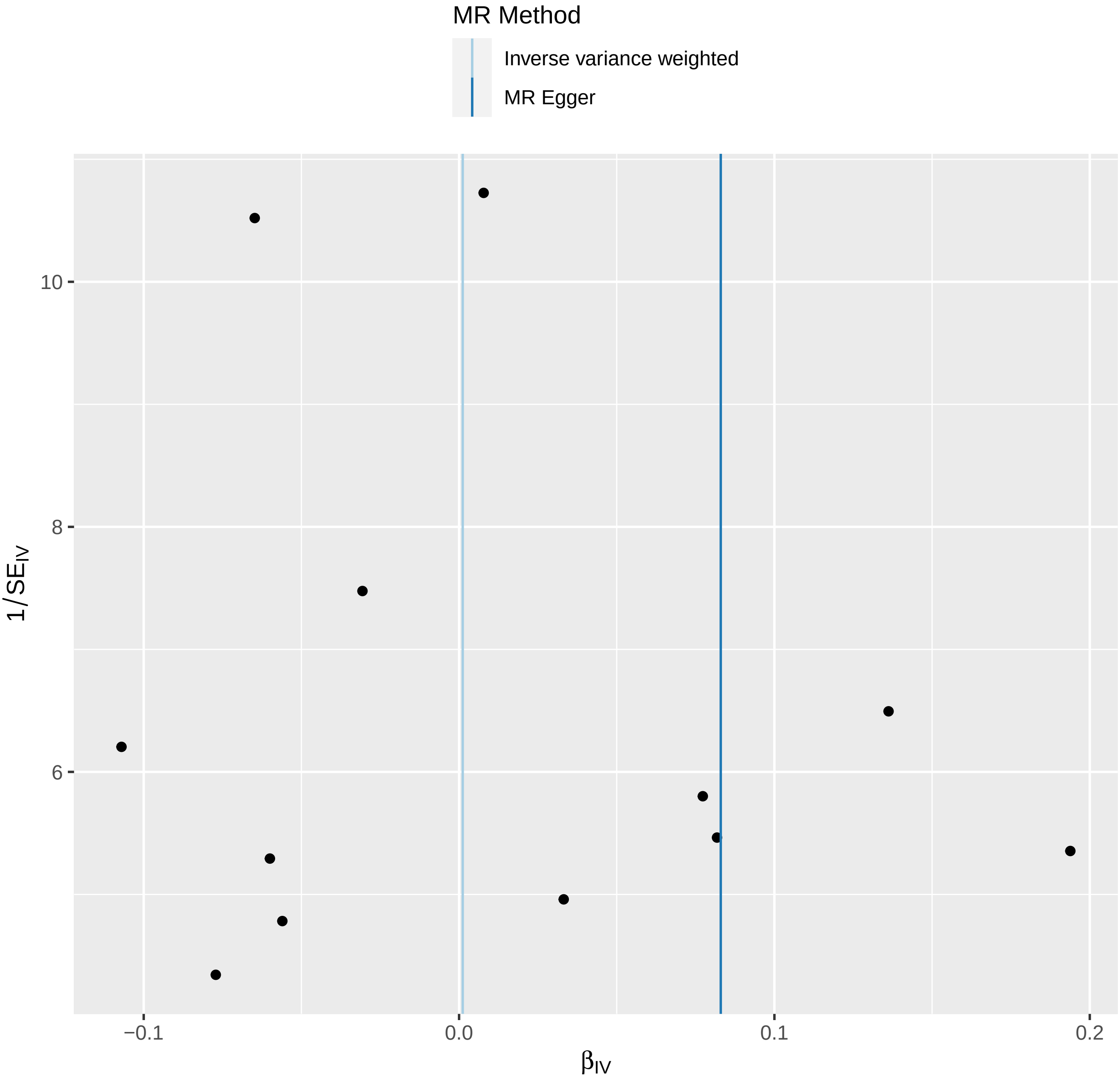

Batch 3−2977 : Selenium on Thymocyte nuclear protein 1

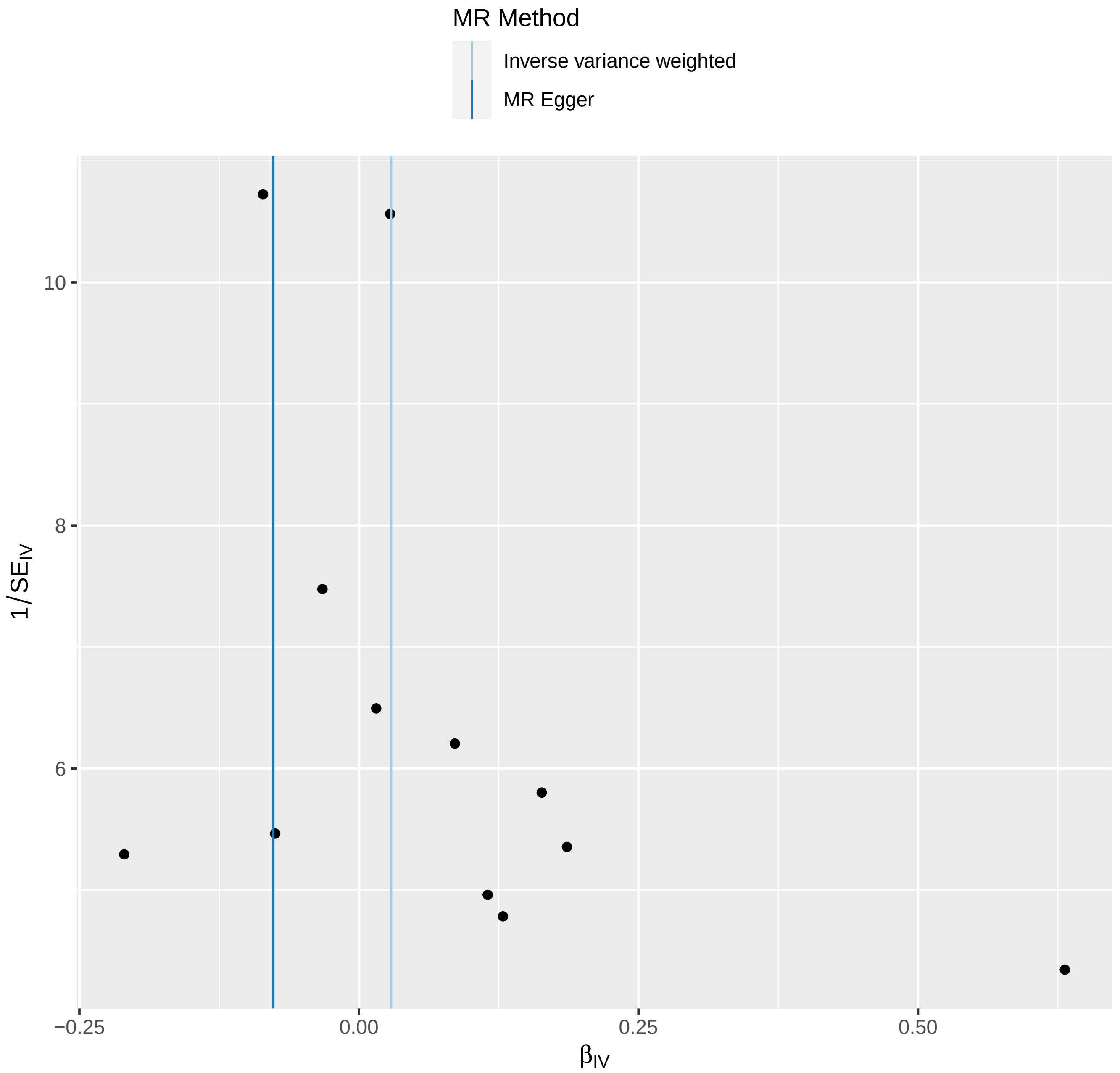

Batch 3−2978 : Selenium on Tyrosine−protein kinase receptor Tie−1, soluble

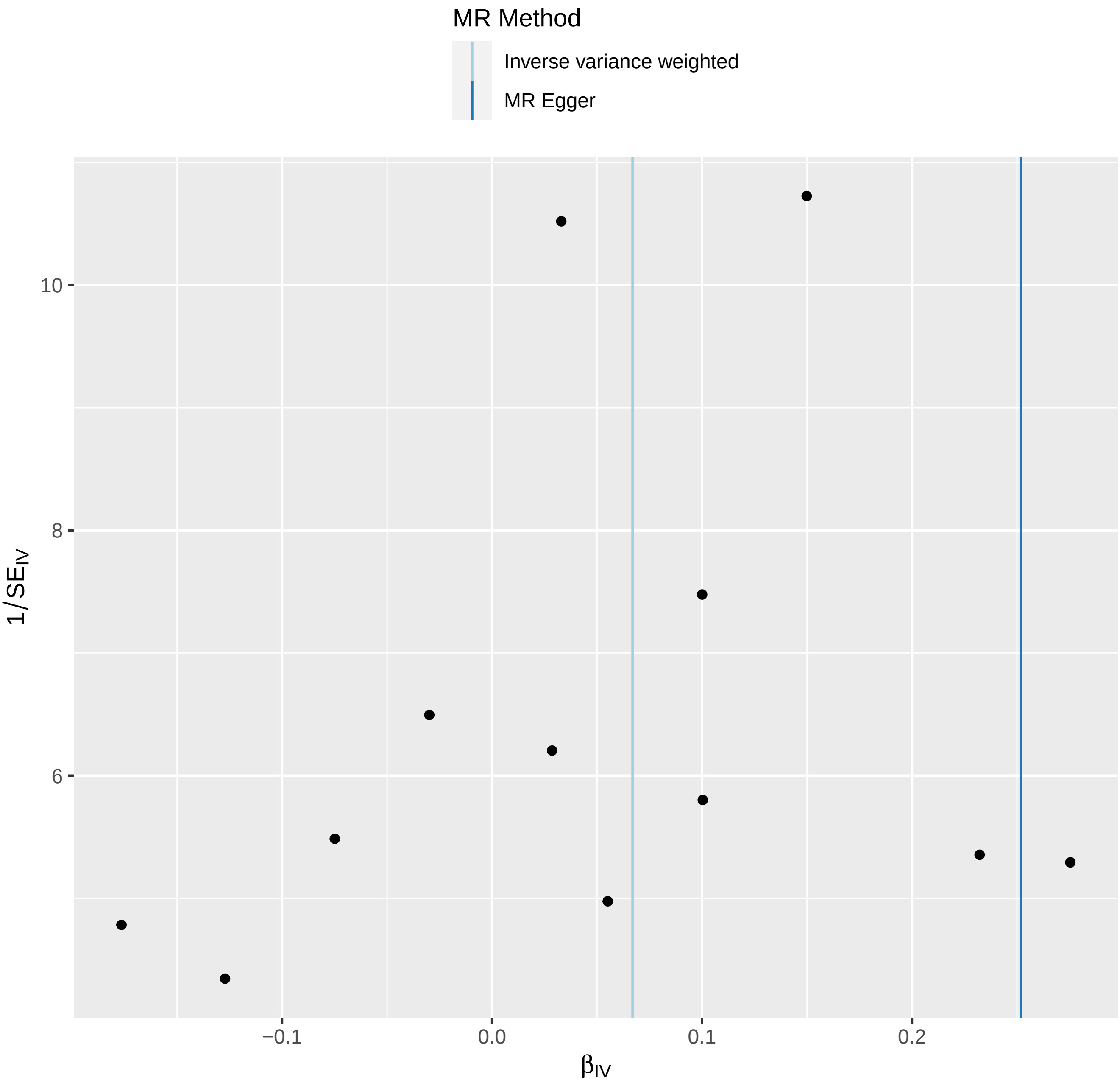

Batch 3−2979 : Selenium on Fructose−2,6−bisphosphatase TIGAR

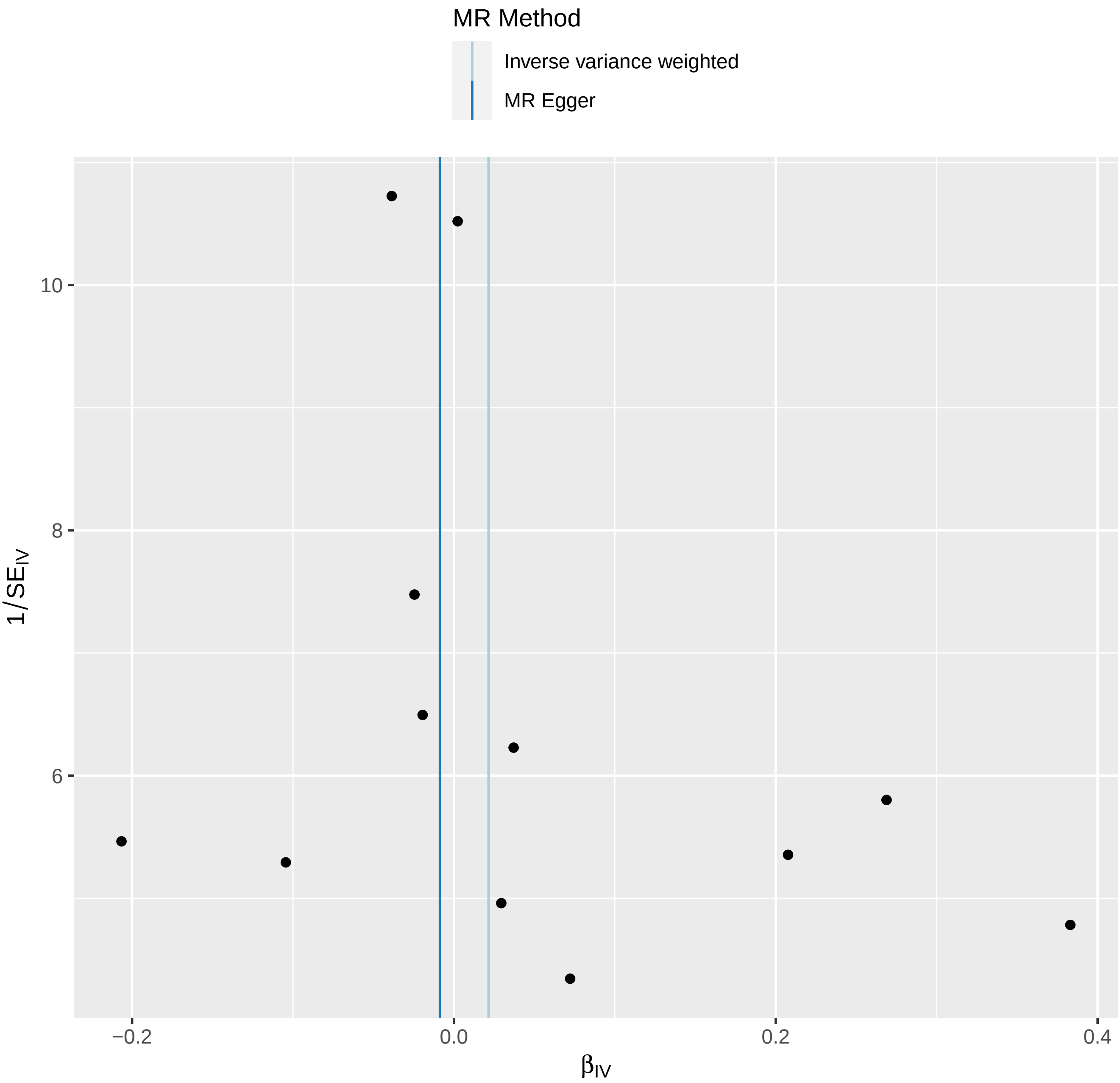

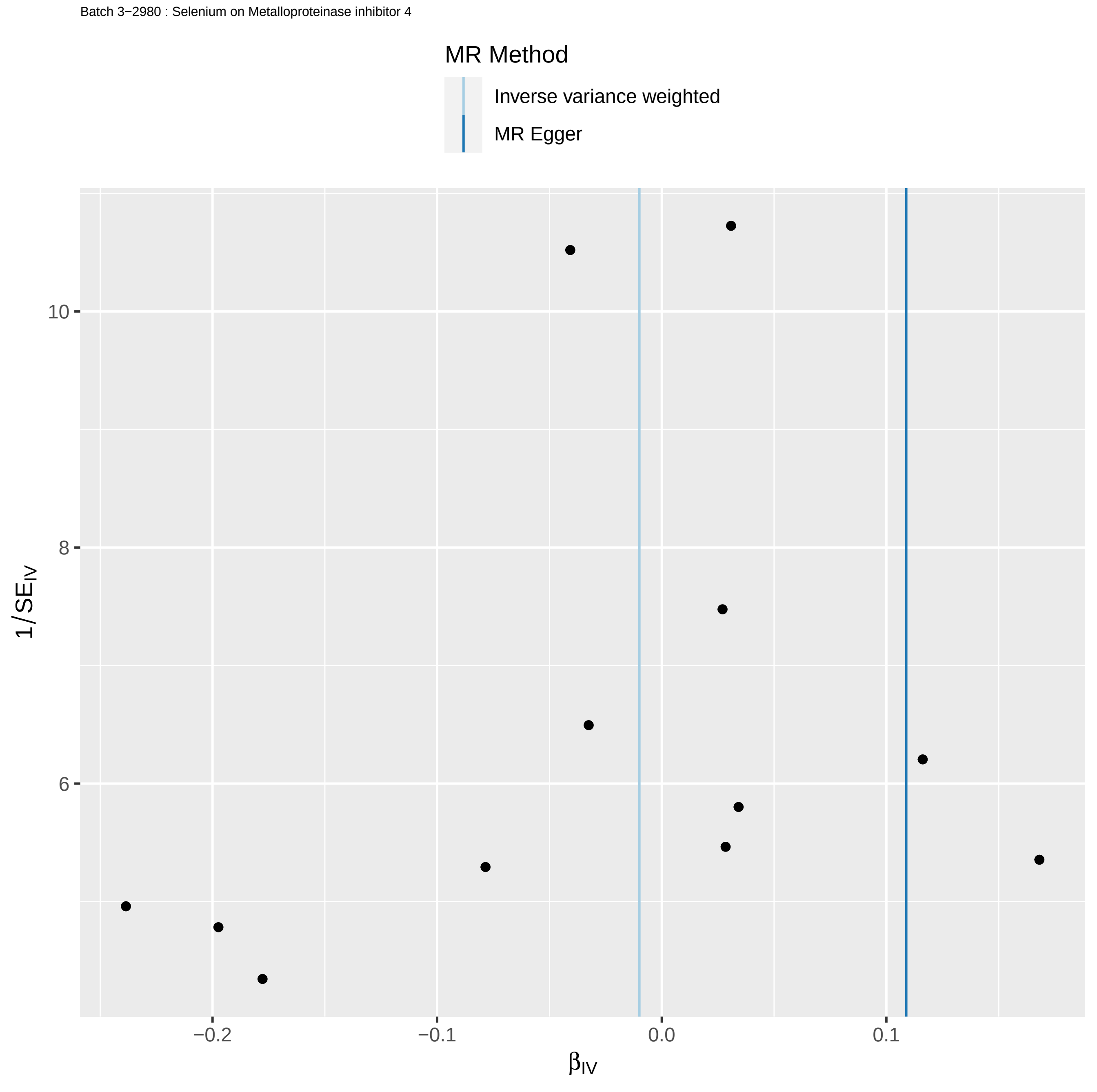

Batch 3−2980 : Selenium on Metalloproteinase inhibitor 4
MR Method
Inverse variance weighted
MR Egger
10
8
6
1/SE_IV
−0.2
−0.1
0.0
0.1
β_IV

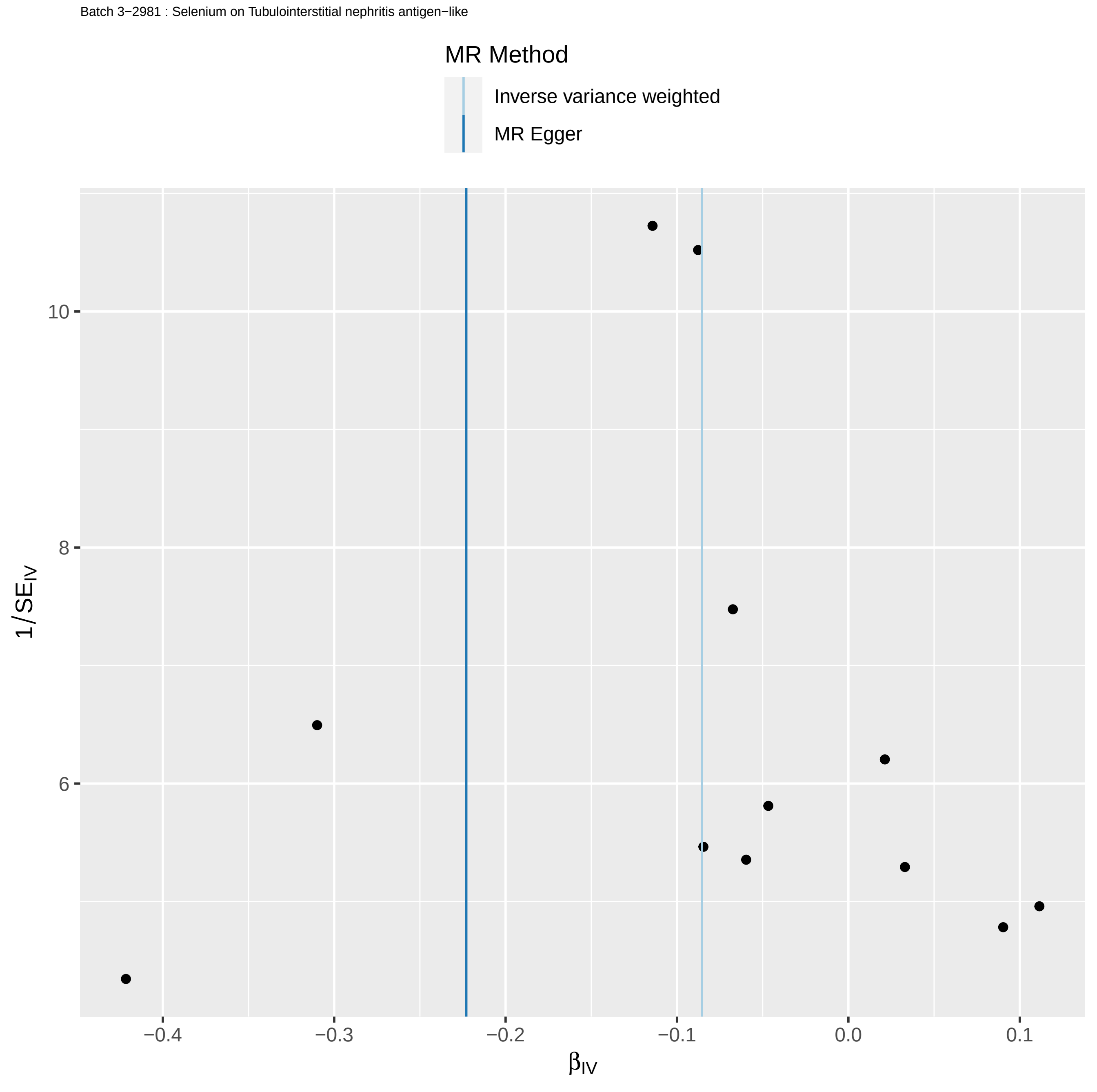

Batch 3−2981 : Selenium on Tubulointerstitial nephritis antigen−like
MR Method
Inverse variance weighted
MR Egger
10
8
6
1/SE_IV
−0.4
−0.3
−0.2
−0.1
0.0
0.1
β_IV

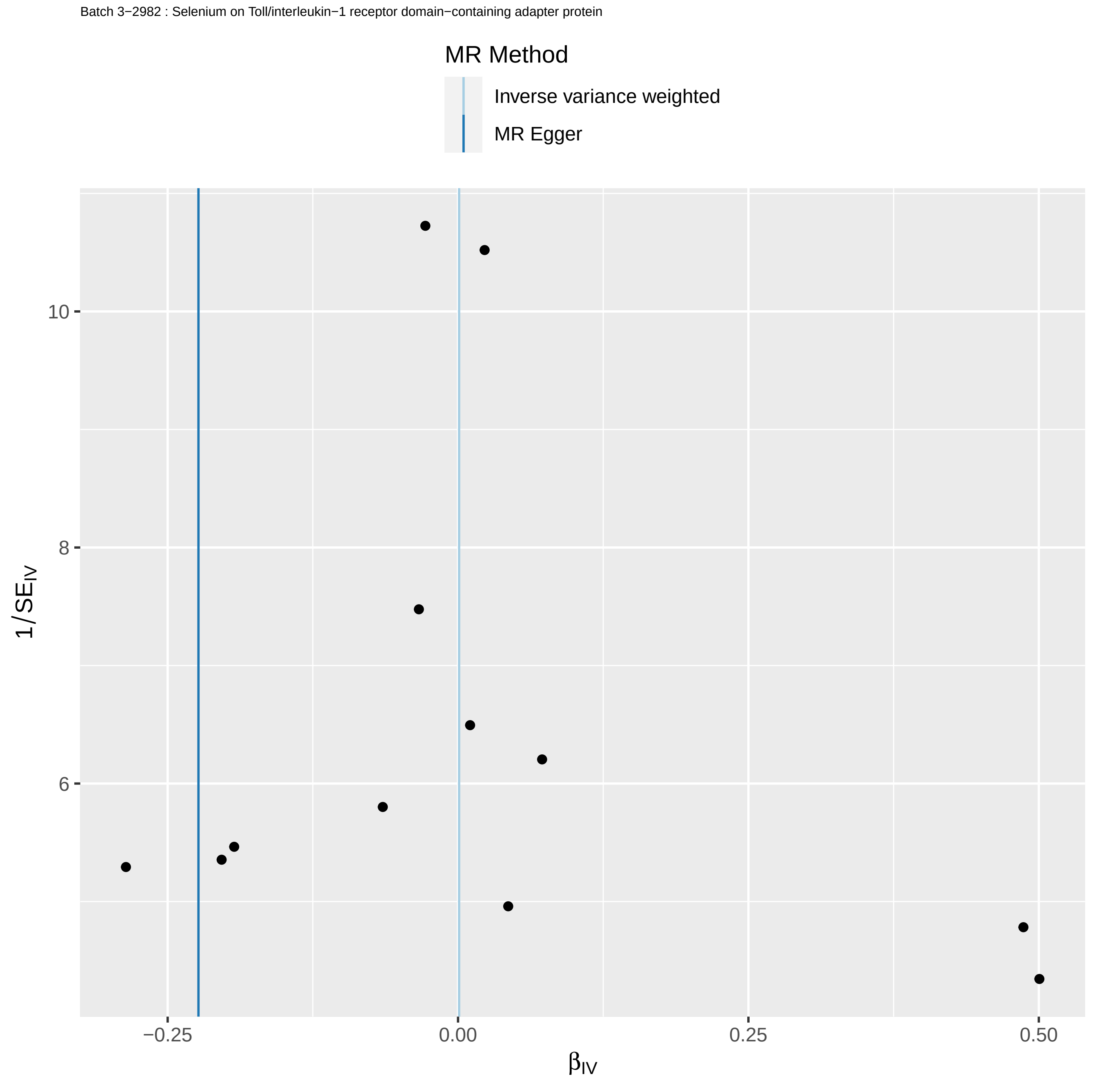

Batch 3−2982 : Selenium on Toll/interleukin−1 receptor domain−containing adapter protein
MR Method
Inverse variance weighted
MR Egger
10
8
6
1/SE_IV
−0.25
0.00
0.25
0.50
β_IV

Batch 3−2983 : Selenium on Tight junction protein ZO−1

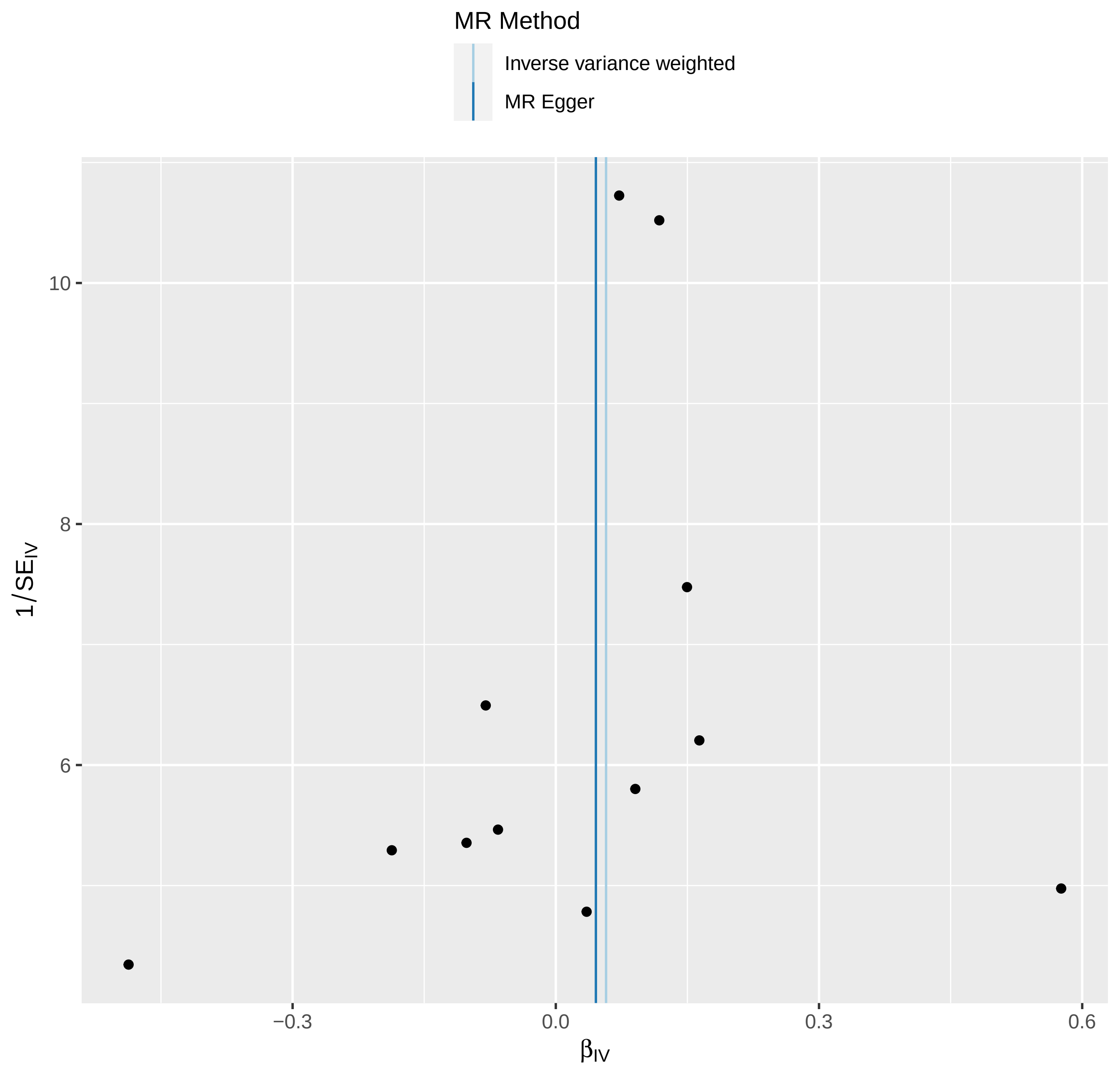

Batch 3−2984 : Selenium on Thymidine kinase, cytosolic

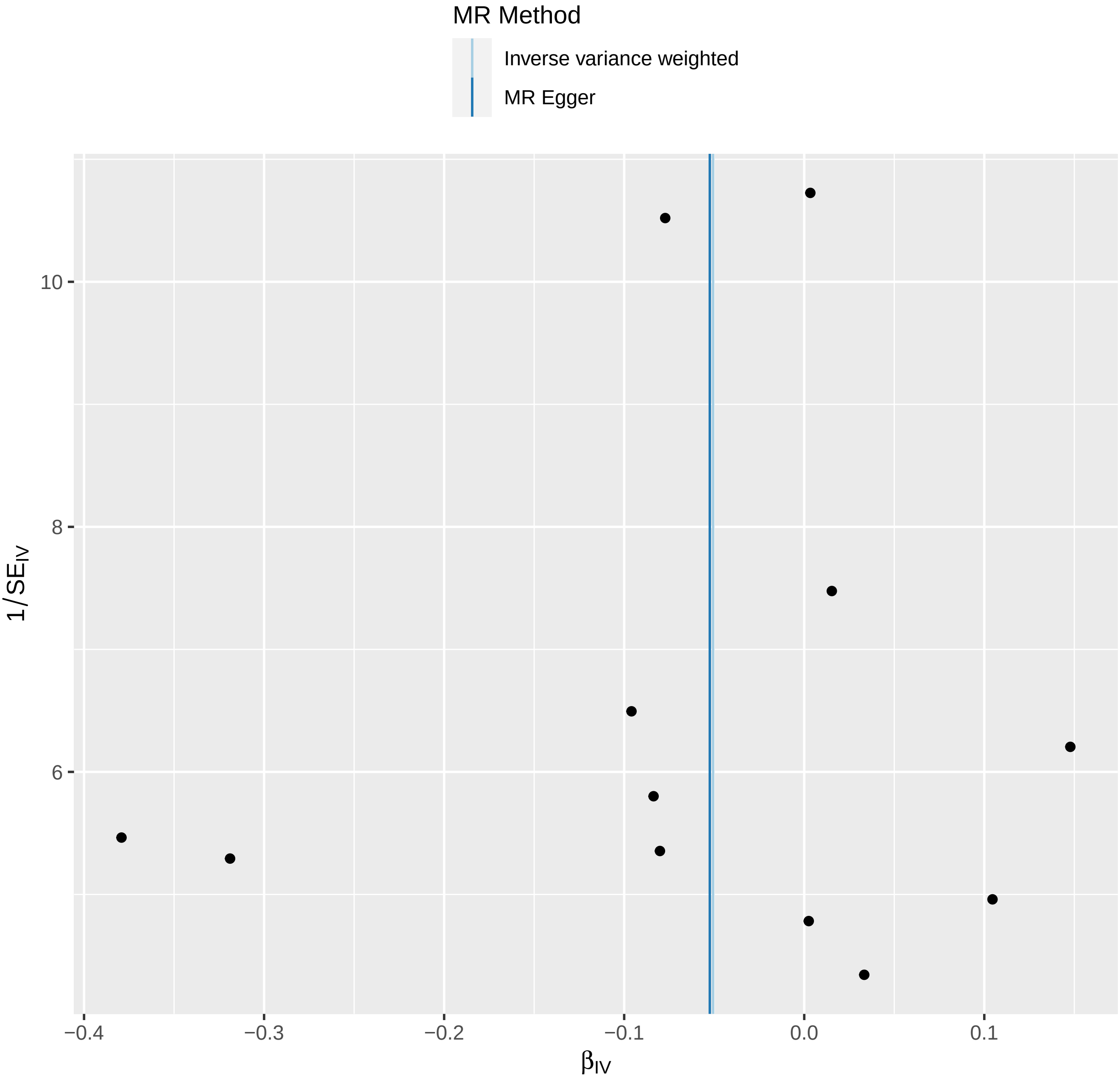

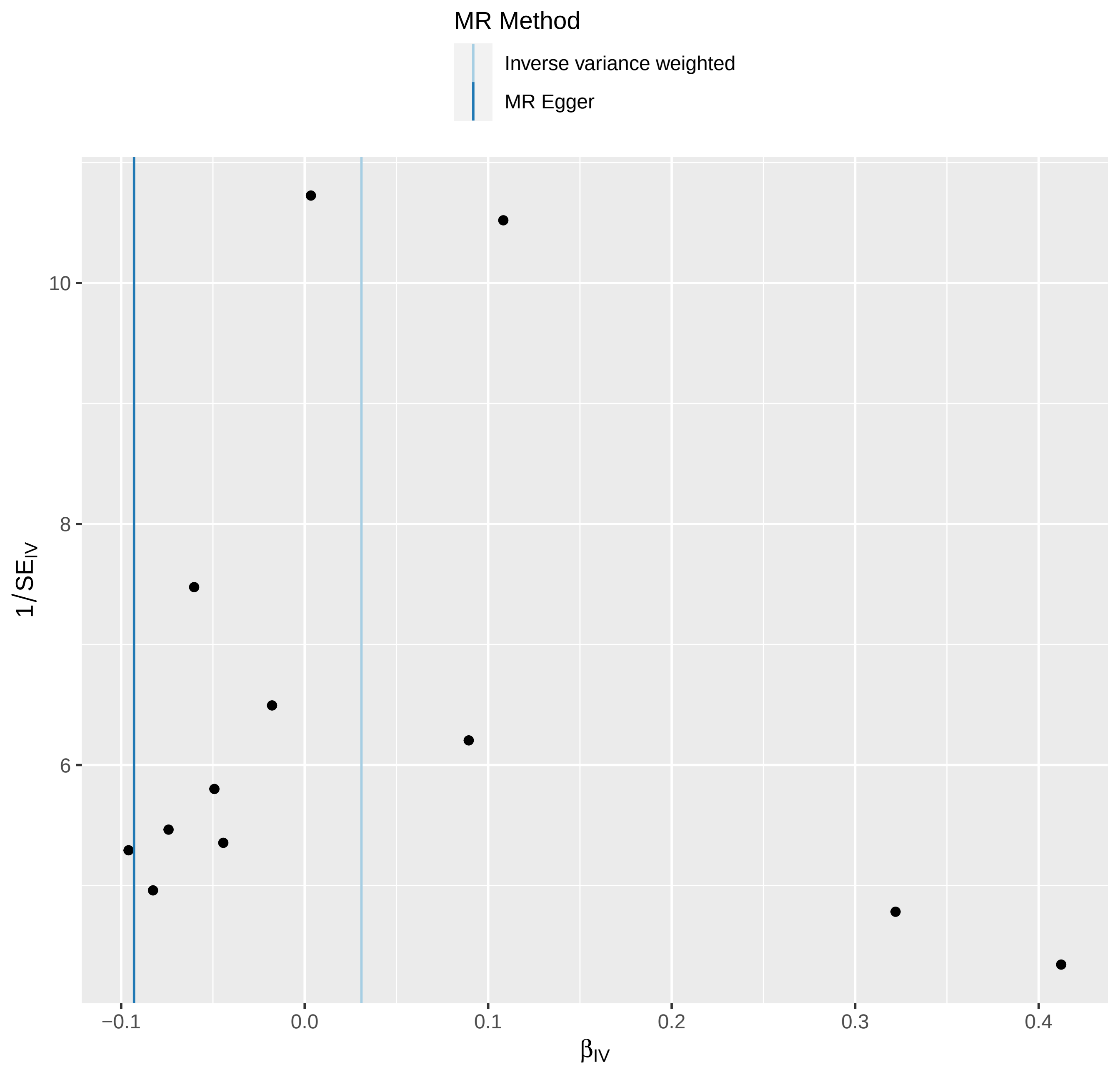
MR Method
Inverse variance weighted
MR Egger
10
8
6
1/SE$_{IV}$
−0.1
0.0
0.1
0.2
0.3
0.4
β_{IV}

Batch 3−2986 : Selenium on Tolloid−like protein 1

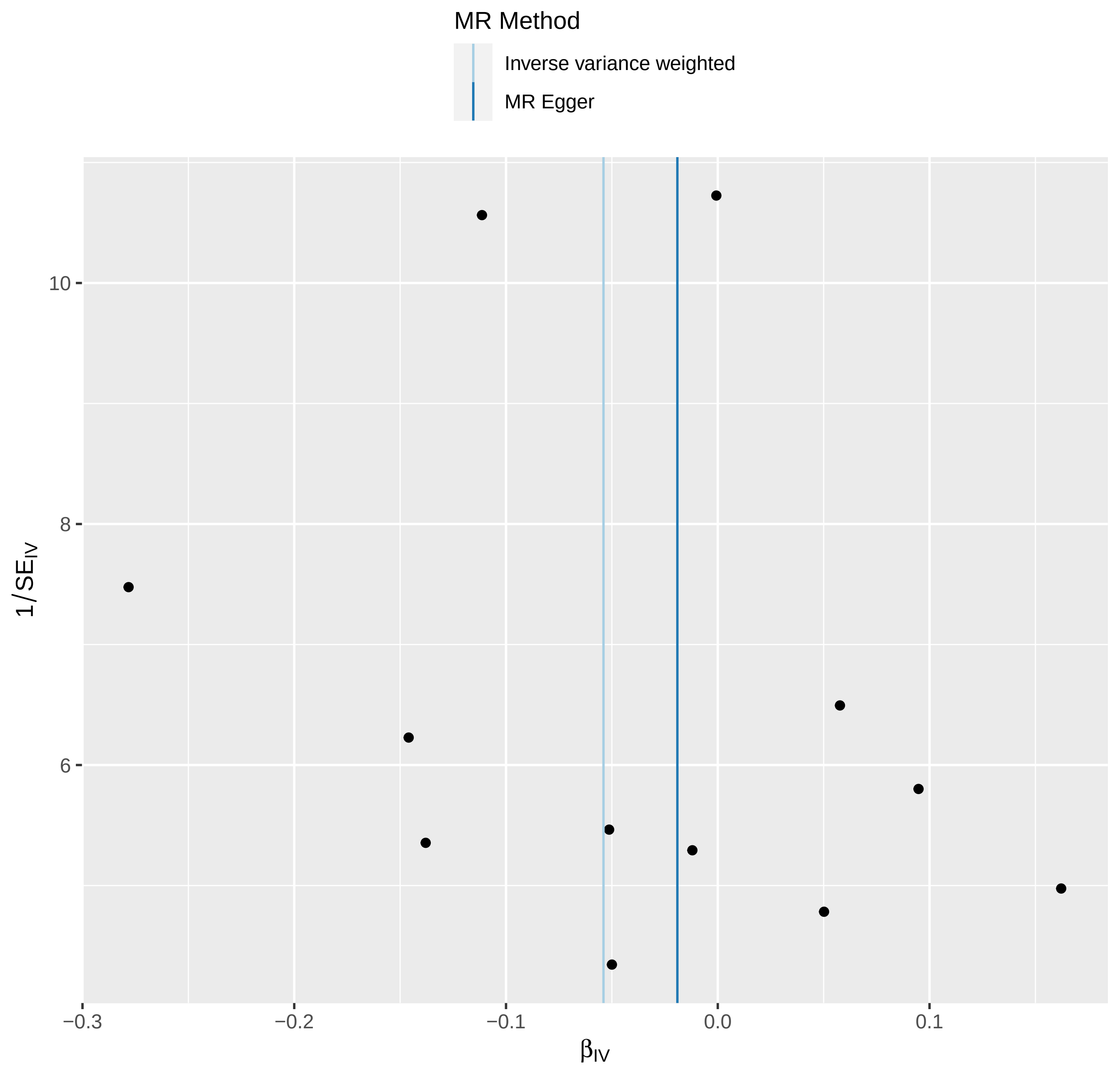

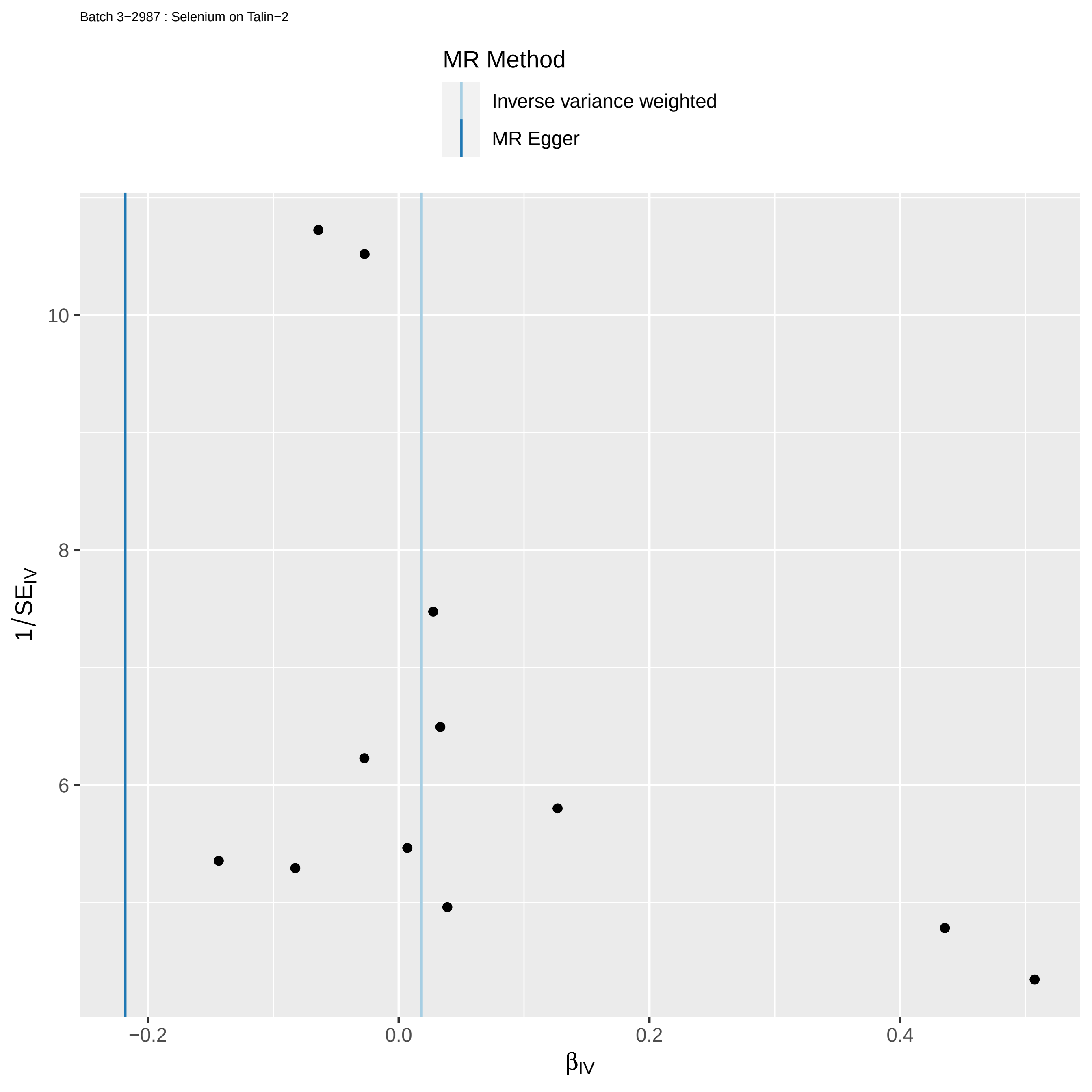

Batch 3−2987 : Selenium on Talin−2
MR Method
Inverse variance weighted
MR Egger
10
8
6
1/SE$_{IV}$
−0.2
0.0
0.2
0.4
β$_{IV}$

Batch 3−2988 : Selenium on Toll−like receptor 1

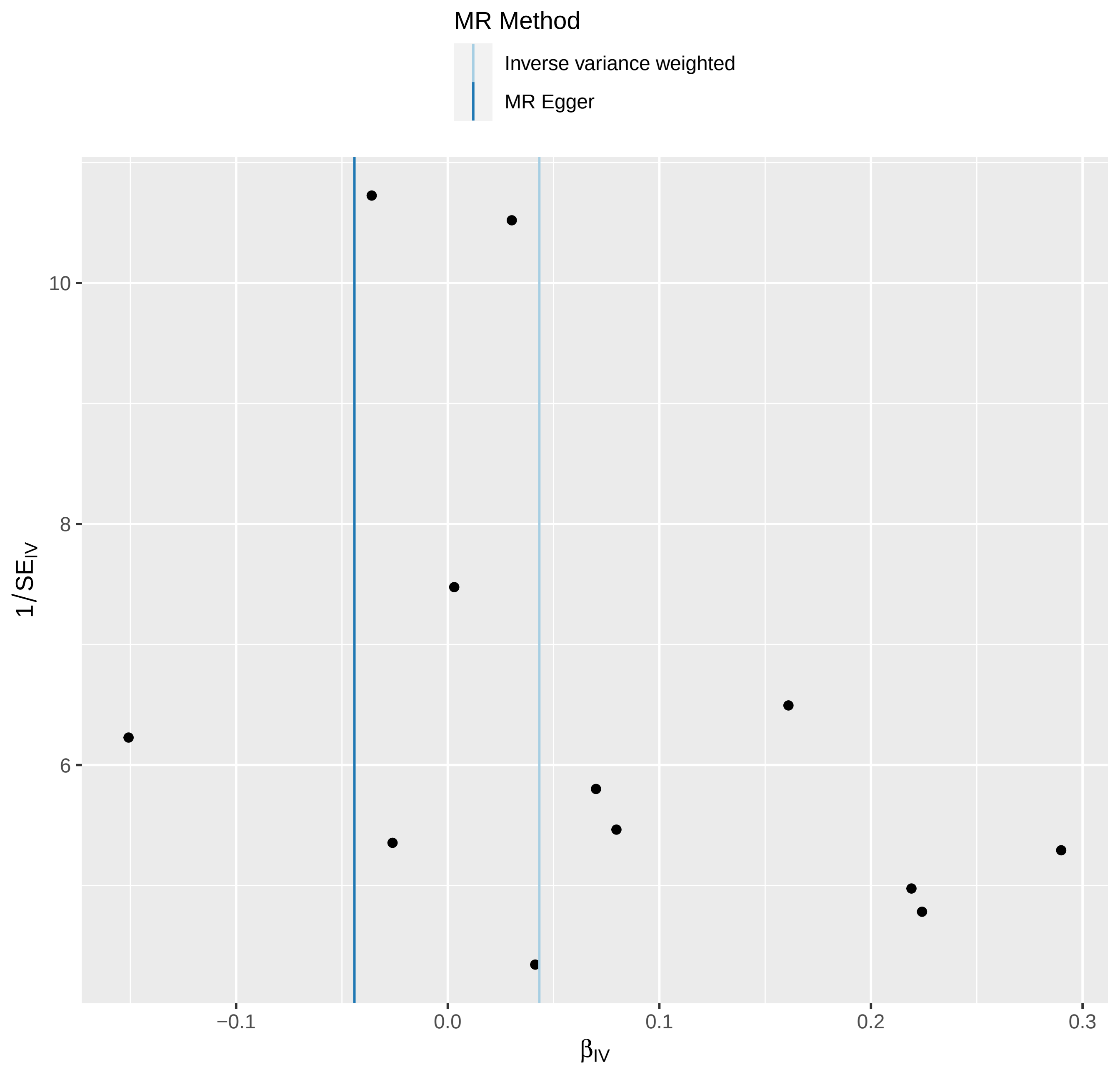

Batch 3−2989 : Selenium on Toll−like receptor 2

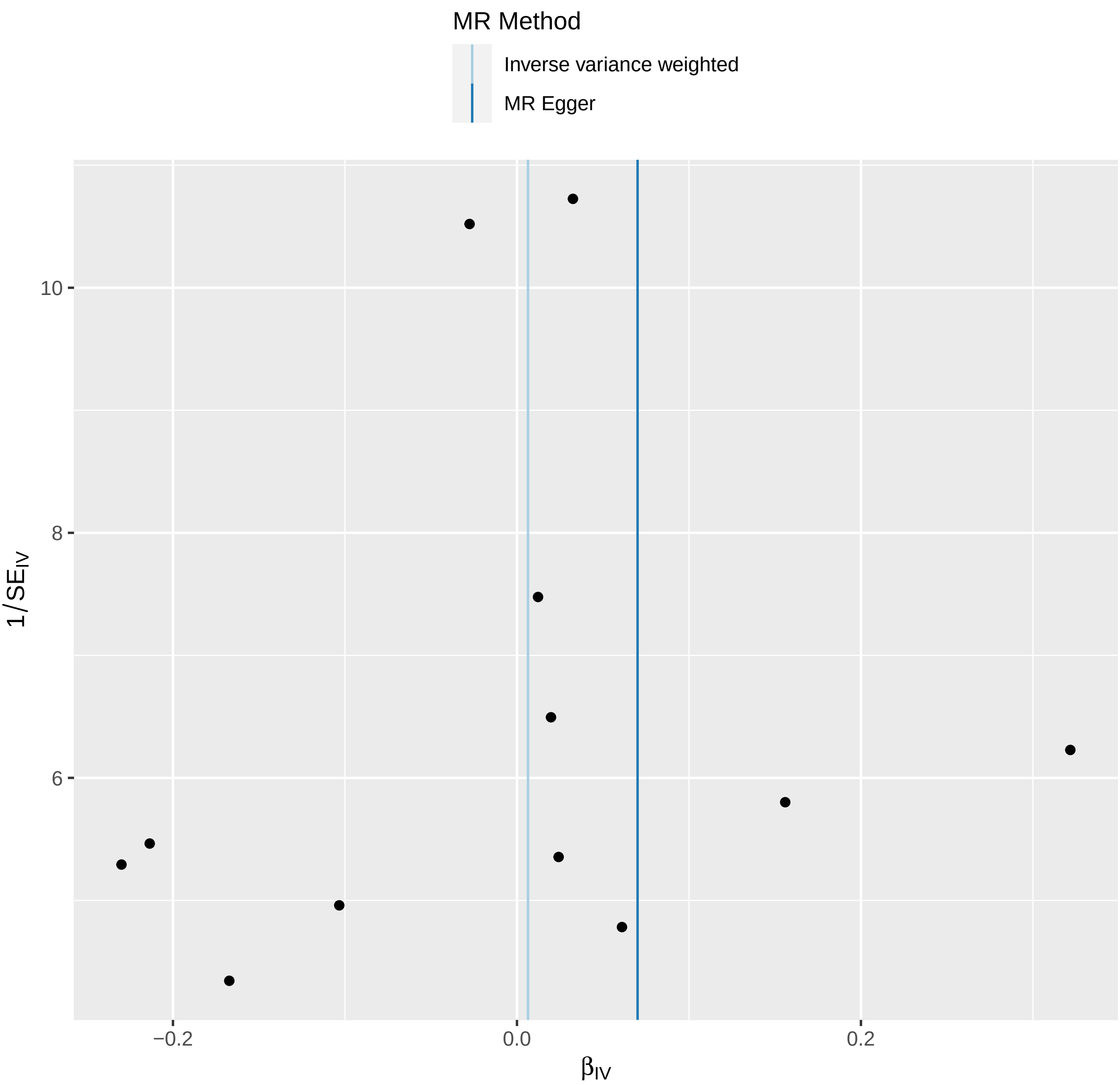

Batch 3−2990 : Selenium on Toll−like receptor 4

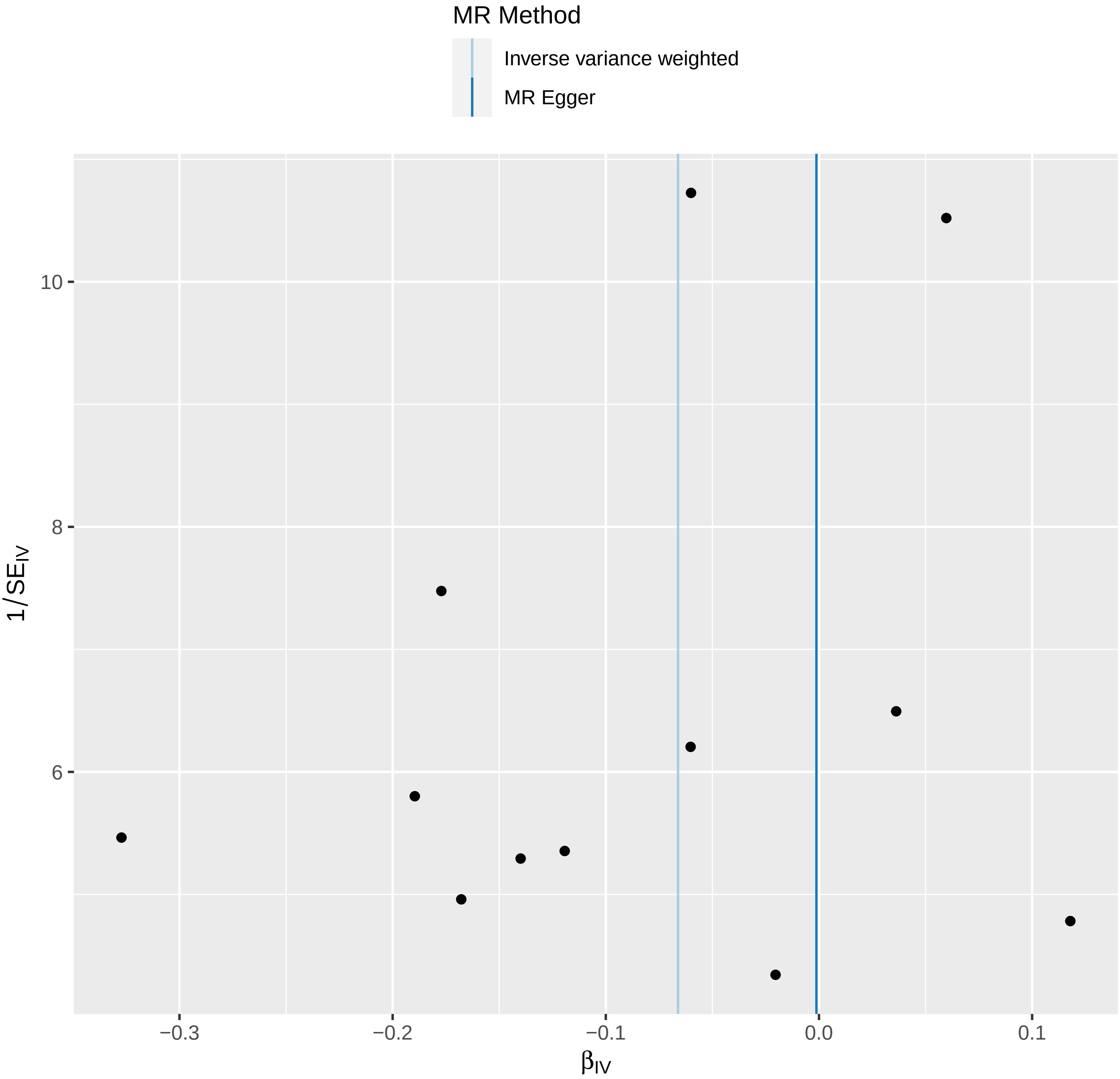

Batch 3−2991 : Selenium on Toll−like receptor 4:Lymphocyte antigen 96 complex

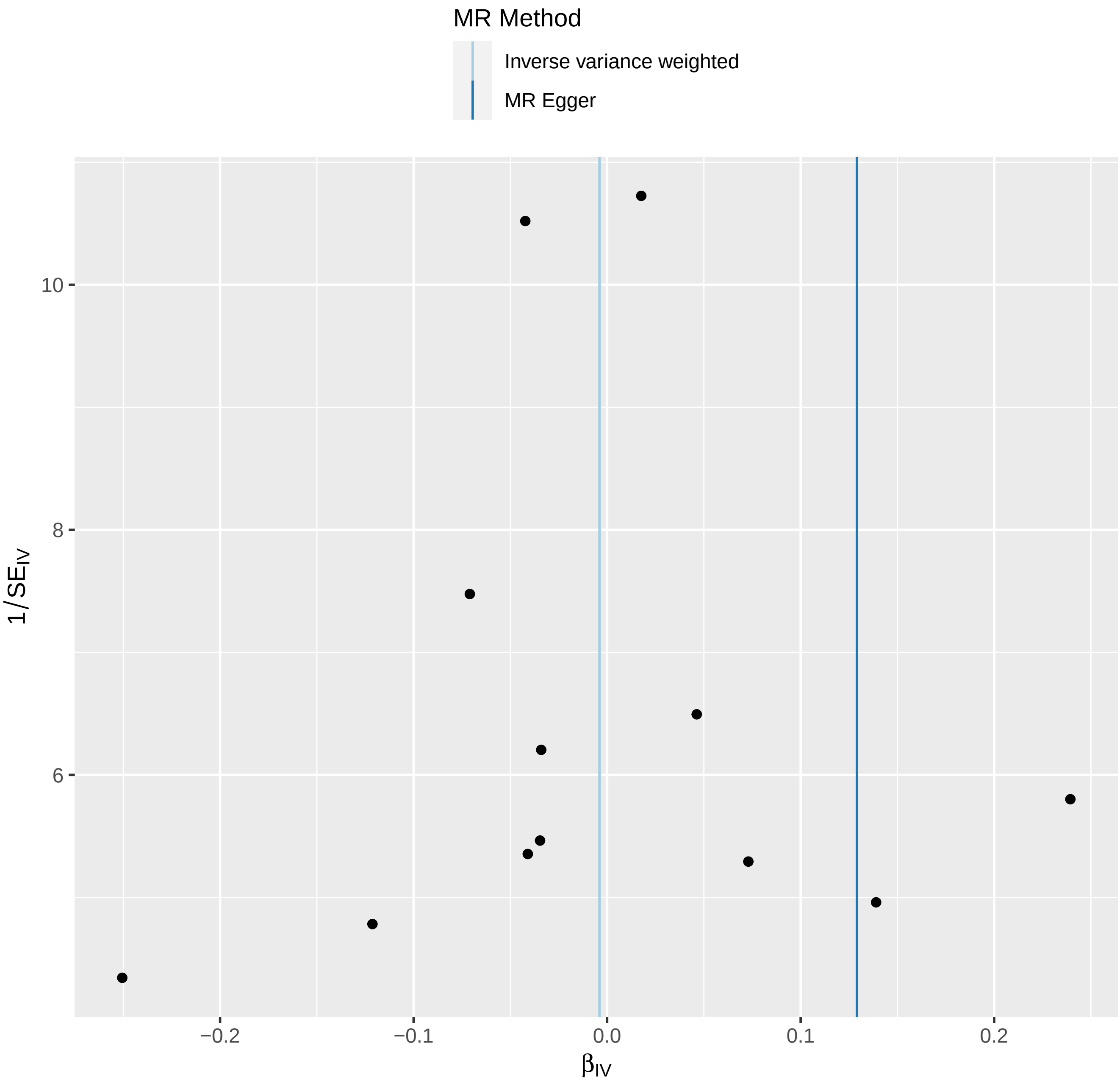

Batch 3−2992 : Selenium on Transmembrane and coiled−coil domain−containing protein 5A

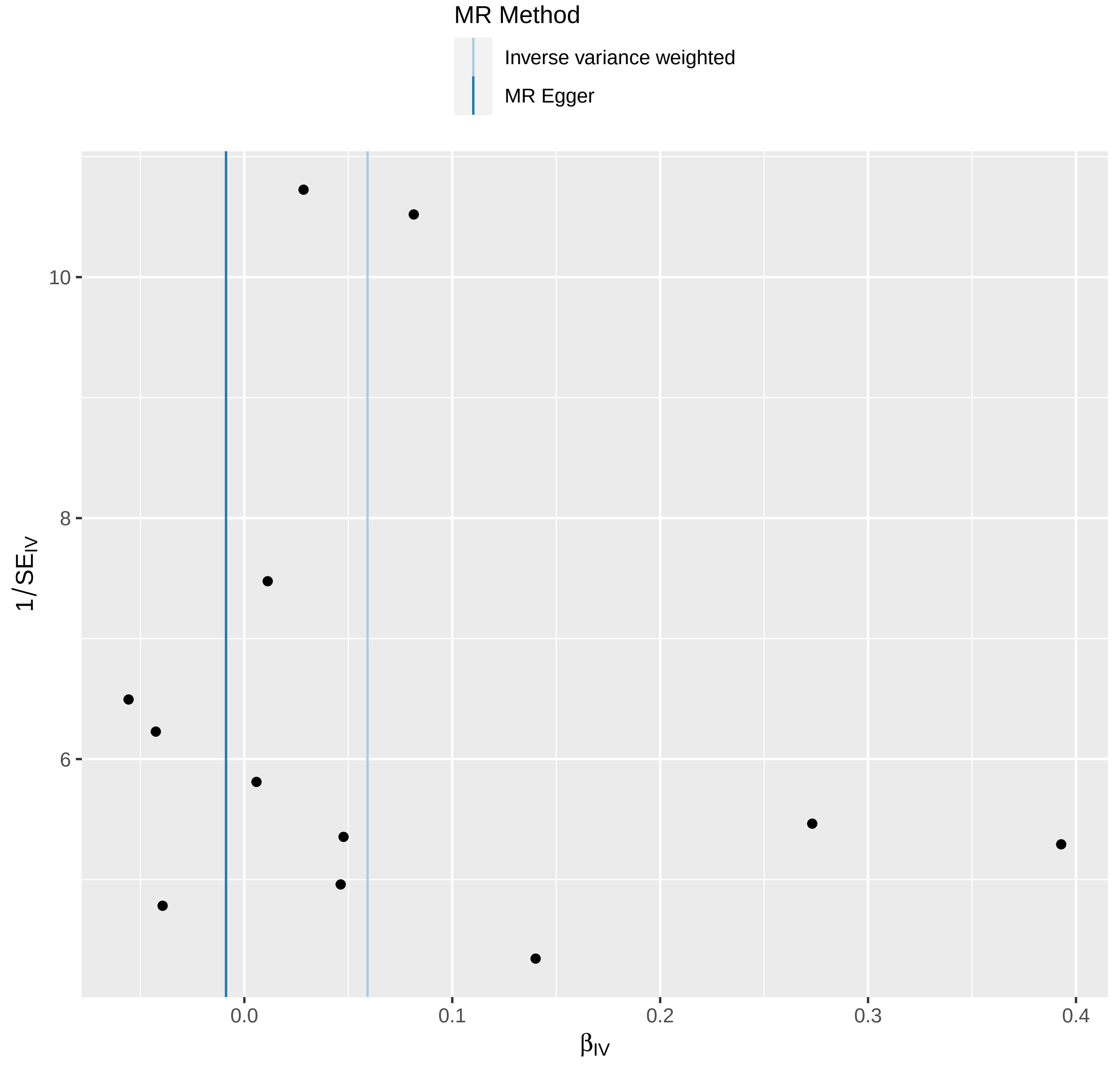

Batch 3−2993 : Selenium on Transmembrane emp24 domain−containing protein 10

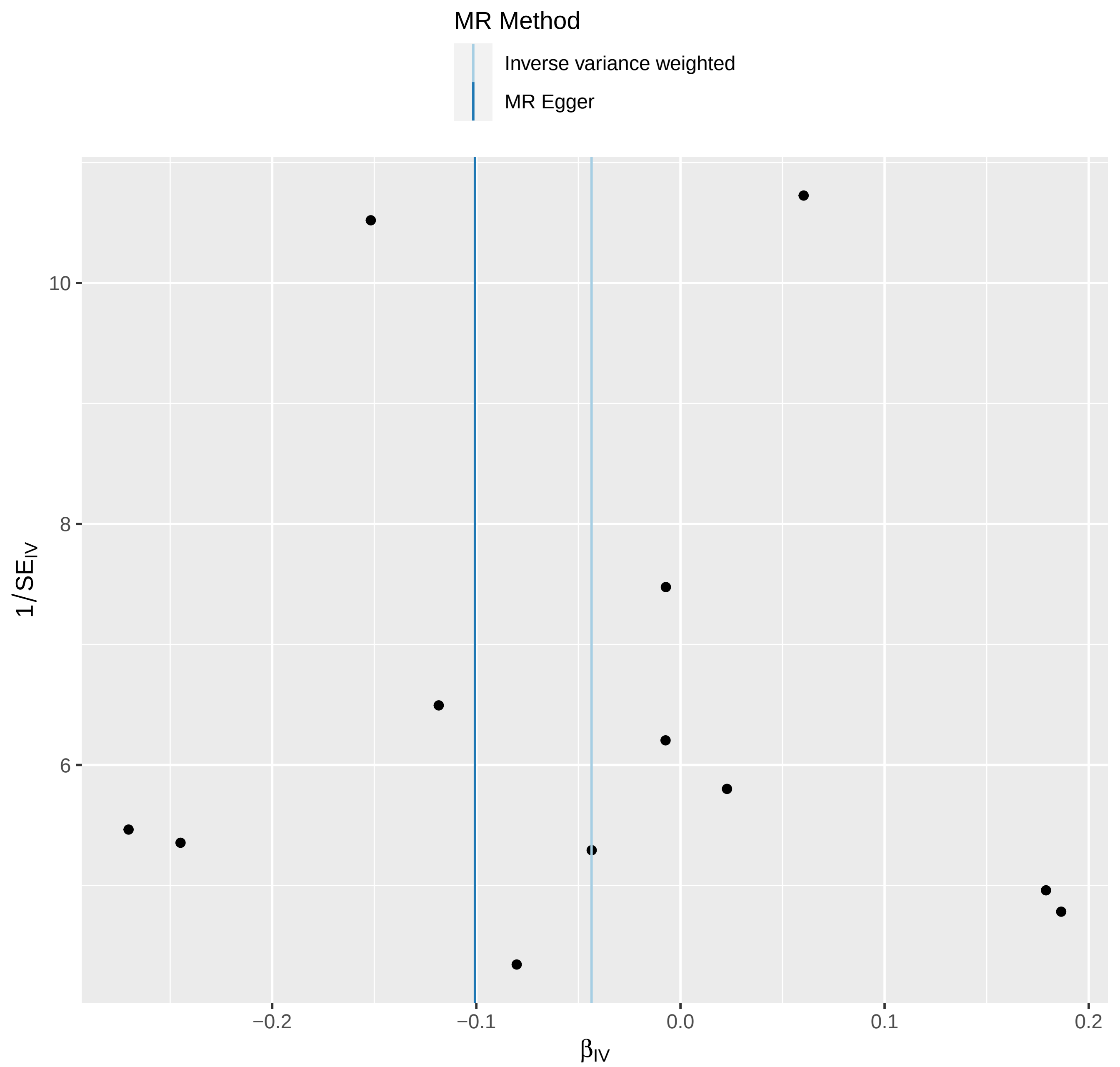

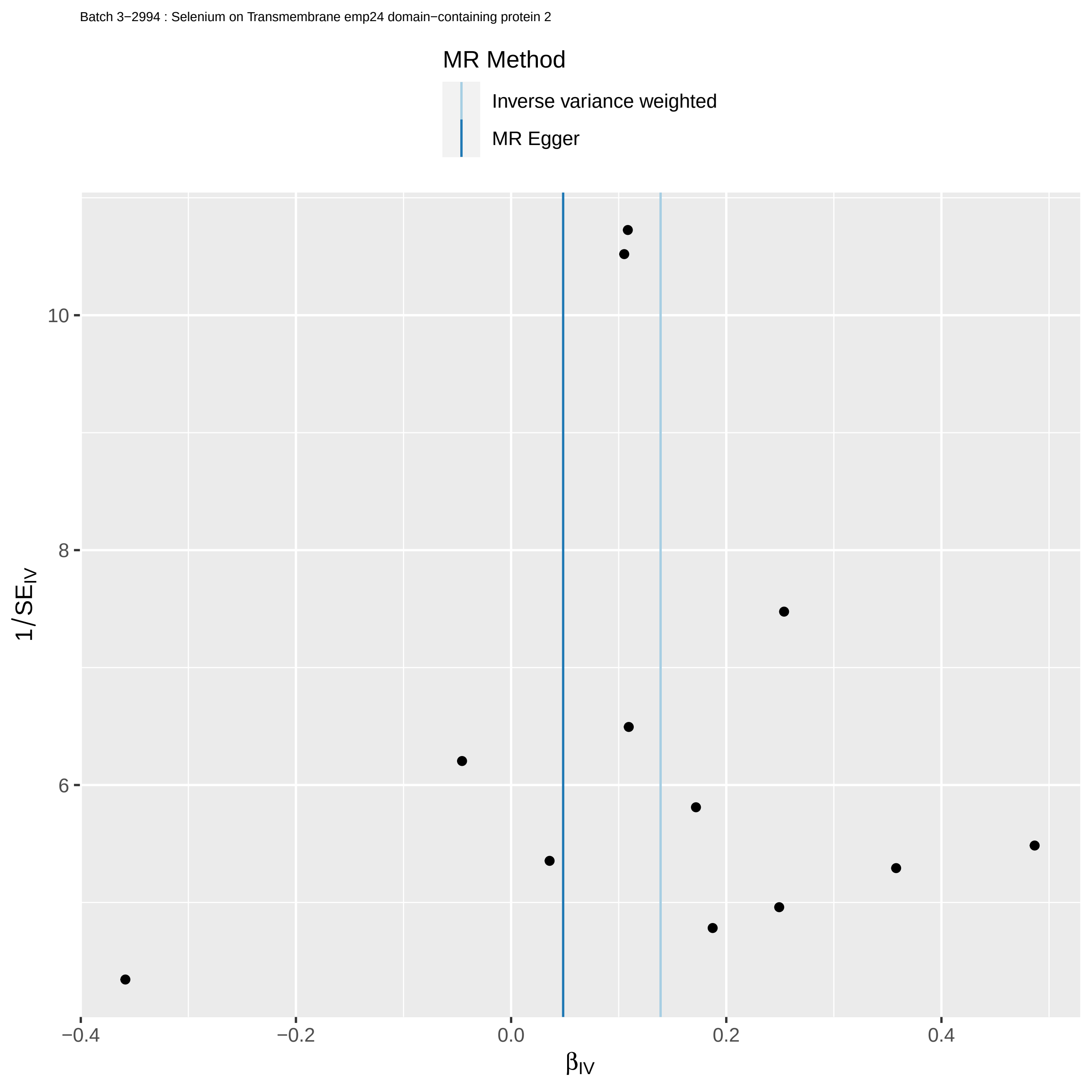
Batch 3−2994 : Selenium on Transmembrane emp24 domain−containing protein 2
MR Method
Inverse variance weighted
MR Egger
10
8
6
1/SE$_{IV}$
−0.4
−0.2
0.0
0.2
0.4
β$_{IV}$

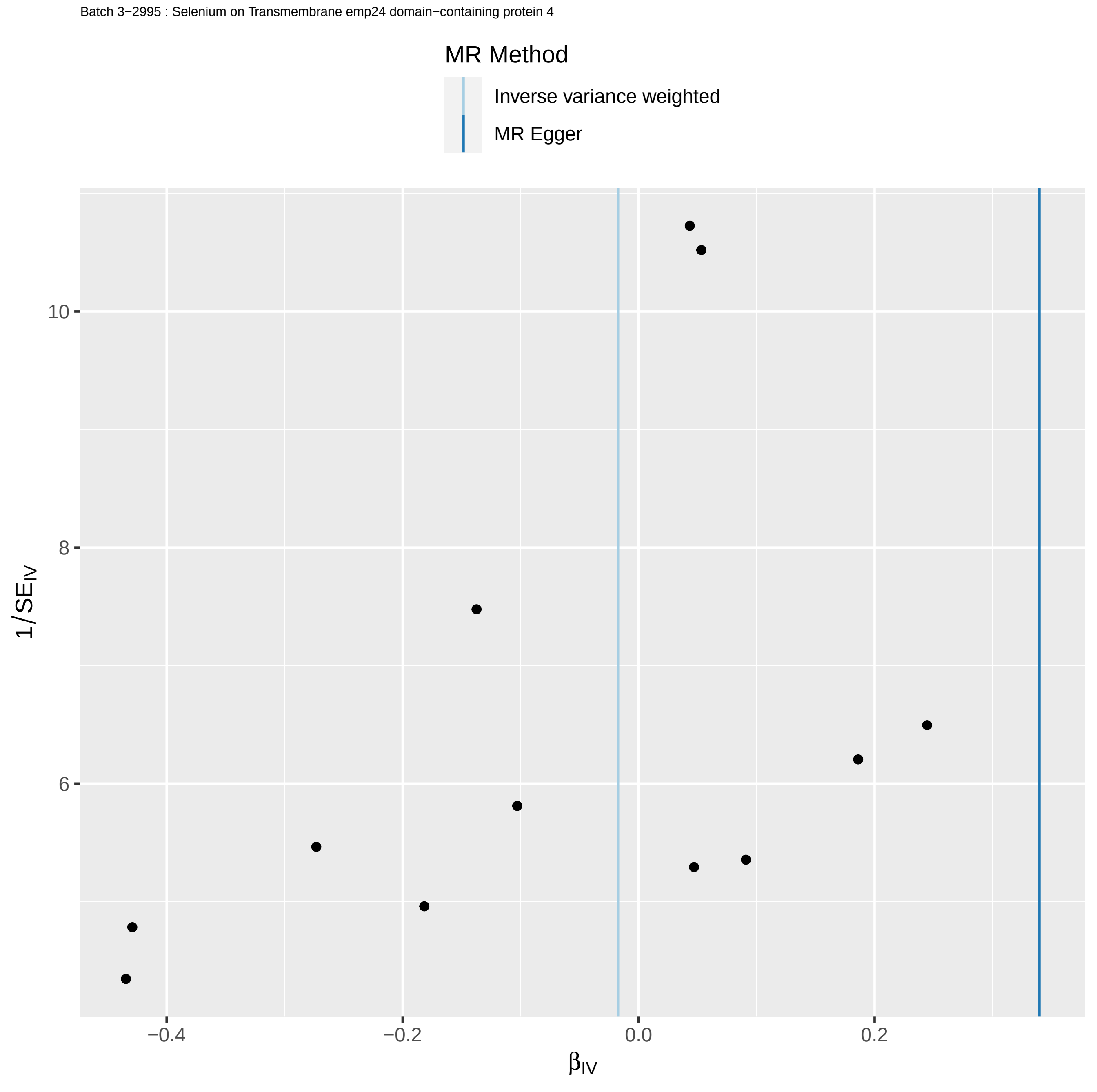
Batch 3−2995 : Selenium on Transmembrane emp24 domain−containing protein 4
MR Method
Inverse variance weighted
MR Egger
10
8
6
$1/SE_{IV}$
−0.4
−0.2
0.0
0.2
β_{IV}

Batch 3−2996 : Selenium on Transmembrane protein 119

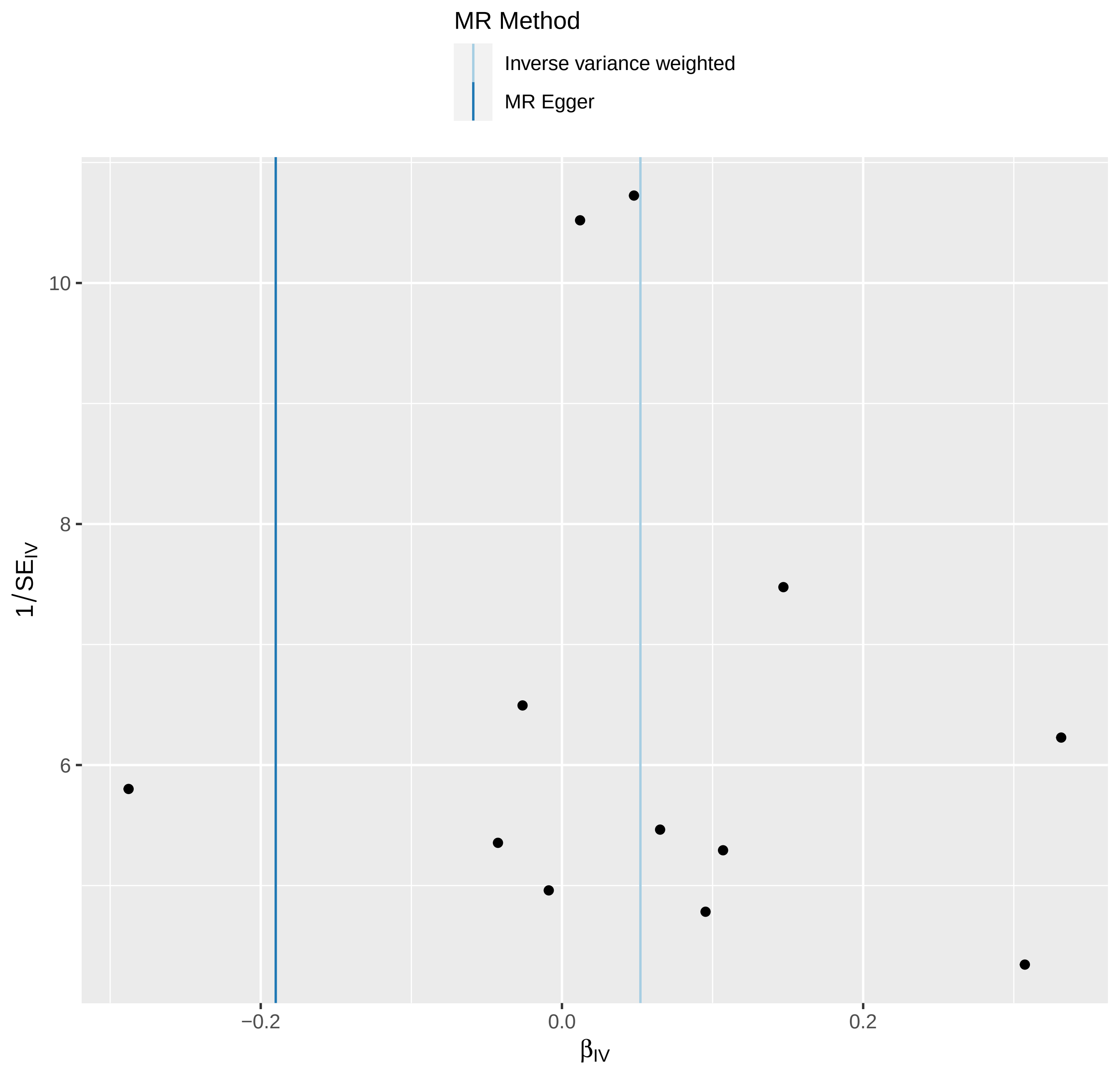

Batch 3−2997 : Selenium on Transmembrane protein 132A

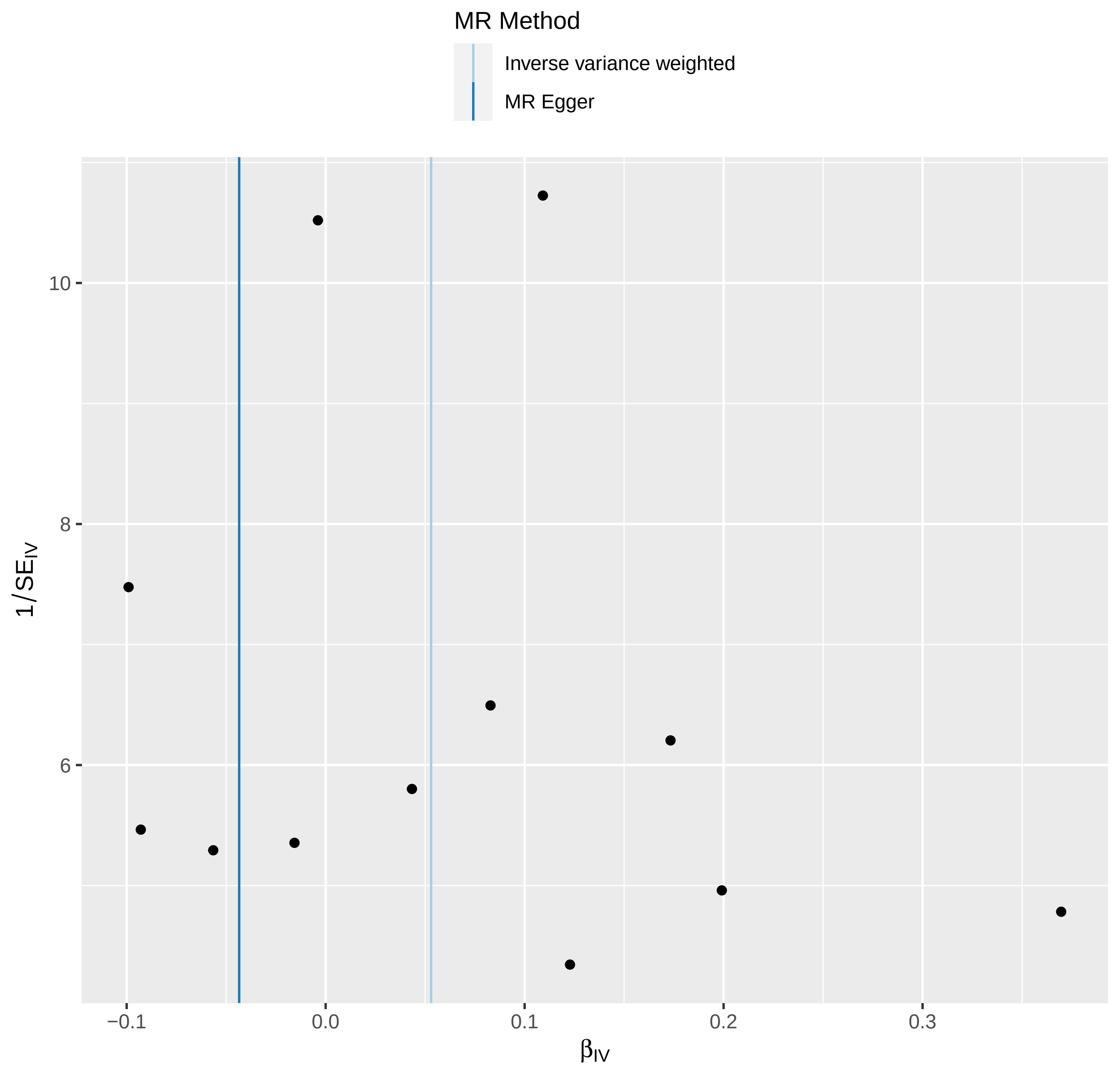

Batch 3−2998 : Selenium on Transmembrane protein 132B

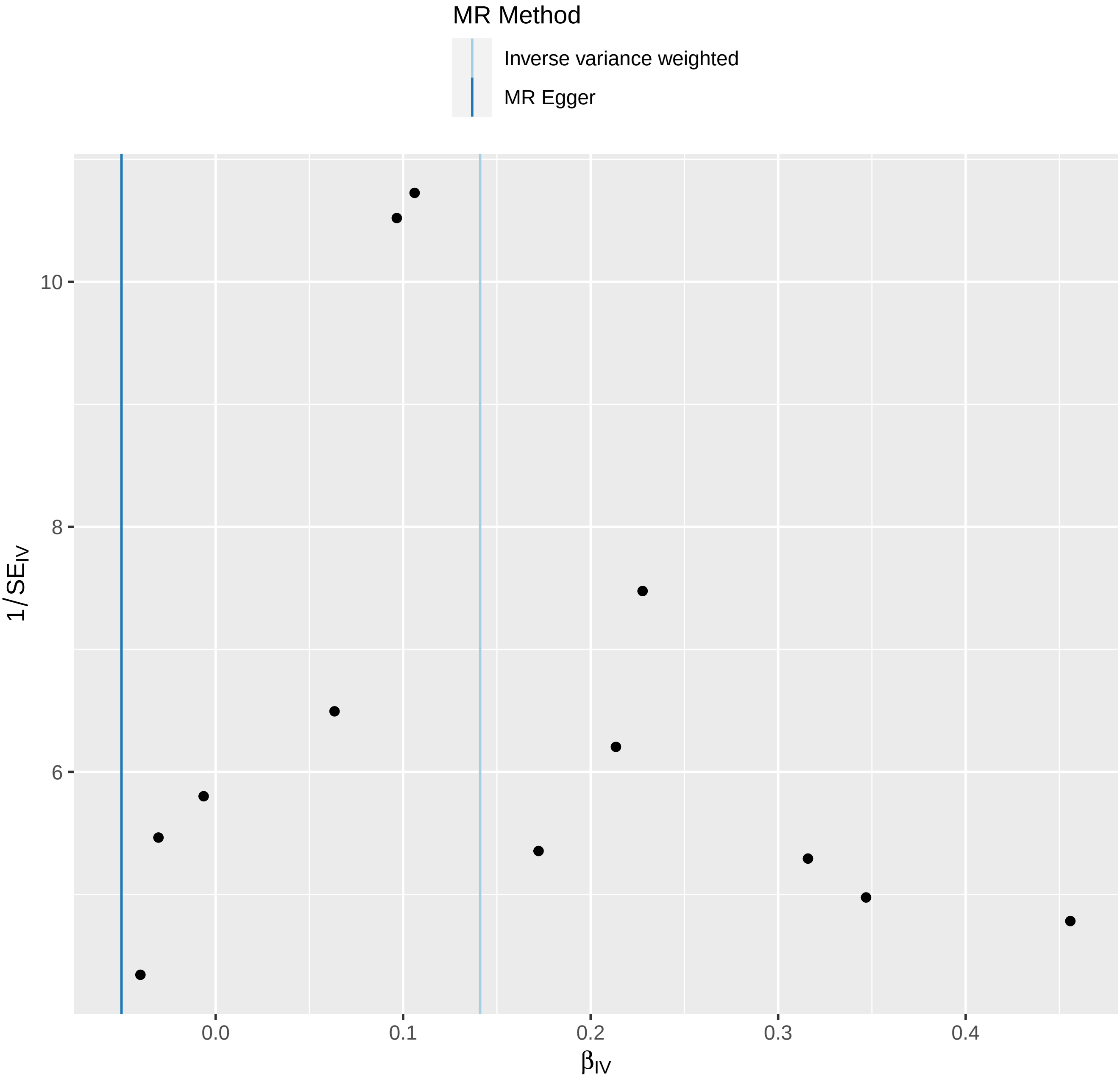

Batch 3−2999 : Selenium on Transmembrane protein 132C

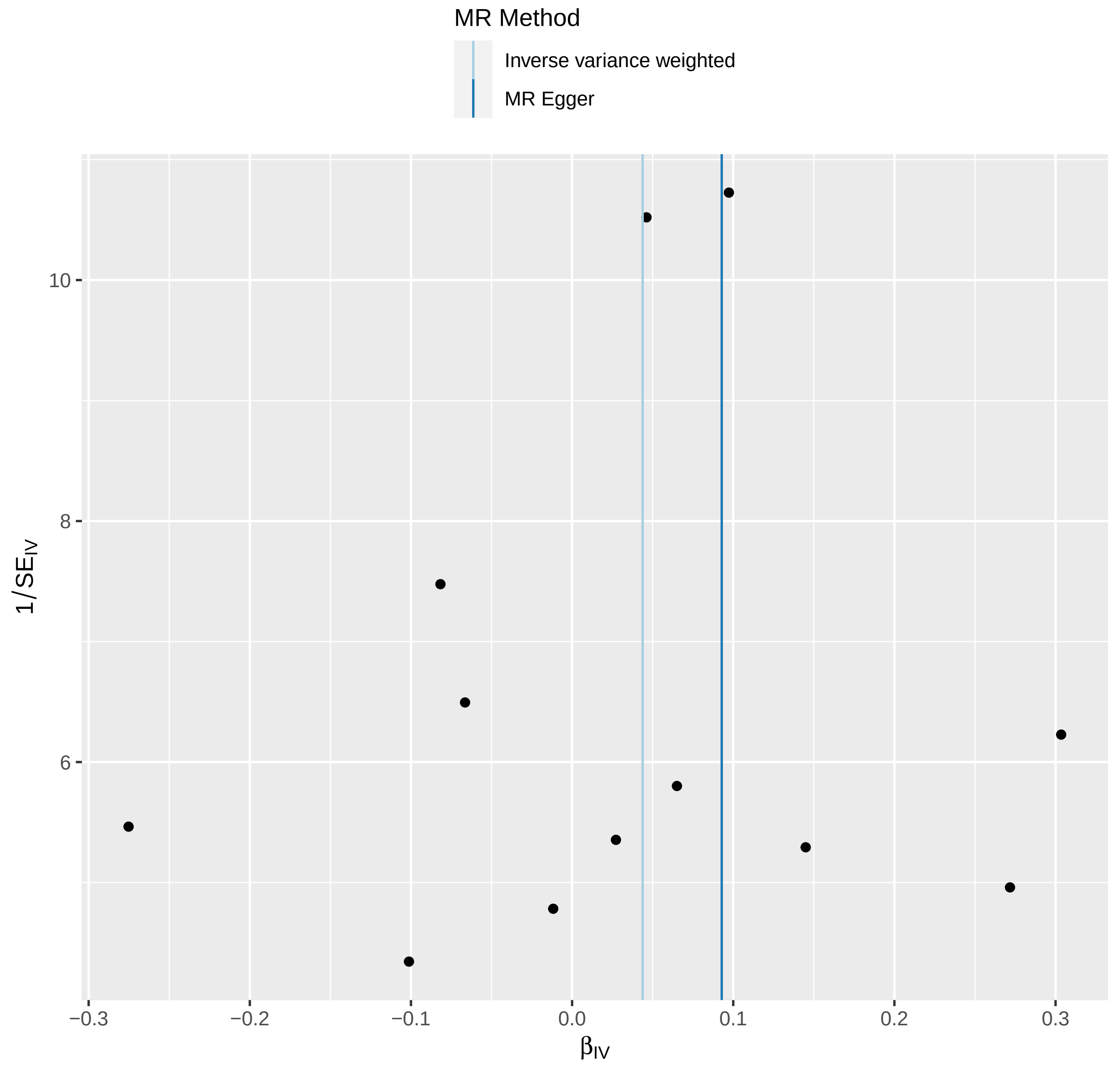

Batch 3−3000 : Selenium on Transmembrane protein 132C

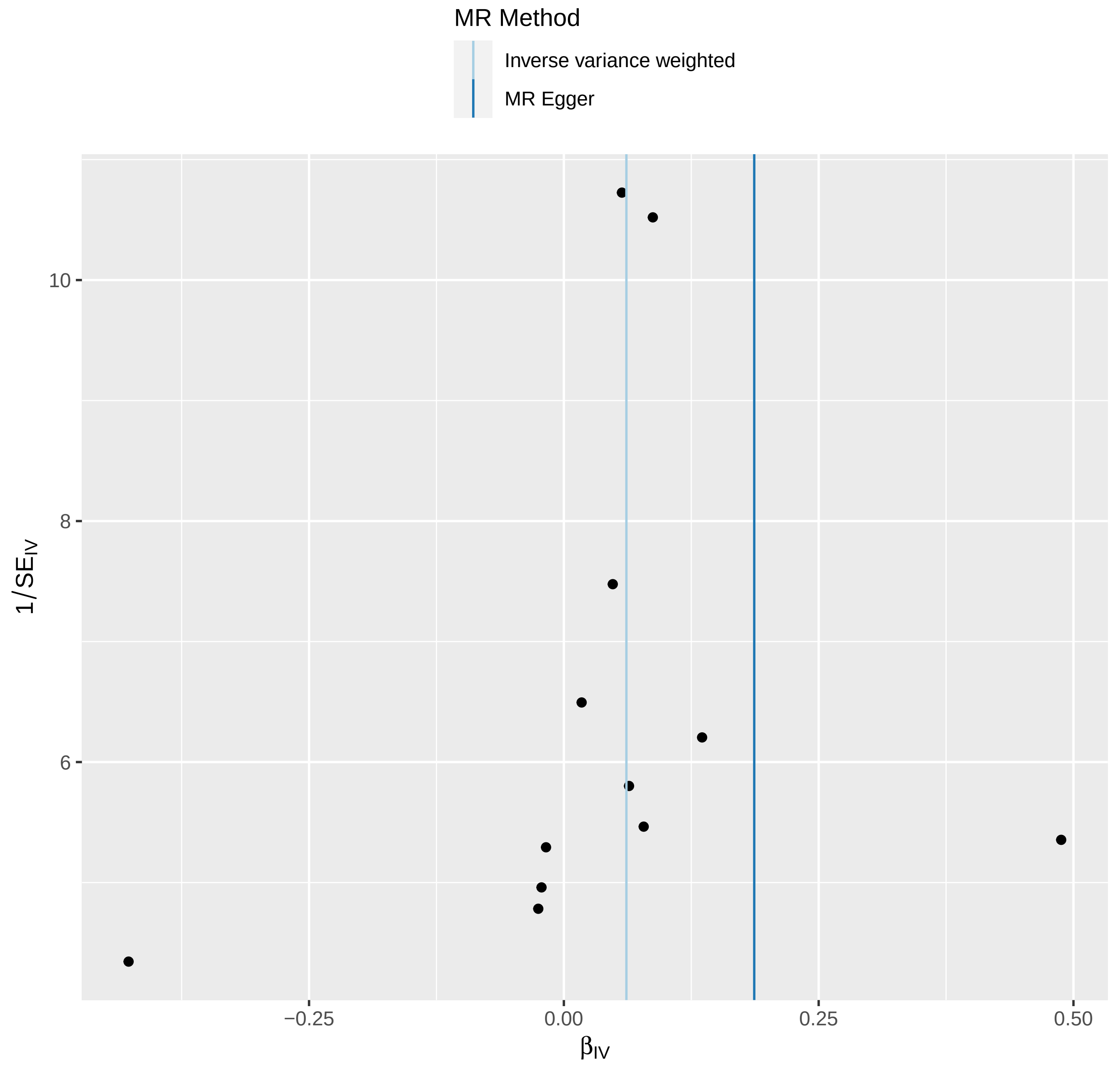

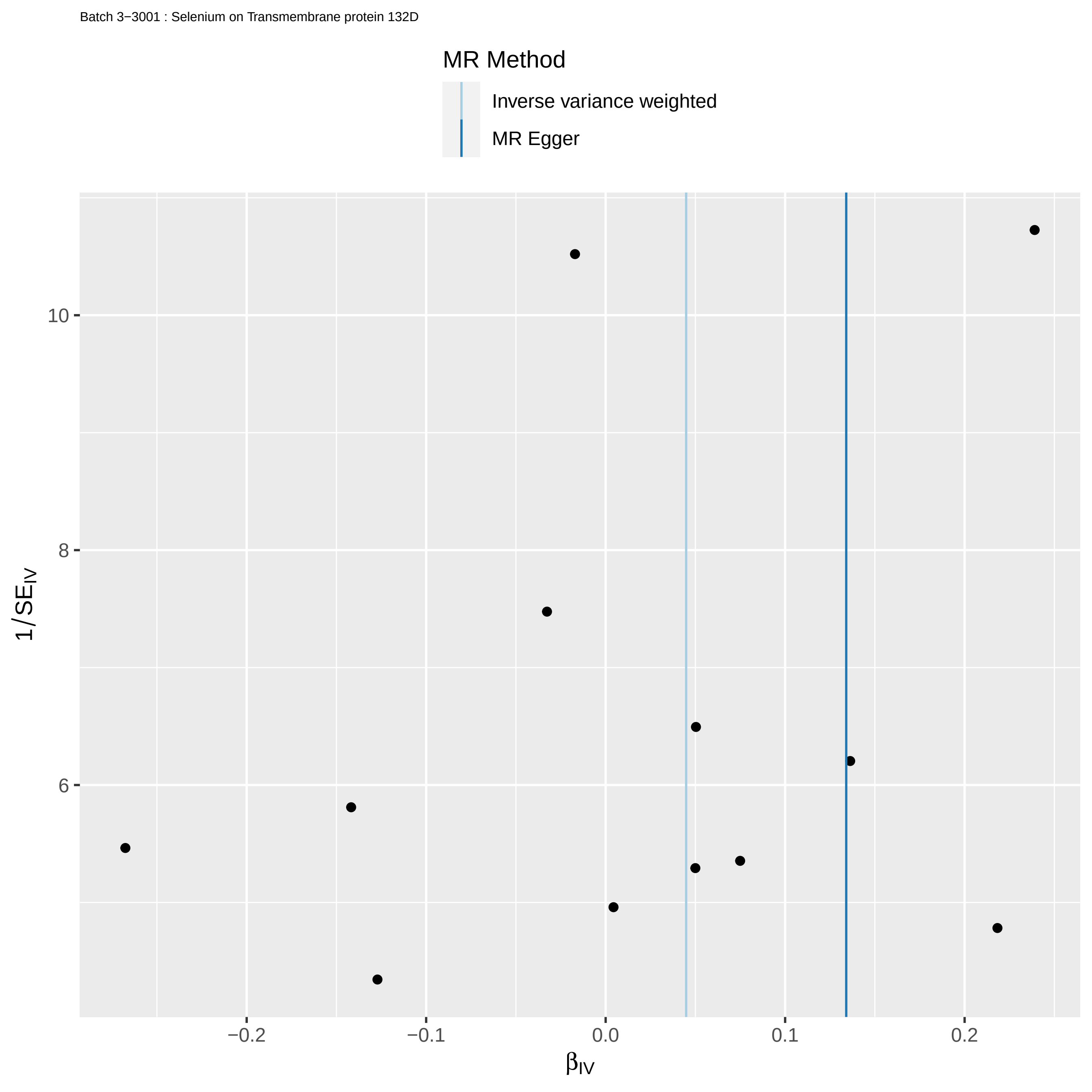

Batch 3−3001 : Selenium on Transmembrane protein 132D
MR Method
Inverse variance weighted
MR Egger
10
8
6
1/SE_IV
−0.2
−0.1
0.0
0.1
0.2
β_IV

Batch 3−3002 : Selenium on Protein kish−A

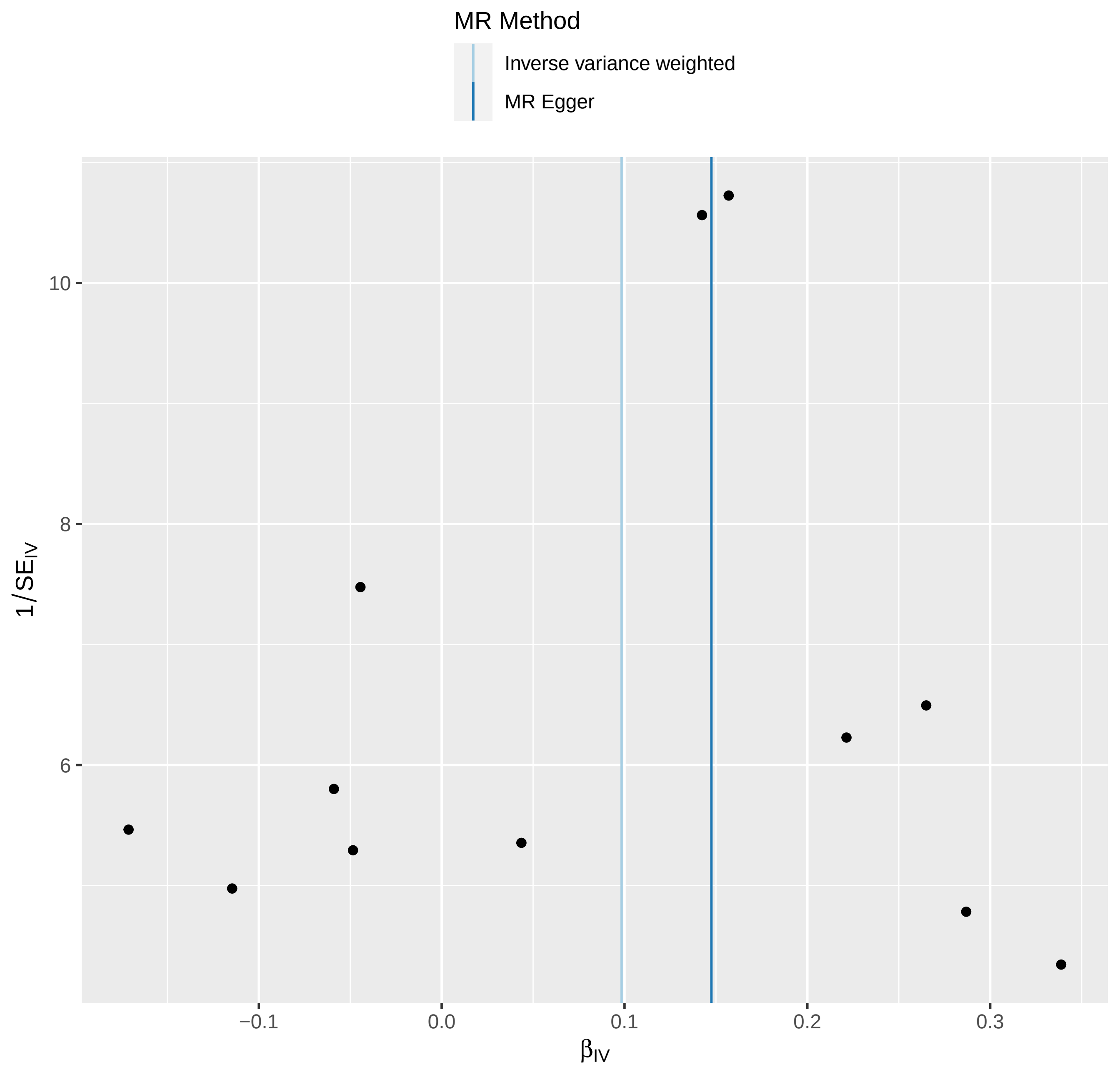

Batch 3−3003 : Selenium on Protein kish−B

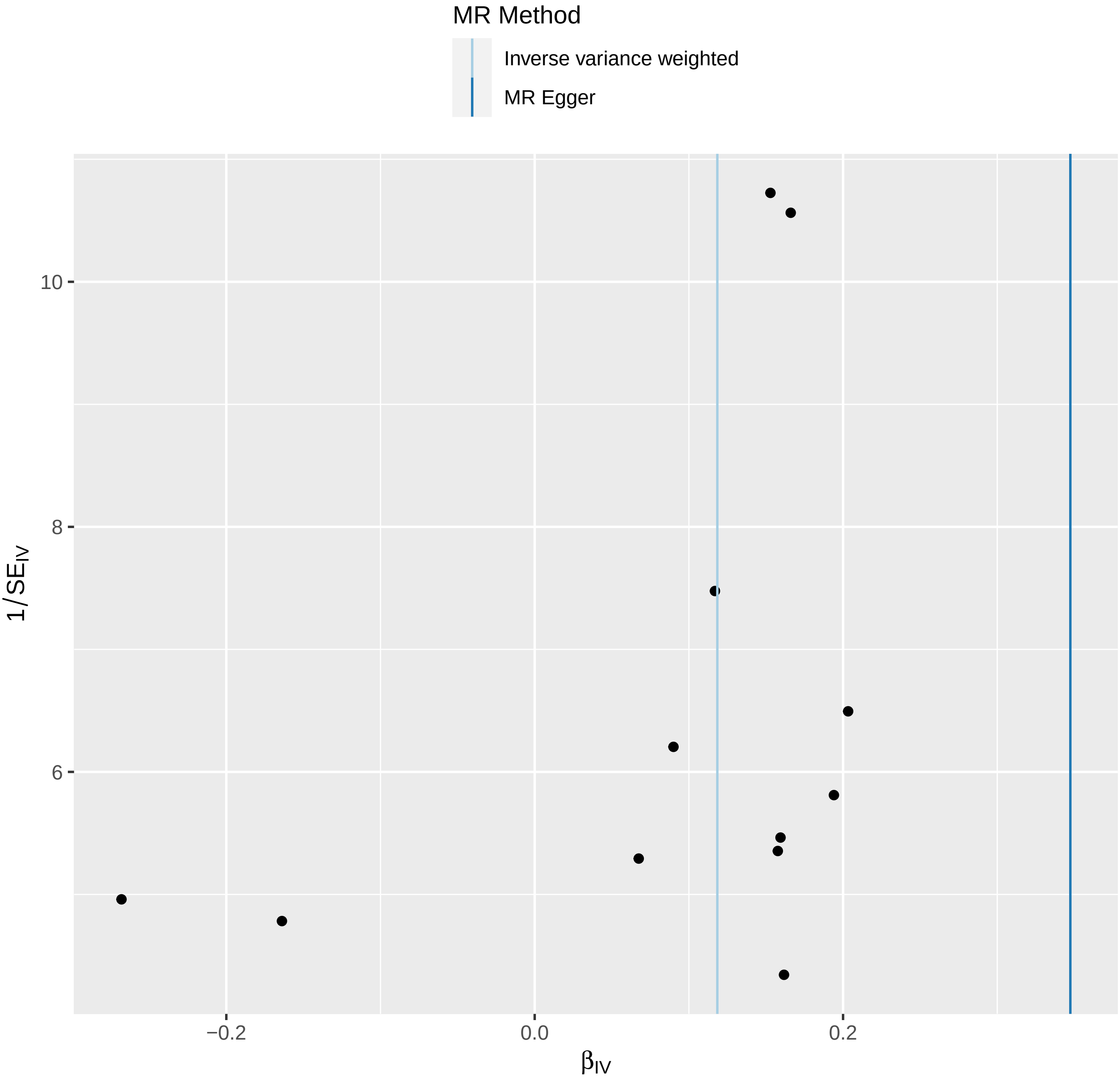

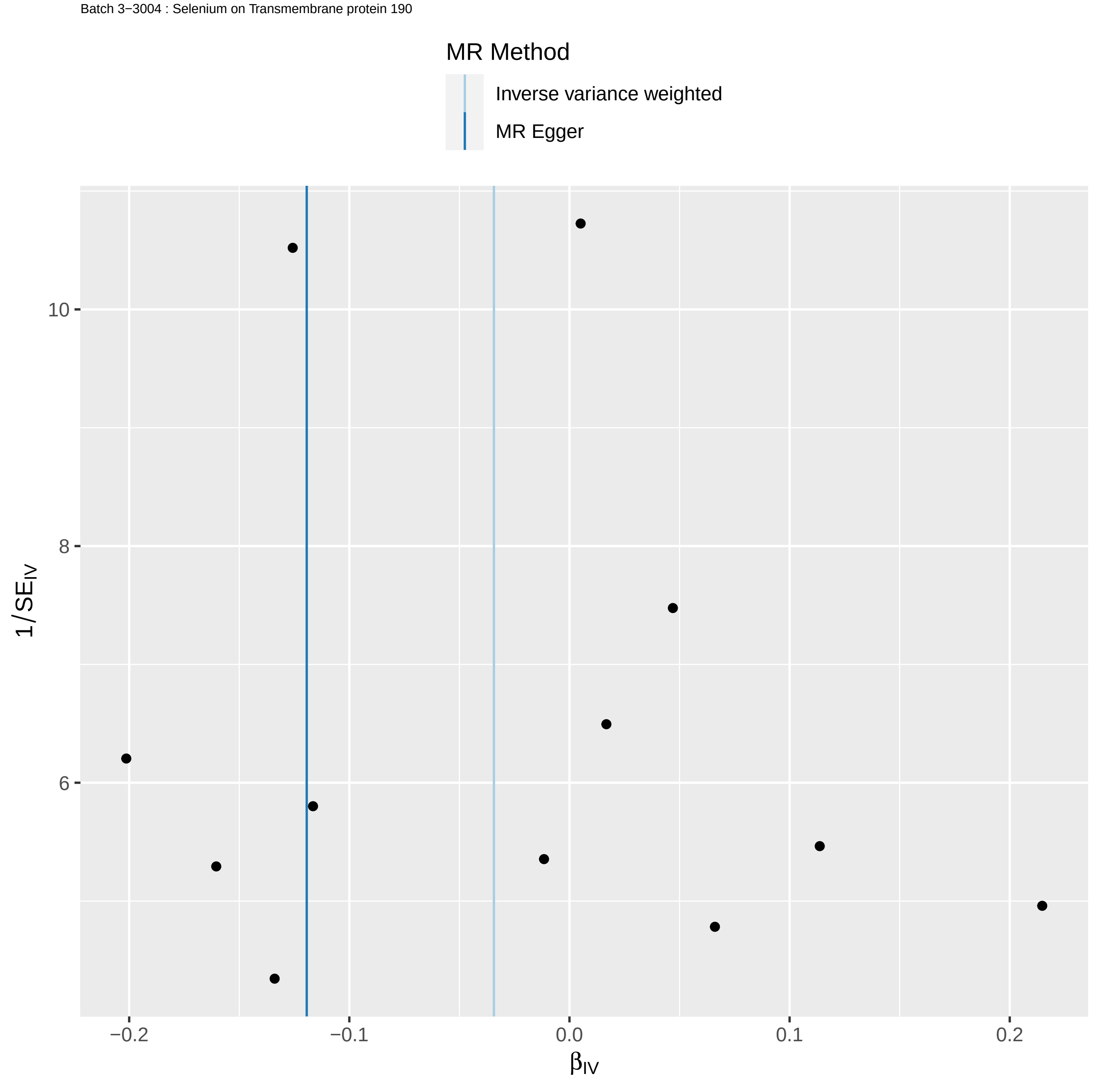

Batch 3−3004 : Selenium on Transmembrane protein 190
MR Method
Inverse variance weighted
MR Egger
10
8
6
1/SE$_{IV}$
−0.2
−0.1
0.0
0.1
0.2
β$_{IV}$

Batch 3−3005 : Selenium on Transmembrane protein 230

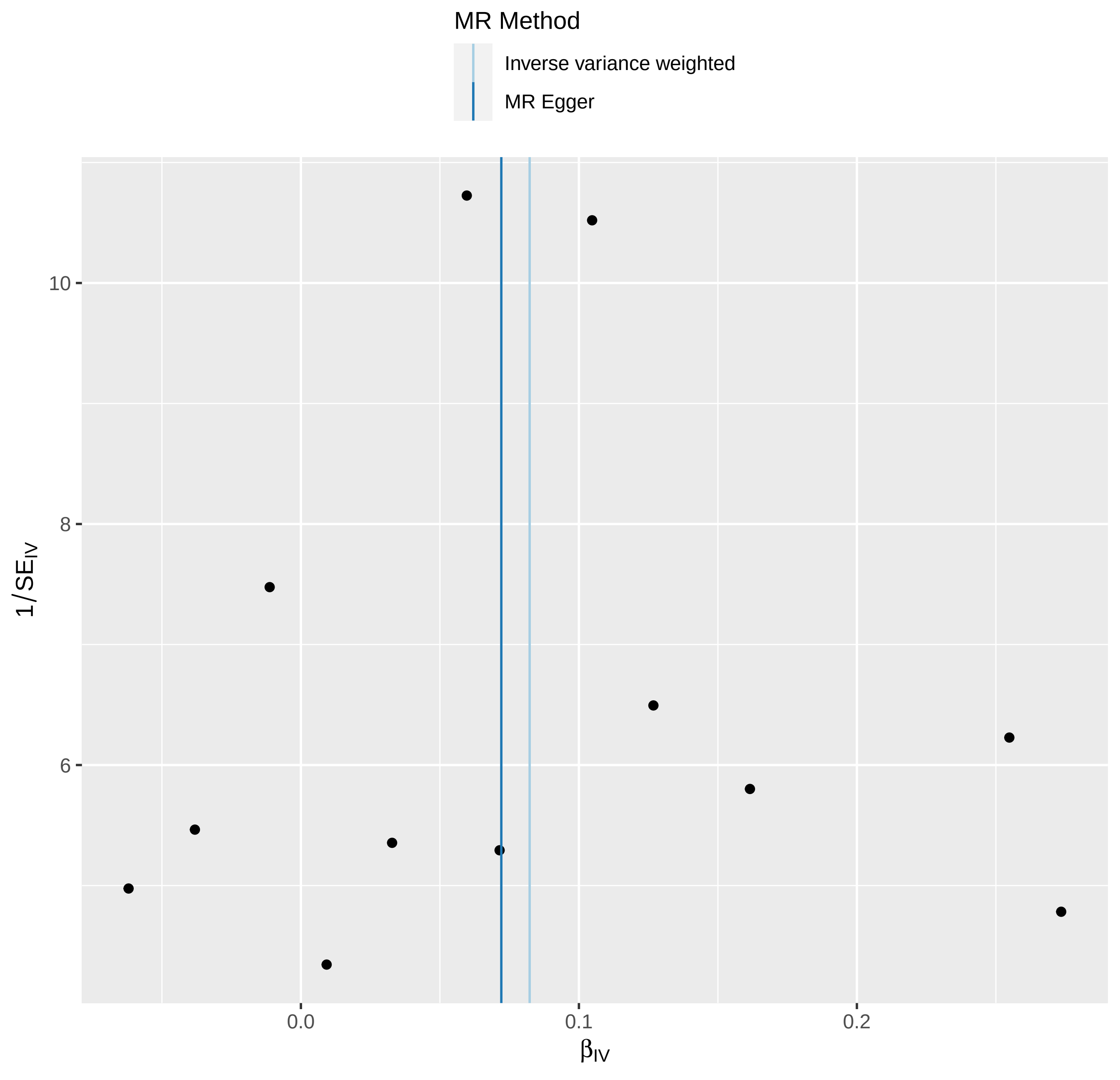

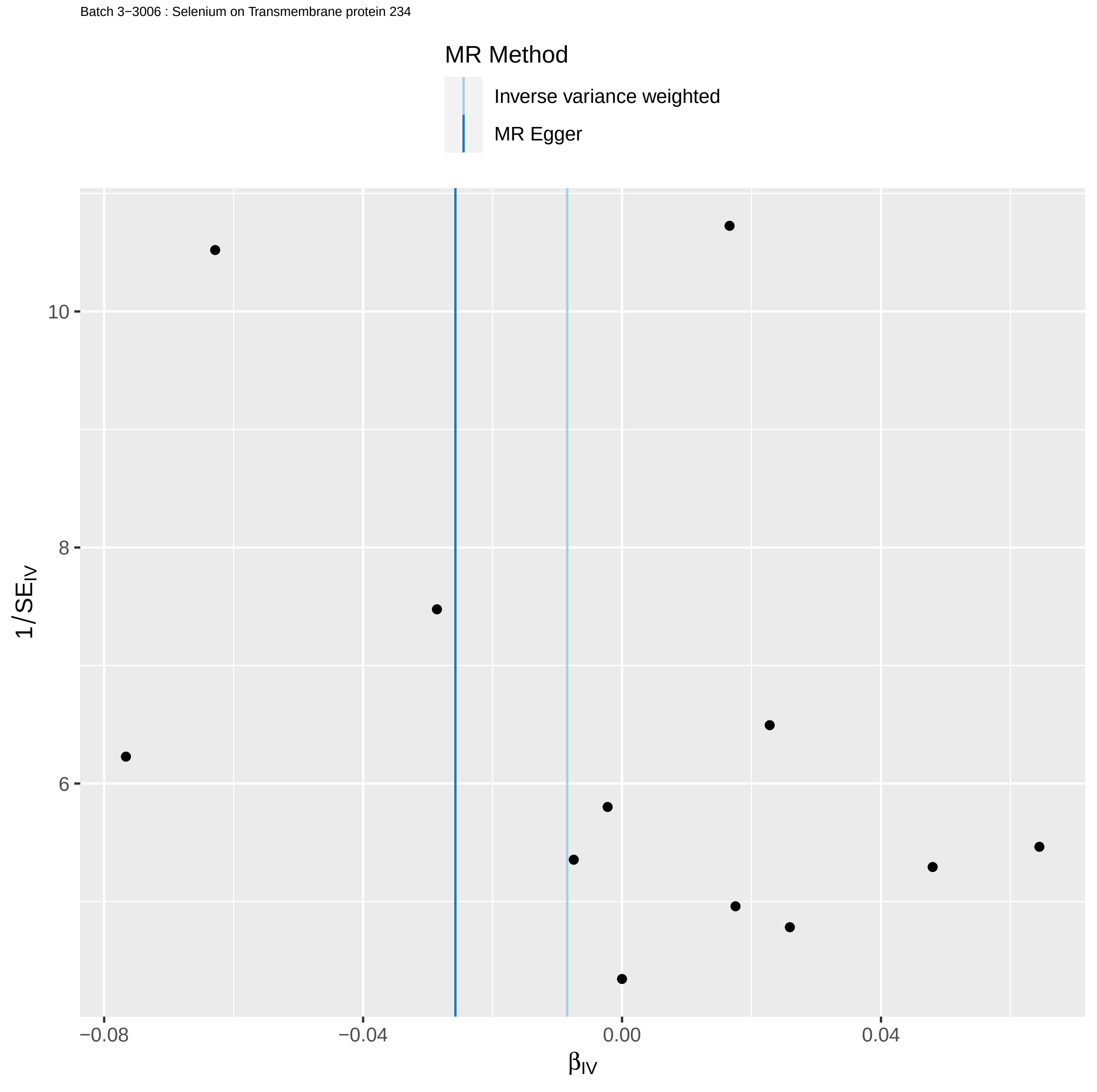

Batch 3−3006 : Selenium on Transmembrane protein 234
MR Method
Inverse variance weighted
MR Egger
10
8
6
1/SE_IV
−0.08
−0.04
0.00
0.04
β_IV

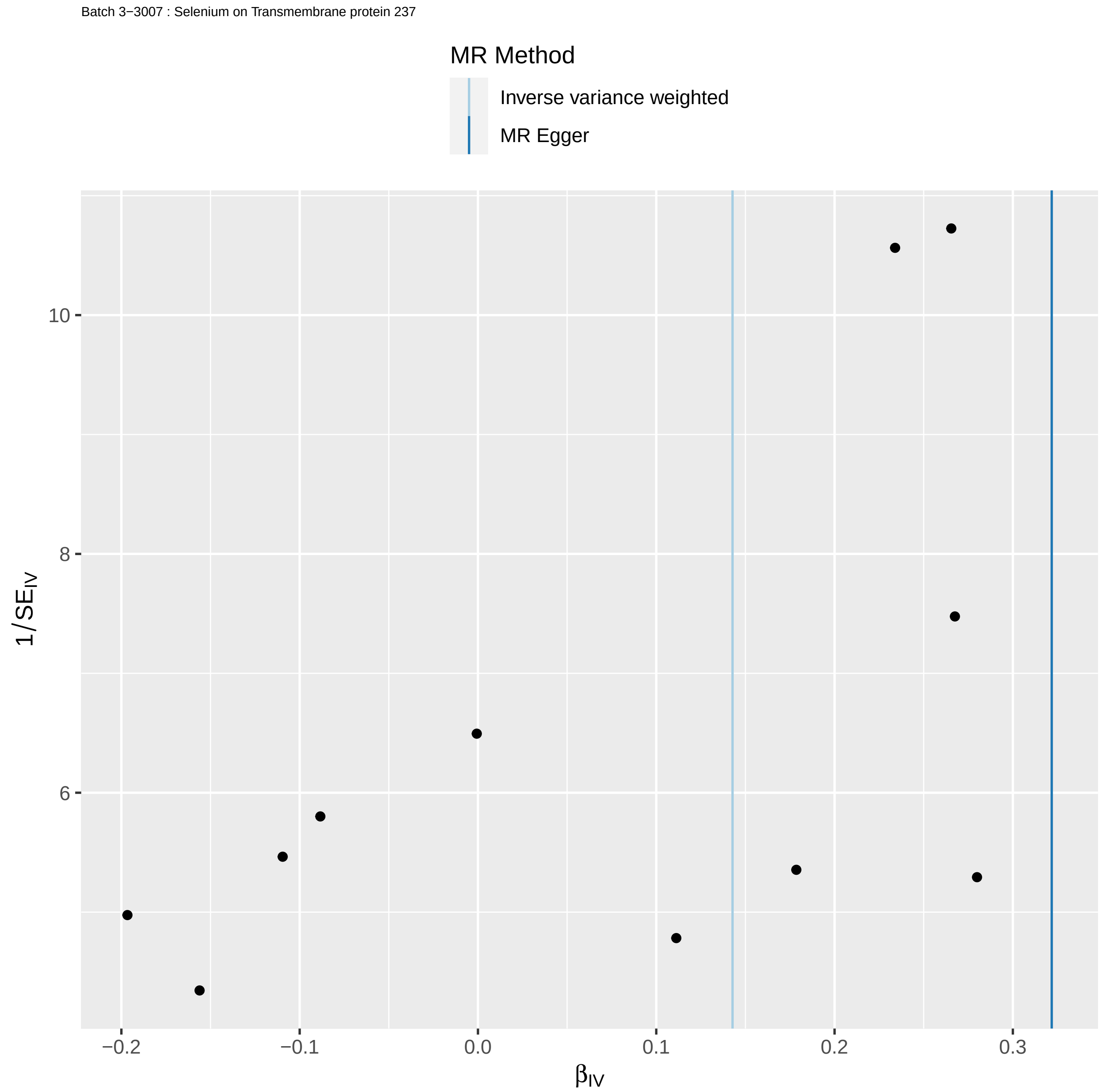
Batch 3−3007 : Selenium on Transmembrane protein 237
MR Method
Inverse variance weighted
MR Egger
10
8
6
$1/SE_{IV}$
−0.2
−0.1
0.0
0.1
0.2
0.3
β_{IV}

Batch 3−3008 : Selenium on Collectrin

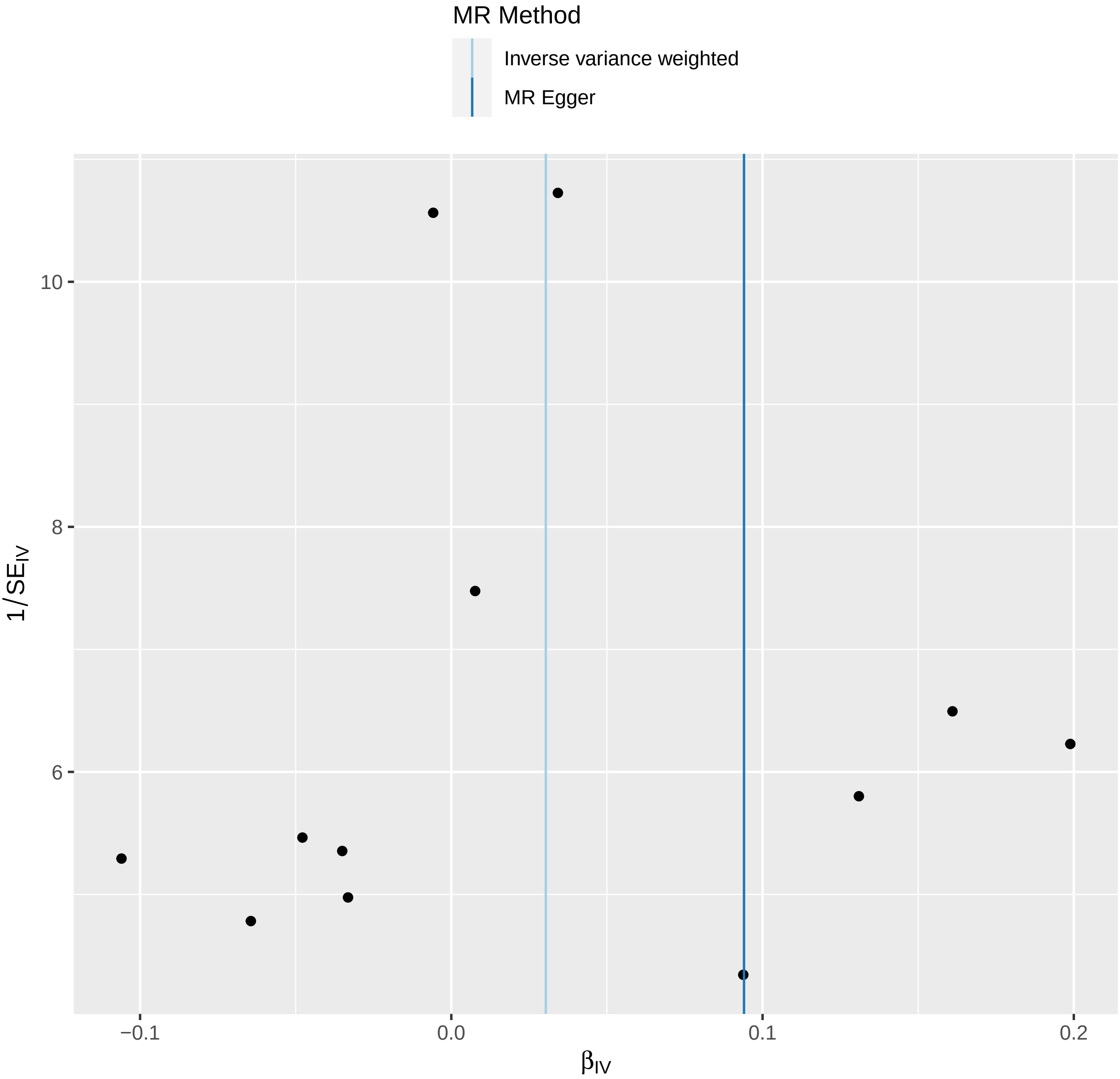

Batch 3−3009 : Selenium on Transmembrane protein 2

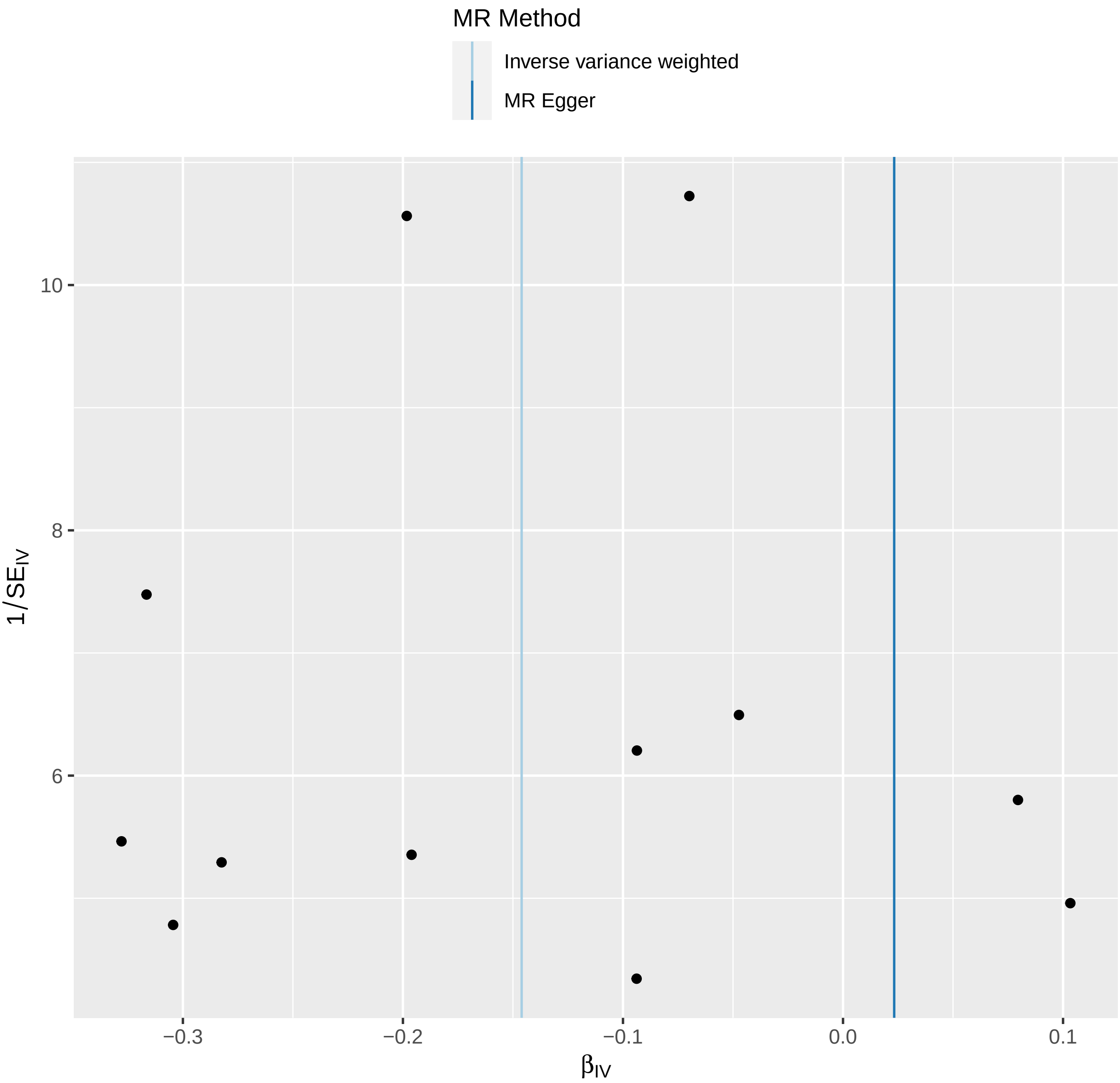

Batch 3−3010 : Selenium on Transmembrane protein 40

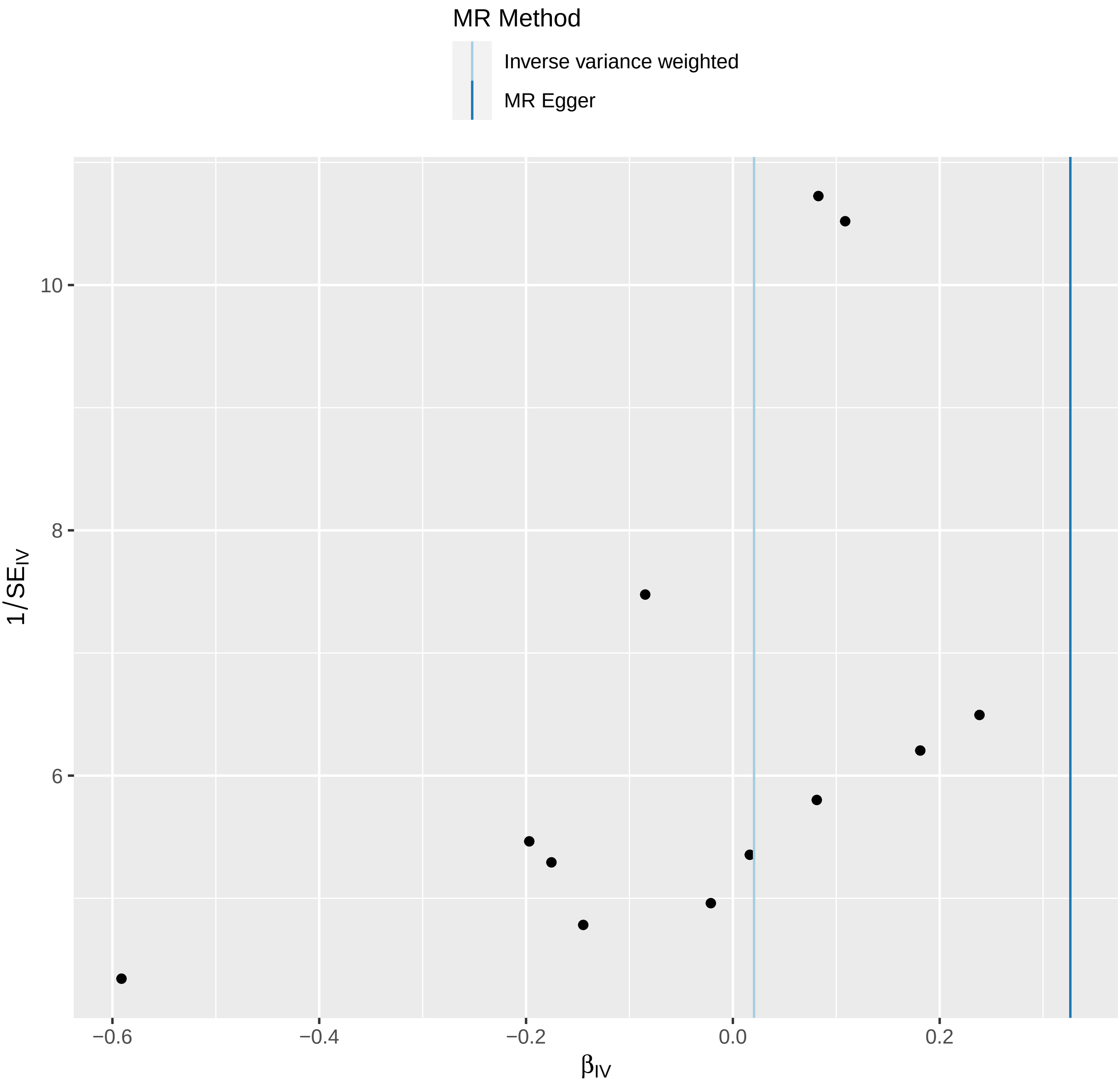

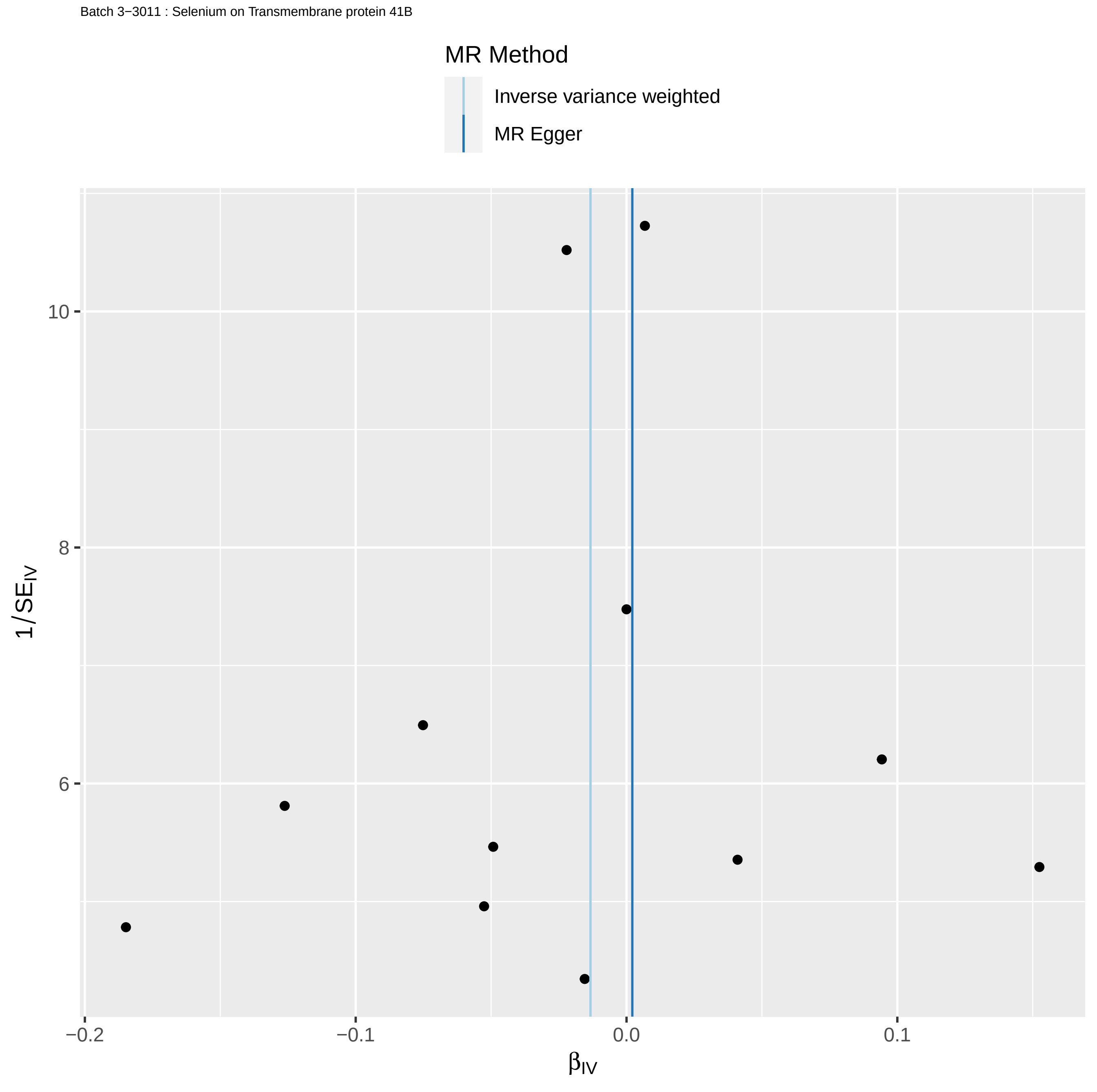

Batch 3−3011 : Selenium on Transmembrane protein 41B
MR Method
Inverse variance weighted
MR Egger
10
8
6
1/SE$_{IV}$
−0.2
−0.1
0.0
0.1
β$_{IV}$

Batch 3−3012 : Selenium on Transmembrane protein 52

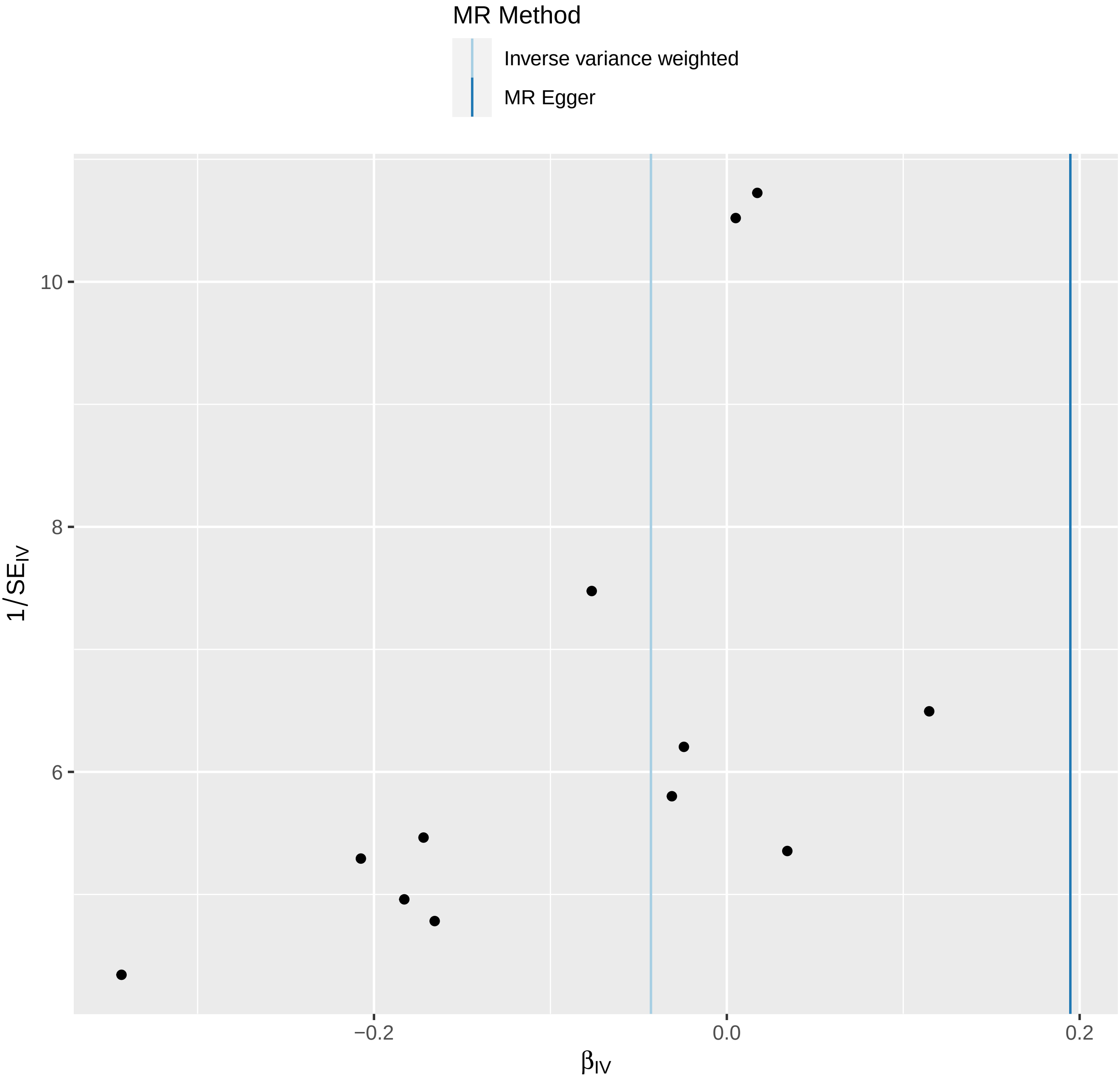

Batch 3−3013 : Selenium on Macoilin

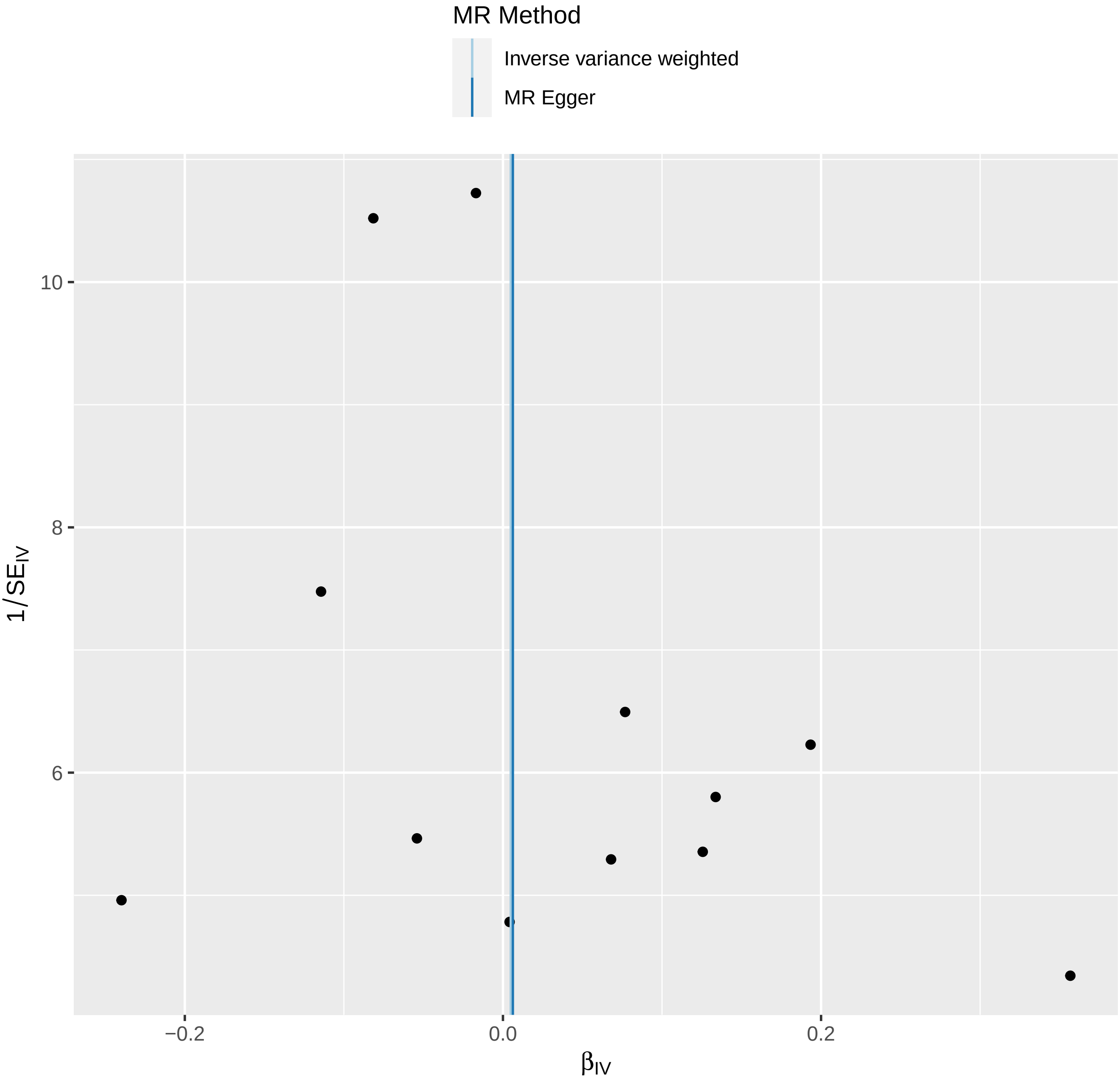

Batch 3−3014 : Selenium on Transmembrane protein 59−like

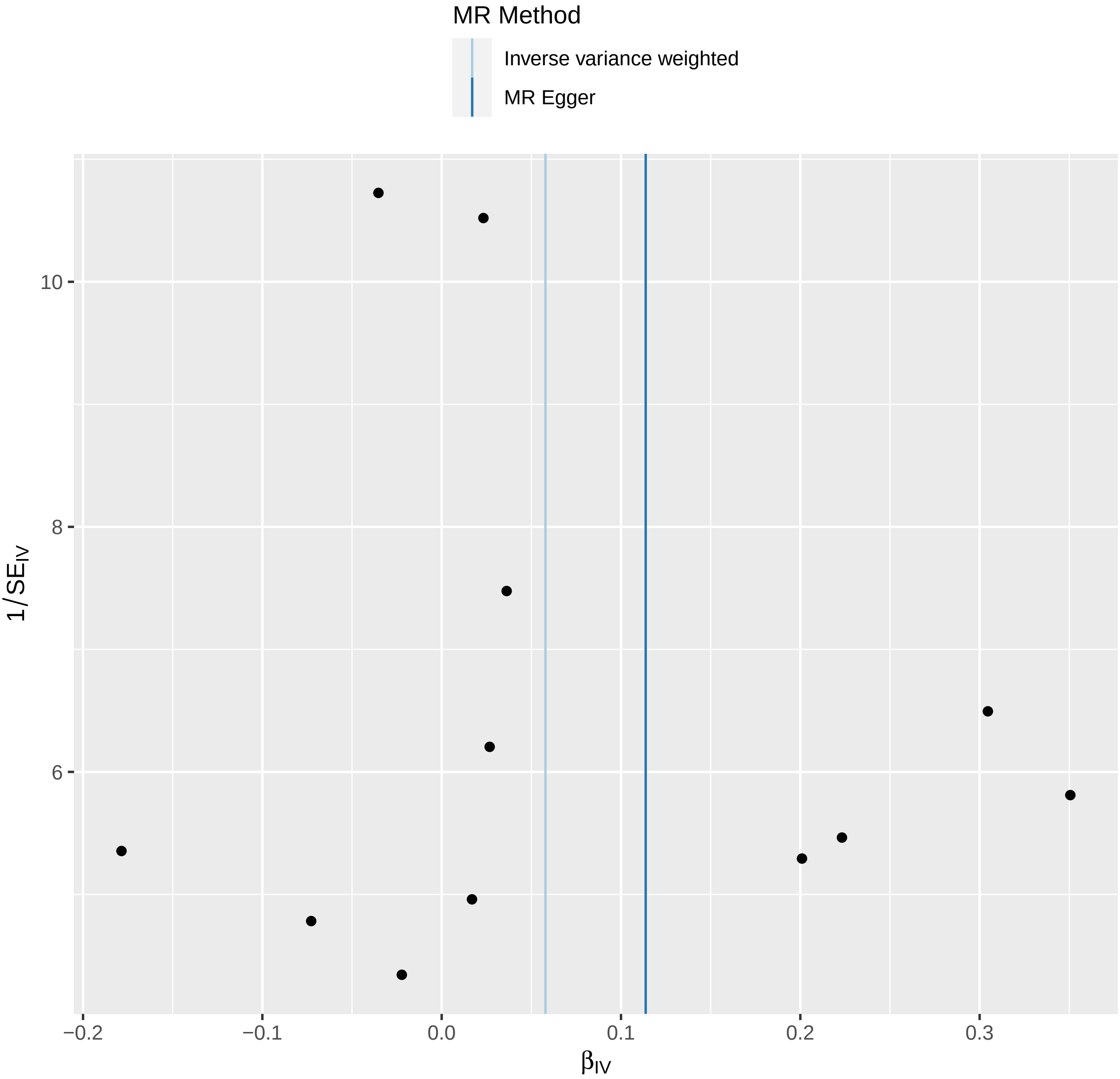

Batch 3−3015 : Selenium on Transmembrane protein 70, mitochondrial

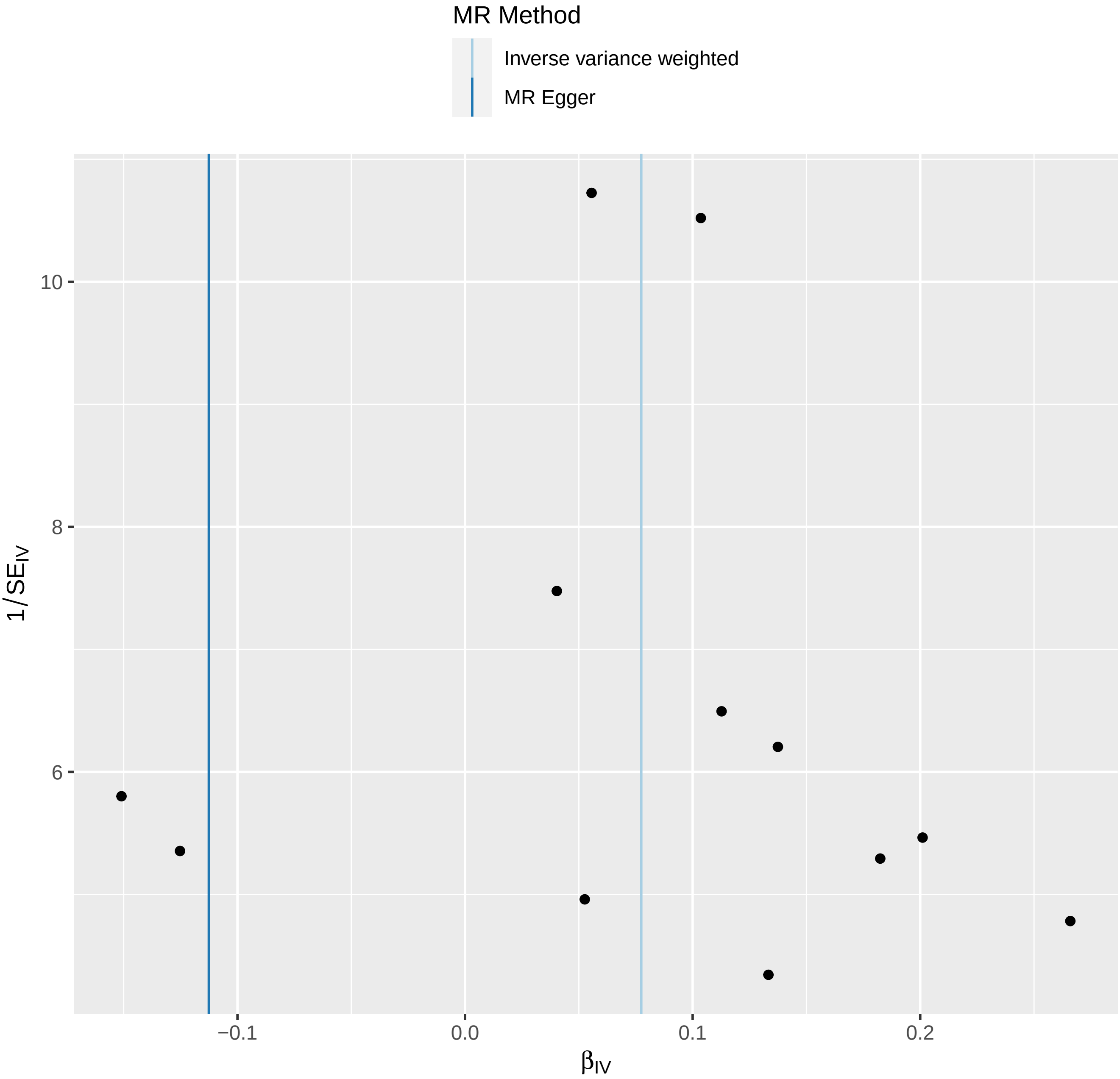

Batch 3−3016 : Selenium on Transmembrane protein 87B

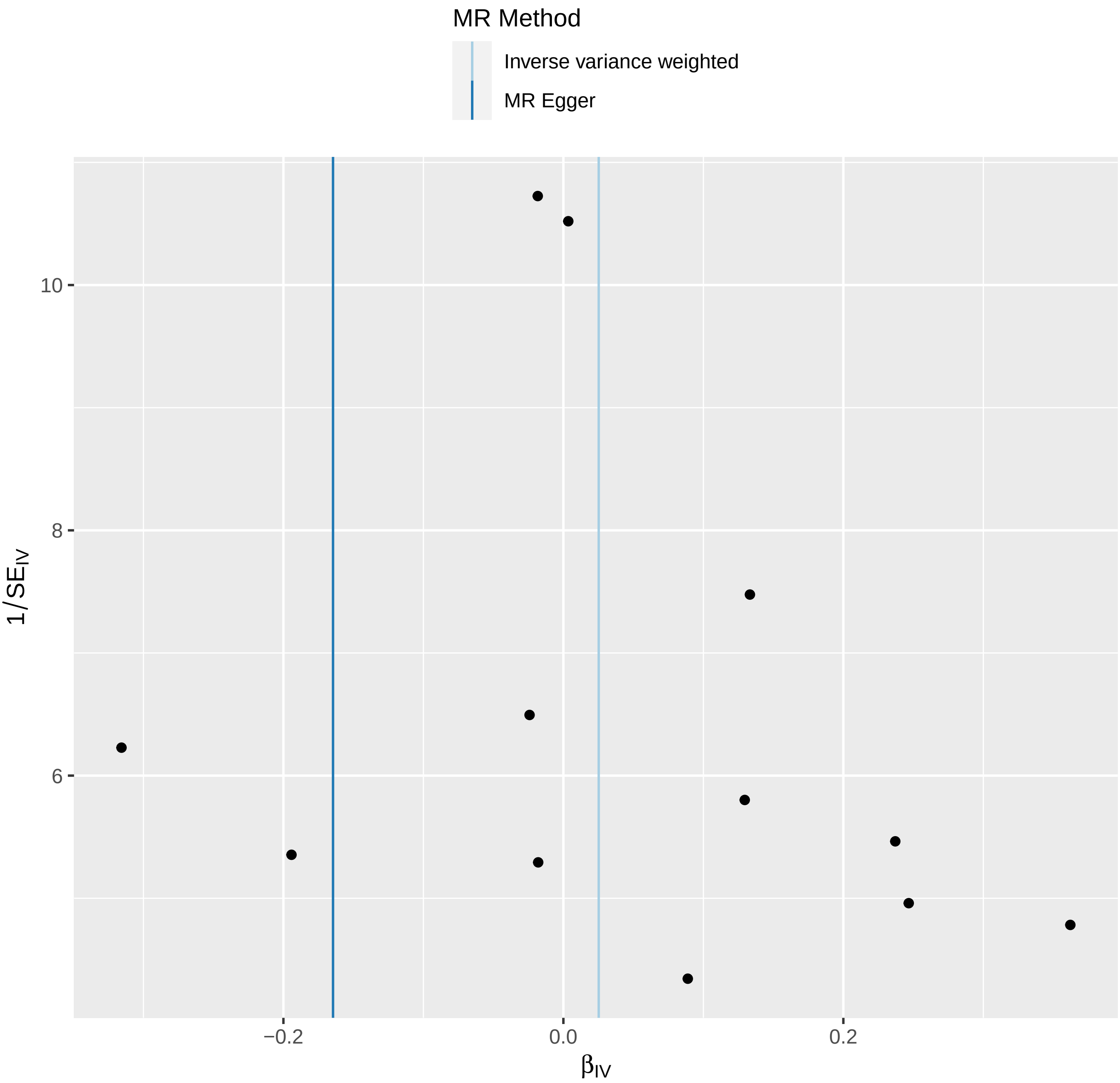

Batch 3−3017 : Selenium on Transmembrane protein 8B

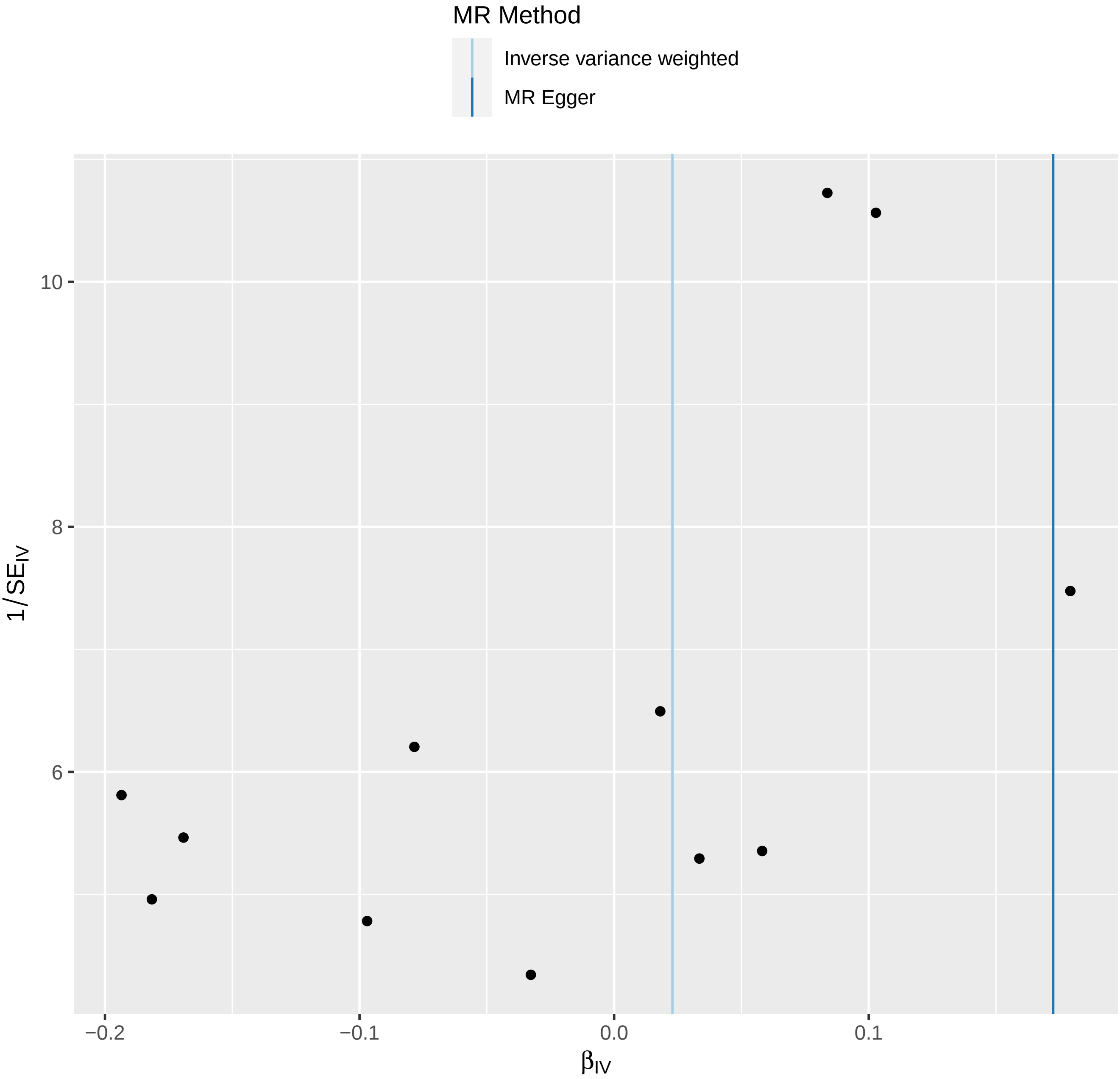

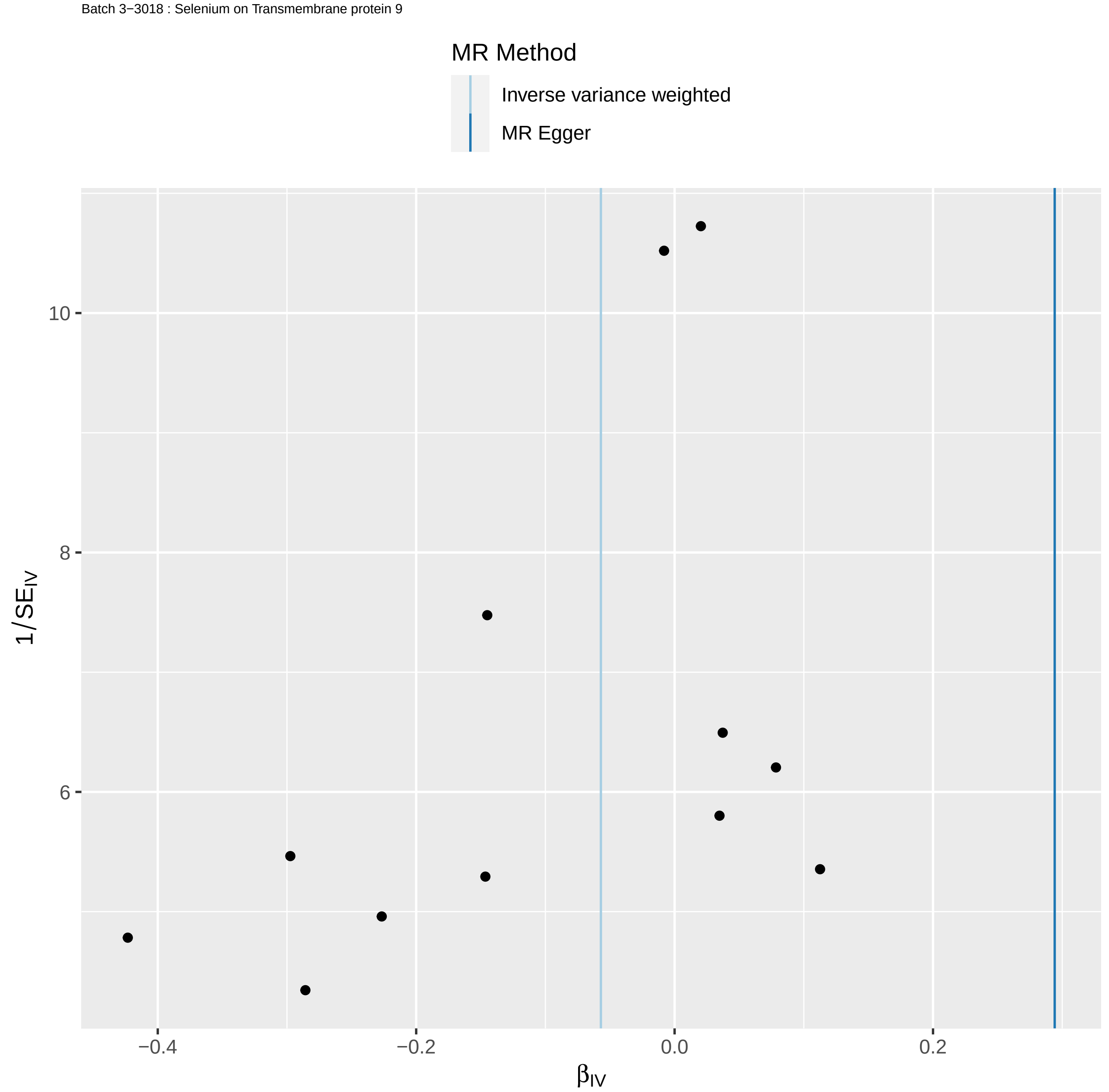

Batch 3−3018 : Selenium on Transmembrane protein 9
MR Method
Inverse variance weighted
MR Egger
10
8
6
1/SE$_{IV}$
−0.4
−0.2
0.0
0.2
β$_{IV}$

Batch 3−3019 : Selenium on Transmembrane inner ear expressed protein

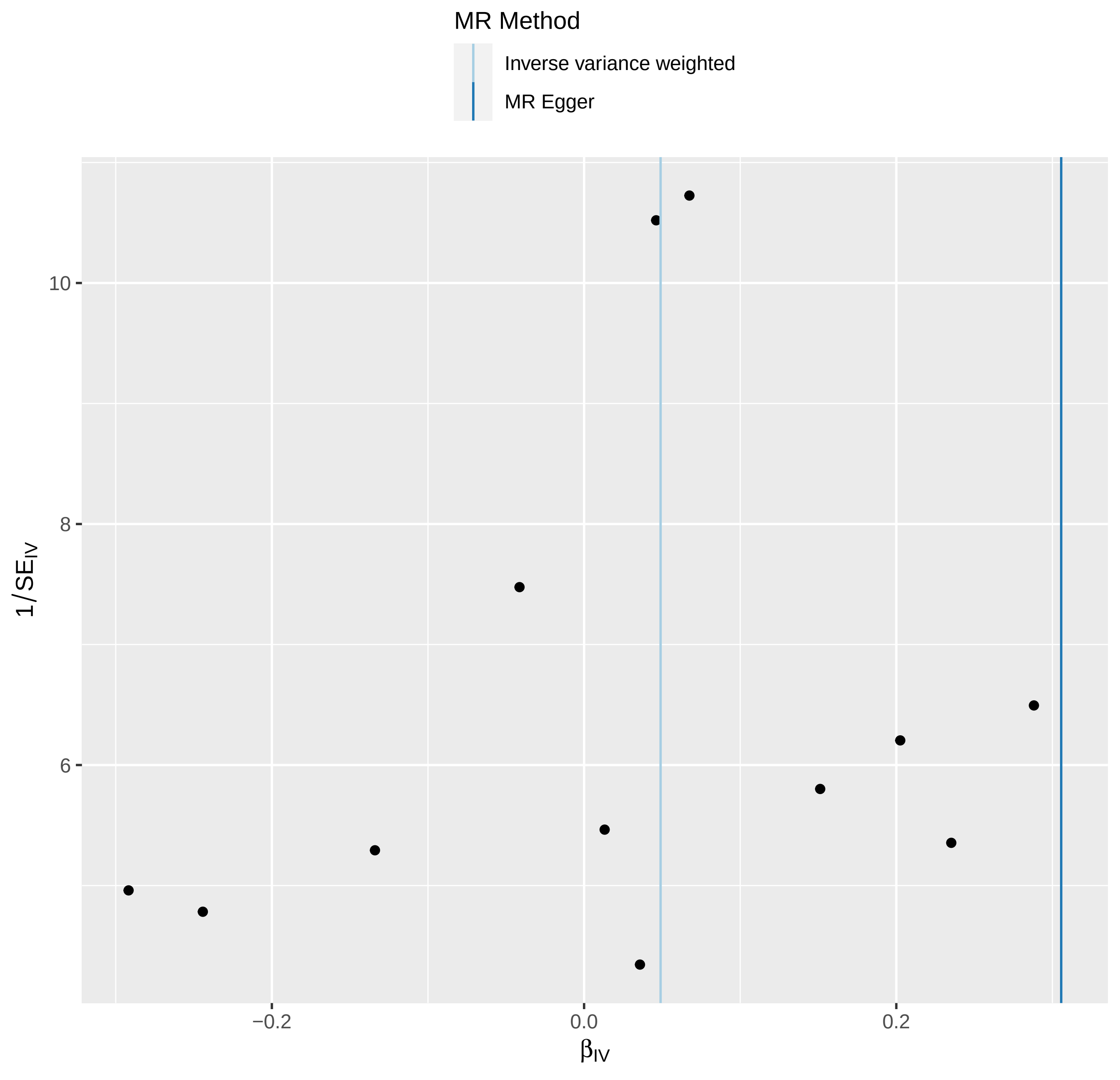

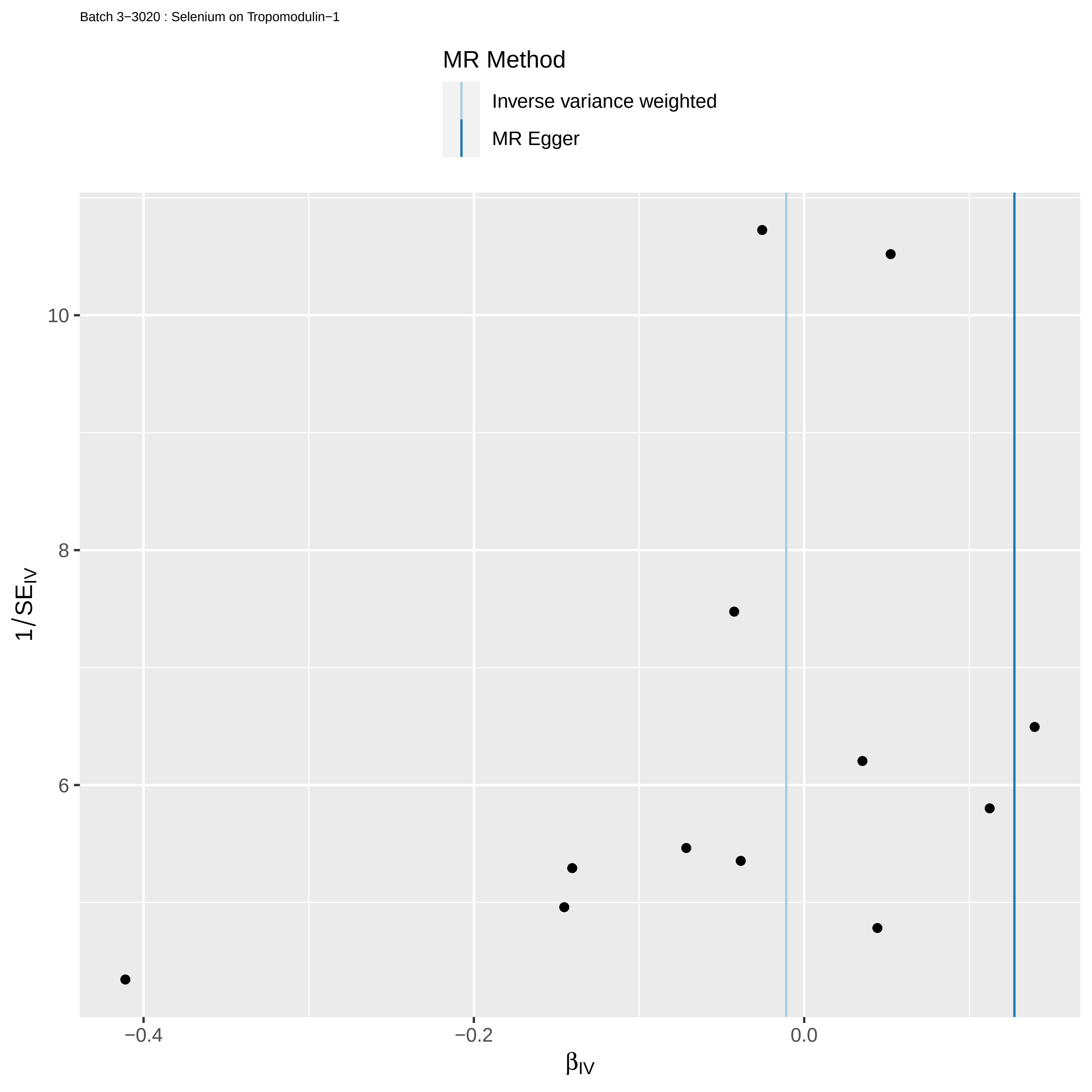

Batch 3−3020 : Selenium on Tropomodulin−1
MR Method
Inverse variance weighted
MR Egger
10
8
6
1/SE$_{IV}$
−0.4
−0.2
0.0
β$_{IV}$

Batch 3−3021 : Selenium on Lamina−associated polypeptide 2, isoforms beta/gamma

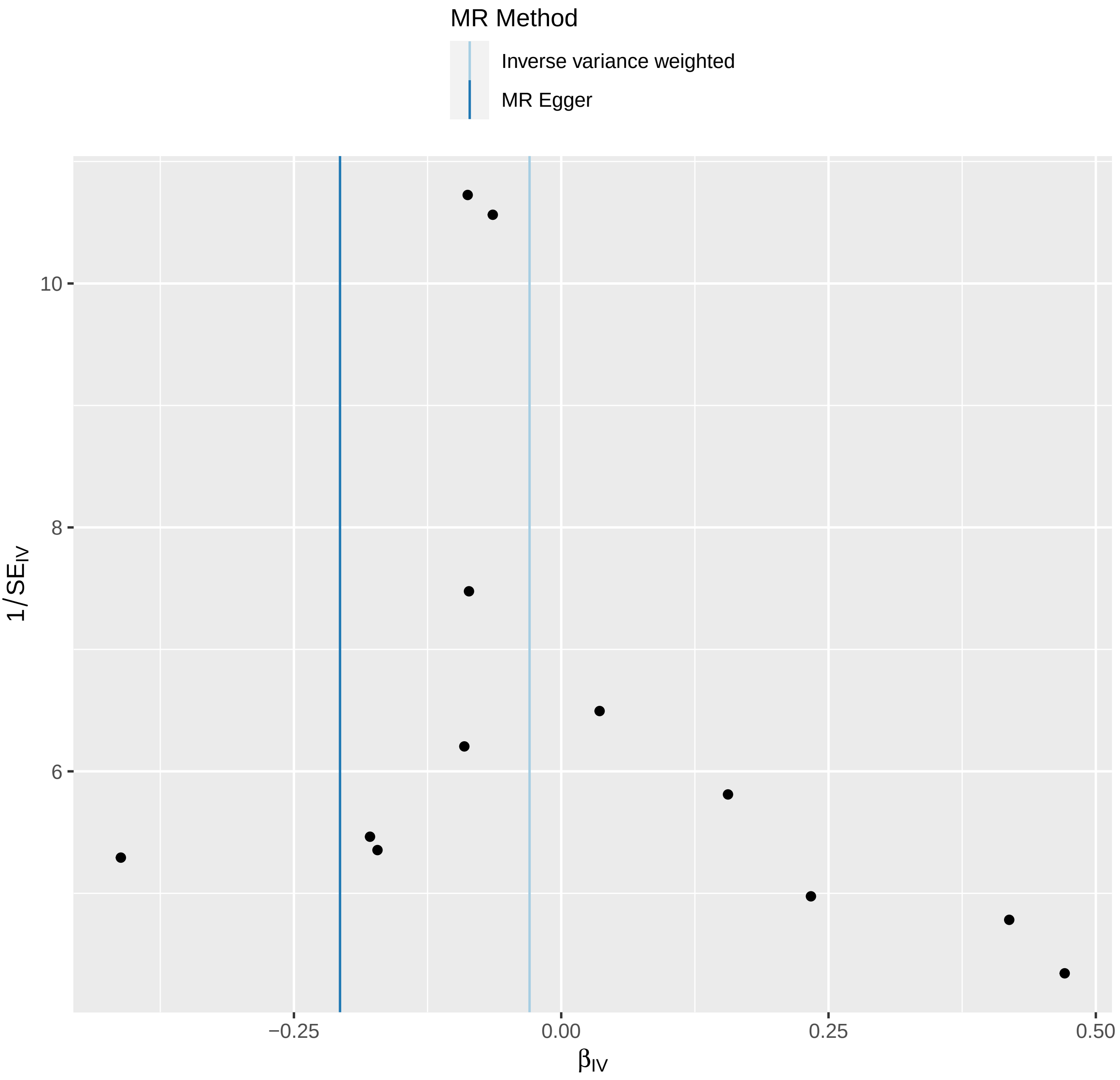

Batch 3−3022 : Selenium on Transmembrane protease serine 11A

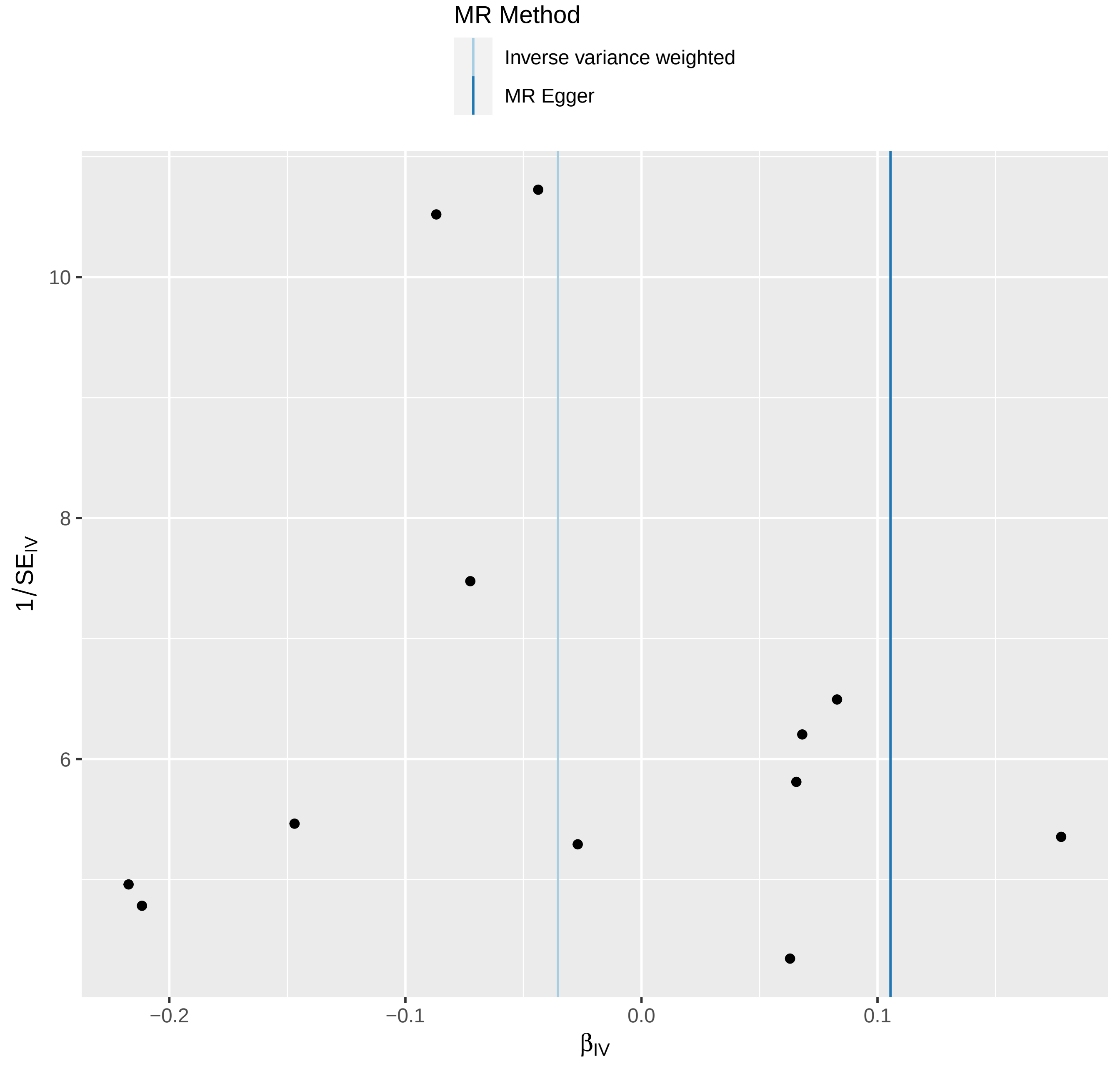

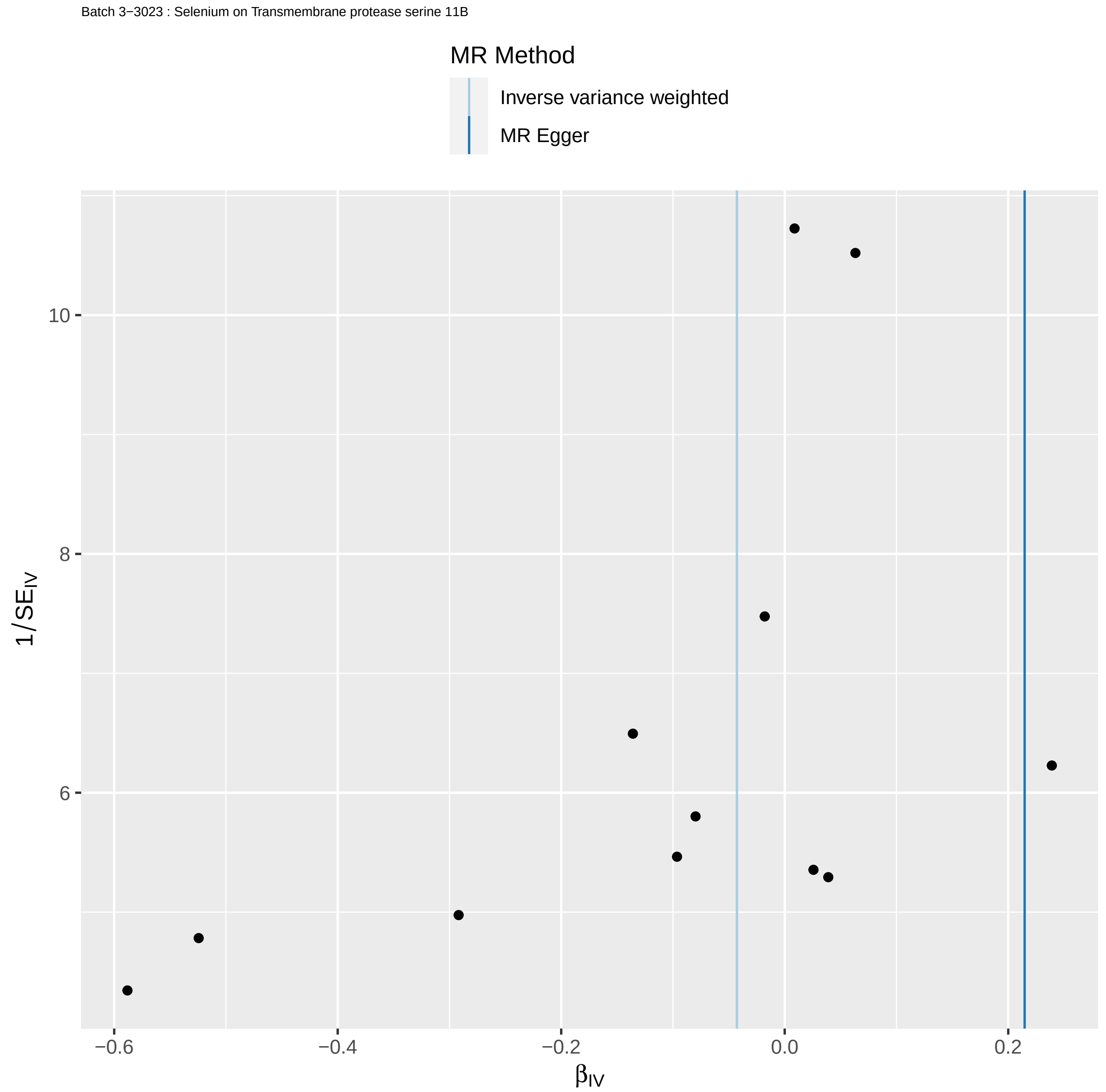

Batch 3−3023 : Selenium on Transmembrane protease serine 11B
MR Method
Inverse variance weighted
MR Egger
10
8
6
1/SE$_{IV}$
−0.6
−0.4
−0.2
0.0
0.2
β$_{IV}$

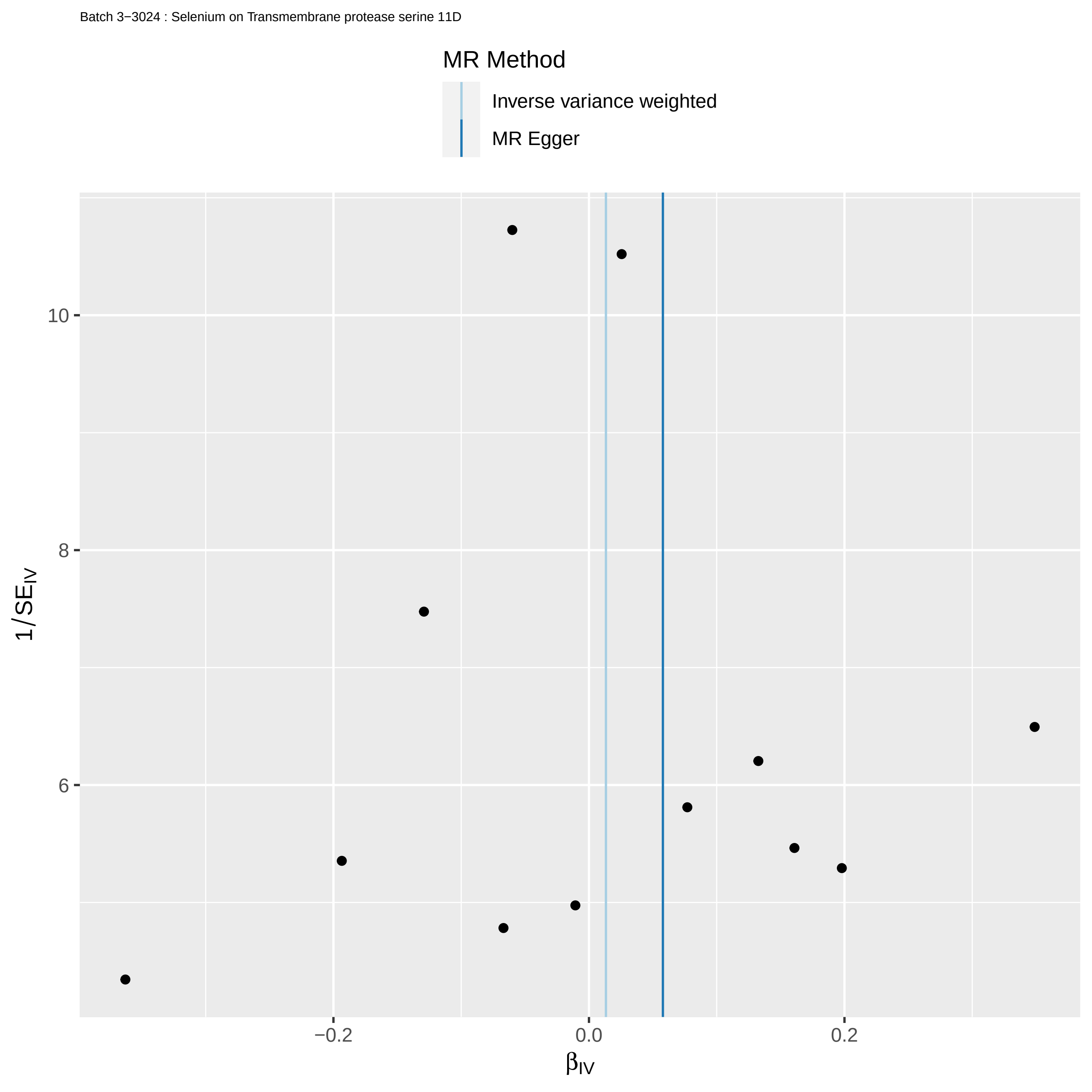

Batch 3−3024 : Selenium on Transmembrane protease serine 11D
MR Method
Inverse variance weighted
MR Egger
10
8
6
1/SE_IV
−0.2
0.0
0.2
β_IV

Batch 3−3025 : Selenium on Enteropeptidase

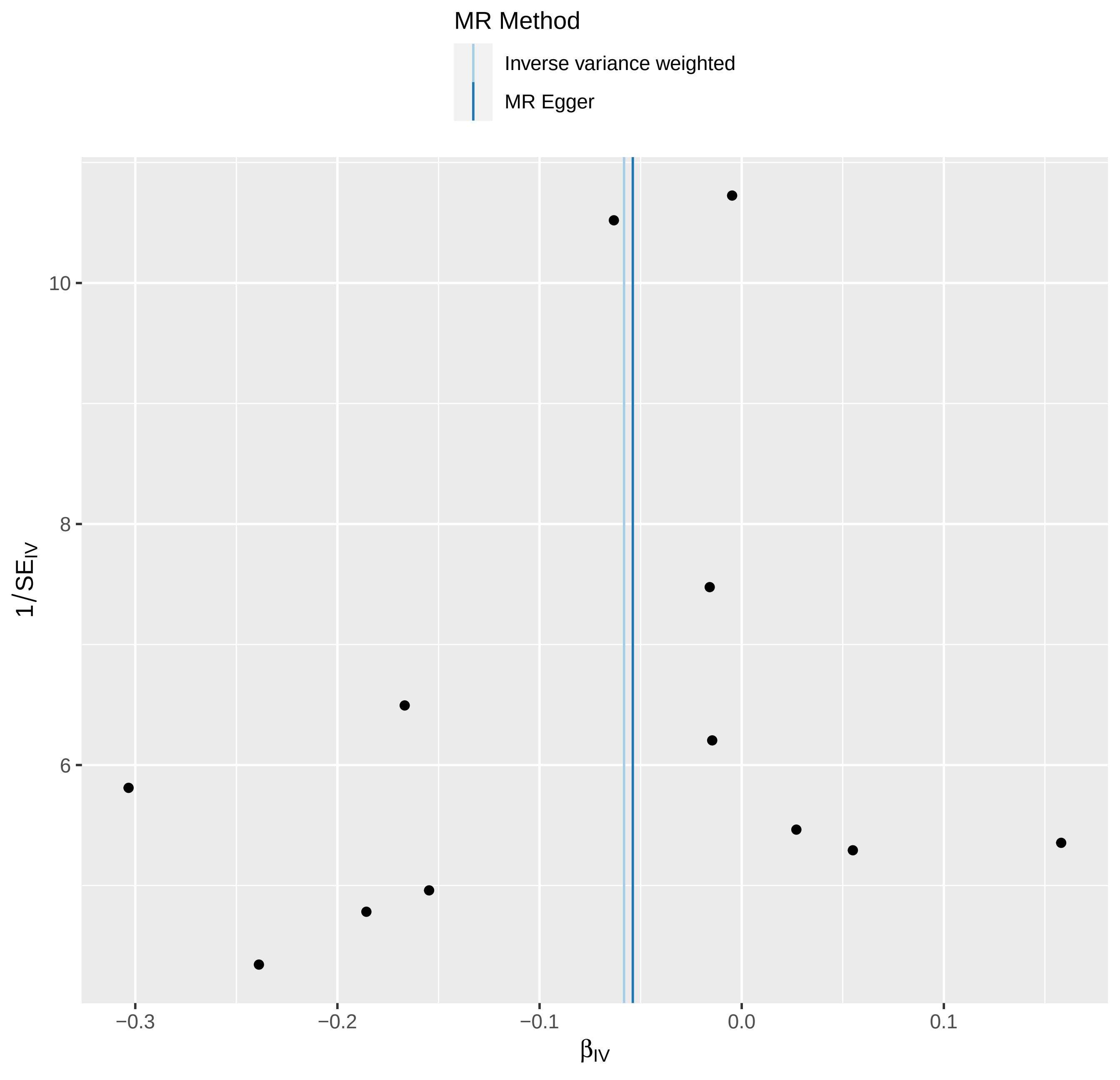

Batch 3−3026 : Selenium on Transmembrane protease serine 5

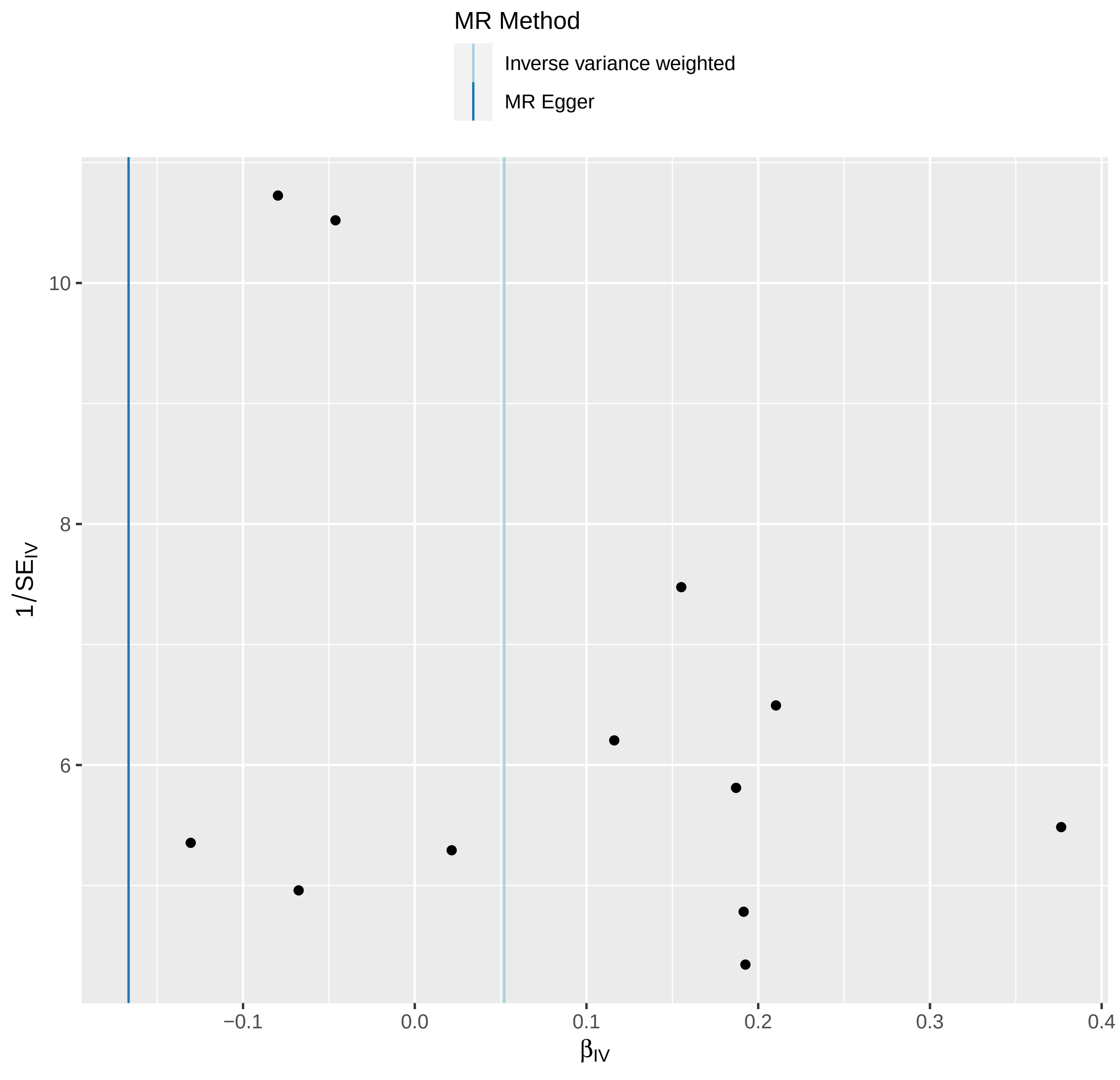

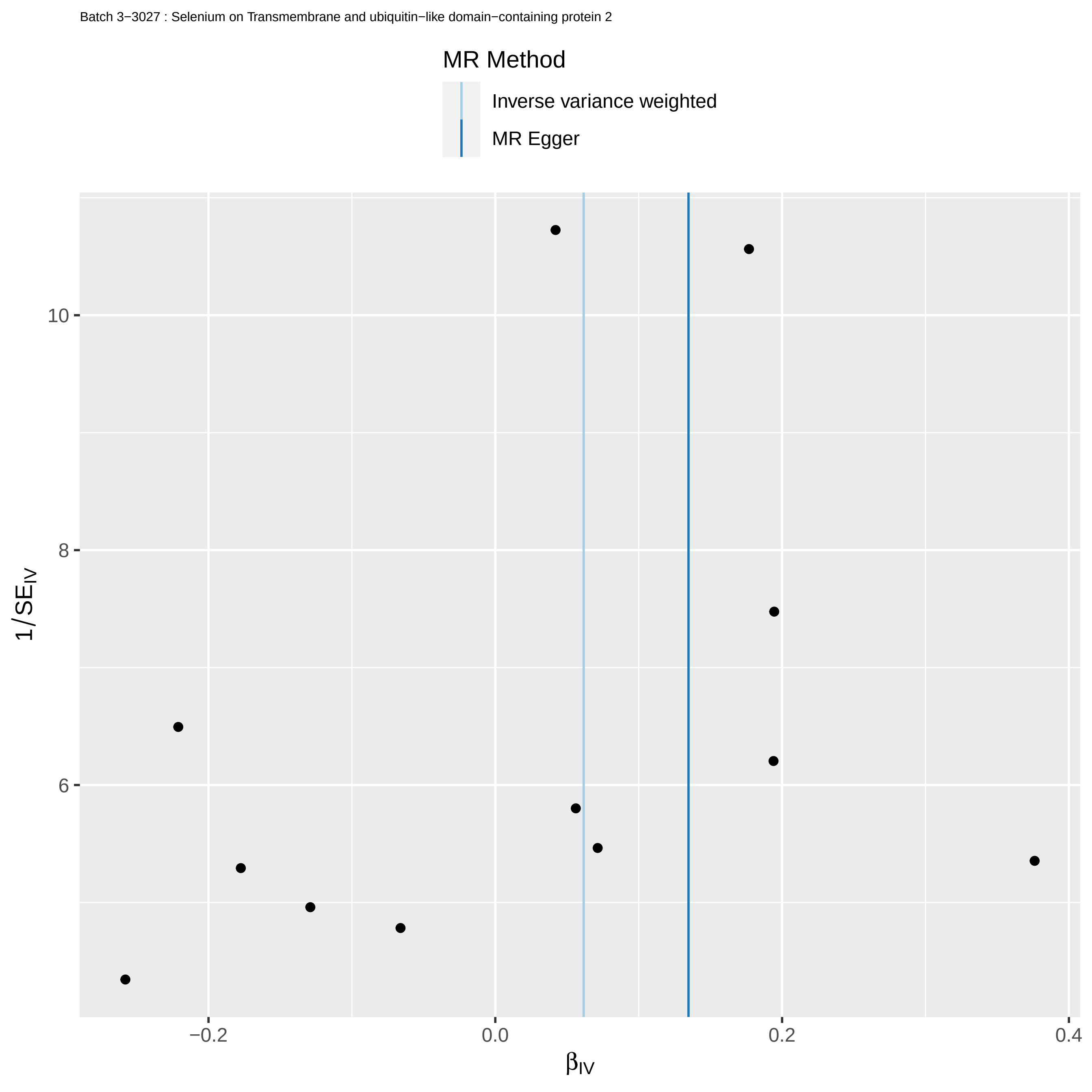
Batch 3−3027 : Selenium on Transmembrane and ubiquitin−like domain−containing protein 2
MR Method
Inverse variance weighted
MR Egger
10
8
6
1/SE_IV
−0.2
0.0
0.2
0.4
β_IV

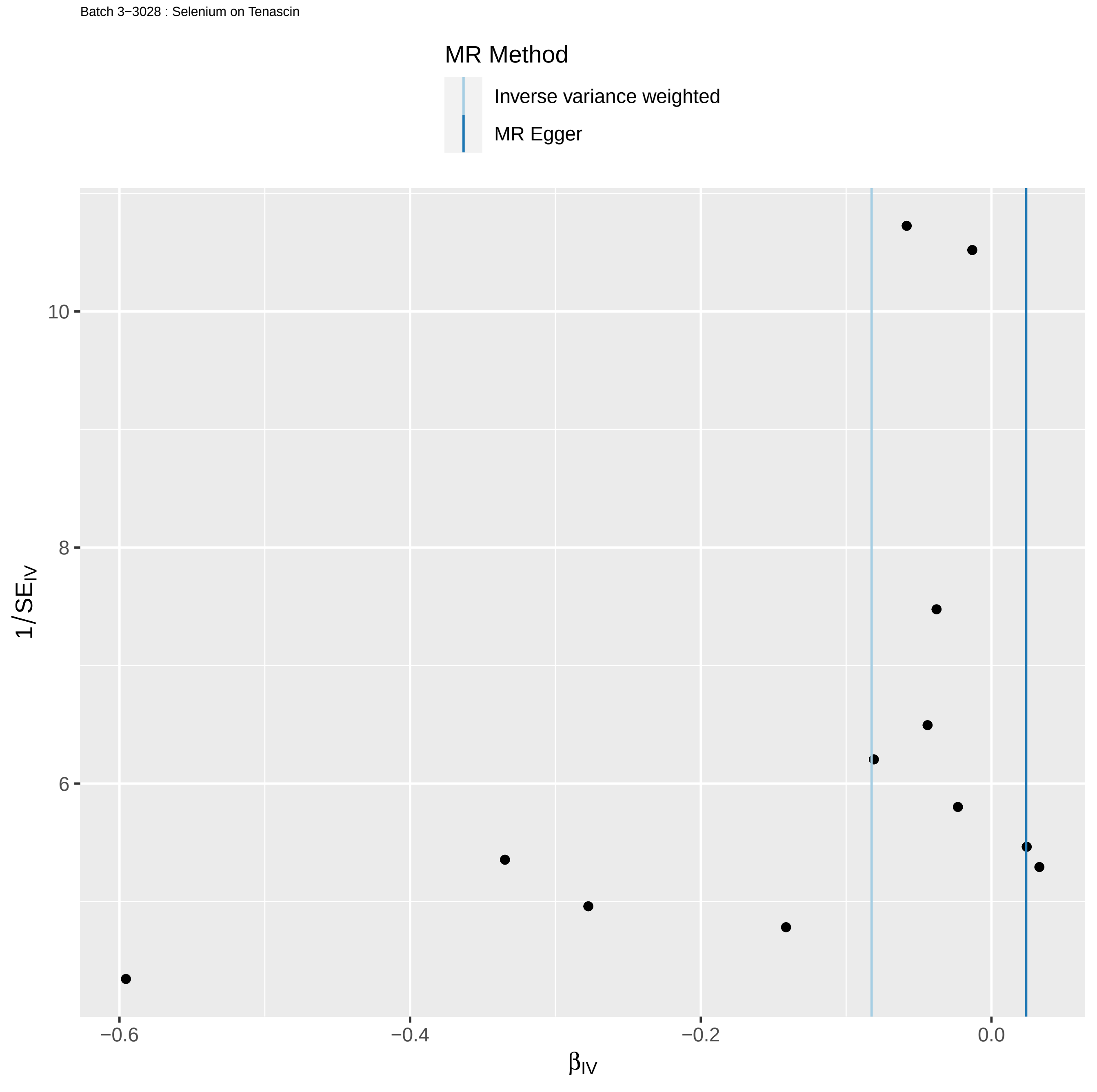

Batch 3−3028 : Selenium on Tenascin
MR Method
Inverse variance weighted
MR Egger
10
8
6
1/SE$_{IV}$
−0.6
−0.4
−0.2
0.0
β$_{IV}$

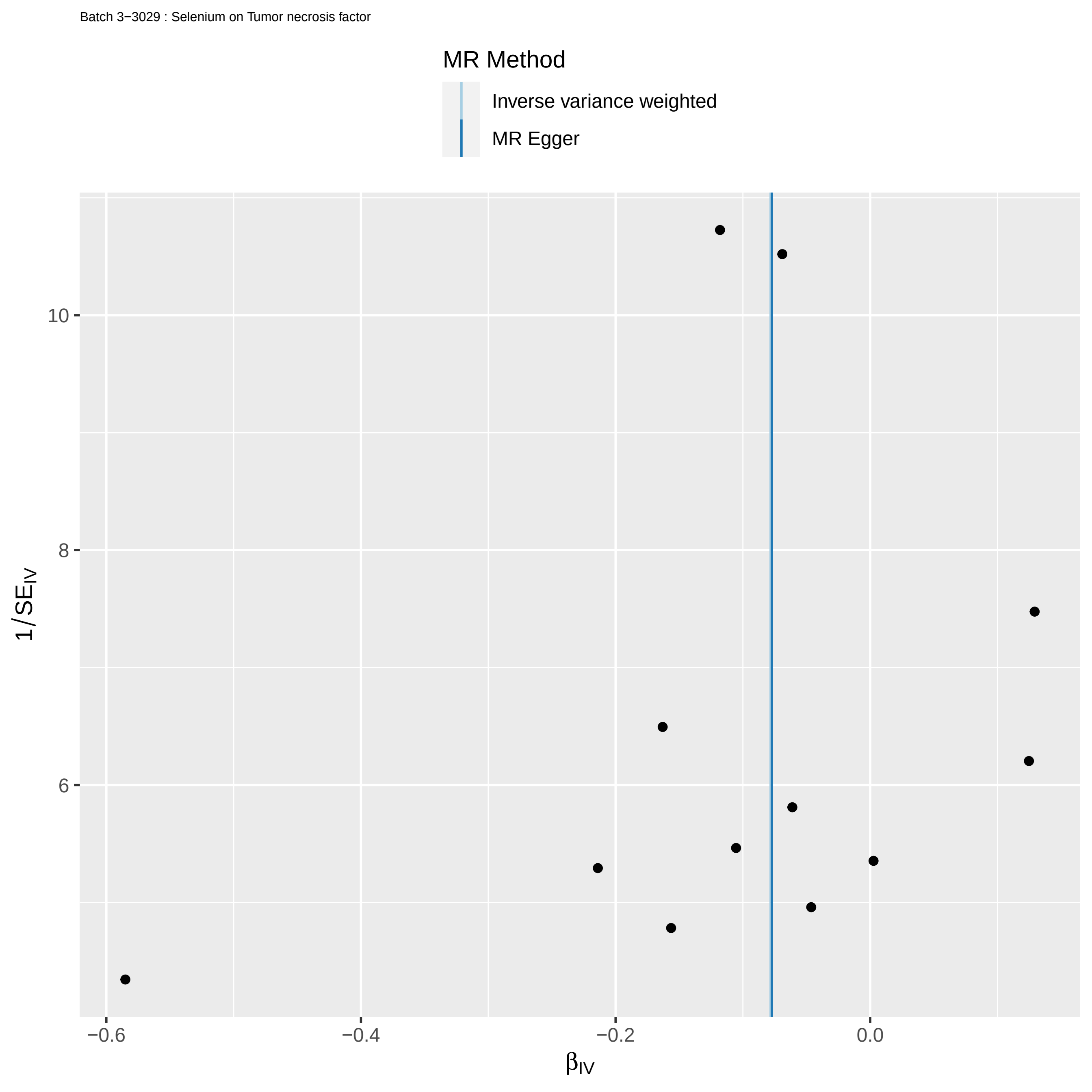

Batch 3−3029 : Selenium on Tumor necrosis factor
MR Method
Inverse variance weighted
MR Egger
10
8
6
1/SE$_{IV}$
−0.6
−0.4
−0.2
0.0
β$_{IV}$

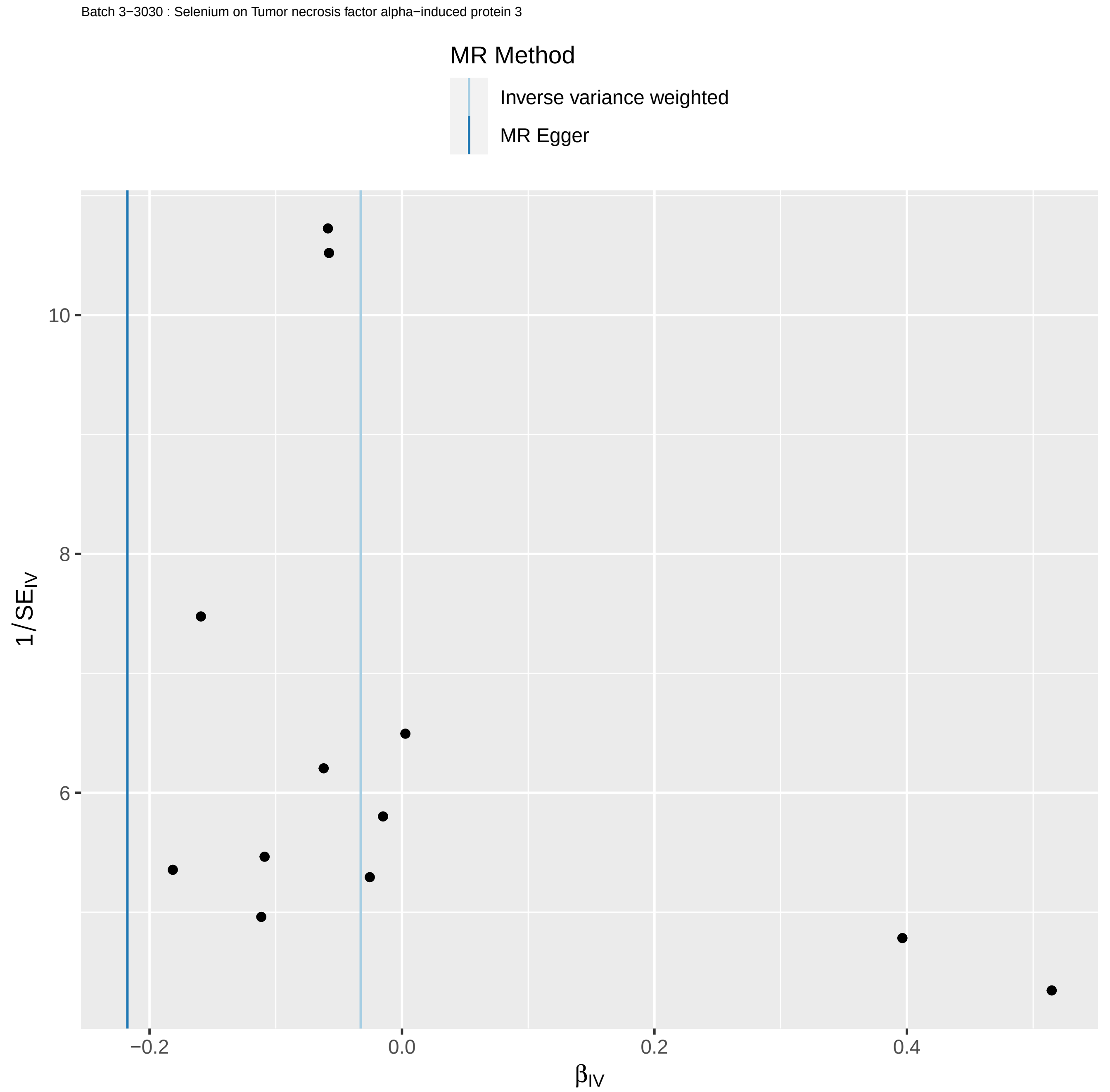
Batch 3−3030 : Selenium on Tumor necrosis factor alpha−induced protein 3
MR Method
Inverse variance weighted
MR Egger
10
8
6
1/SE$_{IV}$
−0.2
0.0
0.2
0.4
β$_{IV}$

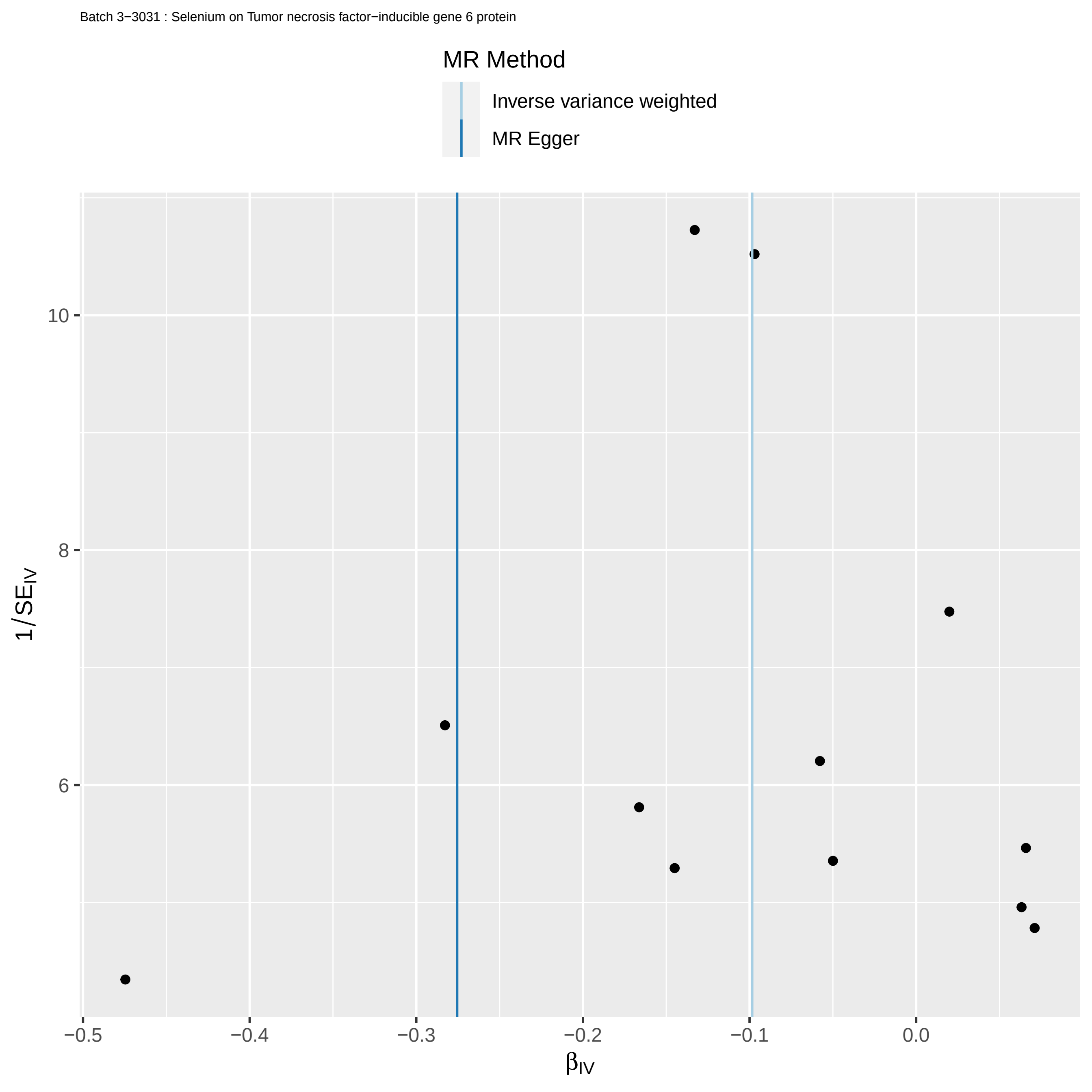
Batch 3−3031 : Selenium on Tumor necrosis factor−inducible gene 6 protein
MR Method
Inverse variance weighted
MR Egger
10
8
6
1/SE_IV
−0.5
−0.4
−0.3
−0.2
−0.1
0.0
β_IV

Batch 3−3032 : Selenium on Tumor necrosis factor receptor superfamily member 10A

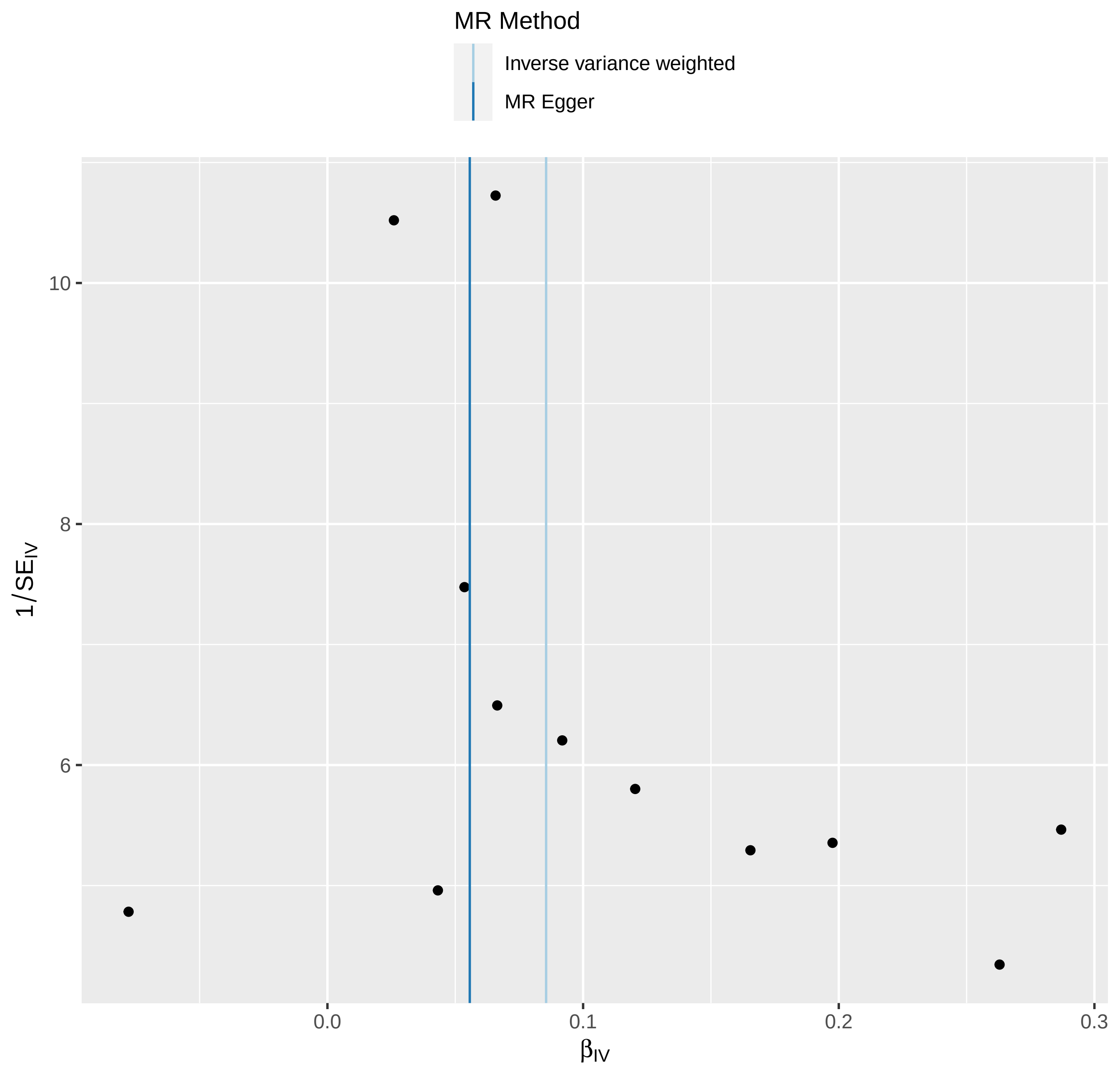

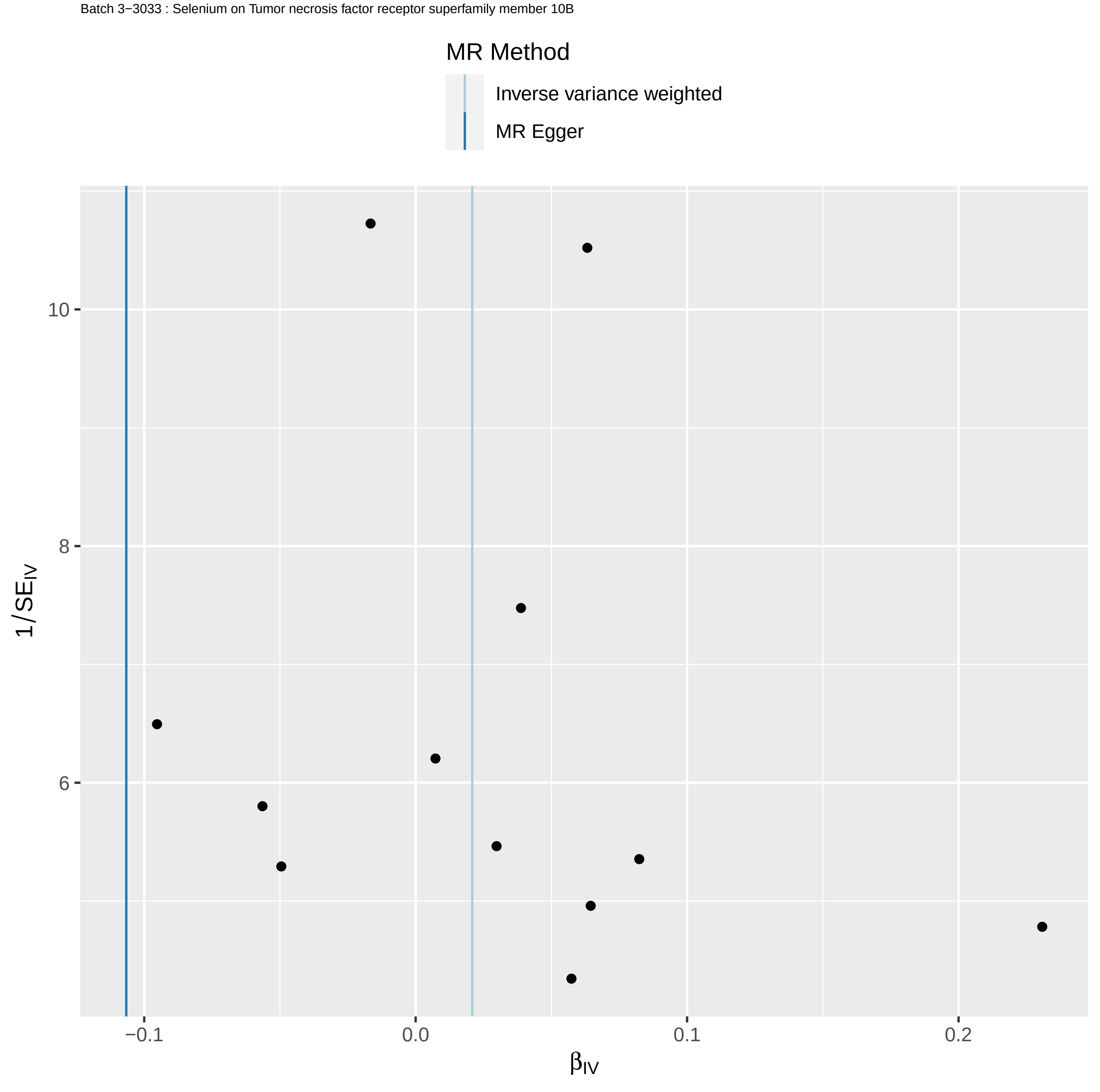

Batch 3−3033 : Selenium on Tumor necrosis factor receptor superfamily member 10B
MR Method
Inverse variance weighted
MR Egger
10
8
6
1/SE$_{IV}$
−0.1
0.0
0.1
0.2
β$_{IV}$

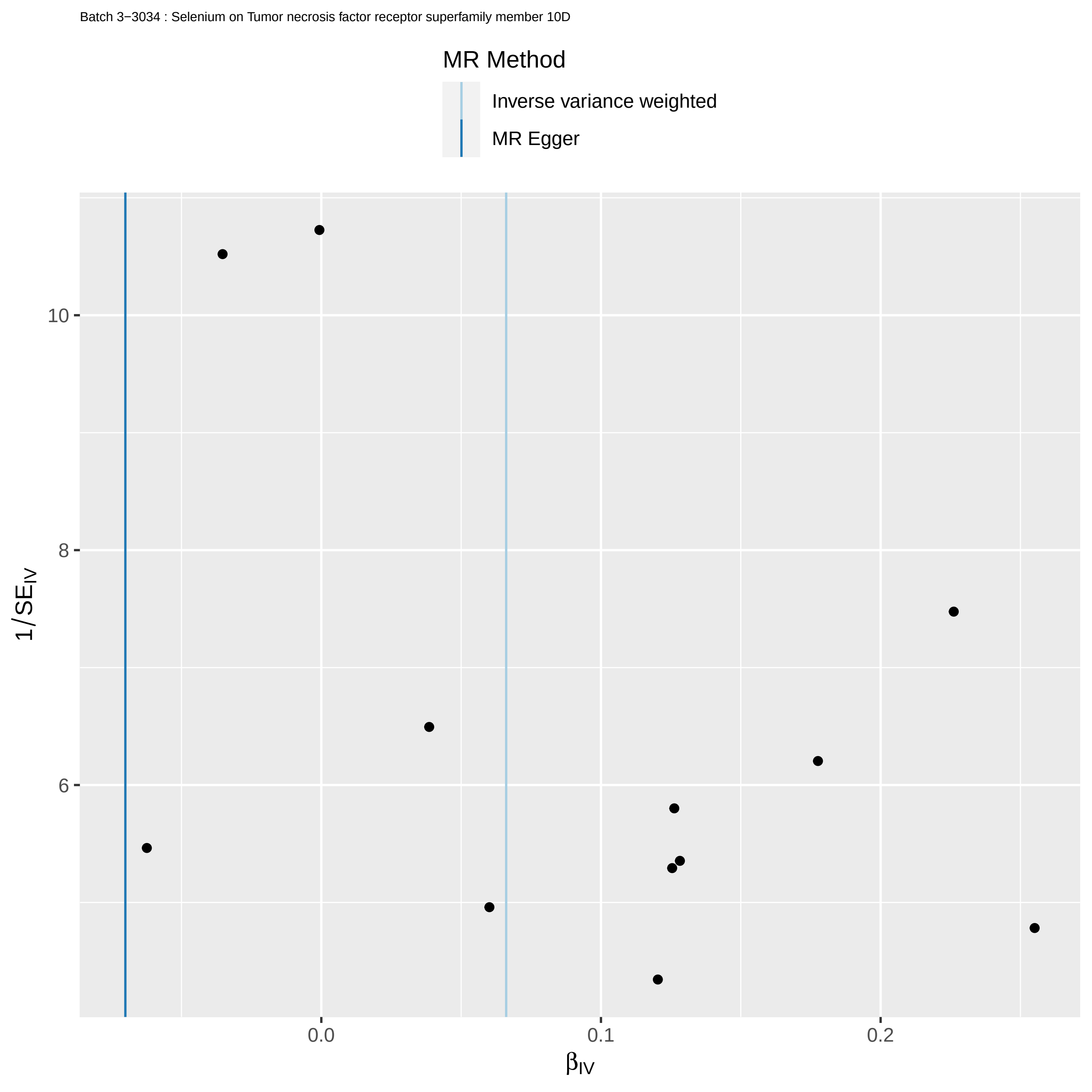

Batch 3−3034 : Selenium on Tumor necrosis factor receptor superfamily member 10D
MR Method
Inverse variance weighted
MR Egger
10
8
6
1/SE$_{IV}$
0.0
0.1
0.2
β$_{IV}$

Batch 3−3035 : Selenium on Tumor necrosis factor receptor superfamily member 11A

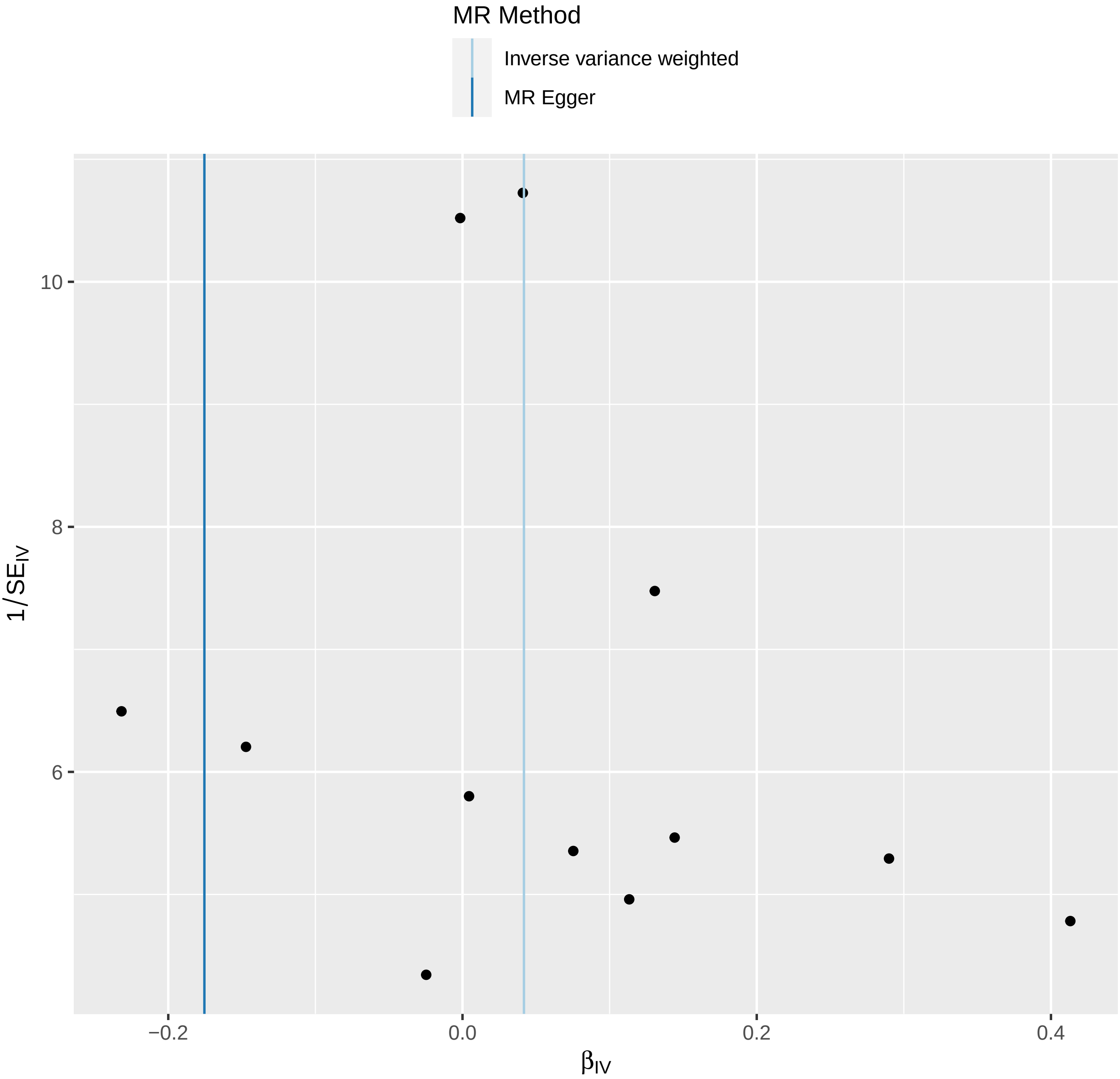

Batch 3−3036 : Selenium on Tumor necrosis factor receptor superfamily member 11A

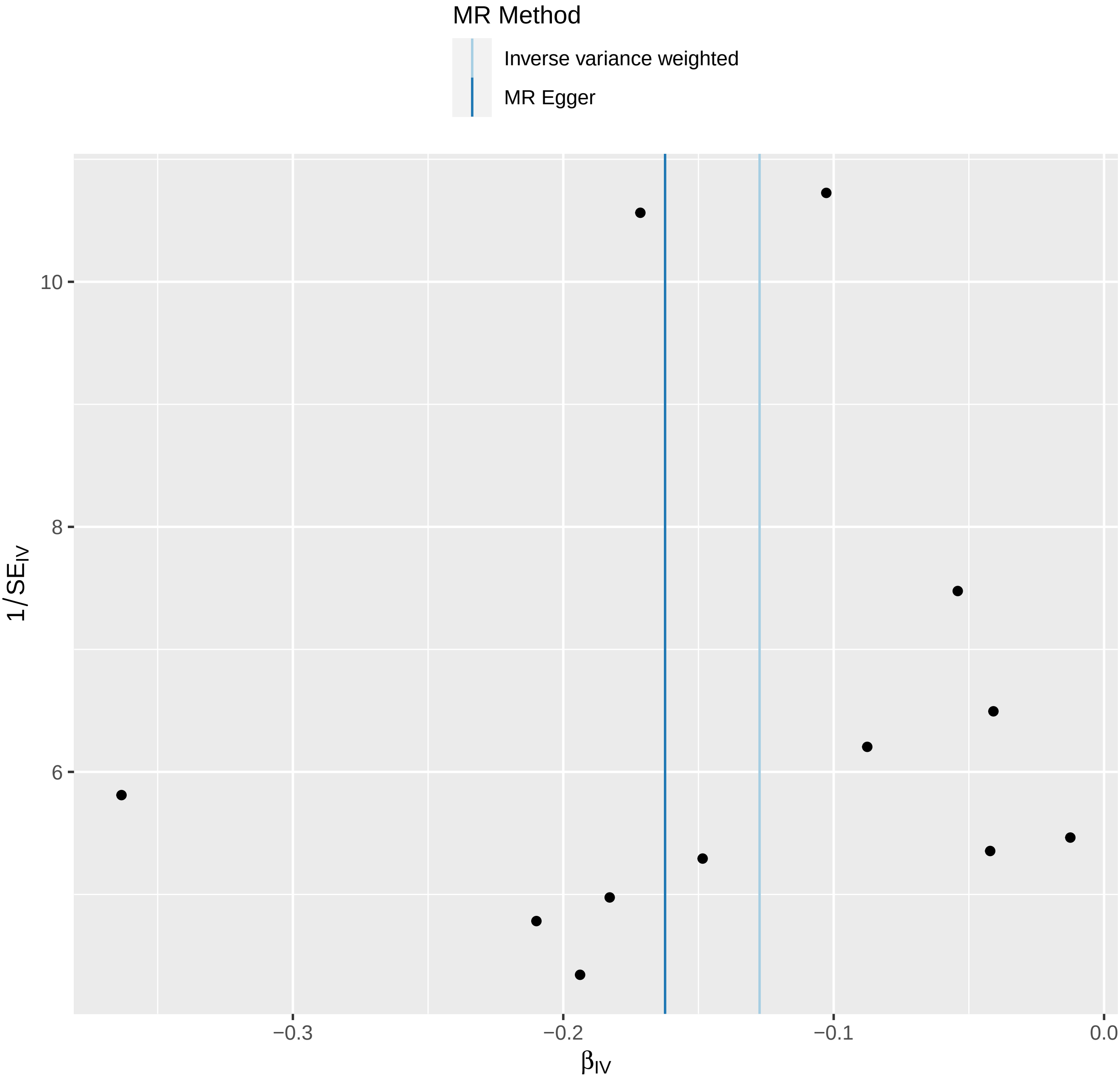

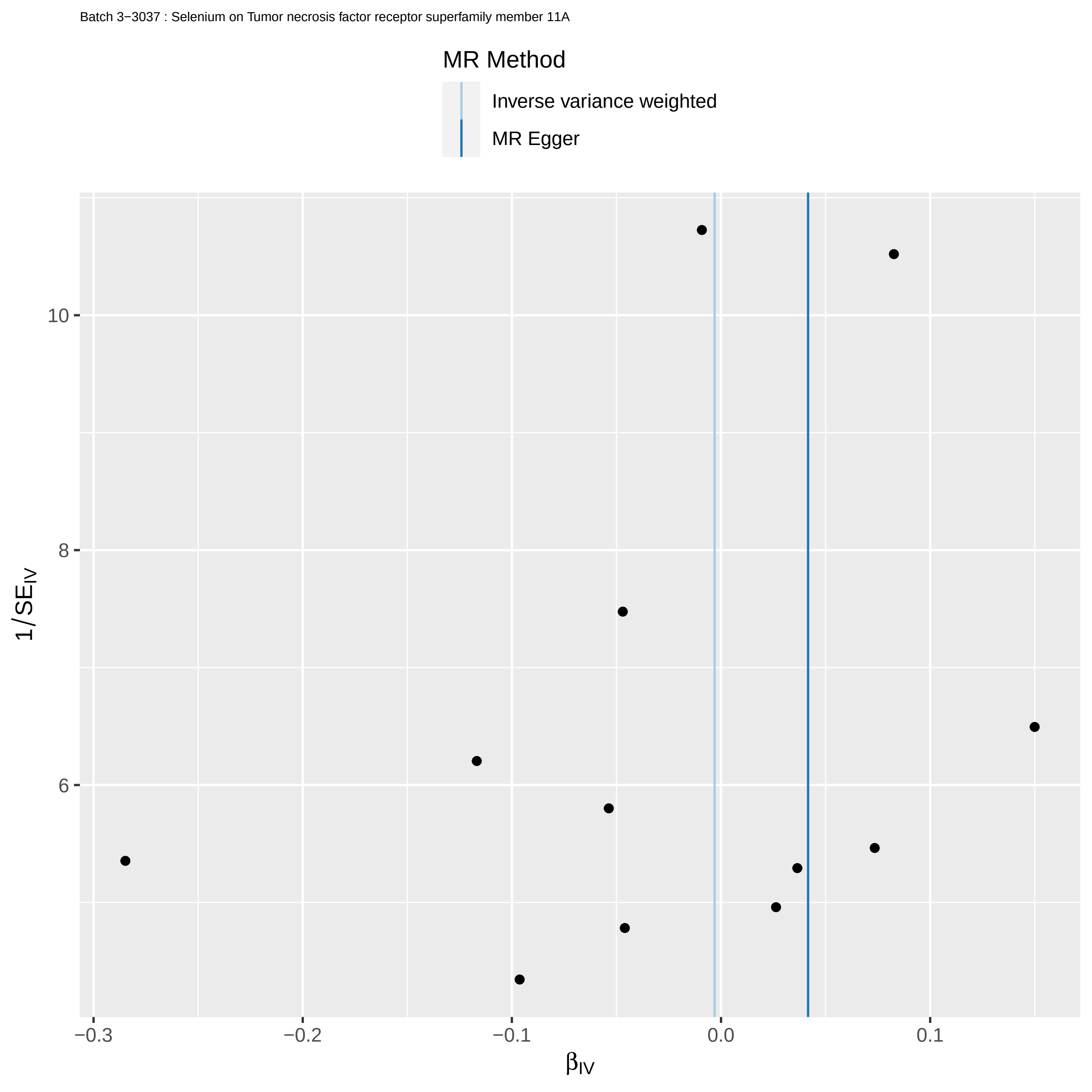
Batch 3−3037 : Selenium on Tumor necrosis factor receptor superfamily member 11A
MR Method
Inverse variance weighted
MR Egger
10
8
6
1/SE$_{IV}$
−0.3
−0.2
−0.1
0.0
0.1
β$_{IV}$

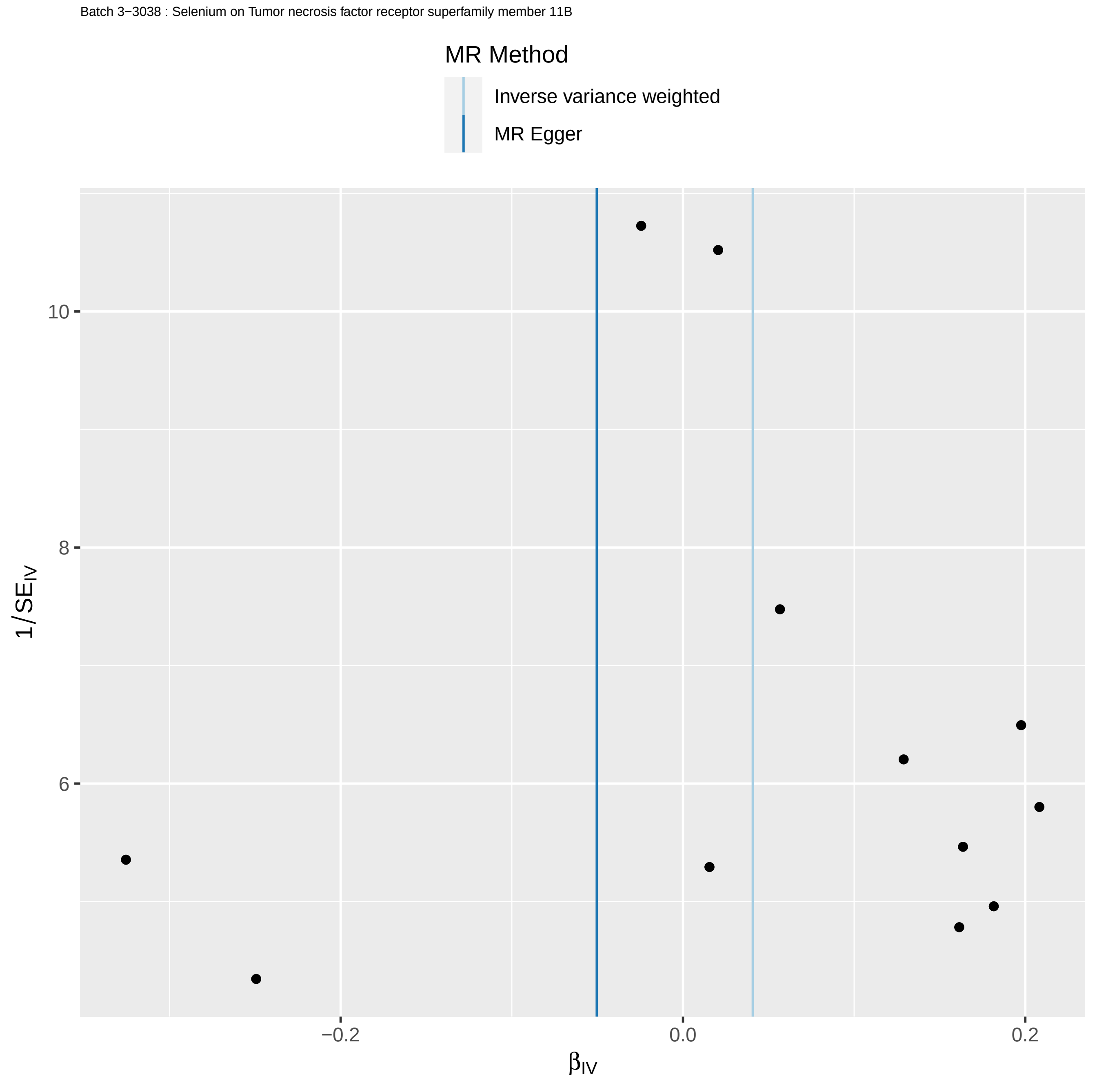
Batch 3−3038 : Selenium on Tumor necrosis factor receptor superfamily member 11B
MR Method
Inverse variance weighted
MR Egger
10
8
6
1/SE$_{IV}$
−0.2
0.0
0.2
β_{IV}

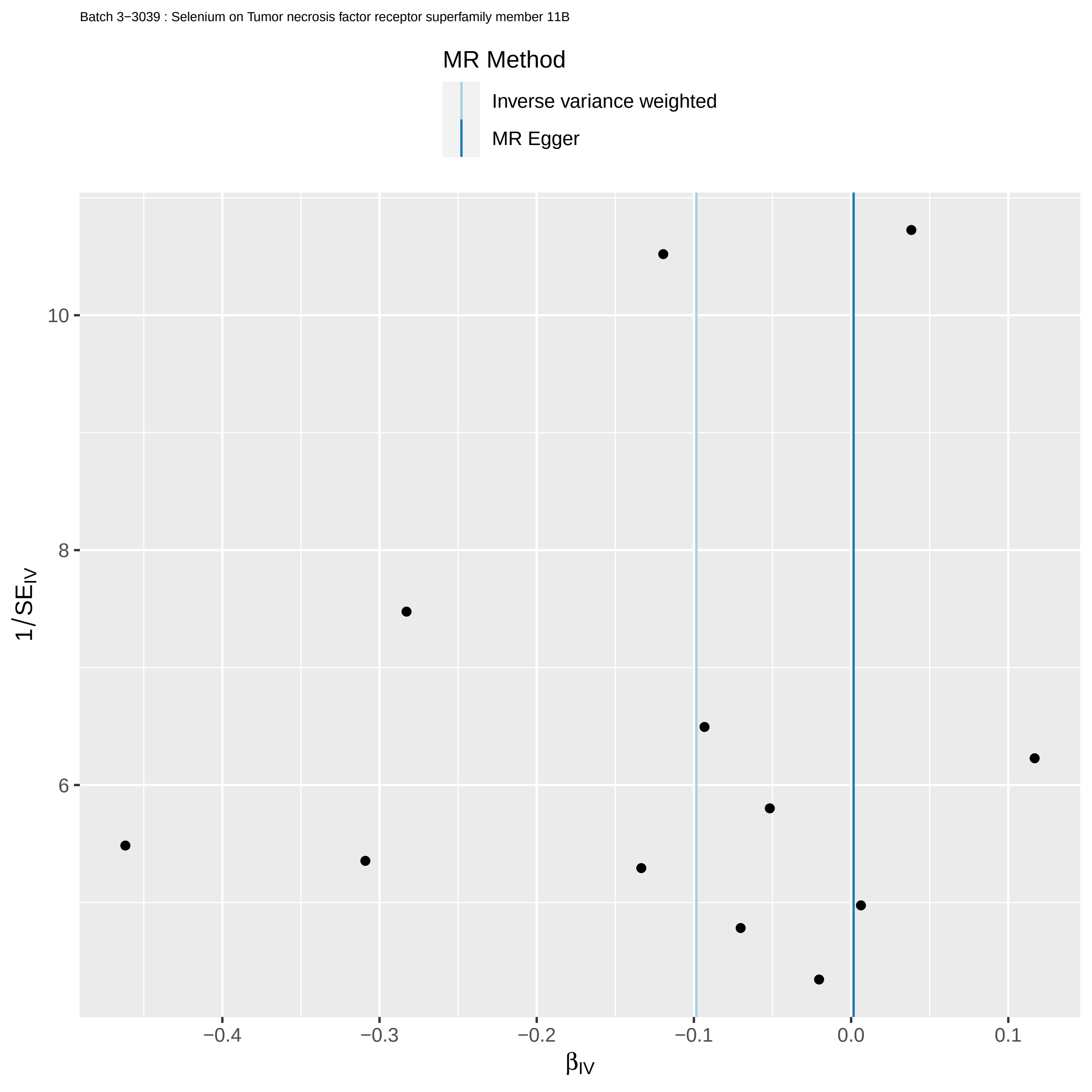

Batch 3−3039 : Selenium on Tumor necrosis factor receptor superfamily member 11B
MR Method
Inverse variance weighted
MR Egger
10
8
6
1/SE_IV
−0.4
−0.3
−0.2
−0.1
0.0
0.1
β_IV

Batch 3−3040 : Selenium on Tumor necrosis factor receptor superfamily member 12A

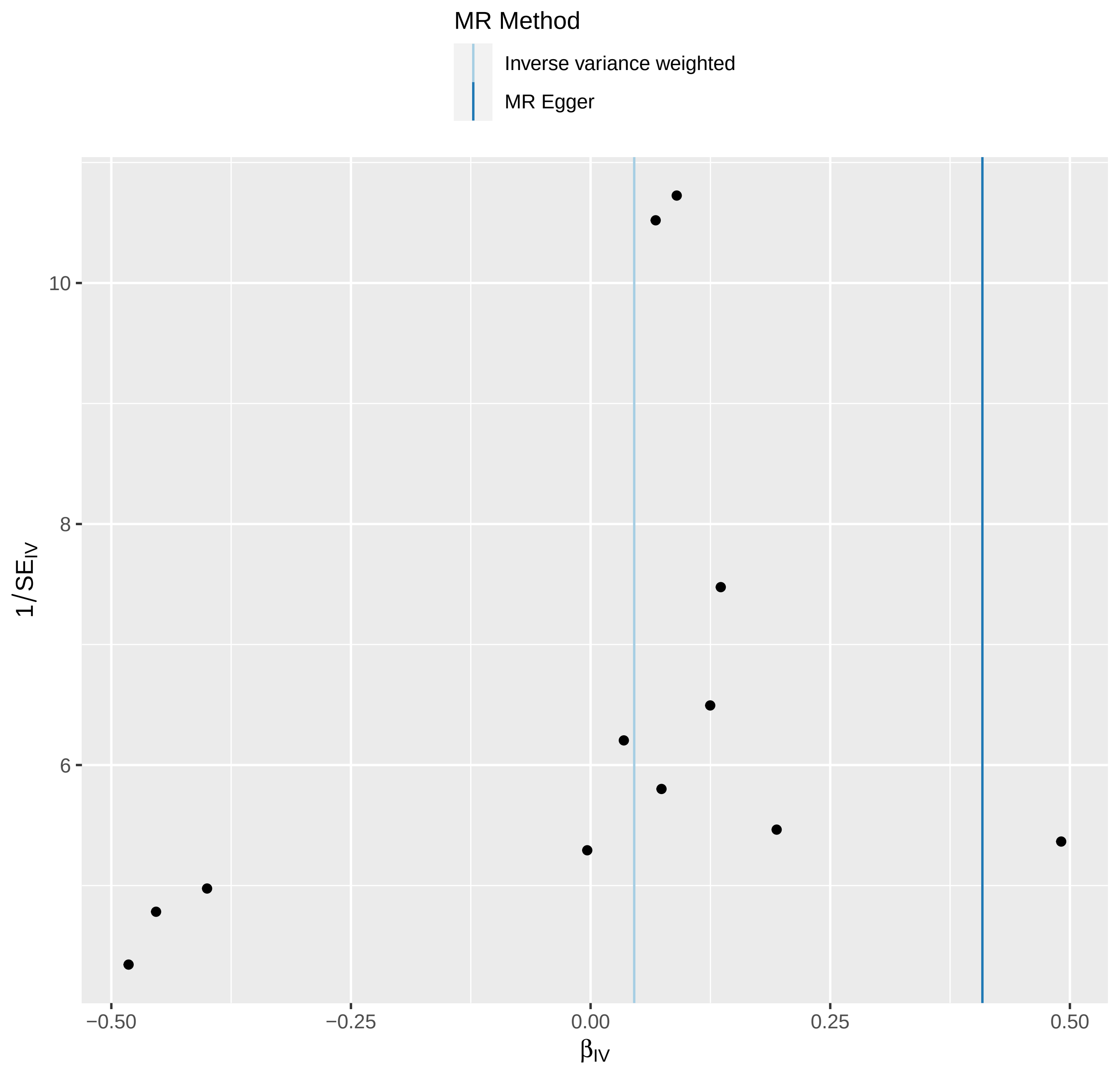

Batch 3−3041 : Selenium on Tumor necrosis factor receptor superfamily member 13B

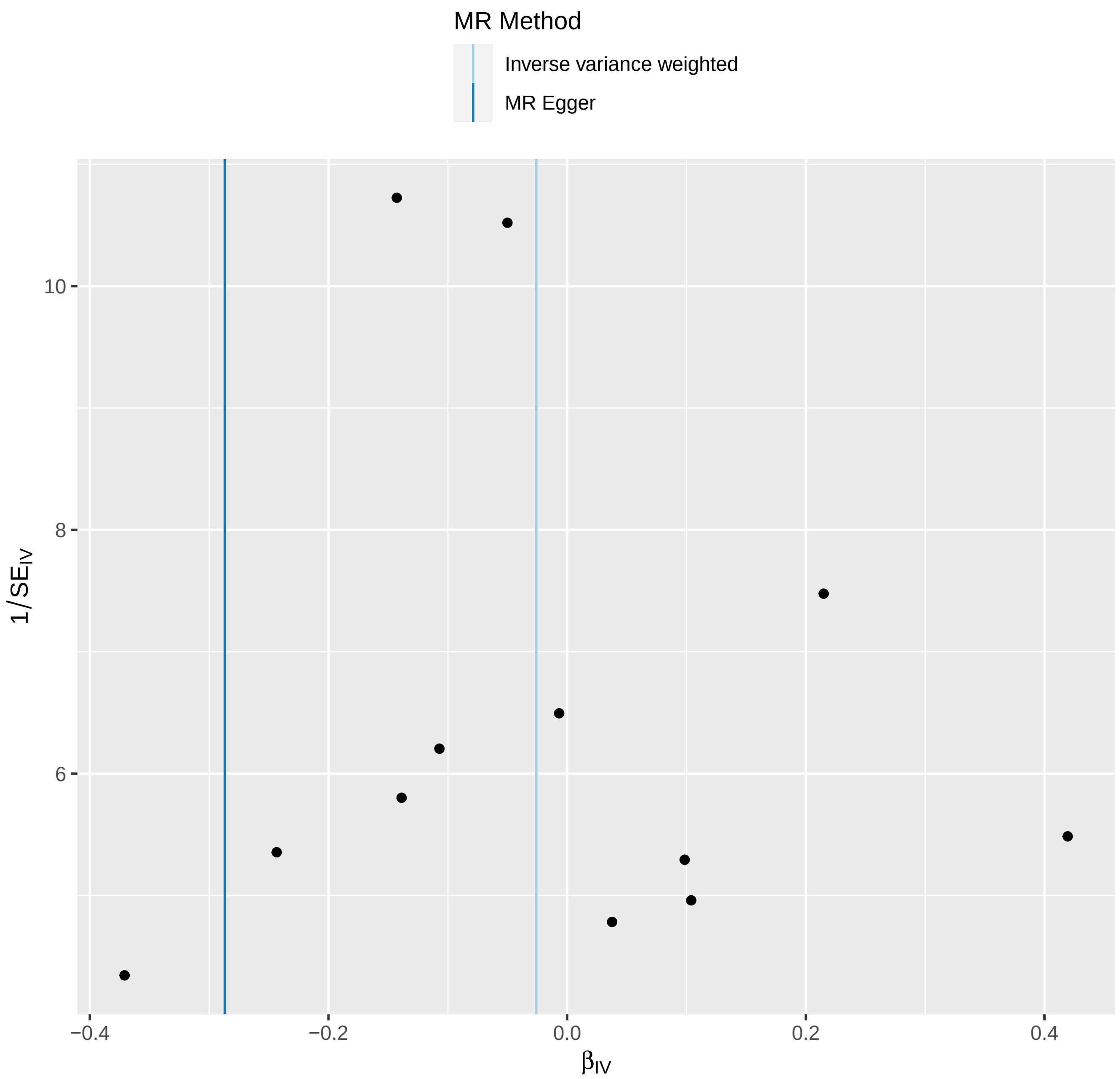

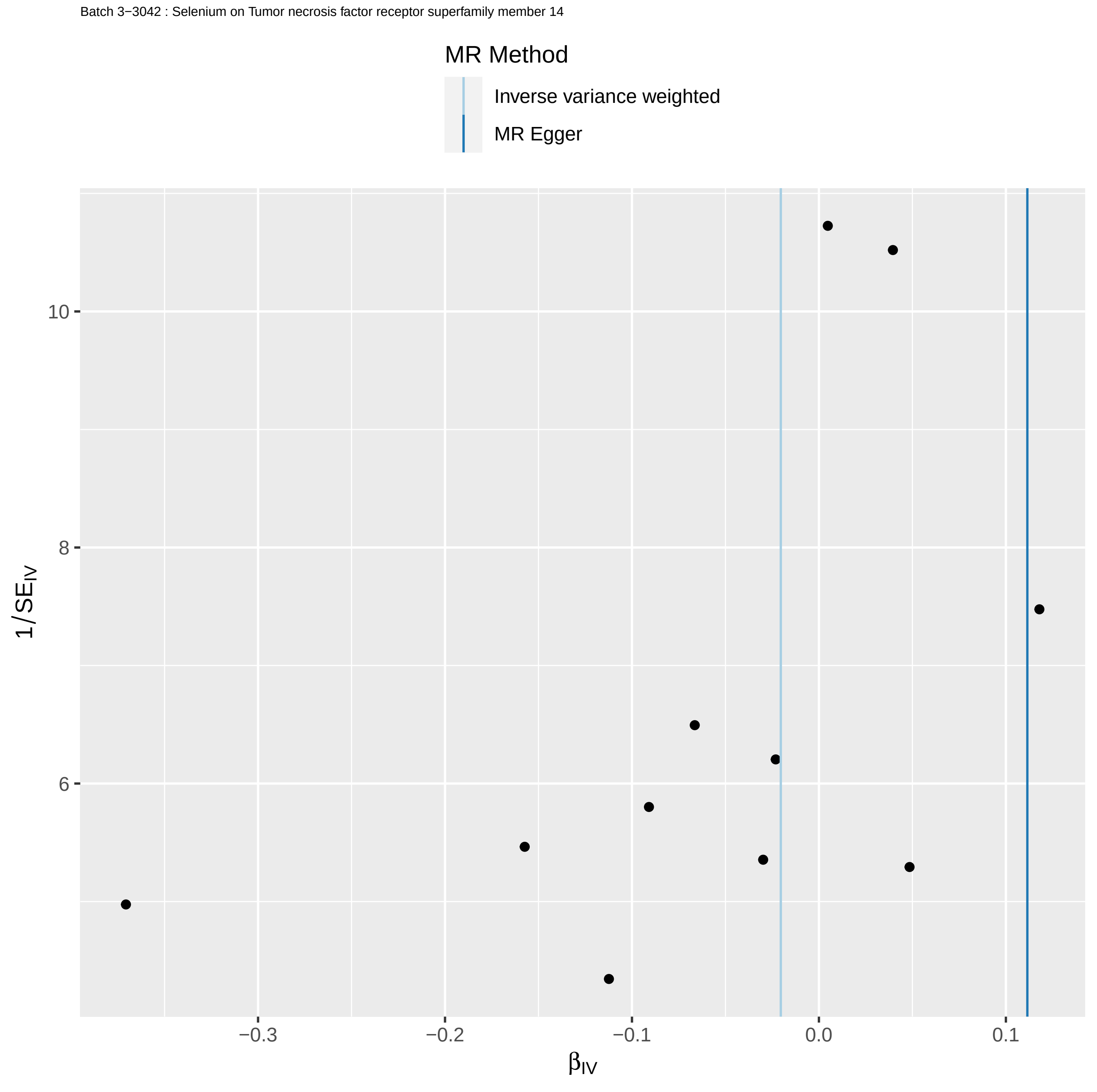

Batch 3−3042 : Selenium on Tumor necrosis factor receptor superfamily member 14
MR Method
Inverse variance weighted
MR Egger
10
8
6
1/SE_IV
−0.3
−0.2
−0.1
0.0
0.1
β_IV

Batch 3−3043 : Selenium on Tumor necrosis factor receptor superfamily member 17

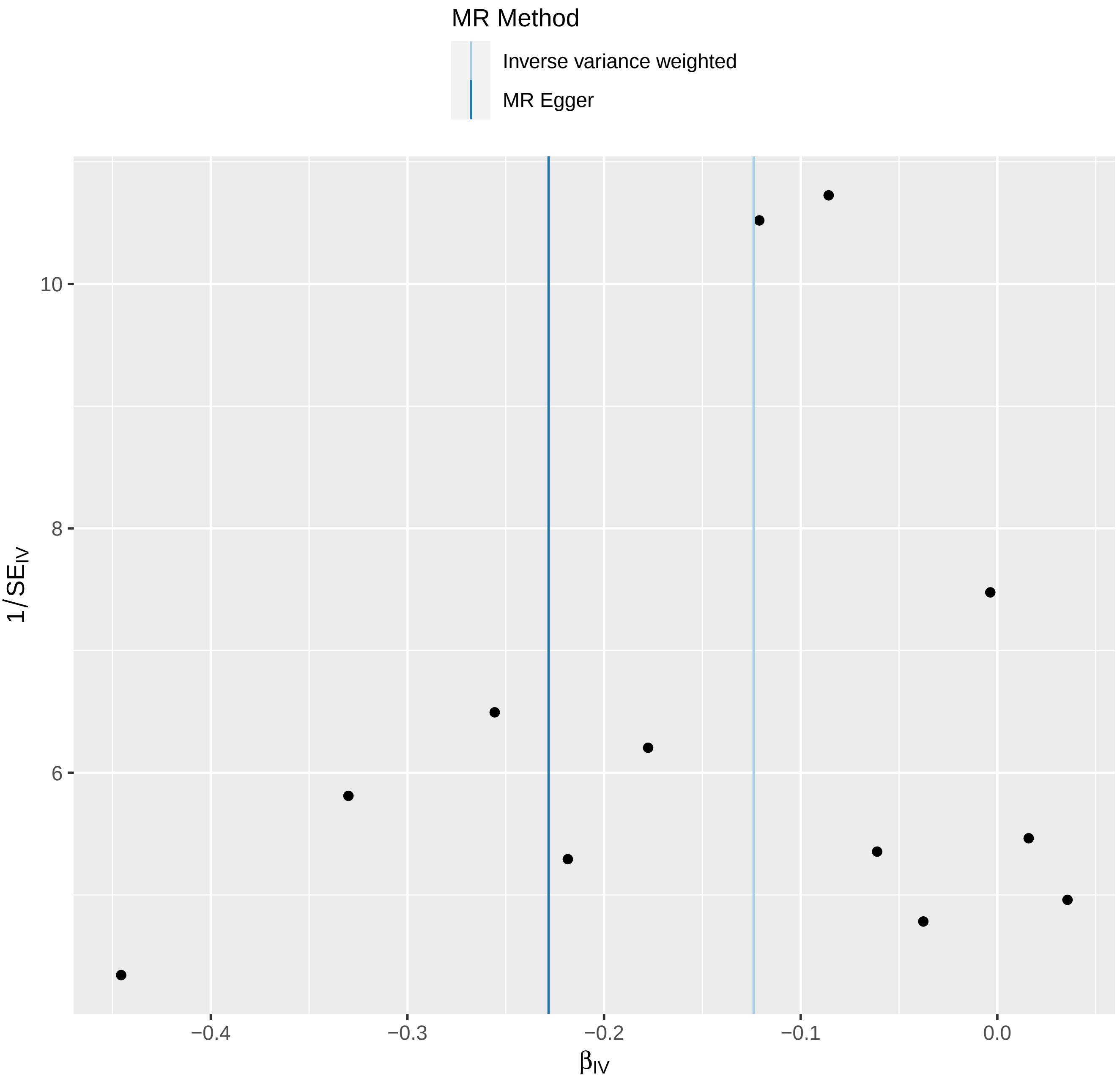

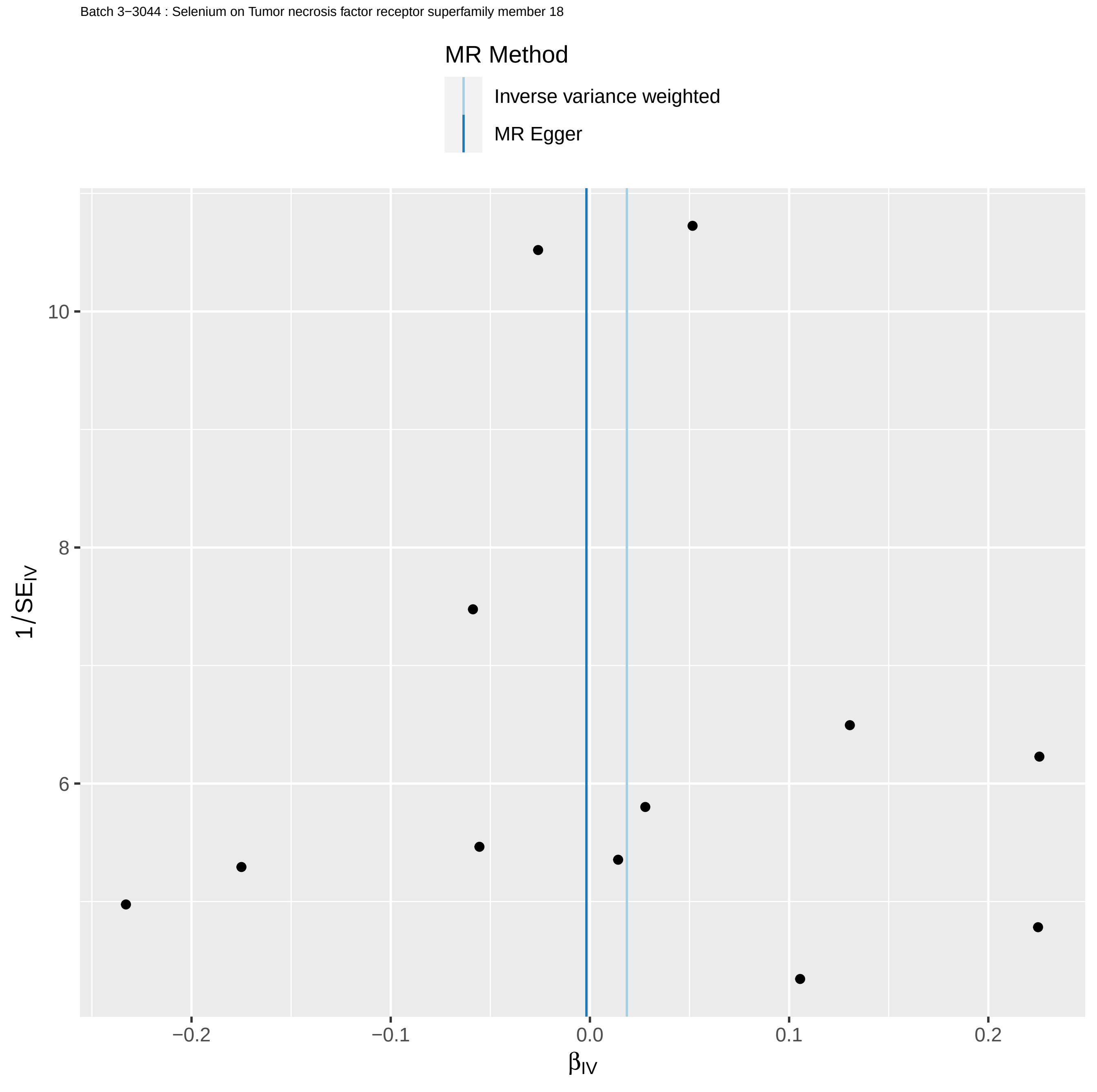
Batch 3−3044 : Selenium on Tumor necrosis factor receptor superfamily member 18
MR Method
Inverse variance weighted
MR Egger
10
8
6
1/SE_IV
−0.2
−0.1
0.0
0.1
0.2
β_IV

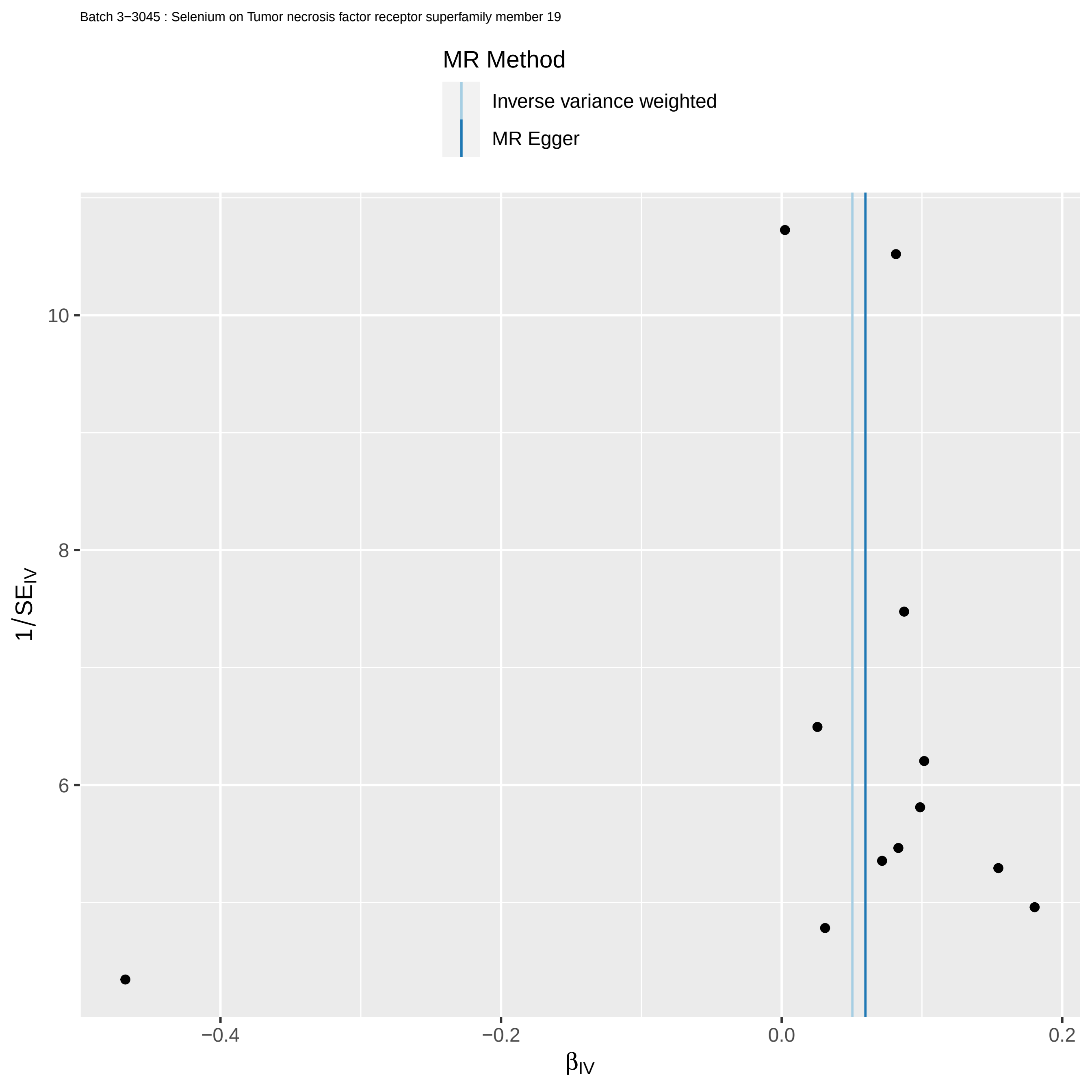
Batch 3−3045 : Selenium on Tumor necrosis factor receptor superfamily member 19
MR Method
Inverse variance weighted
MR Egger
10
8
6
1/SE$_{IV}$
−0.4
−0.2
0.0
0.2
β_{IV}

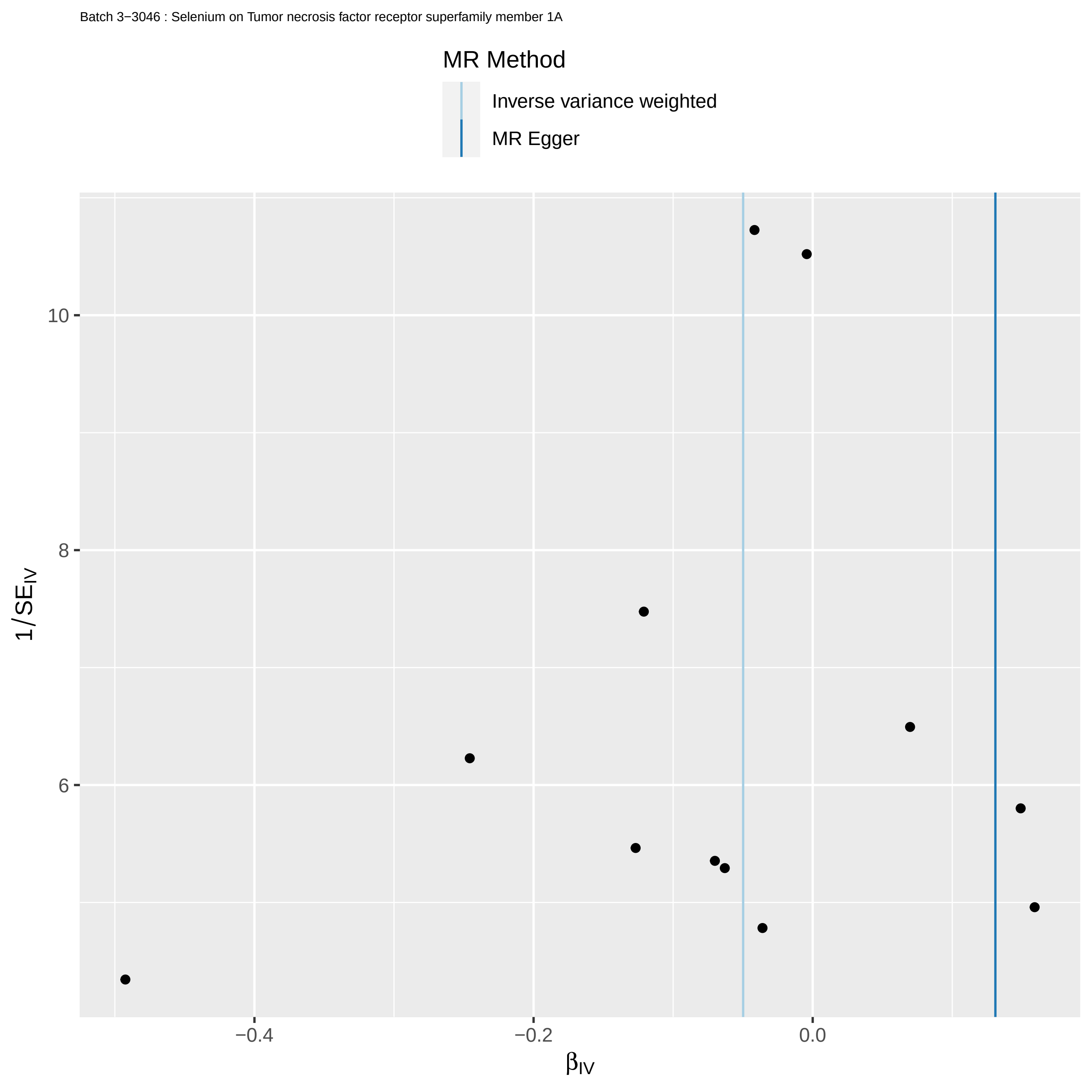

Batch 3−3046 : Selenium on Tumor necrosis factor receptor superfamily member 1A
MR Method
Inverse variance weighted
MR Egger
10
8
6
1/SE_IV
−0.4
−0.2
0.0
β_IV

Batch 3−3047 : Selenium on Tumor necrosis factor receptor superfamily member 1B

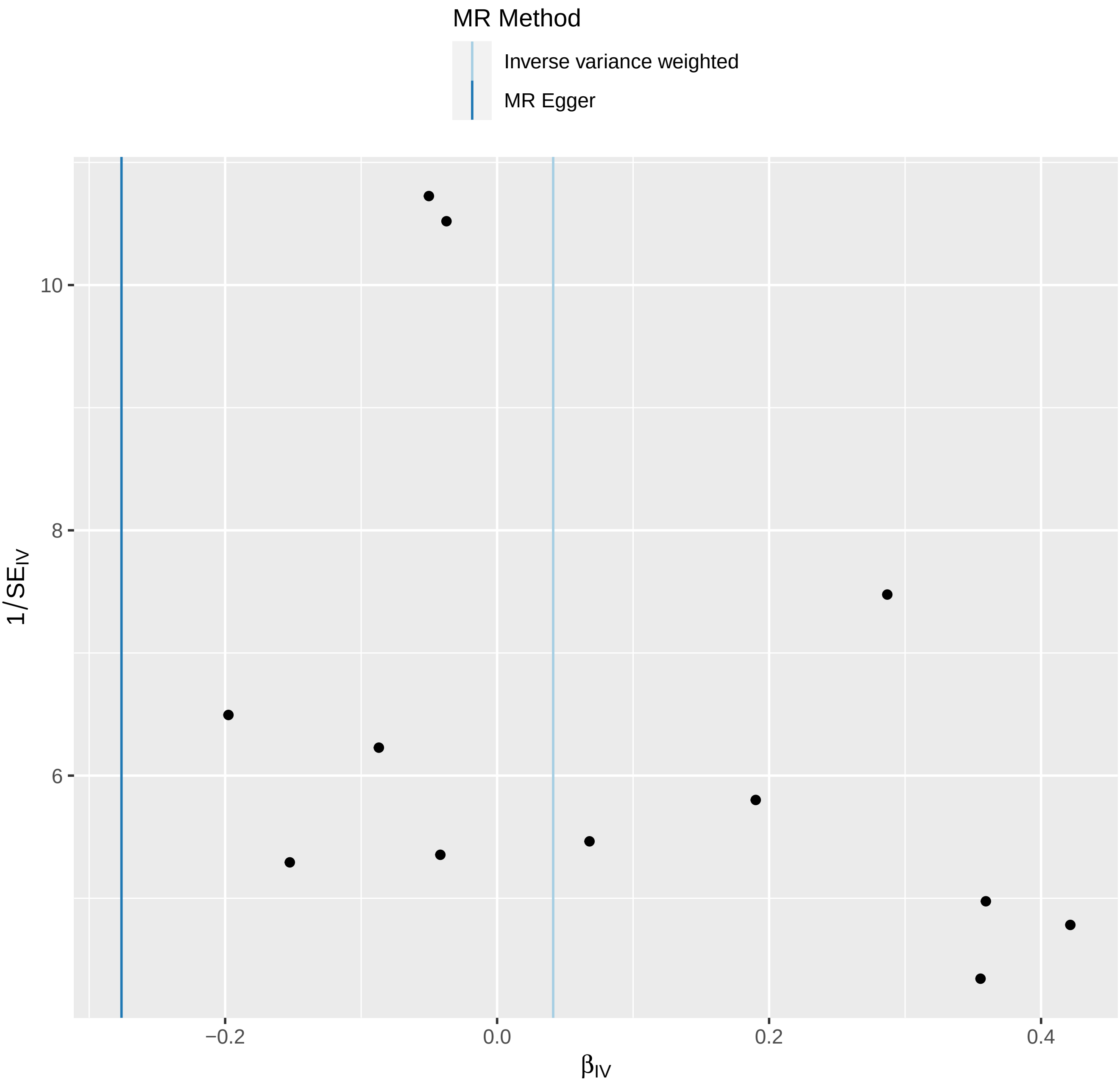

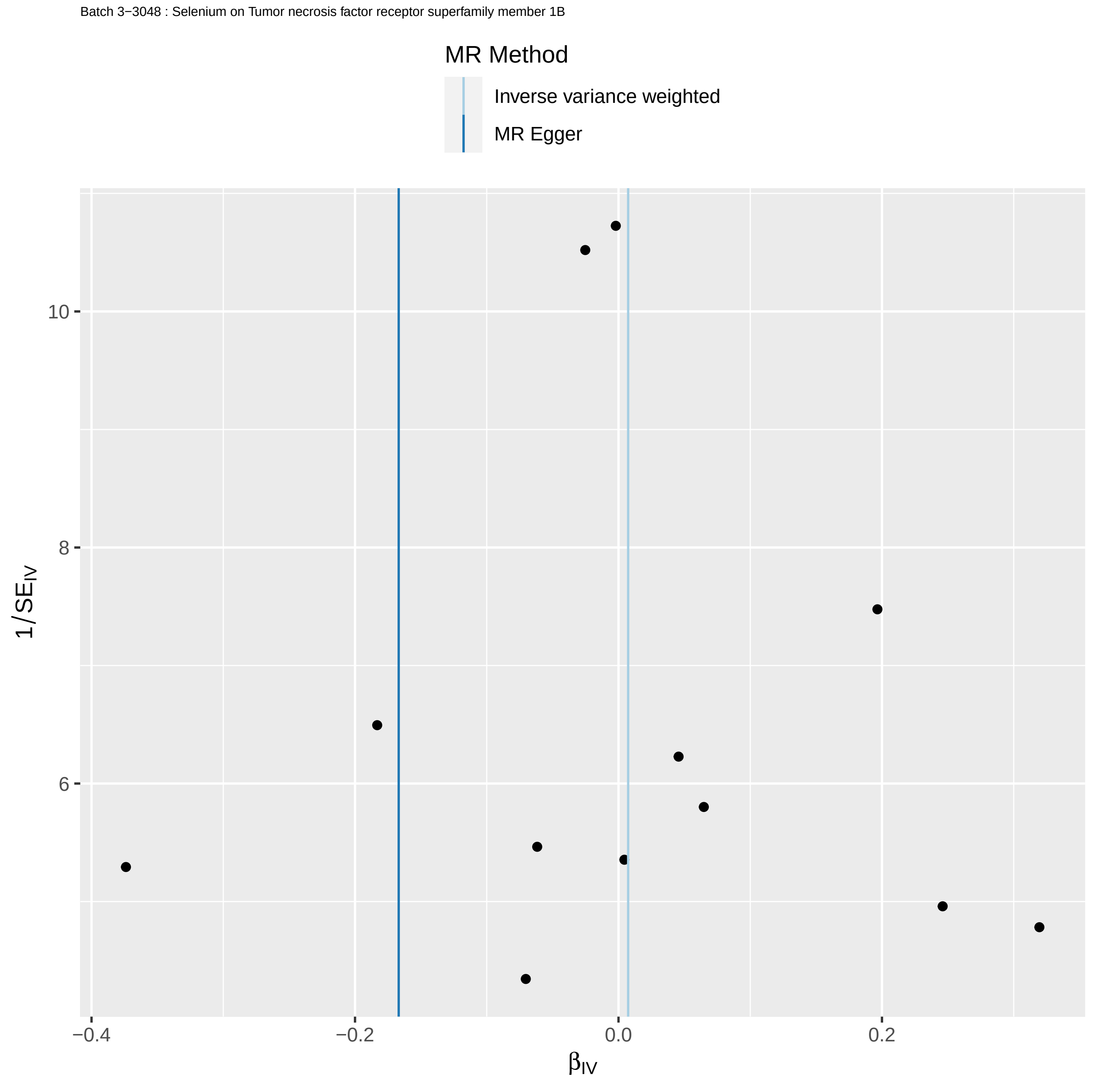

Batch 3−3048 : Selenium on Tumor necrosis factor receptor superfamily member 1B
MR Method
Inverse variance weighted
MR Egger
10
8
6
1/SE_IV
−0.4
−0.2
0.0
0.2
β_IV

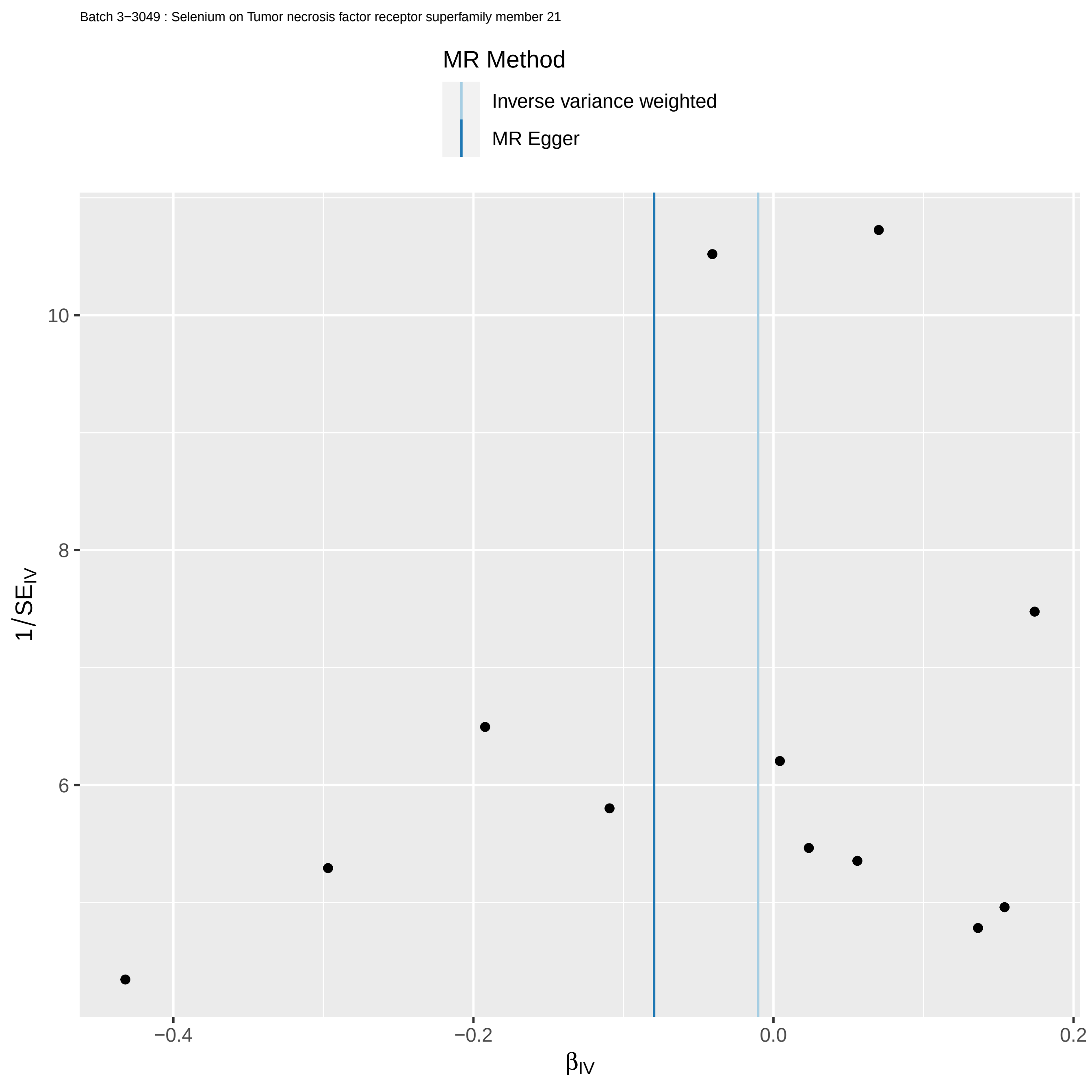
Batch 3−3049 : Selenium on Tumor necrosis factor receptor superfamily member 21
MR Method
Inverse variance weighted
MR Egger
10
8
6
1/SE_IV
−0.4
−0.2
0.0
0.2
β_IV

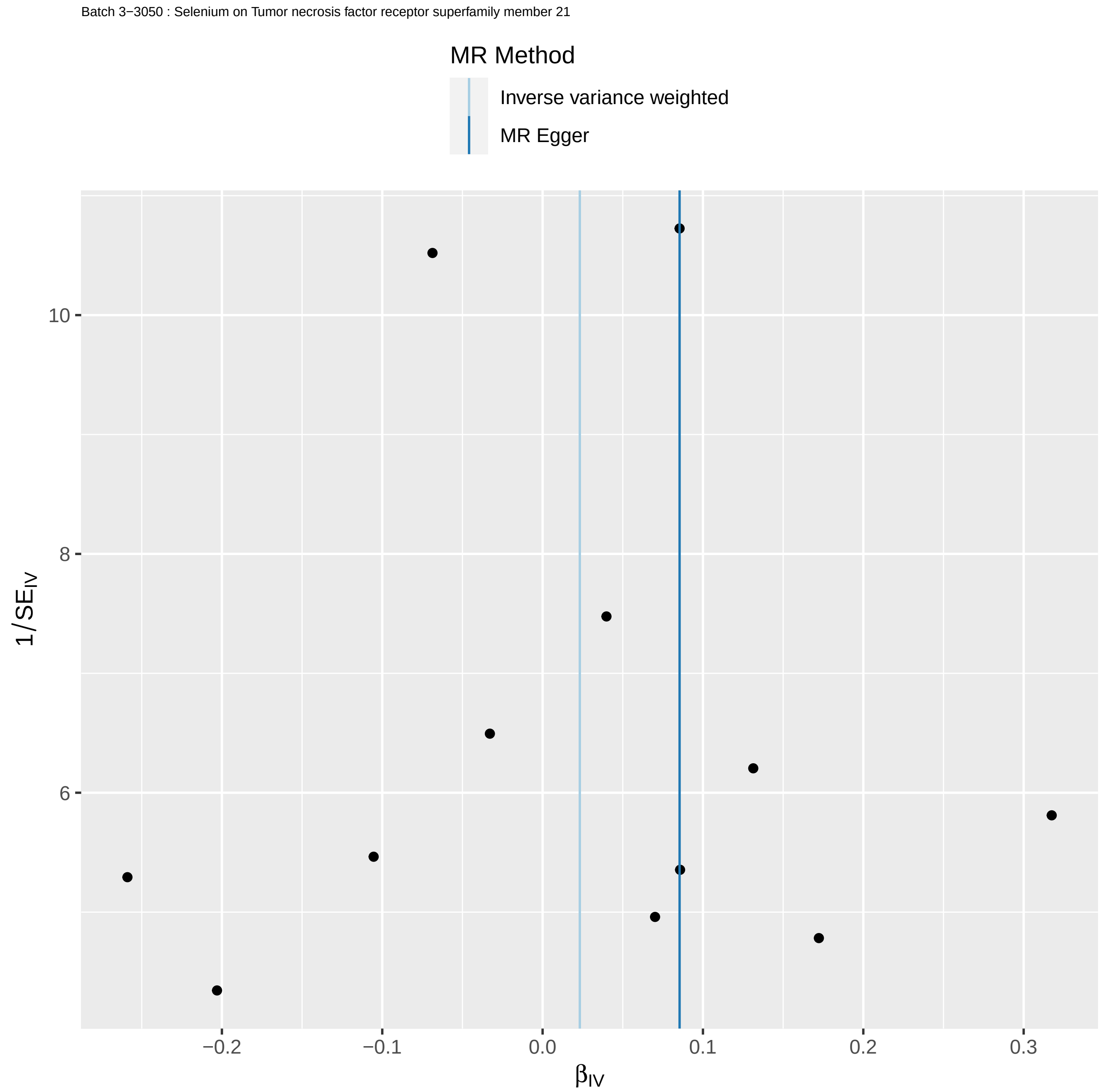
Batch 3−3050 : Selenium on Tumor necrosis factor receptor superfamily member 21
MR Method
Inverse variance weighted
MR Egger
10
8
6
$1/SE_{IV}$
−0.2
−0.1
0.0
0.1
0.2
0.3
β_{IV}

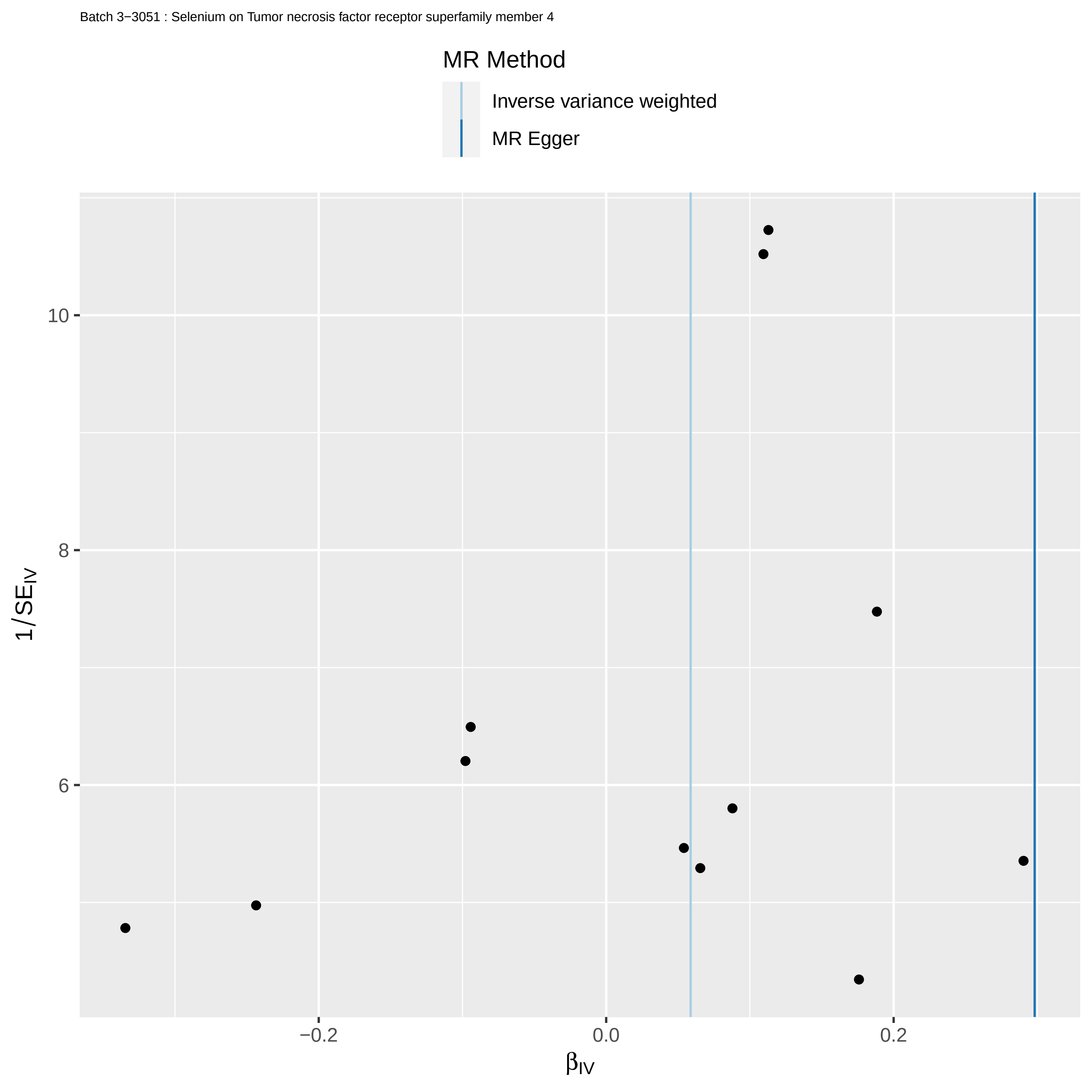
Batch 3−3051 : Selenium on Tumor necrosis factor receptor superfamily member 4
MR Method
Inverse variance weighted
MR Egger
10
8
6
1/SE_IV
−0.2
0.0
0.2
β_IV

Batch 3−3052 : Selenium on Tumor necrosis factor receptor superfamily member 6B

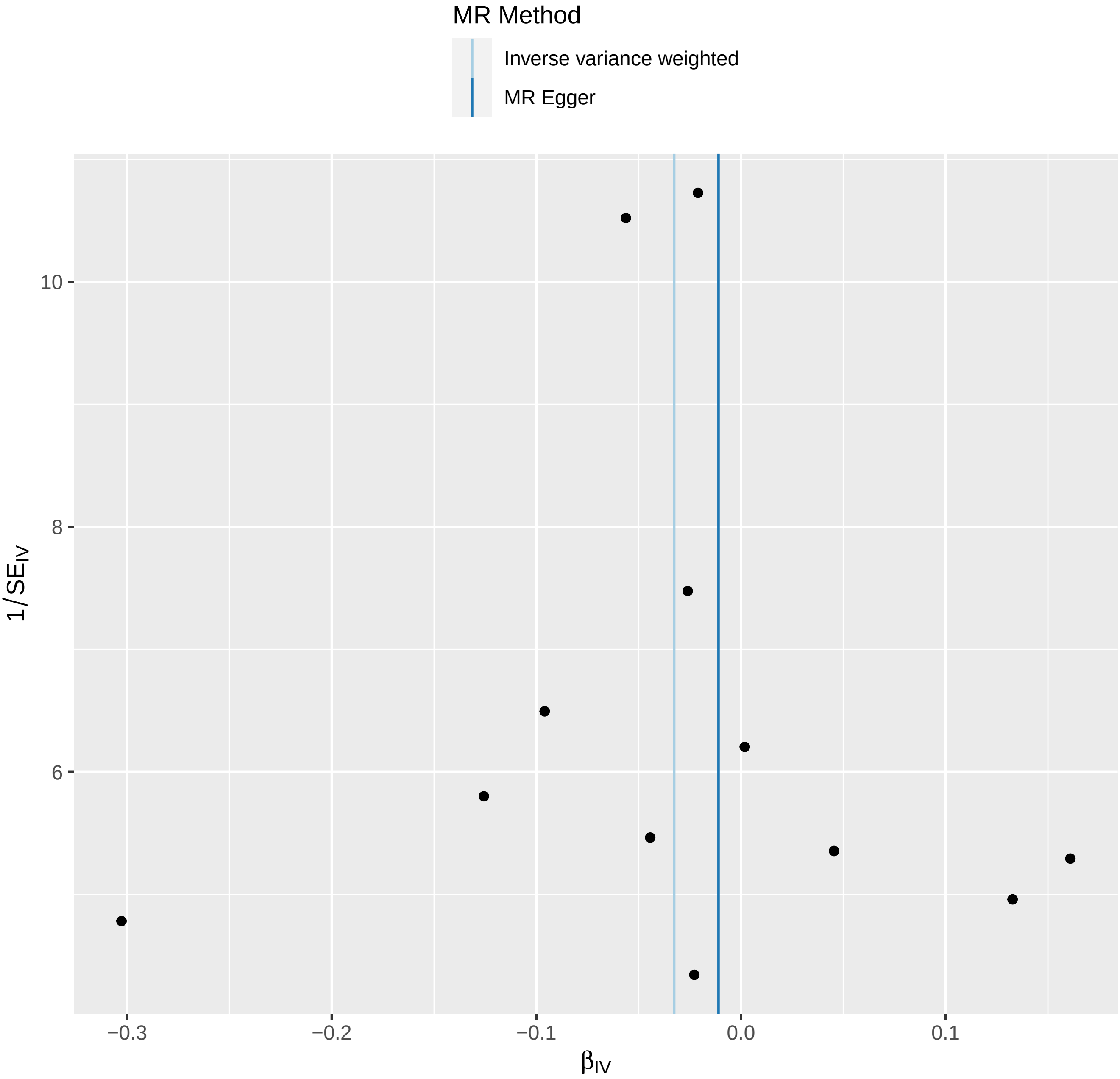

Batch 3−3053 : Selenium on Tumor necrosis factor receptor superfamily member 8

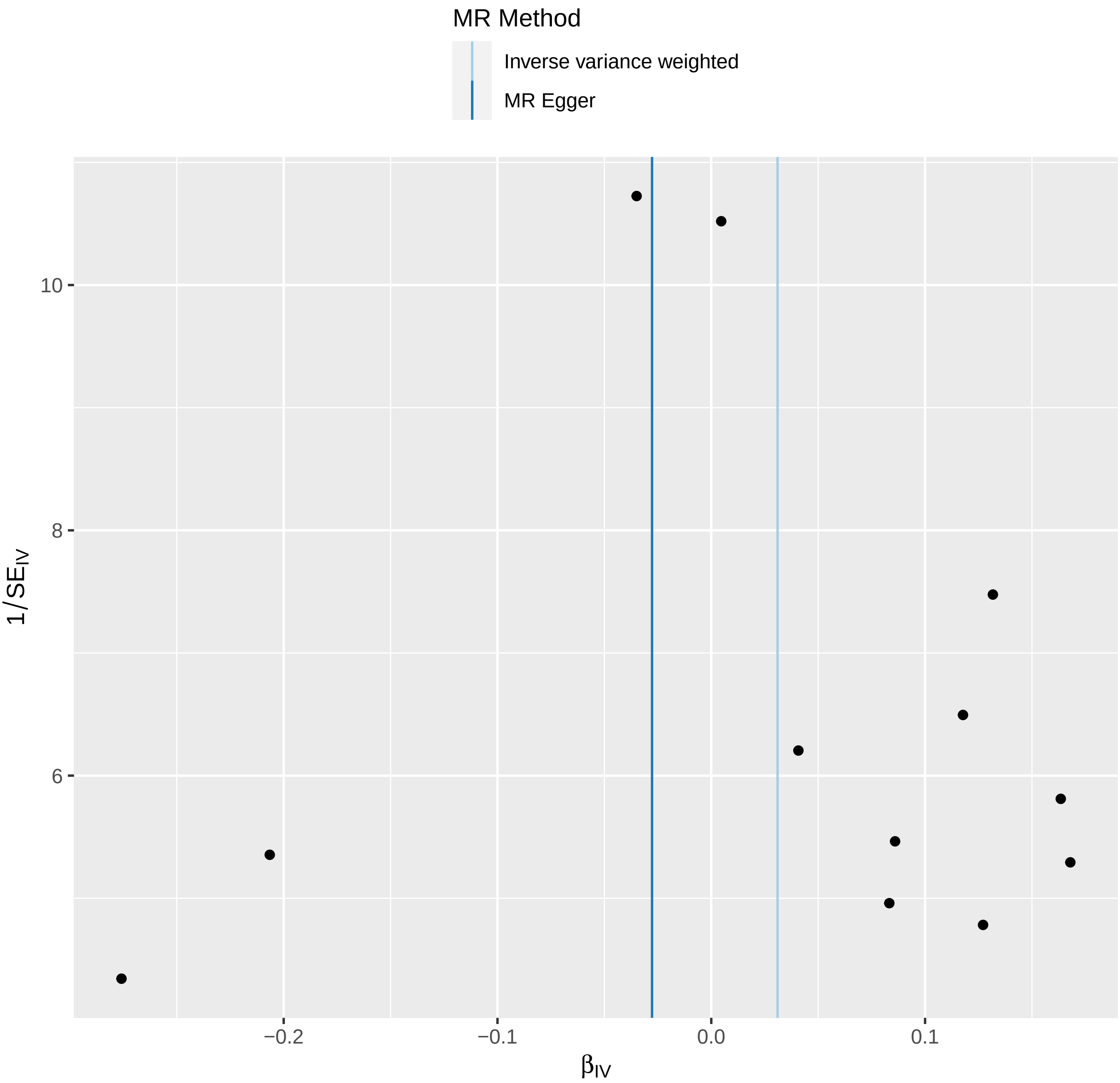

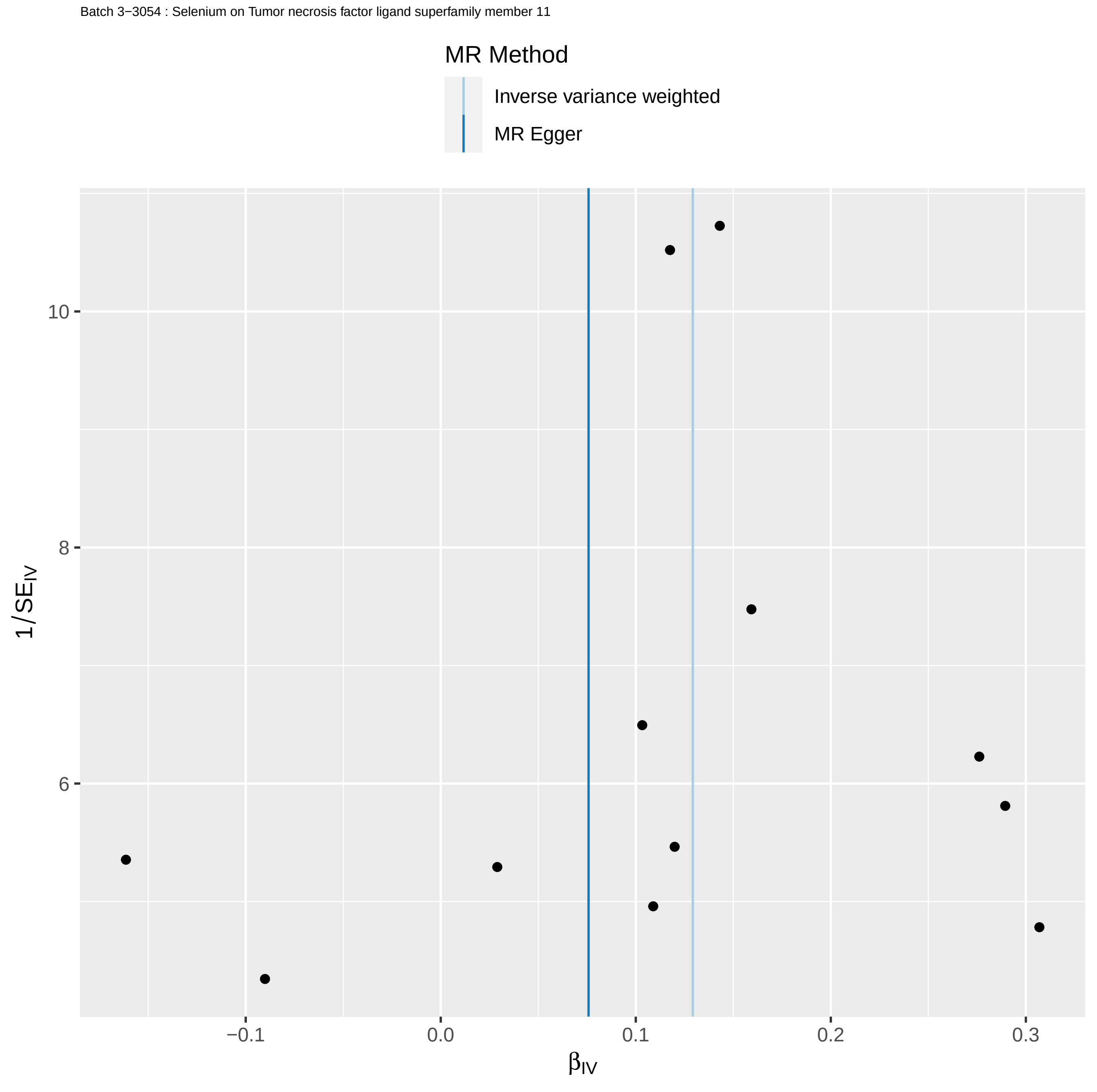

Batch 3−3054 : Selenium on Tumor necrosis factor ligand superfamily member 11
MR Method
Inverse variance weighted
MR Egger
10
8
6
1/SE$_{IV}$
−0.1
0.0
0.1
0.2
0.3
β$_{IV}$

Batch 3−3055 : Selenium on Tumor necrosis factor ligand superfamily member 12

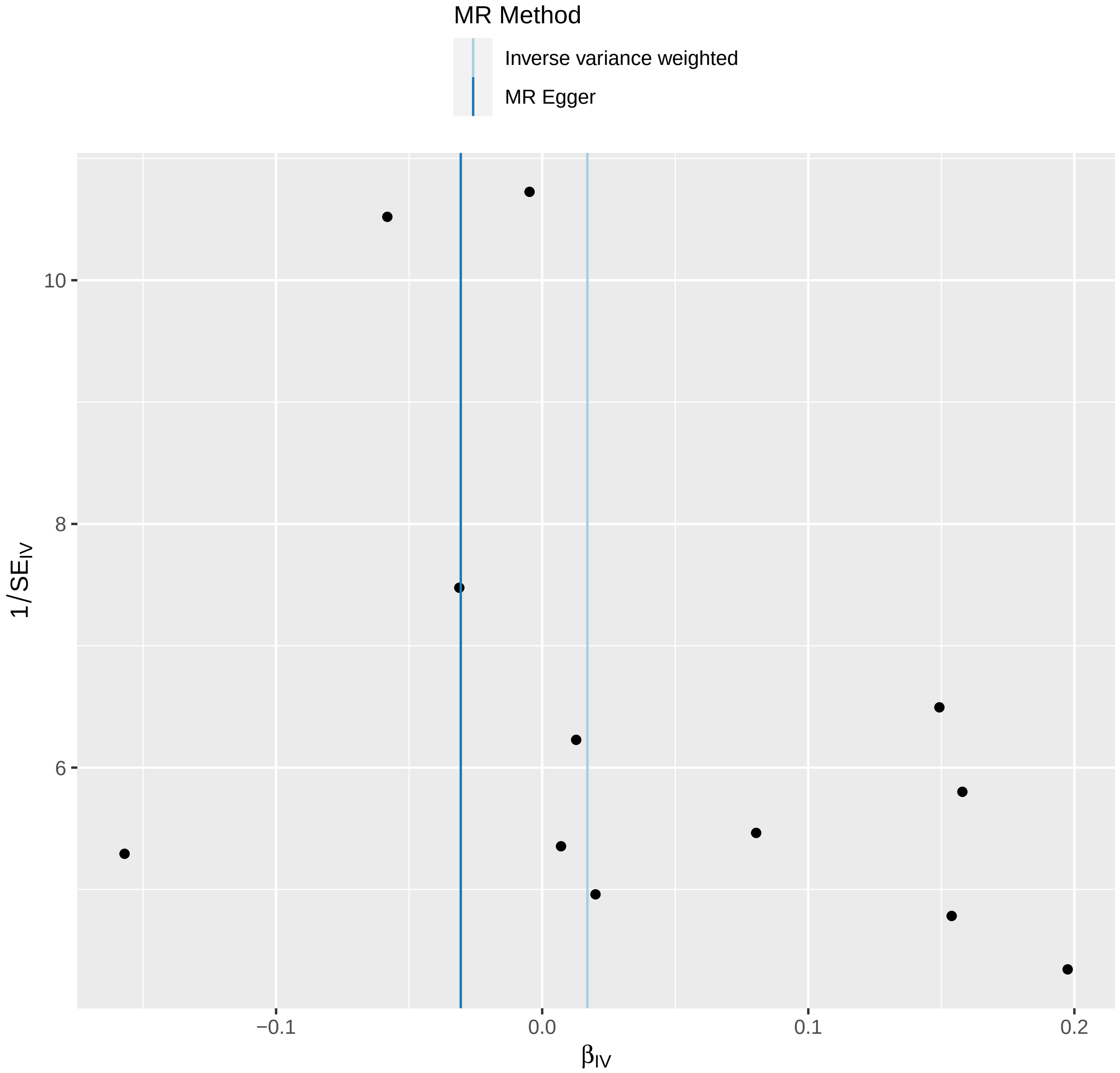

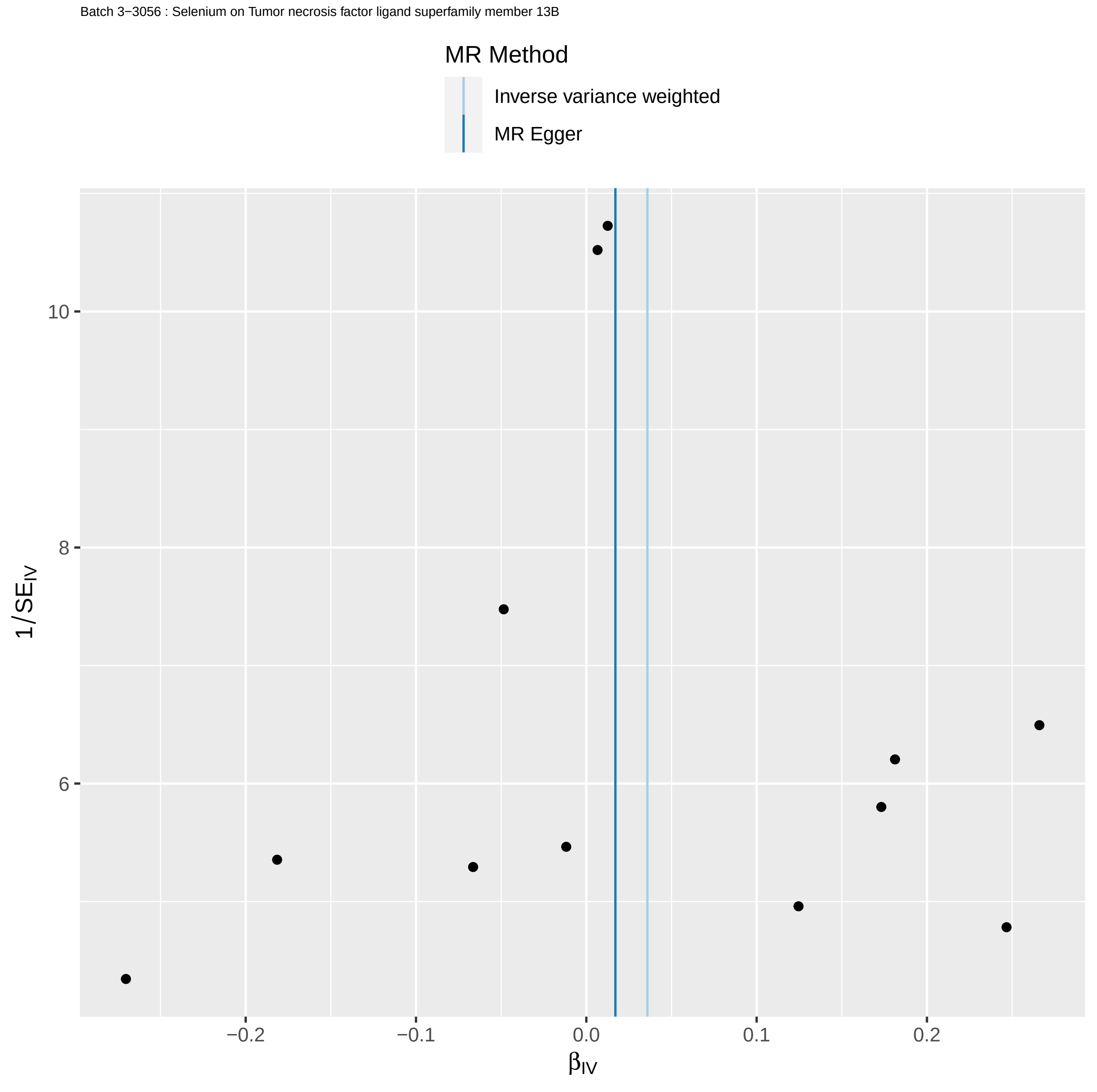
Batch 3−3056 : Selenium on Tumor necrosis factor ligand superfamily member 13B
MR Method
Inverse variance weighted
MR Egger
10
8
6
1/SE$_{IV}$
−0.2
−0.1
0.0
0.1
0.2
β$_{IV}$

Batch 3−3057 : Selenium on Tumor necrosis factor ligand superfamily member 14

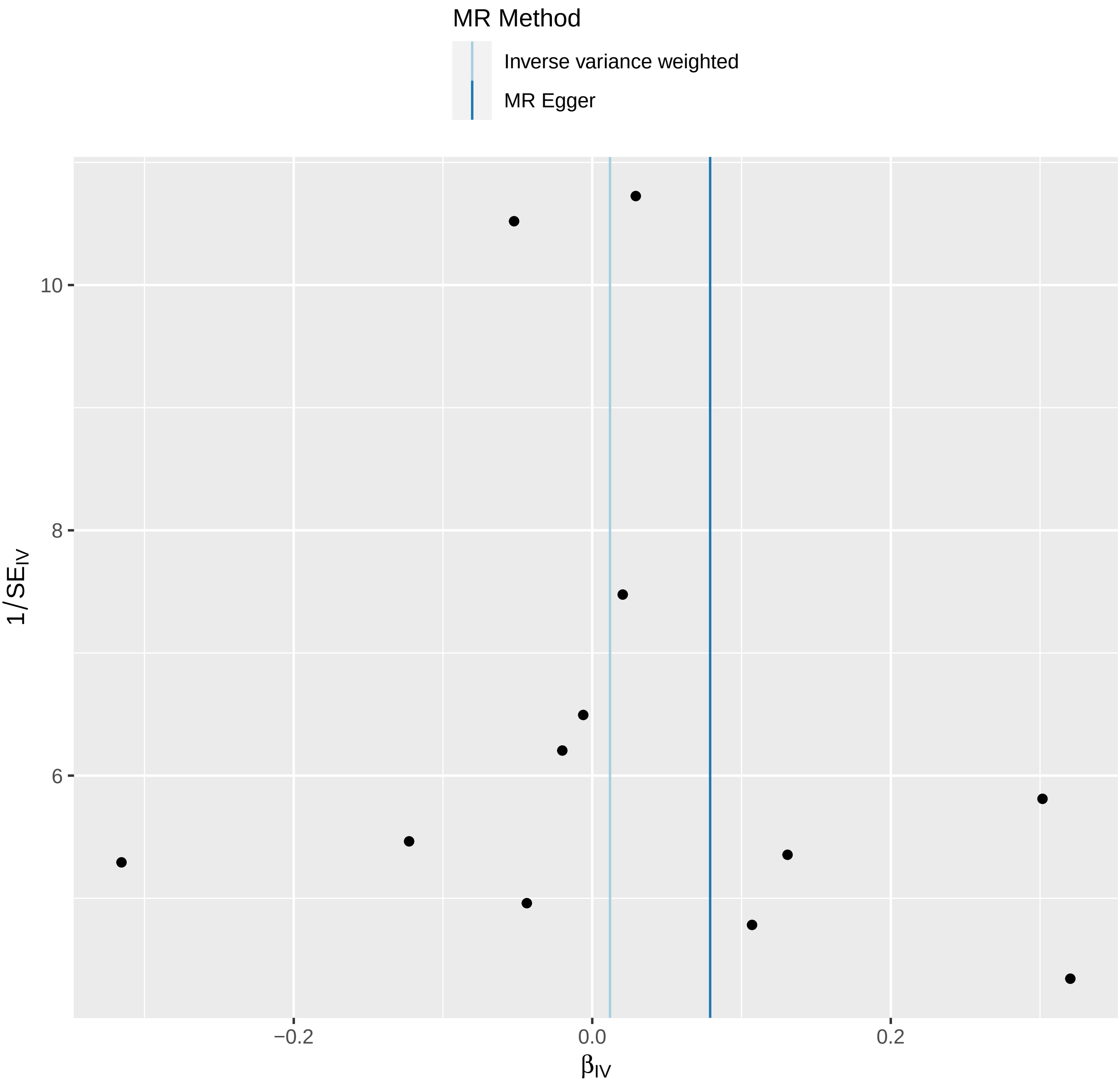

Batch 3−3058 : Selenium on Tumor necrosis factor ligand superfamily member 15

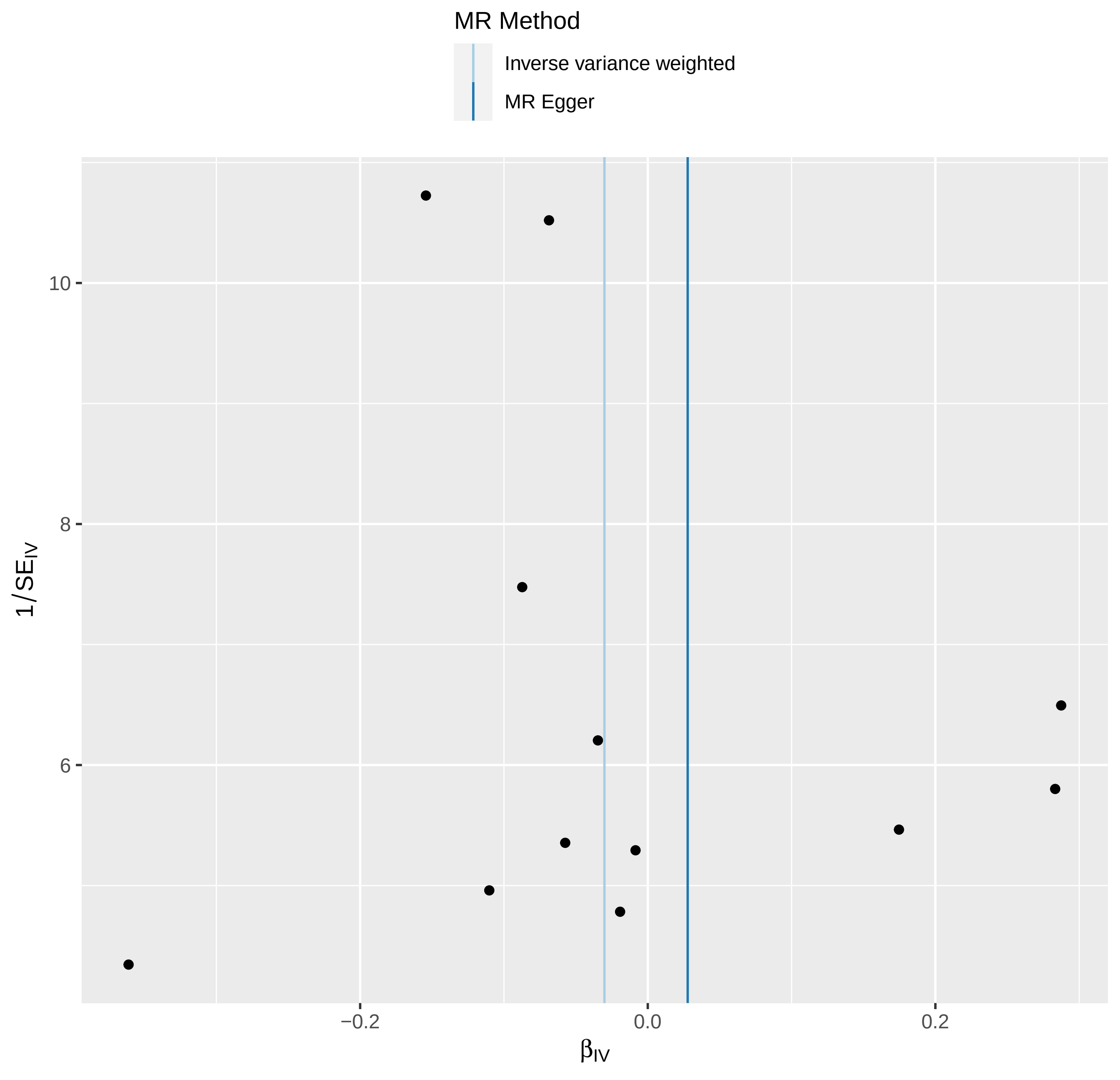

Batch 3−3059 : Selenium on Tumor necrosis factor ligand superfamily member 15

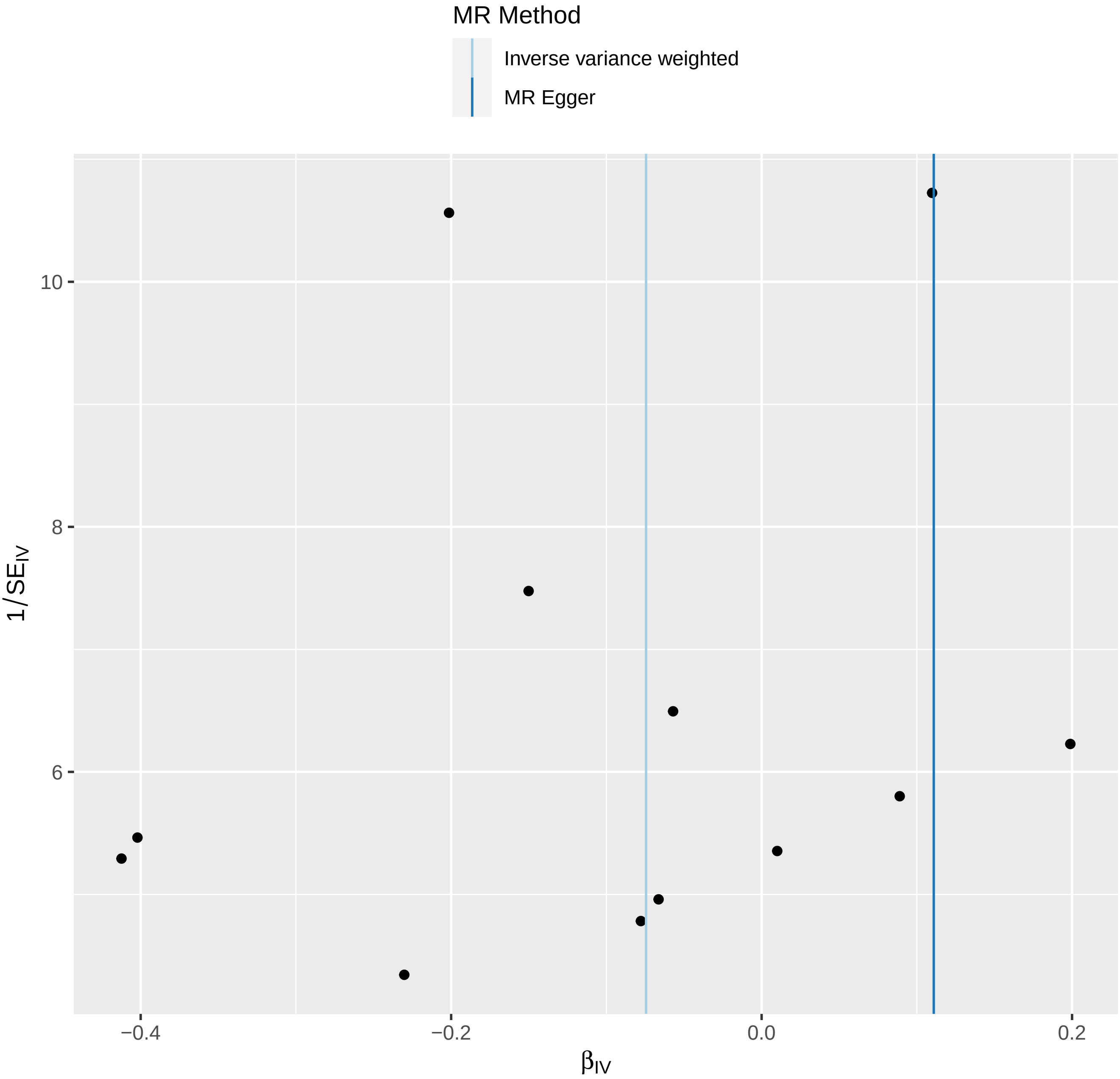

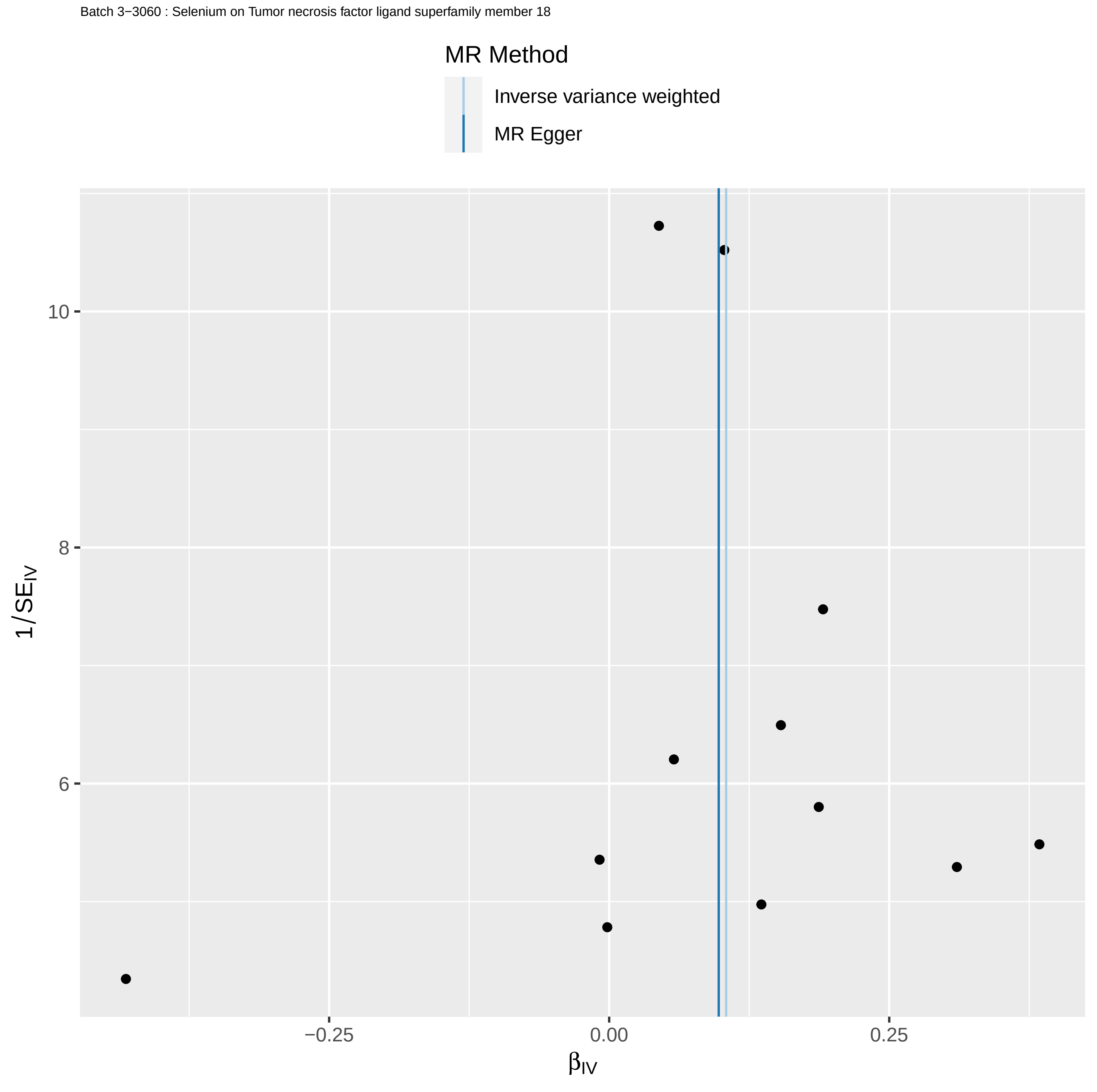

Batch 3−3060 : Selenium on Tumor necrosis factor ligand superfamily member 18
MR Method
Inverse variance weighted
MR Egger
10
8
6
1/SE$_{IV}$
−0.25
0.00
0.25
β$_{IV}$

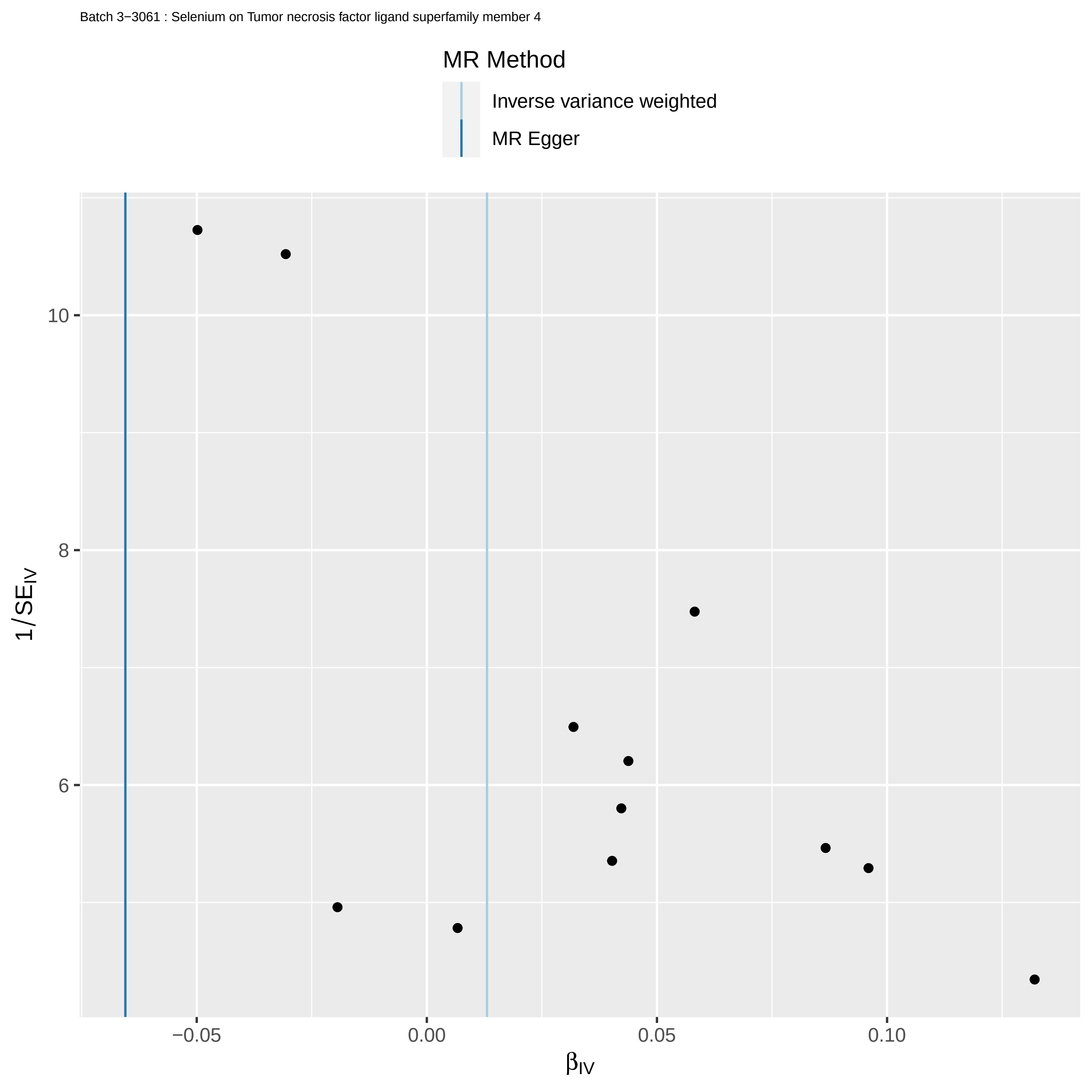

Batch 3−3061 : Selenium on Tumor necrosis factor ligand superfamily member 4
MR Method
Inverse variance weighted
MR Egger
10
8
6
1/SE$_{IV}$
−0.05
0.00
0.05
0.10
β_{IV}

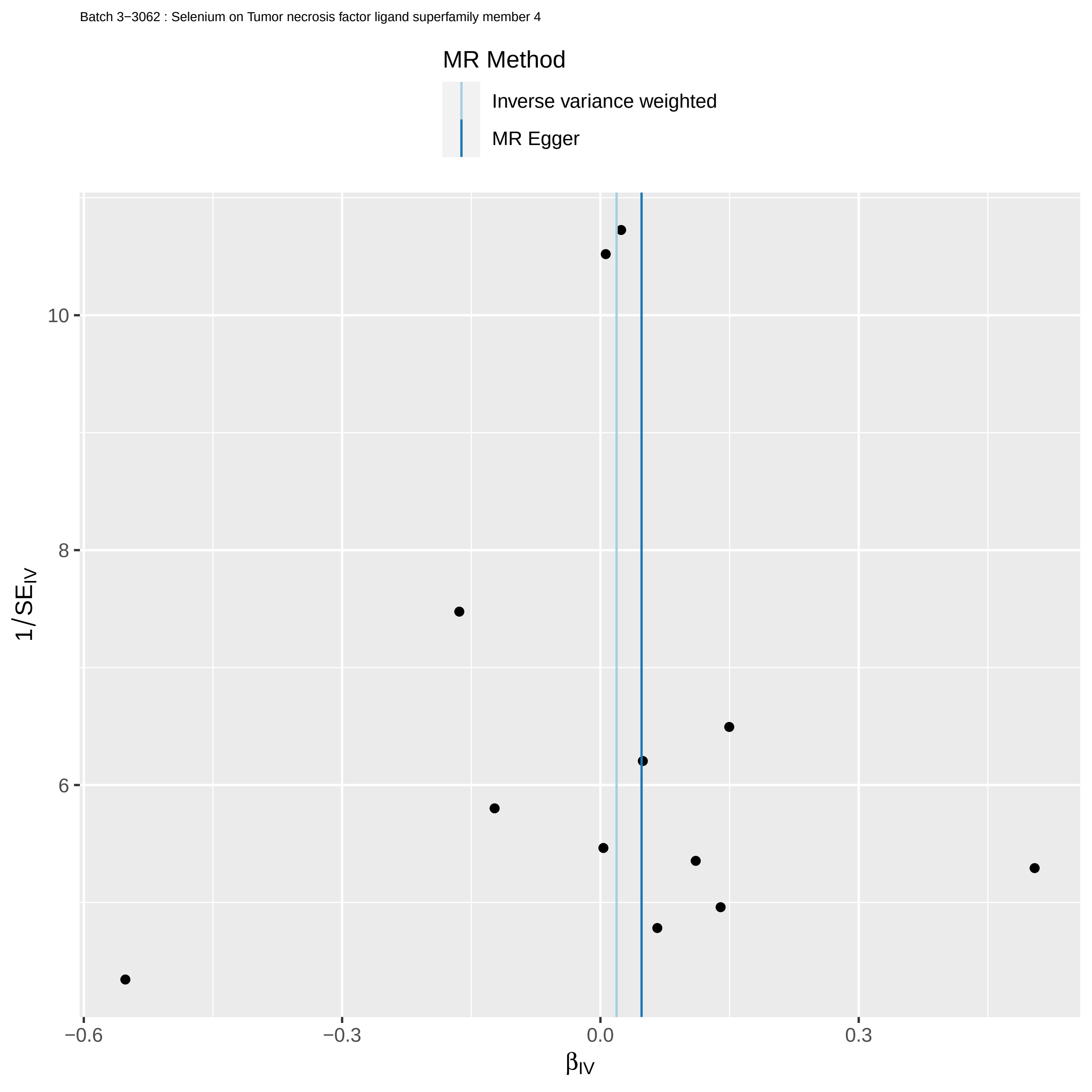
Batch 3−3062 : Selenium on Tumor necrosis factor ligand superfamily member 4
MR Method
Inverse variance weighted
MR Egger
10
8
6
1/SE_IV
−0.6
−0.3
0.0
0.3
β_IV

Batch 3−3063 : Selenium on Tumor necrosis factor ligand superfamily member 8

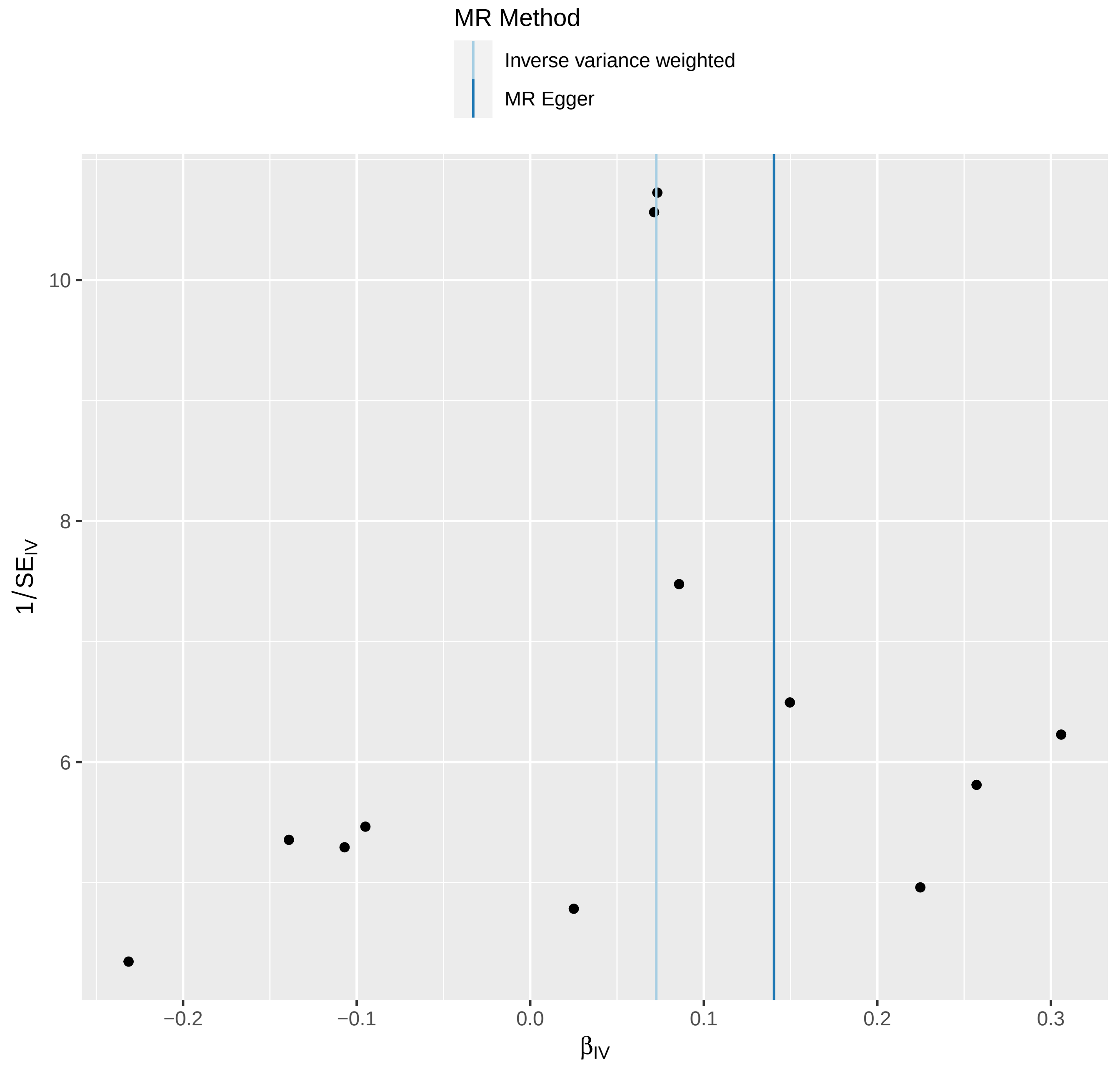

Batch 3−3064 : Selenium on Tumor necrosis factor ligand superfamily member 9

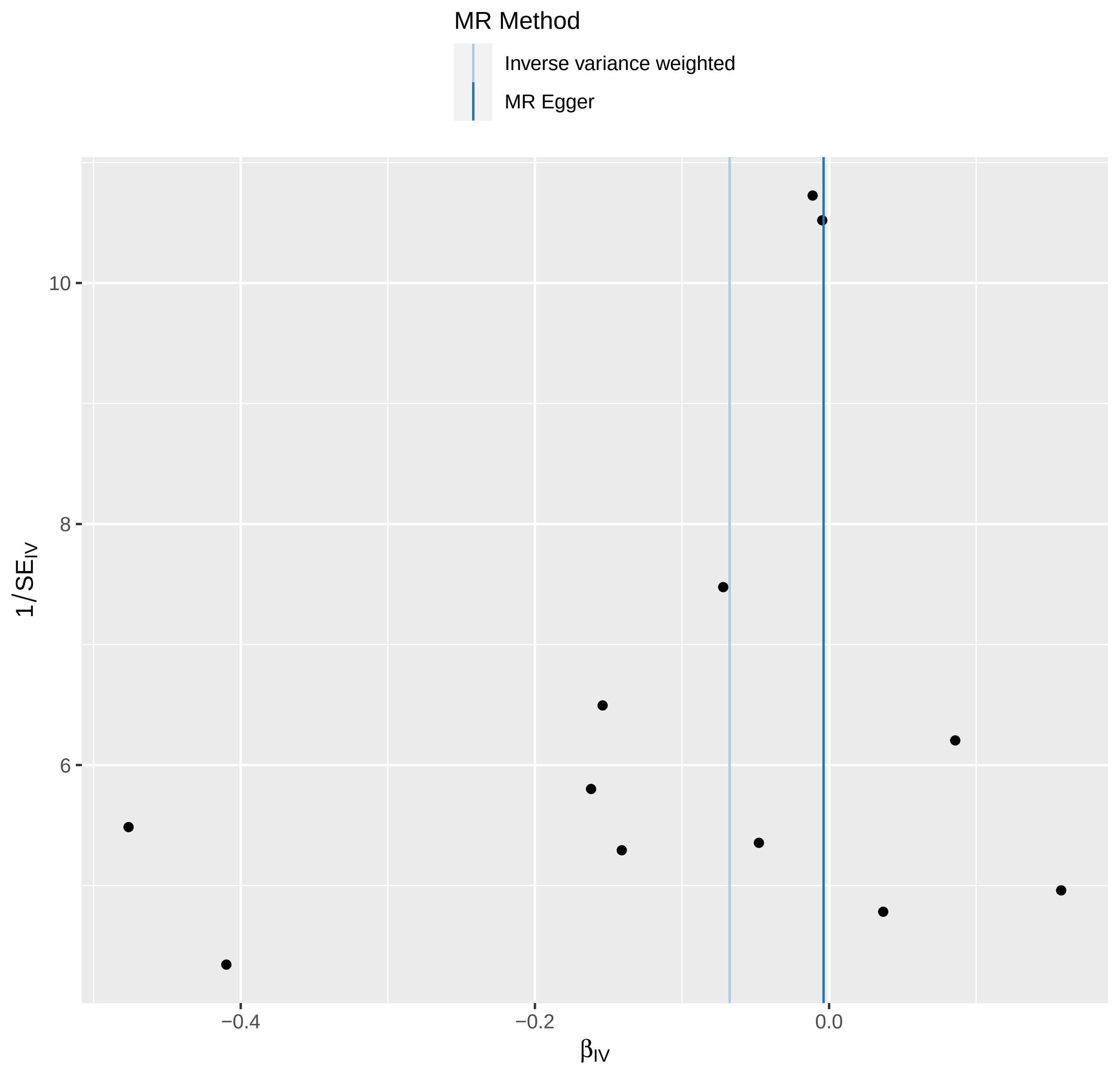

Batch 3−3065 : Selenium on Tenomodulin

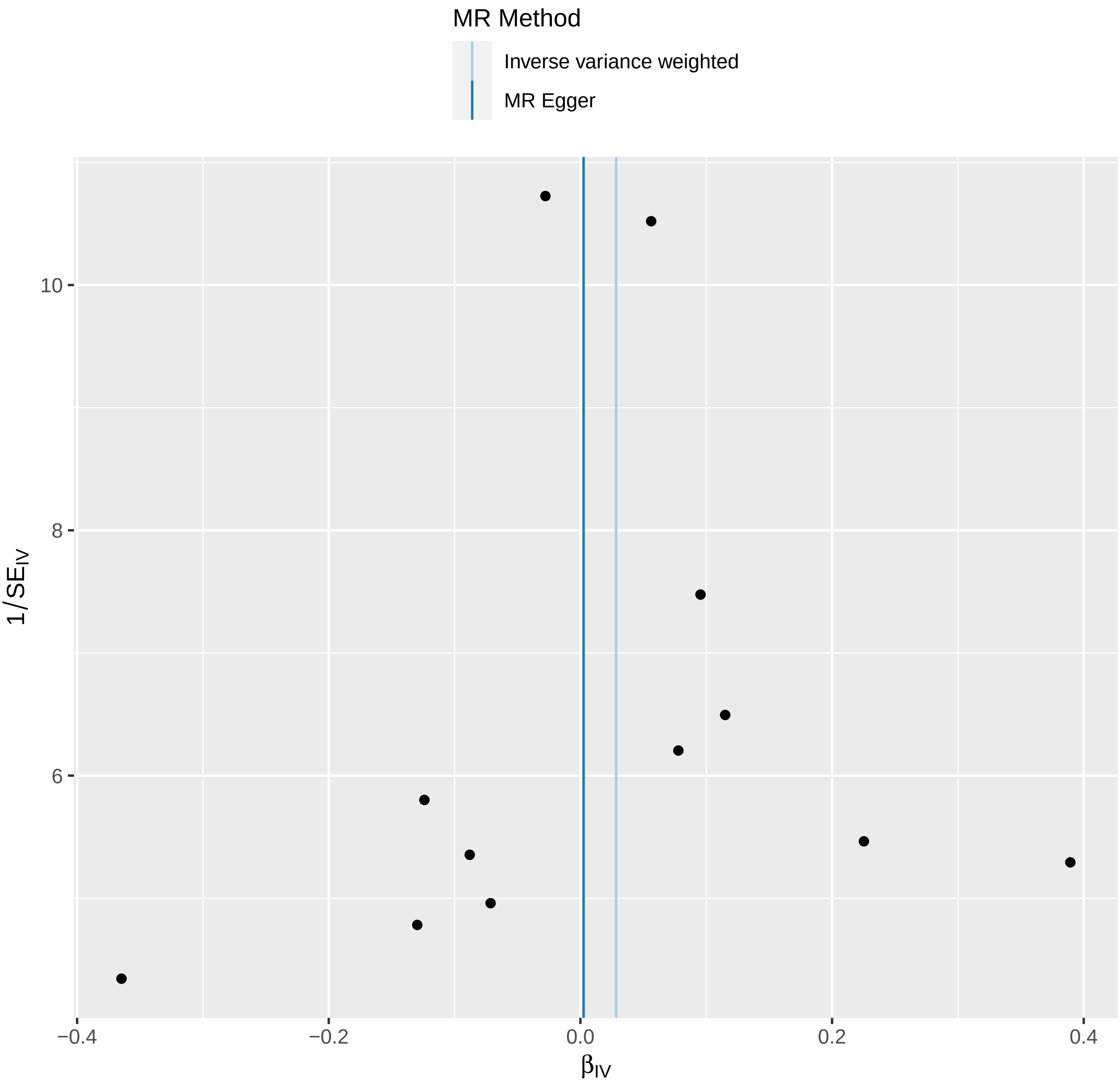

Batch 3−3066 : Selenium on Troponin I, cardiac muscle

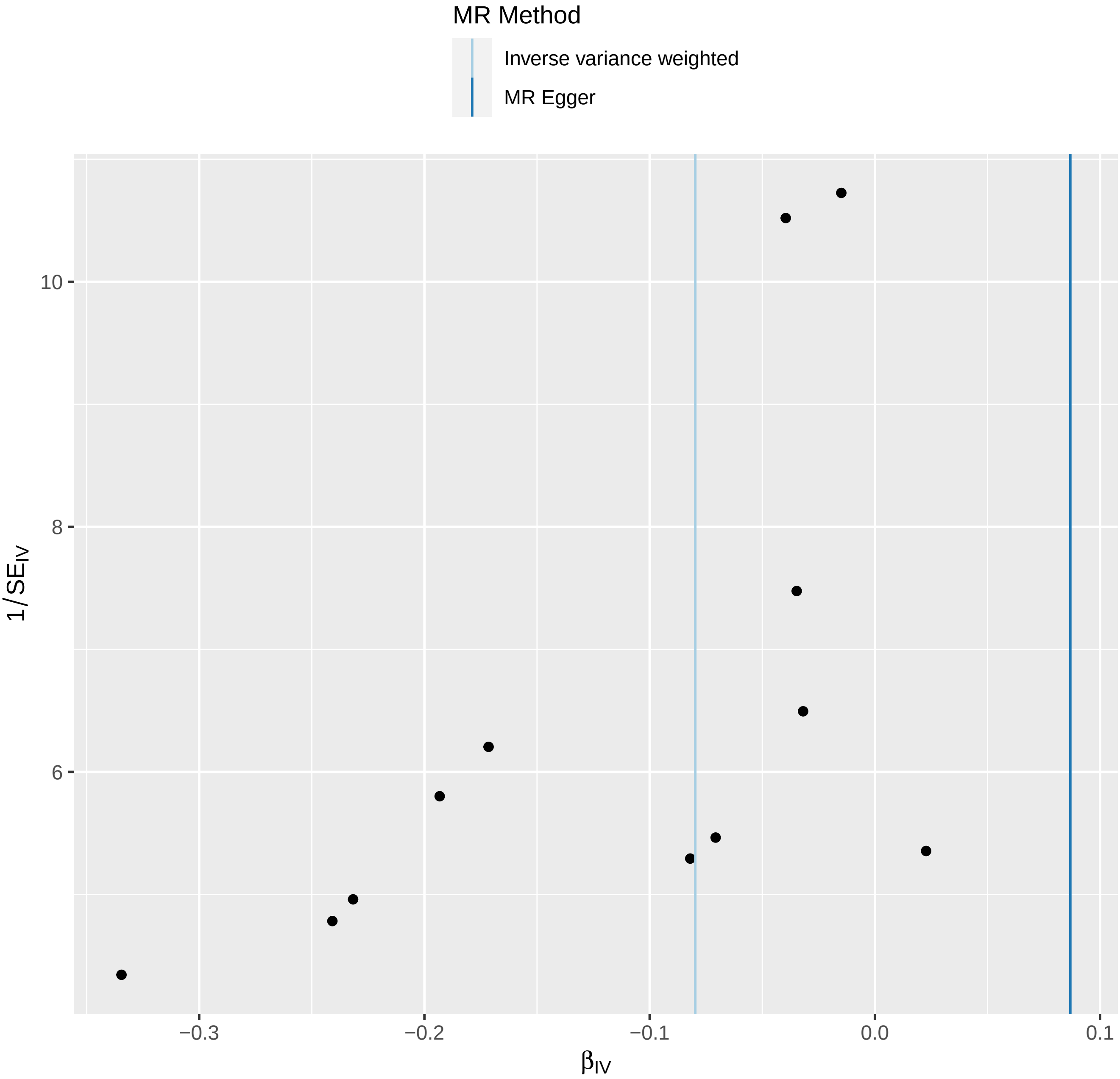

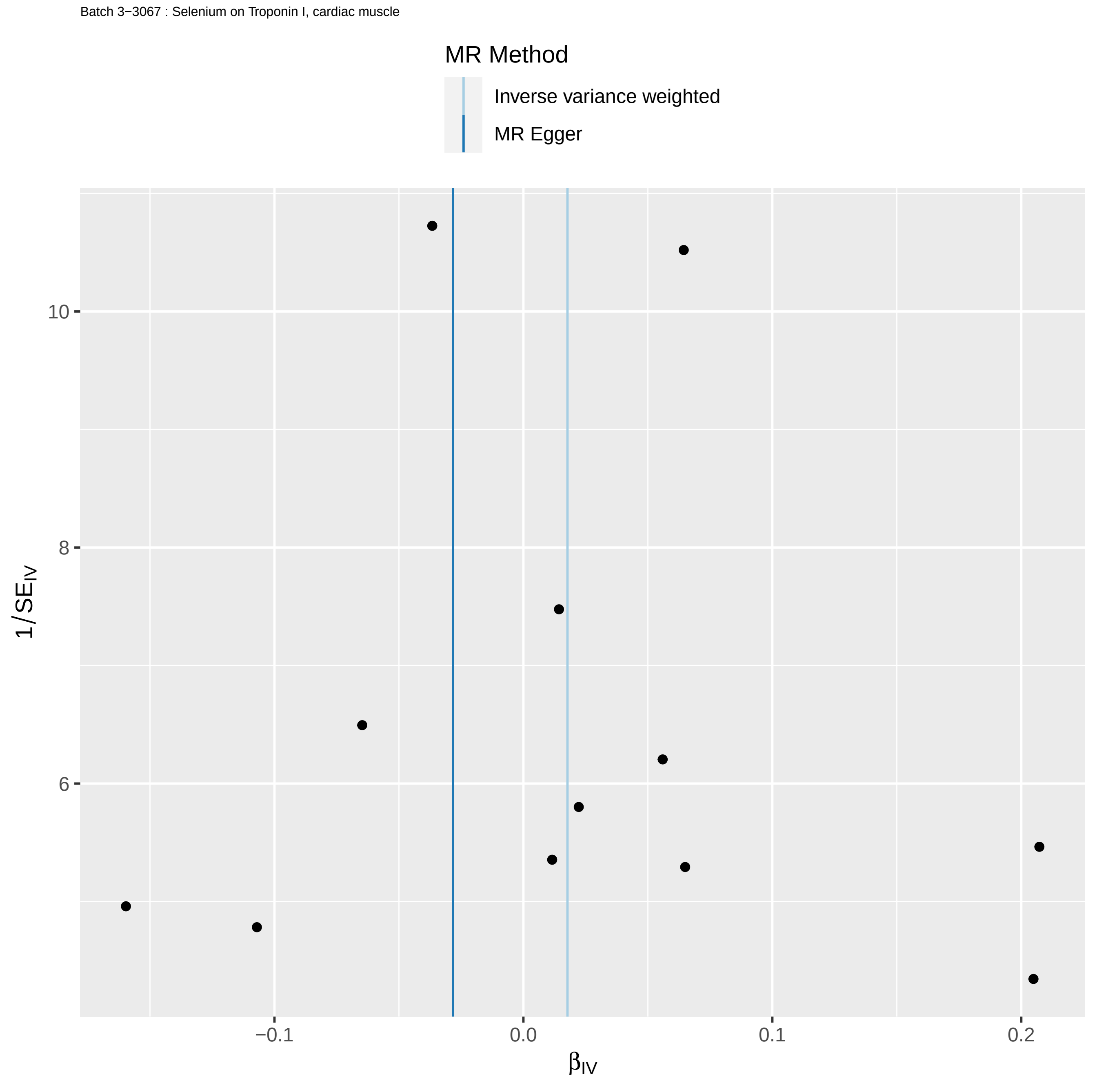
Batch 3−3067 : Selenium on Troponin I, cardiac muscle
MR Method
Inverse variance weighted
MR Egger
10
8
6
1/SE_IV
−0.1
0.0
0.1
0.2
β_IV

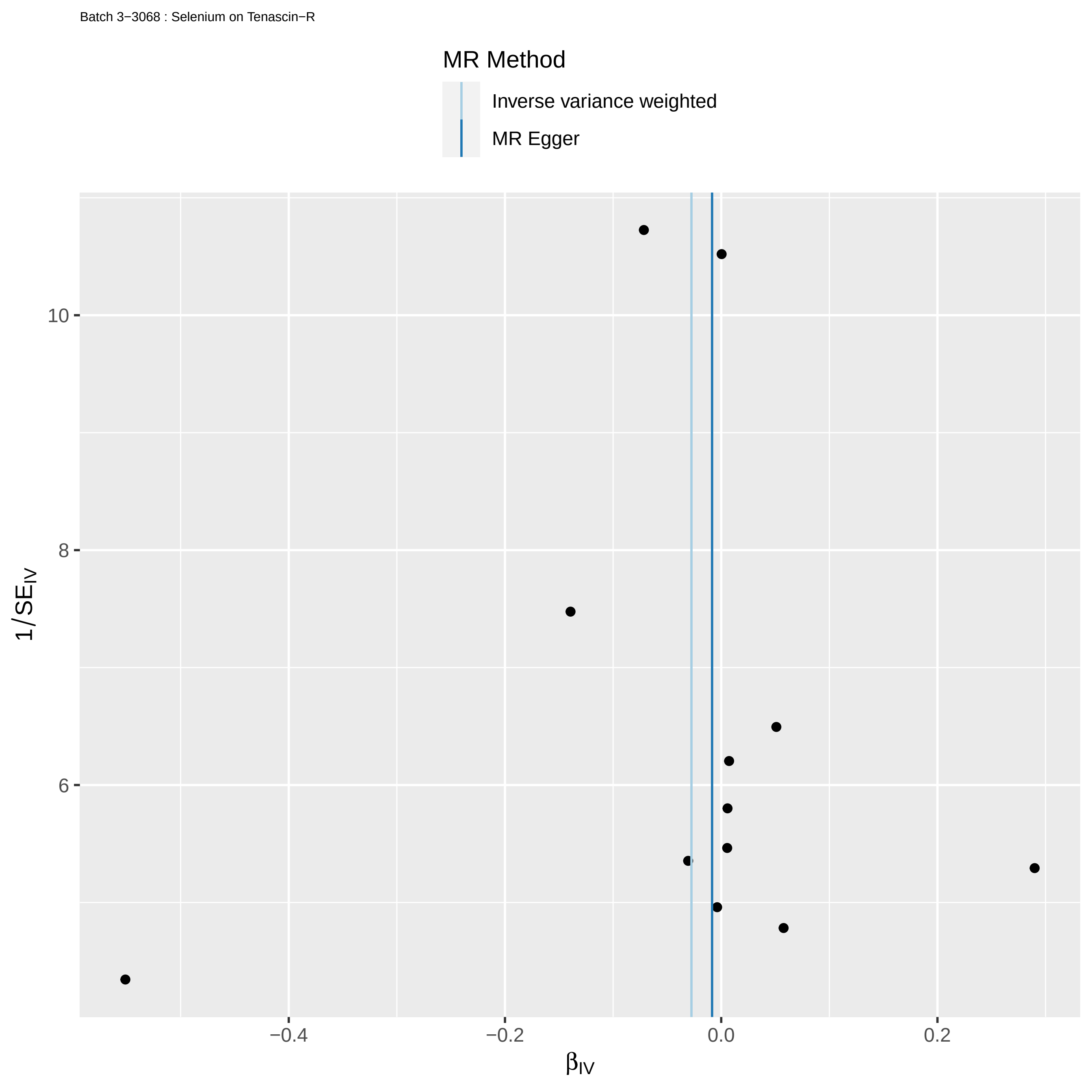

Batch 3−3068 : Selenium on Tenascin−R
MR Method
Inverse variance weighted
MR Egger
10
8
6
1/SE$_{IV}$
−0.4
−0.2
0.0
0.2
β$_{IV}$

Batch 3−3069 : Selenium on Tensin−2

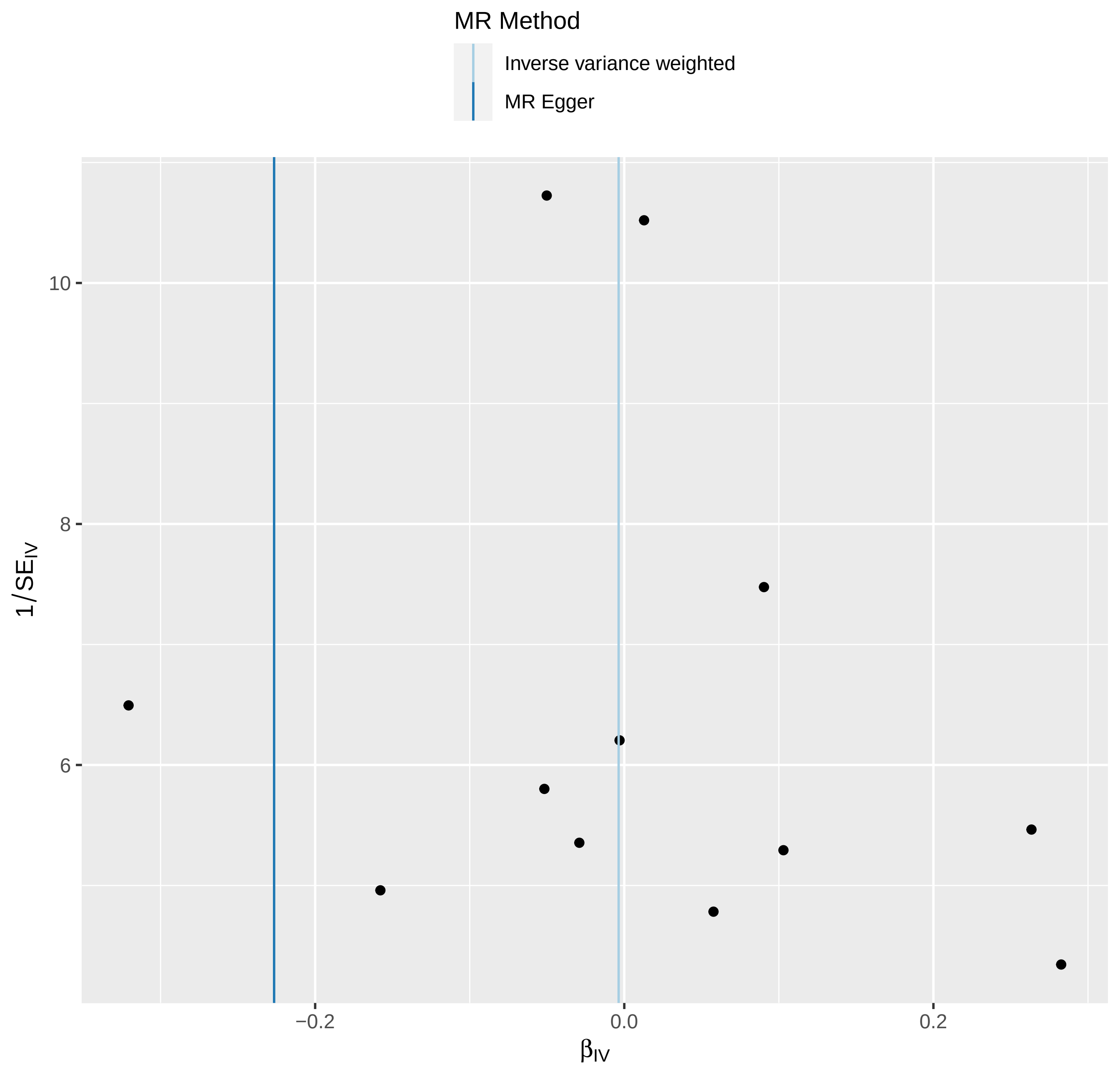

Batch 3−3070 : Selenium on Tensin−4

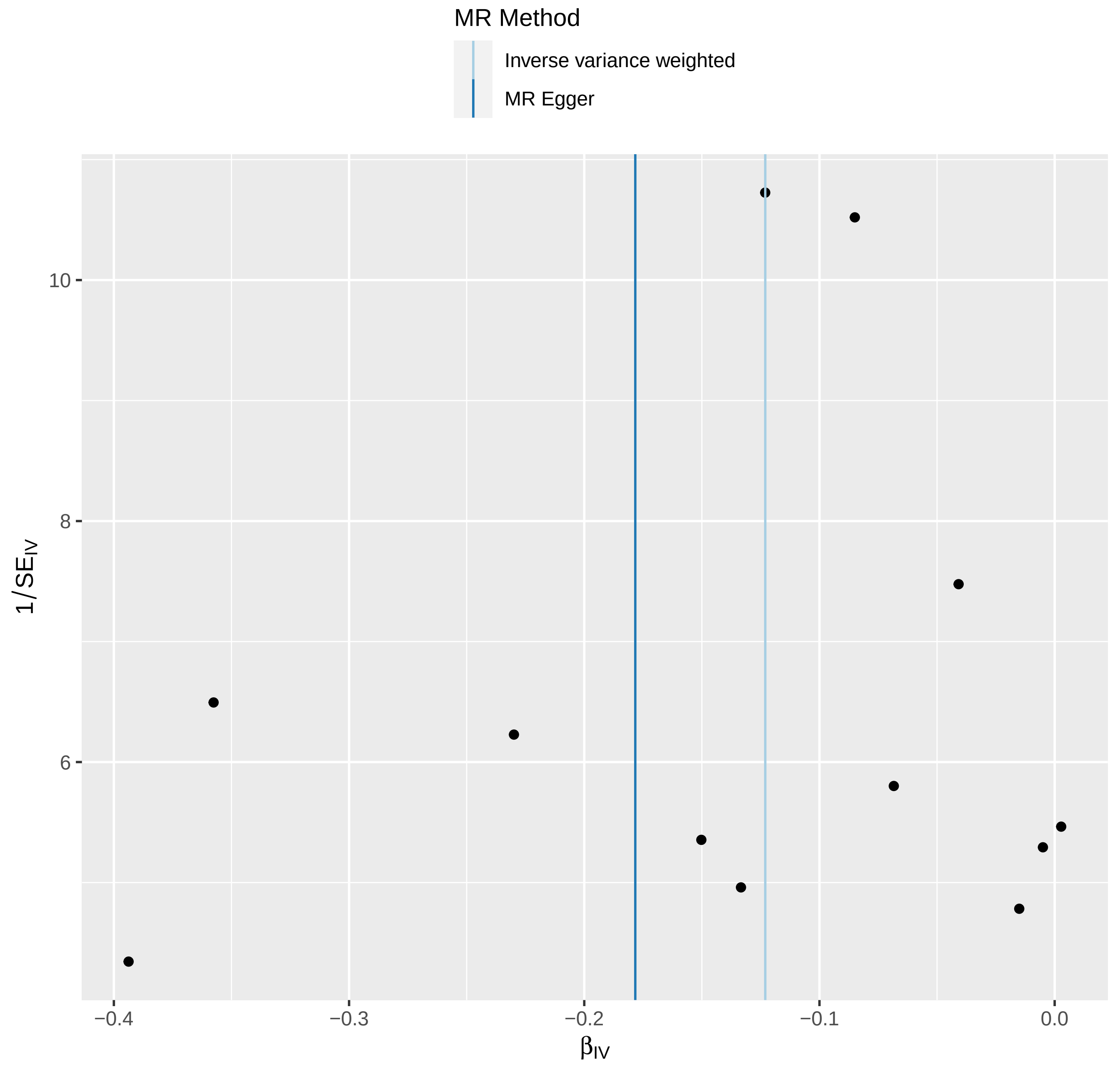

Batch 3−3071 : Selenium on Tenascin−X

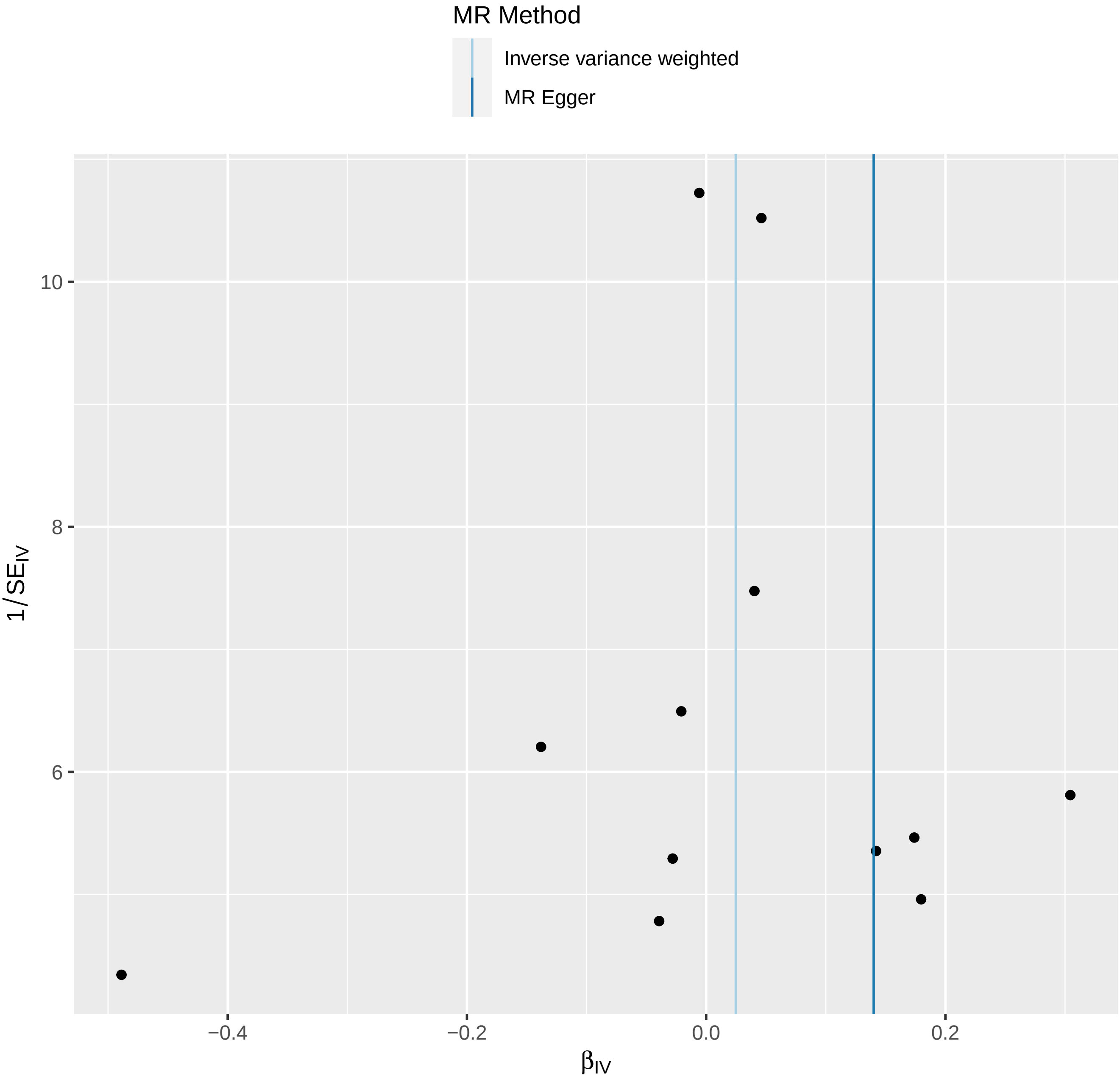

Batch 3−3072 : Selenium on Toll−interacting protein

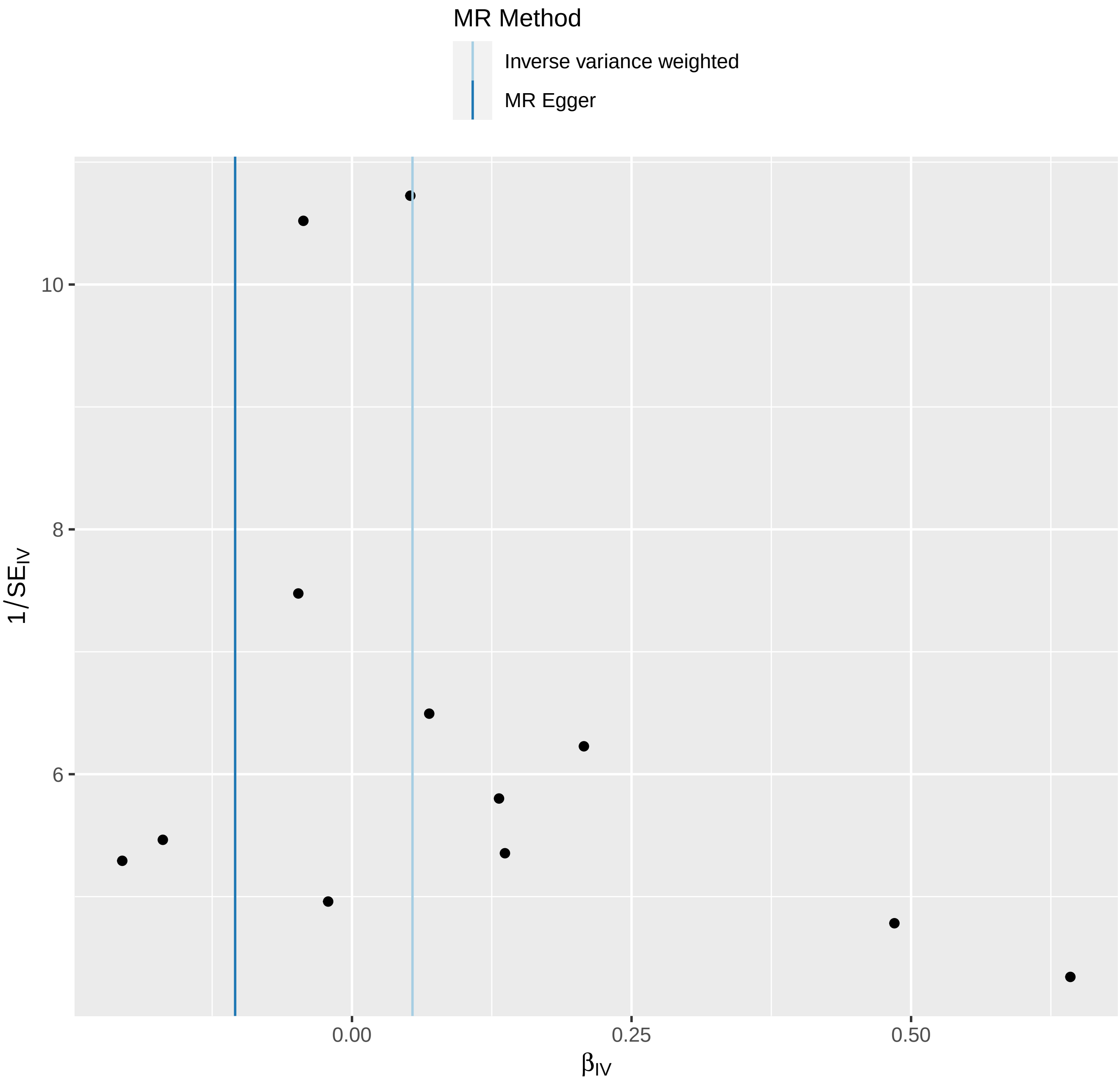

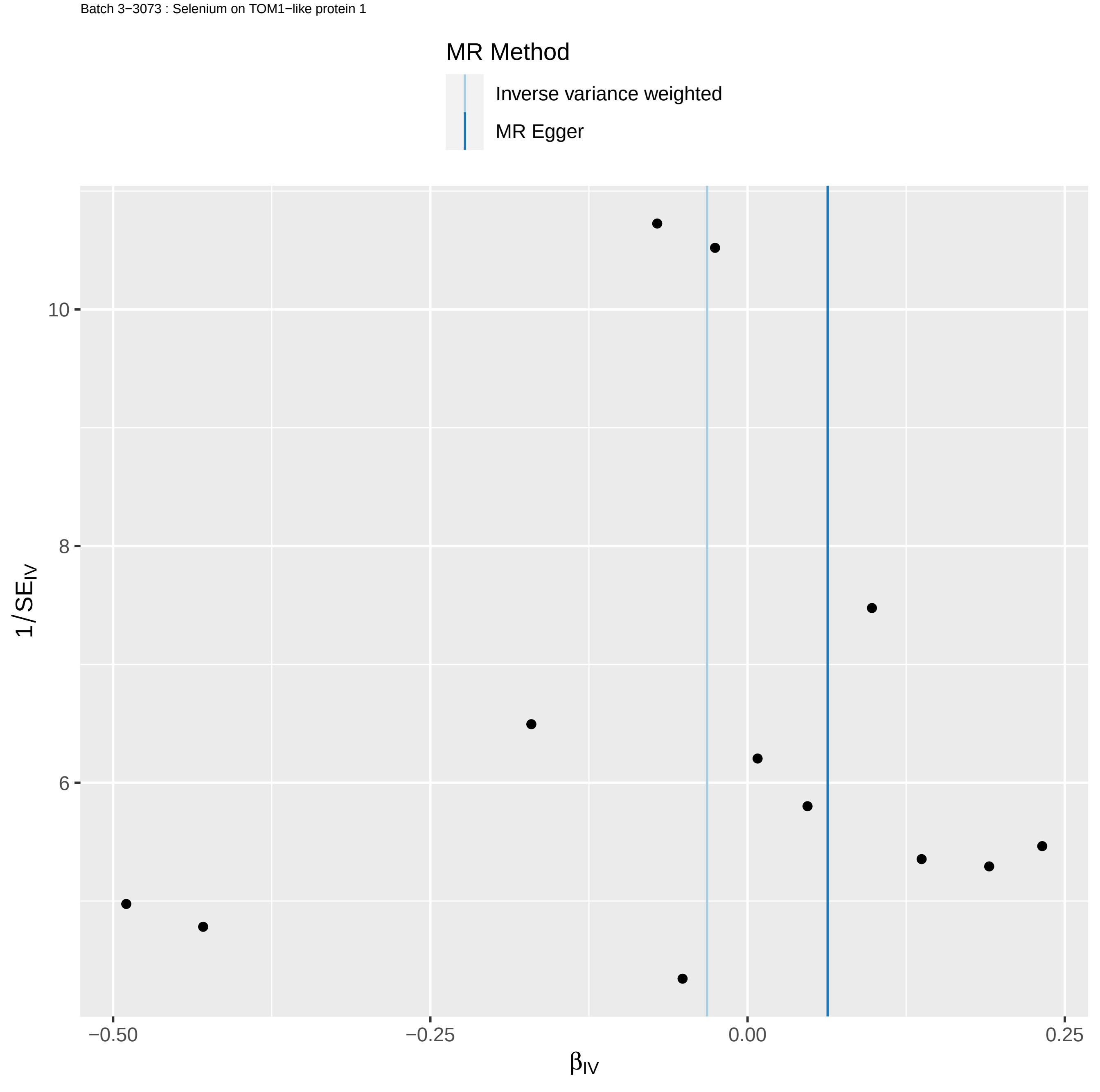

Batch 3−3073 : Selenium on TOM1−like protein 1
MR Method
Inverse variance weighted
MR Egger
10
8
6
1/SE_IV
−0.50
−0.25
0.00
0.25
β_IV

Batch 3−3074 : Selenium on DNA topoisomerase 1

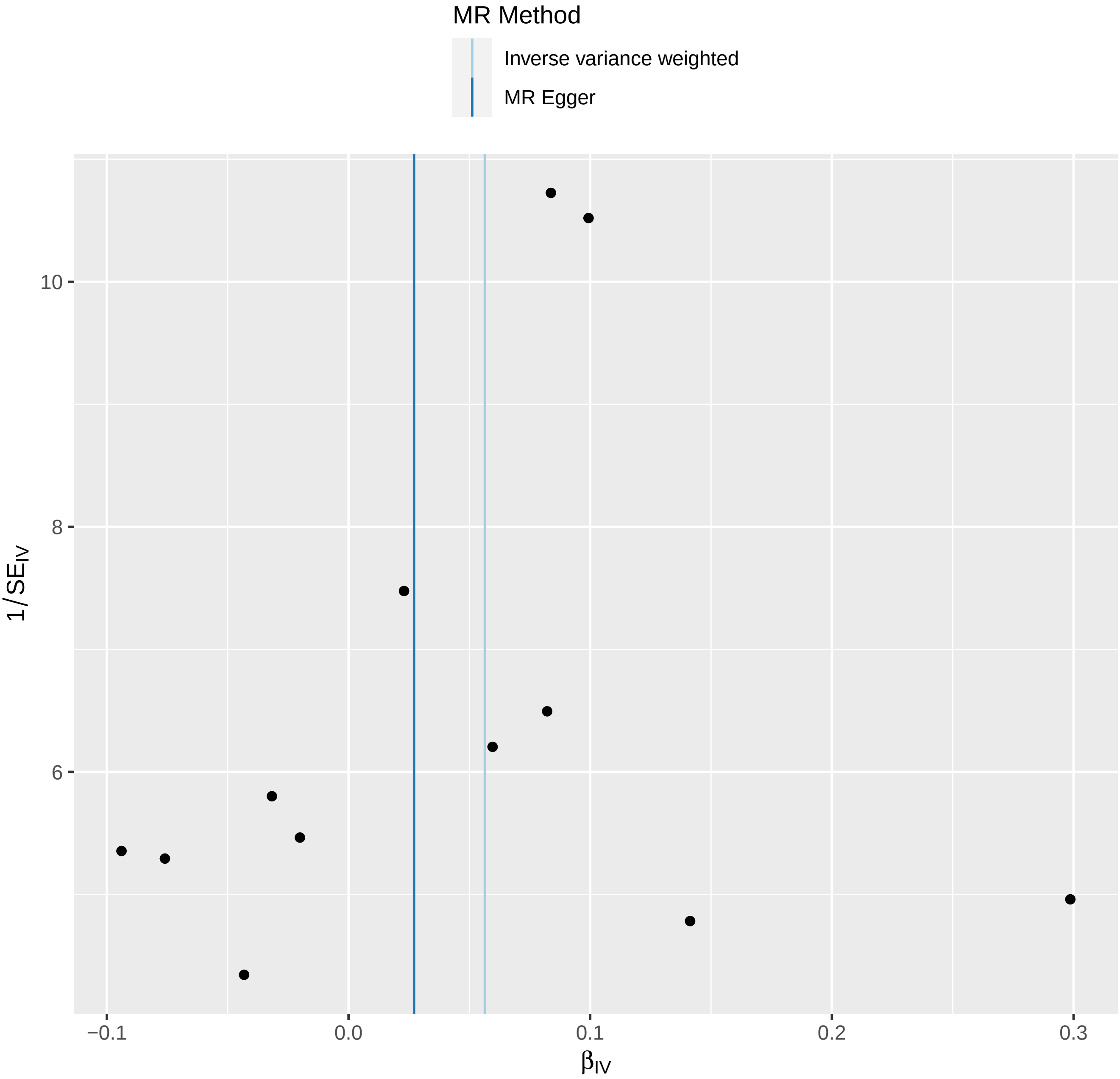

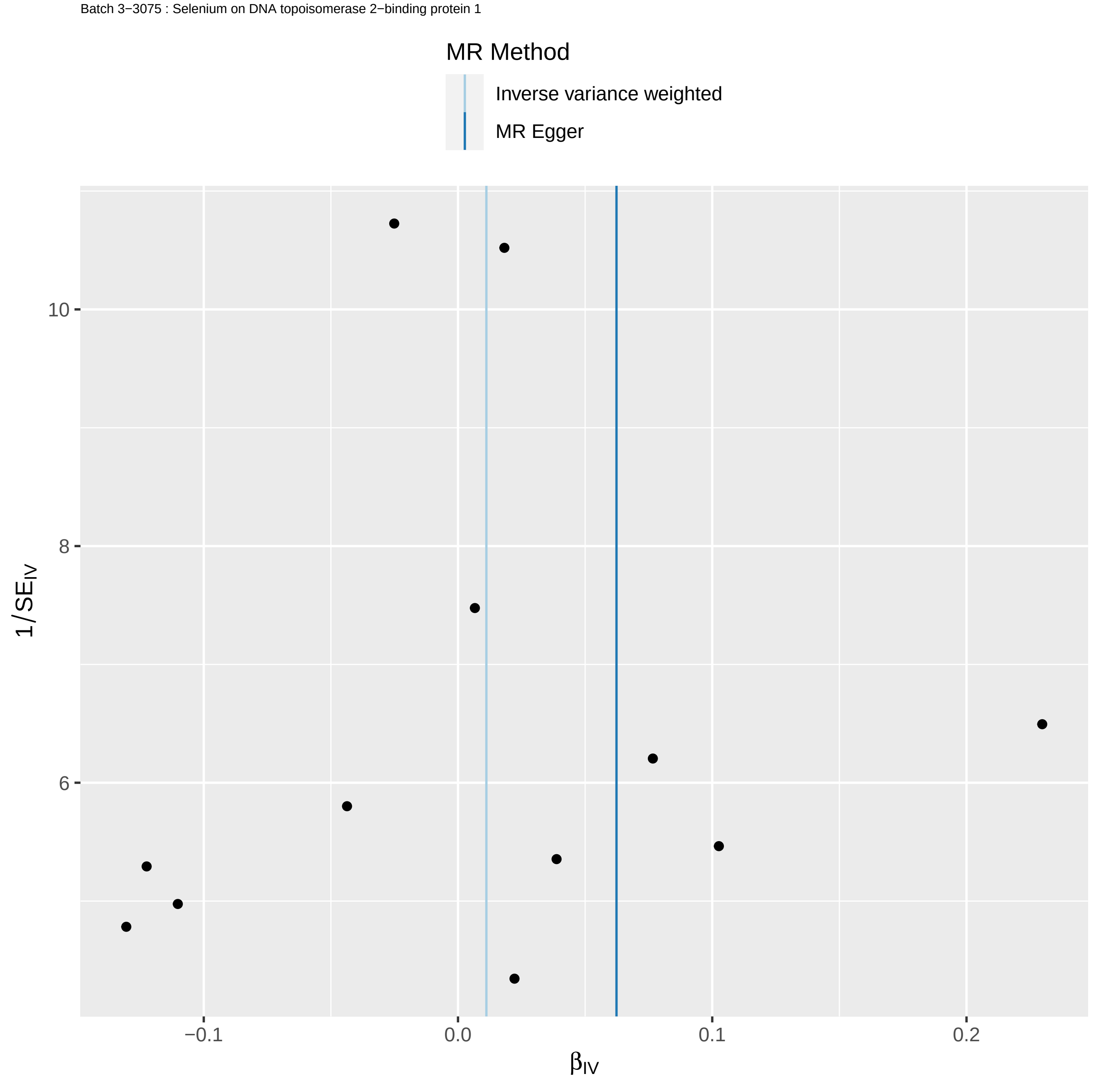

Batch 3−3075 : Selenium on DNA topoisomerase 2−binding protein 1
MR Method
Inverse variance weighted
MR Egger
10
8
6
−0.1
0.0
0.1
0.2
1/SE_IV
β_IV

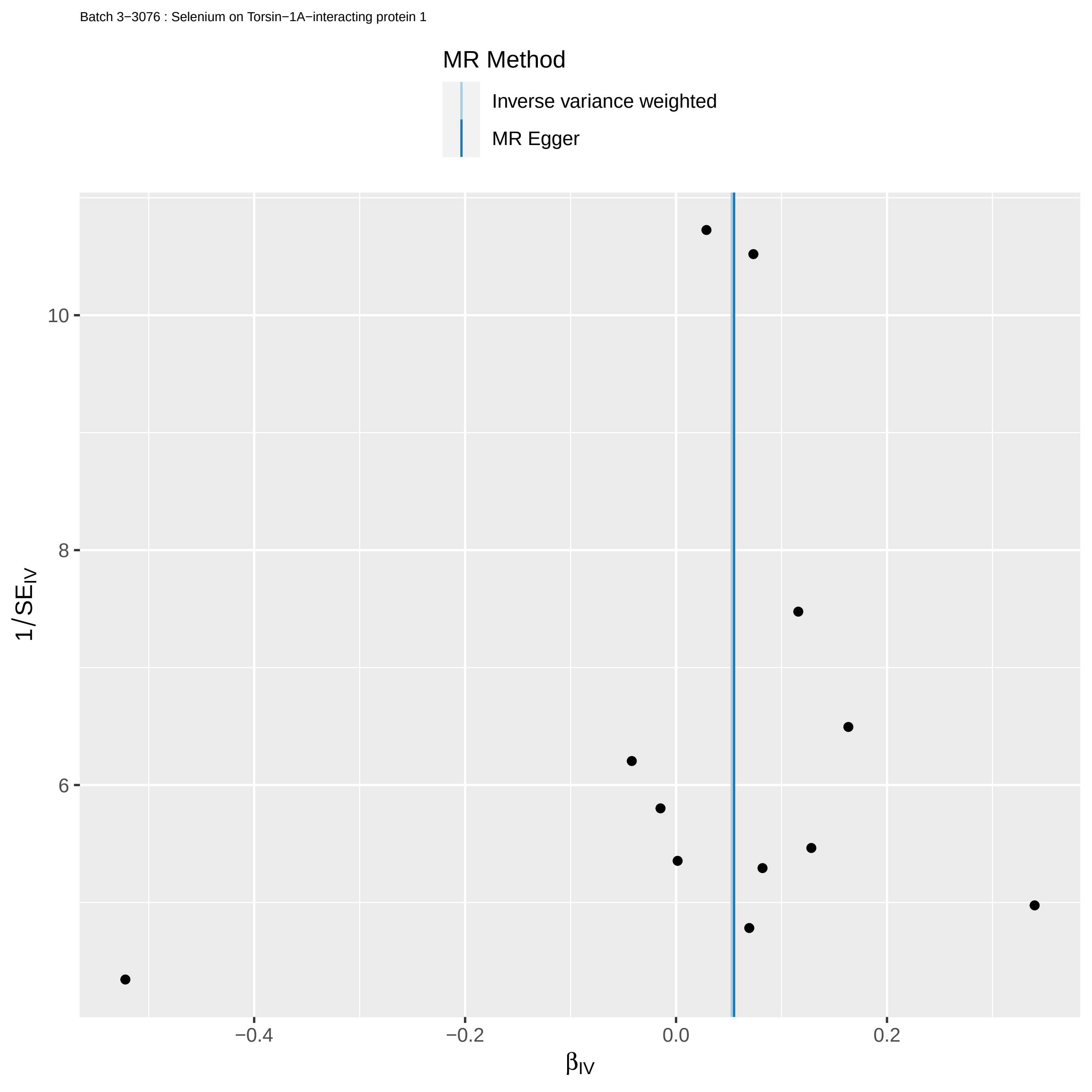

Batch 3−3076 : Selenium on Torsin−1A−interacting protein 1
MR Method
Inverse variance weighted
MR Egger
10
8
6
1/SE_IV
−0.4
−0.2
0.0
0.2
β_IV

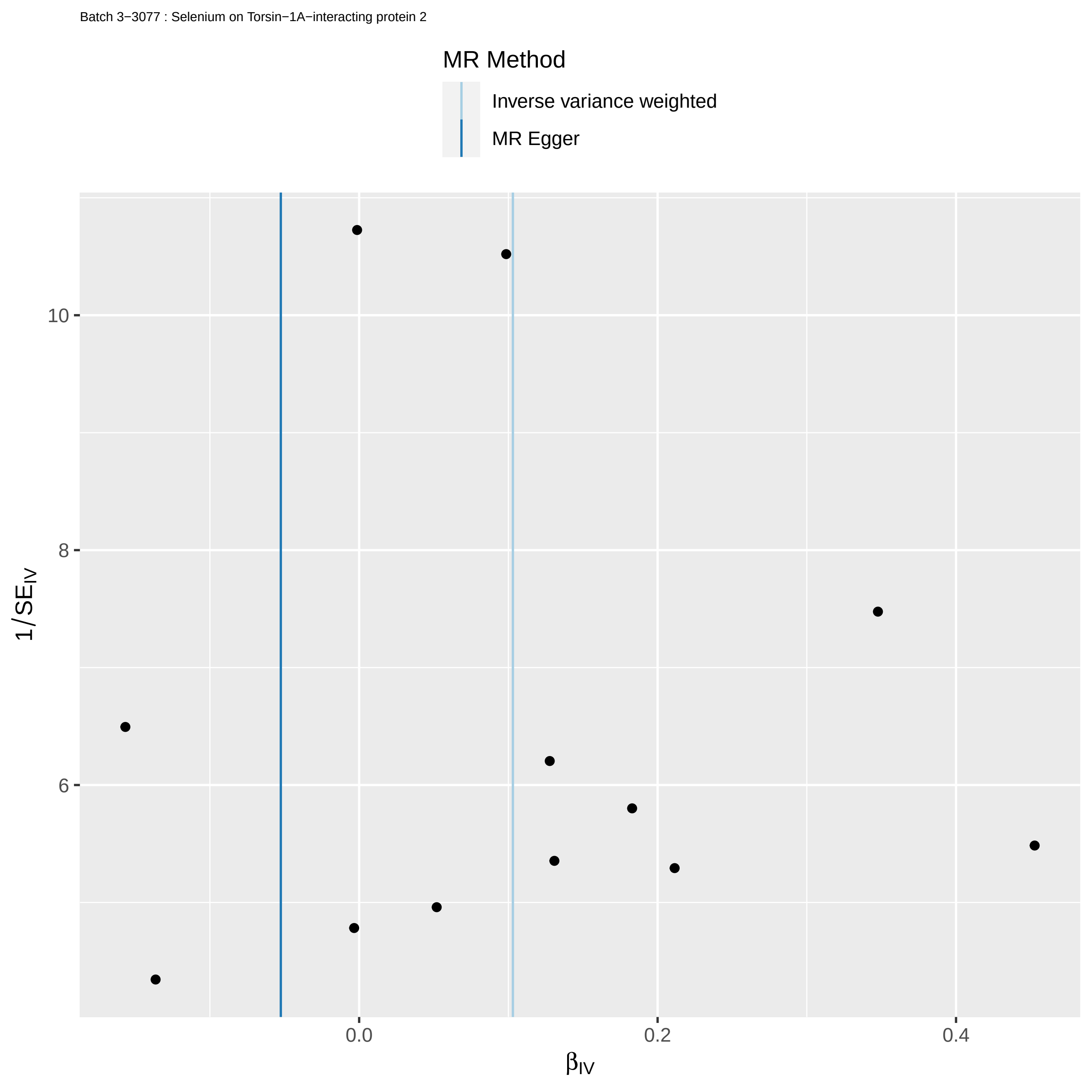

Batch 3−3077 : Selenium on Torsin−1A−interacting protein 2
MR Method
Inverse variance weighted
MR Egger
10
8
6
1/SE$_{IV}$
0.0
0.2
0.4
β$_{IV}$

Batch 3−3078 : Selenium on Torsin−1A−interacting protein 2

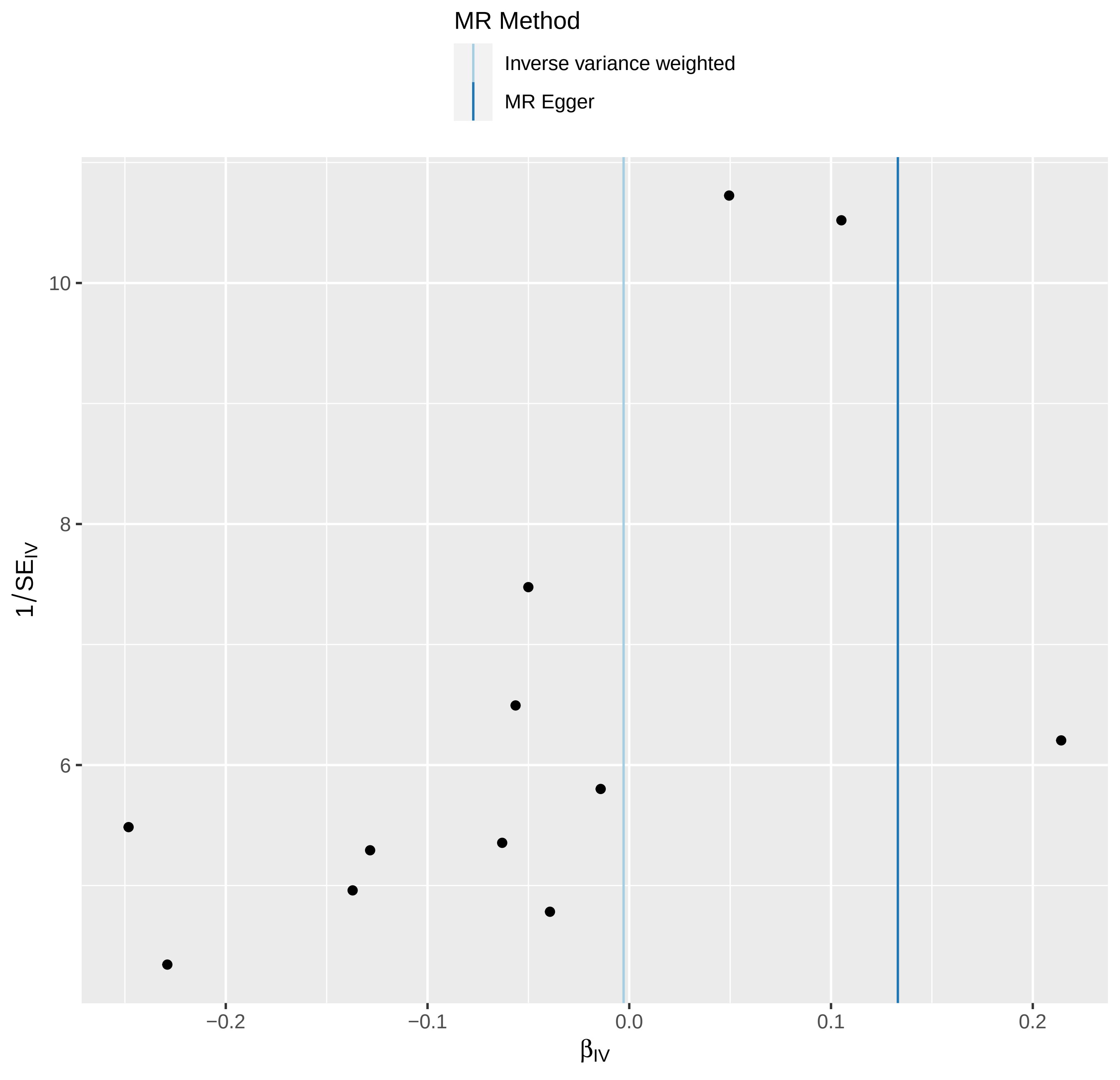

Batch 3−3079 : Selenium on TOX high mobility group box family member 3

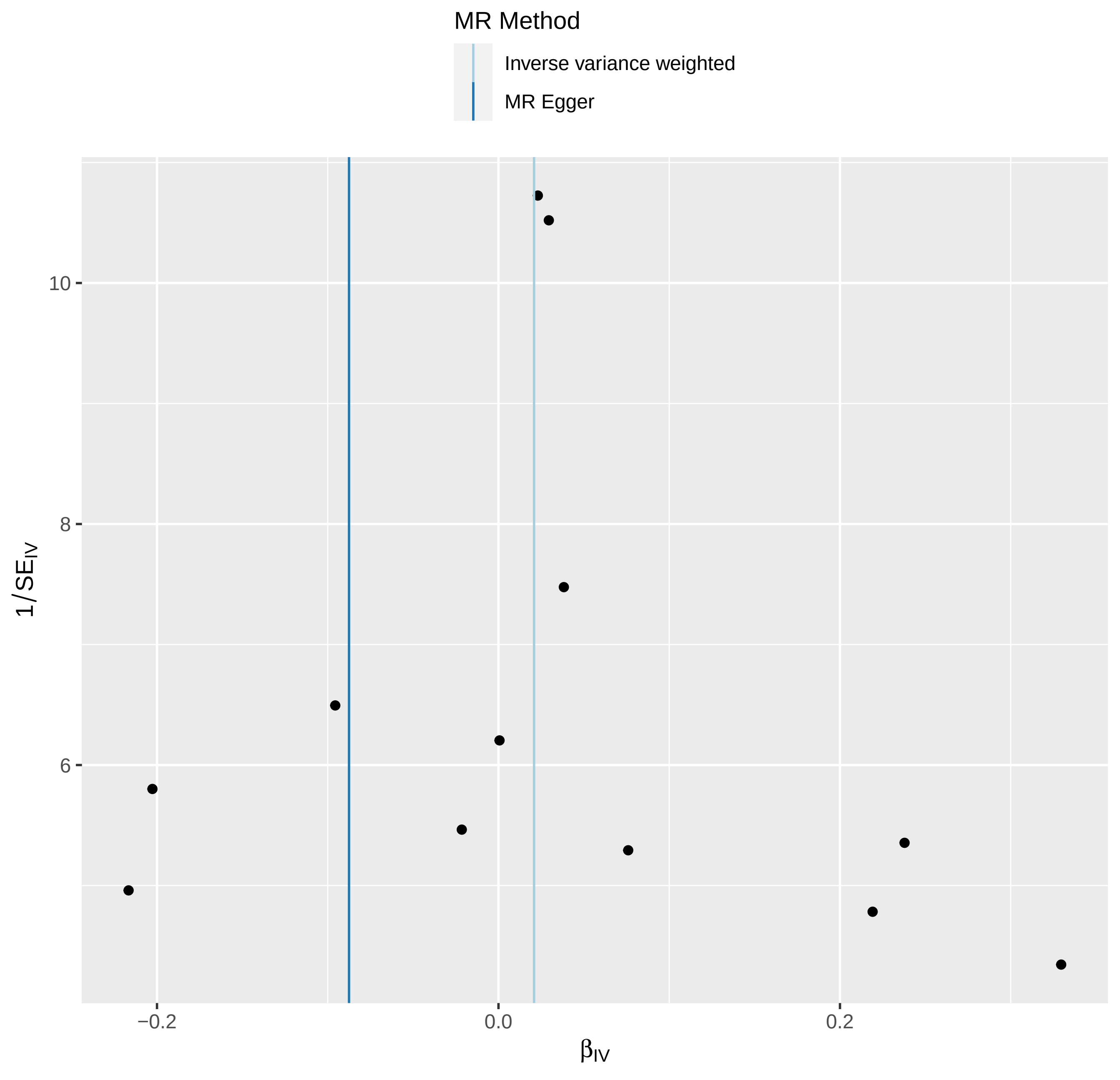

Batch 3−3080 : Selenium on Cellular tumor antigen p53

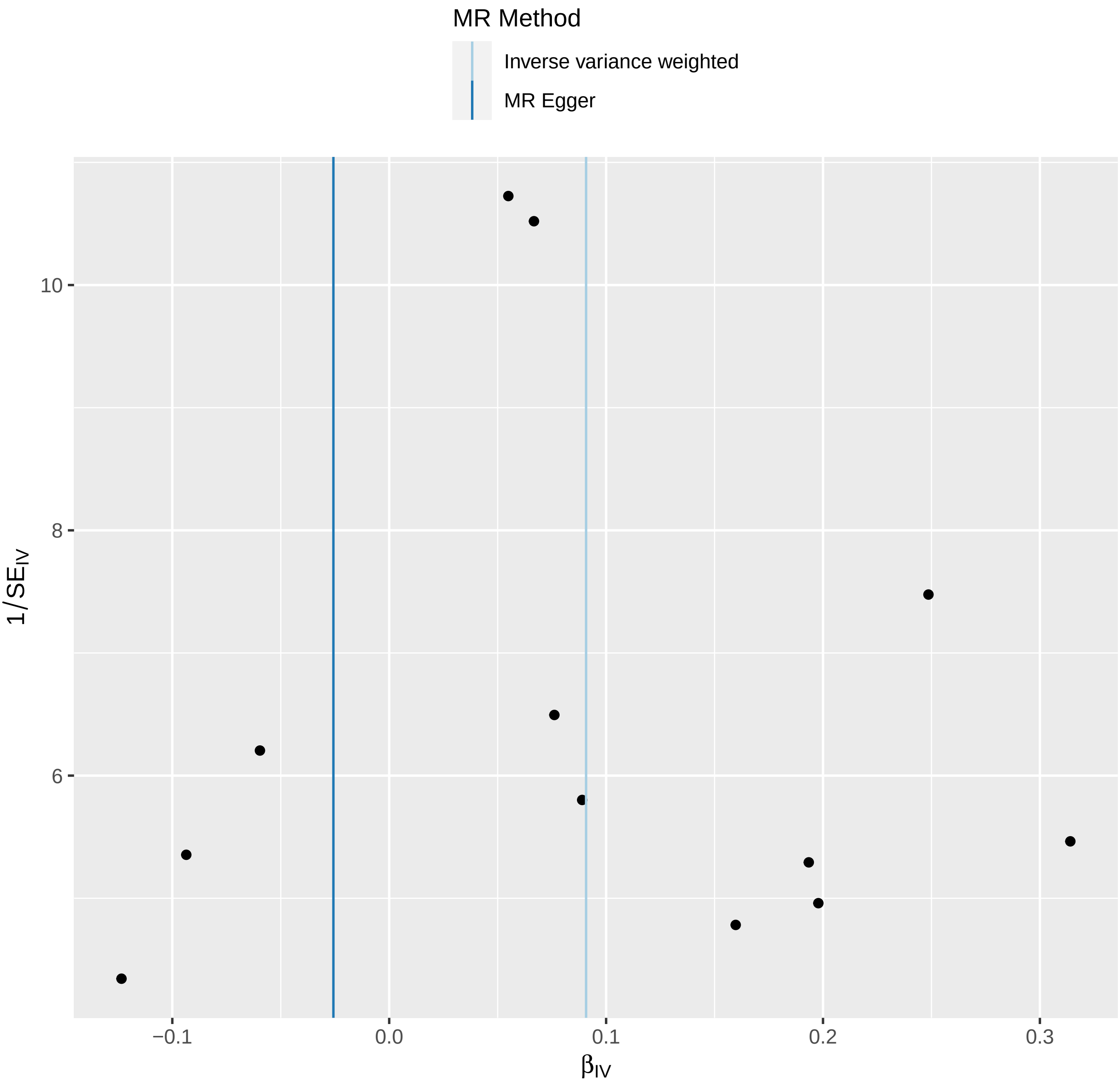

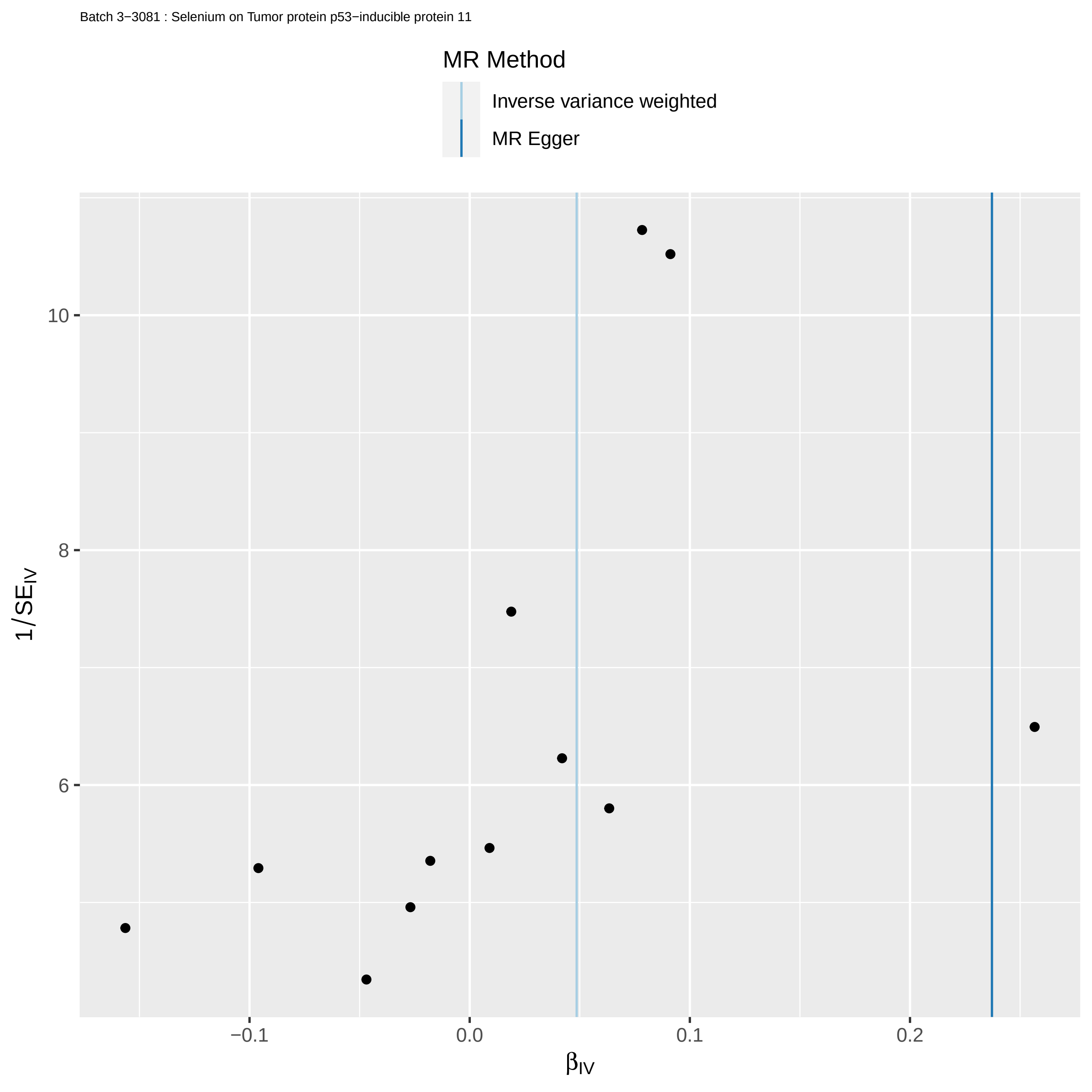
Batch 3−3081 : Selenium on Tumor protein p53−inducible protein 11
MR Method
Inverse variance weighted
MR Egger
10
8
6
1/SE$_{IV}$
−0.1
0.0
0.1
0.2
β_{IV}

Batch 3−3082 : Selenium on Tumor protein 63

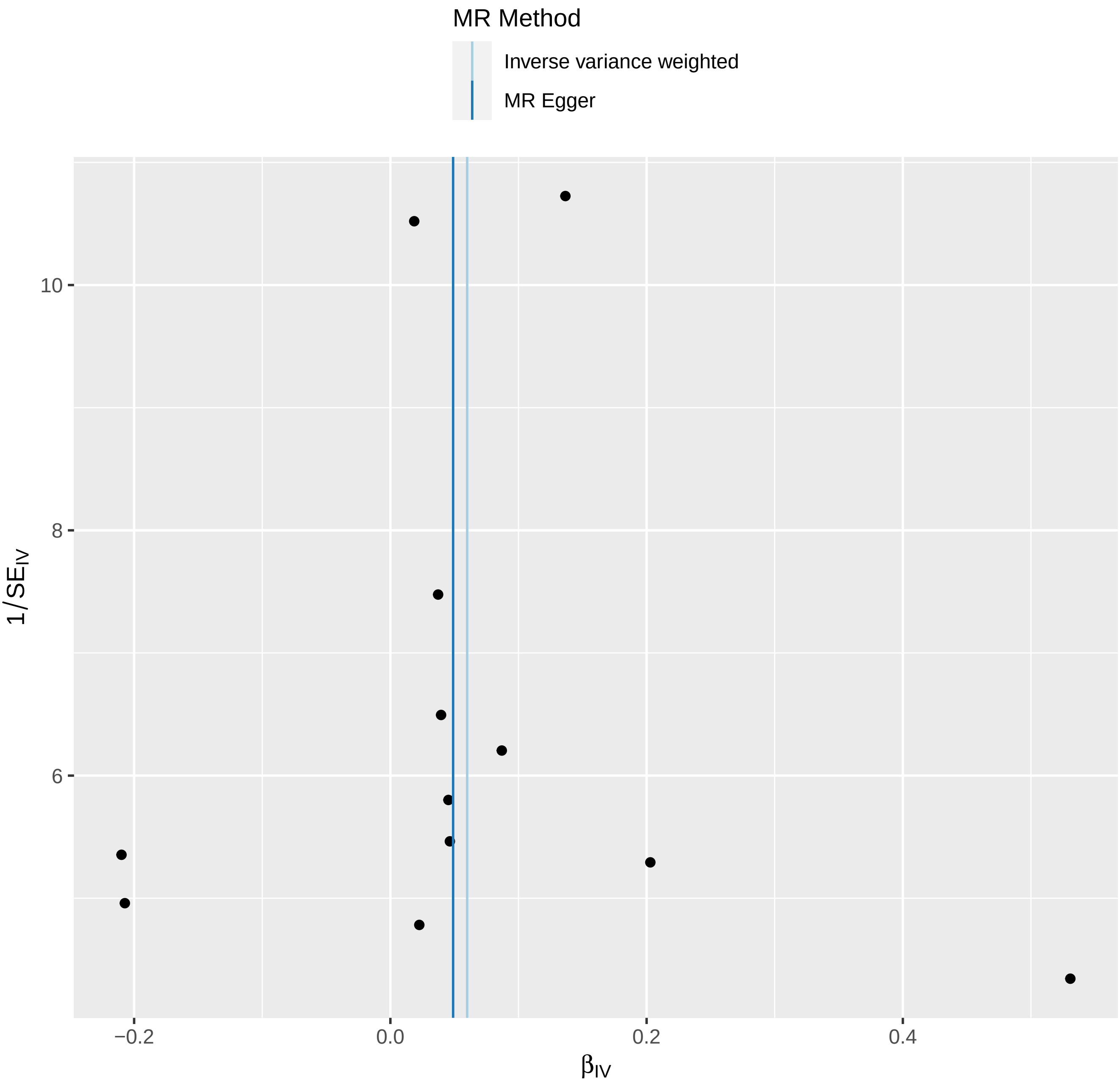

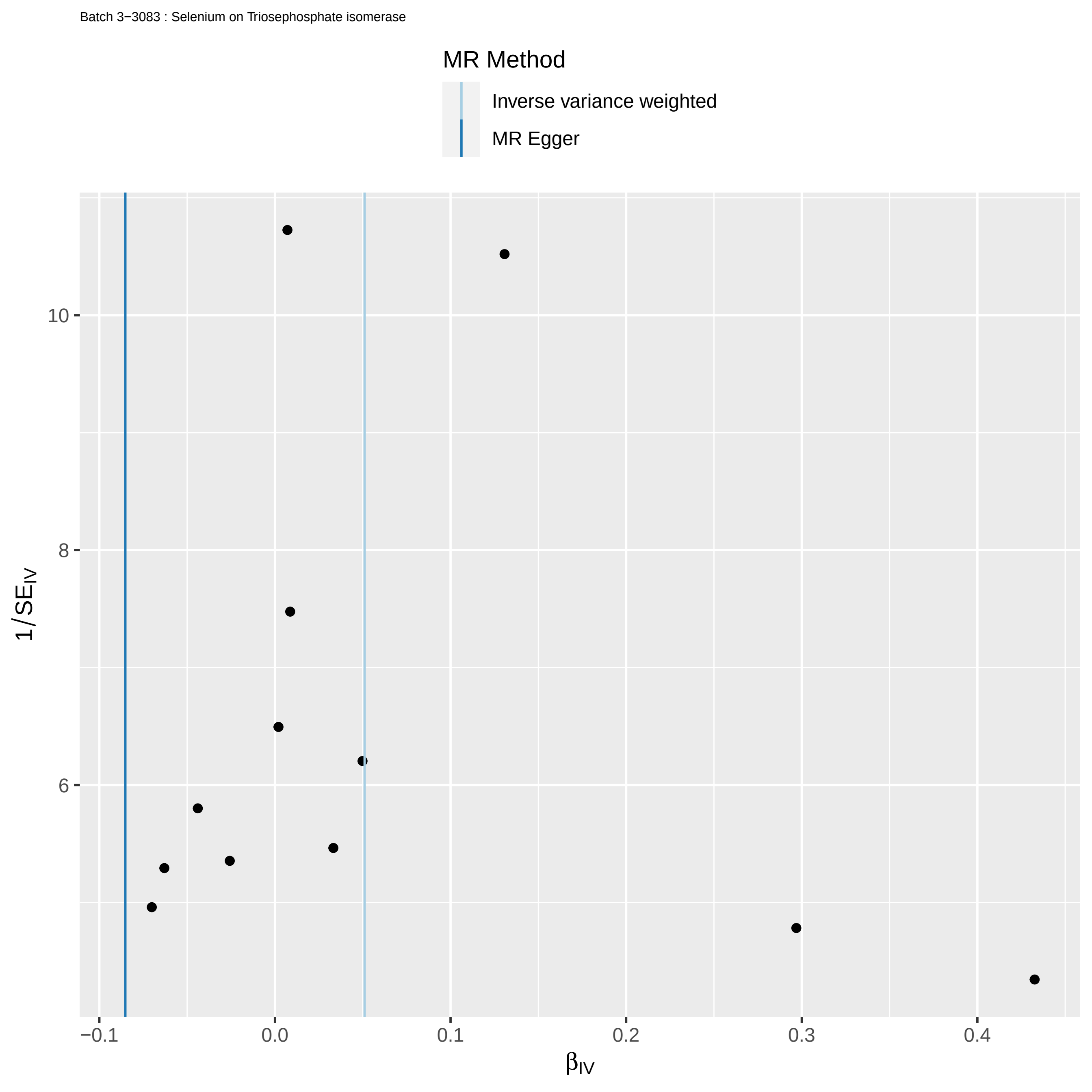

Batch 3−3083 : Selenium on Triosephosphate isomerase
MR Method
Inverse variance weighted
MR Egger
10
8
6
1/SE$_{IV}$
−0.1
0.0
0.1
0.2
0.3
0.4
β$_{IV}$

Batch 3−3084 : Selenium on Thiamin pyrophosphokinase 1

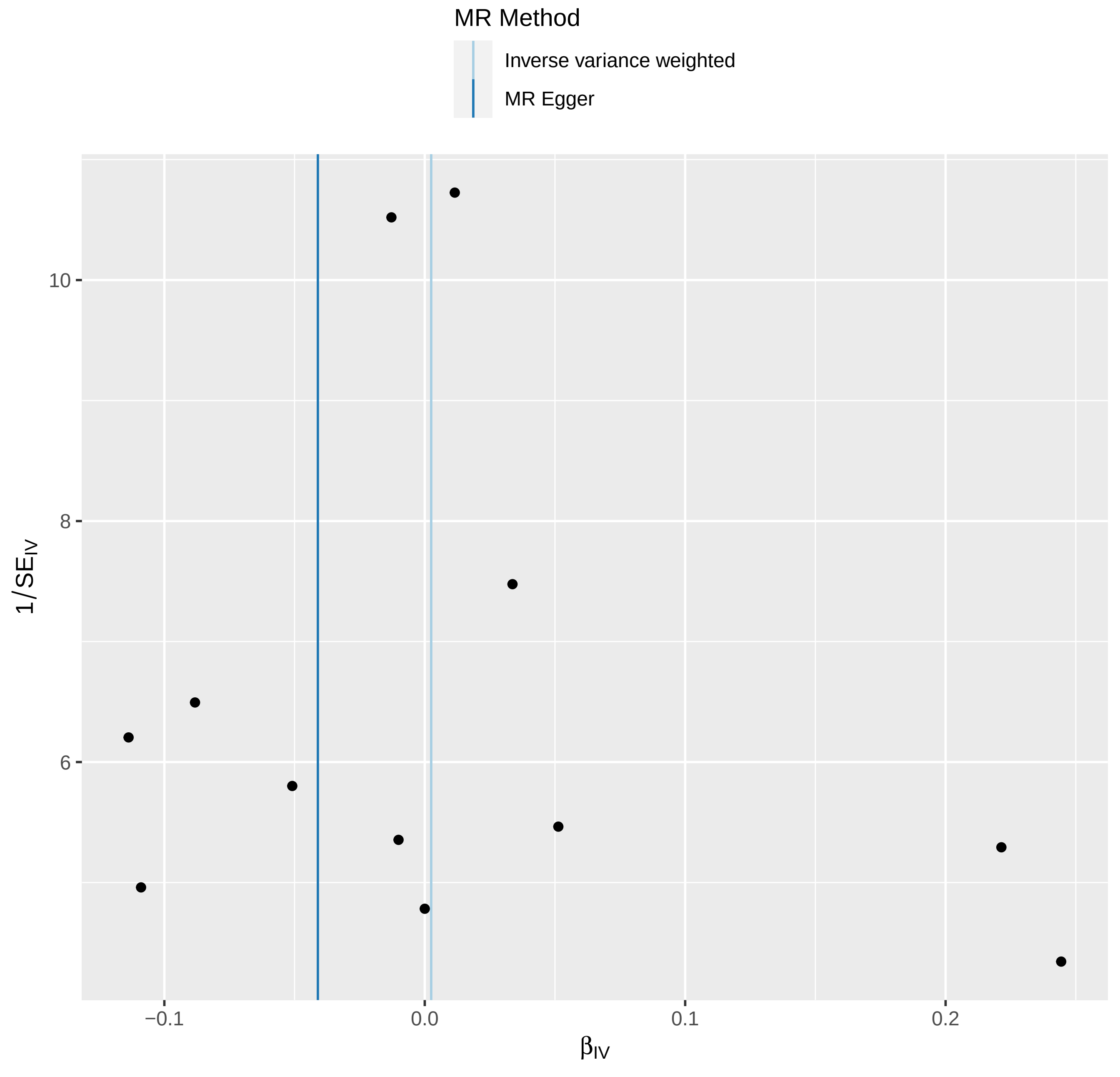

Batch 3−3085 : Selenium on Tropomyosin alpha−1 chain

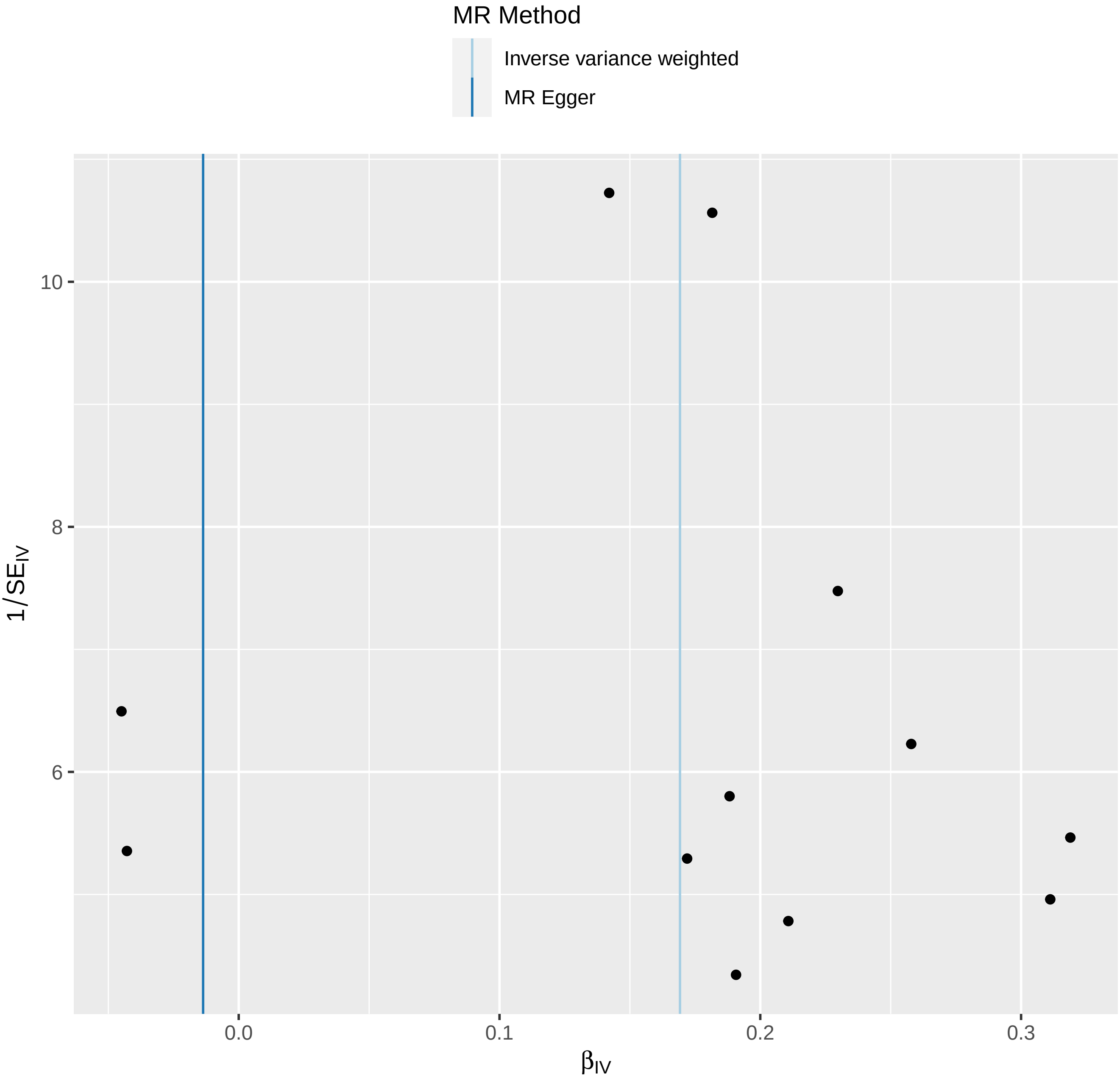

Batch 3−3086 : Selenium on Tropomyosin beta chain

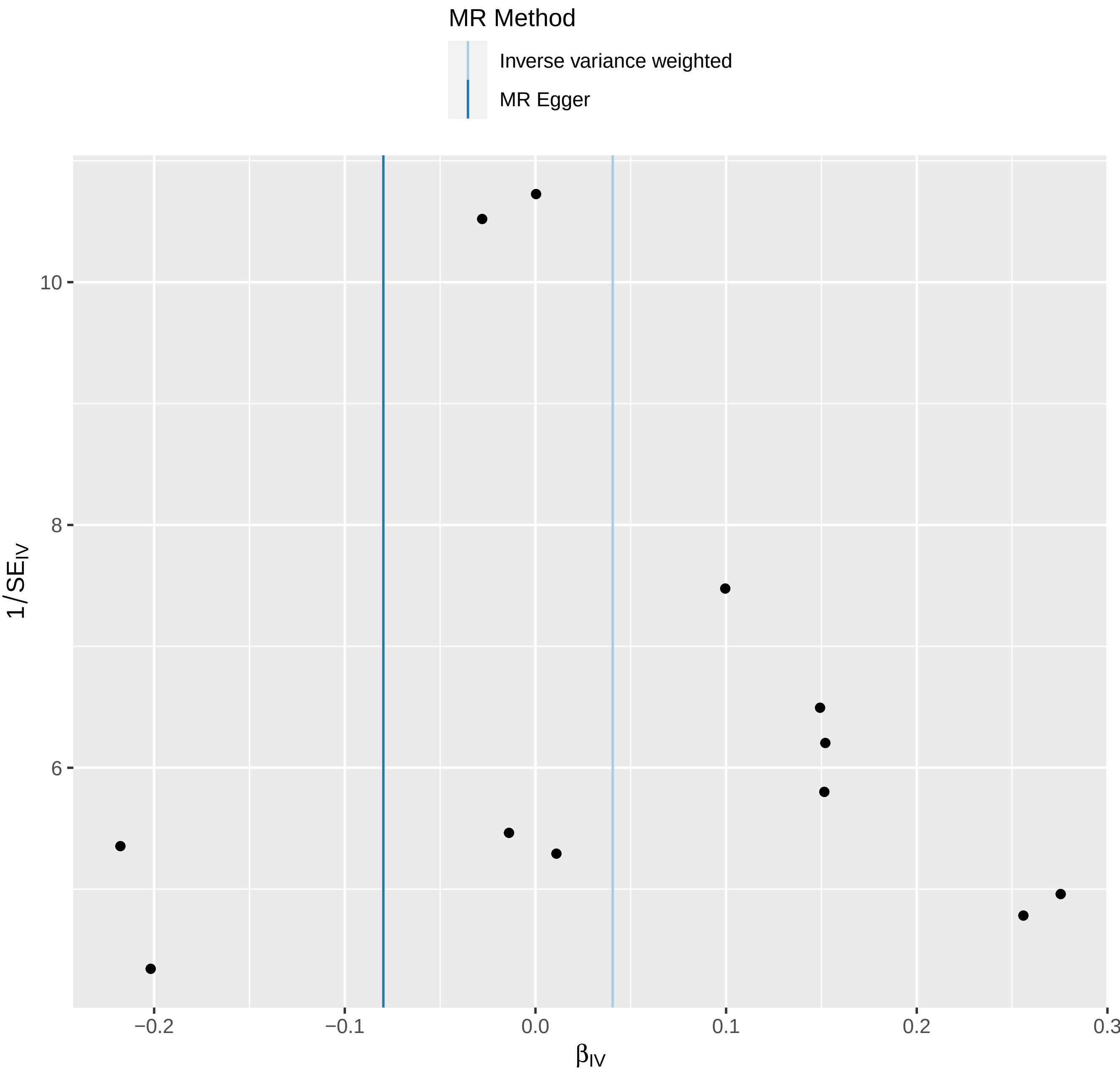

Batch 3−3087 : Selenium on Tropomyosin alpha−3 chain

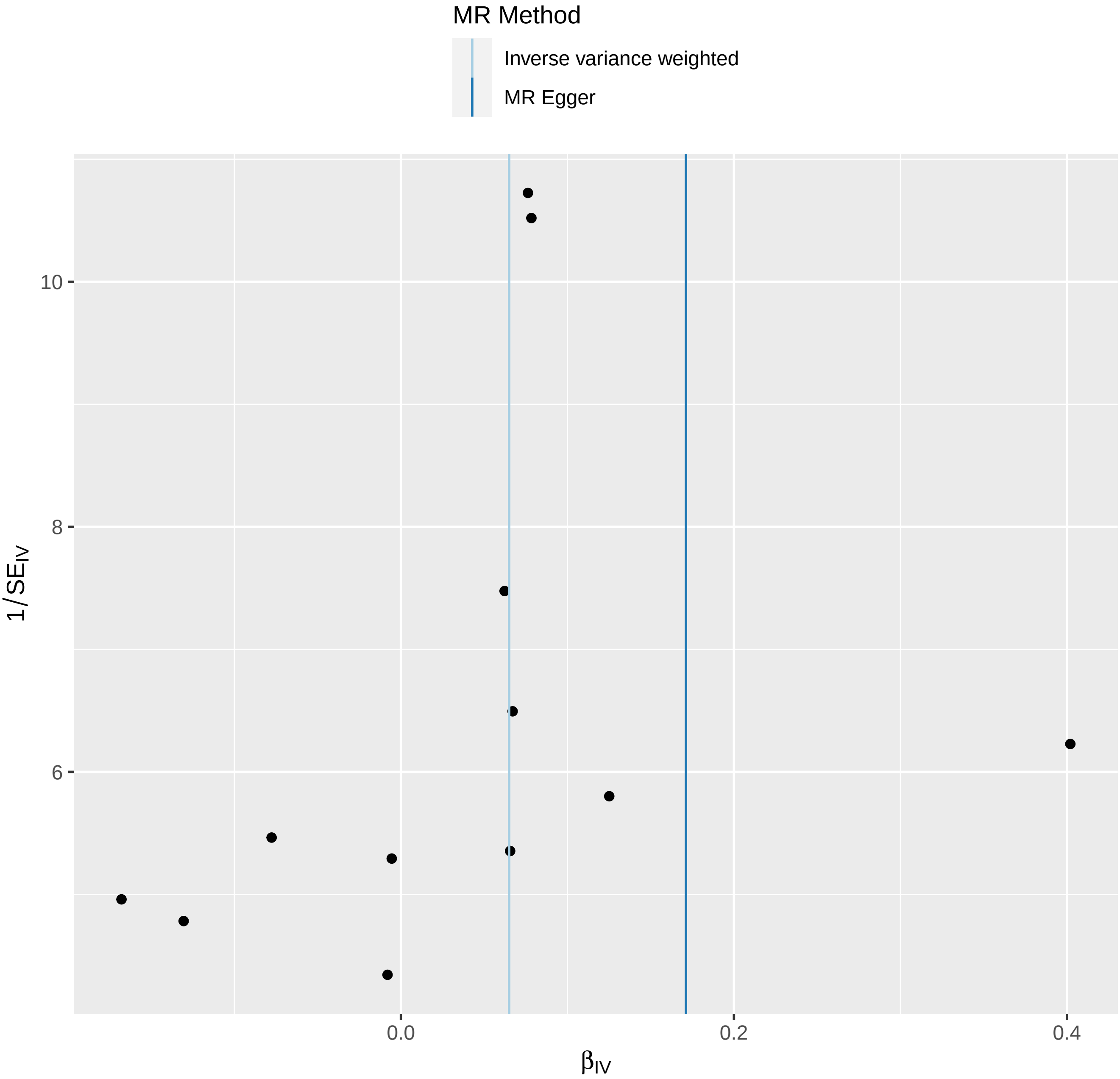

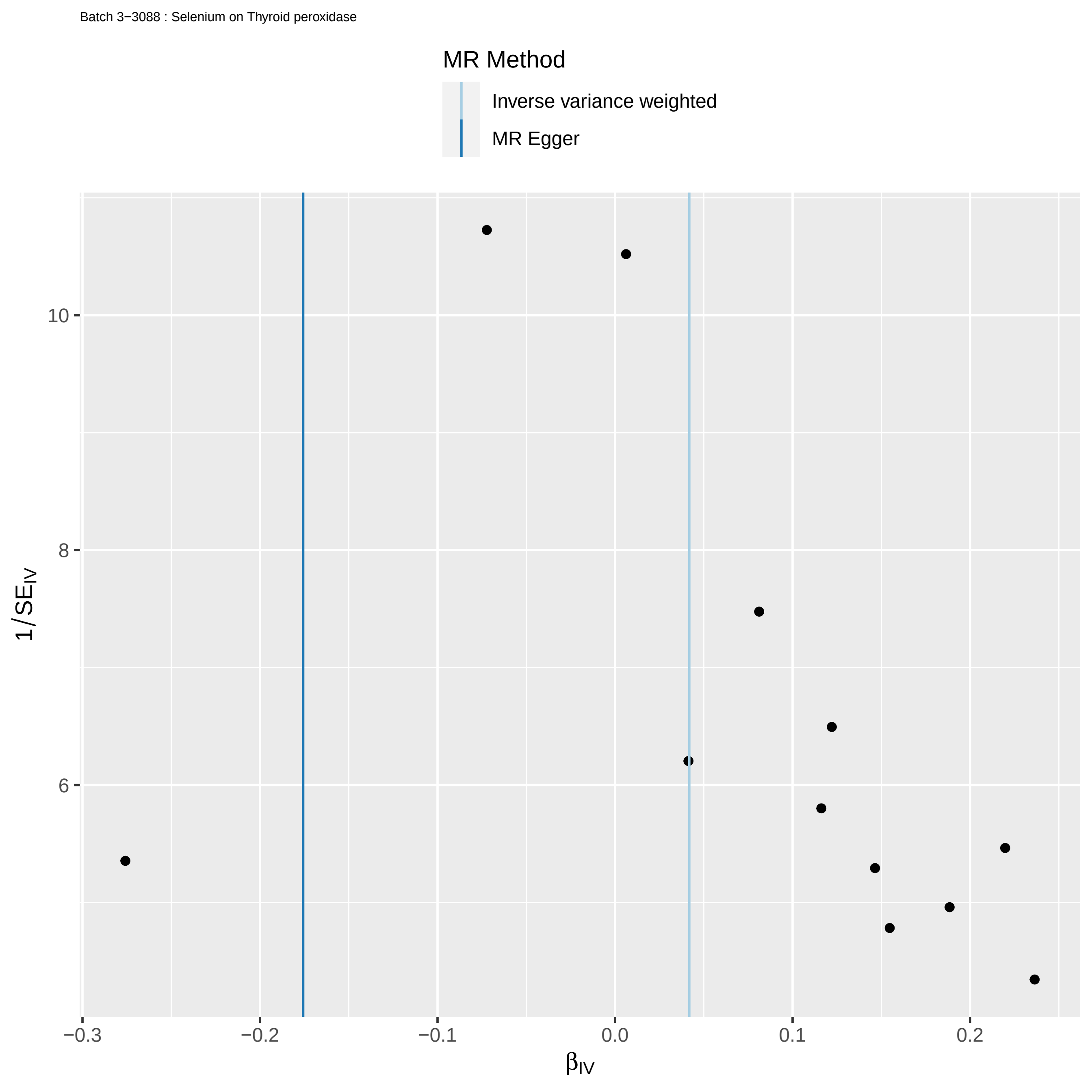
Batch 3−3088 : Selenium on Thyroid peroxidase
MR Method
Inverse variance weighted
MR Egger
10
8
6
1/SE_IV
−0.3
−0.2
−0.1
0.0
0.1
0.2
β_IV

Batch 3−3089 : Selenium on Tubulin polymerization−promoting protein family member 2

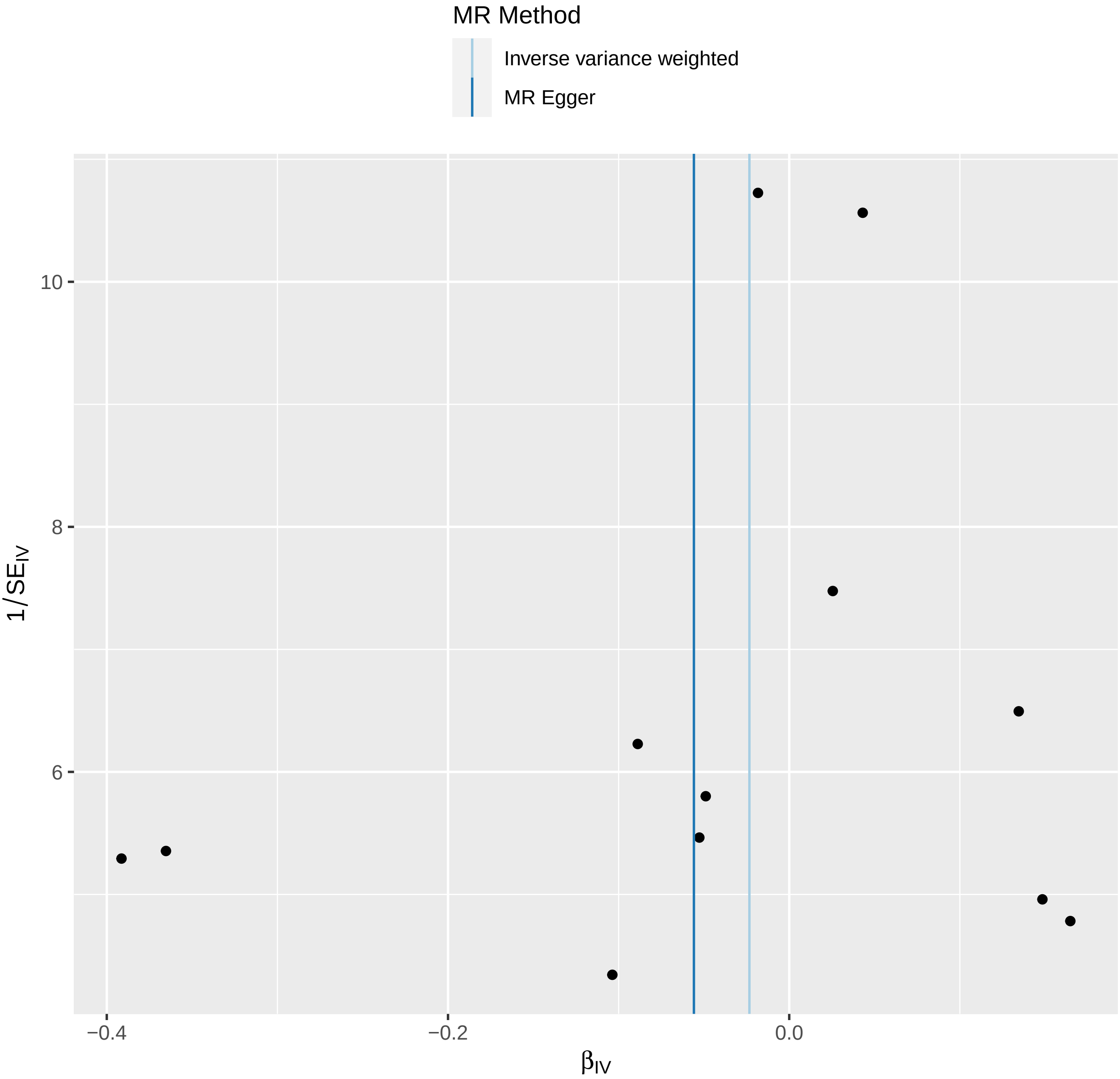

Batch 3−3090 : Selenium on Tryptase beta−2

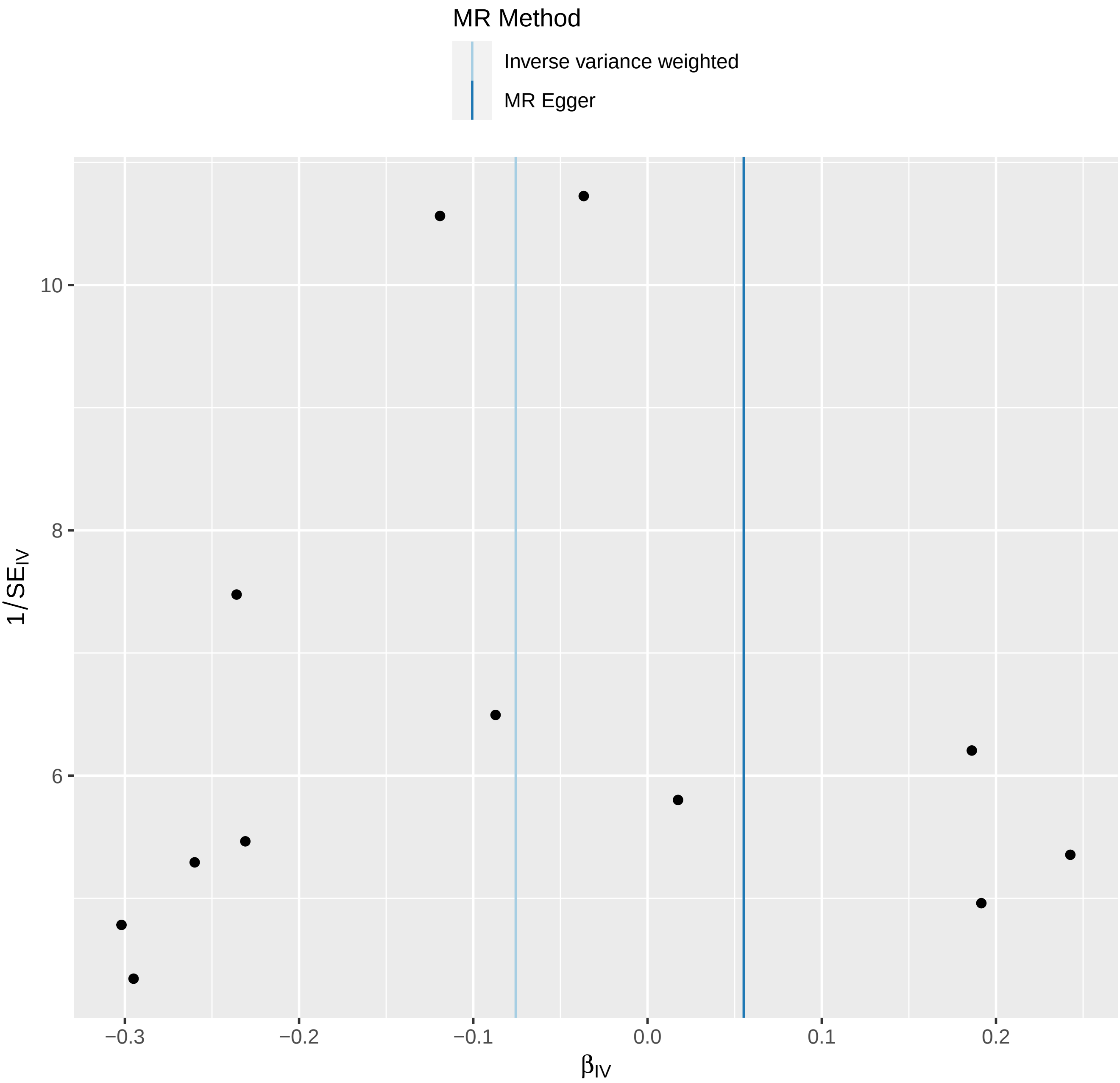

Batch 3−3091 : Selenium on Tryptase gamma

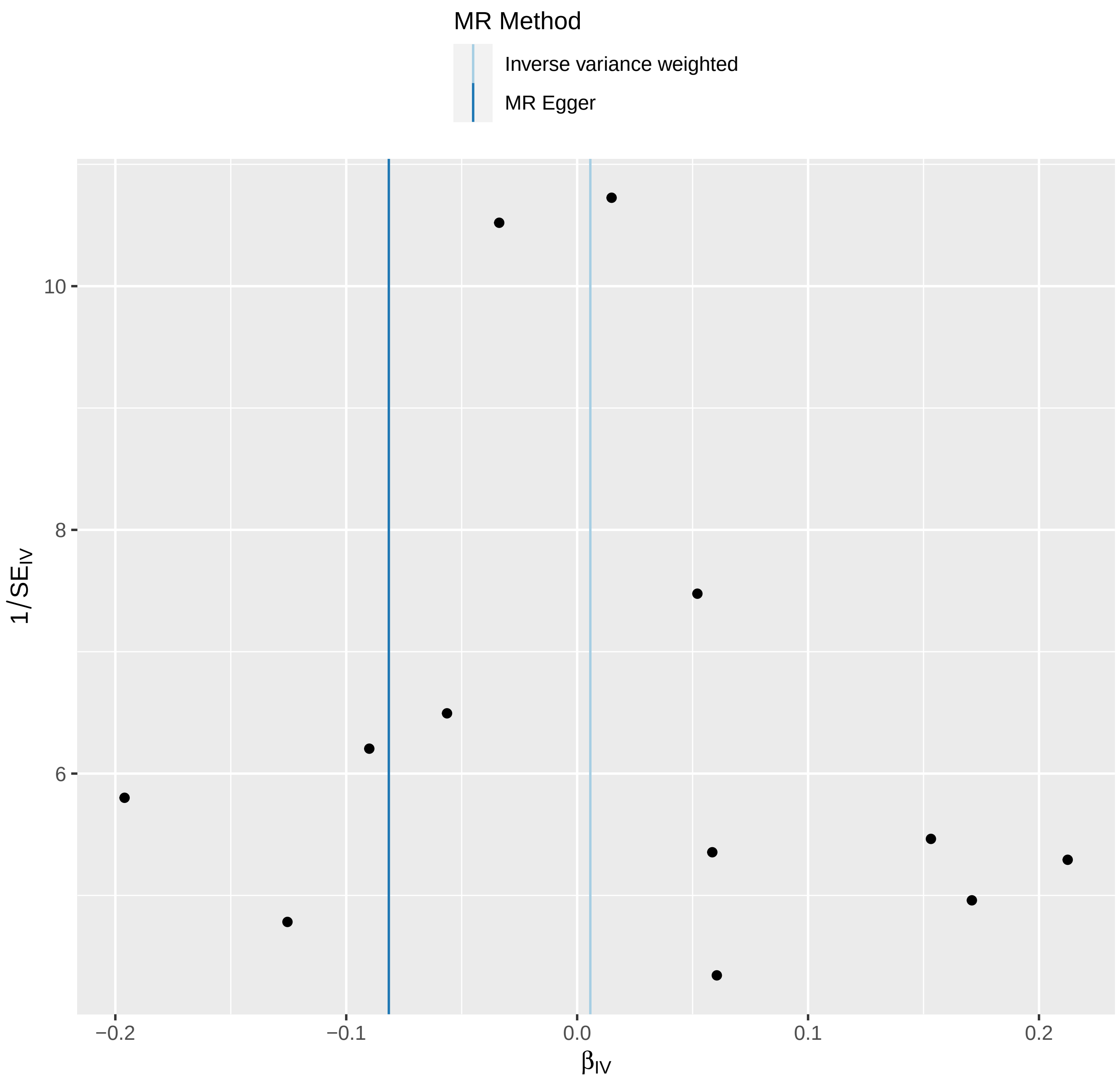

Batch 3−3092 : Selenium on Protein−tyrosine sulfotransferase 1

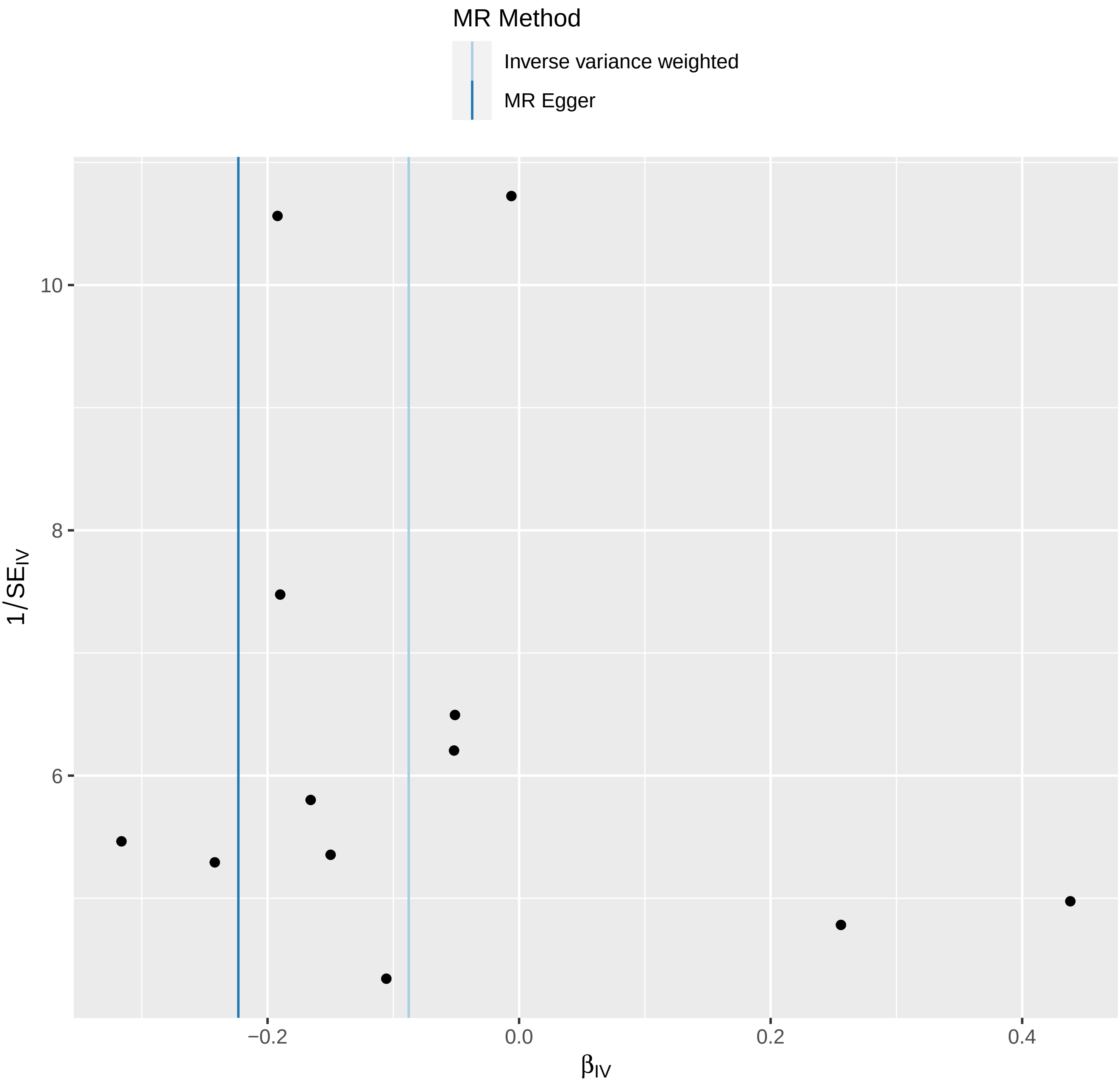

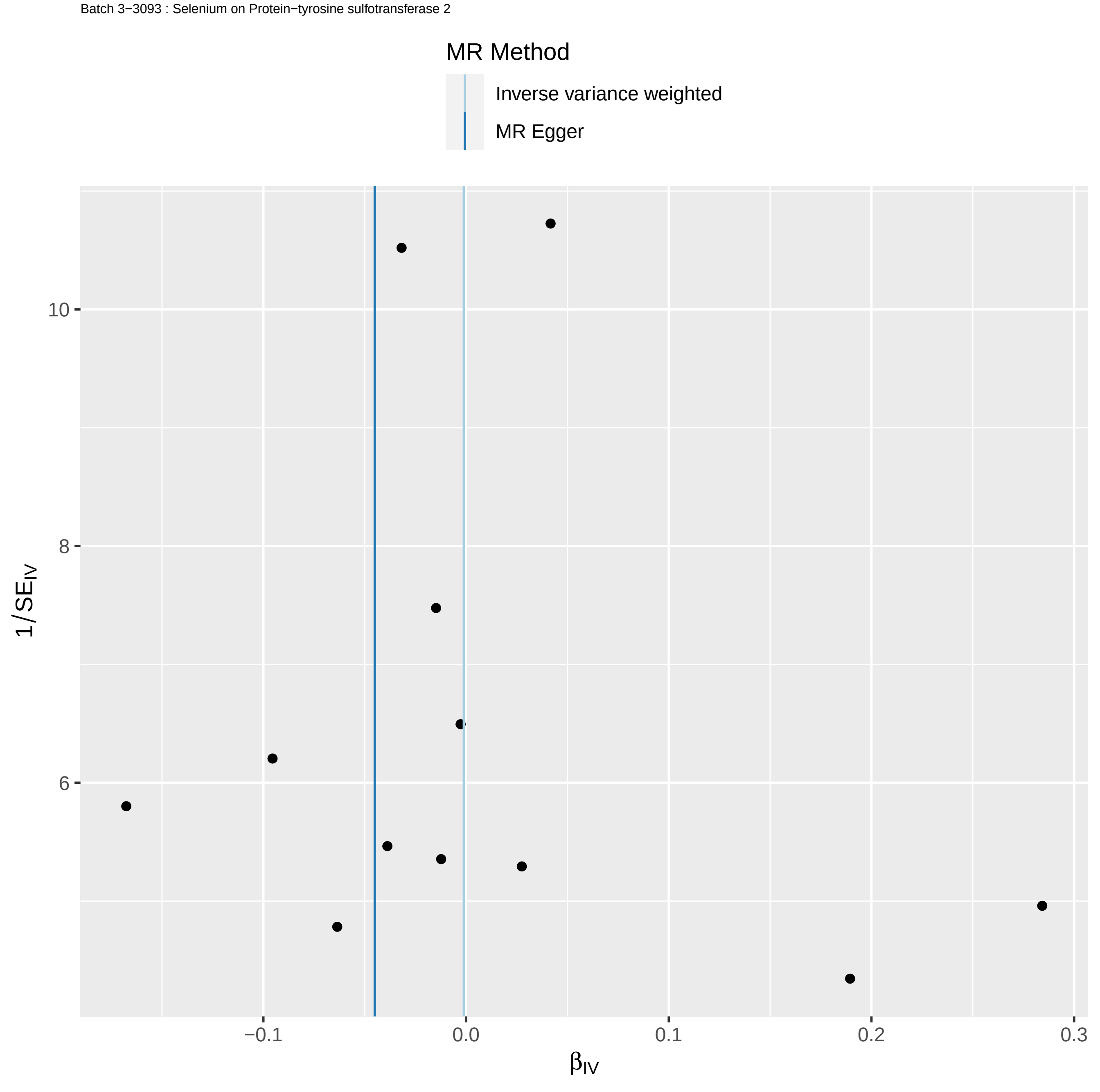
Batch 3−3093 : Selenium on Protein−tyrosine sulfotransferase 2
MR Method
Inverse variance weighted
MR Egger
10
8
6
1/SE_IV
−0.1
0.0
0.1
0.2
0.3
β_IV

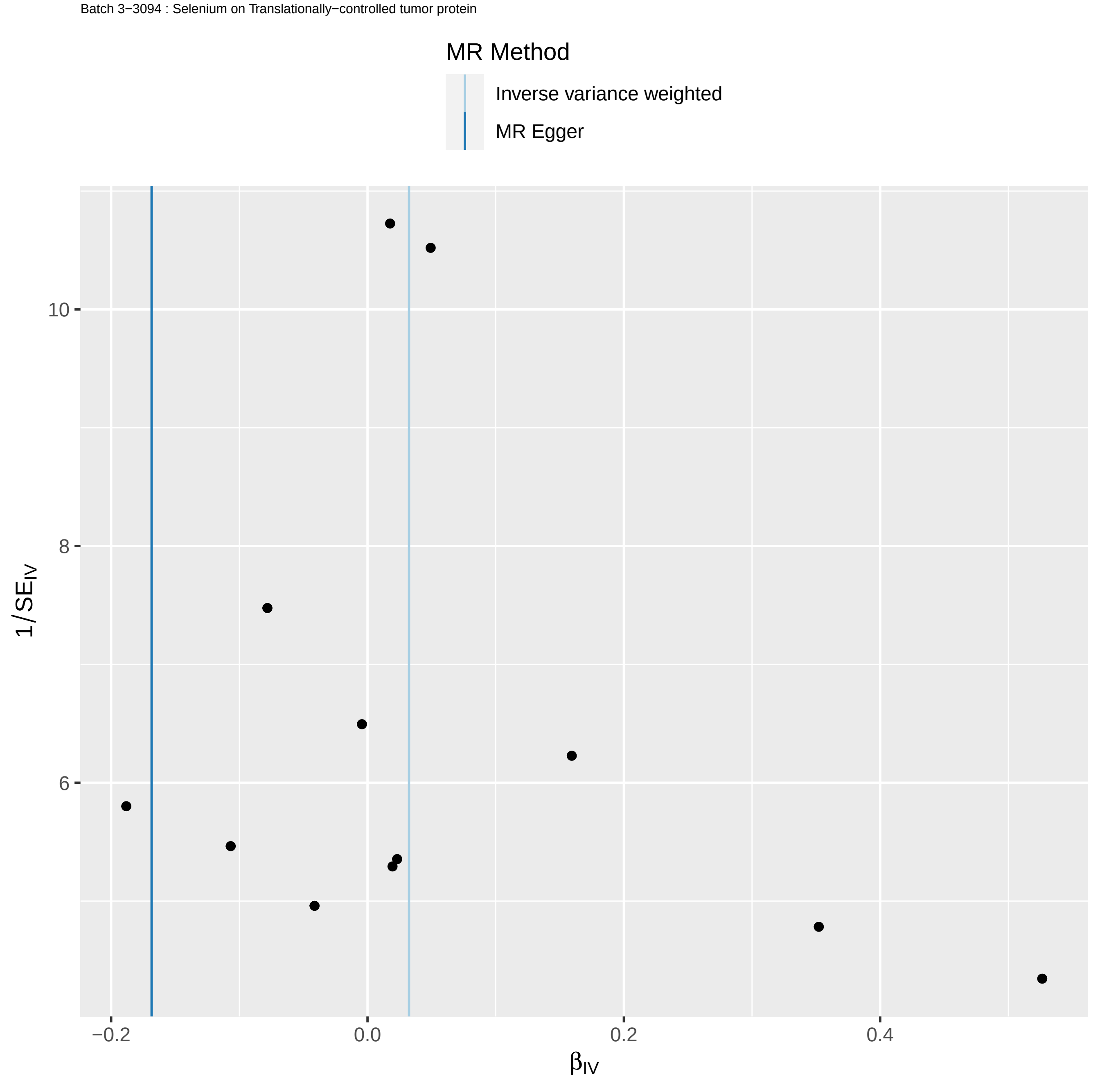
Batch 3−3094 : Selenium on Translationally−controlled tumor protein
MR Method
Inverse variance weighted
MR Egger
10
8
6
1/SE_IV
−0.2
0.0
0.2
0.4
β_IV

Batch 3−3095 : Selenium on Transformer−2 protein homolog beta

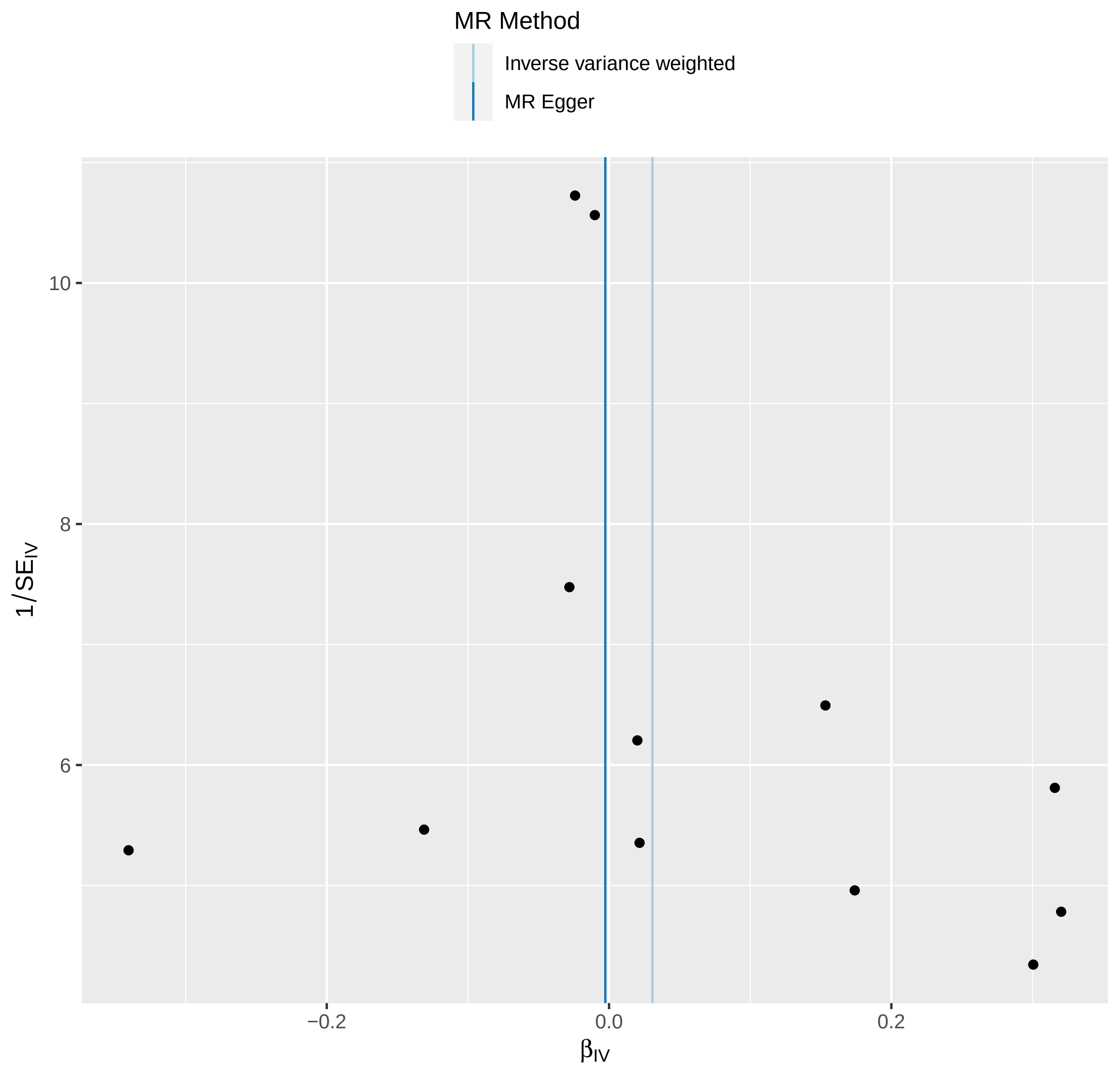

Batch 3−3096 : Selenium on TNF receptor−associated factor 4

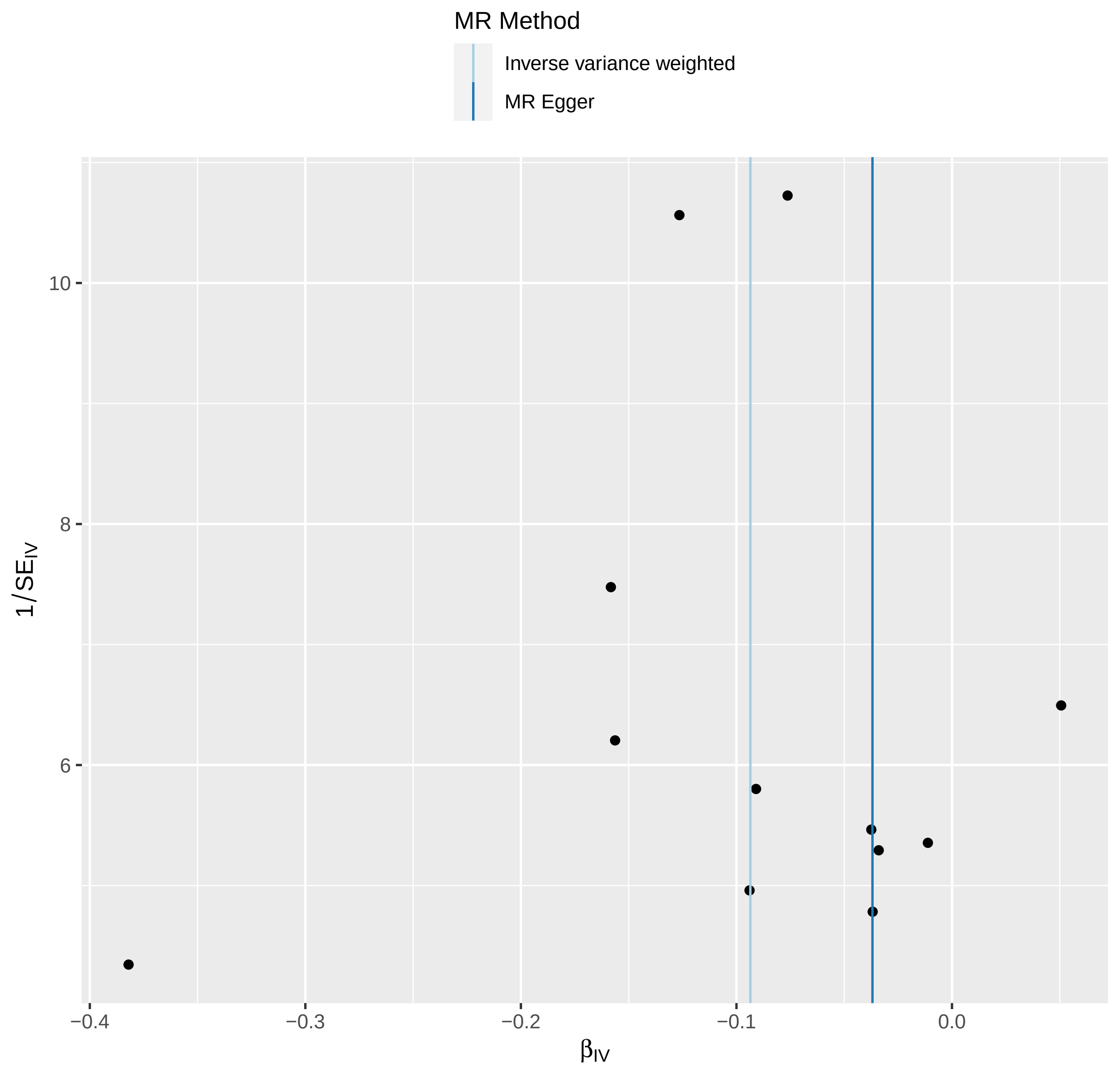

Batch 3−3097 : Selenium on T−cell receptor−associated transmembrane adapter 1

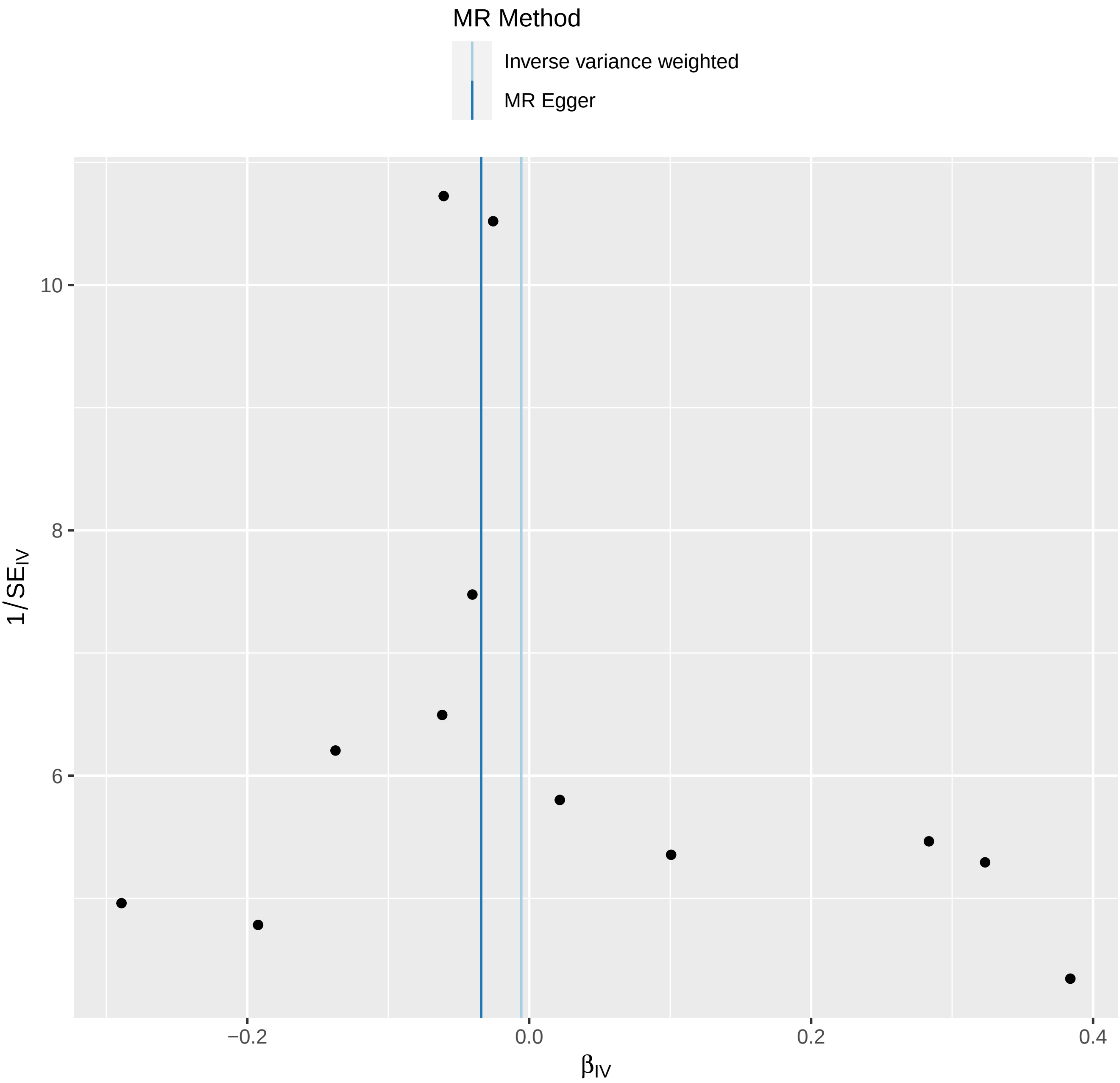

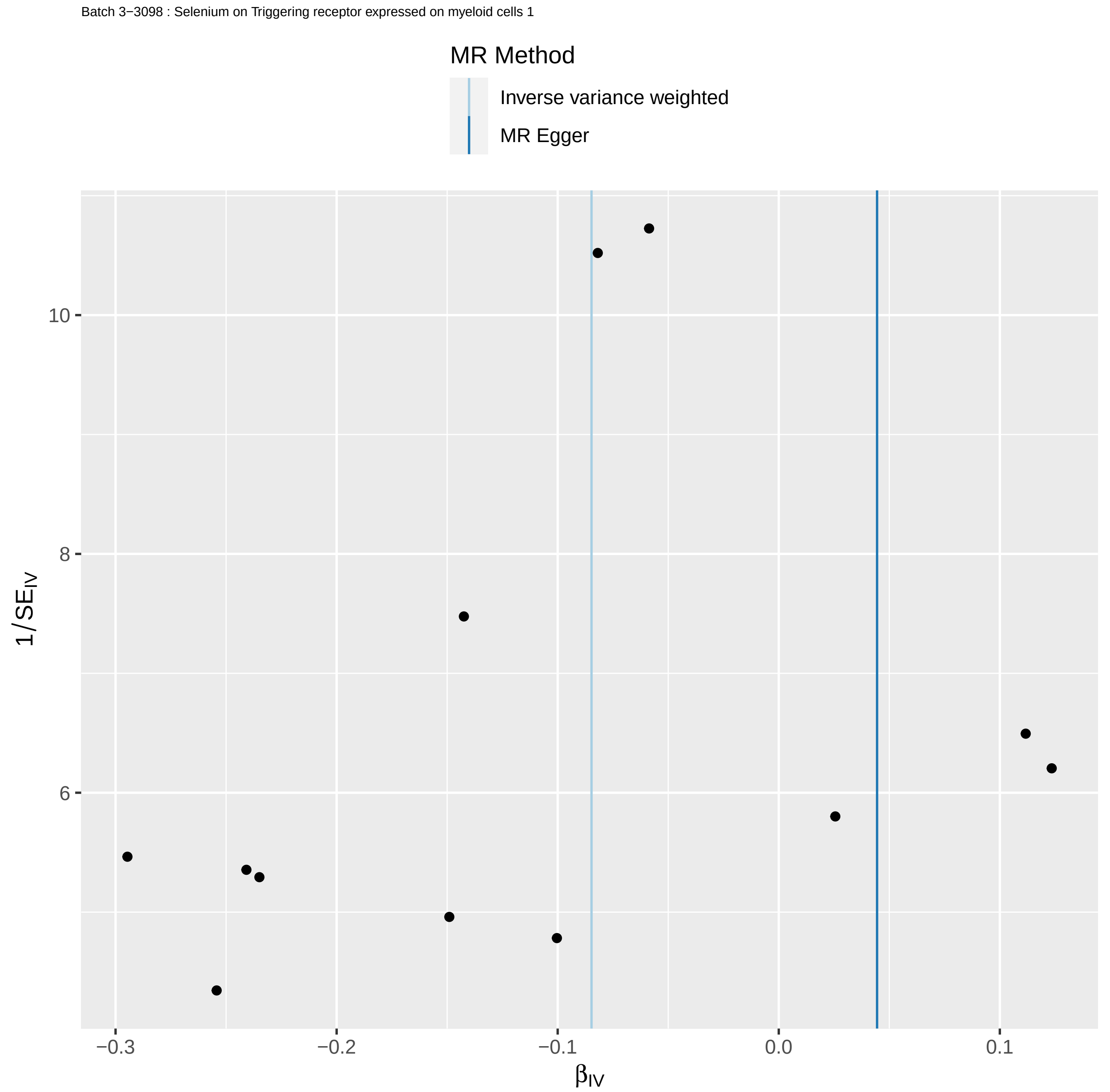

Batch 3−3098 : Selenium on Triggering receptor expressed on myeloid cells 1
MR Method
Inverse variance weighted
MR Egger
10
8
6
1/SE$_{IV}$
−0.3
−0.2
−0.1
0.0
0.1
β$_{IV}$

Batch 3−3099 : Selenium on Trem−like transcript 1 protein

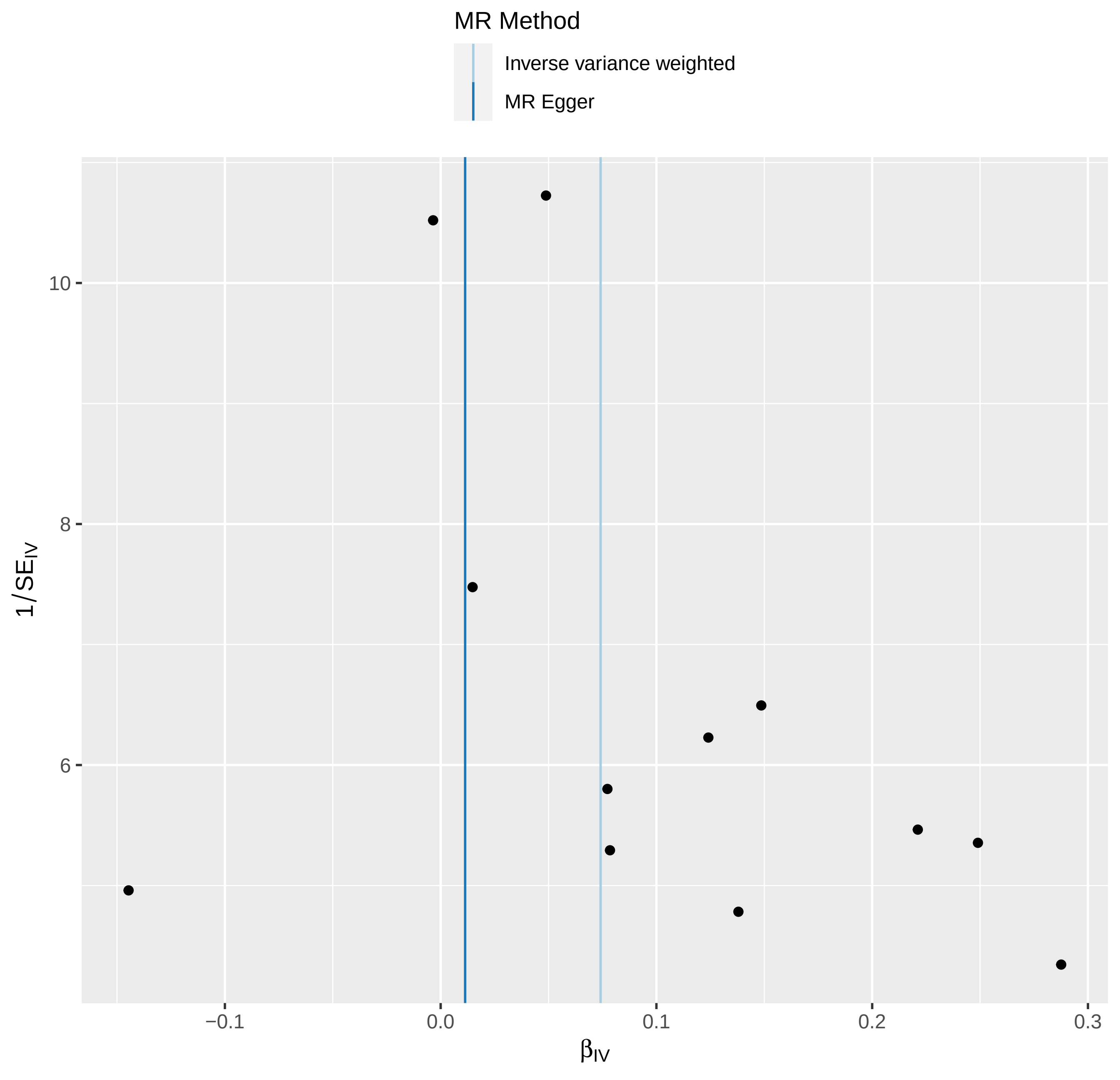

Batch 3−3100 : Selenium on Trem−like transcript 2 protein

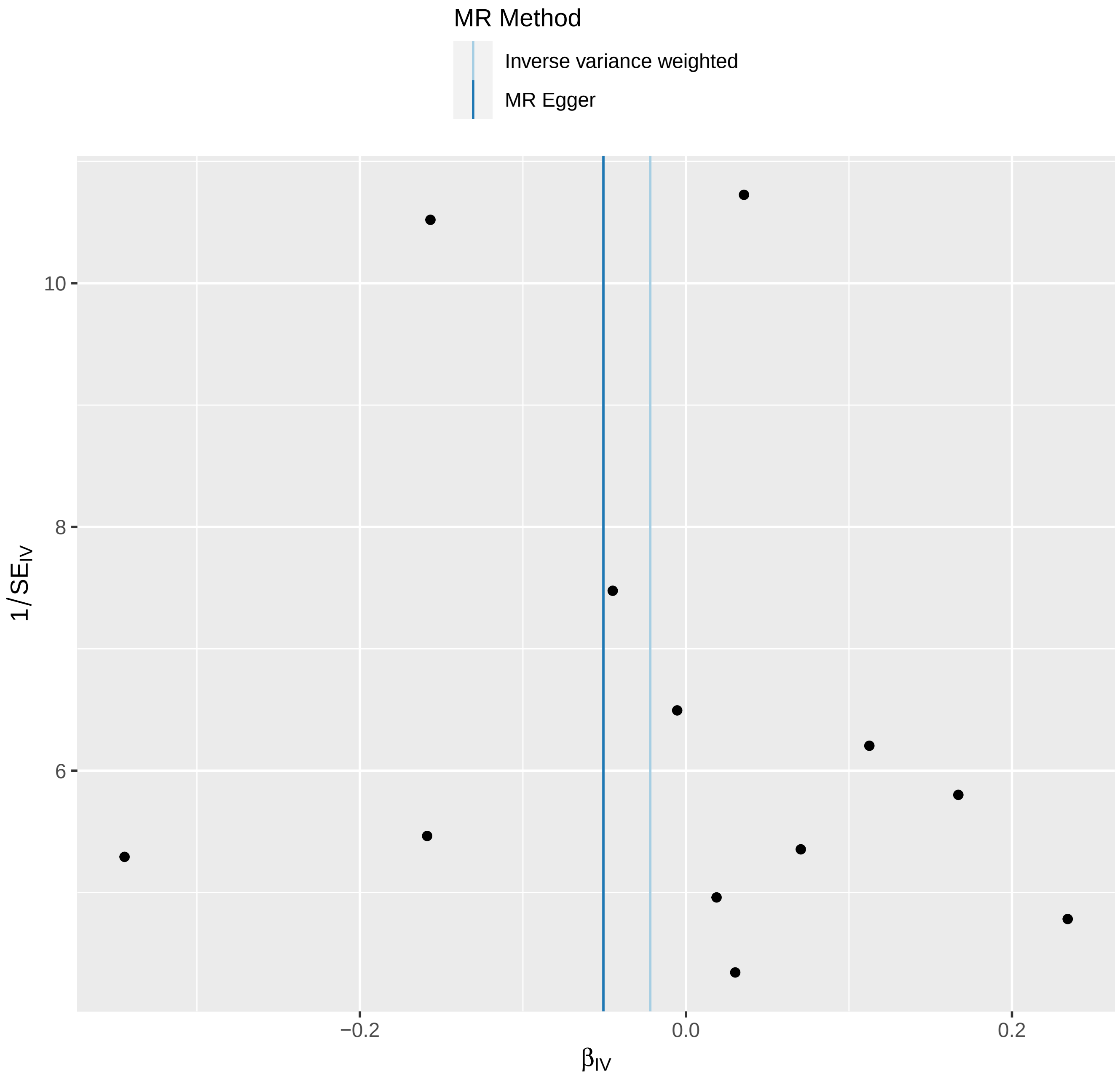

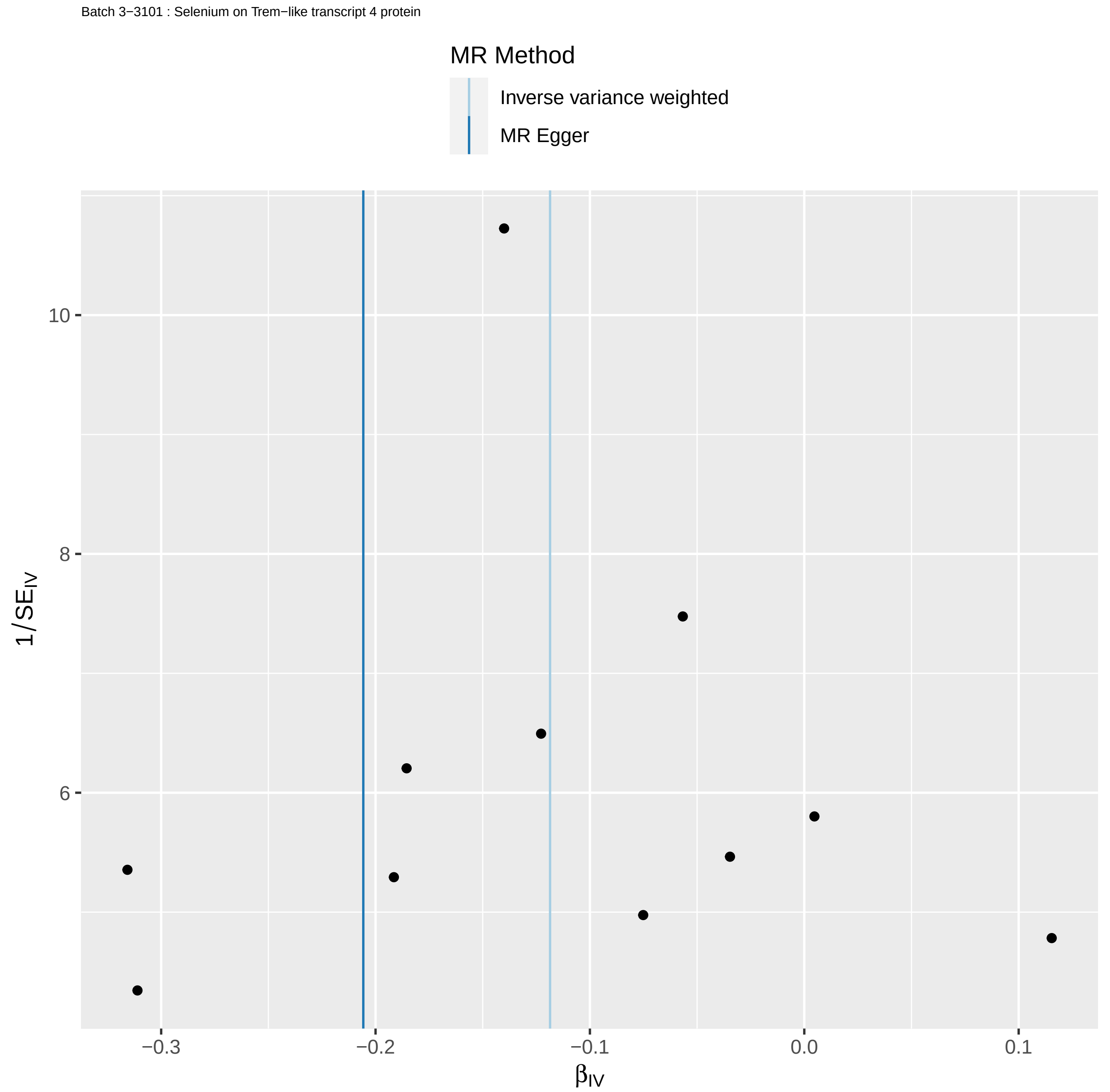
Batch 3−3101 : Selenium on Trem−like transcript 4 protein
MR Method
Inverse variance weighted
MR Egger
10
8
6
1/SE_IV
−0.3
−0.2
−0.1
0.0
0.1
β_IV

Batch 3−3102 : Selenium on Thyrotropin−releasing hormone

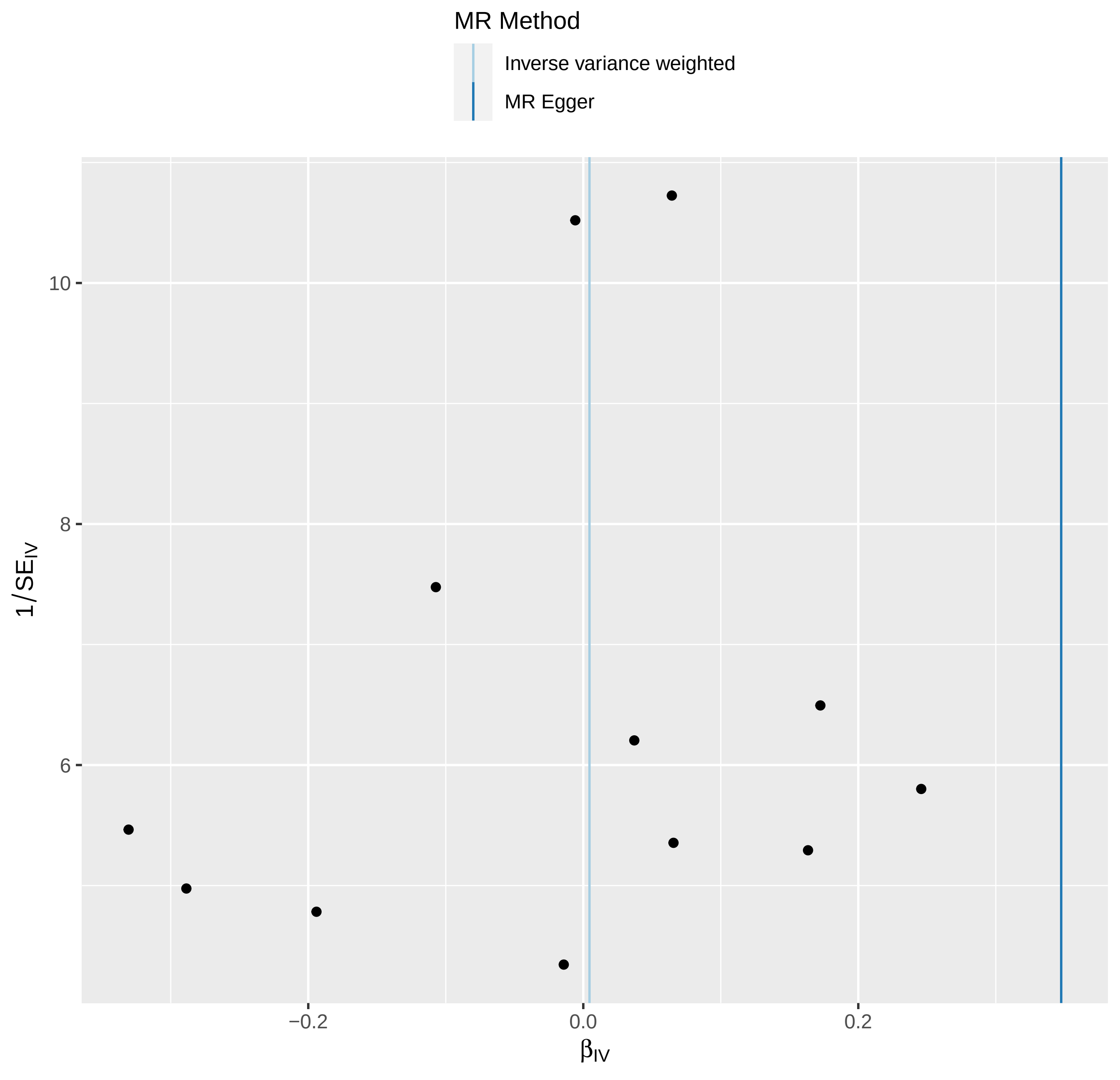

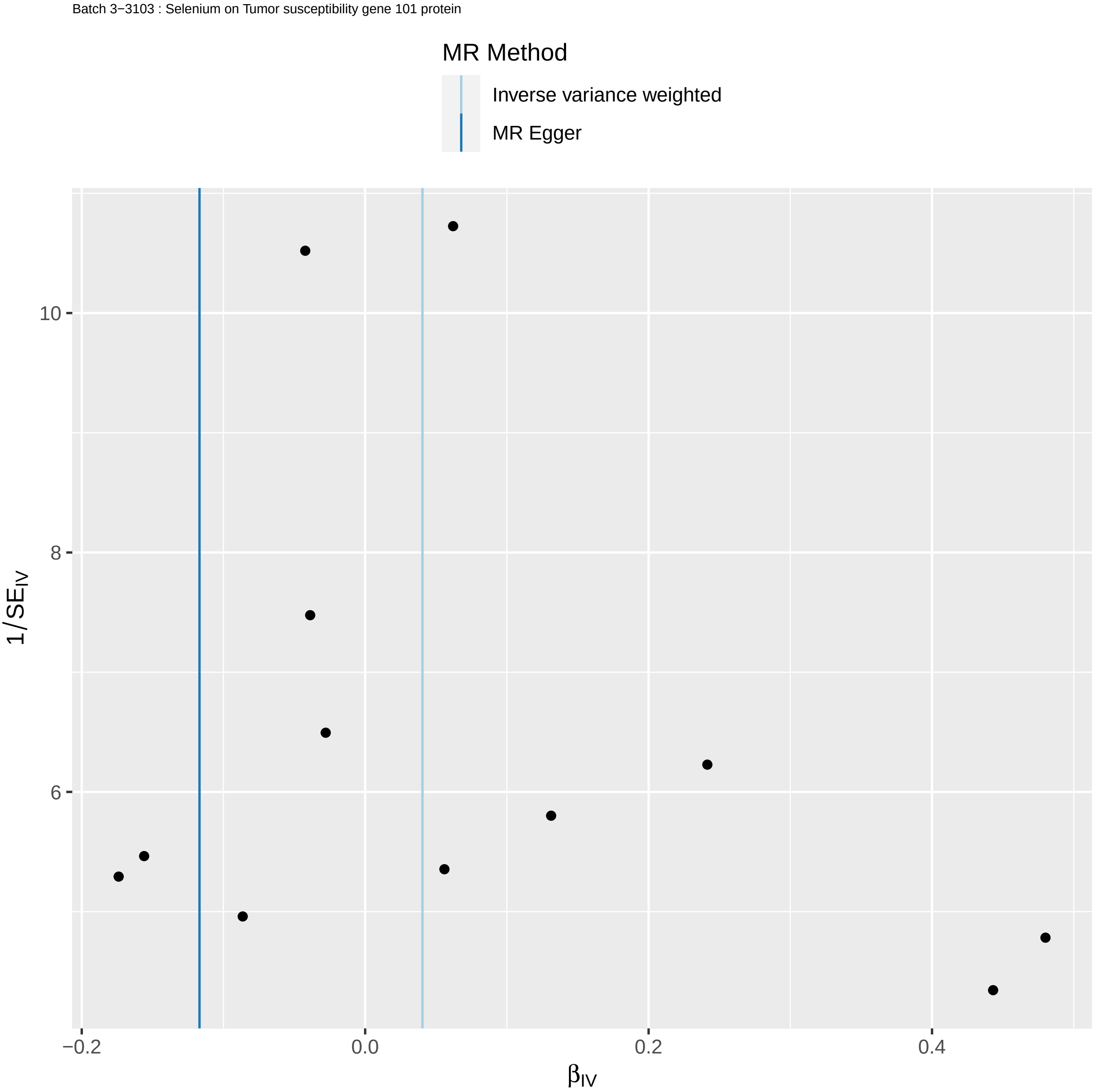
Batch 3−3103 : Selenium on Tumor susceptibility gene 101 protein
MR Method
Inverse variance weighted
MR Egger
10
8
6
$1/SE_{IV}$
−0.2
0.0
0.2
0.4
β_{IV}

Batch 3−3104 : Selenium on Thymic stromal lymphopoietin

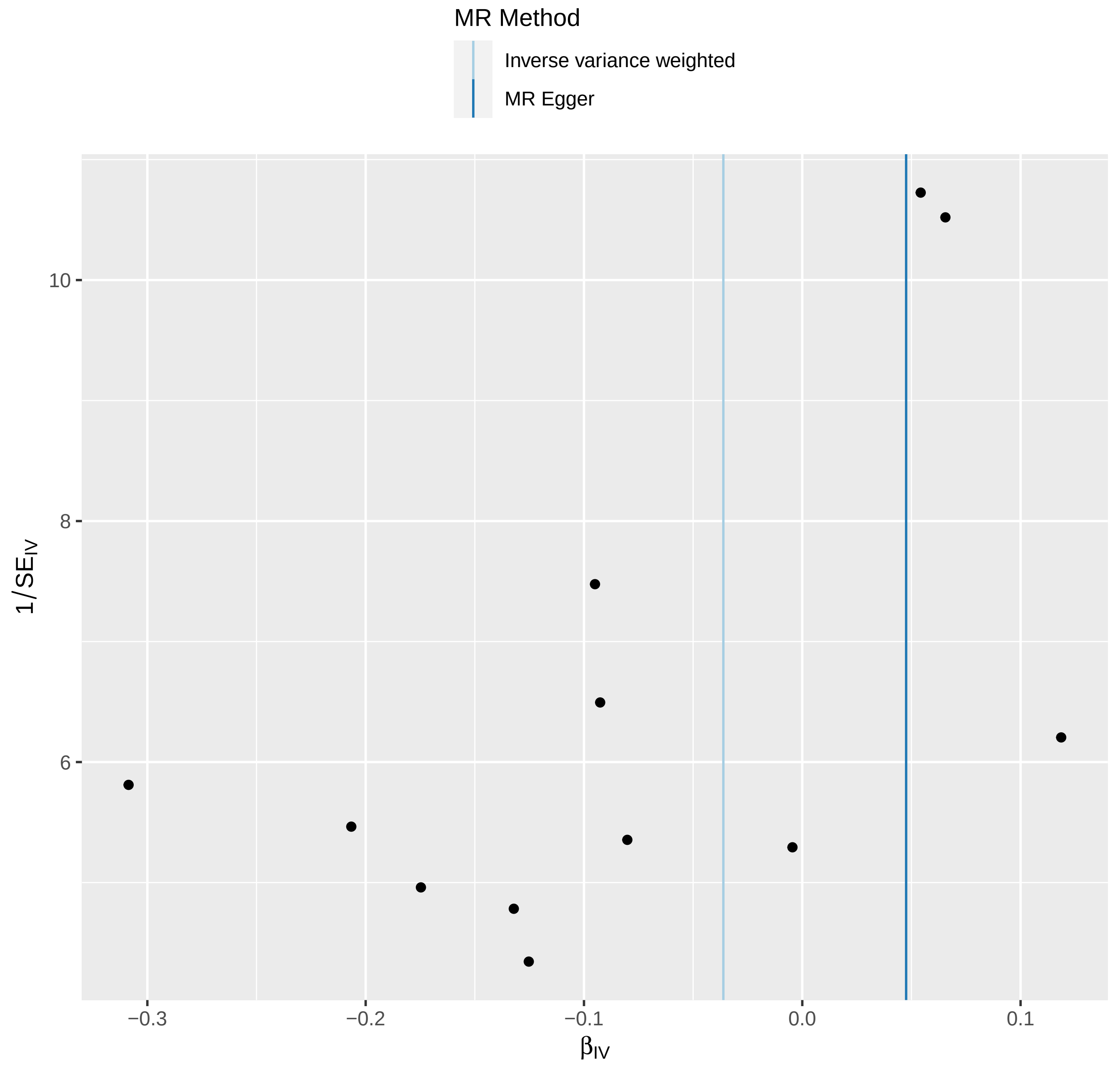

Batch 3−3105 : Selenium on Translin

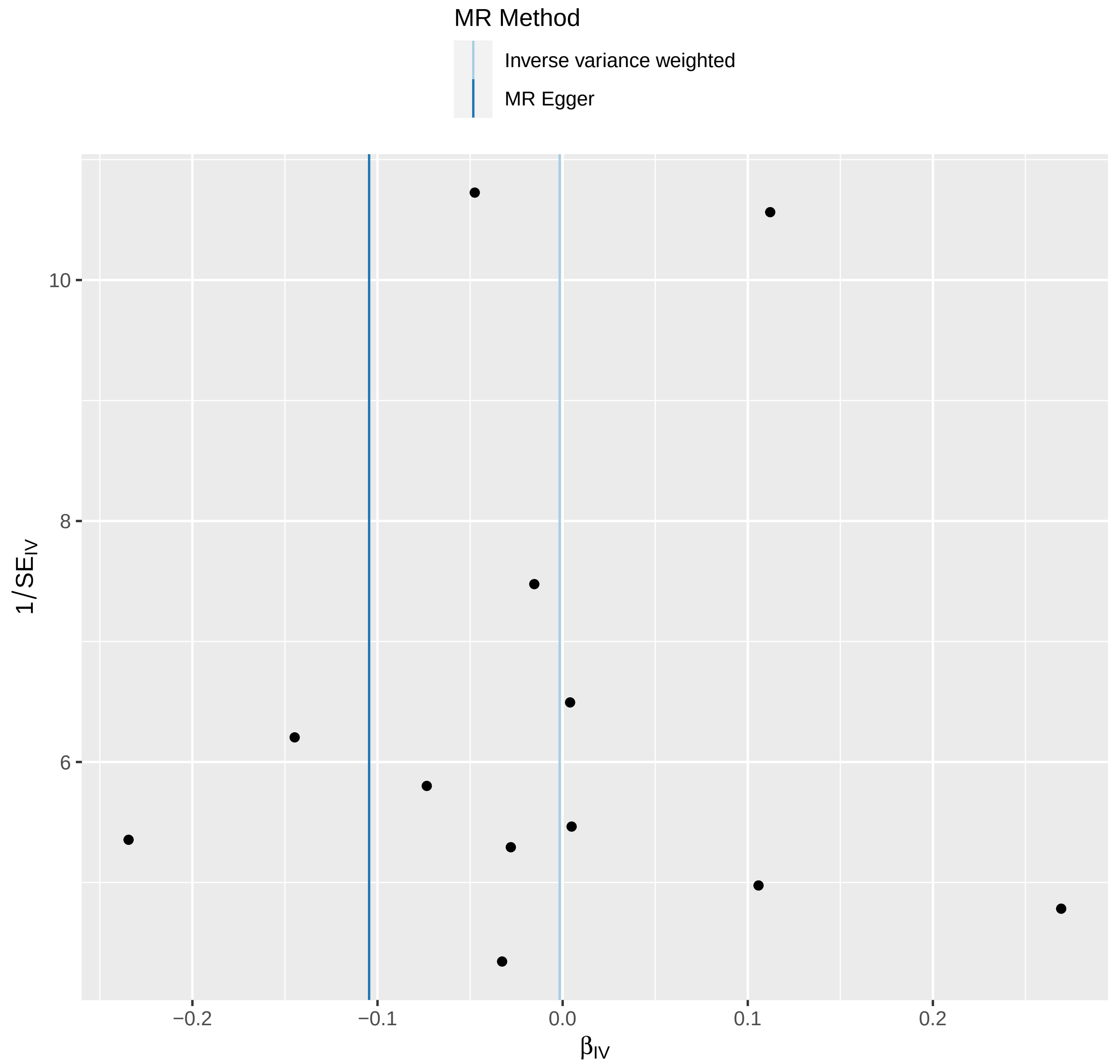

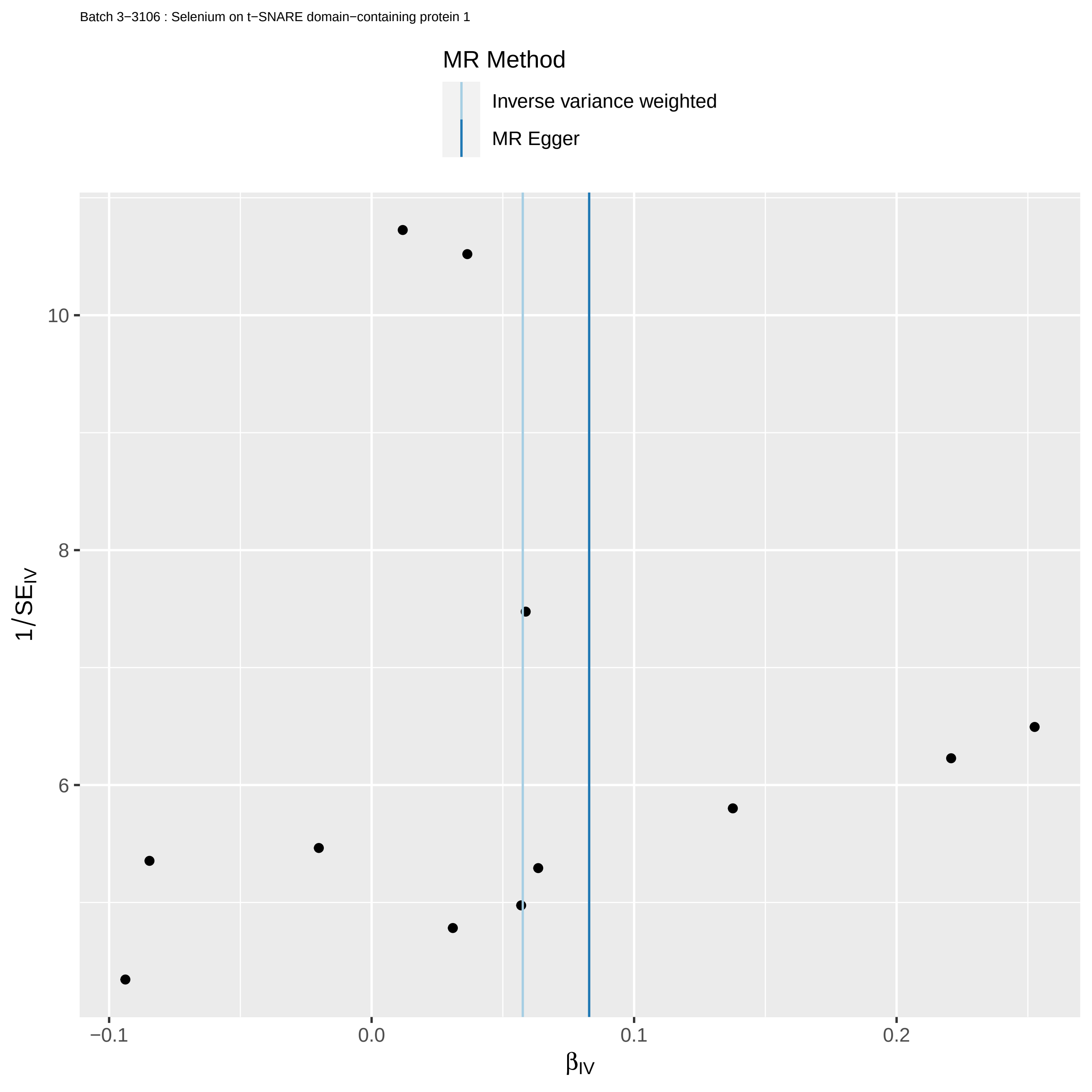

Batch 3−3106 : Selenium on t−SNARE domain−containing protein 1
MR Method
Inverse variance weighted
MR Egger
10
8
6
1/SE_IV
−0.1
0.0
0.1
0.2
β_IV

Batch 3−3107 : Selenium on Ribosome biogenesis protein TSR3 homolog

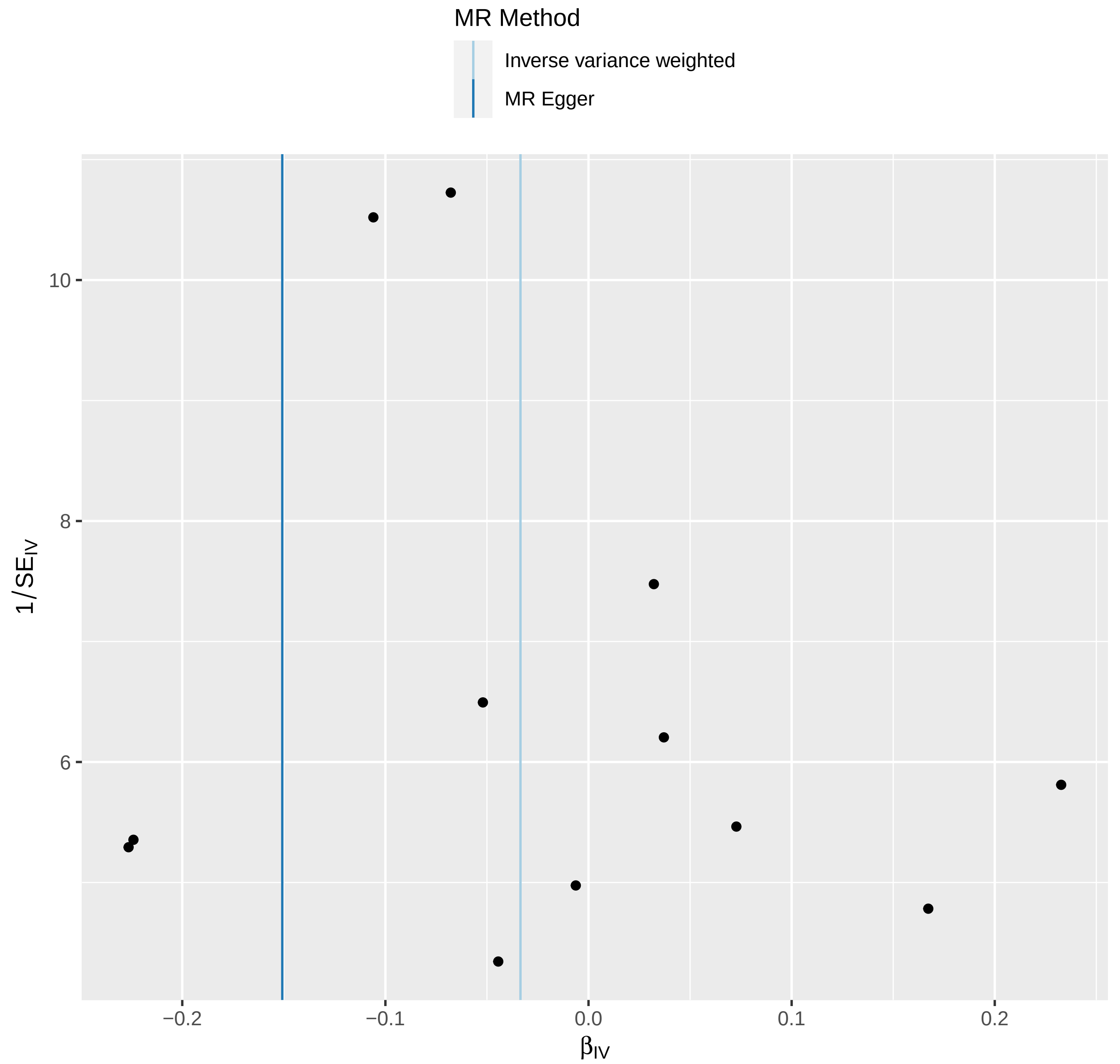

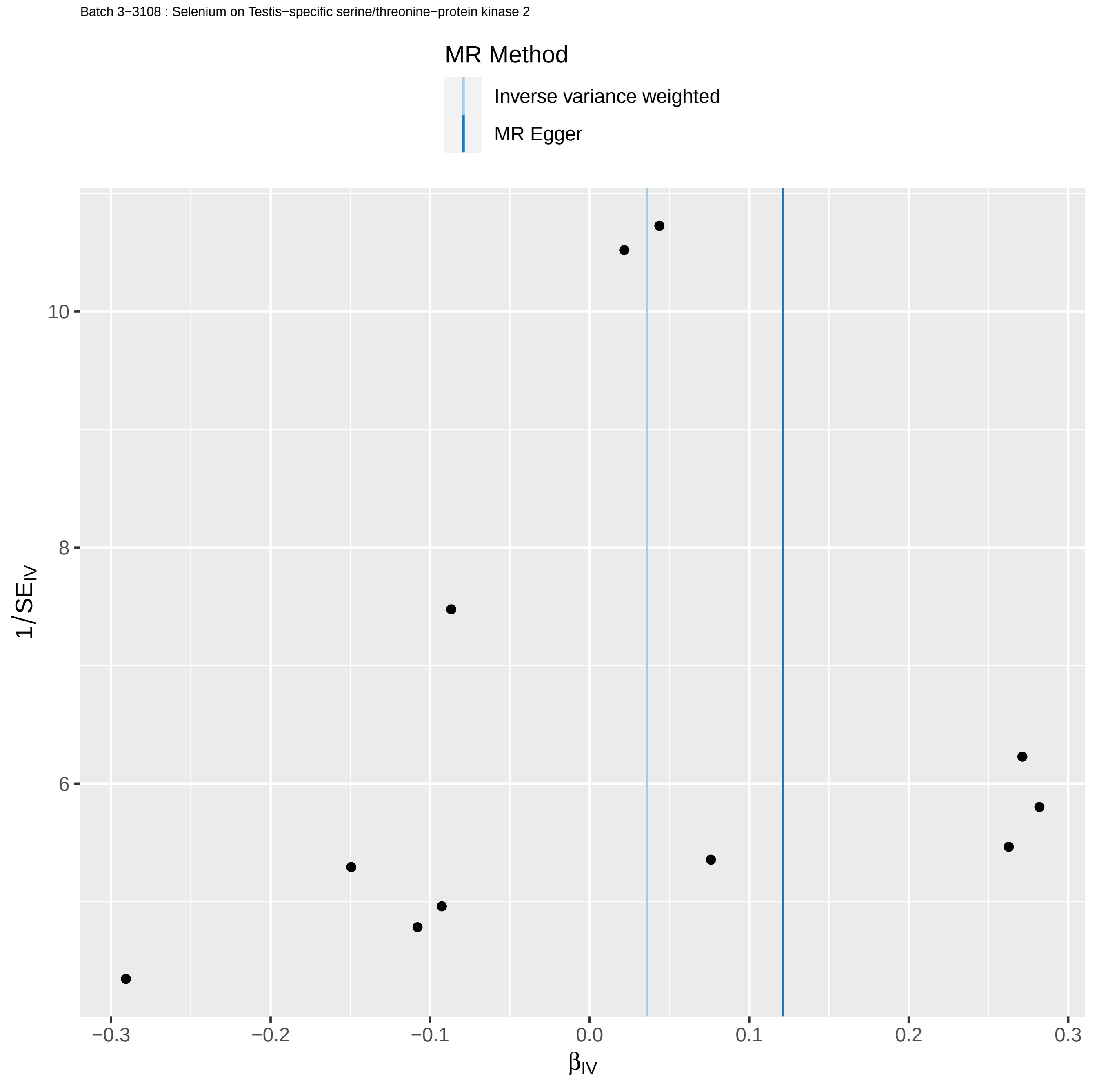
Batch 3−3108 : Selenium on Testis−specific serine/threonine−protein kinase 2
MR Method
Inverse variance weighted
MR Egger
10
8
6
1/SE_IV
−0.3
−0.2
−0.1
0.0
0.1
0.2
0.3
β_IV

Batch 3−3109 : Selenium on Thiosulfate sulfurtransferase

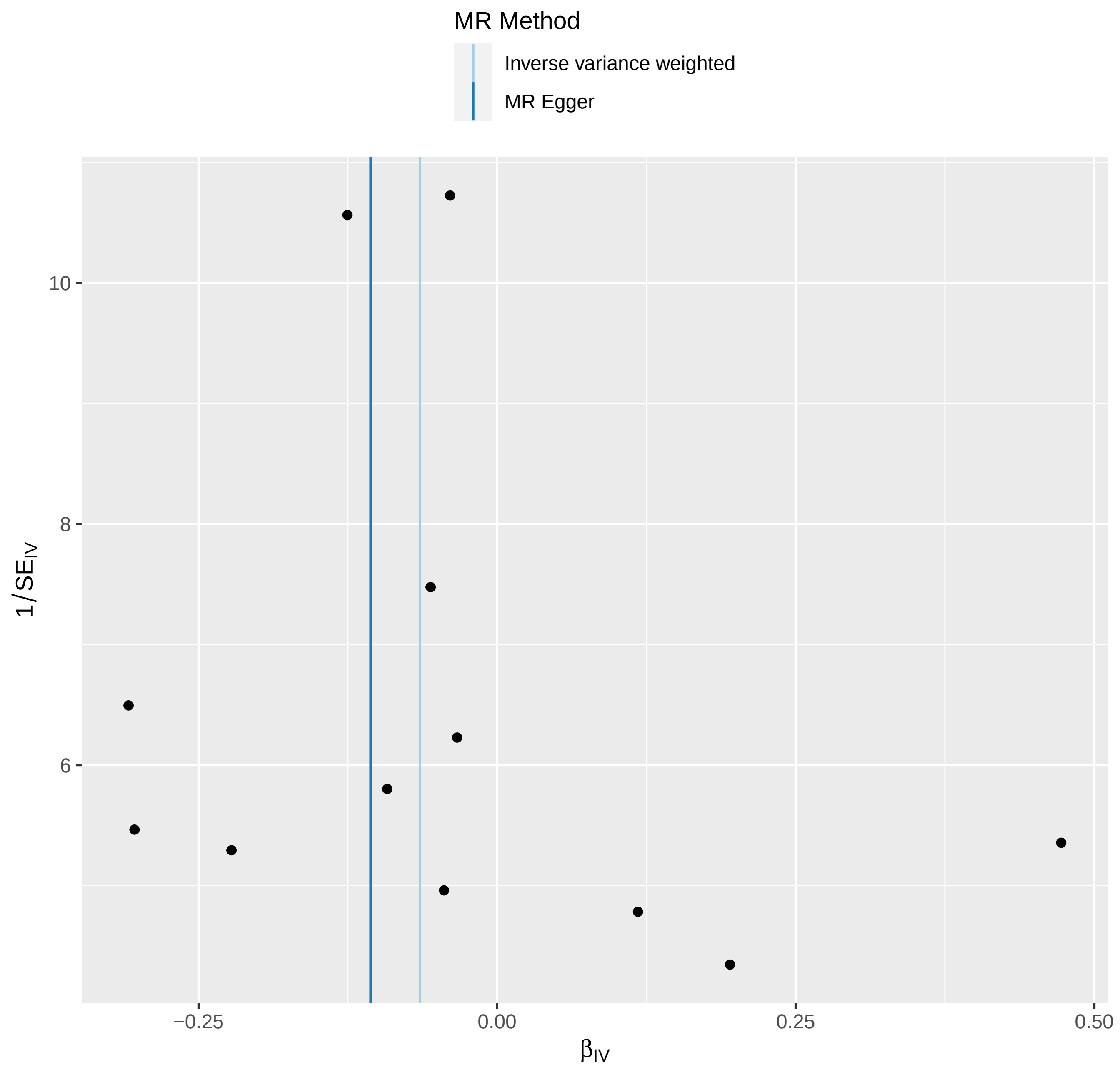

Batch 3−3110 : Selenium on GDP−L−fucose synthase

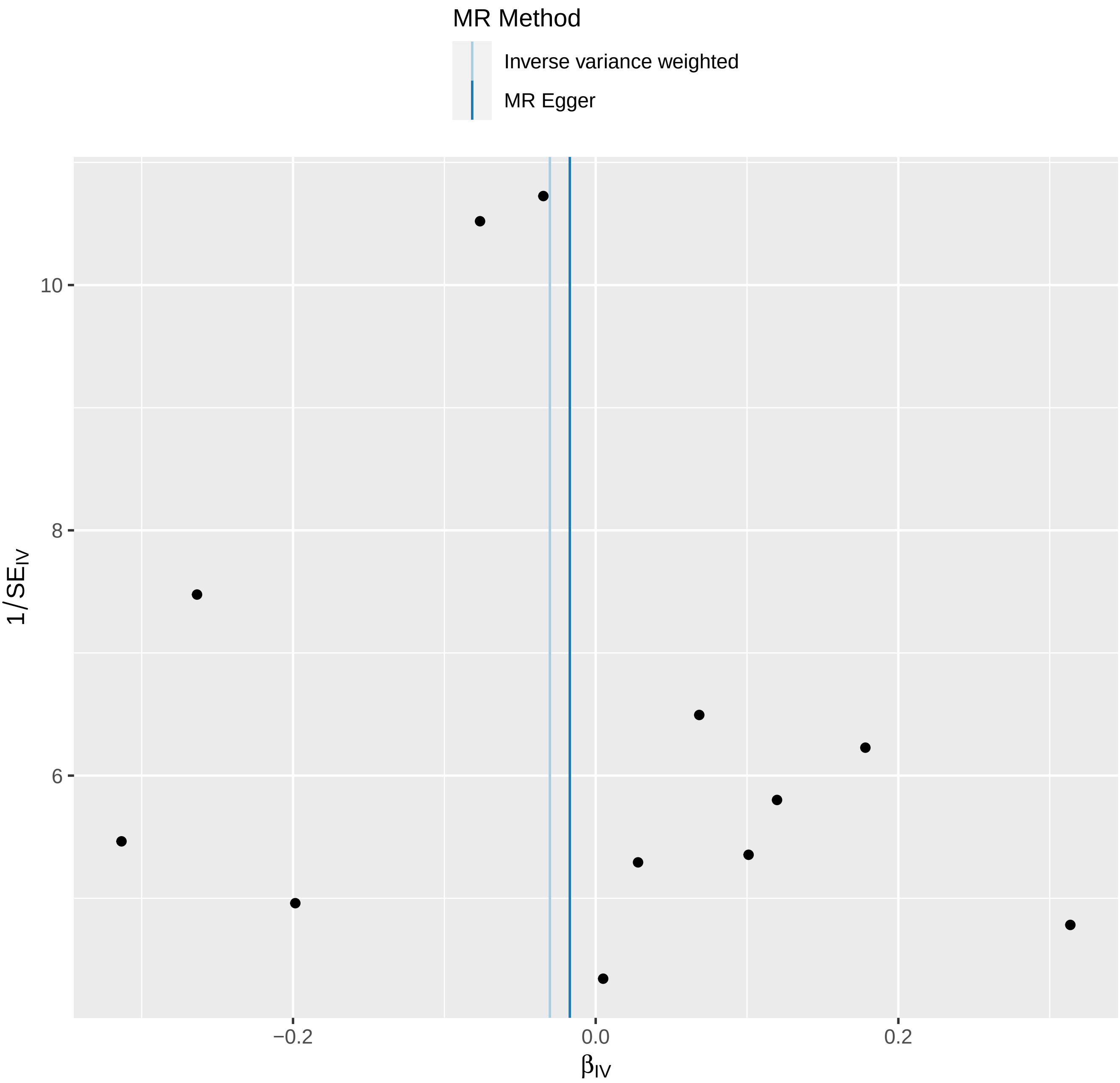

Batch 3−3111 : Selenium on Tetratricopeptide repeat protein 17

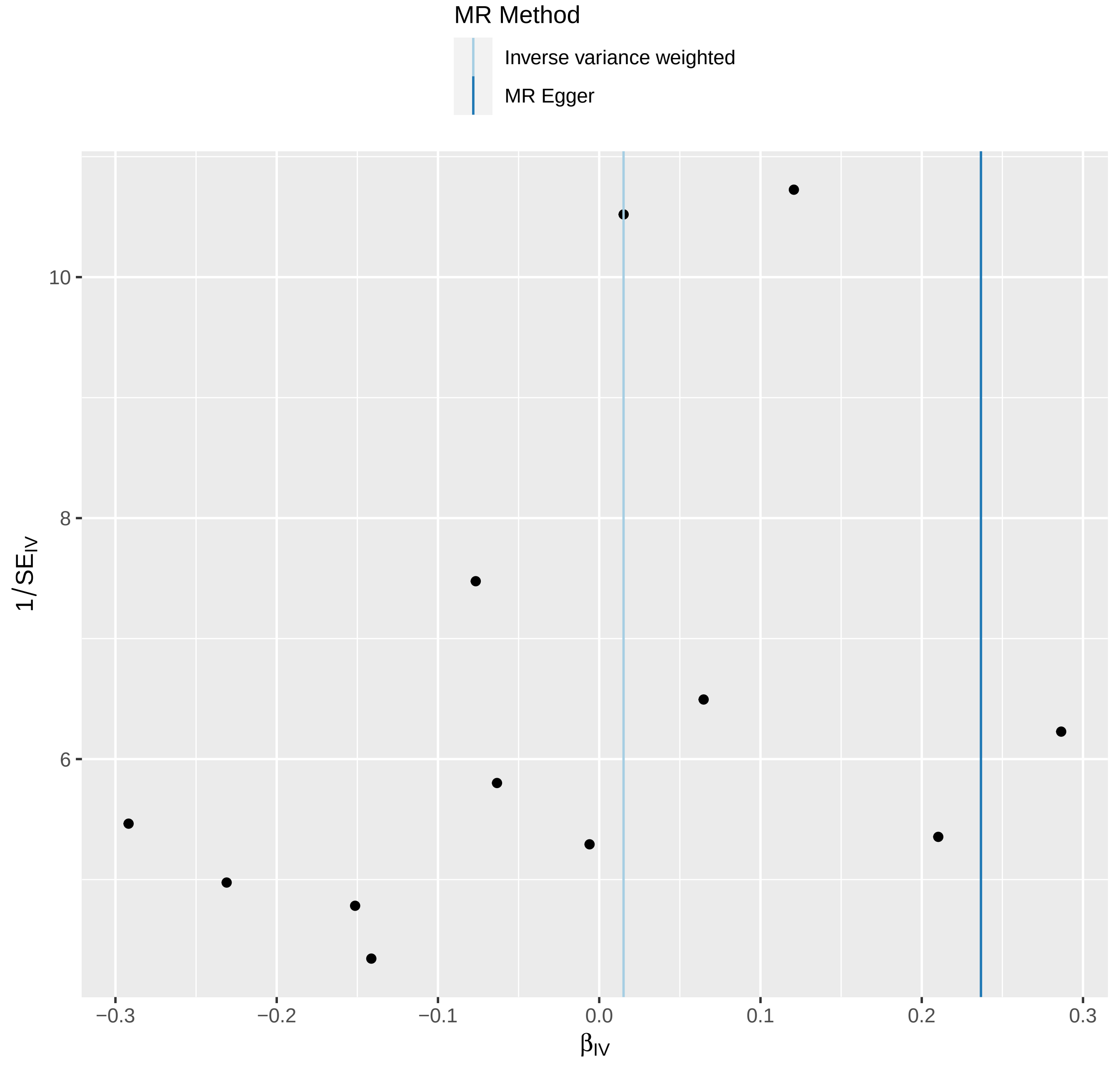

Batch 3−3112 : Selenium on Tetratricopeptide repeat protein 17

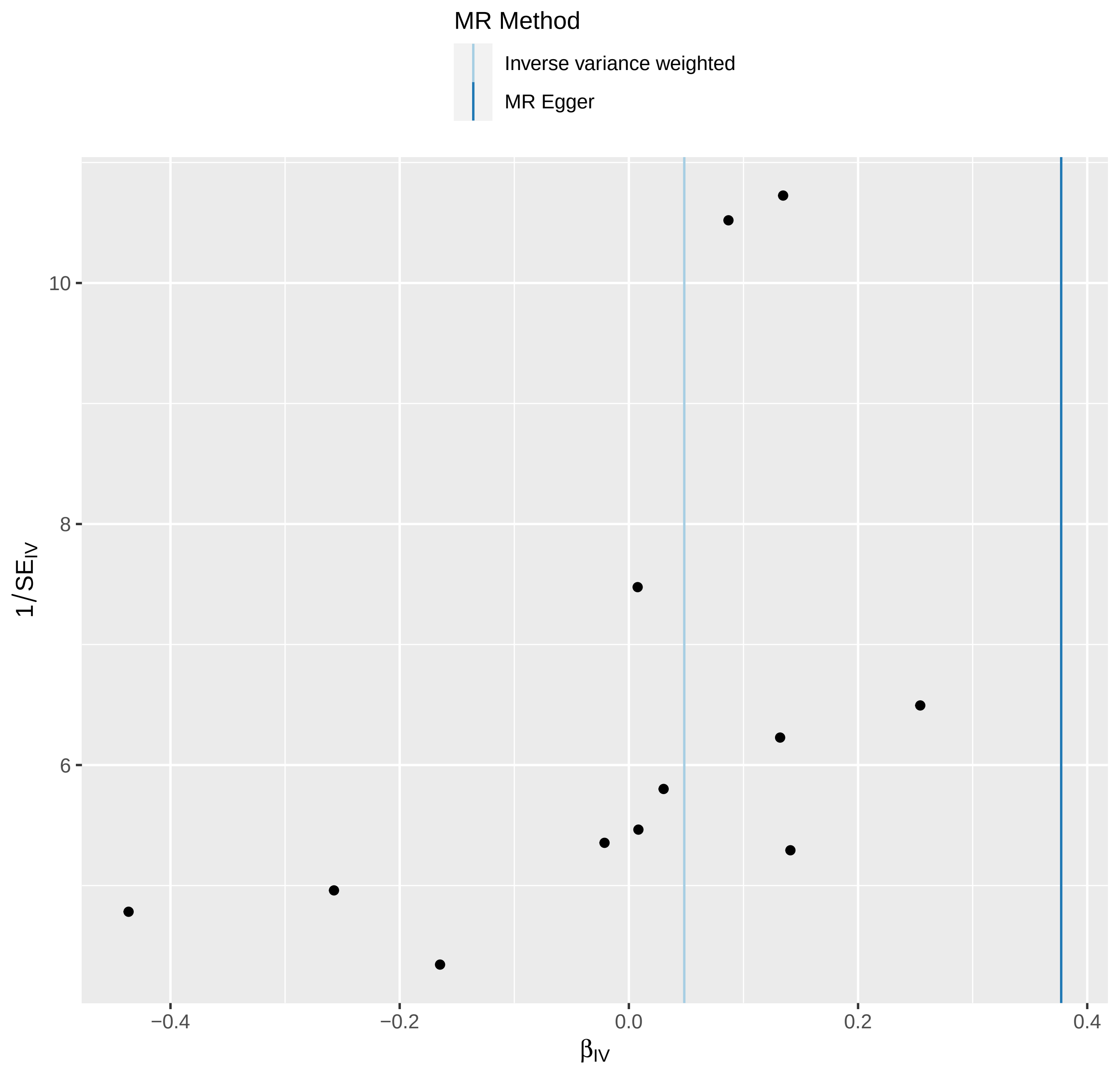

Batch 3−3113 : Selenium on Tetratricopeptide repeat protein 9B

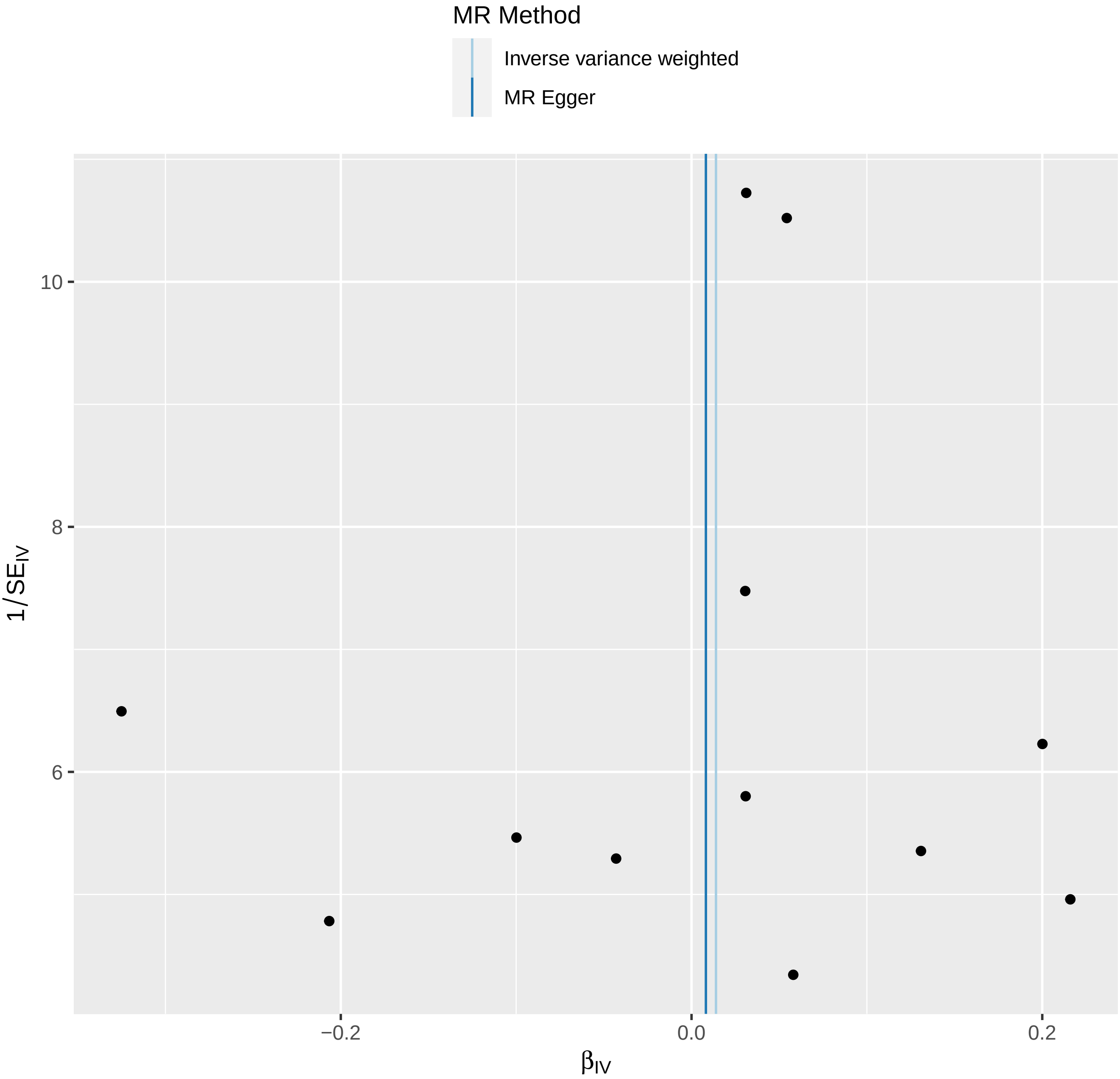

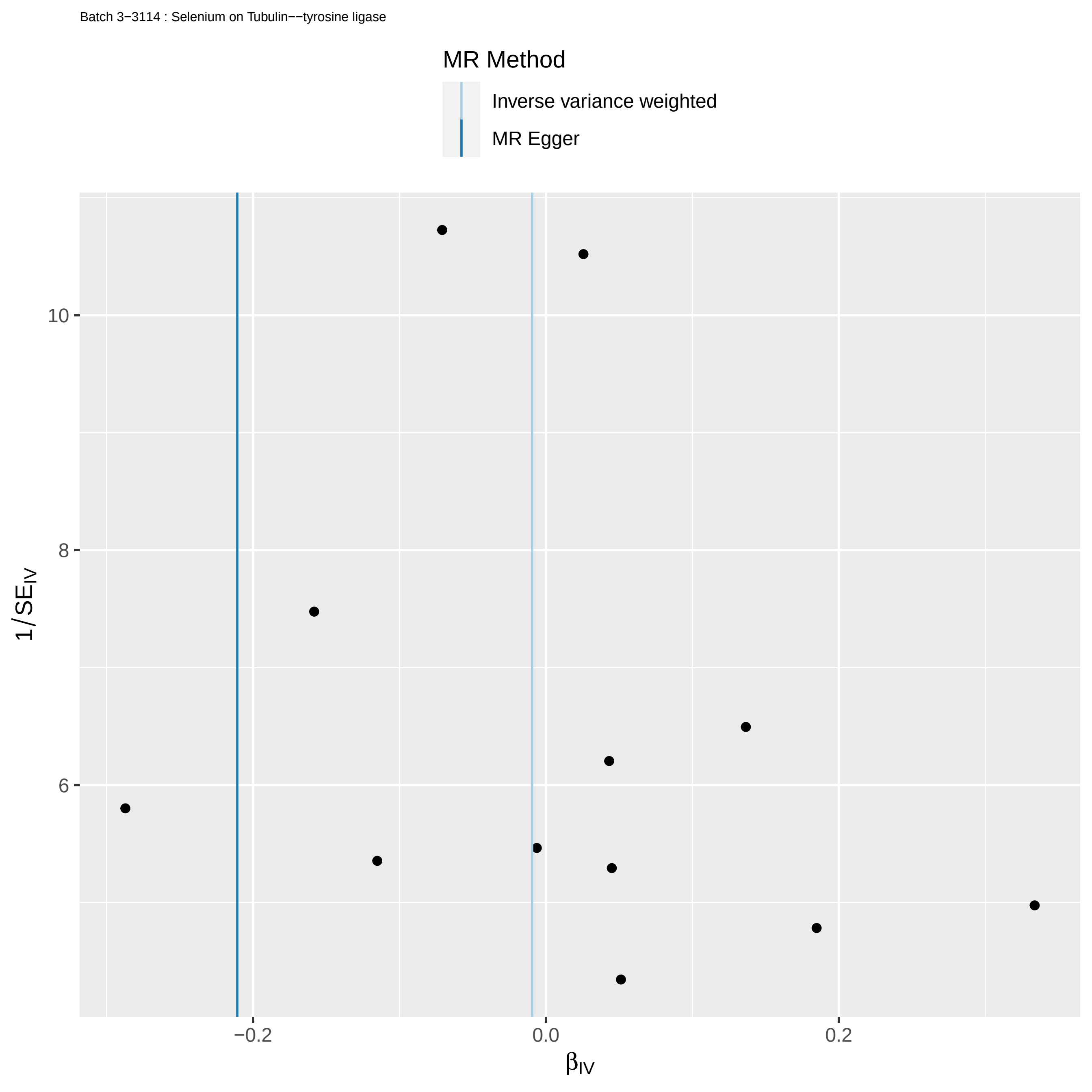

Batch 3−3114 : Selenium on Tubulin−−tyrosine ligase
MR Method
Inverse variance weighted
MR Egger
10
8
6
1/SE$_{IV}$
−0.2
0.0
0.2
β$_{IV}$

Batch 3−3115 : Selenium on TPA−induced transmembrane protein

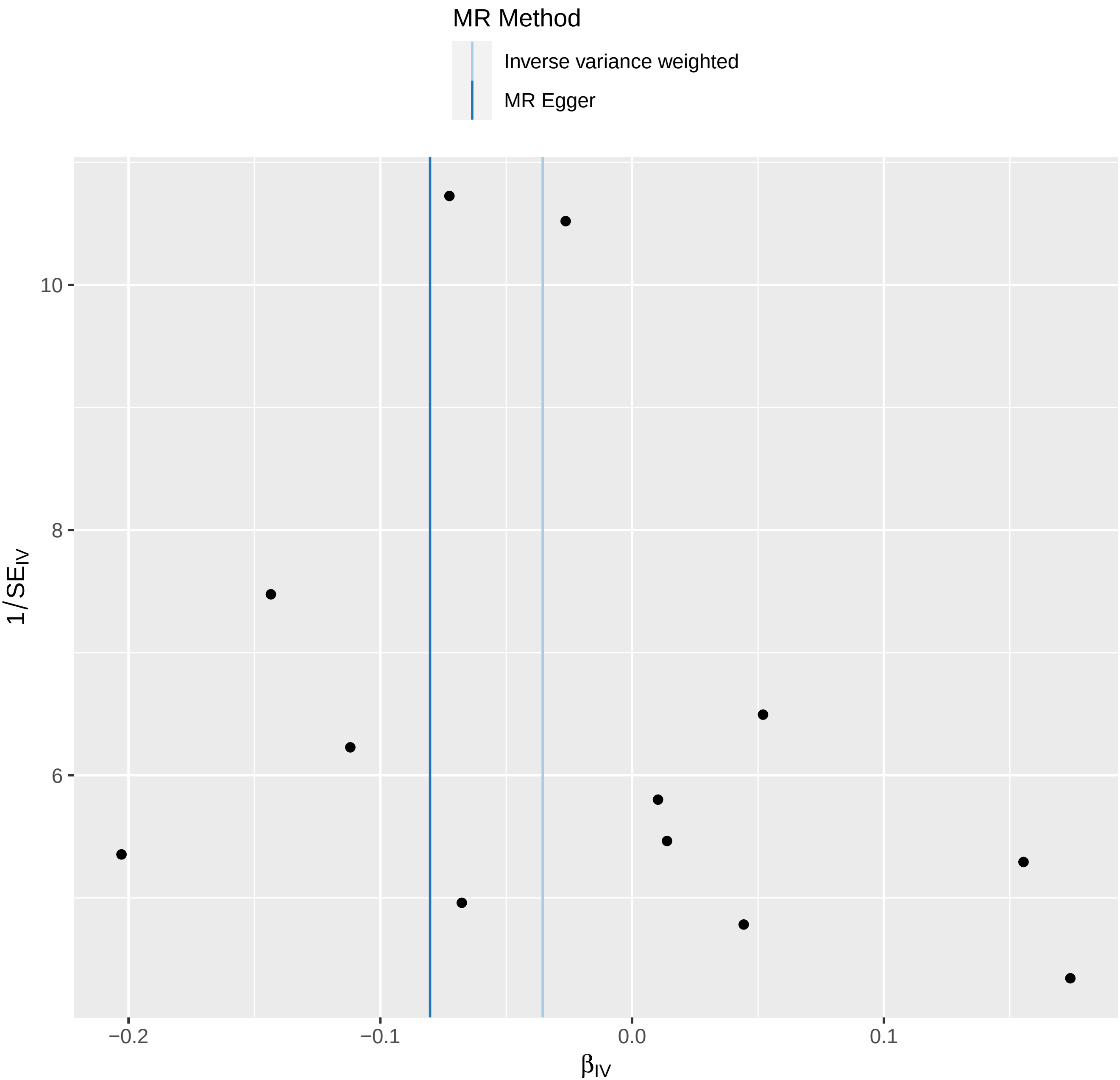

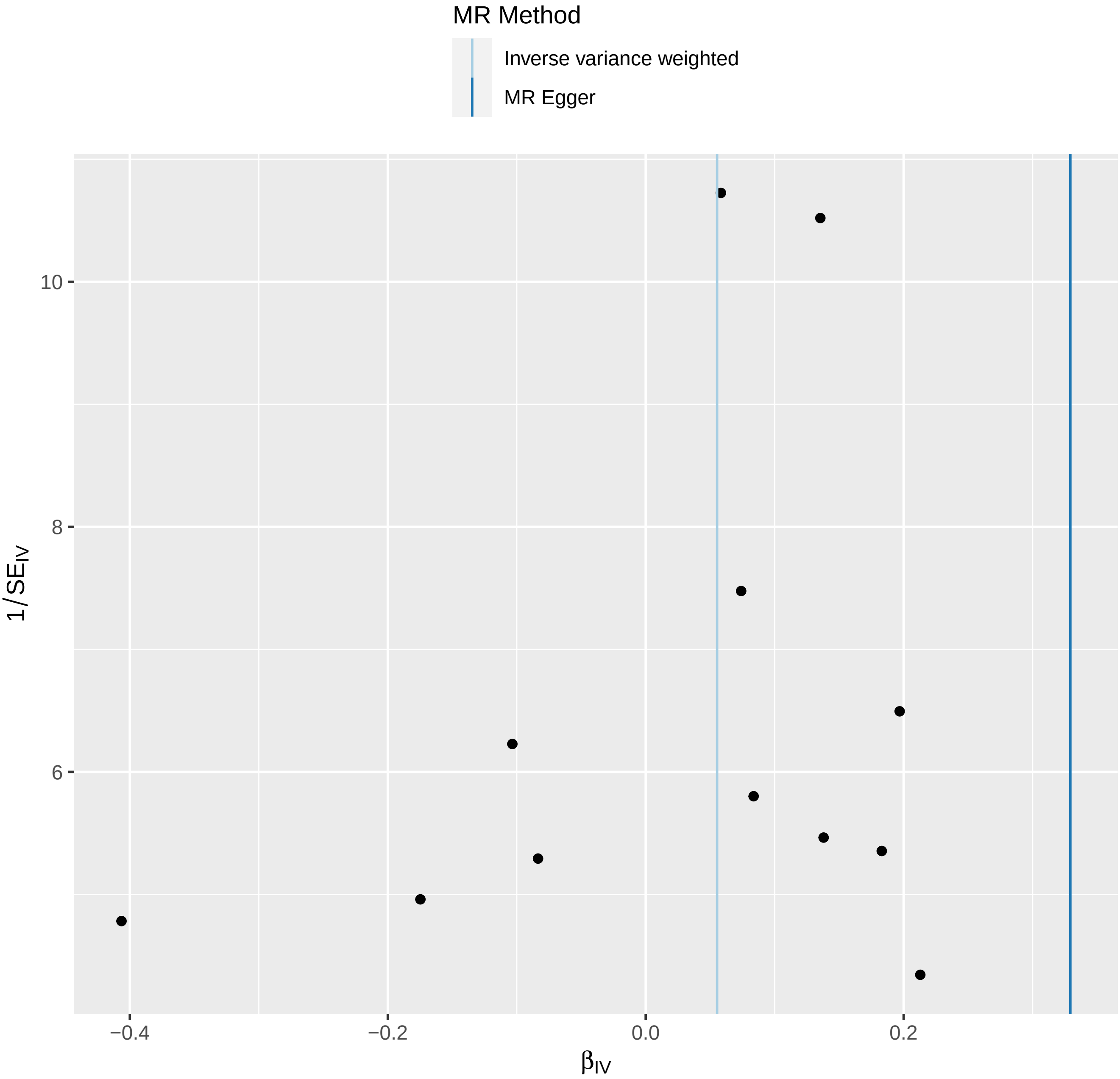
MR Method
Inverse variance weighted
MR Egger
10
8
6
1/SE$_{IV}$
−0.4
−0.2
0.0
0.2
β$_{IV}$

Batch 3−3117 : Selenium on Tuftelin

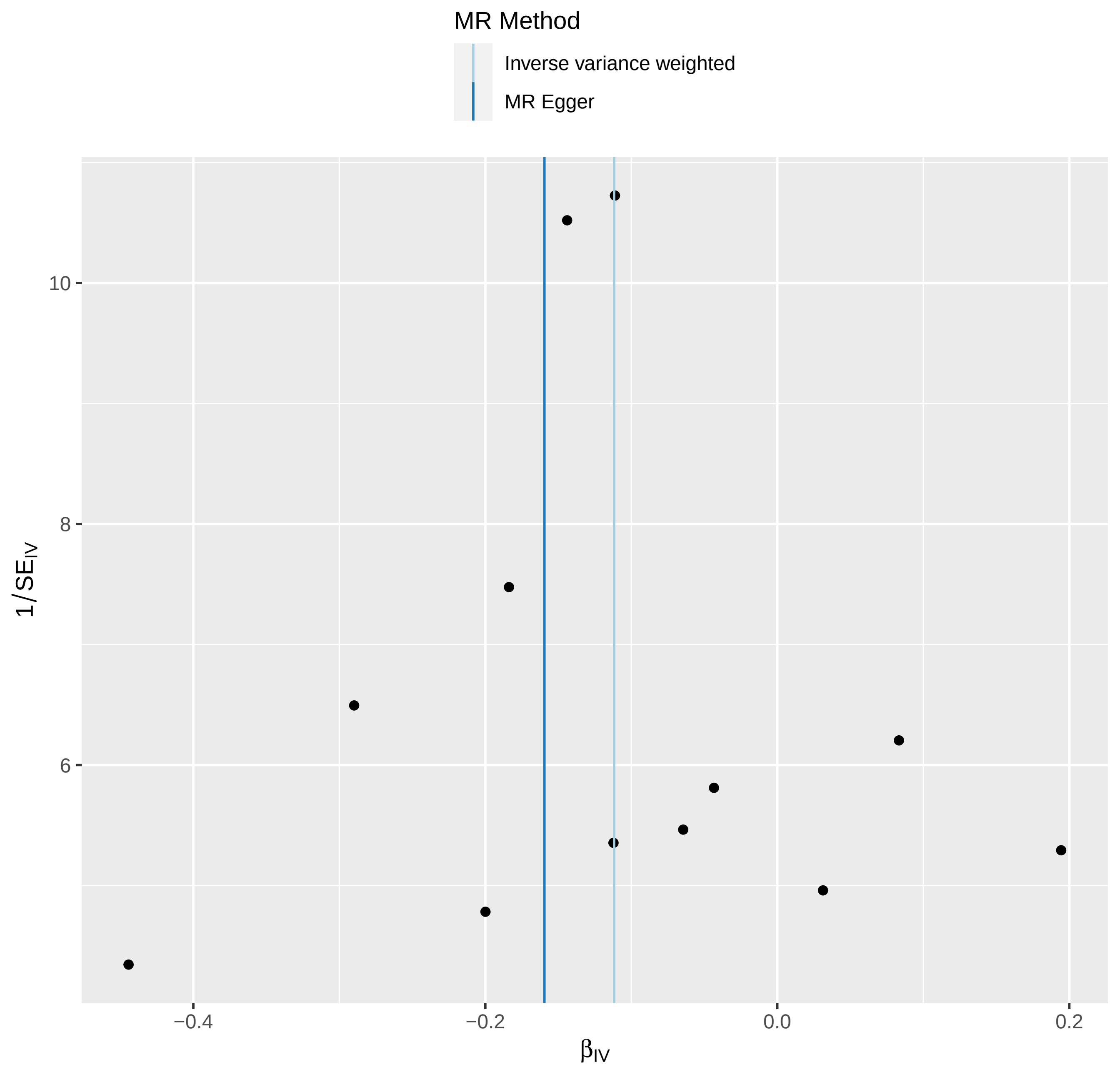

Batch 3−3118 : Selenium on Twinfilin−1

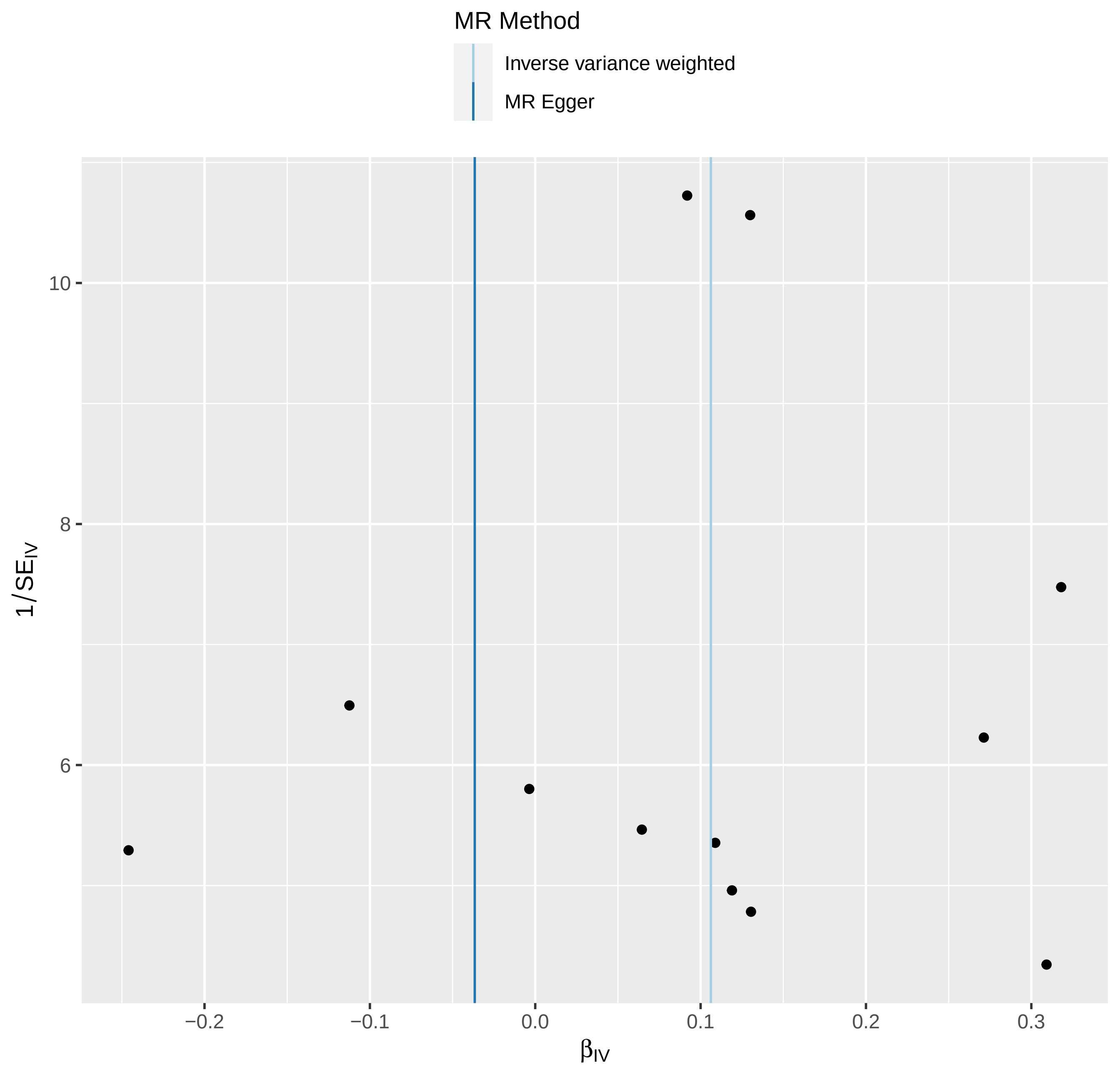

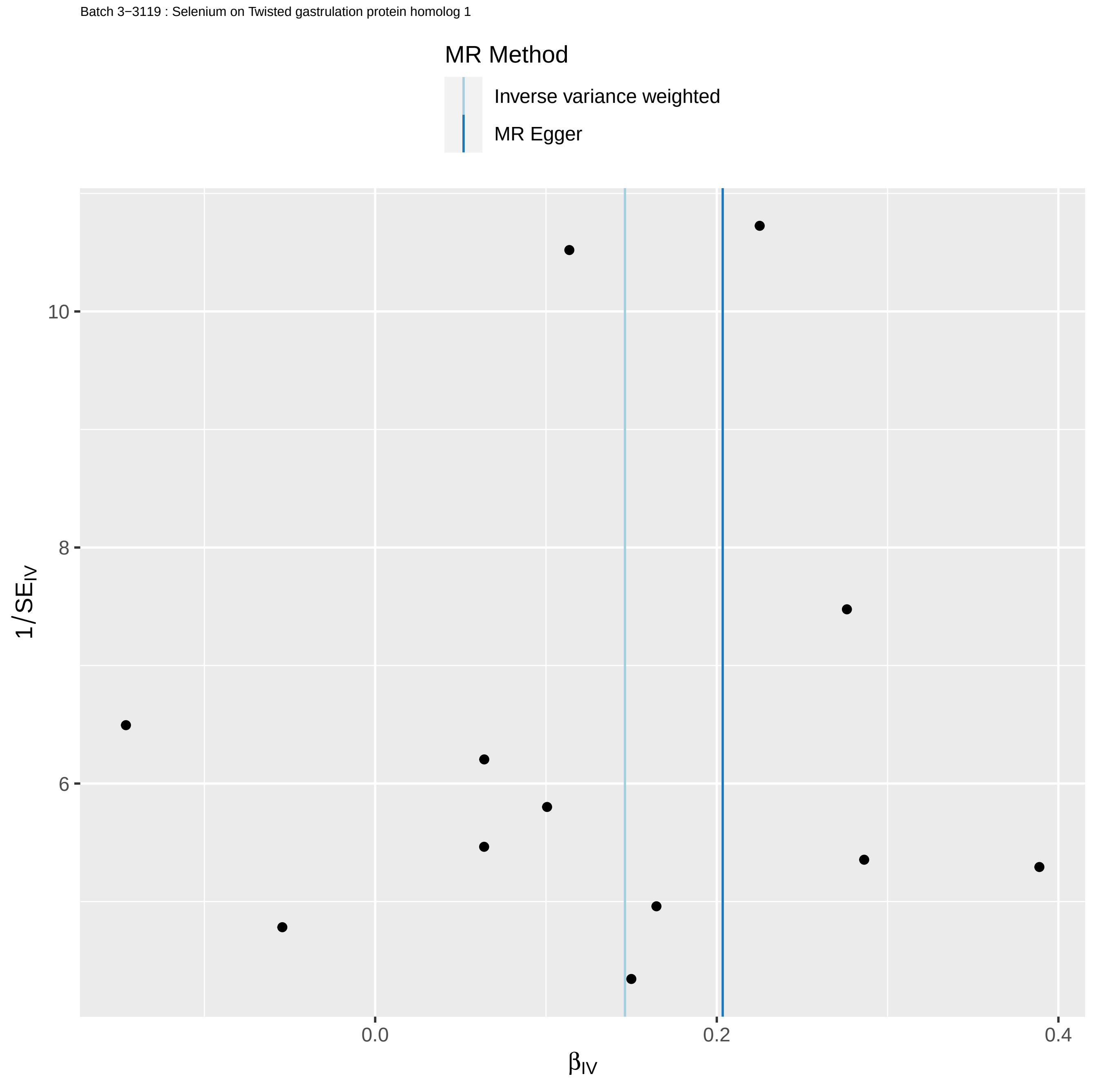

Batch 3−3119 : Selenium on Twisted gastrulation protein homolog 1
MR Method
Inverse variance weighted
MR Egger
10
8
6
1/SE_IV
0.0
0.2
0.4
β_IV

Batch 3−3120 : Selenium on Alpha−taxilin

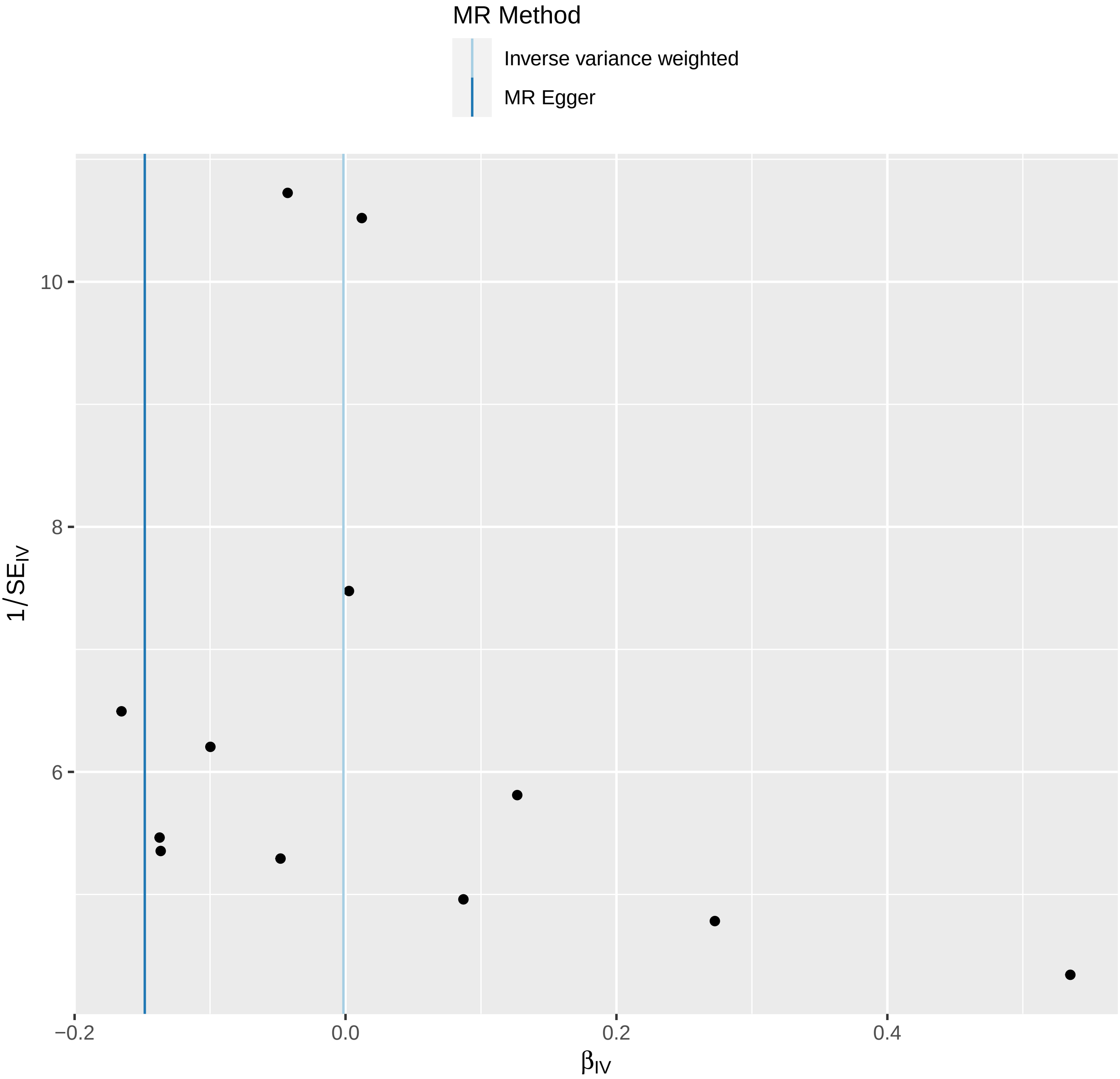

Batch 3−3121 : Selenium on Thioredoxin

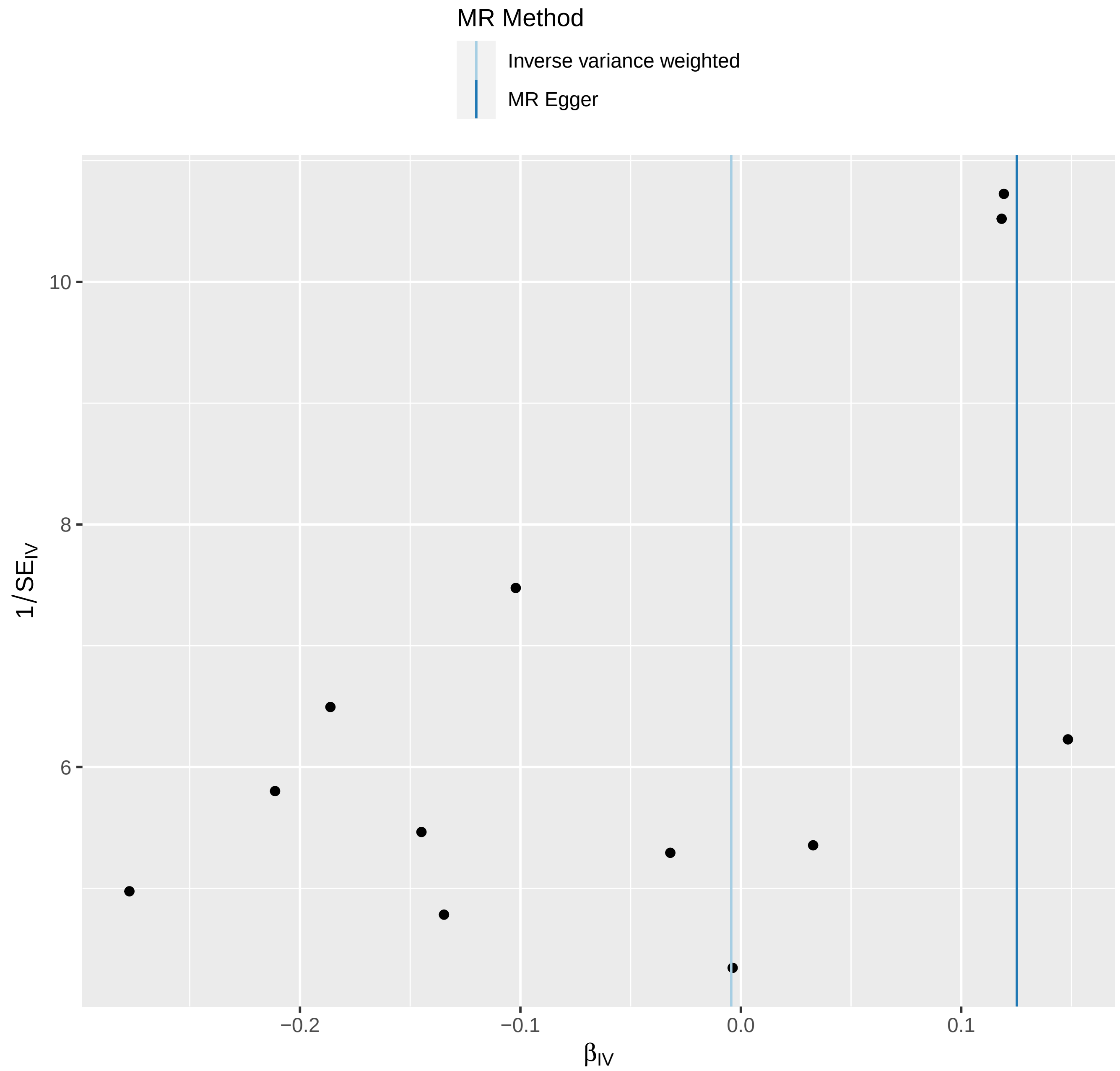

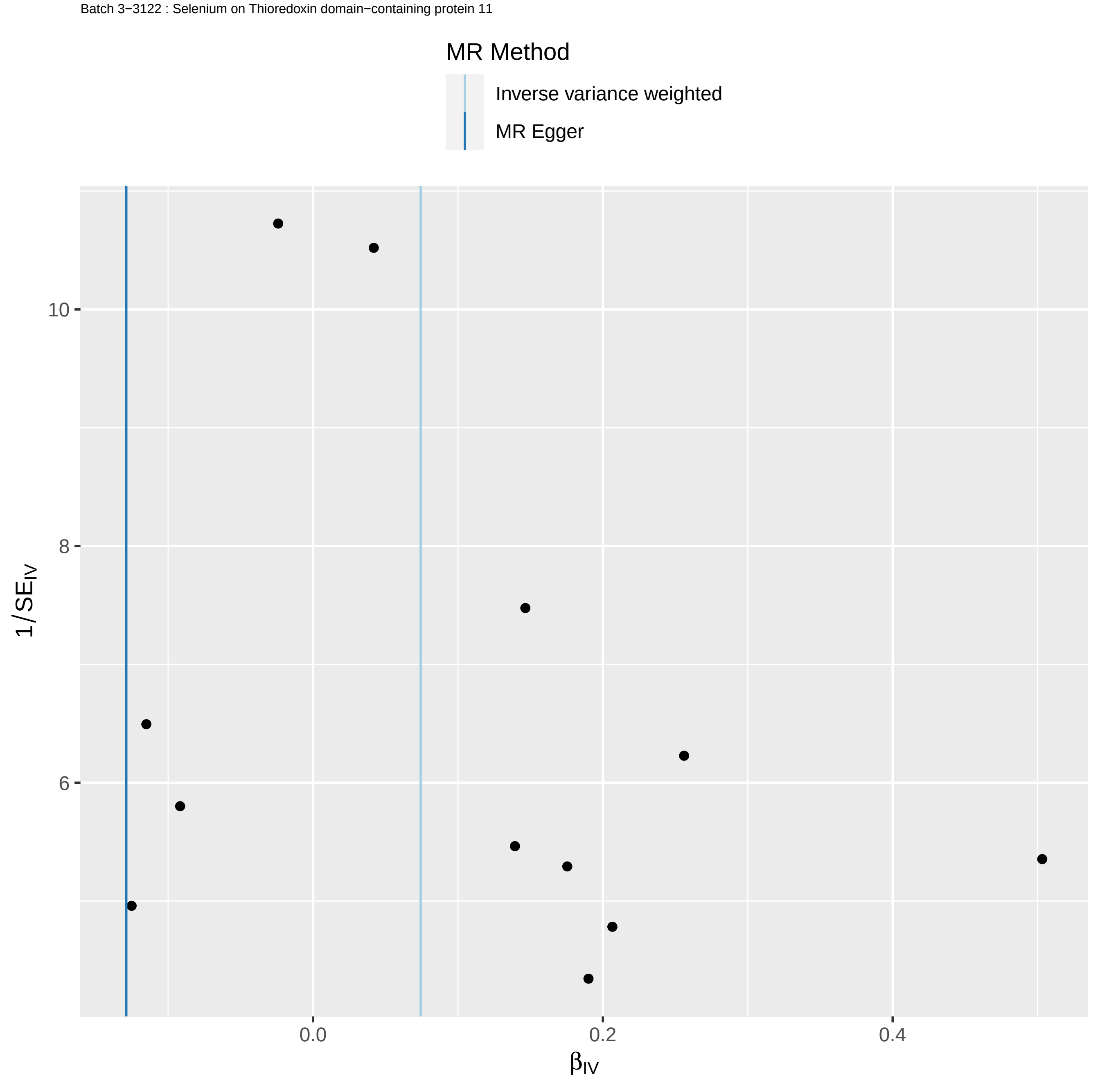
Batch 3−3122 : Selenium on Thioredoxin domain−containing protein 11
MR Method
Inverse variance weighted
MR Egger
10
8
6
1/SE$_{IV}$
0.0
0.2
0.4
β$_{IV}$

Batch 3−3123 : Selenium on Thioredoxin domain−containing protein 12

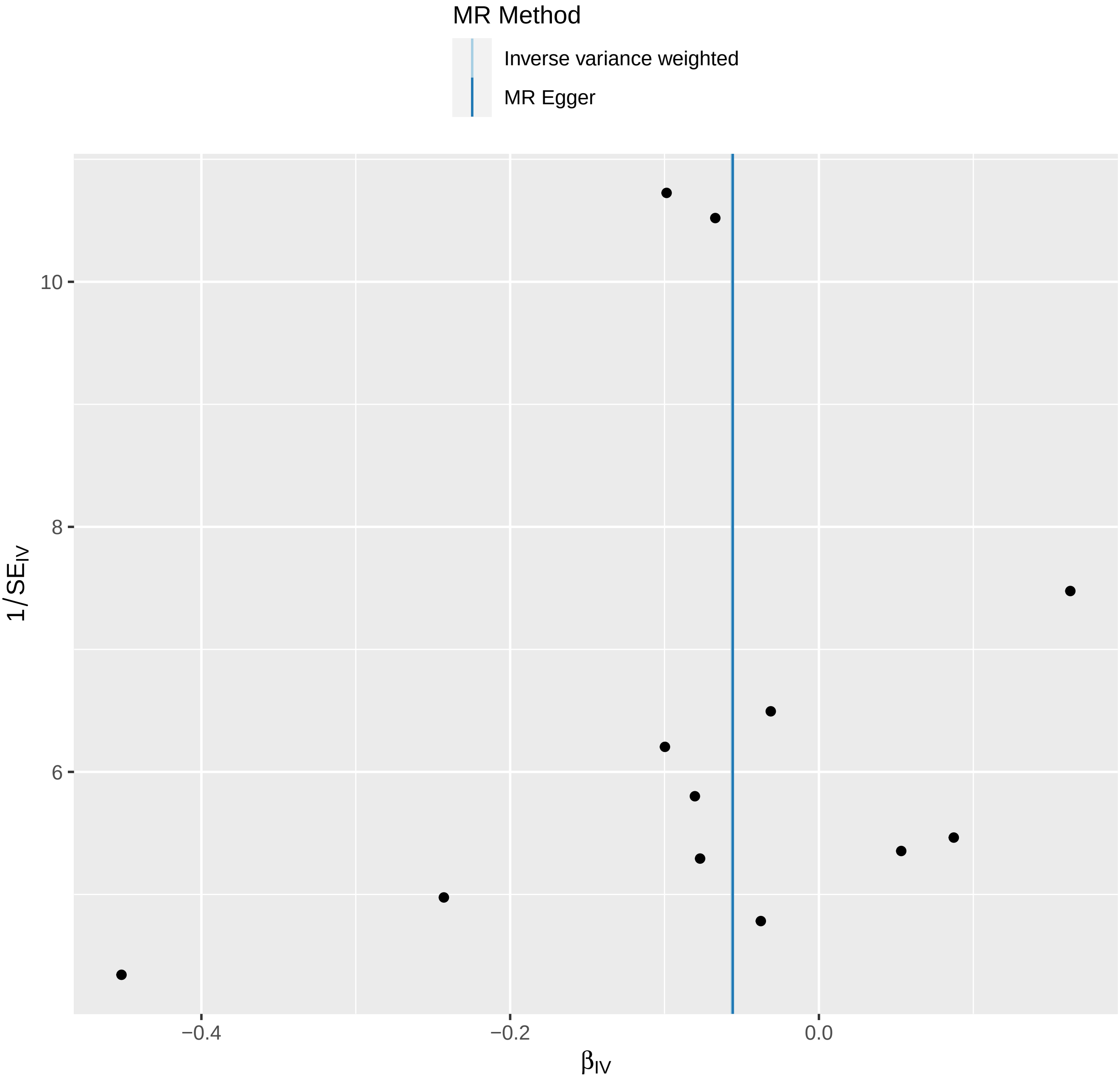

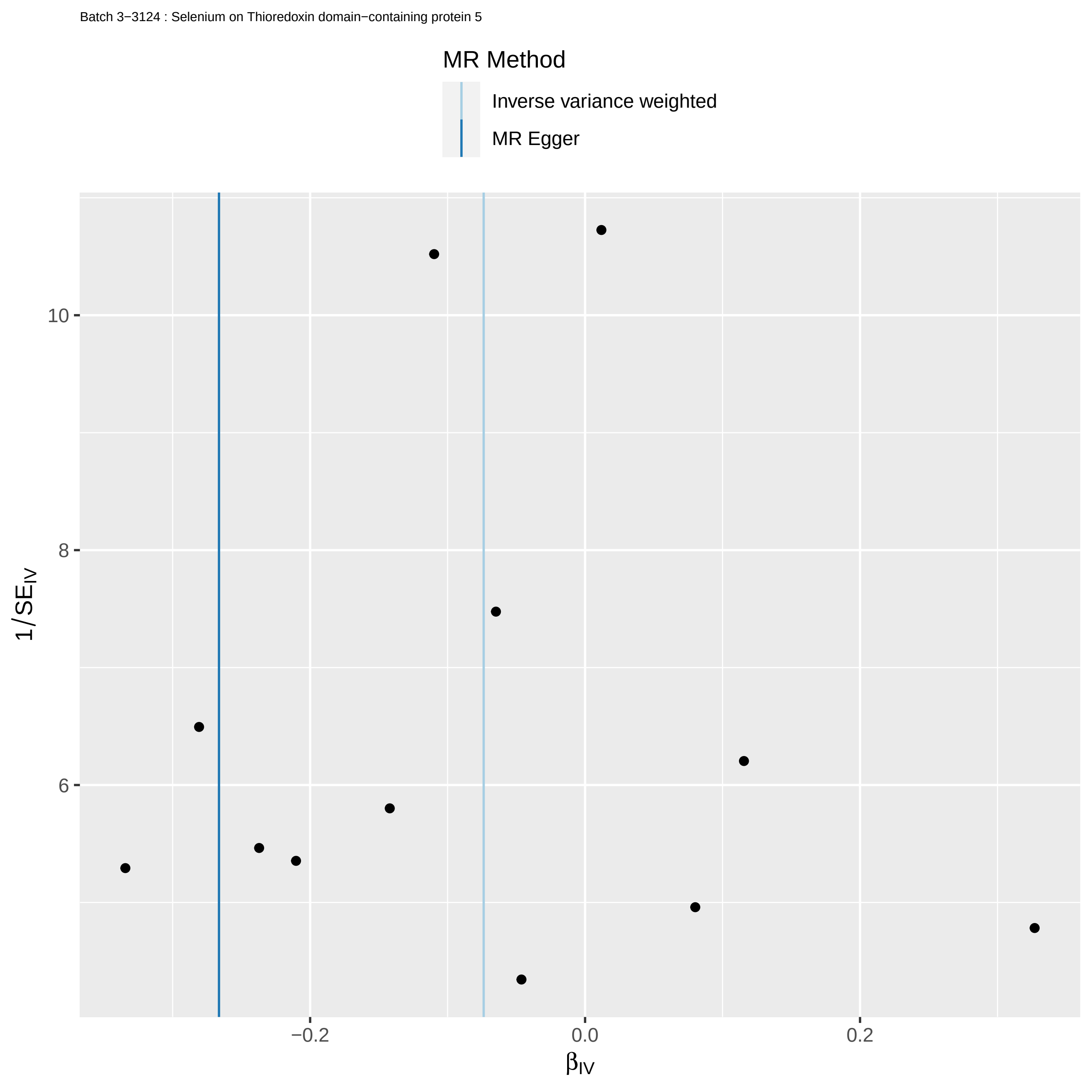
Batch 3−3124 : Selenium on Thioredoxin domain−containing protein 5
MR Method
Inverse variance weighted
MR Egger
10
8
6
1/SE$_{IV}$
−0.2
0.0
0.2
β$_{IV}$

Batch 3−3125 : Selenium on Thioredoxin−interacting protein

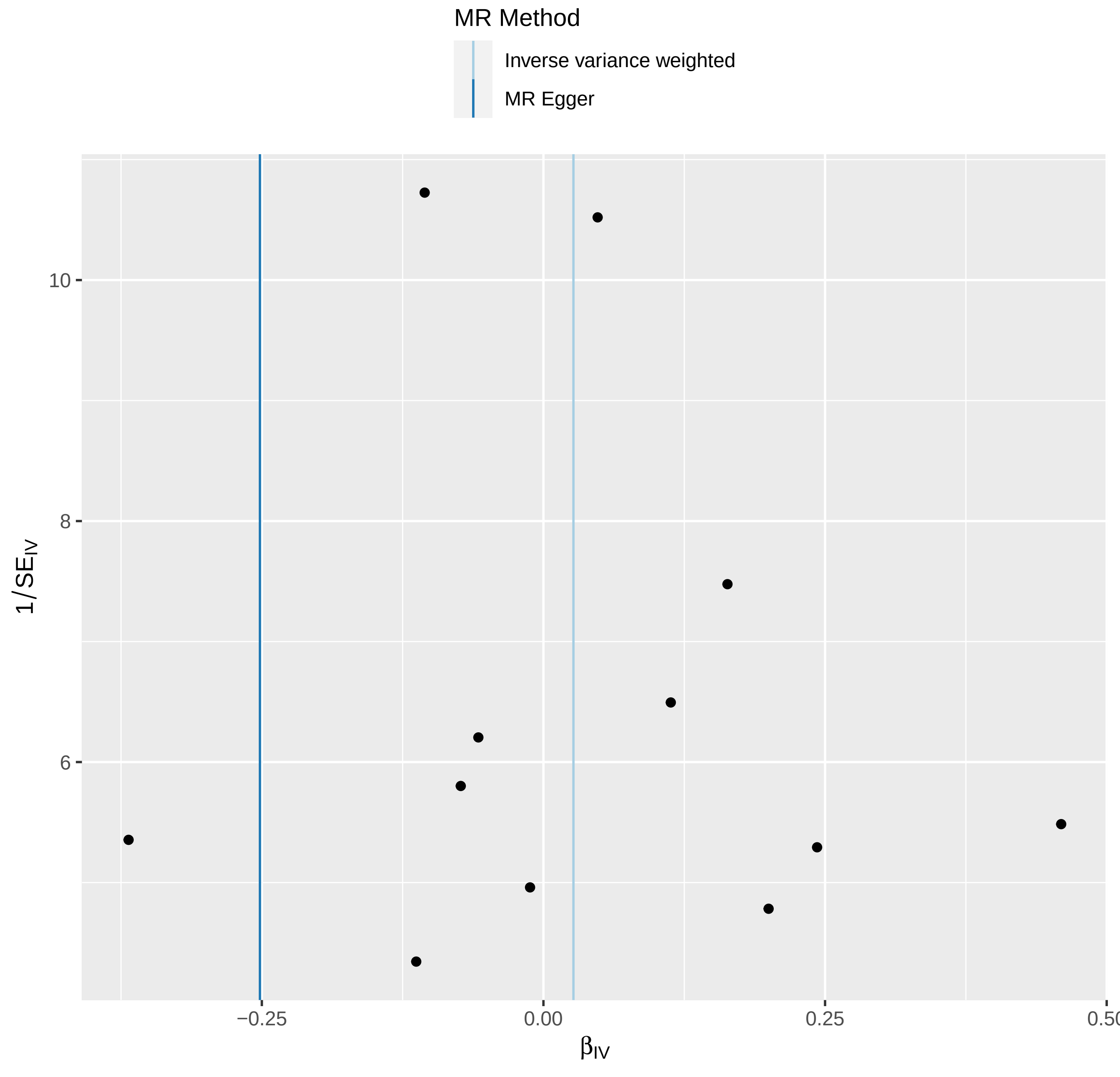

Batch 3−3126 : Selenium on Thioredoxin reductase 1, cytoplasmic

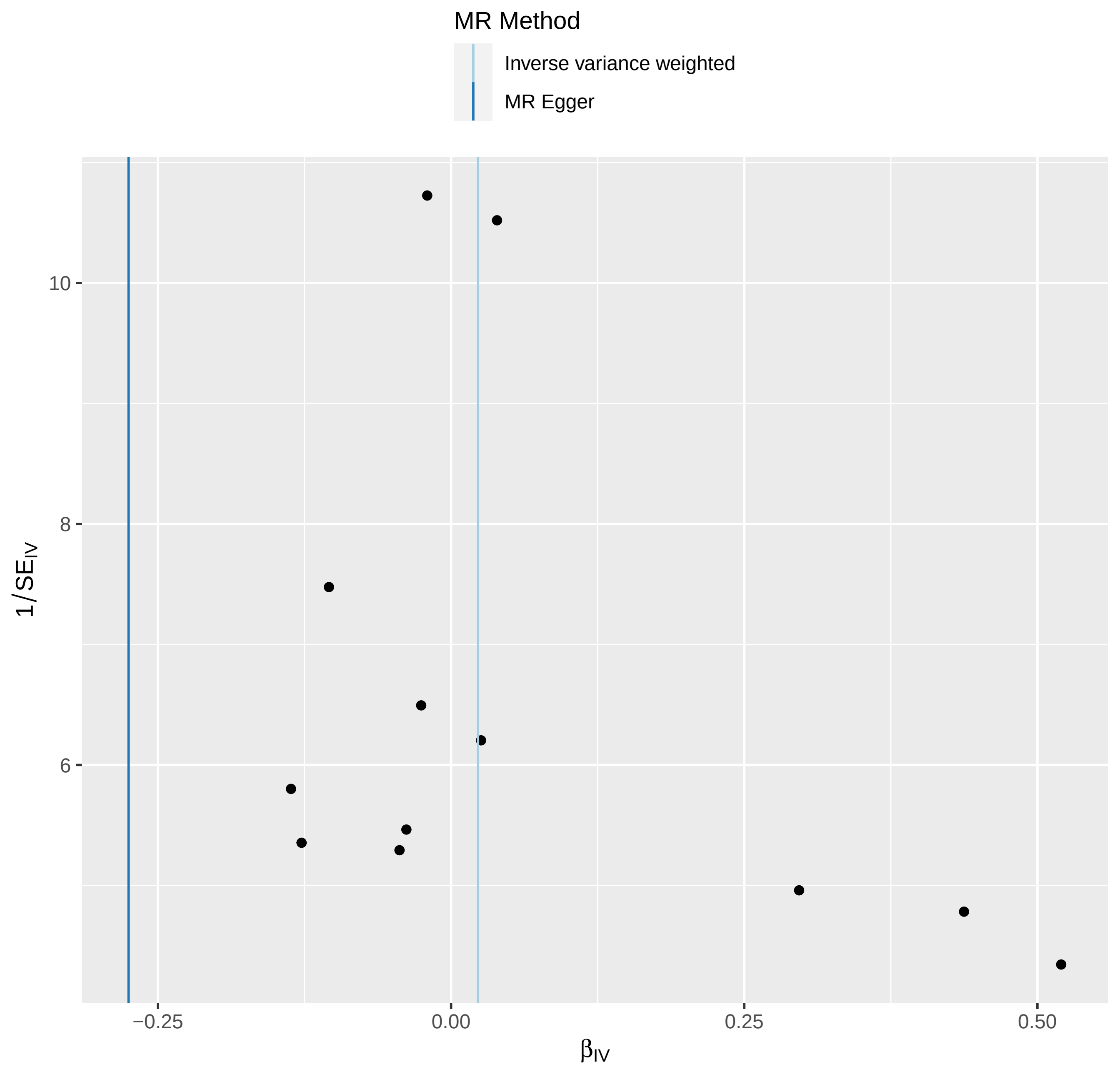

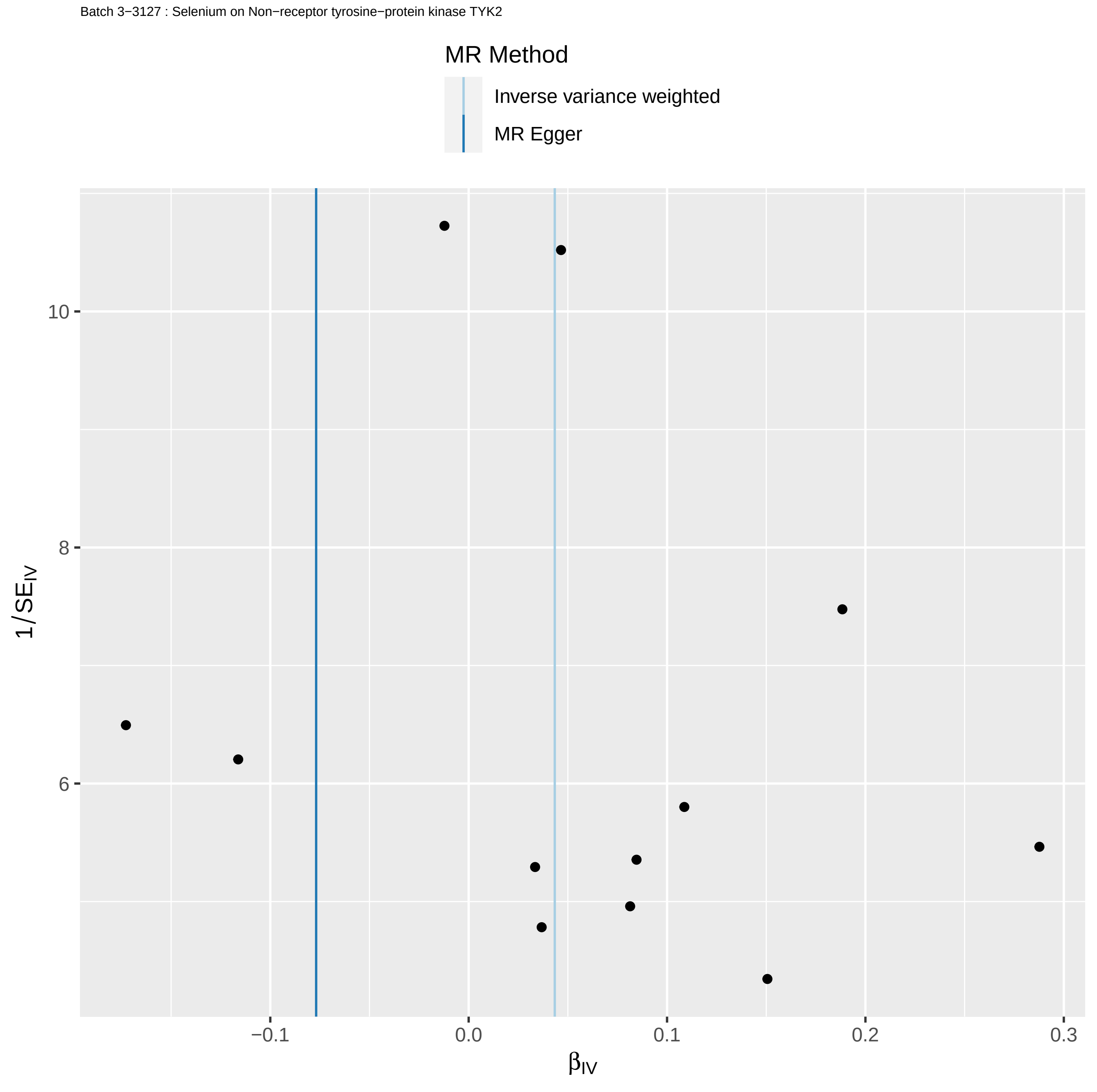

Batch 3−3127 : Selenium on Non−receptor tyrosine−protein kinase TYK2
MR Method
Inverse variance weighted
MR Egger
10
8
6
1/SE$_{IV}$
−0.1
0.0
0.1
0.2
0.3
β$_{IV}$

Batch 3−3128 : Selenium on TYMS opposite strand protein

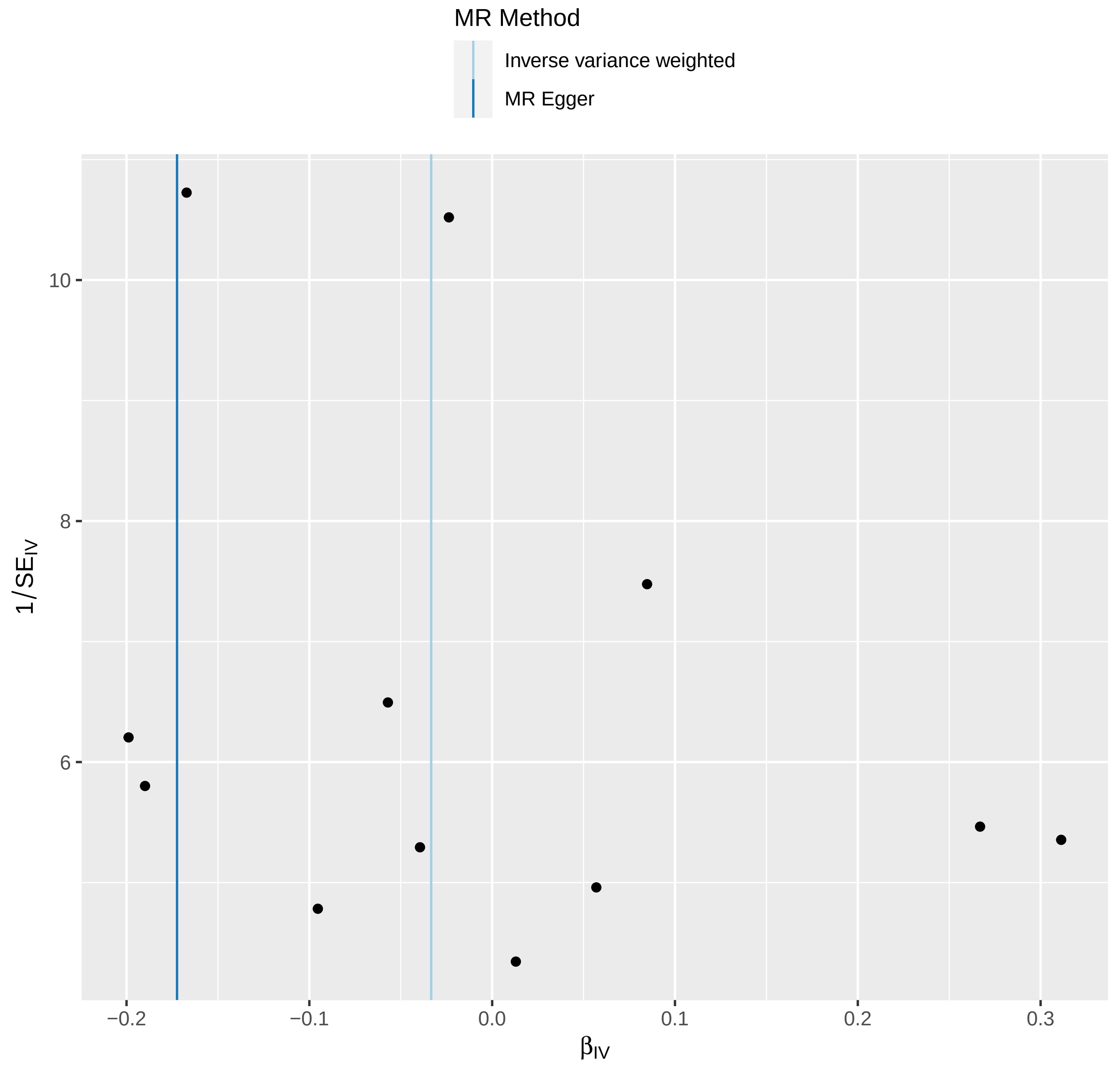

Batch 3−3129 : Selenium on Tyrosine−protein kinase receptor TYRO3

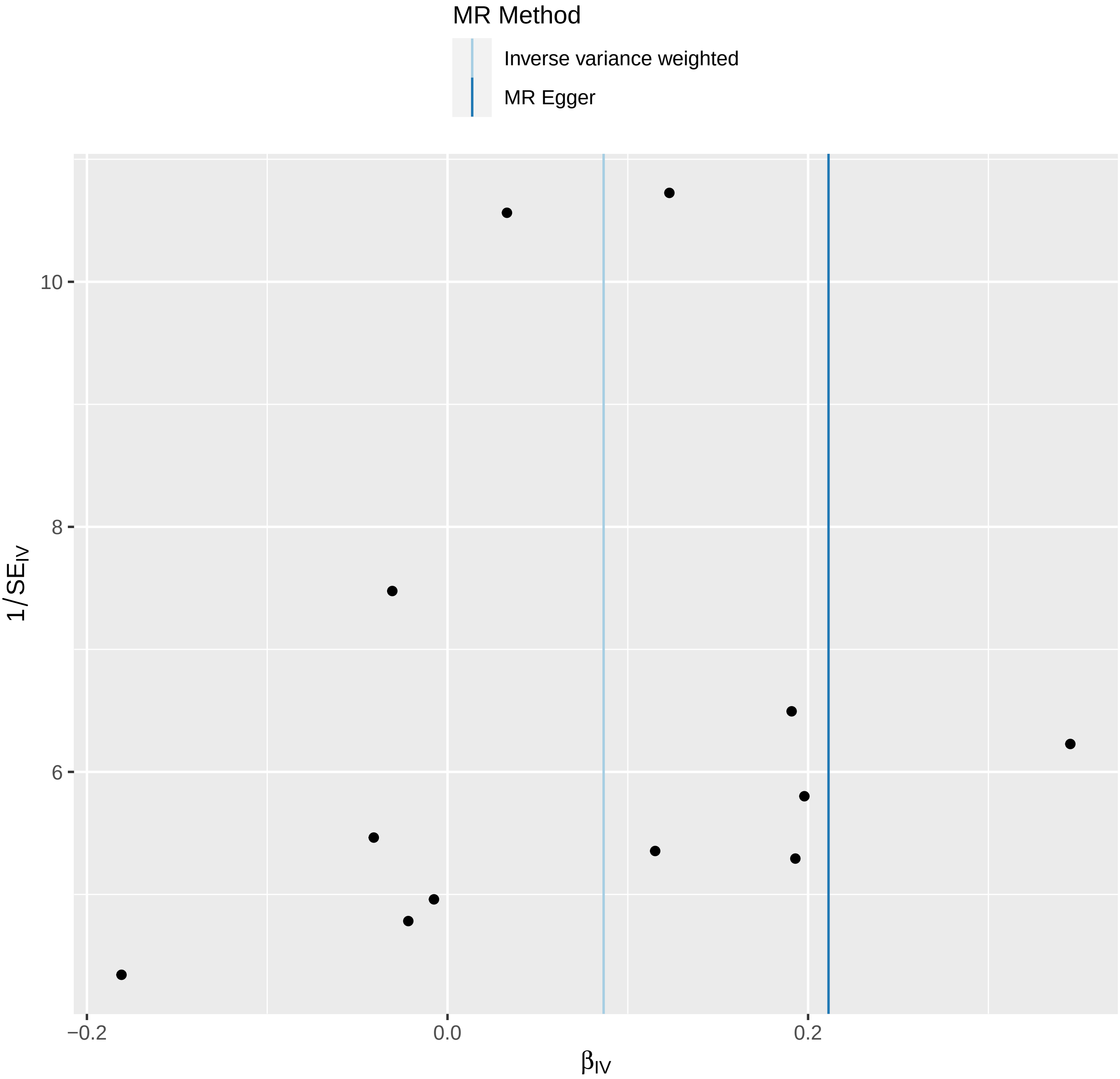

Batch 3−3130 : Selenium on Splicing factor U2AF 65 kDa subunit

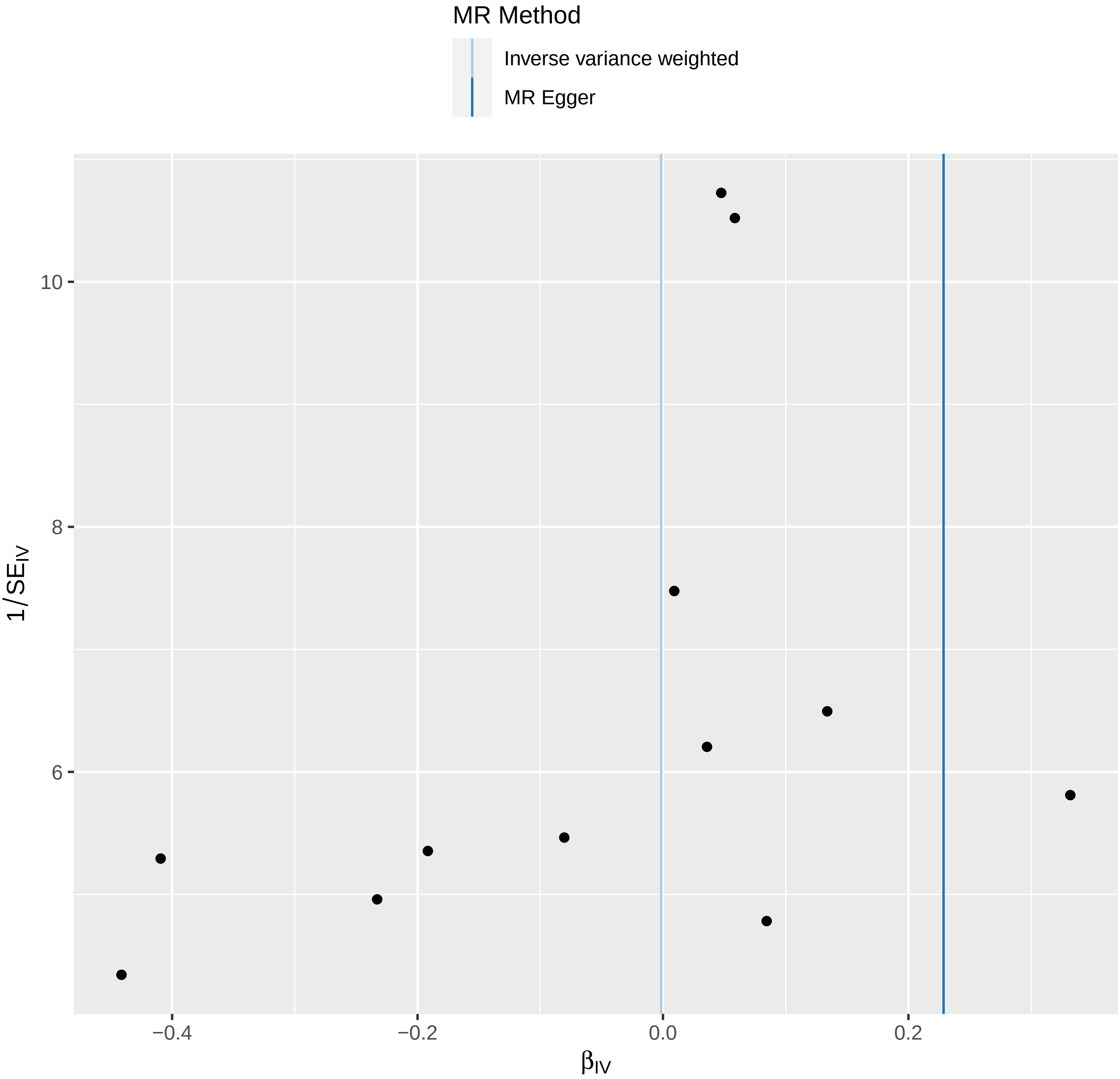

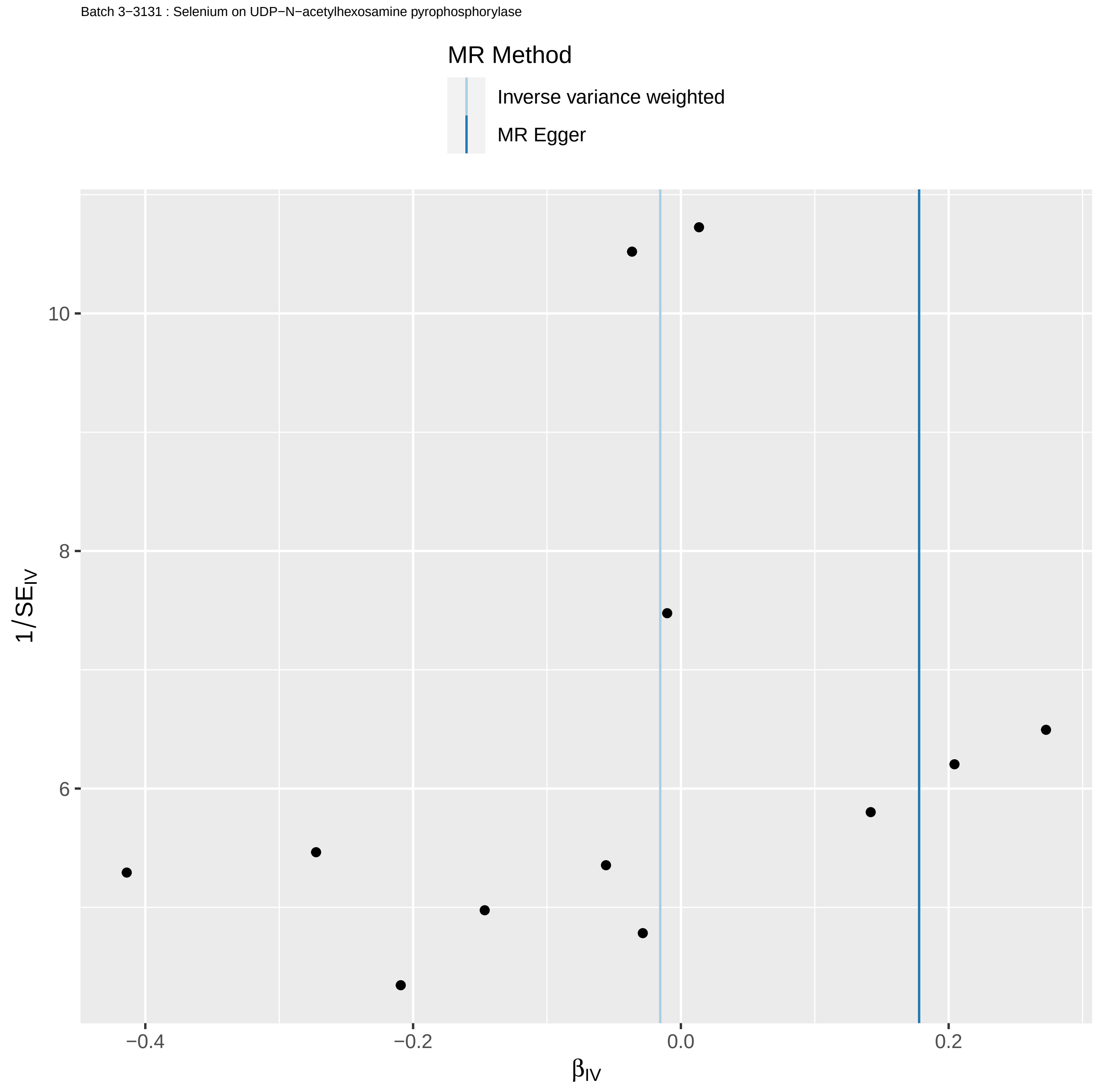
Batch 3−3131 : Selenium on UDP−N−acetylhexosamine pyrophosphorylase
MR Method
Inverse variance weighted
MR Egger
10
8
6
1/SE$_{IV}$
−0.4
−0.2
0.0
0.2
β$_{IV}$

Batch 3−3132 : Selenium on PolyUbiquitin K48−linked

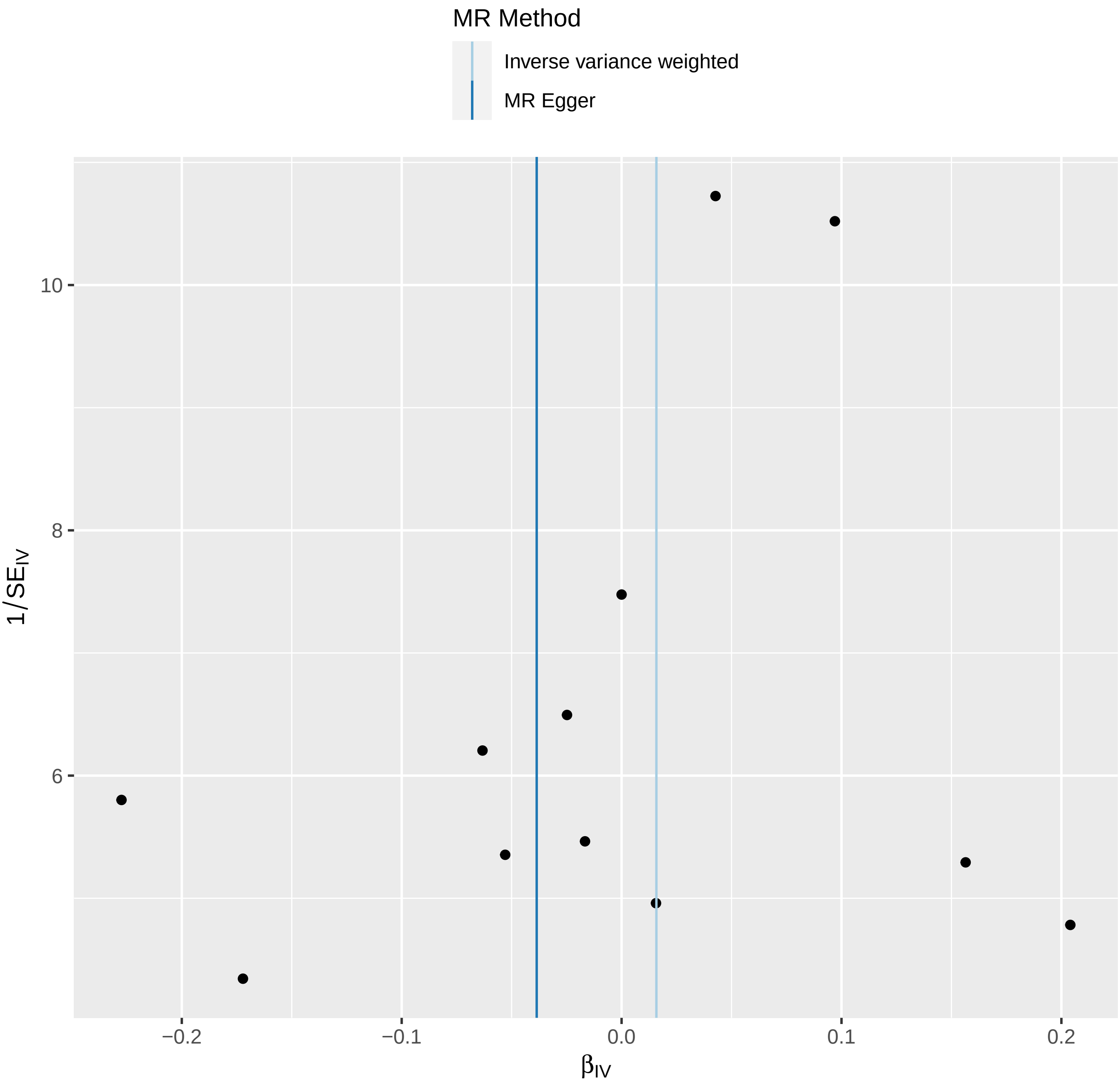

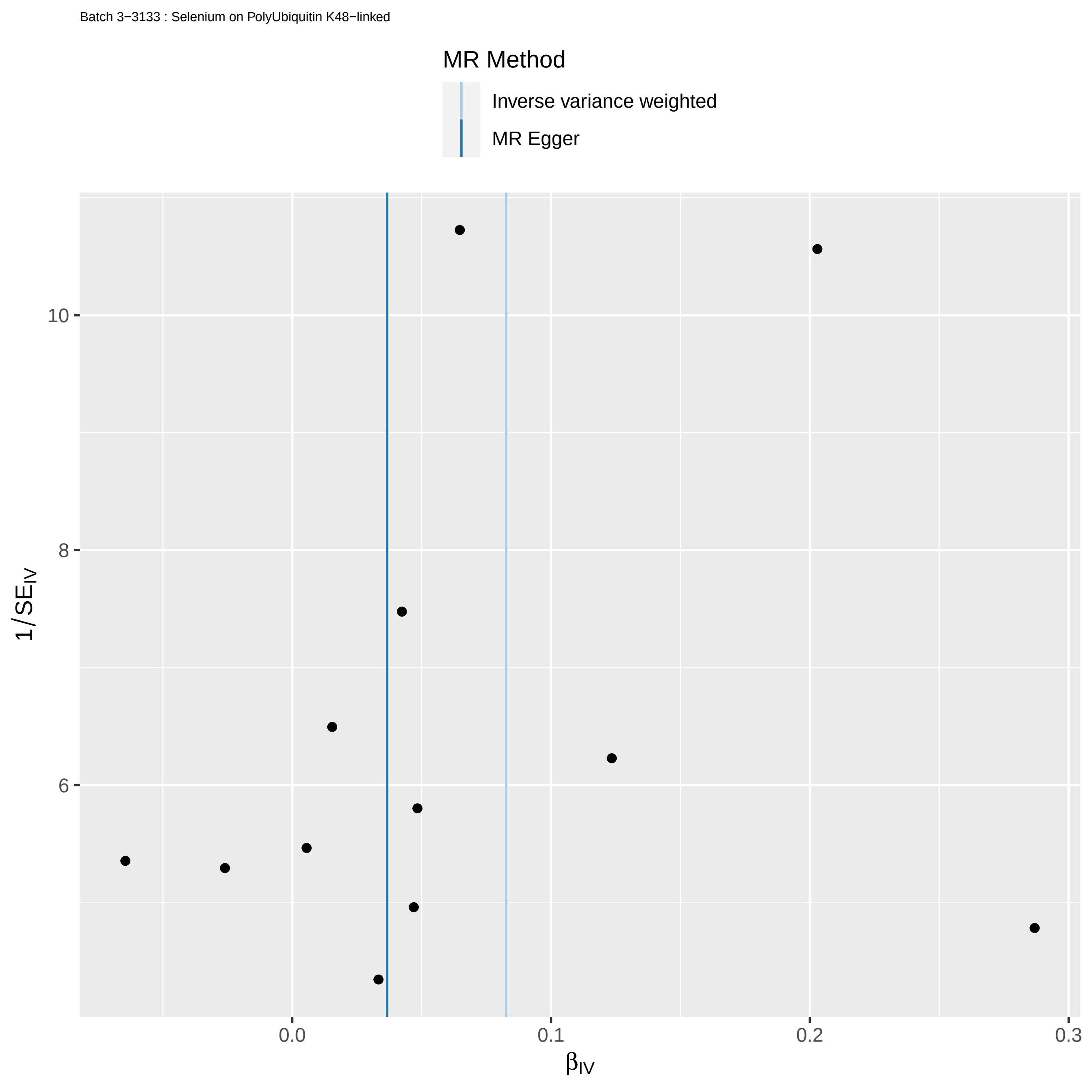

Batch 3−3133 : Selenium on PolyUbiquitin K48−linked
MR Method
Inverse variance weighted
MR Egger
10
8
6
1/SE$_{IV}$
0.0
0.1
0.2
0.3
β$_{IV}$

Batch 3−3134 : Selenium on PolyUbiquitin K63−linked

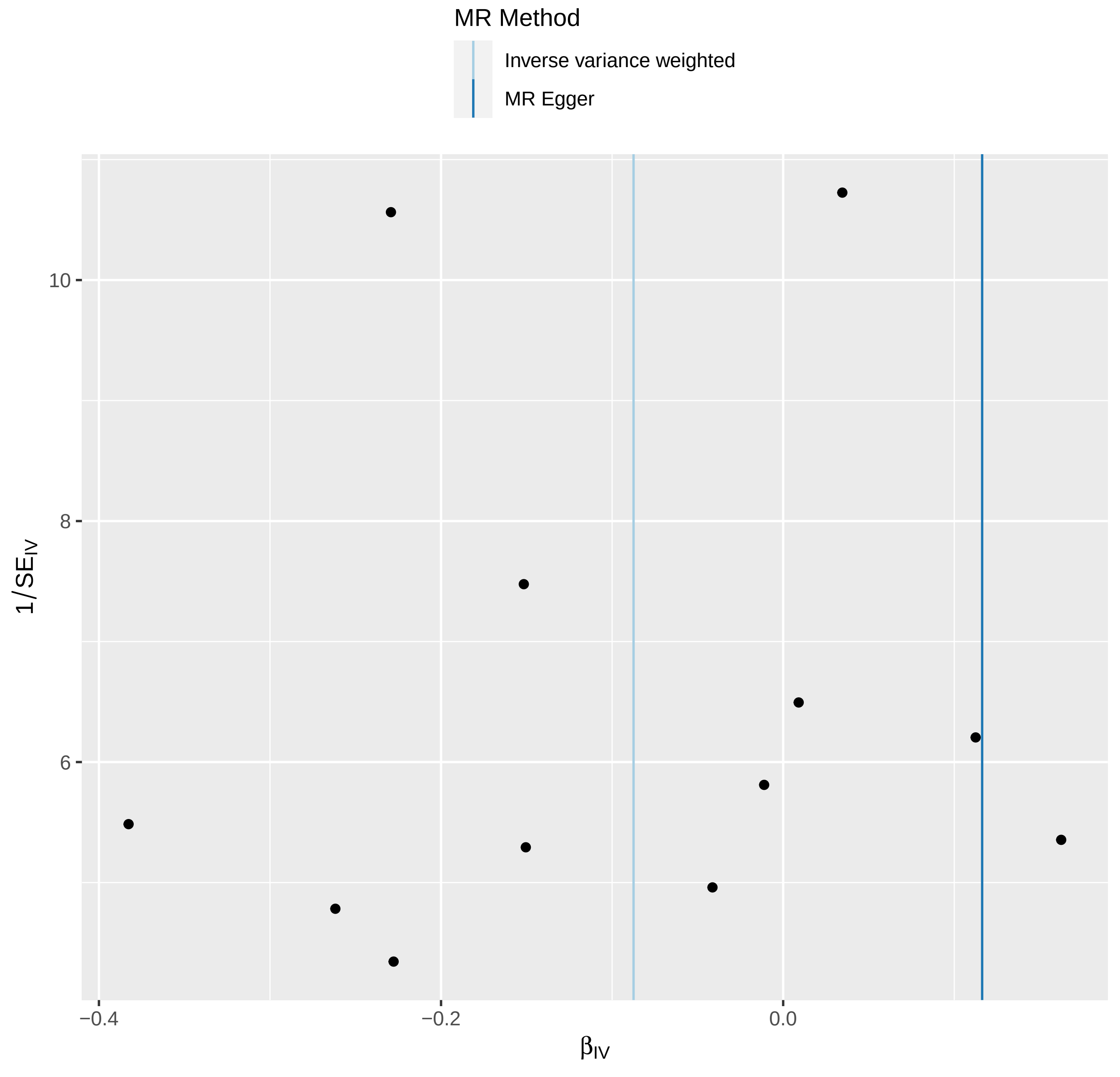

Batch 3−3135 : Selenium on Ubiquitin−conjugating enzyme E2 B

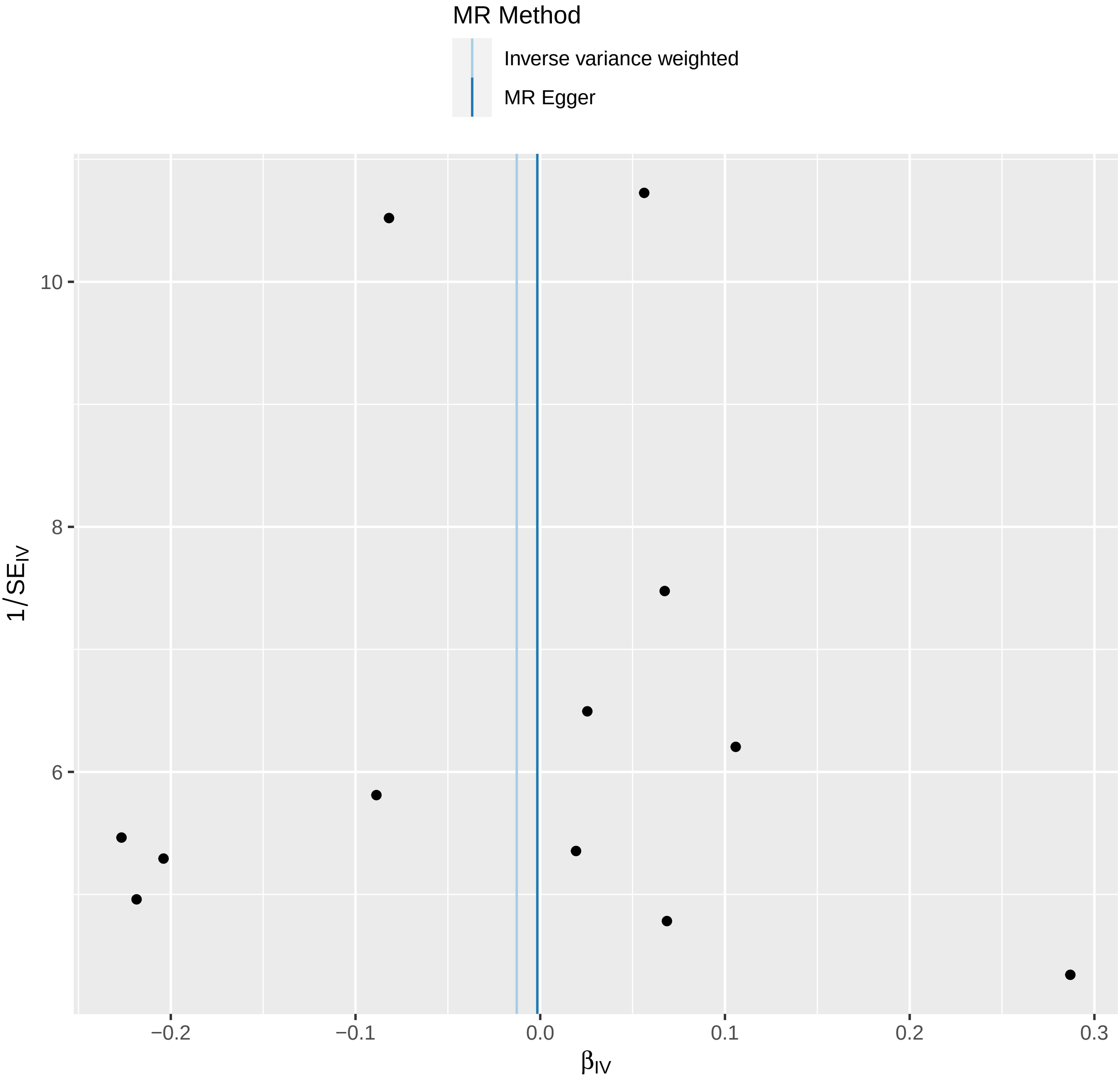

Batch 3−3136 : Selenium on Ubiquitin−conjugating enzyme E2 D4

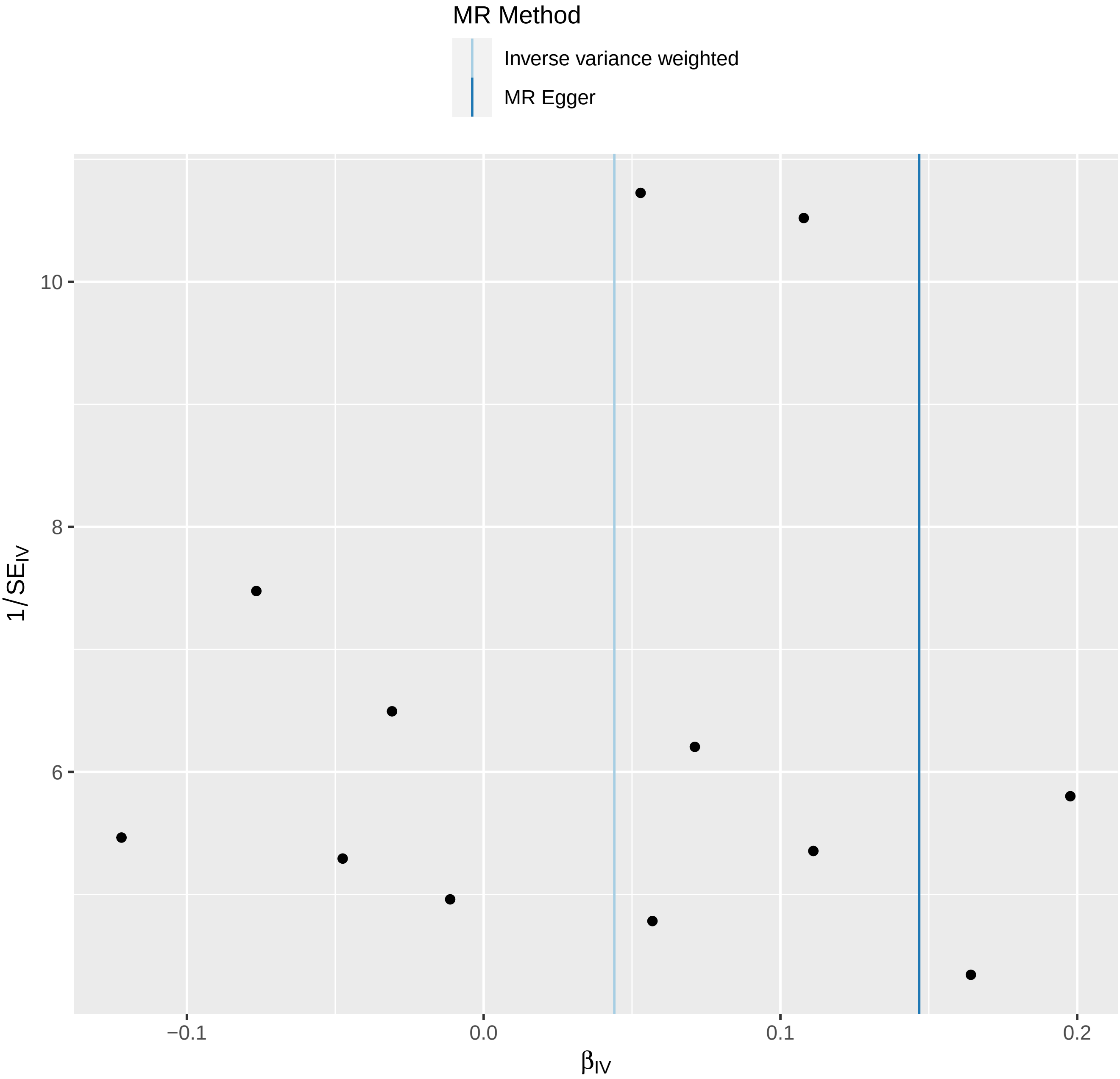

Batch 3−3137 : Selenium on Ubiquitin−conjugating enzyme E2 G2

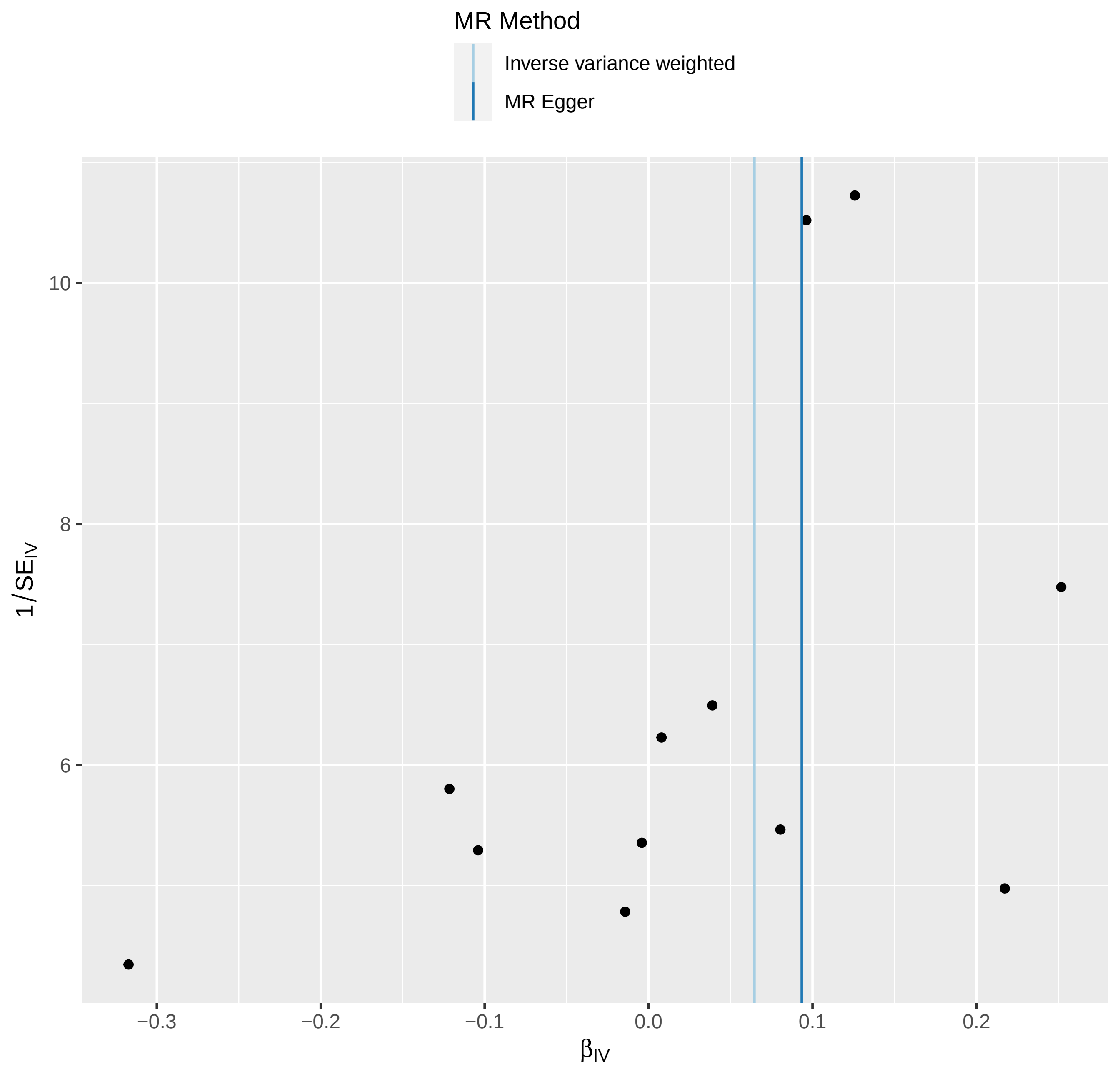

Batch 3−3138 : Selenium on SUMO−conjugating enzyme UBC9

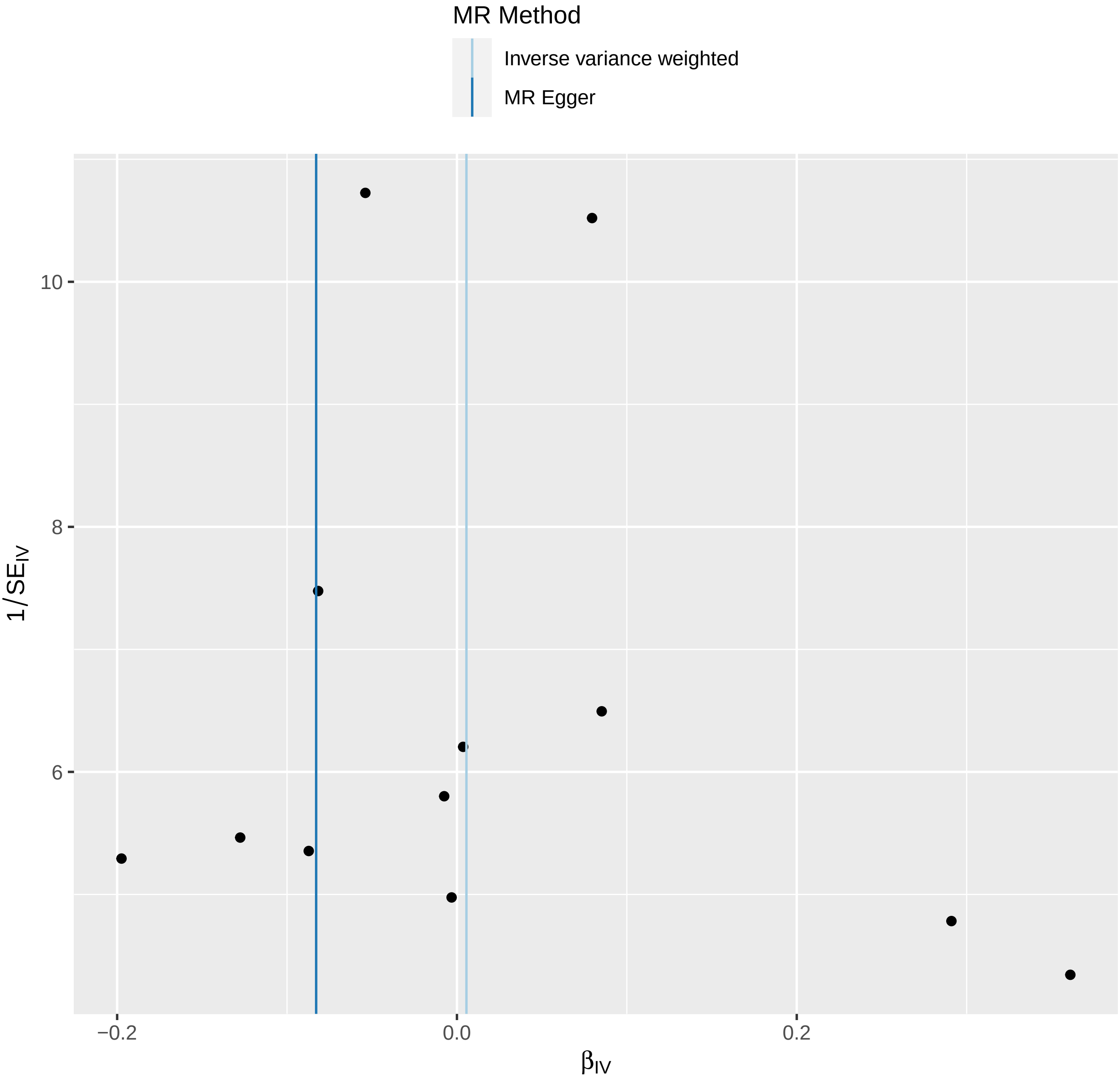

Batch 3−3139 : Selenium on Ubiquitin−conjugating enzyme E2 J1

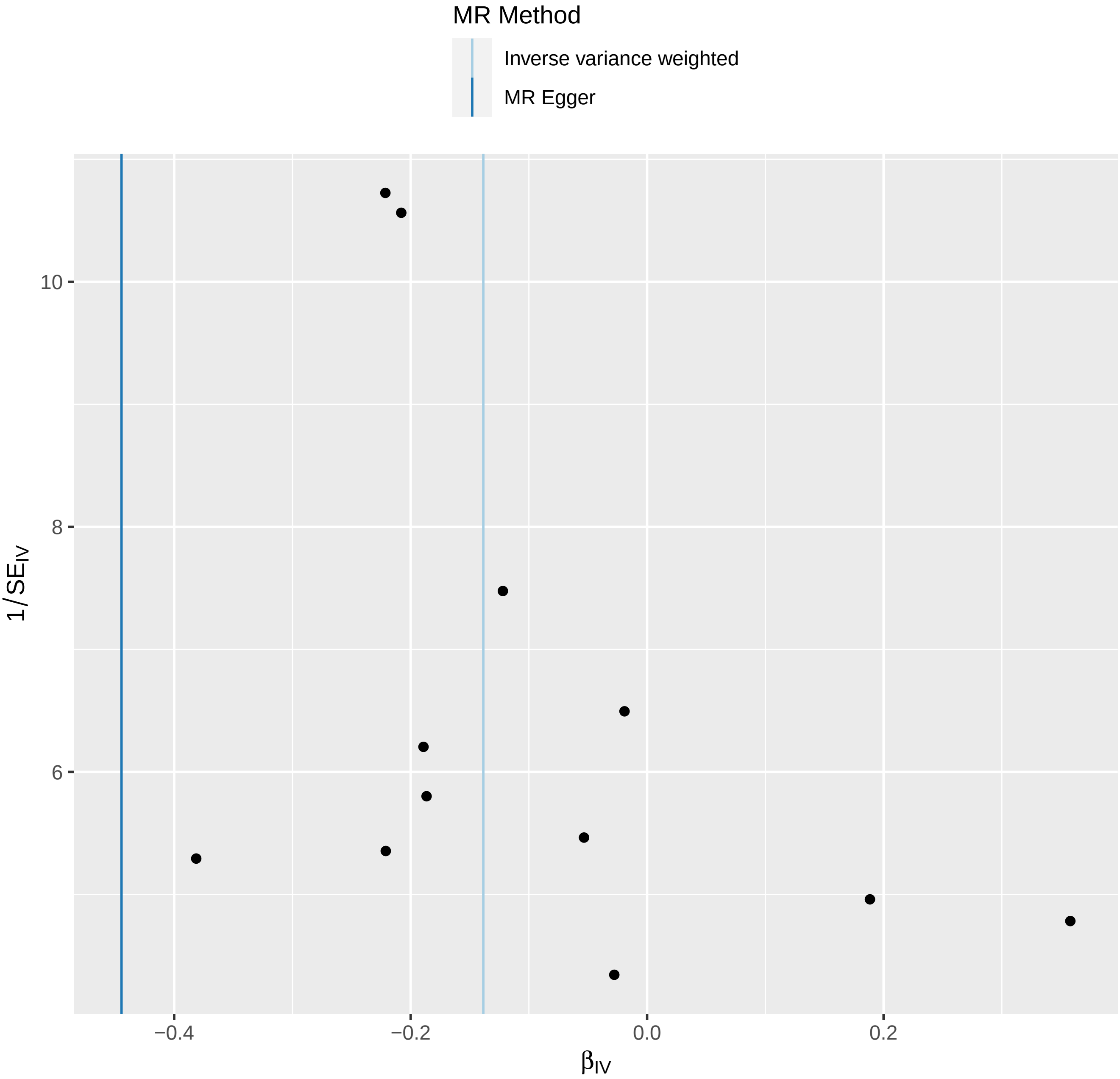

Batch 3−3140 : Selenium on Ubiquitin−conjugating enzyme E2 J2

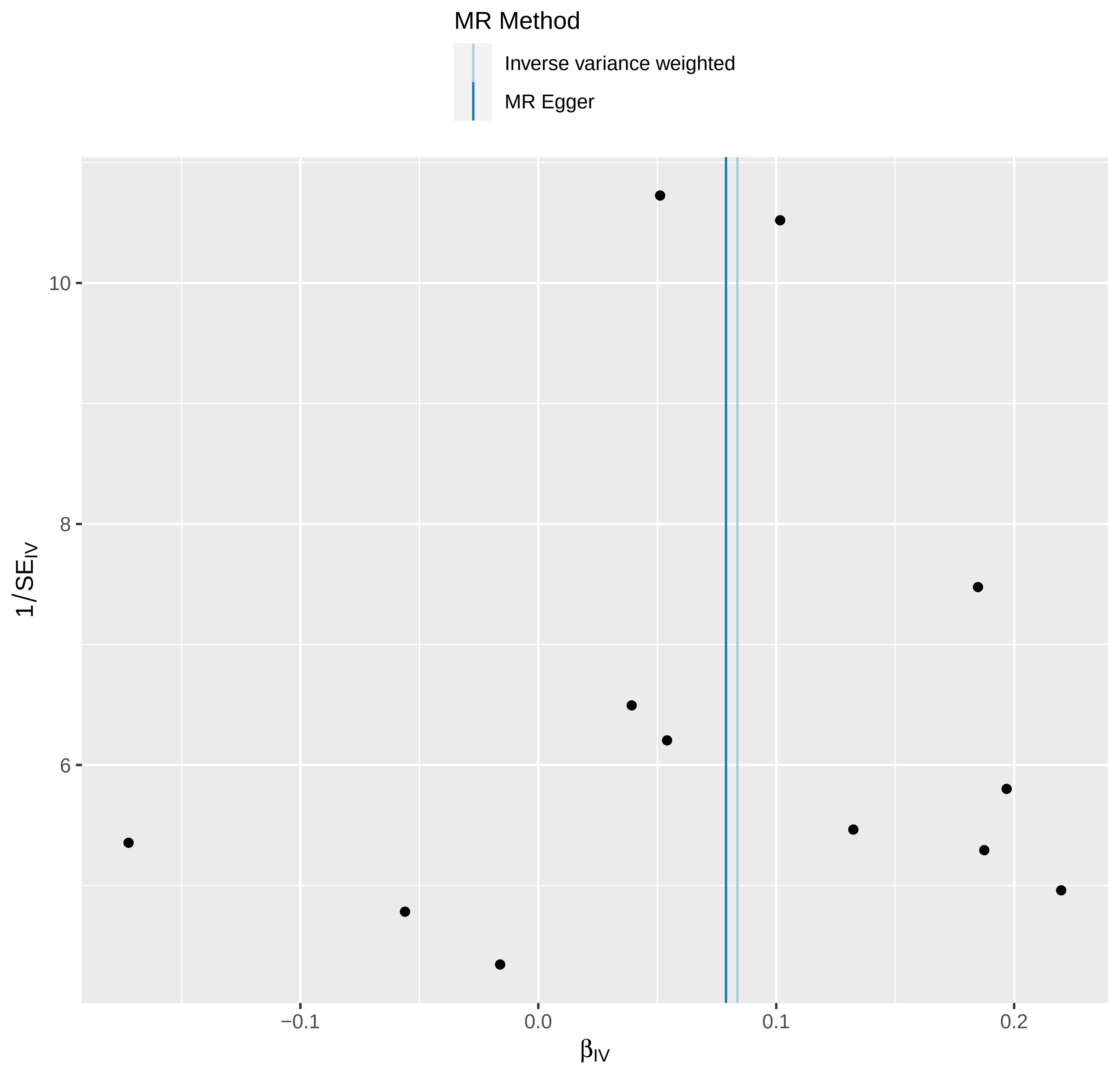

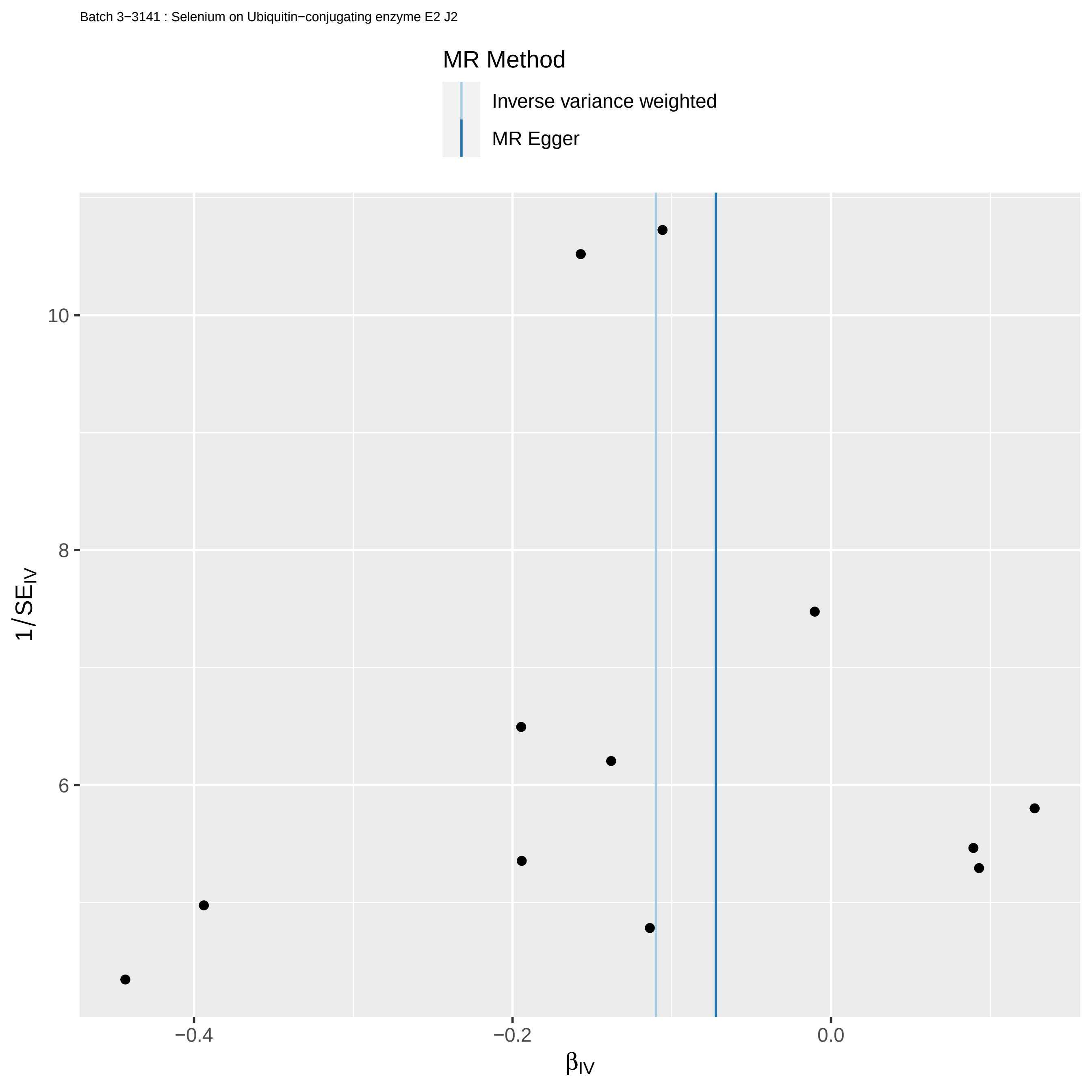
Batch 3−3141 : Selenium on Ubiquitin−conjugating enzyme E2 J2
MR Method
Inverse variance weighted
MR Egger
10
8
6
1/SE_IV
−0.4
−0.2
0.0
β_IV

Batch 3−3142 : Selenium on Ubiquitin−conjugating enzyme E2 L3

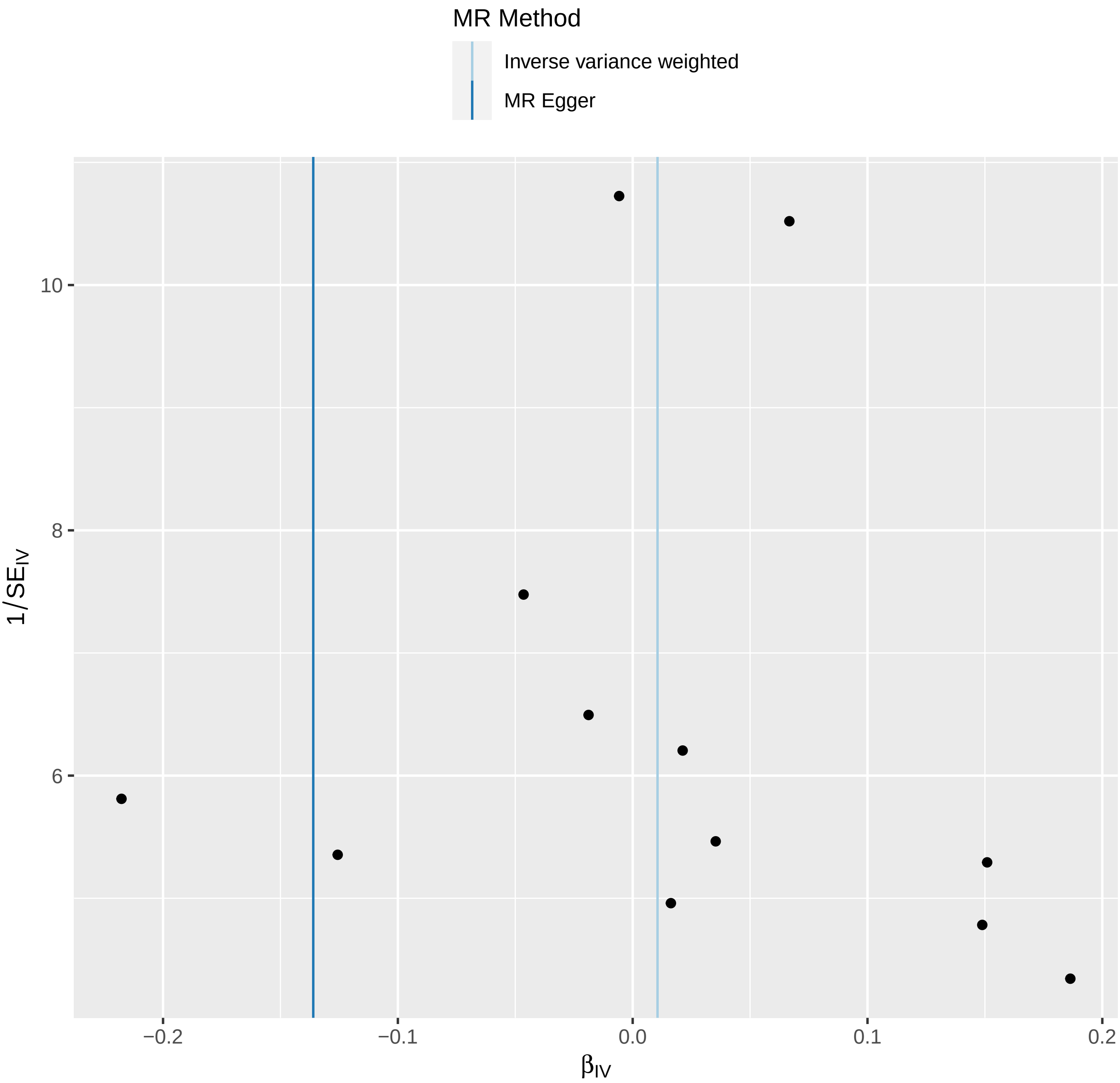

Batch 3−3143 : Selenium on Ubiquitin−conjugating enzyme E2 N

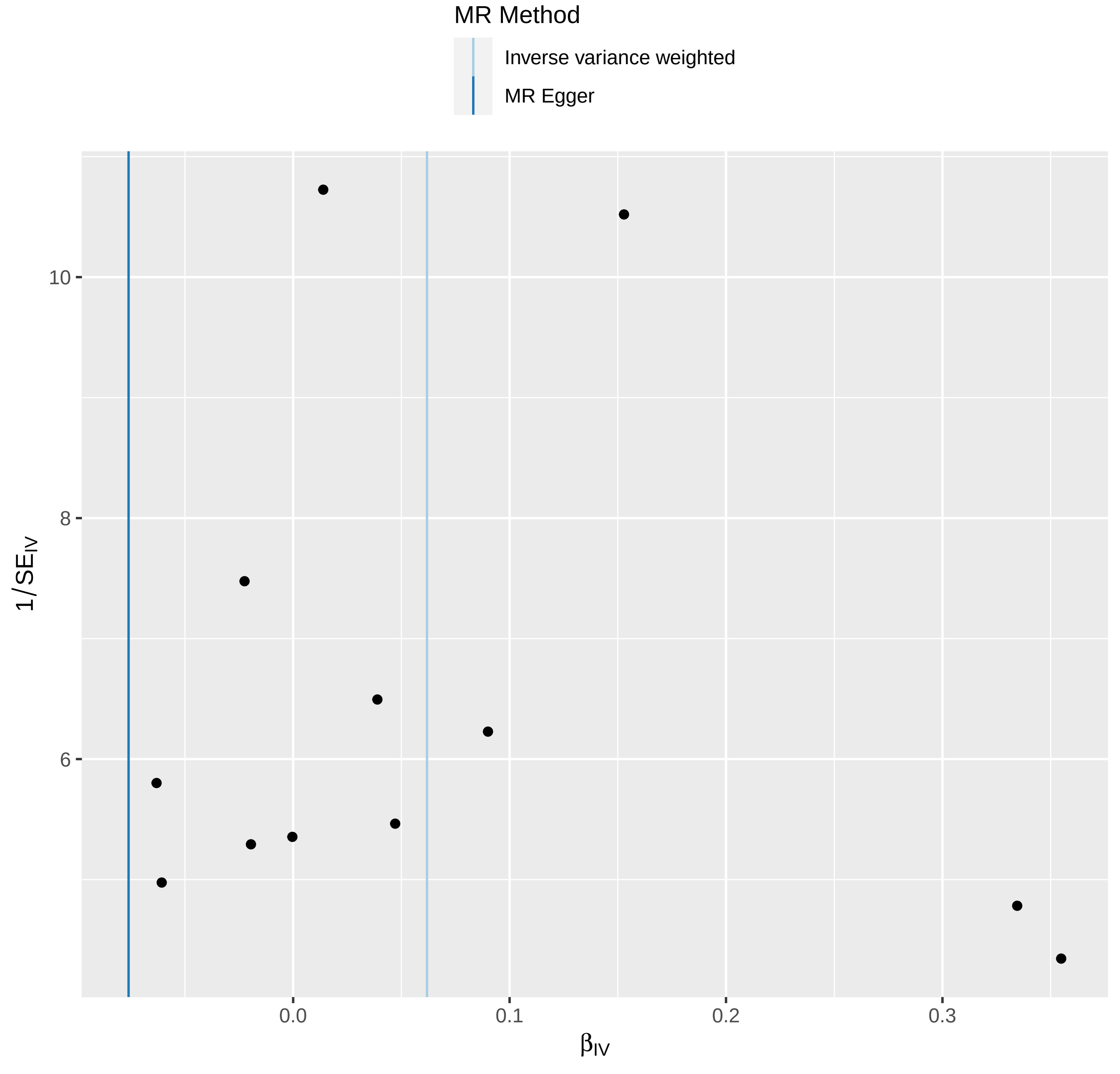

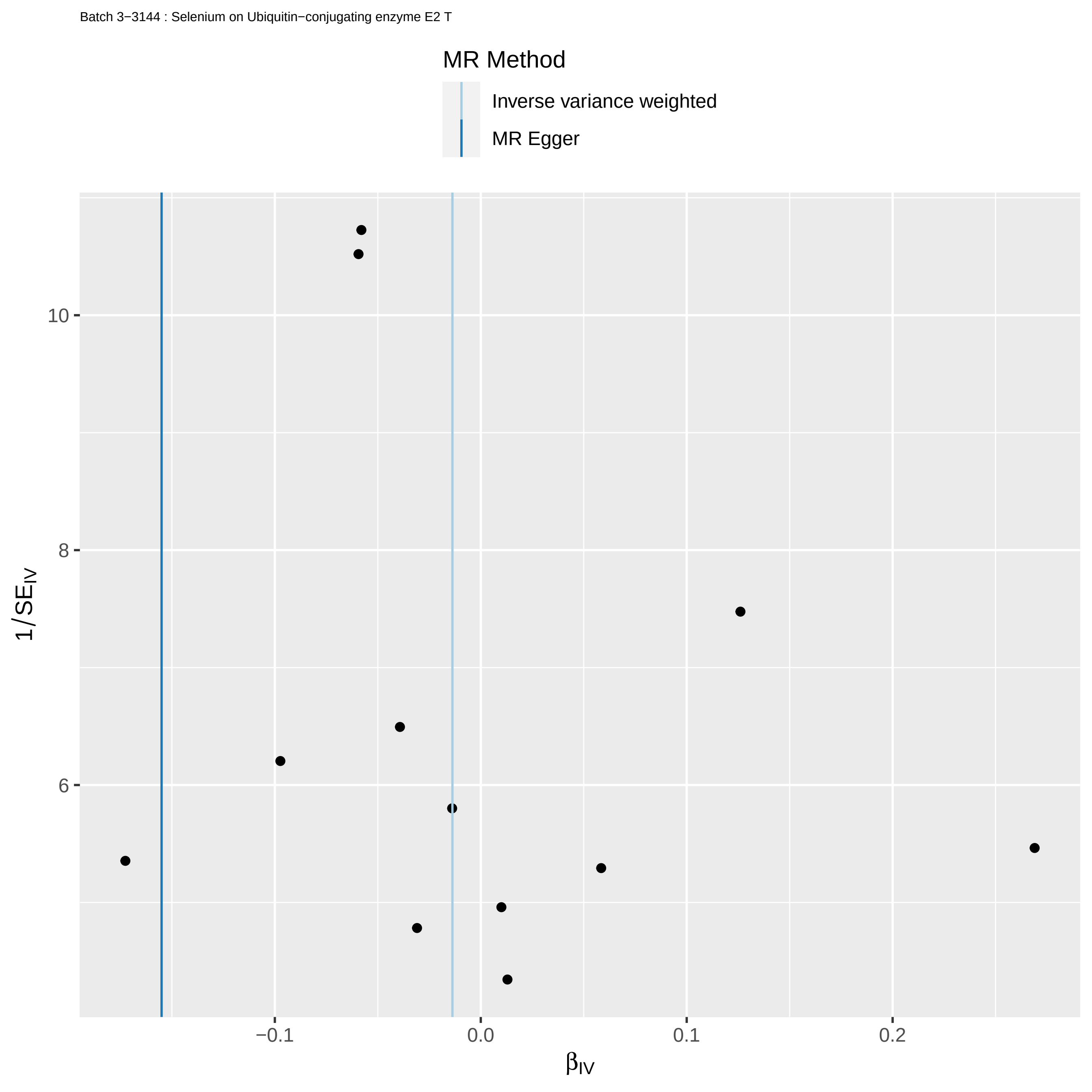

Batch 3−3144 : Selenium on Ubiquitin−conjugating enzyme E2 T
MR Method
Inverse variance weighted
MR Egger
10
8
6
1/SE$_{IV}$
−0.1
0.0
0.1
0.2
β$_{IV}$

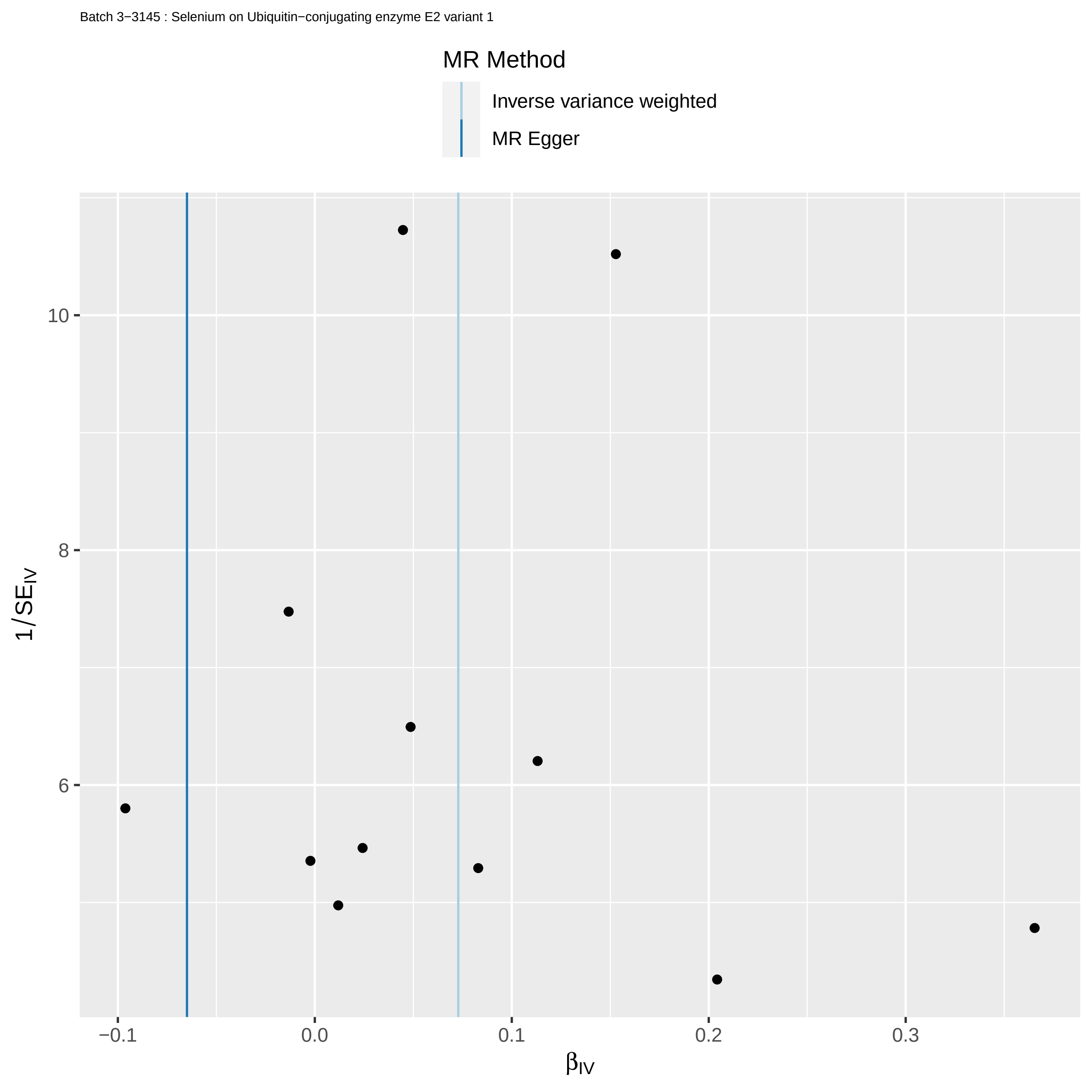

Batch 3−3145 : Selenium on Ubiquitin−conjugating enzyme E2 variant 1
MR Method
Inverse variance weighted
MR Egger
10
8
6
1/SE_IV
−0.1
0.0
0.1
0.2
0.3
β_IV

Batch 3−3146 : Selenium on Ubiquitin−like protein 4A

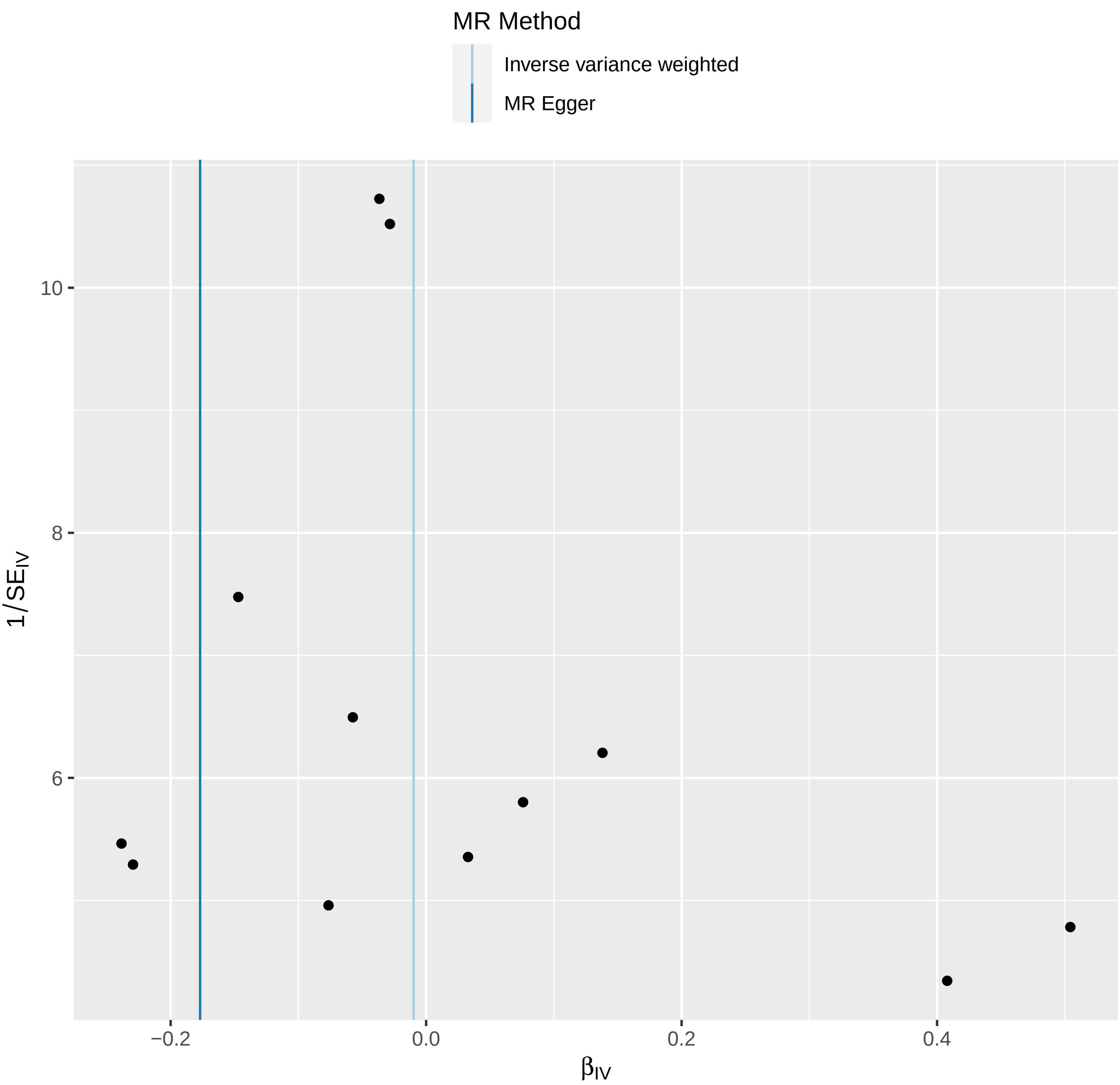

Batch 3−3147 : Selenium on Ubiquilin−4

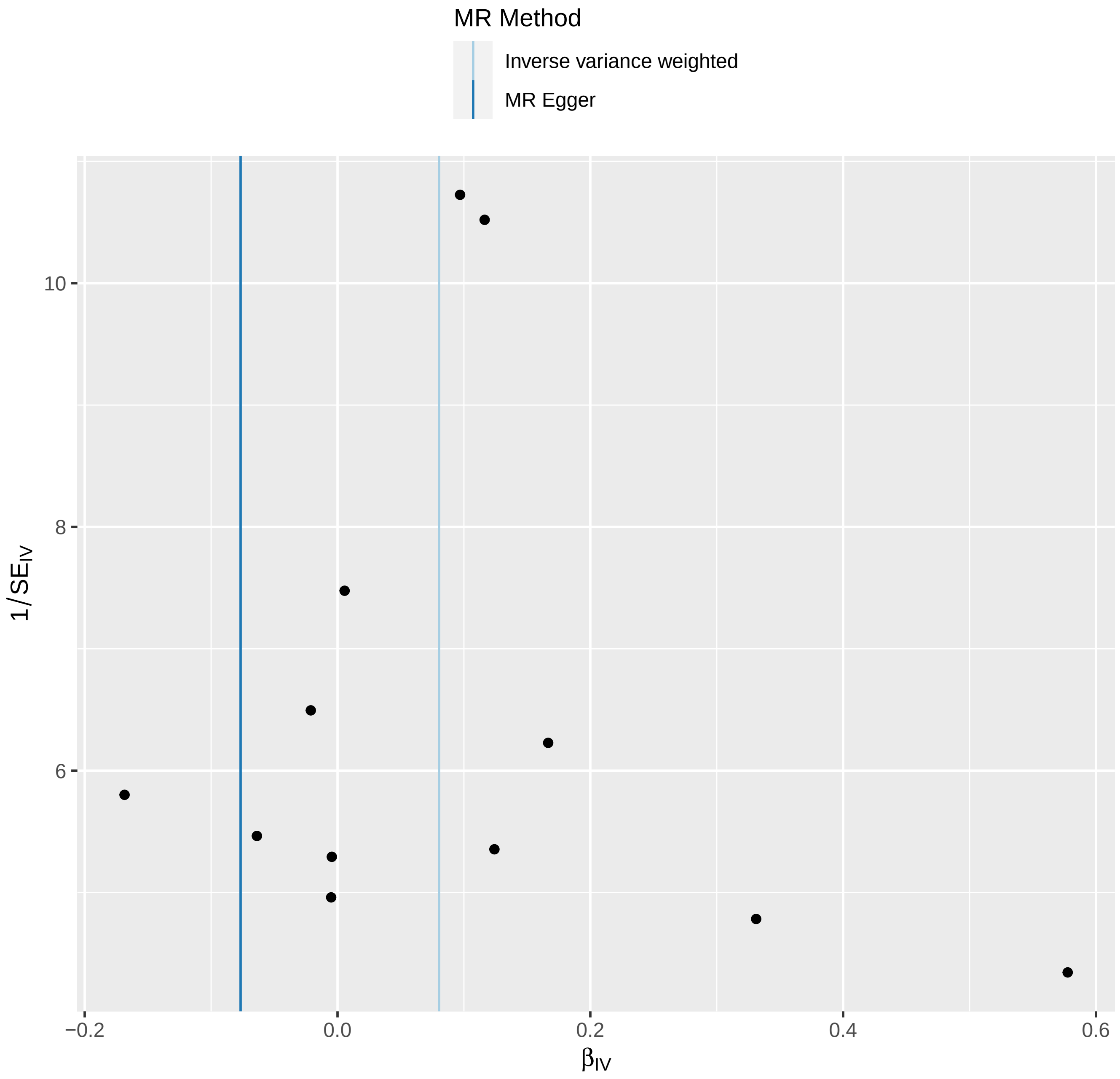

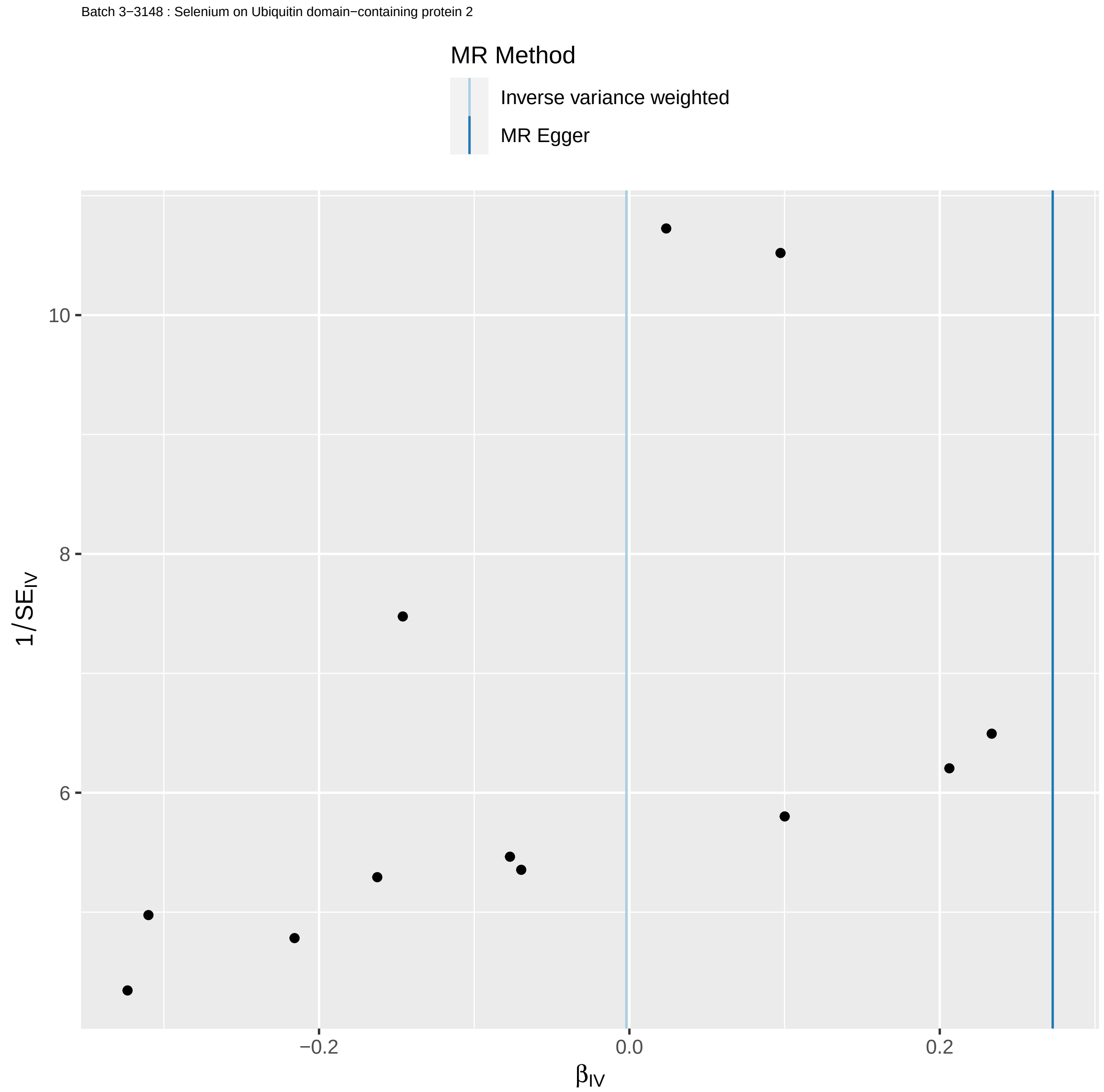

Batch 3−3148 : Selenium on Ubiquitin domain−containing protein 2
MR Method
Inverse variance weighted
MR Egger
10
8
6
1/SE_IV
−0.2
0.0
0.2
β_IV

Batch 3−3149 : Selenium on UBX domain−containing protein 4

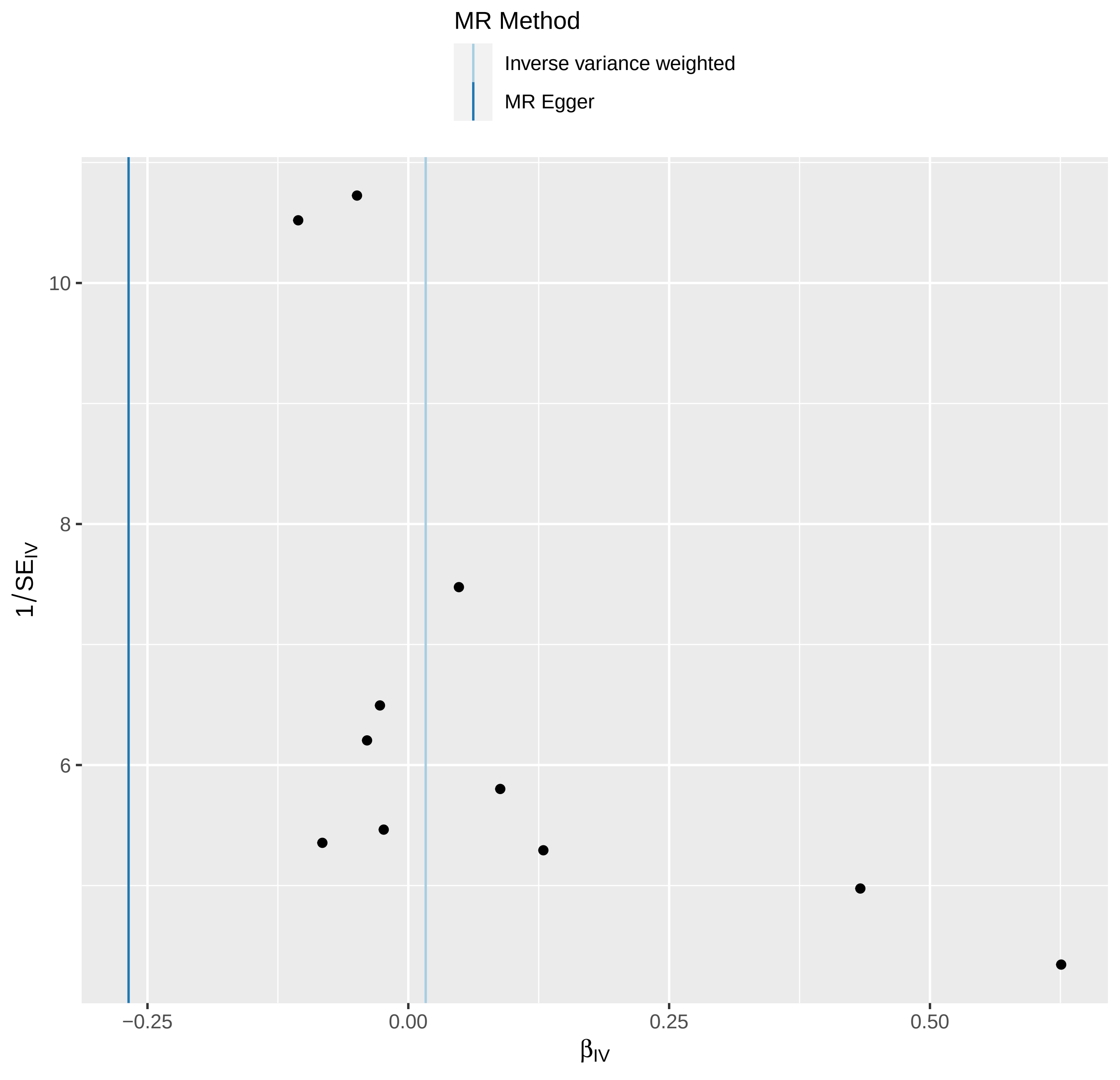

Batch 3−3150 : Selenium on UBX domain−containing protein 4

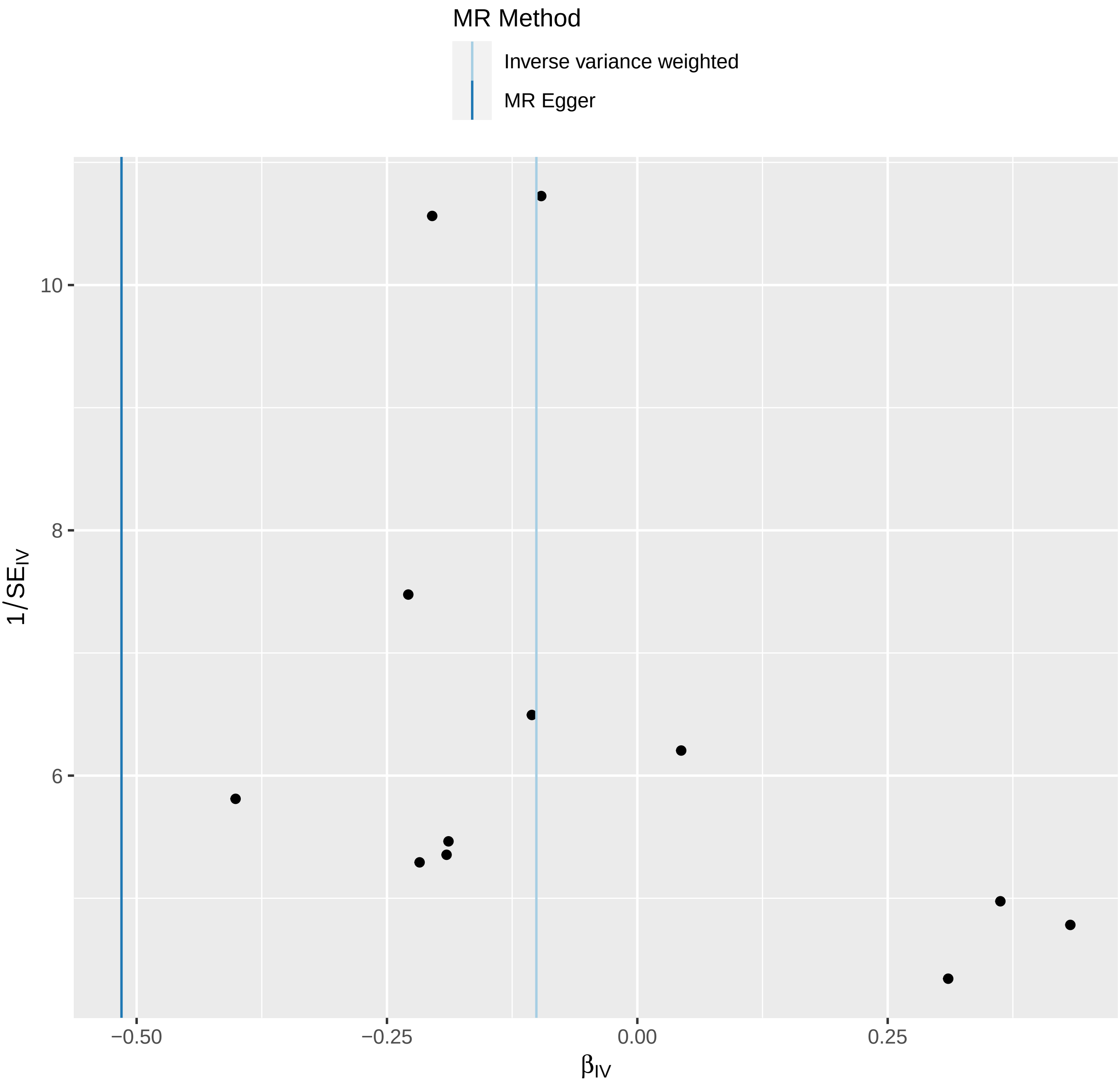

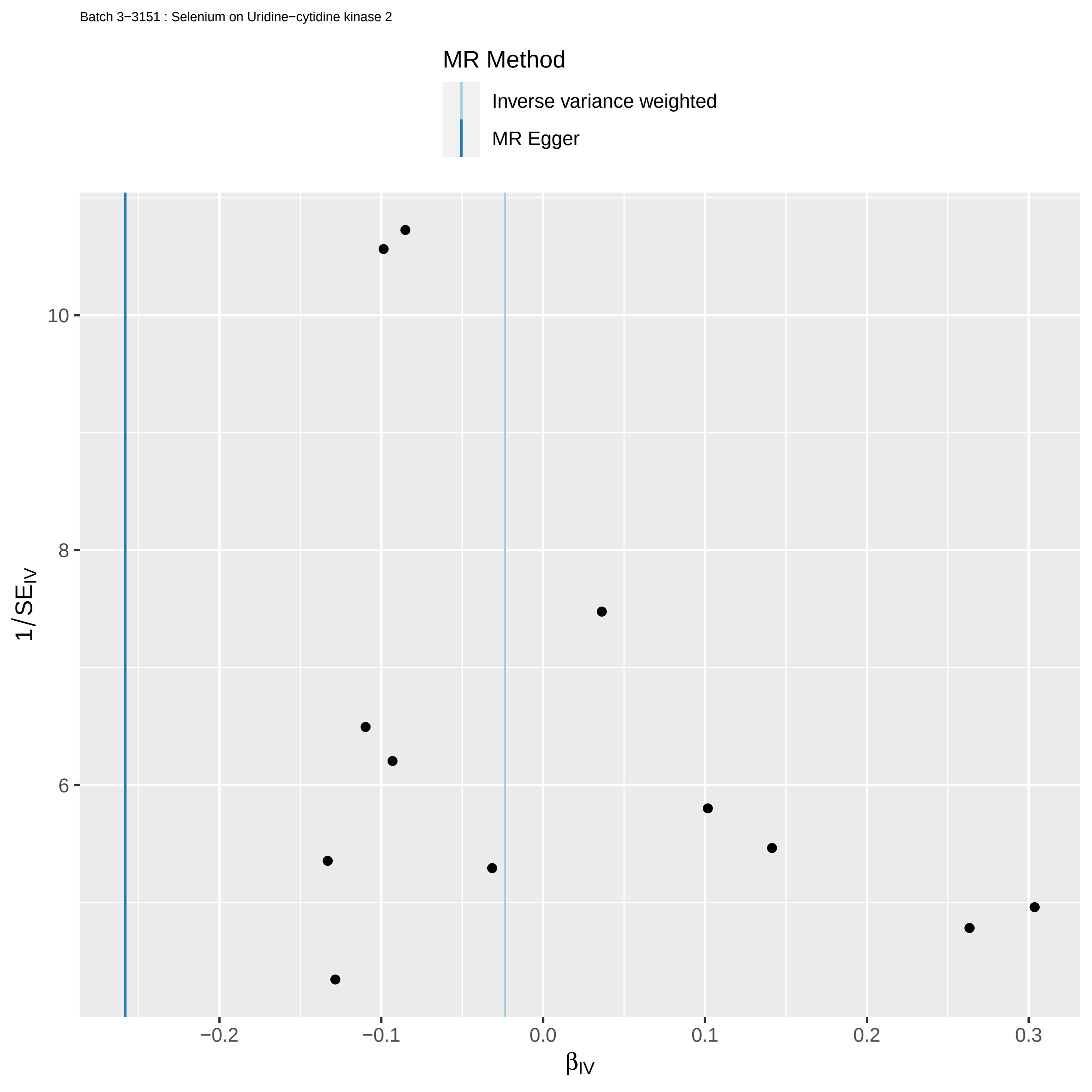

Batch 3−3151 : Selenium on Uridine−cytidine kinase 2
MR Method
Inverse variance weighted
MR Egger
10
8
6
1/SE_IV
−0.2
−0.1
0.0
0.1
0.2
0.3
β_IV

Batch 3−3152 : Selenium on Unique cartilage matrix−associated protein

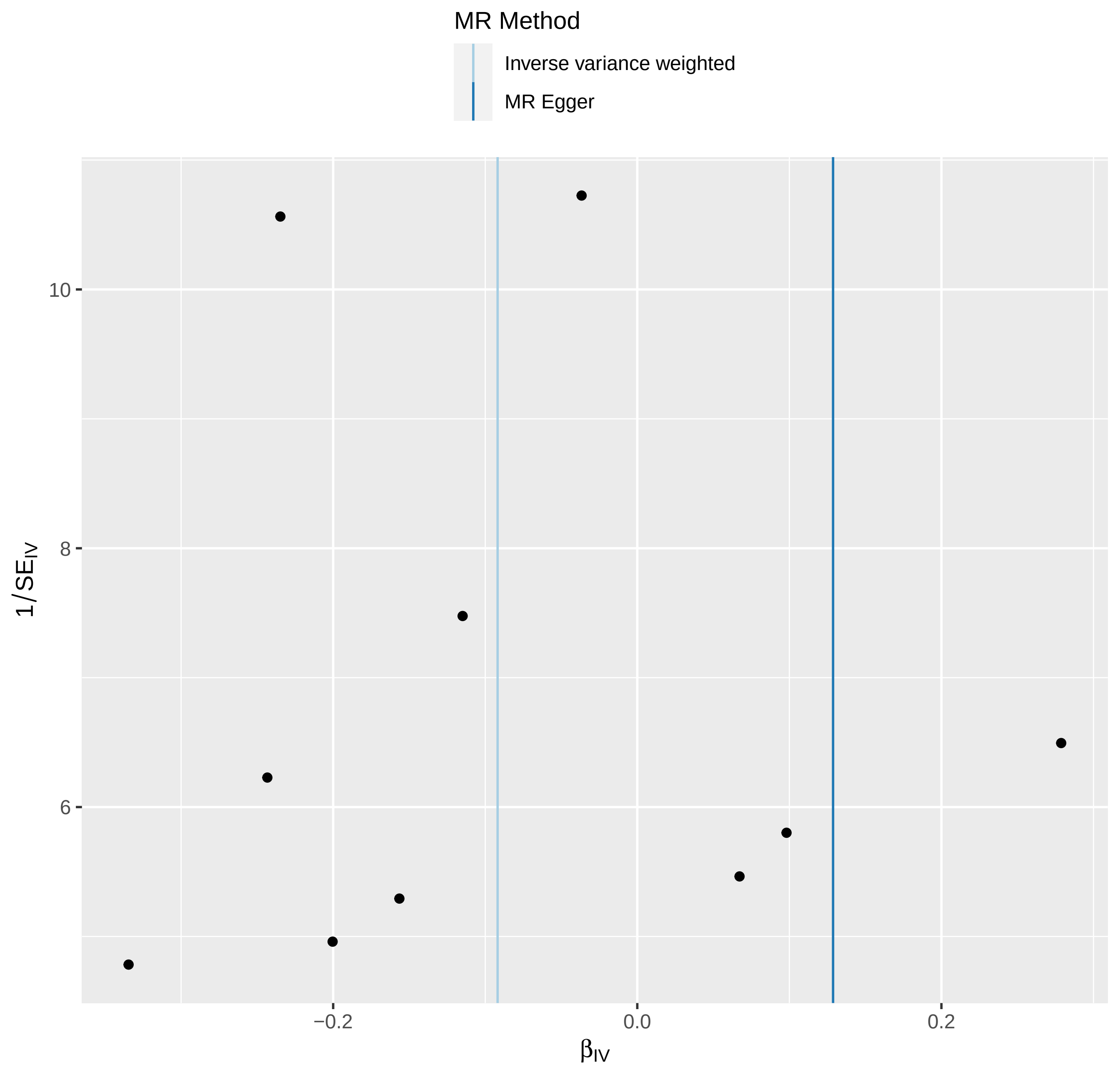

Batch 3−3153 : Selenium on Ubiquitin−fold modifier−conjugating enzyme 1

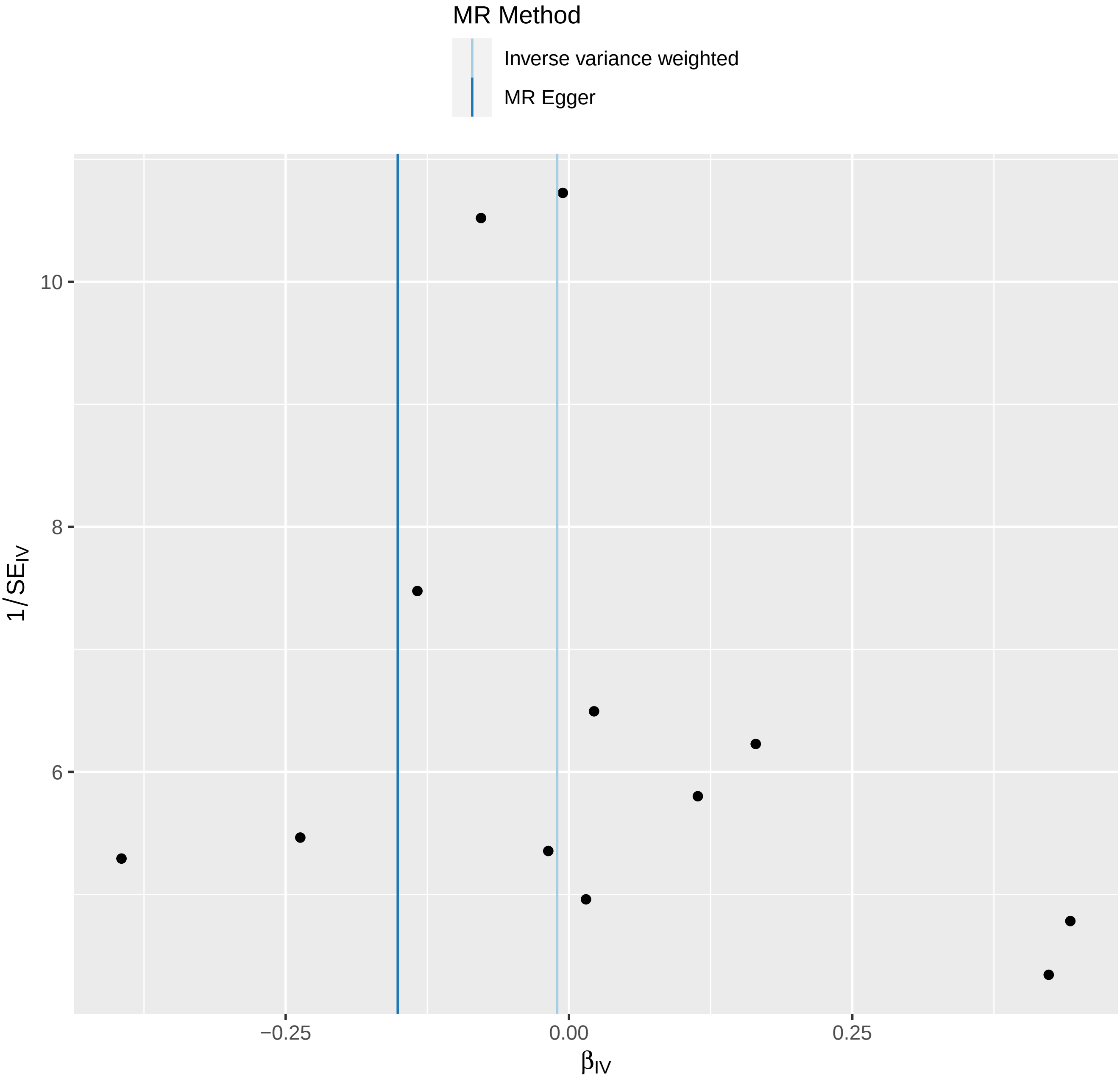

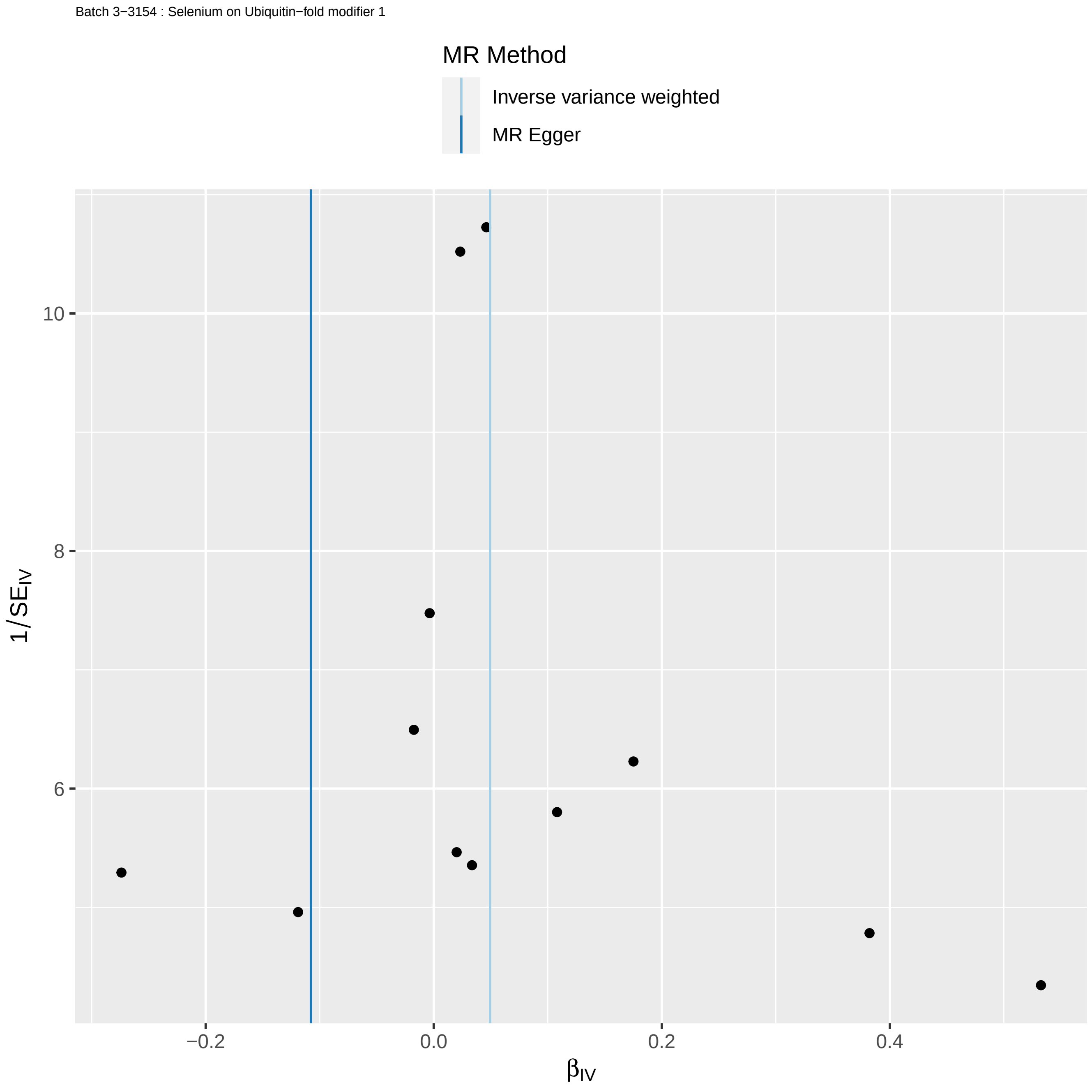
Batch 3−3154 : Selenium on Ubiquitin−fold modifier 1
MR Method
Inverse variance weighted
MR Egger
10
8
6
1/SE_IV
−0.2
0.0
0.2
0.4
β_IV

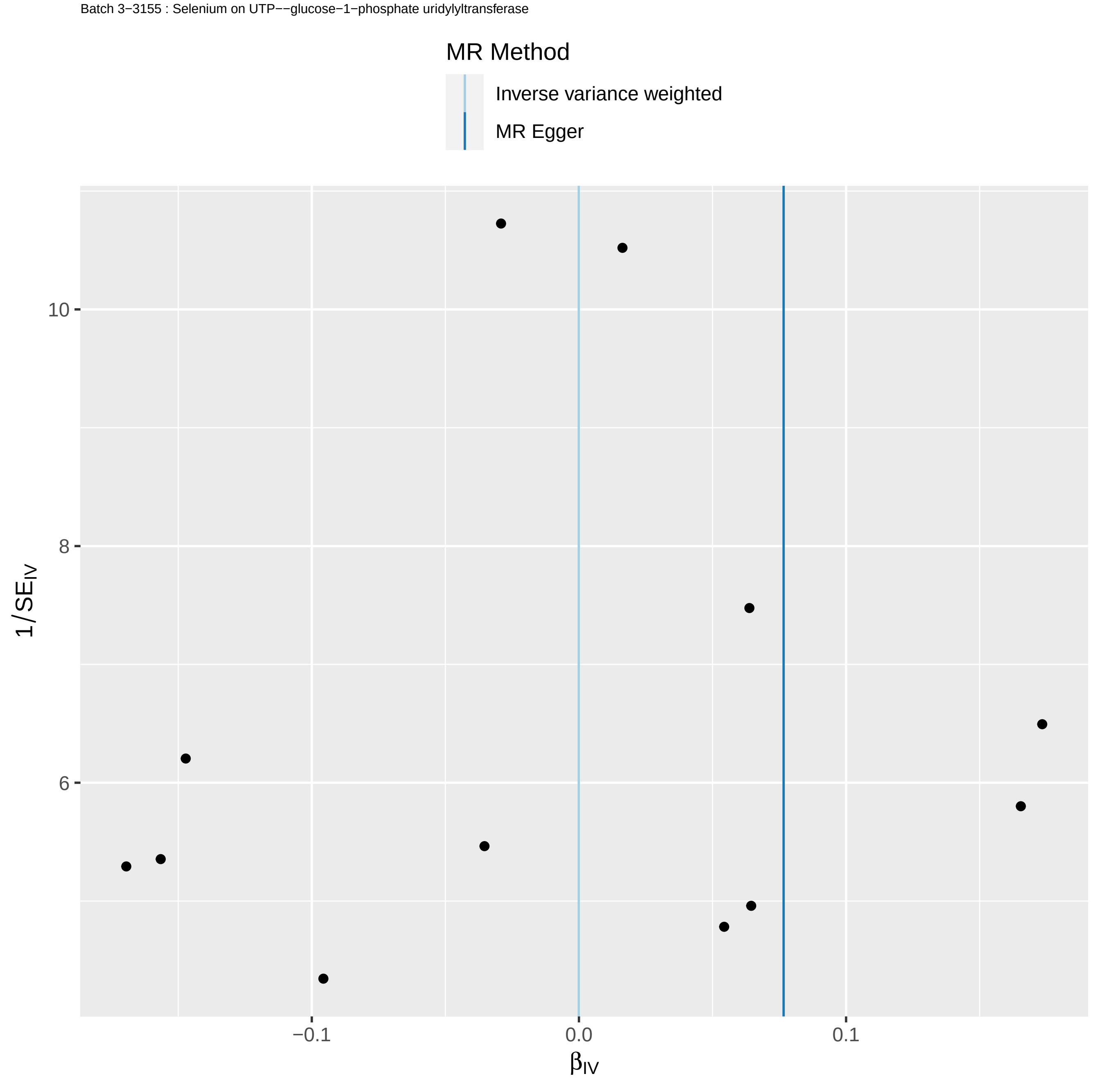
Batch 3−3155 : Selenium on UTP−−glucose−1−phosphate uridylyltransferase
MR Method
Inverse variance weighted
MR Egger
10
8
6
1/SE_IV
−0.1
0.0
0.1
β_IV

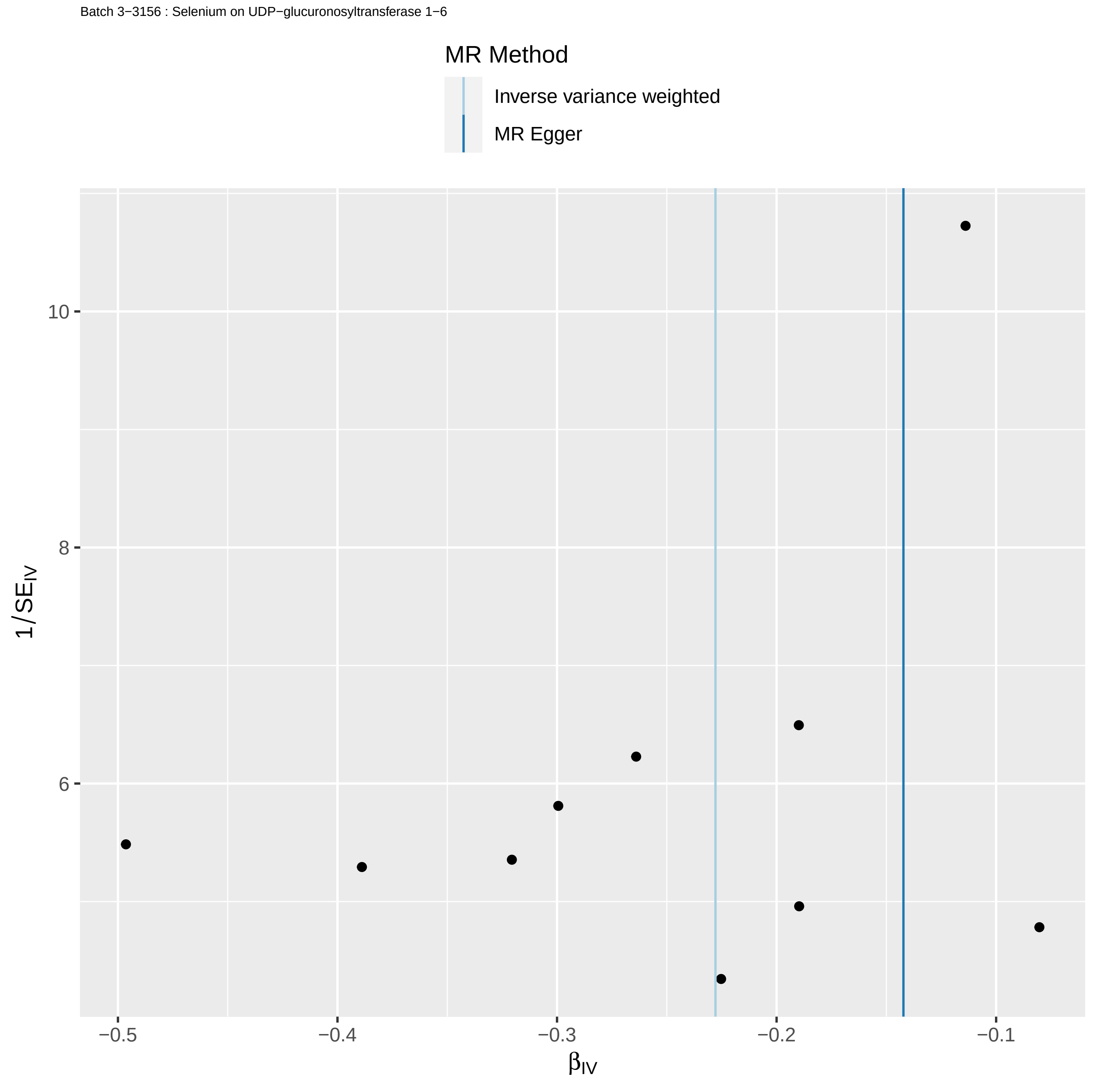

Batch 3−3156 : Selenium on UDP−glucuronosyltransferase 1−6
MR Method
Inverse variance weighted
MR Egger
10
8
6
1/SE$_{IV}$
−0.5
−0.4
−0.3
−0.2
−0.1
β$_{IV}$

Batch 3−3157 : Selenium on UDP−glucuronosyltransferase 1−8

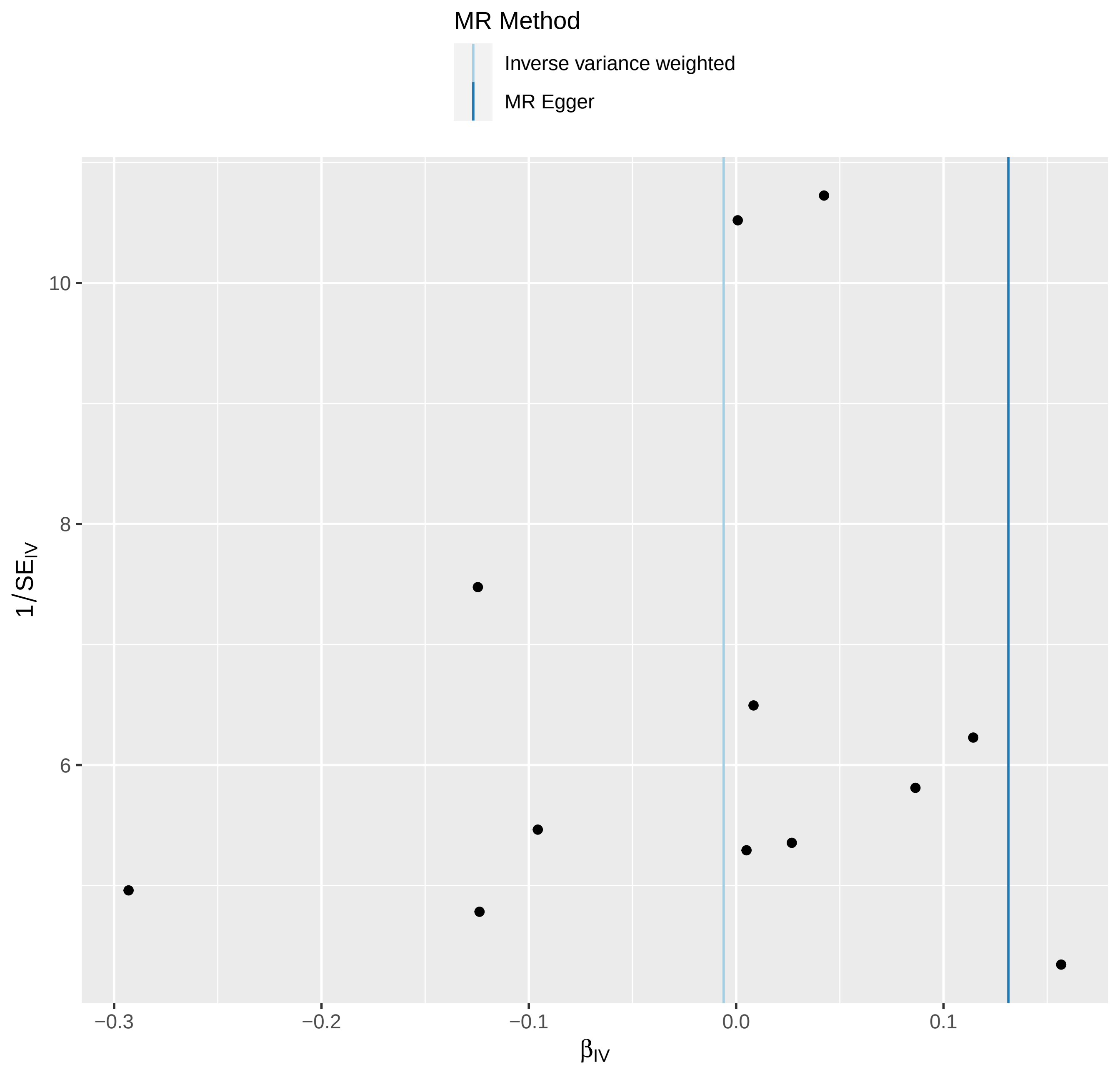

Batch 3−3158 : Selenium on UDP−glucuronosyltransferase 2A1

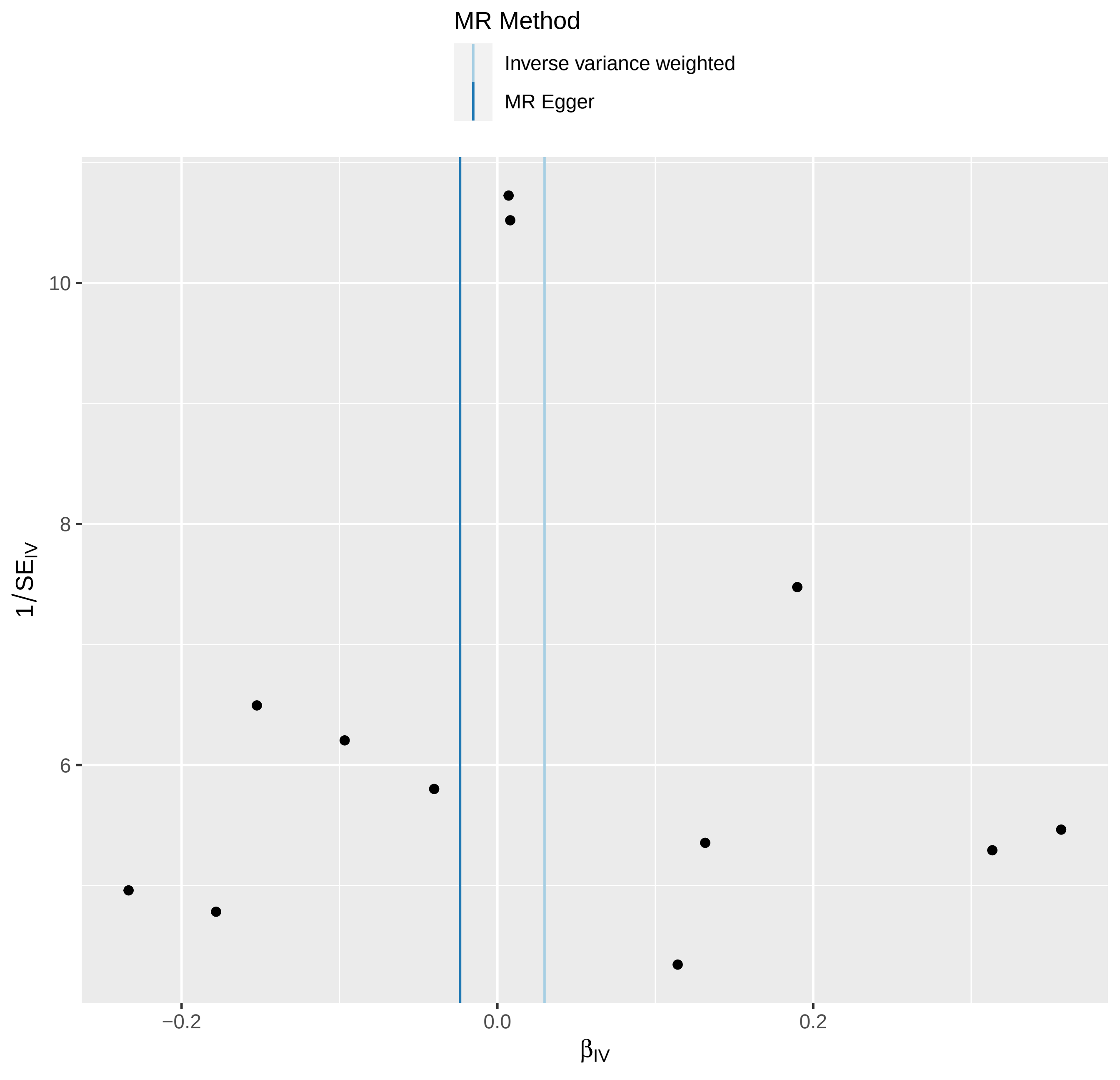

Batch 3−3159 : Selenium on NKG2D ligand 1

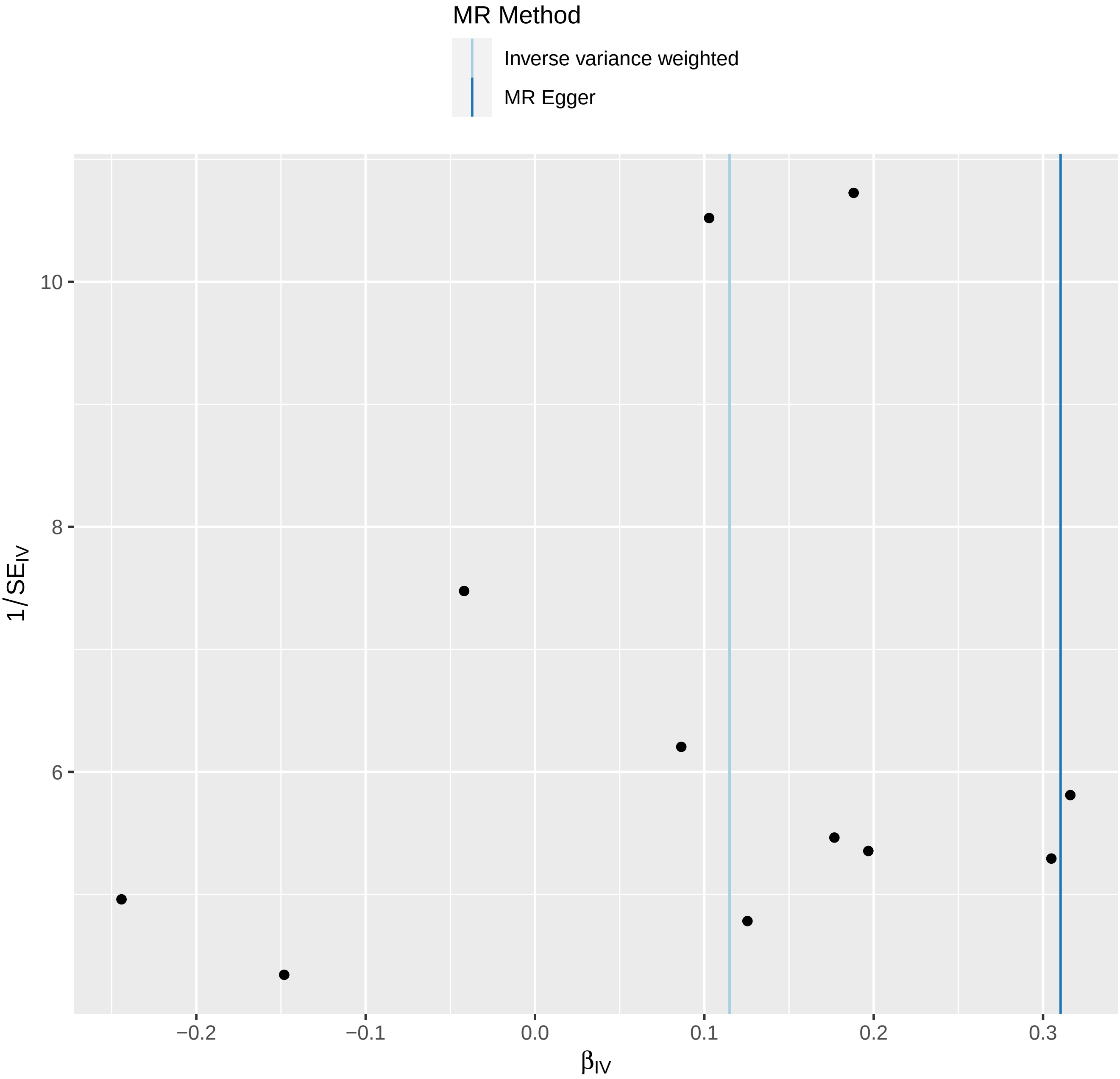

Batch 3−3160 : Selenium on NKG2D ligand 1

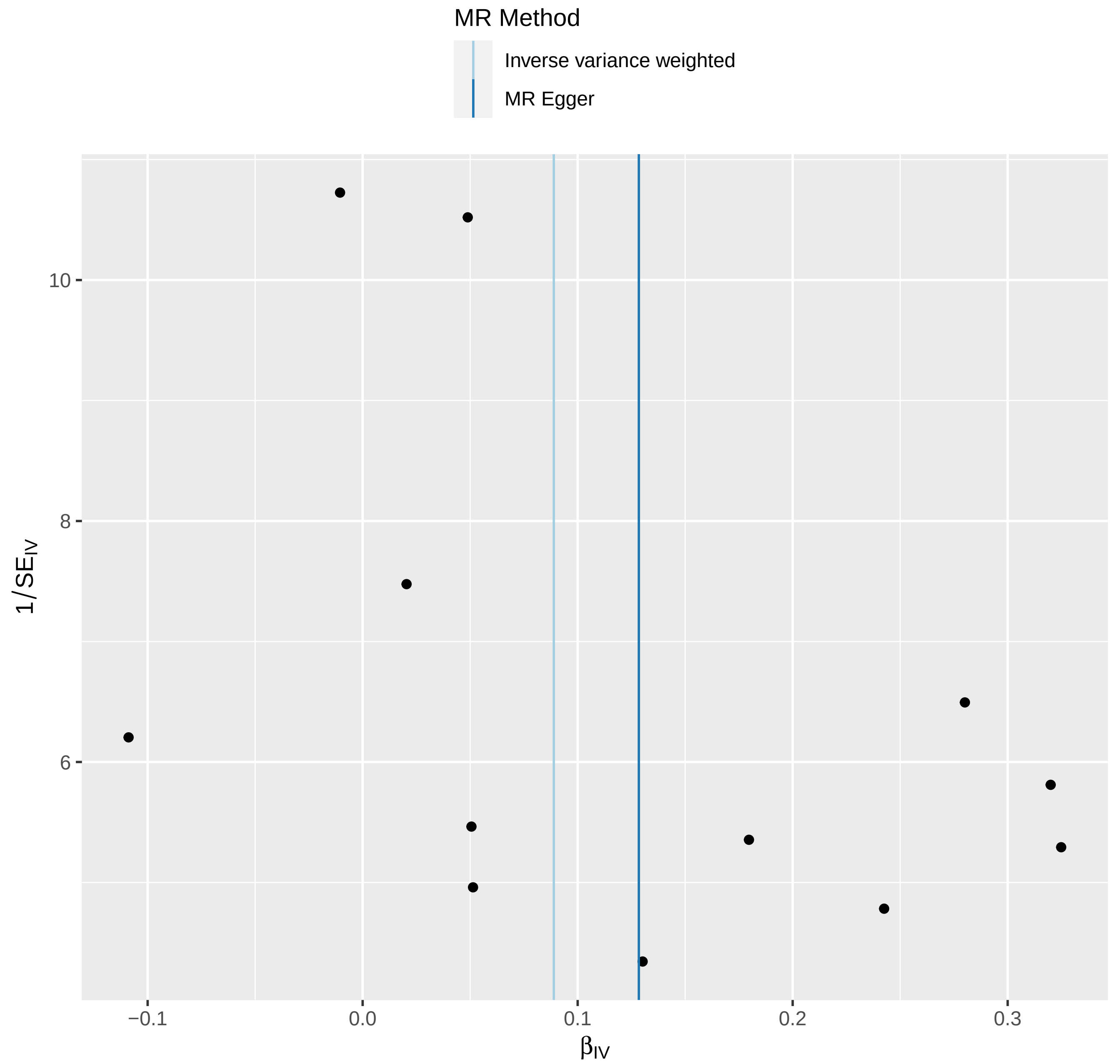

Batch 3−3161 : Selenium on NKG2D ligand 2

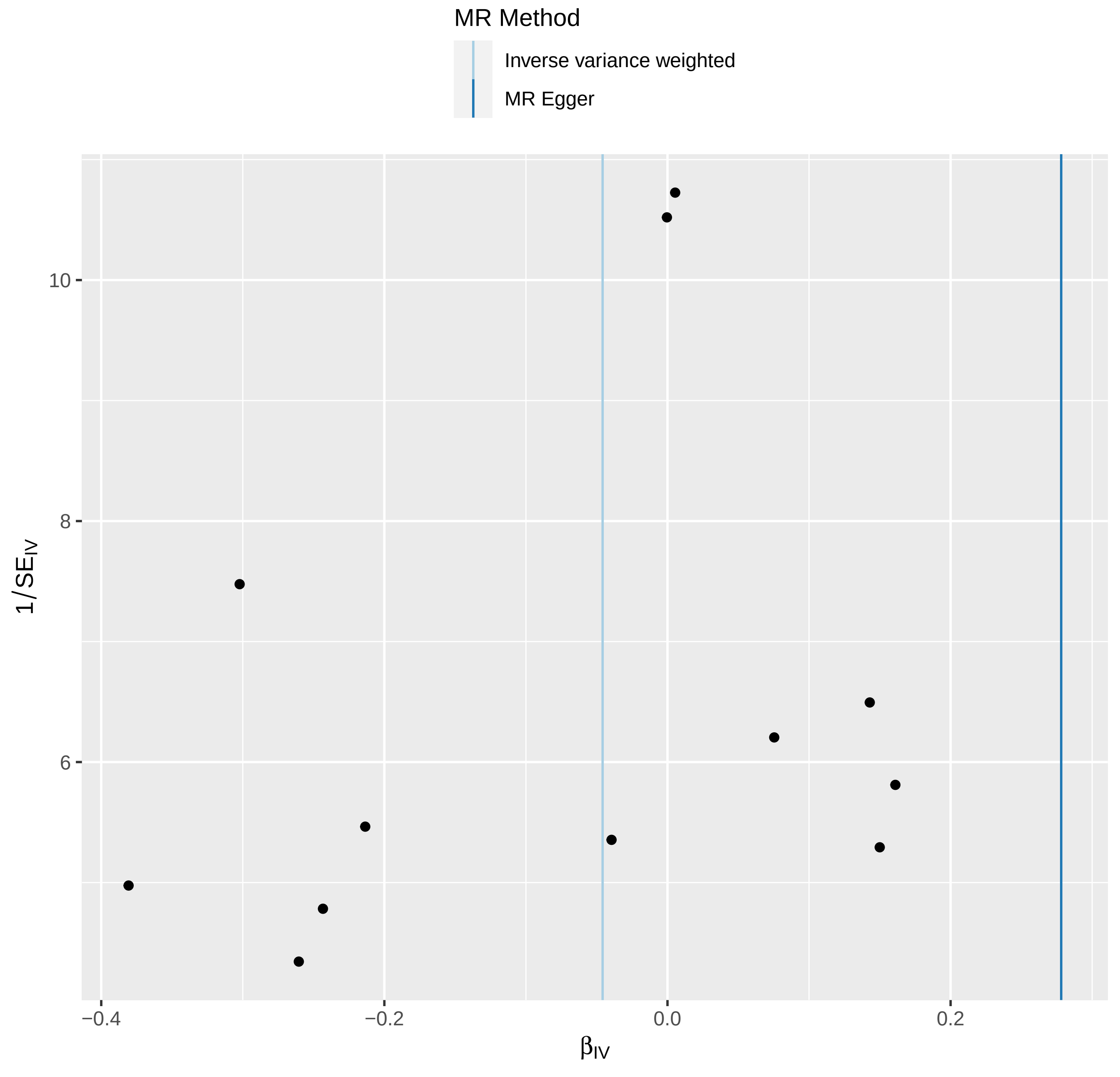

Batch 3−3162 : Selenium on NKG2D ligand 3

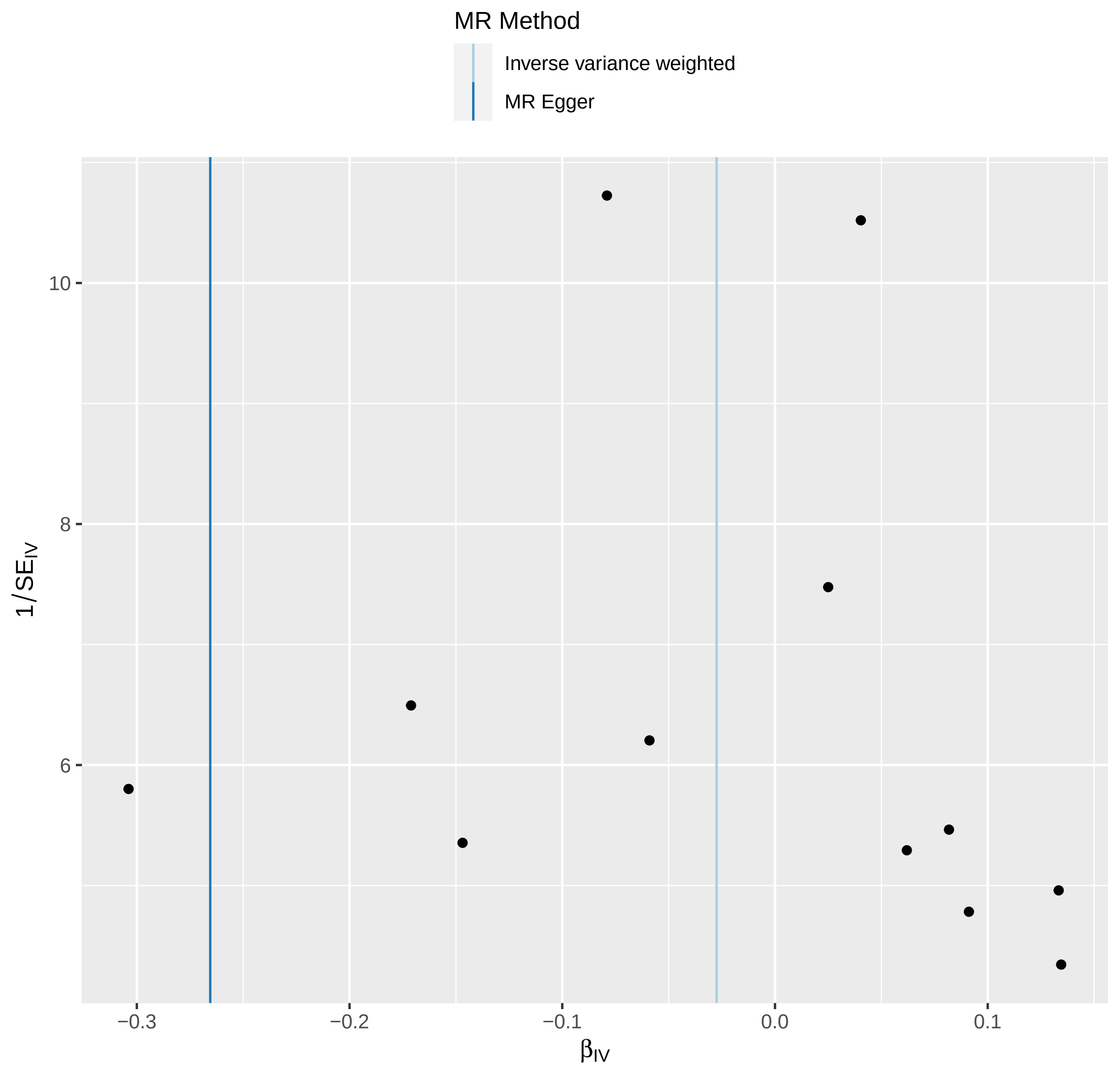

Batch 3−3163 : Selenium on Serine/threonine−protein kinase ULK3

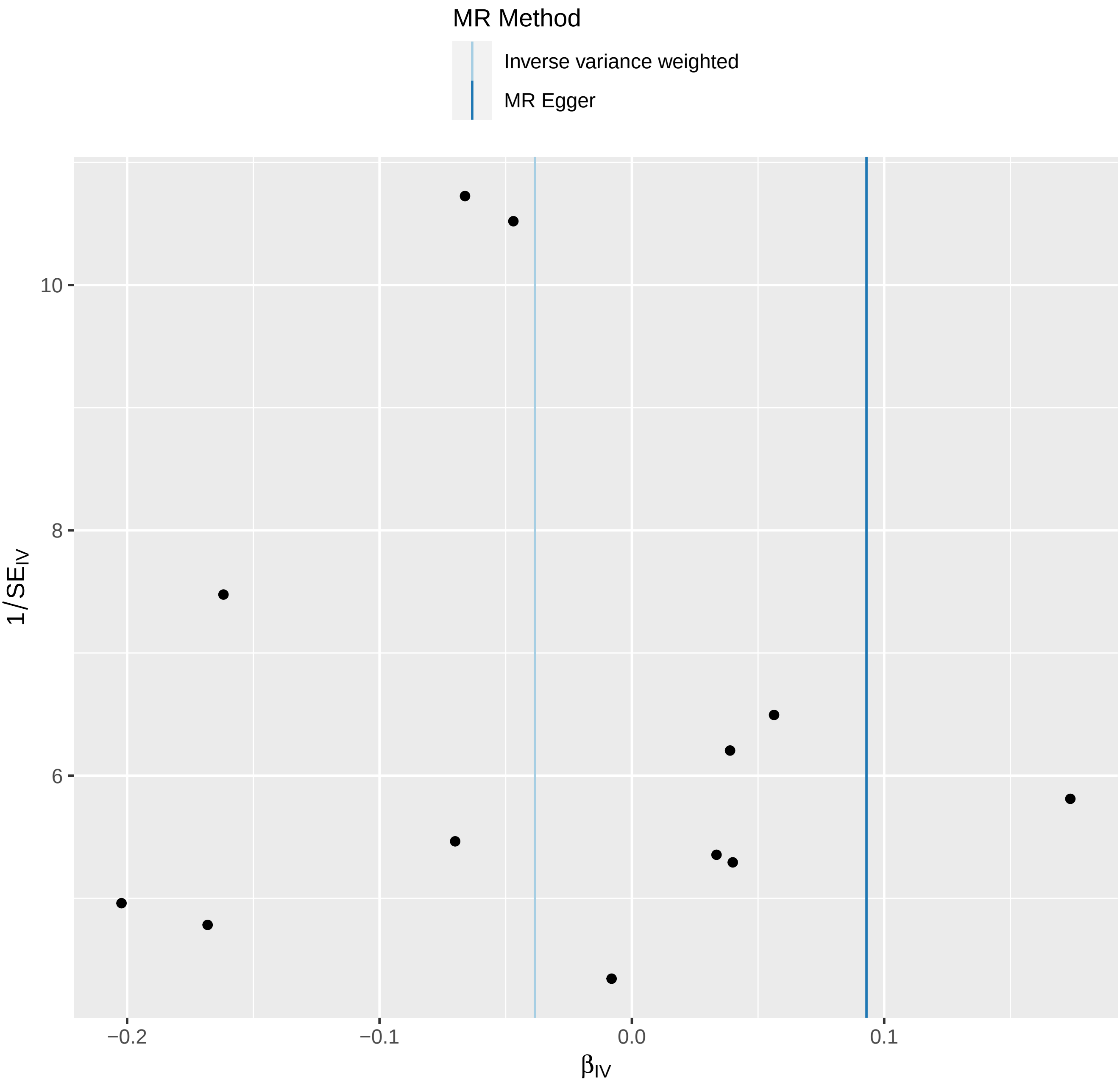

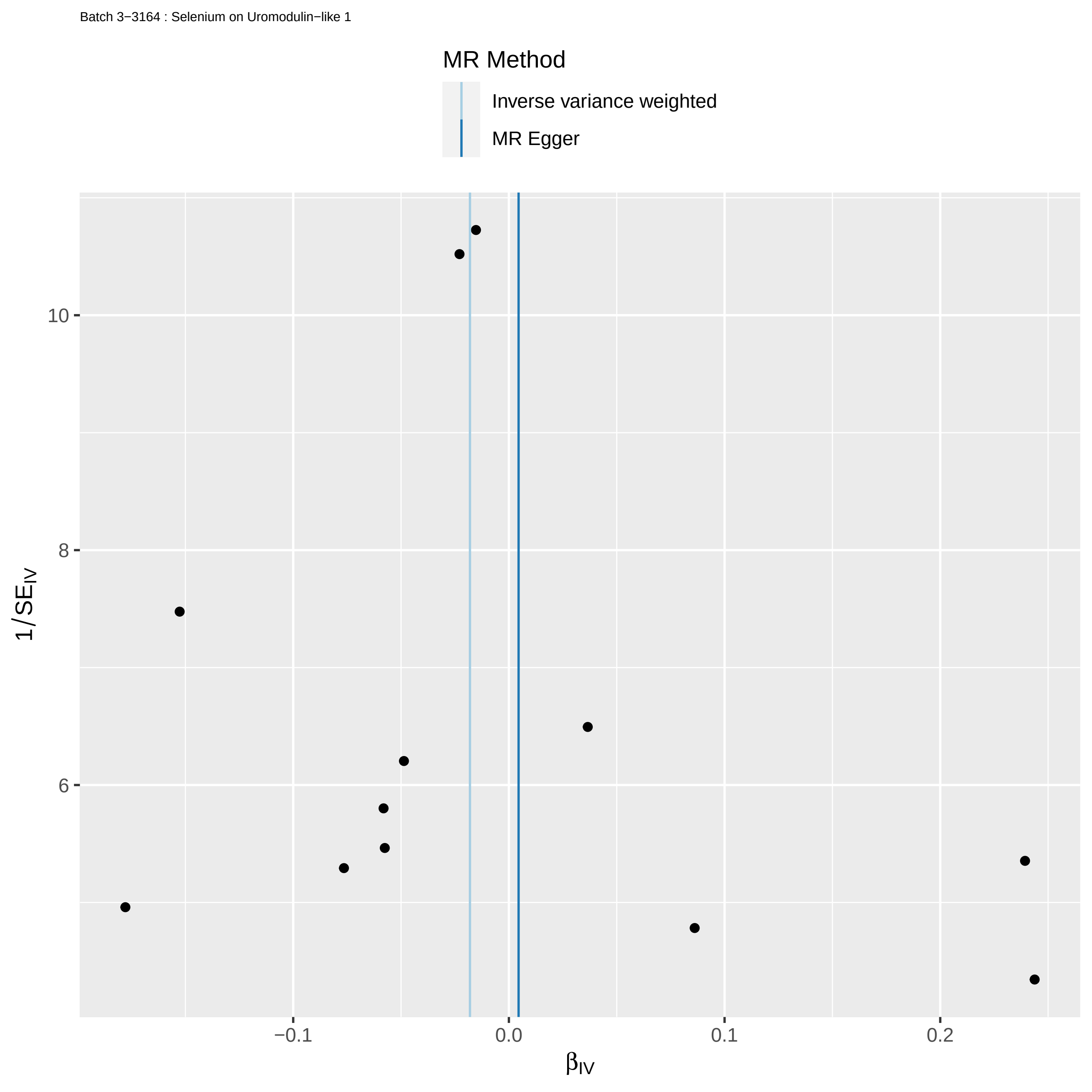
Batch 3−3164 : Selenium on Uromodulin−like 1
MR Method
Inverse variance weighted
MR Egger
10
8
6
1/SE$_{IV}$
−0.1
0.0
0.1
0.2
β$_{IV}$

Batch 3−3165 : Selenium on Protein unc−13 homolog A

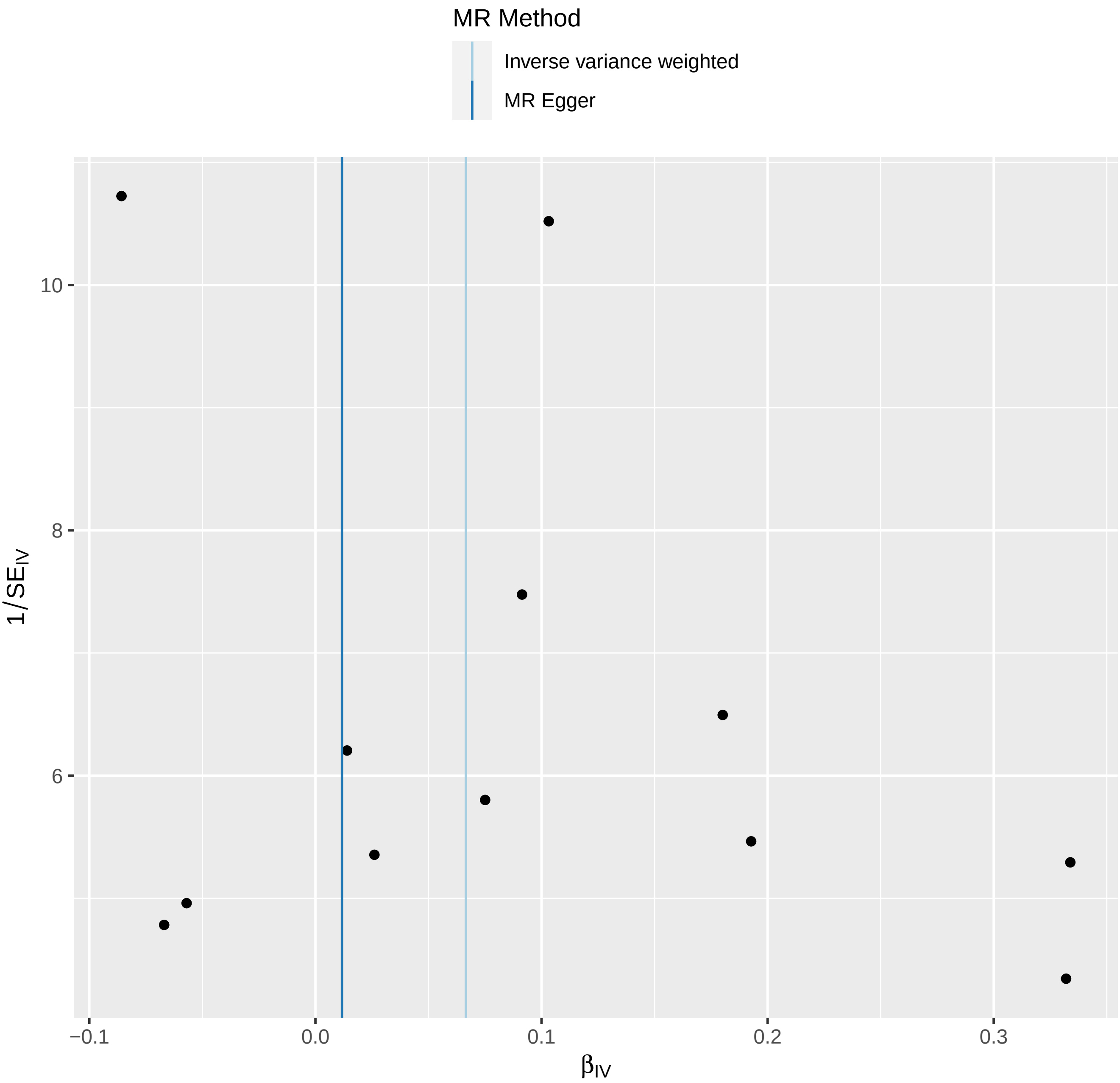

Batch 3−3166 : Selenium on Netrin receptor UNC5A

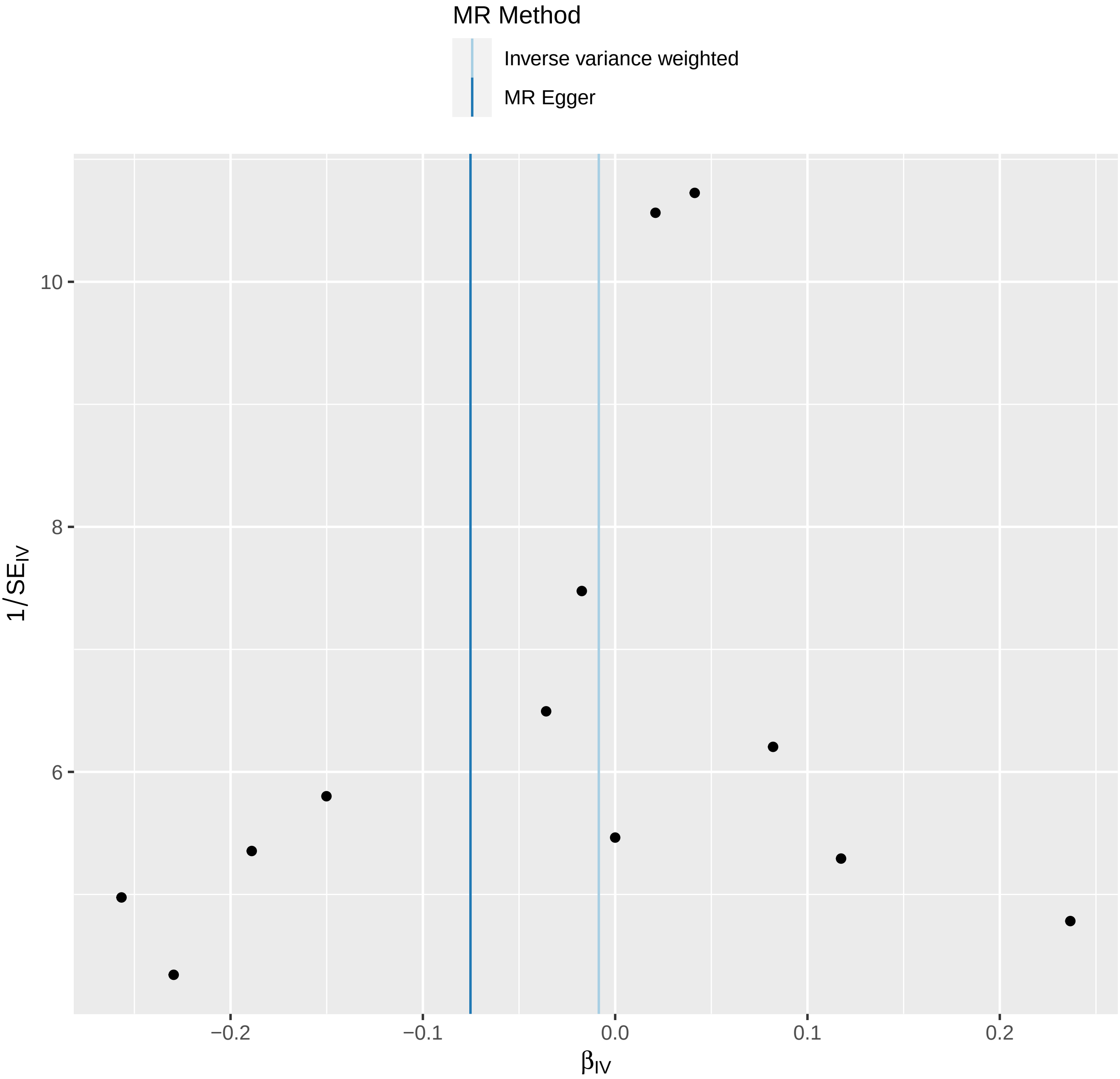

Batch 3−3167 : Selenium on Netrin receptor UNC5B

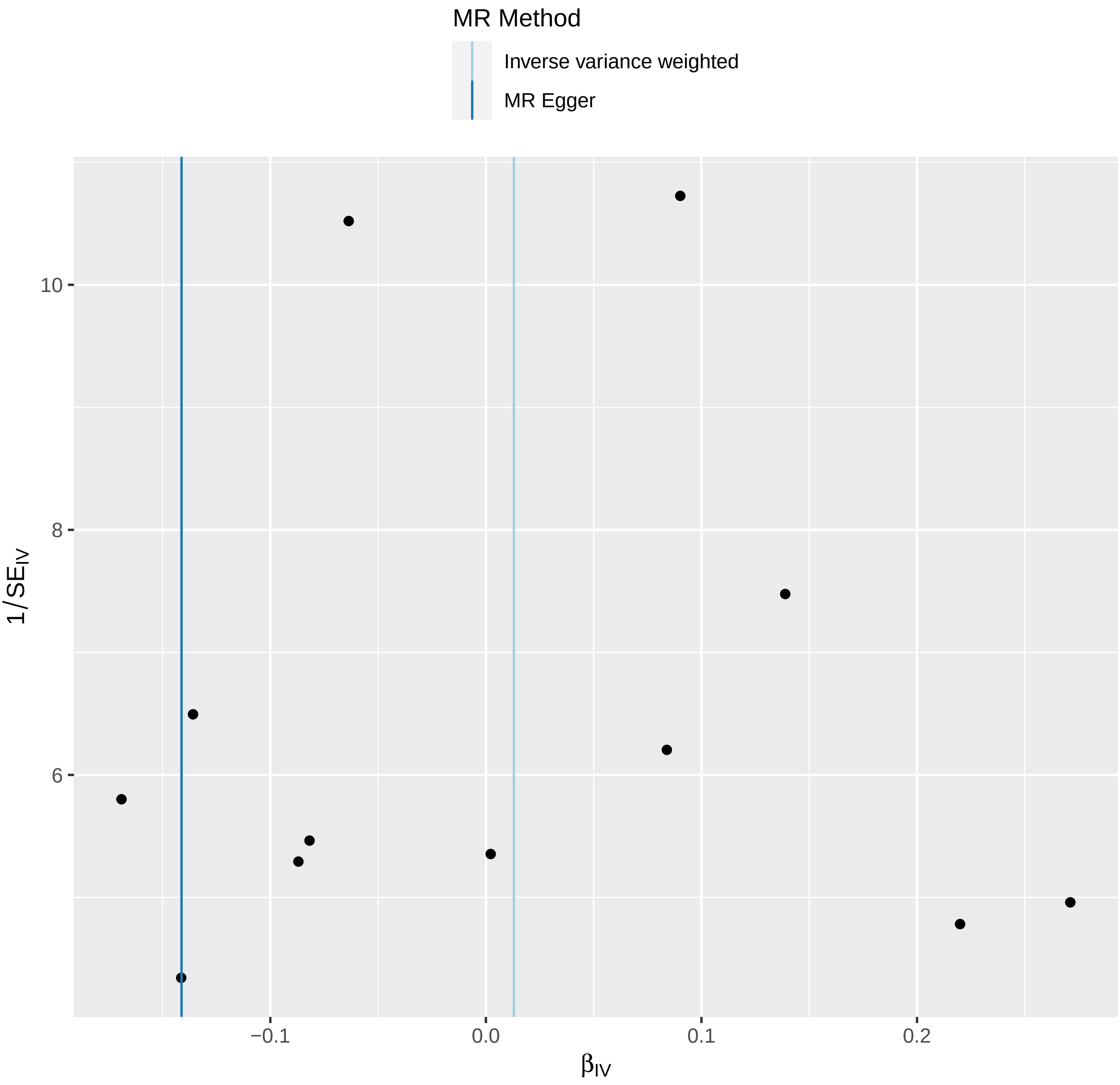

Batch 3−3168 : Selenium on Netrin receptor UNC5C

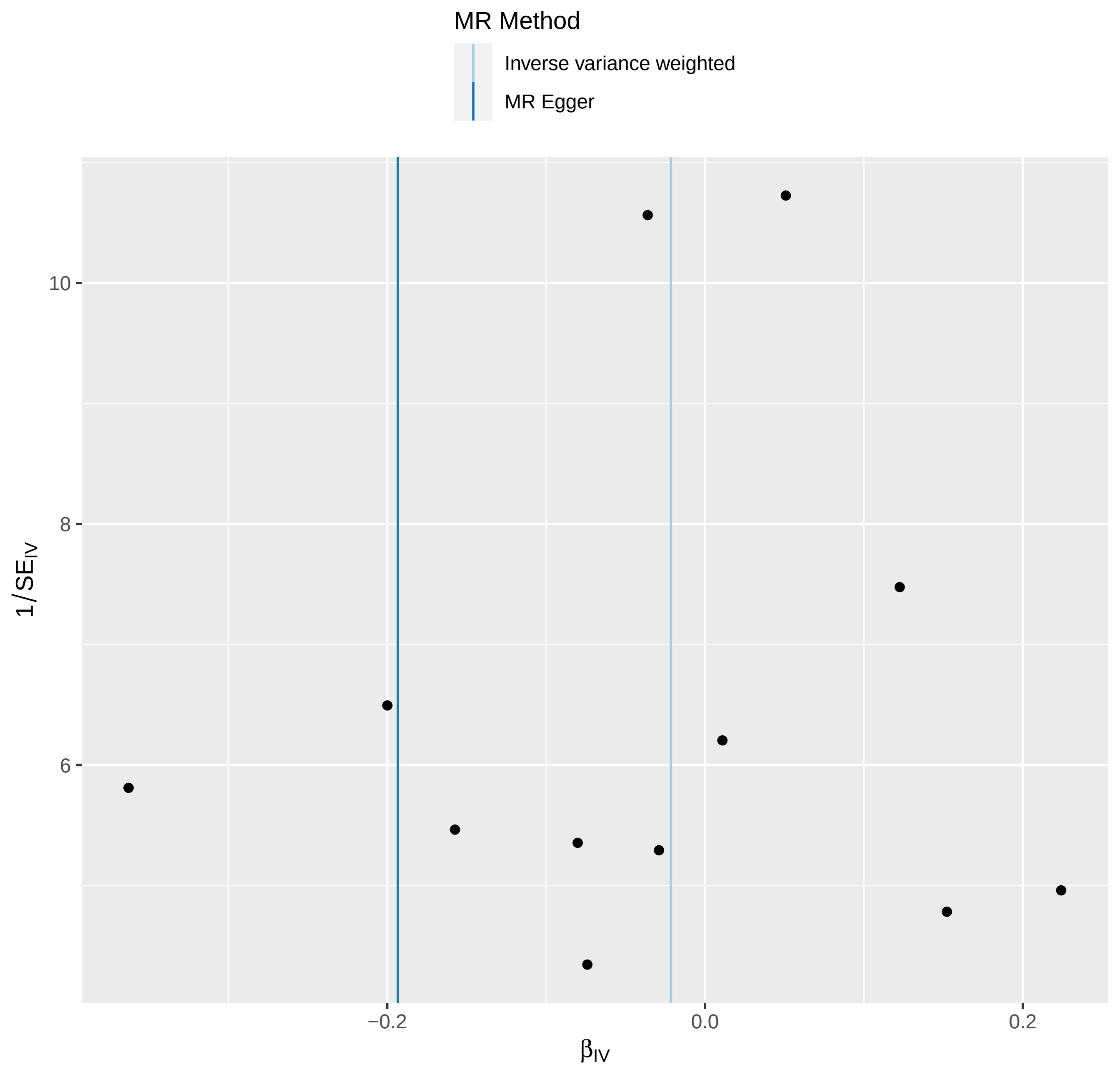

Batch 3−3169 : Selenium on Netrin receptor UNC5D

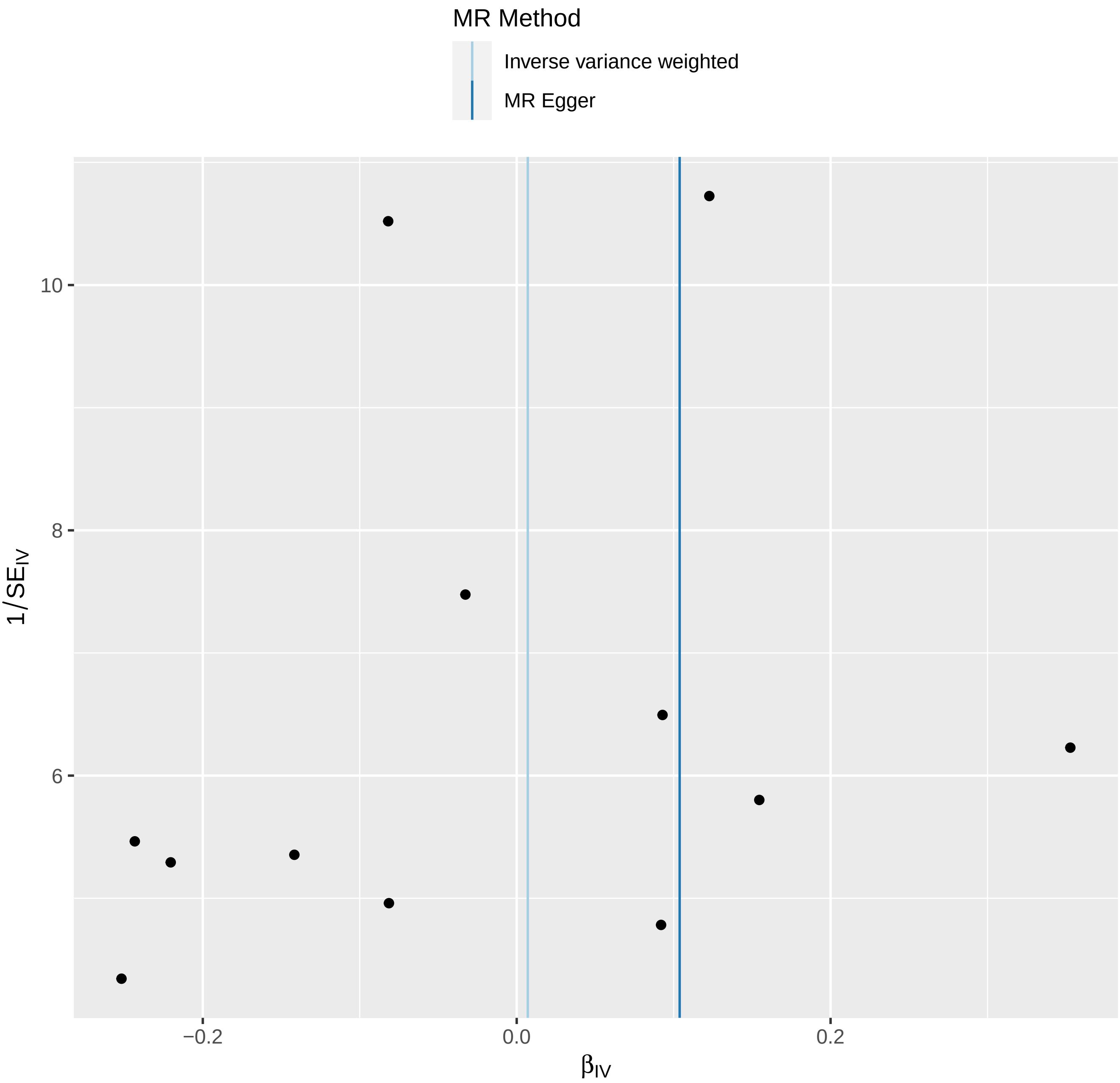

Batch 3−3170 : Selenium on Netrin receptor UNC5D

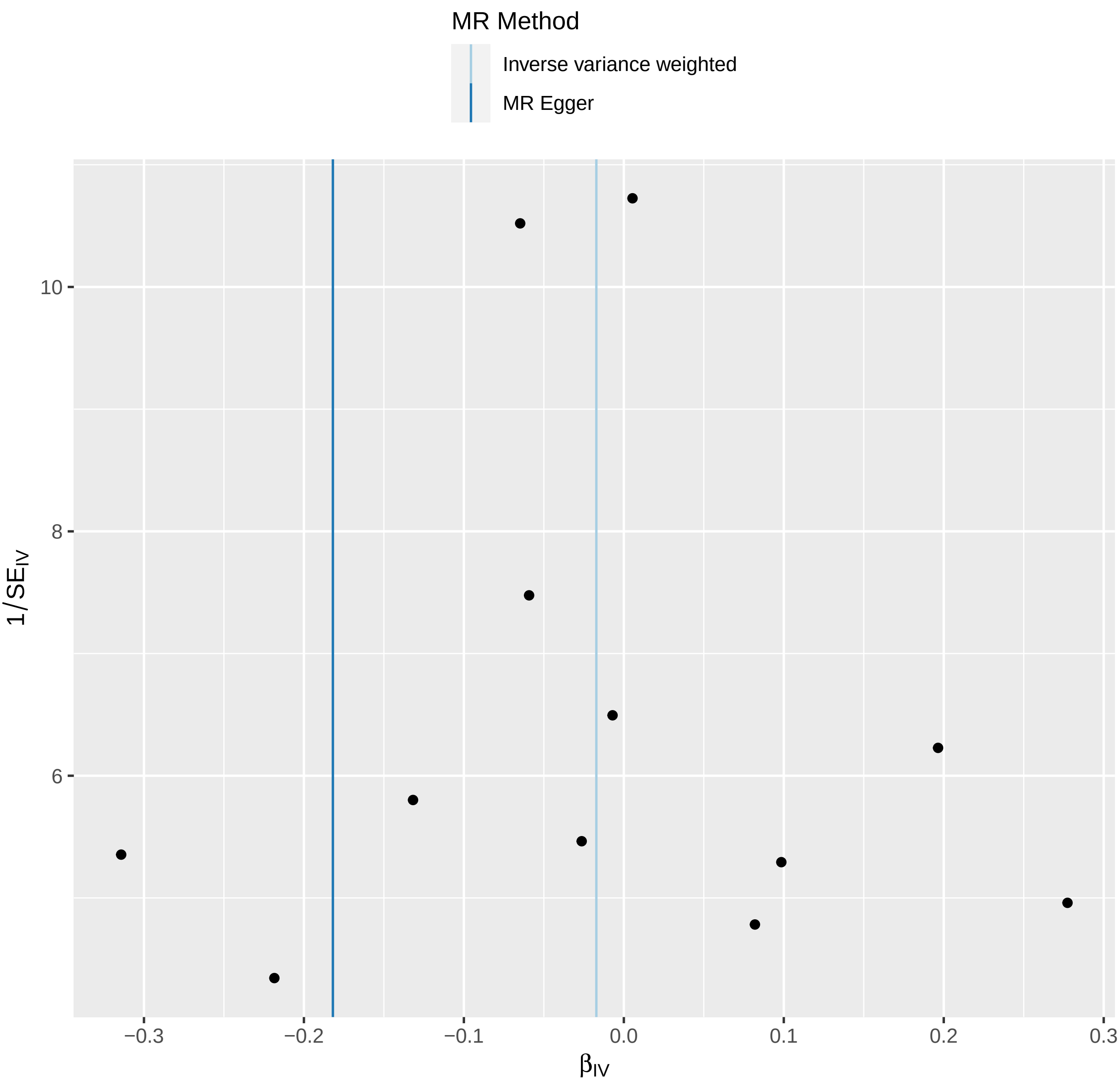

Batch 3−3171 : Selenium on Protein unc−93 homolog B1

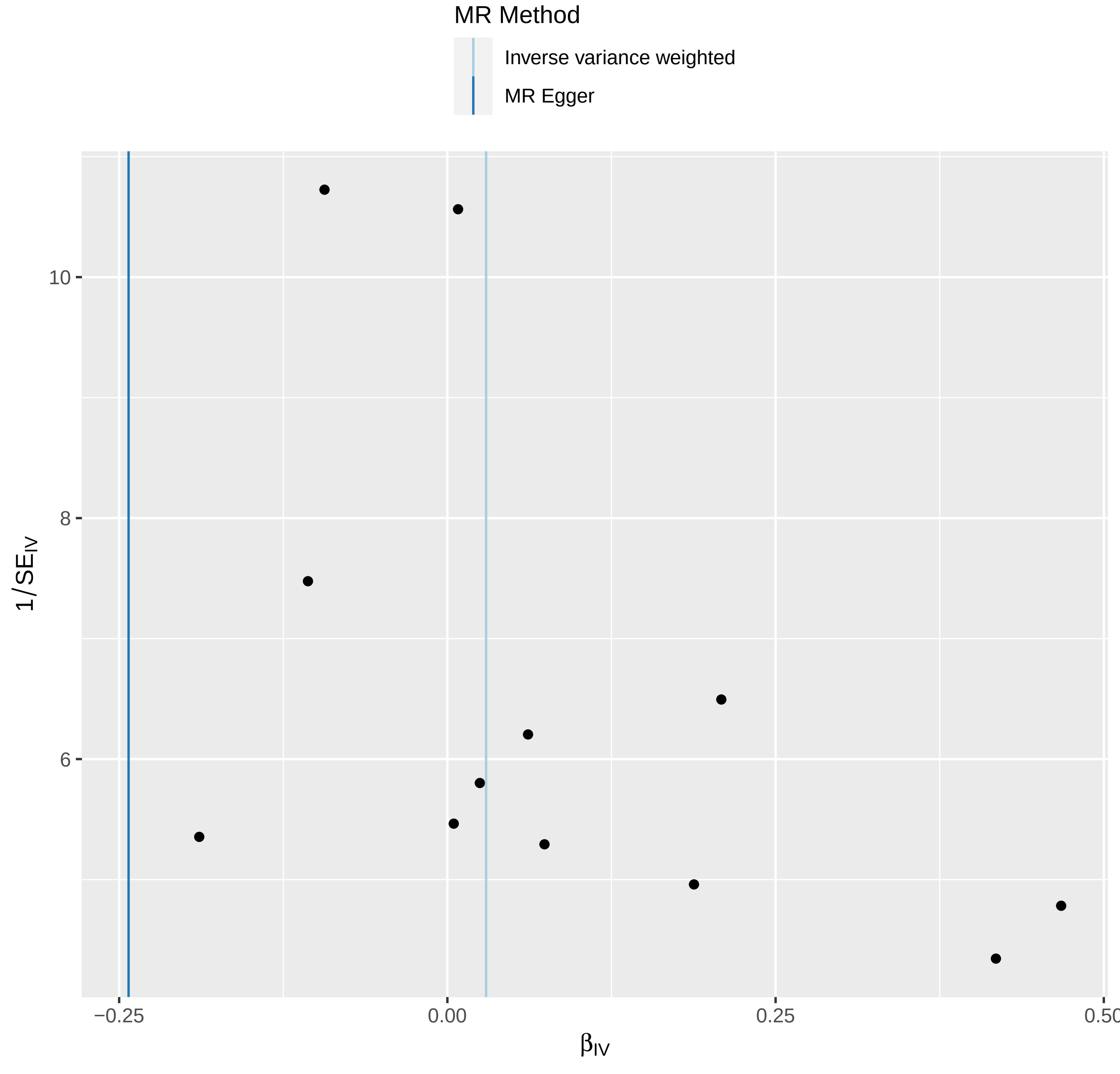

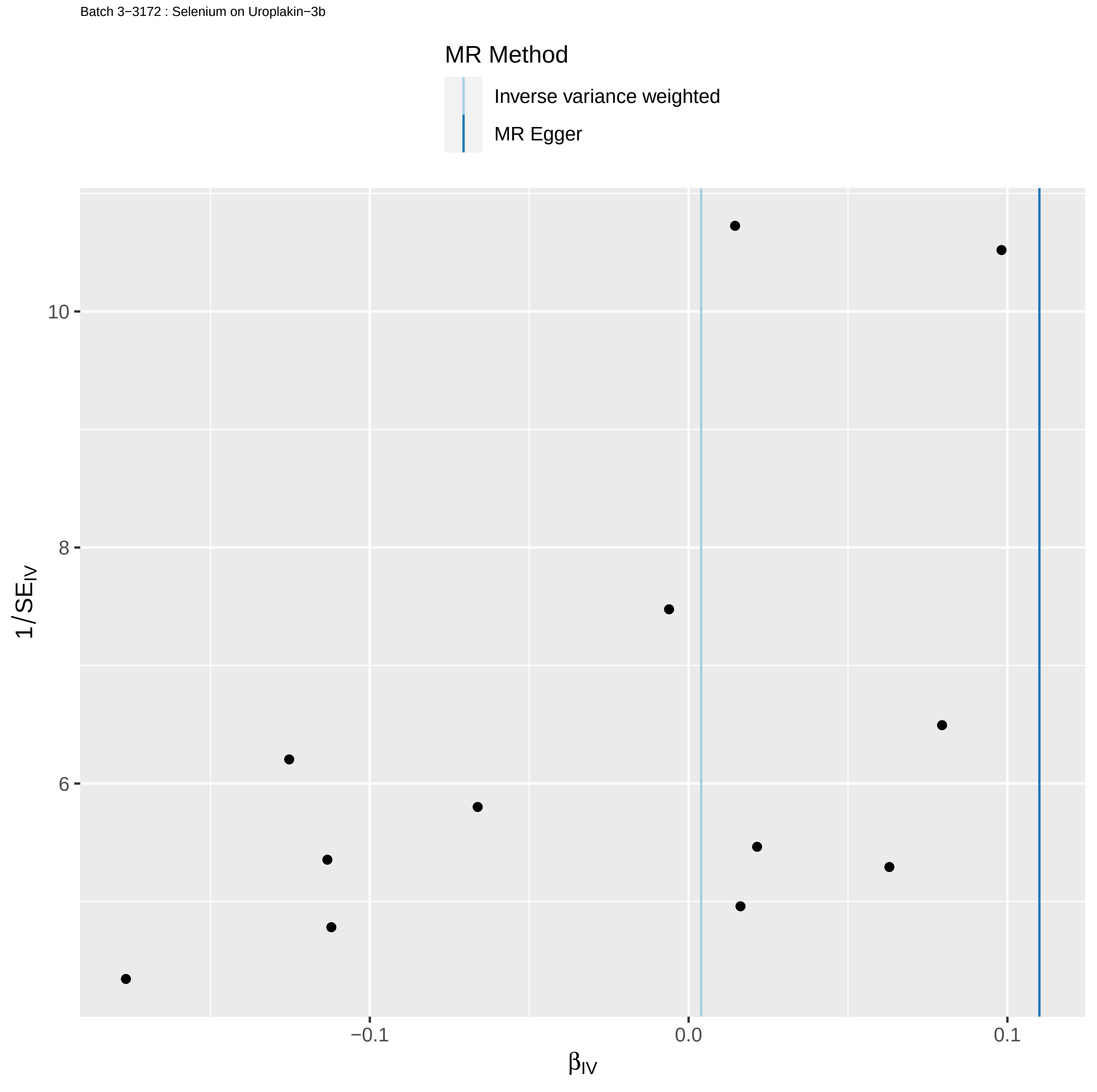

Batch 3−3172 : Selenium on Uroplakin−3b
MR Method
Inverse variance weighted
MR Egger
10
8
6
1/SE$_{IV}$
−0.1
0.0
0.1
β$_{IV}$

Batch 3−3173 : Selenium on Uroplakin−3b−like protein

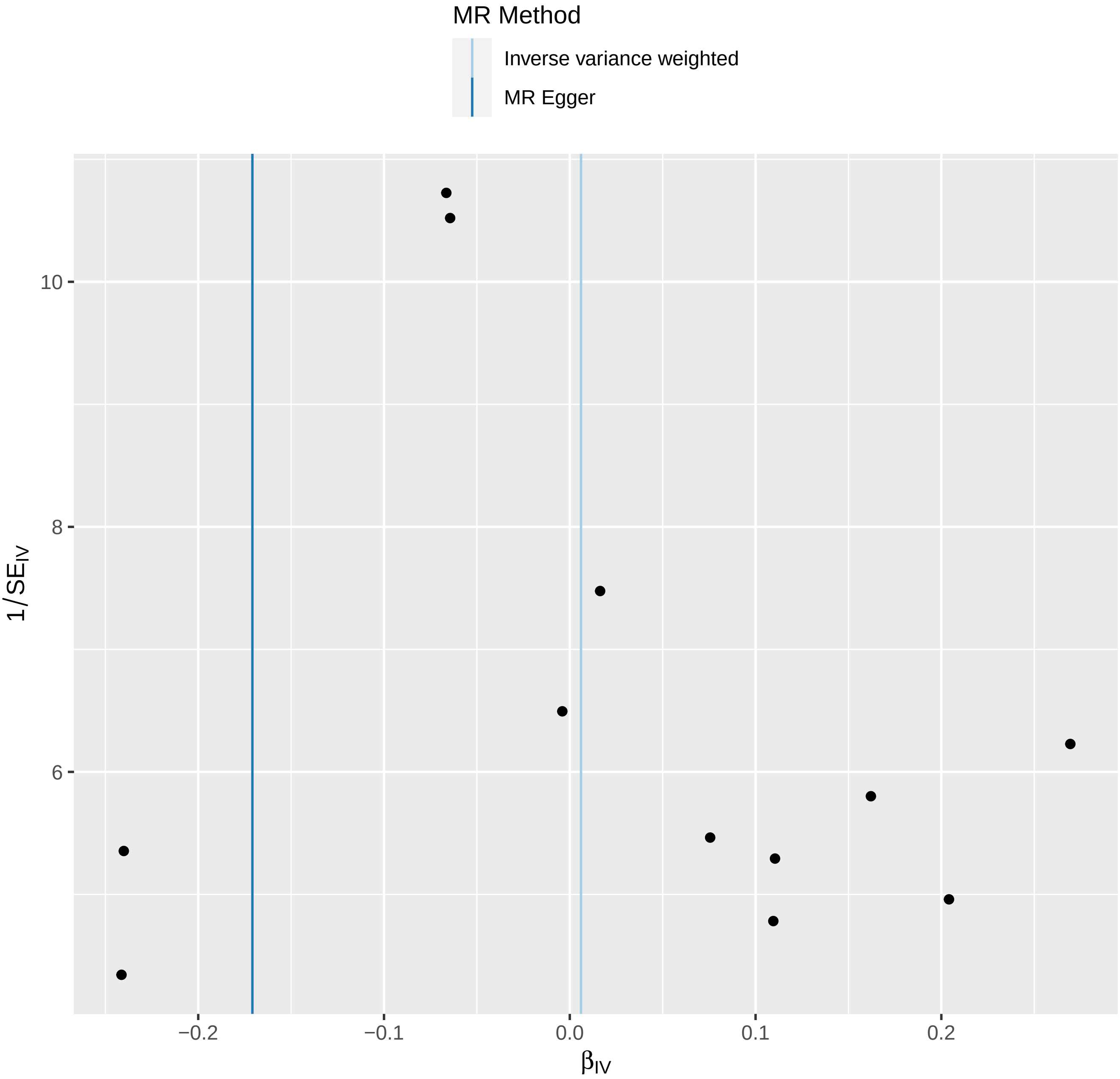

Batch 3−3174 : Selenium on Ubiquinol−cytochrome−c reductase complex assembly factor 3

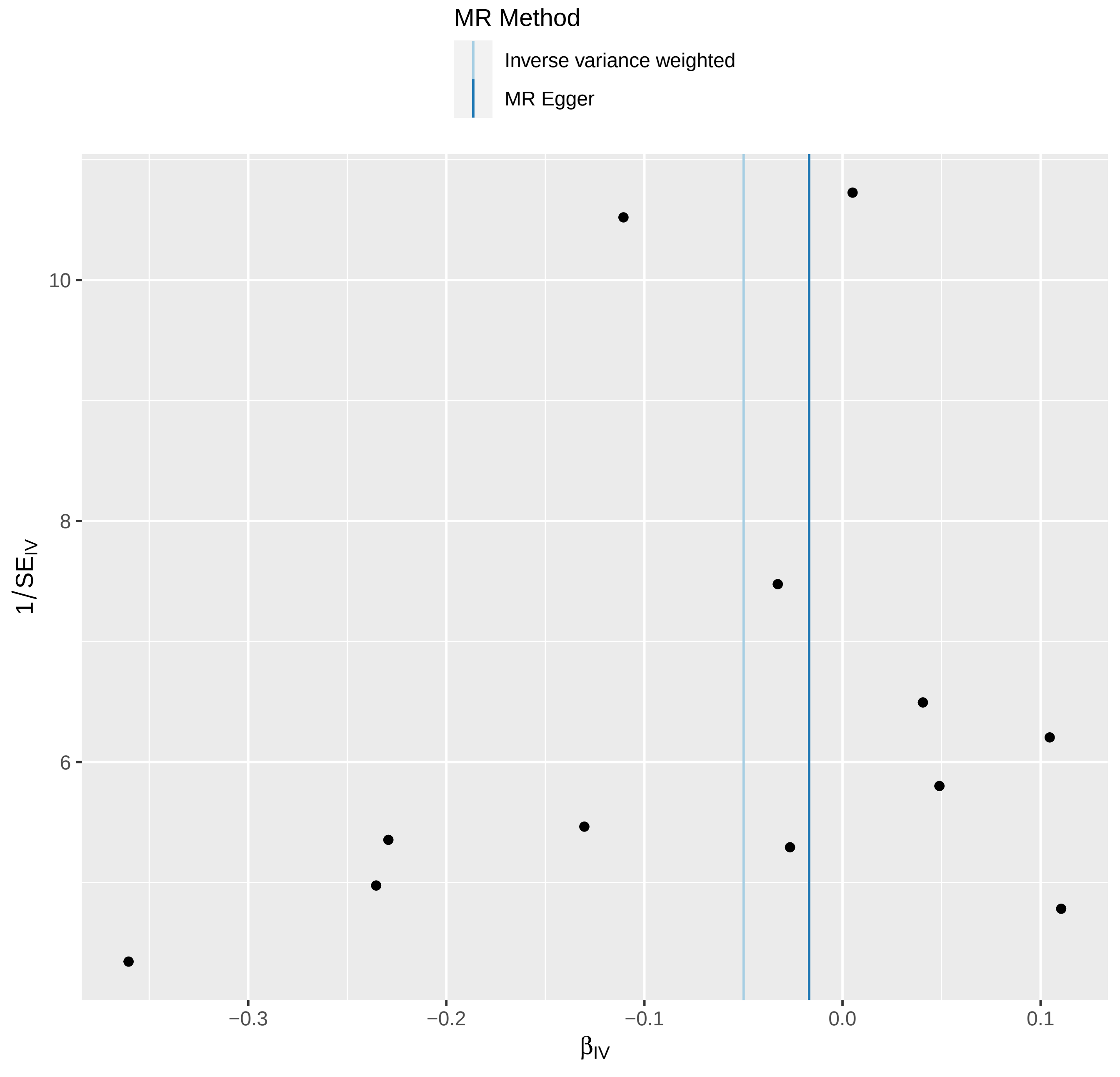

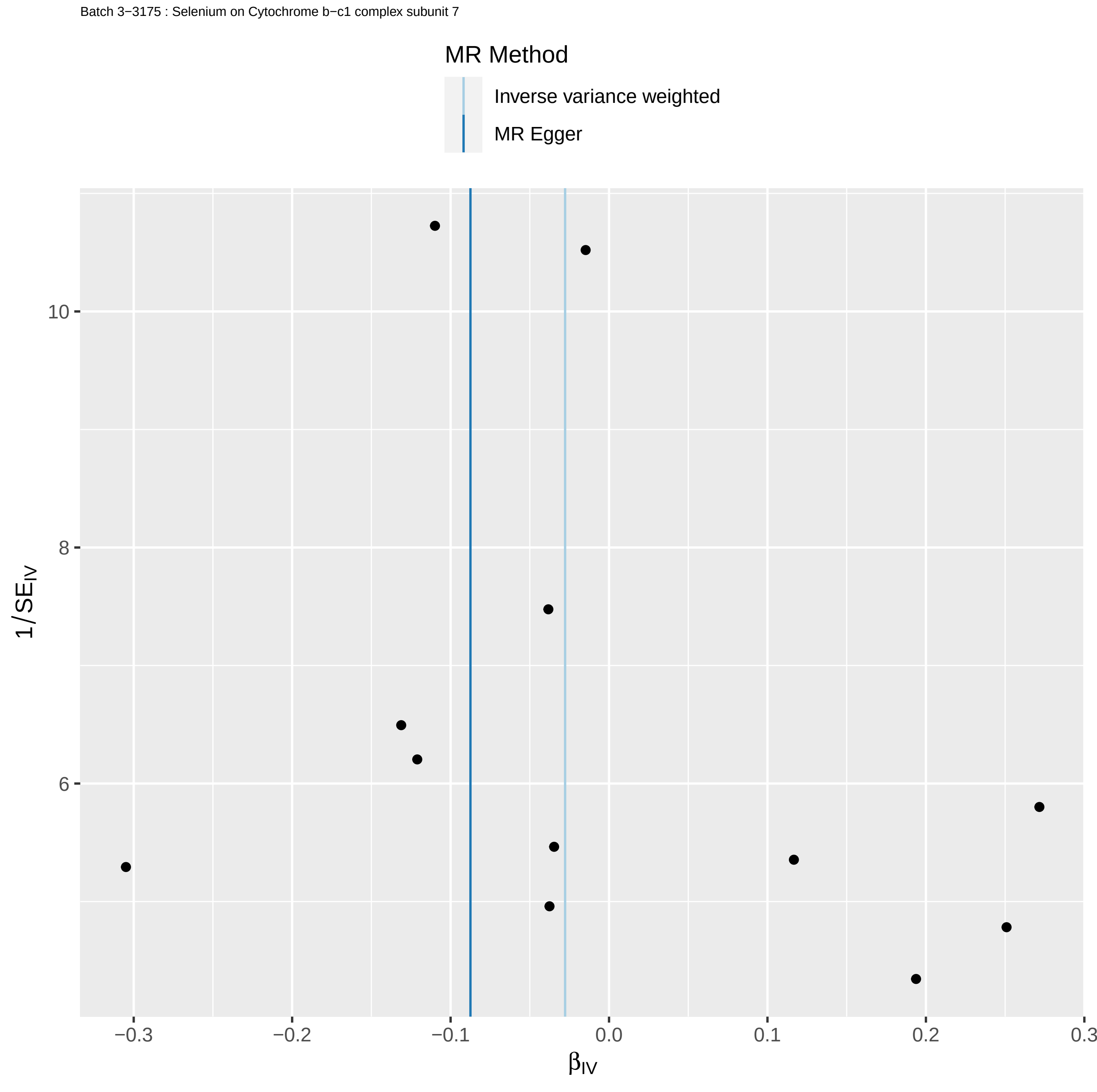
Batch 3−3175 : Selenium on Cytochrome b−c1 complex subunit 7
MR Method
Inverse variance weighted
MR Egger
10
8
6
1/SE$_{IV}$
−0.3
−0.2
−0.1
0.0
0.1
0.2
0.3
β$_{IV}$

Batch 3−3176 : Selenium on Uroporphyrinogen−III synthase

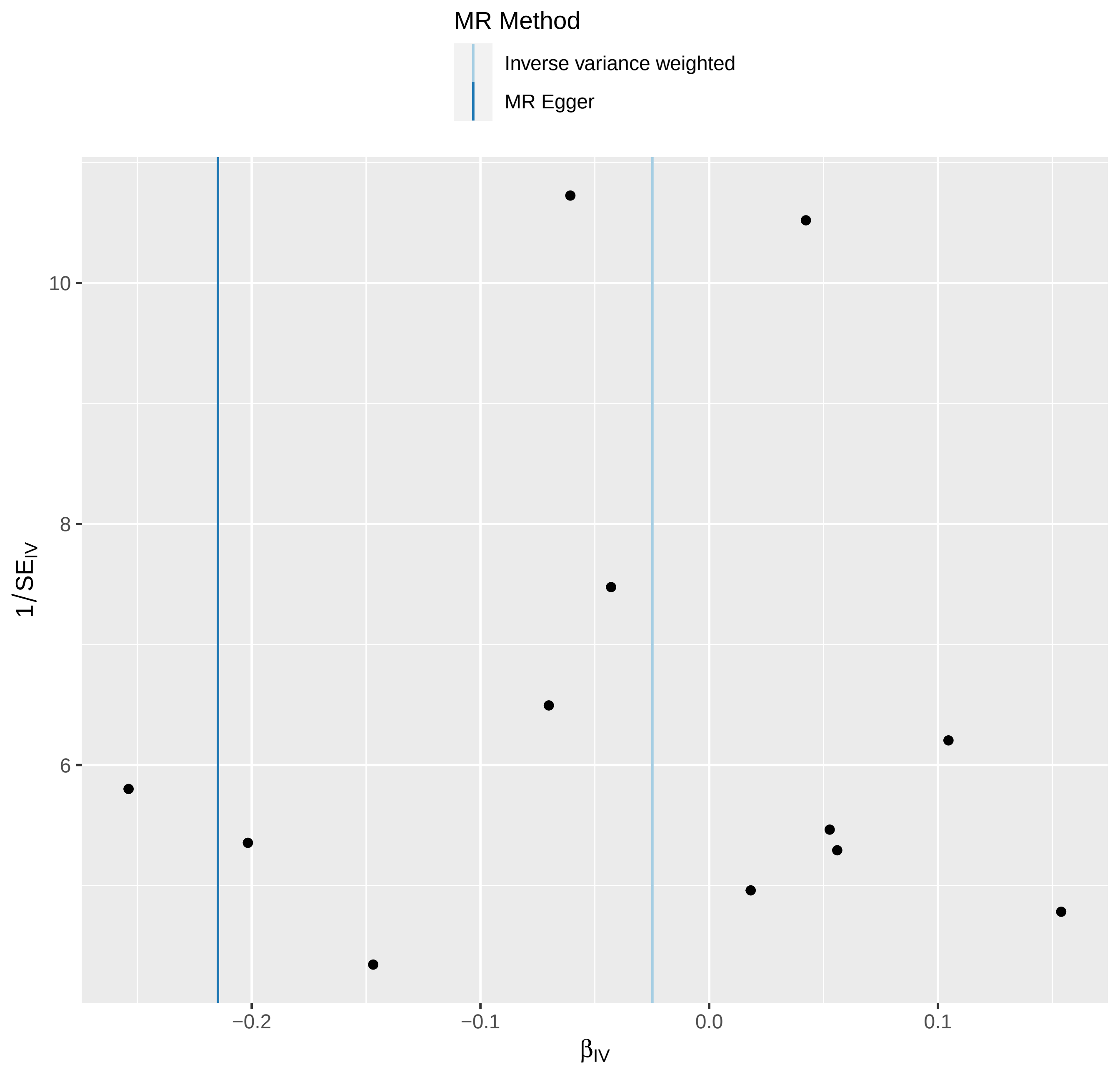

Batch 3−3177 : Selenium on U6 snRNA phosphodiesterase

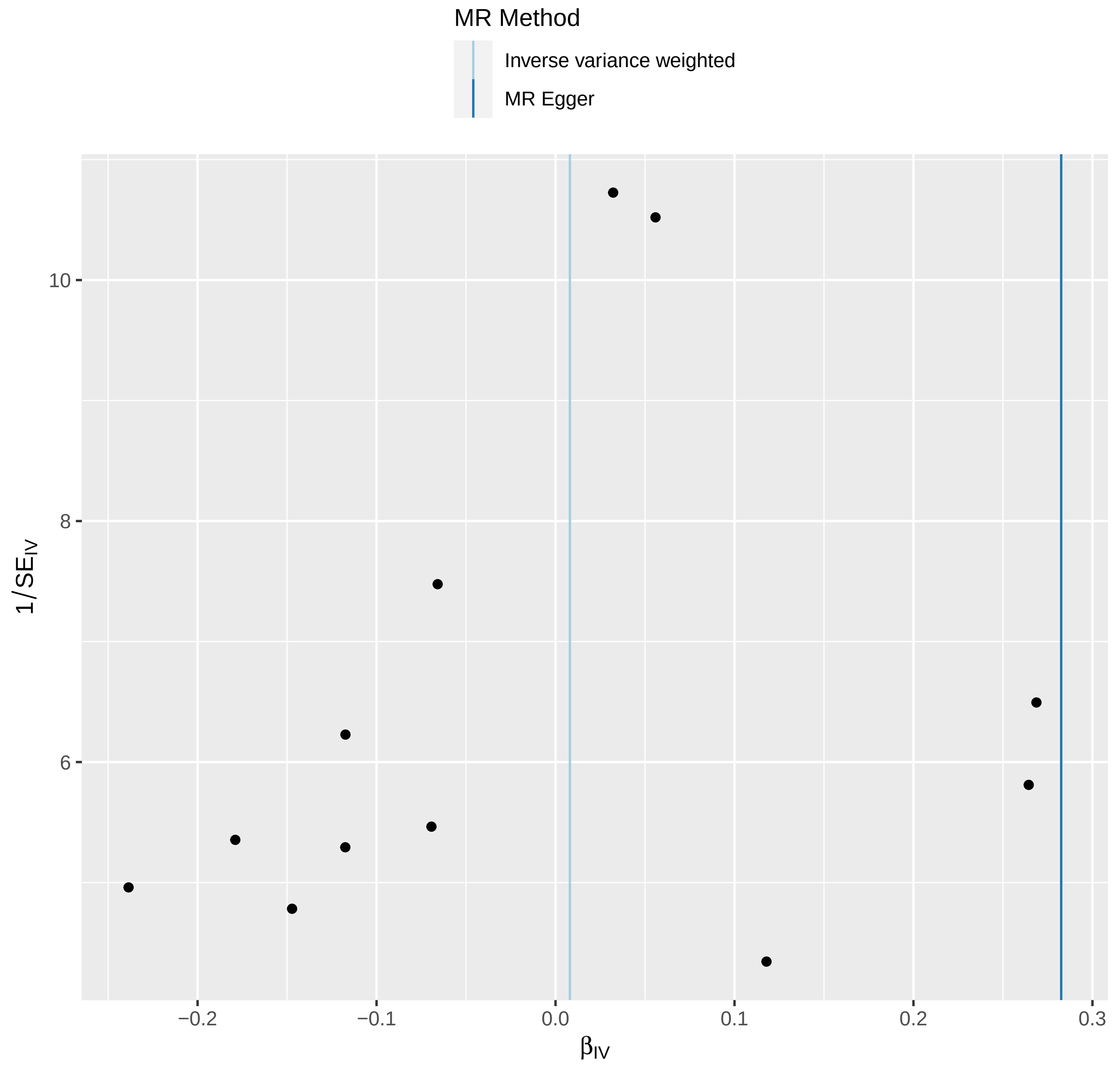

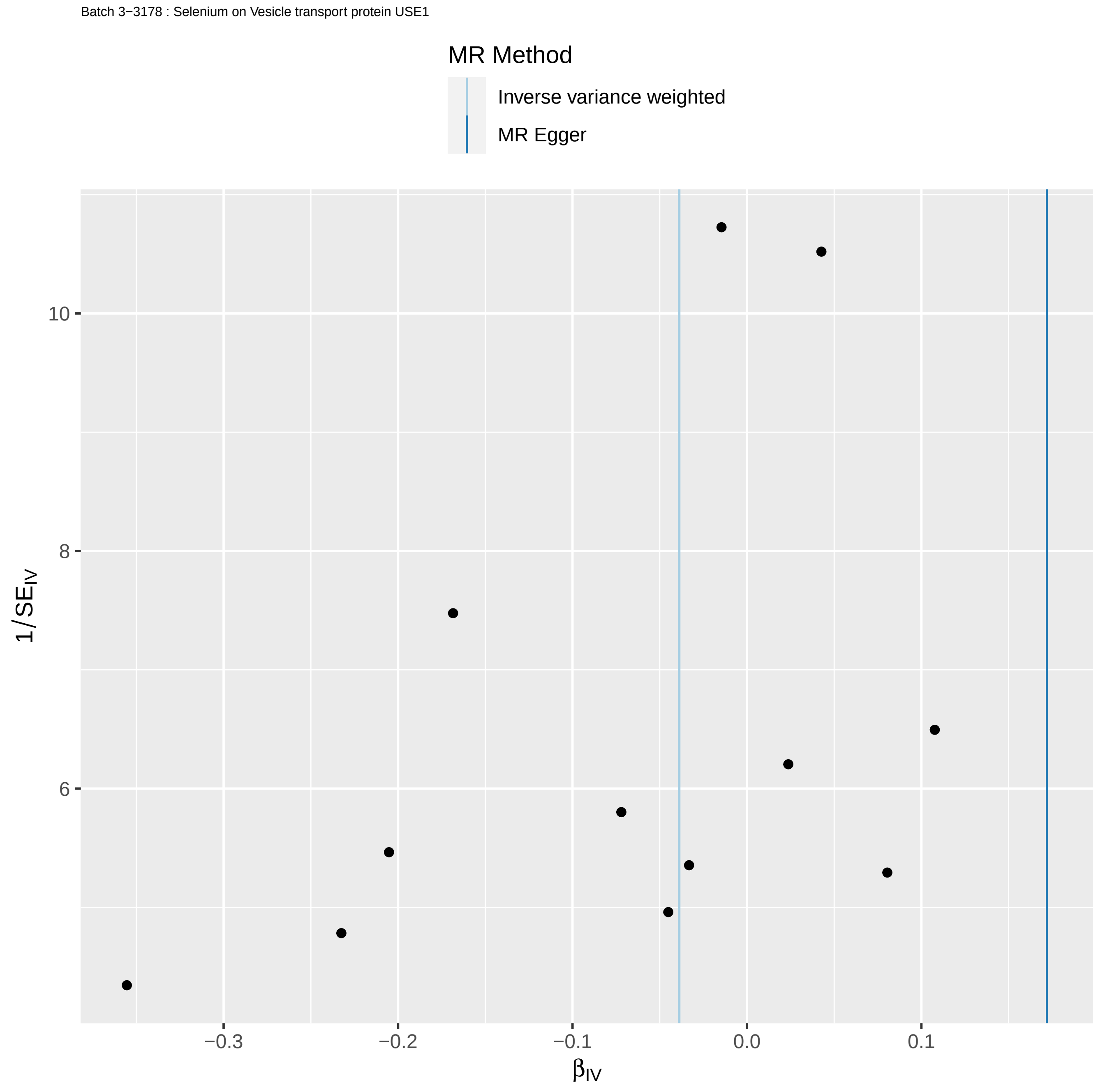
Batch 3−3178 : Selenium on Vesicle transport protein USE1
MR Method
Inverse variance weighted
MR Egger
10
8
6
1/SE_IV
−0.3
−0.2
−0.1
0.0
0.1
β_IV

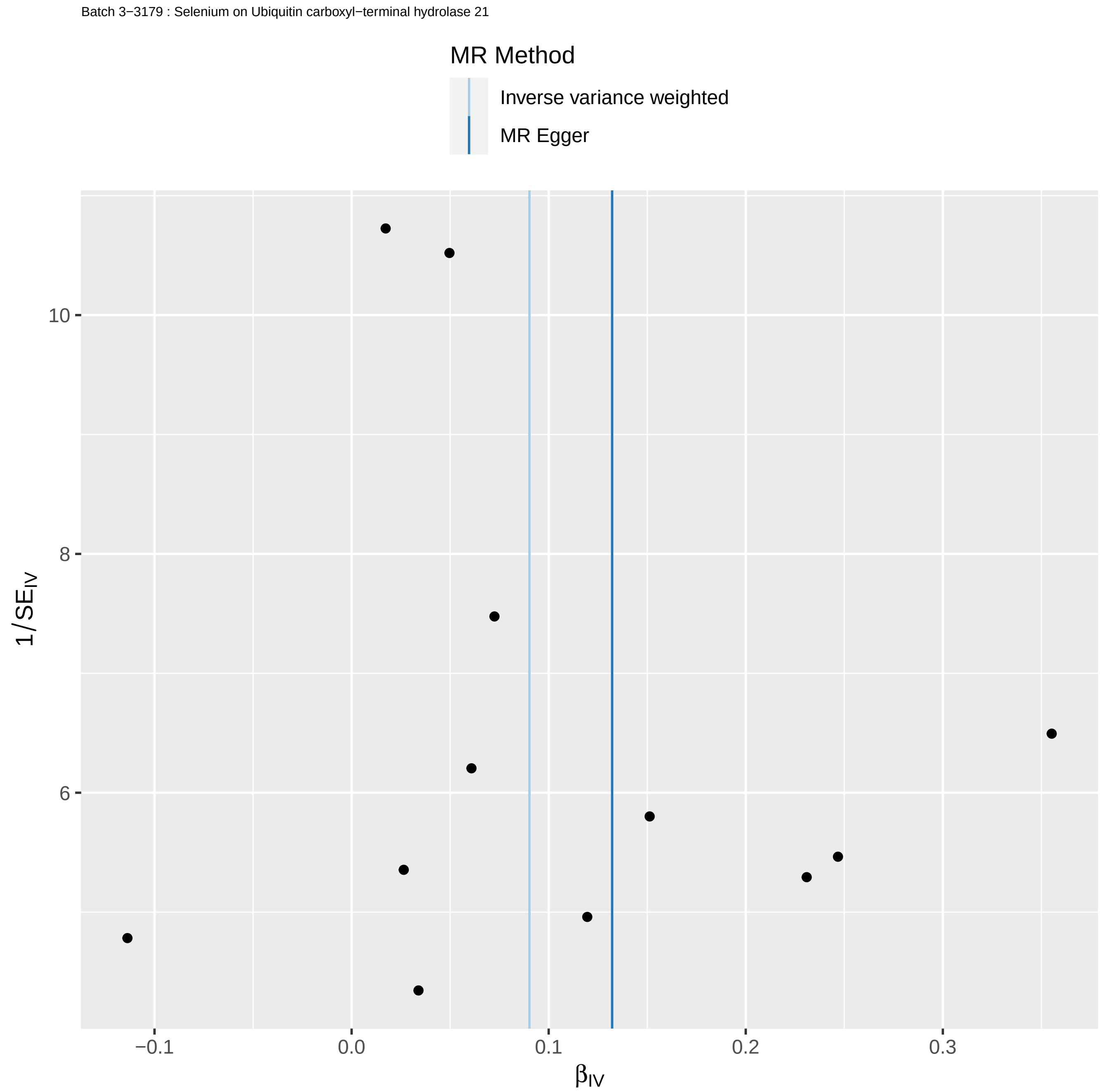

Batch 3−3179 : Selenium on Ubiquitin carboxyl−terminal hydrolase 21
MR Method
Inverse variance weighted
MR Egger
10
8
6
1/SE$_{IV}$
−0.1
0.0
0.1
0.2
0.3
β$_{IV}$

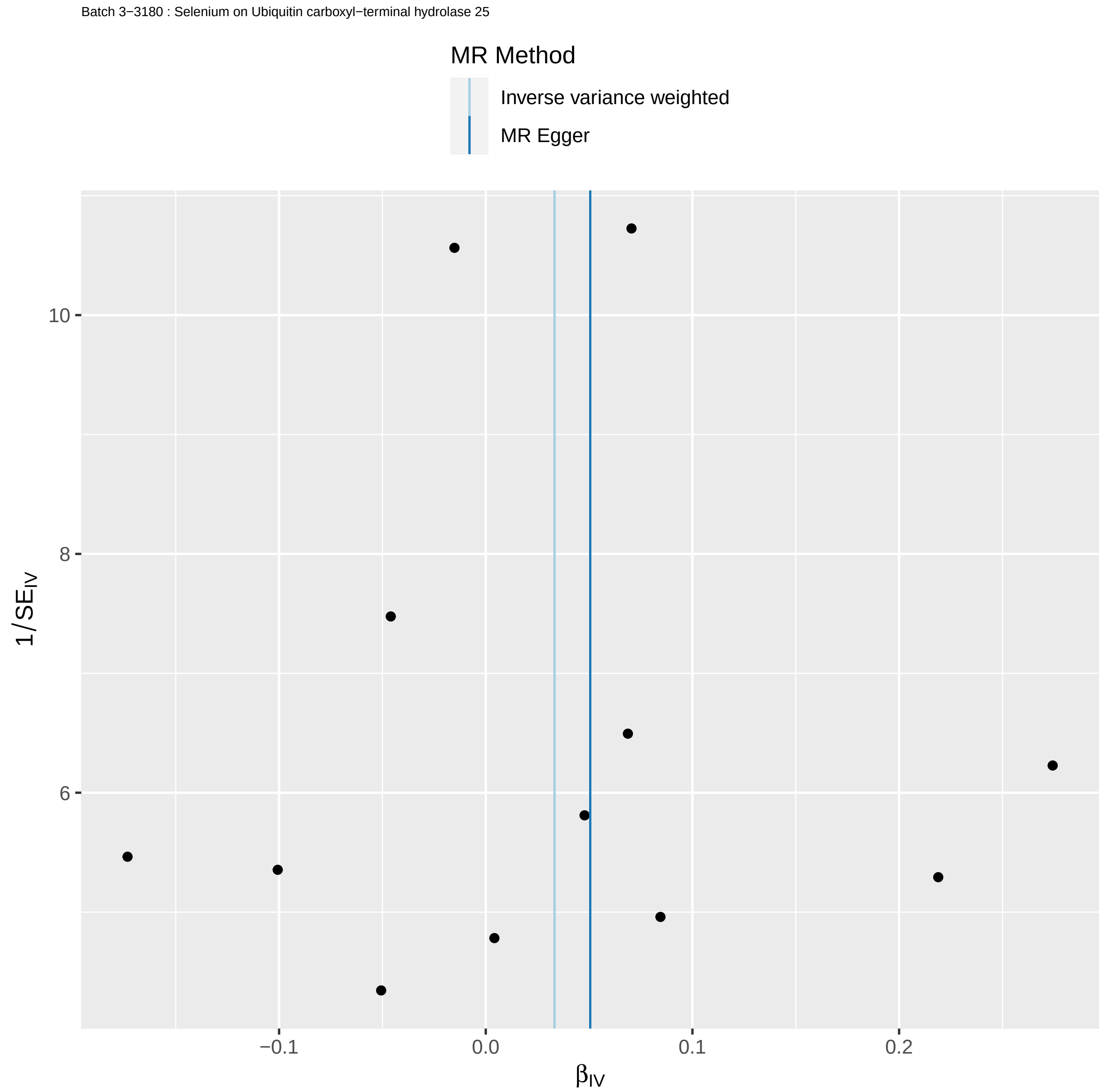

Batch 3−3180 : Selenium on Ubiquitin carboxyl−terminal hydrolase 25
MR Method
Inverse variance weighted
MR Egger
10
8
6
1/SE$_{IV}$
−0.1
0.0
0.1
0.2
β$_{IV}$

Batch 3−3181 : Selenium on Ubiquitin carboxyl−terminal hydrolase 8

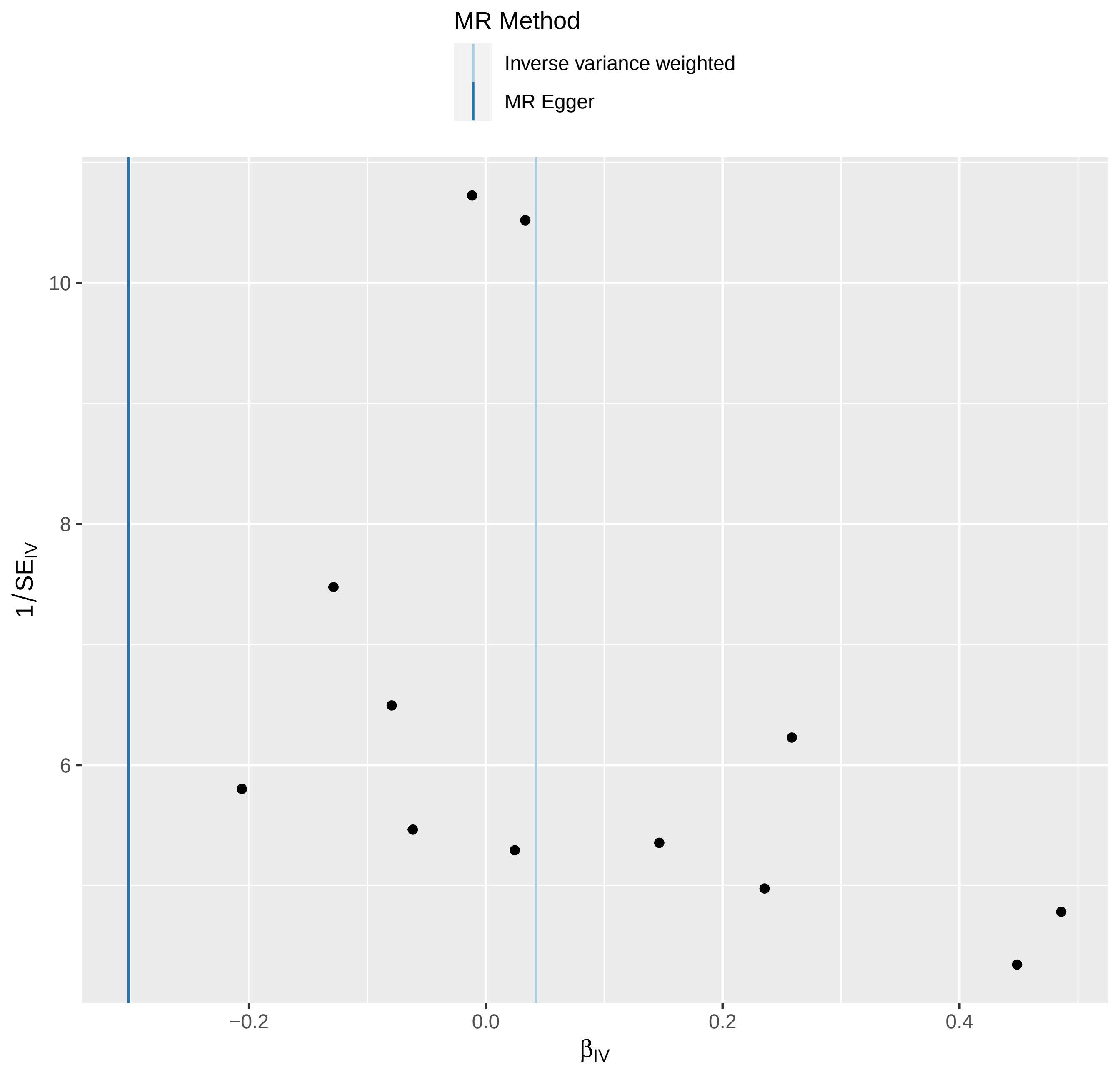

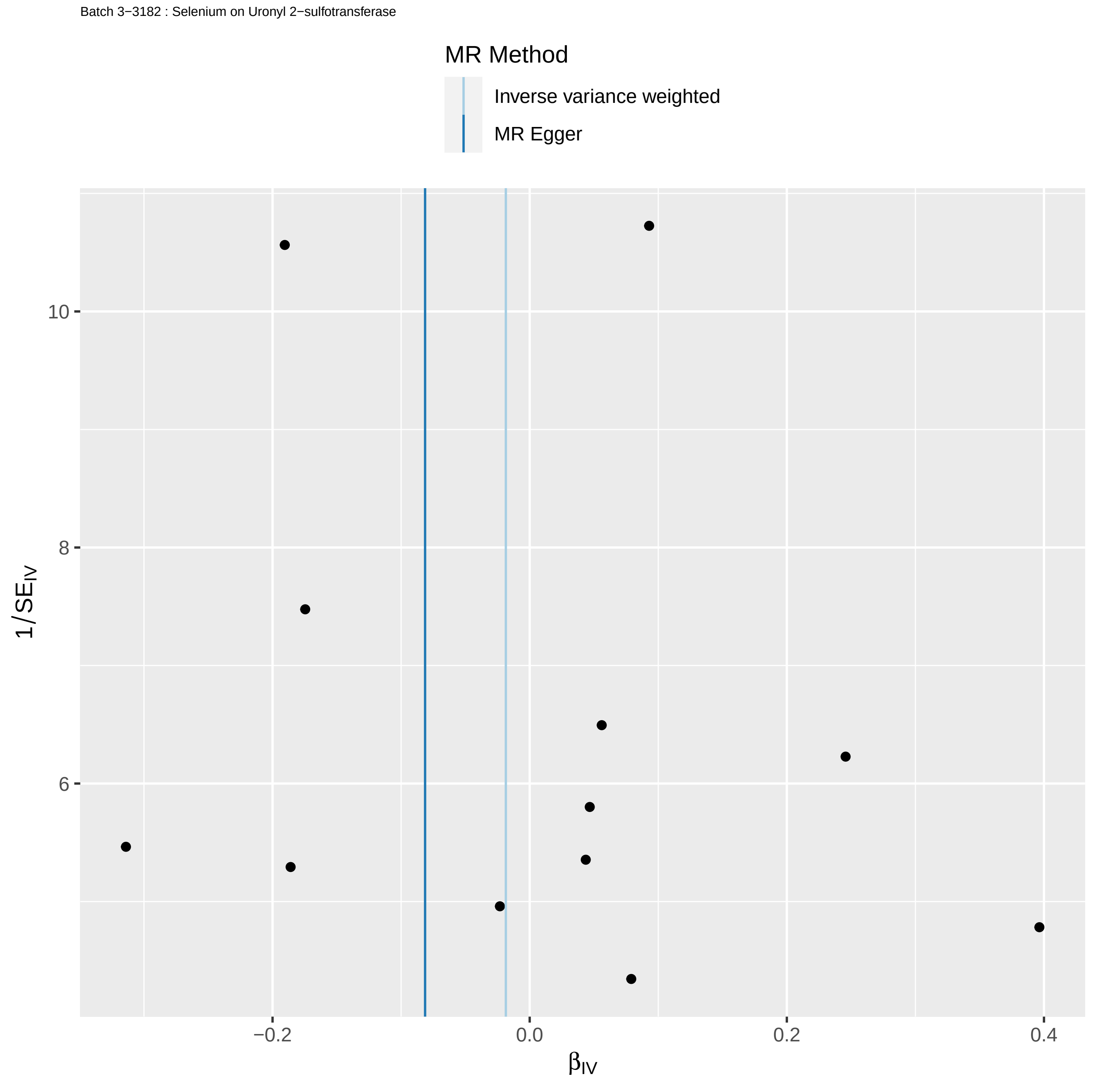
Batch 3−3182 : Selenium on Uronyl 2−sulfotransferase
MR Method
Inverse variance weighted
MR Egger
1/SE_IV
10
8
6
−0.2
0.0
0.2
0.4
β_IV

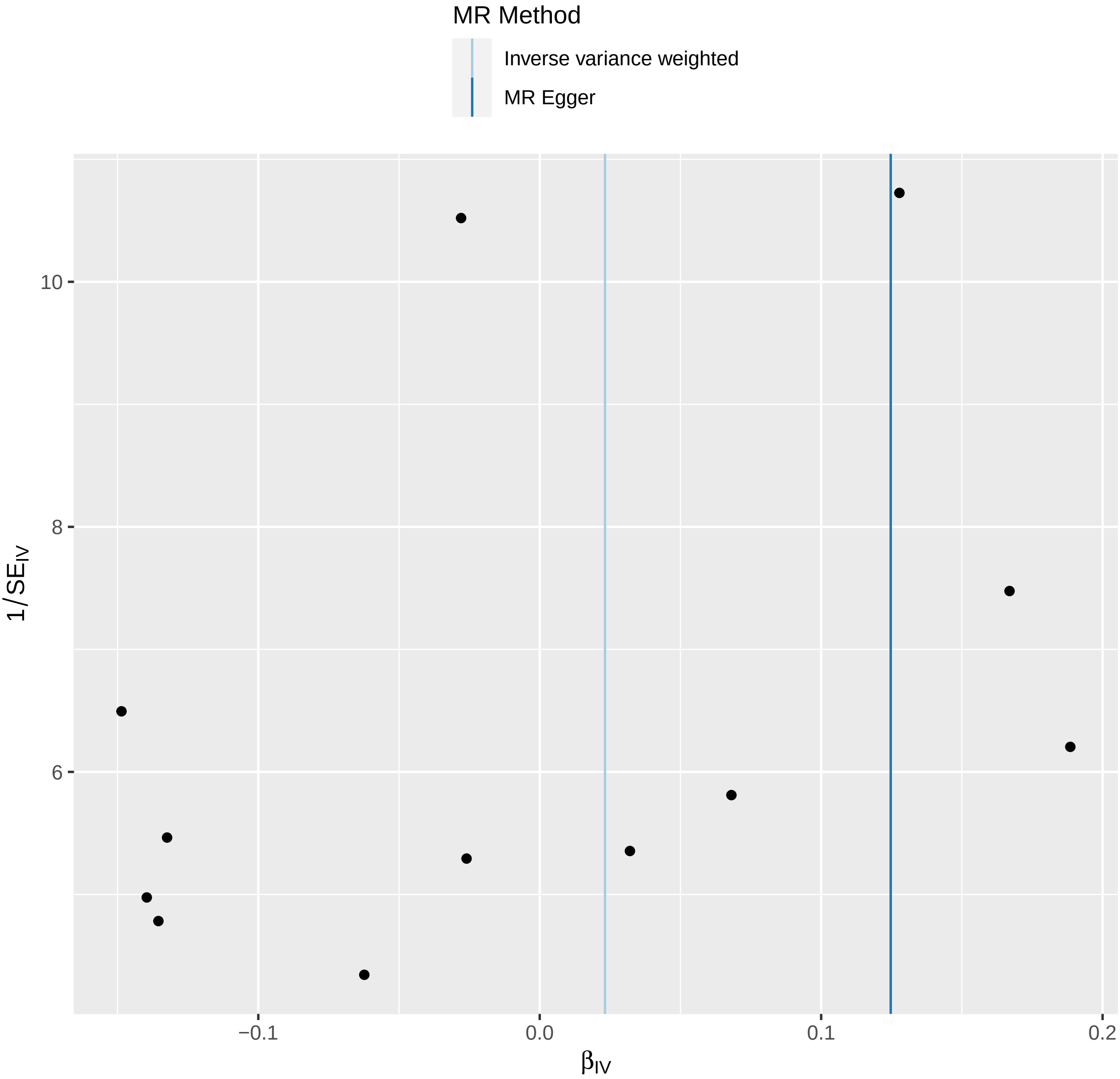
MR Method
Inverse variance weighted
MR Egger
10
8
6
−0.1
0.0
0.1
0.2
$1/SE_{IV}$
β_{IV}

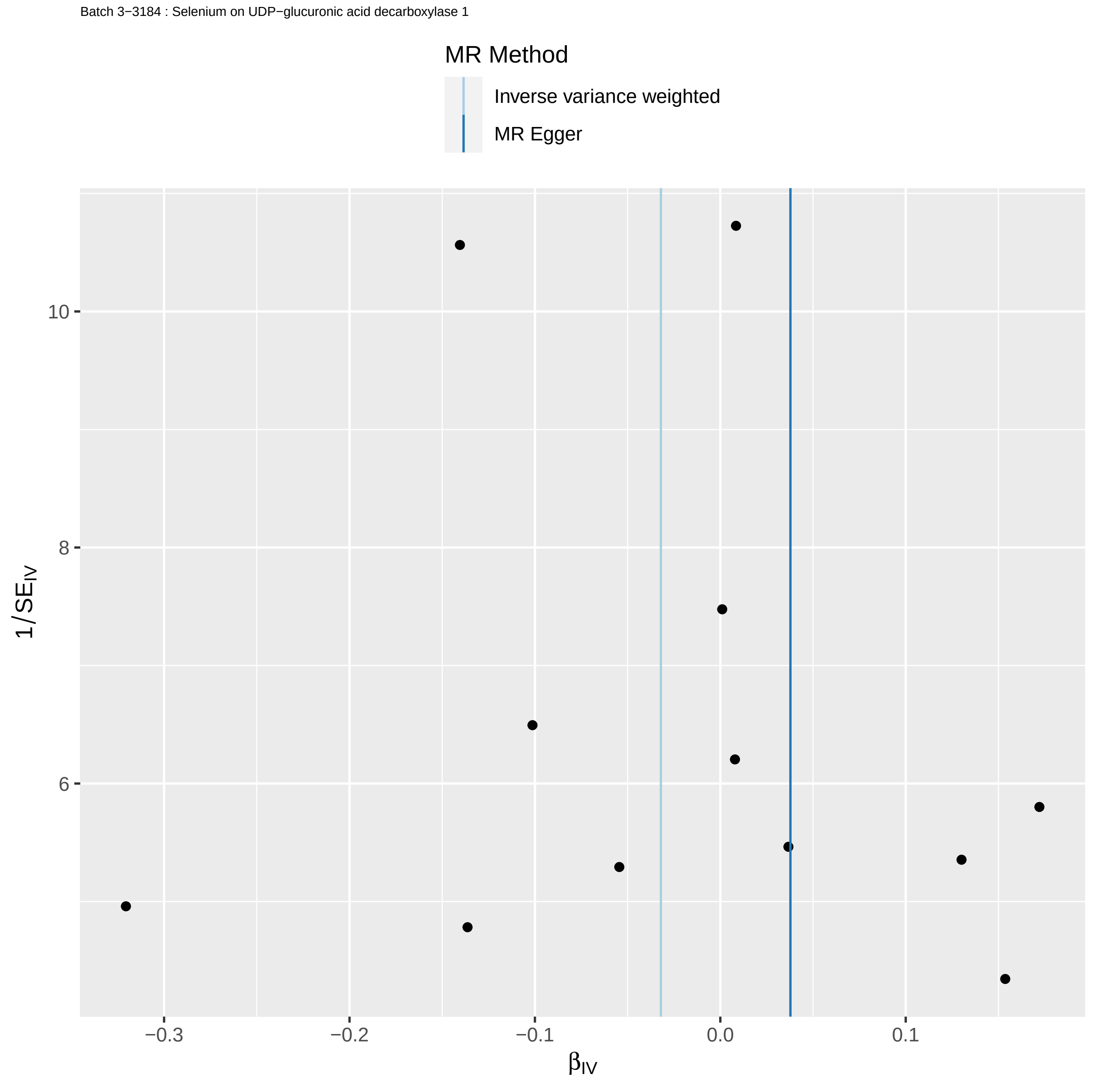

Batch 3−3184 : Selenium on UDP−glucuronic acid decarboxylase 1
MR Method
Inverse variance weighted
MR Egger
10
8
6
1/SE$_{IV}$
−0.3
−0.2
−0.1
0.0
0.1
β$_{IV}$

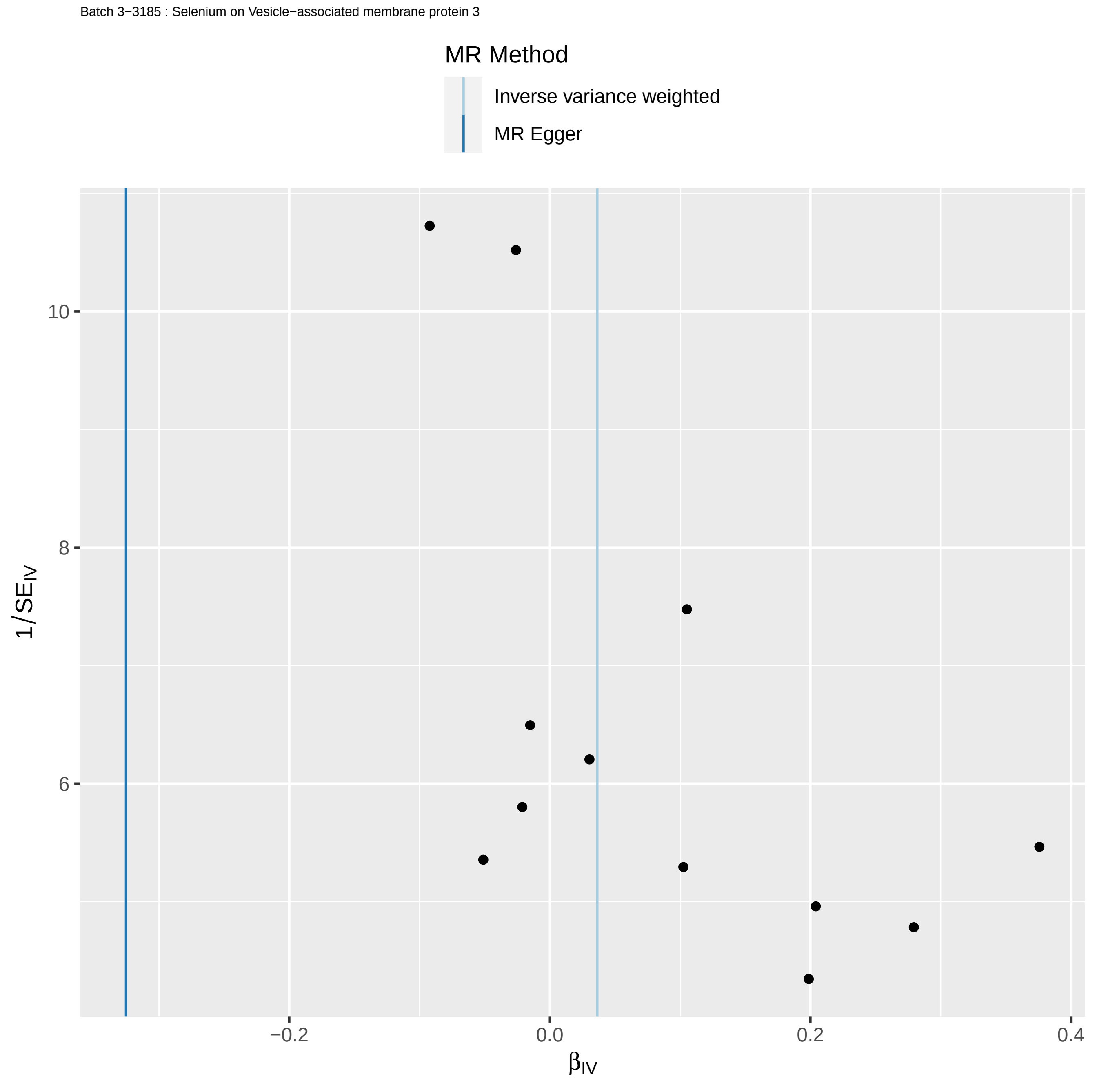
Batch 3−3185 : Selenium on Vesicle−associated membrane protein 3
MR Method
Inverse variance weighted
MR Egger
10
8
6
1/SE$_{IV}$
−0.2
0.0
0.2
0.4
β$_{IV}$

Batch 3−3186 : Selenium on Vesicle−associated membrane protein 4

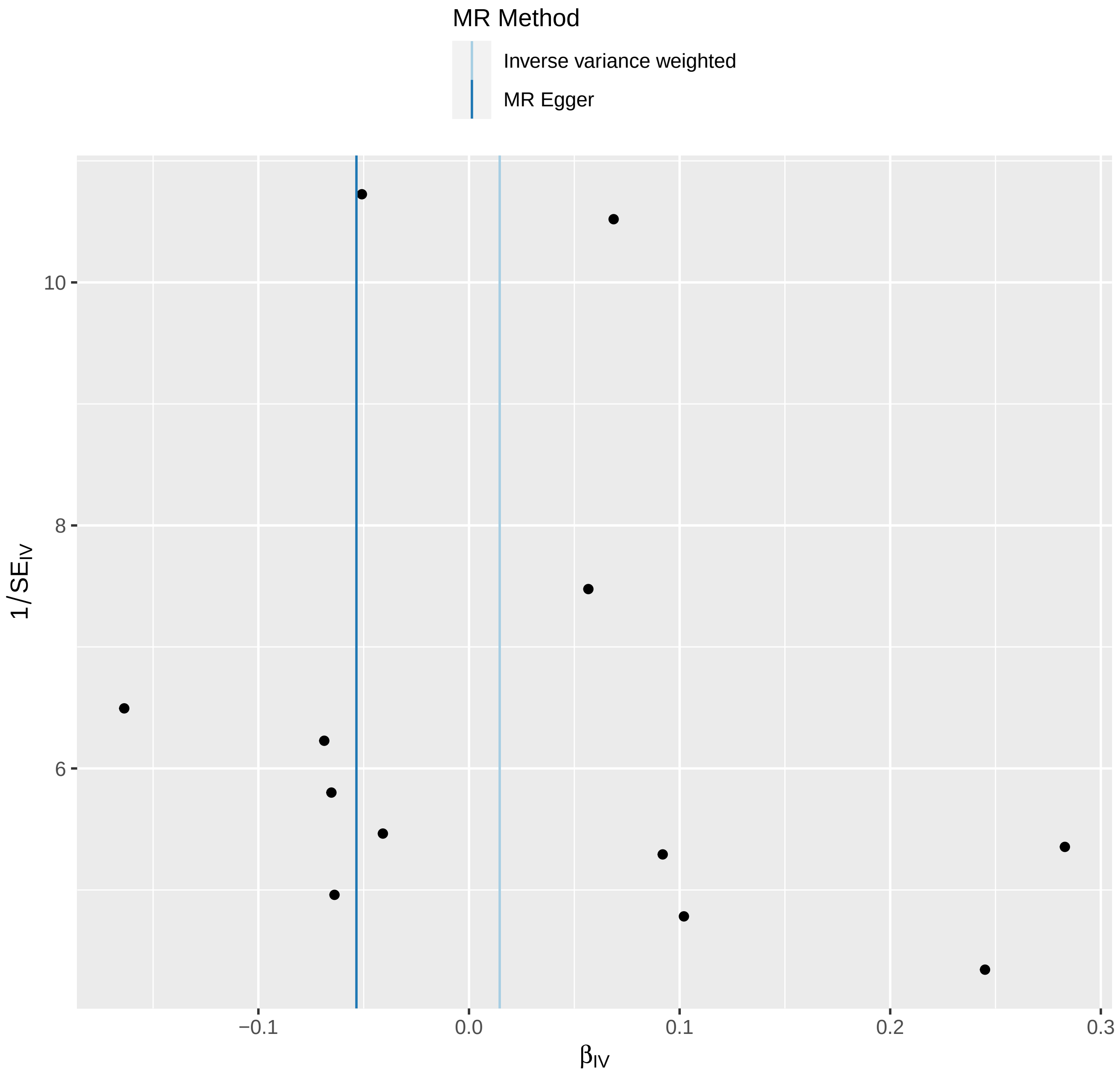

Batch 3−3187 : Selenium on Vesicle−associated membrane protein 5

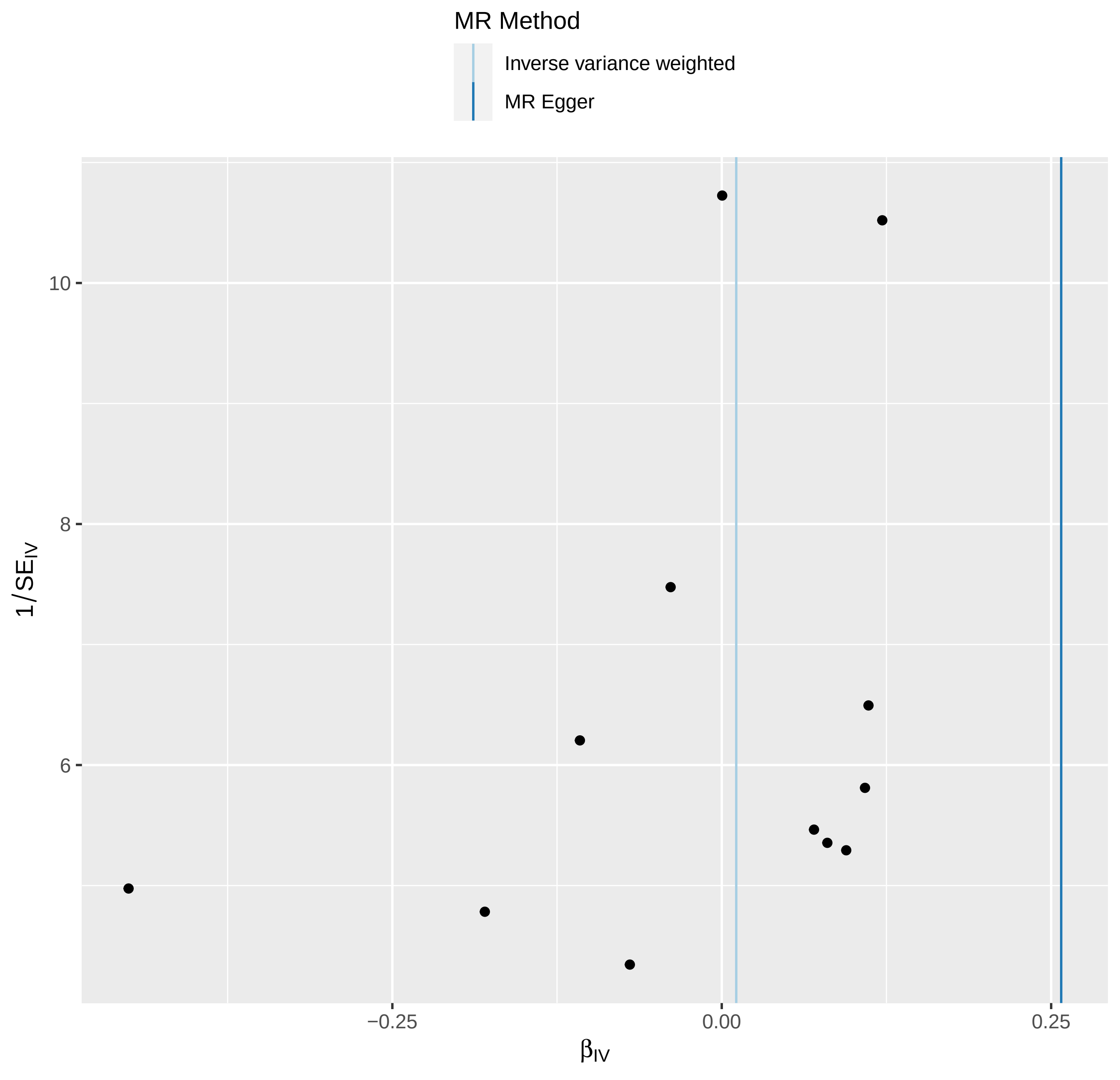

Batch 3−3188 : Selenium on Vesicle−associated membrane protein 8

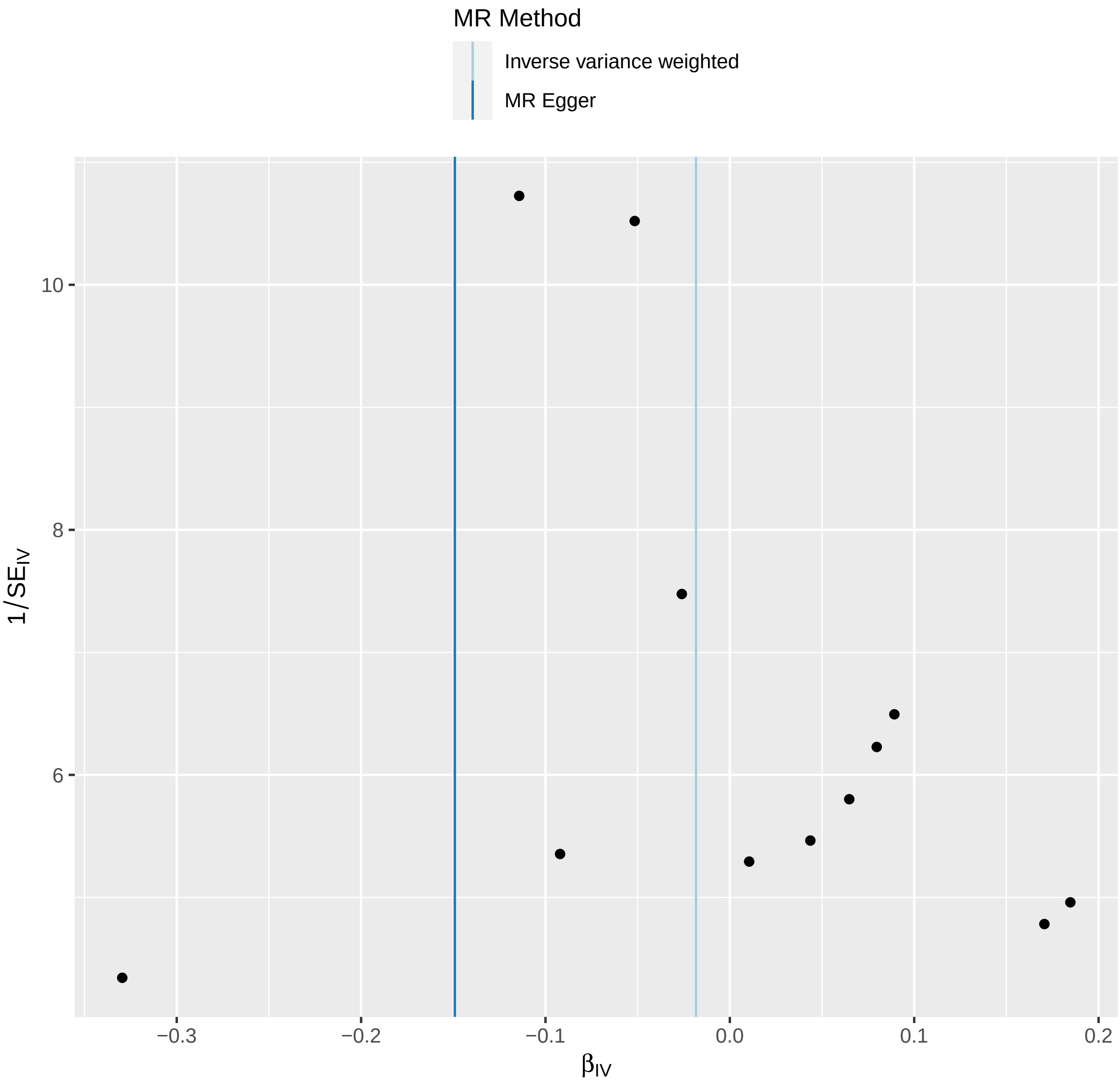

Batch 3−3189 : Selenium on Vesicle−associated membrane protein−associated protein A

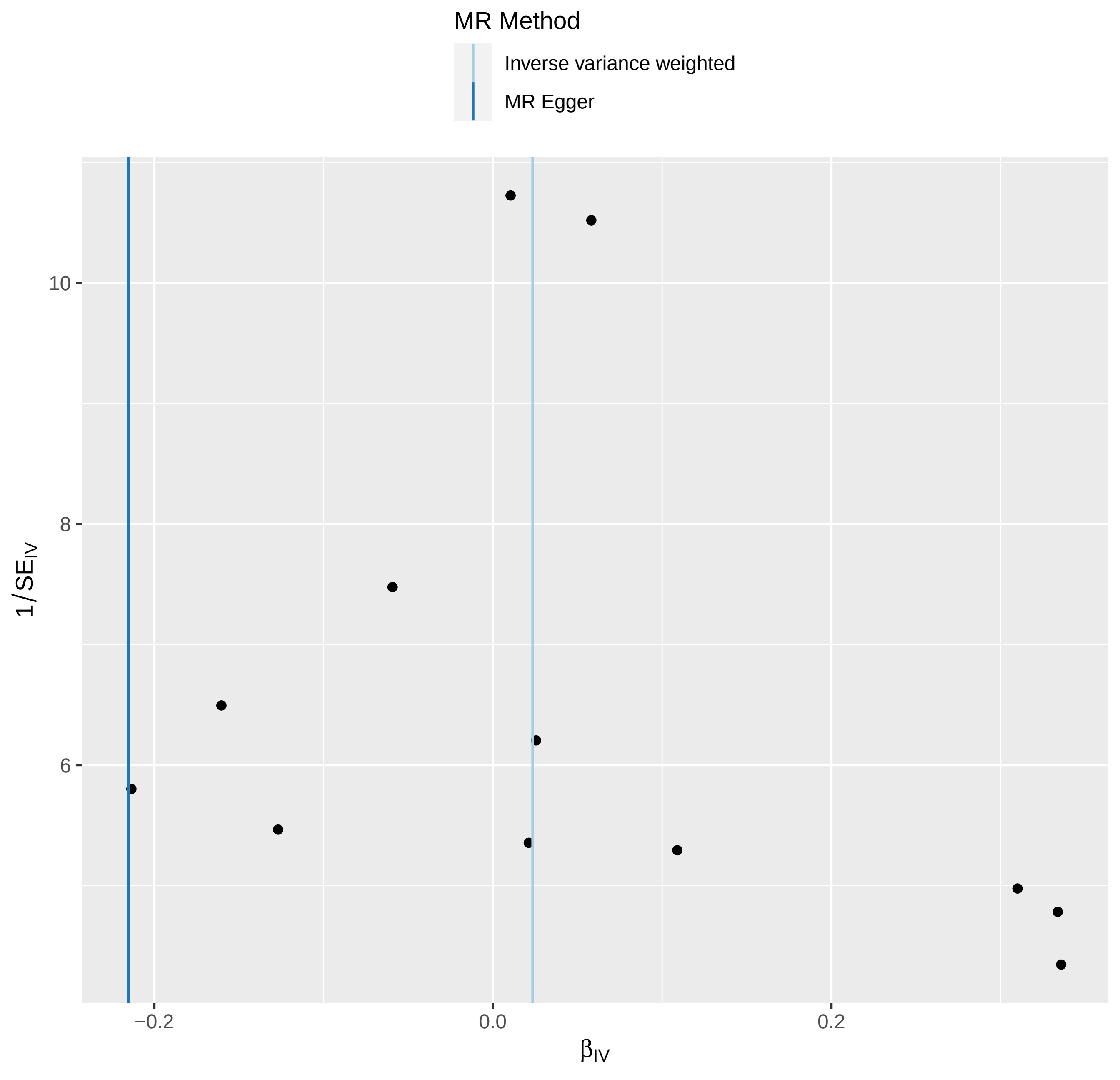

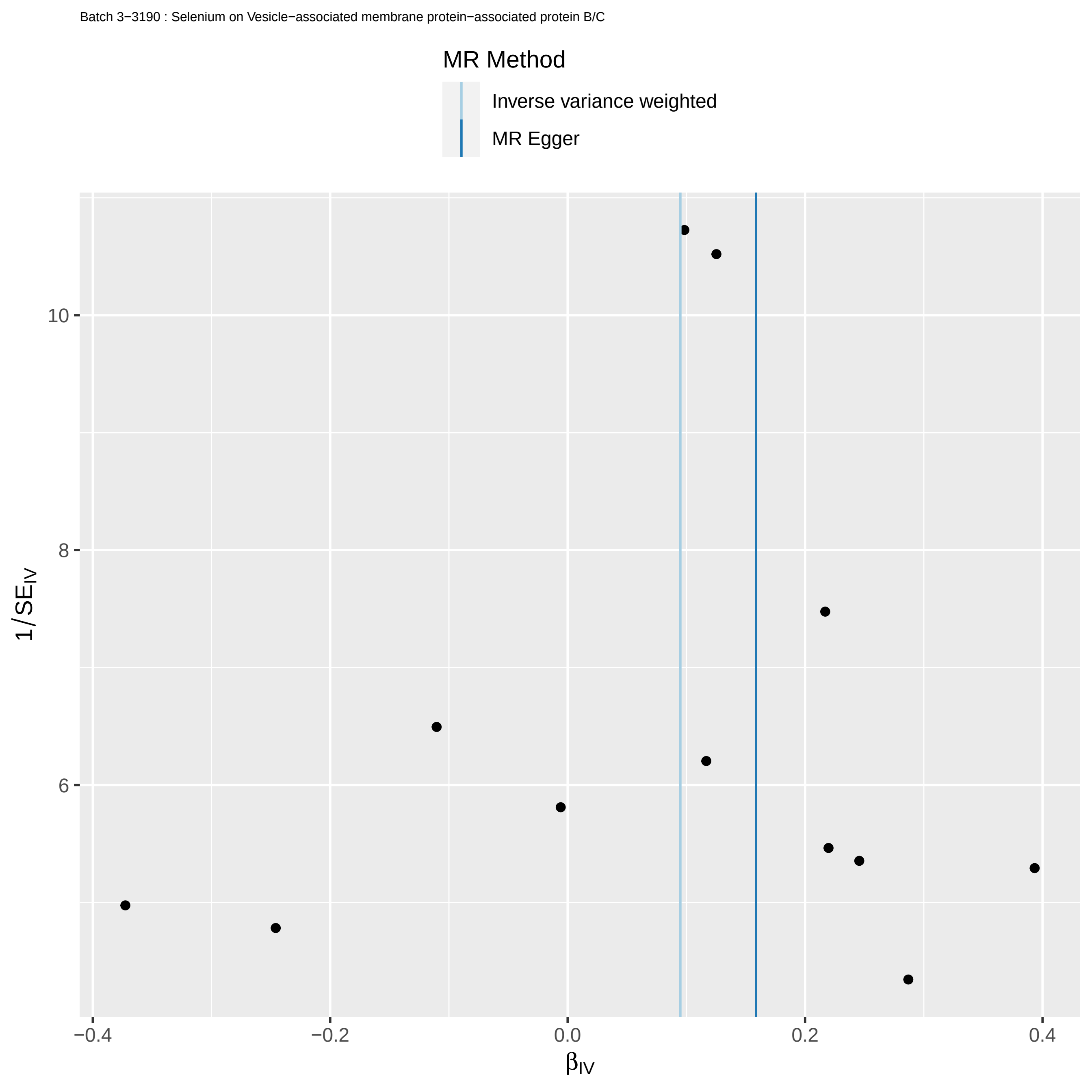
Batch 3−3190 : Selenium on Vesicle−associated membrane protein−associated protein B/C
MR Method
Inverse variance weighted
MR Egger
10
8
6
1/SE$_{IV}$
−0.4
−0.2
0.0
0.2
0.4
β_{IV}

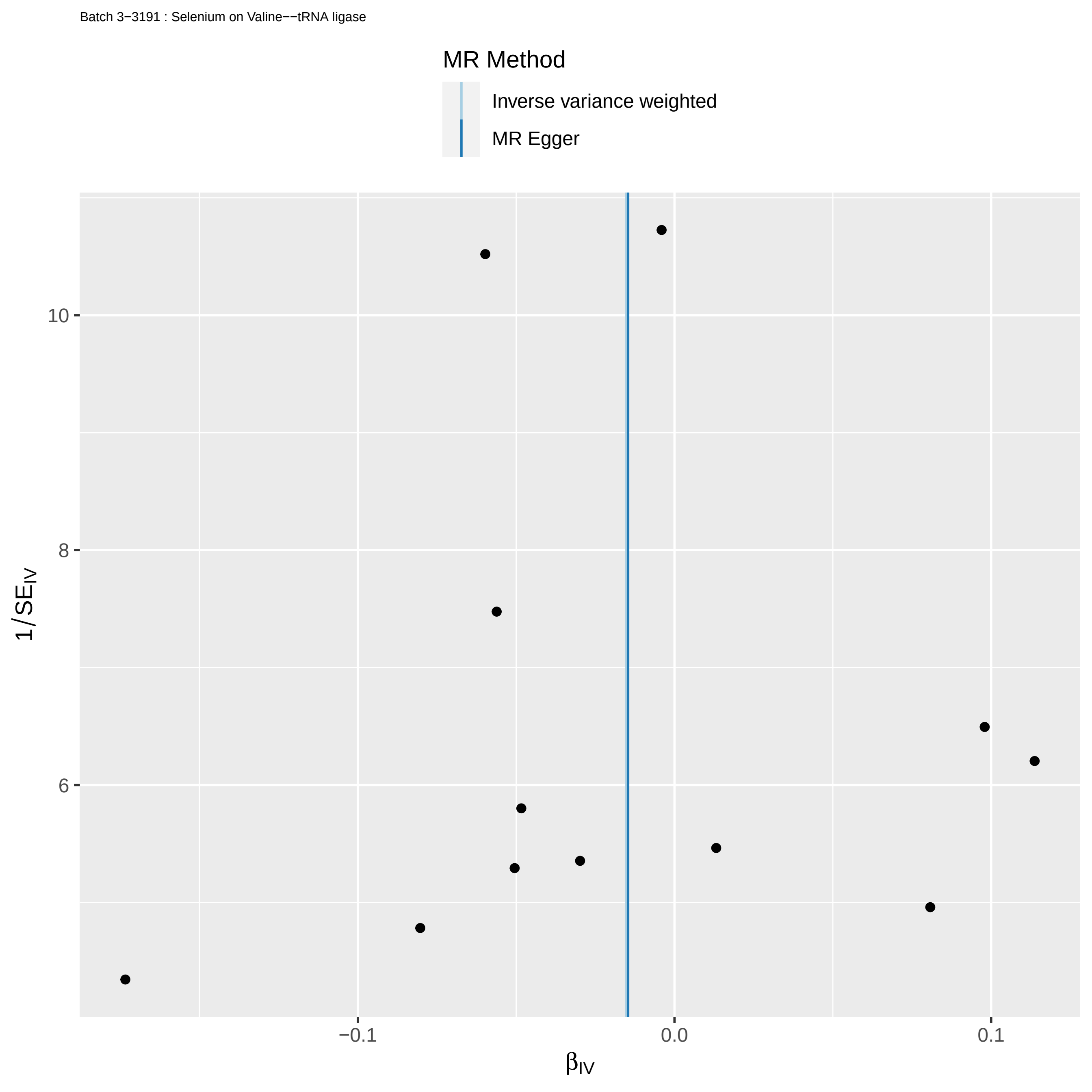

Batch 3−3191 : Selenium on Valine−−tRNA ligase
MR Method
Inverse variance weighted
MR Egger
10
8
6
1/SE_IV
−0.1
0.0
0.1
β_IV

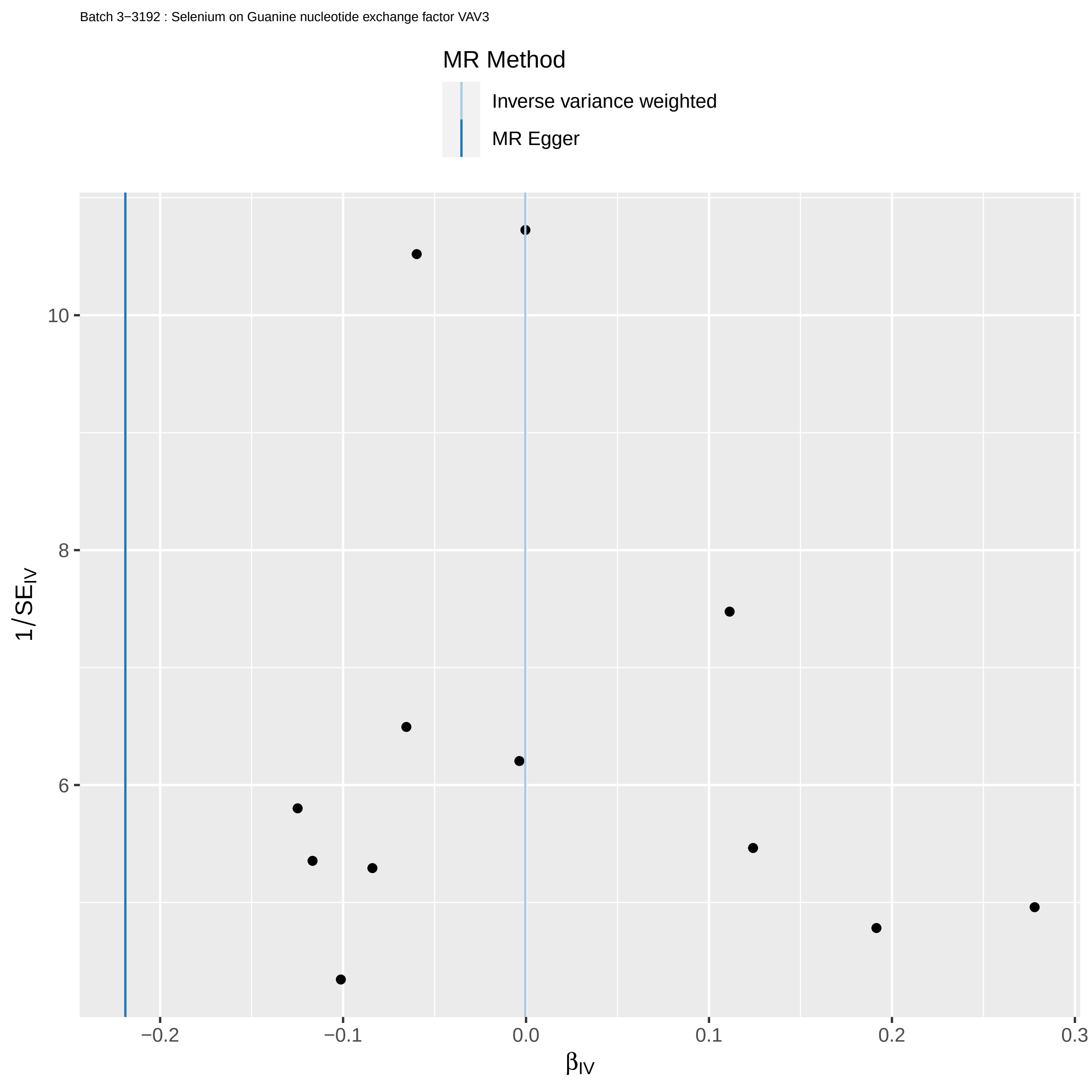

Batch 3−3192 : Selenium on Guanine nucleotide exchange factor VAV3
MR Method
Inverse variance weighted
MR Egger
10
8
6
1/SE_IV
−0.2
−0.1
0.0
0.1
0.2
0.3
β_IV

Batch 3−3193 : Selenium on Vascular cell adhesion protein 1

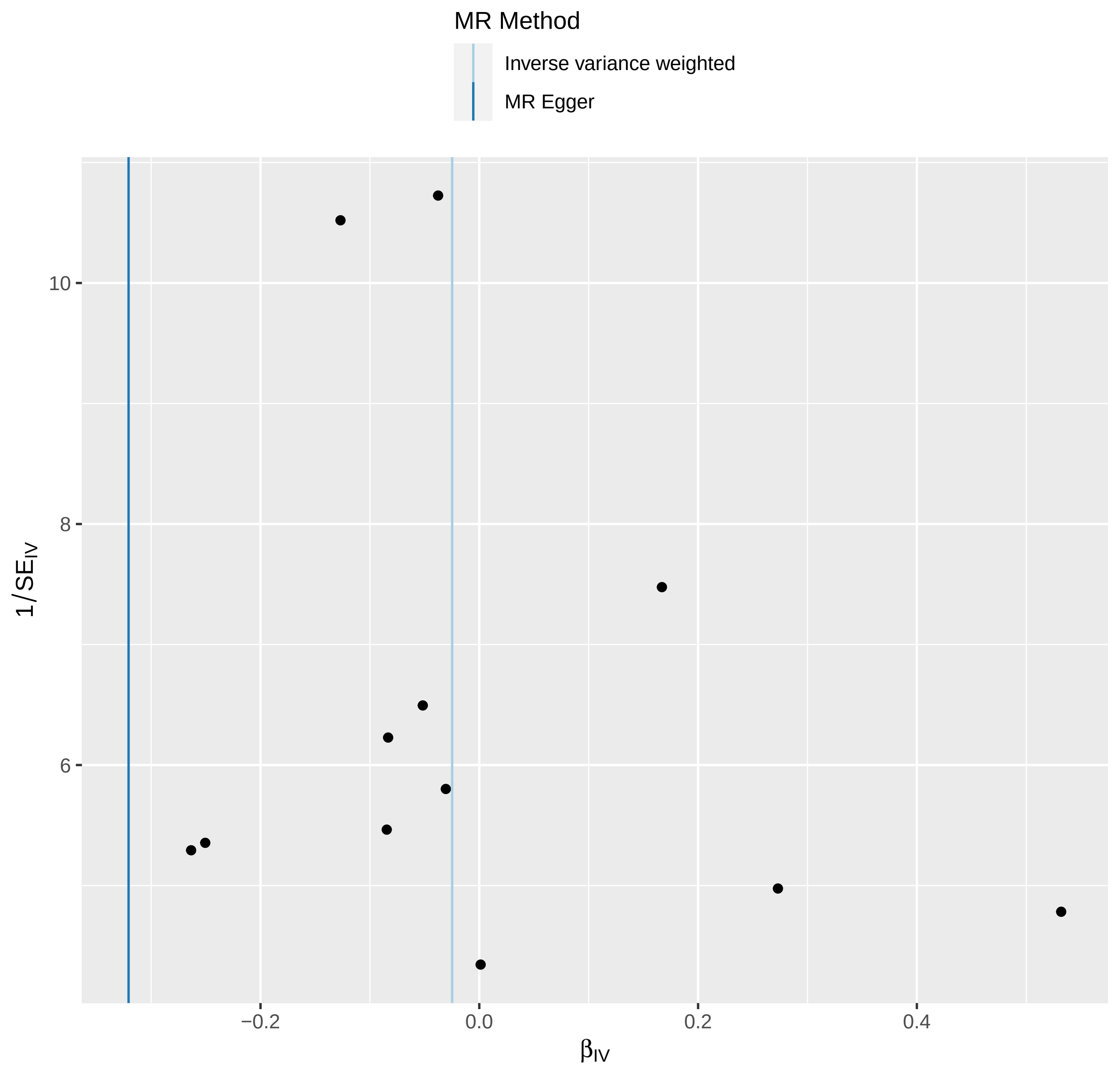

Batch 3−3194 : Selenium on Versican core protein

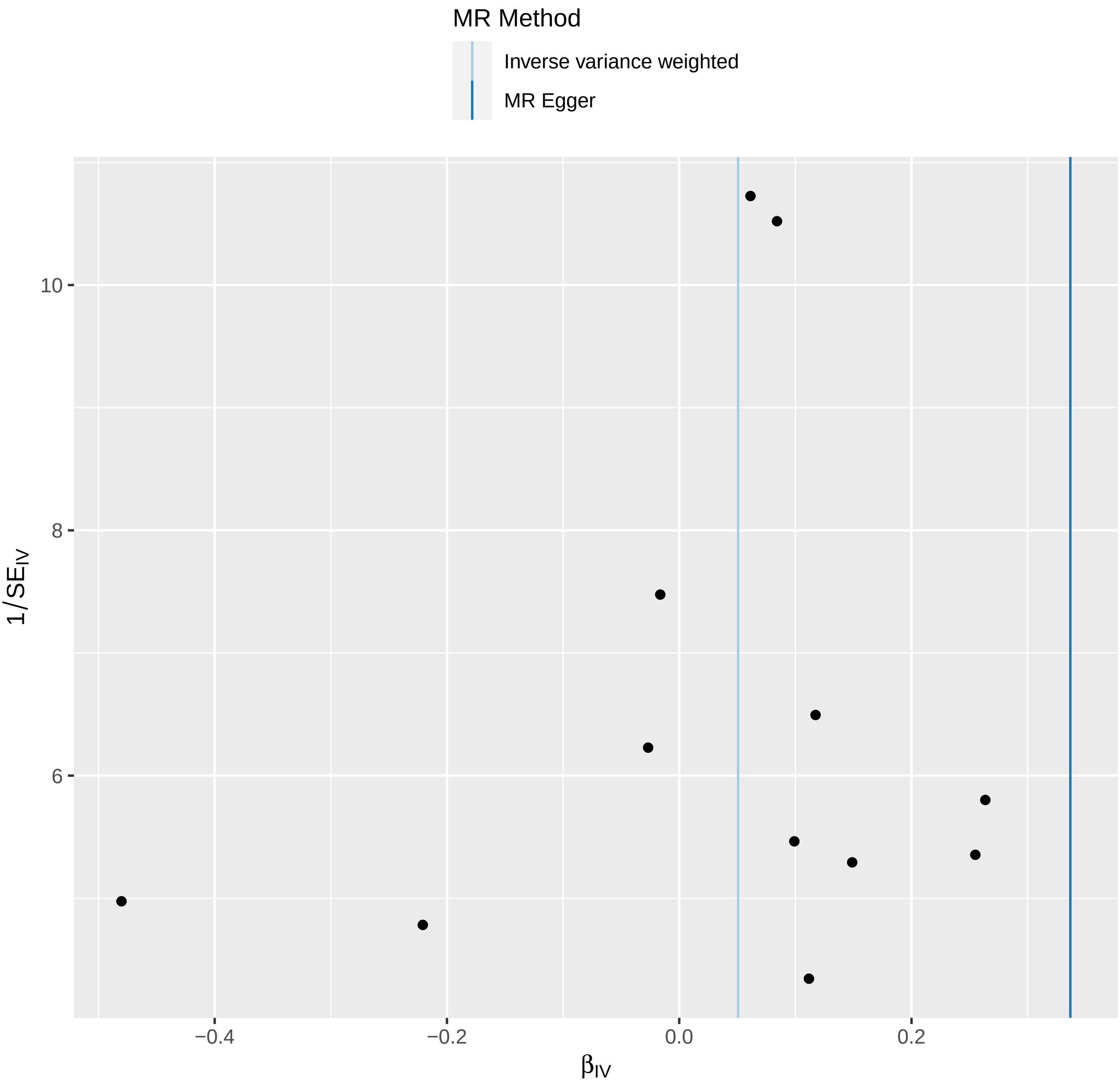

Batch 3−3195 : Selenium on Vinculin

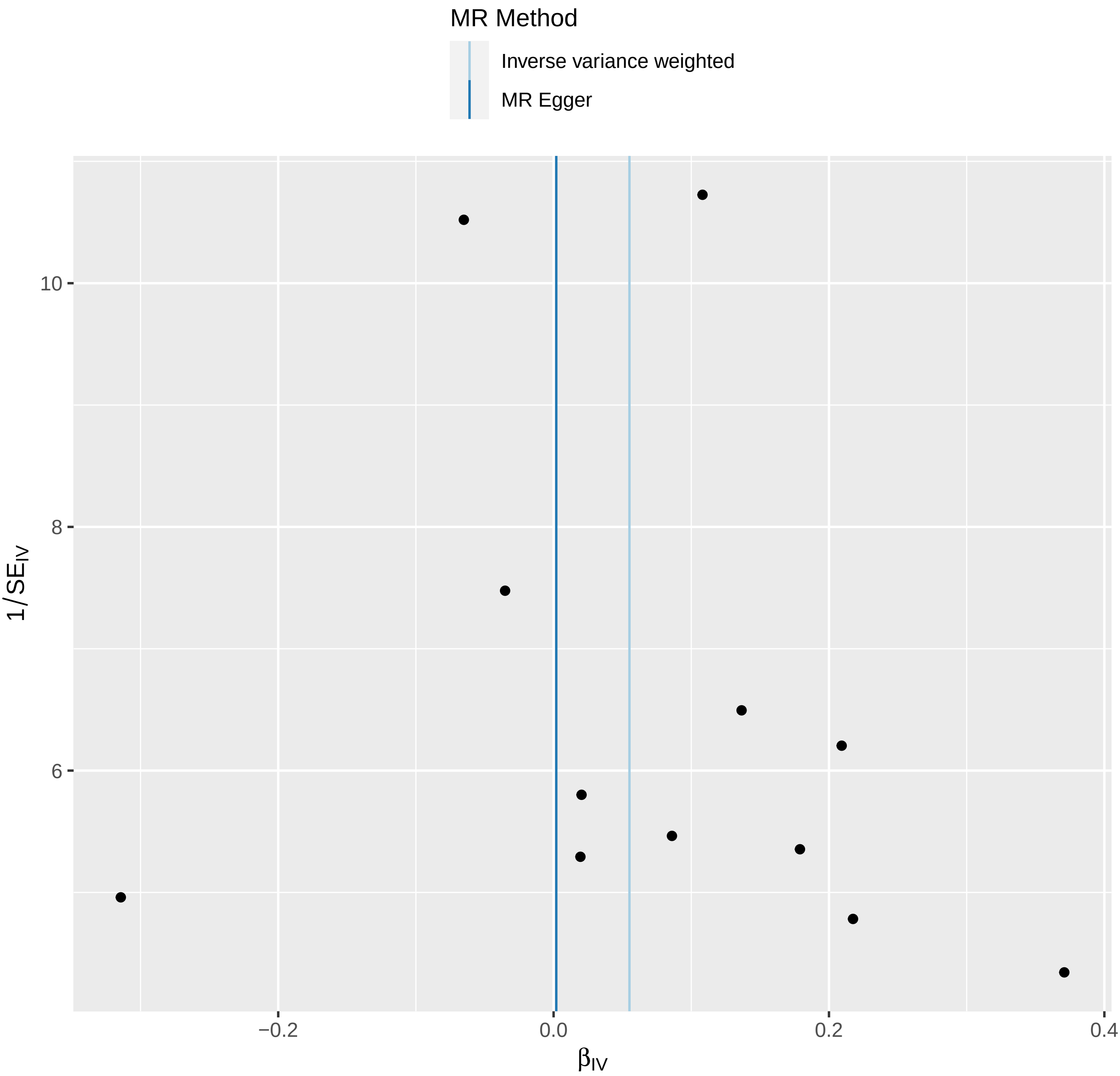

Batch 3−3196 : Selenium on Testis−specific basic protein Y 1

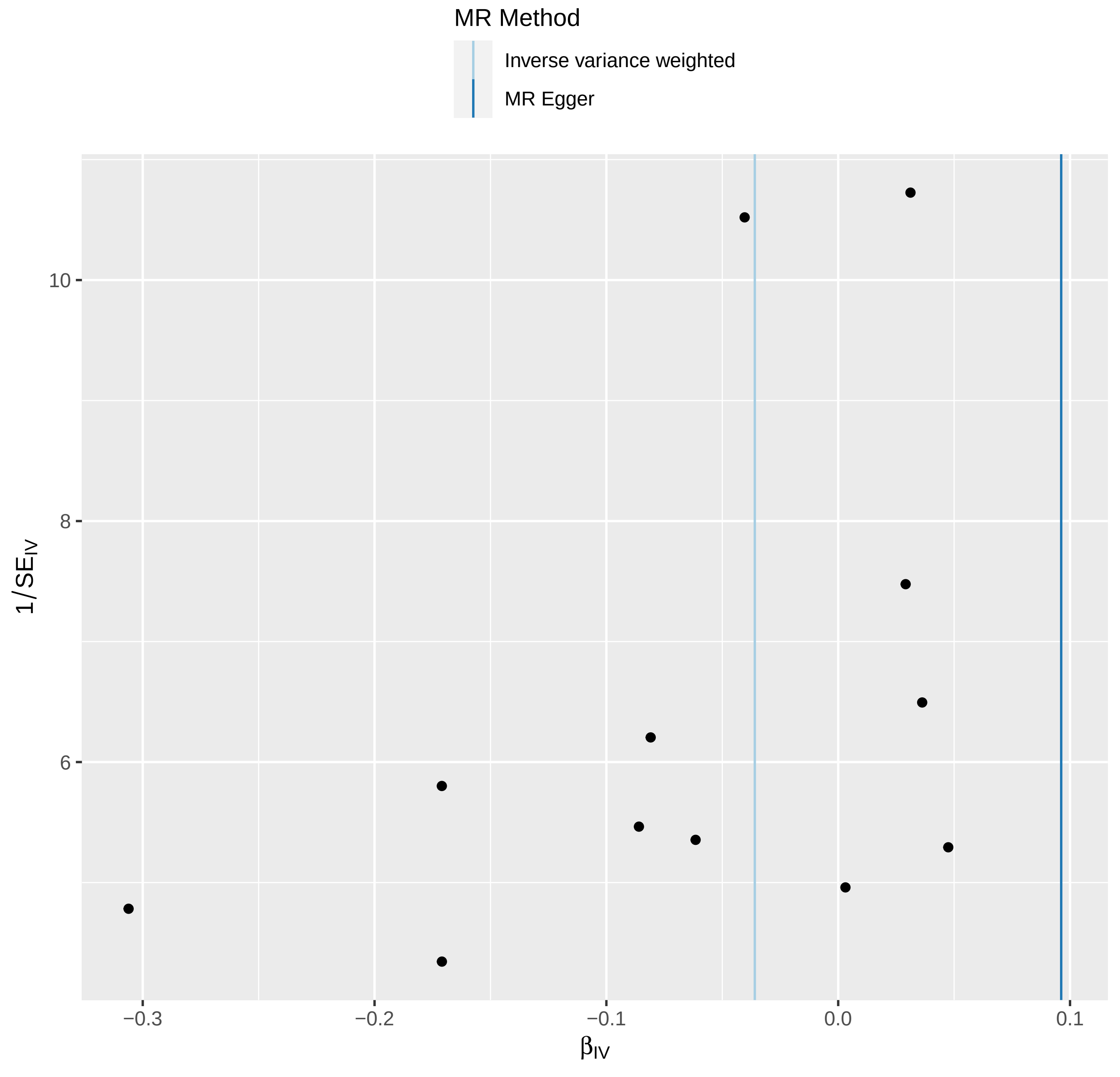

Batch 3−3197 : Selenium on Vascular endothelial growth factor A, isoform 121

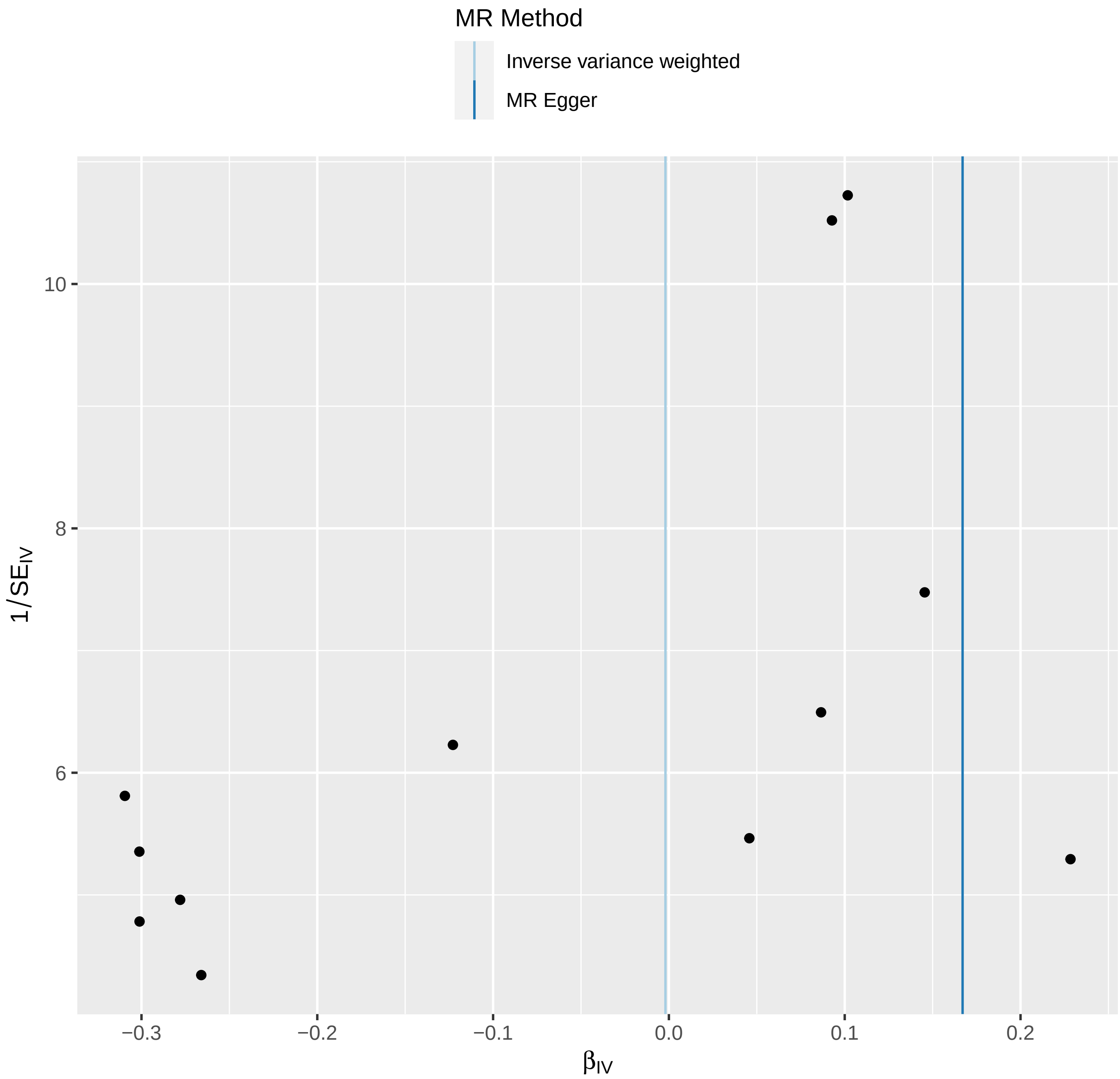

Batch 3−3198 : Selenium on Vascular endothelial growth factor A

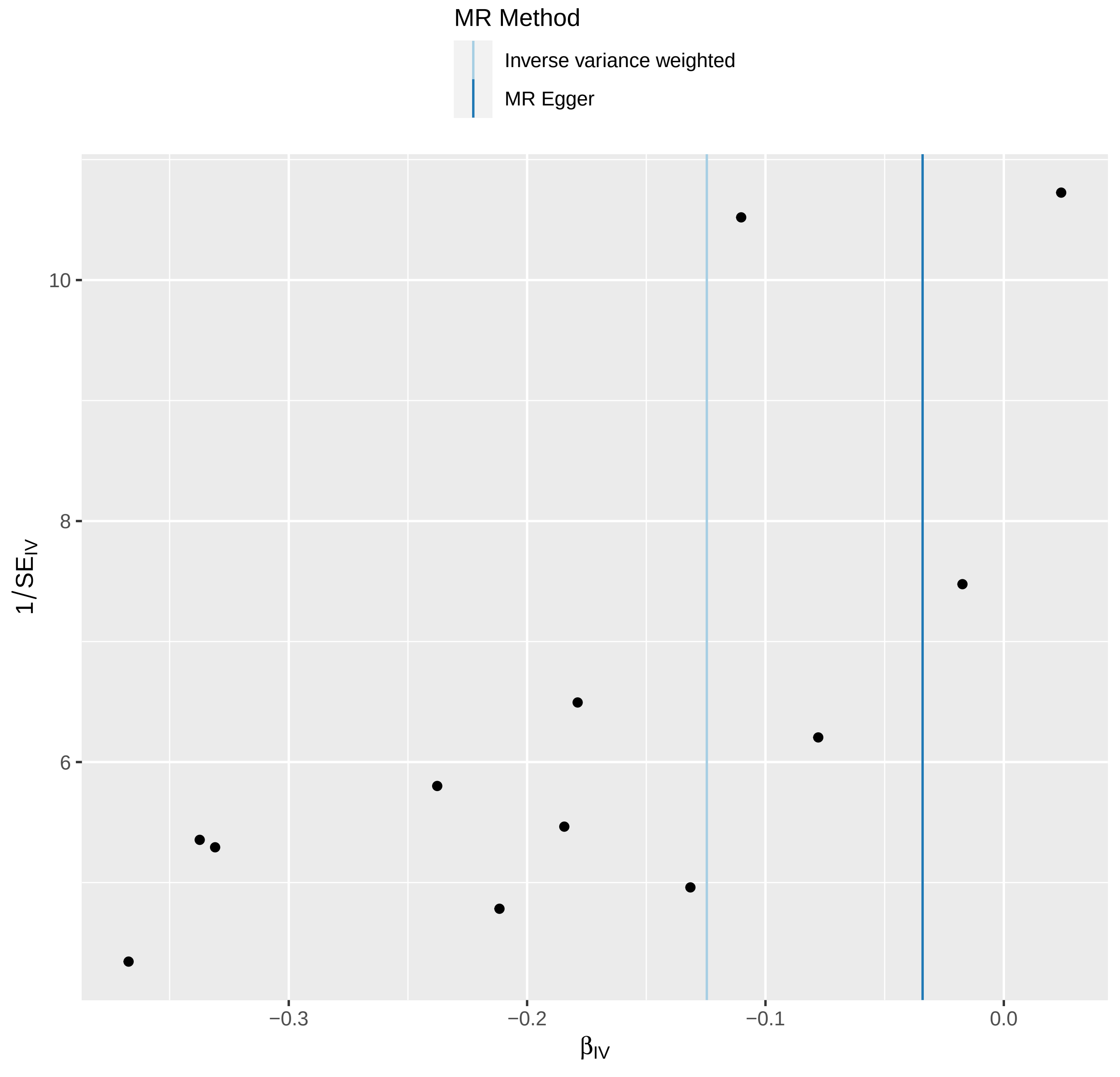

Batch 3−3199 : Selenium on Vascular endothelial growth factor C

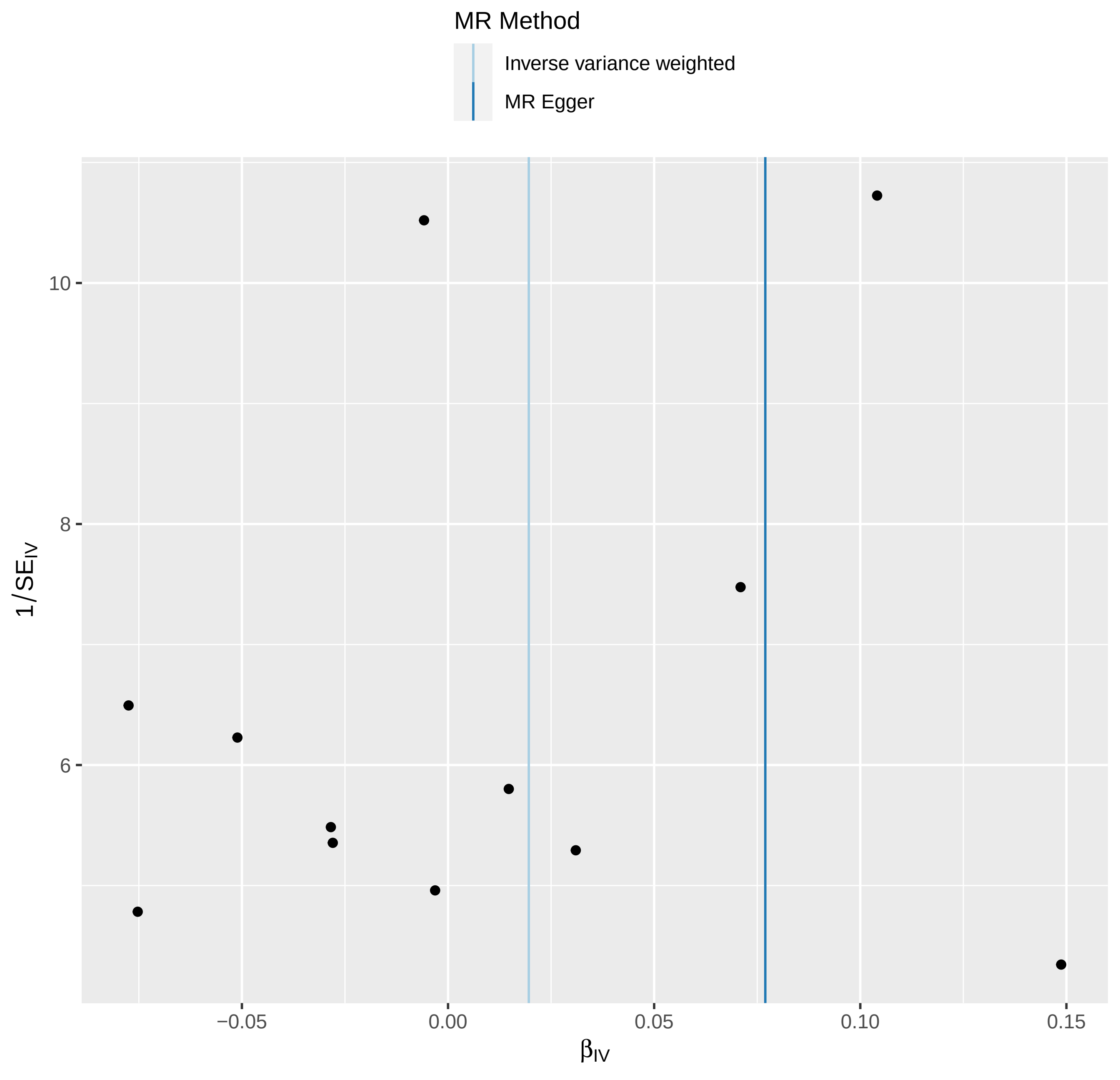

Batch 3−3200 : Selenium on Selenoprotein S

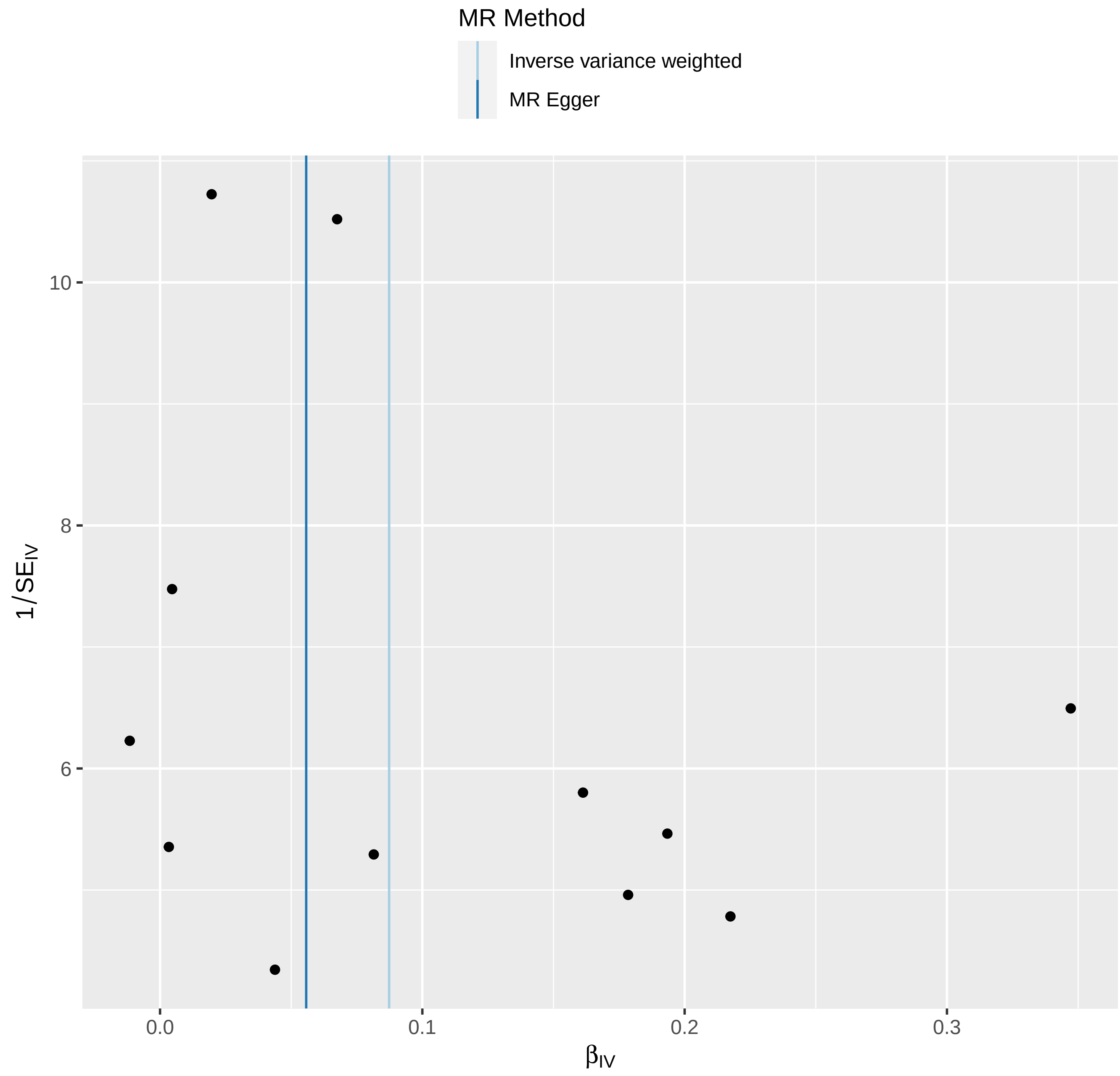

Batch 3−3201 : Selenium on Vitrin

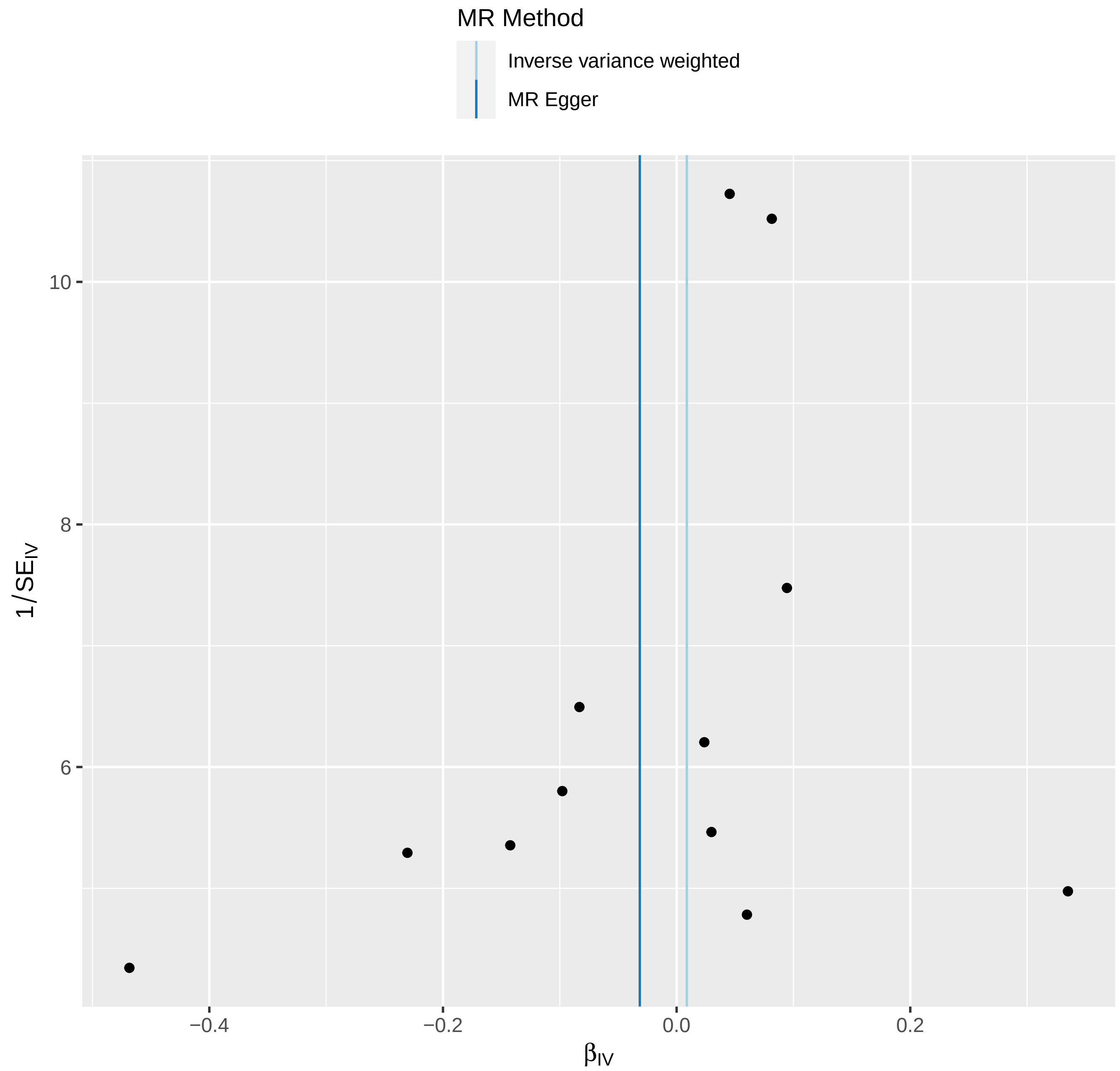

Batch 3−3202 : Selenium on Charged multivesicular body protein 3

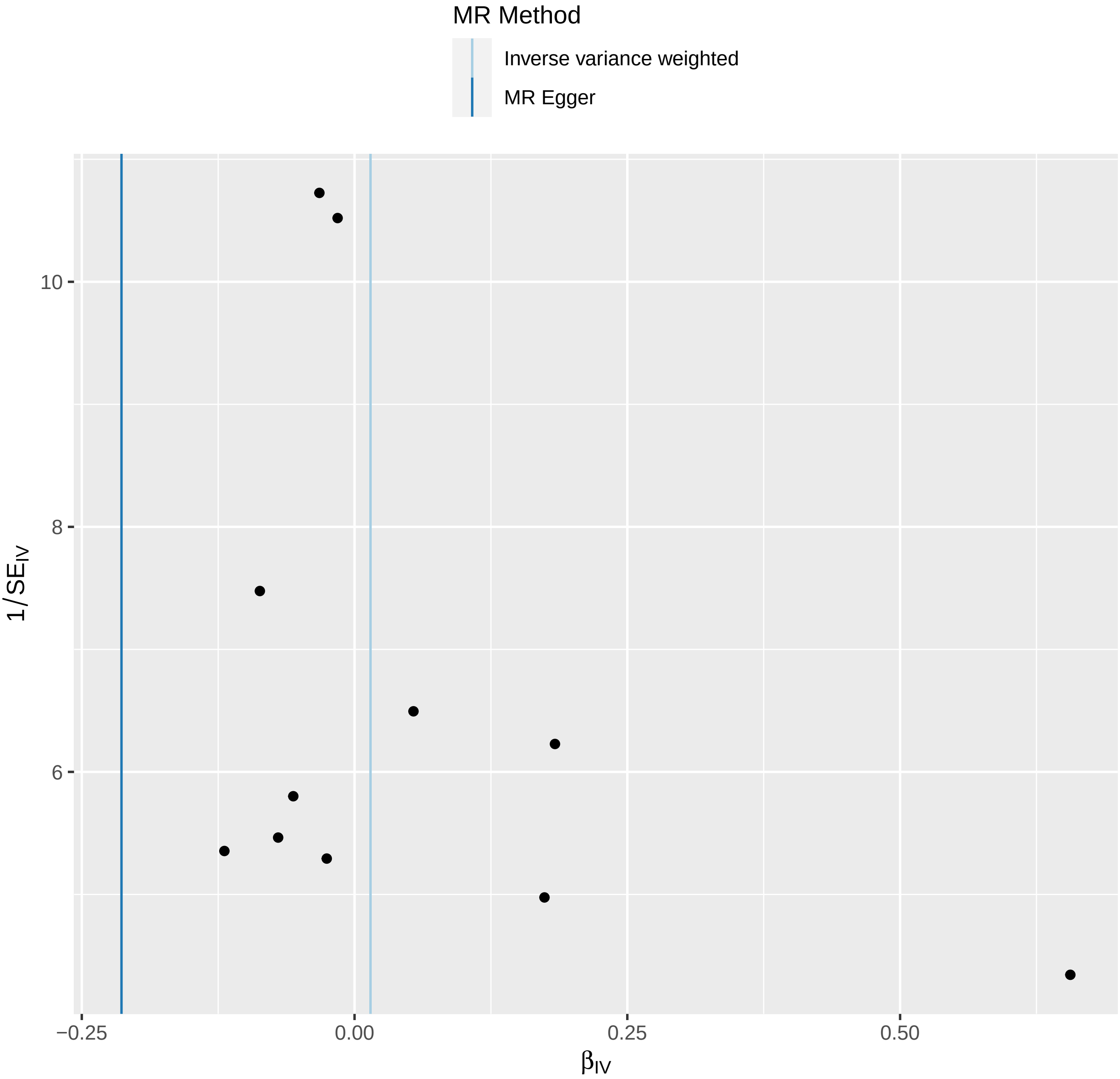

Batch 3−3203 : Selenium on Vacuolar protein sorting−associated protein 29

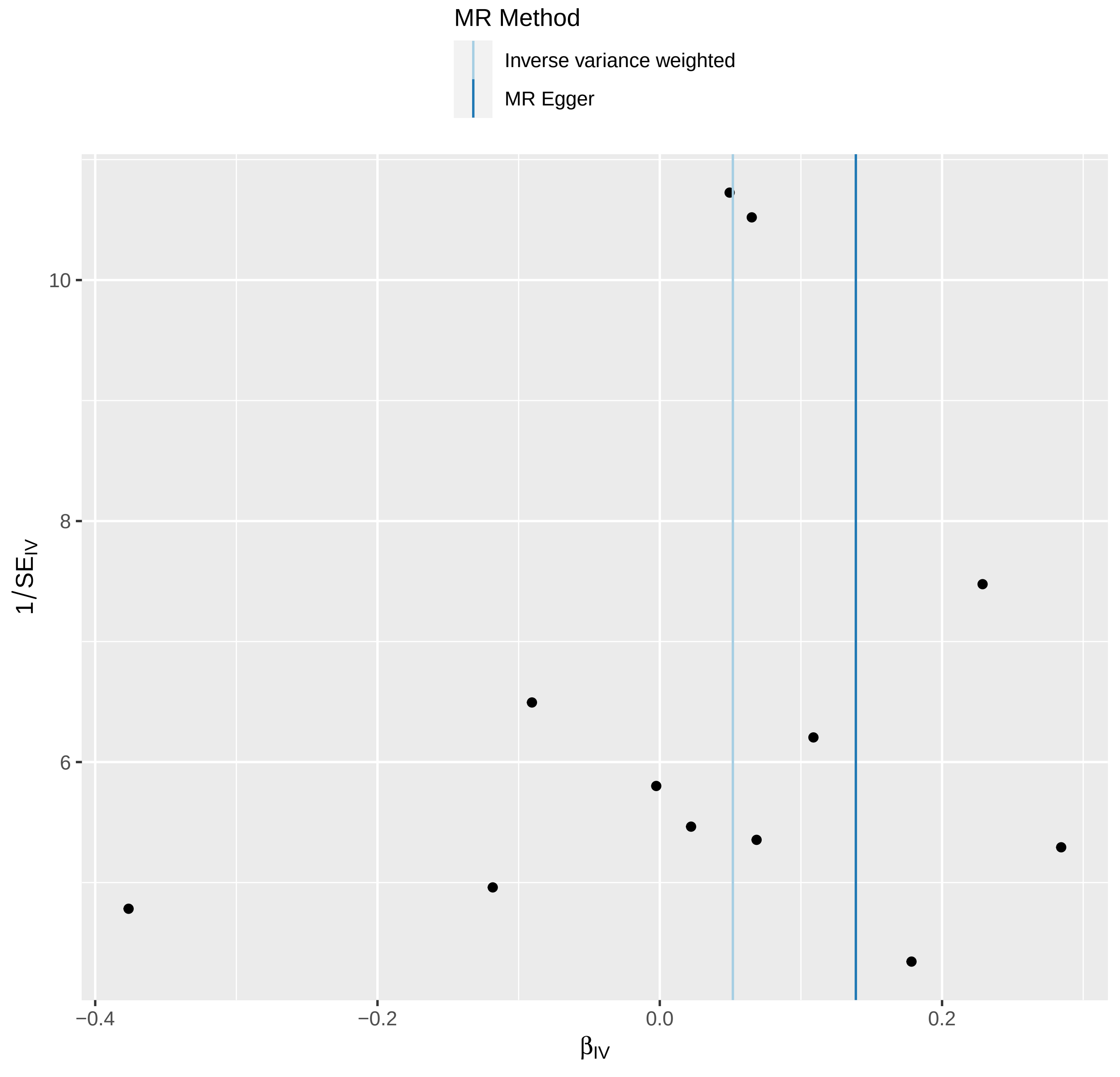

Batch 3−3204 : Selenium on Vacuolar protein sorting−associated protein 4A

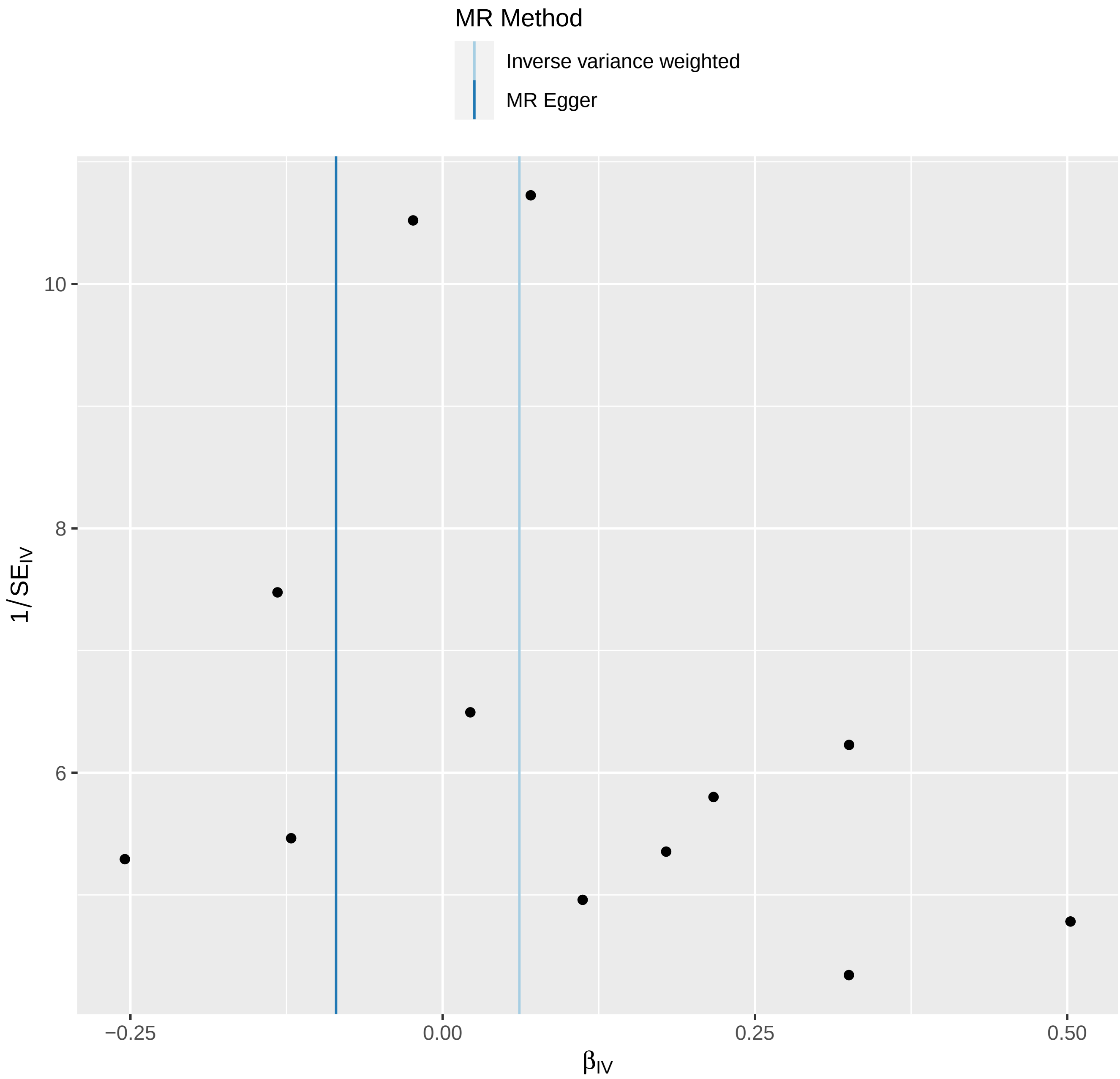

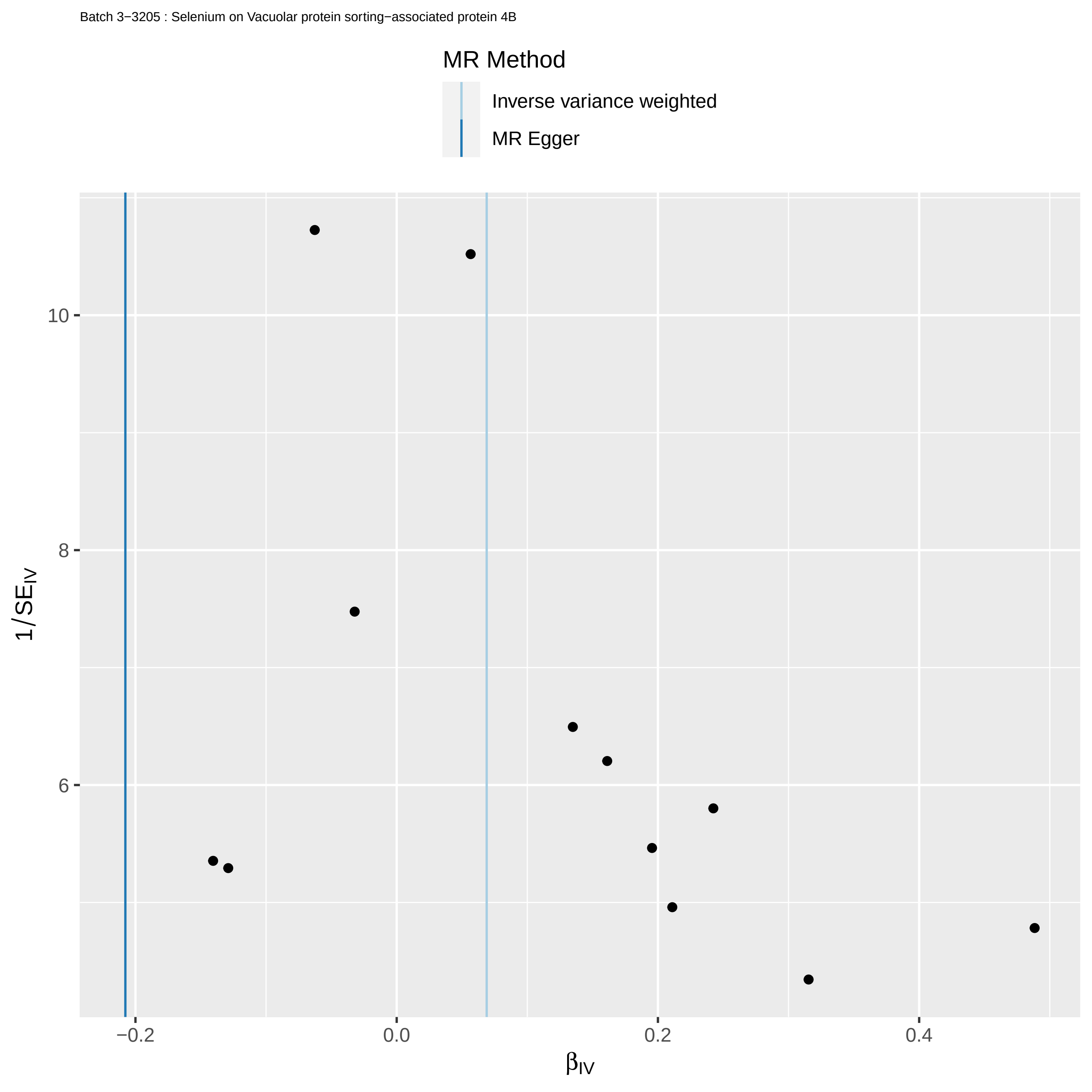
Batch 3−3205 : Selenium on Vacuolar protein sorting−associated protein 4B
MR Method
Inverse variance weighted
MR Egger
10
8
6
1/SE$_{IV}$
−0.2
0.0
0.2
0.4
β_{IV}

Batch 3−3206 : Selenium on Serine/threonine−protein kinase VRK1

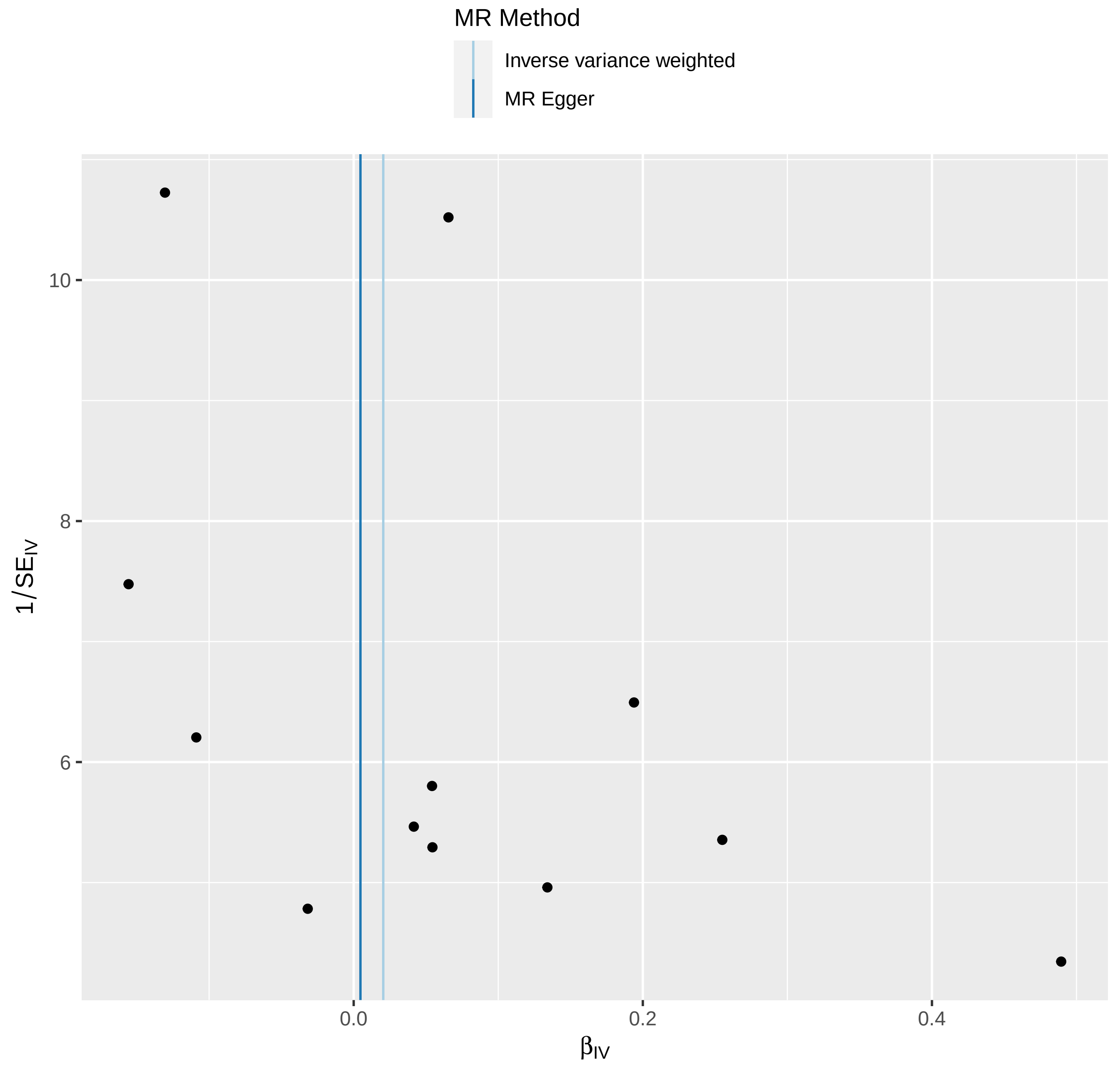

Batch 3−3207 : Selenium on V−set and immunoglobulin domain−containing protein 1

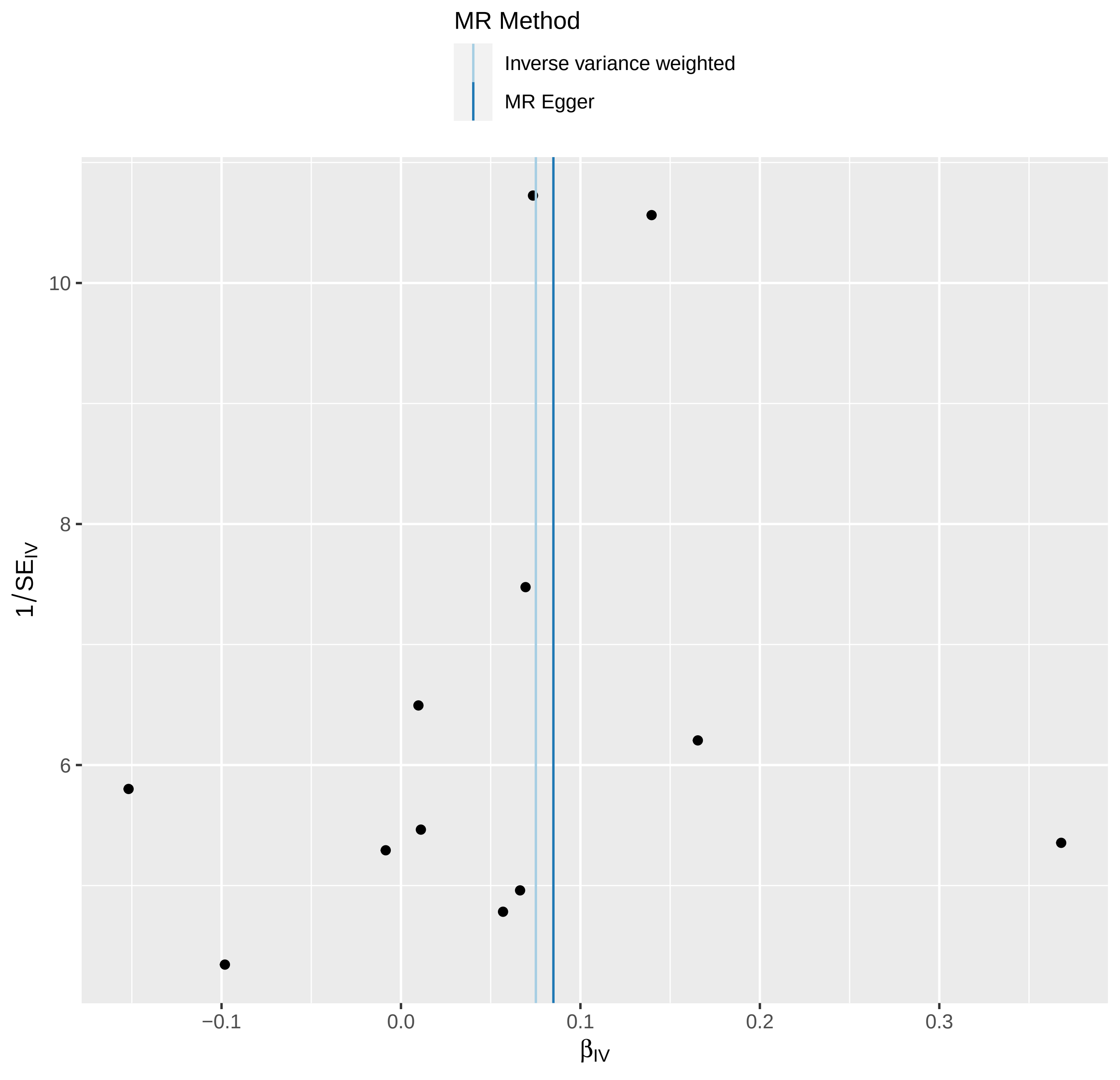

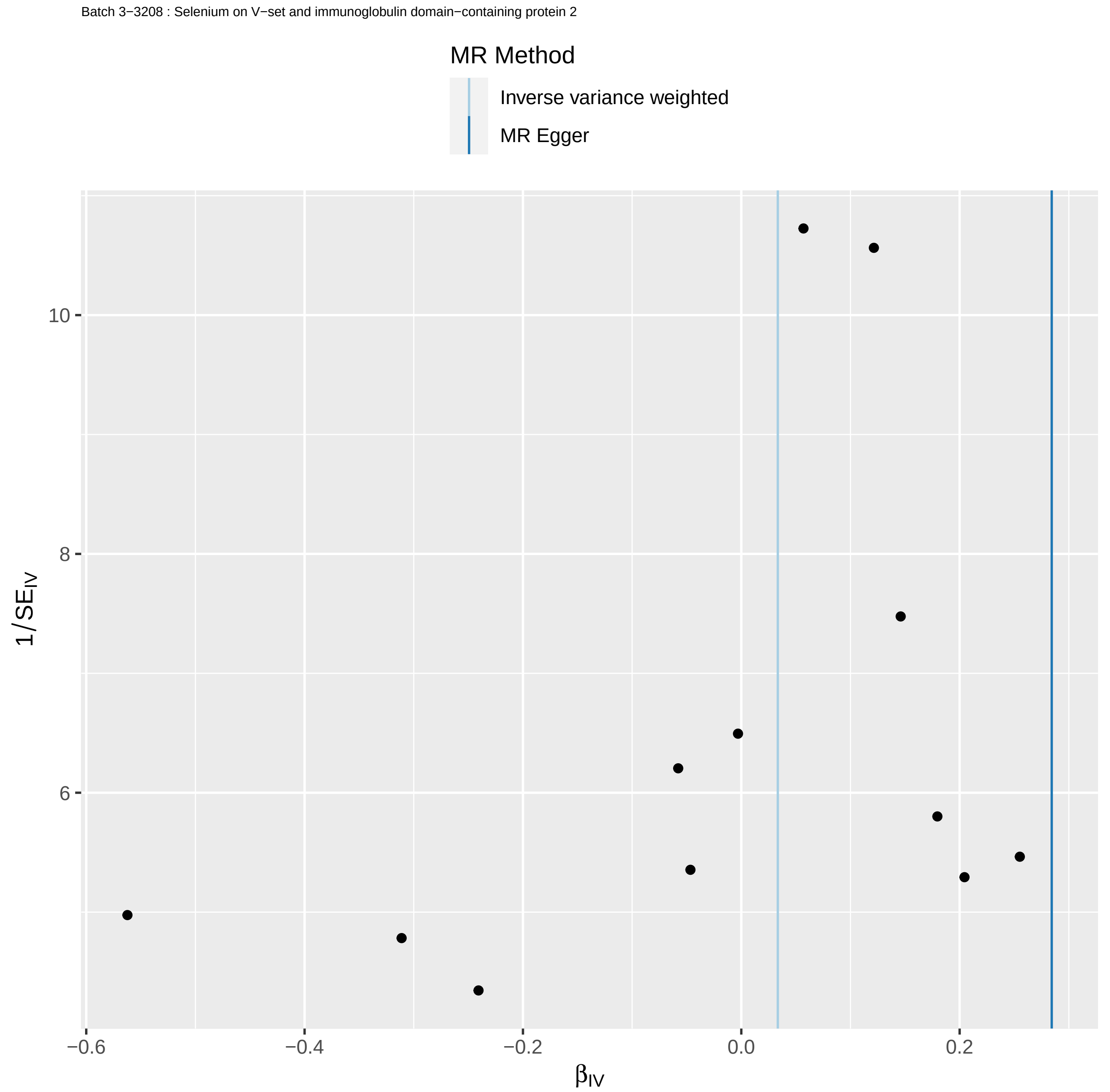
Batch 3−3208 : Selenium on V−set and immunoglobulin domain−containing protein 2
MR Method
Inverse variance weighted
MR Egger
10
8
6
$1/SE_{IV}$
−0.6
−0.4
−0.2
0.0
0.2
β_{IV}

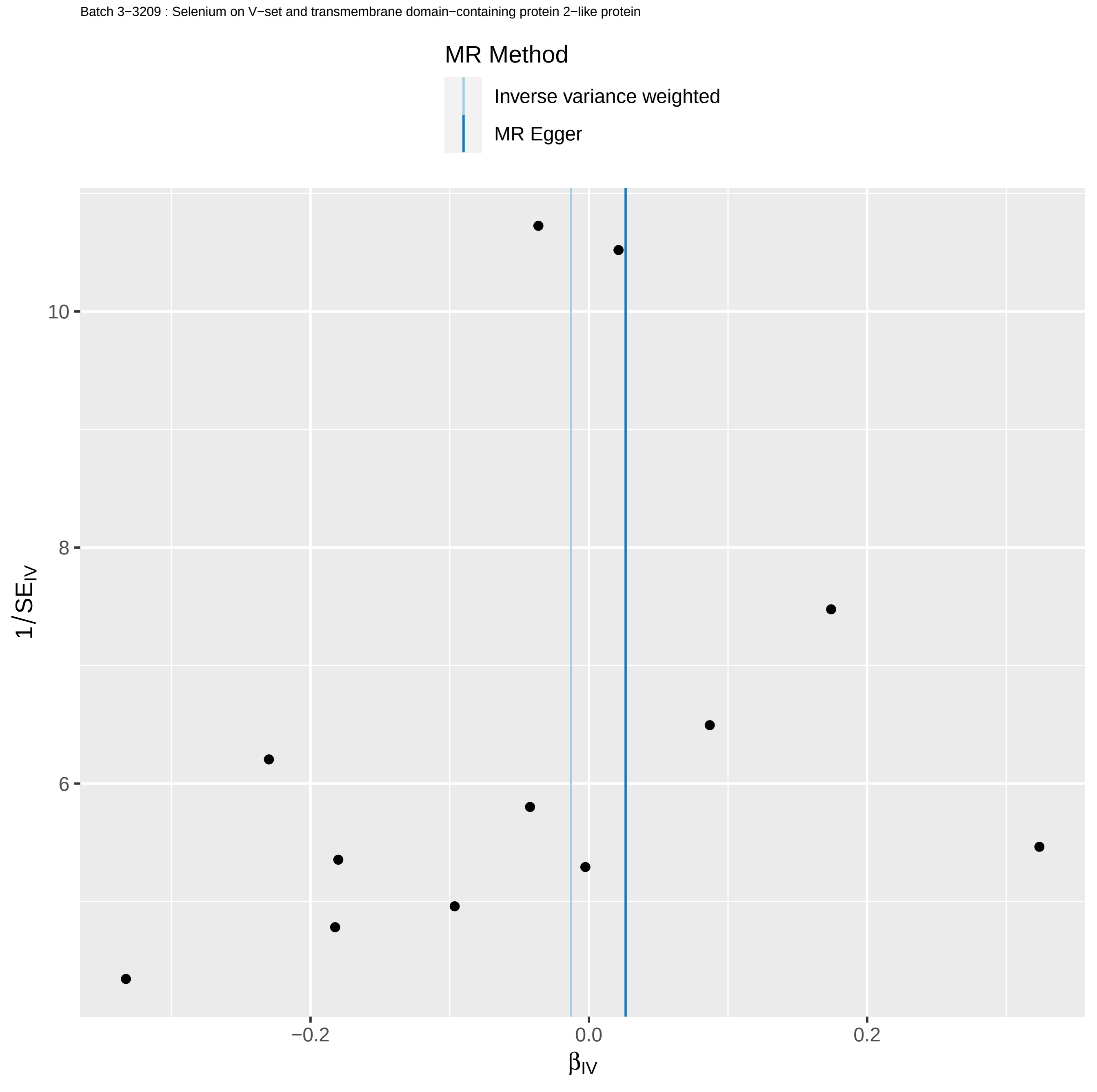

Batch 3−3209 : Selenium on V−set and transmembrane domain−containing protein 2−like protein
MR Method
Inverse variance weighted
MR Egger
10
8
6
1/SE_IV
−0.2
0.0
0.2
β_IV

Batch 3−3210 : Selenium on V−set and transmembrane domain−containing protein 4

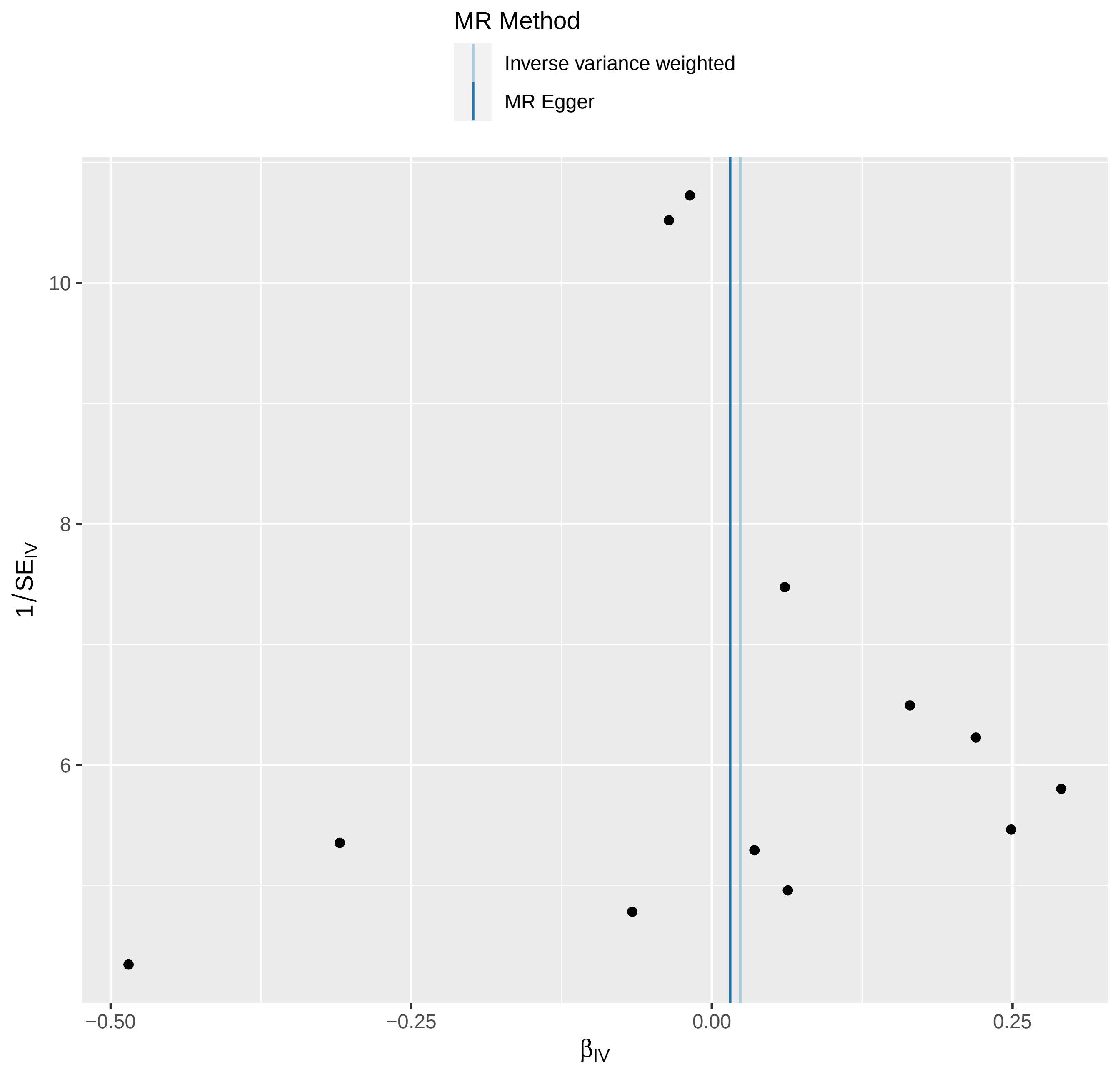

Batch 3−3211 : Selenium on Vacuolar protein sorting−associated protein VTA1 homolog

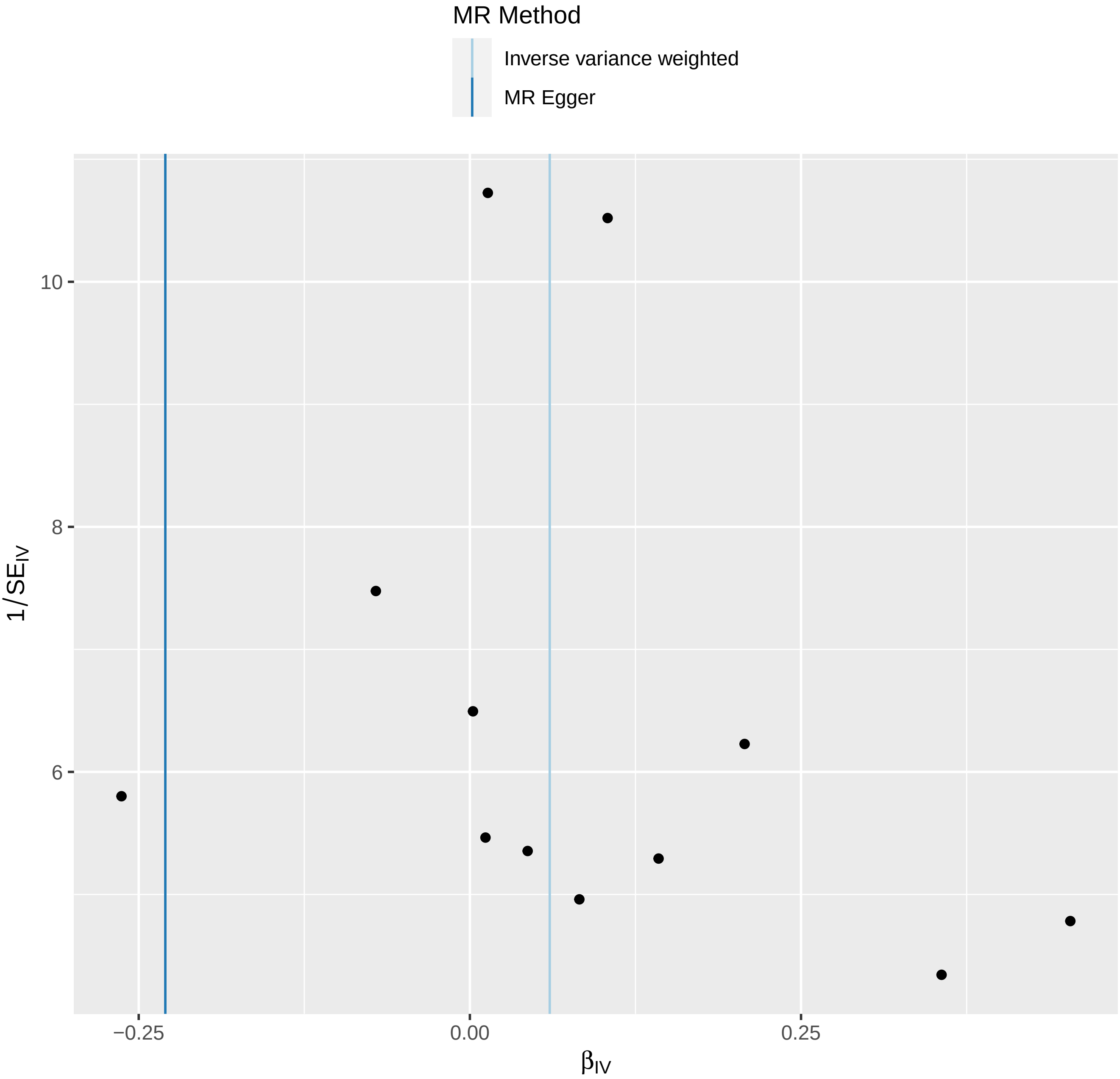

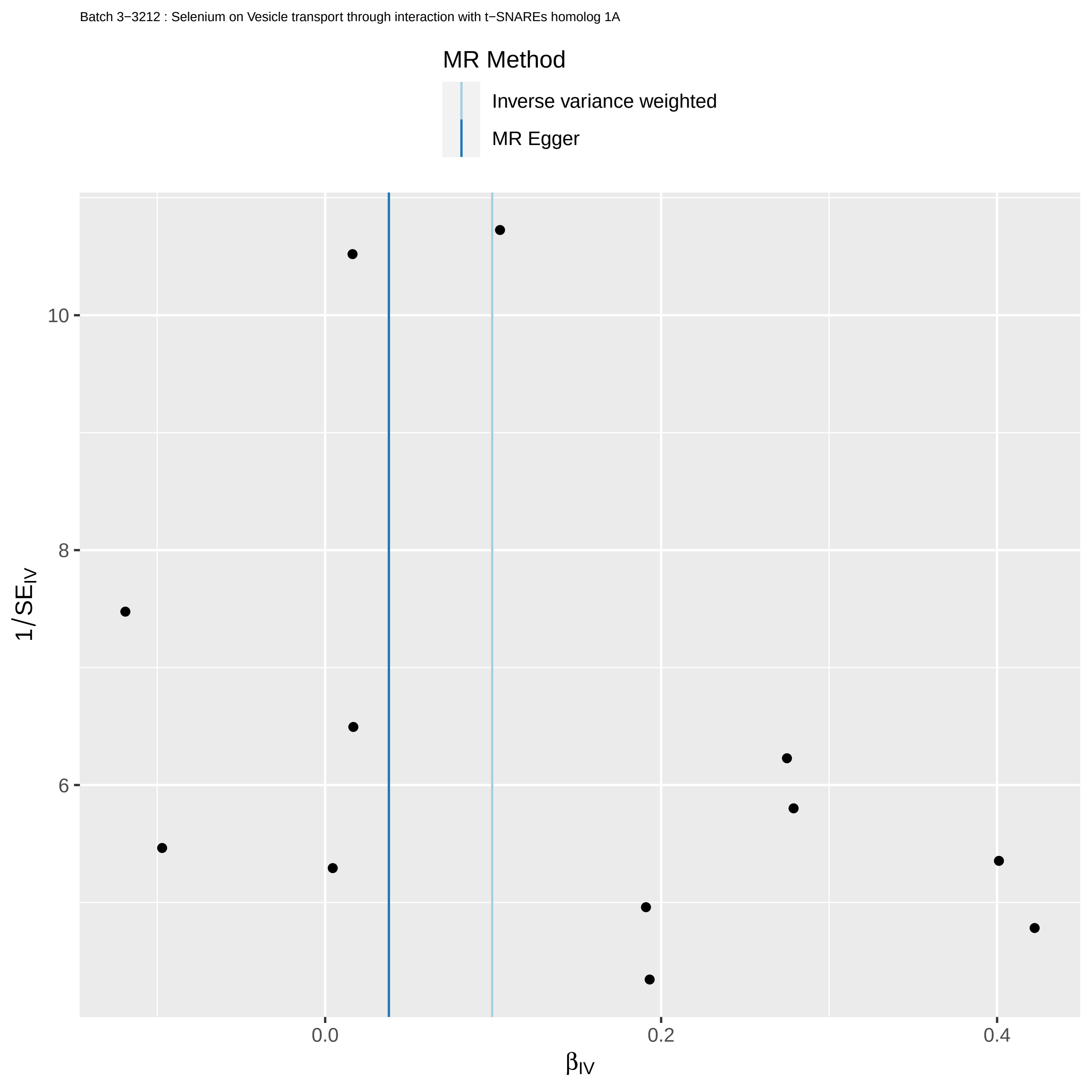

Batch 3−3212 : Selenium on Vesicle transport through interaction with t−SNAREs homolog 1A
MR Method
Inverse variance weighted
MR Egger
10
8
6
1/SE_IV
0.0
0.2
0.4
β_IV

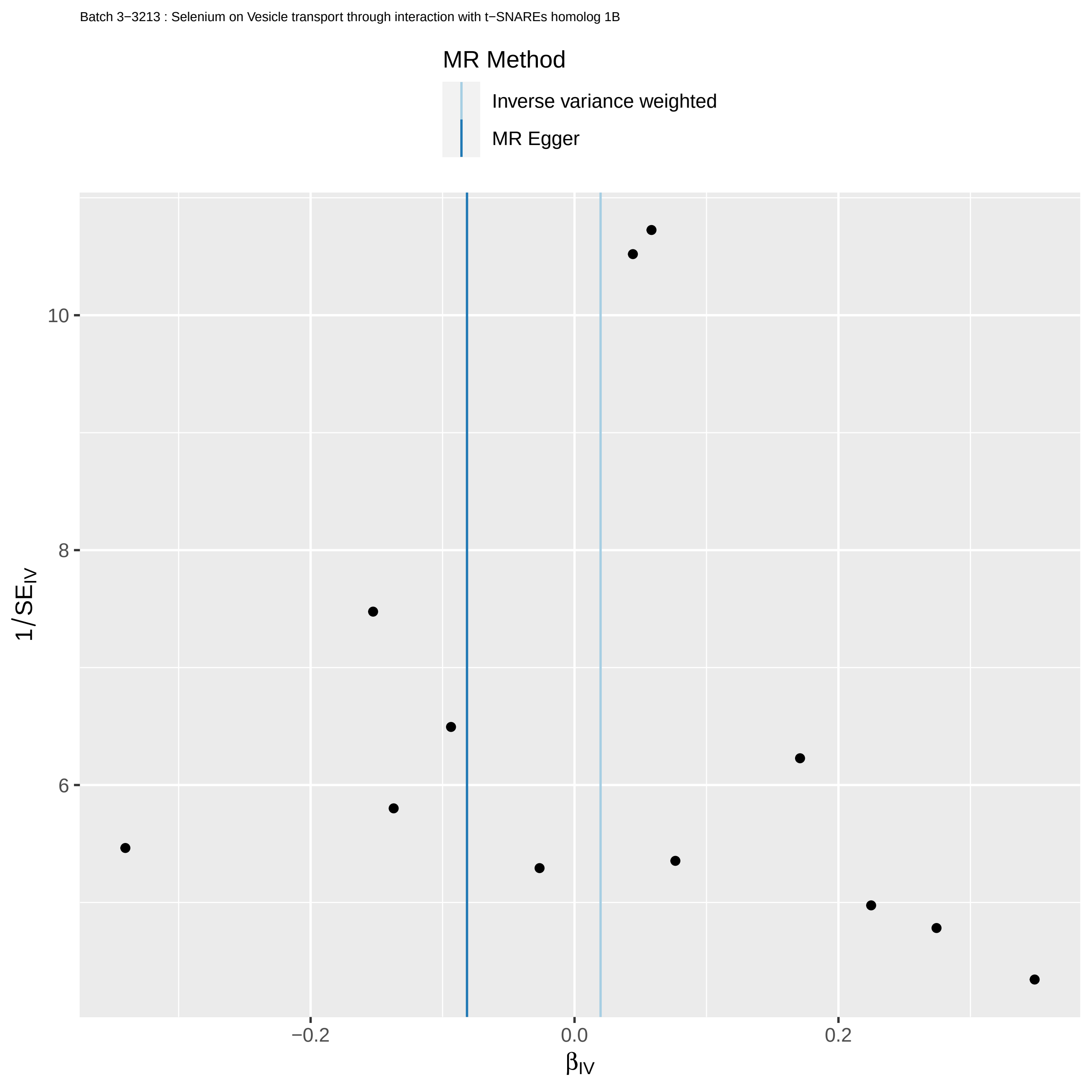
Batch 3−3213 : Selenium on Vesicle transport through interaction with t−SNAREs homolog 1B
MR Method
Inverse variance weighted
MR Egger
10
8
6
1/SE$_{IV}$
−0.2
0.0
0.2
β$_{IV}$

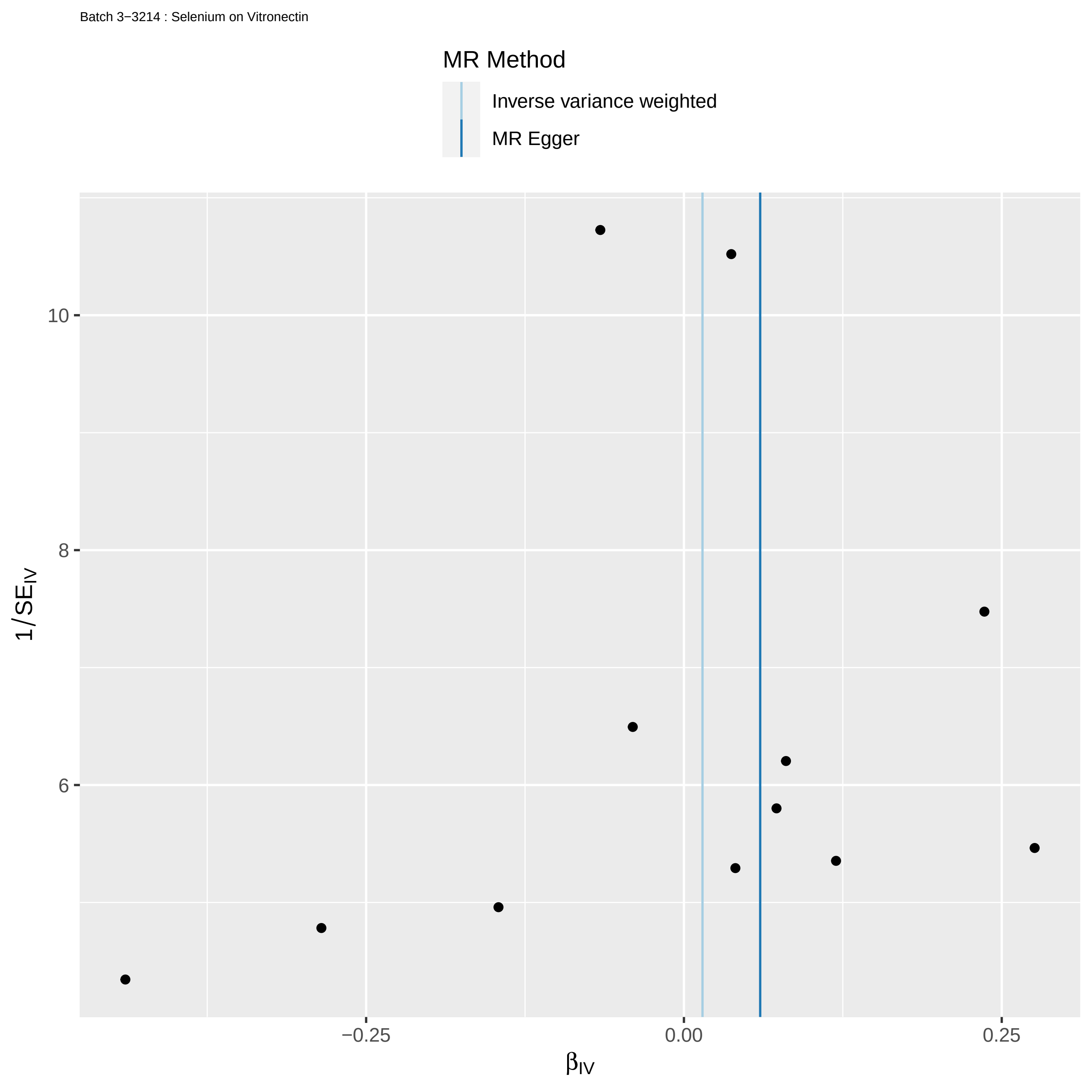

Batch 3−3214 : Selenium on Vitronectin
MR Method
Inverse variance weighted
MR Egger
10
8
6
−0.25
0.00
0.25
$1/SE_{IV}$
β_{IV}

Batch 3−3215 : Selenium on Vitronectin

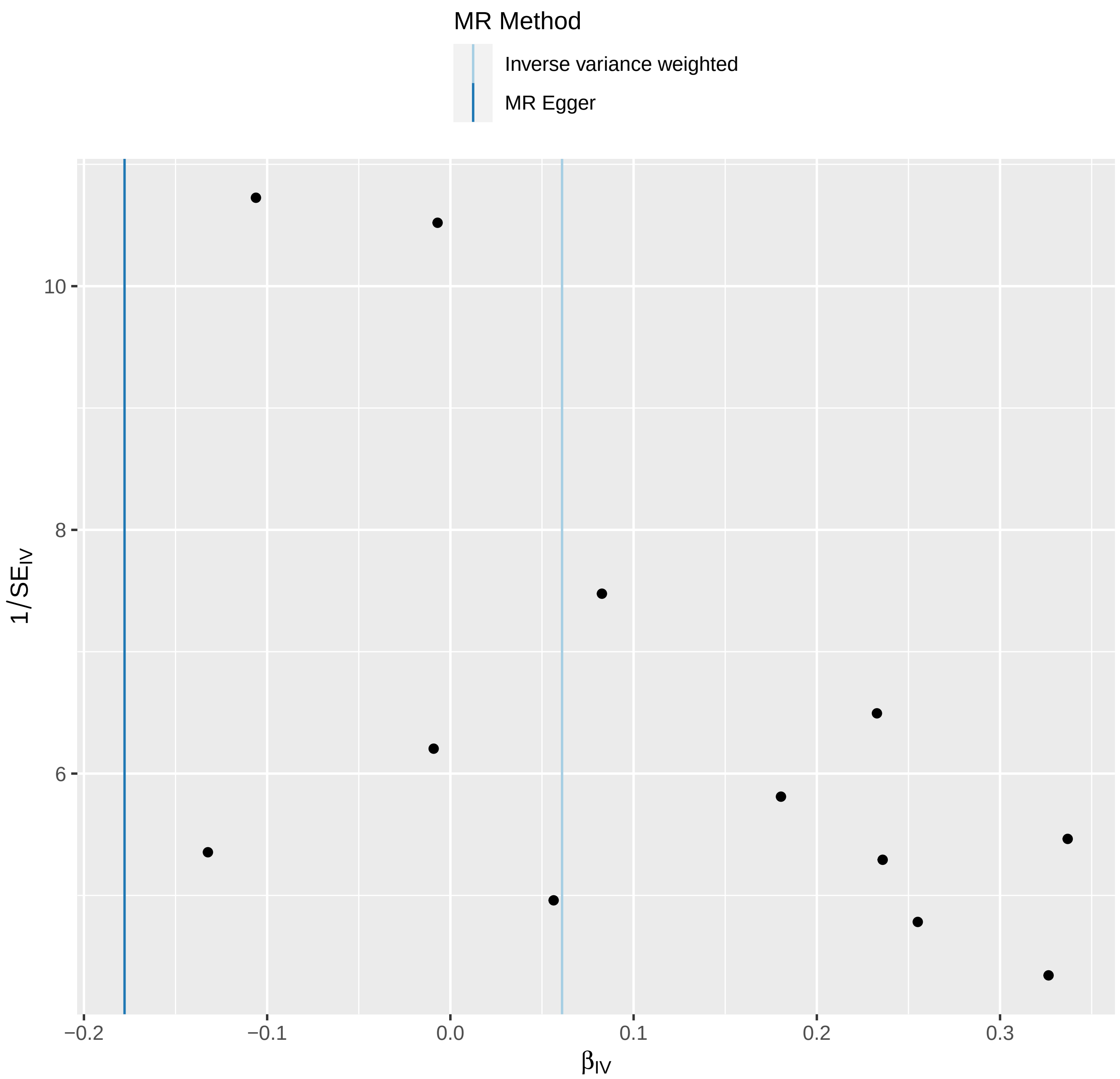

Batch 3−3216 : Selenium on Brorin

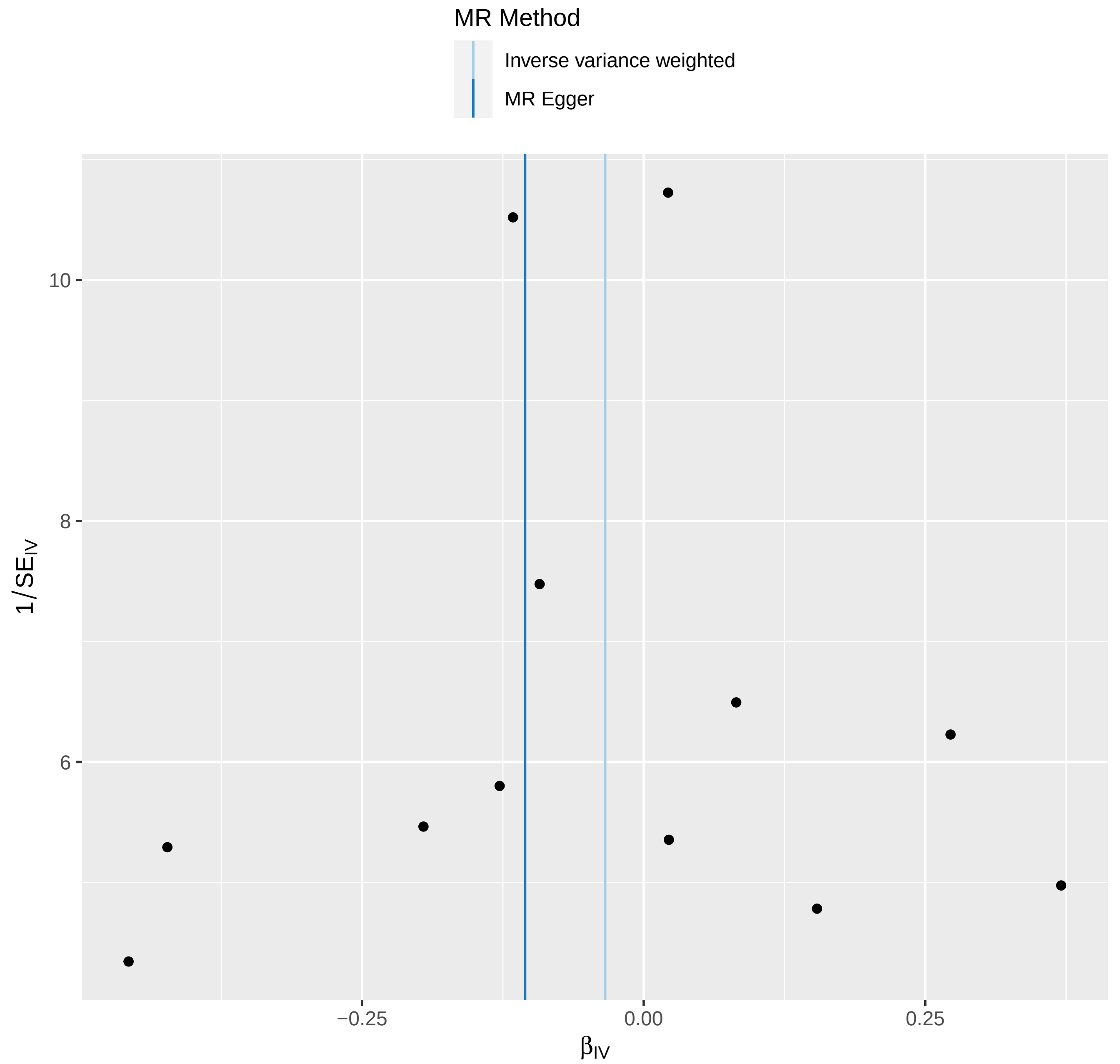

Batch 3−3217 : Selenium on von Willebrand factor C domain−containing protein 2−like

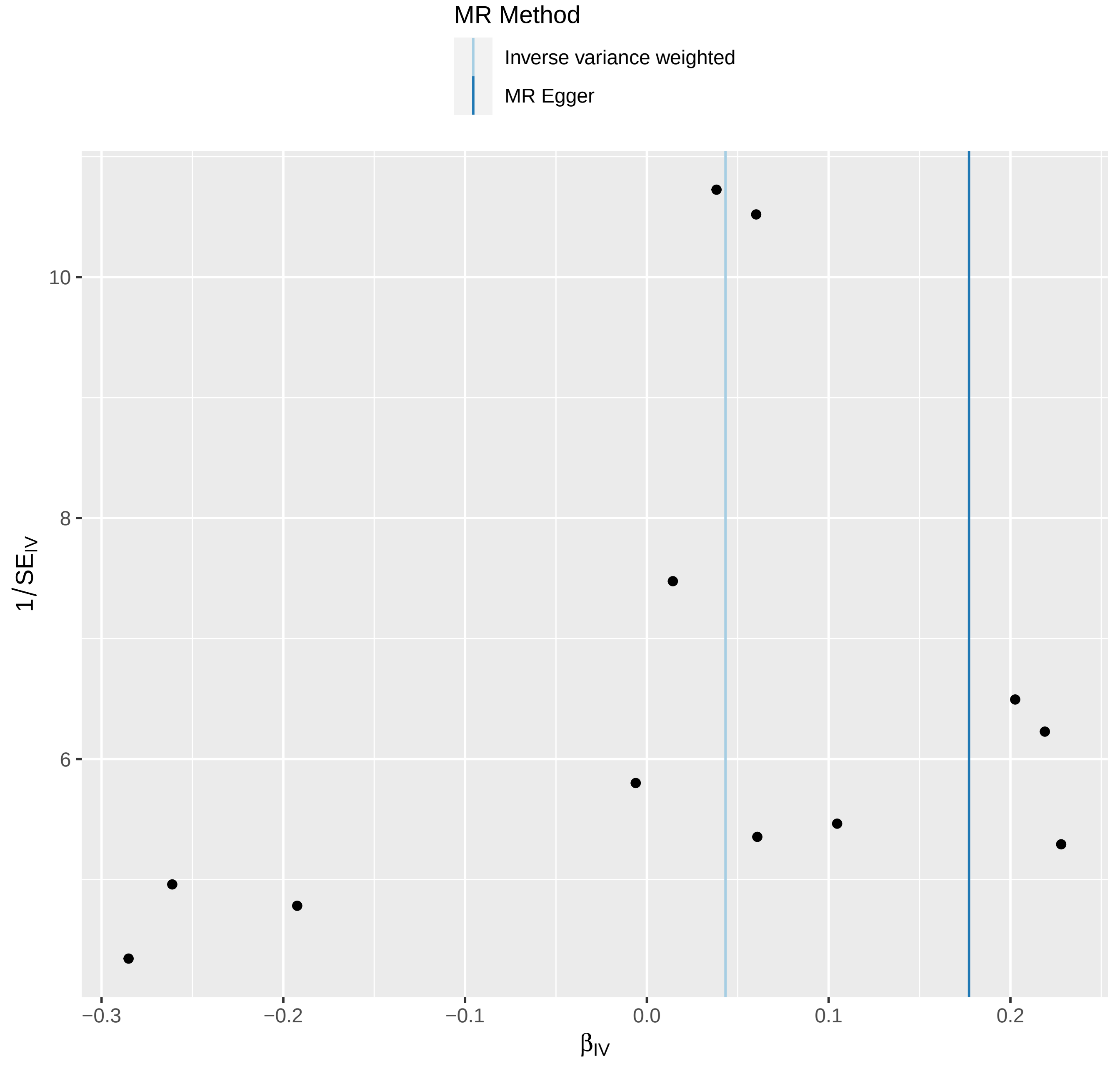

Batch 3−3218 : Selenium on WW domain−binding protein 1

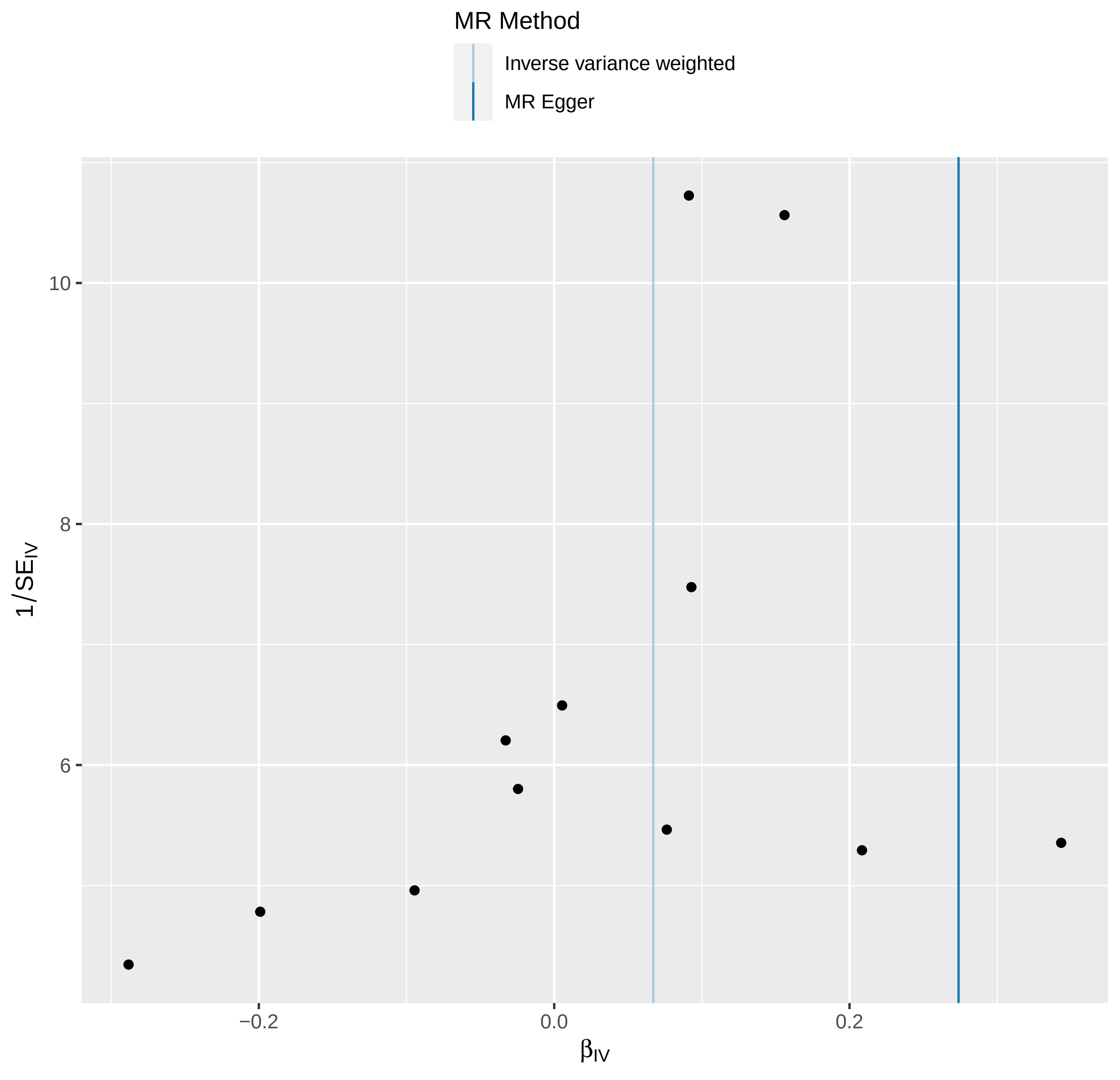

Batch 3−3219 : Selenium on WW domain binding protein 1−like

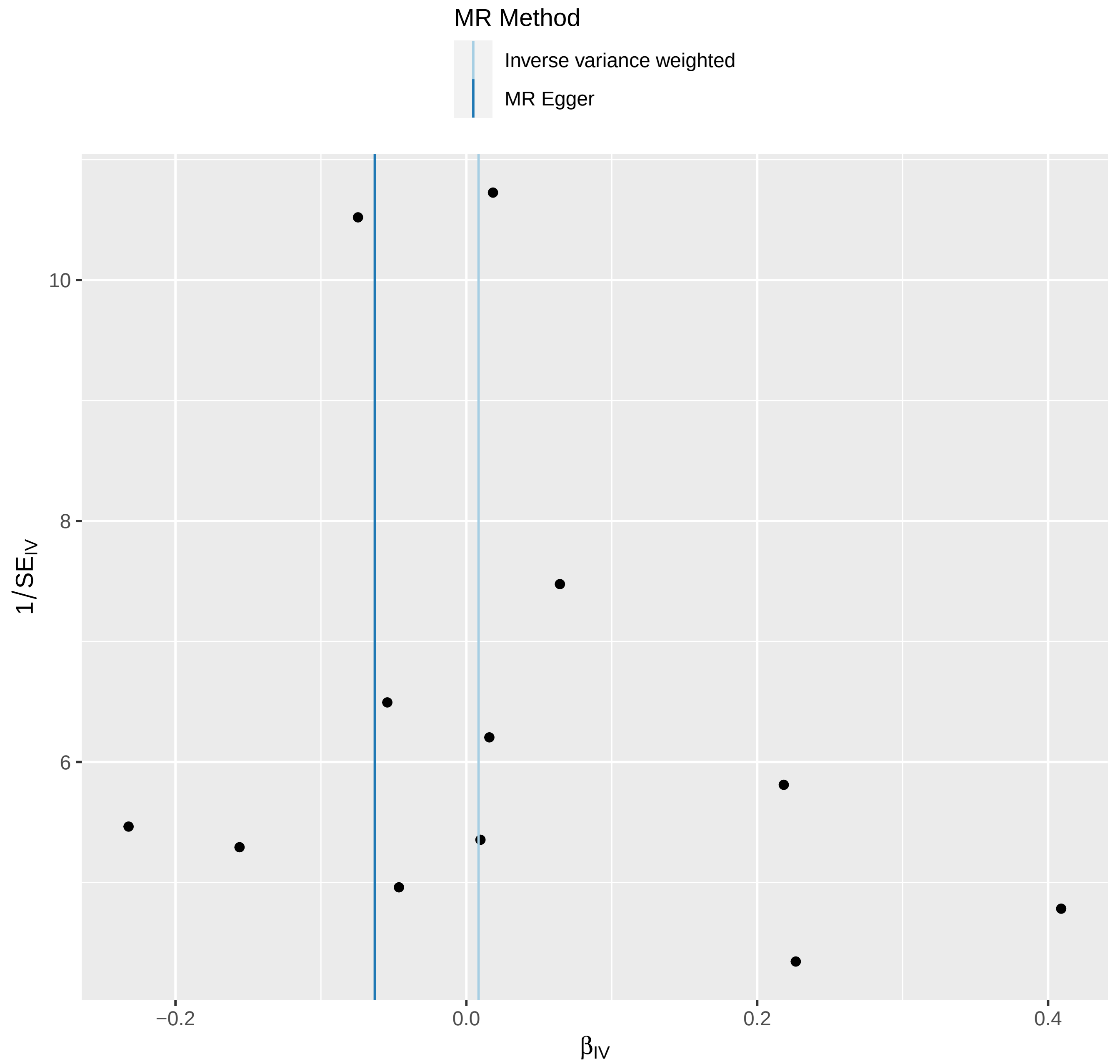

Batch 3−3220 : Selenium on WD repeat−containing protein 1

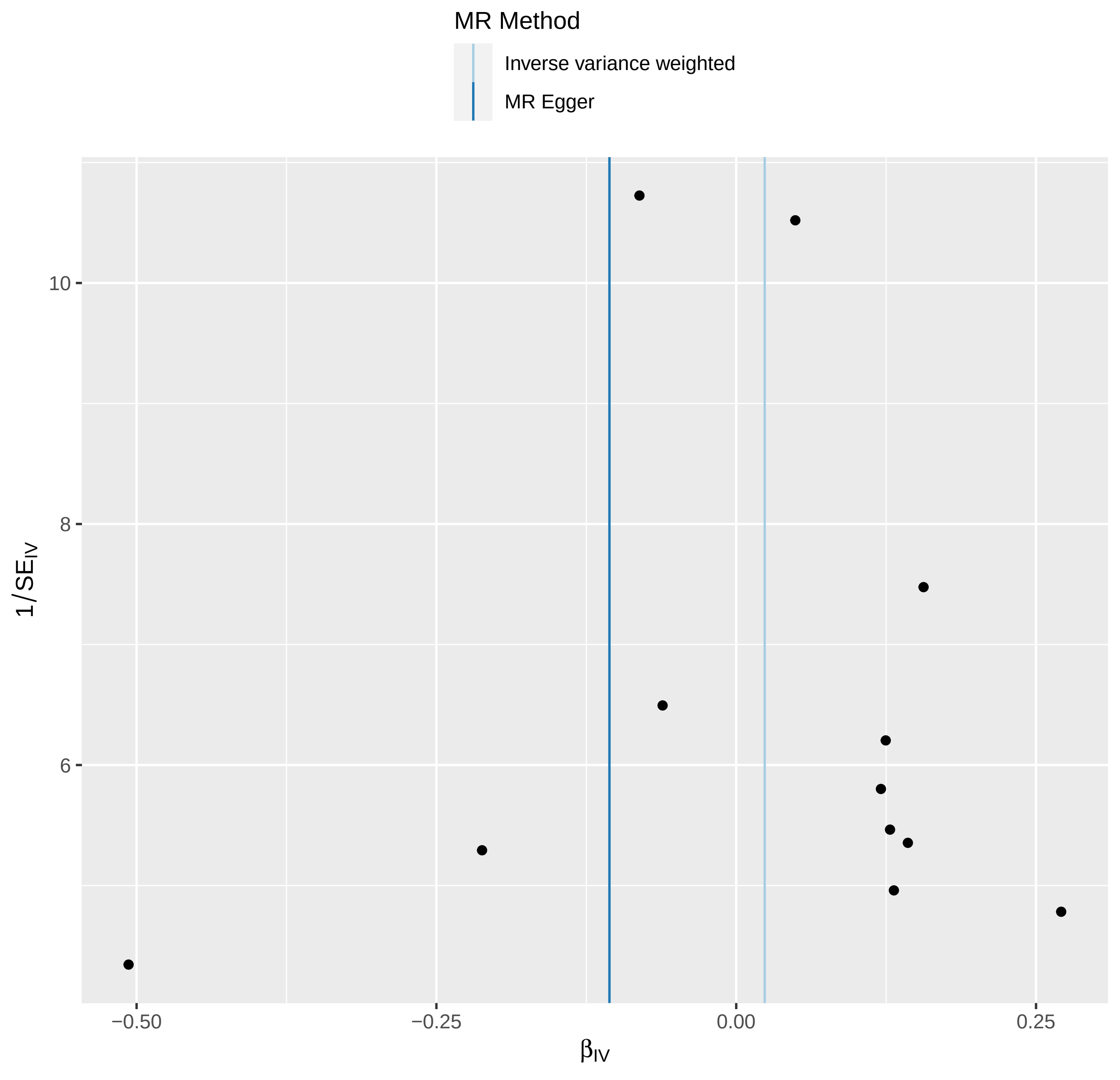

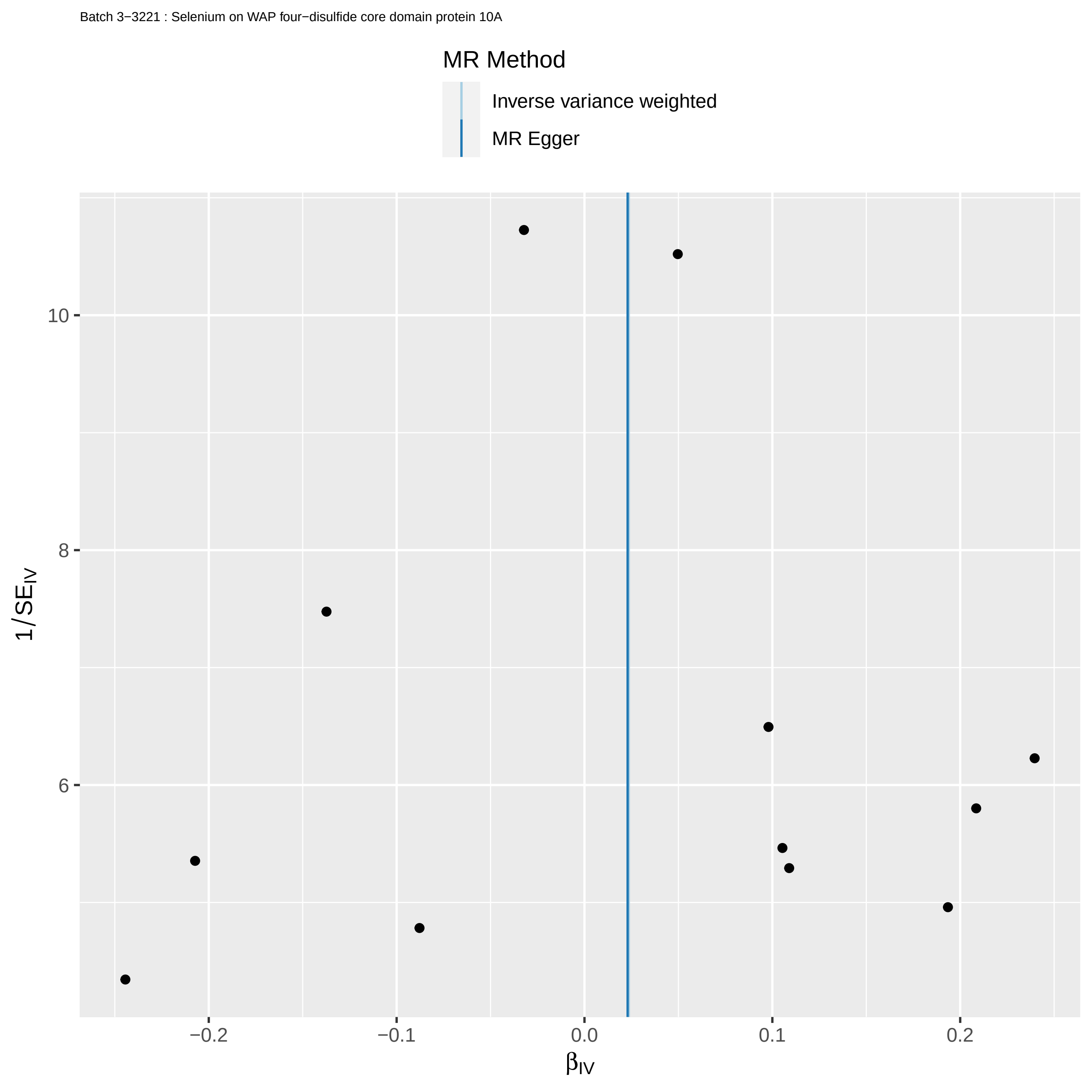
Batch 3−3221 : Selenium on WAP four−disulfide core domain protein 10A
MR Method
Inverse variance weighted
MR Egger
10
8
6
1/SE$_{IV}$
−0.2
−0.1
0.0
0.1
0.2
β$_{IV}$

Batch 3−3222 : Selenium on WAP four−disulfide core domain protein 12

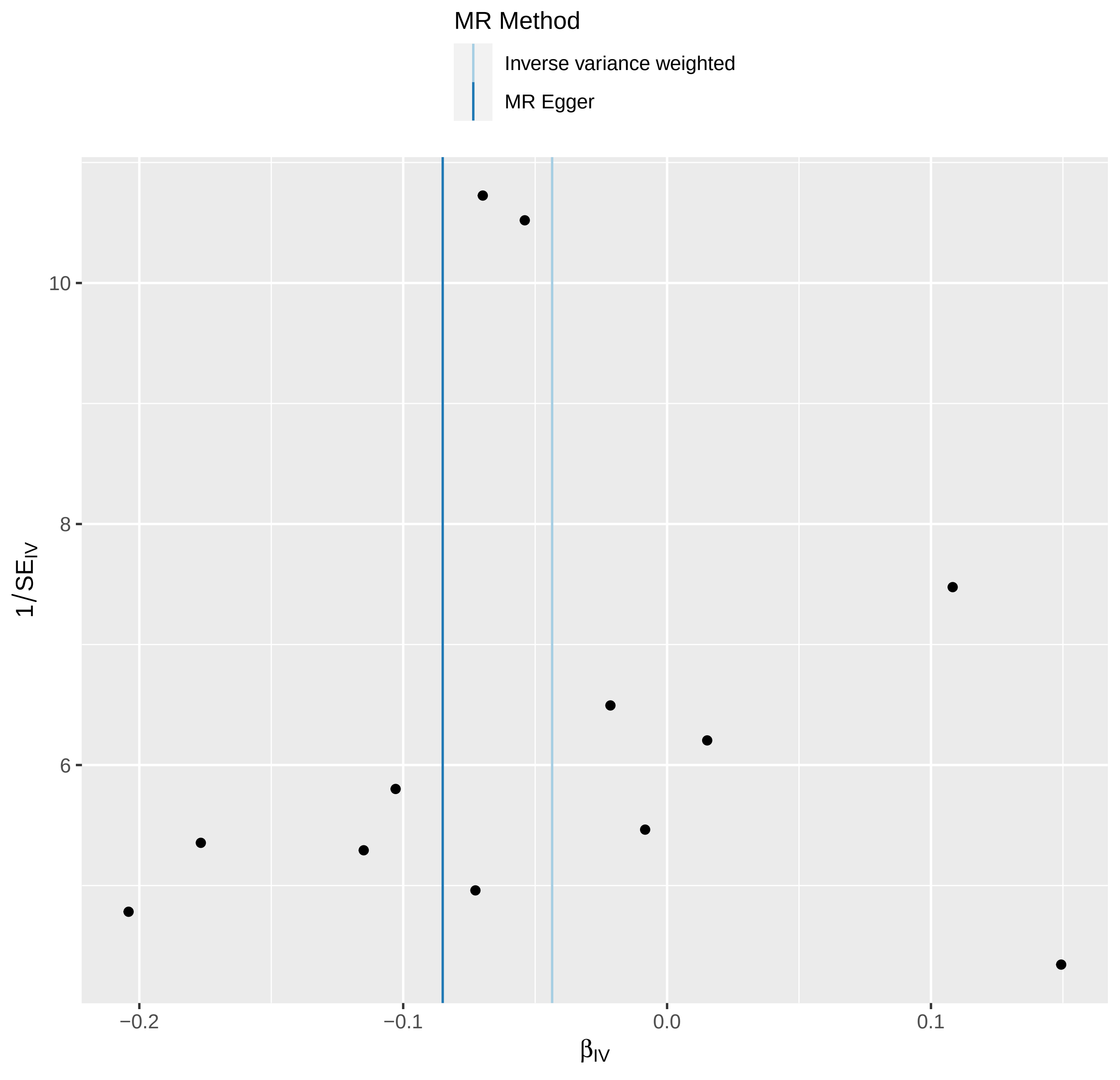

Batch 3−3223 : Selenium on WAP four−disulfide core domain protein 13

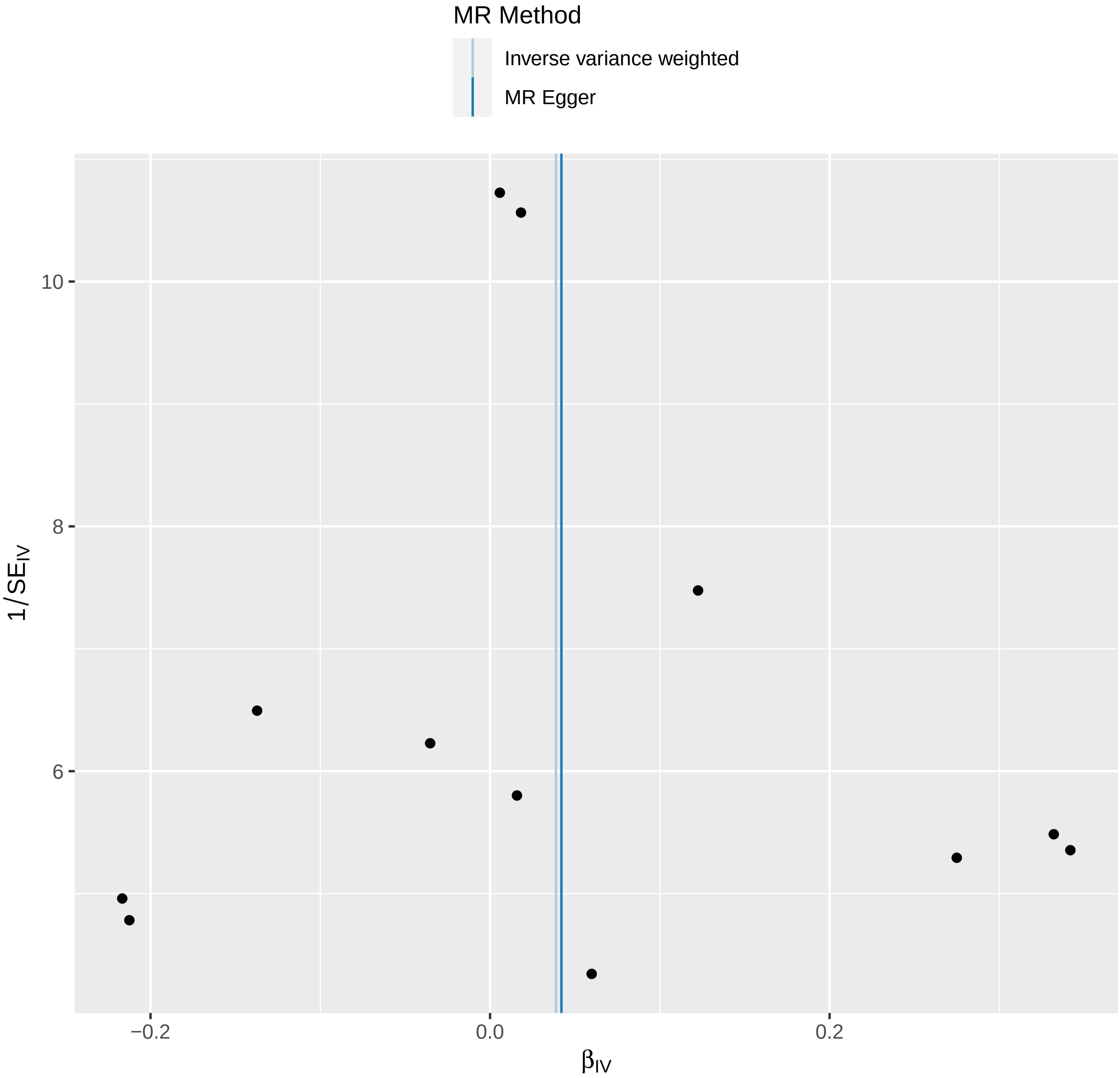

Batch 3−3224 : Selenium on WAP four−disulfide core domain protein 2

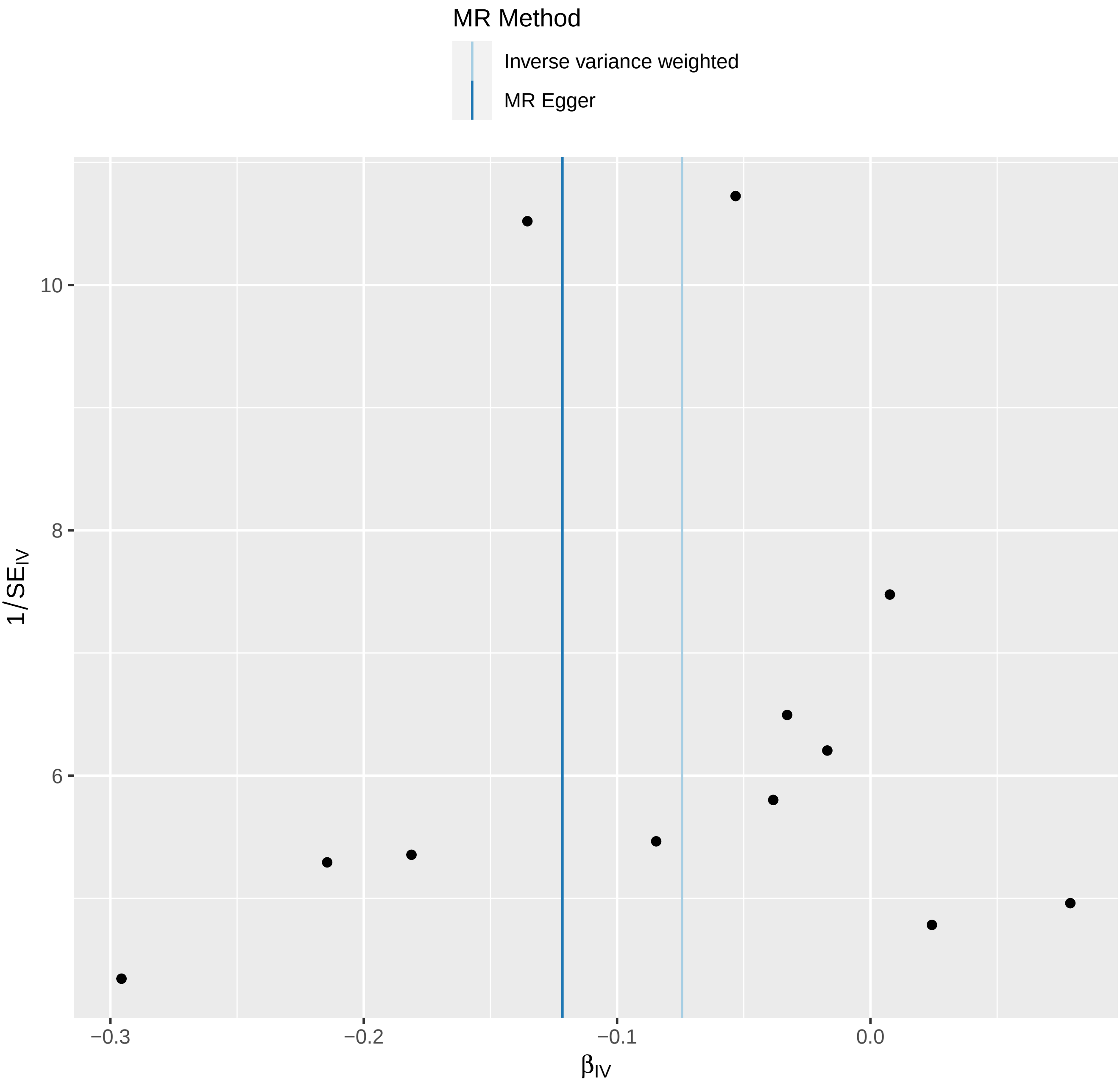

Batch 3−3225 : Selenium on WAP four−disulfide core domain protein 3

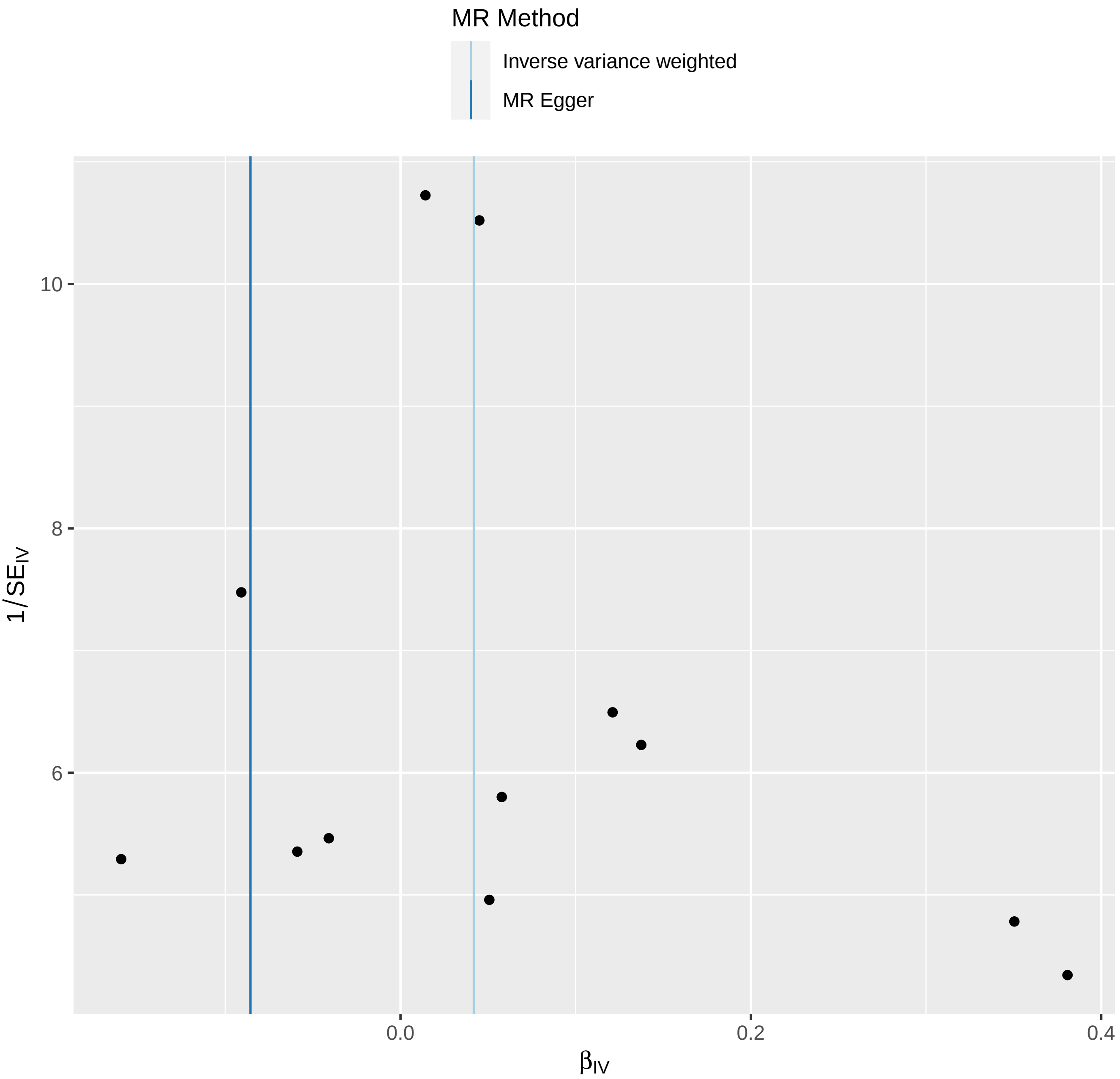

Batch 3−3226 : Selenium on WAP four−disulfide core domain protein 5

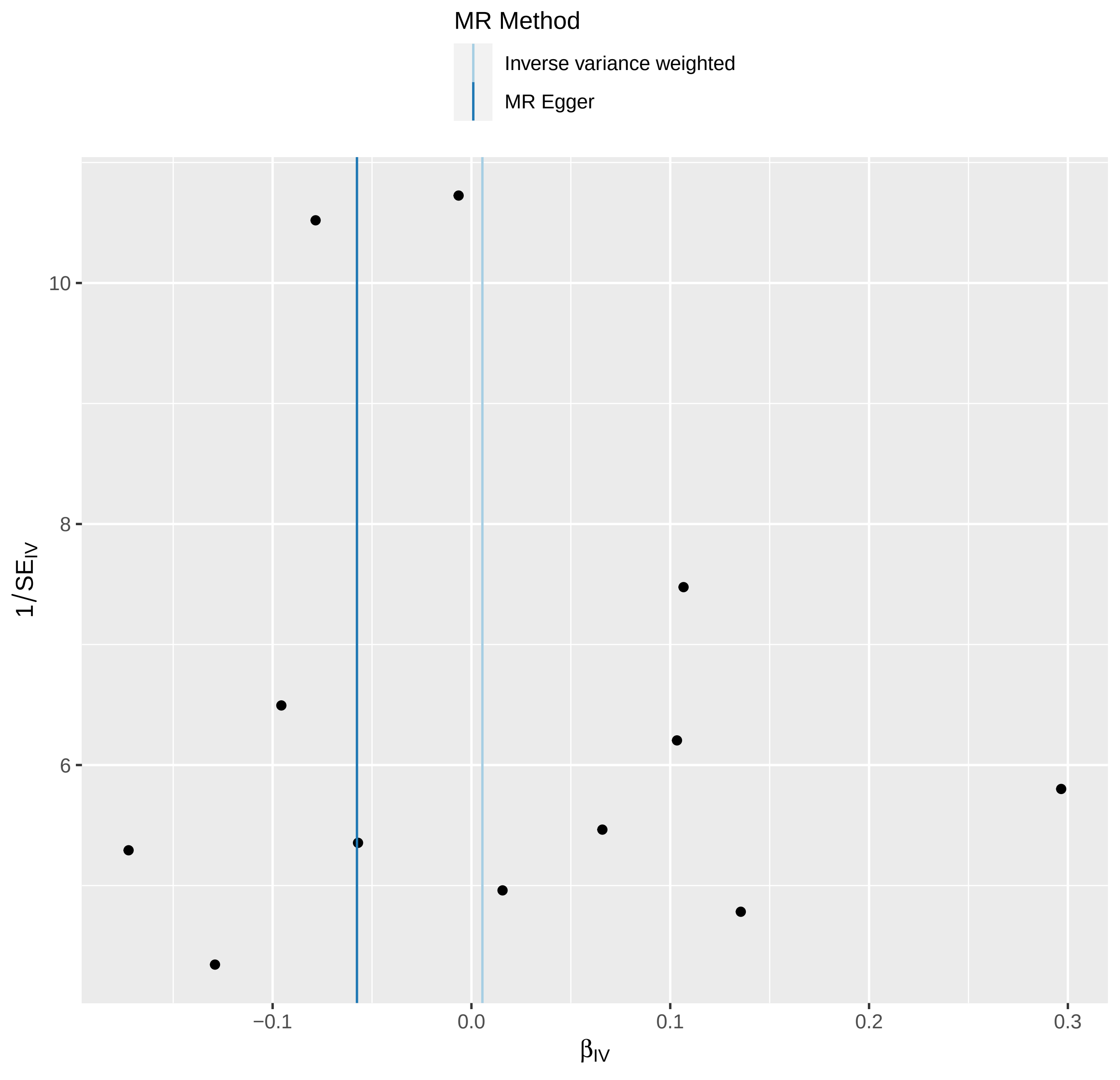

Batch 3−3227 : Selenium on WAP, kazal, immunoglobulin, kunitz and NTR domain−containing protein 1

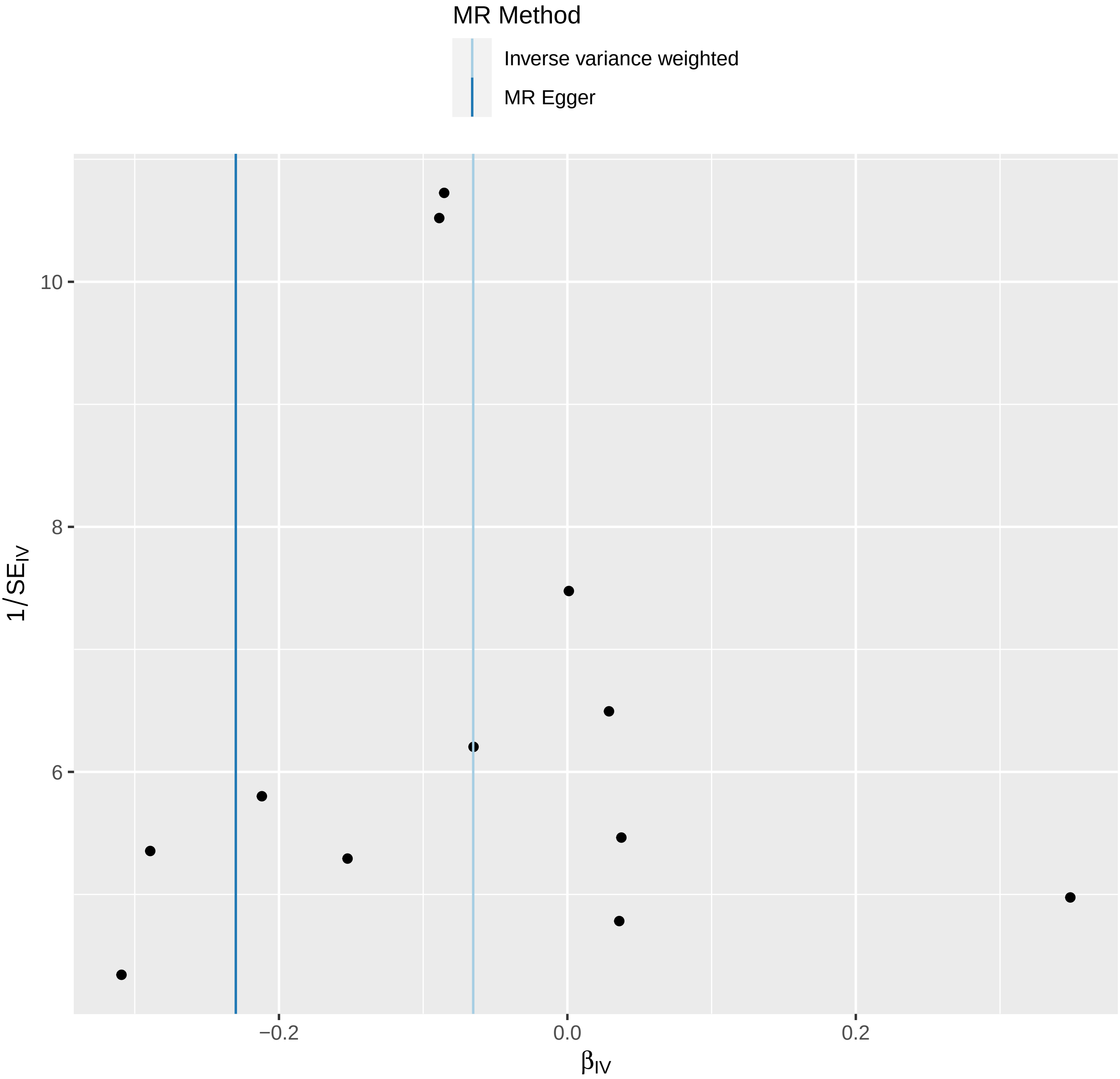

Batch 3−3228 : Selenium on WAP, Kazal, immunoglobulin, Kunitz and NTR domain−containing protein 2

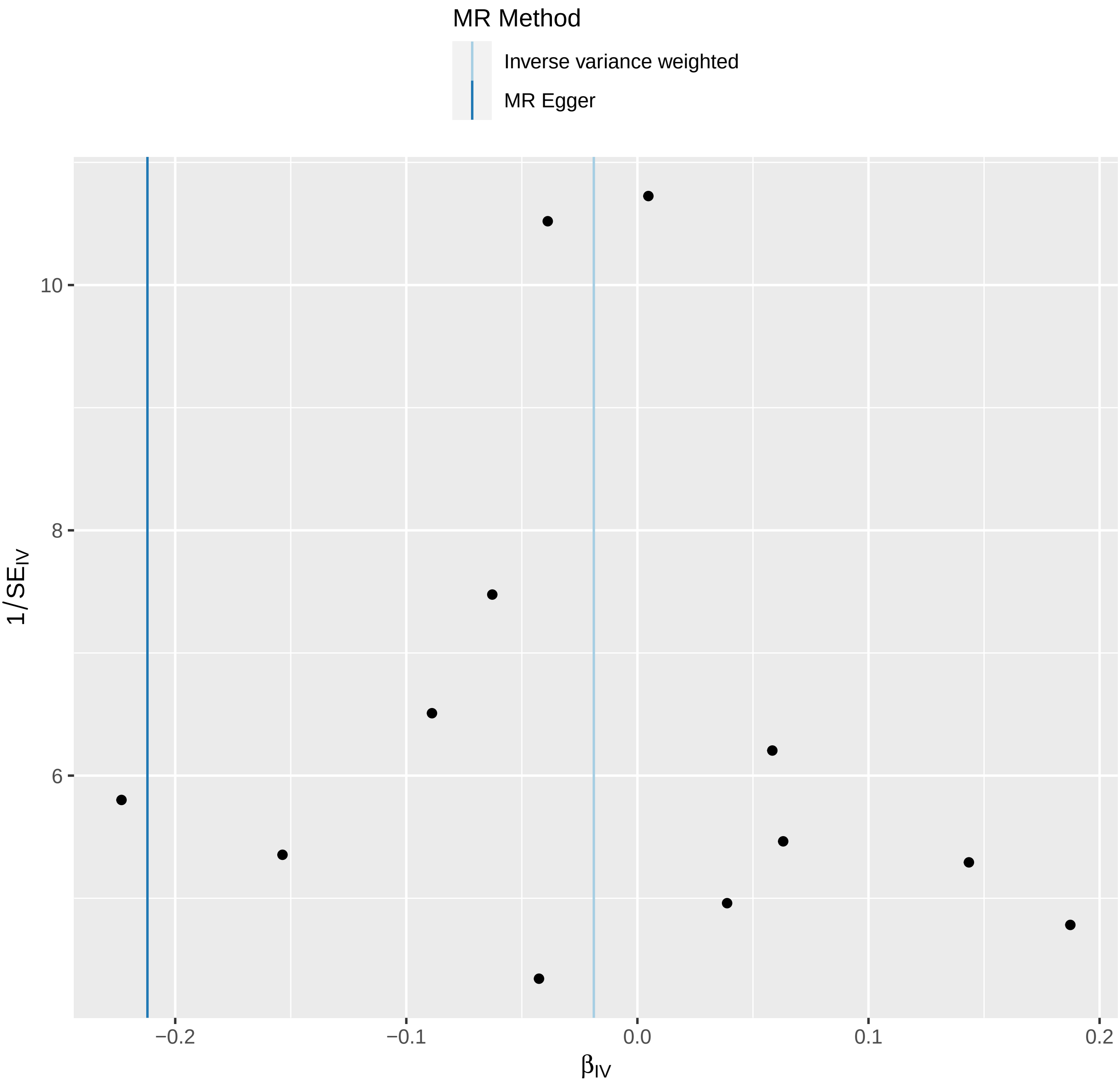

Batch 3−3229 : Selenium on WAP, Kazal, immunoglobulin, Kunitz and NTR domain−containing protein 2

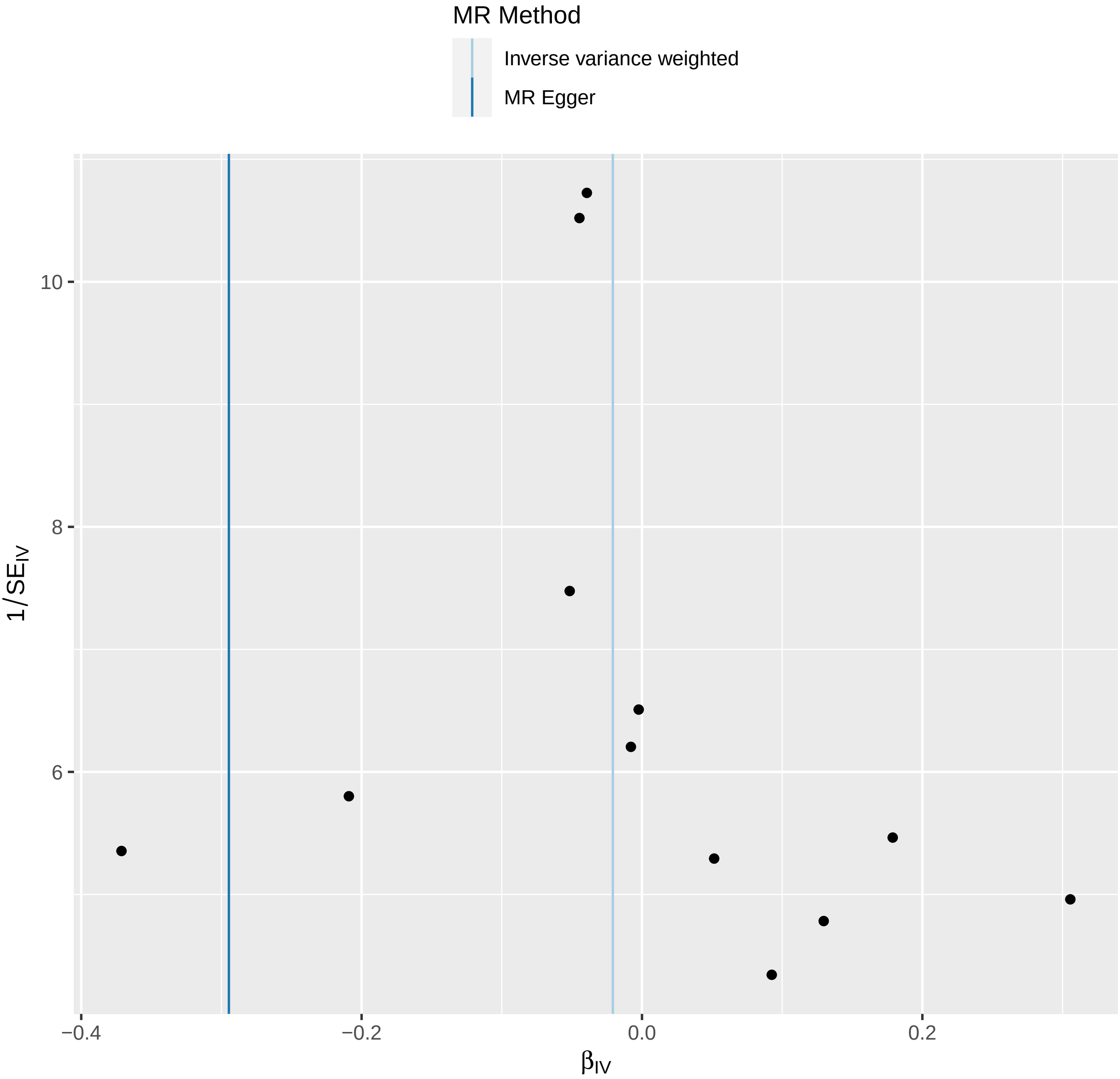

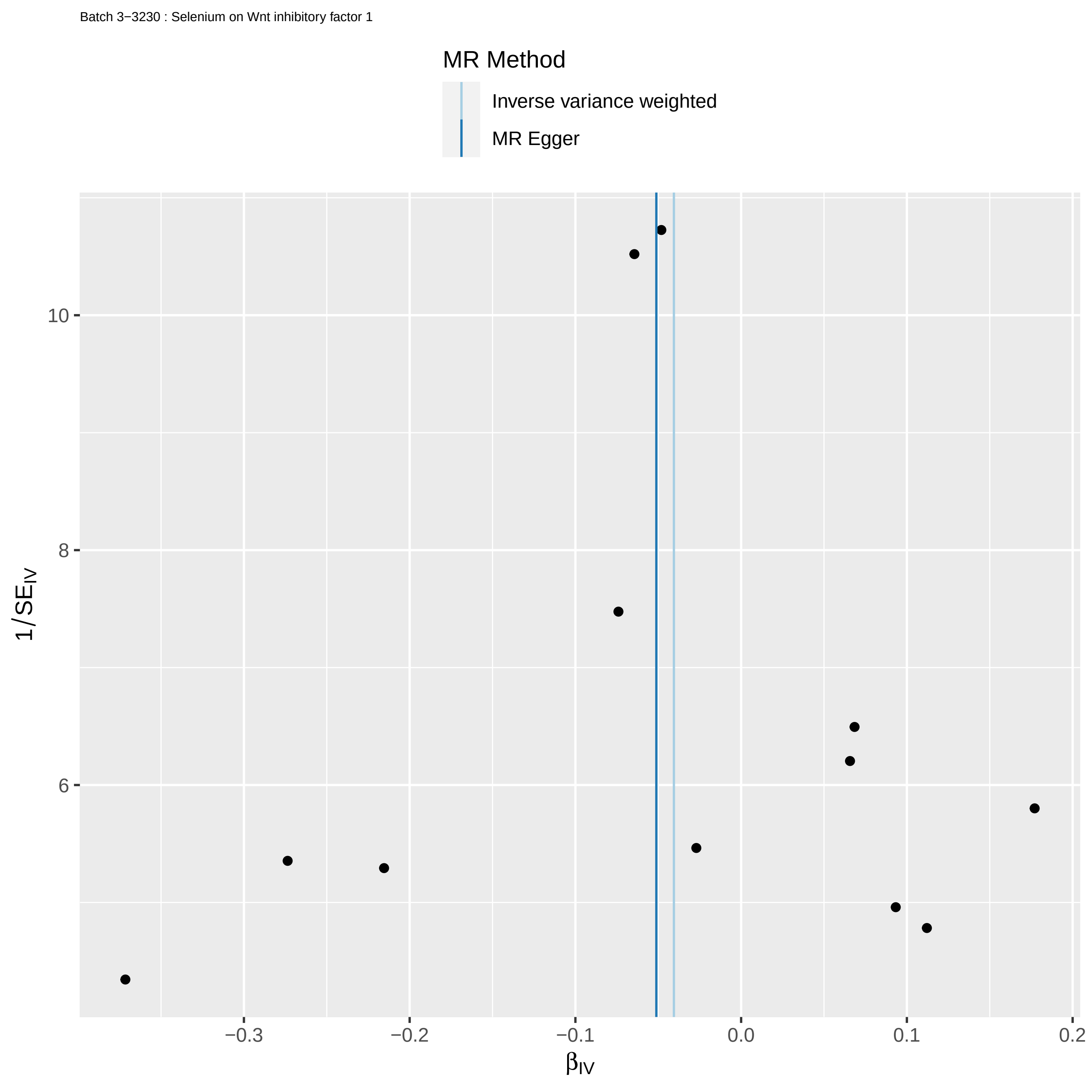

Batch 3−3230 : Selenium on Wnt inhibitory factor 1
MR Method
Inverse variance weighted
MR Egger
10
8
6
1/SE_IV
−0.3
−0.2
−0.1
0.0
0.1
0.2
β_IV

Batch 3−3231 : Selenium on WNT1−inducible−signaling pathway protein 1

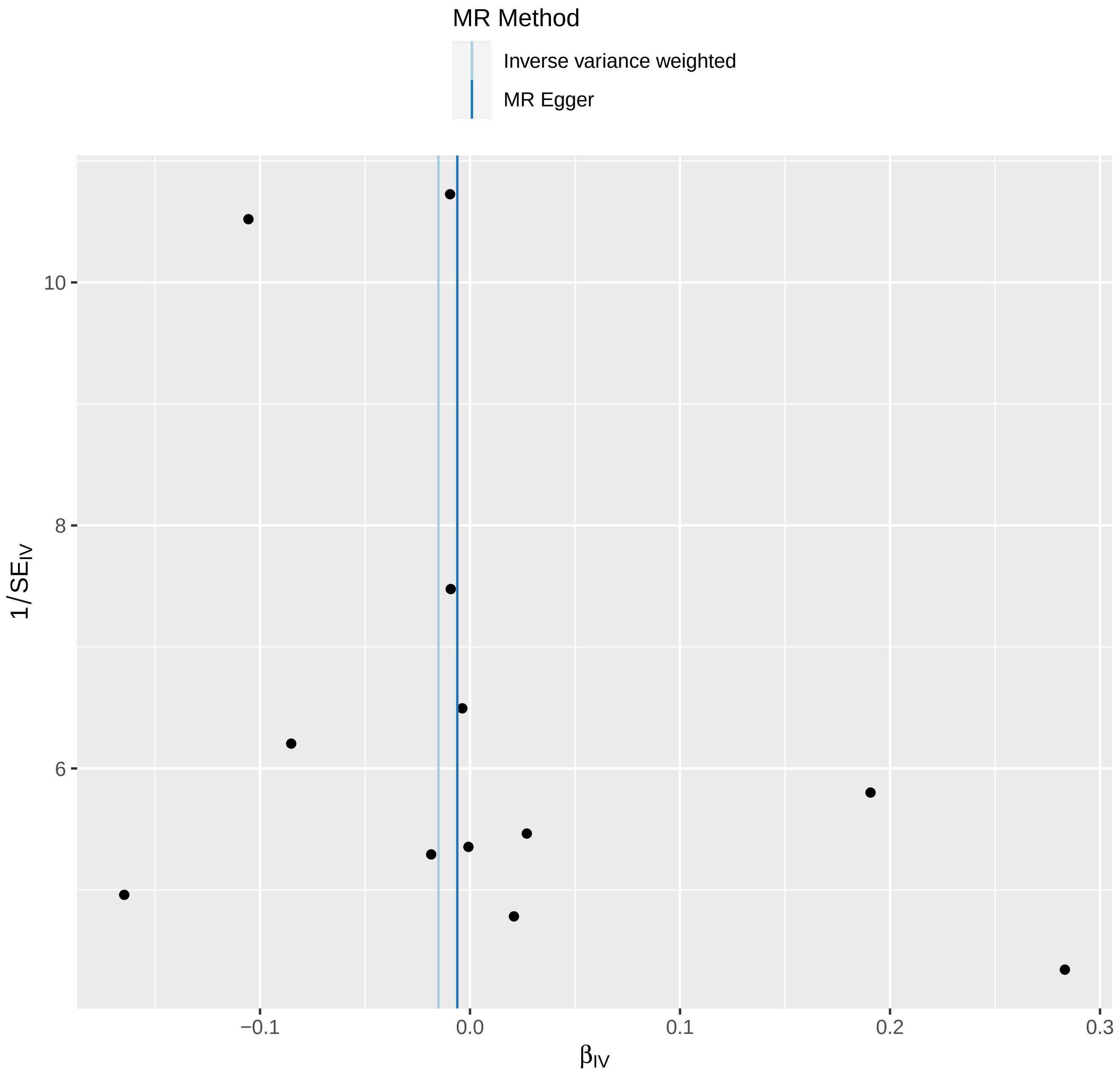

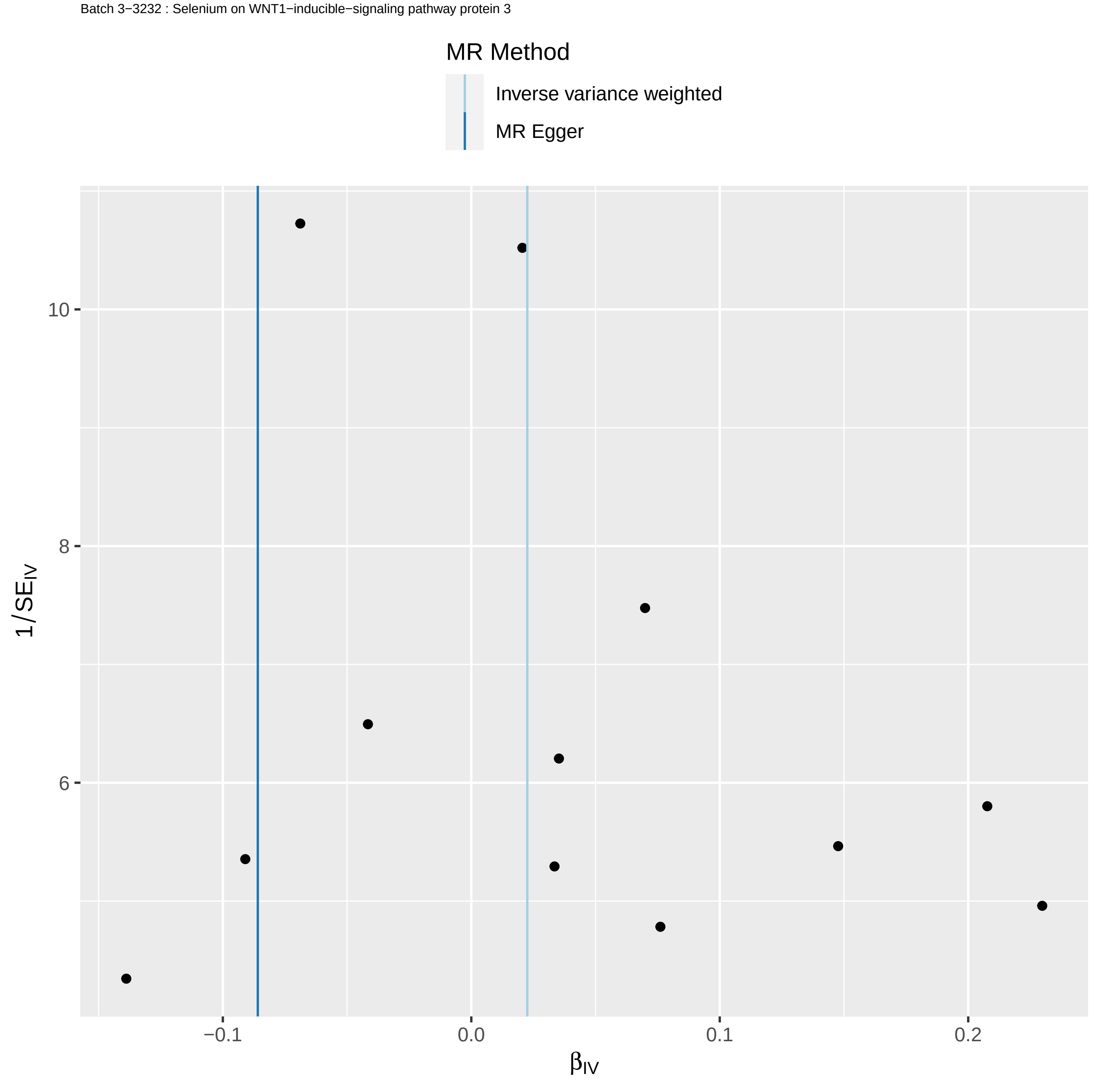

Batch 3−3232 : Selenium on WNT1−inducible−signaling pathway protein 3
MR Method
Inverse variance weighted
MR Egger
10
8
6
1/SE_IV
−0.1
0.0
0.1
0.2
β_IV

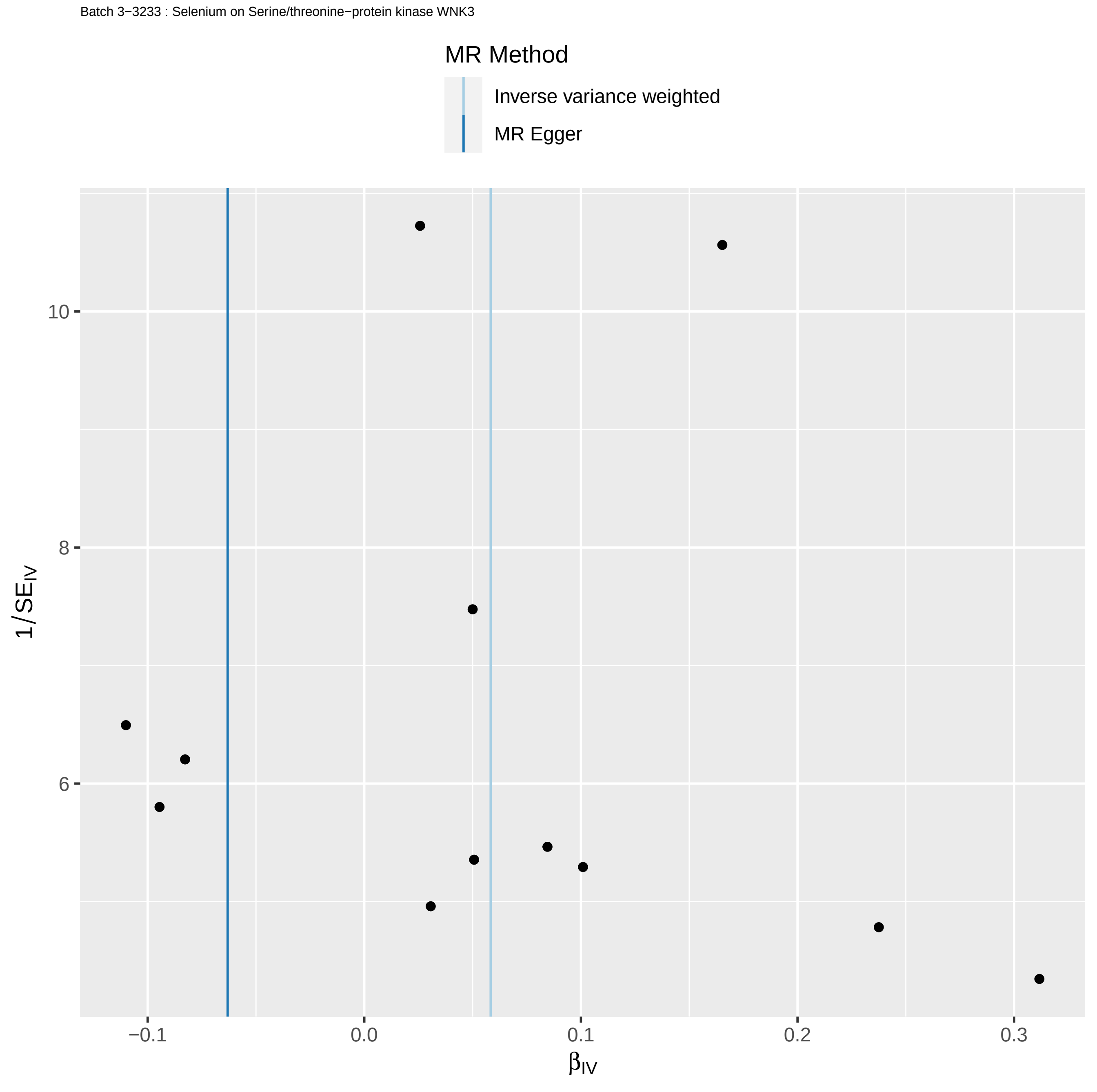

Batch 3−3233 : Selenium on Serine/threonine−protein kinase WNK3
MR Method
Inverse variance weighted
MR Egger
10
8
6
1/SE$_{IV}$
−0.1
0.0
0.1
0.2
0.3
β$_{IV}$

Batch 3−3234 : Selenium on Protein Wnt−7a

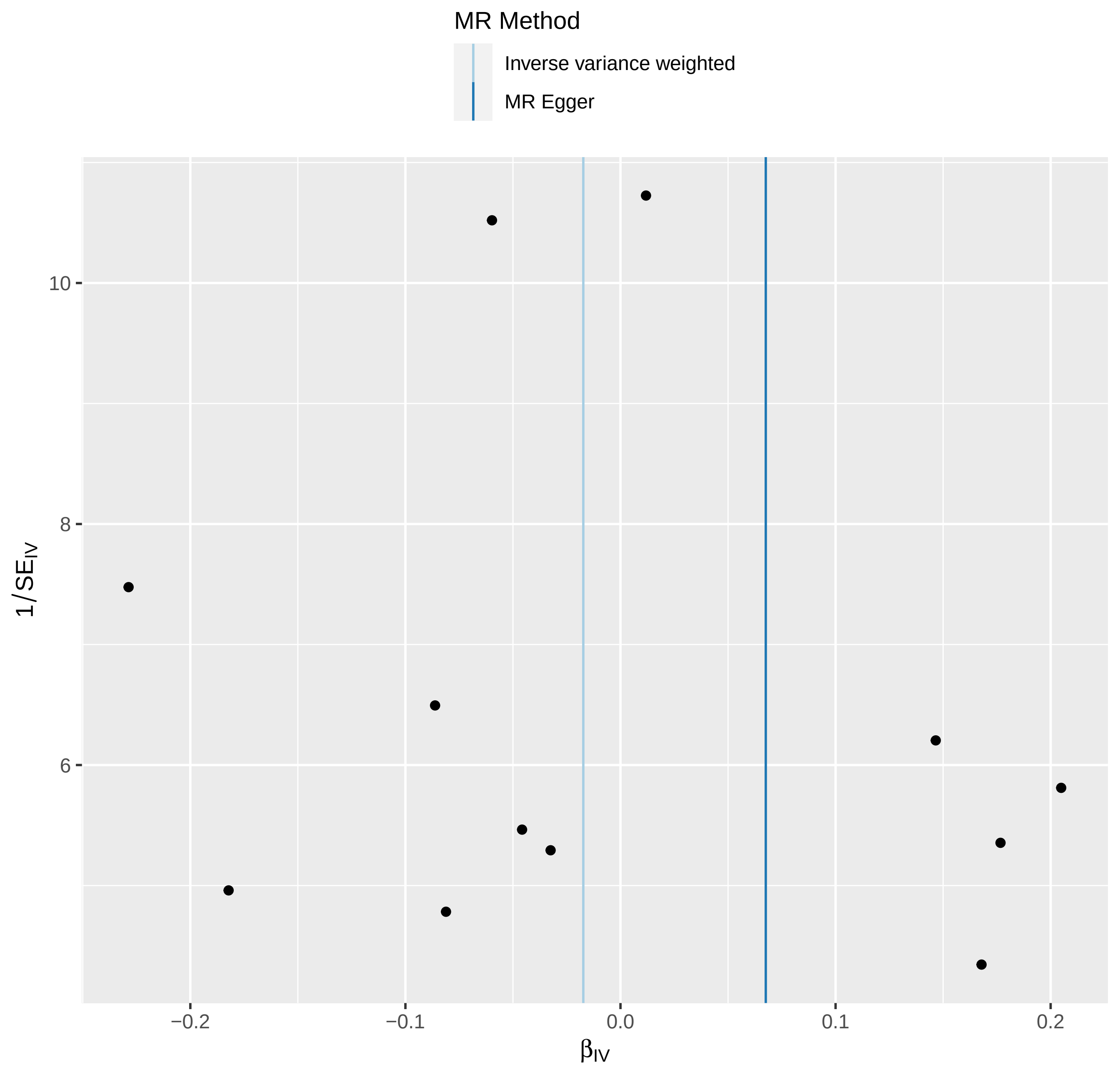

Batch 3−3235 : Selenium on WSC domain−containing protein 2

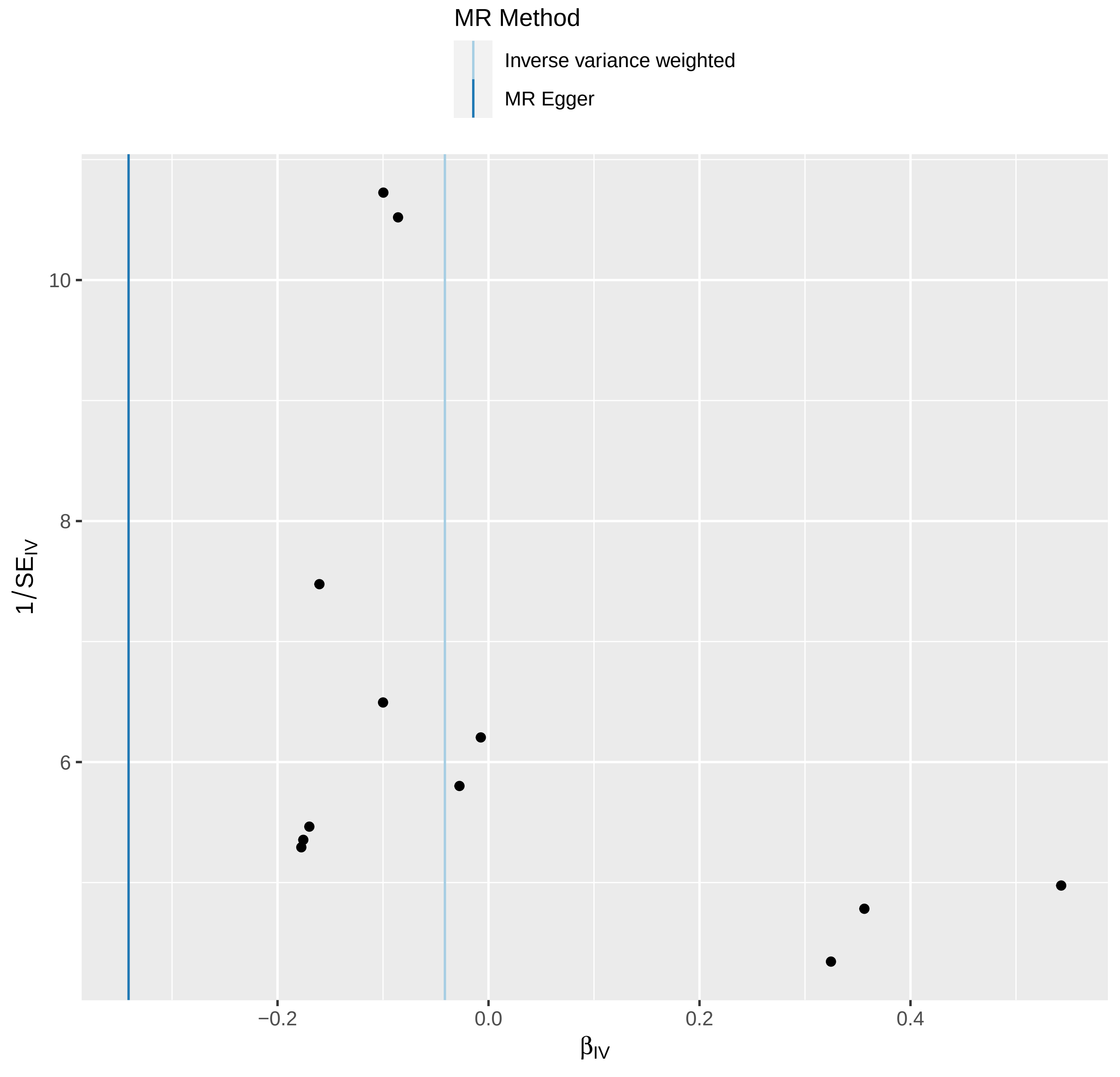

Batch 3−3236 : Selenium on Lymphotactin

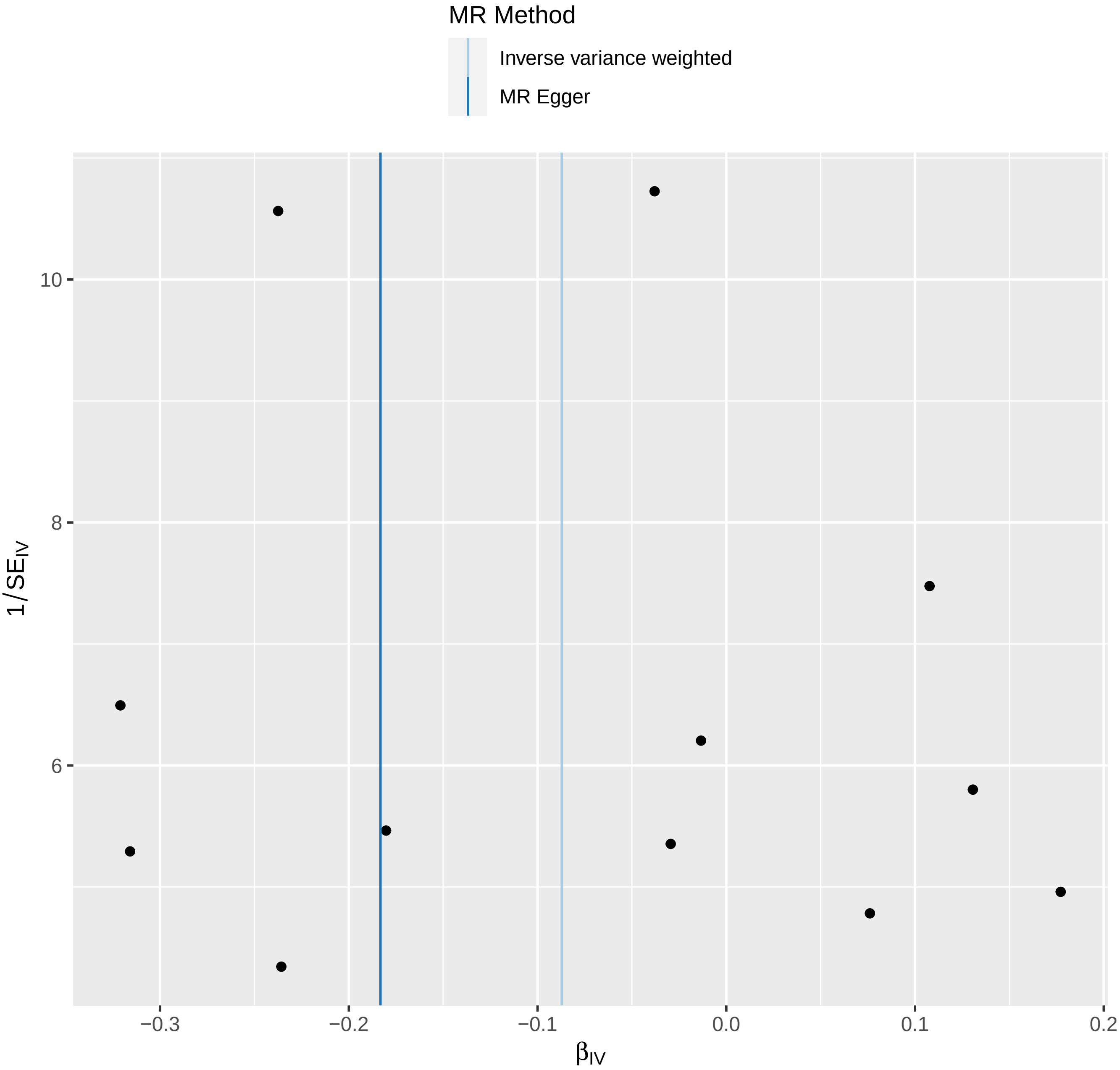

Batch 3−3237 : Selenium on Lymphotactin

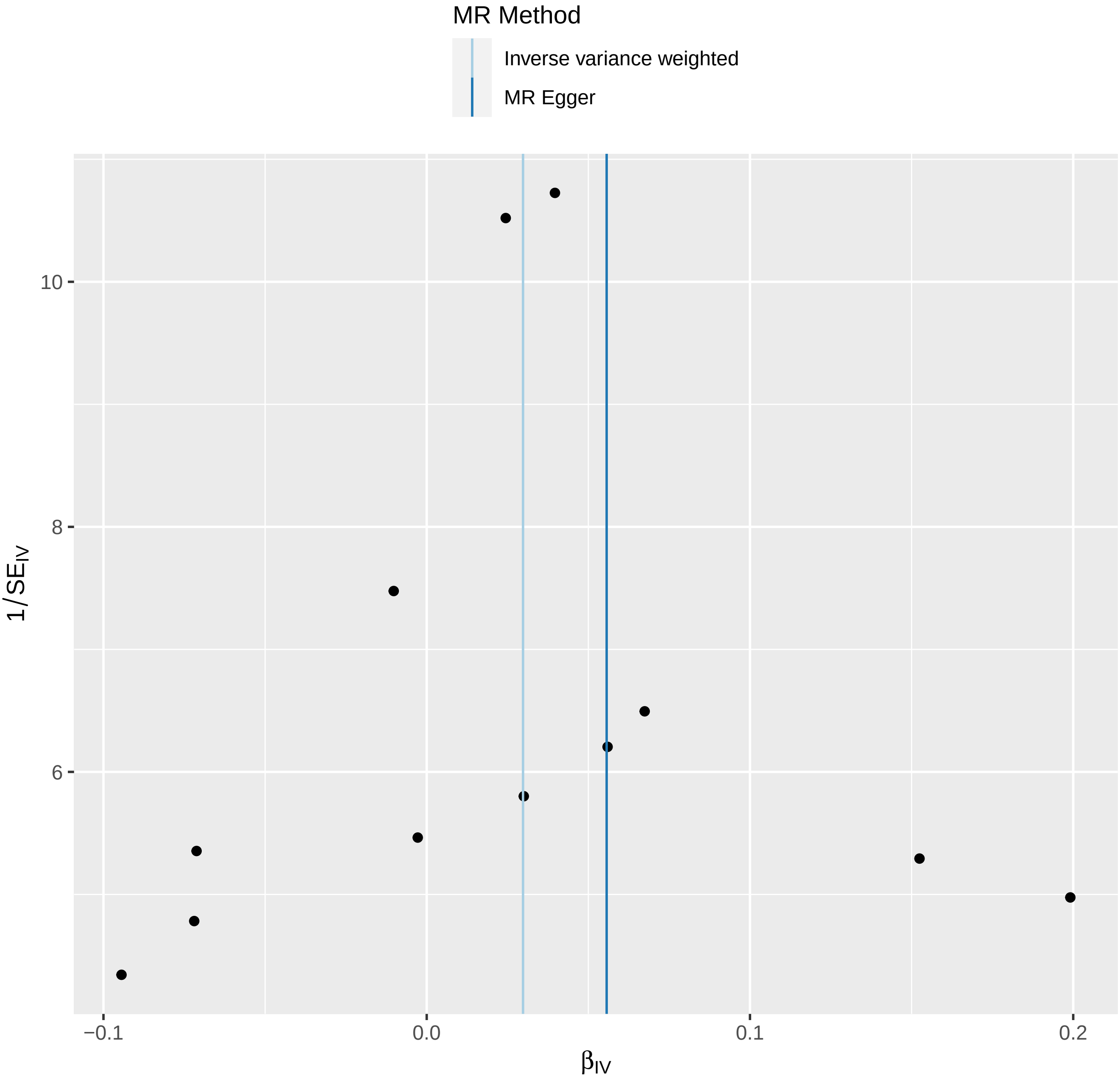

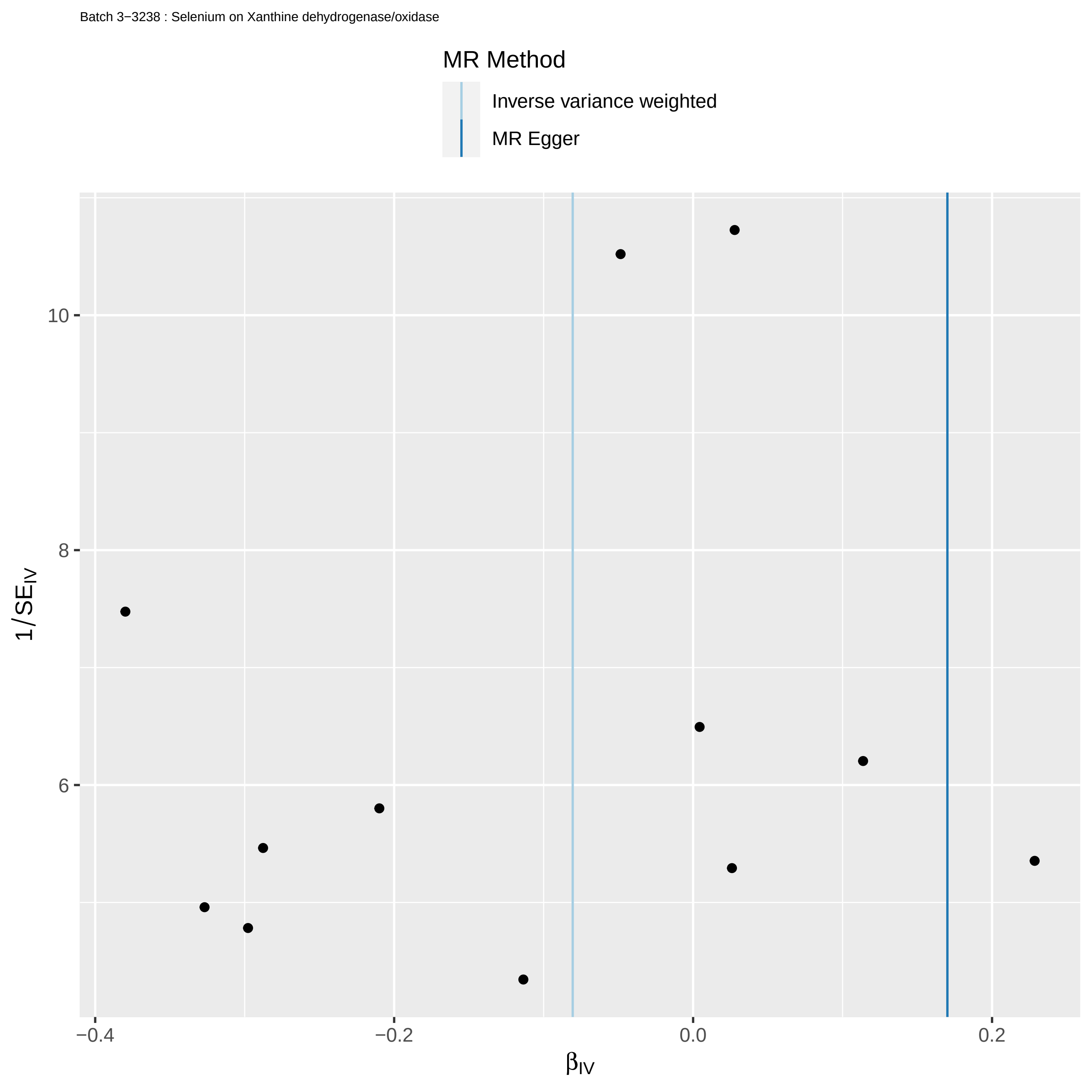

Batch 3−3238 : Selenium on Xanthine dehydrogenase/oxidase
MR Method
Inverse variance weighted
MR Egger
10
8
6
1/SE_IV
−0.4
−0.2
0.0
0.2
β_IV

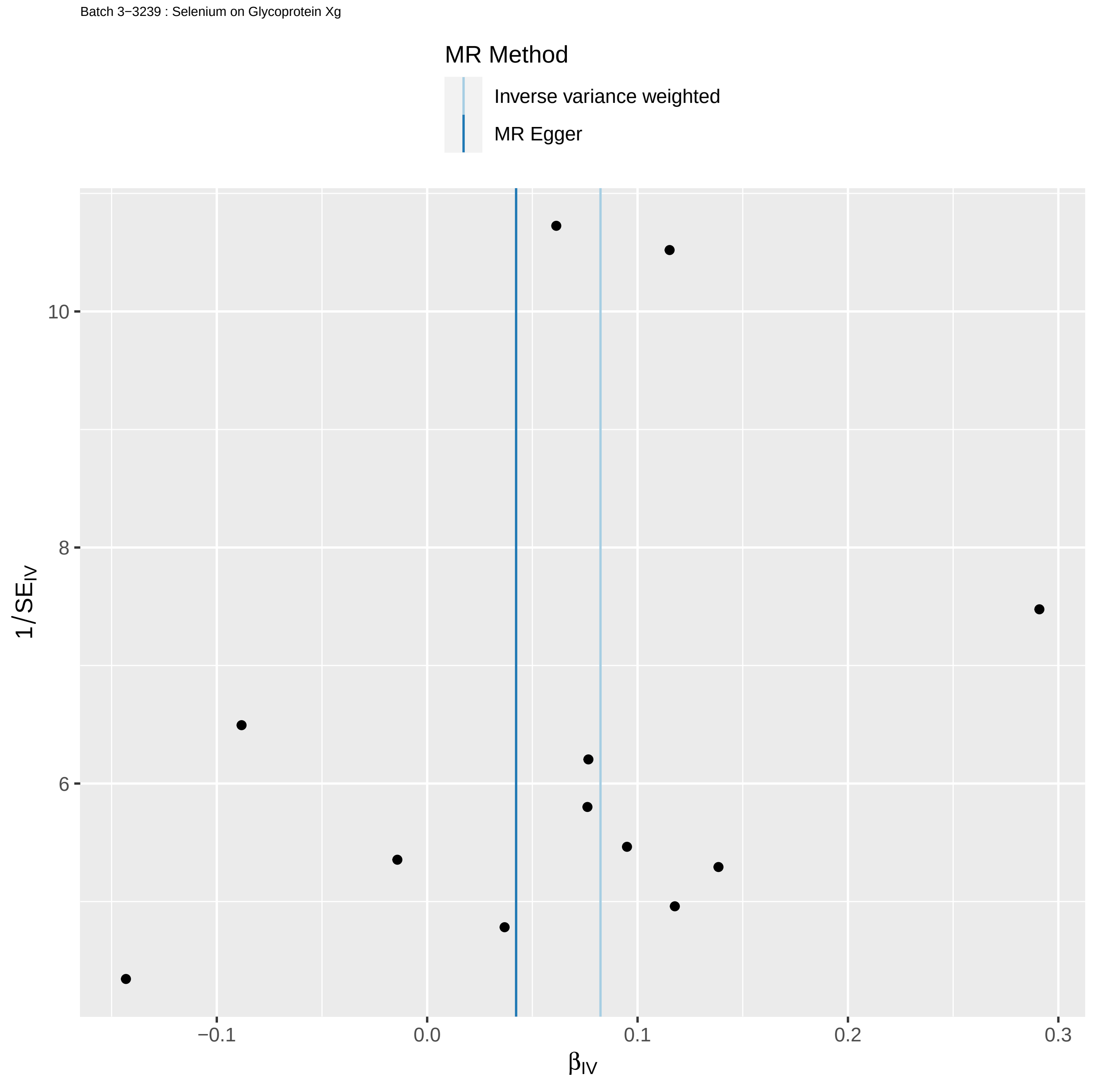
Batch 3−3239 : Selenium on Glycoprotein Xg
MR Method
Inverse variance weighted
MR Egger
10
8
6
1/SE$_{IV}$
−0.1
0.0
0.1
0.2
0.3
β$_{IV}$

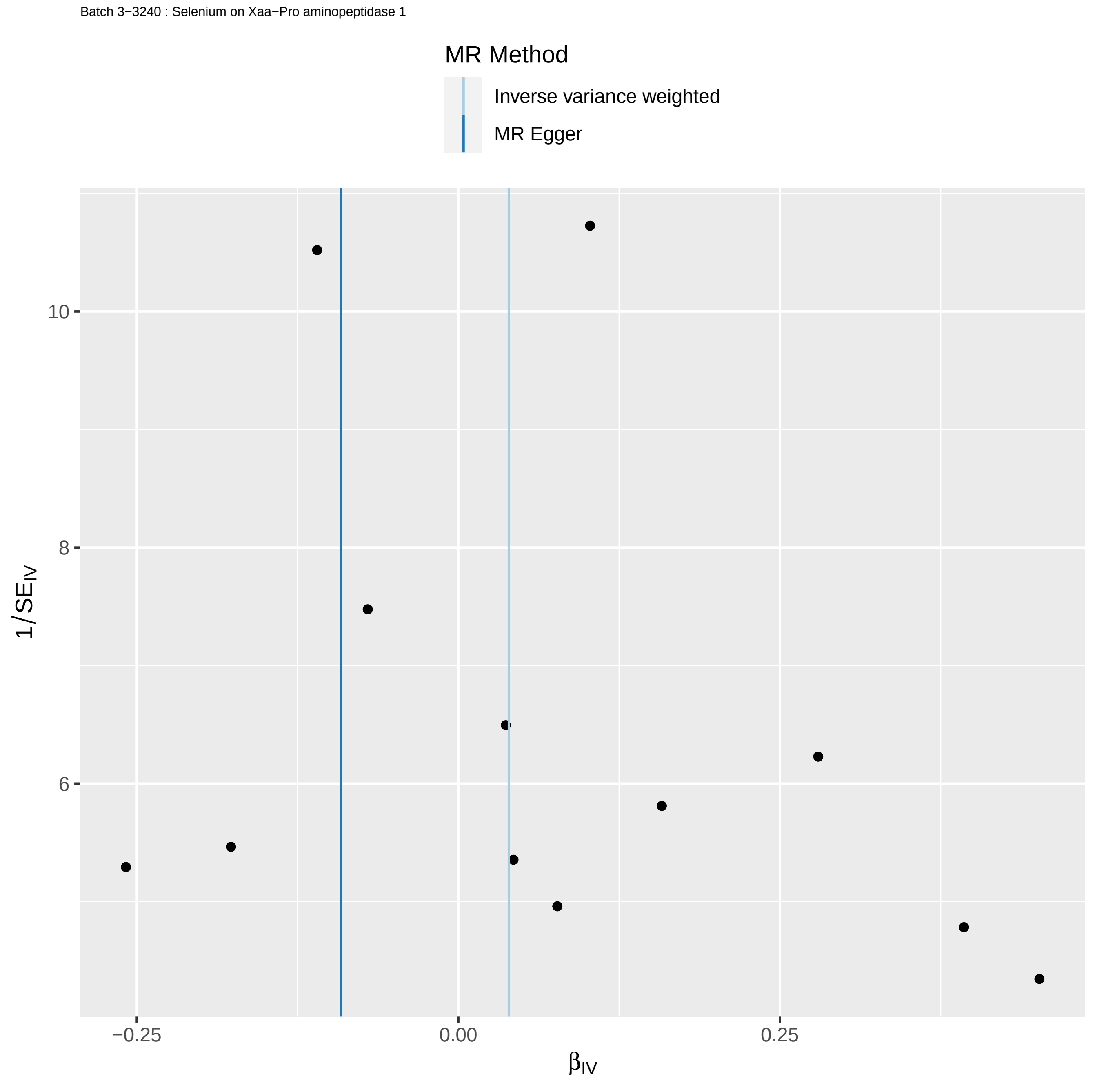

Batch 3−3240 : Selenium on Xaa−Pro aminopeptidase 1
MR Method
Inverse variance weighted
MR Egger
10
8
6
1/SE$_{IV}$
−0.25
0.00
0.25
β$_{IV}$

Batch 3−3241 : Selenium on DNA repair protein XRCC1

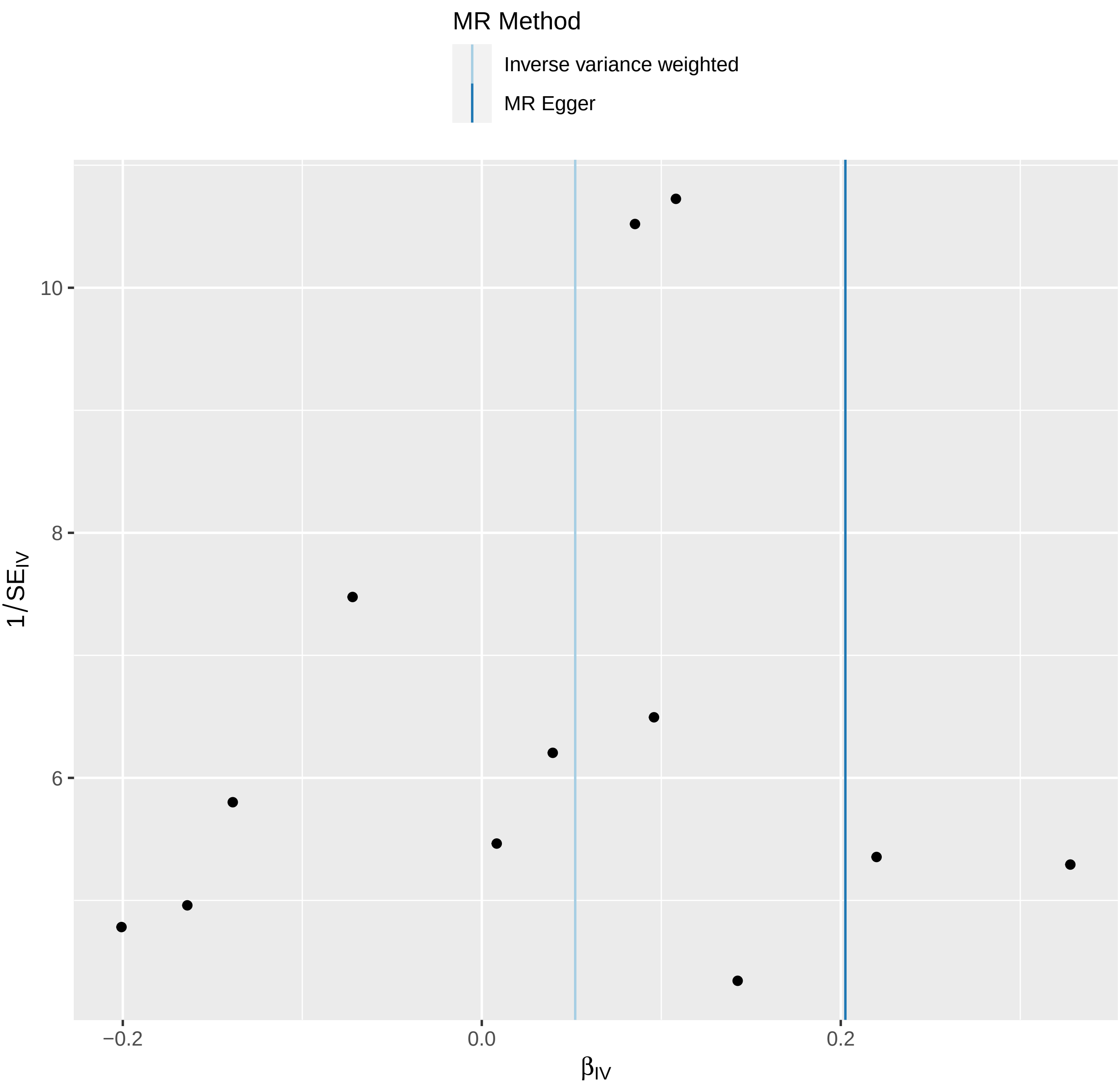

Batch 3−3242 : Selenium on DNA repair protein XRCC4

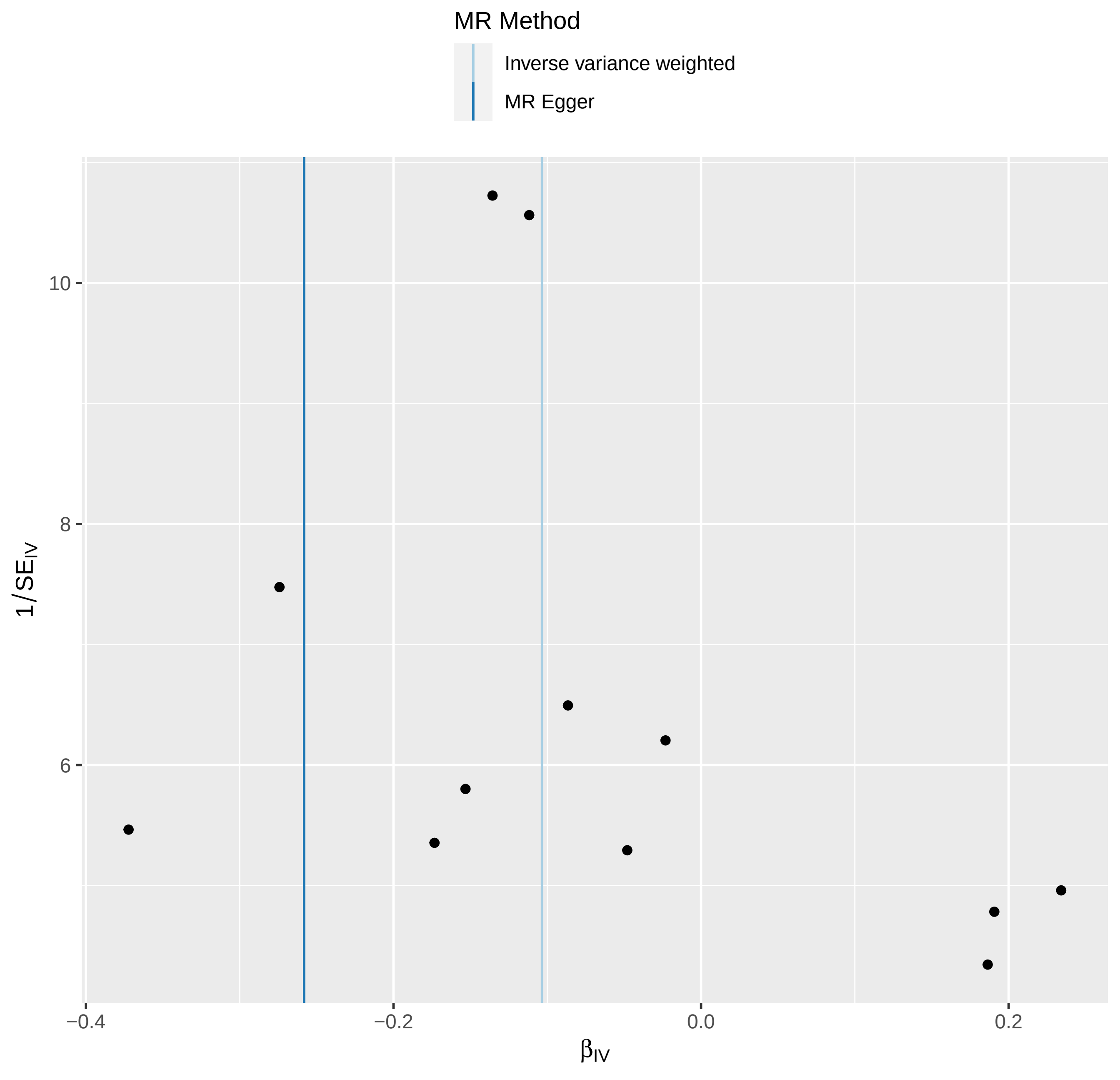

Batch 3−3243 : Selenium on Xyloside xylosyltransferase 1

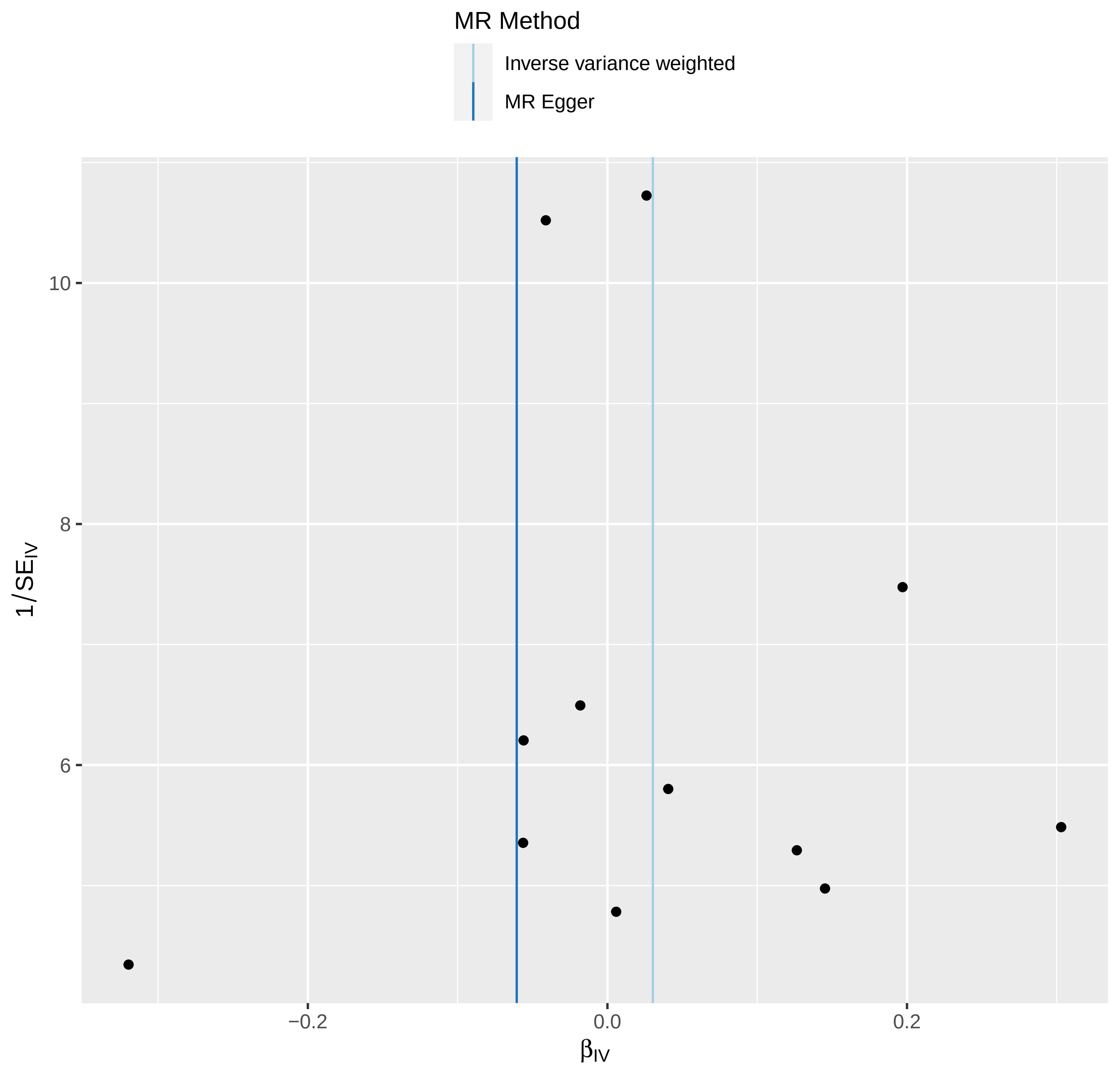

Batch 3−3244 : Selenium on Y−box−binding protein 2

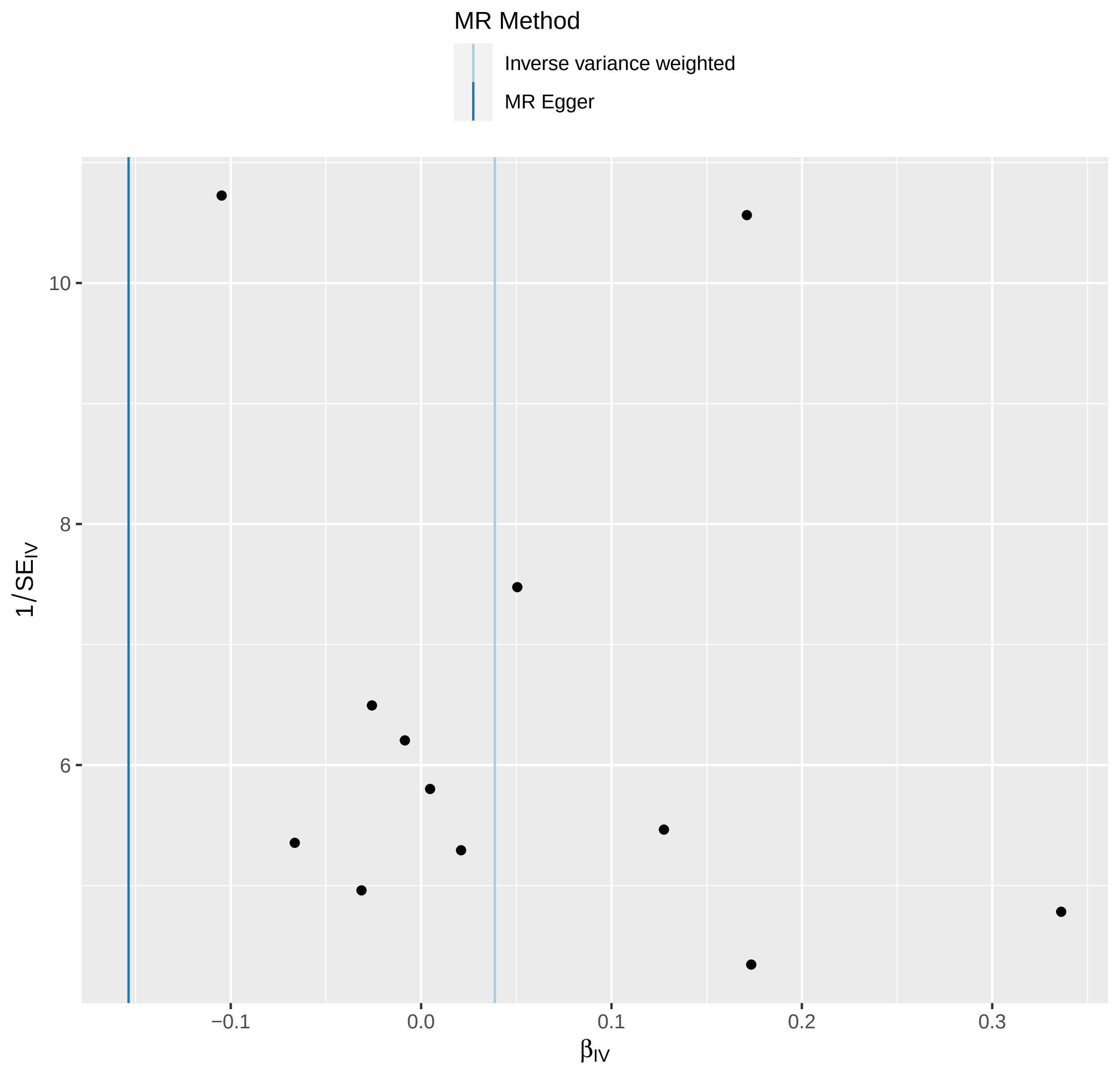

Batch 3−3245 : Selenium on Tyrosine−protein kinase Yes

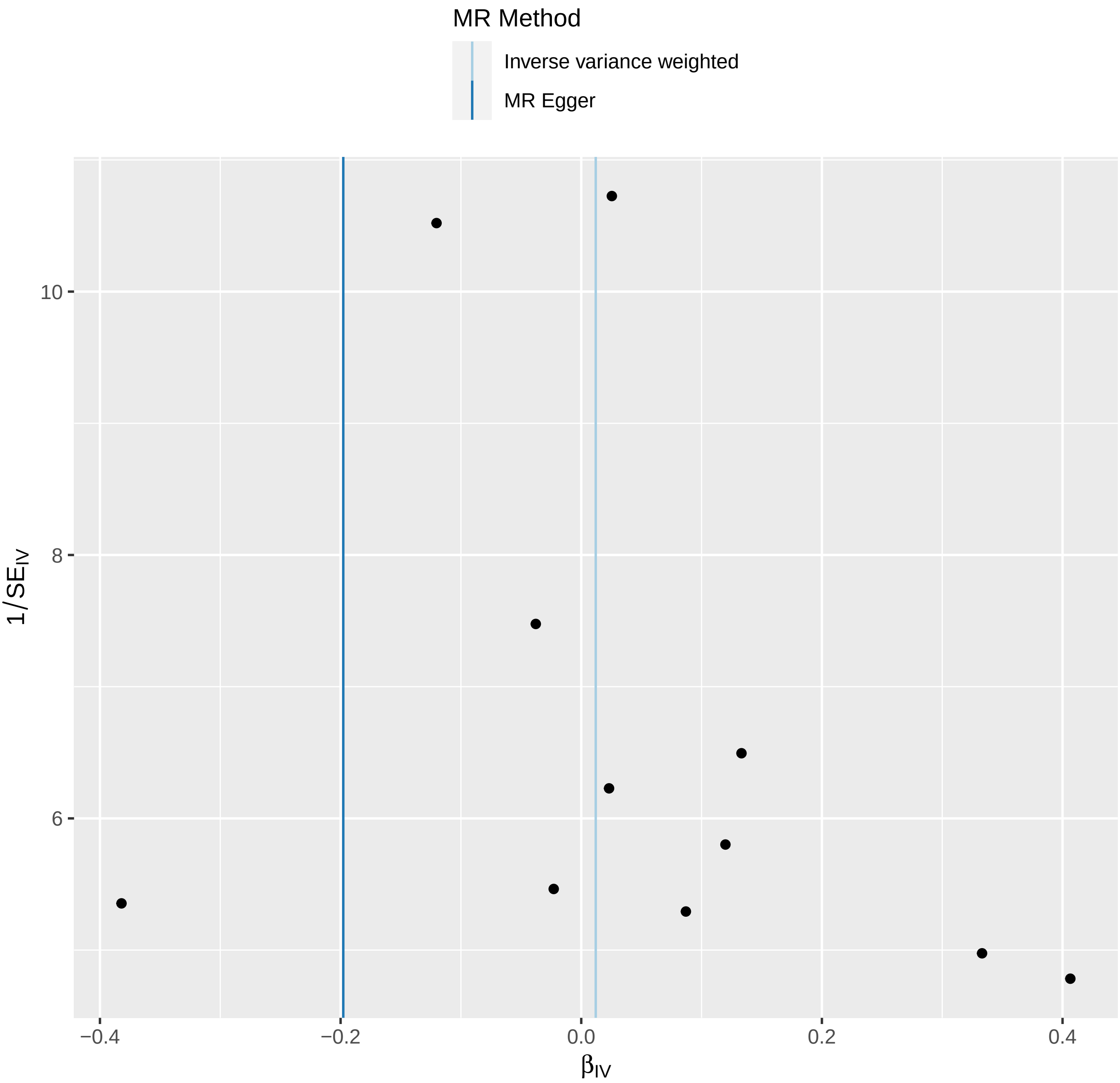

Batch 3−3246 : Selenium on Protein YIPF6

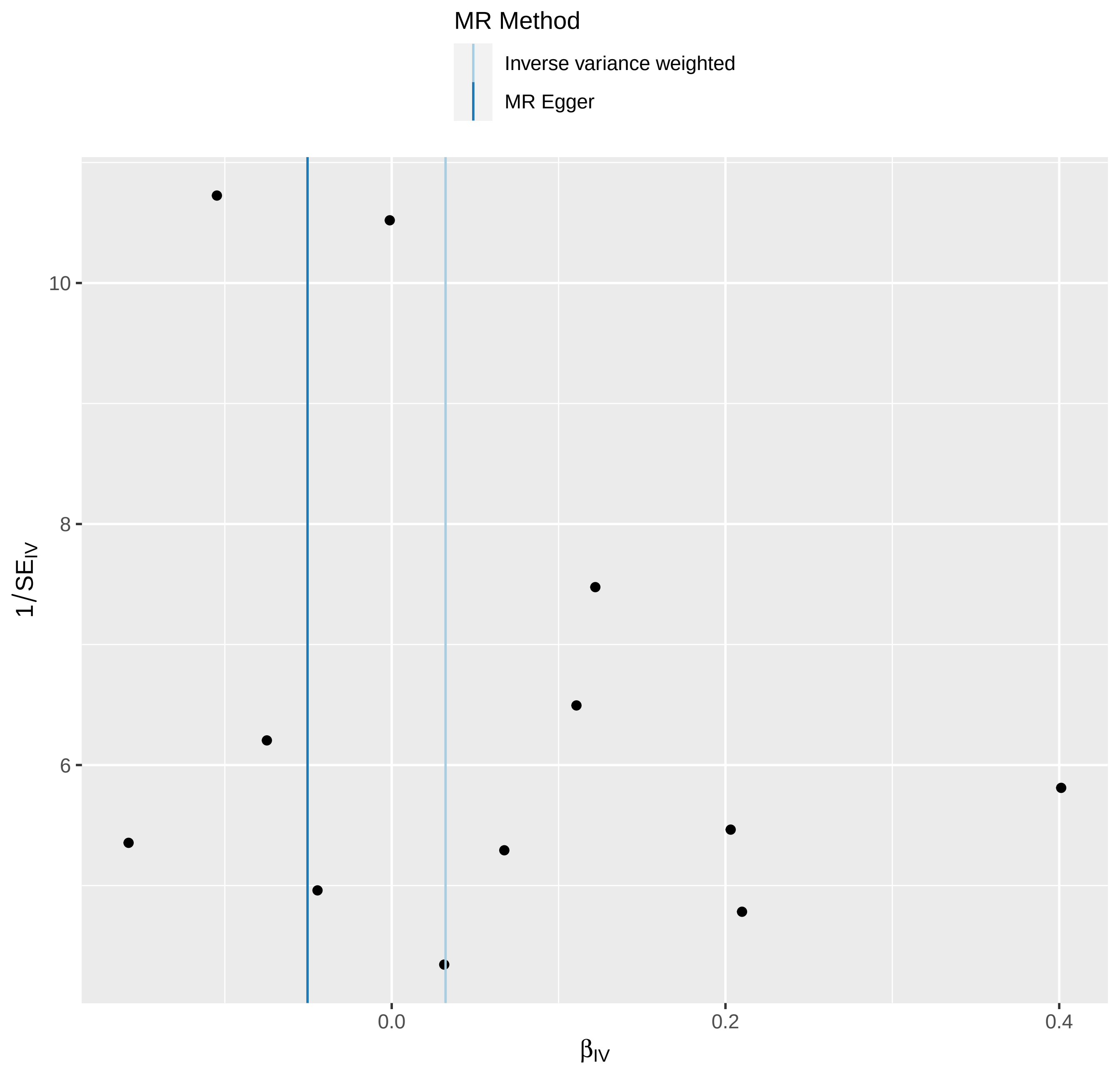

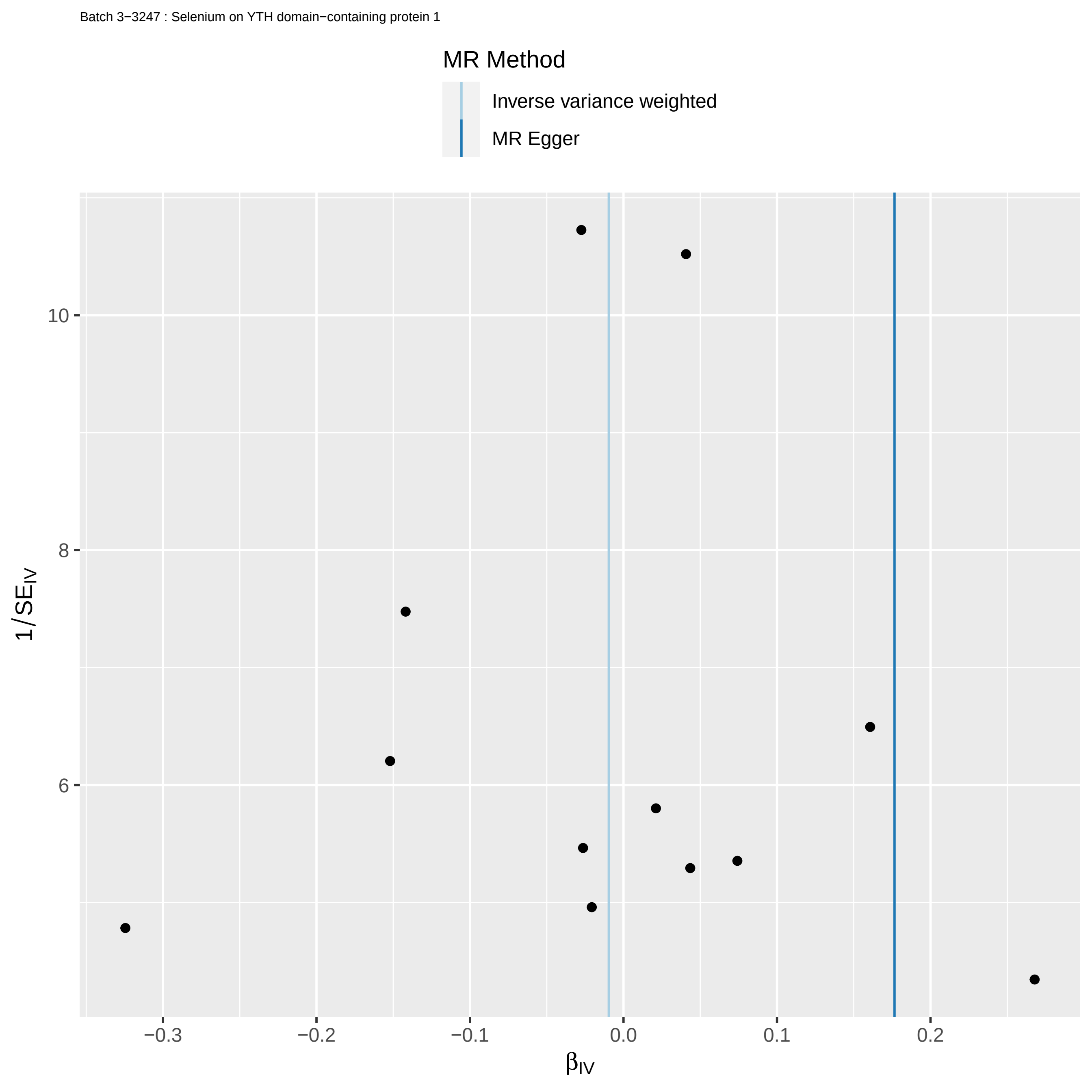

Batch 3−3247 : Selenium on YTH domain−containing protein 1
MR Method
Inverse variance weighted
MR Egger
10
8
6
1/SE$_{IV}$
−0.3
−0.2
−0.1
0.0
0.1
0.2
β_{IV}

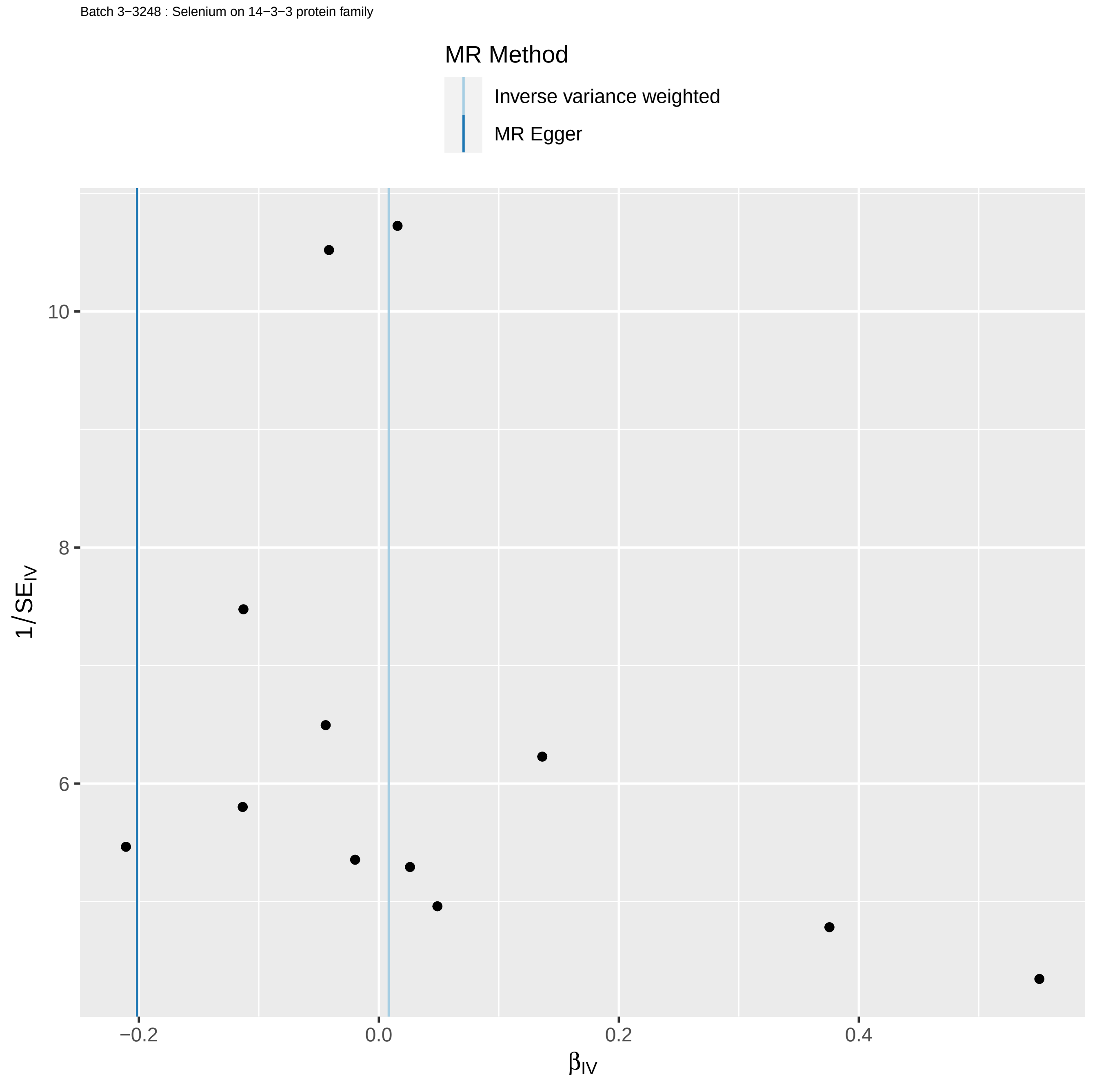

Batch 3−3248 : Selenium on 14−3−3 protein family
MR Method
Inverse variance weighted
MR Egger
10
8
6
1/SE_IV
−0.2
0.0
0.2
0.4
β_IV

Batch 3−3249 : Selenium on 14−3−3 protein epsilon

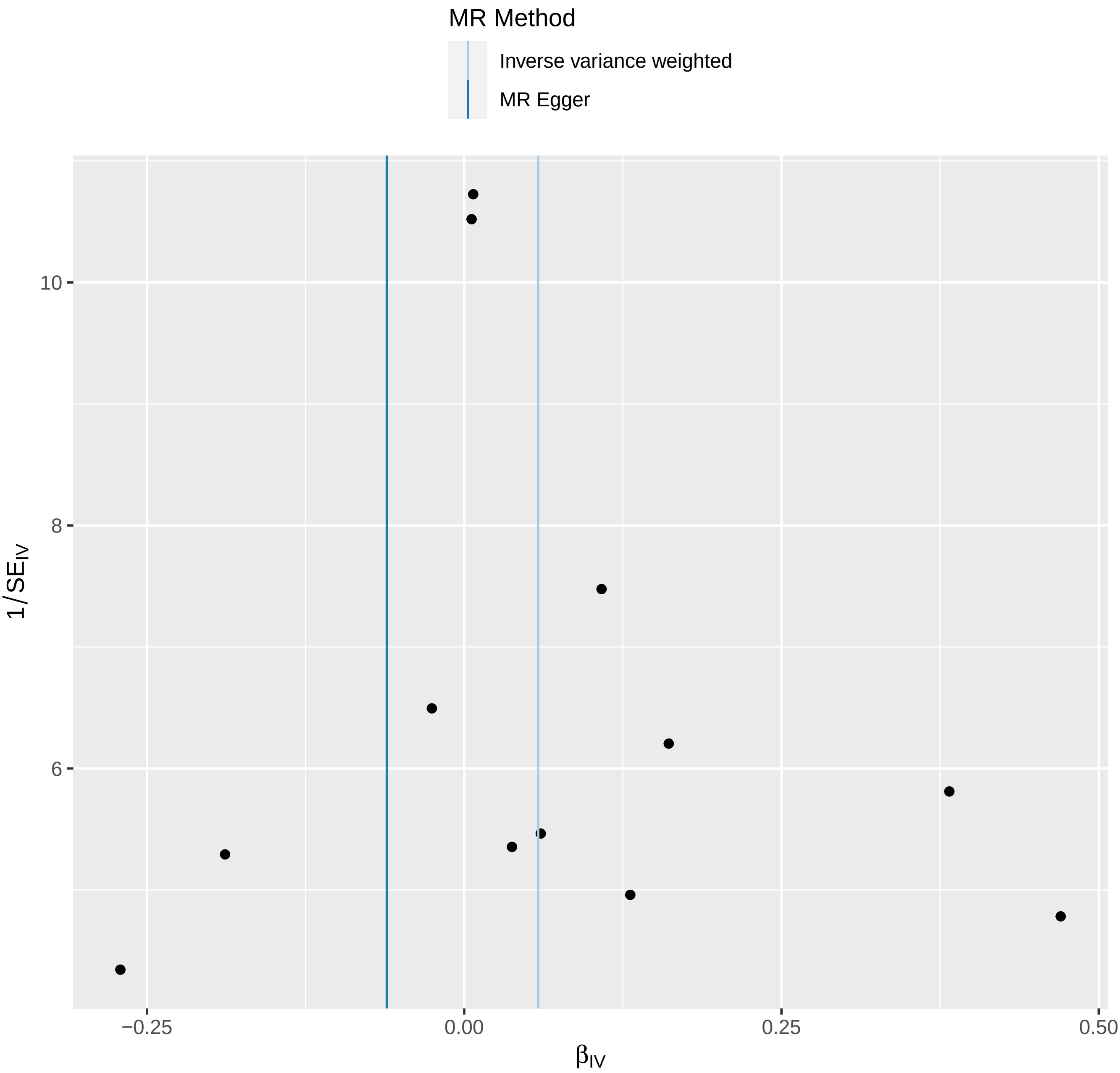

Batch 3−3250 : Selenium on 14−3−3 protein theta

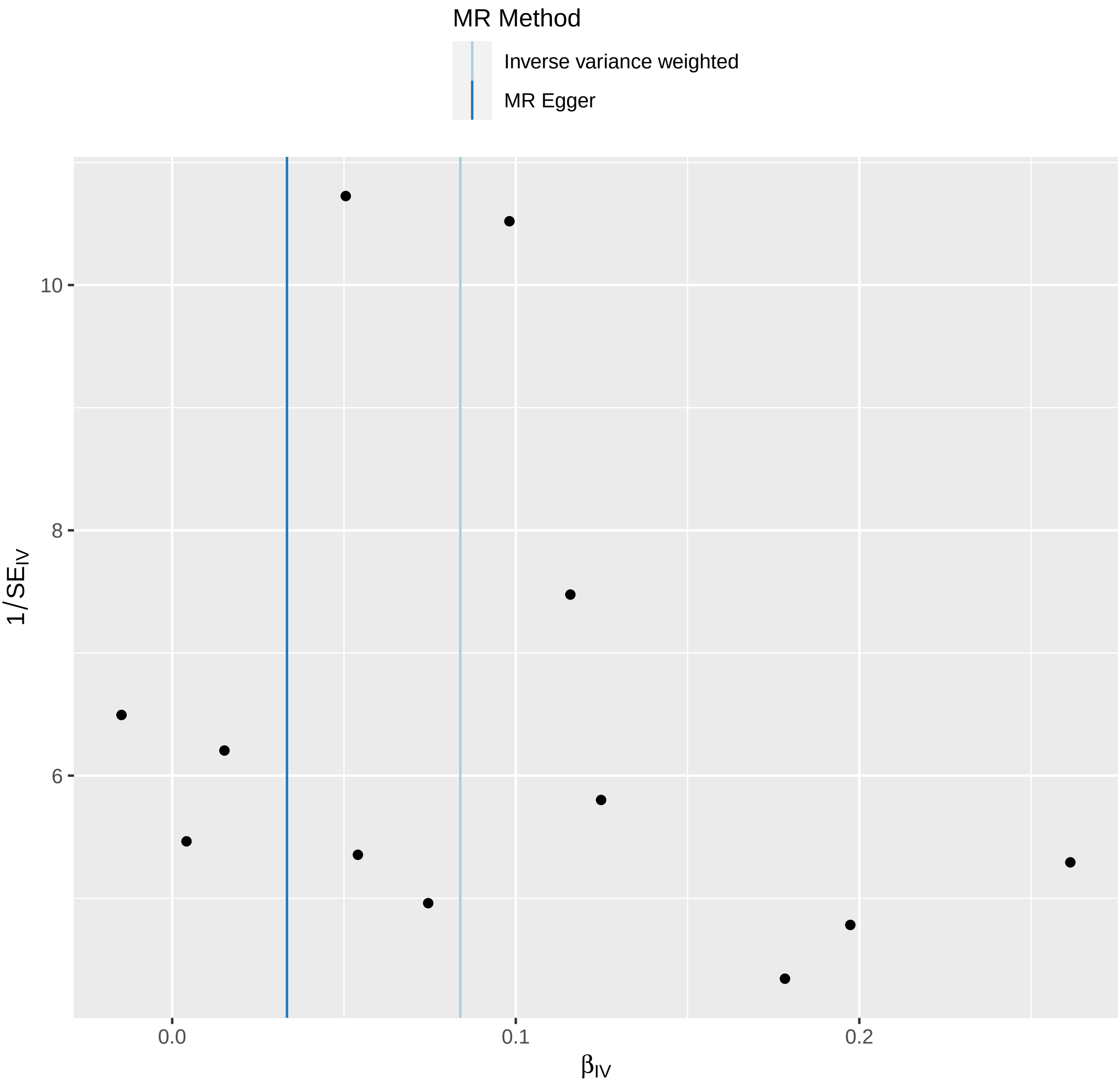

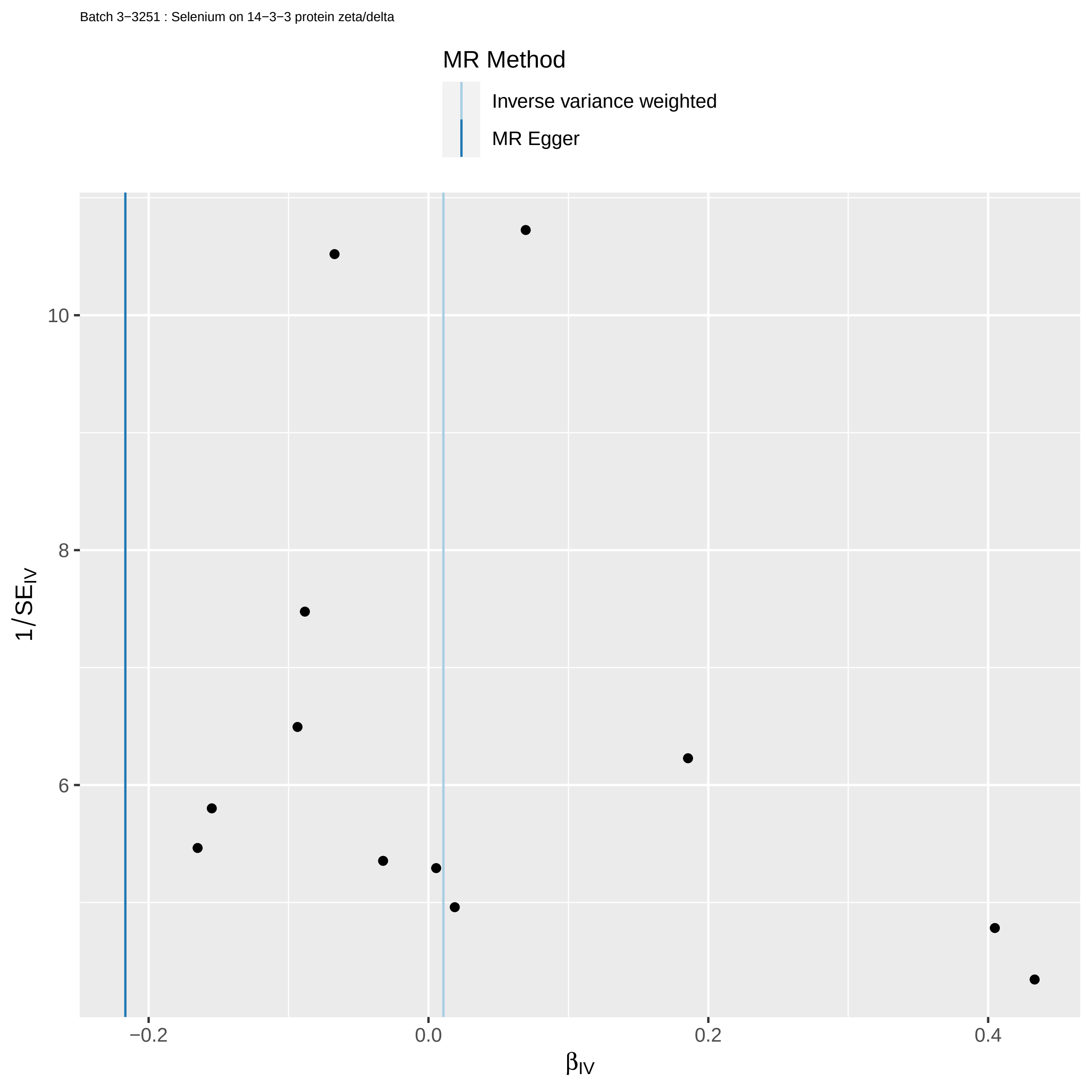

Batch 3−3251 : Selenium on 14−3−3 protein zeta/delta
MR Method
Inverse variance weighted
MR Egger
10
8
6
1/SE_IV
−0.2
0.0
0.2
0.4
β_IV

Batch 3−3252 : Selenium on Tyrosine−protein kinase ZAP−70

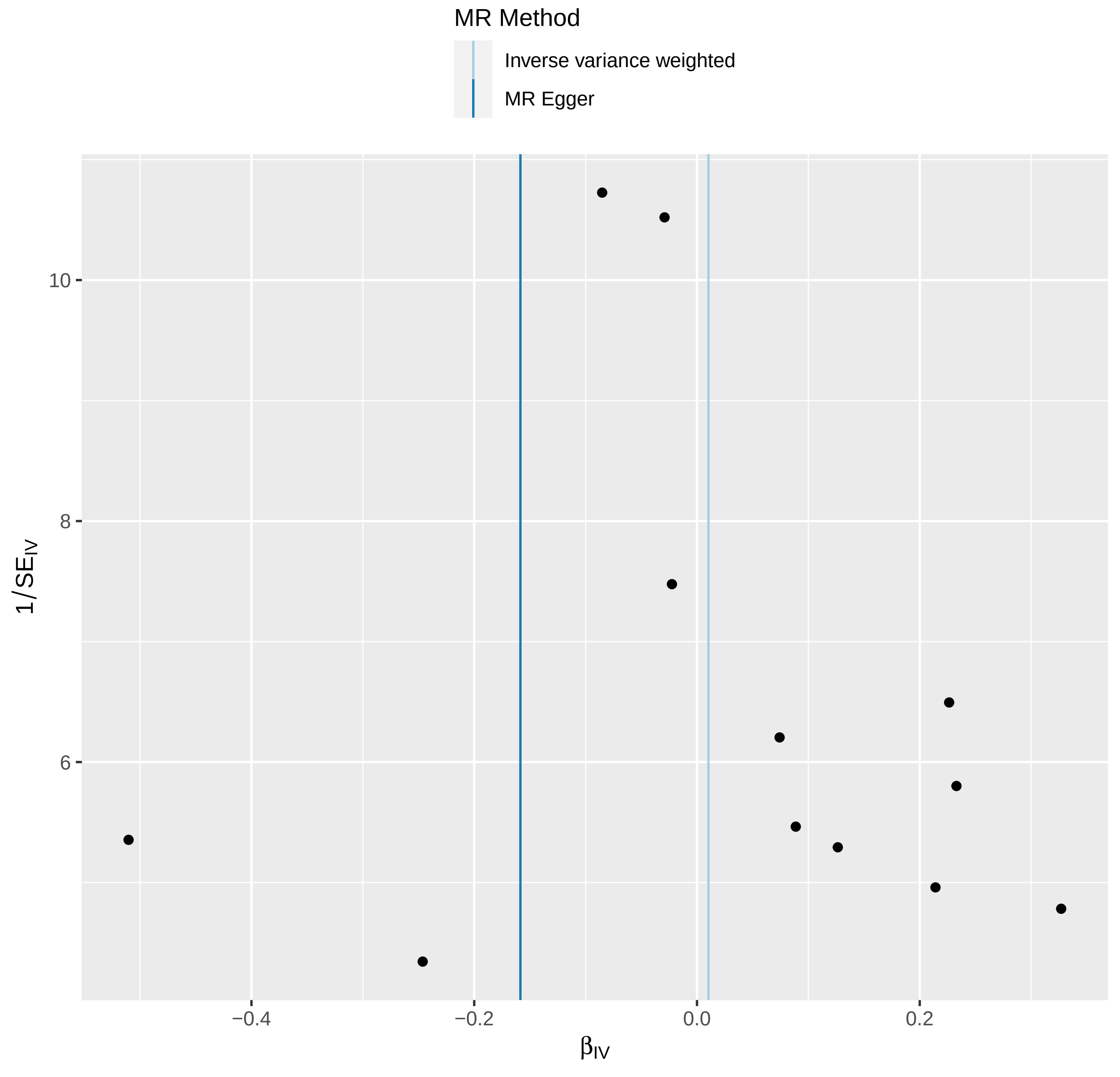

Batch 3−3253 : Selenium on Tyrosine−protein kinase ZAP−70

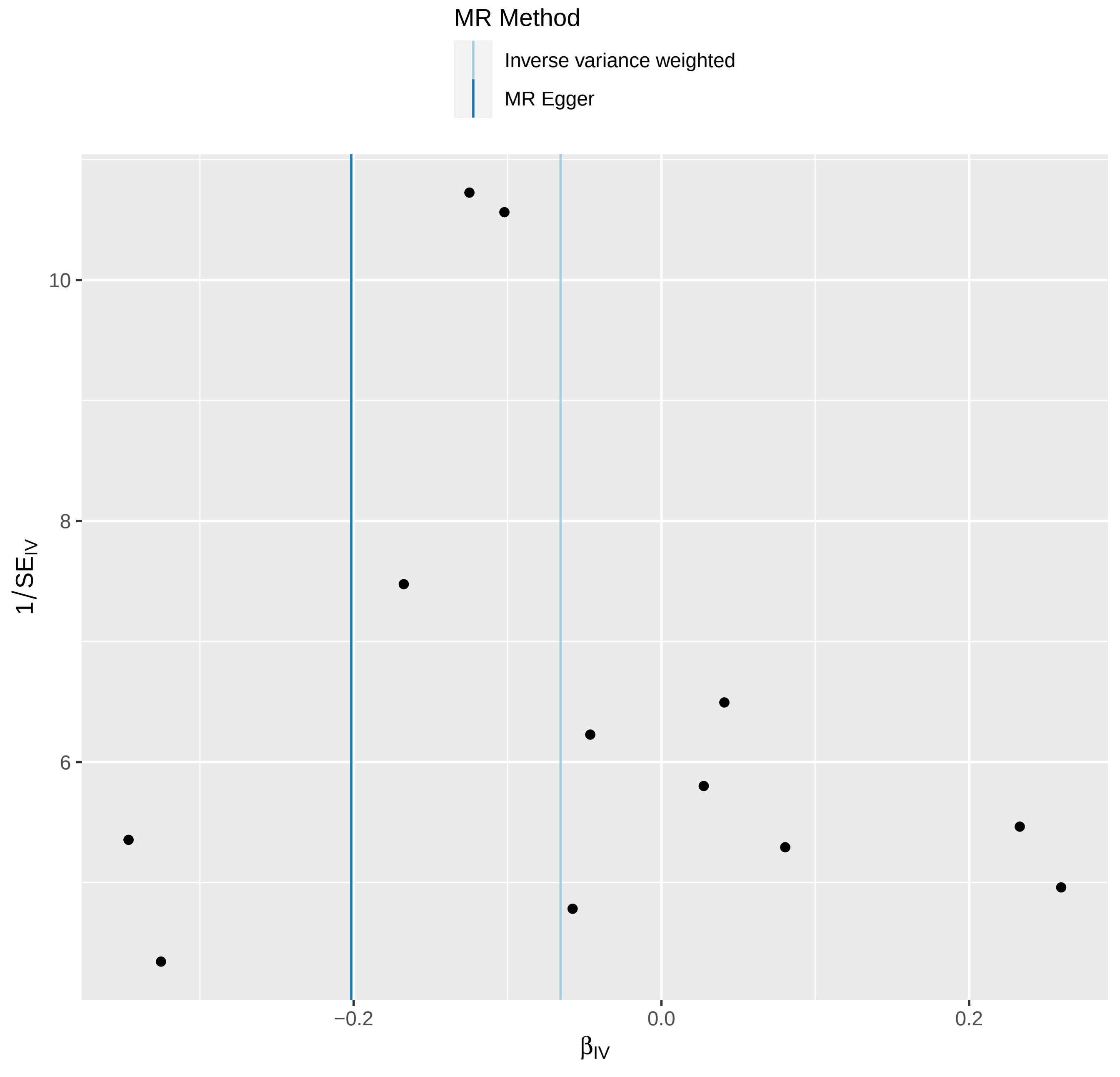

Batch 3−3254 : Selenium on Tyrosine−protein kinase ZAP−70

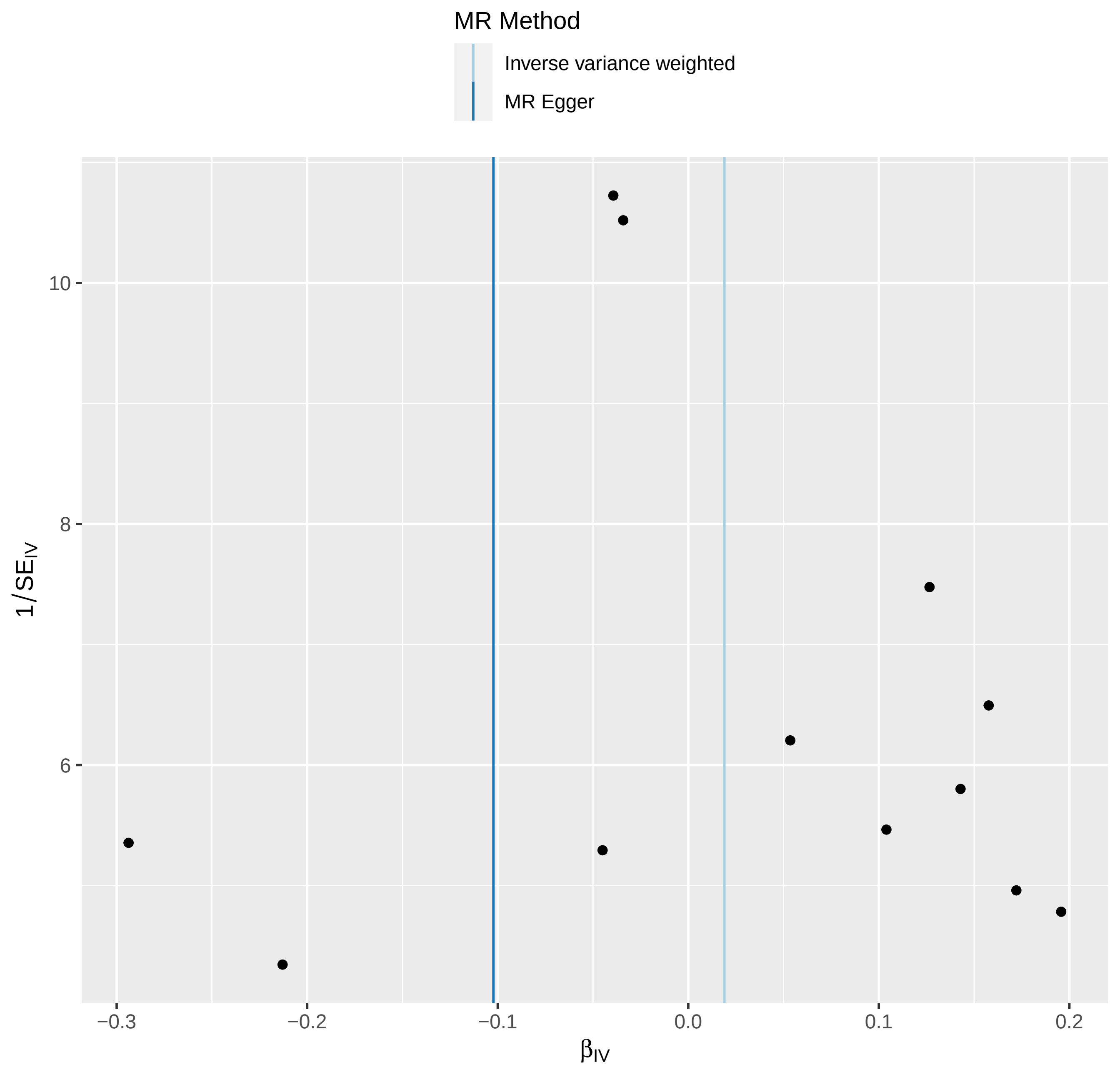

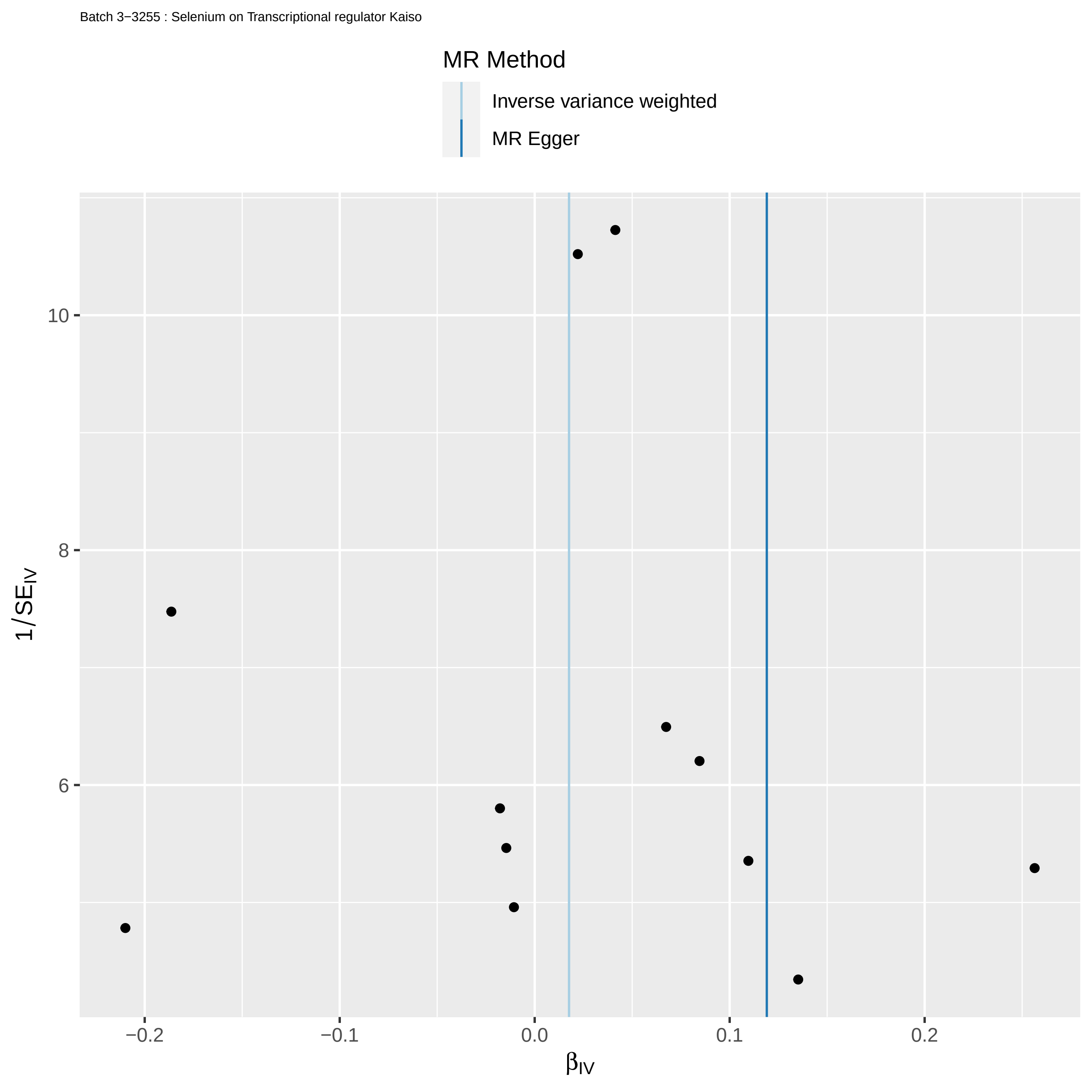
Batch 3−3255 : Selenium on Transcriptional regulator Kaiso
MR Method
Inverse variance weighted
MR Egger
10
8
6
$1/SE_{IV}$
−0.2
−0.1
0.0
0.1
0.2
β_{IV}

Batch 3−3256 : Selenium on E3 ubiquitin−protein ligase ZFP91

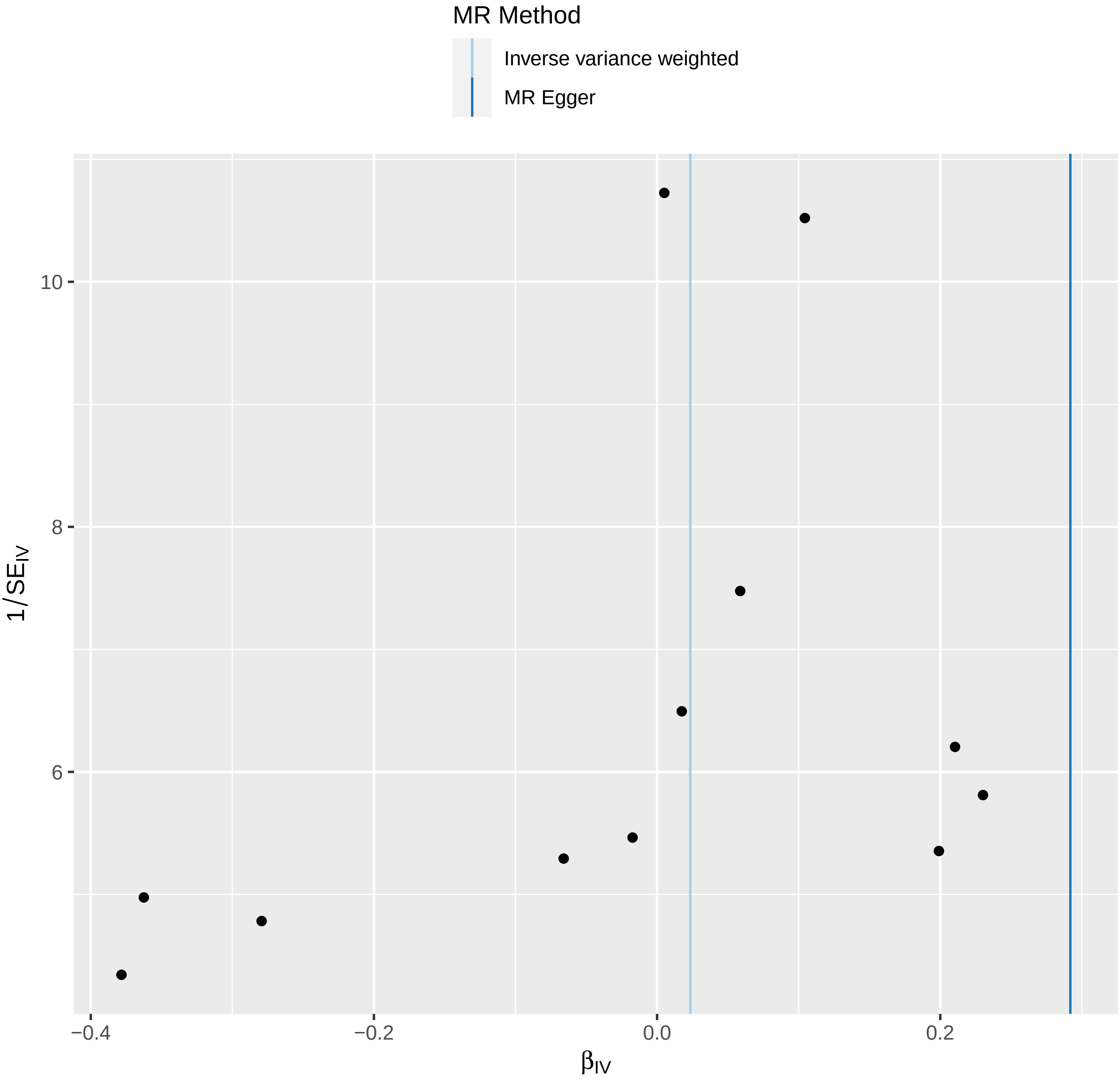

Batch 3−3257 : Selenium on Protrudin

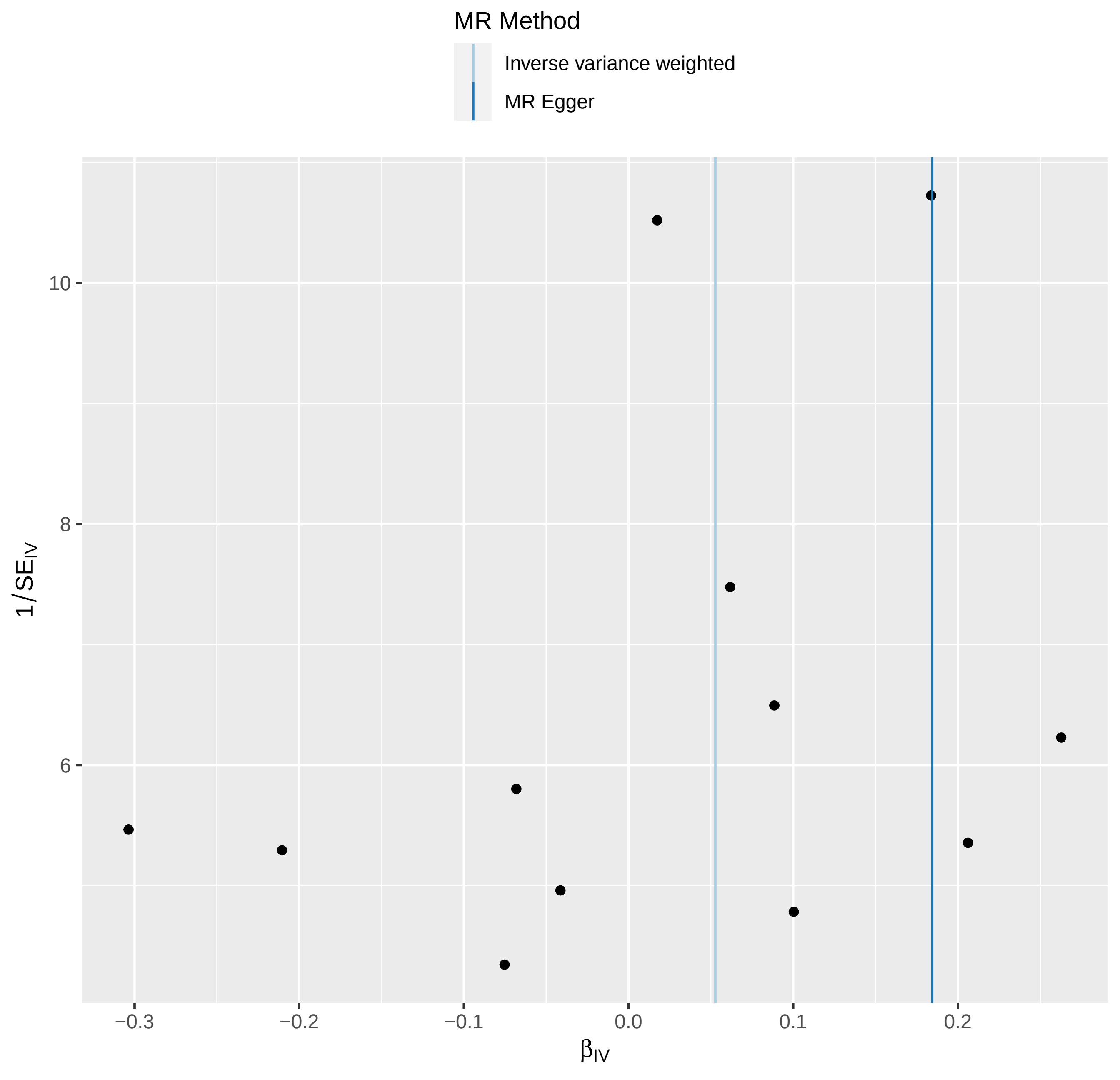

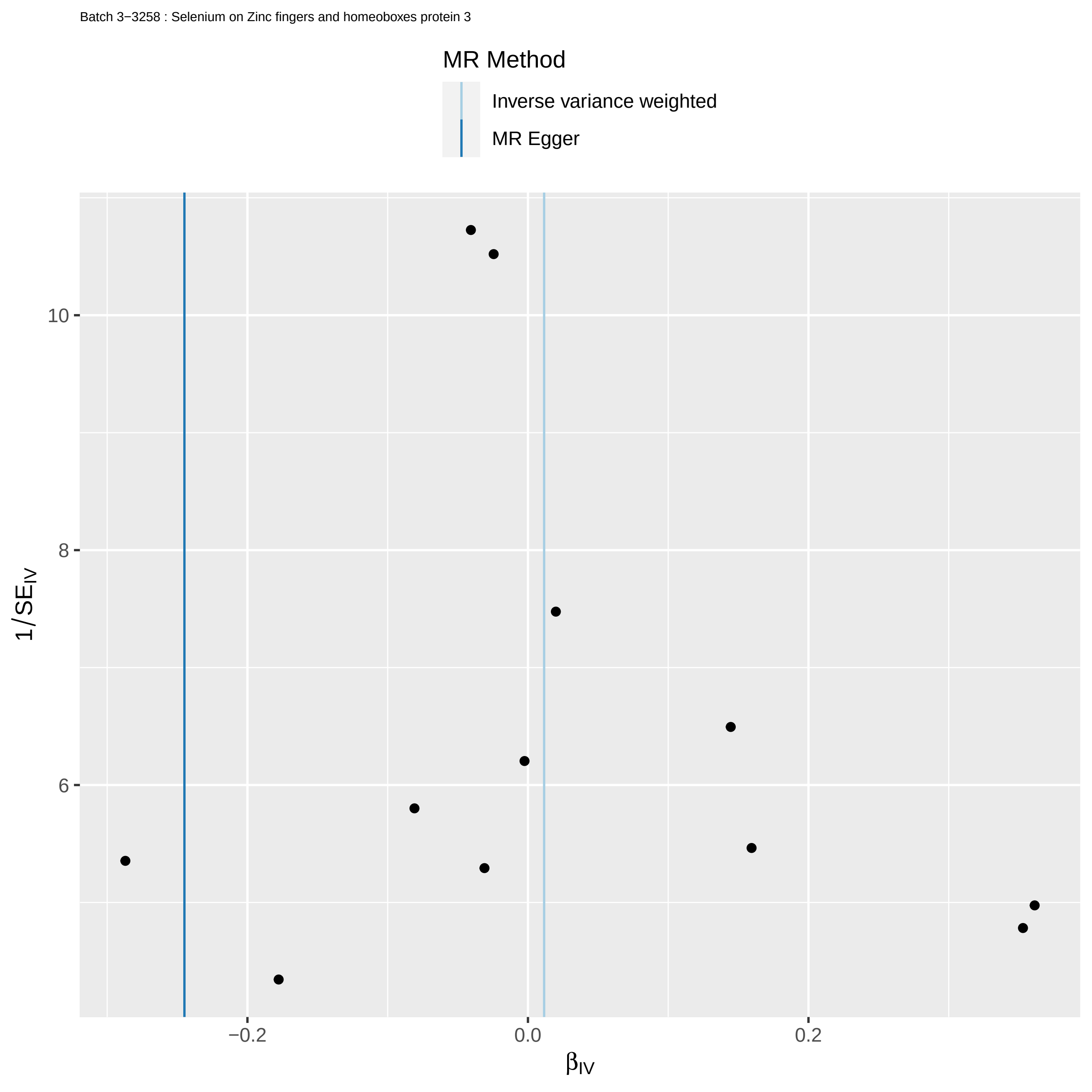

Batch 3−3258 : Selenium on Zinc fingers and homeoboxes protein 3
MR Method
Inverse variance weighted
MR Egger
10
8
6
$1/SE_{IV}$
−0.2
0.0
0.2
β_{IV}

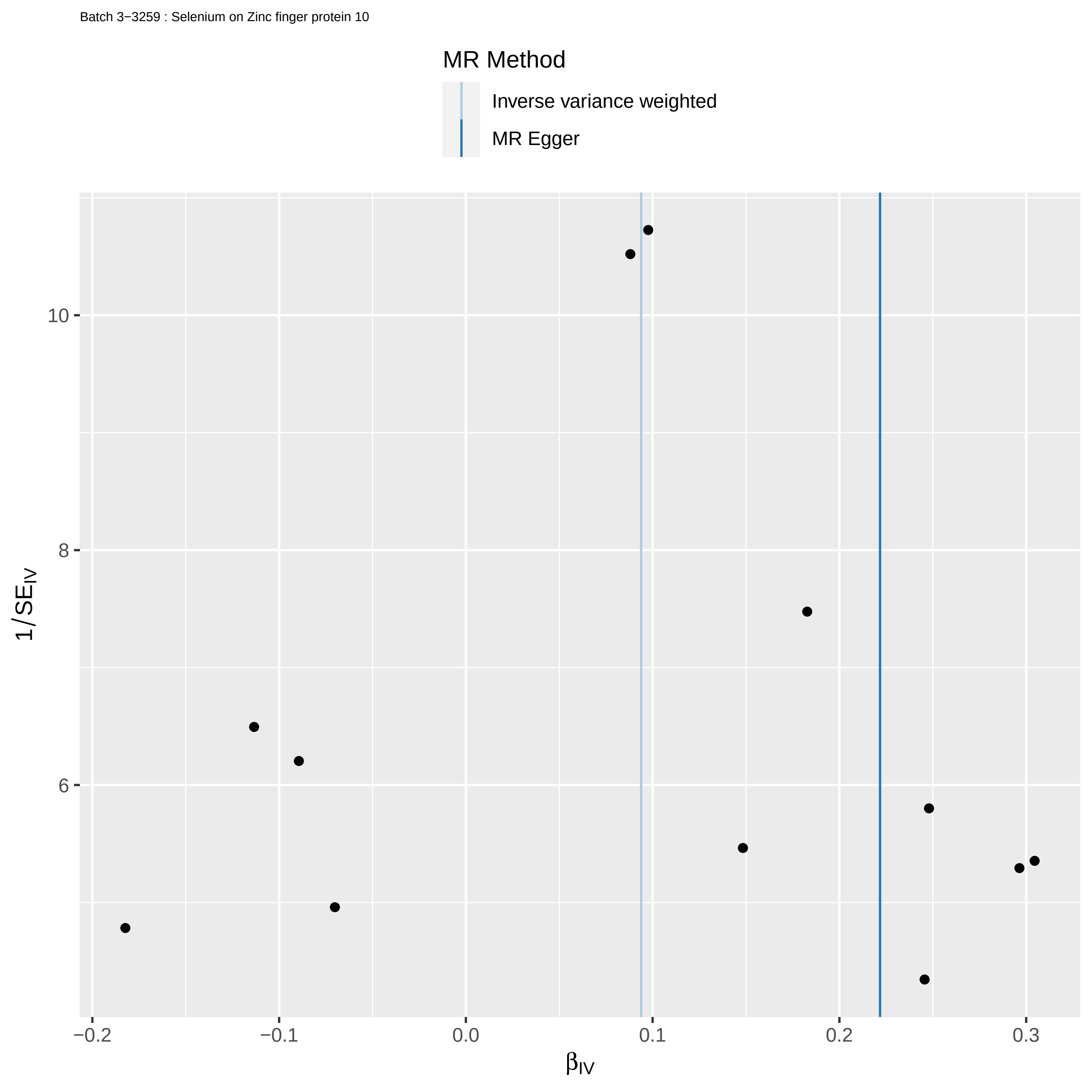

Batch 3−3259 : Selenium on Zinc finger protein 10
MR Method
Inverse variance weighted
MR Egger
10
8
6
1/SE_IV
−0.2
−0.1
0.0
0.1
0.2
0.3
β_IV

Batch 3−3260 : Selenium on Zinc finger protein 134

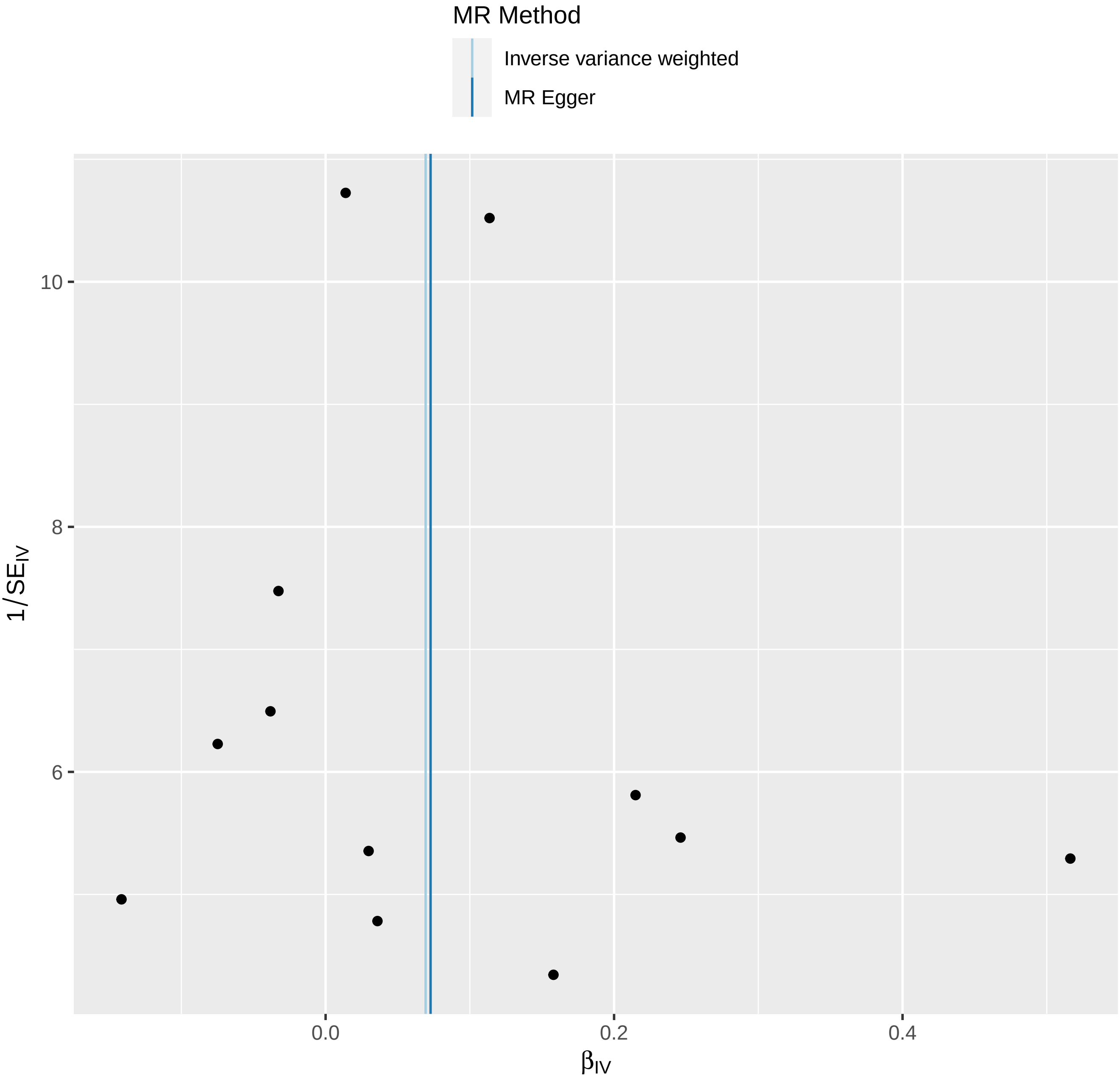

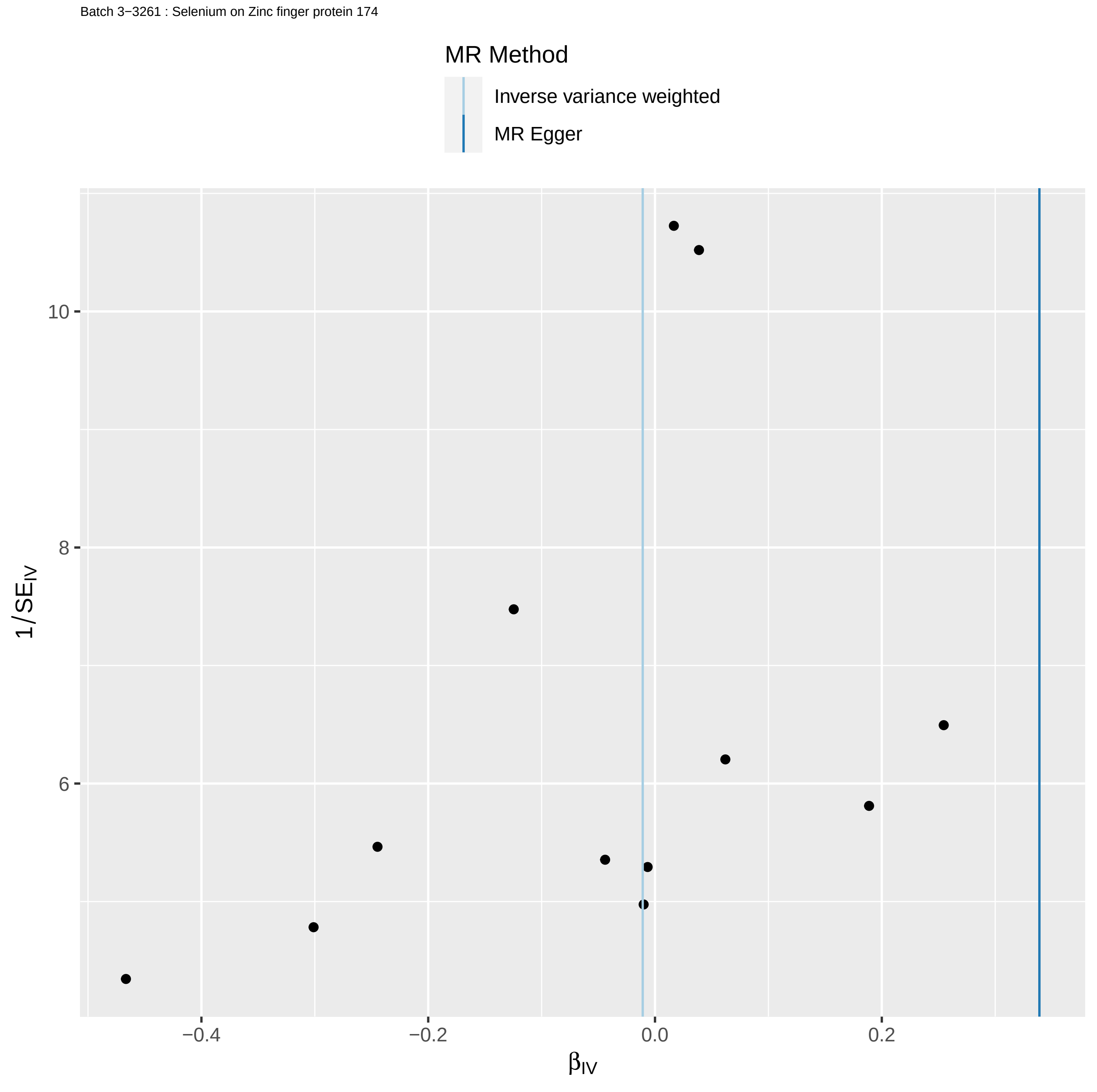

Batch 3−3261 : Selenium on Zinc finger protein 174
MR Method
Inverse variance weighted
MR Egger
10
8
6
1/SE_IV
−0.4
−0.2
0.0
0.2
β_IV

Batch 3−3262 : Selenium on Zinc finger protein 175

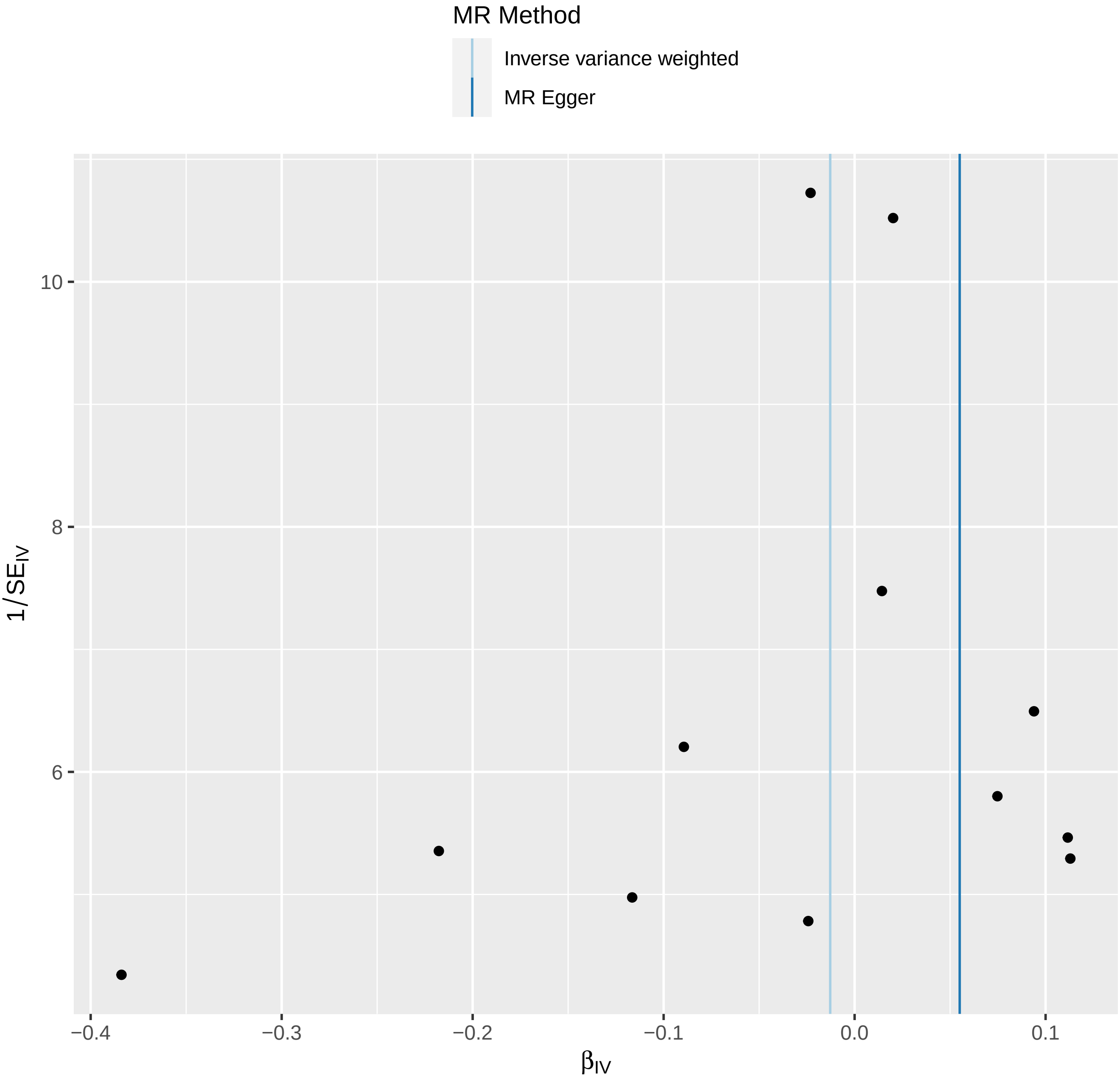

Batch 3−3263 : Selenium on Zinc finger protein 180

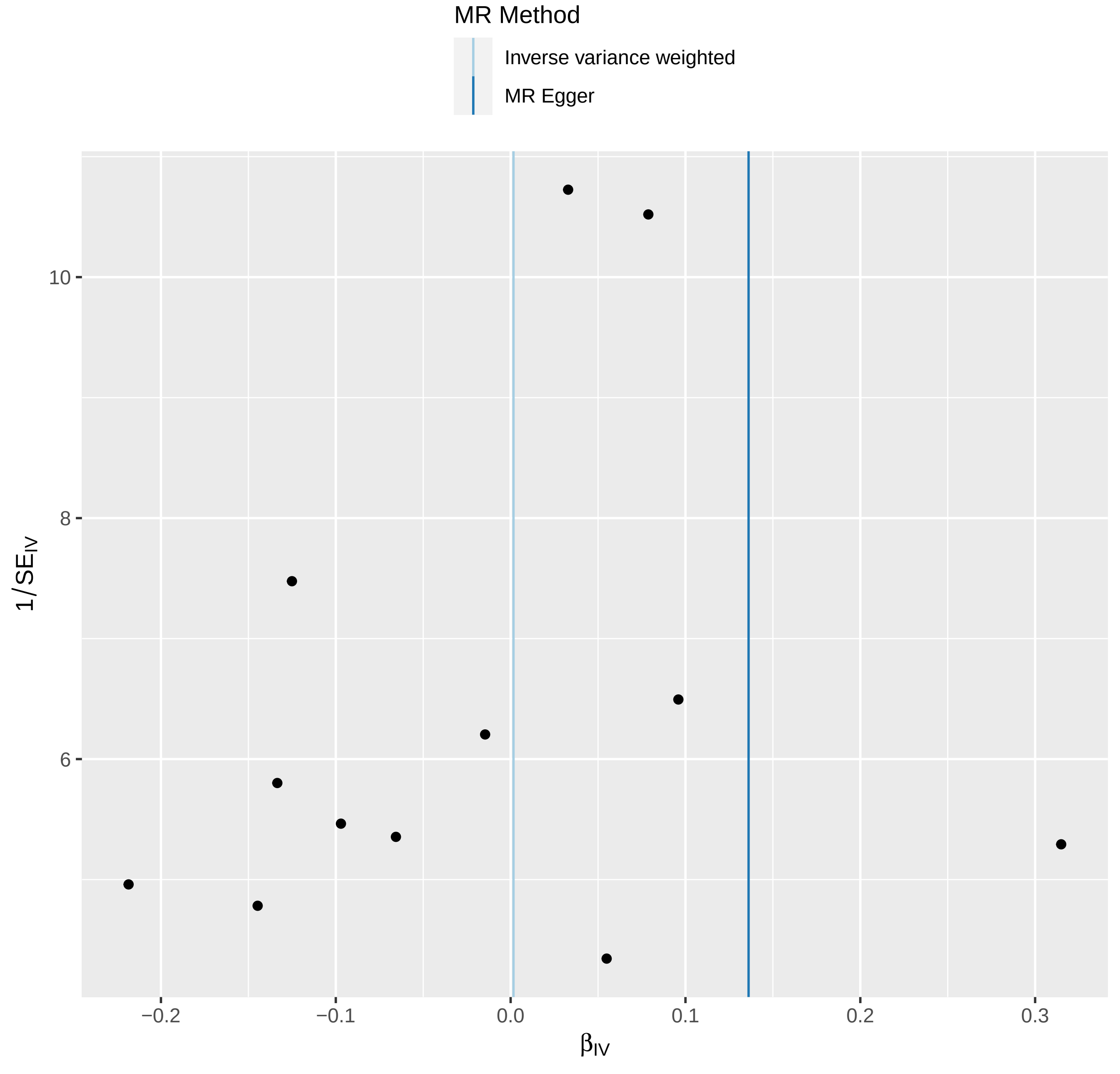

Batch 3−3264 : Selenium on Zinc finger protein 18

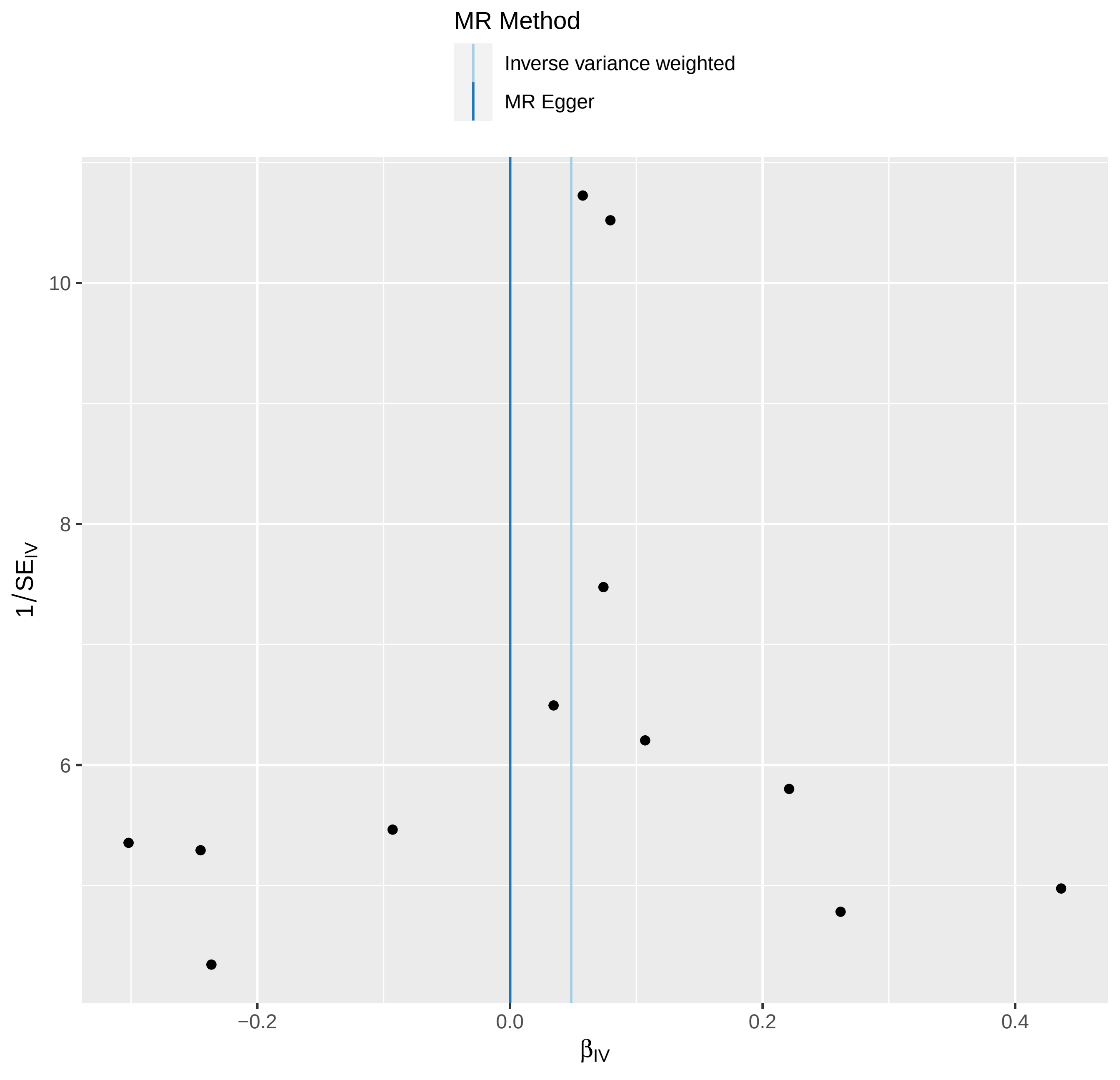

Batch 3−3265 : Selenium on Zinc finger protein 23

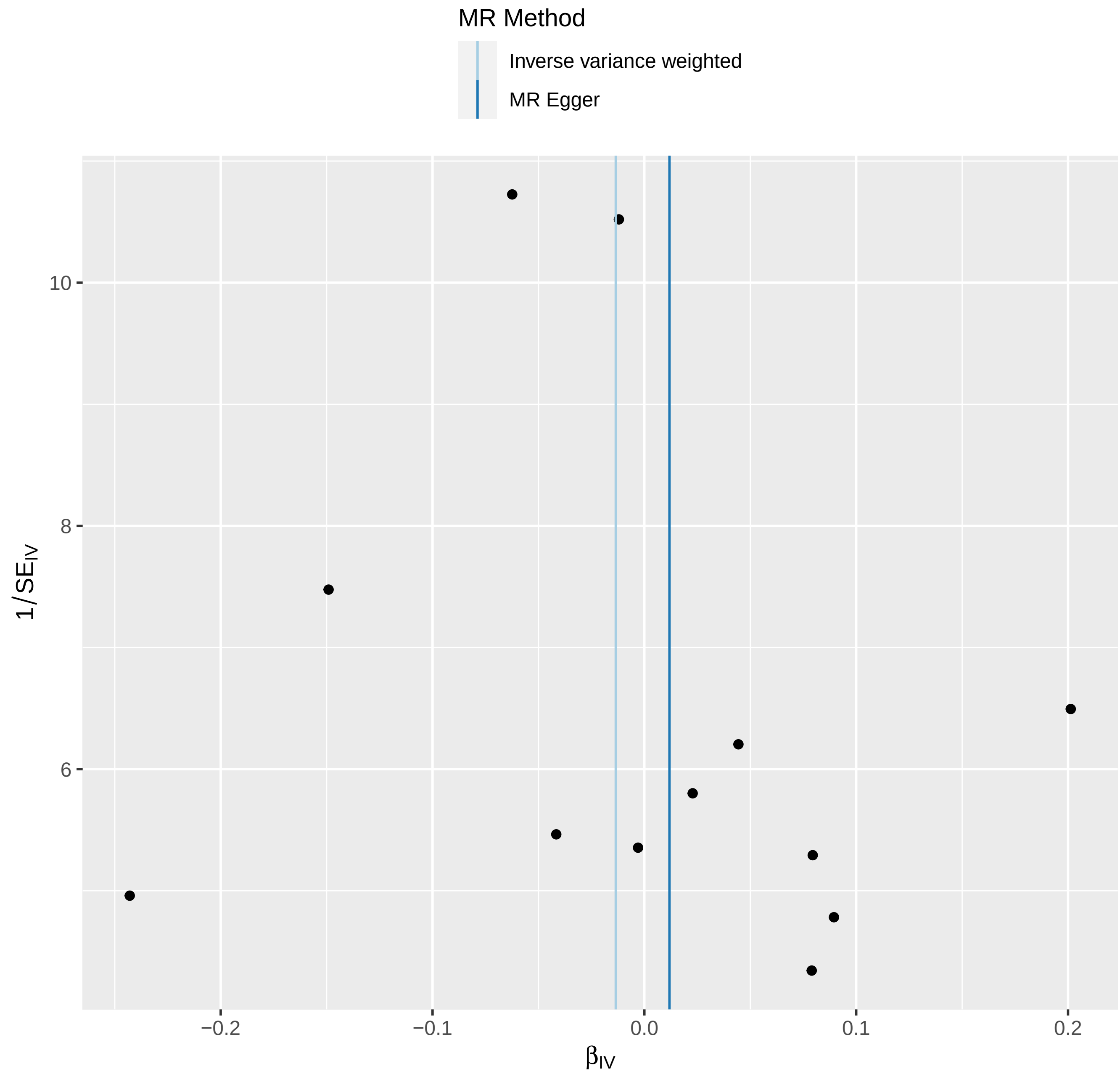

Batch 3−3266 : Selenium on Zinc finger protein 264

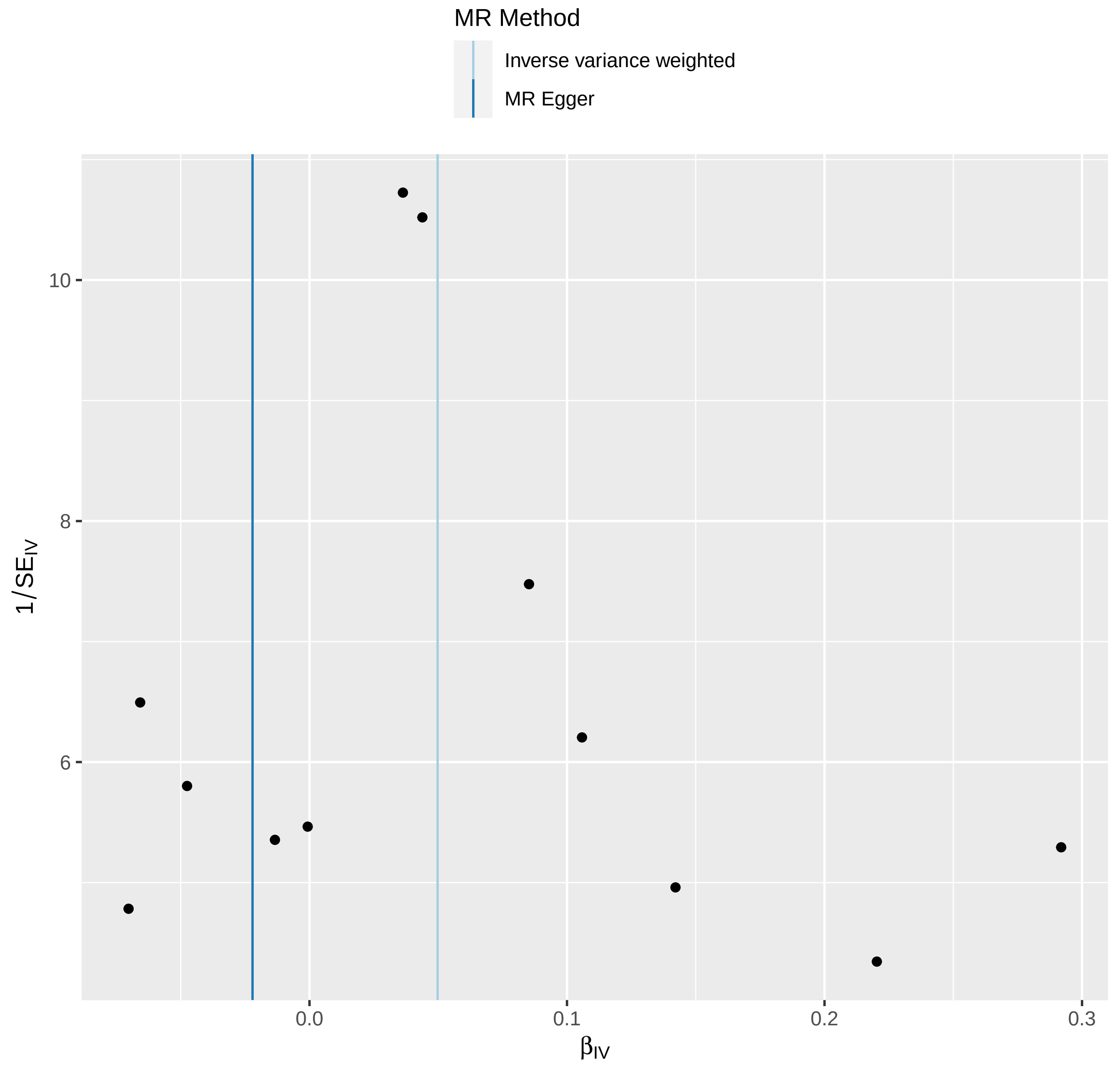

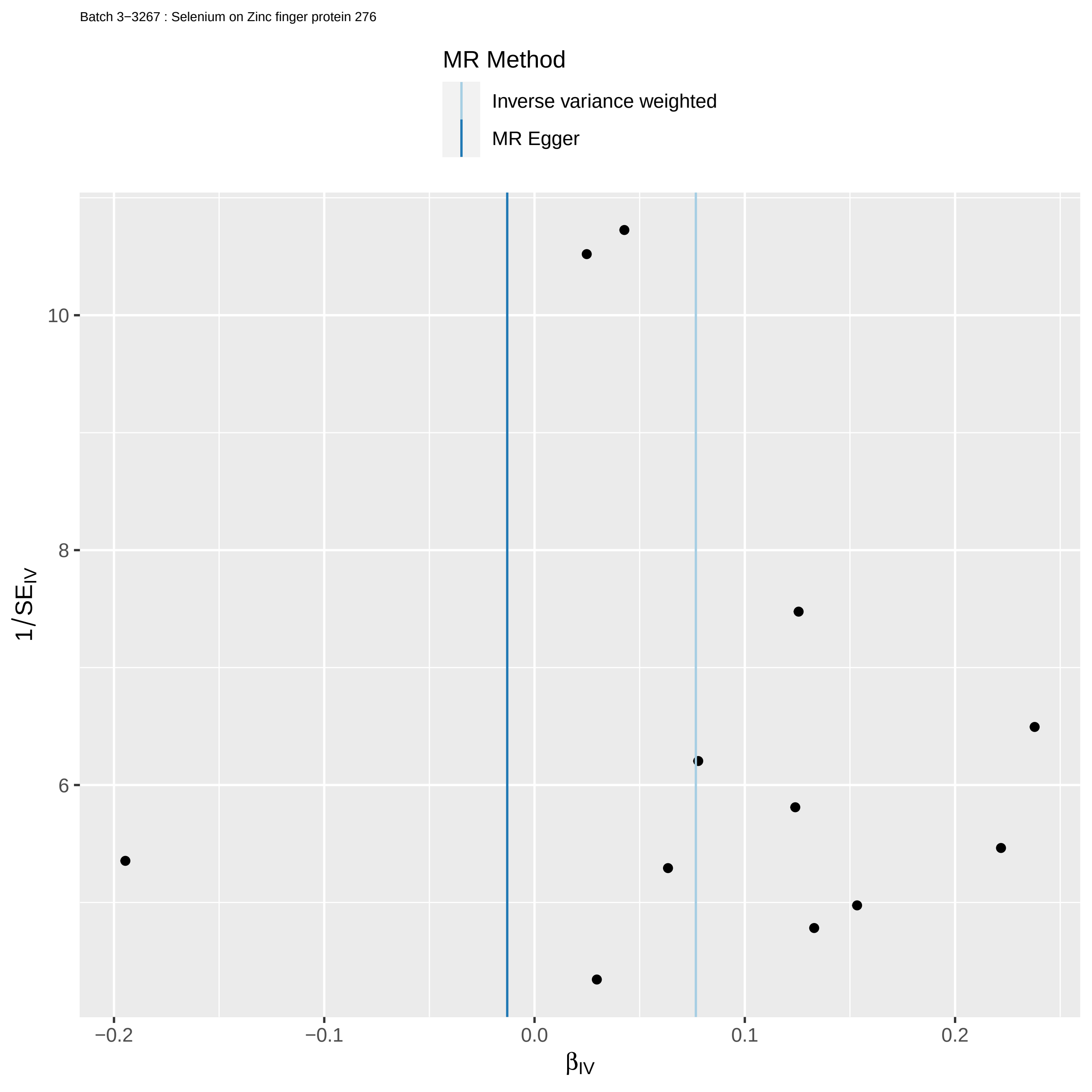

Batch 3−3267 : Selenium on Zinc finger protein 276
MR Method
Inverse variance weighted
MR Egger
10
8
6
$1/SE_{IV}$
−0.2
−0.1
0.0
0.1
0.2
β_{IV}

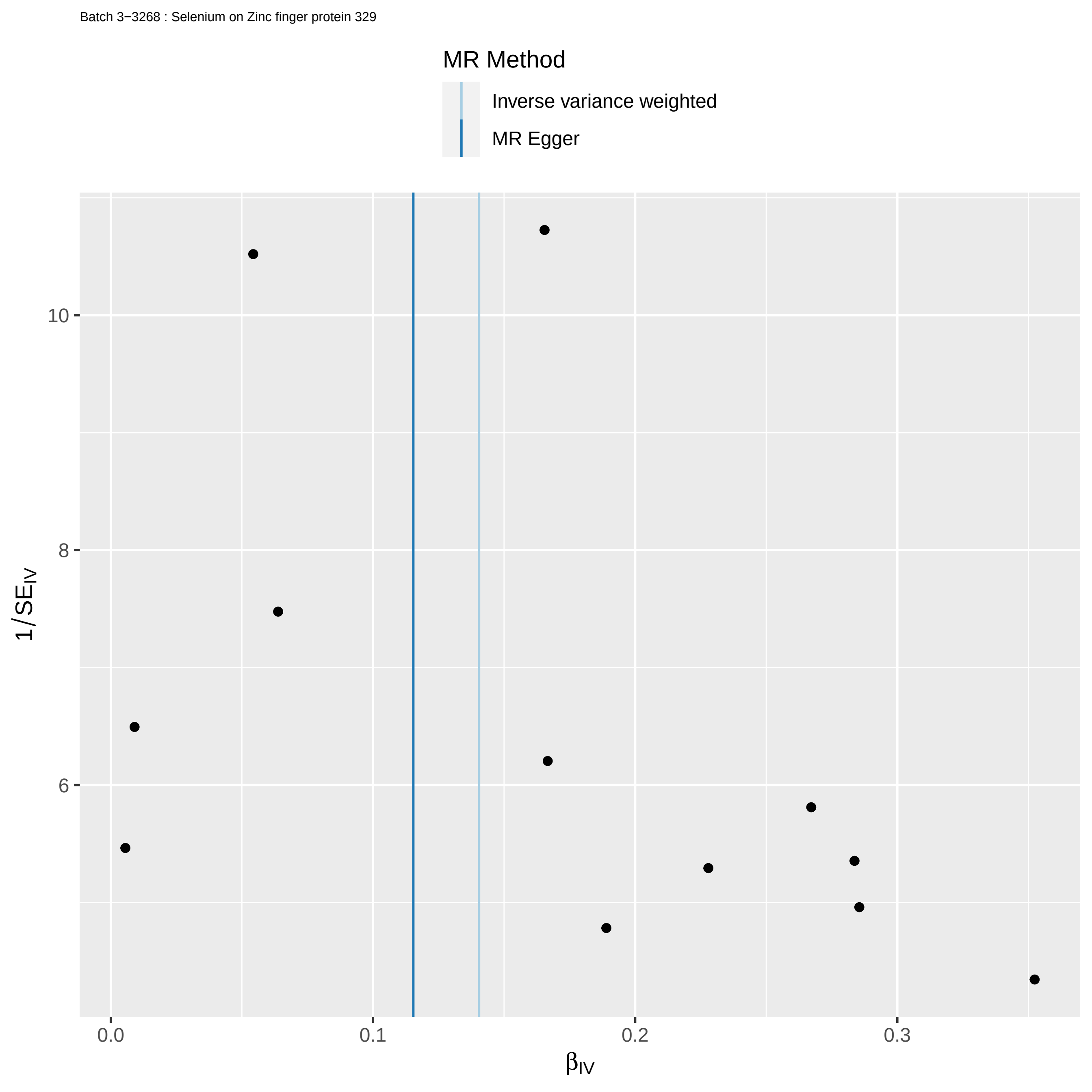
Batch 3−3268 : Selenium on Zinc finger protein 329
MR Method
Inverse variance weighted
MR Egger
10
8
6
$1/SE_{IV}$
0.0
0.1
0.2
0.3
β_{IV}

Batch 3−3269 : Selenium on Zinc finger protein 334

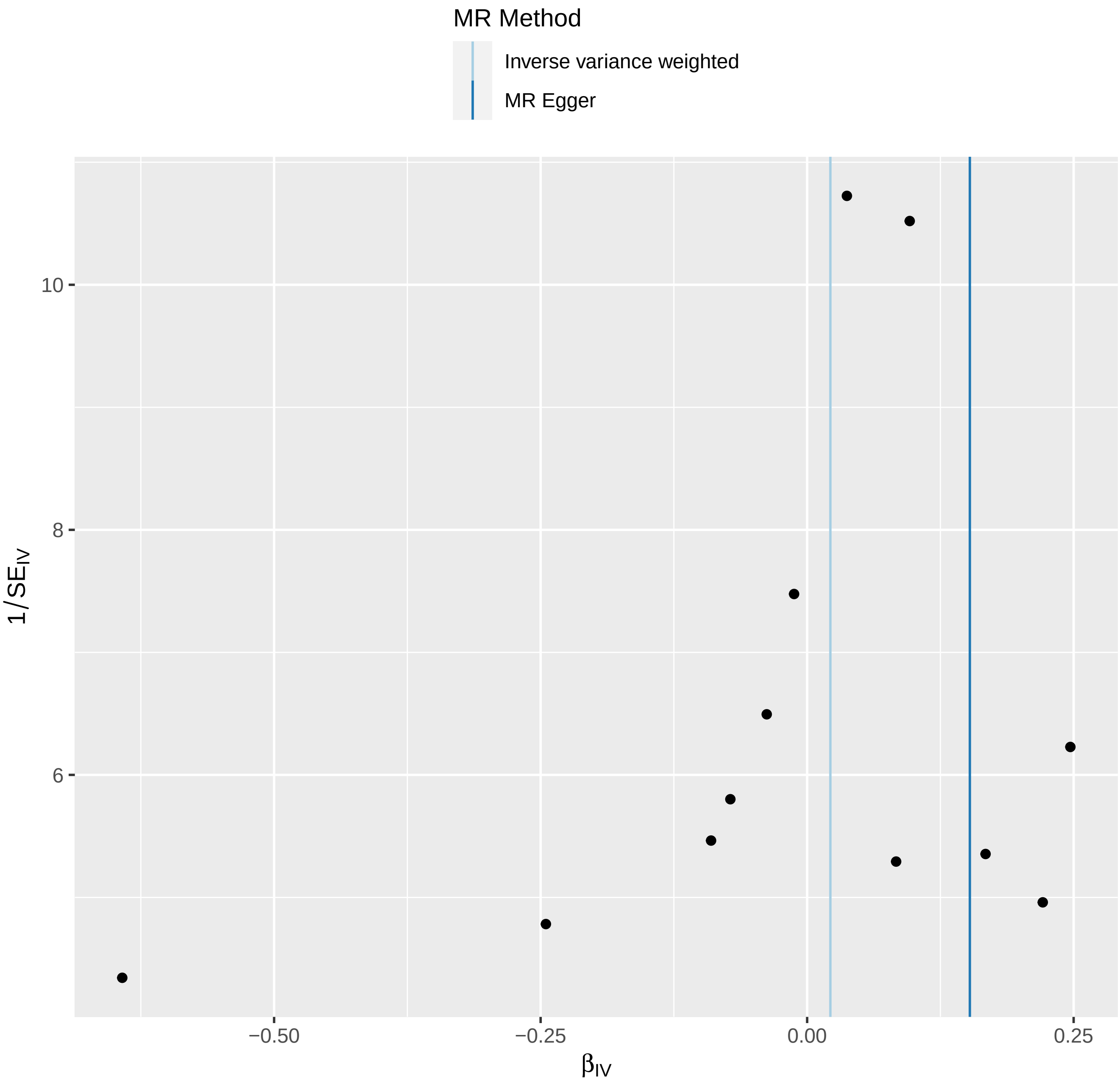

Batch 3−3270 : Selenium on Zinc finger protein 358

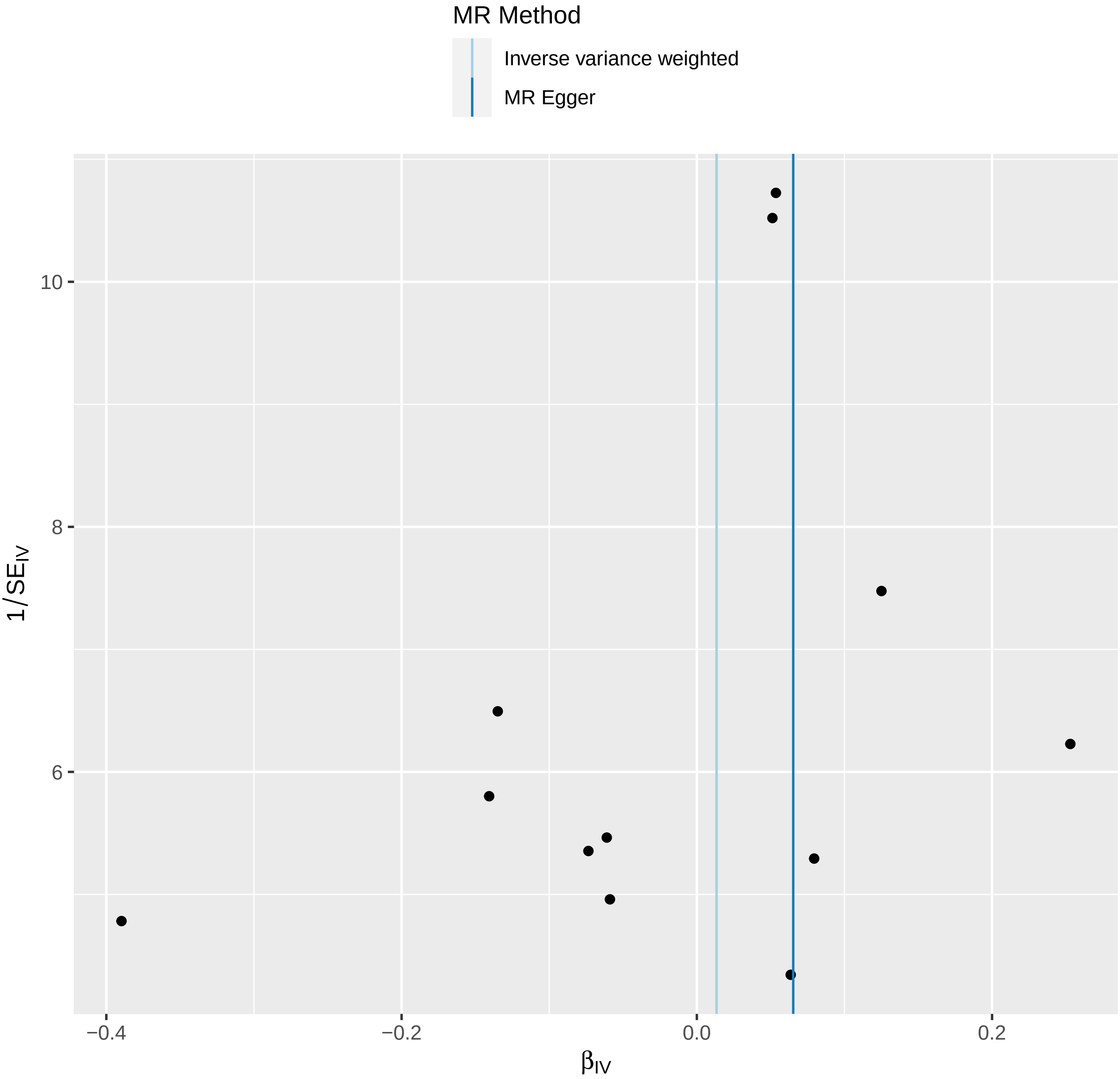

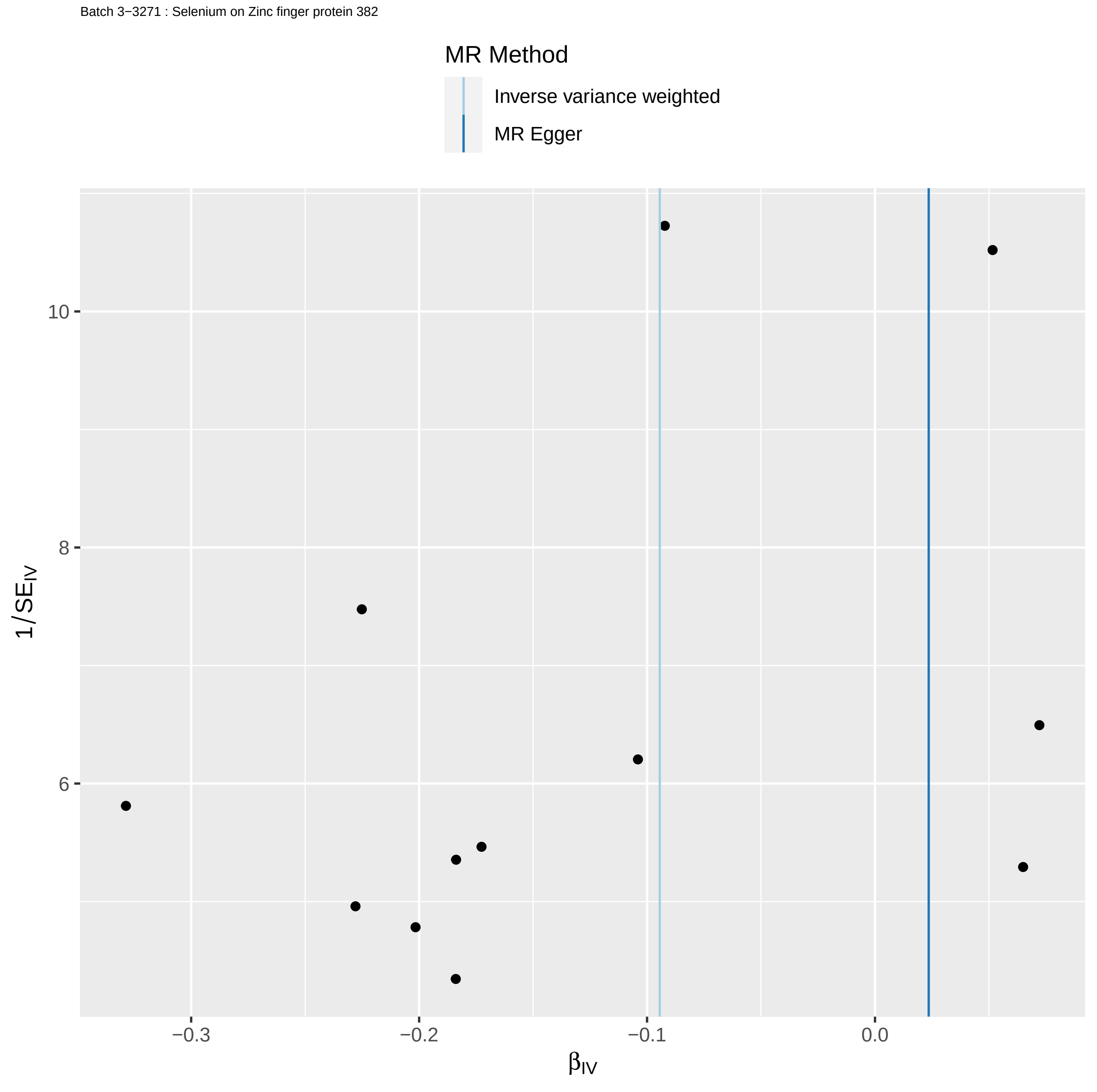

Batch 3−3271 : Selenium on Zinc finger protein 382
MR Method
Inverse variance weighted
MR Egger
10
8
6
1/SE_IV
−0.3
−0.2
−0.1
0.0
β_IV

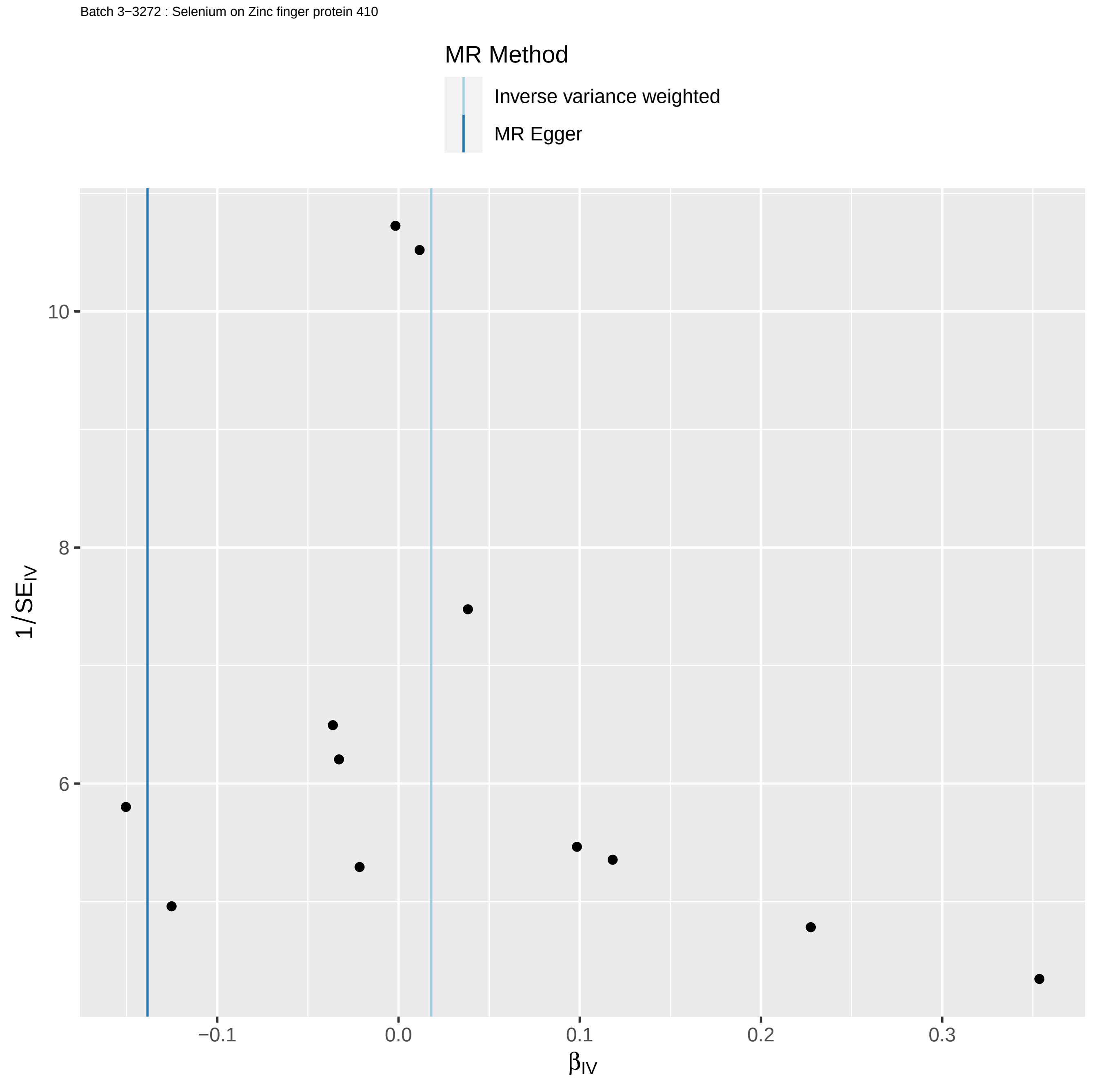
Batch 3−3272 : Selenium on Zinc finger protein 410
MR Method
Inverse variance weighted
MR Egger
10
8
6
1/SE_IV
−0.1
0.0
0.1
0.2
0.3
β_IV

Batch 3−3273 : Selenium on Zinc finger protein 415

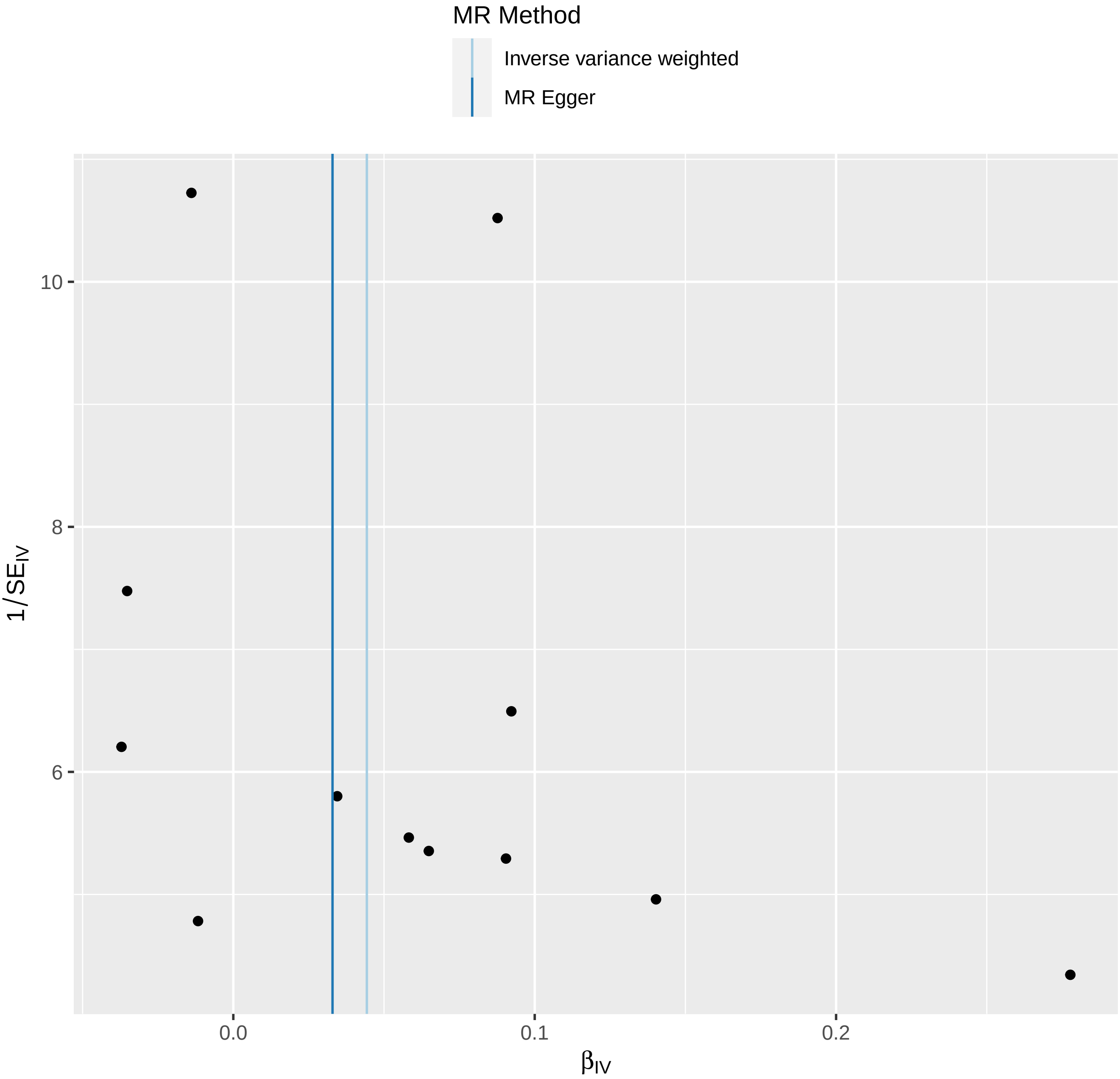

Batch 3−3274 : Selenium on Zinc finger protein 566

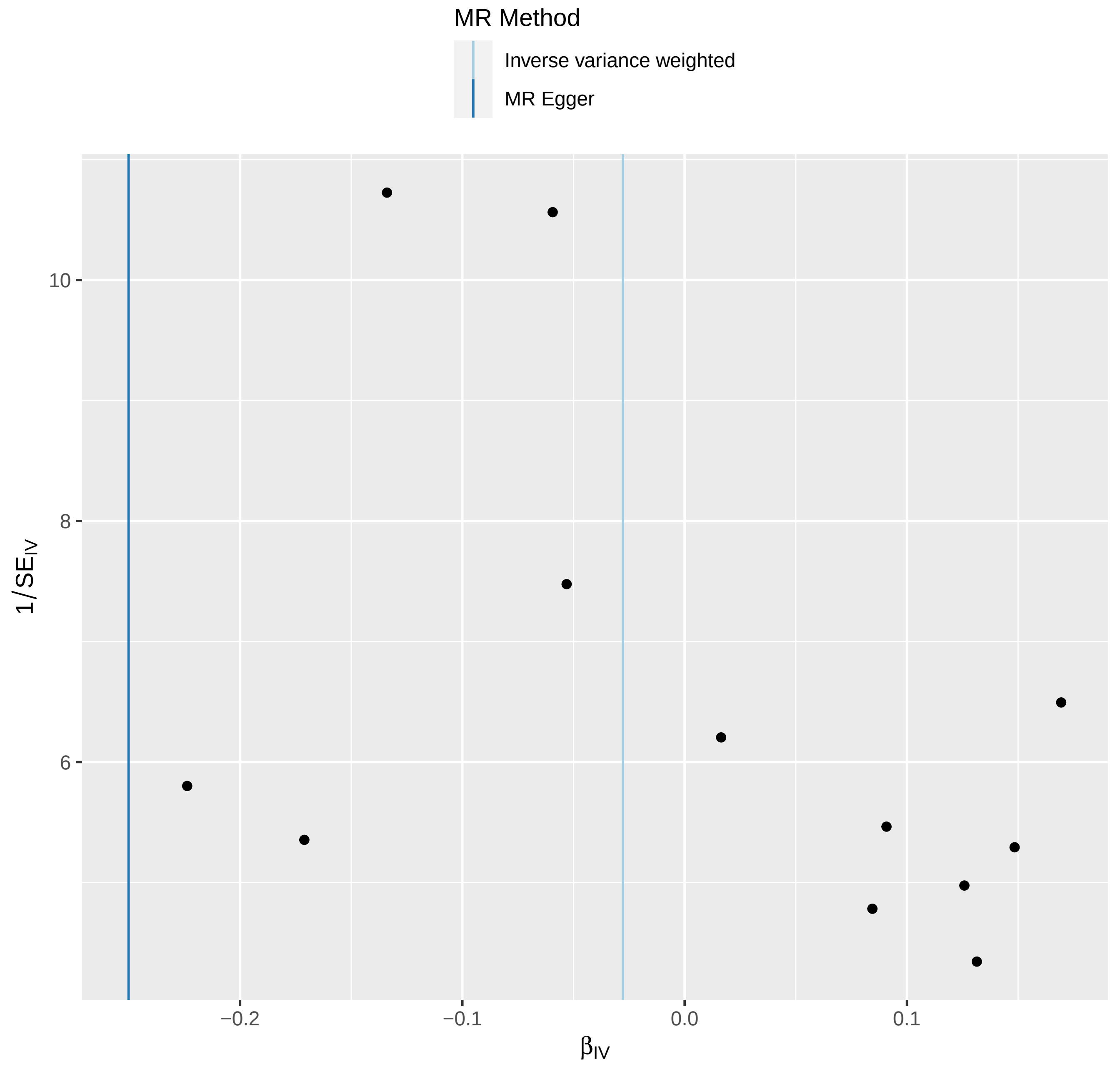

Batch 3−3275 : Selenium on Zinc finger protein 75D

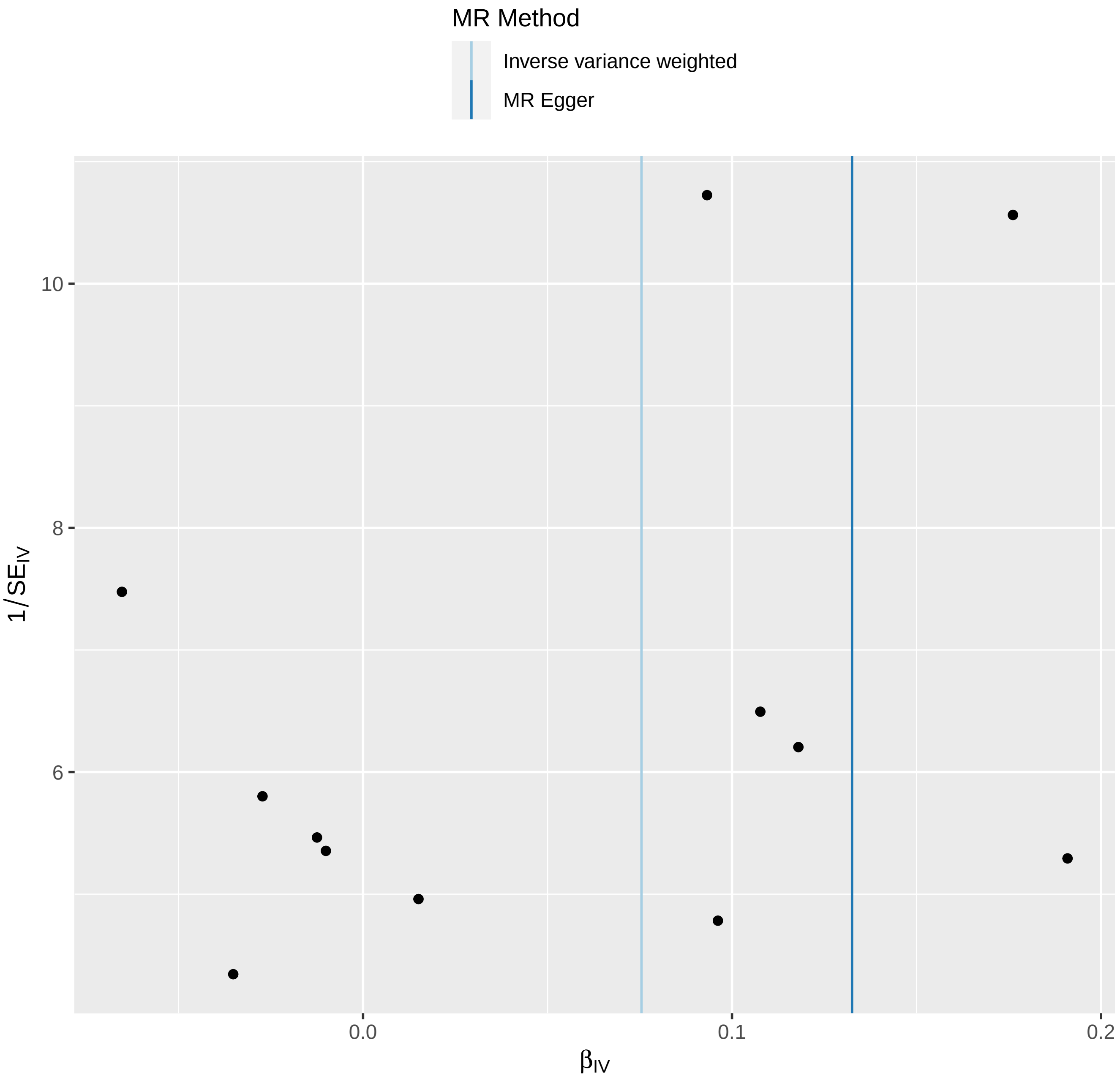

Batch 3−3276 : Selenium on Zinc finger protein 774

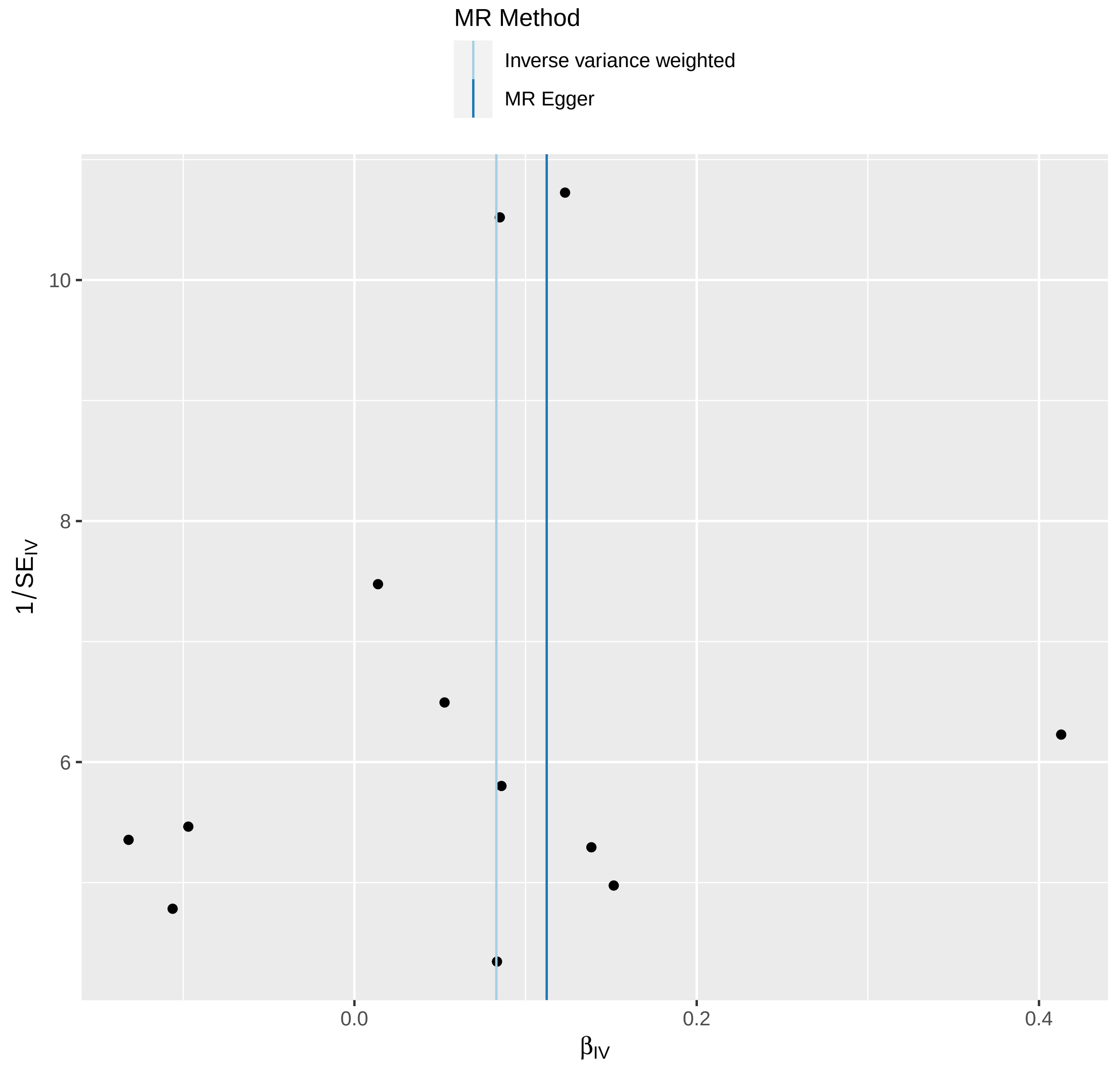

Batch 3−3277 : Selenium on Zinc finger protein 843

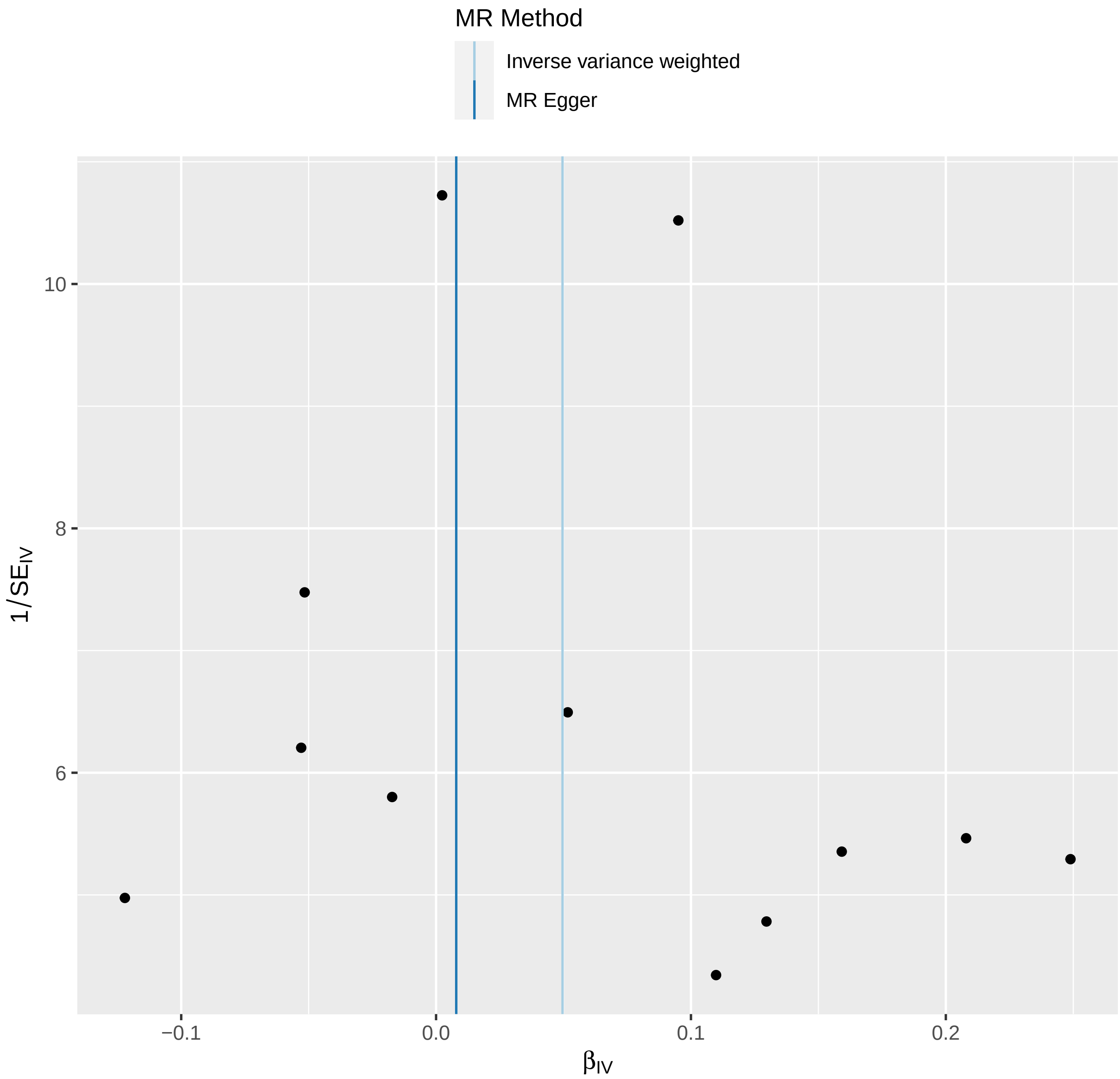

Batch 3−3278 : Selenium on E3 ubiquitin−protein ligase ZNRF3

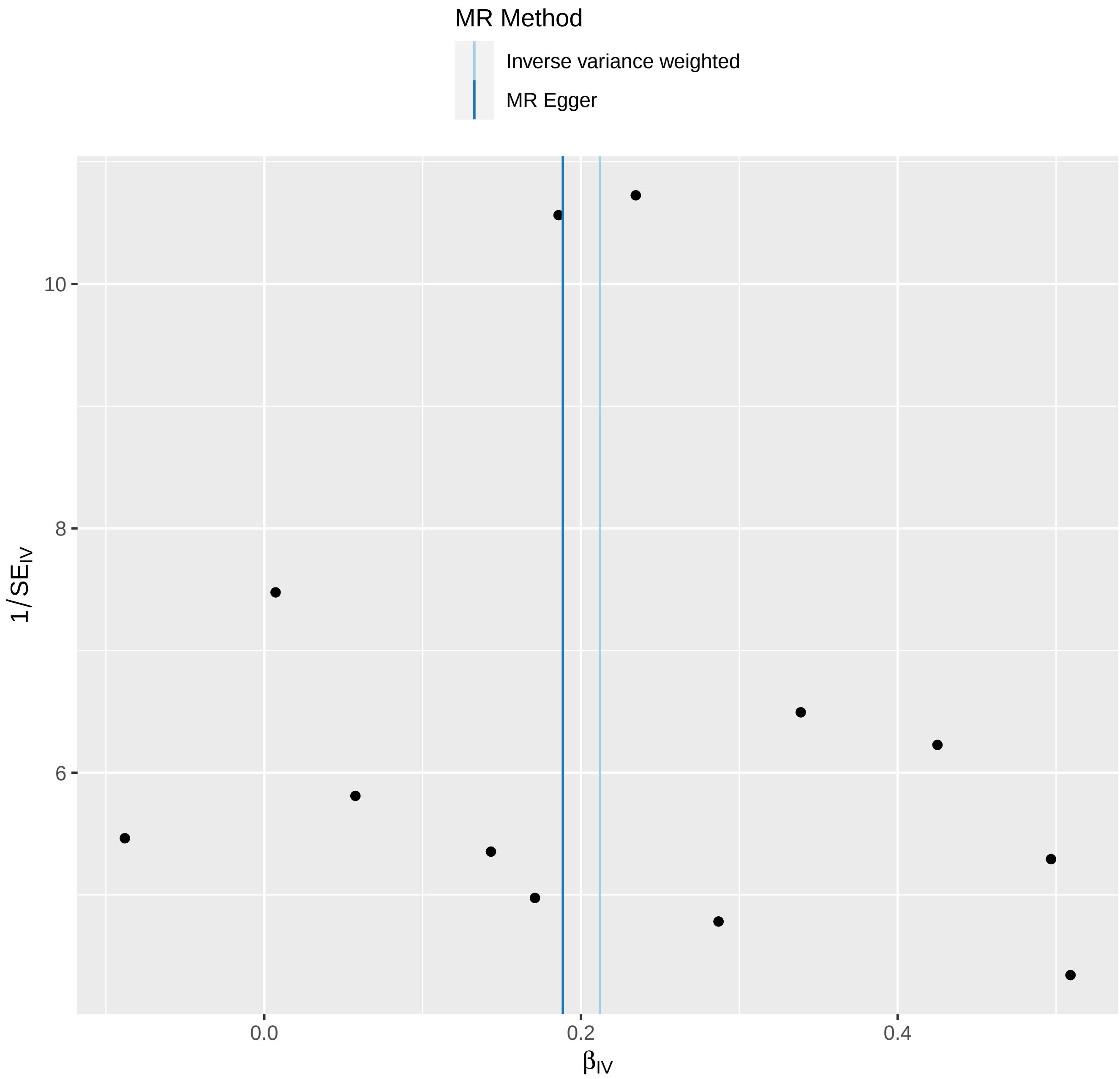

Batch 3−3279 : Selenium on E3 ubiquitin−protein ligase ZNRF3

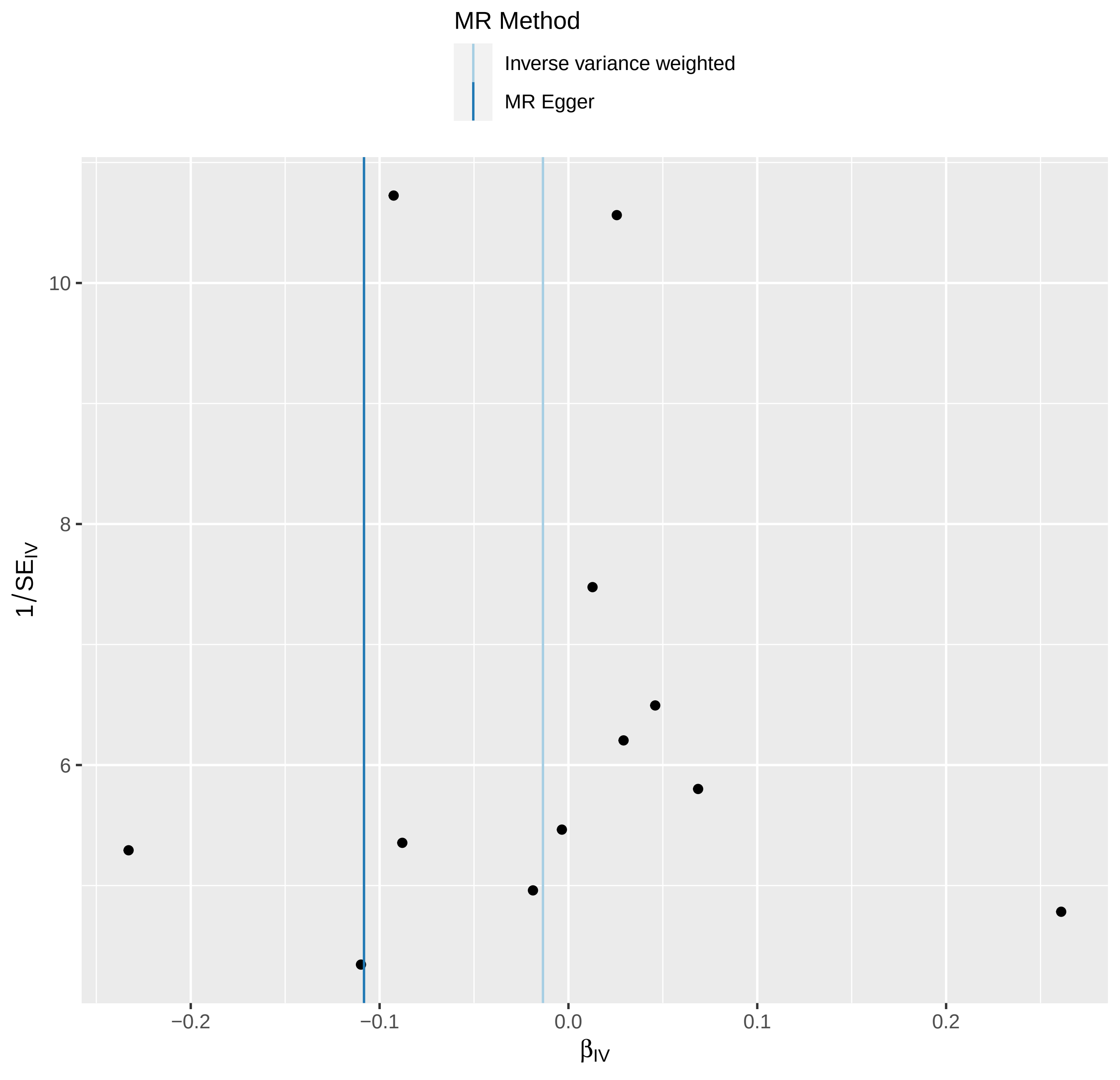

Batch 3−3280 : Selenium on Zinc/RING finger protein 4

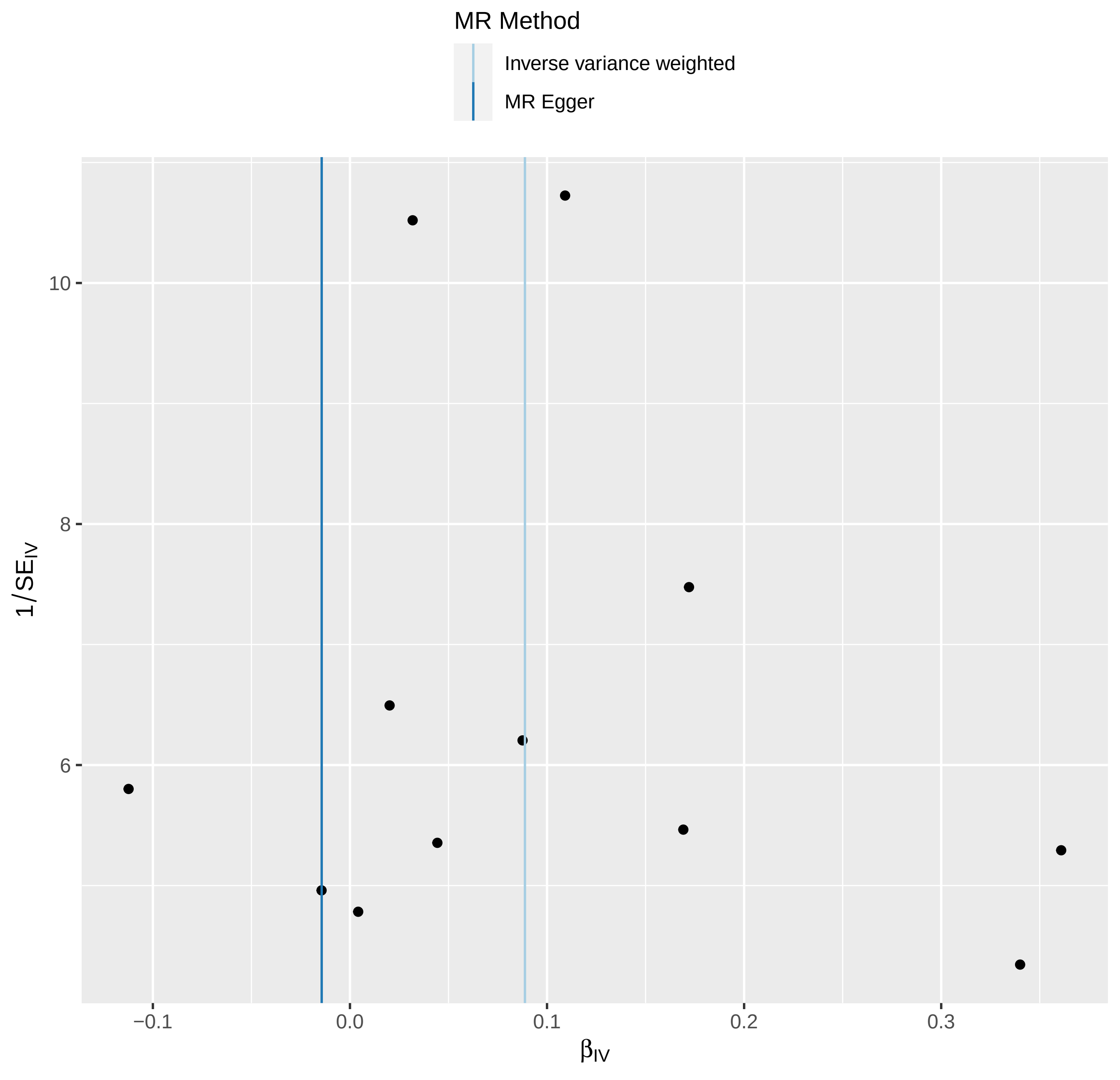

Batch 3−3281 : Selenium on Zona pellucida sperm−binding protein 4

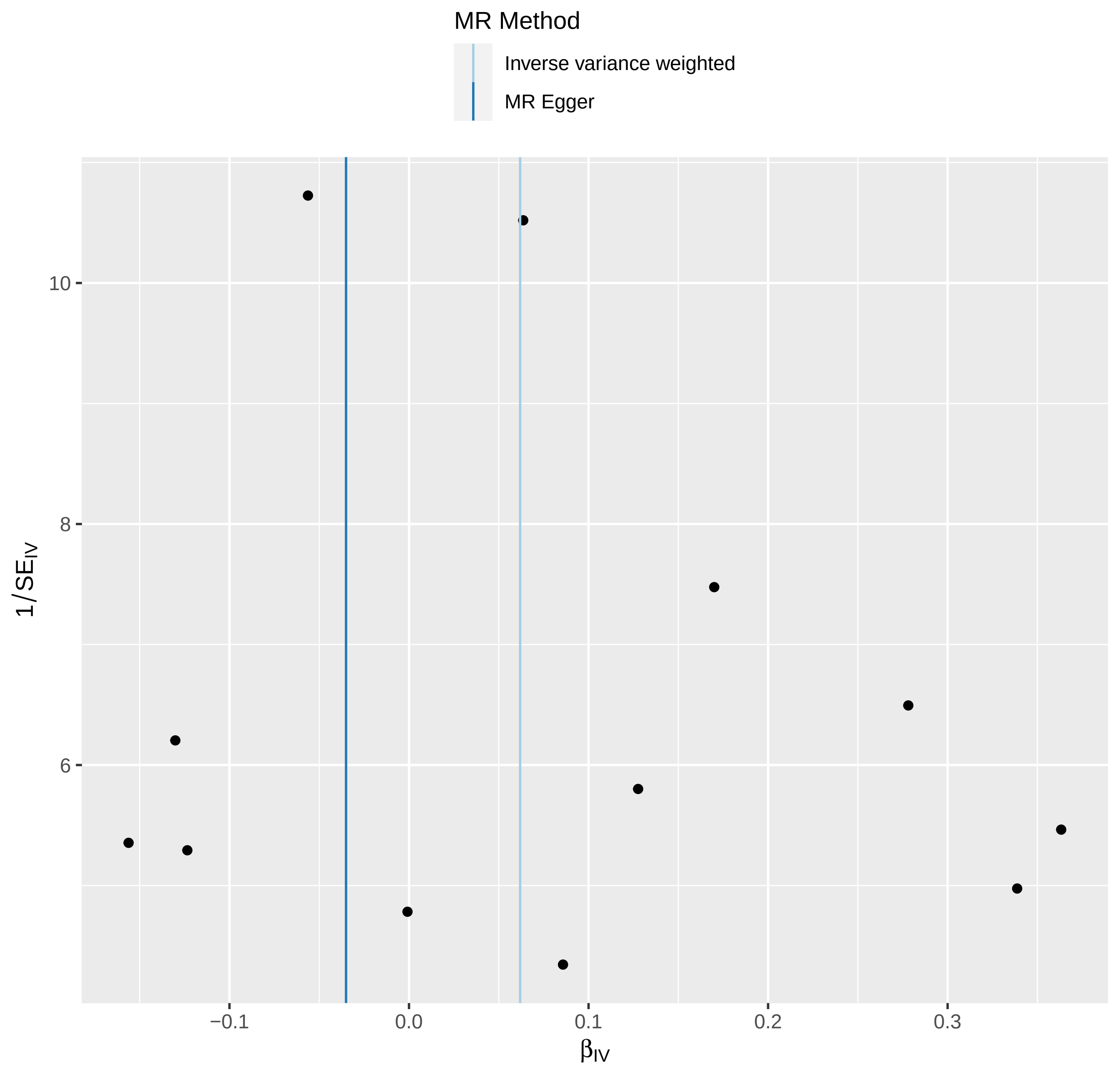

Batch 3−3282 : Selenium on Zona pellucida−like domain−containing protein 1

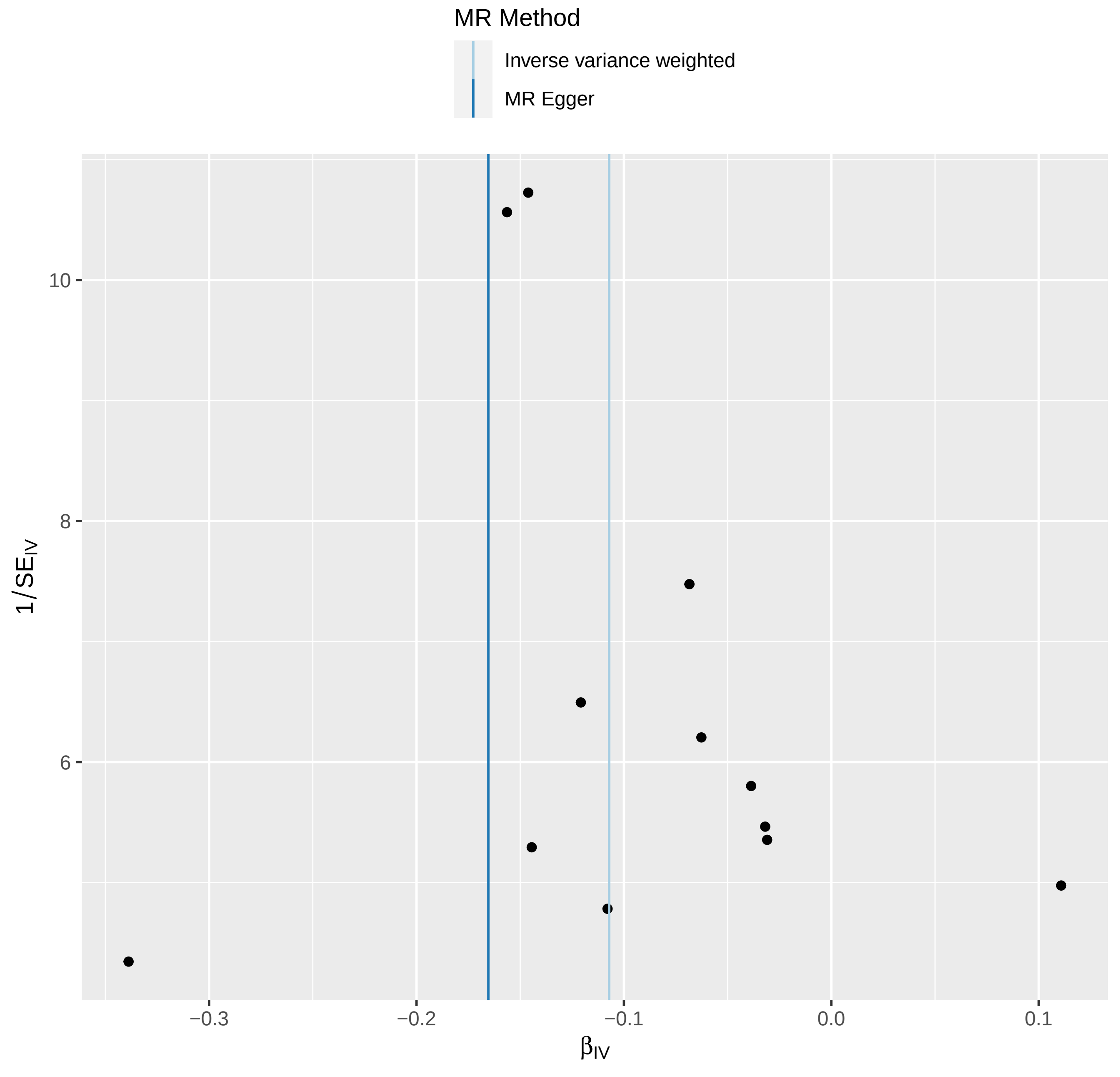

Batch 2−1 : APOBEC1 complementation factor on eGFR

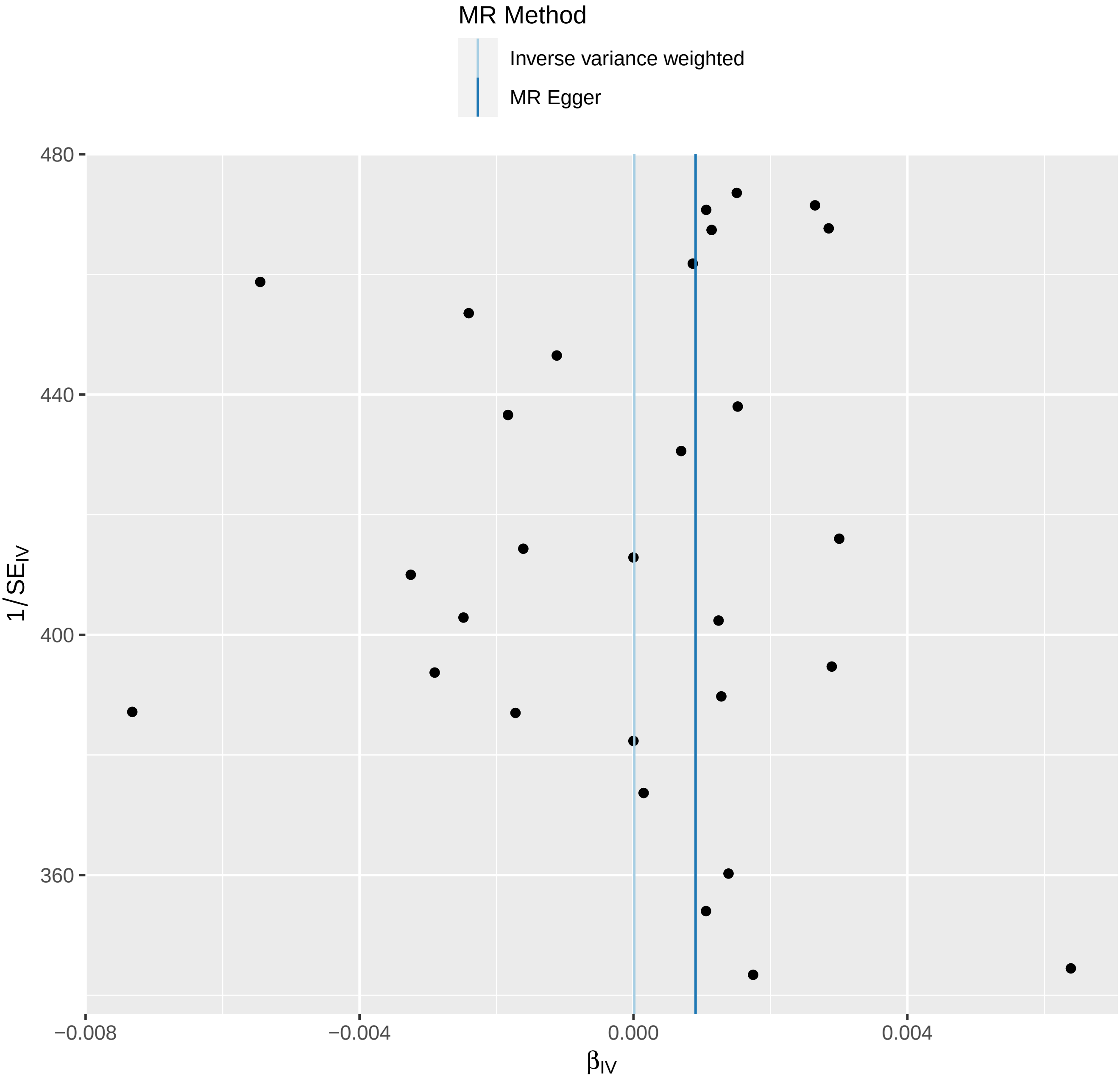

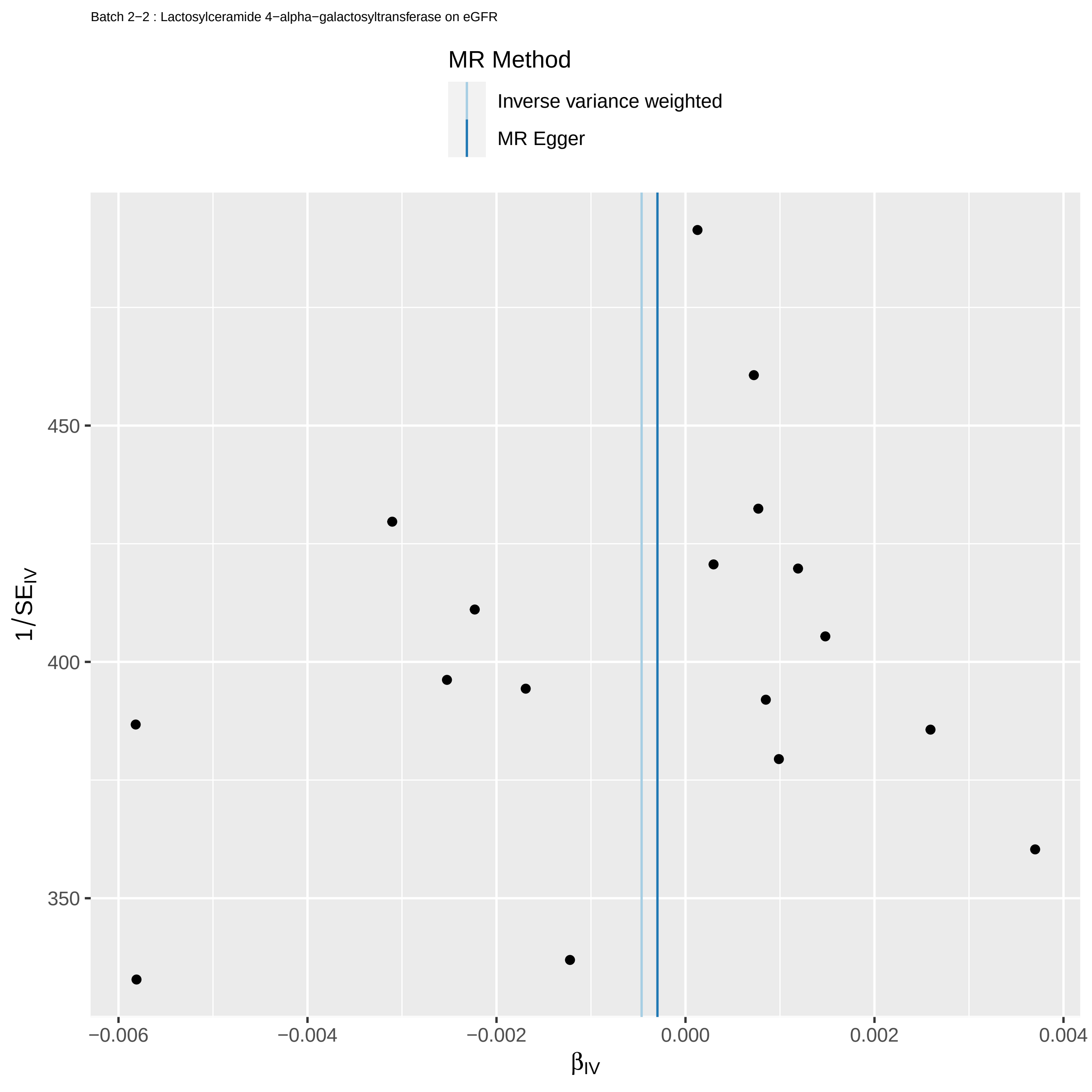
Batch 2−2 : Lactosylceramide 4−alpha−galactosyltransferase on eGFR
MR Method
Inverse variance weighted
MR Egger
450
400
350
1/SE_IV
−0.006
−0.004
−0.002
0.000
0.002
0.004
β_IV

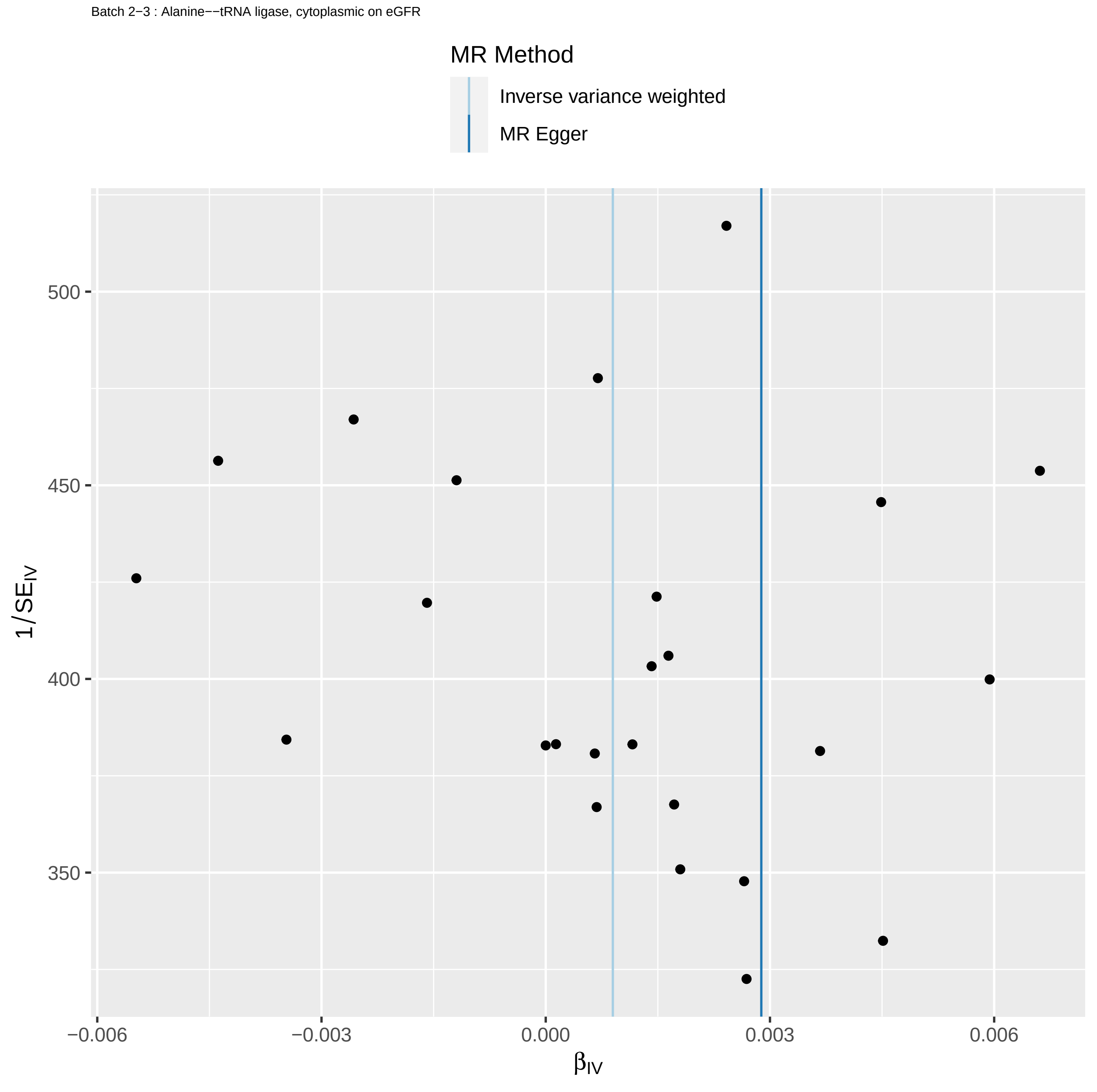
Batch 2−3 : Alanine−−tRNA ligase, cytoplasmic on eGFR
MR Method
Inverse variance weighted
MR Egger
500
450
400
350
1/SE_IV
−0.006
−0.003
0.000
0.003
0.006
β_IV

Batch 2−4 : Multidrug resistance−associated protein 6 on eGFR

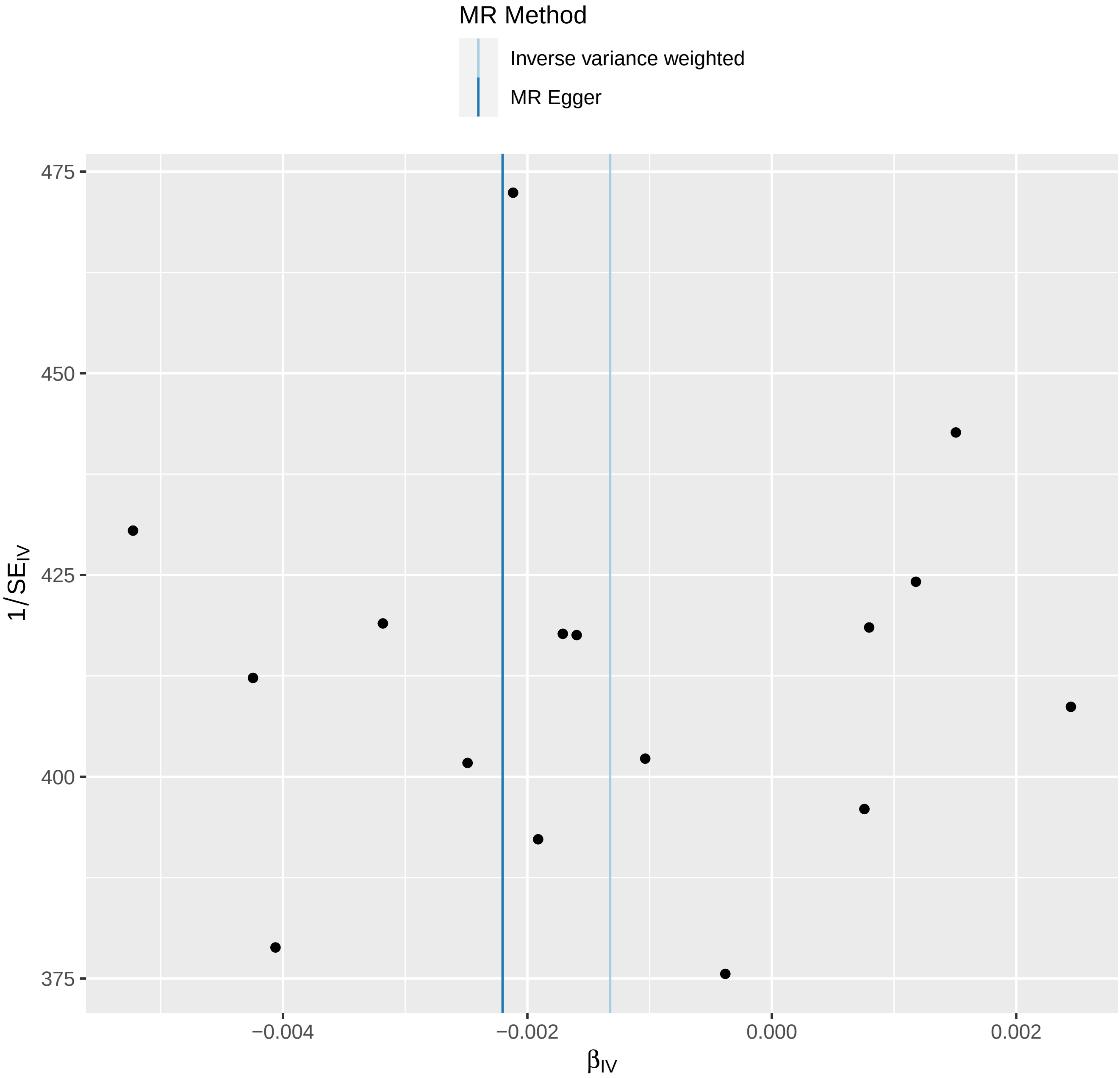

Batch 2−5 : Monoacylglycerol lipase ABHD12 on eGFR

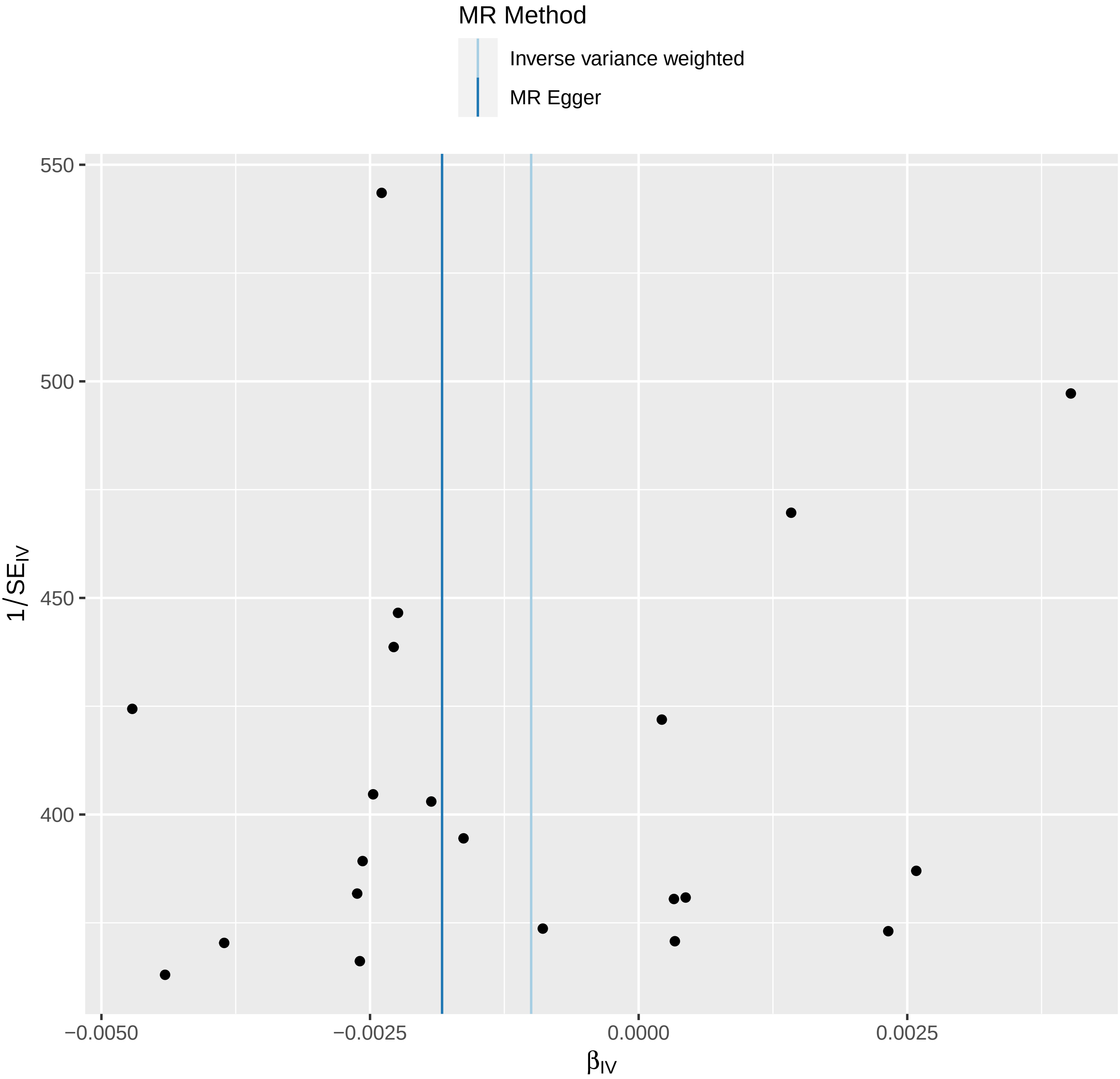

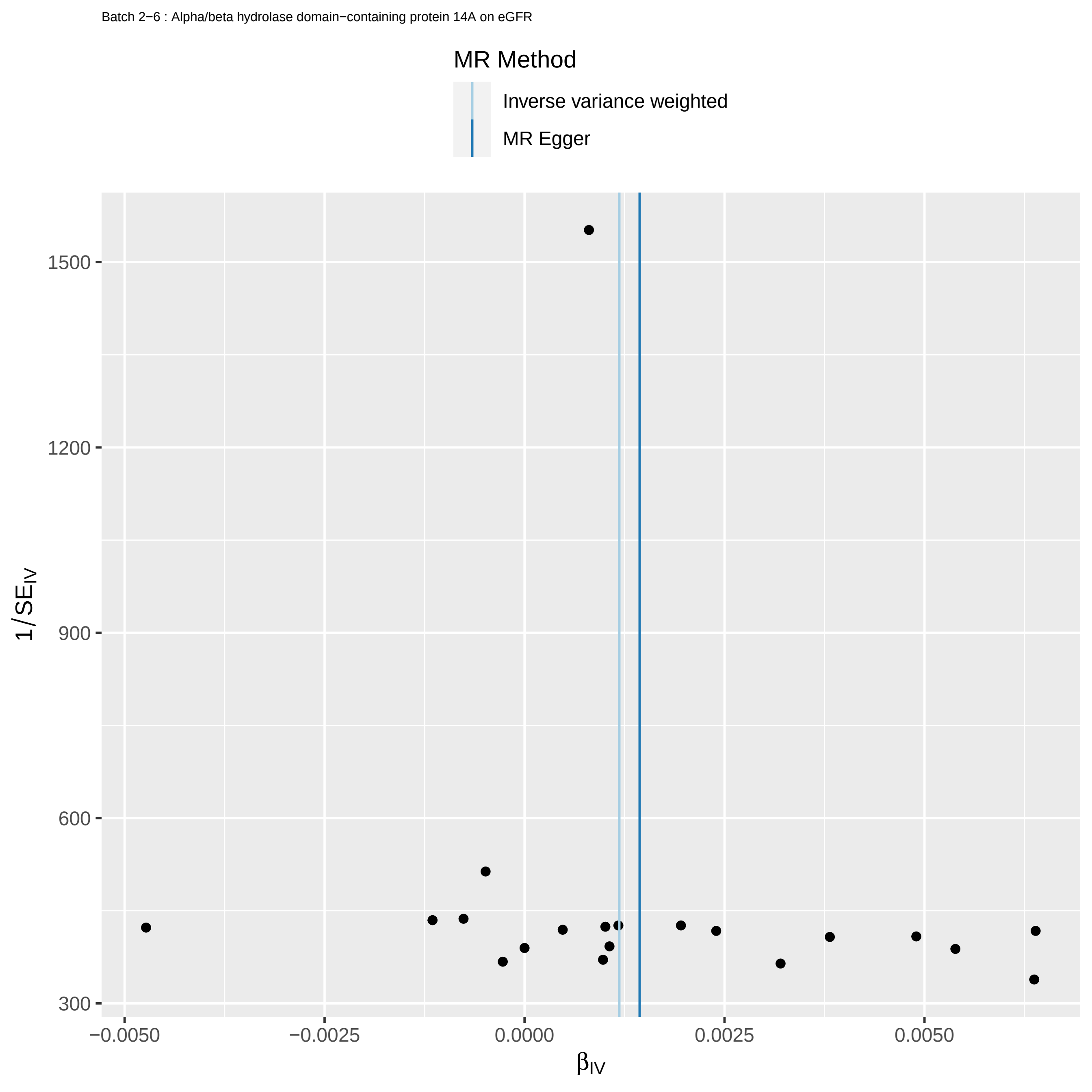

Batch 2−6 : Alpha/beta hydrolase domain−containing protein 14A on eGFR
MR Method
Inverse variance weighted
MR Egger
1500
1200
900
600
300
1/SE_IV
−0.0050
−0.0025
0.0000
0.0025
0.0050
β_IV

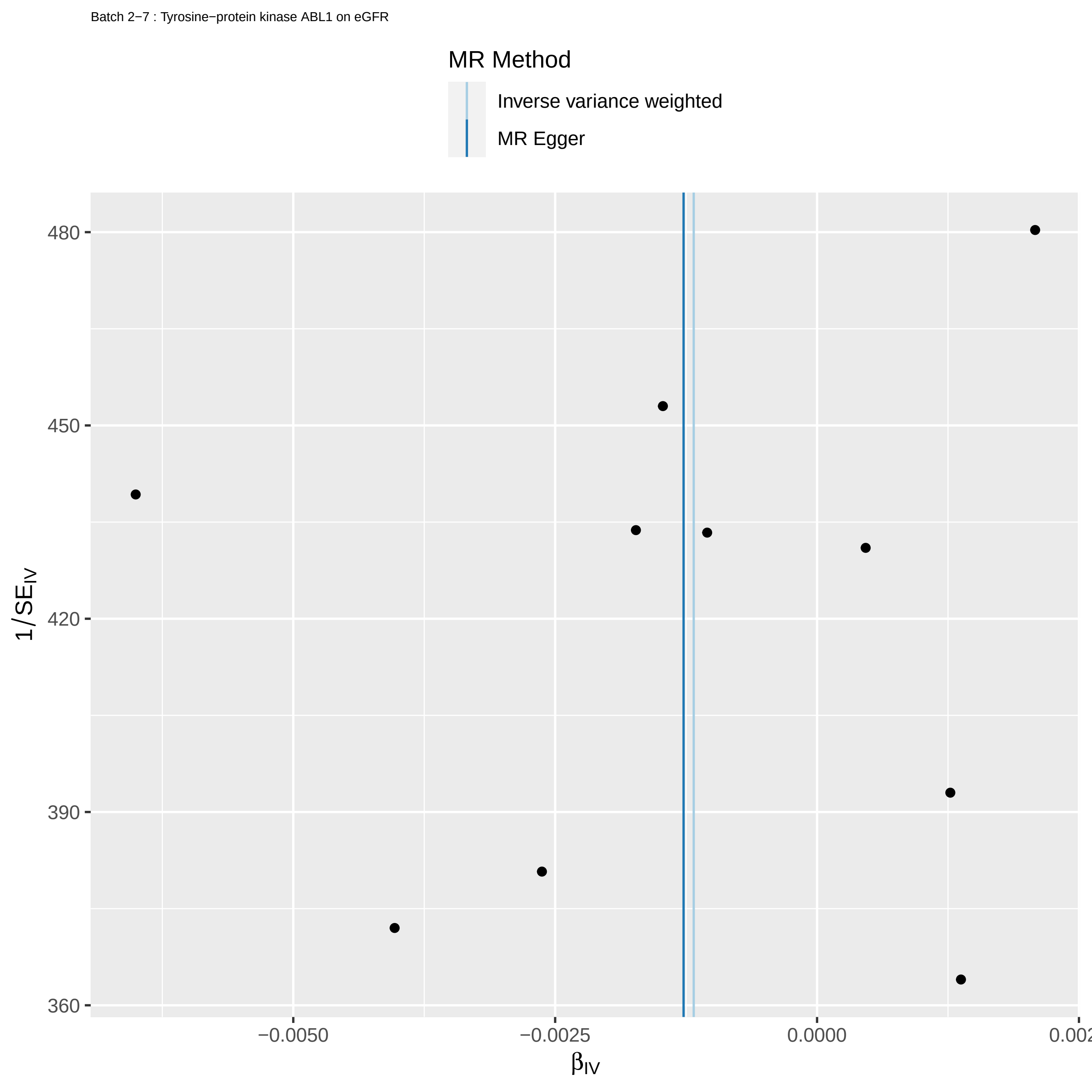

Batch 2−7 : Tyrosine−protein kinase ABL1 on eGFR
MR Method
Inverse variance weighted
MR Egger
480
450
420
390
360
1/SE_IV
−0.0050
−0.0025
0.0000
0.002
β_IV

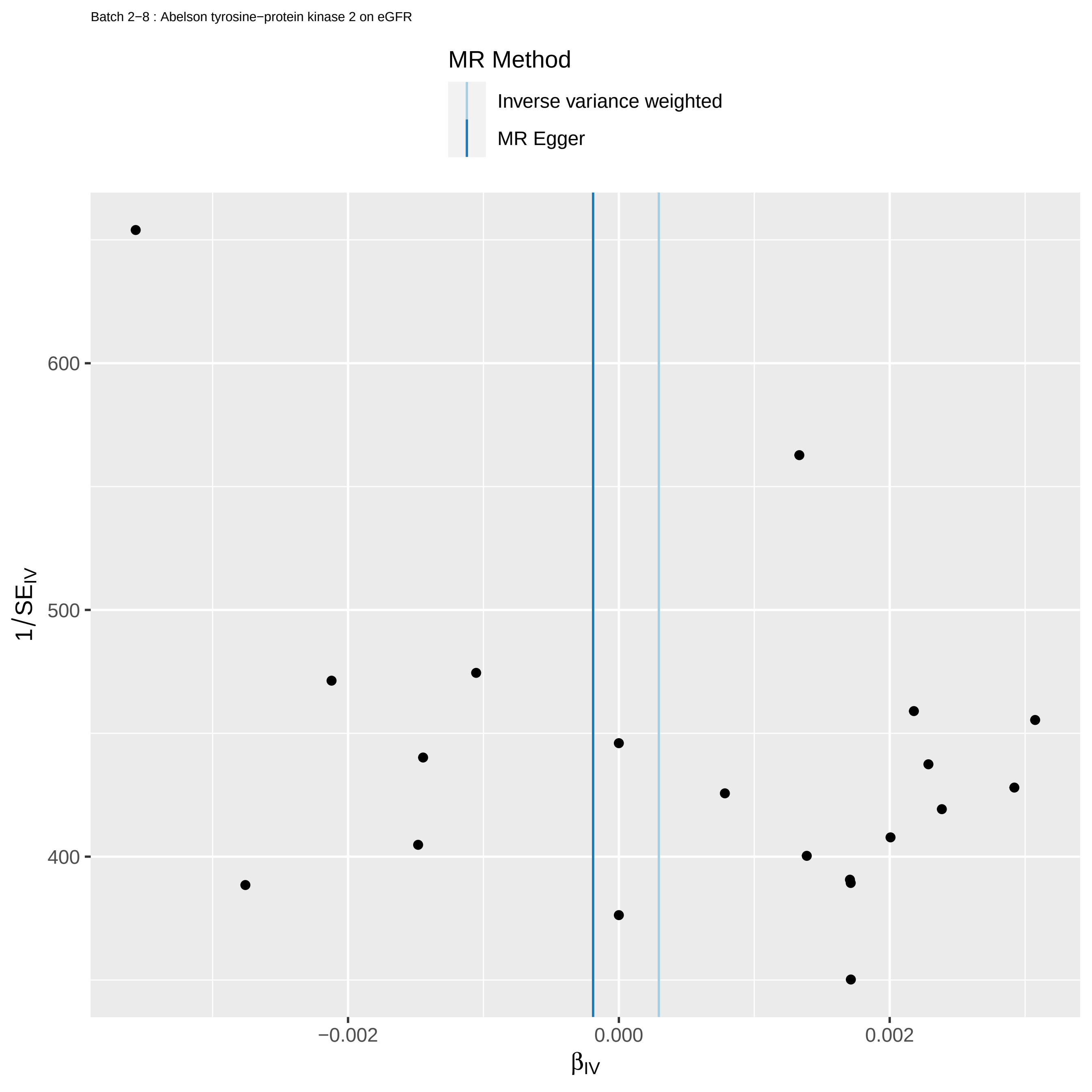

Batch 2−8 : Abelson tyrosine−protein kinase 2 on eGFR
MR Method
Inverse variance weighted
MR Egger
600
500
400
1/SE_IV
−0.002
0.000
0.002
β_IV

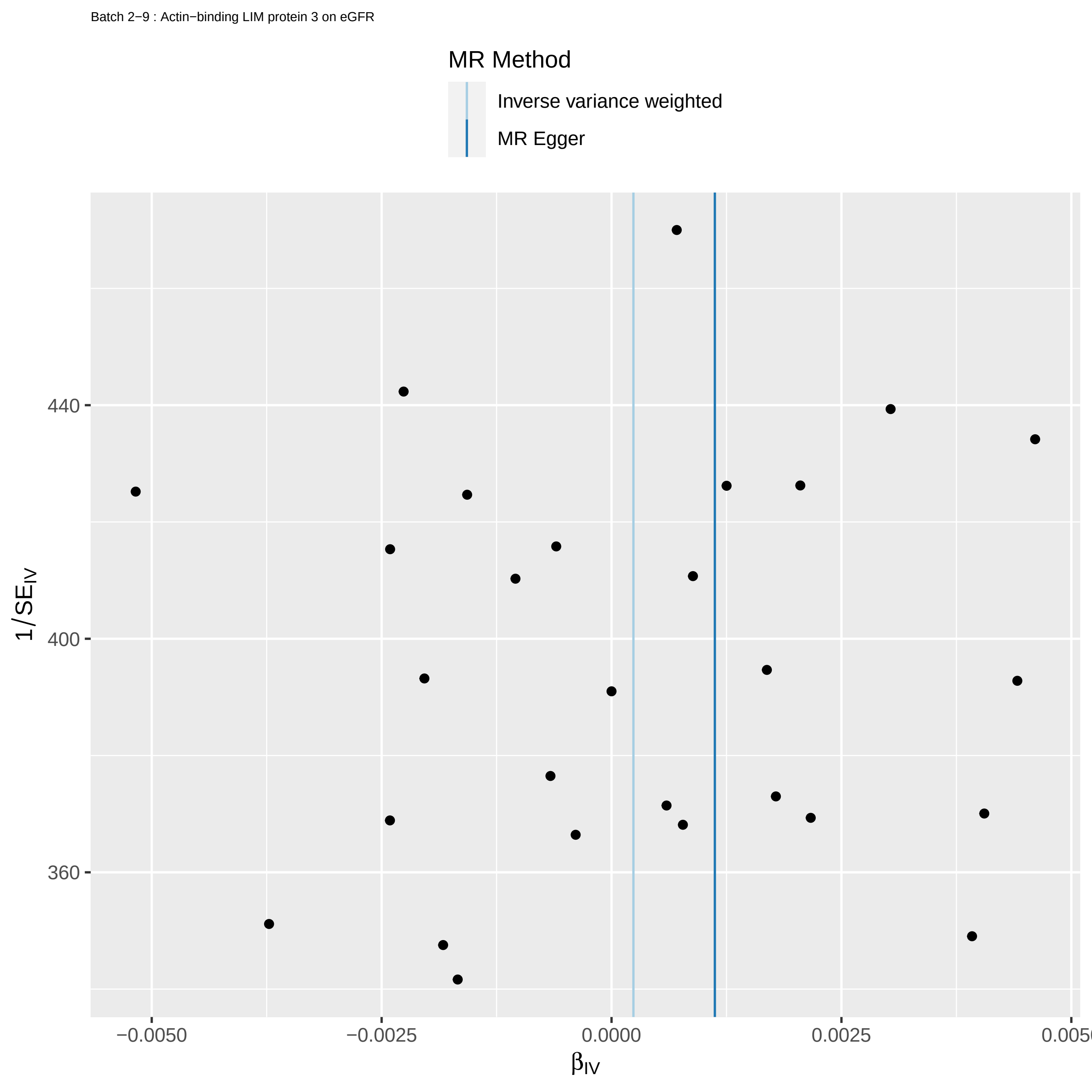
Batch 2−9 : Actin−binding LIM protein 3 on eGFR
MR Method
Inverse variance weighted
MR Egger
440
400
360
1/SE_IV
−0.0050
−0.0025
0.0000
0.0025
0.0050
β_IV

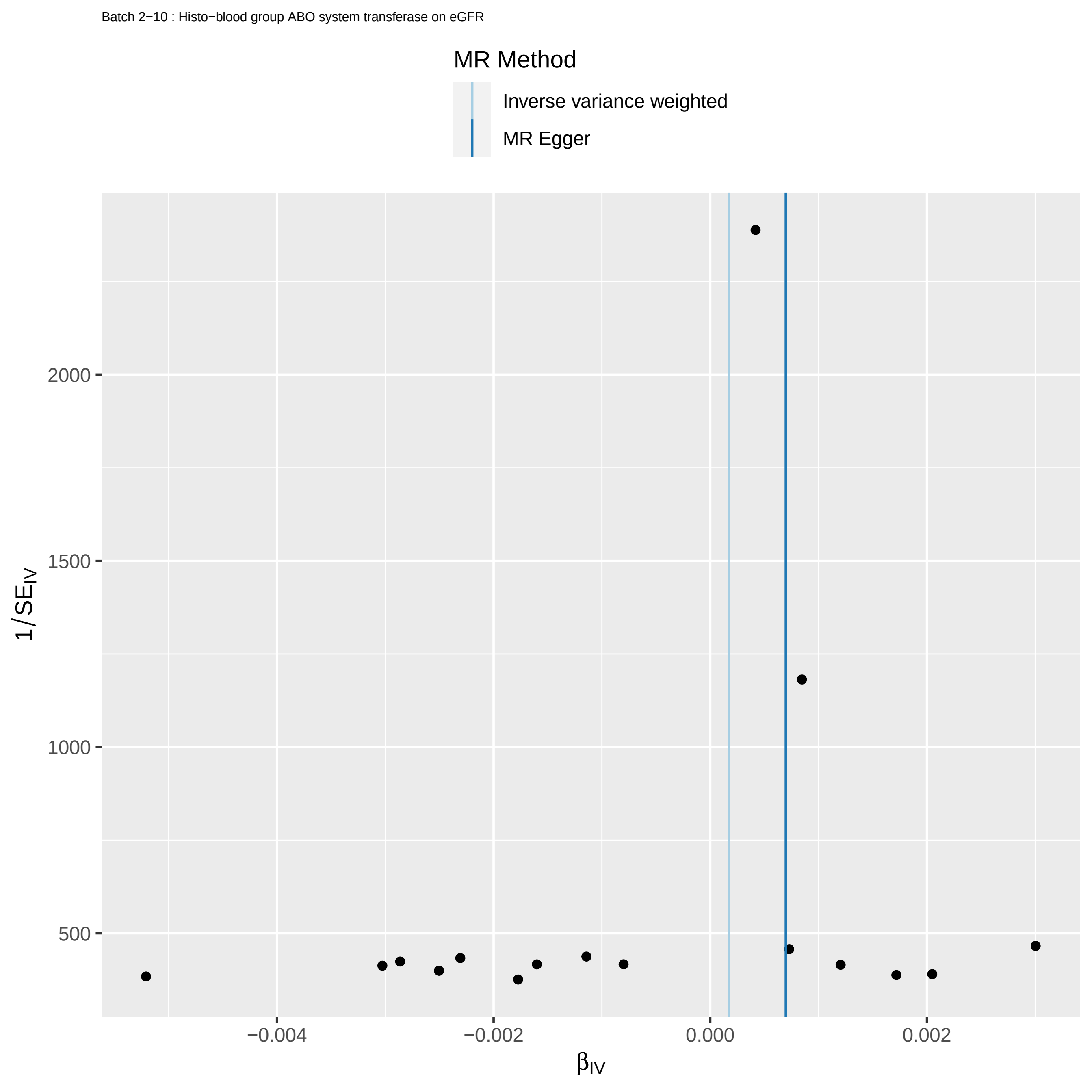

Batch 2−10 : Histo−blood group ABO system transferase on eGFR
MR Method
Inverse variance weighted
MR Egger
2000
1500
1000
500
1/SE$_{IV}$
−0.004
−0.002
0.000
0.002
β_{IV}

Batch 2−11 : Acetyl−CoA carboxylase 2 on eGFR

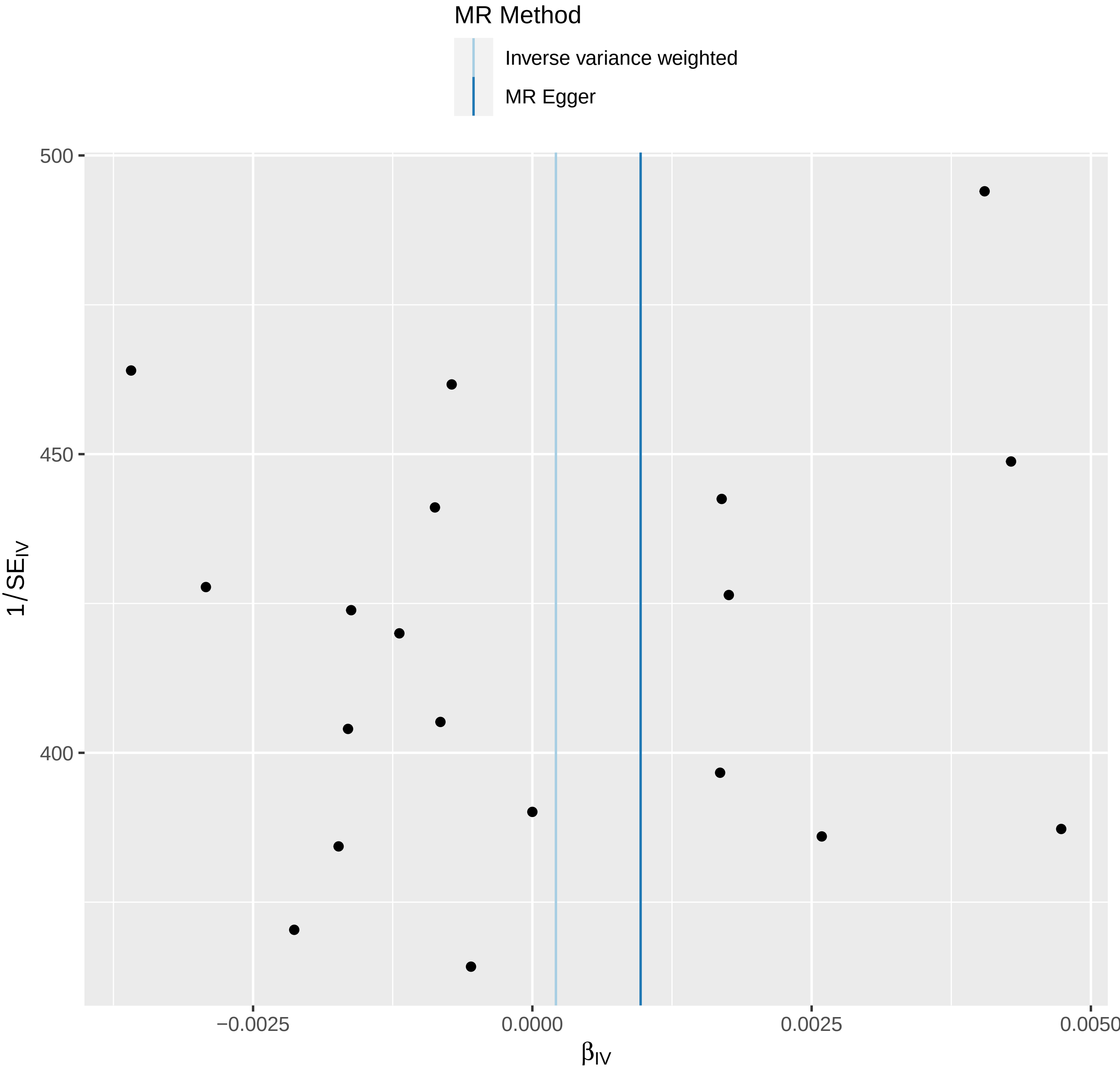

Batch 2−12 : Aggrecan core protein on eGFR

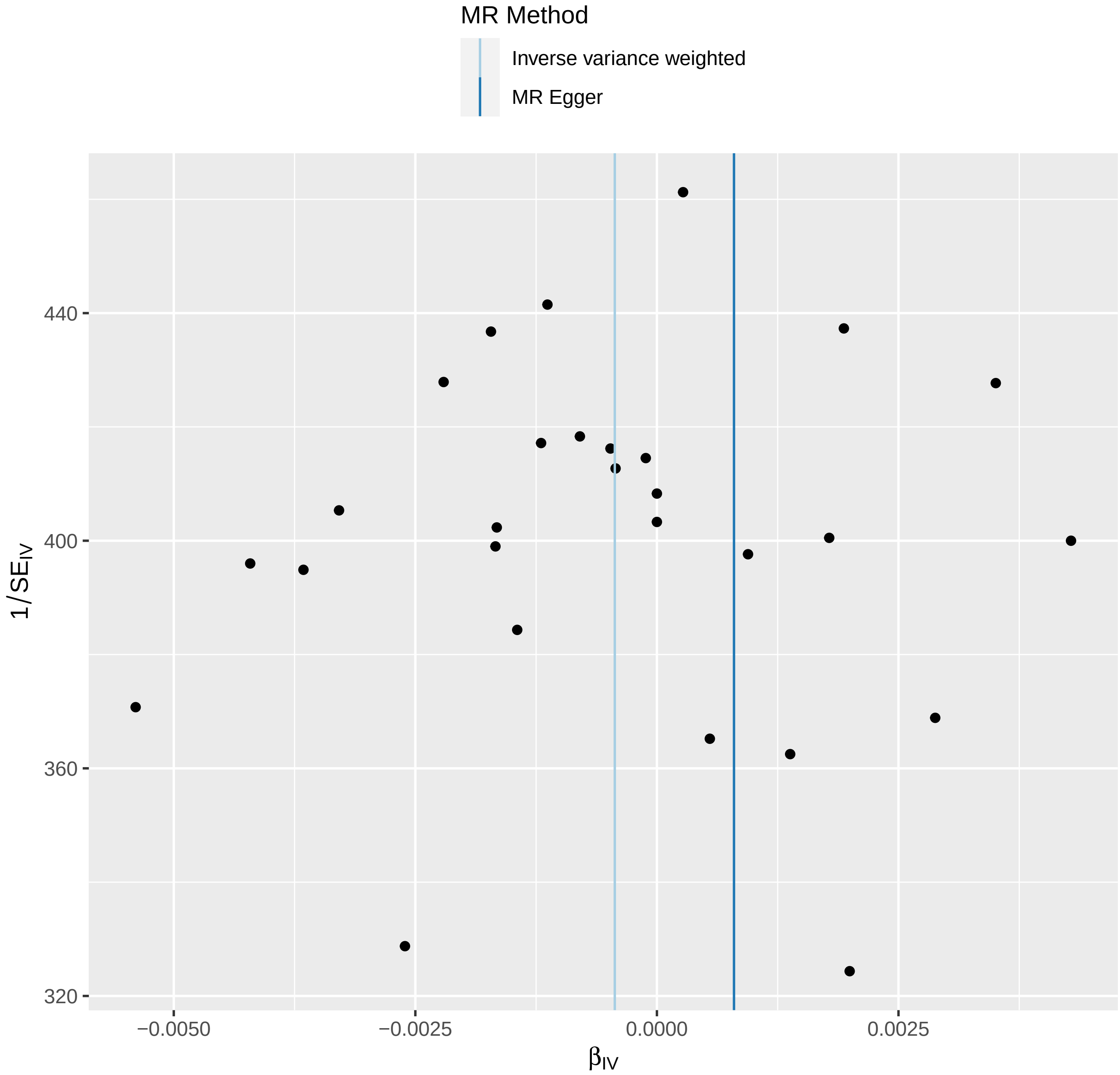

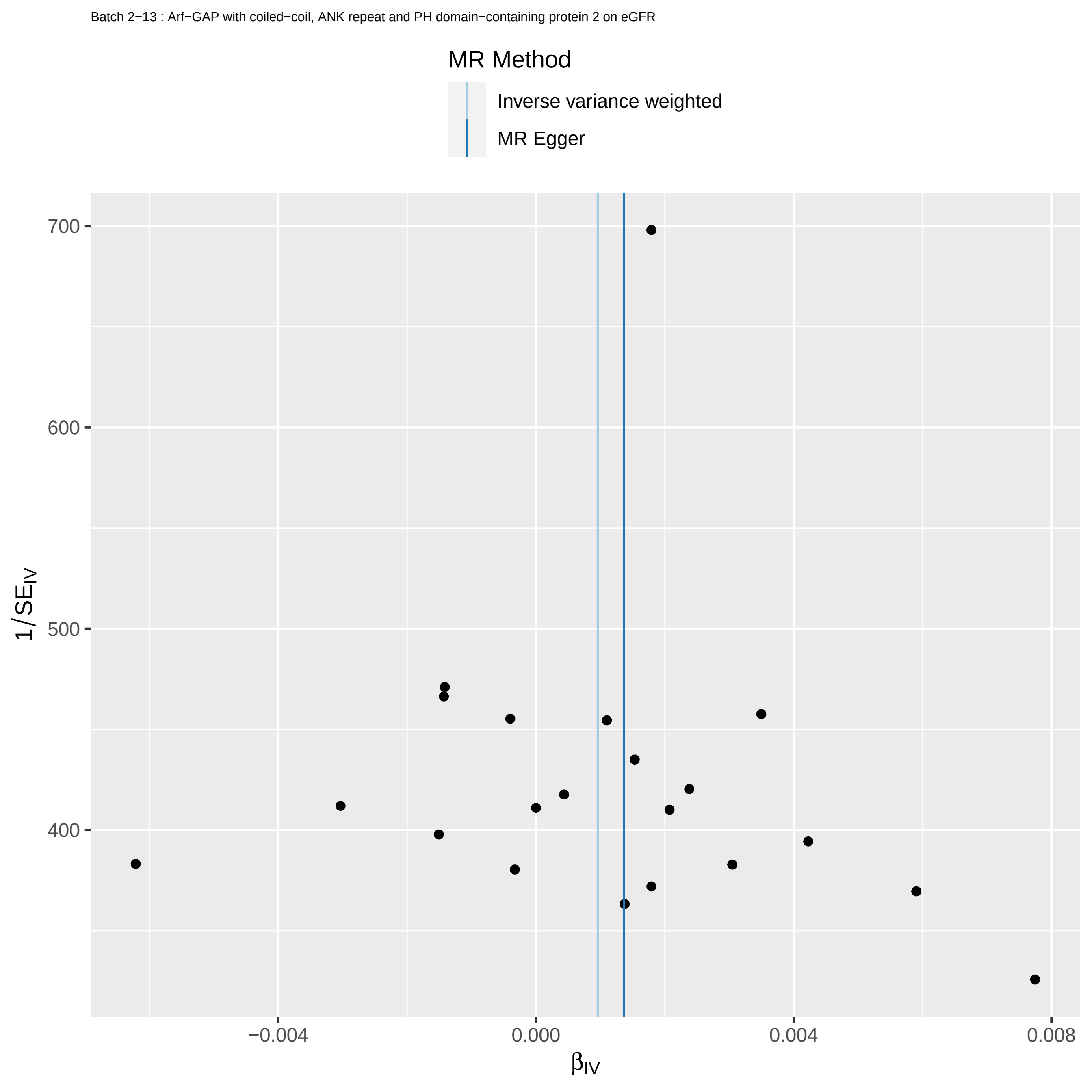

Batch 2−13 : Arf−GAP with coiled−coil, ANK repeat and PH domain−containing protein 2 on eGFR
MR Method
Inverse variance weighted
MR Egger
700
600
500
400
1/SE$_{IV}$
−0.004
0.000
0.004
0.008
β$_{IV}$

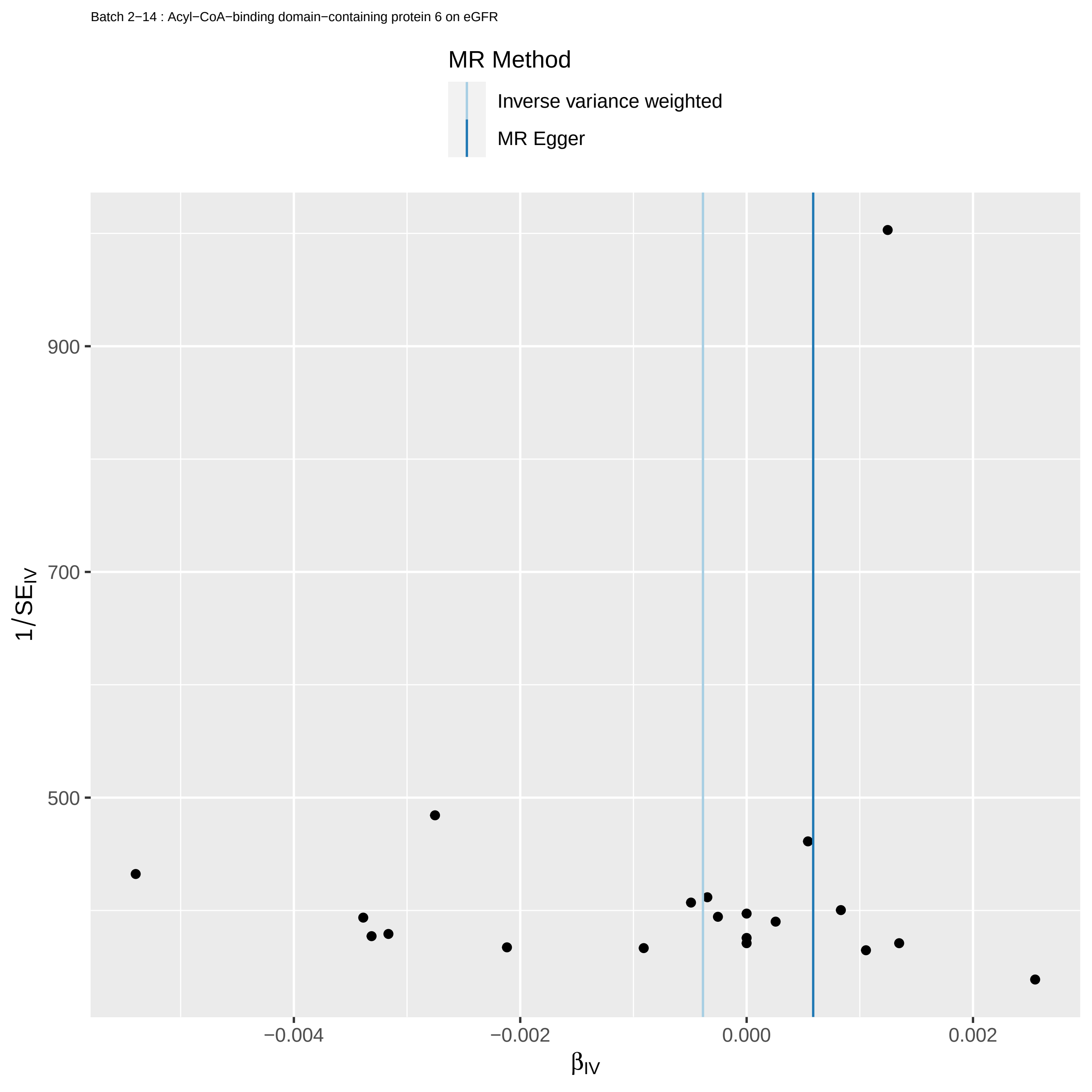

Batch 2−14 : Acyl−CoA−binding domain−containing protein 6 on eGFR
MR Method
Inverse variance weighted
MR Egger
900
700
500
1/SE_IV
−0.004
−0.002
0.000
0.002
β_IV

Batch 2−15 : Acyl−CoA−binding domain−containing protein 7 on eGFR

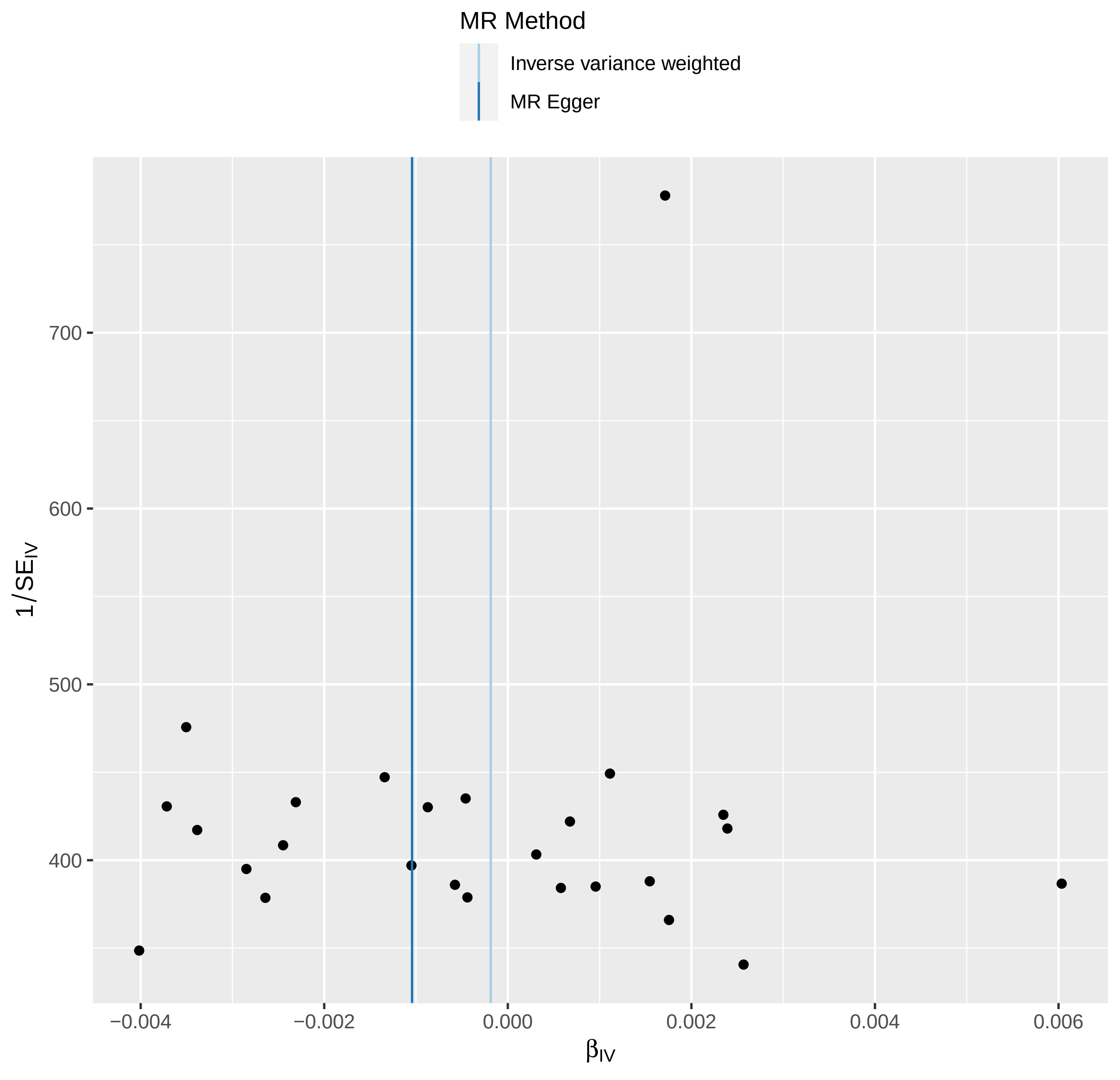

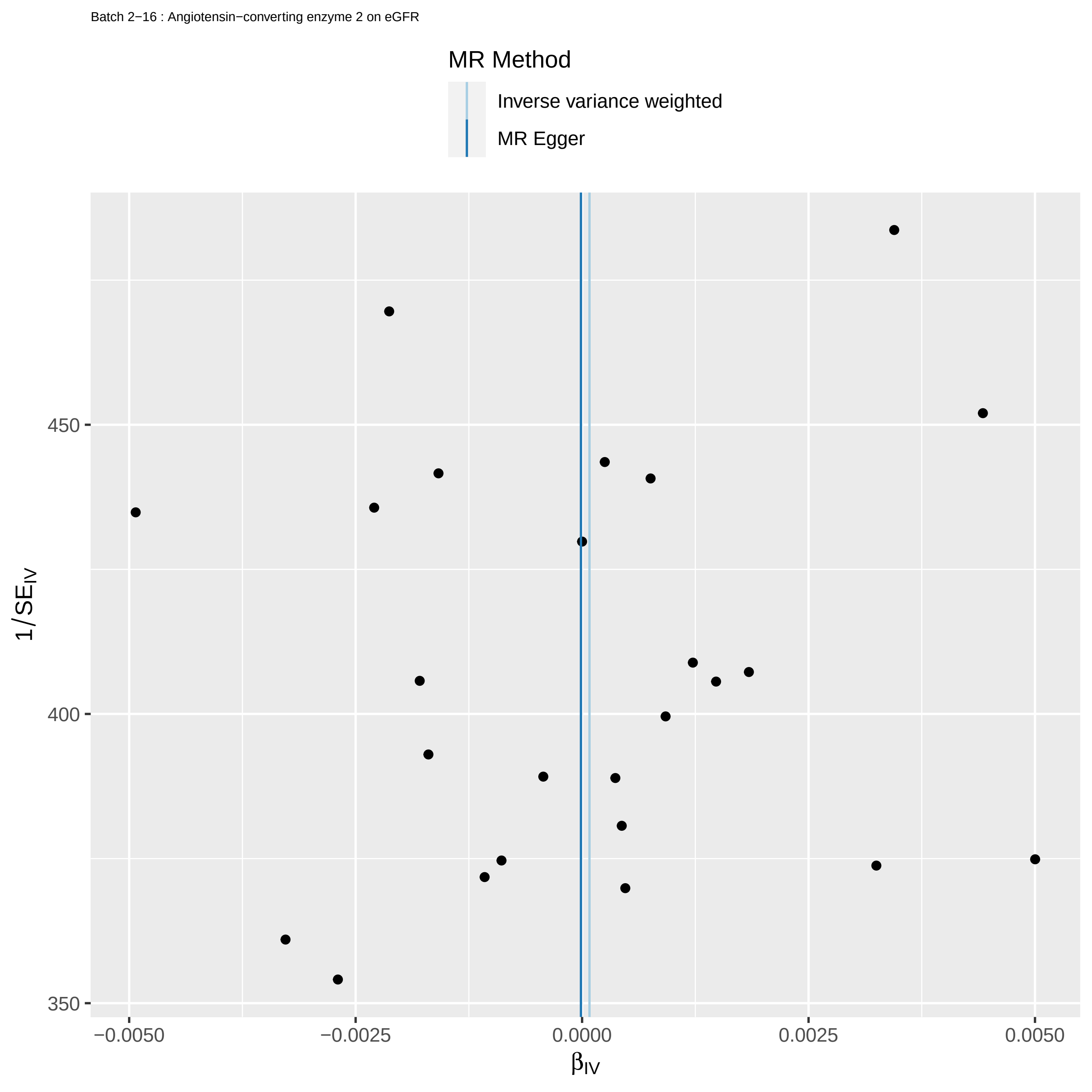

Batch 2−16 : Angiotensin−converting enzyme 2 on eGFR
MR Method
Inverse variance weighted
MR Egger
450
400
350
−0.0050
−0.0025
0.0000
0.0025
0.0050
1/SE_IV
β_IV

Batch 2−17 : ATP−citrate synthase on eGFR

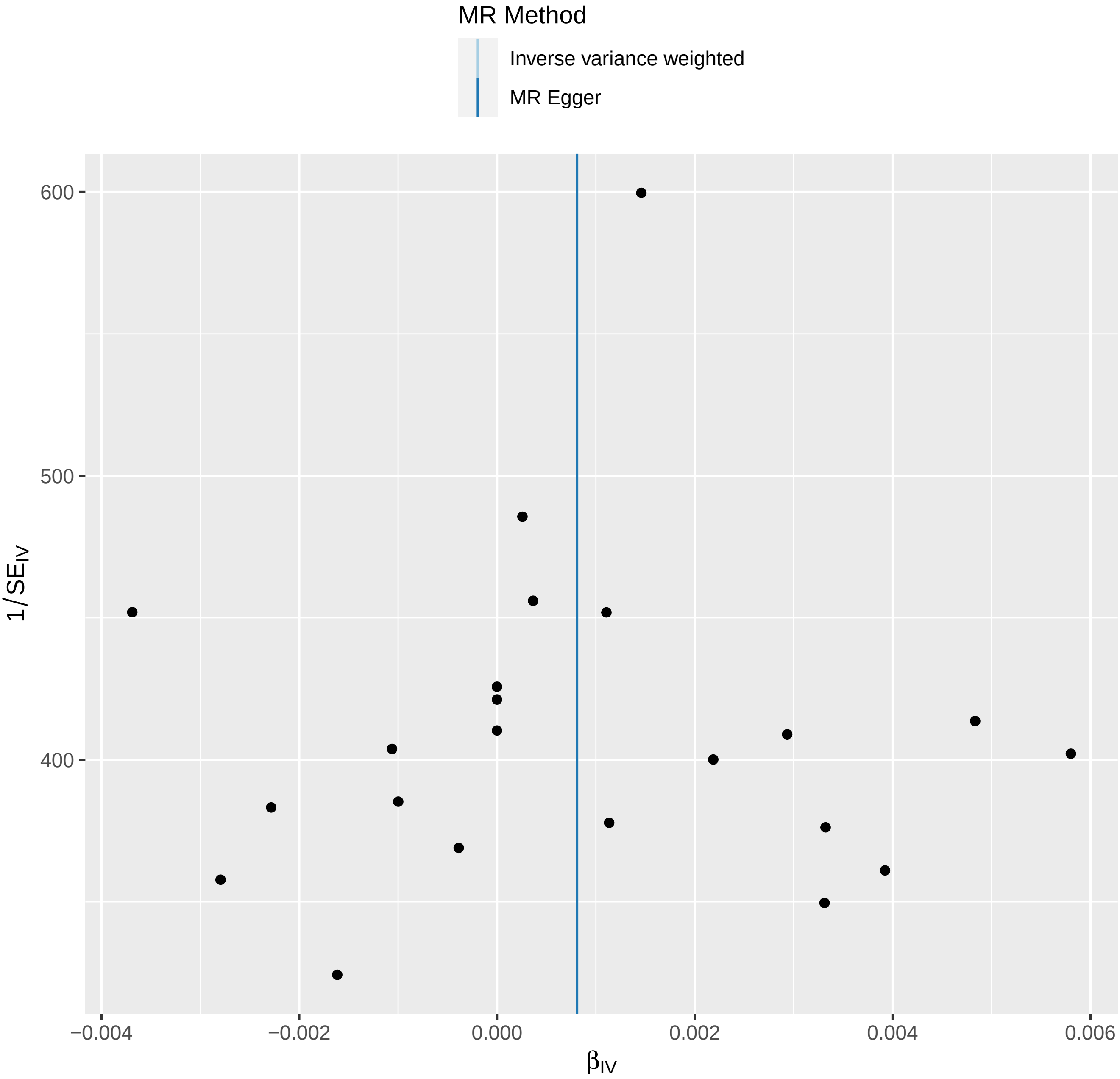

Batch 2−18 : Low molecular weight phosphotyrosine protein phosphatase on eGFR

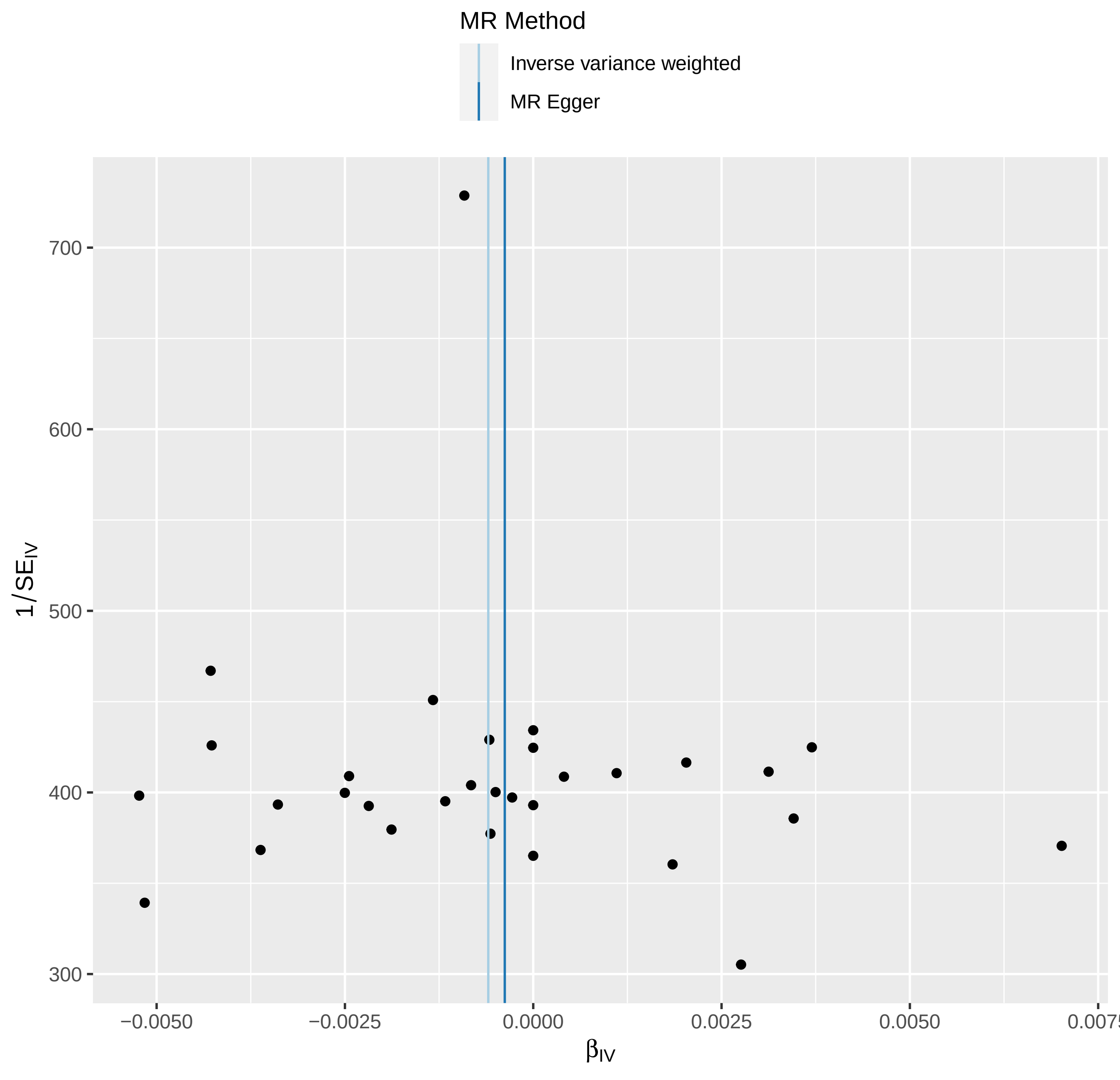

Batch 2−19 : Lysosomal acid phosphatase on eGFR

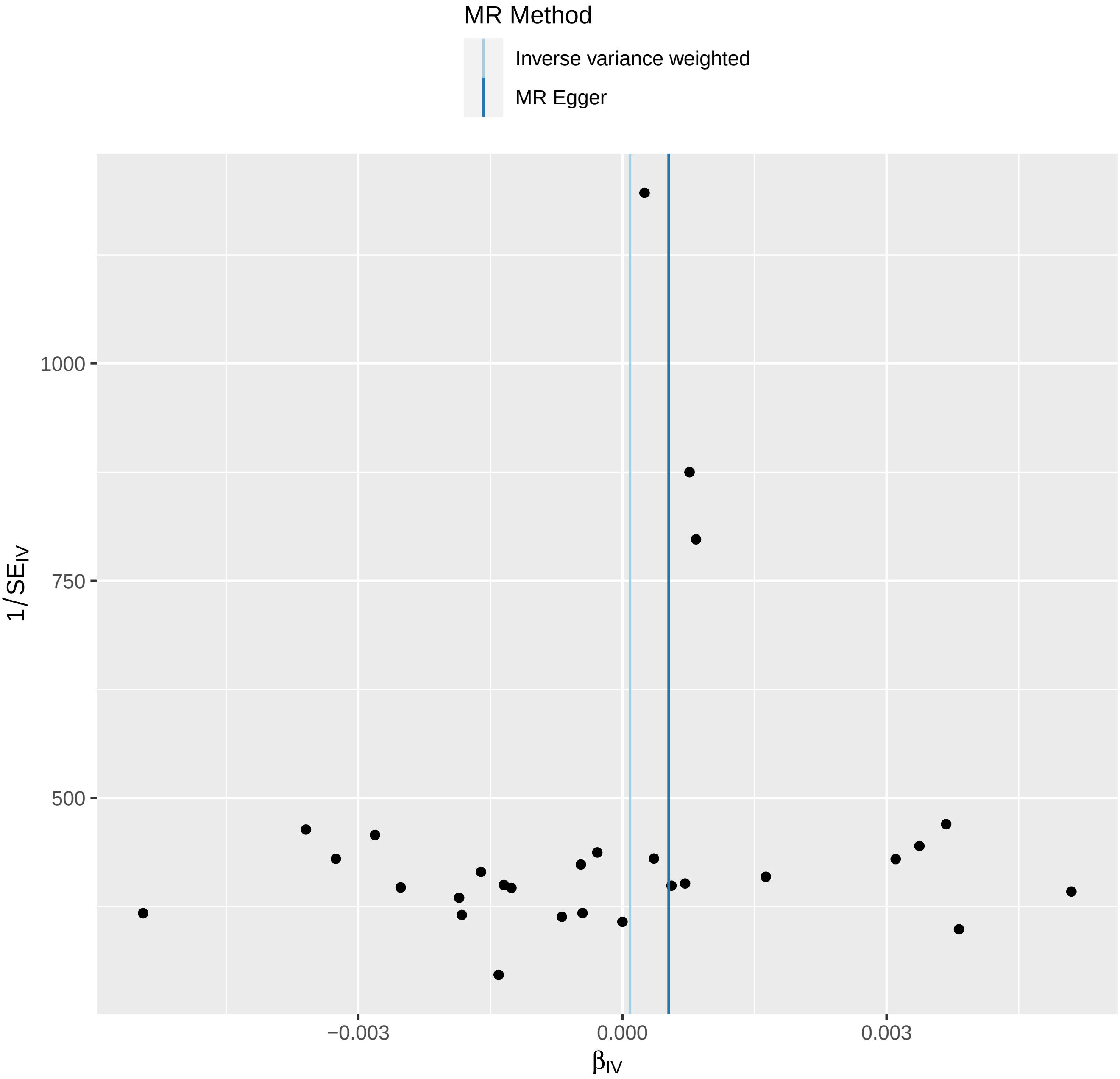

Batch 2−20 : Tartrate−resistant acid phosphatase type 5 on eGFR

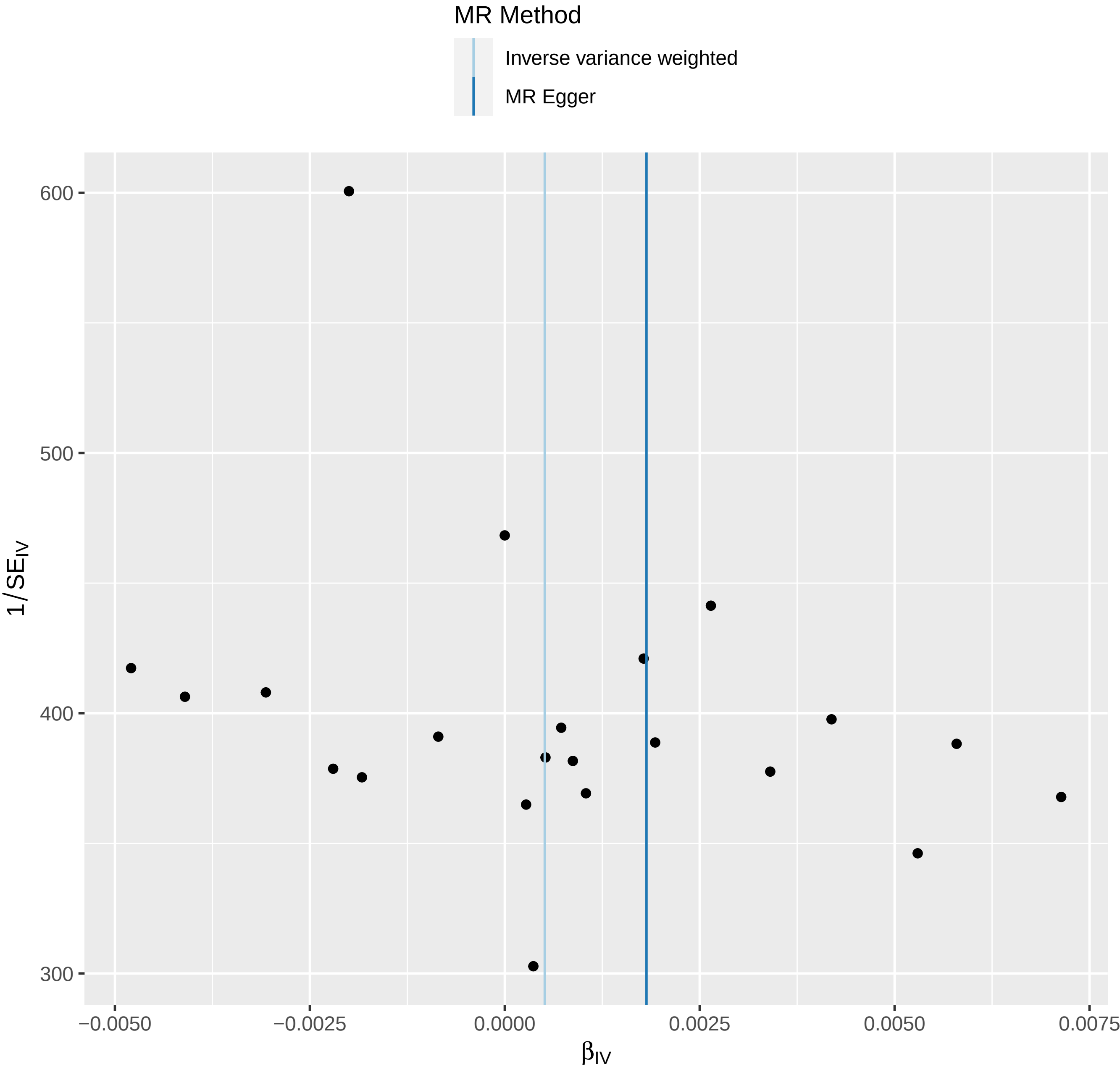

Batch 2−21 : 2−phosphoxylose phosphatase 1 on eGFR

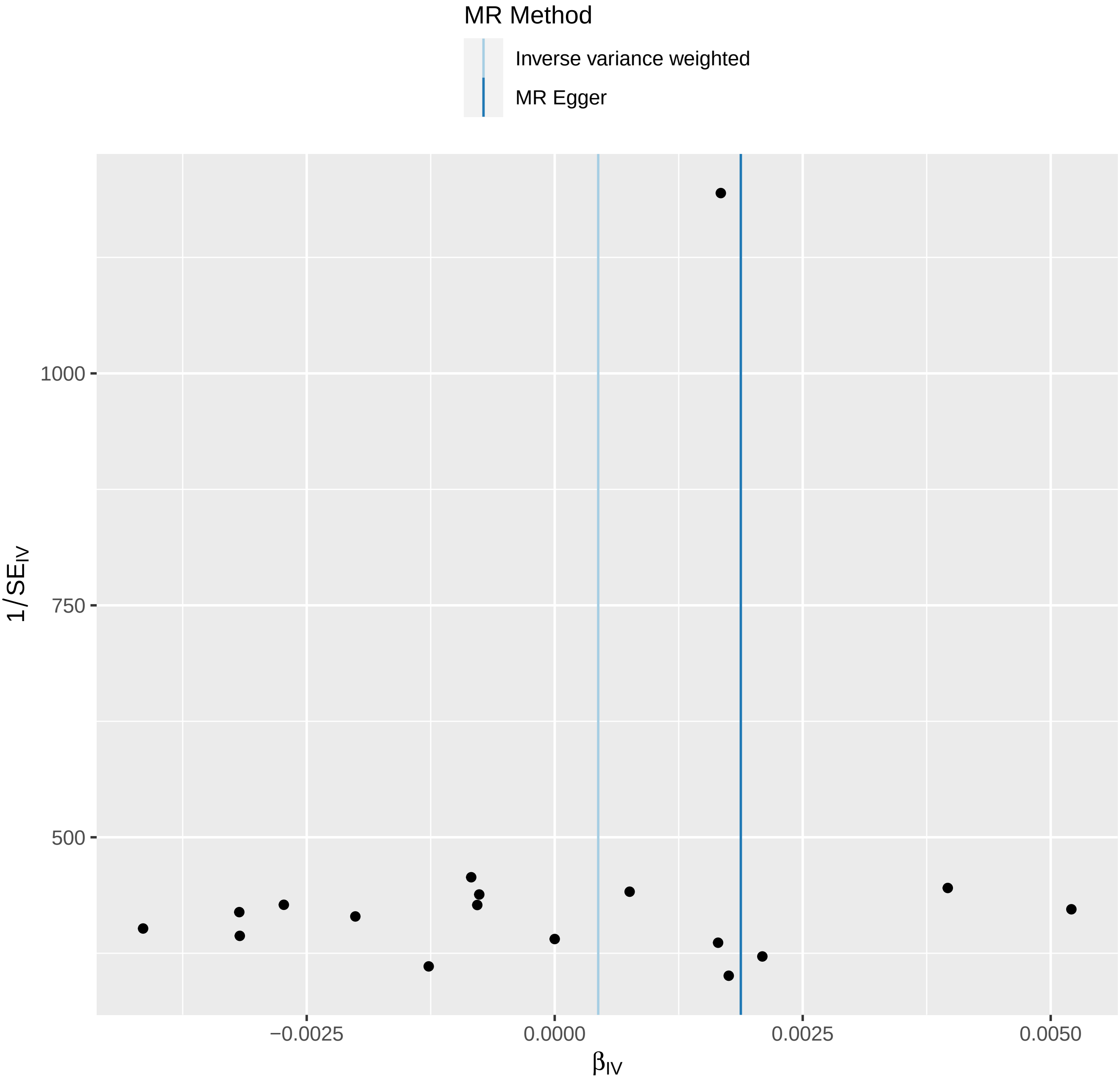

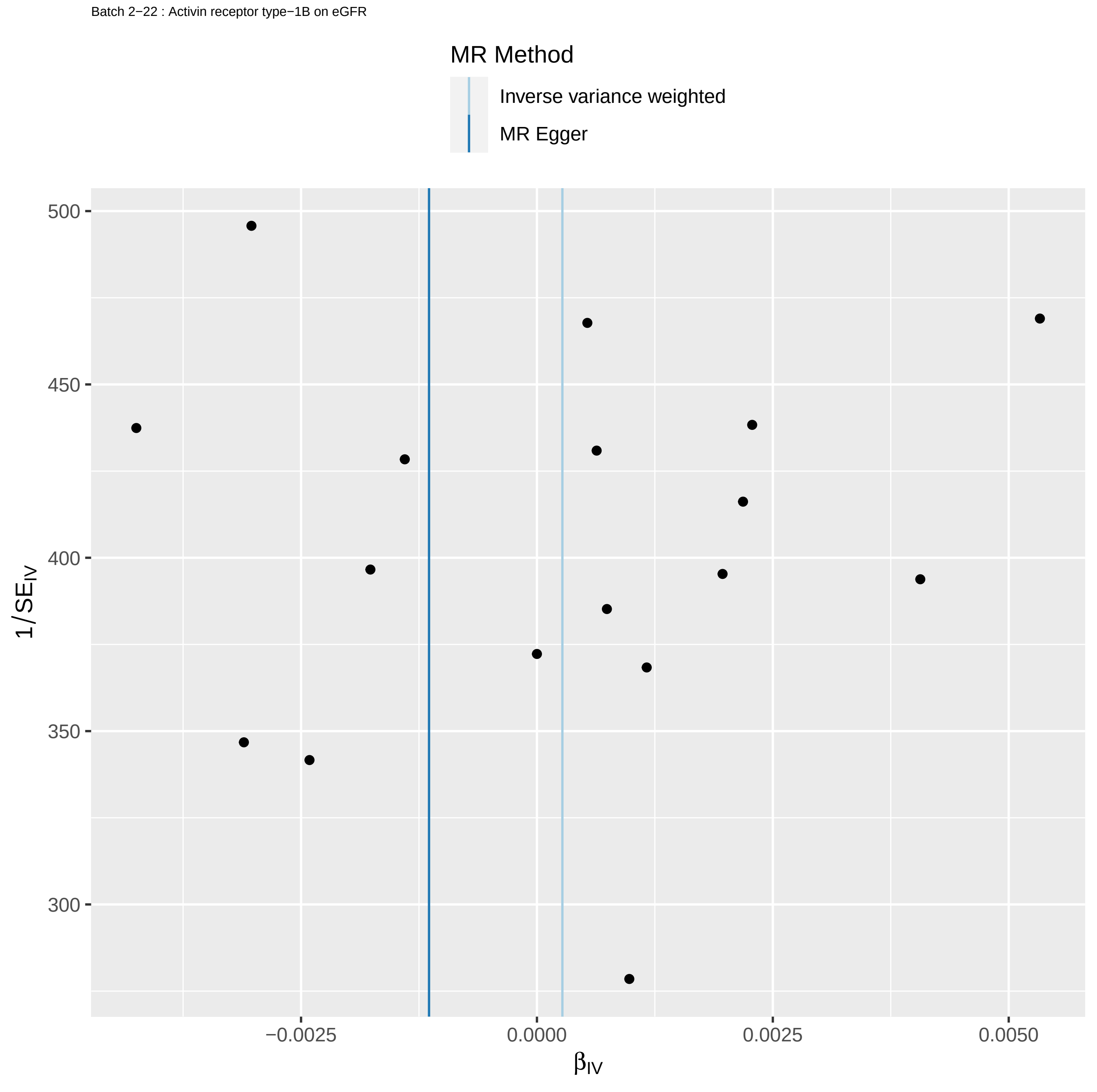

Batch 2−22 : Activin receptor type−1B on eGFR
MR Method
Inverse variance weighted
MR Egger
500
450
400
350
300
1/SE$_{IV}$
−0.0025
0.0000
0.0025
0.0050
β_{IV}

Batch 2−23 : Serine/threonine−protein kinase receptor R3 on eGFR

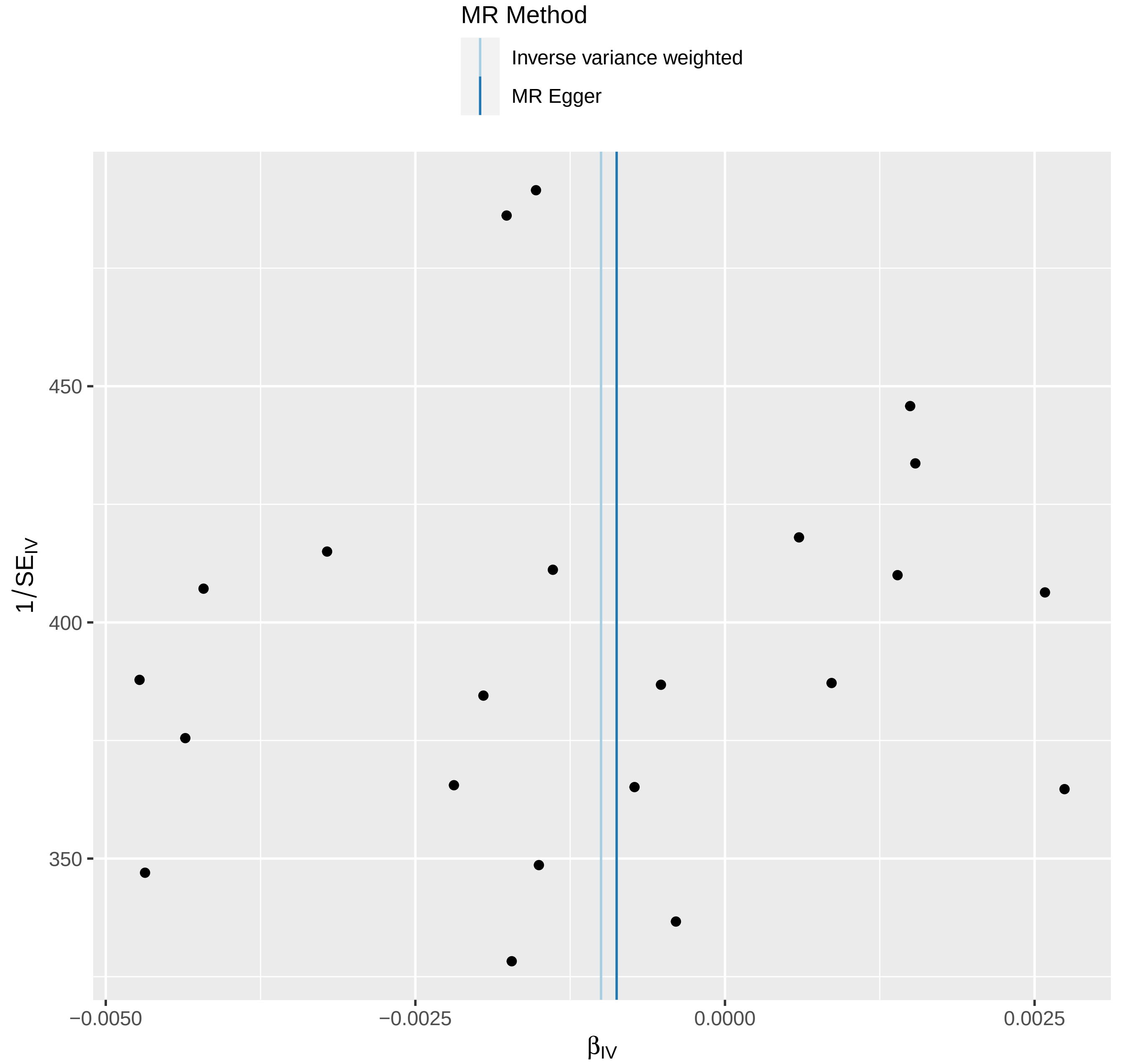

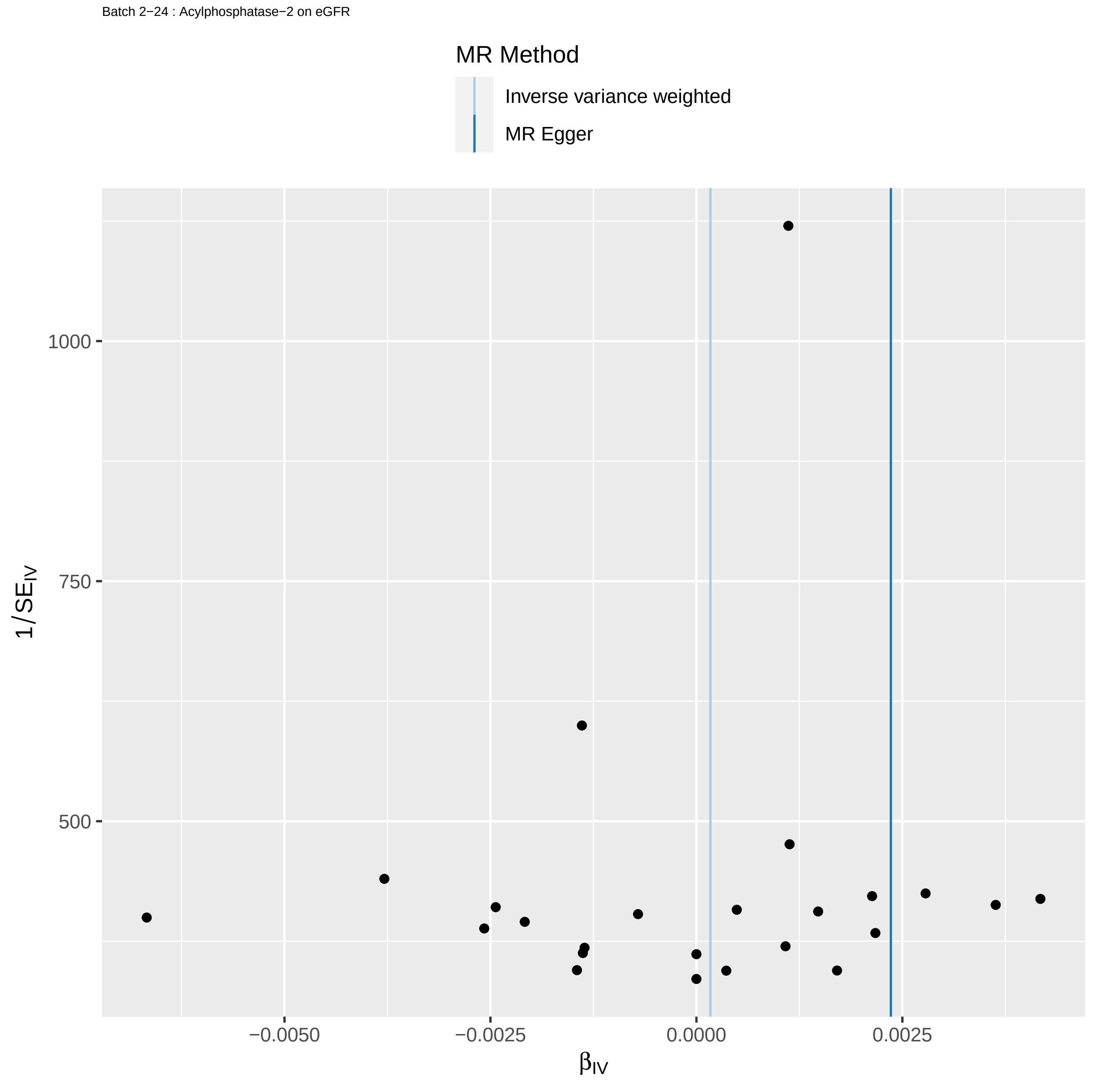
Batch 2−24 : Acylphosphatase−2 on eGFR
MR Method
Inverse variance weighted
MR Egger
1000
750
500
1/SE_IV
−0.0050
−0.0025
0.0000
0.0025
β_IV

Batch 2−25 : Disintegrin and metalloproteinase domain−containing protein 11 on eGFR

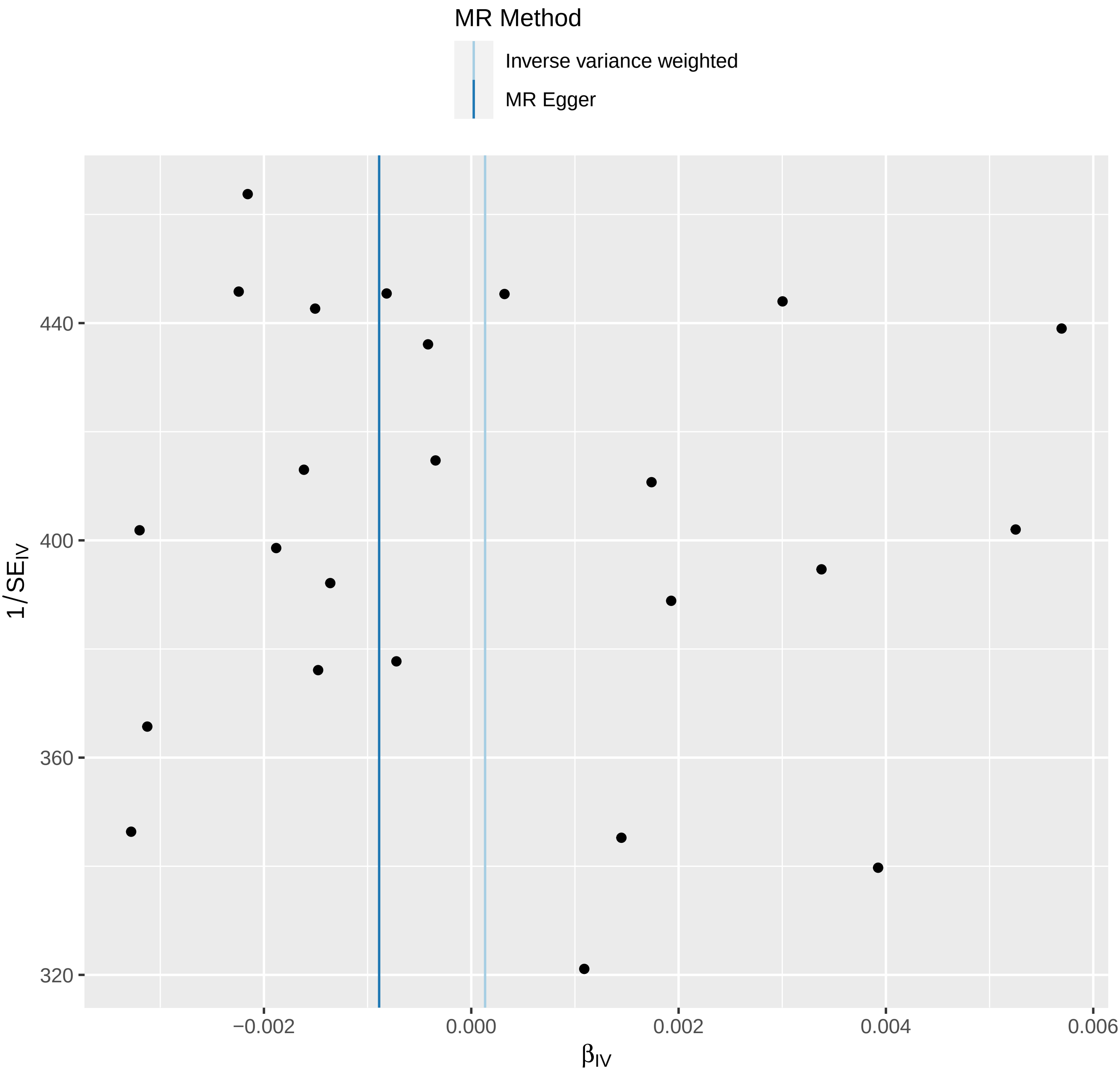

Batch 2−26 : Disintegrin and metalloproteinase domain−containing protein 12 on eGFR

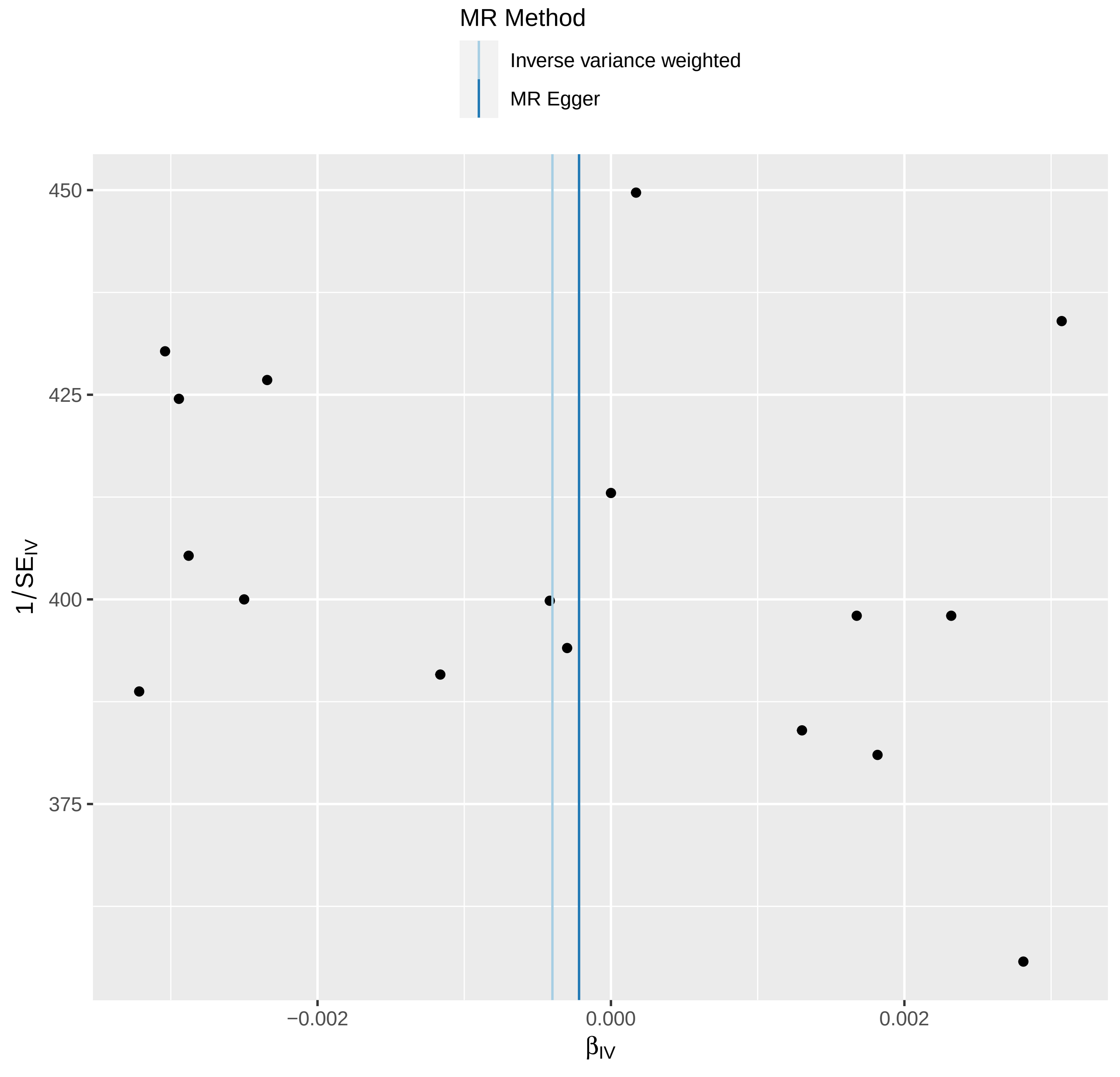

Batch 2−27 : Disintegrin and metalloproteinase domain−containing protein 19 on eGFR

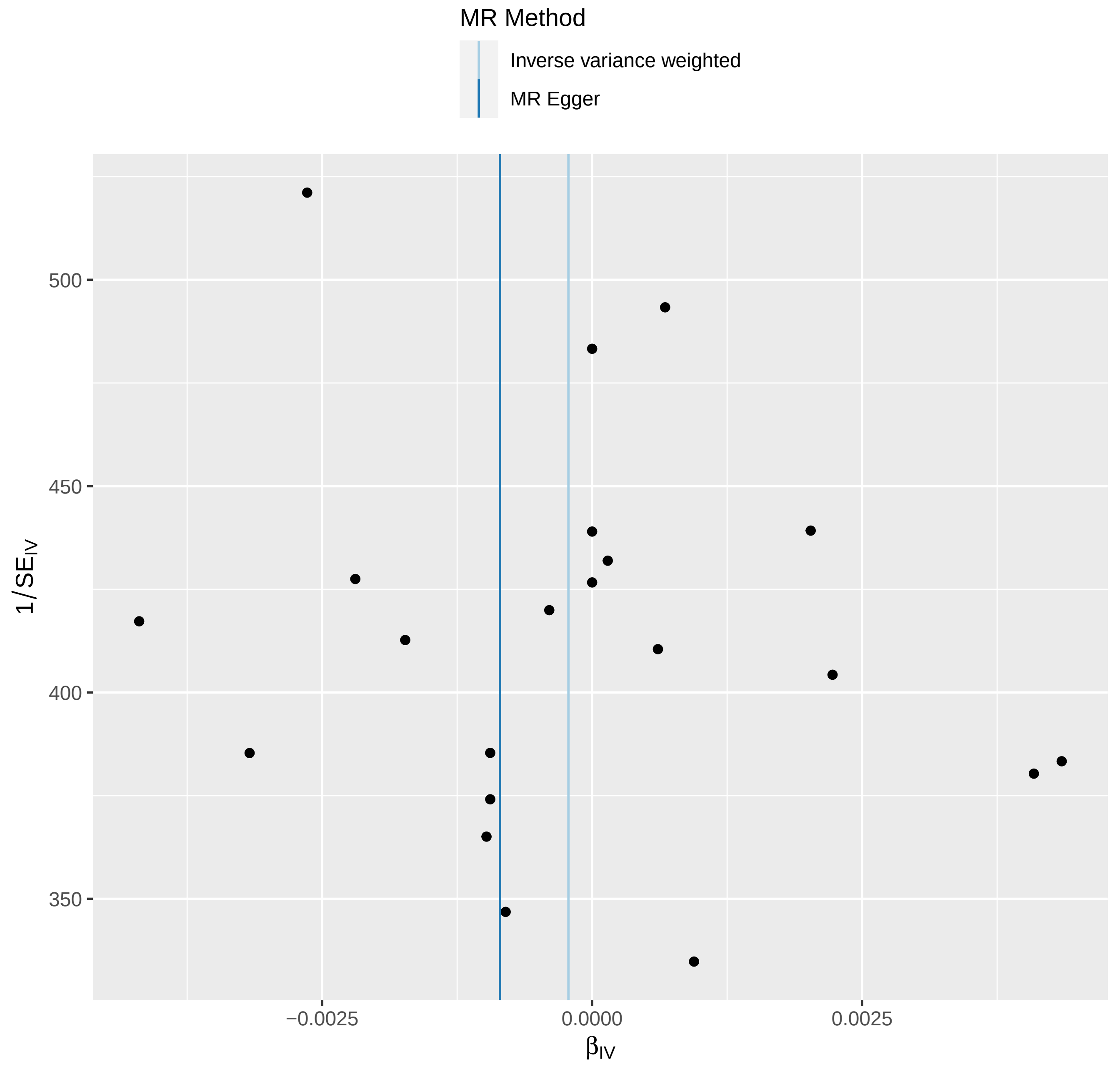

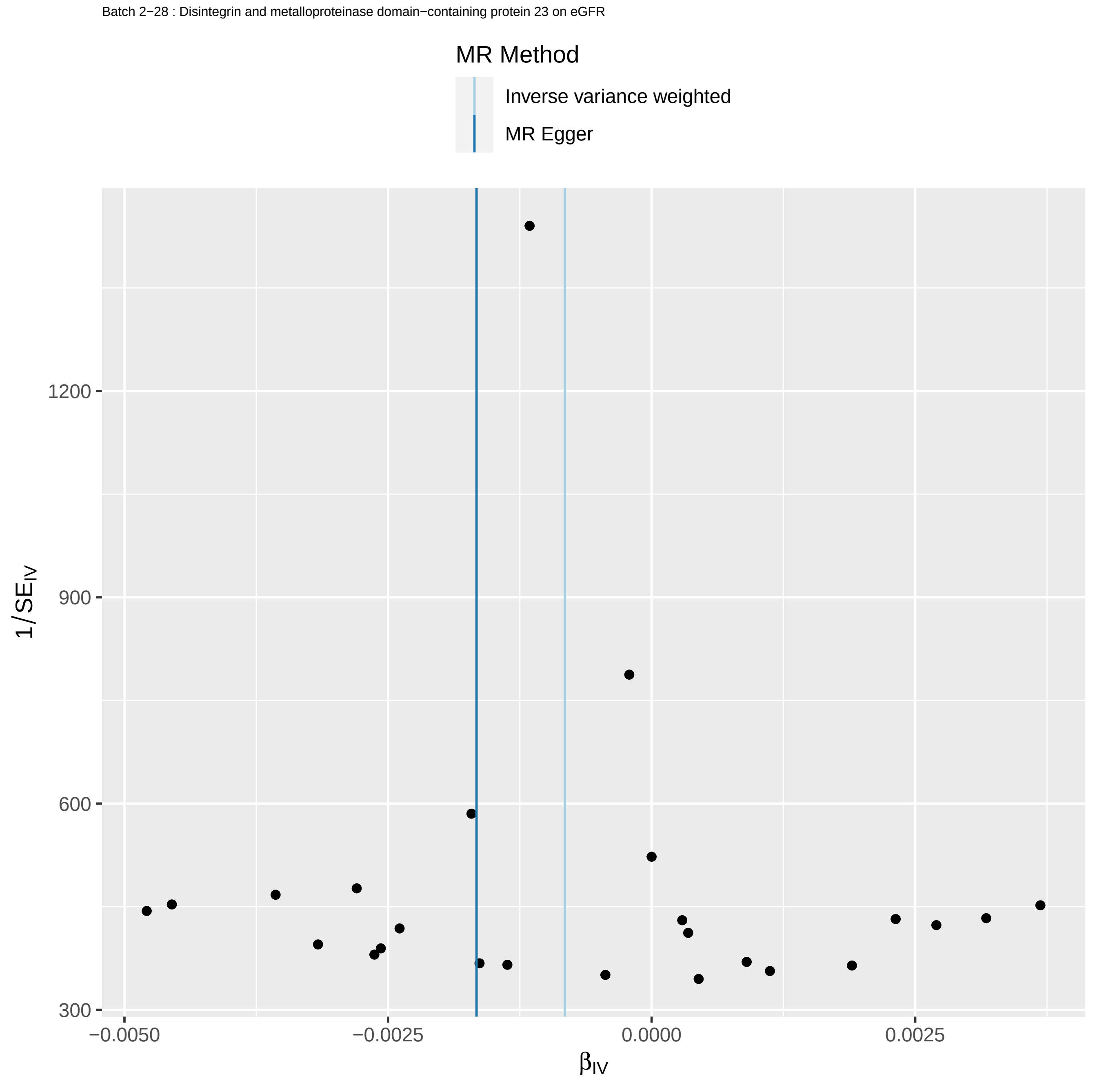
Batch 2−28 : Disintegrin and metalloproteinase domain−containing protein 23 on eGFR
MR Method
Inverse variance weighted
MR Egger
1200
900
600
300
−0.0050
−0.0025
0.0000
0.0025
$1/SE_{IV}$
β_{IV}

Batch 2−29 : Disintegrin and metalloproteinase domain−containing protein 29 on eGFR

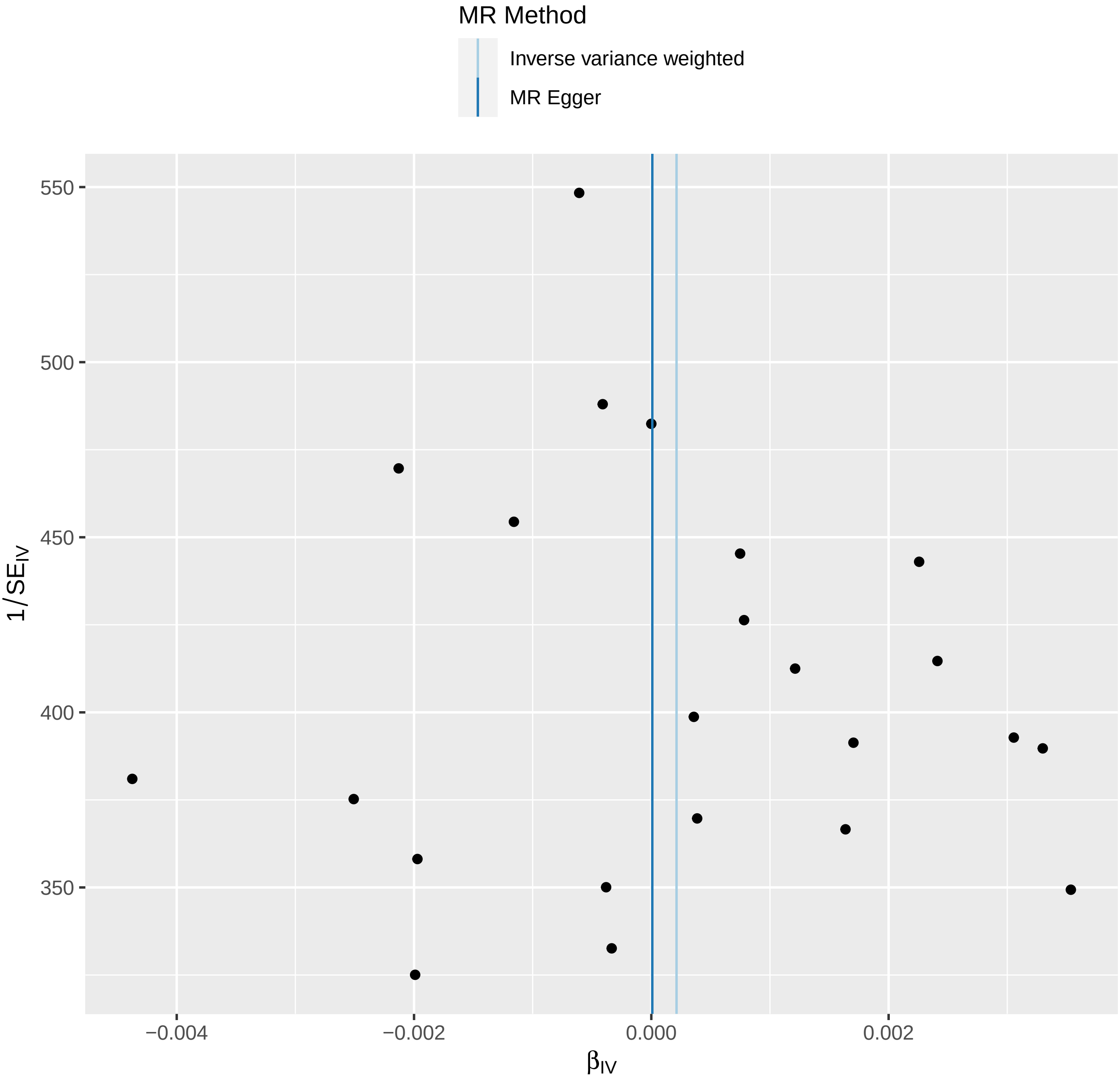

Batch 2−30 : Disintegrin and metalloproteinase domain−containing protein 9 on eGFR

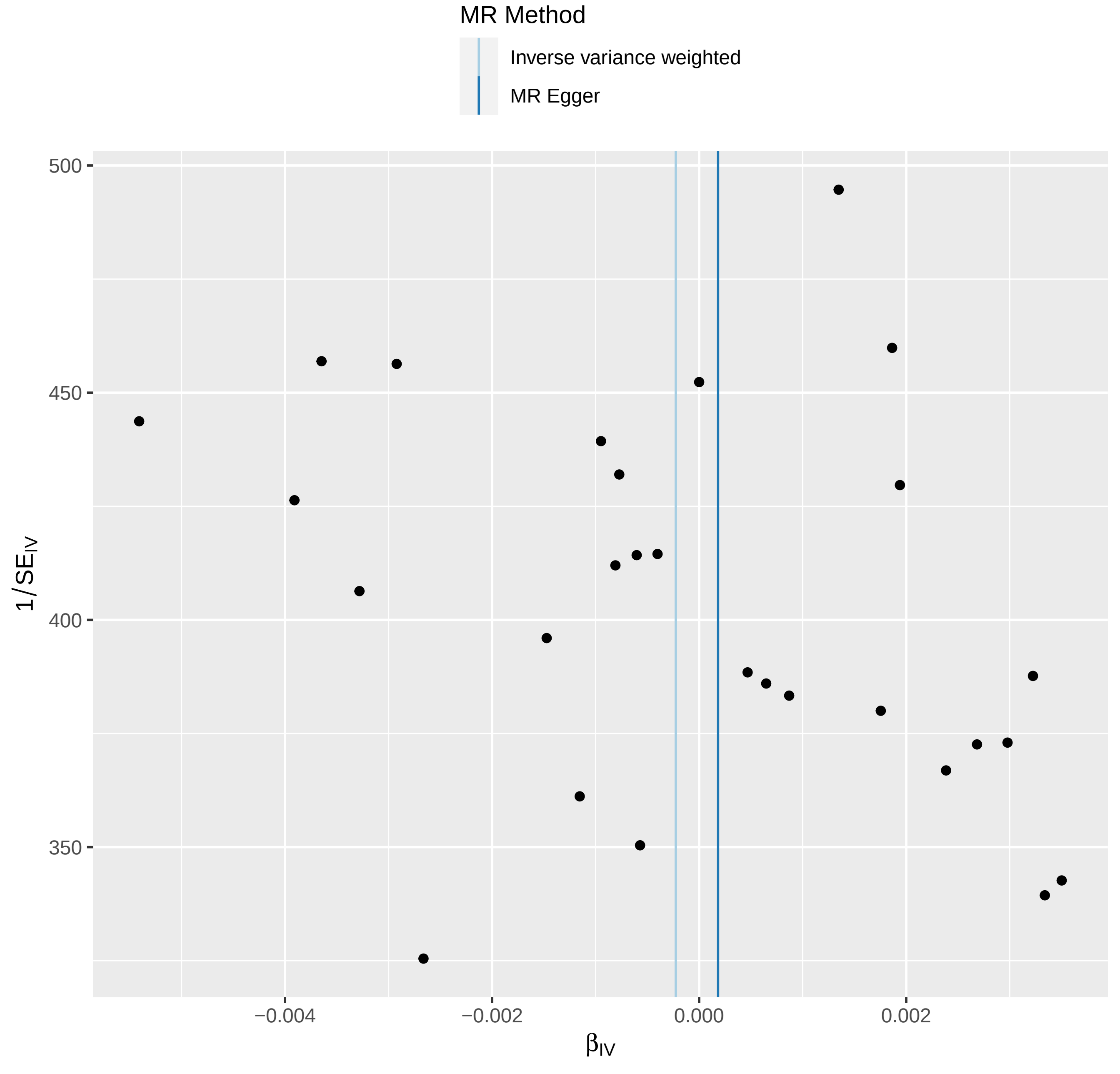

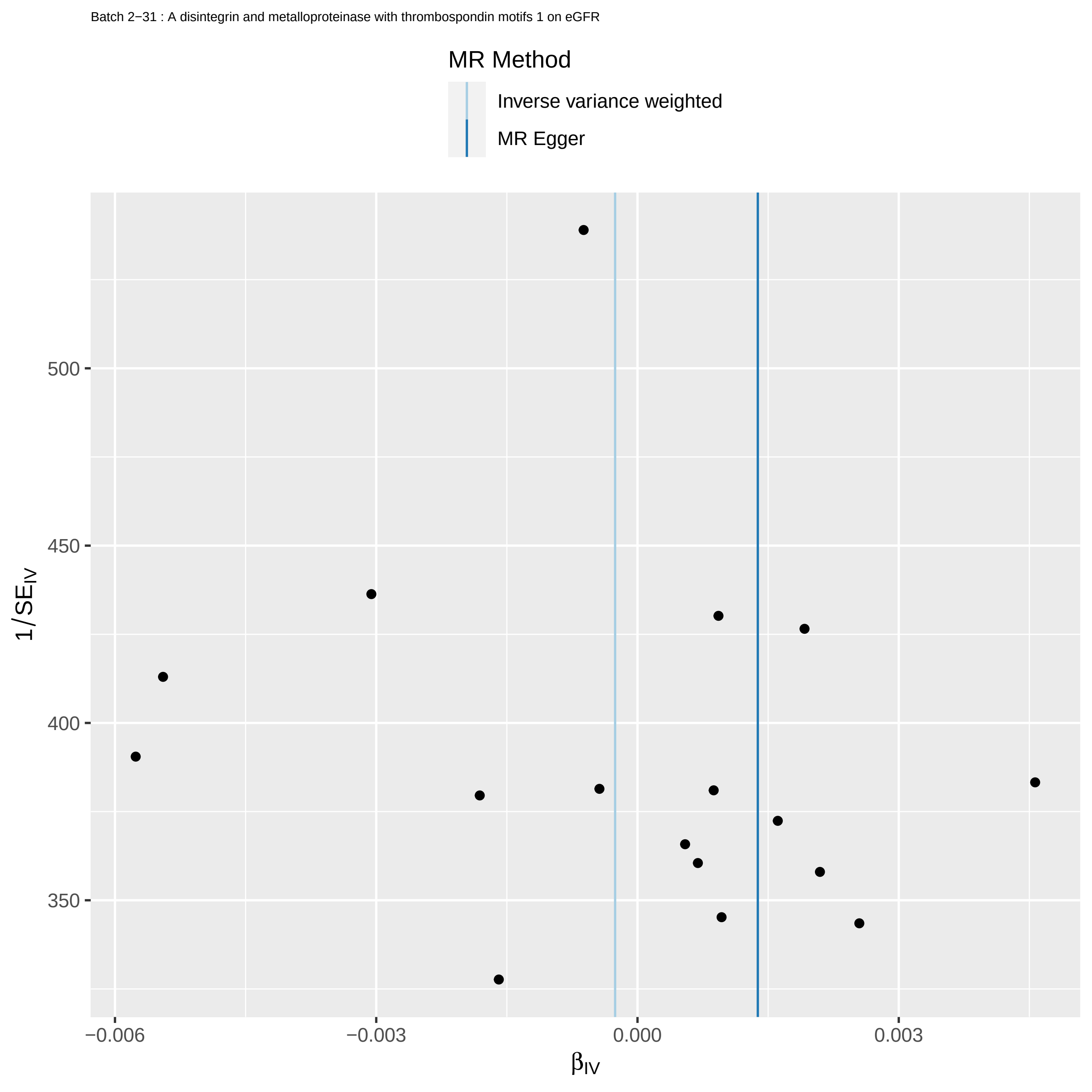
Batch 2−31 : A disintegrin and metalloproteinase with thrombospondin motifs 1 on eGFR
MR Method
Inverse variance weighted
MR Egger
500
450
400
350
1/SE_IV
−0.006
−0.003
0.000
0.003
β_IV

Batch 2−32 : A disintegrin and metalloproteinase with thrombospondin motifs 13 on eGFR

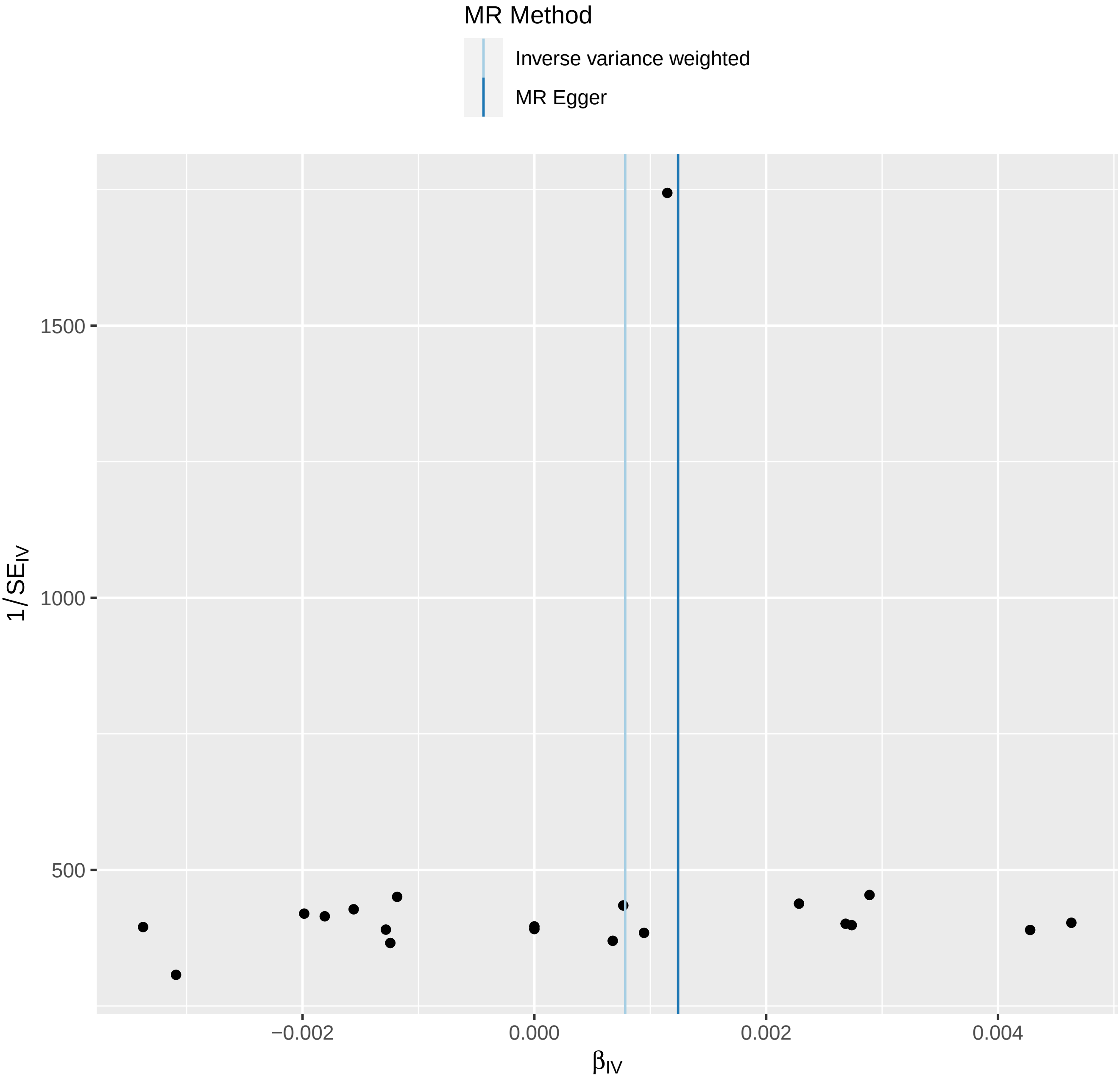

Batch 2−33 : A disintegrin and metalloproteinase with thrombospondin motifs 15 on eGFR

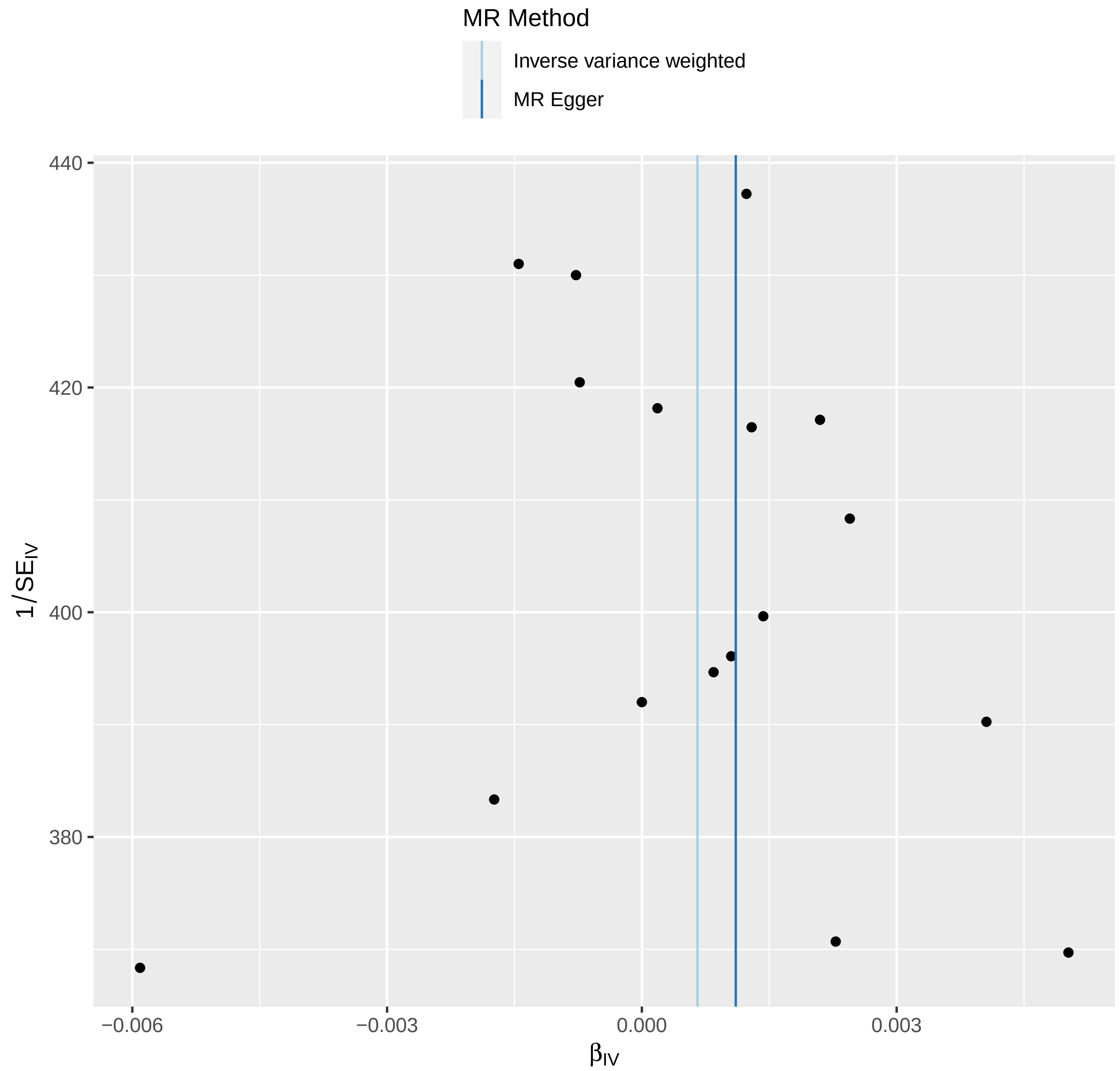

Batch 2−34 : A disintegrin and metalloproteinase with thrombospondin motifs 3 on eGFR

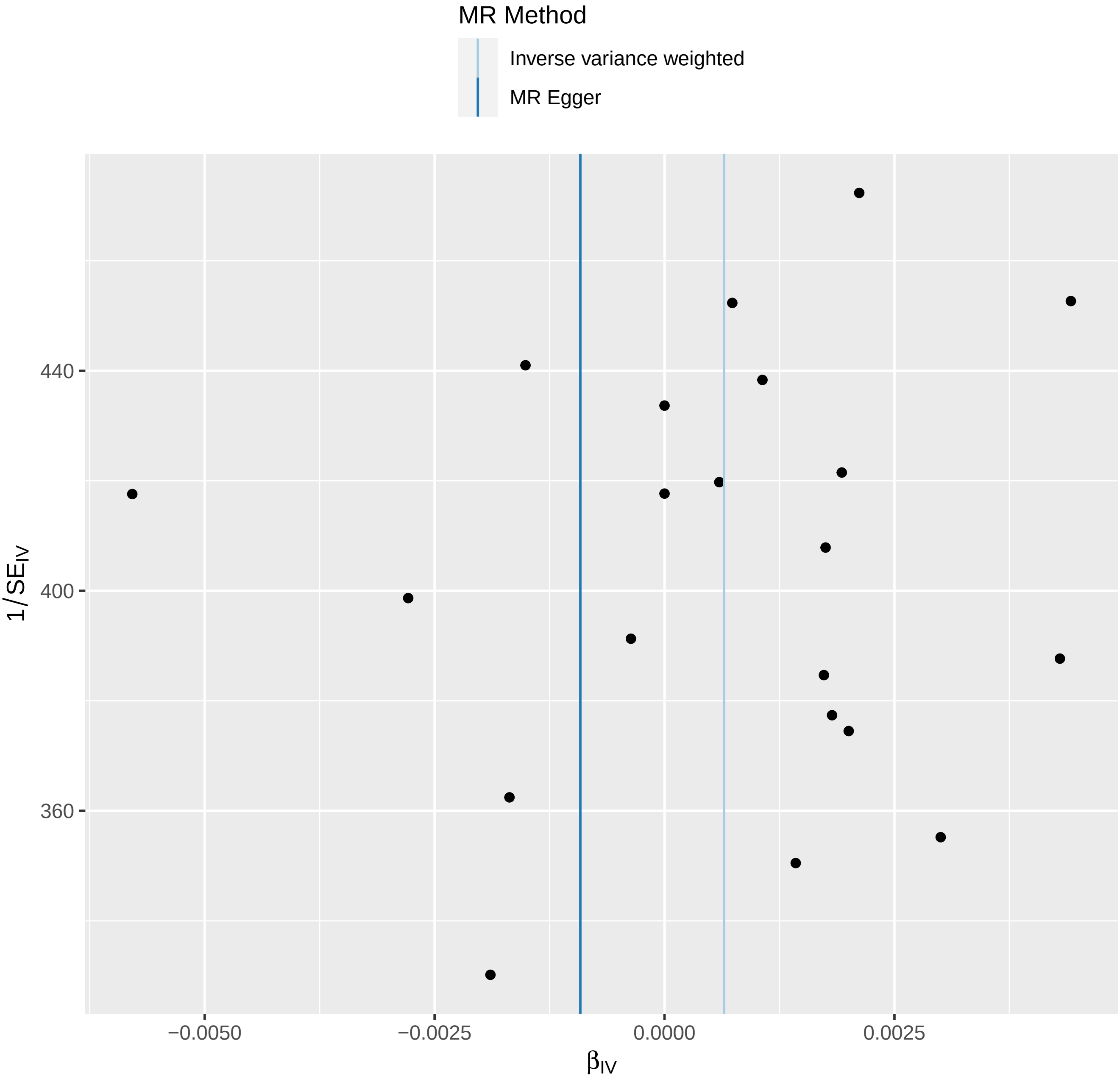

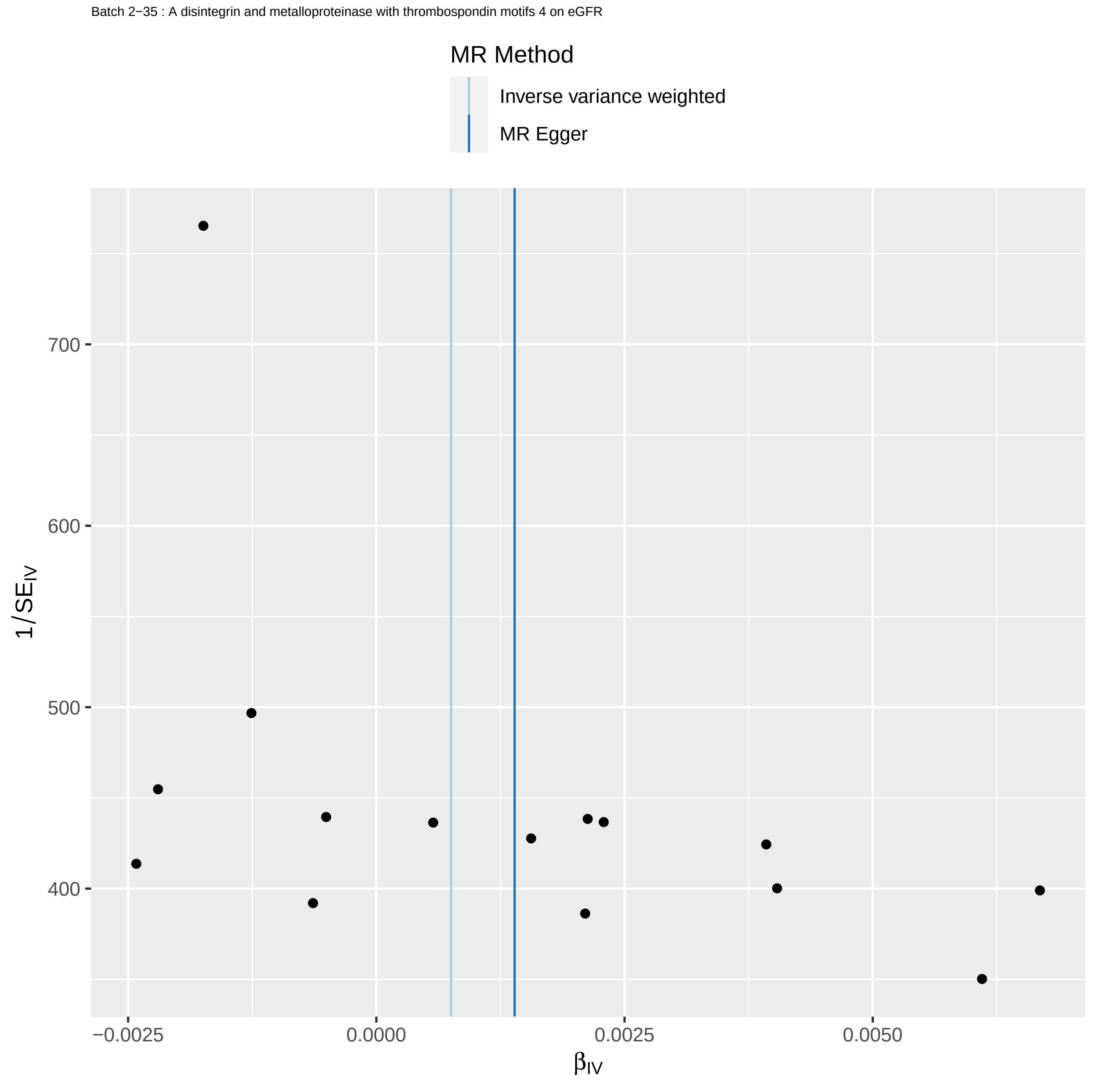

Batch 2−35 : A disintegrin and metalloproteinase with thrombospondin motifs 4 on eGFR
MR Method
Inverse variance weighted
MR Egger
700
600
500
400
1/SE$_{IV}$
−0.0025
0.0000
0.0025
0.0050
β$_{IV}$

Batch 2−36 : A disintegrin and metalloproteinase with thrombospondin motifs 5 on eGFR

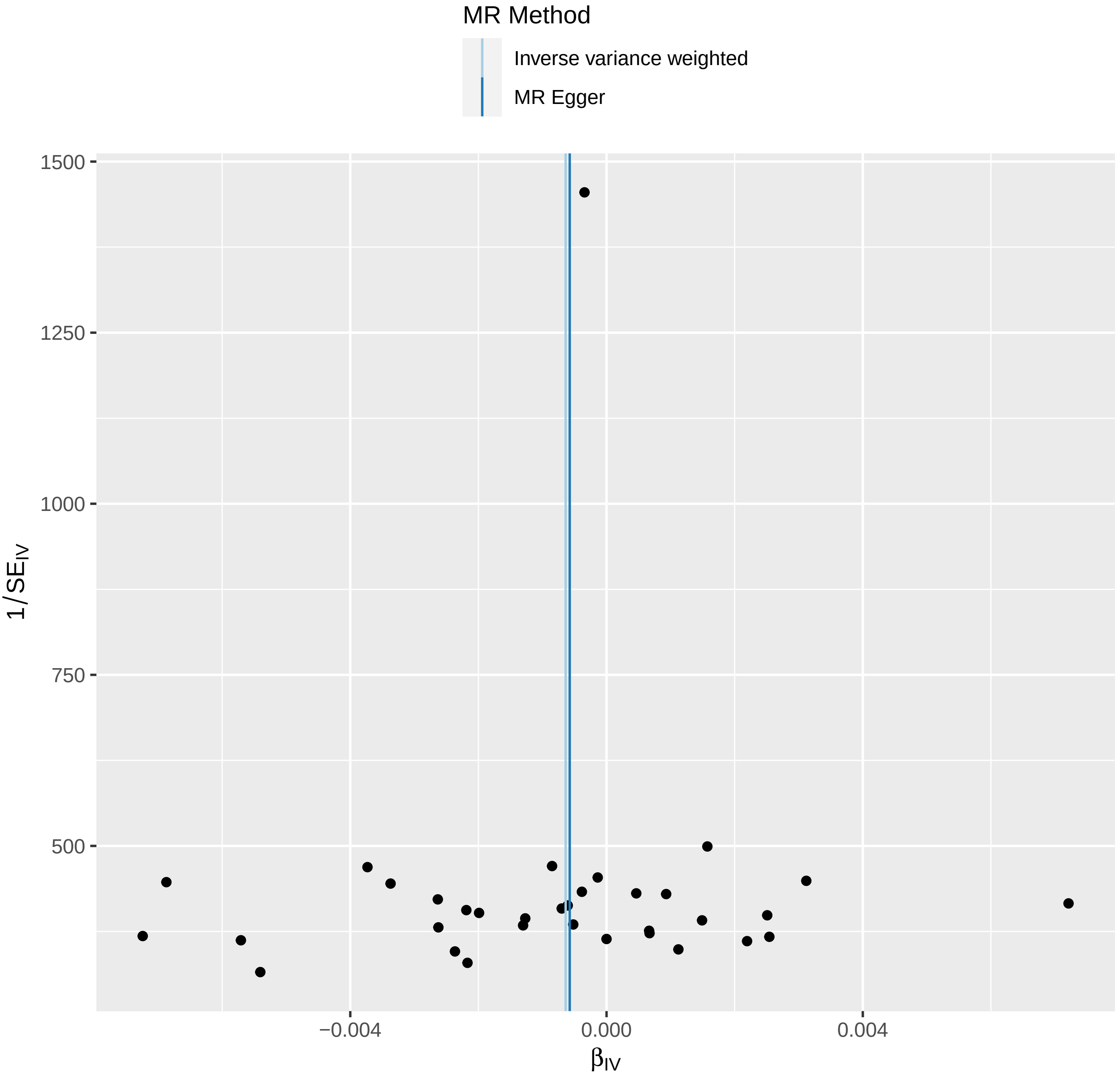

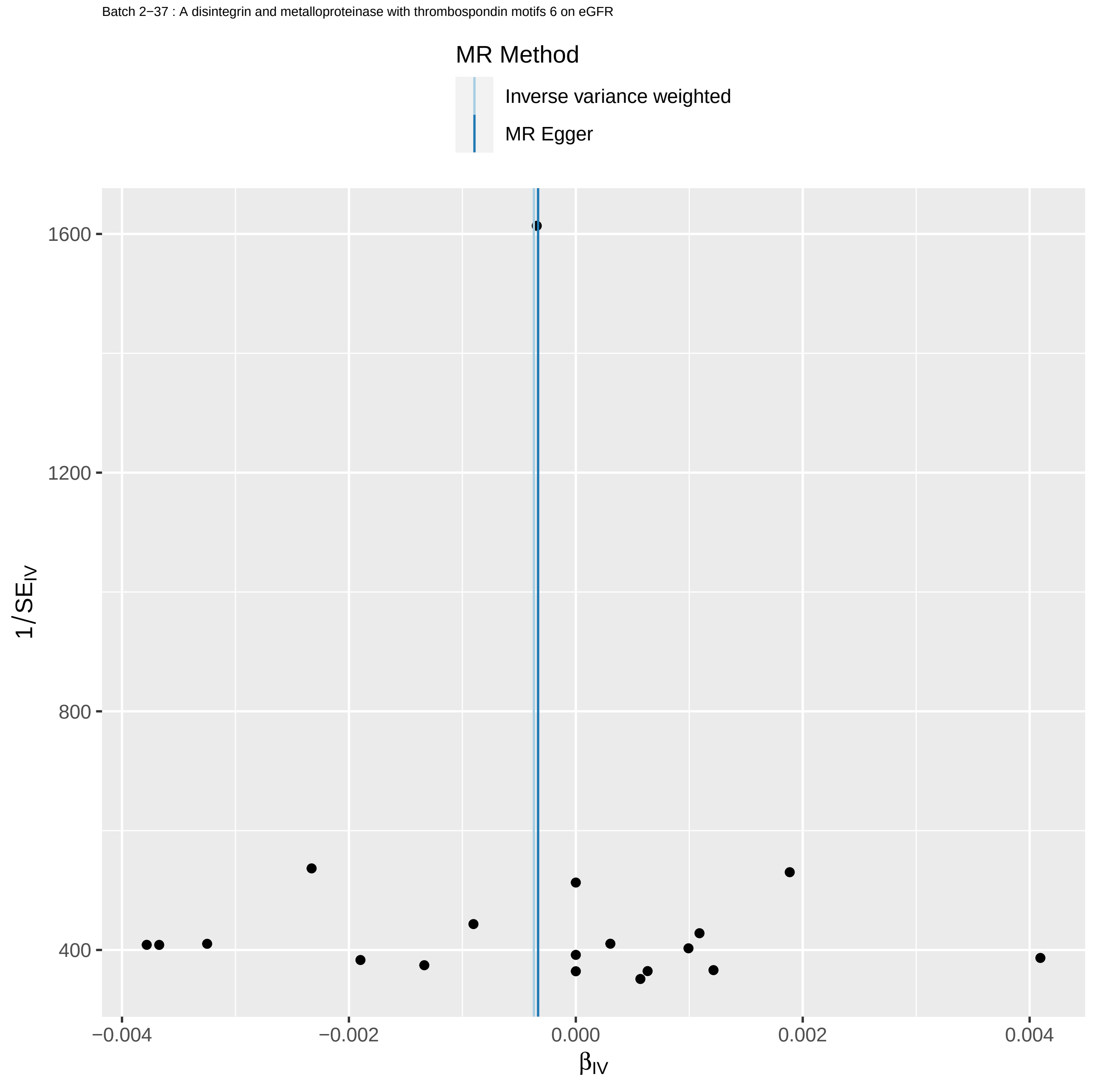

Batch 2−37 : A disintegrin and metalloproteinase with thrombospondin motifs 6 on eGFR
MR Method
Inverse variance weighted
MR Egger
1600
1200
800
400
1/SE_IV
−0.004
−0.002
0.000
0.002
0.004
β_IV

Batch 2−38 : ADAMTS−like protein 1 on eGFR

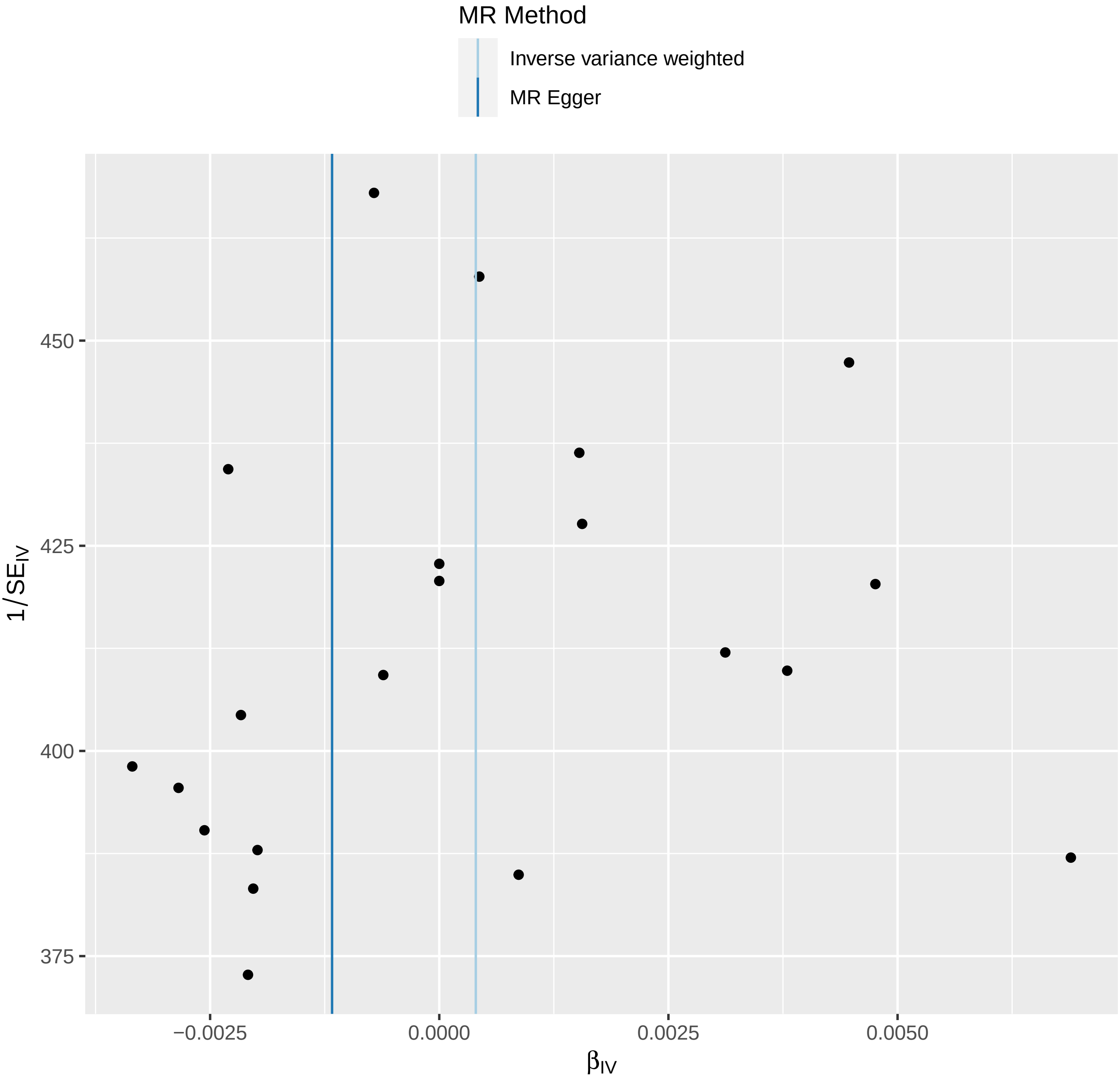

Batch 2−39 : AarF domain−containing protein kinase 4 on eGFR

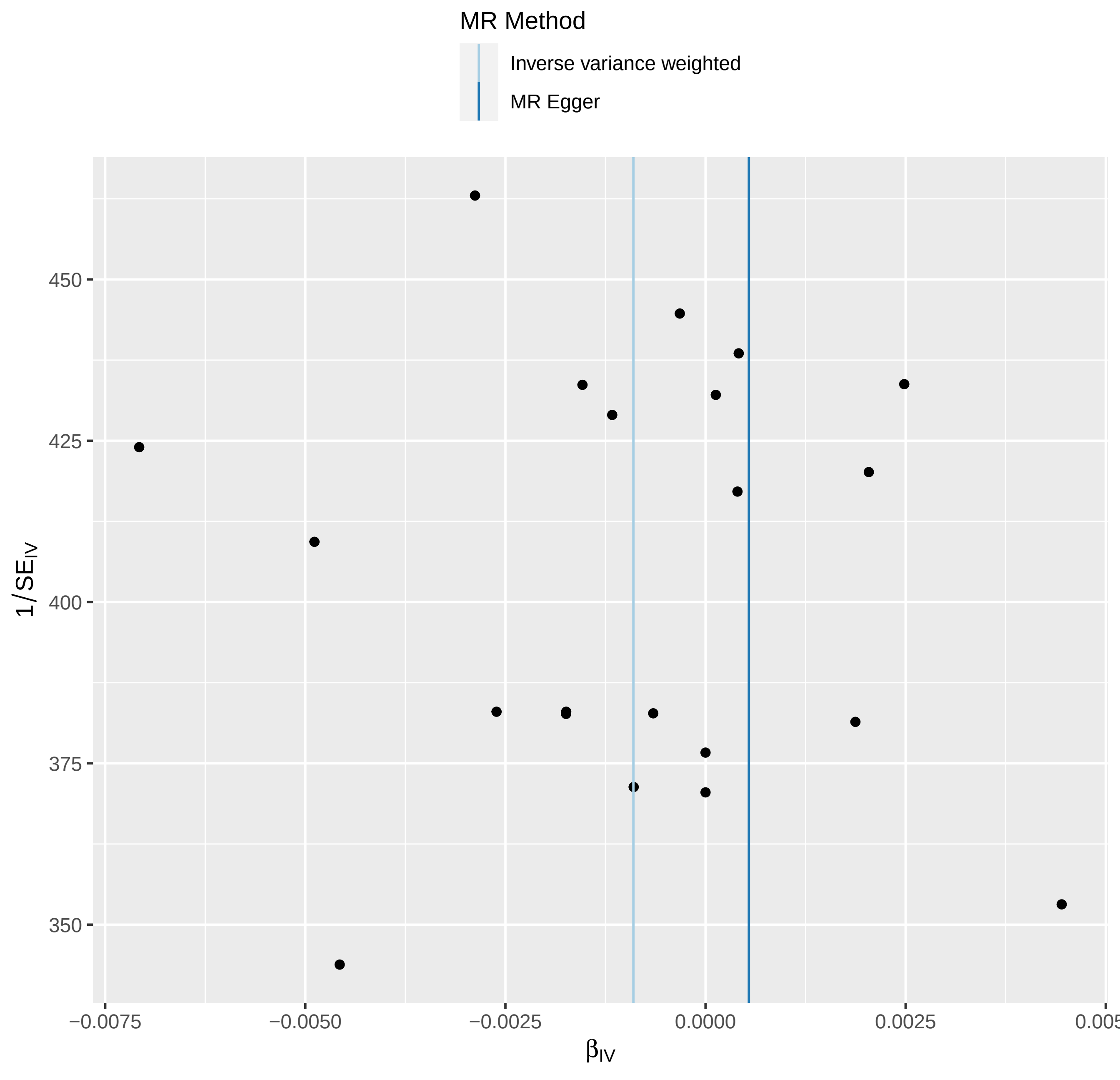

Batch 2−40 : Pituitary adenylate cyclase−activating polypeptide 27 on eGFR

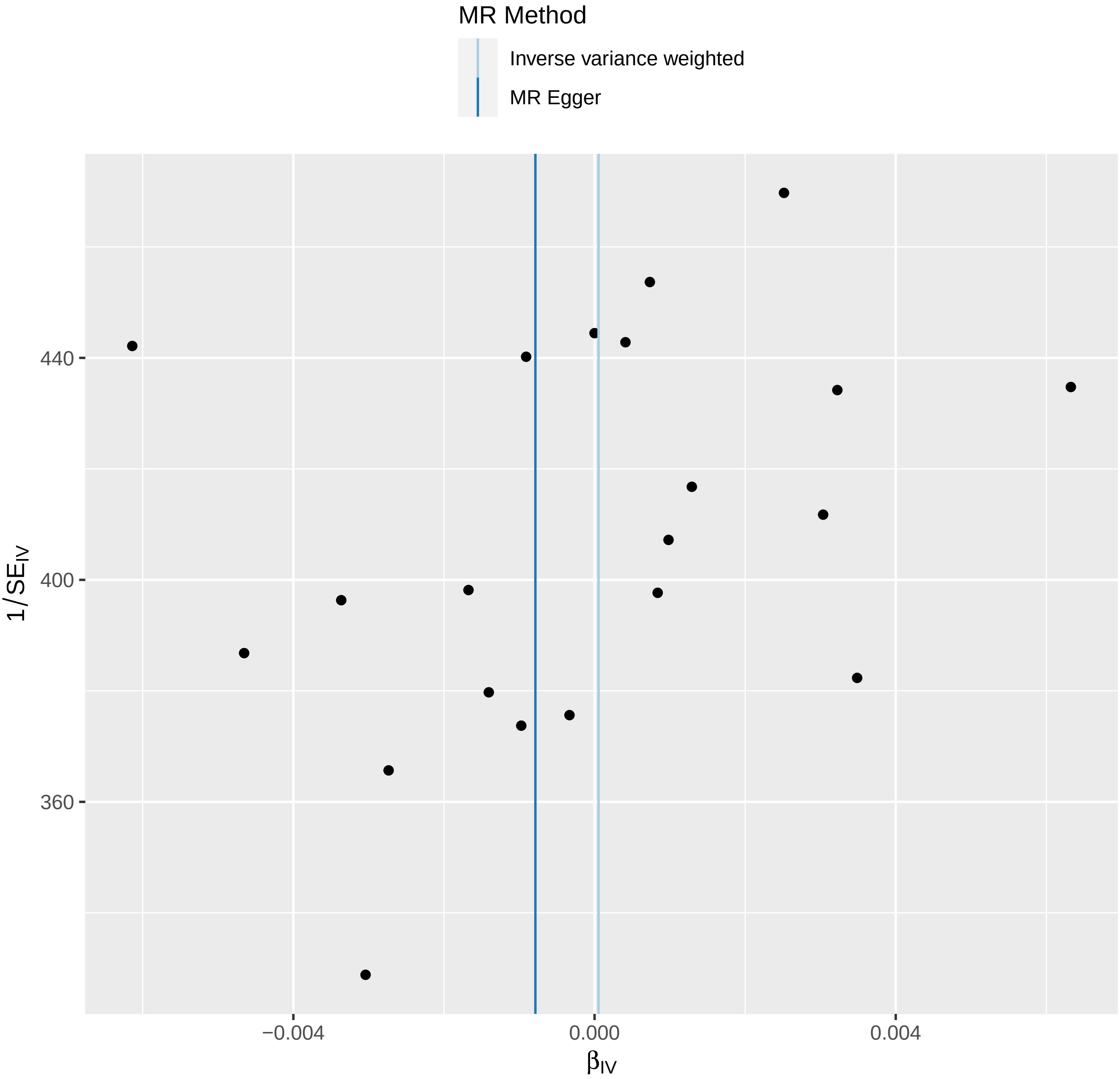

Batch 2−41 : Pituitary adenylate cyclase−activating polypeptide 38 on eGFR

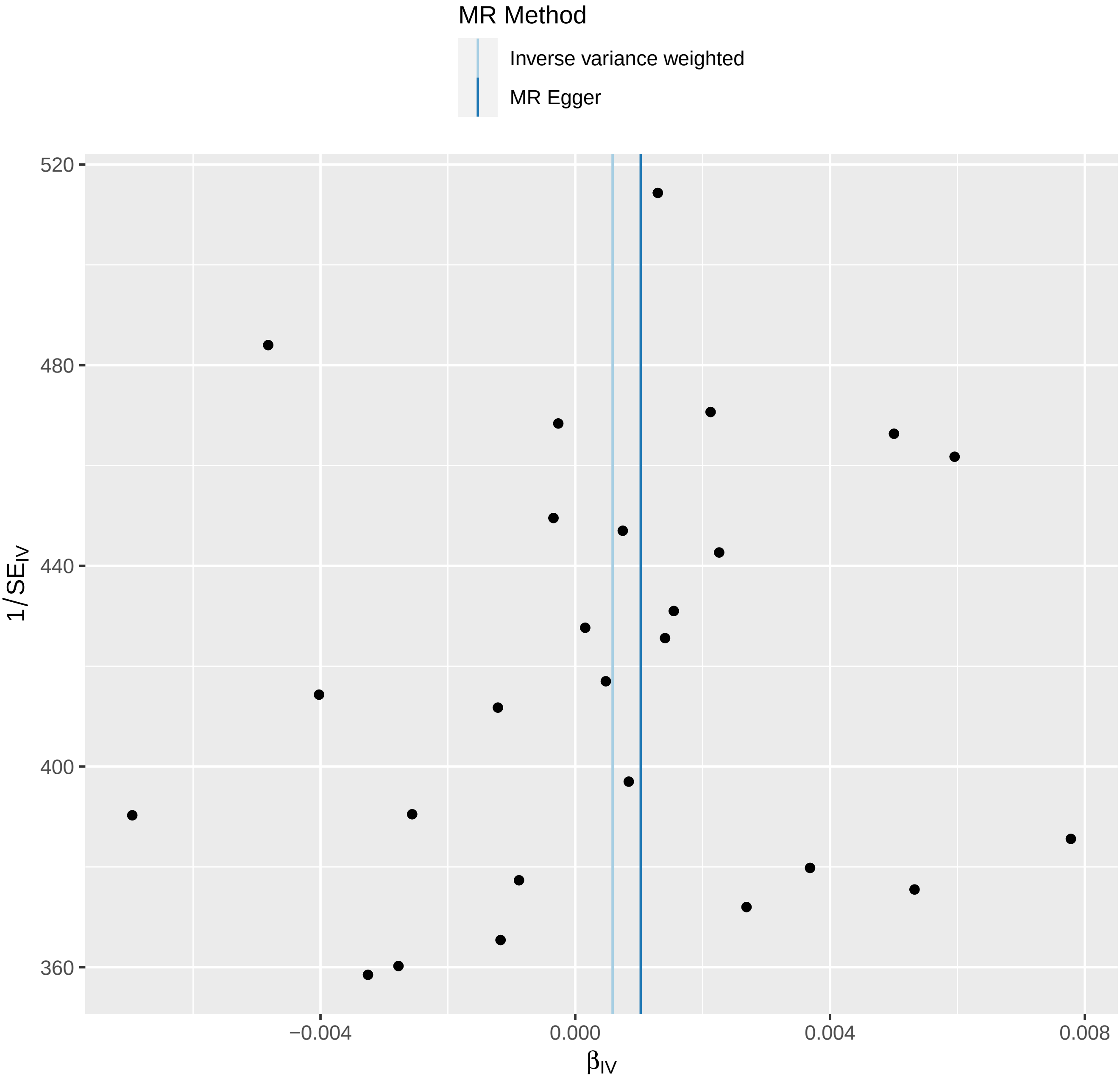

Batch 2−42 : Pituitary adenylate cyclase−activating polypeptide on eGFR

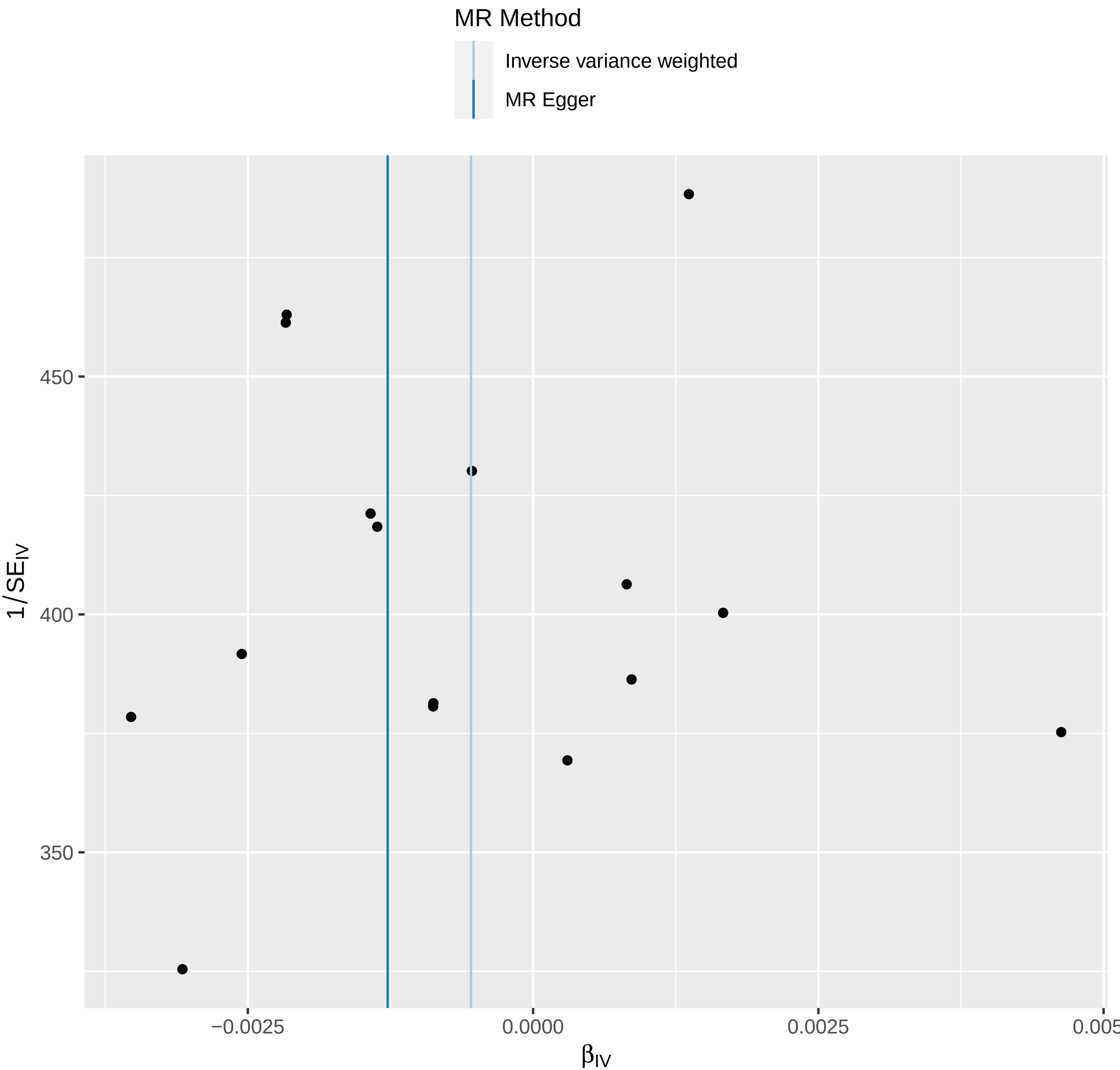

Batch 2−43 : Adhesion G−protein coupled receptor F1 on eGFR

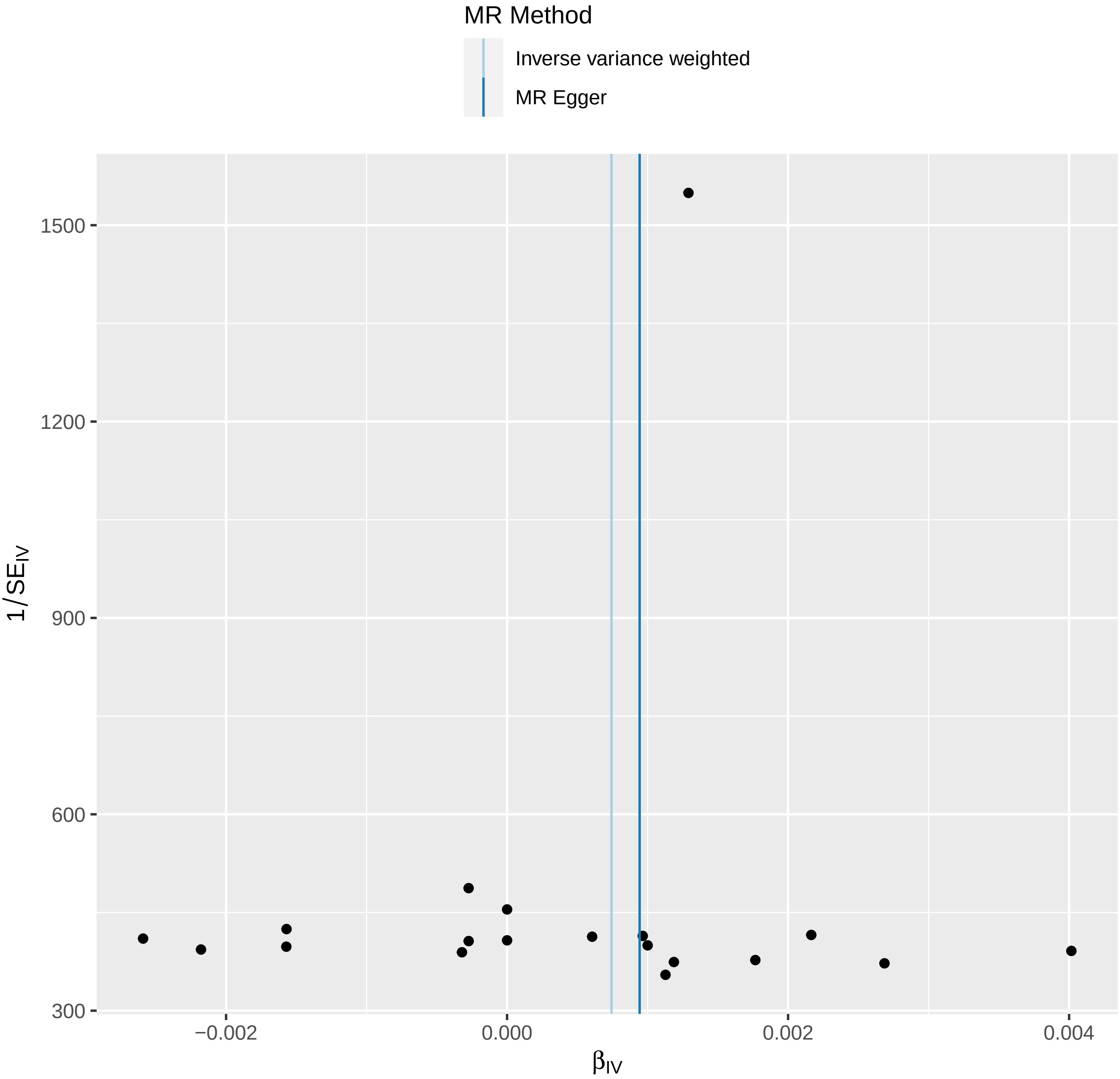

Batch 2−44 : Adhesion G protein−coupled receptor F5 on eGFR

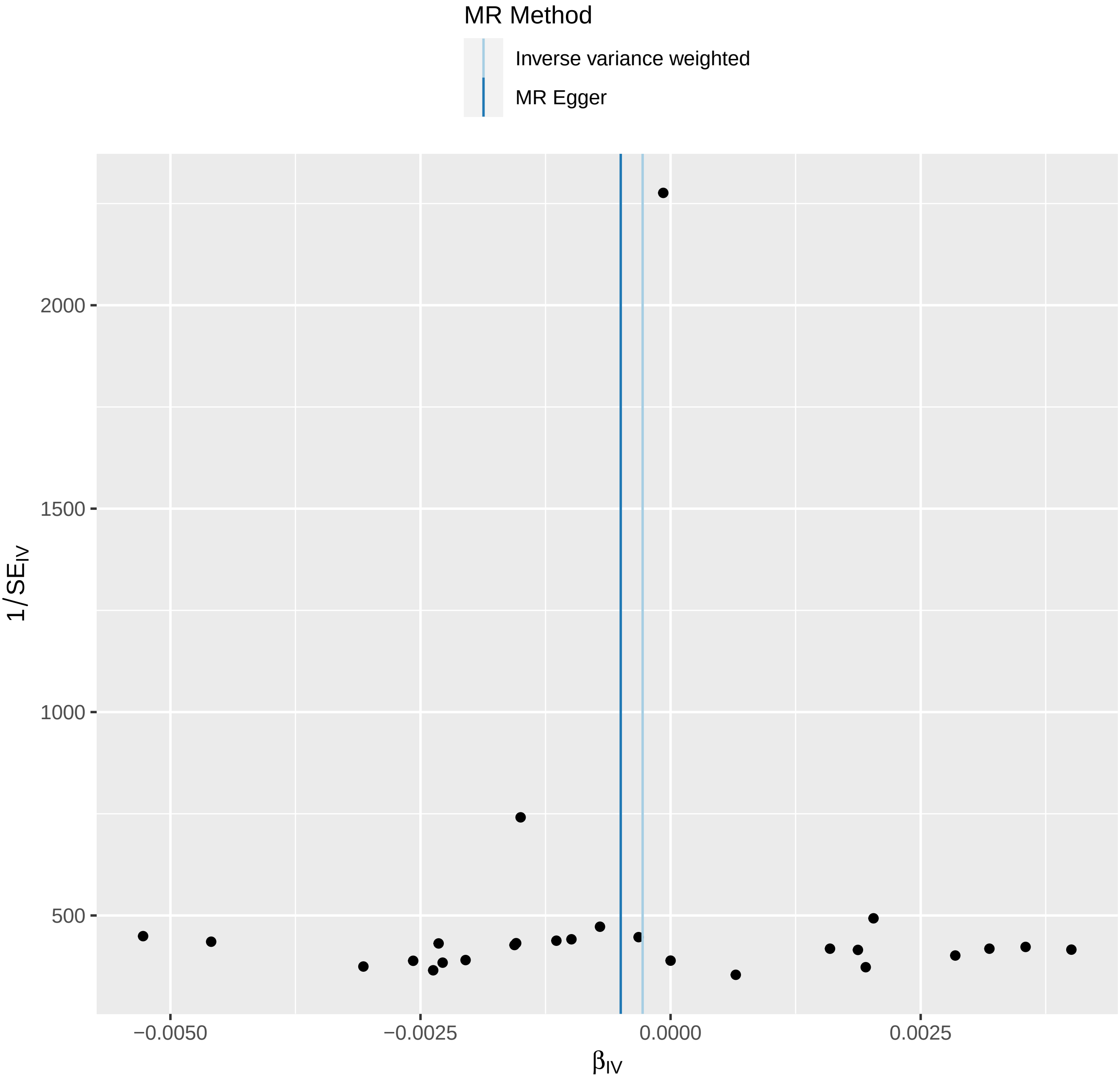

Batch 2−45 : Adhesion G−protein coupled receptor G5 on eGFR

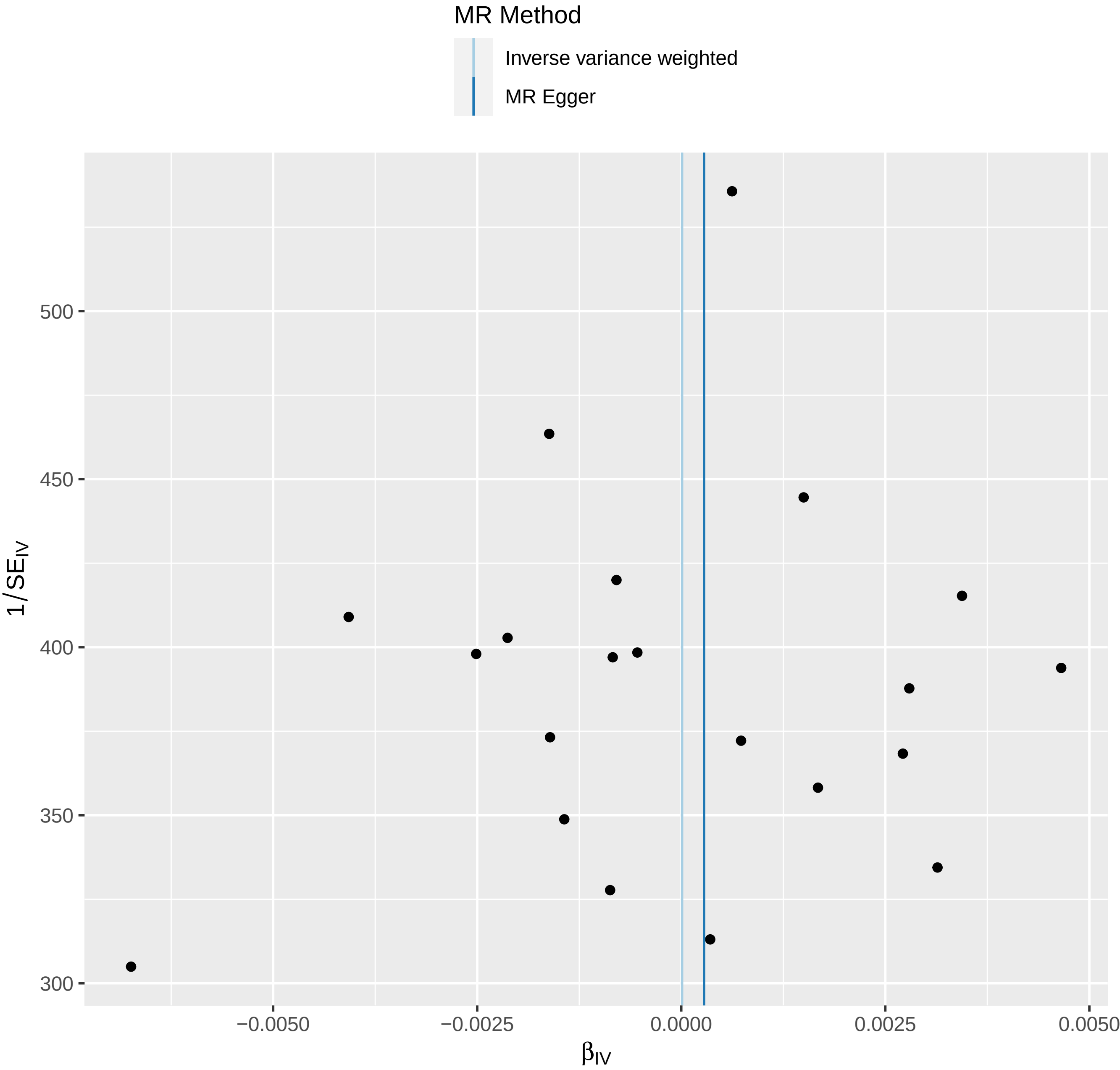

Batch 2−46 : Alcohol dehydrogenase 1B on eGFR

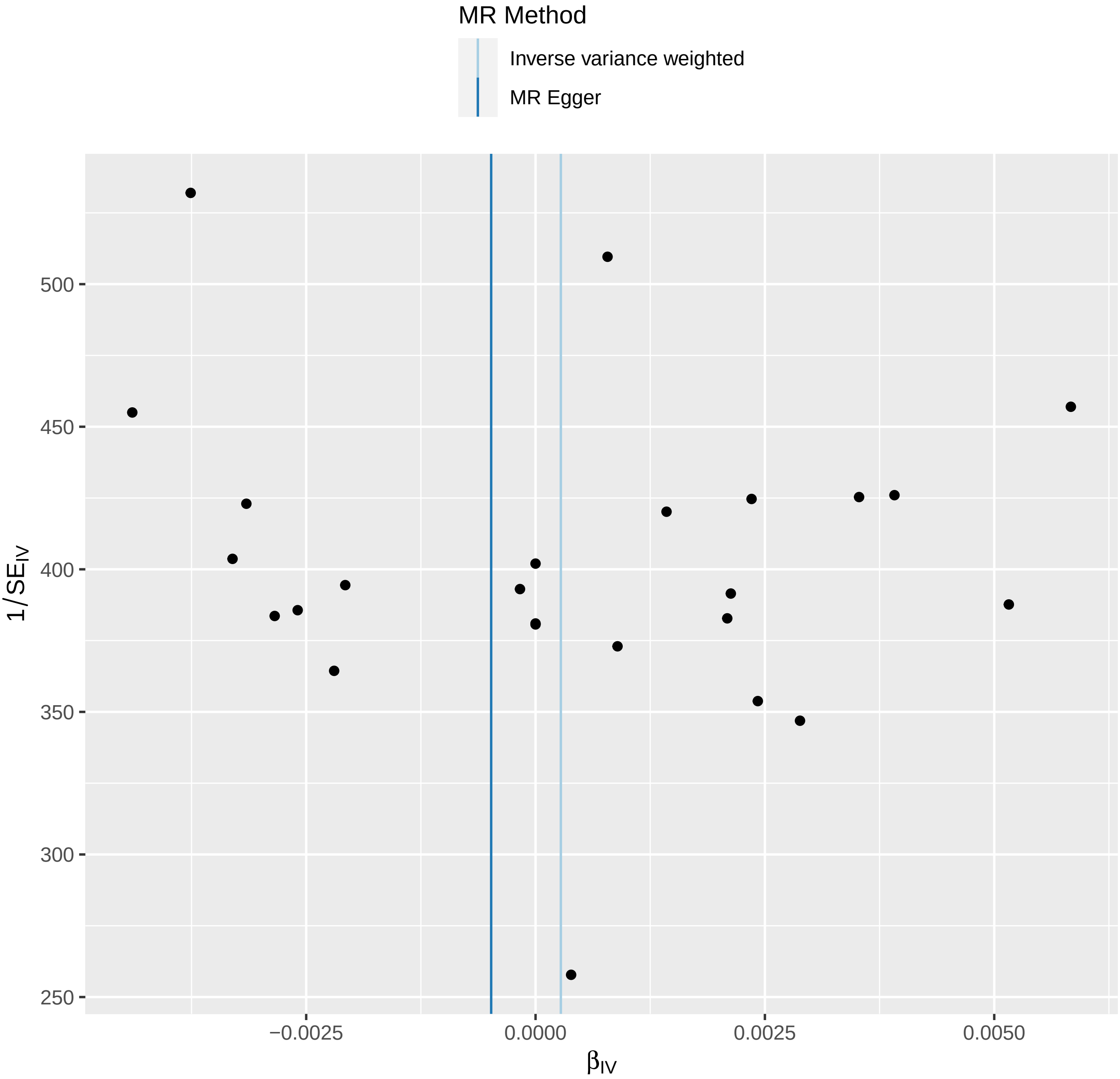

Batch 2−47 : Alcohol dehydrogenase 4 on eGFR

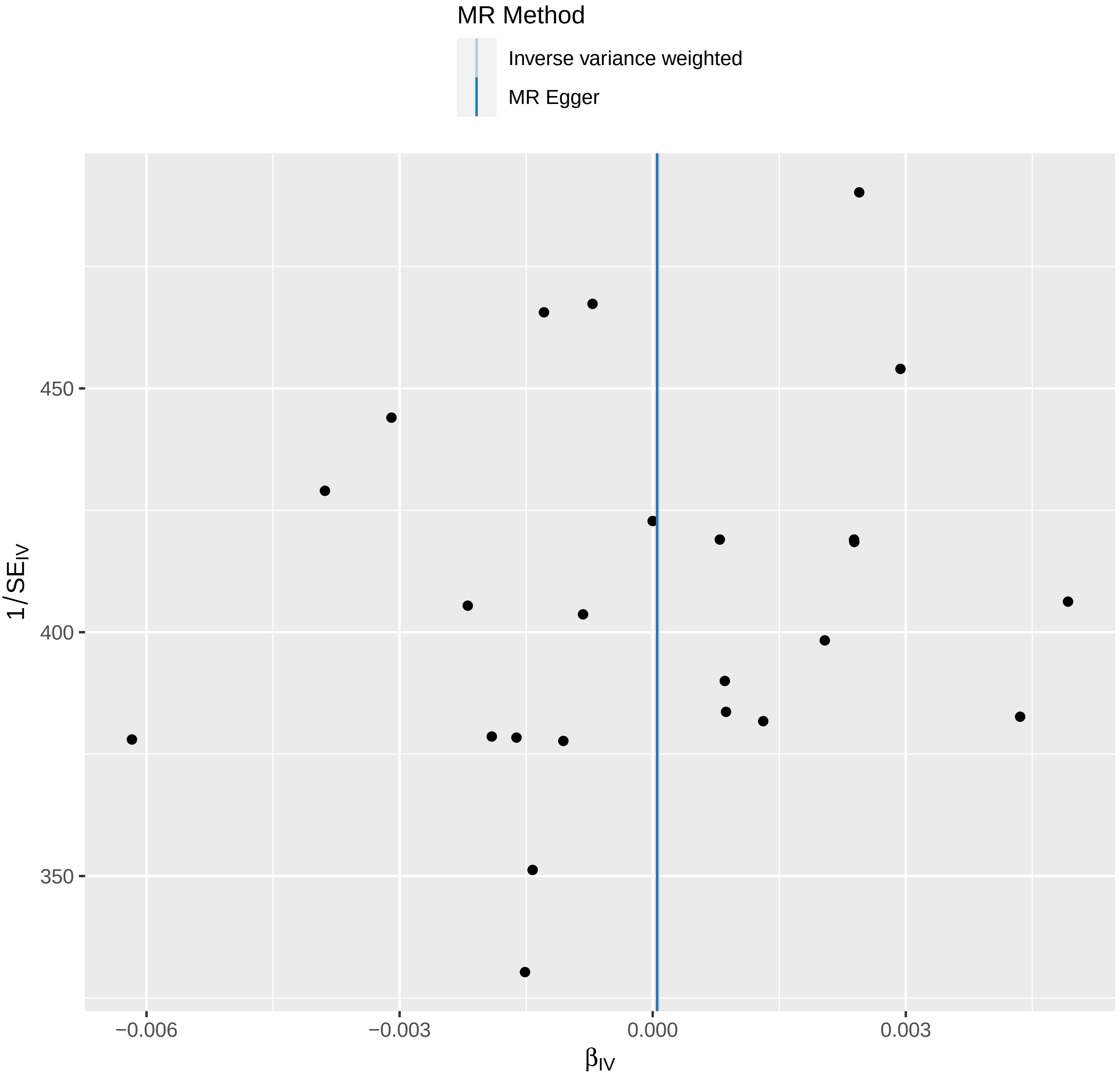

Batch 2−48 : Adrenomedullin on eGFR

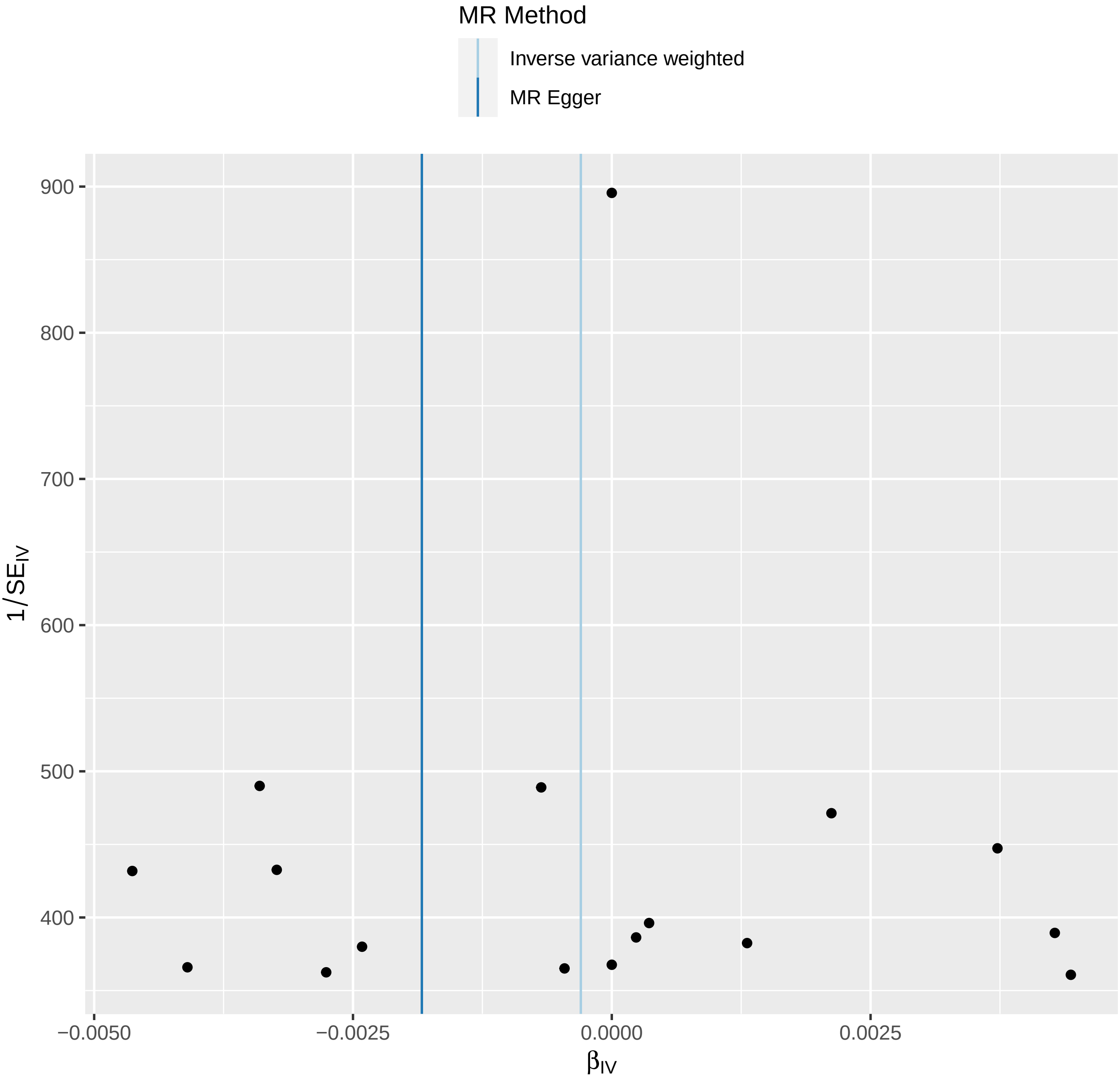

Batch 2−49 : ADP−dependent glucokinase on eGFR

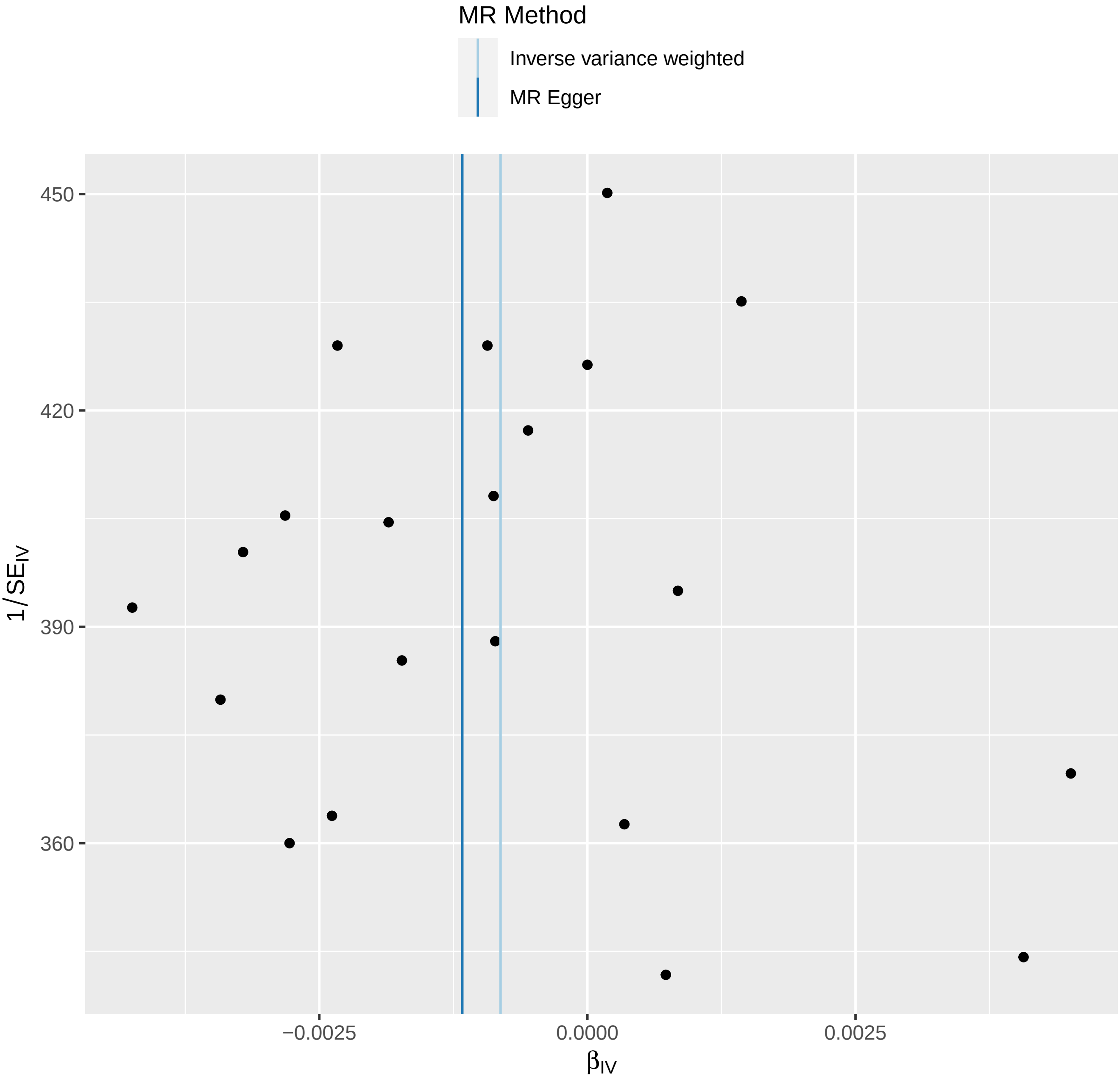

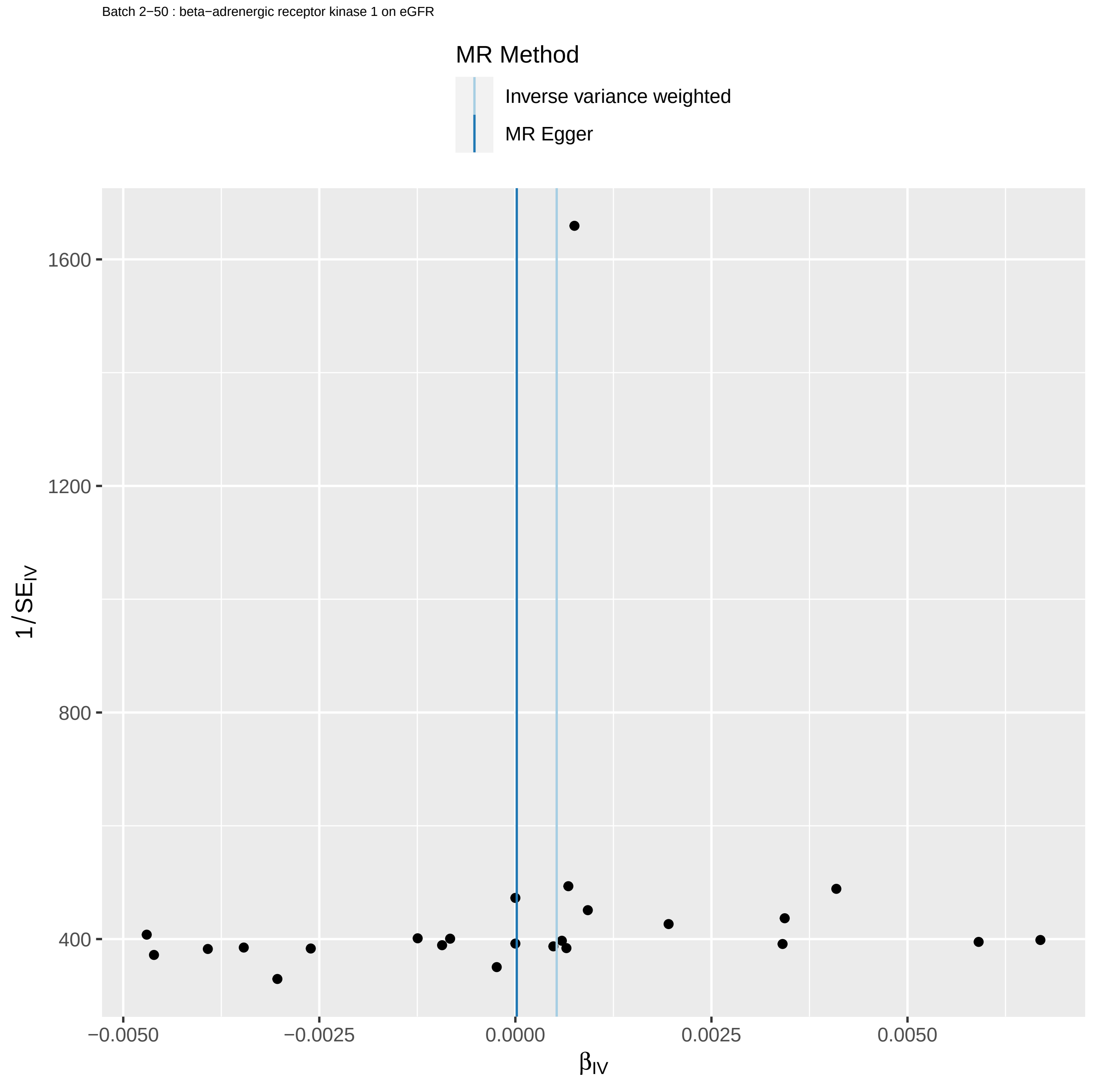
Batch 2−50 : beta−adrenergic receptor kinase 1 on eGFR
MR Method
Inverse variance weighted
MR Egger
1600
1200
800
400
1/SE_IV
−0.0050
−0.0025
0.0000
0.0025
0.0050
β_IV

Batch 2−51 : Adenylosuccinate synthetase isozyme 2 on eGFR

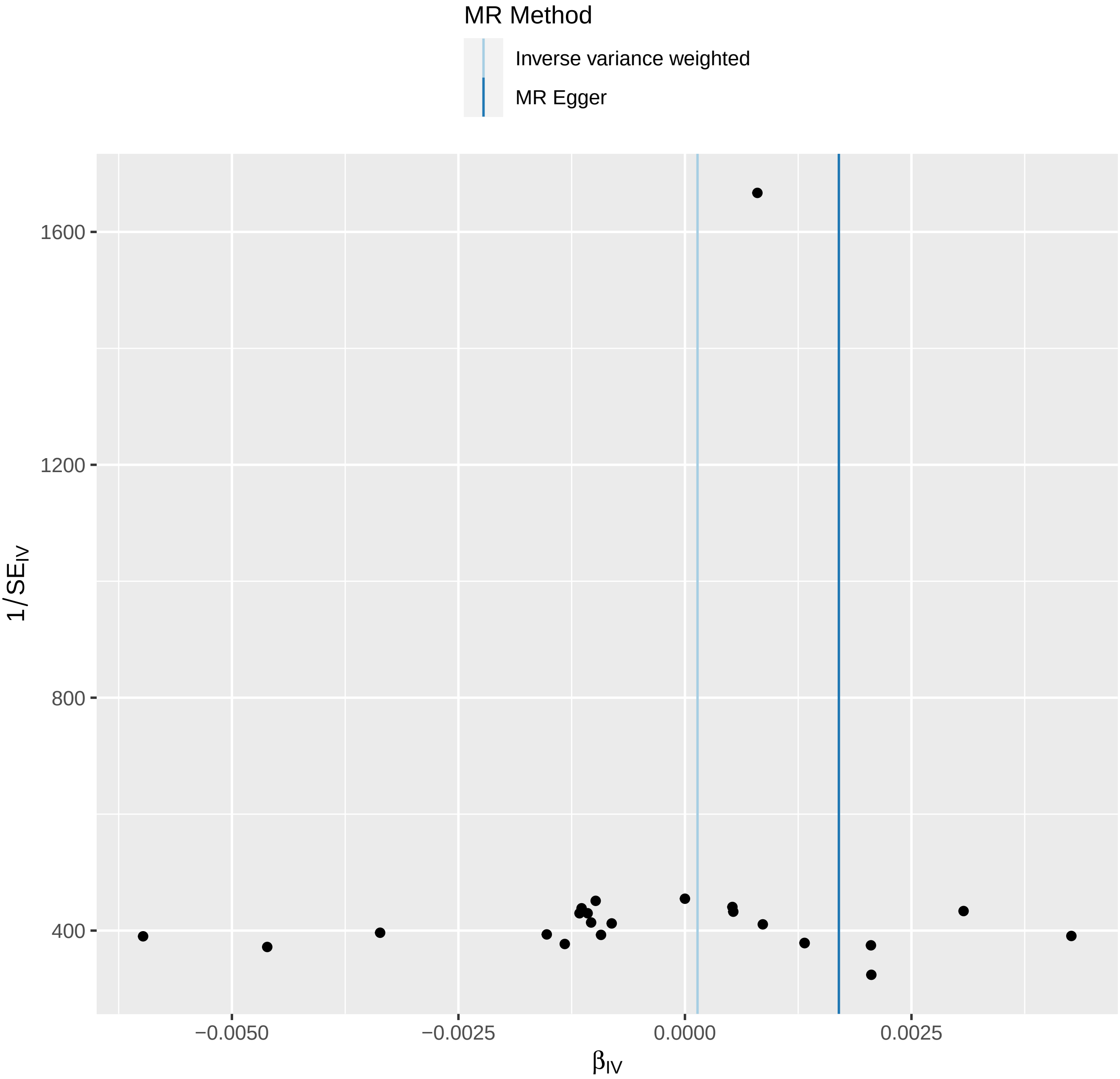

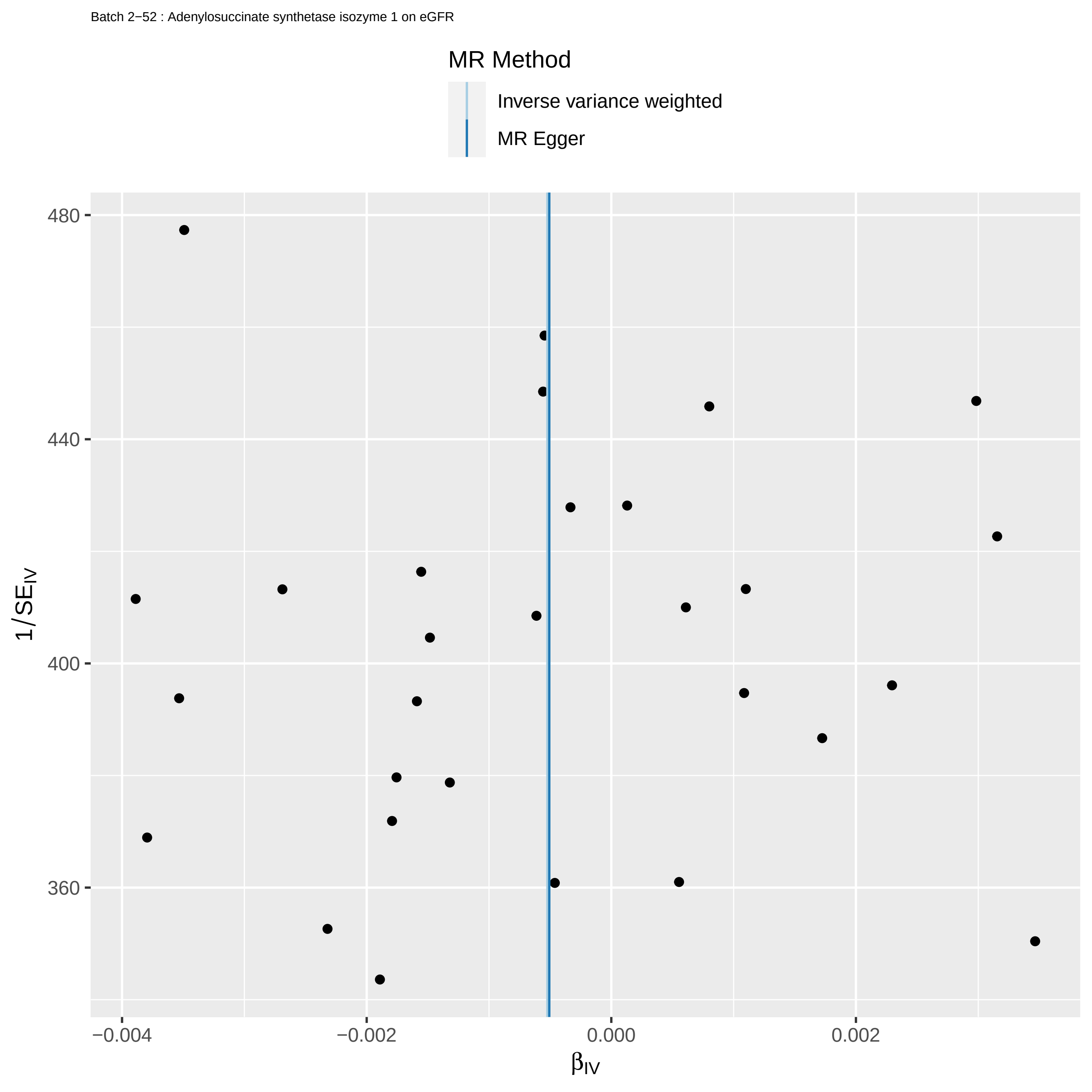

Batch 2−52 : Adenylosuccinate synthetase isozyme 1 on eGFR
MR Method
Inverse variance weighted
MR Egger
480
440
400
360
1/SE$_{IV}$
−0.004
−0.002
0.000
0.002
β$_{IV}$

Batch 2−53 : alpha−Fetoprotein on eGFR

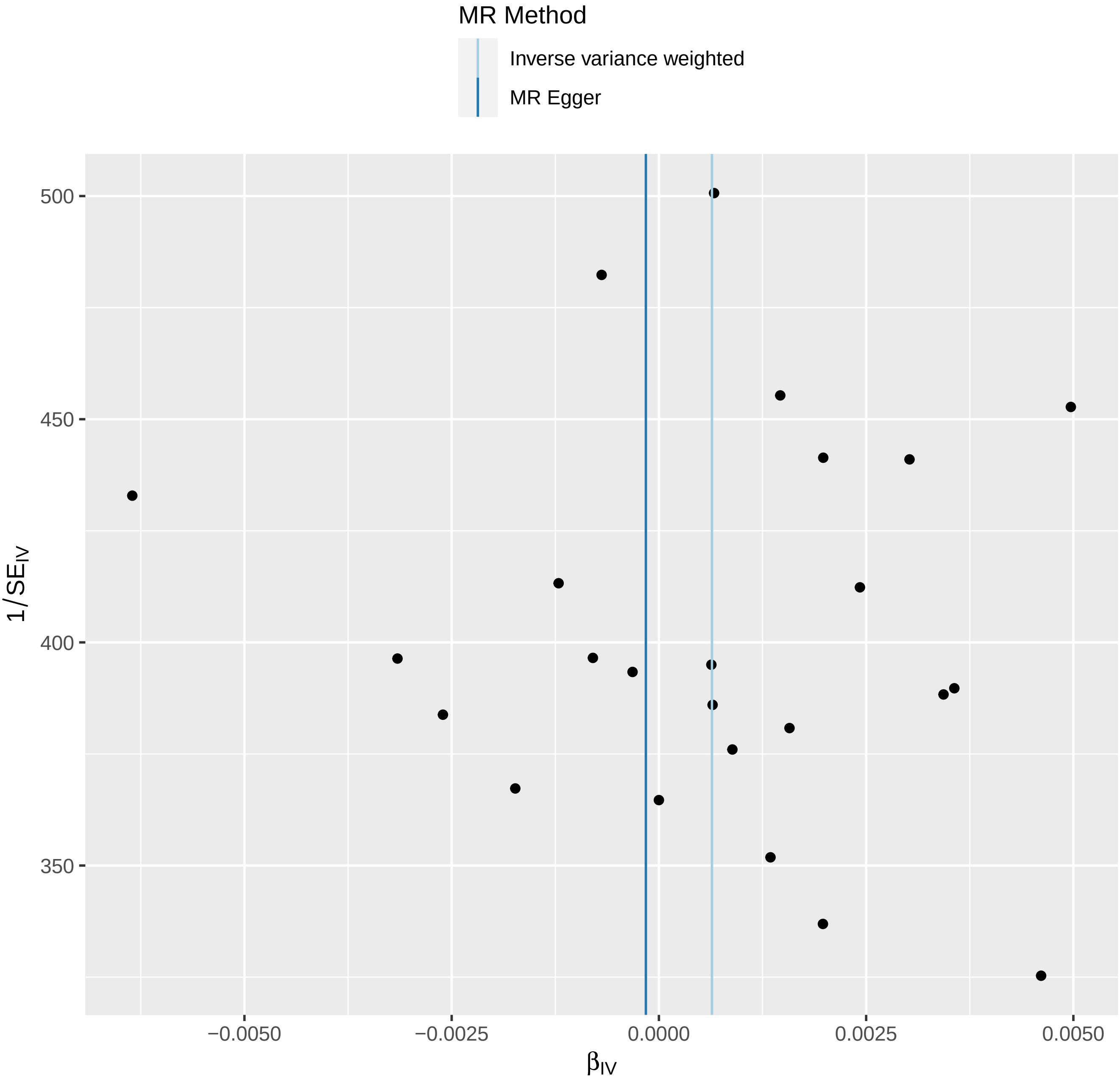

Batch 2−54 : Advanced glycosylation end product−specific receptor, soluble on eGFR

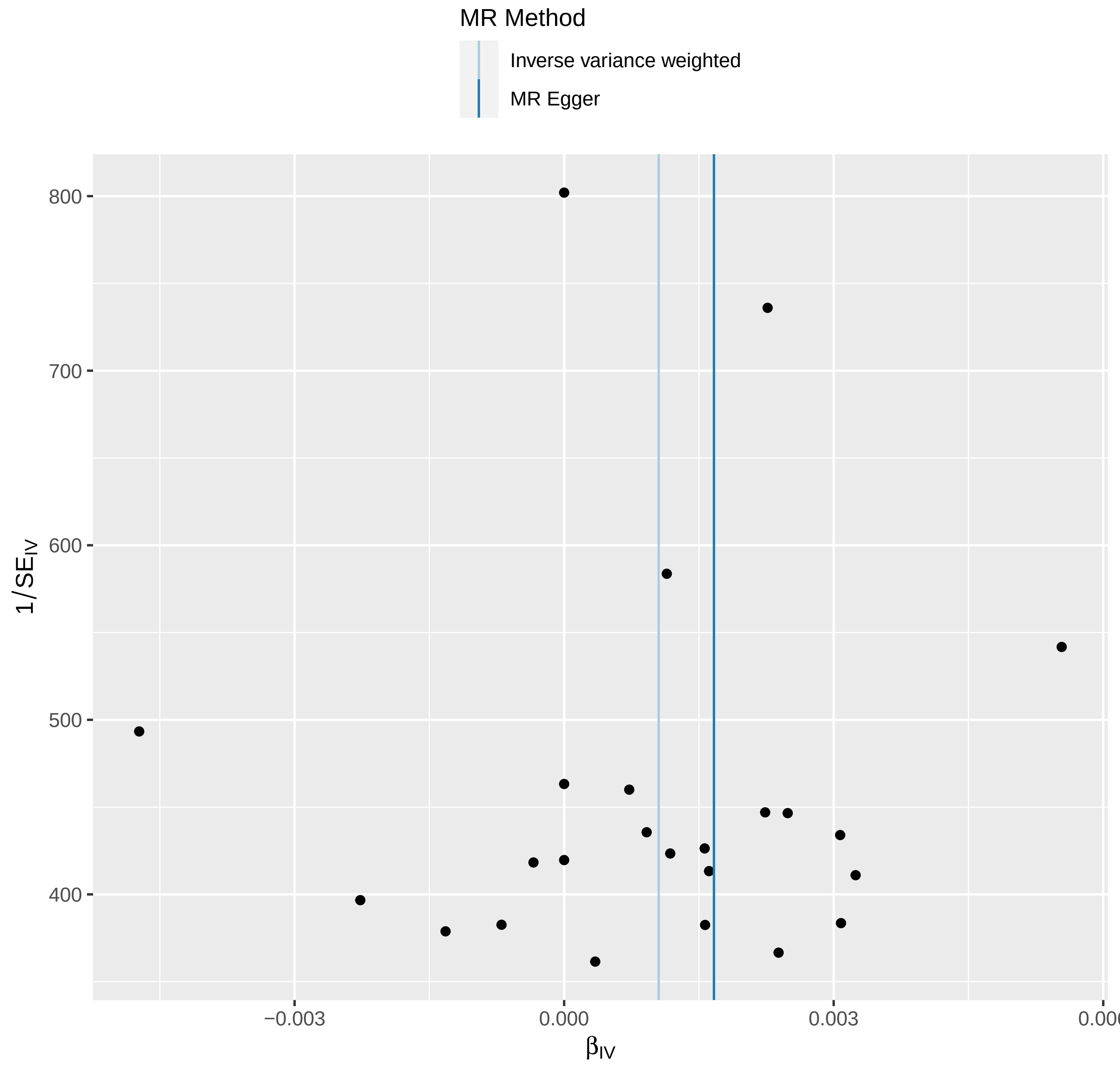

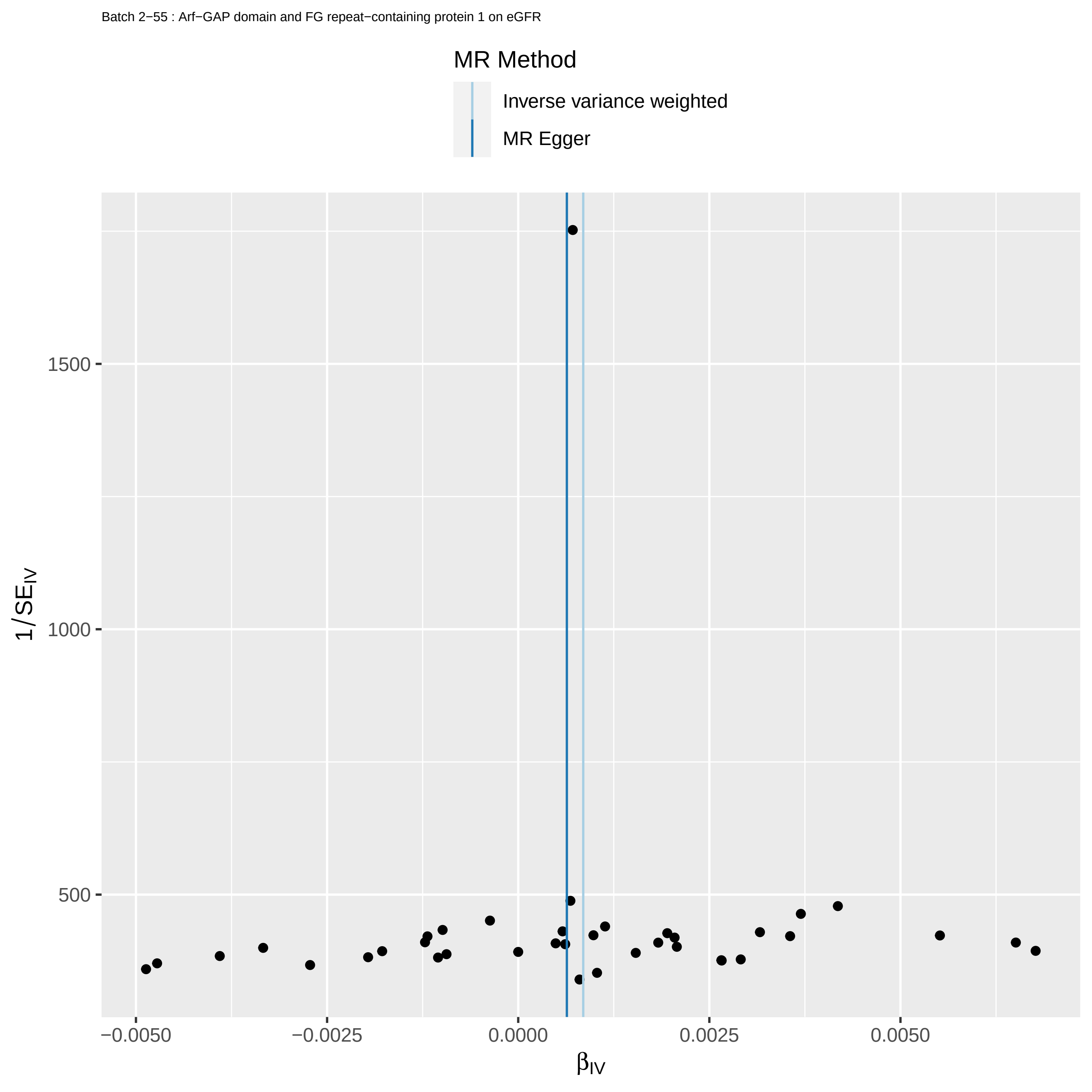
Batch 2−55 : Arf−GAP domain and FG repeat−containing protein 1 on eGFR
MR Method
Inverse variance weighted
MR Egger
1500
1000
500
1/SE_IV
−0.0050
−0.0025
0.0000
0.0025
0.0050
β_IV

Batch 2−56 : Angiogenic factor with G patch and FHA domains 1 on eGFR

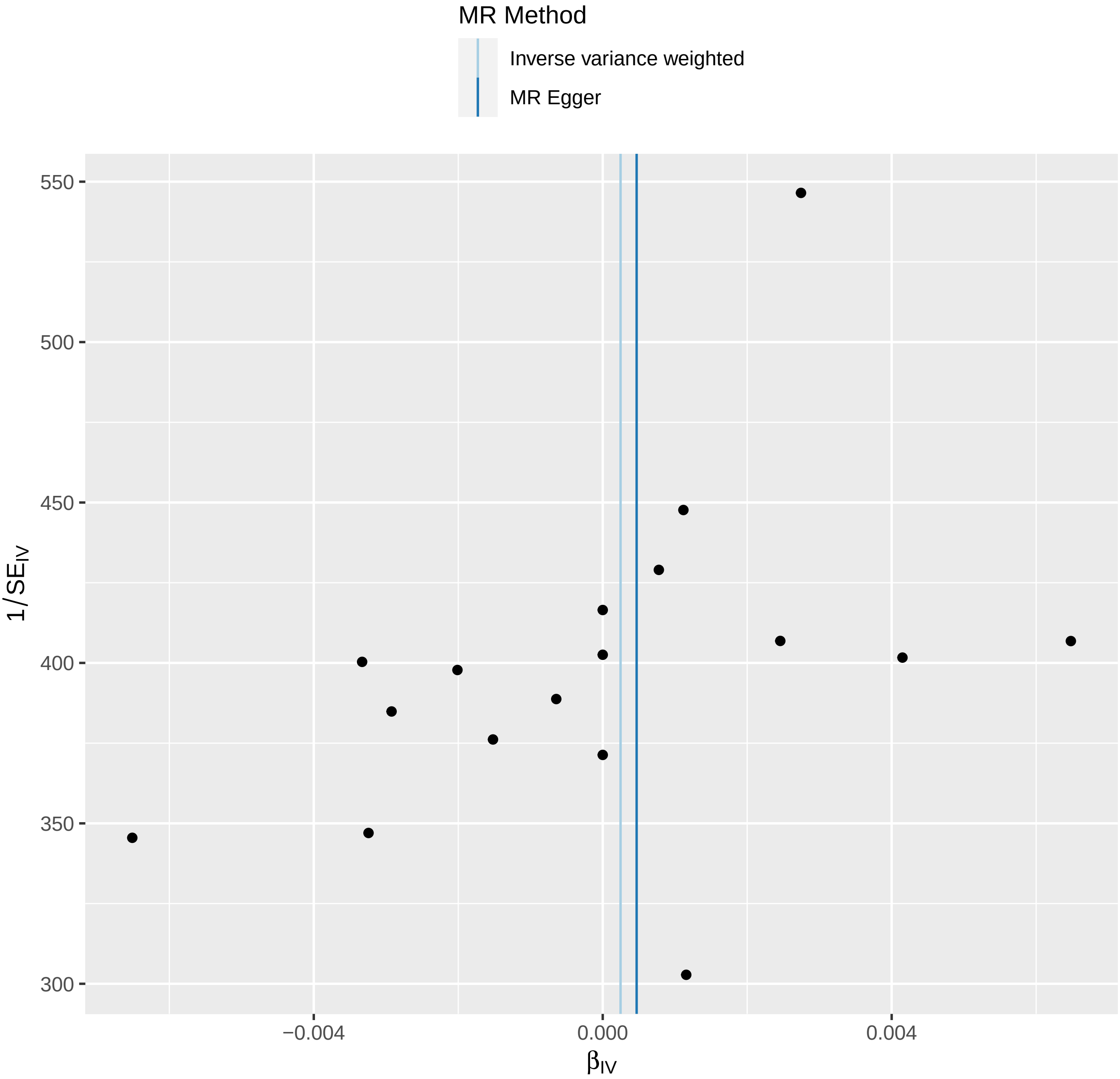

Batch 2−57 : Protein argonaute−1 on eGFR

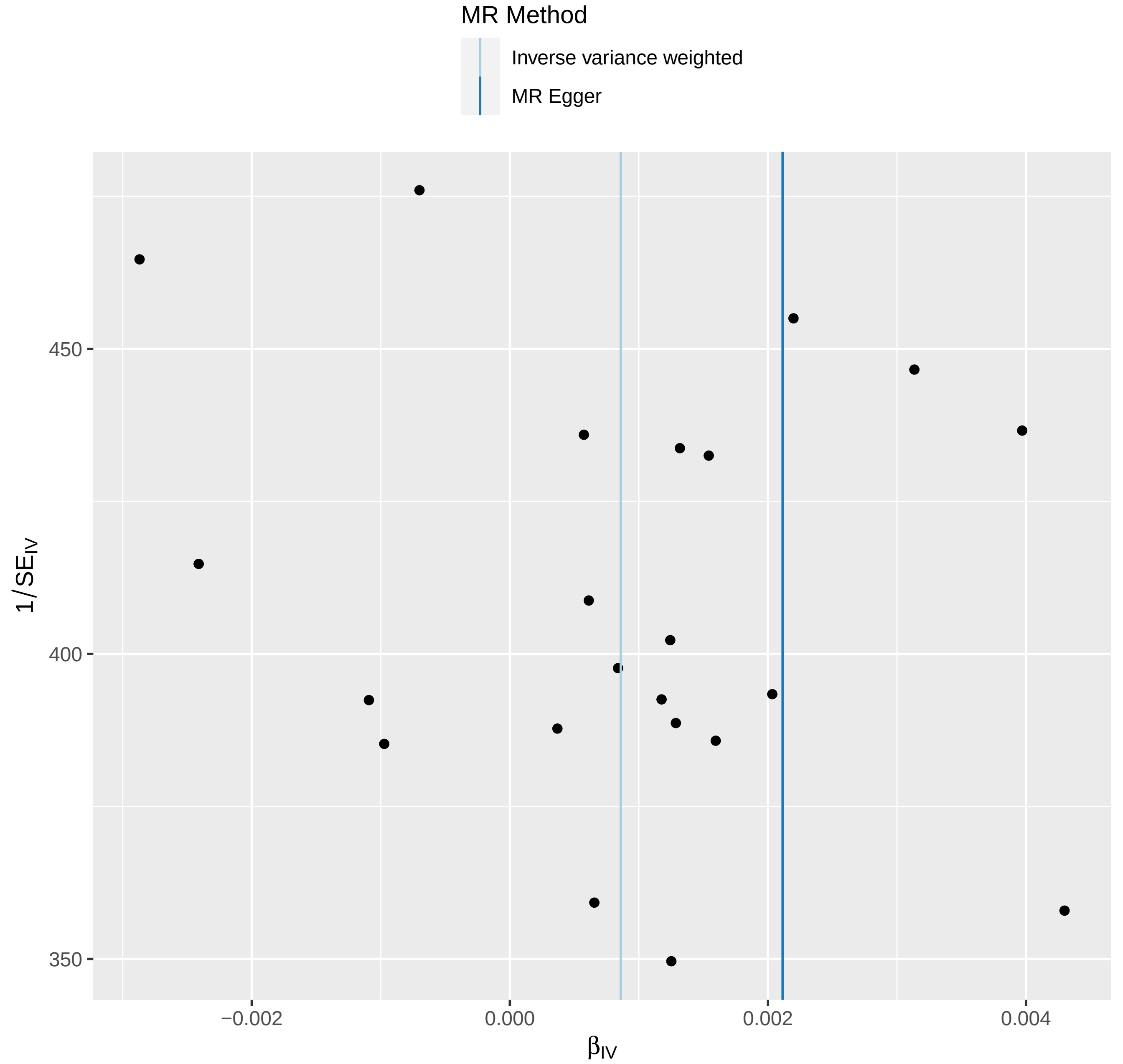

Batch 2−58 : Anterior gradient protein 2 homolog on eGFR

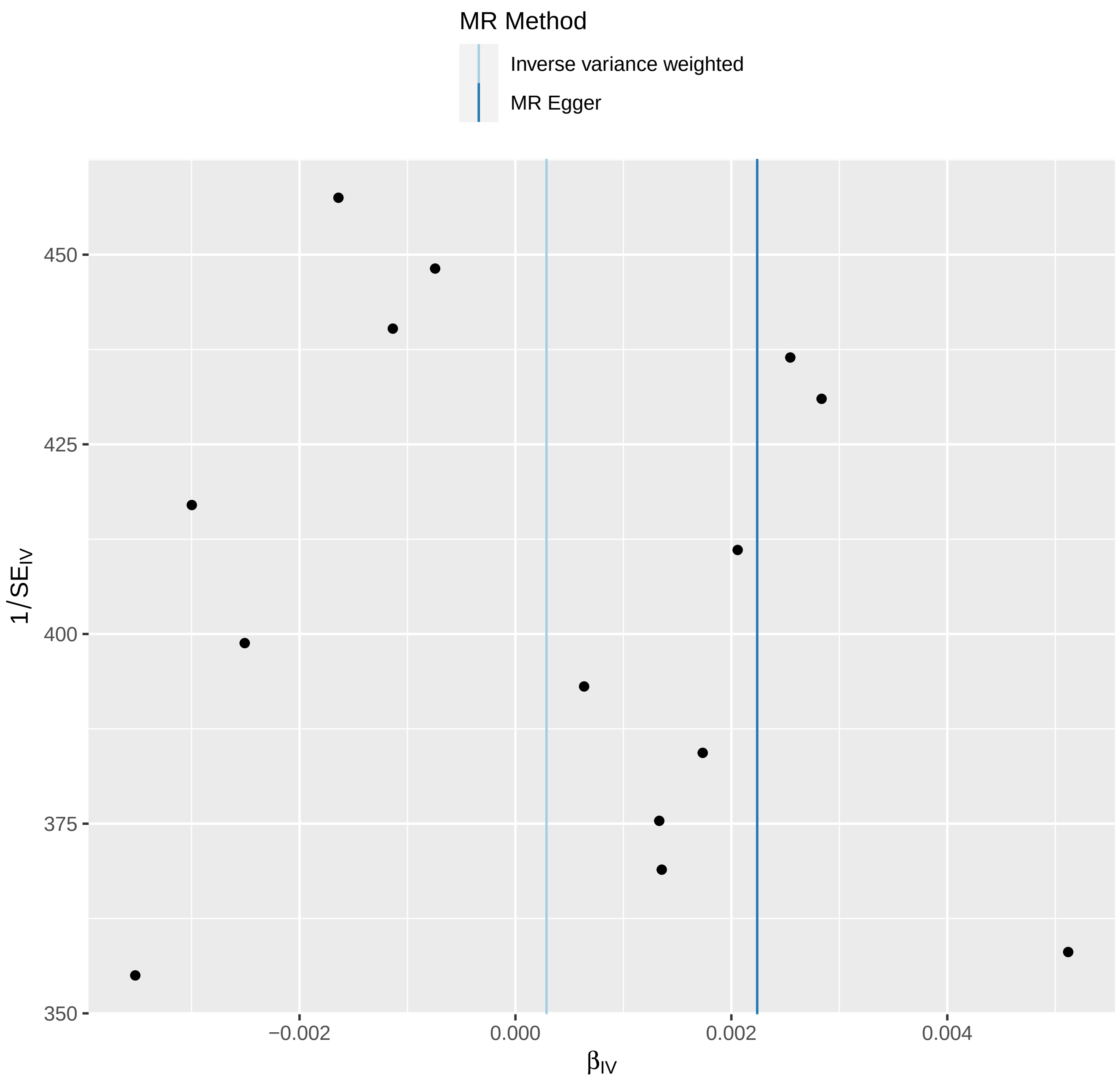

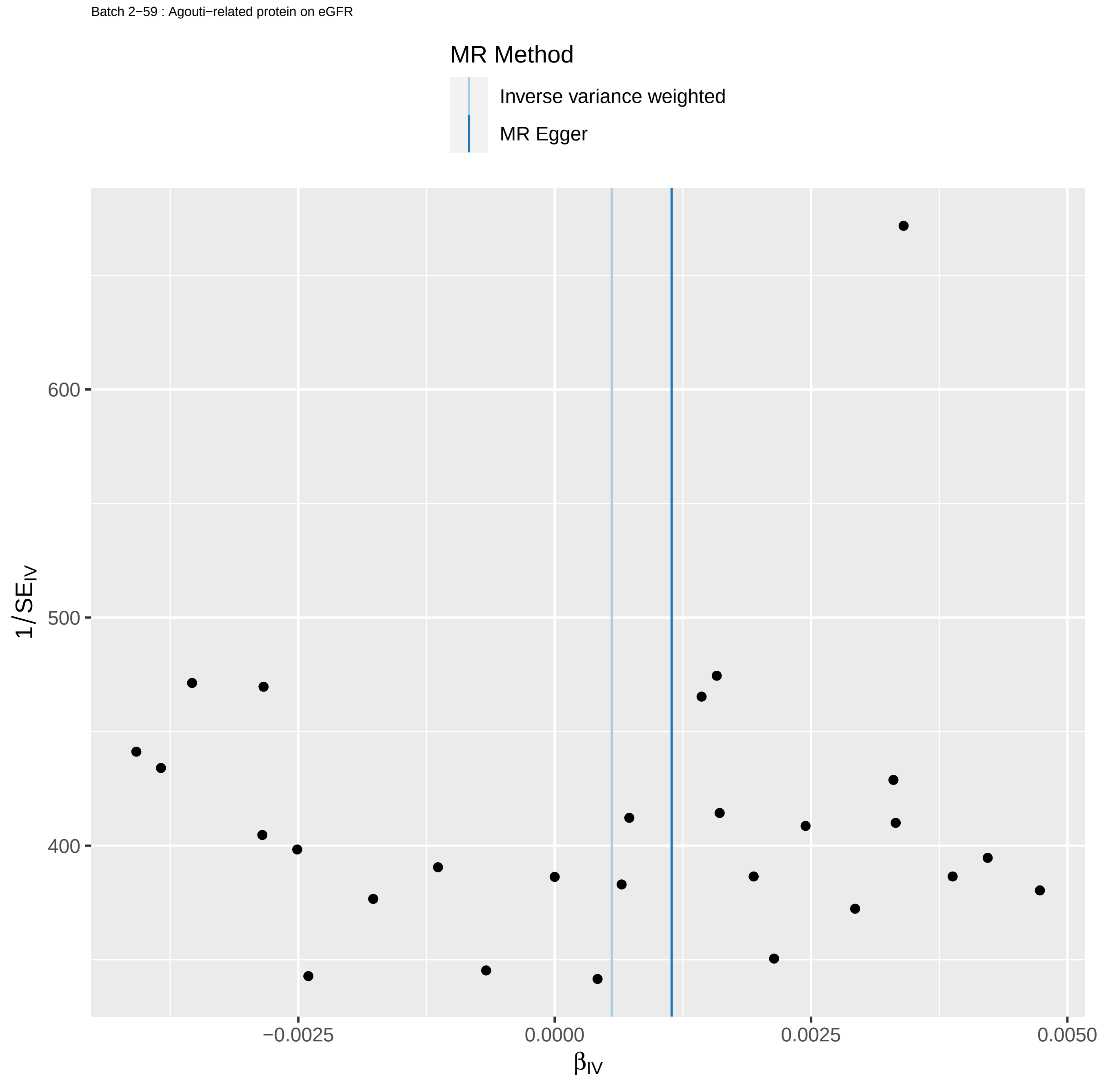
Batch 2−59 : Agouti−related protein on eGFR
MR Method
Inverse variance weighted
MR Egger
600
500
400
1/SE_IV
−0.0025
0.0000
0.0025
0.0050
β_IV

Batch 2−60 : Activator of 90 kDa heat shock protein ATPase homolog 1 on eGFR

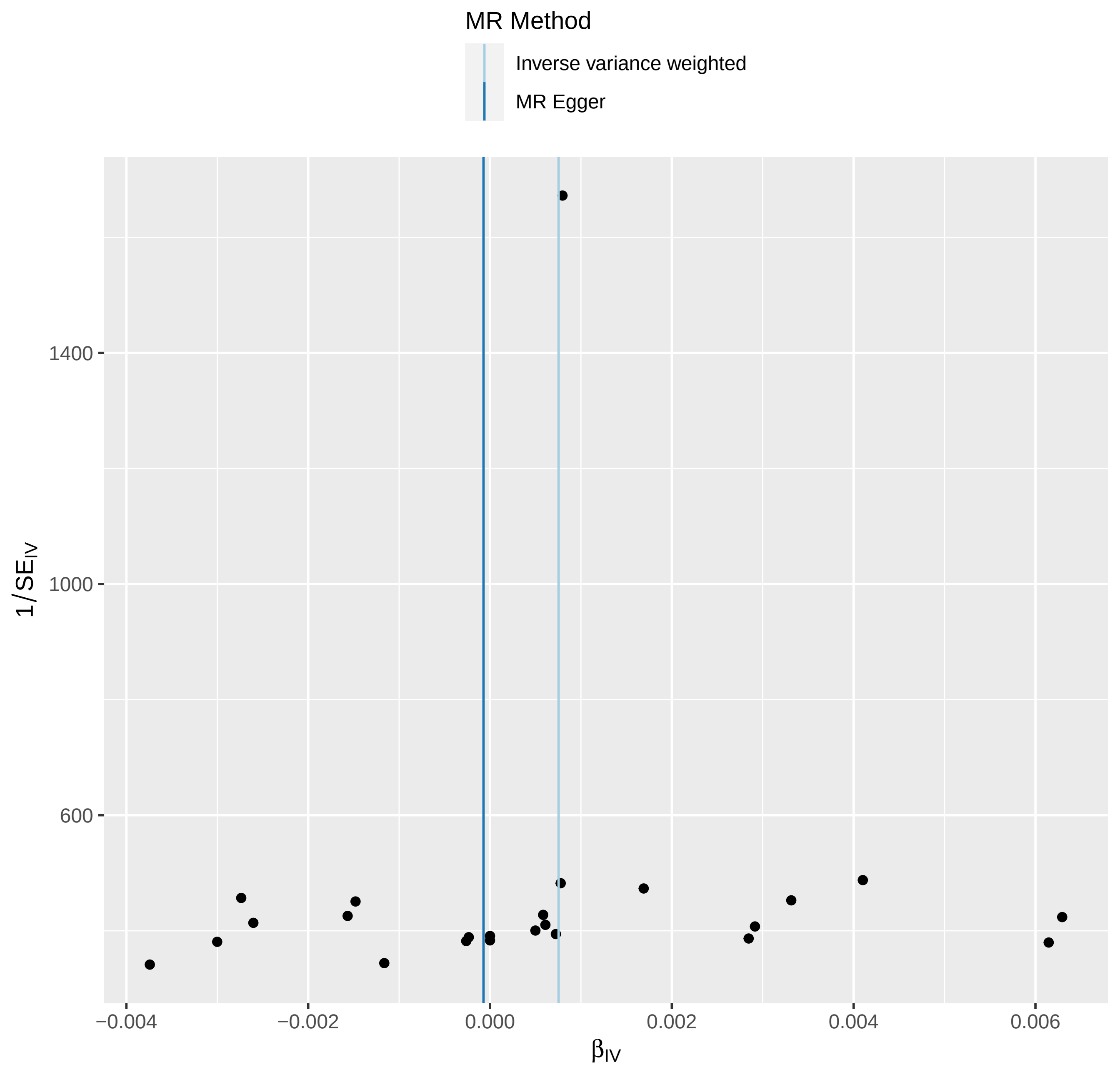

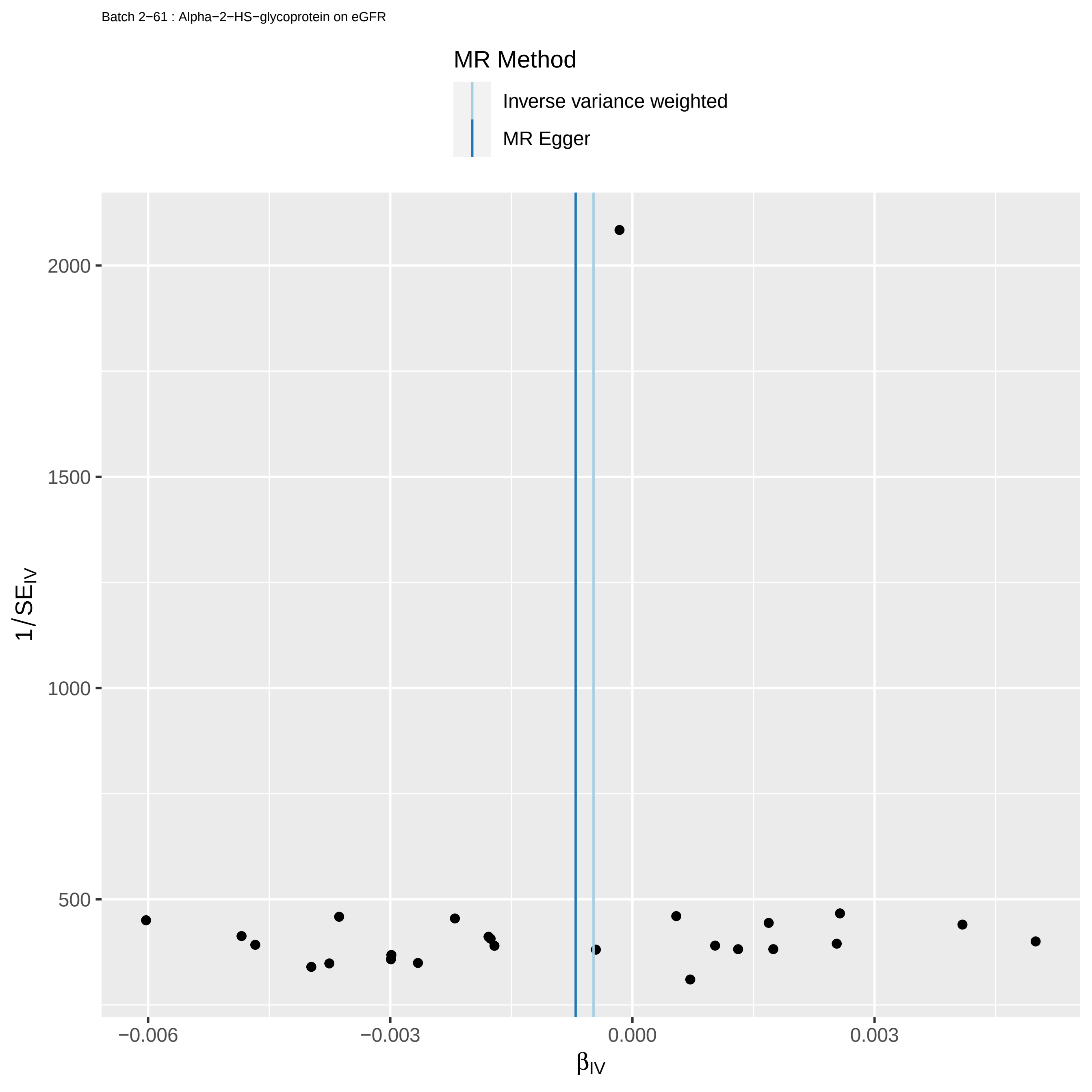
Batch 2−61 : Alpha−2−HS−glycoprotein on eGFR
MR Method
Inverse variance weighted
MR Egger
2000
1500
1000
500
1/SE_IV
−0.006
−0.003
0.000
0.003
β_IV

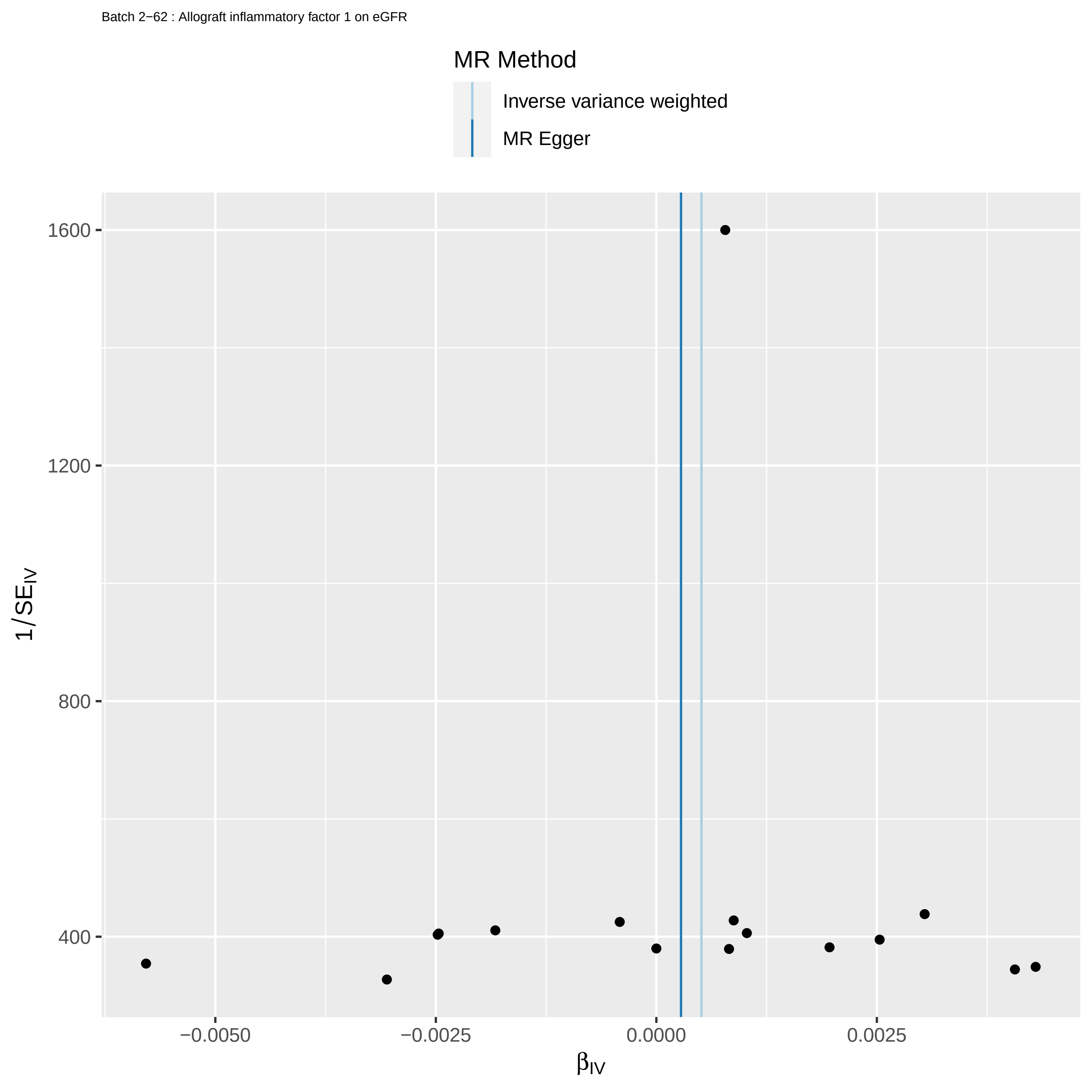

Batch 2−62 : Allograft inflammatory factor 1 on eGFR
MR Method
Inverse variance weighted
MR Egger
1600
1200
800
400
1/SE$_{IV}$
−0.0050
−0.0025
0.0000
0.0025
β$_{IV}$

Batch 2−63 : Apoptosis−inducing factor 1, mitochondrial on eGFR

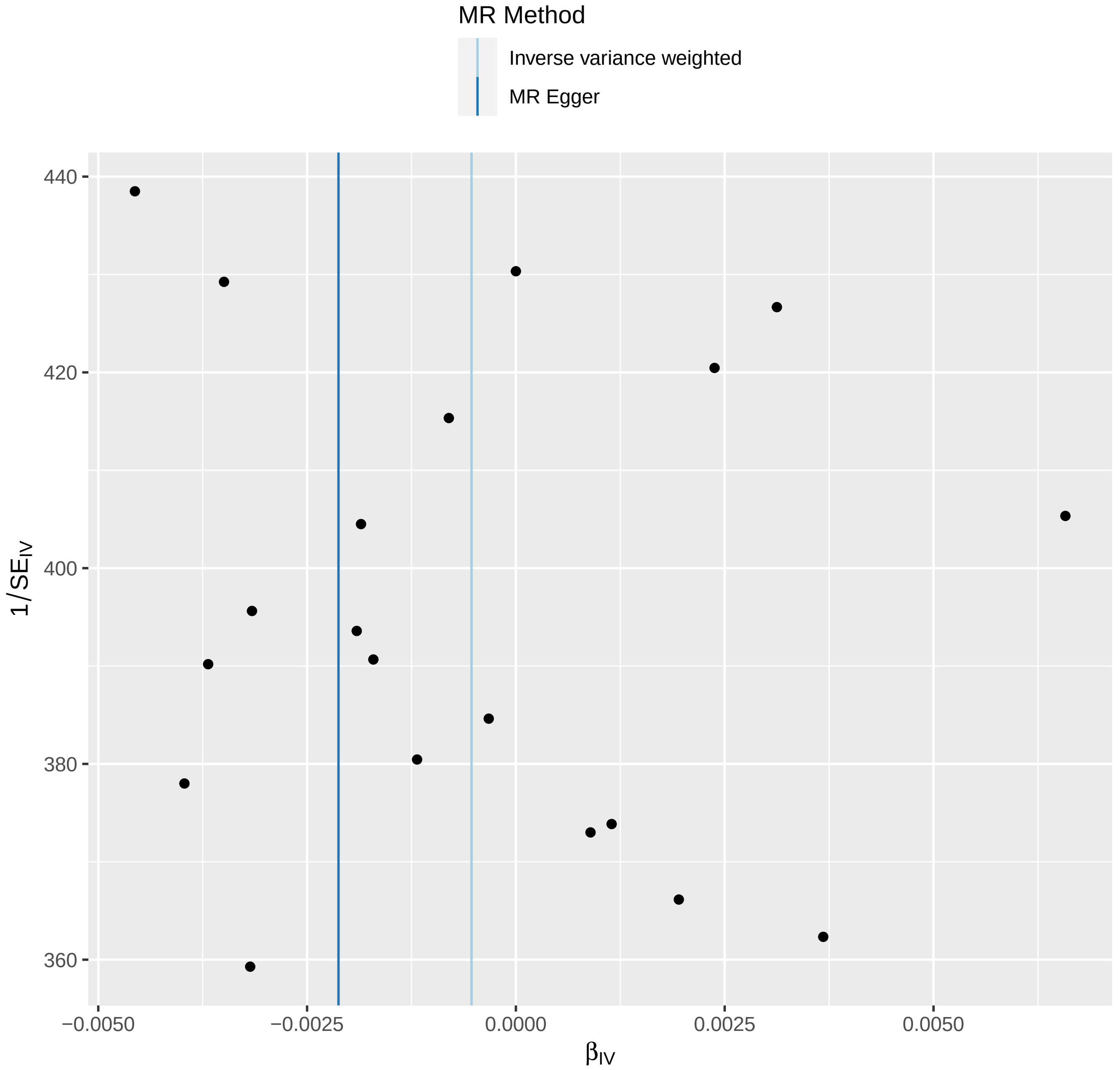

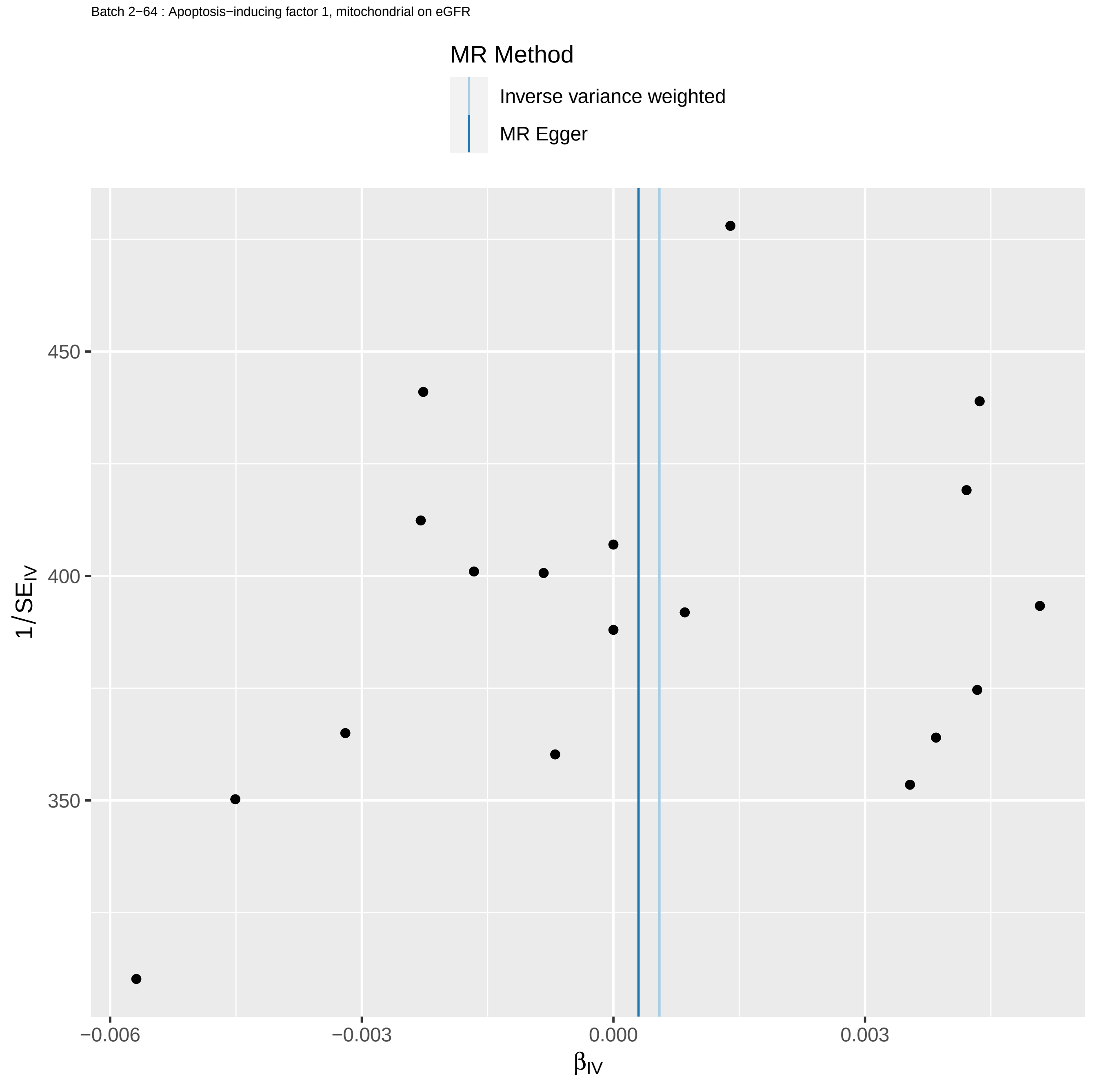
Batch 2−64 : Apoptosis−inducing factor 1, mitochondrial on eGFR
MR Method
Inverse variance weighted
MR Egger
450
400
350
1/SE_IV
−0.006
−0.003
0.000
0.003
β_IV

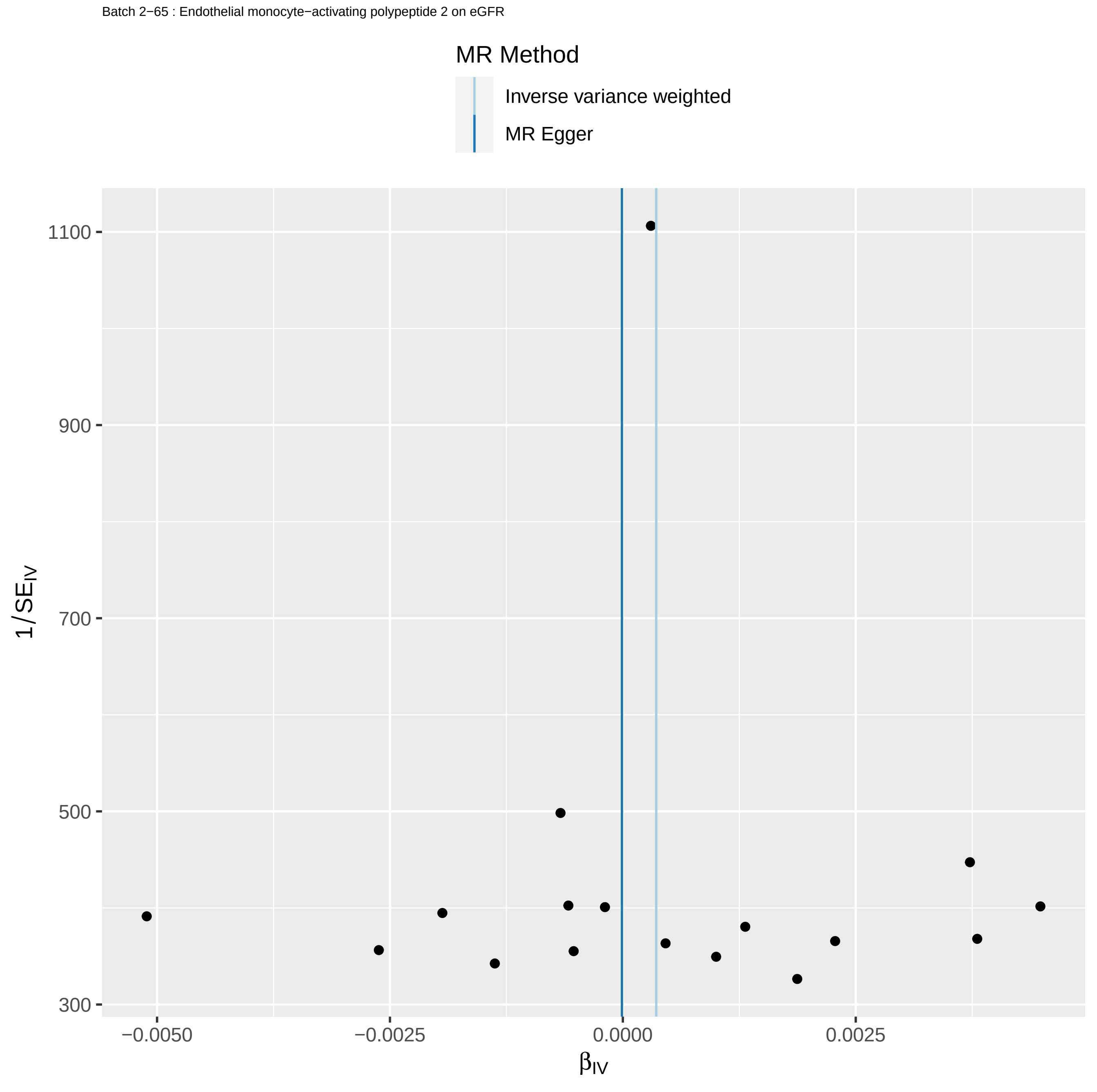

Batch 2−65 : Endothelial monocyte−activating polypeptide 2 on eGFR
MR Method
Inverse variance weighted
MR Egger
1100
900
700
500
300
−0.0050
−0.0025
0.0000
0.0025
$1/SE_{IV}$
β_{IV}

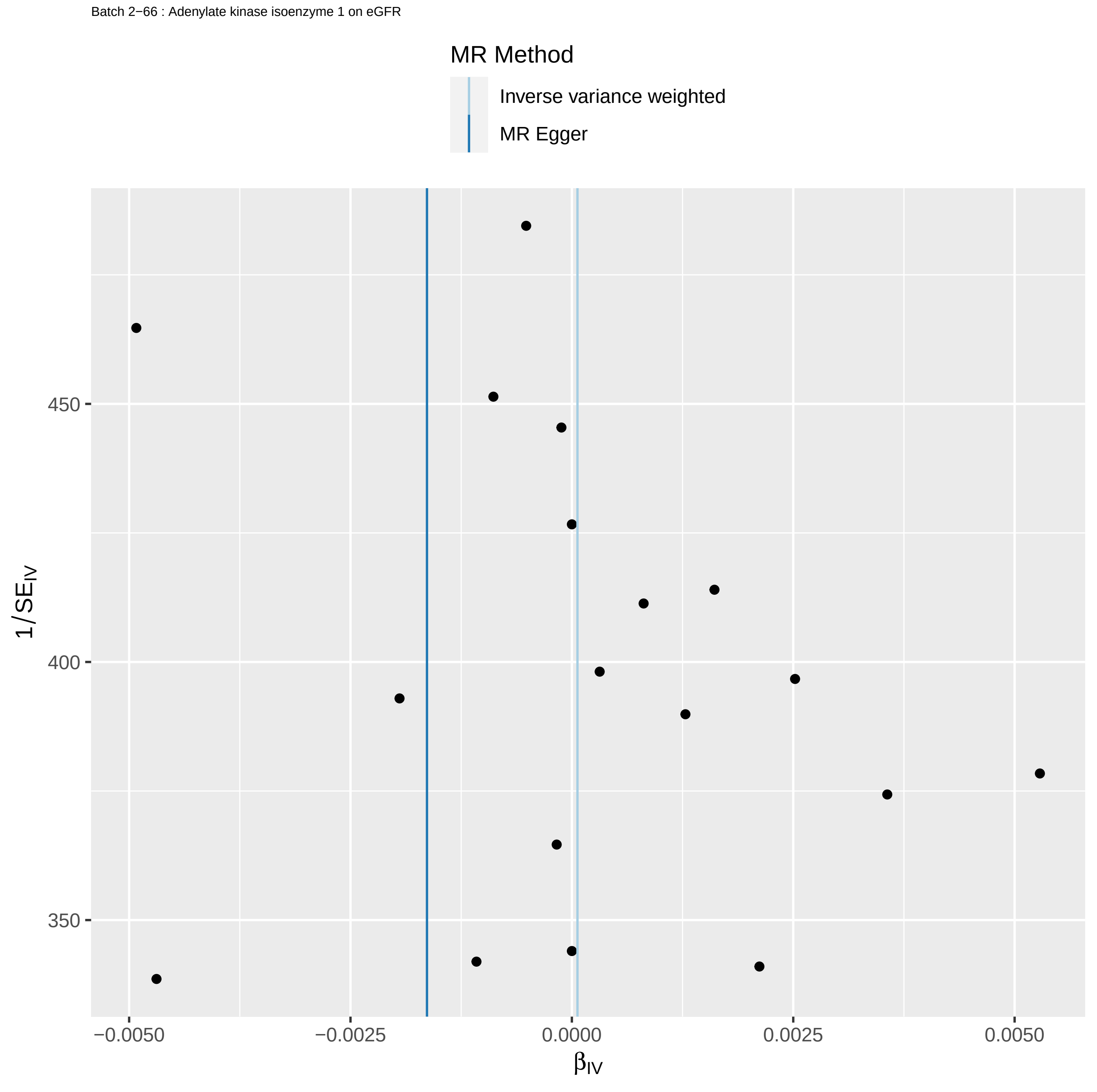
Batch 2−66 : Adenylate kinase isoenzyme 1 on eGFR
MR Method
Inverse variance weighted
MR Egger
450
400
350
1/SE_IV
−0.0050
−0.0025
0.0000
0.0025
0.0050
β_IV

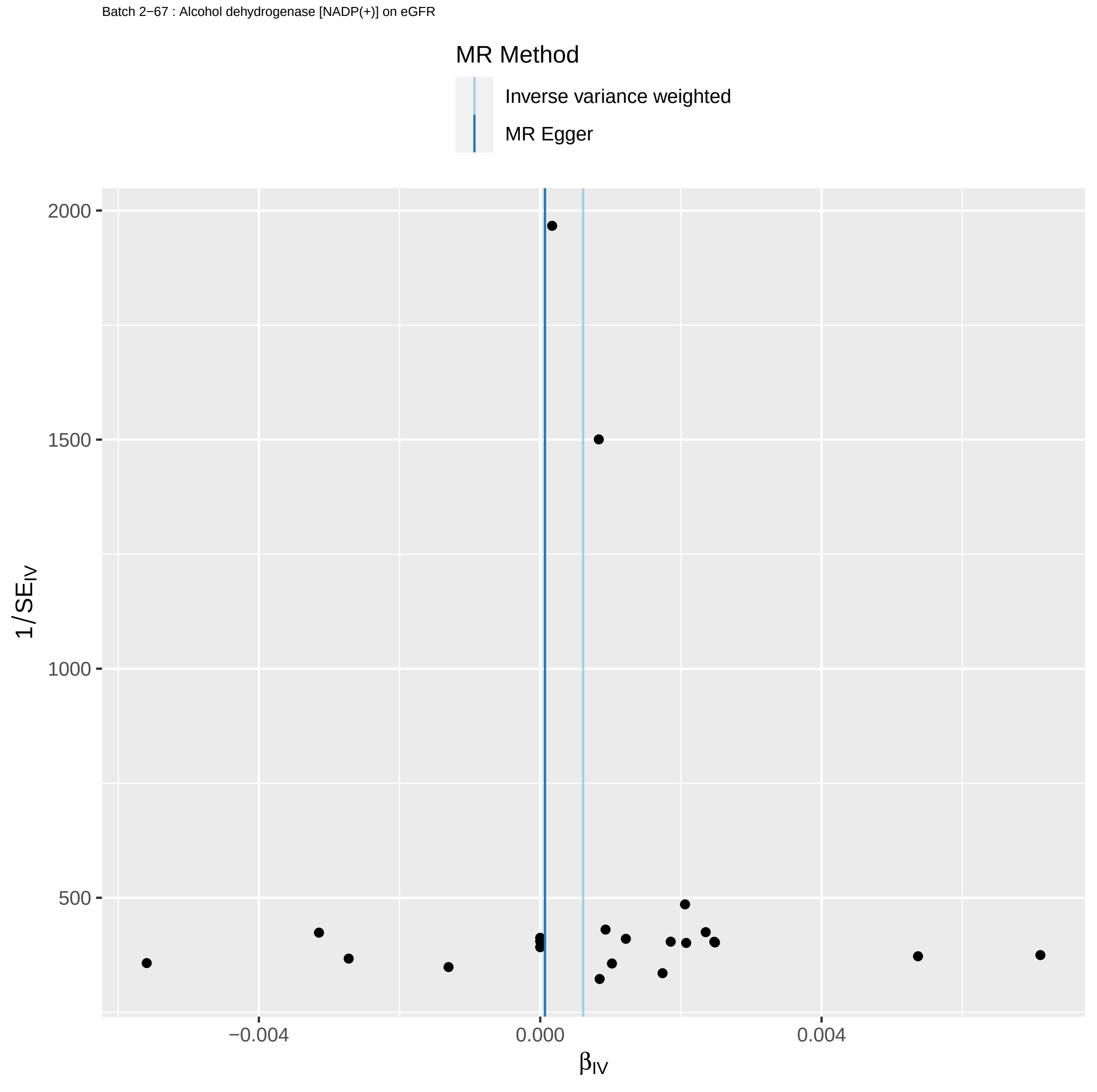
Batch 2−67 : Alcohol dehydrogenase [NADP(+)] on eGFR
MR Method
Inverse variance weighted
MR Egger
2000
1500
1000
500
1/SE_IV
−0.004
0.000
0.004
β_IV

Batch 2−68 : Aldose reductase on eGFR

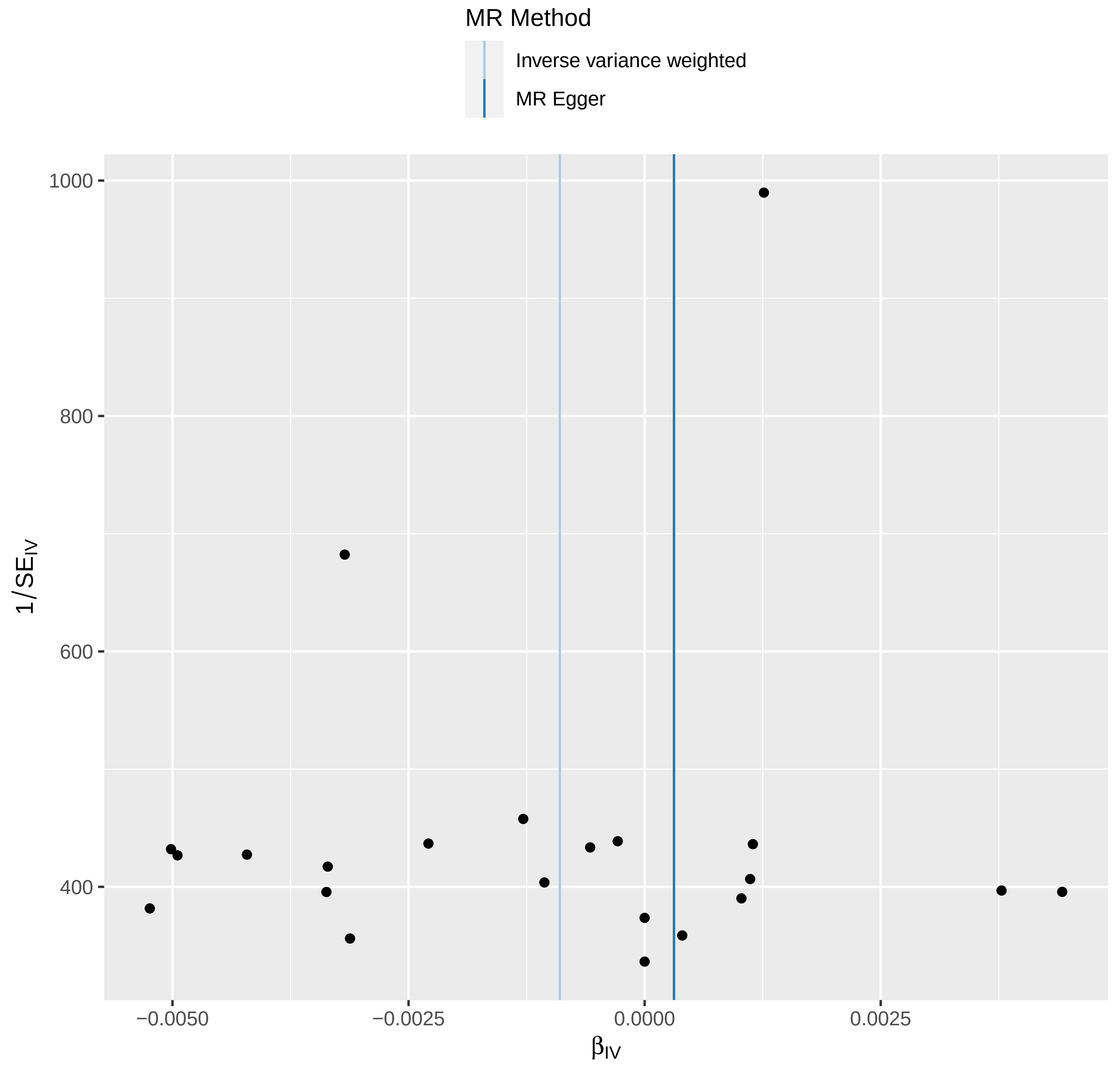

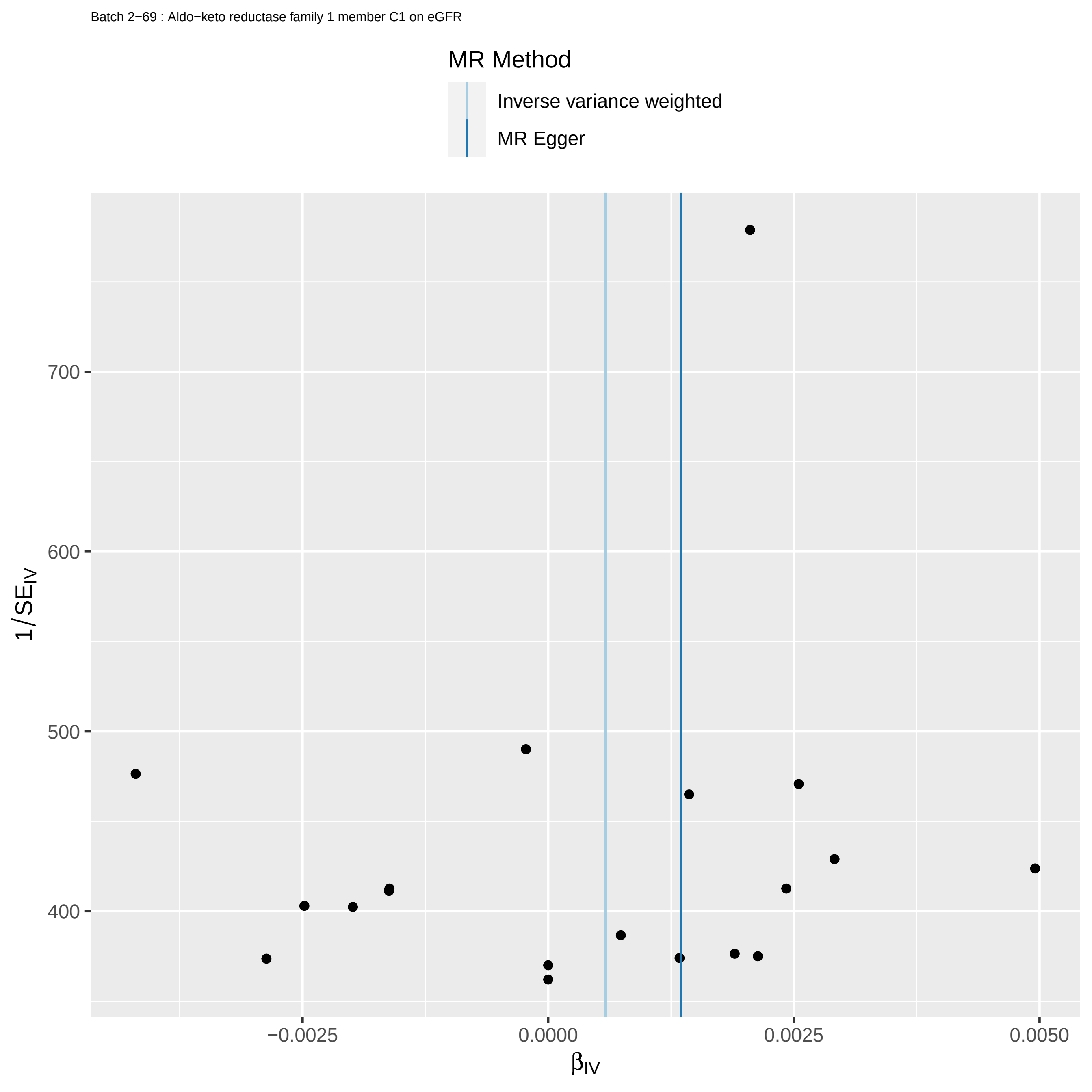

Batch 2−69 : Aldo−keto reductase family 1 member C1 on eGFR
MR Method
Inverse variance weighted
MR Egger
700
600
500
400
1/SE$_{IV}$
−0.0025
0.0000
0.0025
0.0050
β_{IV}

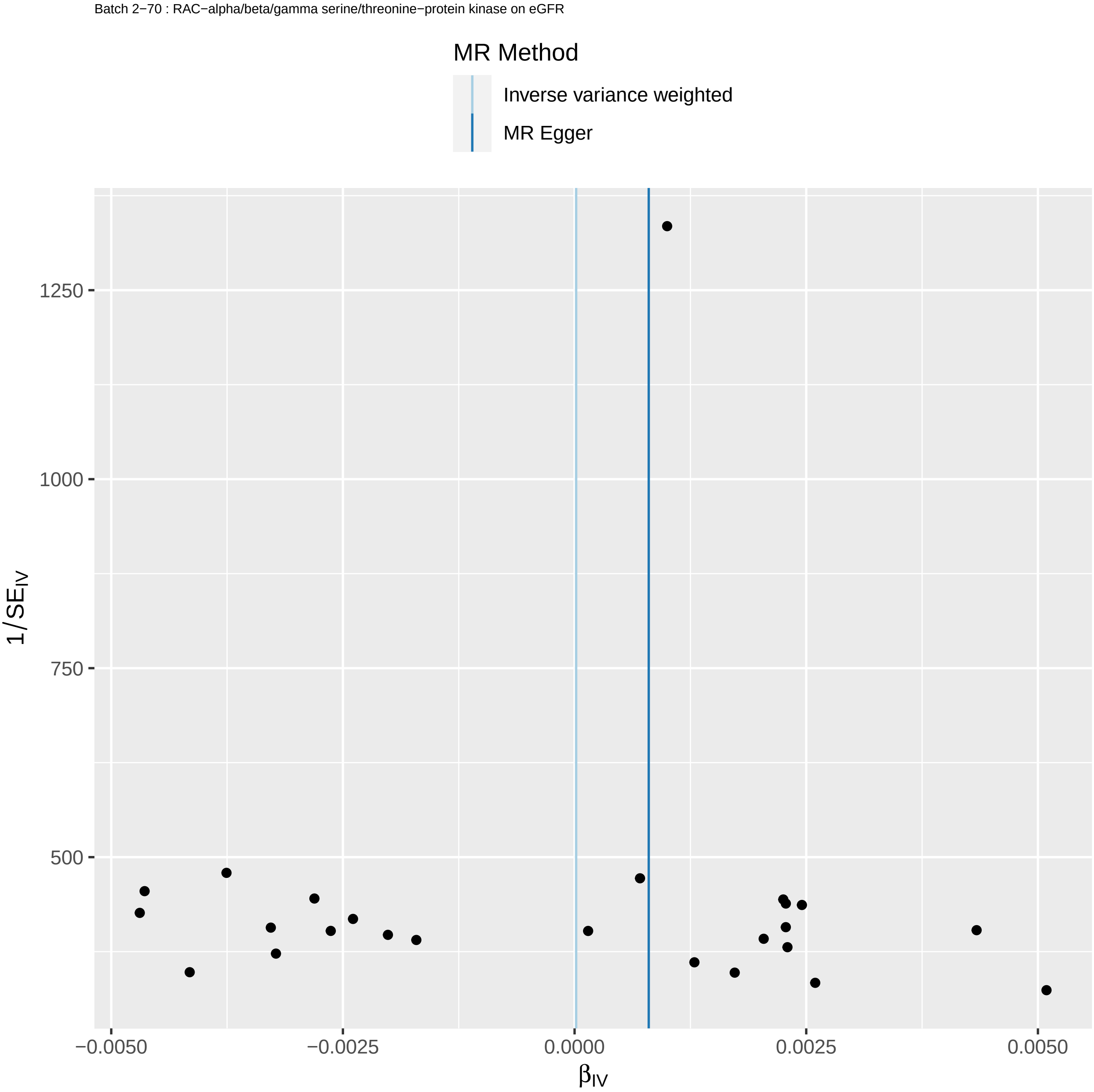
Batch 2−70 : RAC−alpha/beta/gamma serine/threonine−protein kinase on eGFR
MR Method
Inverse variance weighted
MR Egger
1250
1000
750
500
1/SE_IV
−0.0050
−0.0025
0.0000
0.0025
0.0050
β_IV

Batch 2−71 : RAC−alpha/beta/gamma serine/threonine−protein kinase on eGFR

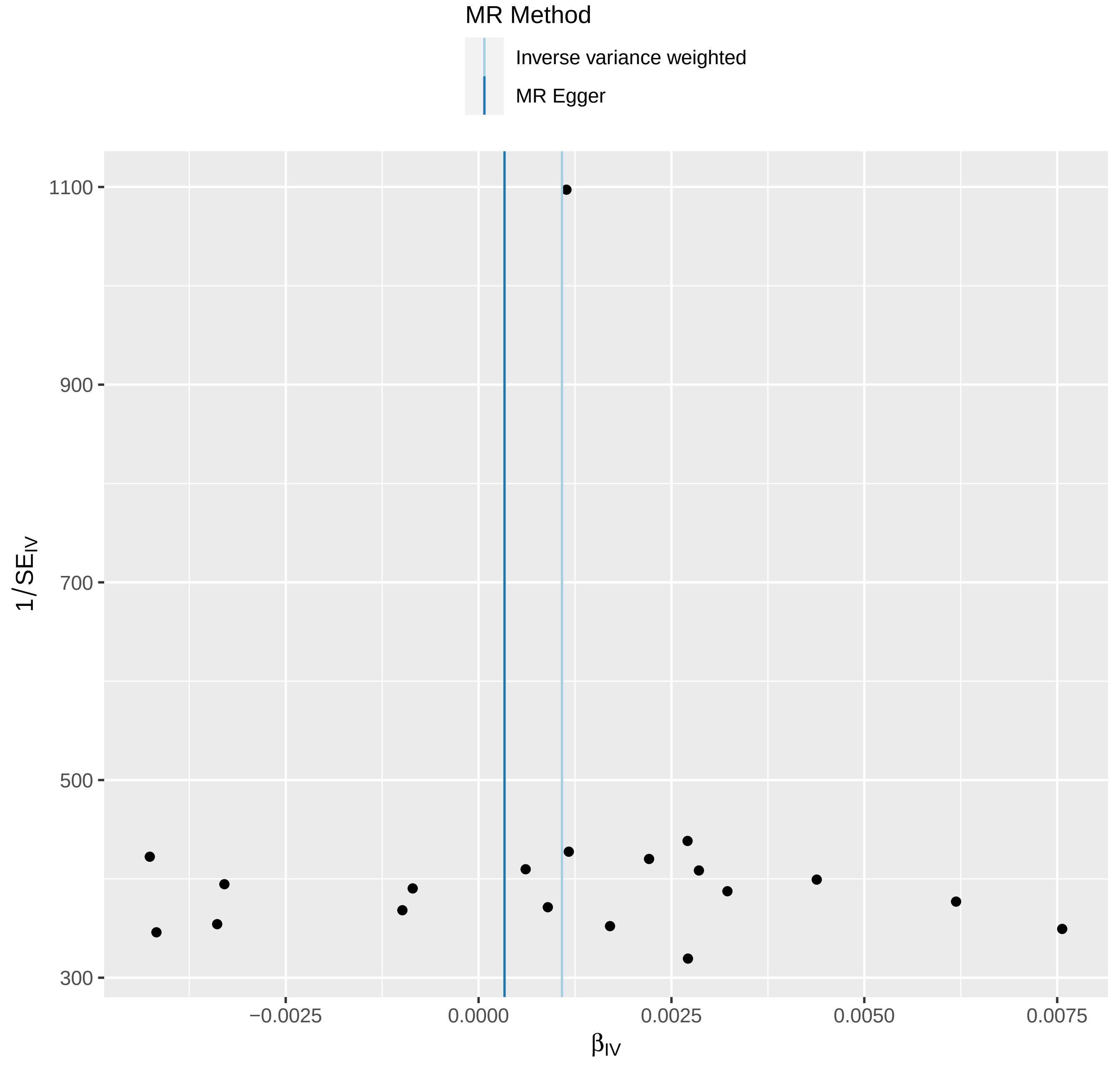

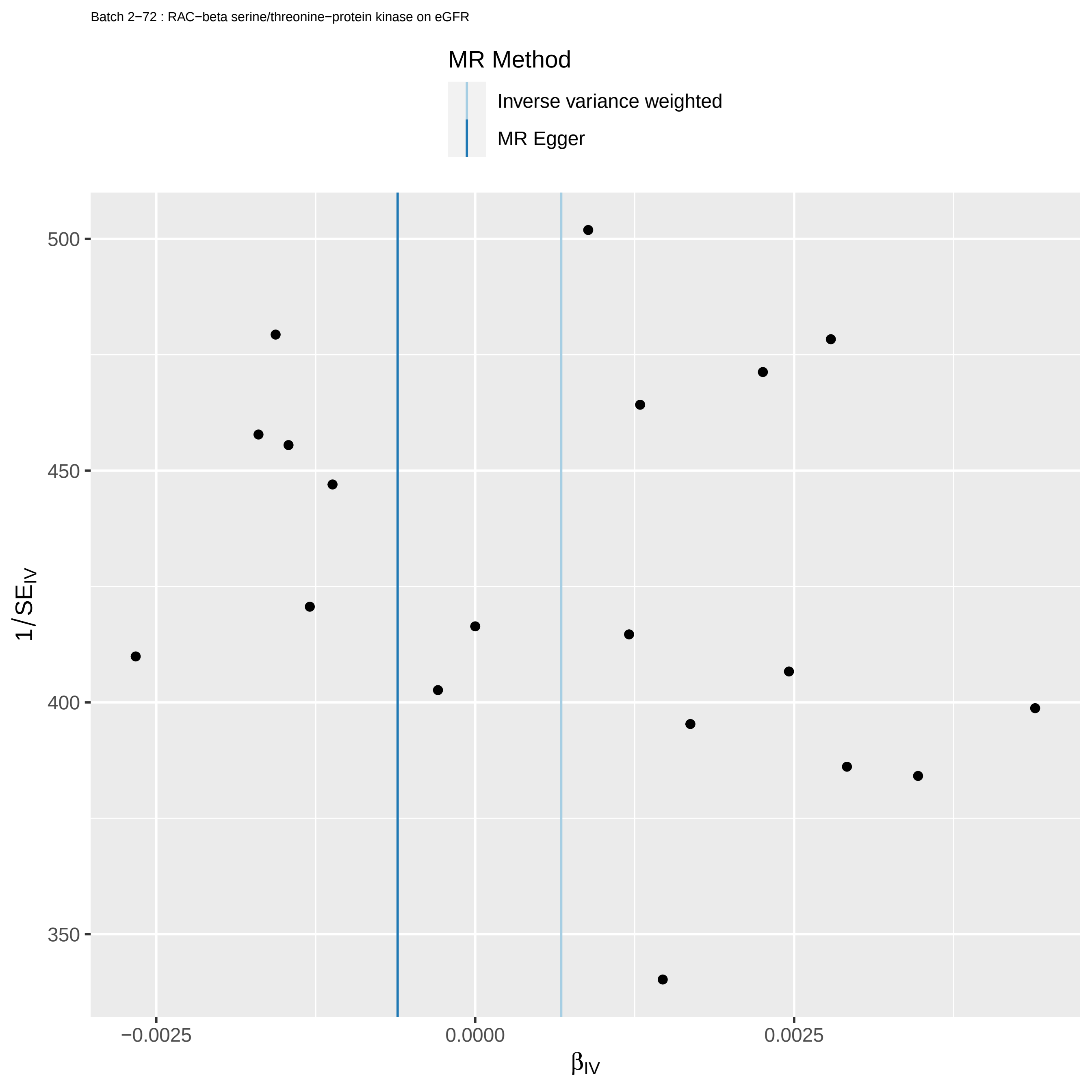
Batch 2−72 : RAC−beta serine/threonine−protein kinase on eGFR
MR Method
Inverse variance weighted
MR Egger
500
450
400
350
1/SE_IV
−0.0025
0.0000
0.0025
β_IV

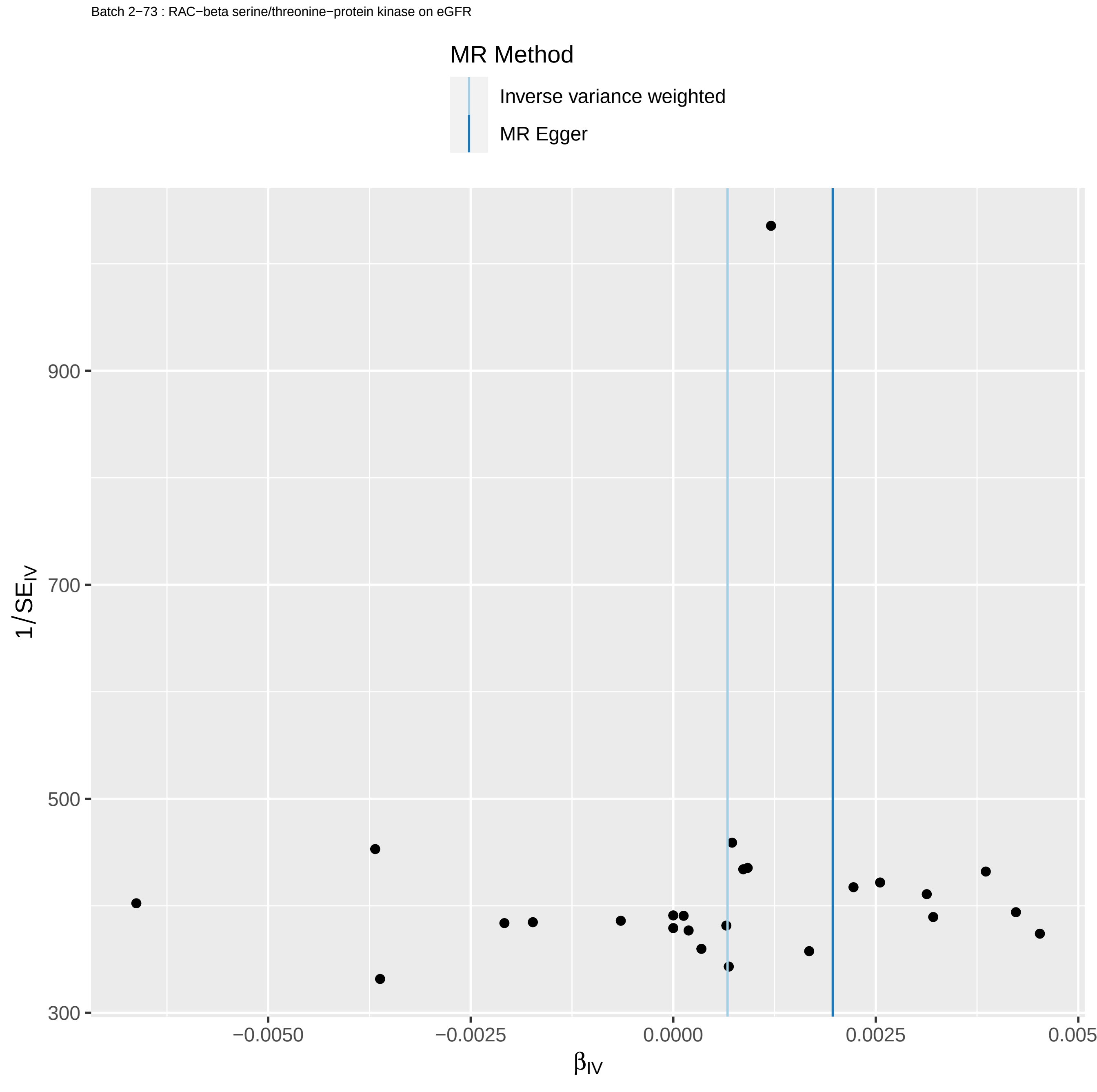

Batch 2−73 : RAC−beta serine/threonine−protein kinase on eGFR
MR Method
Inverse variance weighted
MR Egger
900
700
500
300
1/SE_IV
−0.0050
−0.0025
0.0000
0.0025
0.005
β_IV

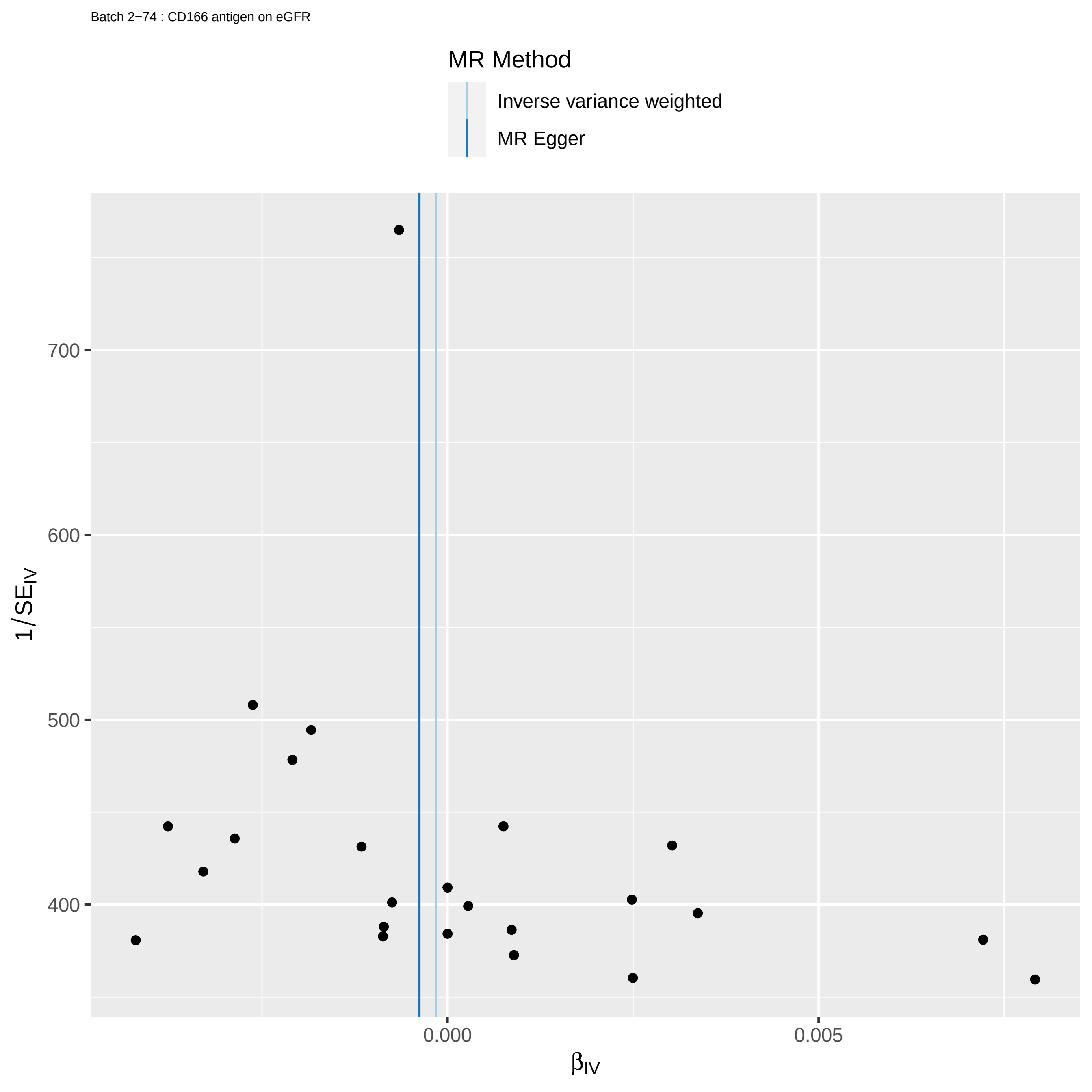

Batch 2−74 : CD166 antigen on eGFR
MR Method
Inverse variance weighted
MR Egger
700
600
500
400
1/SE$_{IV}$
0.000
0.005
β$_{IV}$

Batch 2−75 : Aldehyde dehydrogenase, dimeric NADP−preferring on eGFR

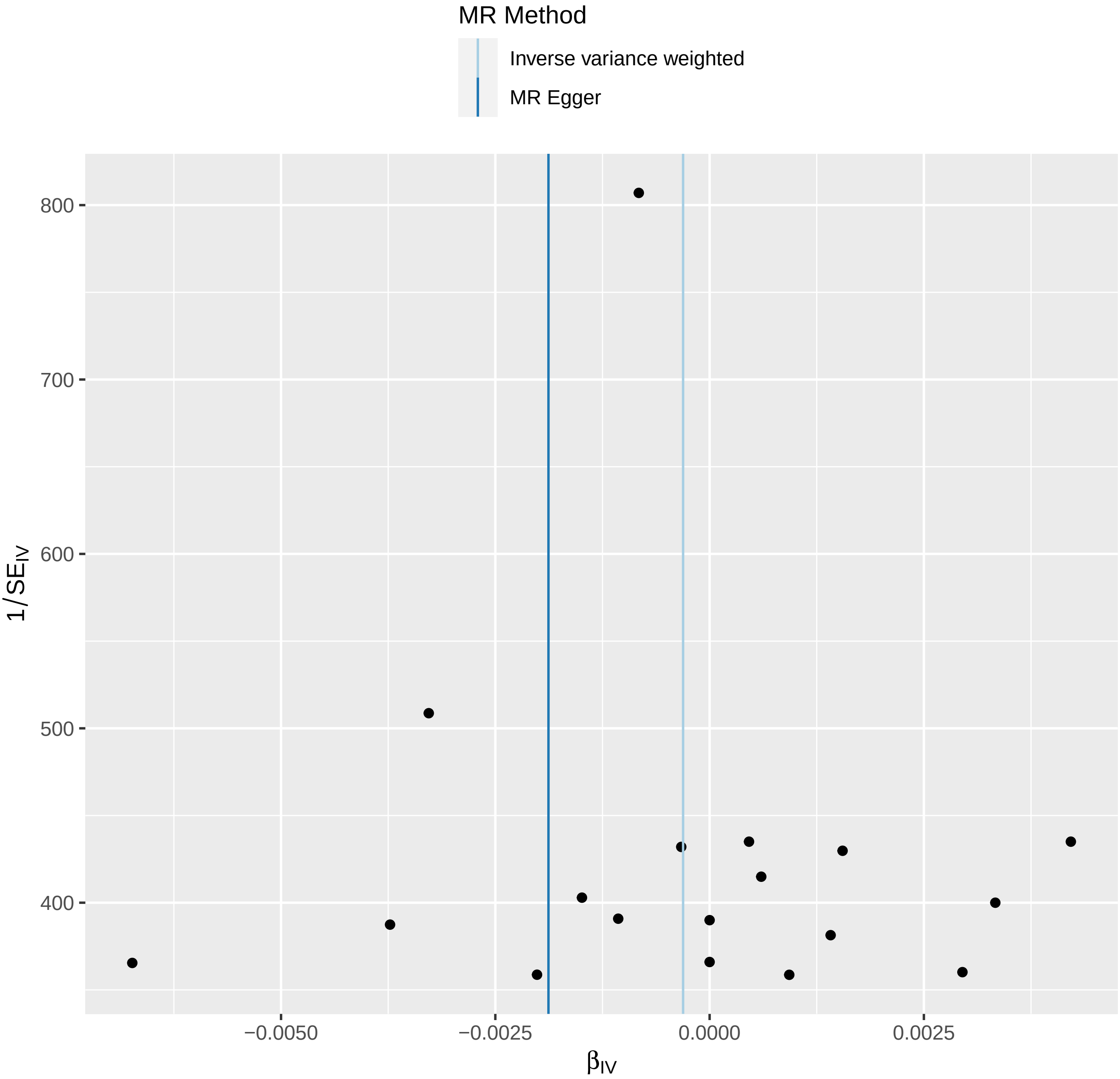

Batch 2−76 : Aldehyde dehydrogenase family 3 member B1 on eGFR

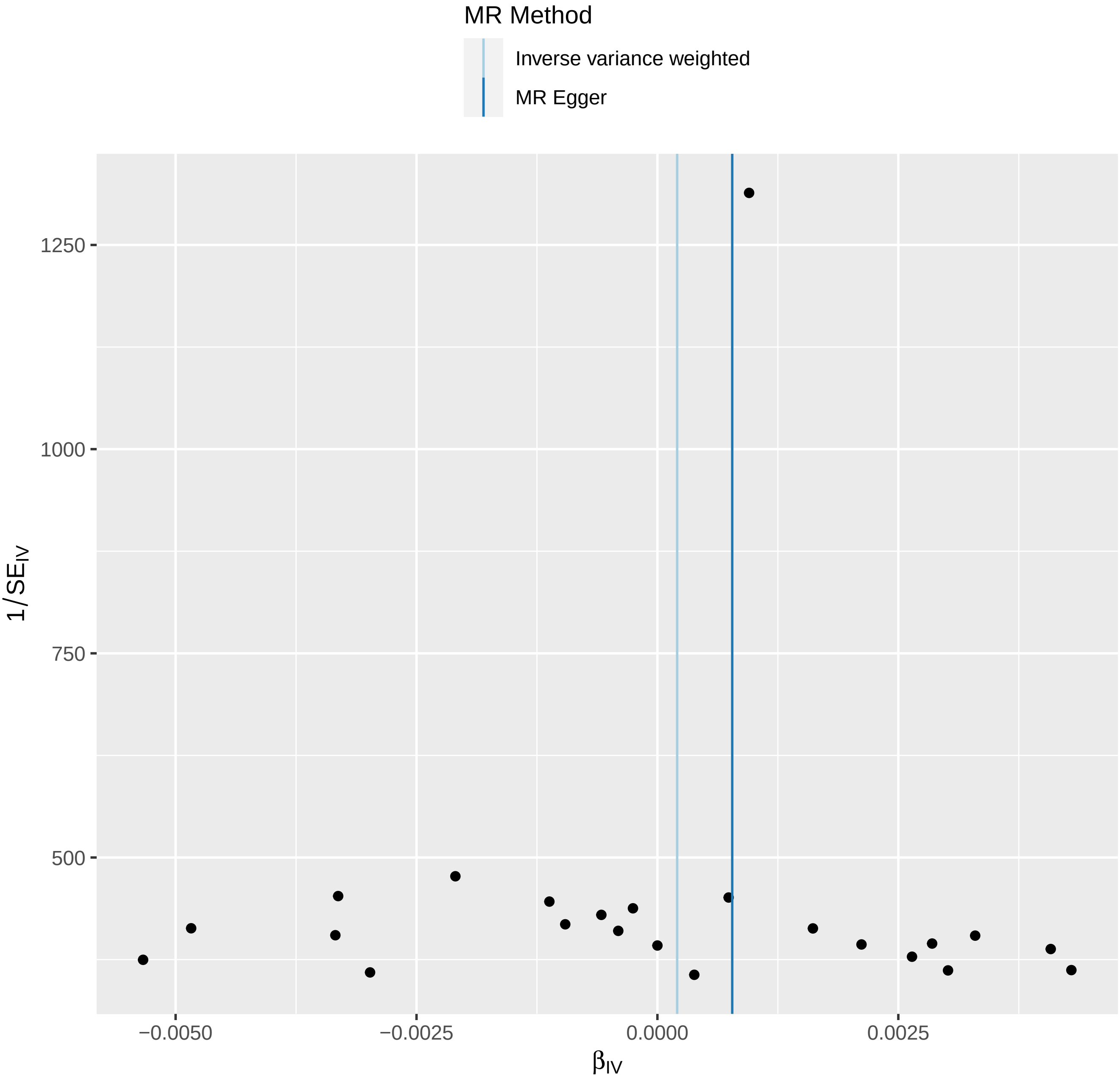

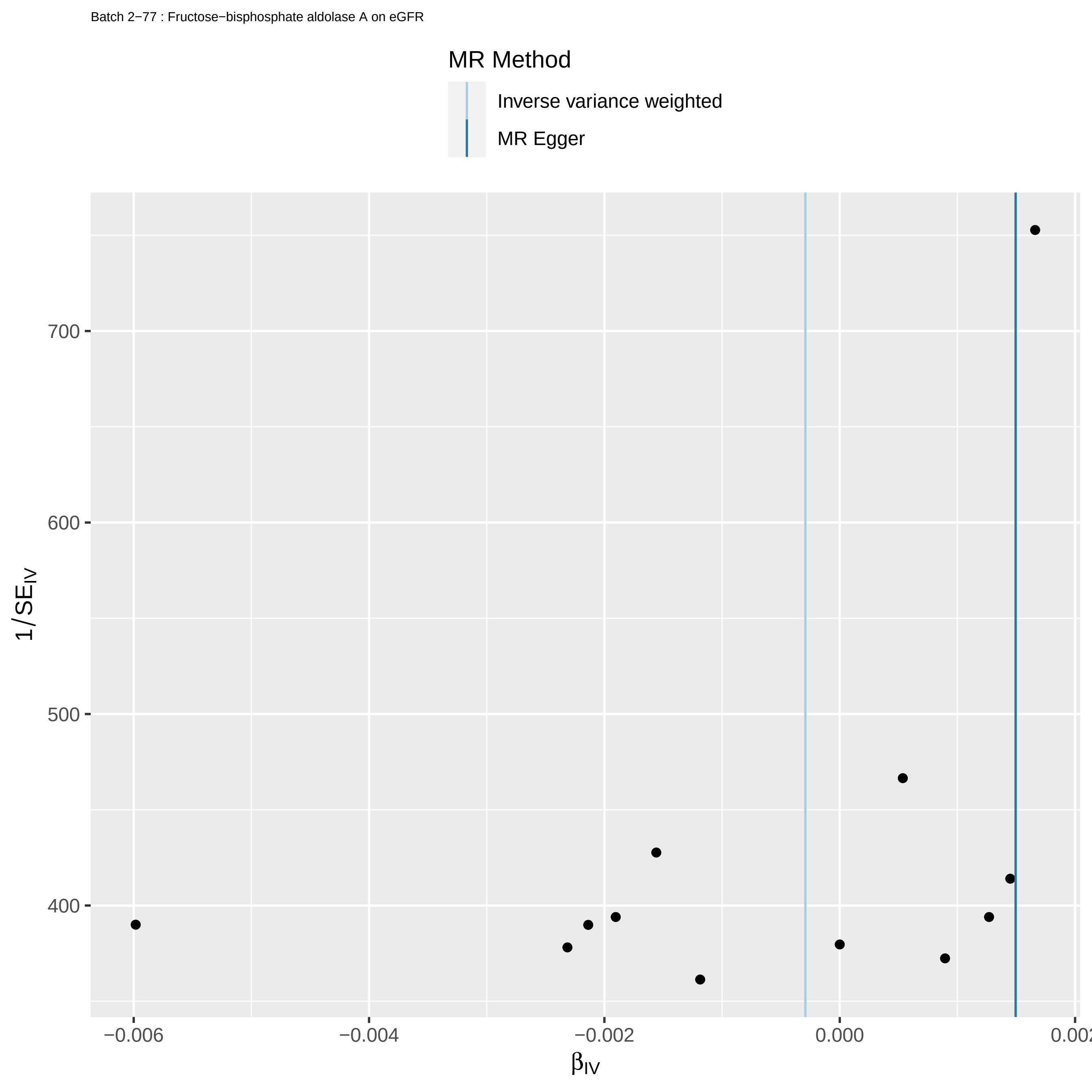

Batch 2−77 : Fructose−bisphosphate aldolase A on eGFR
MR Method
Inverse variance weighted
MR Egger
700
600
500
400
1/SE$_{IV}$
−0.006
−0.004
−0.002
0.000
0.002
β$_{IV}$

Batch 2−78 : Fructose−bisphosphate aldolase C on eGFR

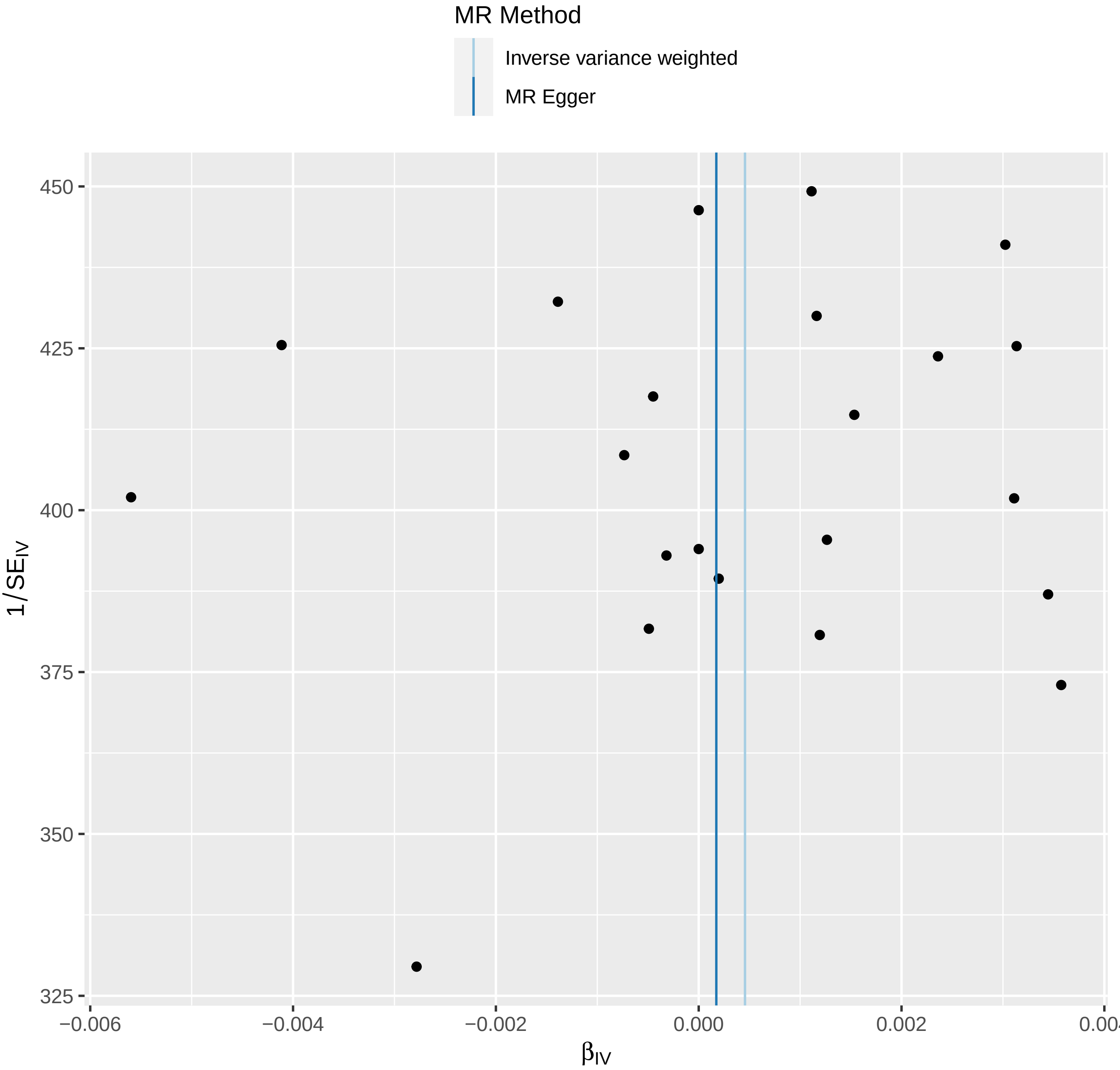

Batch 2−79 : RNA demethylase ALKBH5 on eGFR

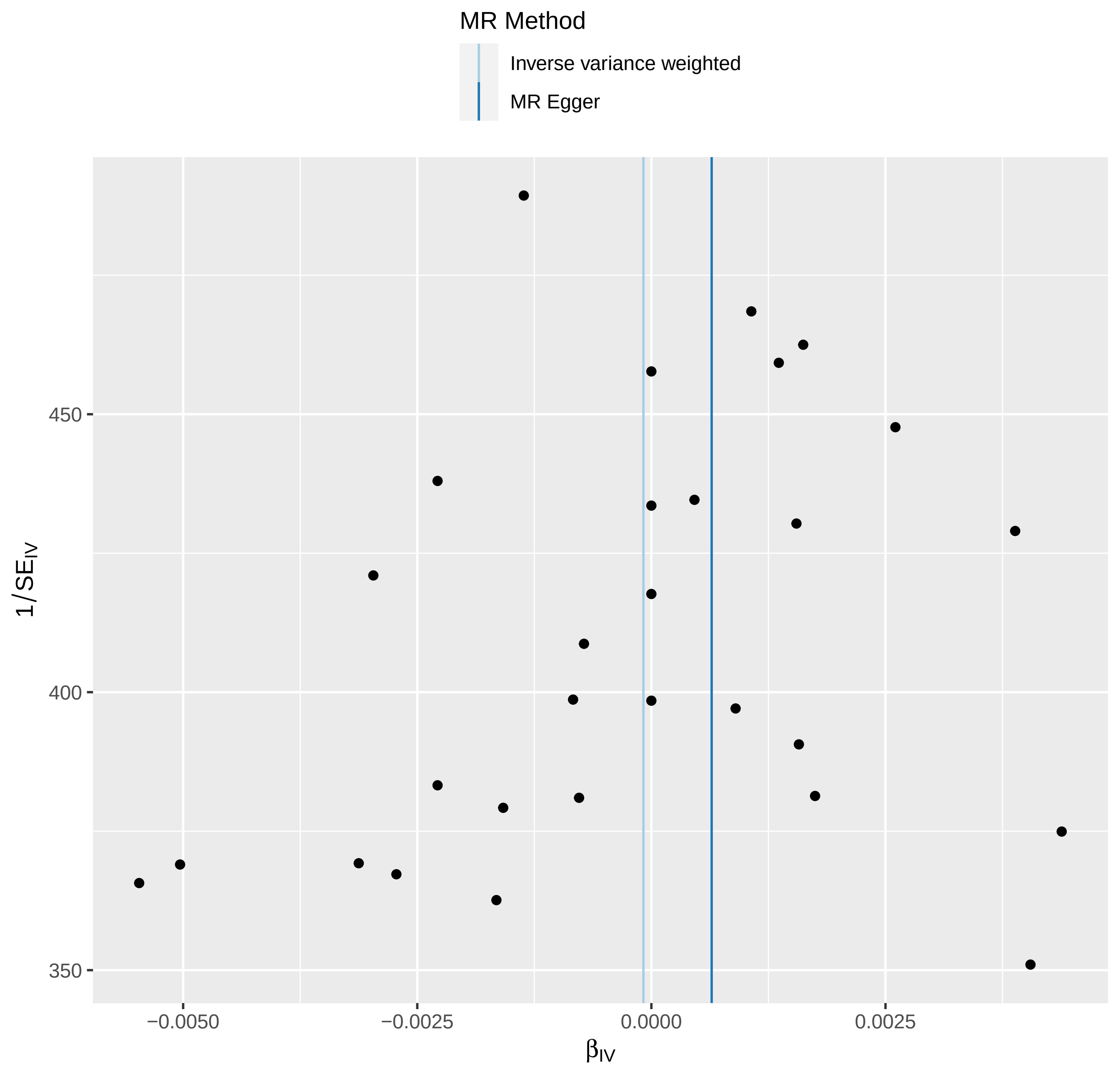

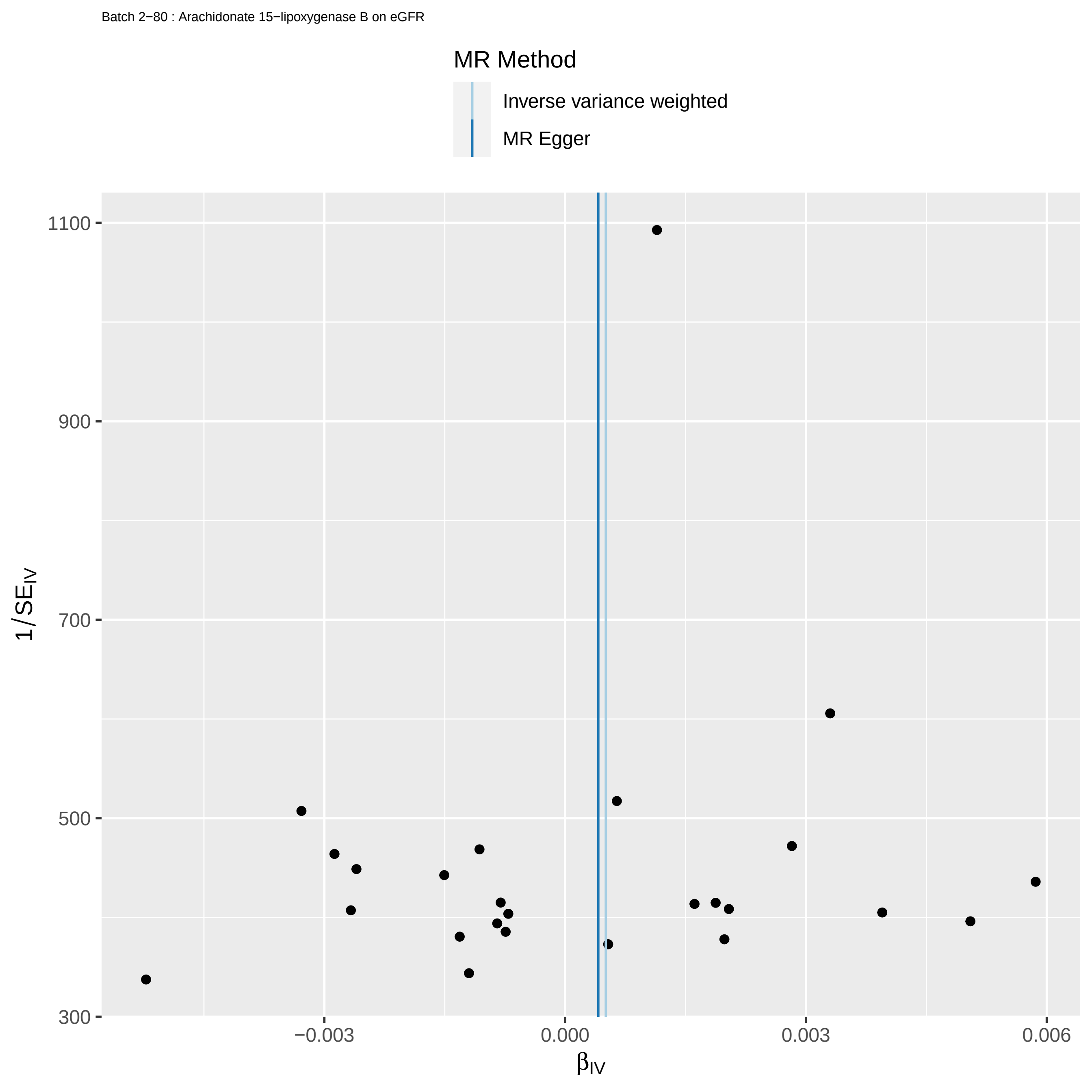

Batch 2−80 : Arachidonate 15−lipoxygenase B on eGFR
MR Method
Inverse variance weighted
MR Egger
1100
900
700
500
300
1/SE$_{IV}$
−0.003
0.000
0.003
0.006
β$_{IV}$

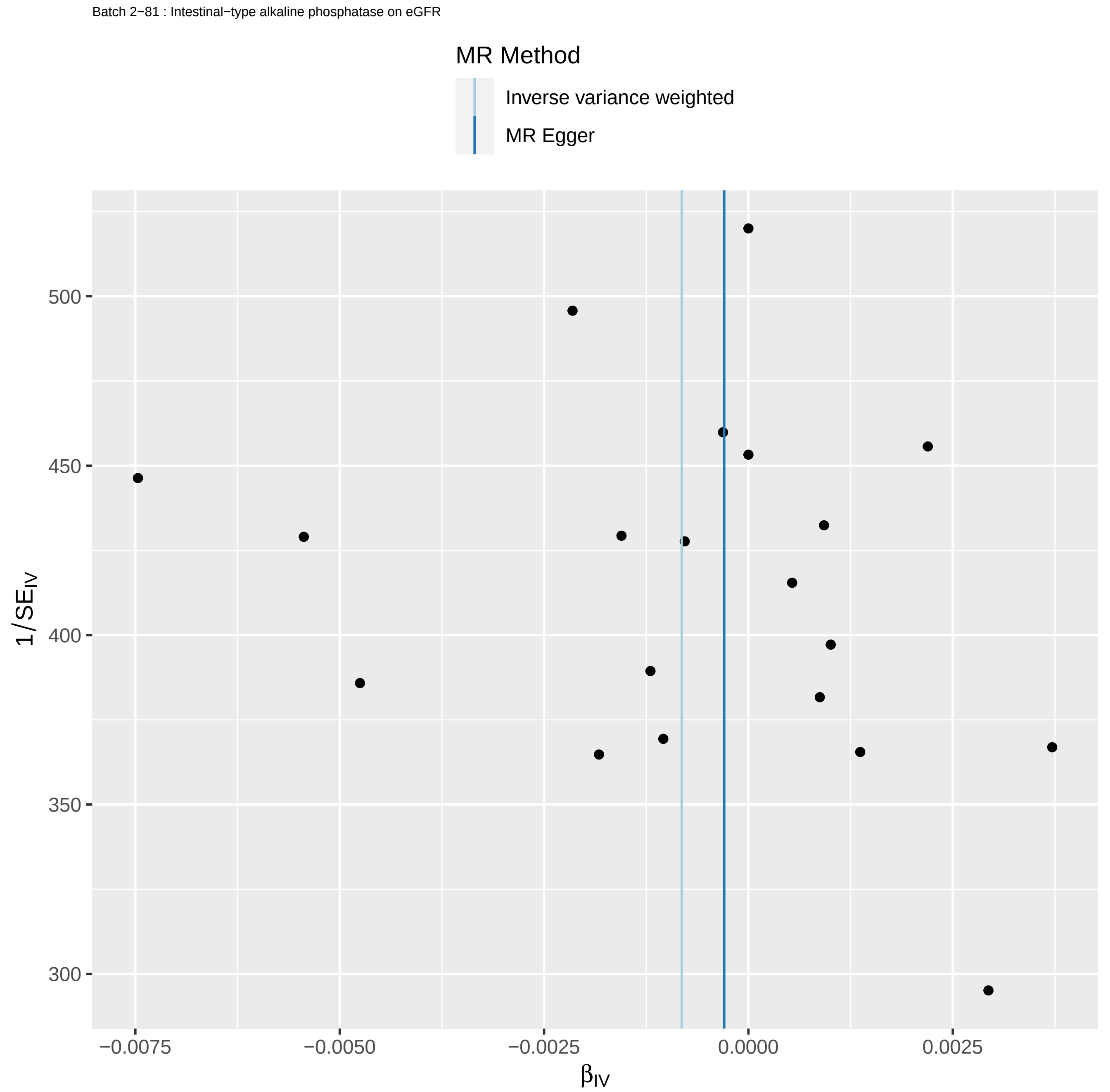

Batch 2−81 : Intestinal−type alkaline phosphatase on eGFR
MR Method
Inverse variance weighted
MR Egger
500
450
400
350
300
1/SE_IV
−0.0075
−0.0050
−0.0025
0.0000
0.0025
β_IV

Batch 2−82 : Ameloblastin on eGFR

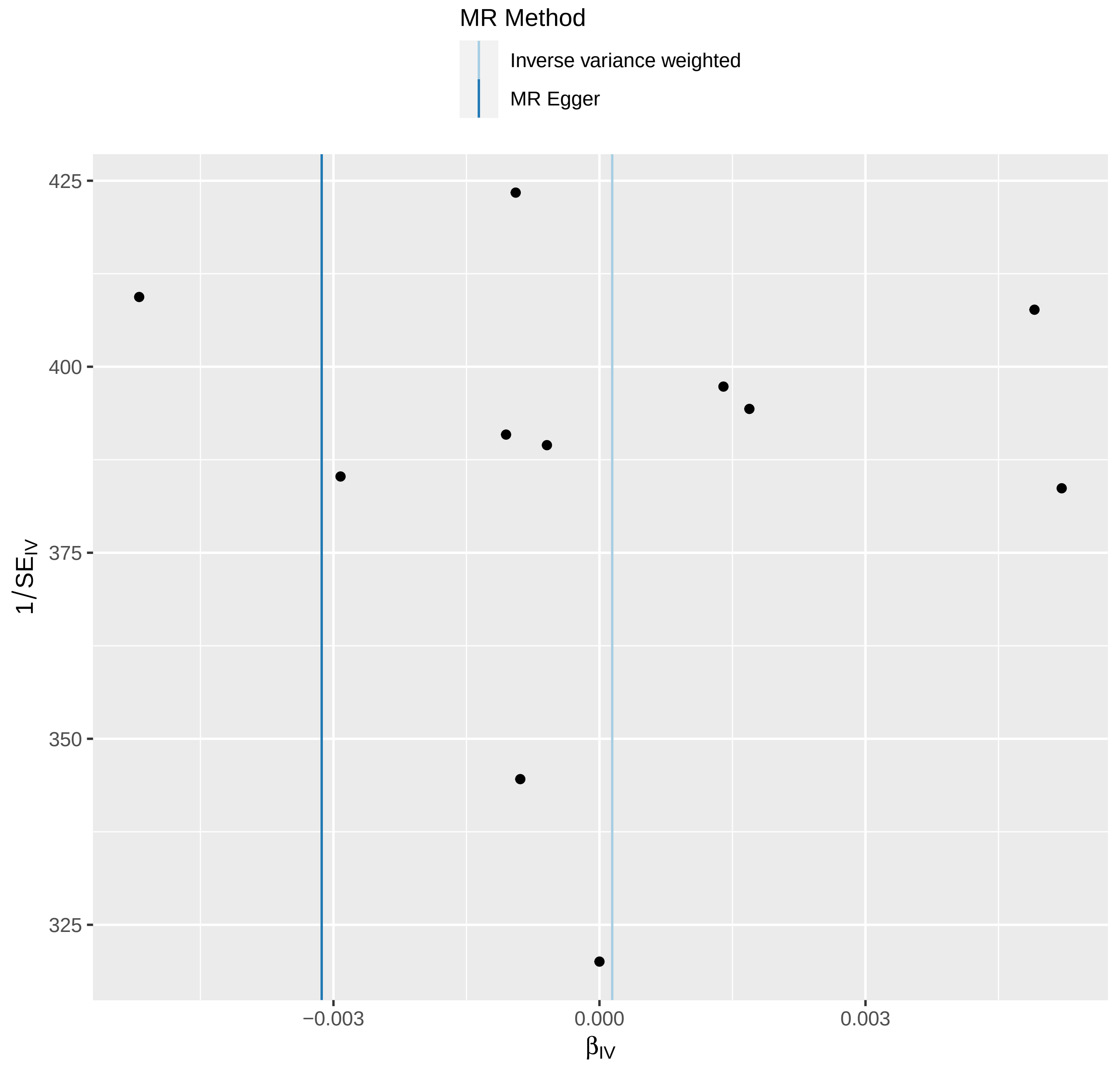

Batch 2−83 : Amelogenin, X isoform on eGFR

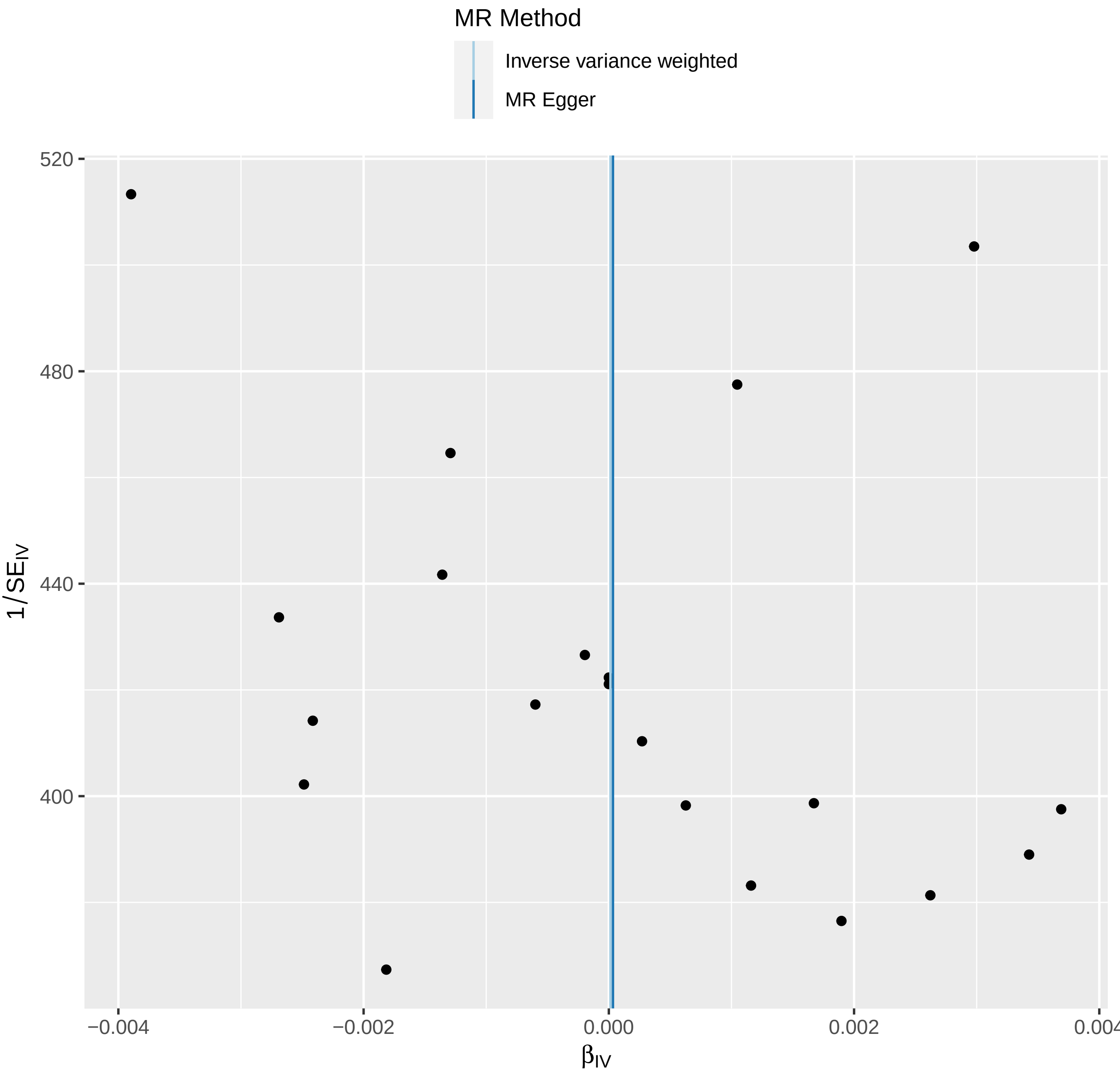

Batch 2−84 : Muellerian−inhibiting factor on eGFR

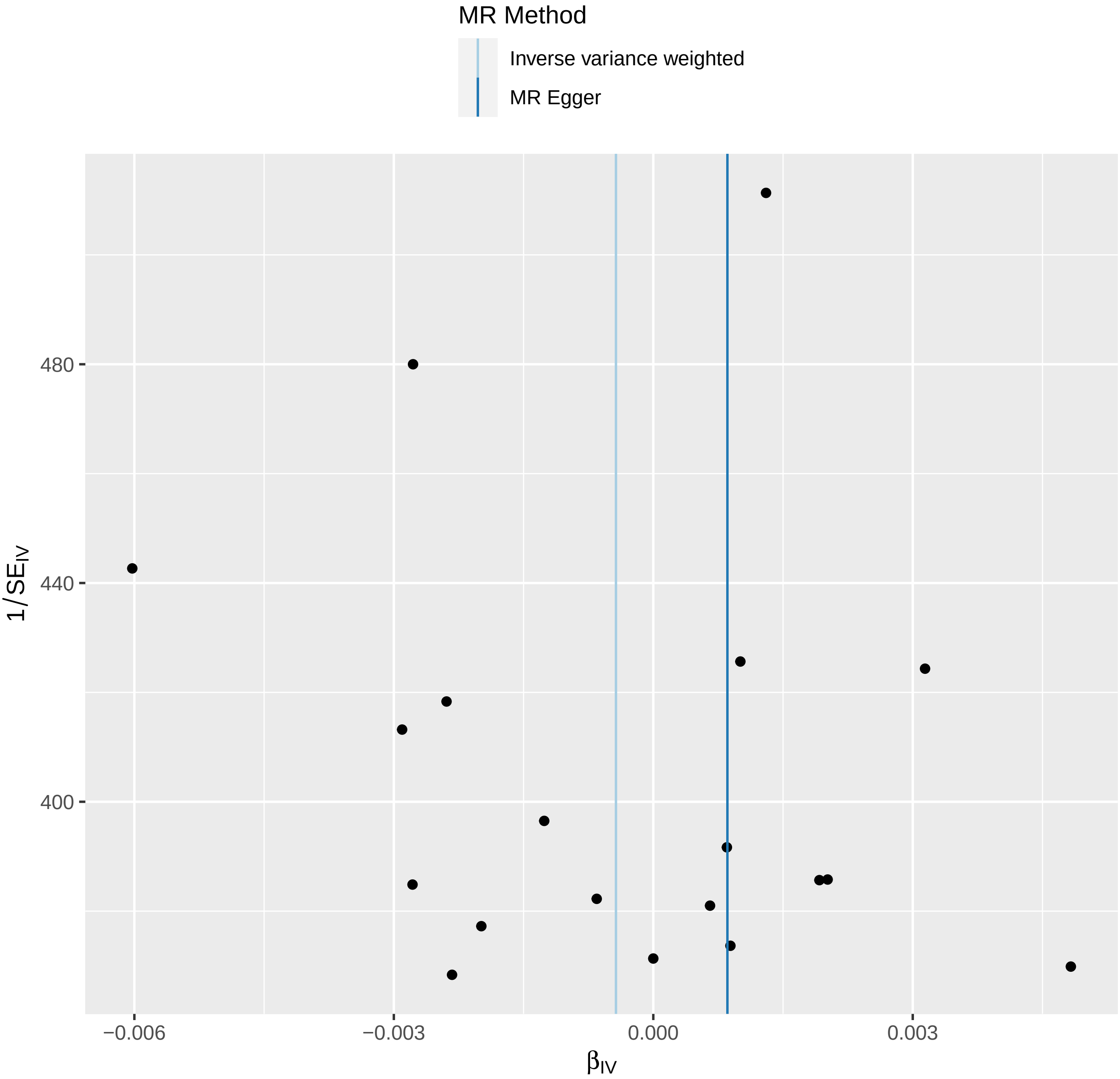

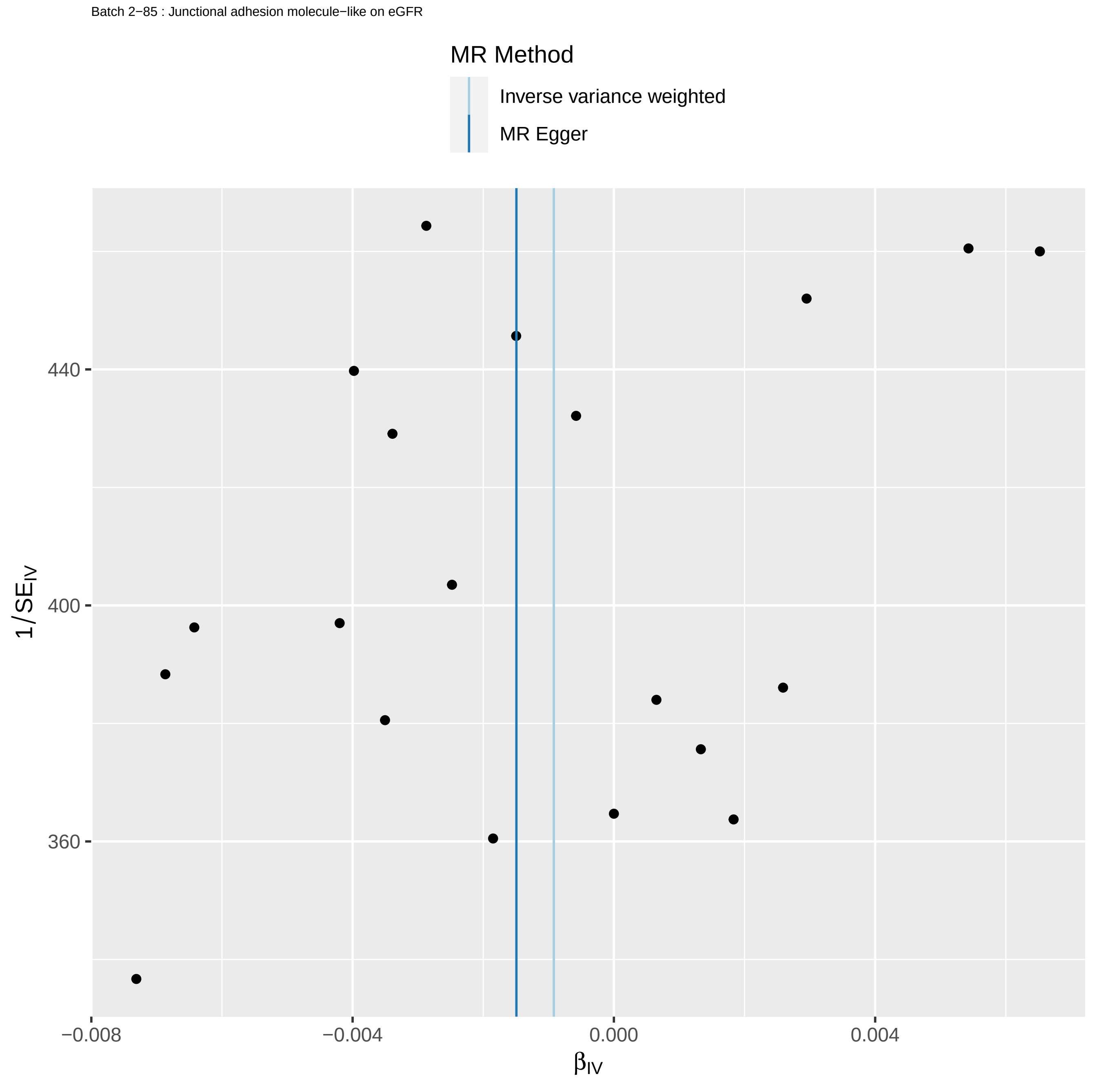
Batch 2−85 : Junctional adhesion molecule−like on eGFR
MR Method
Inverse variance weighted
MR Egger
440
400
360
1/SE_IV
−0.008
−0.004
0.000
0.004
β_IV

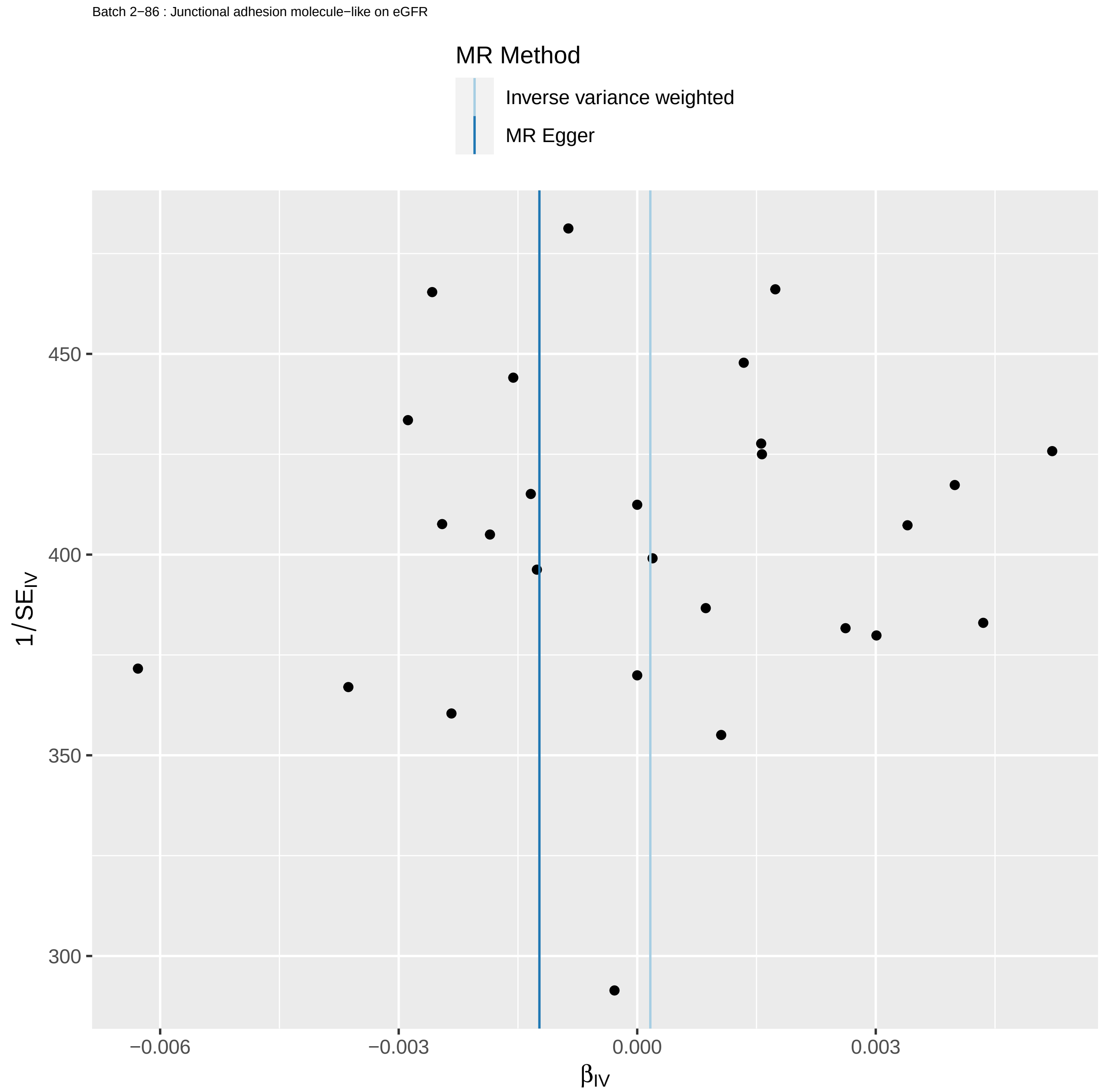

Batch 2−86 : Junctional adhesion molecule−like on eGFR
MR Method
Inverse variance weighted
MR Egger
450
400
350
300
1/SE_IV
−0.006
−0.003
0.000
0.003
β_IV

Batch 2−87 : Amphoterin−induced protein 1 on eGFR

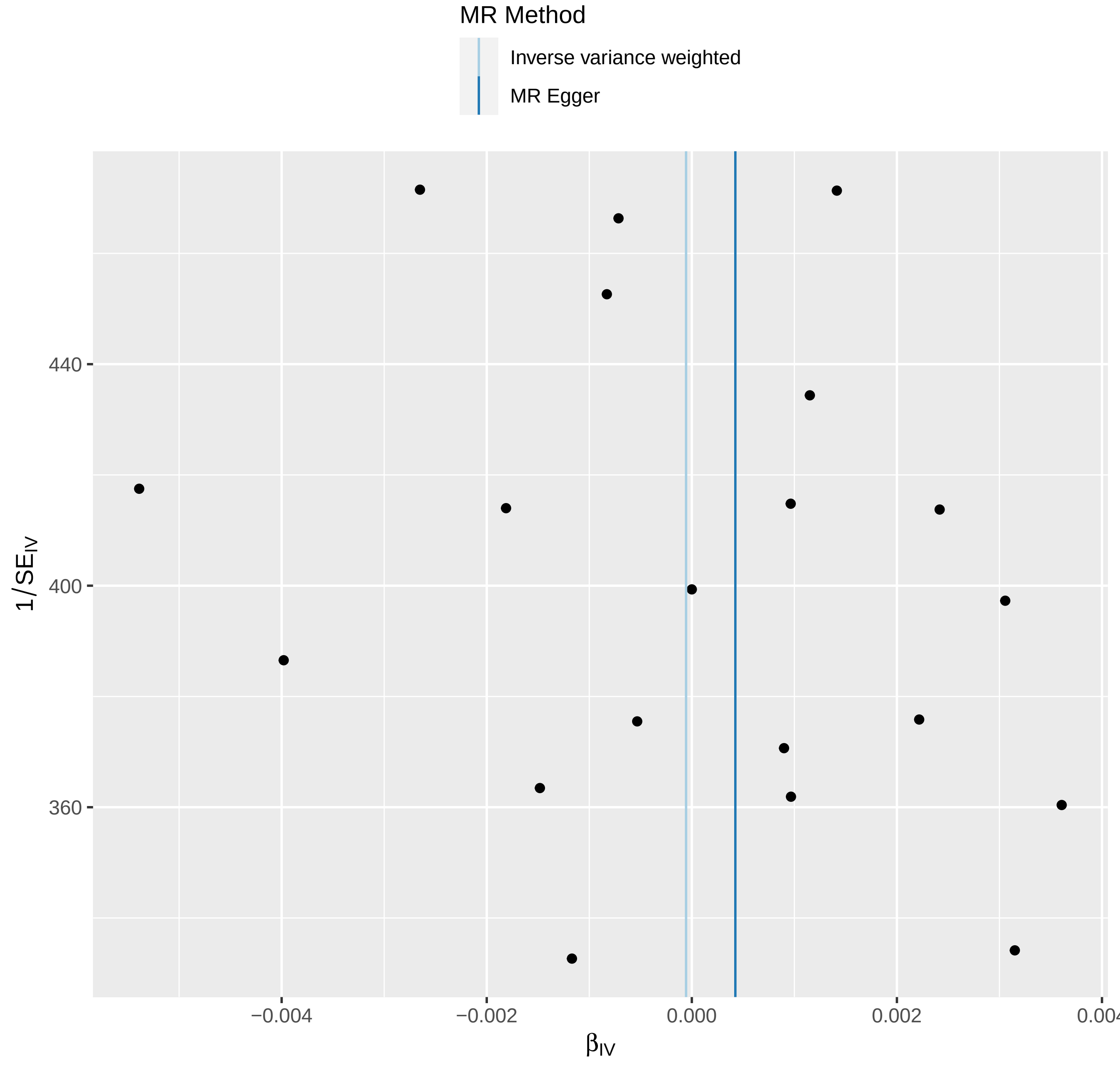

Batch 2−88 : Protein amnionless on eGFR

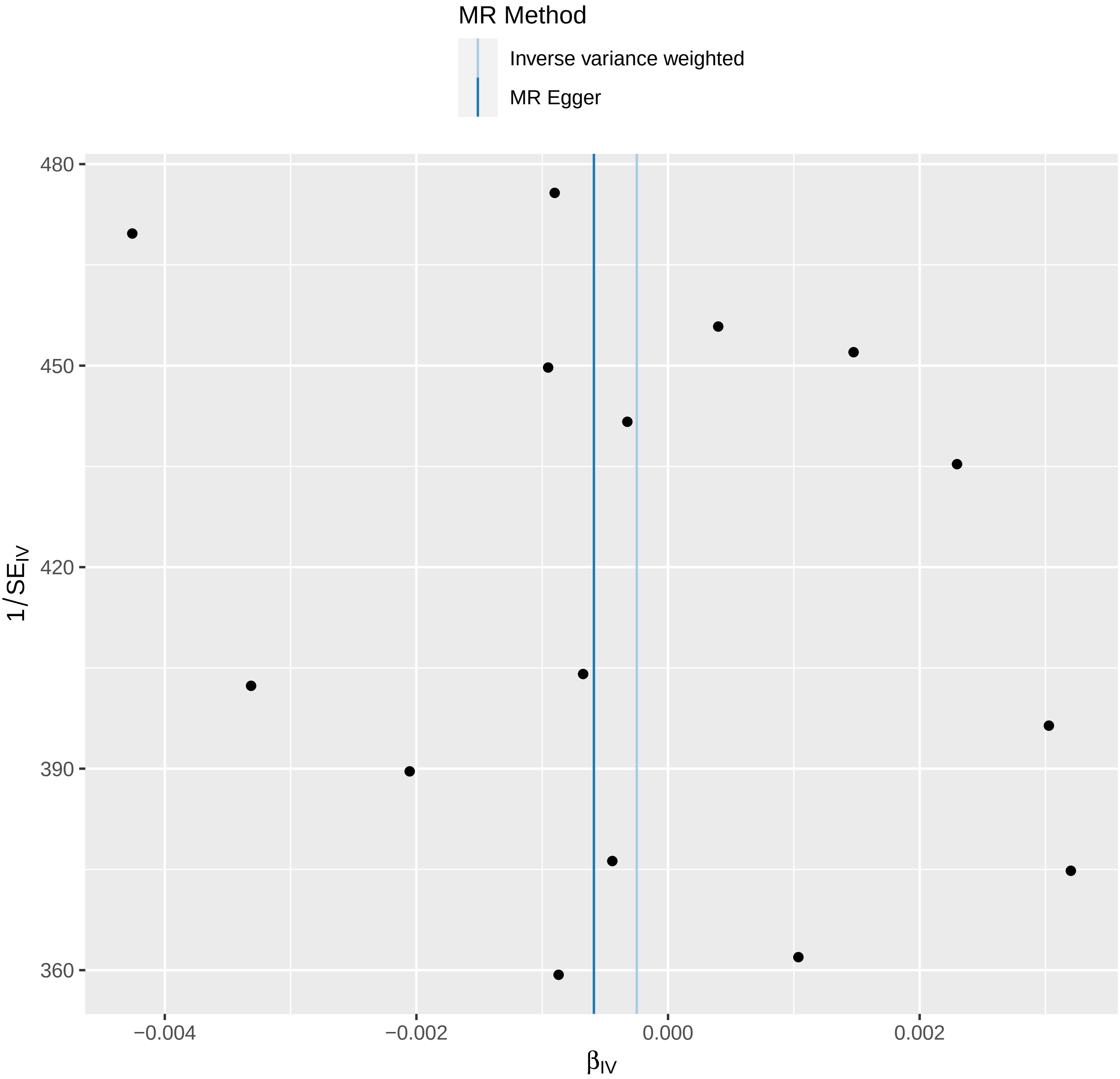

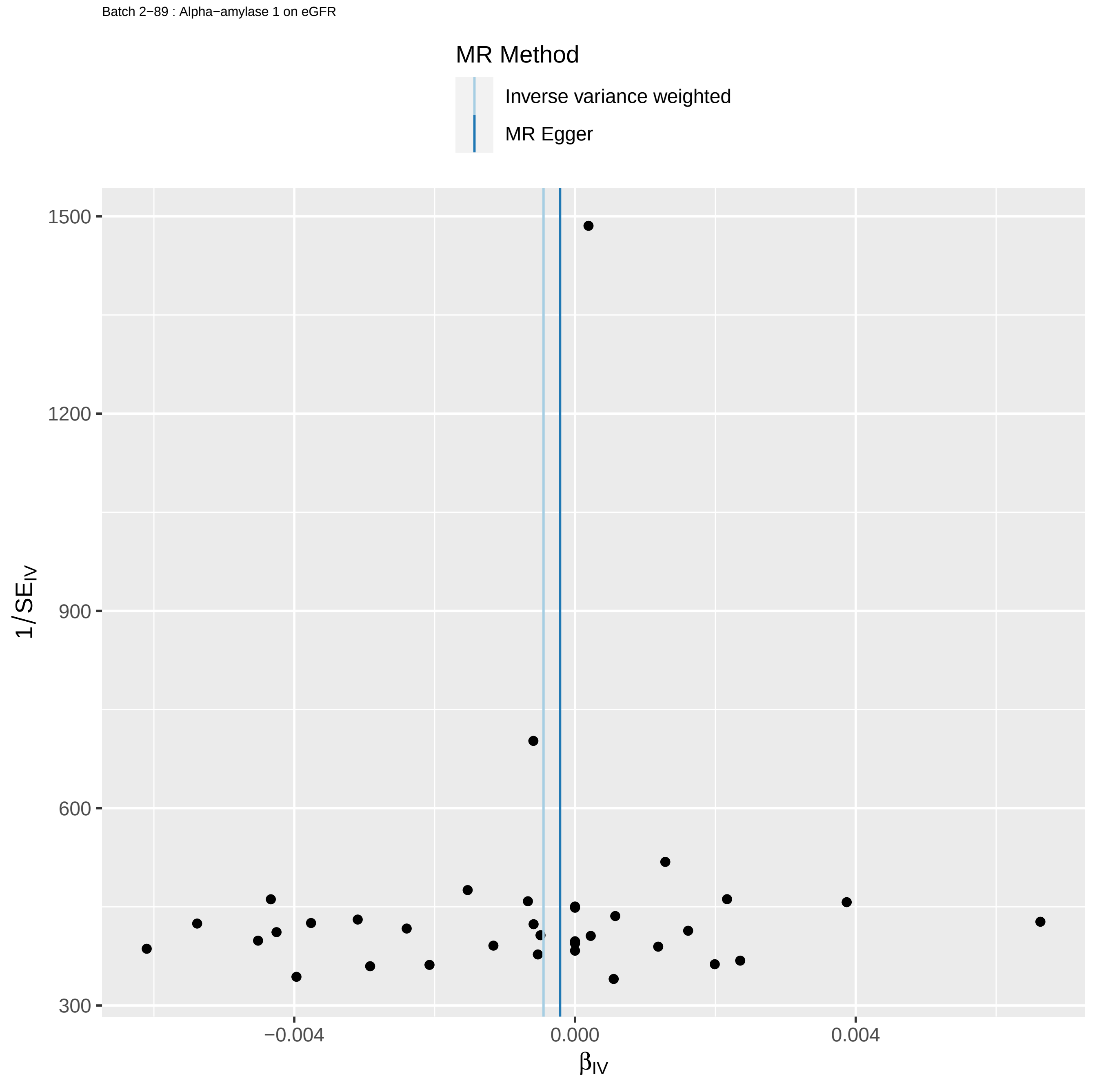
Batch 2−89 : Alpha−amylase 1 on eGFR
MR Method
Inverse variance weighted
MR Egger
1500
1200
900
600
300
1/SE$_{IV}$
−0.004
0.000
0.004
β_{IV}

Batch 2−90 : Anaphase−promoting complex subunit 10 on eGFR

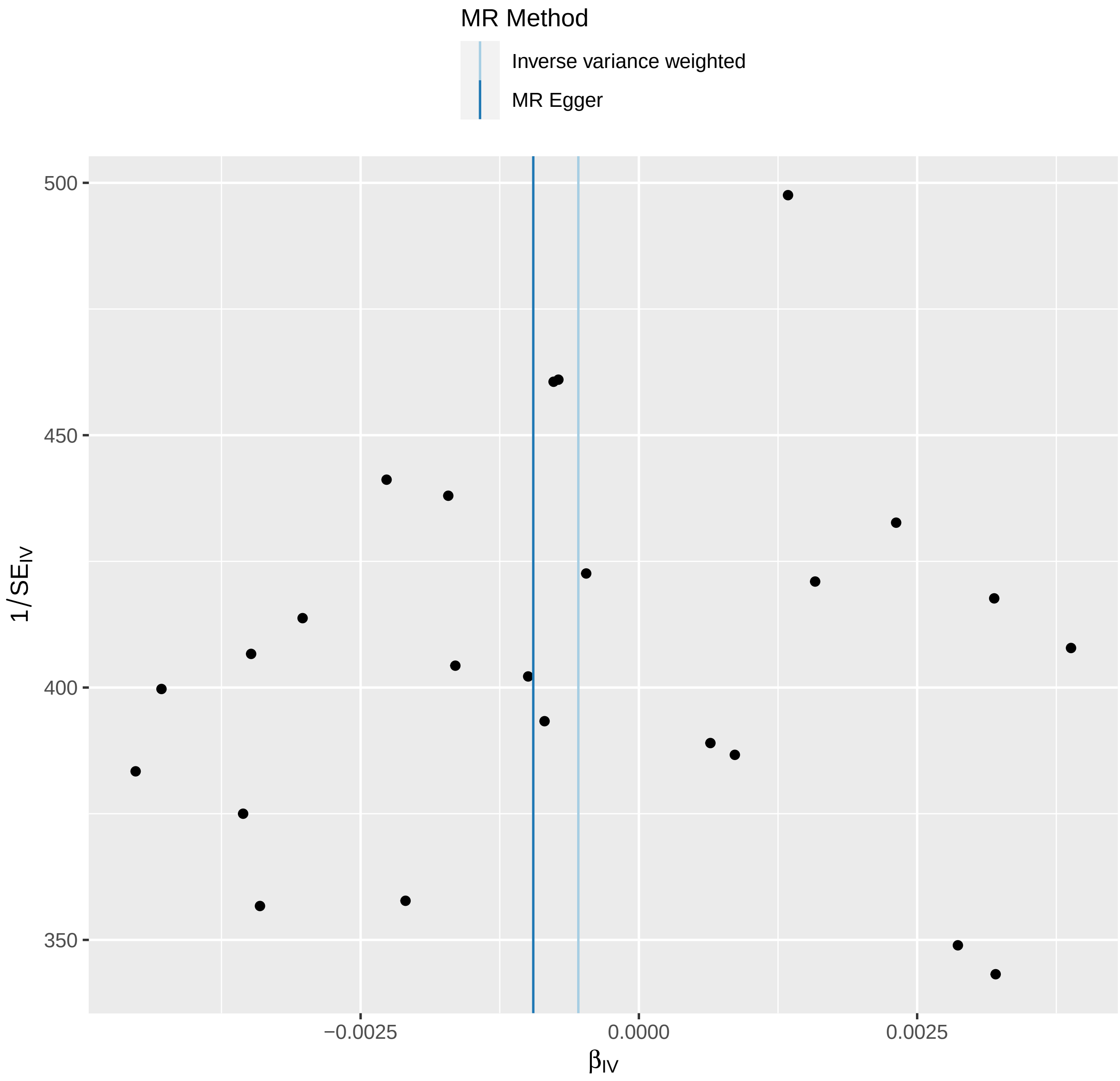

Batch 2−91 : Anaphase−promoting complex subunit 7 on eGFR

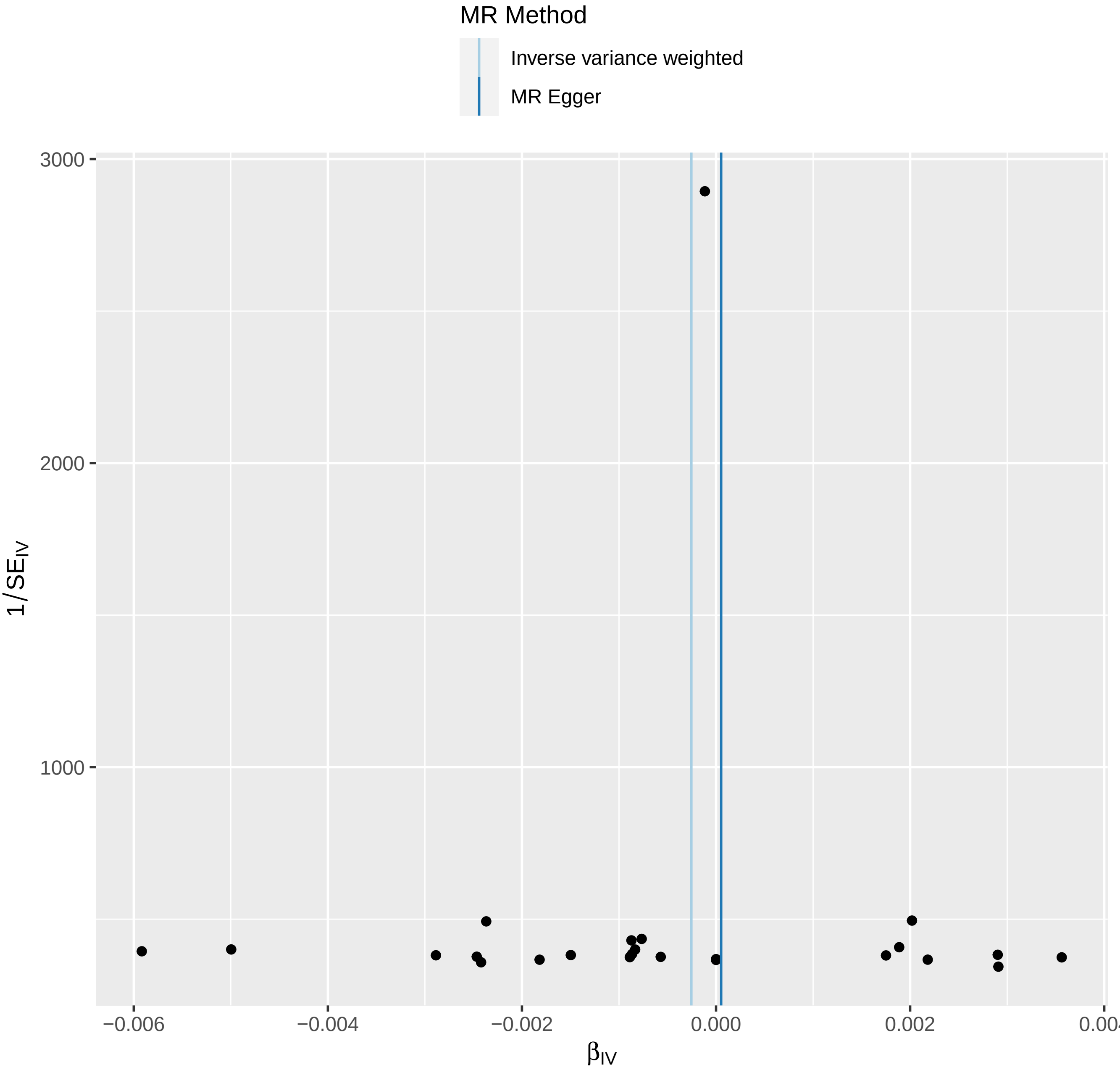

Batch 2−92 : Angiopoietin−1 on eGFR

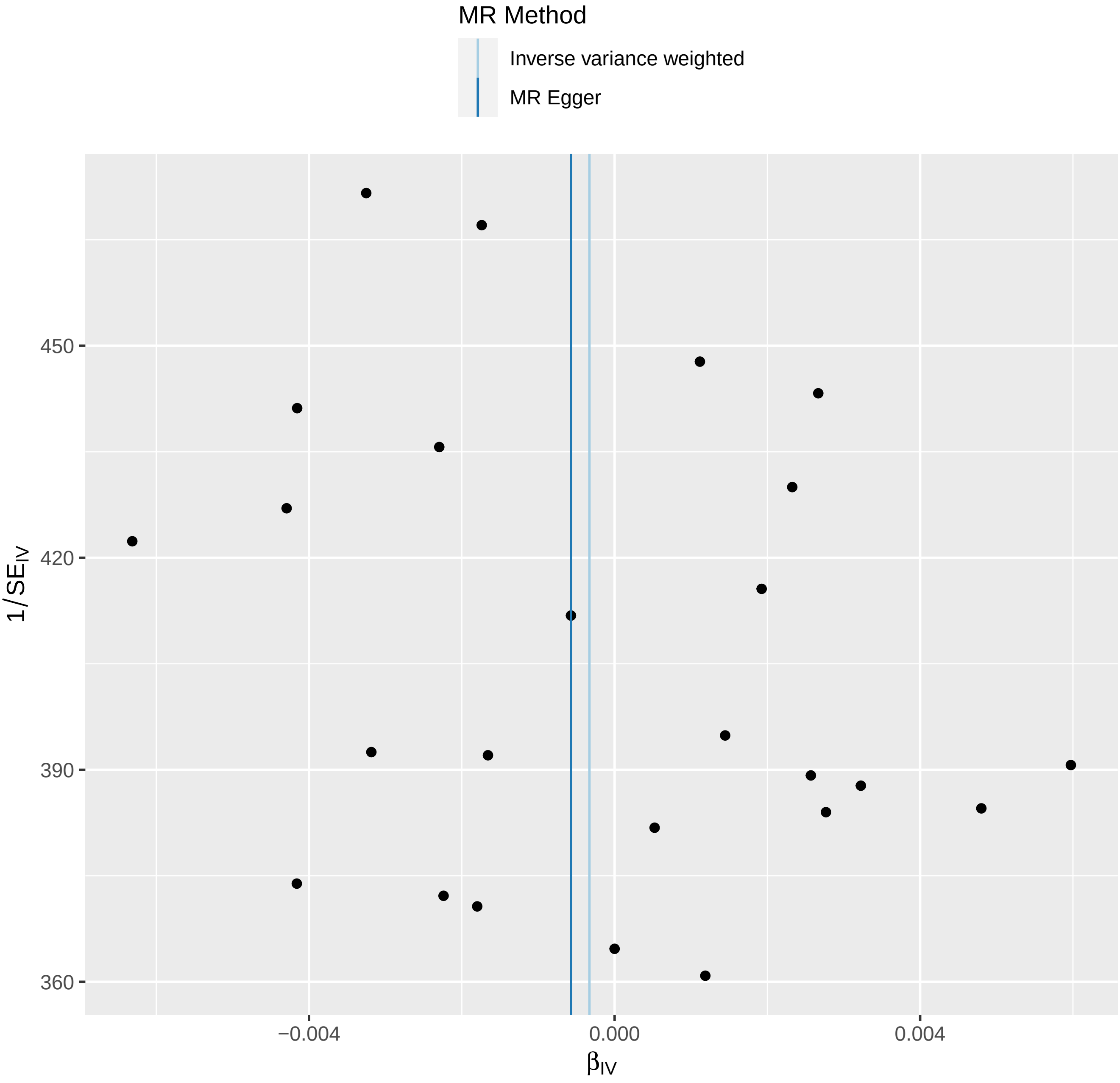

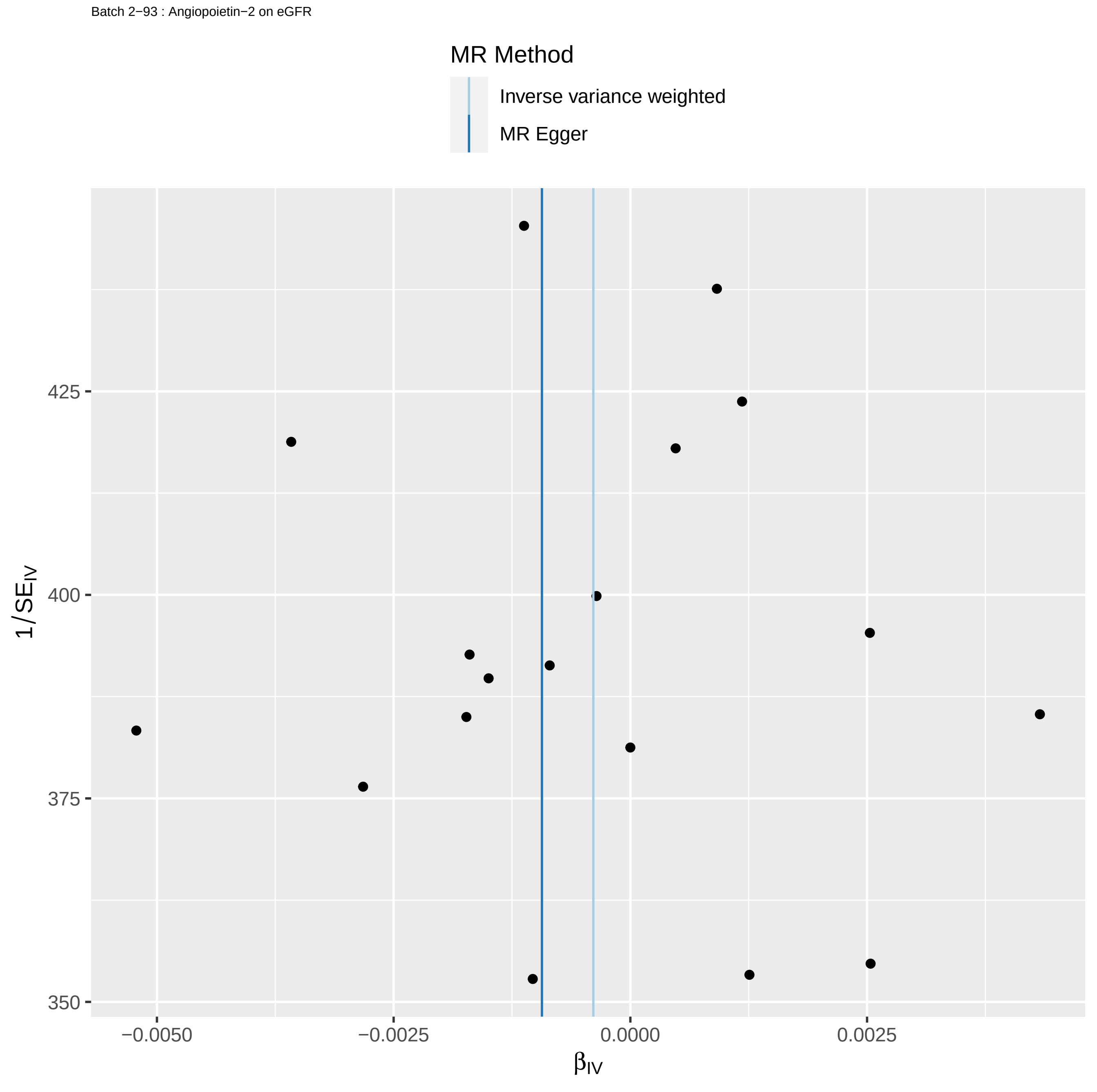
Batch 2−93 : Angiopoietin−2 on eGFR
MR Method
Inverse variance weighted
MR Egger
425
400
375
350
1/SE_IV
−0.0050
−0.0025
0.0000
0.0025
β_IV

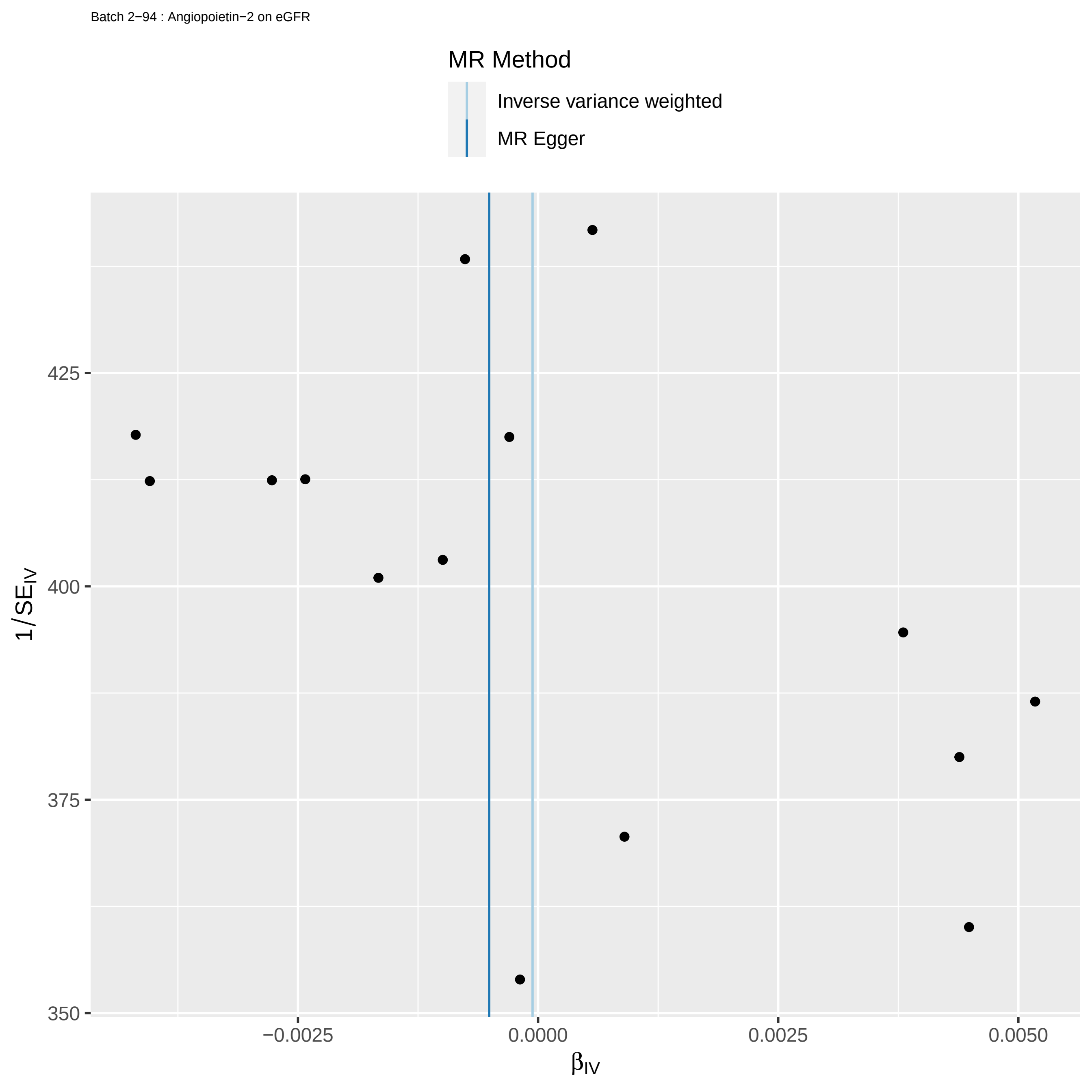

Batch 2−94 : Angiopoietin−2 on eGFR
MR Method
Inverse variance weighted
MR Egger
1/SE_IV
425
400
375
350
−0.0025
0.0000
0.0025
0.0050
β_IV

Batch 2−95 : Angiopoietin−4 on eGFR

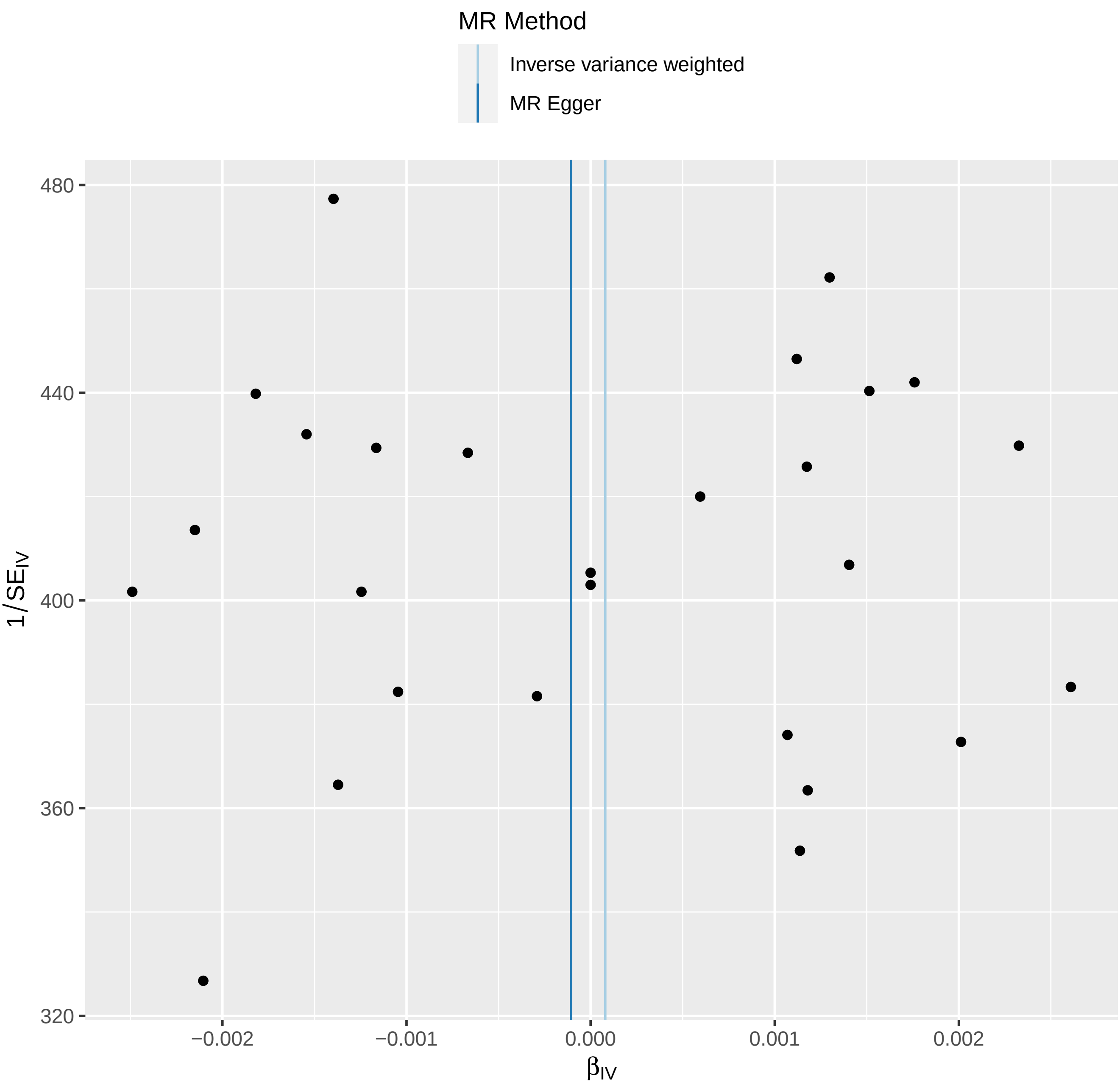

Batch 2−96 : Angiopoietin−related protein 1 on eGFR

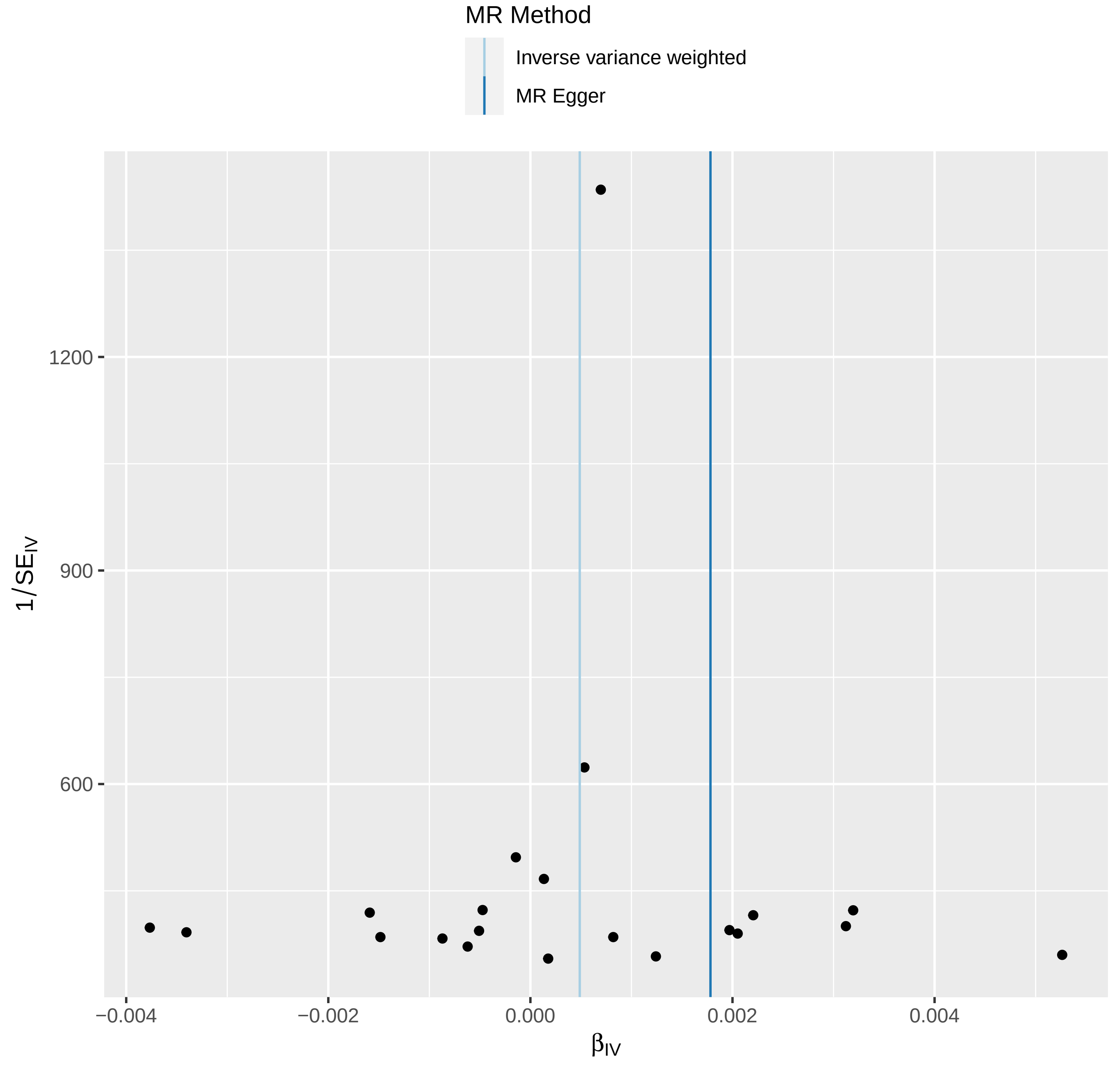

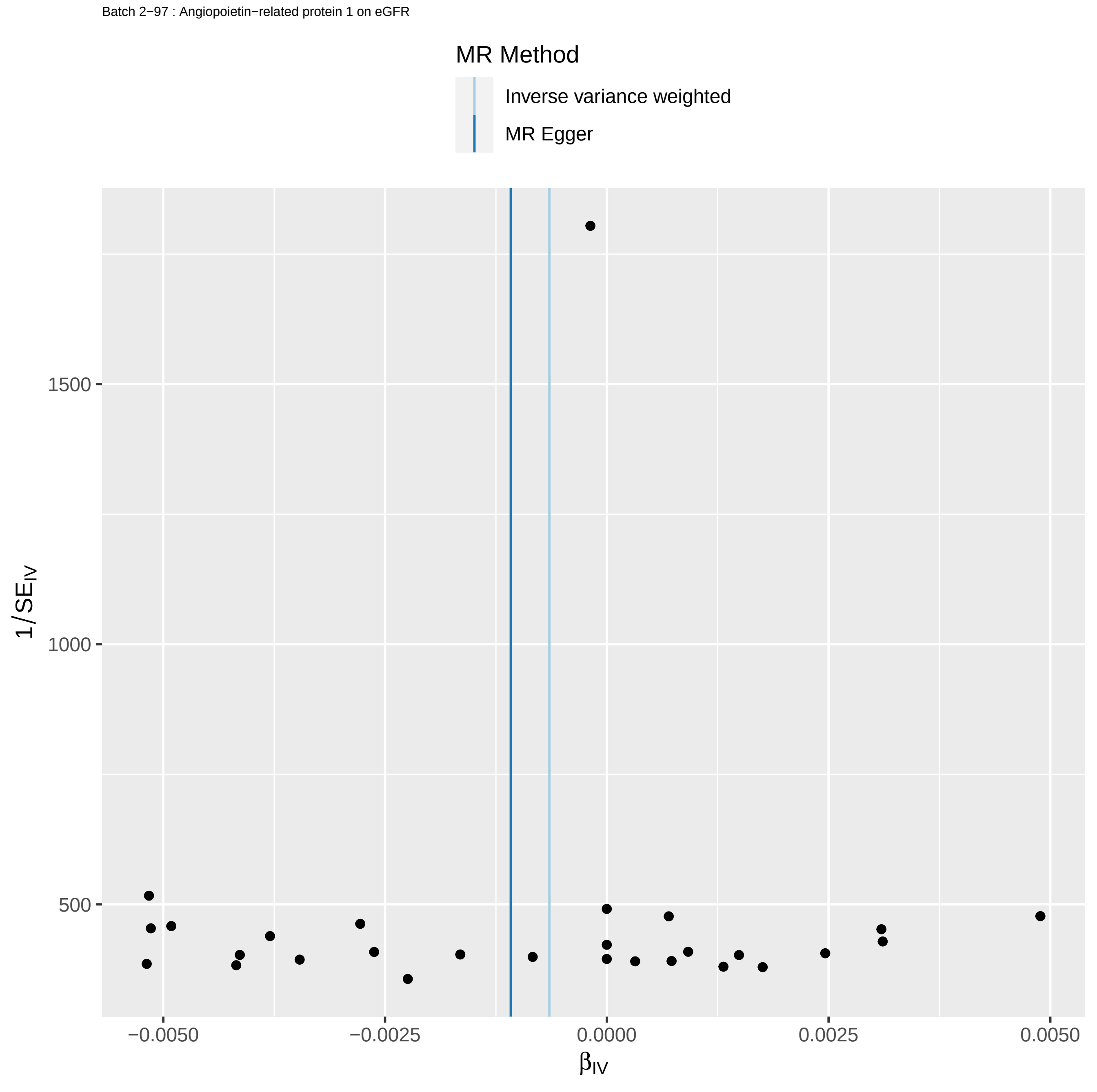

Batch 2−97 : Angiopoietin−related protein 1 on eGFR
MR Method
Inverse variance weighted
MR Egger
1500
1000
500
1/SE$_{IV}$
−0.0050
−0.0025
0.0000
0.0025
0.0050
β$_{IV}$

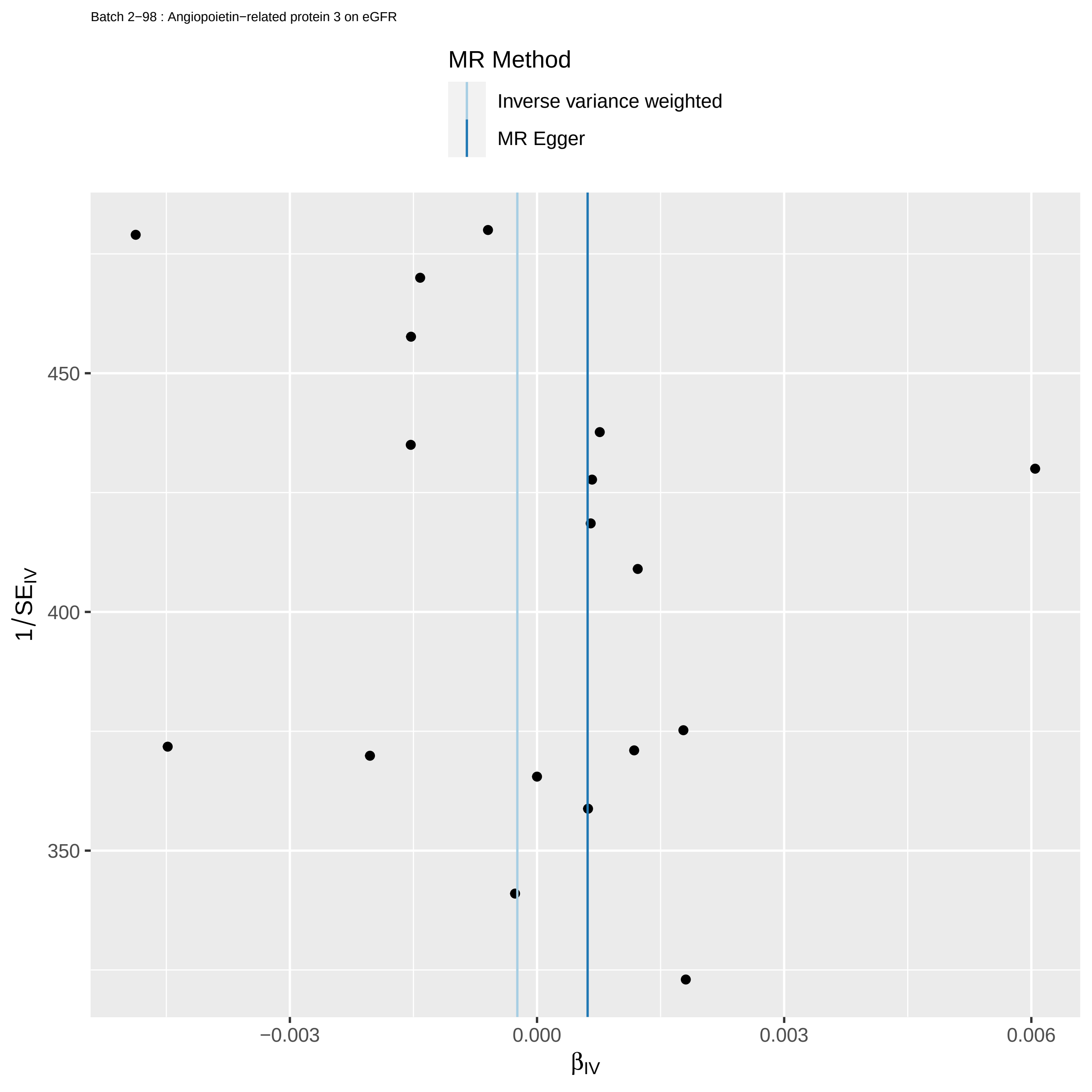

Batch 2−98 : Angiopoietin−related protein 3 on eGFR
MR Method
Inverse variance weighted
MR Egger
450
400
350
1/SE$_{IV}$
−0.003
0.000
0.003
0.006
β$_{IV}$

Batch 2−99 : Angiopoietin−related protein 4 on eGFR

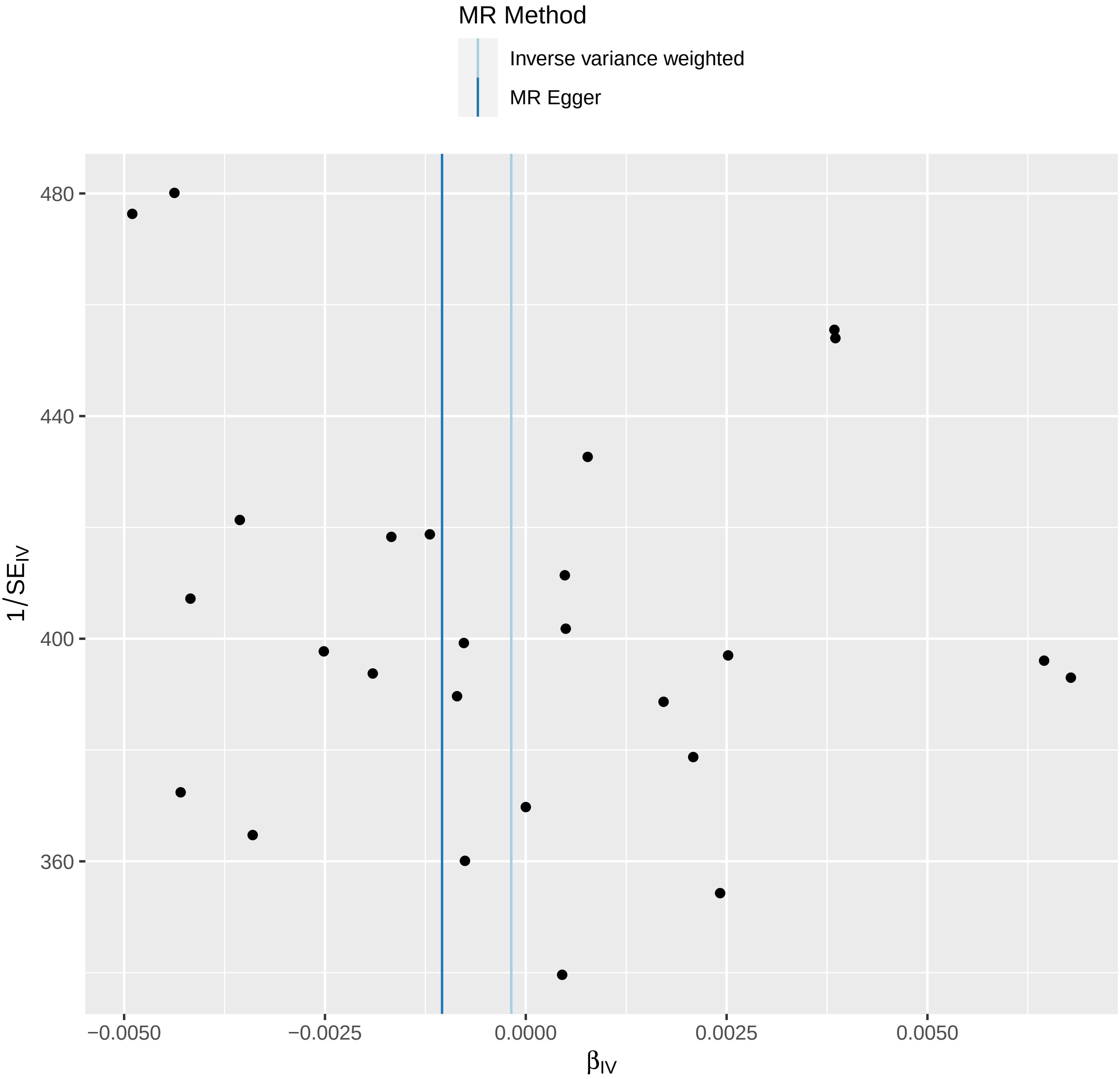

Batch 2−100 : Angiopoietin−related protein 7 on eGFR

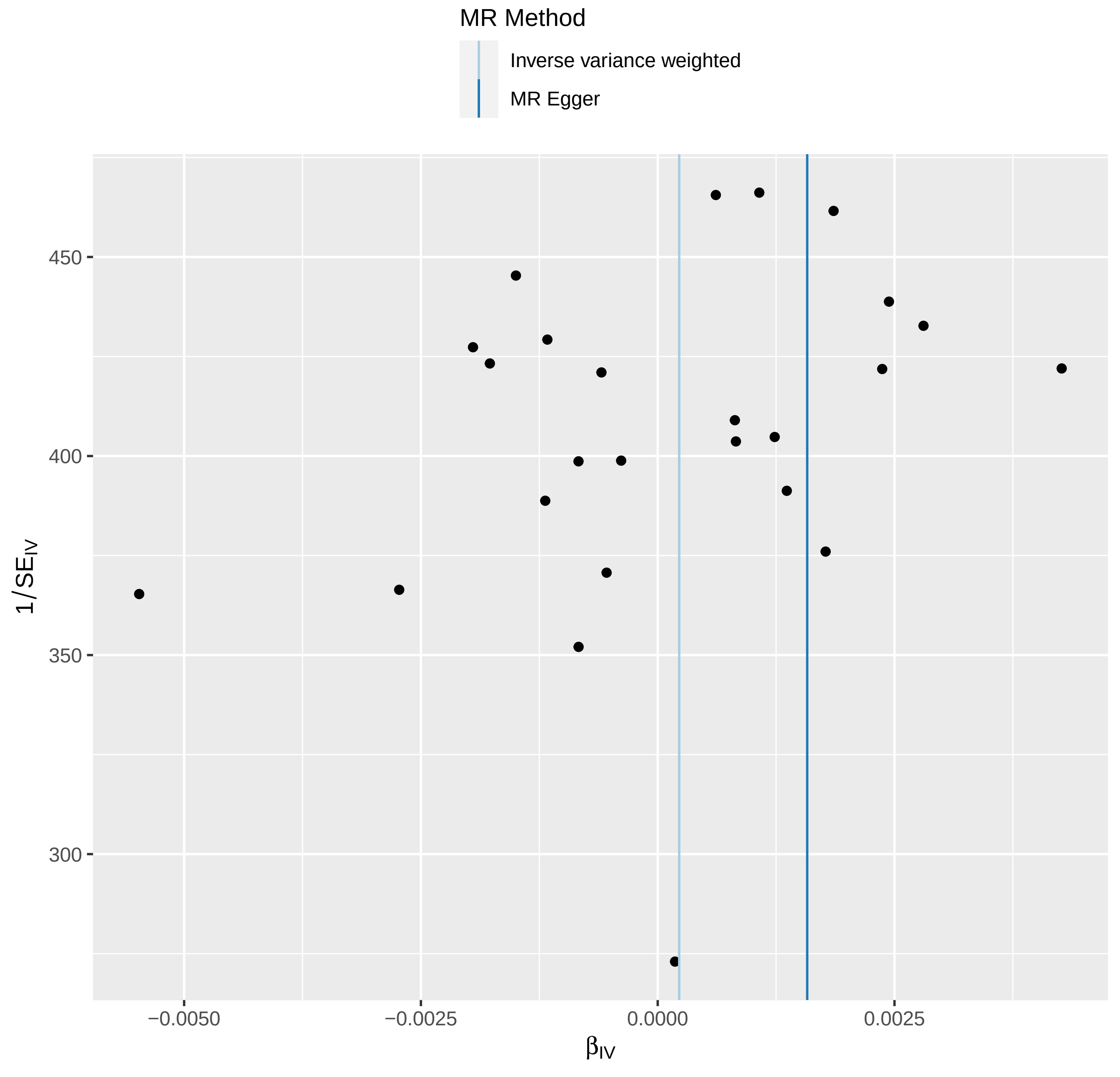

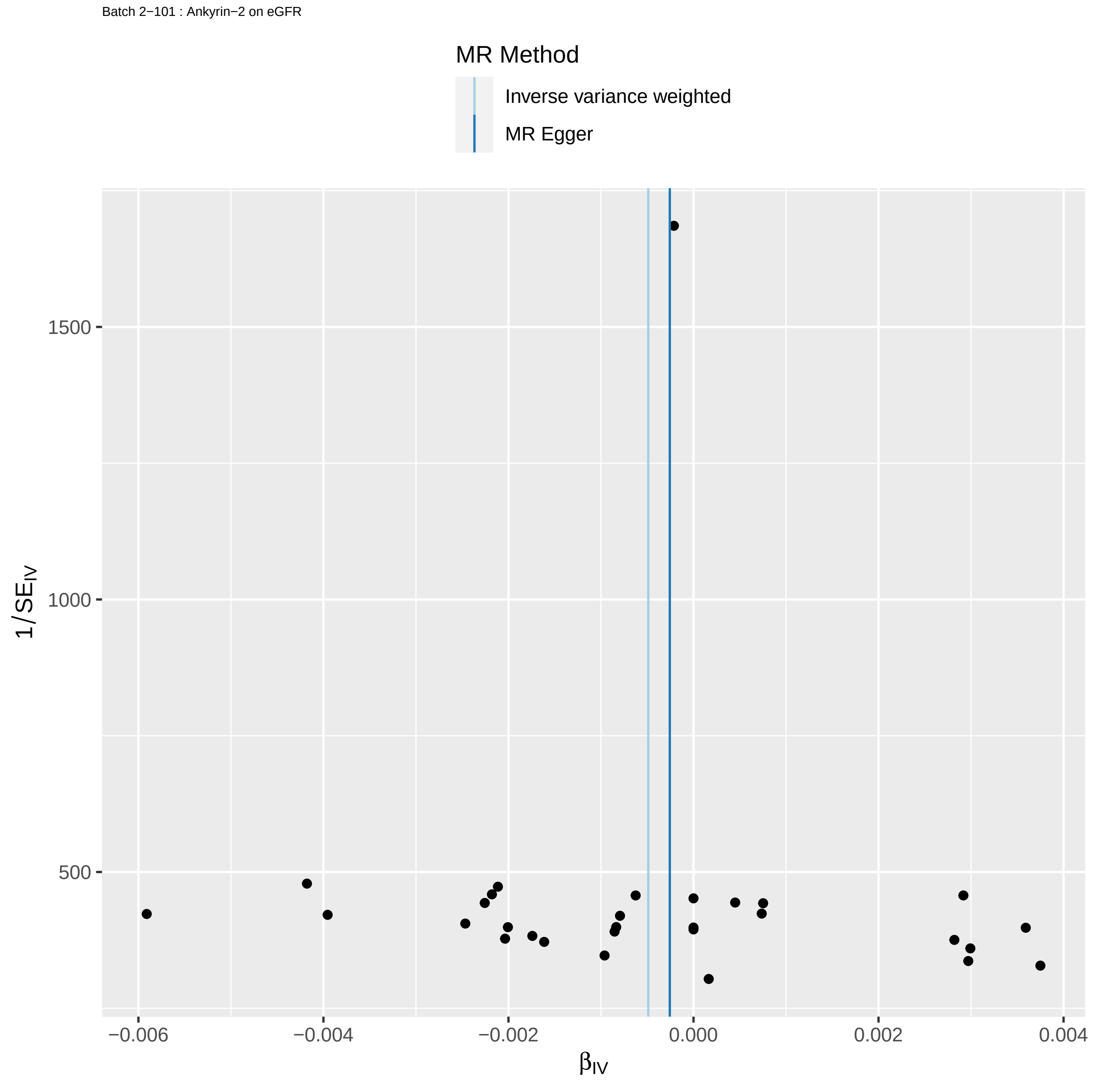

Batch 2−101 : Ankyrin−2 on eGFR
MR Method
Inverse variance weighted
MR Egger
1500
1000
500
1/SE$_{IV}$
−0.006
−0.004
−0.002
0.000
0.002
0.004
β$_{IV}$

Batch 2−102 : Ankyrin repeat domain−containing protein 27 on eGFR

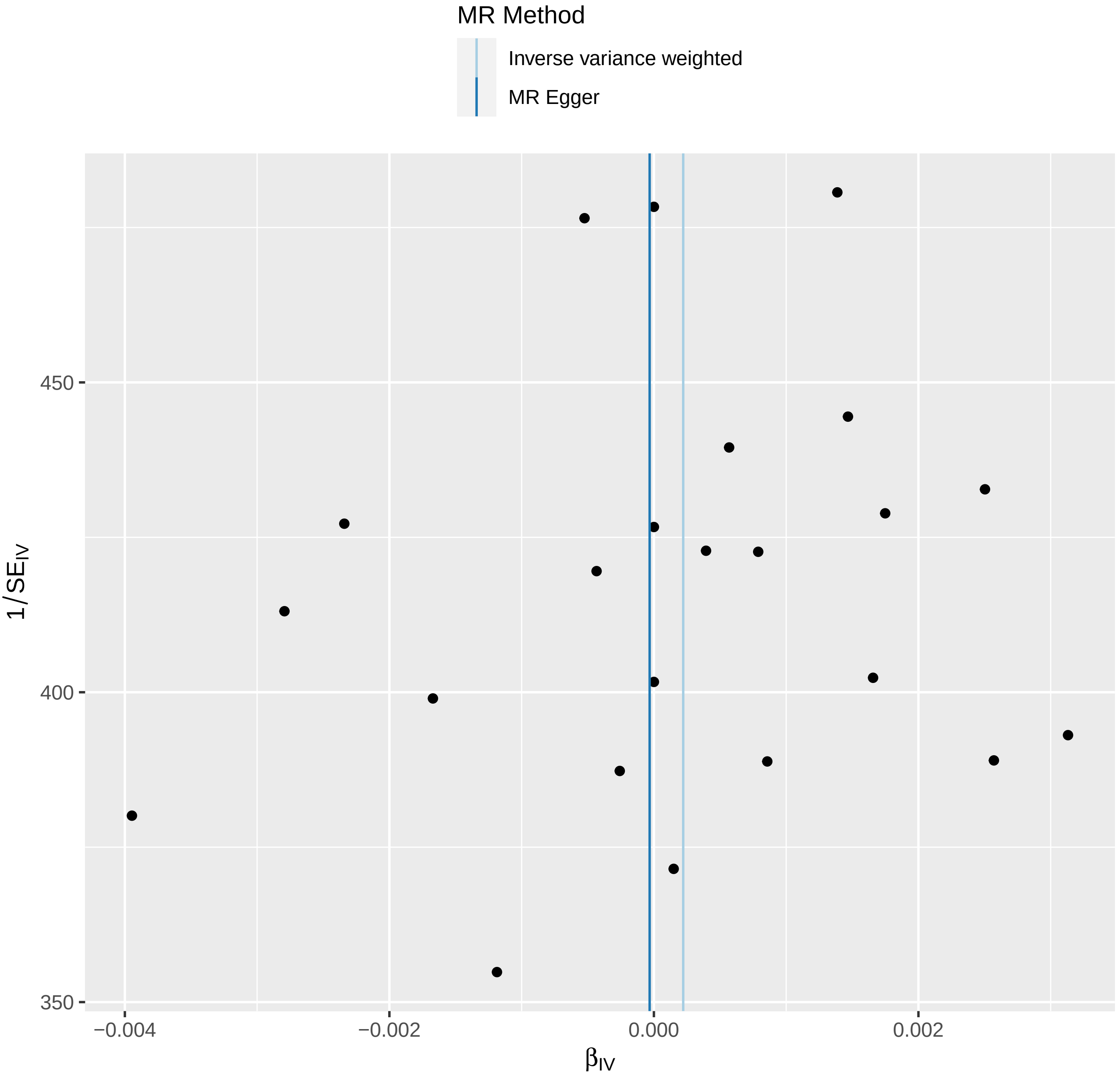

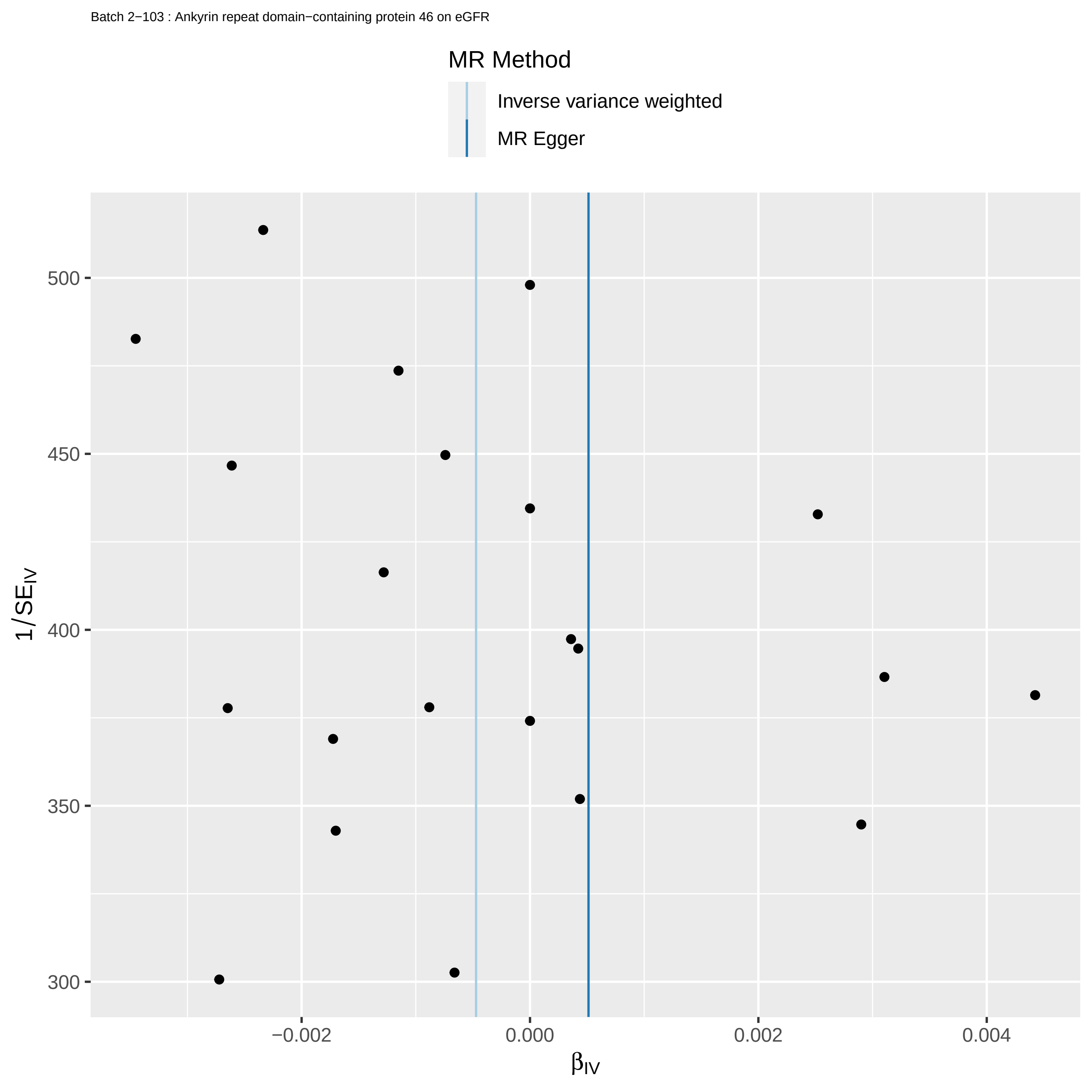

Batch 2−103 : Ankyrin repeat domain−containing protein 46 on eGFR
MR Method
Inverse variance weighted
MR Egger
500
450
400
350
300
1/SE_IV
−0.002
0.000
0.002
0.004
β_IV

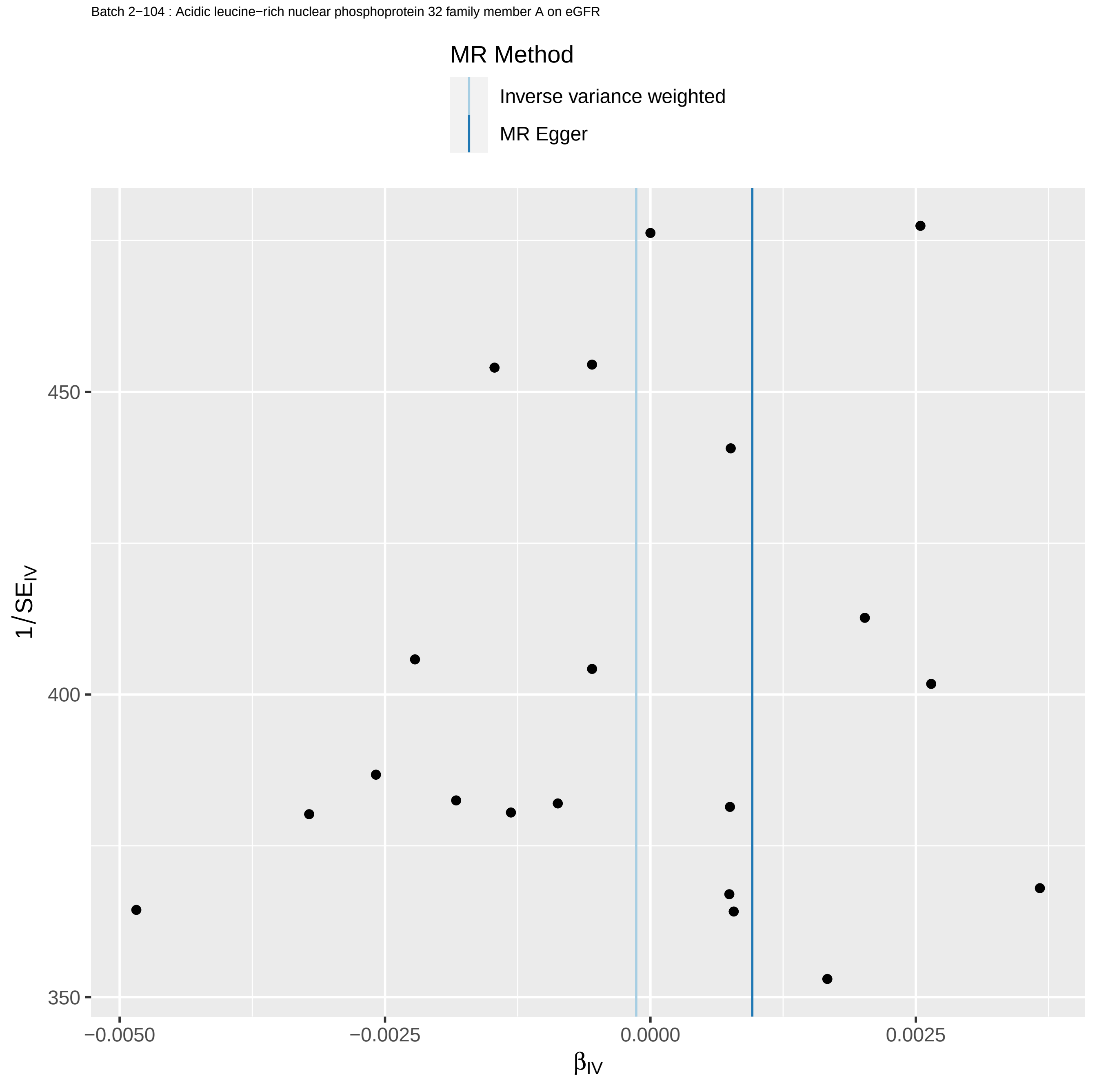
Batch 2−104 : Acidic leucine−rich nuclear phosphoprotein 32 family member A on eGFR
MR Method
Inverse variance weighted
MR Egger
450
400
350
1/SE_IV
−0.0050
−0.0025
0.0000
0.0025
β_IV

Batch 2−105 : Acidic leucine−rich nuclear phosphoprotein 32 family member B on eGFR

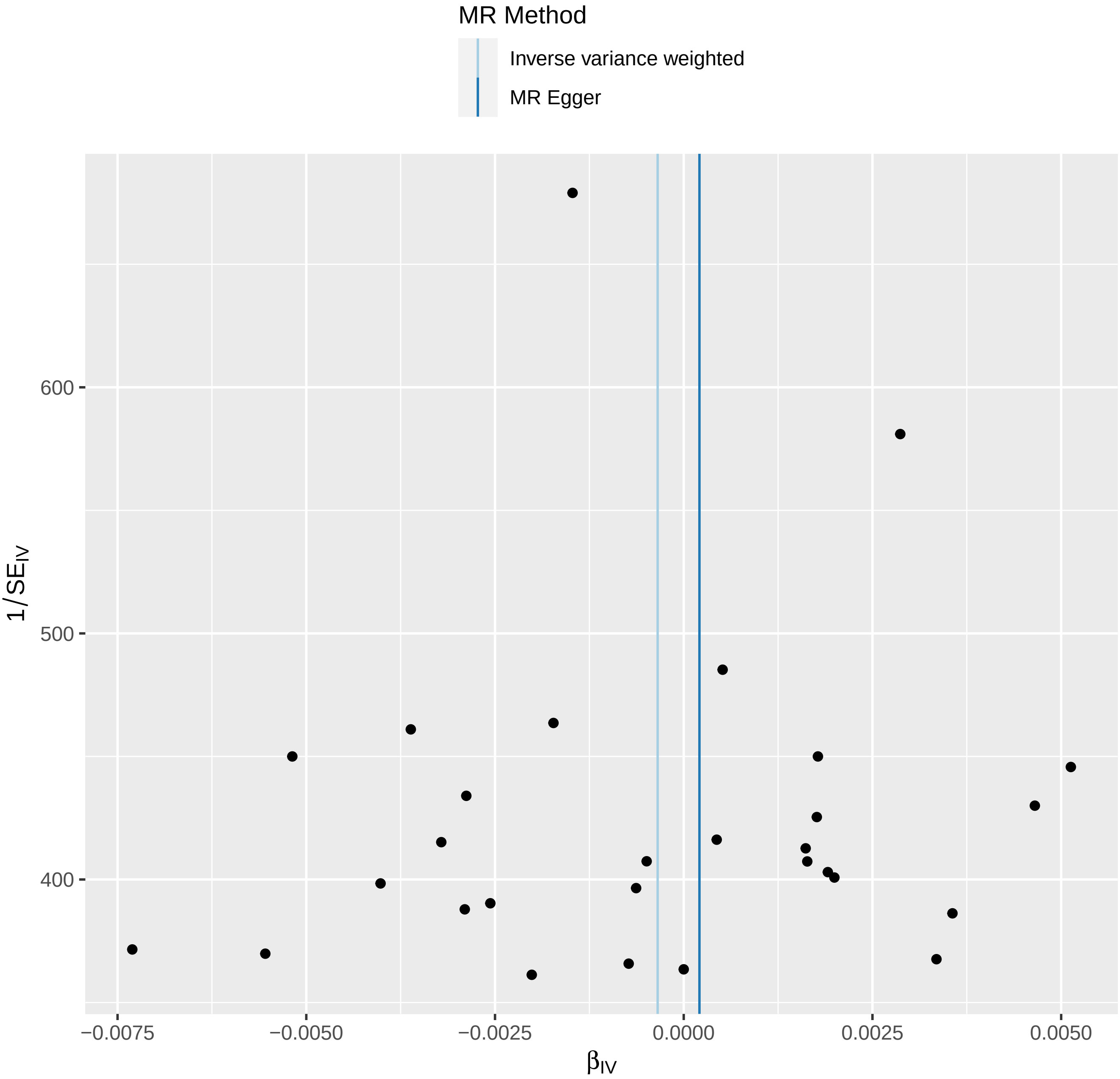

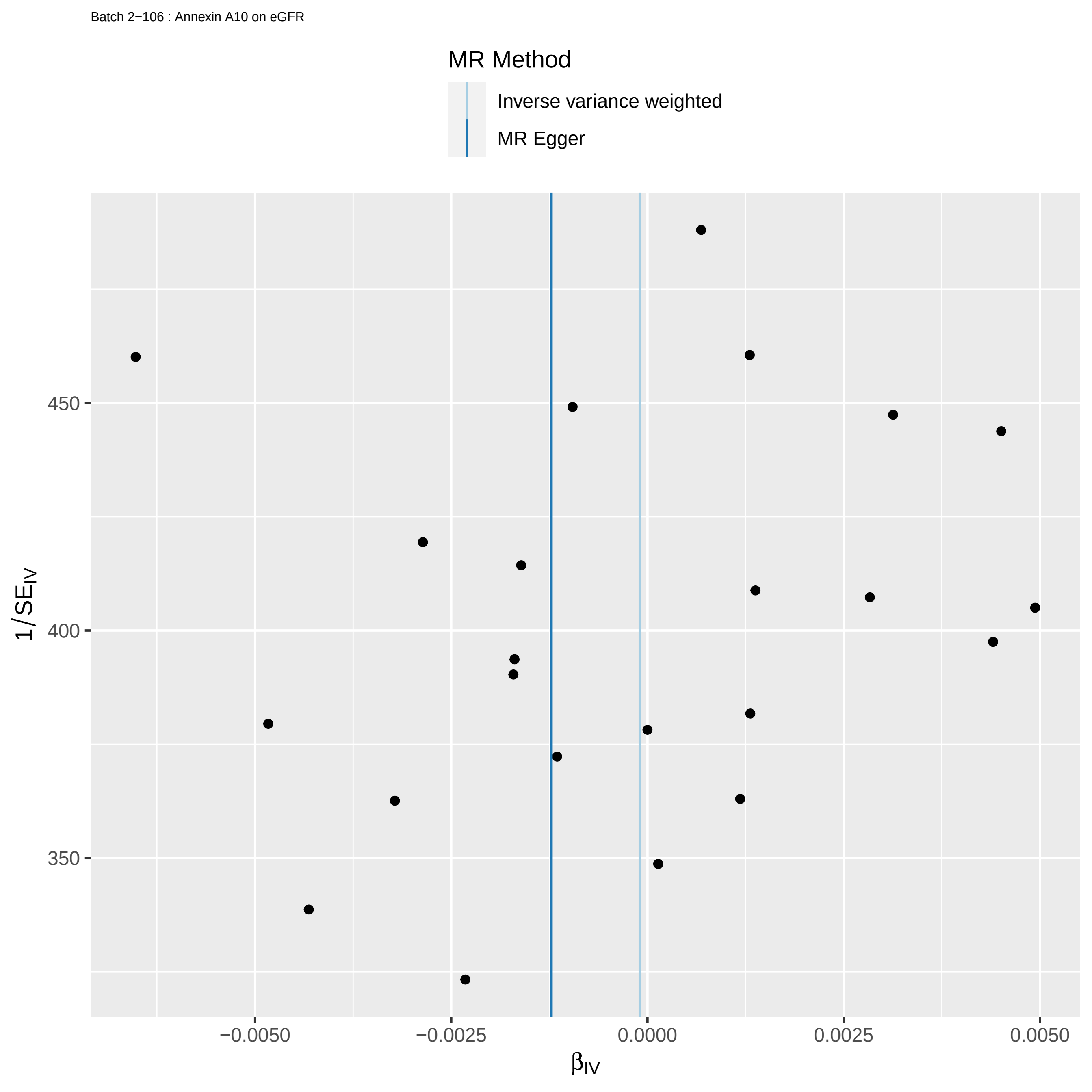
Batch 2−106 : Annexin A10 on eGFR
MR Method
Inverse variance weighted
MR Egger
450
400
350
1/SE$_{IV}$
−0.0050
−0.0025
0.0000
0.0025
0.0050
β$_{IV}$

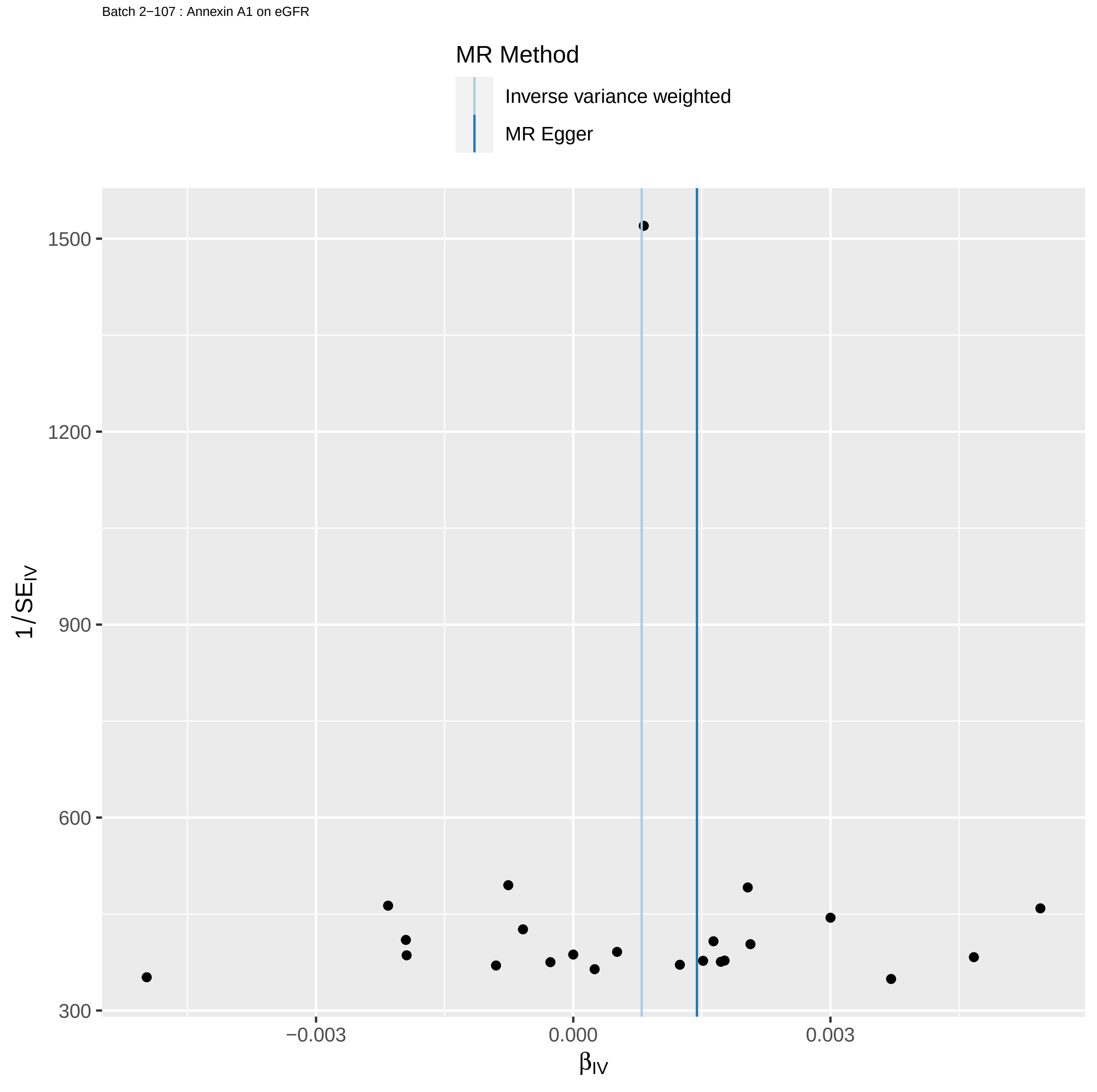
Batch 2−107 : Annexin A1 on eGFR
MR Method
Inverse variance weighted
MR Egger
1500
1200
900
600
300
1/SE_IV
−0.003
0.000
0.003
β_IV

Batch 2−108 : Annexin A2 on eGFR

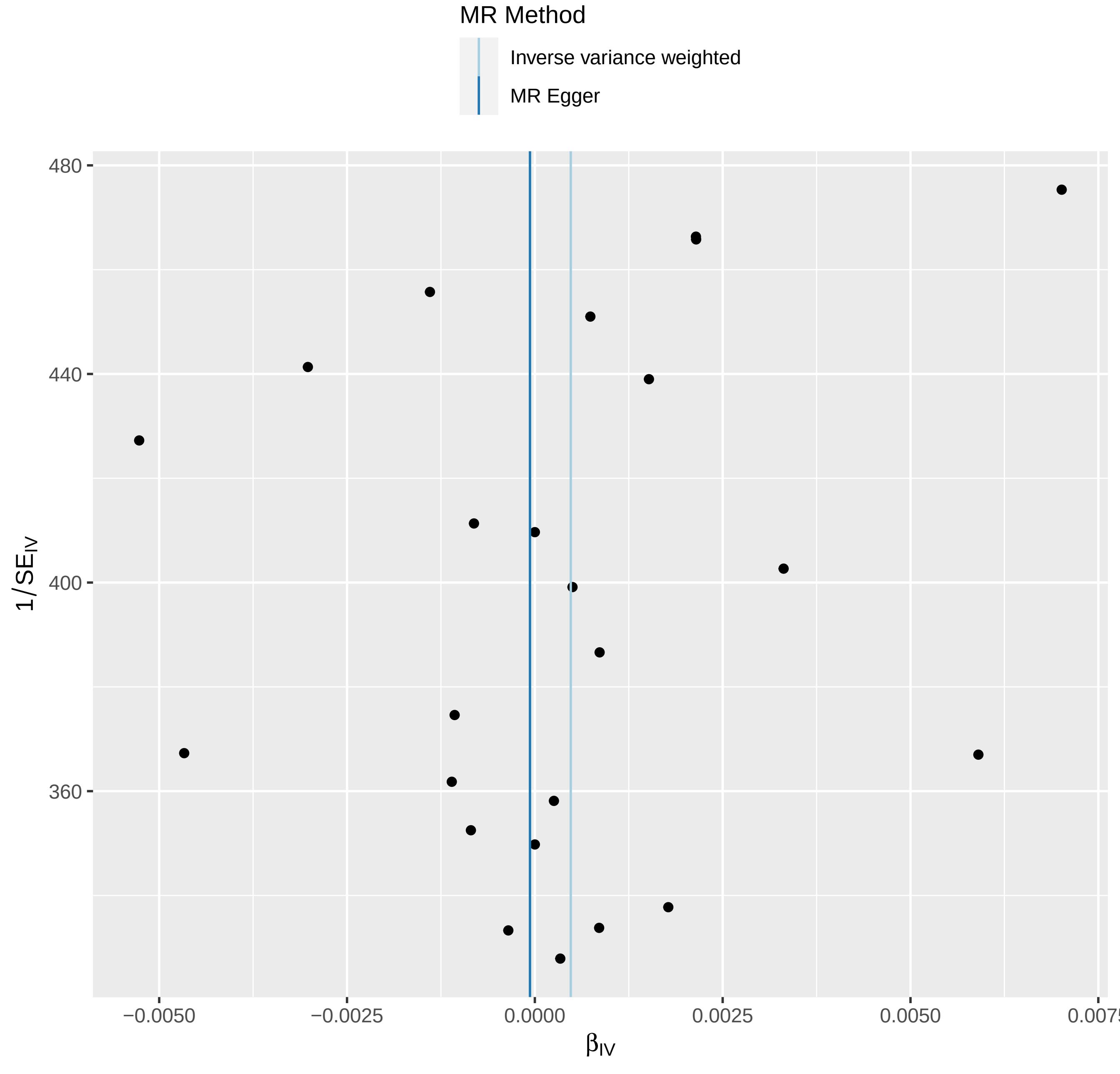

Batch 2−109 : Annexin A2 on eGFR

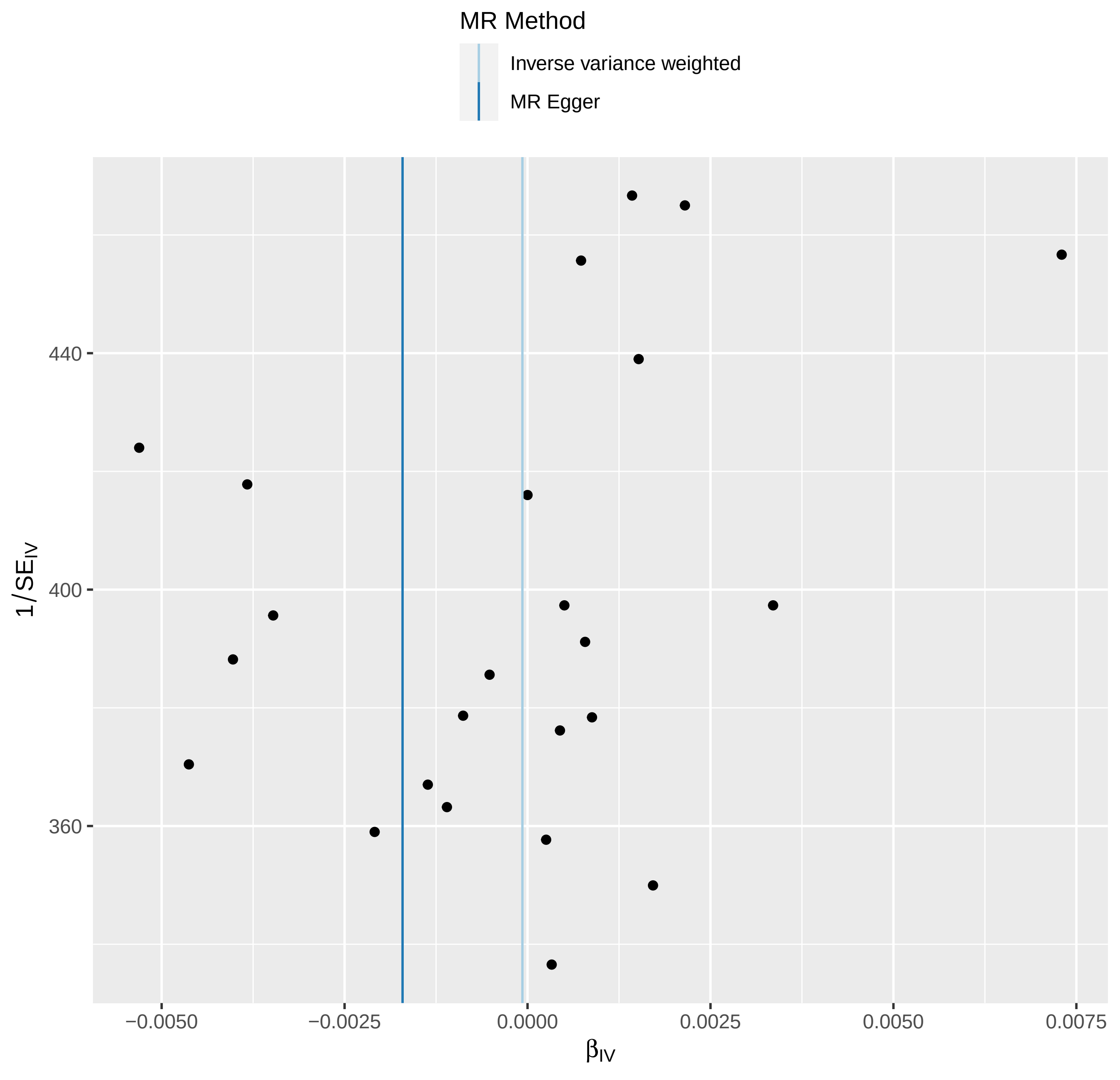

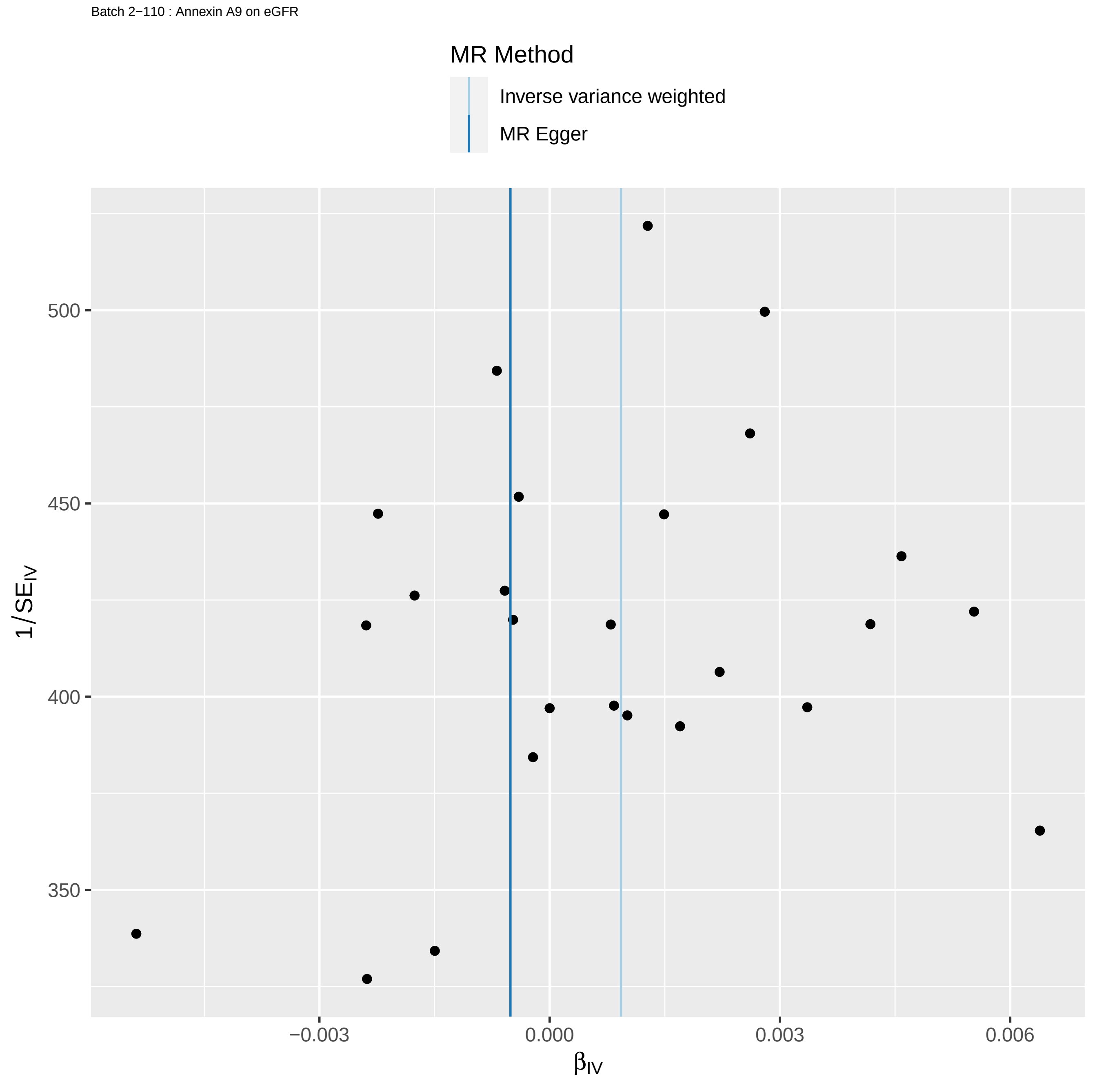
Batch 2−110 : Annexin A9 on eGFR
MR Method
Inverse variance weighted
MR Egger
500
450
400
350
1/SE$_{IV}$
−0.003
0.000
0.003
0.006
β_{IV}

Batch 2−111 : Retina−specific copper amine oxidase on eGFR

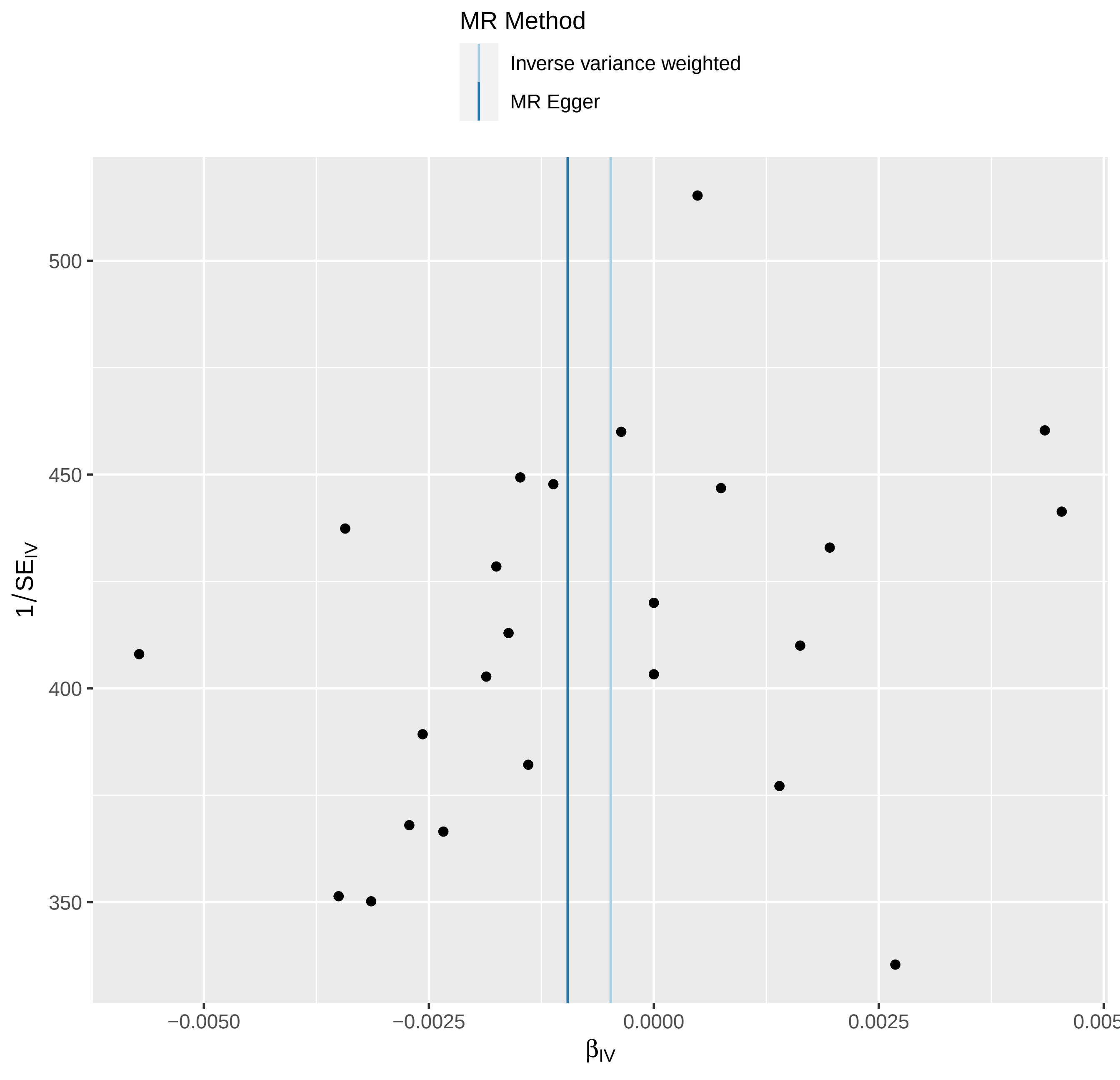

Batch 2−112 : AP−4 complex subunit mu−1 on eGFR

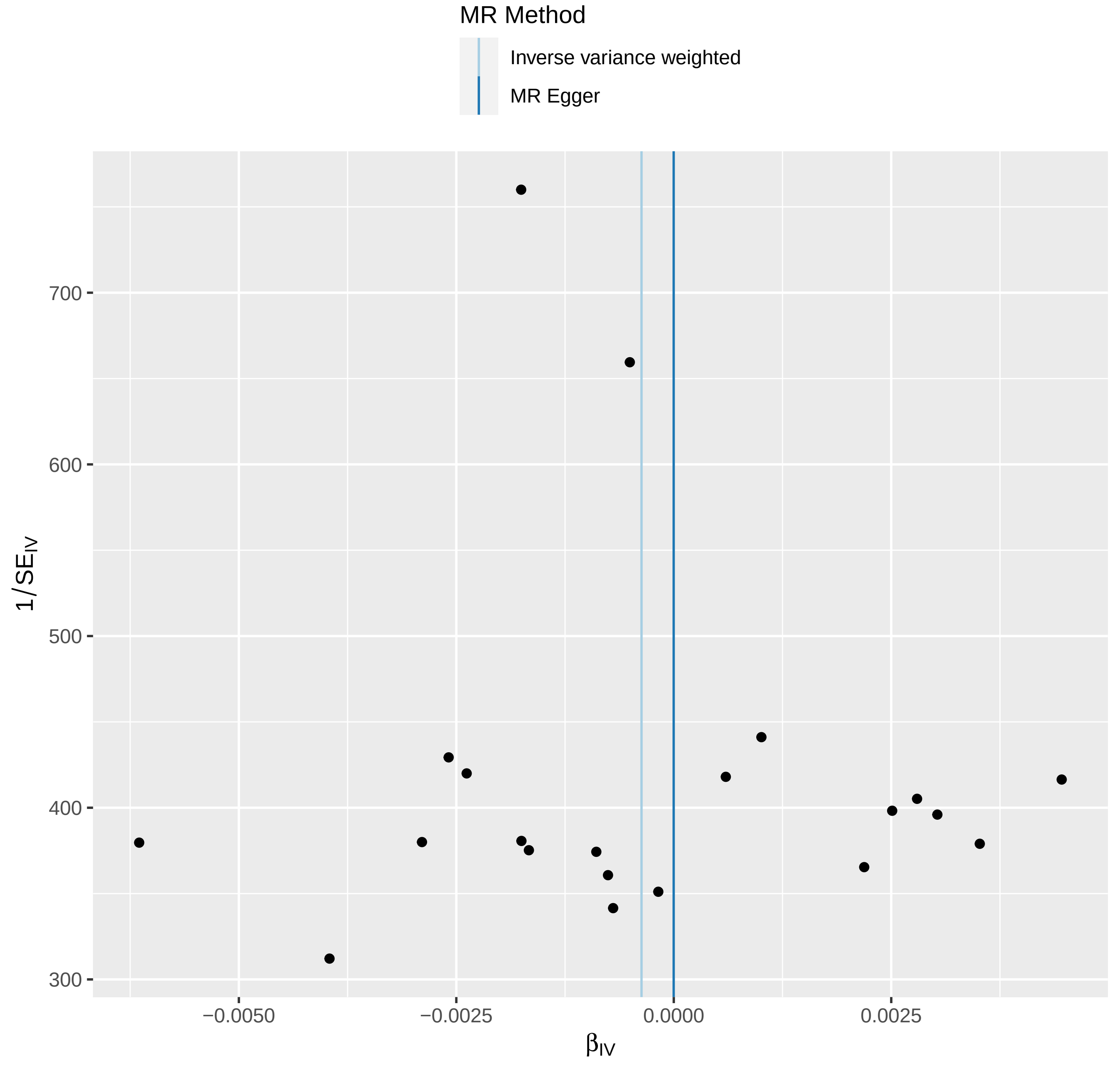

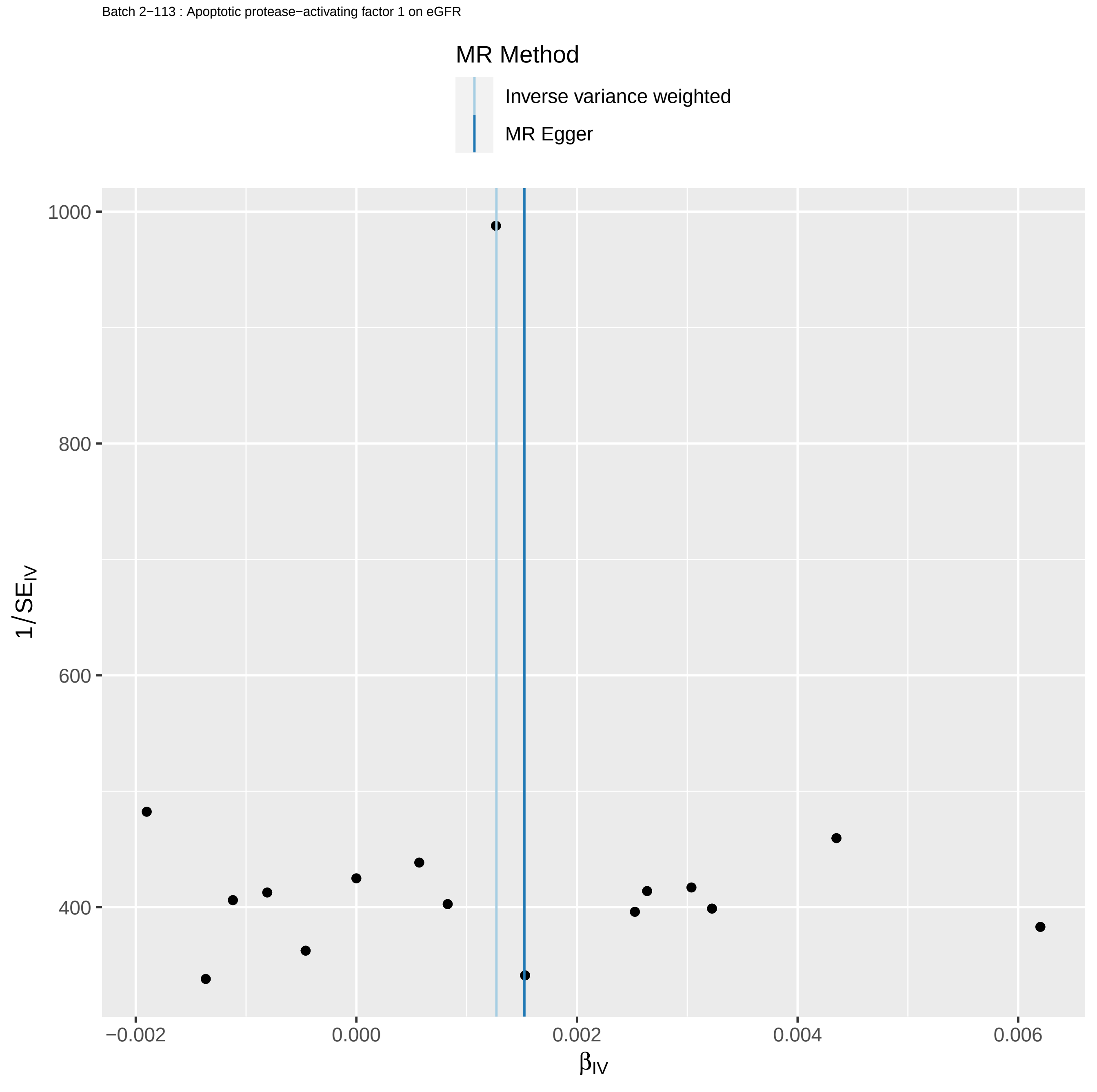
Batch 2−113 : Apoptotic protease−activating factor 1 on eGFR
MR Method
Inverse variance weighted
MR Egger
1000
800
600
400
1/SE$_{IV}$
−0.002
0.000
0.002
0.004
0.006
β$_{IV}$

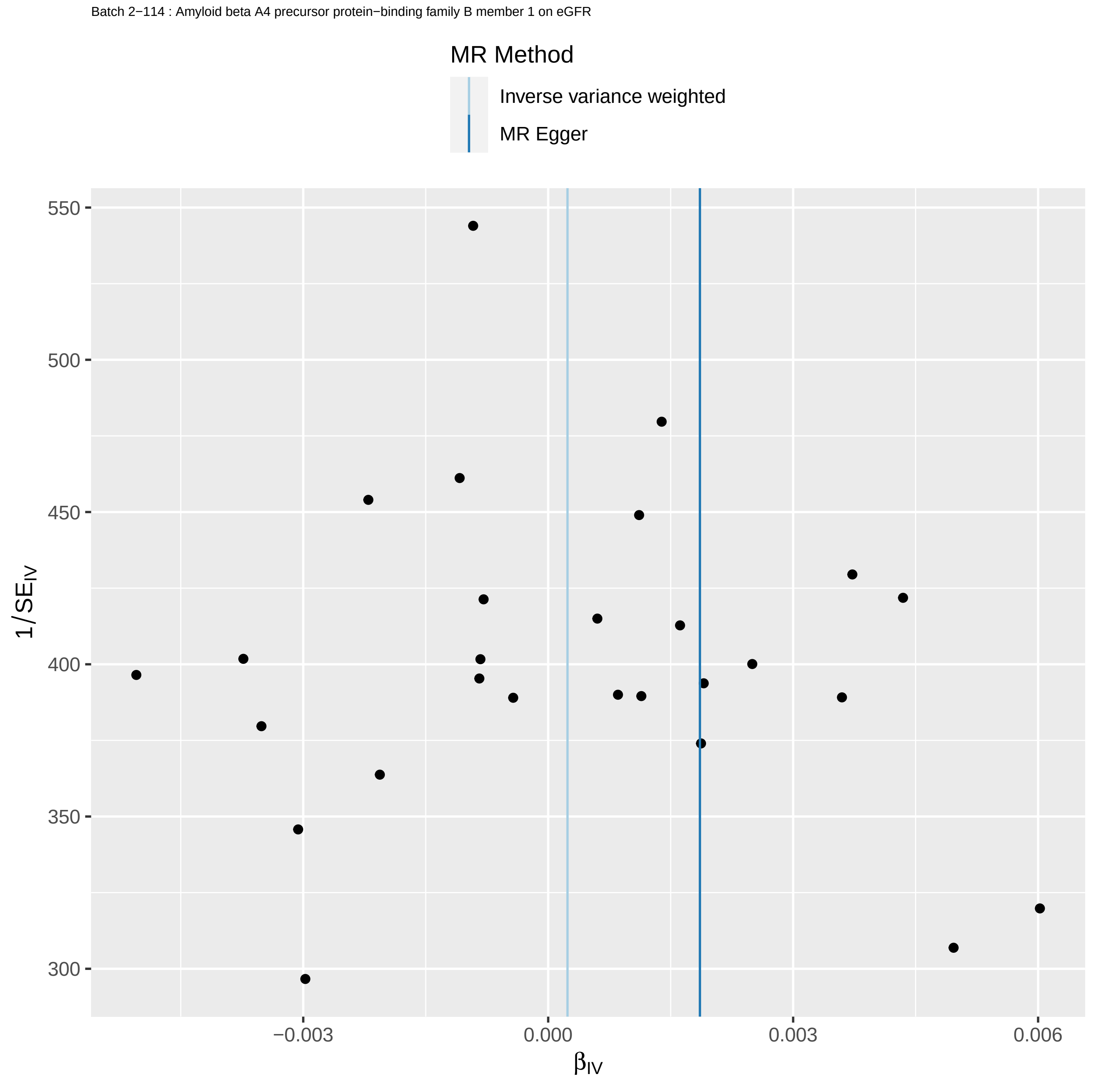

Batch 2−114 : Amyloid beta A4 precursor protein−binding family B member 1 on eGFR
MR Method
Inverse variance weighted
MR Egger
550
500
450
400
350
300
1/SE_IV
−0.003
0.000
0.003
0.006
β_IV

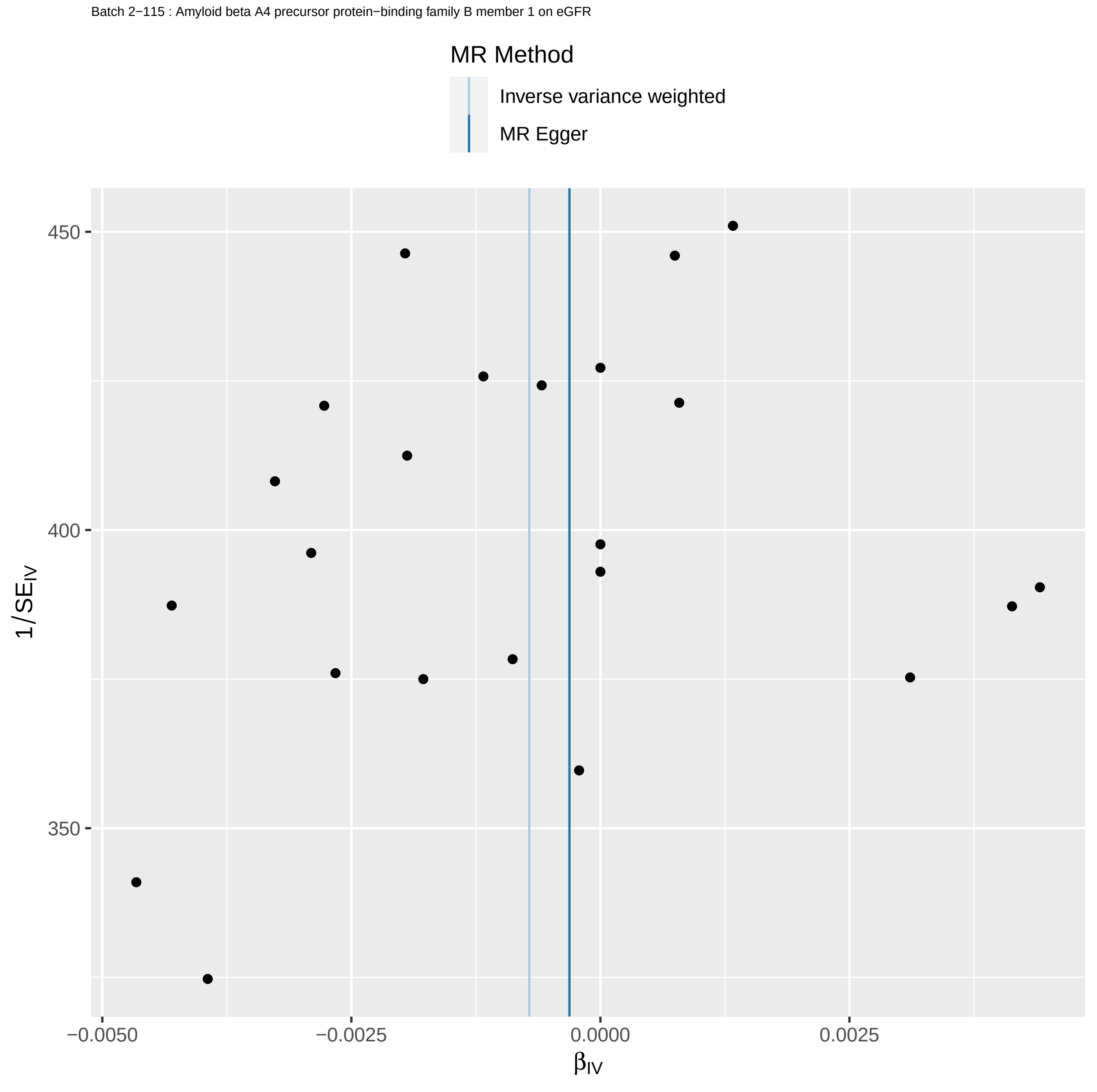

Batch 2−115 : Amyloid beta A4 precursor protein−binding family B member 1 on eGFR
MR Method
Inverse variance weighted
MR Egger
450
400
350
1/SE_IV
−0.0050
−0.0025
0.0000
0.0025
β_IV

Batch 2−116 : Amyloid beta A4 precursor protein−binding family B member 2 on eGFR

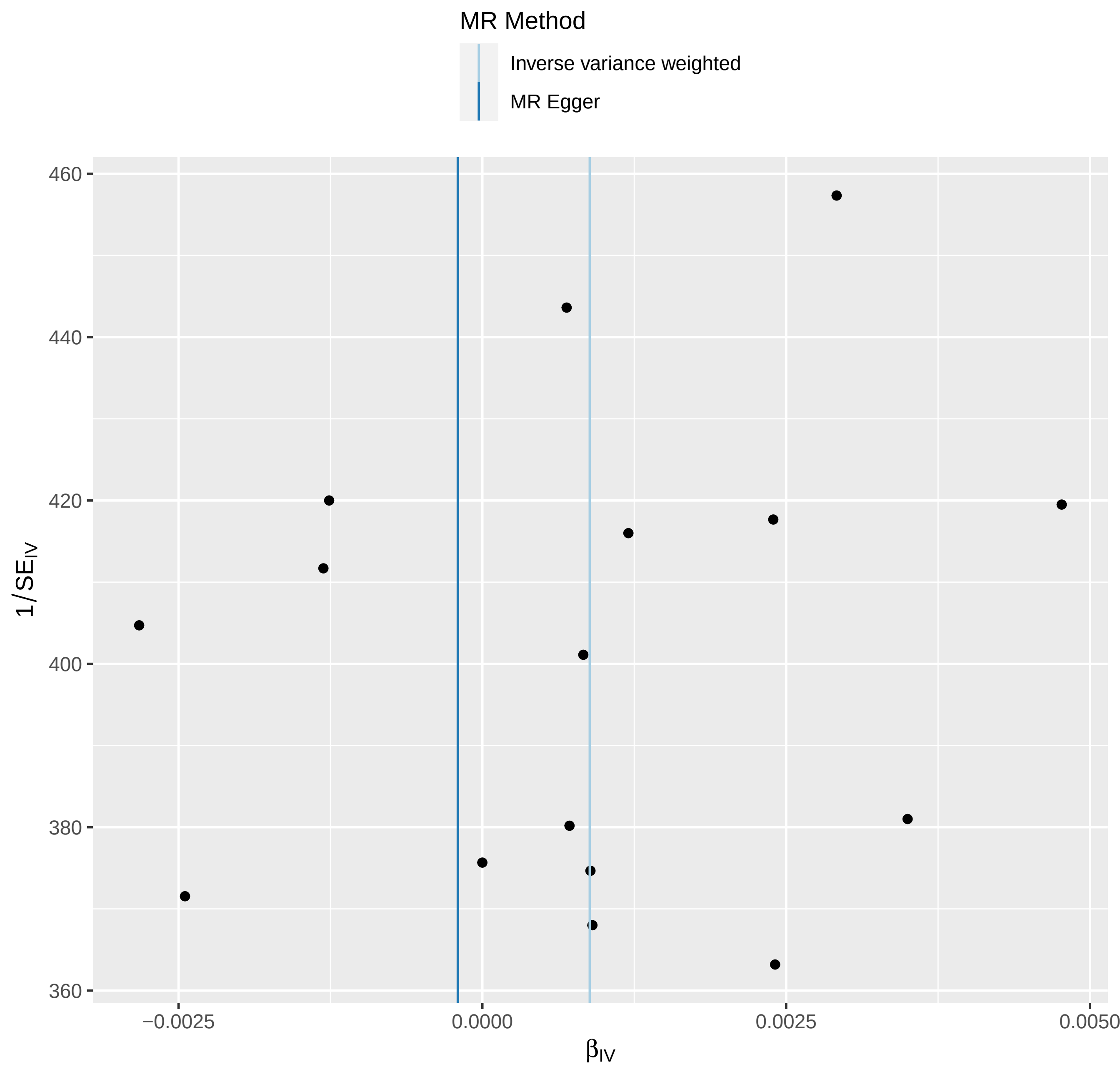

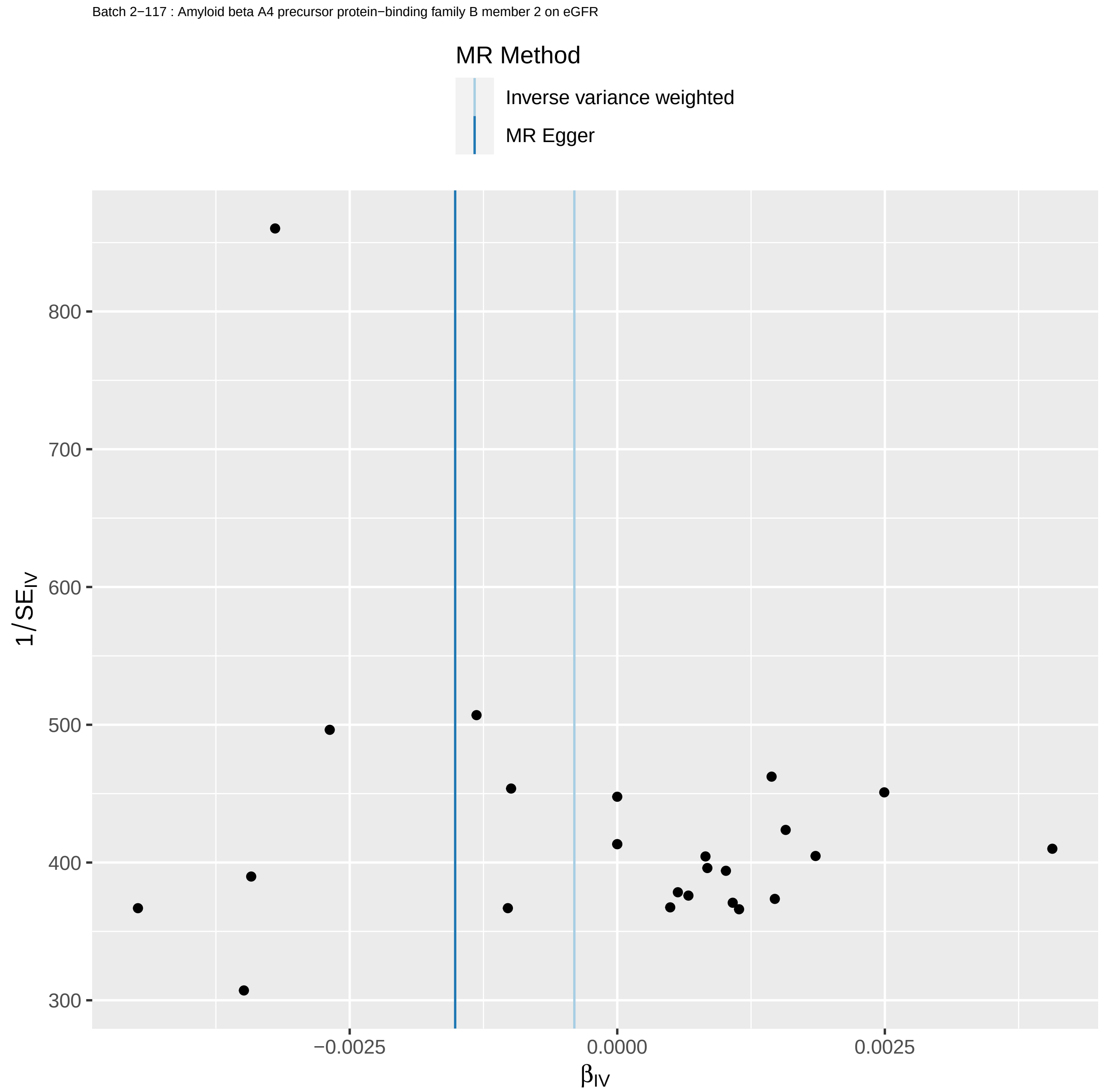
Batch 2−117 : Amyloid beta A4 precursor protein−binding family B member 2 on eGFR
MR Method
Inverse variance weighted
MR Egger
800
700
600
500
400
300
1/SE_IV
−0.0025
0.0000
0.0025
β_IV

Batch 2−118 : Amyloid beta A4 precursor protein−binding family B member 3 on eGFR

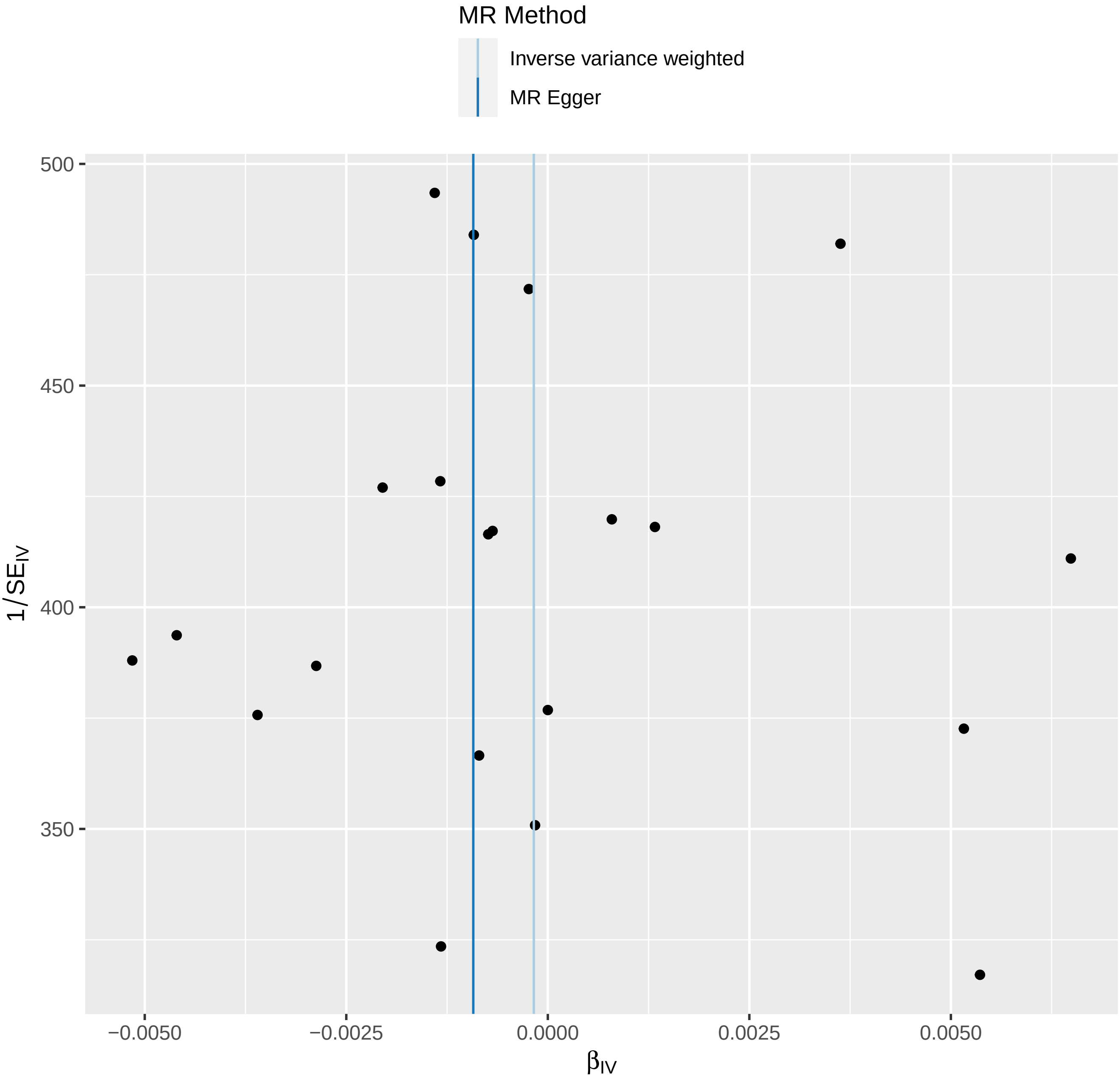

Batch 2−119 : Protein APCDD1 on eGFR

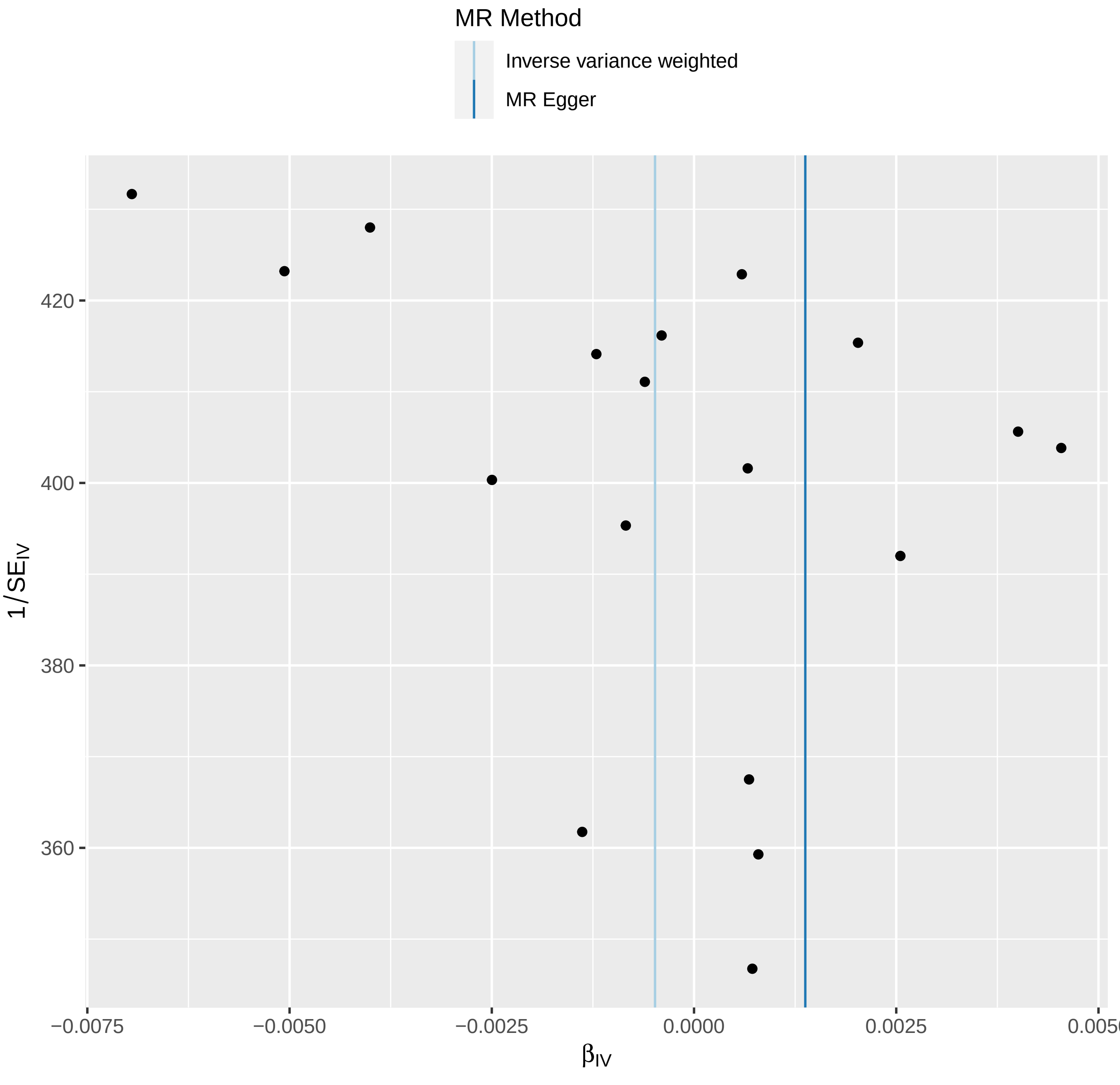

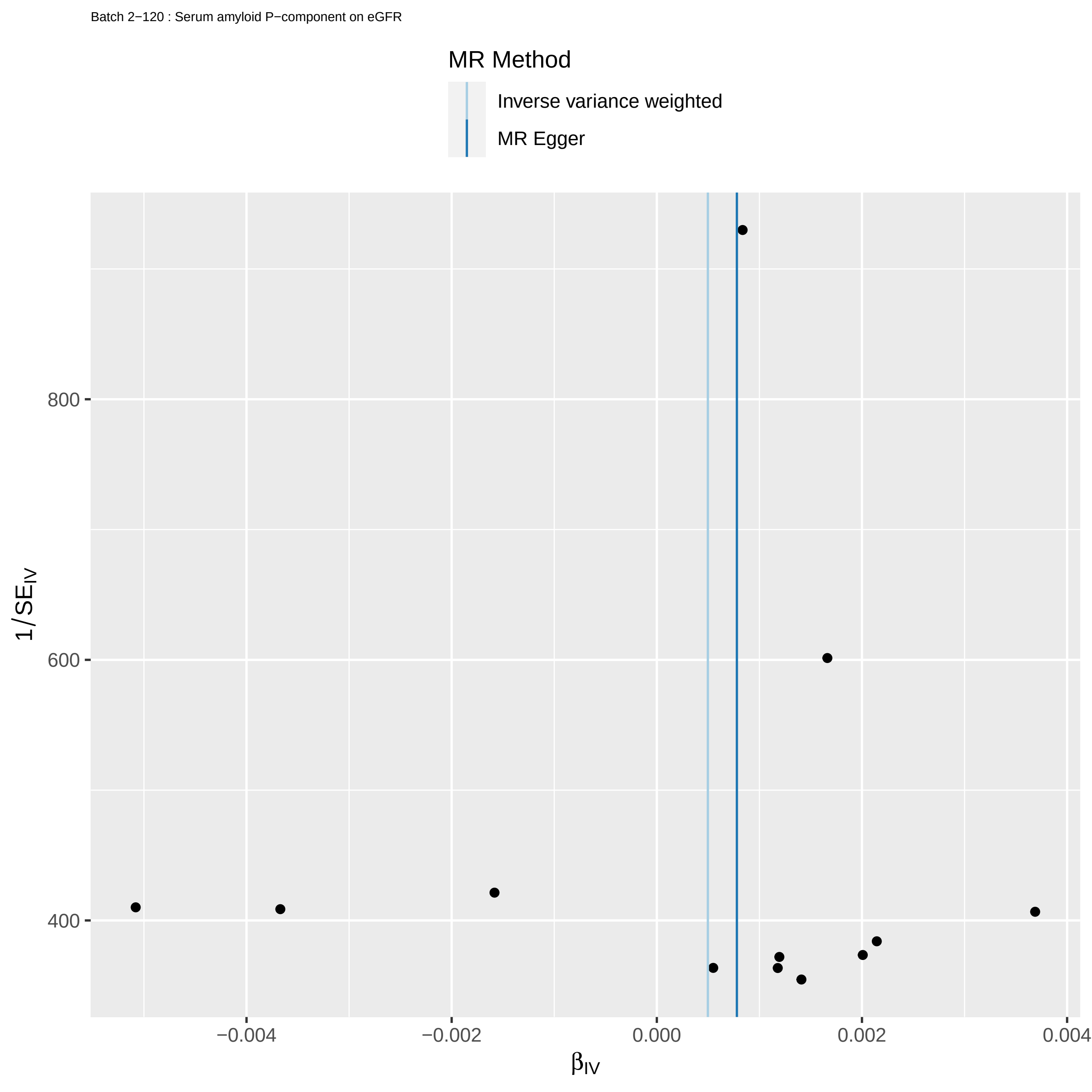
Batch 2−120 : Serum amyloid P−component on eGFR
MR Method
Inverse variance weighted
MR Egger
800
600
400
1/SE_IV
−0.004
−0.002
0.000
0.002
0.004
β_IV

Batch 2−121 : DNA−(apurinic or apyrimidinic site) lyase on eGFR

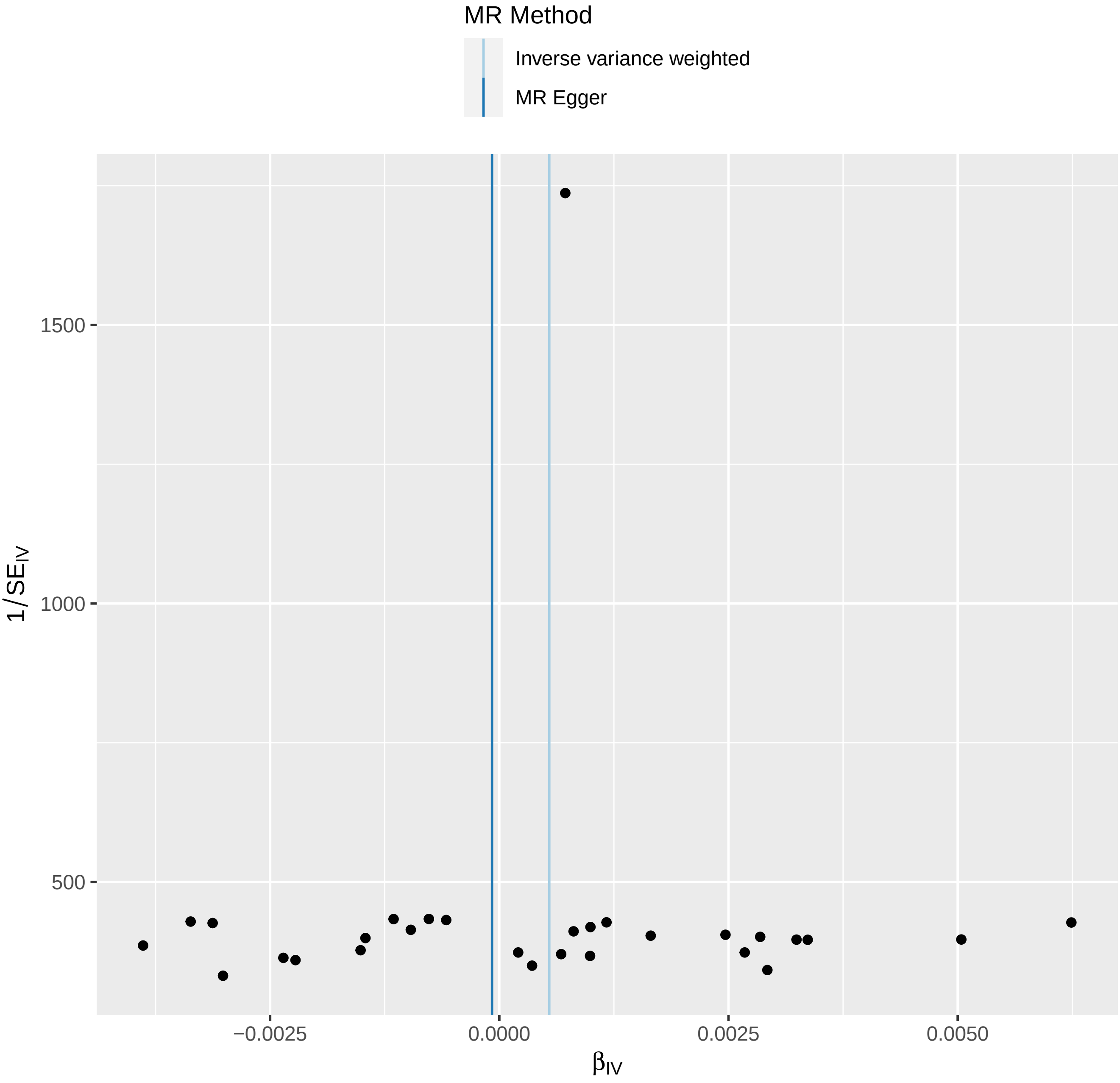

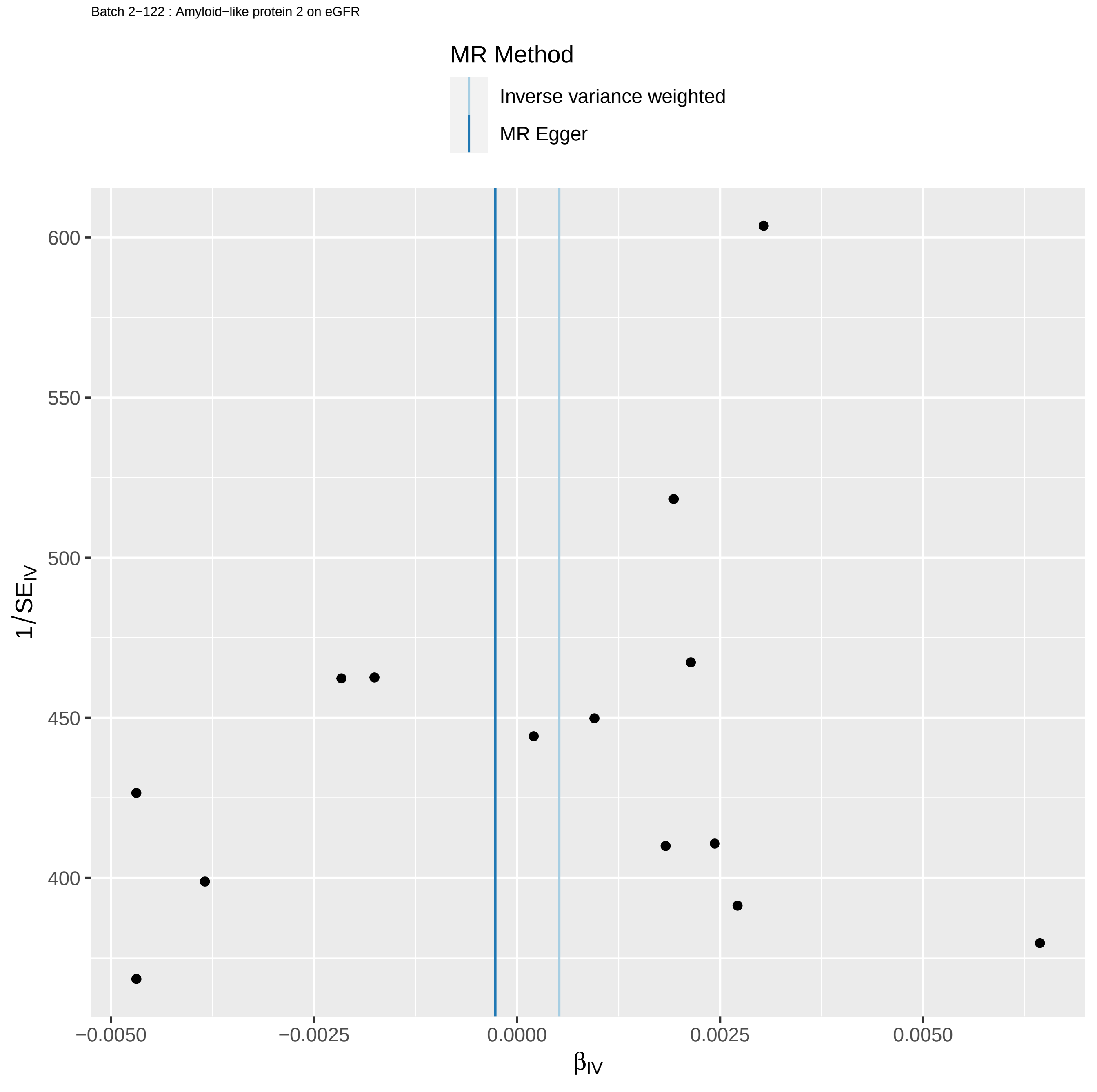

Batch 2−122 : Amyloid−like protein 2 on eGFR
MR Method
Inverse variance weighted
MR Egger
600
550
500
450
400
1/SE_IV
−0.0050
−0.0025
0.0000
0.0025
0.0050
β_IV

Batch 2−123 : Amyloid−like protein 2 on eGFR

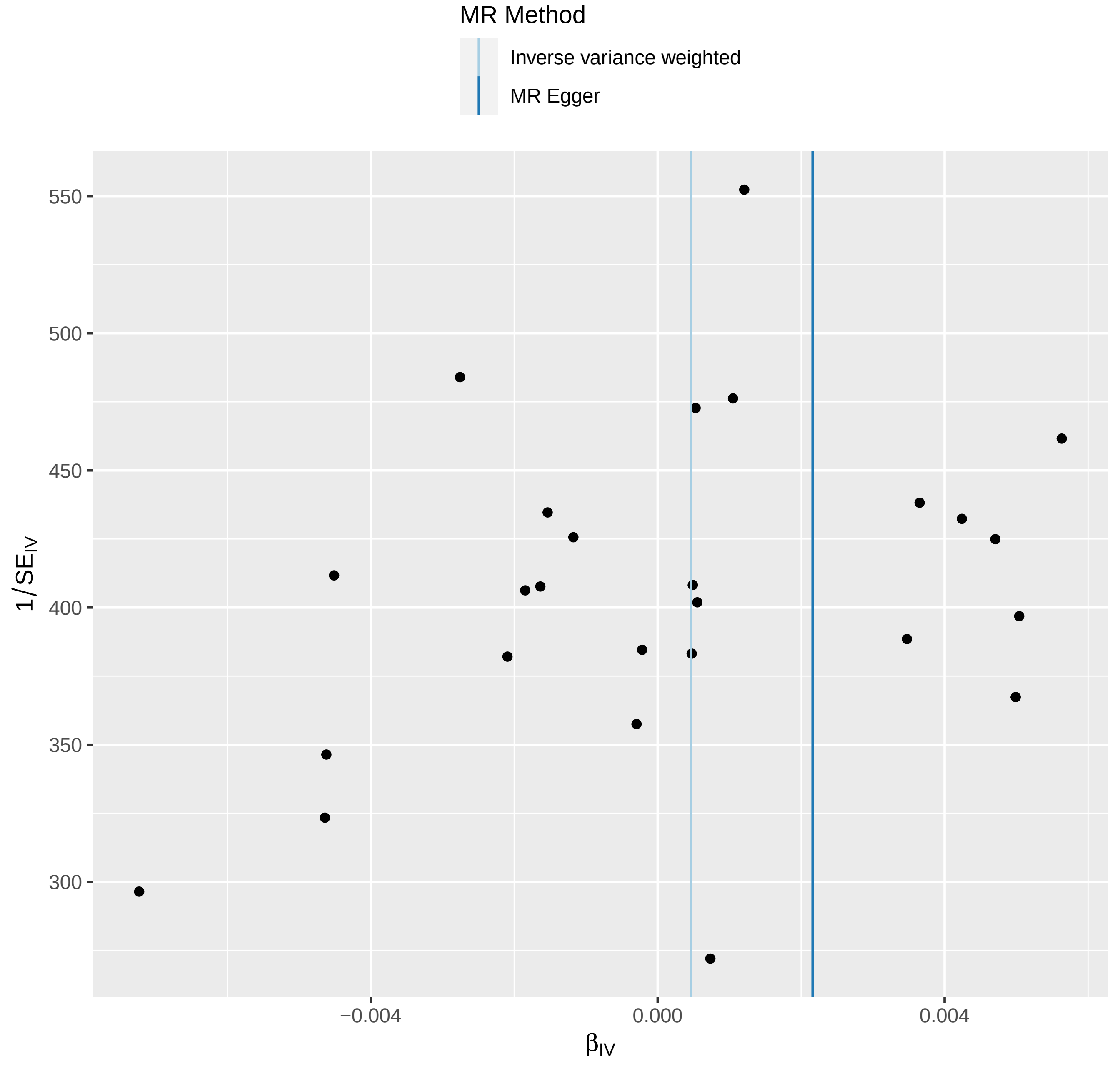

Batch 2−124 : Adipocyte plasma membrane−associated protein on eGFR

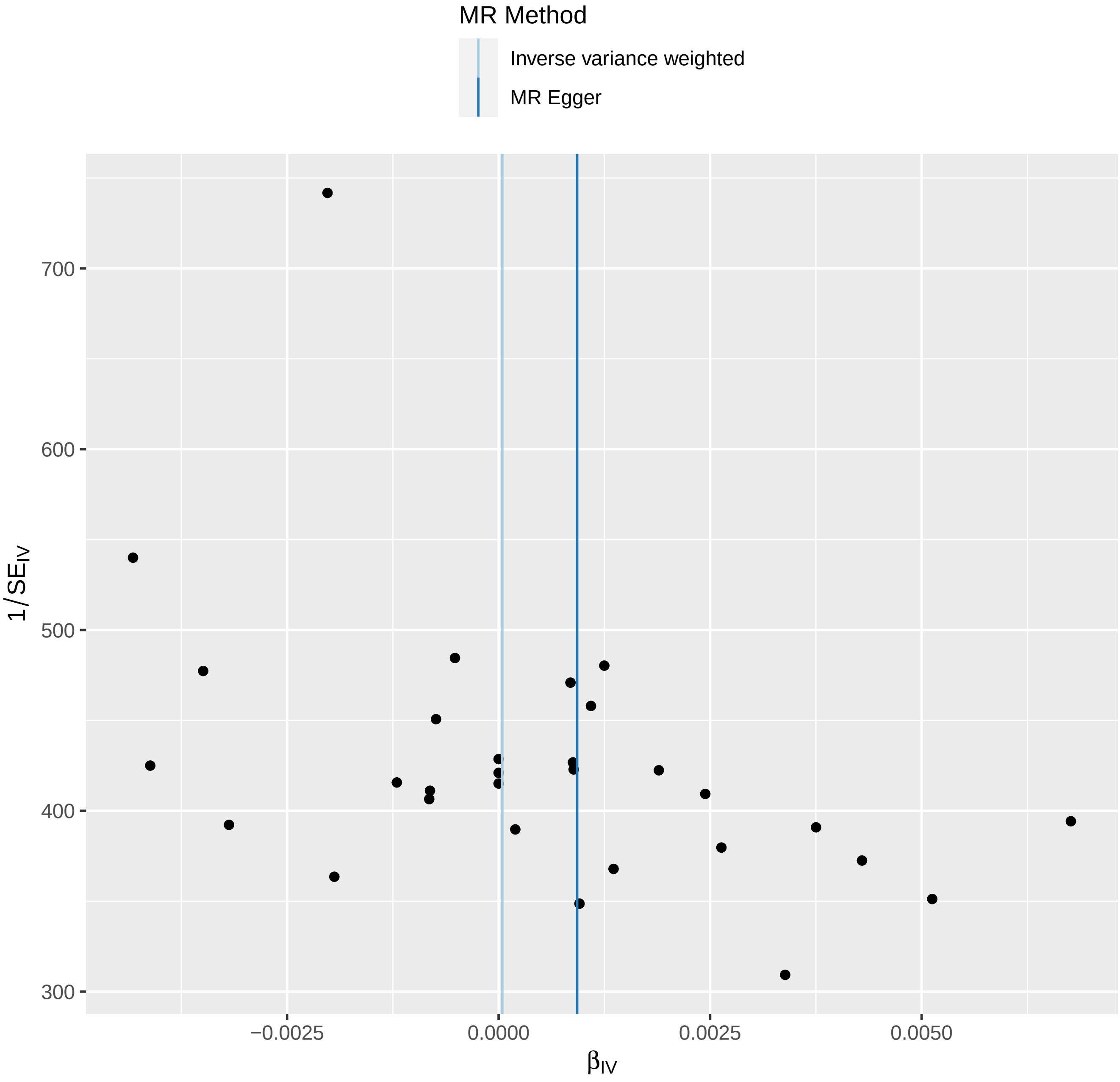

Batch 2−125 : Apolipoprotein A−V on eGFR

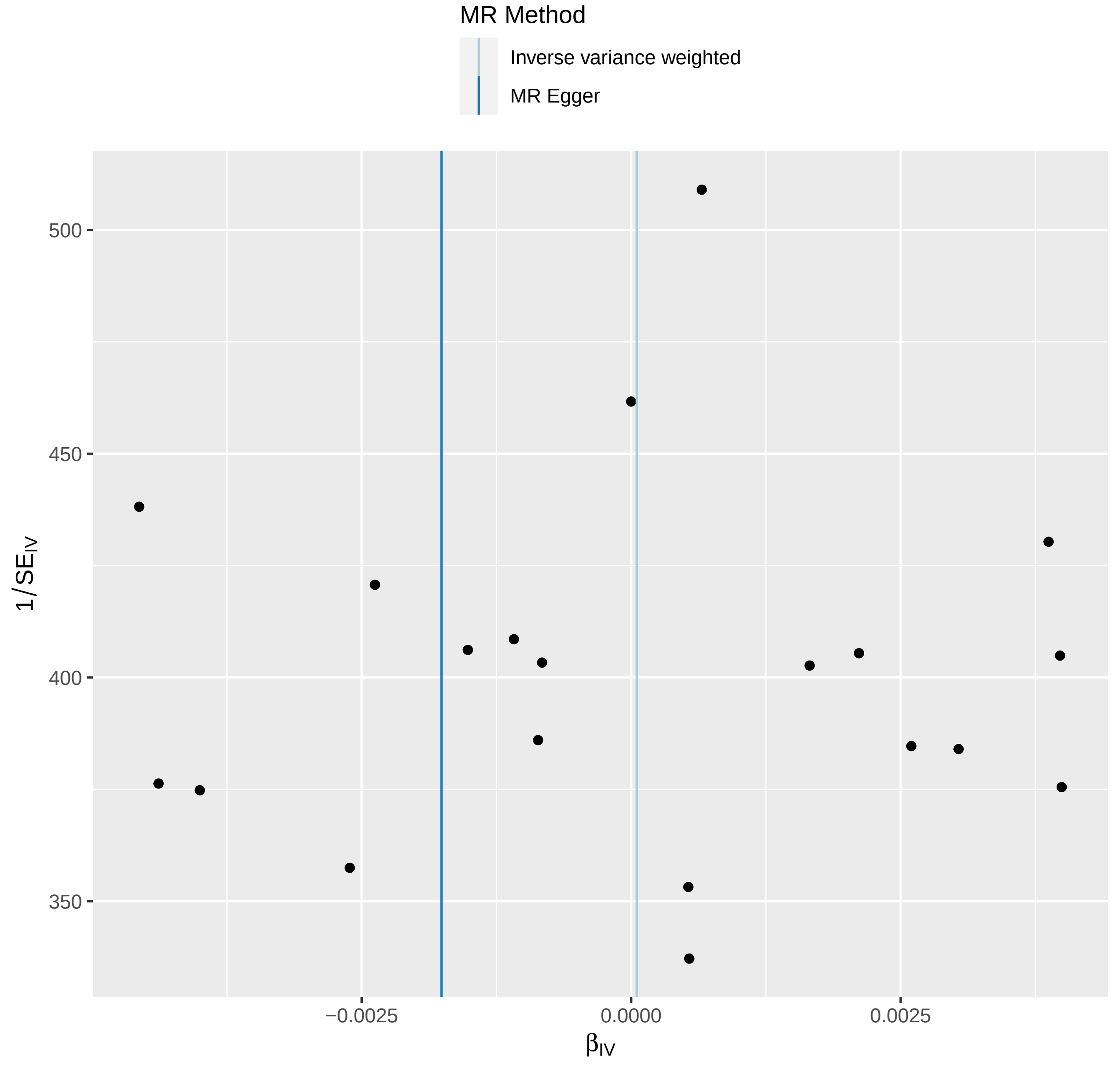

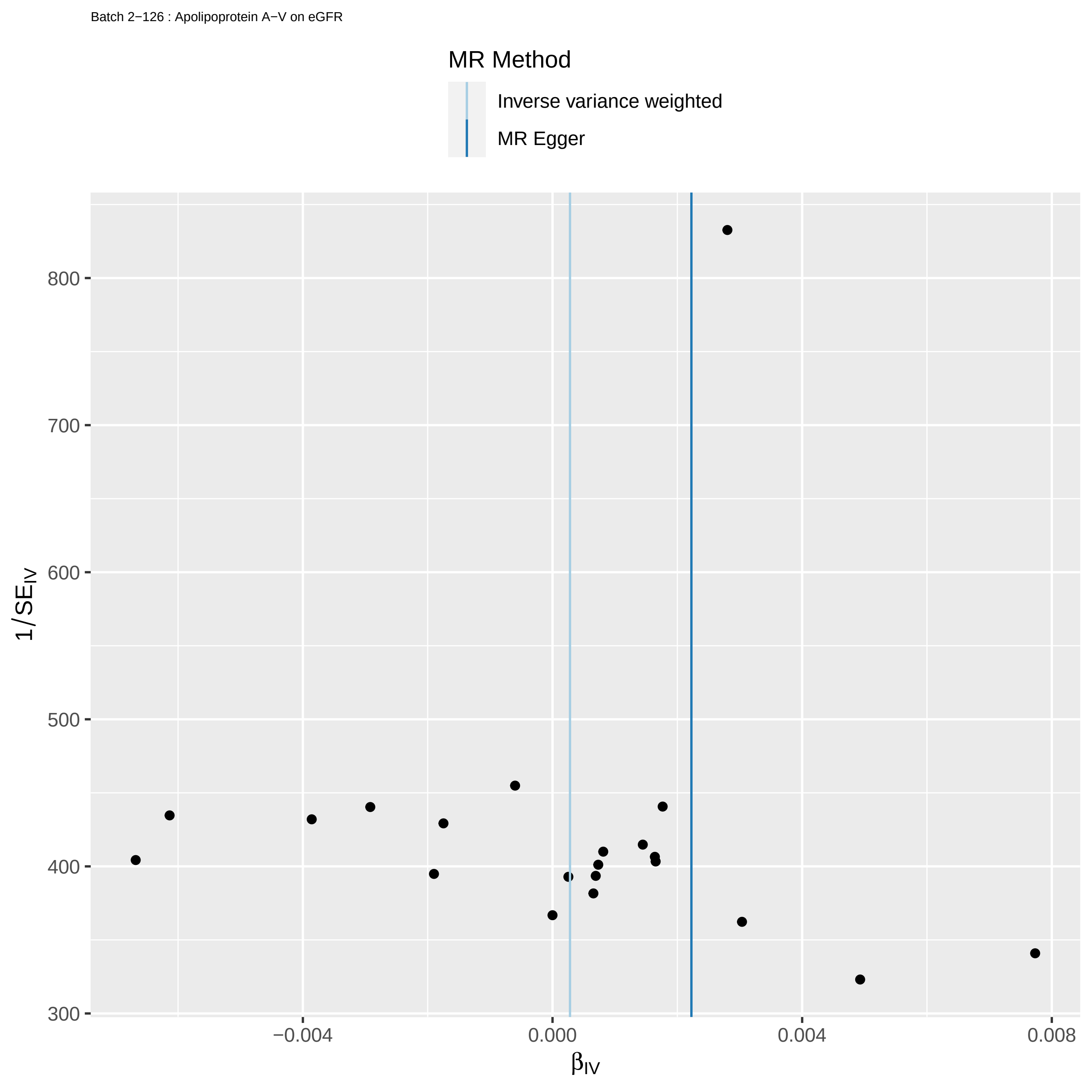

Batch 2−126 : Apolipoprotein A−V on eGFR
MR Method
Inverse variance weighted
MR Egger
800
700
600
500
400
300
1/SE_IV
−0.004
0.000
0.004
0.008
β_IV

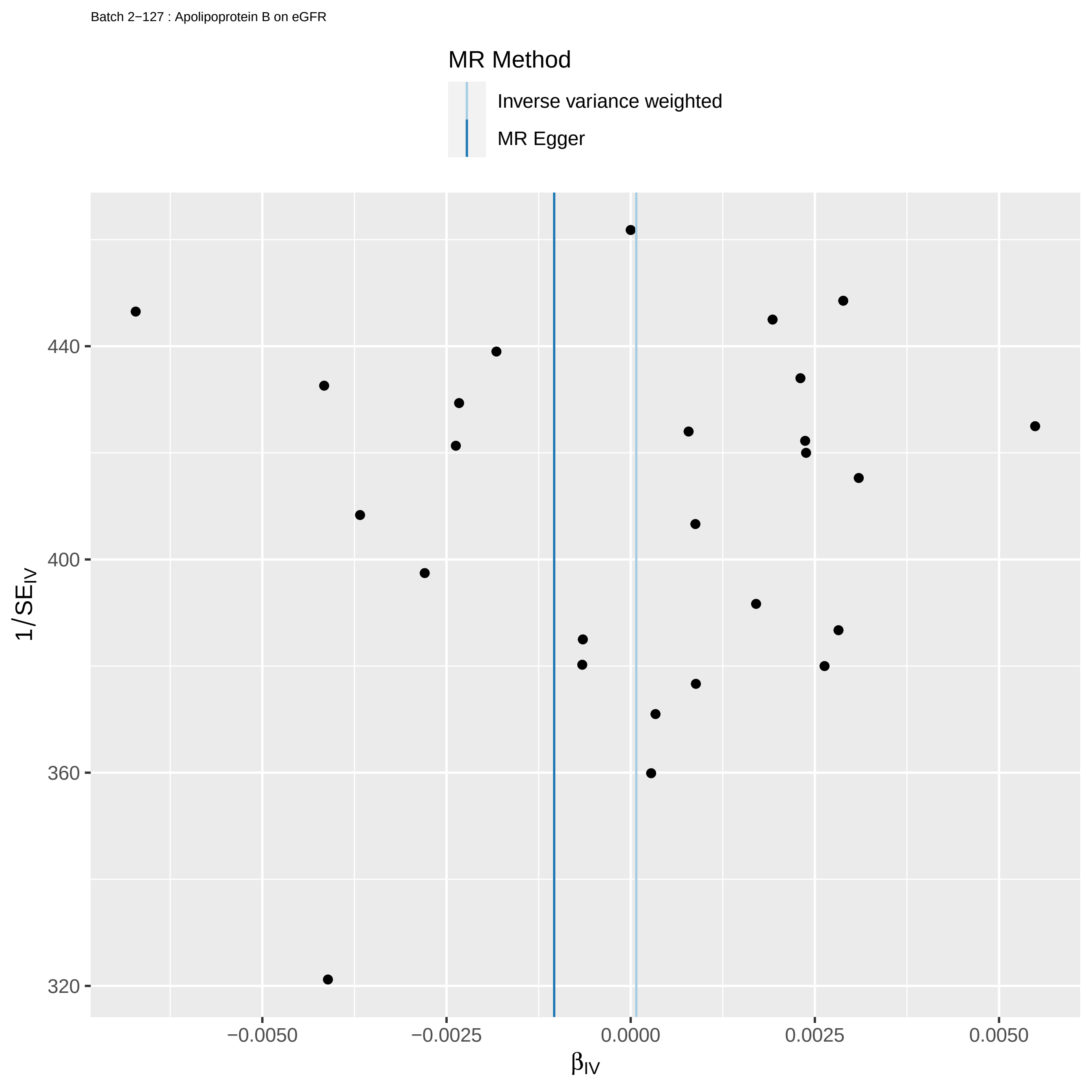

Batch 2−127 : Apolipoprotein B on eGFR
MR Method
Inverse variance weighted
MR Egger
440
400
360
320
1/SE$_{IV}$
−0.0050
−0.0025
0.0000
0.0025
0.0050
β$_{IV}$

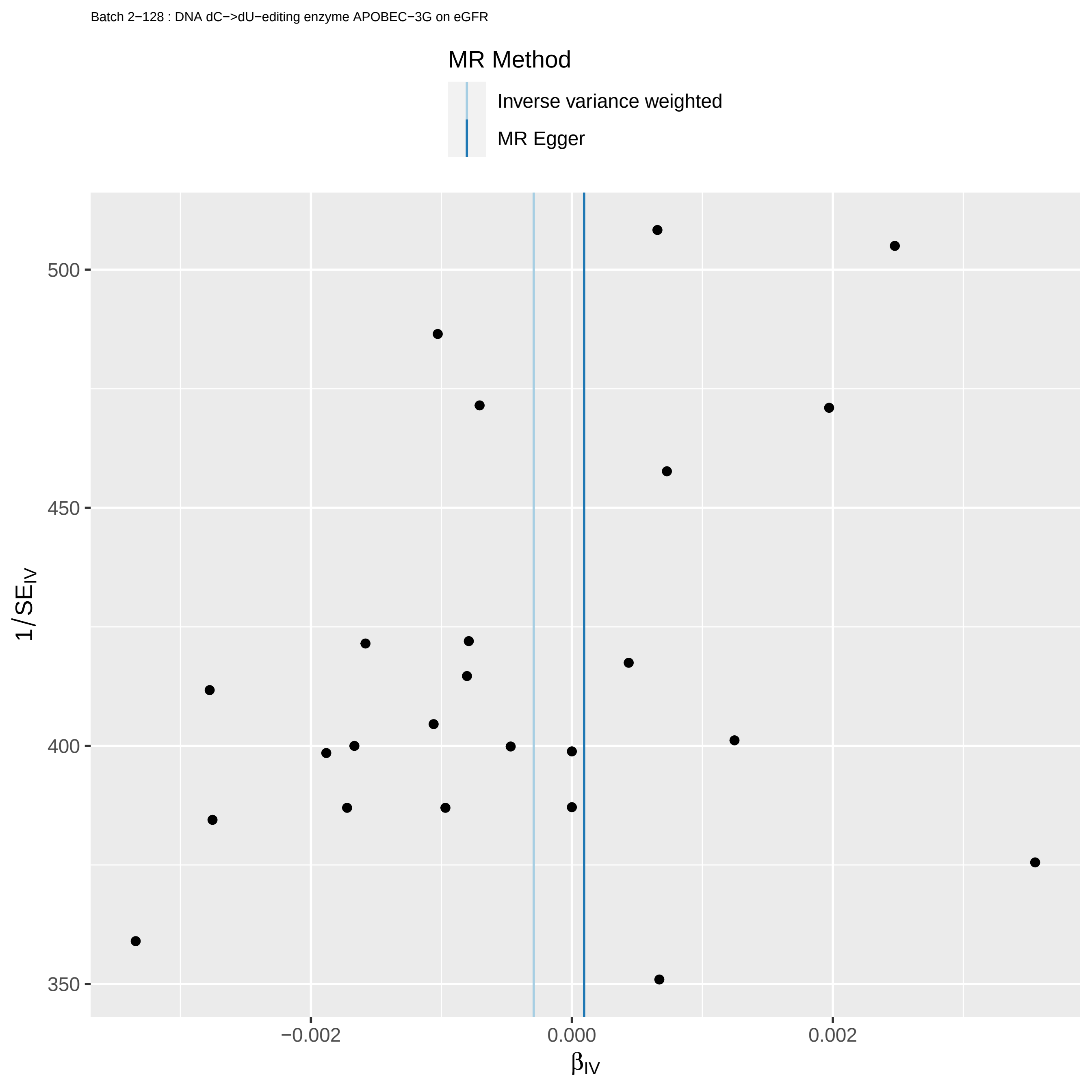
Batch 2−128 : DNA dC−>dU−editing enzyme APOBEC−3G on eGFR
MR Method
Inverse variance weighted
MR Egger
500
450
400
350
1/SE_IV
−0.002
0.000
0.002
β_IV

Batch 2−129 : Apolipoprotein D on eGFR

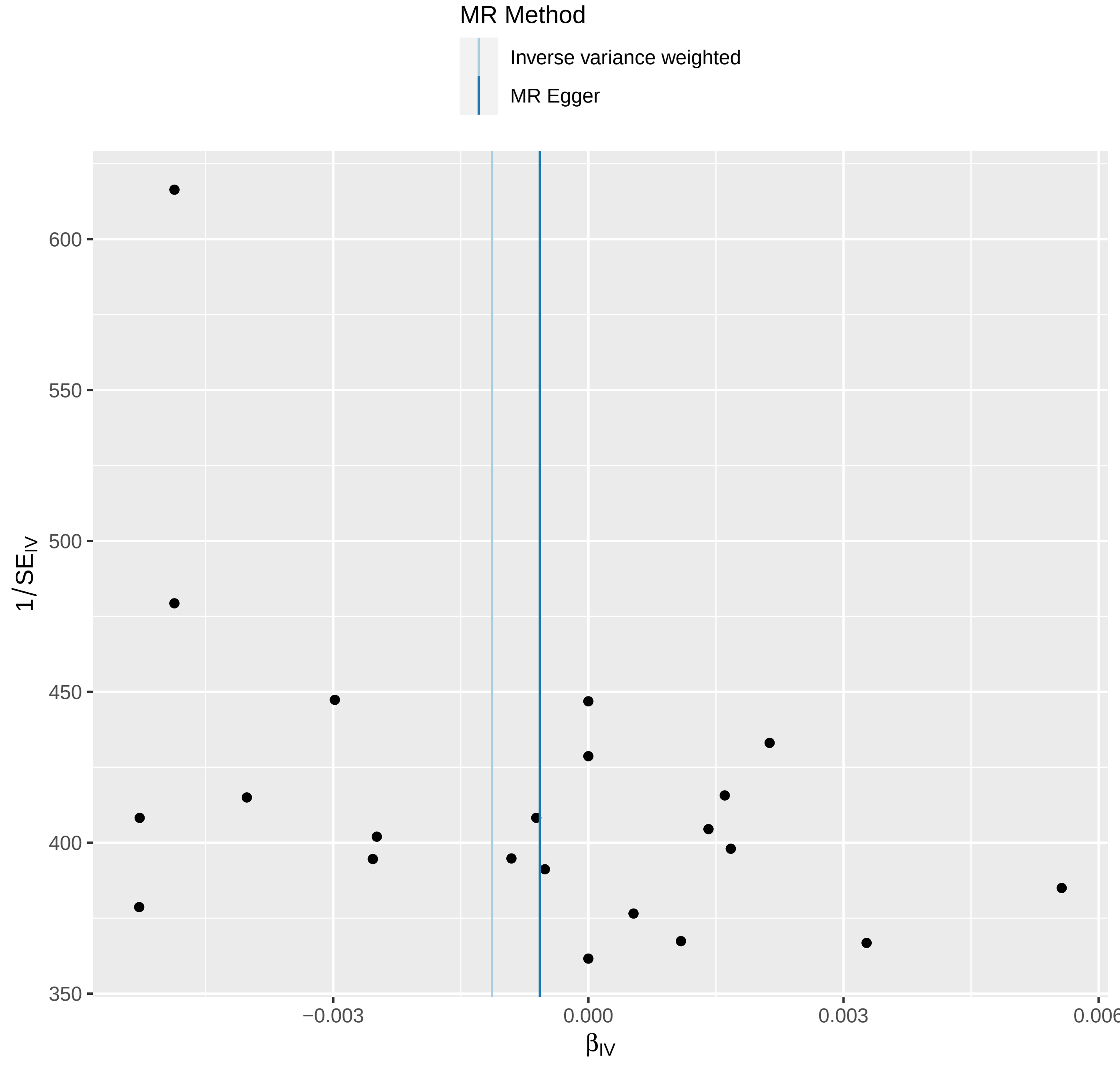

Batch 2−130 : Apolipoprotein D on eGFR

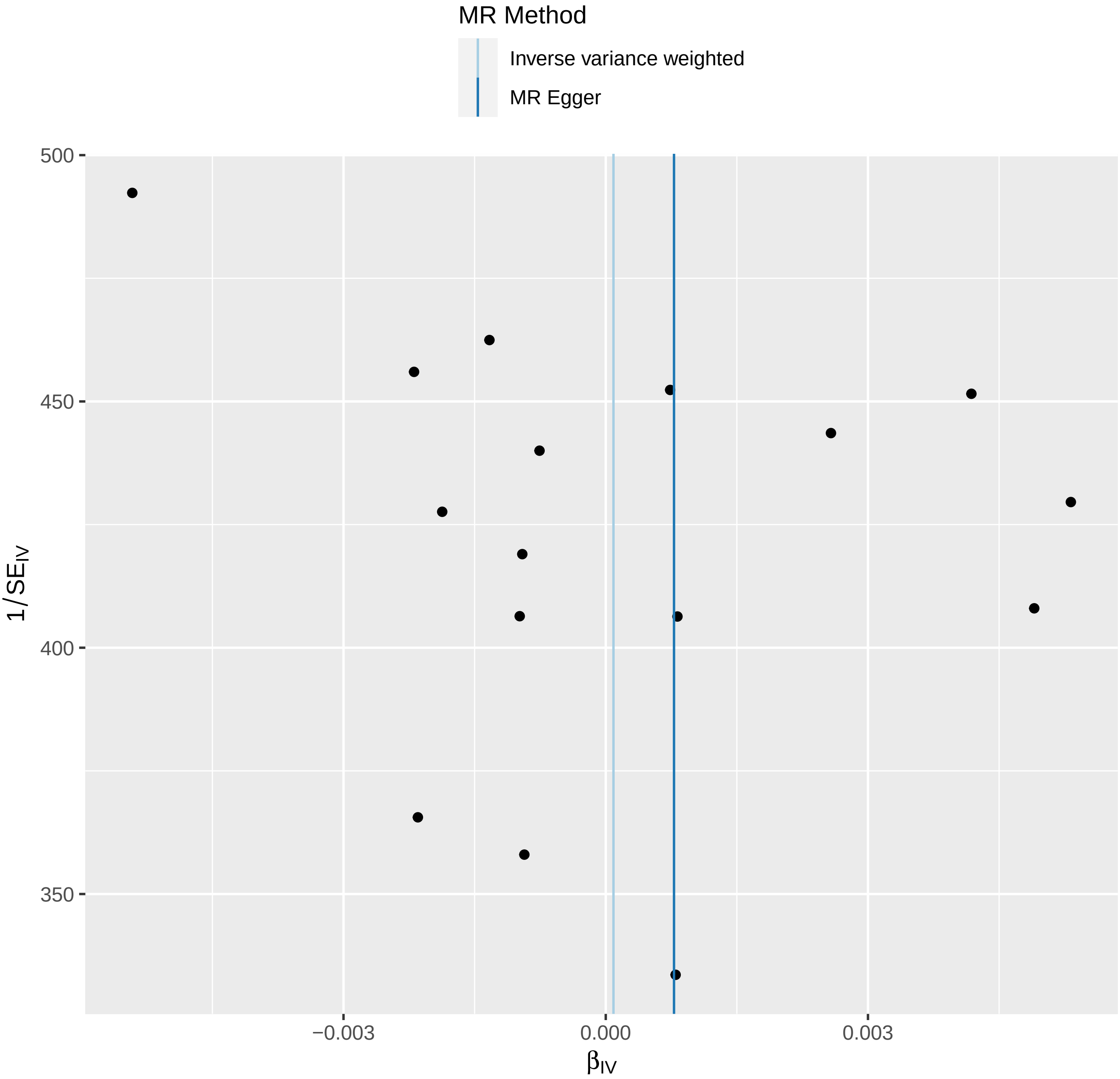

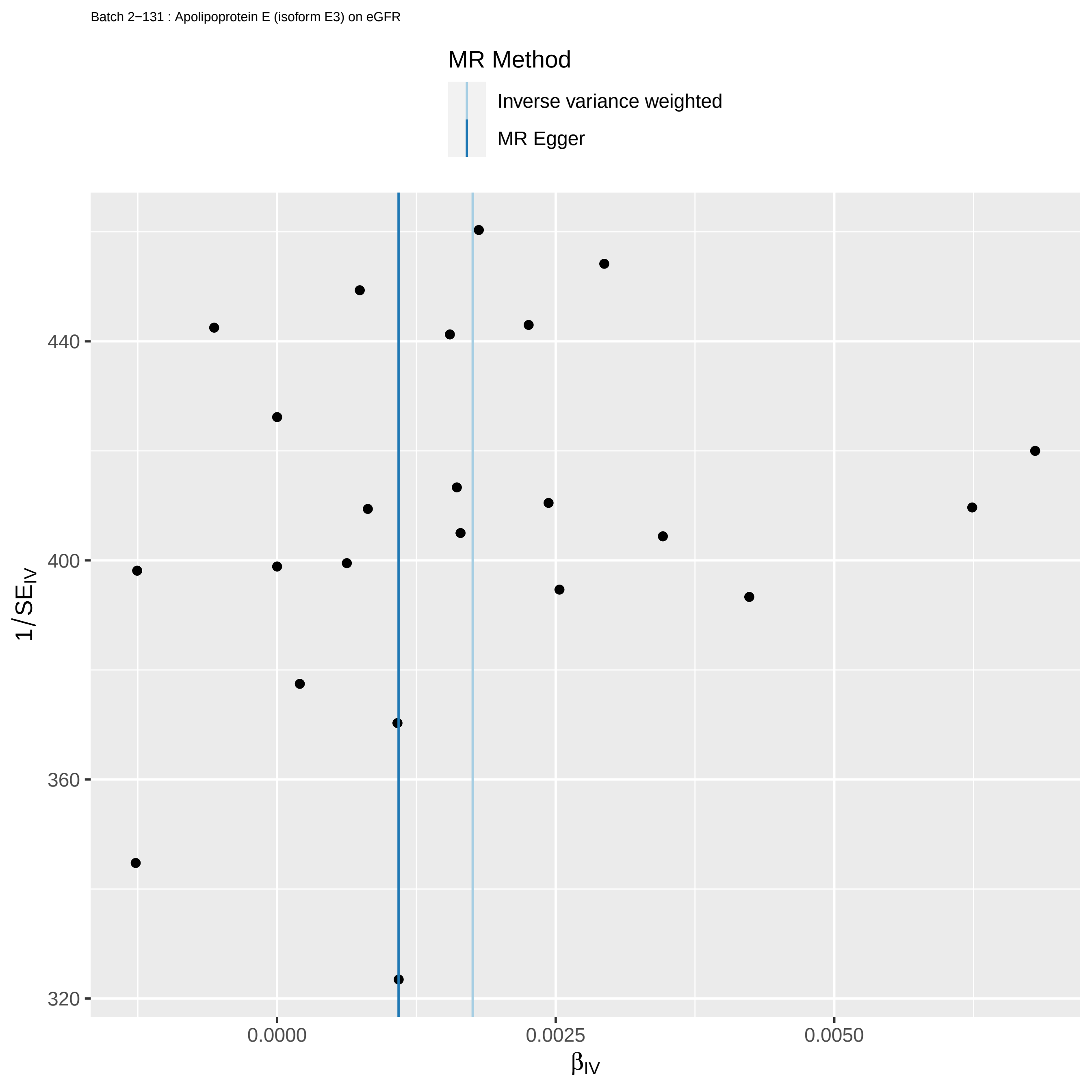

Batch 2−131 : Apolipoprotein E (isoform E3) on eGFR
MR Method
Inverse variance weighted
MR Egger
440
400
360
320
1/SE$_{IV}$
0.0000
0.0025
0.0050
β_{IV}

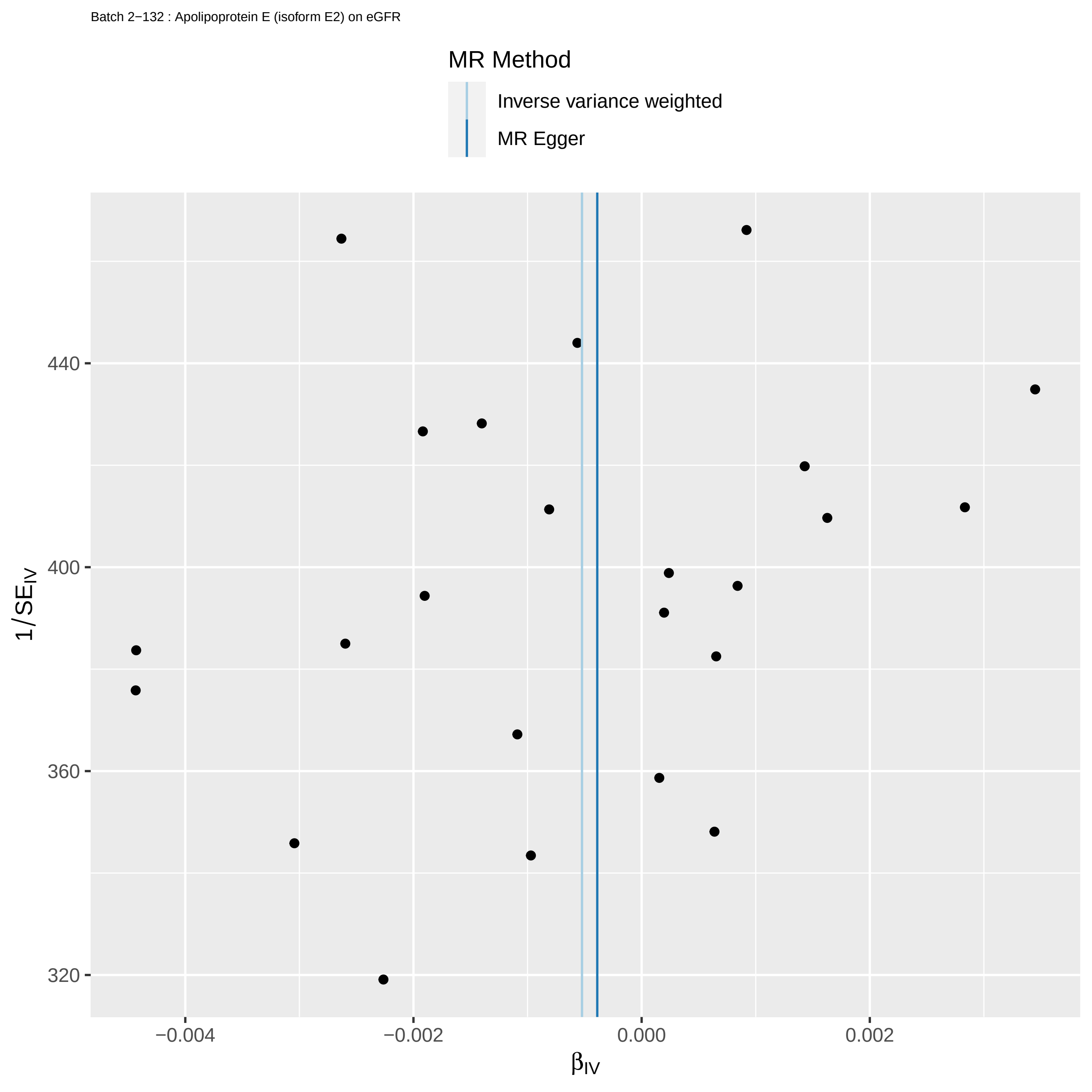

Batch 2−132 : Apolipoprotein E (isoform E2) on eGFR
MR Method
Inverse variance weighted
MR Egger
440
400
360
320
1/SE_IV
−0.004
−0.002
0.000
0.002
β_IV

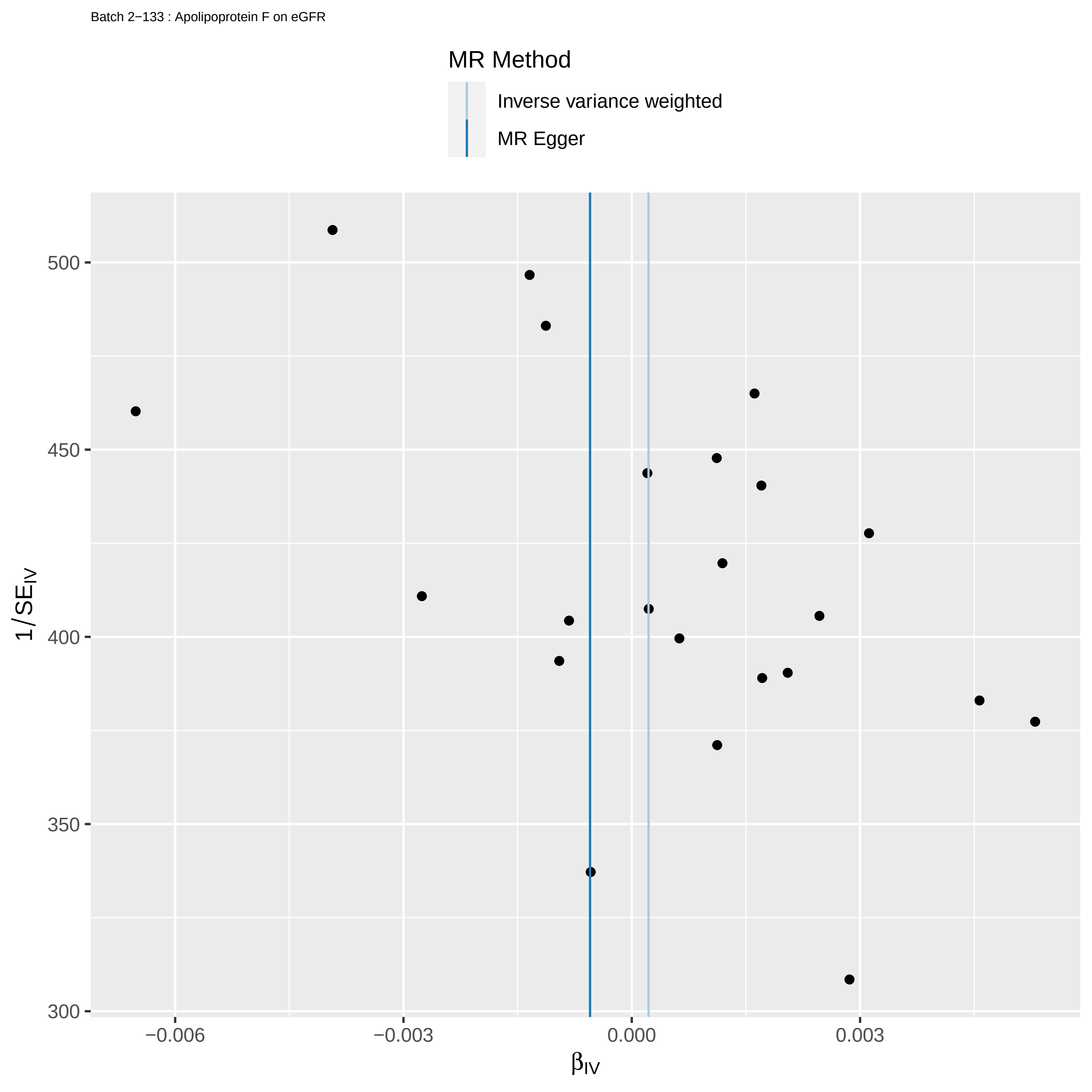
Batch 2−133 : Apolipoprotein F on eGFR
MR Method
Inverse variance weighted
MR Egger
500
450
400
350
300
1/SE_IV
−0.006
−0.003
0.000
0.003
β_IV

Batch 2−134 : Apolipoprotein L1 on eGFR

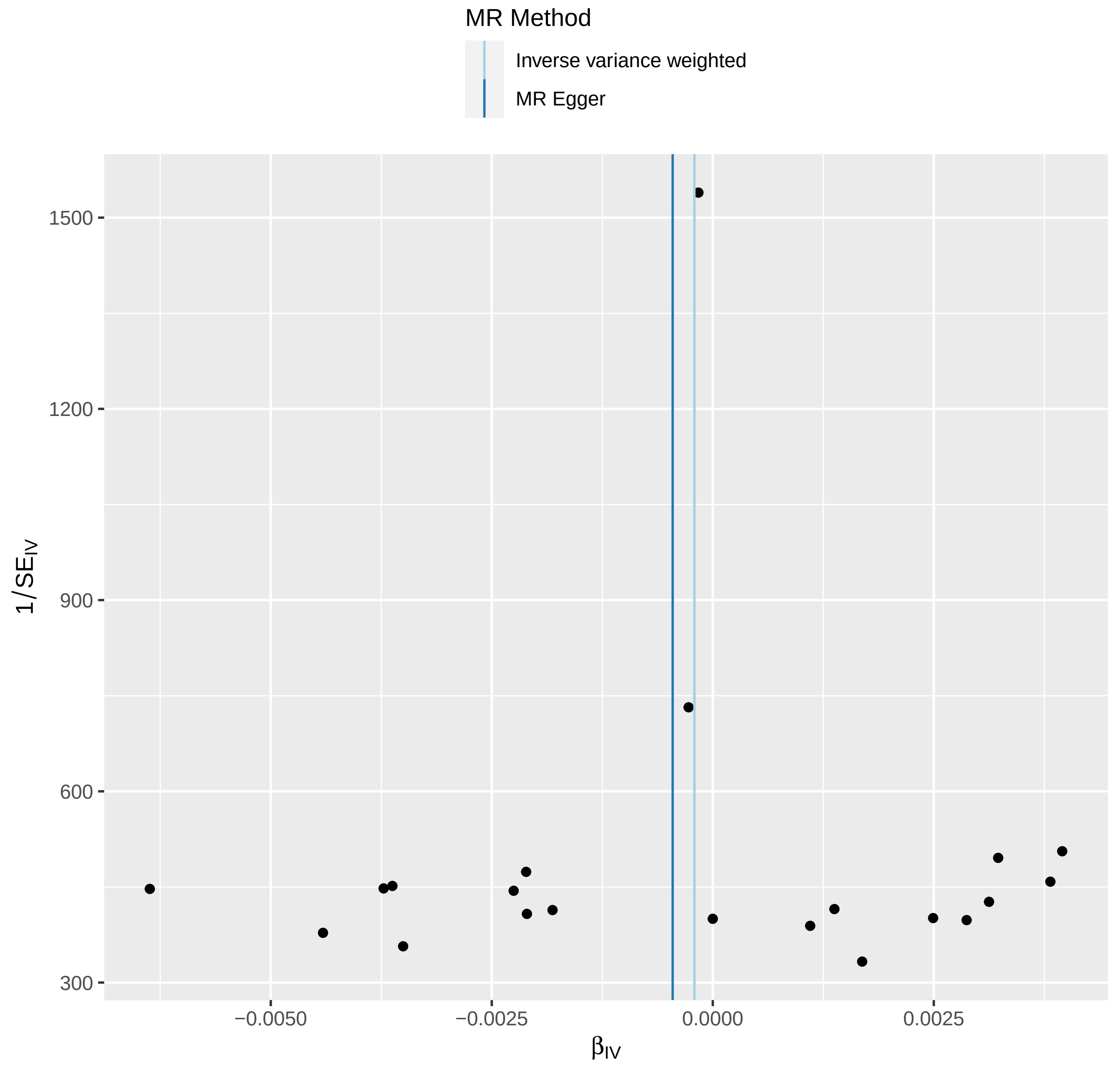

Batch 2−135 : Apolipoprotein L1 on eGFR

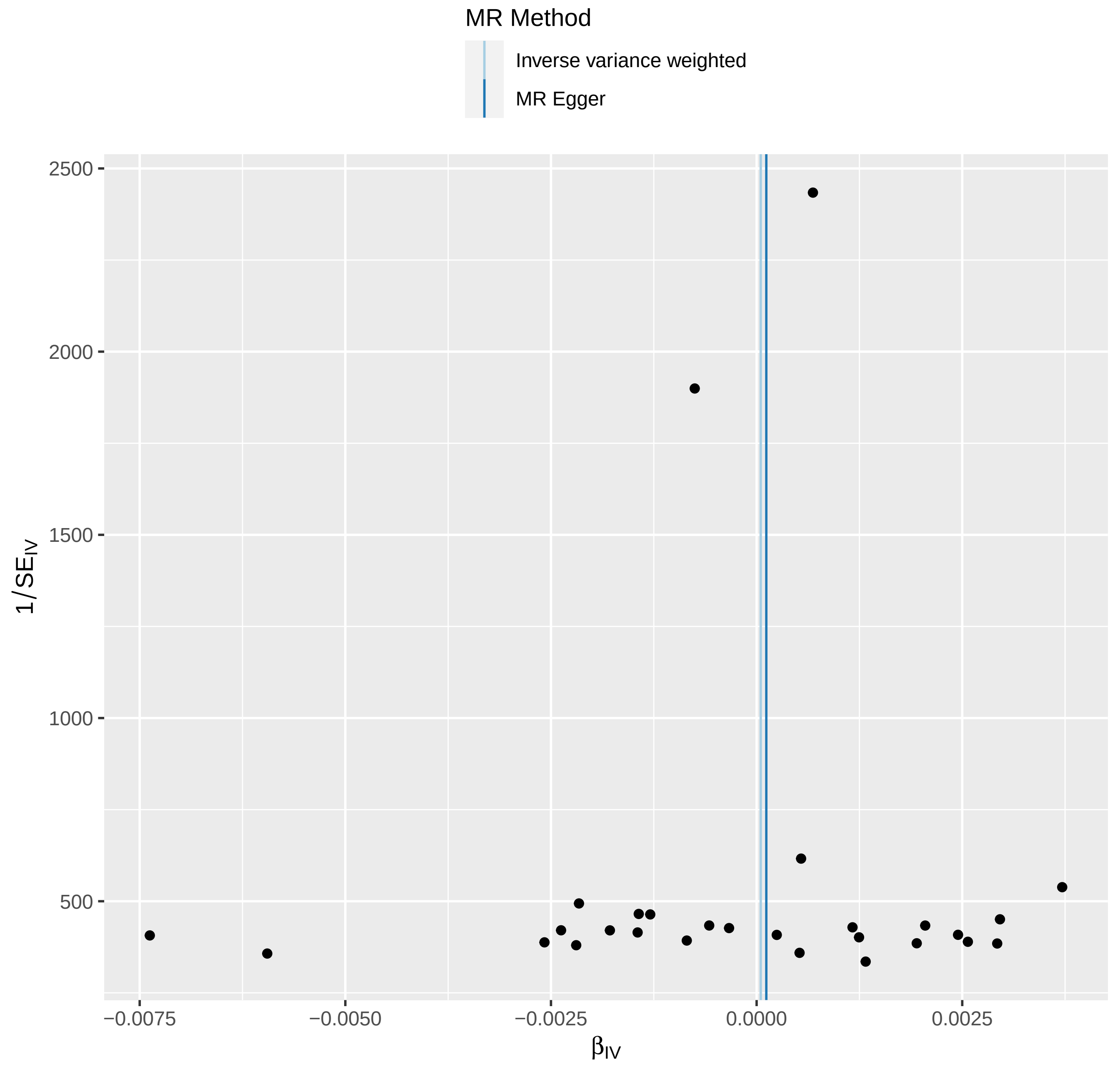

Batch 2−136 : Apolipoprotein M on eGFR

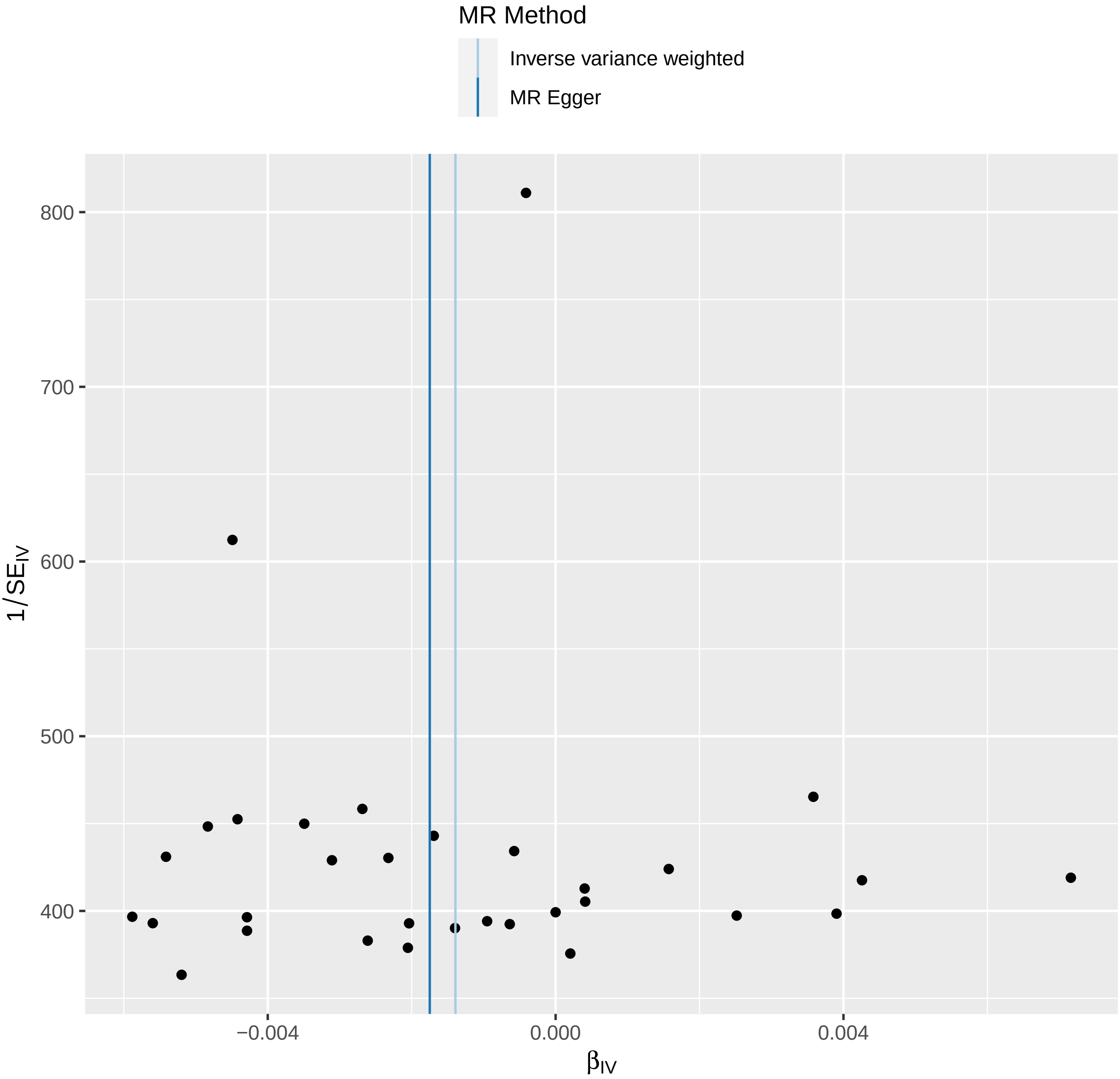

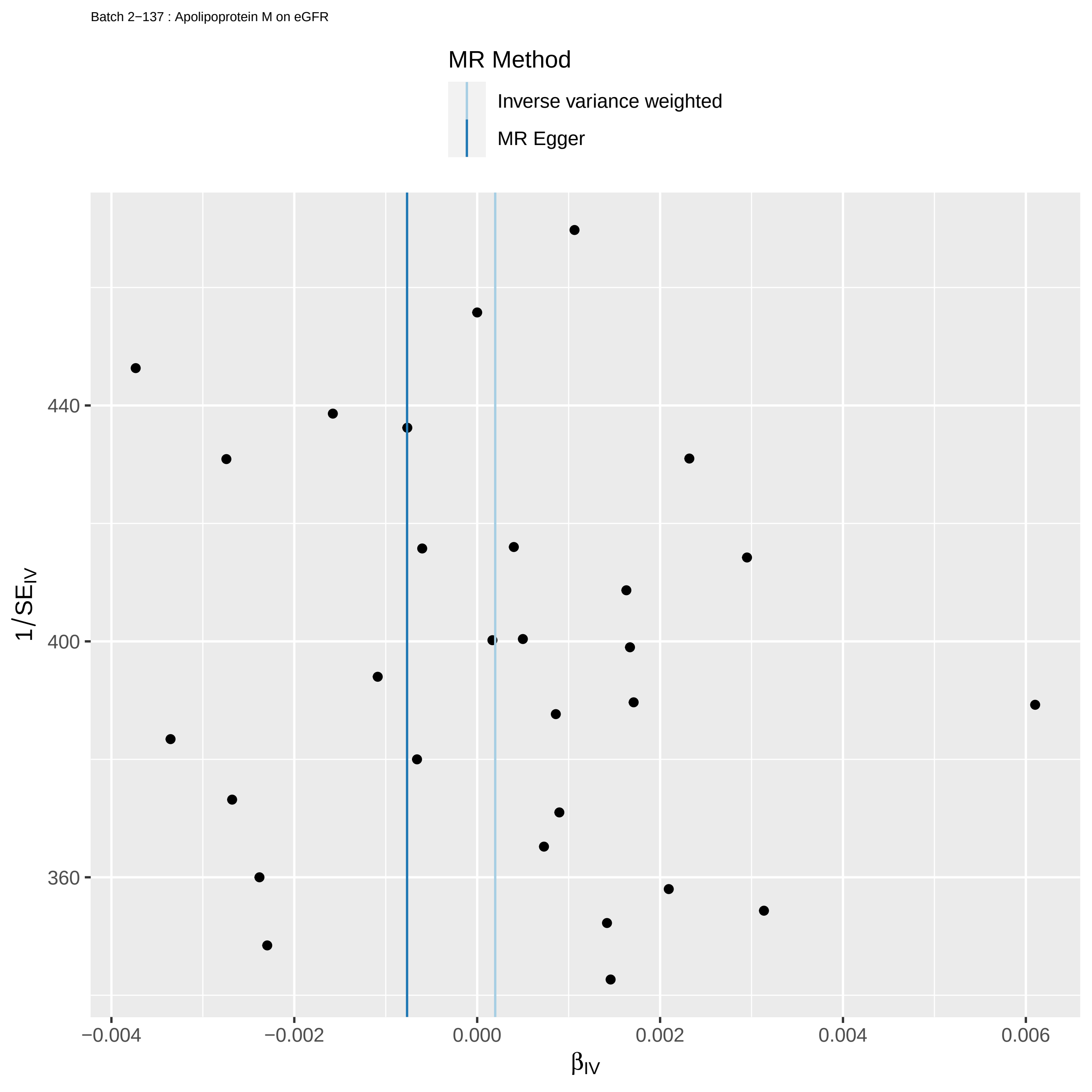

Batch 2−137 : Apolipoprotein M on eGFR
MR Method
Inverse variance weighted
MR Egger
440
400
360
1/SE$_{IV}$
−0.004
−0.002
0.000
0.002
0.004
0.006
β$_{IV}$

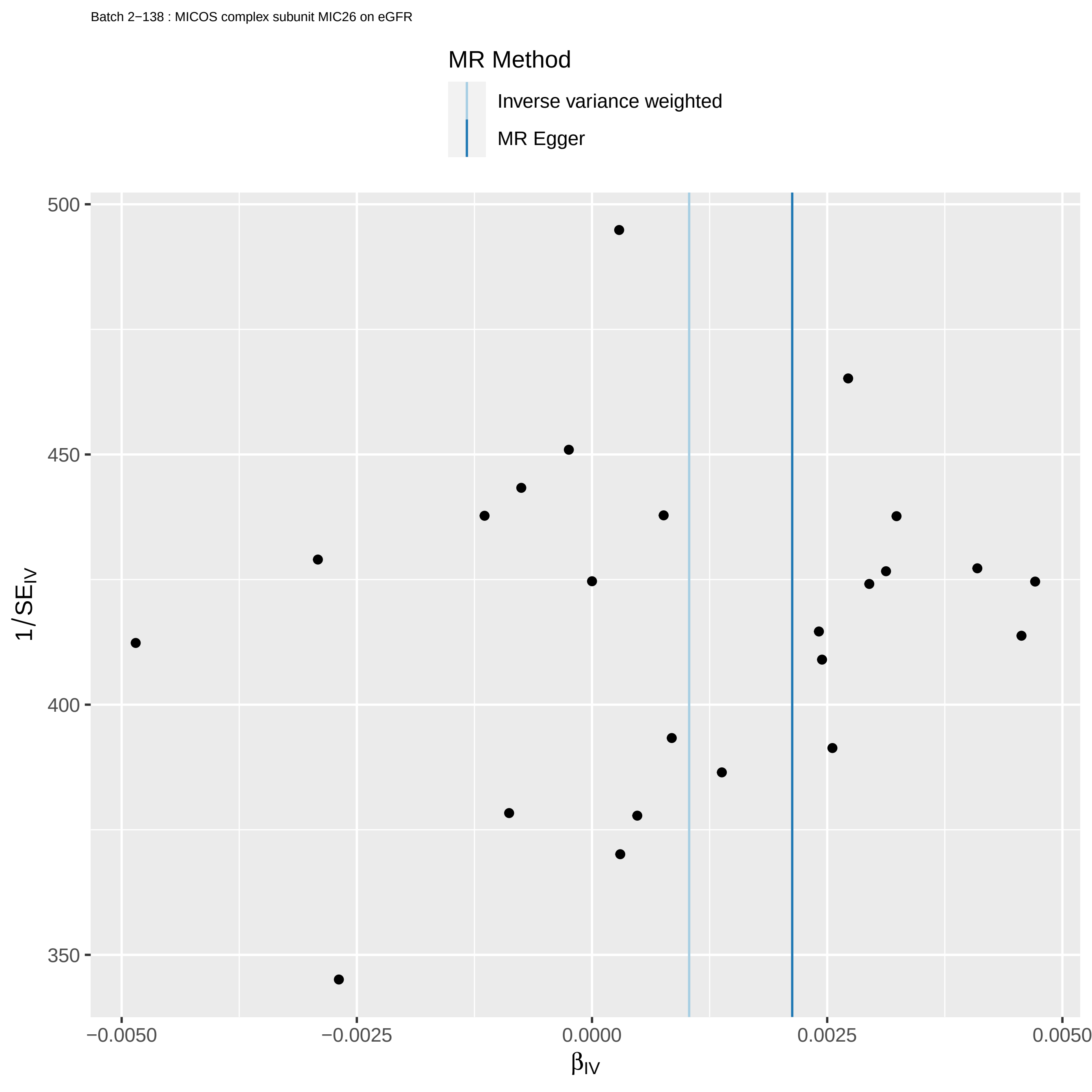
Batch 2−138 : MICOS complex subunit MIC26 on eGFR
MR Method
Inverse variance weighted
MR Egger
500
450
400
350
1/SE_IV
−0.0050
−0.0025
0.0000
0.0025
0.0050
β_IV

Batch 2−139 : Amyloid beta A4 protein on eGFR

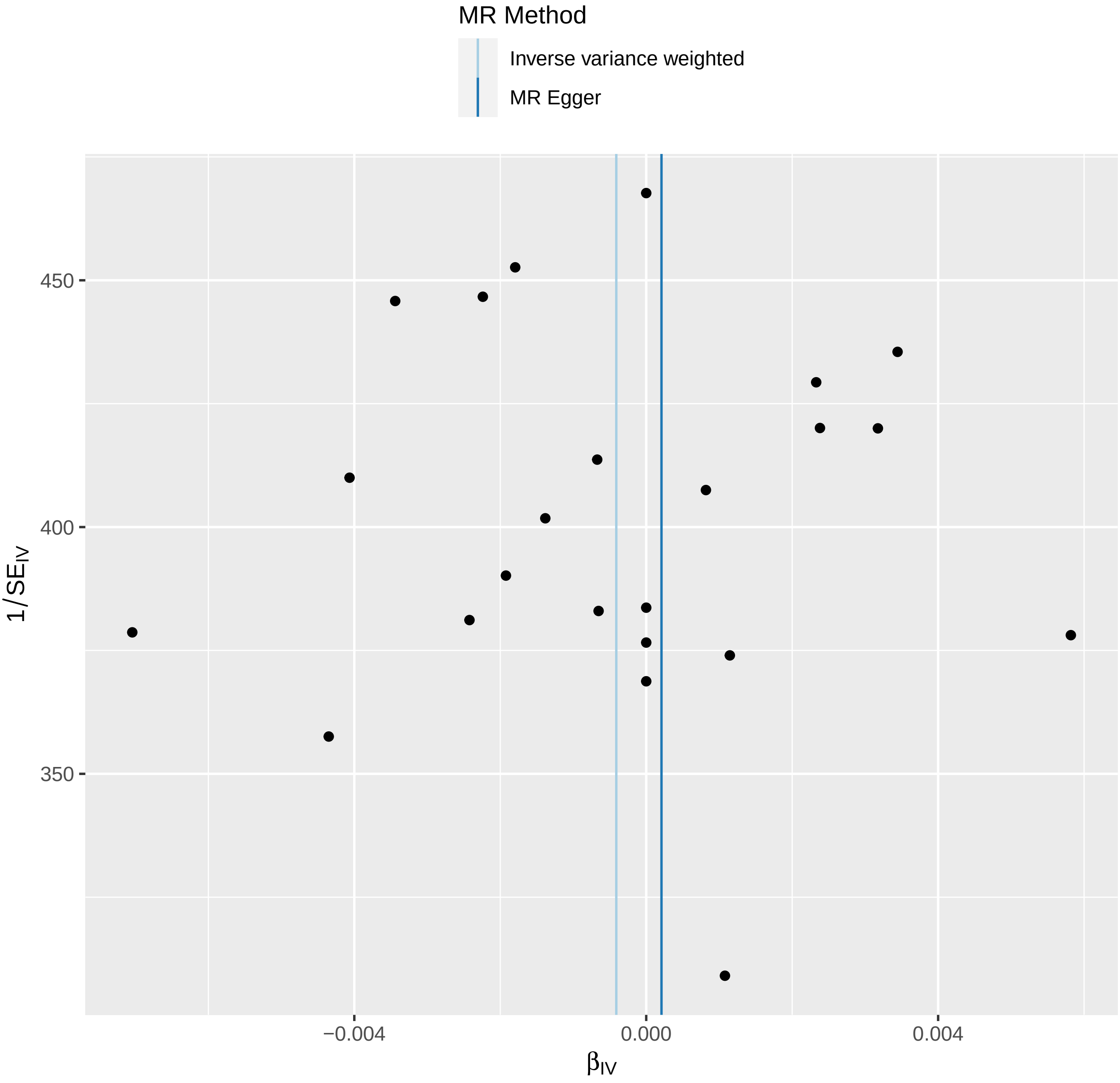

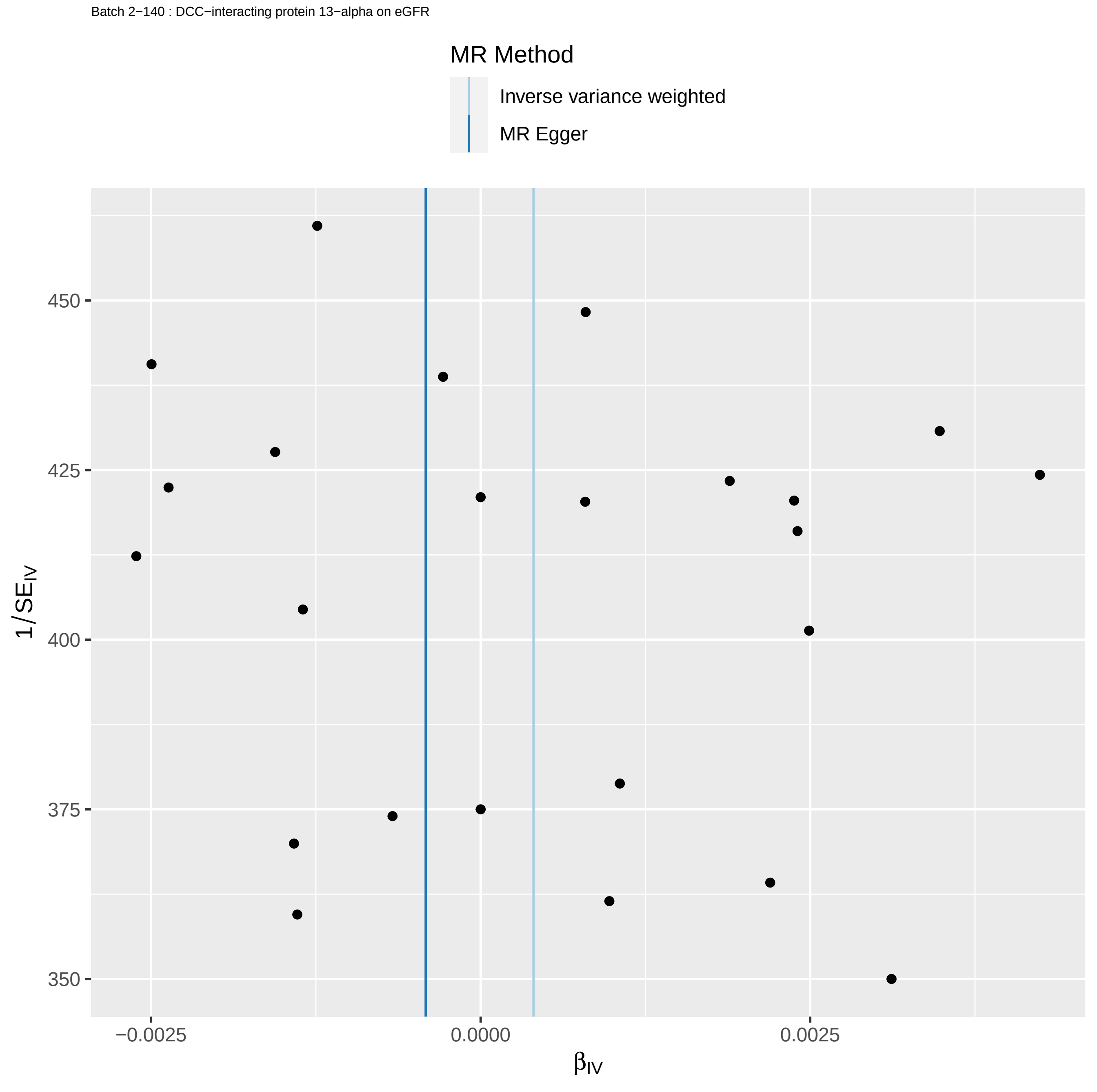
Batch 2−140 : DCC−interacting protein 13−alpha on eGFR
MR Method
Inverse variance weighted
MR Egger
450
425
400
375
350
1/SE_IV
−0.0025
0.0000
0.0025
β_IV

Batch 2−141 : Amphiregulin on eGFR

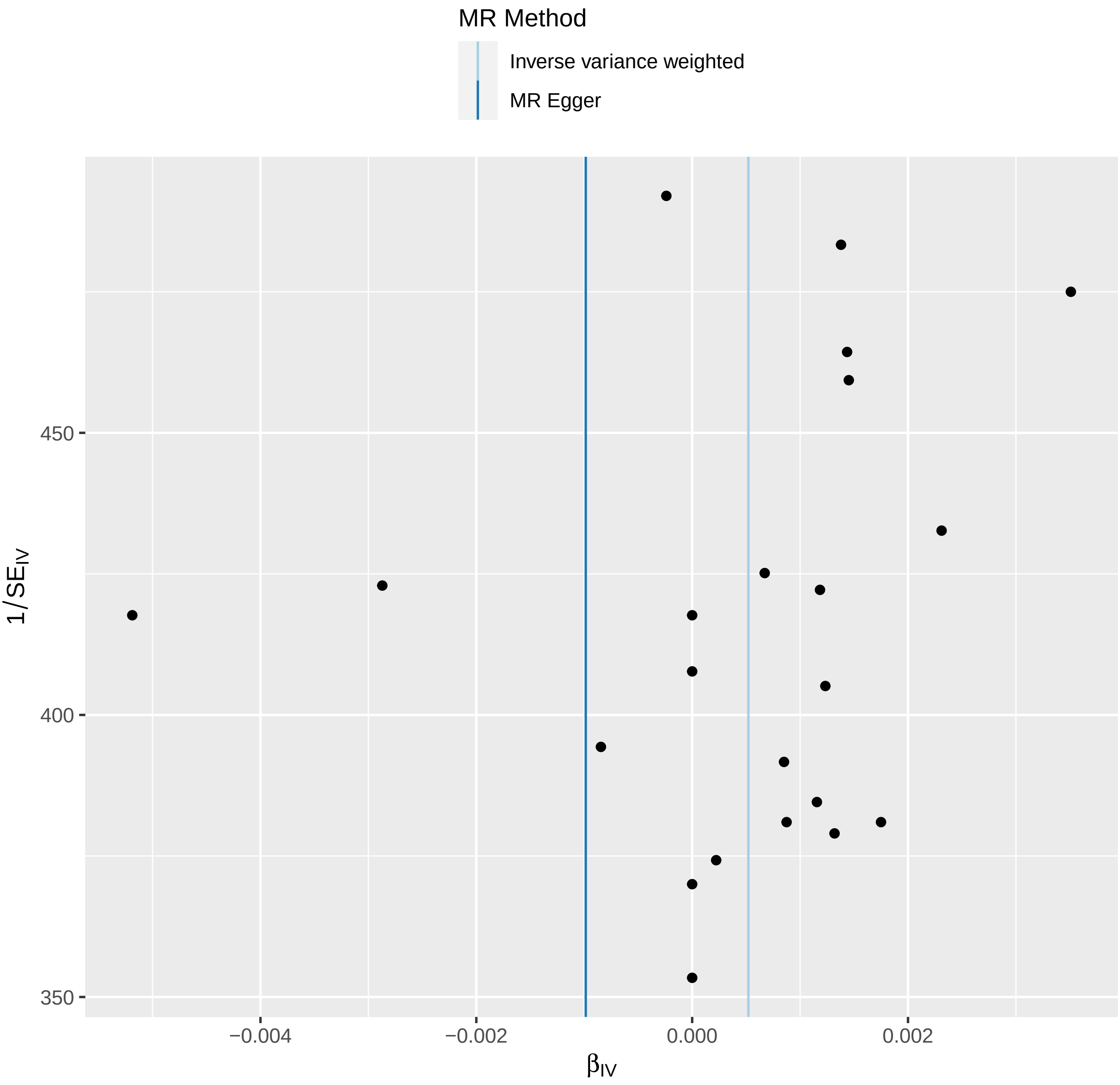

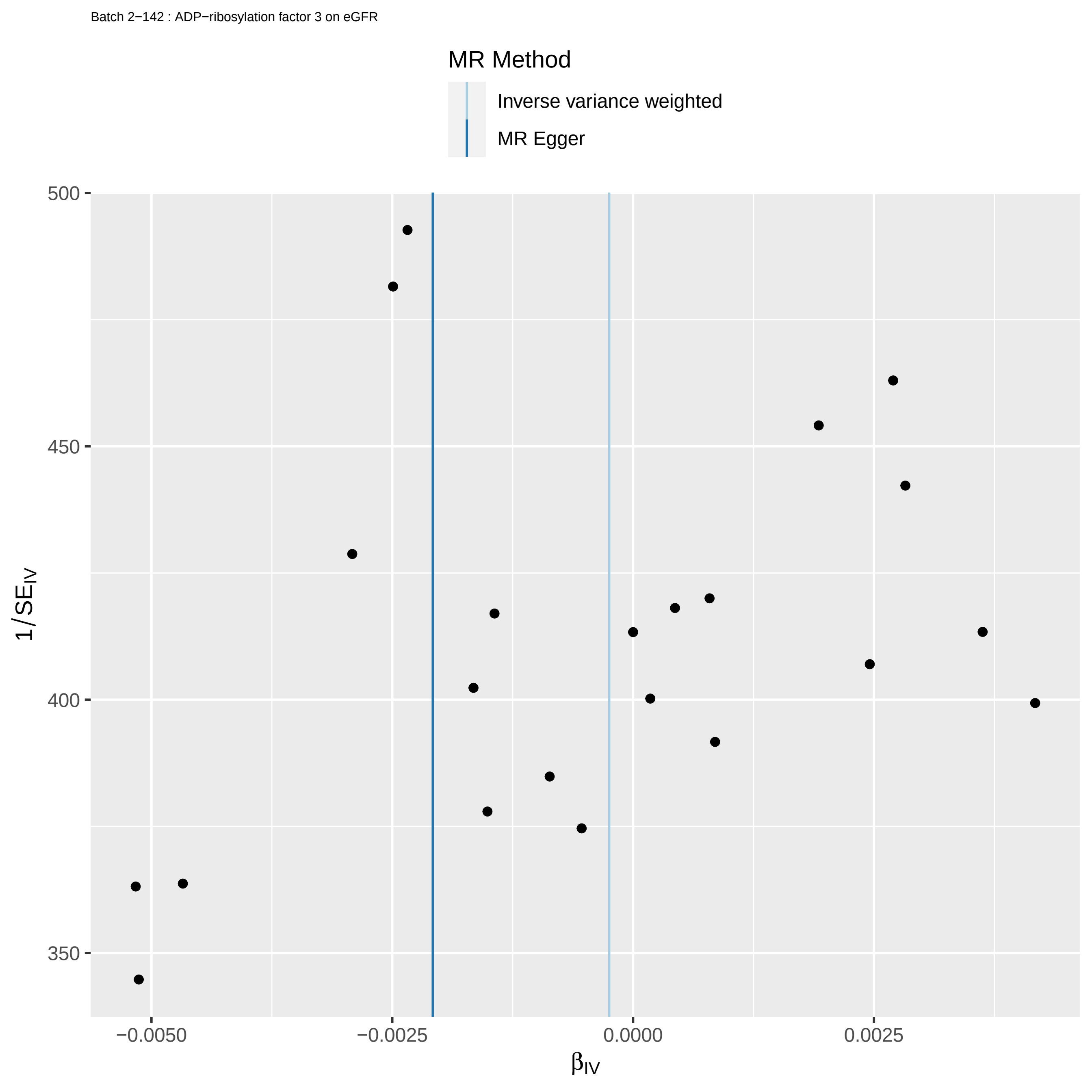

Batch 2−142 : ADP−ribosylation factor 3 on eGFR
MR Method
Inverse variance weighted
MR Egger
500
450
400
350
1/SE_IV
−0.0050
−0.0025
0.0000
0.0025
β_IV

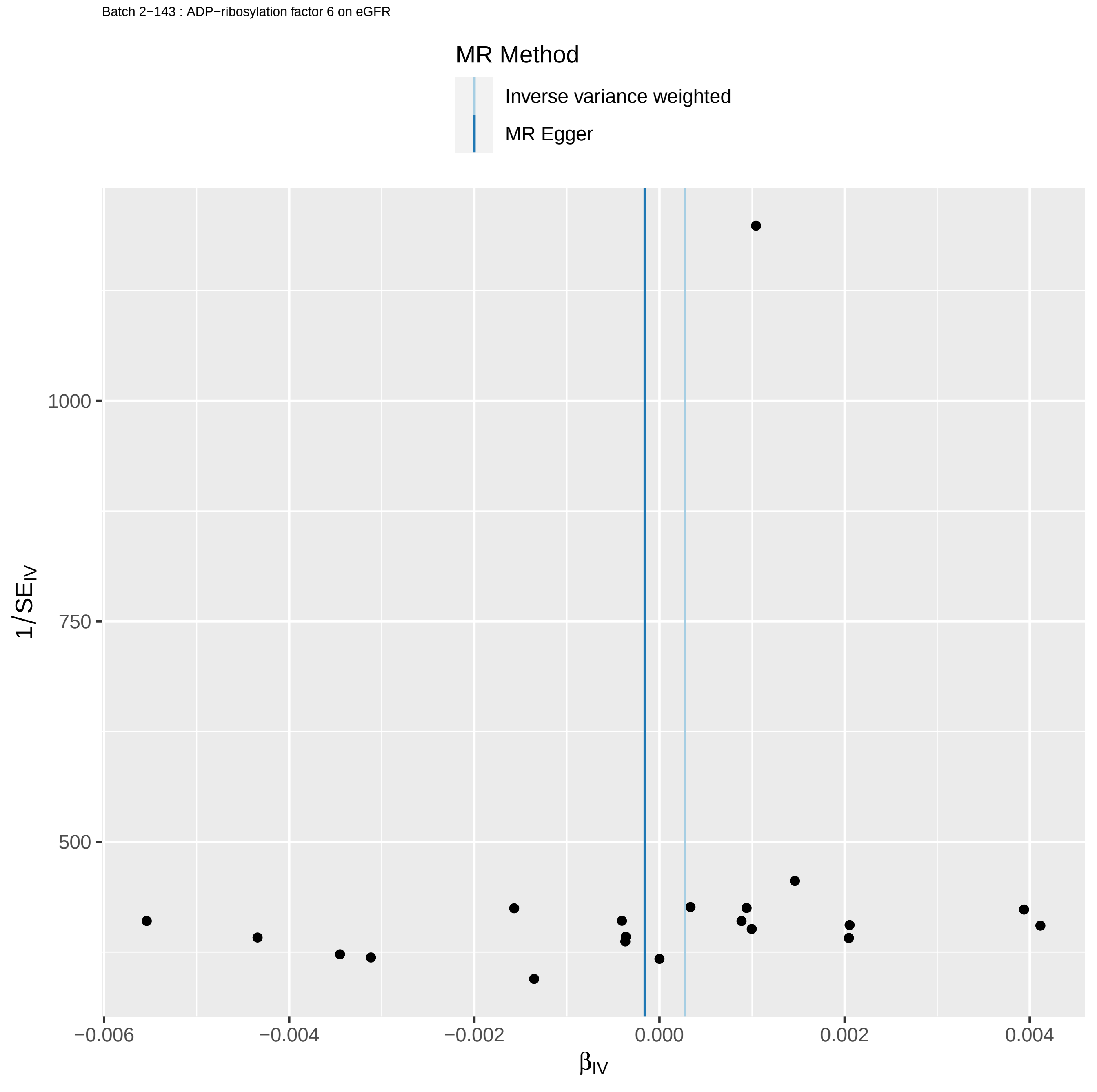

Batch 2−143 : ADP−ribosylation factor 6 on eGFR
MR Method
Inverse variance weighted
MR Egger
1000
750
500
1/SE_IV
−0.006
−0.004
−0.002
0.000
0.002
0.004
β_IV

Batch 2−144 : ADP−ribosylation factor GTPase−activating protein 2 on eGFR

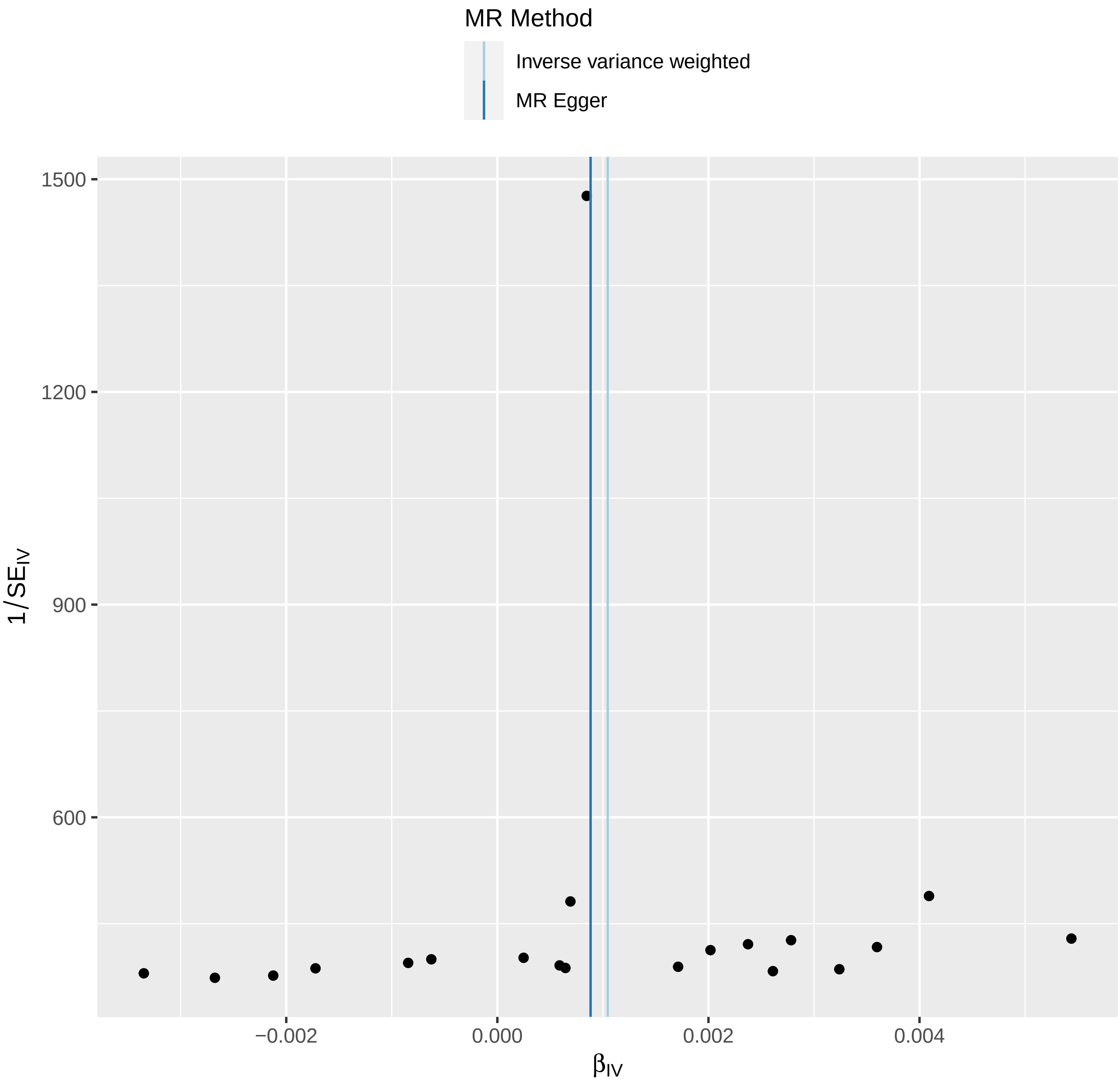

Batch 2−145 : Arfaptin−1 on eGFR

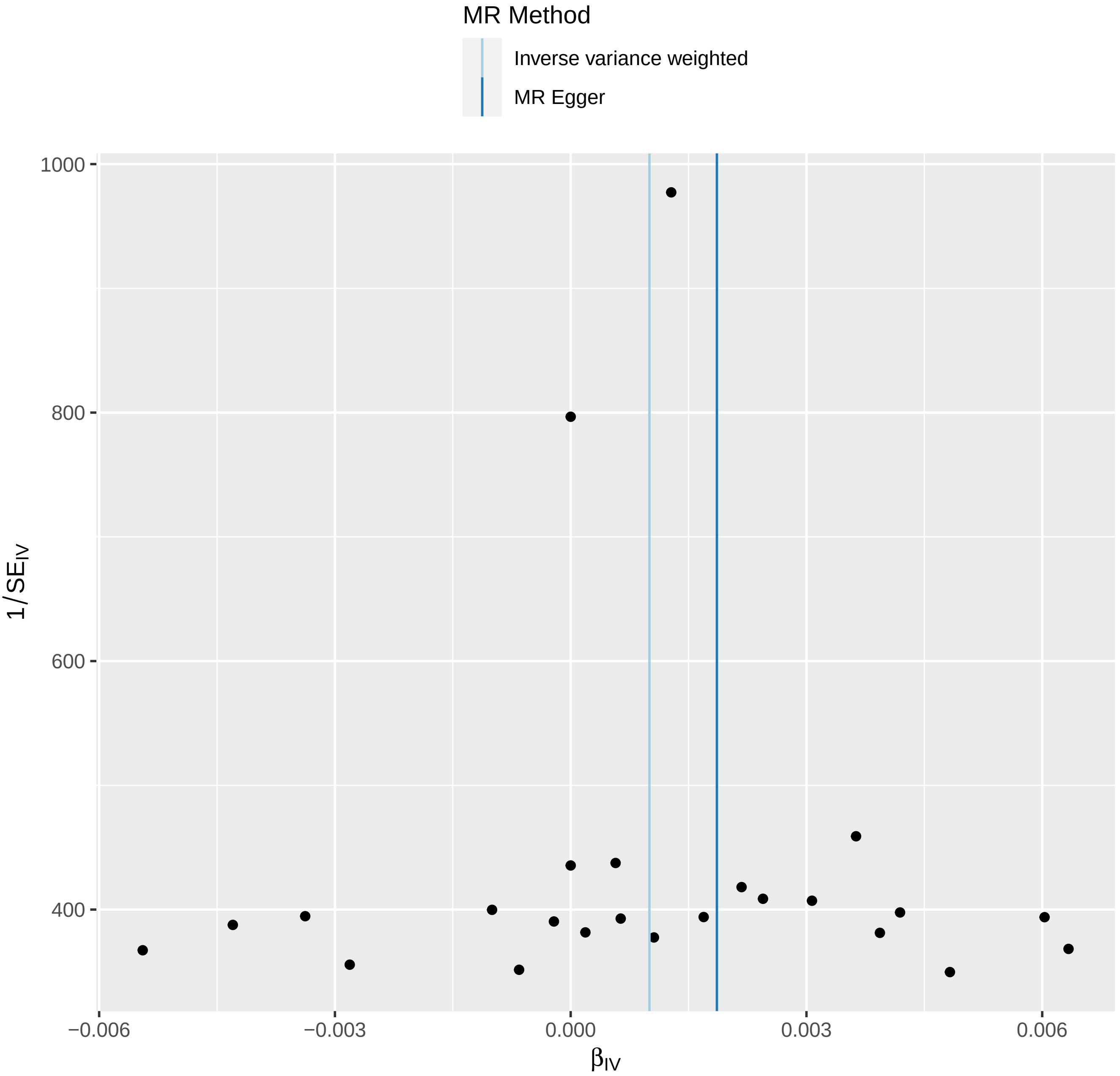

Batch 2−146 : Arfaptin−2 on eGFR

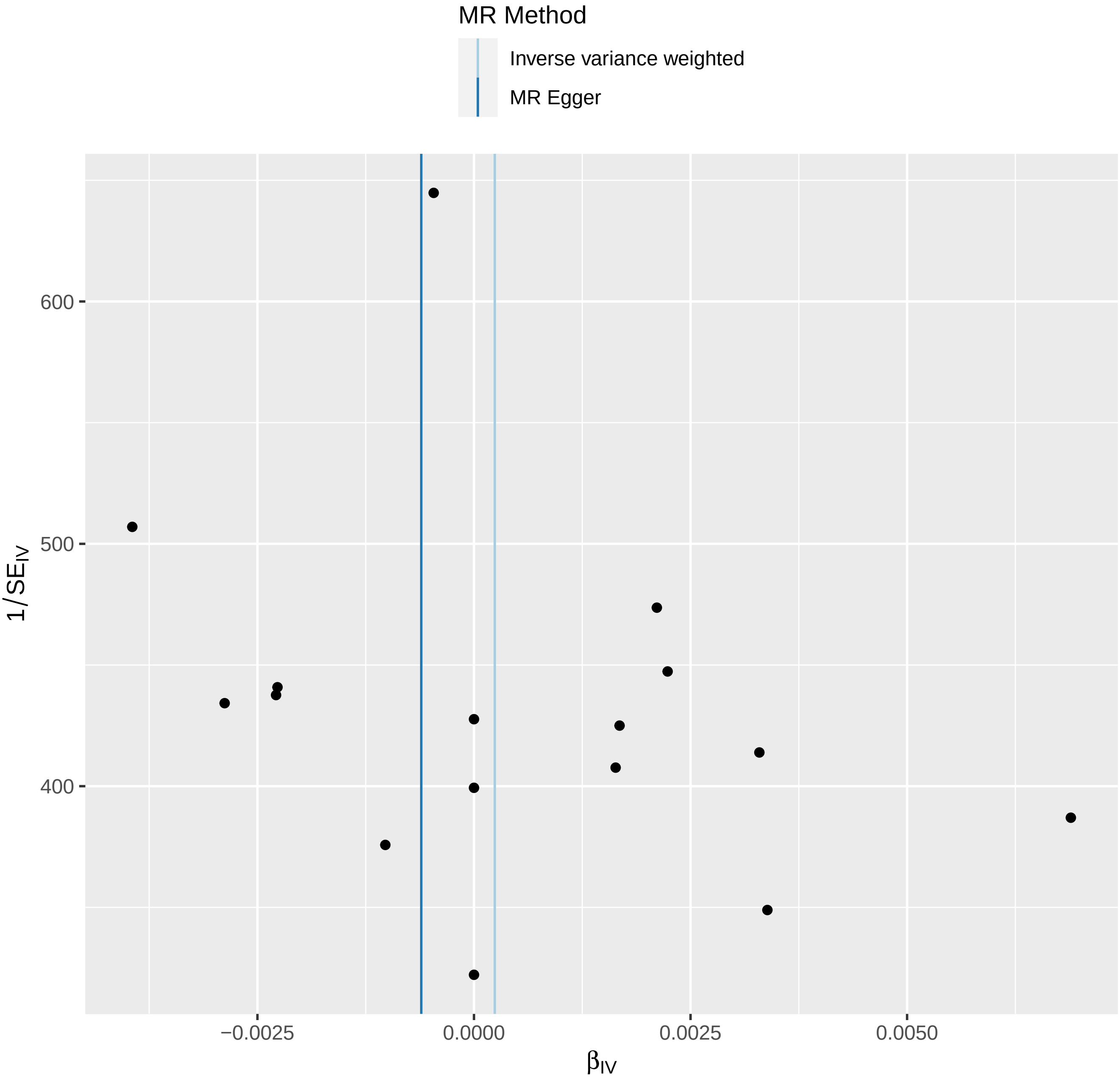

Batch 2−147 : Rho GTPase−activating protein 1 on eGFR

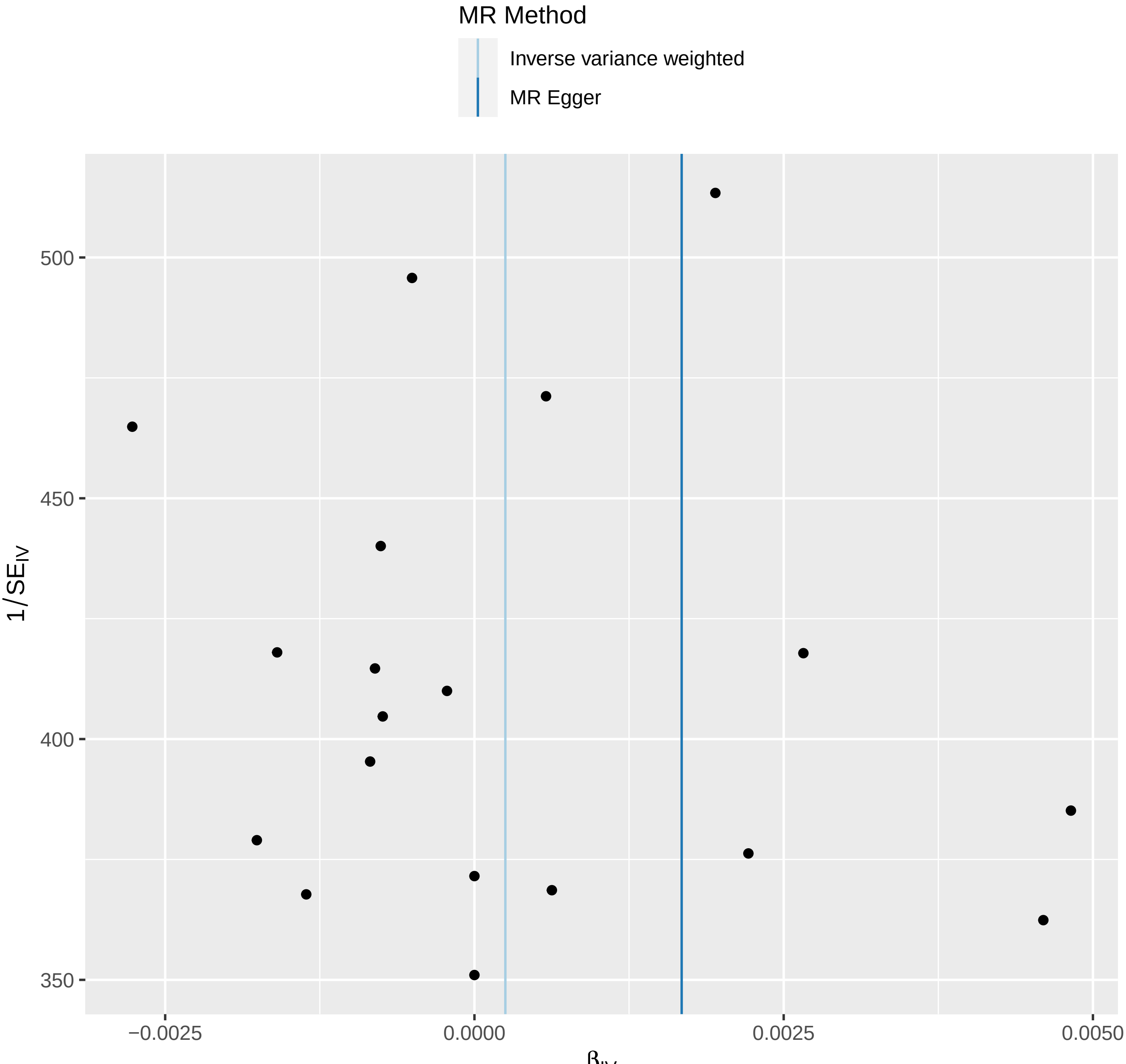

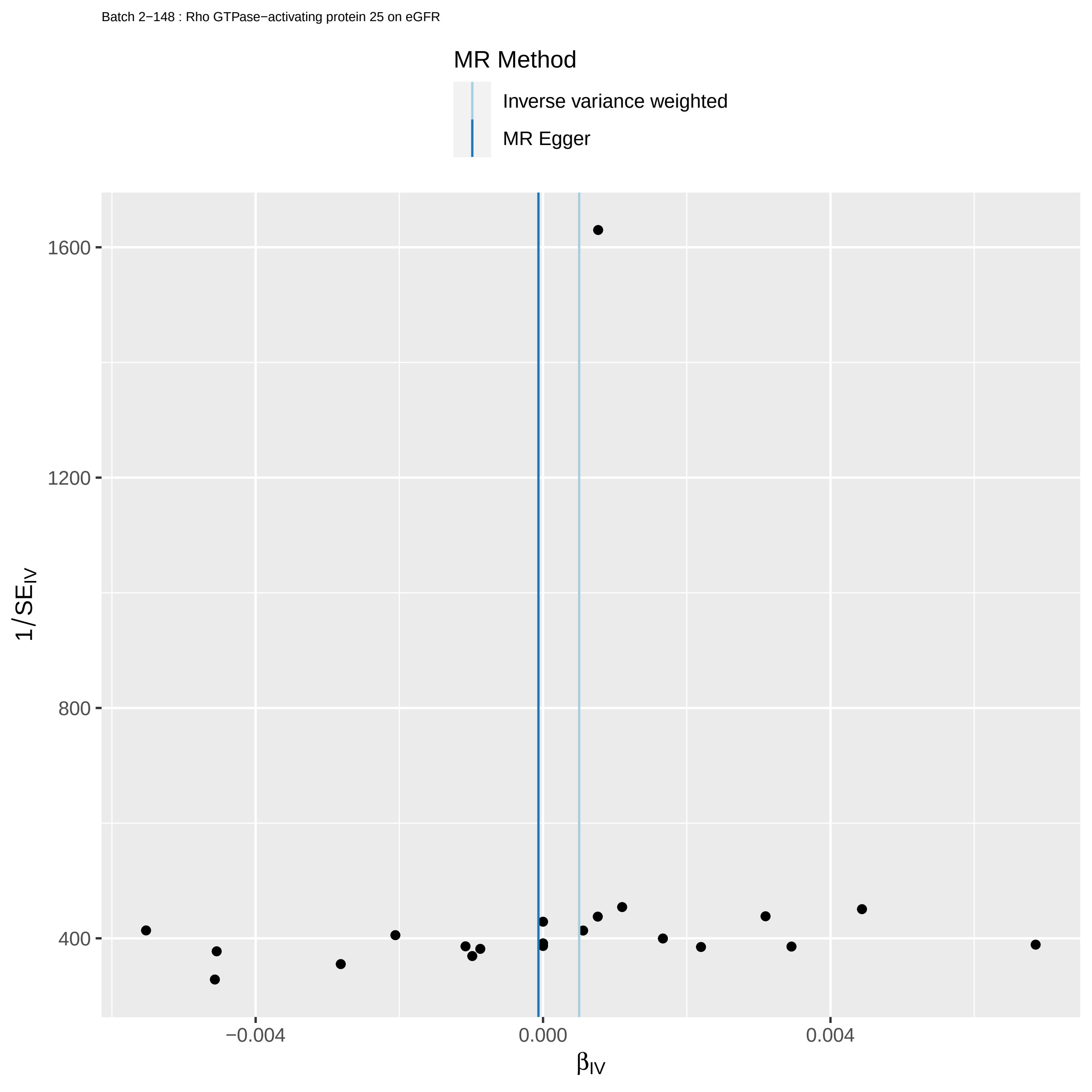

Batch 2−148 : Rho GTPase−activating protein 25 on eGFR
MR Method
Inverse variance weighted
MR Egger
1600
1200
800
400
1/SE$_{IV}$
−0.004
0.000
0.004
β_{IV}

Batch 2−149 : Rho GTPase−activating protein 30 on eGFR

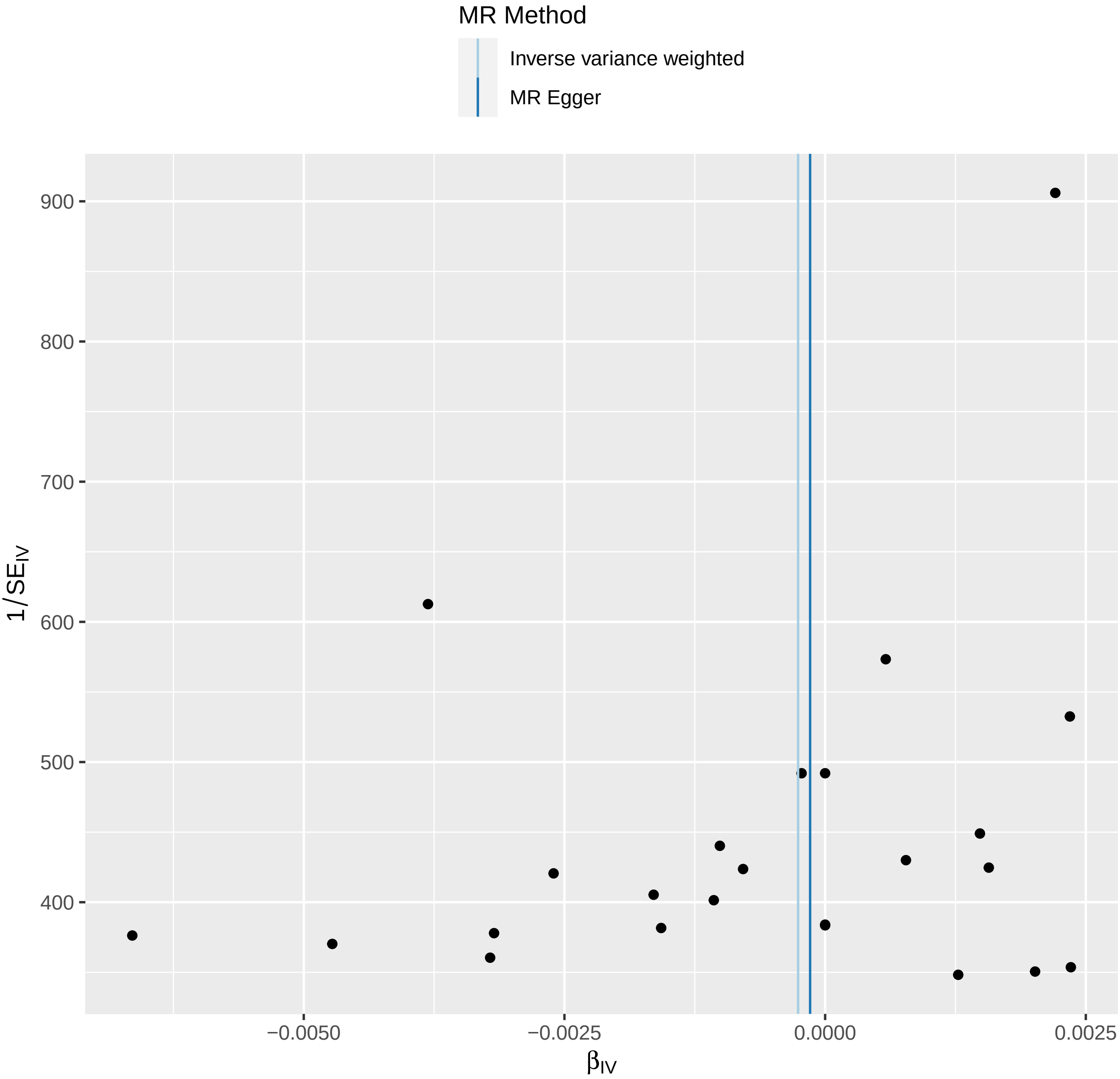

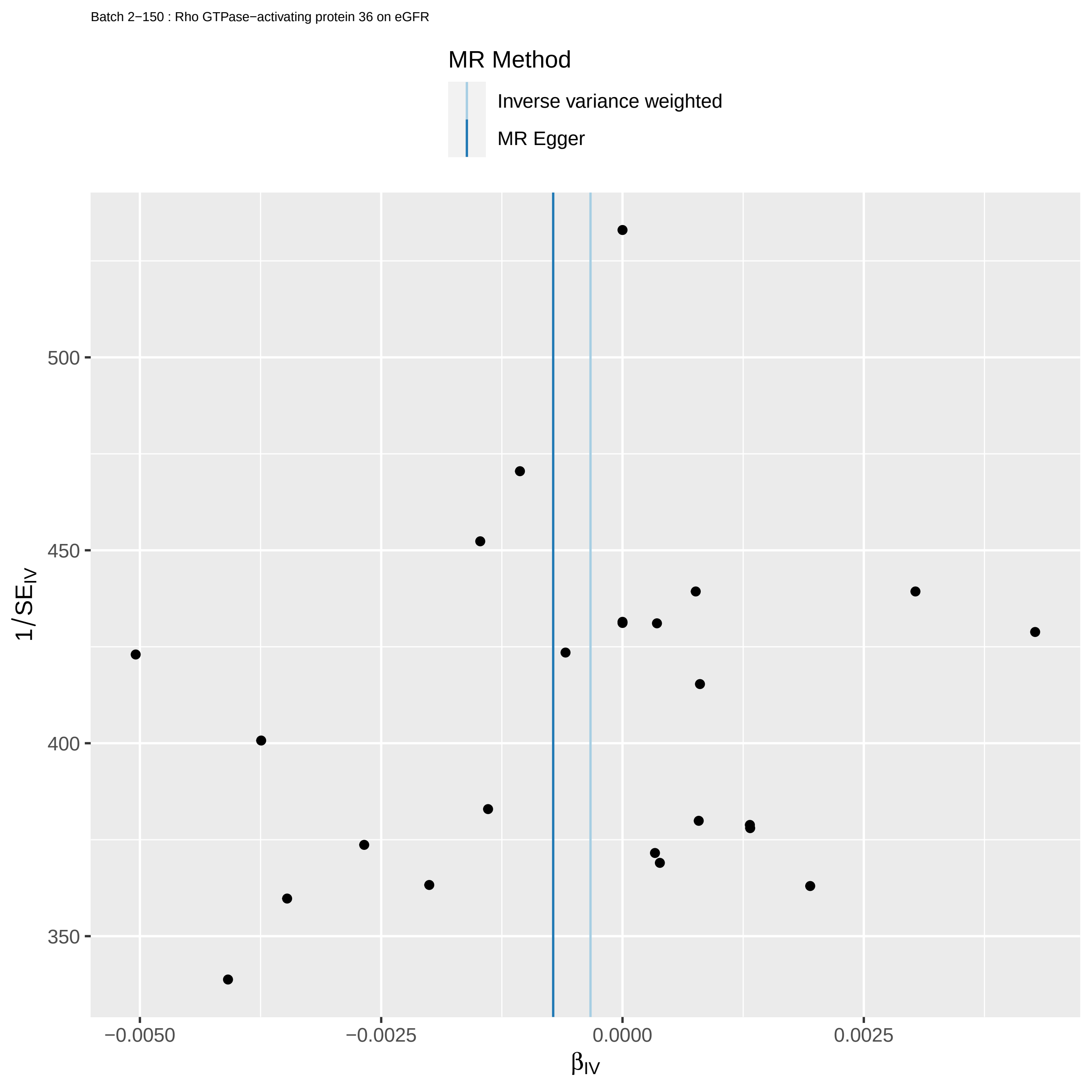
Batch 2−150 : Rho GTPase−activating protein 36 on eGFR
MR Method
Inverse variance weighted
MR Egger
1/SE_IV
500
450
400
350
−0.0050
−0.0025
0.0000
0.0025
β_IV

Batch 2−151 : Rho GTPase−activating protein 5 on eGFR

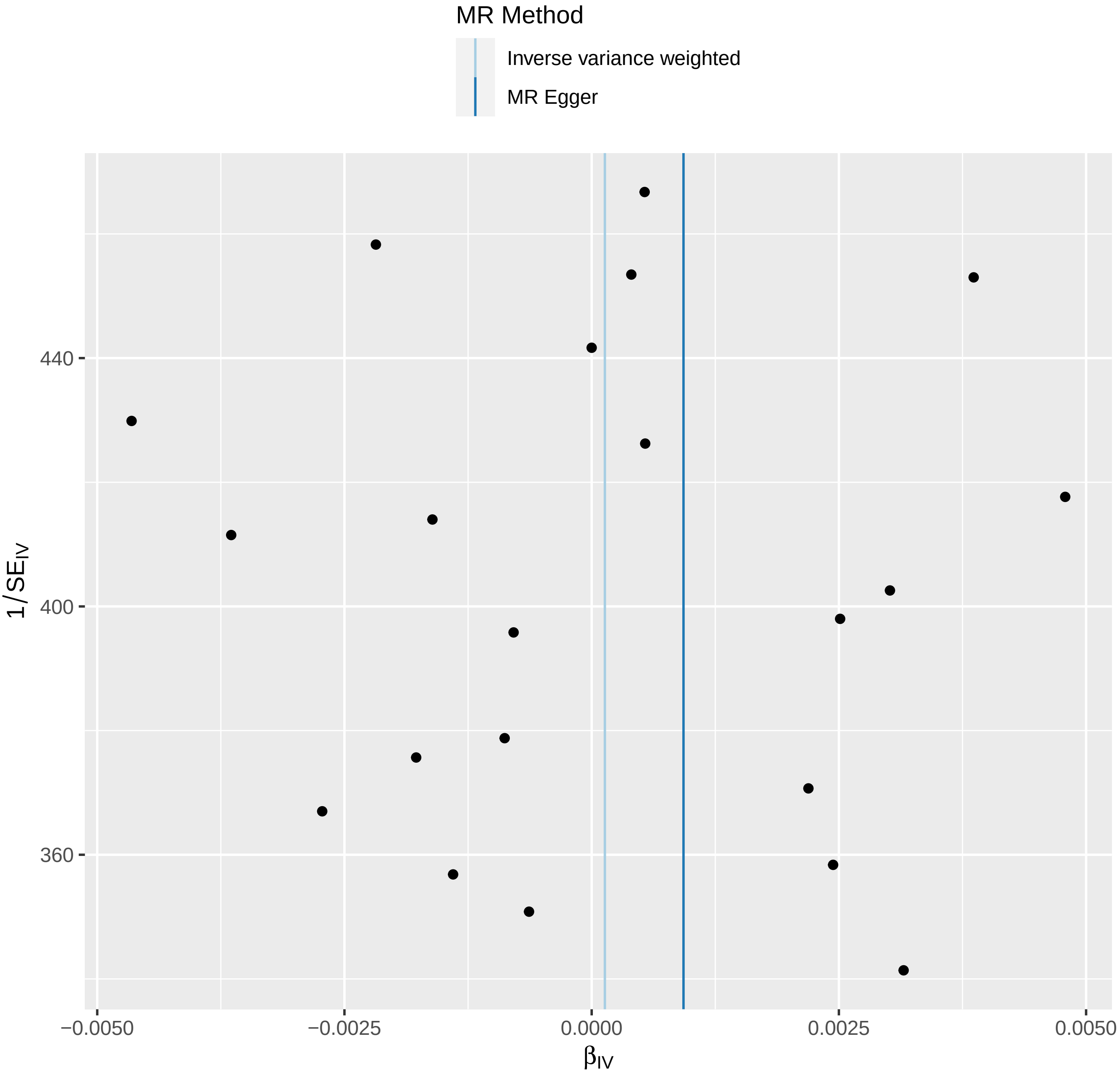

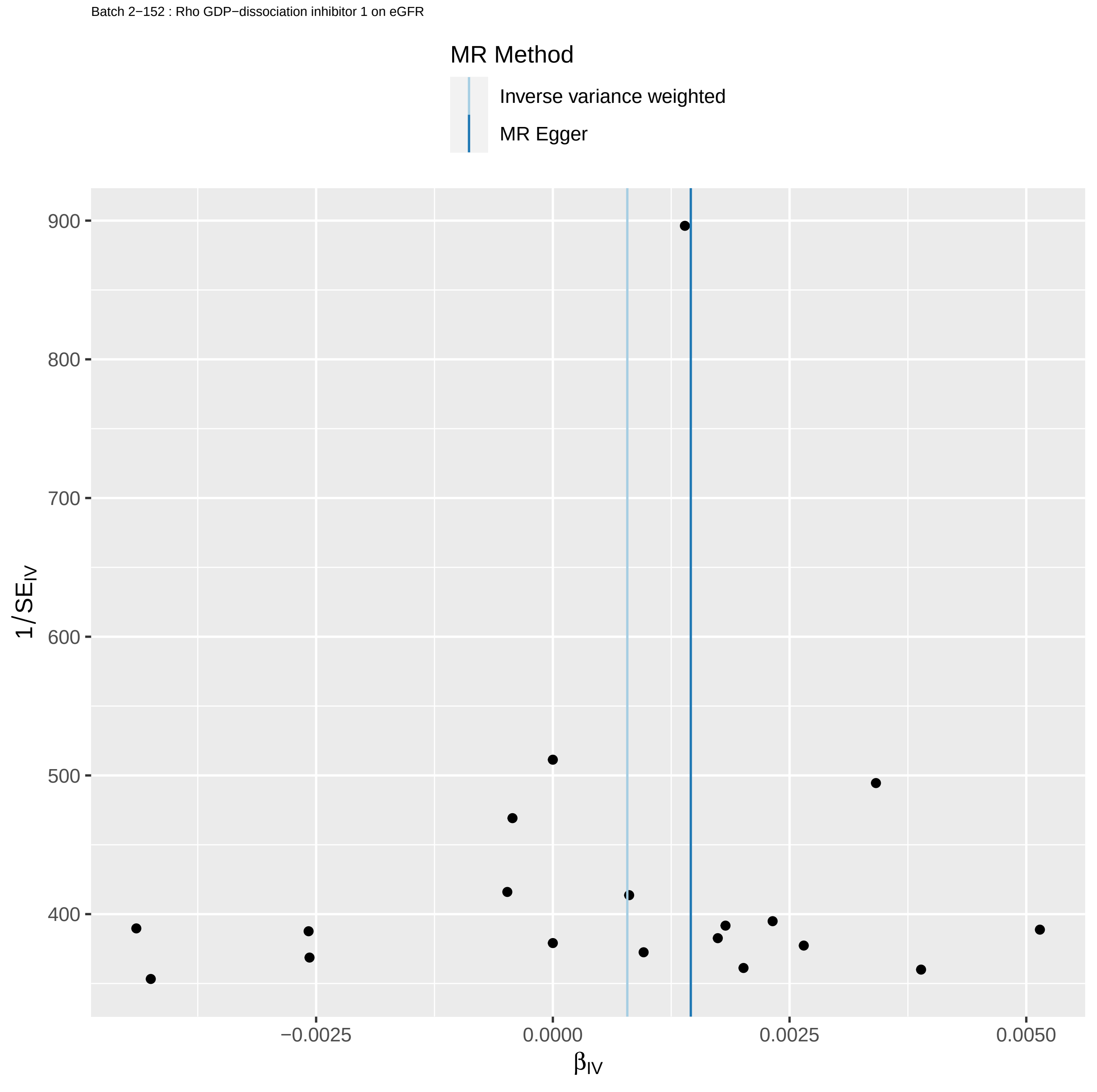
Batch 2−152 : Rho GDP−dissociation inhibitor 1 on eGFR
MR Method
Inverse variance weighted
MR Egger
900
800
700
600
500
400
1/SE_IV
−0.0025
0.0000
0.0025
0.0050
β_IV

Batch 2−153 : Rho GDP−dissociation inhibitor 2 on eGFR

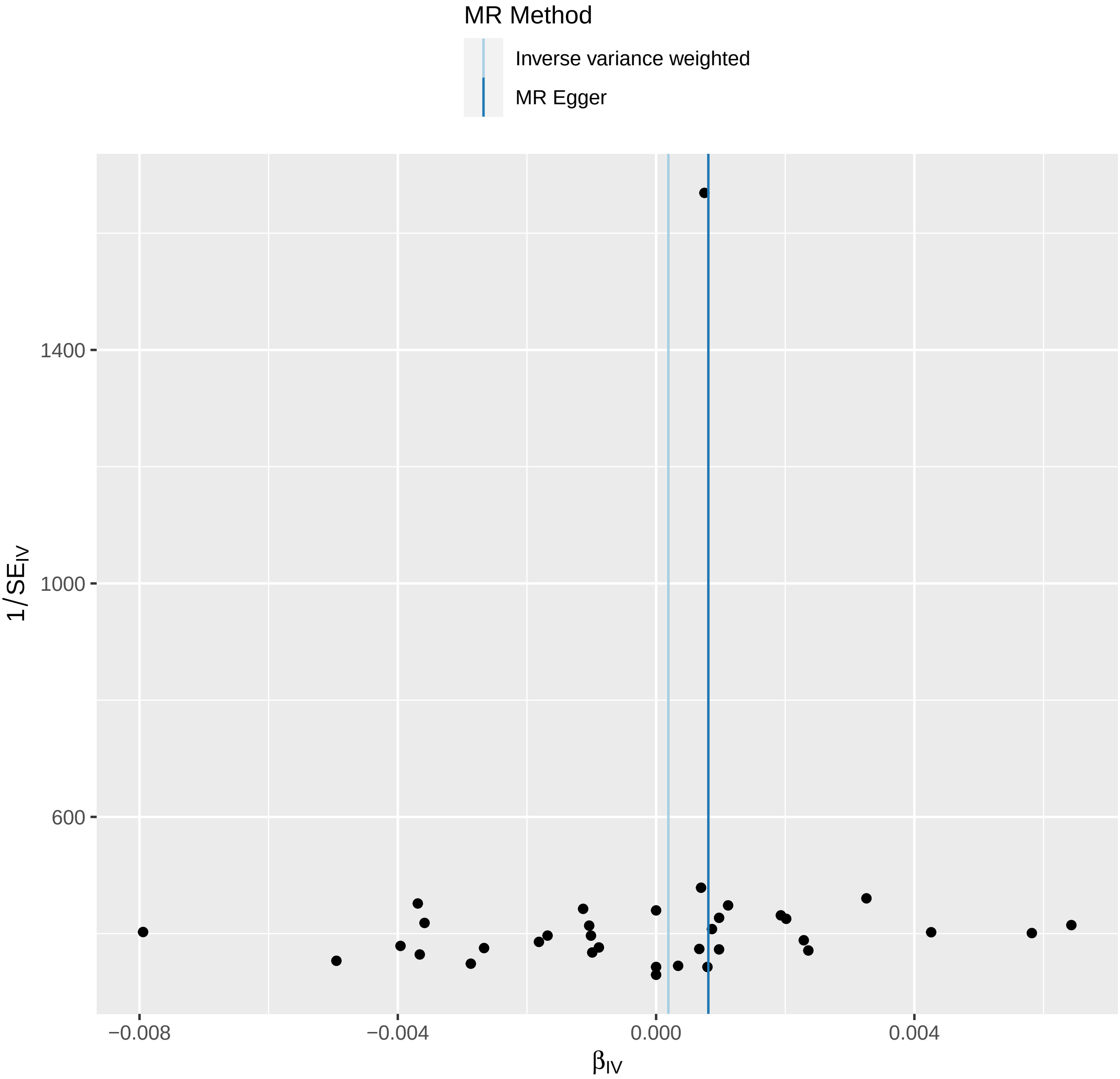

Batch 2−154 : Rho guanine nucleotide exchange factor 10 on eGFR

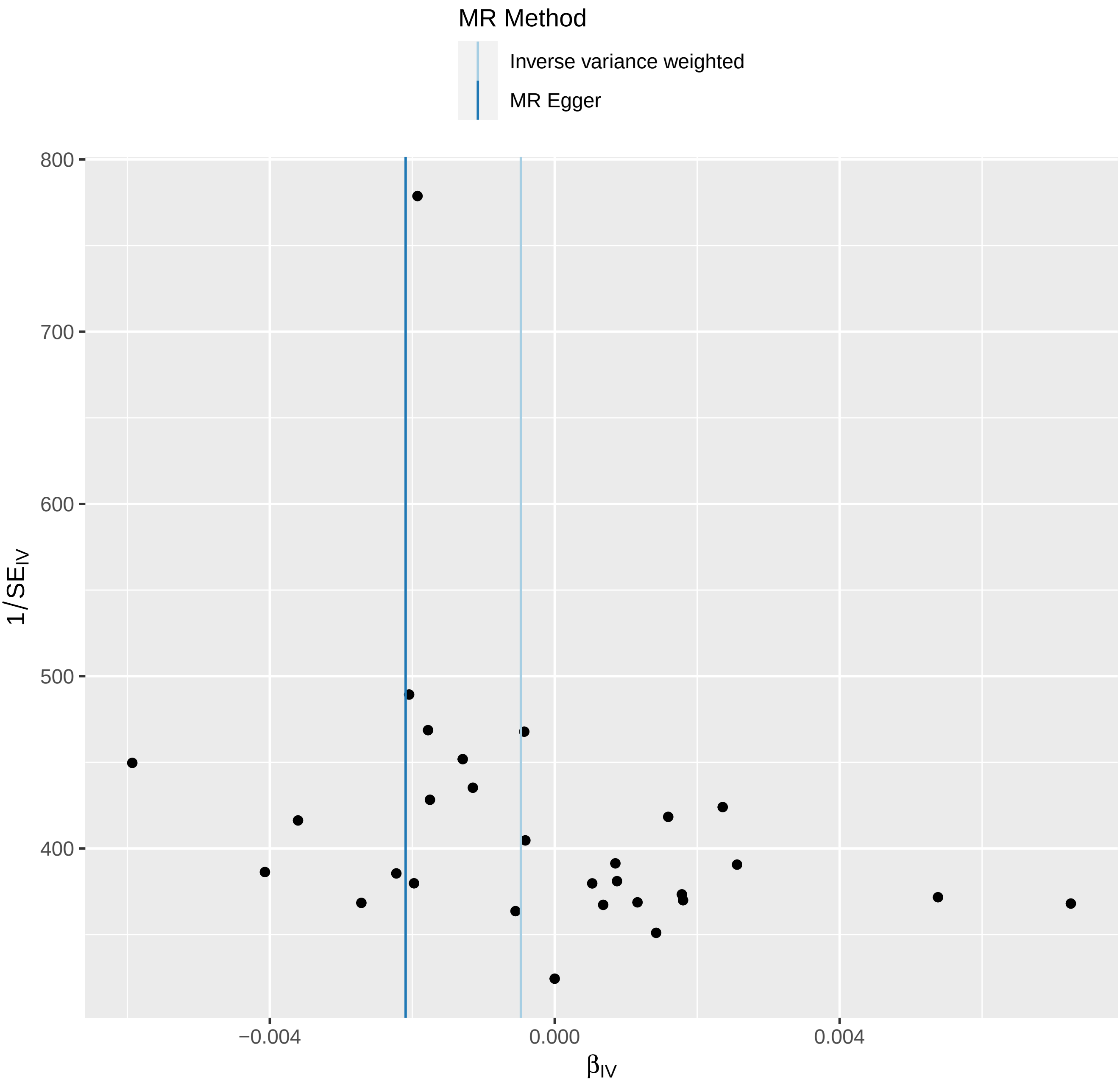

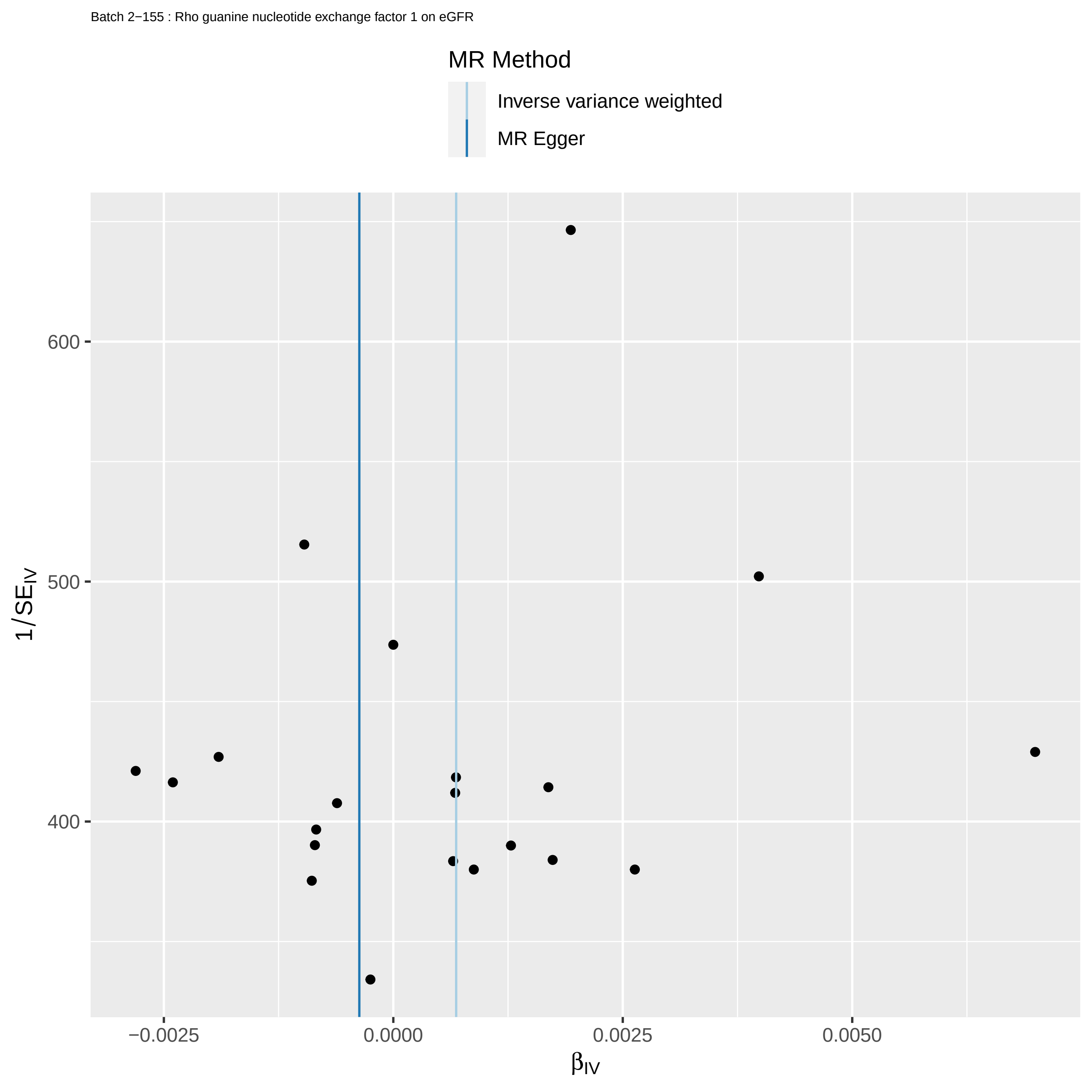

Batch 2−155 : Rho guanine nucleotide exchange factor 1 on eGFR
MR Method
Inverse variance weighted
MR Egger
600
500
400
1/SE_IV
−0.0025
0.0000
0.0025
0.0050
β_IV

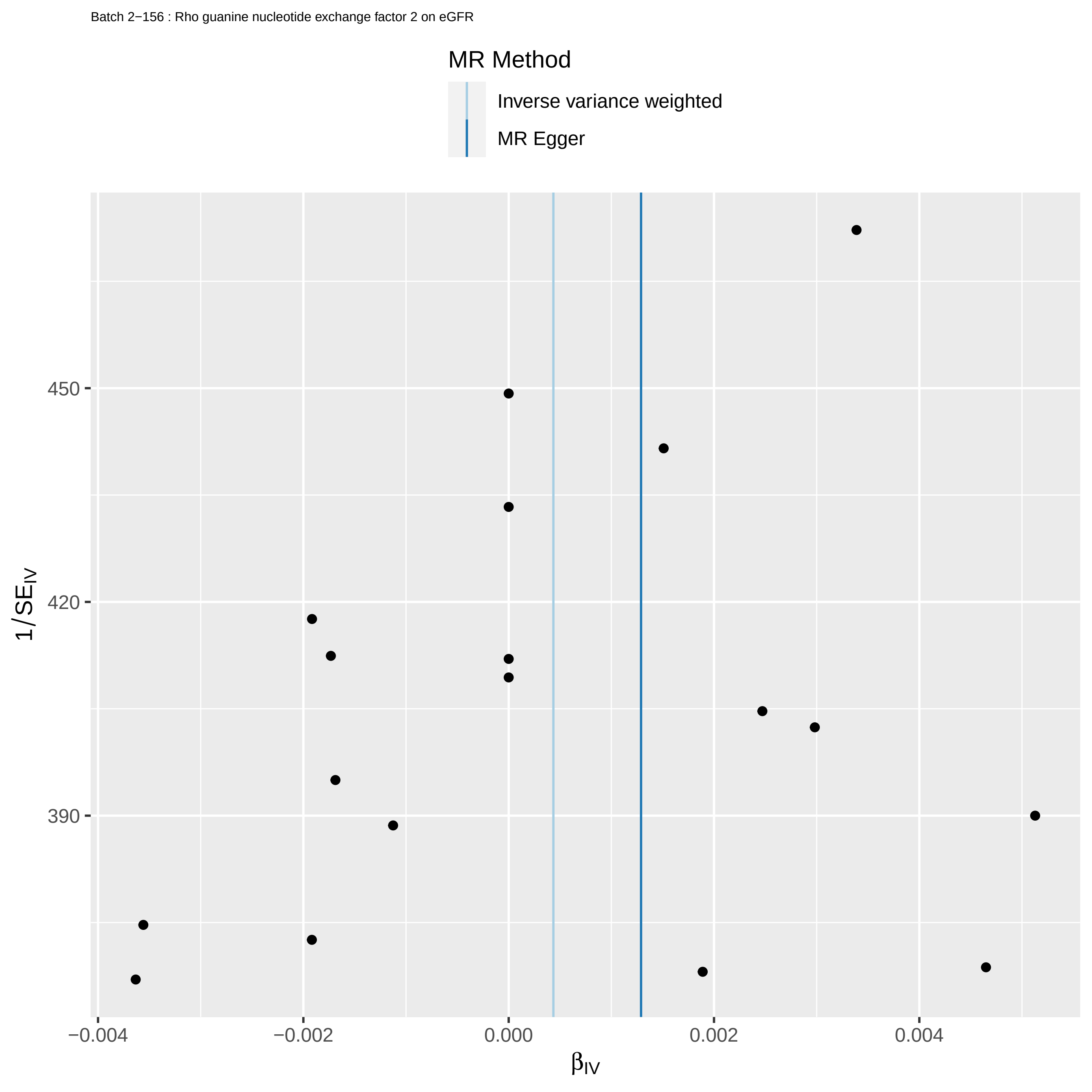
Batch 2−156 : Rho guanine nucleotide exchange factor 2 on eGFR
MR Method
Inverse variance weighted
MR Egger
450
420
390
1/SE_IV
−0.004
−0.002
0.000
0.002
0.004
β_IV

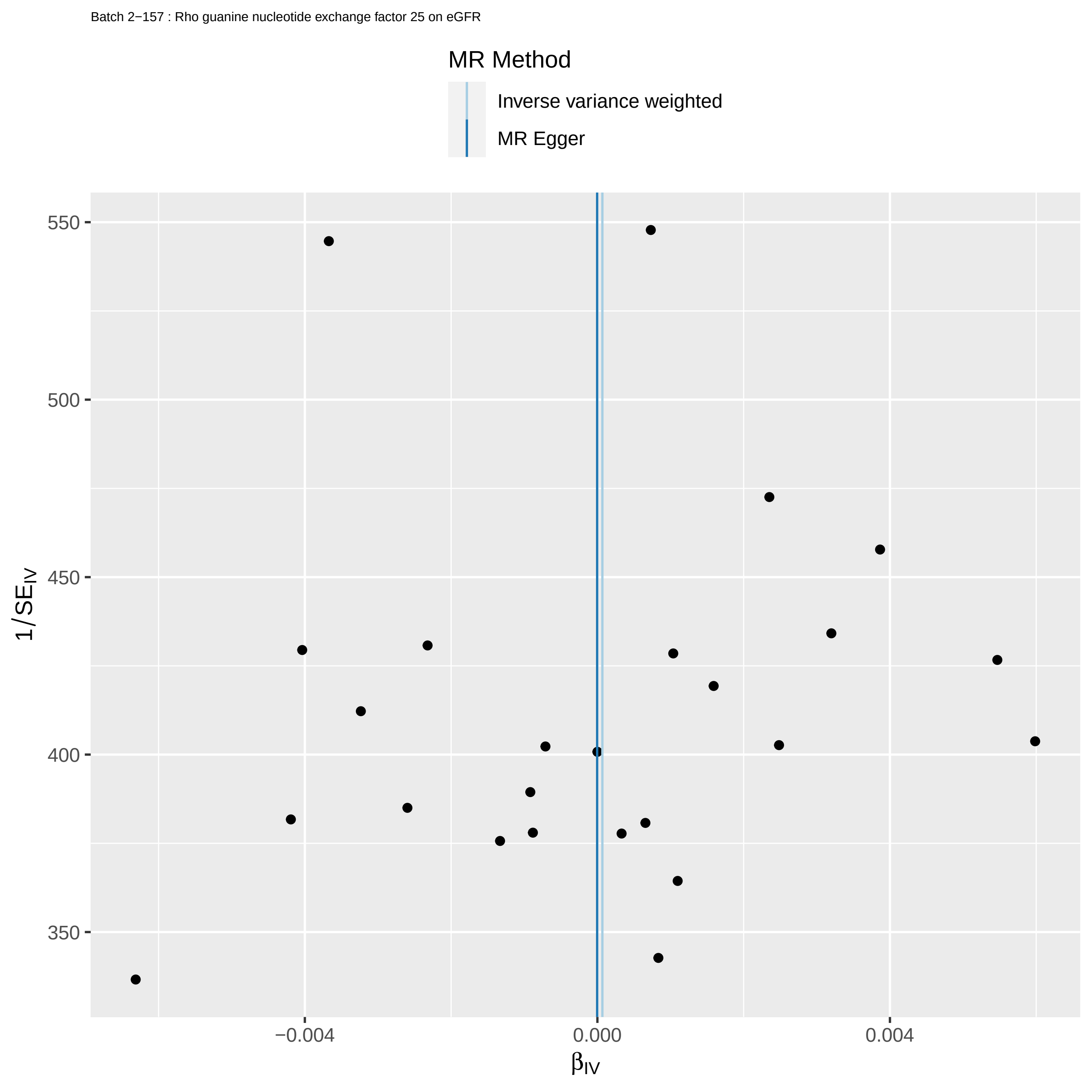

Batch 2−157 : Rho guanine nucleotide exchange factor 25 on eGFR
MR Method
Inverse variance weighted
MR Egger
550
500
450
400
350
1/SE_IV
−0.004
0.000
0.004
β_IV

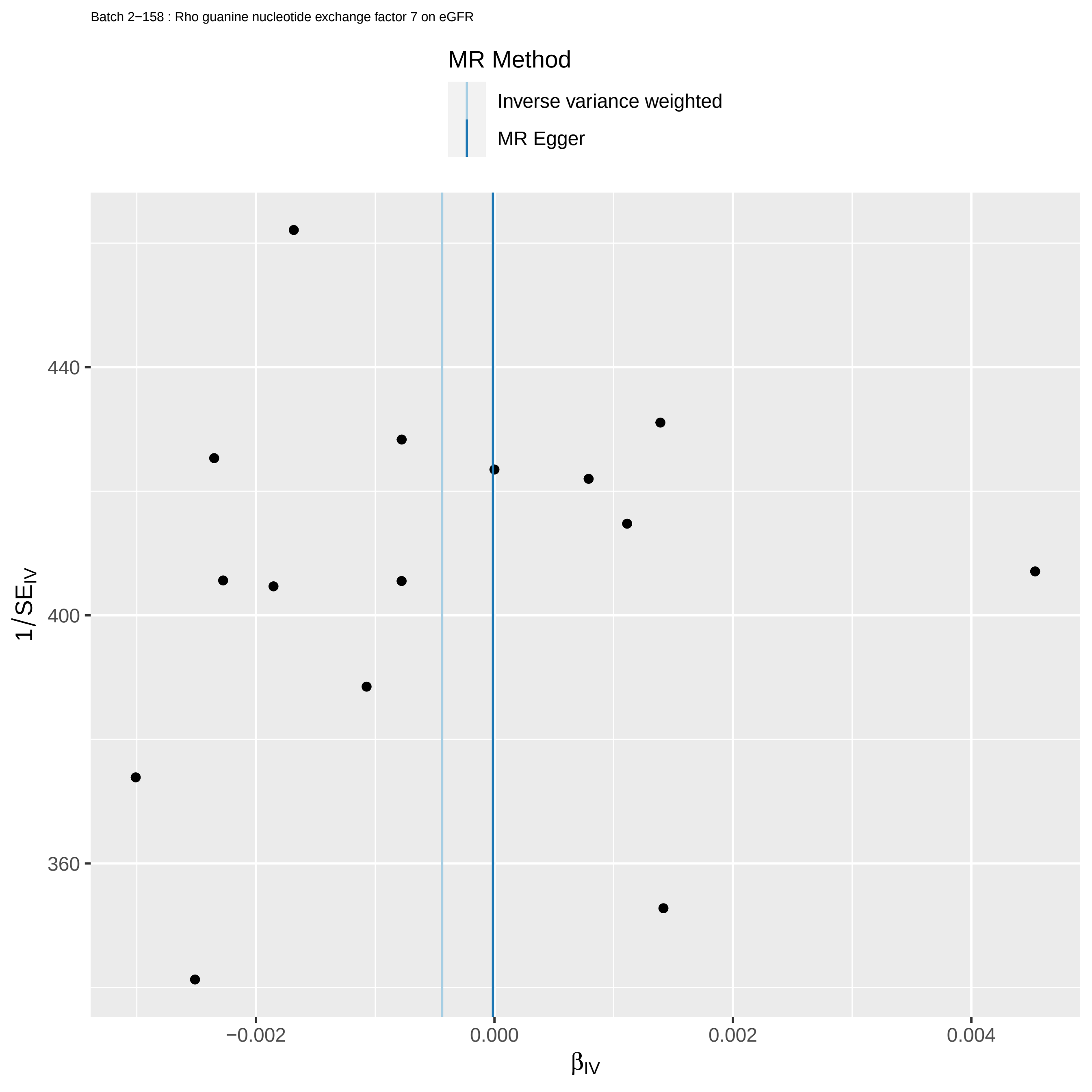
Batch 2−158 : Rho guanine nucleotide exchange factor 7 on eGFR
MR Method
Inverse variance weighted
MR Egger
440
400
360
1/SE$_{IV}$
−0.002
0.000
0.002
0.004
β$_{IV}$

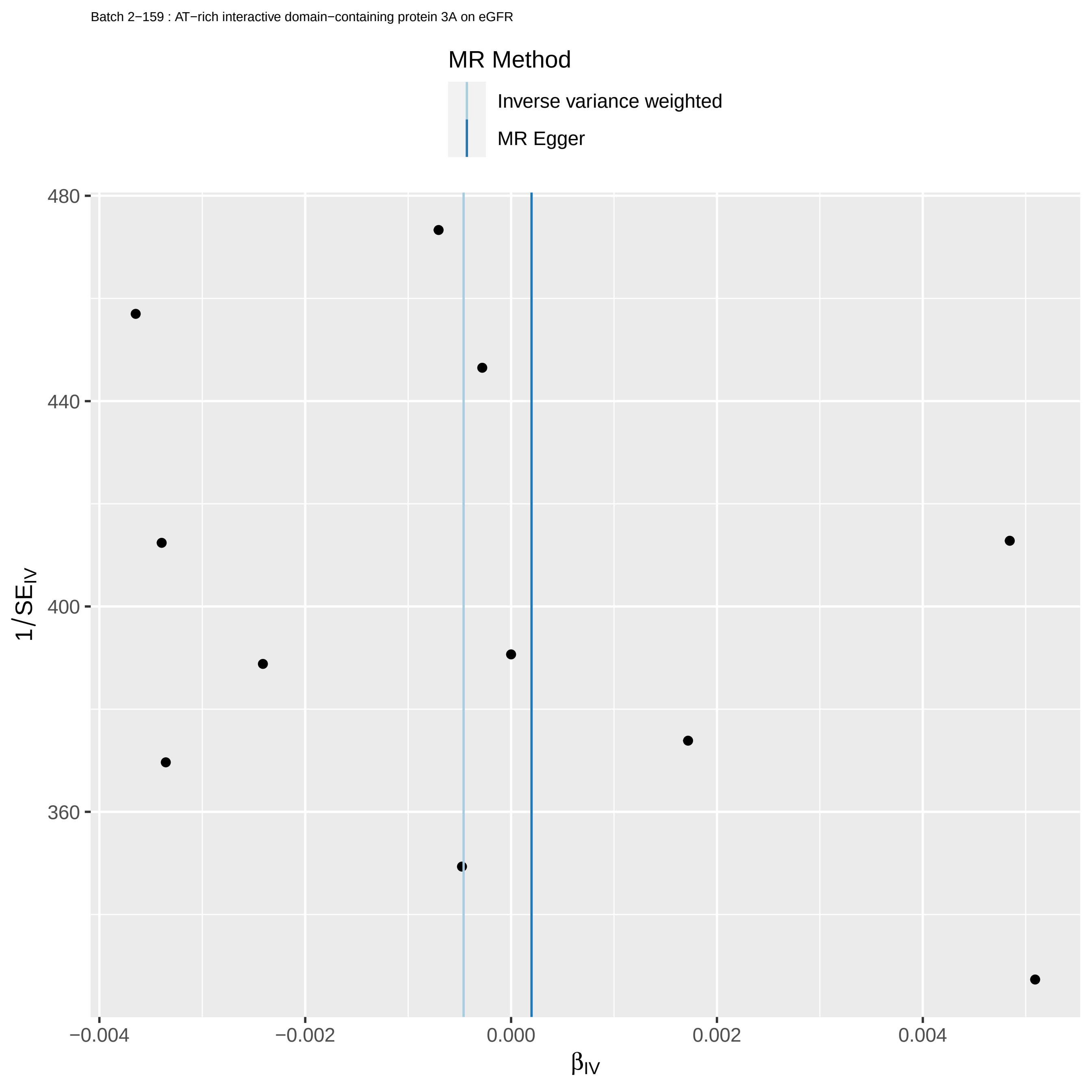
Batch 2−159 : AT−rich interactive domain−containing protein 3A on eGFR
MR Method
Inverse variance weighted
MR Egger
480
440
400
360
1/SE$_{IV}$
−0.004
−0.002
0.000
0.002
0.004
β$_{IV}$

Batch 2−160 : ADP−ribosylation factor−like protein 11 on eGFR

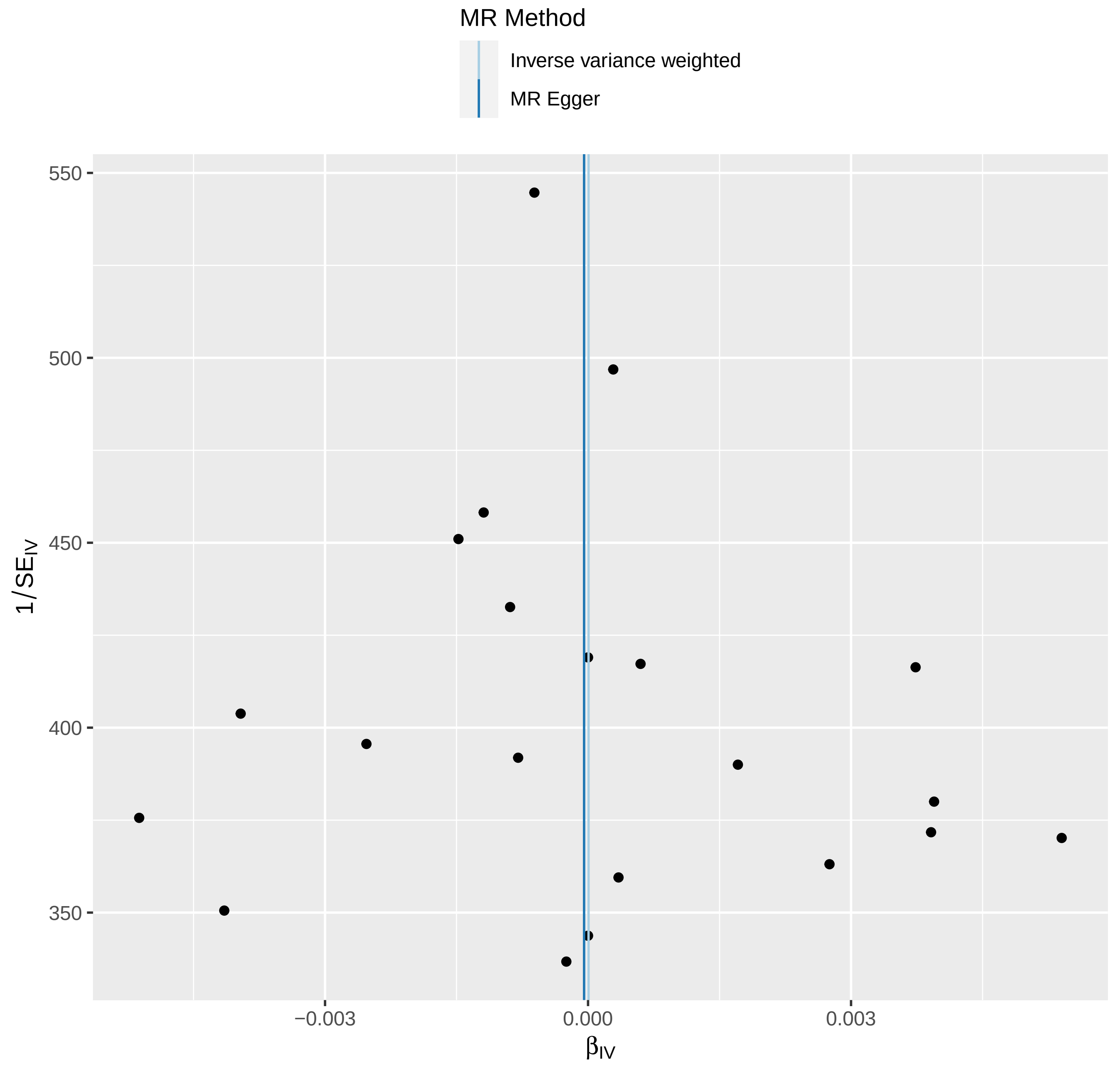

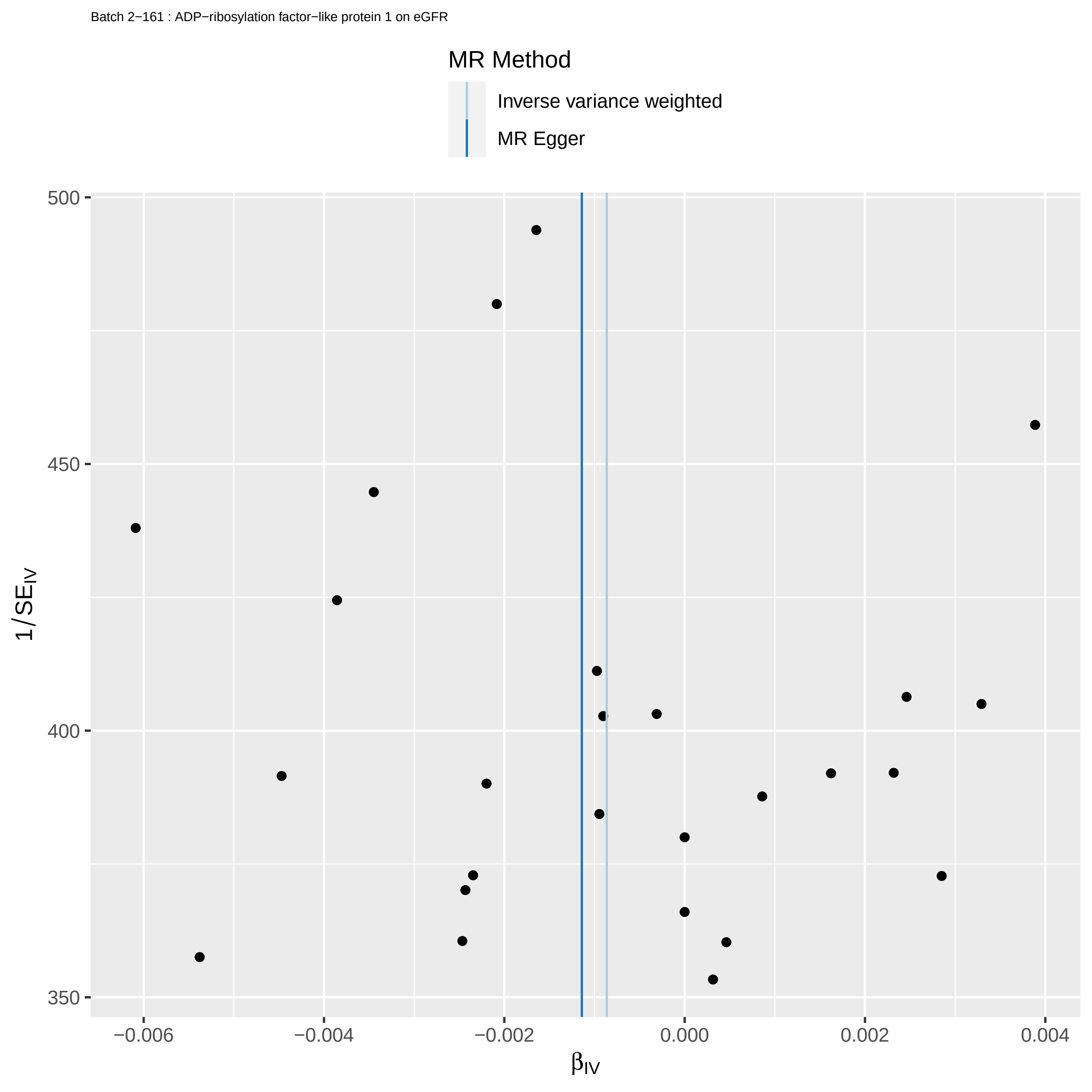

Batch 2−161 : ADP−ribosylation factor−like protein 1 on eGFR
MR Method
Inverse variance weighted
MR Egger
500
450
400
350
1/SE_IV
−0.006
−0.004
−0.002
0.000
0.002
0.004
β_IV

Batch 2−162 : ADP−ribosylation factor−like protein 3 on eGFR

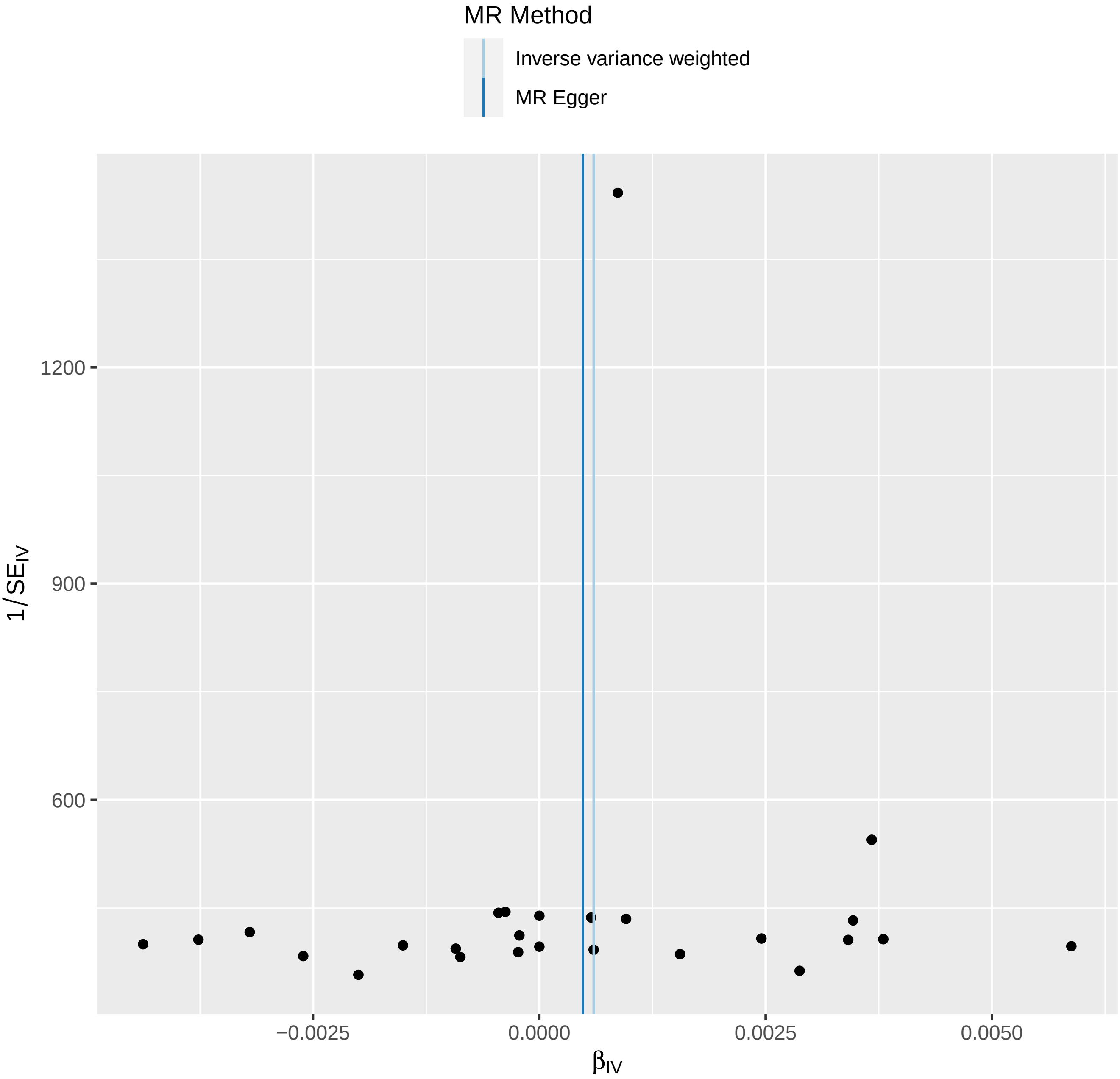

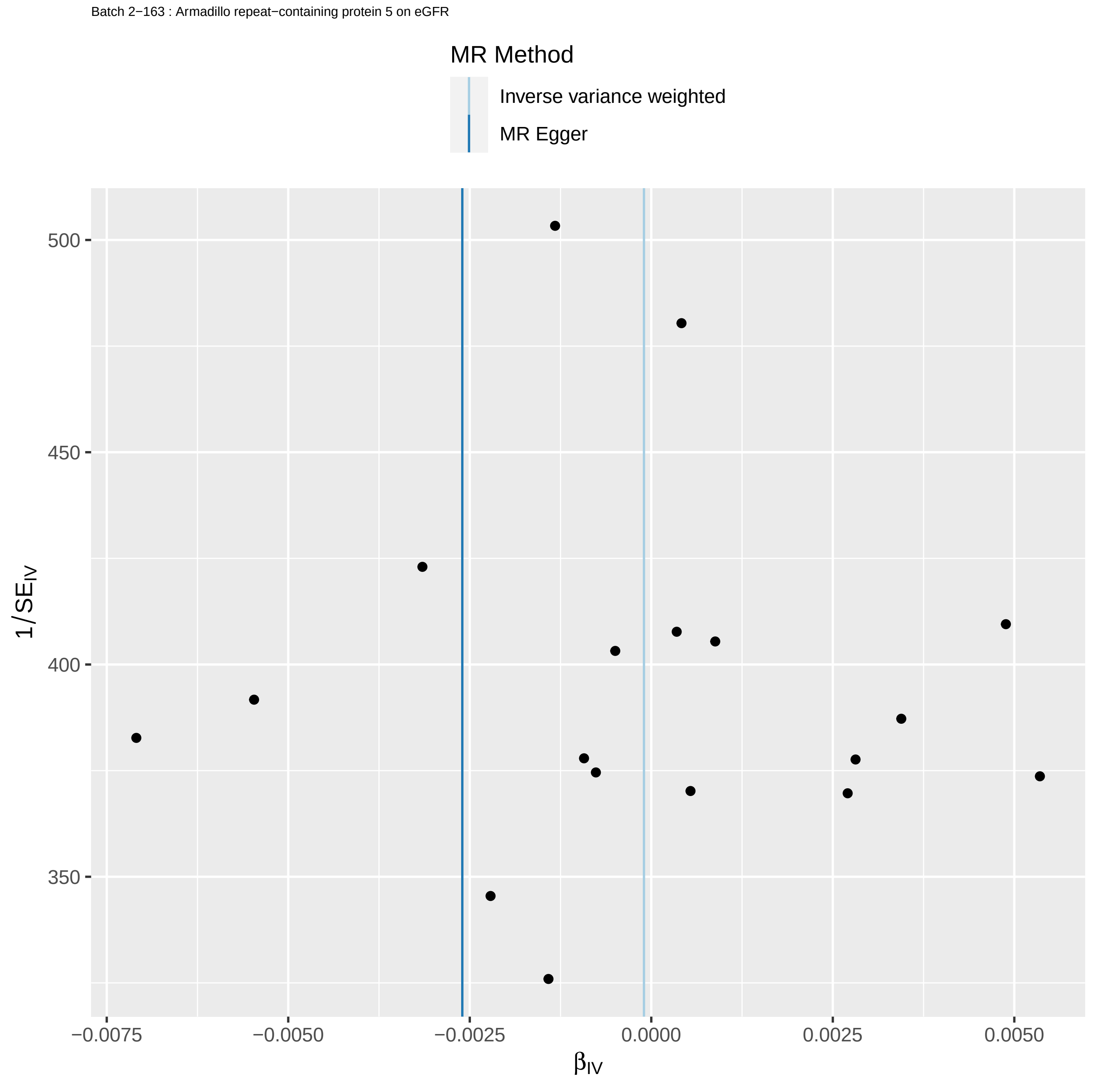
Batch 2−163 : Armadillo repeat−containing protein 5 on eGFR
MR Method
Inverse variance weighted
MR Egger
500
450
400
350
1/SE_IV
−0.0075
−0.0050
−0.0025
0.0000
0.0025
0.0050
β_IV

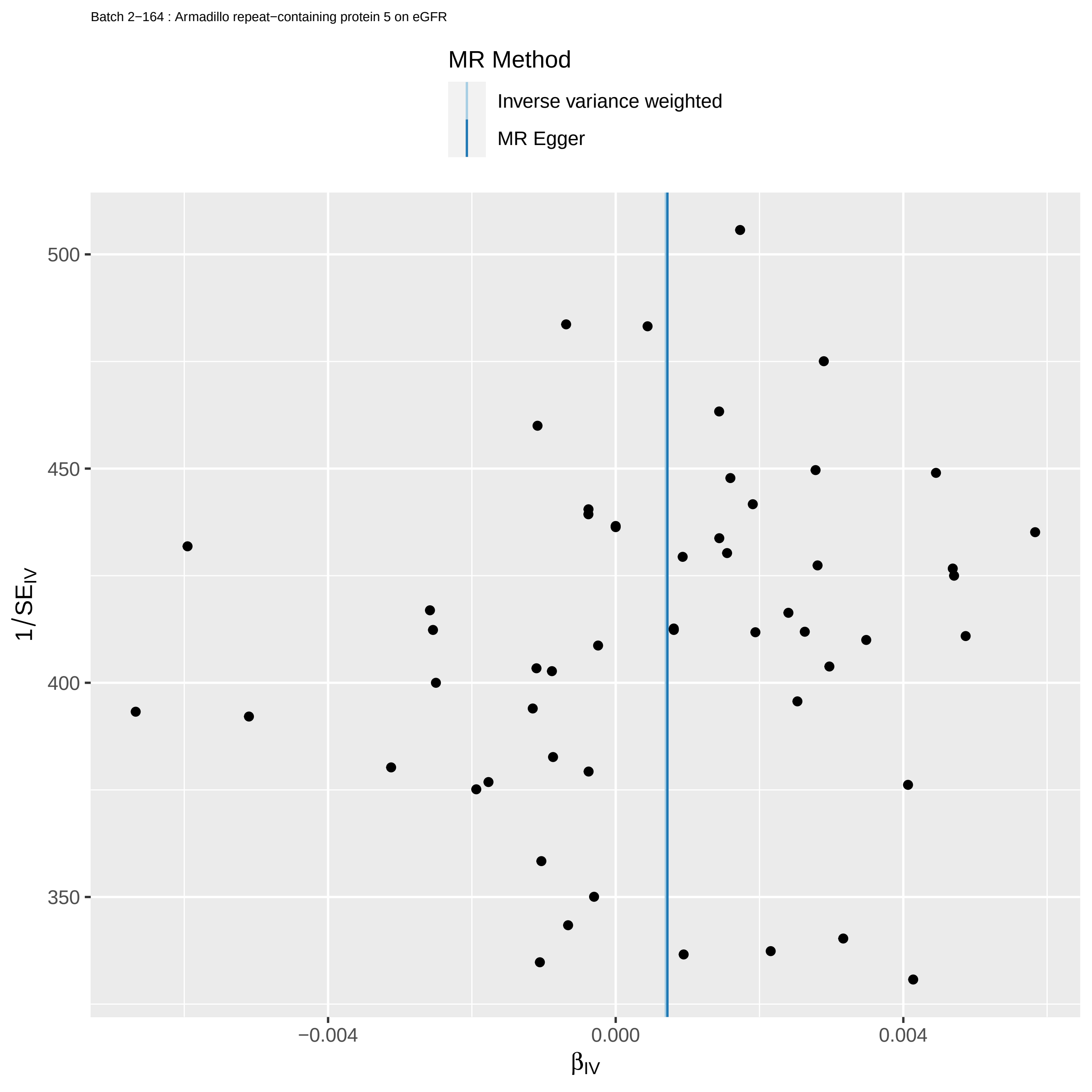

Batch 2−164 : Armadillo repeat−containing protein 5 on eGFR
MR Method
Inverse variance weighted
MR Egger
500
450
400
350
1/SE_IV
−0.004
0.000
0.004
β_IV

Batch 2−165 : Armadillo repeat−containing protein 5 on eGFR

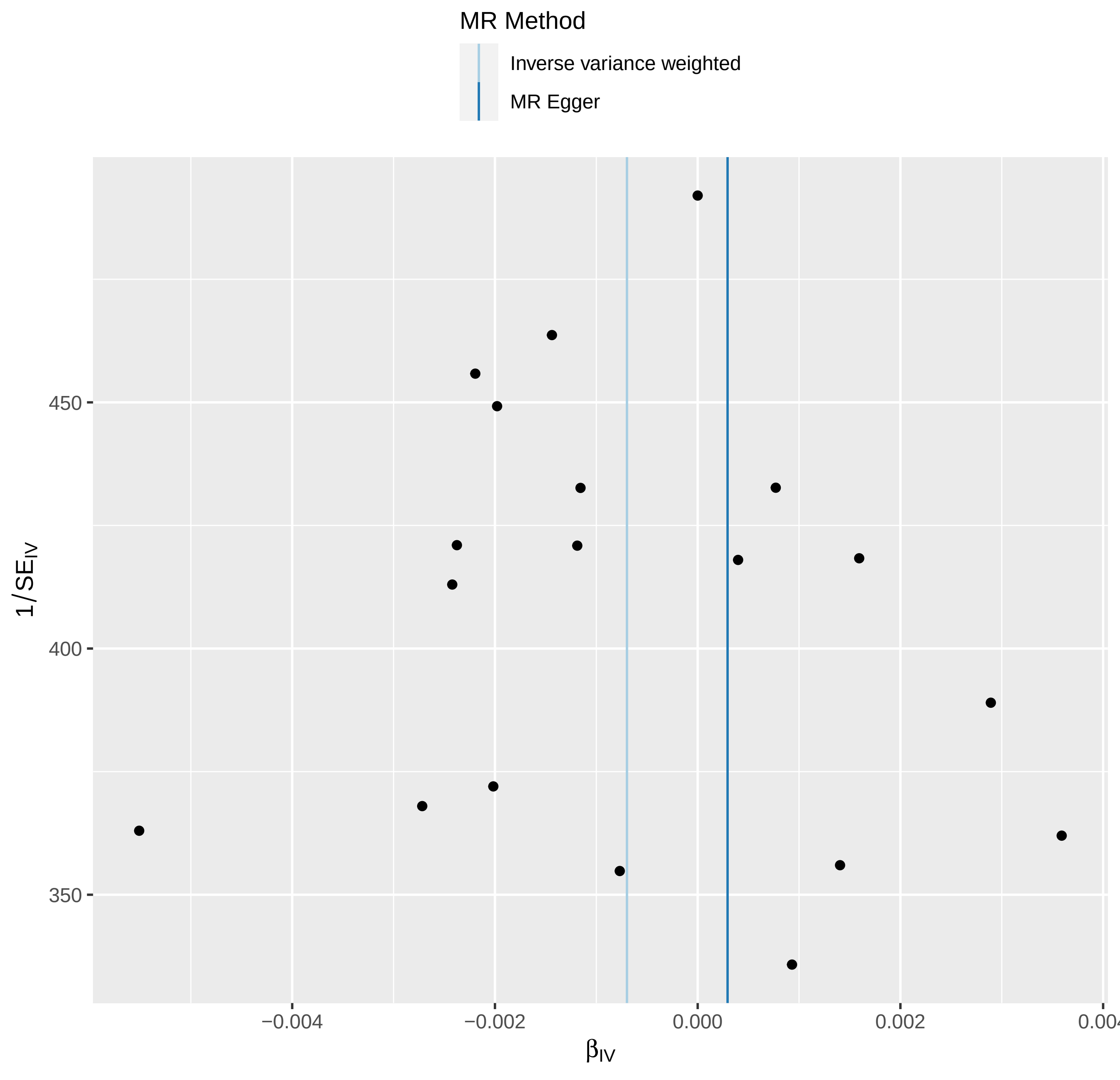

Batch 2−166 : Actin−related protein 2/3 complex subunit 3 on eGFR

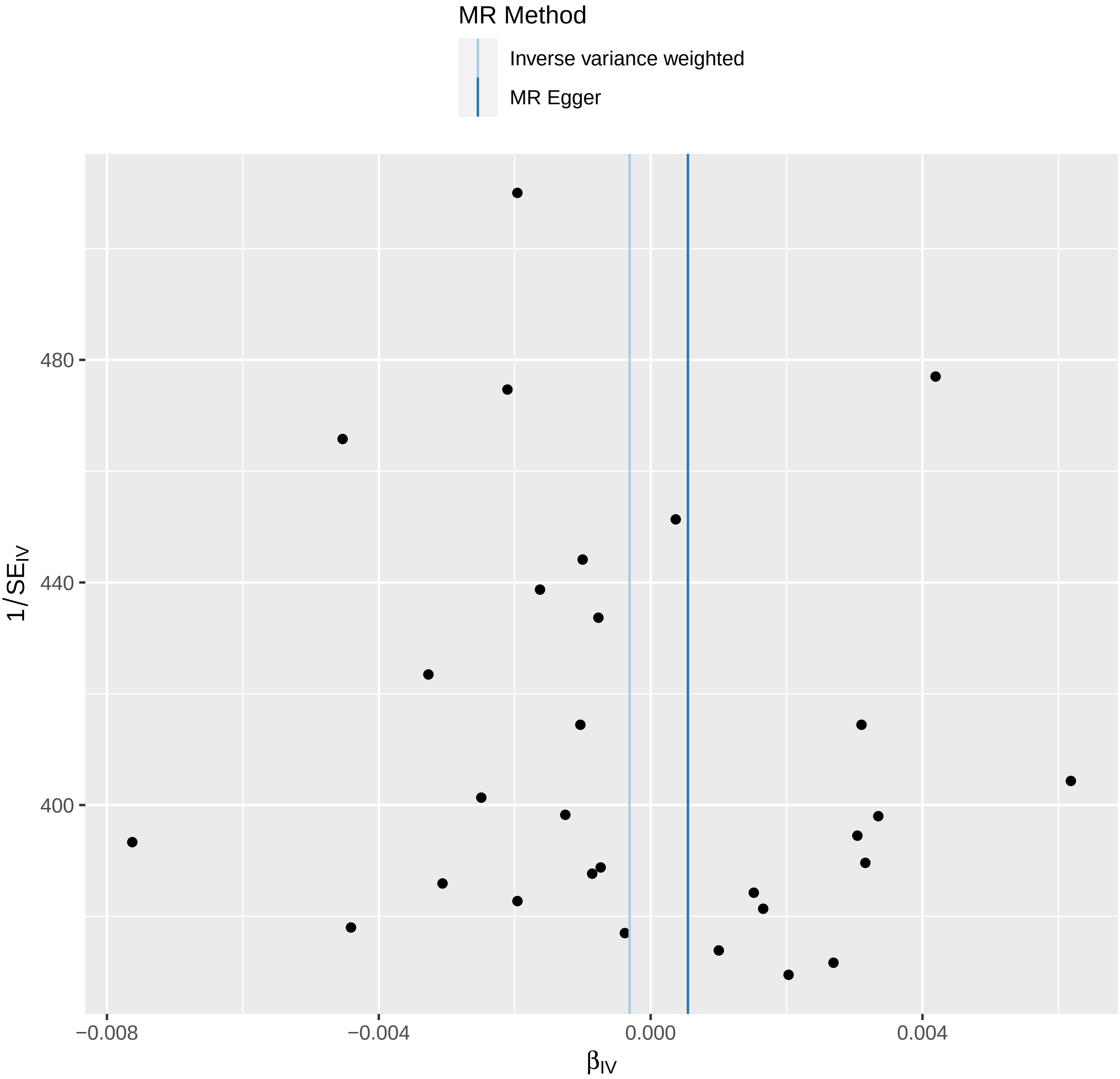

Batch 2−167 : cAMP−regulated phosphoprotein 19 on eGFR

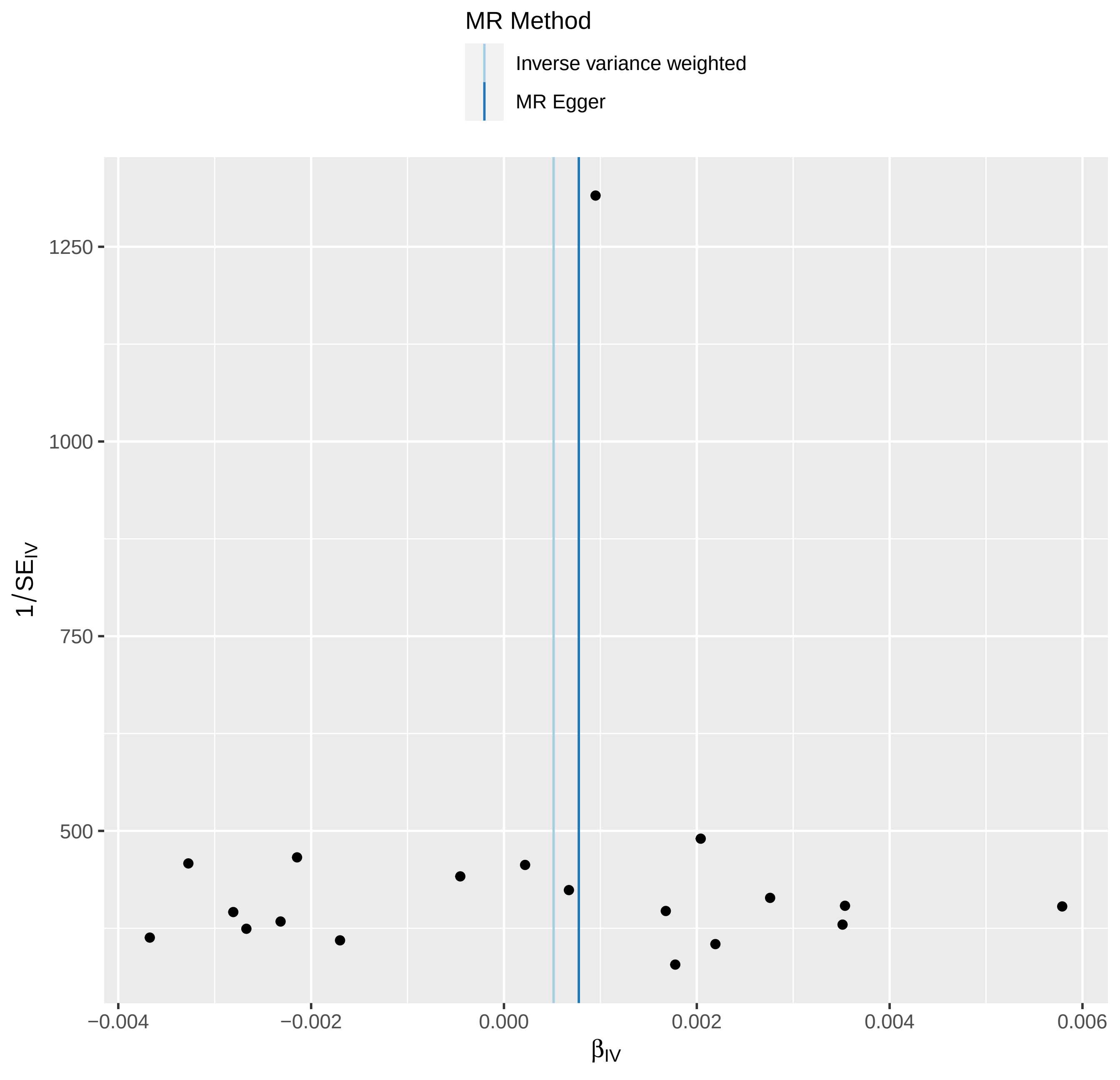

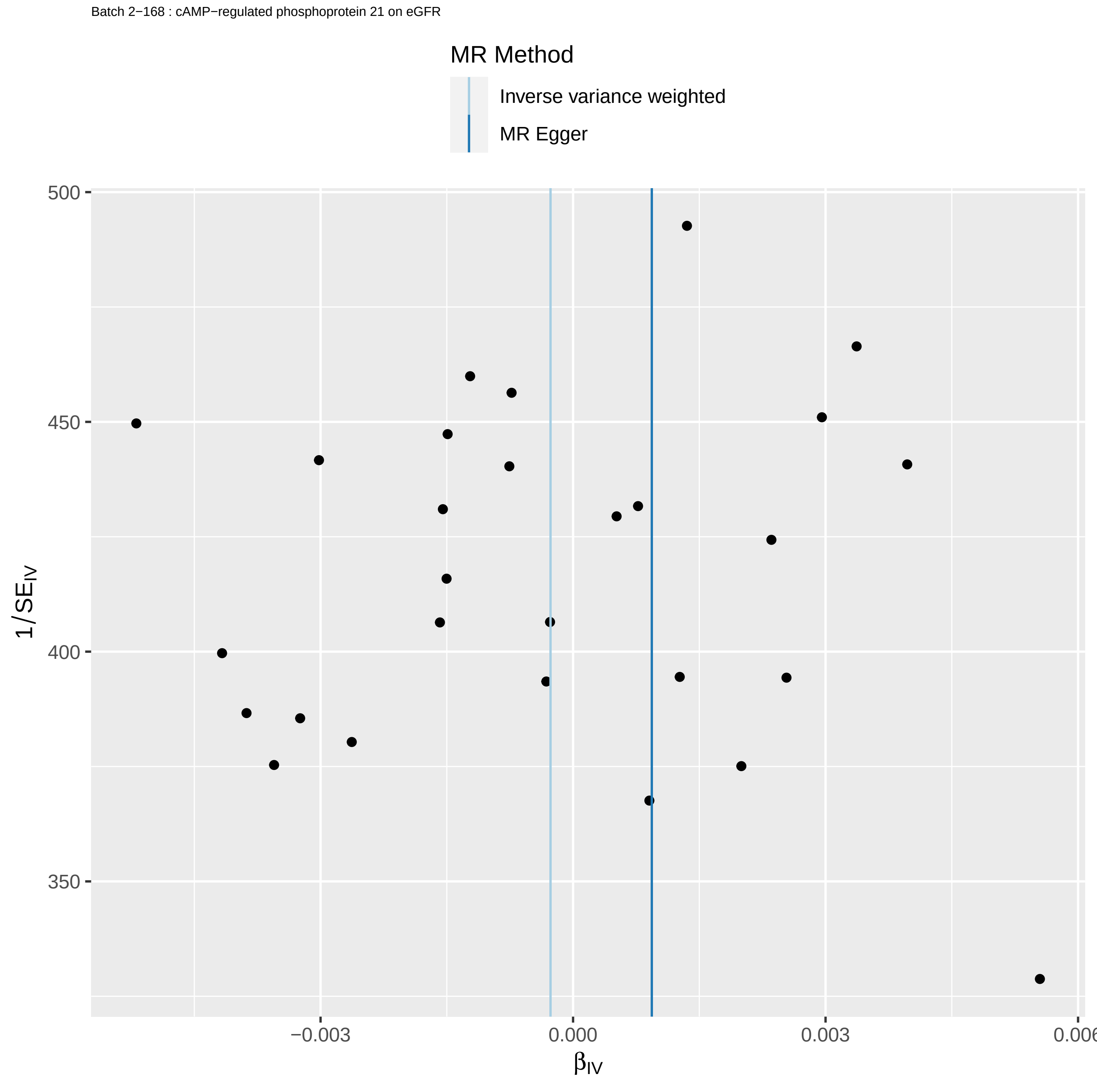

Batch 2−168 : cAMP−regulated phosphoprotein 21 on eGFR
MR Method
Inverse variance weighted
MR Egger
500
450
400
350
1/SE_IV
−0.003
0.000
0.003
0.006
β_IV

Batch 2−169 : Beta−arrestin−1 on eGFR

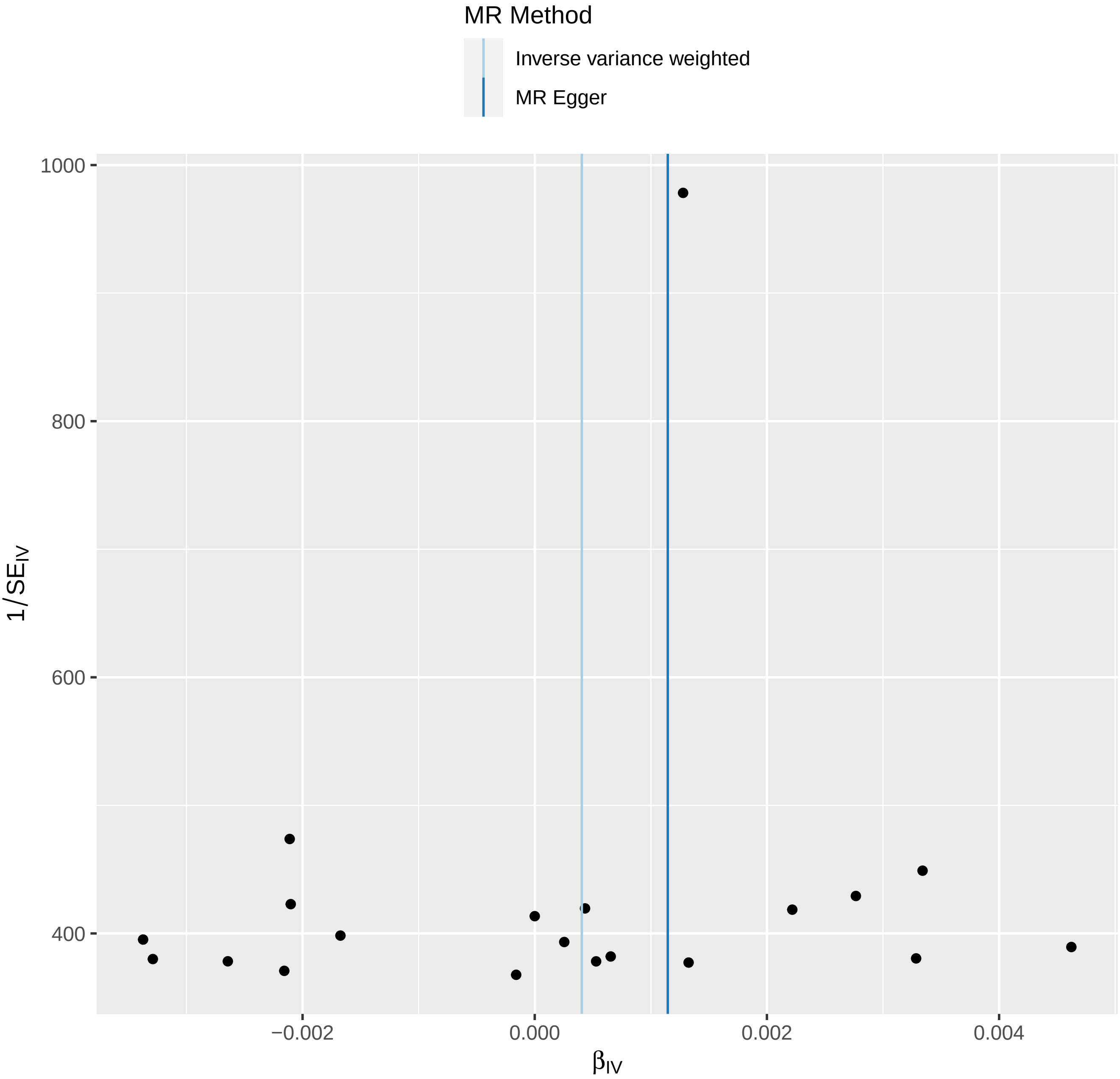

Batch 2−170 : Arrestin domain−containing protein 3 on eGFR

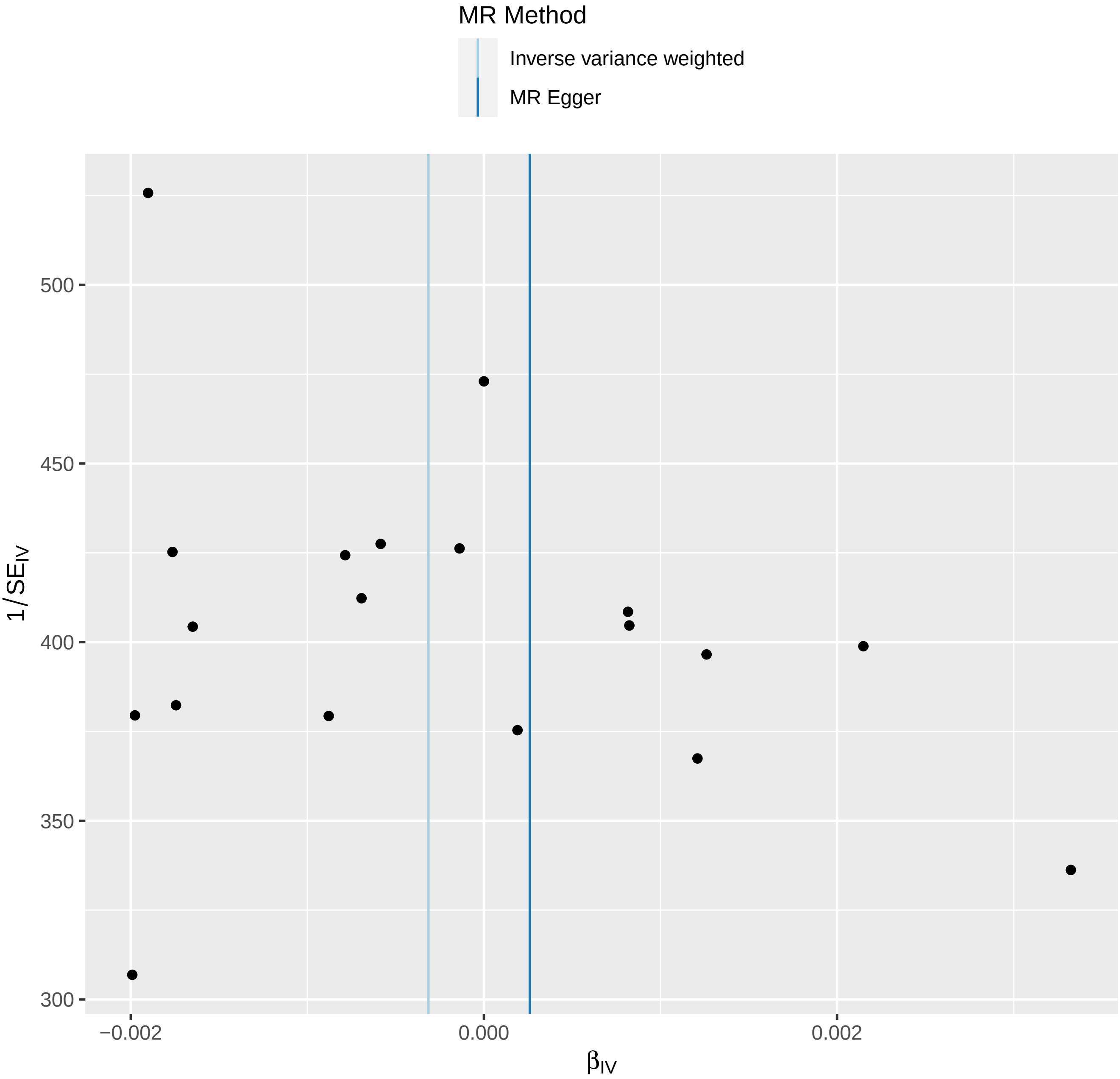

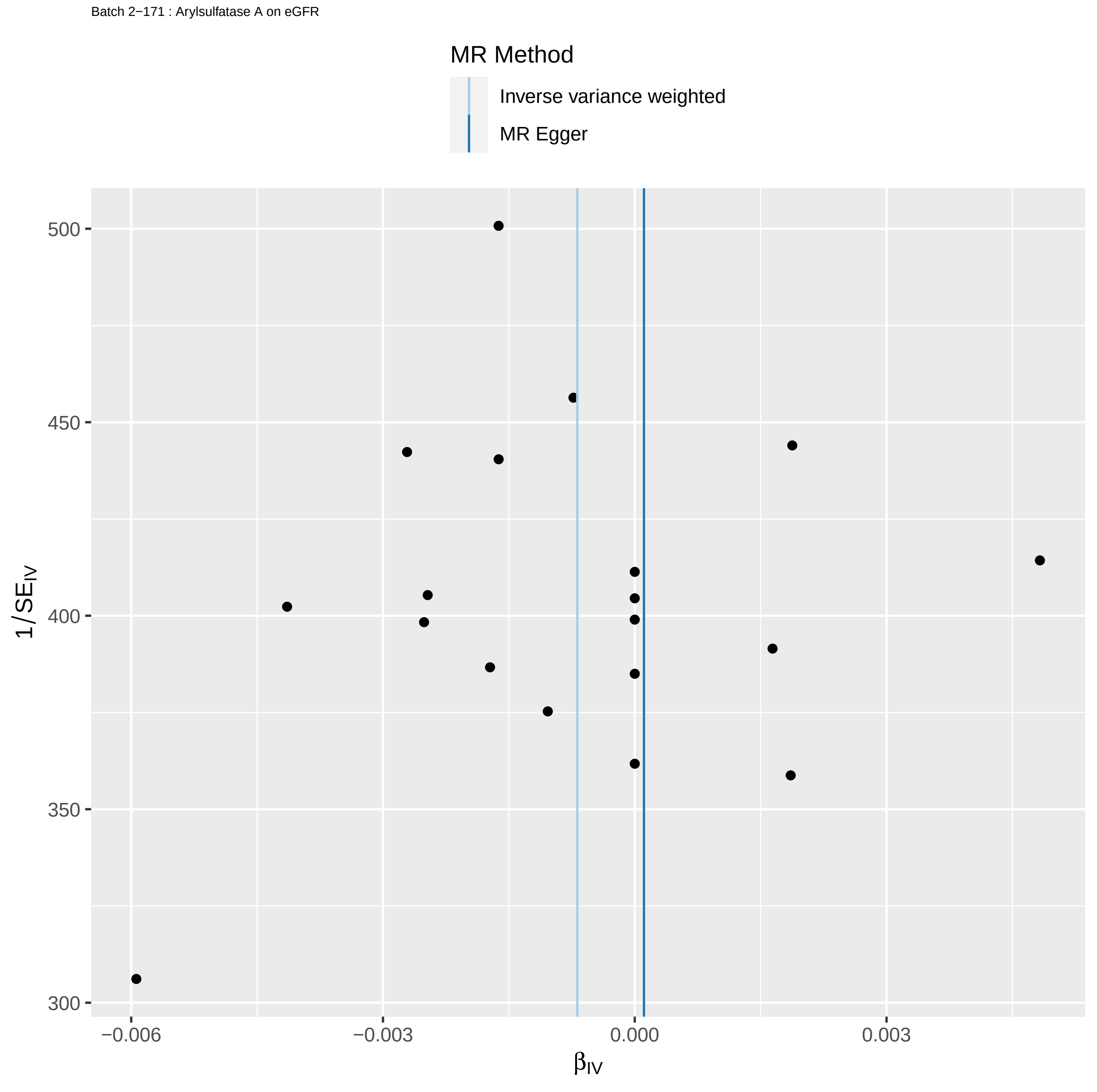

Batch 2−171 : Arylsulfatase A on eGFR
MR Method
Inverse variance weighted
MR Egger
1/SE_IV
500
450
400
350
300
−0.006
−0.003
0.000
0.003
β_IV

Batch 2−172 : Arylsulfatase B on eGFR

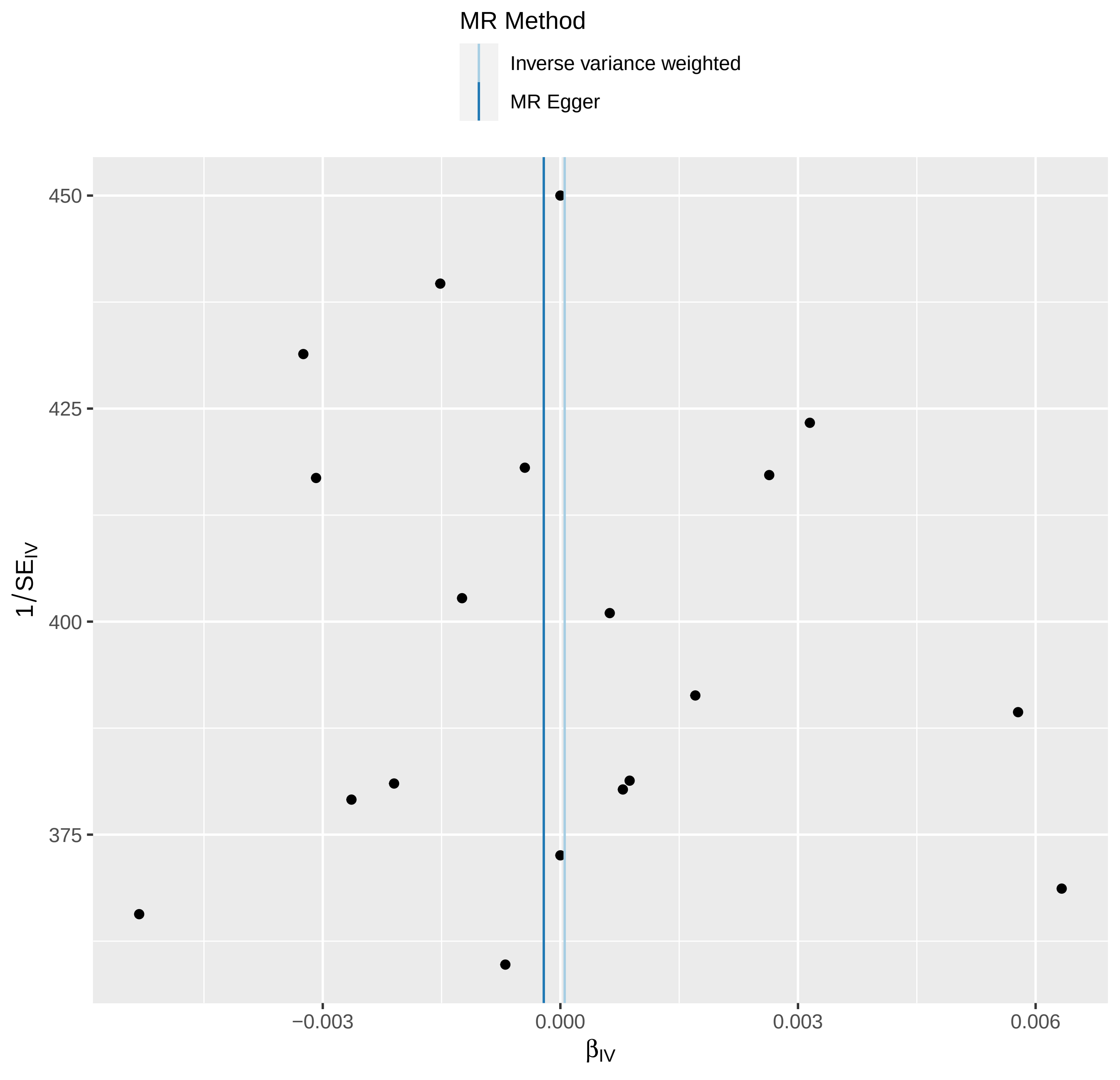

Batch 2−173 : Arylsulfatase K on eGFR

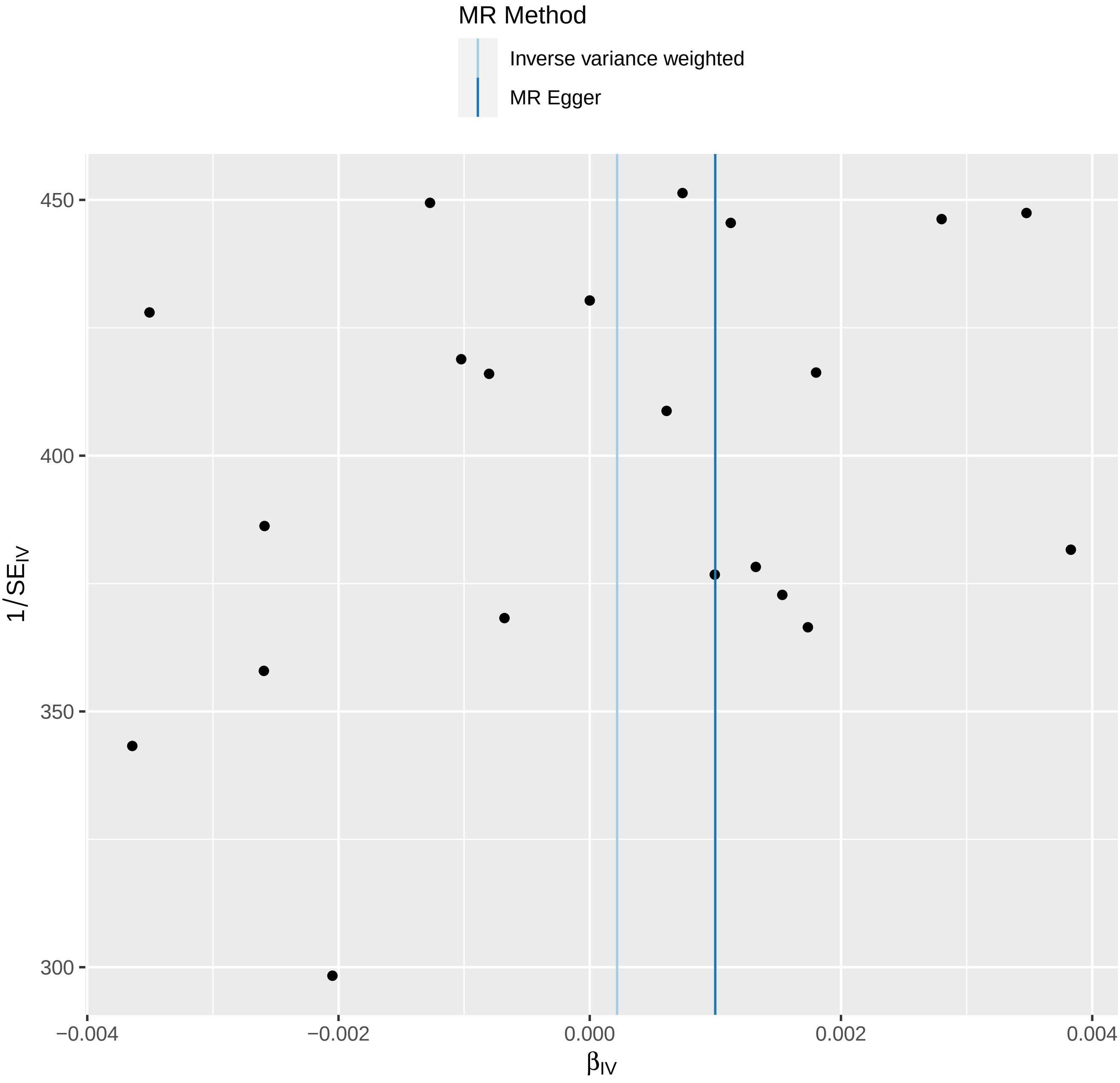

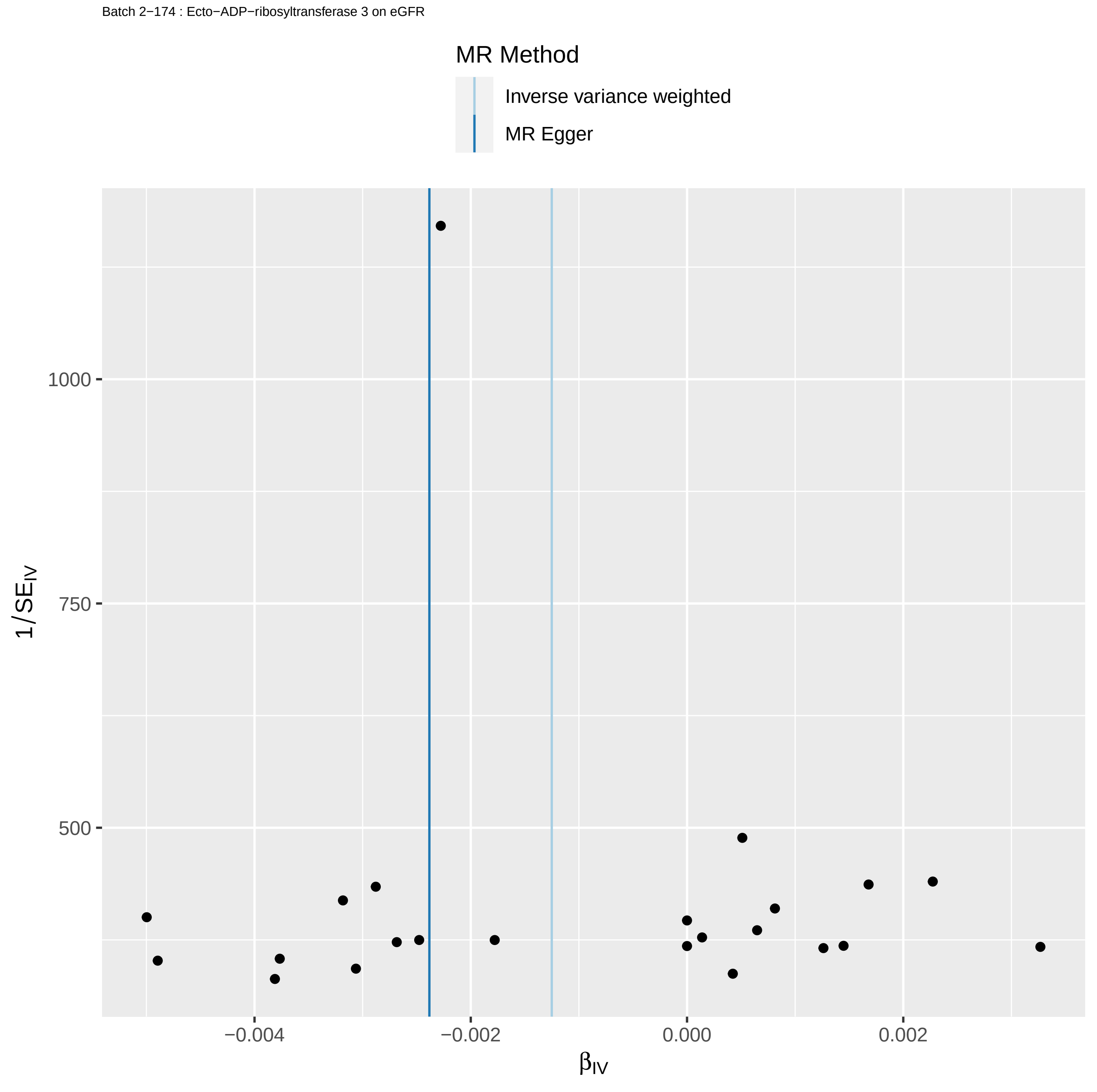
Batch 2−174 : Ecto−ADP−ribosyltransferase 3 on eGFR
MR Method
Inverse variance weighted
MR Egger
1000
750
500
1/SE_IV
−0.004
−0.002
0.000
0.002
β_IV

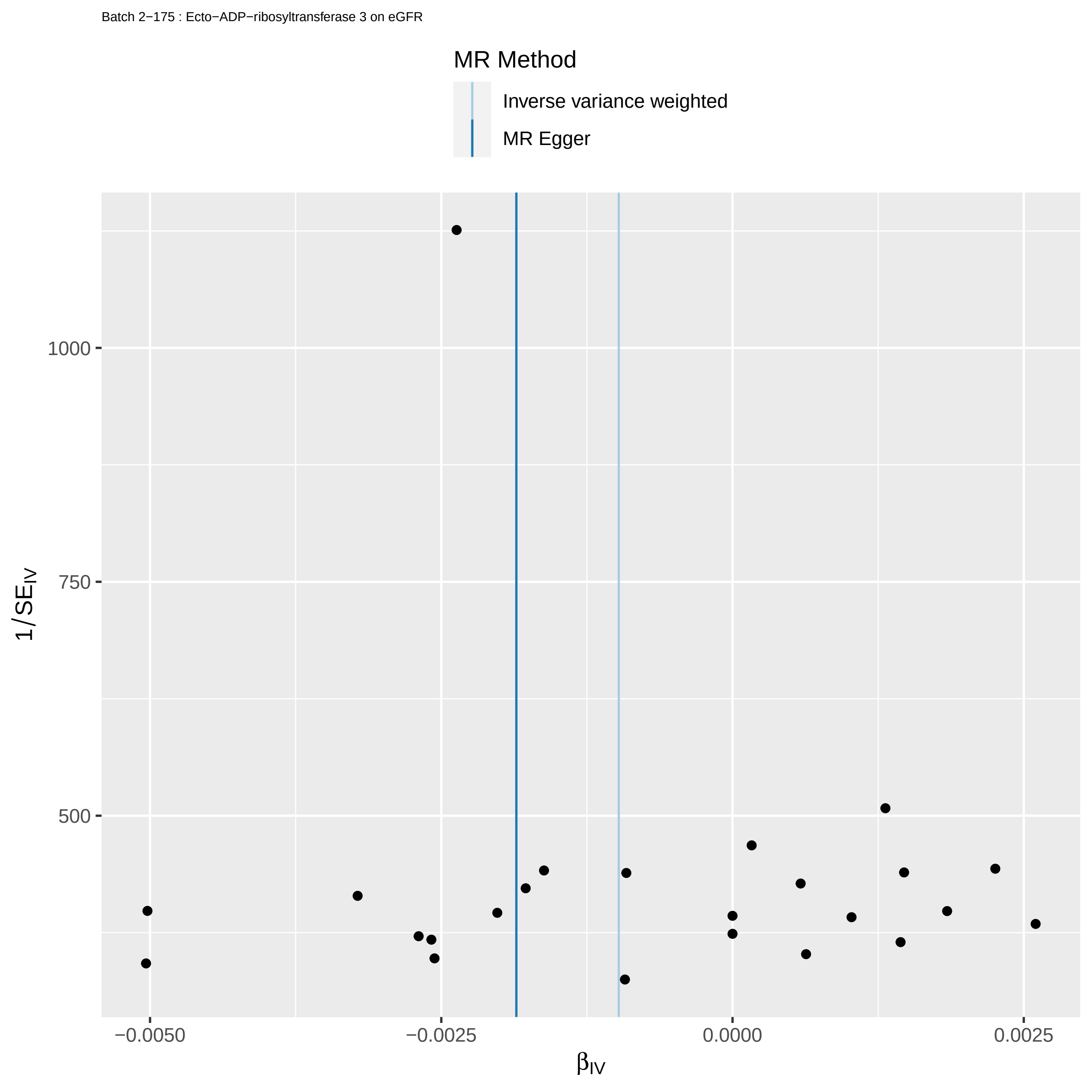

Batch 2−175 : Ecto−ADP−ribosyltransferase 3 on eGFR
MR Method
Inverse variance weighted
MR Egger
1000
750
500
1/SE$_{IV}$
−0.0050
−0.0025
0.0000
0.0025
β$_{IV}$

Batch 2−176 : Ecto−ADP−ribosyltransferase 4 on eGFR

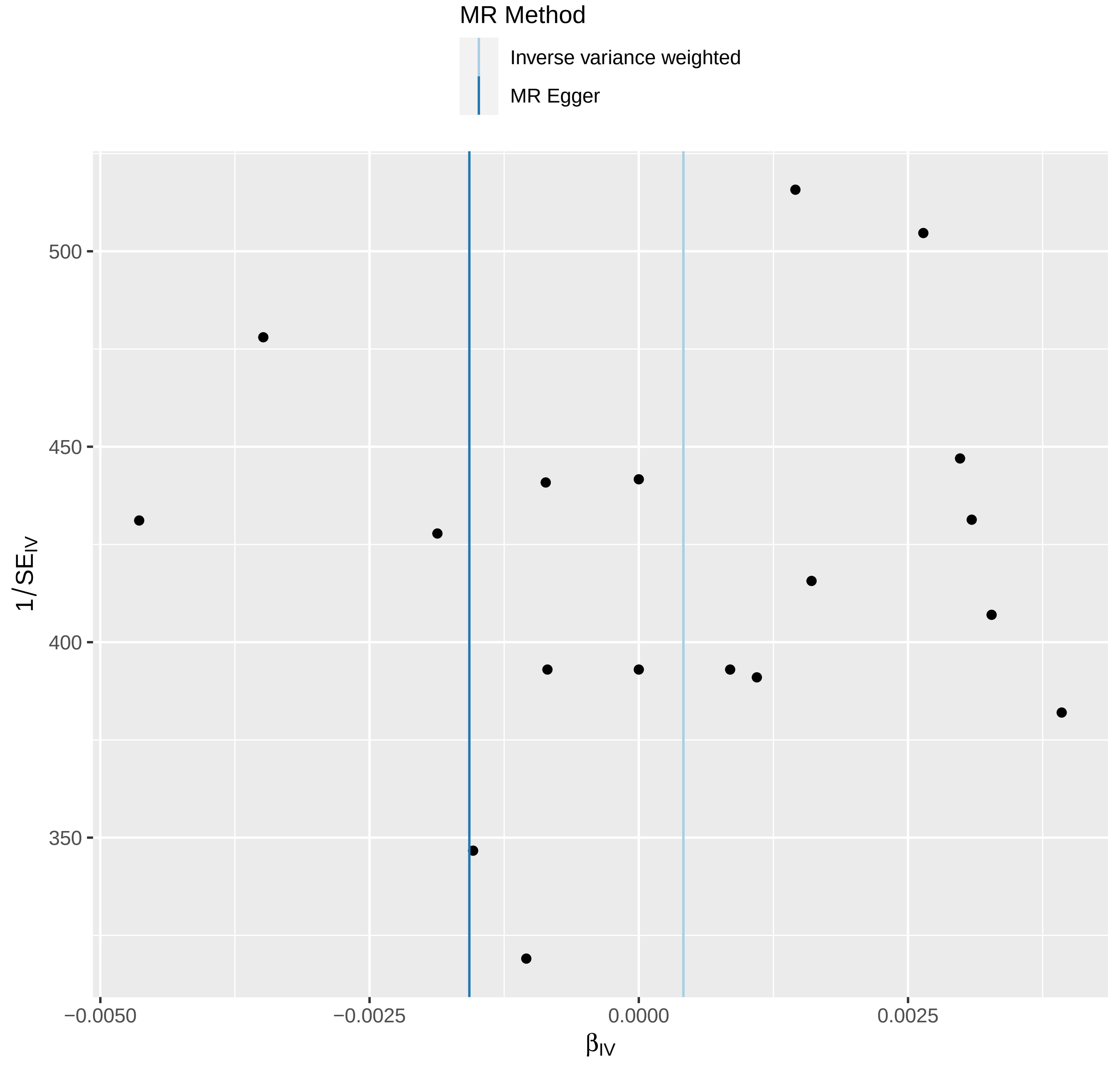

Batch 2−177 : Artemin on eGFR

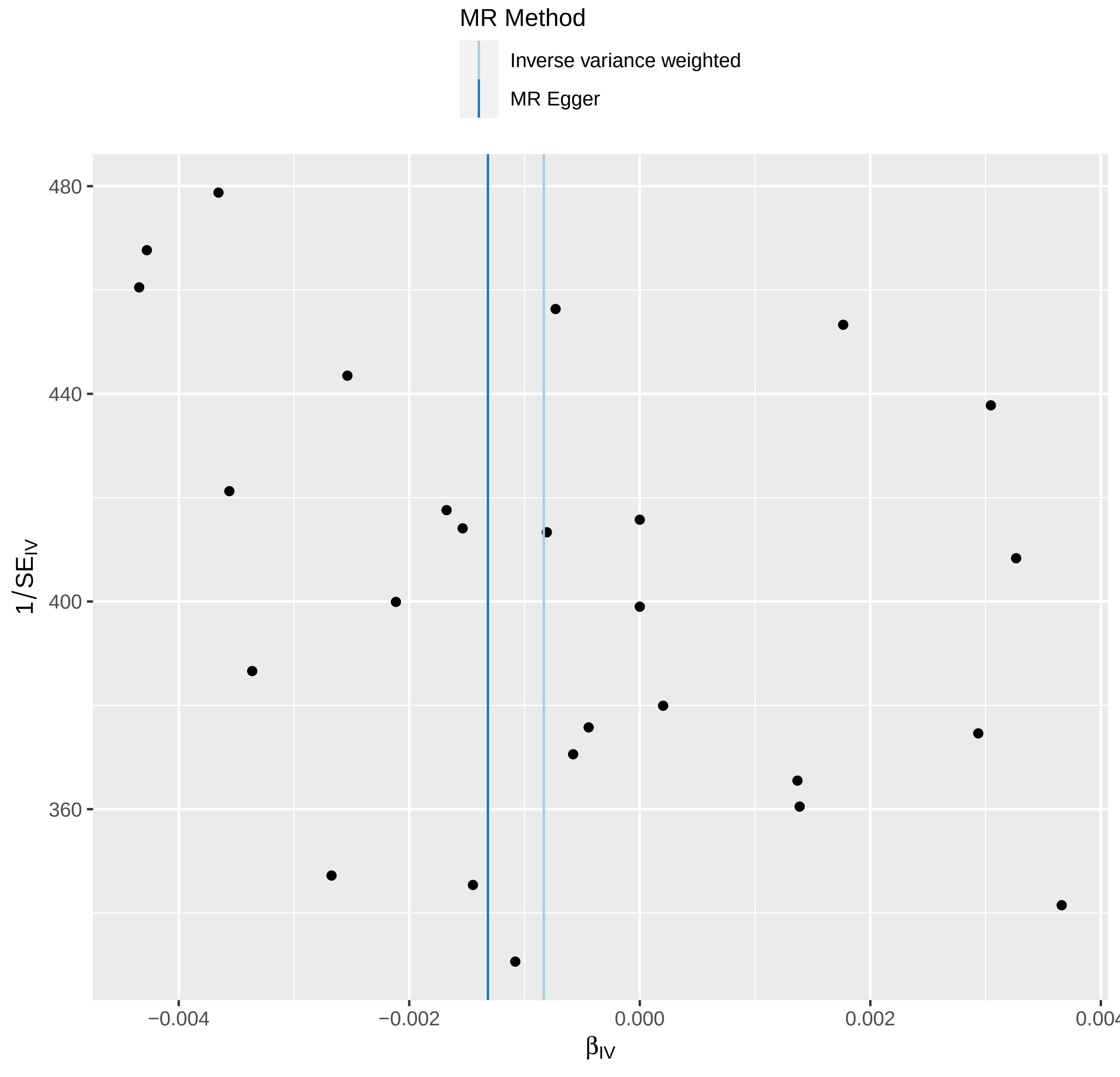

Batch 2−178 : Acid ceramidase on eGFR

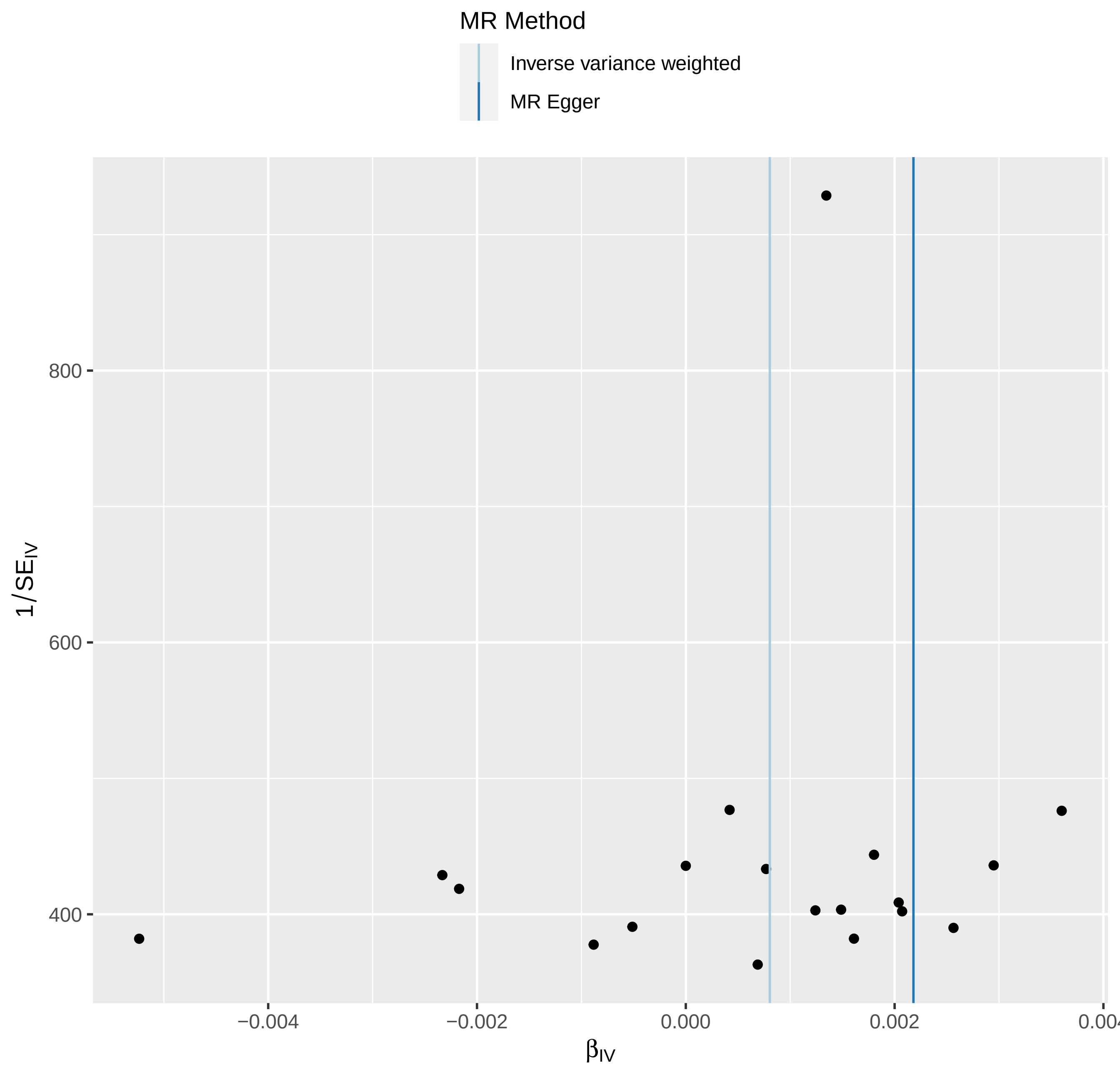

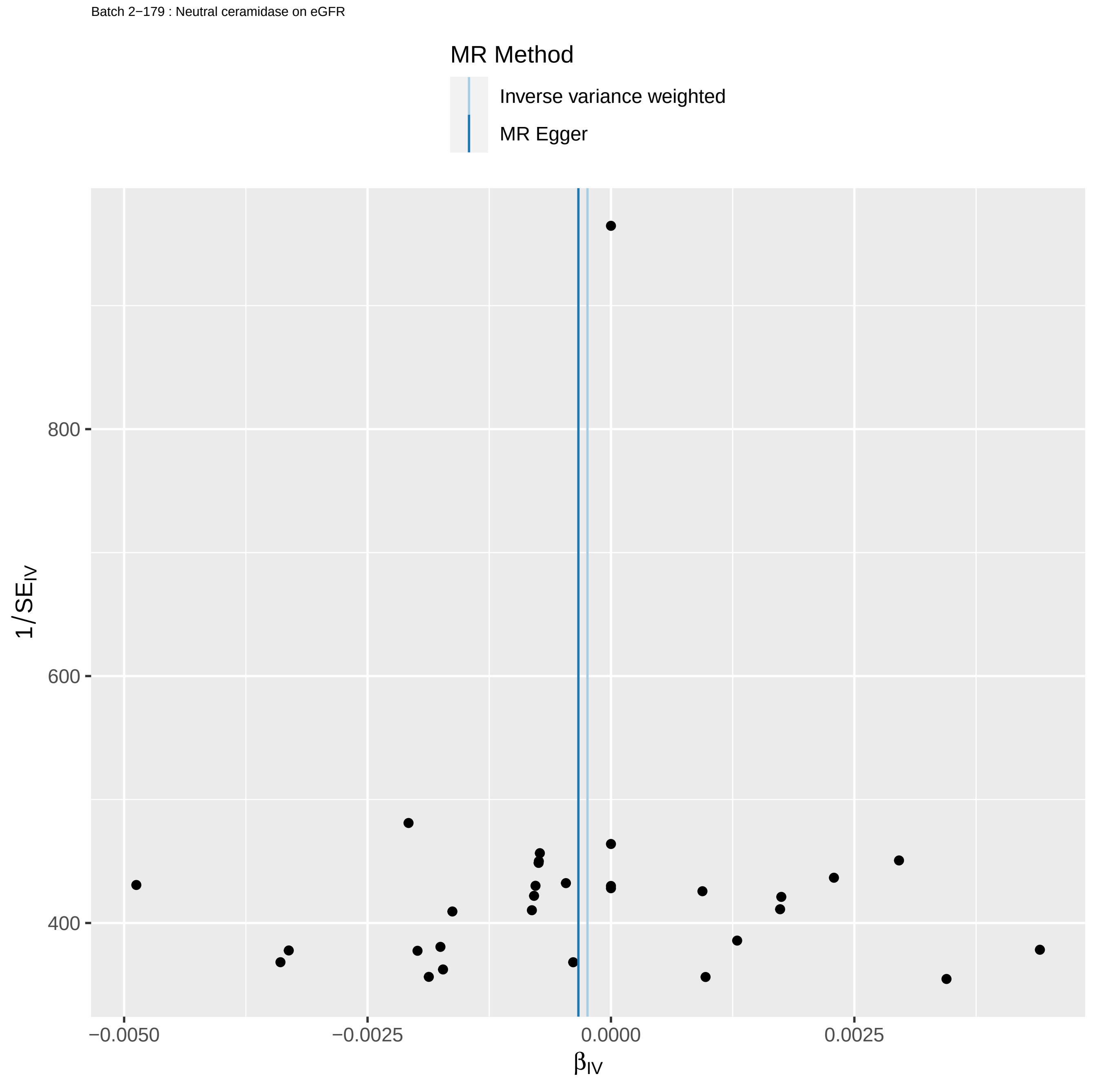

Batch 2−179 : Neutral ceramidase on eGFR
MR Method
Inverse variance weighted
MR Egger
800
600
400
1/SE_IV
−0.0050
−0.0025
0.0000
0.0025
β_IV

Batch 2−180 : Arf−GAP with SH3 domain, ANK repeat and PH domain−containing protein 2 on eGFR

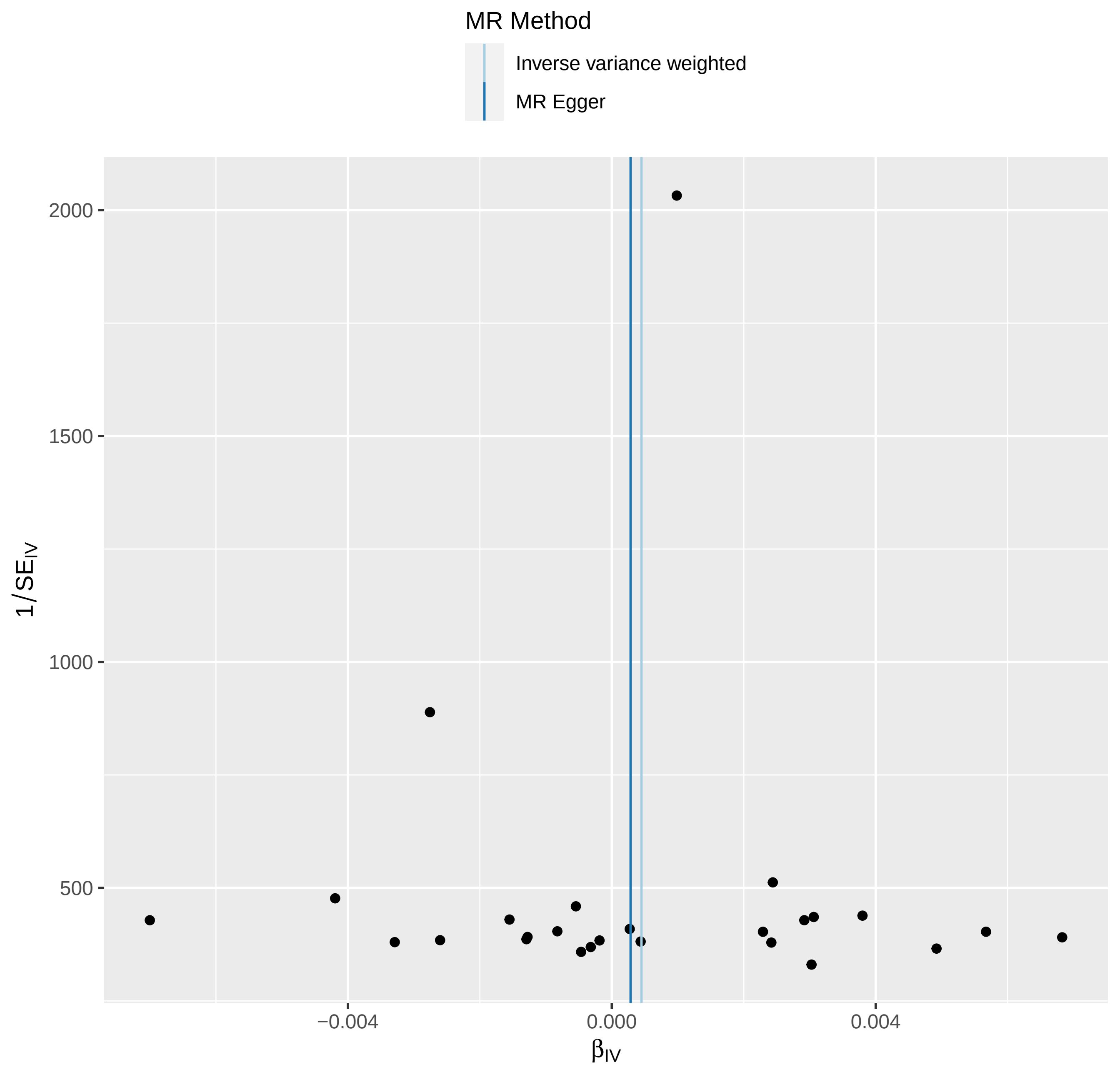

Batch 2−181 : Activating signal cointegrator 1 complex subunit 1 on eGFR

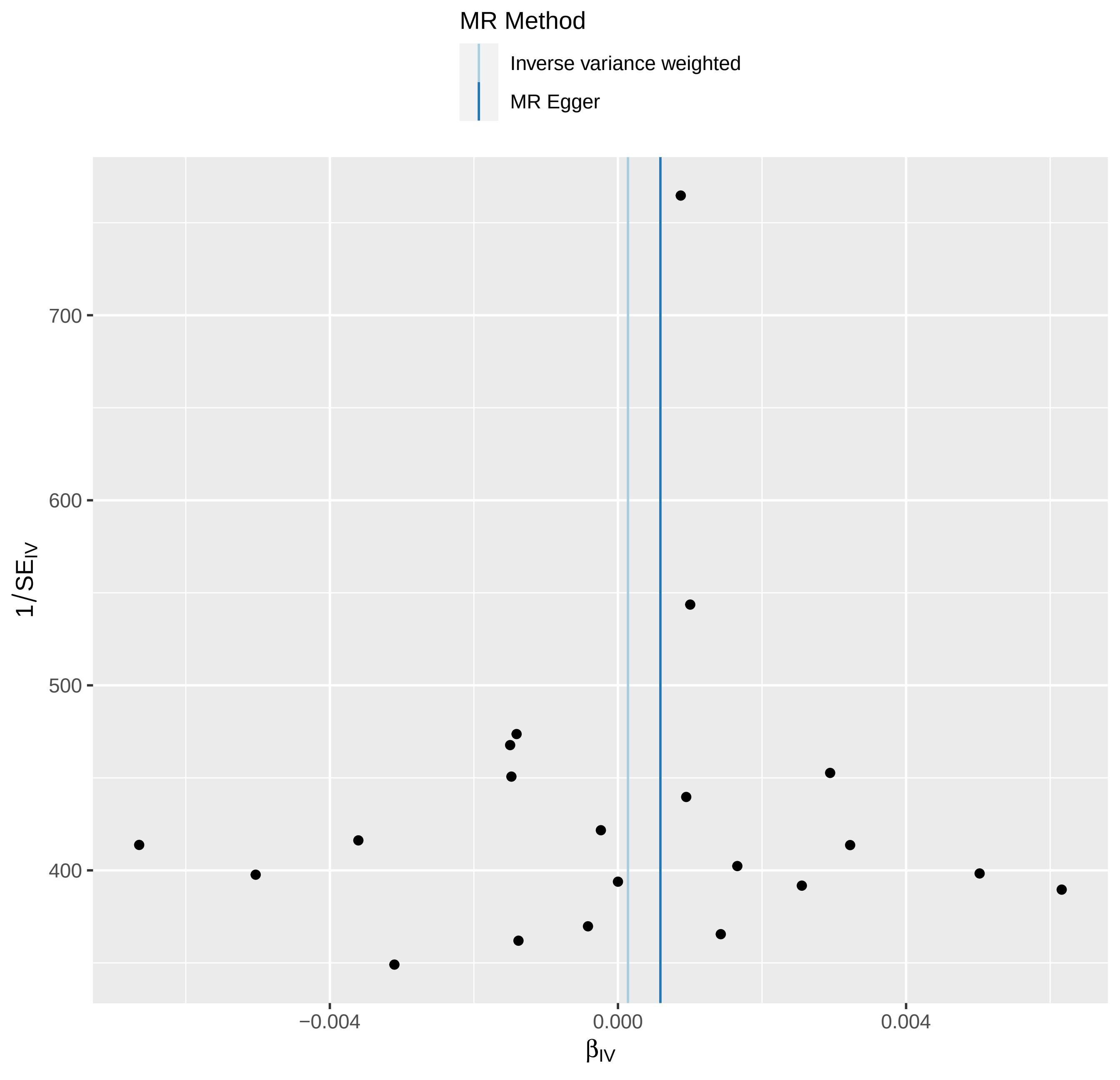

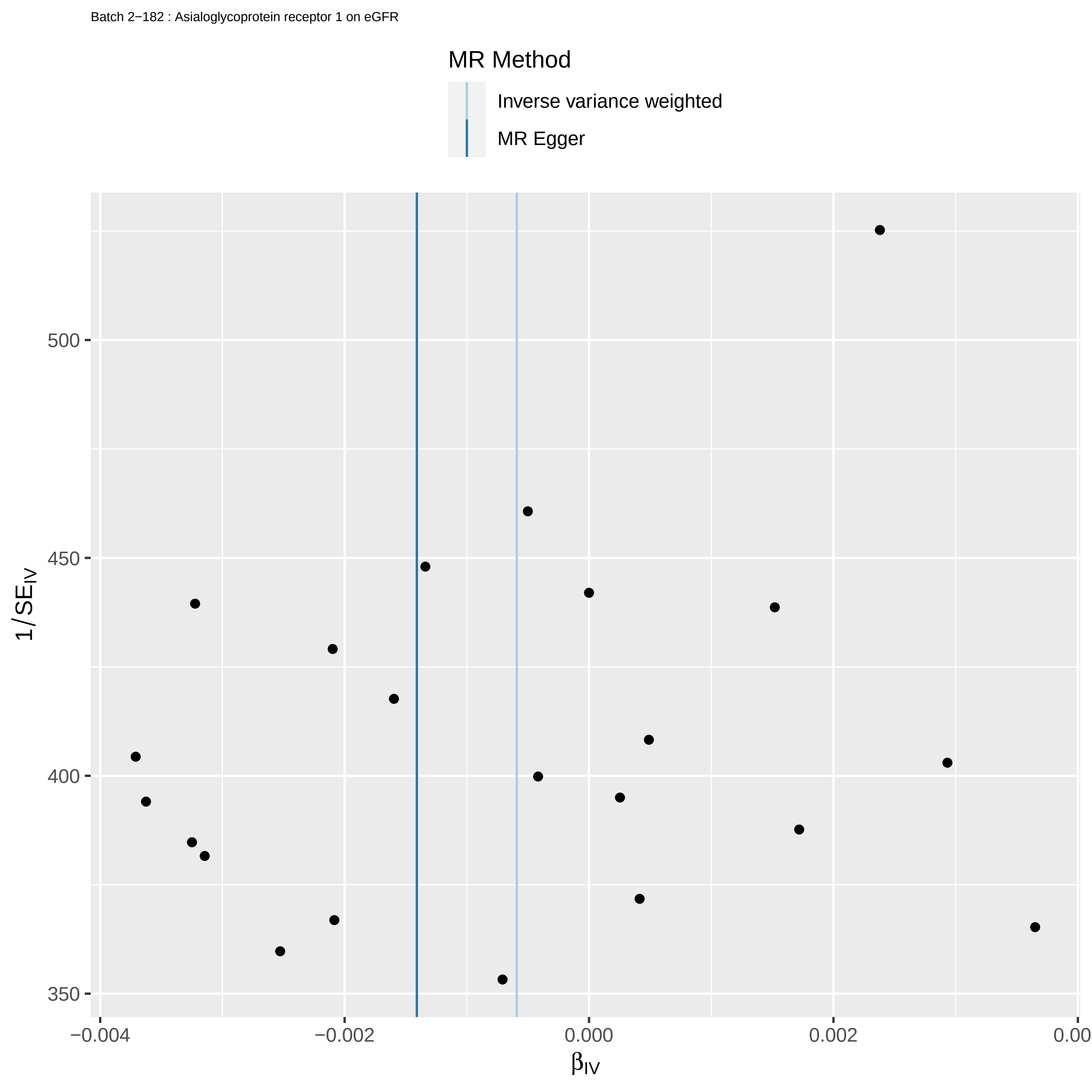
Batch 2−182 : Asialoglycoprotein receptor 1 on eGFR
MR Method
Inverse variance weighted
MR Egger
500
450
400
350
1/SE_IV
−0.004
−0.002
0.000
0.002
β_IV

Batch 2−183 : Asialoglycoprotein receptor 2 on eGFR

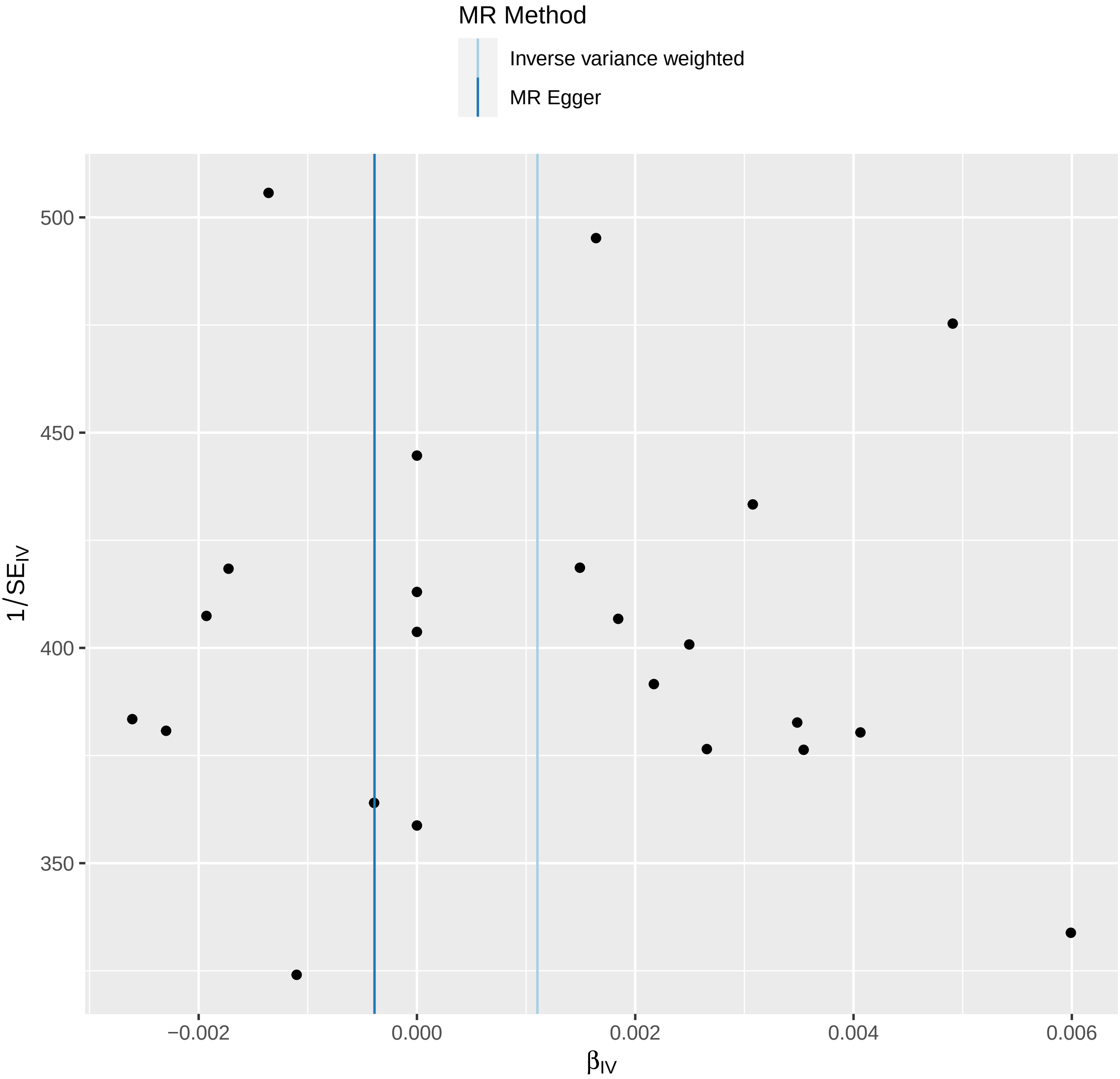

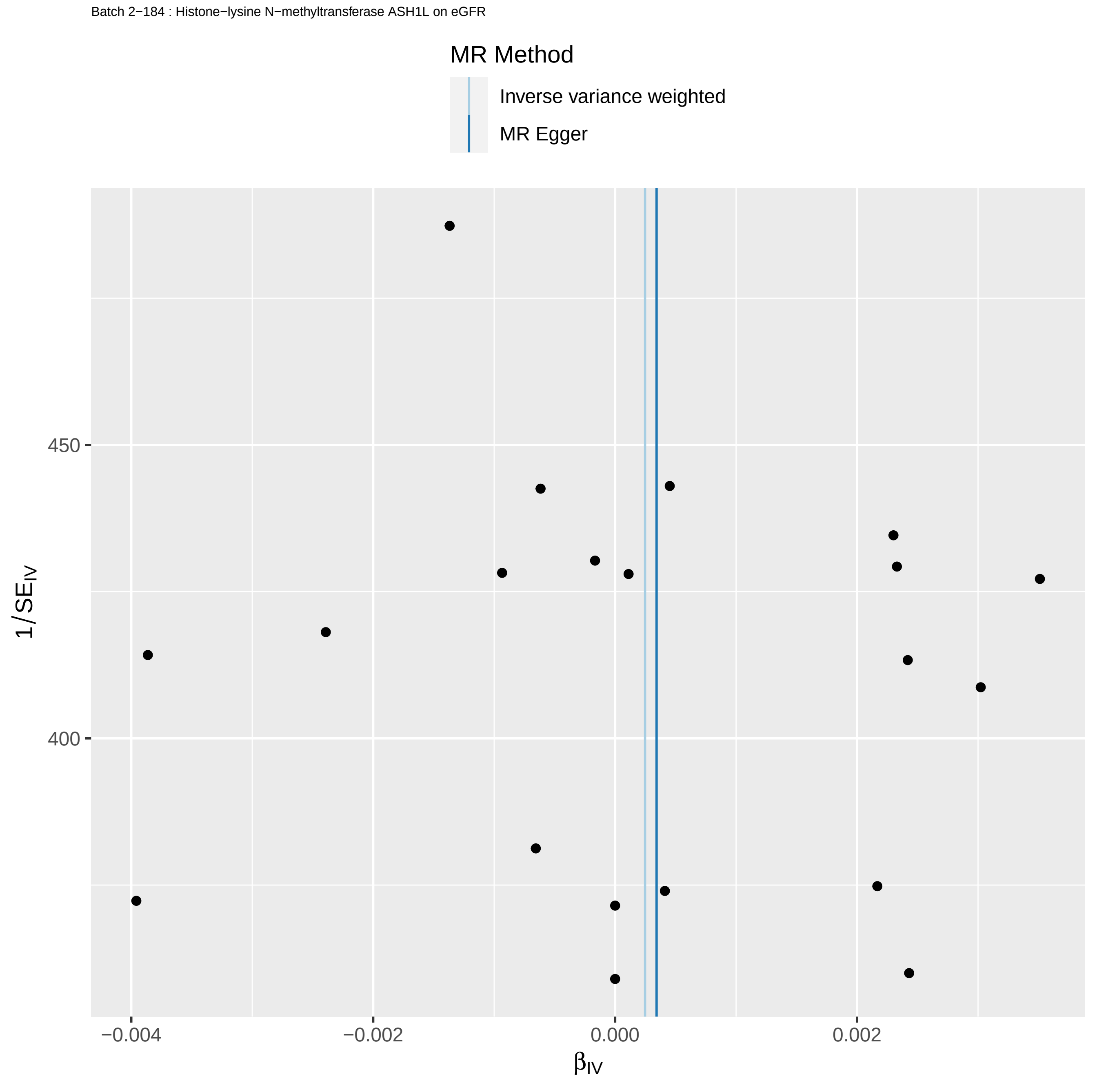
Batch 2−184 : Histone−lysine N−methyltransferase ASH1L on eGFR
MR Method
Inverse variance weighted
MR Egger
450
400
1/SE_IV
−0.004
−0.002
0.000
0.002
β_IV

Batch 2−185 : Set1/Ash2 histone methyltransferase complex subunit ASH2 on eGFR

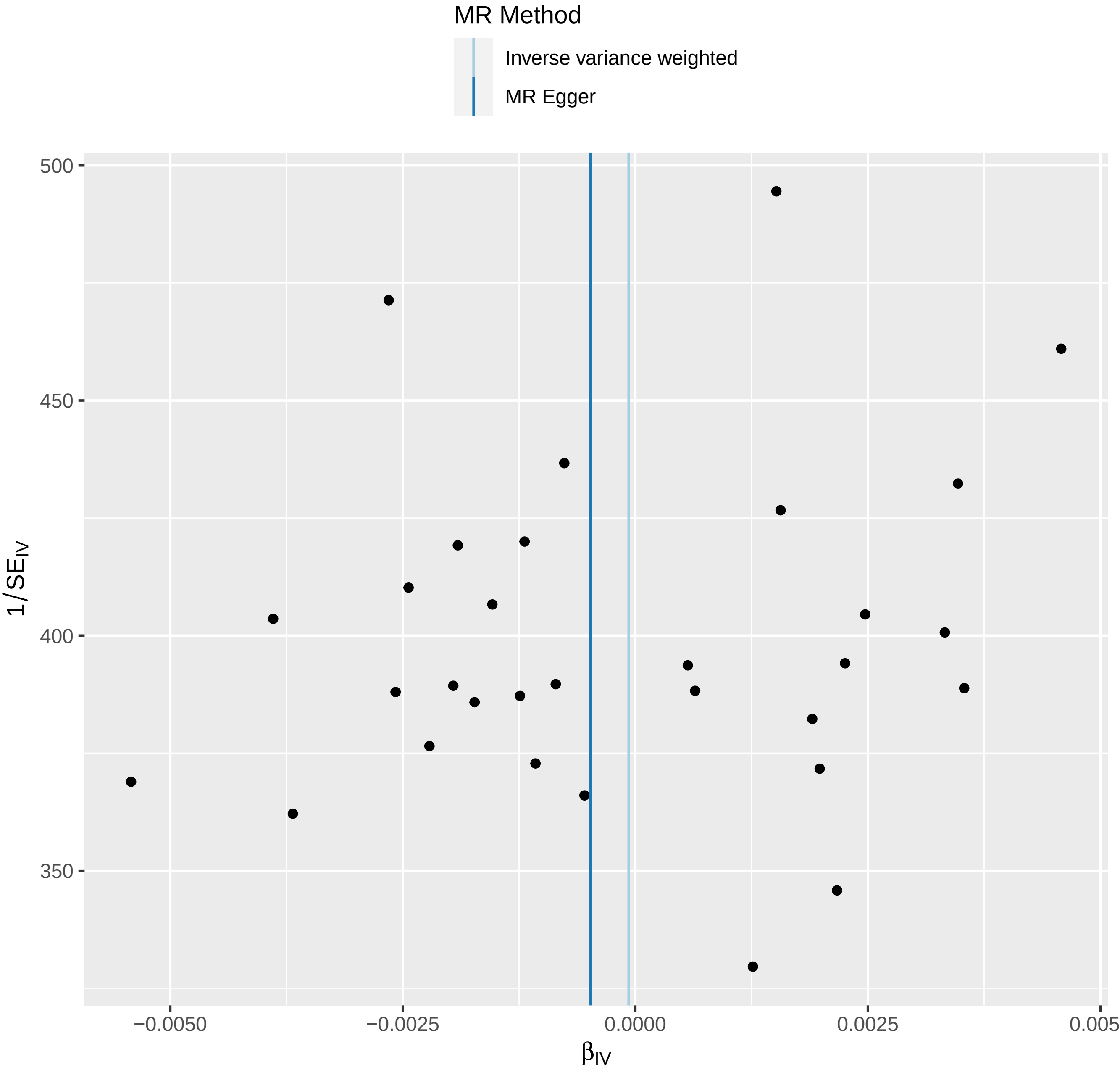

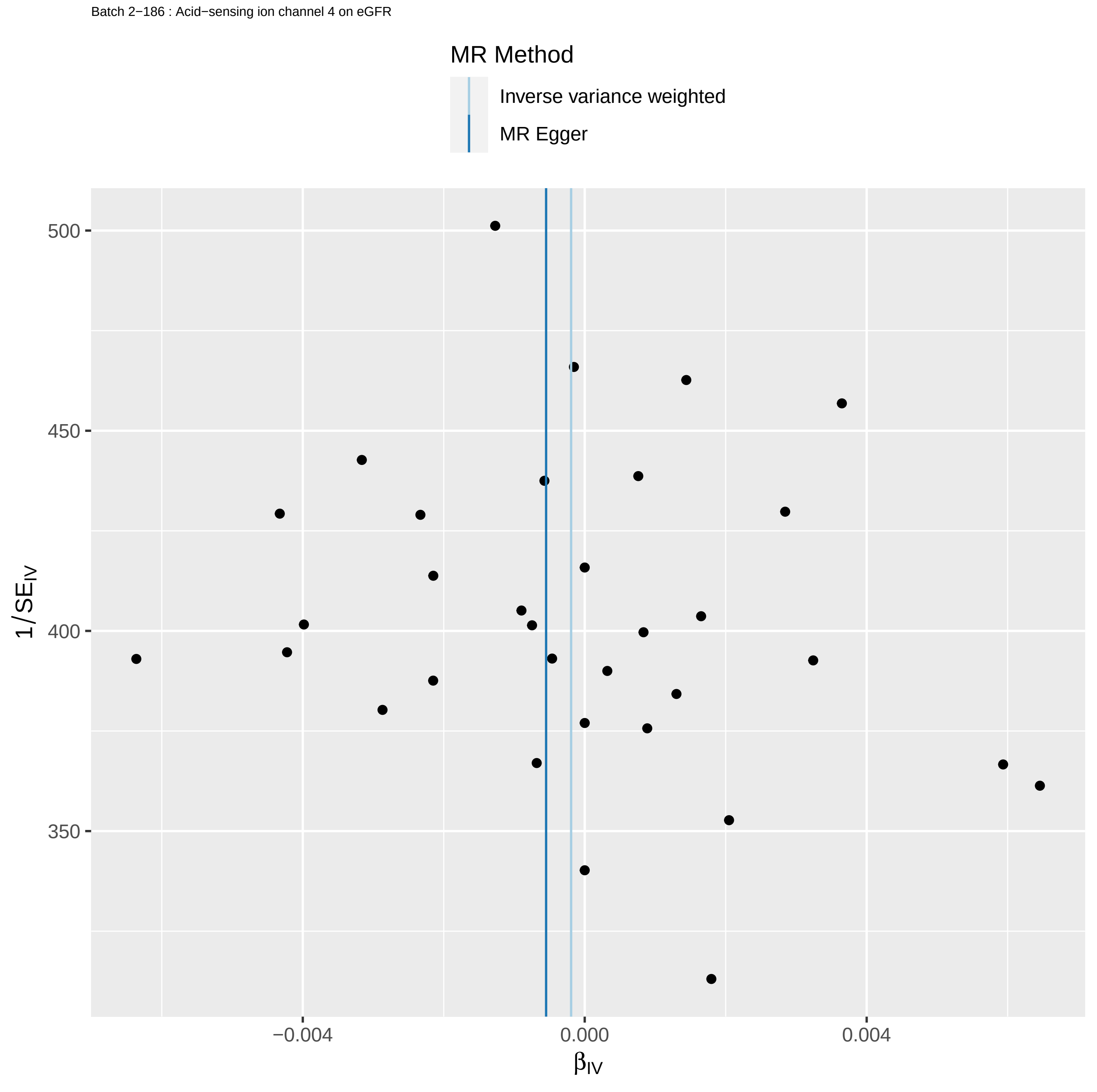
Batch 2−186 : Acid−sensing ion channel 4 on eGFR
MR Method
Inverse variance weighted
MR Egger
500
450
400
350
1/SE_IV
−0.004
0.000
0.004
β_IV

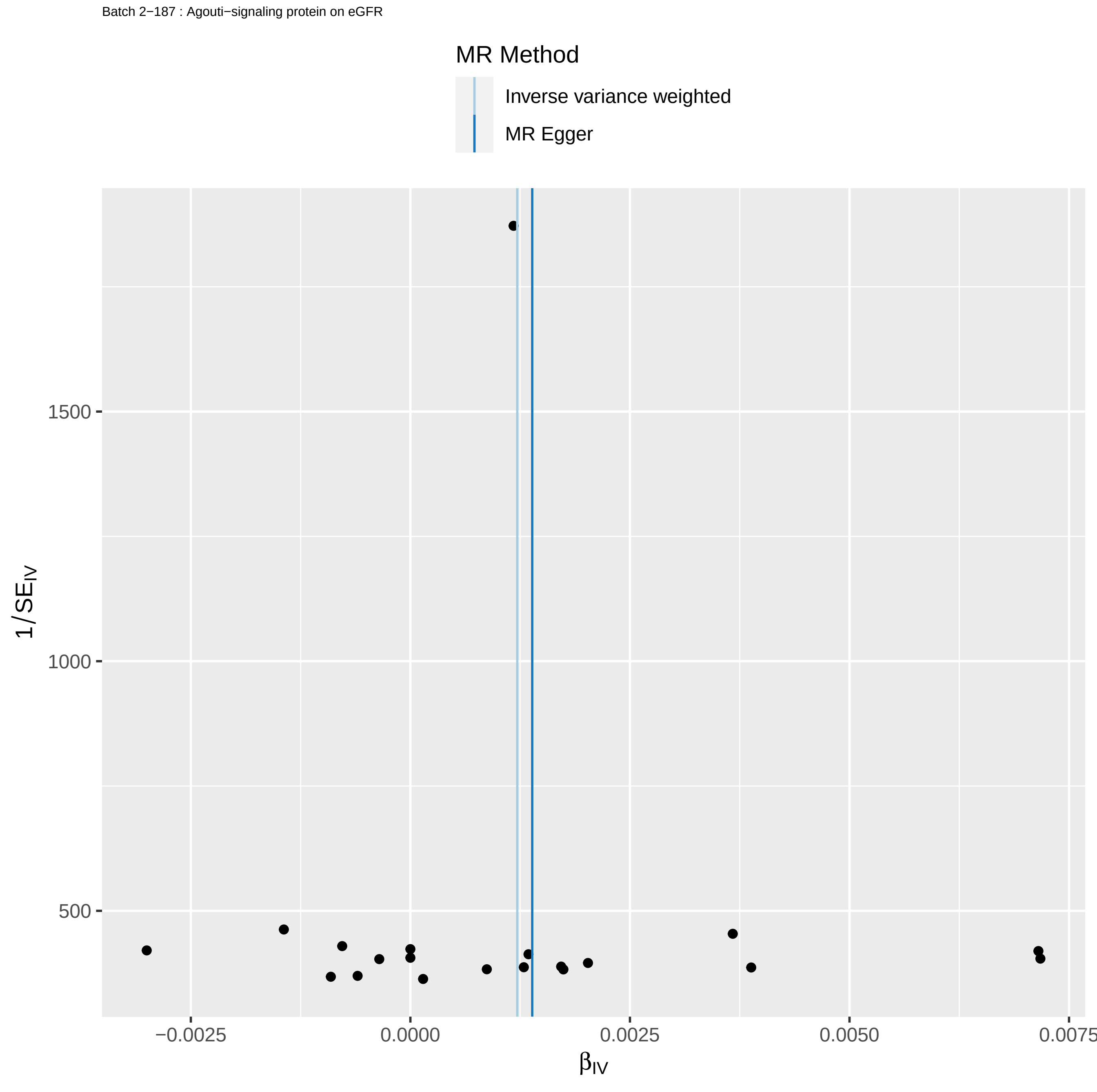
Batch 2−187 : Agouti−signaling protein on eGFR
MR Method
Inverse variance weighted
MR Egger
1500
1000
500
1/SE_IV
−0.0025
0.0000
0.0025
0.0050
0.0075
β_IV

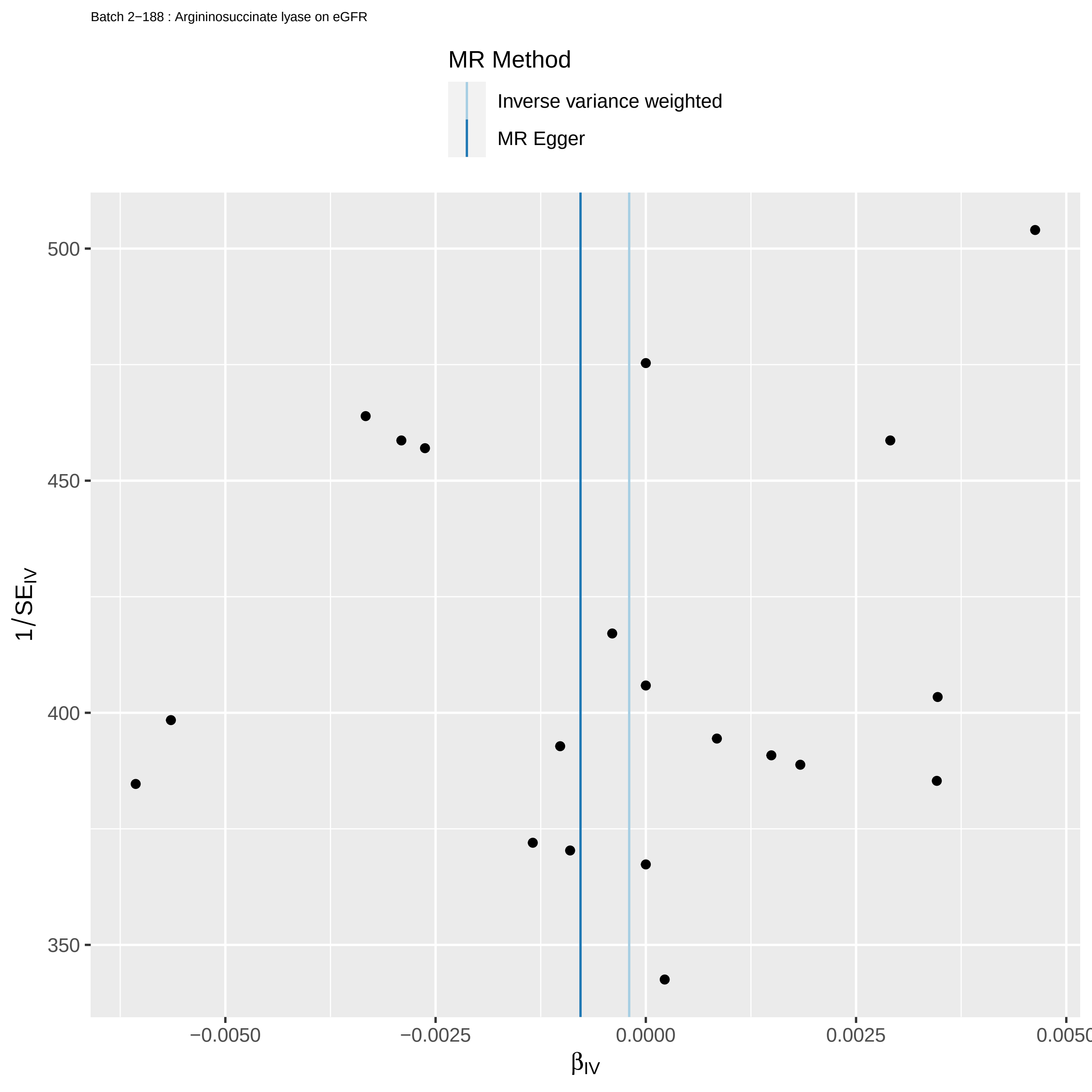

Batch 2−188 : Argininosuccinate lyase on eGFR
MR Method
Inverse variance weighted
MR Egger
500
450
400
350
1/SE_IV
−0.0050
−0.0025
0.0000
0.0025
0.0050
β_IV

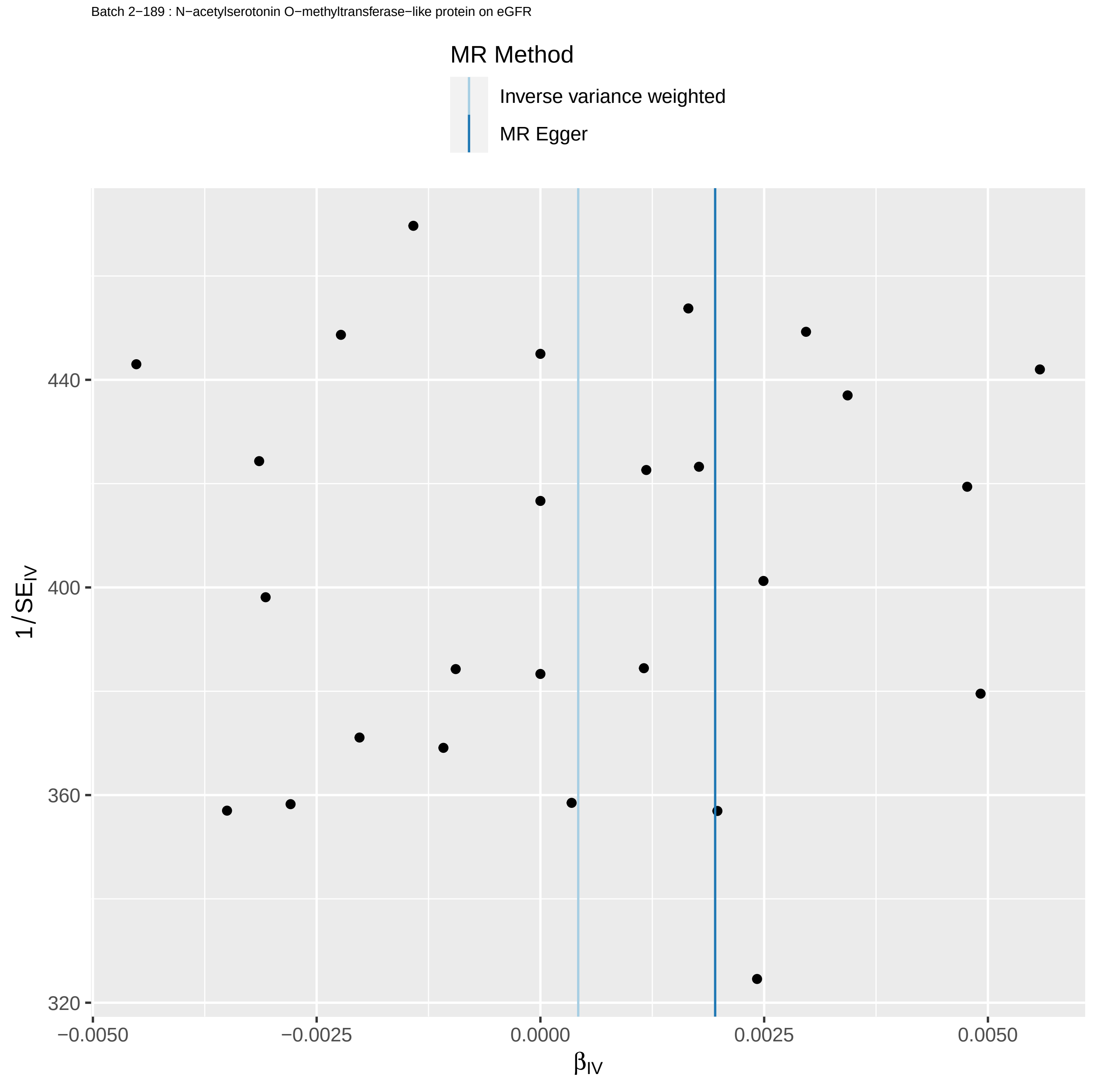

Batch 2−189 : N−acetylserotonin O−methyltransferase−like protein on eGFR
MR Method
Inverse variance weighted
MR Egger
440
400
360
320
1/SE_IV
−0.0050
−0.0025
0.0000
0.0025
0.0050
β_IV

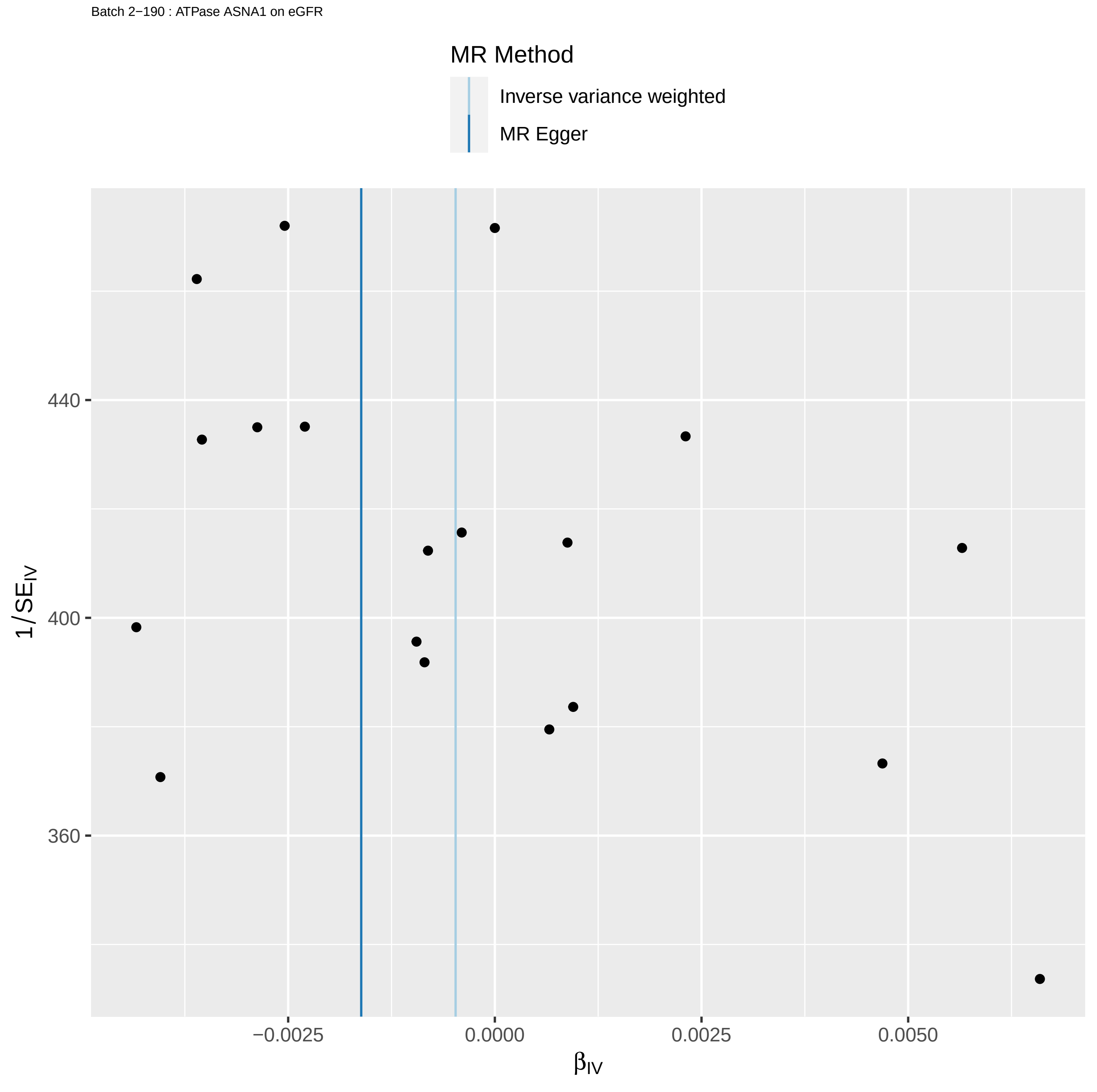
Batch 2−190 : ATPase ASNA1 on eGFR
MR Method
Inverse variance weighted
MR Egger
440
400
360
1/SE_IV
−0.0025
0.0000
0.0025
0.0050
β_IV

Batch 2−191 : Aspartyl/asparaginyl beta−hydroxylase on eGFR

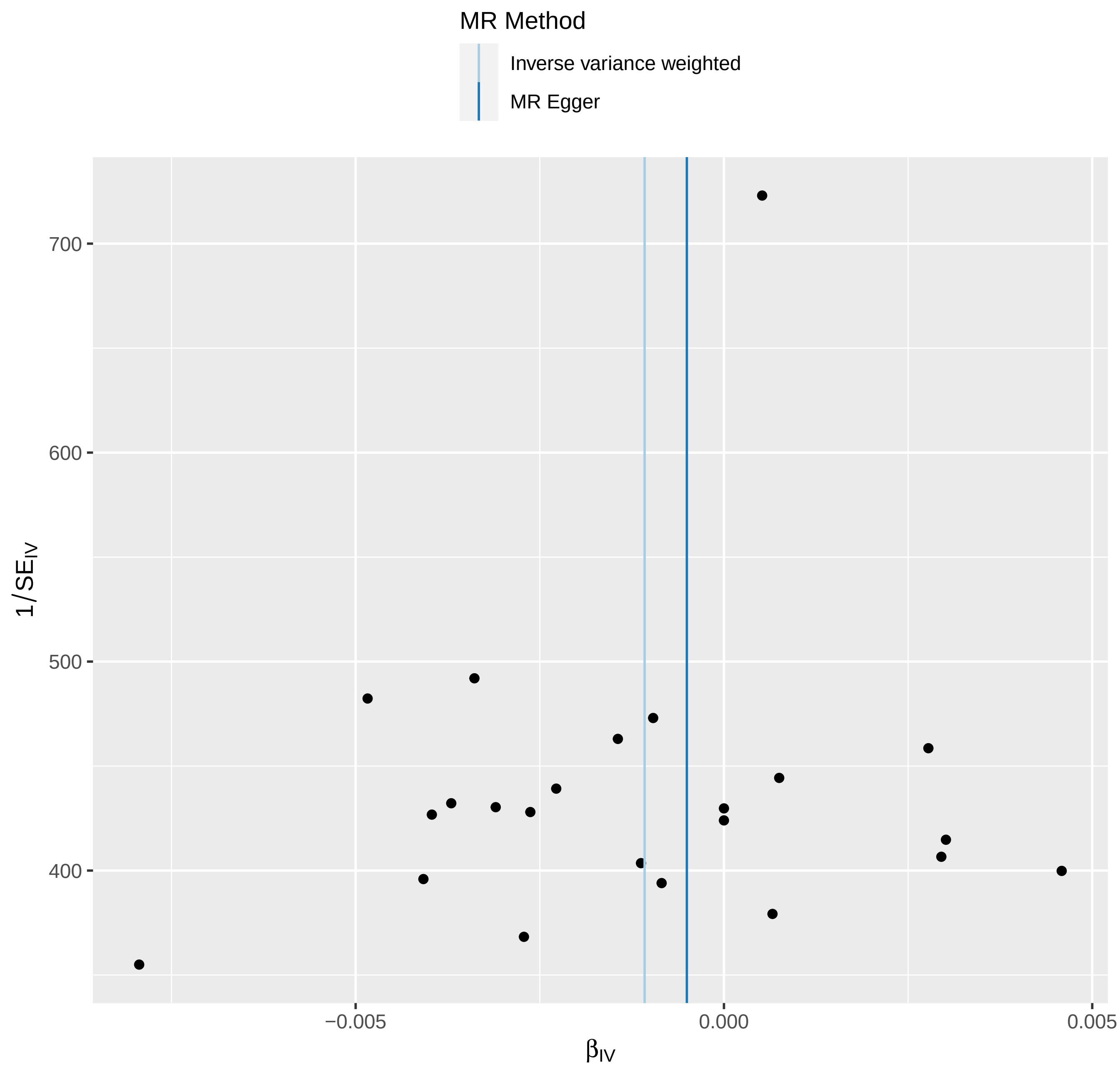

Batch 2−192 : Asporin on eGFR

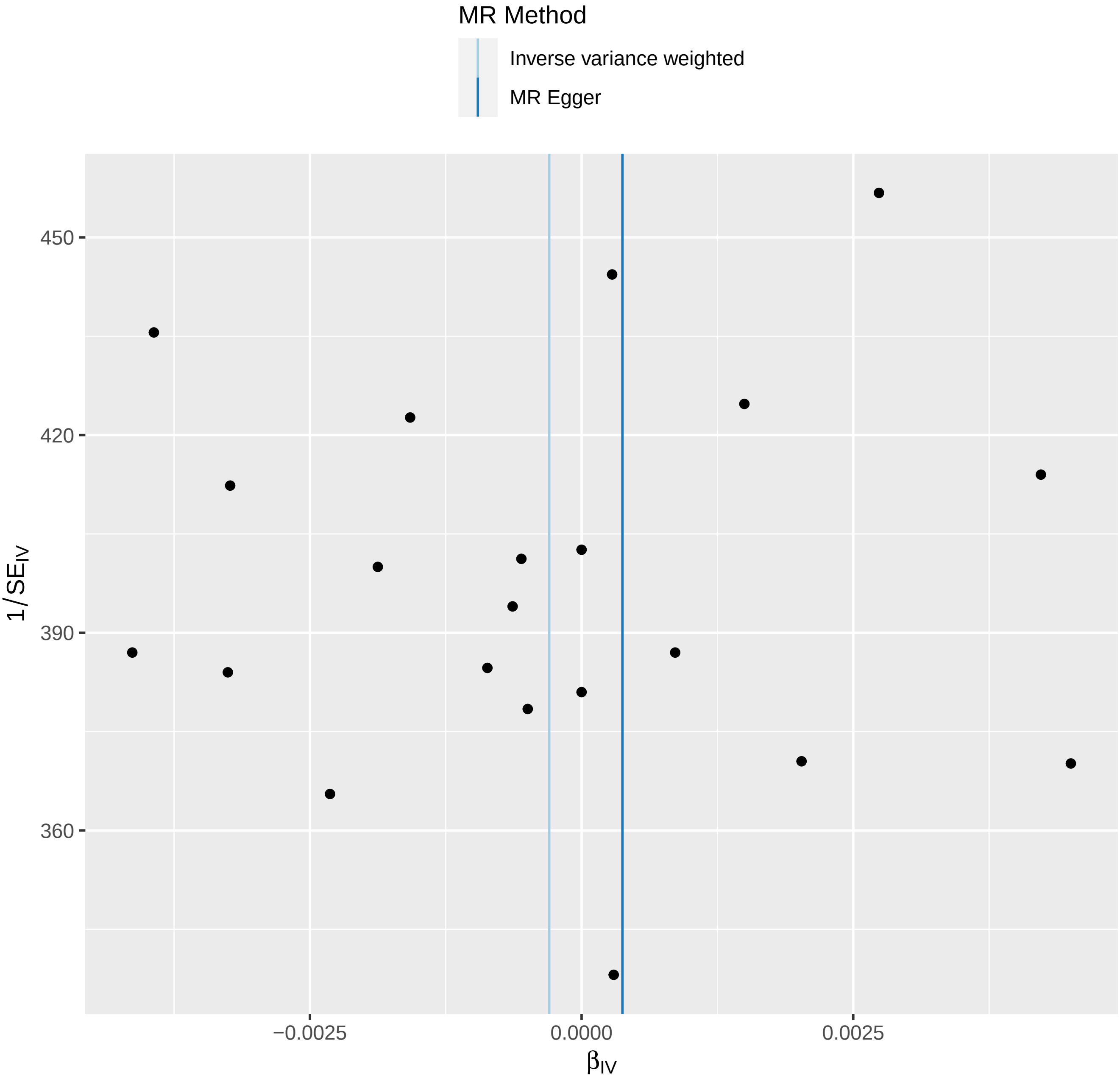

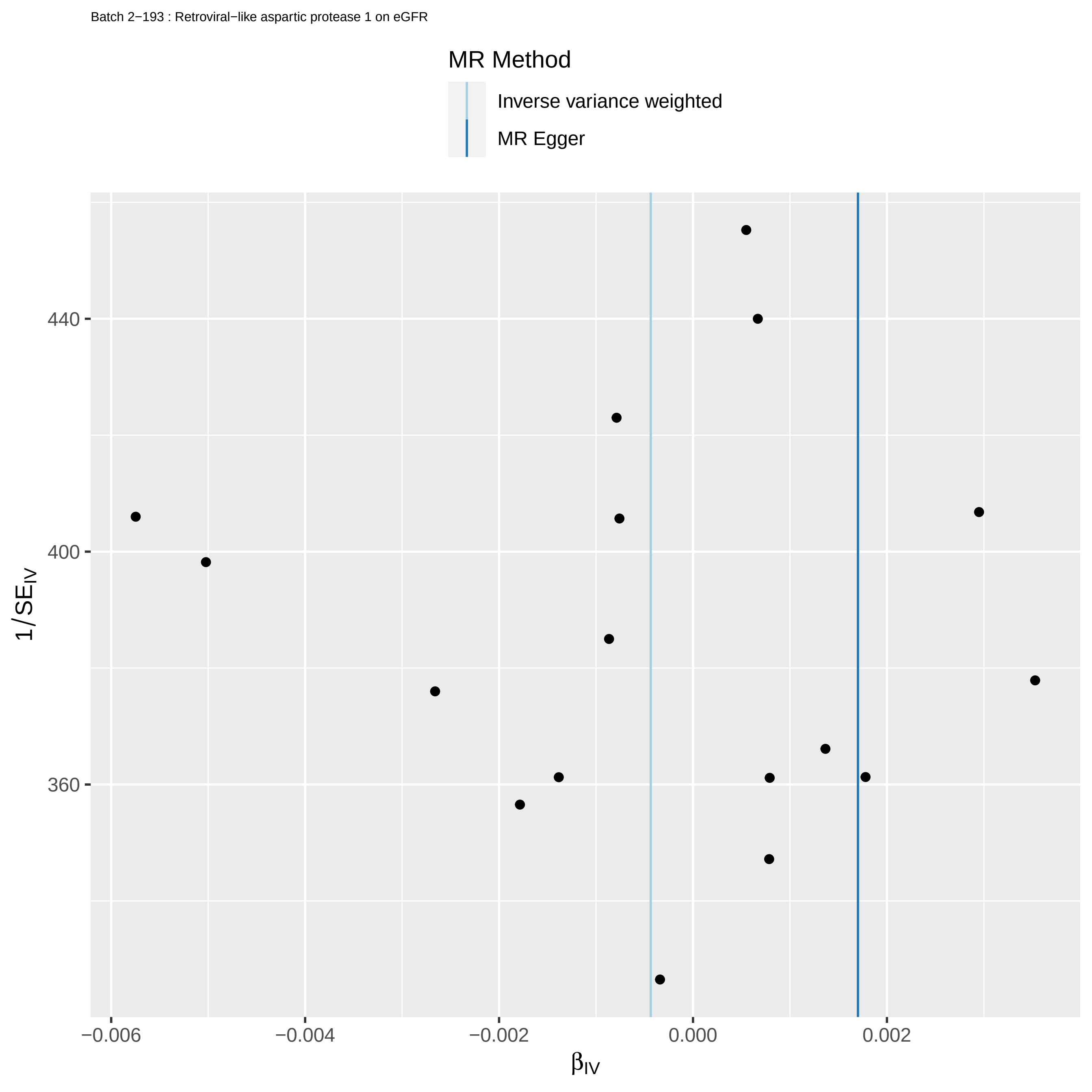

Batch 2−193 : Retroviral−like aspartic protease 1 on eGFR
MR Method
Inverse variance weighted
MR Egger
440
400
360
1/SE$_{IV}$
−0.006
−0.004
−0.002
0.000
0.002
β$_{IV}$

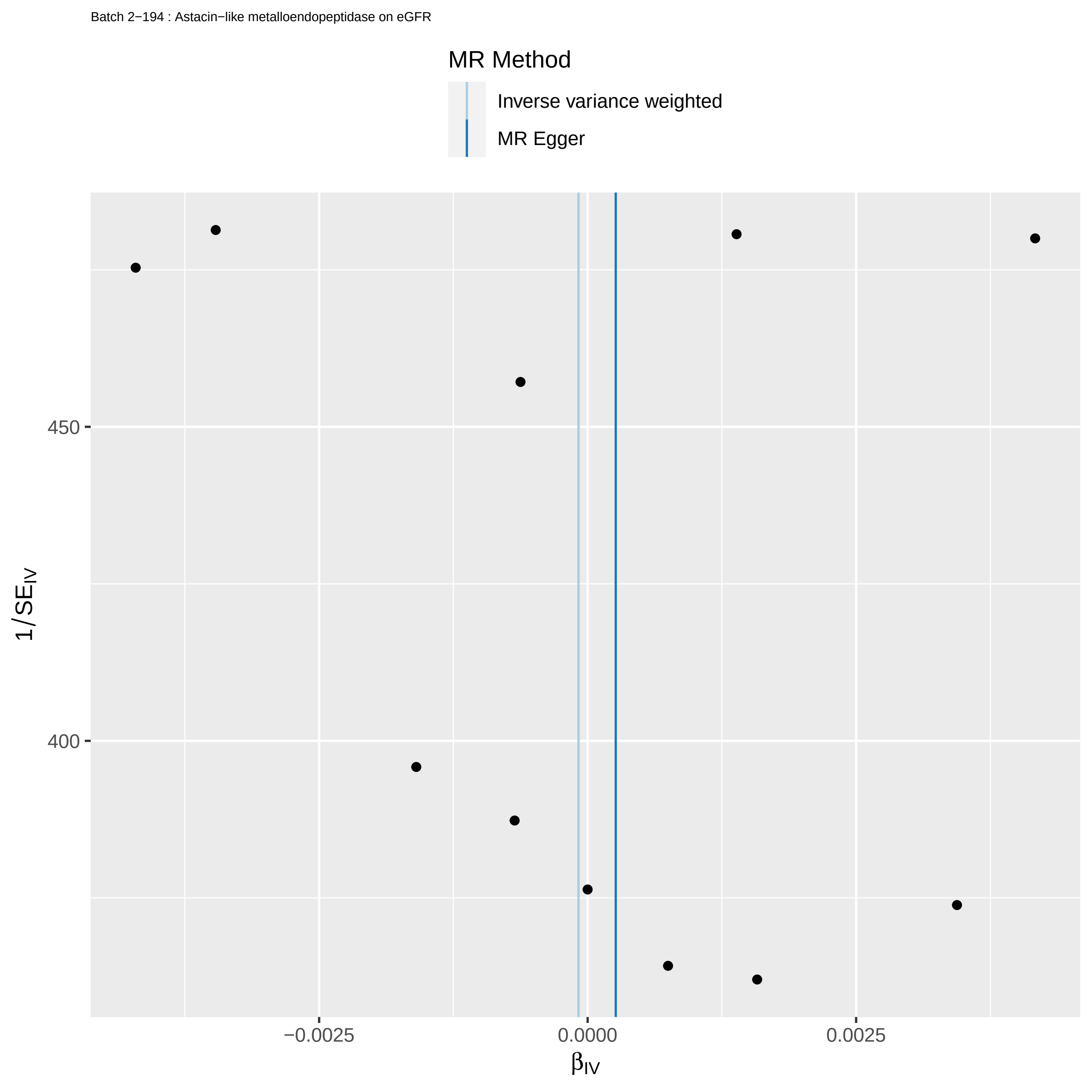

Batch 2−194 : Astacin−like metalloendopeptidase on eGFR
MR Method
Inverse variance weighted
MR Egger
450
400
1/SE$_{IV}$
−0.0025
0.0000
0.0025
β$_{IV}$

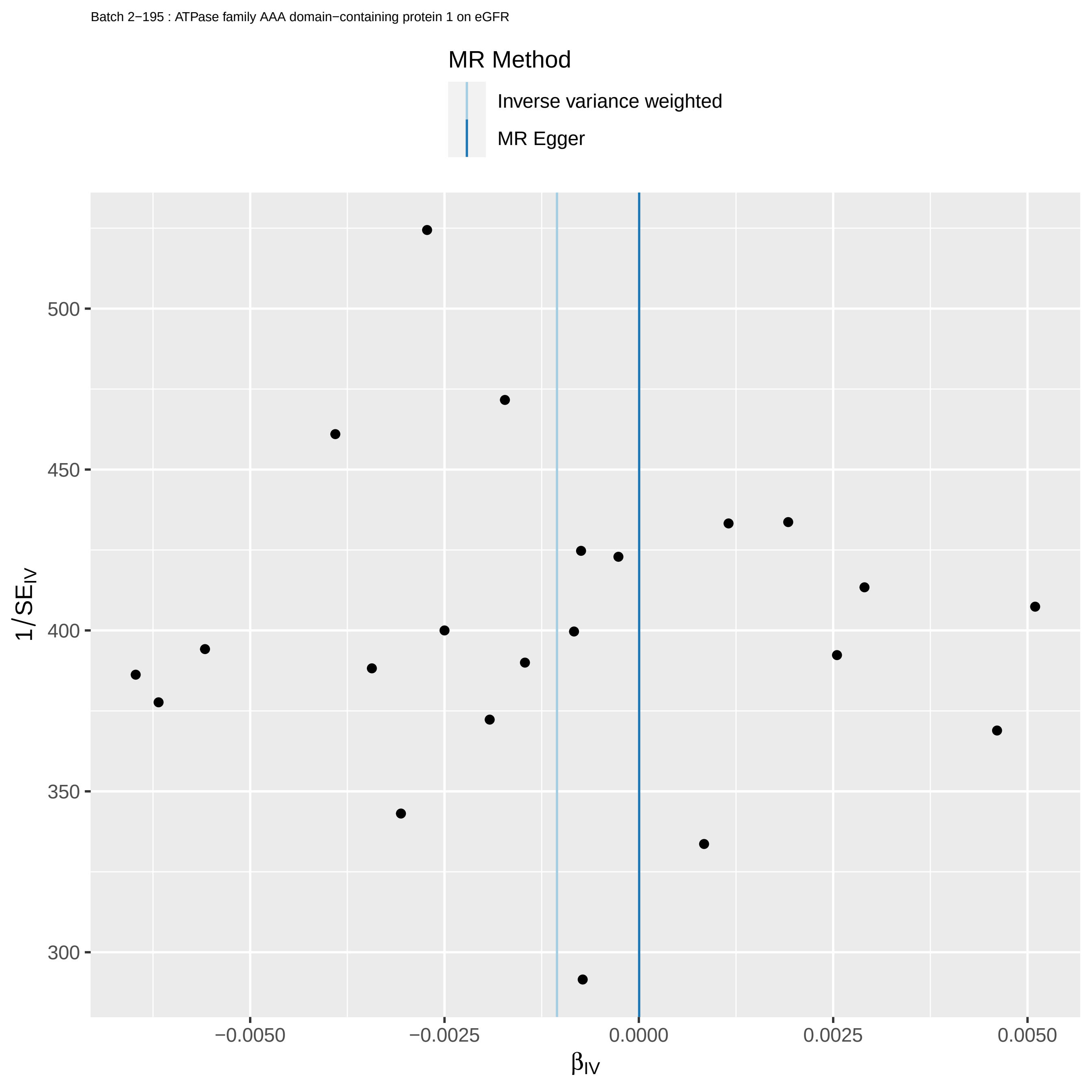
Batch 2−195 : ATPase family AAA domain−containing protein 1 on eGFR
MR Method
Inverse variance weighted
MR Egger
500
450
400
350
300
1/SE_IV
−0.0050
−0.0025
0.0000
0.0025
0.0050
β_IV

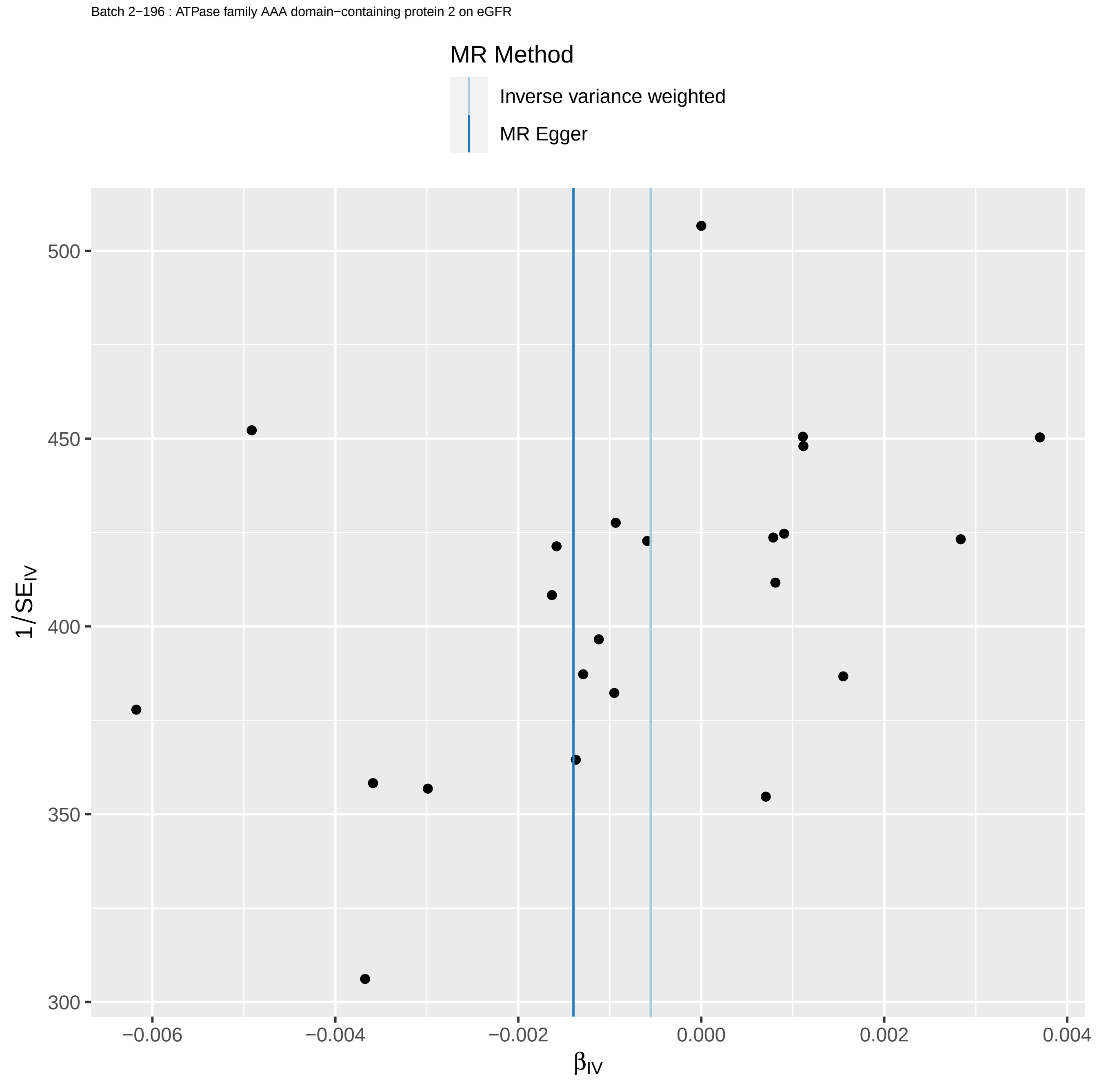
Batch 2−196 : ATPase family AAA domain−containing protein 2 on eGFR
MR Method
Inverse variance weighted
MR Egger
500
450
400
350
300
1/SE_IV
−0.006
−0.004
−0.002
0.000
0.002
0.004
β_IV

Batch 2−197 : Cyclic AMP−dependent transcription factor ATF−6 alpha on eGFR

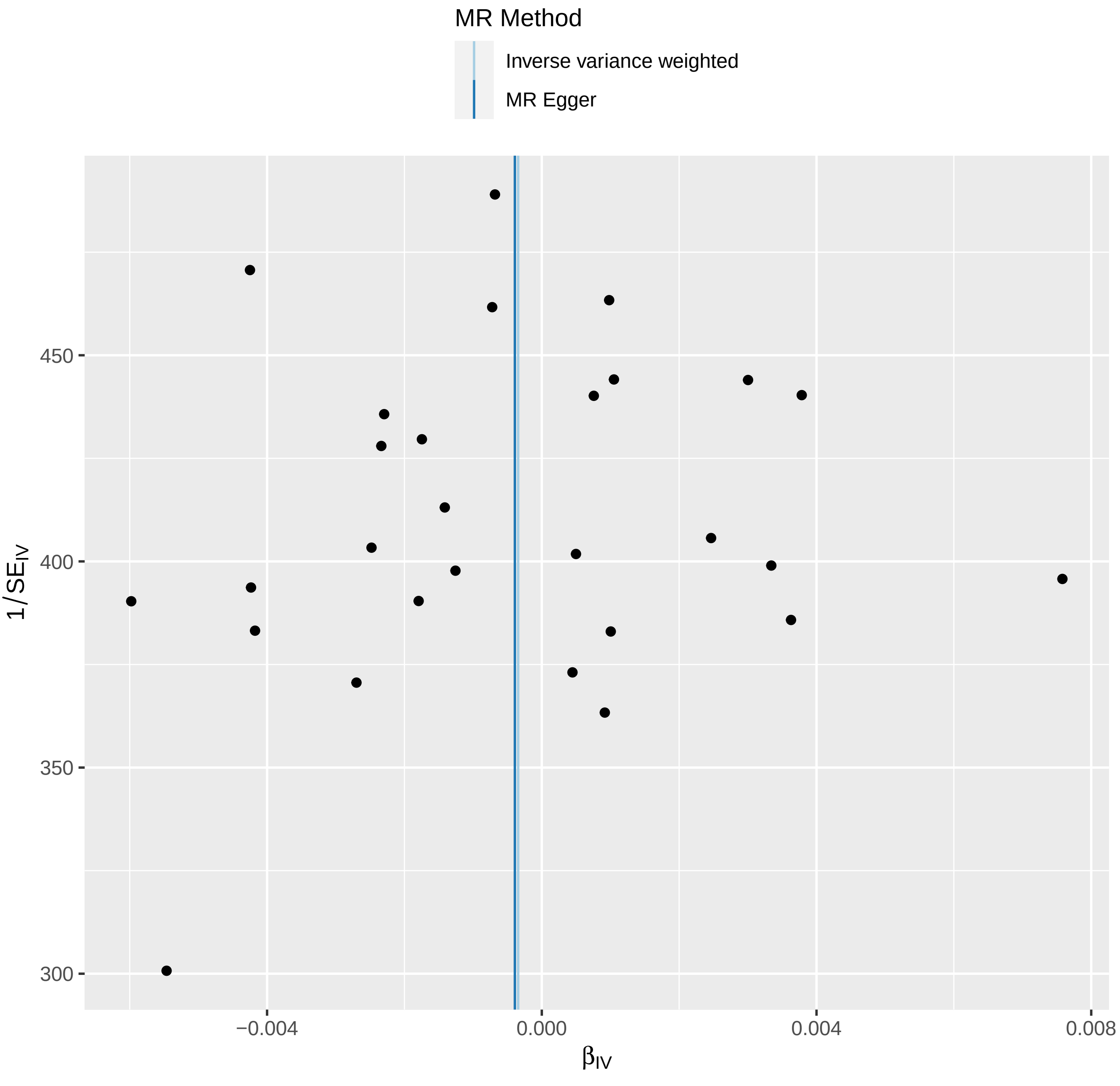

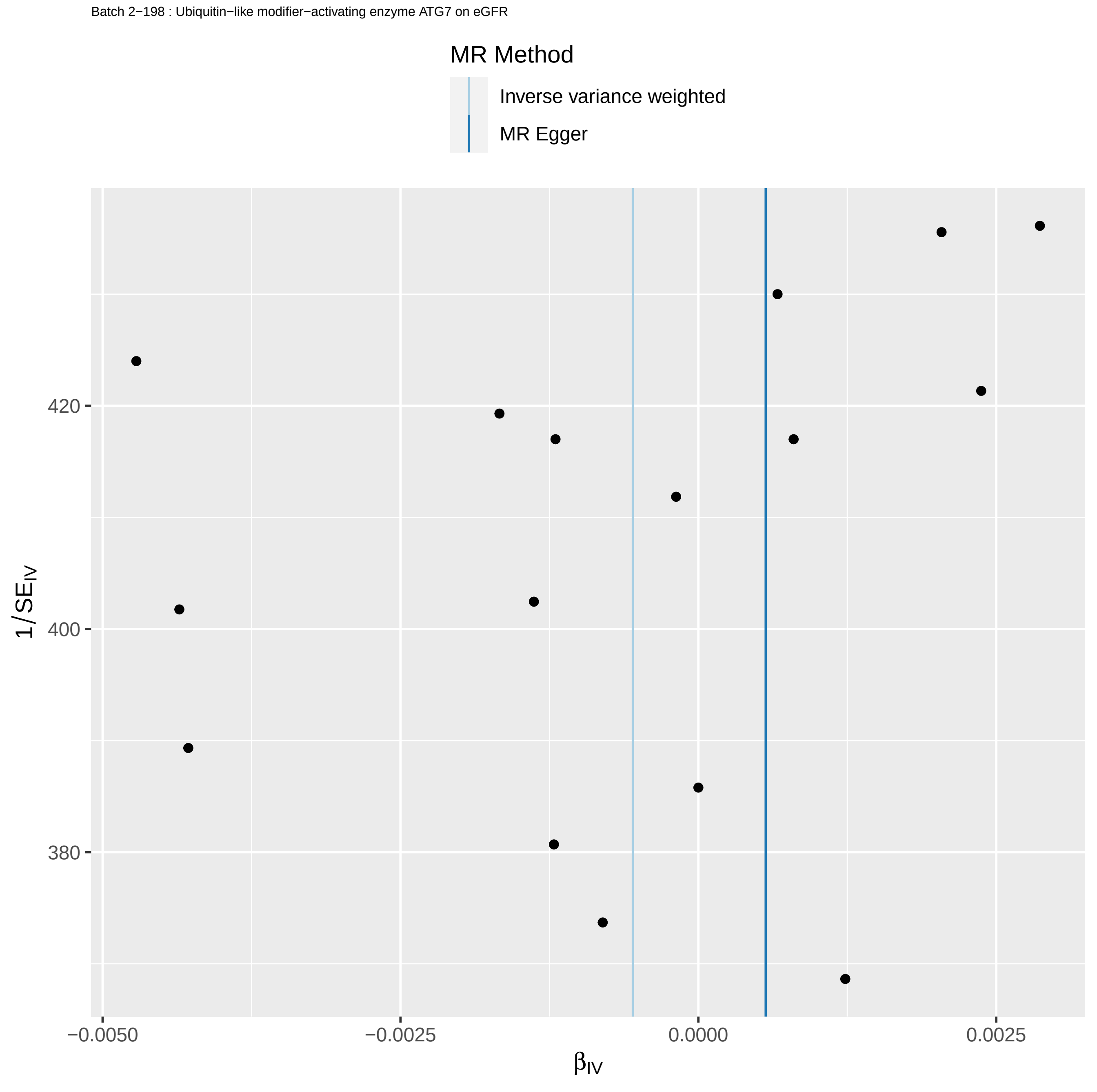
Batch 2−198 : Ubiquitin−like modifier−activating enzyme ATG7 on eGFR
MR Method
Inverse variance weighted
MR Egger
420
400
380
1/SE$_{IV}$
−0.0050
−0.0025
0.0000
0.0025
β_{IV}

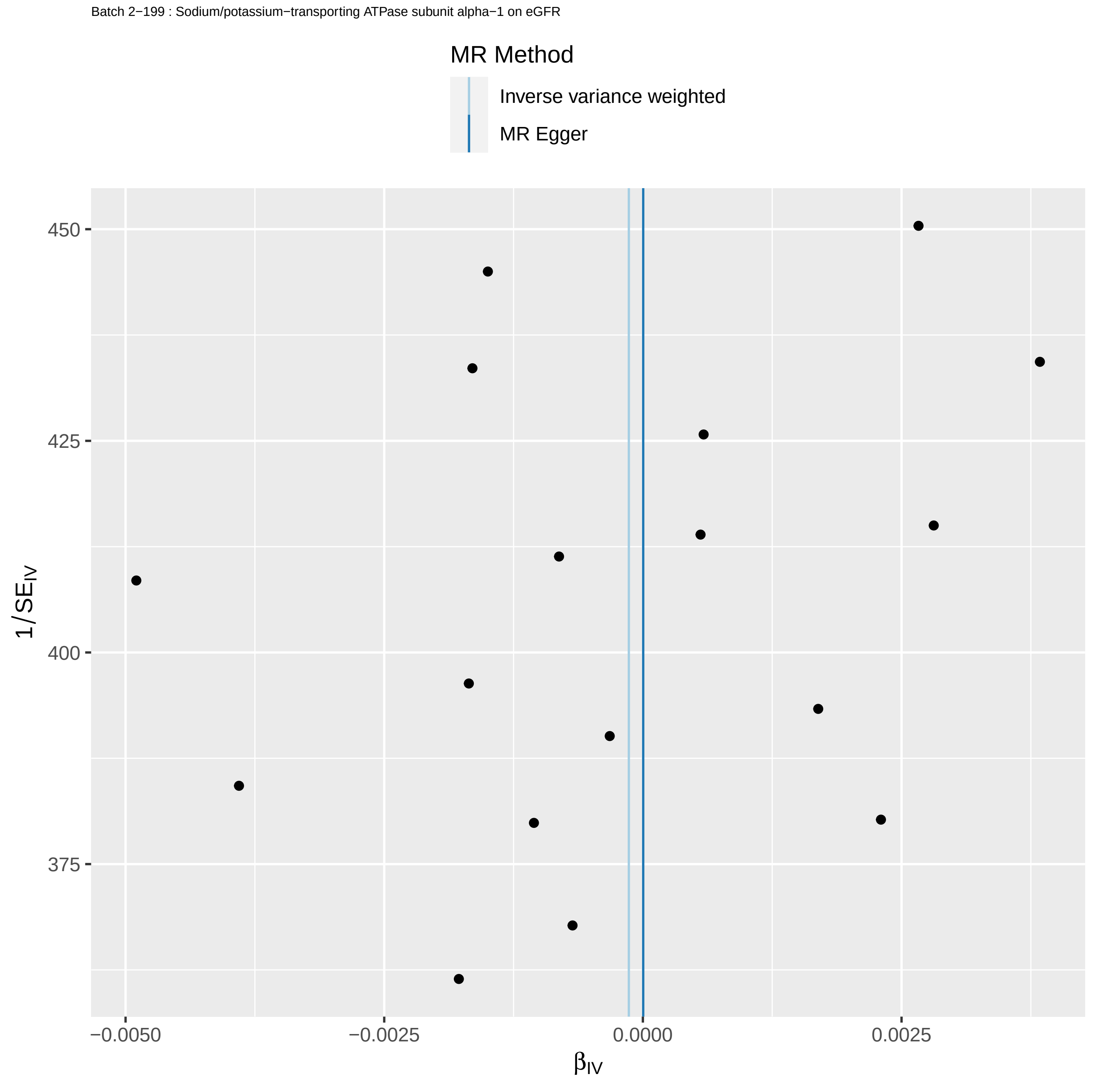
Batch 2−199 : Sodium/potassium−transporting ATPase subunit alpha−1 on eGFR
MR Method
Inverse variance weighted
MR Egger
450
425
400
375
1/SE_IV
−0.0050
−0.0025
0.0000
0.0025
β_IV

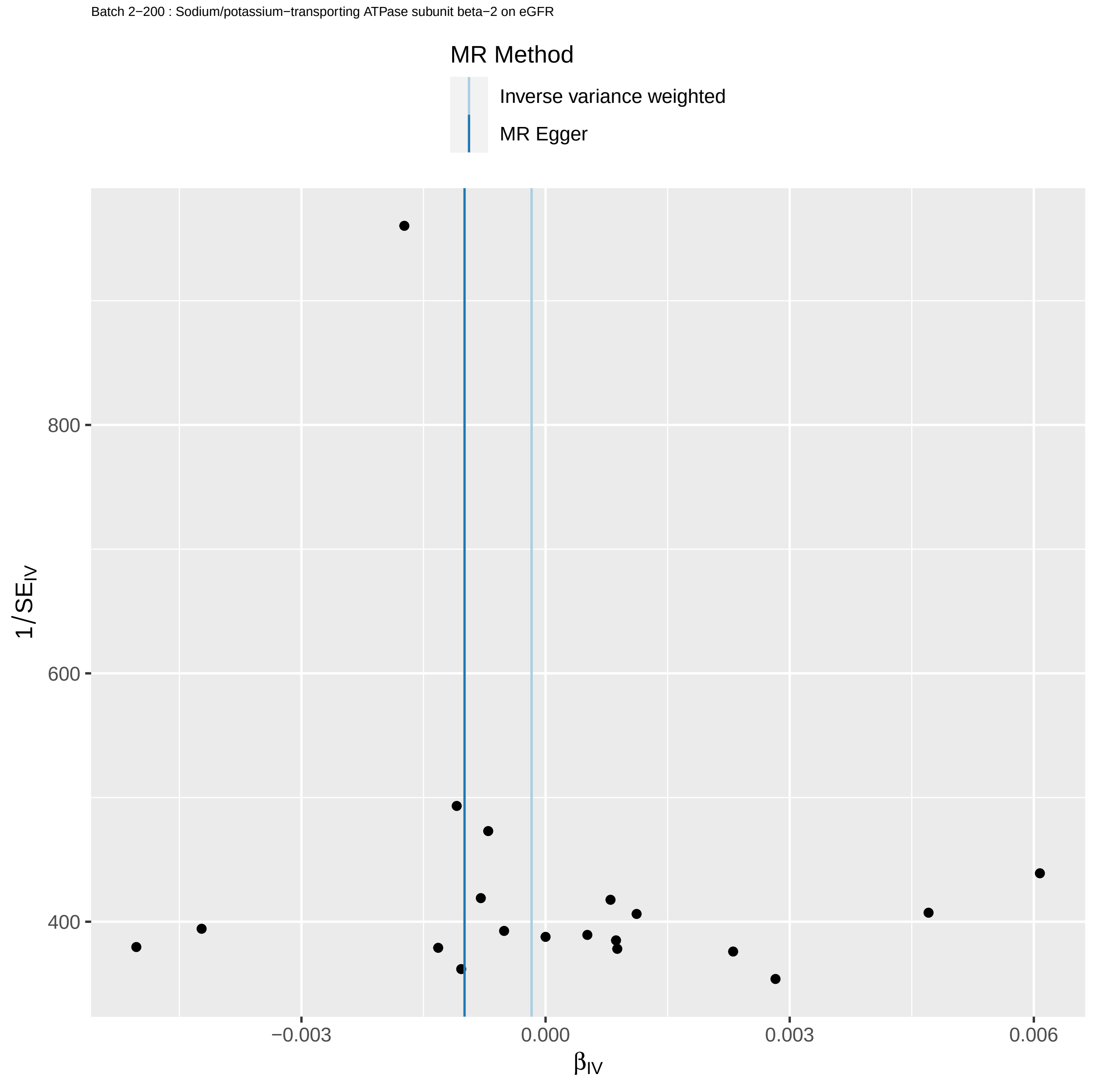

Batch 2−200 : Sodium/potassium−transporting ATPase subunit beta−2 on eGFR
MR Method
Inverse variance weighted
MR Egger
800
600
400
1/SE$_{IV}$
−0.003
0.000
0.003
0.006
β$_{IV}$

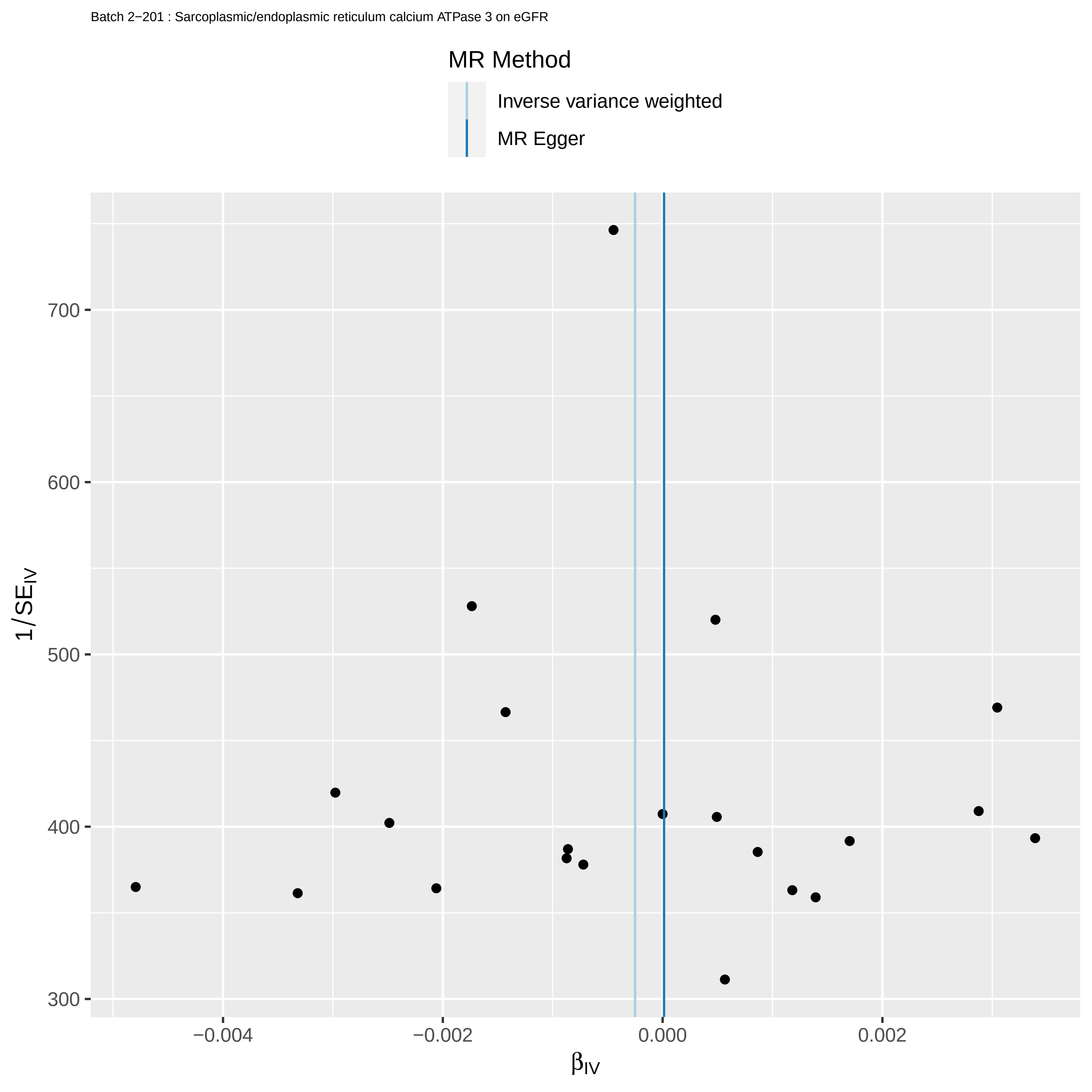

Batch 2−201 : Sarcoplasmic/endoplasmic reticulum calcium ATPase 3 on eGFR
MR Method
Inverse variance weighted
MR Egger
700
600
500
400
300
1/SE_IV
−0.004
−0.002
0.000
0.002
β_IV

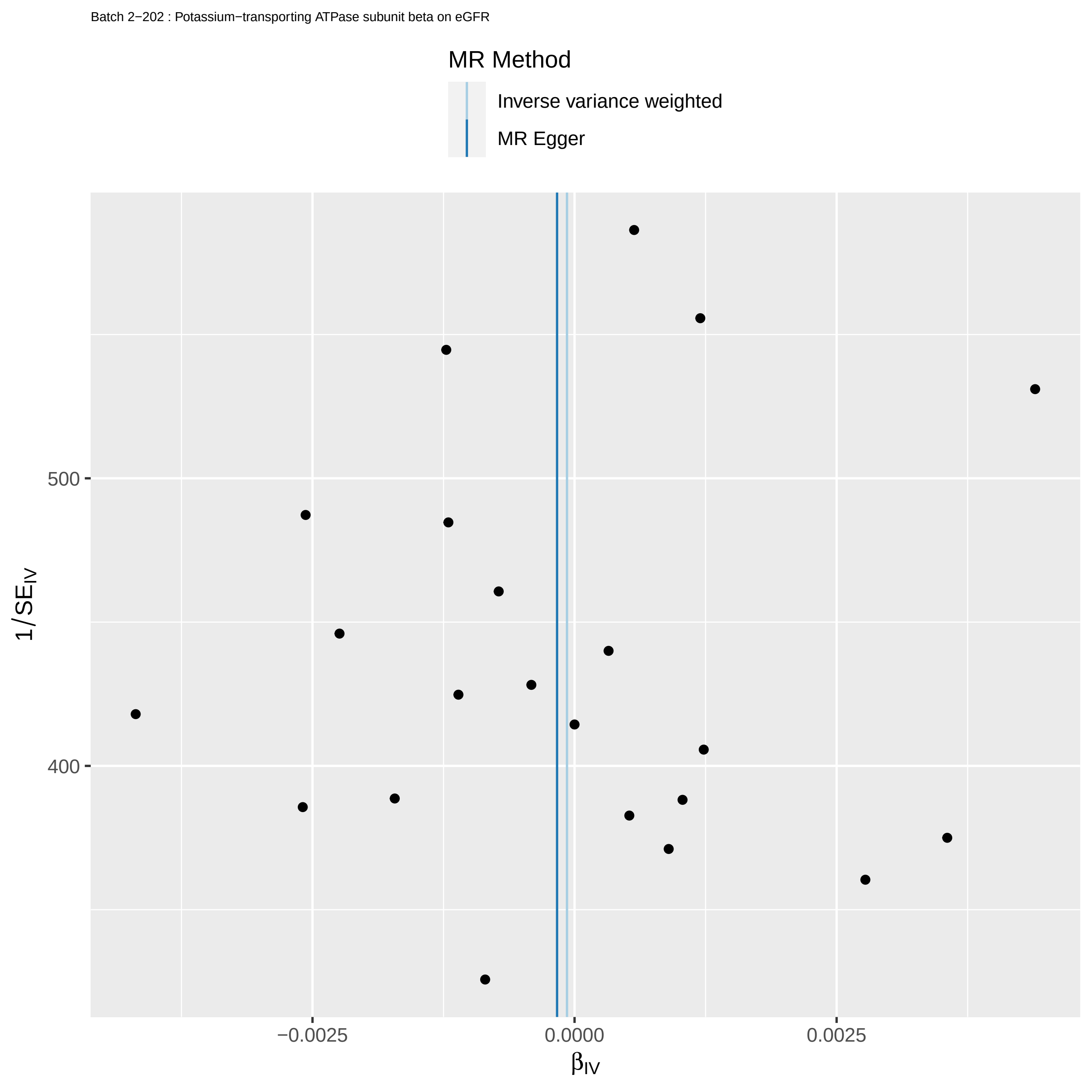

Batch 2−202 : Potassium−transporting ATPase subunit beta on eGFR
MR Method
Inverse variance weighted
MR Egger
500
400
1/SE_IV
−0.0025
0.0000
0.0025
β_IV

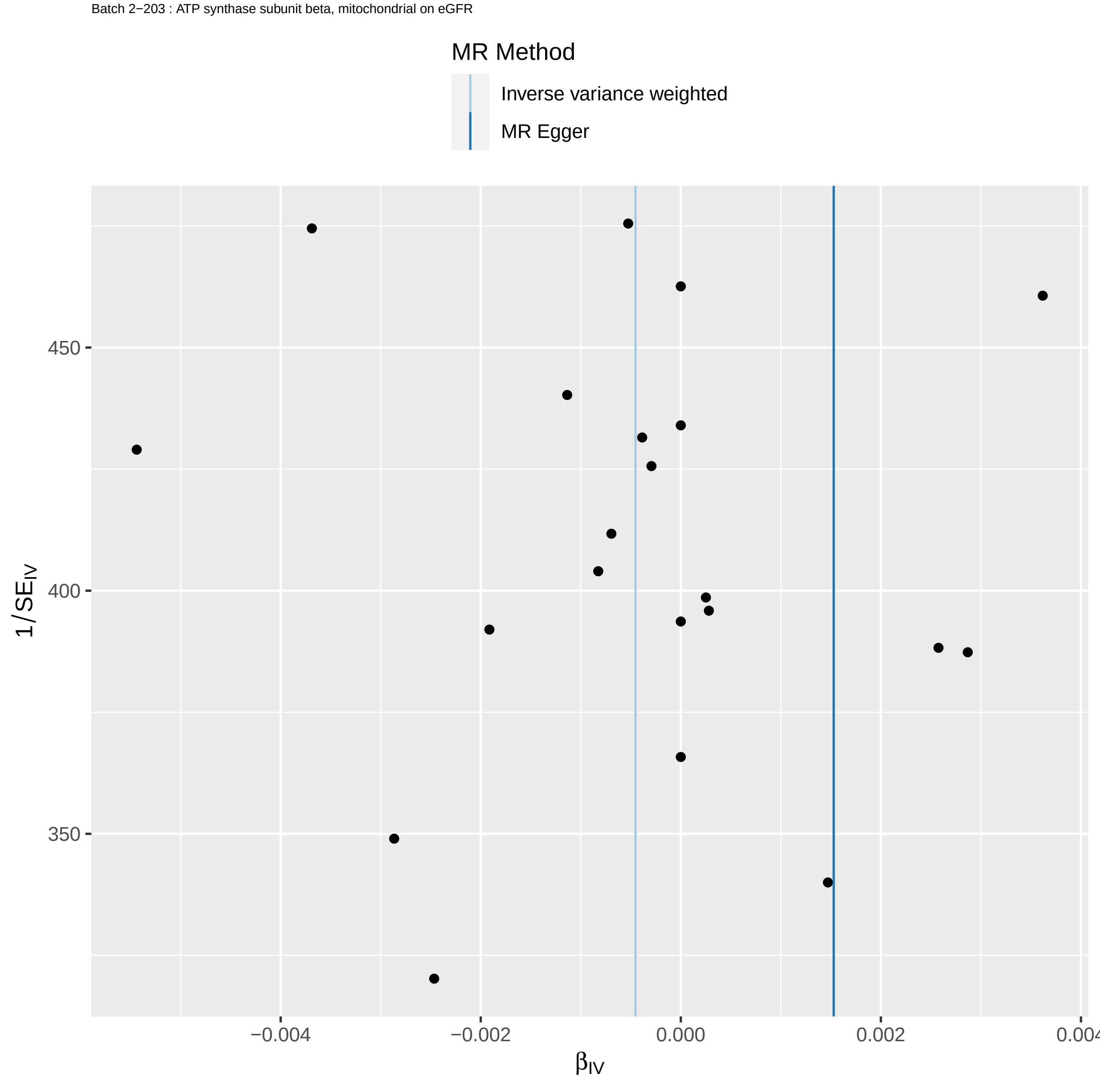

Batch 2−203 : ATP synthase subunit beta, mitochondrial on eGFR
MR Method
Inverse variance weighted
MR Egger
450
400
350
1/SE_IV
−0.004
−0.002
0.000
0.002
0.004
β_IV

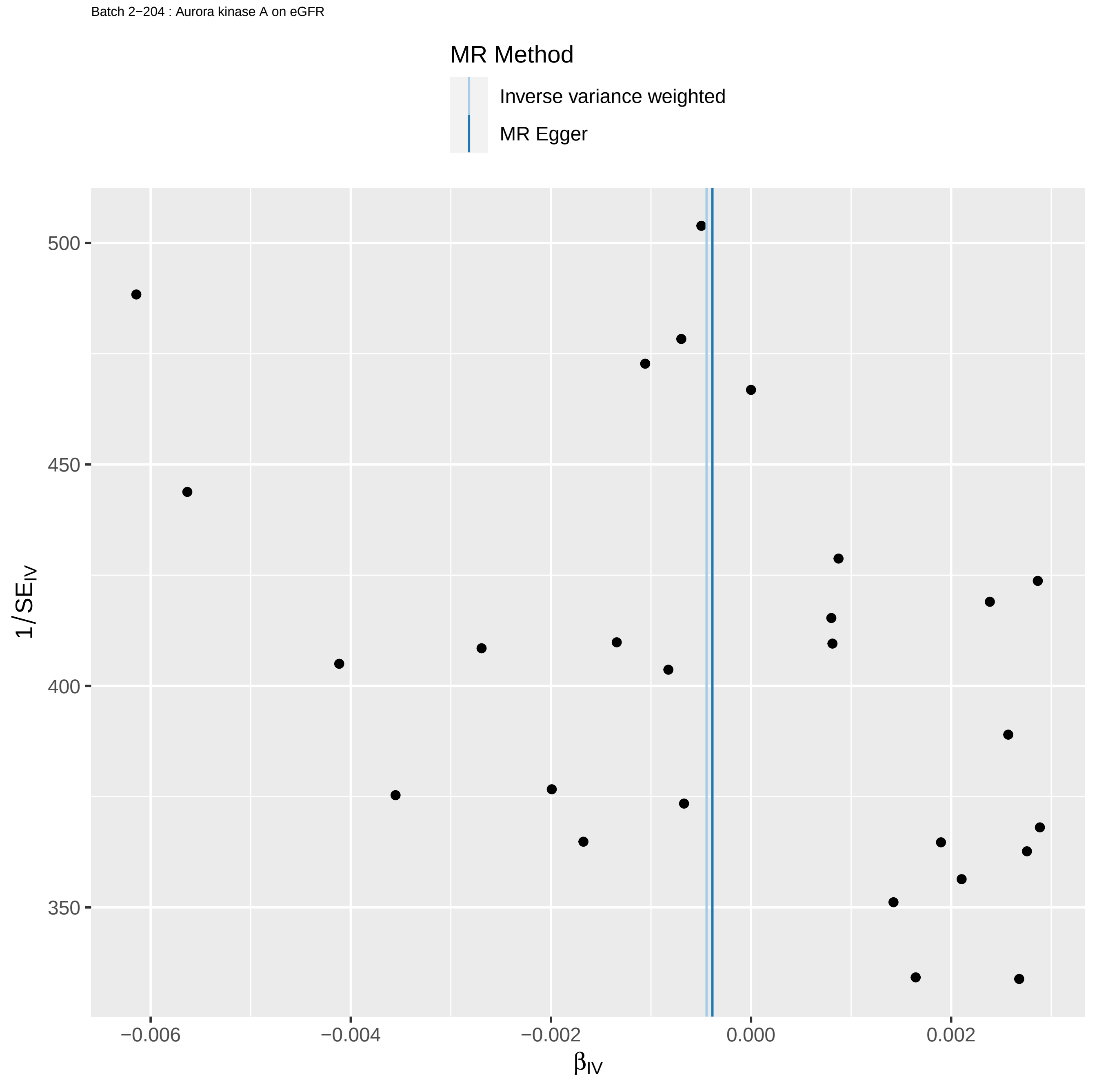
Batch 2−204 : Aurora kinase A on eGFR
MR Method
Inverse variance weighted
MR Egger
500
450
400
350
1/SE_IV
−0.006
−0.004
−0.002
0.000
0.002
β_IV

Batch 2−205 : Aurora kinase B on eGFR

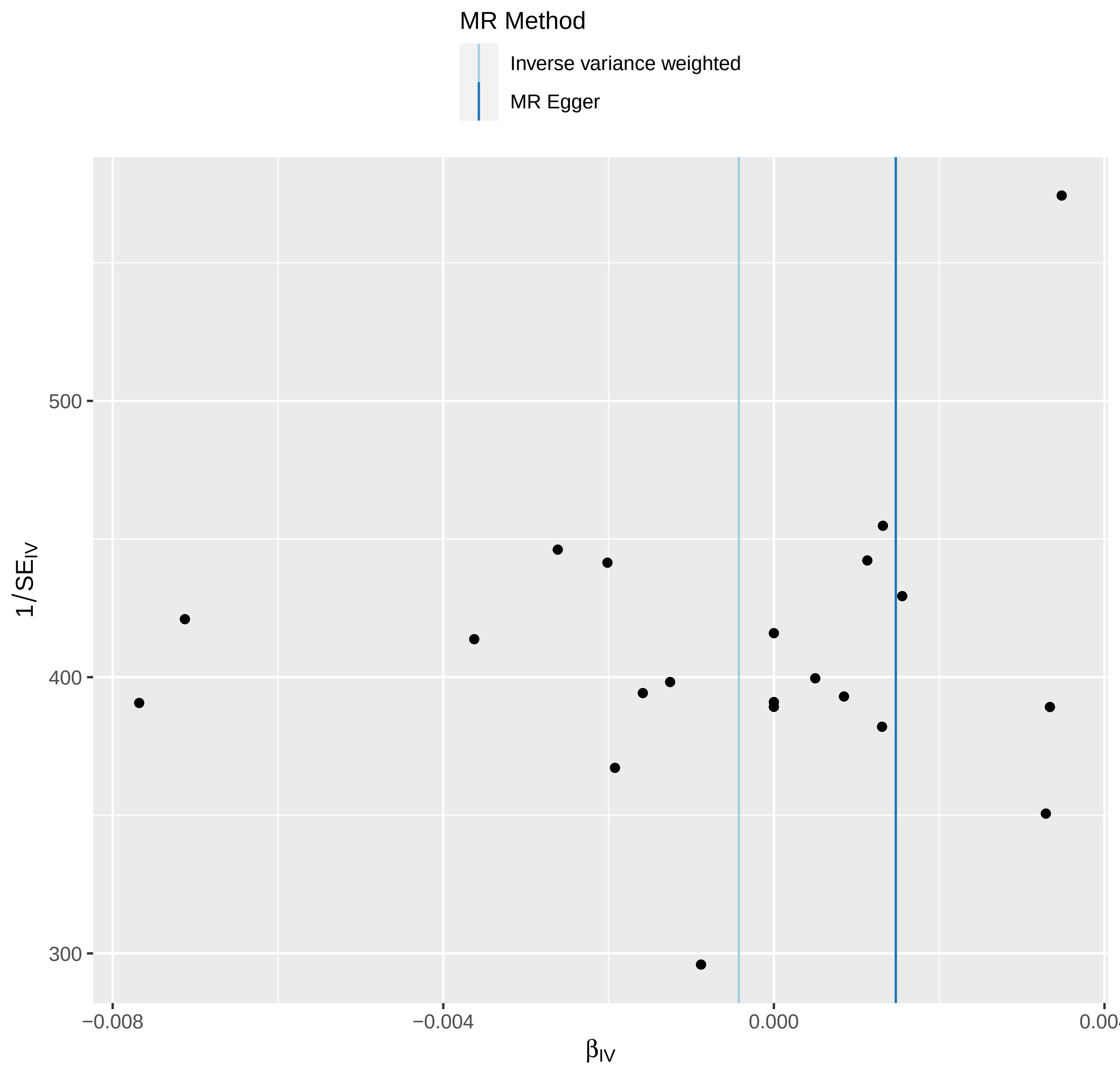

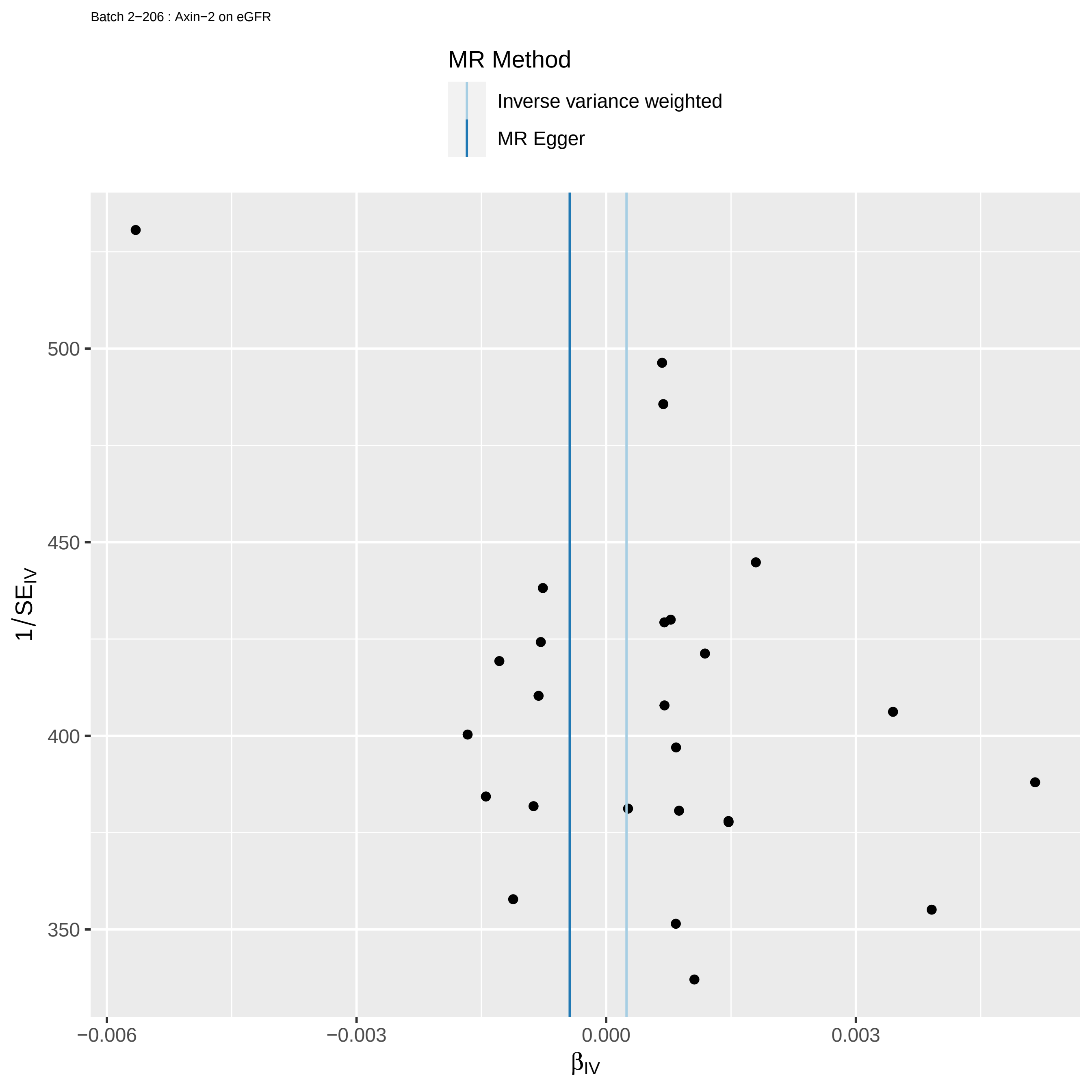
Batch 2−206 : Axin−2 on eGFR
MR Method
Inverse variance weighted
MR Egger
500
450
400
350
1/SE$_{IV}$
−0.006
−0.003
0.000
0.003
β$_{IV}$

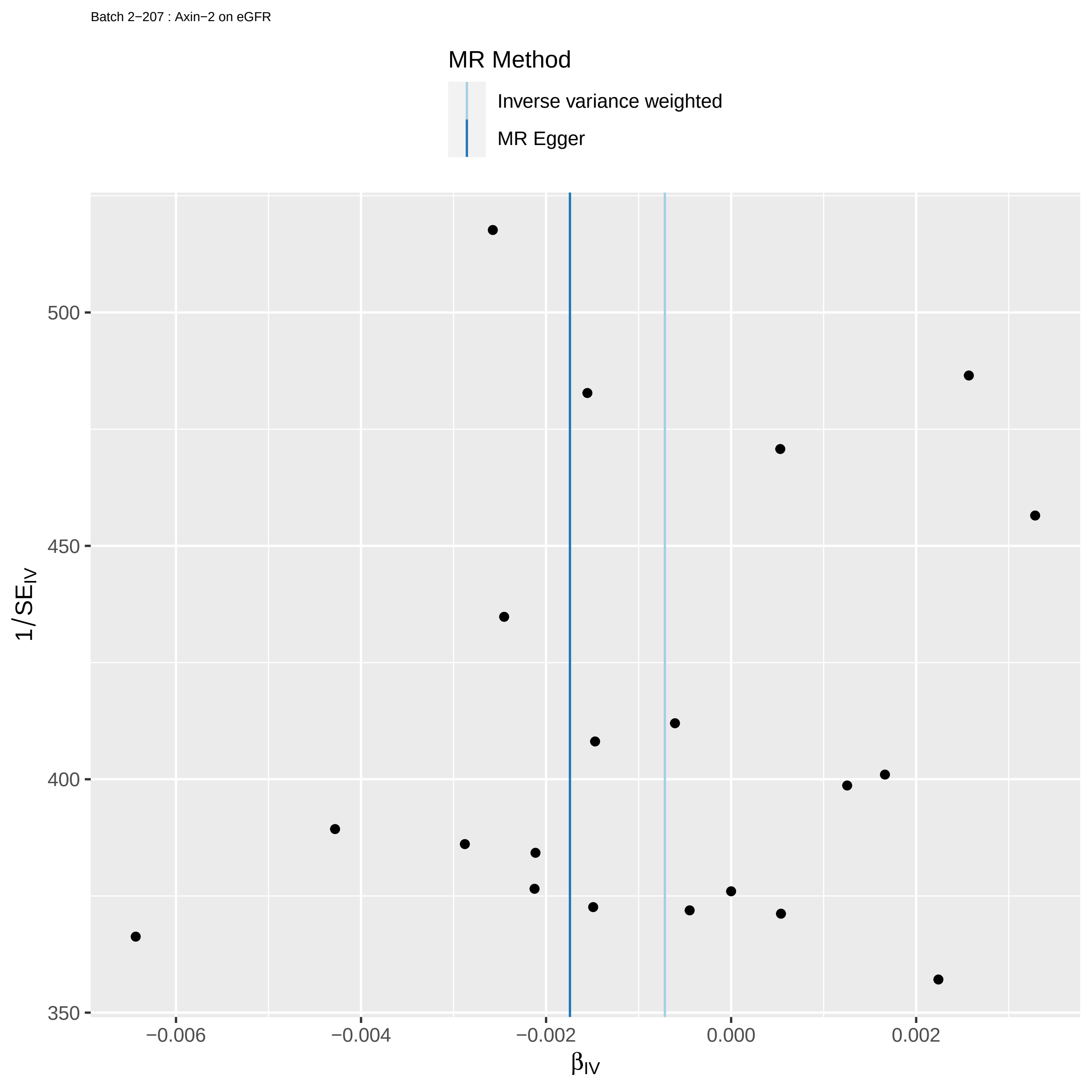

Batch 2−207 : Axin−2 on eGFR
MR Method
Inverse variance weighted
MR Egger
500
450
400
350
1/SE$_{IV}$
−0.006
−0.004
−0.002
0.000
0.002
β$_{IV}$

Batch 2−208 : Zinc−alpha−2−glycoprotein on eGFR

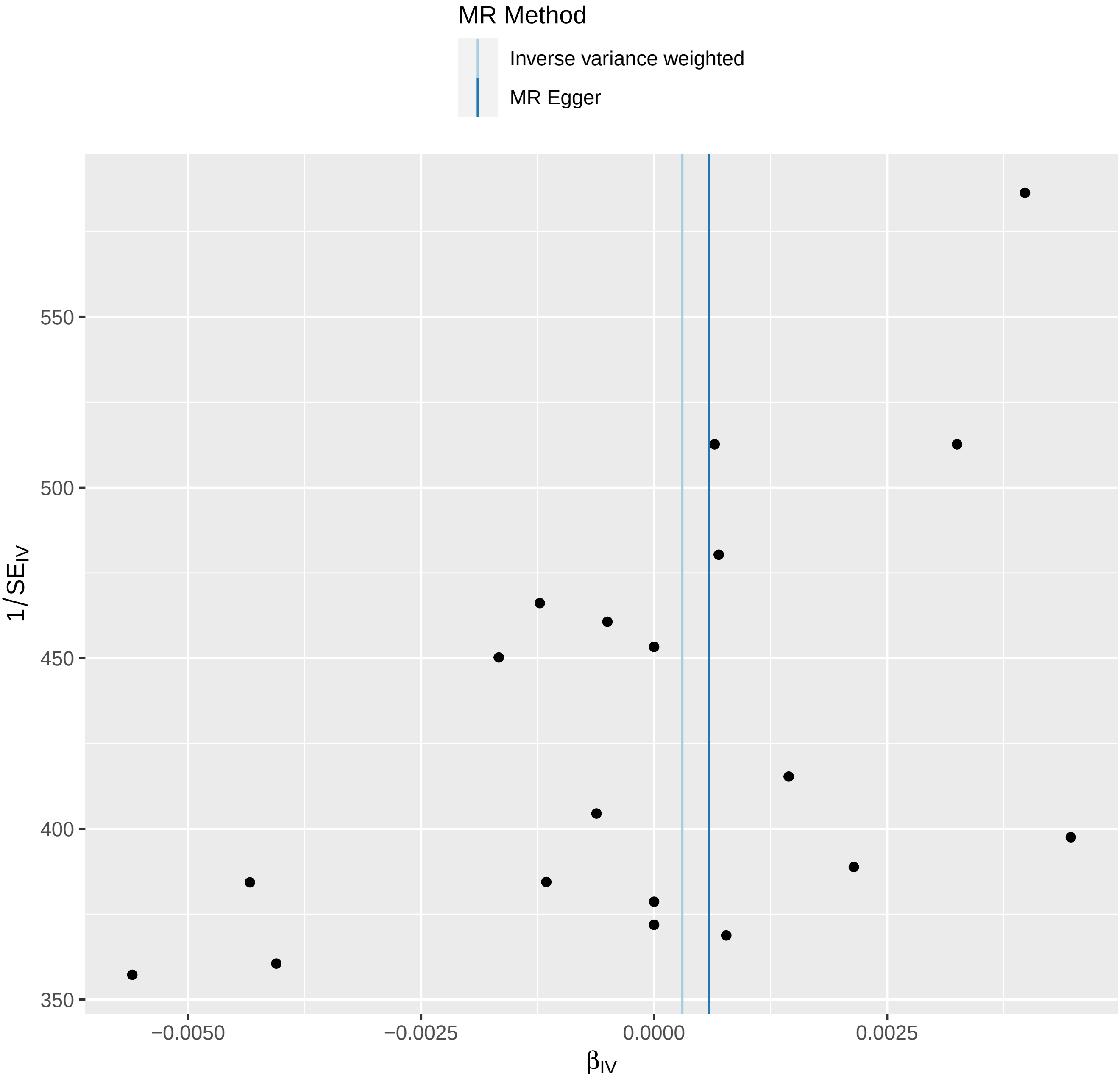

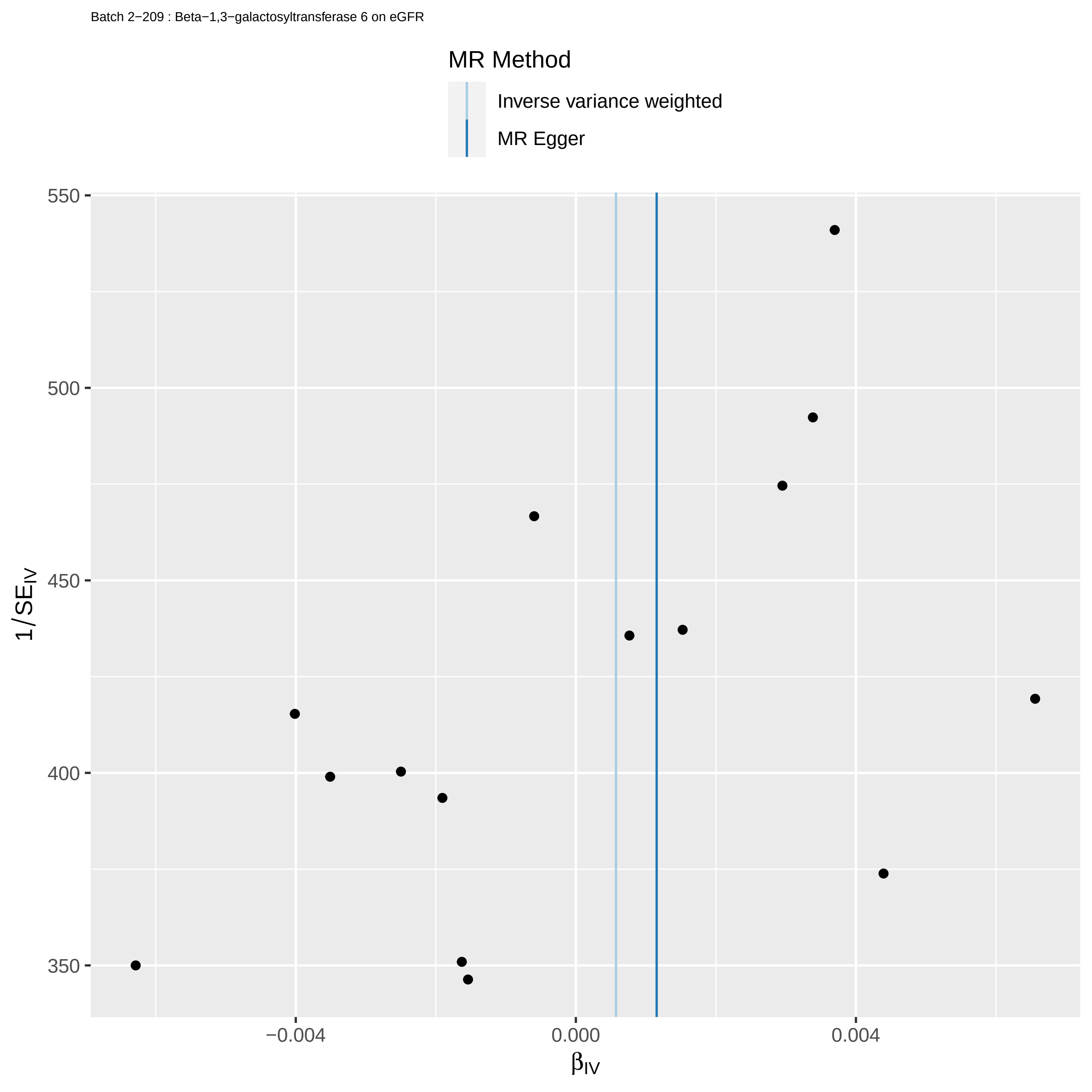
Batch 2−209 : Beta−1,3−galactosyltransferase 6 on eGFR
MR Method
Inverse variance weighted
MR Egger
550
500
450
400
350
1/SE_IV
−0.004
0.000
0.004
β_IV

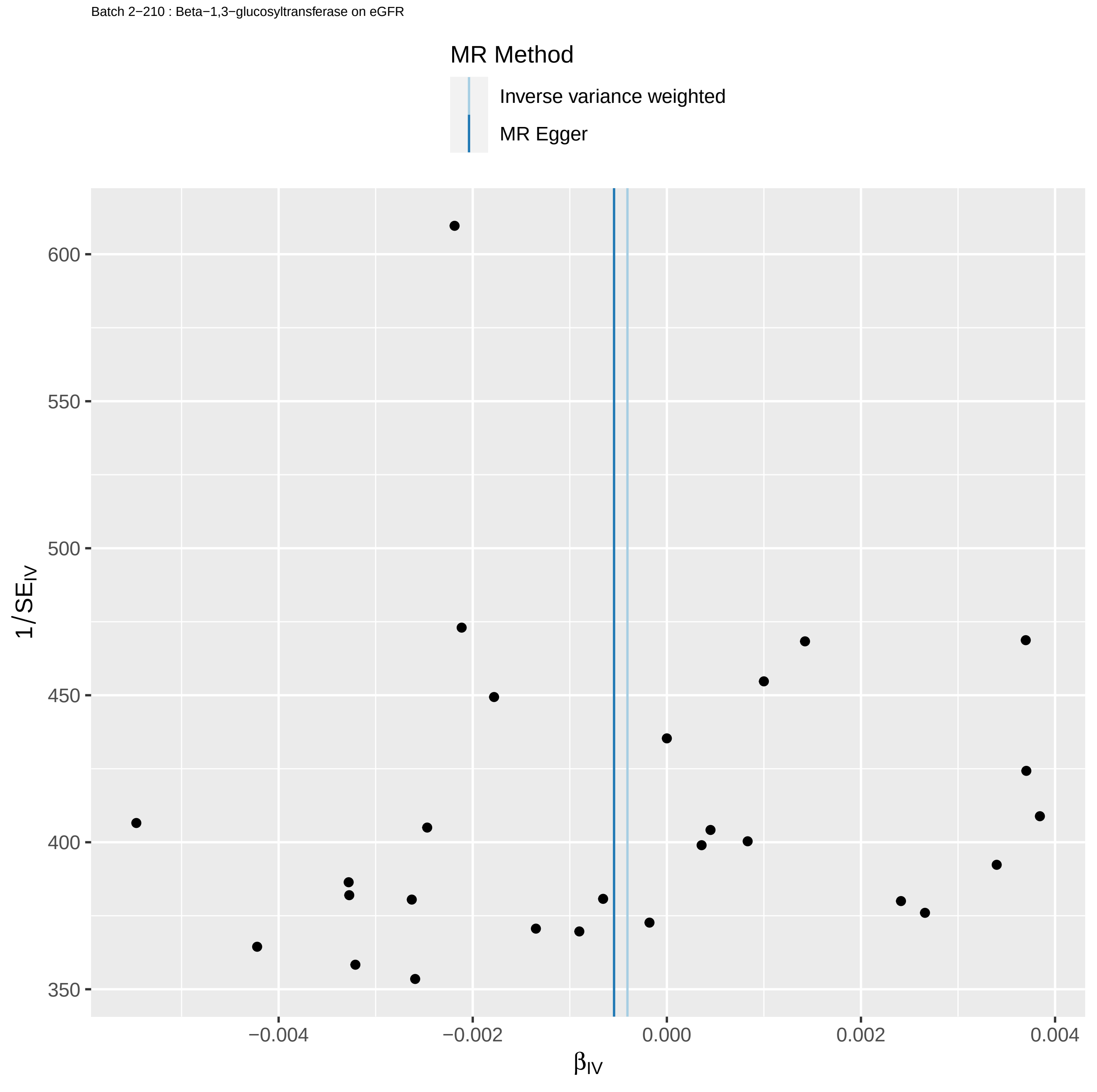
Batch 2−210 : Beta−1,3−glucosyltransferase on eGFR
MR Method
Inverse variance weighted
MR Egger
600
550
500
450
400
350
1/SE_IV
−0.004
−0.002
0.000
0.002
0.004
β_IV

Batch 2−211 : Galactosylgalactosylxylosylprotein 3−beta−glucuronosyltransferase 3 on eGFR

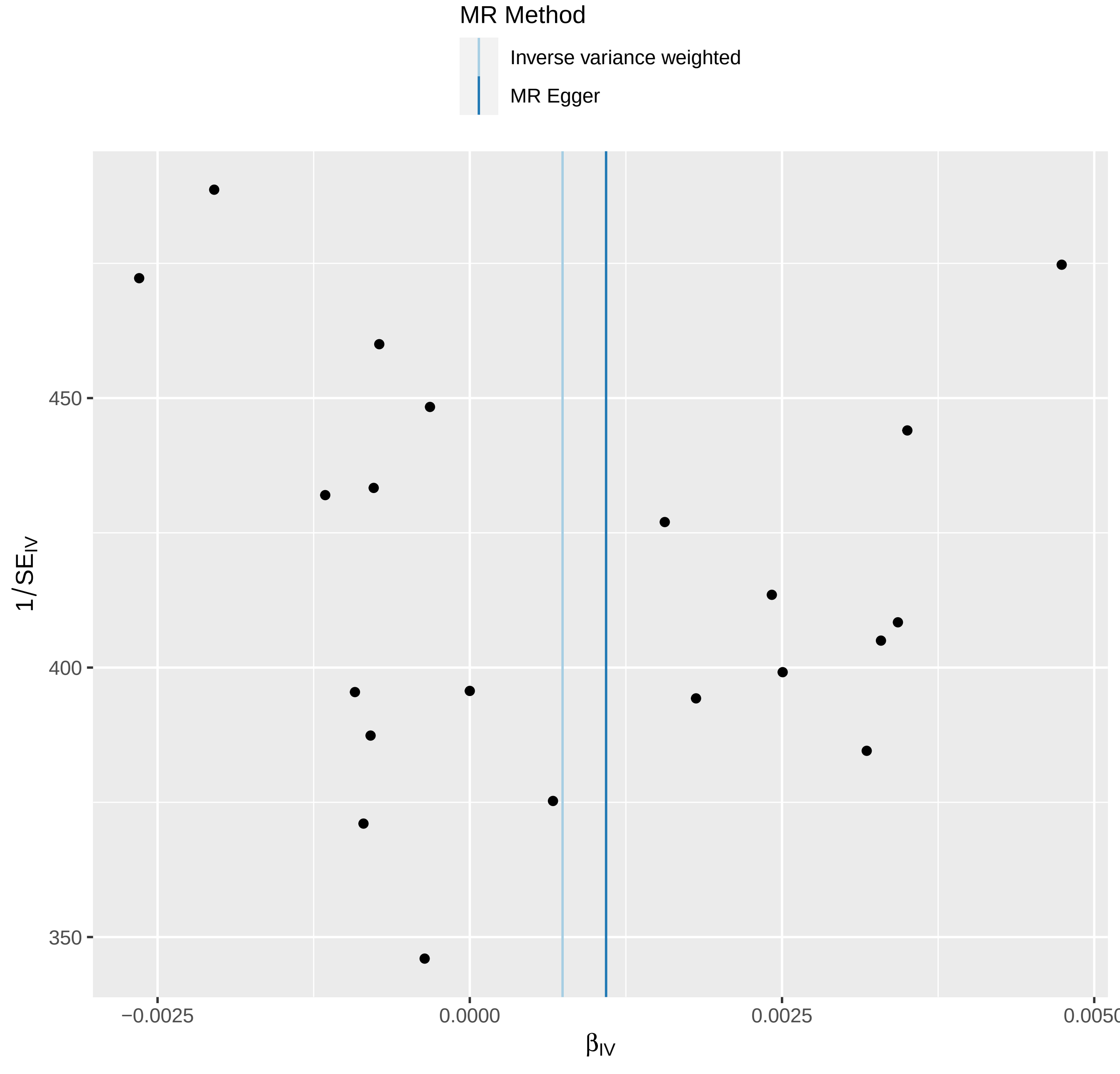

Batch 2−212 : Beta−1,4−glucuronyltransferase 1 on eGFR

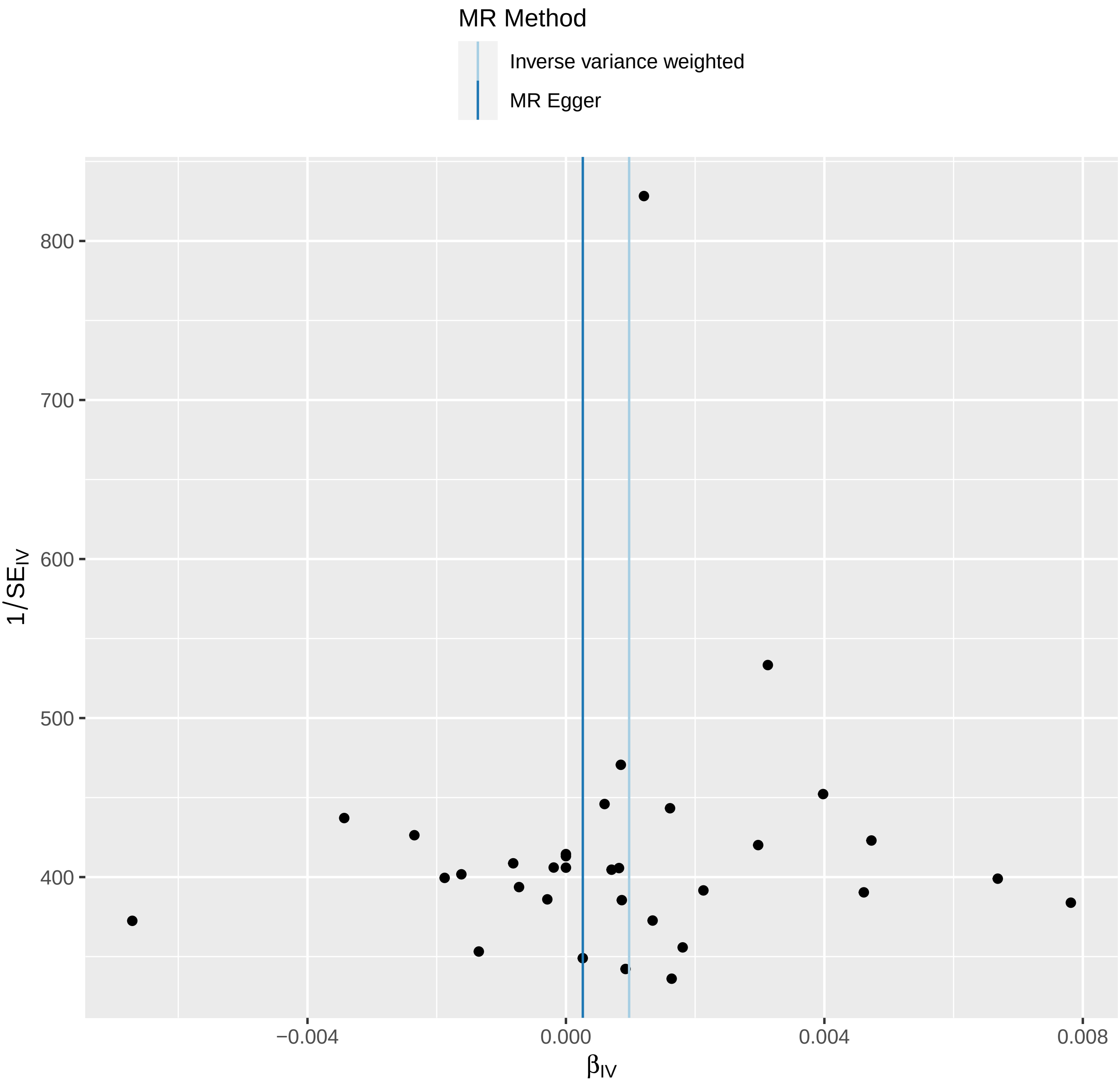

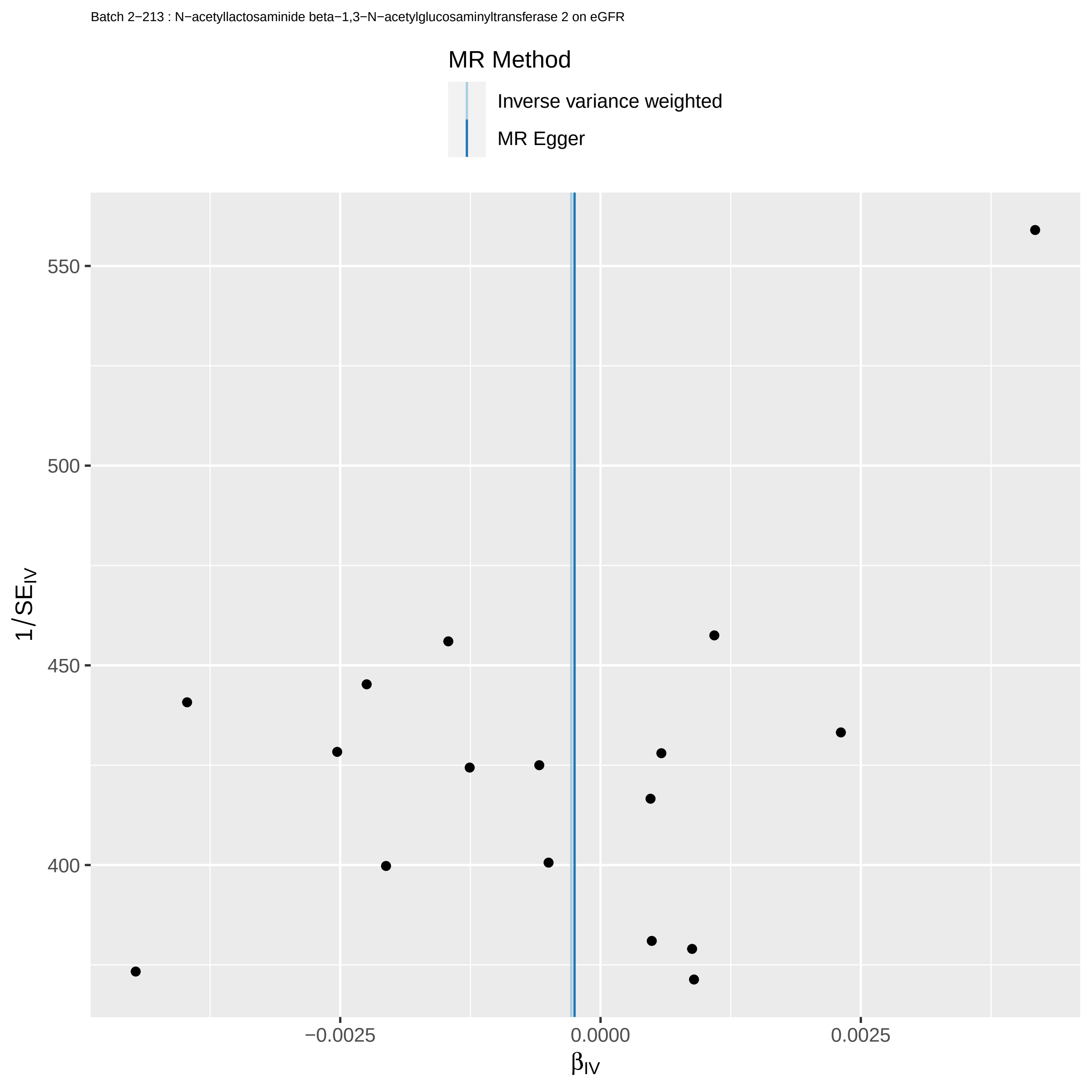

Batch 2−213 : N−acetyllactosaminide beta−1,3−N−acetylglucosaminyltransferase 2 on eGFR
MR Method
Inverse variance weighted
MR Egger
550
500
450
400
1/SE$_{IV}$
−0.0025
0.0000
0.0025
β_{IV}

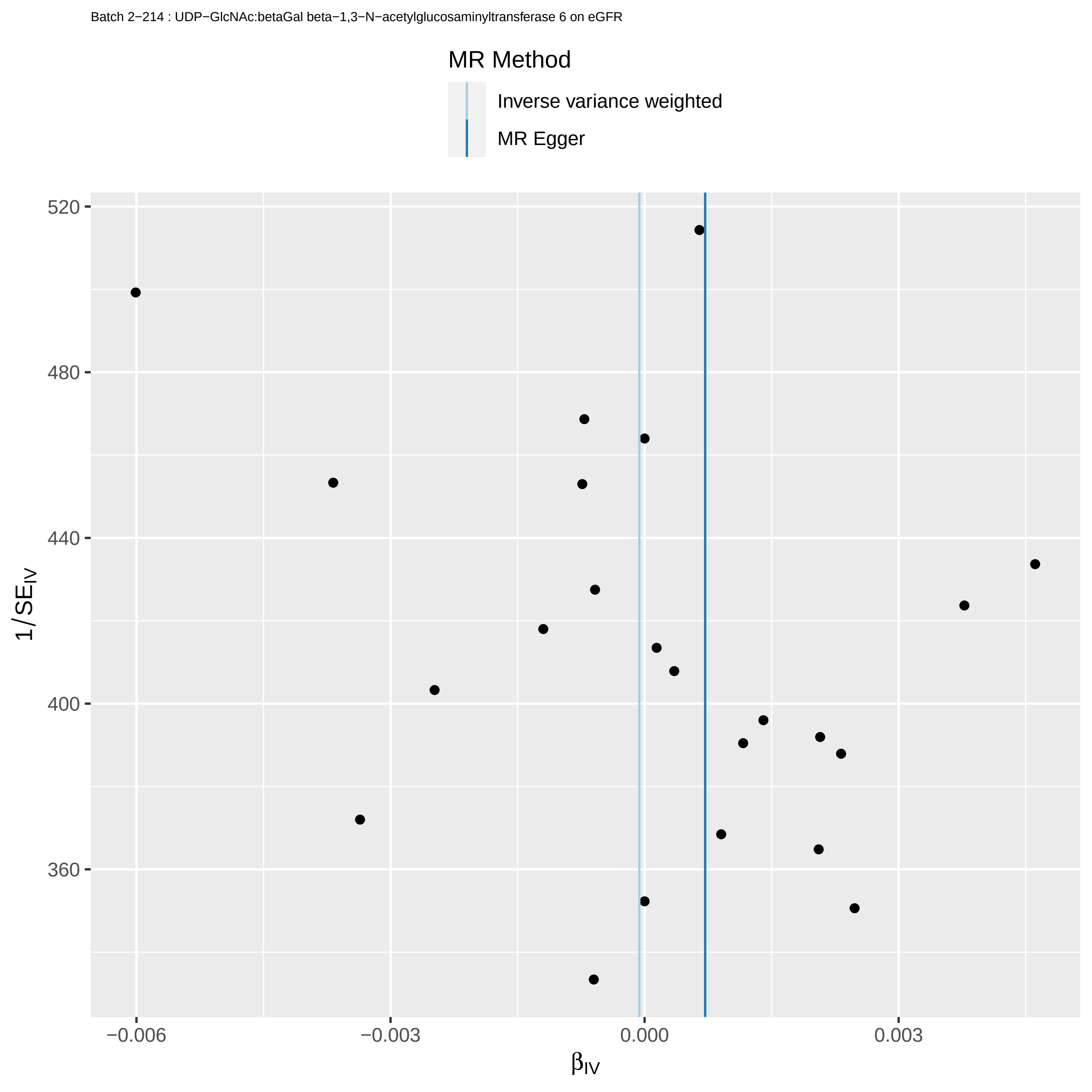

Batch 2−214 : UDP−GlcNAc:betaGal beta−1,3−N−acetylglucosaminyltransferase 6 on eGFR
MR Method
Inverse variance weighted
MR Egger
520
480
440
400
360
1/SE_IV
−0.006
−0.003
0.000
0.003
β_IV

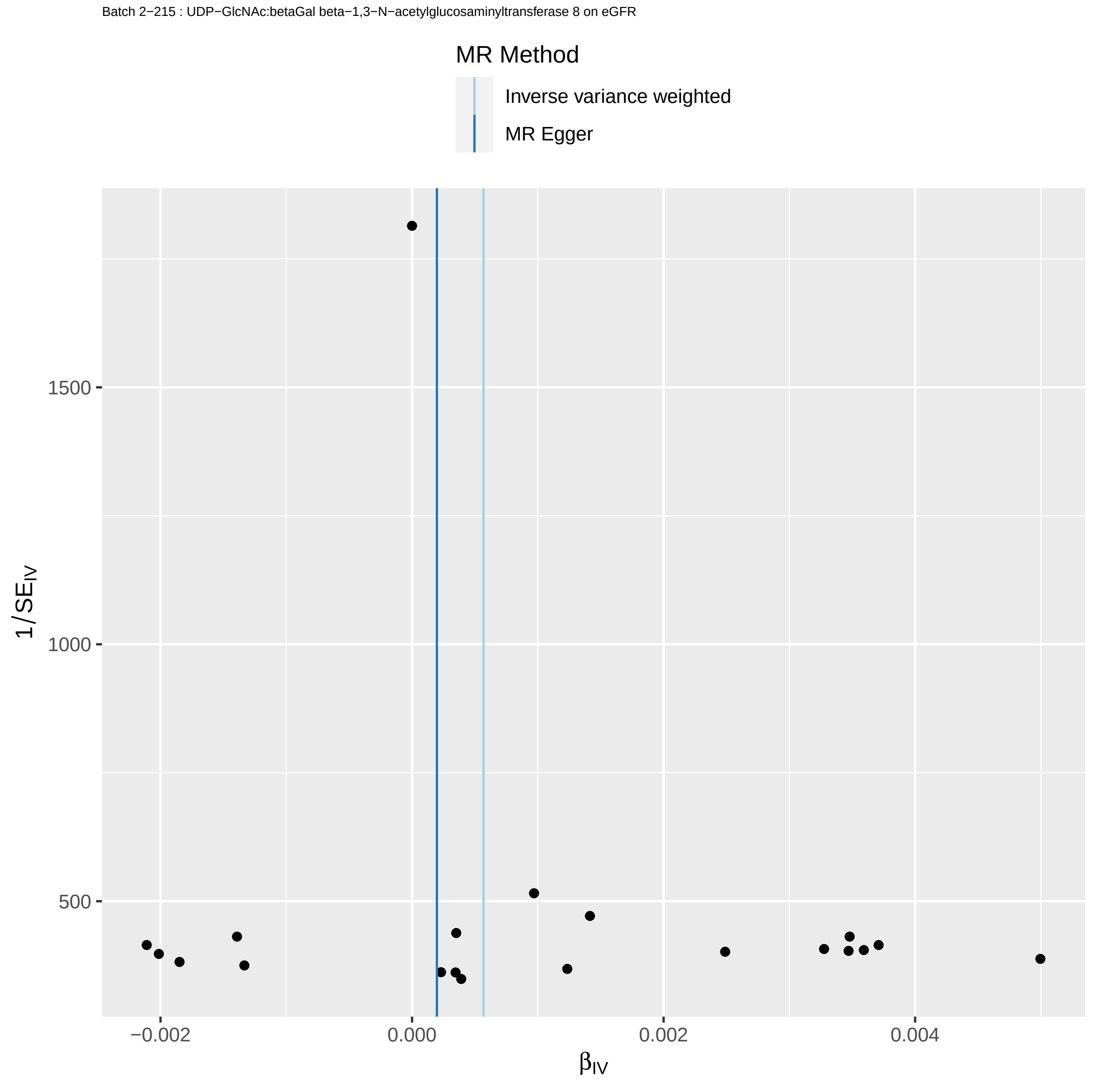

Batch 2−215 : UDP−GlcNAc:betaGal beta−1,3−N−acetylglucosaminyltransferase 8 on eGFR
MR Method
Inverse variance weighted
MR Egger
1500
1000
500
1/SE$_{IV}$
−0.002
0.000
0.002
0.004
β$_{IV}$

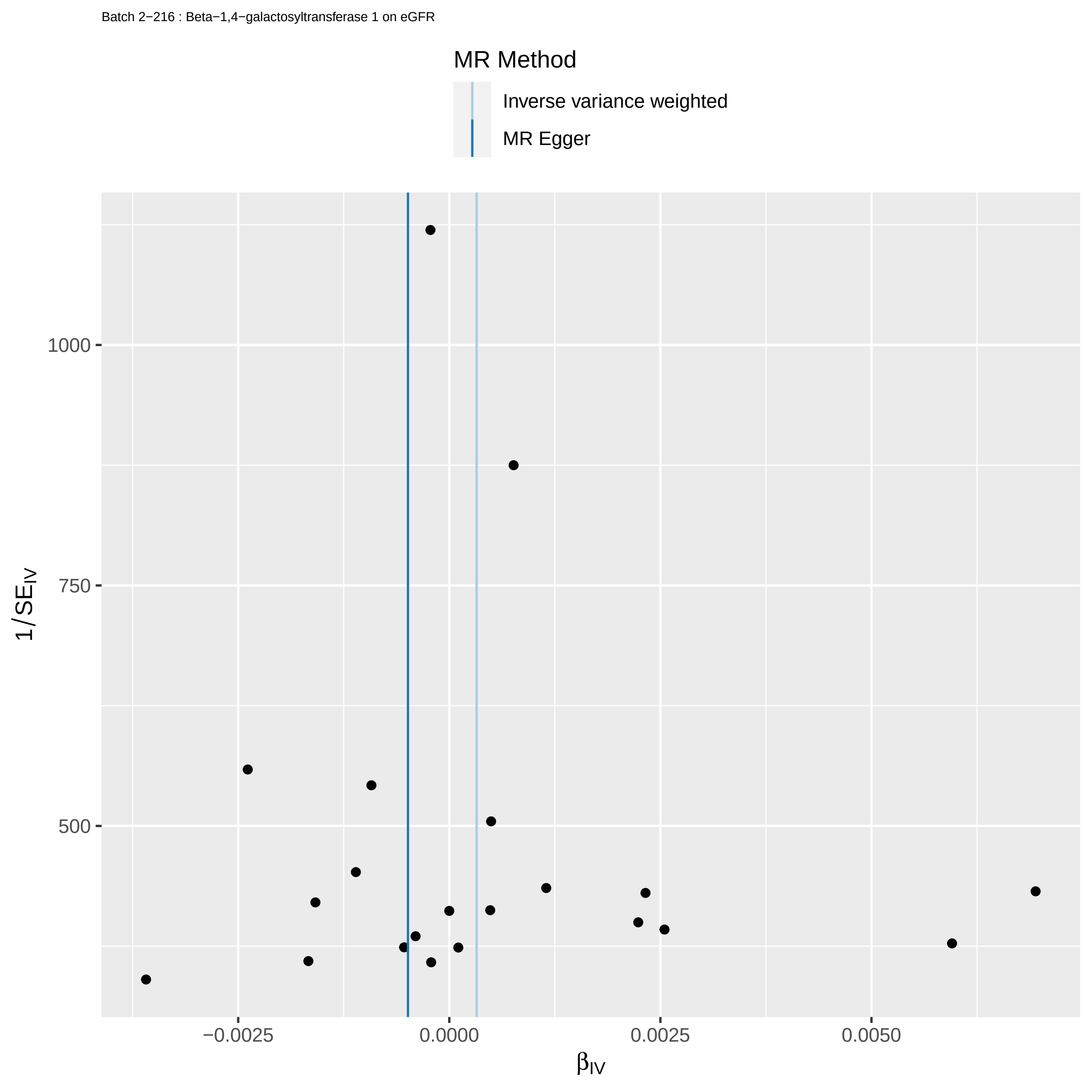
Batch 2−216 : Beta−1,4−galactosyltransferase 1 on eGFR
MR Method
Inverse variance weighted
MR Egger
1000
750
500
1/SE$_{IV}$
−0.0025
0.0000
0.0025
0.0050
β$_{IV}$

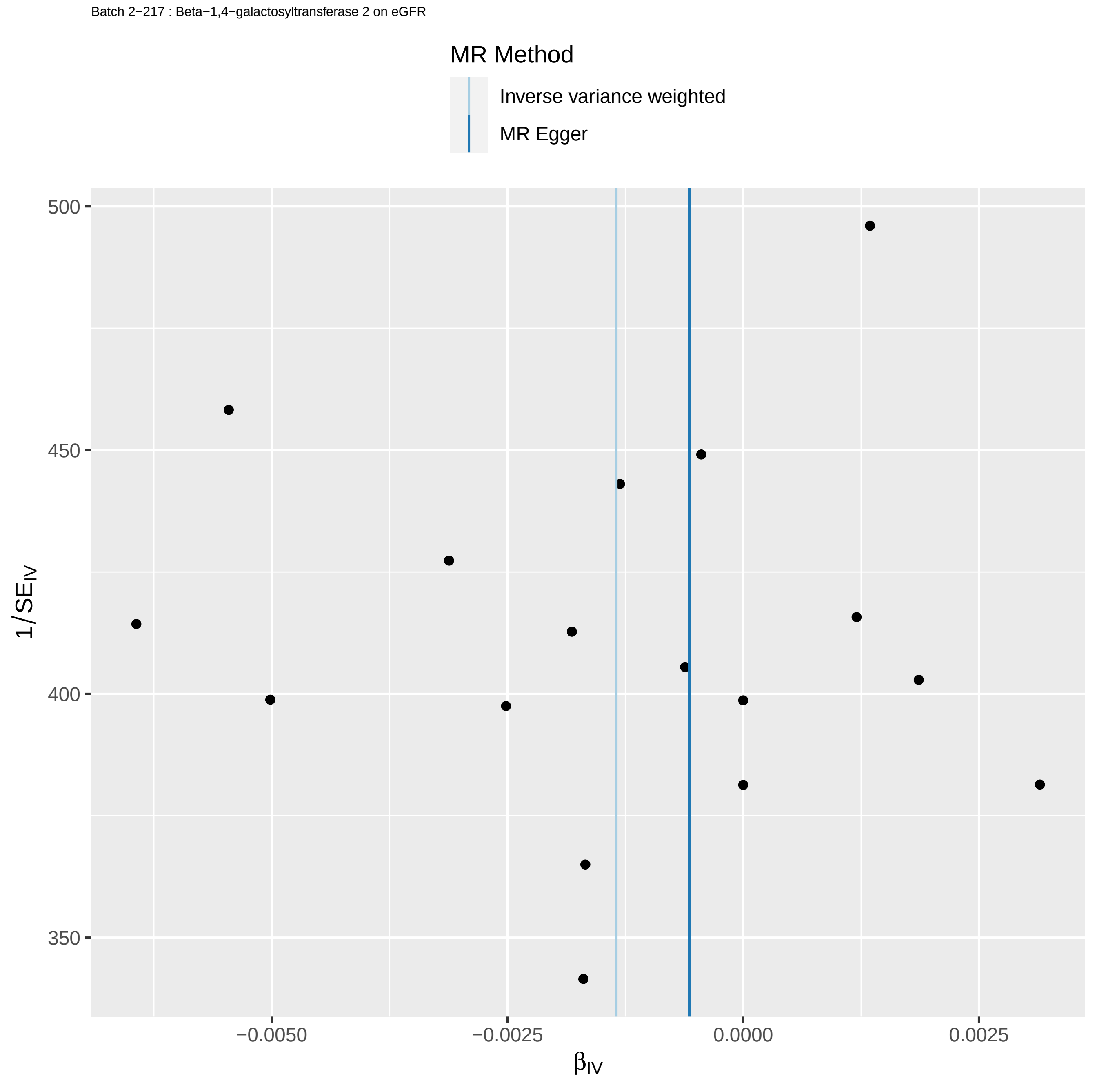

Batch 2−217 : Beta−1,4−galactosyltransferase 2 on eGFR
MR Method
Inverse variance weighted
MR Egger
500
450
400
350
1/SE_IV
−0.0050
−0.0025
0.0000
0.0025
β_IV

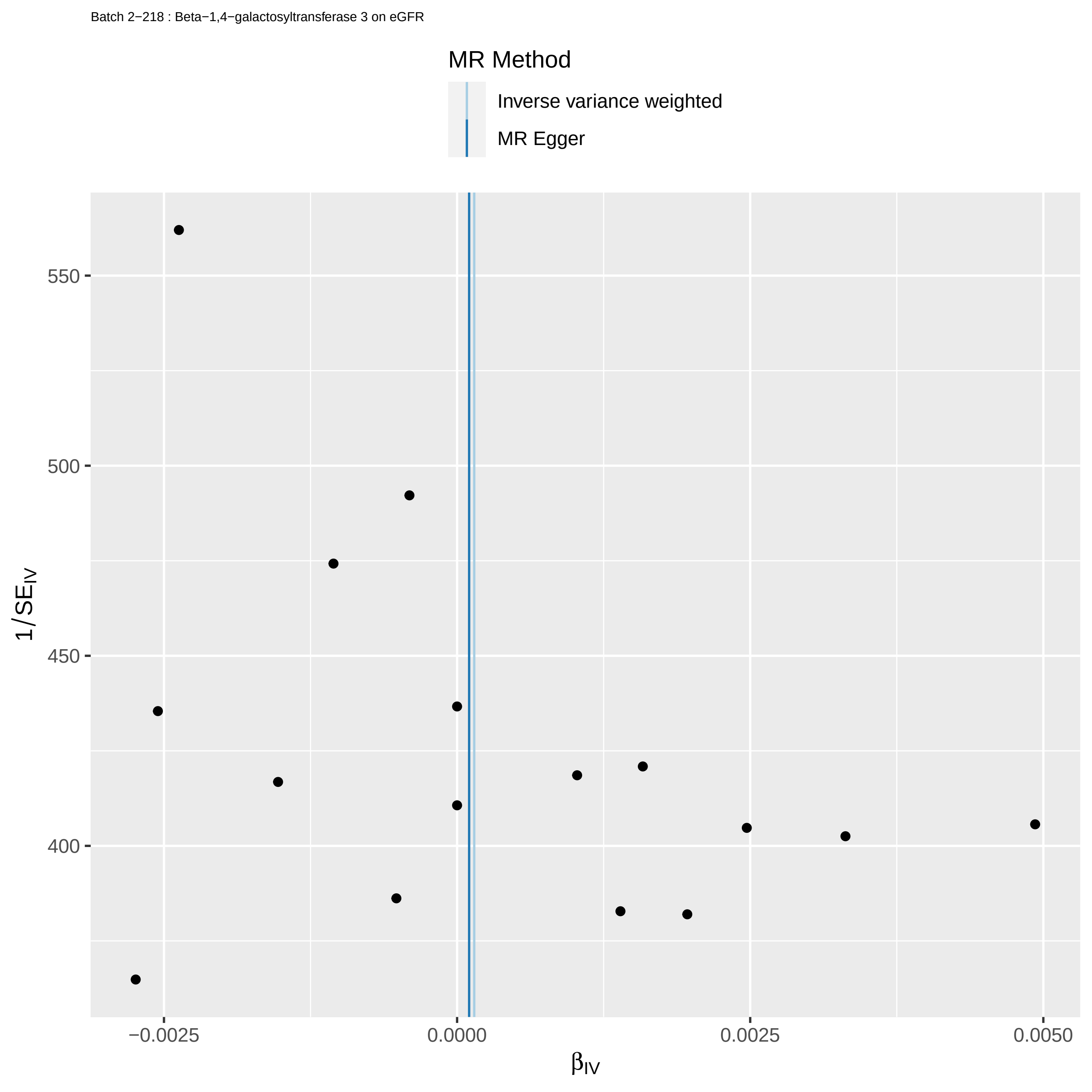

Batch 2−218 : Beta−1,4−galactosyltransferase 3 on eGFR
MR Method
Inverse variance weighted
MR Egger
550
500
450
400
1/SE$_{IV}$
−0.0025
0.0000
0.0025
0.0050
β$_{IV}$

Batch 2−219 : Beta−1,4−galactosyltransferase 5 on eGFR

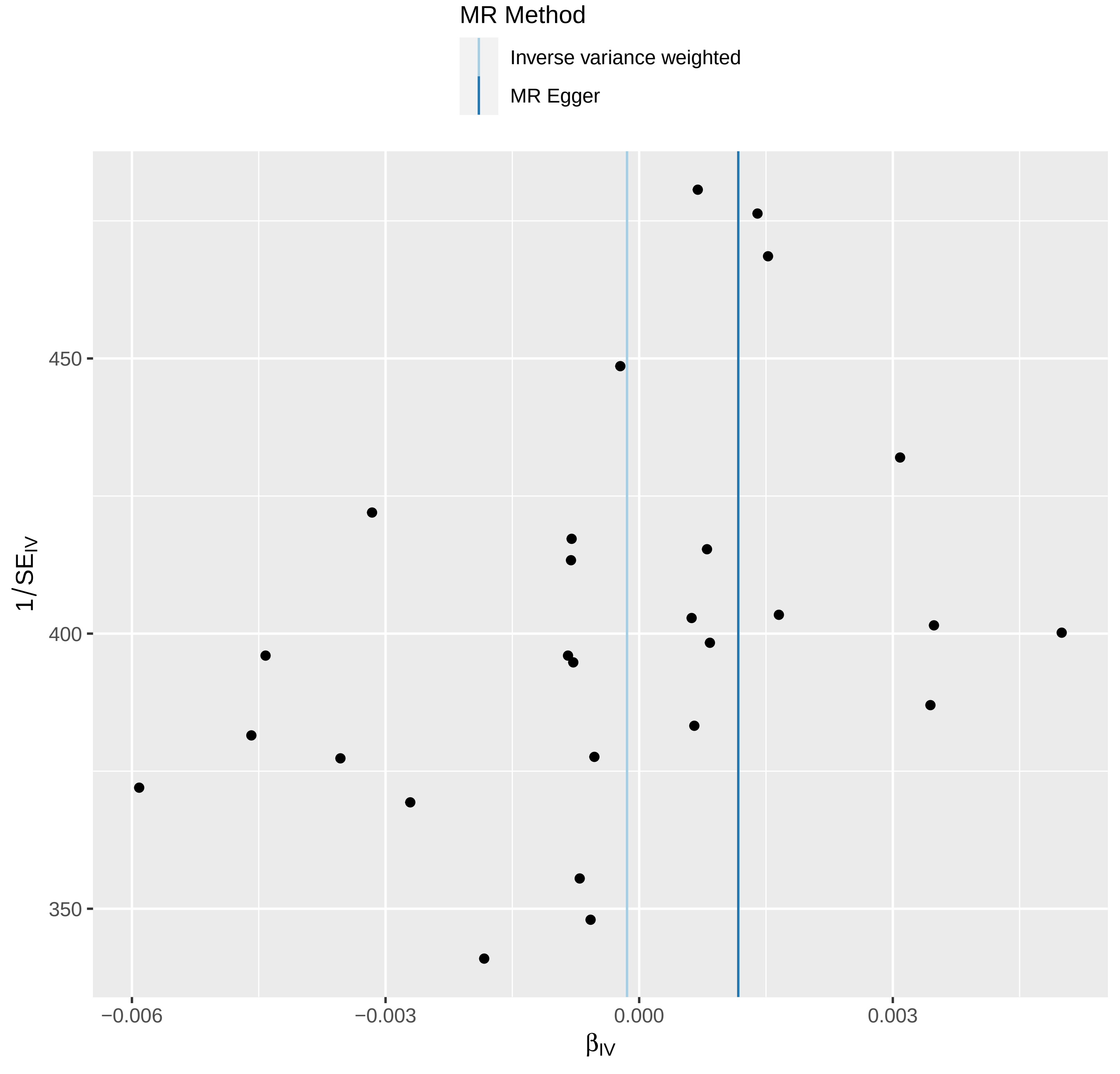

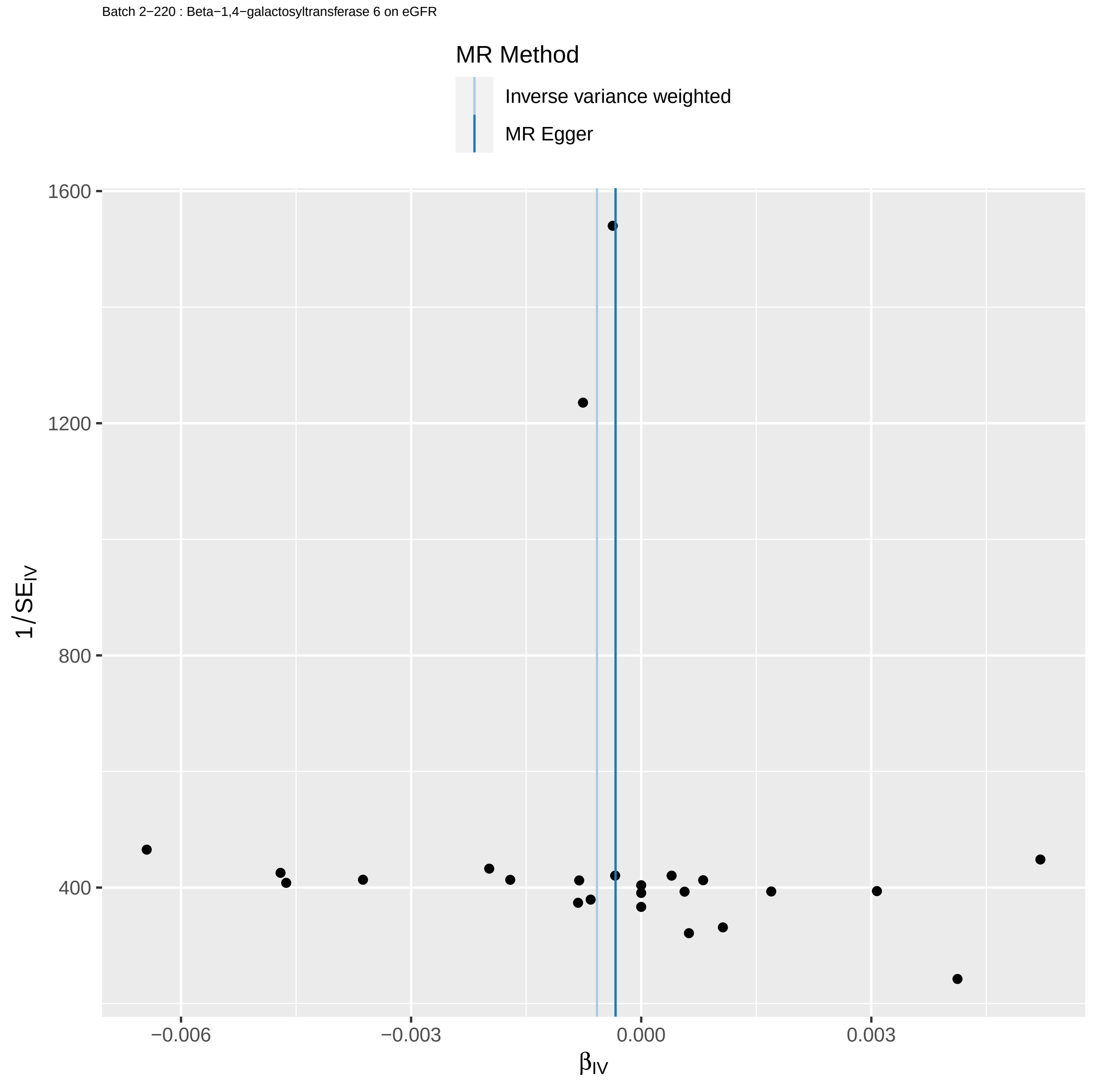
Batch 2−220 : Beta−1,4−galactosyltransferase 6 on eGFR
MR Method
Inverse variance weighted
MR Egger
1600
1200
800
400
1/SE$_{IV}$
−0.006
−0.003
0.000
0.003
β$_{IV}$

Batch 2−221 : Beta−1,4−galactosyltransferase 7 on eGFR

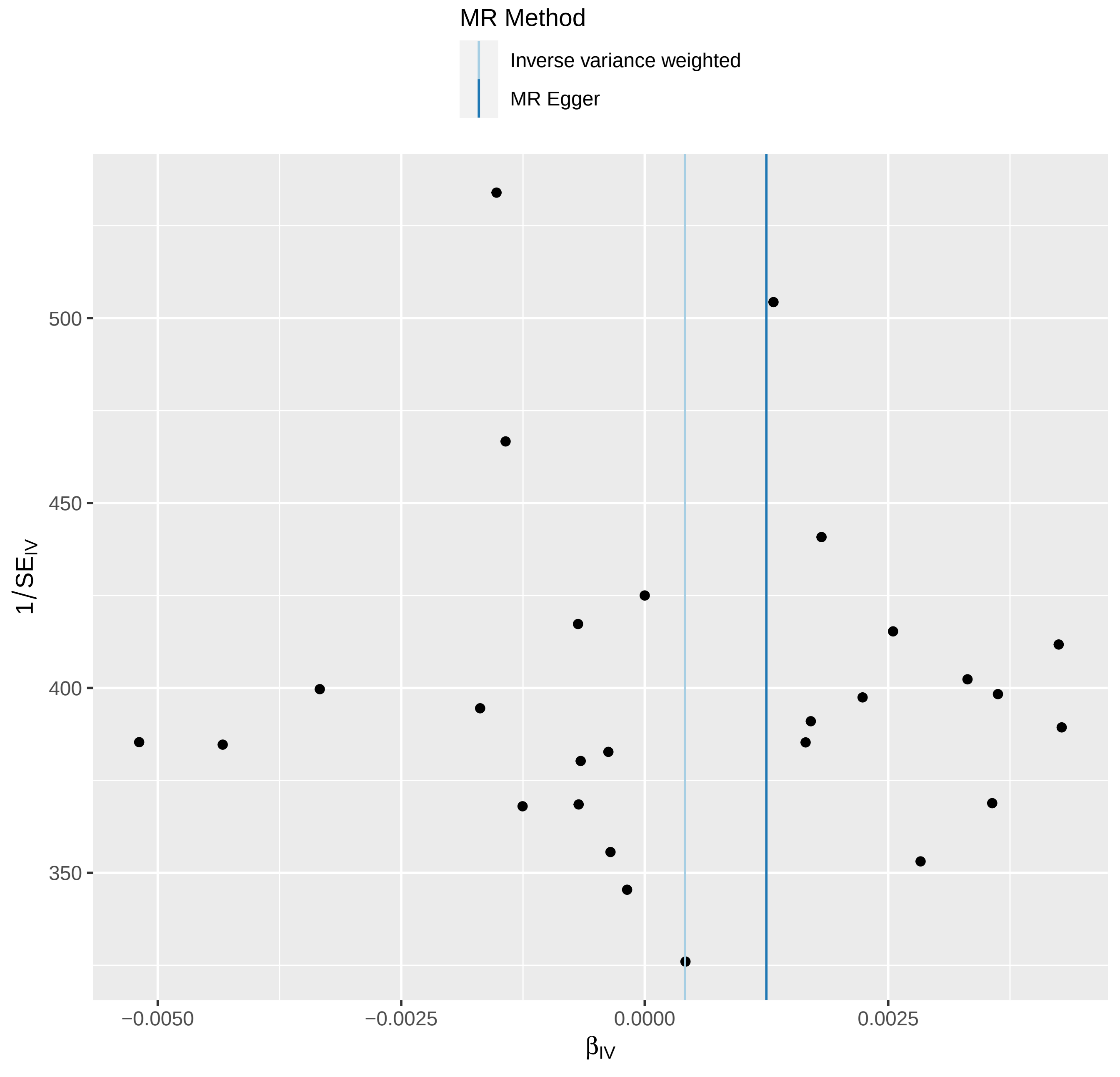

Batch 2−222 : Transcription regulator protein BACH1 on eGFR

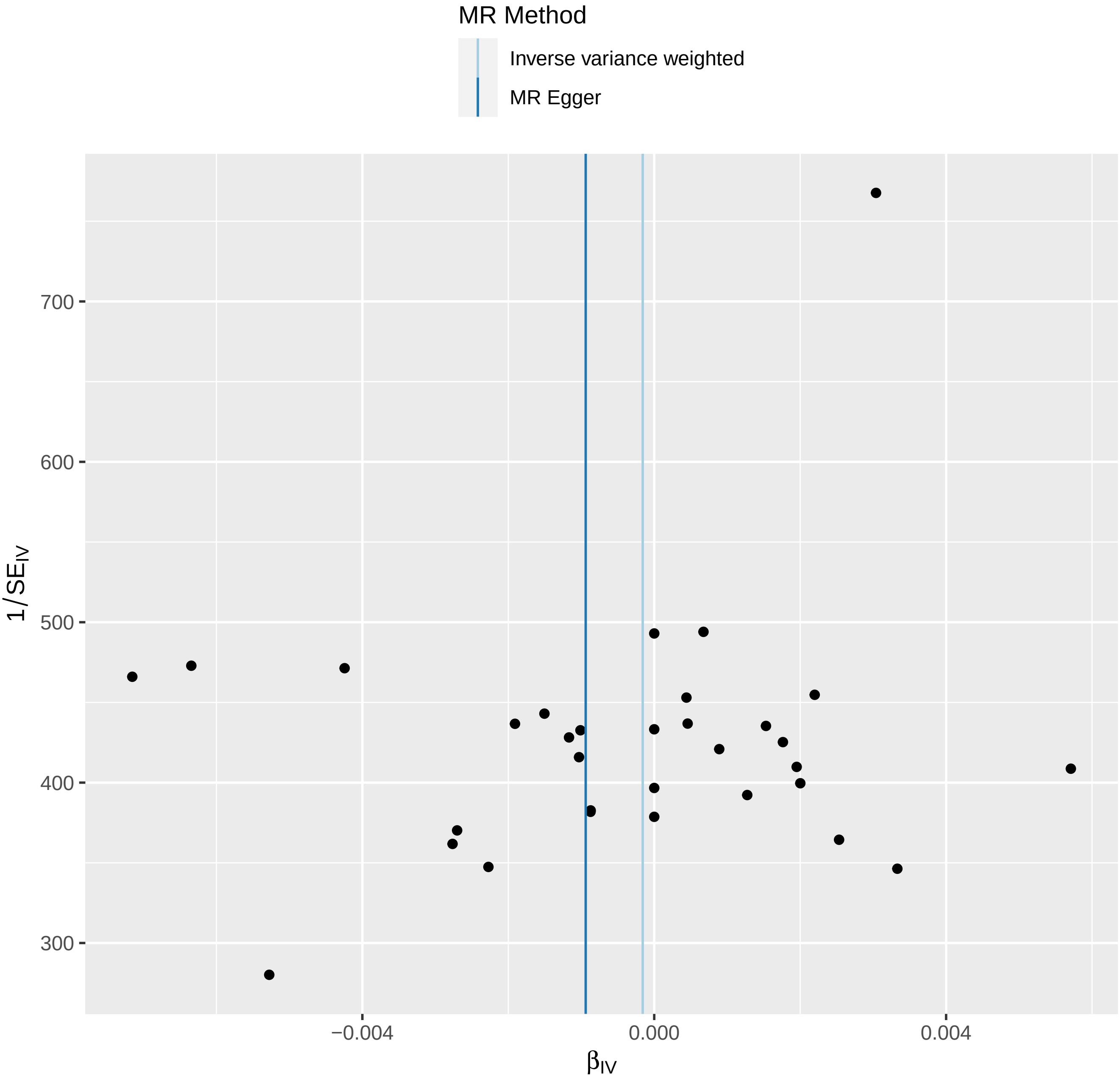

Batch 2−223 : Transcription regulator protein BACH2 on eGFR

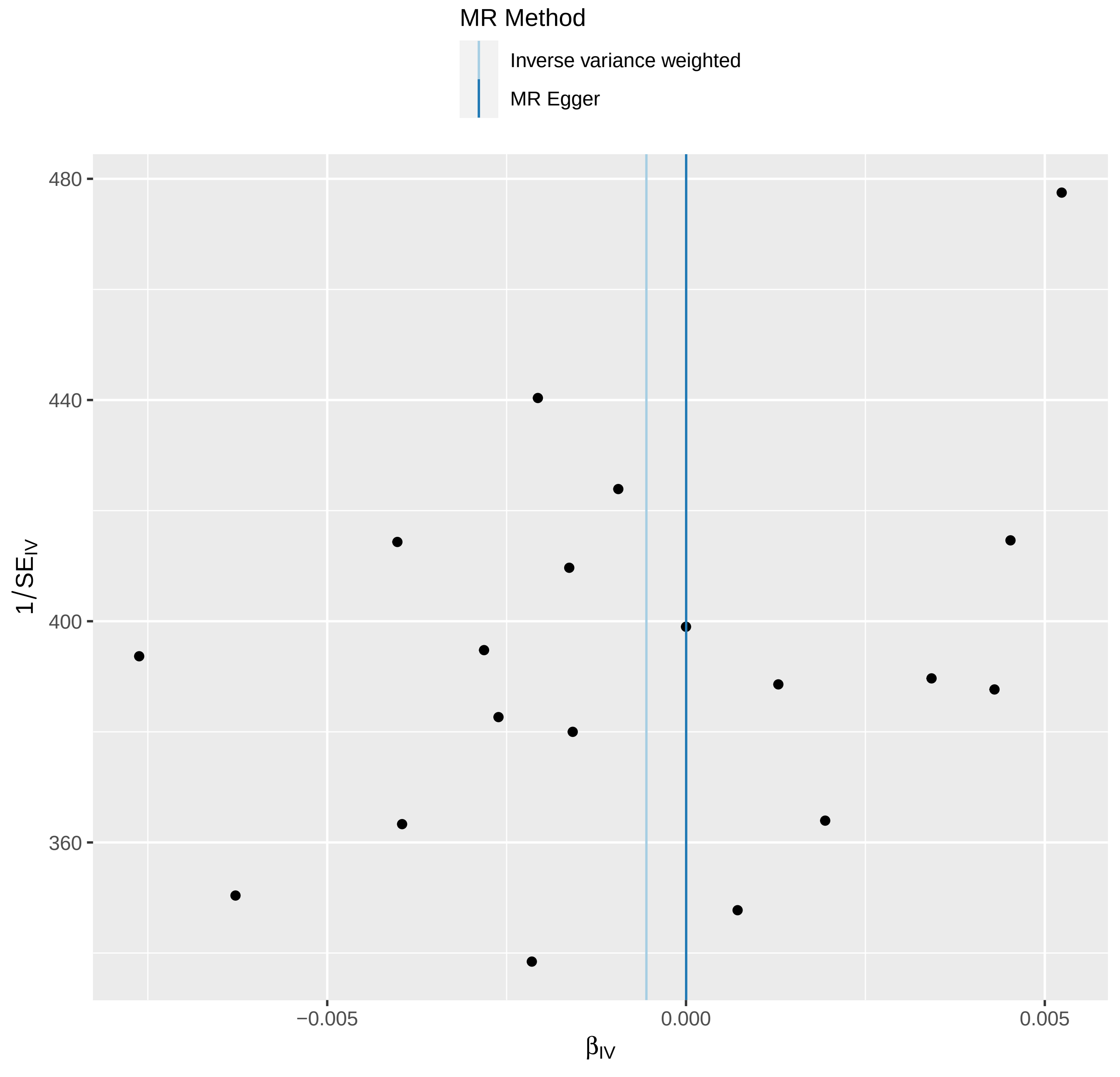

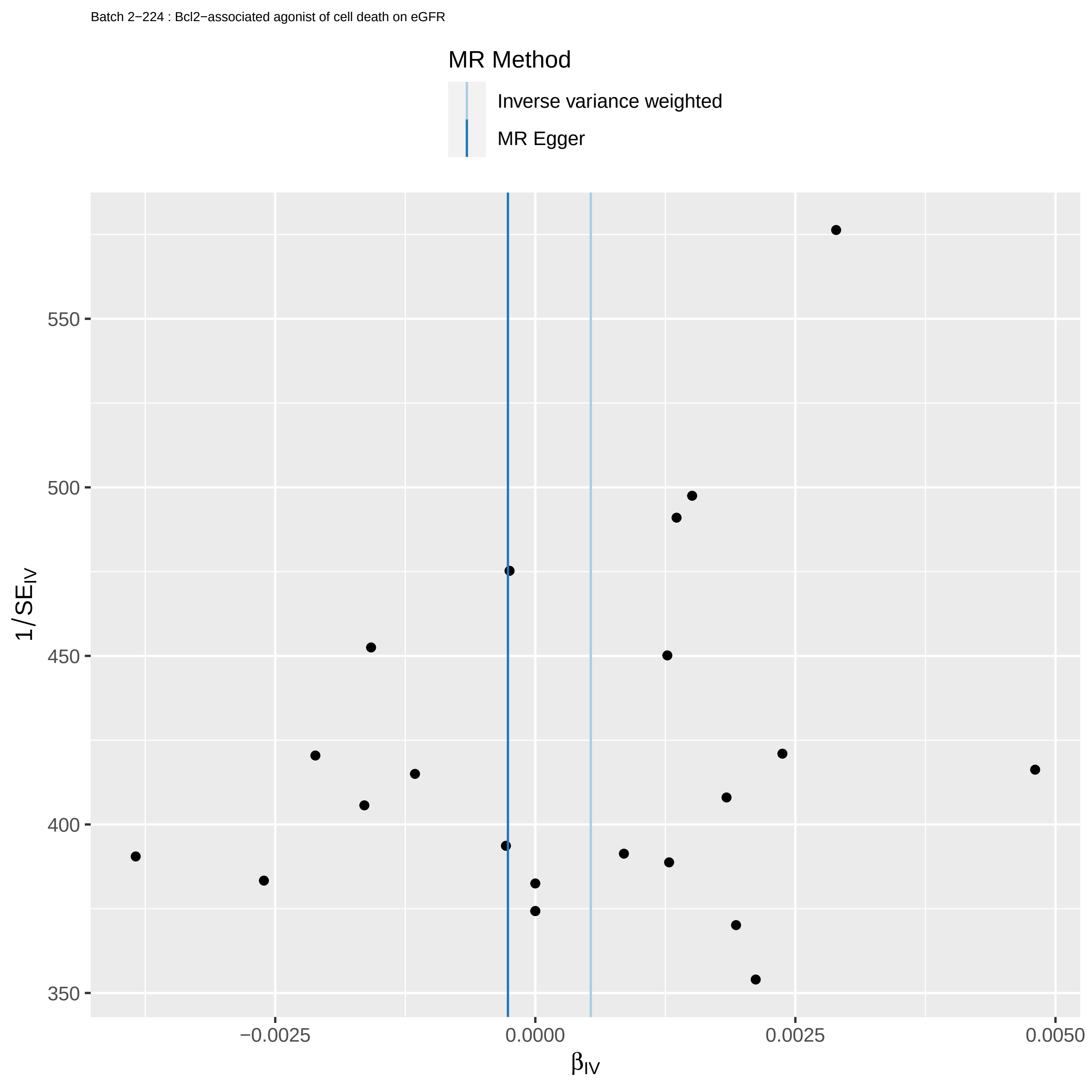

Batch 2−224 : Bcl2−associated agonist of cell death on eGFR
MR Method
Inverse variance weighted
MR Egger
550
500
450
400
350
1/SE_IV
−0.0025
0.0000
0.0025
0.0050
β_IV

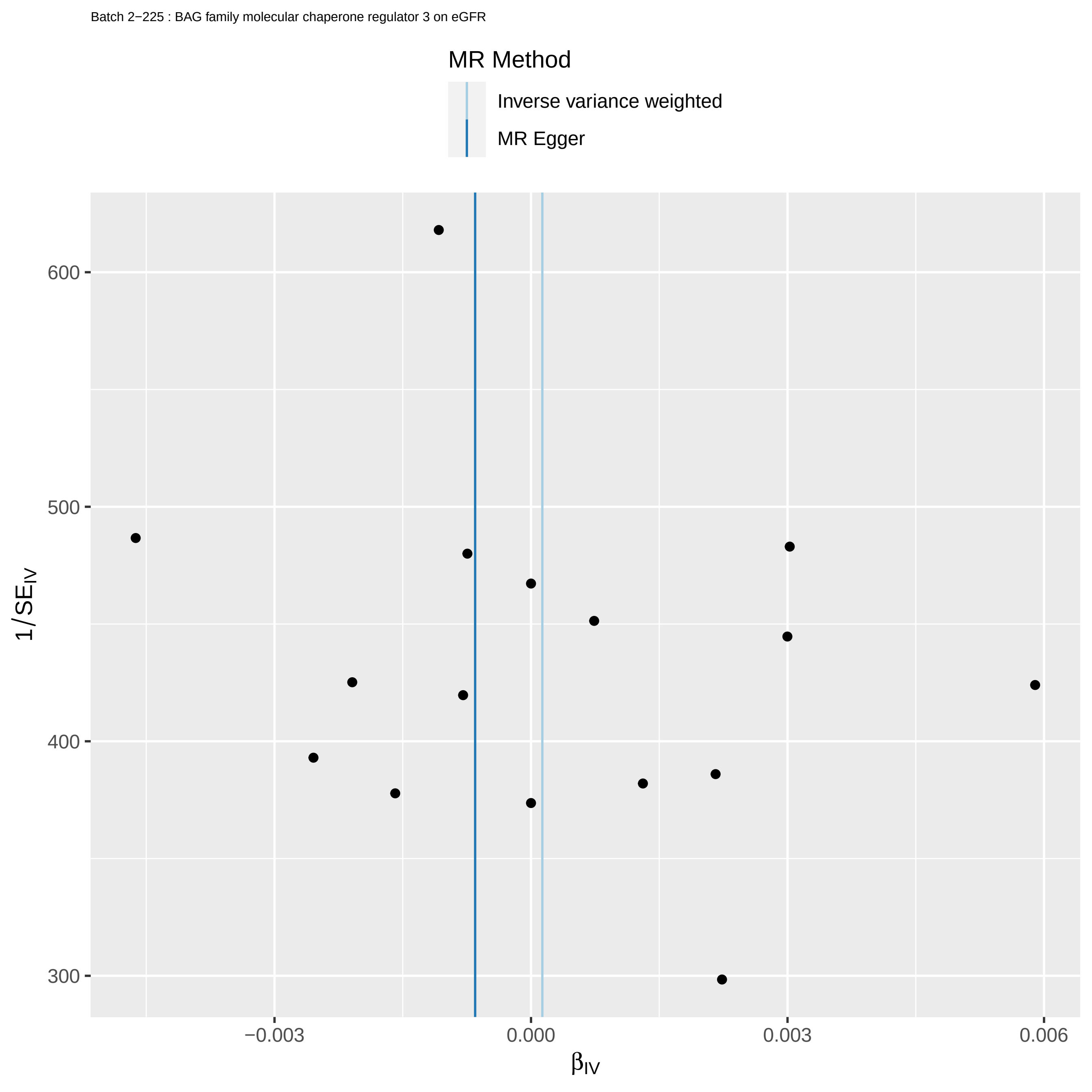
Batch 2−225 : BAG family molecular chaperone regulator 3 on eGFR
MR Method
Inverse variance weighted
MR Egger
600
500
400
300
1/SE_IV
−0.003
0.000
0.003
0.006
β_IV

Batch 2−226 : BAG family molecular chaperone regulator 4 on eGFR

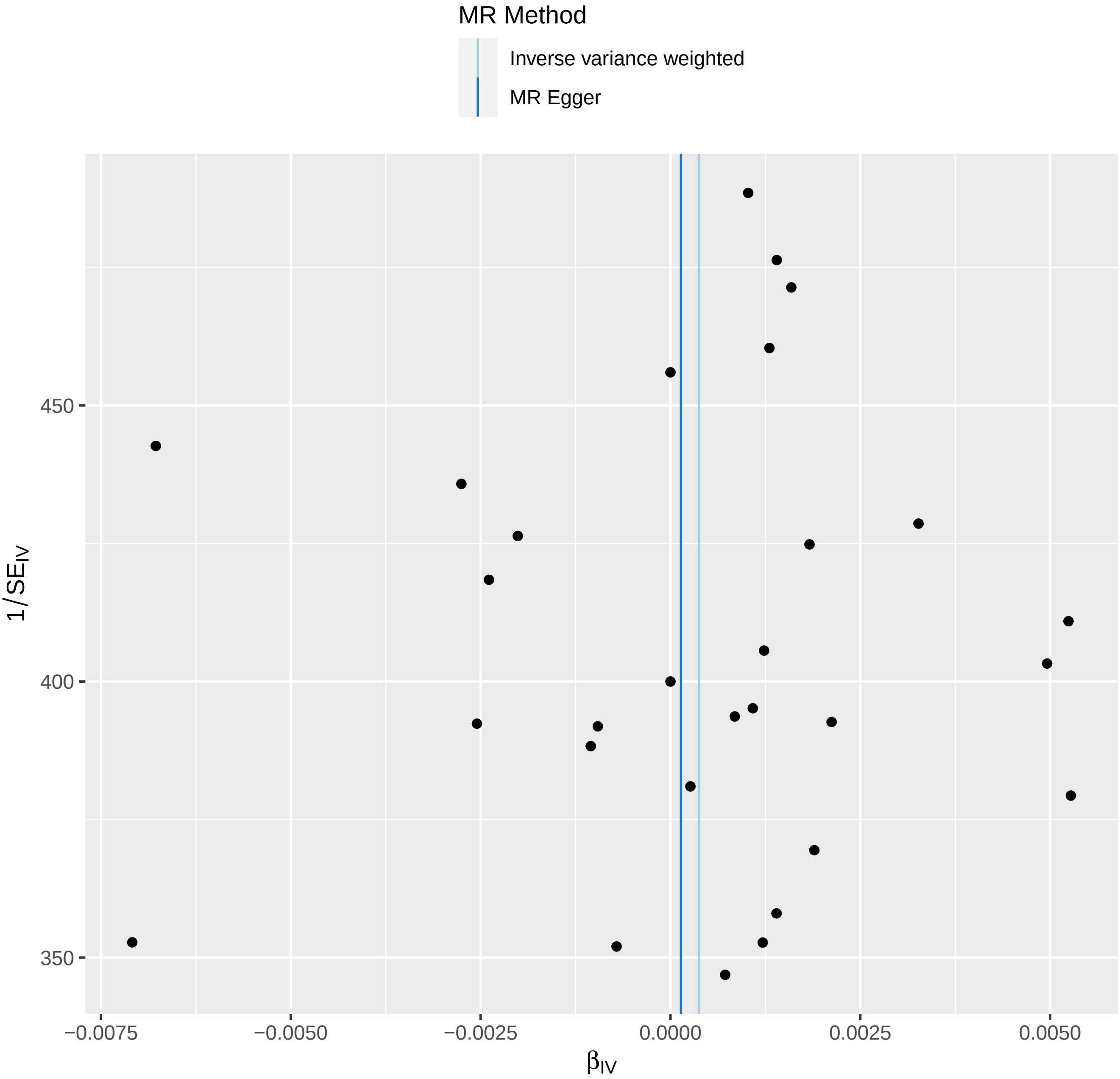

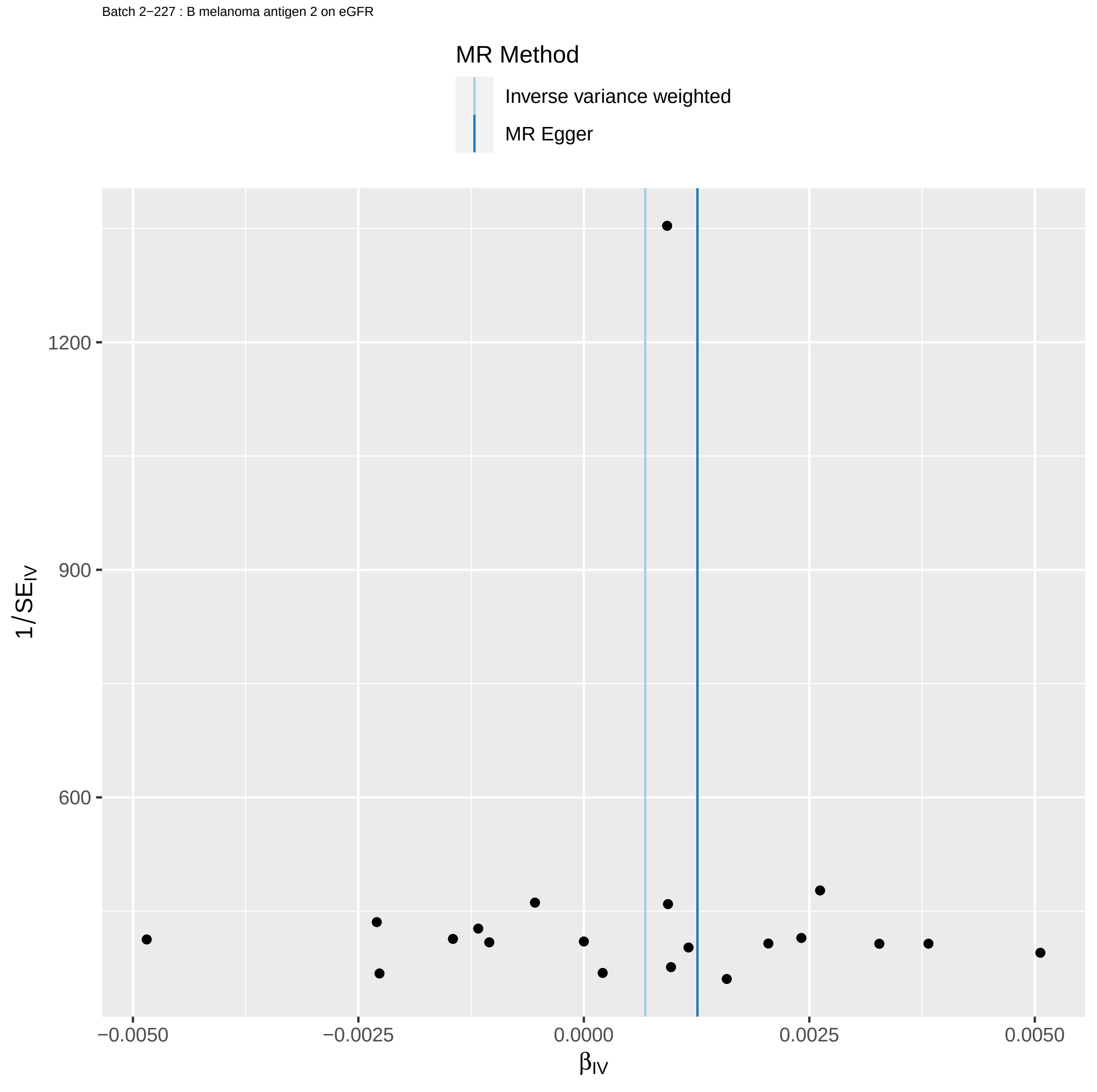

Batch 2−227 : B melanoma antigen 2 on eGFR
MR Method
Inverse variance weighted
MR Egger
1200
900
600
$1/SE_{IV}$
−0.0050
−0.0025
0.0000
0.0025
0.0050
β_{IV}

Batch 2−228 : B melanoma antigen 3 on eGFR

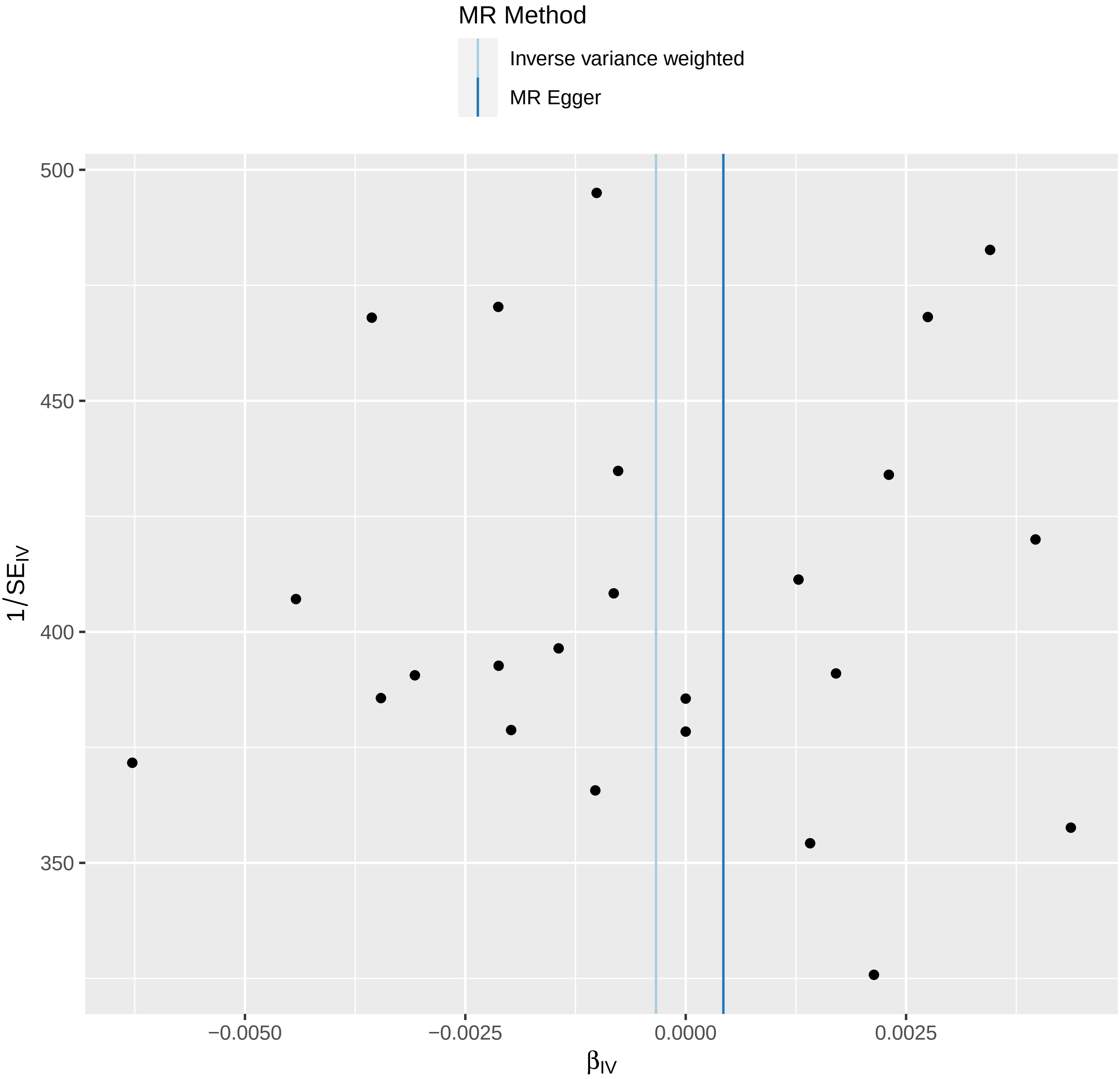

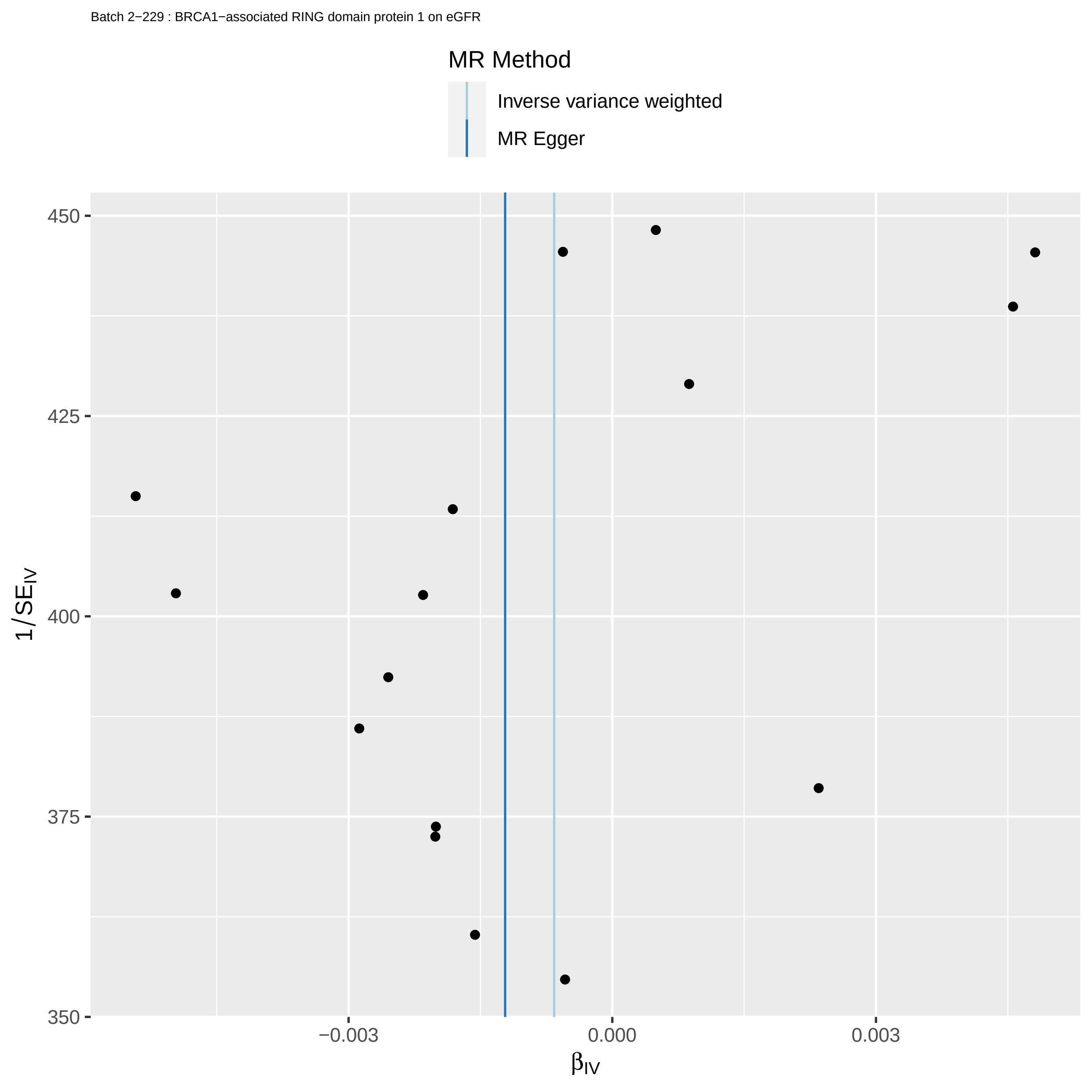
Batch 2−229 : BRCA1−associated RING domain protein 1 on eGFR
MR Method
Inverse variance weighted
MR Egger
450
425
400
375
350
1/SE$_{IV}$
−0.003
0.000
0.003
β$_{IV}$

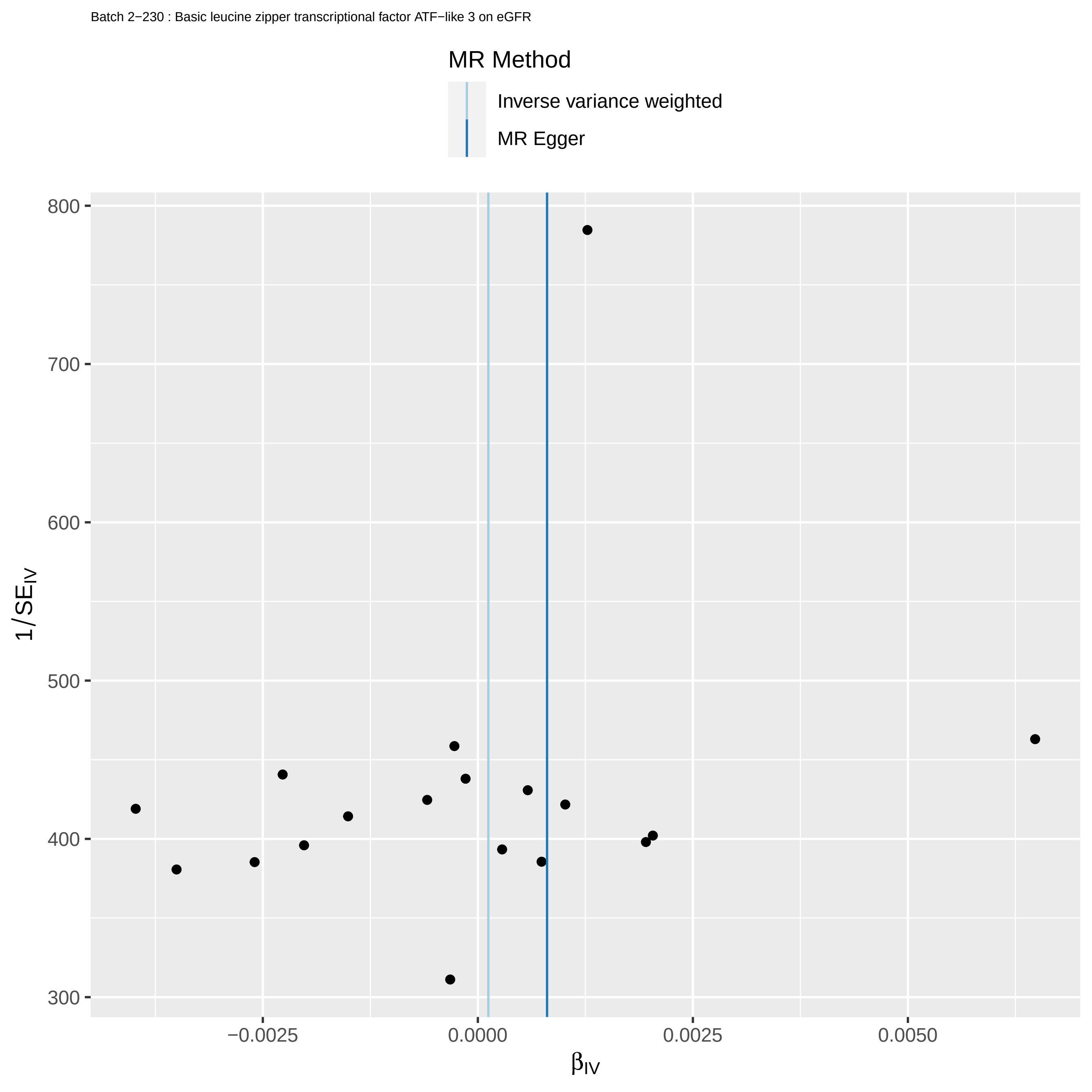

Batch 2−230 : Basic leucine zipper transcriptional factor ATF−like 3 on eGFR
MR Method
Inverse variance weighted
MR Egger
800
700
600
500
400
300
−0.0025
0.0000
0.0025
0.0050
1/SE_IV
β_IV

Batch 2−231 : Basal Cell Adhesion Molecule on eGFR

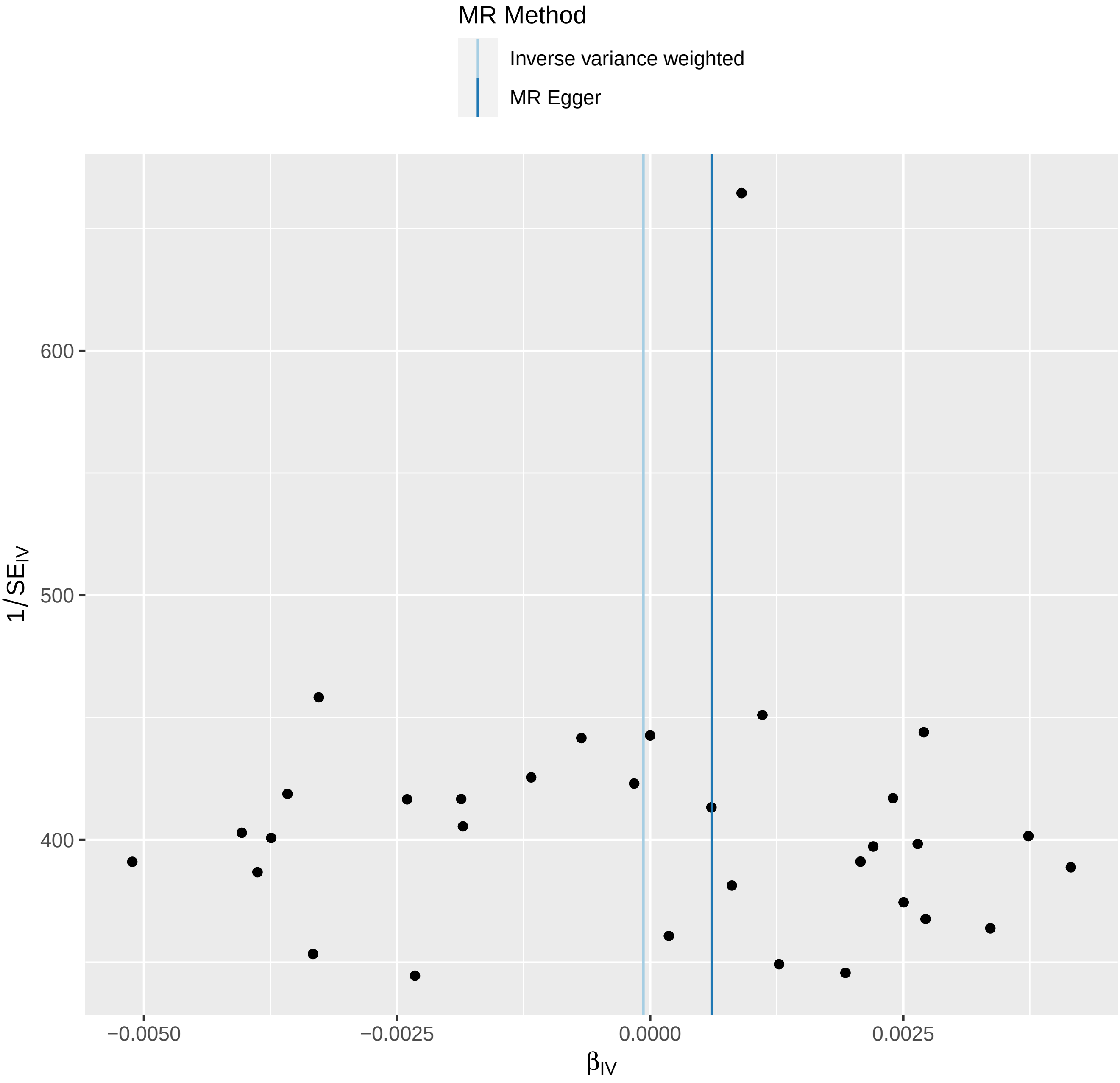

Batch 2−232 : Brevican core protein on eGFR

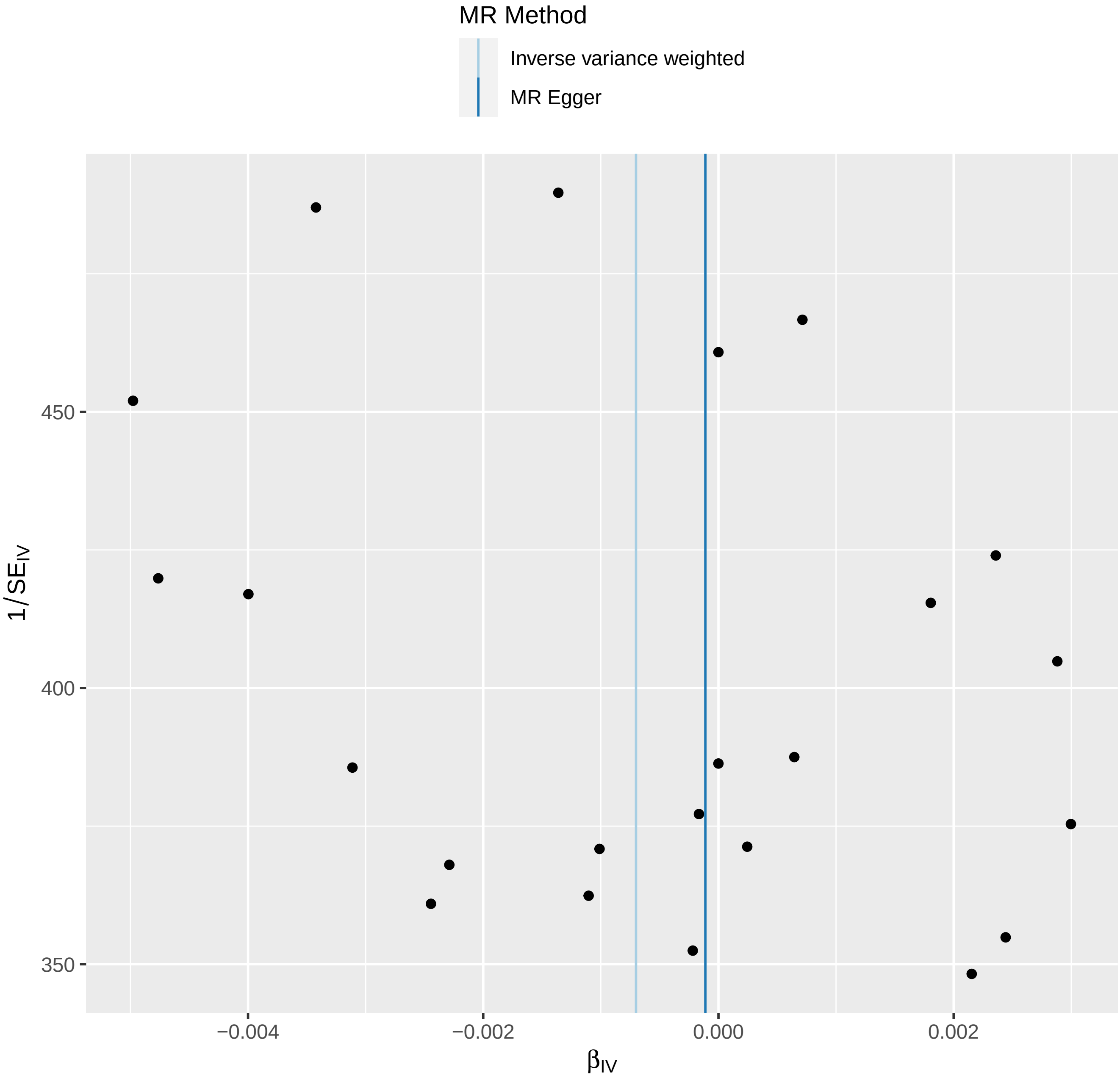

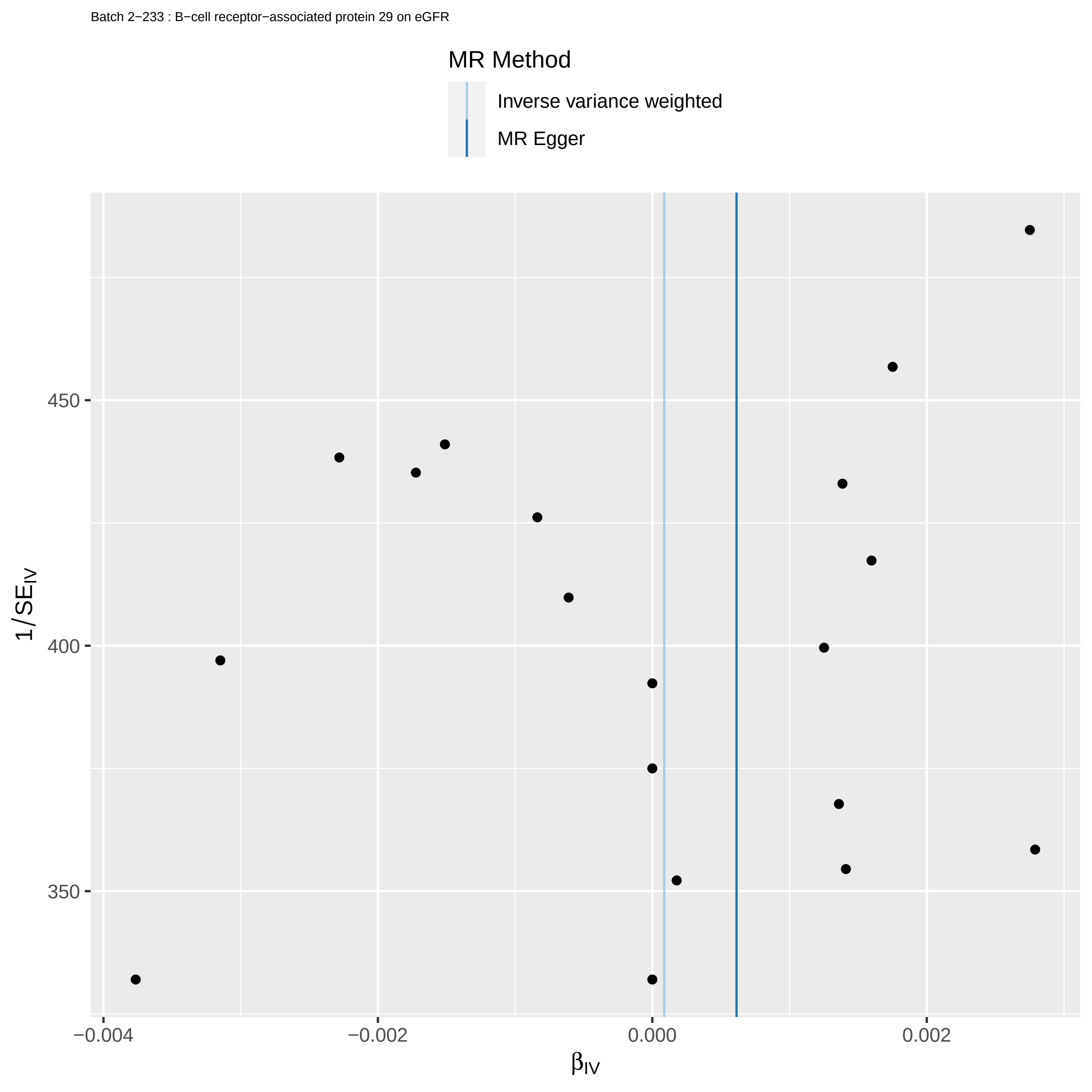
Batch 2−233 : B−cell receptor−associated protein 29 on eGFR
MR Method
Inverse variance weighted
MR Egger
450
400
350
−0.004
−0.002
0.000
0.002
1/SE_IV
β_IV

Batch 2−234 : Breast cancer anti−estrogen resistance protein 3 on eGFR

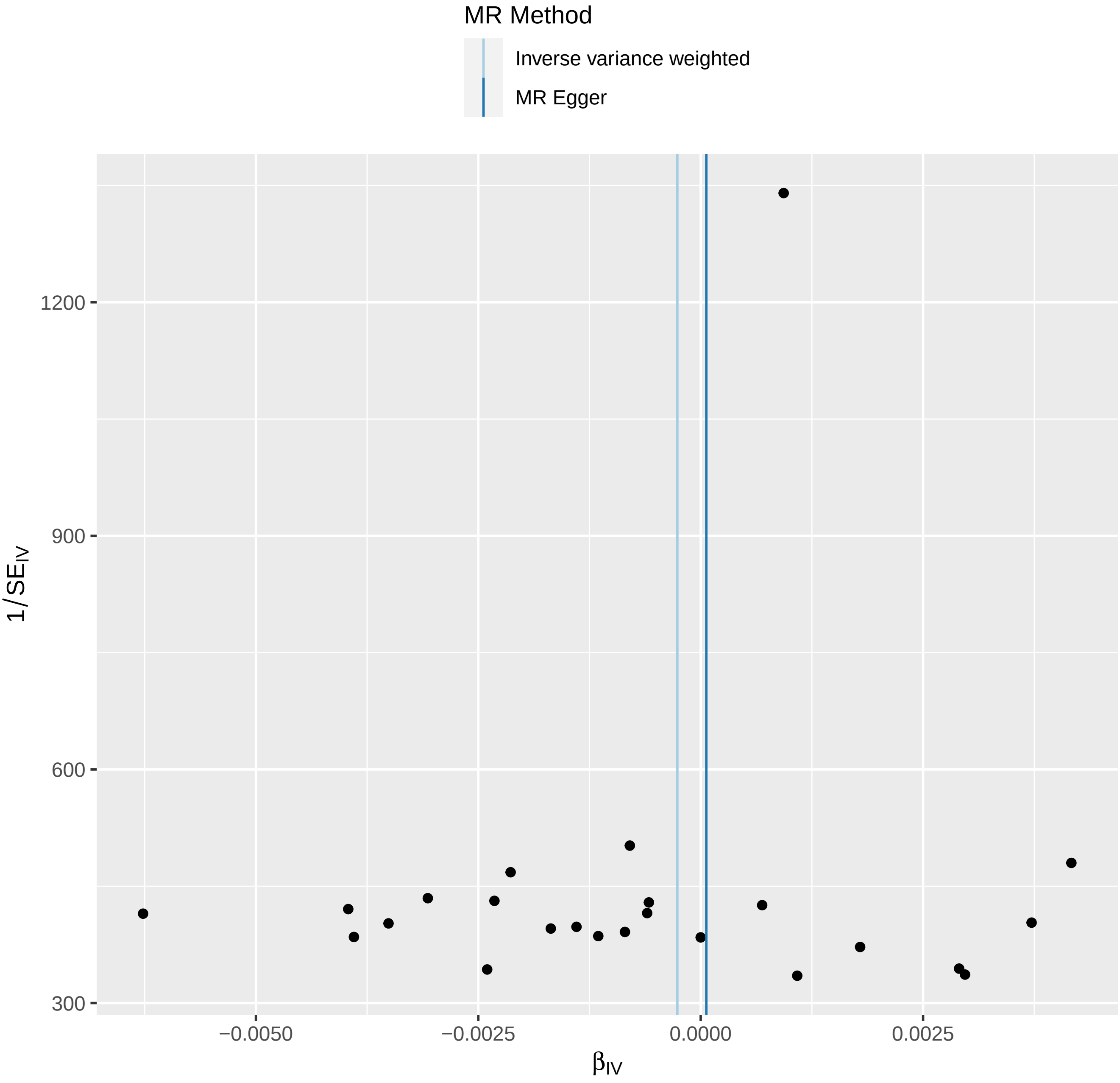

Batch 2−235 : B−cell lymphoma/leukemia 10 on eGFR

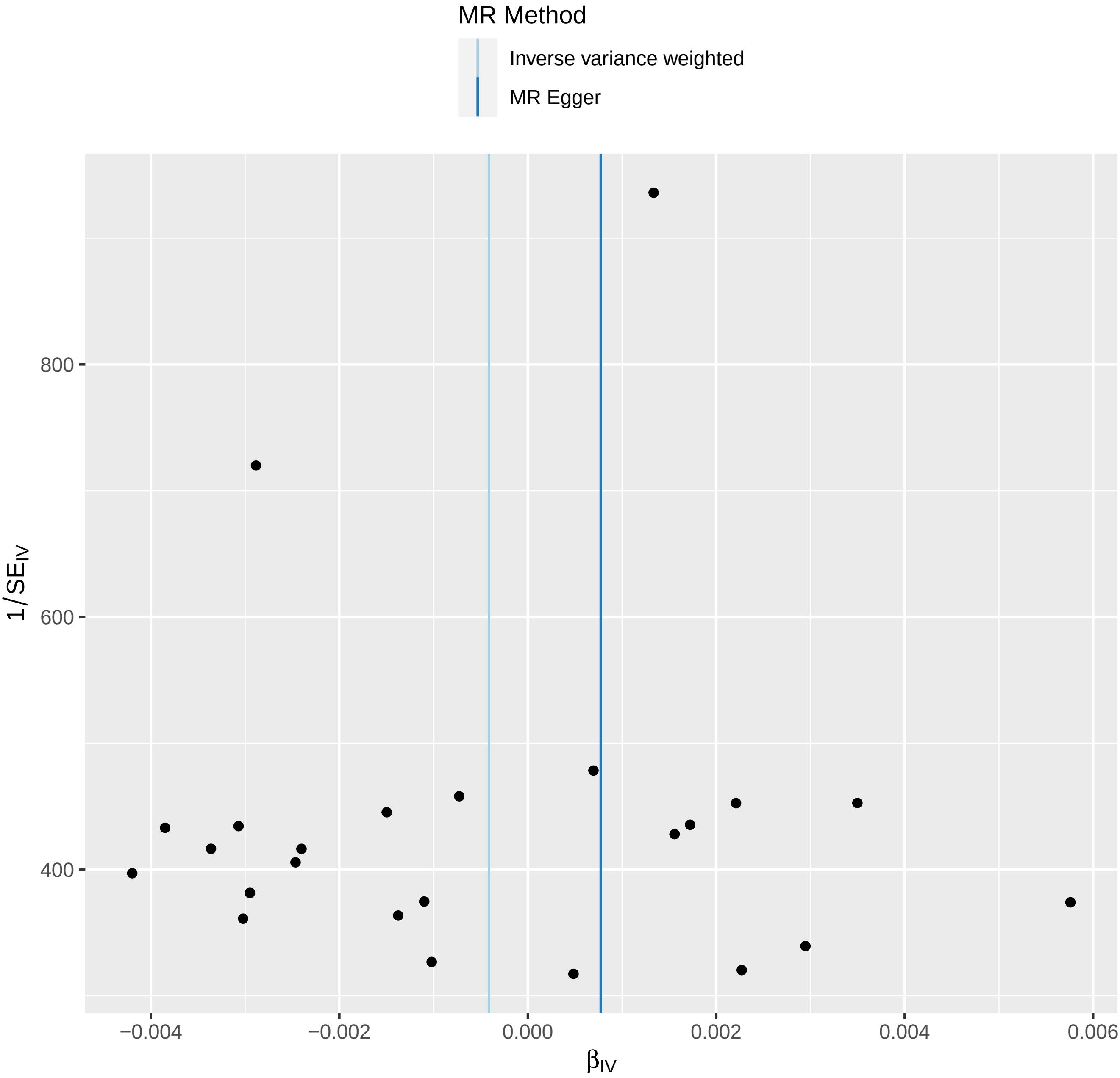

Batch 2−236 : Apoptosis regulator Bcl−2 on eGFR

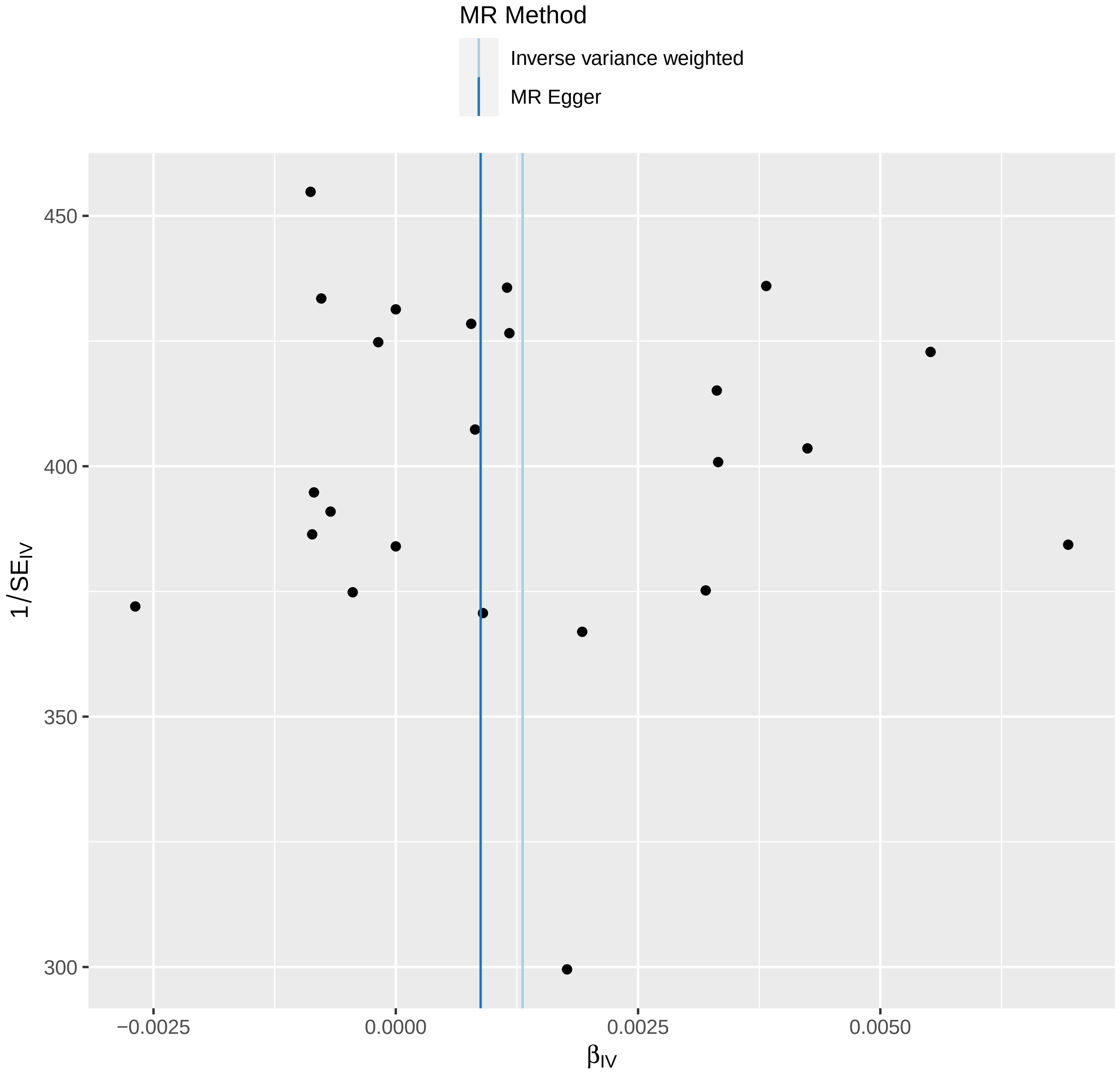

Batch 2−237 : Bcl−2−related protein A1 on eGFR

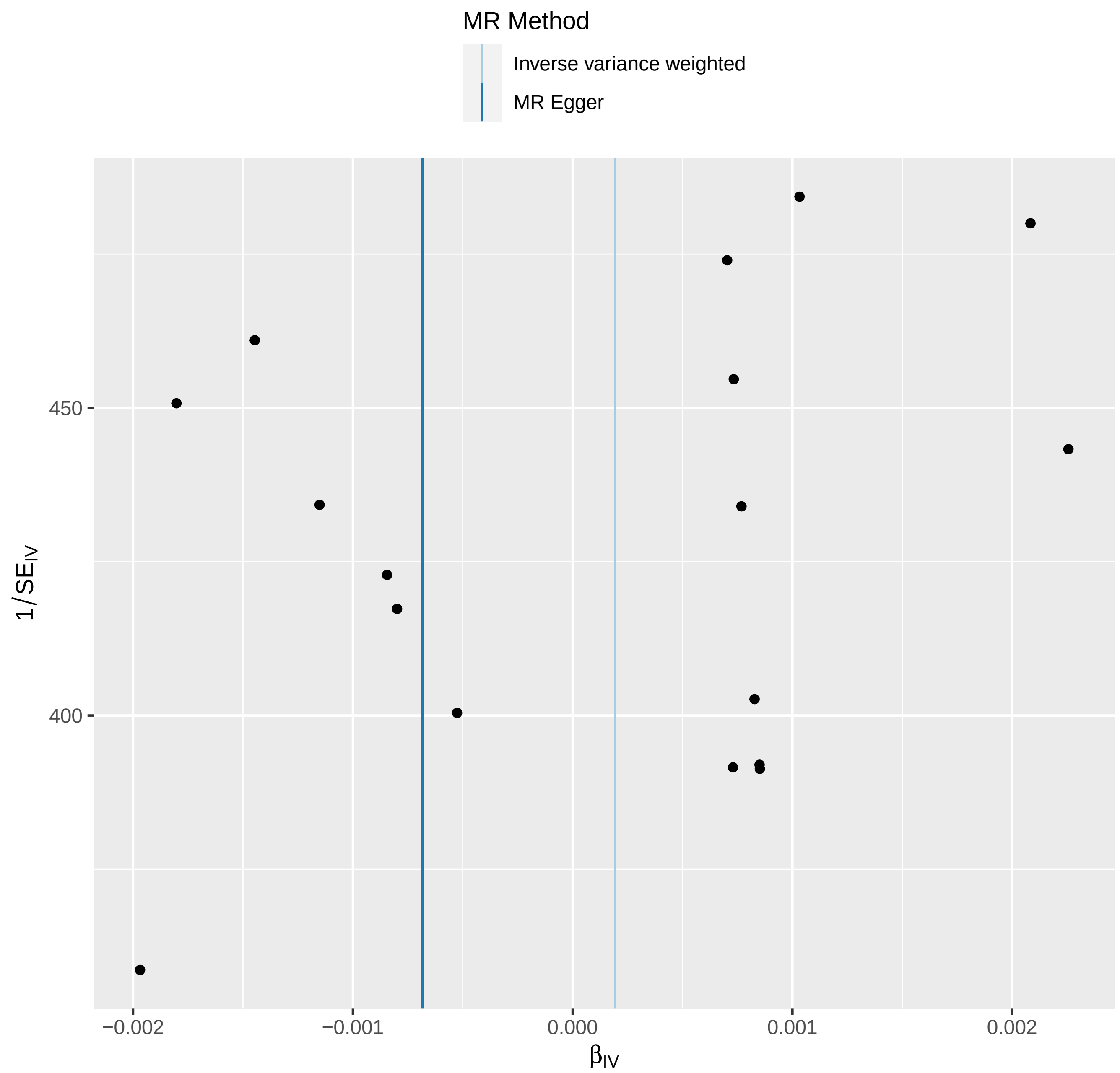

Batch 2−238 : Bcl−2−like protein 1 on eGFR

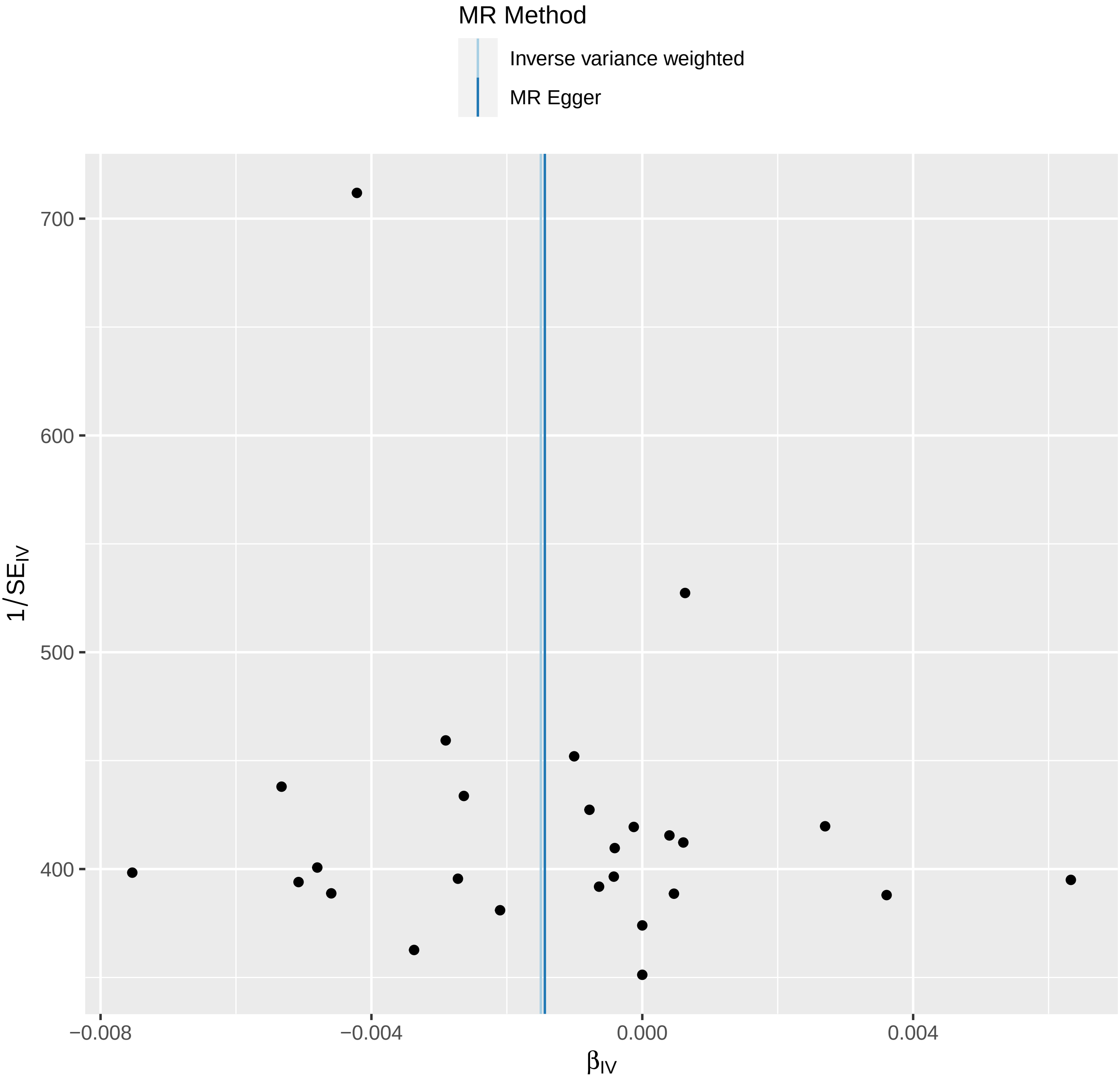

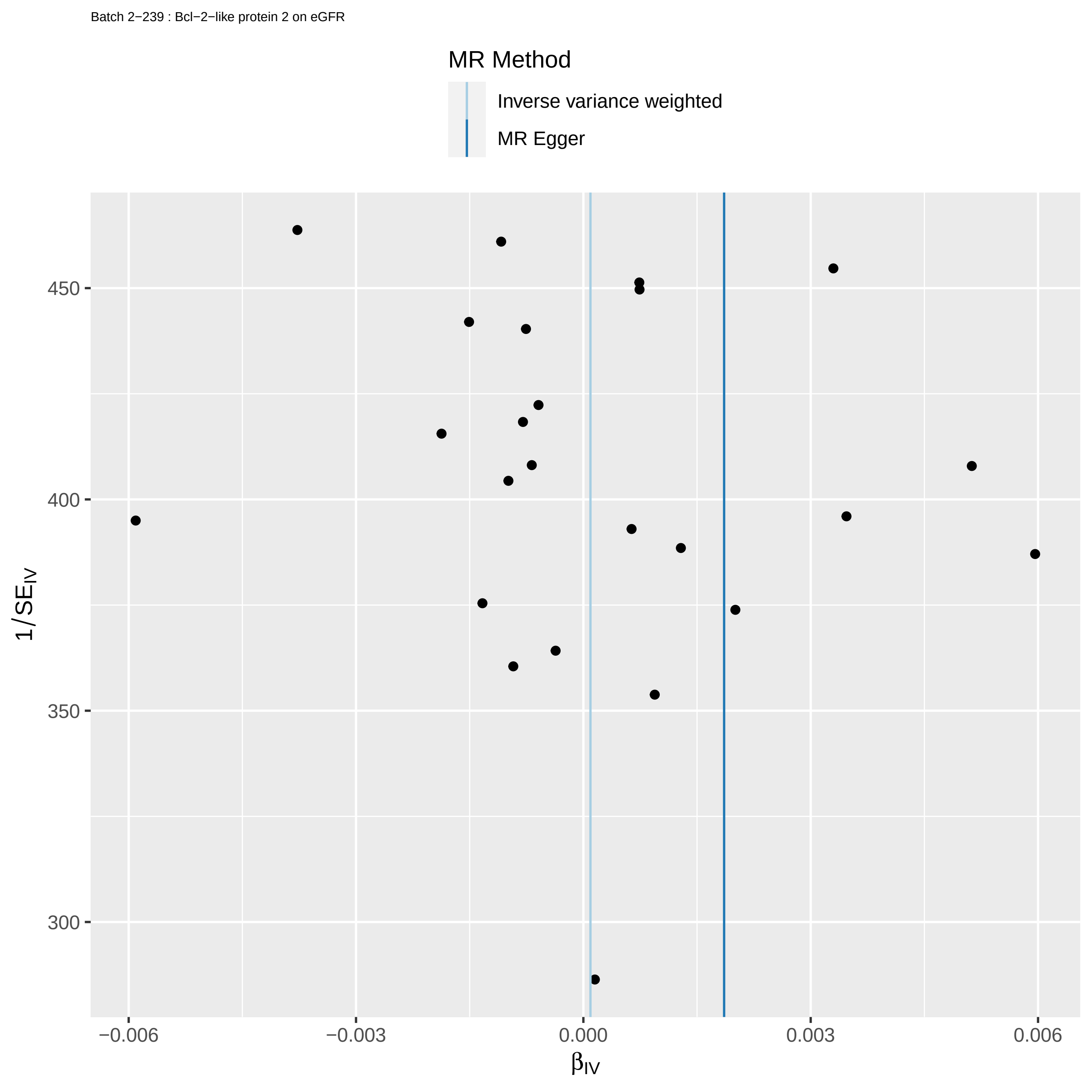

Batch 2−239 : Bcl−2−like protein 2 on eGFR
MR Method
Inverse variance weighted
MR Egger
1/SE$_{IV}$
450
400
350
300
−0.006
−0.003
0.000
0.003
0.006
β$_{IV}$

Batch 2−240 : Bcl−2−like protein 2 on eGFR

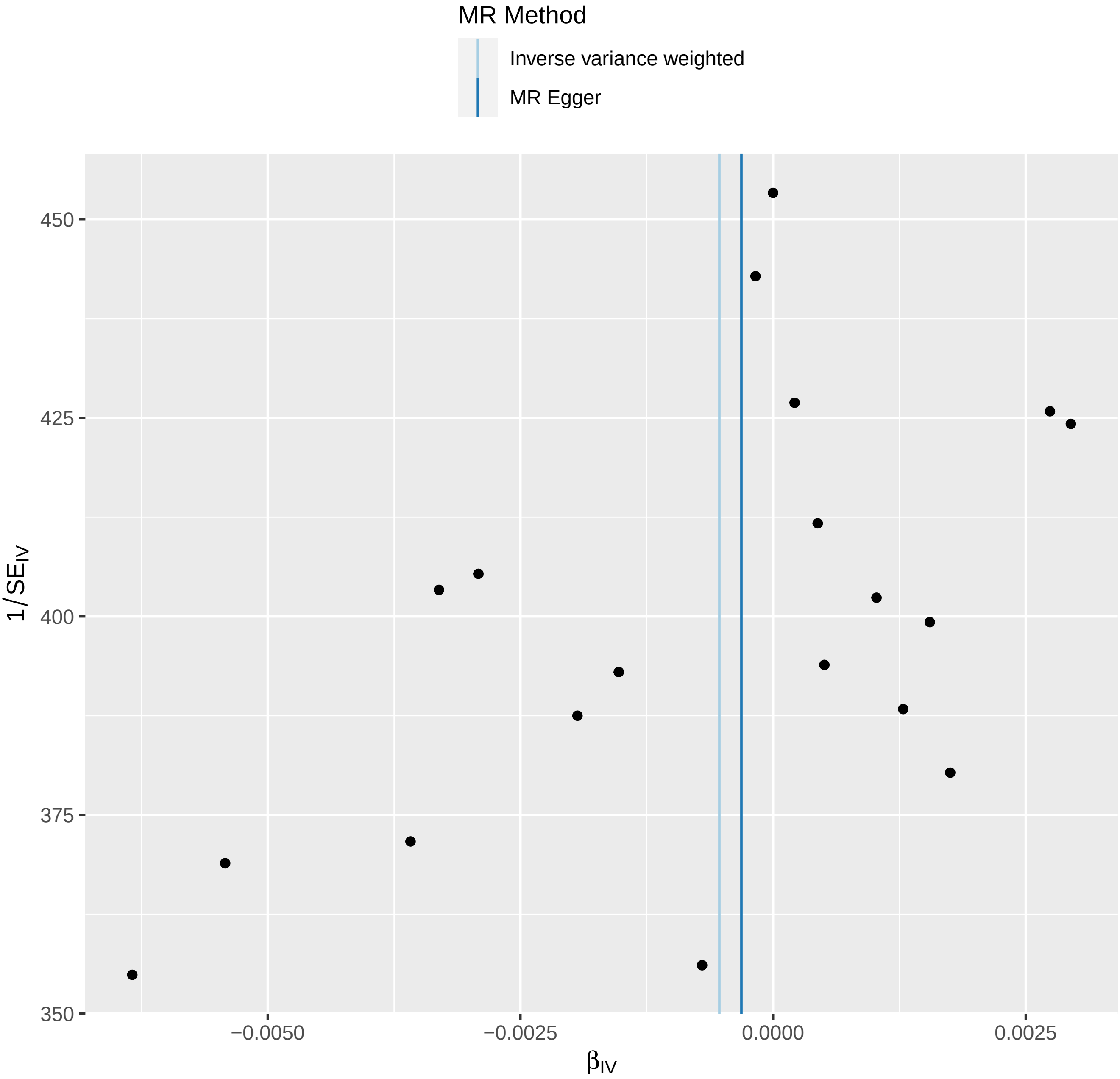

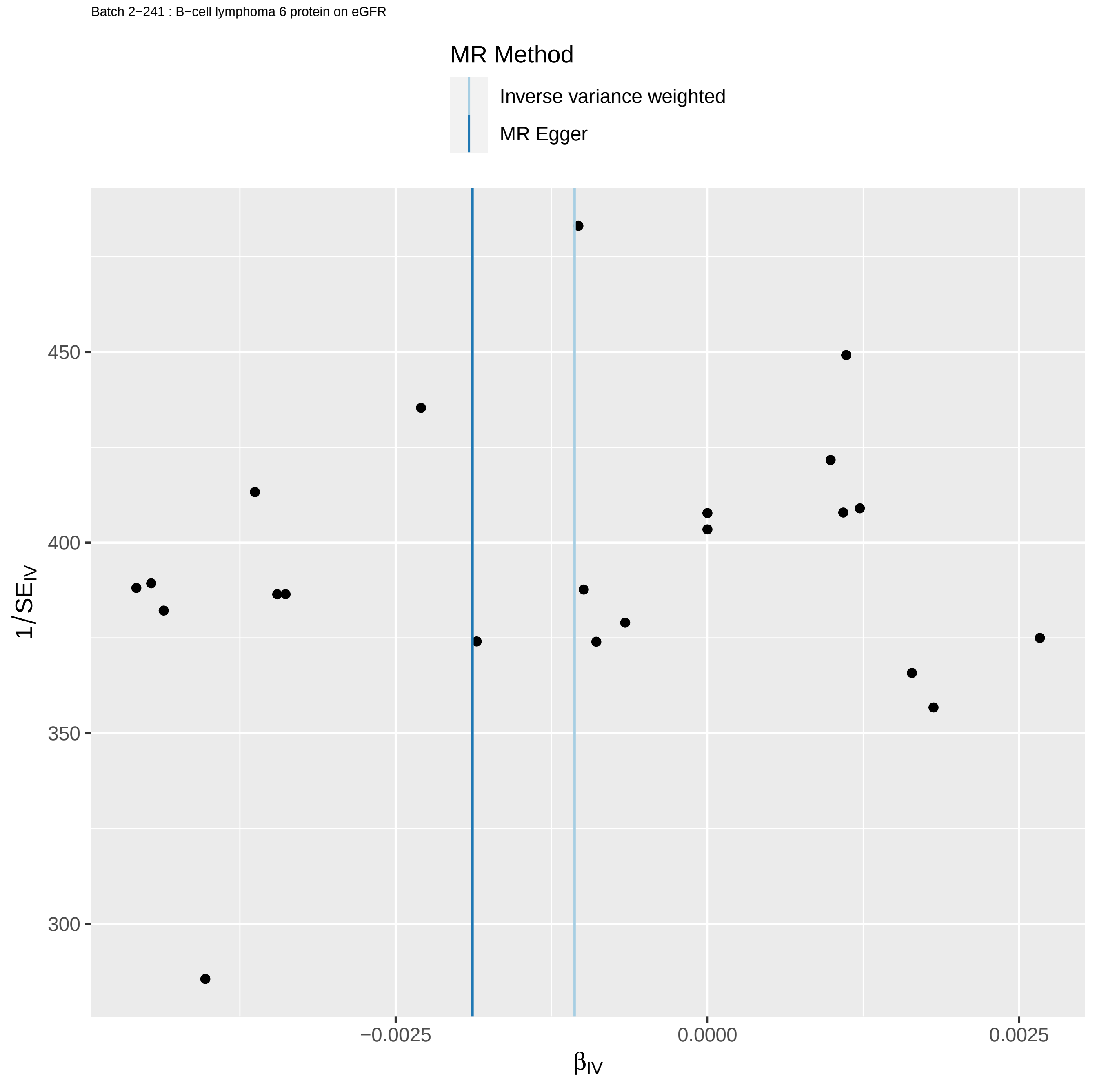
Batch 2−241 : B−cell lymphoma 6 protein on eGFR
MR Method
Inverse variance weighted
MR Egger
450
400
350
300
1/SE$_{IV}$
−0.0025
0.0000
0.0025
β$_{IV}$

Batch 2−242 : Brain−derived neurotrophic factor on eGFR

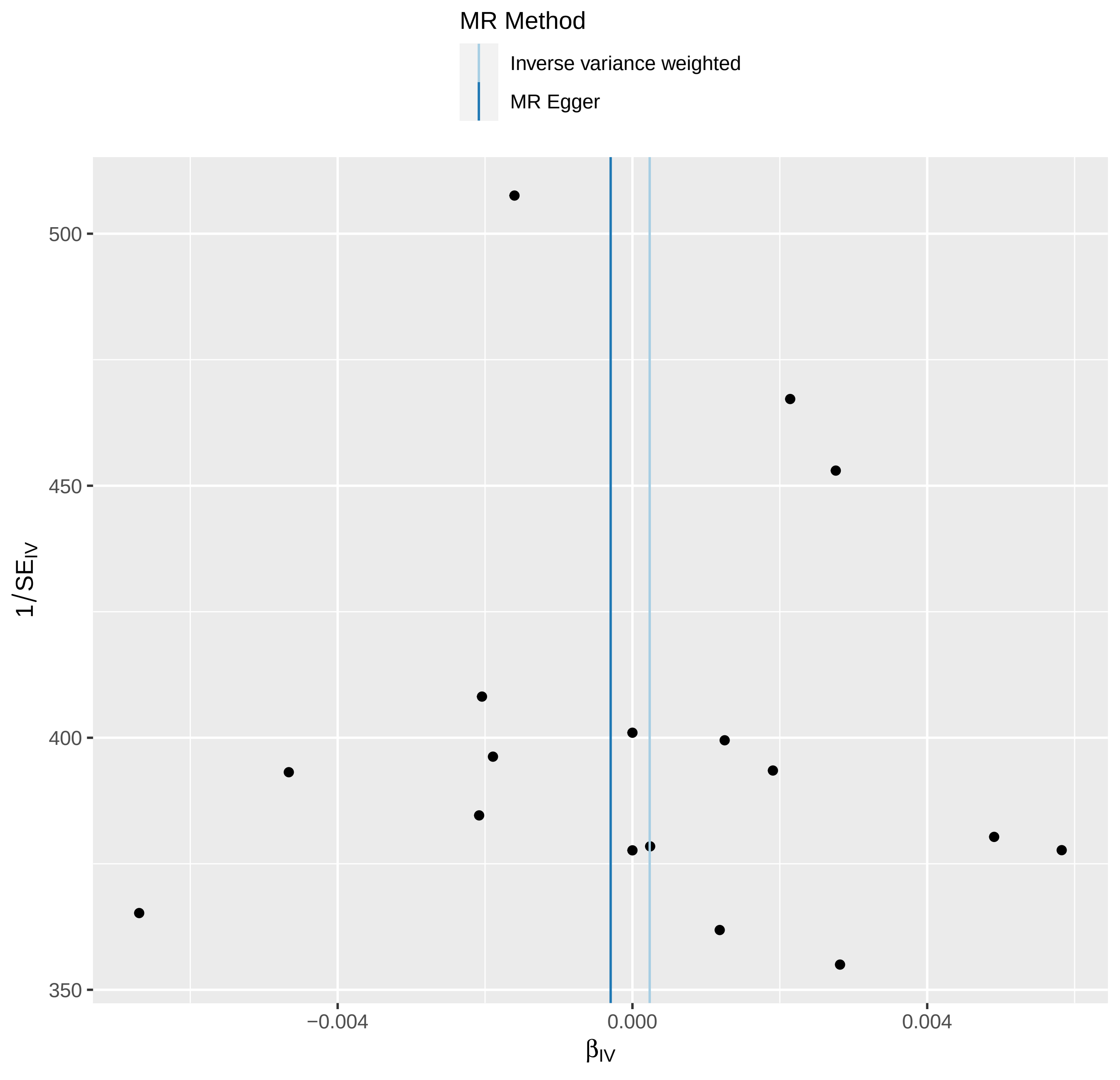

Batch 2−243 : Transcription factor TFIIIB component B" homolog on eGFR

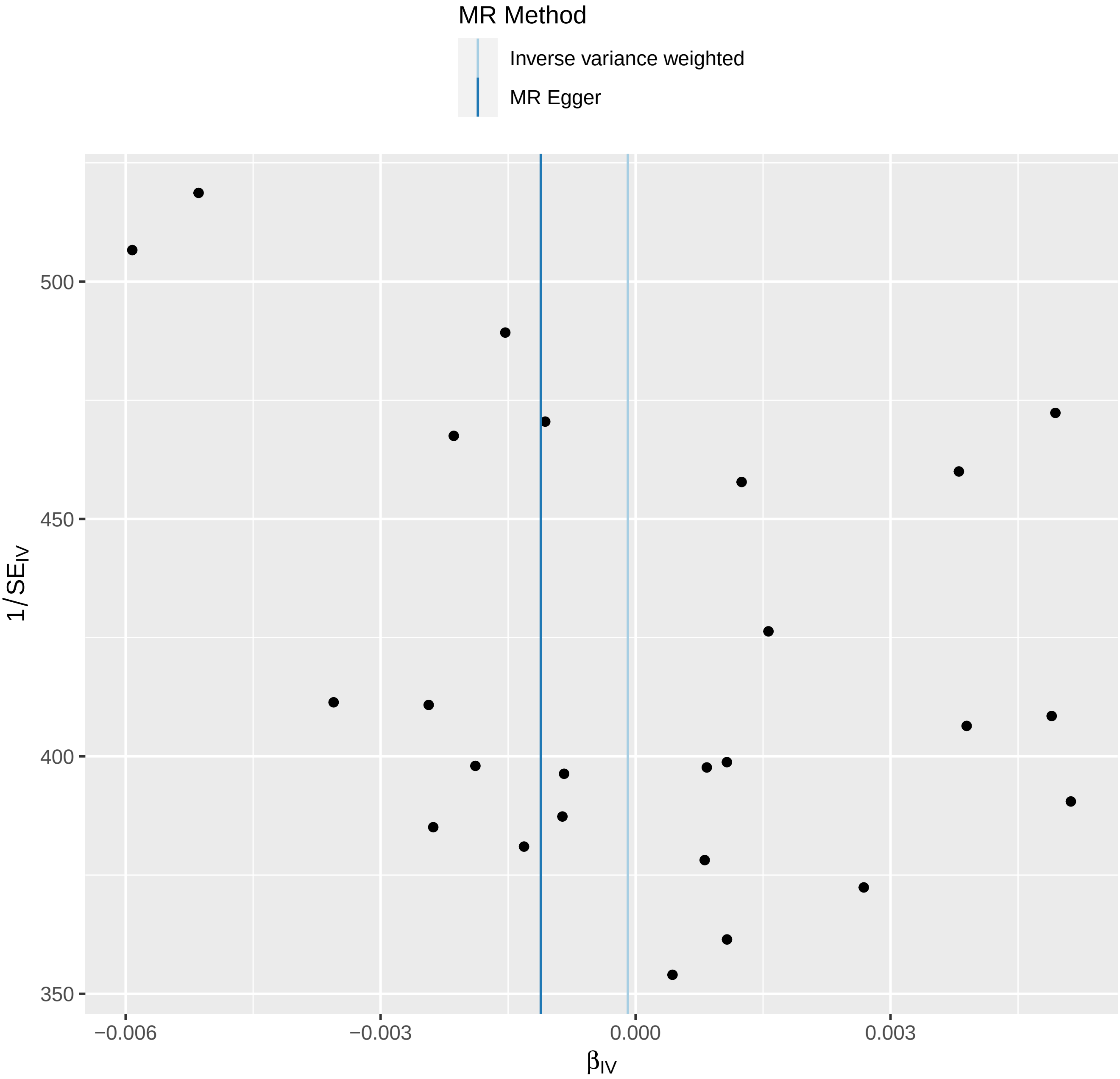

Batch 2−244 : Beclin−1 on eGFR

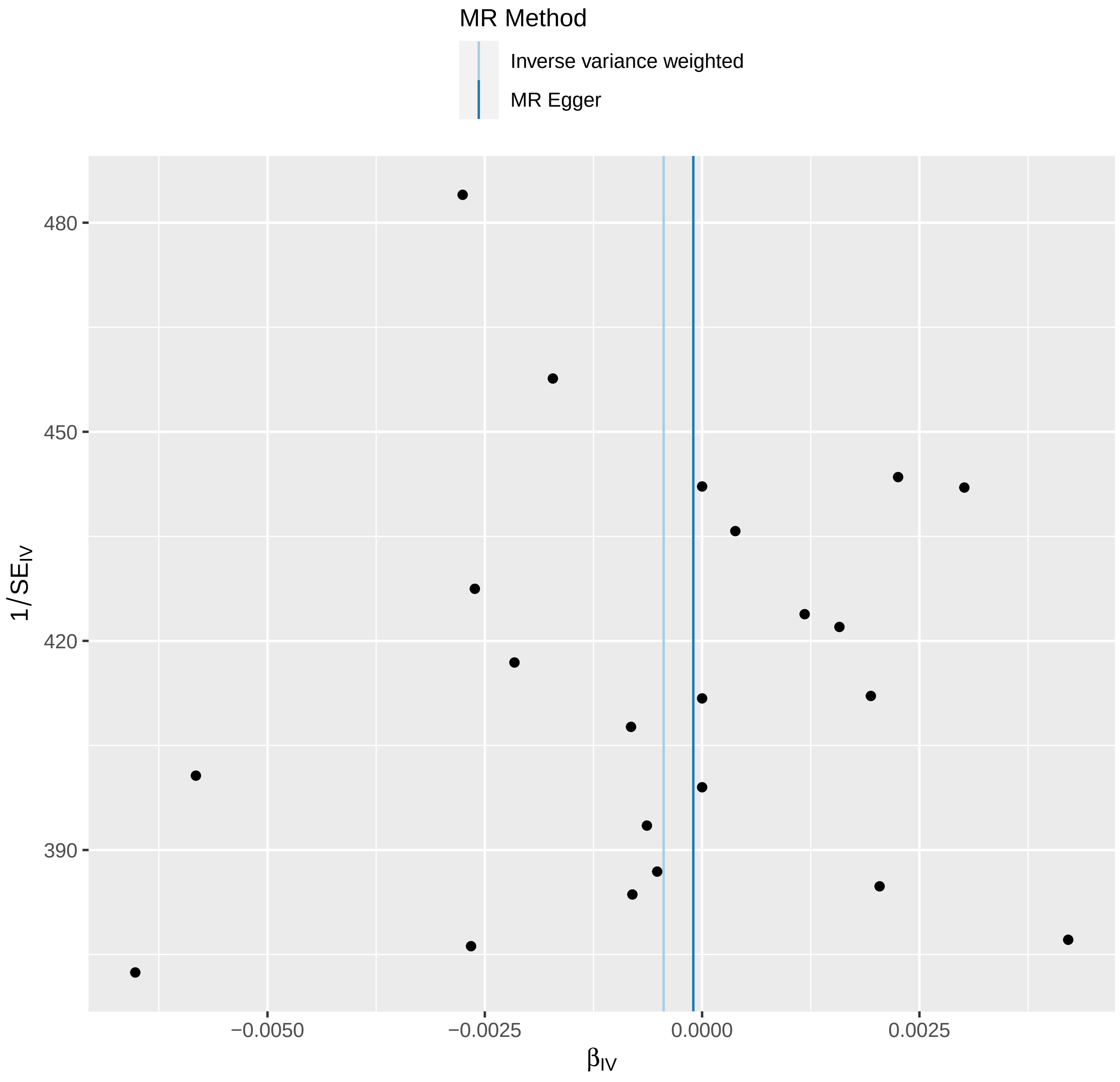

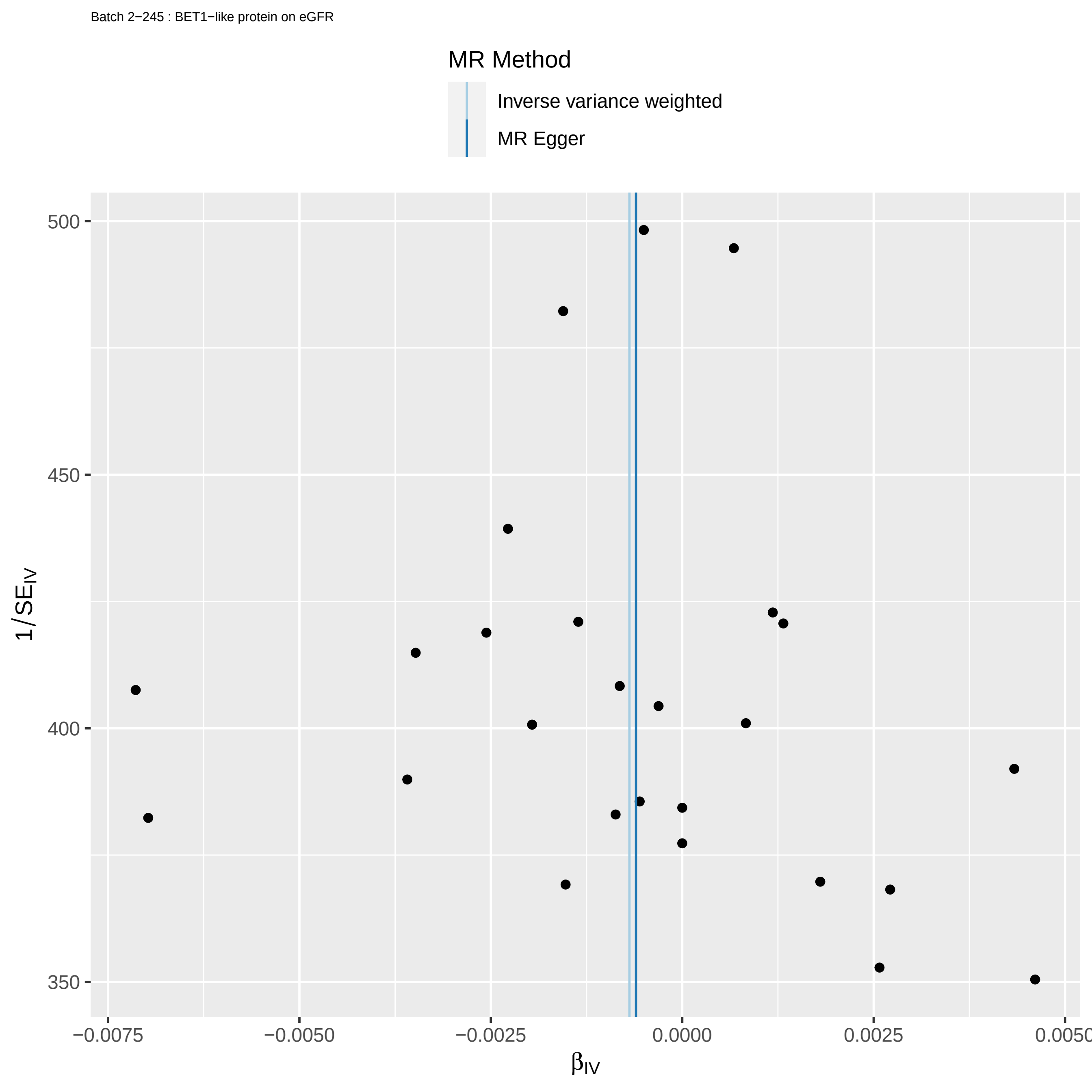

Batch 2−245 : BET1−like protein on eGFR
MR Method
Inverse variance weighted
MR Egger
500
450
400
350
1/SE_IV
−0.0075
−0.0050
−0.0025
0.0000
0.0025
0.0050
β_IV

Batch 2−246 : Osteocalcin on eGFR

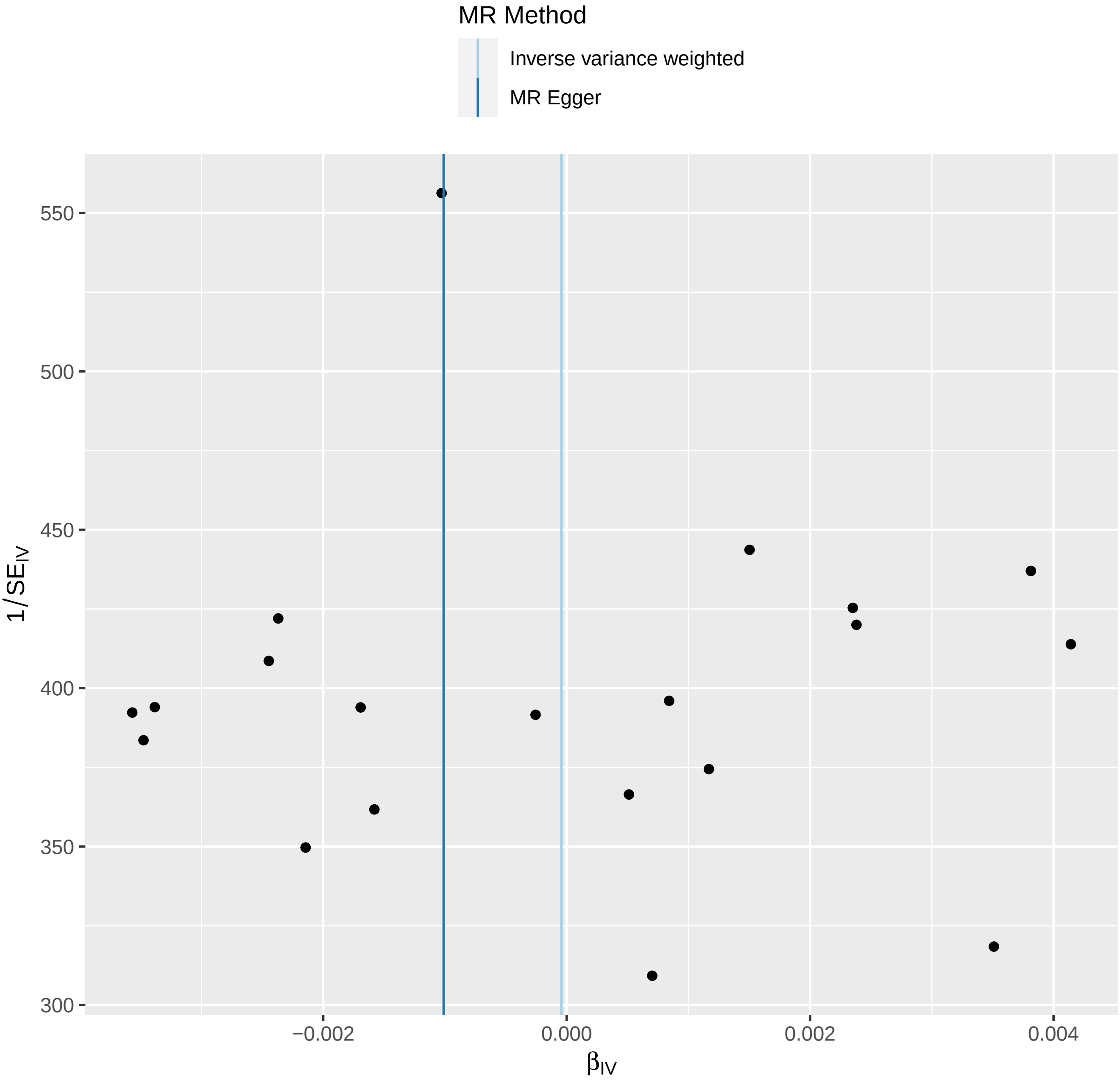

Batch 2−247 : Biglycan on eGFR

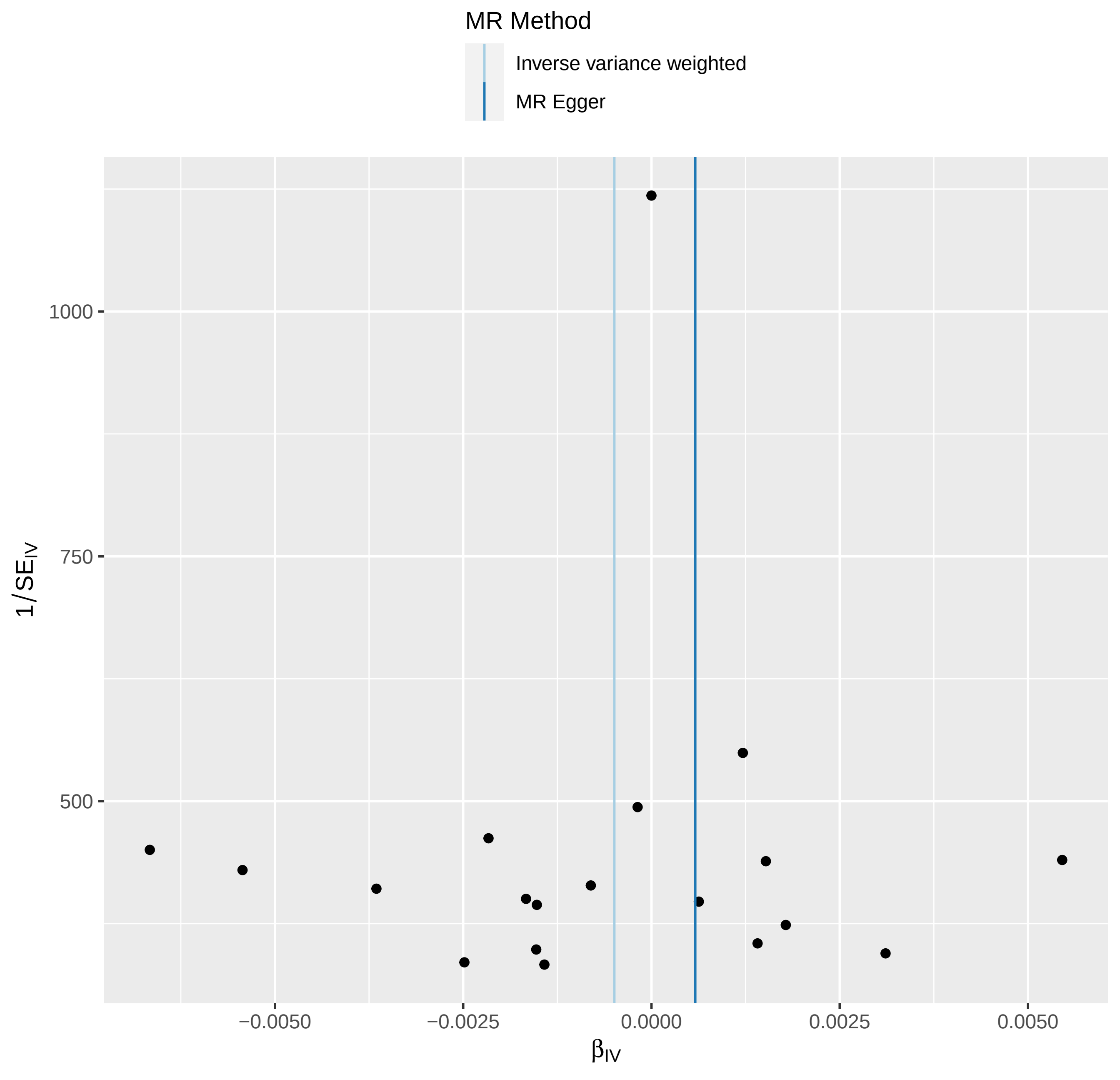

Batch 2−248 : Biglycan on eGFR

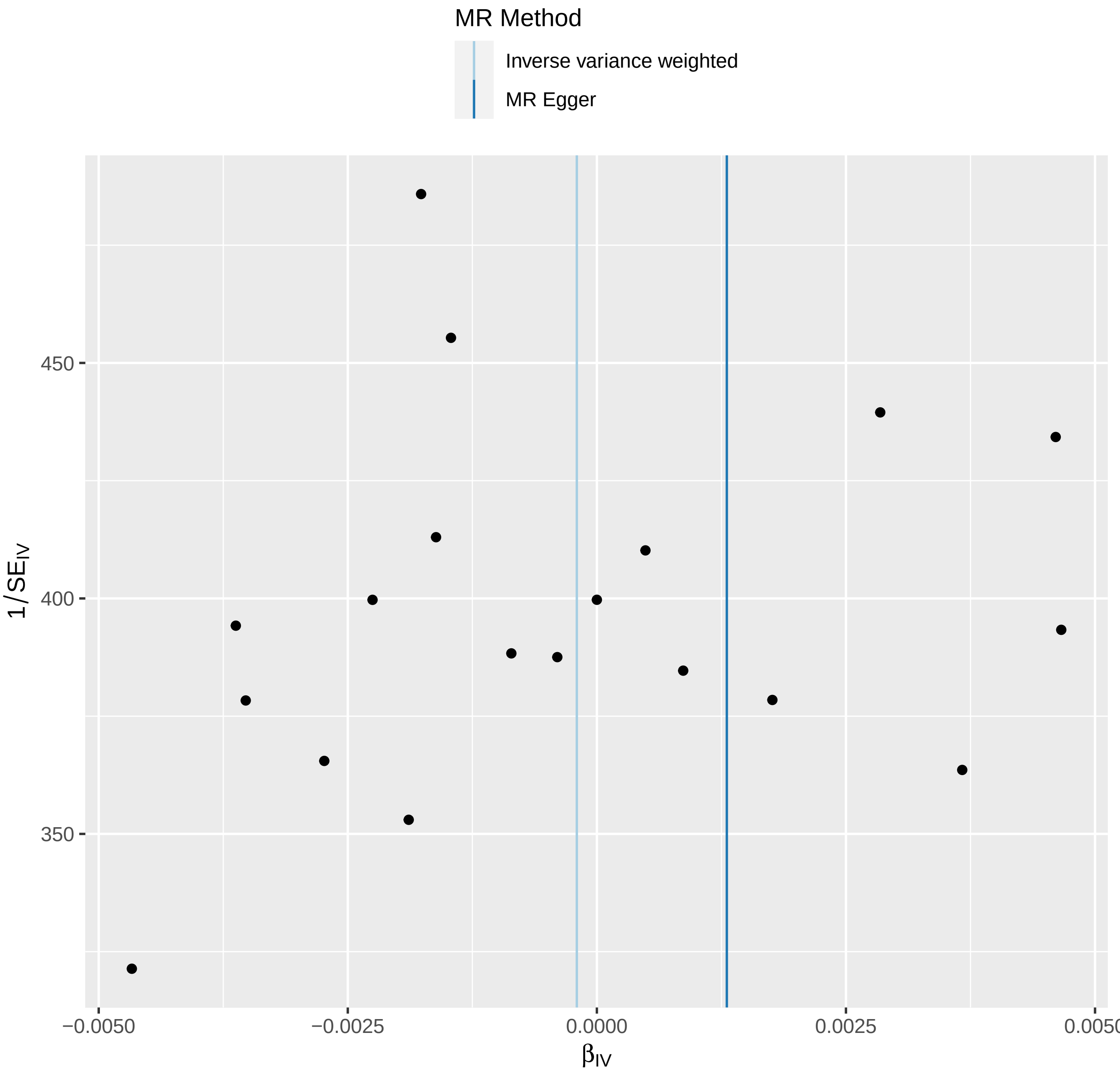

Batch 2−249 : BH3−interacting domain death agonist on eGFR

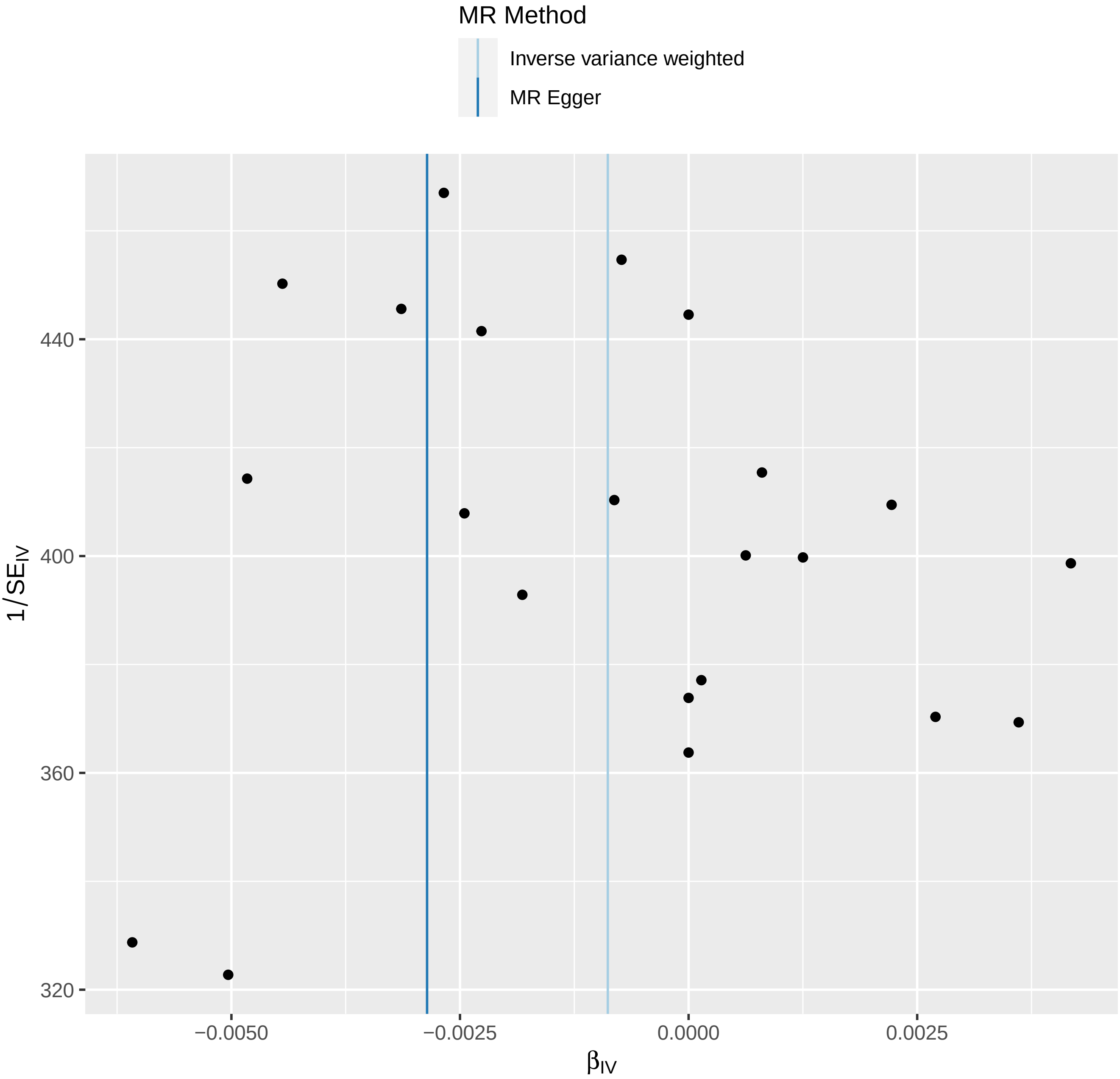

Batch 2−250 : Myc box−dependent−interacting protein 1 on eGFR

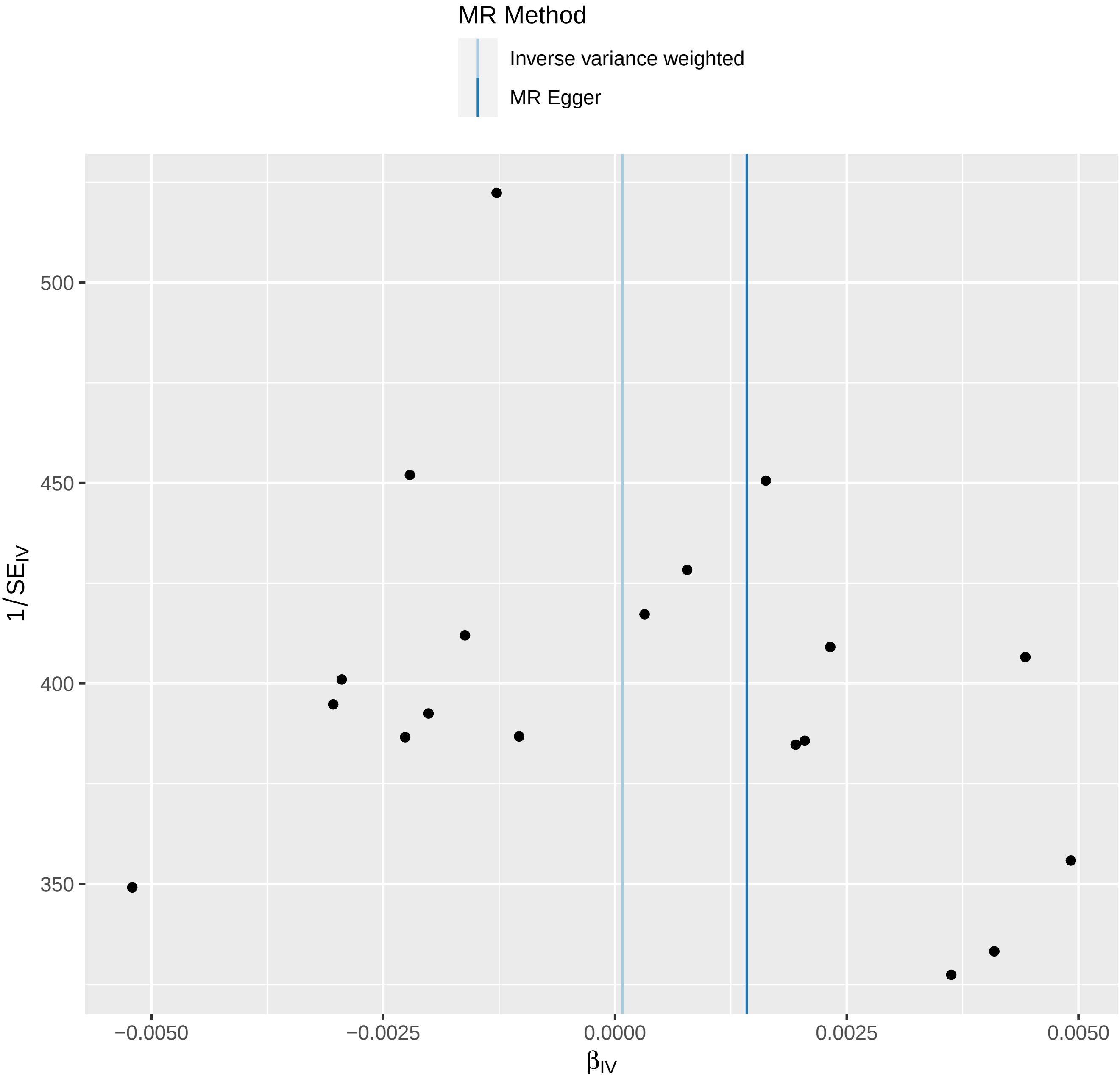

Batch 2−251 : Baculoviral IAP repeat−containing protein 3 on eGFR

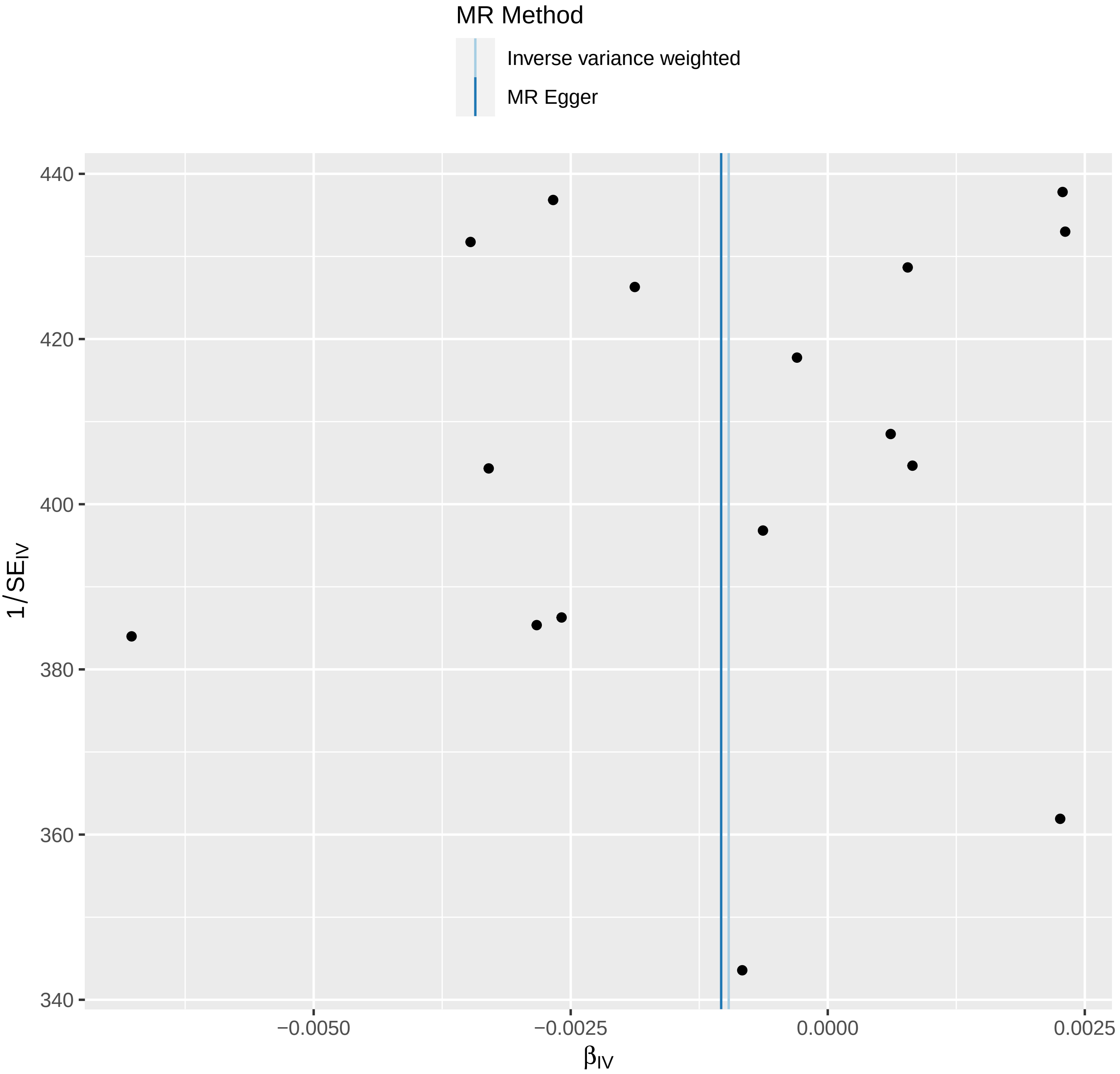

Batch 2−252 : Baculoviral IAP repeat−containing protein 5 on eGFR

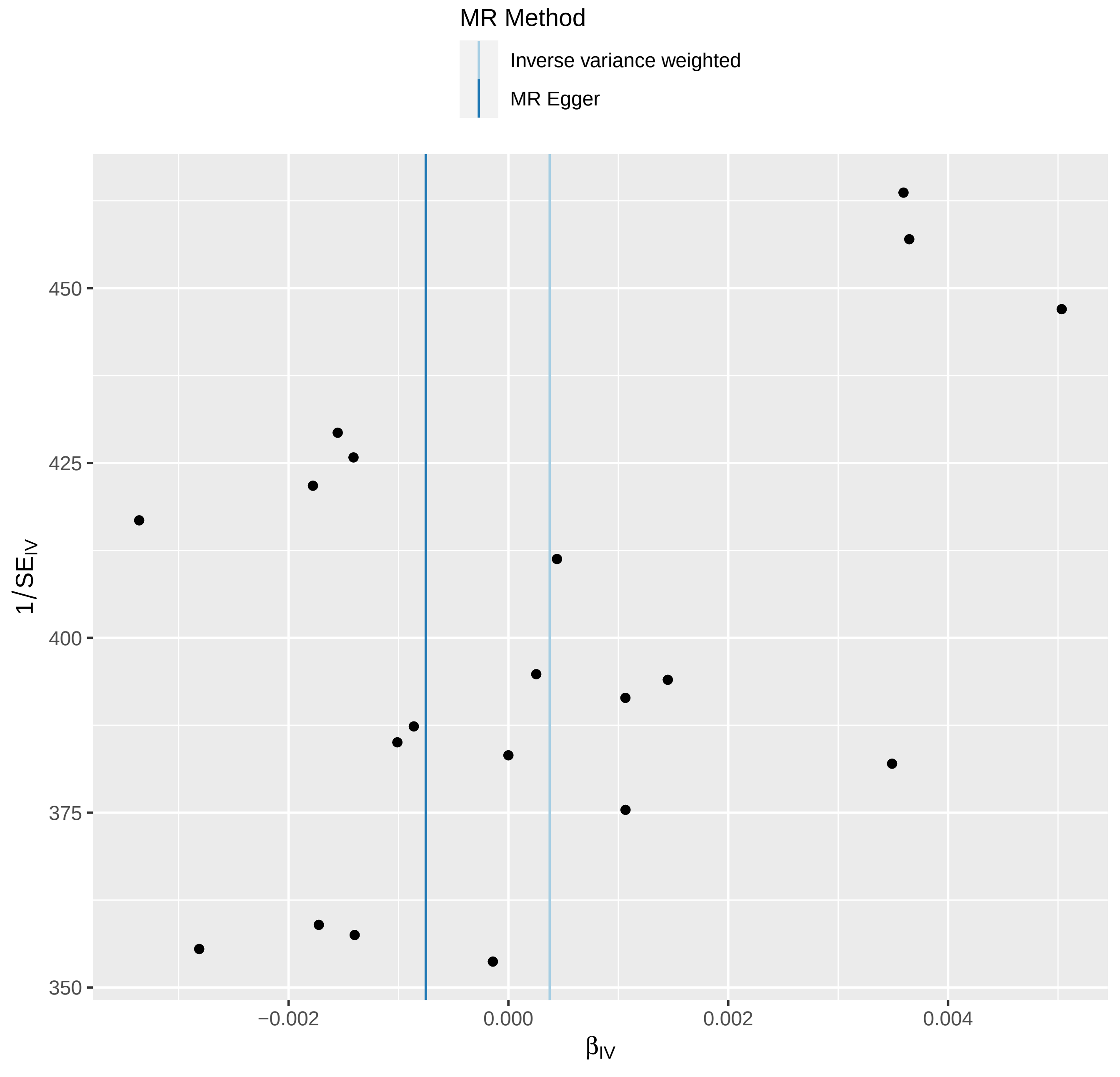

Batch 2−253 : Biliverdin reductase A on eGFR

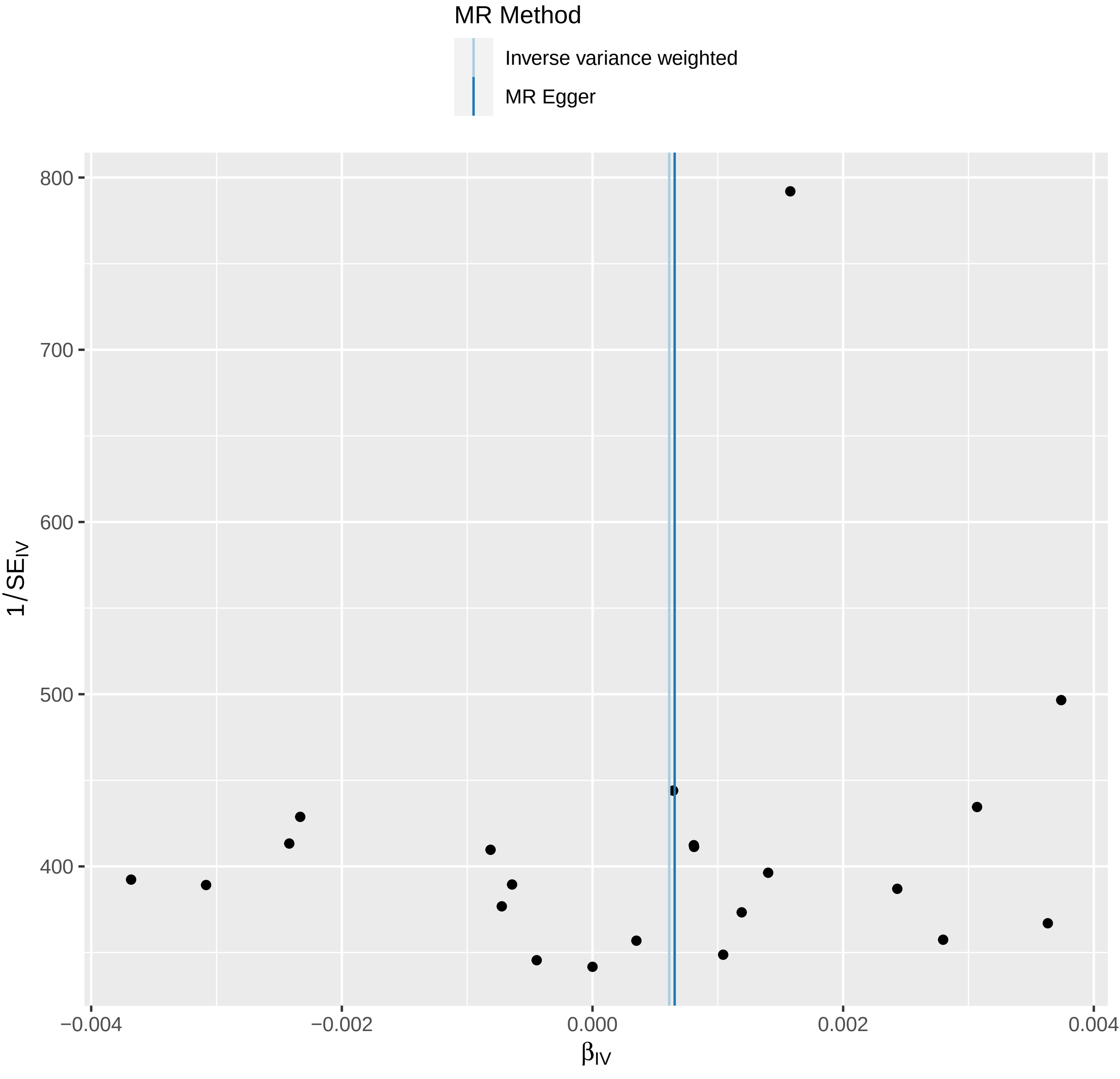

Batch 2−254 : Bone morphogenetic protein 10 on eGFR

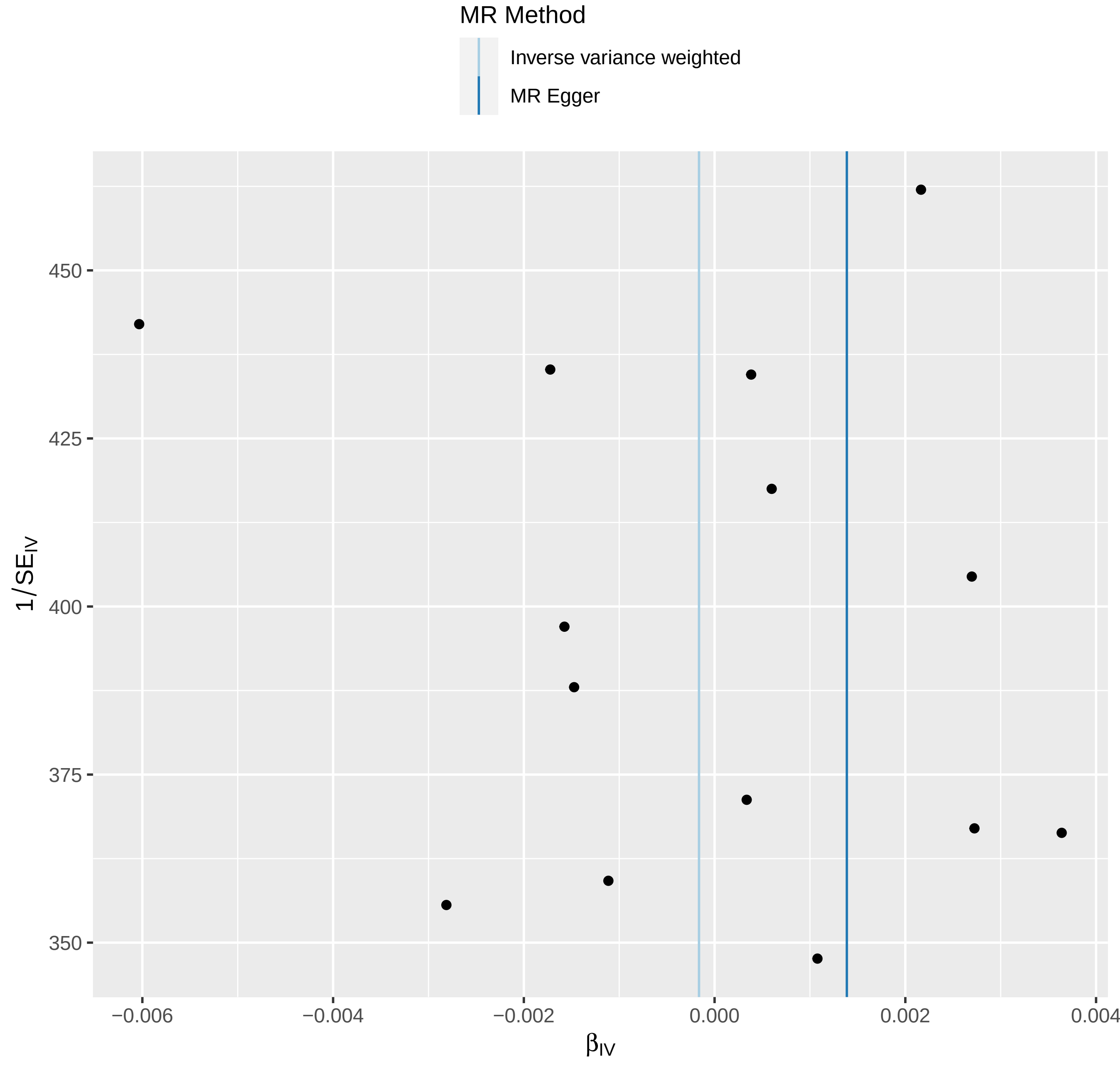

Batch 2−255 : Bone morphogenetic protein 6 on eGFR

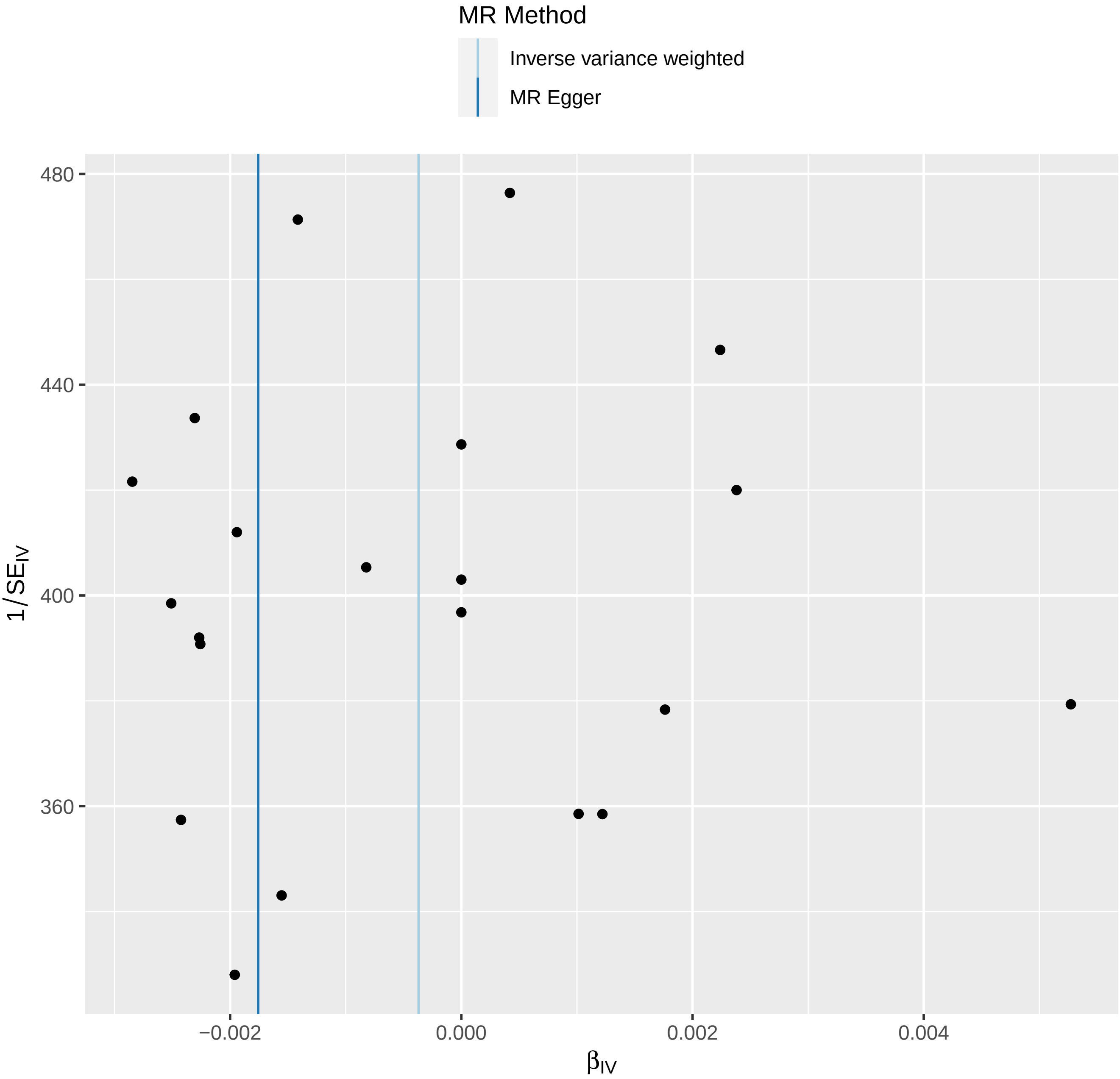

Batch 2−256 : Bone morphogenetic protein 6 on eGFR

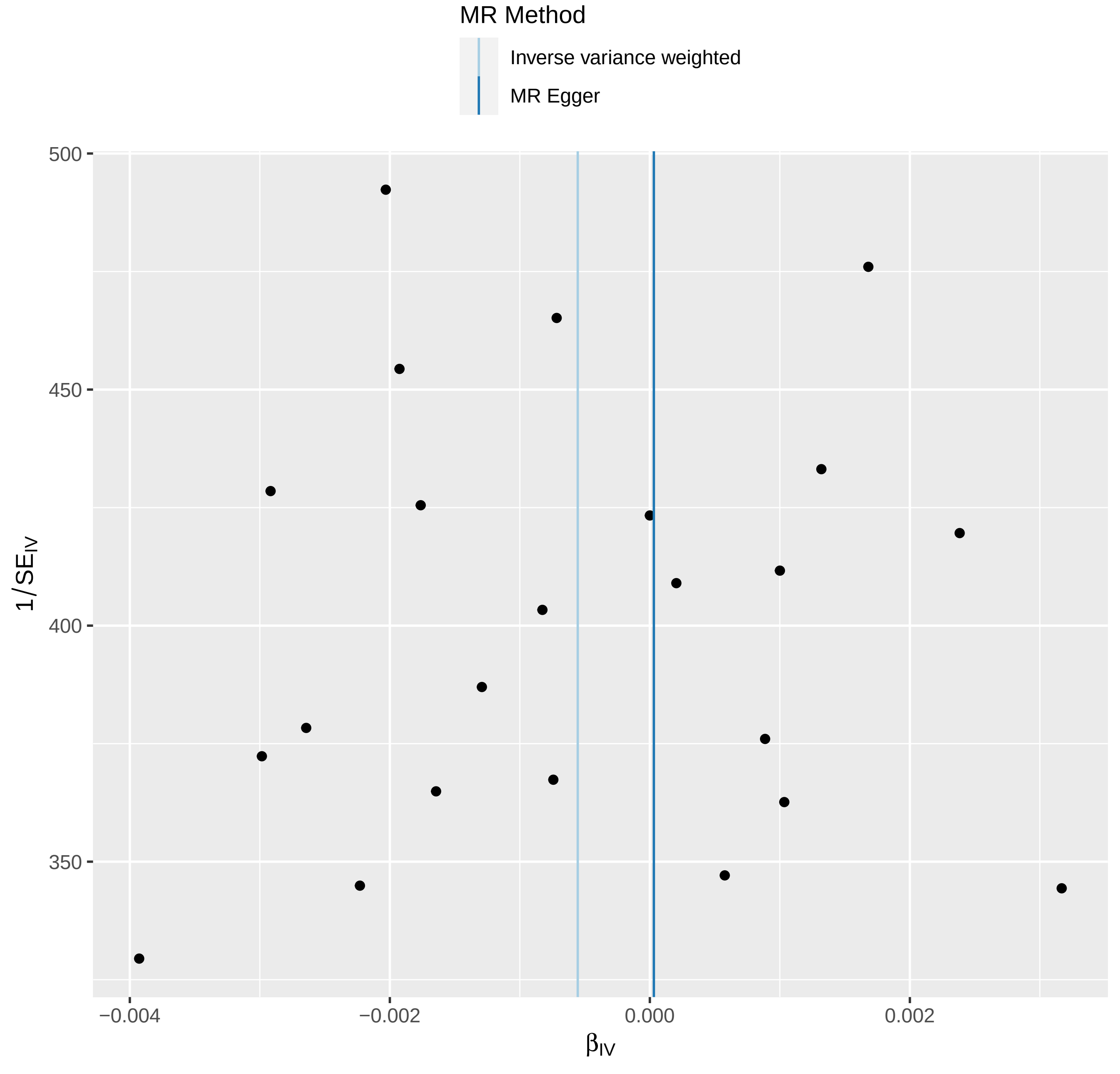

Batch 2−257 : Bone morphogenetic protein 7 on eGFR

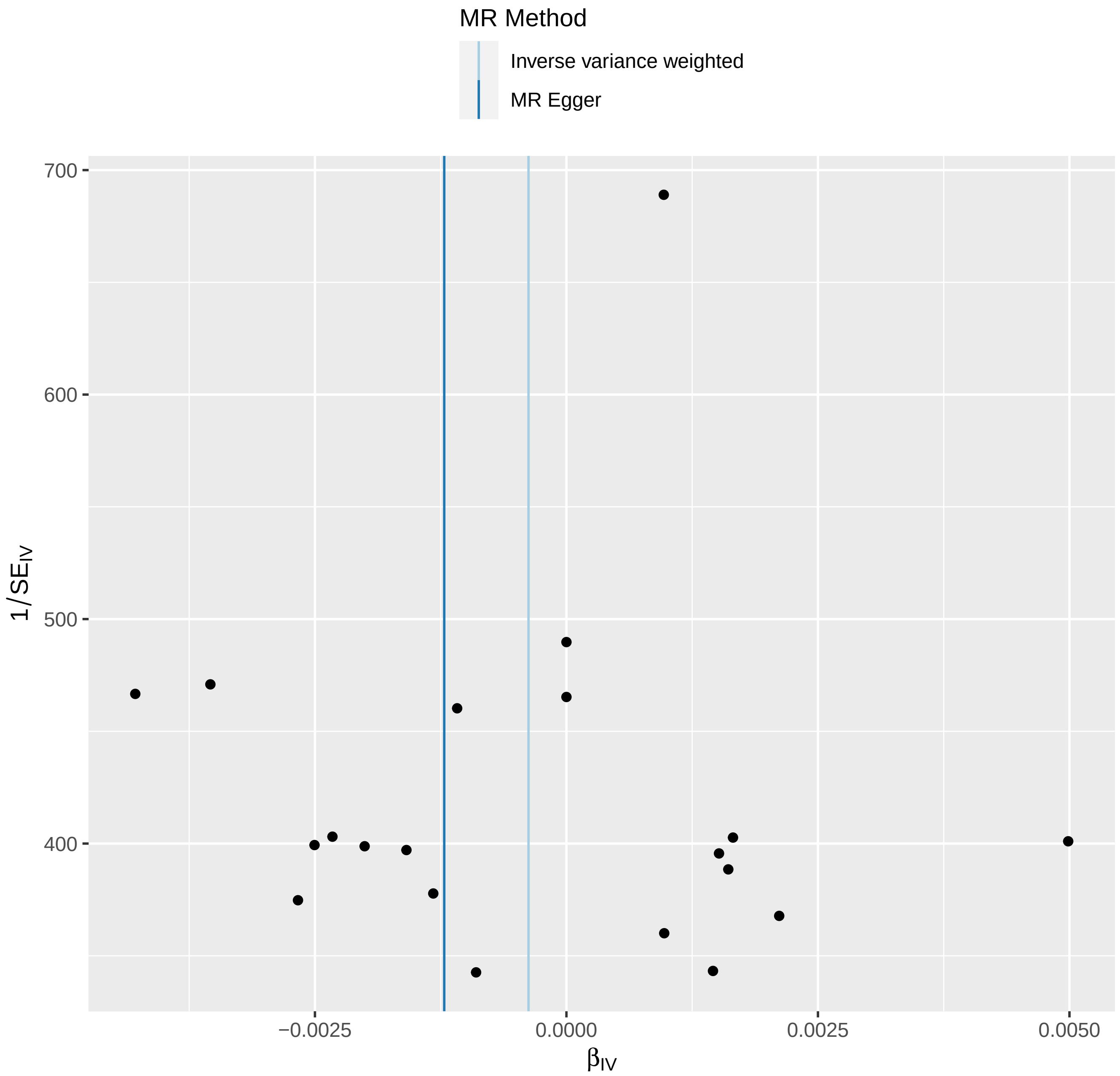

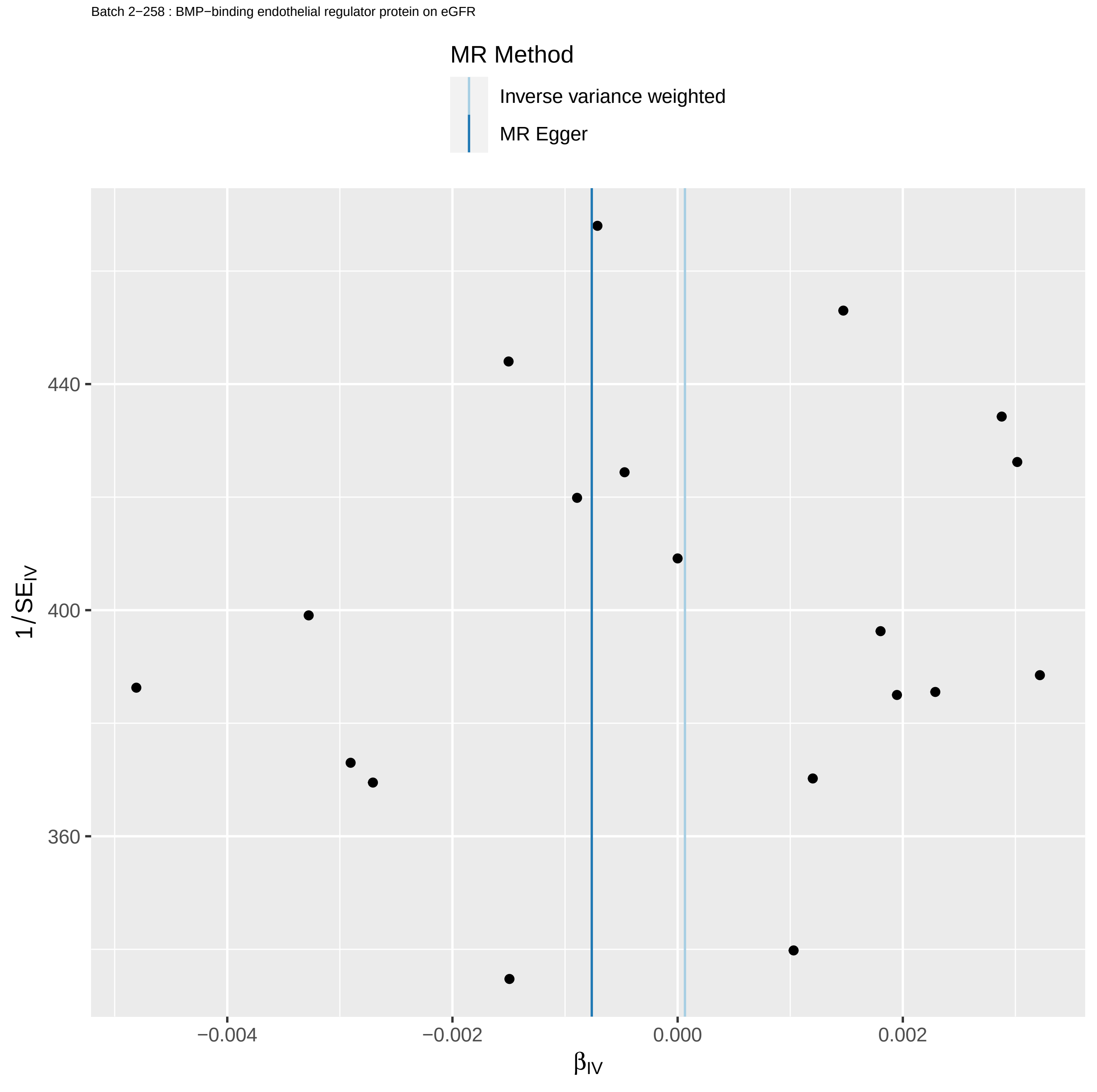

Batch 2−258 : BMP−binding endothelial regulator protein on eGFR
MR Method
Inverse variance weighted
MR Egger
440
400
360
1/SE_IV
−0.004
−0.002
0.000
0.002
β_IV

Batch 2−259 : Bone morphogenetic protein receptor type−1A on eGFR

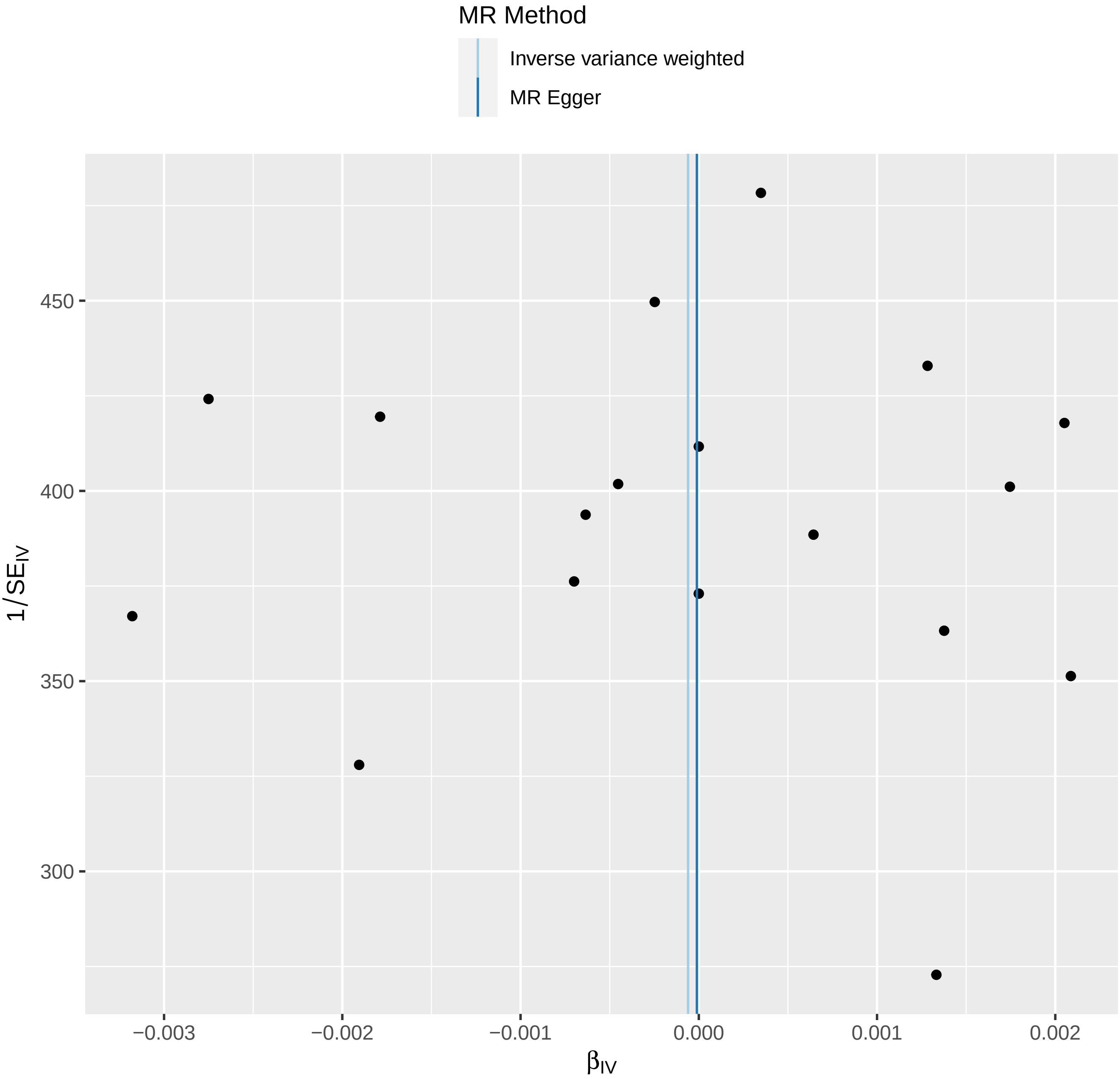

Batch 2−260 : Cytoplasmic tyrosine−protein kinase BMX on eGFR

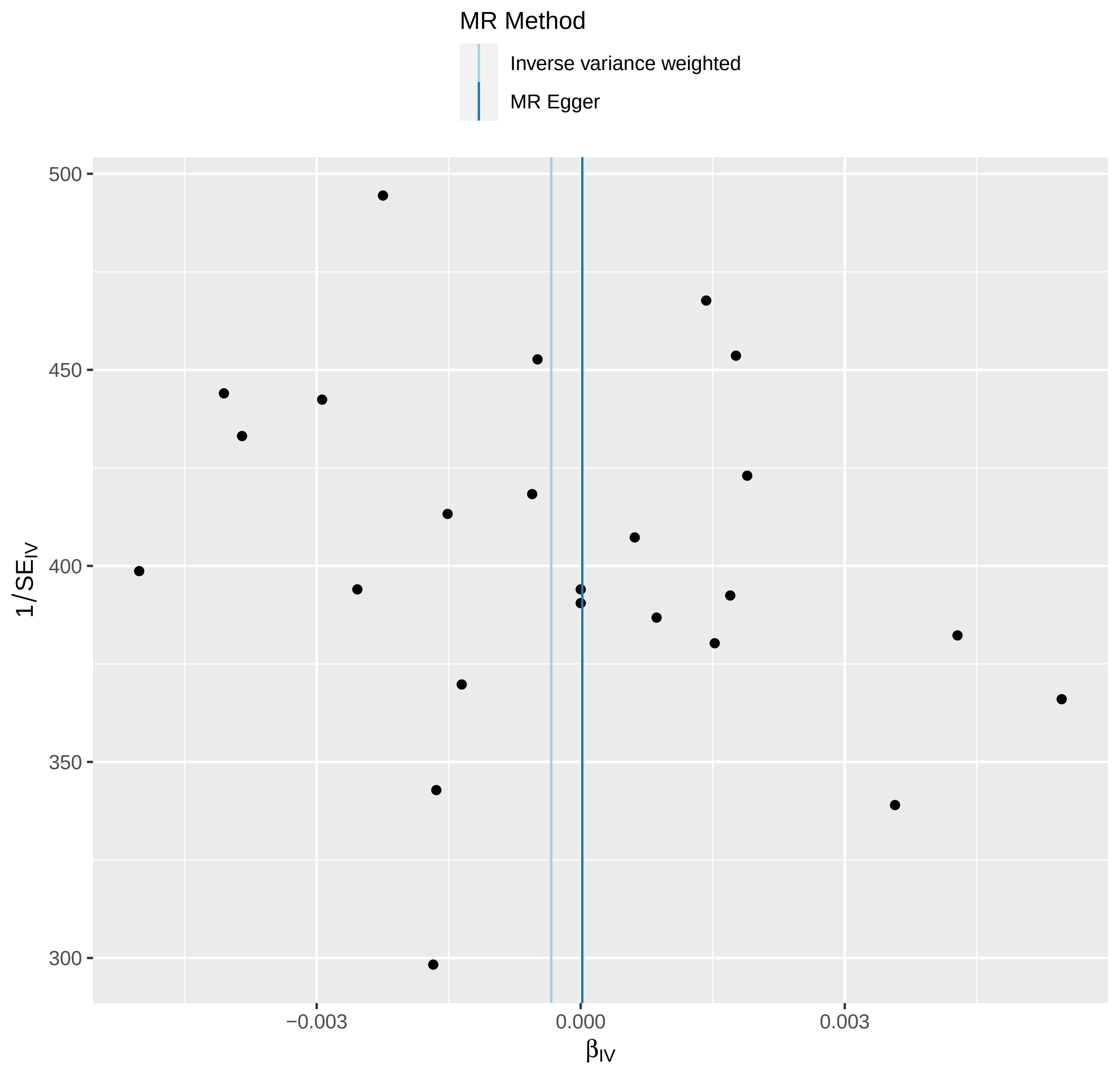

Batch 2−261 : BCL2/adenovirus E1B 19 kDa protein−interacting protein 3 on eGFR

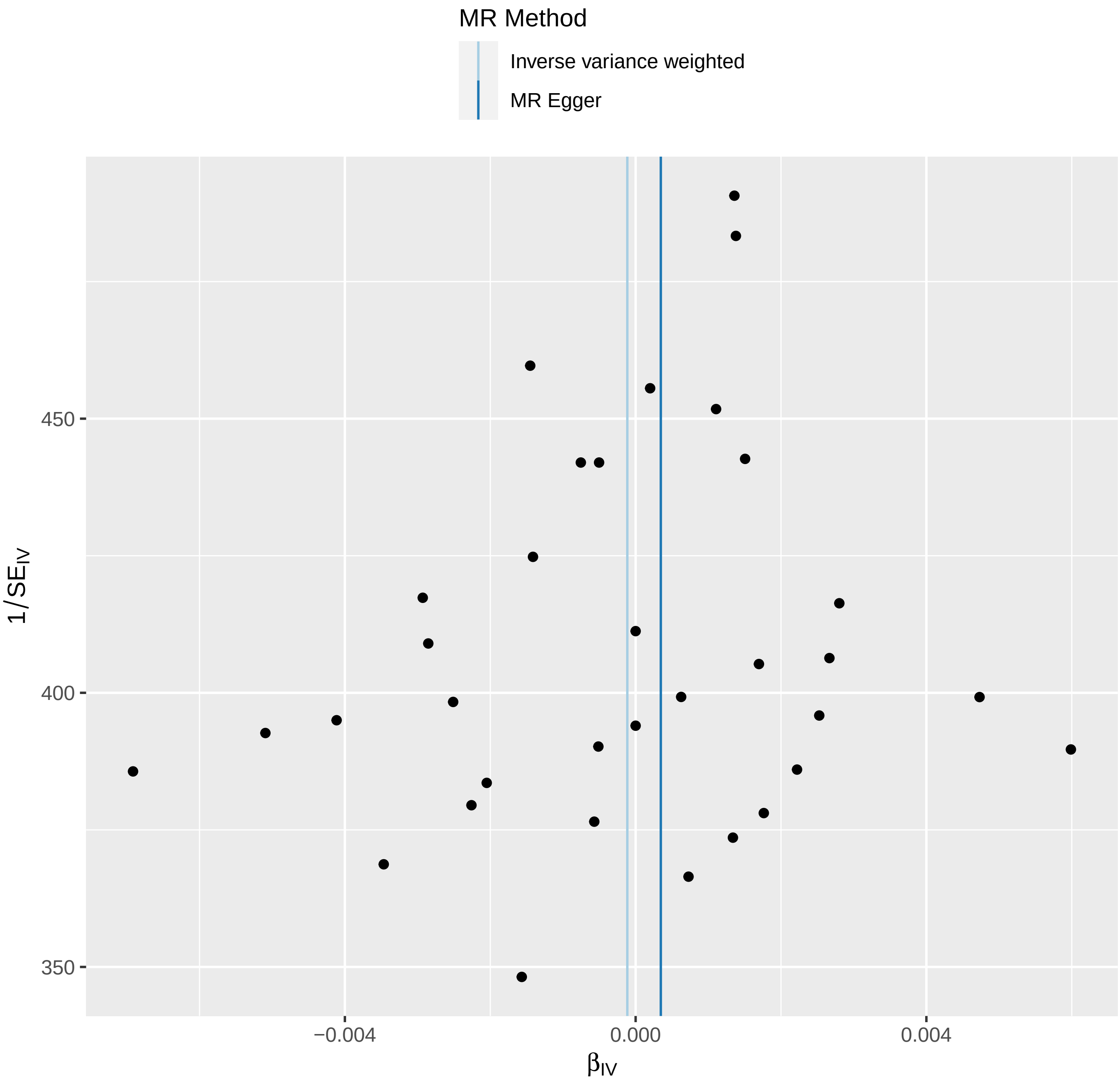

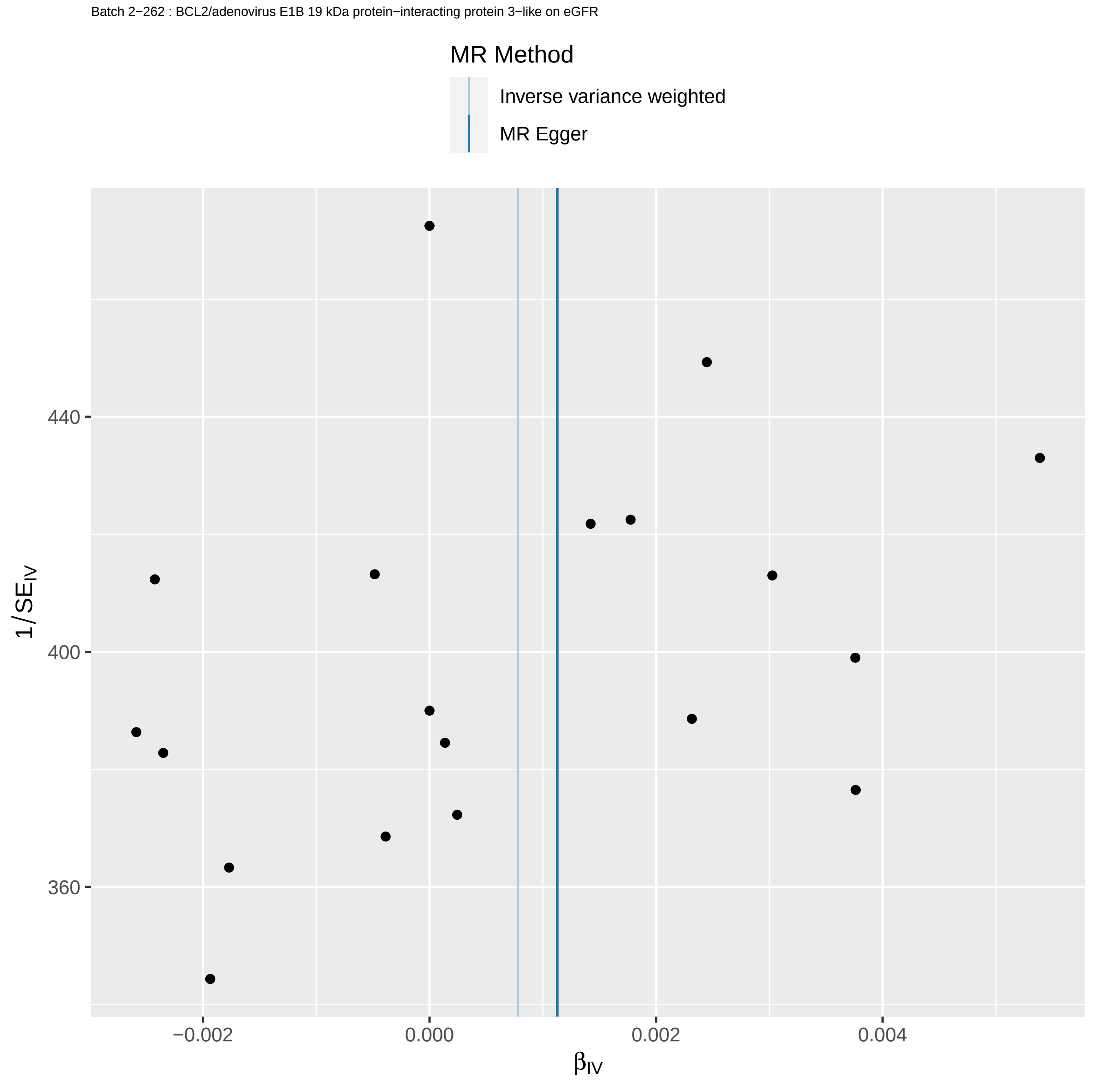

Batch 2−262 : BCL2/adenovirus E1B 19 kDa protein−interacting protein 3−like on eGFR
MR Method
Inverse variance weighted
MR Egger
440
400
360
−0.002
0.000
0.002
0.004
$1/SE_{IV}$
β_{IV}

Batch 2−263 : Brother of CDO on eGFR

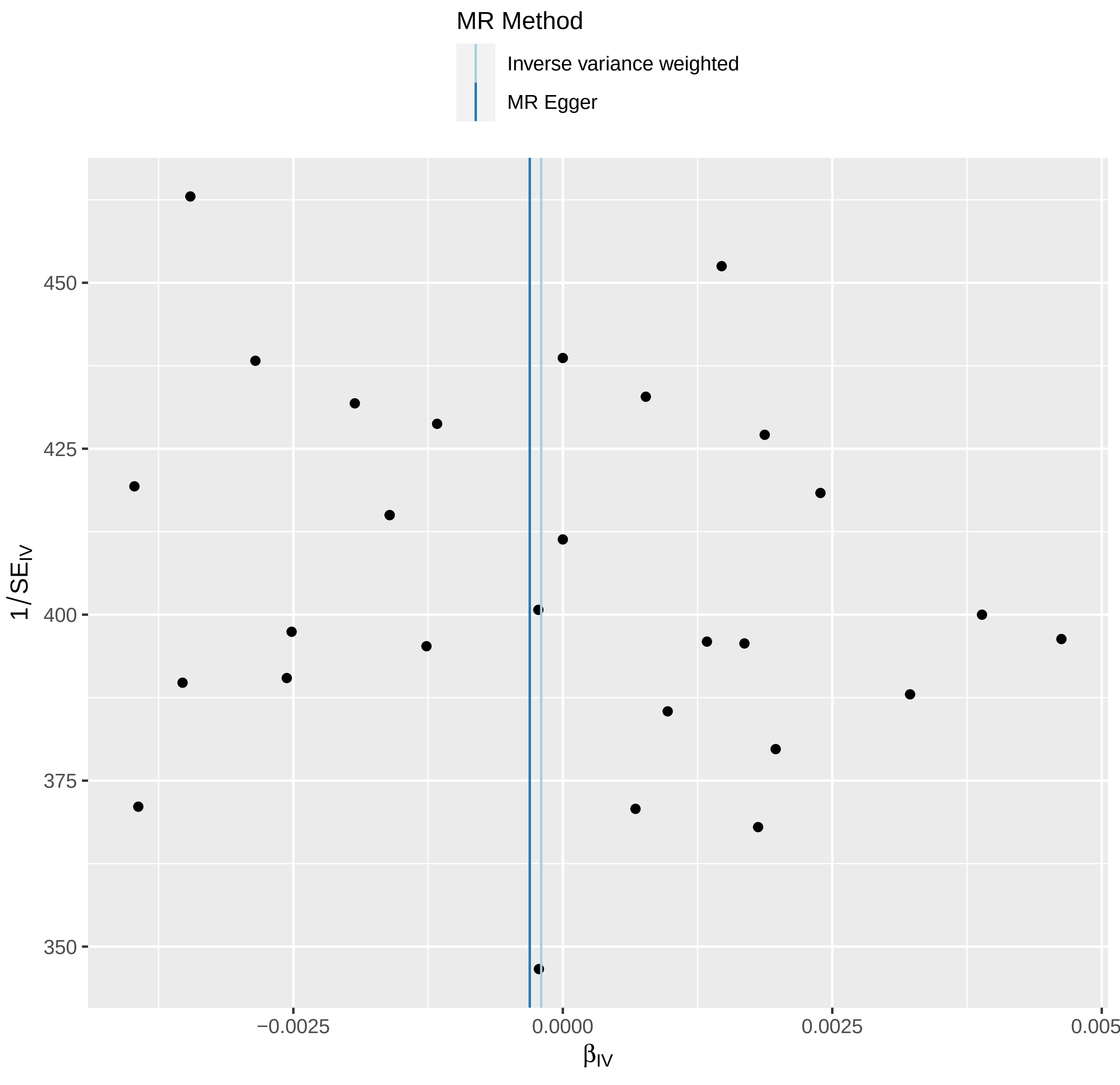

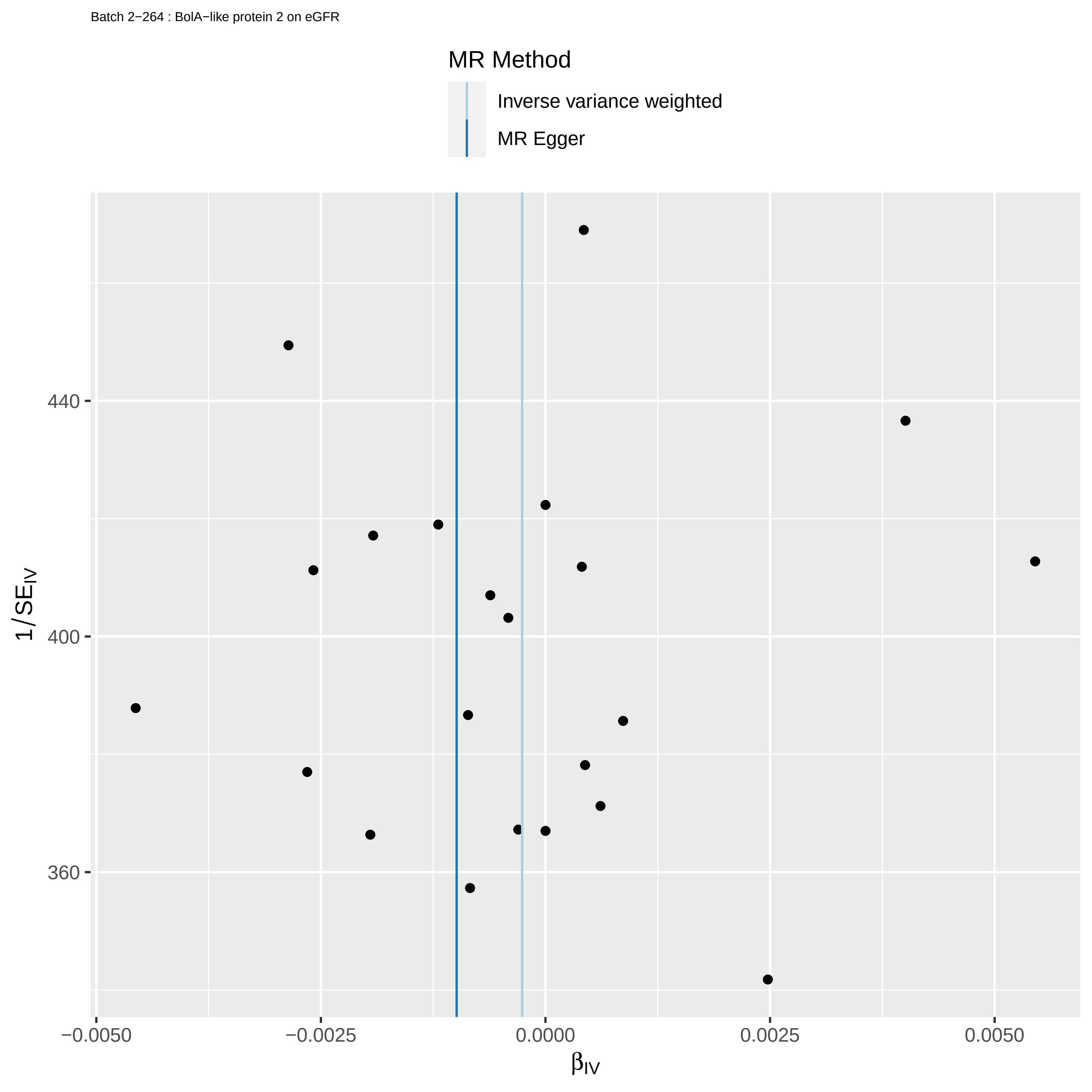
Batch 2−264 : BolA−like protein 2 on eGFR
MR Method
Inverse variance weighted
MR Egger
440
400
360
1/SE_IV
−0.0050
−0.0025
0.0000
0.0025
0.0050
β_IV

Batch 2−265 : Bisphosphoglycerate mutase on eGFR

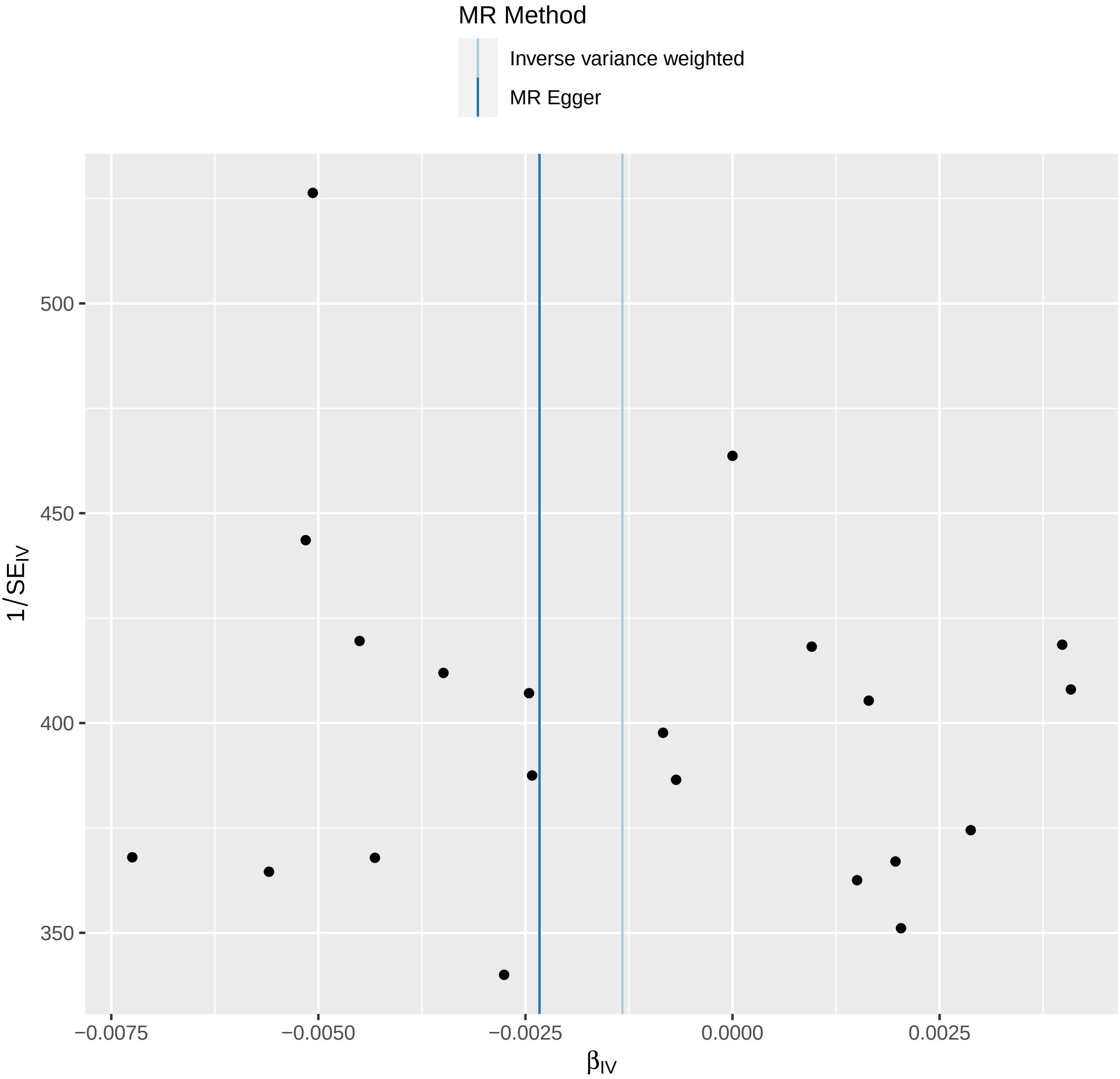

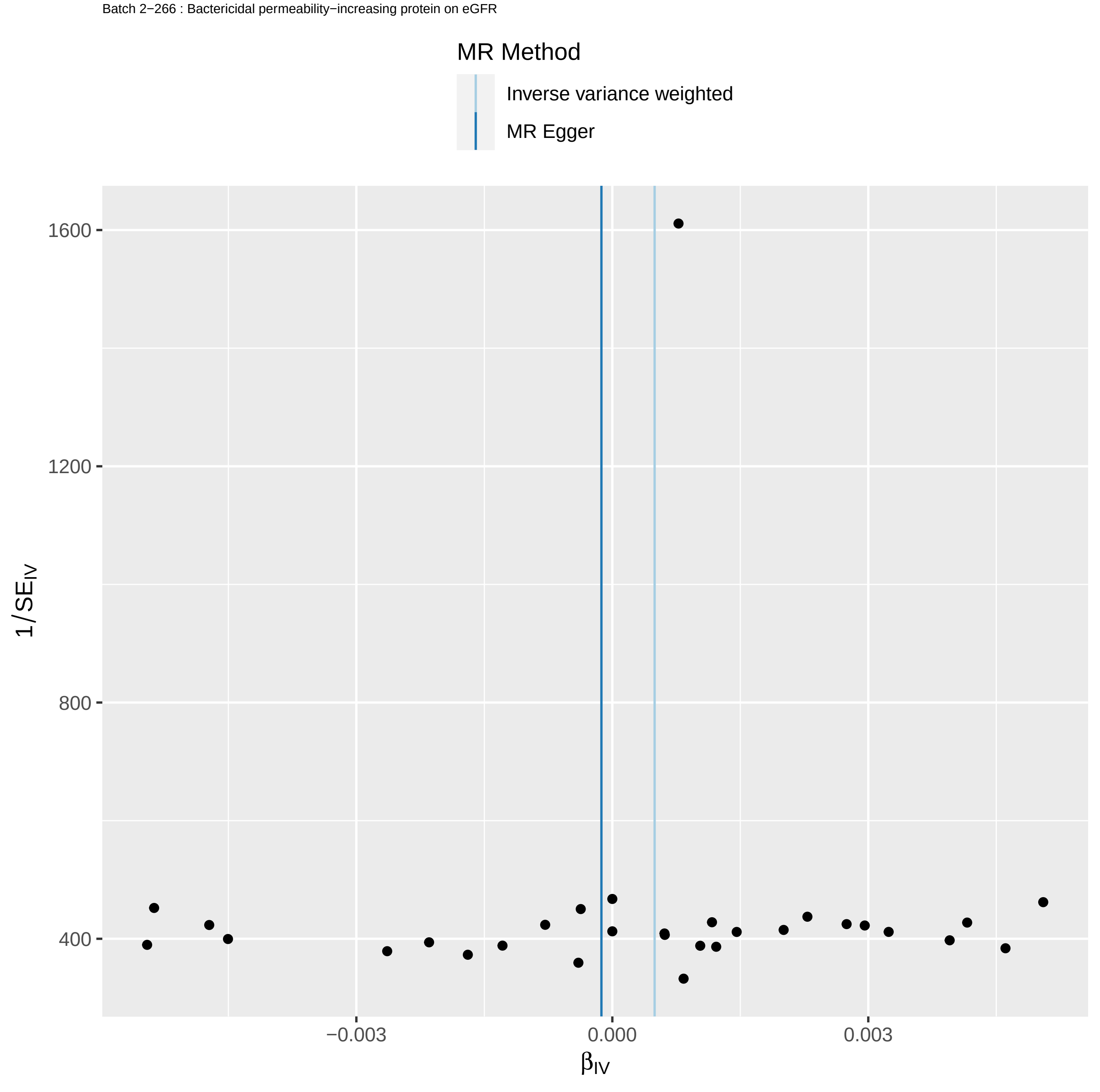
Batch 2−266 : Bactericidal permeability−increasing protein on eGFR
MR Method
Inverse variance weighted
MR Egger
1600
1200
800
400
1/SE_IV
−0.003
0.000
0.003
β_IV

Batch 2−267 : BPI fold−containing family A member 1 on eGFR

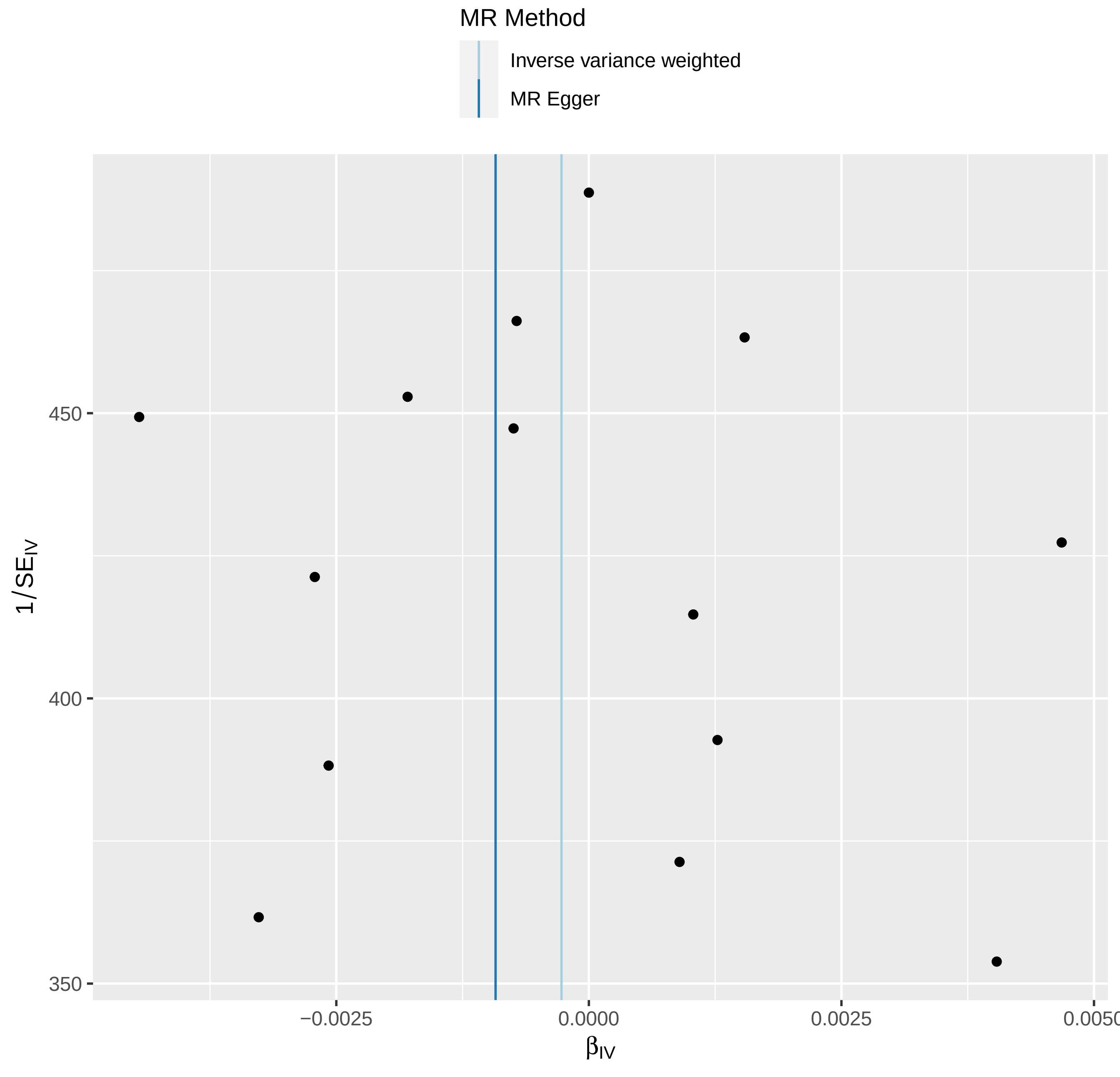

Batch 2−268 : BPI fold−containing family B member 1 on eGFR

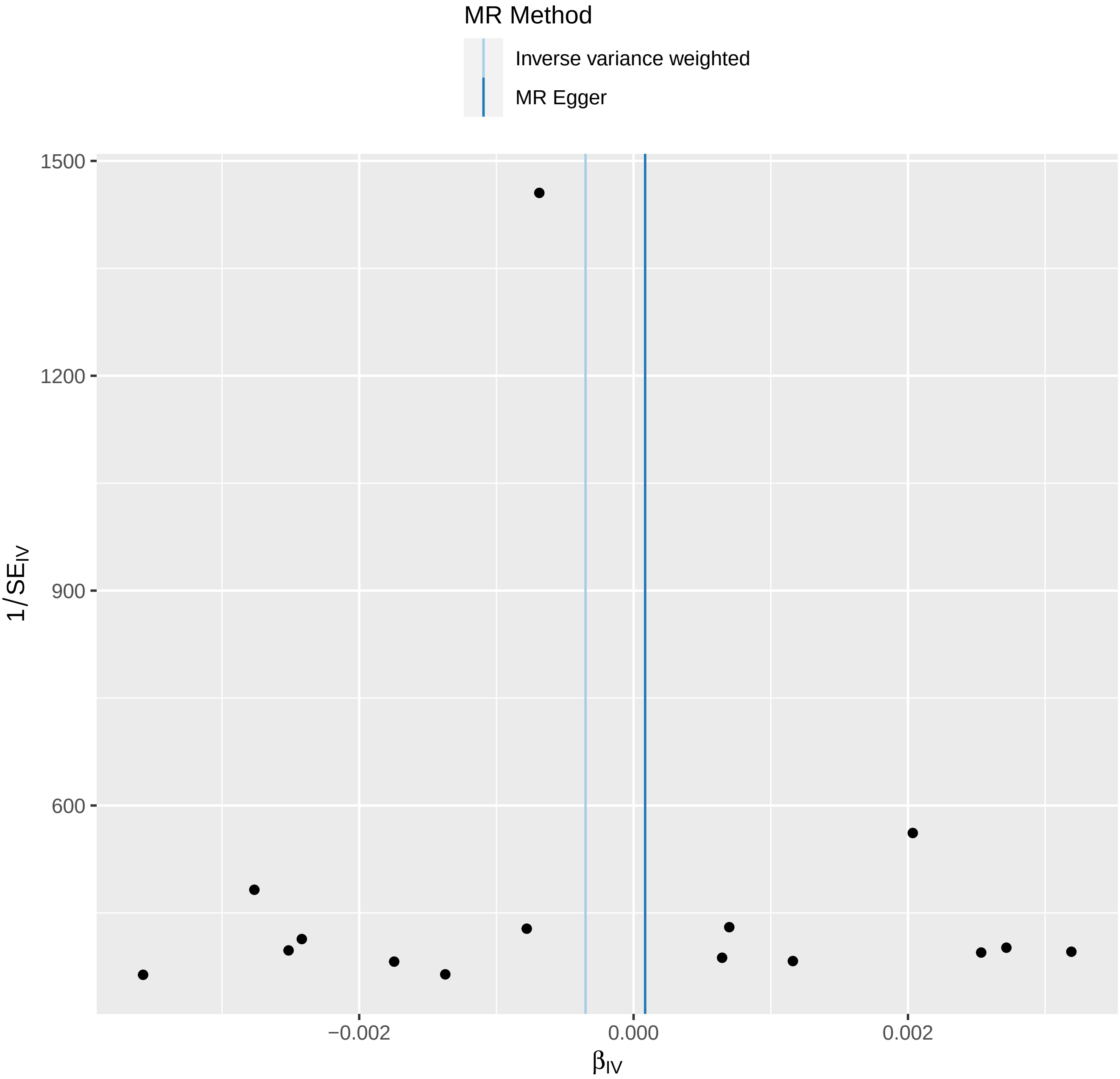

Batch 2−269 : Bromodomain−containing protein 1 on eGFR

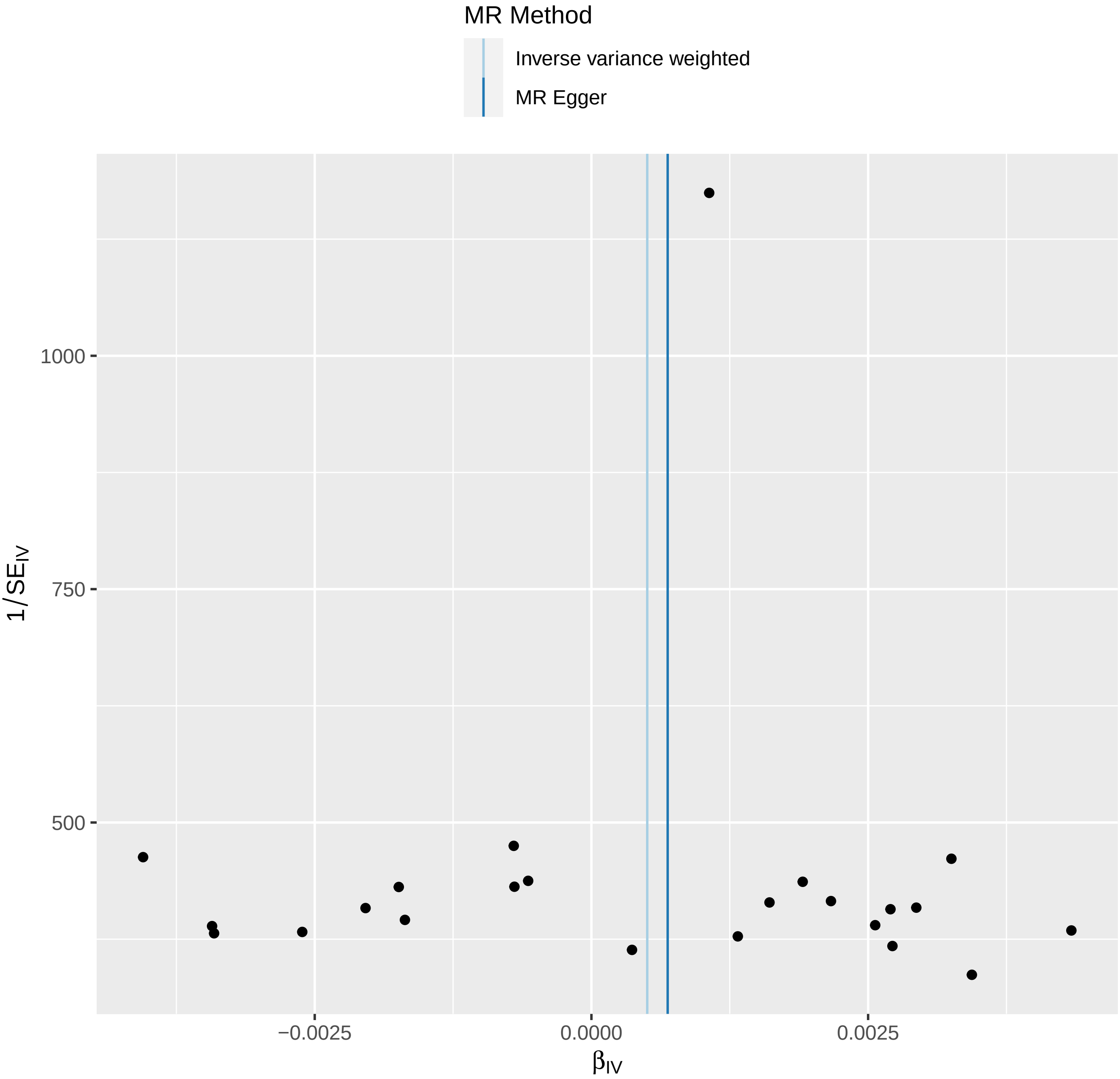

Batch 2−270 : Bromodomain testis−specific protein on eGFR

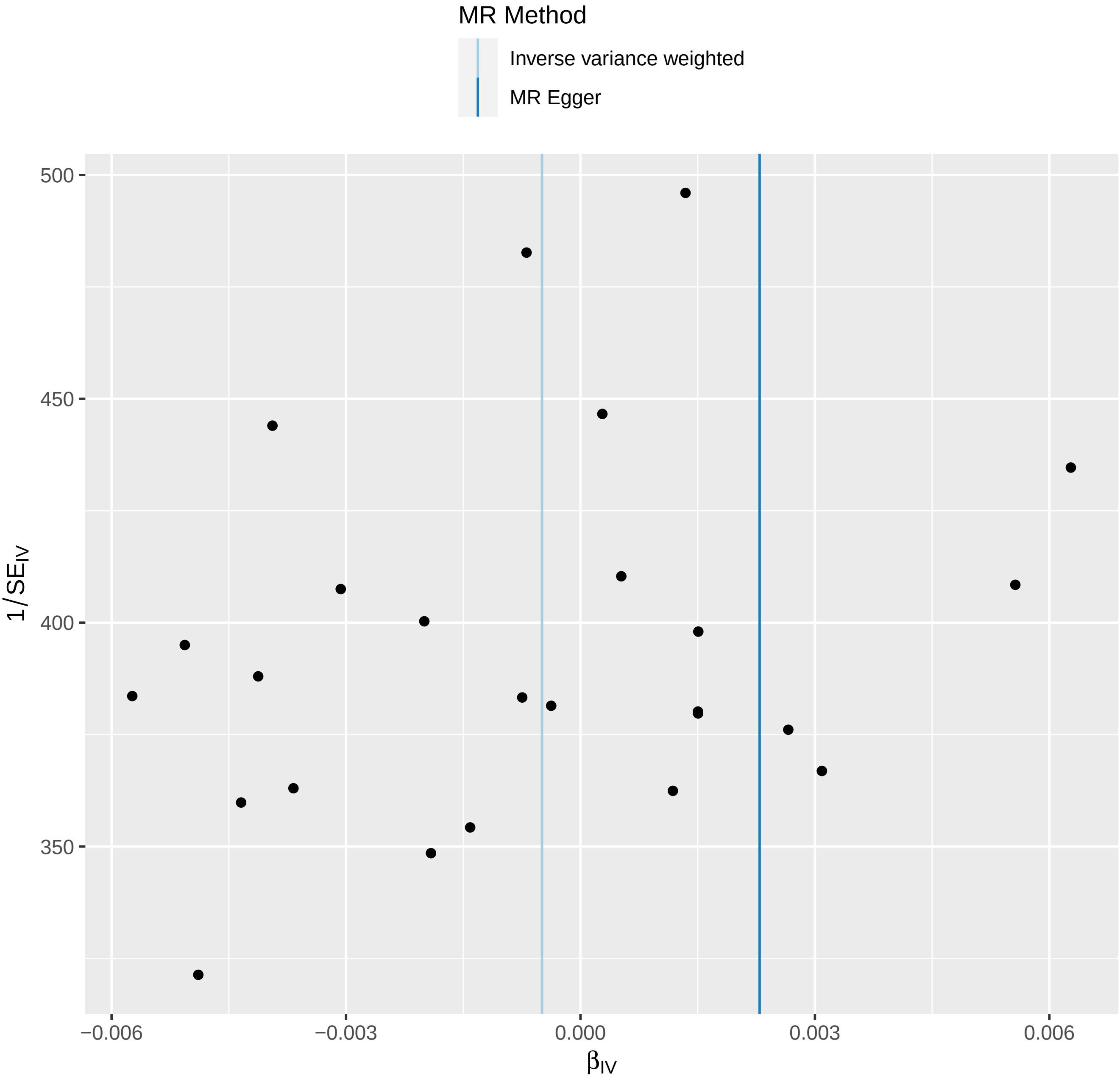

Batch 2−271 : Transcription factor IIIB 90 kDa subunit on eGFR

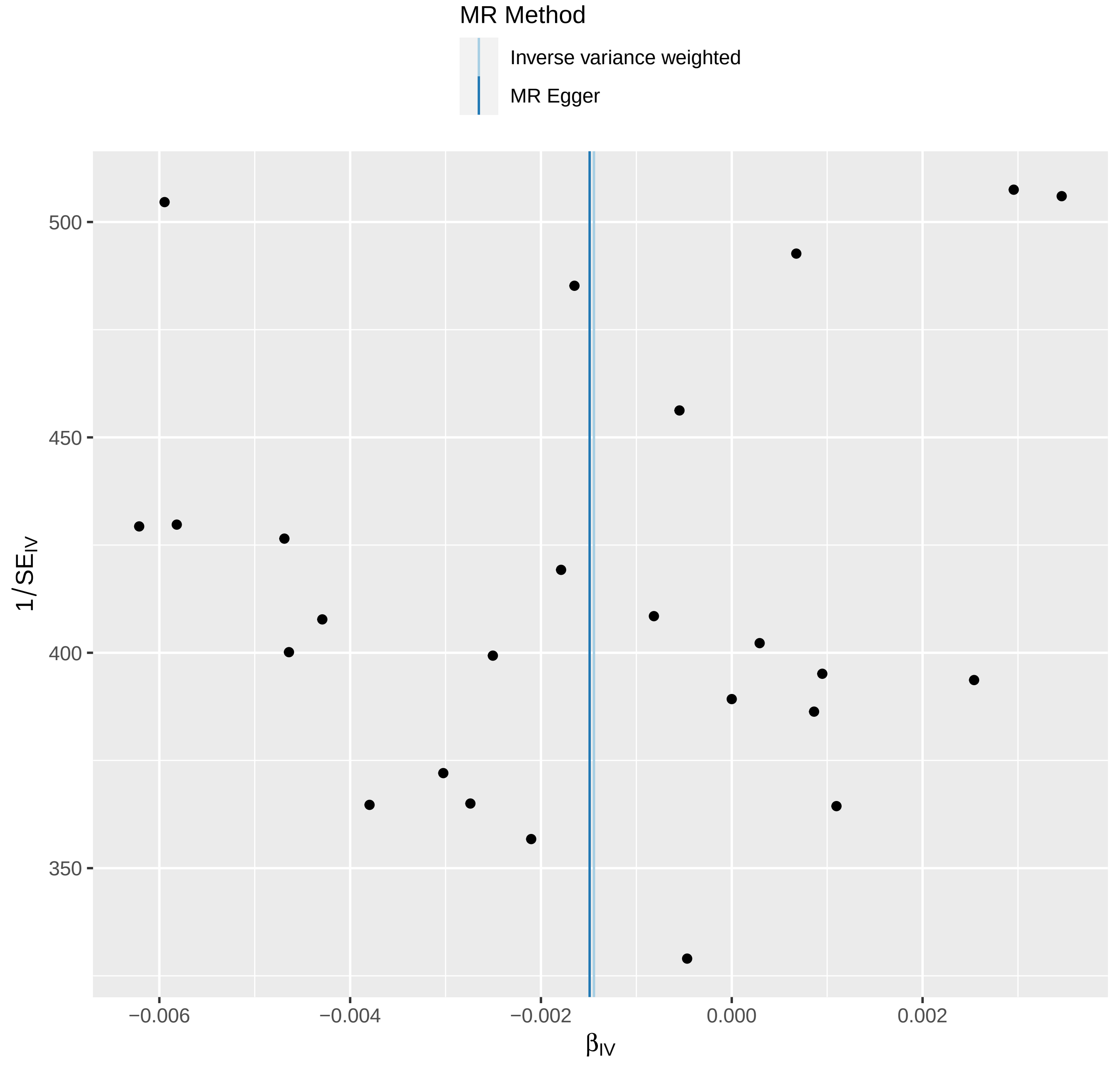

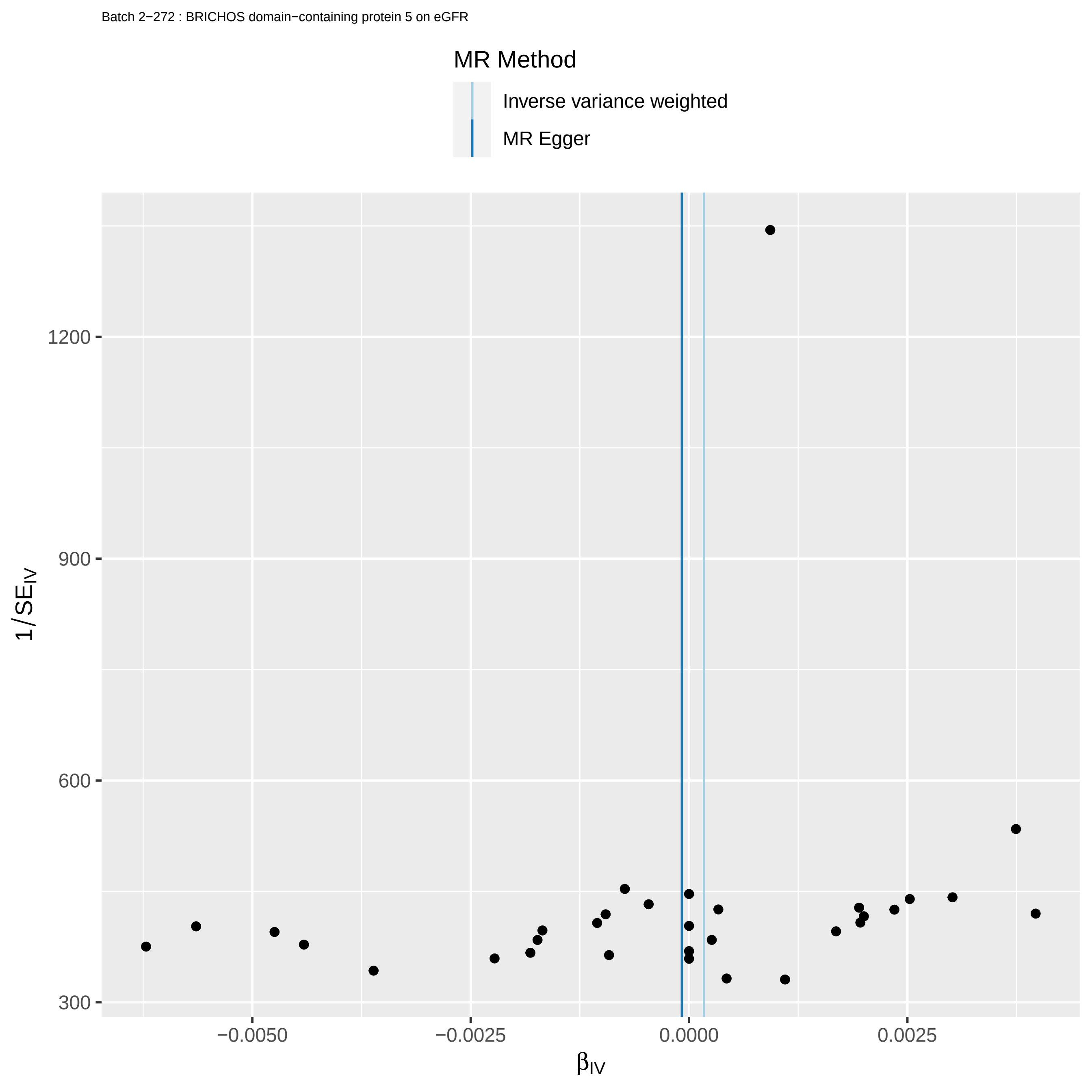
Batch 2−272 : BRICHOS domain−containing protein 5 on eGFR
MR Method
Inverse variance weighted
MR Egger
1200
900
600
300
1/SE_IV
−0.0050
−0.0025
0.0000
0.0025
β_IV

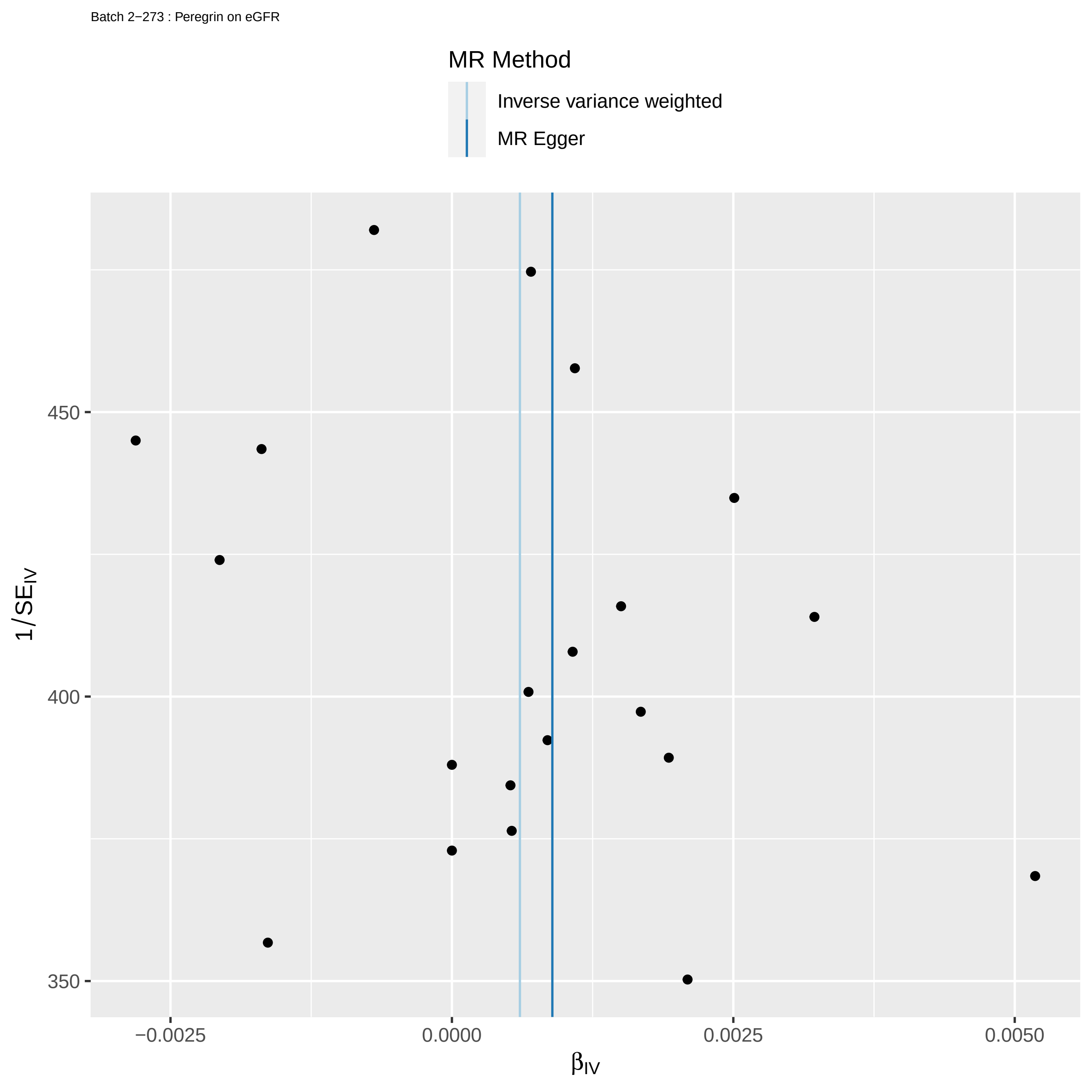
Batch 2−273 : Peregrin on eGFR
MR Method
Inverse variance weighted
MR Egger
1/SE_IV
450
400
350
−0.0025
0.0000
0.0025
0.0050
β_IV

Batch 2−274 : Serine/threonine−protein kinase BRSK2 on eGFR

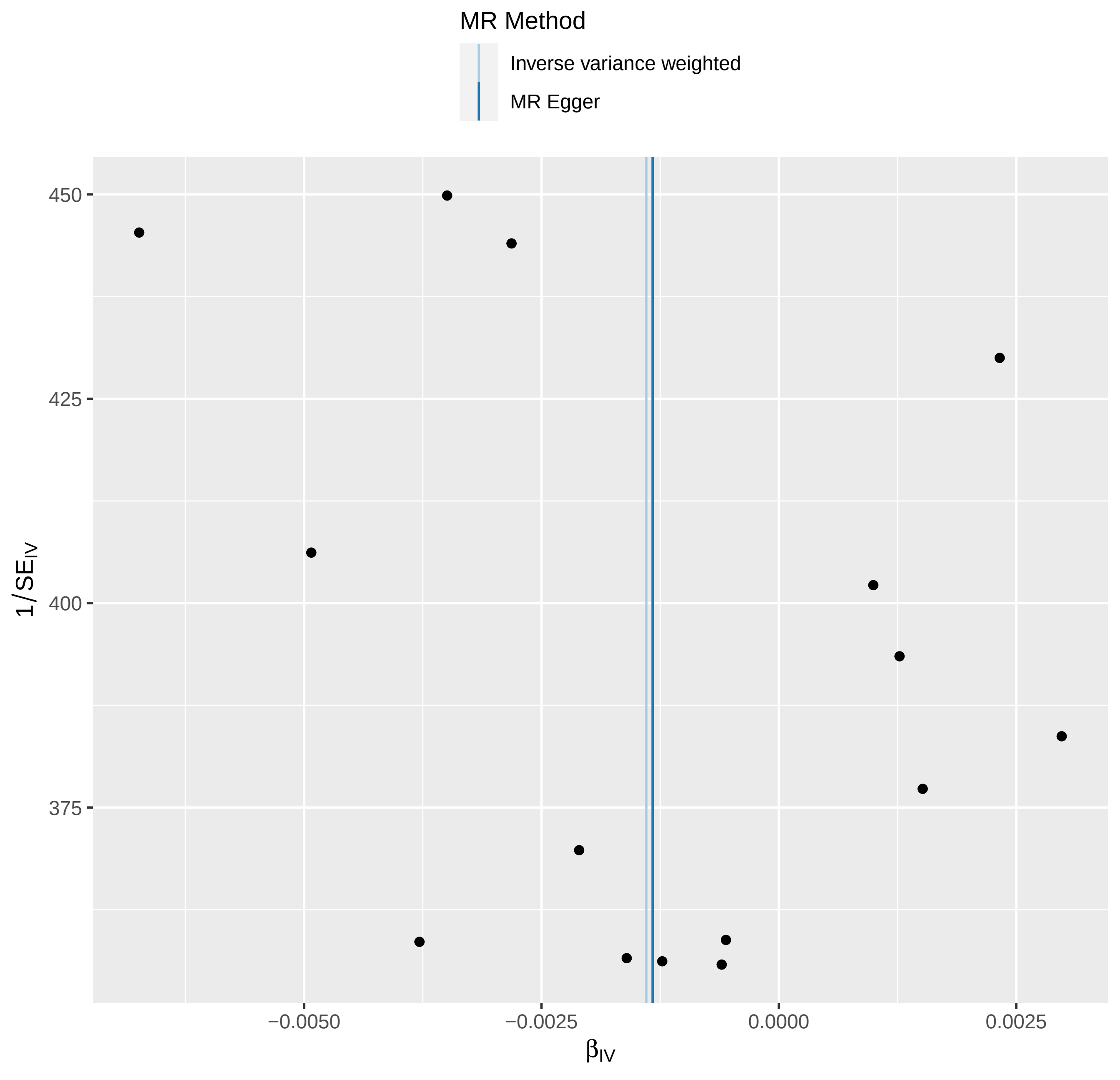

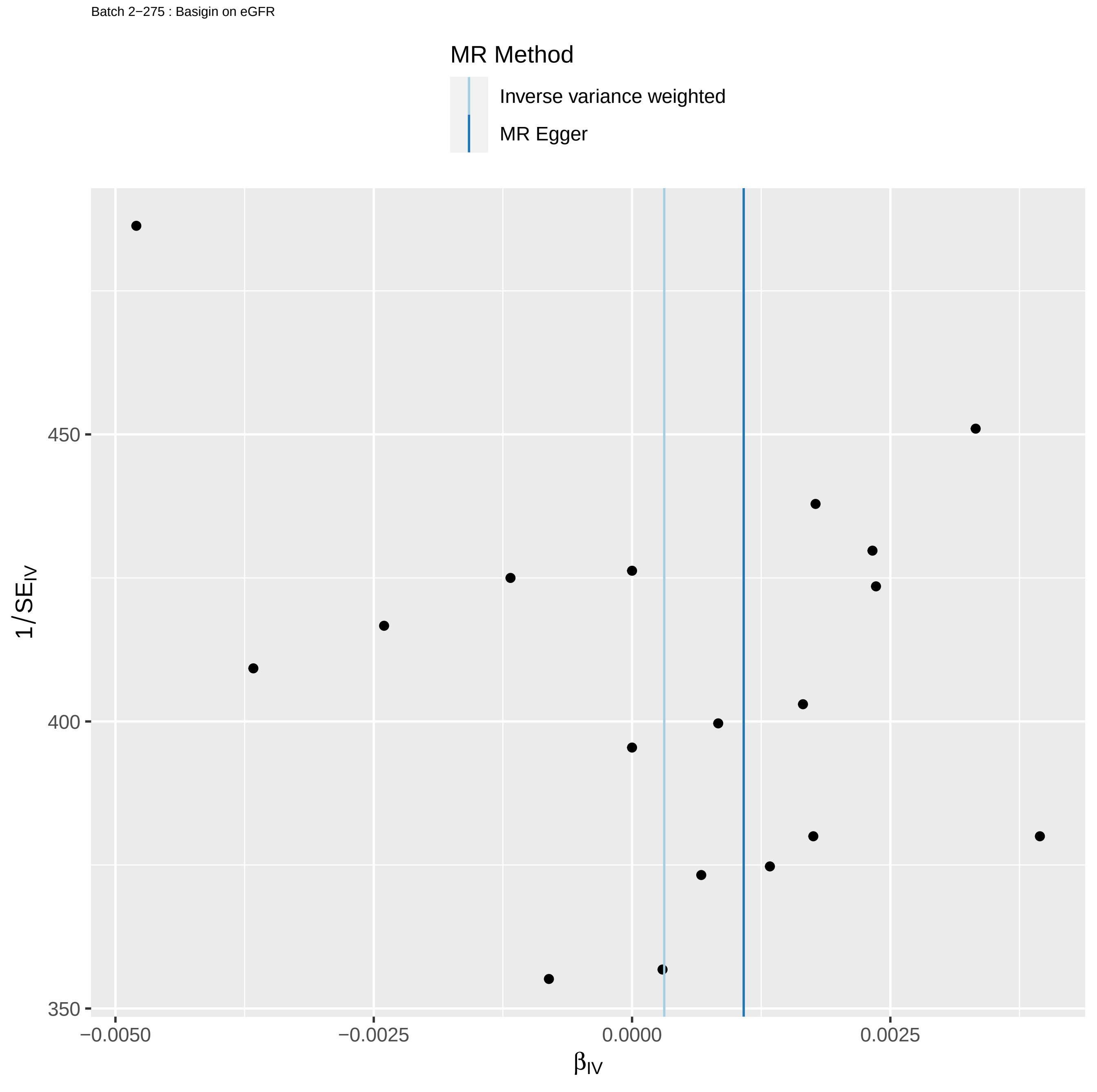

Batch 2−275 : Basigin on eGFR
MR Method
Inverse variance weighted
MR Egger
450
400
350
−0.0050
−0.0025
0.0000
0.0025
1/SE_IV
β_IV

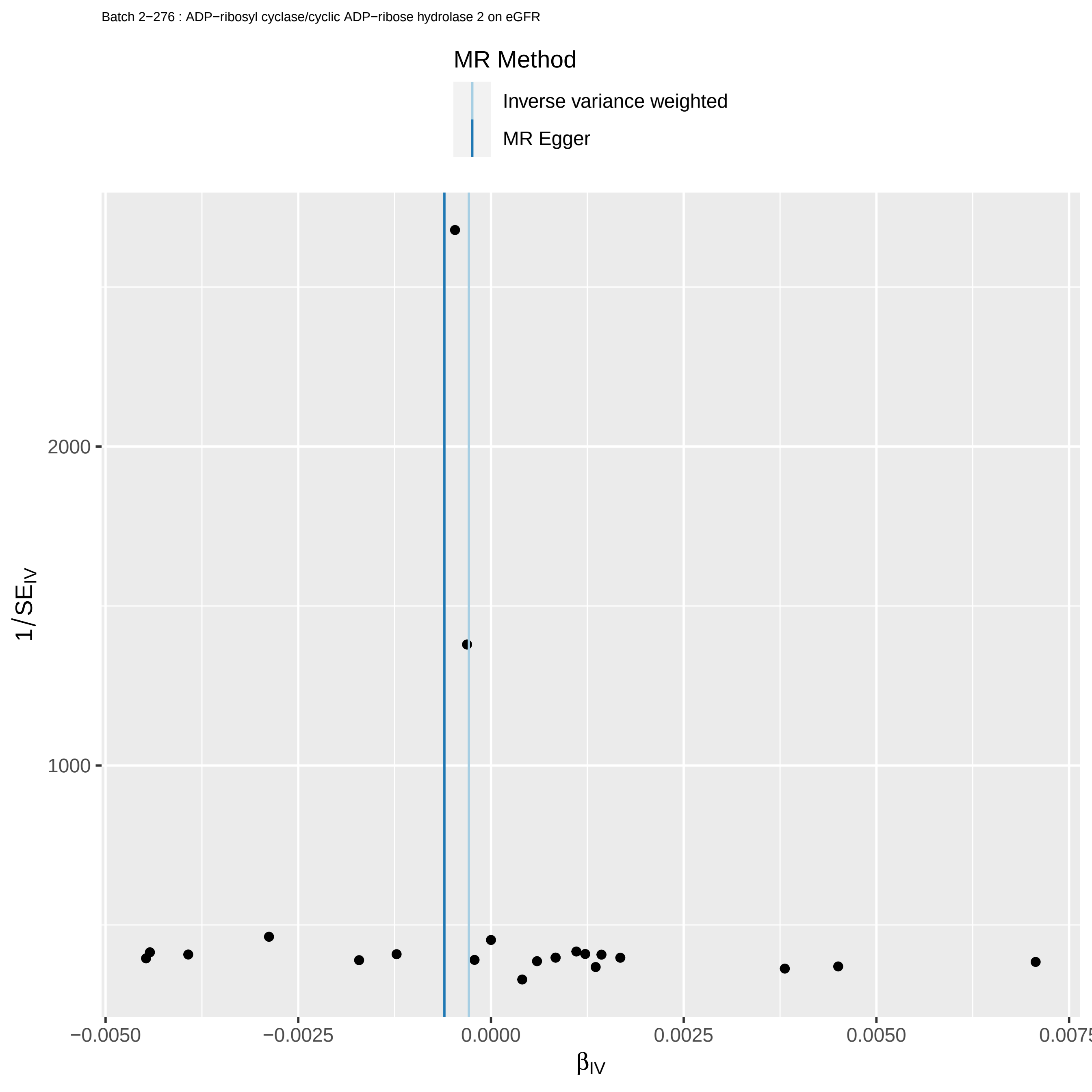

Batch 2−276 : ADP−ribosyl cyclase/cyclic ADP−ribose hydrolase 2 on eGFR
MR Method
Inverse variance weighted
MR Egger
2000
1000
1/SE_IV
−0.0050
−0.0025
0.0000
0.0025
0.0050
0.0075
β_IV

Batch 2−277 : Tyrosine−protein kinase BTK on eGFR

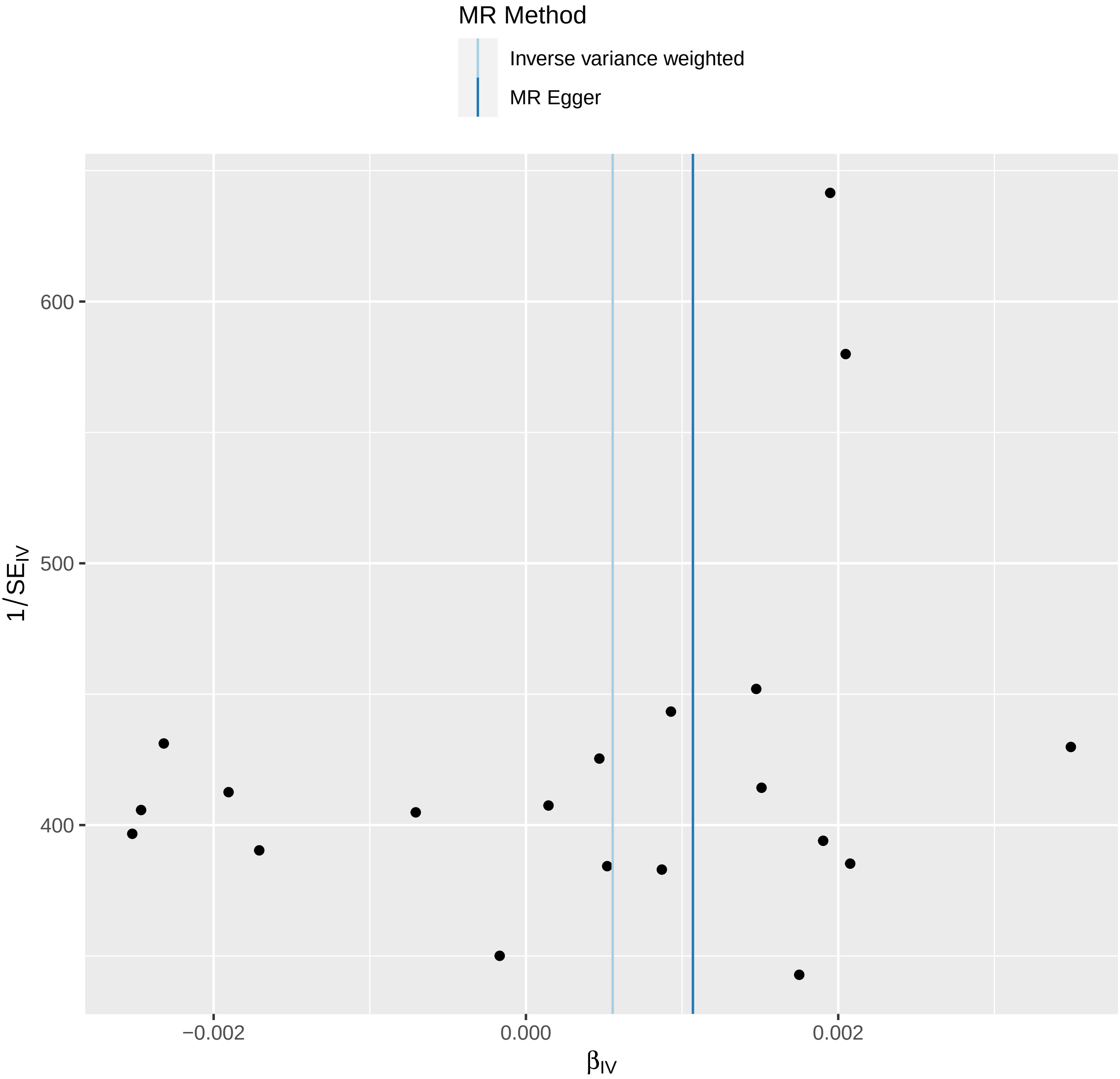

Batch 2−278 : Butyrophilin−like protein 9 on eGFR

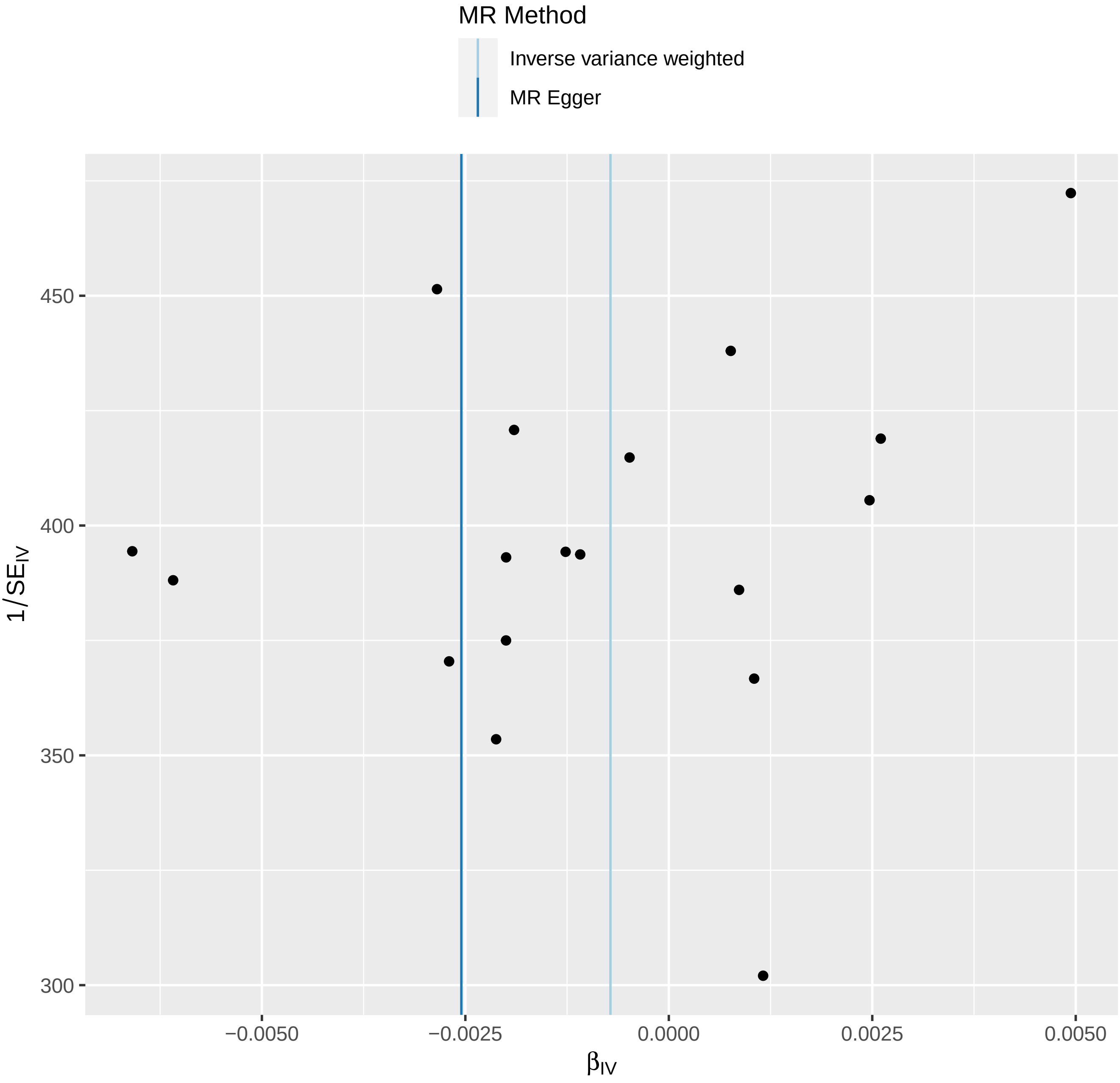

Batch 2−279 : Mitotic checkpoint serine/threonine−protein kinase BUB1 on eGFR

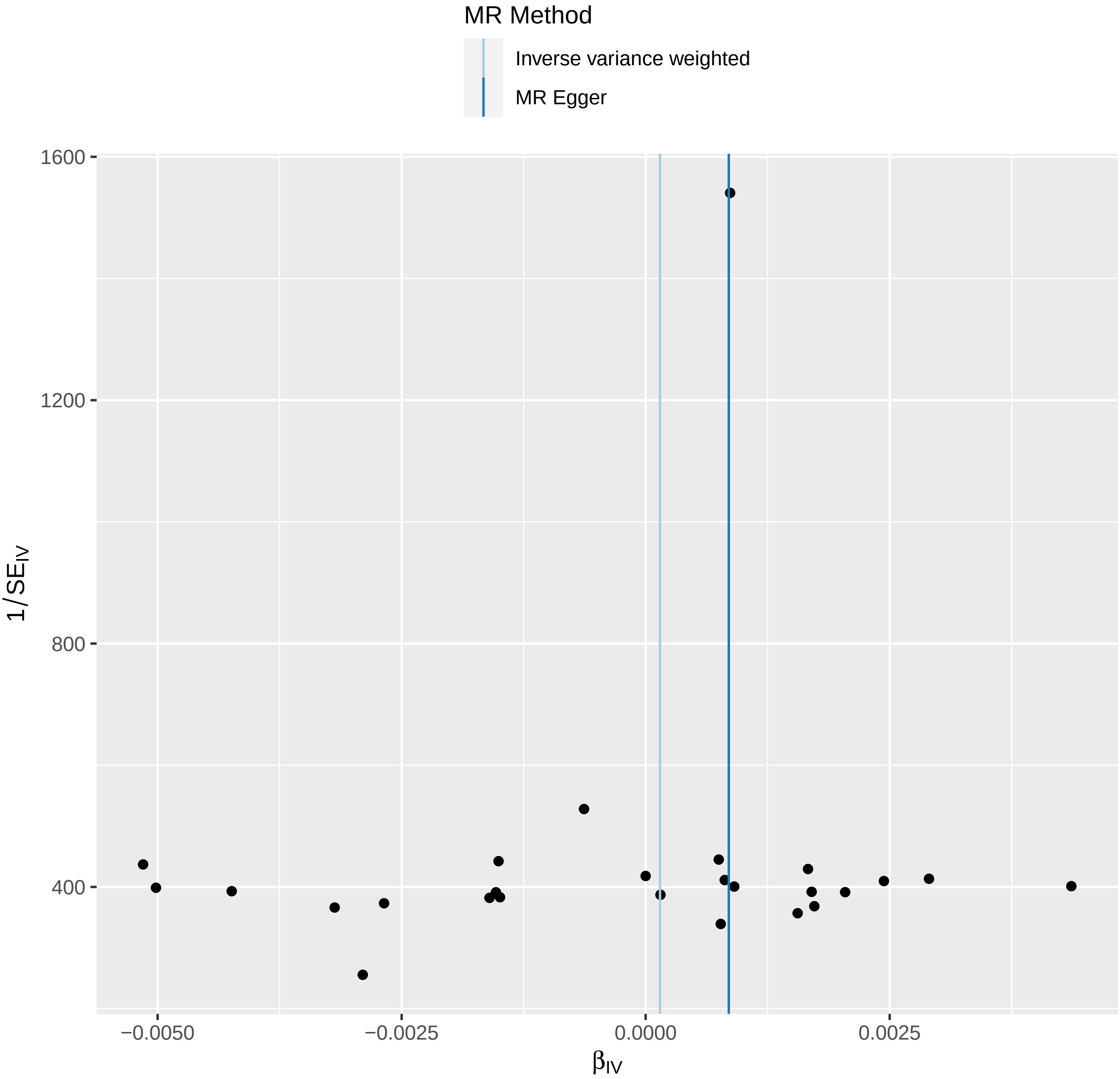

Batch 2−280 : Uncharacterized protein C10orf35 on eGFR

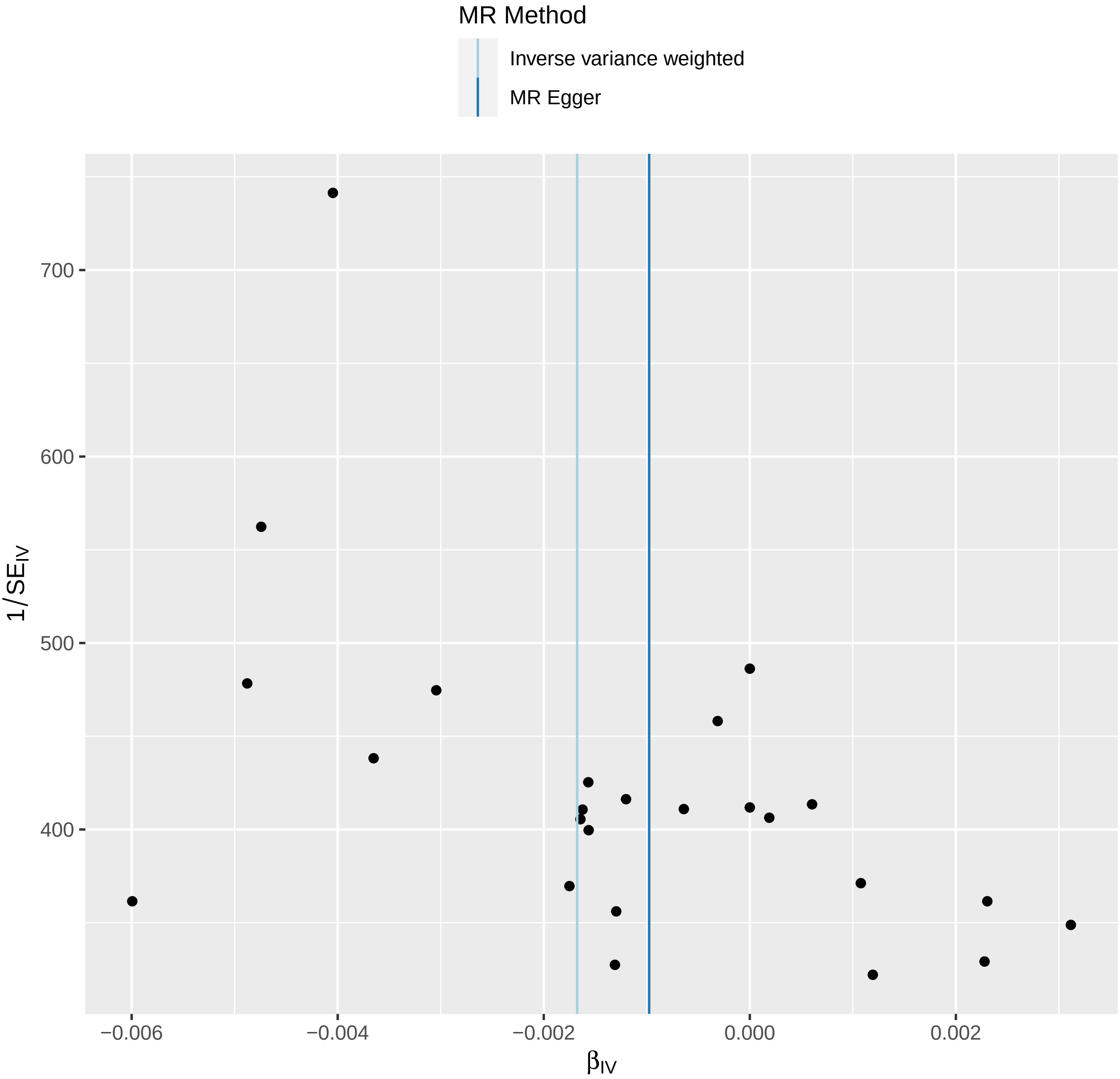

Batch 2−281 : Platelet receptor Gi24 on eGFR

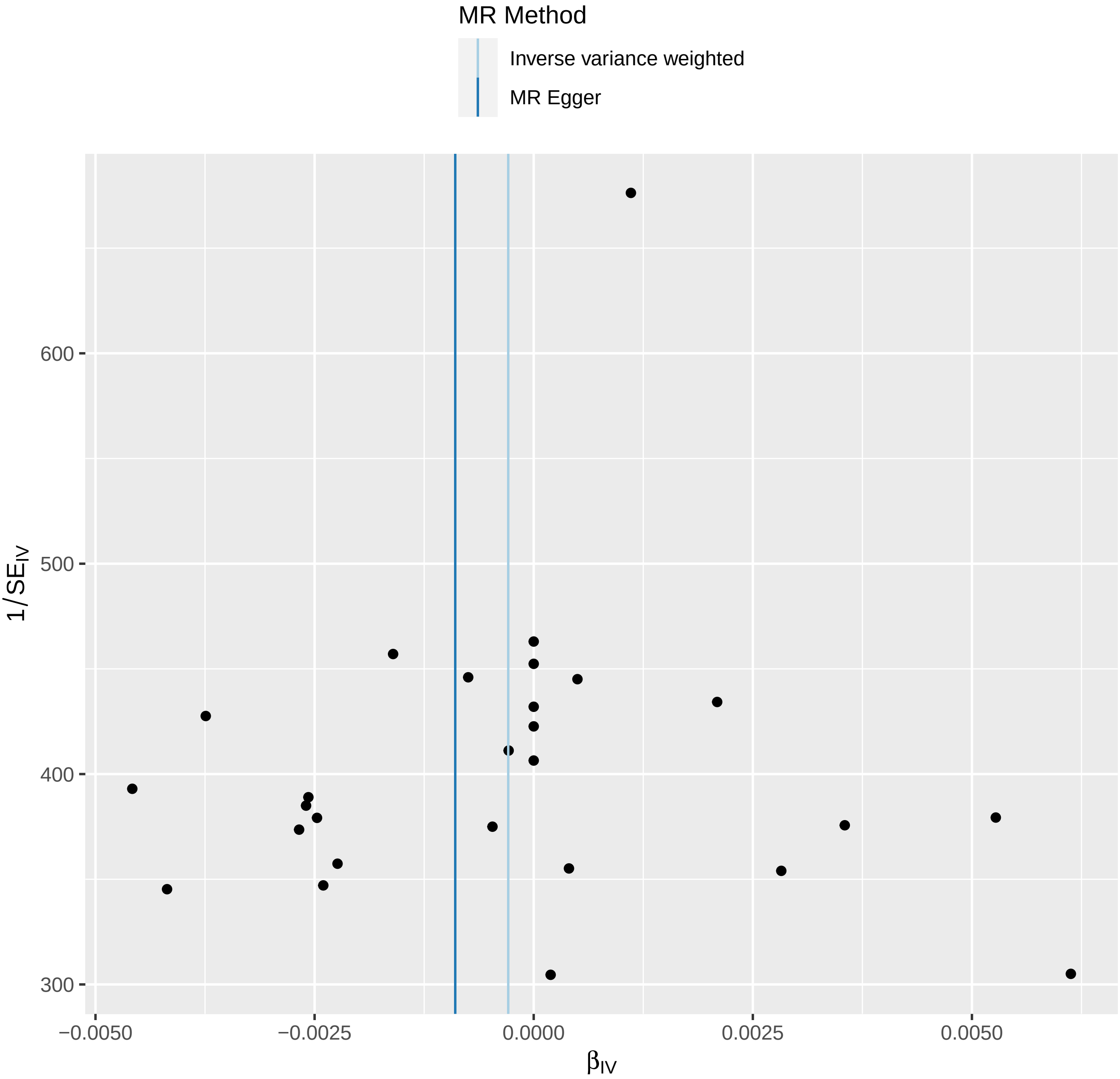

Batch 2−282 : UPF0696 protein C11orf68 on eGFR

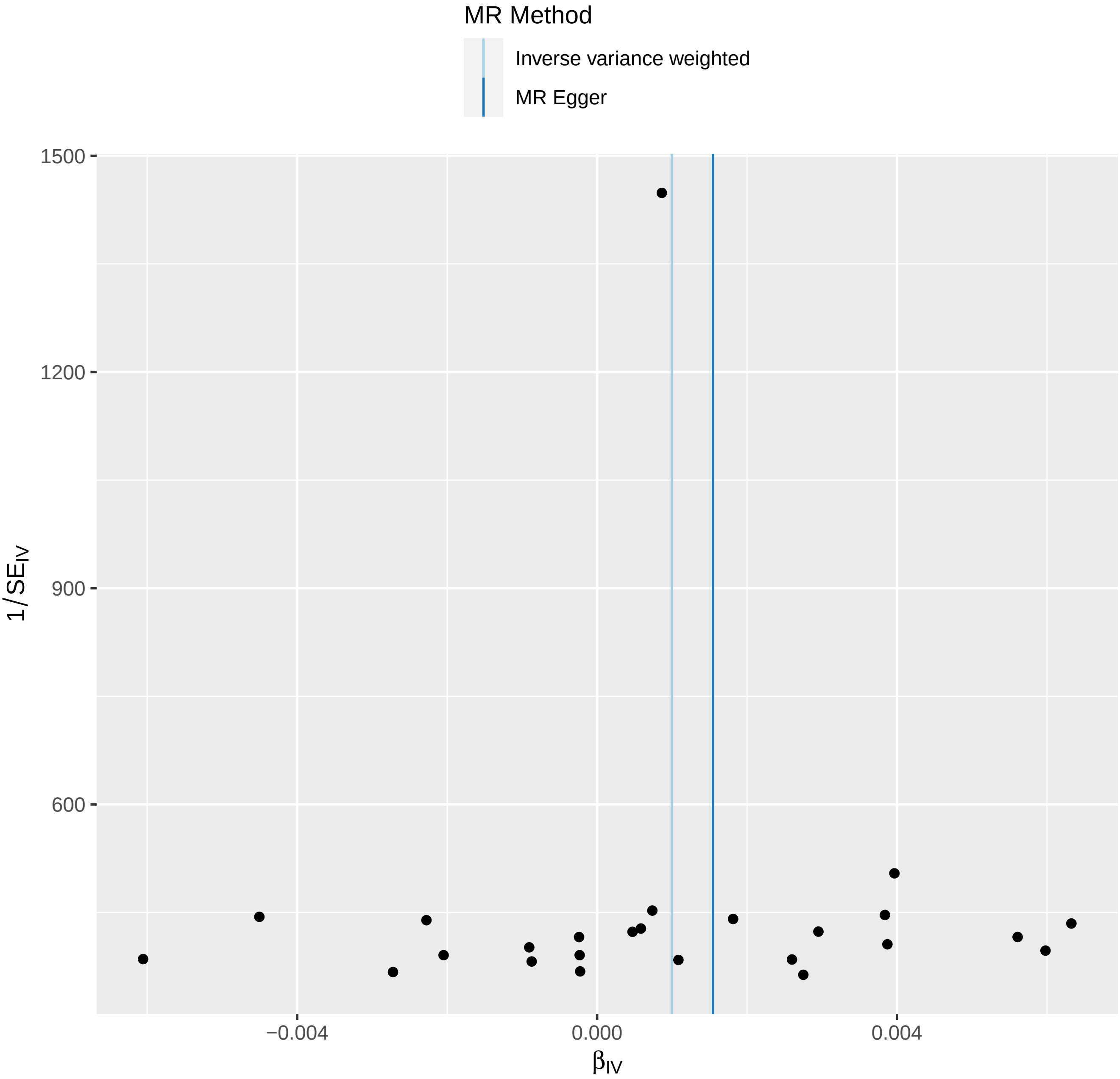

Batch 2−283 : UPF0454 protein C12orf49 on eGFR

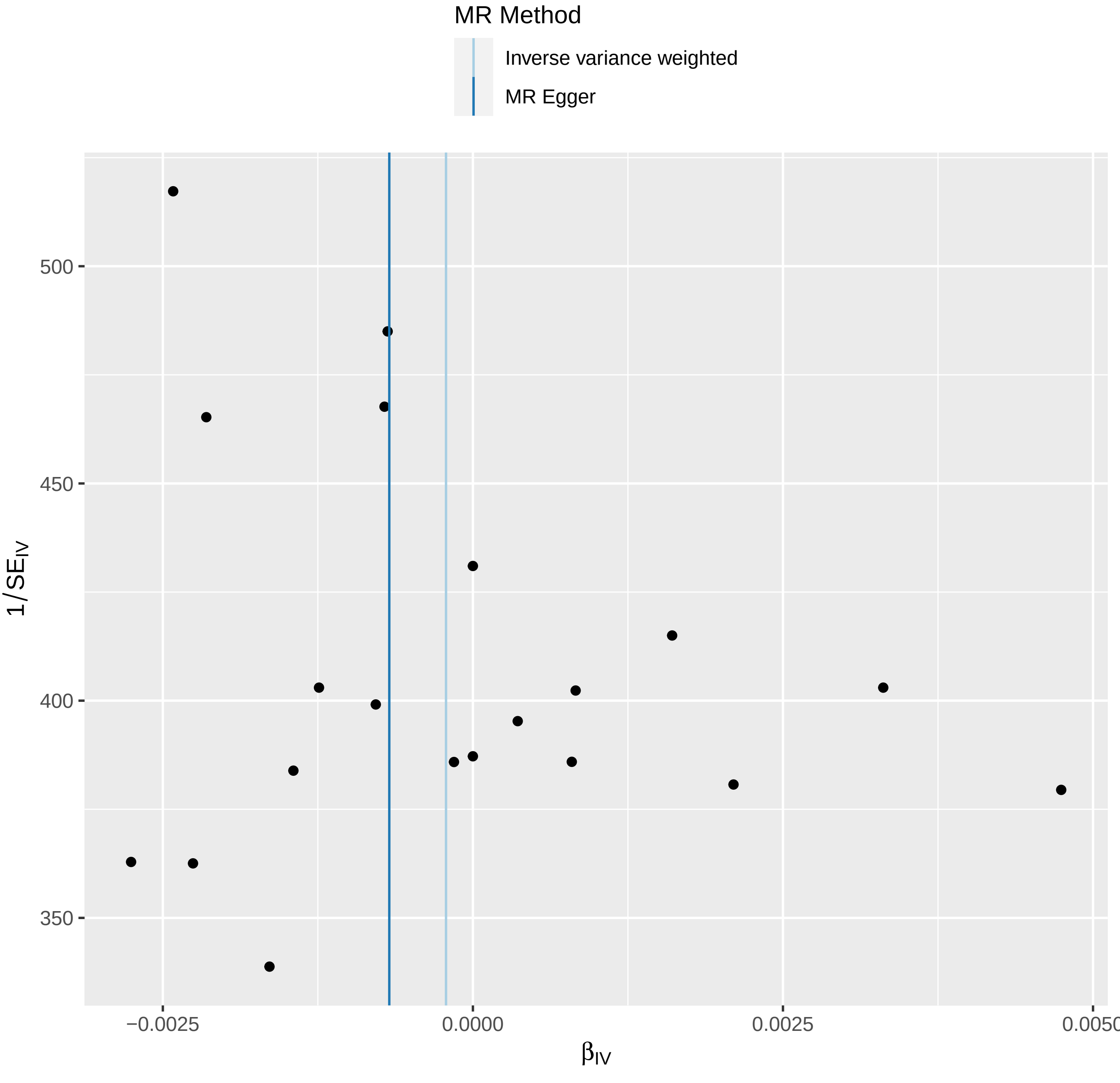

Batch 2−284 : Uncharacterized protein C14orf93 on eGFR

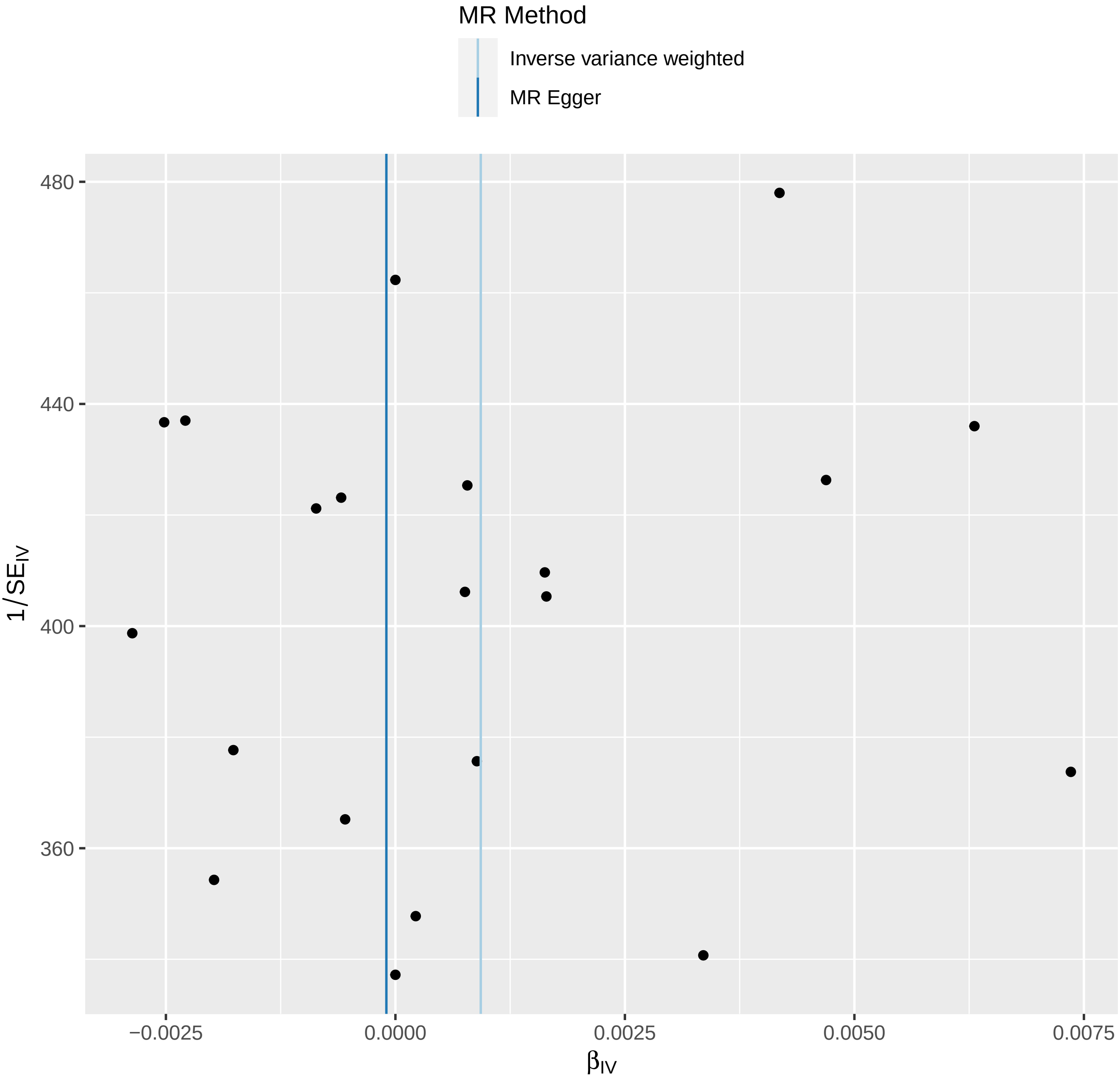

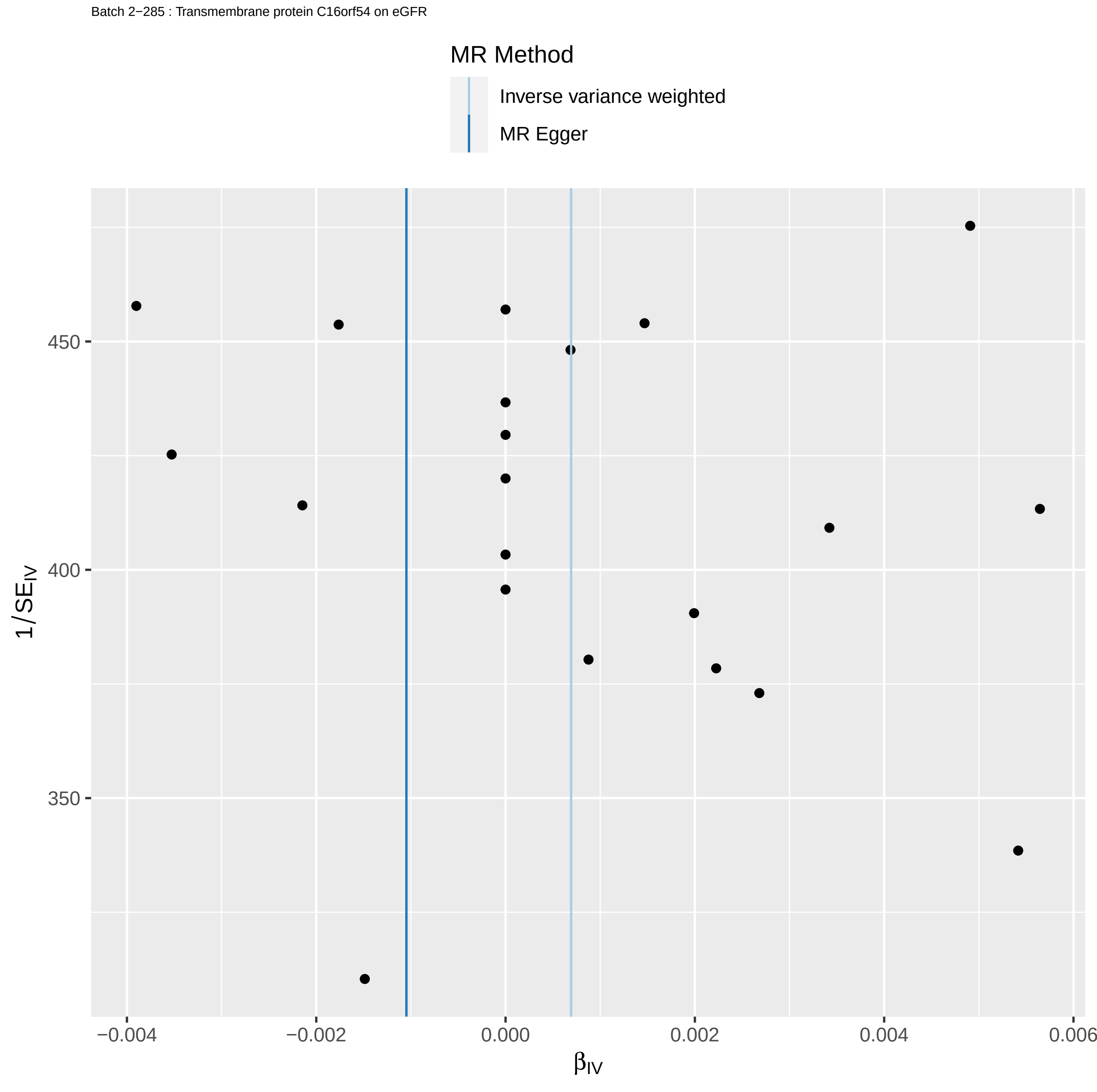

Batch 2−285 : Transmembrane protein C16orf54 on eGFR
MR Method
Inverse variance weighted
MR Egger
450
400
350
1/SE$_{IV}$
−0.004
−0.002
0.000
0.002
0.004
0.006
β$_{IV}$

Batch 2−286 : Uncharacterized protein C17orf67 on eGFR

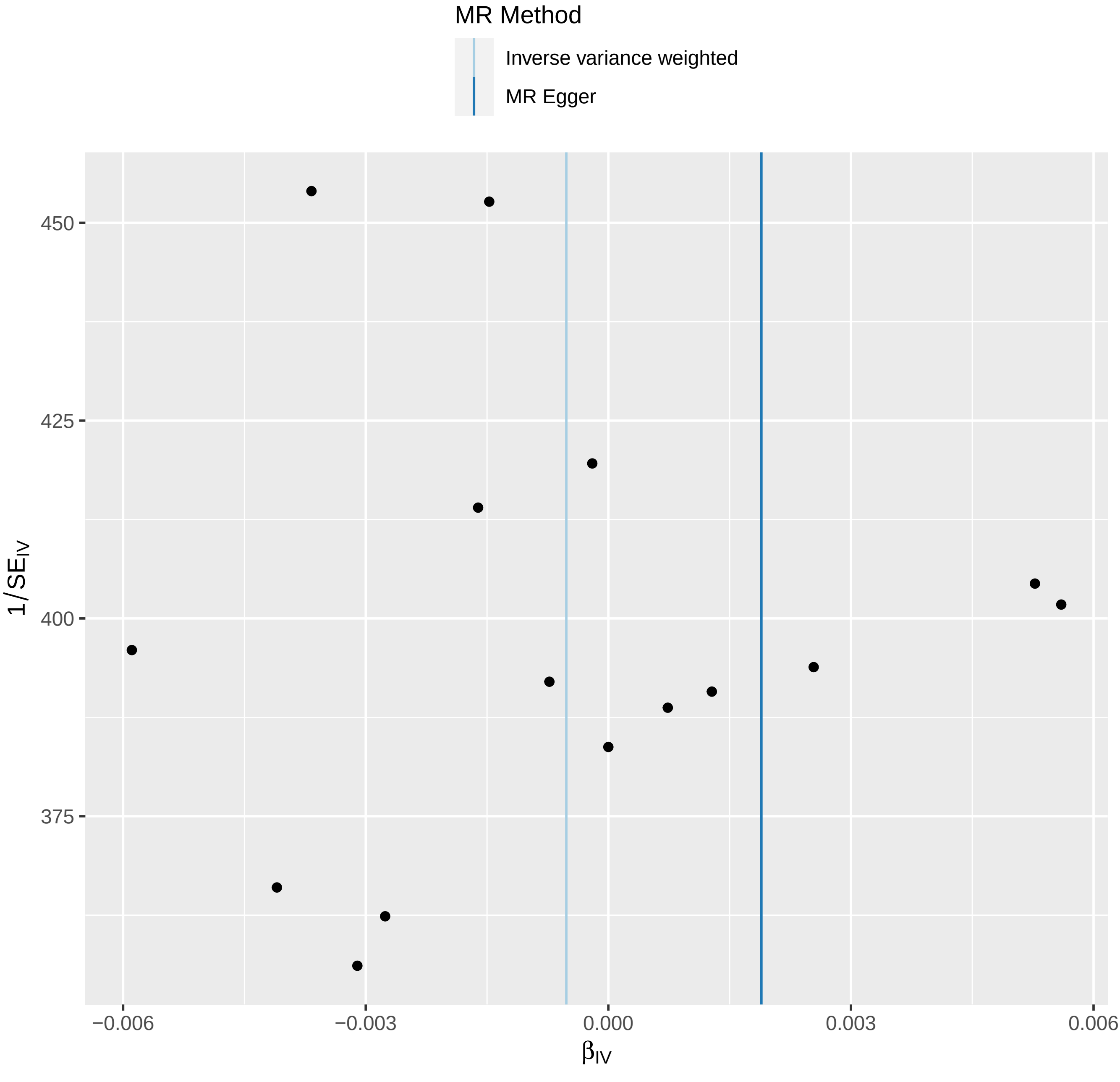

Batch 2−287 : Uncharacterized protein C17orf78 on eGFR

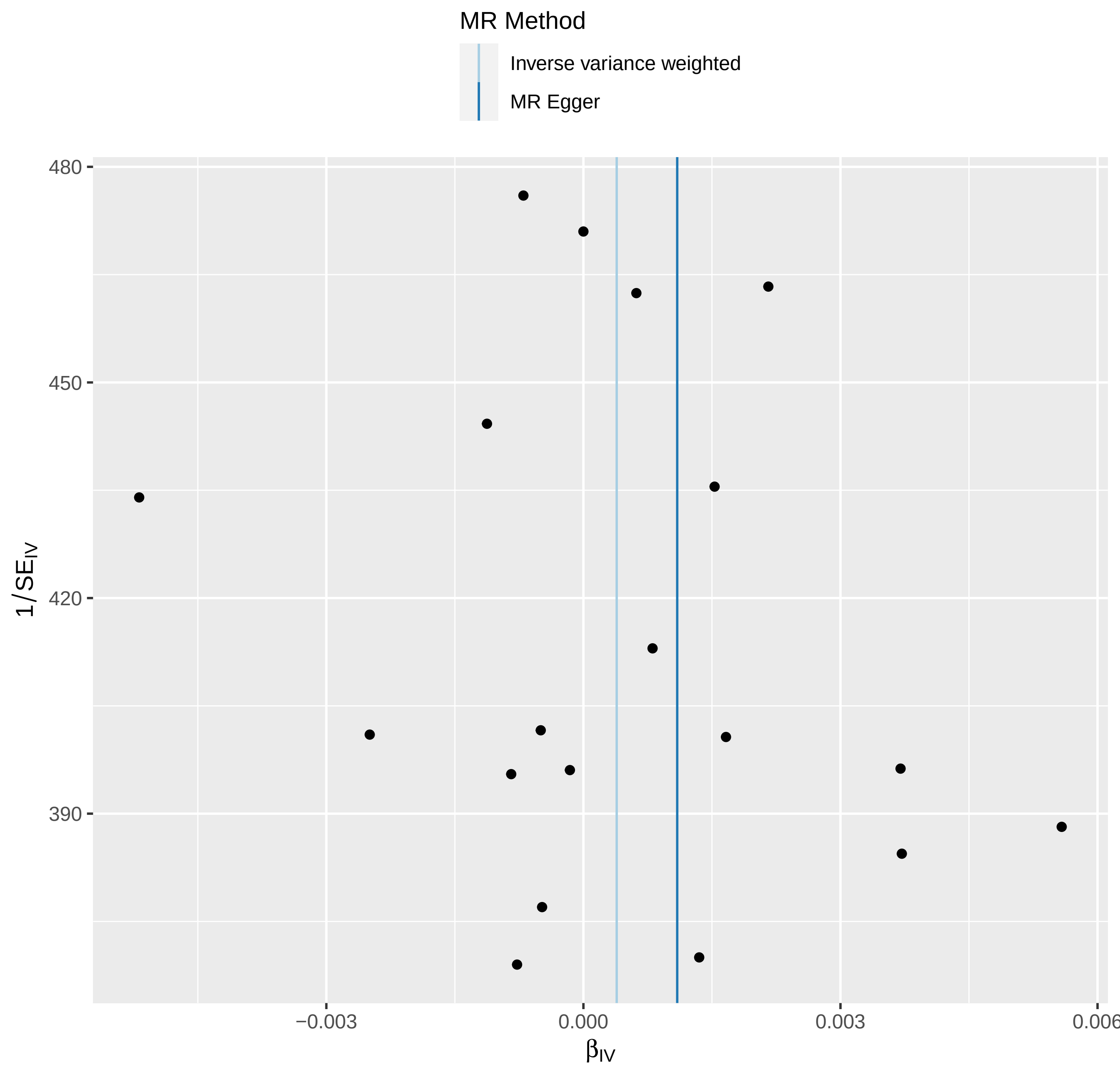

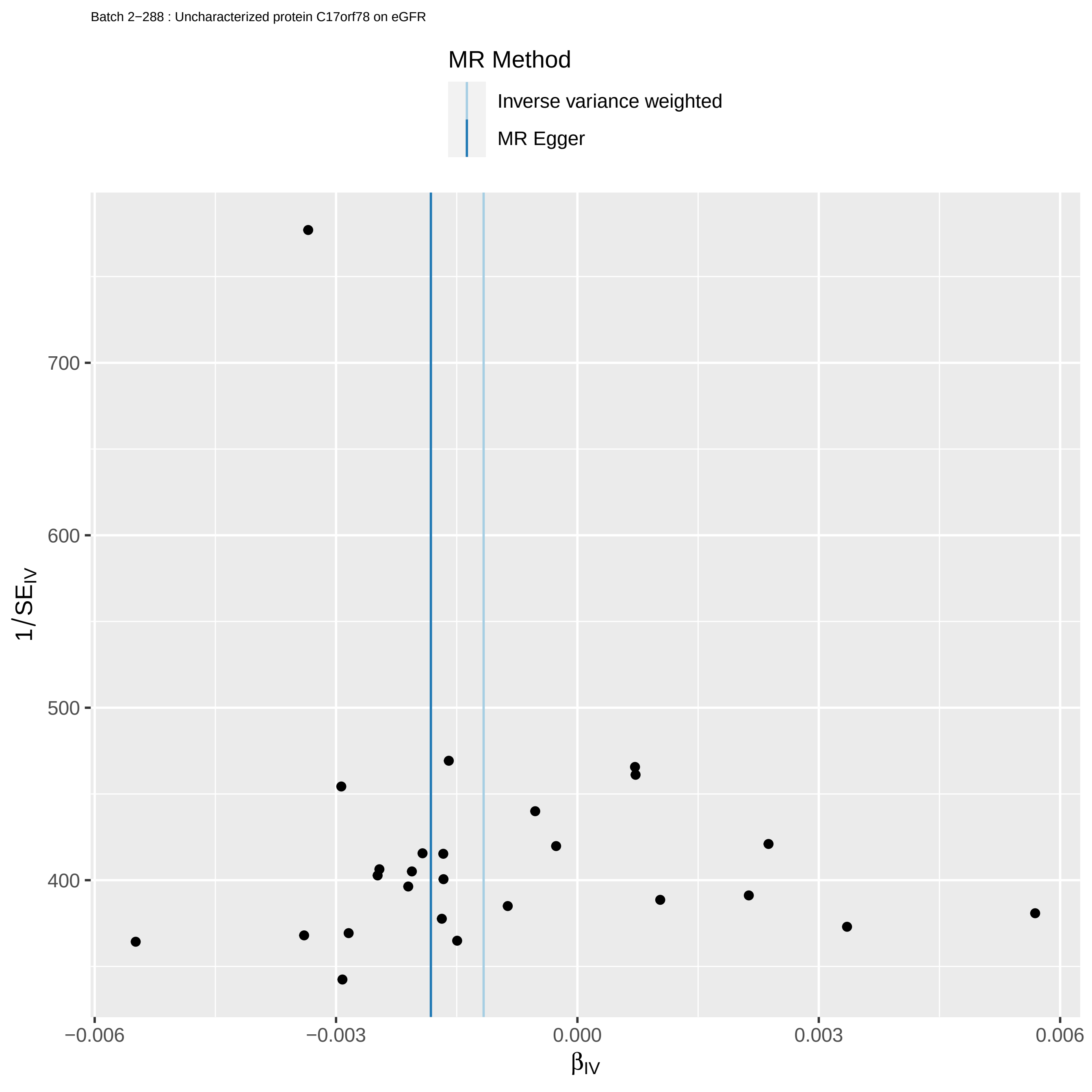

Batch 2−288 : Uncharacterized protein C17orf78 on eGFR
MR Method
Inverse variance weighted
MR Egger
700
600
500
400
1/SE$_{IV}$
−0.006
−0.003
0.000
0.003
0.006
β_{IV}

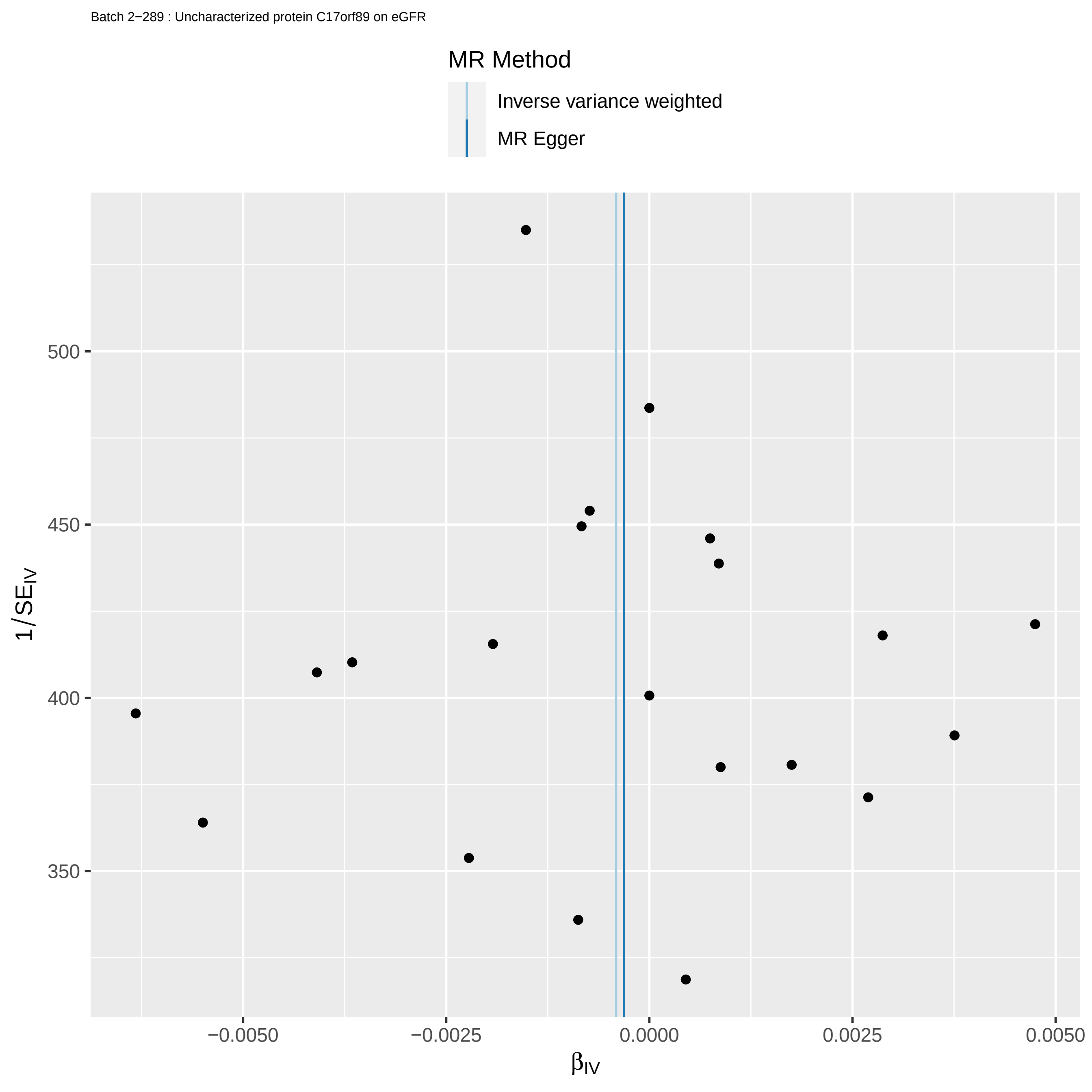

Batch 2−289 : Uncharacterized protein C17orf89 on eGFR
MR Method
Inverse variance weighted
MR Egger
500
450
400
350
1/SE_IV
−0.0050
−0.0025
0.0000
0.0025
0.0050
β_IV

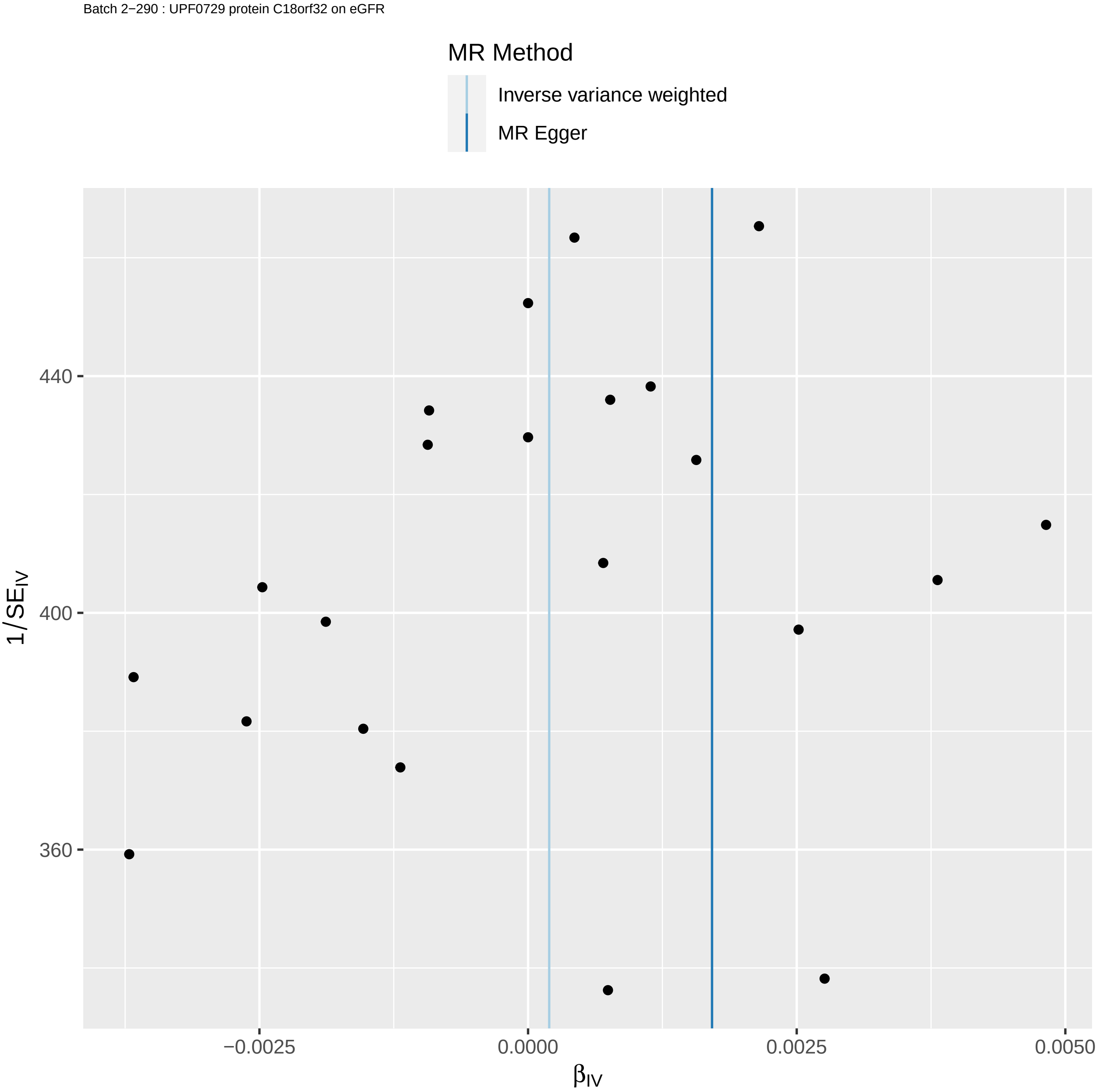

Batch 2−290 : UPF0729 protein C18orf32 on eGFR
MR Method
Inverse variance weighted
MR Egger
440
400
360
1/SE$_{IV}$
−0.0025
0.0000
0.0025
0.0050
β$_{IV}$

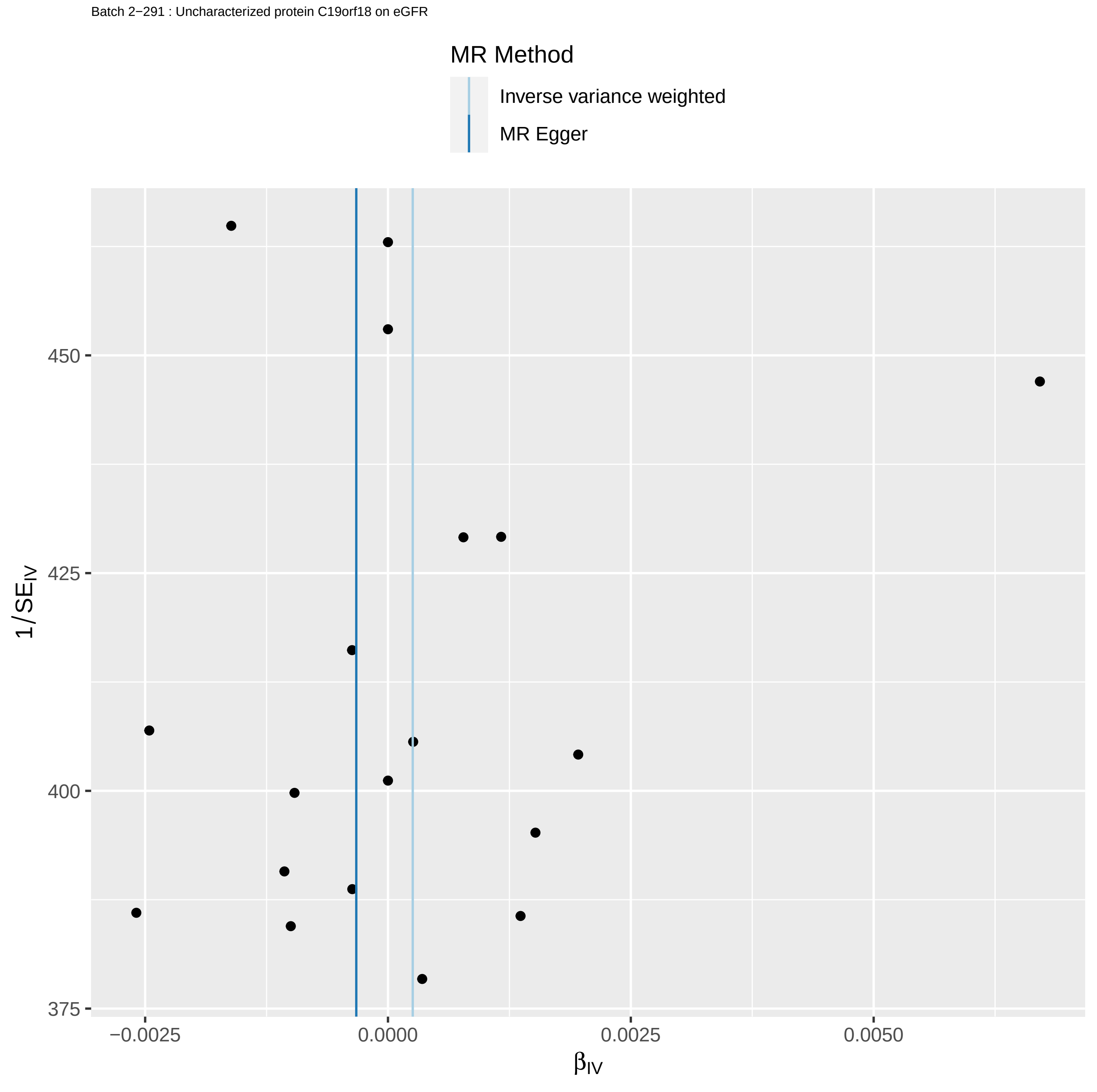

Batch 2−291 : Uncharacterized protein C19orf18 on eGFR
MR Method
Inverse variance weighted
MR Egger
450
425
400
375
1/SE_IV
−0.0025
0.0000
0.0025
0.0050
β_IV

Batch 2−292 : Angiopoietin−like protein 8 on eGFR

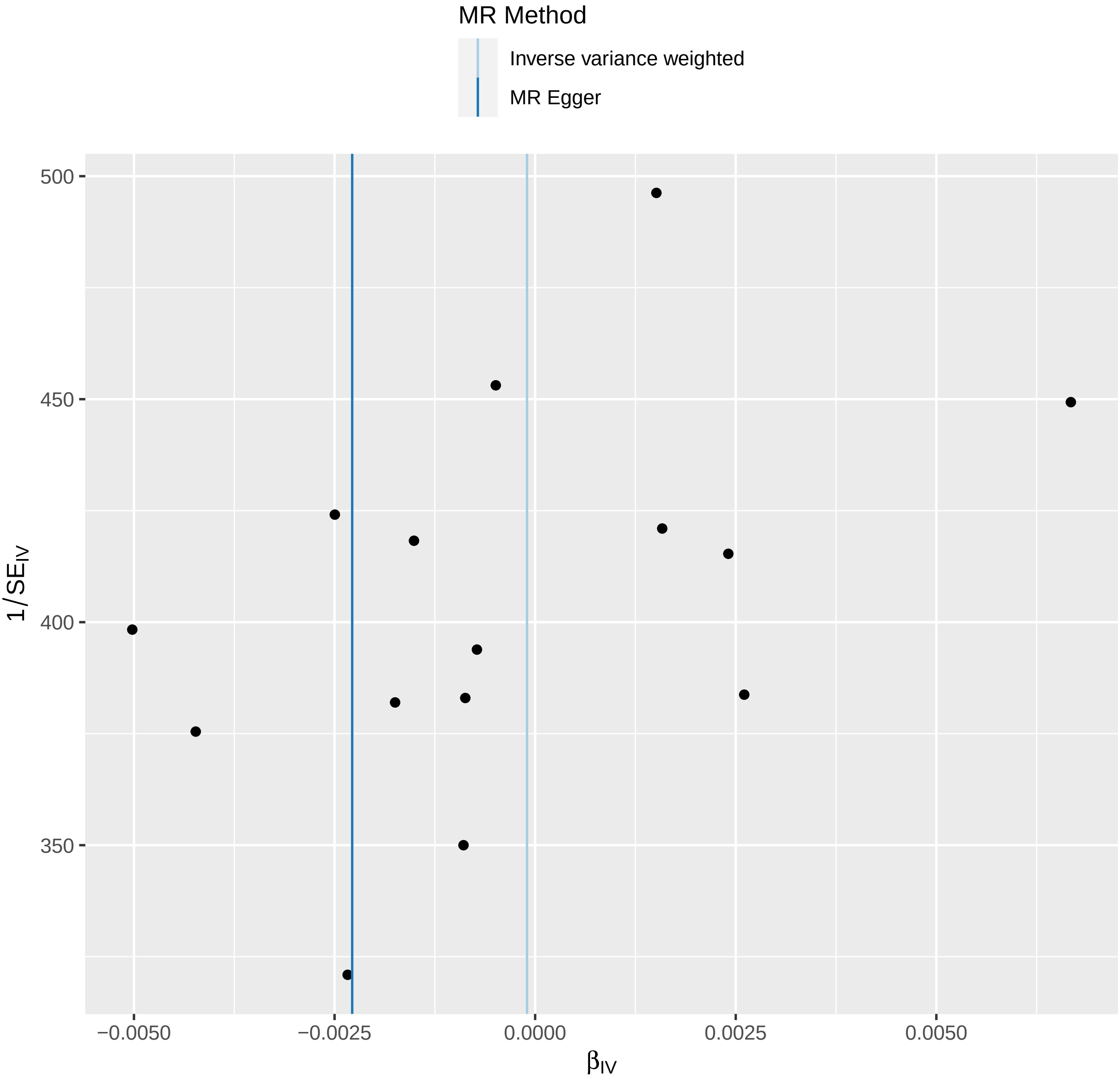

Batch 2−293 : Nuclear nucleic acid−binding protein C1D on eGFR

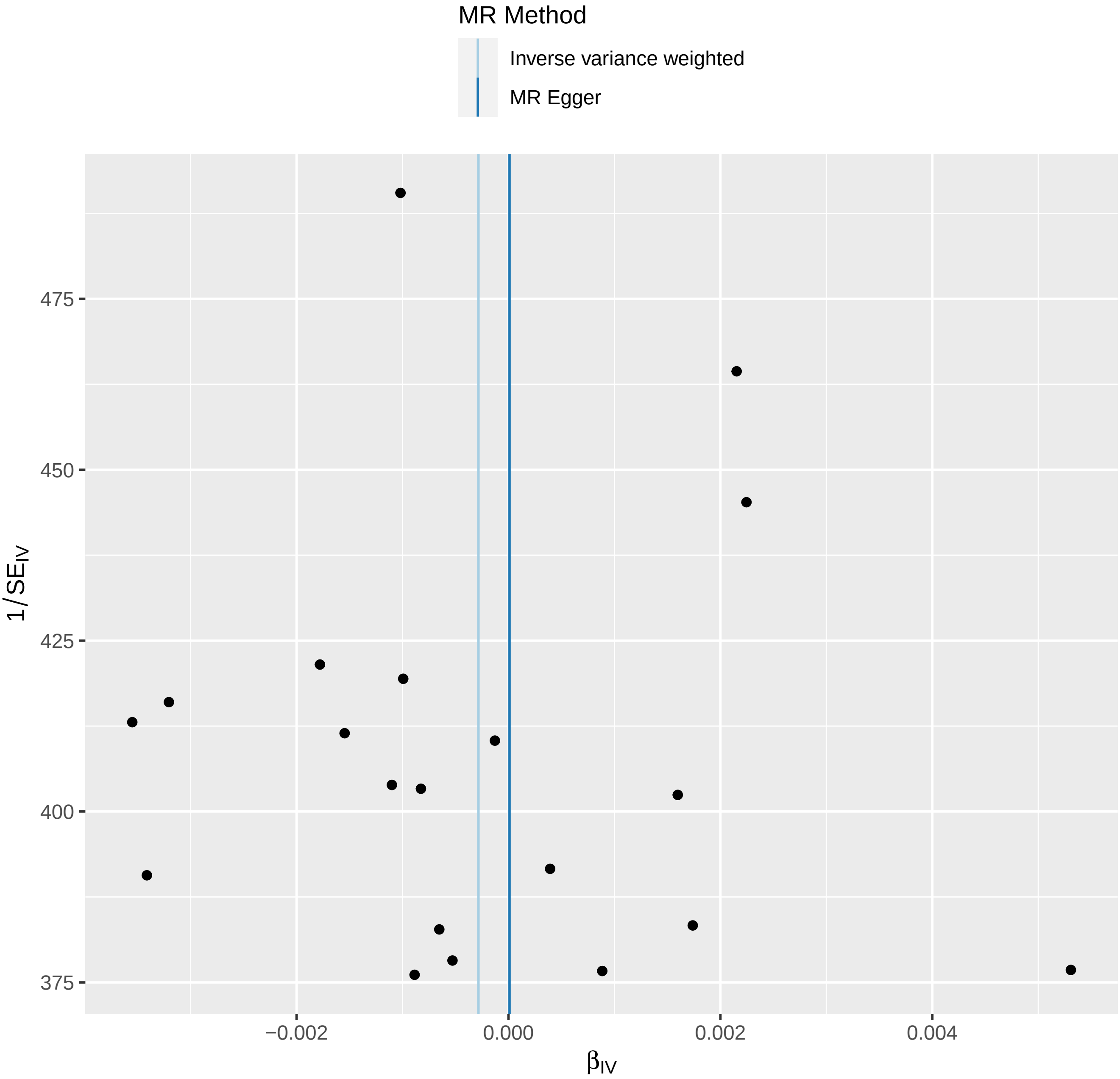

Batch 2−294 : C1GALT1−specific chaperone 1 on eGFR

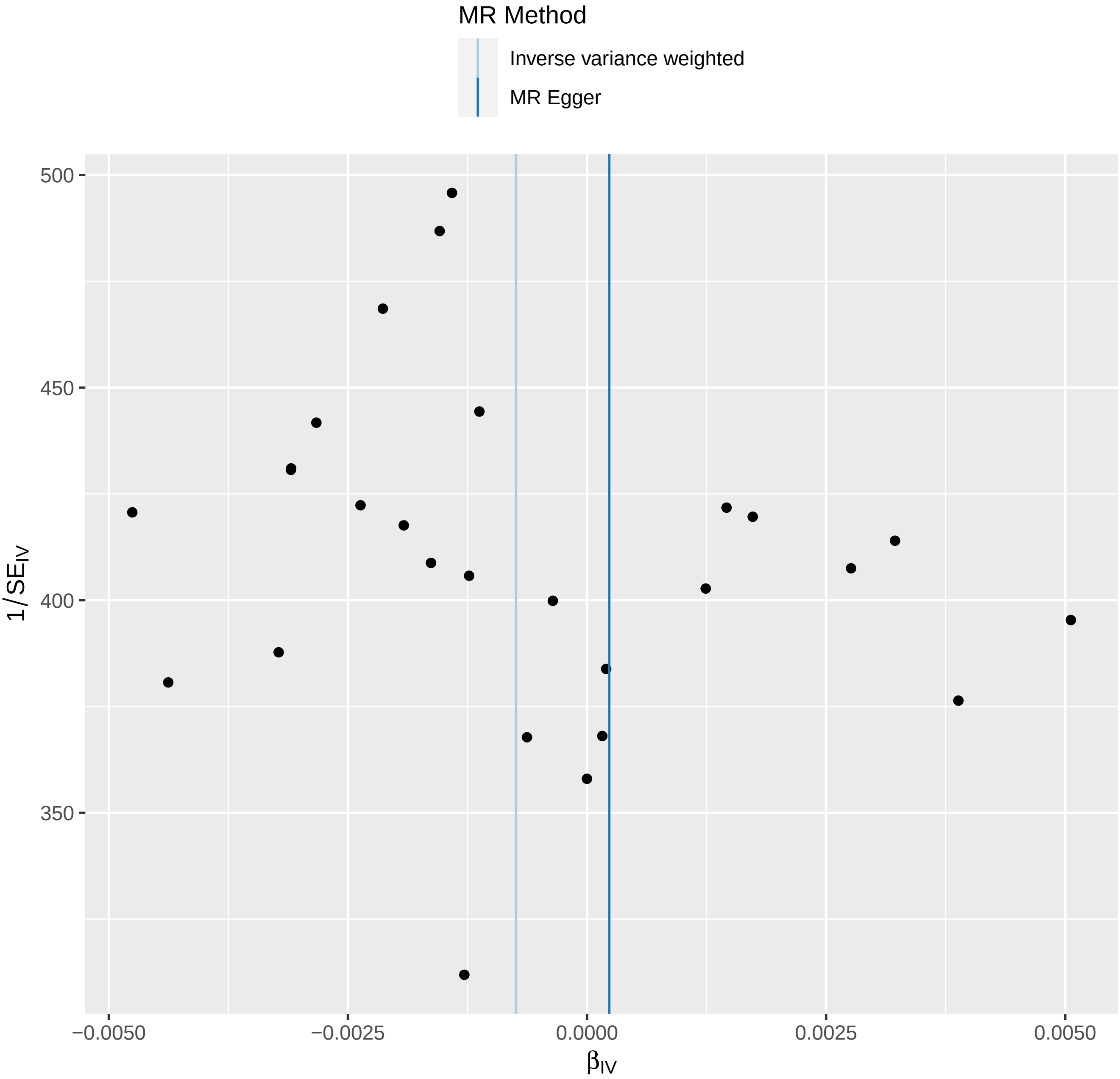

Batch 2−295 : Uncharacterized protein C1orf115 on eGFR

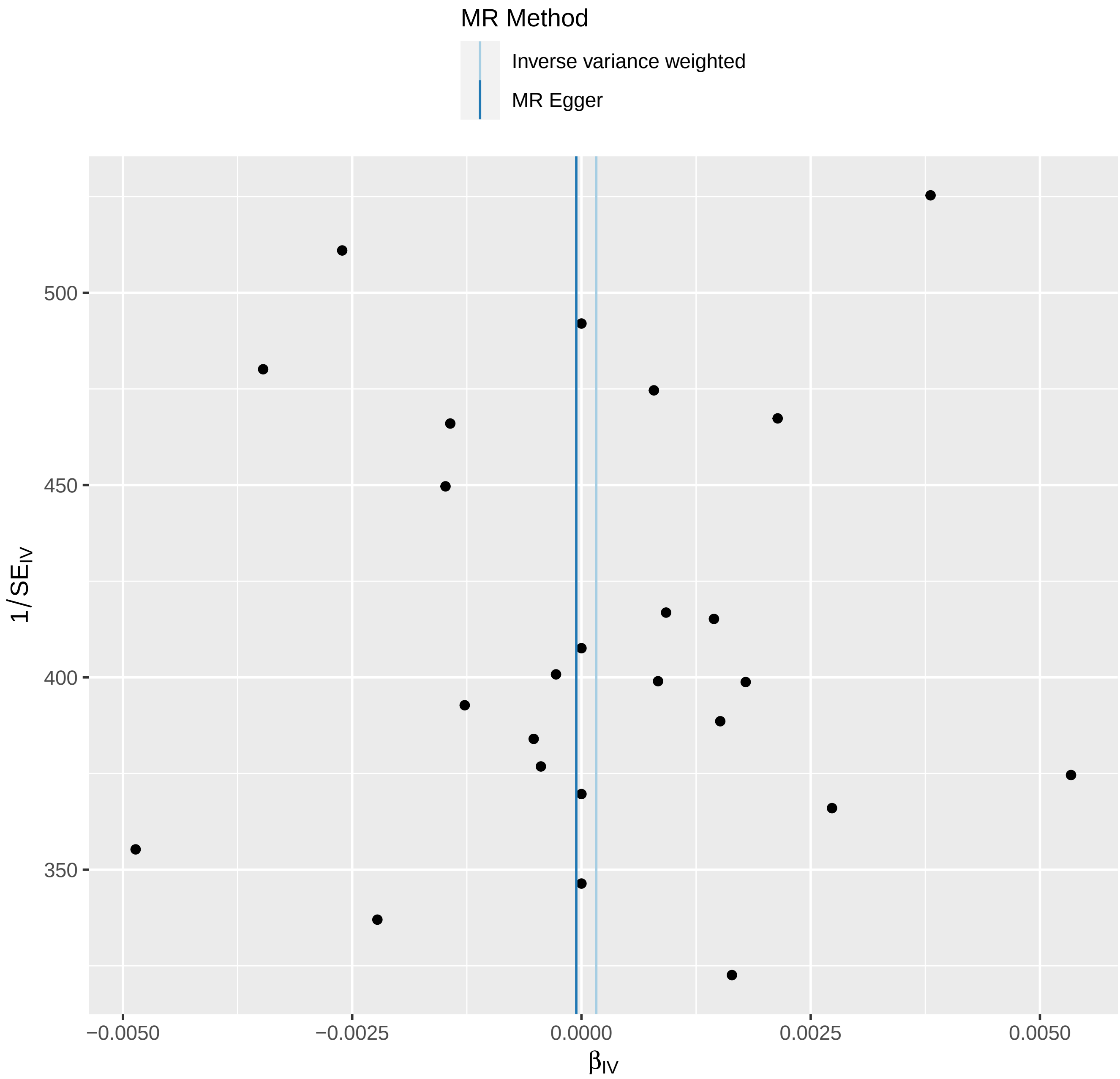

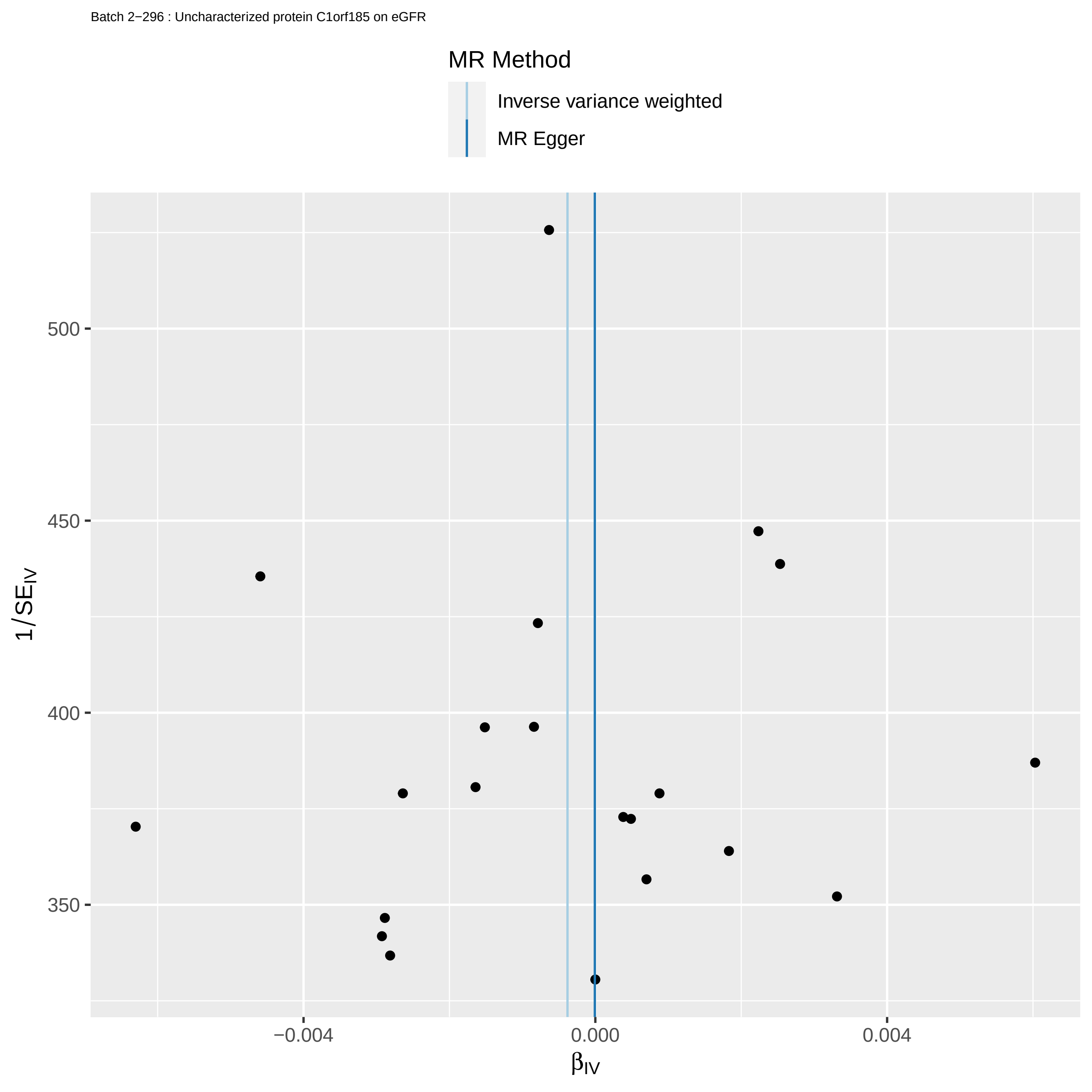

Batch 2−296 : Uncharacterized protein C1orf185 on eGFR
MR Method
Inverse variance weighted
MR Egger
500
450
400
350
1/SE_IV
−0.004
0.000
0.004
β_IV

Batch 2−297 : Uncharacterized protein C1orf198 on eGFR

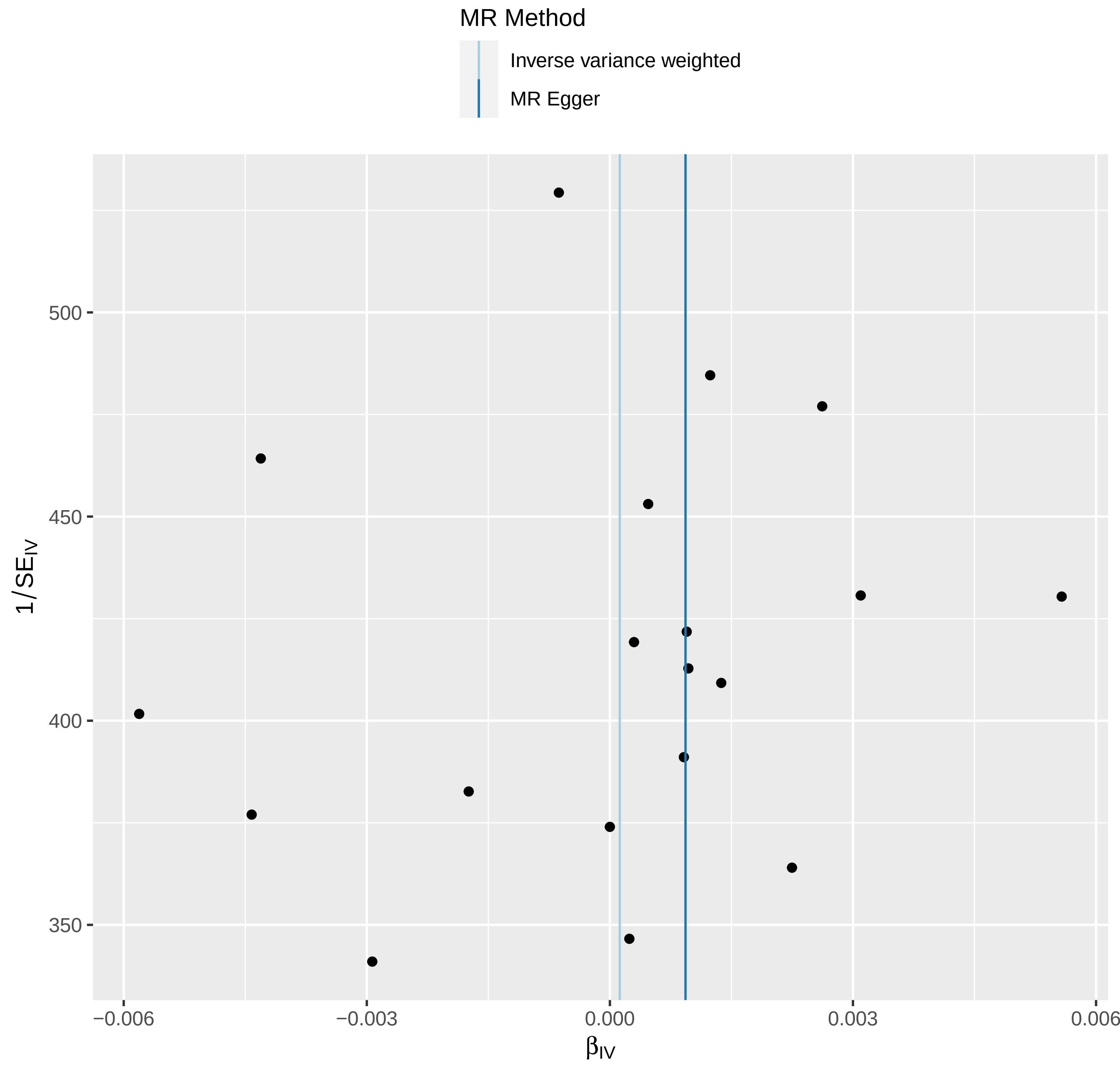

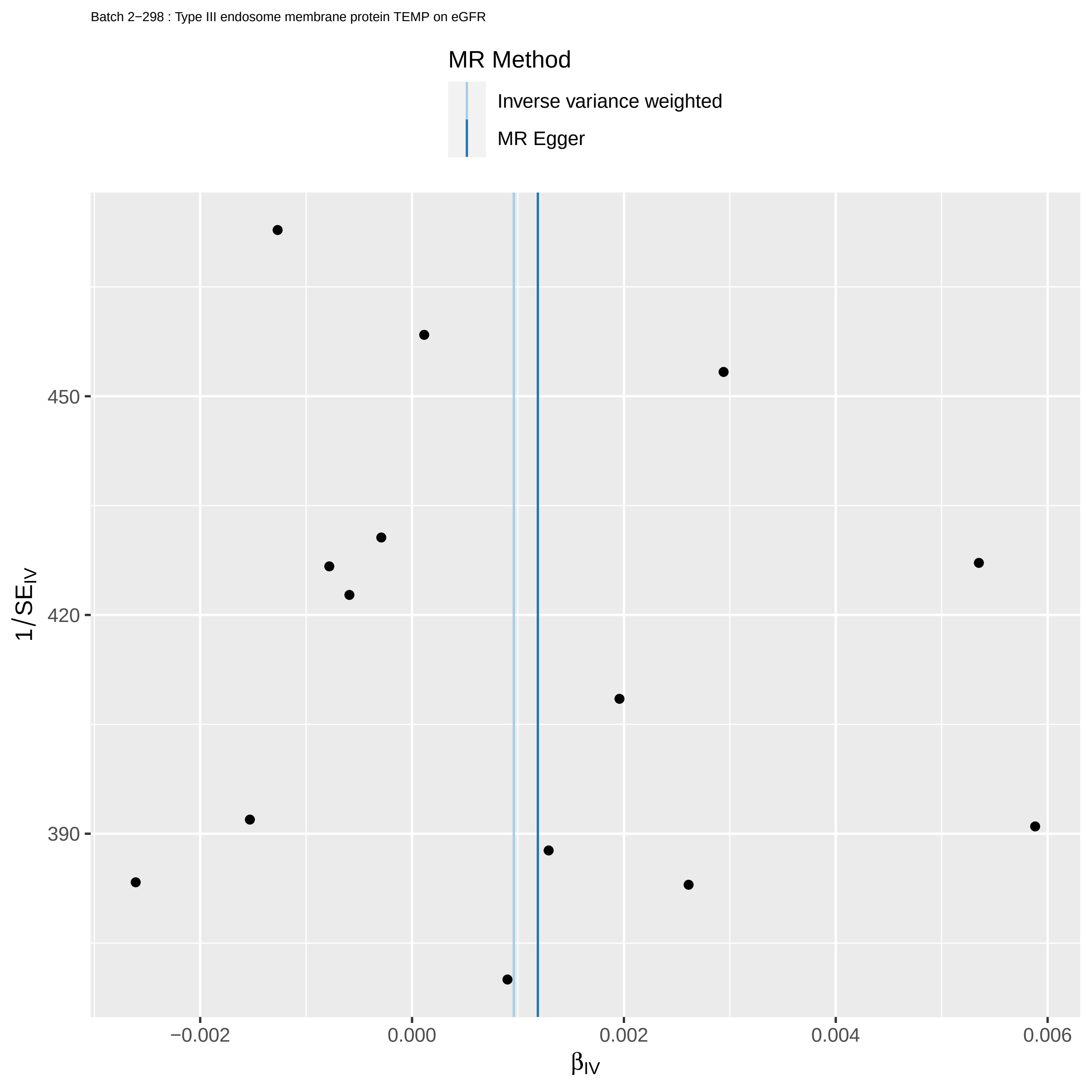
Batch 2−298 : Type III endosome membrane protein TEMP on eGFR
MR Method
Inverse variance weighted
MR Egger
450
420
390
1/SE$_{IV}$
−0.002
0.000
0.002
0.004
0.006
β$_{IV}$

Batch 2−299 : Uncharacterized protein C1orf226 on eGFR

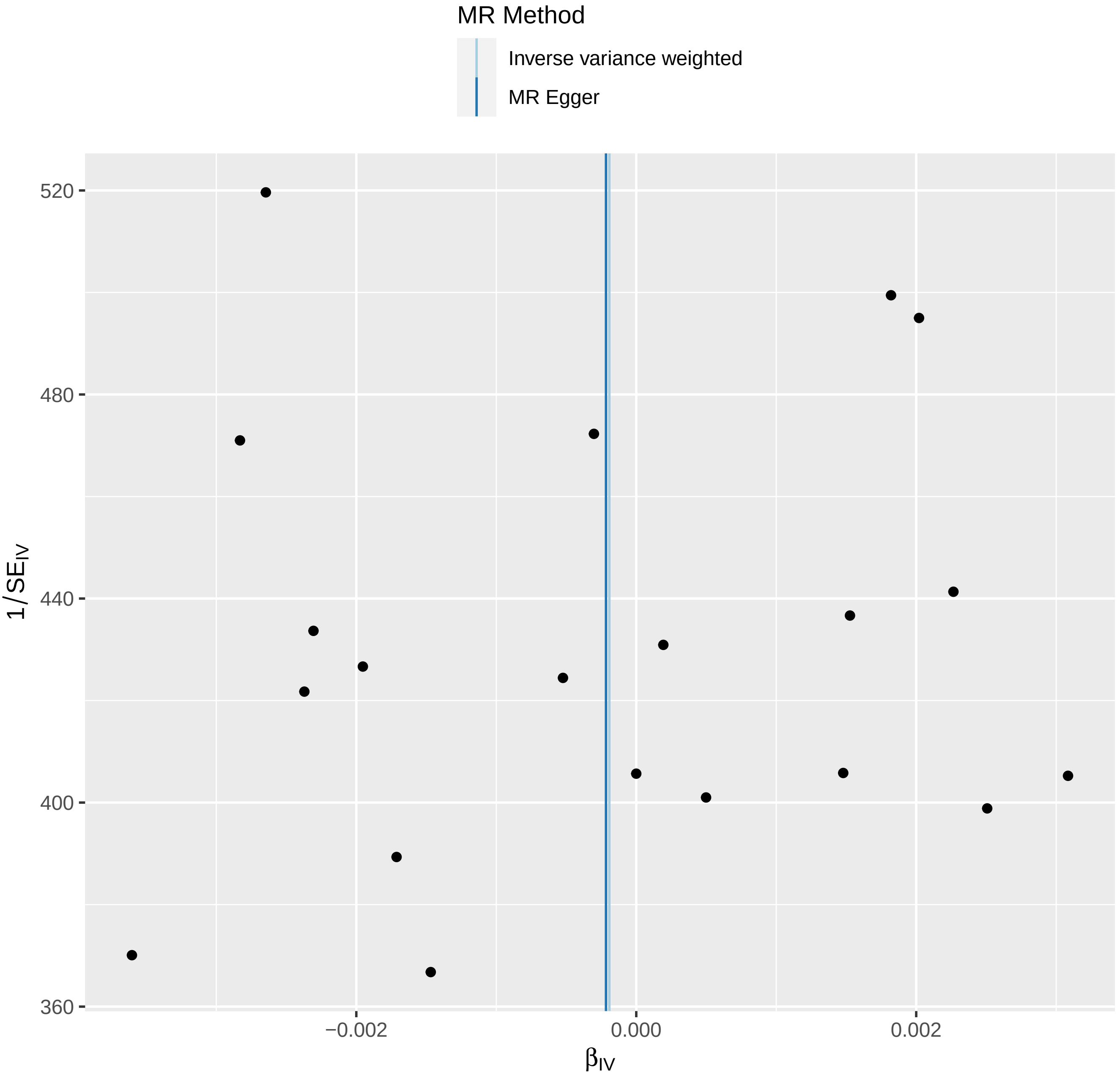

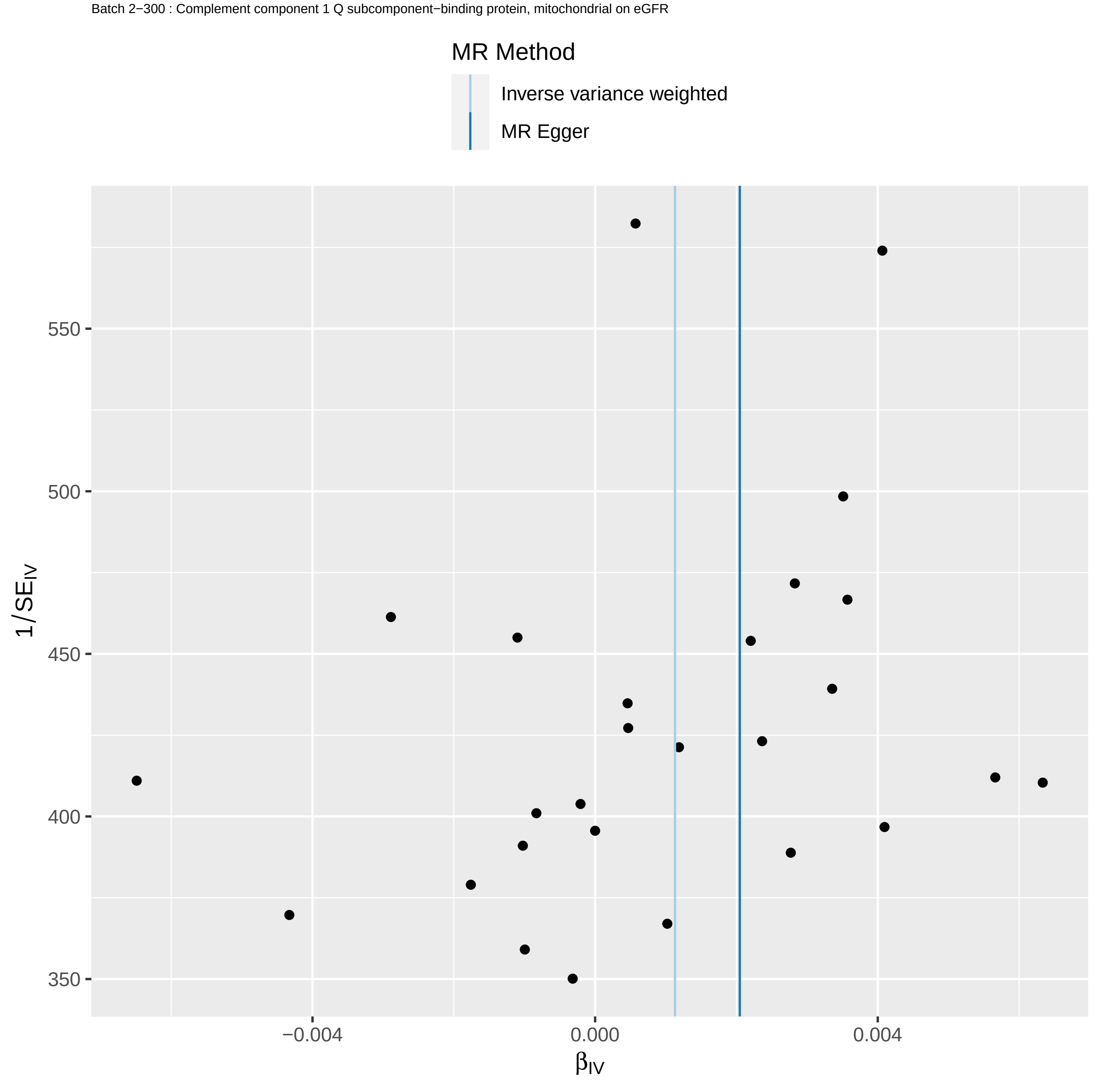

Batch 2−300 : Complement component 1 Q subcomponent−binding protein, mitochondrial on eGFR
MR Method
Inverse variance weighted
MR Egger
550
500
450
400
350
1/SE_IV
−0.004
0.000
0.004
β_IV

Batch 2−301 : Complement C1q subcomponent subunit C on eGFR

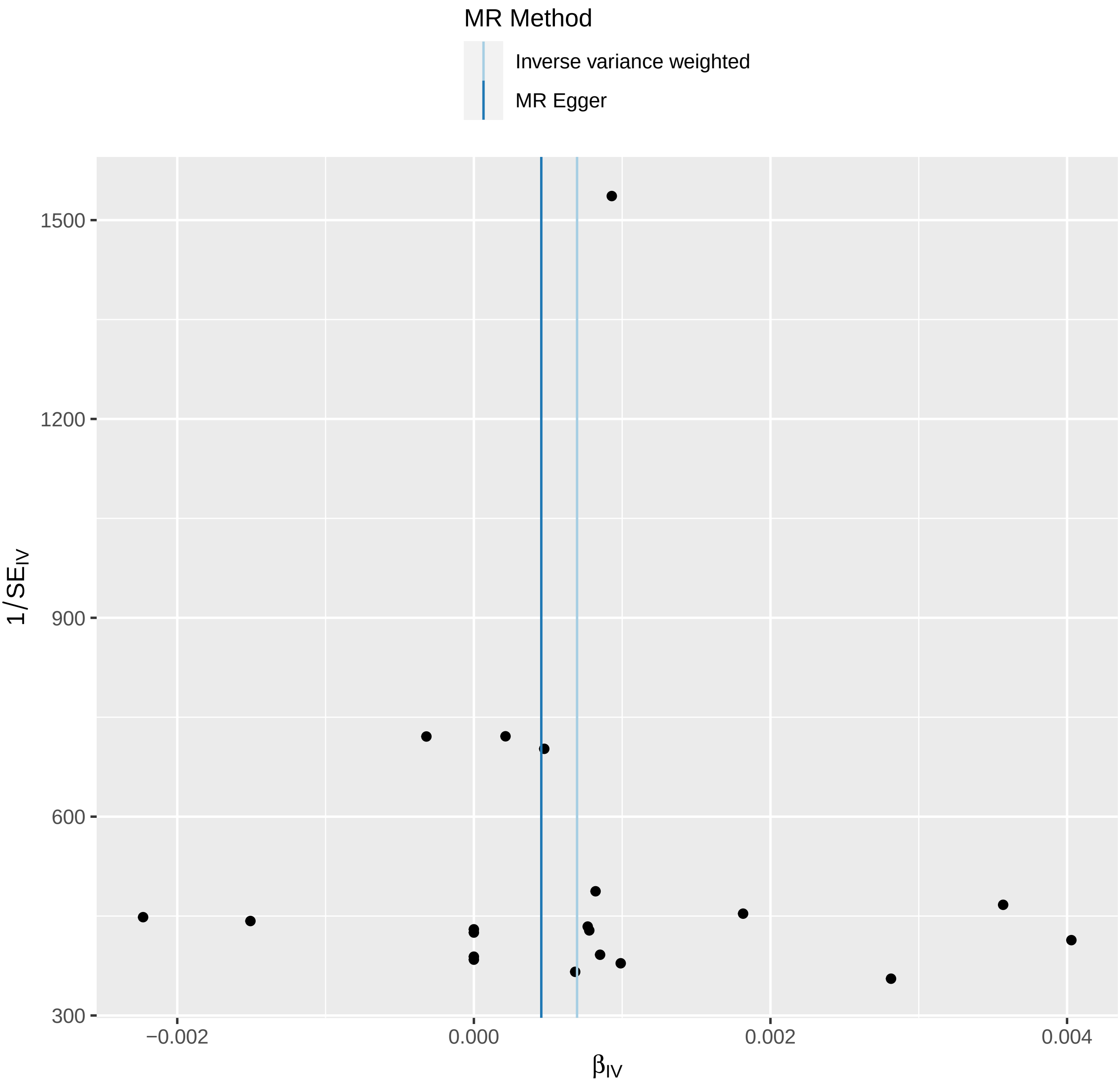

Batch 2−302 : Complement C1q−like protein 4 on eGFR

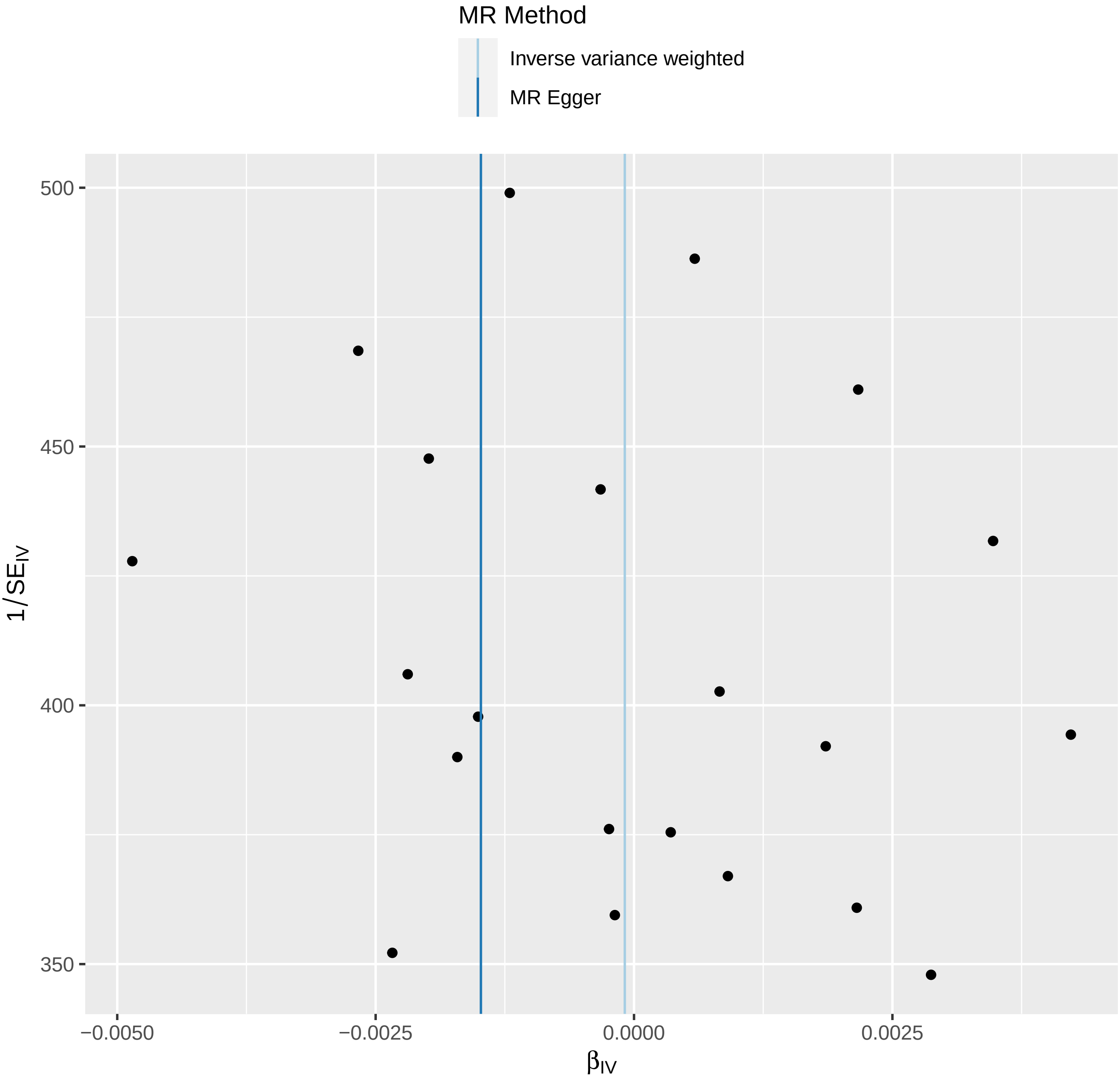

Batch 2−303 : Complement C1q tumor necrosis factor−related protein 1 on eGFR

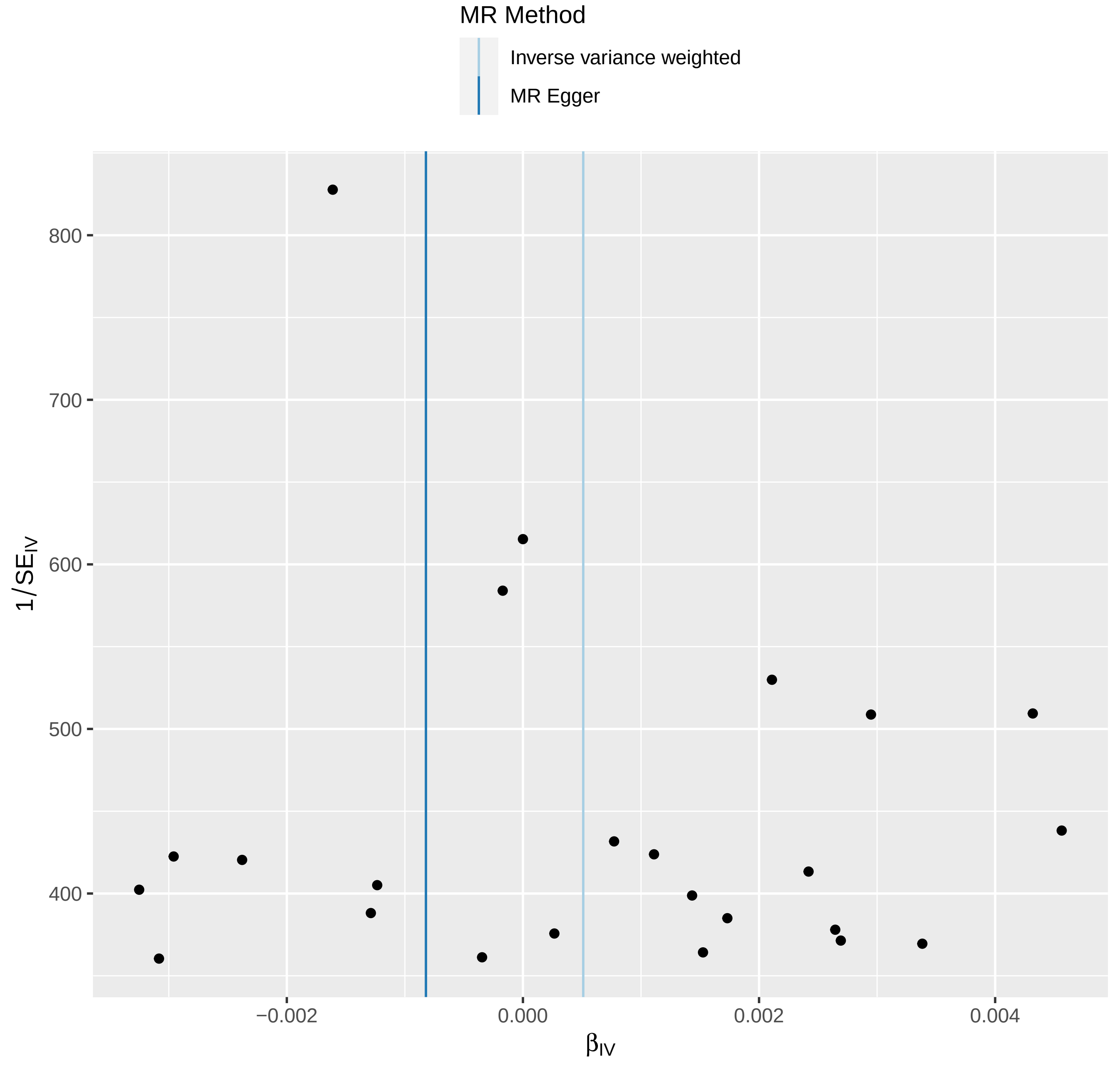

Batch 2−304 : Complement C1q tumor necrosis factor−related protein 3 on eGFR

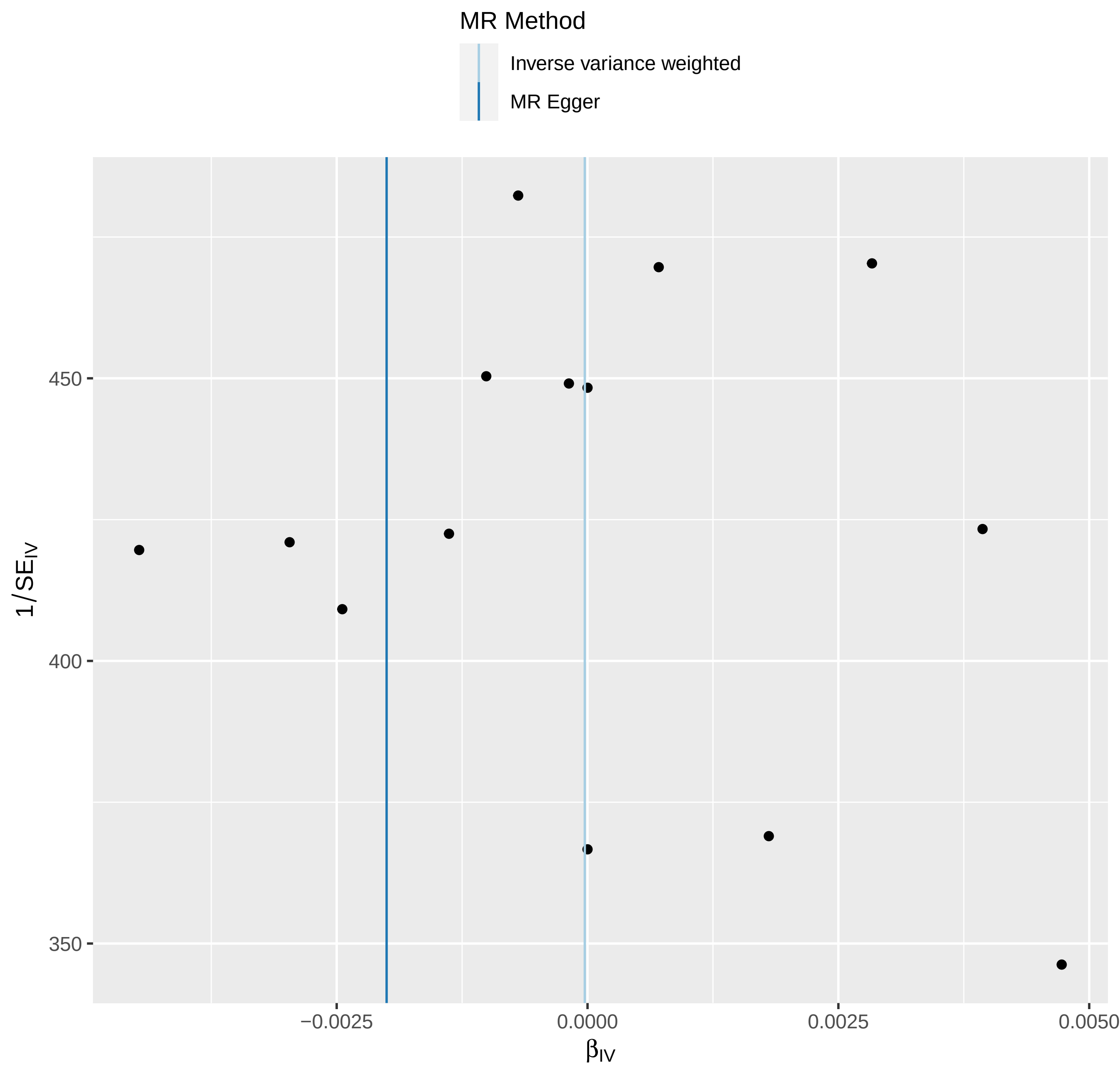

Batch 2−305 : Complement C1q tumor necrosis factor−related protein 5 on eGFR

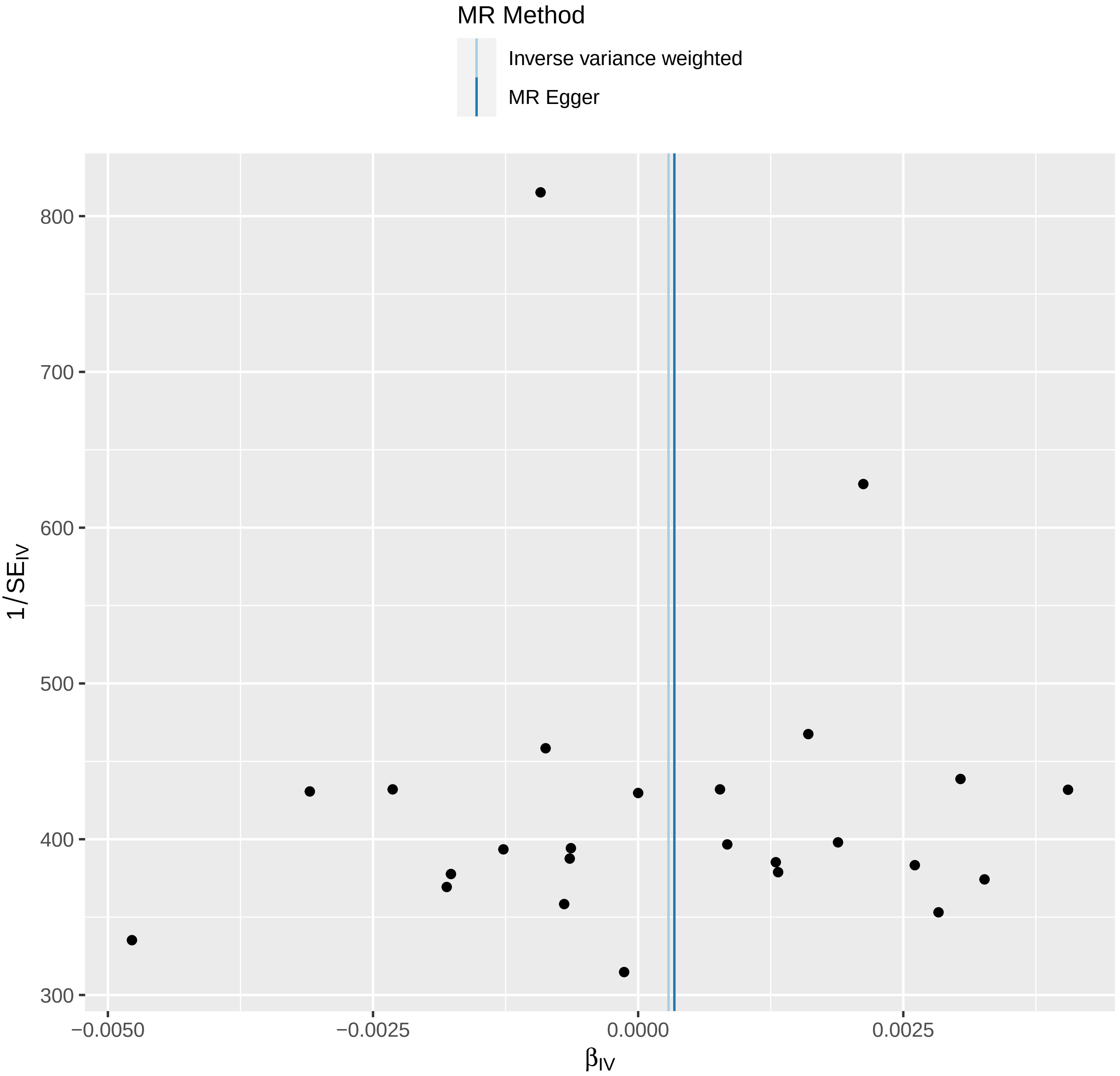

Batch 2−306 : Complement C1q and tumor necrosis factor−related protein 9A on eGFR

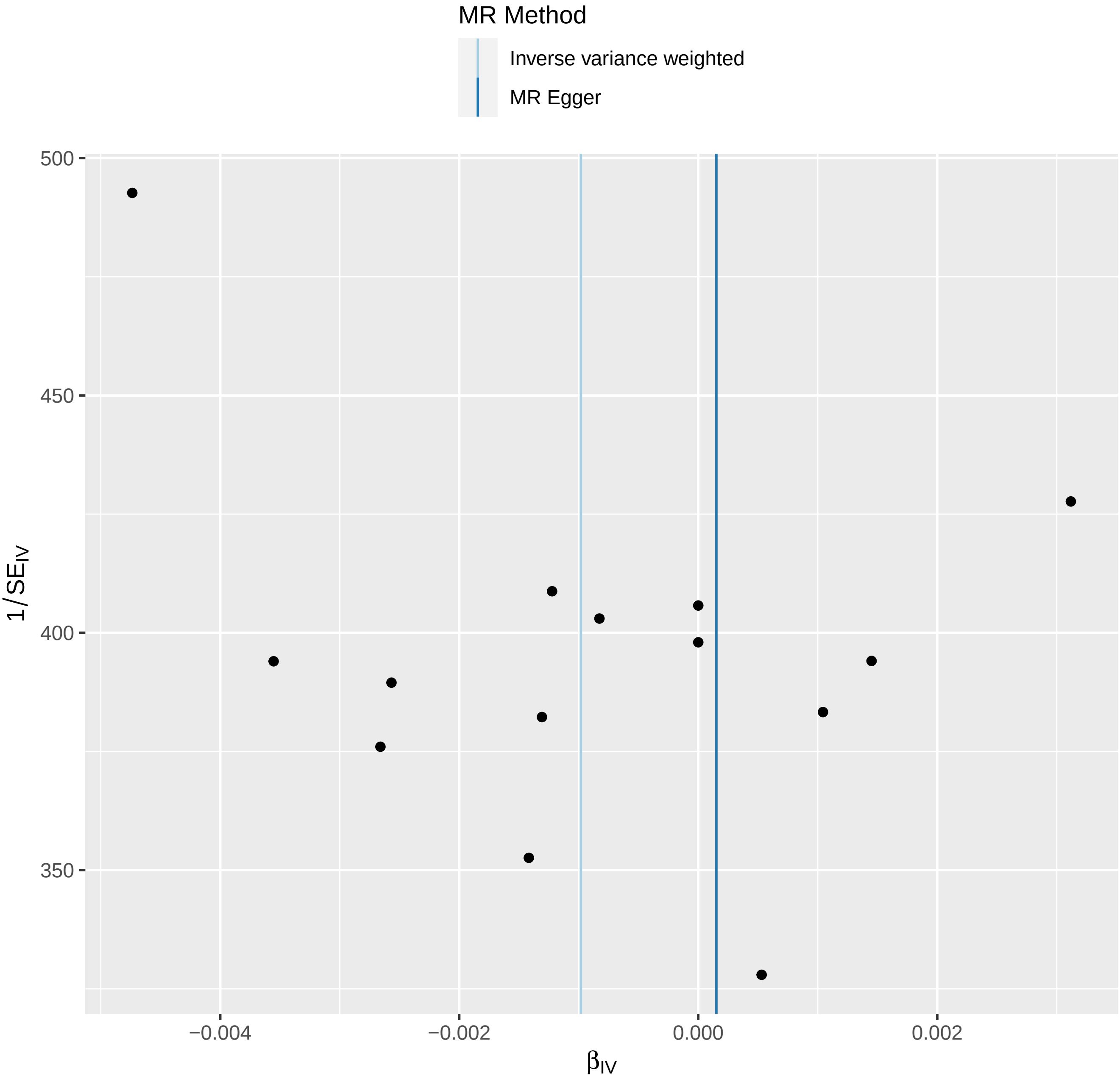

Batch 2−307 : Complement C1r subcomponent−like protein on eGFR

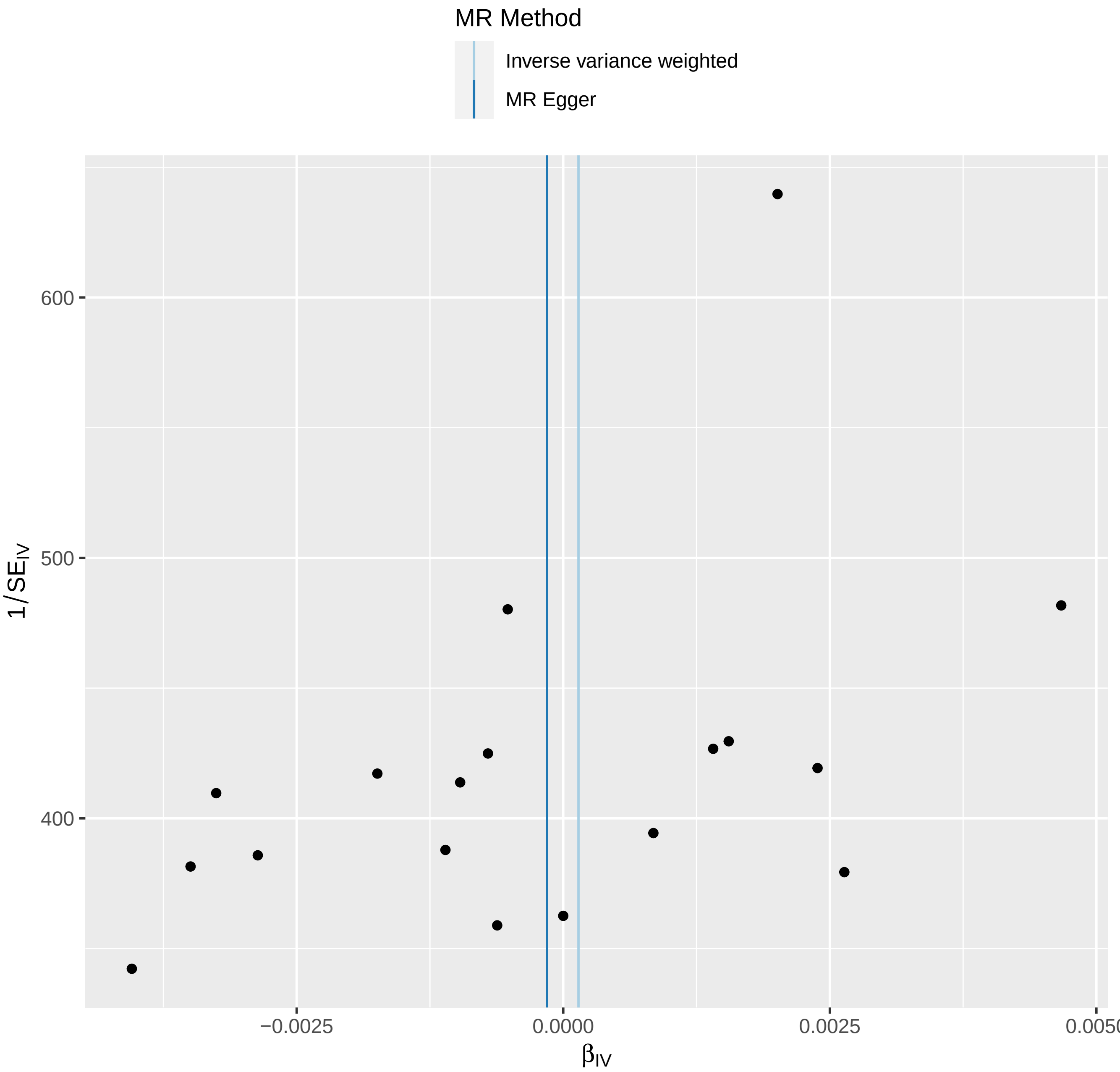

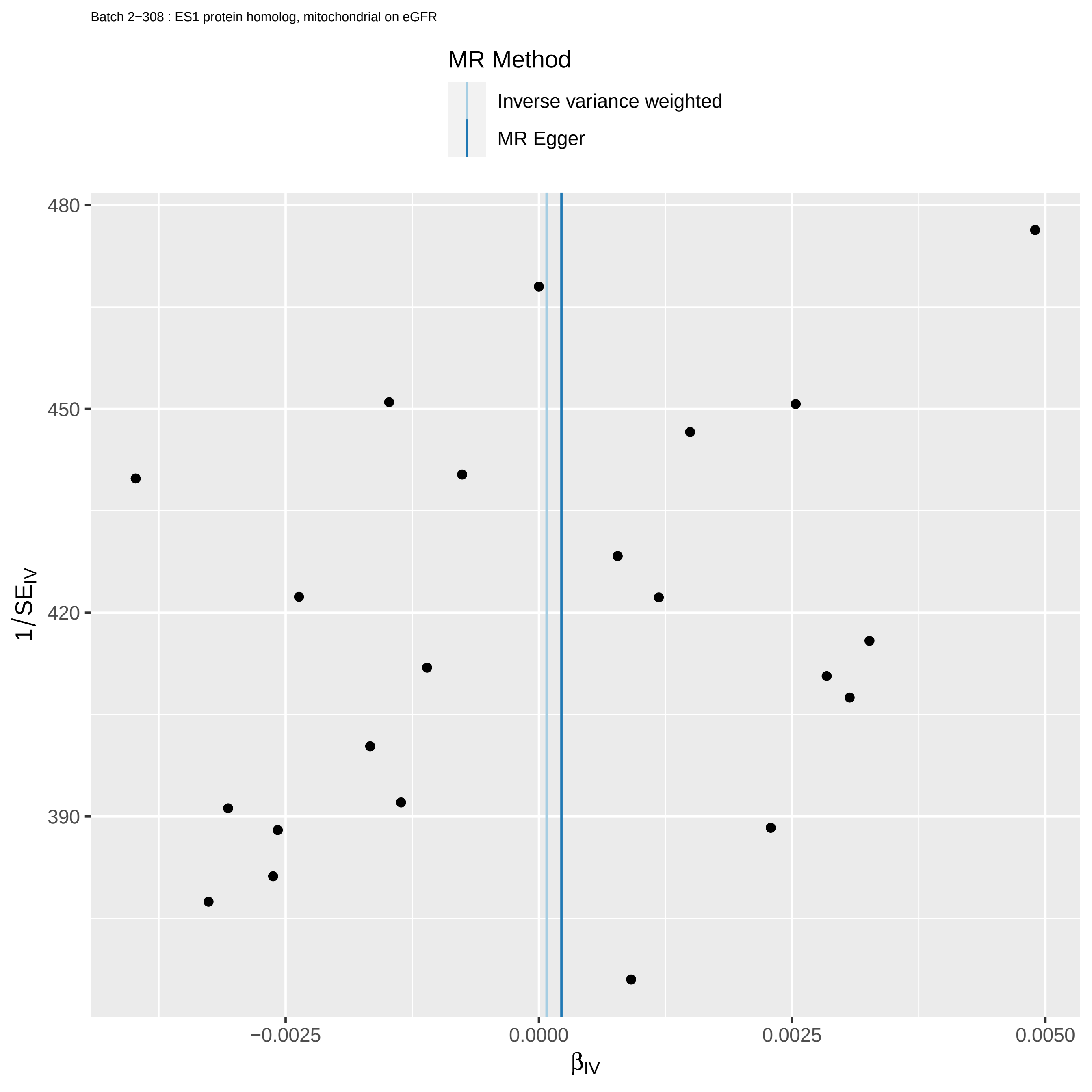

Batch 2−308 : ES1 protein homolog, mitochondrial on eGFR
MR Method
Inverse variance weighted
MR Egger
480
450
420
390
1/SE_IV
−0.0025
0.0000
0.0025
0.0050
β_IV

Batch 2−309 : Uncharacterized protein C22orf15 on eGFR

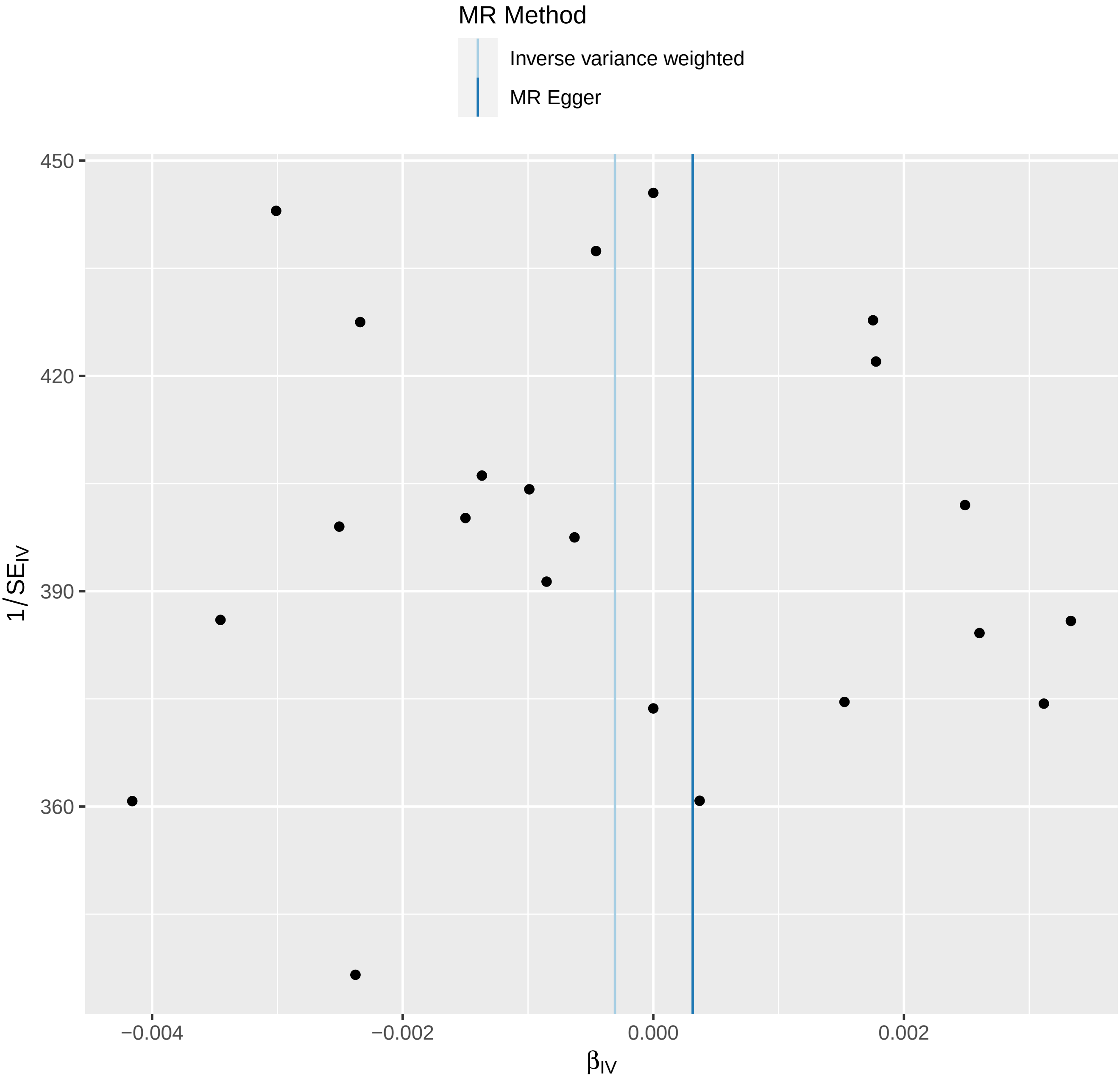

Batch 2−310 : Augurin on eGFR

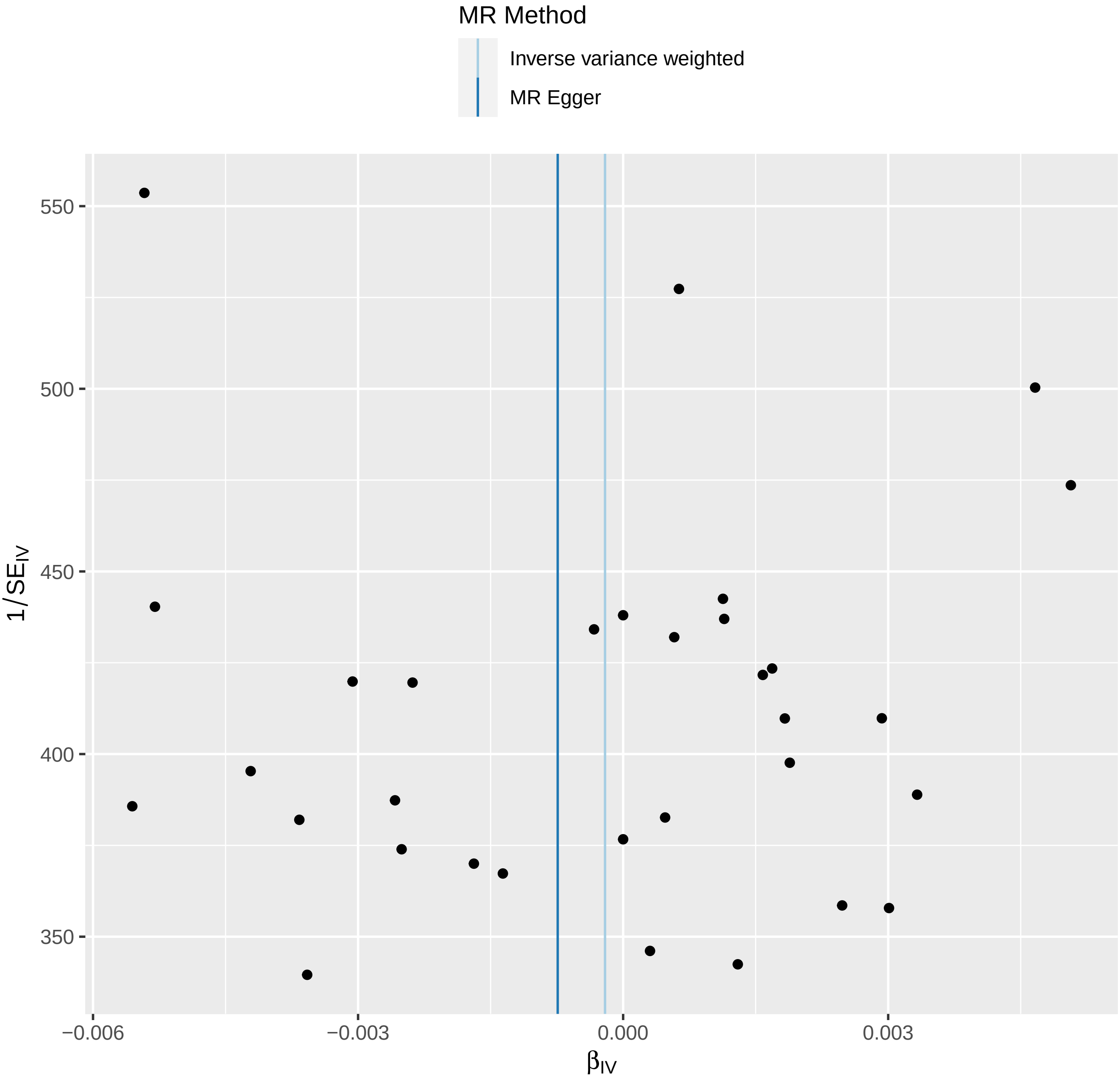

Batch 2−311 : Uncharacterized protein C2orf66 on eGFR

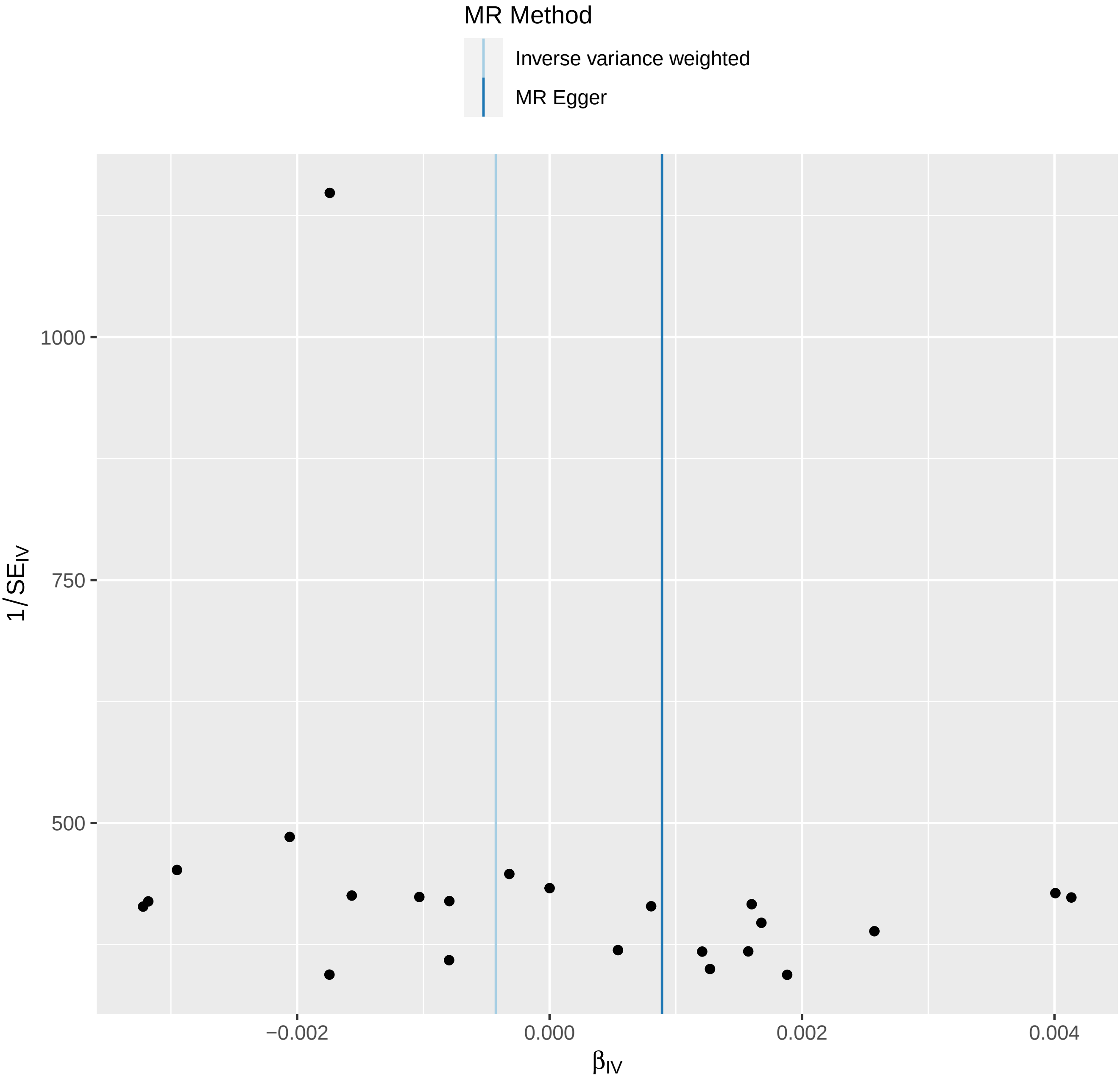

Batch 2−312 : Uncharacterized protein C2orf82 on eGFR

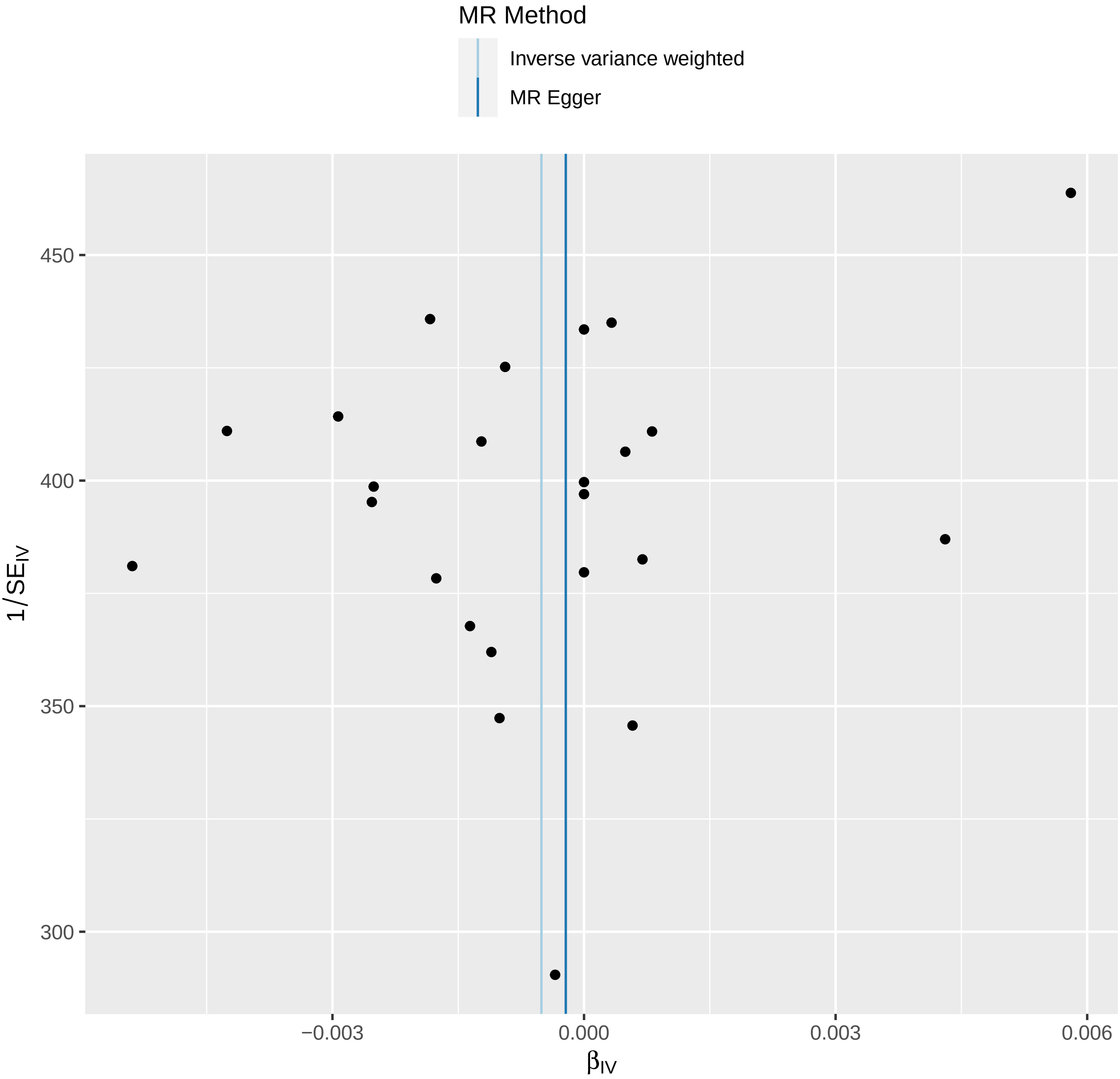

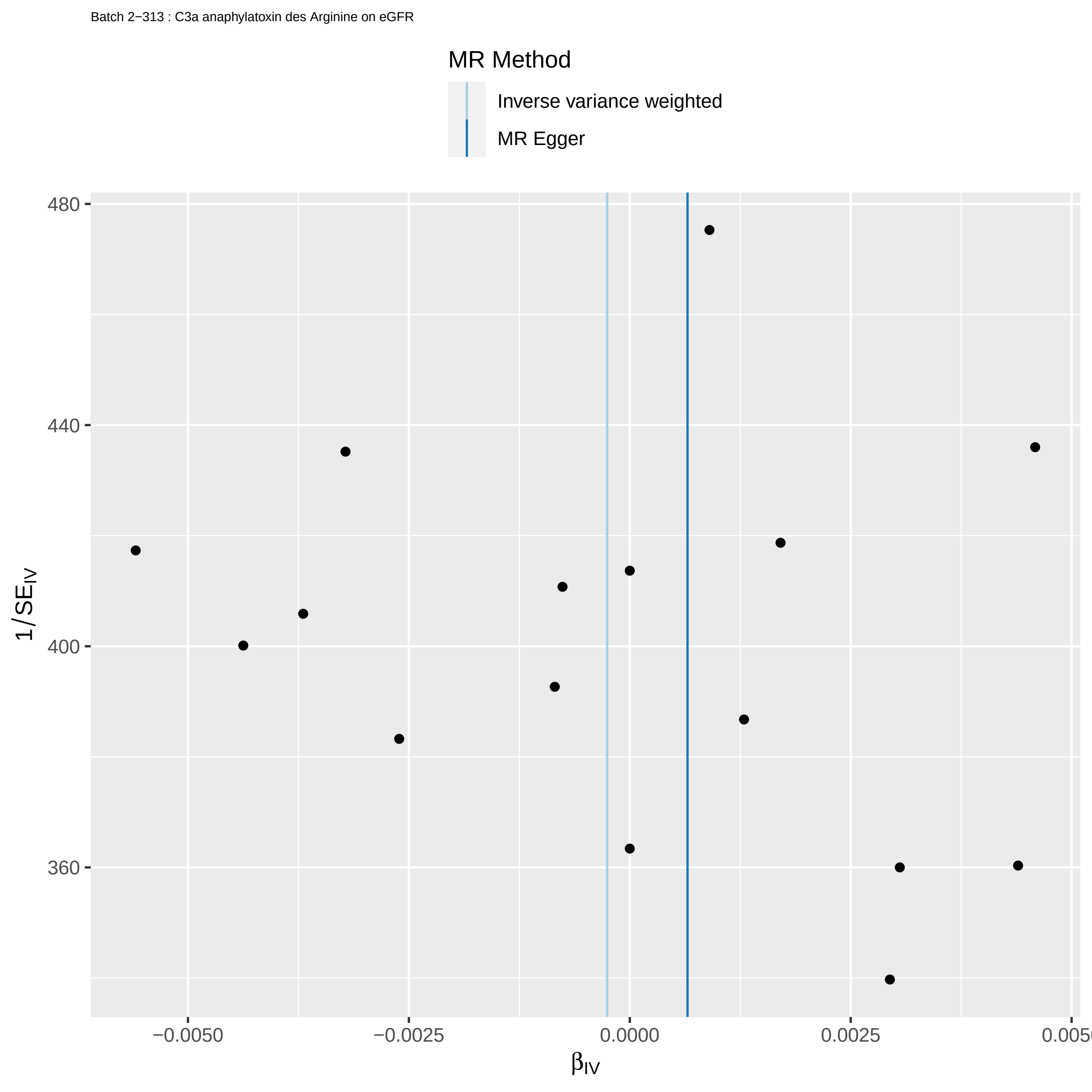

Batch 2−313 : C3a anaphylatoxin des Arginine on eGFR
MR Method
Inverse variance weighted
MR Egger
480
440
400
360
1/SE_IV
−0.0050
−0.0025
0.0000
0.0025
0.0050
β_IV

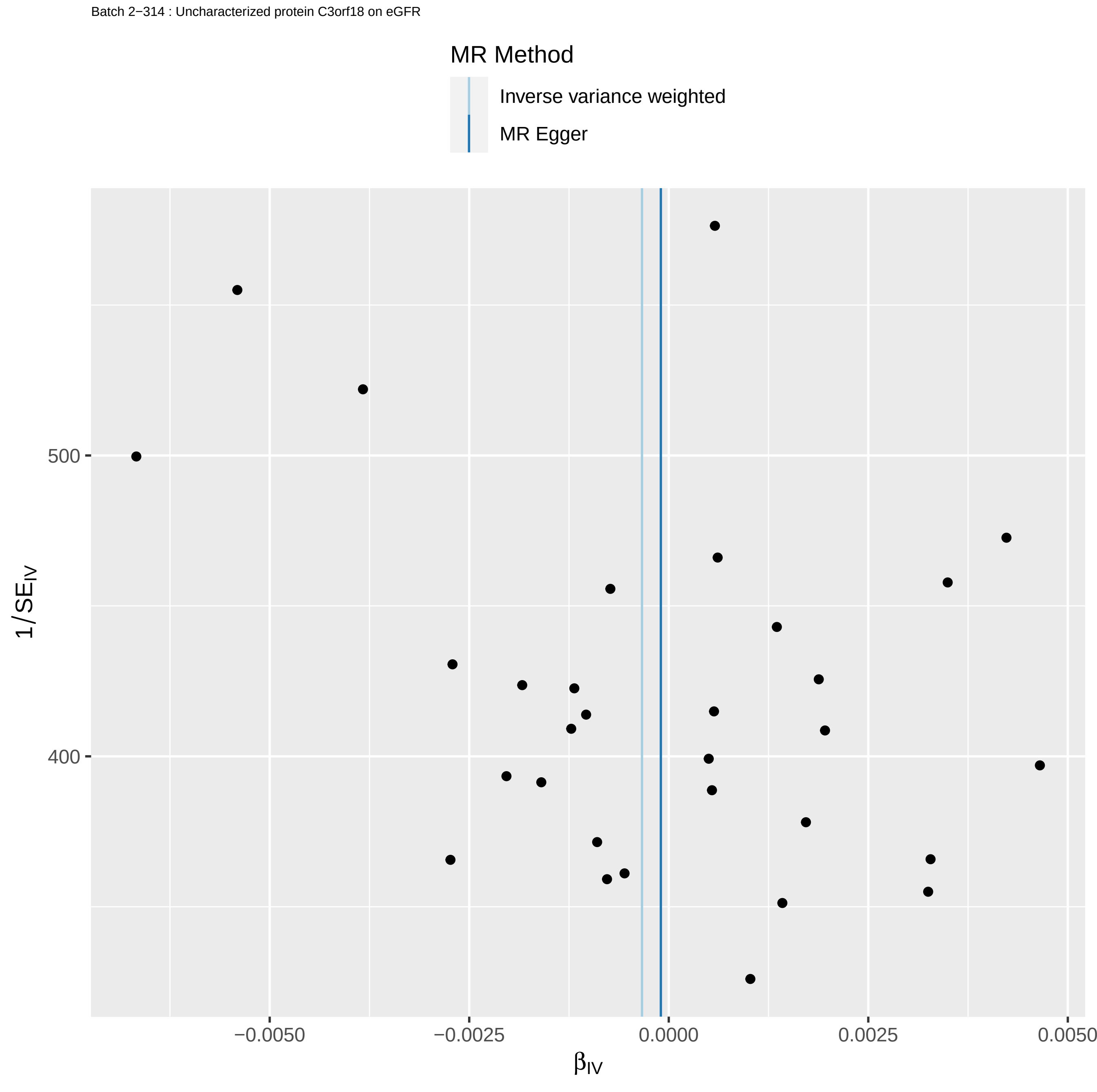
Batch 2−314 : Uncharacterized protein C3orf18 on eGFR
MR Method
Inverse variance weighted
MR Egger
500
400
1/SE_IV
−0.0050
−0.0025
0.0000
0.0025
0.0050
β_IV

Batch 2−315 : Complement C4 on eGFR

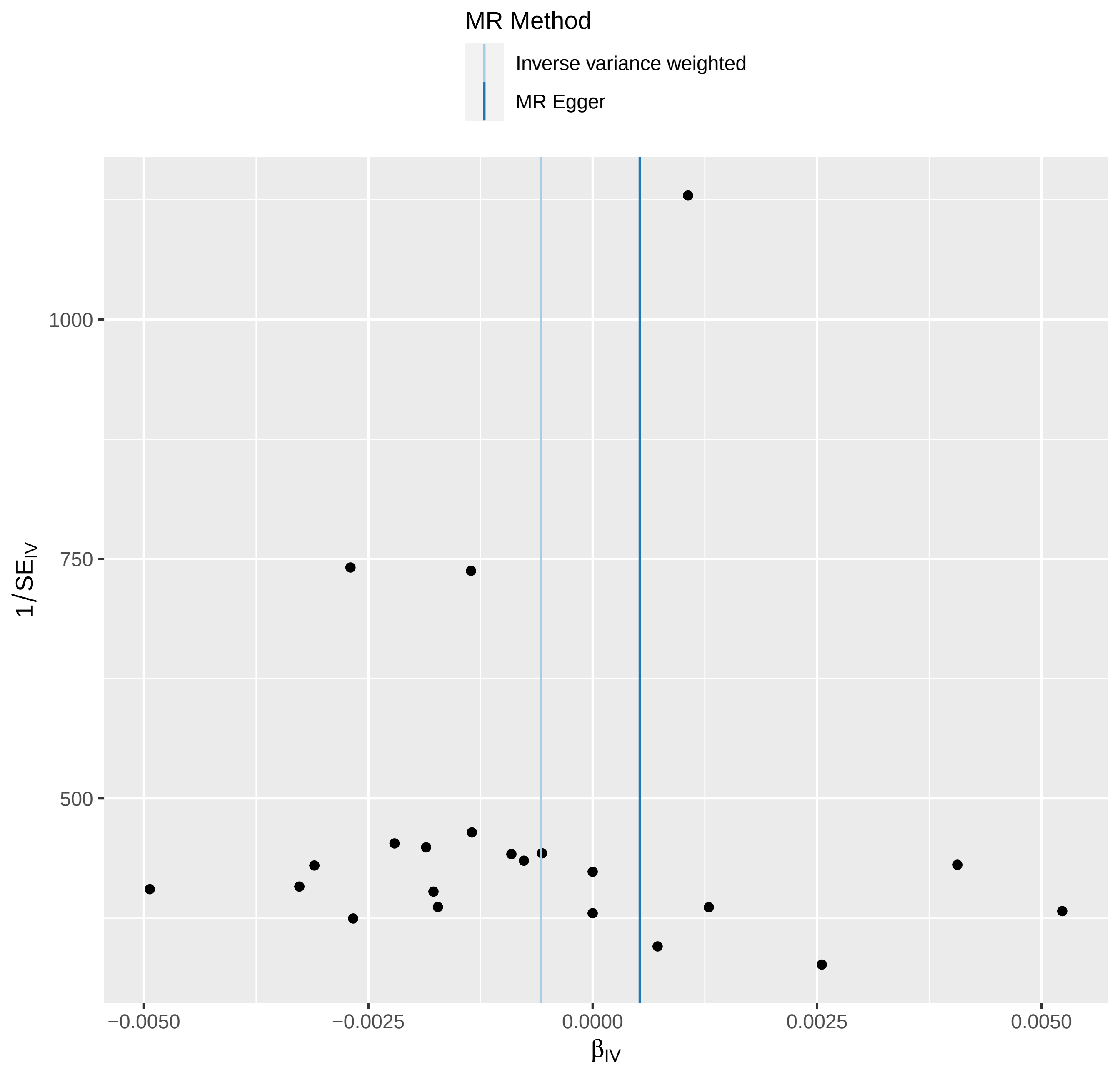

Batch 2−316 : Protein CEI on eGFR

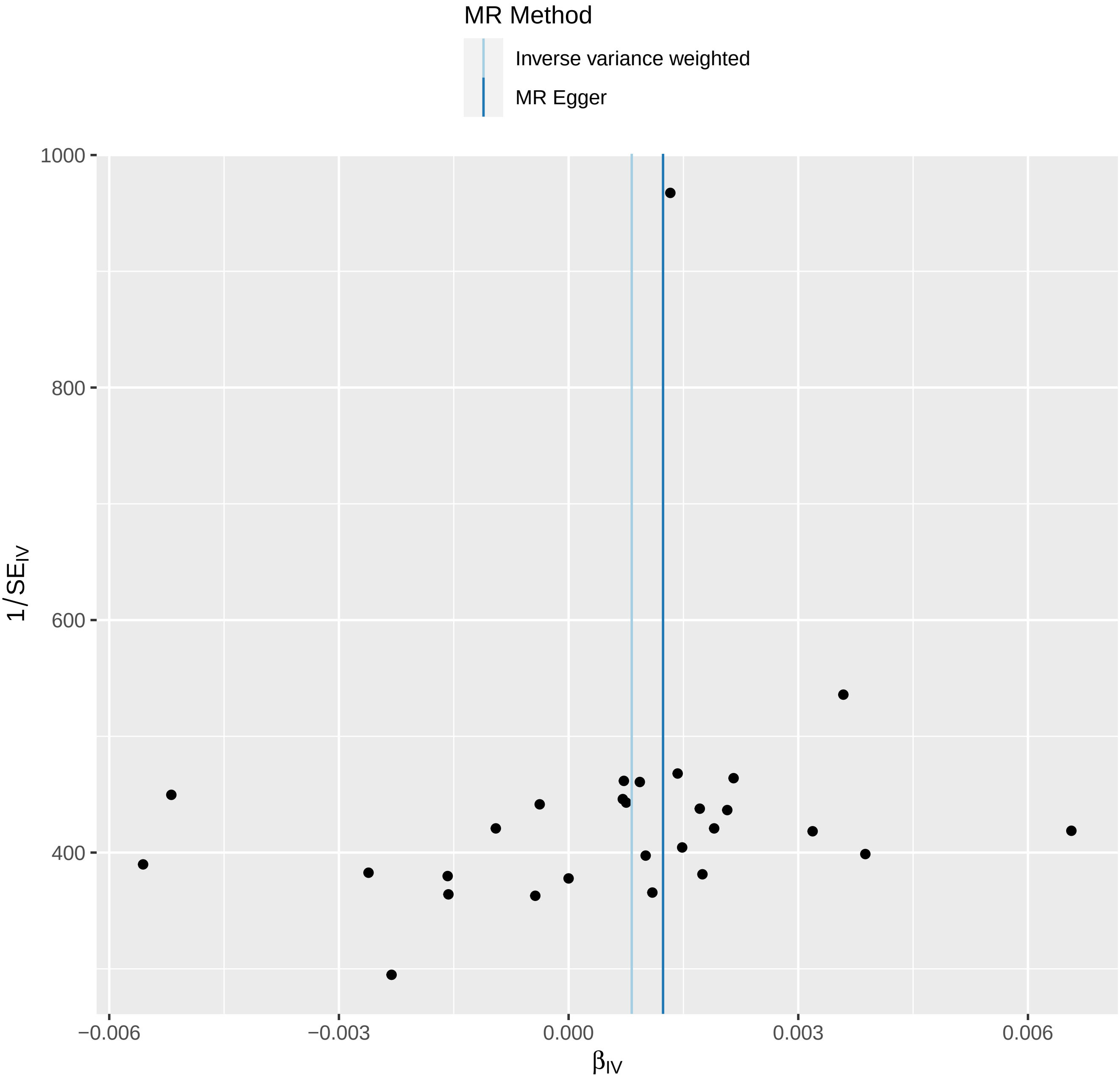

Batch 2−317 : Glutaredoxin−like protein C5orf63 on eGFR

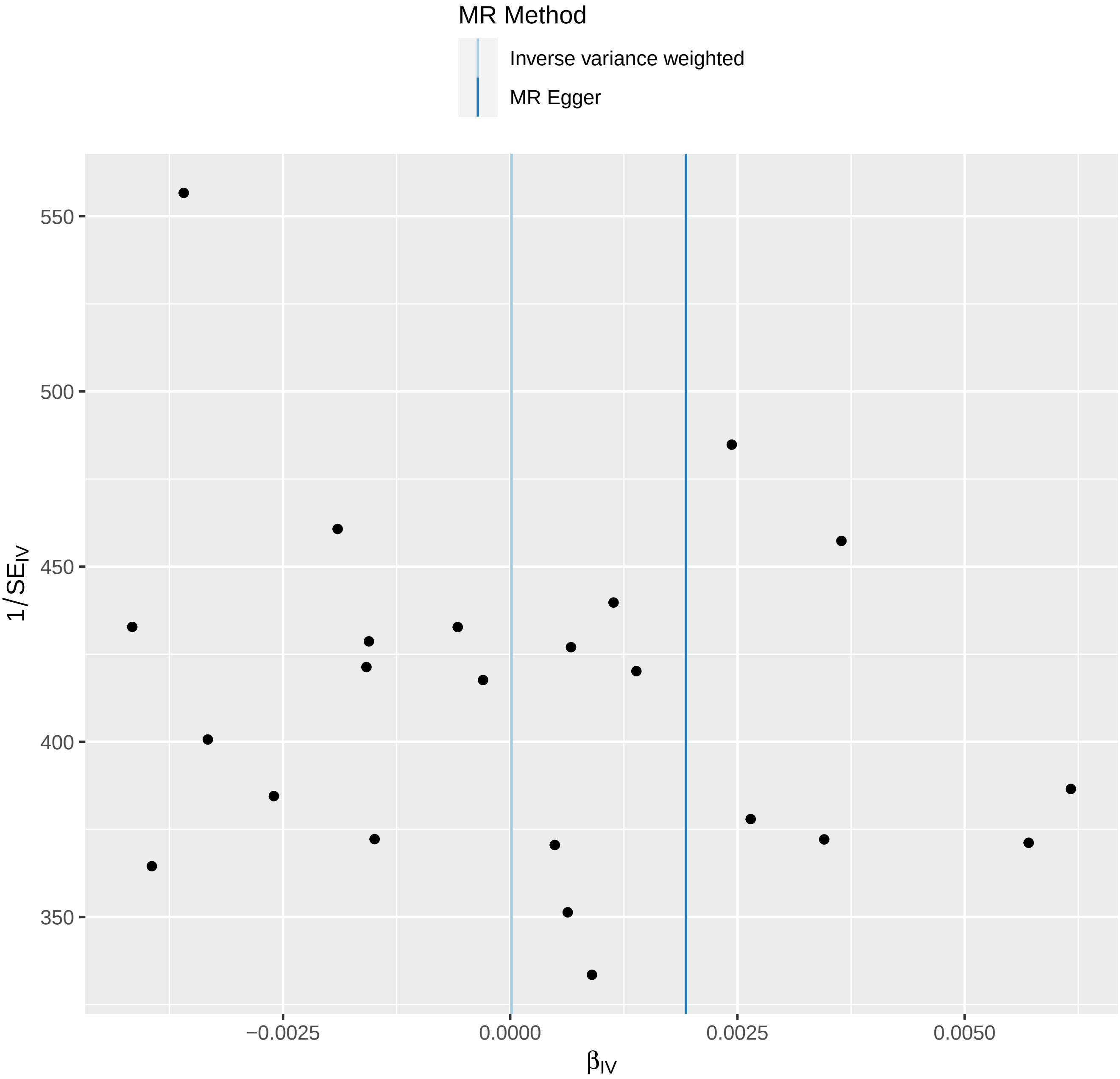

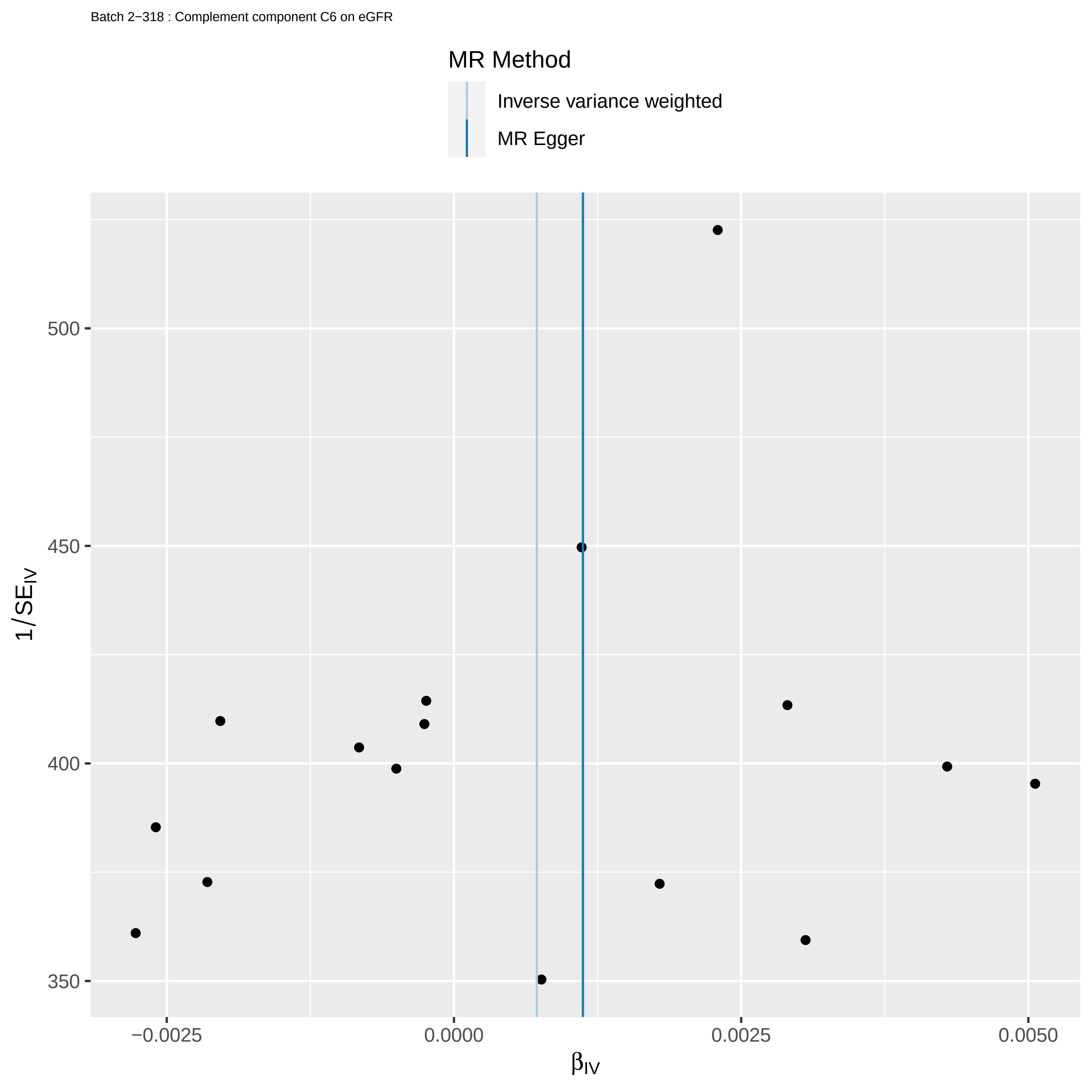

Batch 2−318 : Complement component C6 on eGFR
MR Method
Inverse variance weighted
MR Egger
500
450
400
350
1/SE_IV
−0.0025
0.0000
0.0025
0.0050
β_IV

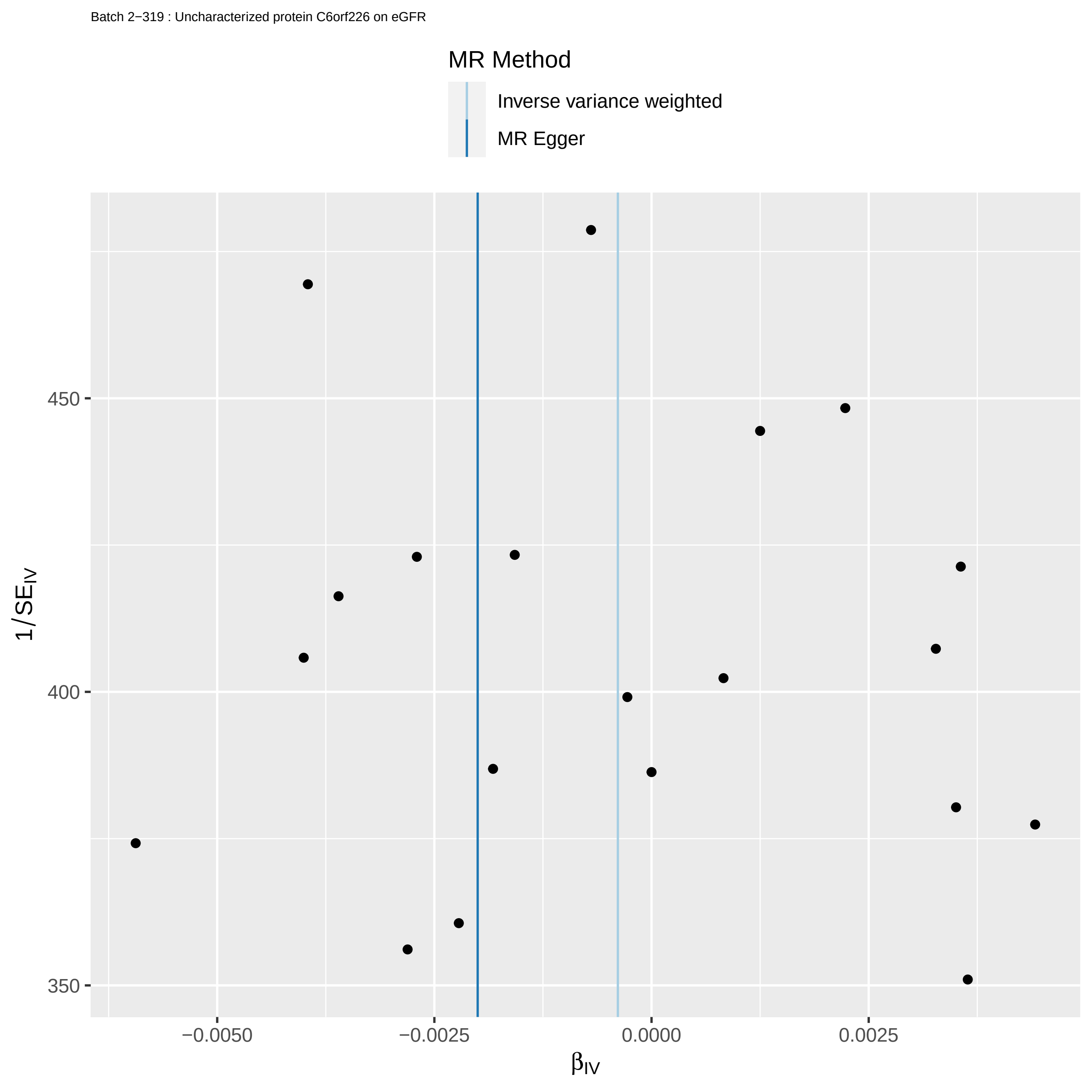

Batch 2−319 : Uncharacterized protein C6orf226 on eGFR
MR Method
Inverse variance weighted
MR Egger
450
400
350
1/SE$_{IV}$
−0.0050
−0.0025
0.0000
0.0025
β$_{IV}$

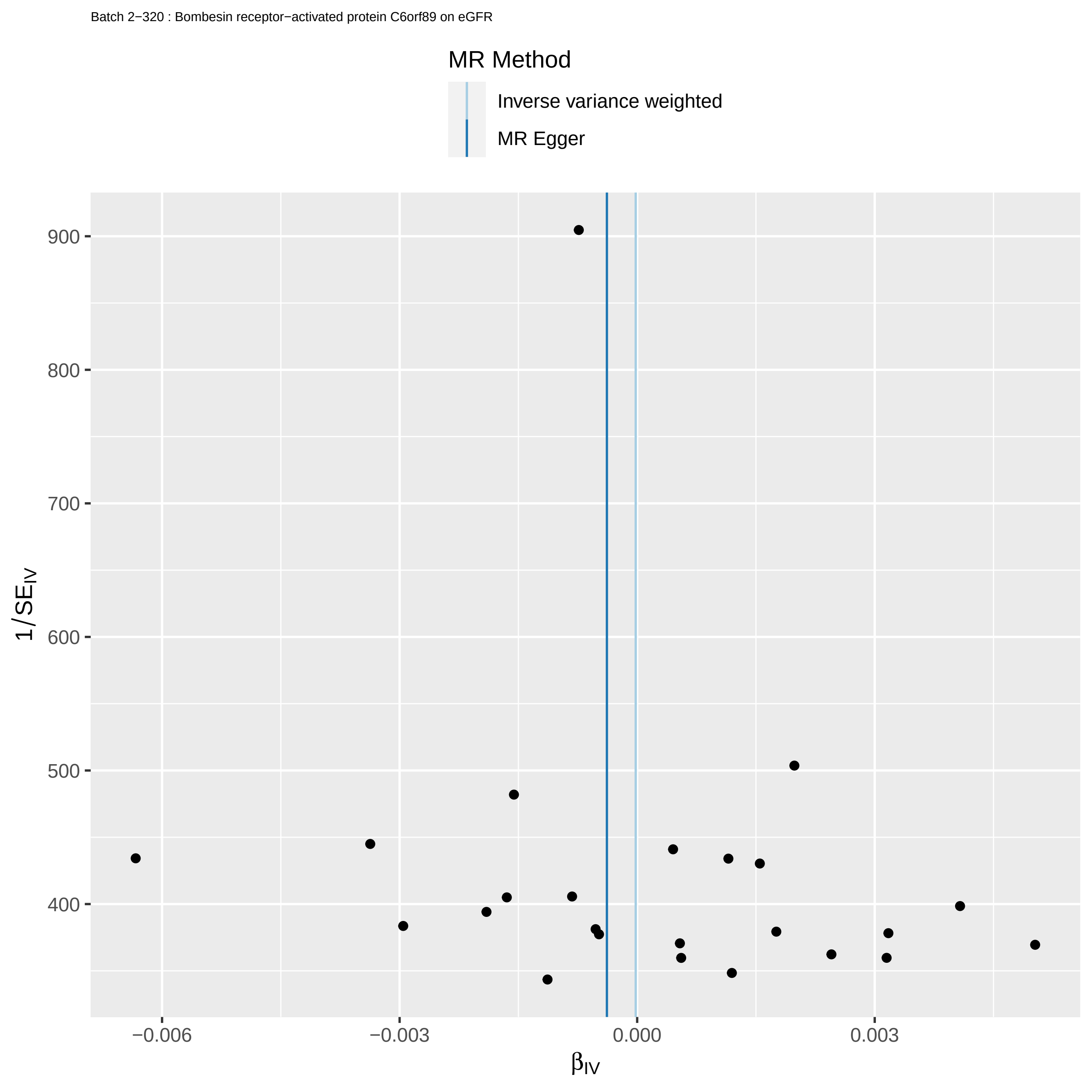
Batch 2−320 : Bombesin receptor−activated protein C6orf89 on eGFR
MR Method
Inverse variance weighted
MR Egger
900
800
700
600
500
400
1/SE_IV
−0.006
−0.003
0.000
0.003
β_IV

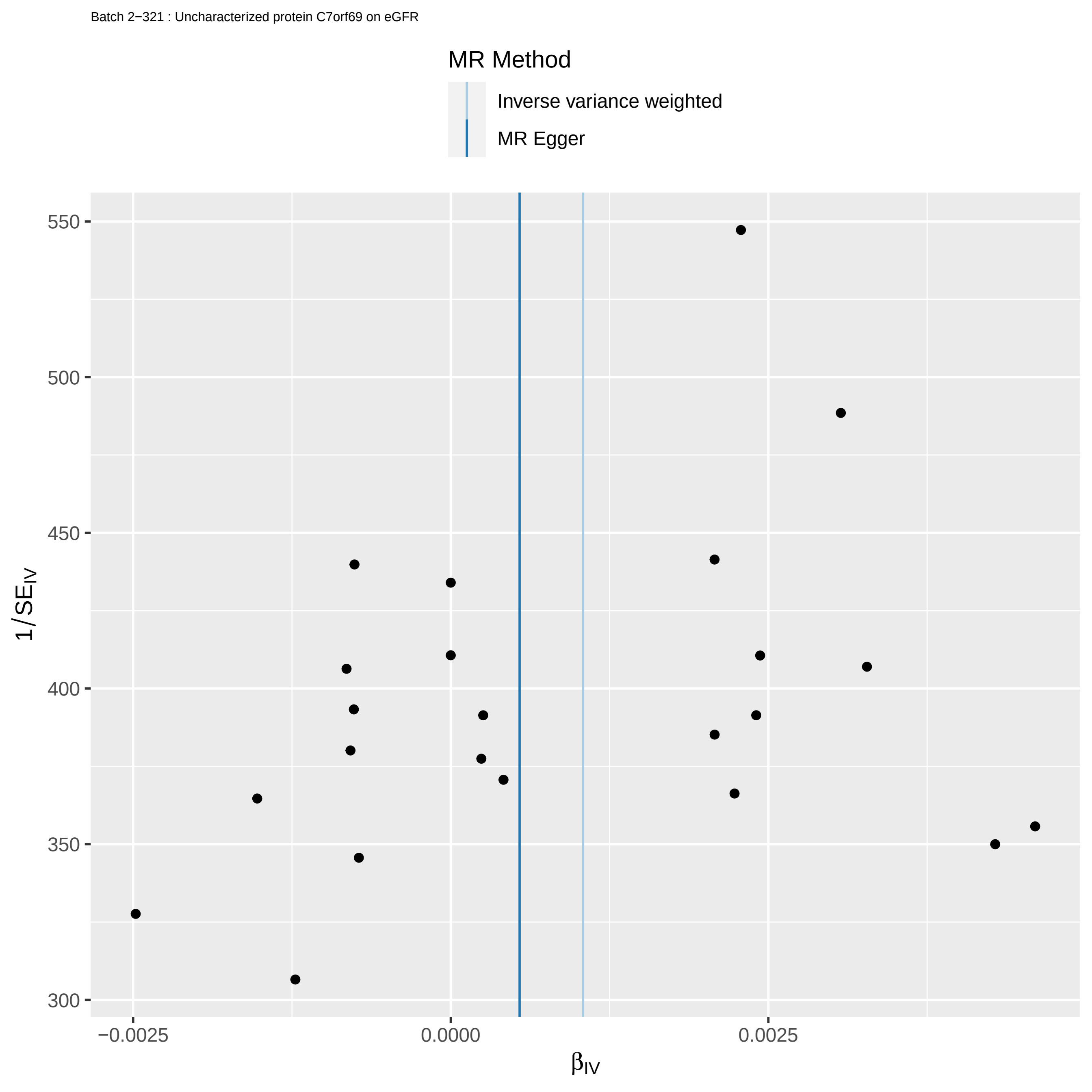

Batch 2−321 : Uncharacterized protein C7orf69 on eGFR
MR Method
Inverse variance weighted
MR Egger
550
500
450
400
350
300
1/SE_IV
−0.0025
0.0000
0.0025
β_IV

Batch 2−322 : Complement component C8 on eGFR

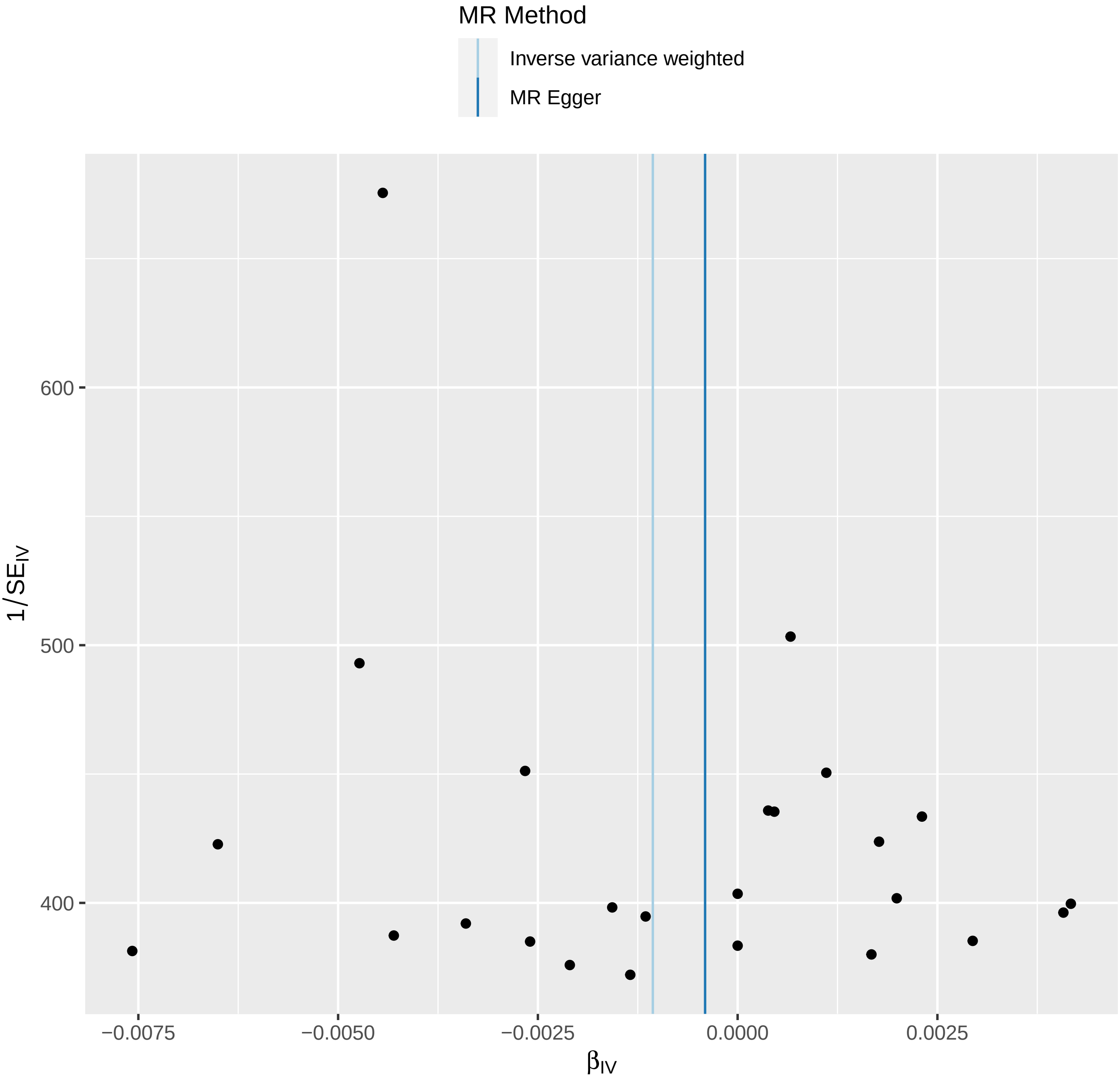

Batch 2−323 : UPF0488 protein C8orf33 on eGFR

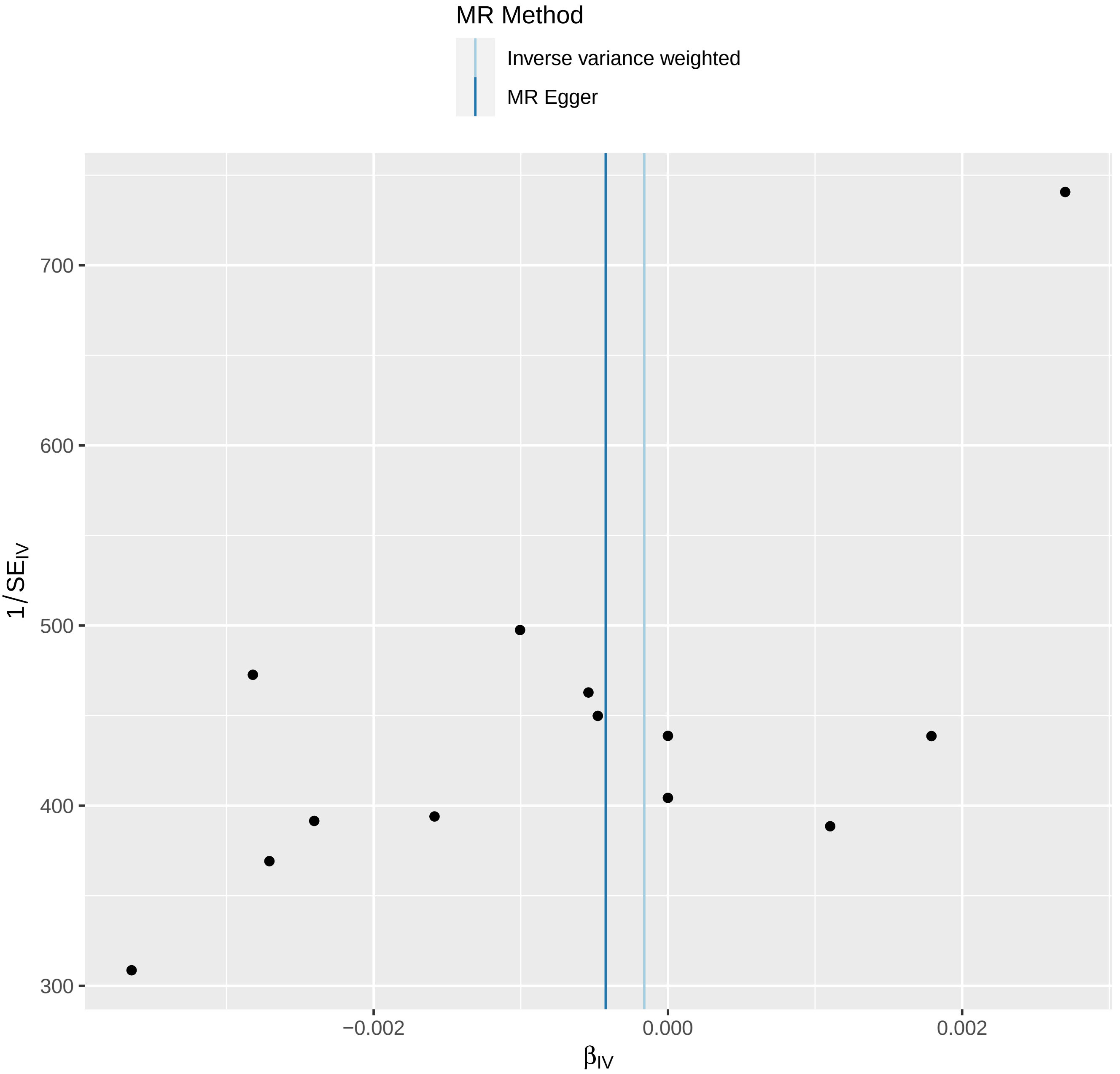

Batch 2−324 : Complement component C9 on eGFR

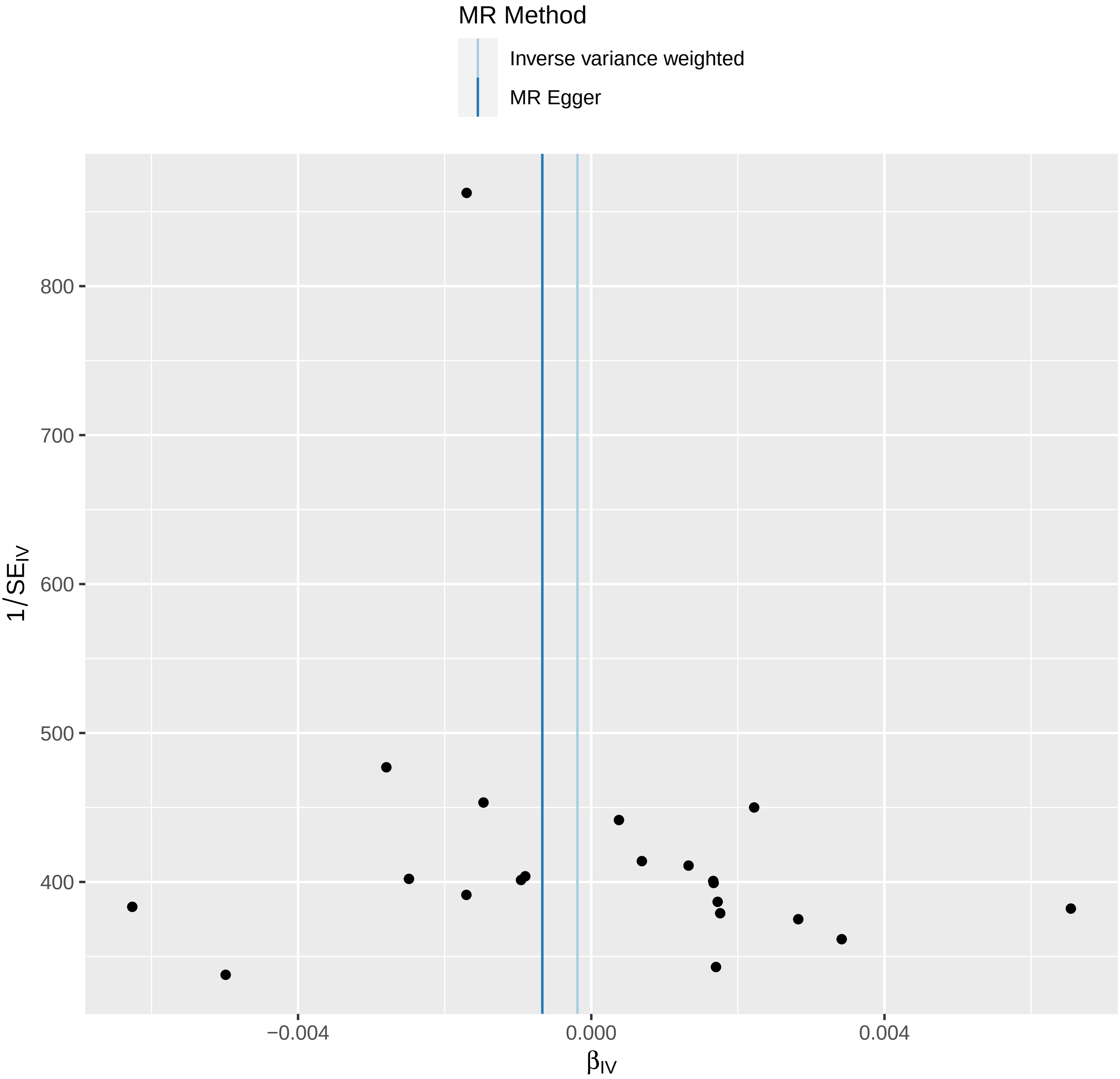

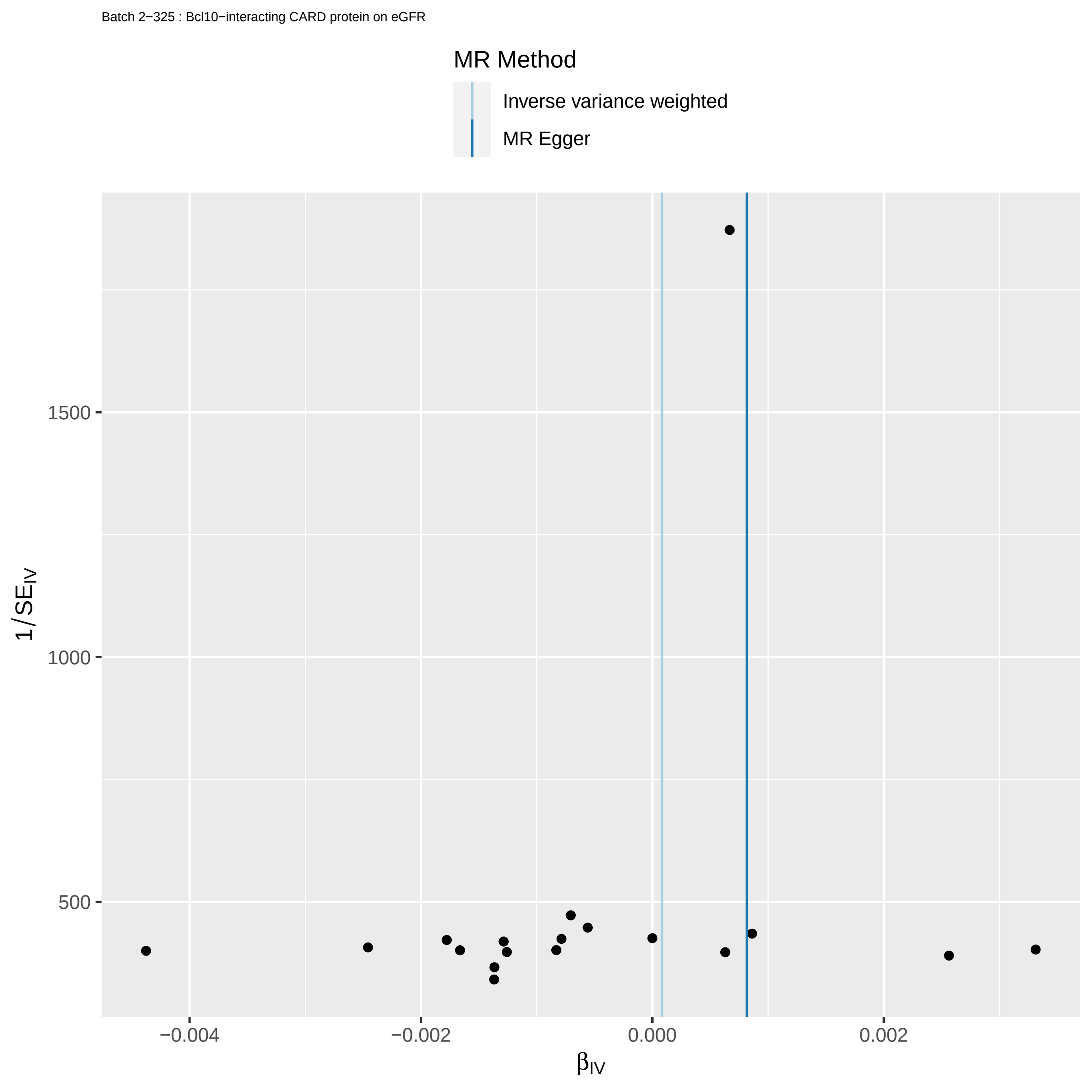
Batch 2−325 : Bcl10−interacting CARD protein on eGFR
MR Method
Inverse variance weighted
MR Egger
1500
1000
500
1/SE_IV
−0.004
−0.002
0.000
0.002
β_IV

Batch 2−326 : Carbonic anhydrase−related protein 10 on eGFR

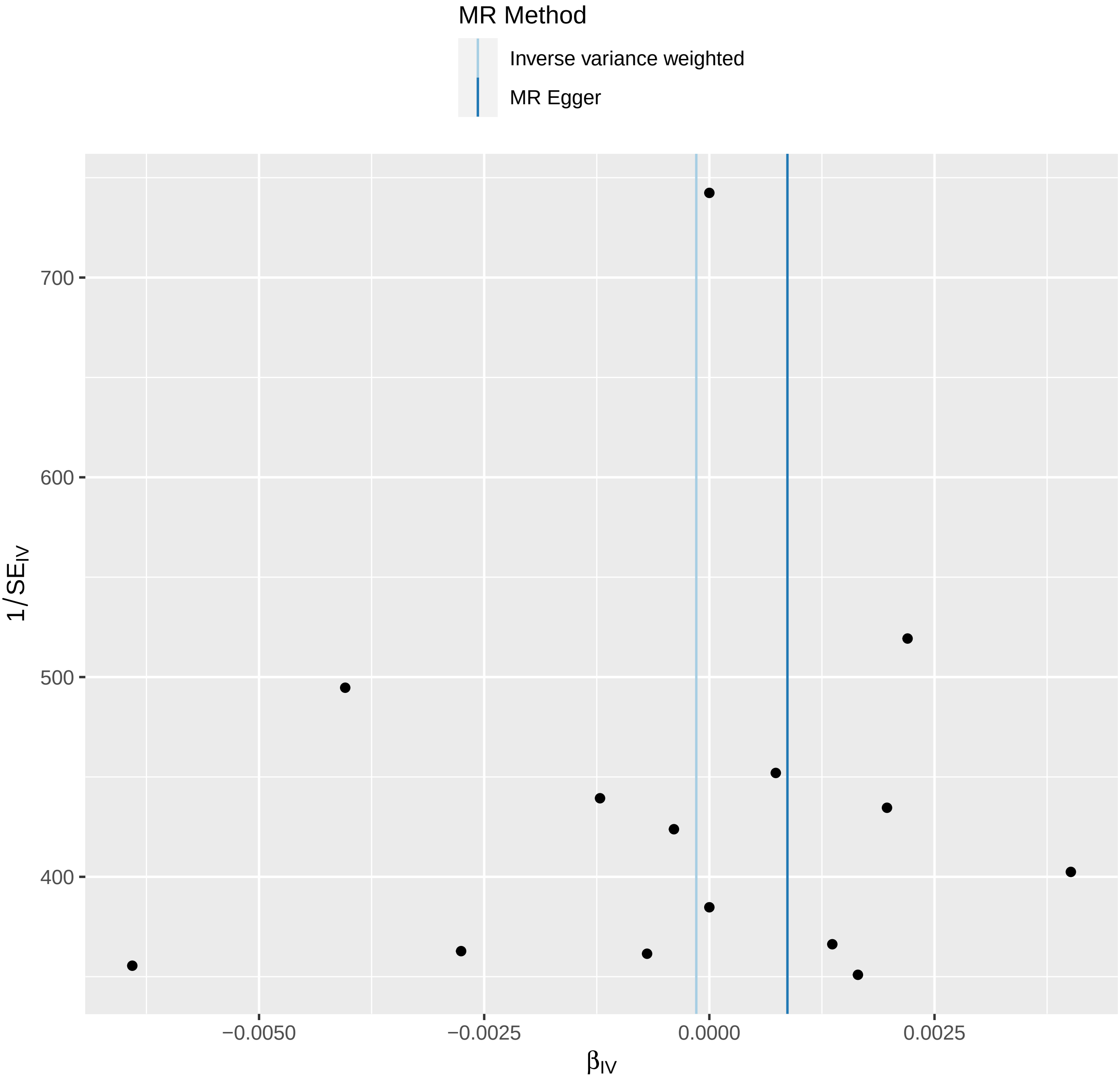

Batch 2−327 : Carbonic anhydrase−related protein 10 on eGFR

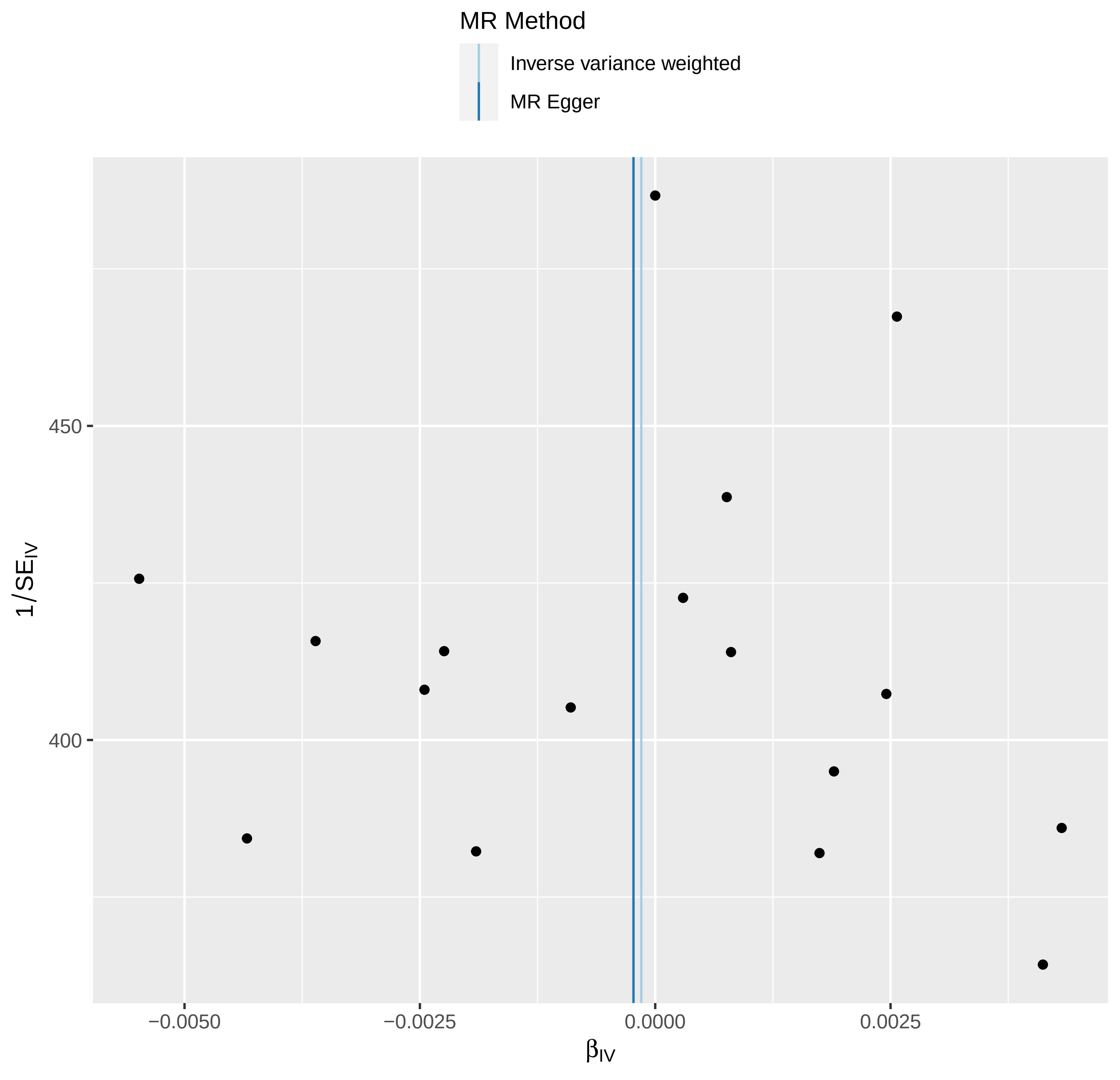

Batch 2−328 : Carbonic anhydrase 1 on eGFR

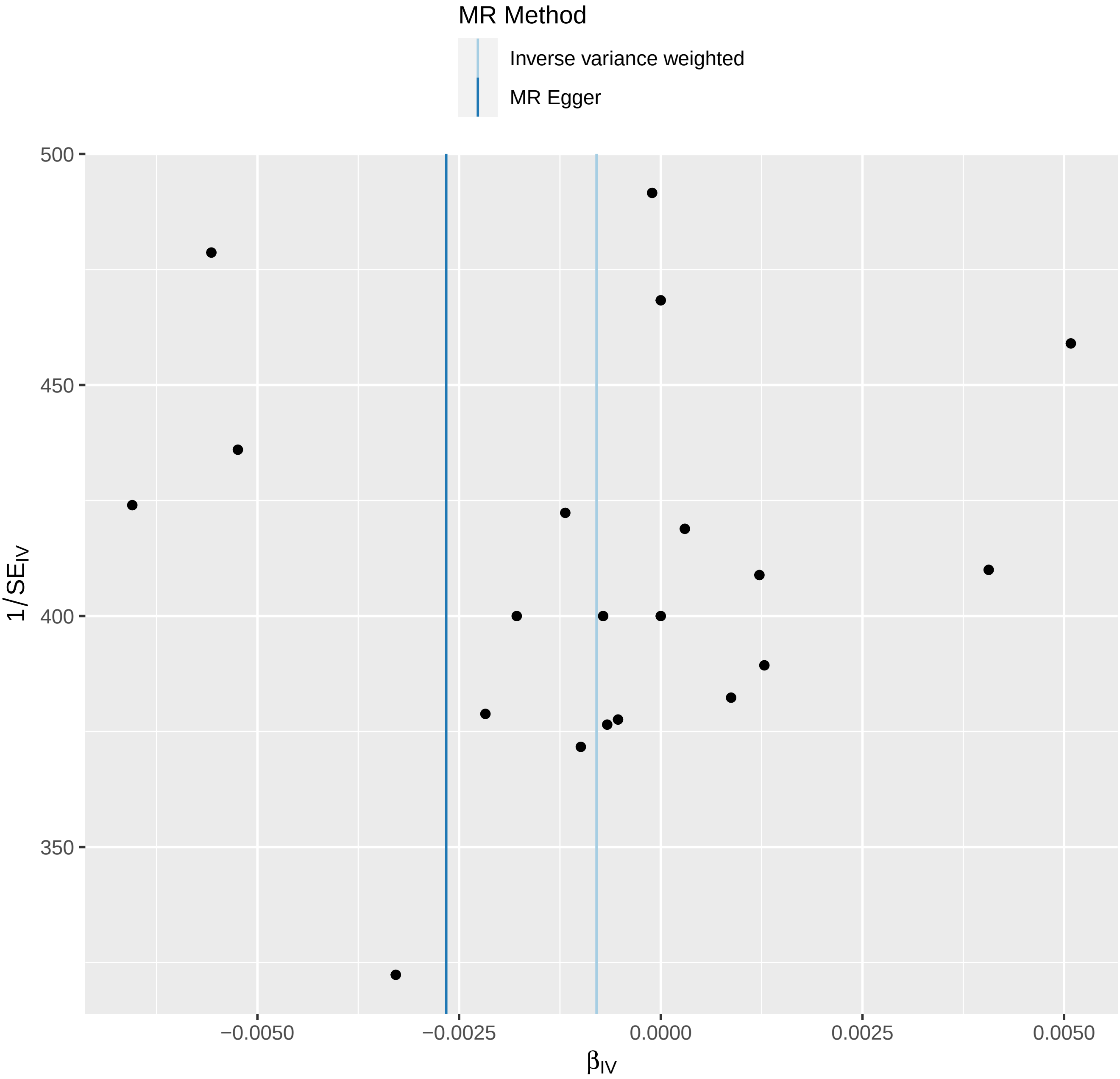

Batch 2−329 : Carbonic anhydrase 2 on eGFR

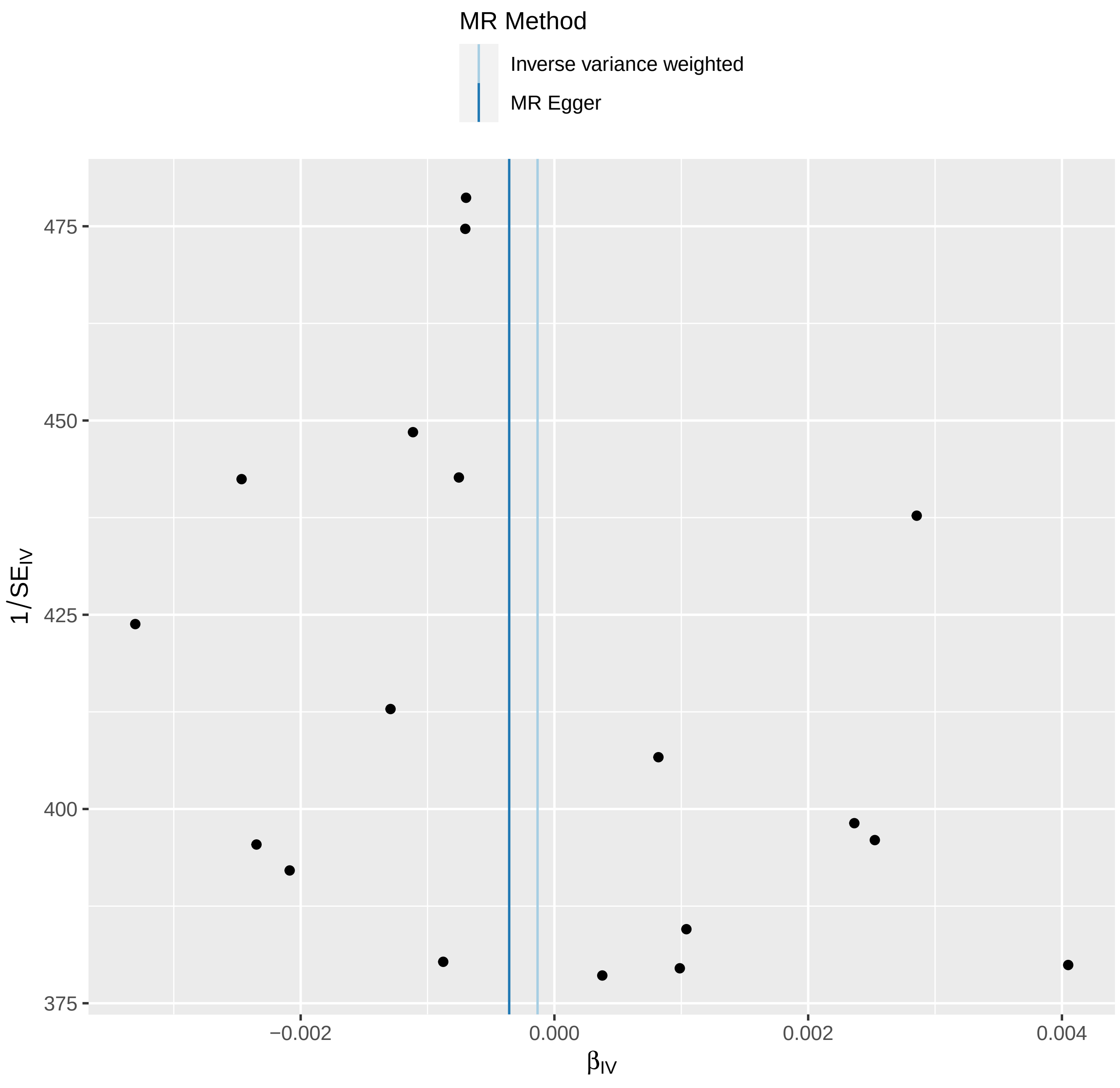

Batch 2−330 : Carbonic anhydrase 3 on eGFR

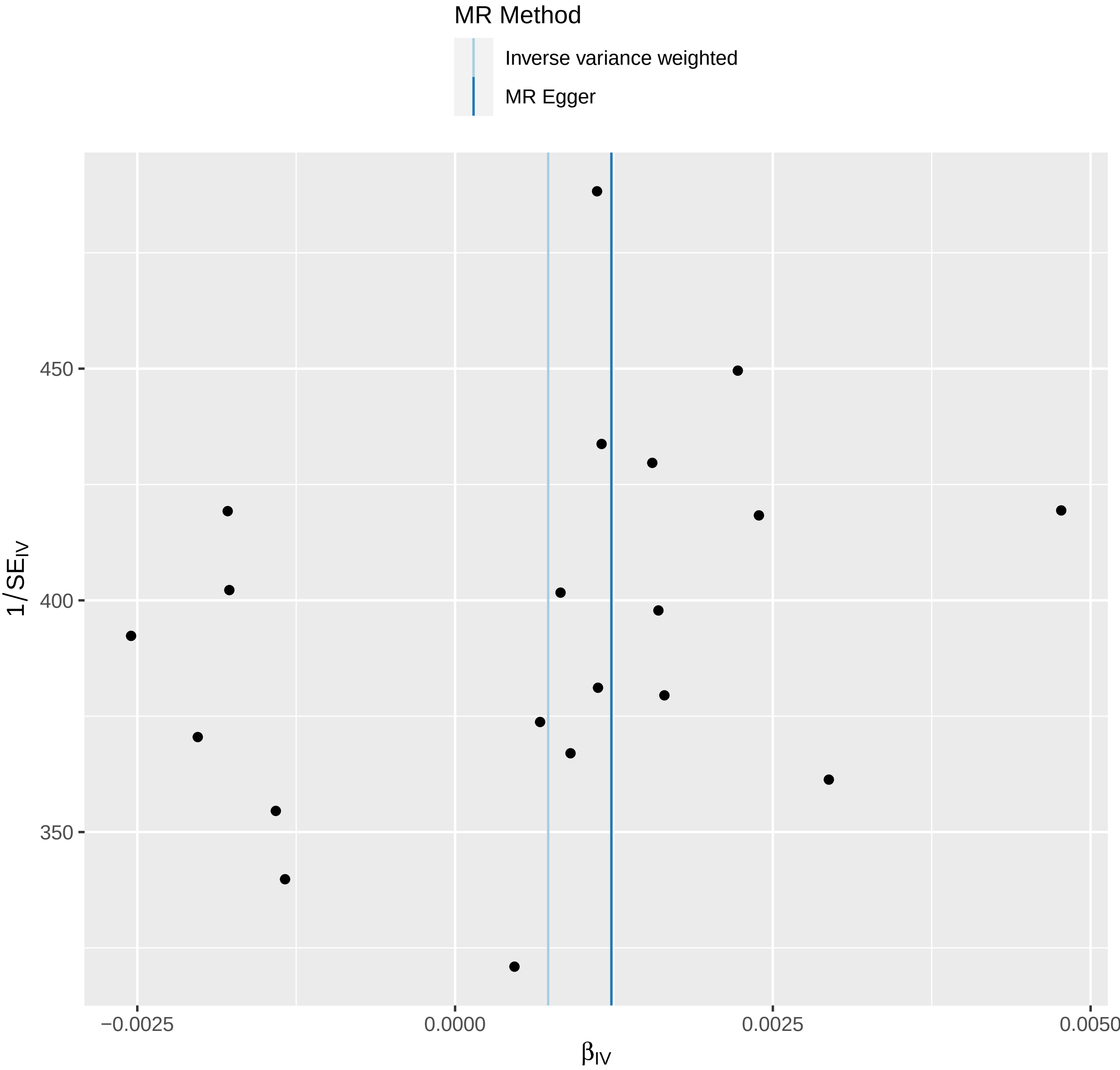

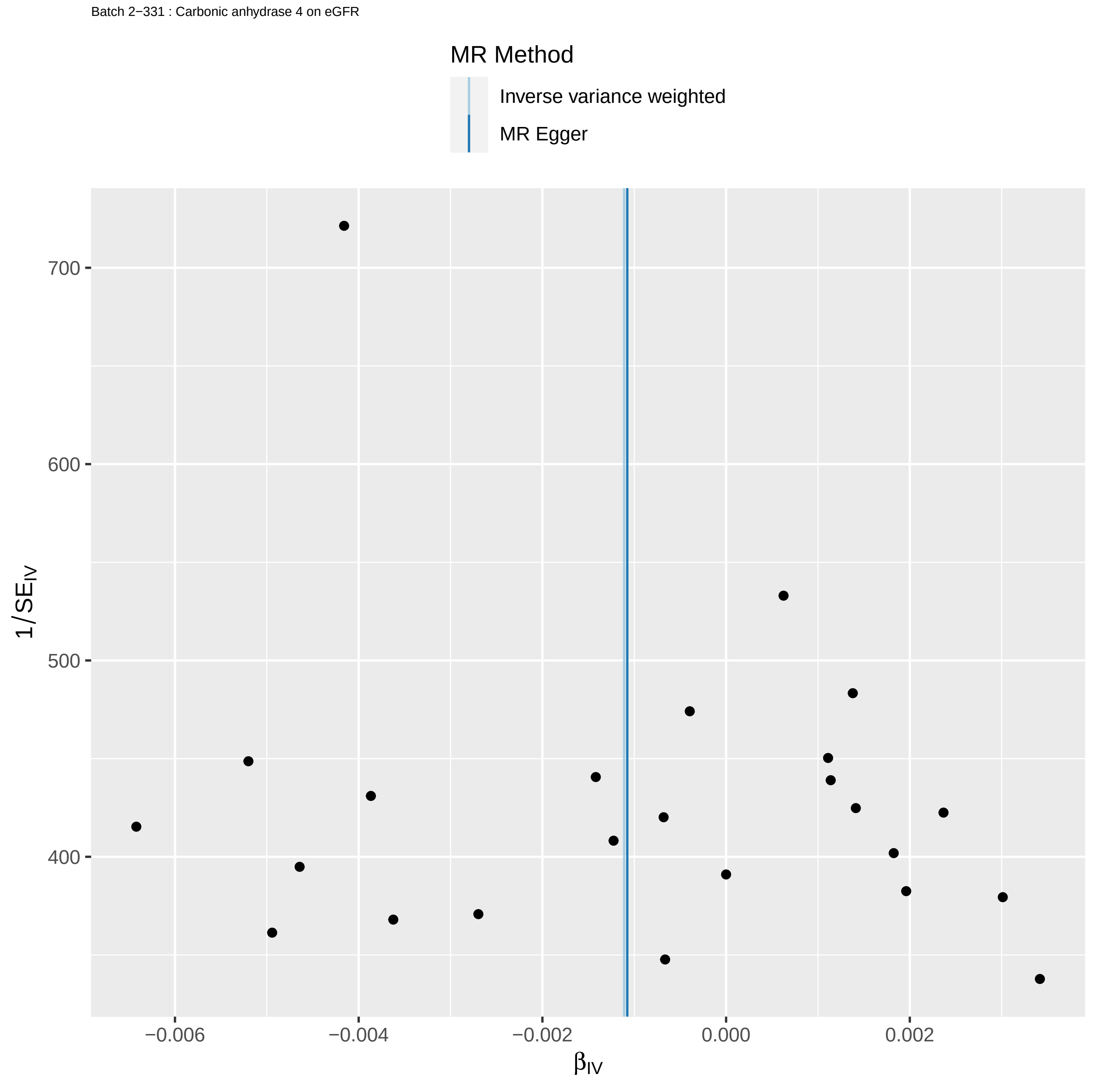
Batch 2−331 : Carbonic anhydrase 4 on eGFR
MR Method
Inverse variance weighted
MR Egger
700
600
500
400
−0.006
−0.004
−0.002
0.000
0.002
1/SE_IV
β_IV

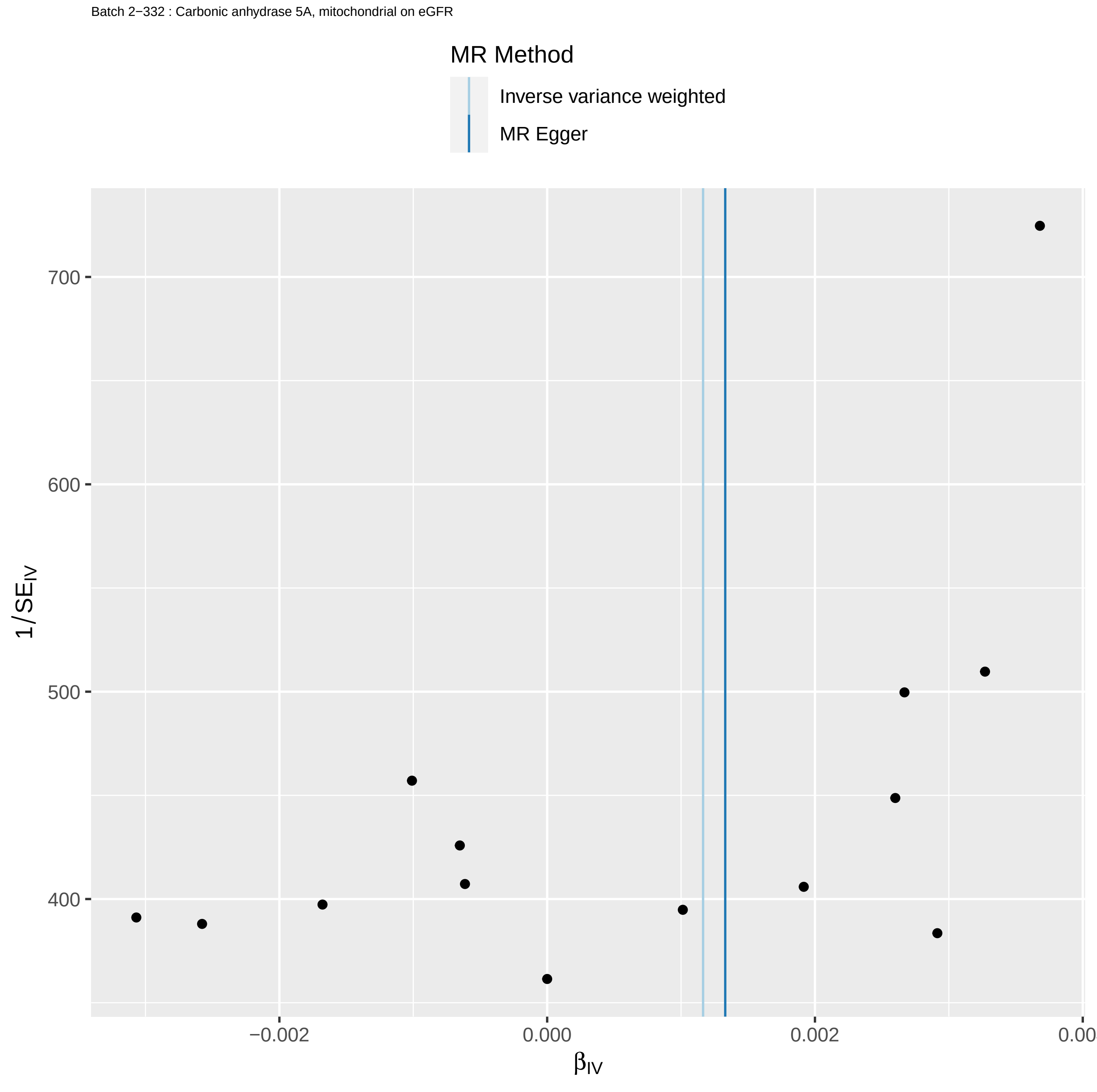

Batch 2−332 : Carbonic anhydrase 5A, mitochondrial on eGFR
MR Method
Inverse variance weighted
MR Egger
700
600
500
400
−0.002
0.000
0.002
1/SE_IV
β_IV

Batch 2−333 : Carbonic anhydrase 6 on eGFR

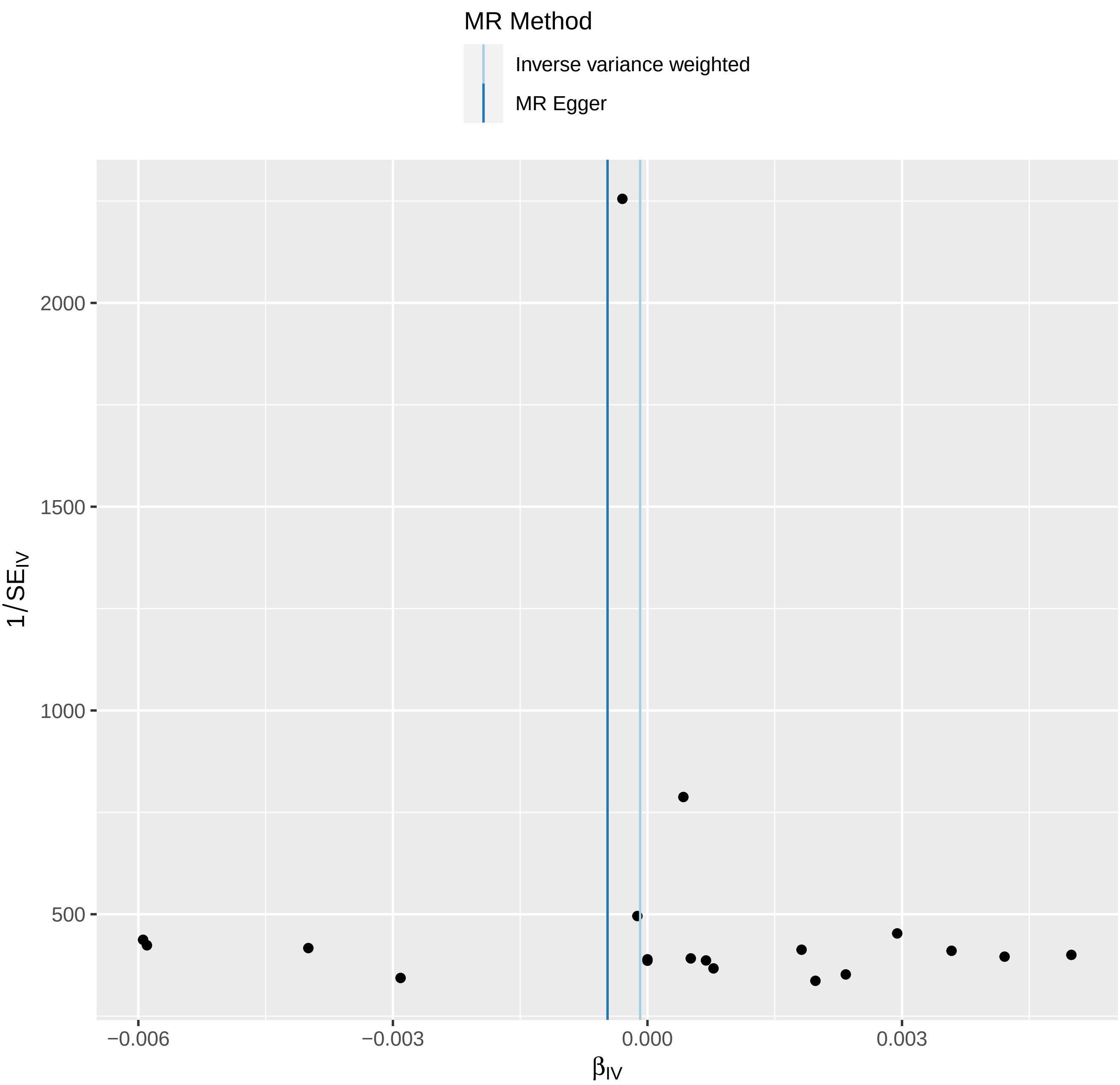

Batch 2−334 : Carbonic anhydrase 9 on eGFR

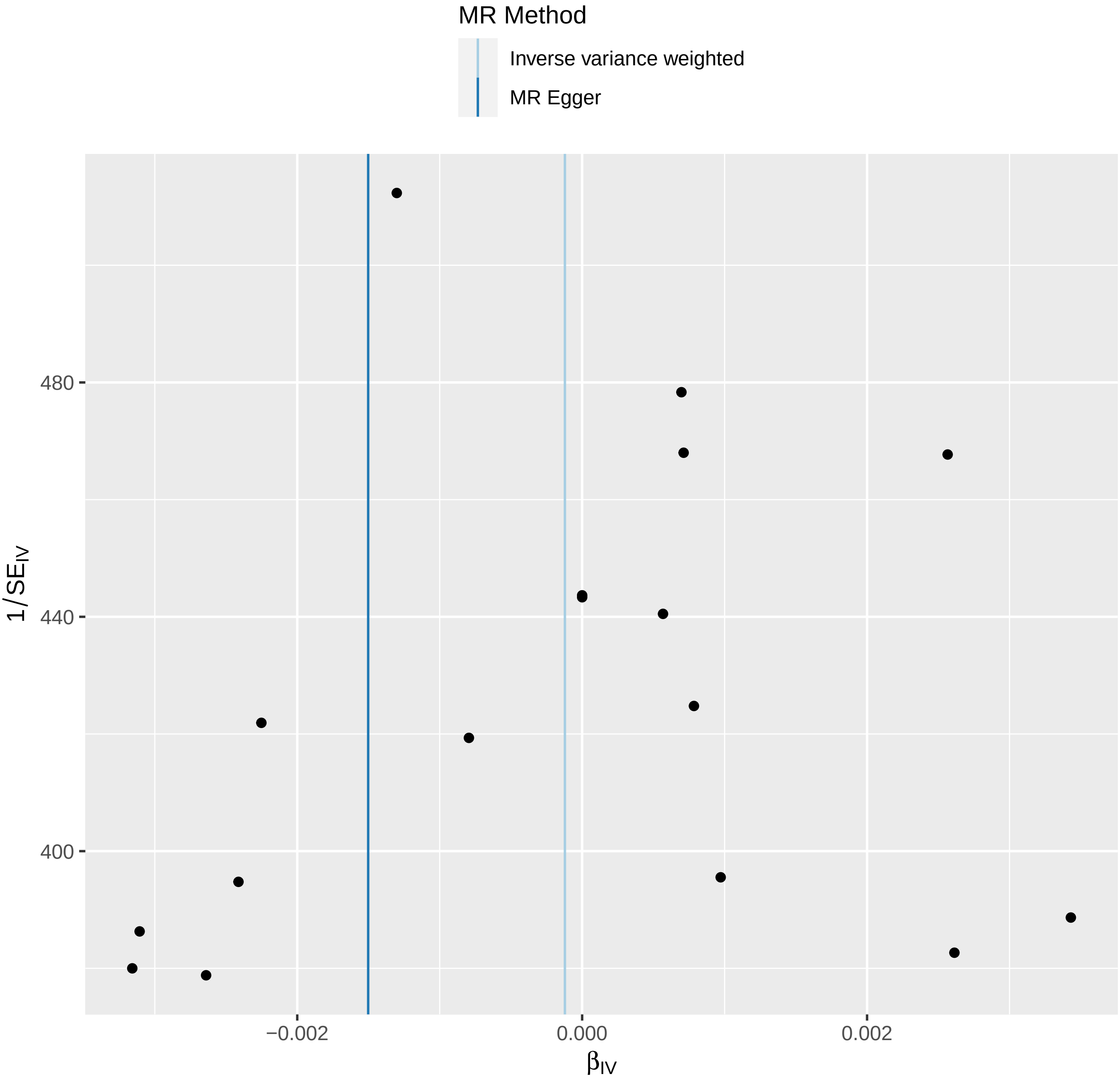

Batch 2−335 : Voltage−dependent calcium channel subunit alpha−2/delta−3 on eGFR

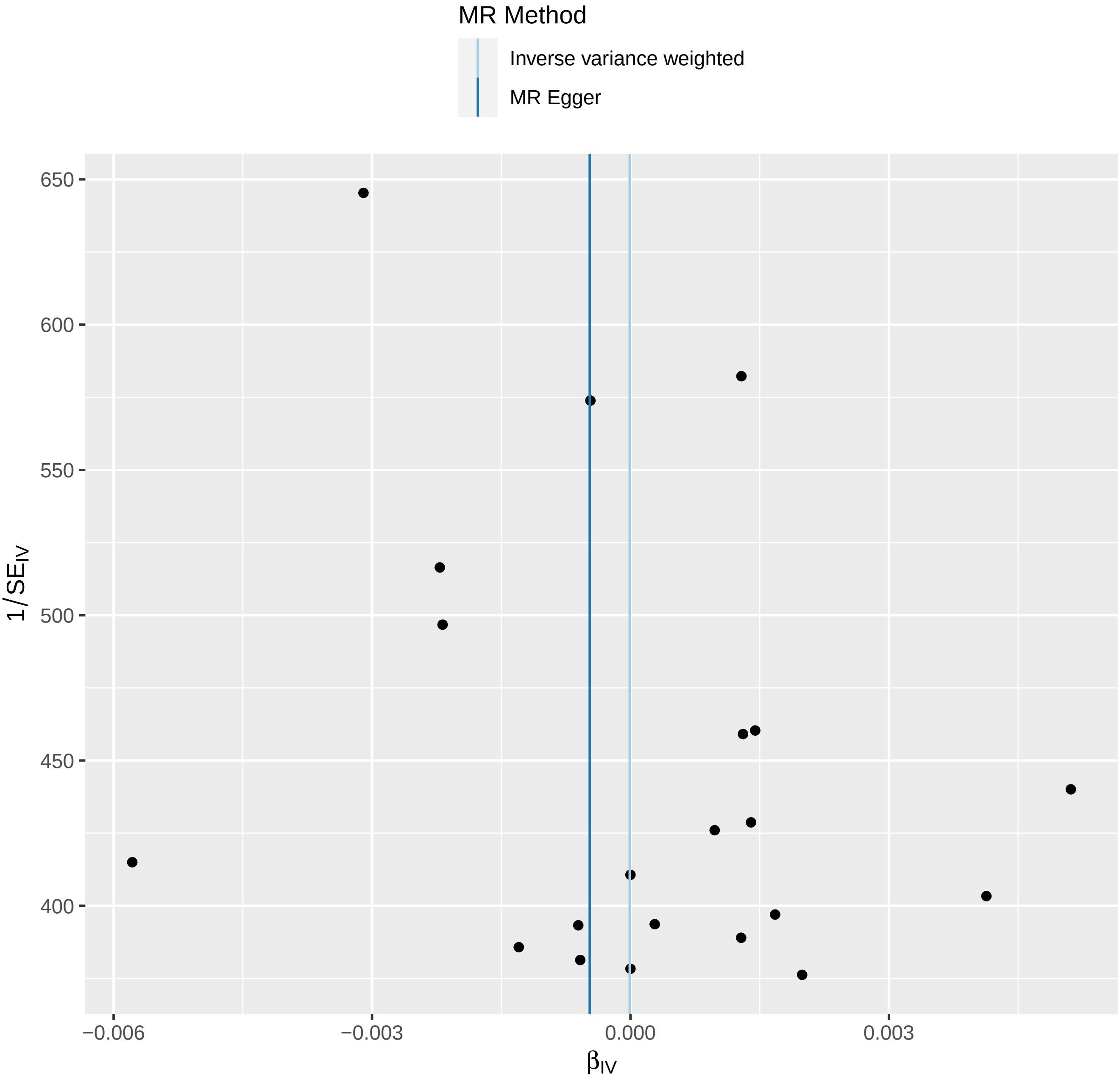

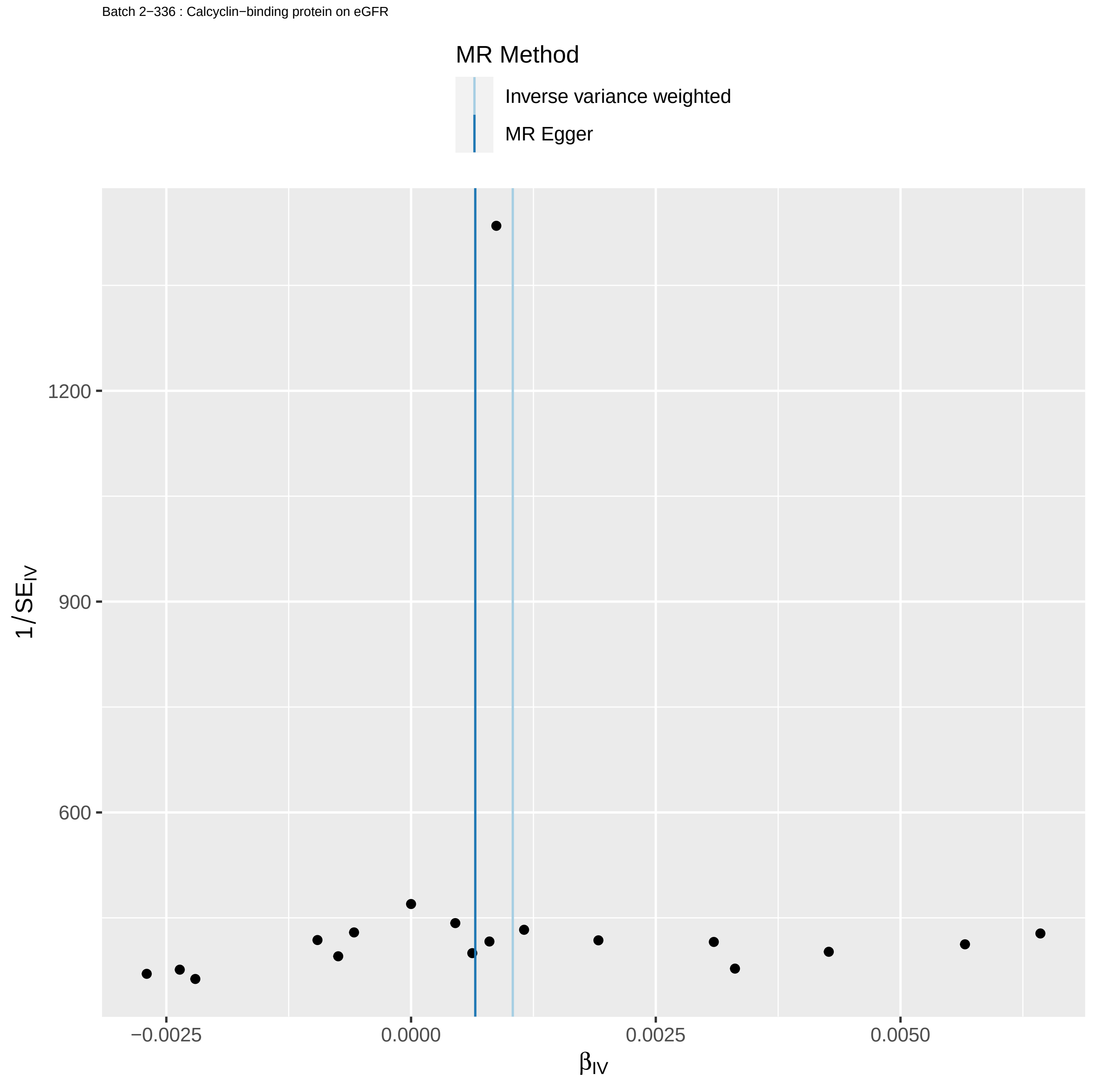
Batch 2−336 : Calcyclin−binding protein on eGFR
MR Method
Inverse variance weighted
MR Egger
1200
900
600
−0.0025
0.0000
0.0025
0.0050
$1/SE_{IV}$
β_{IV}

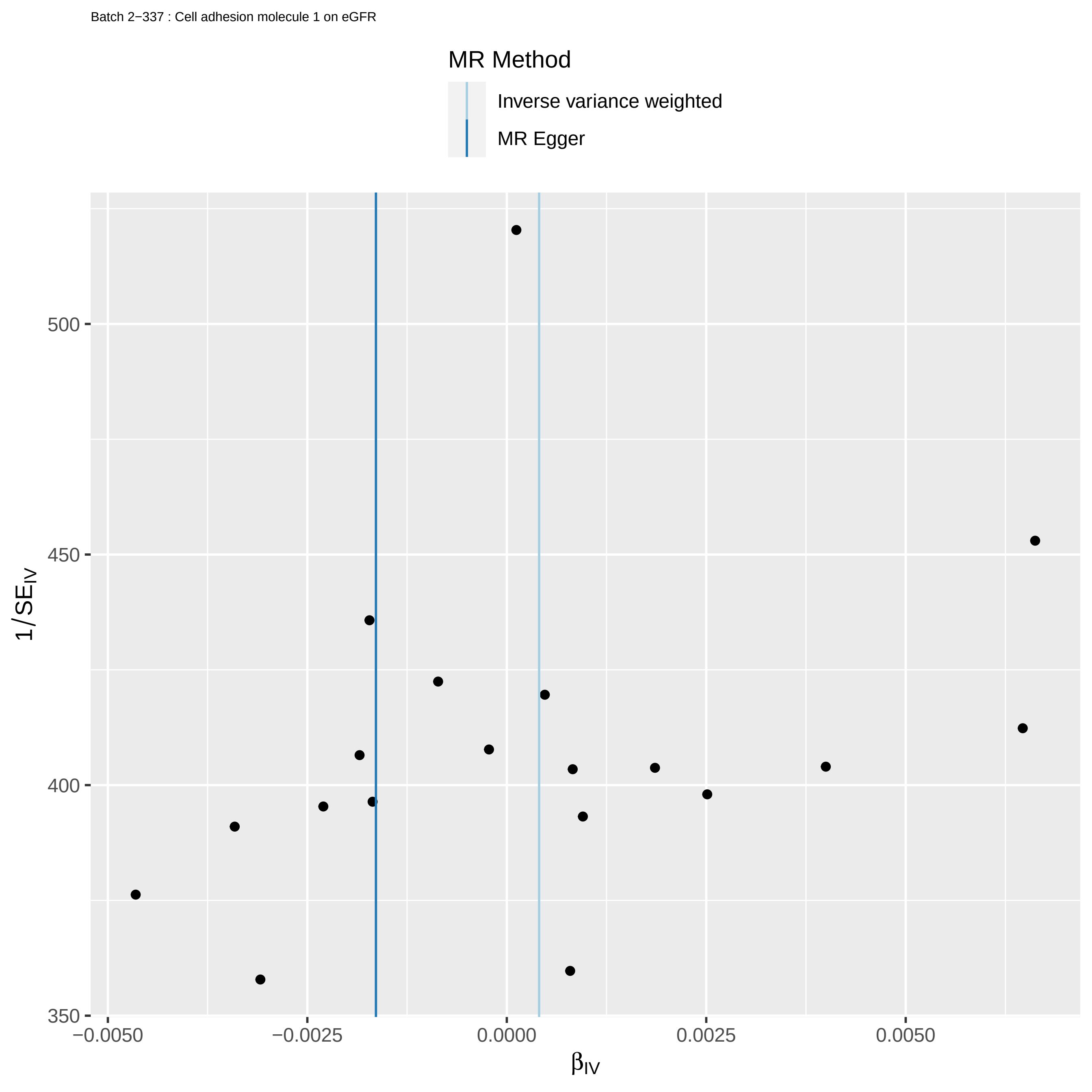
Batch 2−337 : Cell adhesion molecule 1 on eGFR
MR Method
Inverse variance weighted
MR Egger
500
450
400
350
1/SE$_{IV}$
−0.0050
−0.0025
0.0000
0.0025
0.0050
β$_{IV}$

Batch 2−338 : Cell adhesion molecule 3 on eGFR

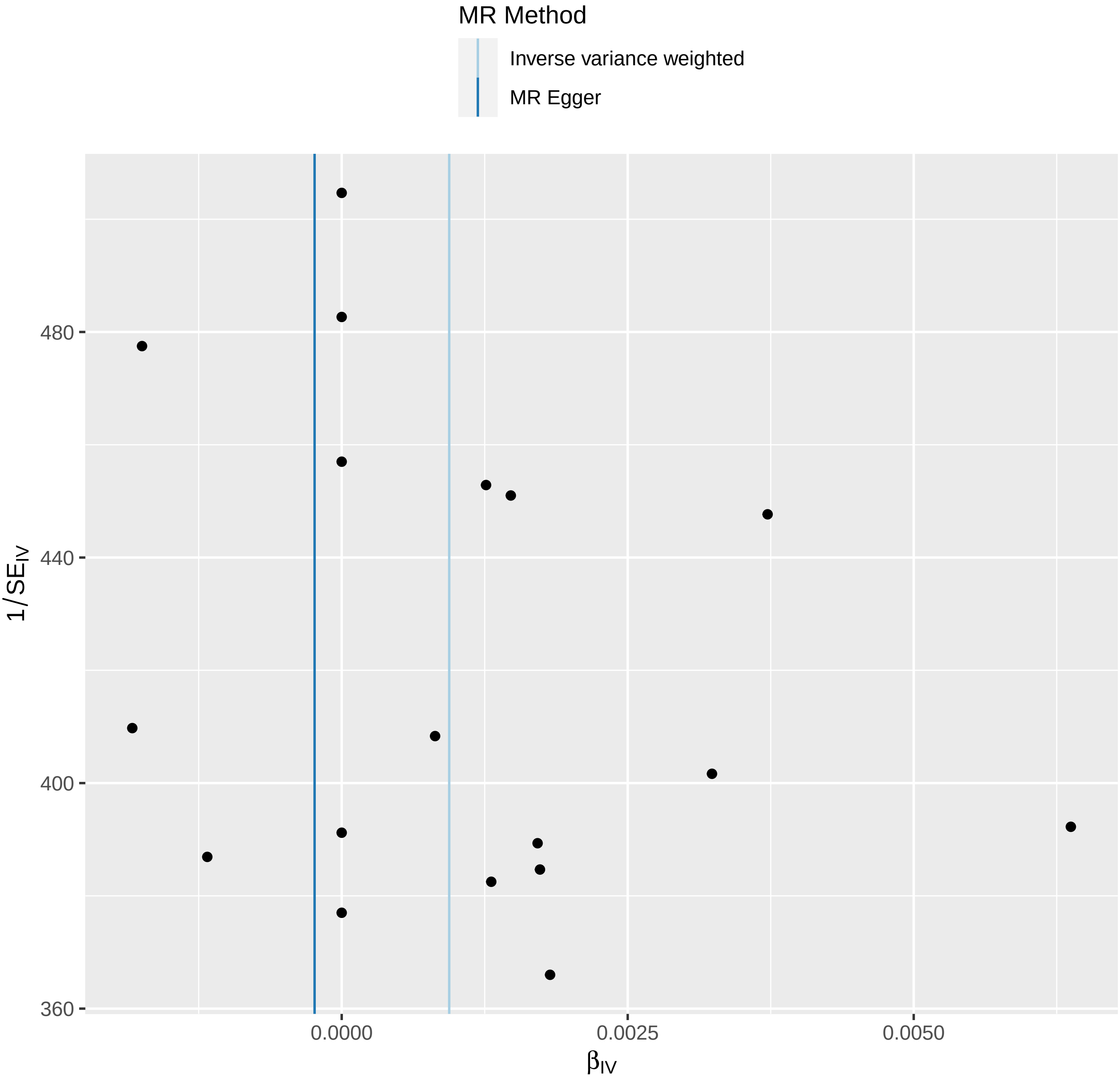

Batch 2−339 : Cell adhesion molecule 4 on eGFR

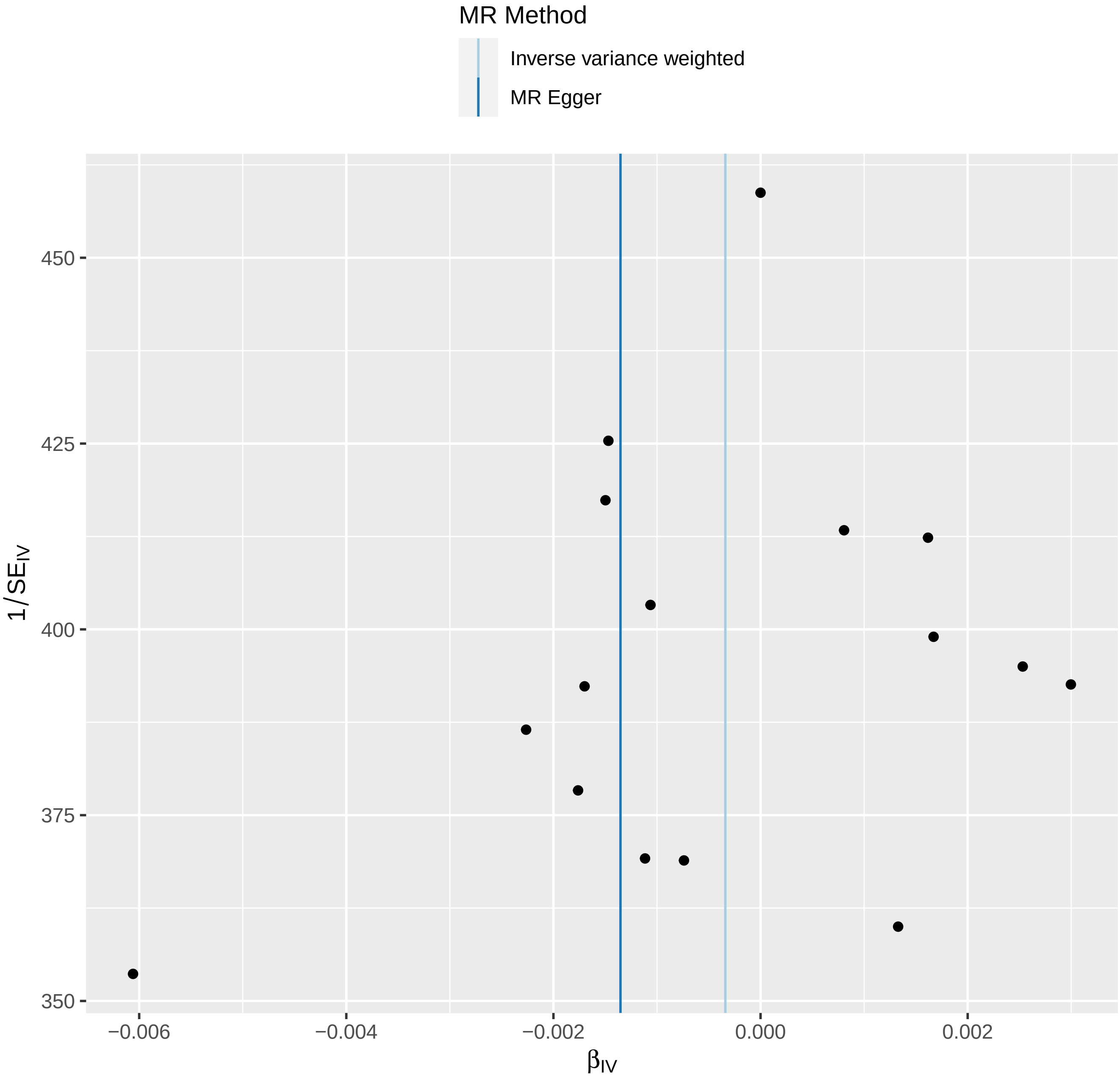

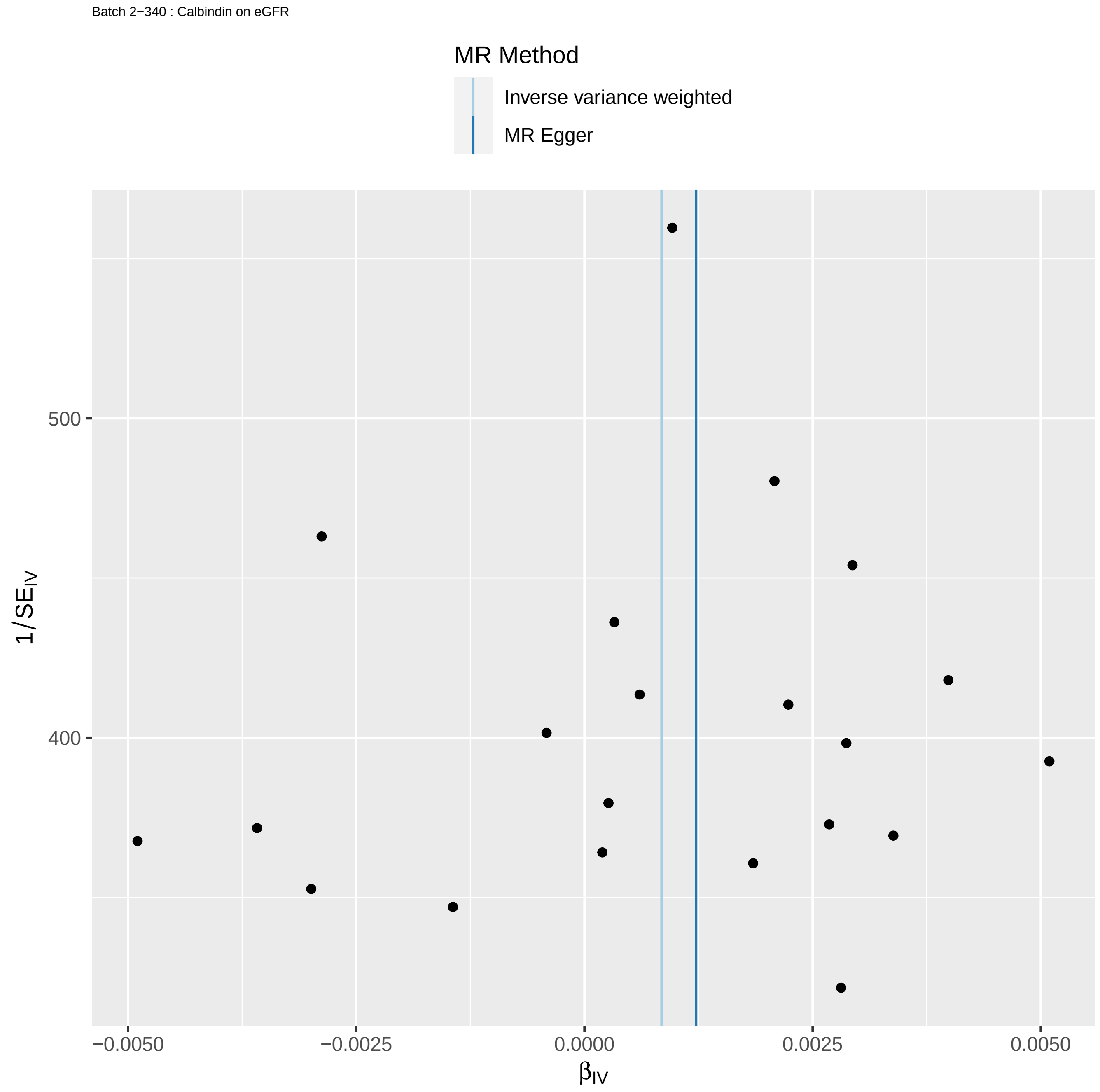
Batch 2−340 : Calbindin on eGFR
MR Method
Inverse variance weighted
MR Egger
500
400
1/SE$_{IV}$
−0.0050
−0.0025
0.0000
0.0025
0.0050
β$_{IV}$

Batch 2−341 : Procalcitonin on eGFR

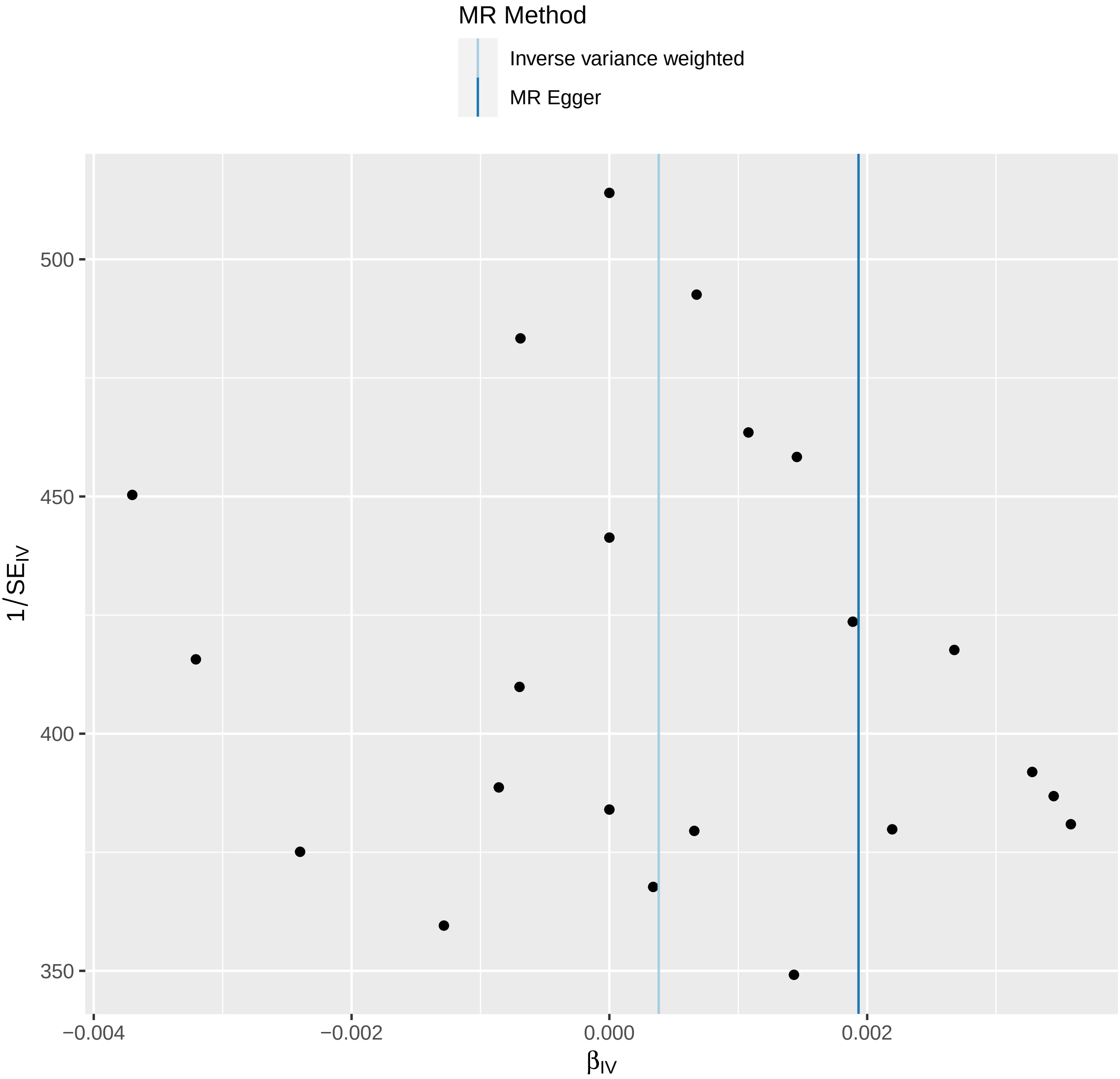

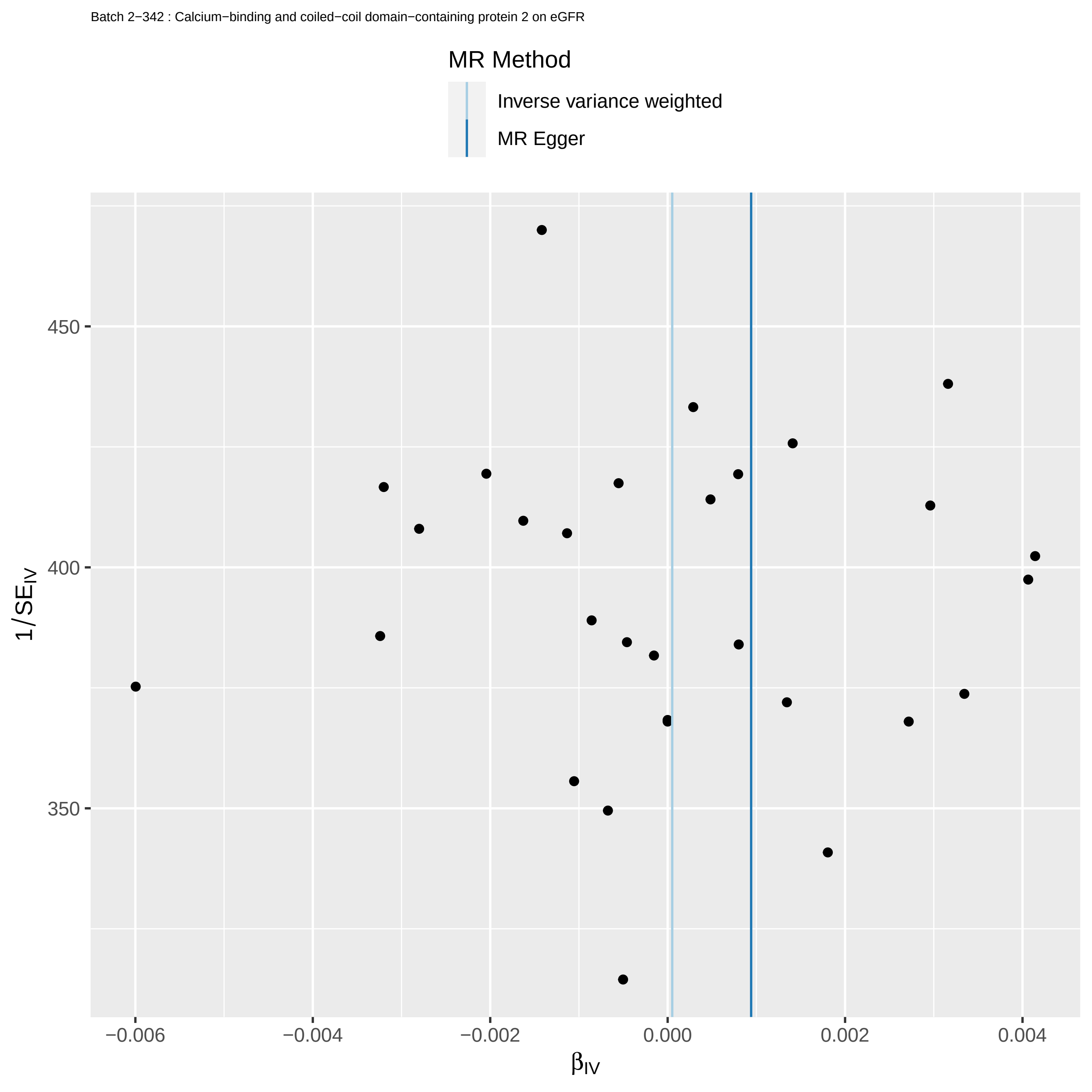
Batch 2−342 : Calcium−binding and coiled−coil domain−containing protein 2 on eGFR
MR Method
Inverse variance weighted
MR Egger
450
400
350
1/SE$_{IV}$
−0.006
−0.004
−0.002
0.000
0.002
0.004
β$_{IV}$

Batch 2−343 : Calcium−binding protein 8 on eGFR

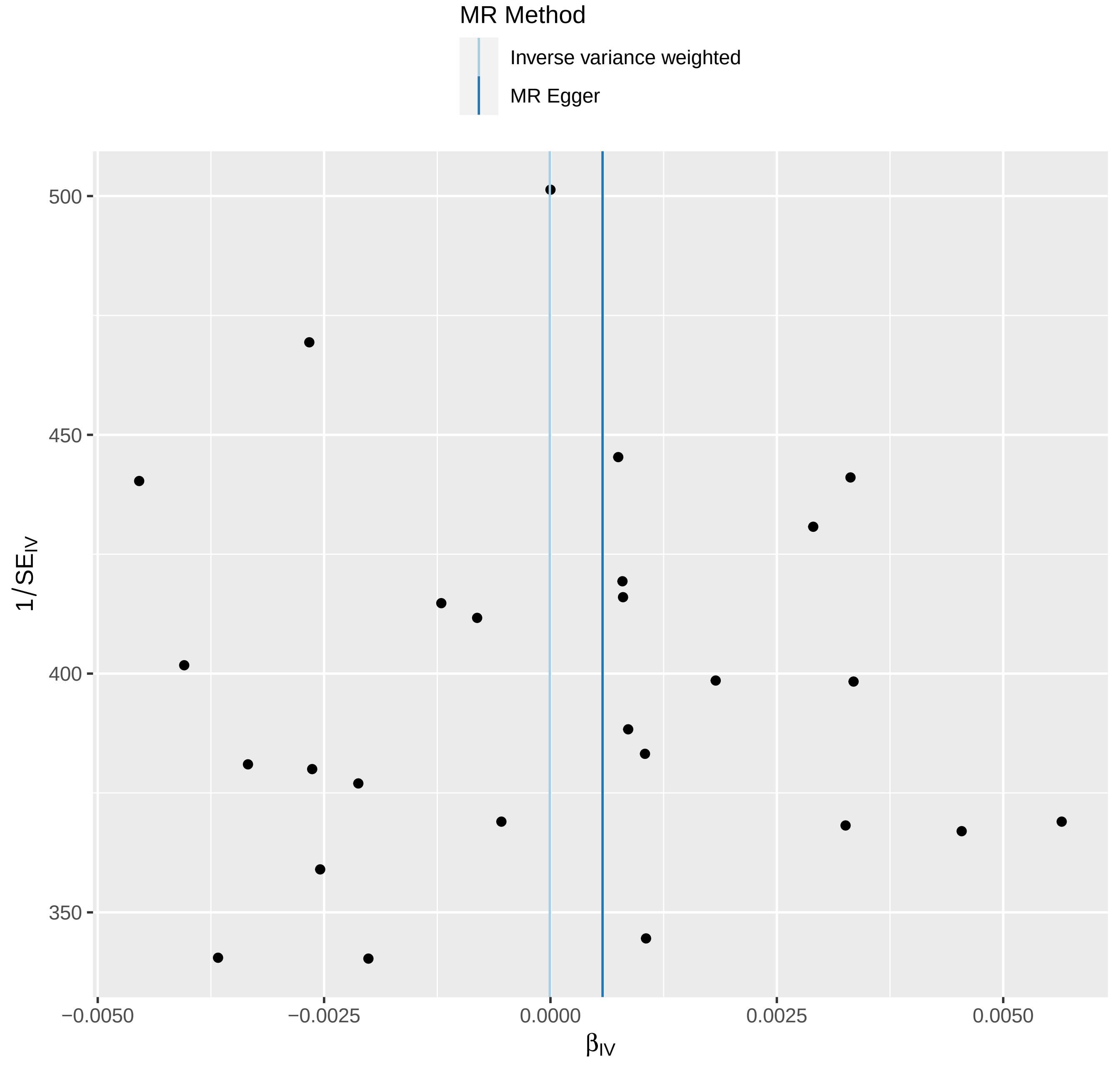

Batch 2−344 : Calreticulin on eGFR

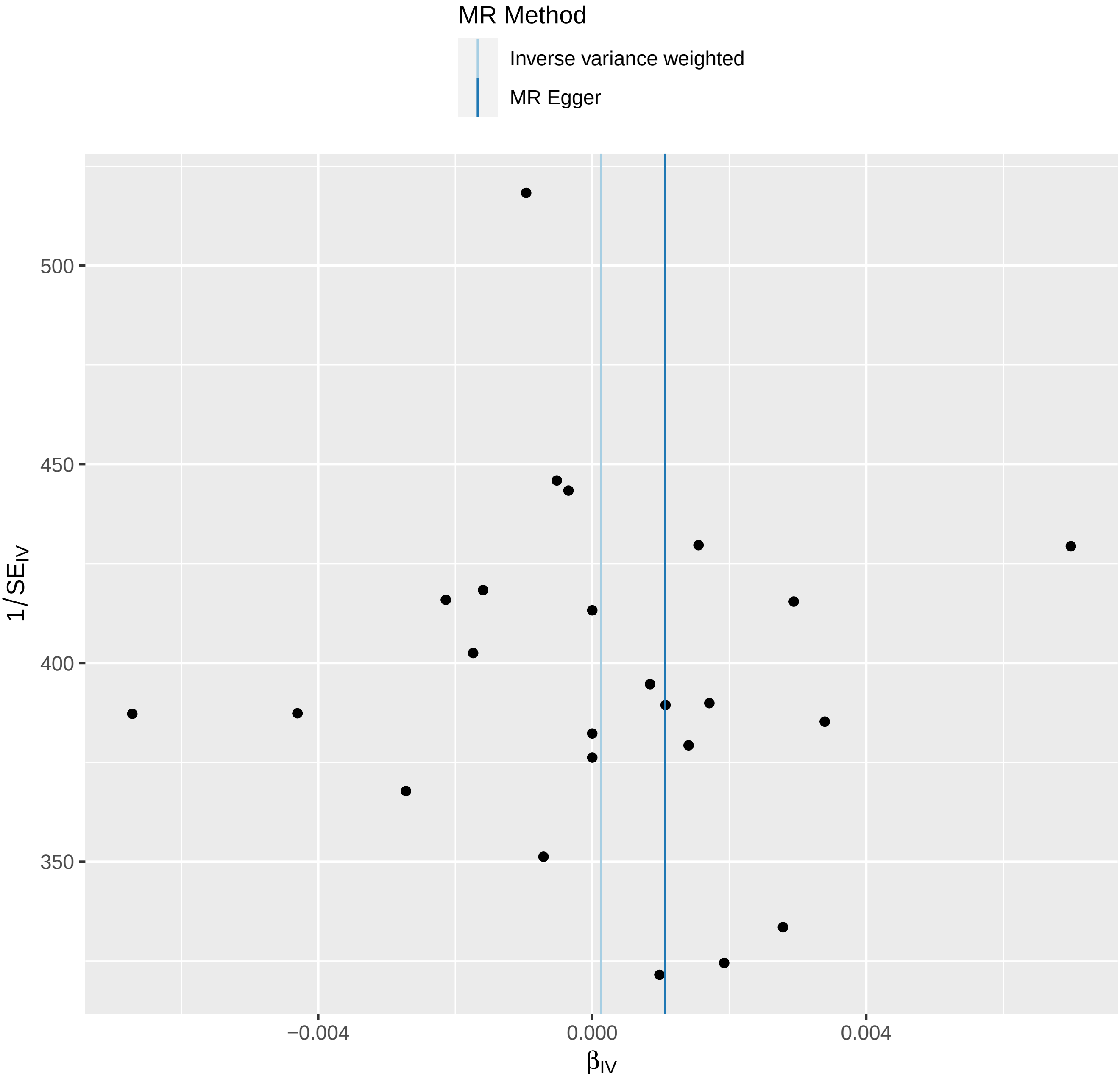

Batch 2−345 : Neuron−specific vesicular protein calcyon on eGFR

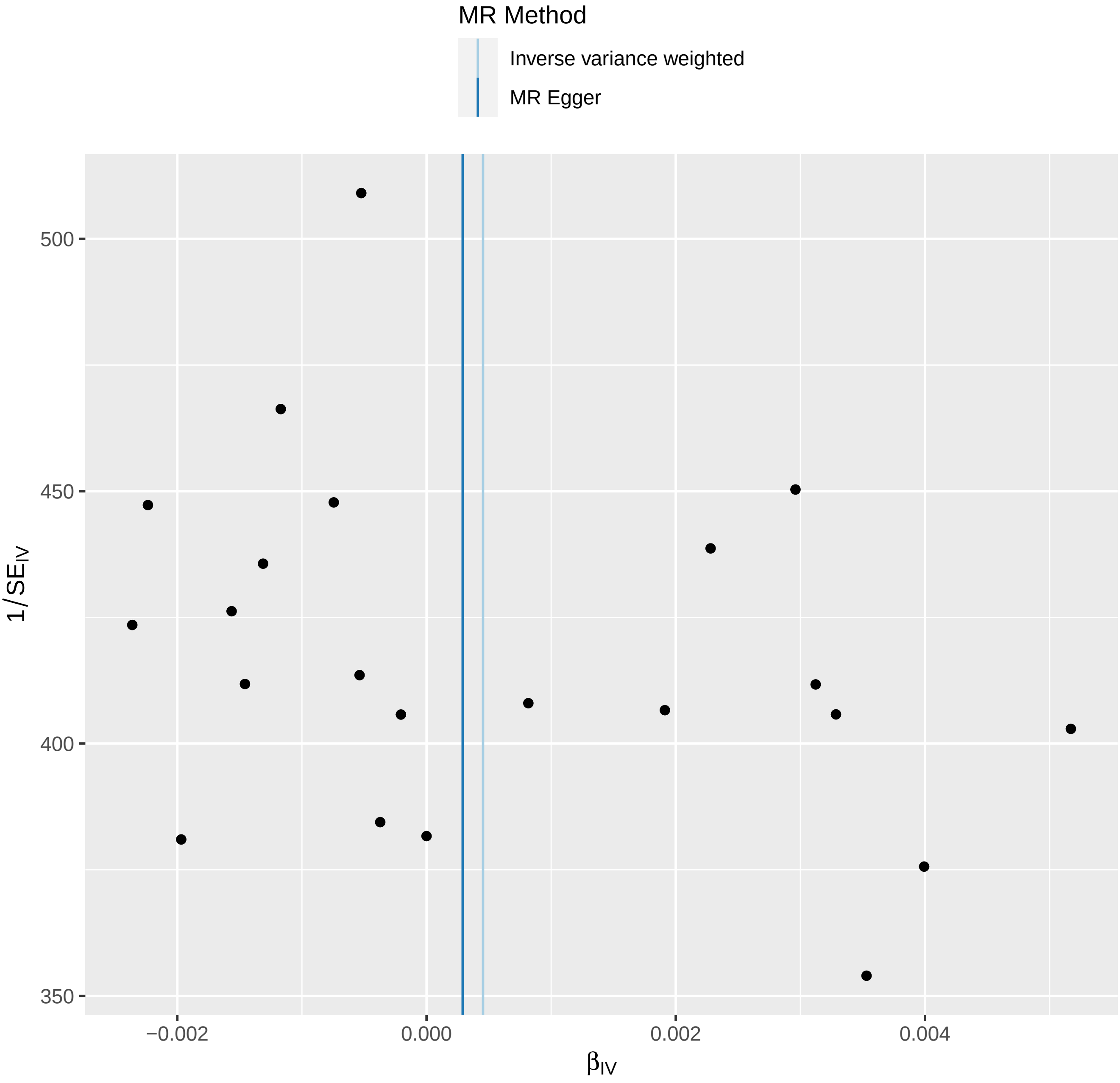

Batch 2−346 : Calcium/calmodulin−dependent protein kinase type 1 on eGFR

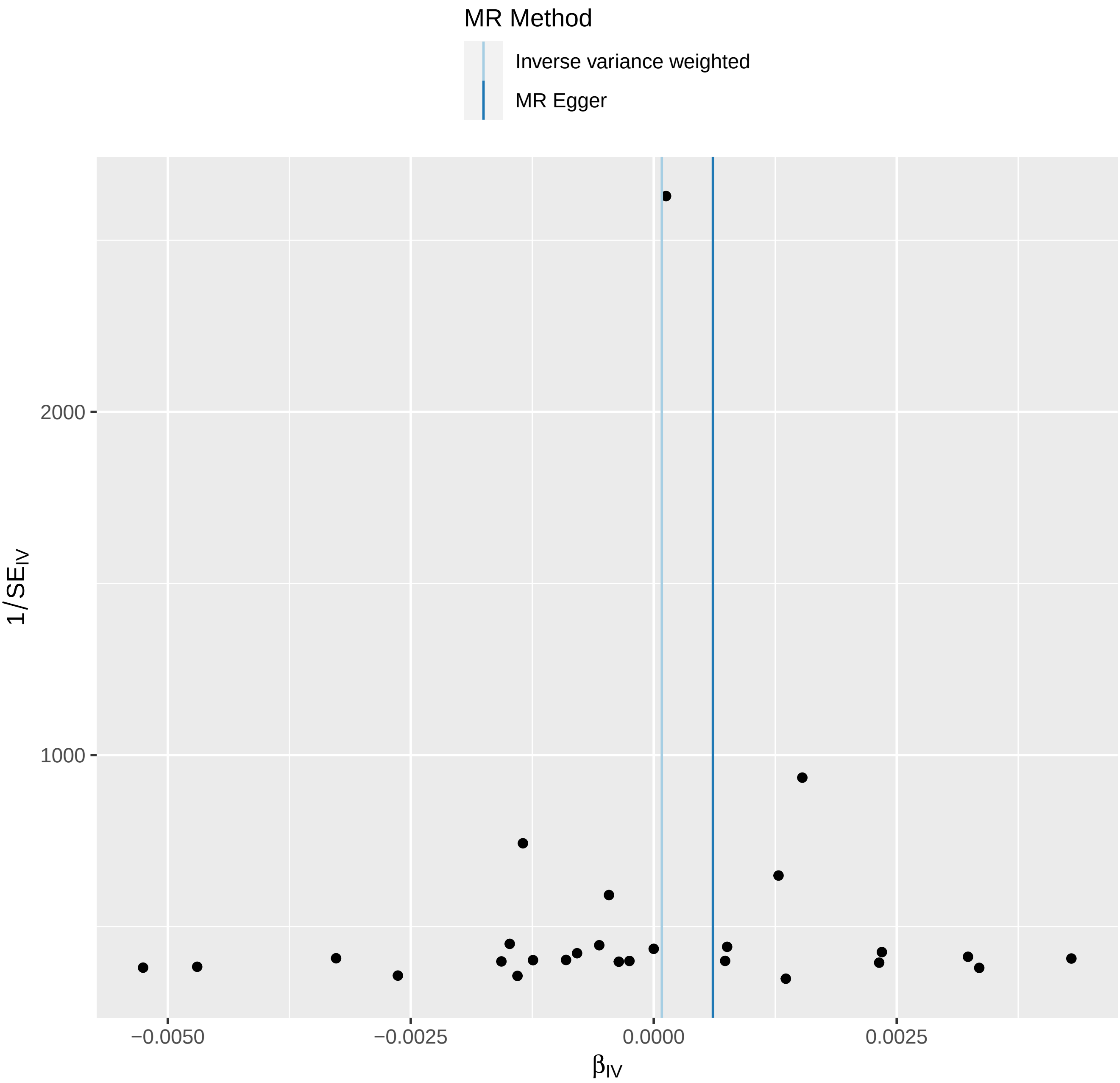

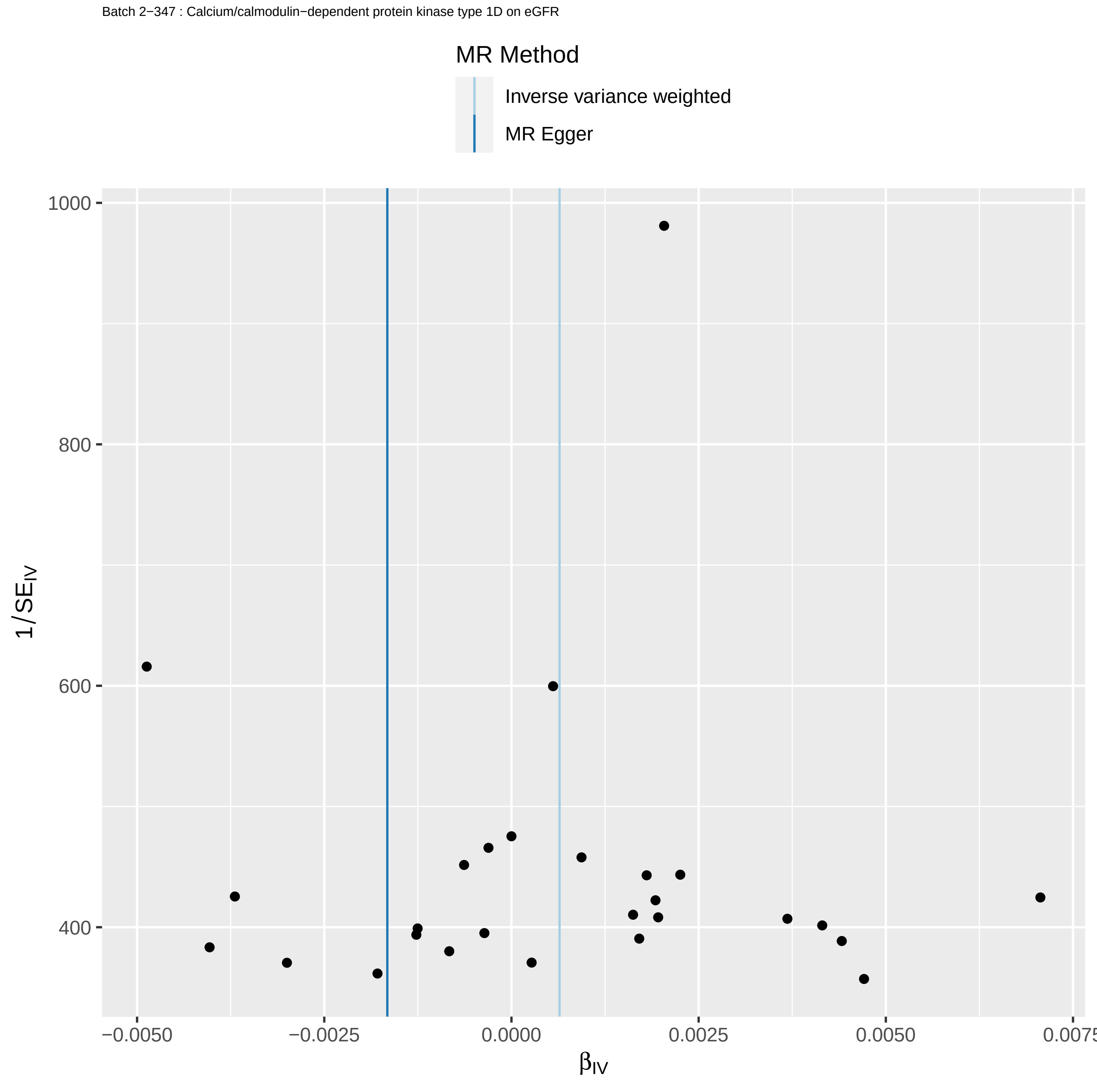

Batch 2−347 : Calcium/calmodulin−dependent protein kinase type 1D on eGFR
MR Method
Inverse variance weighted
MR Egger
1000
800
600
400
1/SE_IV
−0.0050
−0.0025
0.0000
0.0025
0.0050
0.0075
β_IV

Batch 2−348 : Calcium/calmodulin−dependent protein kinase type II subunit alpha on eGFR

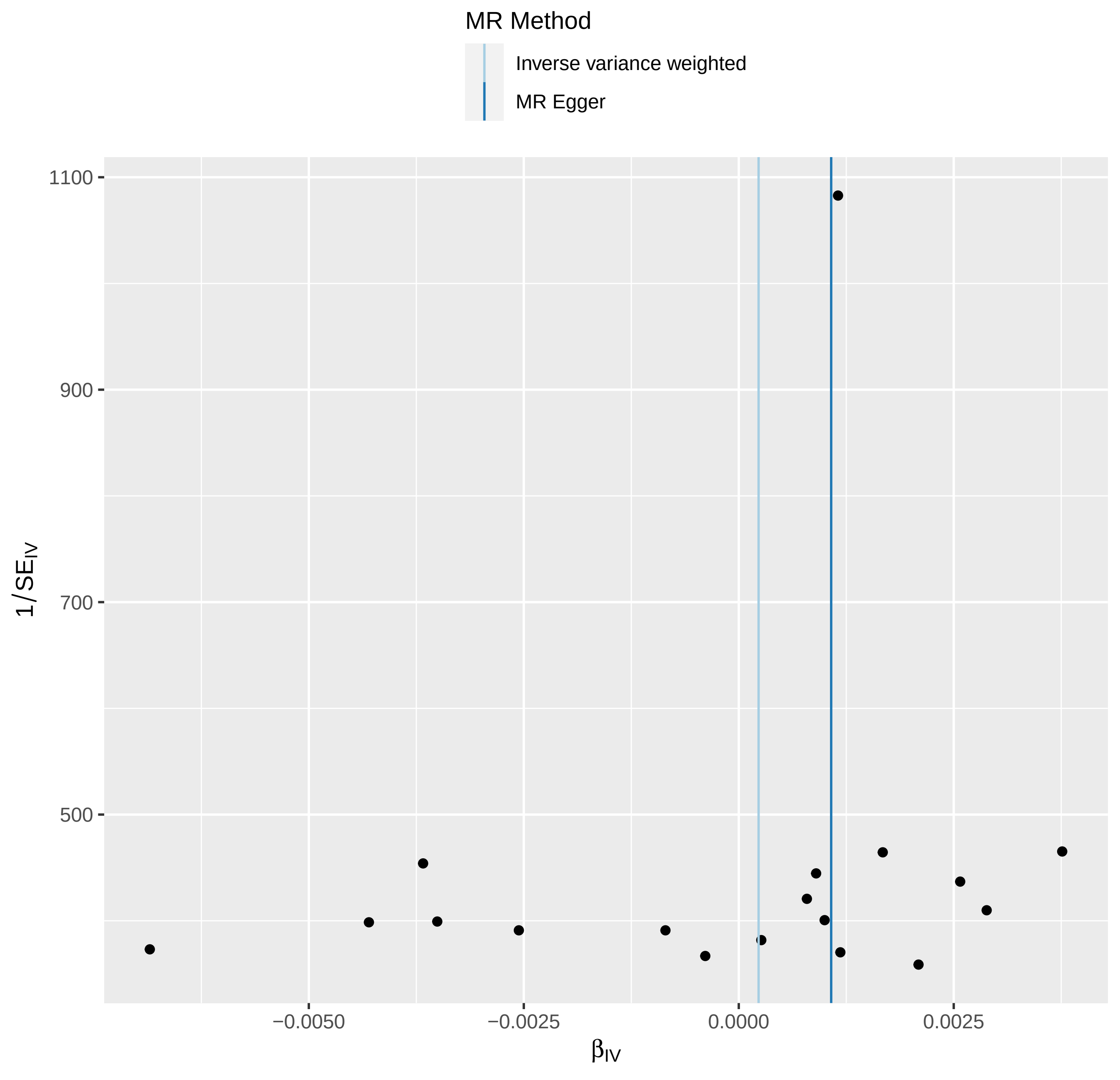

Batch 2−349 : Calcium/calmodulin−dependent protein kinase type II subunit beta on eGFR

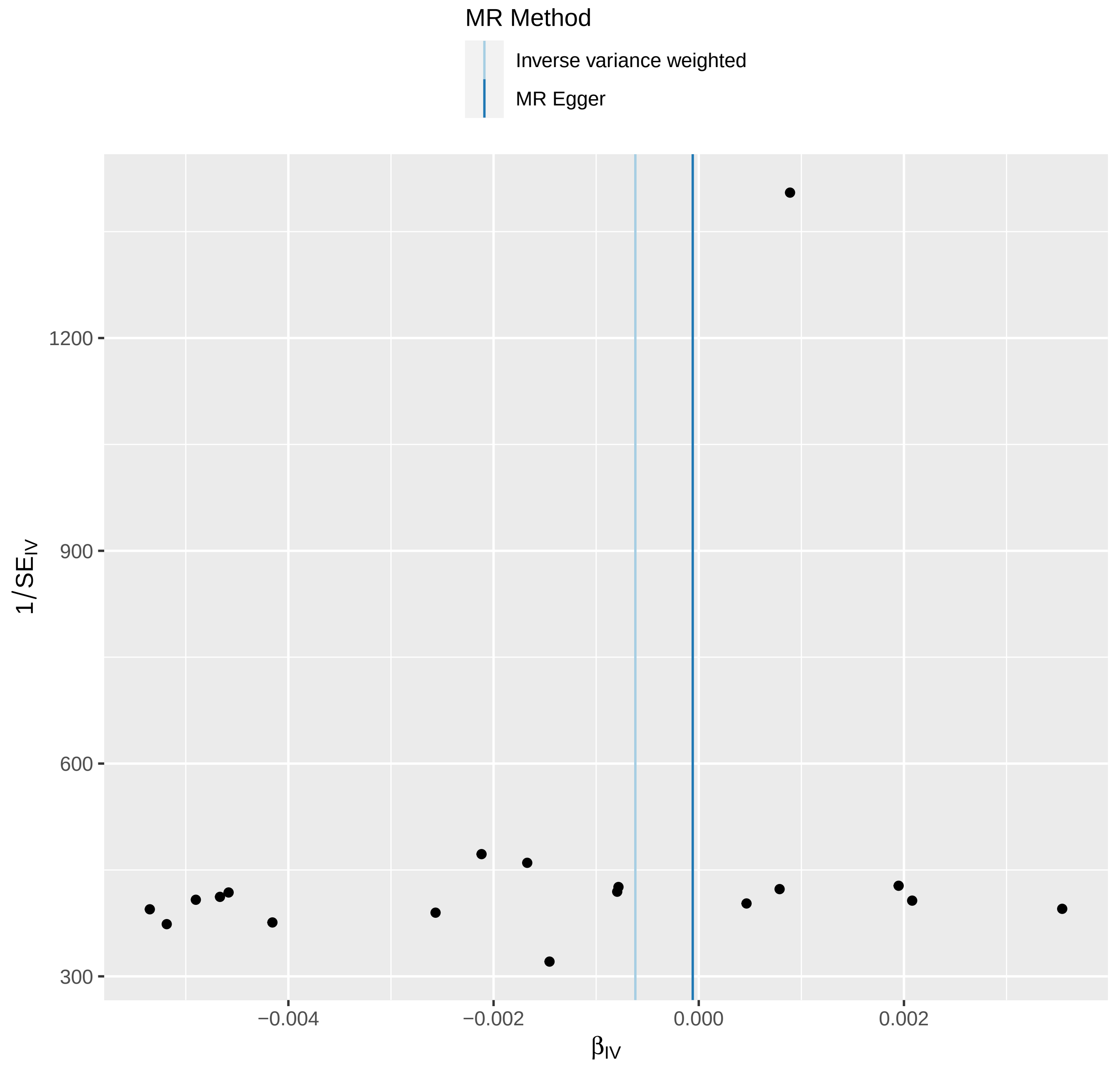

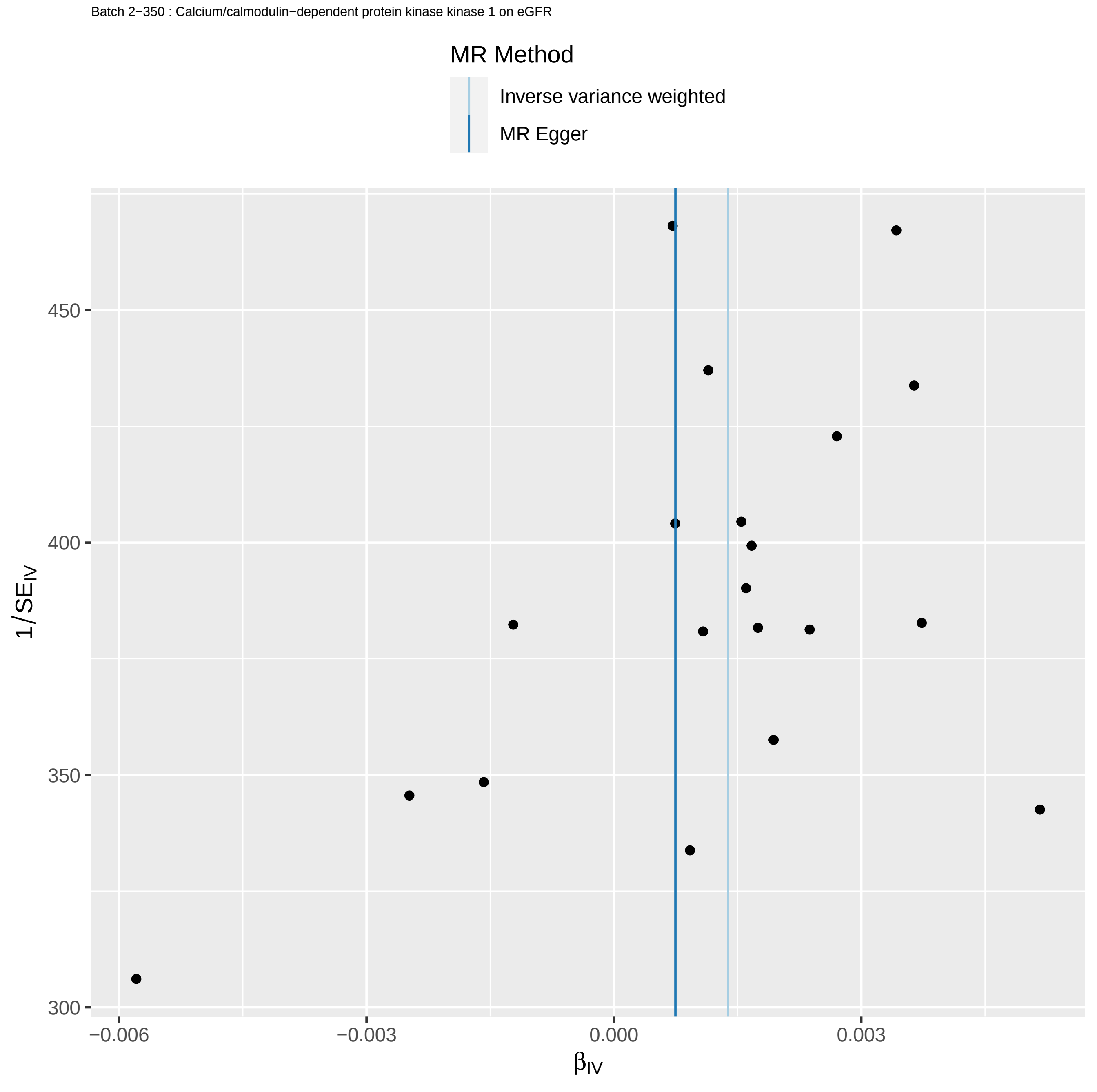
Batch 2−350 : Calcium/calmodulin−dependent protein kinase kinase 1 on eGFR
MR Method
Inverse variance weighted
MR Egger
450
400
350
300
1/SE_IV
−0.006
−0.003
0.000
0.003
β_IV

Batch 2−351 : Cullin−associated NEDD8−dissociated protein 1 on eGFR

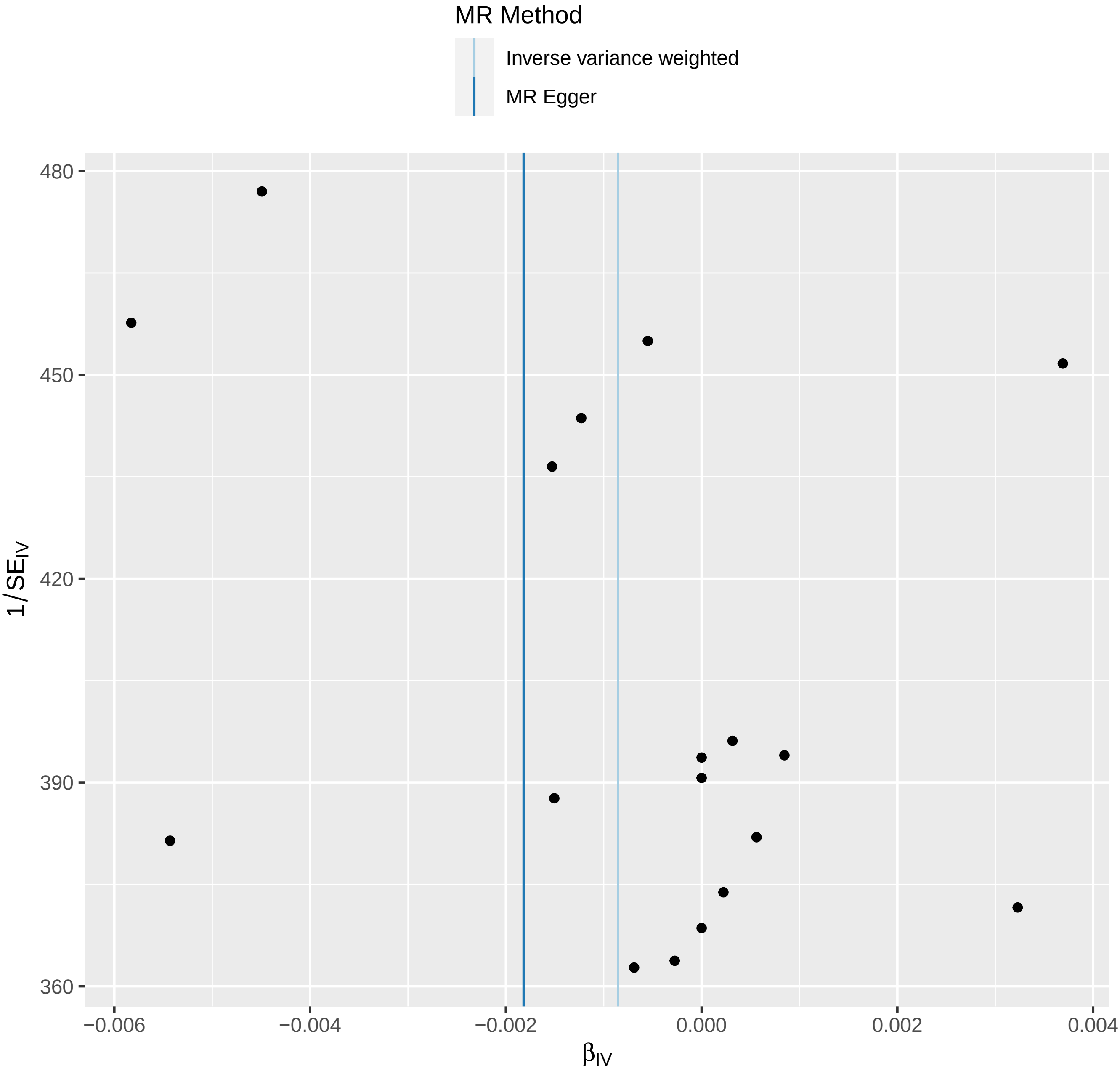

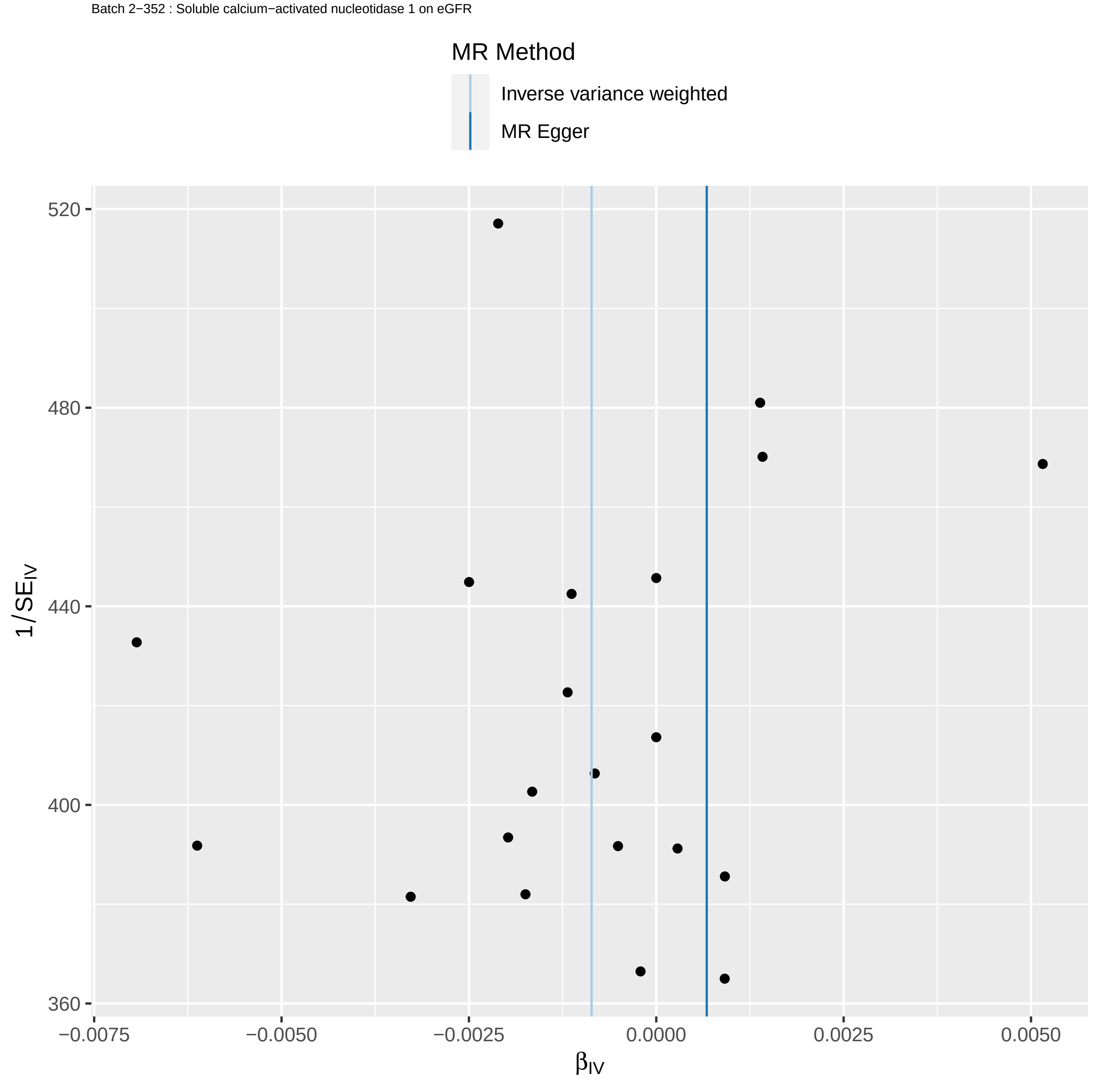

Batch 2−352 : Soluble calcium−activated nucleotidase 1 on eGFR
MR Method
Inverse variance weighted
MR Egger
520
480
440
400
360
1/SE_IV
−0.0075
−0.0050
−0.0025
0.0000
0.0025
0.0050
β_IV

Batch 2−353 : Calnexin on eGFR

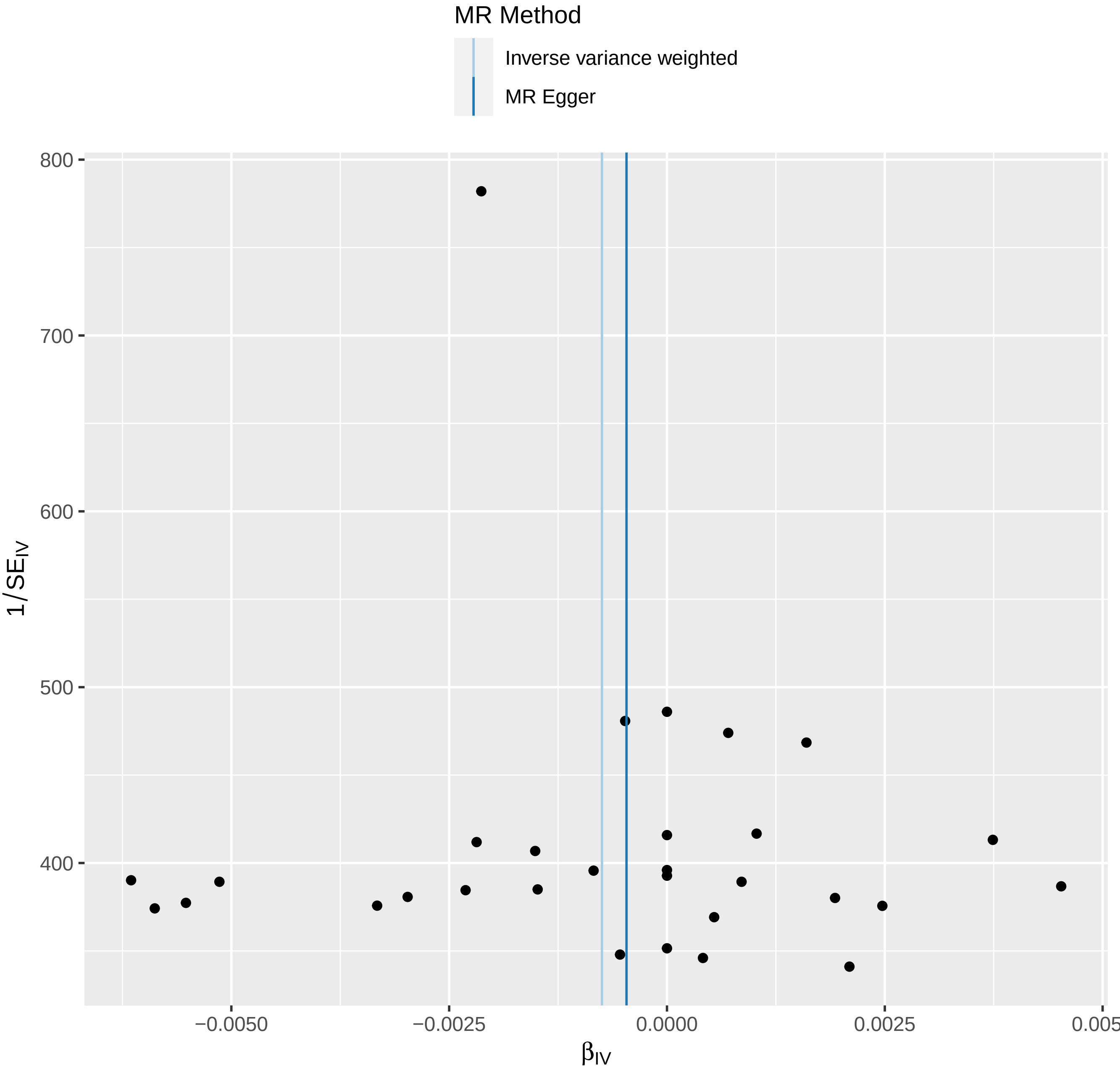

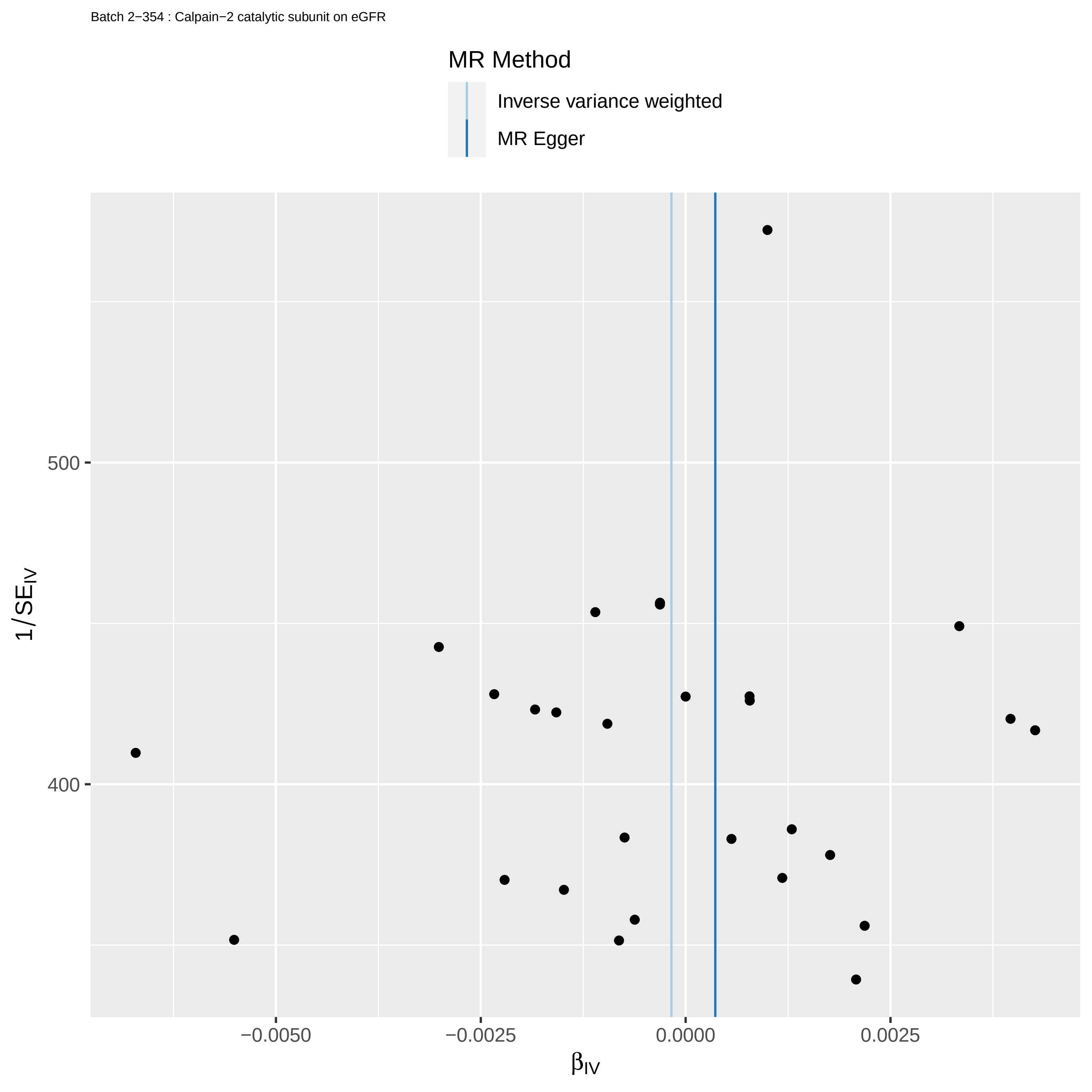

Batch 2−354 : Calpain−2 catalytic subunit on eGFR
MR Method
Inverse variance weighted
MR Egger
1/SE_IV
500
400
−0.0050
−0.0025
0.0000
0.0025
β_IV

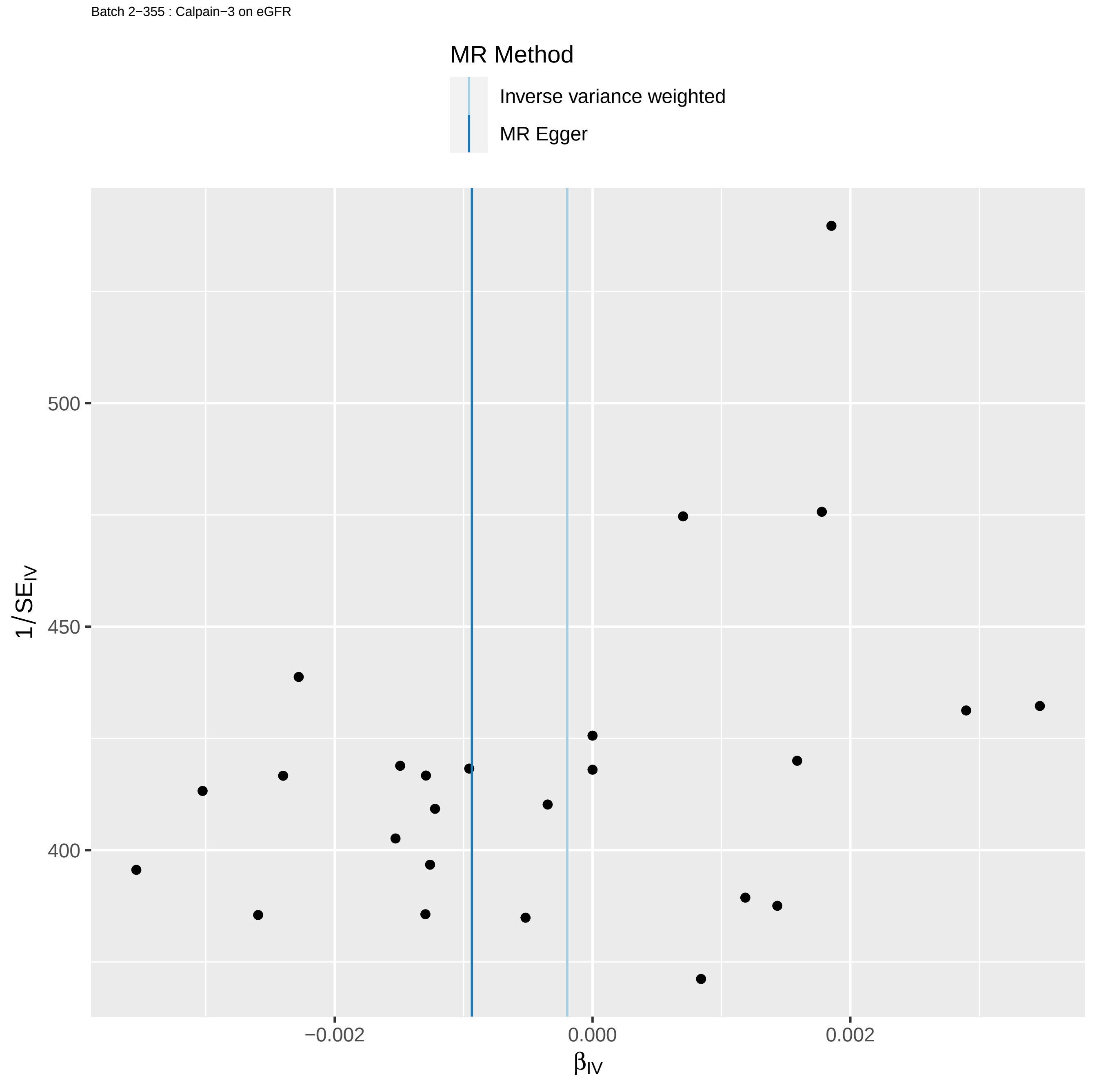

Batch 2−355 : Calpain−3 on eGFR
MR Method
Inverse variance weighted
MR Egger
500
450
400
1/SE$_{IV}$
−0.002
0.000
0.002
β$_{IV}$

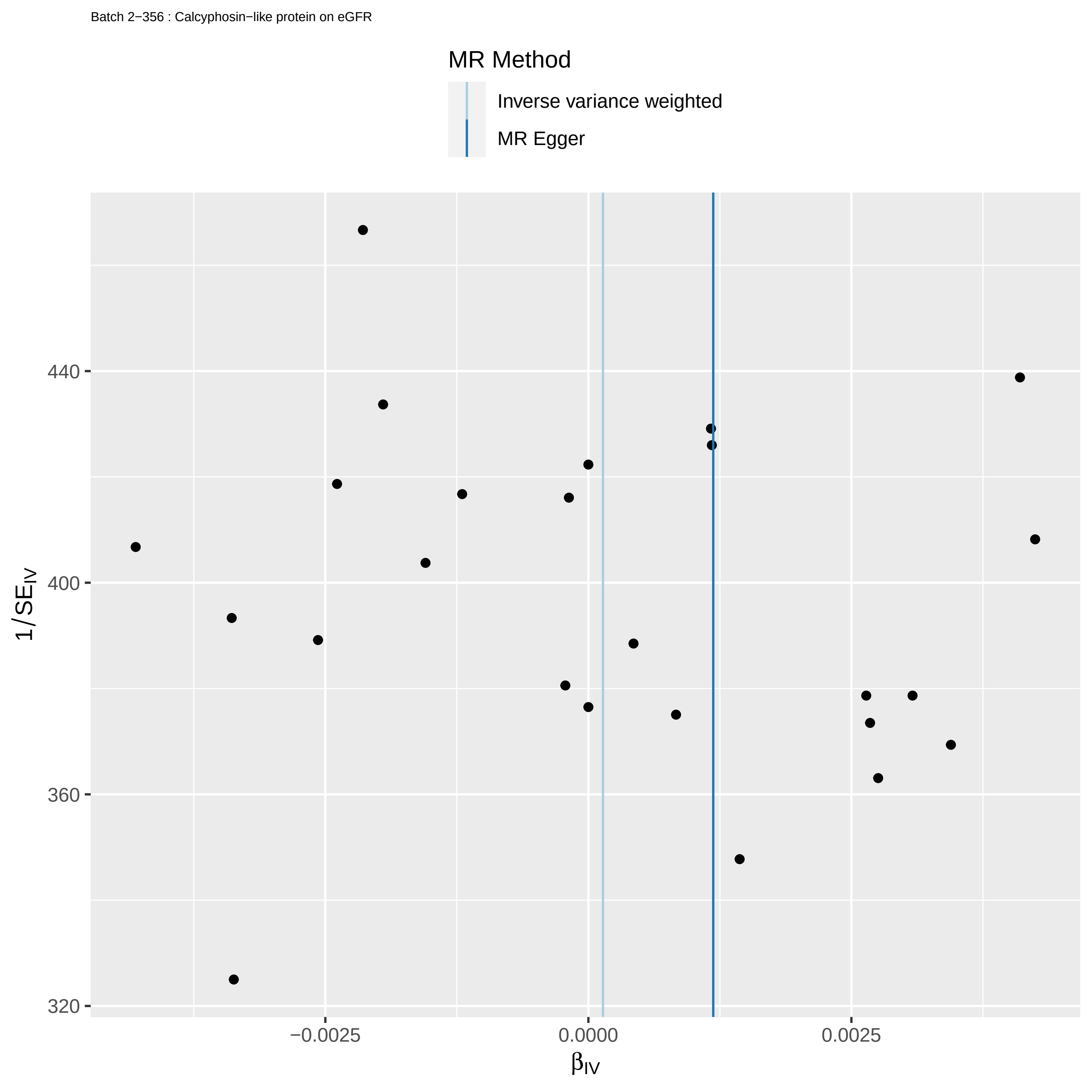
Batch 2−356 : Calcyphosin−like protein on eGFR
MR Method
Inverse variance weighted
MR Egger
440
400
360
320
1/SE$_{IV}$
−0.0025
0.0000
0.0025
β$_{IV}$

Batch 2−357 : Calcium−regulated heat stable protein 1 on eGFR

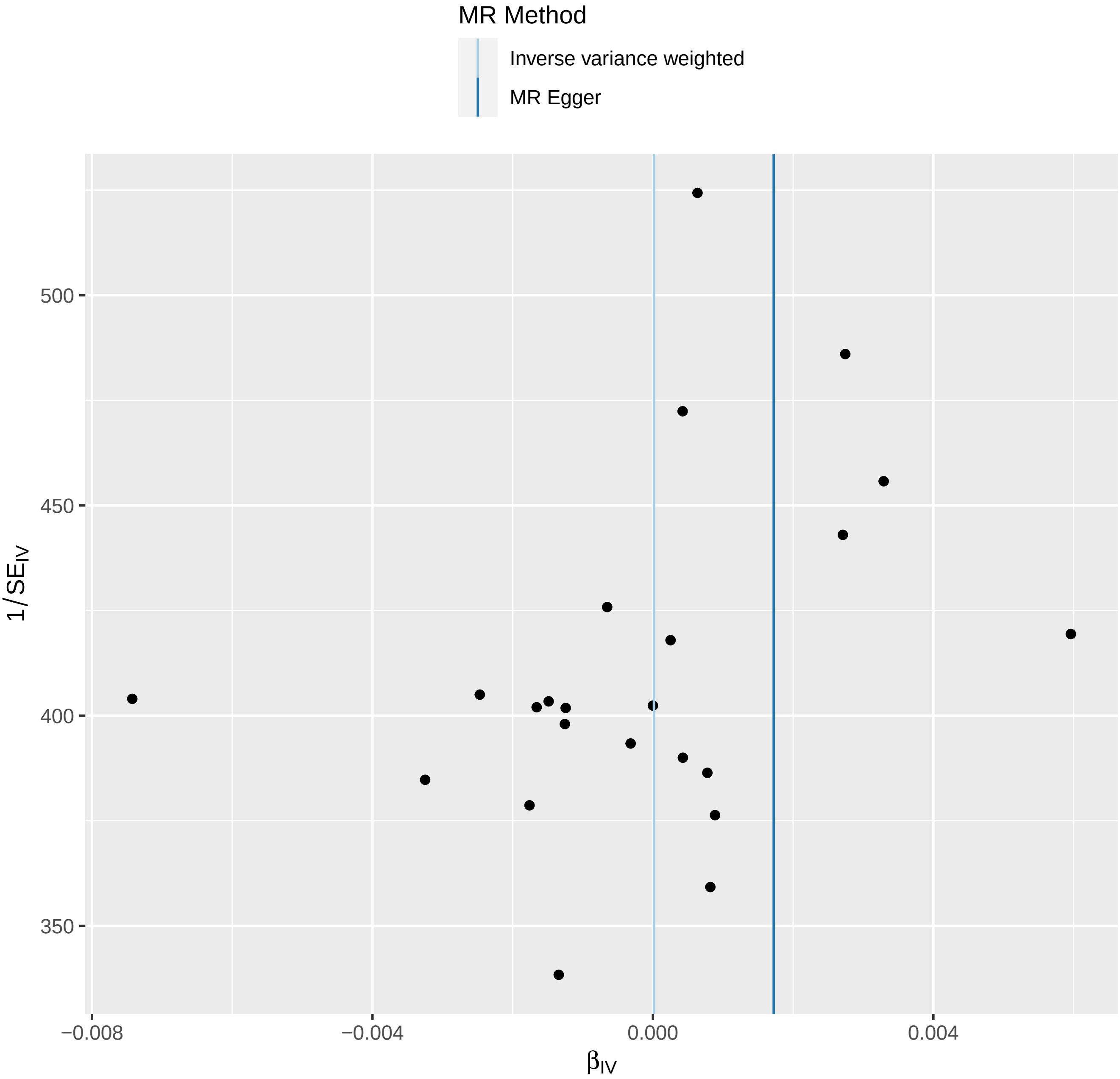

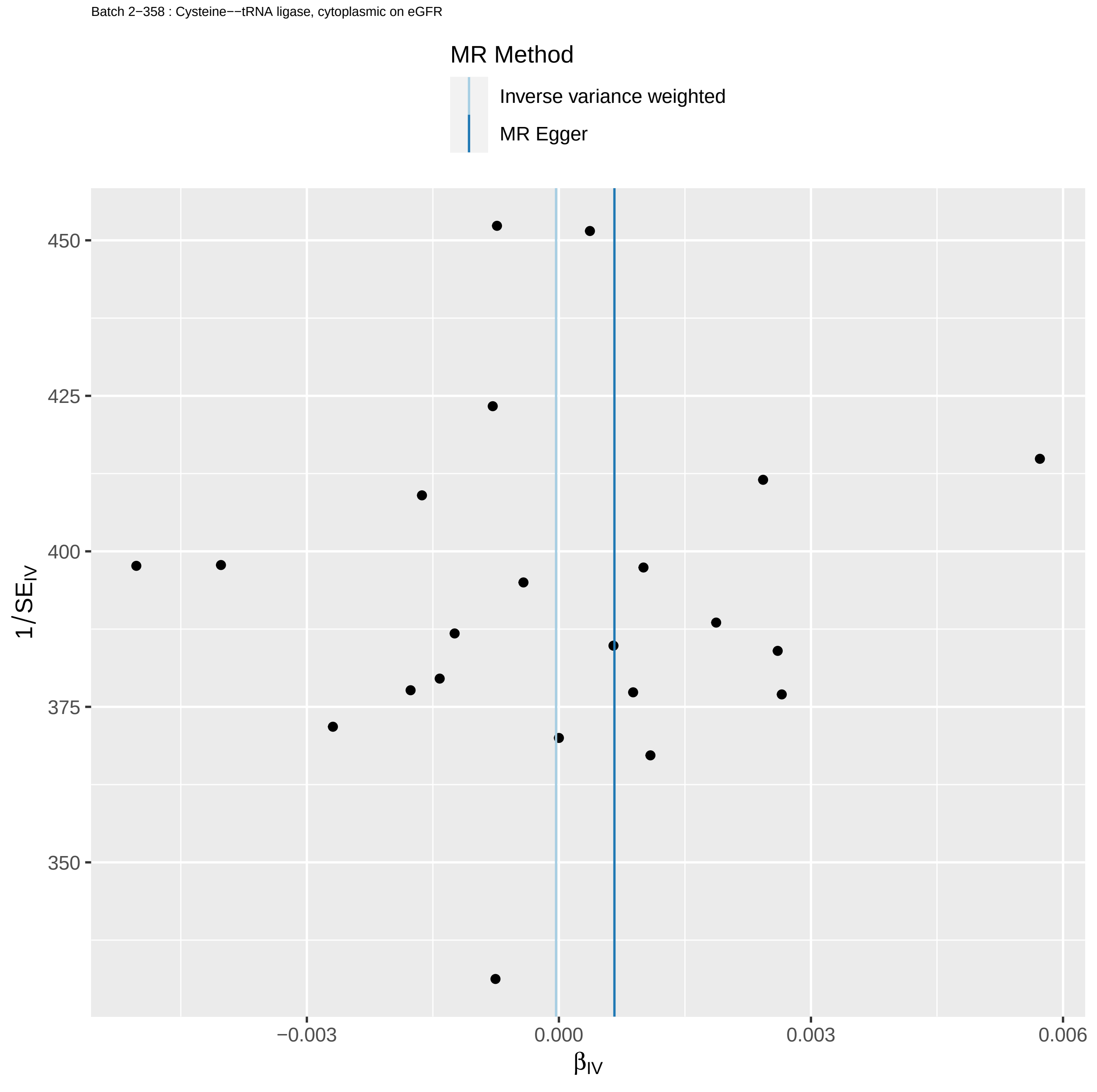

Batch 2−358 : Cysteine−−tRNA ligase, cytoplasmic on eGFR
MR Method
Inverse variance weighted
MR Egger
450
425
400
375
350
1/SE_IV
−0.003
0.000
0.003
0.006
β_IV

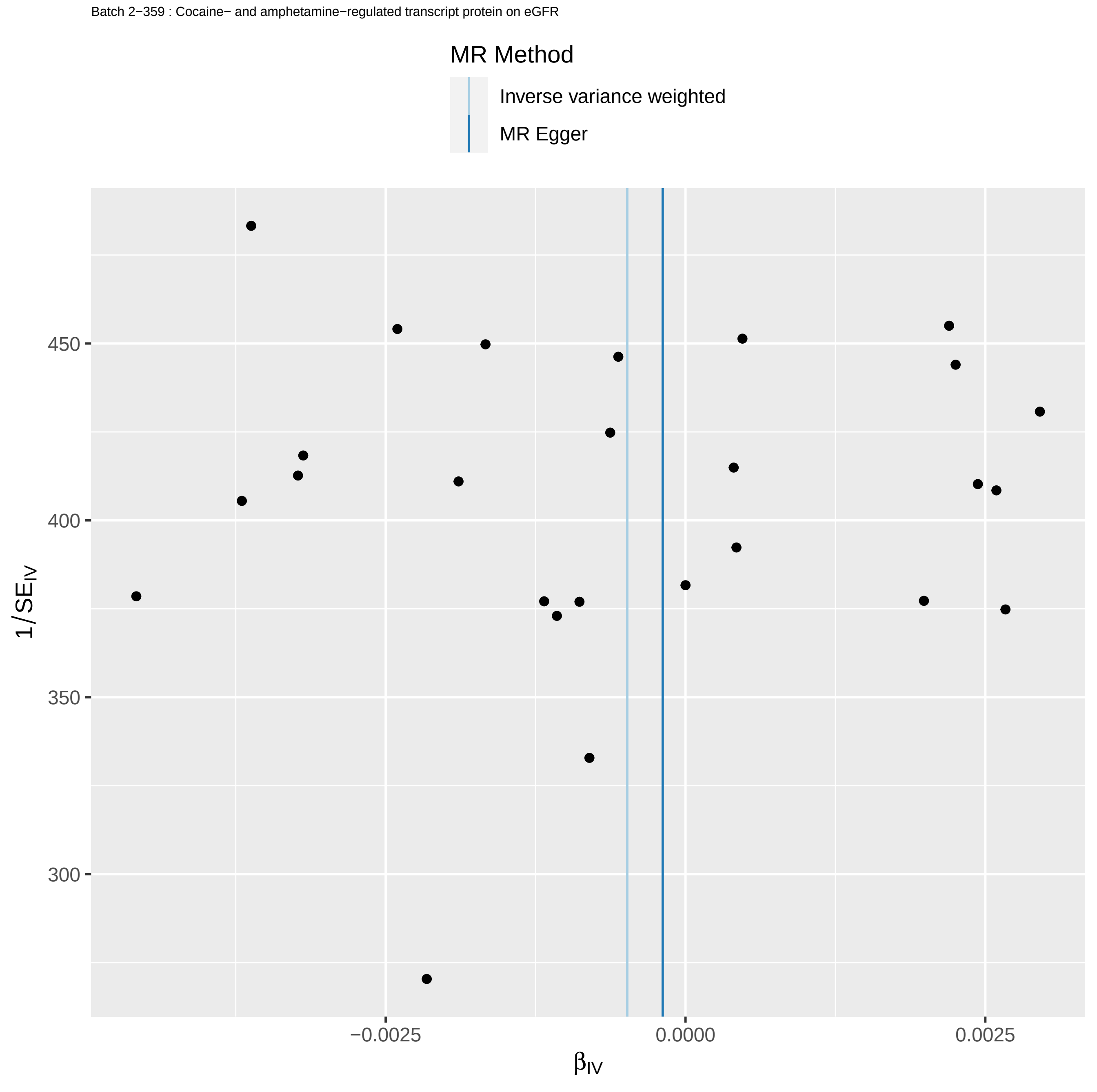

Batch 2−359 : Cocaine− and amphetamine−regulated transcript protein on eGFR
MR Method
Inverse variance weighted
MR Egger
450
400
350
300
1/SE_IV
−0.0025
0.0000
0.0025
β_IV

Batch 2−360 : Protein CASC4 on eGFR

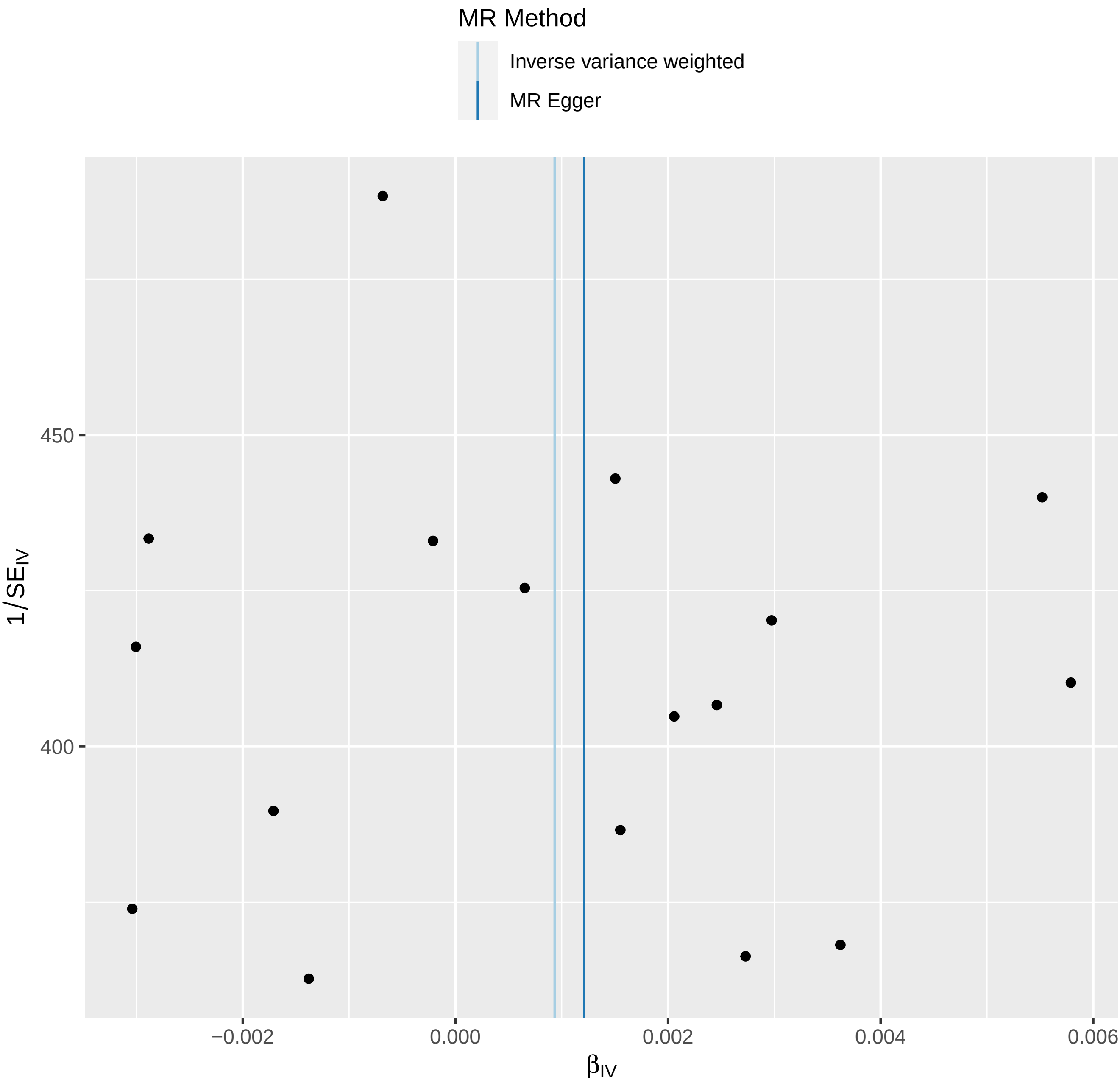

Batch 2−361 : Protein CASC4 on eGFR

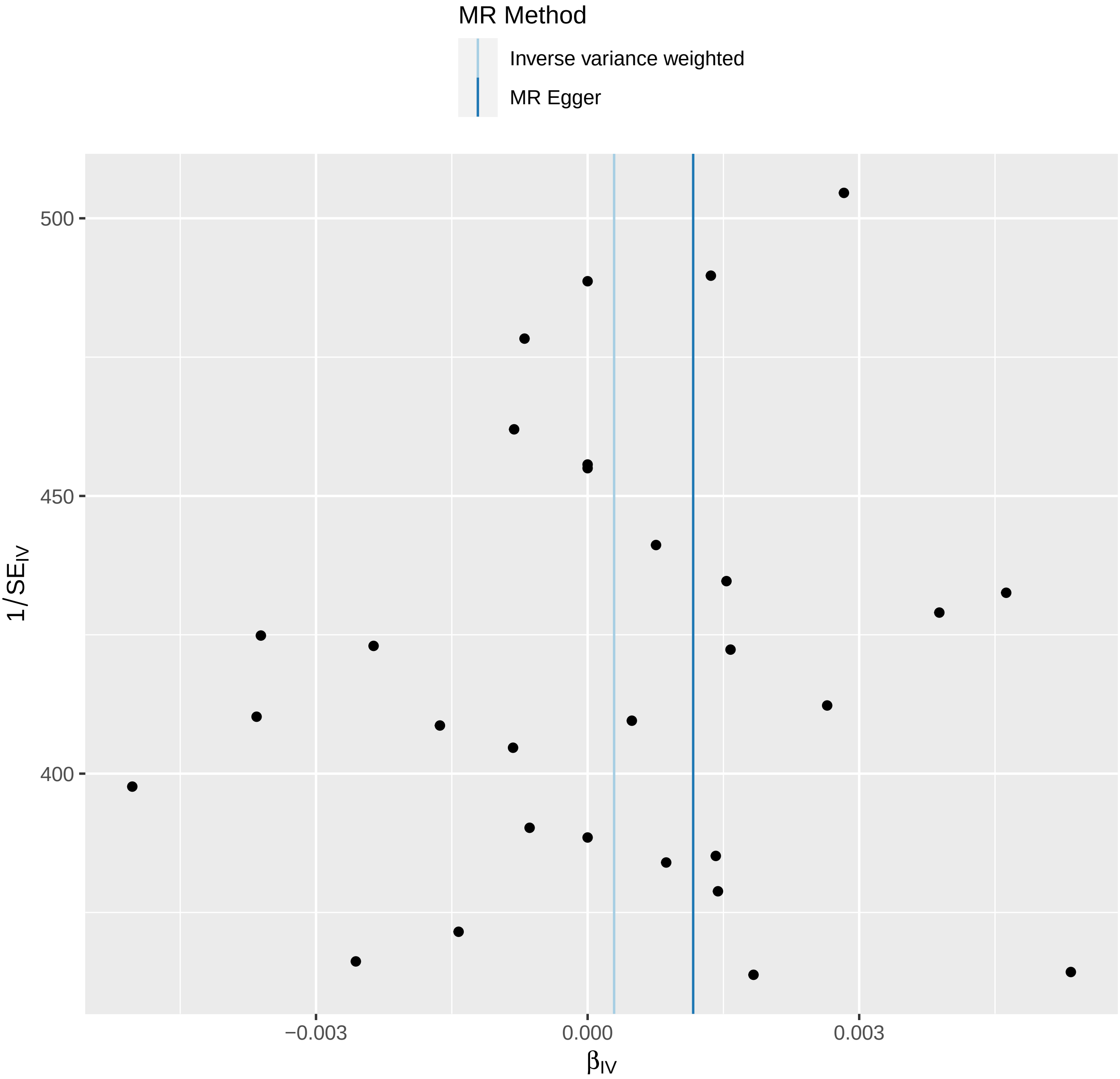

Batch 2−362 : Caspase−10 on eGFR

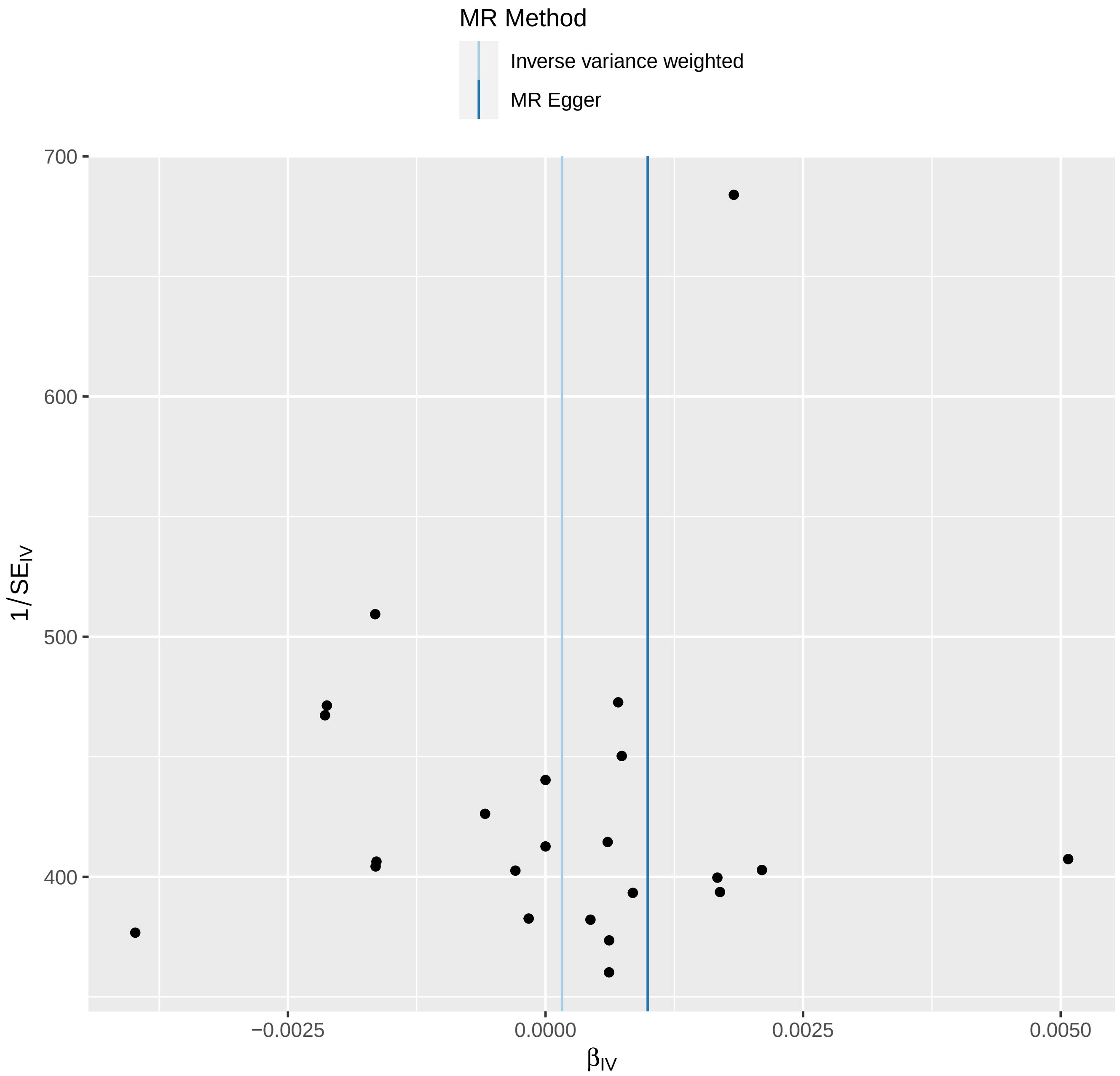

Batch 2−363 : Caspase−2 on eGFR

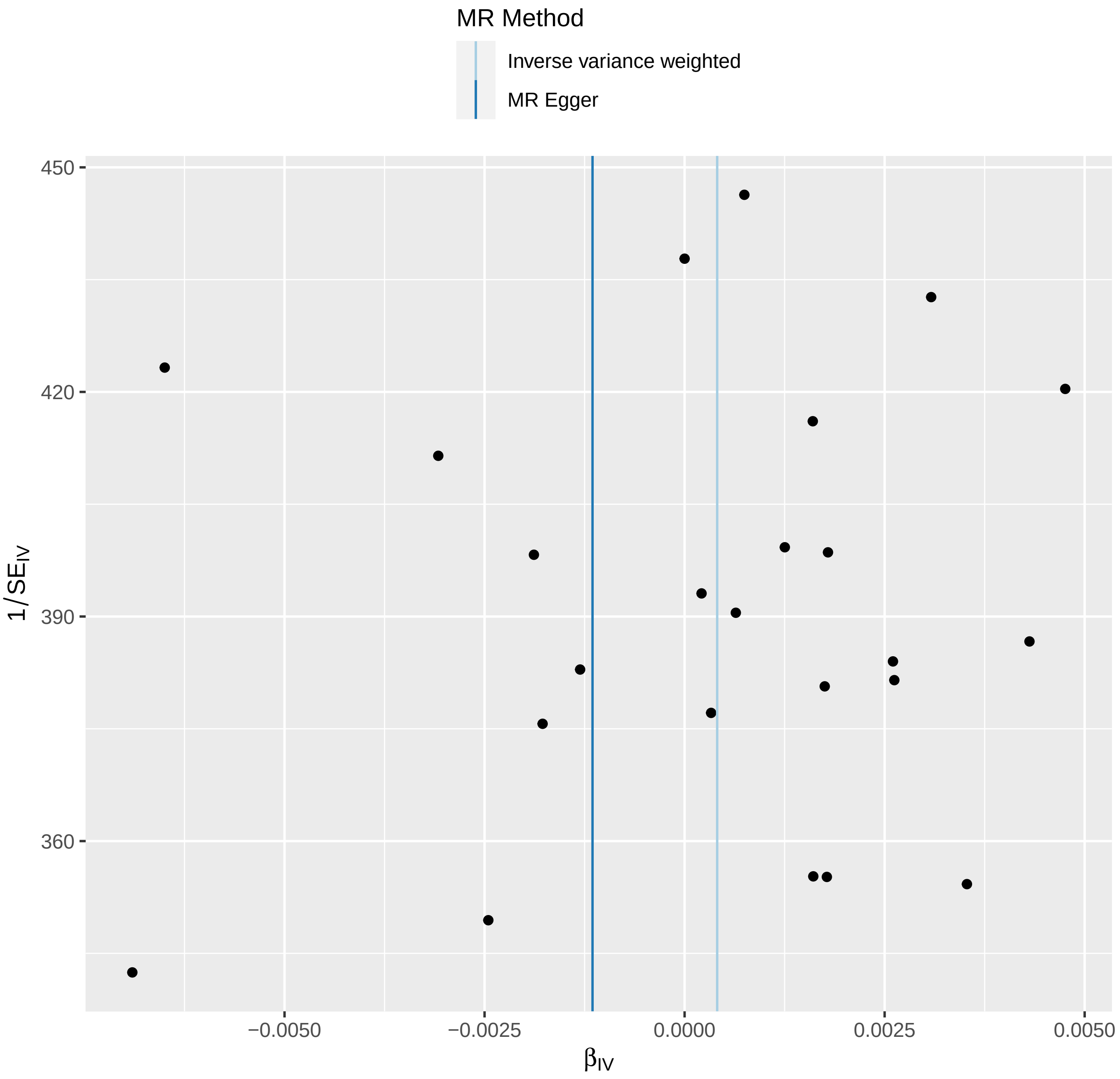

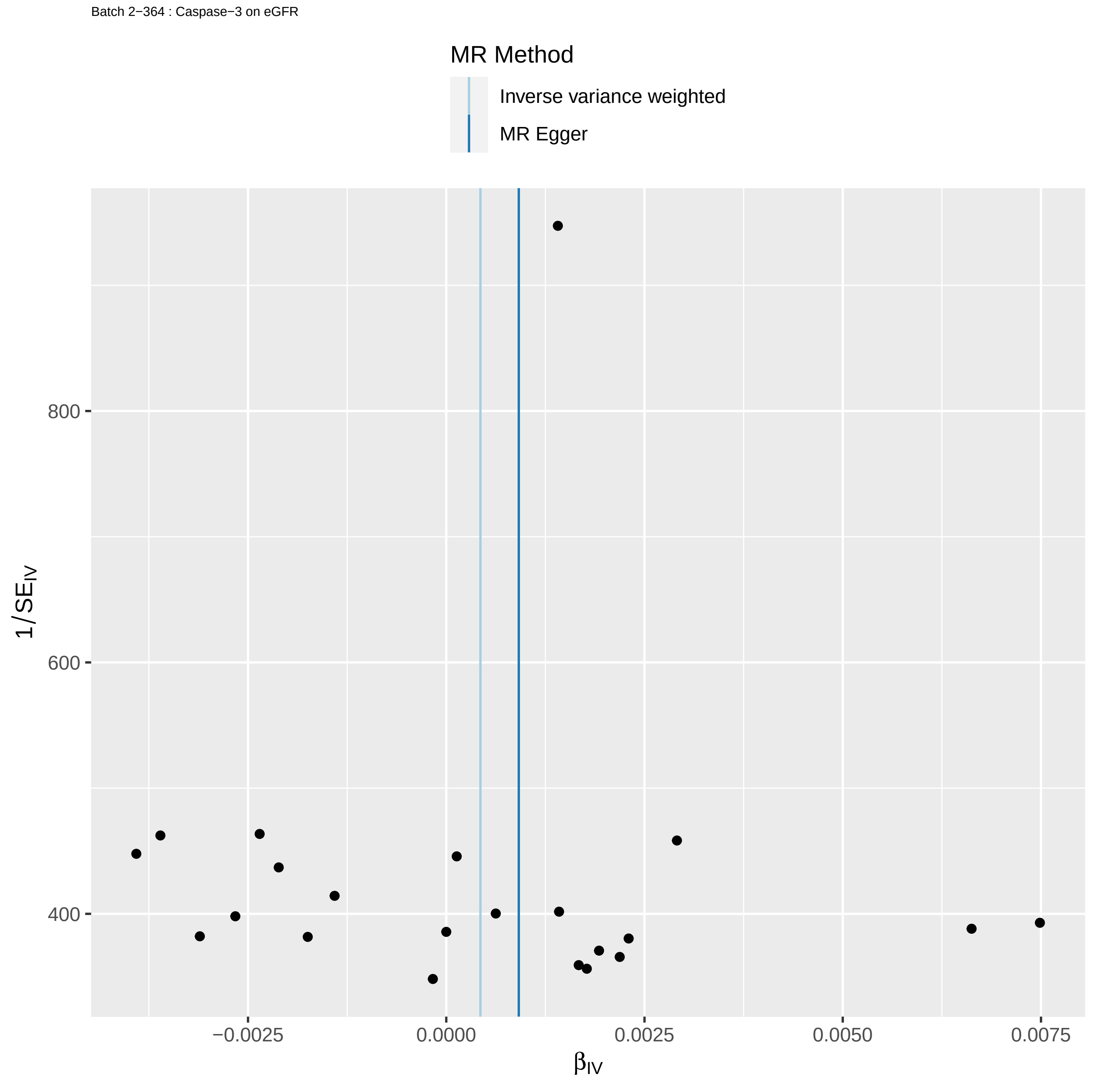

Batch 2−364 : Caspase−3 on eGFR
MR Method
Inverse variance weighted
MR Egger
800
600
400
1/SE_IV
−0.0025
0.0000
0.0025
0.0050
0.0075
β_IV

Batch 2−365 : Cas scaffolding protein family member 4 on eGFR

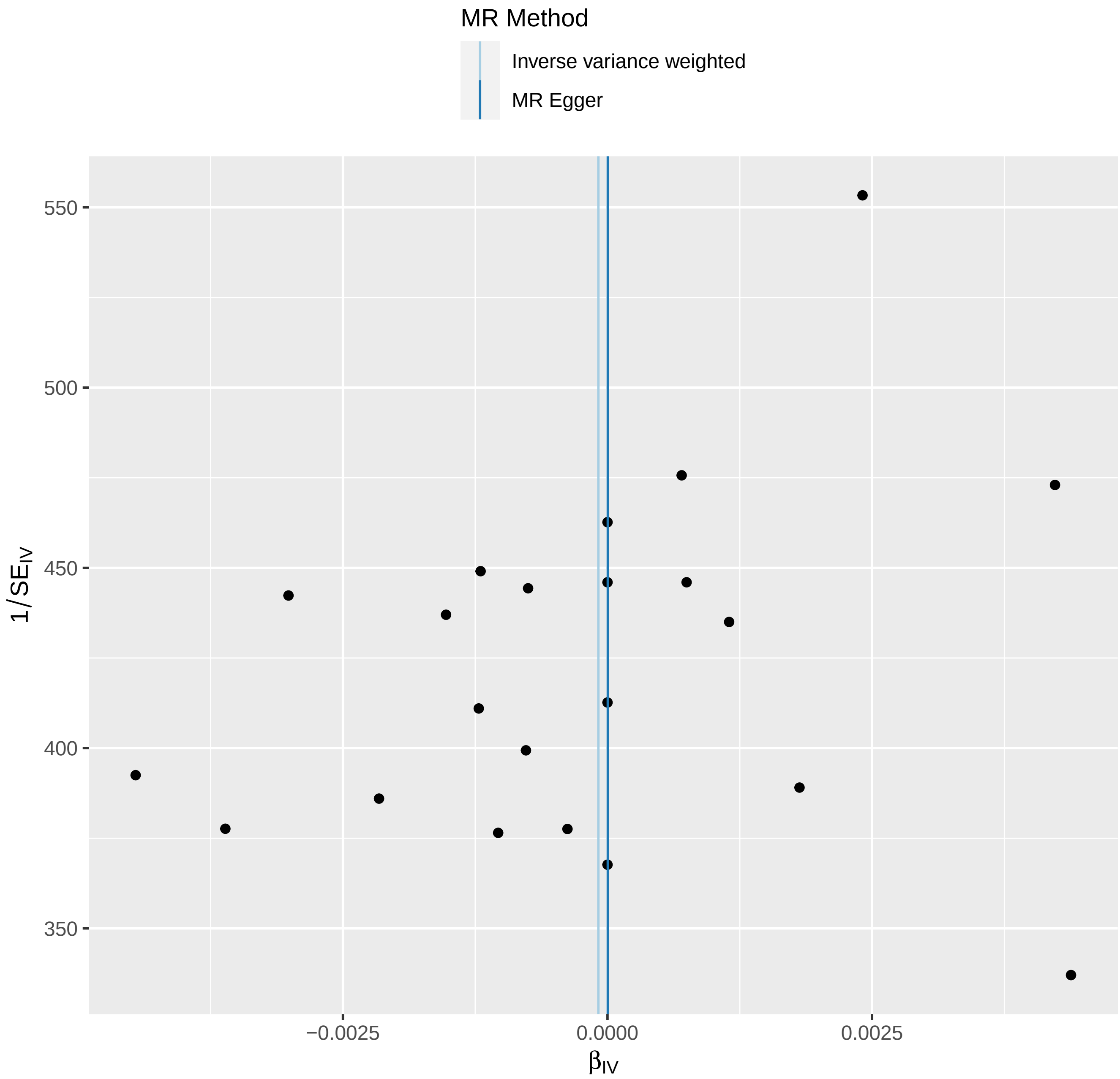

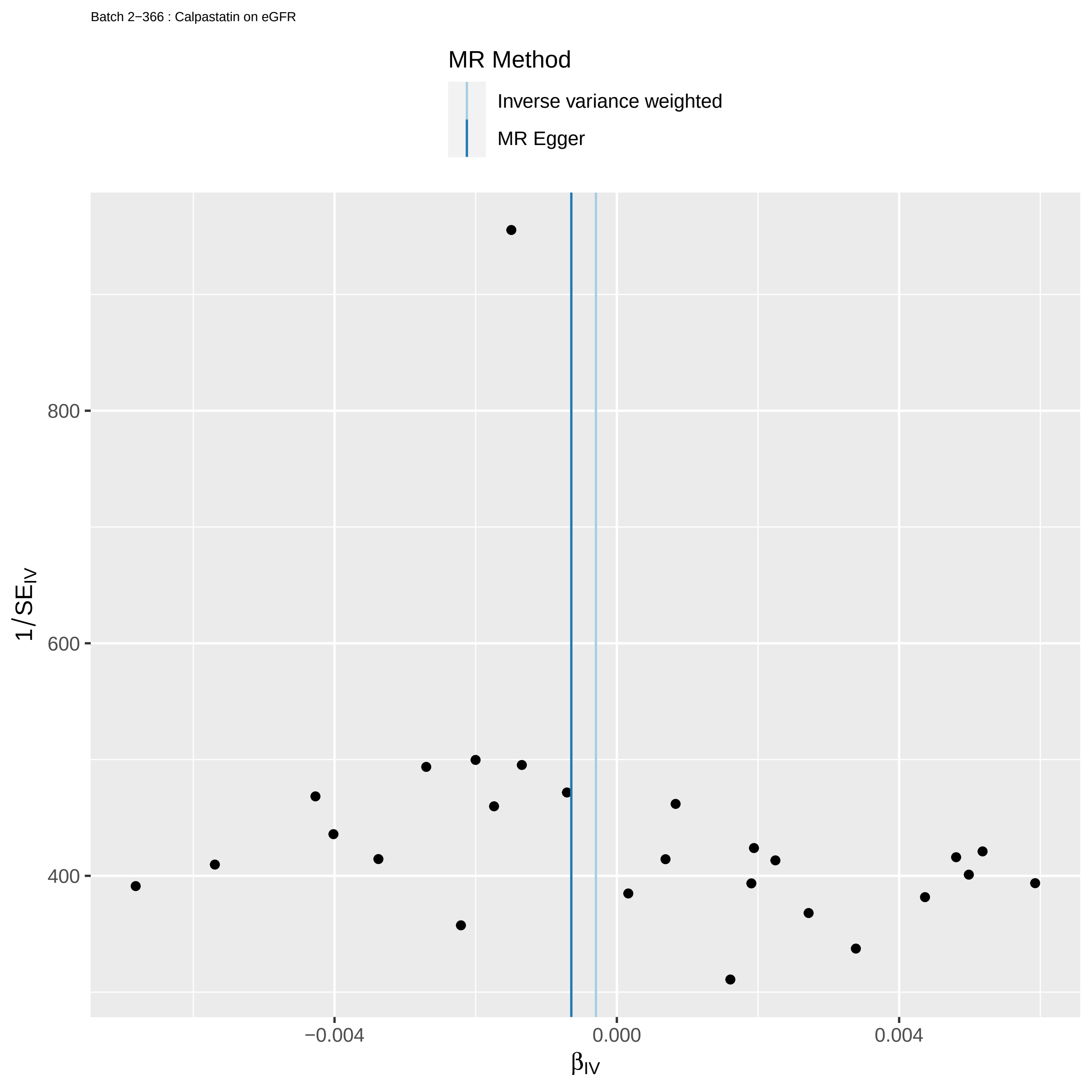

Batch 2−366 : Calpastatin on eGFR
MR Method
Inverse variance weighted
MR Egger
800
600
400
1/SE_IV
−0.004
0.000
0.004
β_IV

Batch 2−367 : Catalase on eGFR

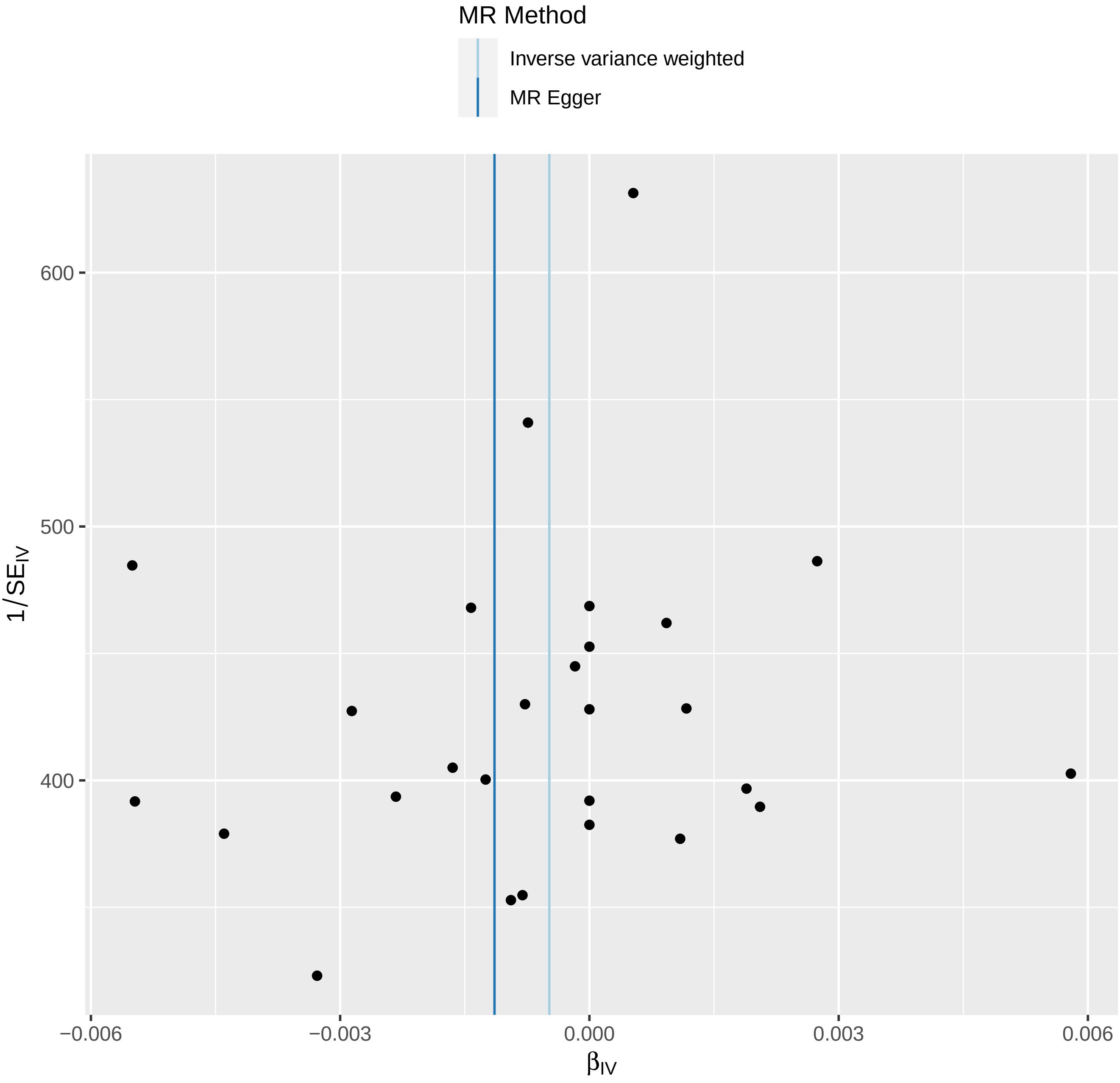

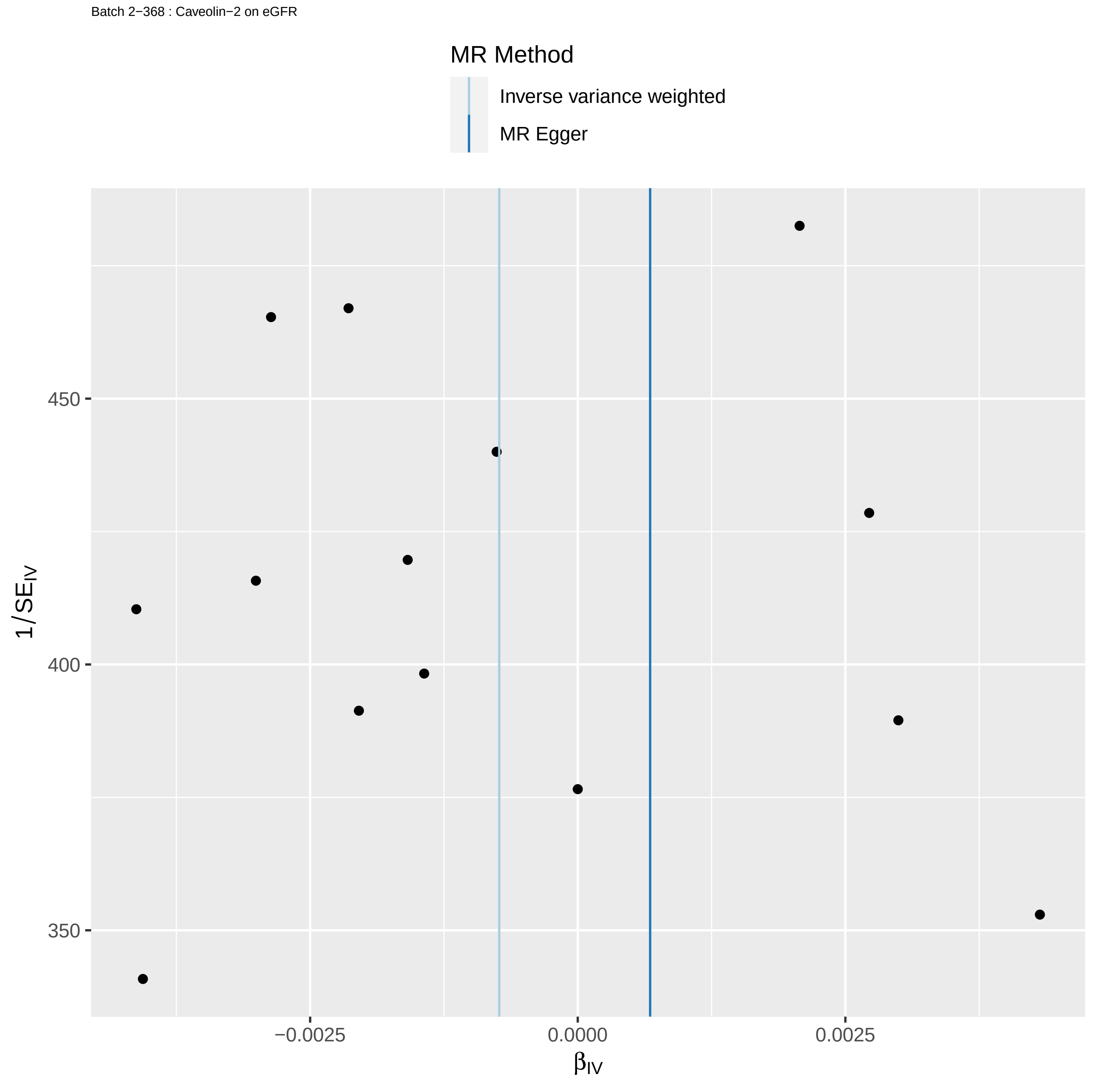
Batch 2−368 : Caveolin−2 on eGFR
MR Method
Inverse variance weighted
MR Egger
450
400
350
1/SE_IV
−0.0025
0.0000
0.0025
β_IV

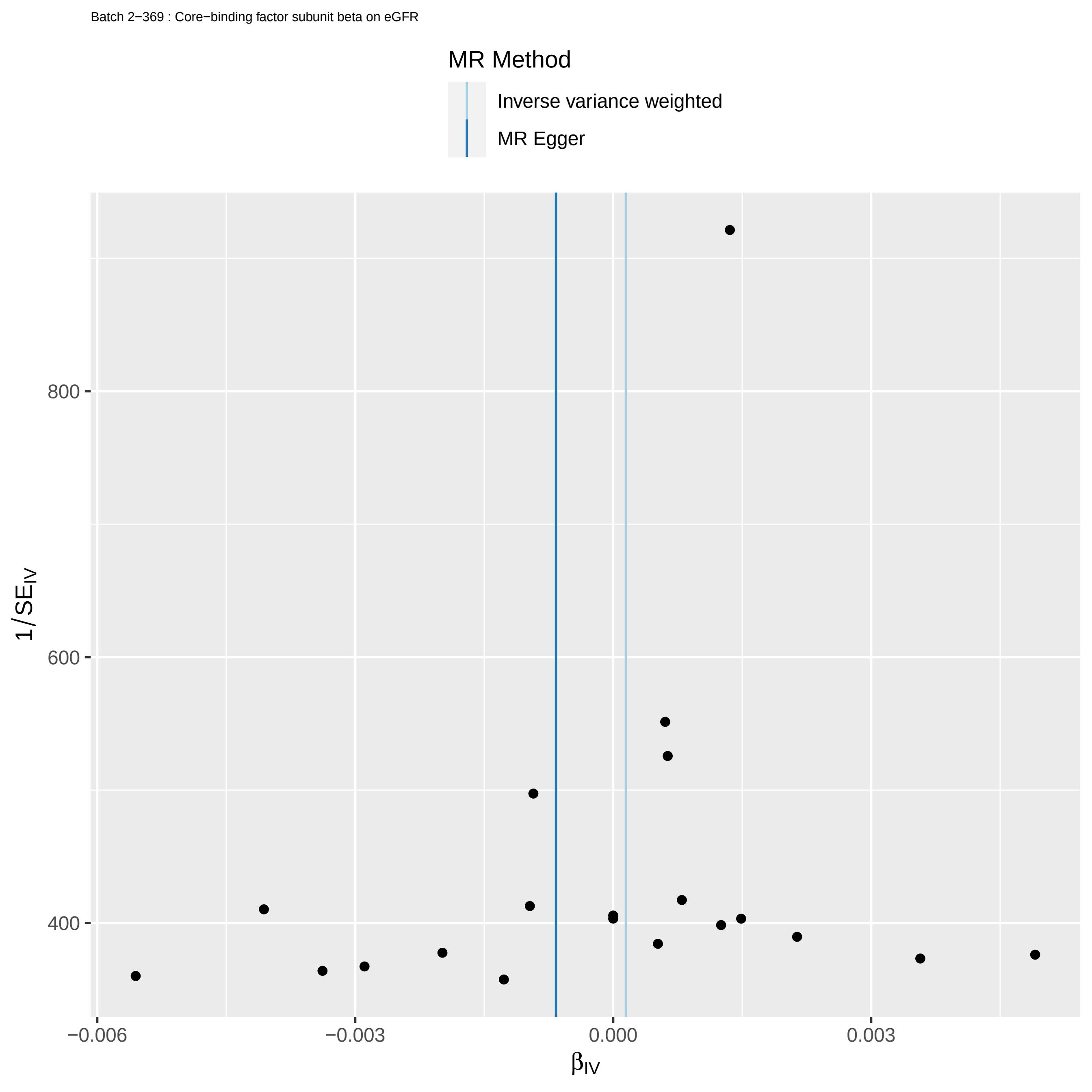

Batch 2−369 : Core−binding factor subunit beta on eGFR
MR Method
Inverse variance weighted
MR Egger
1/SE$_{IV}$
800
600
400
−0.006
−0.003
0.000
0.003
β$_{IV}$

Batch 2−370 : E3 ubiquitin−protein ligase CBL on eGFR

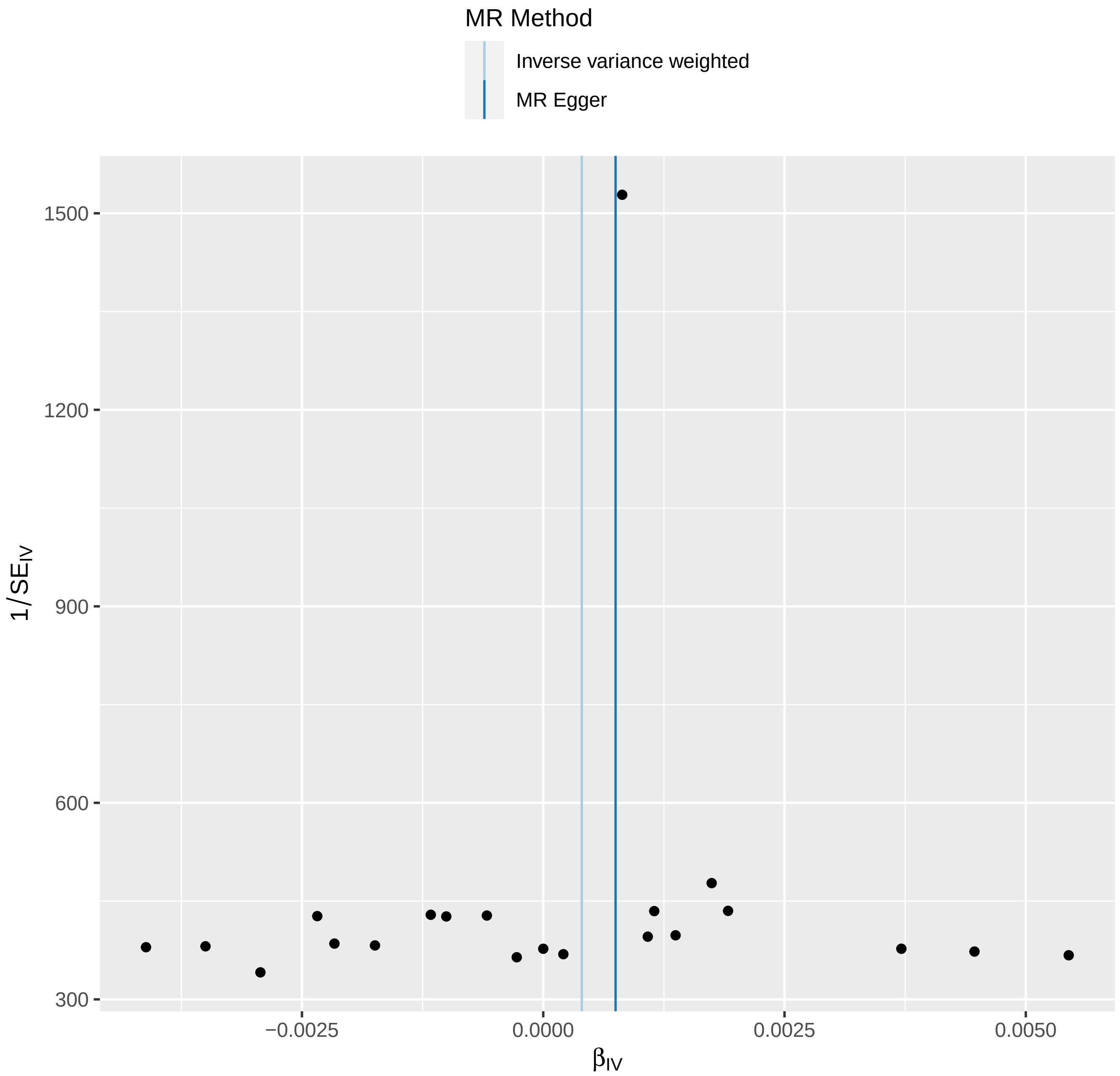

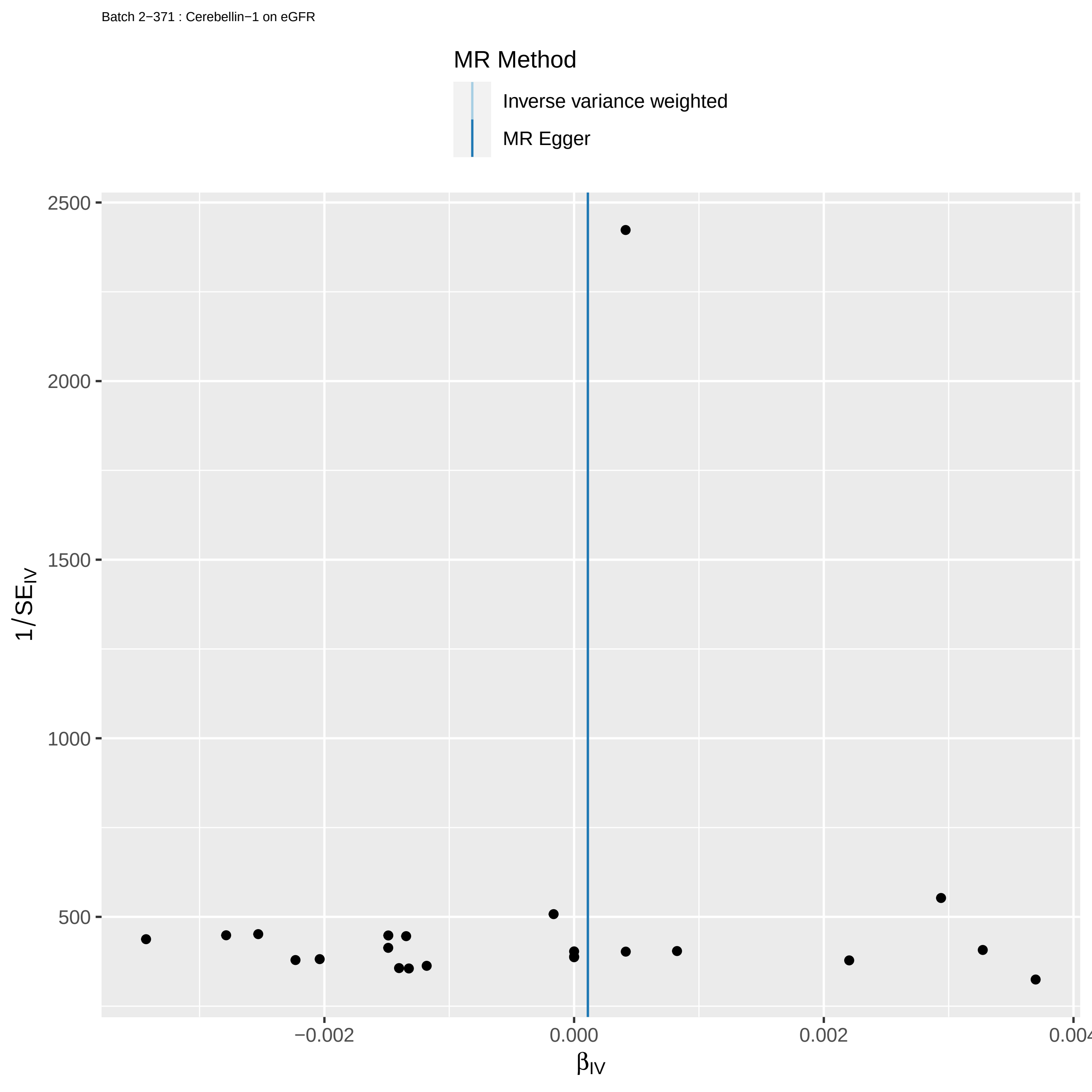

Batch 2−371 : Cerebellin−1 on eGFR
MR Method
Inverse variance weighted
MR Egger
2500
2000
1500
1000
500
1/SE_IV
−0.002
0.000
0.002
0.004
β_IV

Batch 2−372 : Cerebellin−4 on eGFR

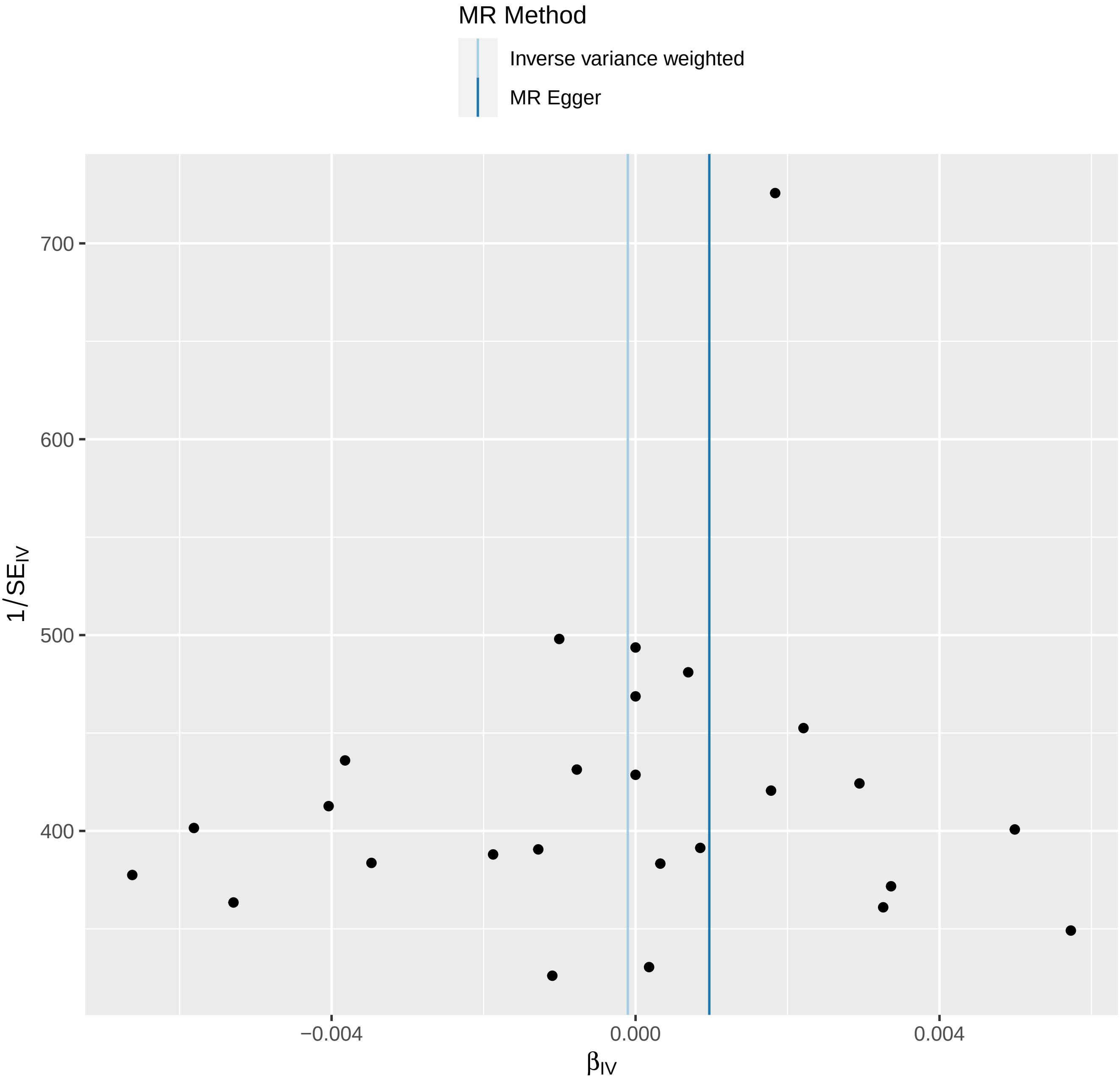

Batch 2−373 : Carbonyl reductase [NADPH] 1 on eGFR

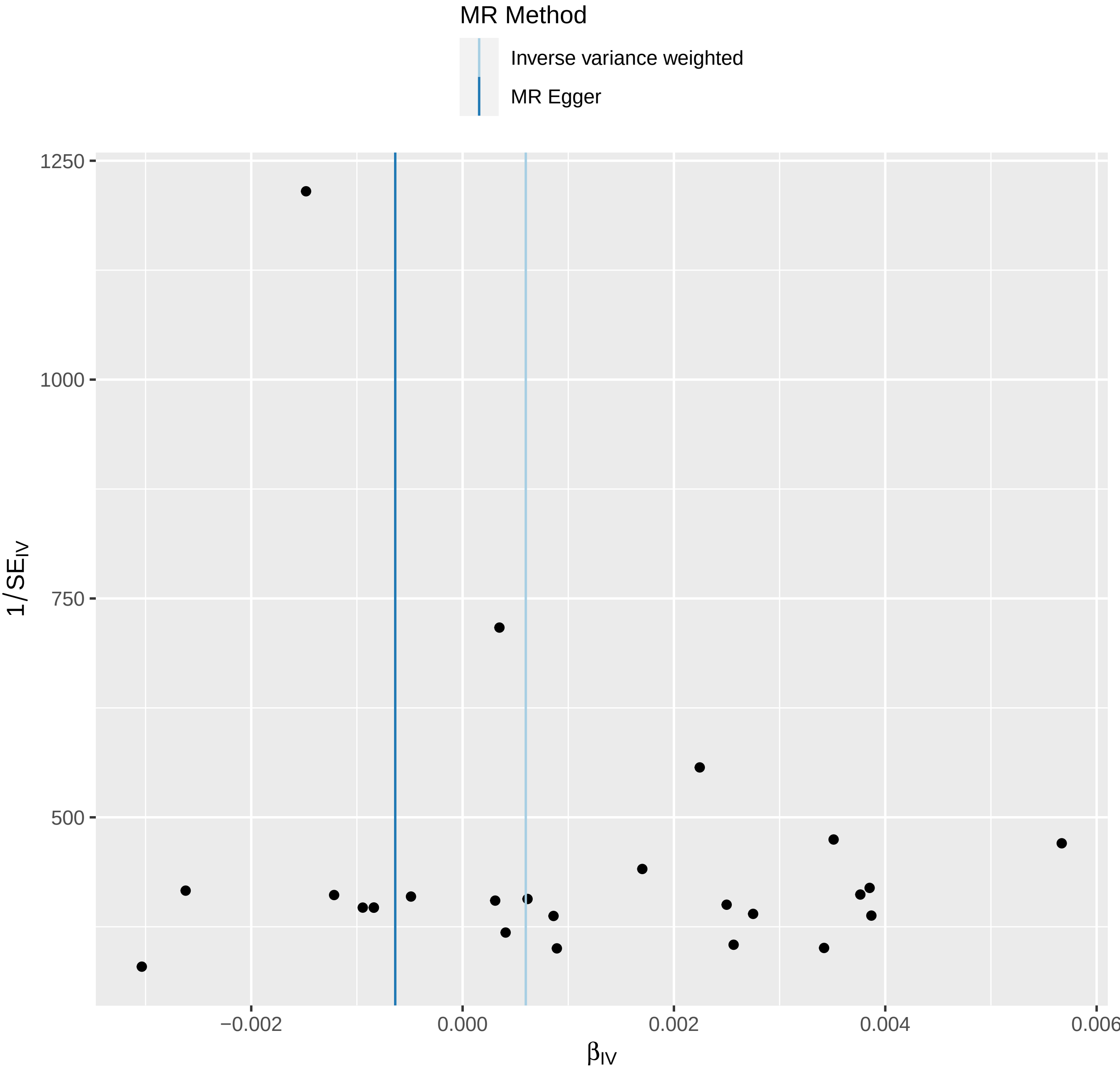

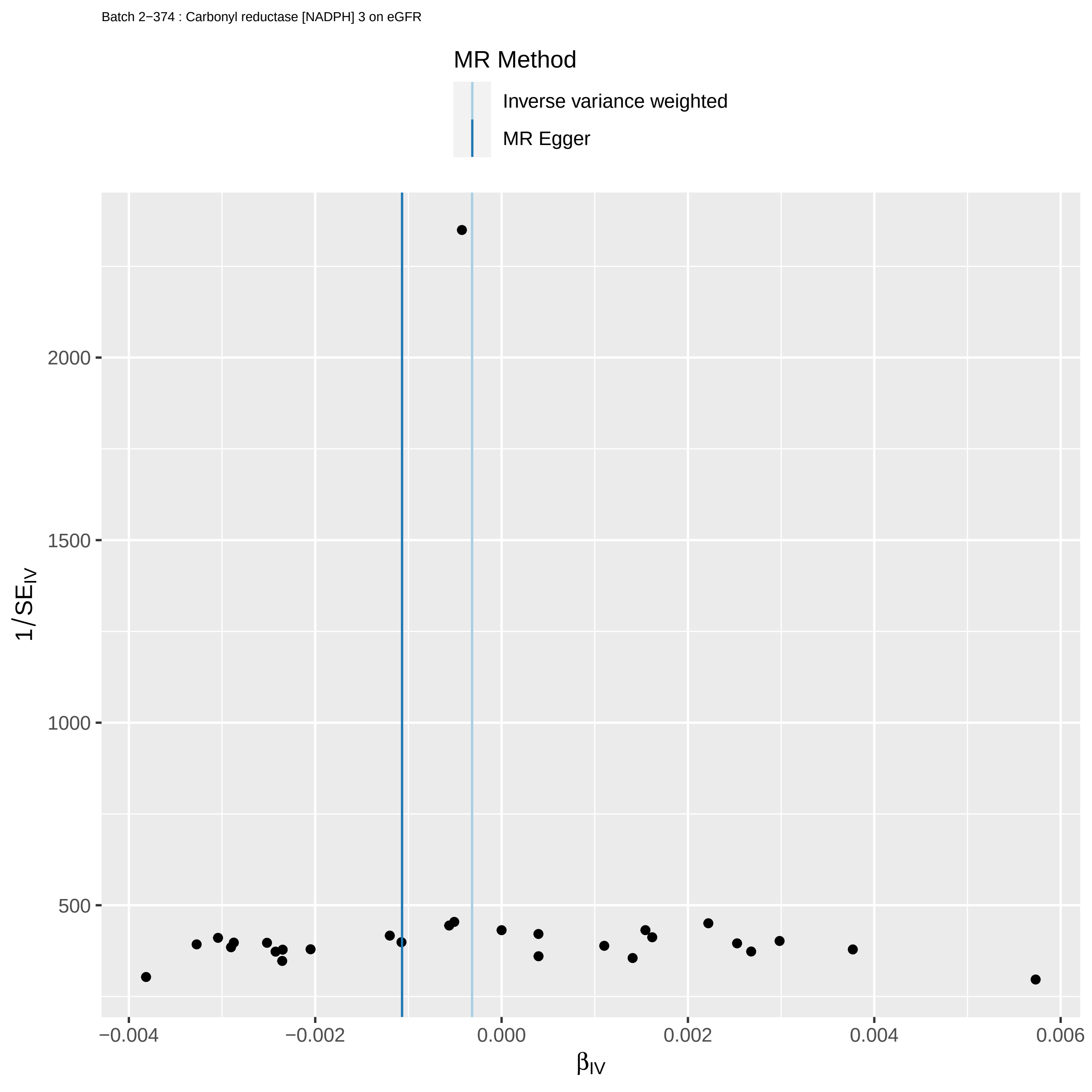
Batch 2−374 : Carbonyl reductase [NADPH] 3 on eGFR
MR Method
Inverse variance weighted
MR Egger
2000
1500
1000
500
1/SE_IV
−0.004
−0.002
0.000
0.002
0.004
0.006
β_IV

Batch 2−375 : Cystathionine beta−synthase on eGFR

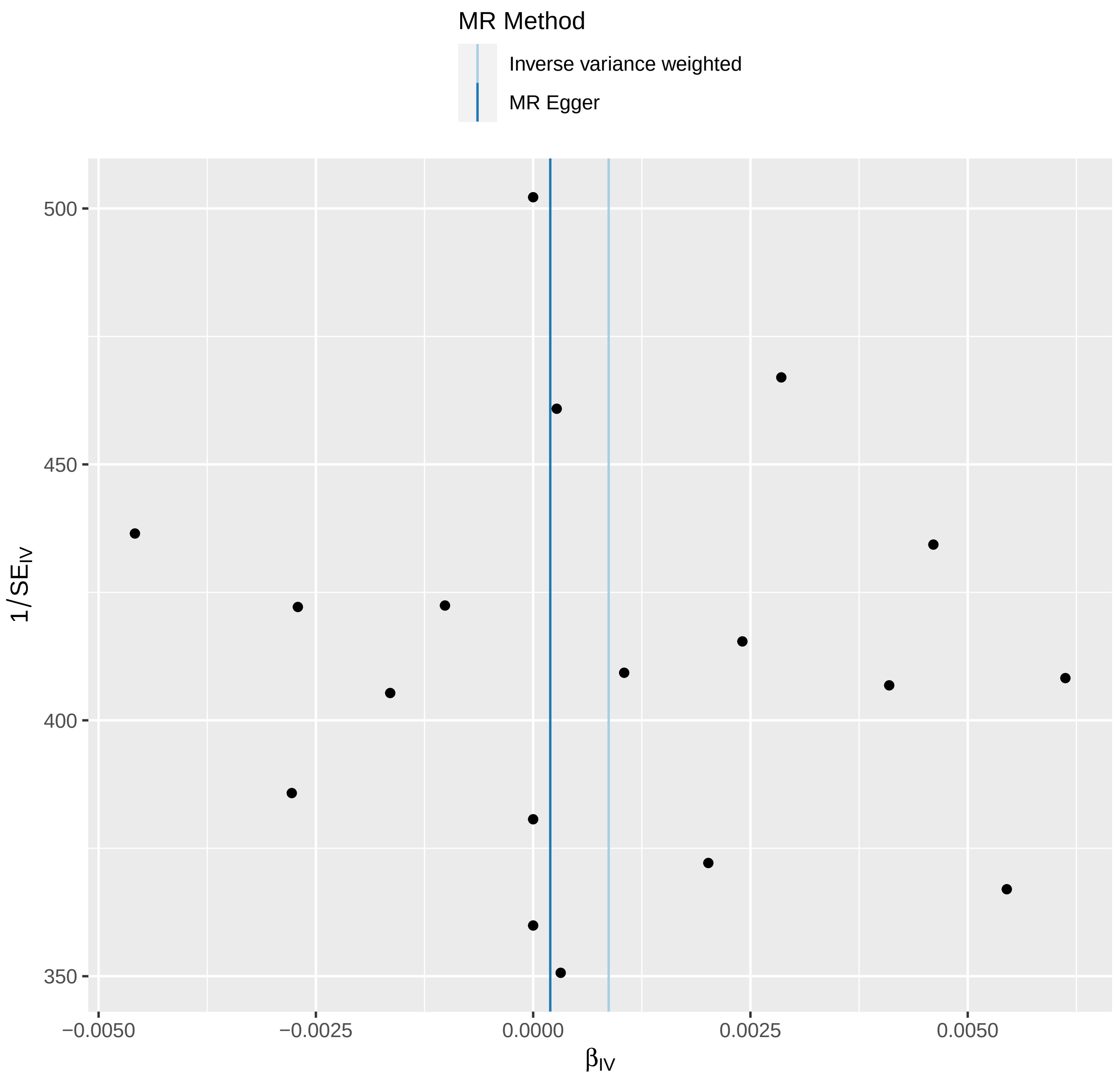

Batch 2−376 : Chromobox protein homolog 5 on eGFR

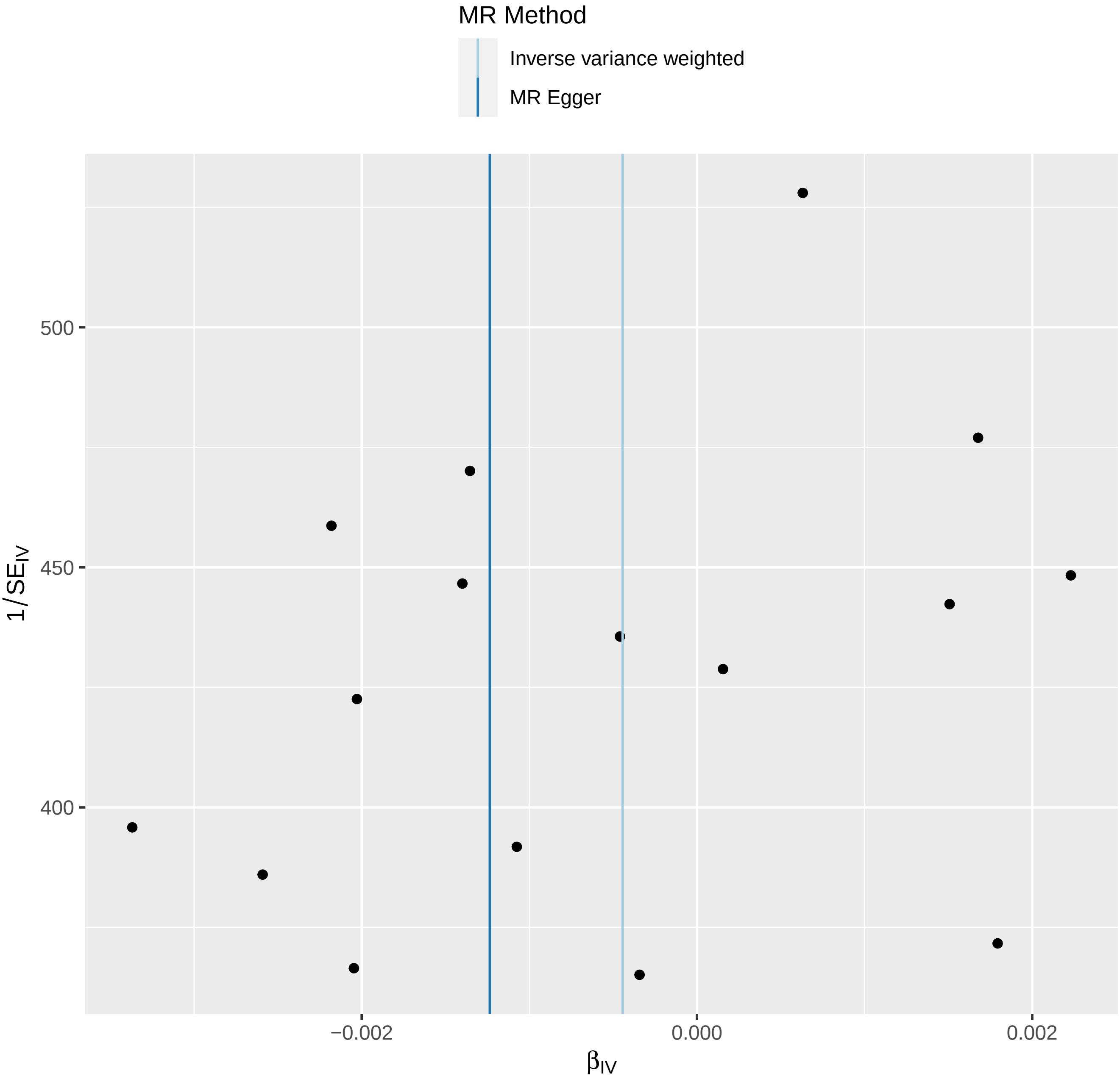

Batch 2−377 : Chromobox protein homolog 7 on eGFR

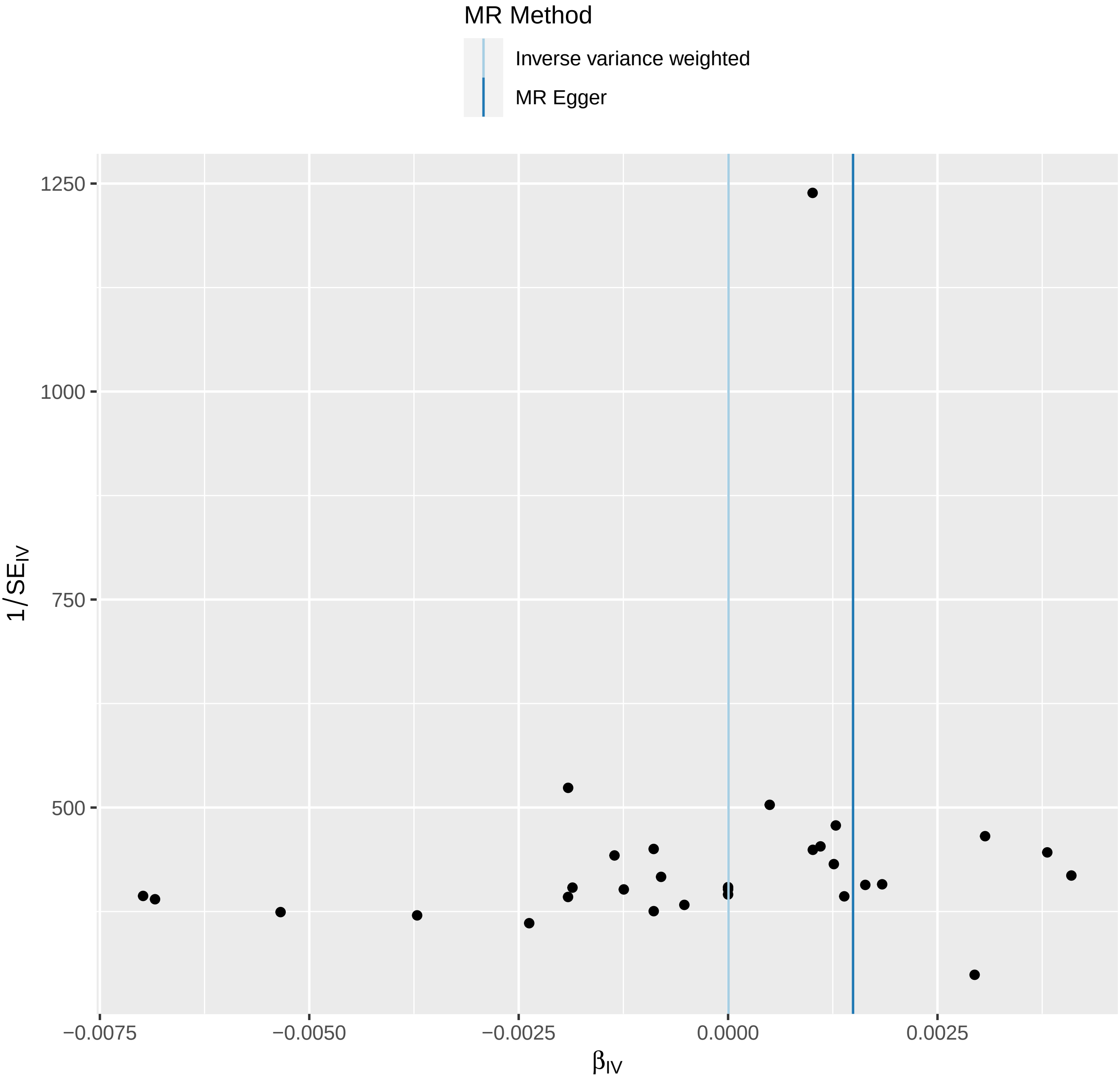

Batch 2−378 : Kynurenine−−oxoglutarate transaminase 3 on eGFR

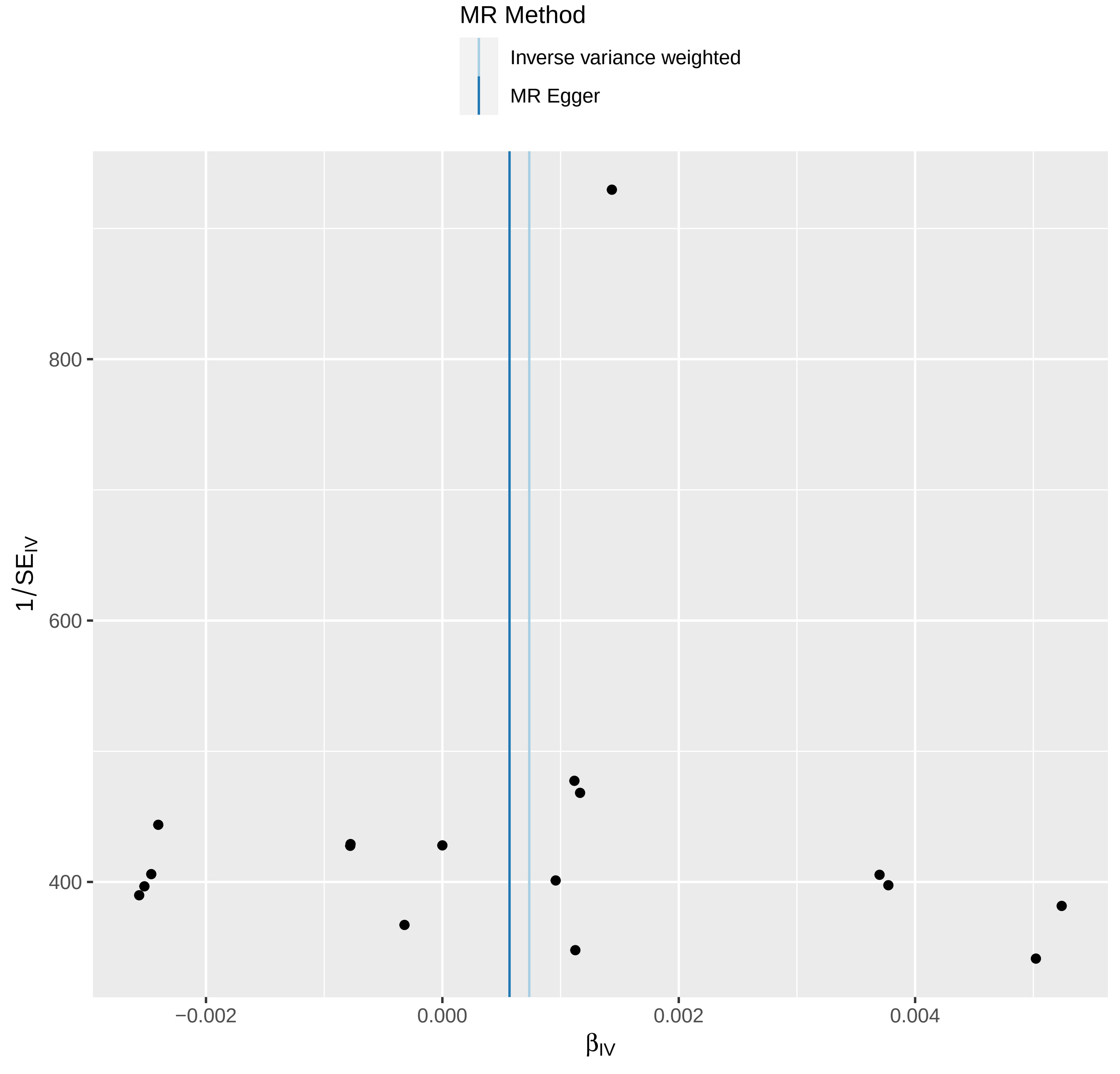

Batch 2−379 : SAGA−associated factor 29 homolog on eGFR

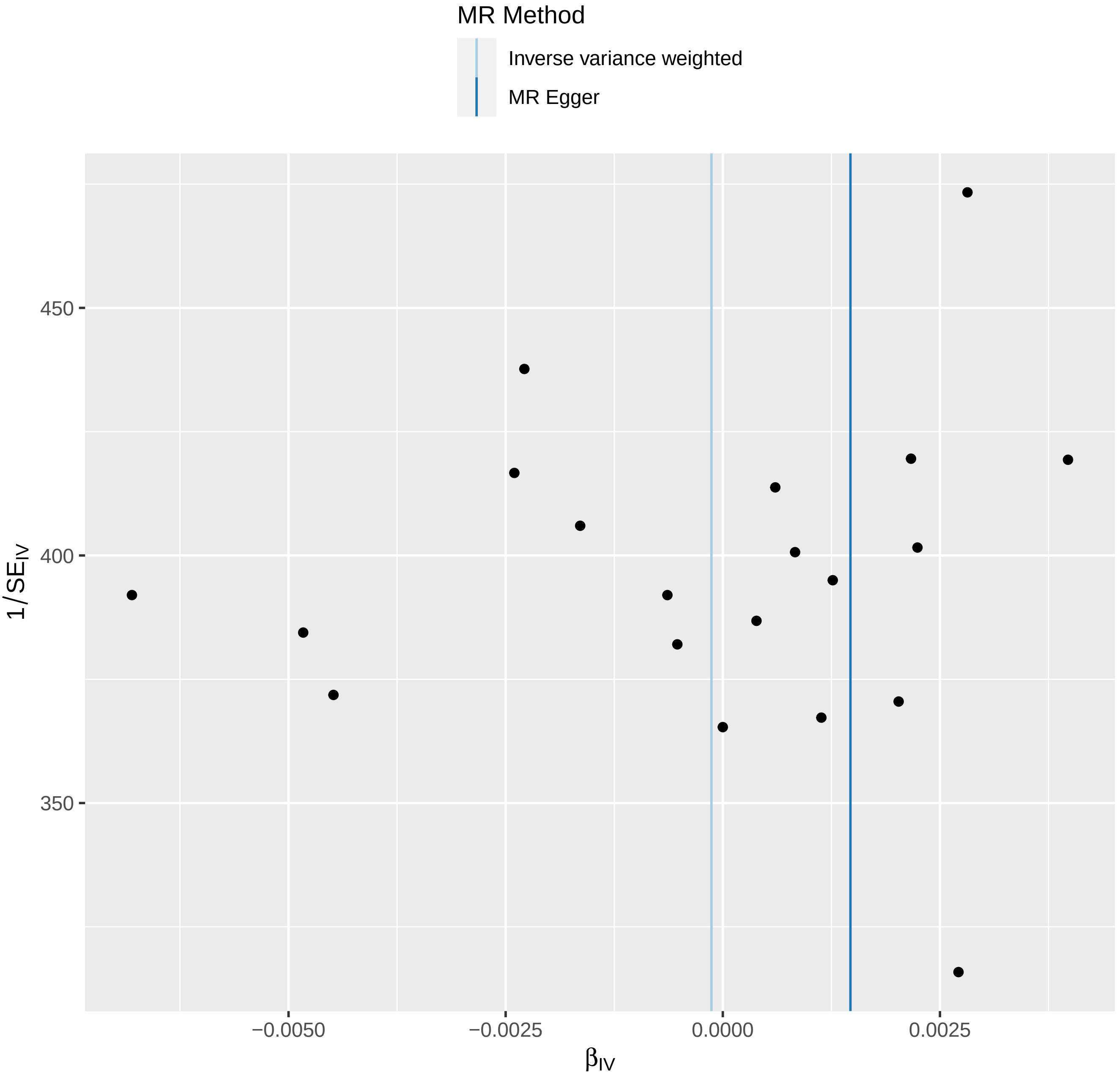

Batch 2−380 : Coiled−coil domain−containing protein 126 on eGFR

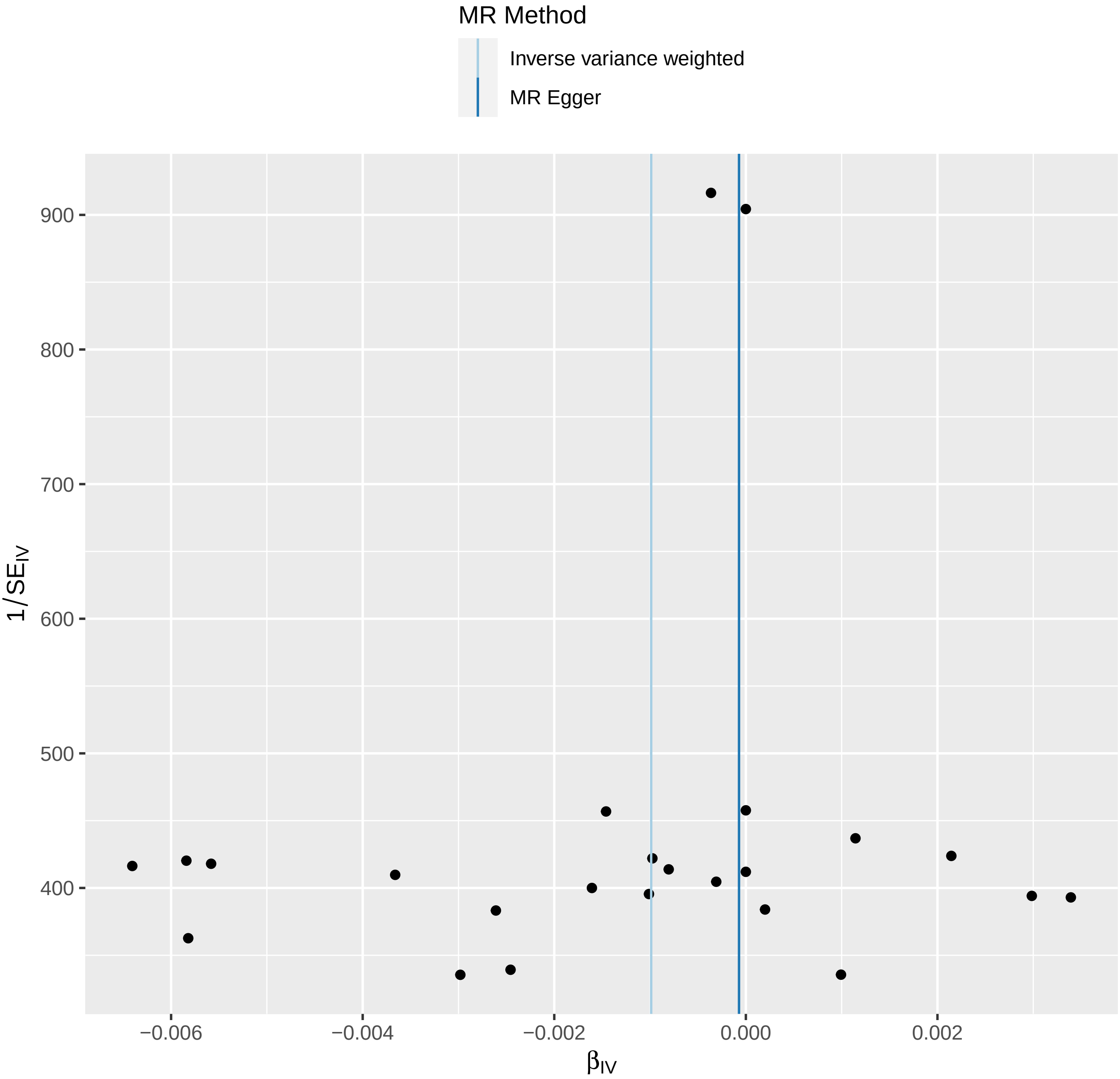

Batch 2−381 : Coiled−coil domain−containing protein 134 on eGFR

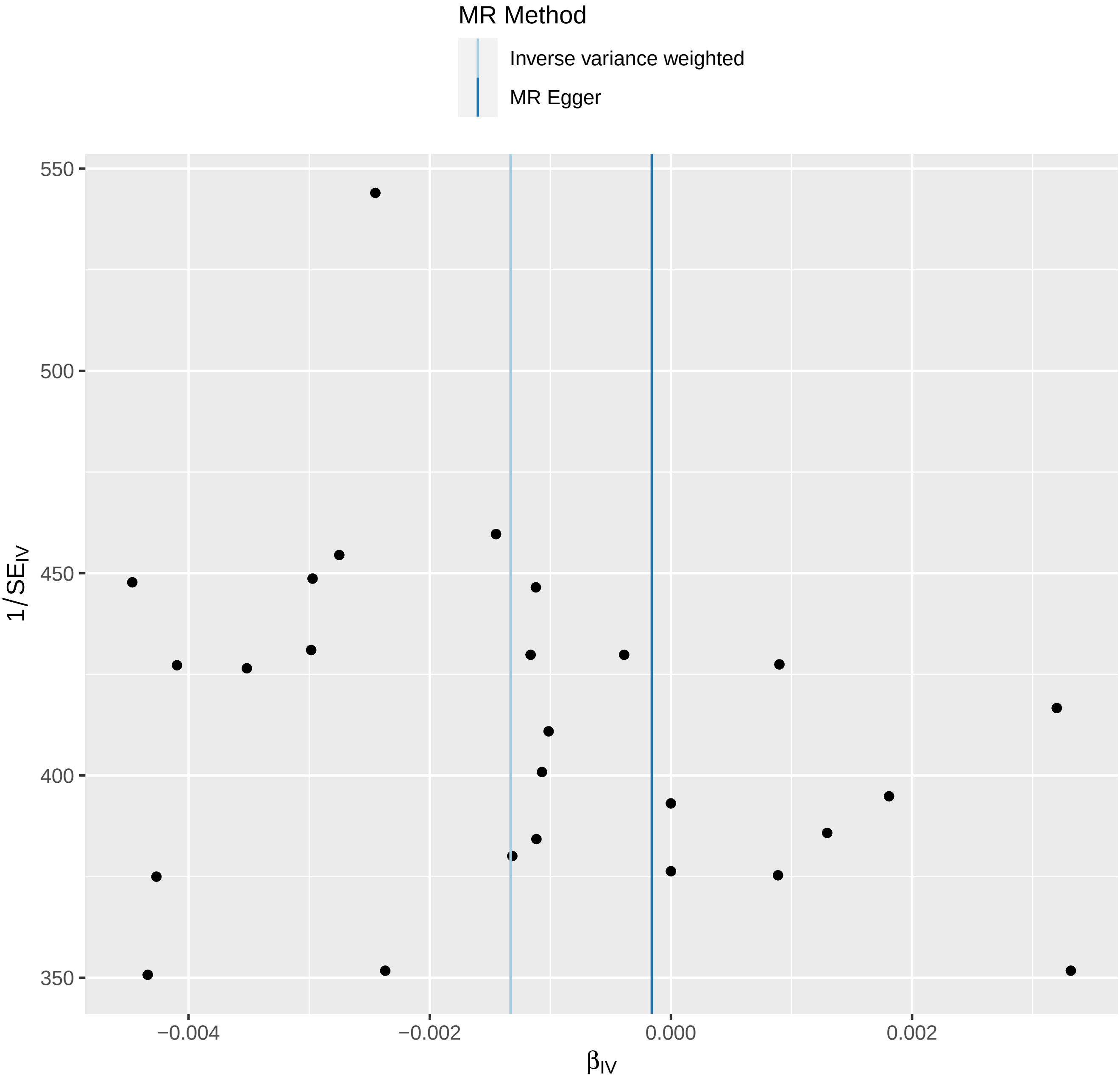

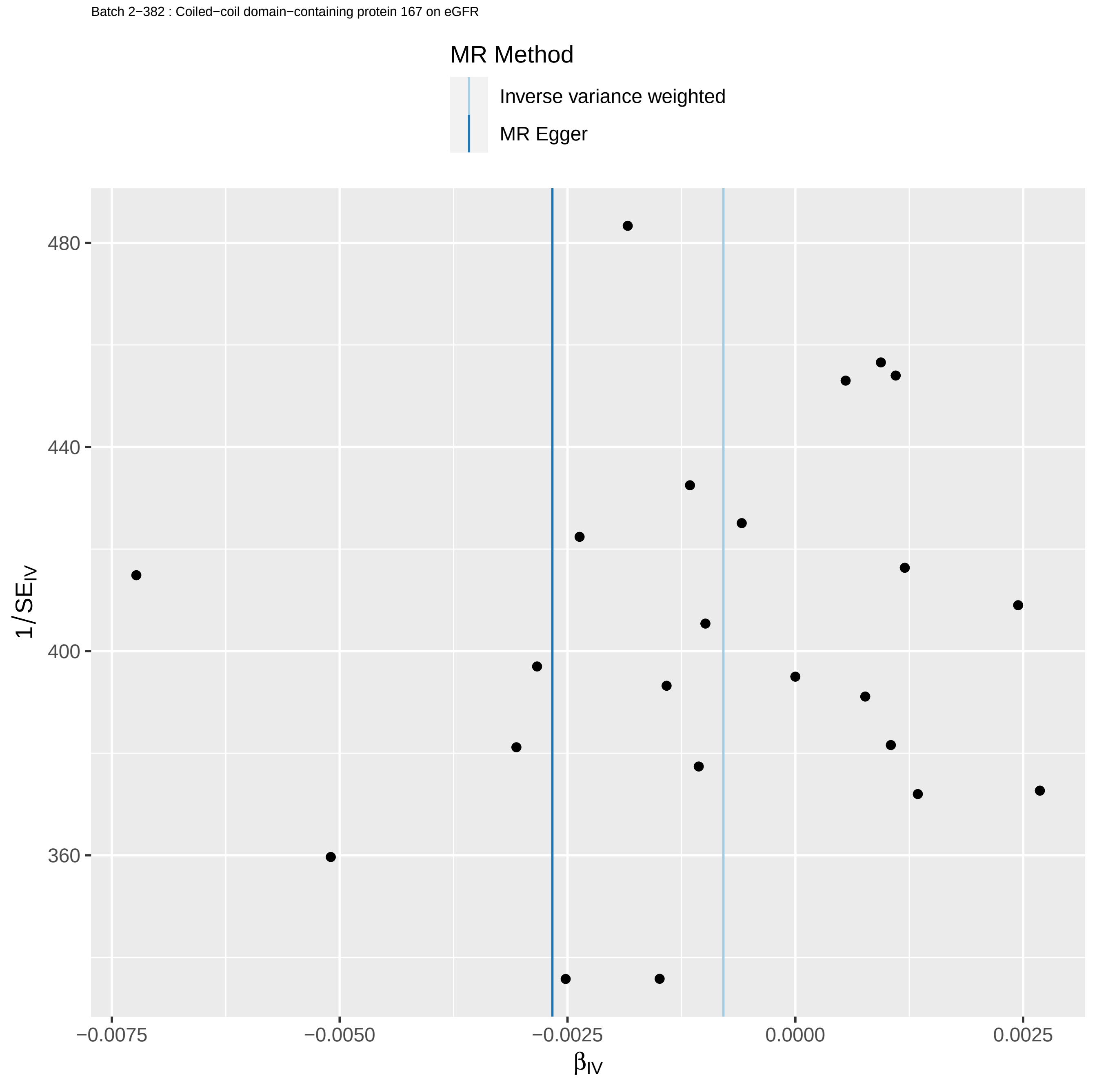

Batch 2−382 : Coiled−coil domain−containing protein 167 on eGFR
MR Method
Inverse variance weighted
MR Egger
480
440
400
360
1/SE_IV
−0.0075
−0.0050
−0.0025
0.0000
0.0025
β_IV

Batch 2−383 : Coiled−coil domain−containing protein 51 on eGFR

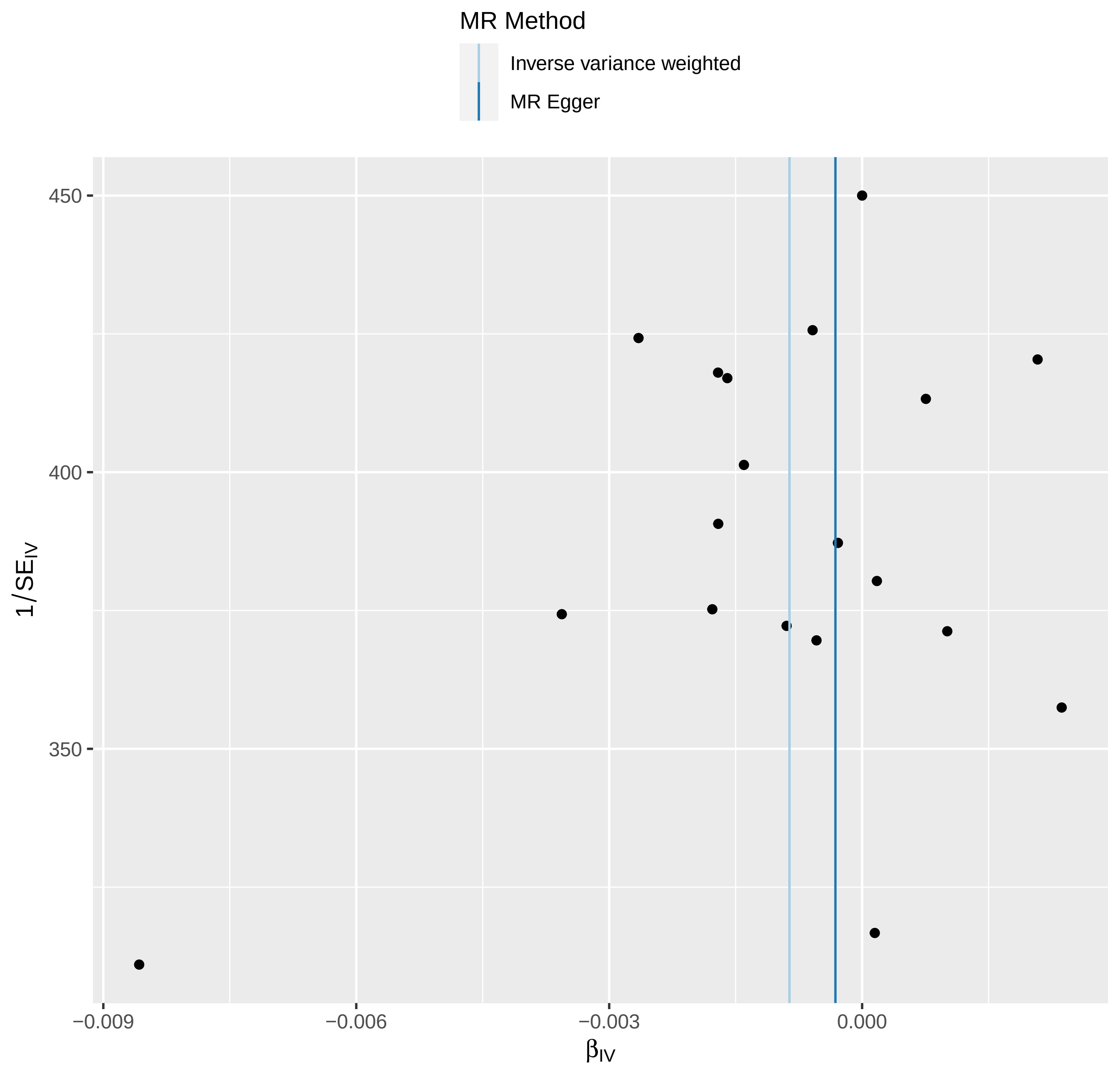

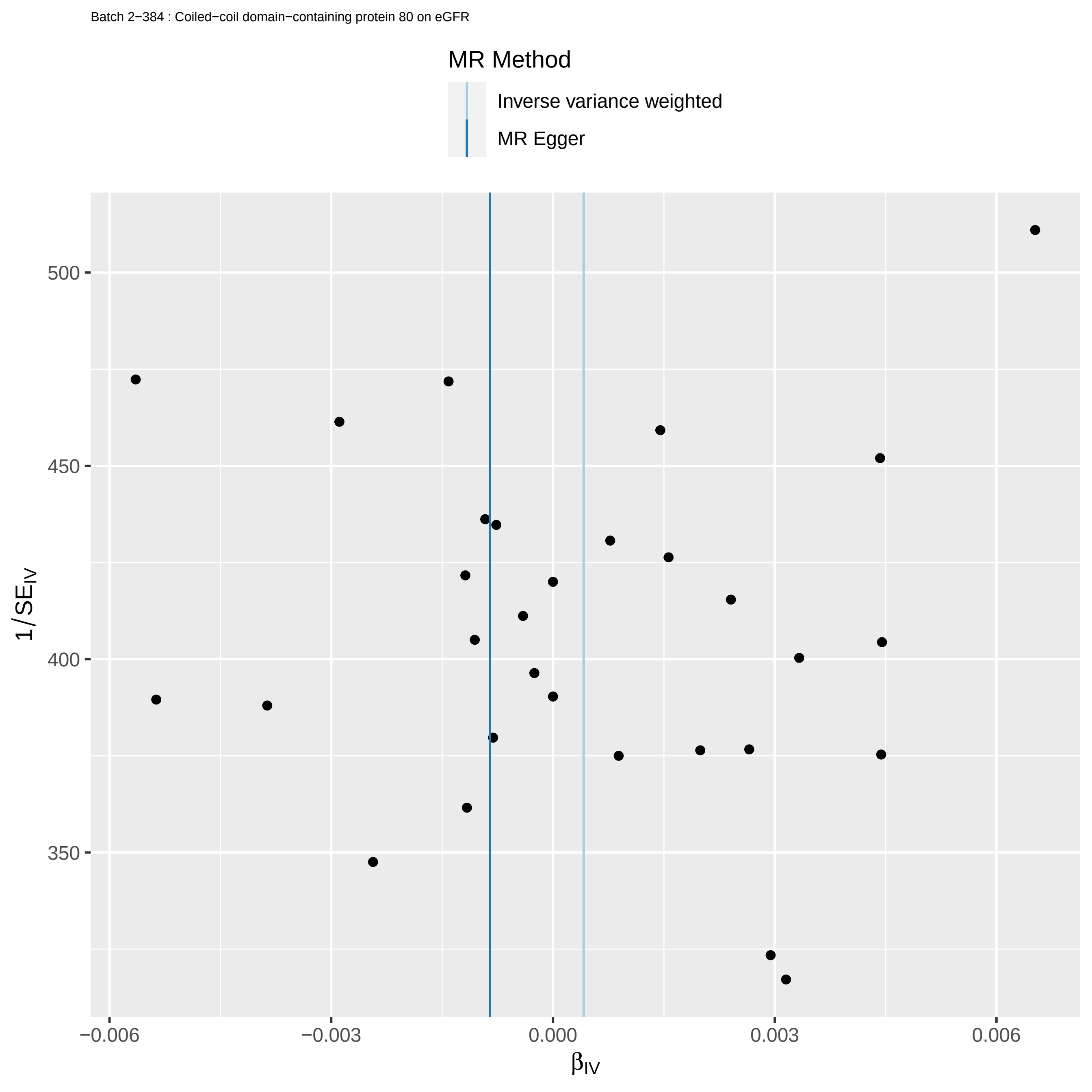

Batch 2−384 : Coiled−coil domain−containing protein 80 on eGFR
MR Method
Inverse variance weighted
MR Egger
500
450
400
350
1/SE_IV
−0.006
−0.003
0.000
0.003
0.006
β_IV

Batch 2−385 : Coiled−coil domain−containing protein 90B, mitochondrial on eGFR

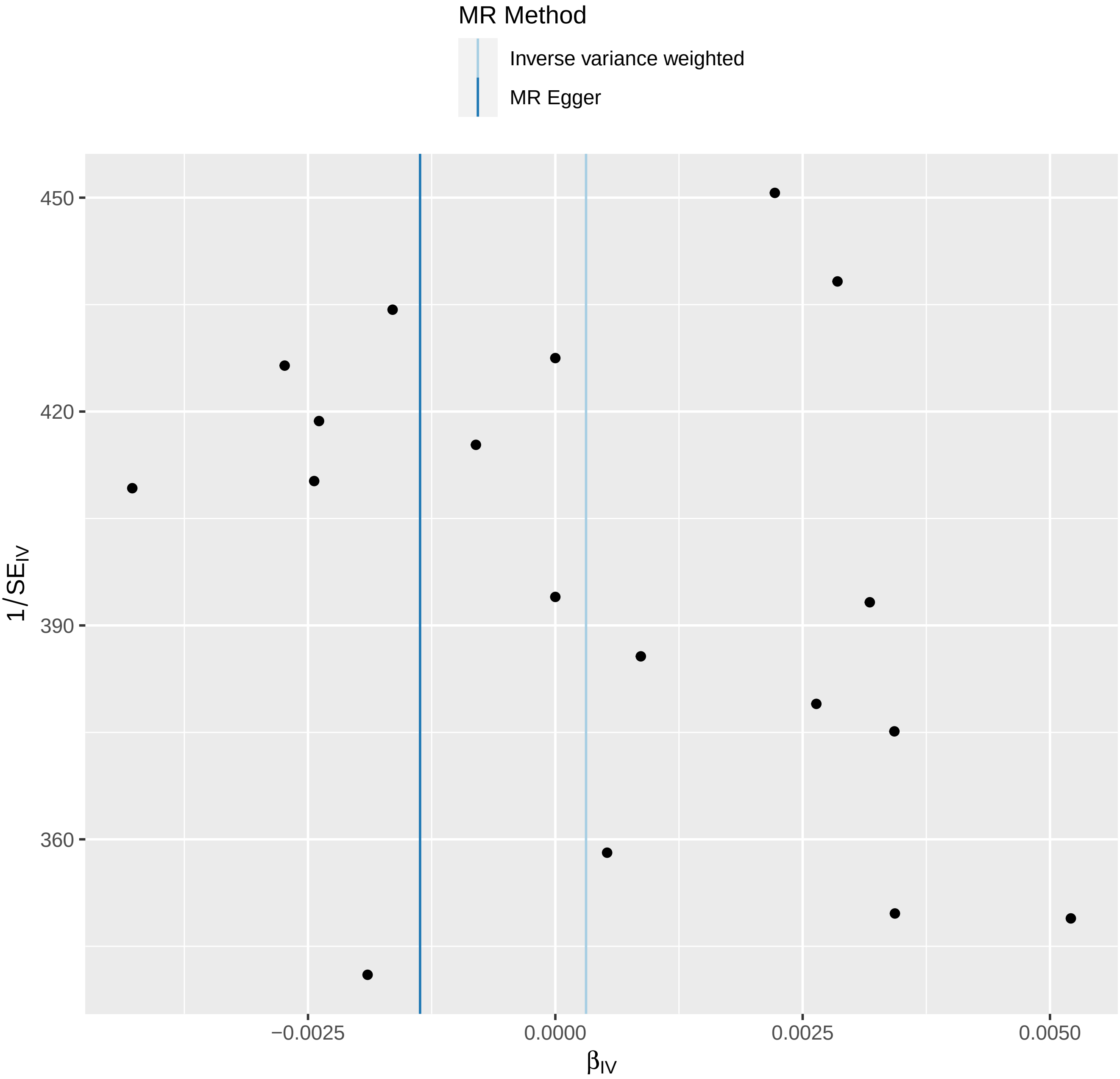

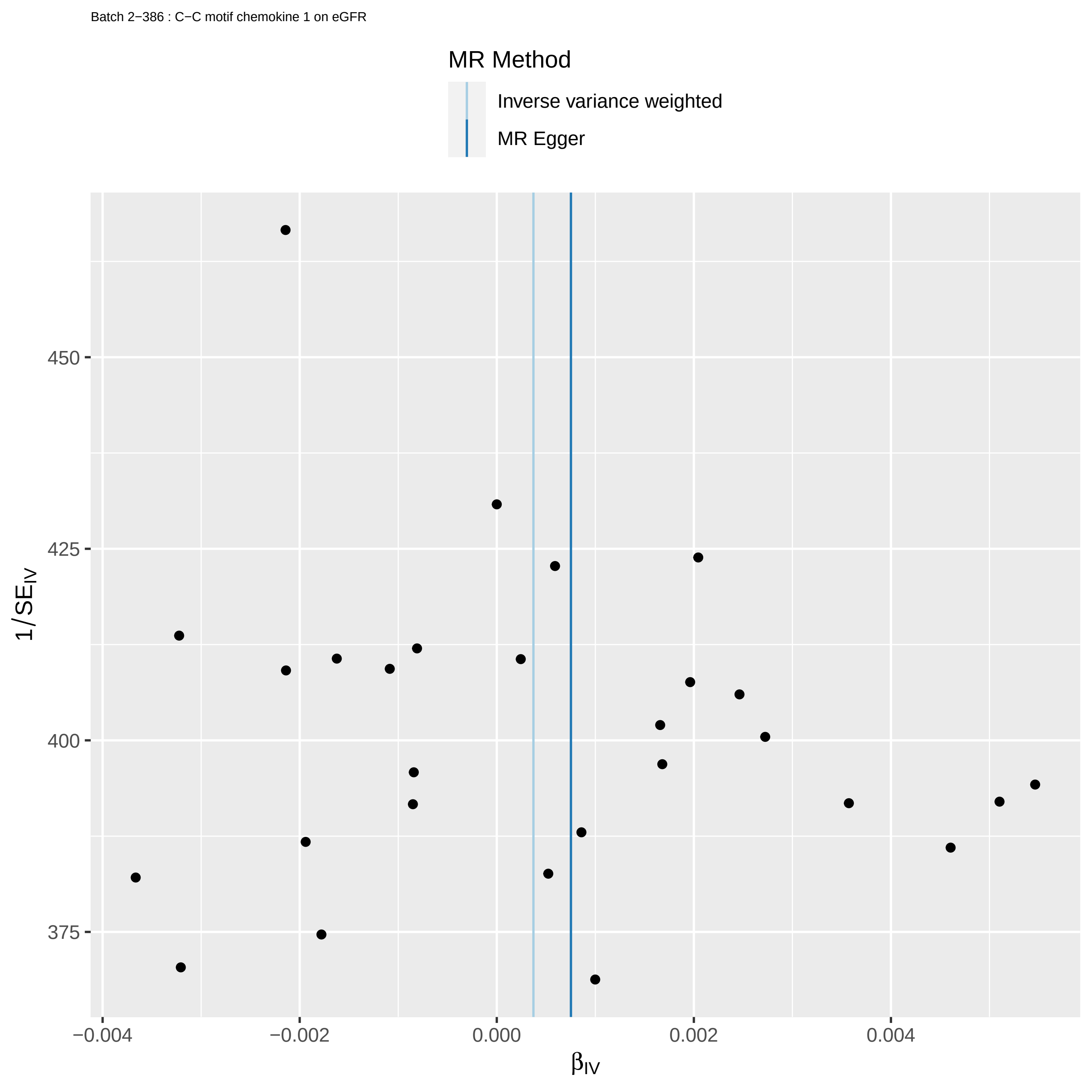
Batch 2−386 : C−C motif chemokine 1 on eGFR
MR Method
Inverse variance weighted
MR Egger
1/SE_IV
450
425
400
375
−0.004
−0.002
0.000
0.002
0.004
β_IV

Batch 2−387 : Eotaxin on eGFR

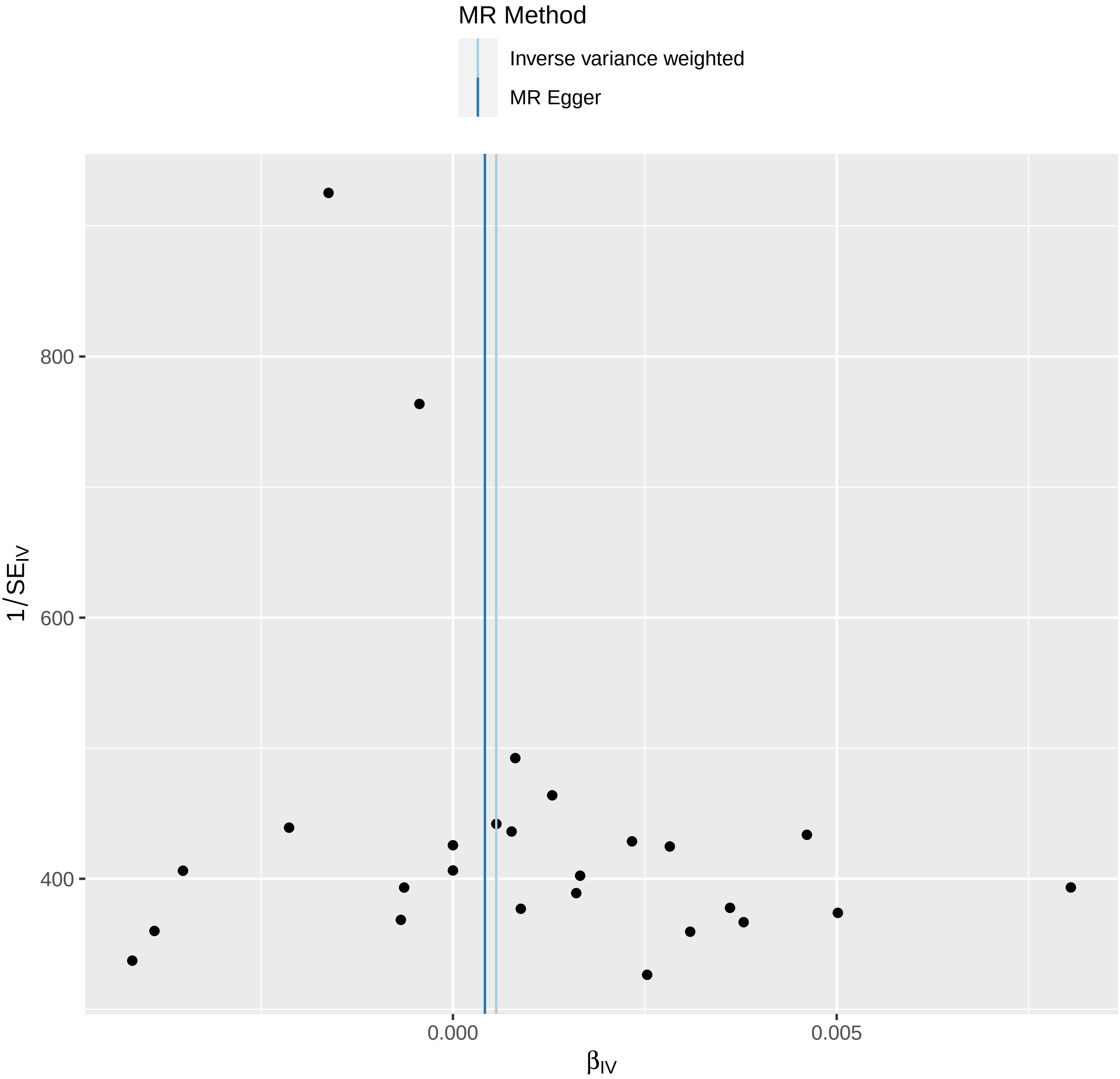

Batch 2−388 : C−C motif chemokine 1 on eGFR

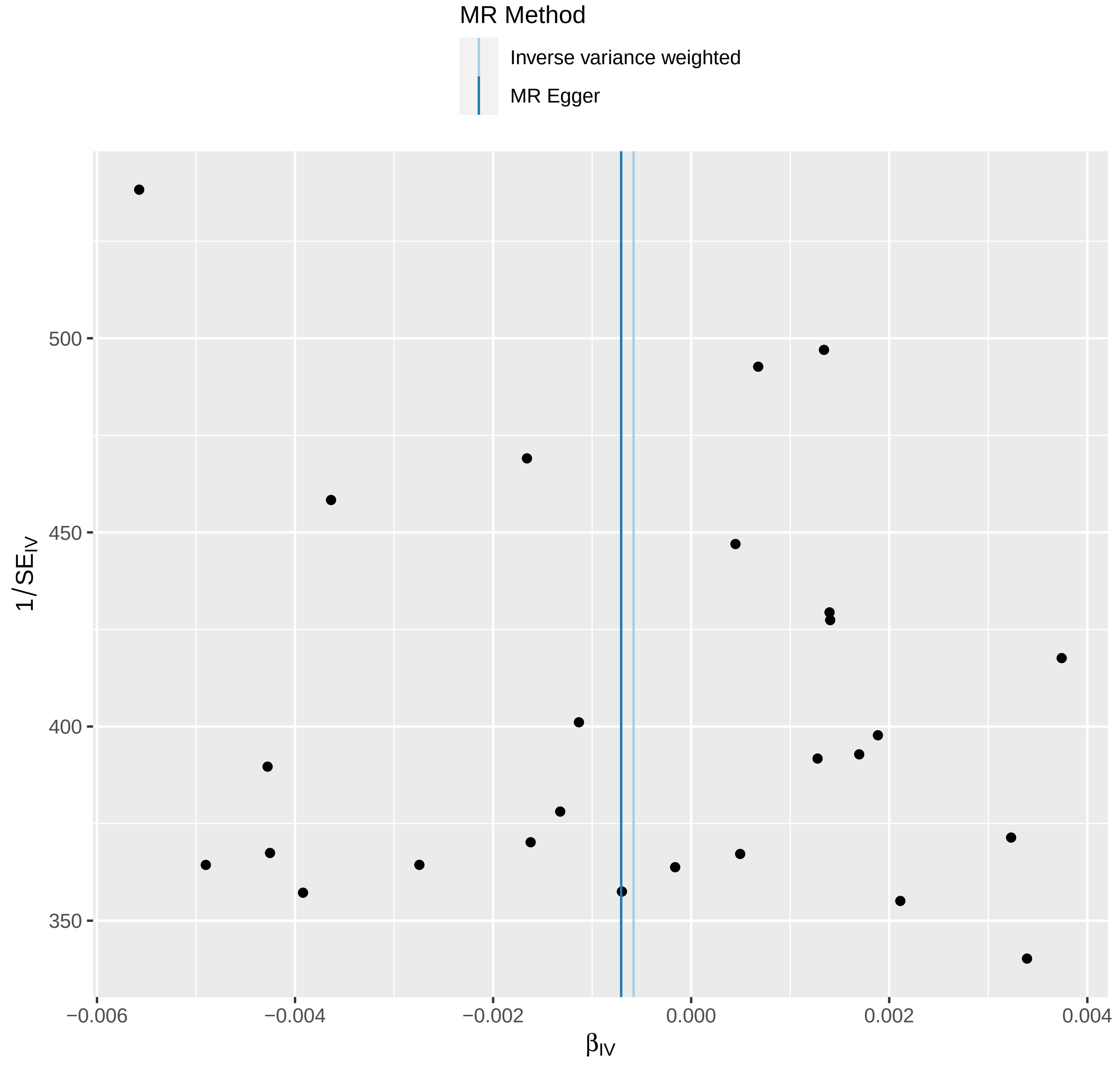

Batch 2−389 : C−C motif chemokine 13 on eGFR

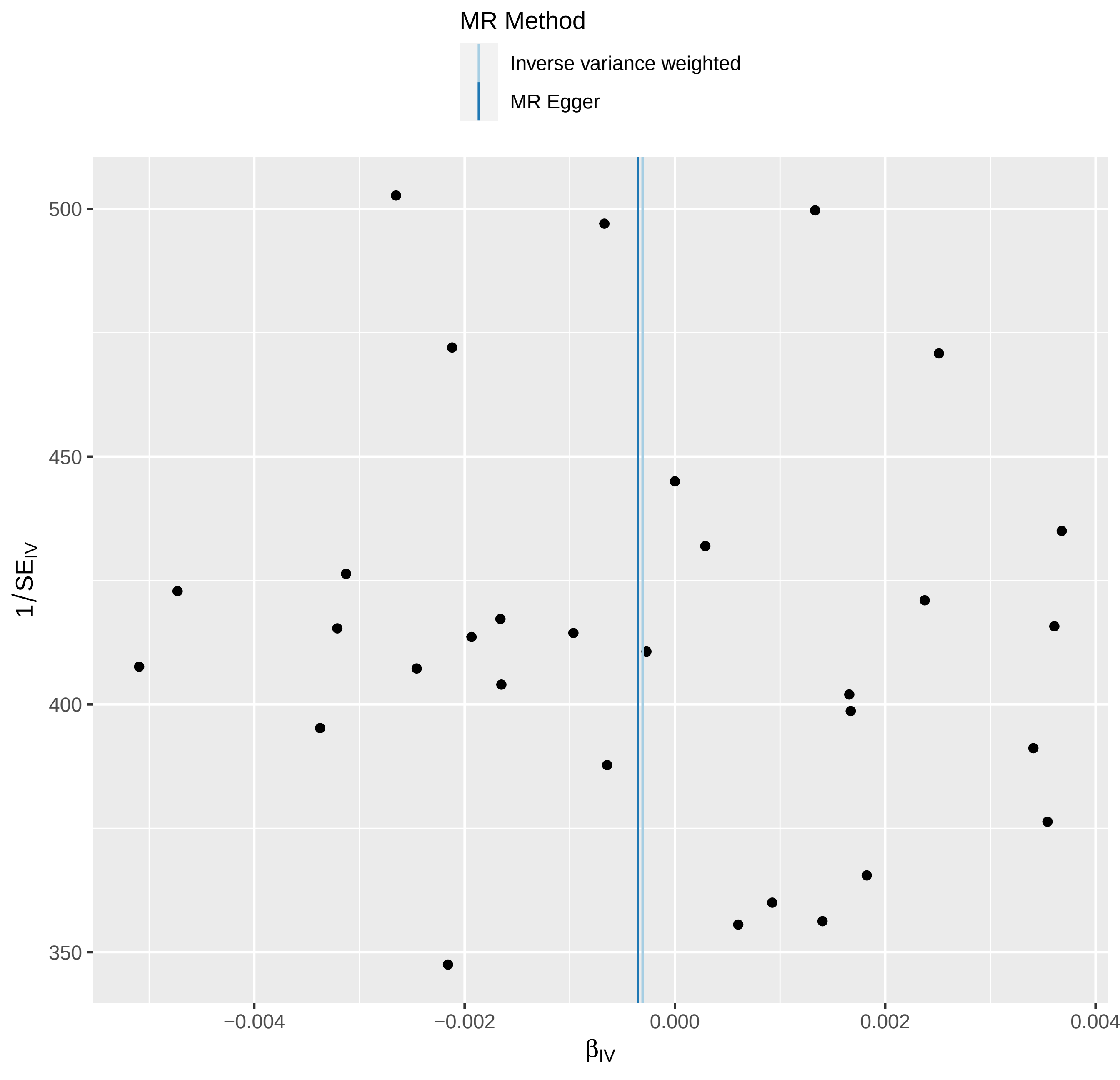

Batch 2−390 : C−C motif chemokine 14 on eGFR

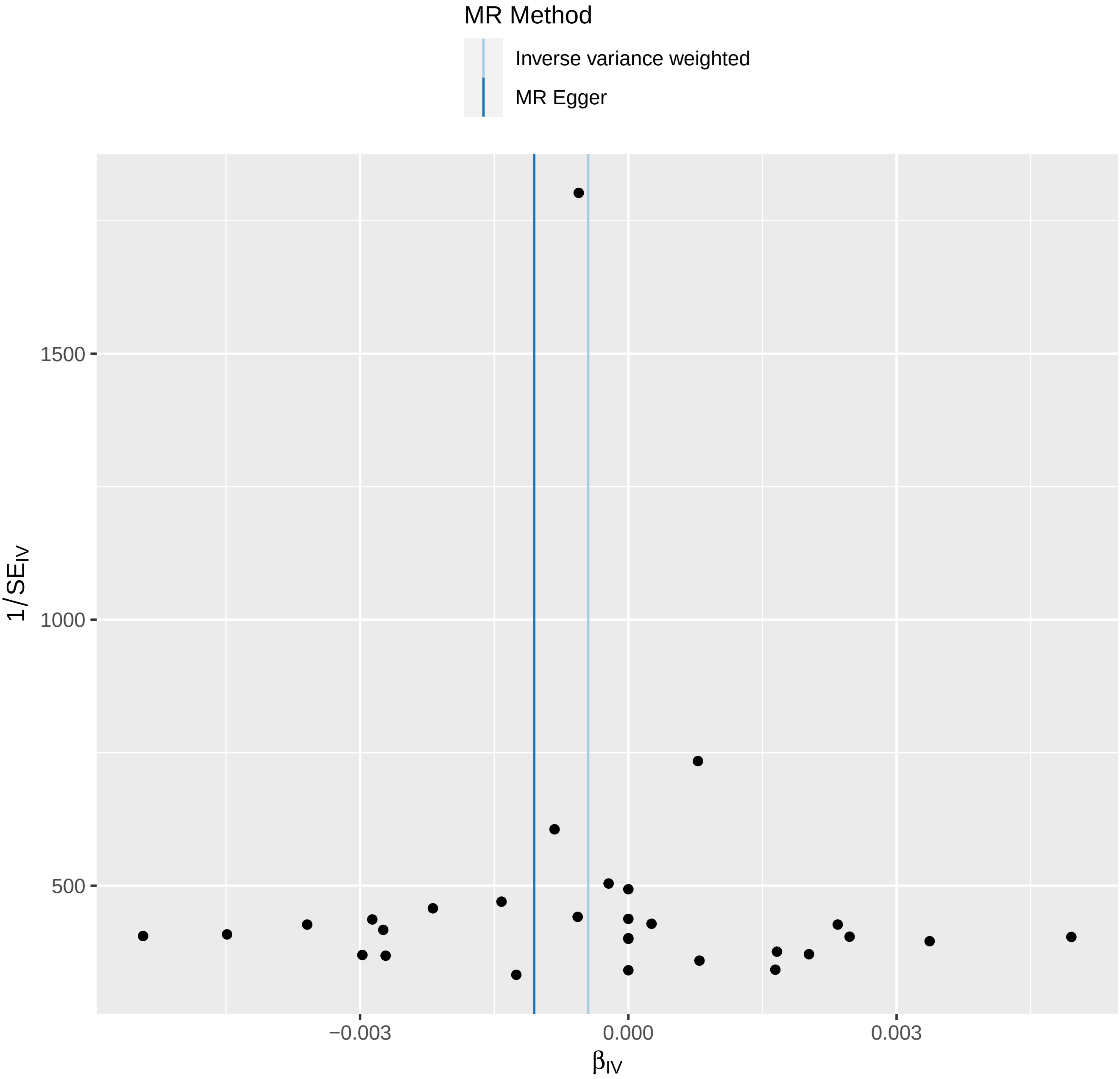

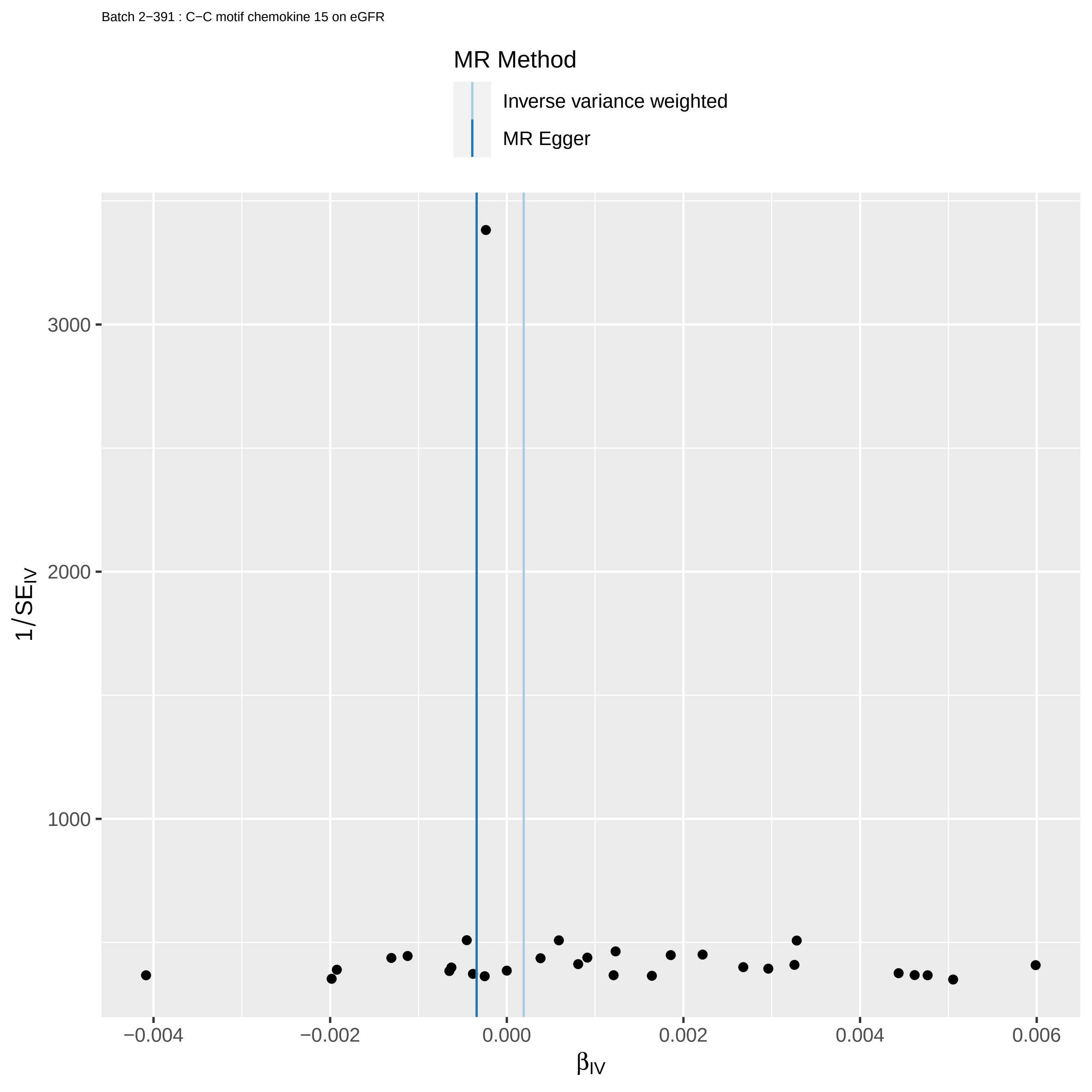

Batch 2−391 : C−C motif chemokine 15 on eGFR
MR Method
Inverse variance weighted
MR Egger
3000
2000
1000
1/SE_IV
−0.004
−0.002
0.000
0.002
0.004
0.006
β_IV

Batch 2−392 : C−C motif chemokine 15 on eGFR

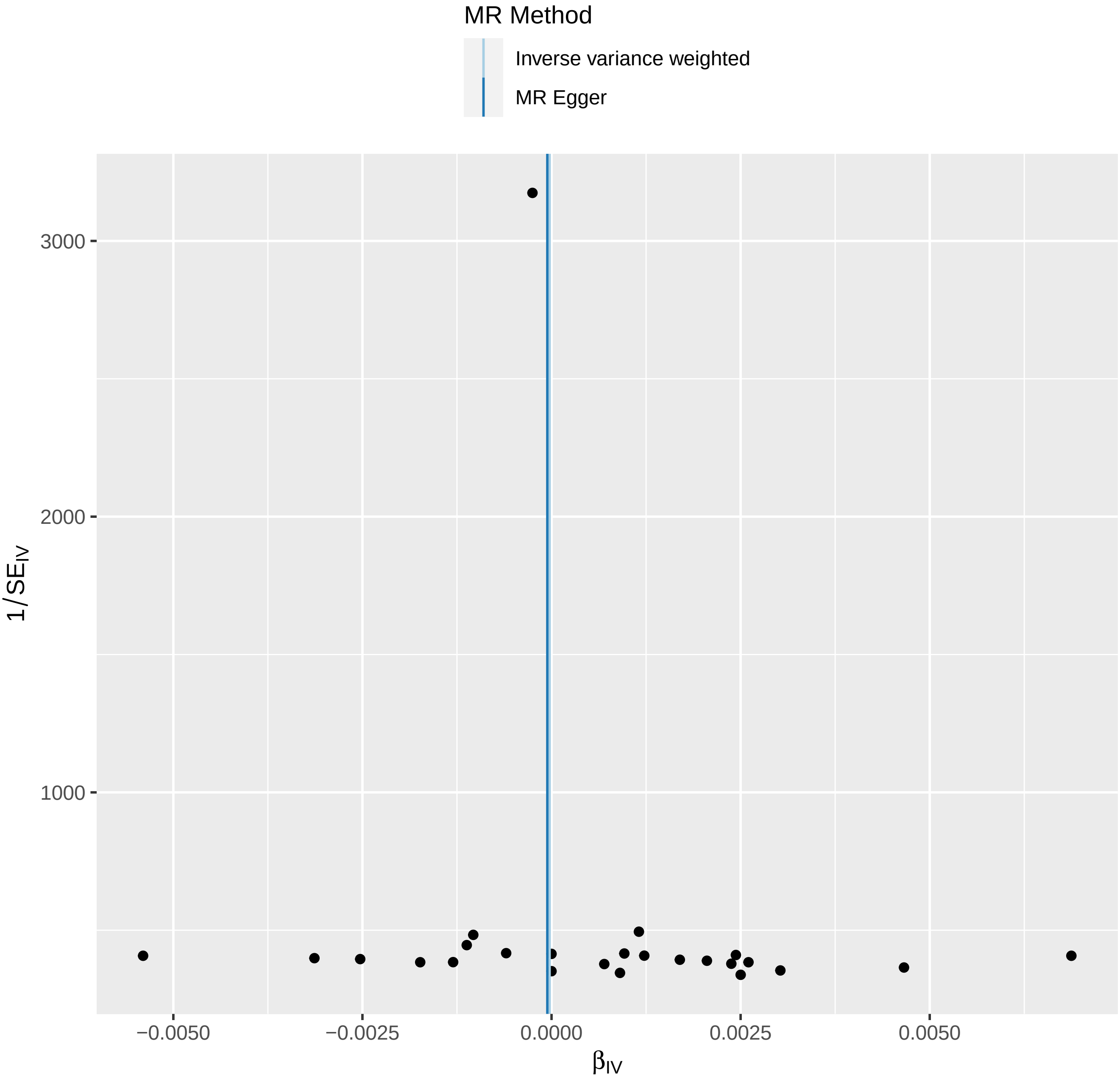

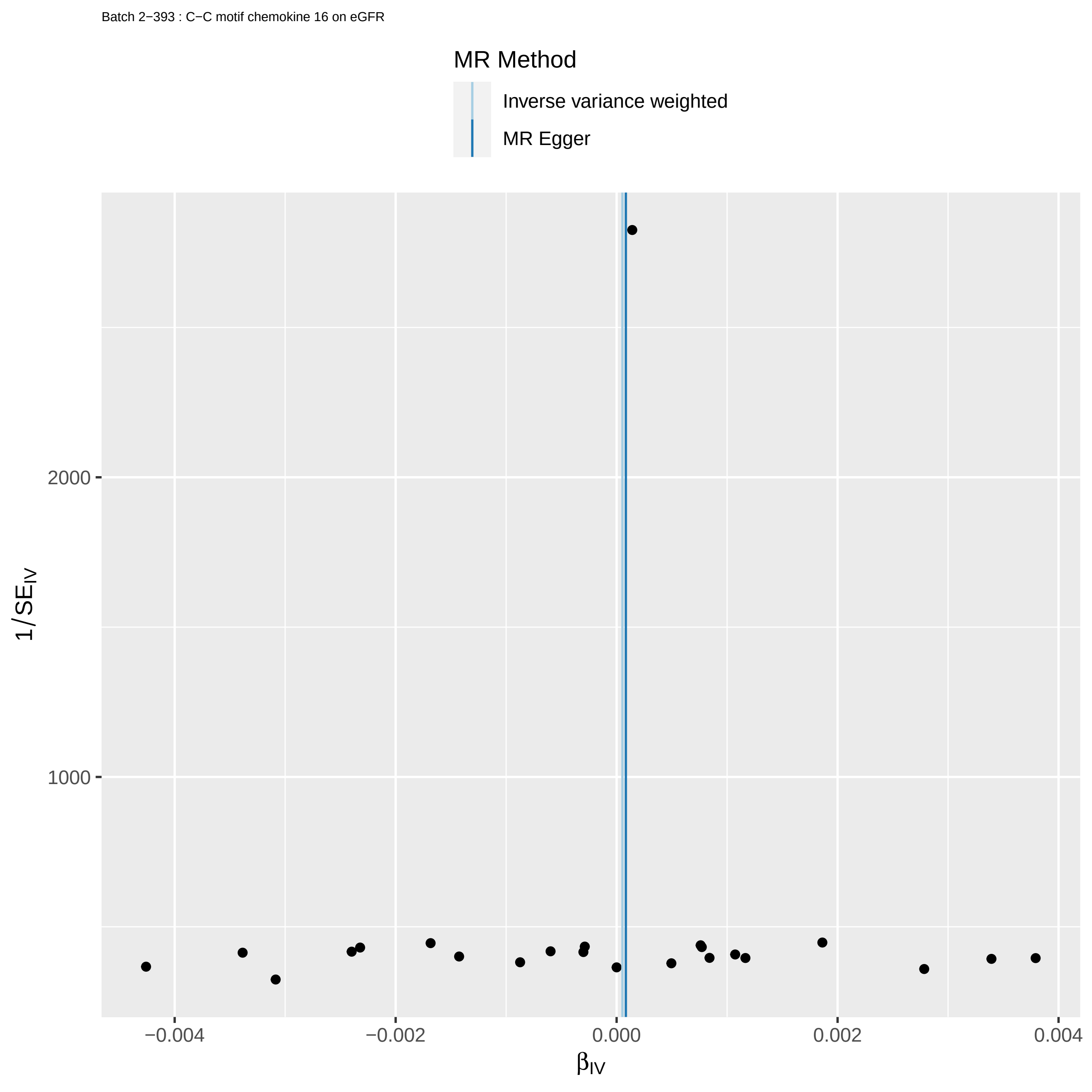
Batch 2−393 : C−C motif chemokine 16 on eGFR
MR Method
Inverse variance weighted
MR Egger
2000
1000
1/SE$_{IV}$
−0.004
−0.002
0.000
0.002
0.004
β$_{IV}$

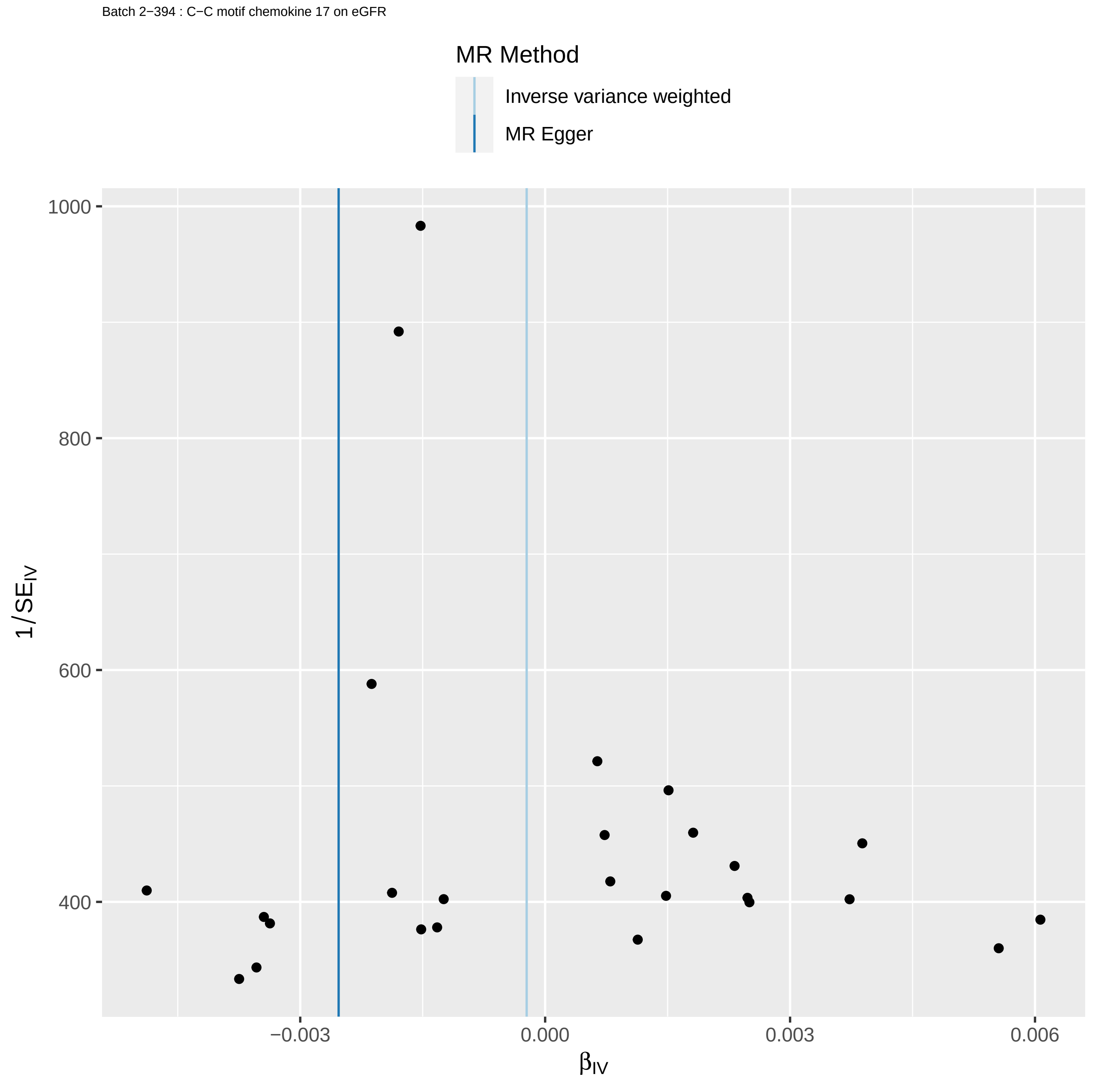
Batch 2−394 : C−C motif chemokine 17 on eGFR
MR Method
Inverse variance weighted
MR Egger
1000
800
600
400
1/SE_IV
−0.003
0.000
0.003
0.006
β_IV

Batch 2−395 : C−C motif chemokine 19 on eGFR

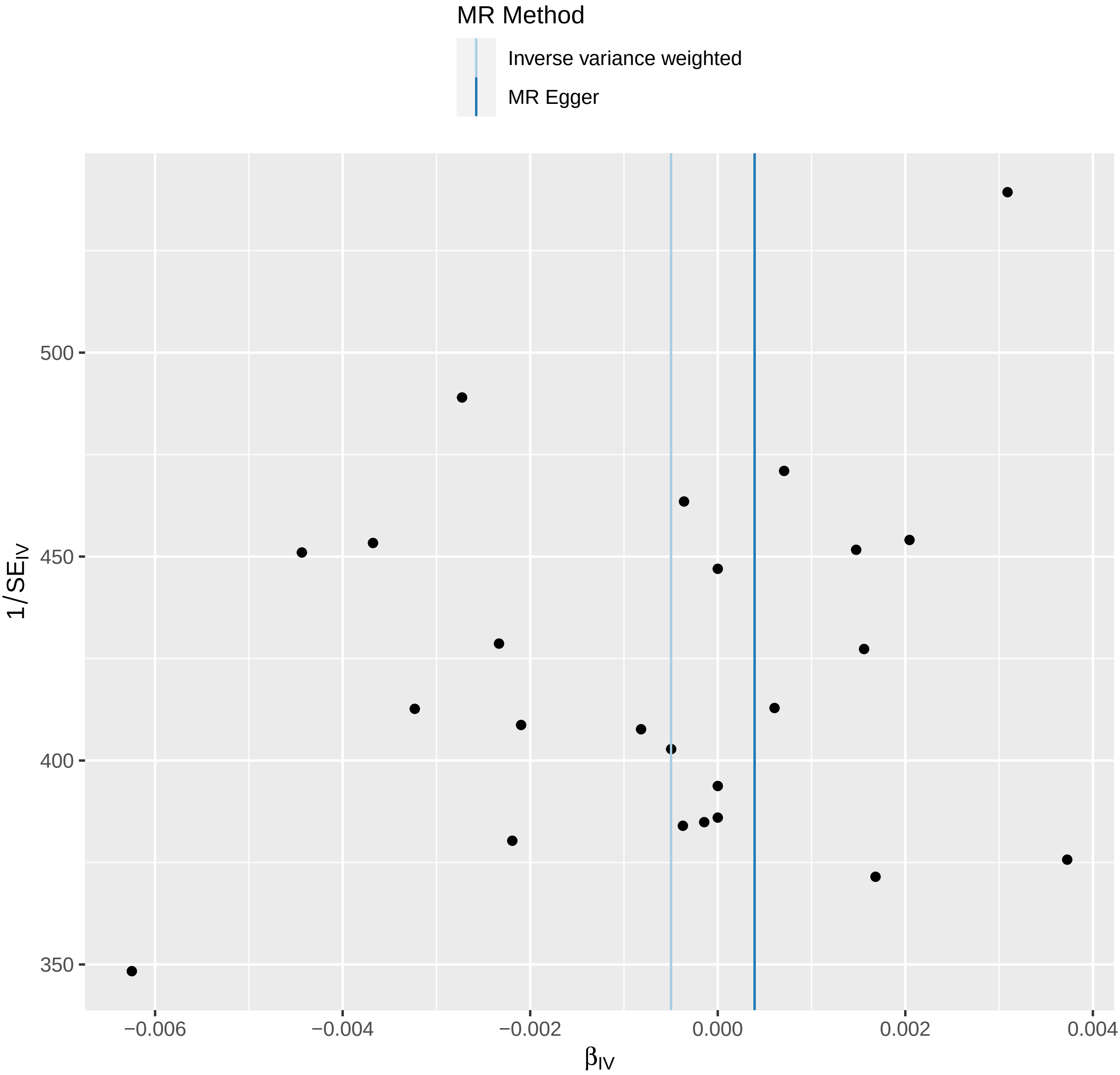

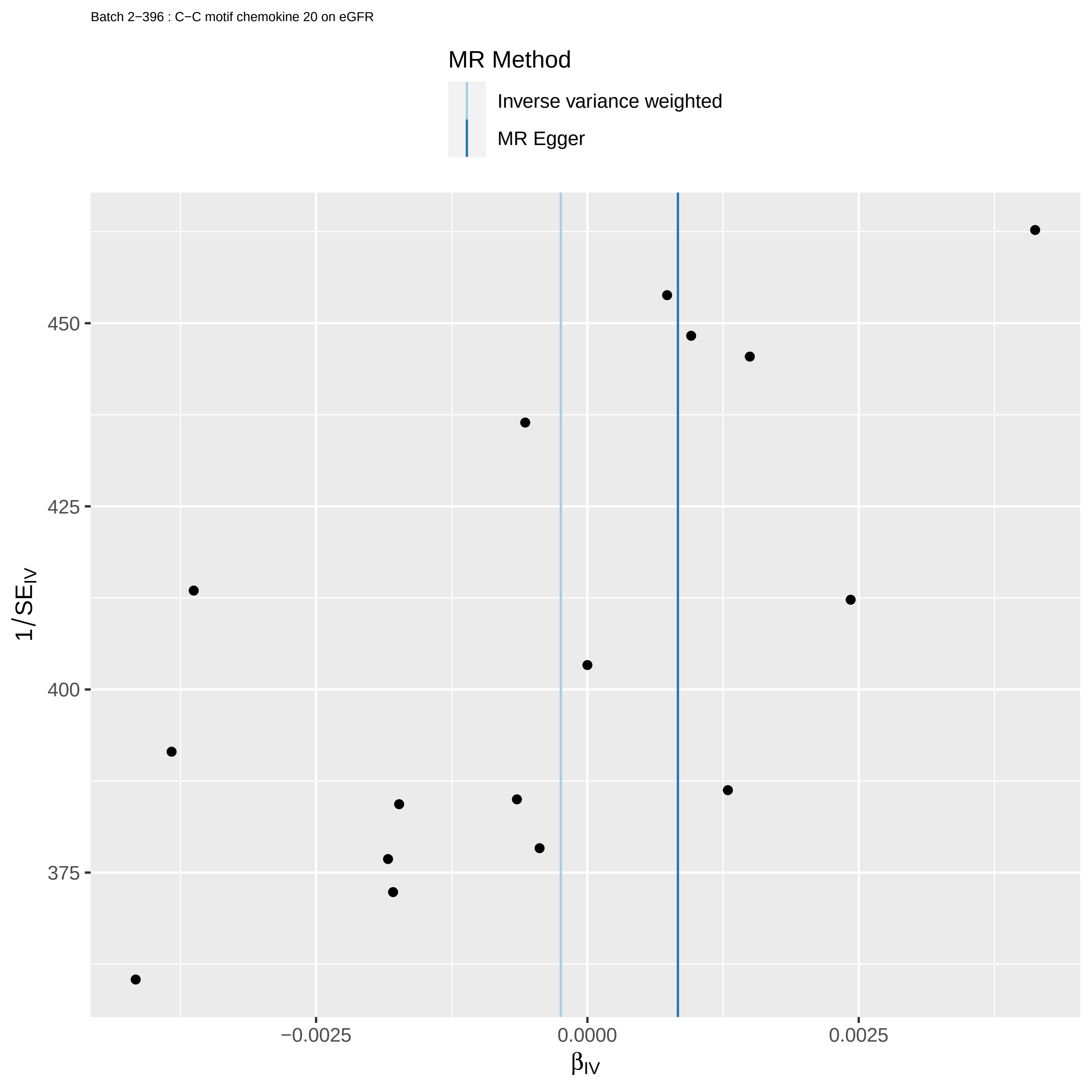
Batch 2−396 : C−C motif chemokine 20 on eGFR
MR Method
Inverse variance weighted
MR Egger
450
425
400
375
1/SE_IV
−0.0025
0.0000
0.0025
β_IV

Batch 2−397 : C−C motif chemokine 21 on eGFR

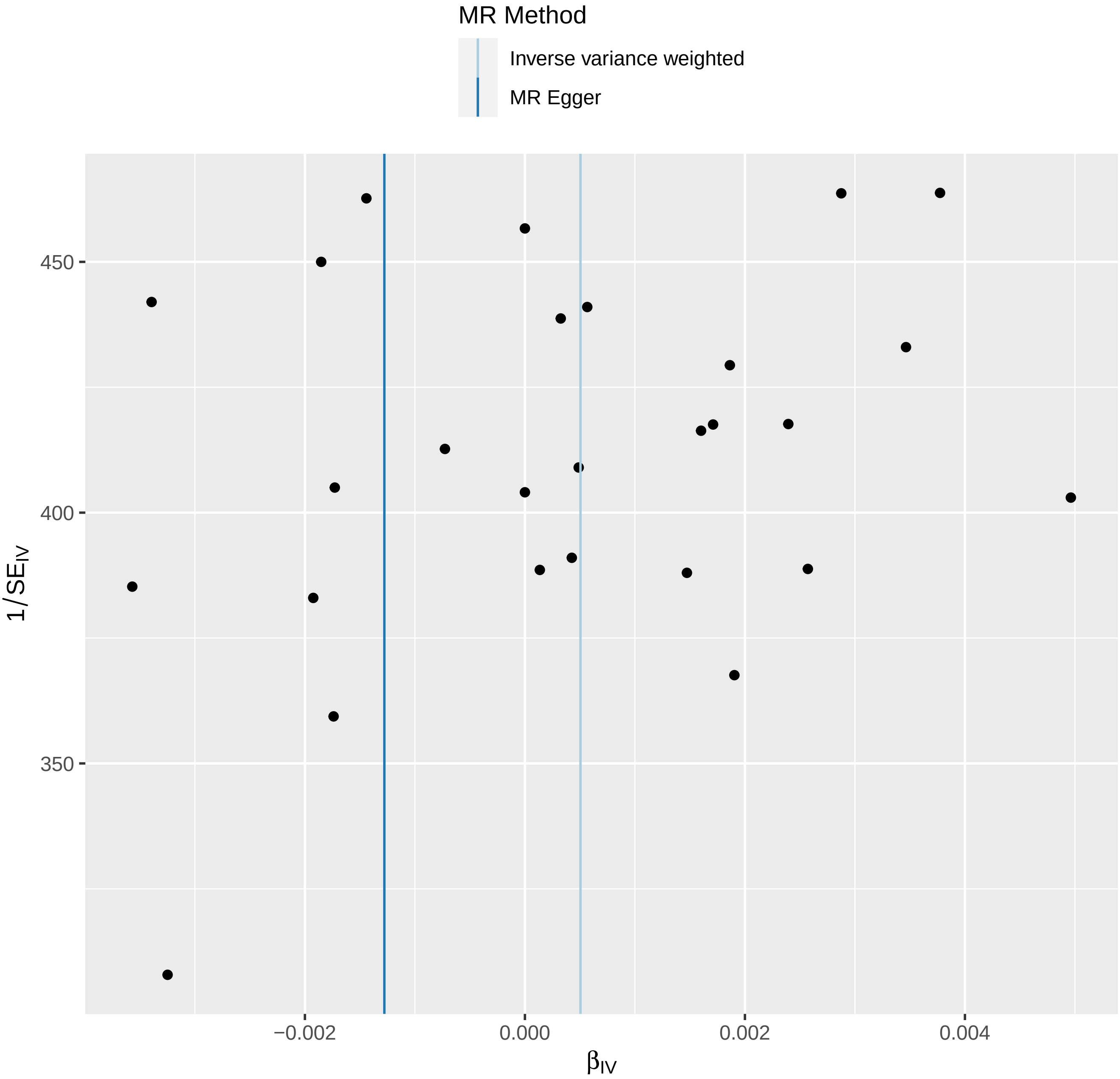

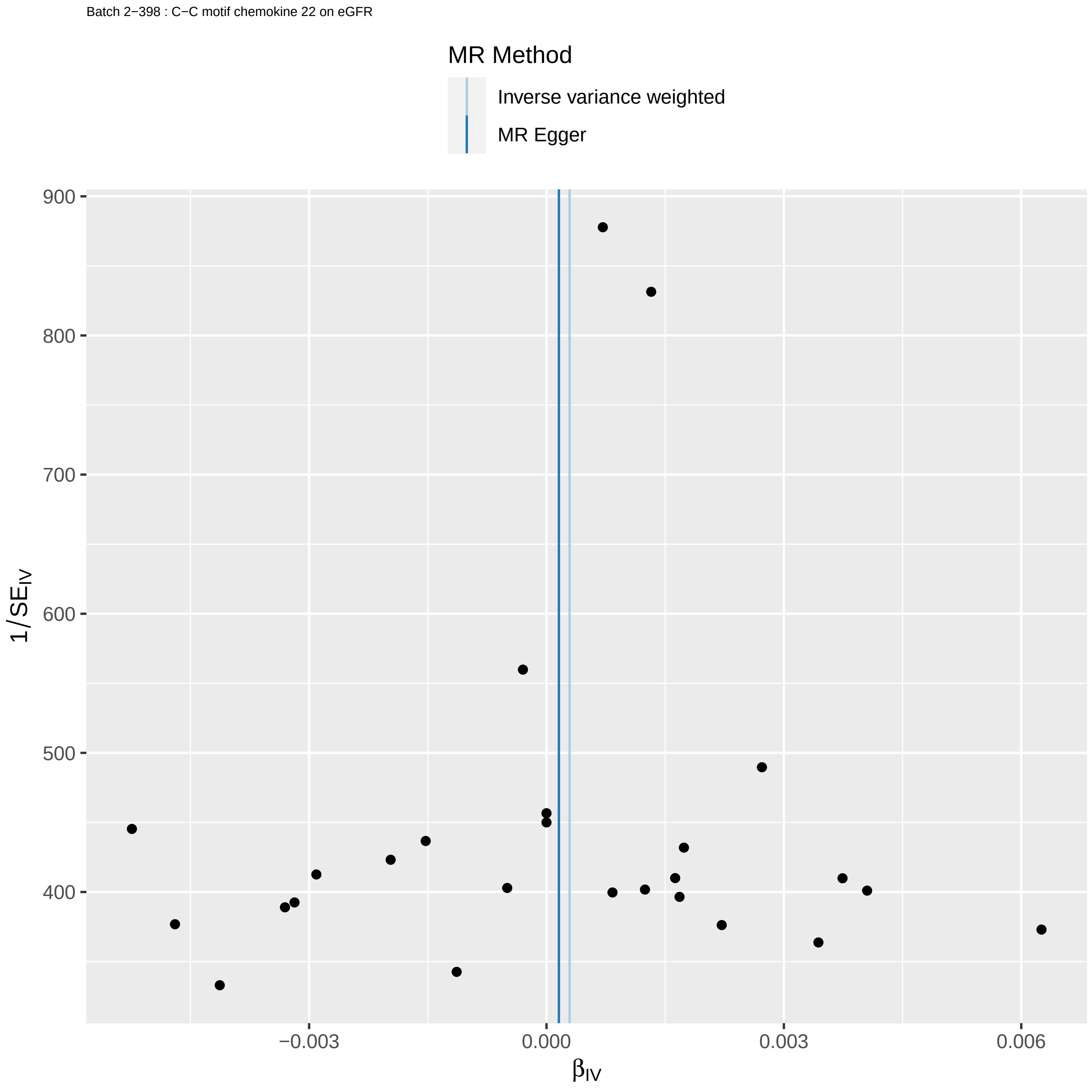
Batch 2−398 : C−C motif chemokine 22 on eGFR
MR Method
Inverse variance weighted
MR Egger
900
800
700
600
500
400
1/SE_IV
−0.003
0.000
0.003
0.006
β_IV

Batch 2−399 : C−C motif chemokine 23 on eGFR

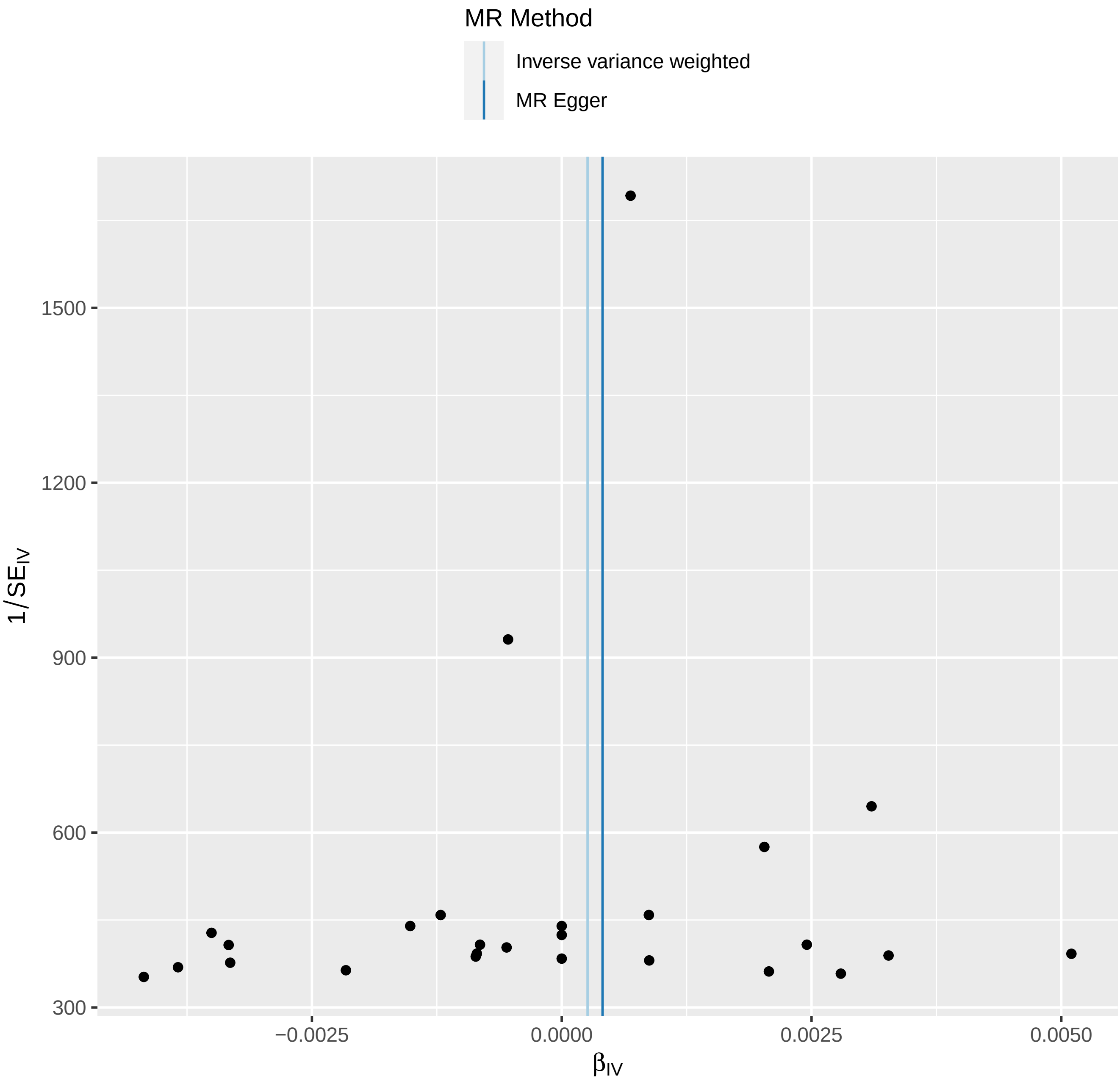

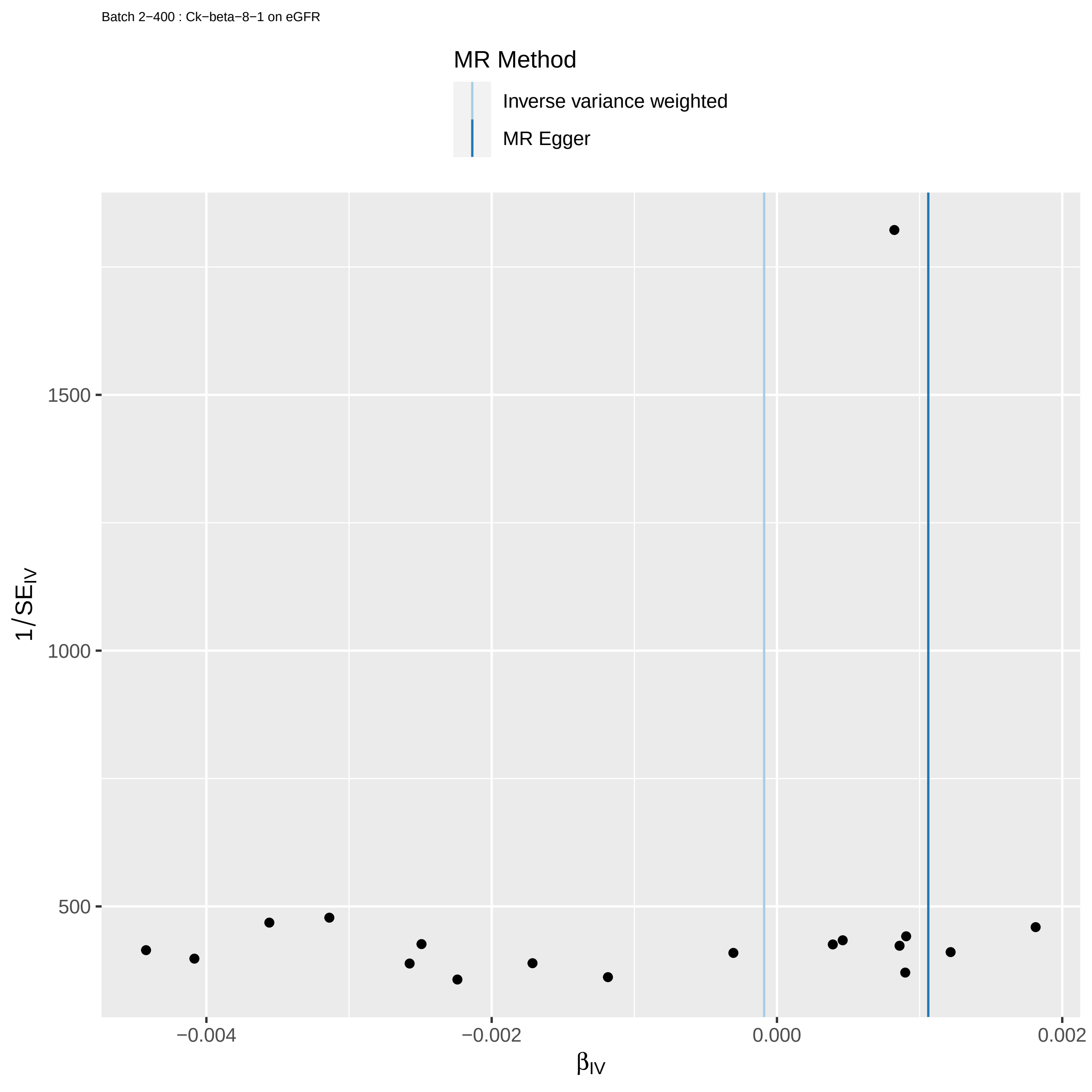

Batch 2−400 : Ck−beta−8−1 on eGFR
MR Method
Inverse variance weighted
MR Egger
1/SE$_{IV}$
1500
1000
500
−0.004
−0.002
0.000
0.002
β$_{IV}$

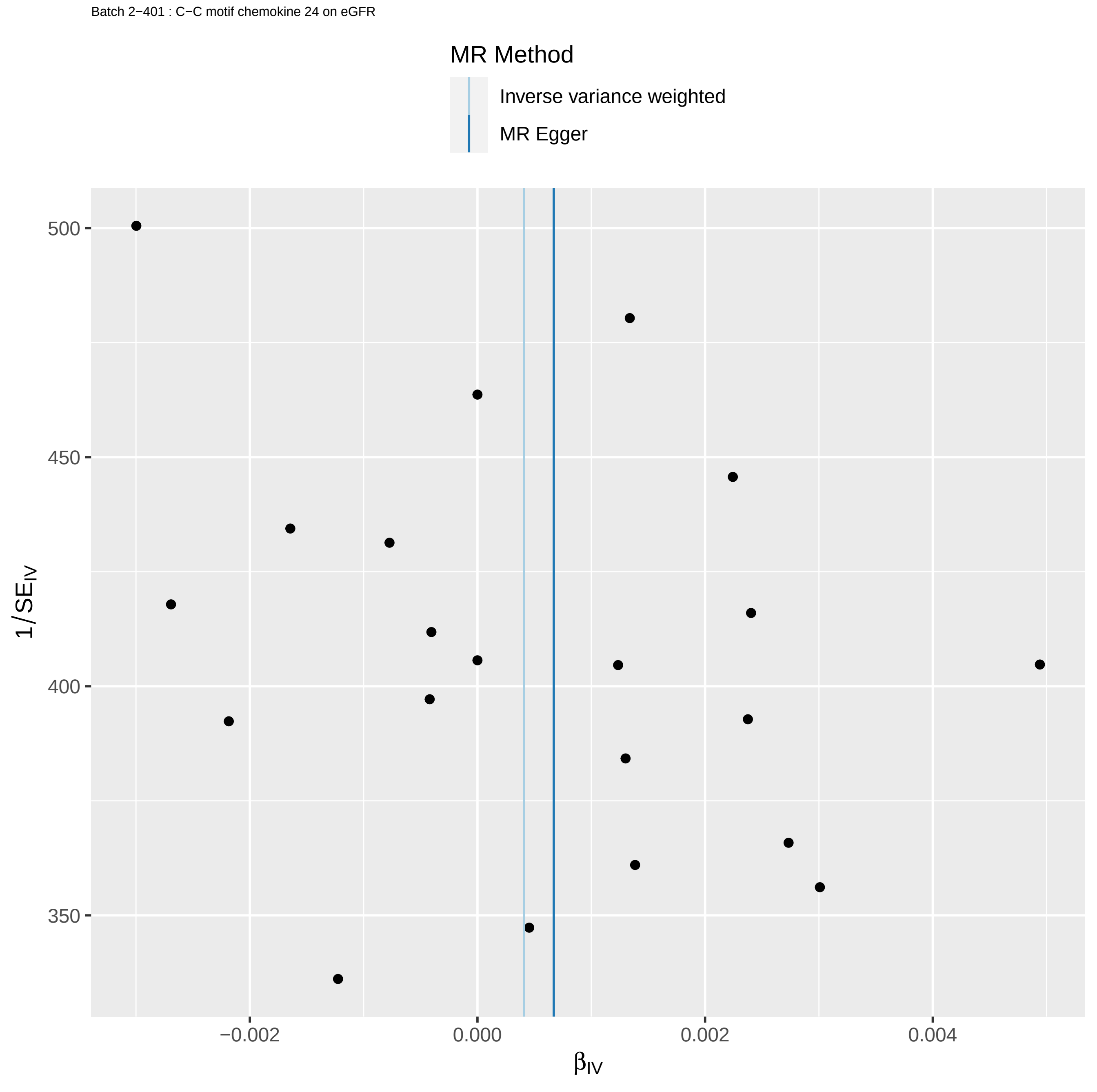
Batch 2−401 : C−C motif chemokine 24 on eGFR
MR Method
Inverse variance weighted
MR Egger
500
450
400
350
1/SE_IV
−0.002
0.000
0.002
0.004
β_IV

Batch 2−402 : C−C motif chemokine 25 on eGFR

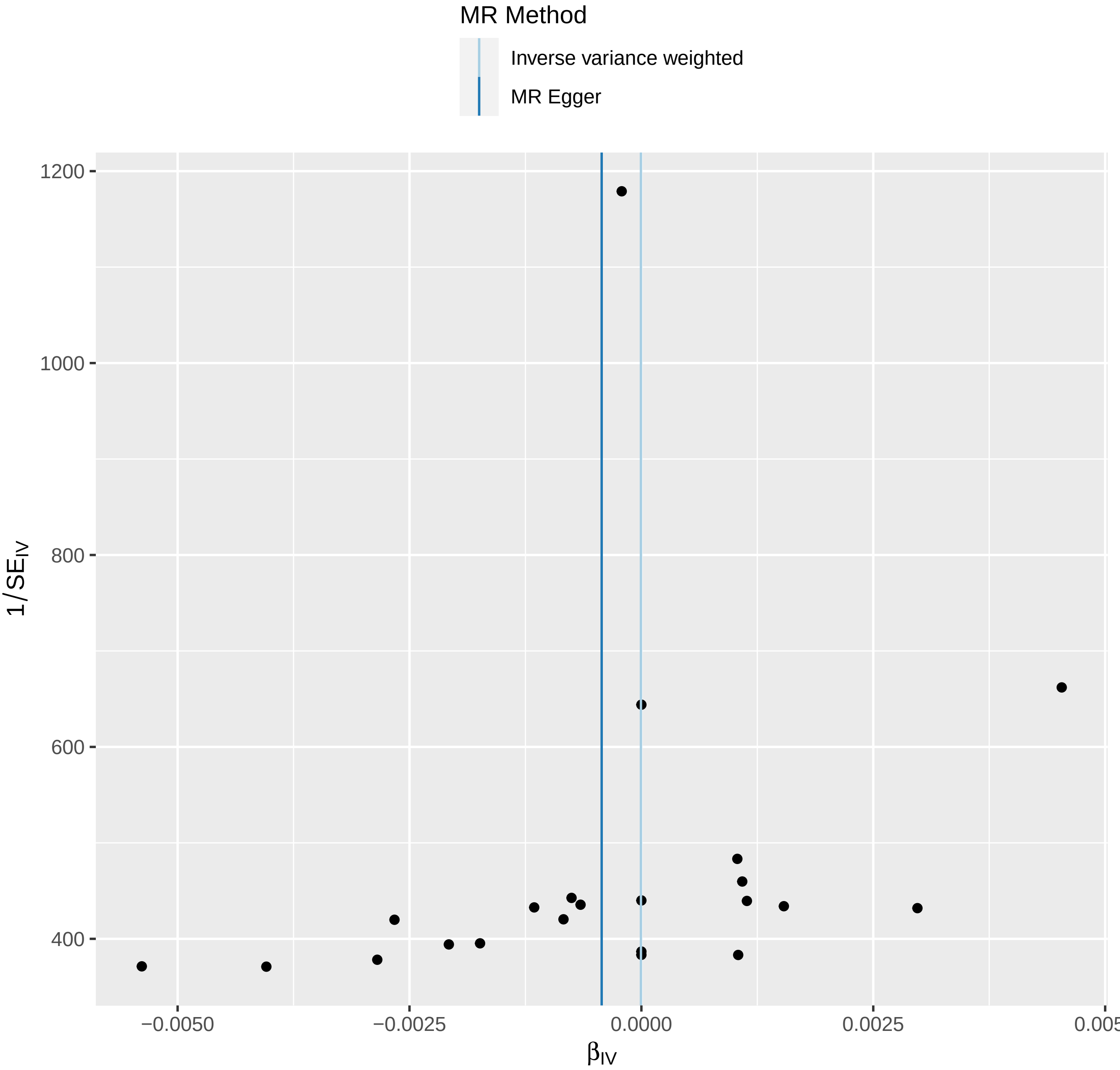

Batch 2−403 : C−C motif chemokine 25 on eGFR

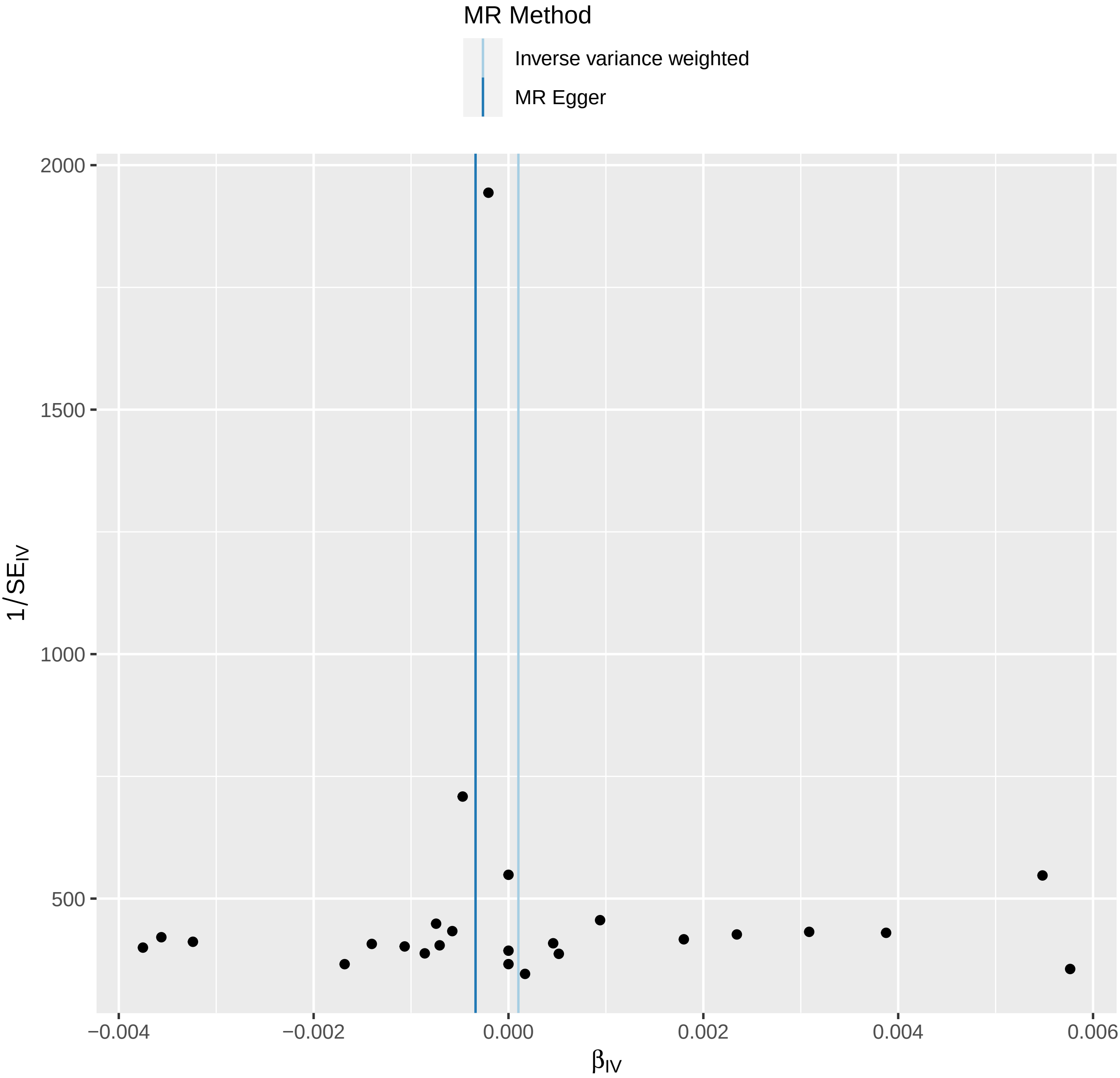

Batch 2−404 : C−C motif chemokine 26 on eGFR

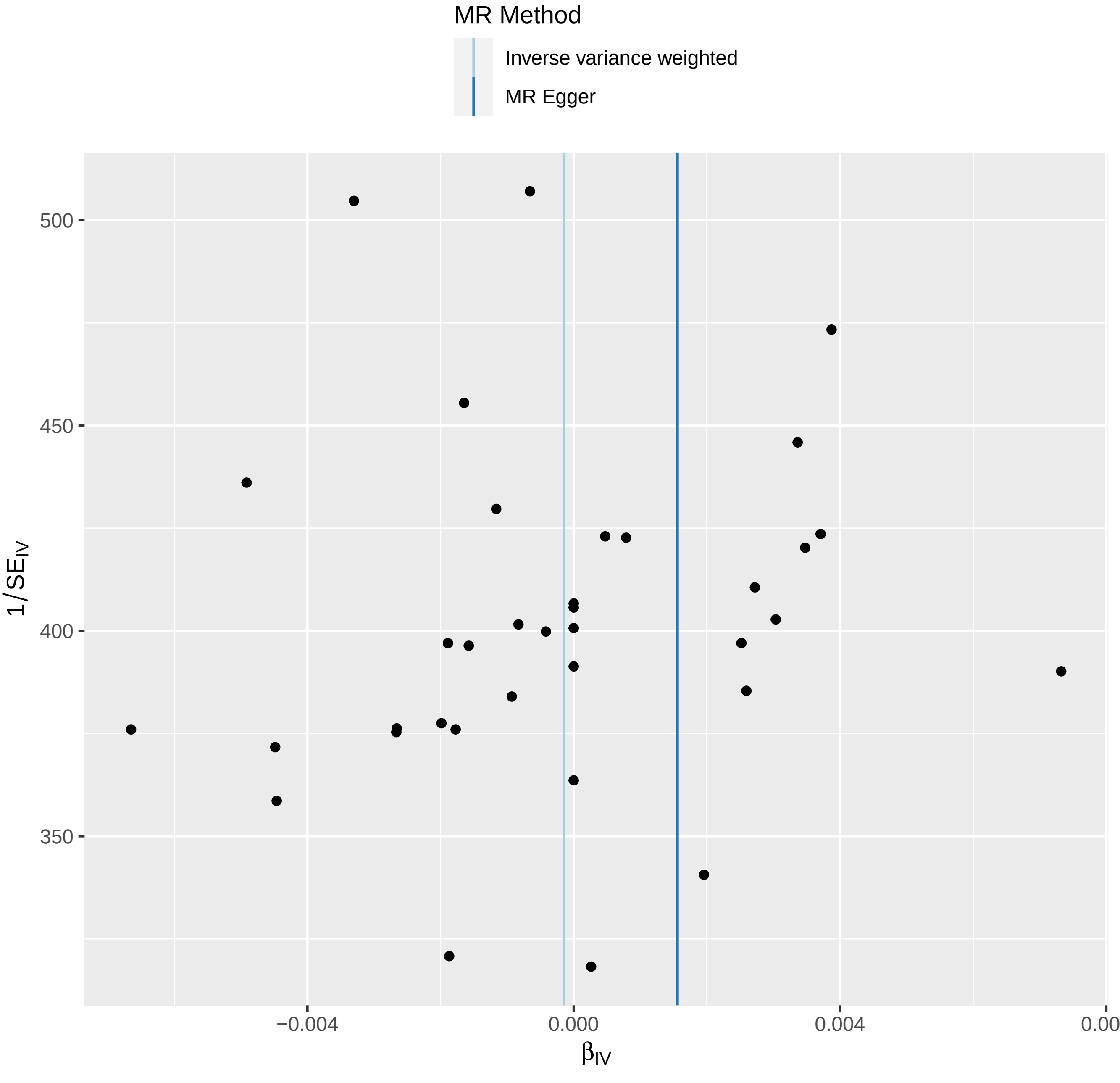

Batch 2−405 : C−C motif chemokine 27 on eGFR

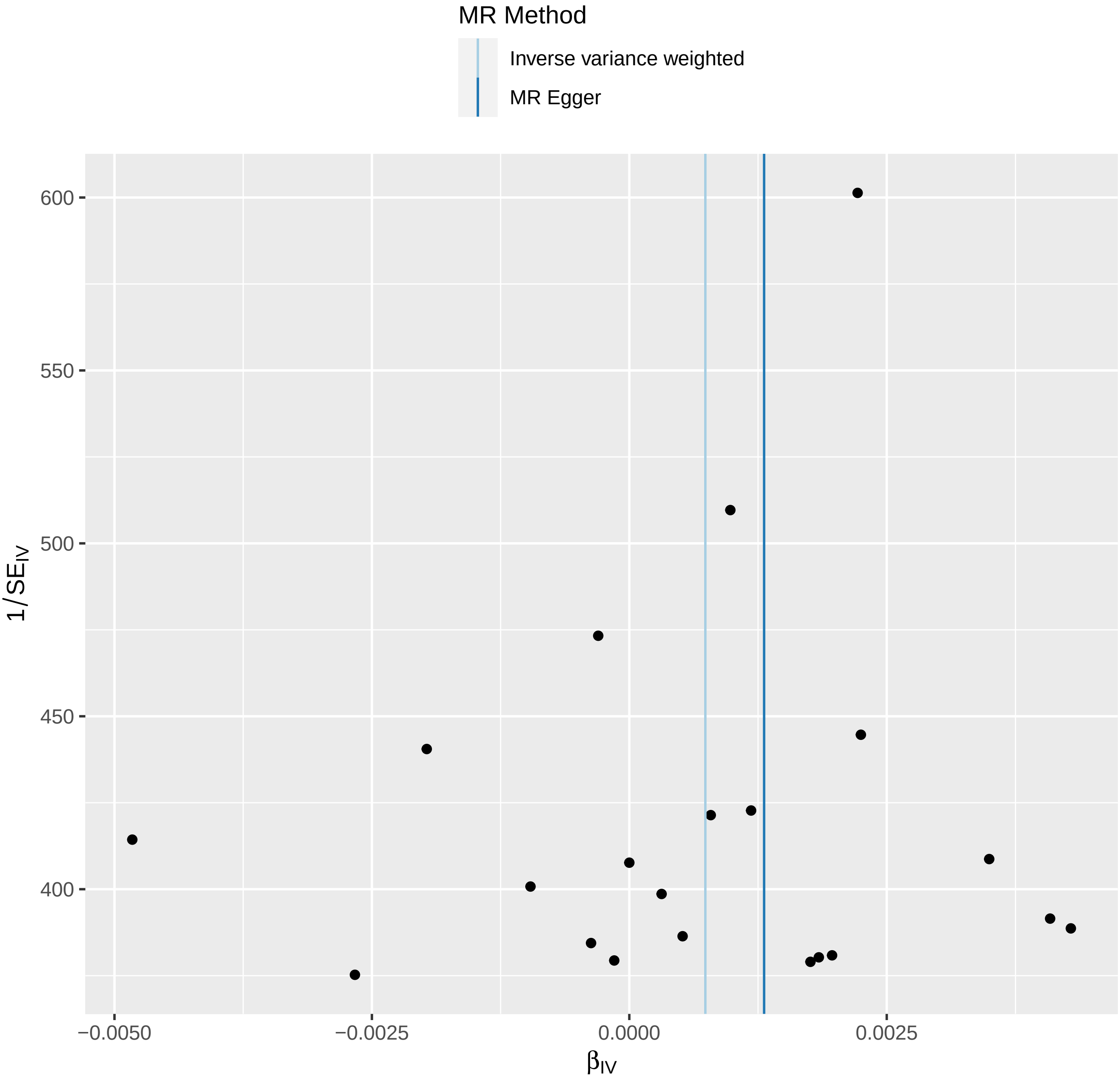

Batch 2−406 : C−C motif chemokine 28 on eGFR

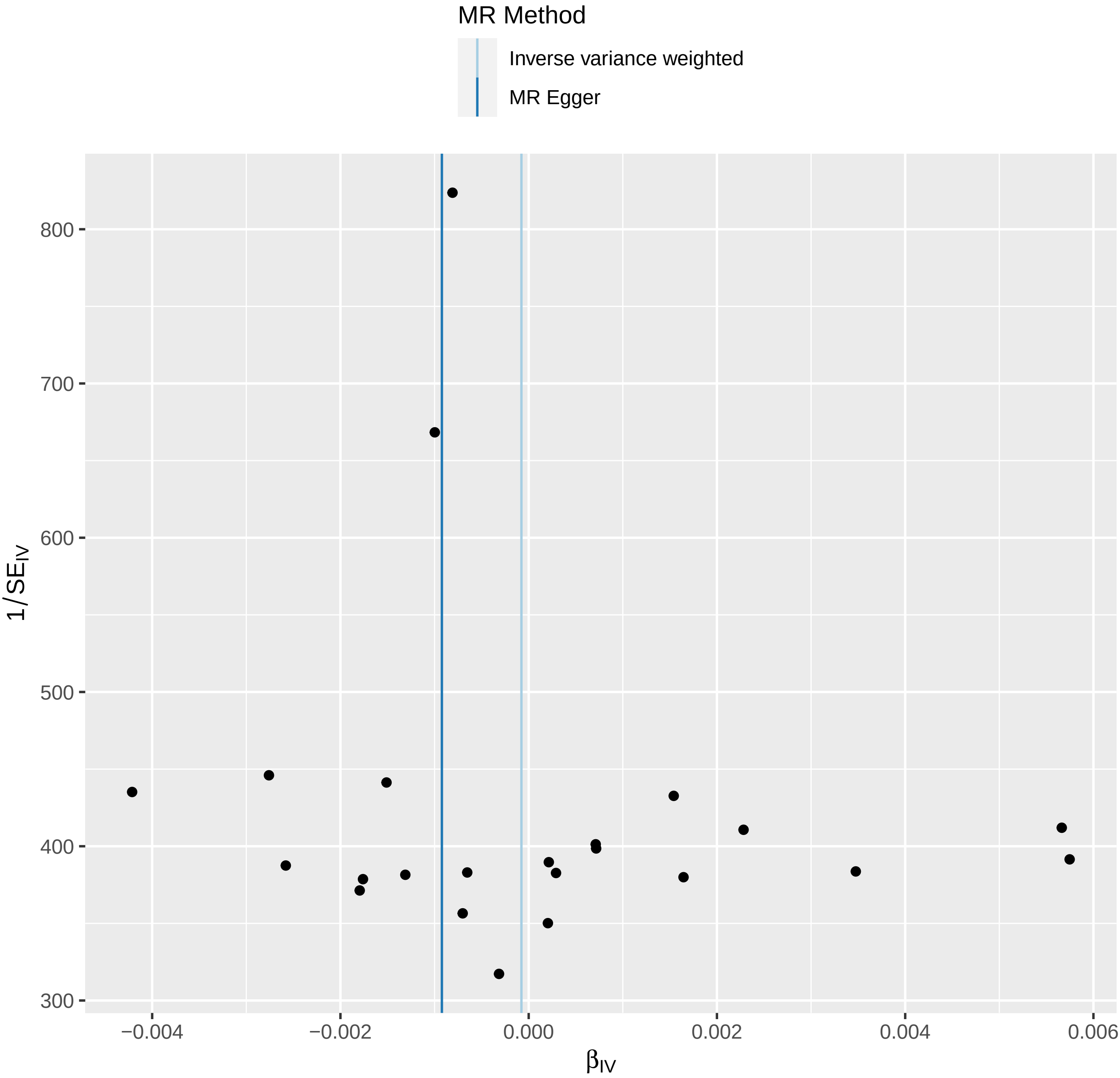

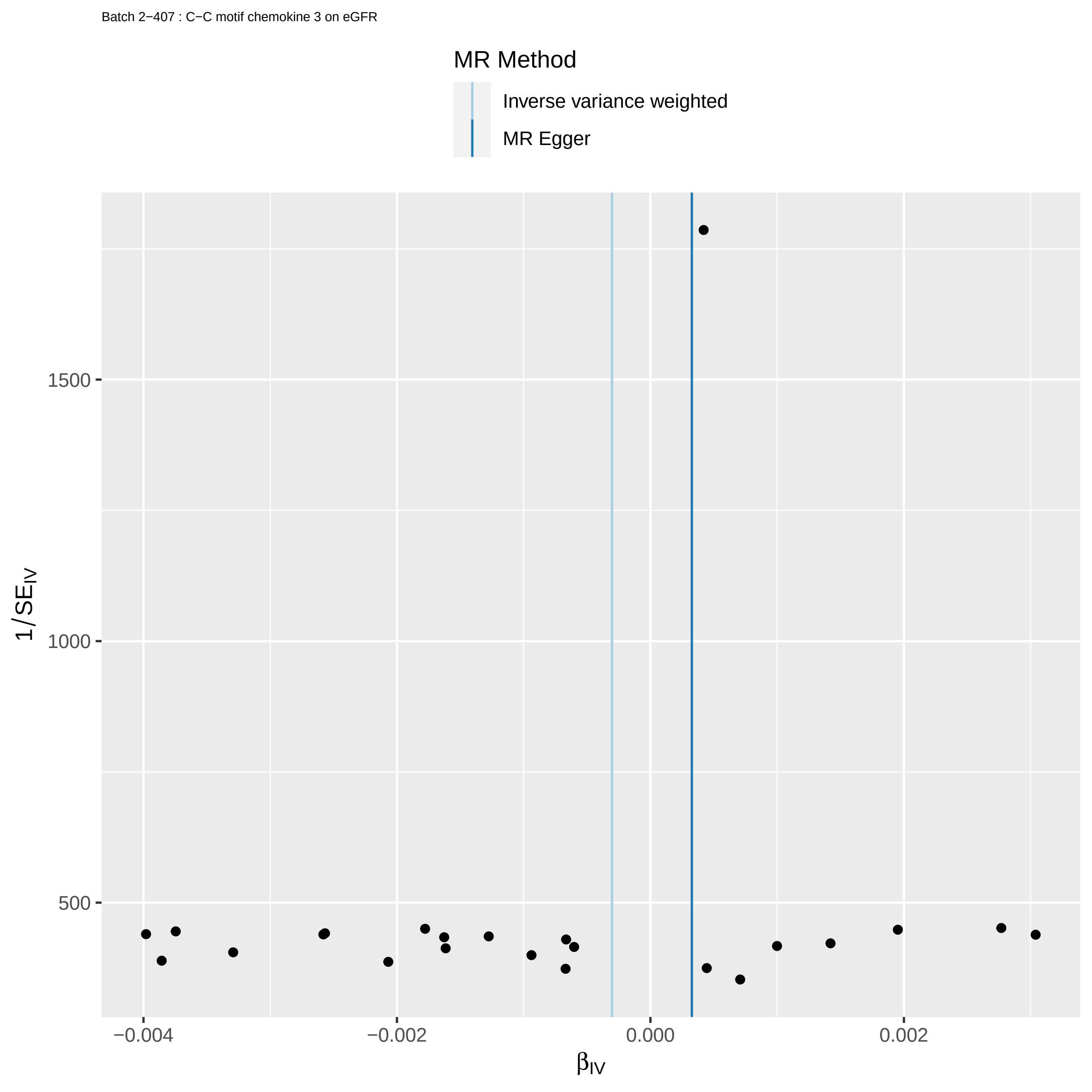
Batch 2−407 : C−C motif chemokine 3 on eGFR
MR Method
Inverse variance weighted
MR Egger
1500
1000
500
1/SE$_{IV}$
−0.004
−0.002
0.000
0.002
β$_{IV}$

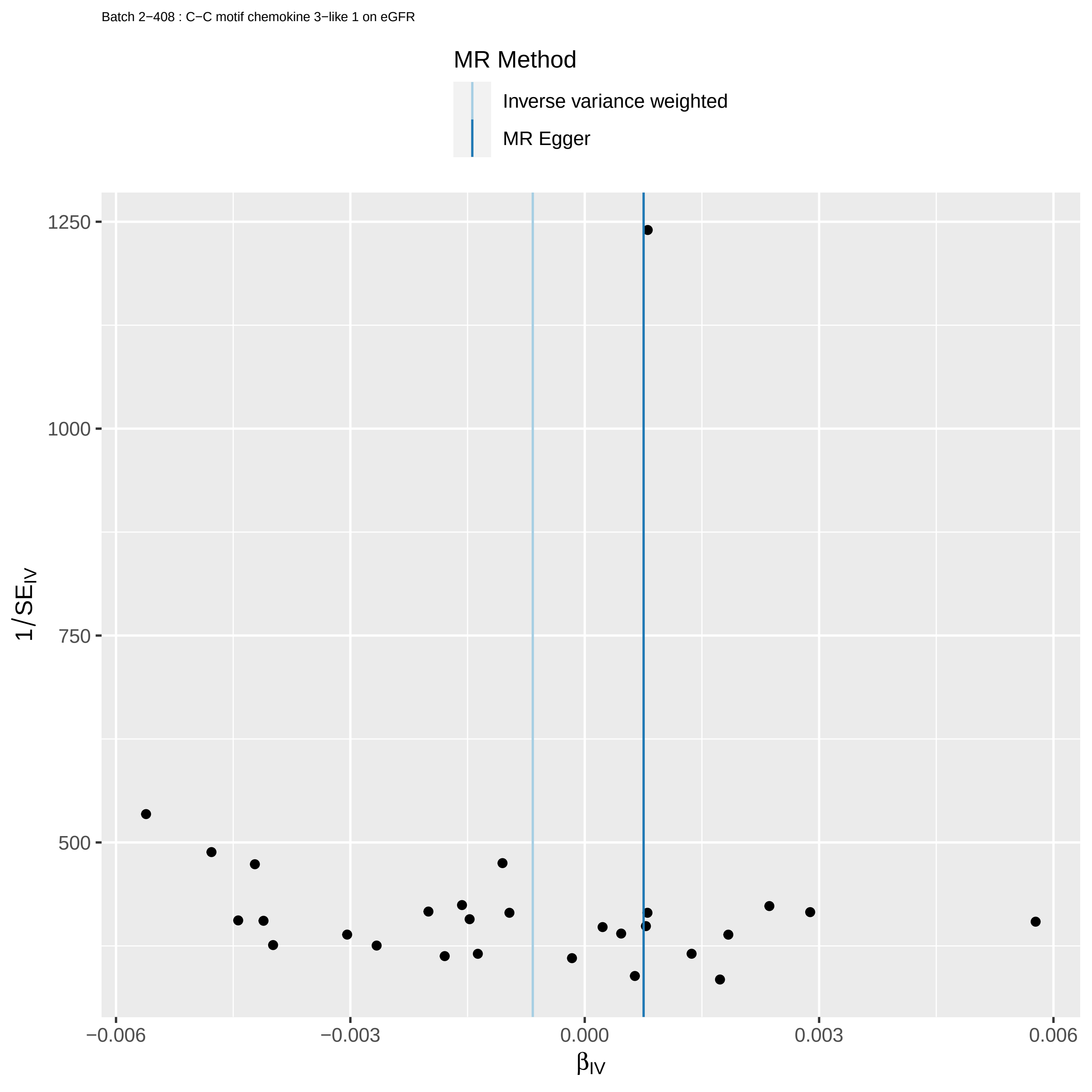
Batch 2−408 : C−C motif chemokine 3−like 1 on eGFR
MR Method
Inverse variance weighted
MR Egger
1250
1000
750
500
1/SE$_{IV}$
−0.006
−0.003
0.000
0.003
0.006
β$_{IV}$

Batch 2−409 : C−C motif chemokine 5 on eGFR

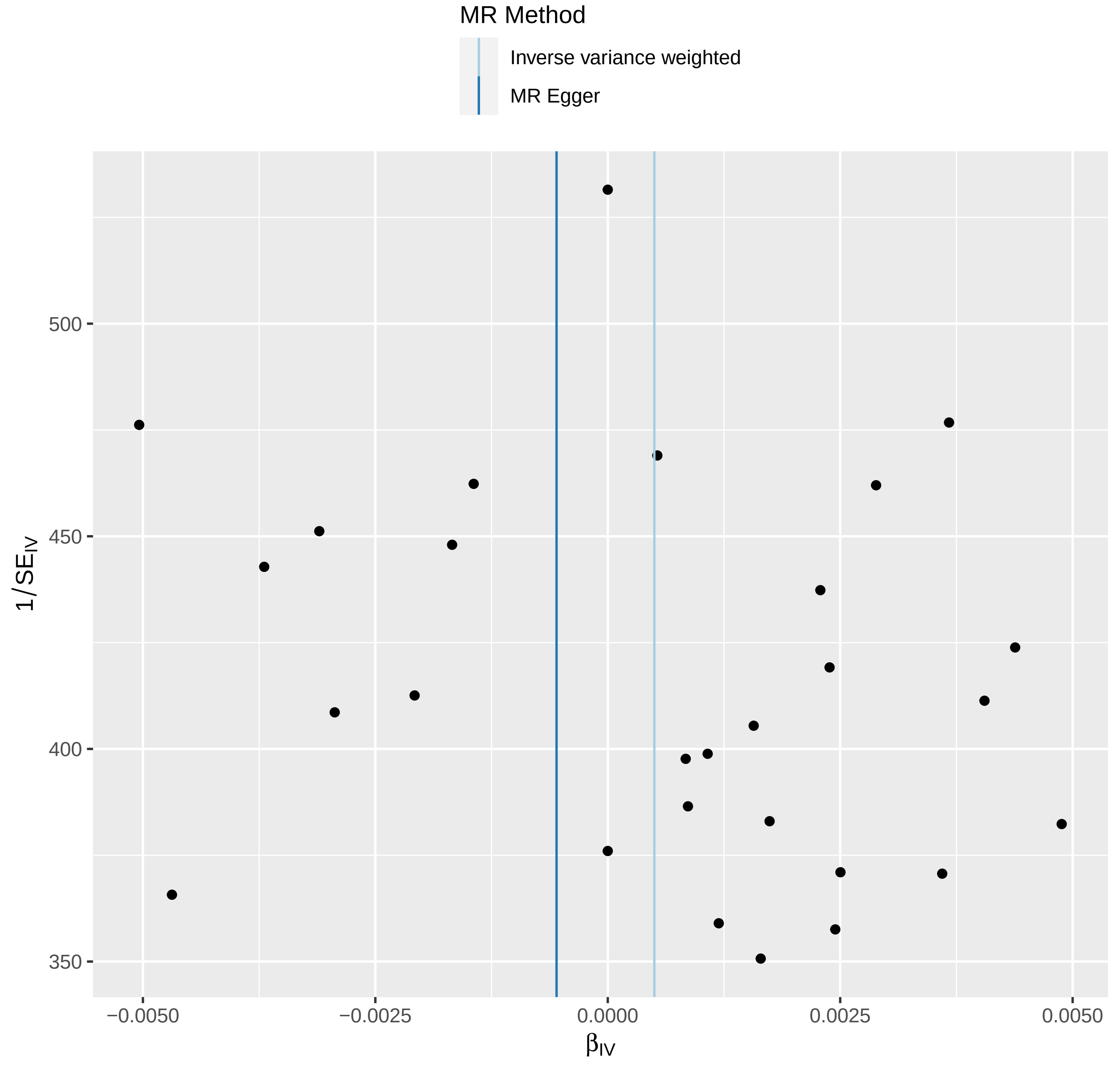

Batch 2−410 : C−C motif chemokine 7 on eGFR

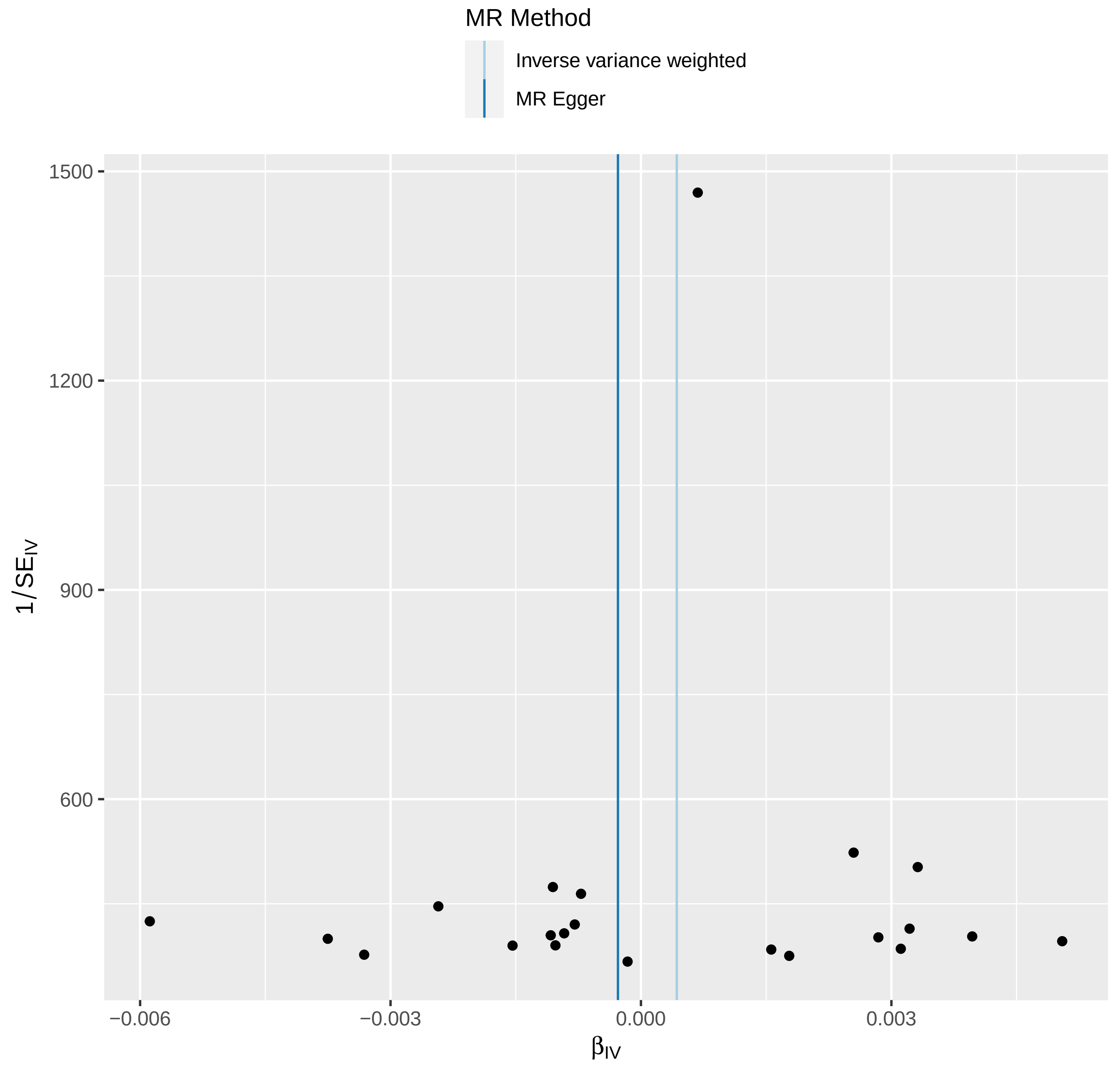

Batch 2−411 : C−C motif chemokine 8 on eGFR

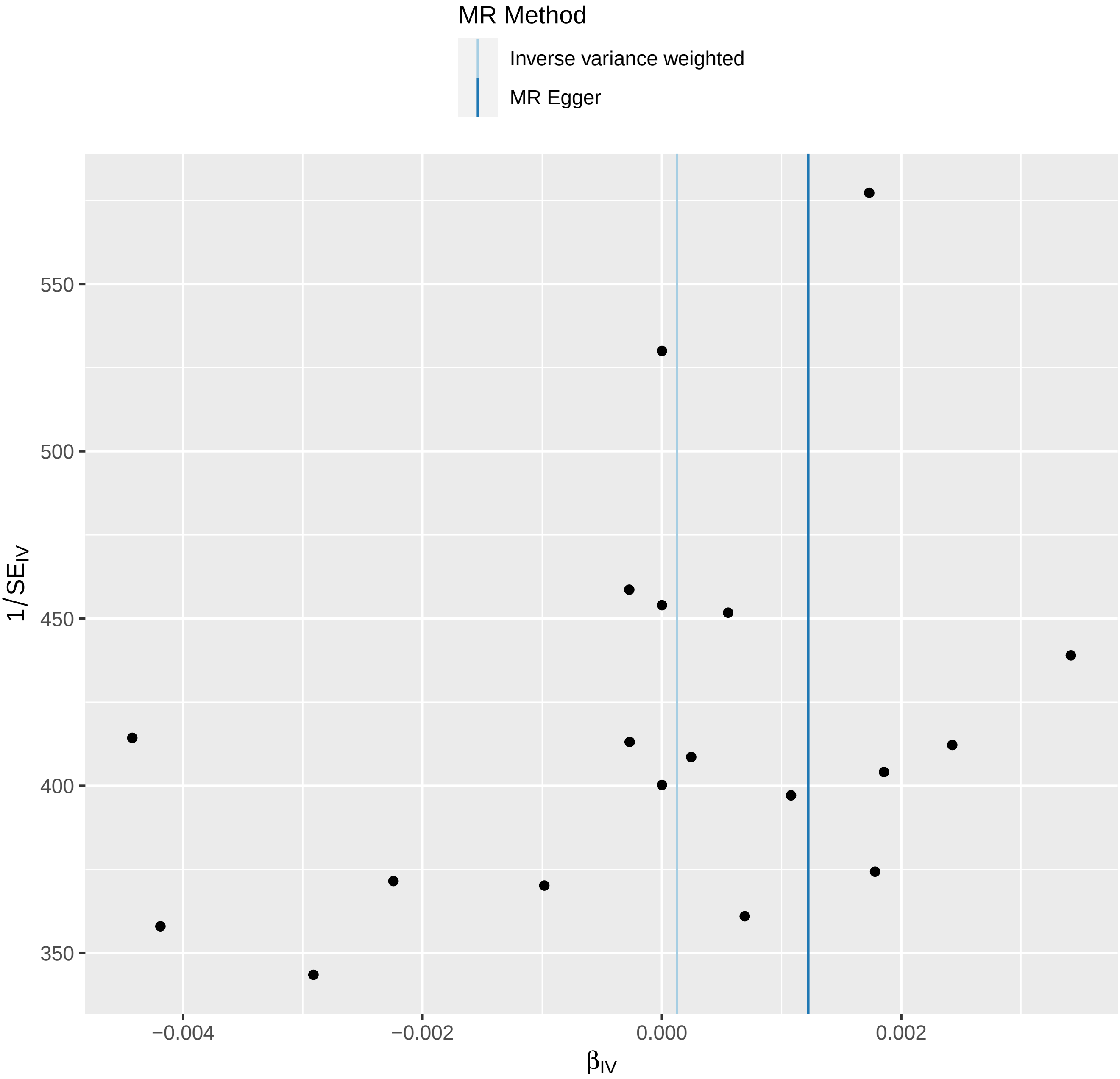

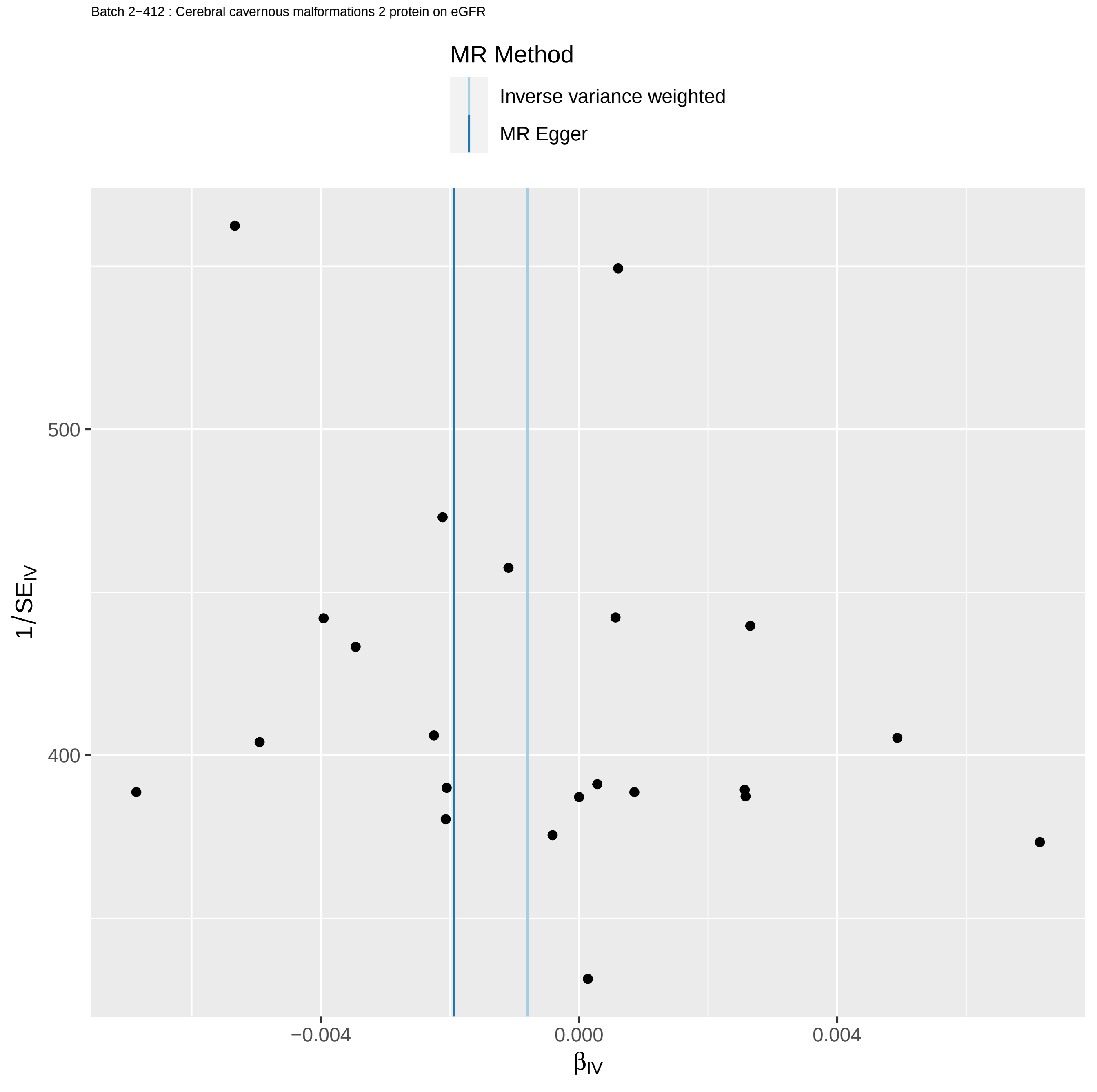
Batch 2−412 : Cerebral cavernous malformations 2 protein on eGFR
MR Method
Inverse variance weighted
MR Egger
500
400
1/SE_IV
−0.004
0.000
0.004
β_IV

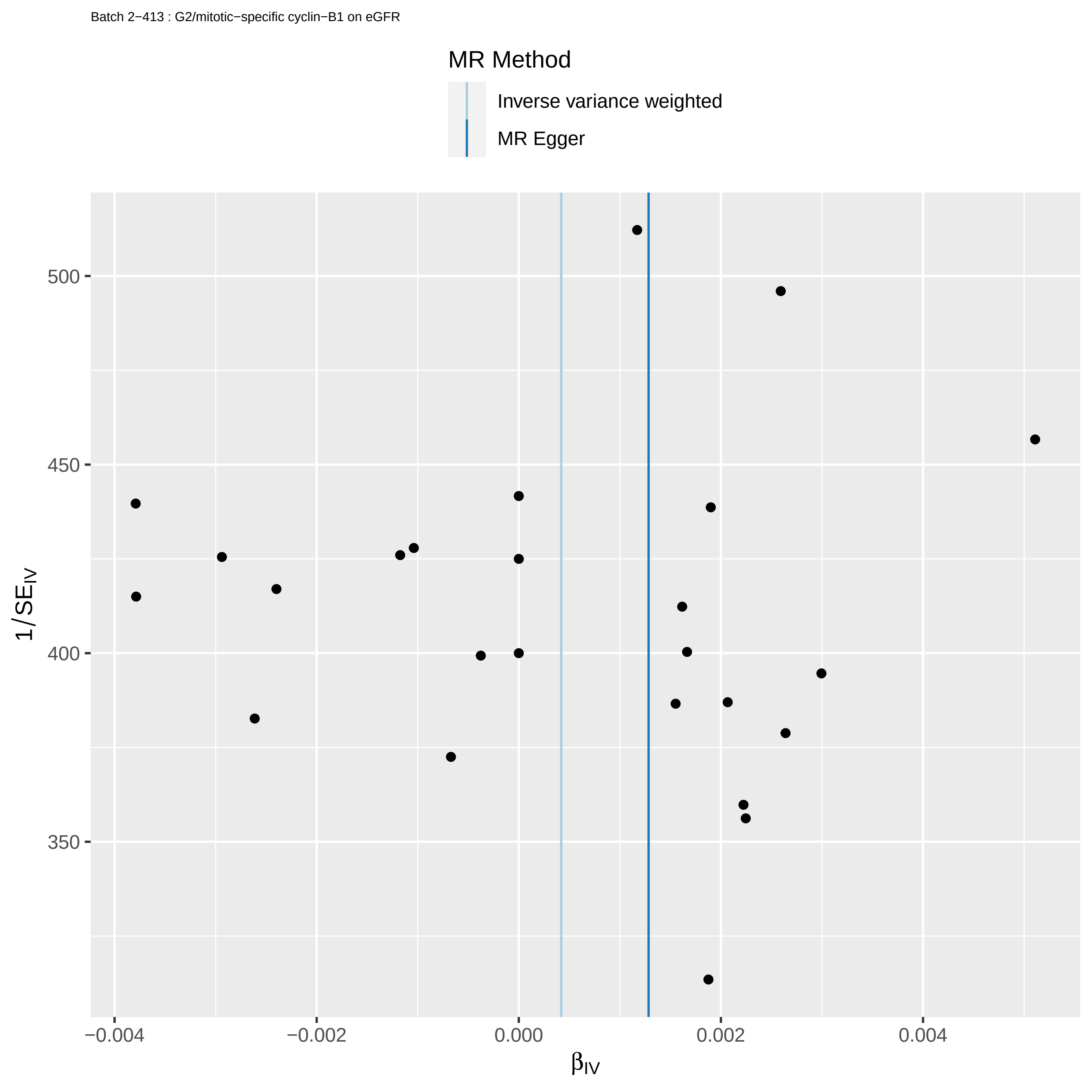

Batch 2−413 : G2/mitotic−specific cyclin−B1 on eGFR
MR Method
Inverse variance weighted
MR Egger
500
450
400
350
1/SE$_{IV}$
−0.004
−0.002
0.000
0.002
0.004
β$_{IV}$

Batch 2−414 : E3 ubiquitin−protein ligase CCNB1IP1 on eGFR

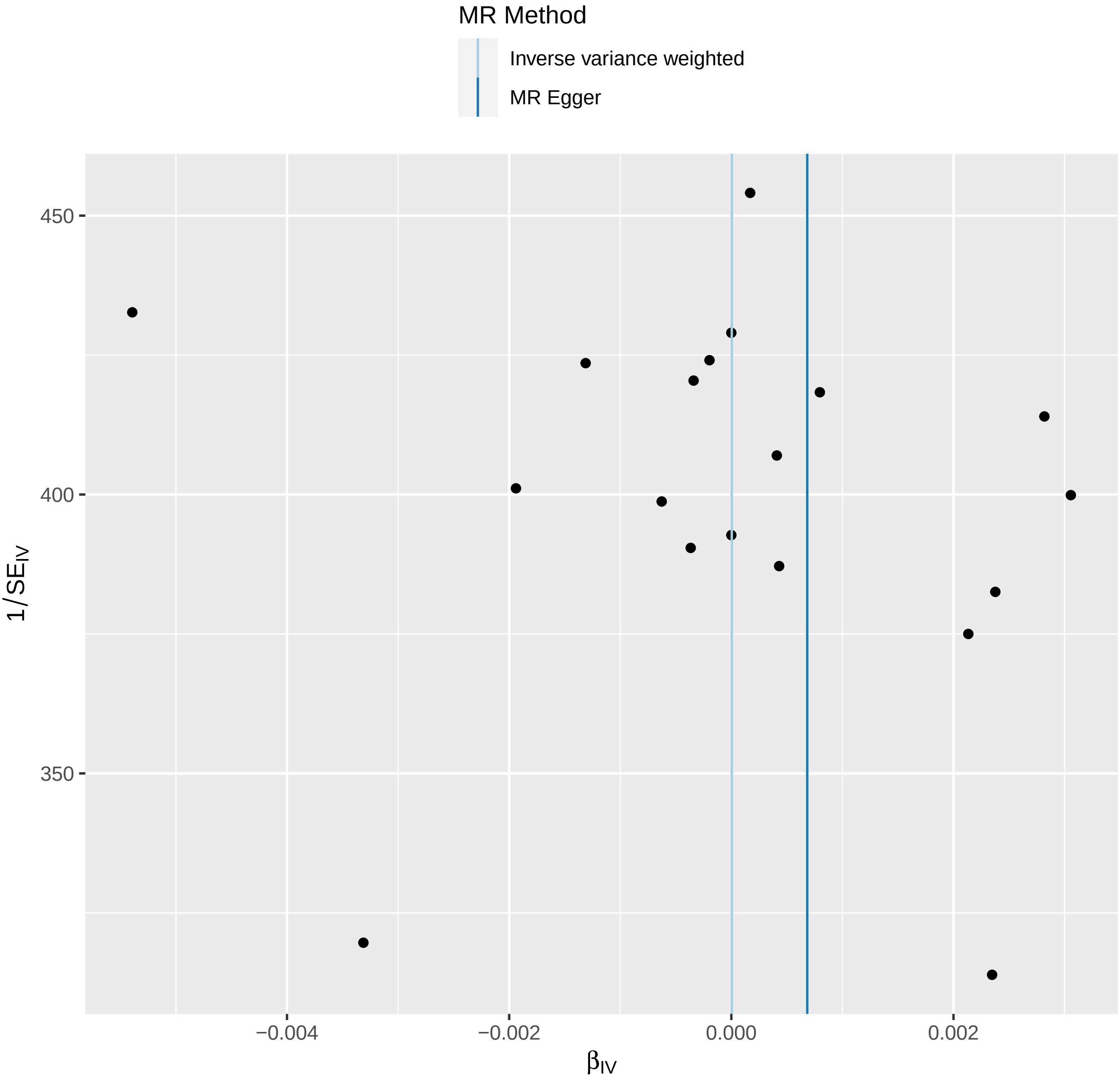

Batch 2−415 : Cyclin−C on eGFR

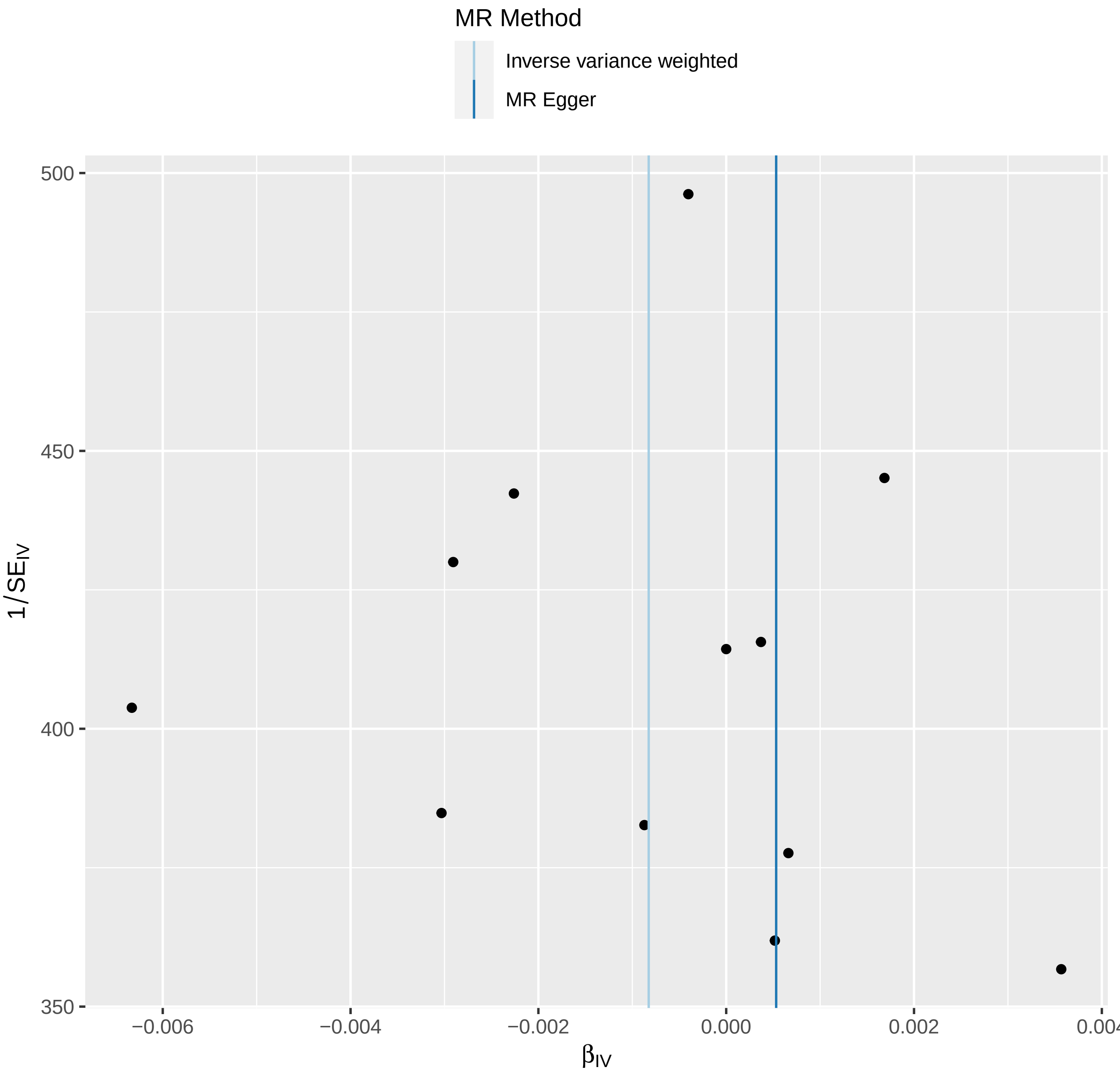

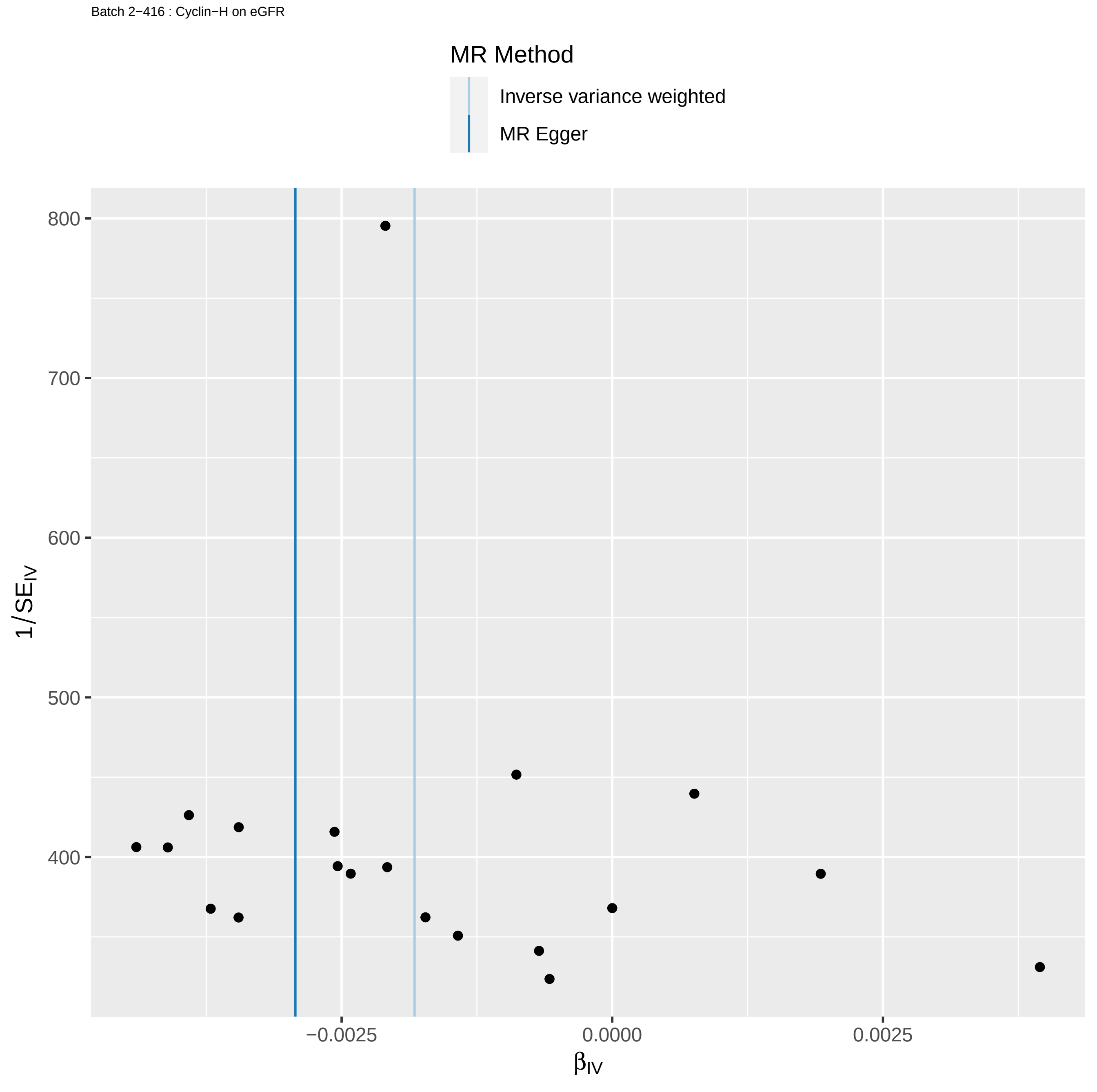

Batch 2−416 : Cyclin−H on eGFR
MR Method
Inverse variance weighted
MR Egger
800
700
600
500
400
1/SE$_{IV}$
−0.0025
0.0000
0.0025
β$_{IV}$

Batch 2−417 : Cell cycle progression protein 1 on eGFR

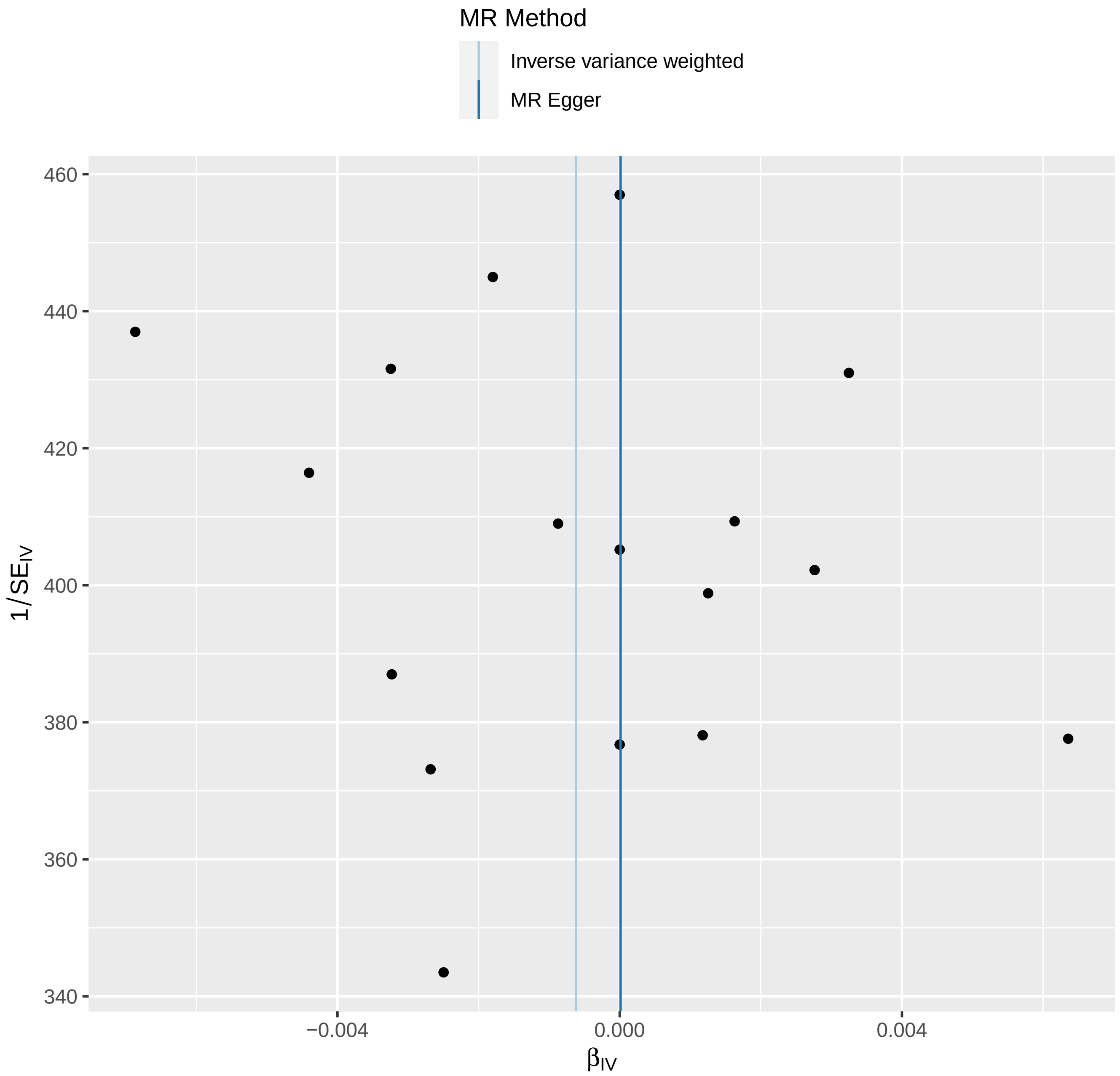

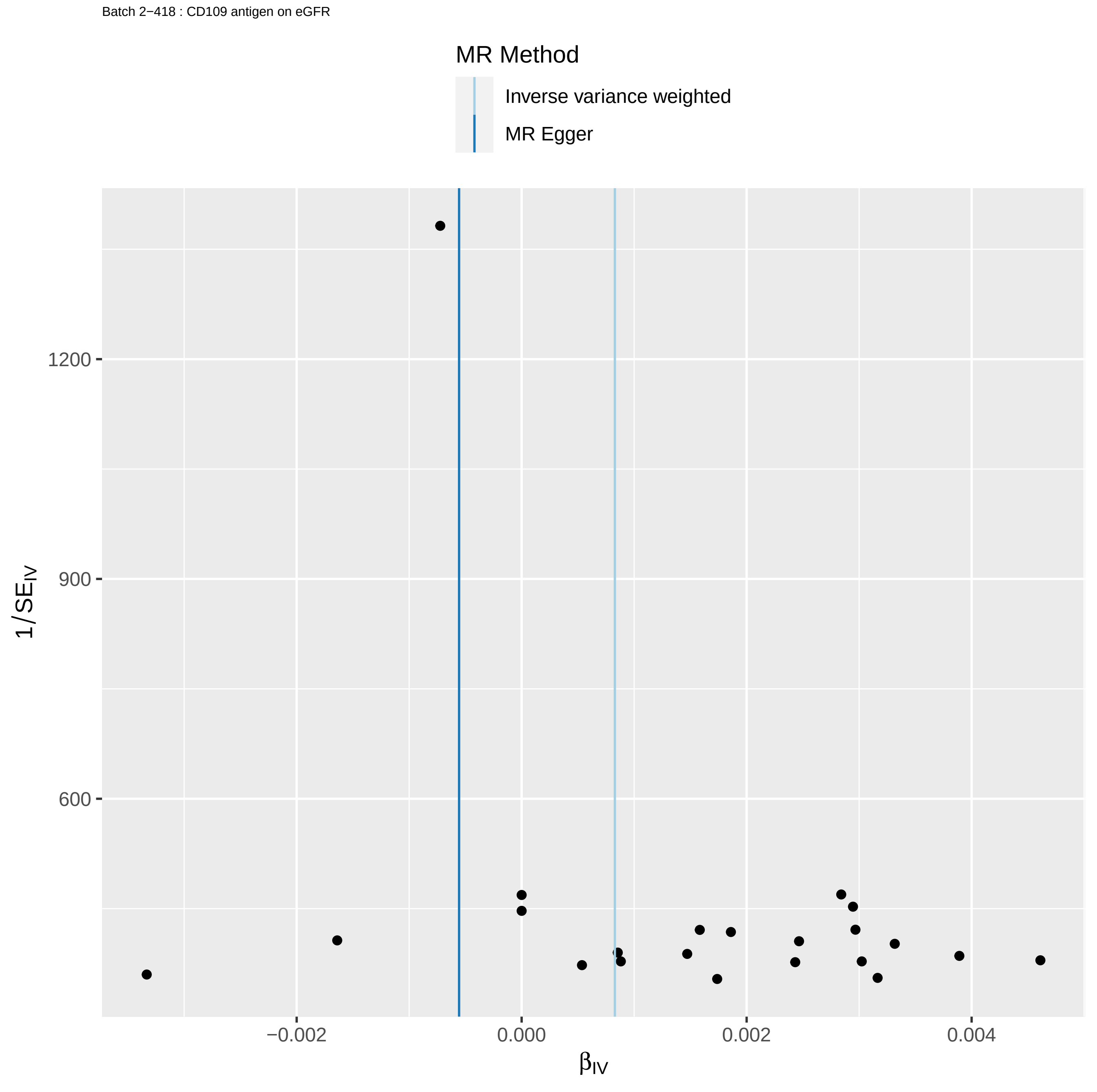

Batch 2−418 : CD109 antigen on eGFR
MR Method
Inverse variance weighted
MR Egger
1200
900
600
1/SE$_{IV}$
−0.002
0.000
0.002
0.004
β_{IV}

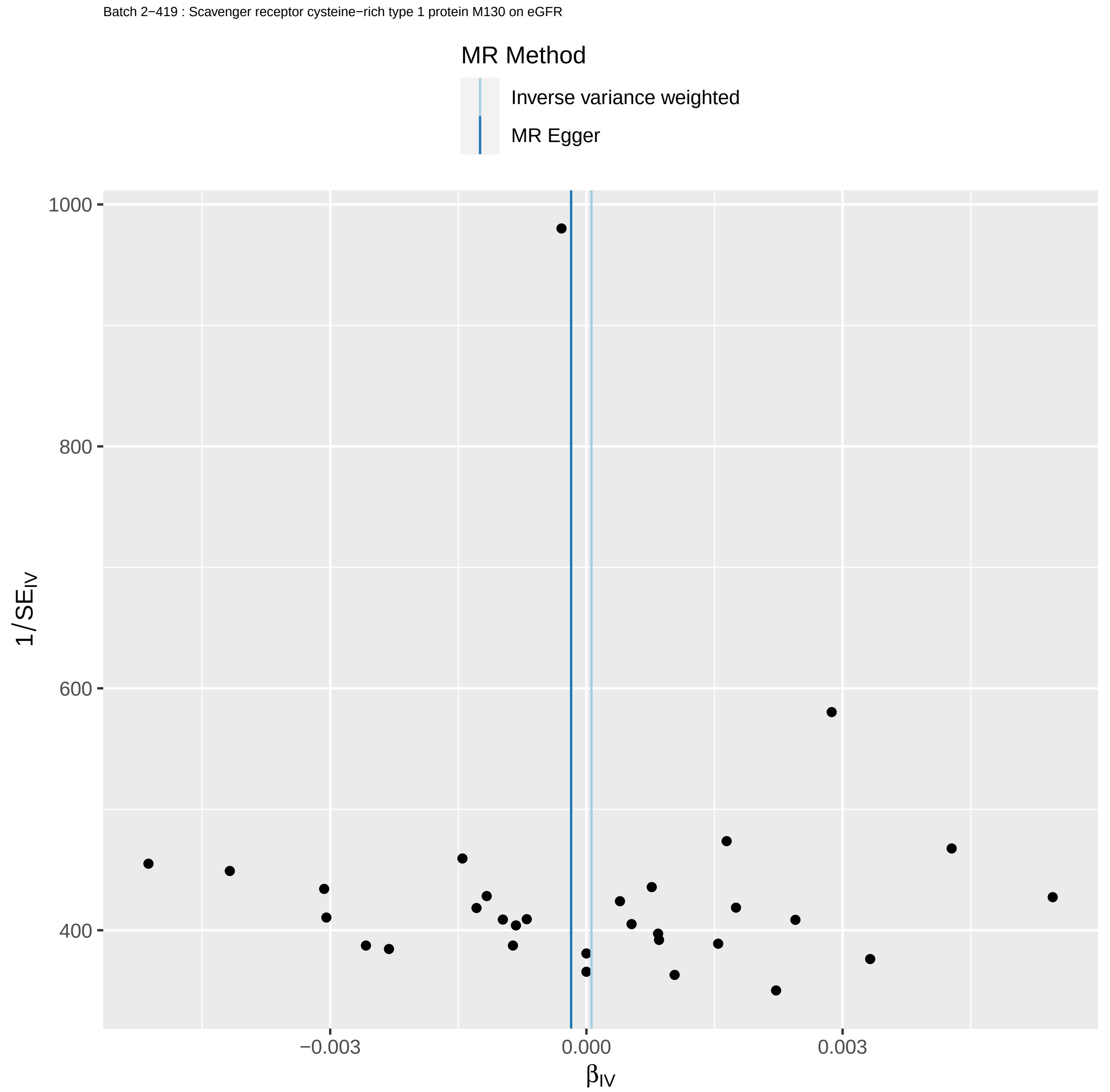
Batch 2−419 : Scavenger receptor cysteine−rich type 1 protein M130 on eGFR
MR Method
Inverse variance weighted
MR Egger
1000
800
600
400
1/SE_IV
−0.003
0.000
0.003
β_IV

Batch 2−420 : CD177 antigen on eGFR

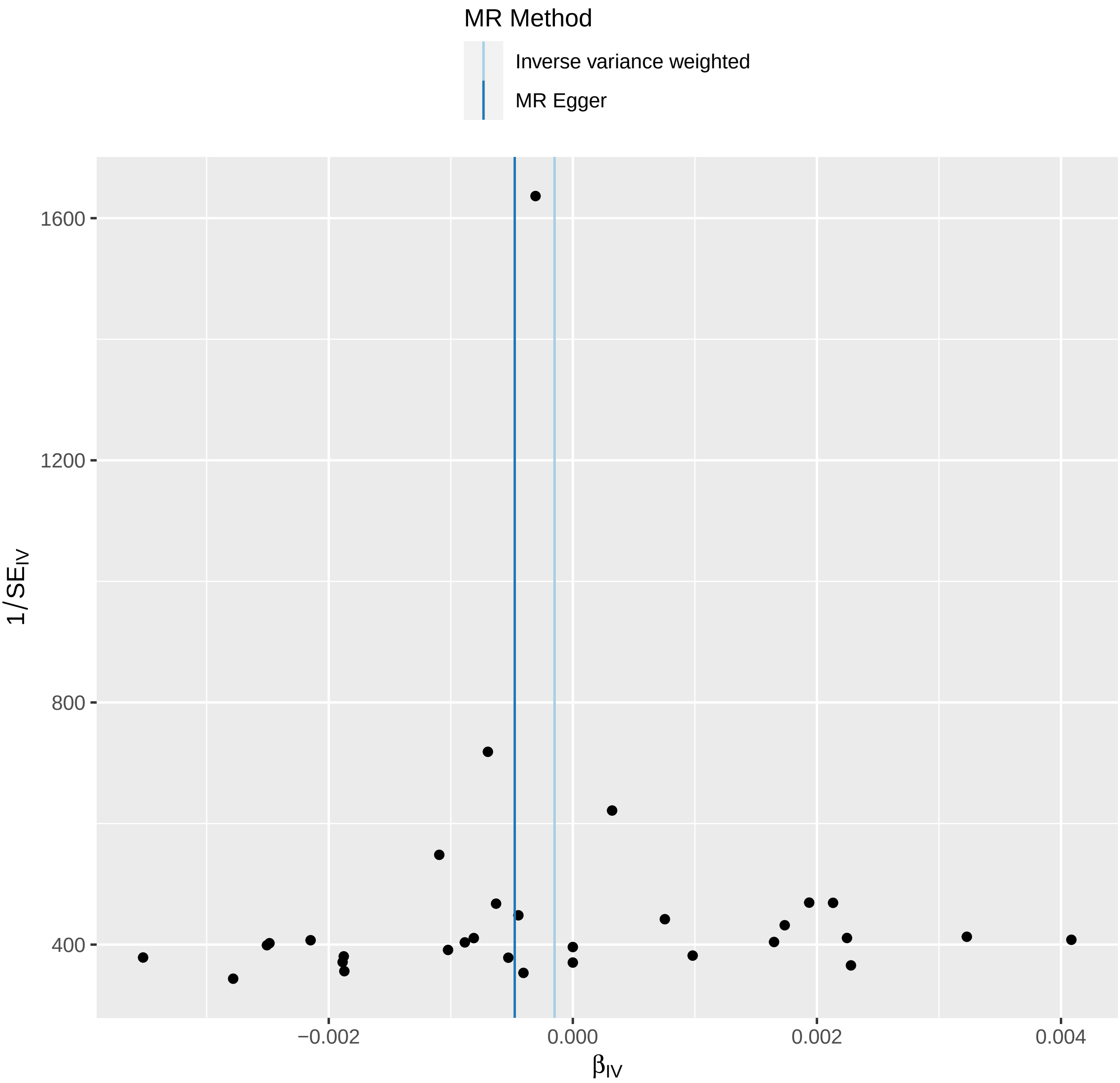

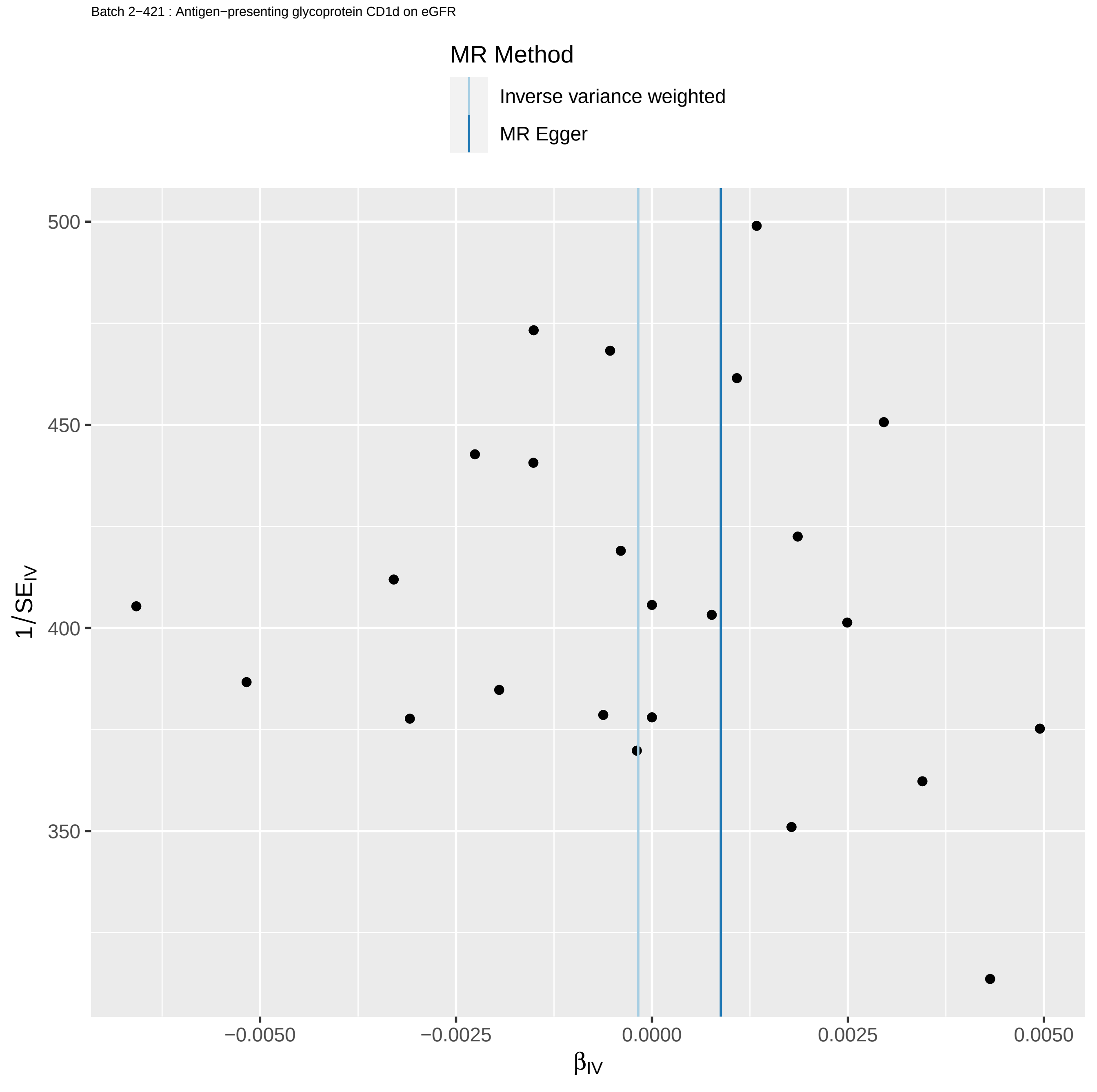

Batch 2−421 : Antigen−presenting glycoprotein CD1d on eGFR
MR Method
Inverse variance weighted
MR Egger
500
450
400
350
1/SE_IV
−0.0050
−0.0025
0.0000
0.0025
0.0050
β_IV

Batch 2−422 : OX−2 membrane glycoprotein on eGFR

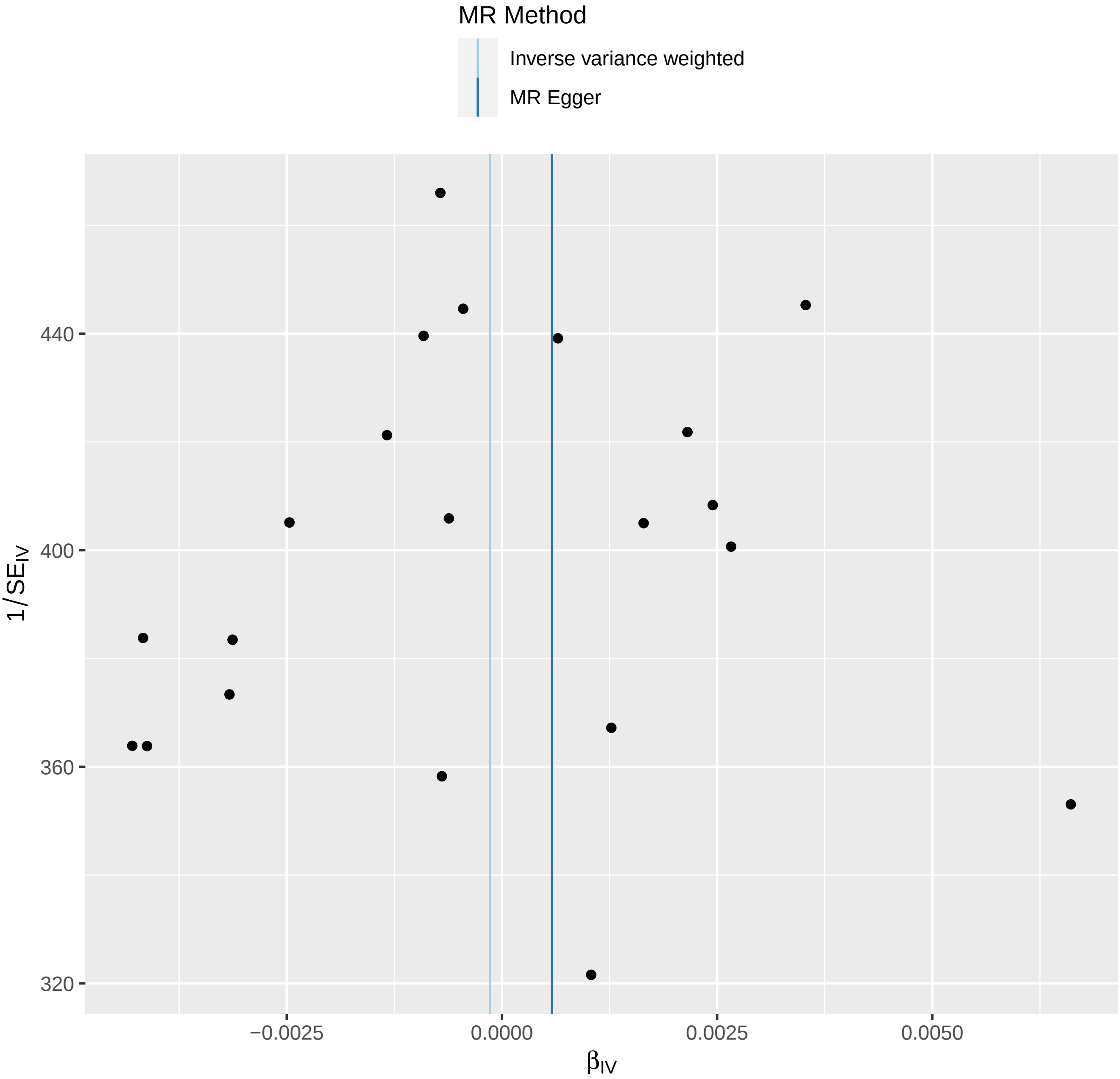

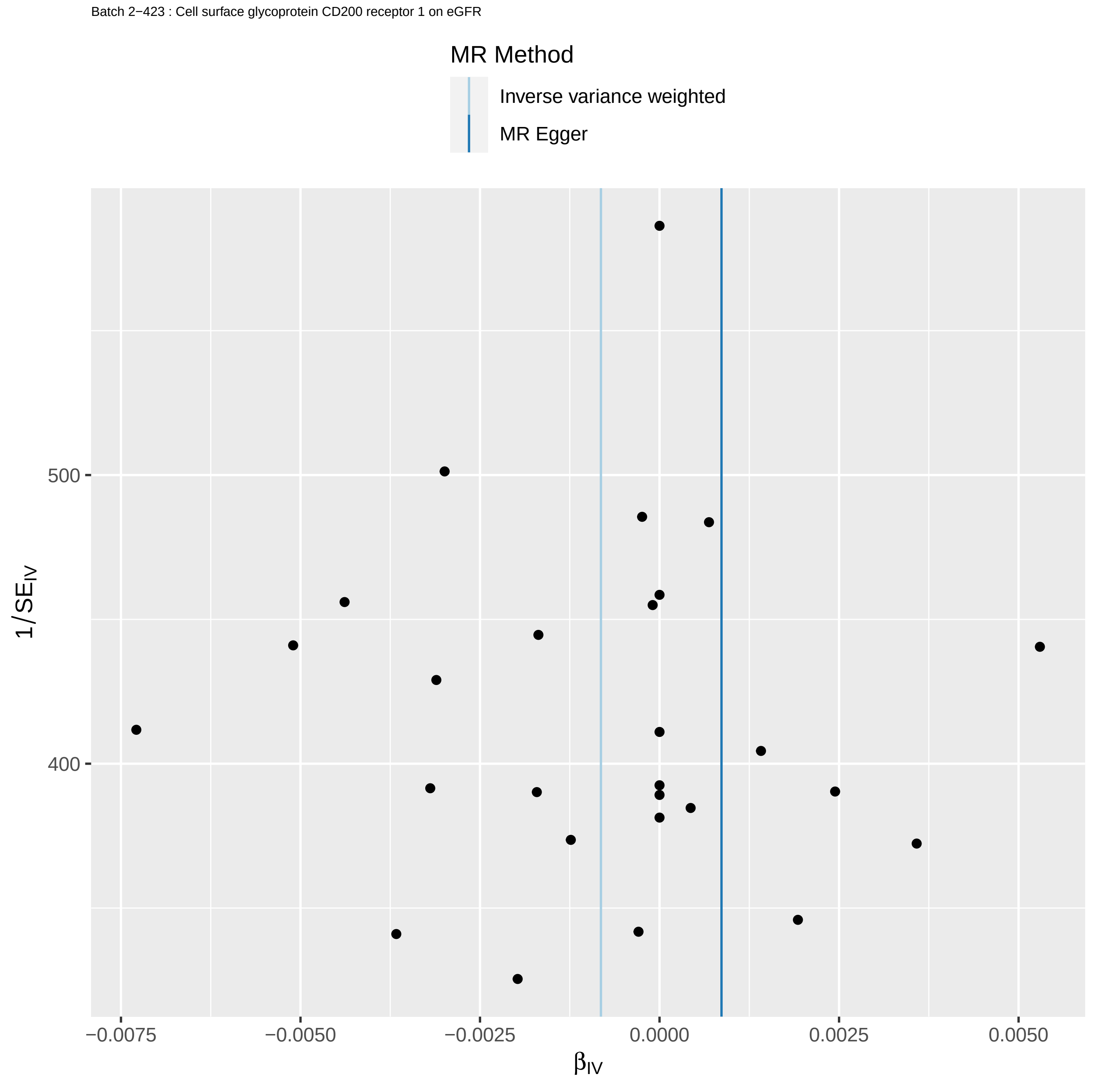
Batch 2−423 : Cell surface glycoprotein CD200 receptor 1 on eGFR
MR Method
Inverse variance weighted
MR Egger
1/SE_IV
500
400
β_IV
−0.0075
−0.0050
−0.0025
0.0000
0.0025
0.0050

Batch 2−424 : Cell surface glycoprotein CD200 receptor 2 on eGFR

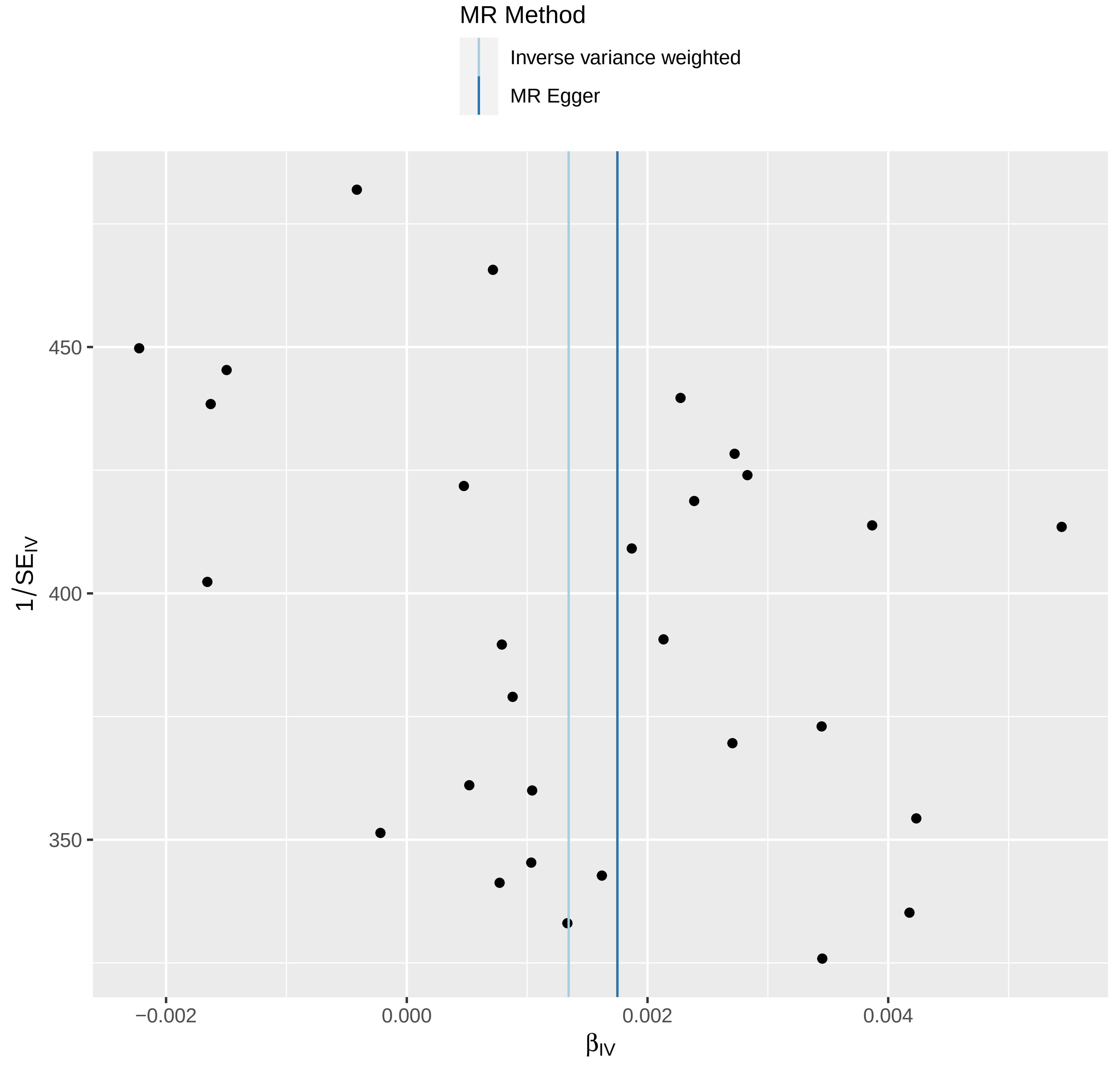

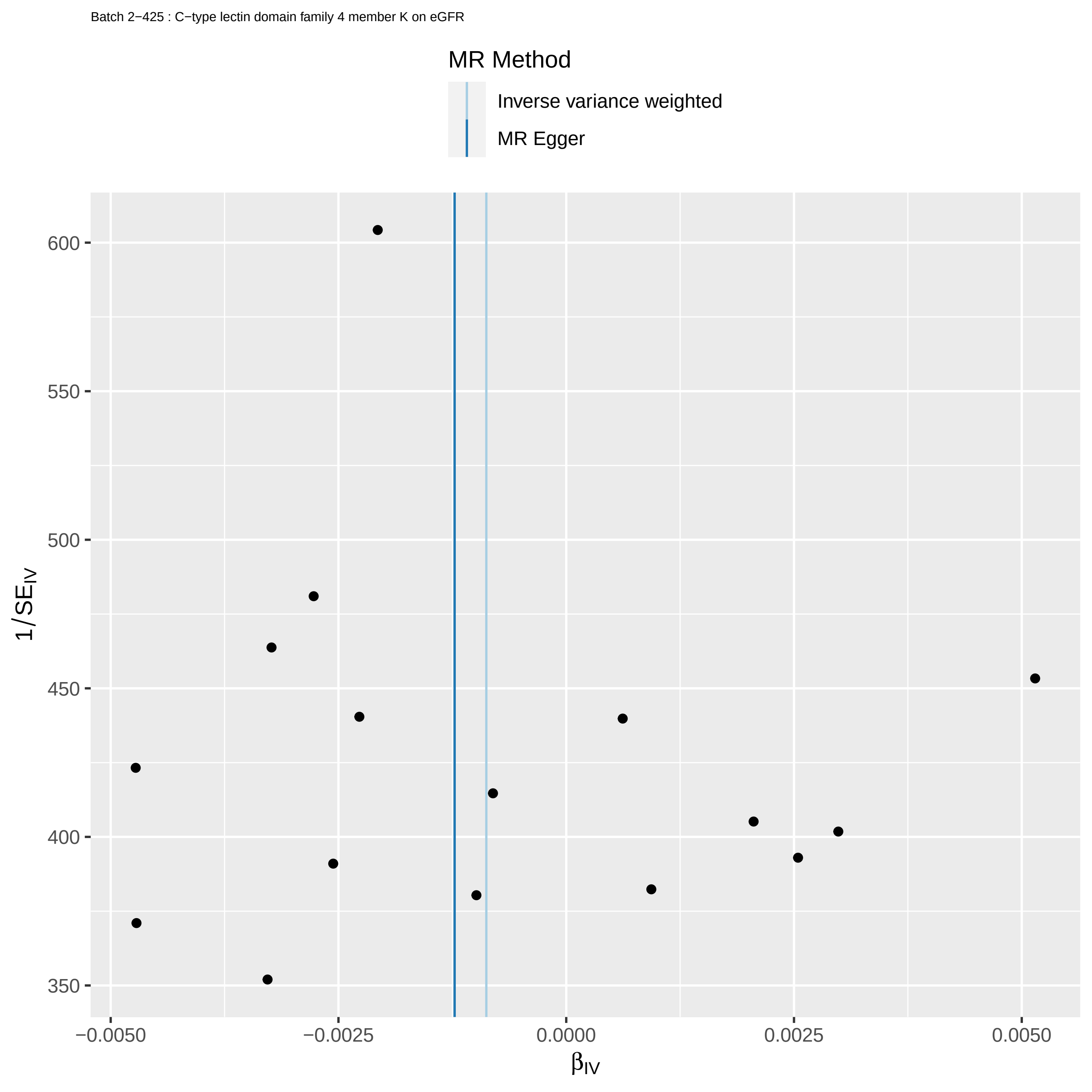
Batch 2−425 : C−type lectin domain family 4 member K on eGFR
MR Method
Inverse variance weighted
MR Egger
600
550
500
450
400
350
1/SE_IV
−0.0050
−0.0025
0.0000
0.0025
0.0050
β_IV

Batch 2−426 : CD209 antigen on eGFR

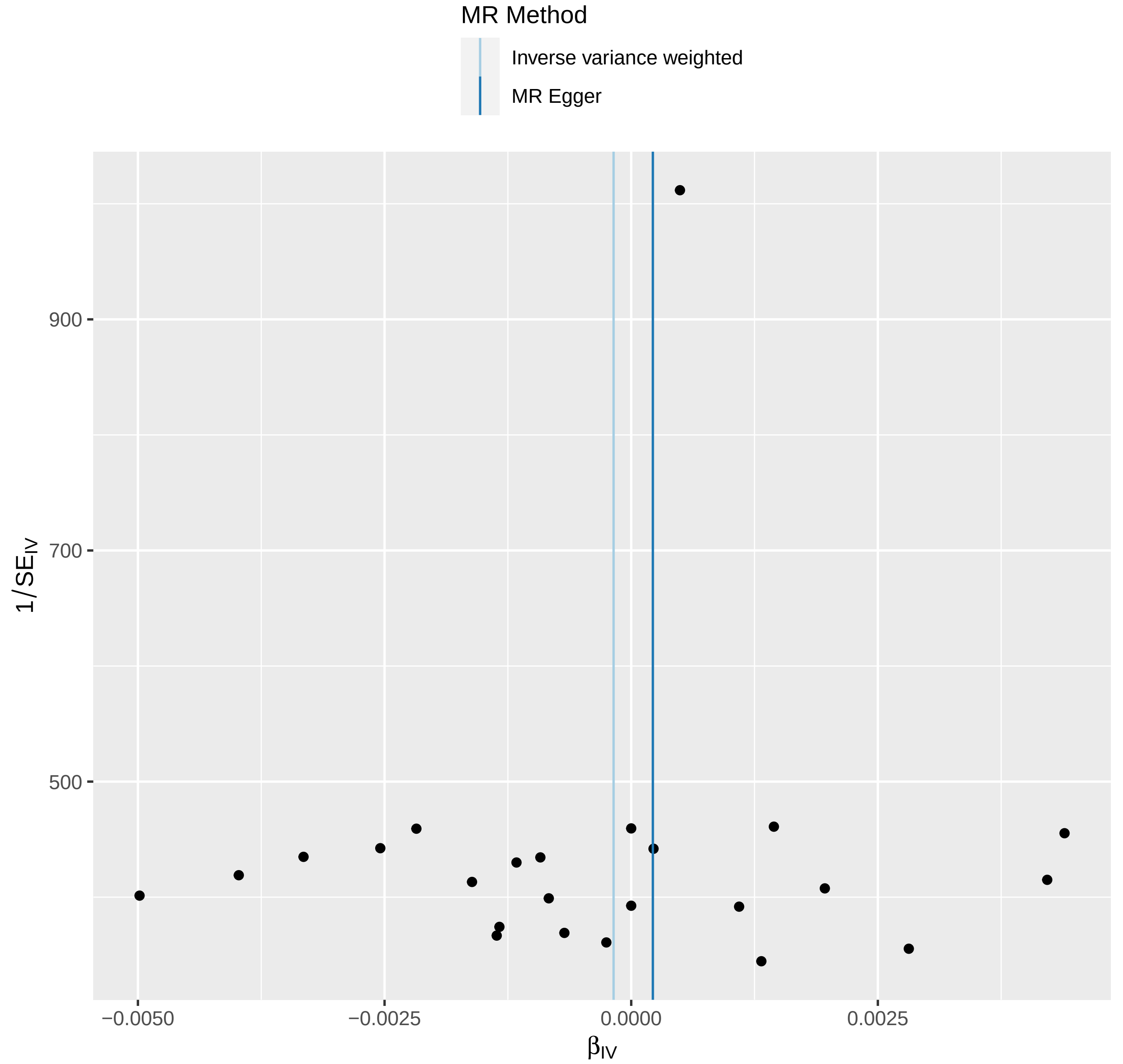

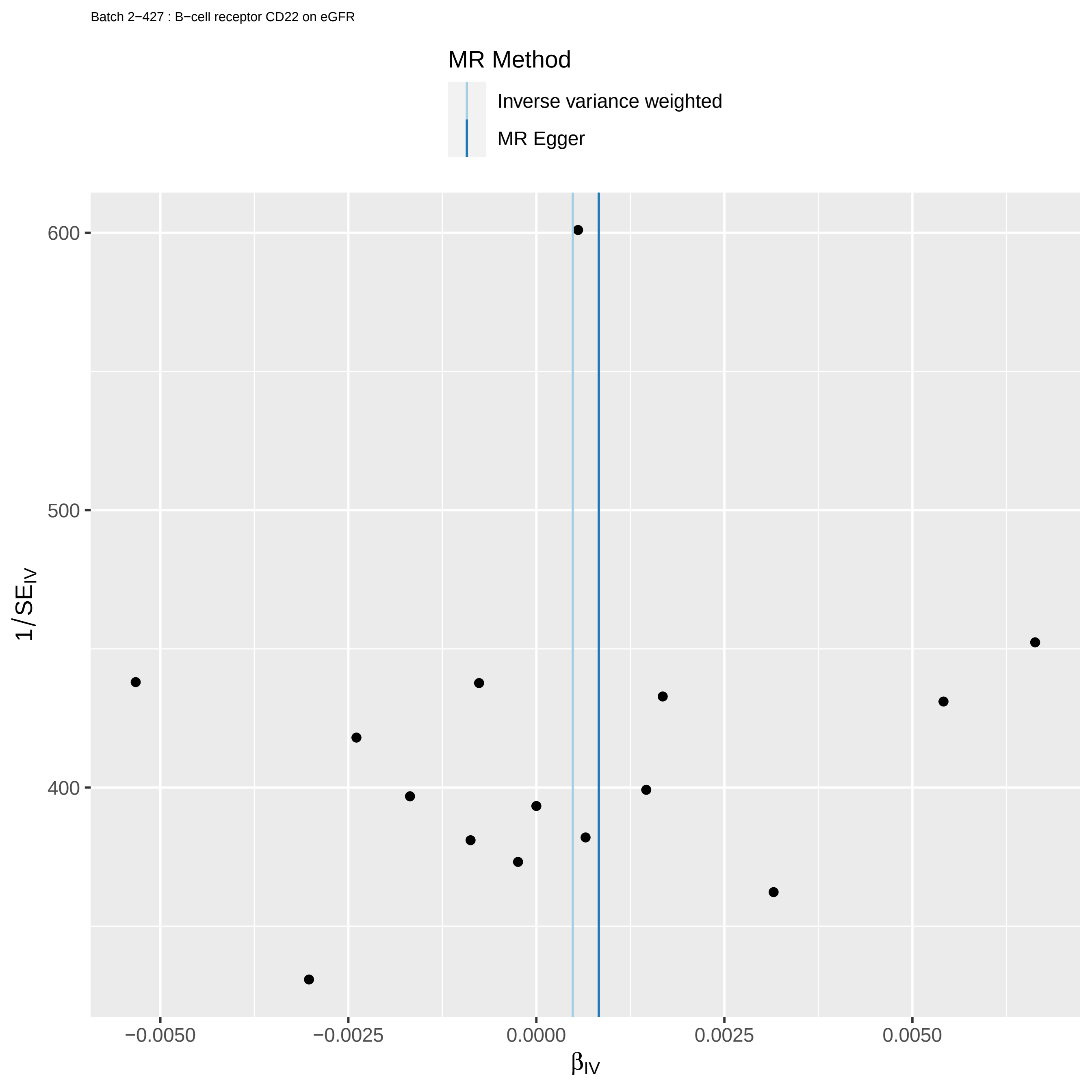

Batch 2−427 : B−cell receptor CD22 on eGFR
MR Method
Inverse variance weighted
MR Egger
600
500
400
1/SE$_{IV}$
−0.0050
−0.0025
0.0000
0.0025
0.0050
β$_{IV}$

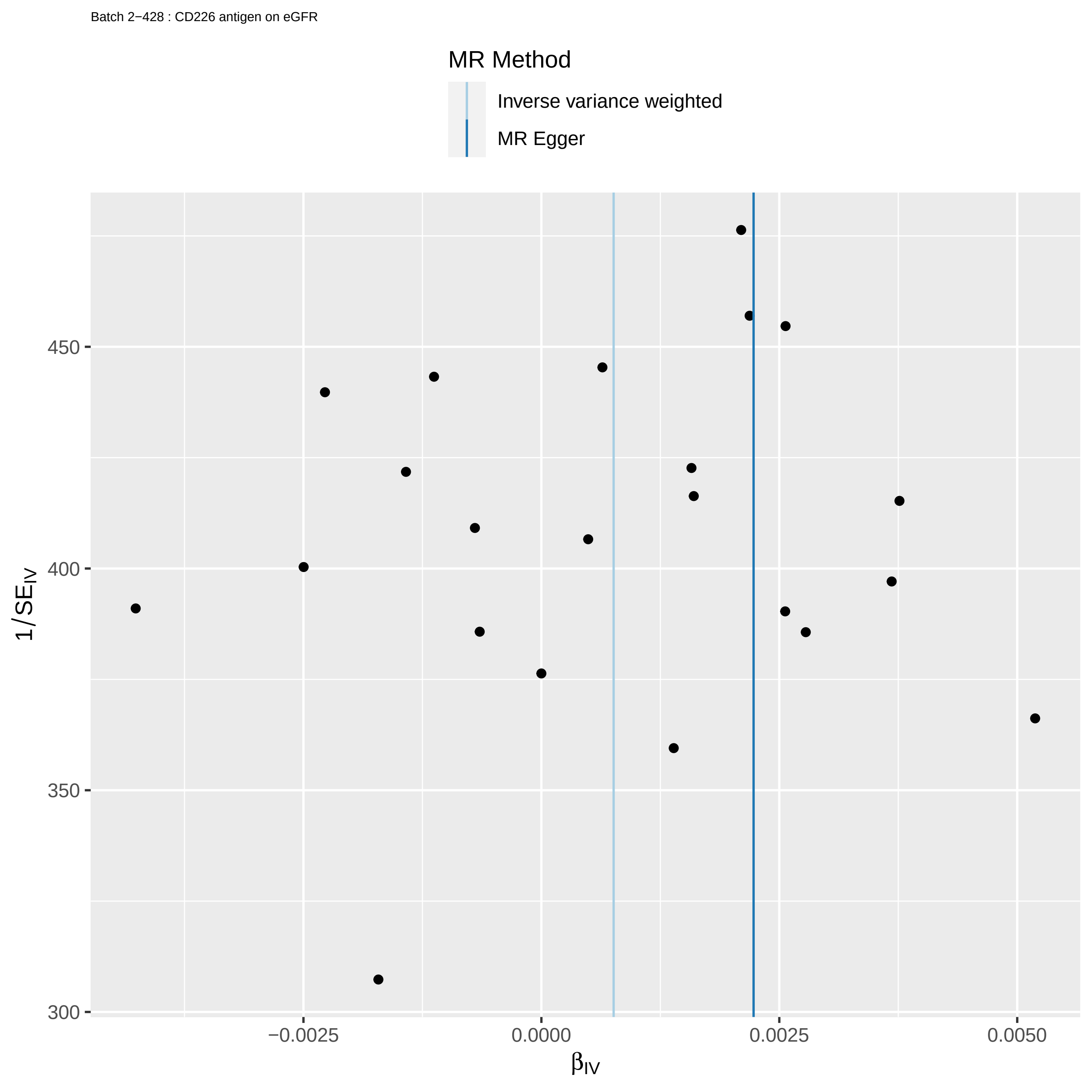

Batch 2−428 : CD226 antigen on eGFR
MR Method
Inverse variance weighted
MR Egger
450
400
350
300
1/SE$_{IV}$
−0.0025
0.0000
0.0025
0.0050
β$_{IV}$

Batch 2−429 : CD226 antigen on eGFR

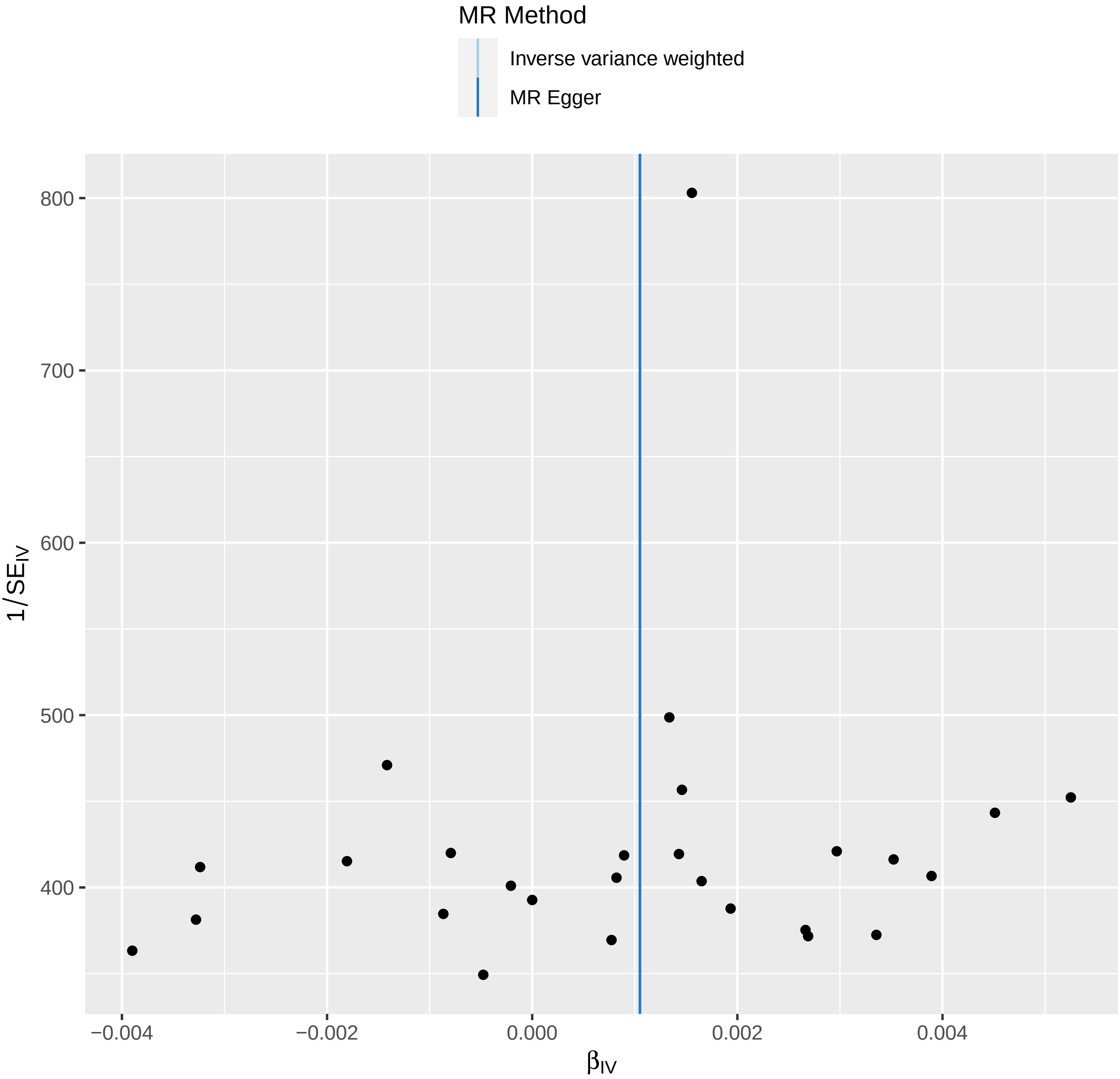

Batch 2−430 : T−cell surface antigen CD2 on eGFR

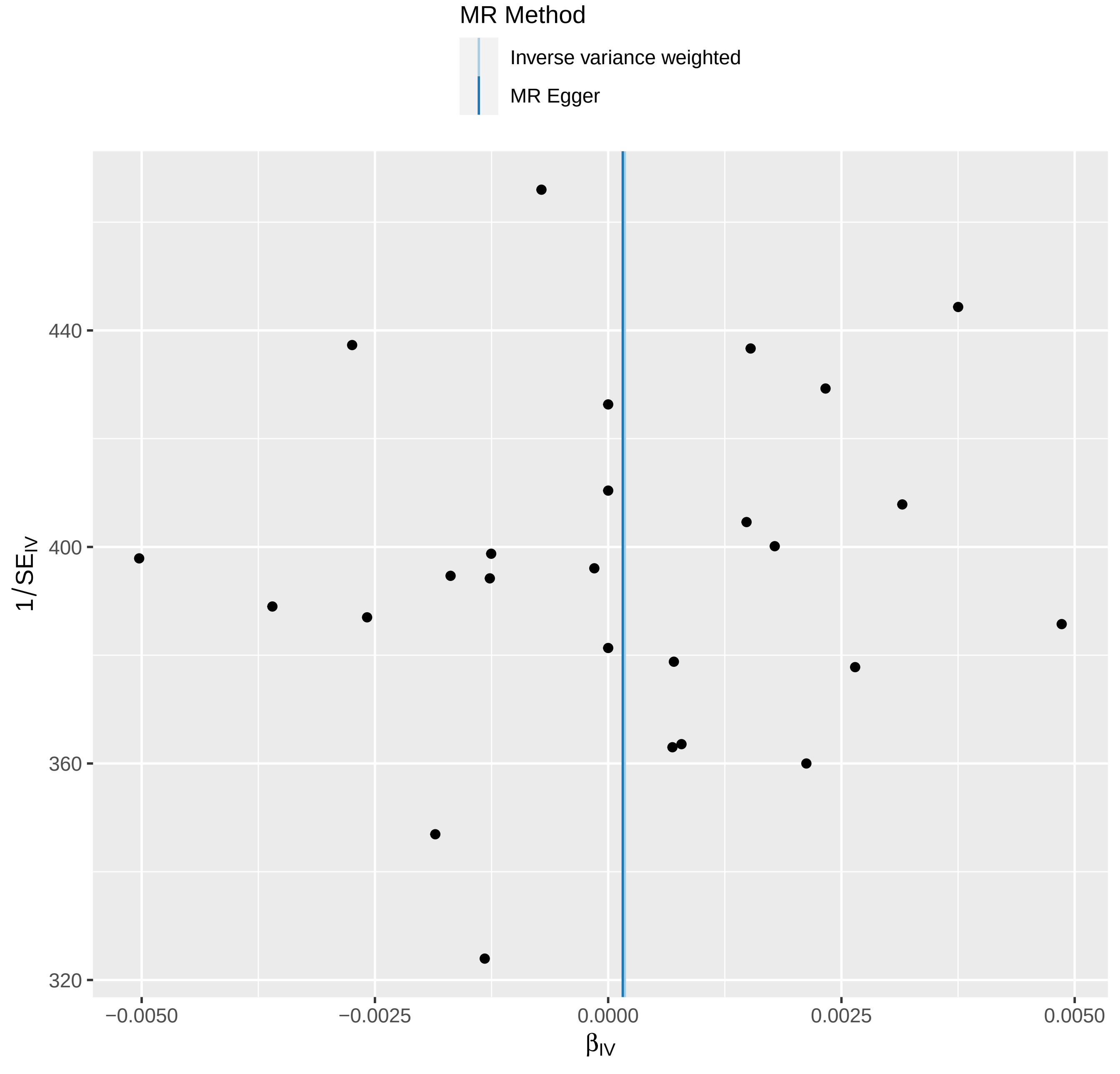

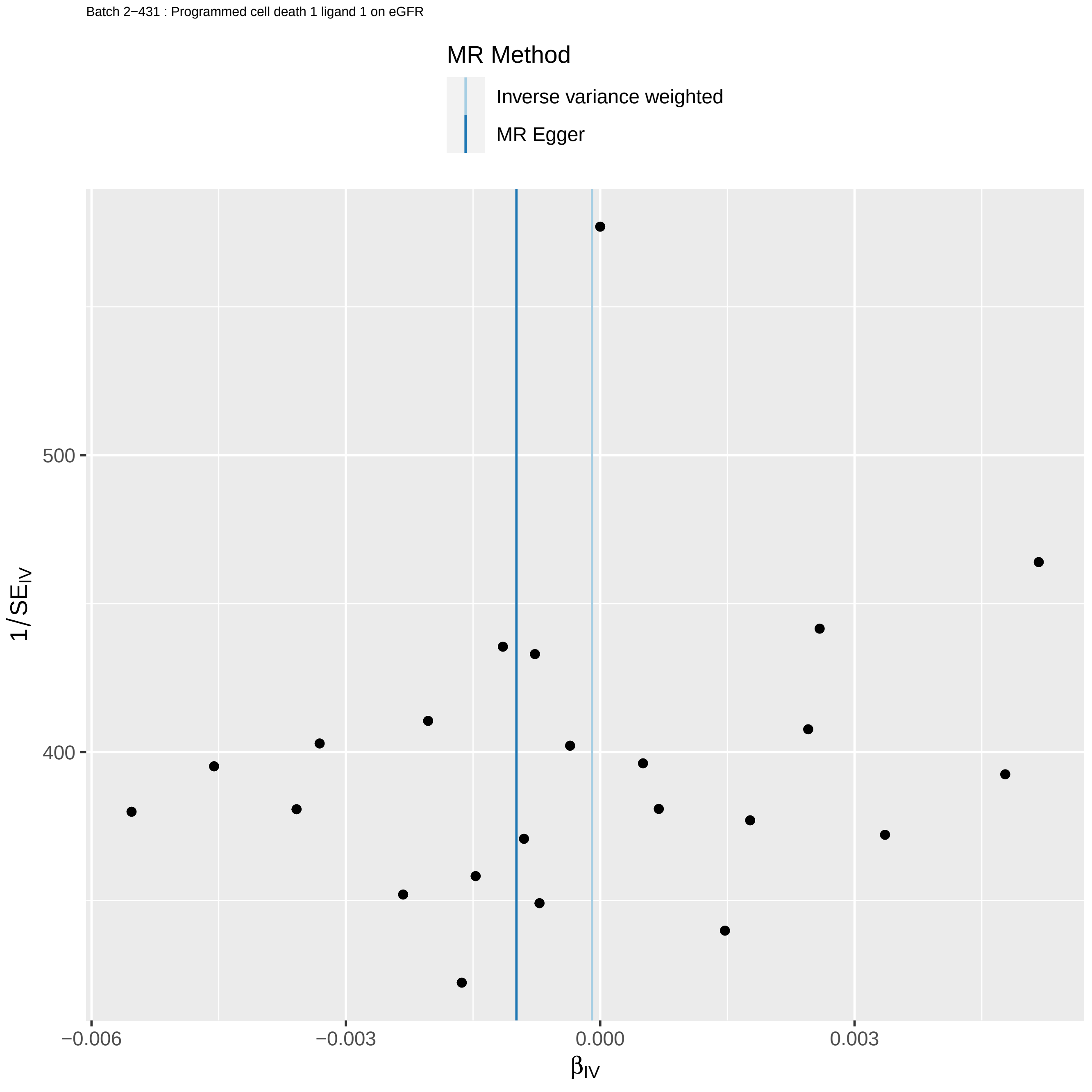

Batch 2−431 : Programmed cell death 1 ligand 1 on eGFR
MR Method
Inverse variance weighted
MR Egger
500
400
1/SE_IV
−0.006
−0.003
0.000
0.003
β_IV

Batch 2−432 : CMRF35−like molecule 8 on eGFR

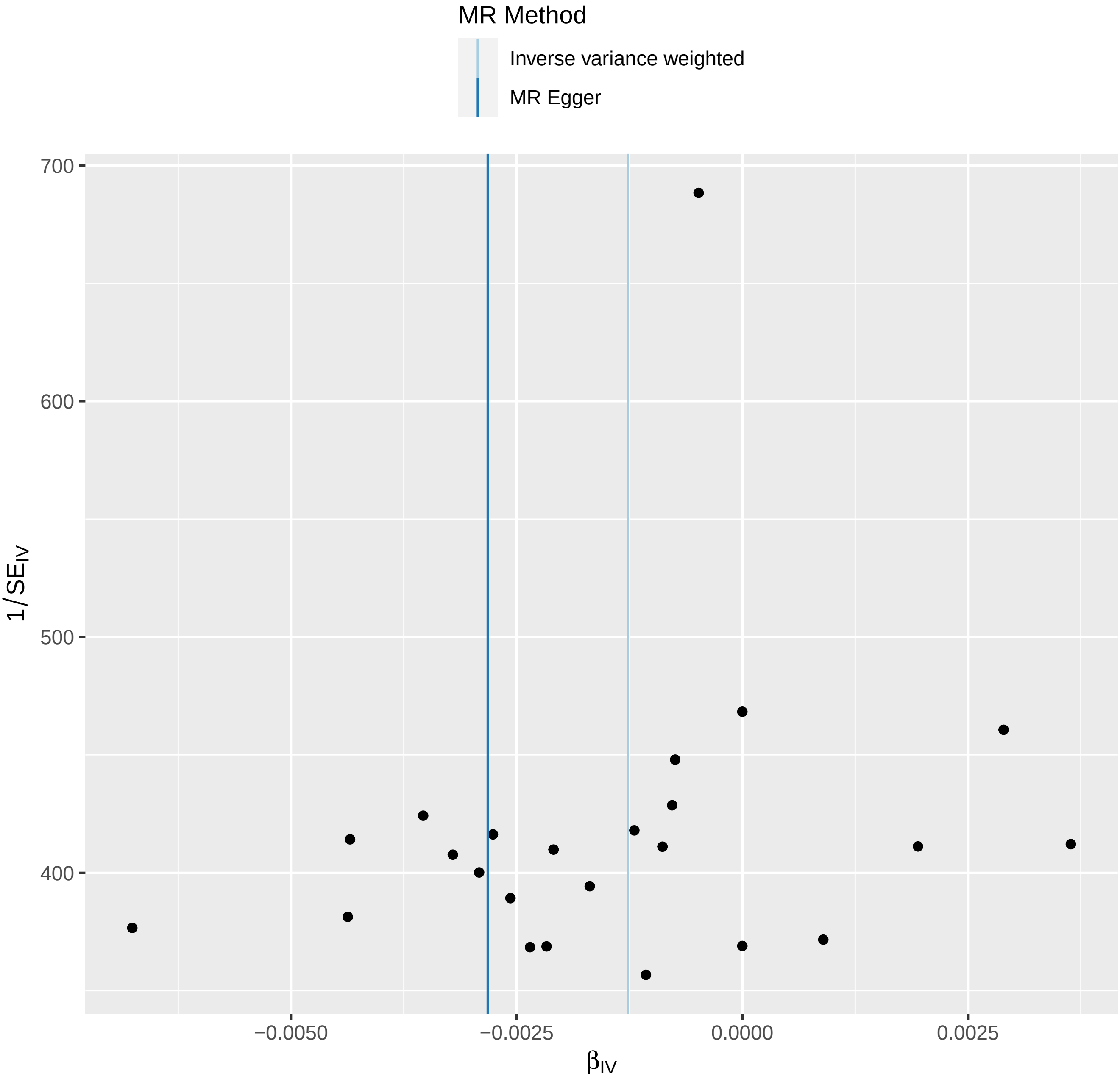

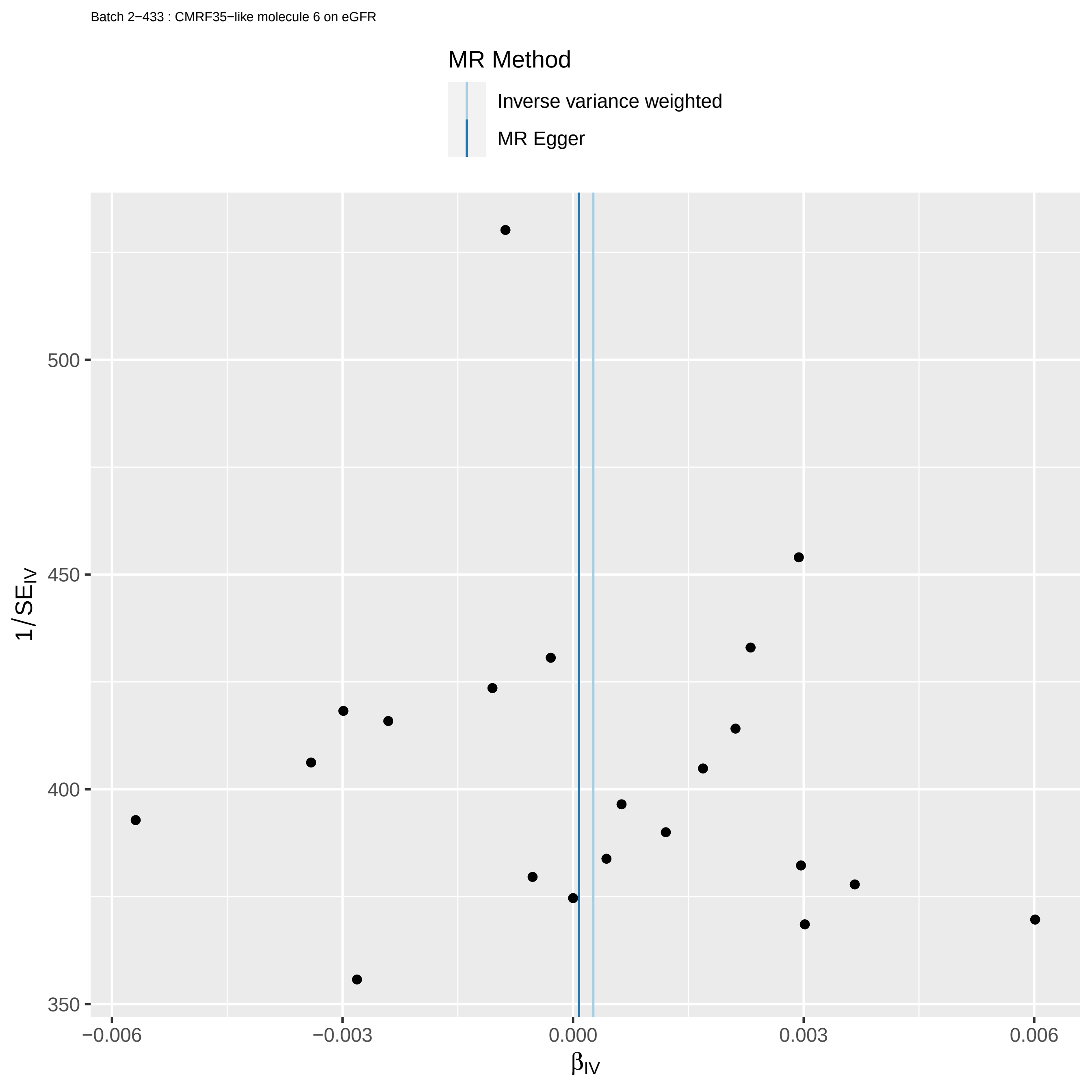
Batch 2−433 : CMRF35−like molecule 6 on eGFR
MR Method
Inverse variance weighted
MR Egger
500
450
400
350
1/SE_IV
−0.006
−0.003
0.000
0.003
0.006
β_IV

Batch 2−434 : CMRF35−like molecule 6 on eGFR

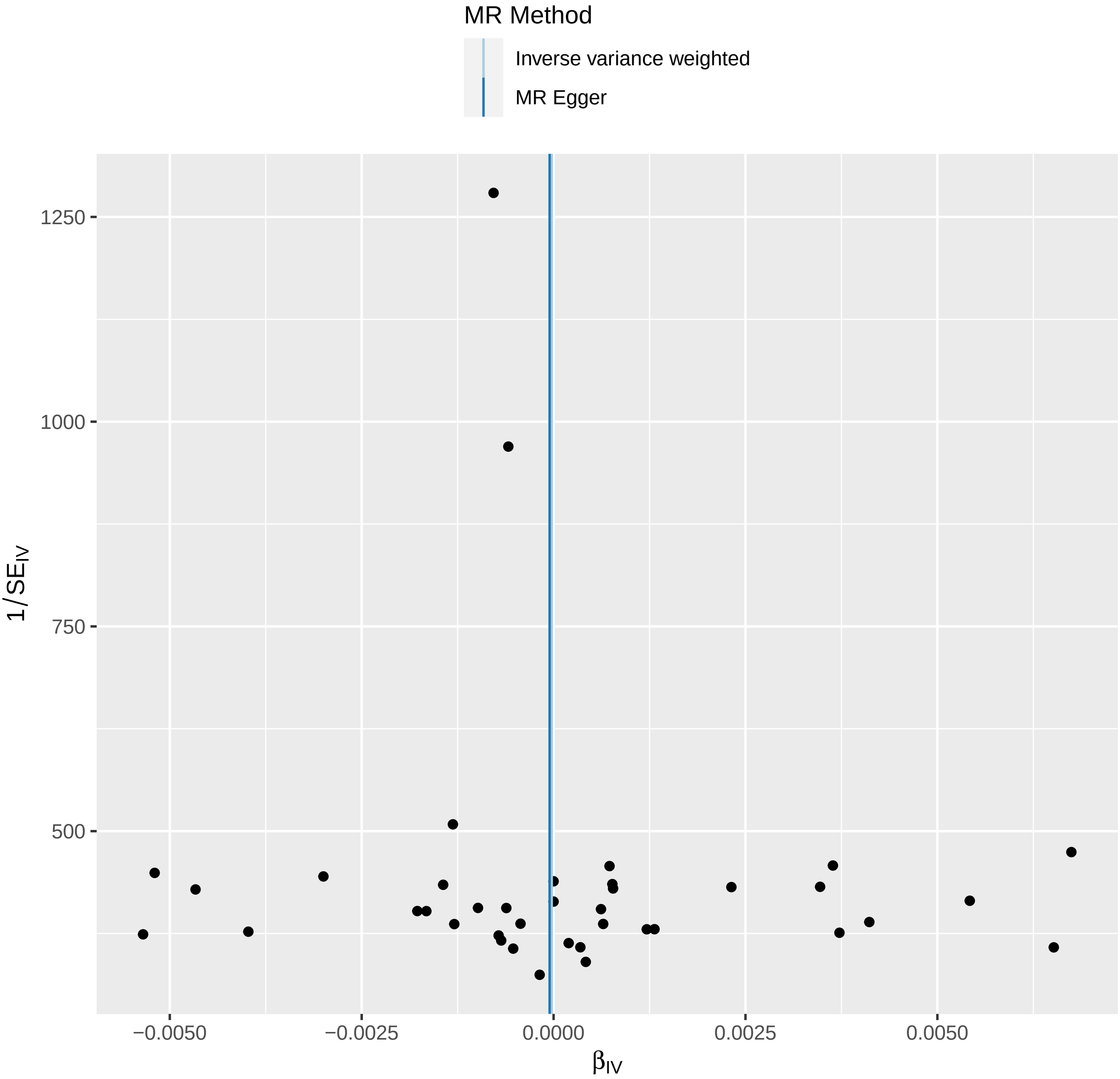

Batch 2−435 : CMRF35−like molecule 2 on eGFR

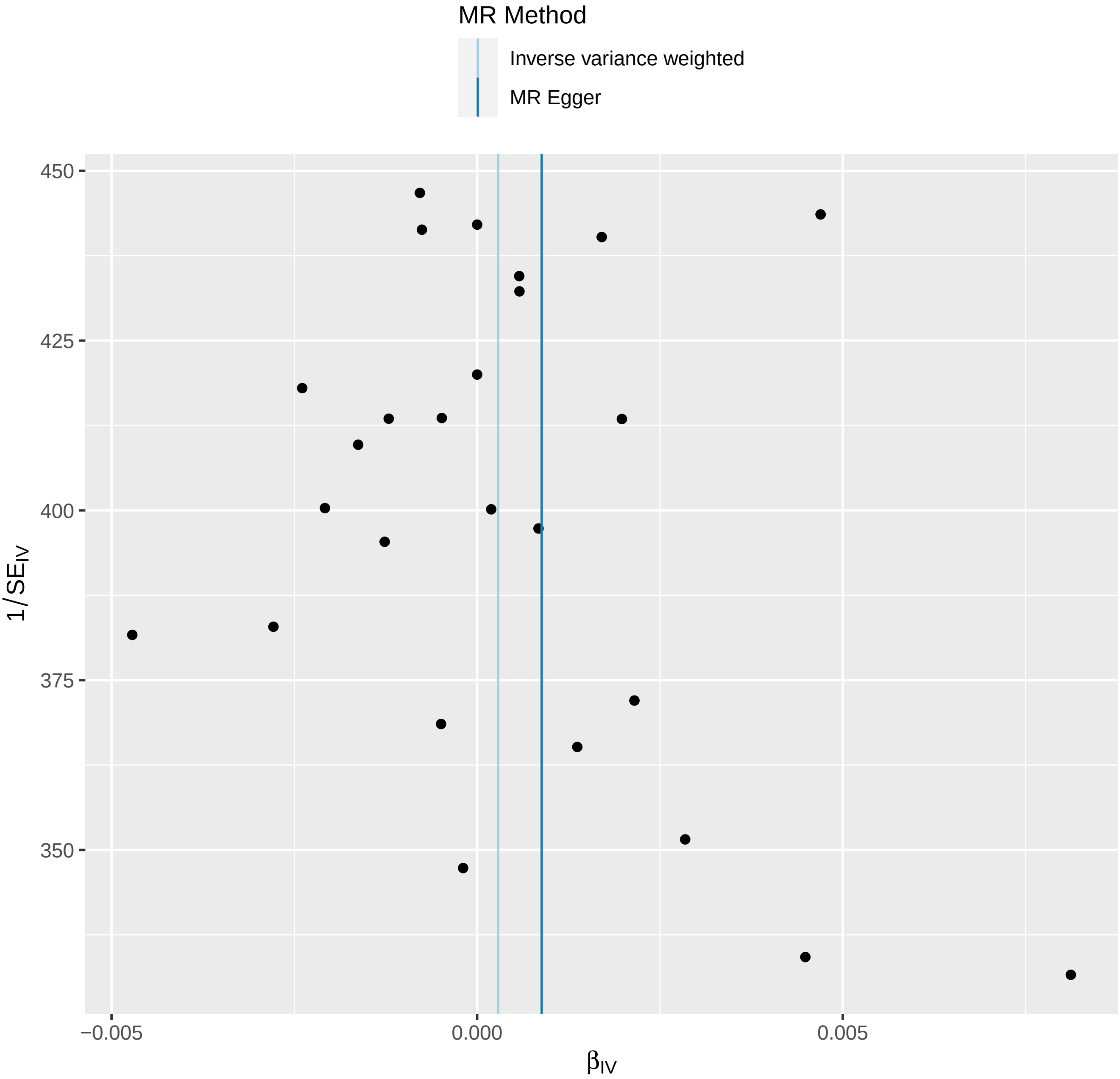

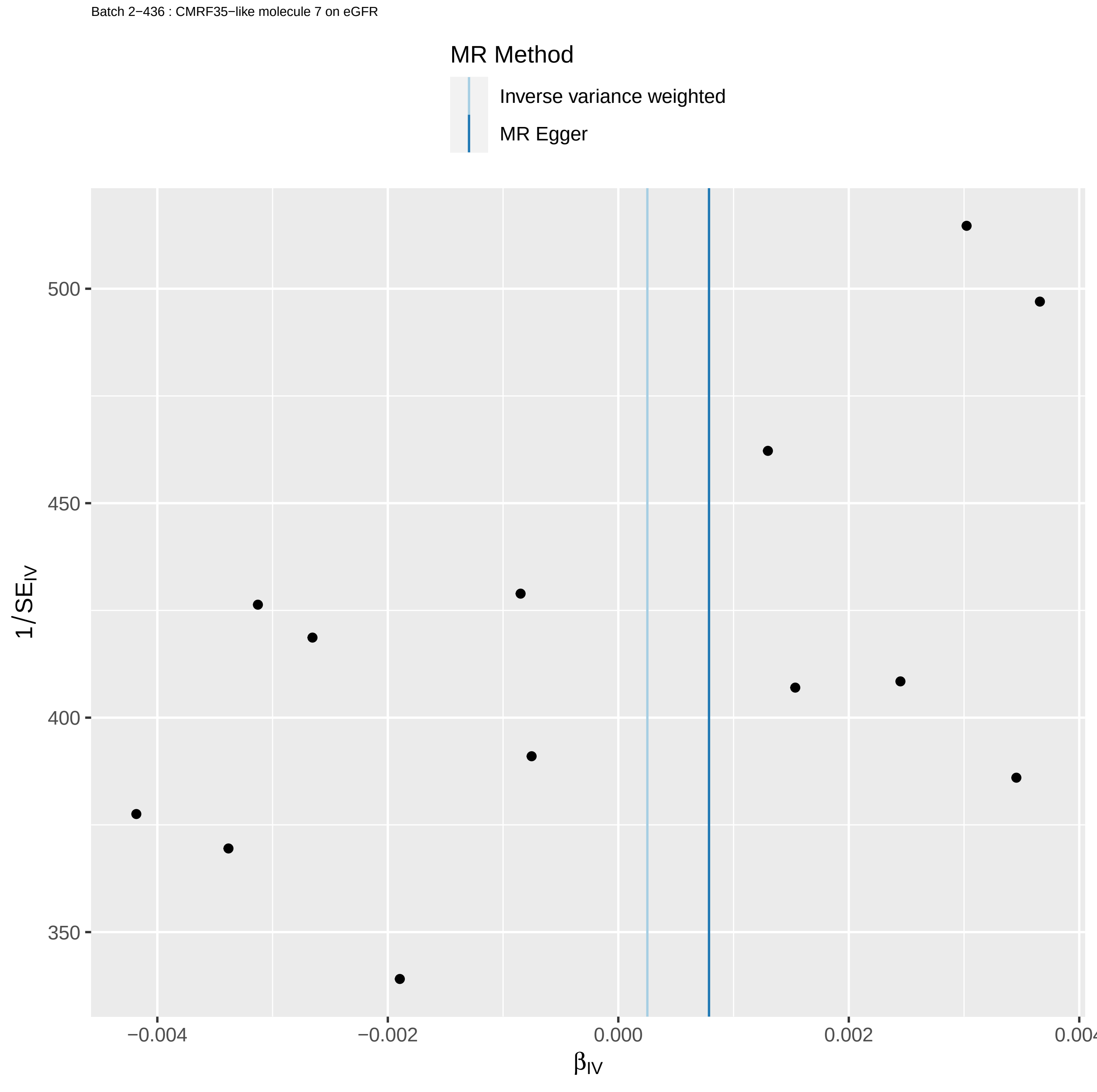
Batch 2−436 : CMRF35−like molecule 7 on eGFR
MR Method
Inverse variance weighted
MR Egger
500
450
400
350
1/SE_IV
−0.004
−0.002
0.000
0.002
0.004
β_IV

Batch 2−437 : CMRF35−like molecule 1 on eGFR

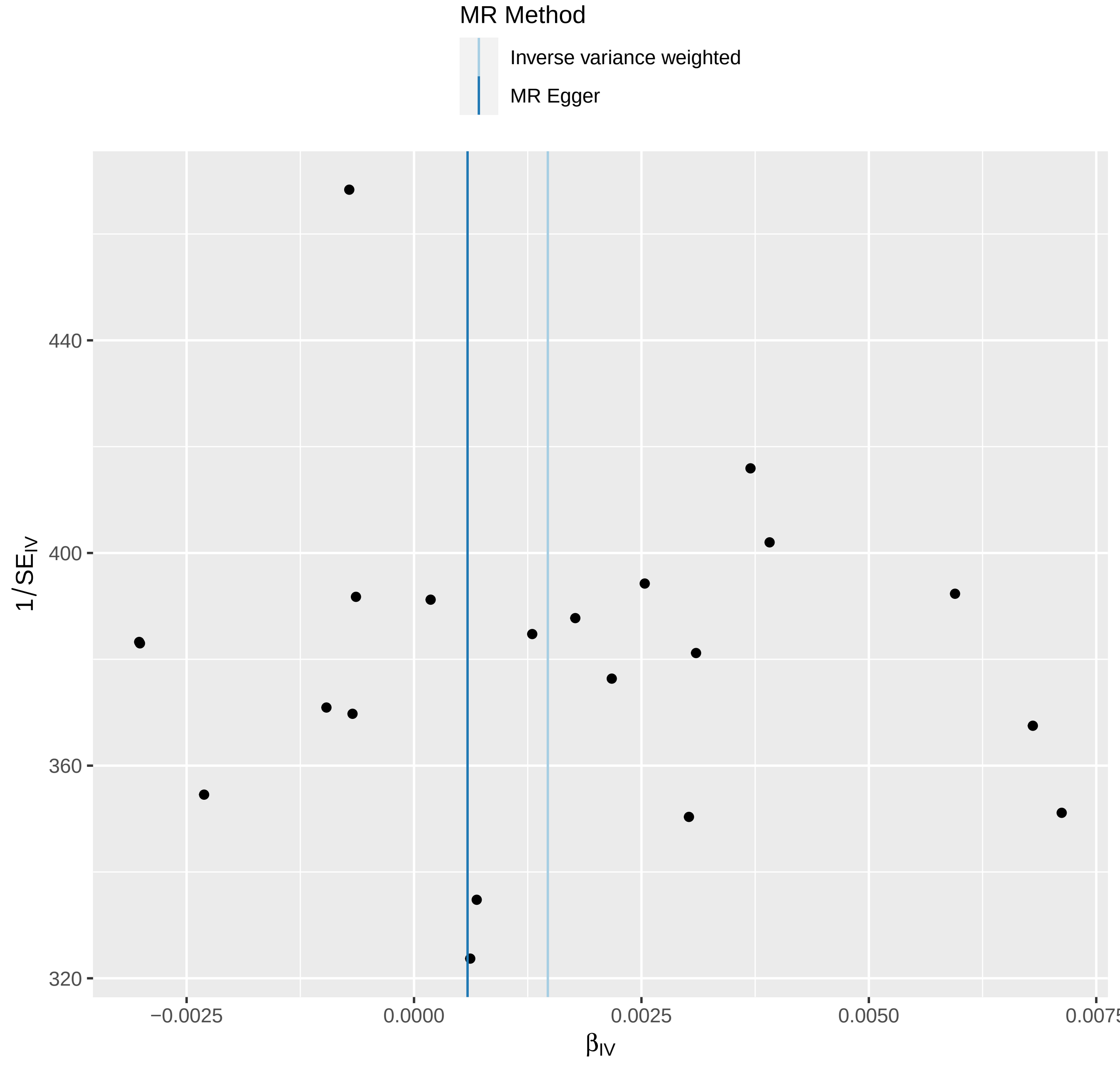

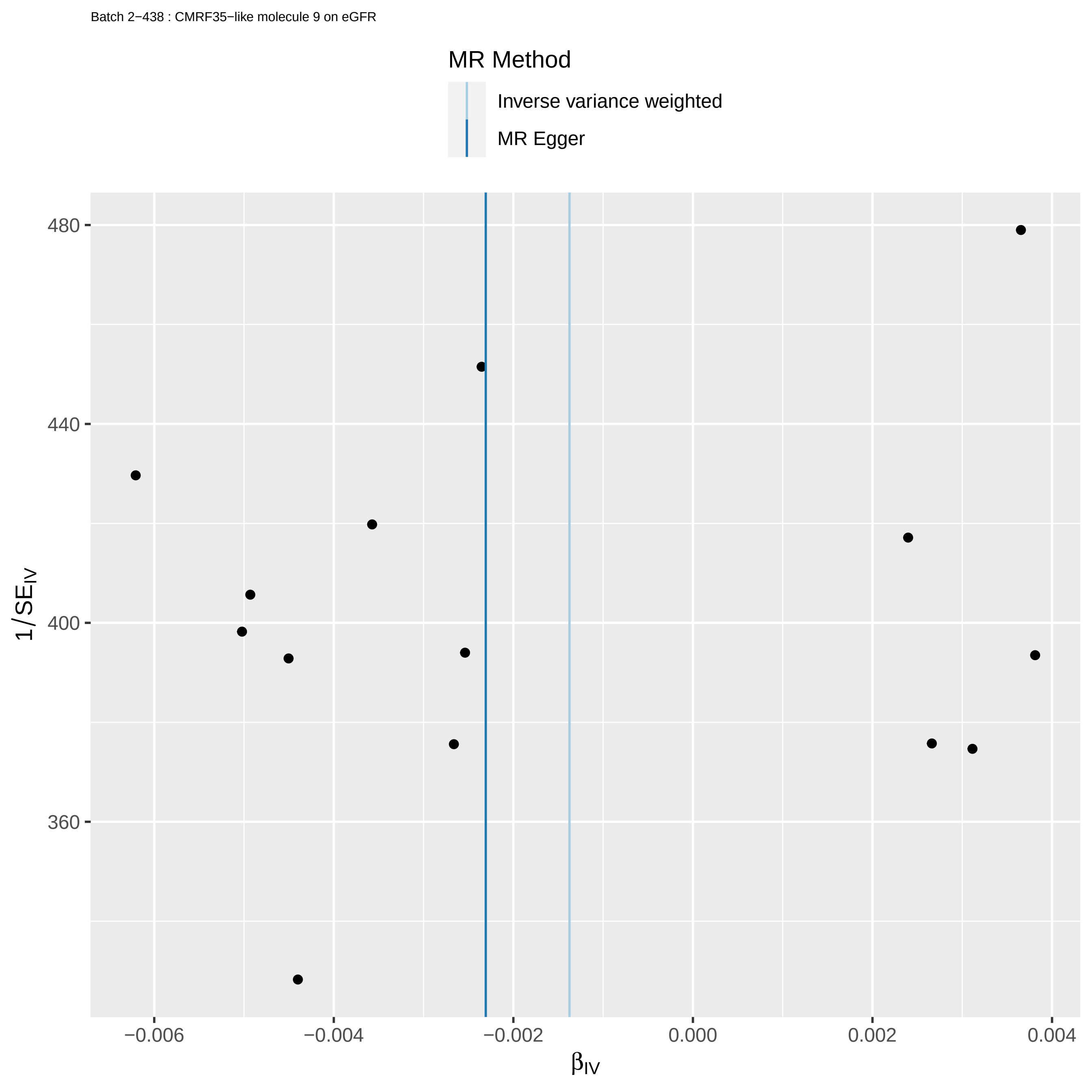

Batch 2−438 : CMRF35−like molecule 9 on eGFR
MR Method
Inverse variance weighted
MR Egger
480
440
400
360
1/SE_IV
−0.006
−0.004
−0.002
0.000
0.002
0.004
β_IV

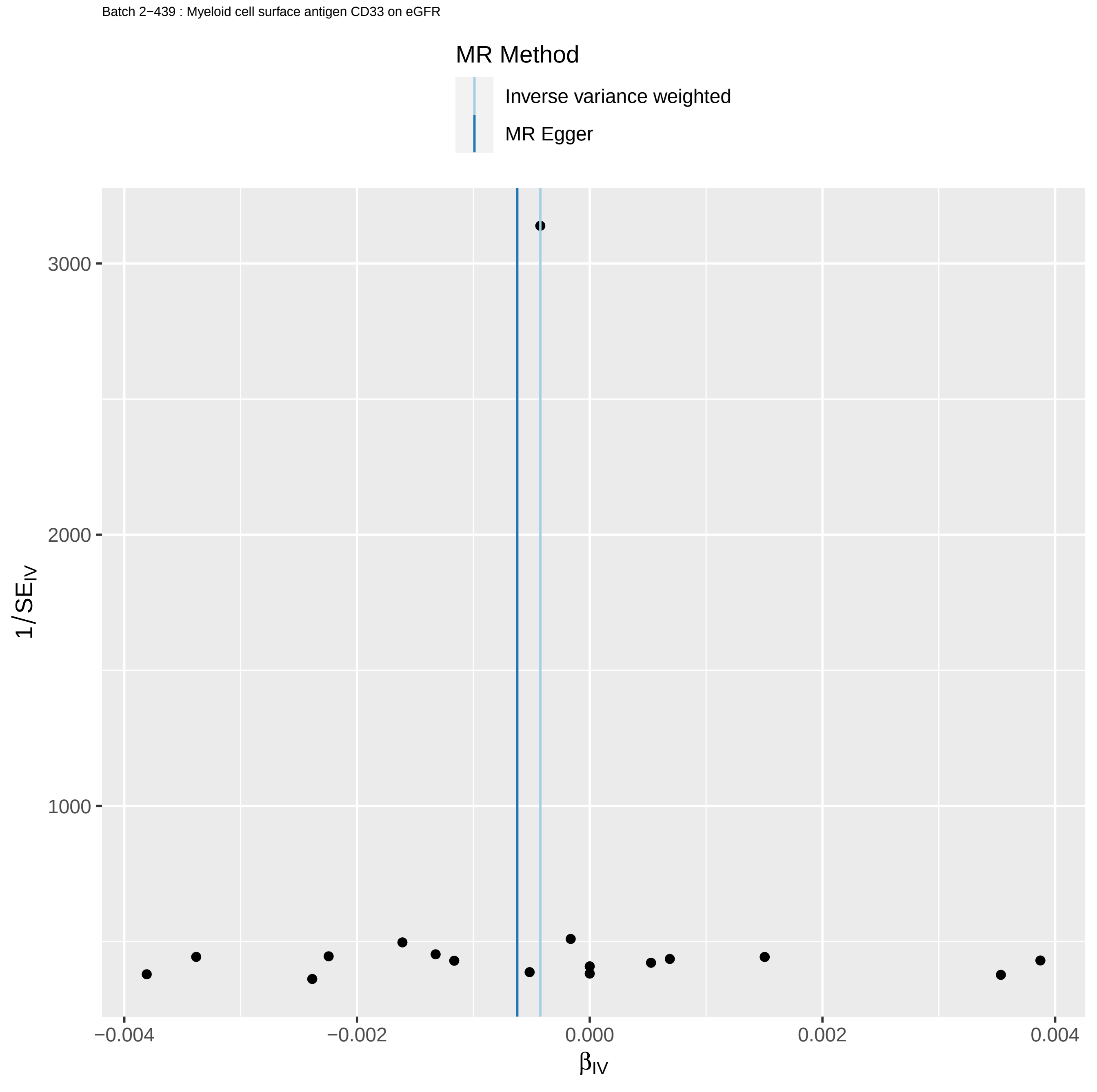
Batch 2−439 : Myeloid cell surface antigen CD33 on eGFR
MR Method
Inverse variance weighted
MR Egger
3000
2000
1000
1/SE$_{IV}$
−0.004
−0.002
0.000
0.002
0.004
β$_{IV}$

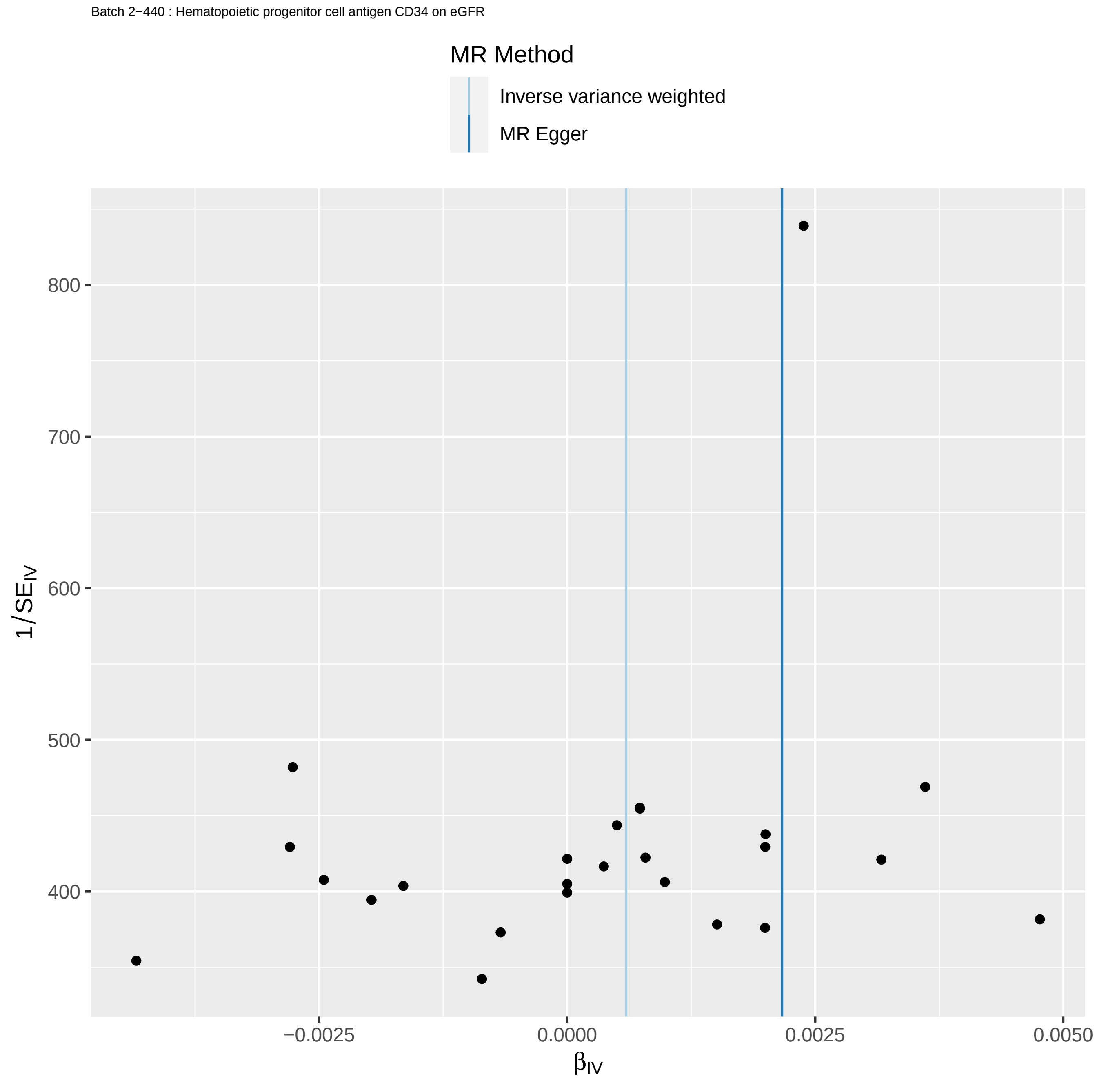

Batch 2−440 : Hematopoietic progenitor cell antigen CD34 on eGFR
MR Method
Inverse variance weighted
MR Egger
800
700
600
500
400
1/SE_IV
−0.0025
0.0000
0.0025
0.0050
β_IV

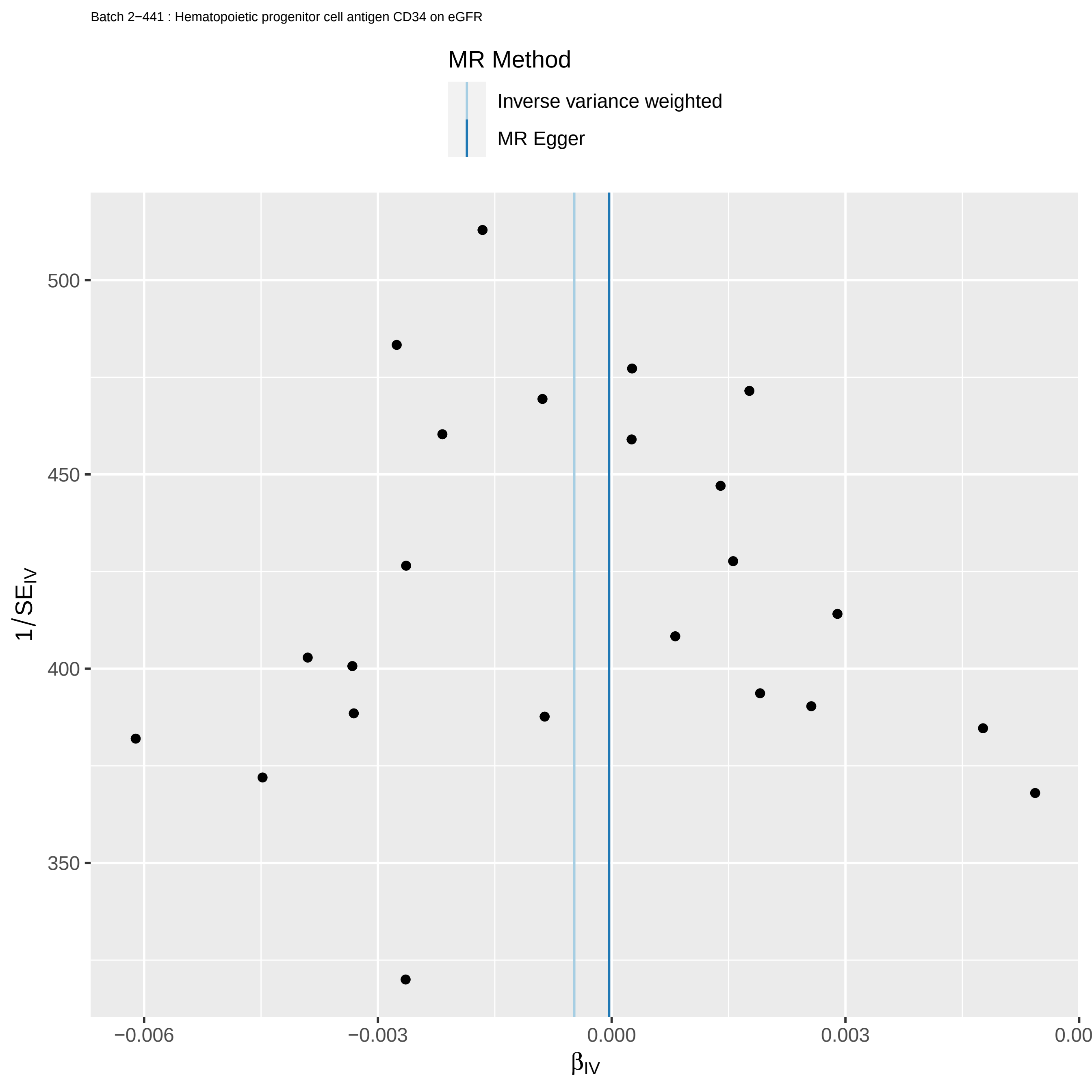
Batch 2−441 : Hematopoietic progenitor cell antigen CD34 on eGFR
MR Method
Inverse variance weighted
MR Egger
500
450
400
350
1/SE_IV
−0.006
−0.003
0.000
0.003
β_IV

Batch 2−442 : Platelet glycoprotein 4 on eGFR

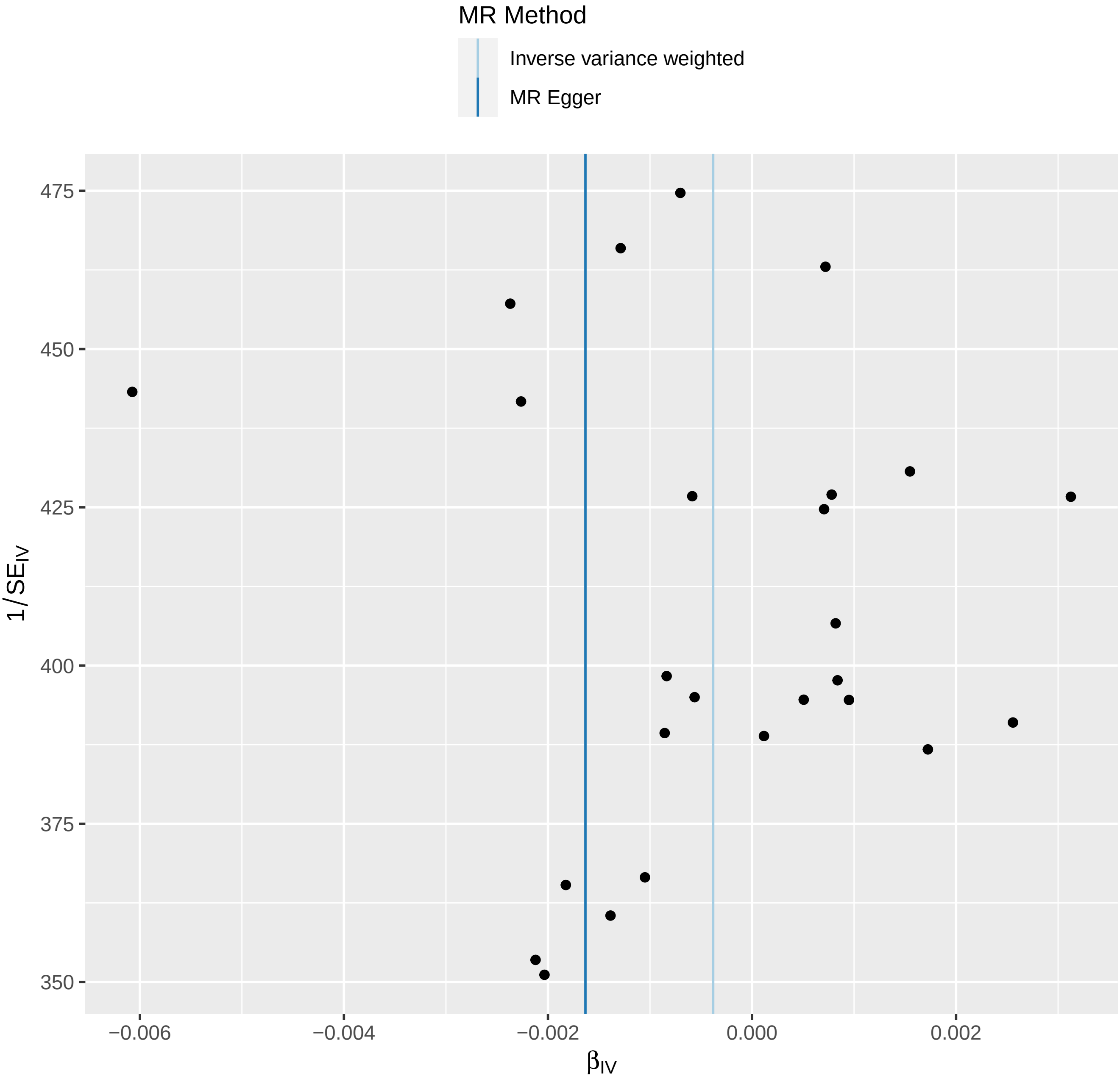

Batch 2−443 : ADP−ribosyl cyclase/cyclic ADP−ribose hydrolase 1 on eGFR

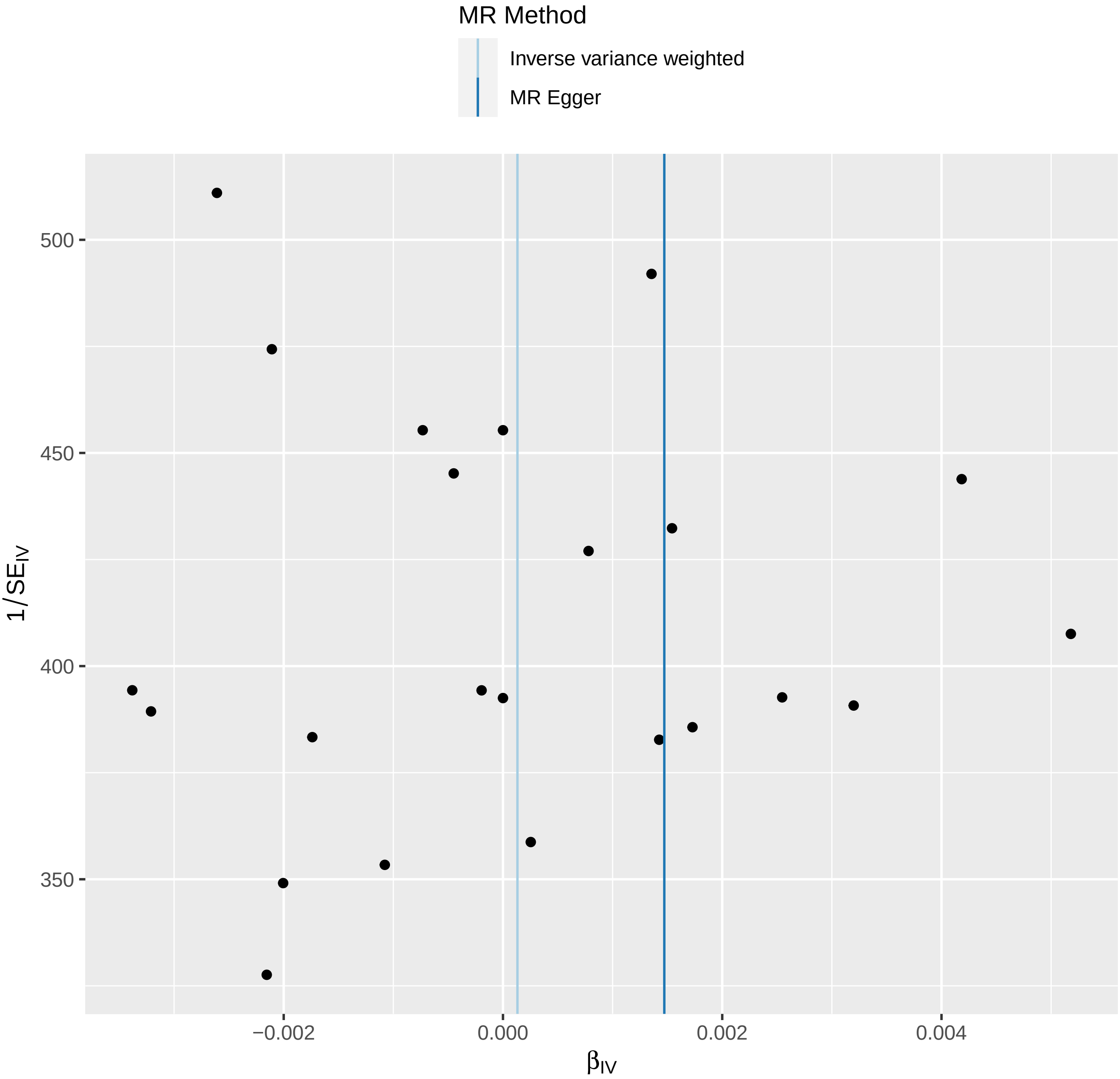

Batch 2−444 : ADP−ribosyl cyclase/cyclic ADP−ribose hydrolase 1 on eGFR

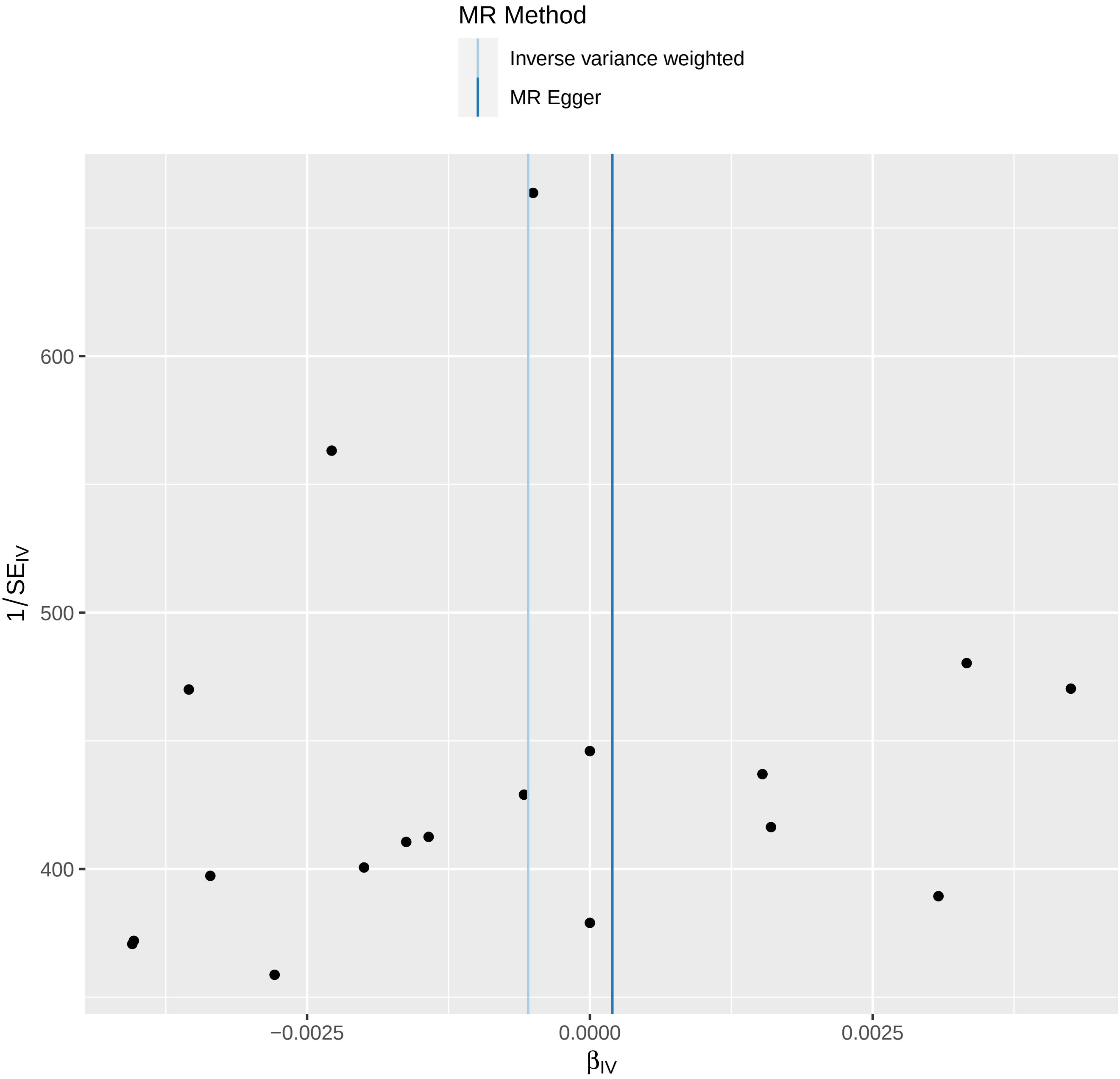

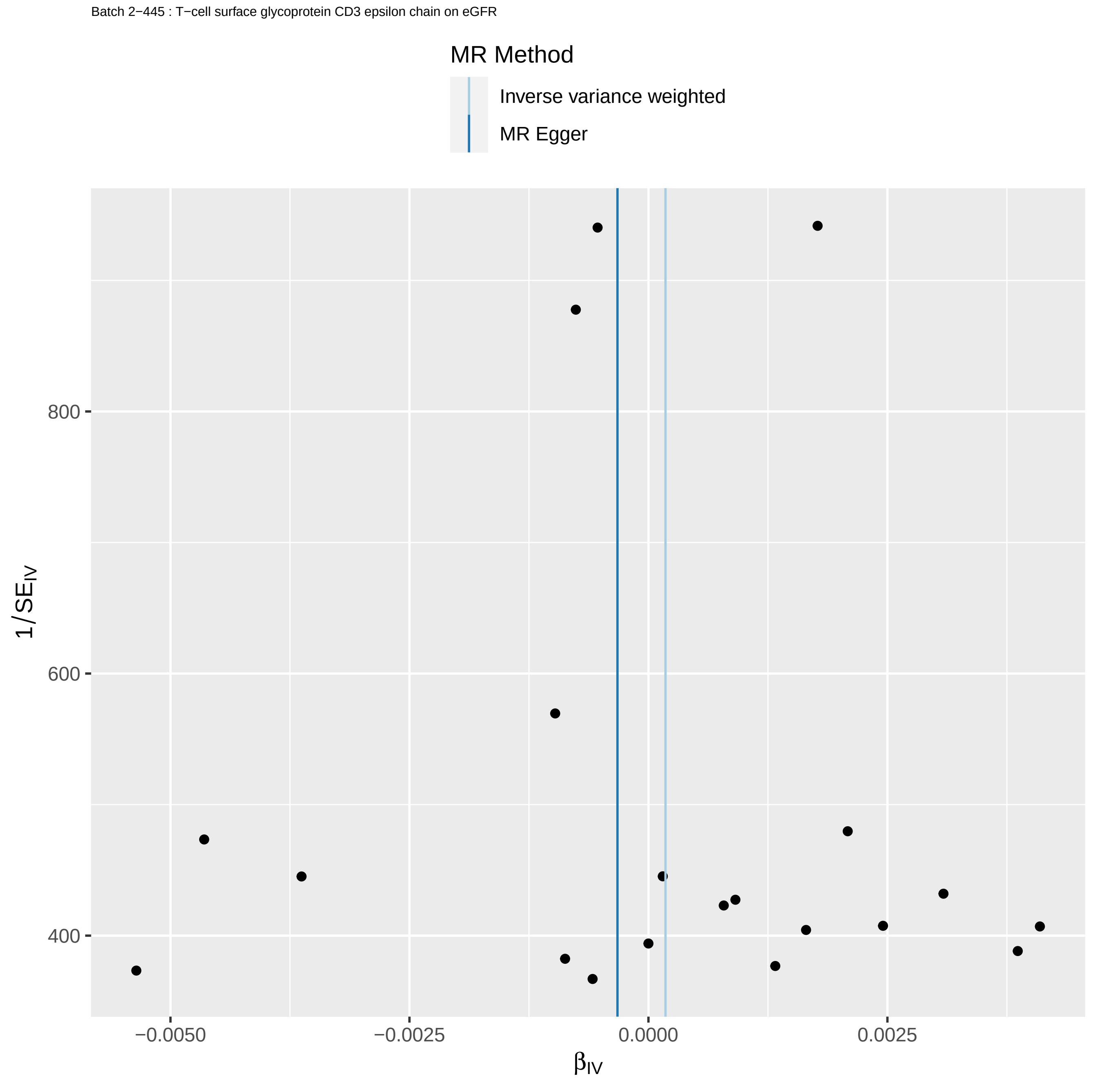

Batch 2−445 : T−cell surface glycoprotein CD3 epsilon chain on eGFR
MR Method
Inverse variance weighted
MR Egger
800
600
400
1/SE$_{IV}$
−0.0050
−0.0025
0.0000
0.0025
β$_{IV}$

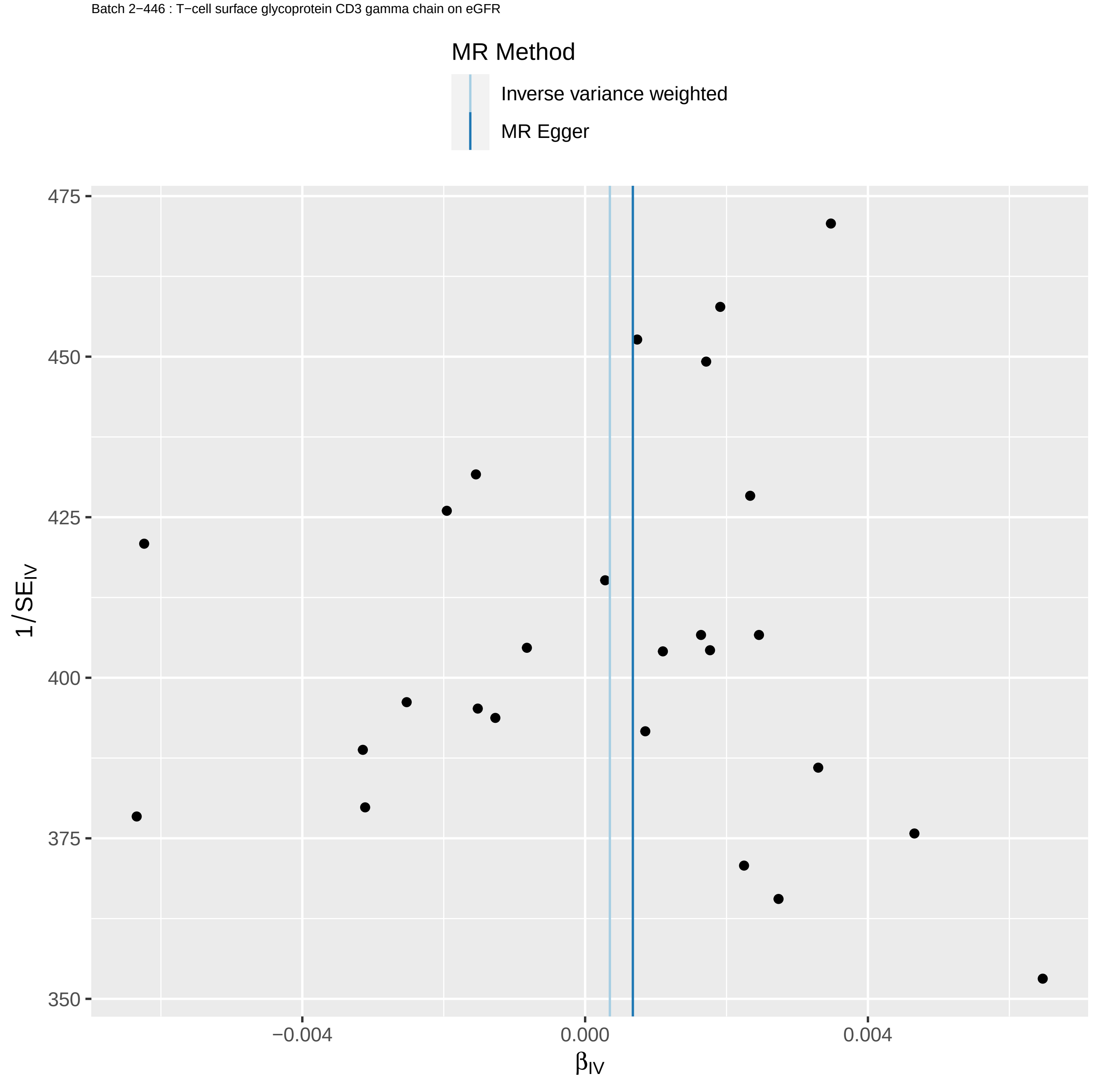
Batch 2−446 : T−cell surface glycoprotein CD3 gamma chain on eGFR
MR Method
Inverse variance weighted
MR Egger
475
450
425
400
375
350
1/SE_IV
−0.004
0.000
0.004
β_IV

Batch 2−447 : CD40 ligand on eGFR

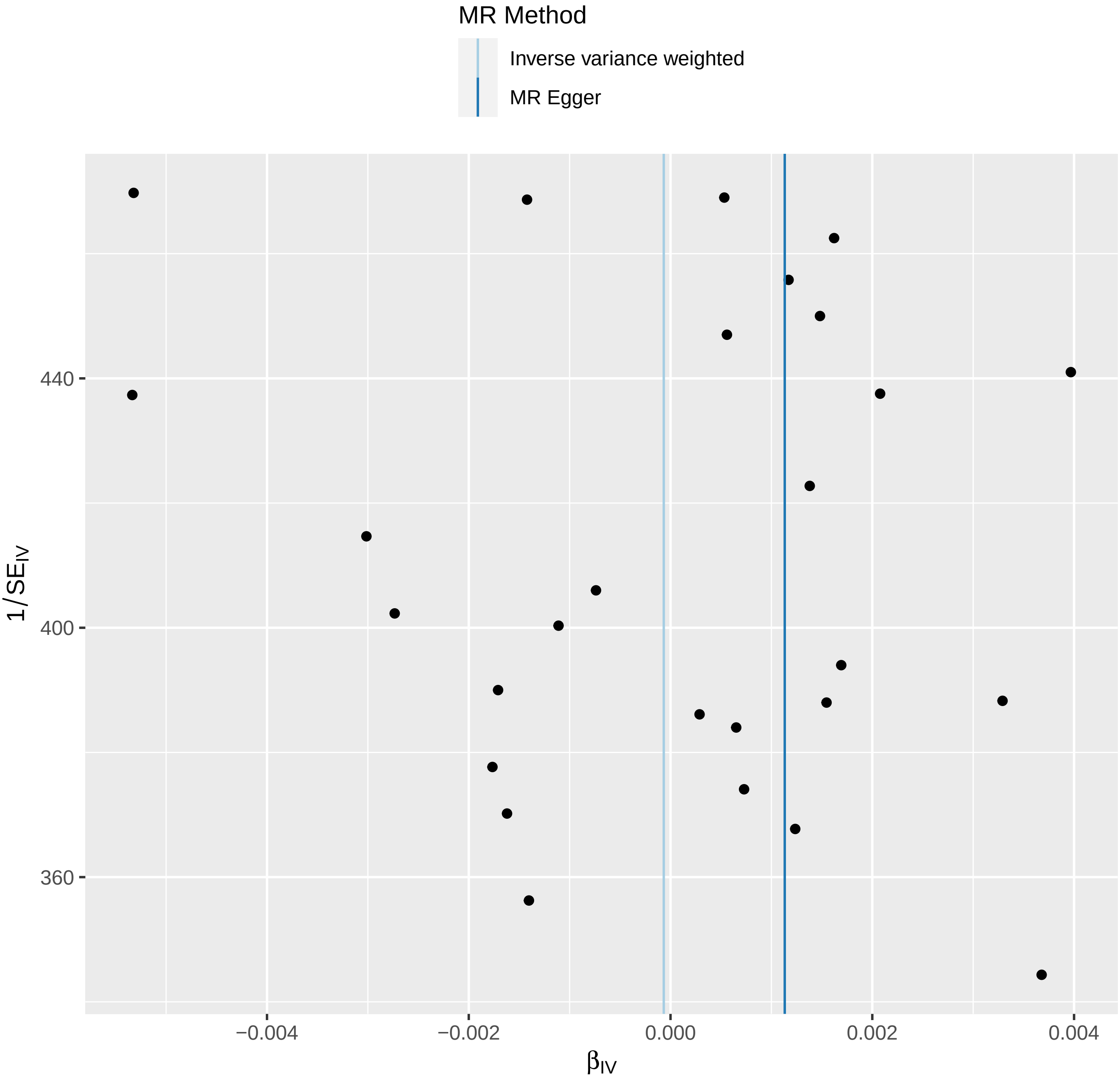

Batch 2−448 : T−cell surface glycoprotein CD4 on eGFR

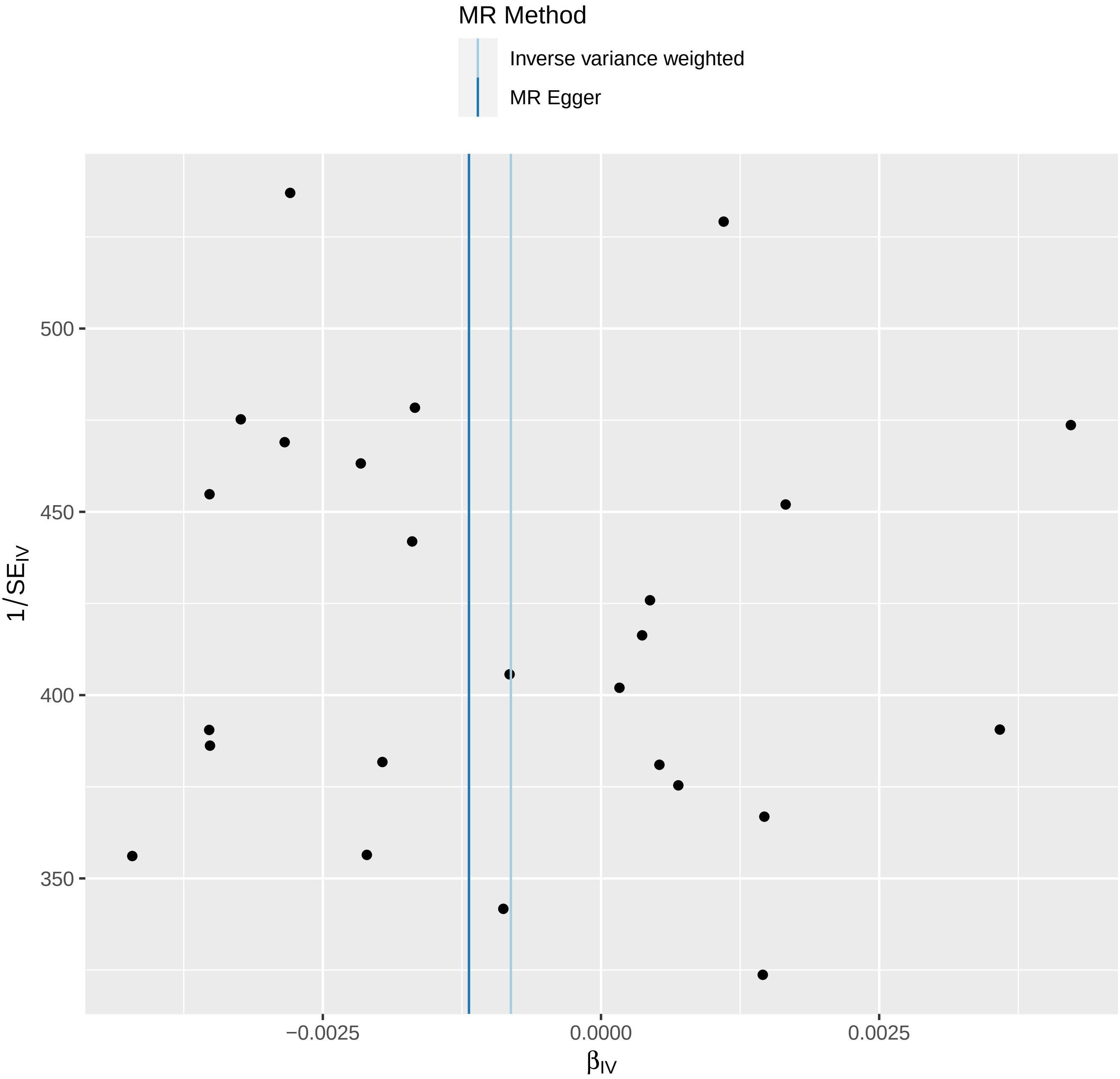

Batch 2−449 : CD44 antigen on eGFR

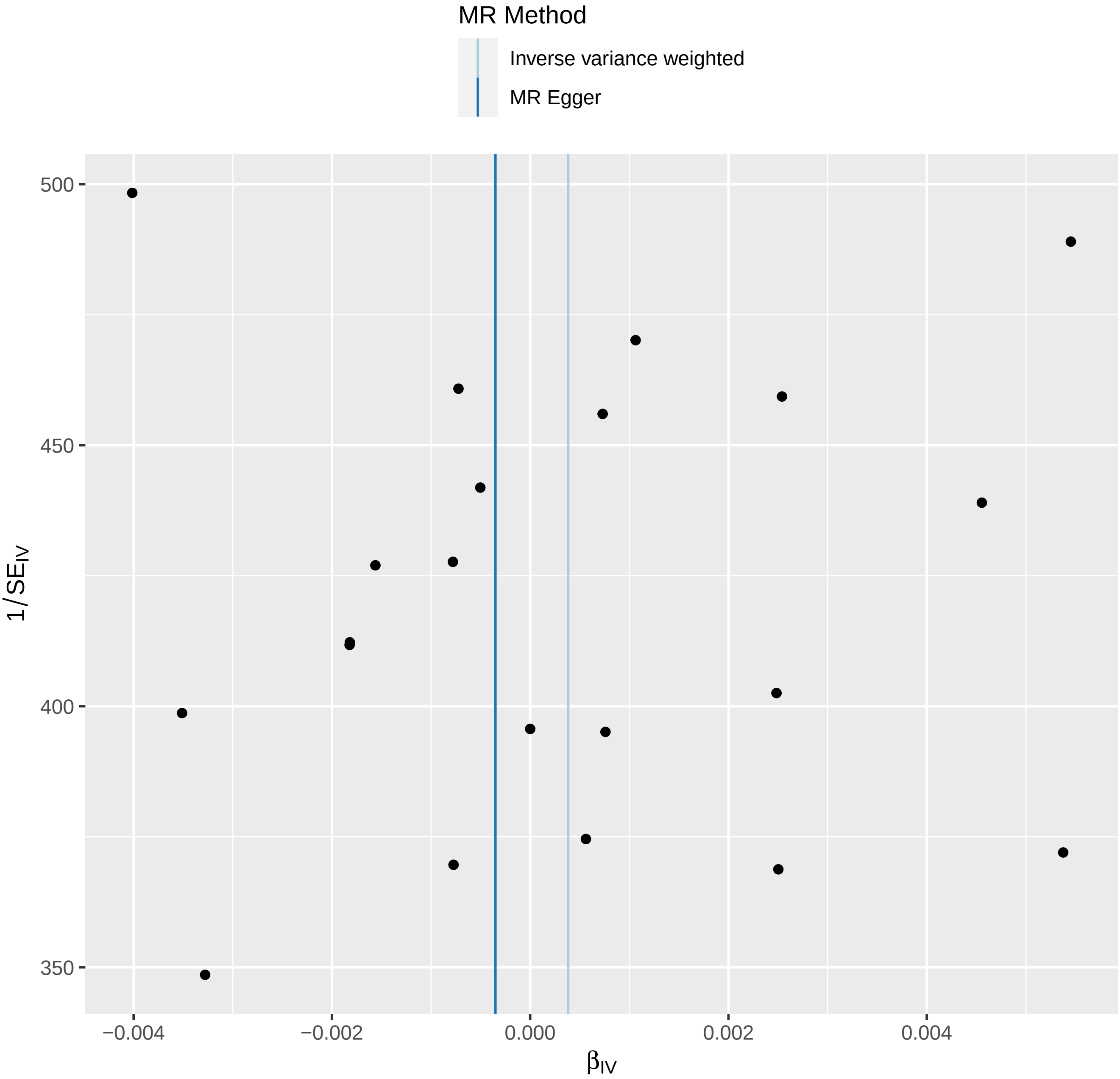

Batch 2−450 : Leukocyte surface antigen CD47 on eGFR

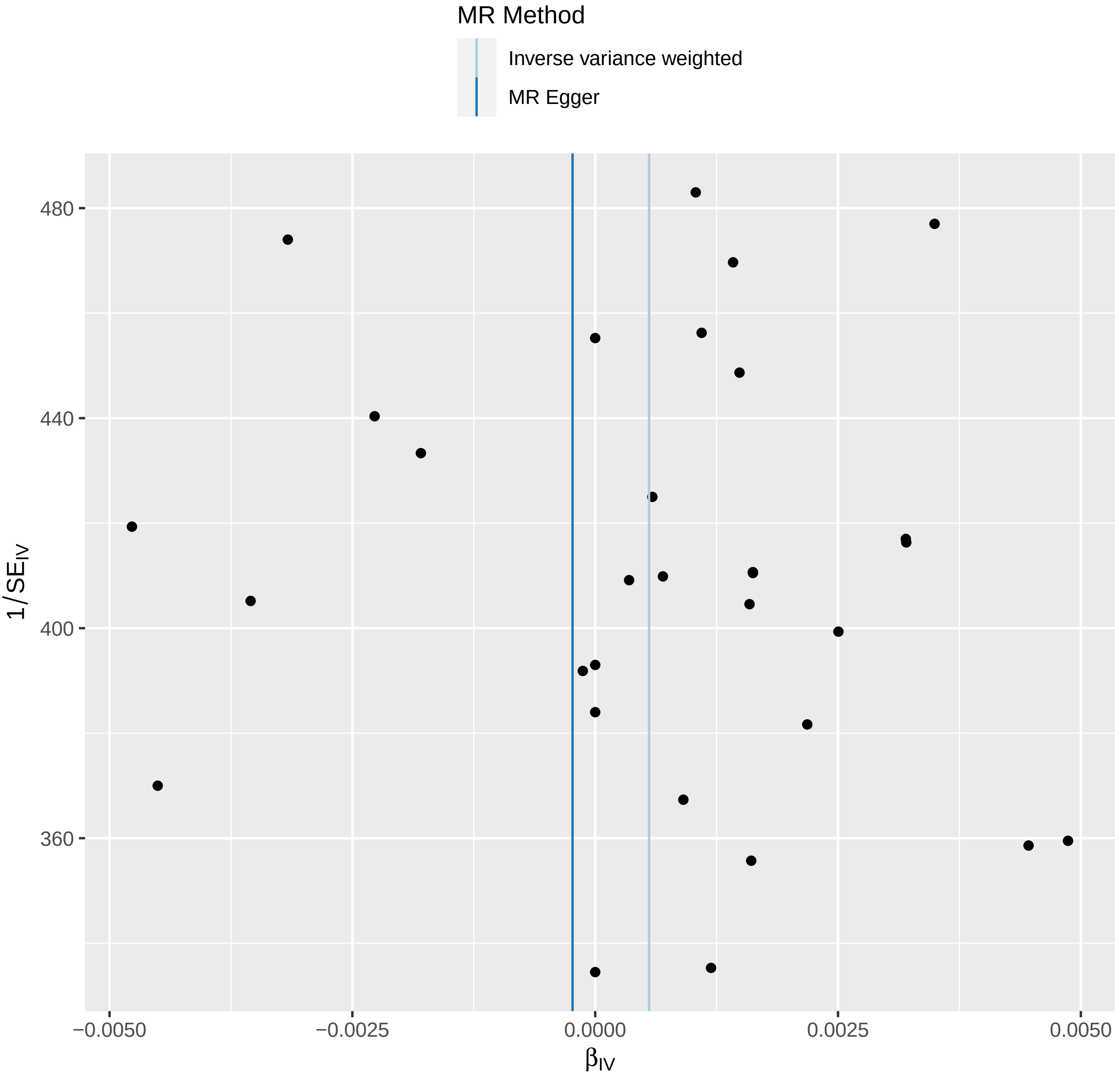

Batch 2−451 : CD48 antigen on eGFR

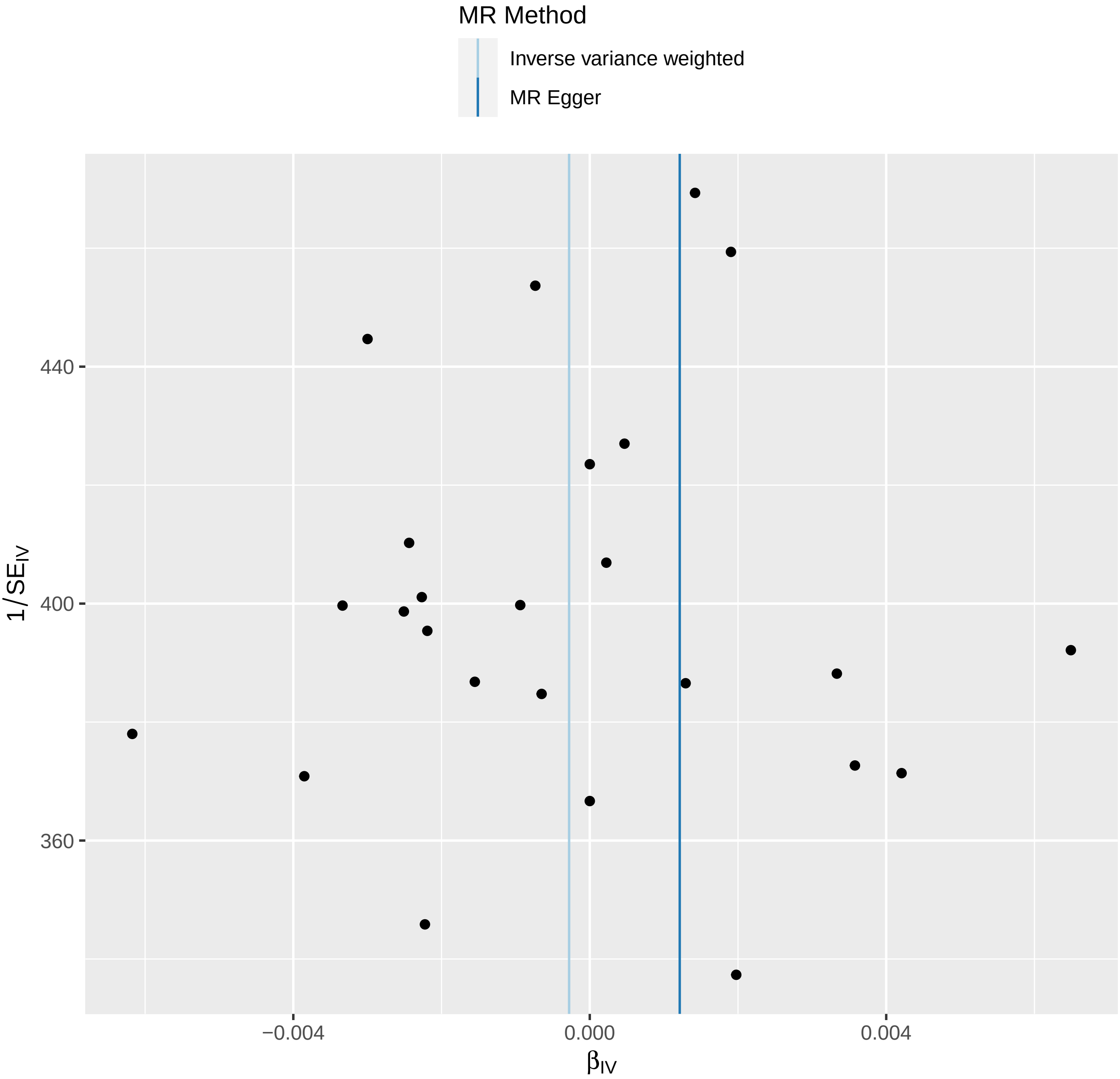

Batch 2−452 : T−cell surface glycoprotein CD5 on eGFR

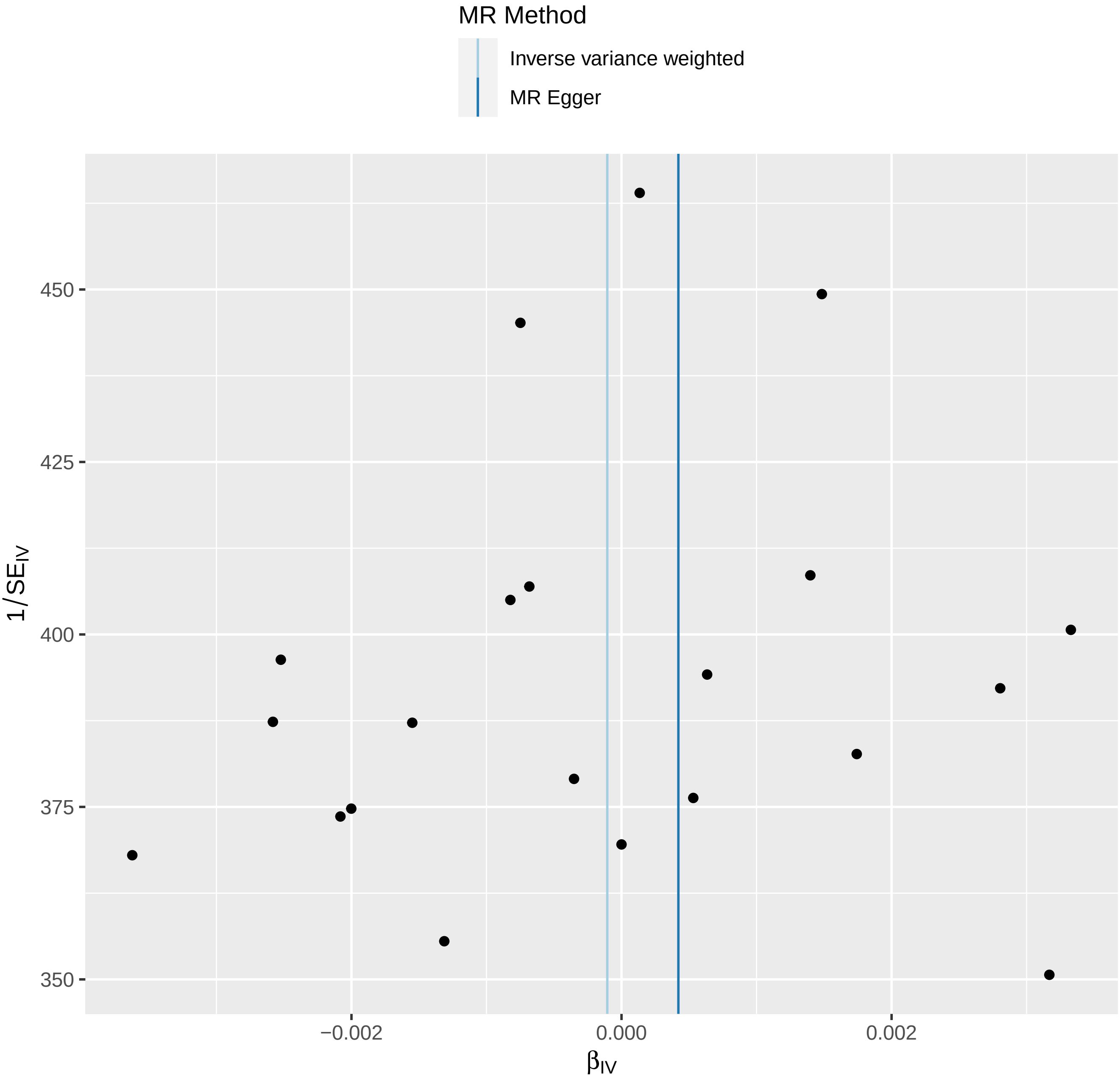

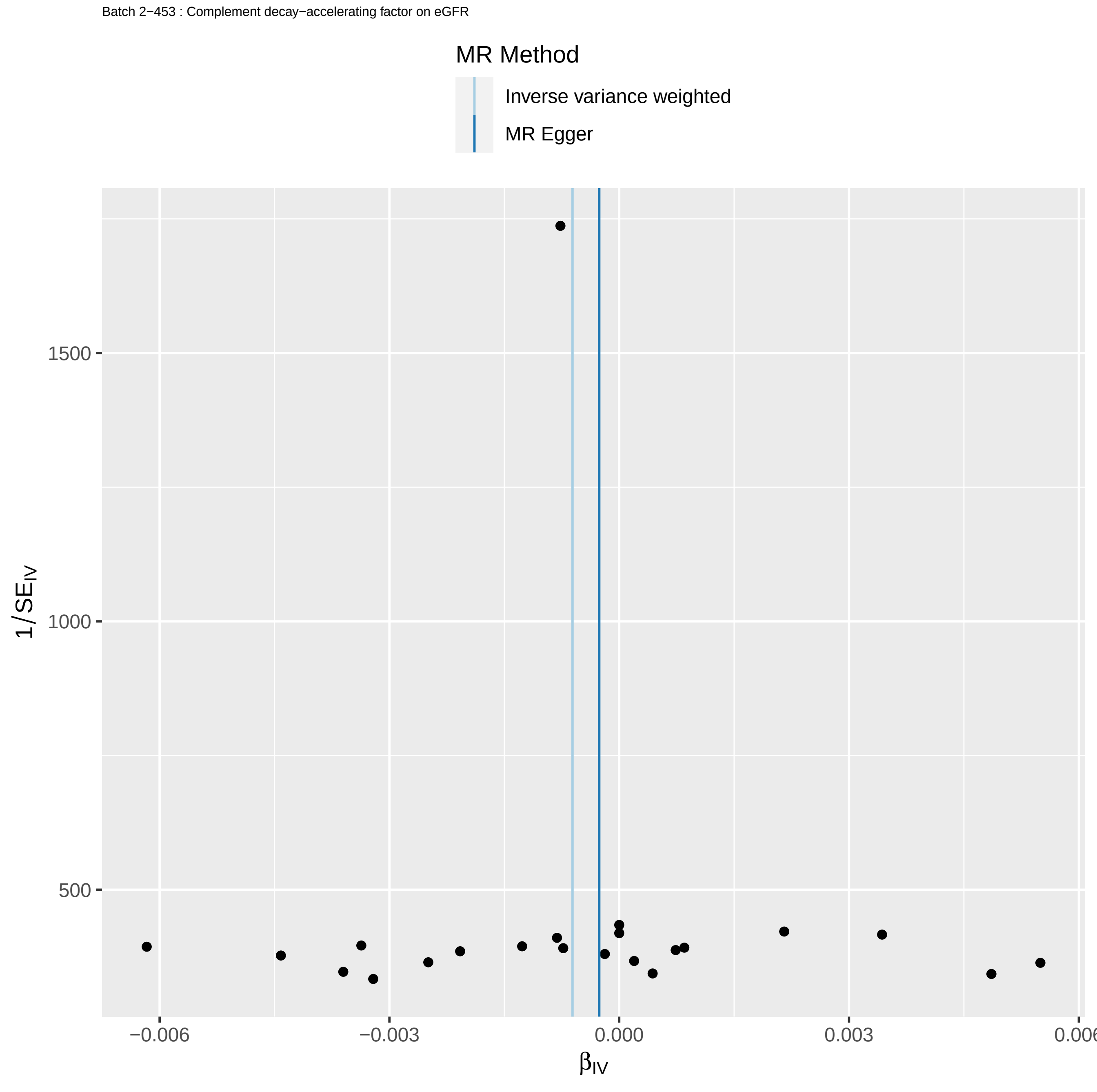
Batch 2−453 : Complement decay−accelerating factor on eGFR
MR Method
Inverse variance weighted
MR Egger
1500
1000
500
1/SE_IV
−0.006
−0.003
0.000
0.003
0.006
β_IV

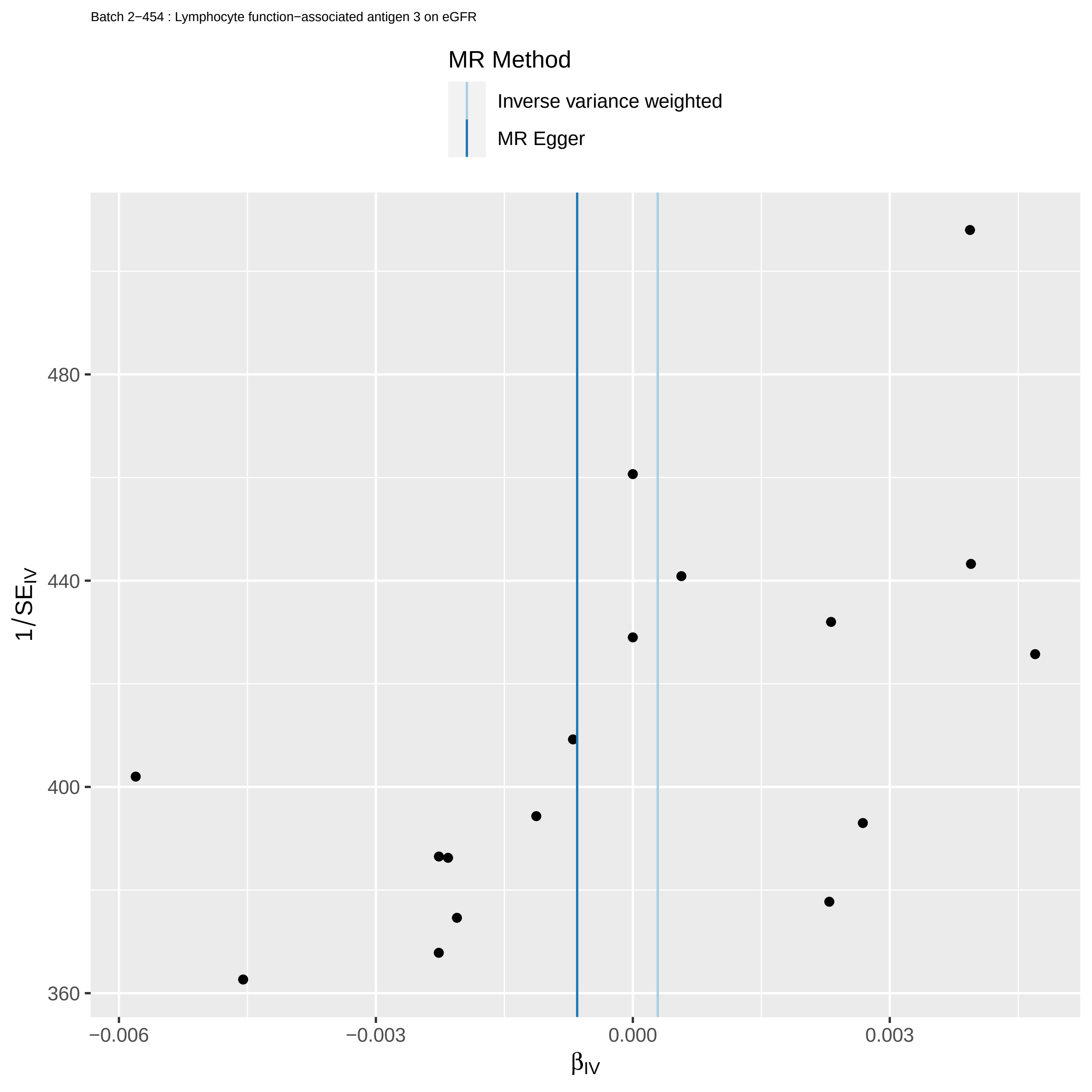
Batch 2−454 : Lymphocyte function−associated antigen 3 on eGFR
MR Method
Inverse variance weighted
MR Egger
480
440
400
360
1/SE$_{IV}$
−0.006
−0.003
0.000
0.003
β_{IV}

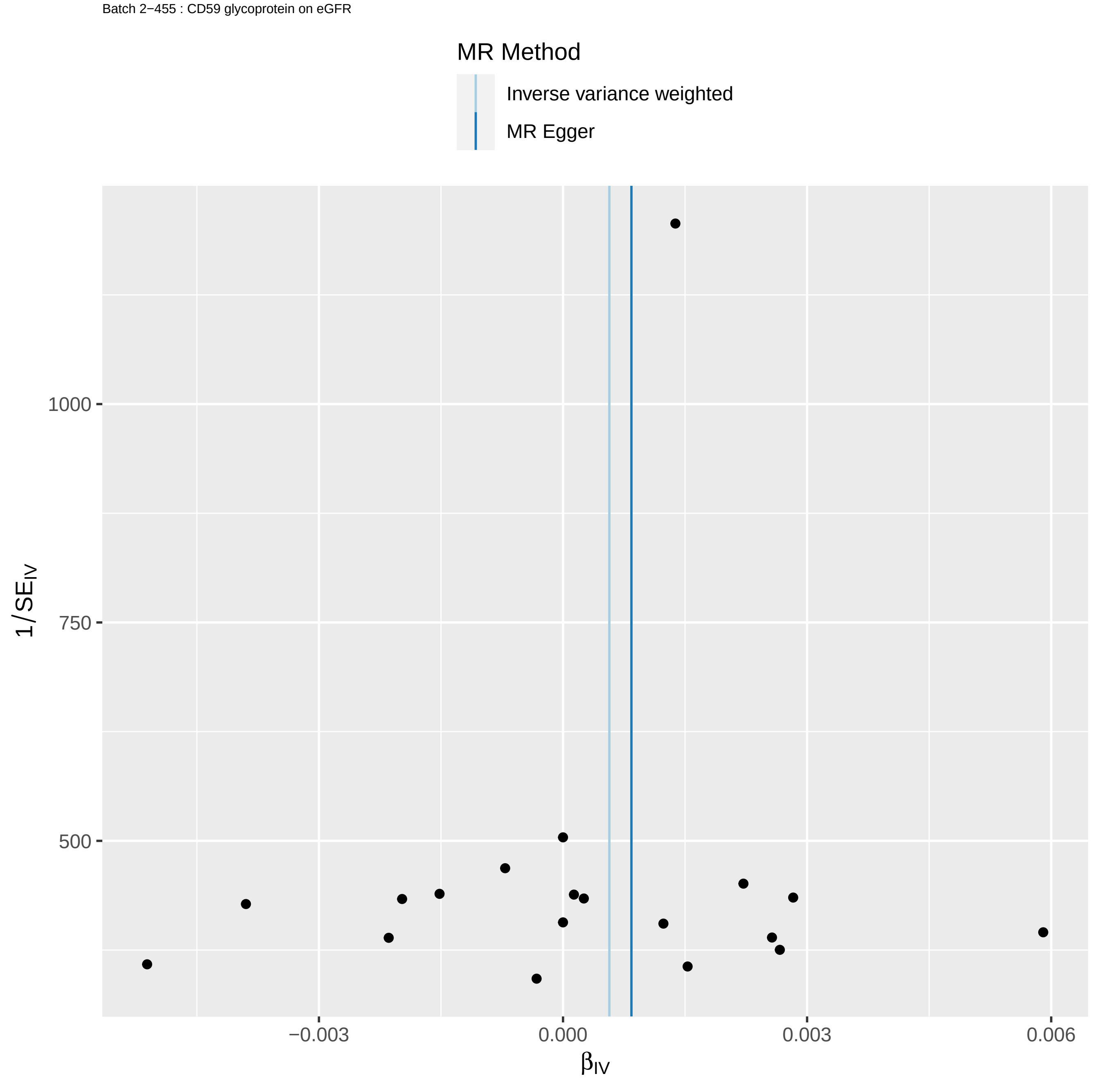
Batch 2−455 : CD59 glycoprotein on eGFR
MR Method
Inverse variance weighted
MR Egger
1000
750
500
1/SE$_{IV}$
−0.003
0.000
0.003
0.006
β$_{IV}$

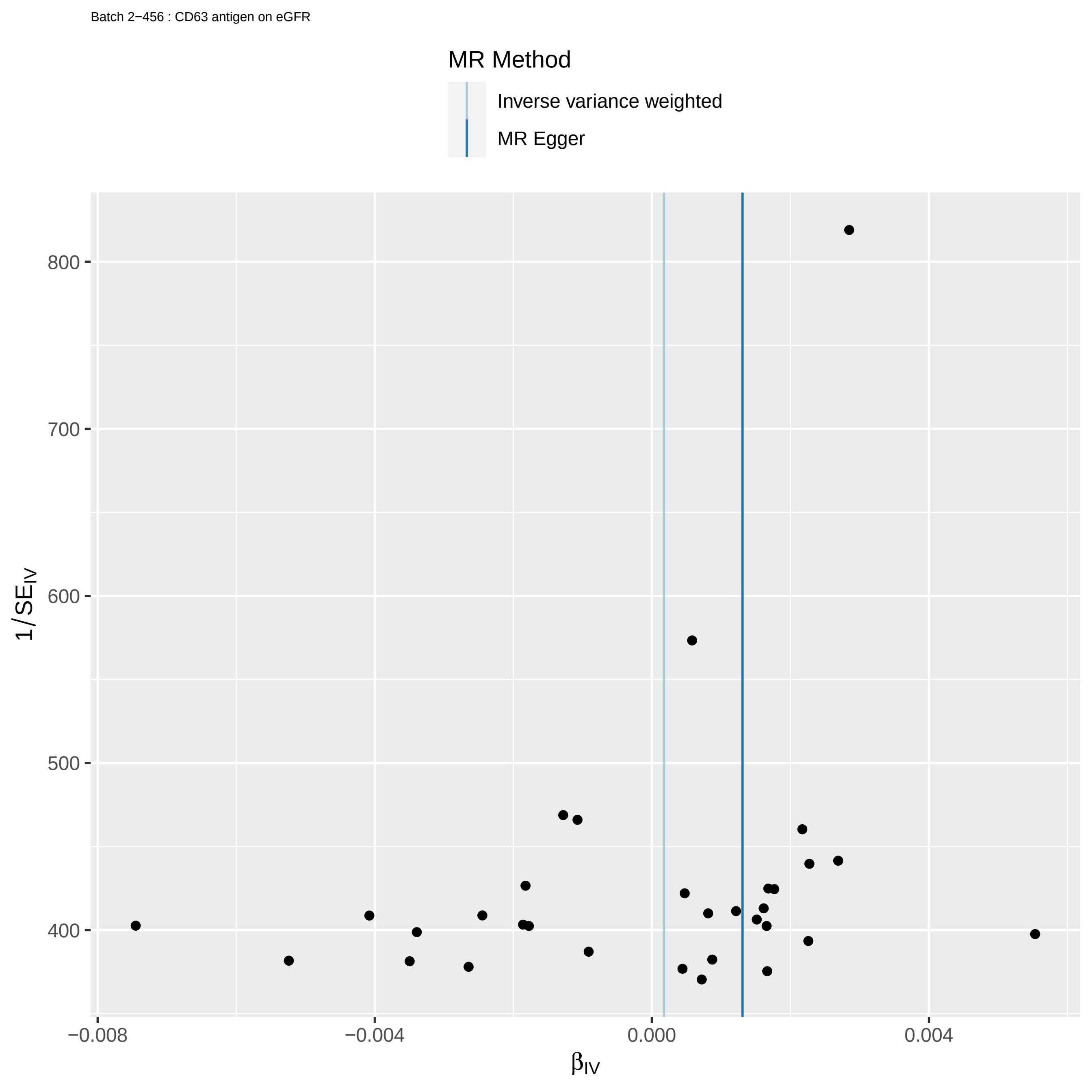

Batch 2−456 : CD63 antigen on eGFR
MR Method
Inverse variance weighted
MR Egger
800
700
600
500
400
1/SE$_{IV}$
−0.008
−0.004
0.000
0.004
β_{IV}

Batch 2−457 : CD70 antigen on eGFR

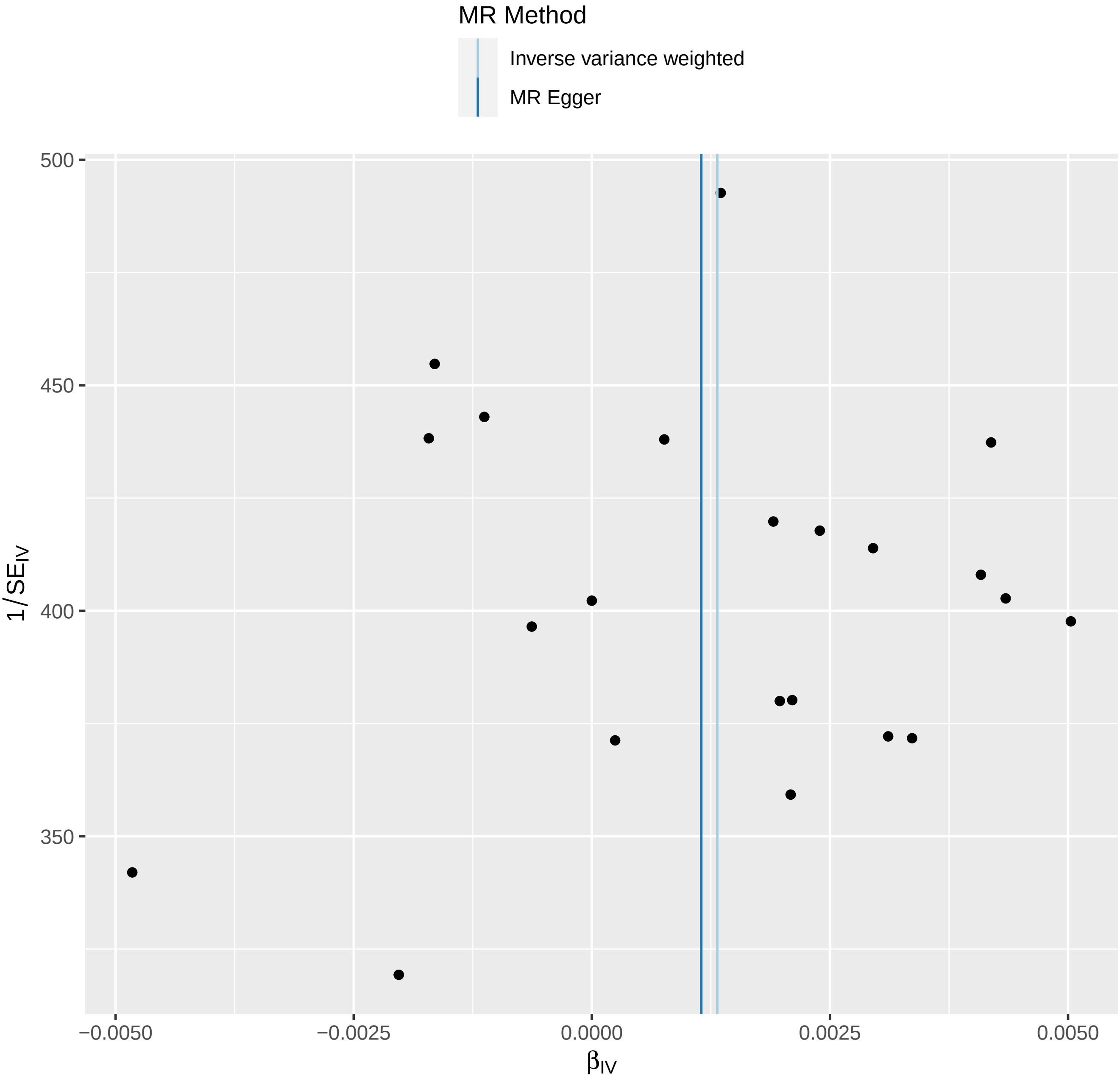

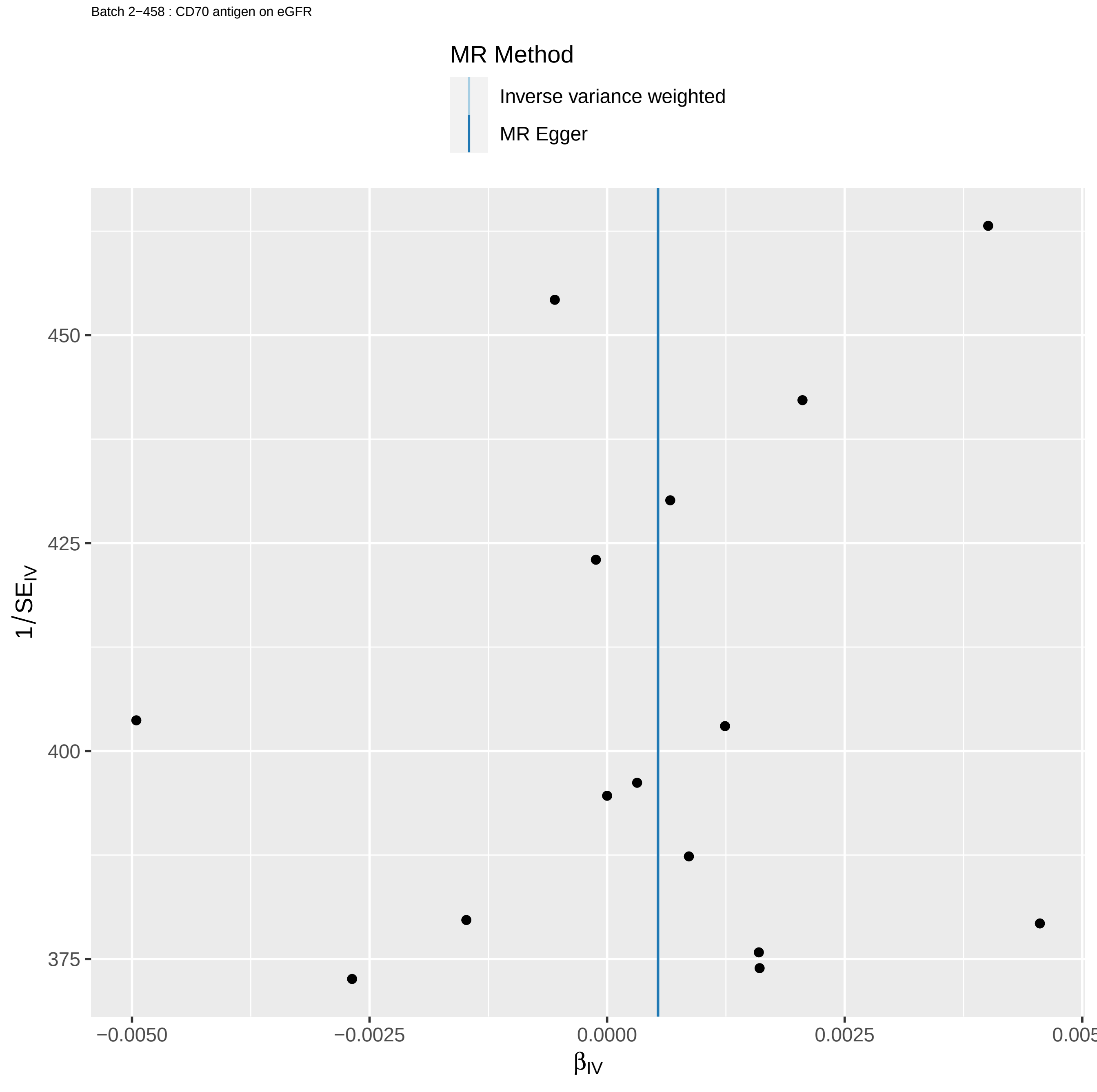

Batch 2−458 : CD70 antigen on eGFR
MR Method
Inverse variance weighted
MR Egger
450
425
400
375
1/SE$_{IV}$
−0.0050
−0.0025
0.0000
0.0025
0.005
β$_{IV}$

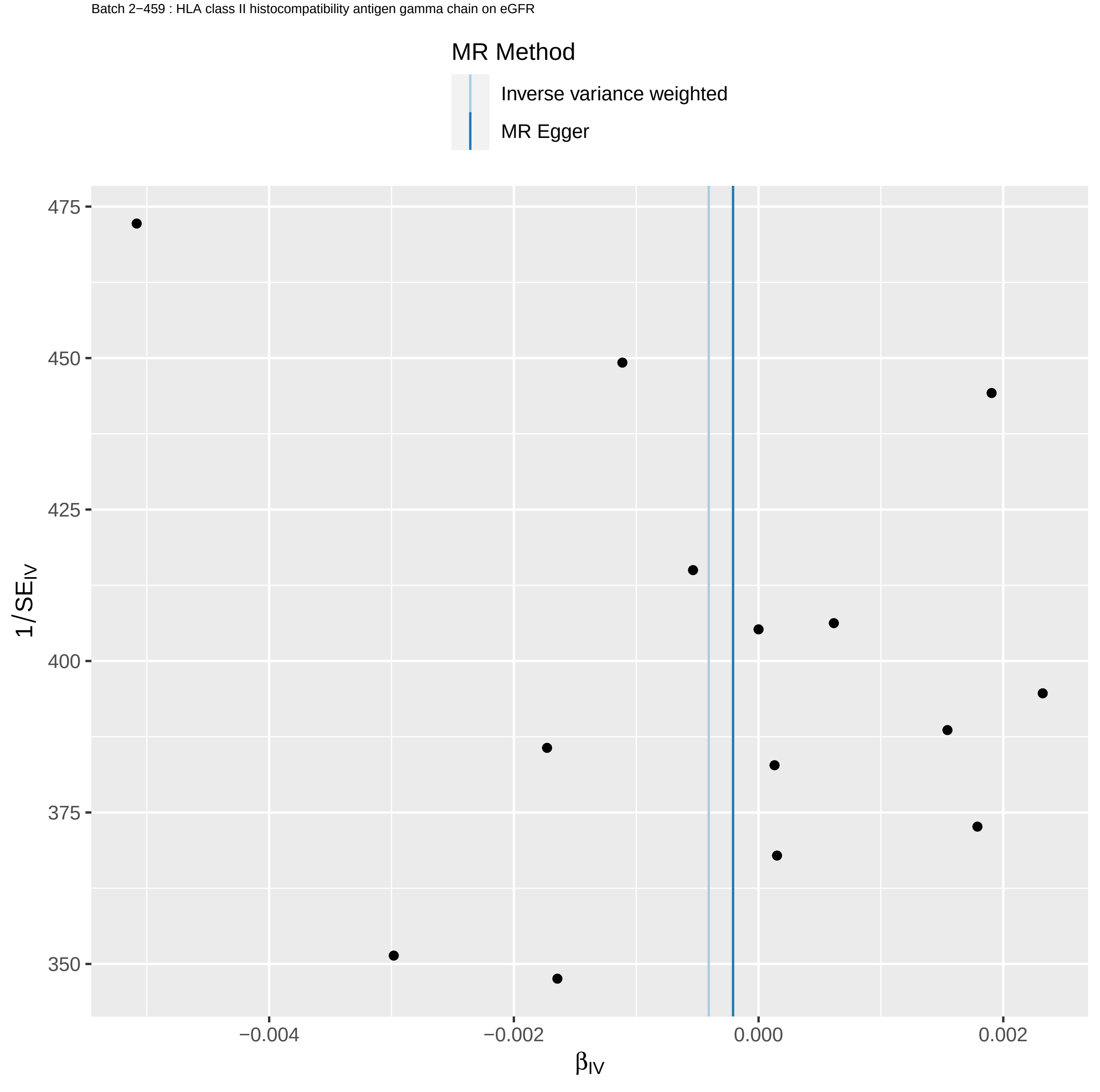

Batch 2−459 : HLA class II histocompatibility antigen gamma chain on eGFR
MR Method
Inverse variance weighted
MR Egger
475
450
425
400
375
350
1/SE_IV
−0.004
−0.002
0.000
0.002
β_IV

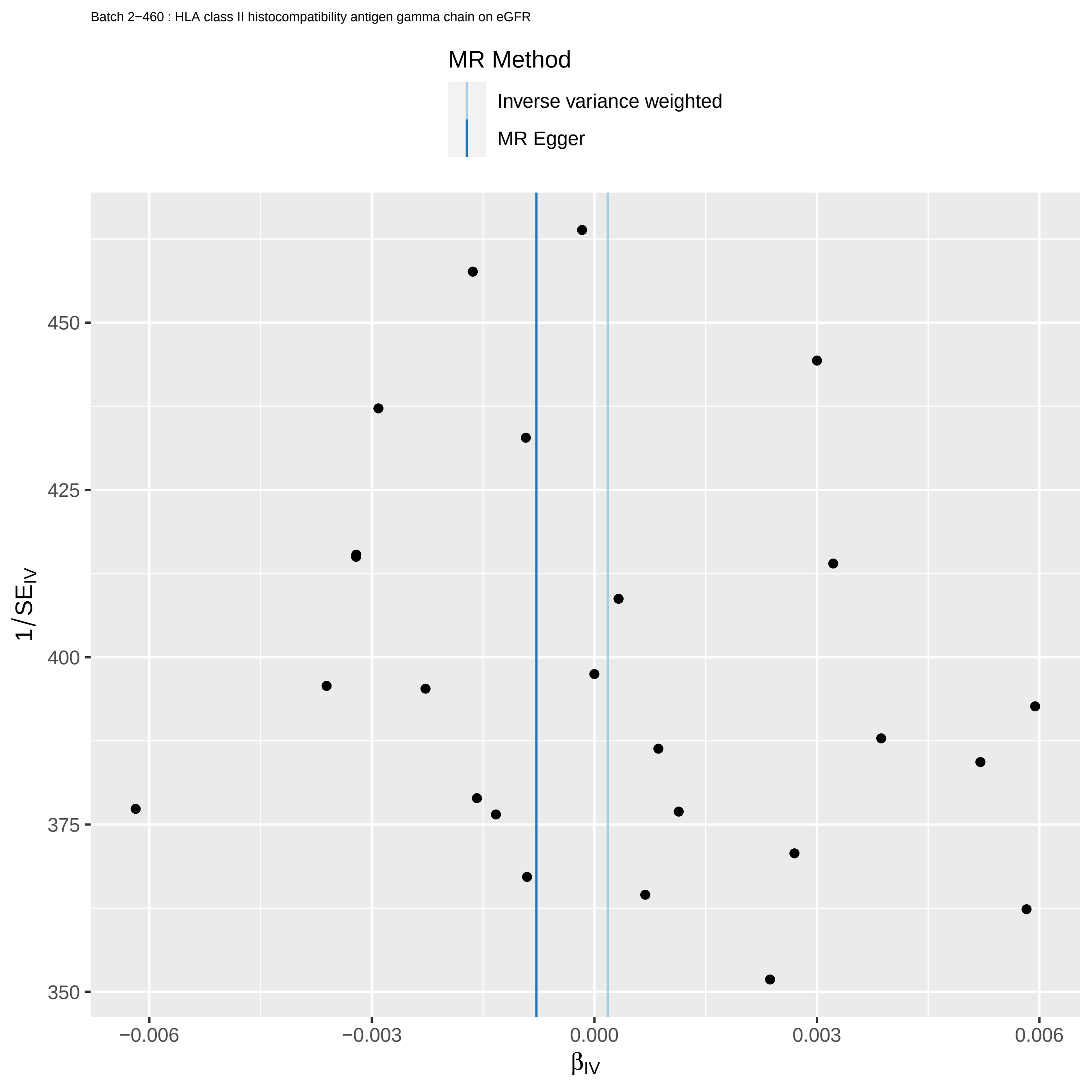

Batch 2−460 : HLA class II histocompatibility antigen gamma chain on eGFR
MR Method
Inverse variance weighted
MR Egger
450
425
400
375
350
1/SE$_{IV}$
−0.006
−0.003
0.000
0.003
0.006
β$_{IV}$

Batch 2−461 : B−cell antigen receptor complex−associated protein alpha chain on eGFR

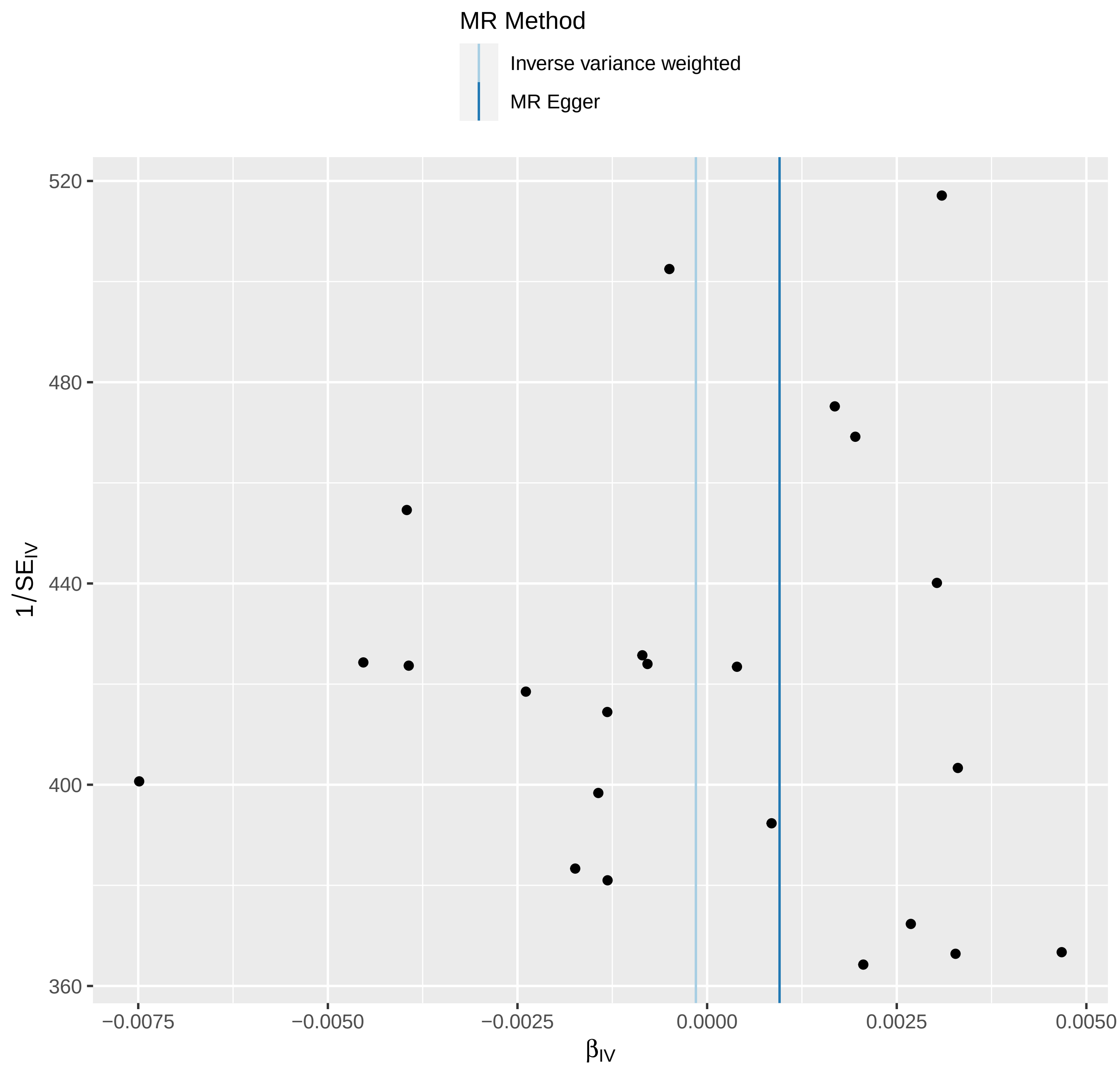

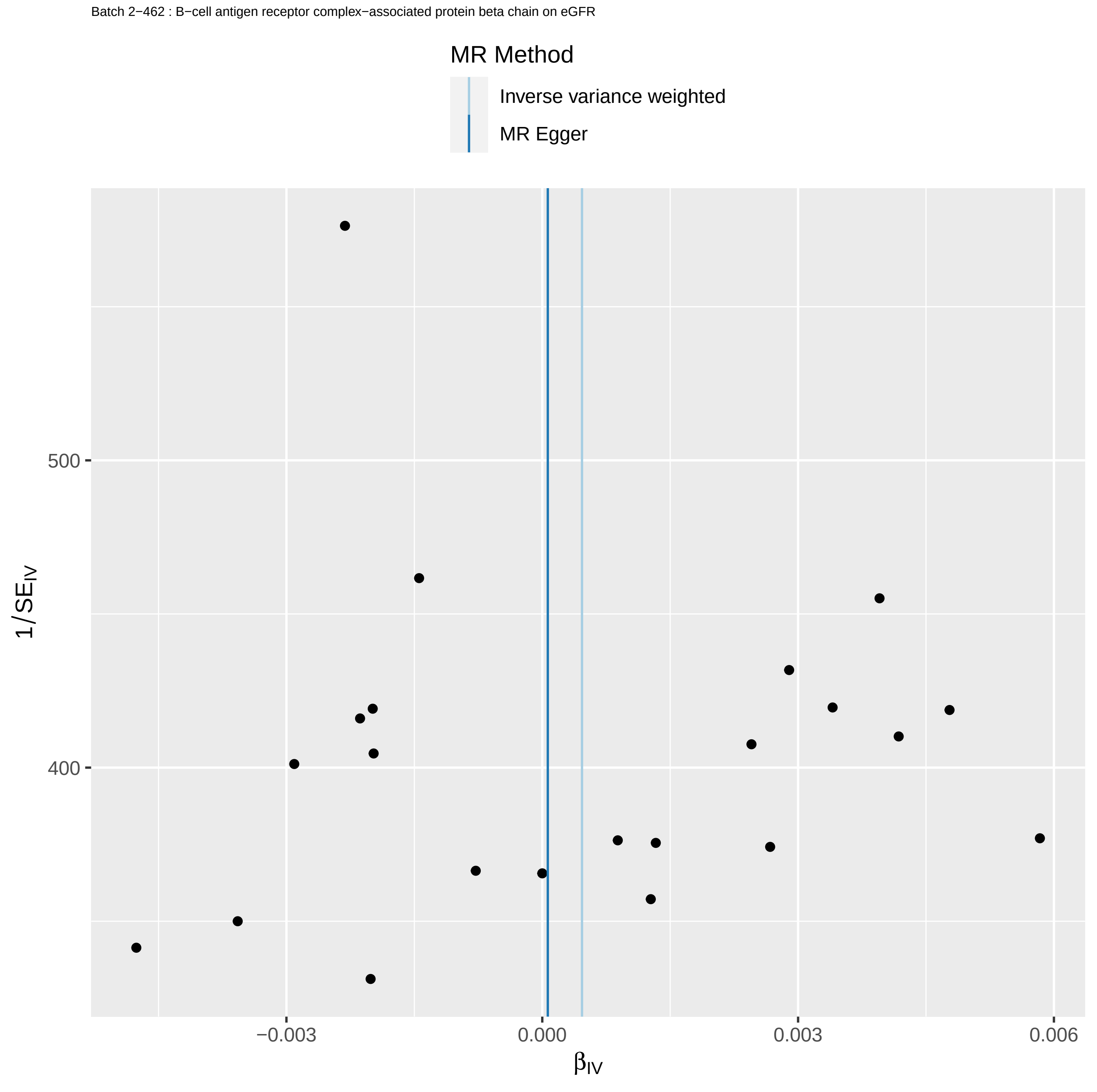

Batch 2−462 : B−cell antigen receptor complex−associated protein beta chain on eGFR
MR Method
Inverse variance weighted
MR Egger
500
400
$1/SE_{IV}$
−0.003
0.000
0.003
0.006
β_{IV}

Batch 2−463 : T−lymphocyte activation antigen CD80 on eGFR

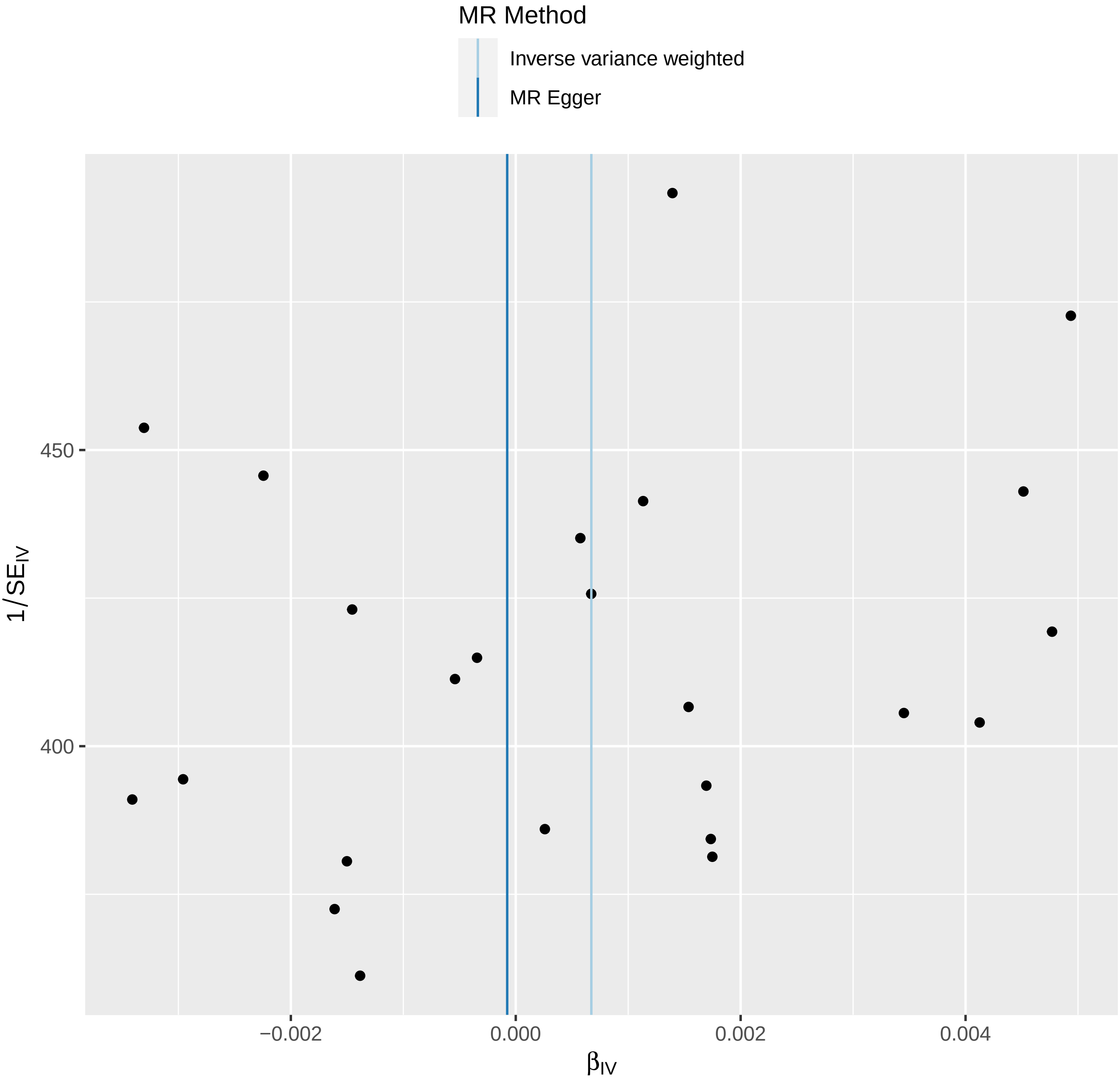

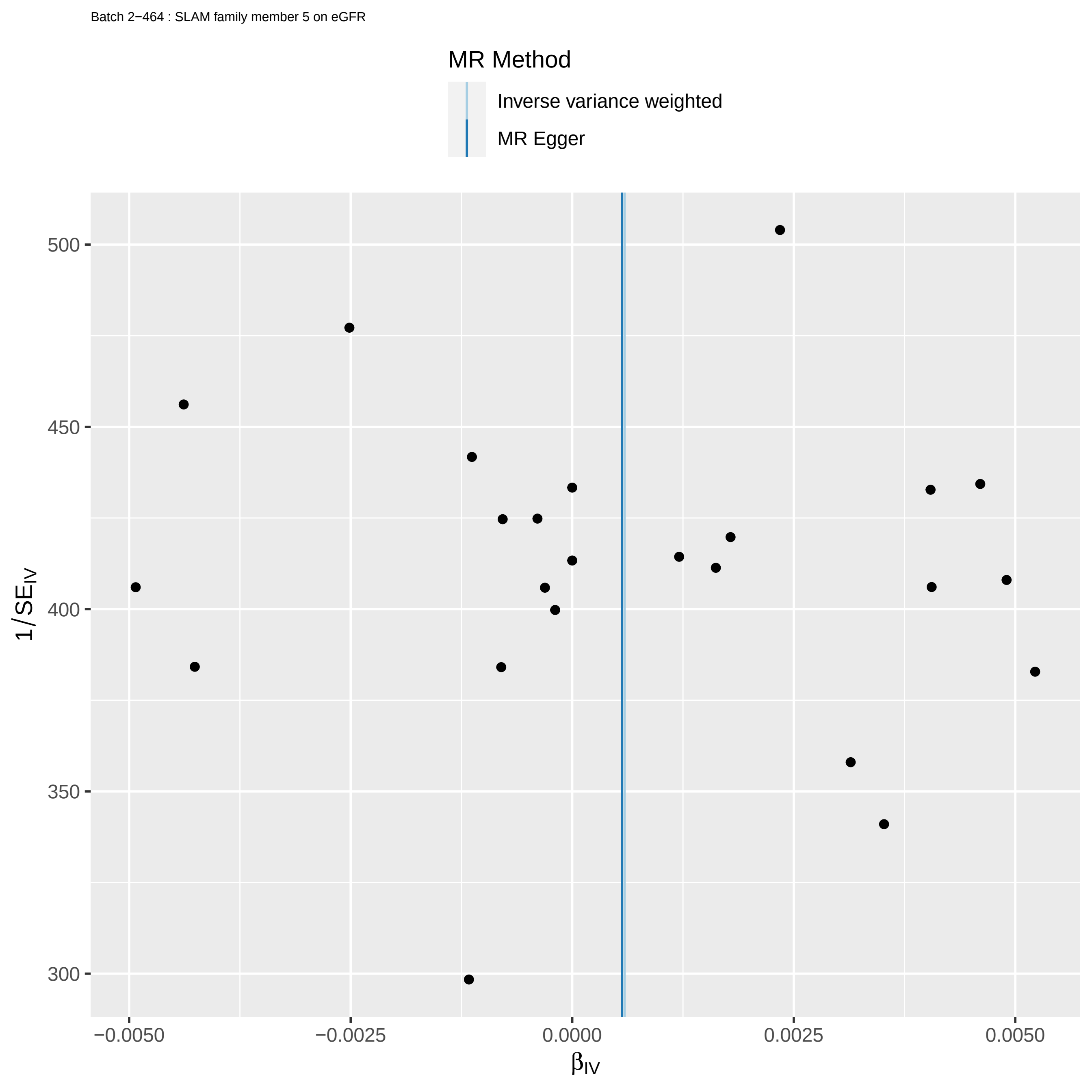

Batch 2−464 : SLAM family member 5 on eGFR
MR Method
Inverse variance weighted
MR Egger
500
450
400
350
300
1/SE$_{IV}$
−0.0050
−0.0025
0.0000
0.0025
0.0050
β$_{IV}$

Batch 2−465 : SLAM family member 5 on eGFR

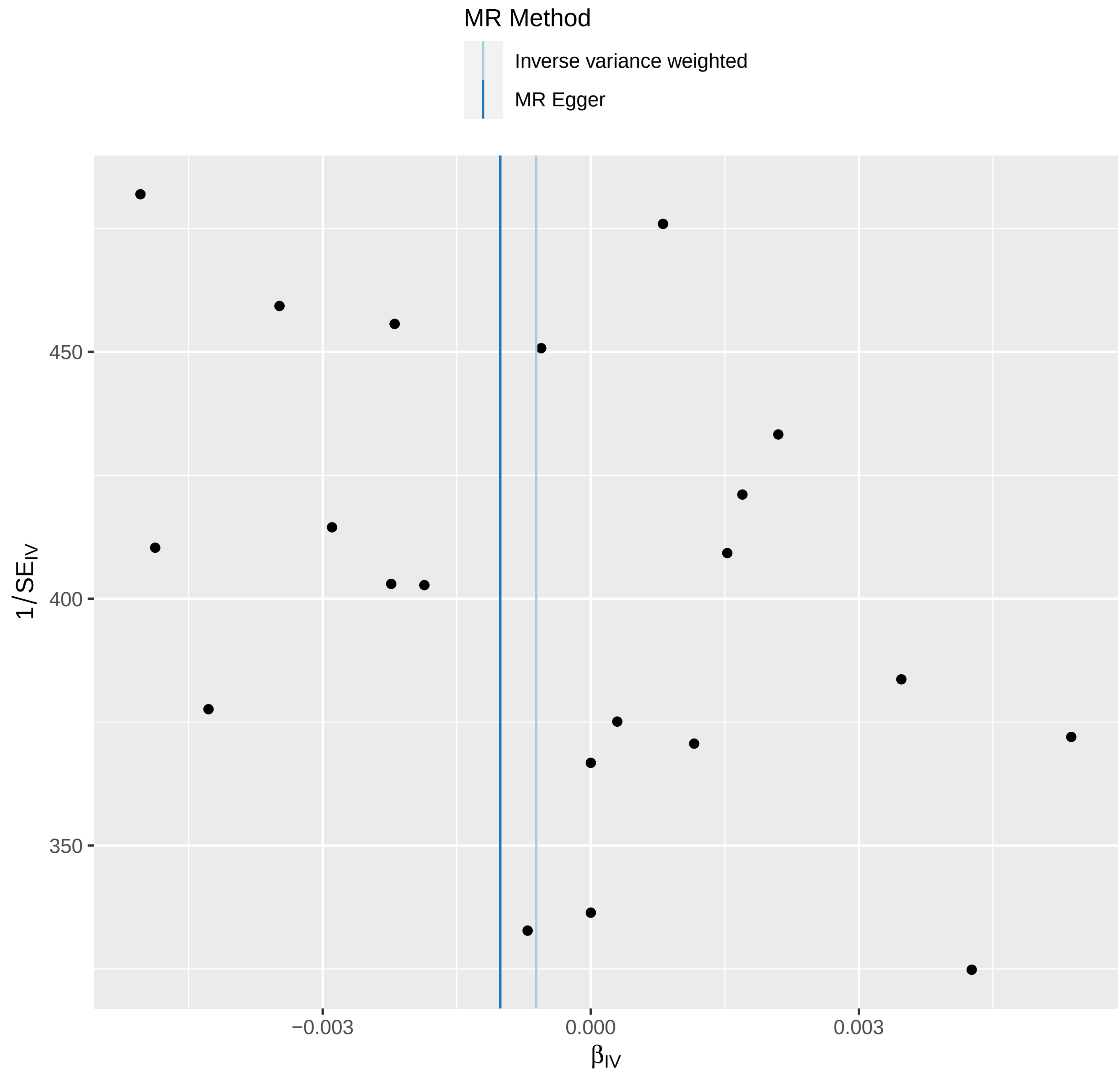

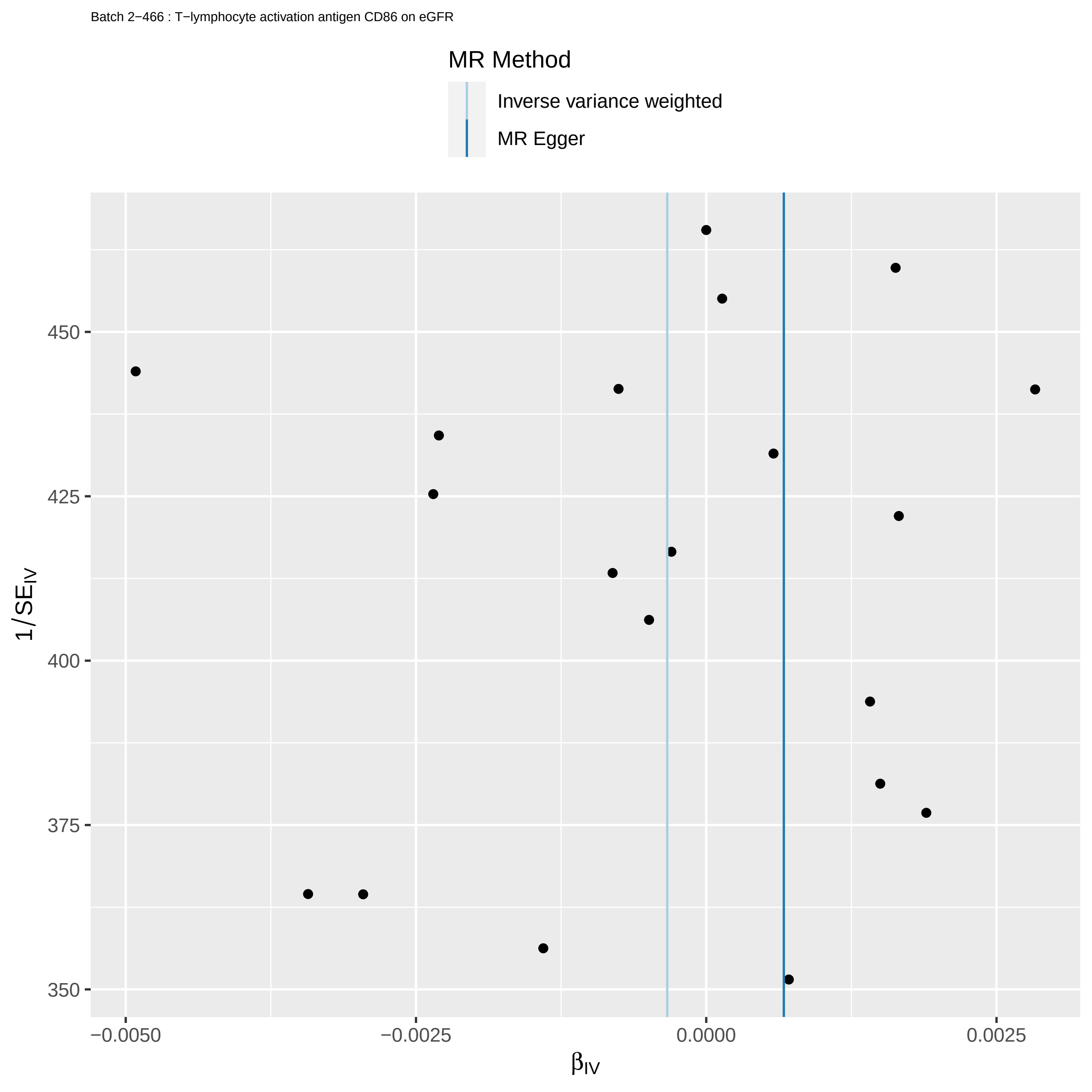

Batch 2−466 : T−lymphocyte activation antigen CD86 on eGFR
MR Method
Inverse variance weighted
MR Egger
450
425
400
375
350
1/SE_IV
−0.0050
−0.0025
0.0000
0.0025
β_IV

Batch 2−467 : T−cell surface glycoprotein CD8 beta chain on eGFR

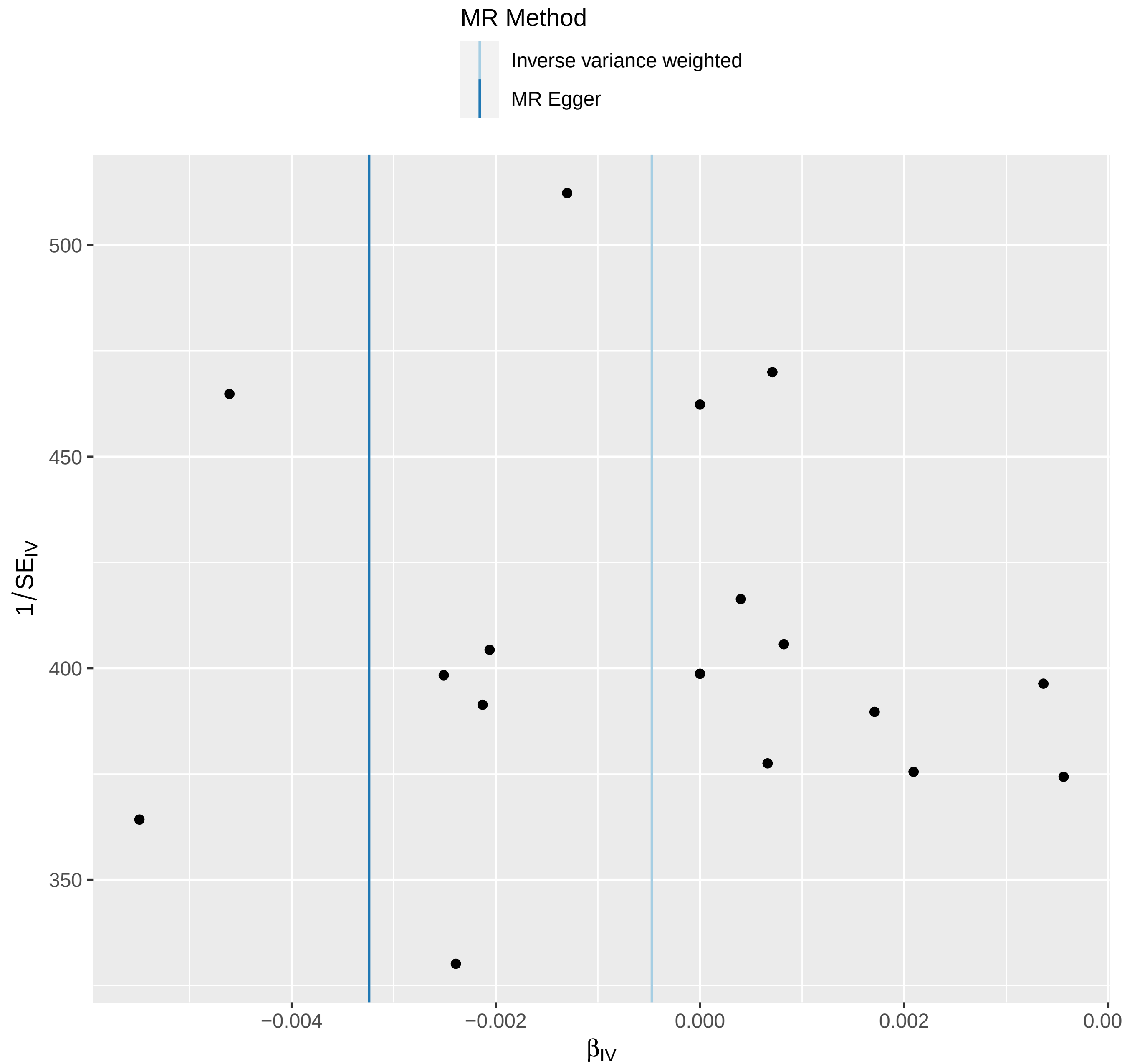

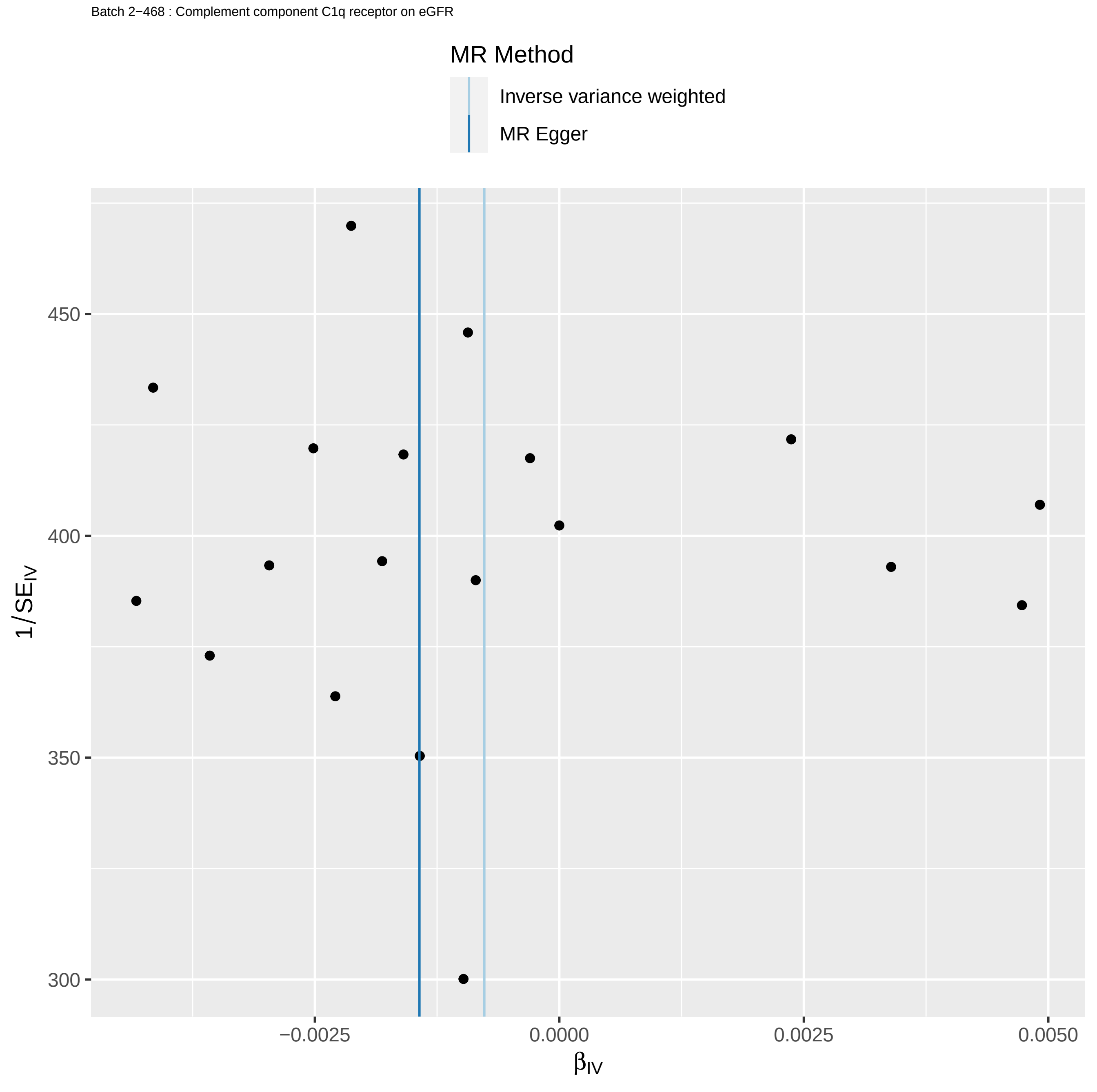

Batch 2−468 : Complement component C1q receptor on eGFR
MR Method
Inverse variance weighted
MR Egger
450
400
350
300
1/SE_IV
−0.0025
0.0000
0.0025
0.0050
β_IV

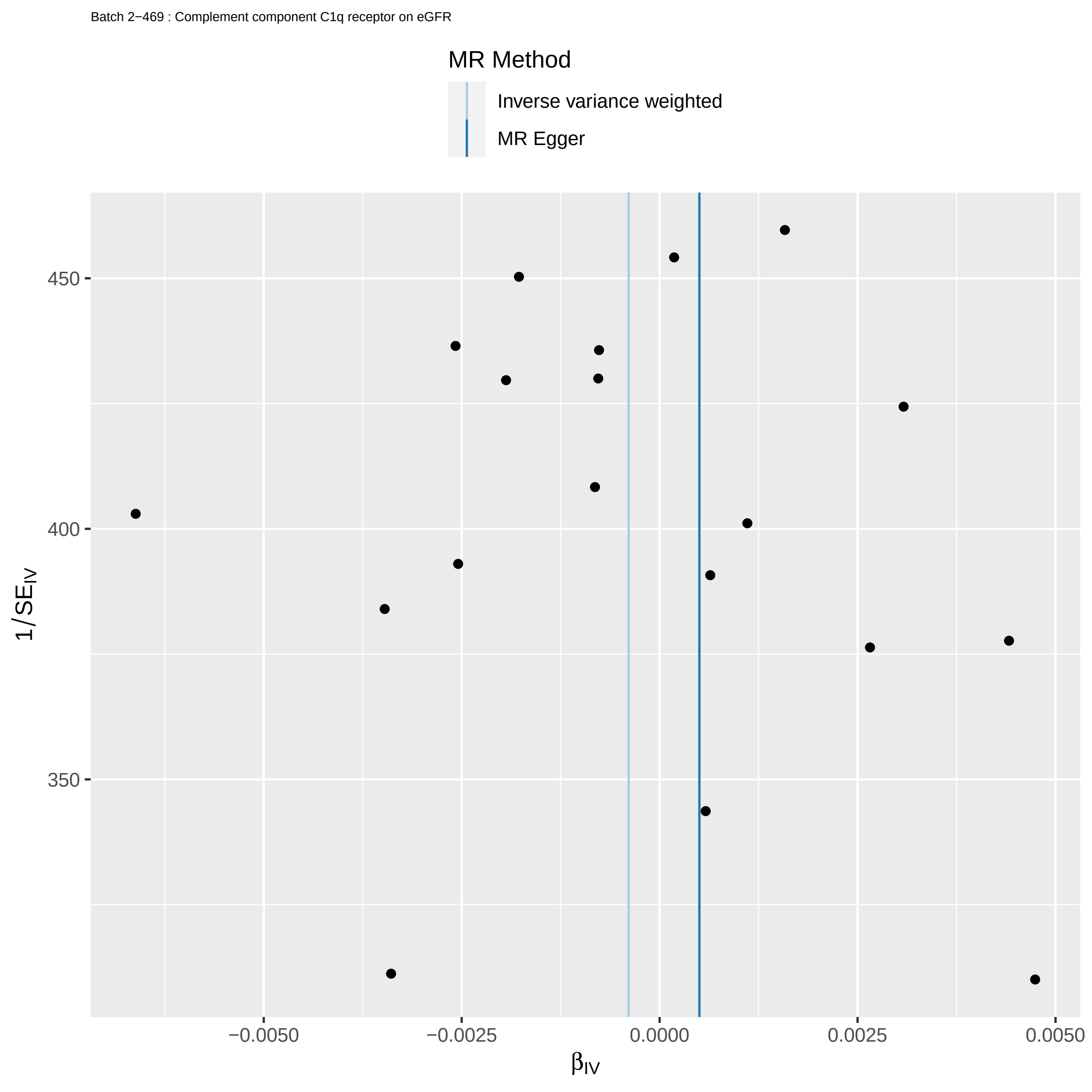
Batch 2−469 : Complement component C1q receptor on eGFR
MR Method
Inverse variance weighted
MR Egger
450
400
350
1/SE_IV
−0.0050
−0.0025
0.0000
0.0025
0.0050
β_IV

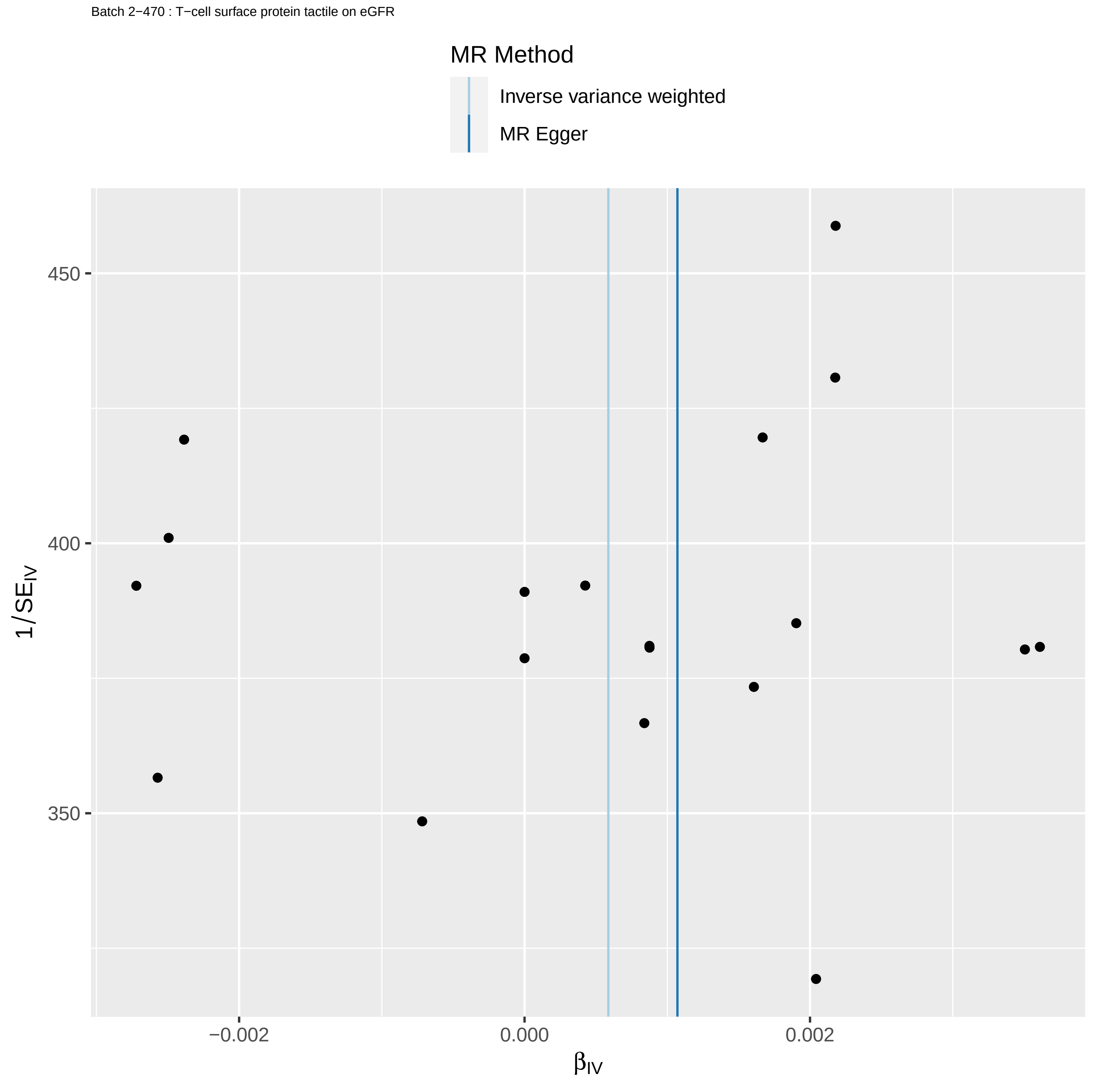
Batch 2−470 : T−cell surface protein tactile on eGFR
MR Method
Inverse variance weighted
MR Egger
450
400
350
1/SE_IV
−0.002
0.000
0.002
β_IV

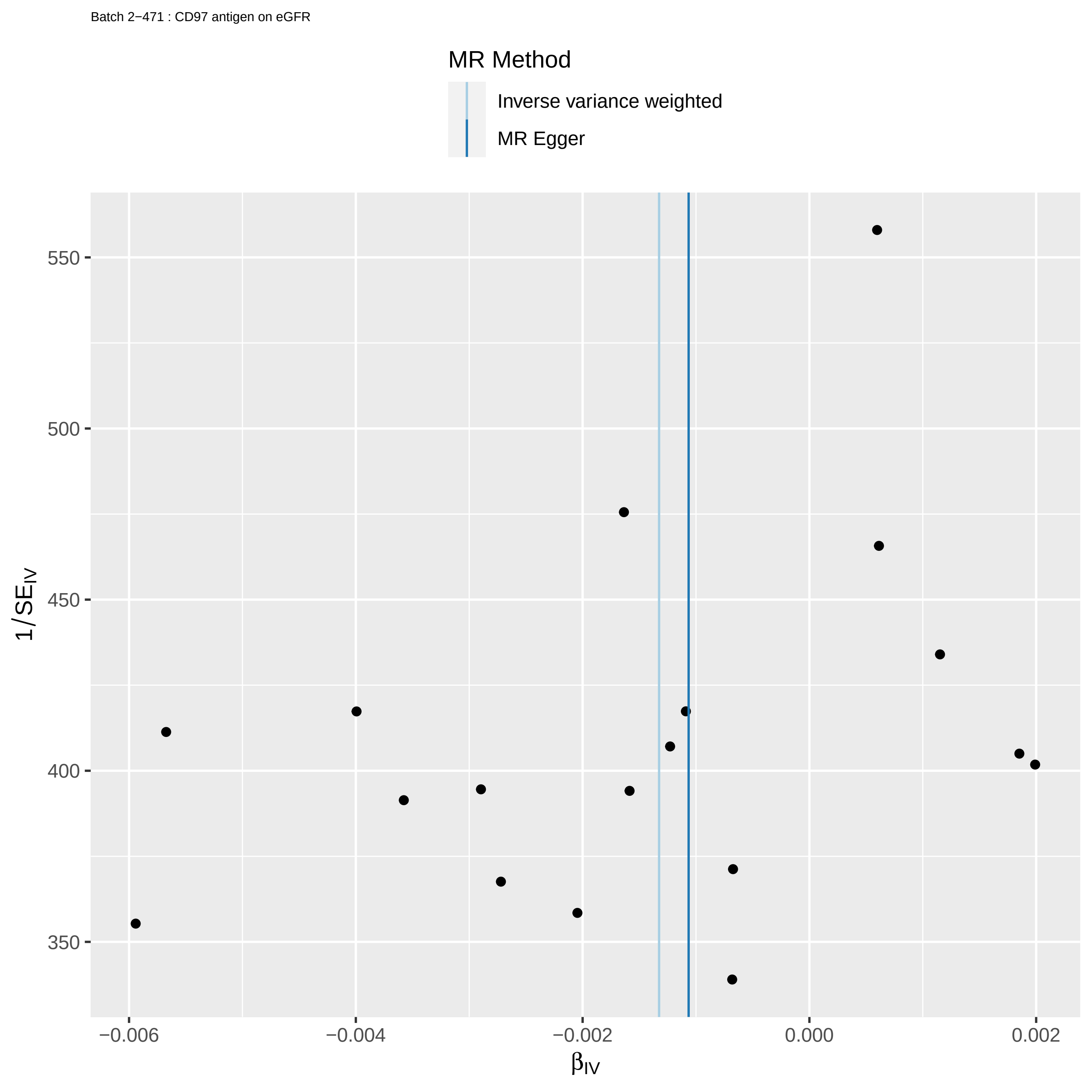
Batch 2−471 : CD97 antigen on eGFR
MR Method
Inverse variance weighted
MR Egger
550
500
450
400
350
1/SE_IV
−0.006
−0.004
−0.002
0.000
0.002
β_IV

Batch 2−472 : M−phase inducer phosphatase 2 on eGFR

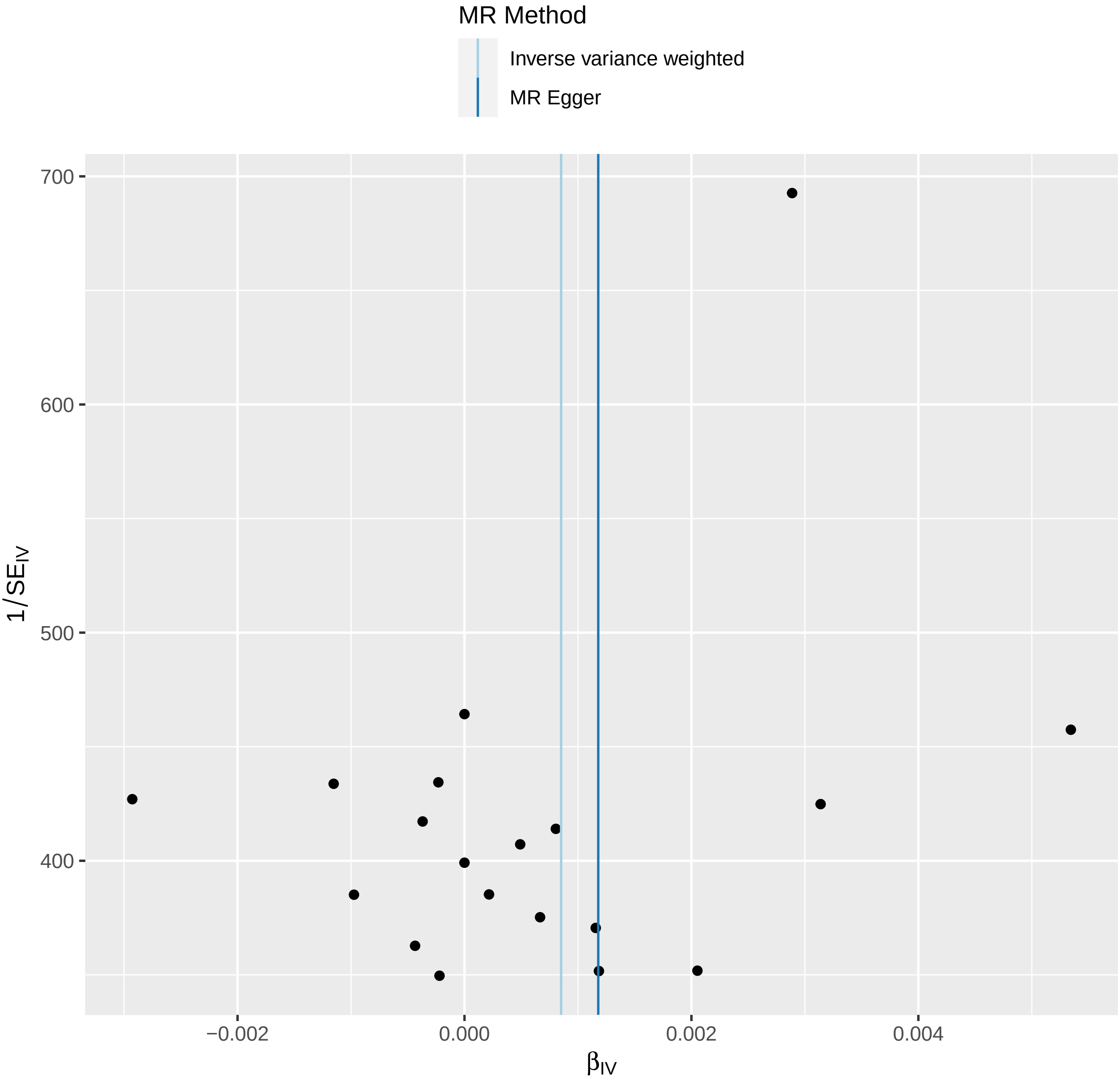

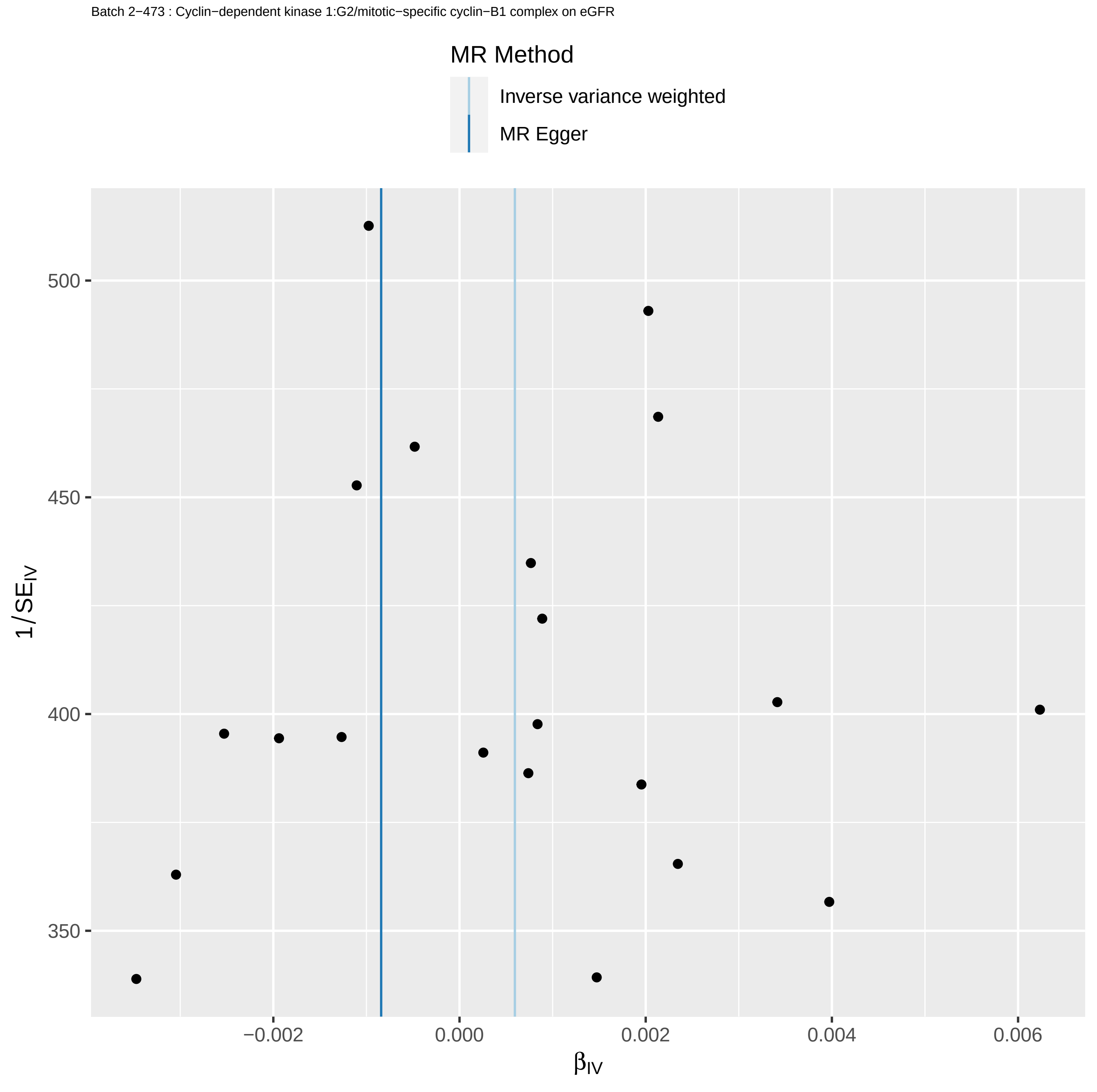

Batch 2−473 : Cyclin−dependent kinase 1:G2/mitotic−specific cyclin−B1 complex on eGFR
MR Method
Inverse variance weighted
MR Egger
500
450
400
350
1/SE$_{IV}$
−0.002
0.000
0.002
0.004
0.006
β$_{IV}$

Batch 2−474 : Cell division control protein 42 homolog on eGFR

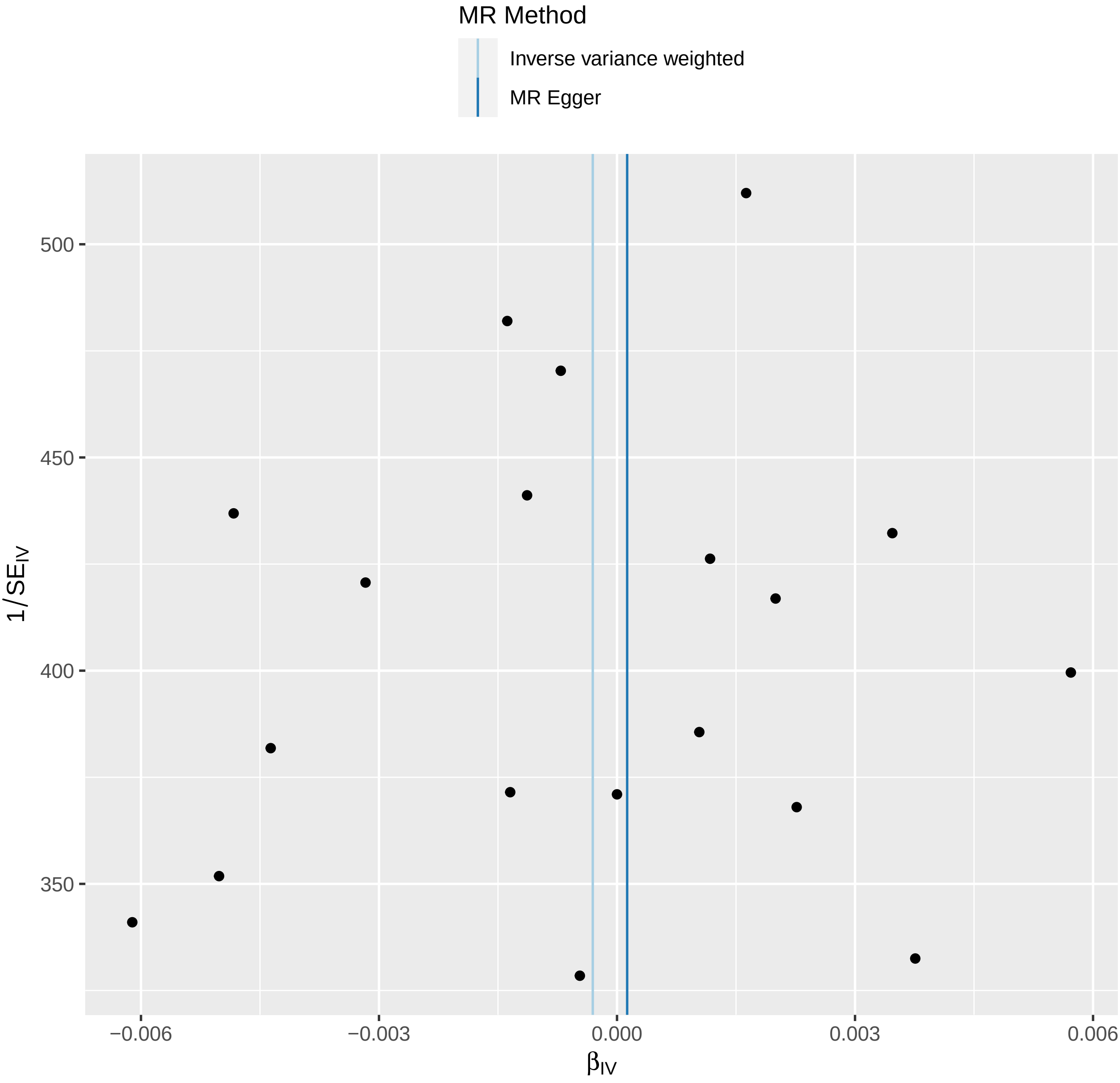

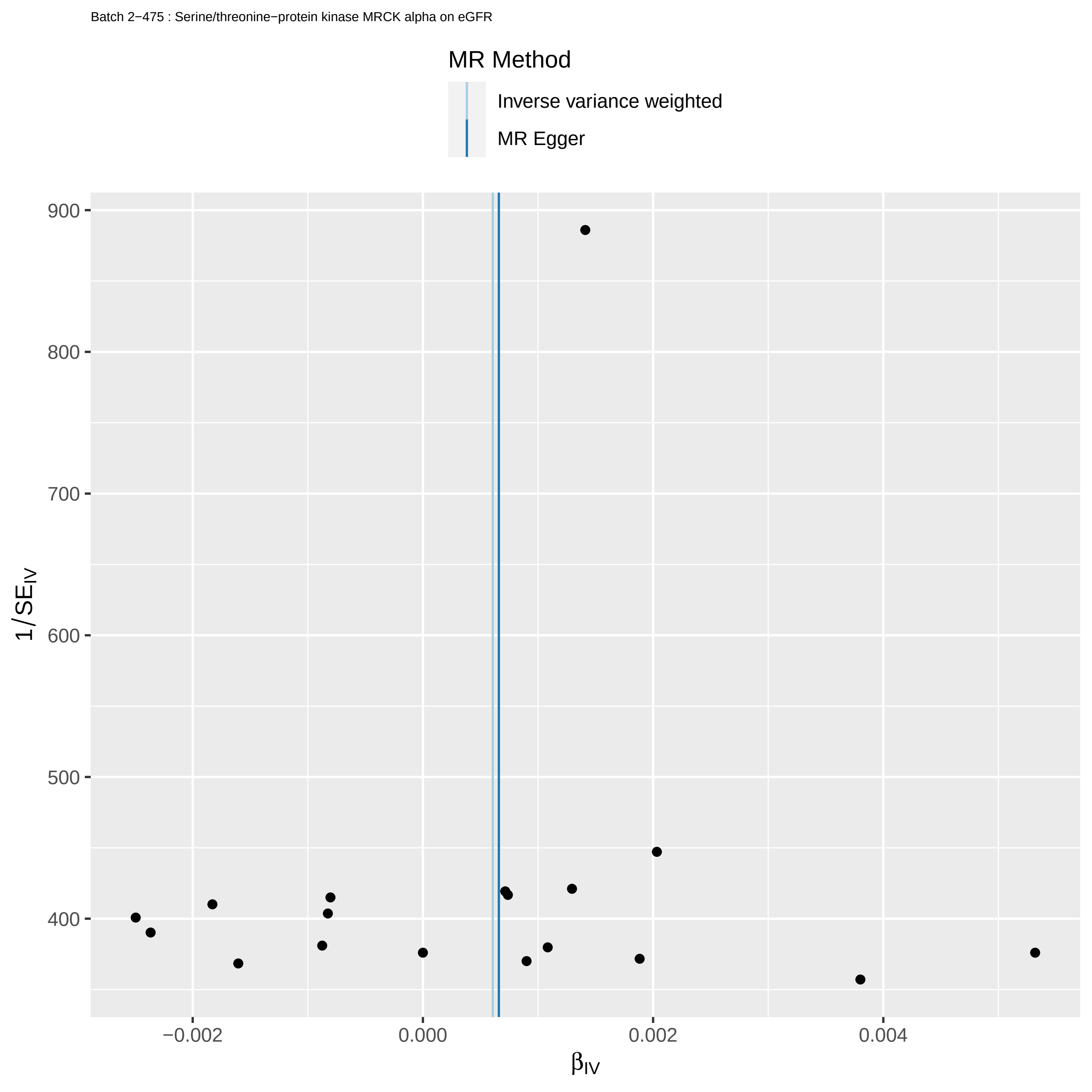
Batch 2−475 : Serine/threonine−protein kinase MRCK alpha on eGFR
MR Method
Inverse variance weighted
MR Egger
900
800
700
600
500
400
1/SE_IV
−0.002
0.000
0.002
0.004
β_IV

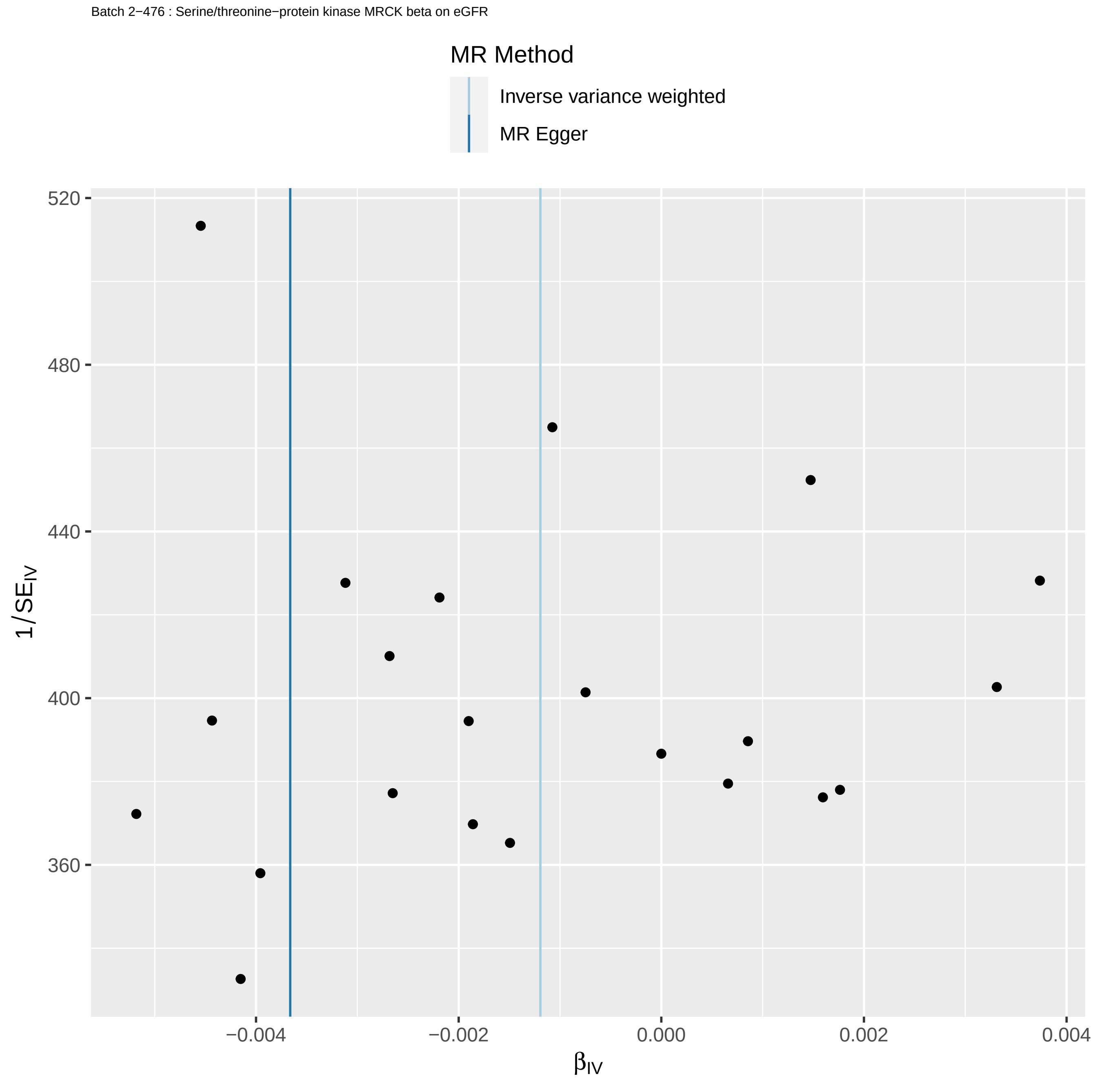
Batch 2−476 : Serine/threonine−protein kinase MRCK beta on eGFR
MR Method
Inverse variance weighted
MR Egger
520
480
440
400
360
1/SE_IV
−0.004
−0.002
0.000
0.002
0.004
β_IV

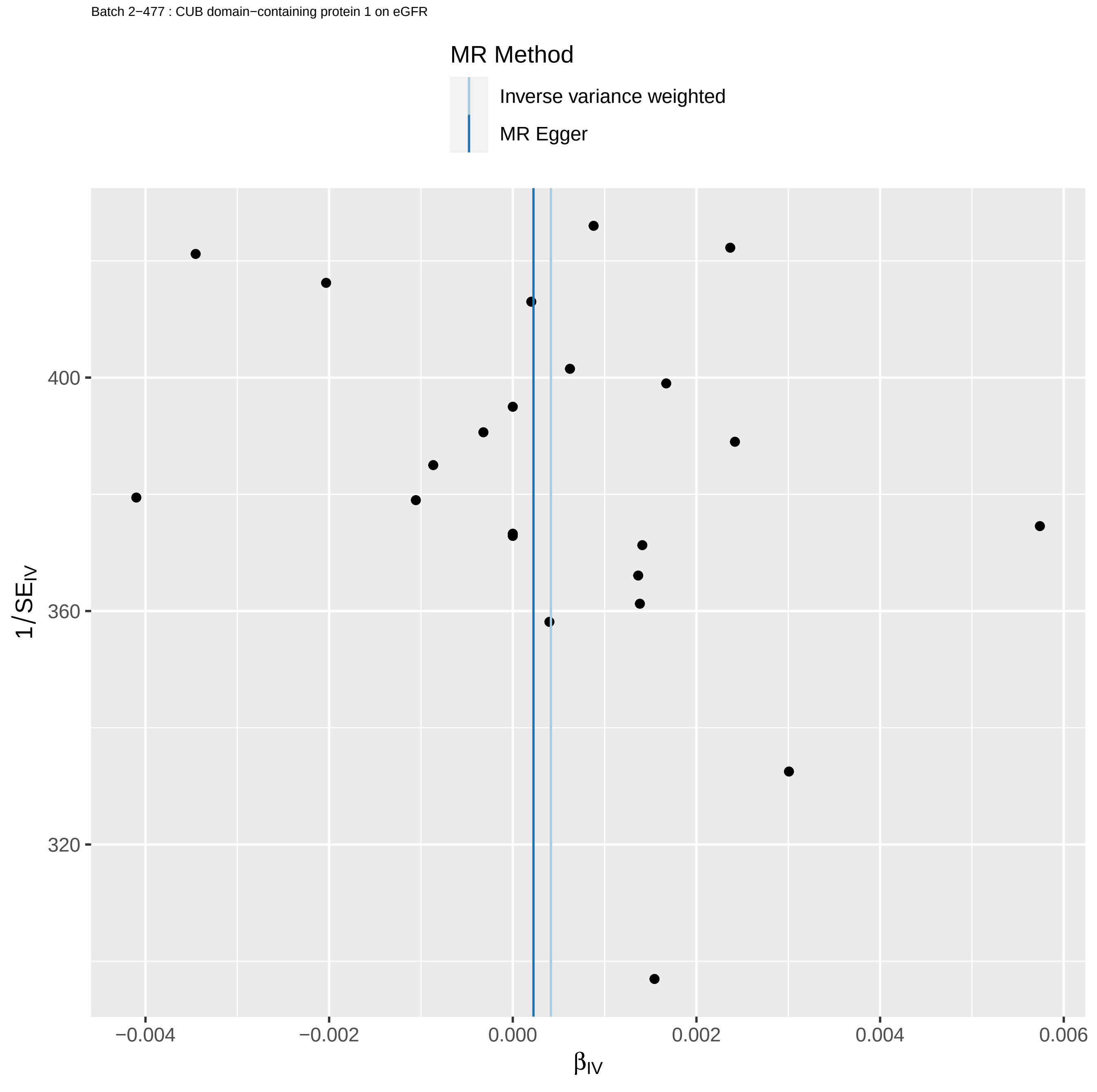

Batch 2−477 : CUB domain−containing protein 1 on eGFR
MR Method
Inverse variance weighted
MR Egger
400
360
320
1/SE$_{IV}$
−0.004
−0.002
0.000
0.002
0.004
0.006
β$_{IV}$

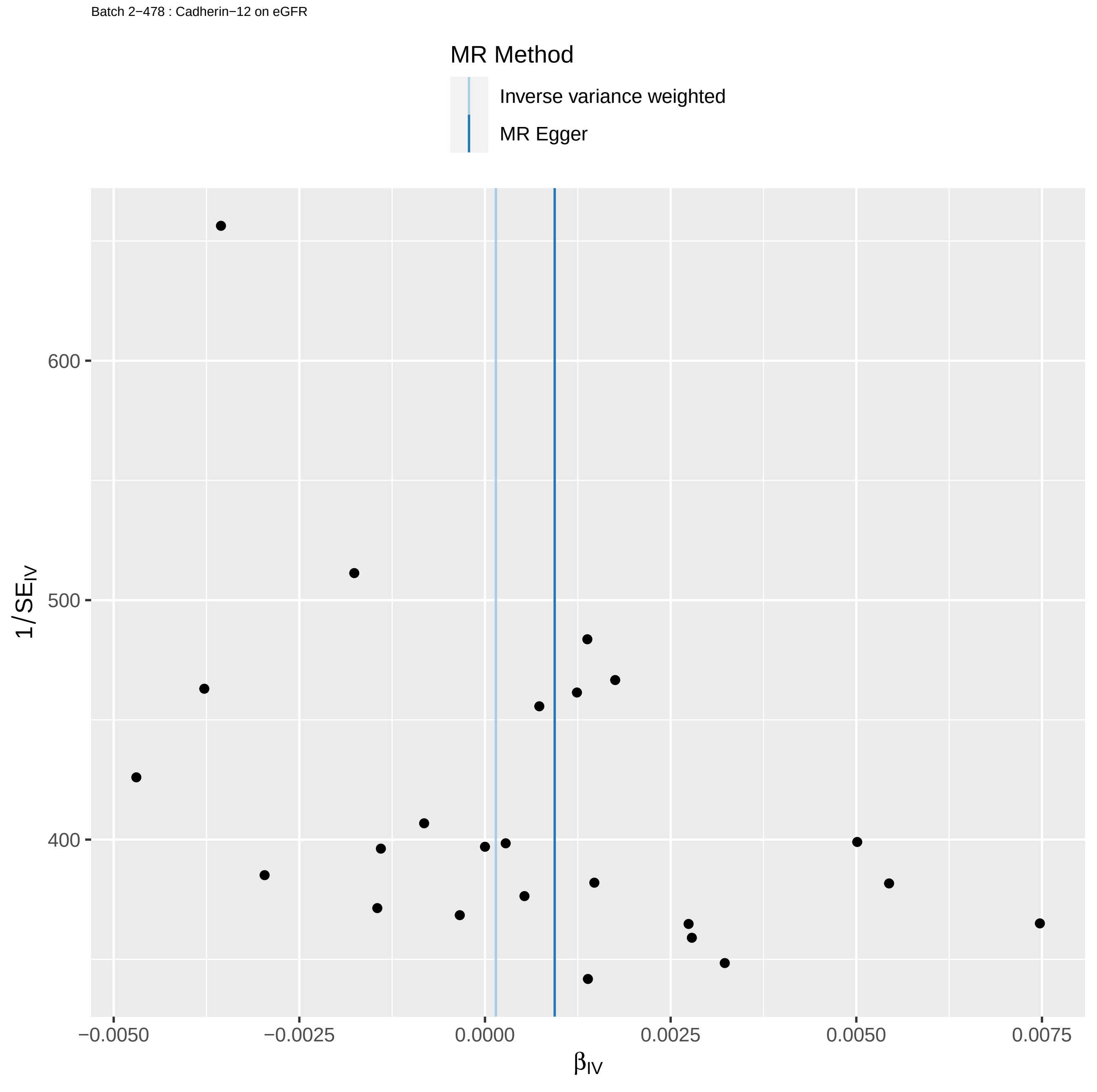

Batch 2−478 : Cadherin−12 on eGFR
MR Method
Inverse variance weighted
MR Egger
600
500
400
1/SE$_{IV}$
−0.0050
−0.0025
0.0000
0.0025
0.0050
0.0075
β$_{IV}$

Batch 2−479 : Cadherin−12 on eGFR

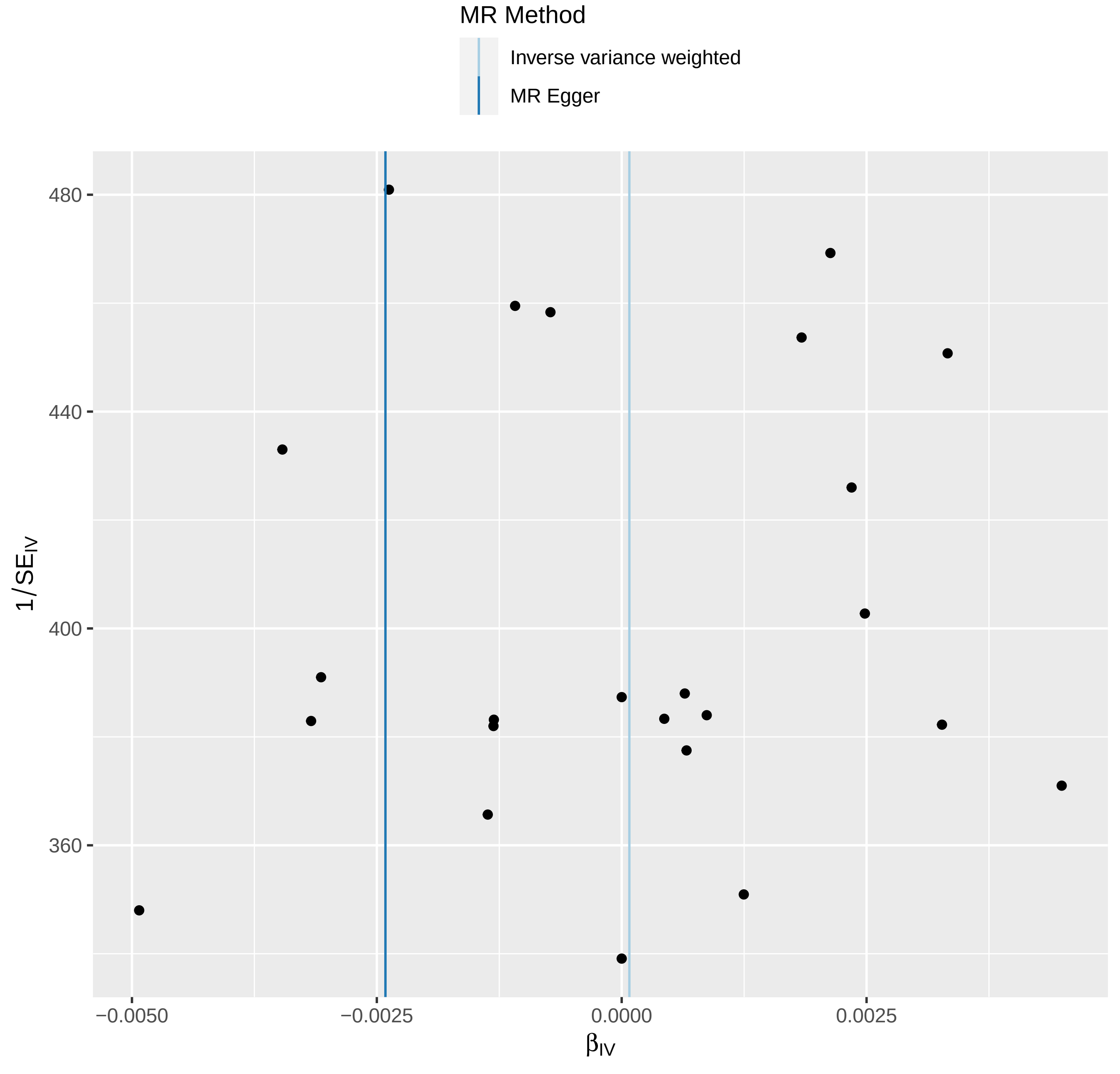

Batch 2−480 : Cadherin−1 on eGFR

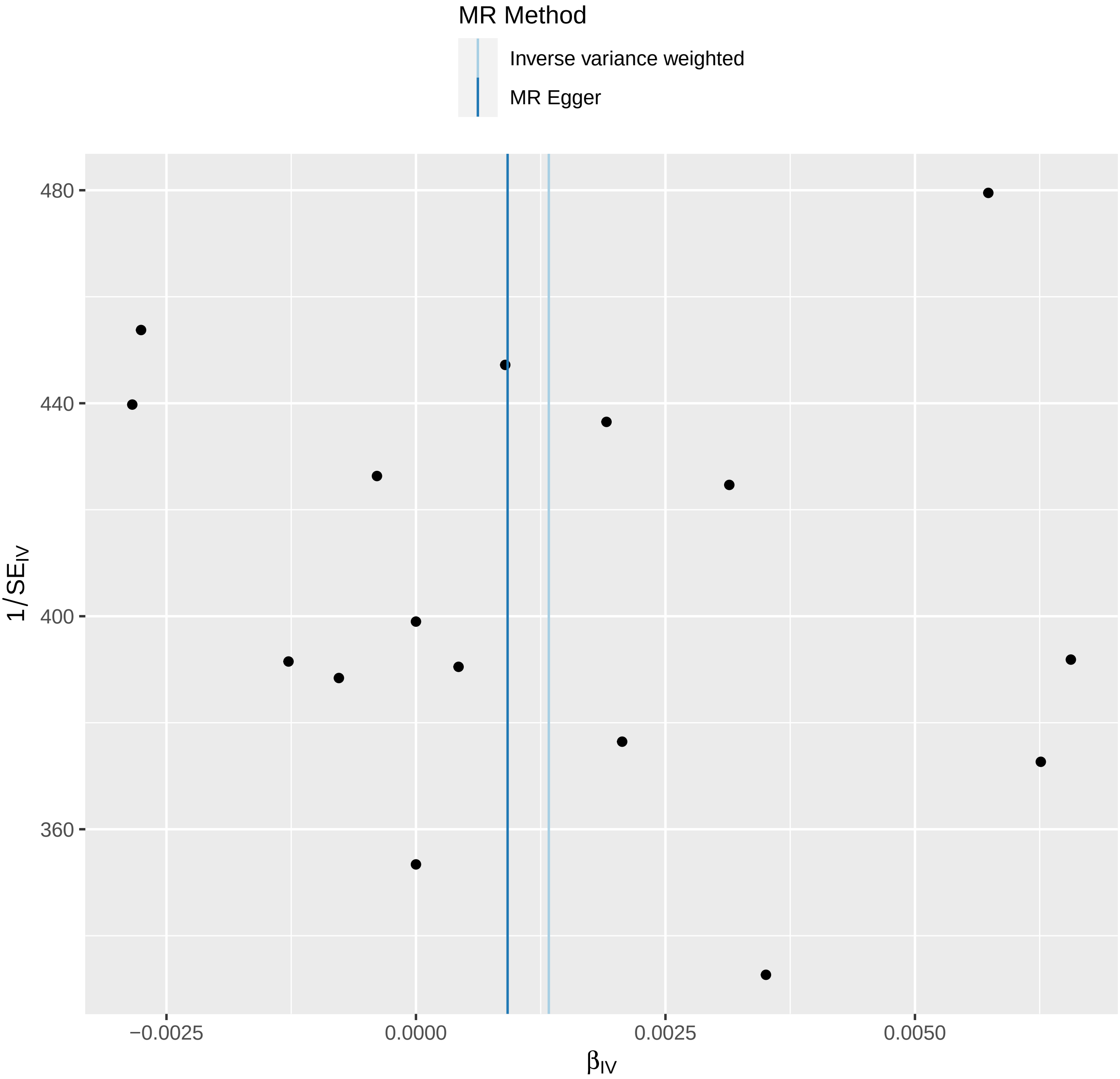

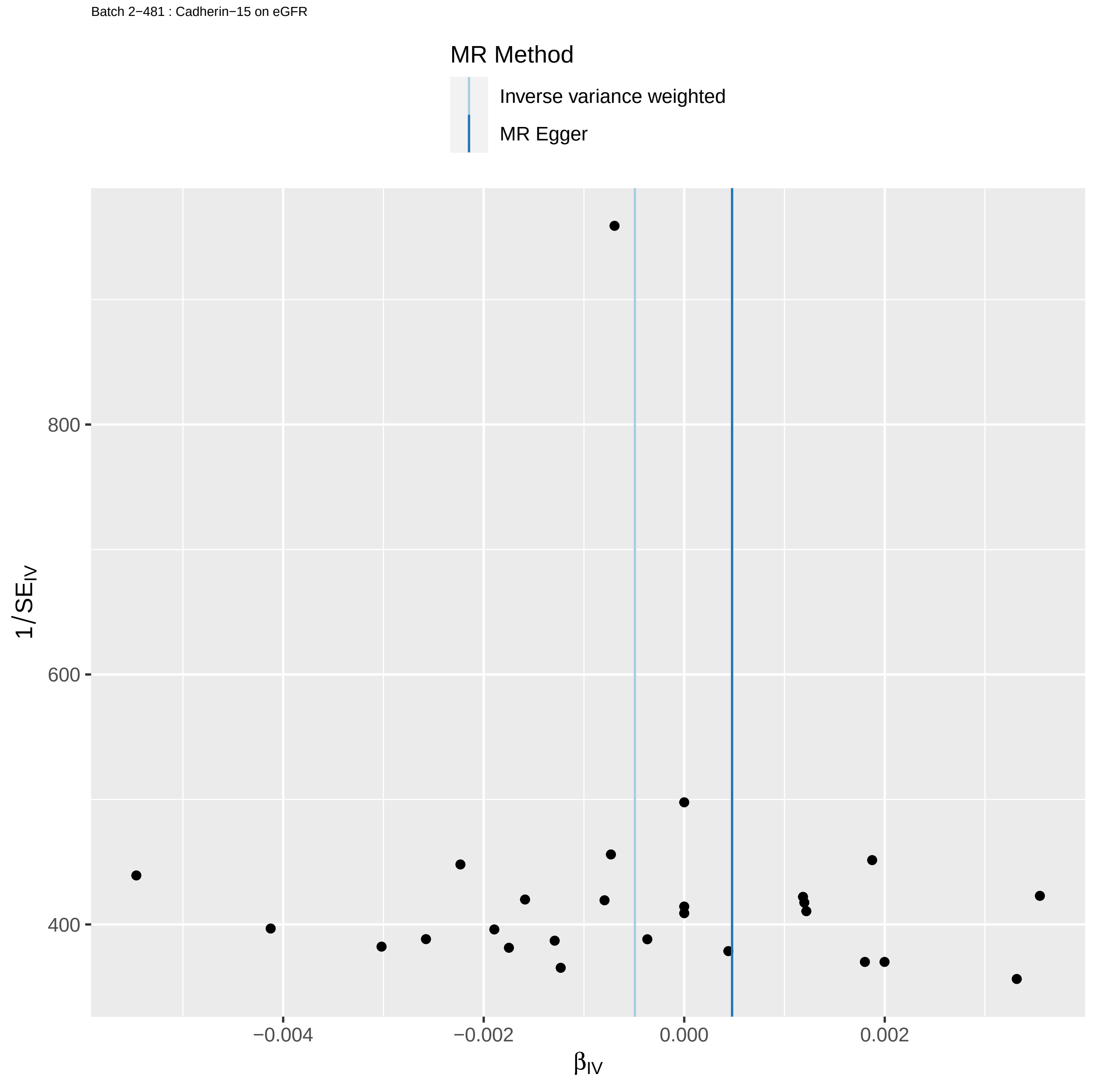
Batch 2−481 : Cadherin−15 on eGFR
MR Method
Inverse variance weighted
MR Egger
800
600
400
1/SE$_{IV}$
−0.004
−0.002
0.000
0.002
β$_{IV}$

Batch 2−482 : Cadherin−15 on eGFR

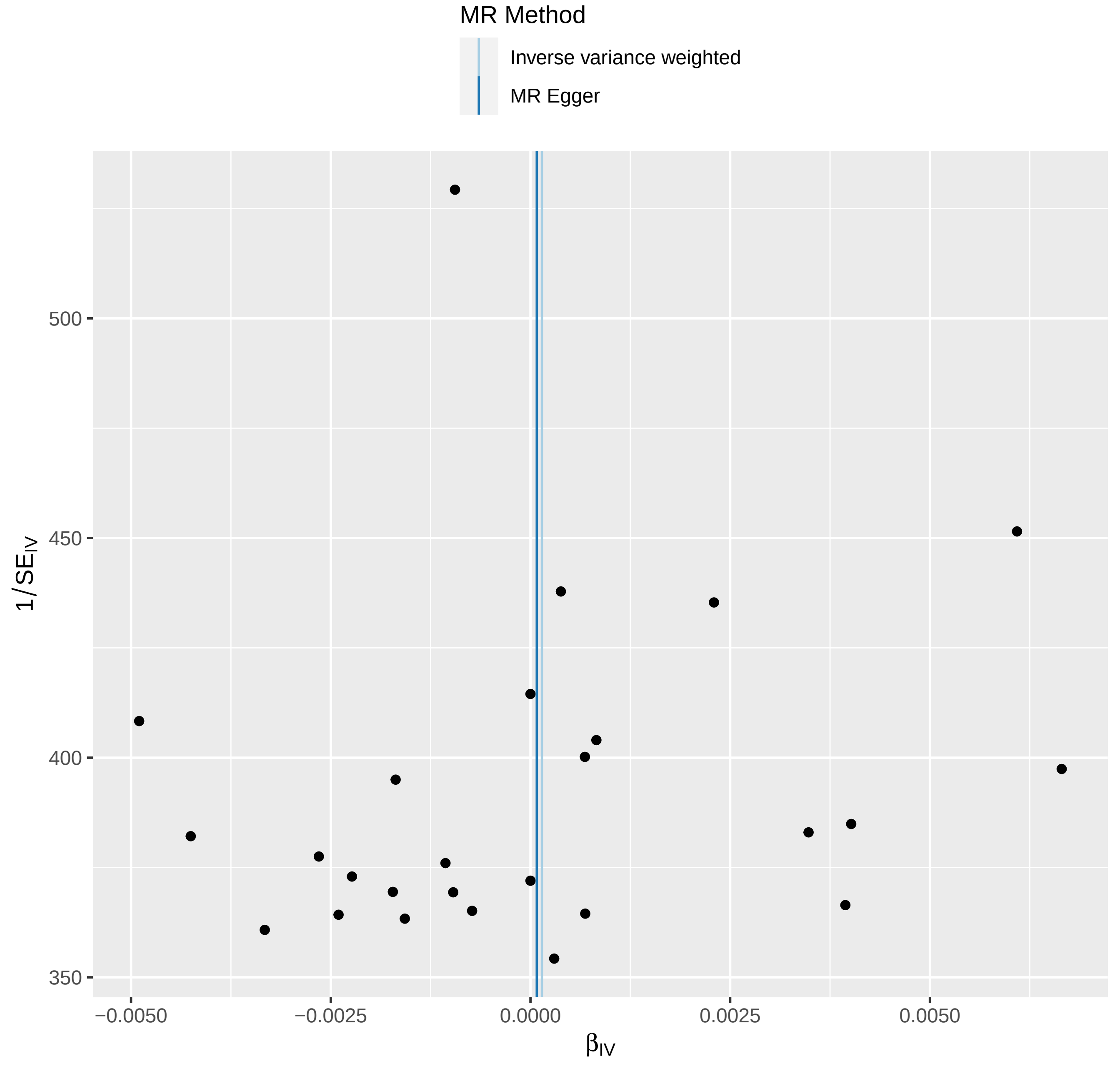

Batch 2−483 : Cadherin−2 on eGFR

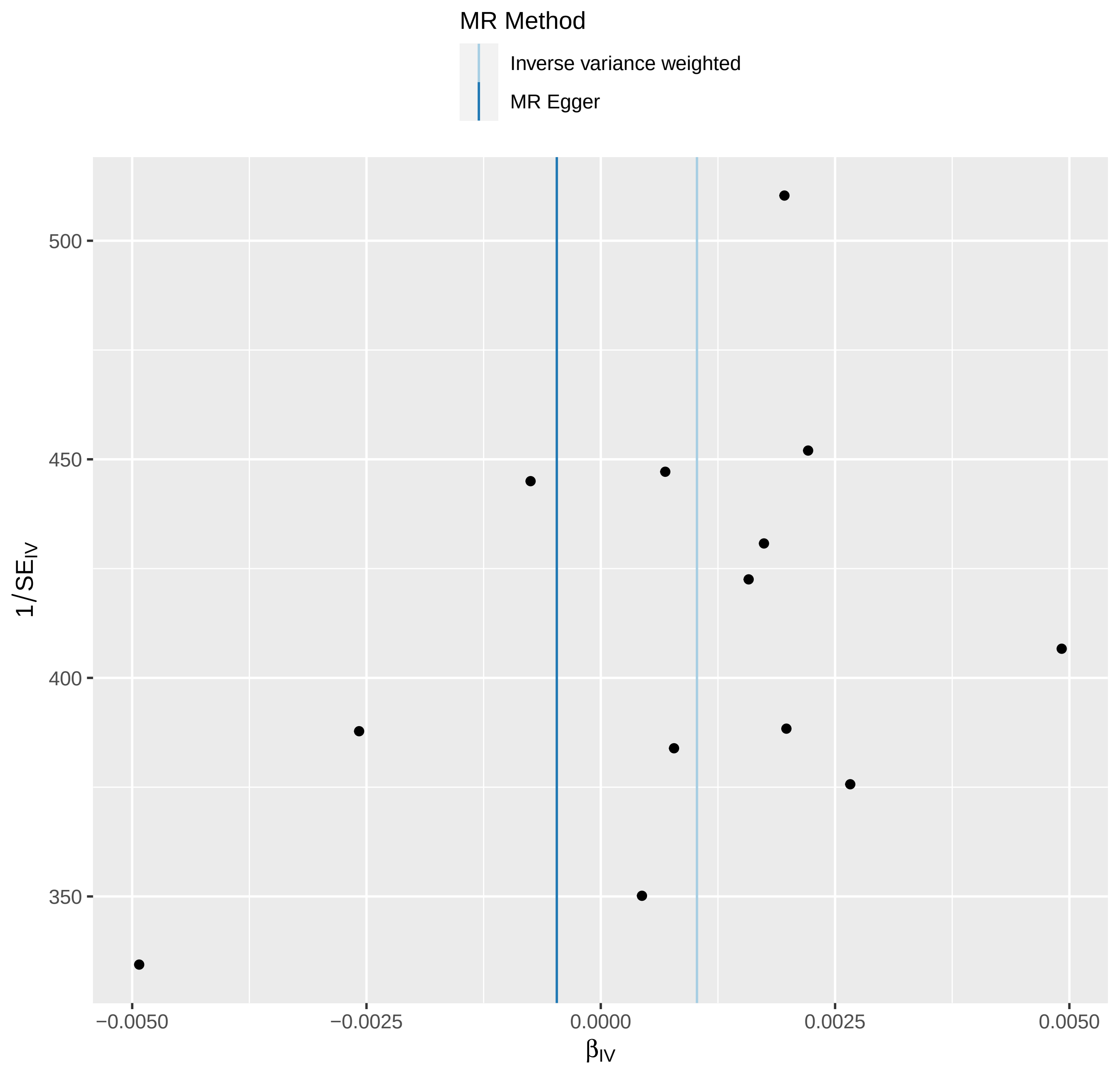

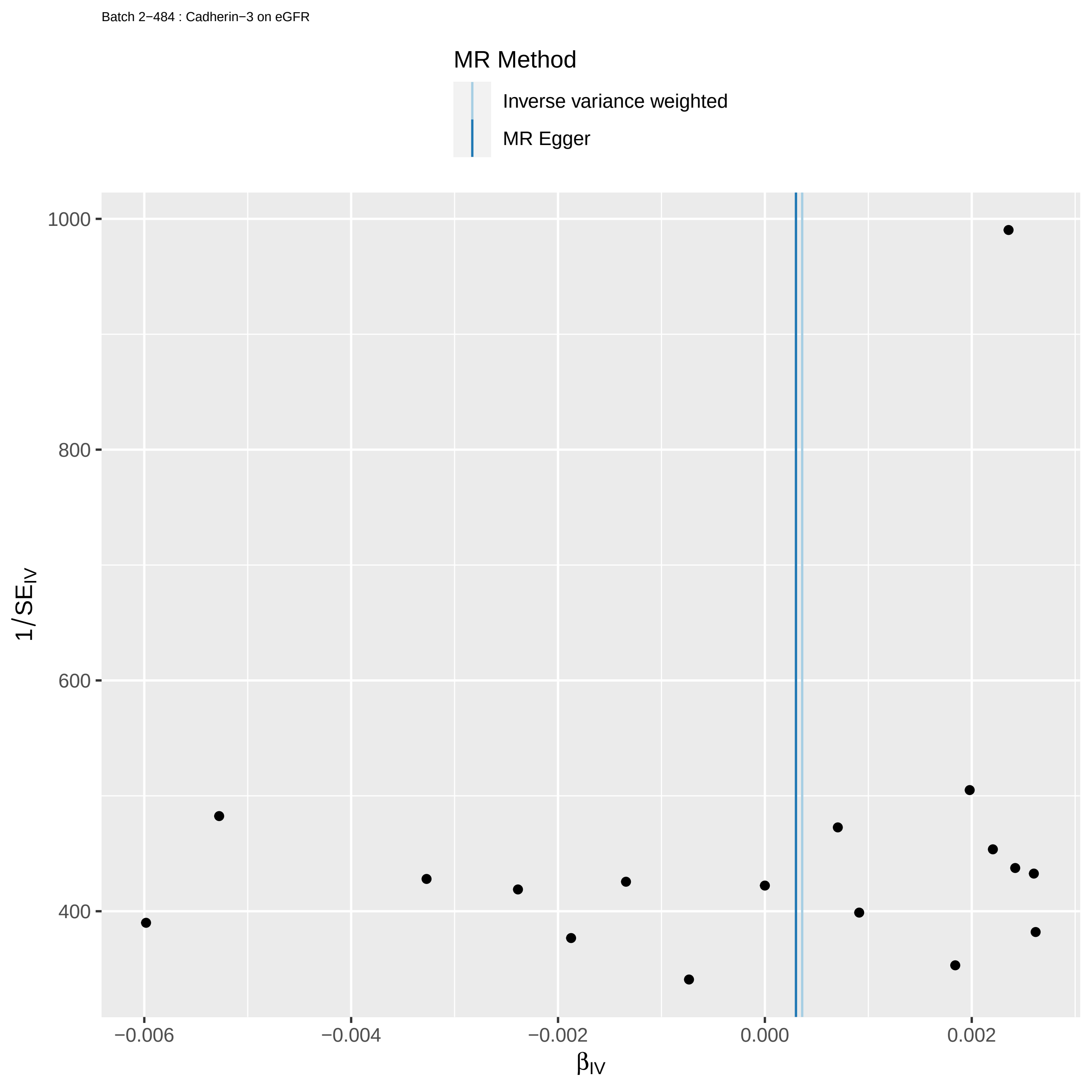

Batch 2−484 : Cadherin−3 on eGFR
MR Method
Inverse variance weighted
MR Egger
1000
800
600
400
1/SE_IV
−0.006
−0.004
−0.002
0.000
0.002
β_IV

Batch 2−485 : Cadherin−5 on eGFR

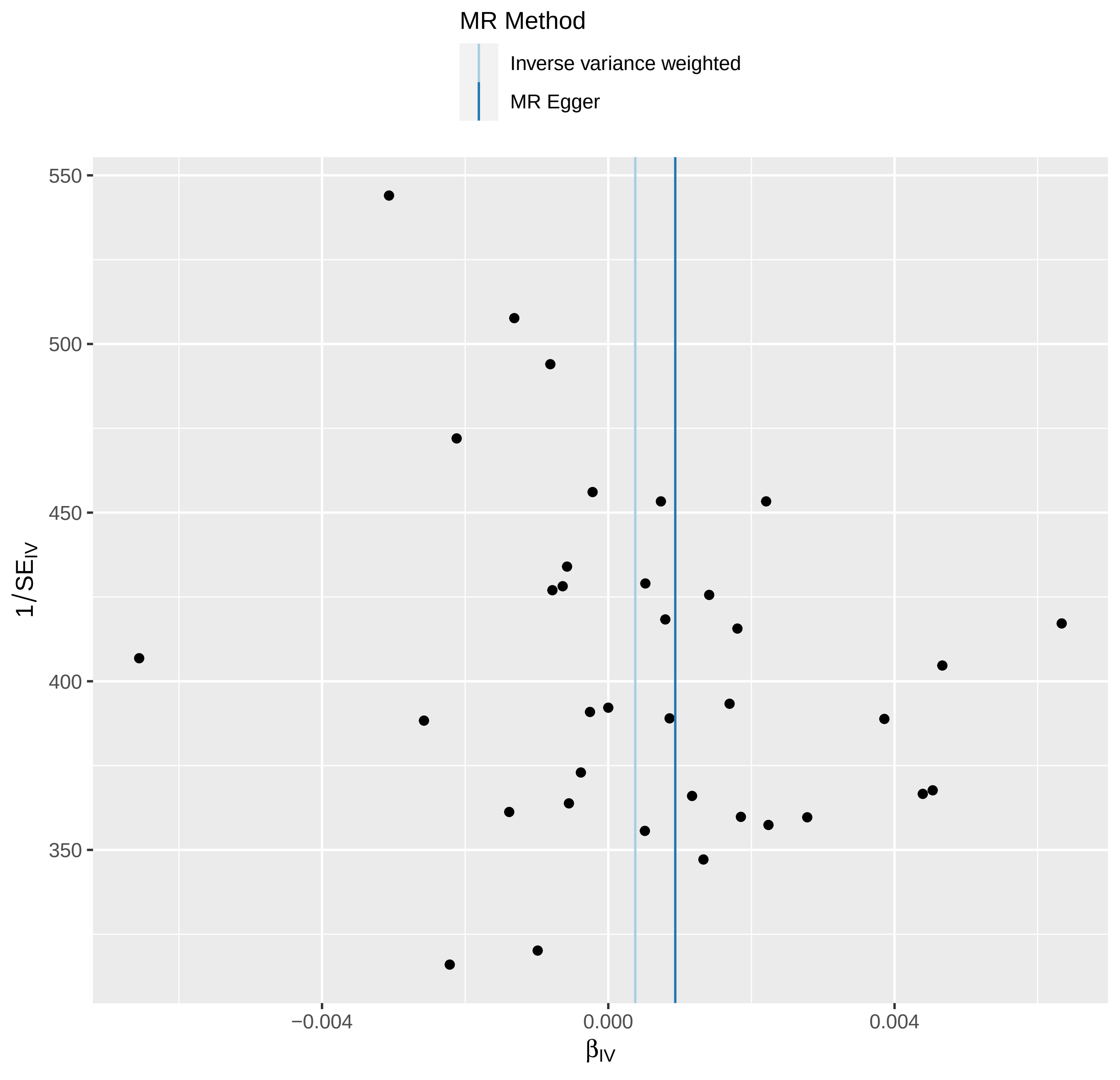

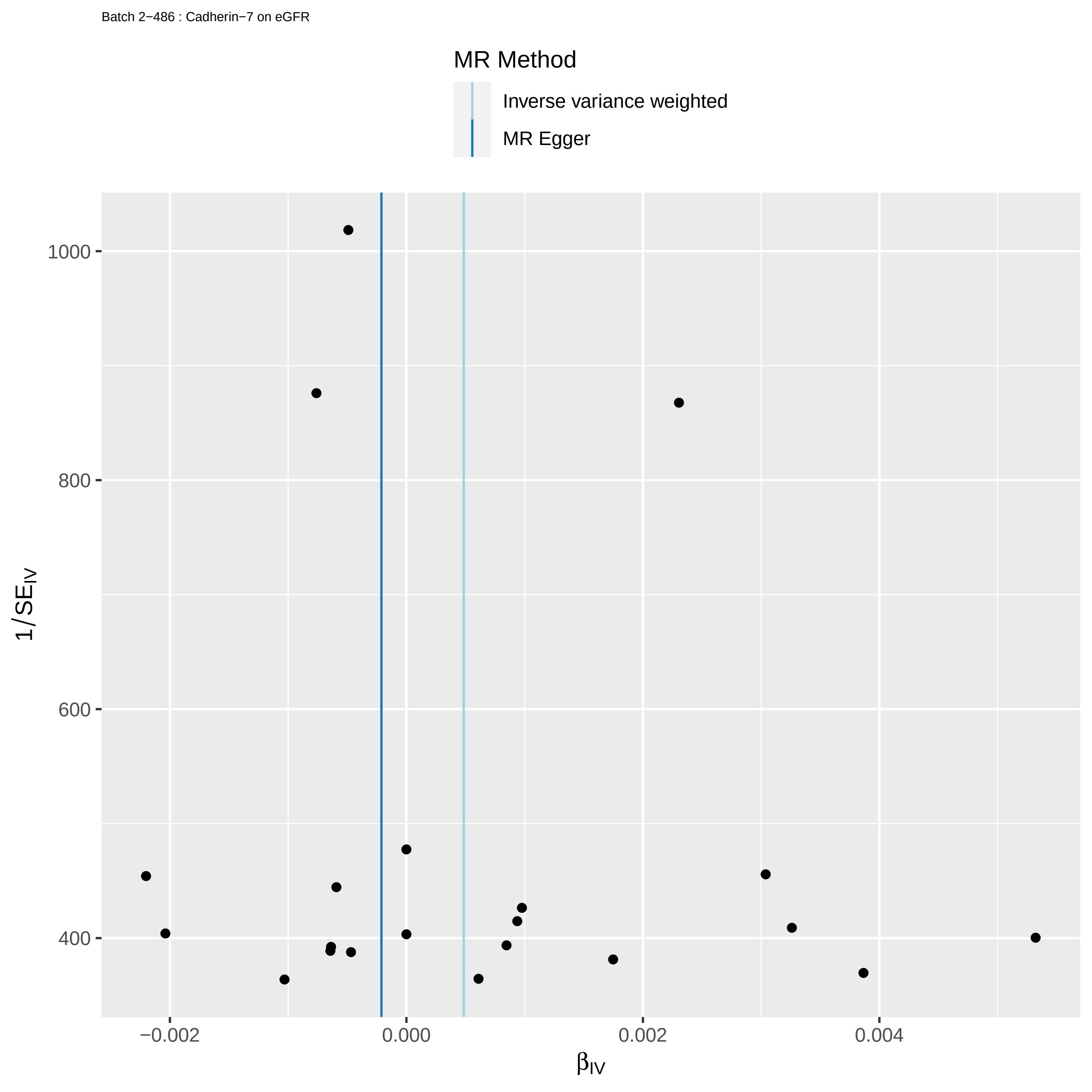

Batch 2−486 : Cadherin−7 on eGFR
MR Method
Inverse variance weighted
MR Egger
1000
800
600
400
1/SE$_{IV}$
−0.002
0.000
0.002
0.004
β_{IV}

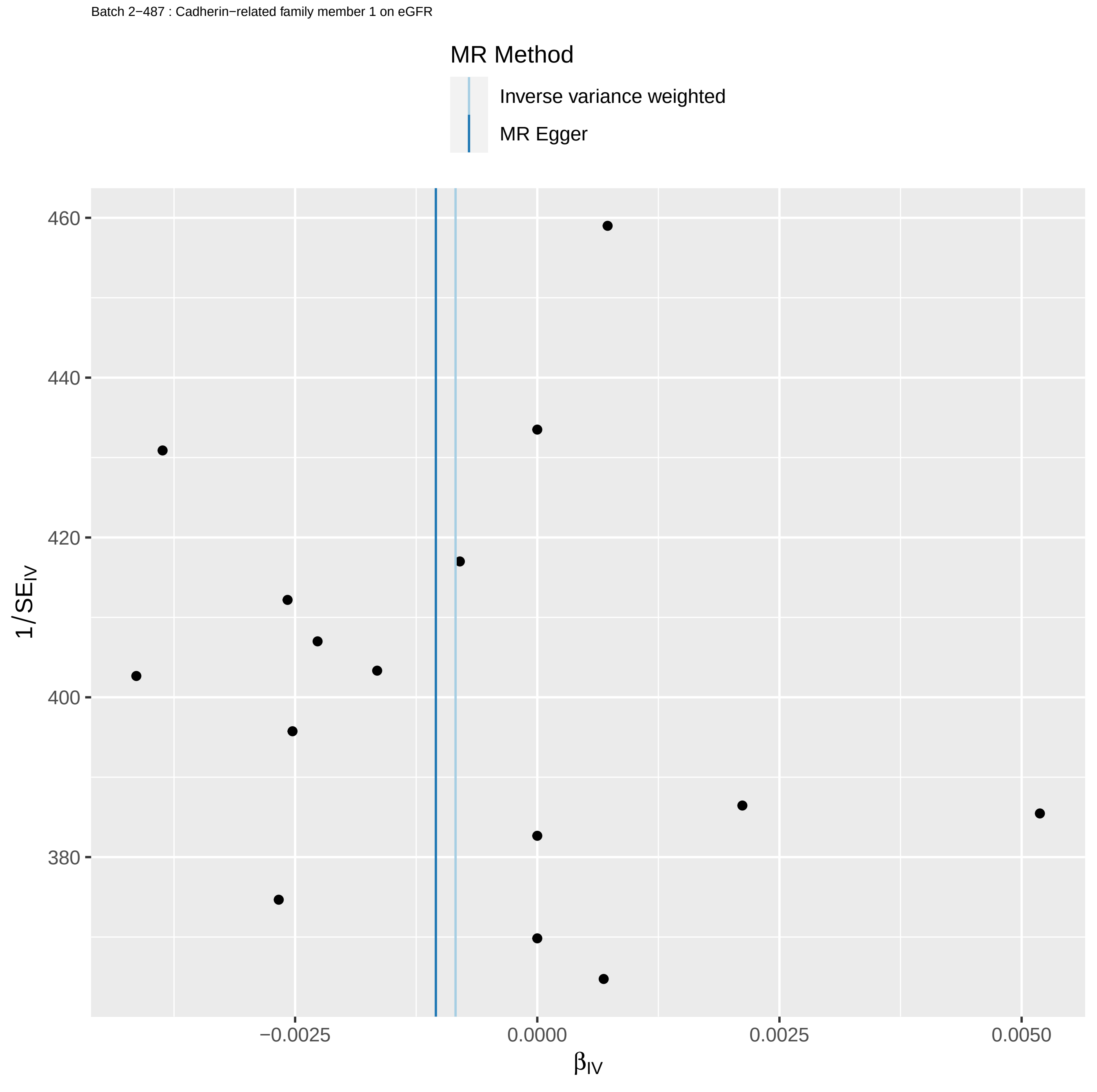
Batch 2−487 : Cadherin−related family member 1 on eGFR
MR Method
Inverse variance weighted
MR Egger
460
440
420
400
380
1/SE$_{IV}$
−0.0025
0.0000
0.0025
0.0050
β$_{IV}$

Batch 2−488 : Cadherin−related family member 3 on eGFR

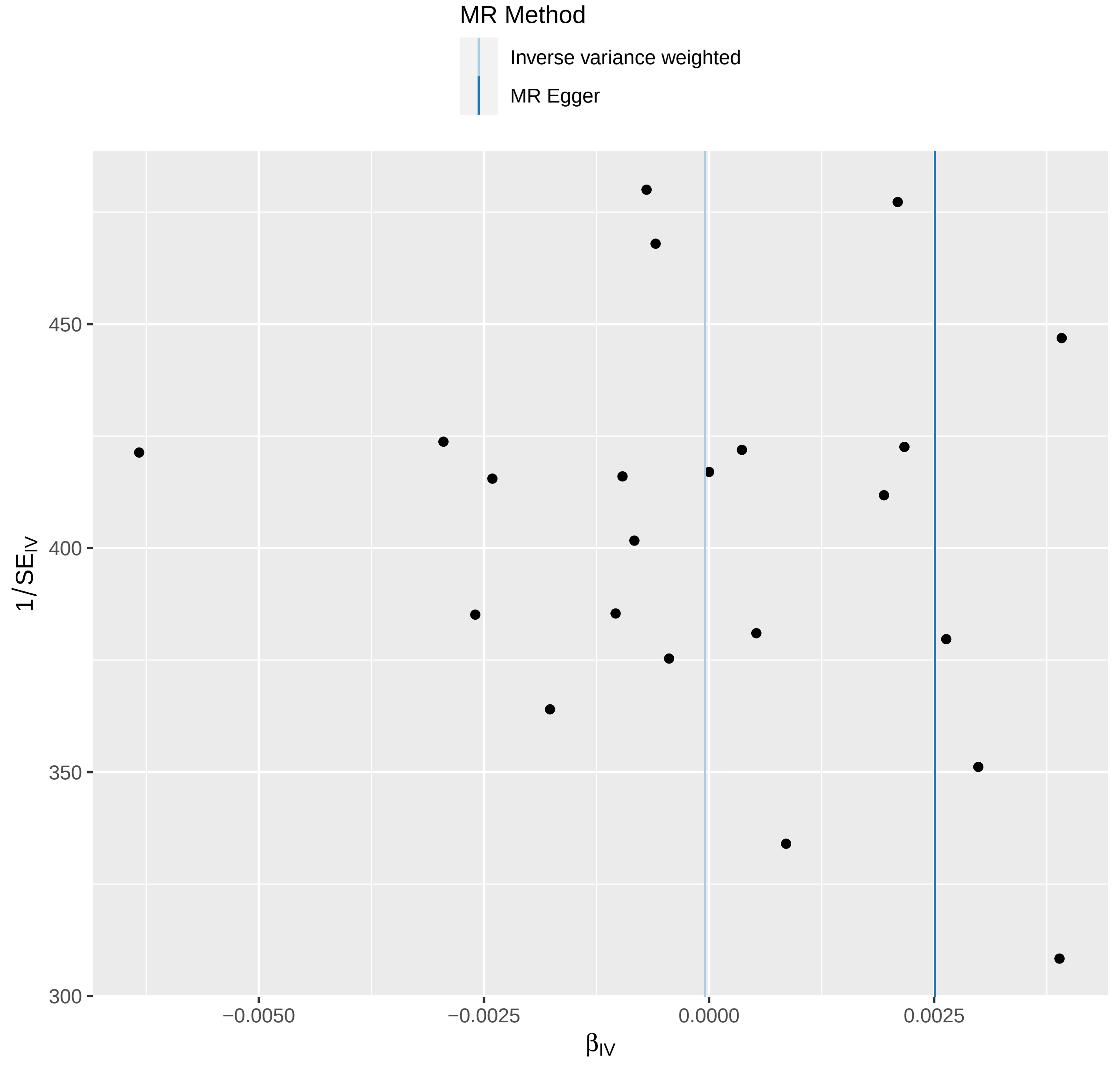

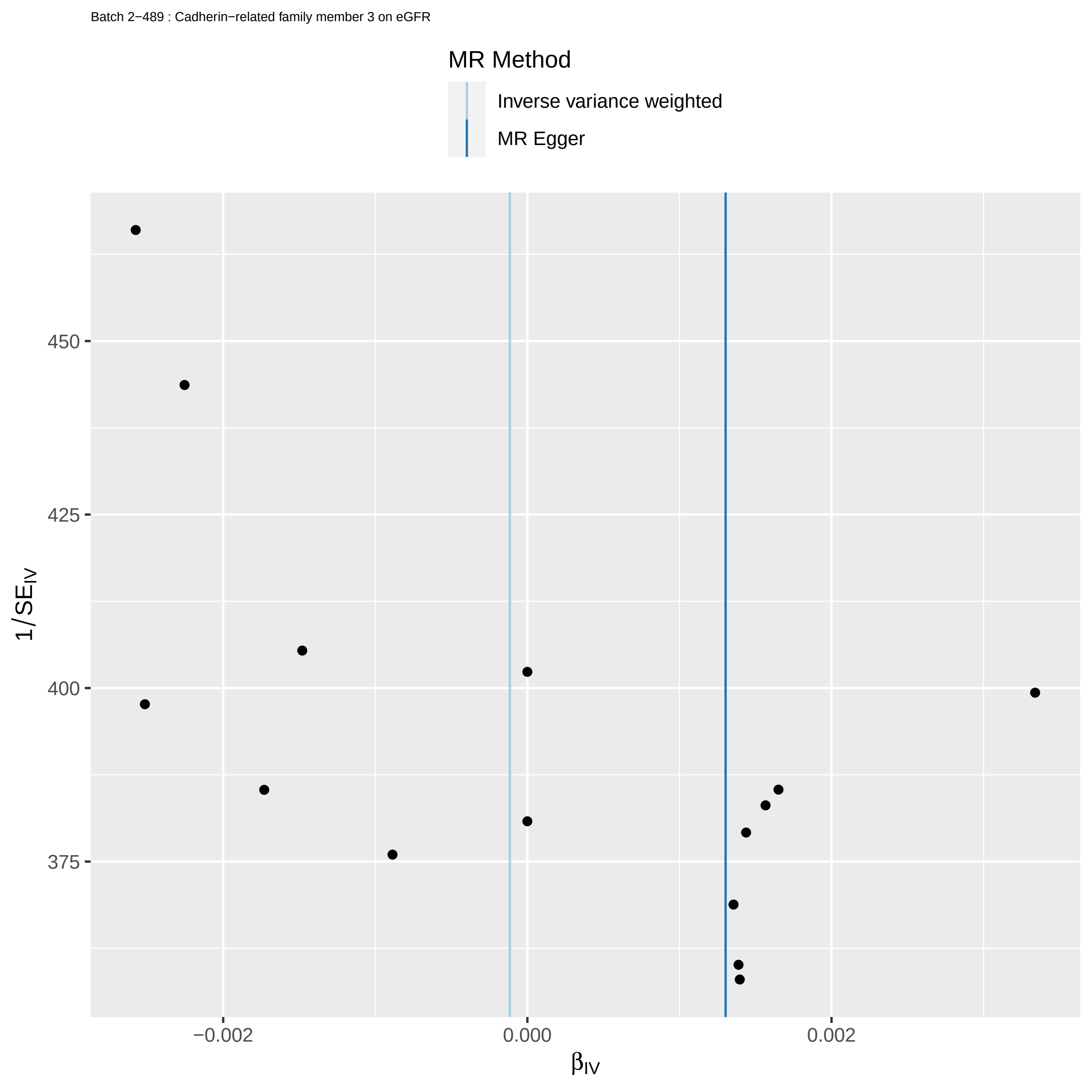
Batch 2−489 : Cadherin−related family member 3 on eGFR
MR Method
Inverse variance weighted
MR Egger
450
425
400
375
1/SE_IV
−0.002
0.000
0.002
β_IV

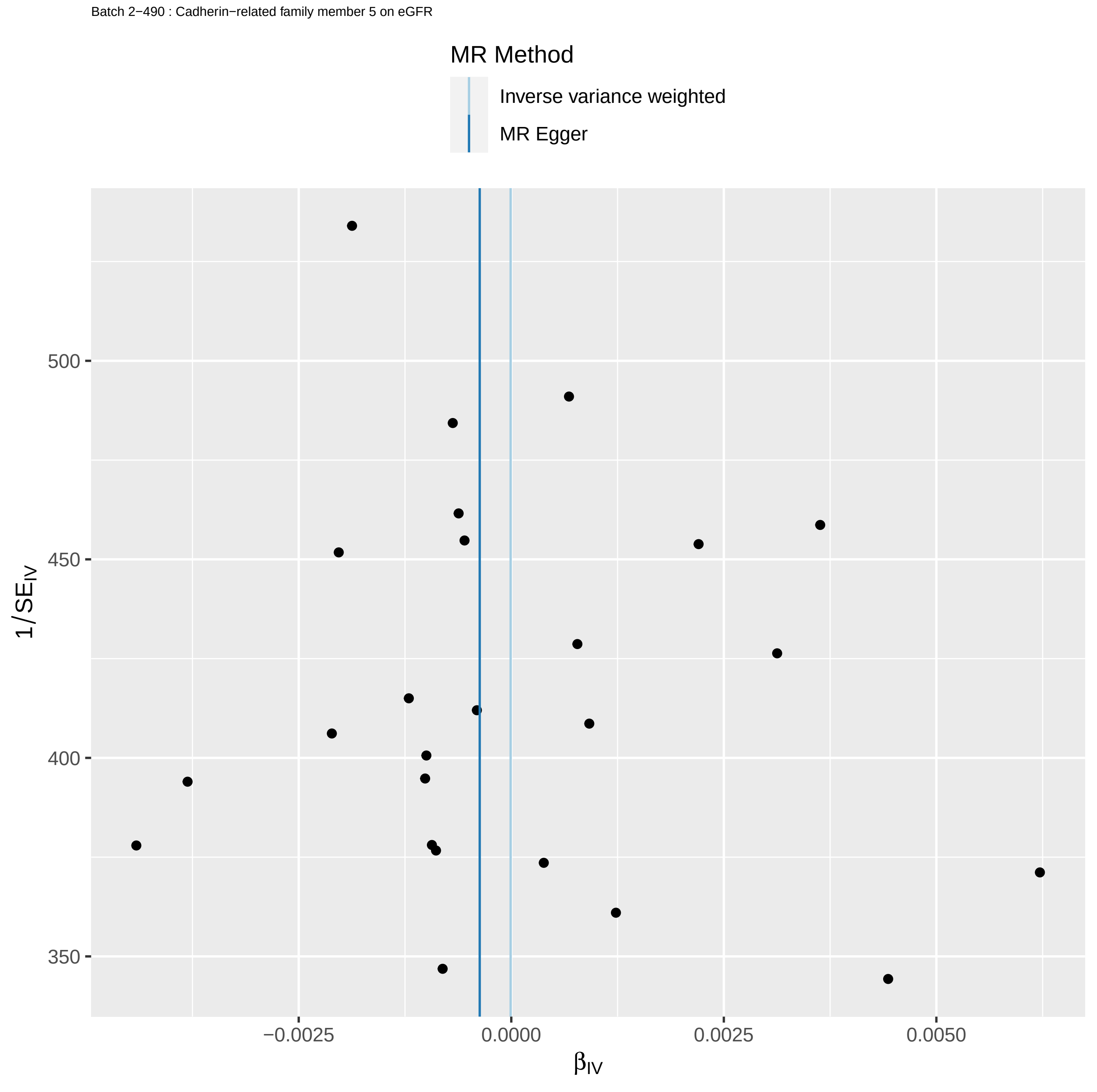

Batch 2−490 : Cadherin−related family member 5 on eGFR
MR Method
Inverse variance weighted
MR Egger
500
450
400
350
1/SE_IV
−0.0025
0.0000
0.0025
0.0050
β_IV

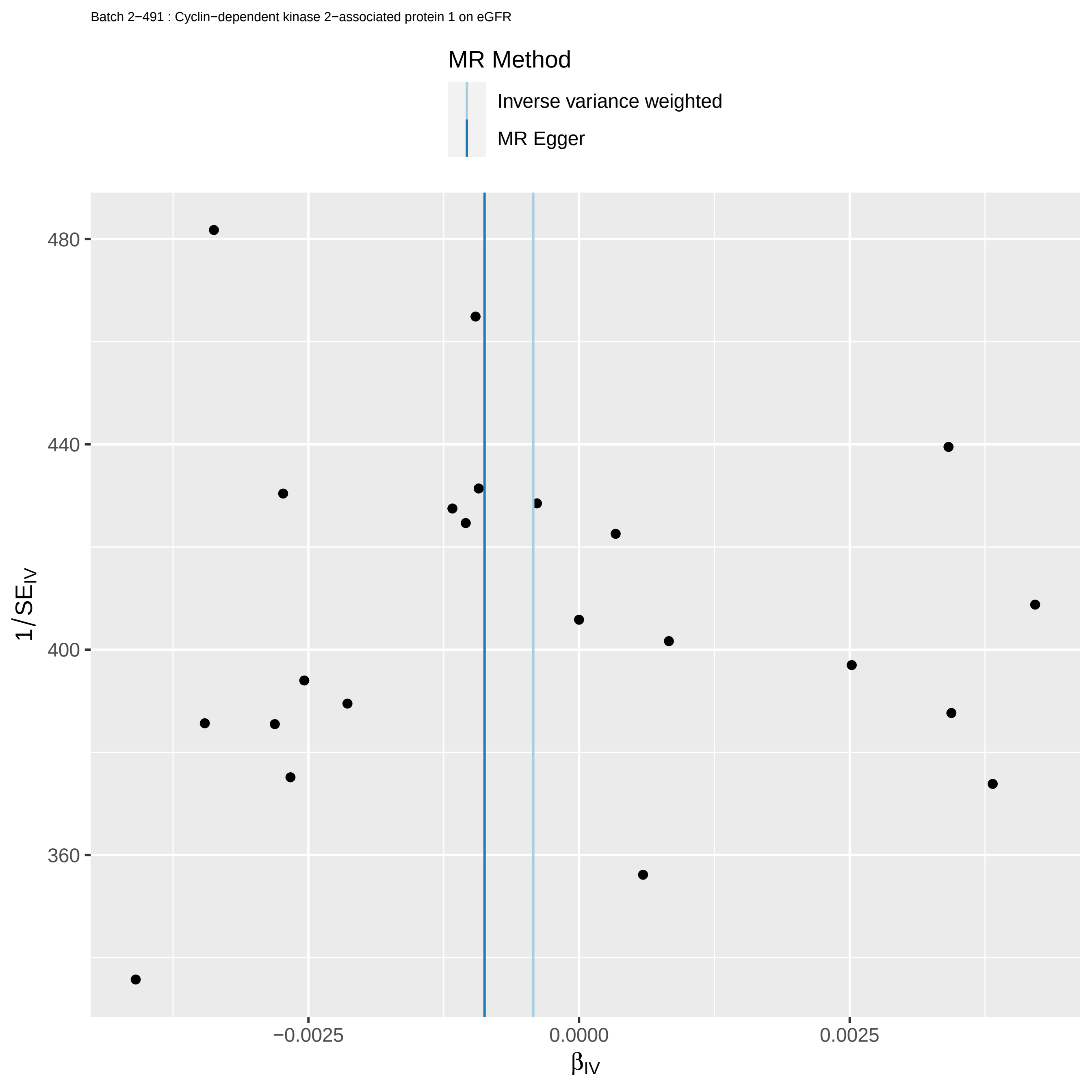
Batch 2−491 : Cyclin−dependent kinase 2−associated protein 1 on eGFR
MR Method
Inverse variance weighted
MR Egger
480
440
400
360
1/SE_IV
−0.0025
0.0000
0.0025
β_IV

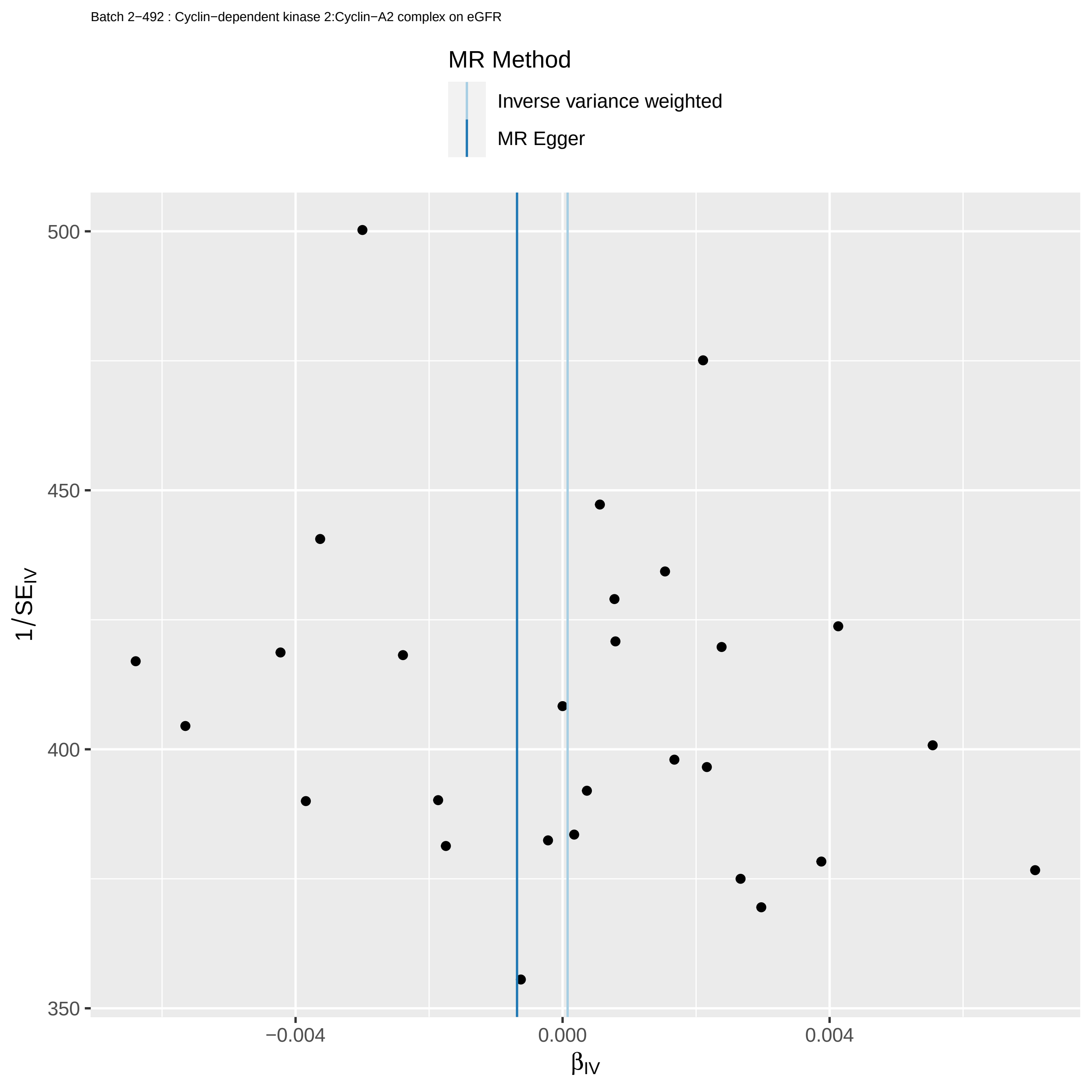

Batch 2−492 : Cyclin−dependent kinase 2:Cyclin−A2 complex on eGFR
MR Method
Inverse variance weighted
MR Egger
500
450
400
350
1/SE$_{IV}$
−0.004
0.000
0.004
β$_{IV}$

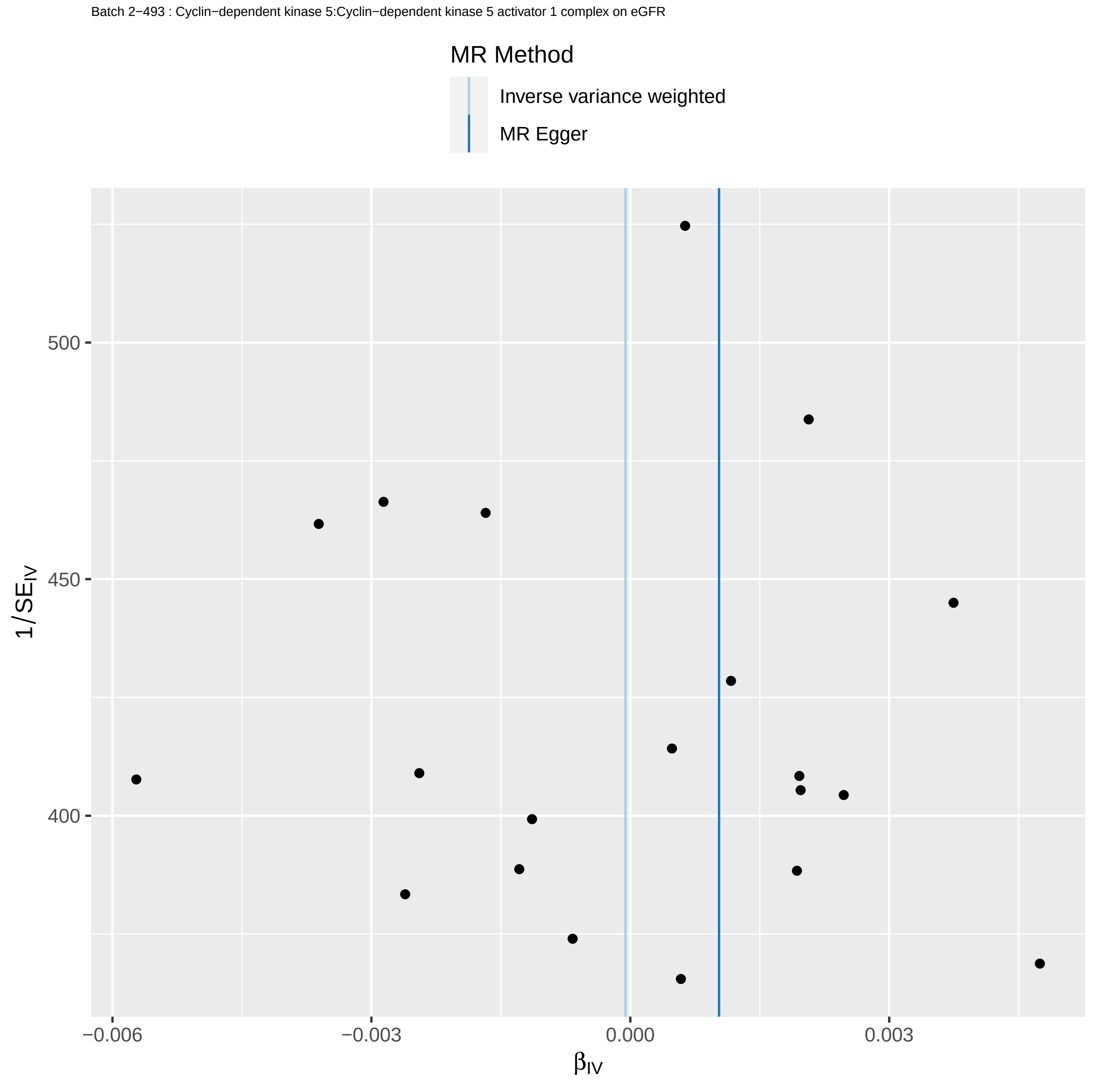
Batch 2−493 : Cyclin−dependent kinase 5:Cyclin−dependent kinase 5 activator 1 complex on eGFR
MR Method
Inverse variance weighted
MR Egger
500
450
400
1/SE_IV
−0.006
−0.003
0.000
0.003
β_IV

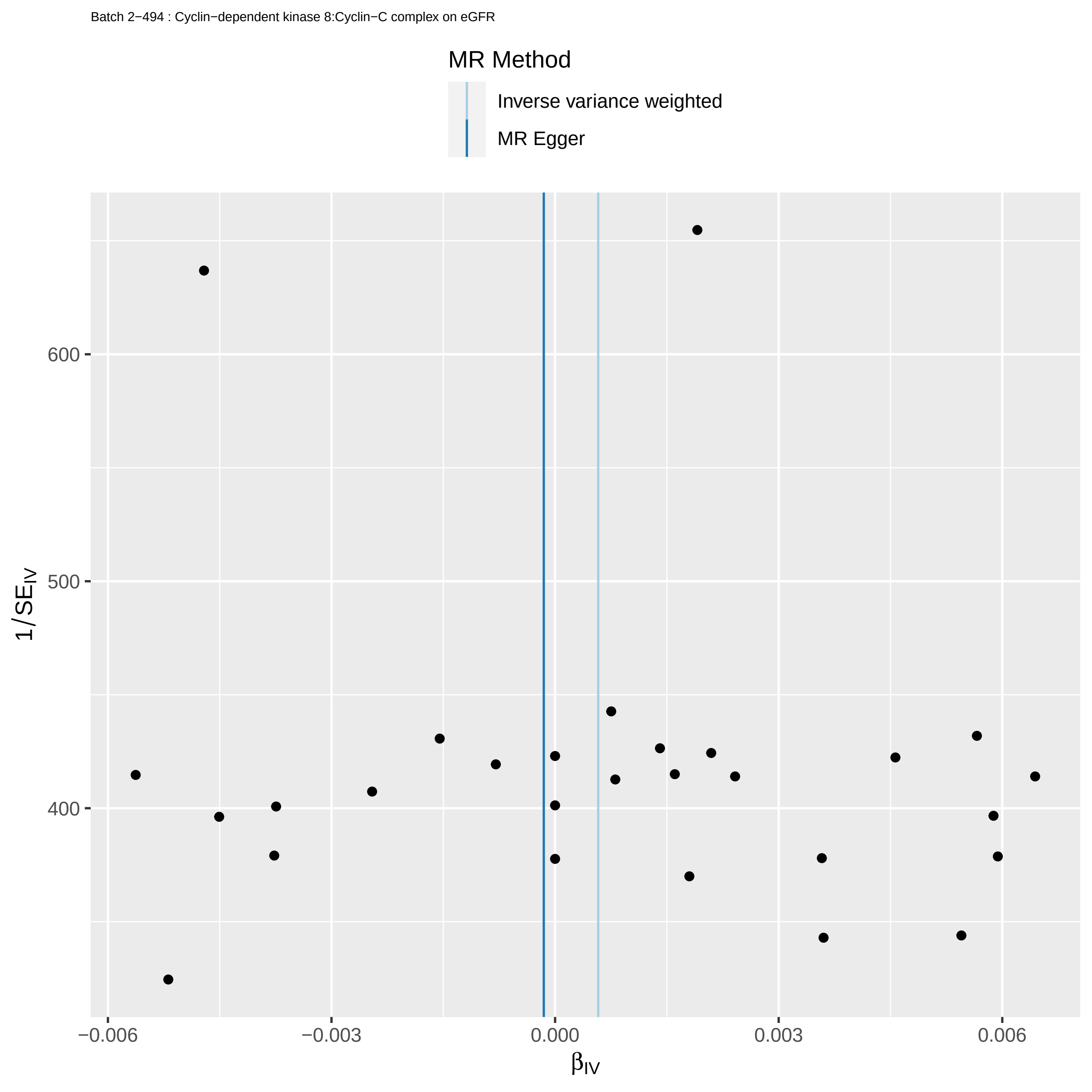

Batch 2−494 : Cyclin−dependent kinase 8:Cyclin−C complex on eGFR
MR Method
Inverse variance weighted
MR Egger
600
500
400
1/SE$_{IV}$
−0.006
−0.003
0.000
0.003
0.006
β_{IV}

Batch 2−495 : Cyclin−dependent kinase inhibitor 1B on eGFR

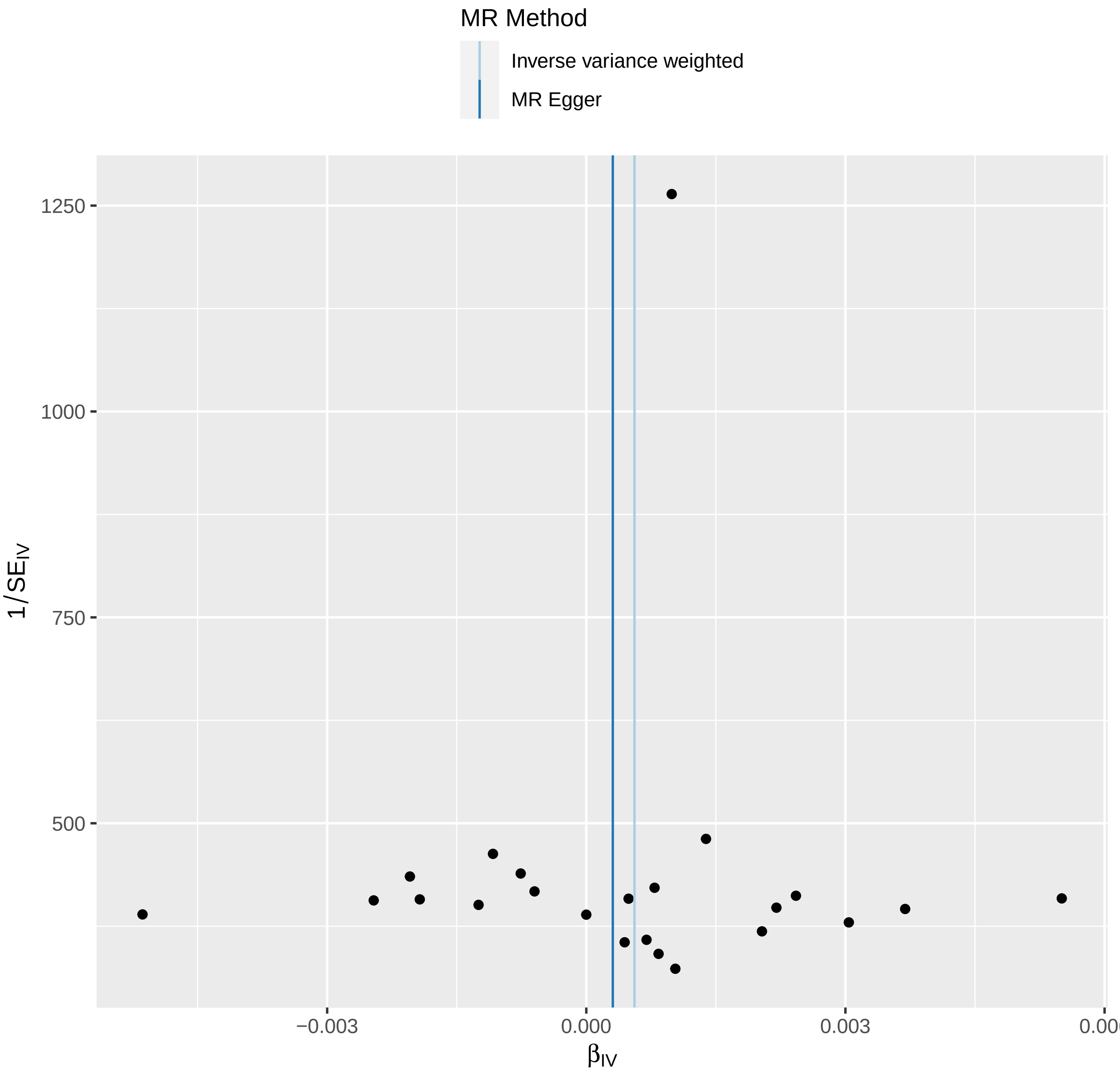

Batch 2−496 : Cyclin−dependent kinase 4 inhibitor B on eGFR

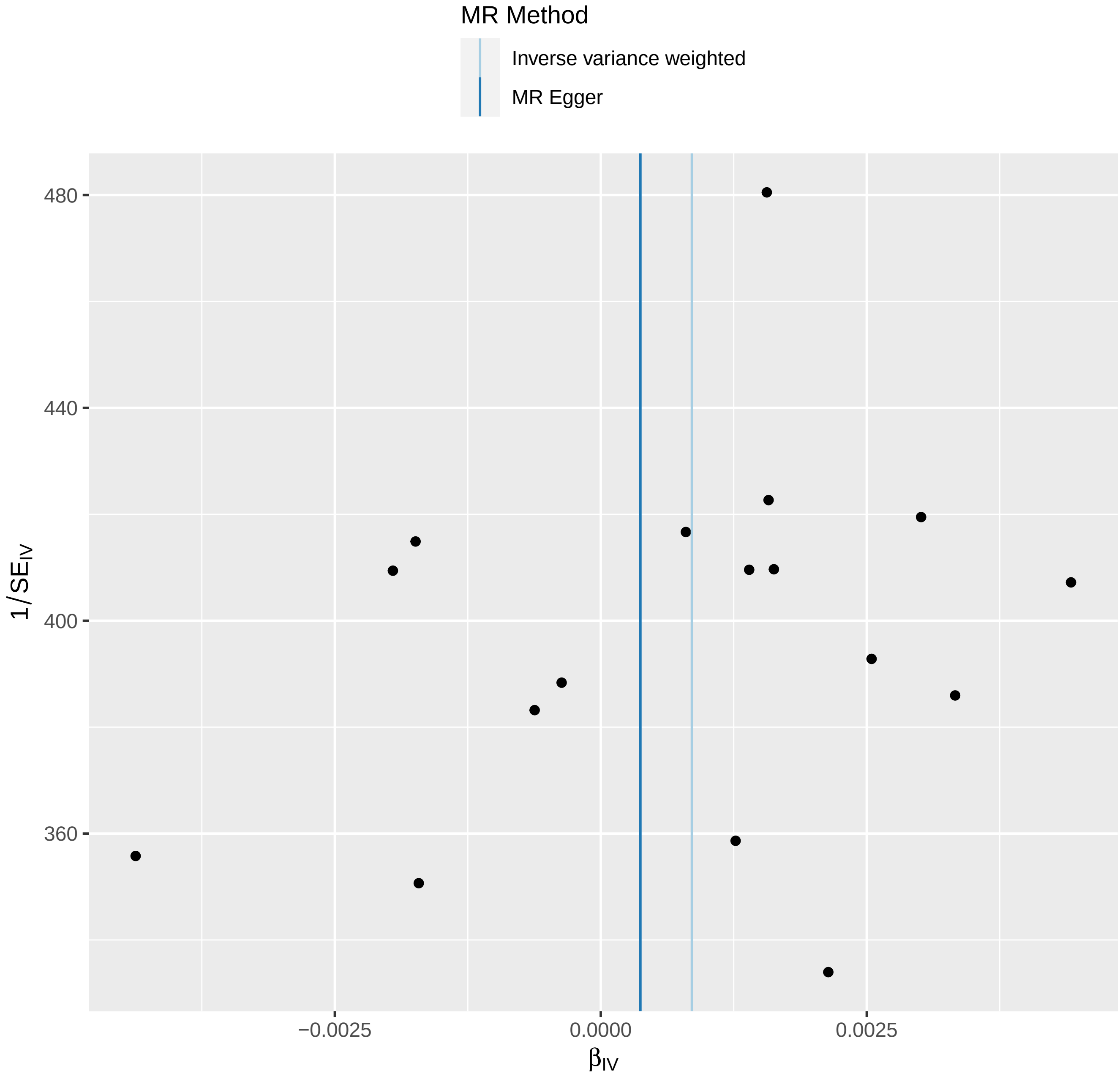

Batch 2−497 : Cyclin−dependent kinase inhibitor 3 on eGFR

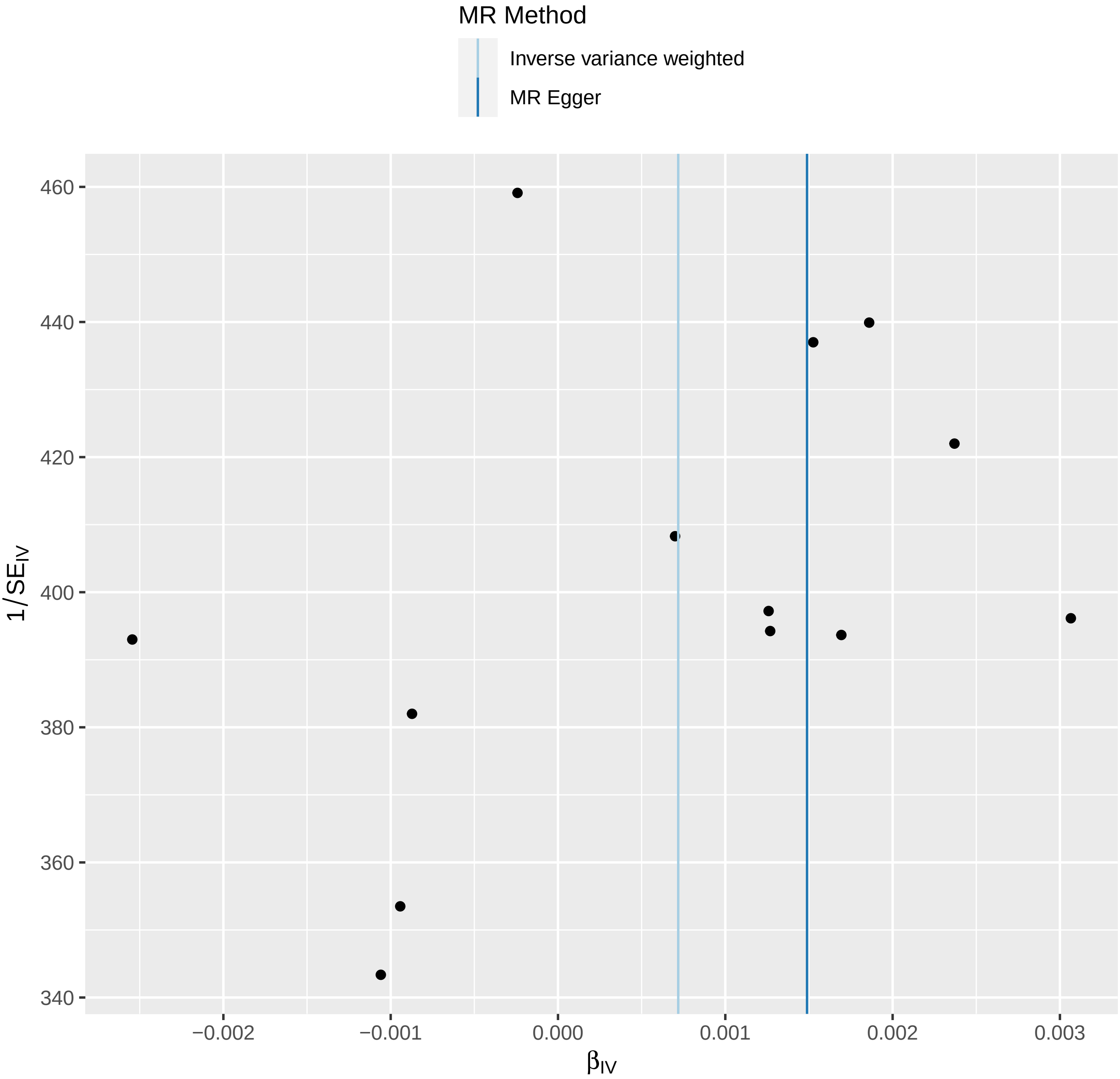

Batch 2−498 : Cerebral dopamine neurotrophic factor on eGFR

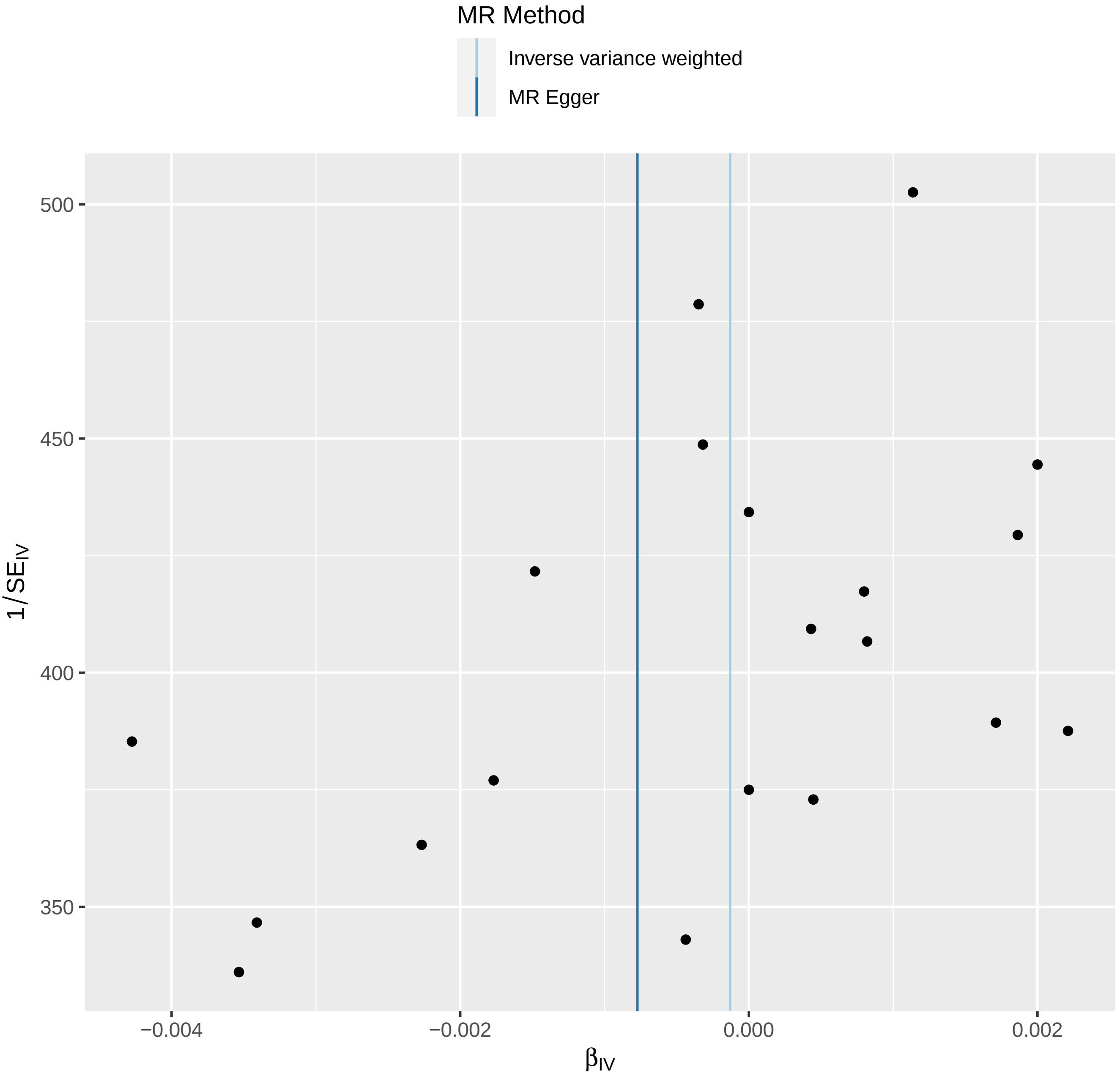

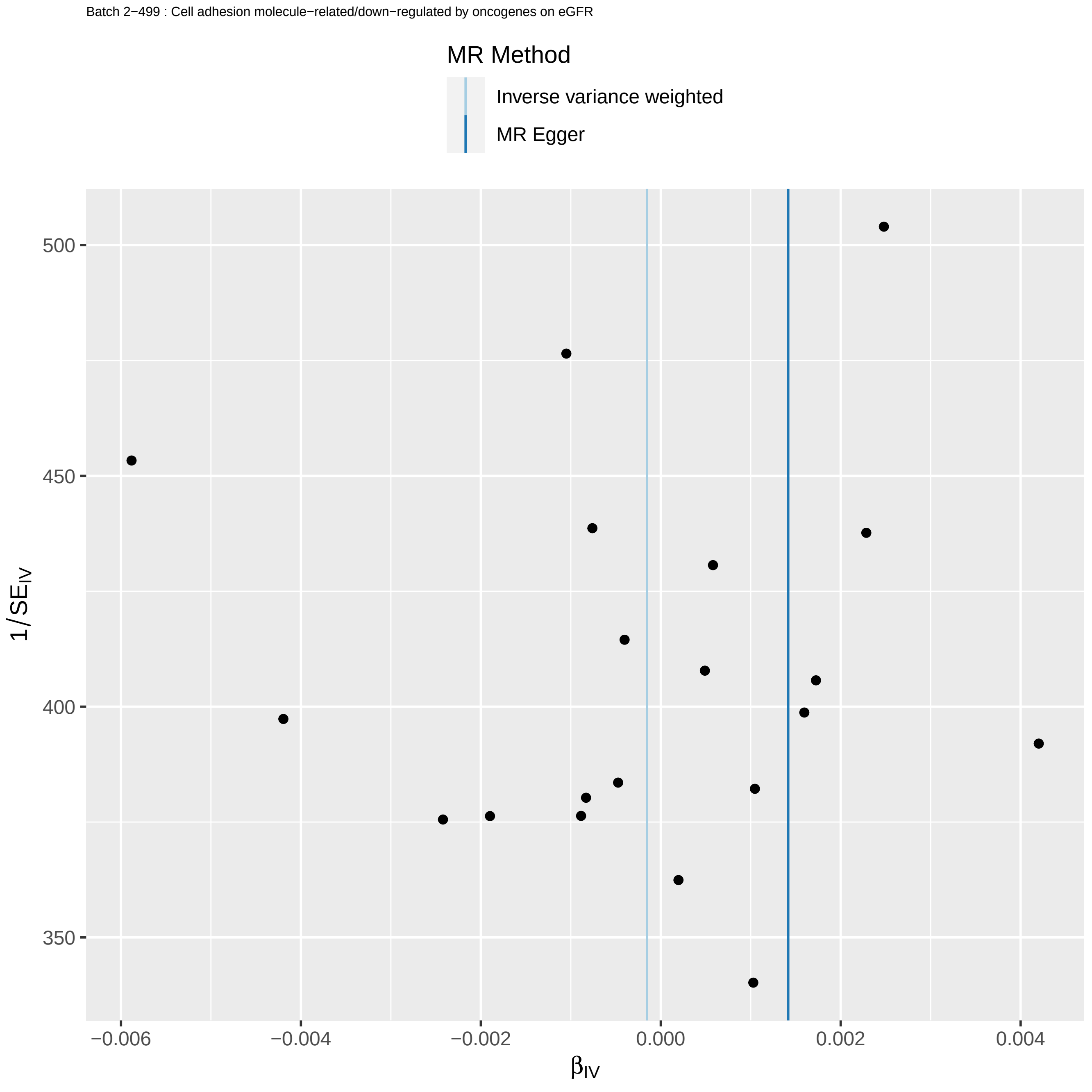
Batch 2−499 : Cell adhesion molecule−related/down−regulated by oncogenes on eGFR
MR Method
Inverse variance weighted
MR Egger
500
450
400
350
1/SE_IV
−0.006
−0.004
−0.002
0.000
0.002
0.004
β_IV

Batch 2−500 : Corneodesmosin on eGFR

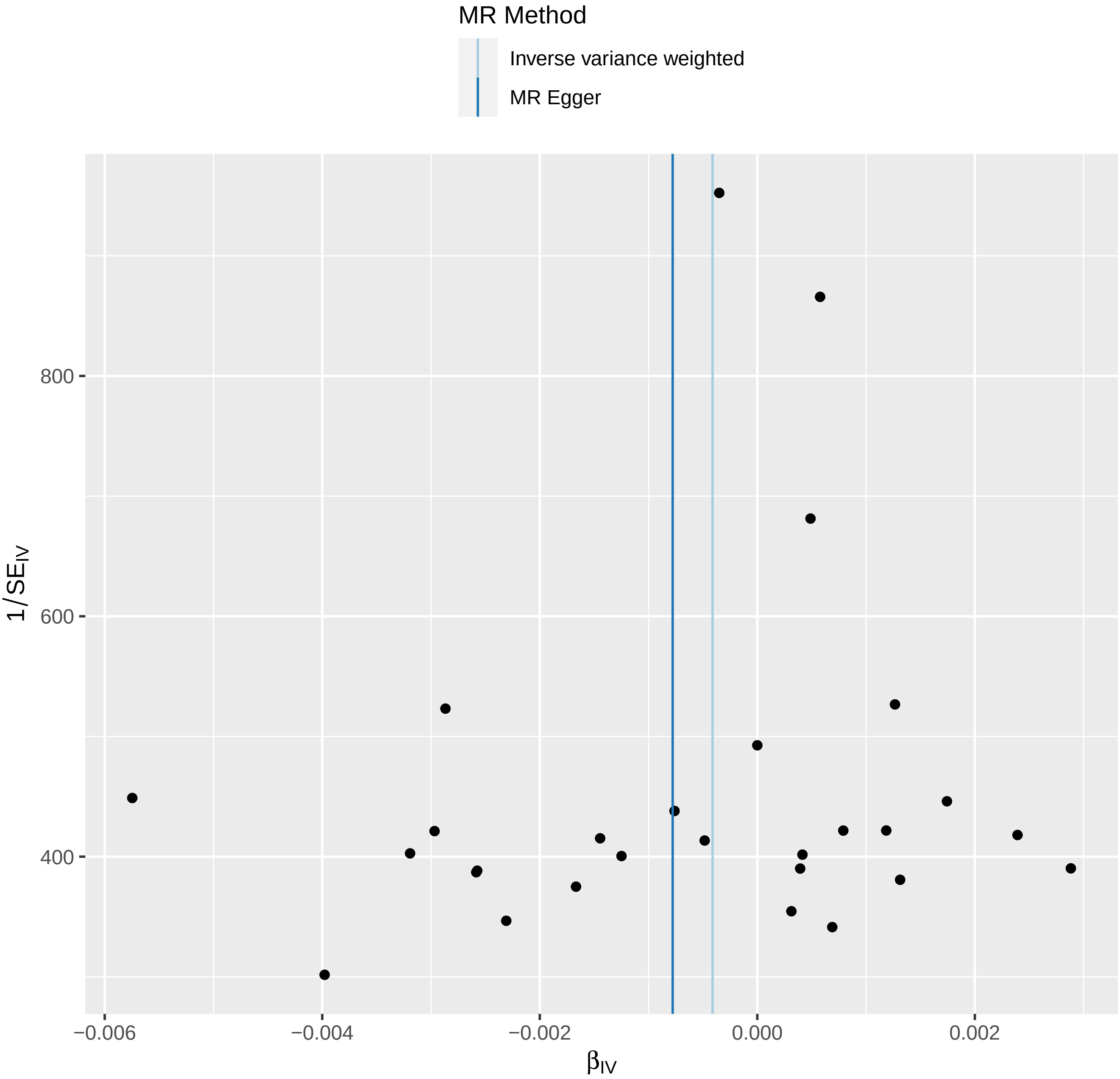

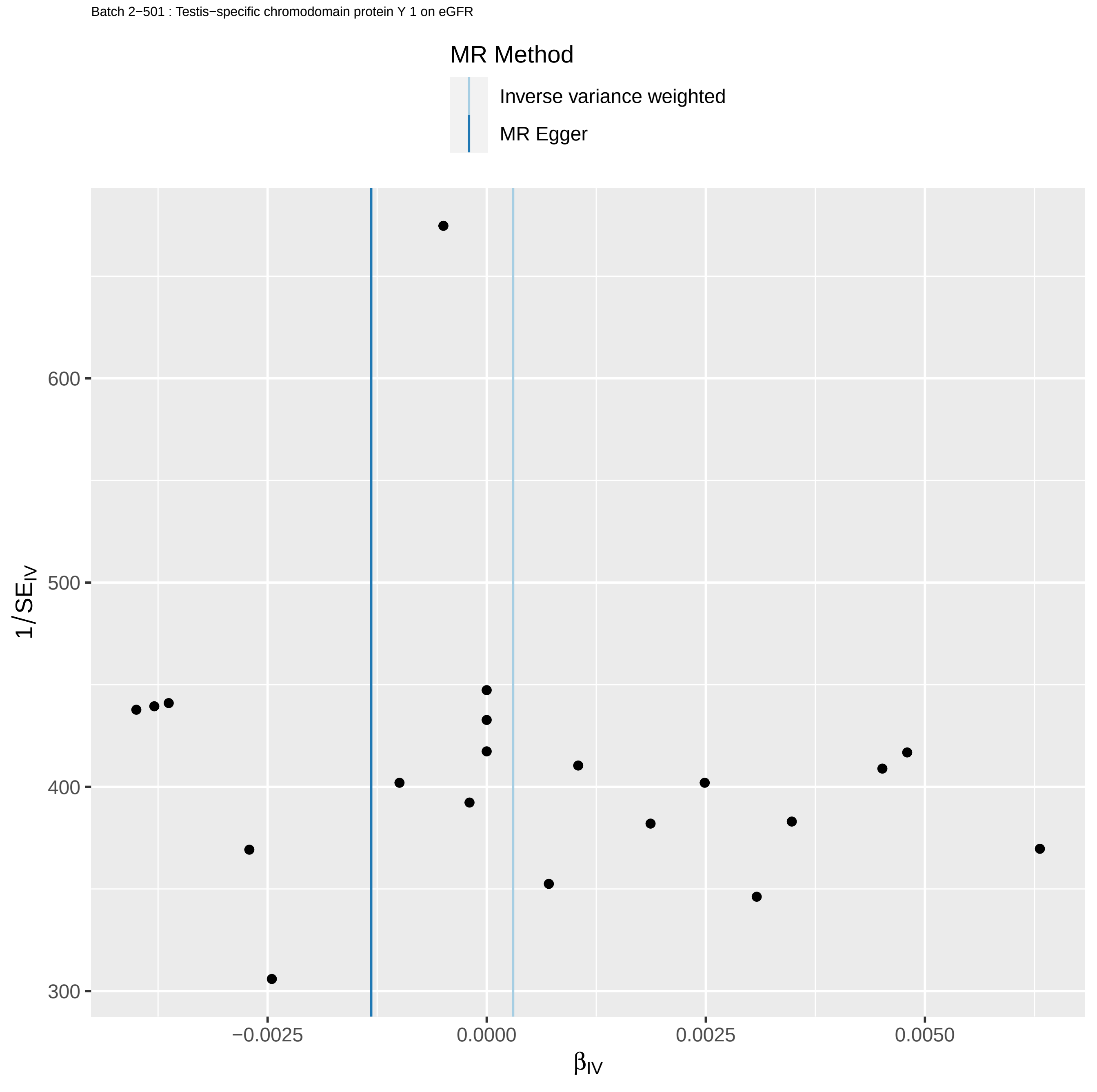

Batch 2−501 : Testis−specific chromodomain protein Y 1 on eGFR
MR Method
Inverse variance weighted
MR Egger
600
500
400
300
1/SE$_{IV}$
−0.0025
0.0000
0.0025
0.0050
β$_{IV}$

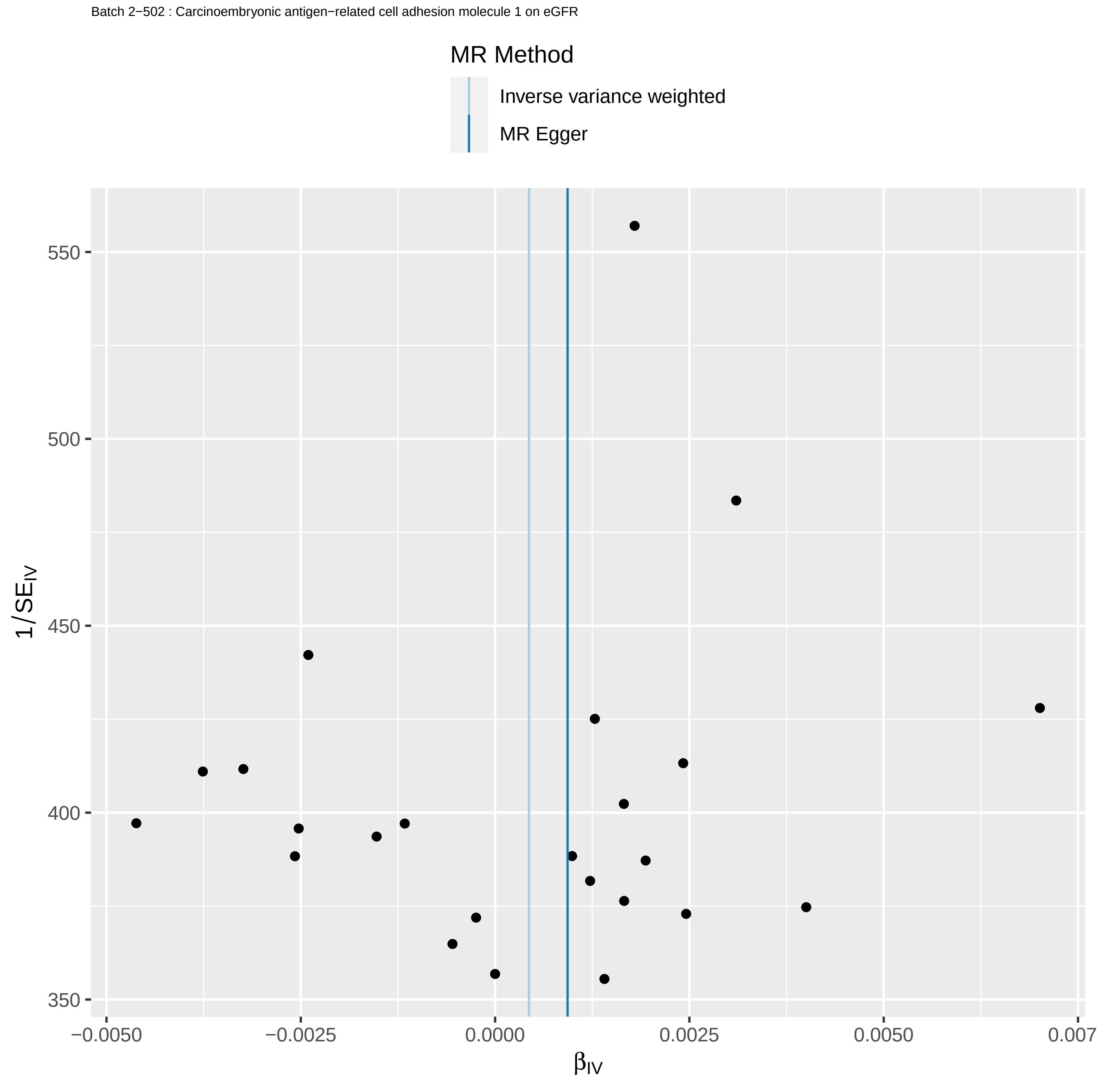

Batch 2−502 : Carcinoembryonic antigen−related cell adhesion molecule 1 on eGFR
MR Method
Inverse variance weighted
MR Egger
550
500
450
400
350
1/SE_IV
−0.0050
−0.0025
0.0000
0.0025
0.0050
0.007
β_IV

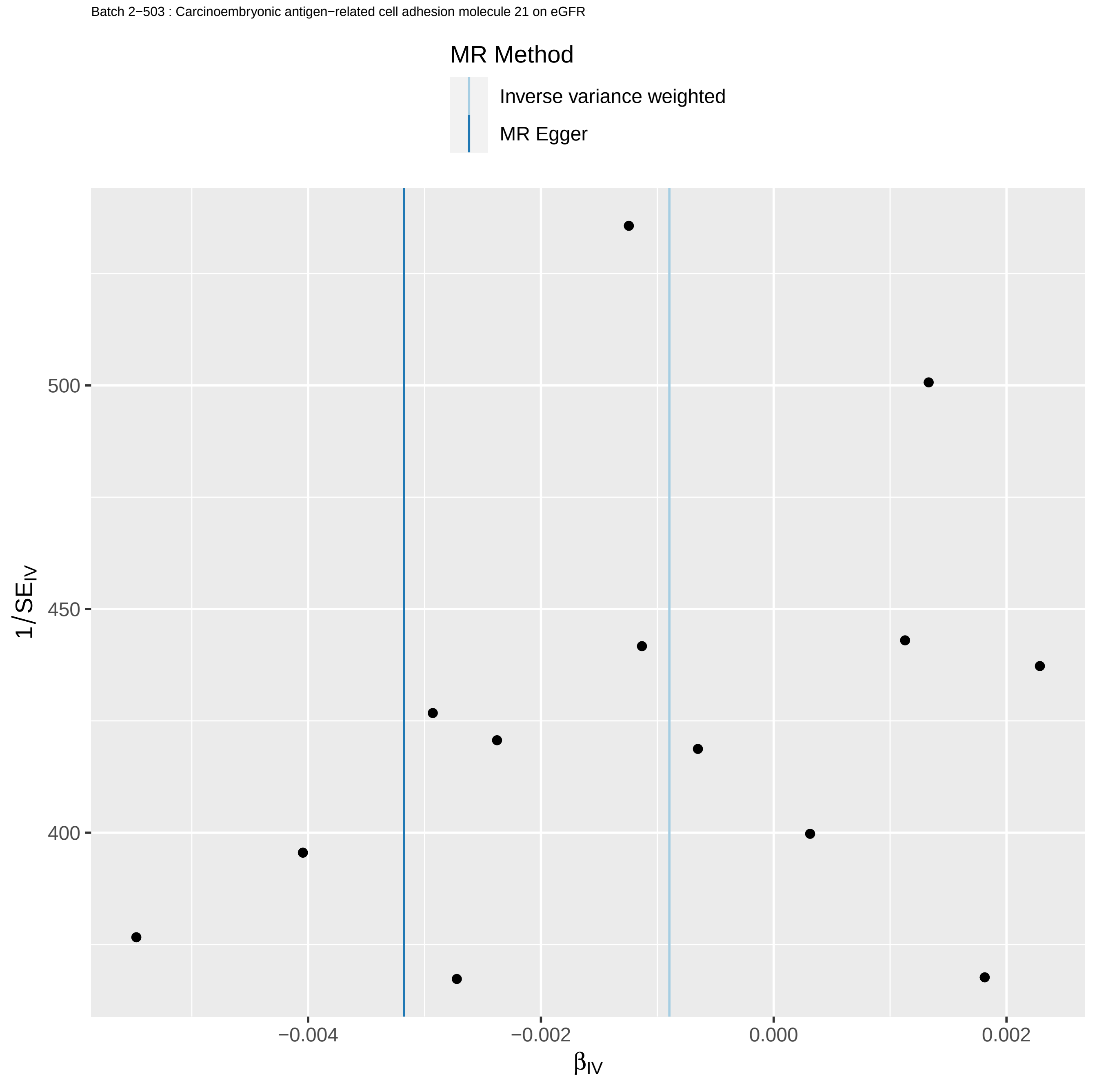

Batch 2−503 : Carcinoembryonic antigen−related cell adhesion molecule 21 on eGFR
MR Method
Inverse variance weighted
MR Egger
500
450
400
1/SE_IV
−0.004
−0.002
0.000
0.002
β_IV

Batch 2−504 : Carcinoembryonic antigen−related cell adhesion molecule 4 on eGFR

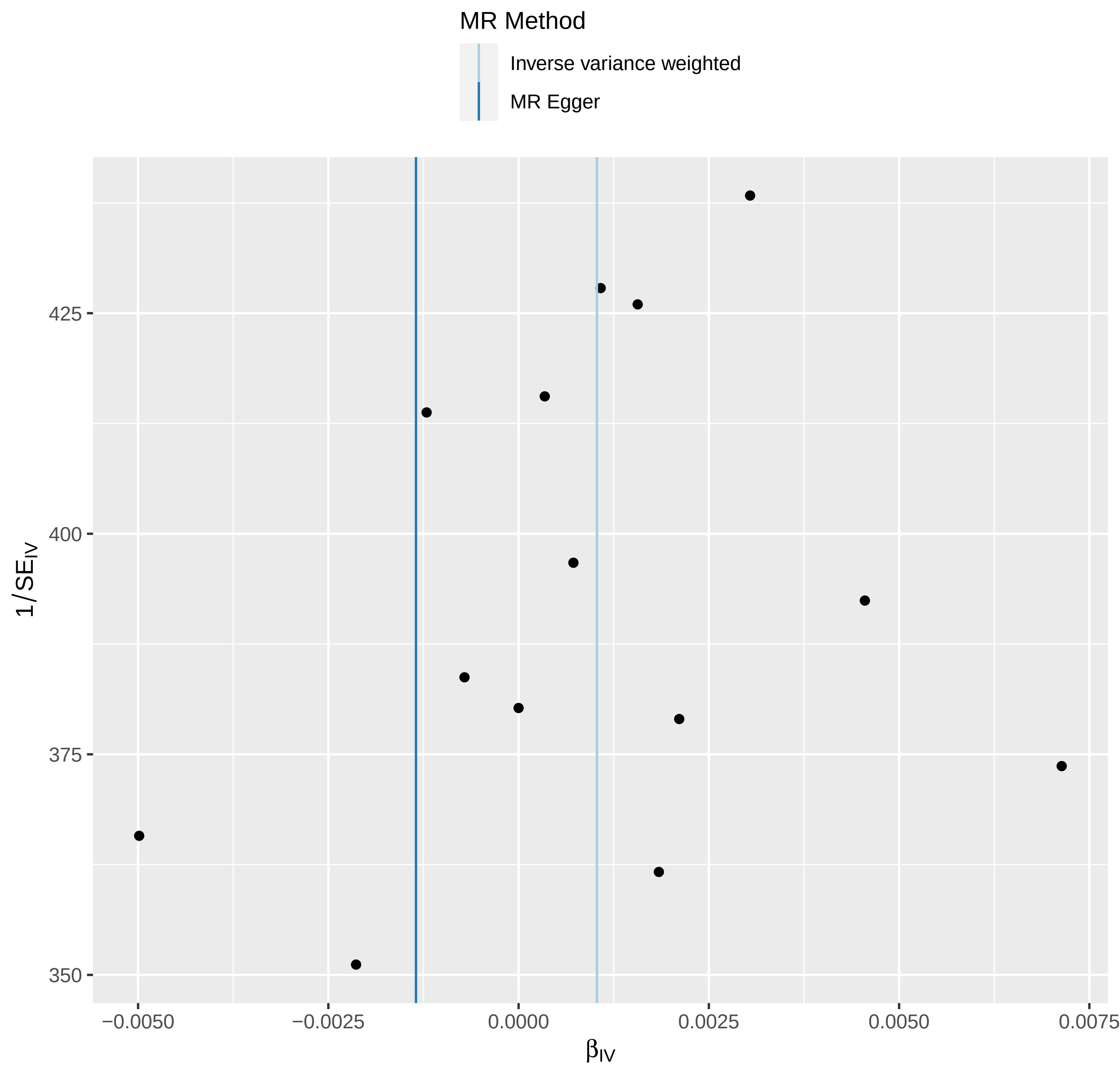

Batch 2−505 : CCAAT/enhancer−binding protein beta on eGFR

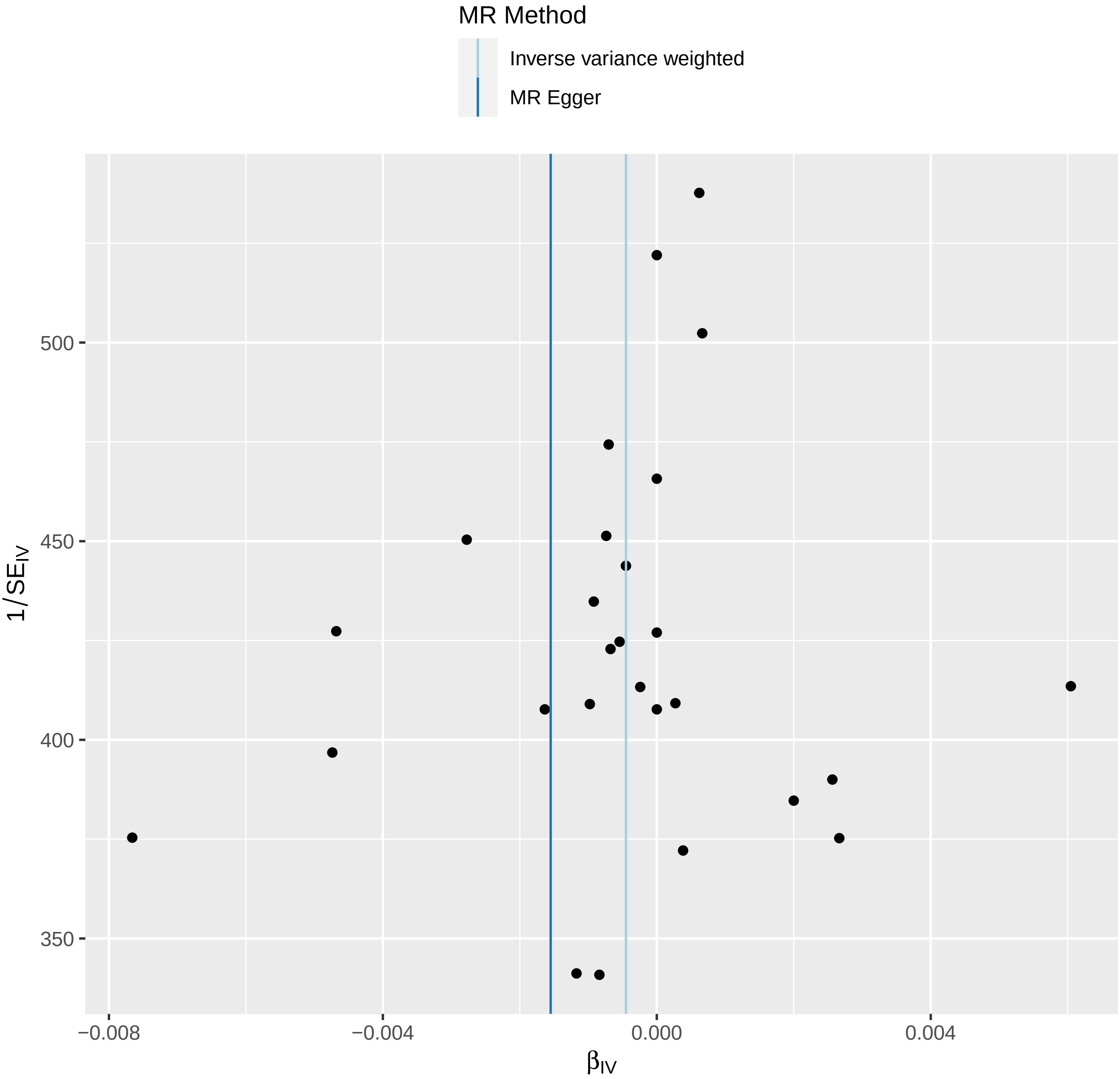

Batch 2−506 : Adenosine deaminase CECR1 on eGFR

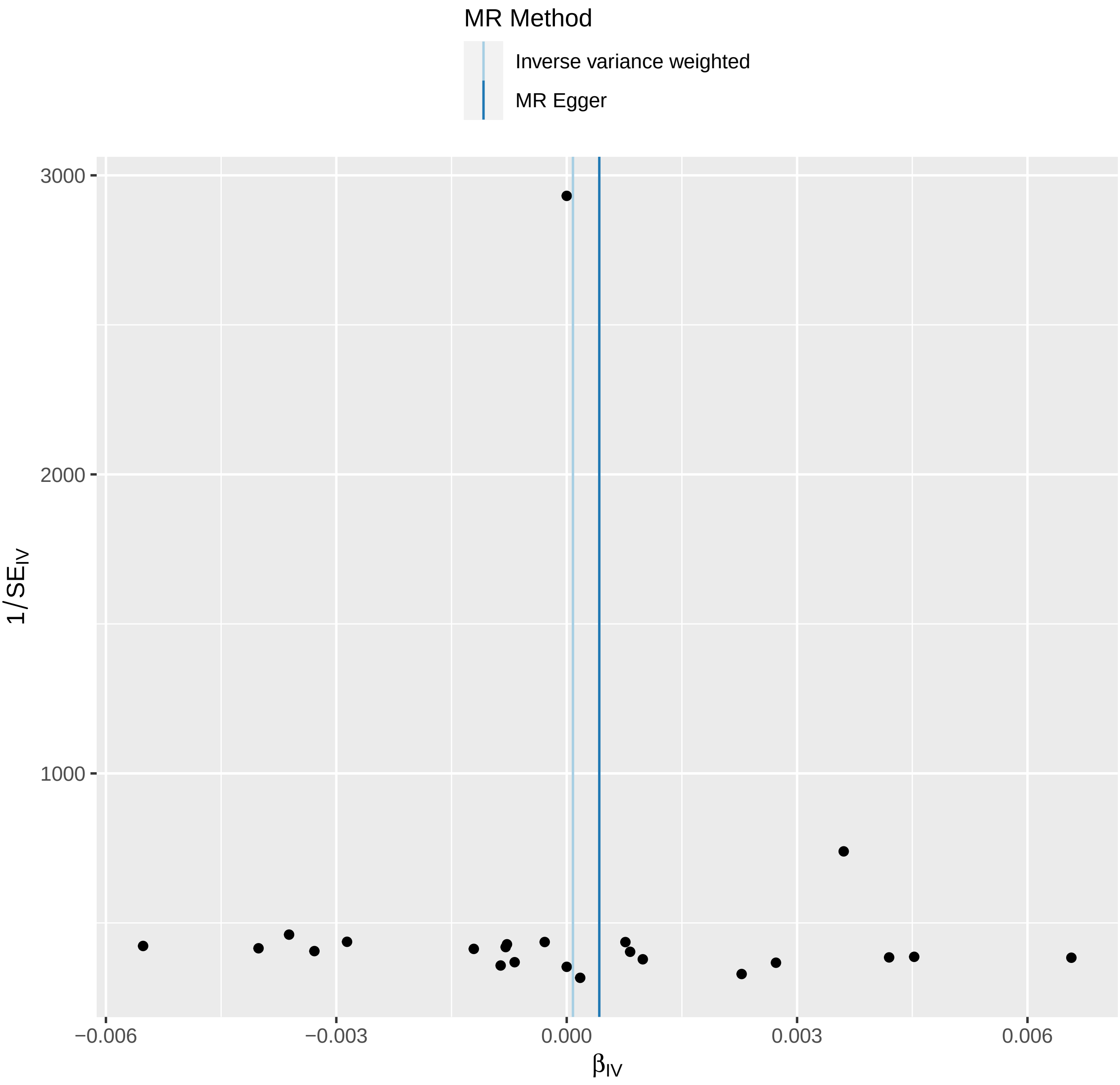

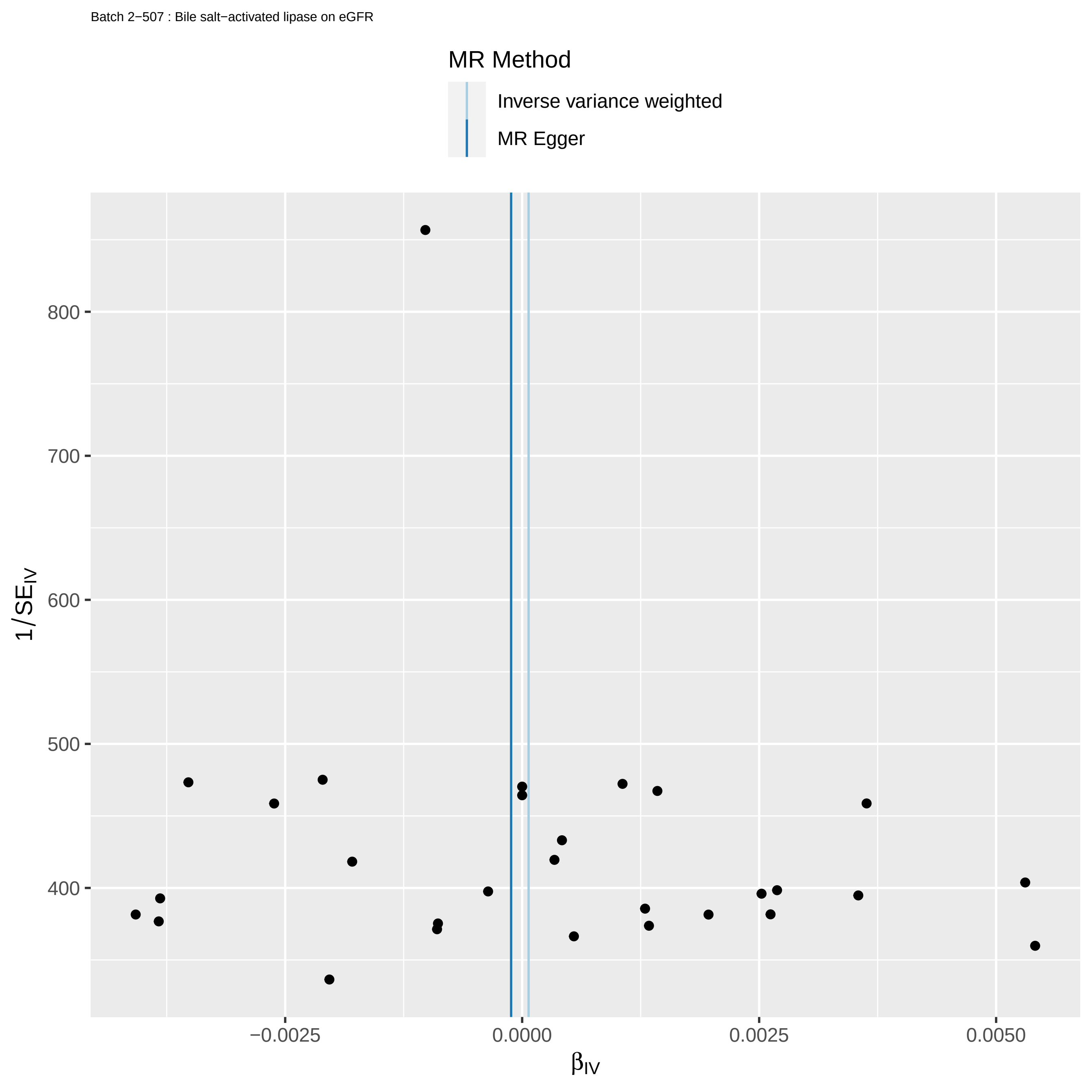

Batch 2−507 : Bile salt−activated lipase on eGFR
MR Method
Inverse variance weighted
MR Egger
800
700
600
500
400
1/SE$_{IV}$
−0.0025
0.0000
0.0025
0.0050
β_{IV}

Batch 2−508 : Chymotrypsin−like elastase family member 2A on eGFR

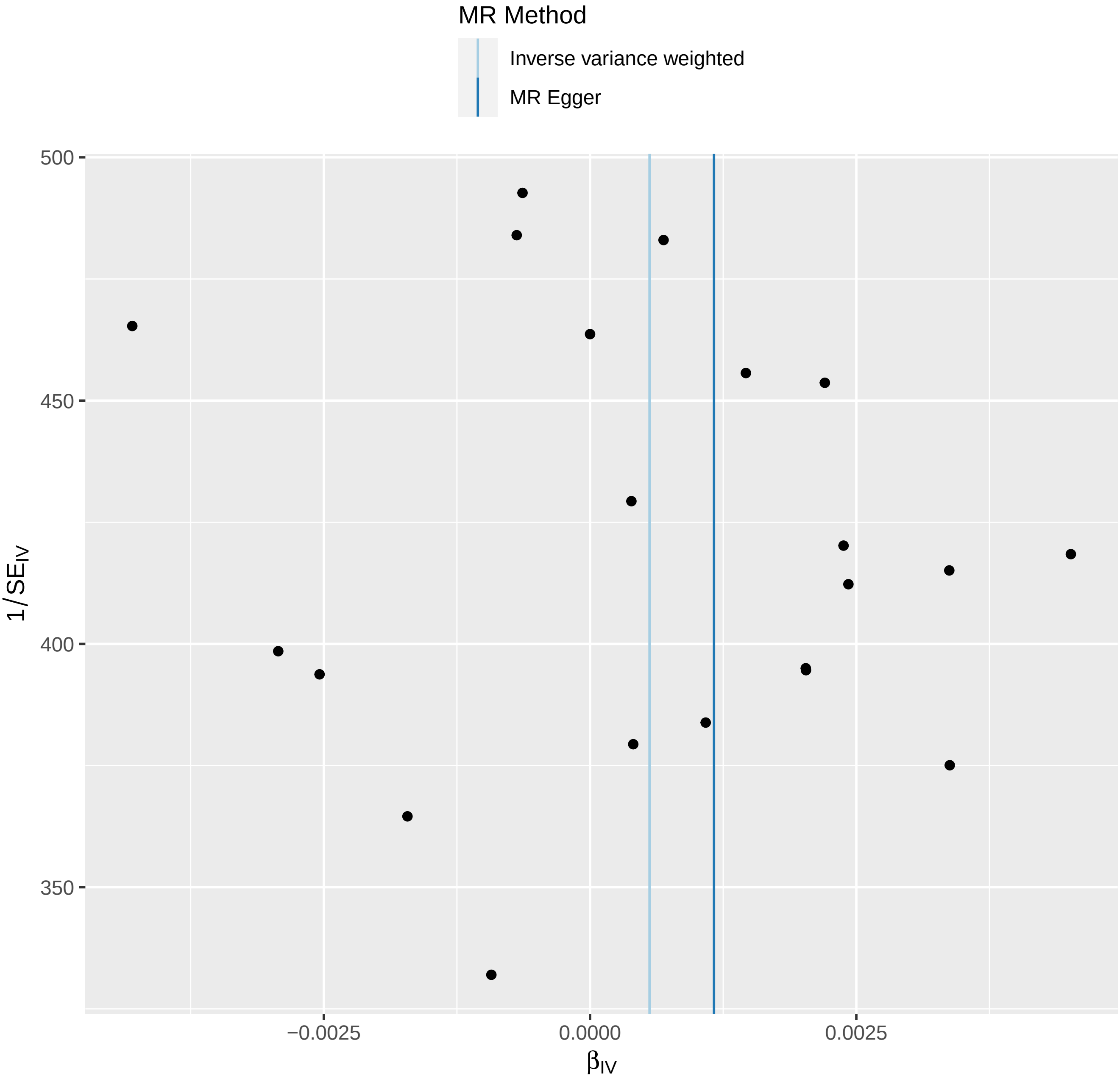

Batch 2−509 : Chymotrypsin−like elastase family member 3B on eGFR

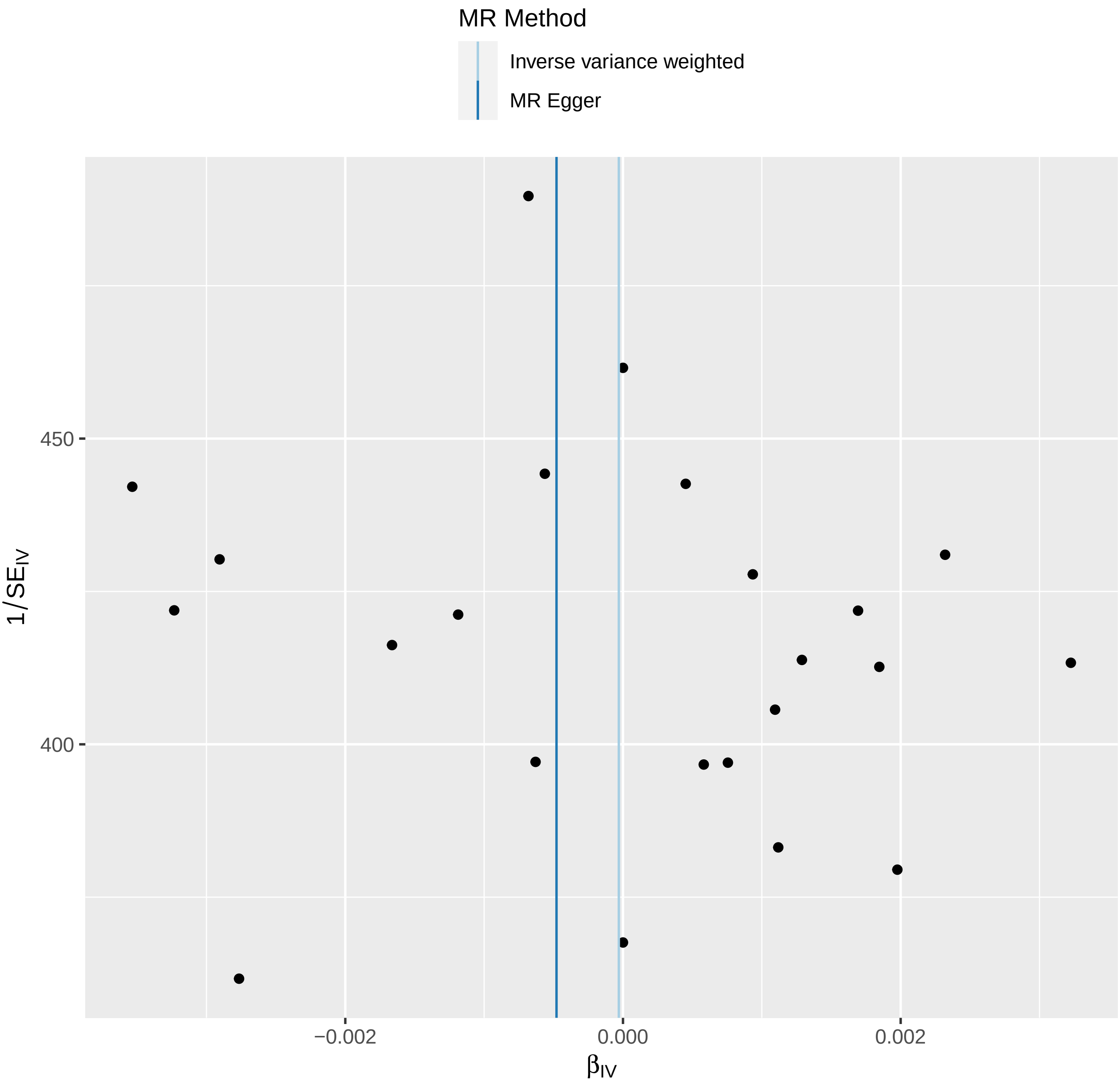

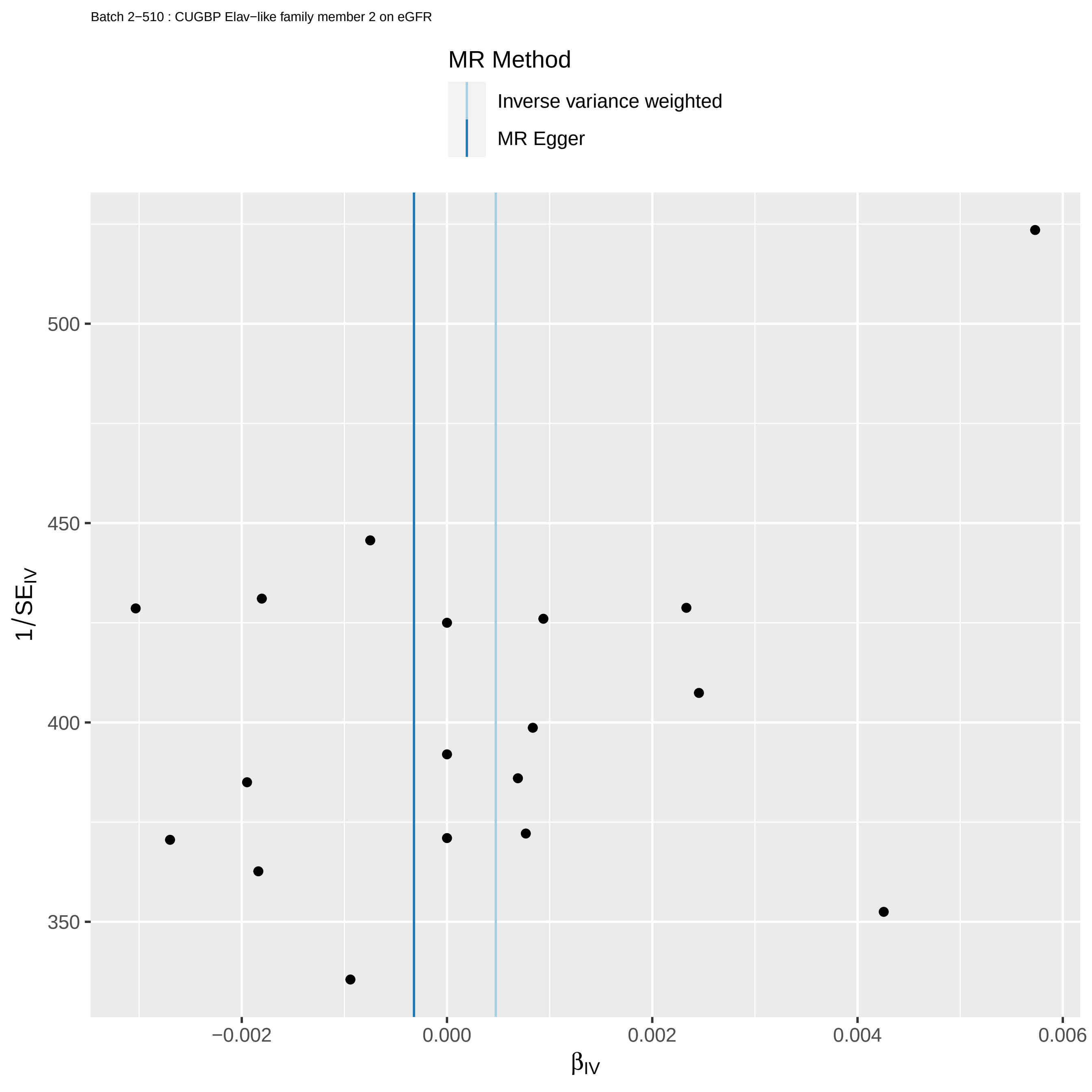

Batch 2−510 : CUGBP Elav−like family member 2 on eGFR
MR Method
Inverse variance weighted
MR Egger
500
450
400
350
1/SE_IV
−0.002
0.000
0.002
0.004
0.006
β_IV

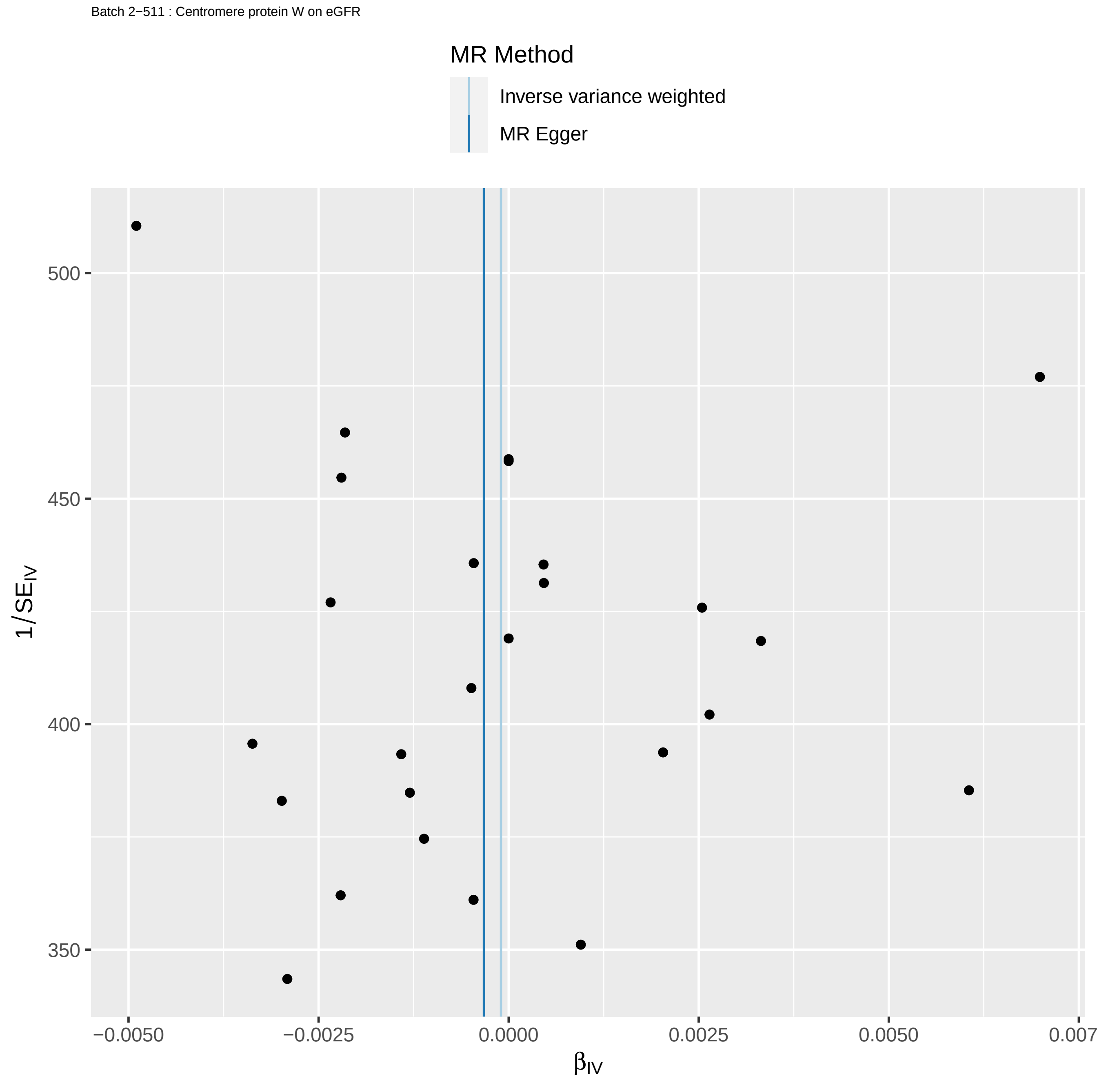

Batch 2−511 : Centromere protein W on eGFR
MR Method
Inverse variance weighted
MR Egger
500
450
400
350
1/SE$_{IV}$
−0.0050
−0.0025
0.0000
0.0025
0.0050
0.007
β$_{IV}$

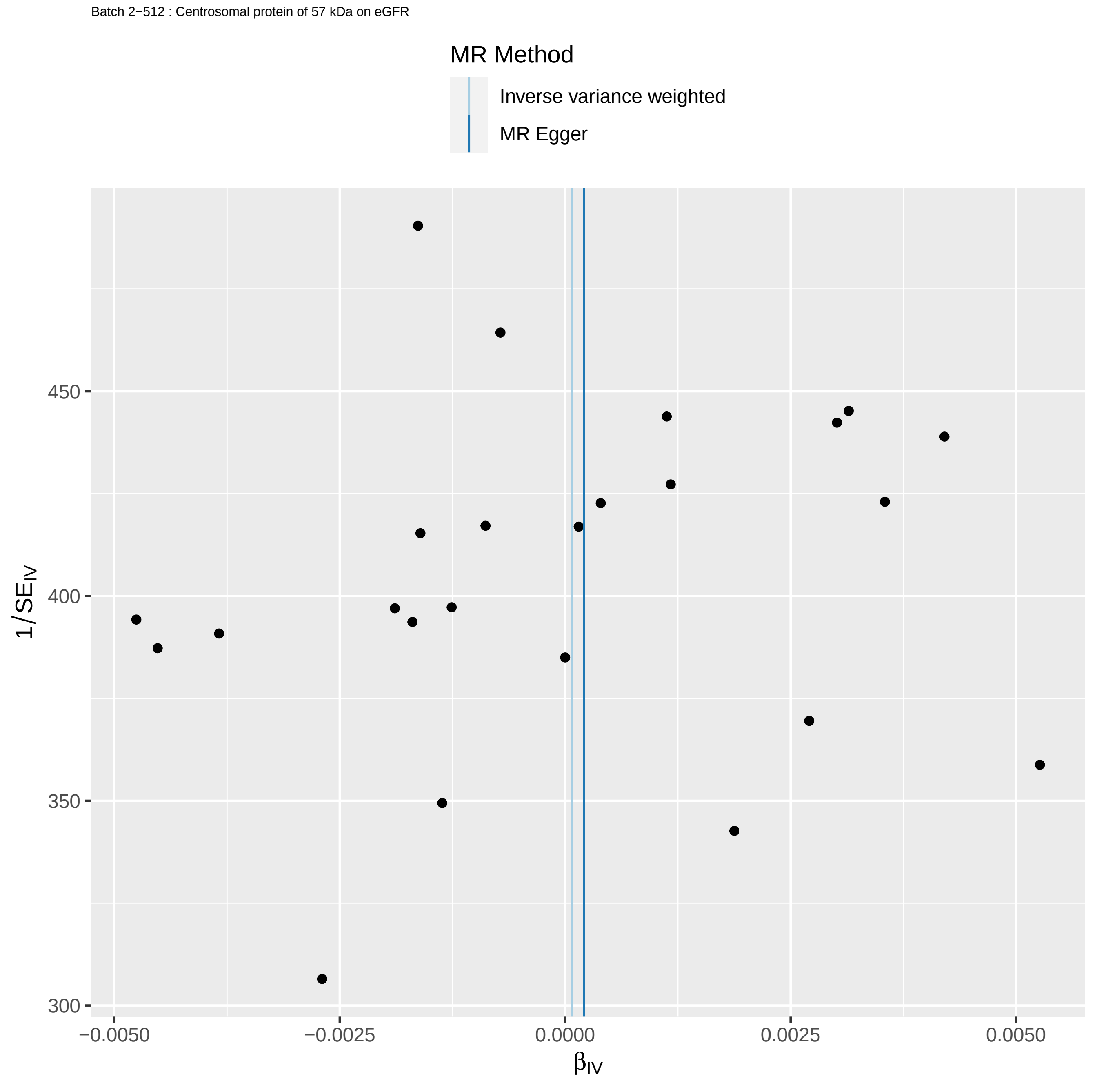

Batch 2−512 : Centrosomal protein of 57 kDa on eGFR
MR Method
Inverse variance weighted
MR Egger
450
400
350
300
−0.0050
−0.0025
0.0000
0.0025
0.0050
$1/SE_{IV}$
β_{IV}

Batch 2−513 : Centrosomal protein of 57 kDa on eGFR

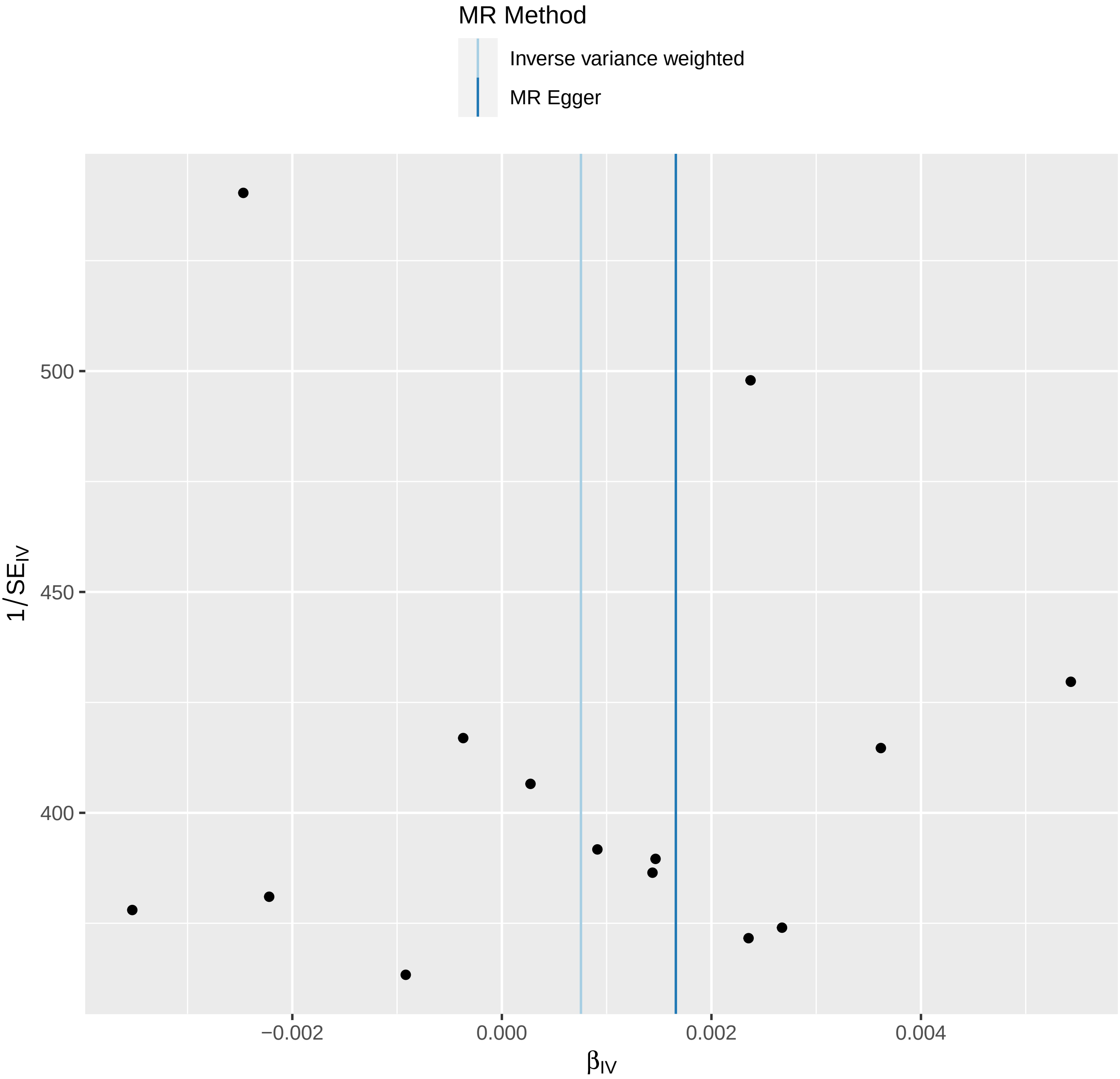

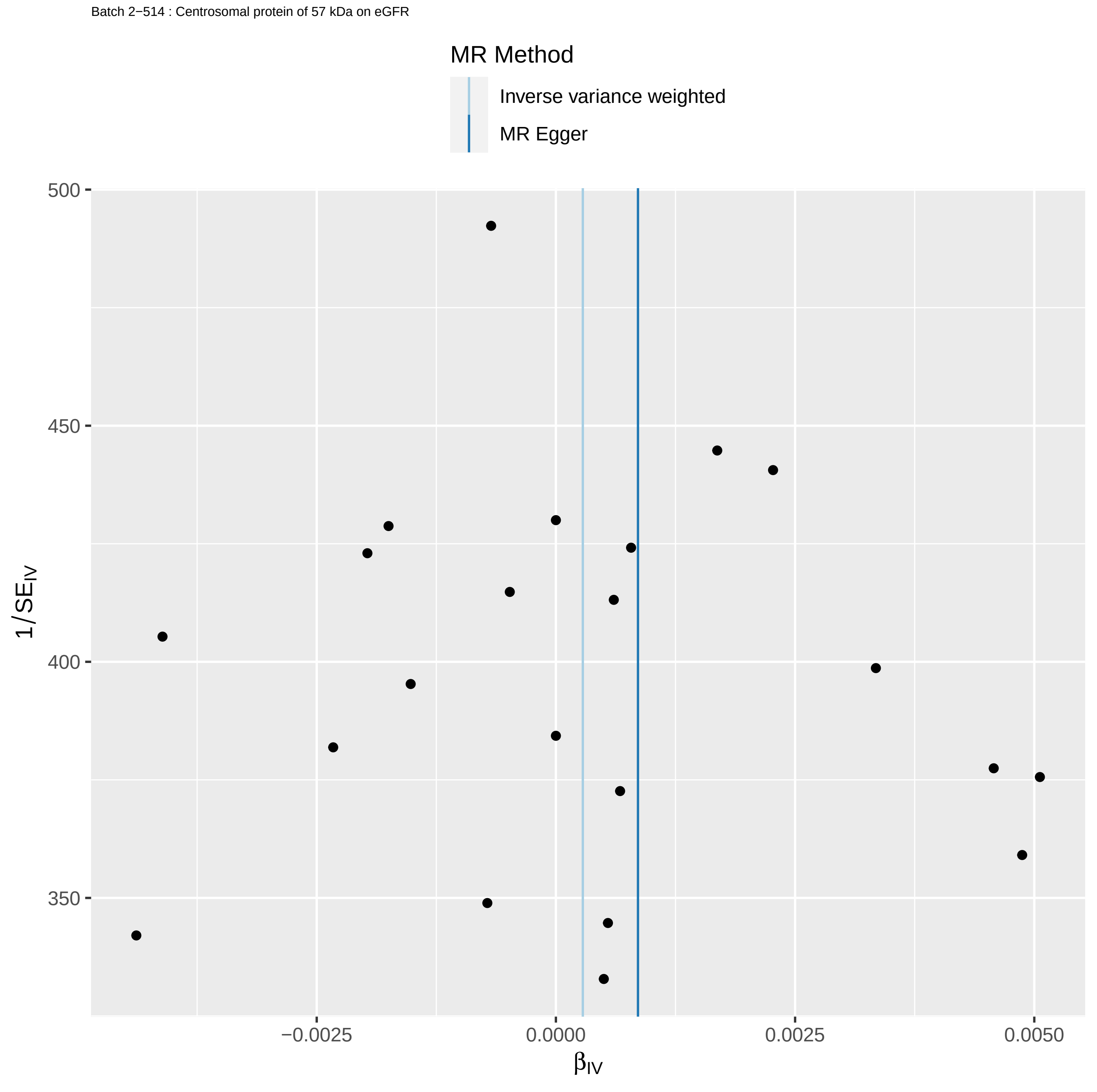

Batch 2−514 : Centrosomal protein of 57 kDa on eGFR
MR Method
Inverse variance weighted
MR Egger
500
450
400
350
1/SE$_{IV}$
−0.0025
0.0000
0.0025
0.0050
β$_{IV}$

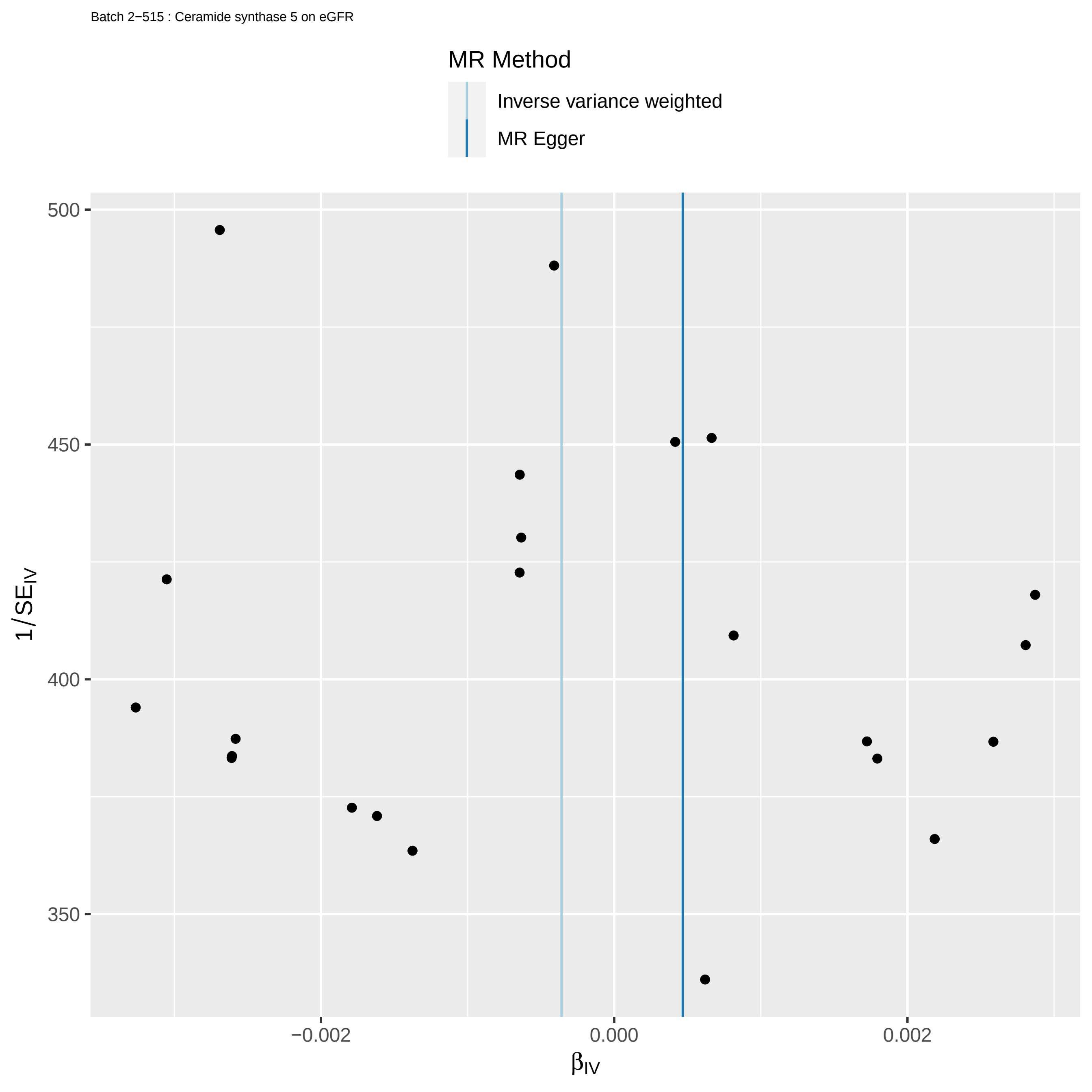
Batch 2−515 : Ceramide synthase 5 on eGFR
MR Method
Inverse variance weighted
MR Egger
500
450
400
350
1/SE_IV
−0.002
0.000
0.002
β_IV

Batch 2−516 : Centrin−2 on eGFR

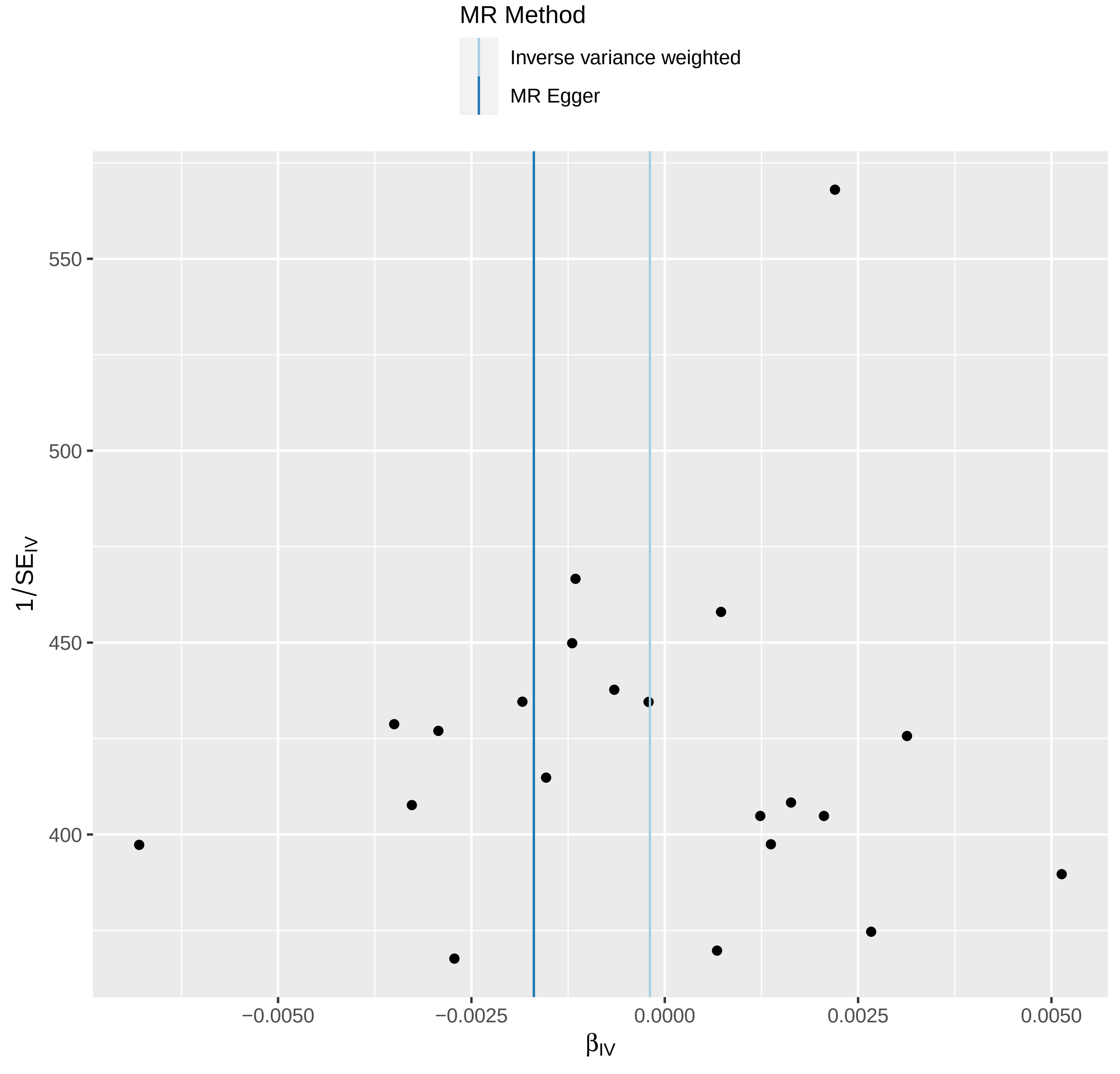

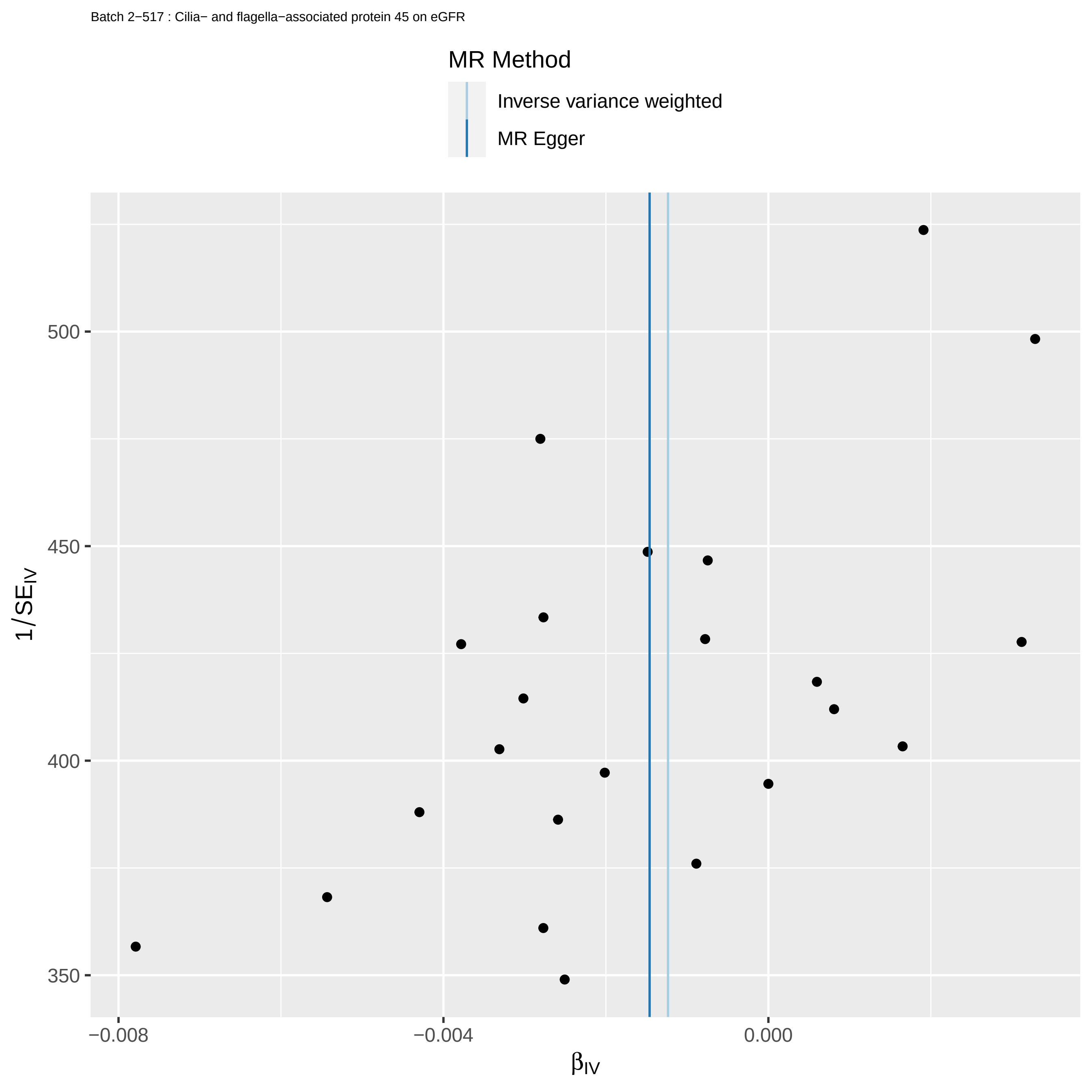

Batch 2−517 : Cilia− and flagella−associated protein 45 on eGFR
MR Method
Inverse variance weighted
MR Egger
500
450
400
350
1/SE_IV
−0.008
−0.004
0.000
β_IV

Batch 2−518 : Complement factor B on eGFR

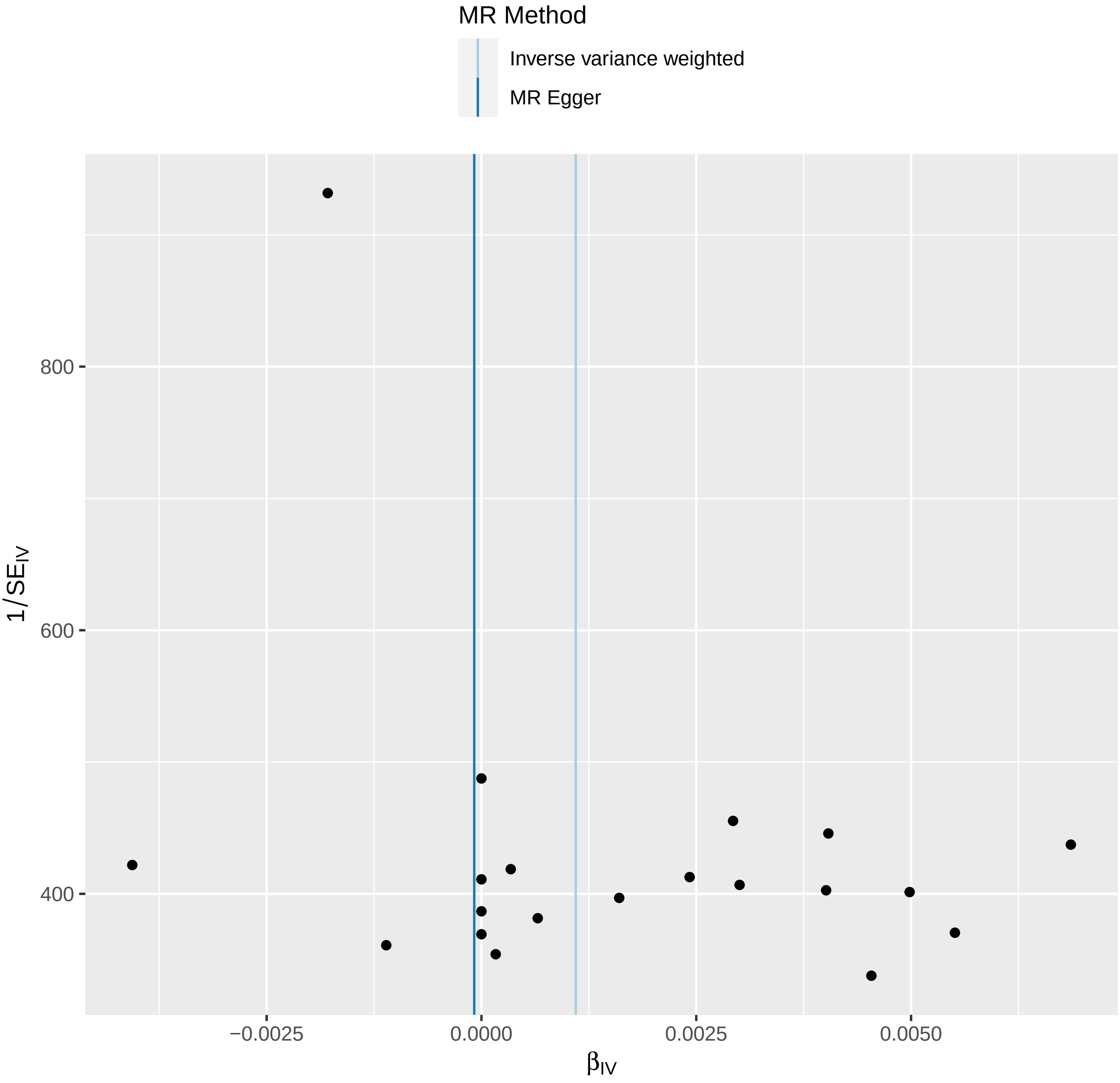

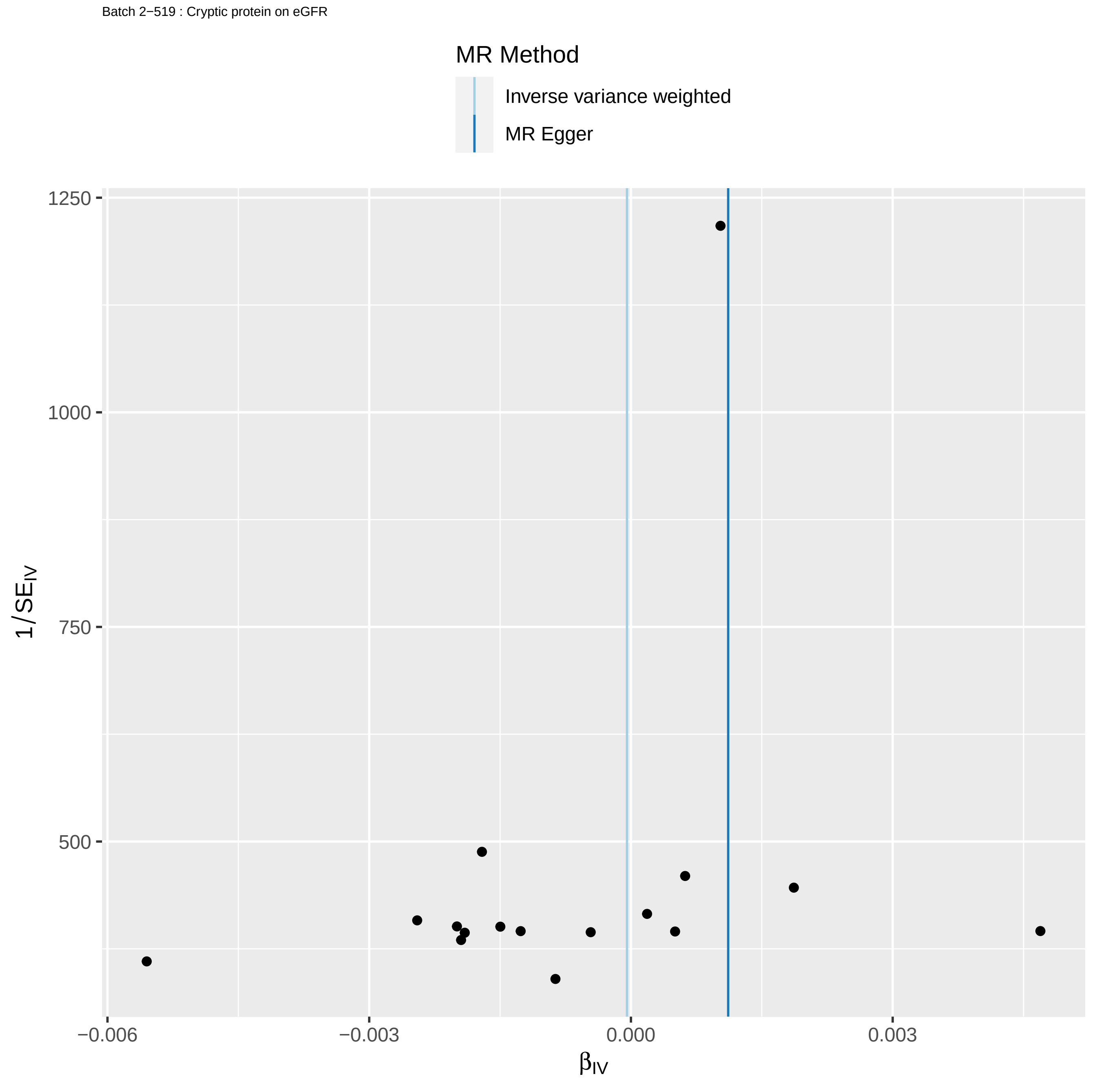

Batch 2−519 : Cryptic protein on eGFR
MR Method
Inverse variance weighted
MR Egger
1250
1000
750
500
1/SE$_{IV}$
−0.006
−0.003
0.000
0.003
β$_{IV}$

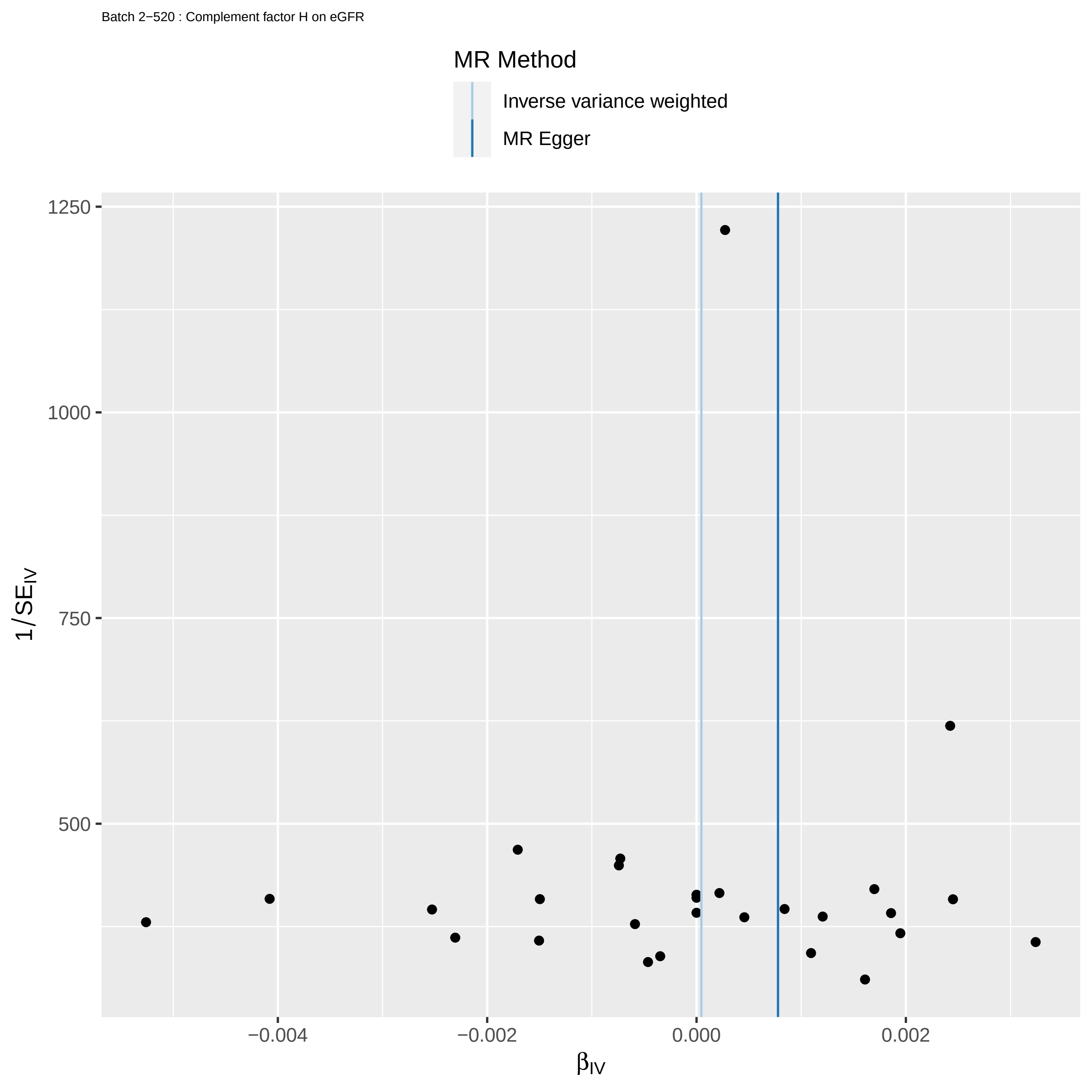
Batch 2−520 : Complement factor H on eGFR
MR Method
Inverse variance weighted
MR Egger
1250
1000
750
500
1/SE$_{IV}$
−0.004
−0.002
0.000
0.002
β$_{IV}$

Batch 2−521 : Complement factor H−related protein 5 on eGFR

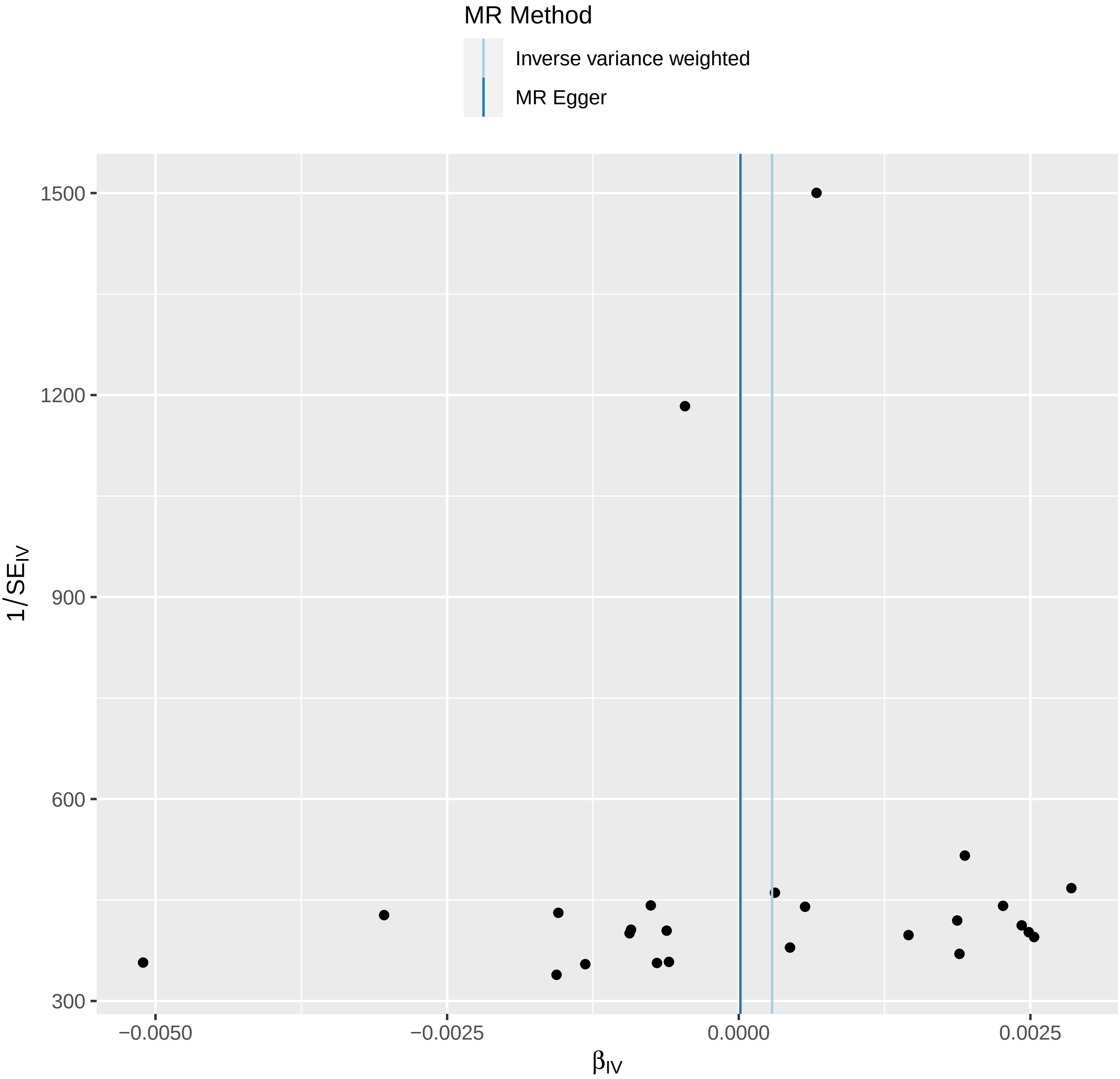

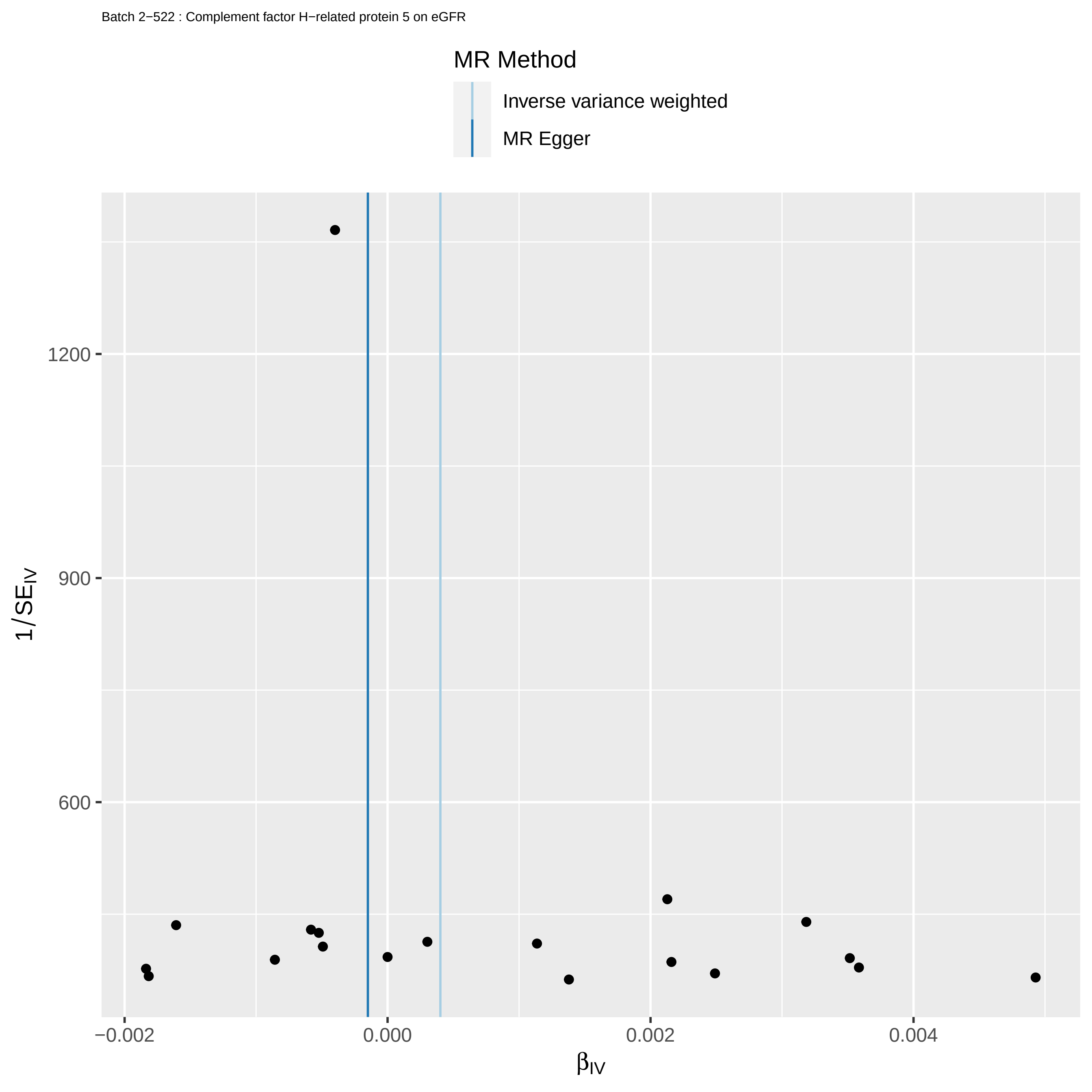
Batch 2−522 : Complement factor H−related protein 5 on eGFR
MR Method
Inverse variance weighted
MR Egger
1200
900
600
1/SE_IV
−0.002
0.000
0.002
0.004
β_IV

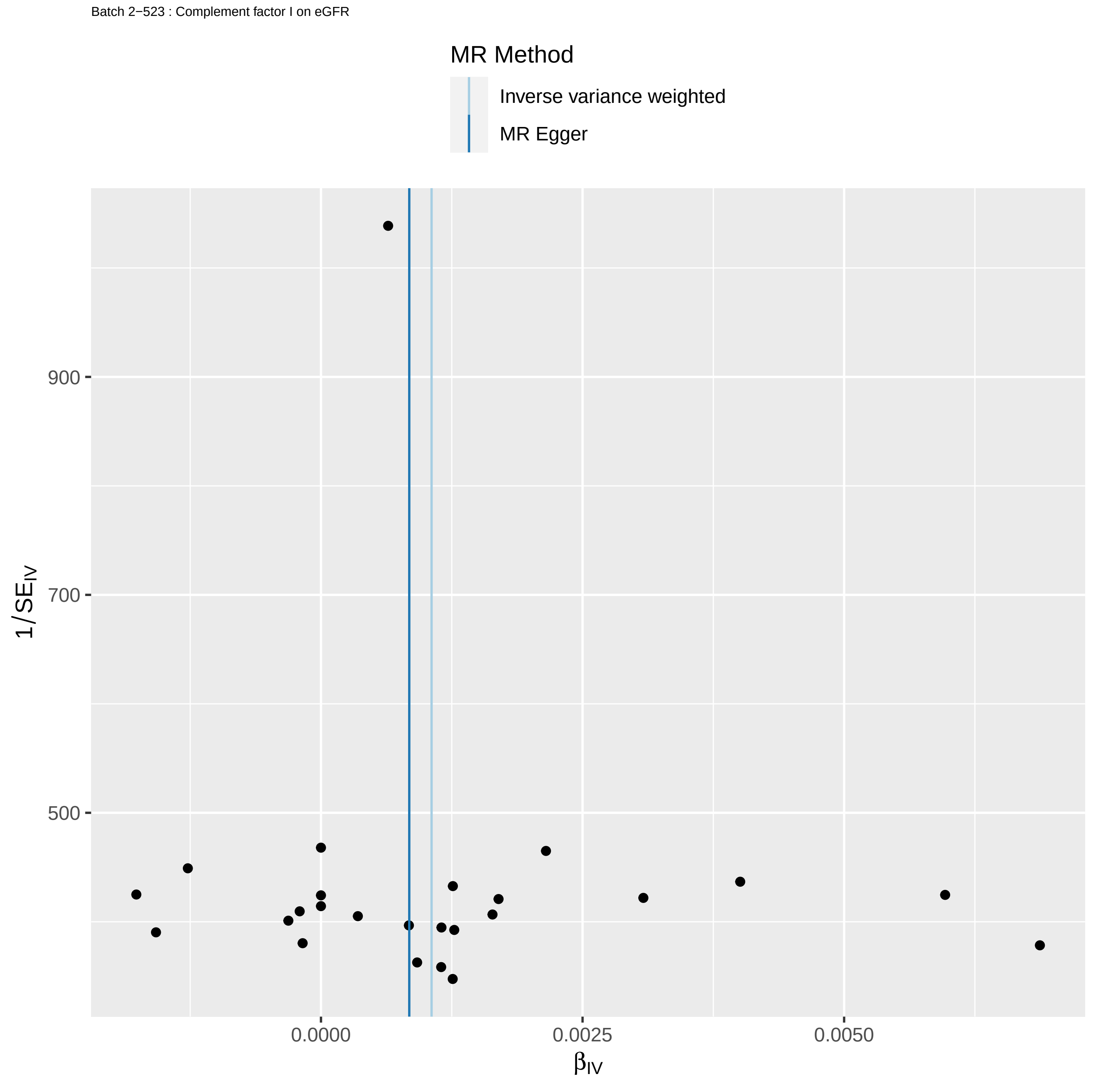

Batch 2−523 : Complement factor I on eGFR
MR Method
Inverse variance weighted
MR Egger
900
700
500
1/SE_IV
0.0000
0.0025
0.0050
β_IV

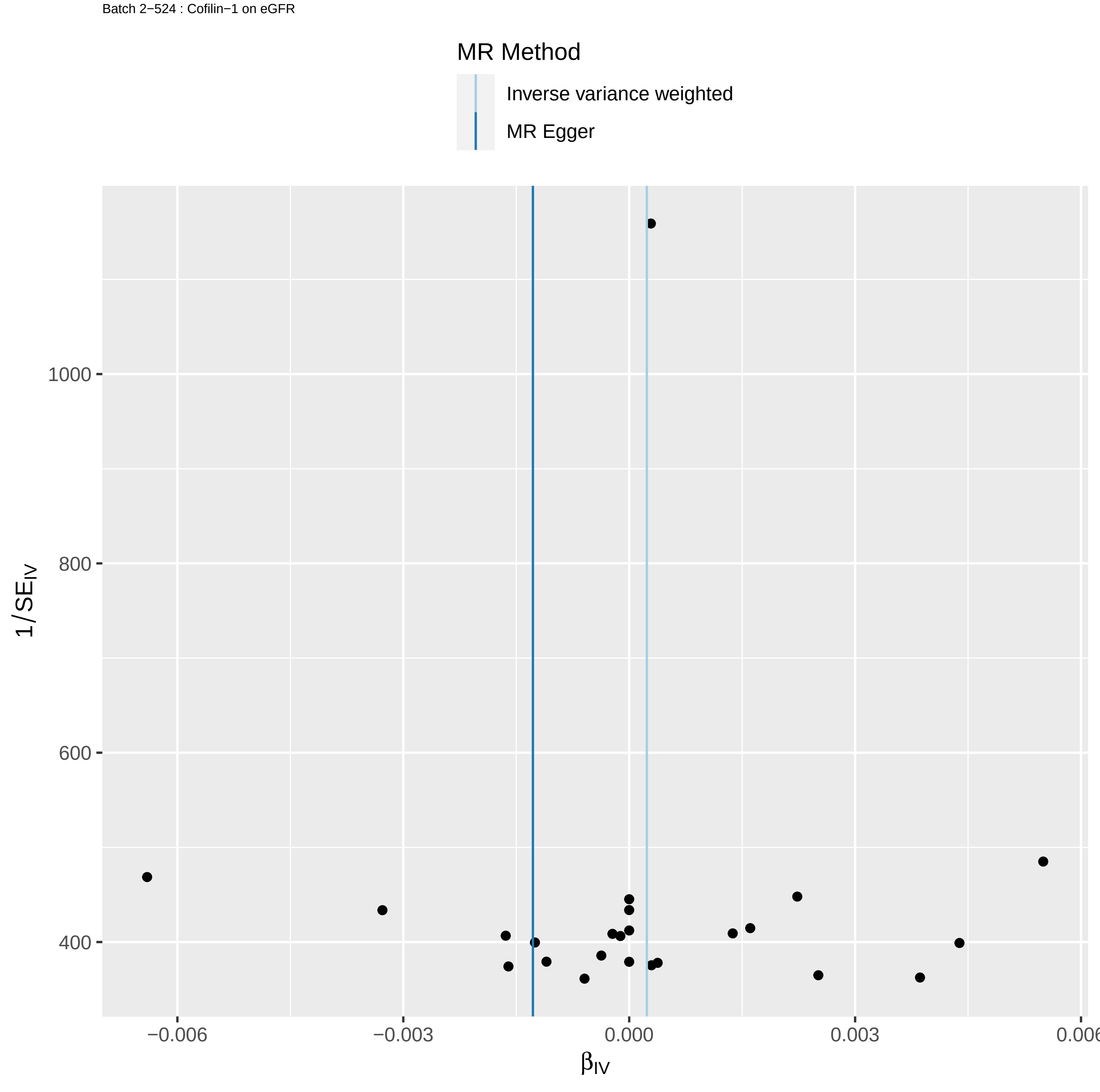

Batch 2−524 : Cofilin−1 on eGFR
MR Method
Inverse variance weighted
MR Egger
1000
800
600
400
1/SE_IV
−0.006
−0.003
0.000
0.003
0.006
β_IV

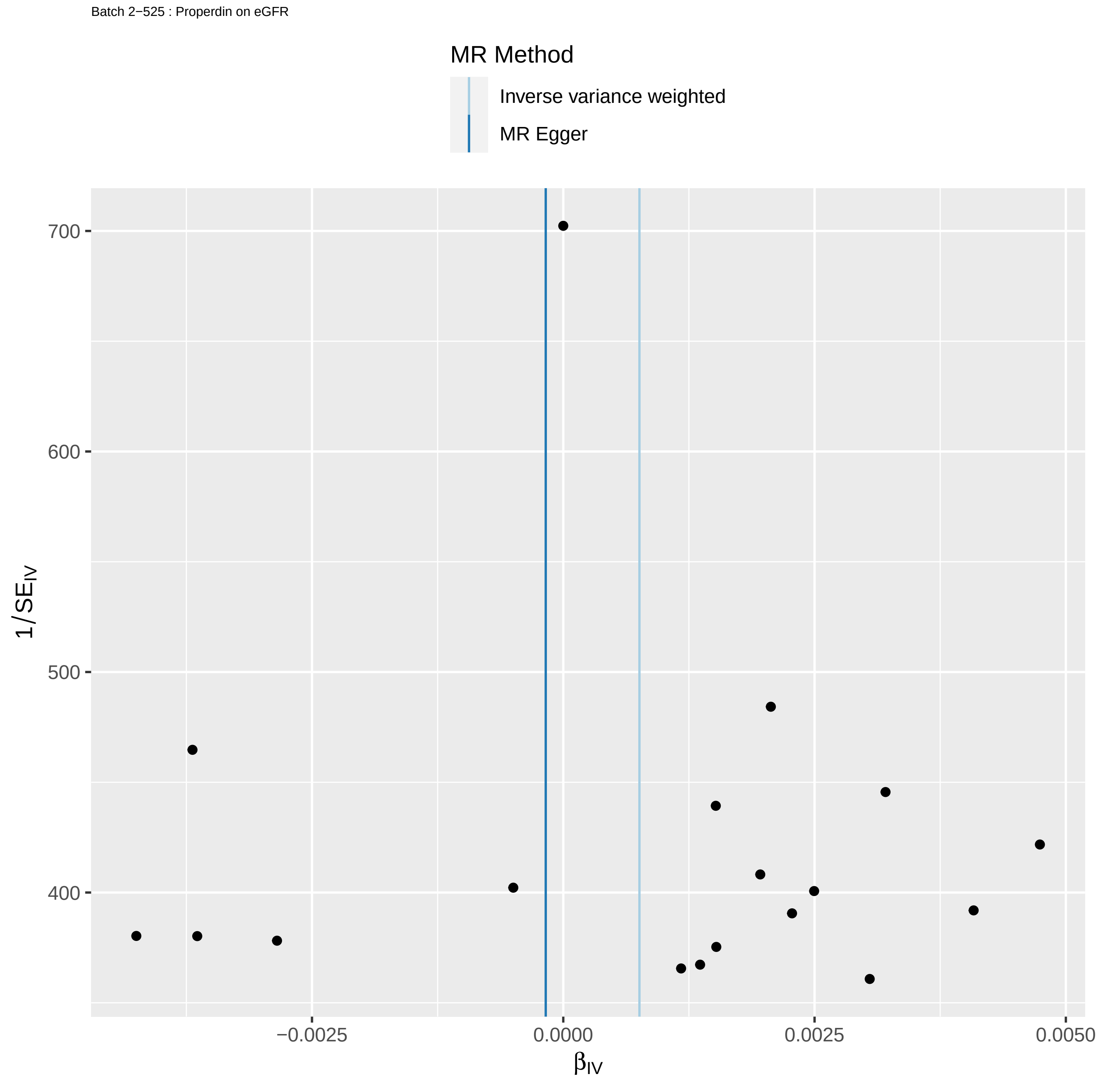

Batch 2−525 : Properdin on eGFR
MR Method
Inverse variance weighted
MR Egger
700
600
500
400
1/SE_IV
−0.0025
0.0000
0.0025
0.0050
β_IV

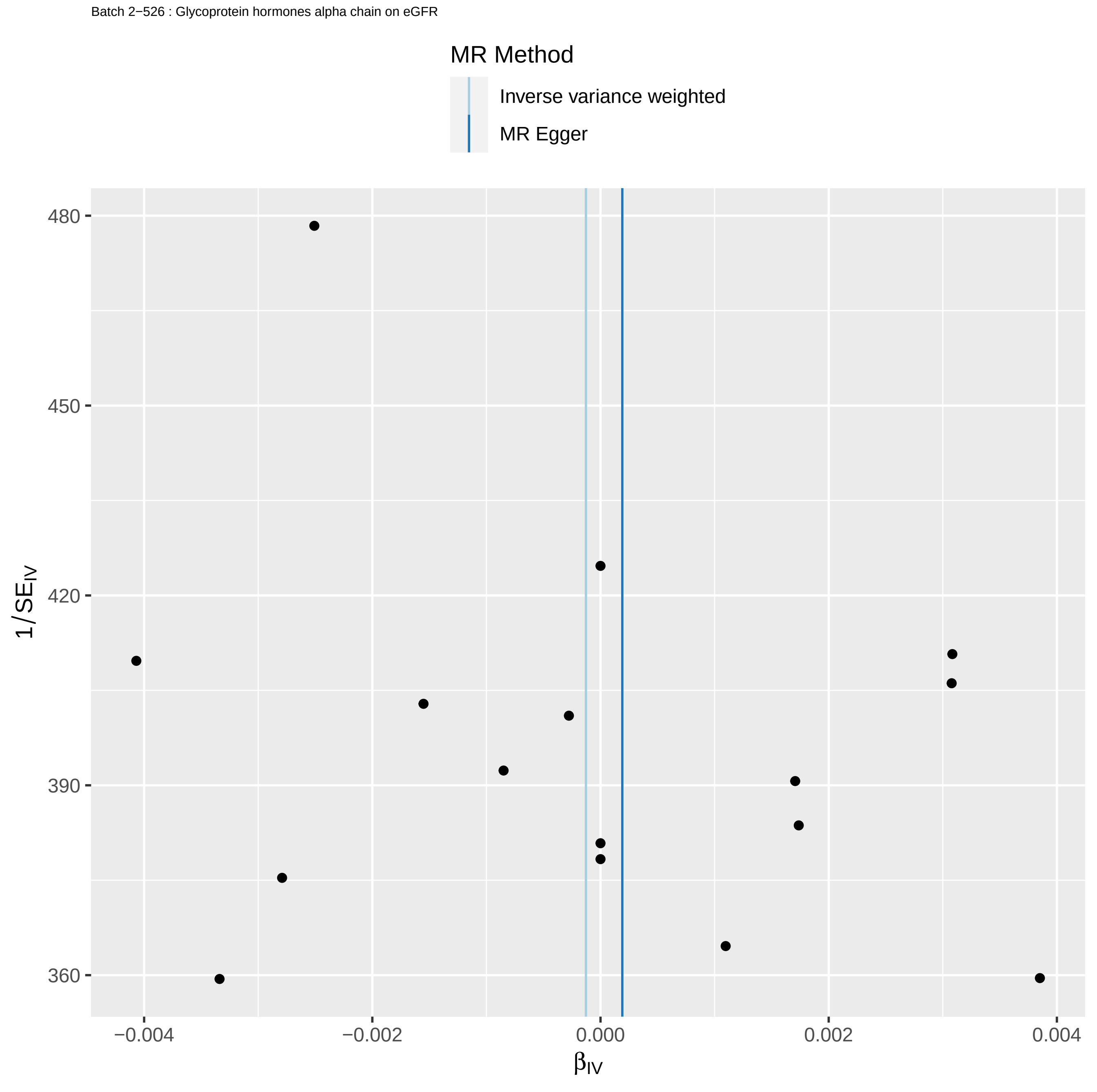

Batch 2−526 : Glycoprotein hormones alpha chain on eGFR
MR Method
Inverse variance weighted
MR Egger
480
450
420
390
360
1/SE_IV
−0.004
−0.002
0.000
0.002
0.004
β_IV

Batch 2−527 : Human Chorionic Gonadotropin on eGFR

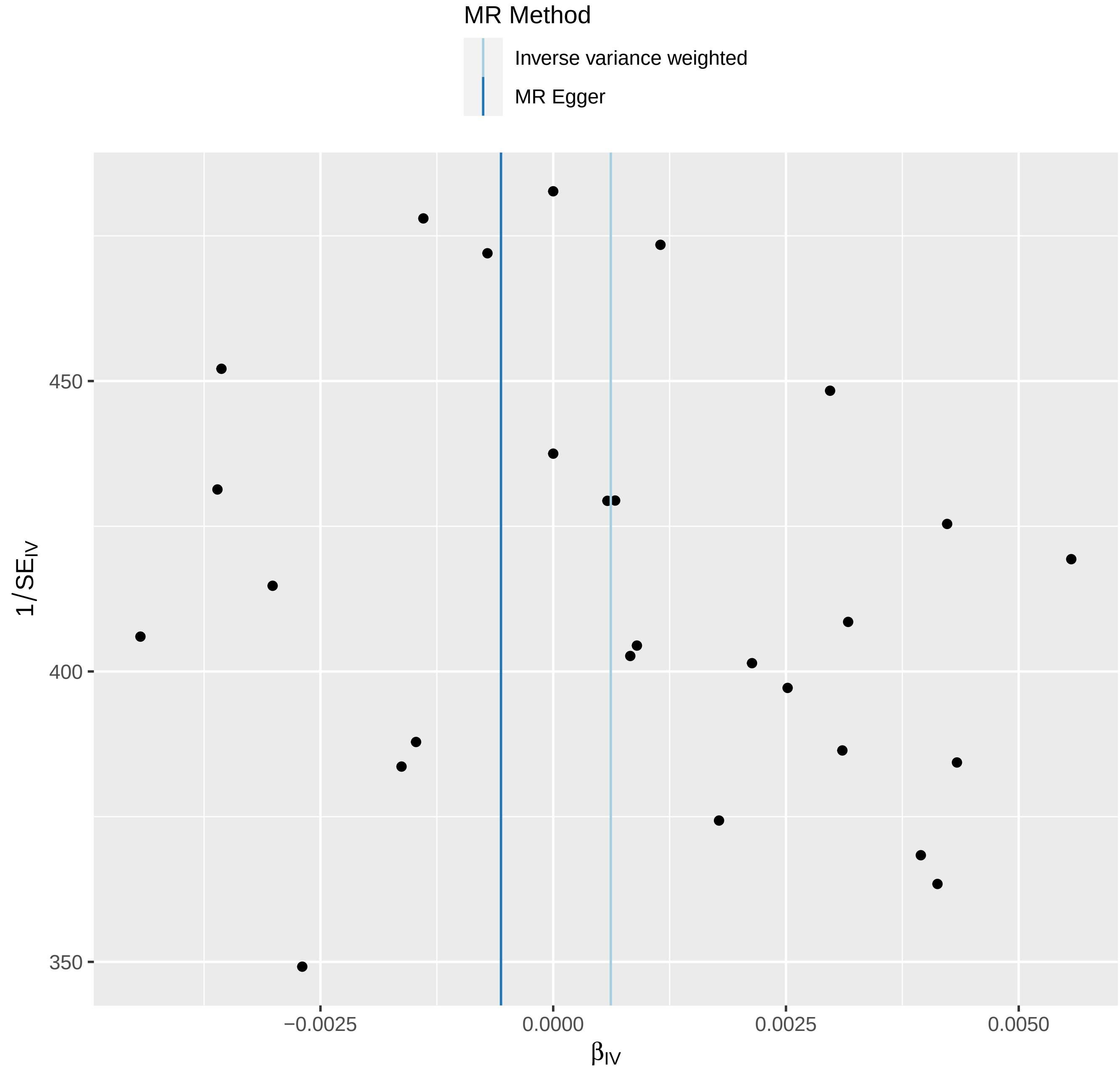

Batch 2−528 : Follicle stimulating hormone on eGFR

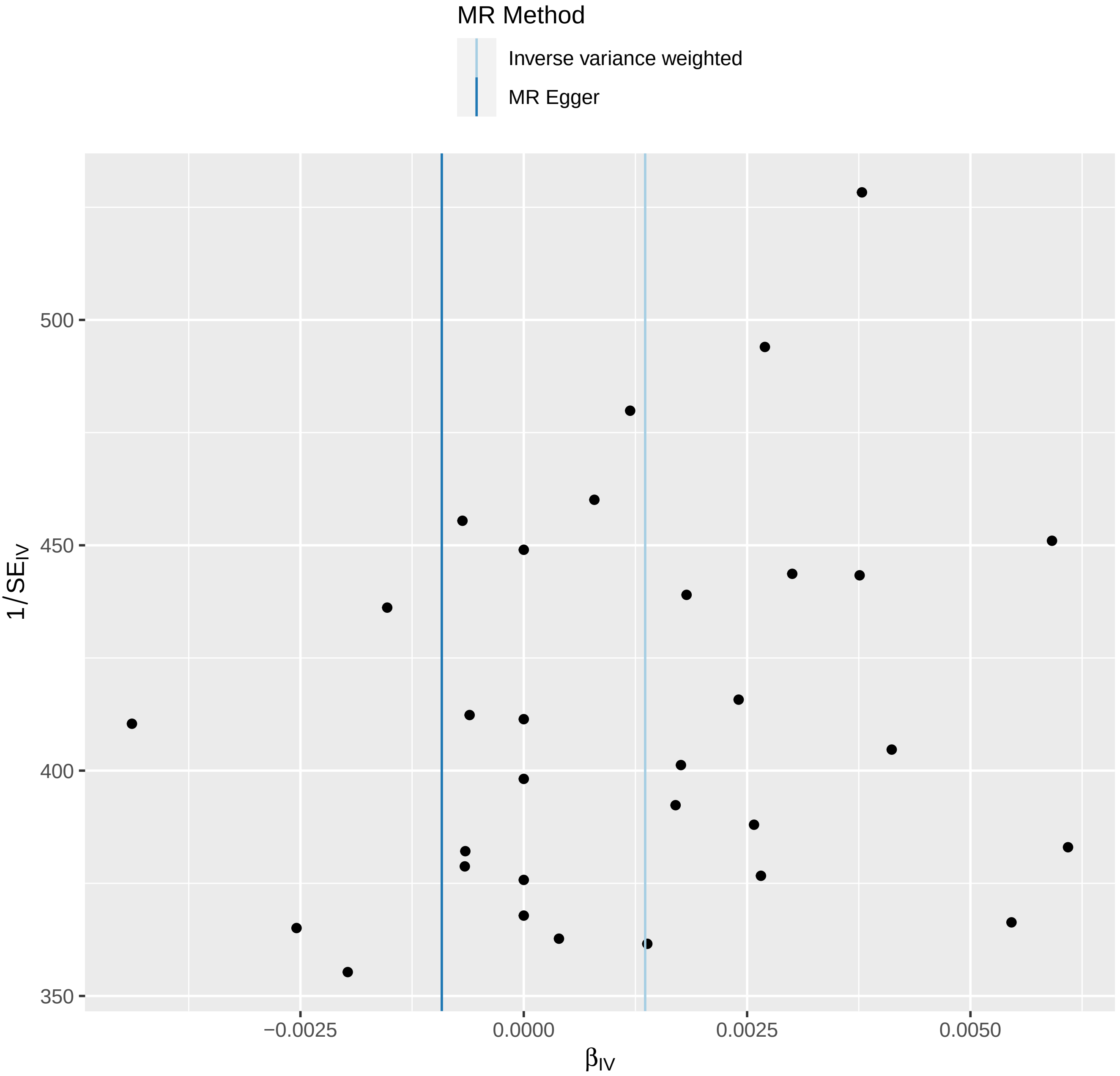

Batch 2−529 : Luteinizing hormone on eGFR

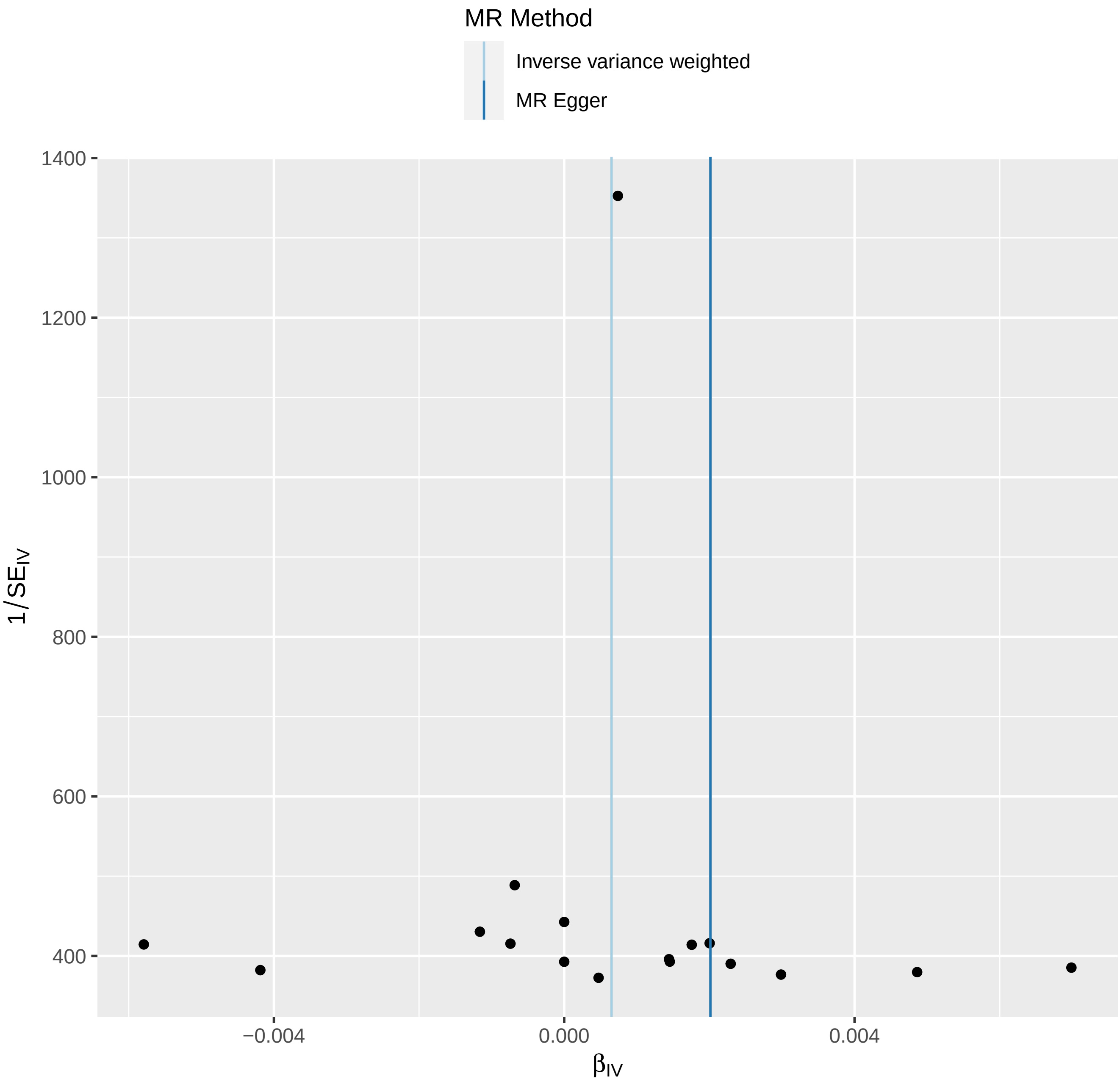

Batch 2−530 : Thyroid Stimulating Hormone on eGFR

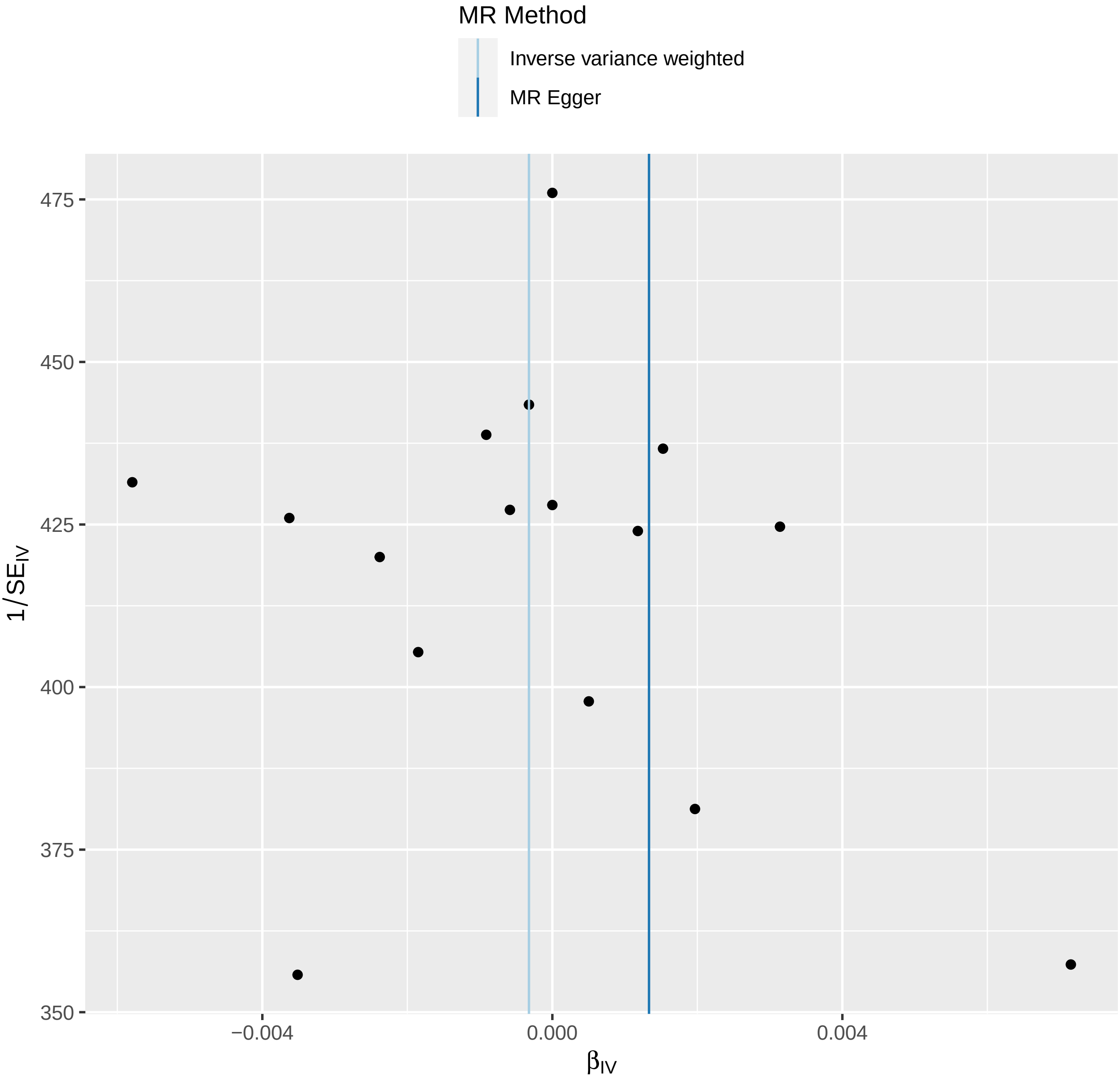

Batch 2−531 : Choriogonadotropin subunit beta variant 2 on eGFR

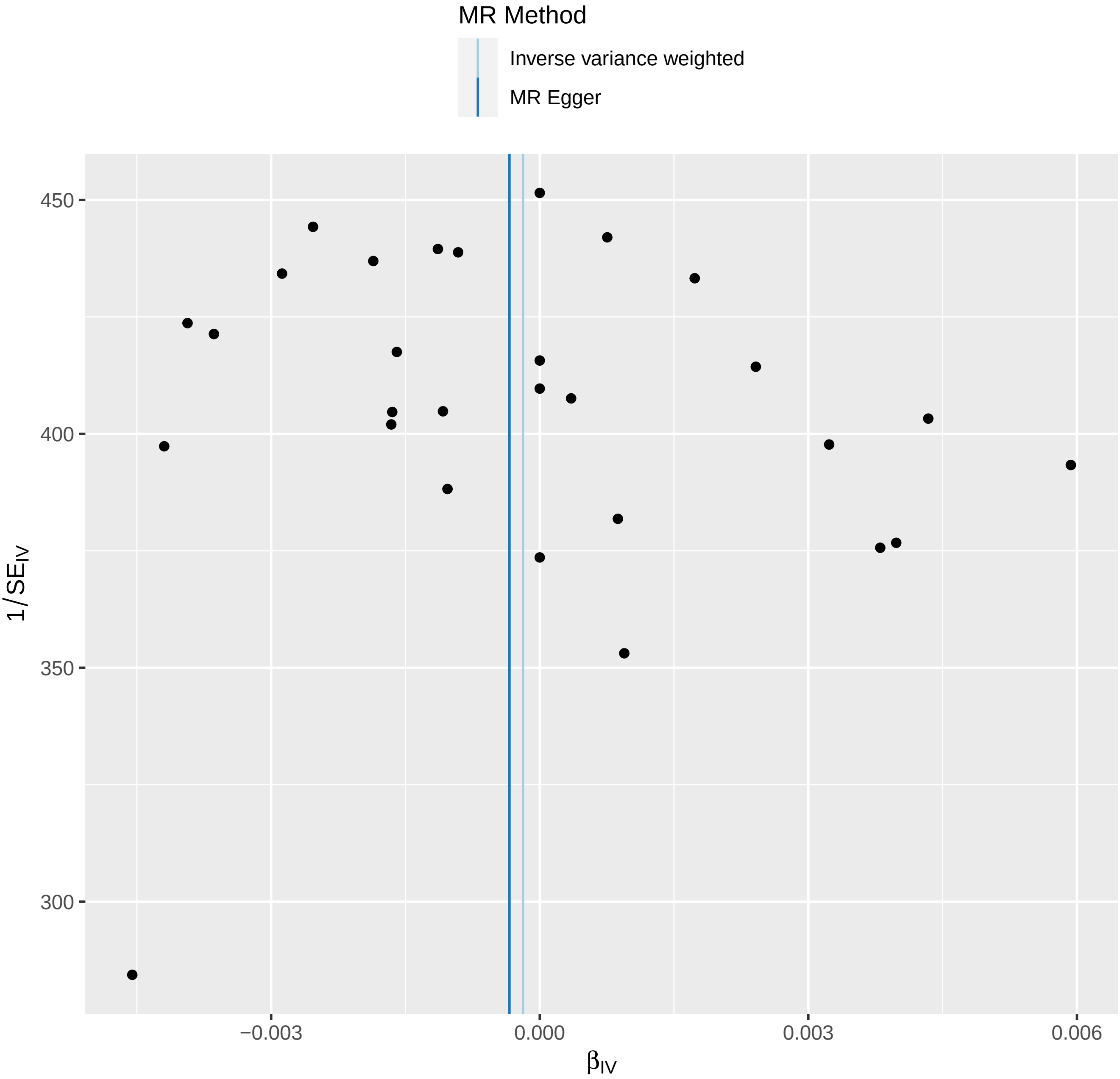

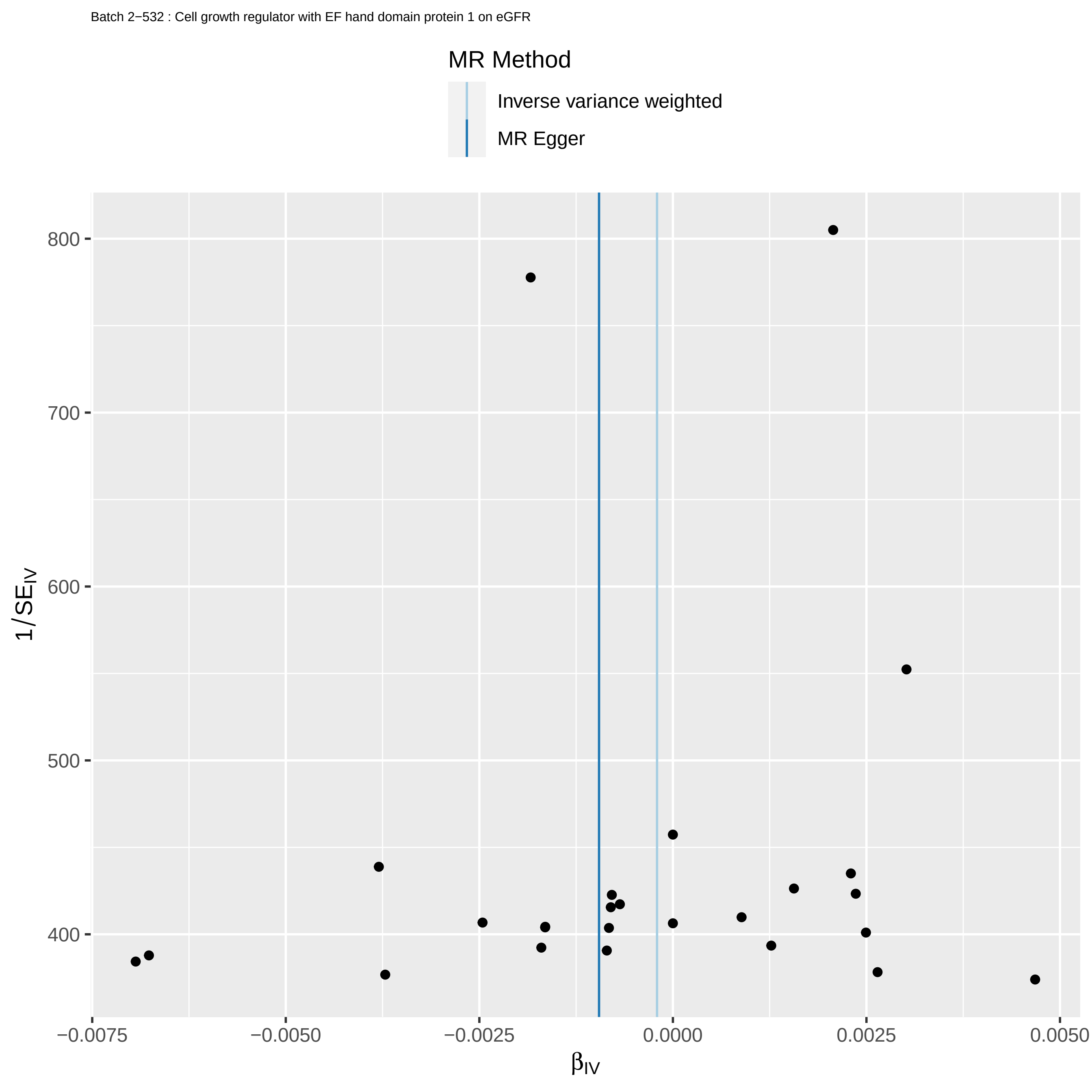

Batch 2−532 : Cell growth regulator with EF hand domain protein 1 on eGFR
MR Method
Inverse variance weighted
MR Egger
800
700
600
500
400
1/SE$_{IV}$
−0.0075
−0.0050
−0.0025
0.0000
0.0025
0.0050
β$_{IV}$

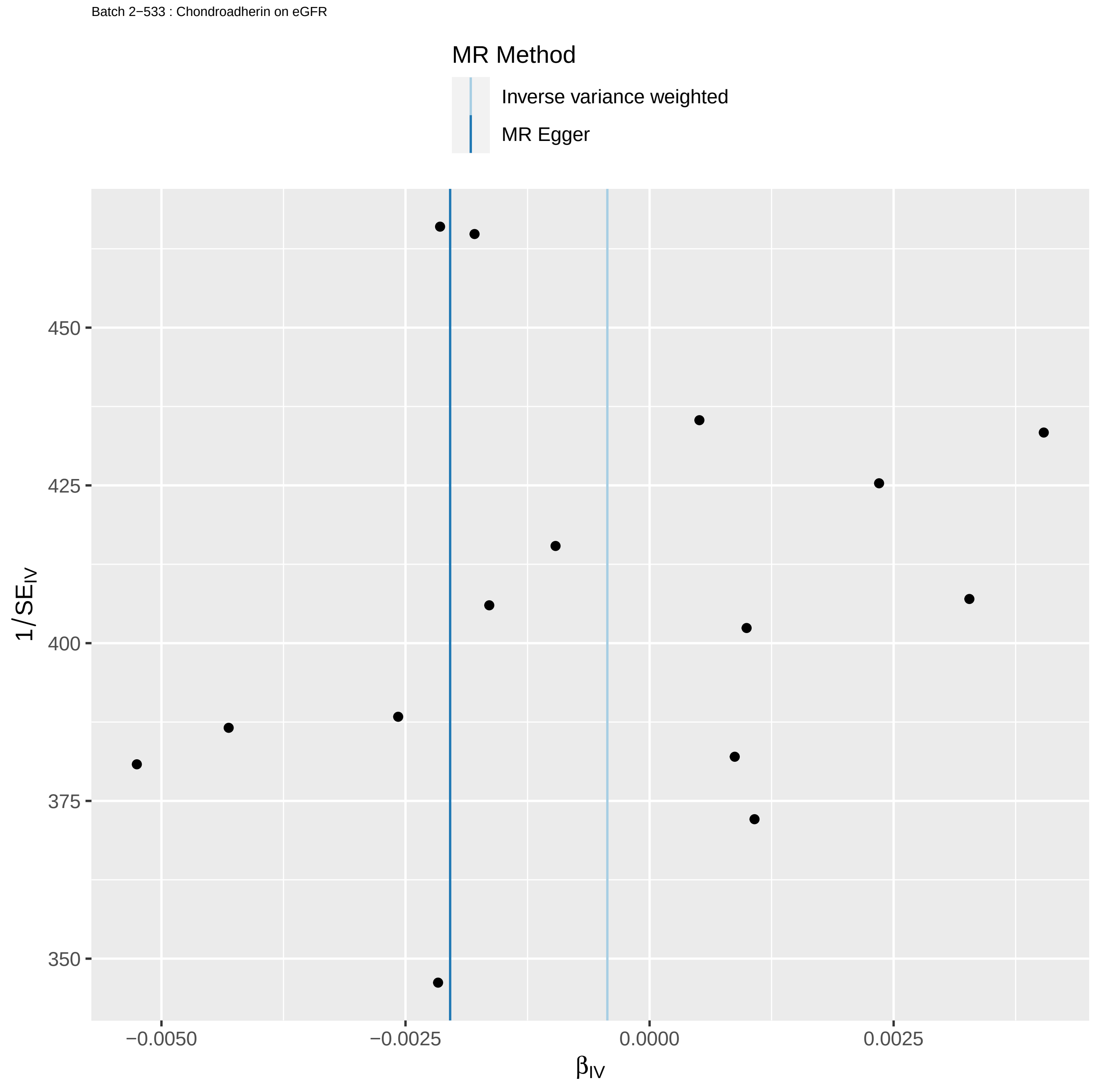
Batch 2−533 : Chondroadherin on eGFR
MR Method
Inverse variance weighted
MR Egger
1/SE_IV
450
425
400
375
350
β_IV
−0.0050
−0.0025
0.0000
0.0025

Batch 2−534 : Coiled−coil−helix−coiled−coil−helix domain−containing protein 10, mitochondrial on eGFR

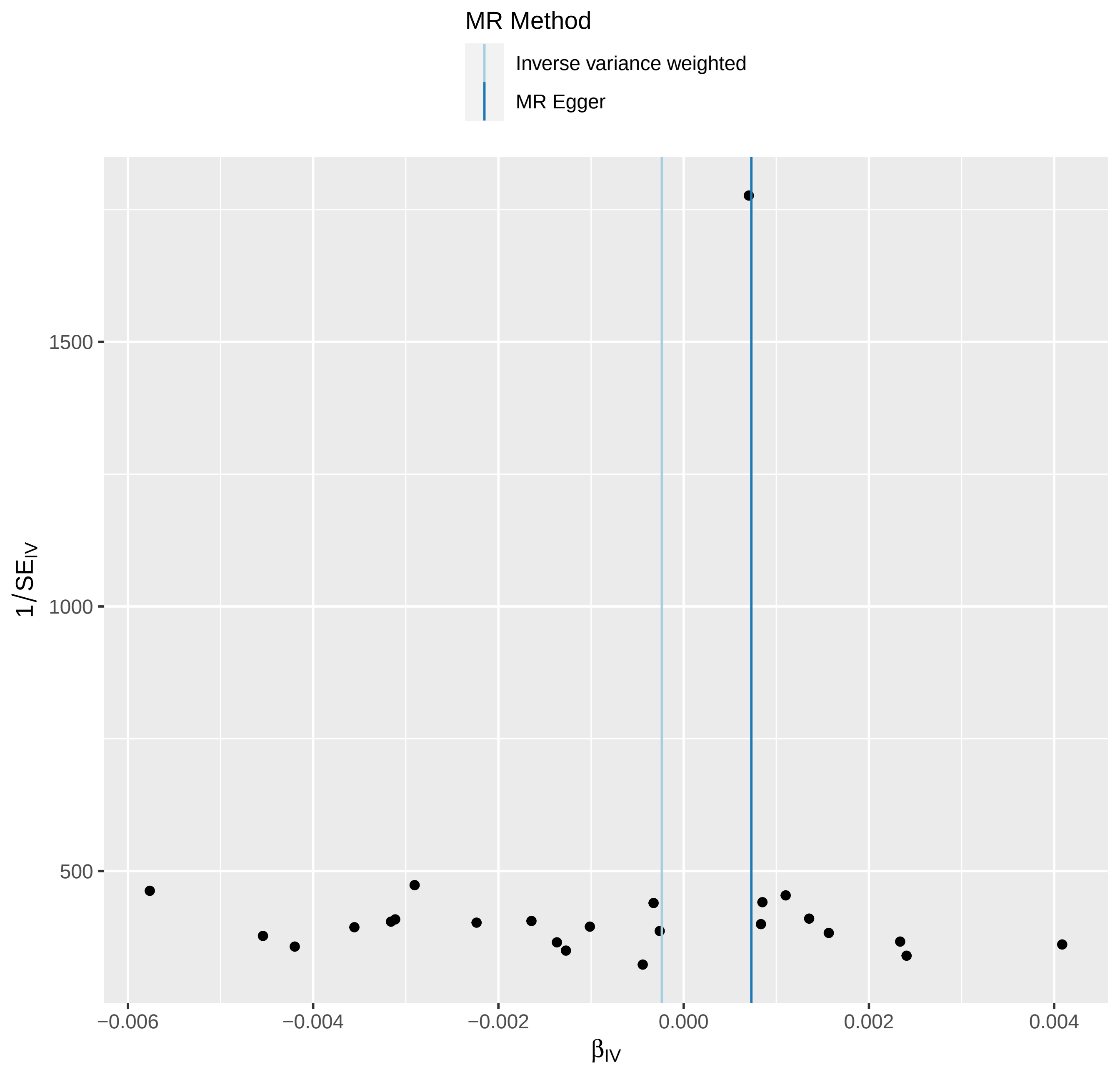

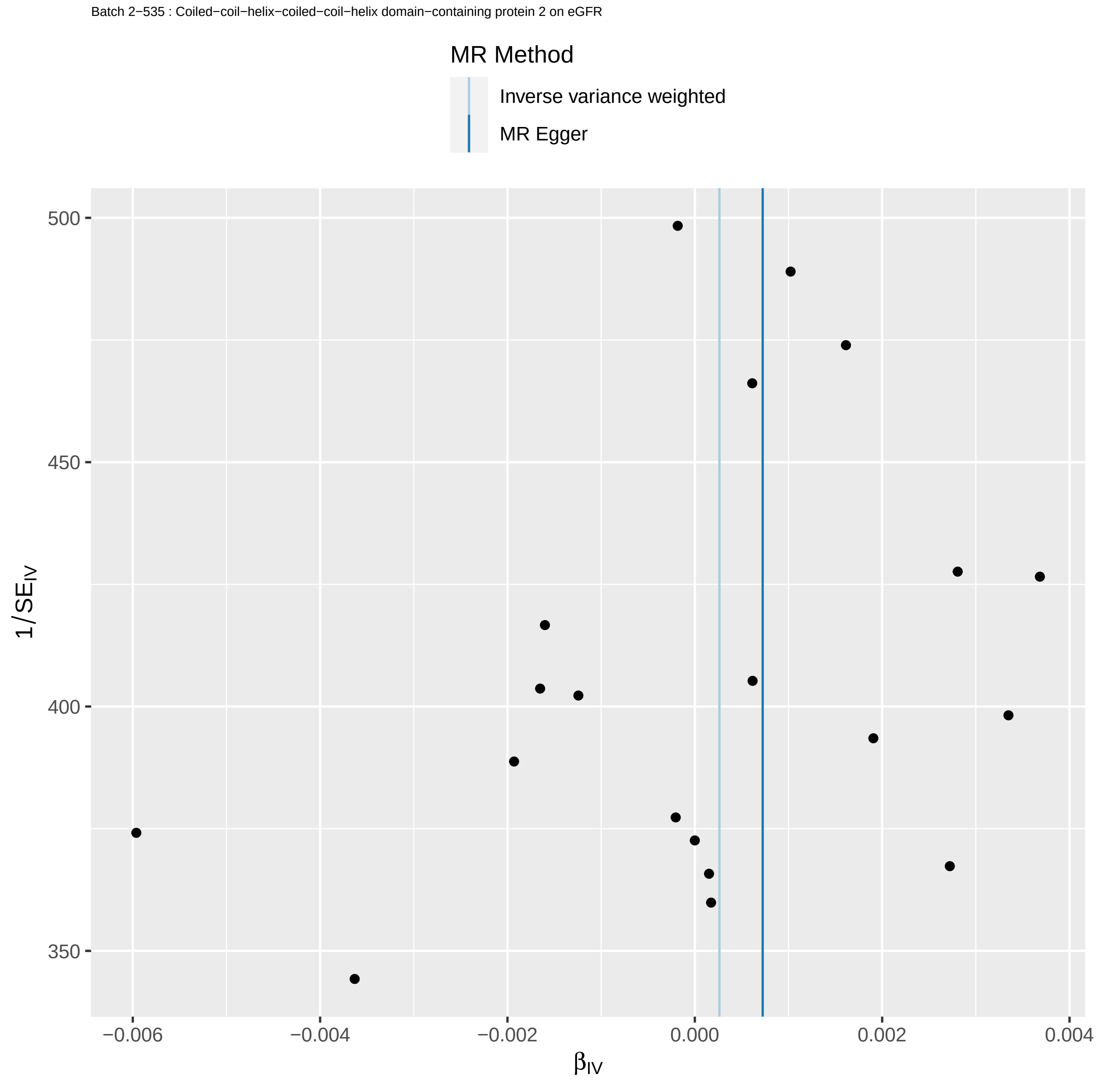

Batch 2−535 : Coiled−coil−helix−coiled−coil−helix domain−containing protein 2 on eGFR
MR Method
Inverse variance weighted
MR Egger
500
450
400
350
$1/SE_{IV}$
−0.006
−0.004
−0.002
0.000
0.002
0.004
β_{IV}

Batch 2−536 : Chromodomain−helicase−DNA−binding protein 7 on eGFR

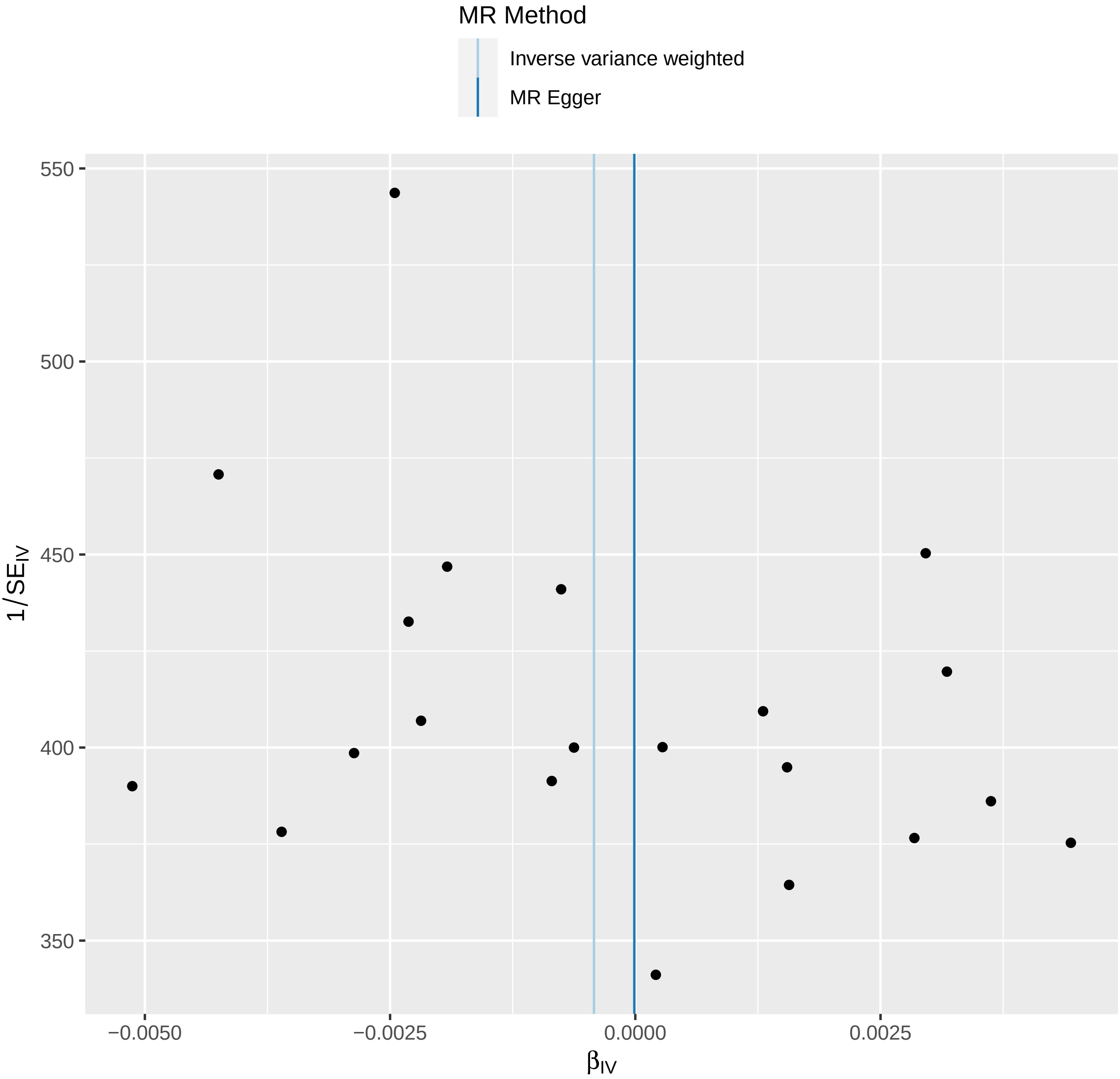

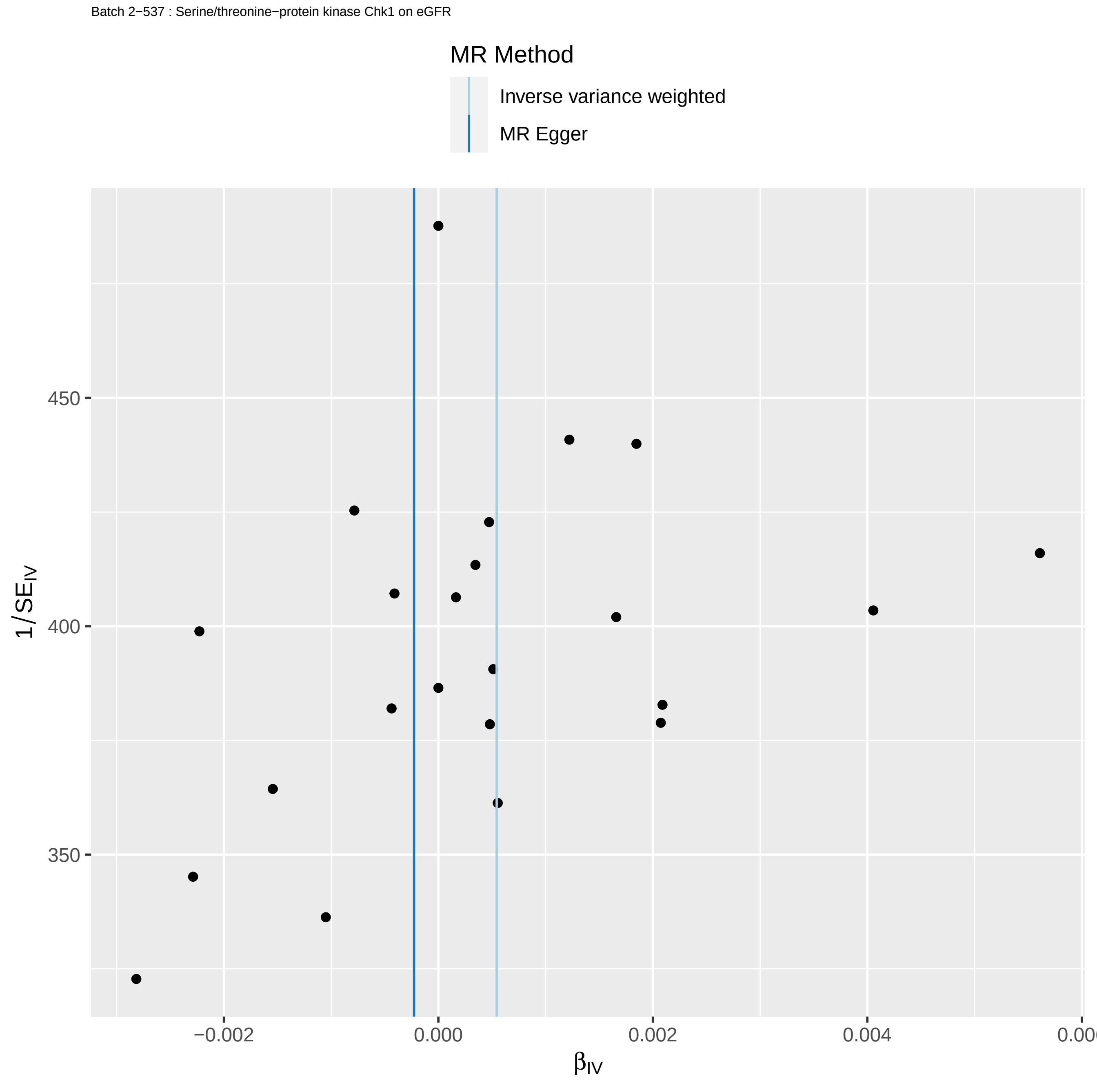

Batch 2−537 : Serine/threonine−protein kinase Chk1 on eGFR
MR Method
Inverse variance weighted
MR Egger
450
400
350
1/SE$_{IV}$
−0.002
0.000
0.002
0.004
β$_{IV}$

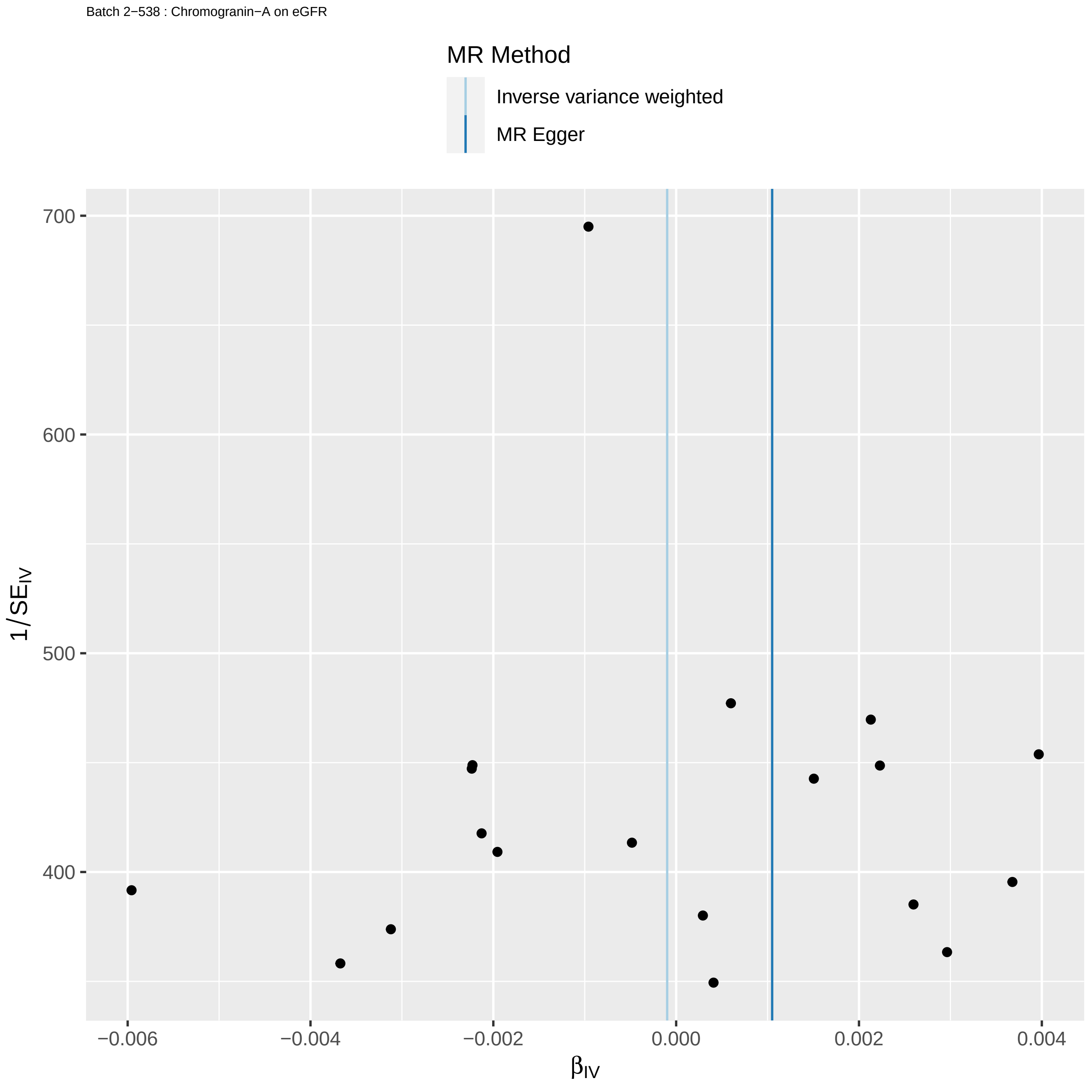

Batch 2−538 : Chromogranin−A on eGFR
MR Method
Inverse variance weighted
MR Egger
700
600
500
400
1/SE_IV
−0.006
−0.004
−0.002
0.000
0.002
0.004
β_IV

Batch 2−539 : Secretogranin−1 on eGFR

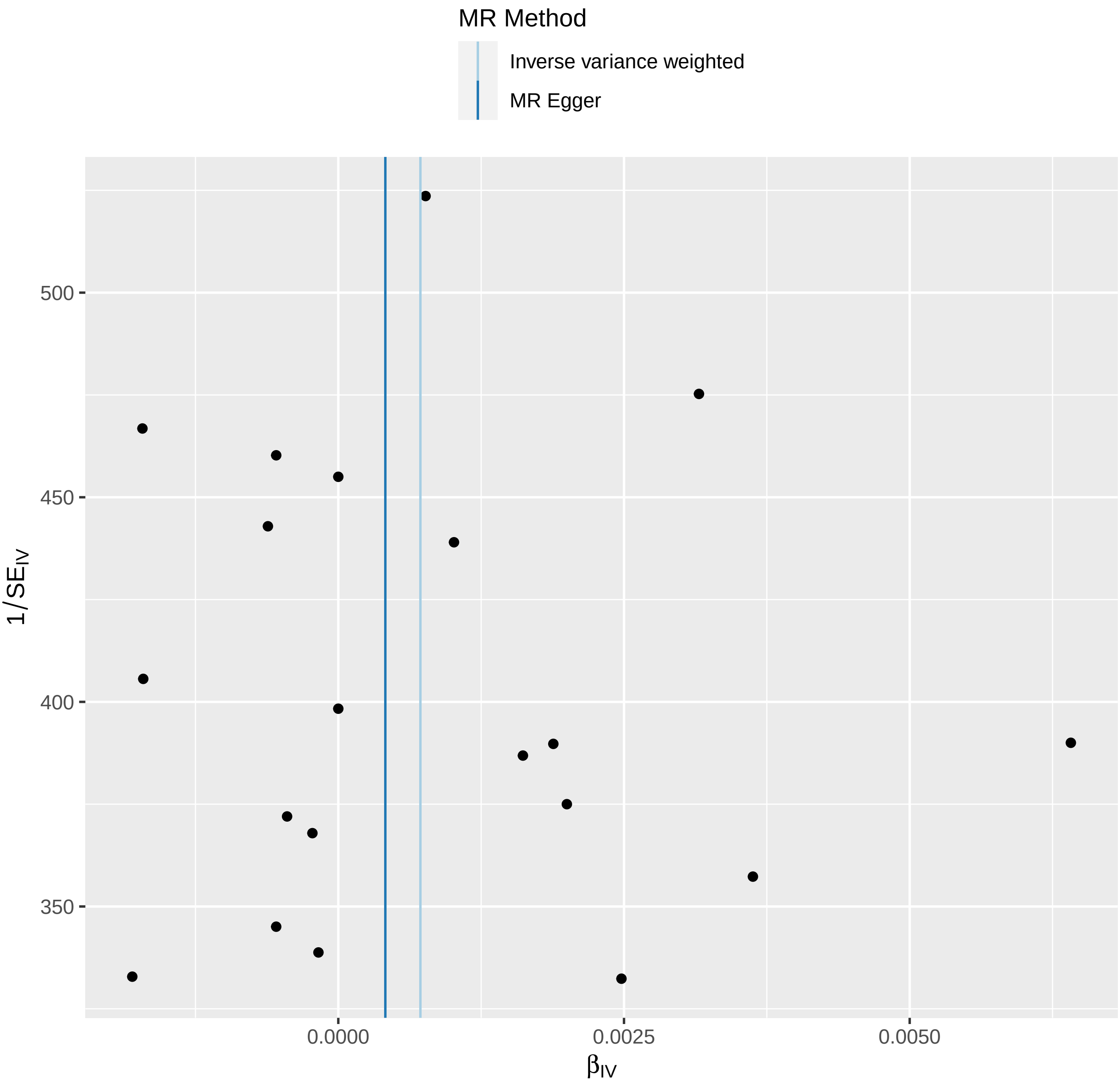

Batch 2−540 : Chitinase−3−like protein 1 on eGFR

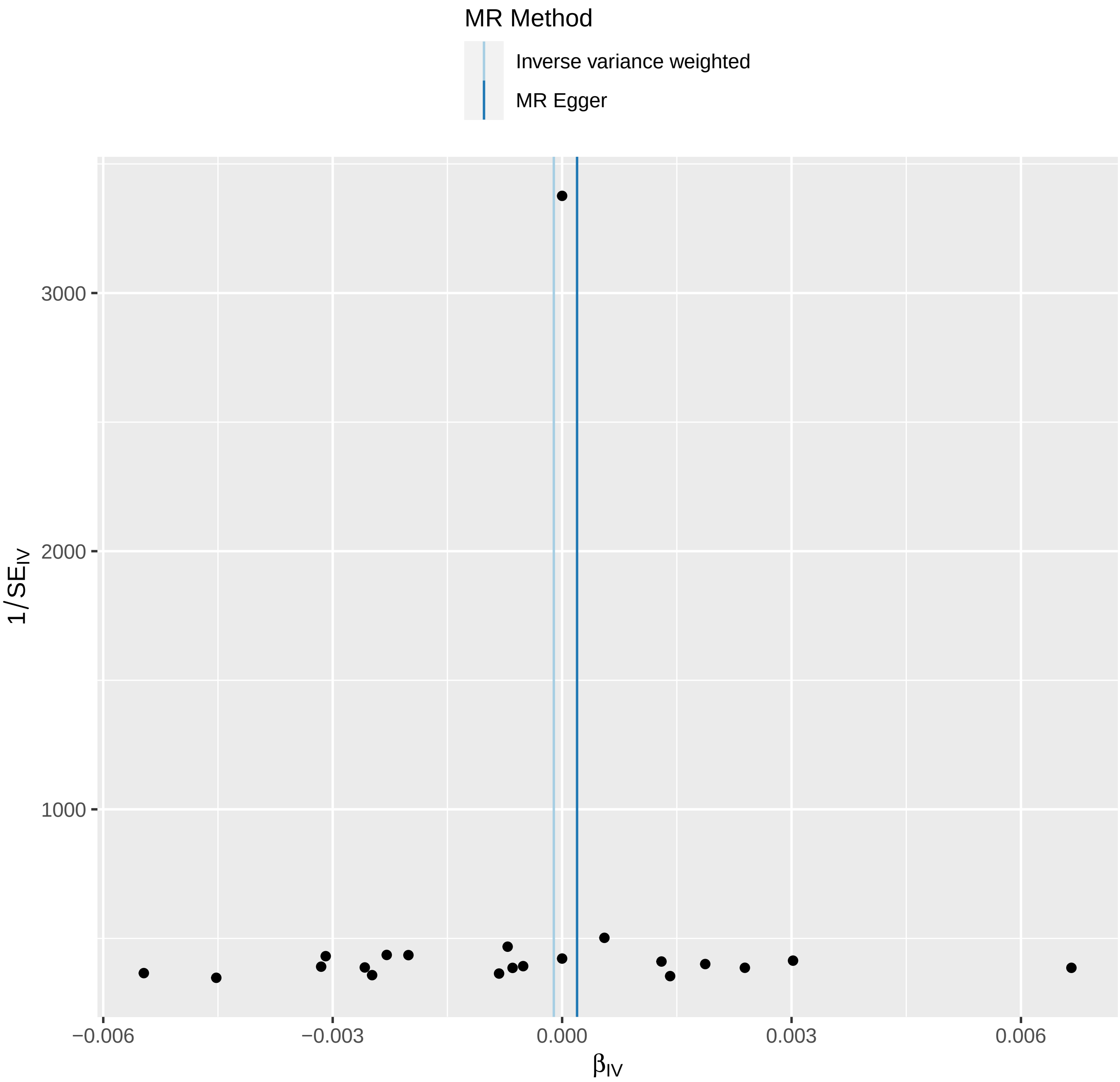

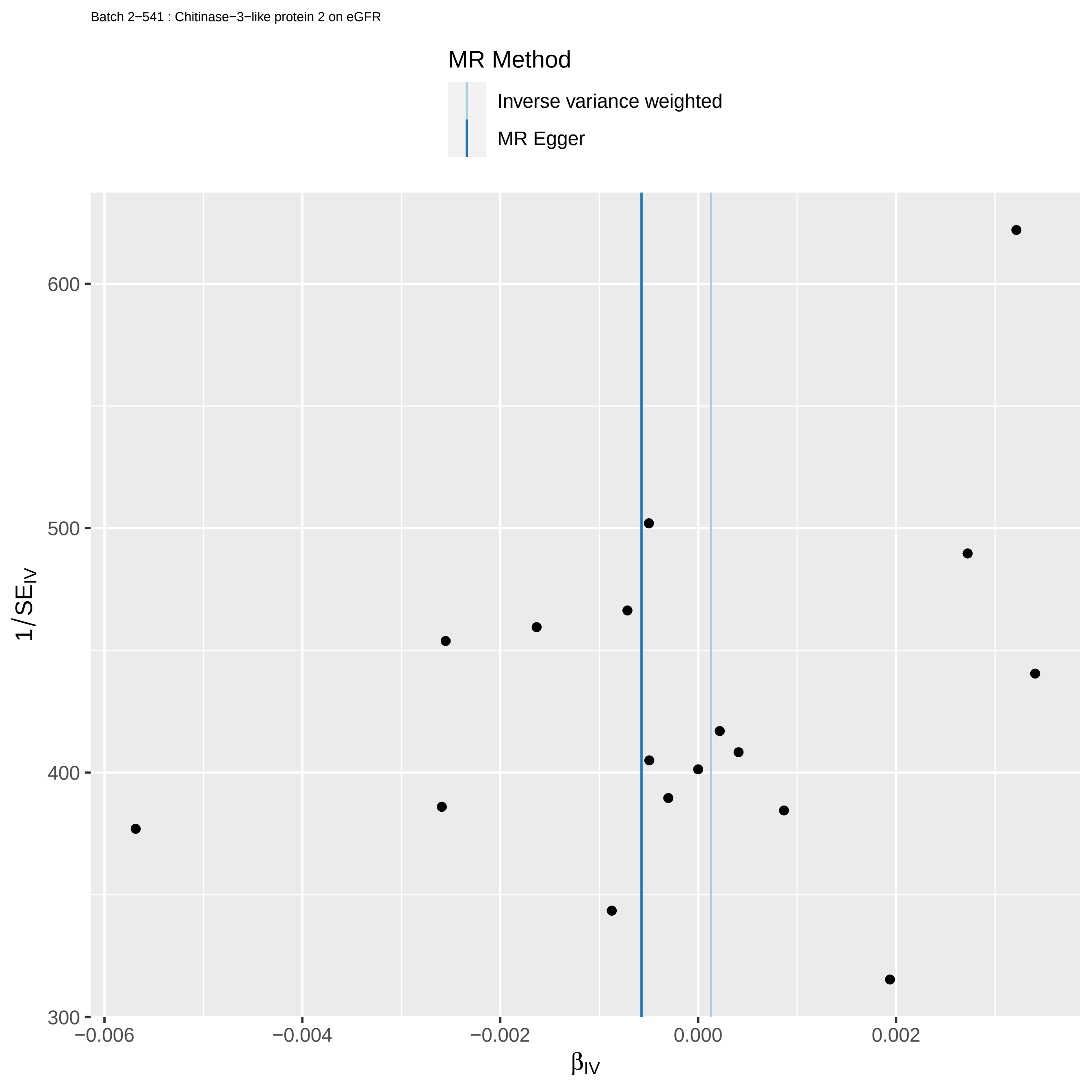
Batch 2−541 : Chitinase−3−like protein 2 on eGFR
MR Method
Inverse variance weighted
MR Egger
600
500
400
300
1/SE_IV
−0.006
−0.004
−0.002
0.000
0.002
β_IV

Batch 2−542 : Cysteine−rich hydrophobic domain−containing protein 2 on eGFR

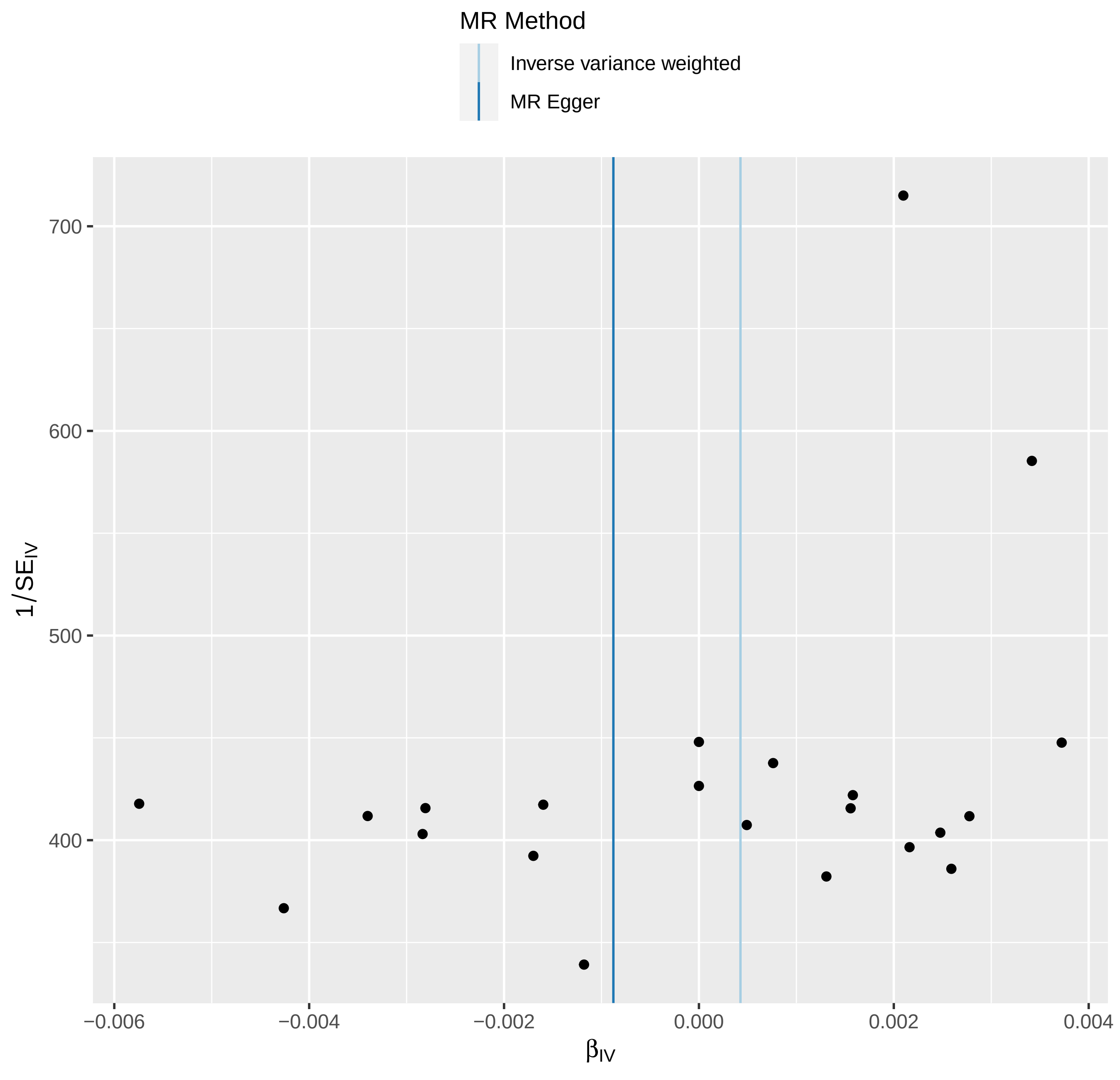

Batch 2−543 : Chitotriosidase−1 on eGFR

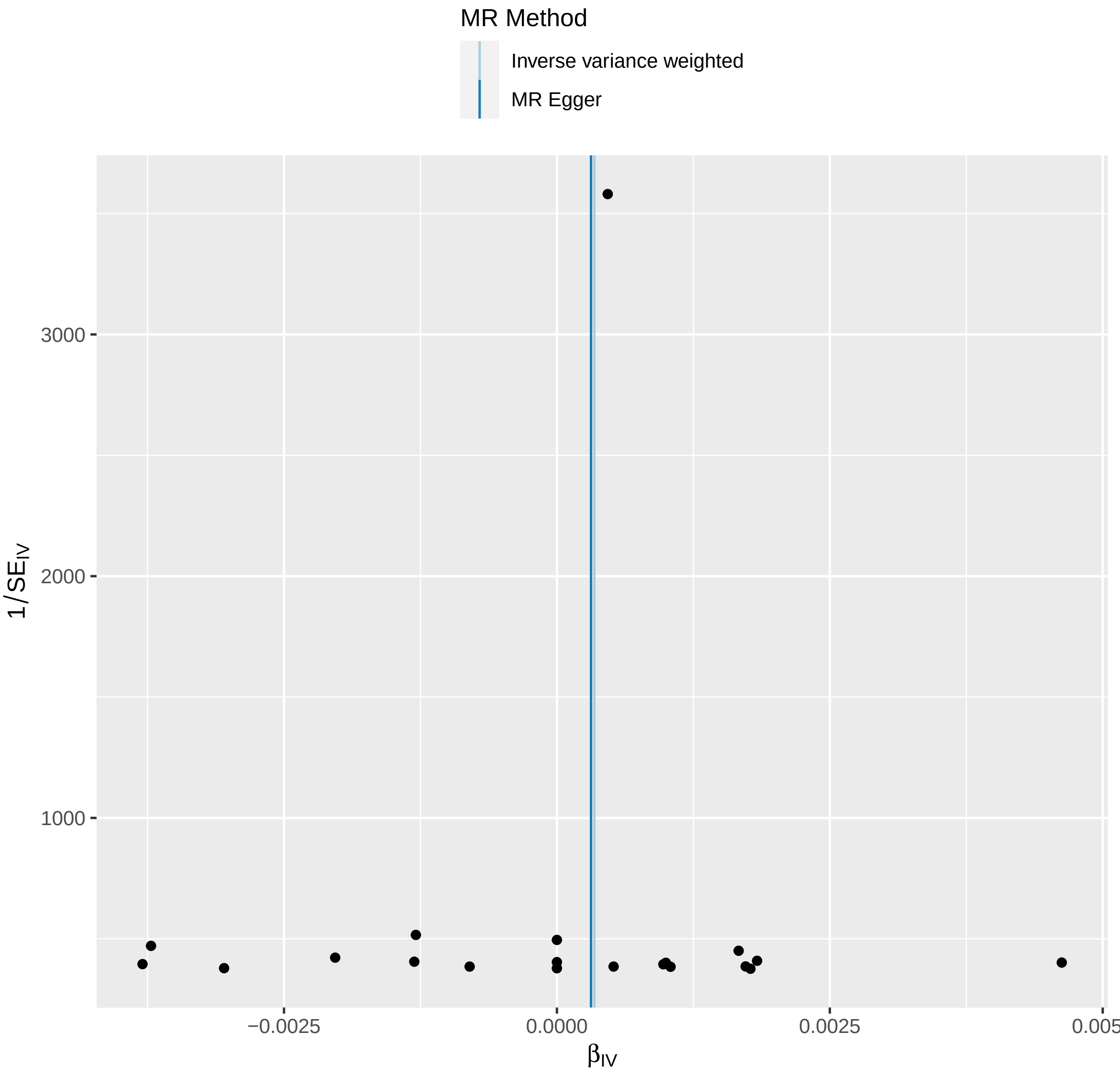

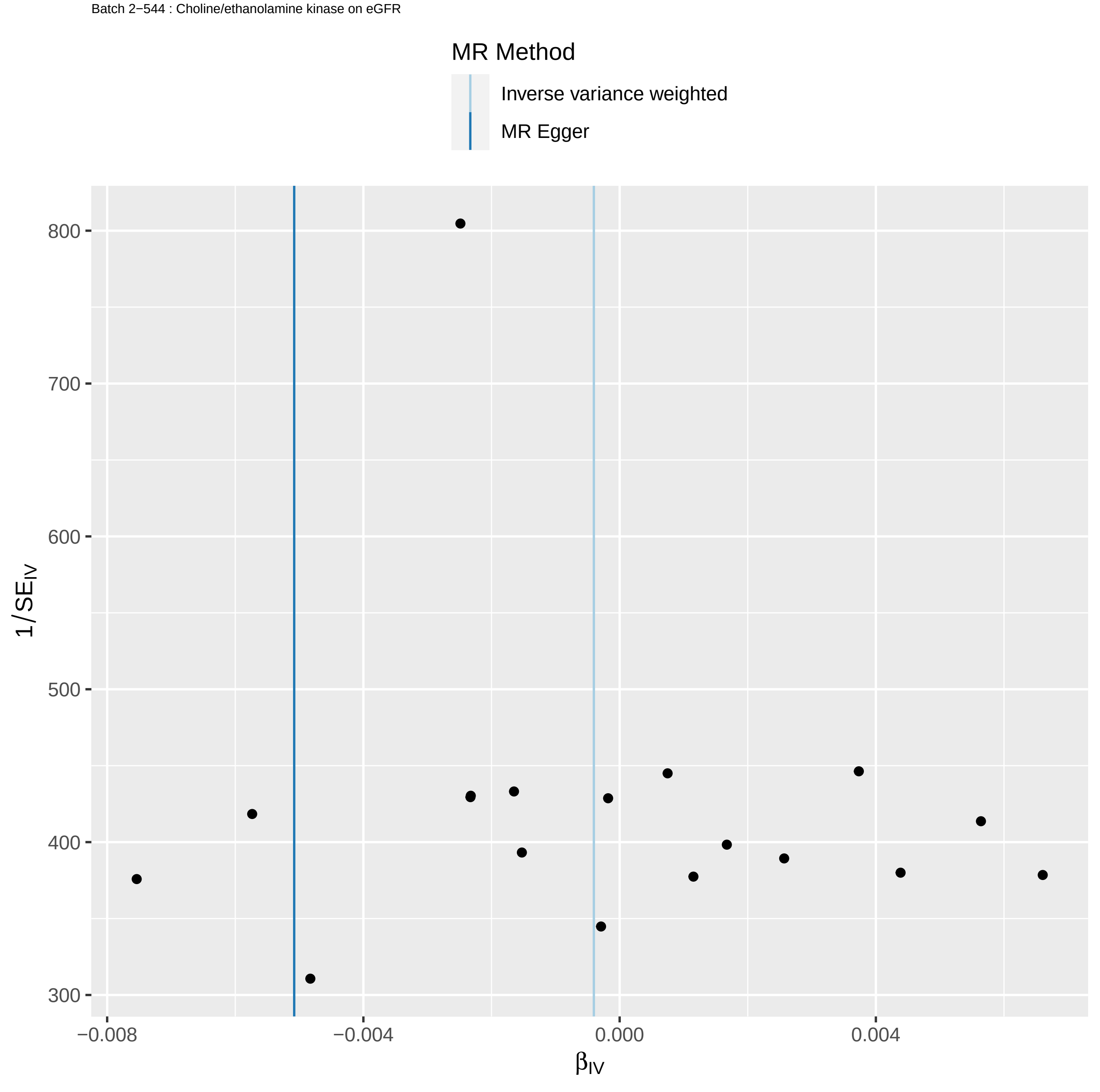

Batch 2−544 : Choline/ethanolamine kinase on eGFR
MR Method
Inverse variance weighted
MR Egger
800
700
600
500
400
300
1/SE$_{IV}$
−0.008
−0.004
0.000
0.004
β$_{IV}$

Batch 2−545 : Neural cell adhesion molecule L1−like protein on eGFR

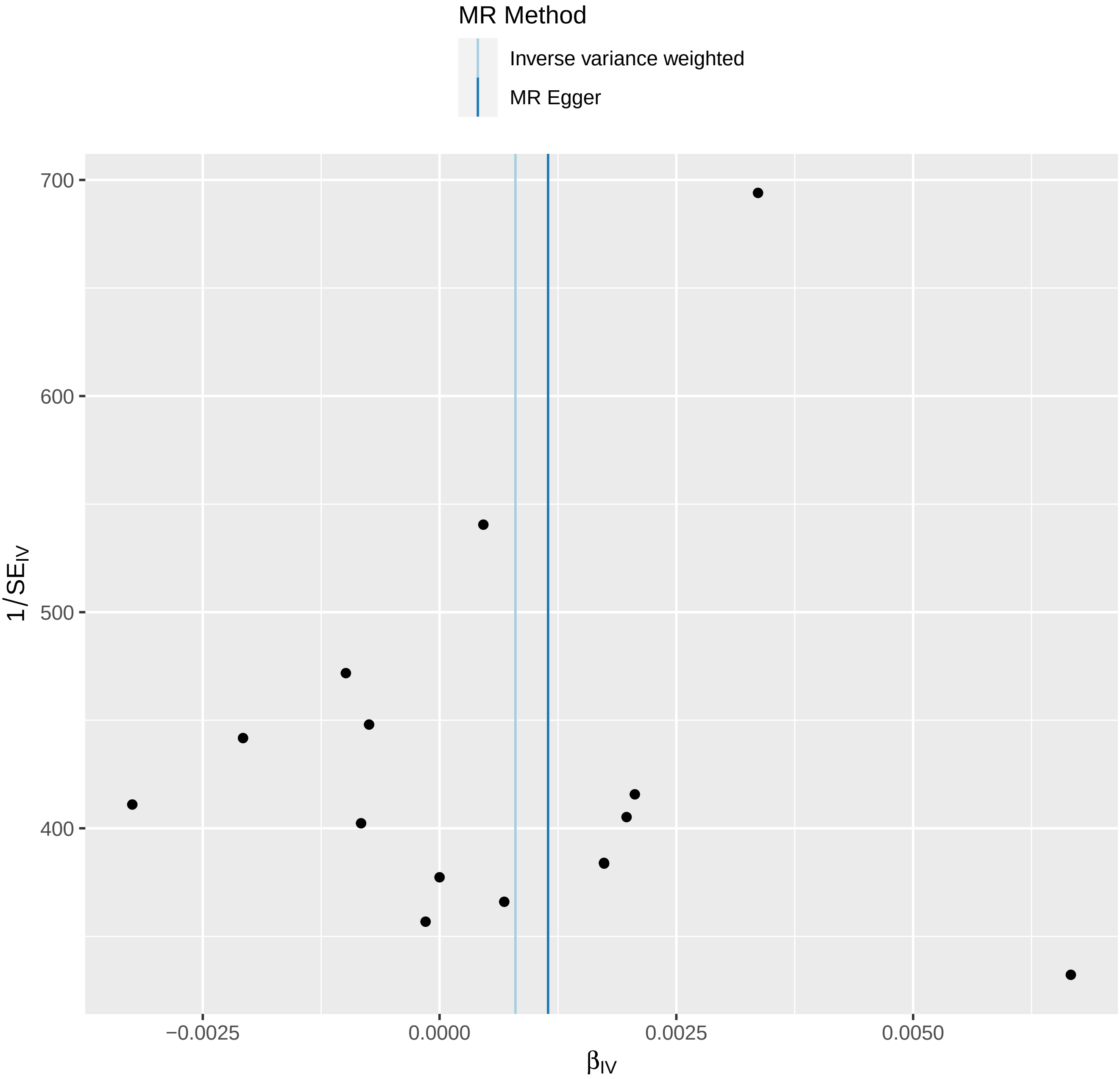

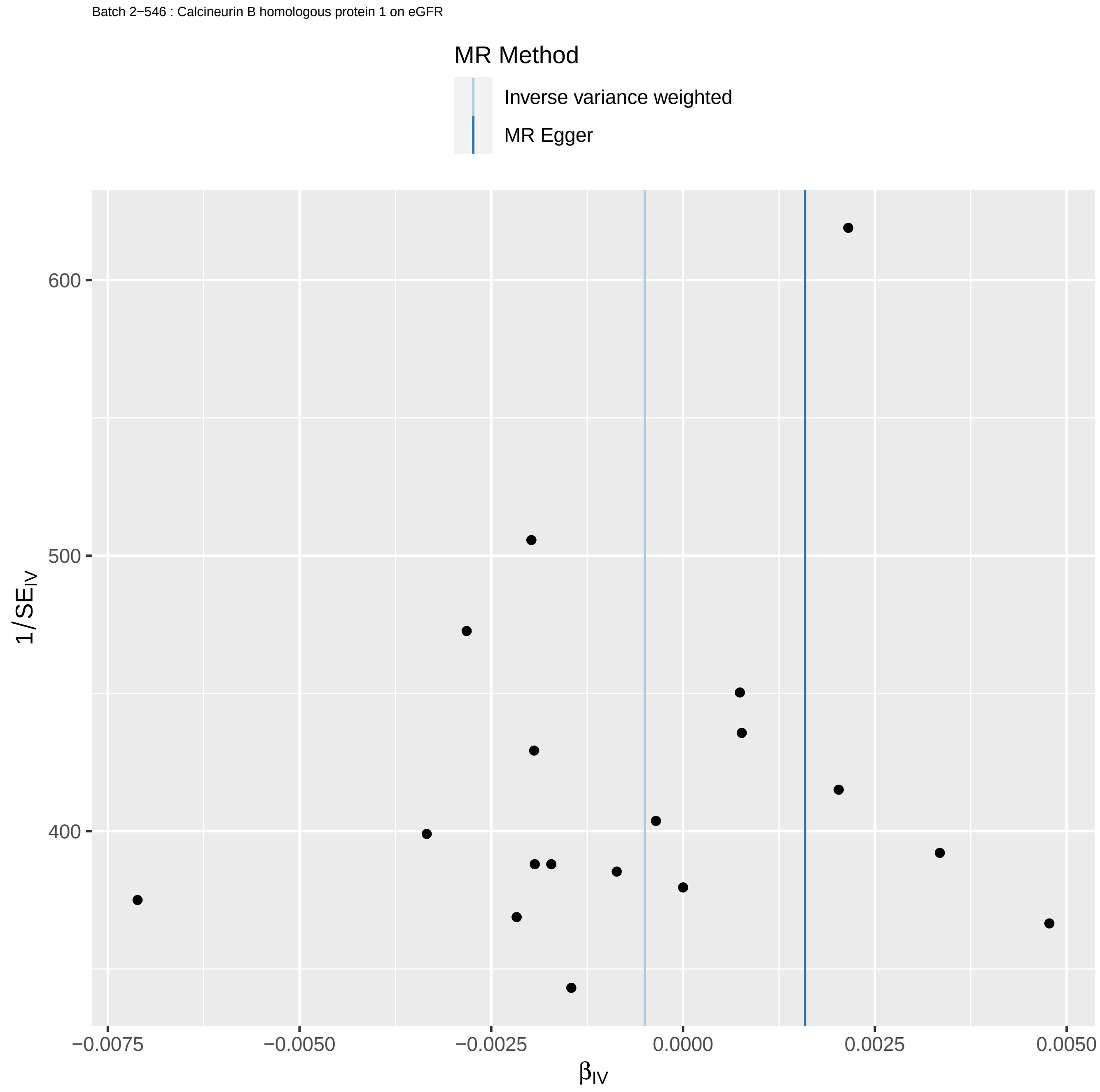
Batch 2−546 : Calcineurin B homologous protein 1 on eGFR
MR Method
Inverse variance weighted
MR Egger
600
500
400
1/SE$_{IV}$
−0.0075
−0.0050
−0.0025
0.0000
0.0025
0.0050
β$_{IV}$

Batch 2−547 : Chordin on eGFR

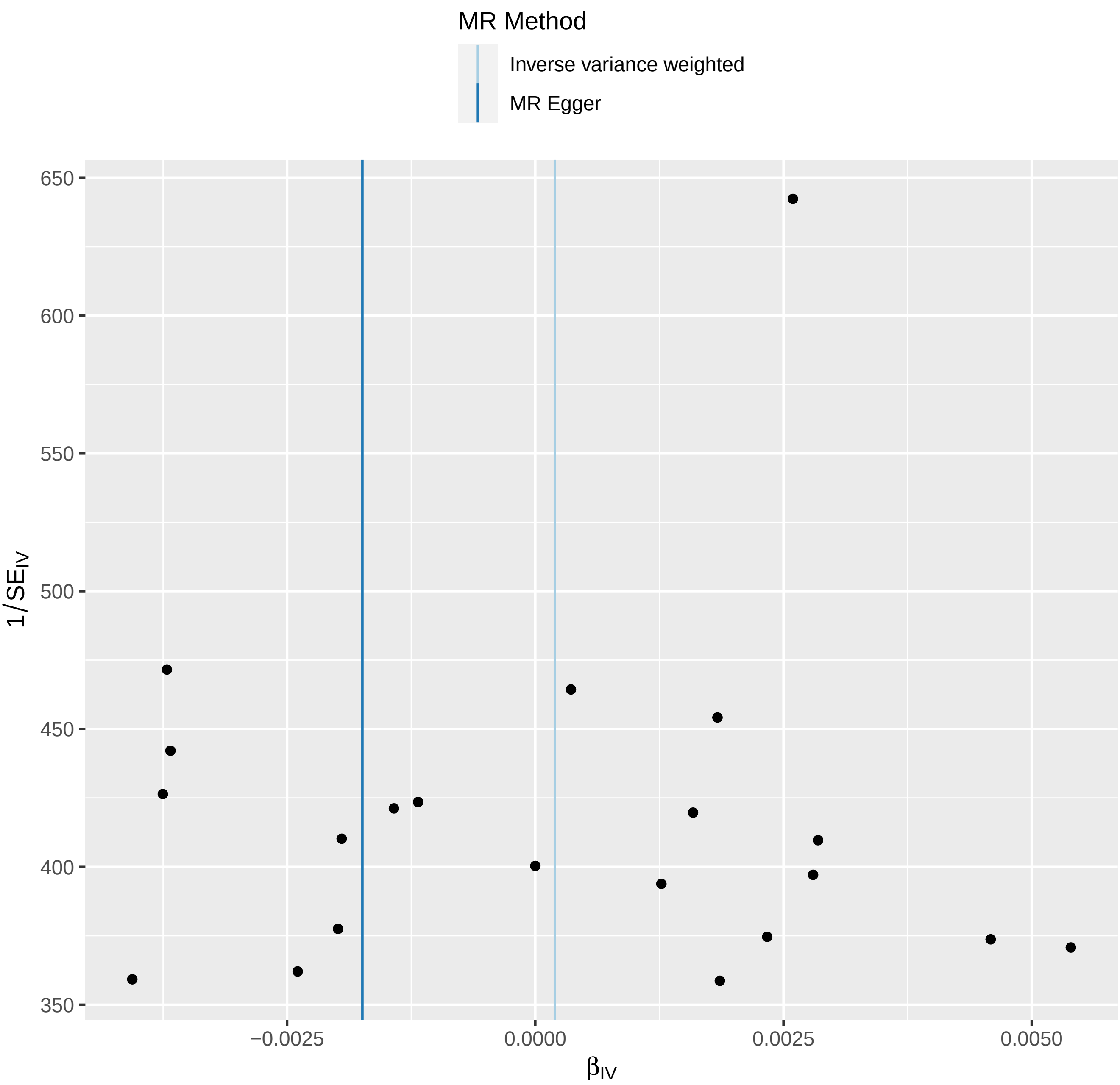

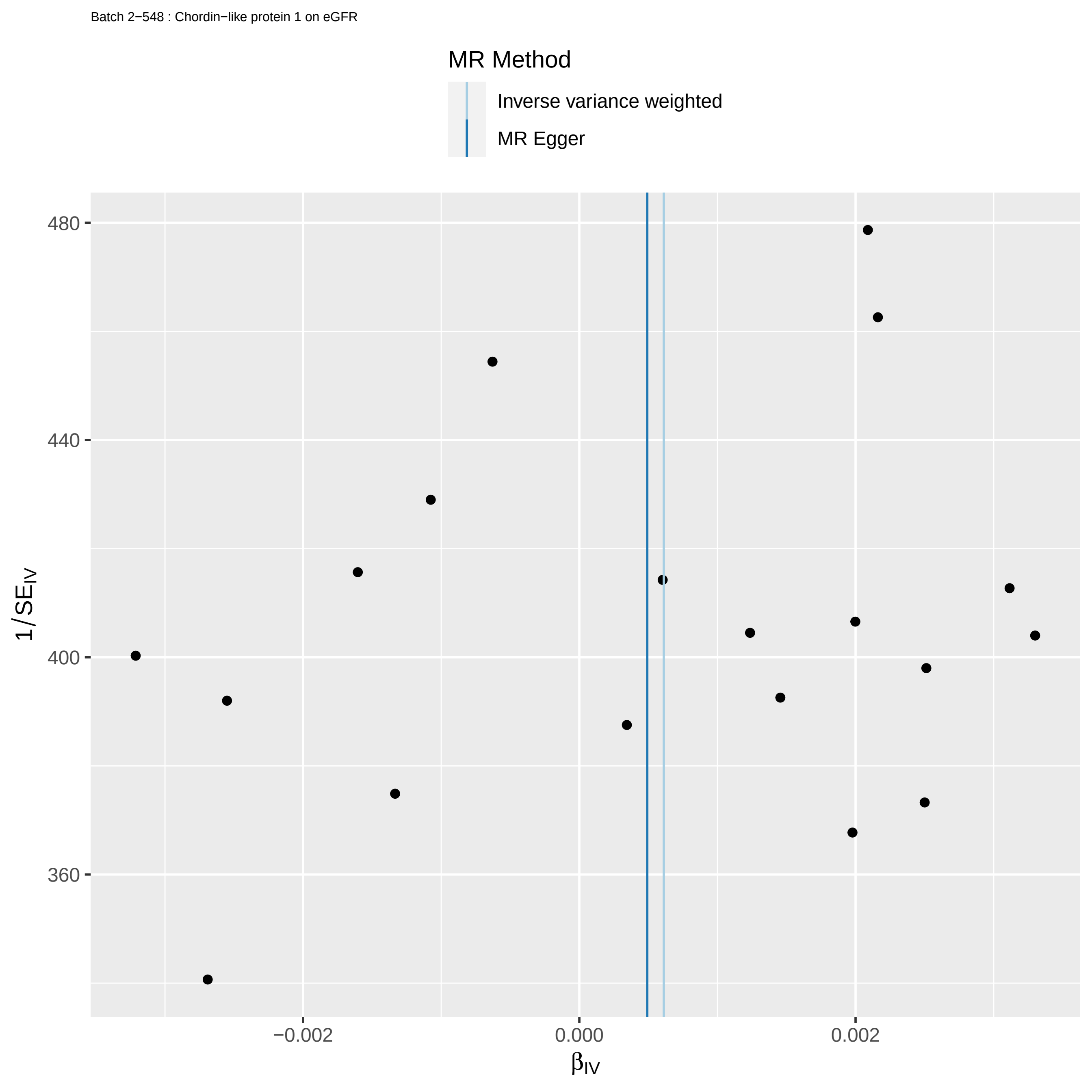

Batch 2−548 : Chordin−like protein 1 on eGFR
MR Method
Inverse variance weighted
MR Egger
480
440
400
360
1/SE_IV
−0.002
0.000
0.002
β_IV

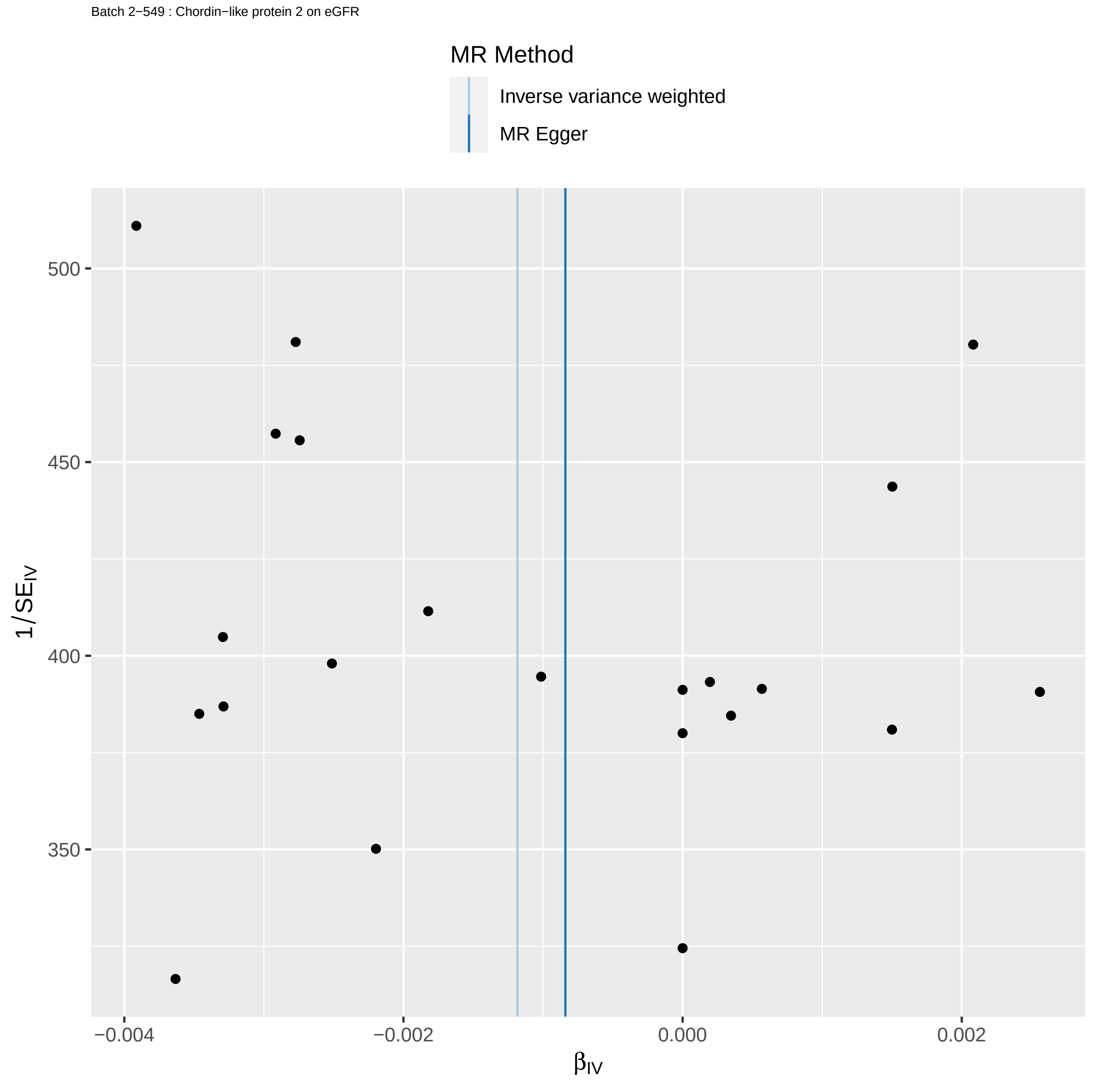

Batch 2−549 : Chordin−like protein 2 on eGFR
MR Method
Inverse variance weighted
MR Egger
500
450
400
350
1/SE_IV
−0.004
−0.002
0.000
0.002
β_IV

Batch 2−550 : Carbohydrate sulfotransferase 11 on eGFR

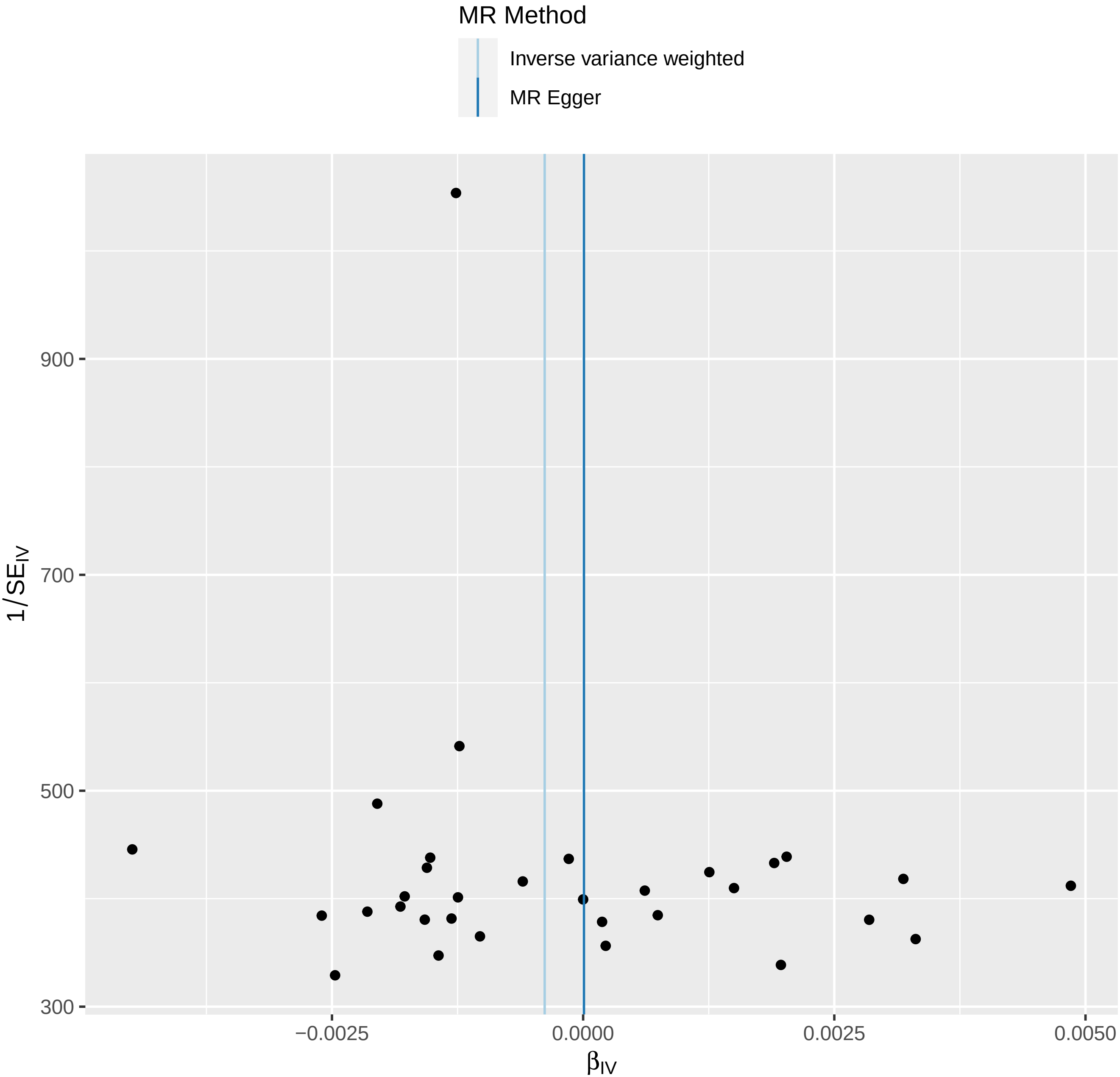

Batch 2−551 : Carbohydrate sulfotransferase 14 on eGFR

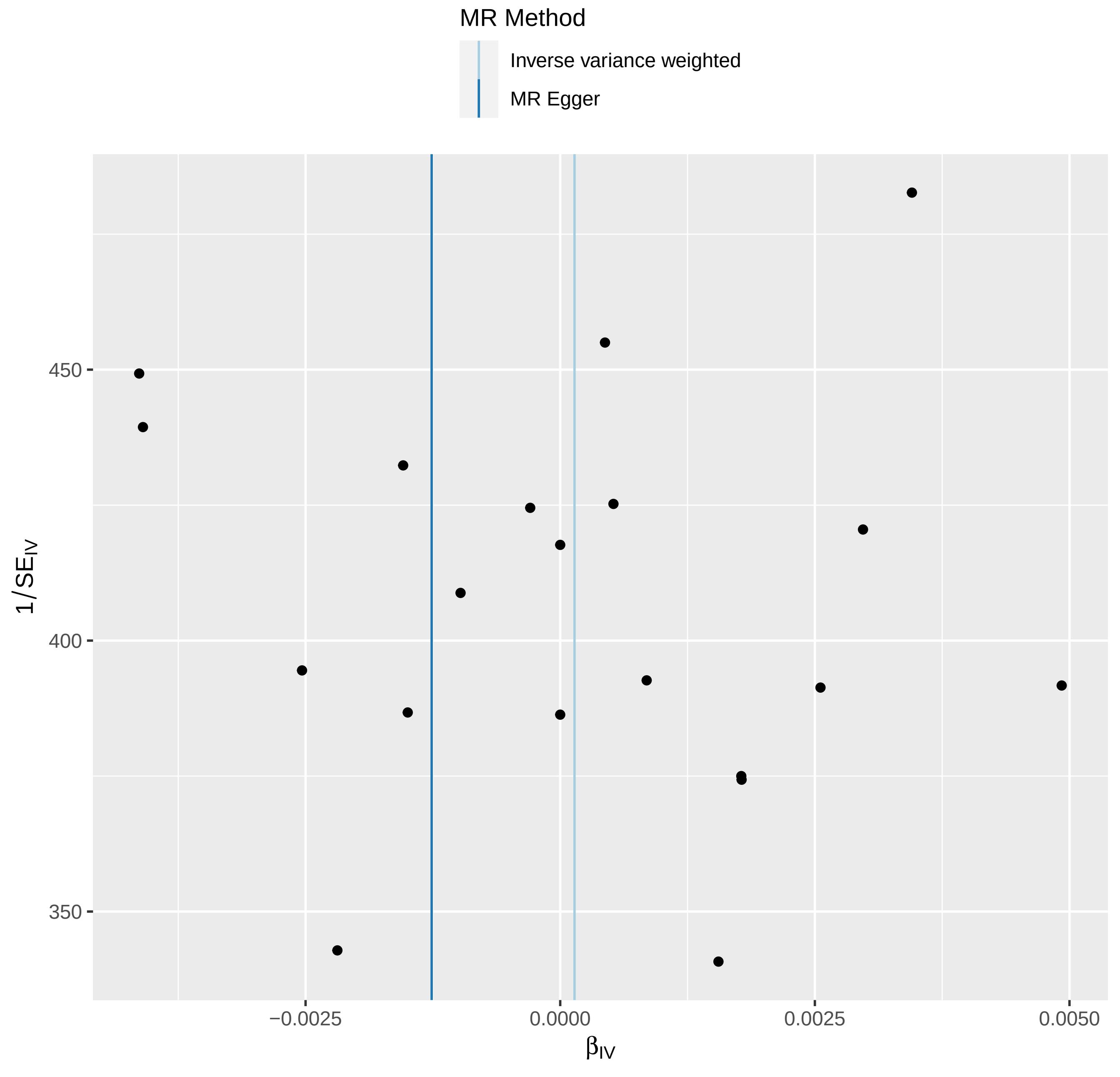

Batch 2−552 : Carbohydrate sulfotransferase 15 on eGFR

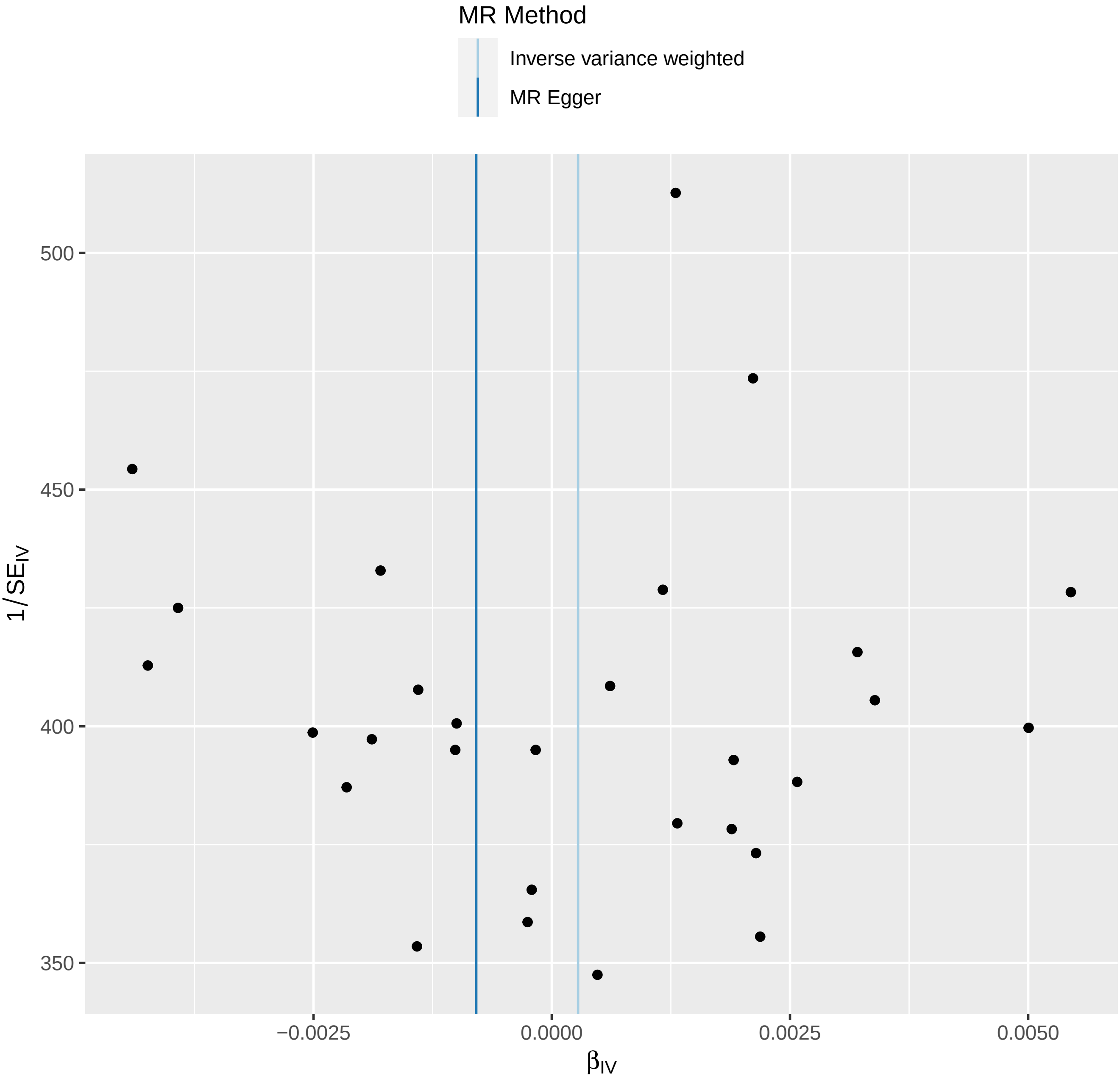

Batch 2−553 : Carbohydrate sulfotransferase 15 on eGFR

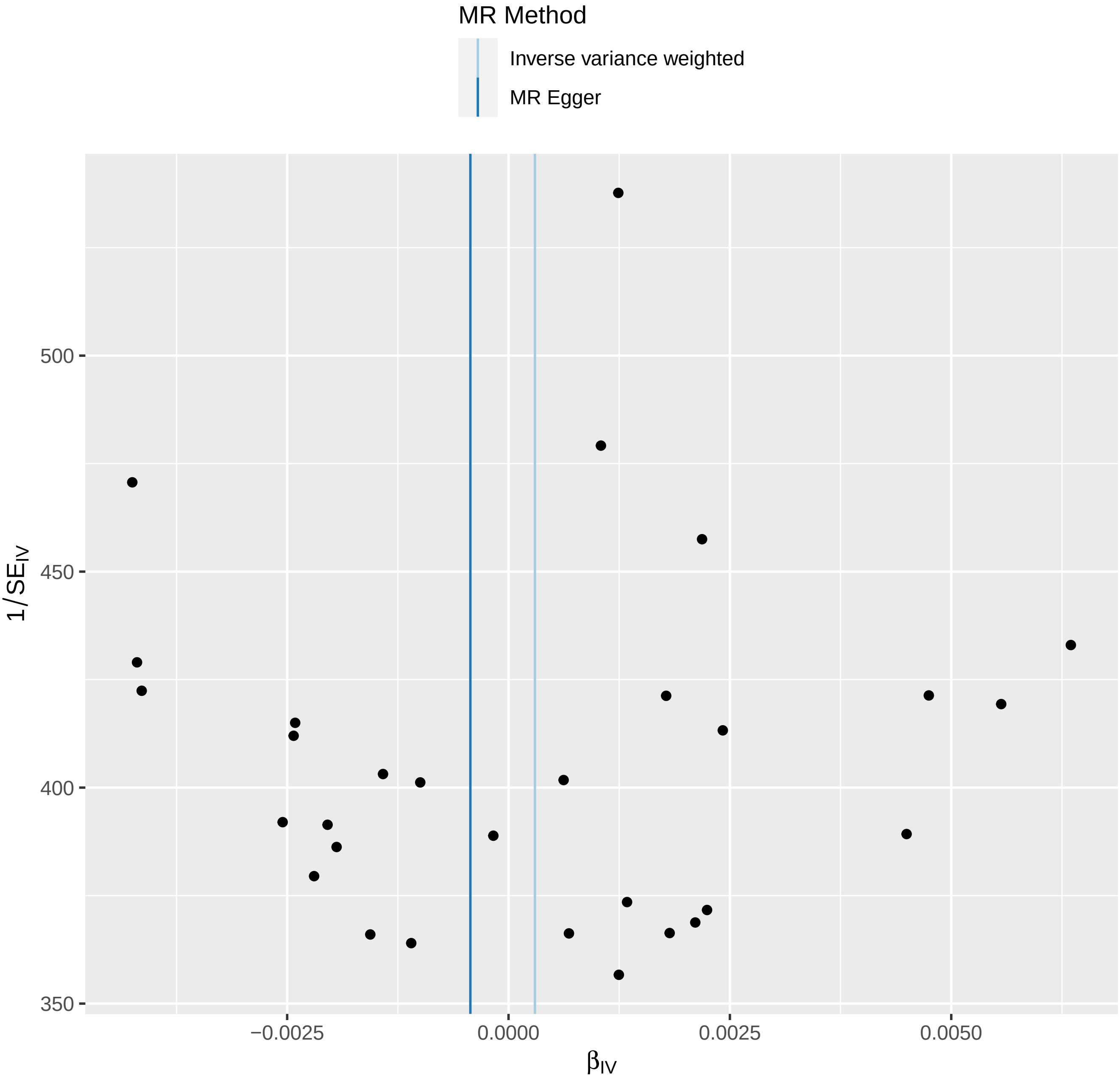

Batch 2−554 : Carbohydrate sulfotransferase 1 on eGFR

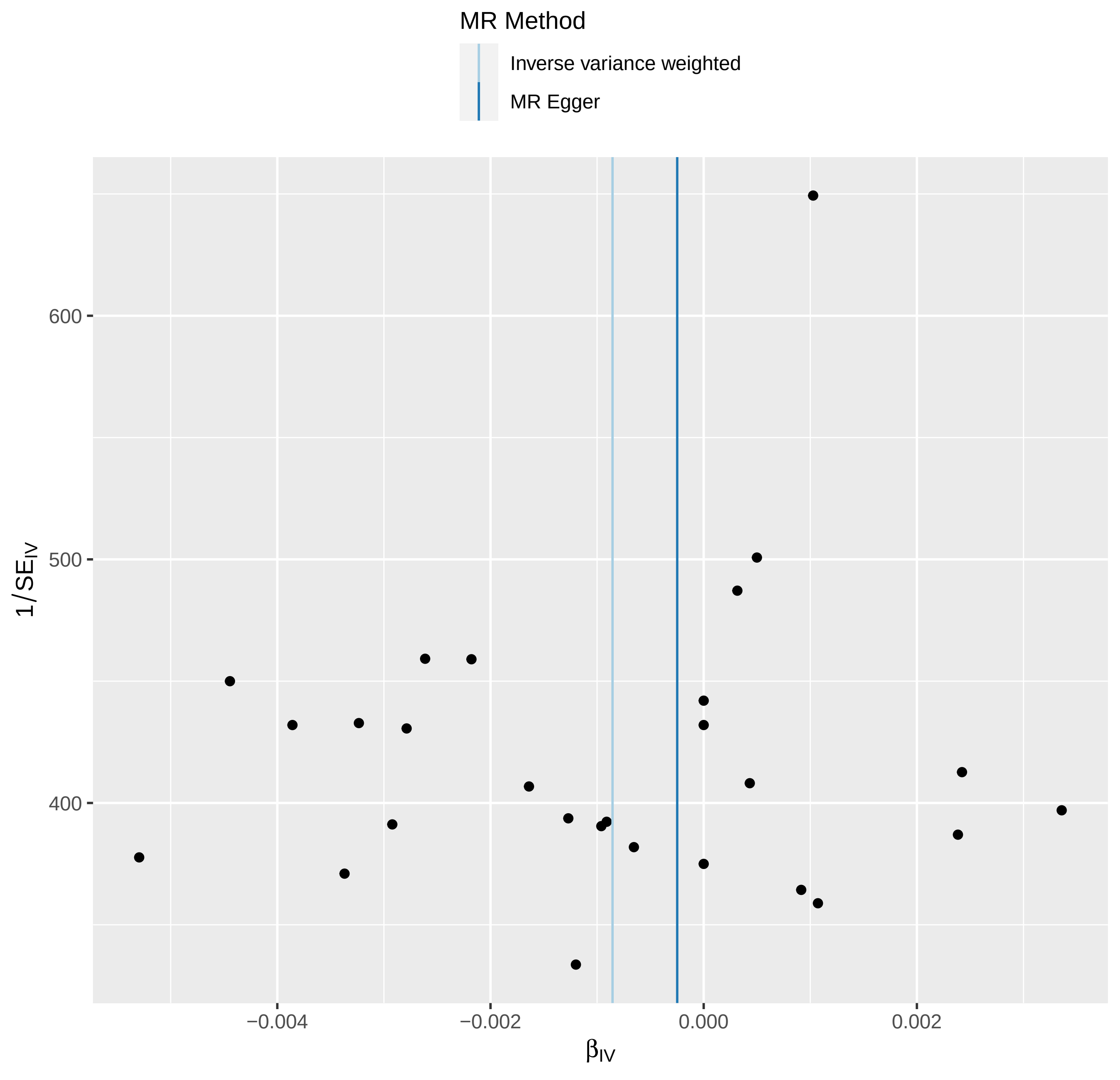

Batch 2−555 : Carbohydrate sulfotransferase 2 on eGFR

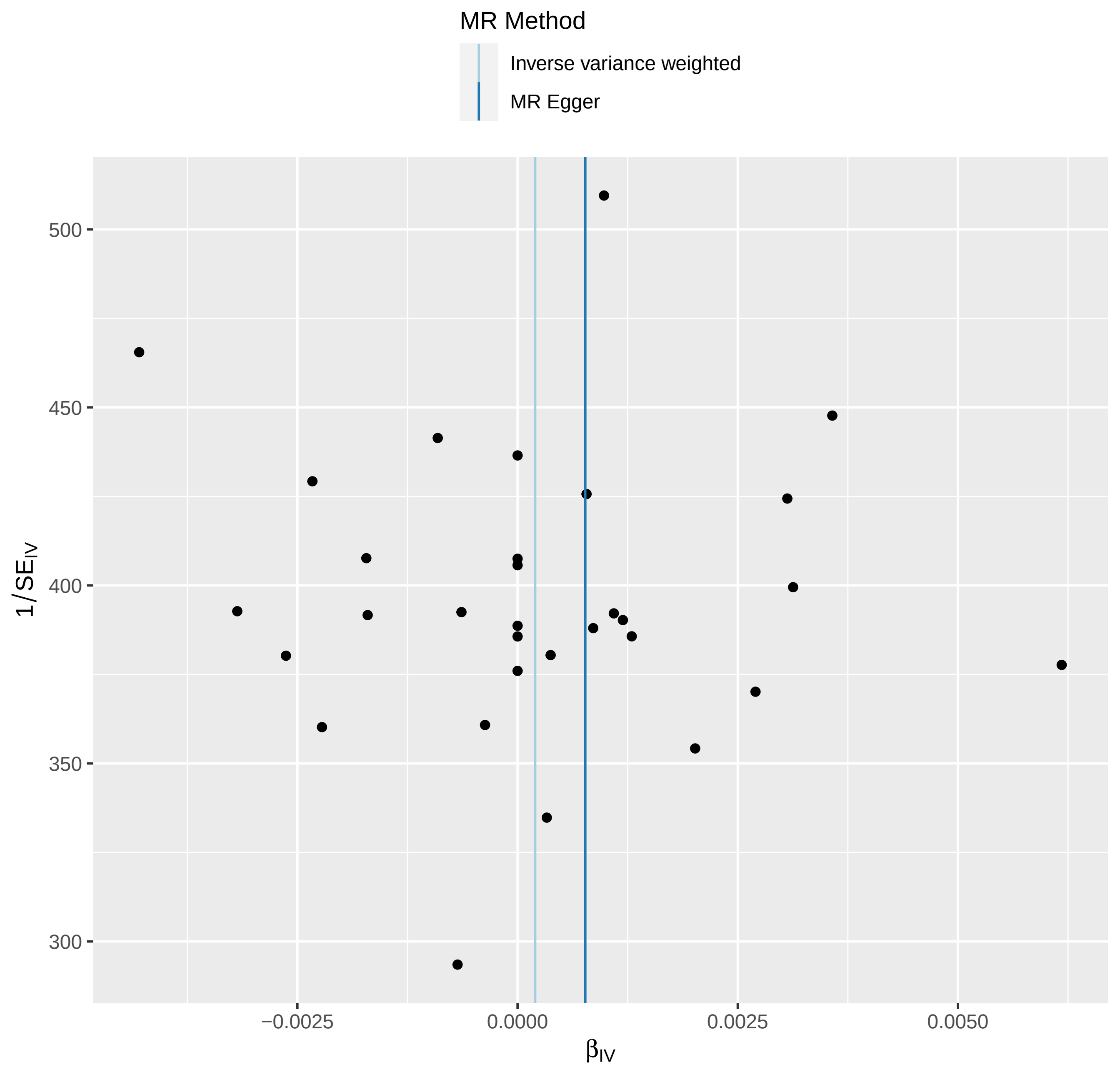

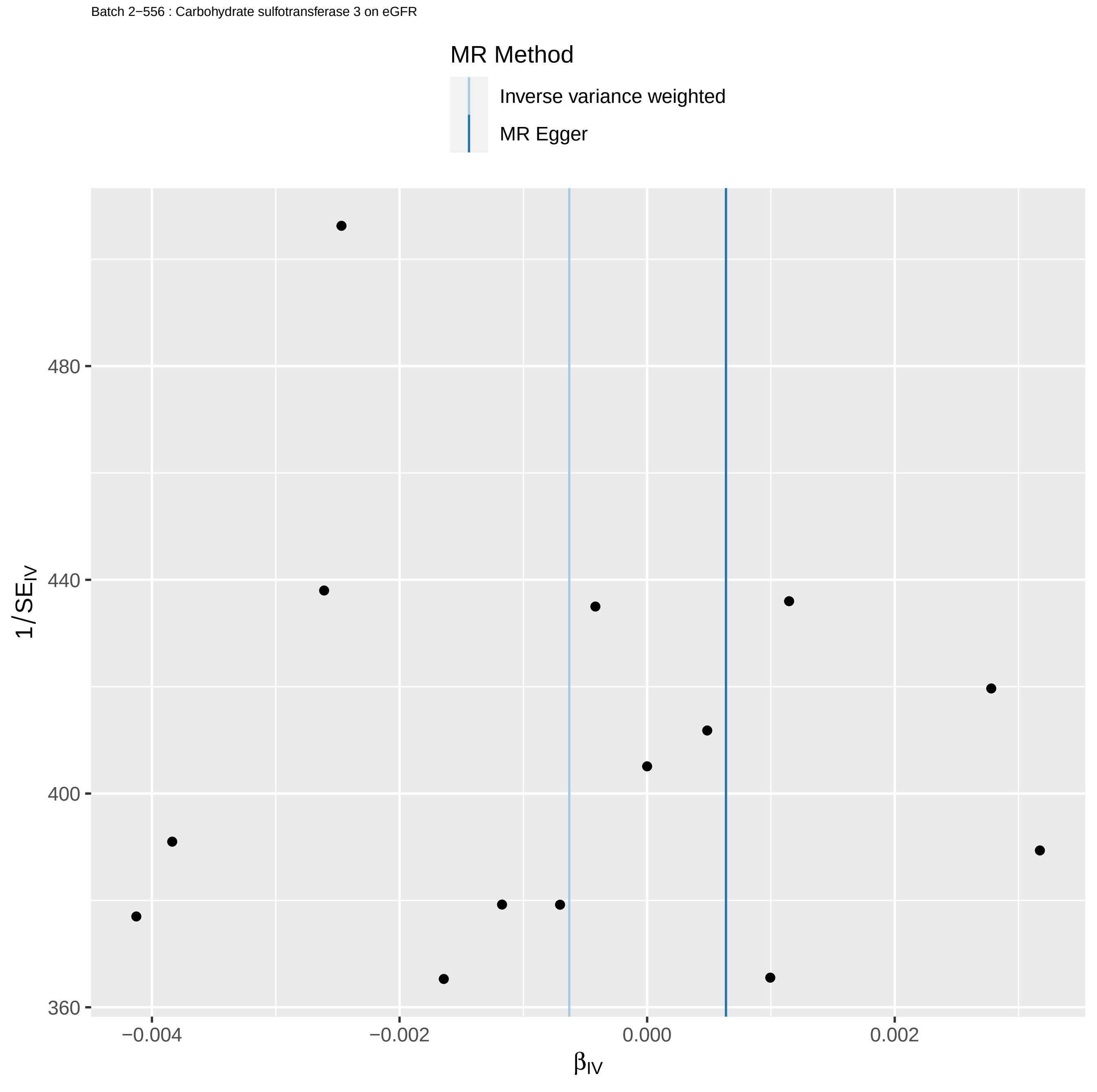

Batch 2−556 : Carbohydrate sulfotransferase 3 on eGFR
MR Method
Inverse variance weighted
MR Egger
480
440
400
360
1/SE_IV
−0.004
−0.002
0.000
0.002
β_IV

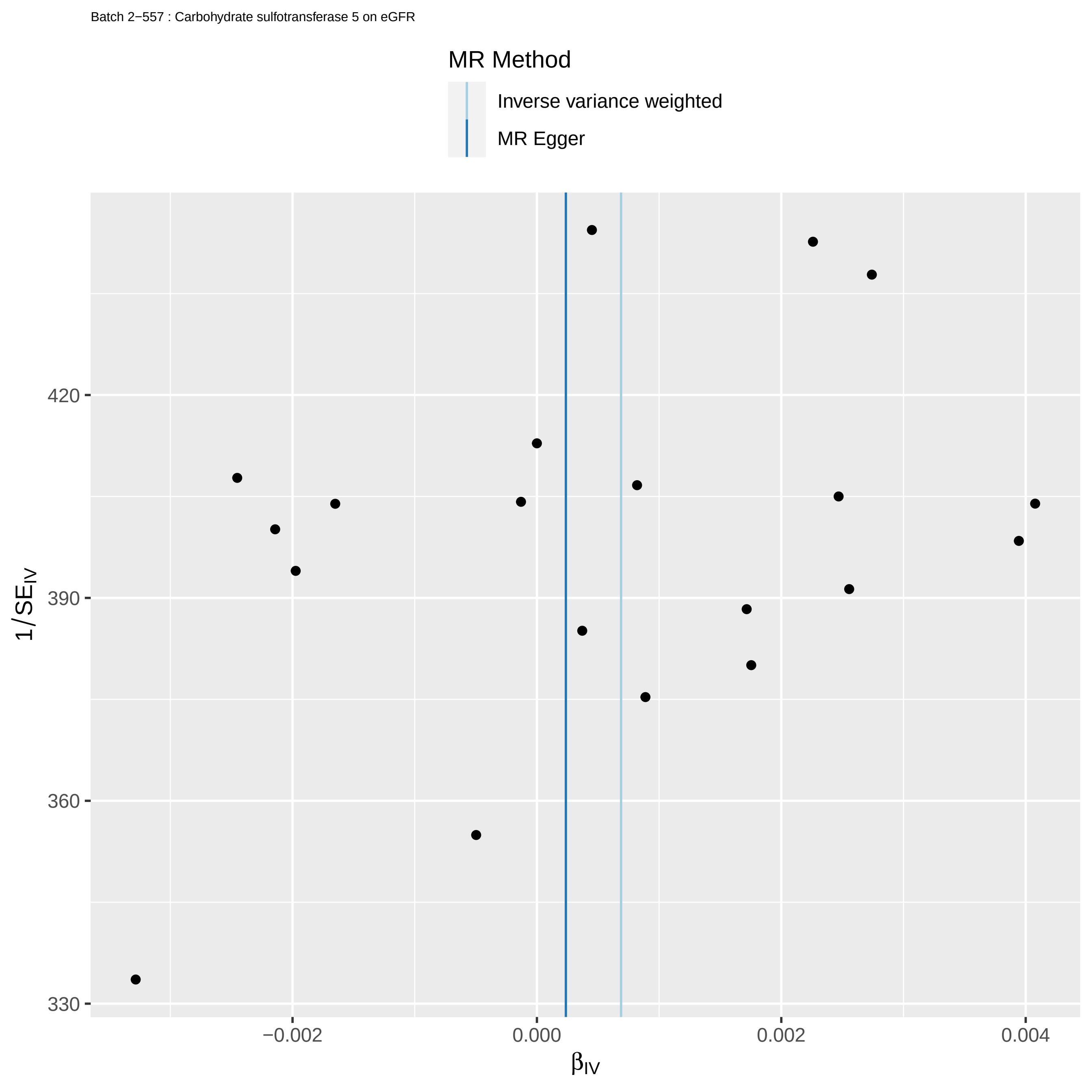
Batch 2−557 : Carbohydrate sulfotransferase 5 on eGFR
MR Method
Inverse variance weighted
MR Egger
420
390
360
330
1/SE_IV
−0.002
0.000
0.002
0.004
β_IV

Batch 2−558 : Carbohydrate sulfotransferase 6 on eGFR

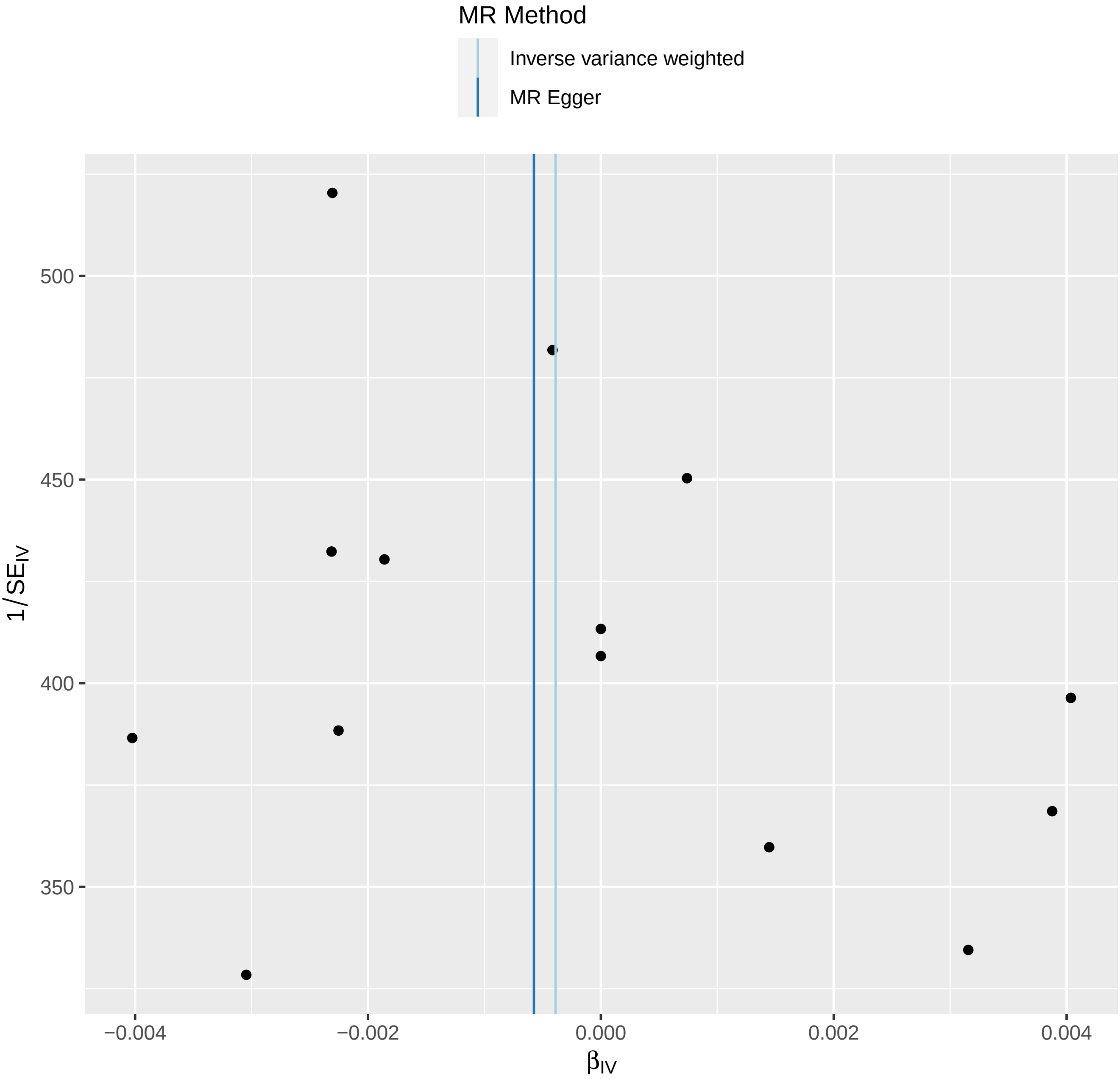

Batch 2−559 : Carbohydrate sulfotransferase 9 on eGFR

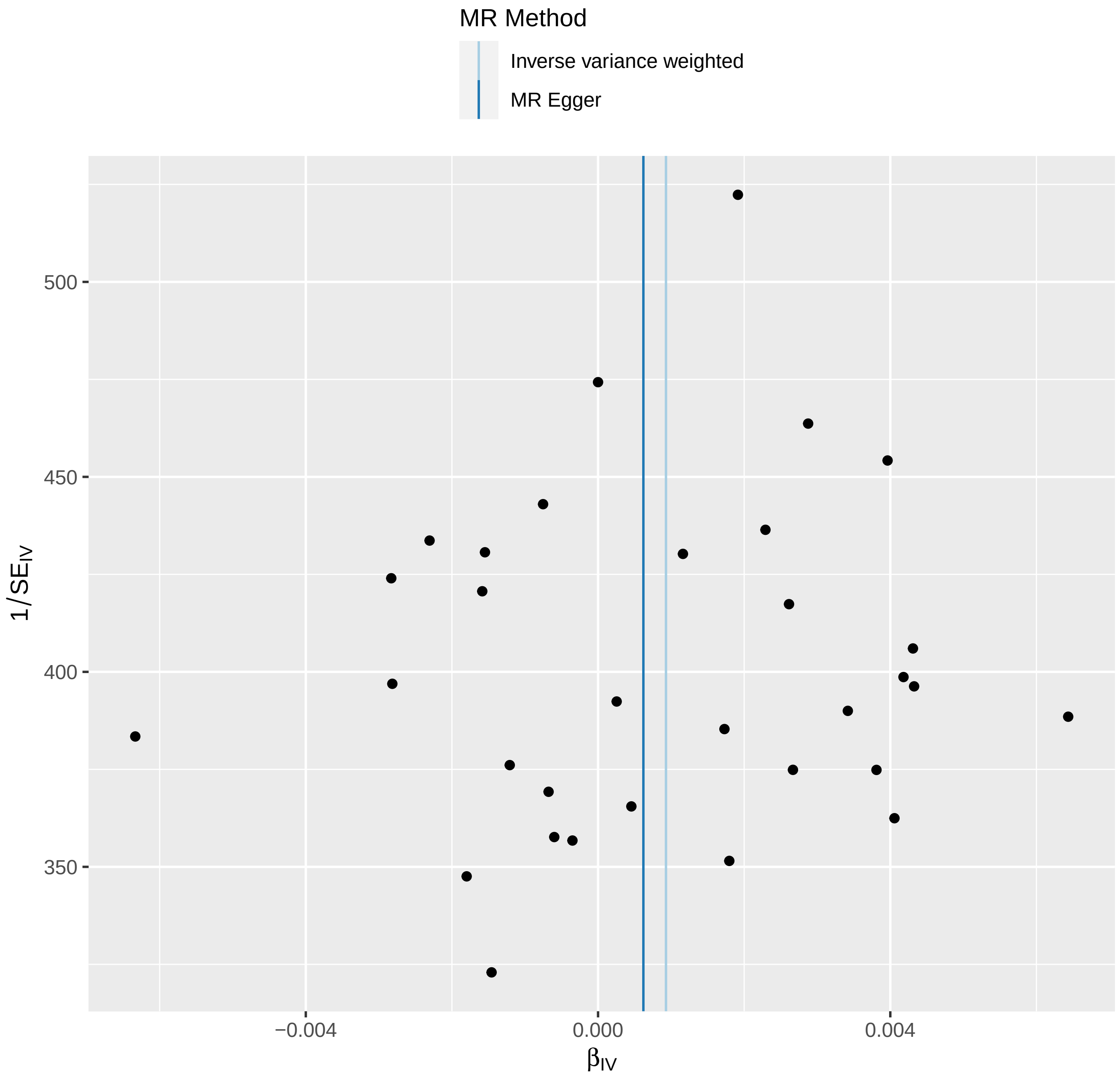

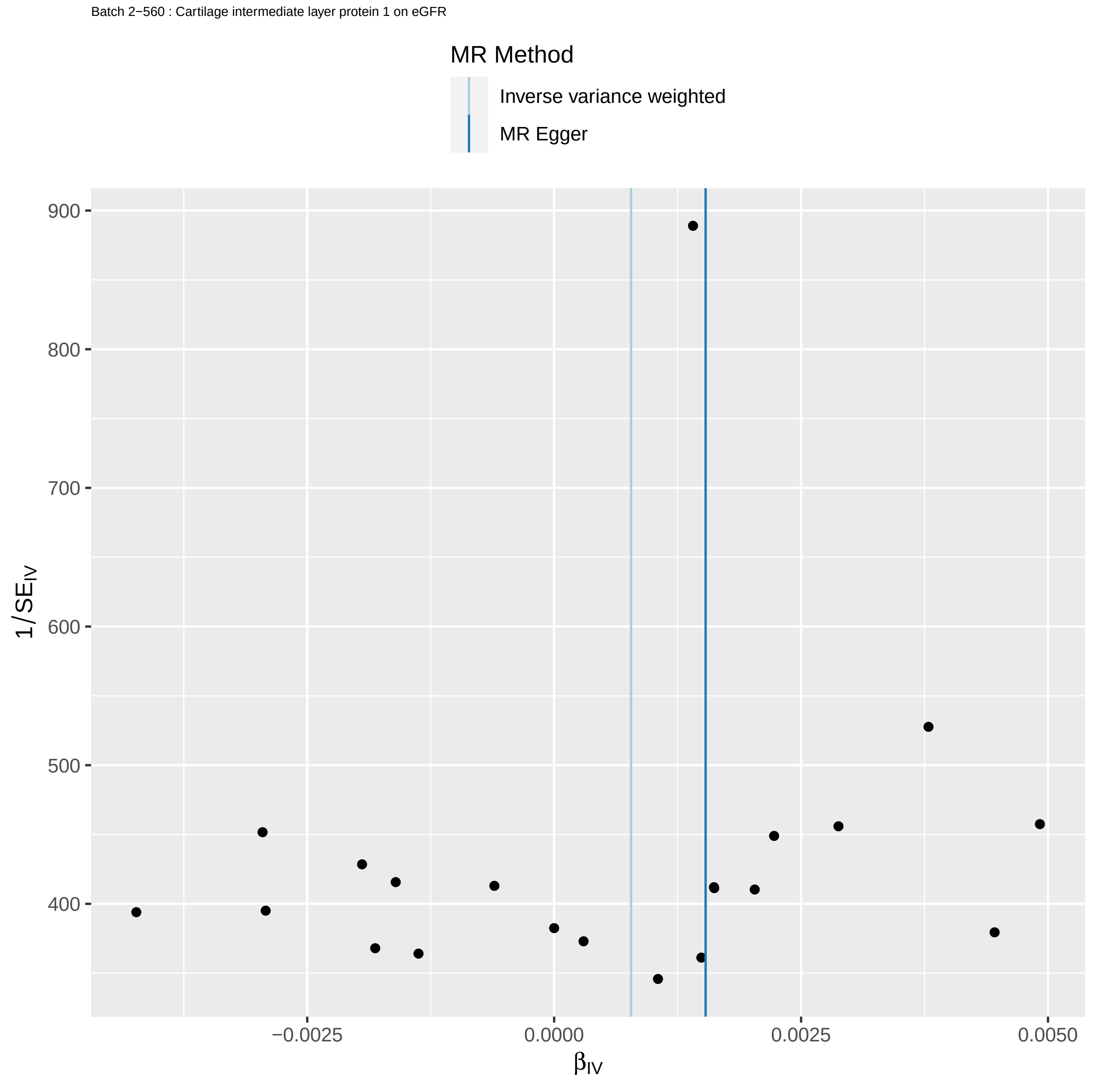
Batch 2−560 : Cartilage intermediate layer protein 1 on eGFR
MR Method
Inverse variance weighted
MR Egger
900
800
700
600
500
400
1/SE_IV
−0.0025
0.0000
0.0025
0.0050
β_IV

Batch 2−561 : Cold−inducible RNA−binding protein on eGFR

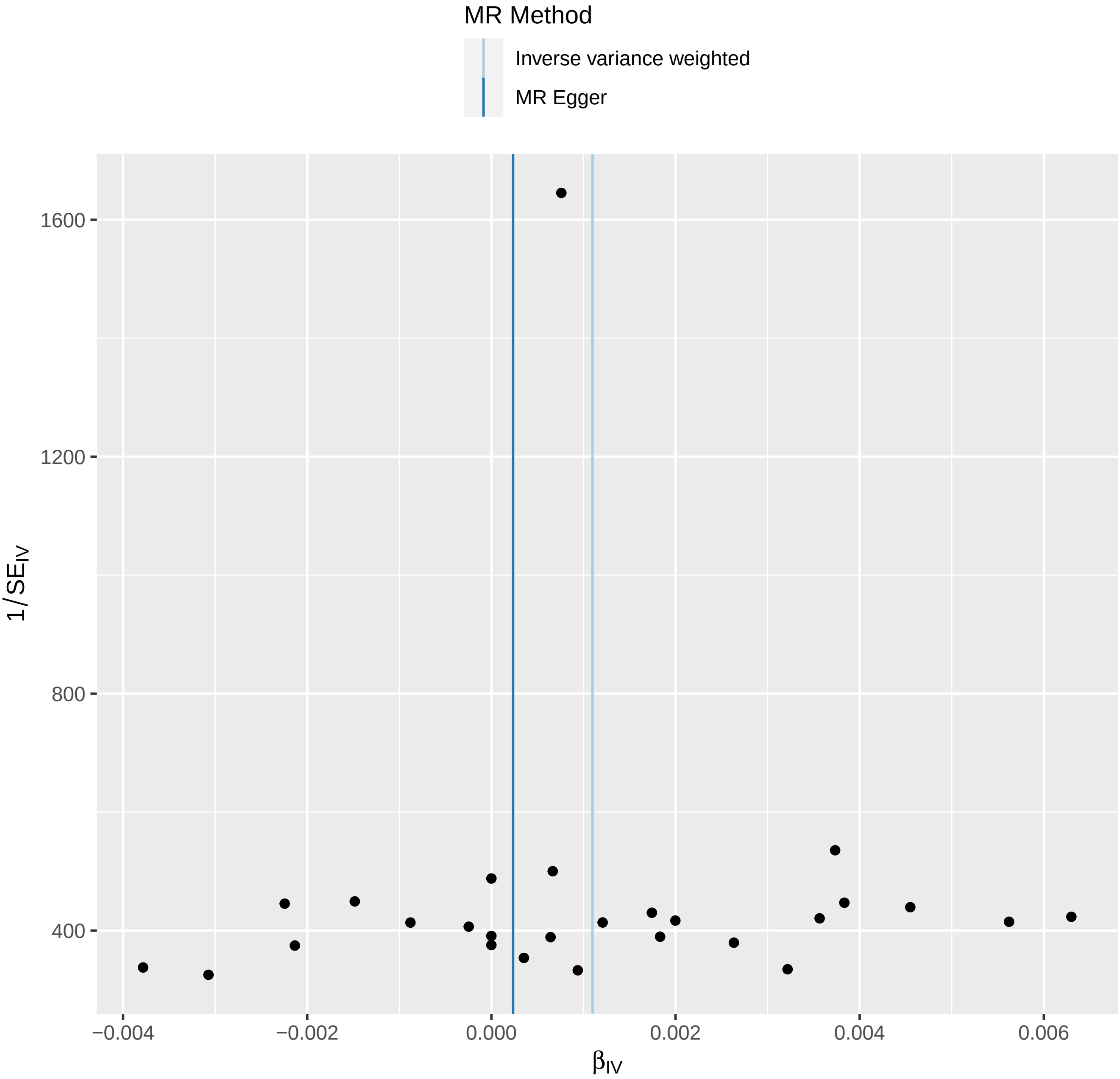

Batch 2−562 : CDGSH iron−sulfur domain−containing protein 1 on eGFR

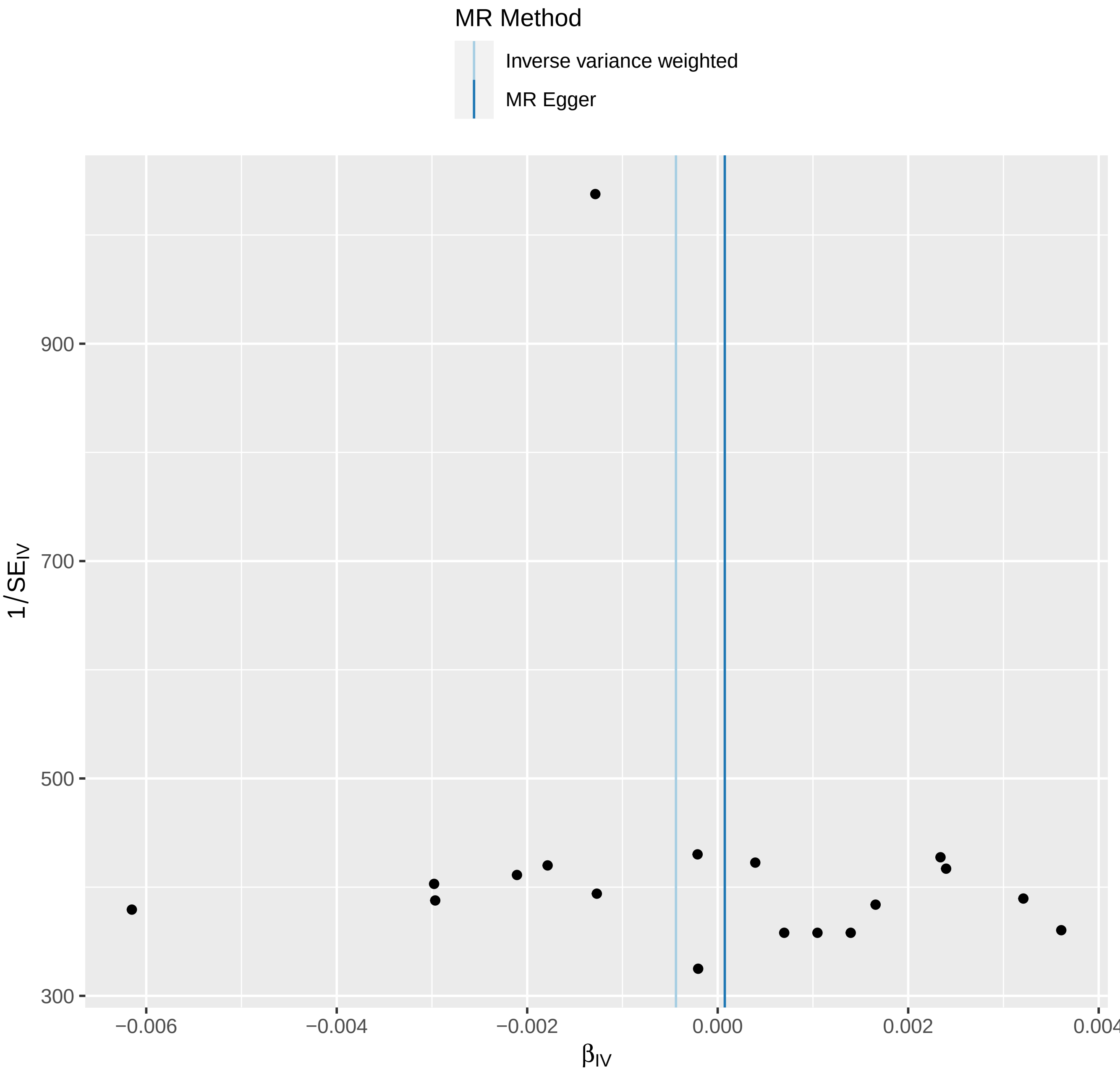

Batch 2−563 : CDGSH iron−sulfur domain−containing protein 2 on eGFR

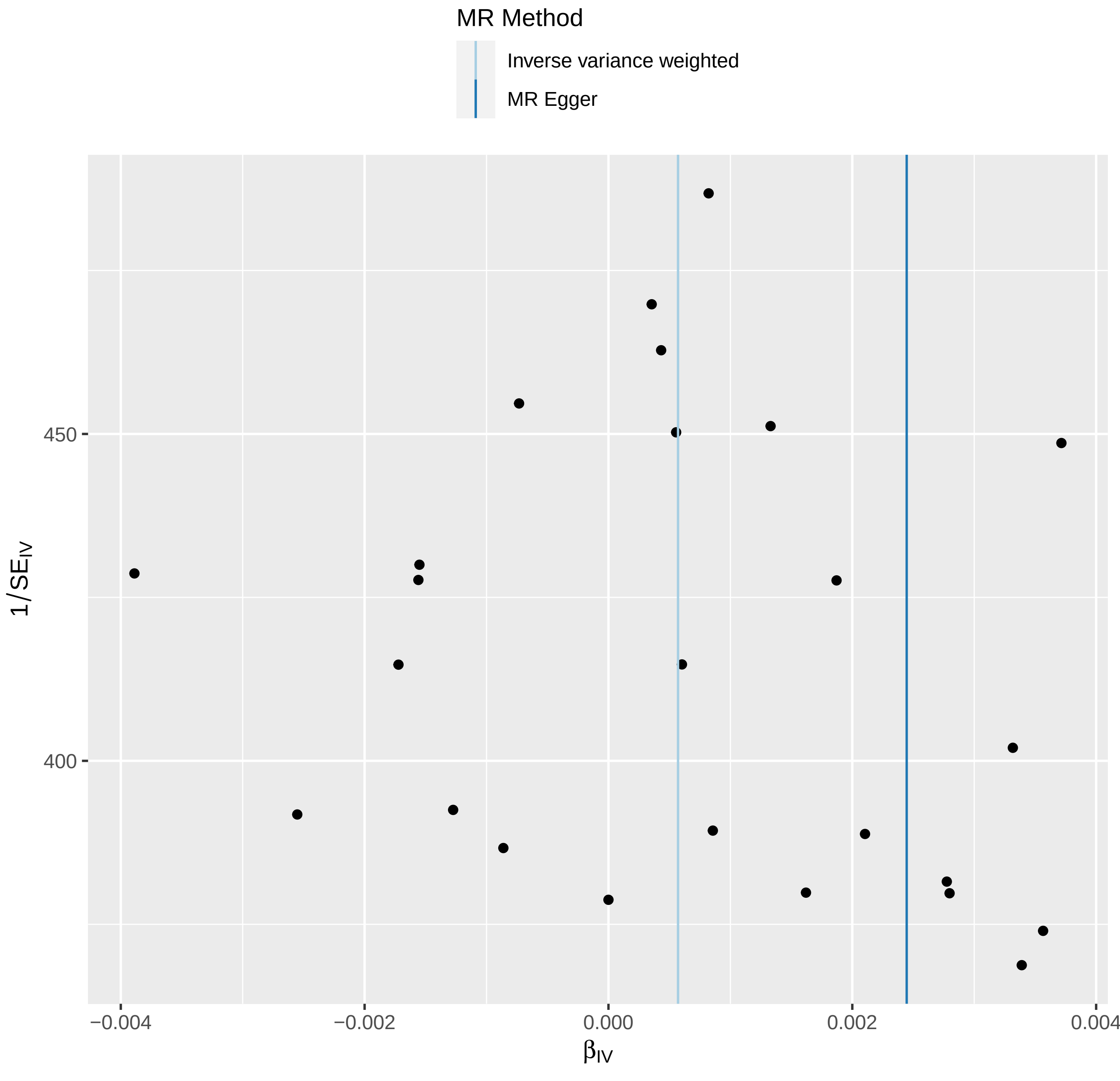

Batch 2−564 : Cytoskeleton−associated protein 2 on eGFR

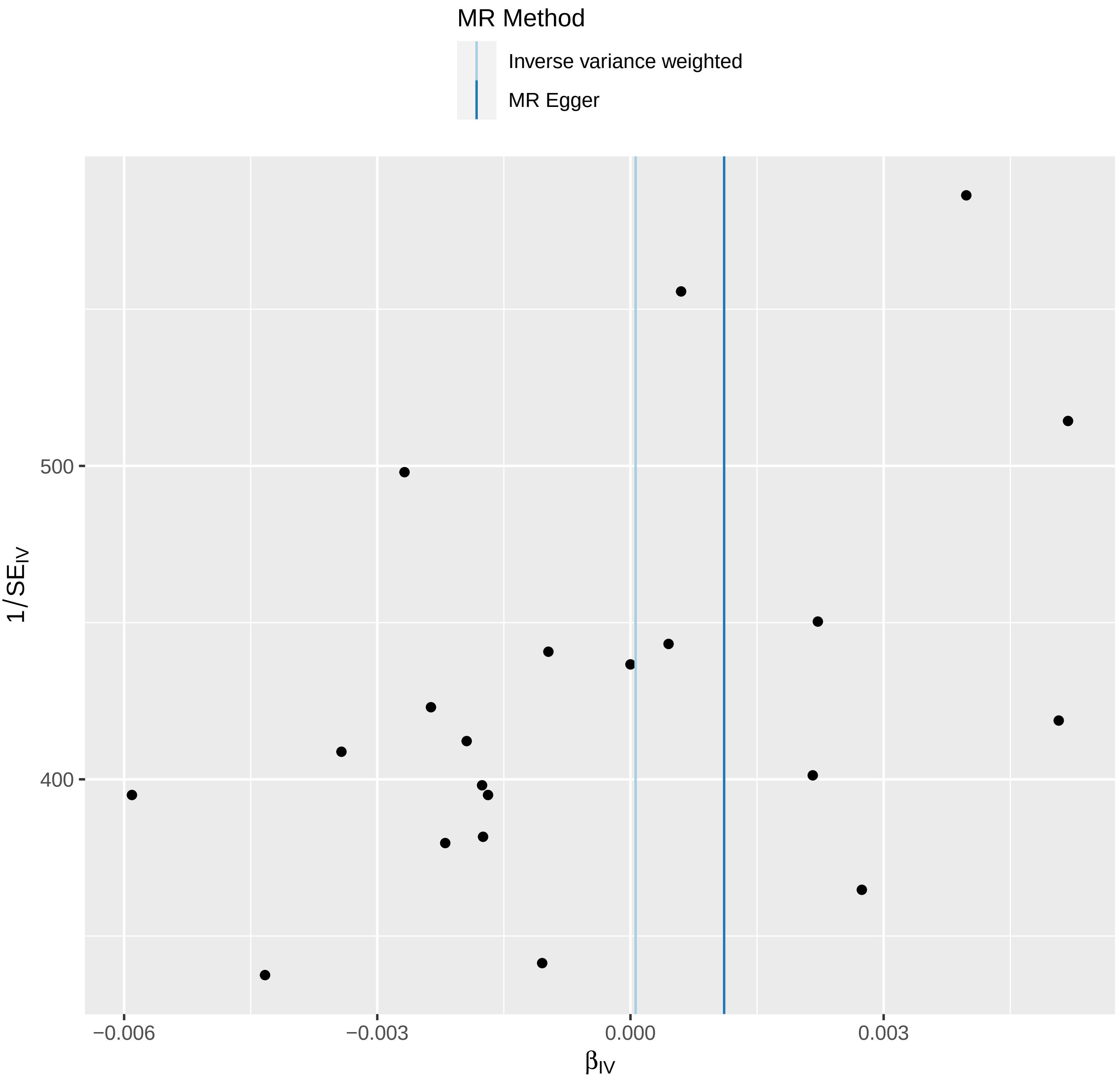

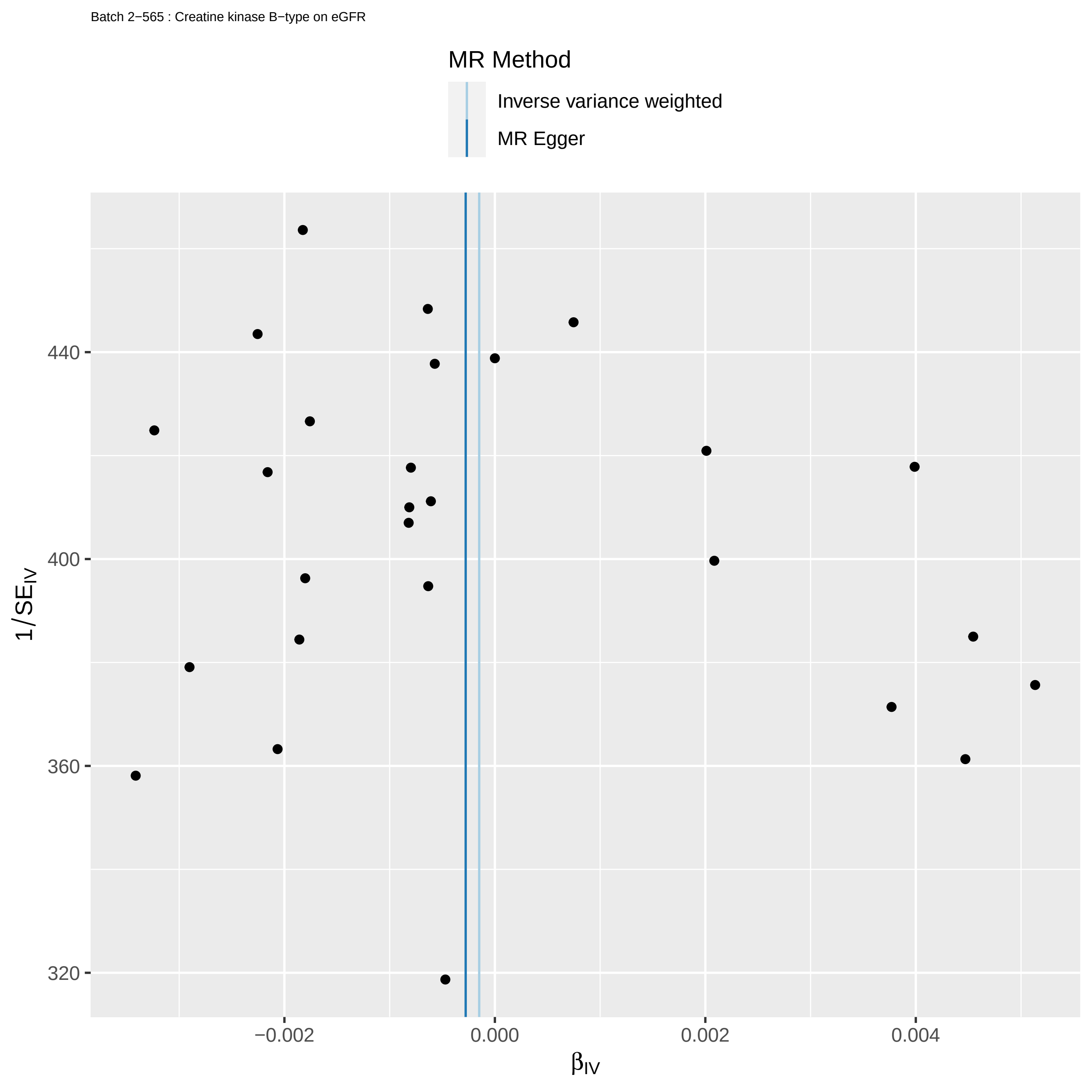

Batch 2−565 : Creatine kinase B−type on eGFR
MR Method
Inverse variance weighted
MR Egger
440
400
360
320
1/SE$_{IV}$
−0.002
0.000
0.002
0.004
β$_{IV}$

Batch 2−566 : Creatine kinase M−type on eGFR

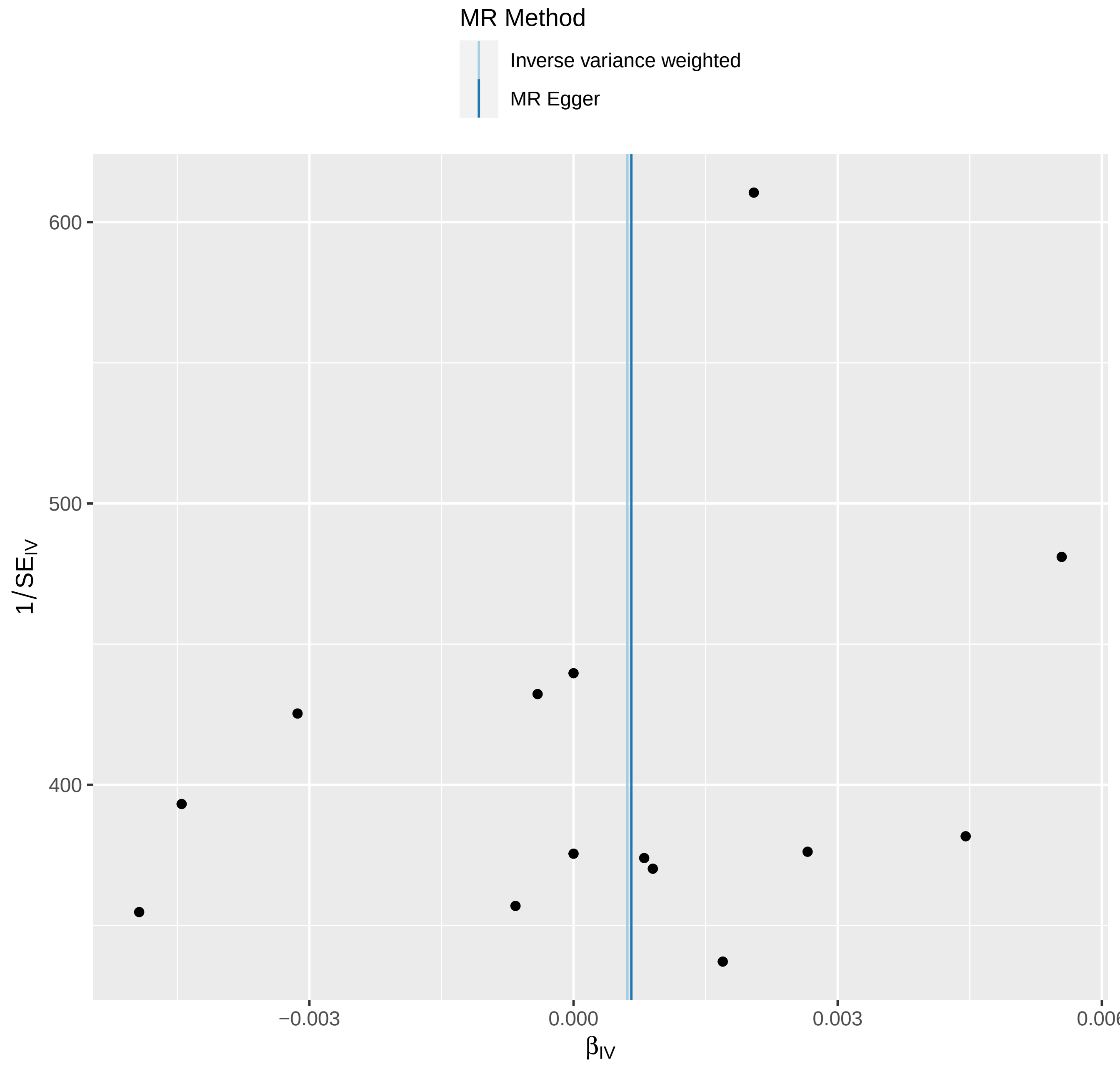

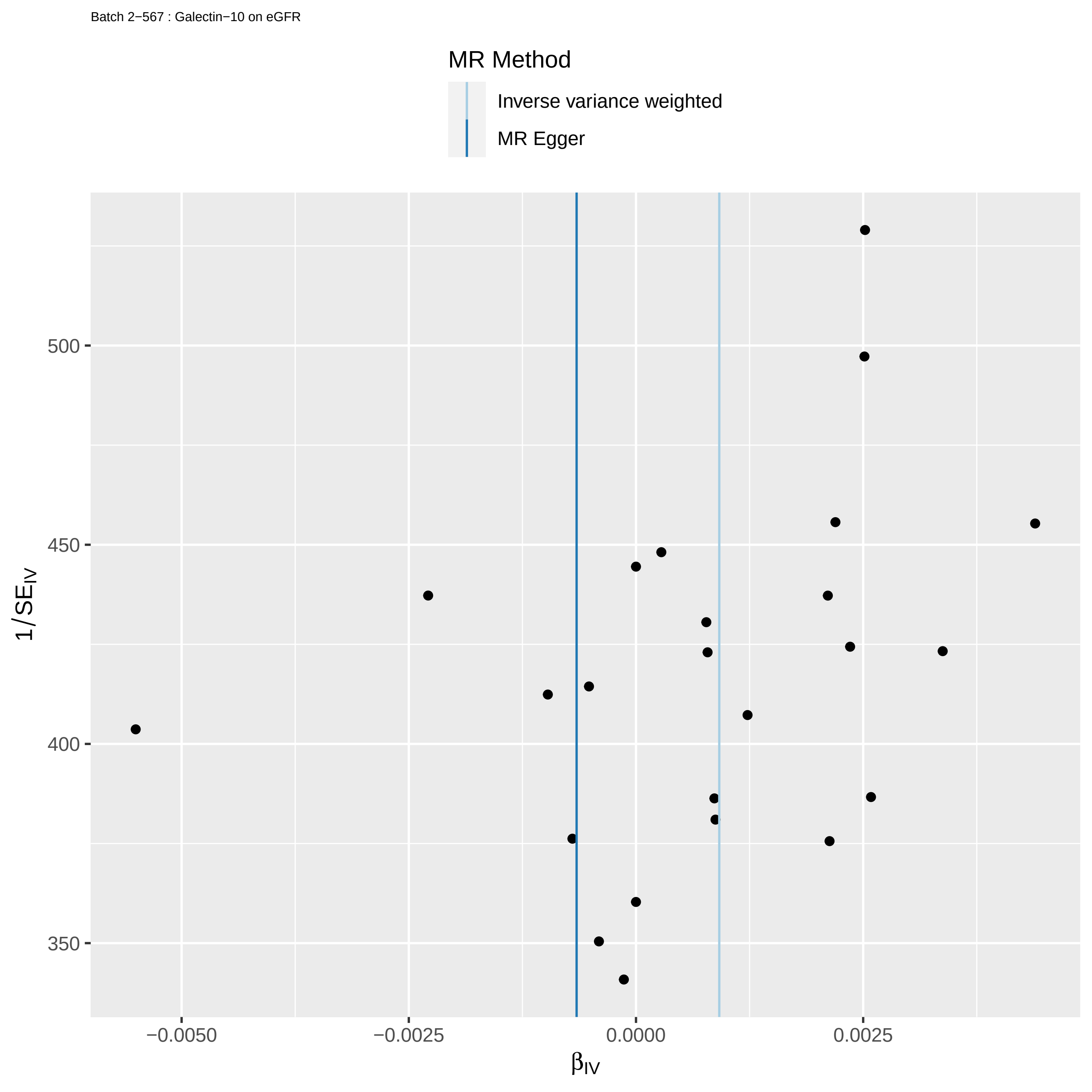

Batch 2−567 : Galectin−10 on eGFR
MR Method
Inverse variance weighted
MR Egger
500
450
400
350
1/SE$_{IV}$
−0.0050
−0.0025
0.0000
0.0025
β$_{IV}$

Batch 2−568 : C−type lectin domain family 10 member A on eGFR

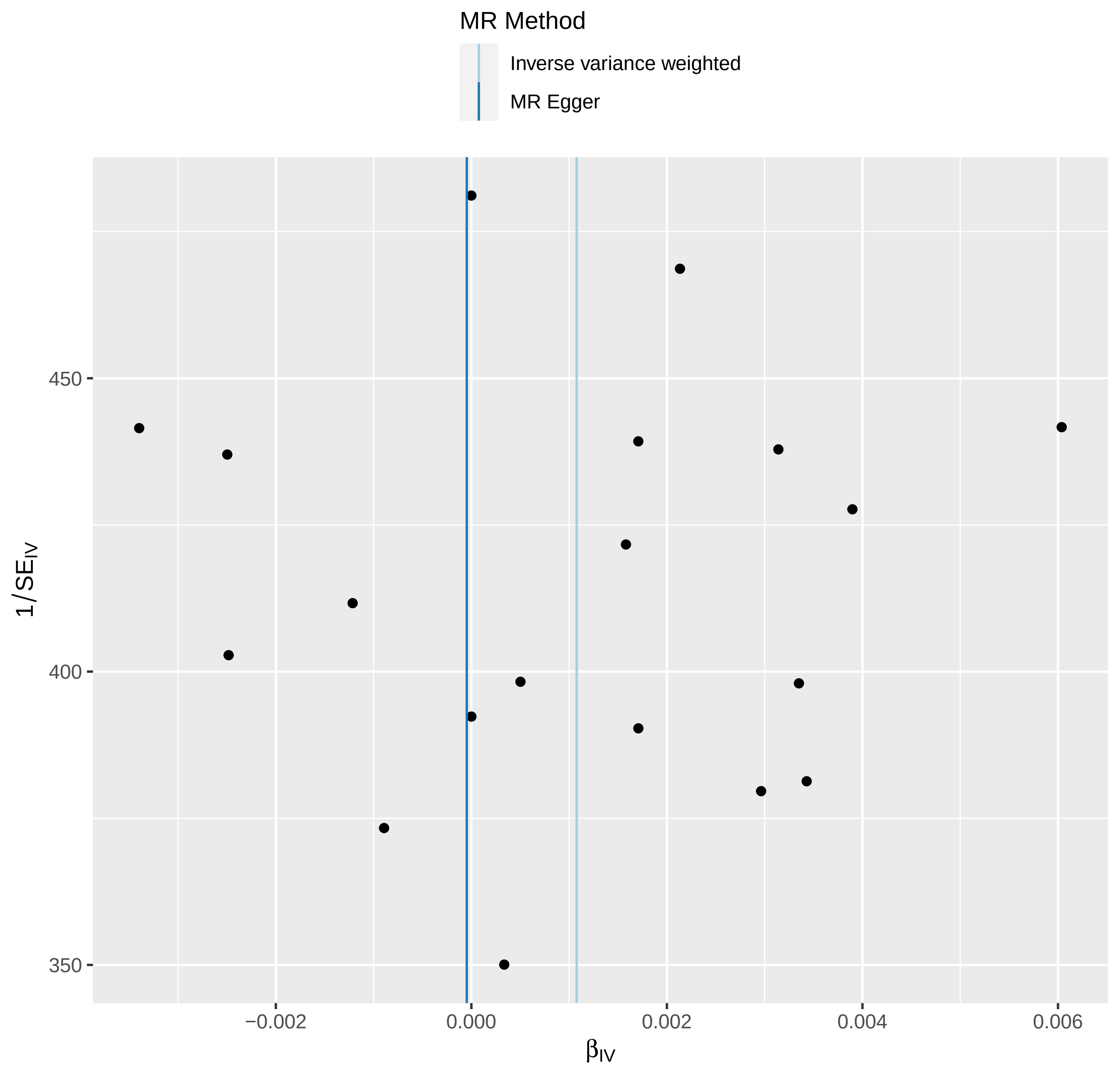

Batch 2−569 : Stem Cell Growth Factor−beta on eGFR

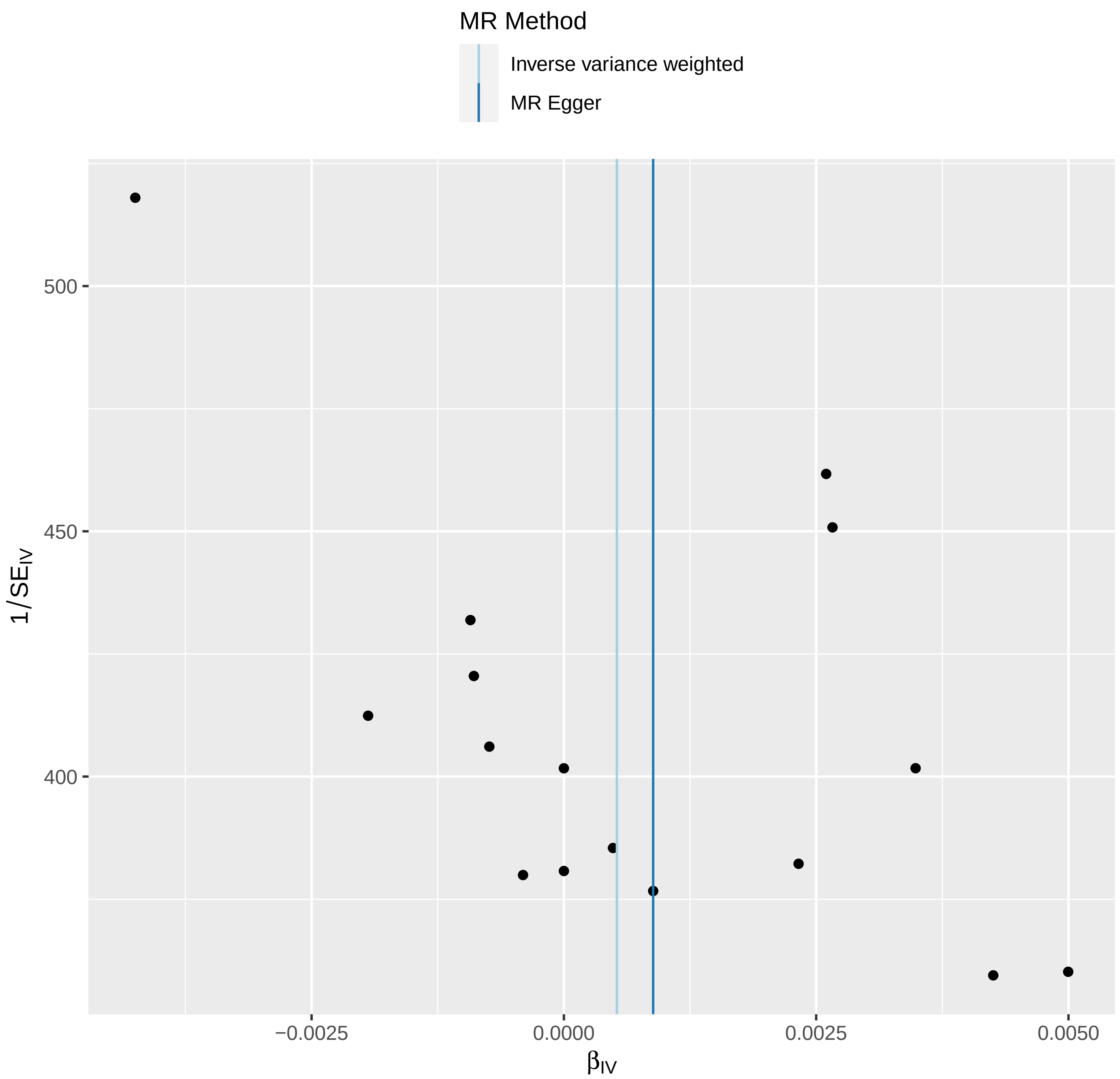

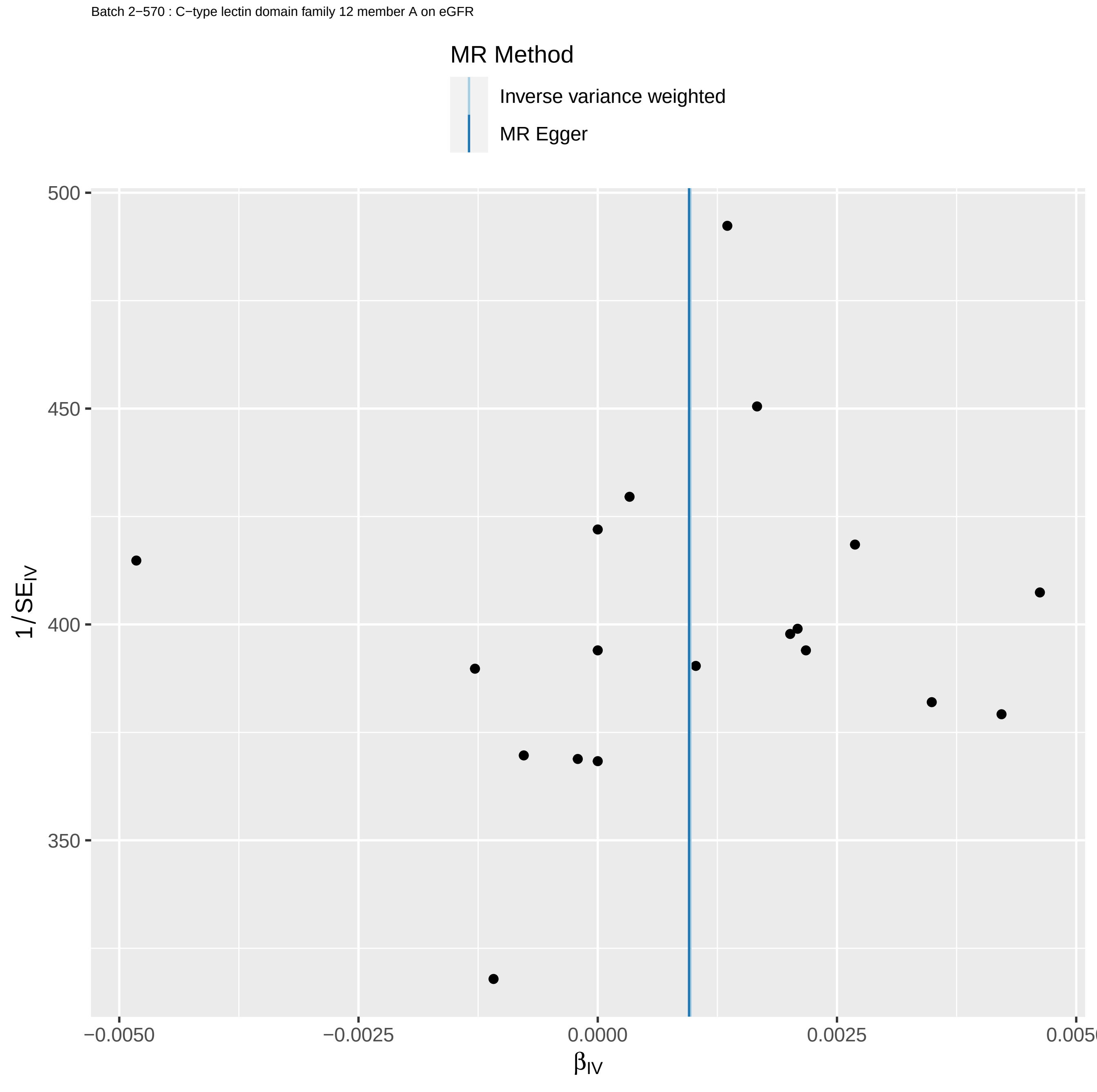

Batch 2−570 : C−type lectin domain family 12 member A on eGFR
MR Method
Inverse variance weighted
MR Egger
500
450
400
350
1/SE$_{IV}$
−0.0050
−0.0025
0.0000
0.0025
β$_{IV}$

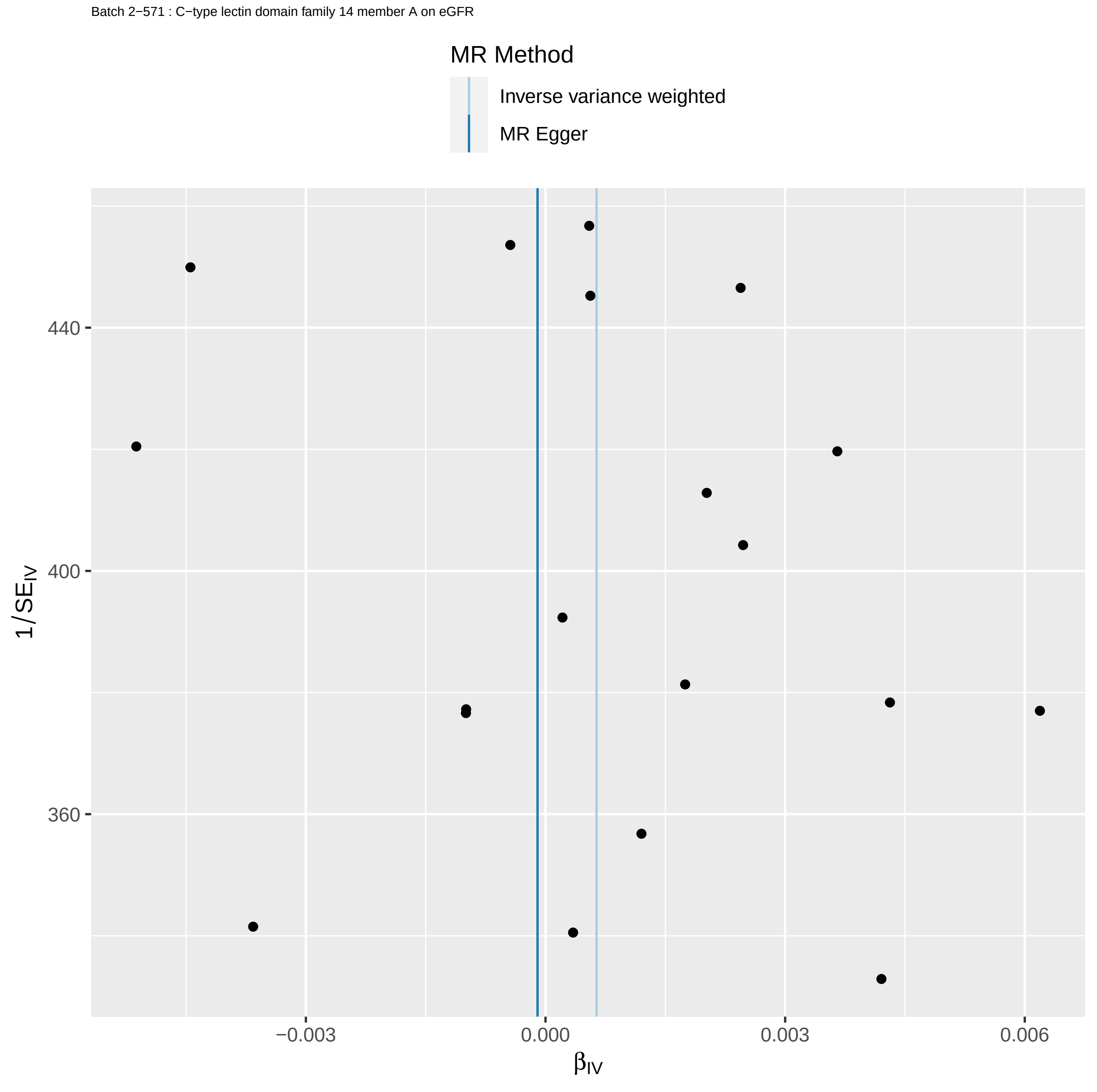
Batch 2−571 : C−type lectin domain family 14 member A on eGFR
MR Method
Inverse variance weighted
MR Egger
440
400
360
1/SE_IV
−0.003
0.000
0.003
0.006
β_IV

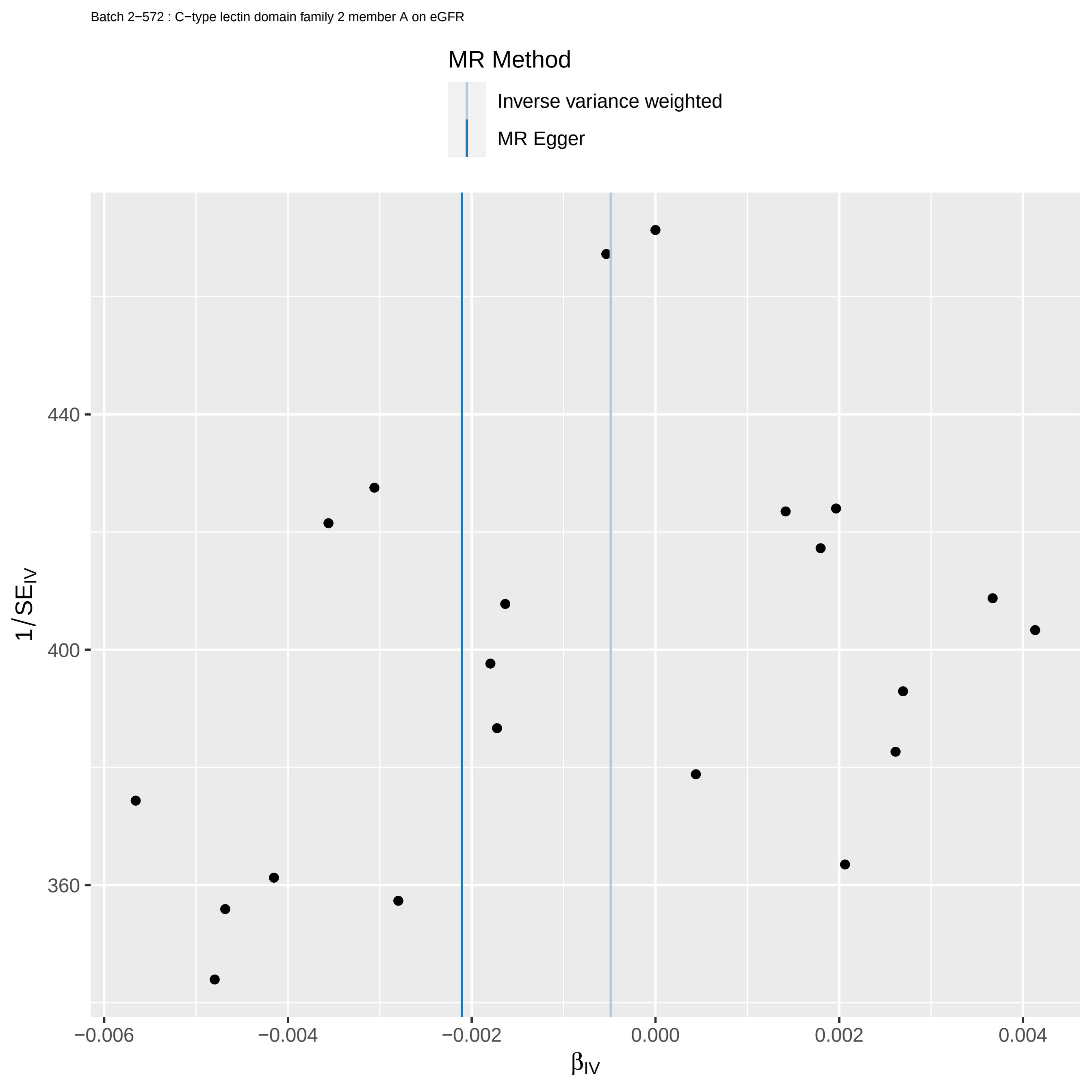
Batch 2−572 : C−type lectin domain family 2 member A on eGFR
MR Method
Inverse variance weighted
MR Egger
440
400
360
$1/SE_{IV}$
−0.006
−0.004
−0.002
0.000
0.002
0.004
β_{IV}

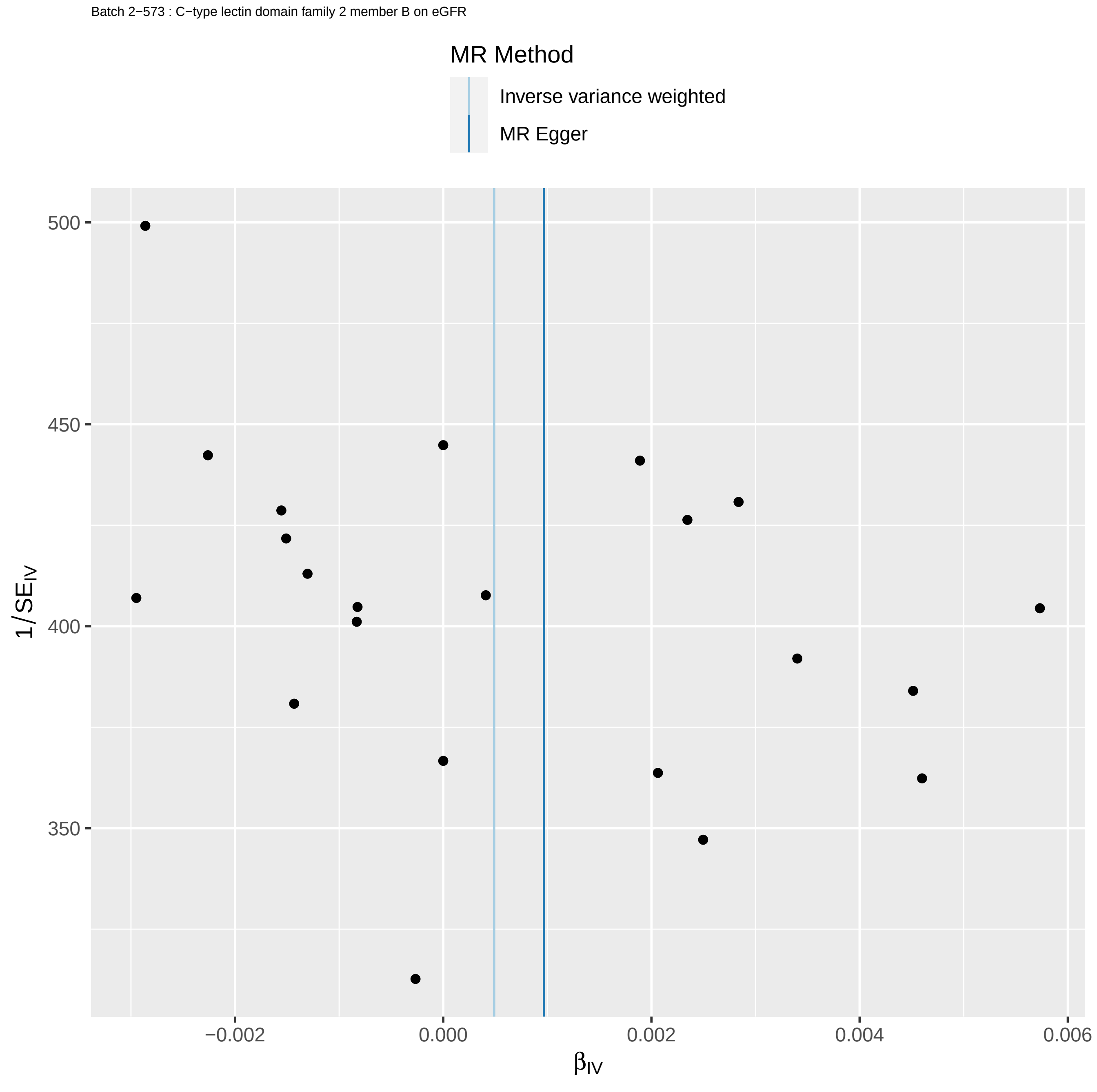

Batch 2−573 : C−type lectin domain family 2 member B on eGFR
MR Method
Inverse variance weighted
MR Egger
500
450
400
350
1/SE_IV
−0.002
0.000
0.002
0.004
0.006
β_IV

Batch 2−574 : C−type lectin domain family 2 member D on eGFR

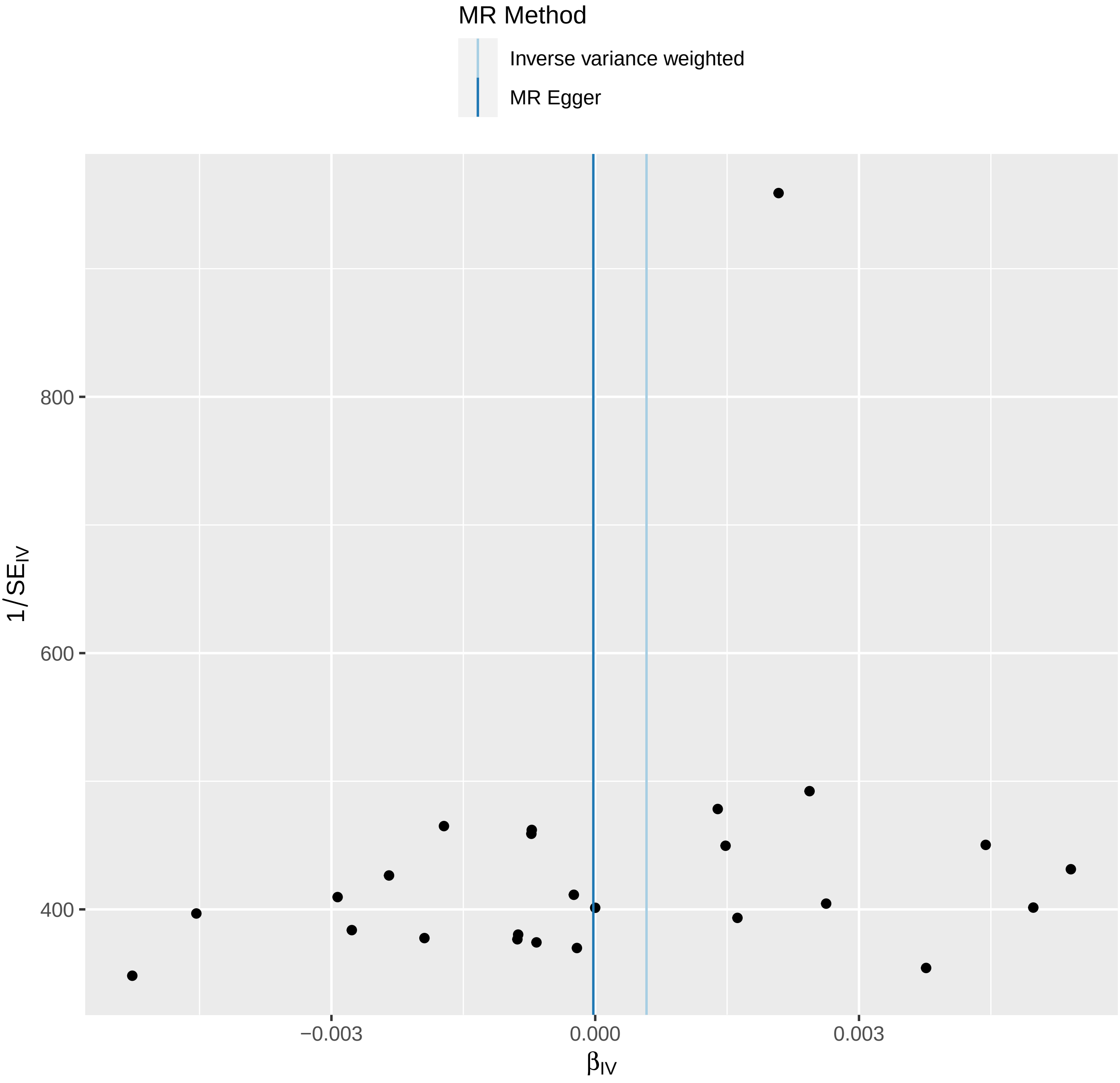

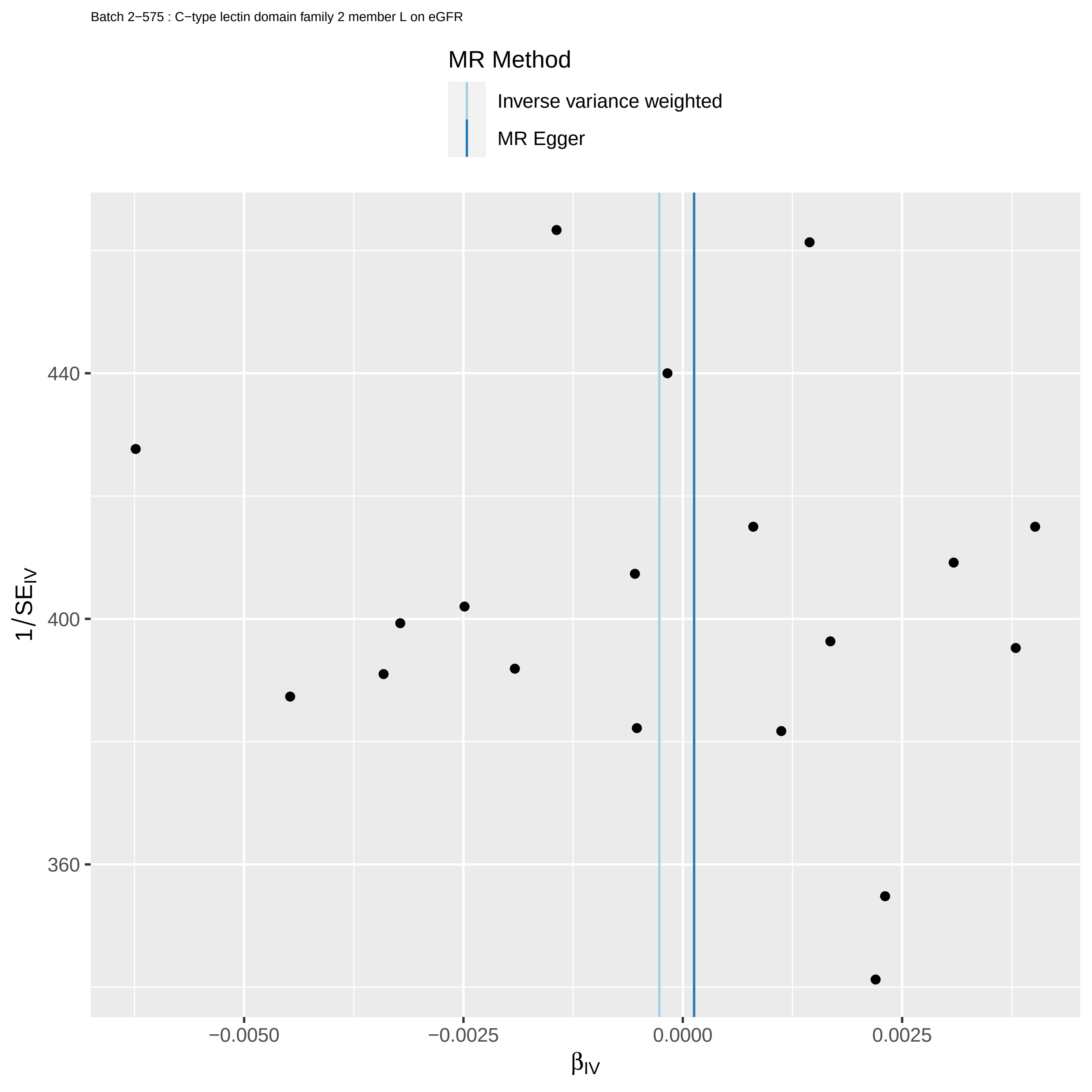

Batch 2−575 : C−type lectin domain family 2 member L on eGFR
MR Method
Inverse variance weighted
MR Egger
440
400
360
1/SE_IV
−0.0050
−0.0025
0.0000
0.0025
β_IV

Batch 2−576 : C−type lectin domain family 4 member D on eGFR

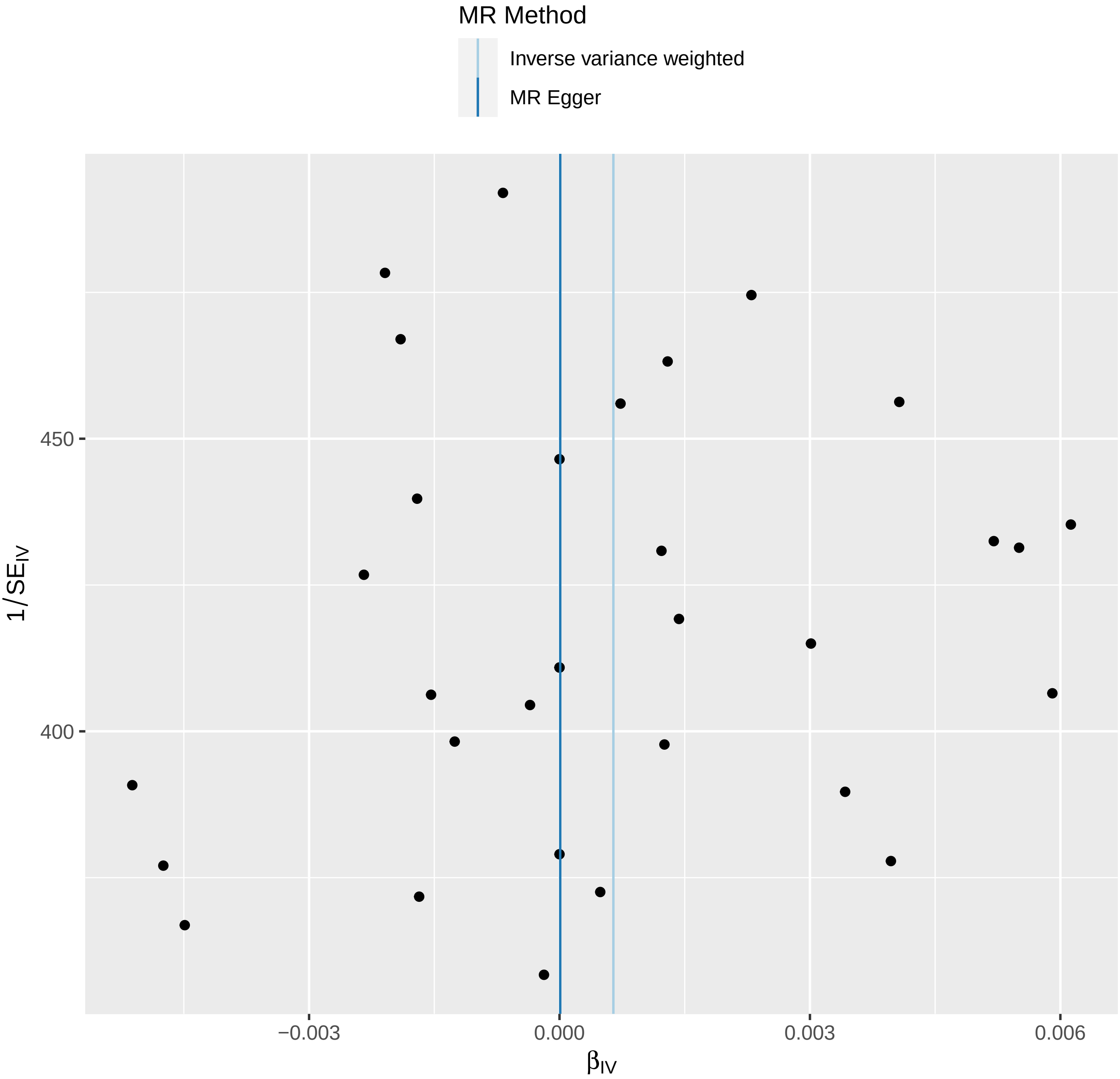

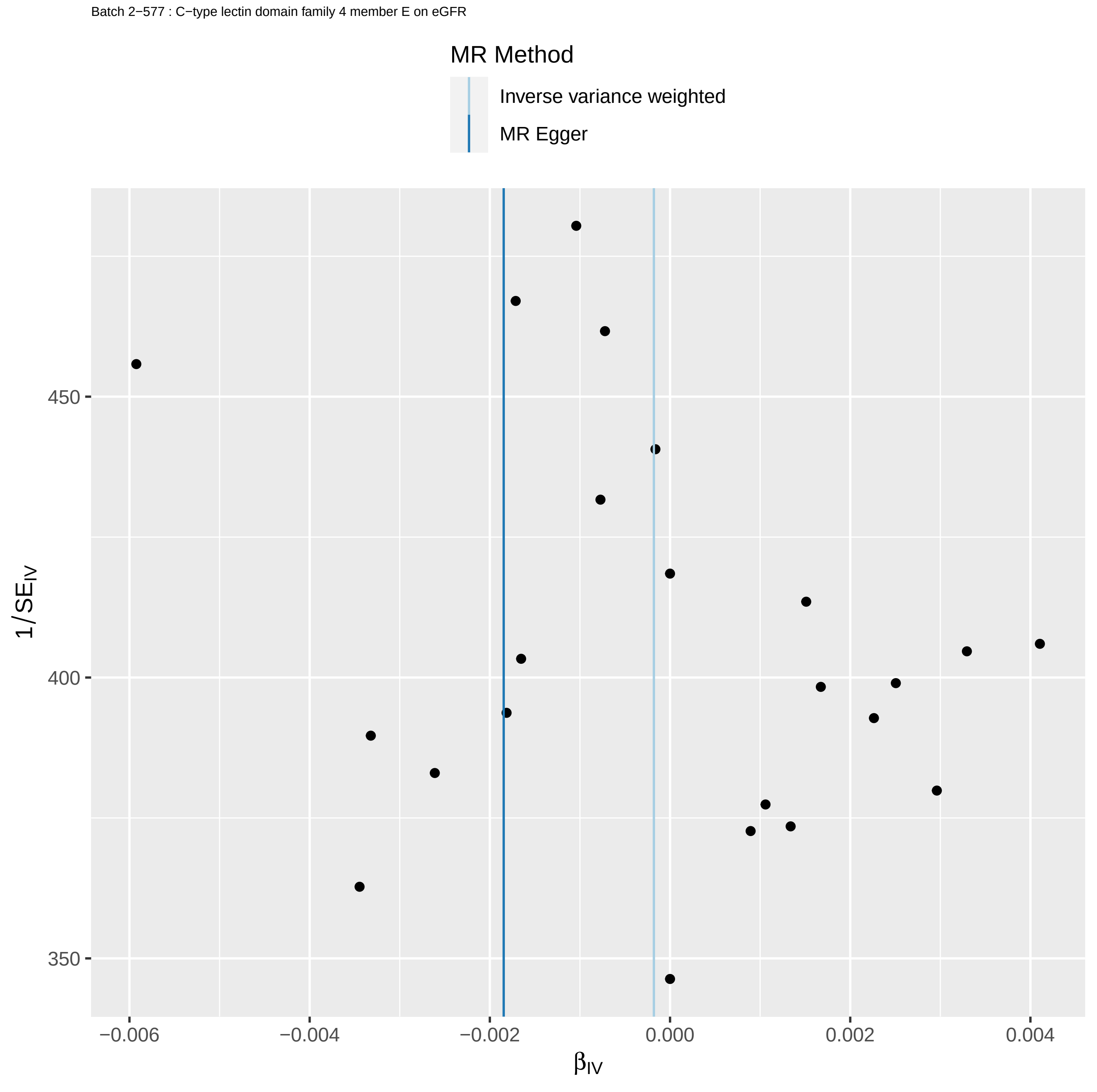

Batch 2−577 : C−type lectin domain family 4 member E on eGFR
MR Method
Inverse variance weighted
MR Egger
450
400
350
1/SE_IV
−0.006
−0.004
−0.002
0.000
0.002
0.004
β_IV

Batch 2−578 : C−type lectin domain family 4 member M on eGFR

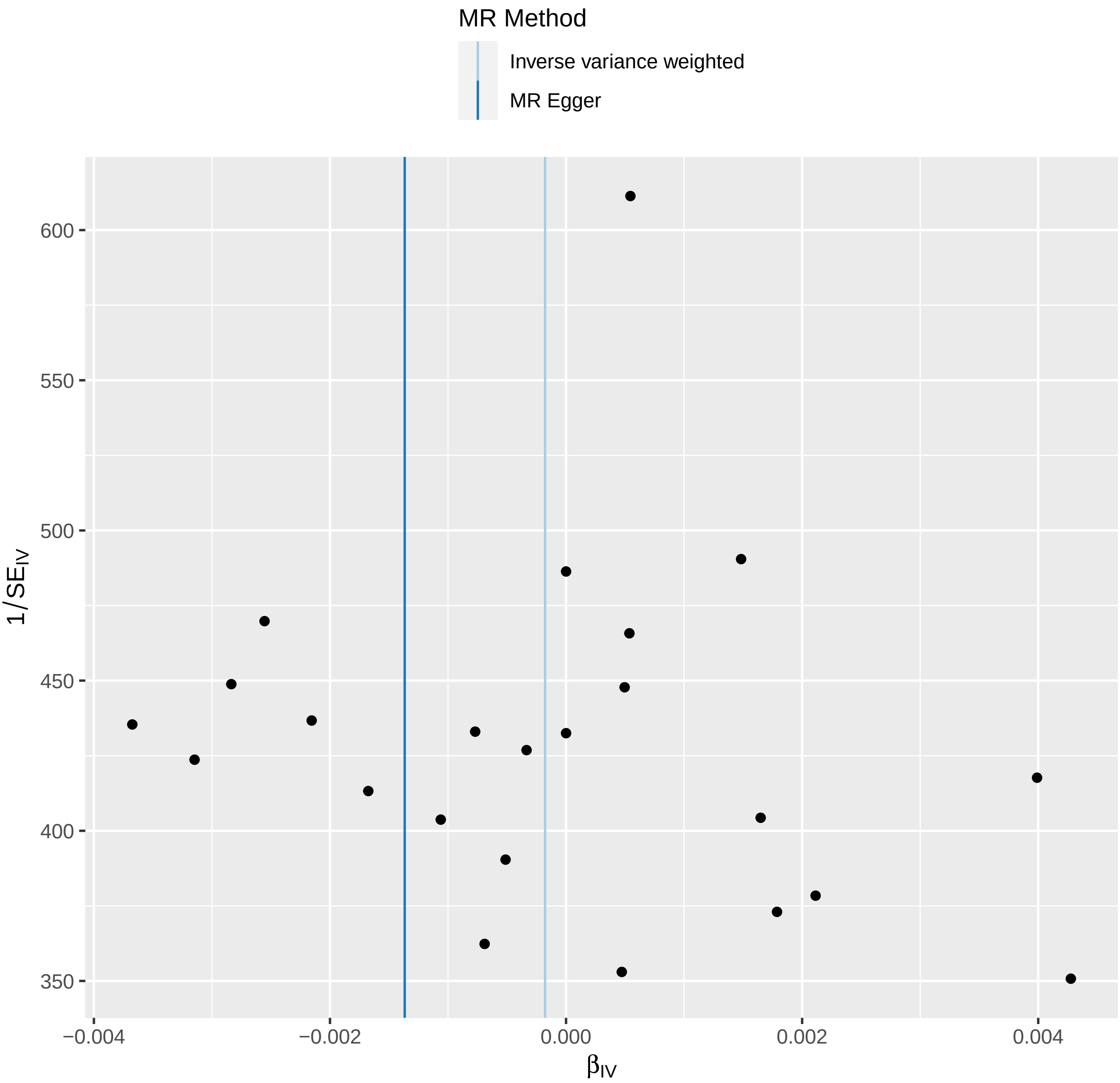

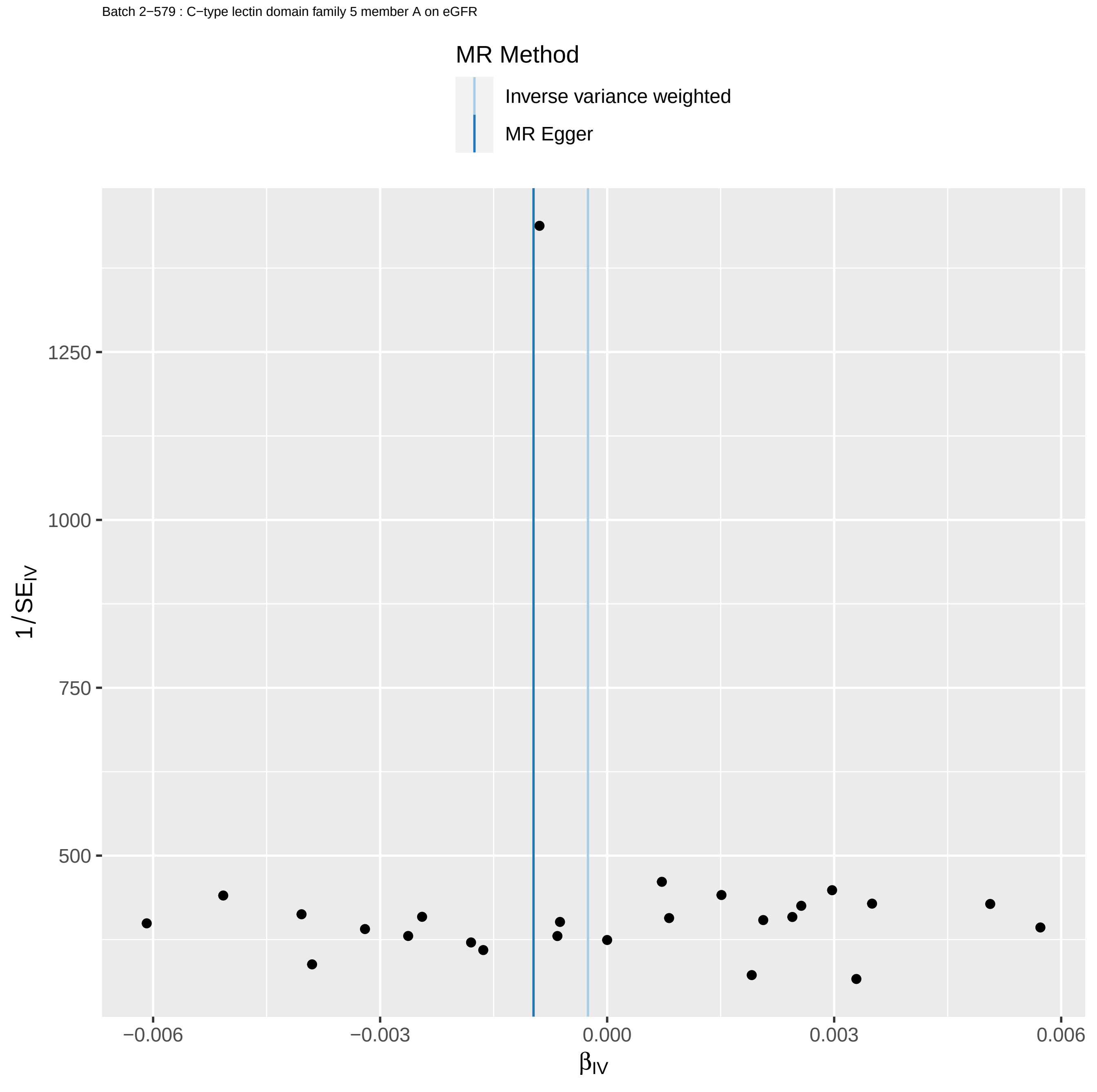

Batch 2−579 : C−type lectin domain family 5 member A on eGFR
MR Method
Inverse variance weighted
MR Egger
1250
1000
750
500
1/SE$_{IV}$
−0.006
−0.003
0.000
0.003
0.006
β$_{IV}$

Batch 2−580 : C−type lectin domain family 6 member A on eGFR

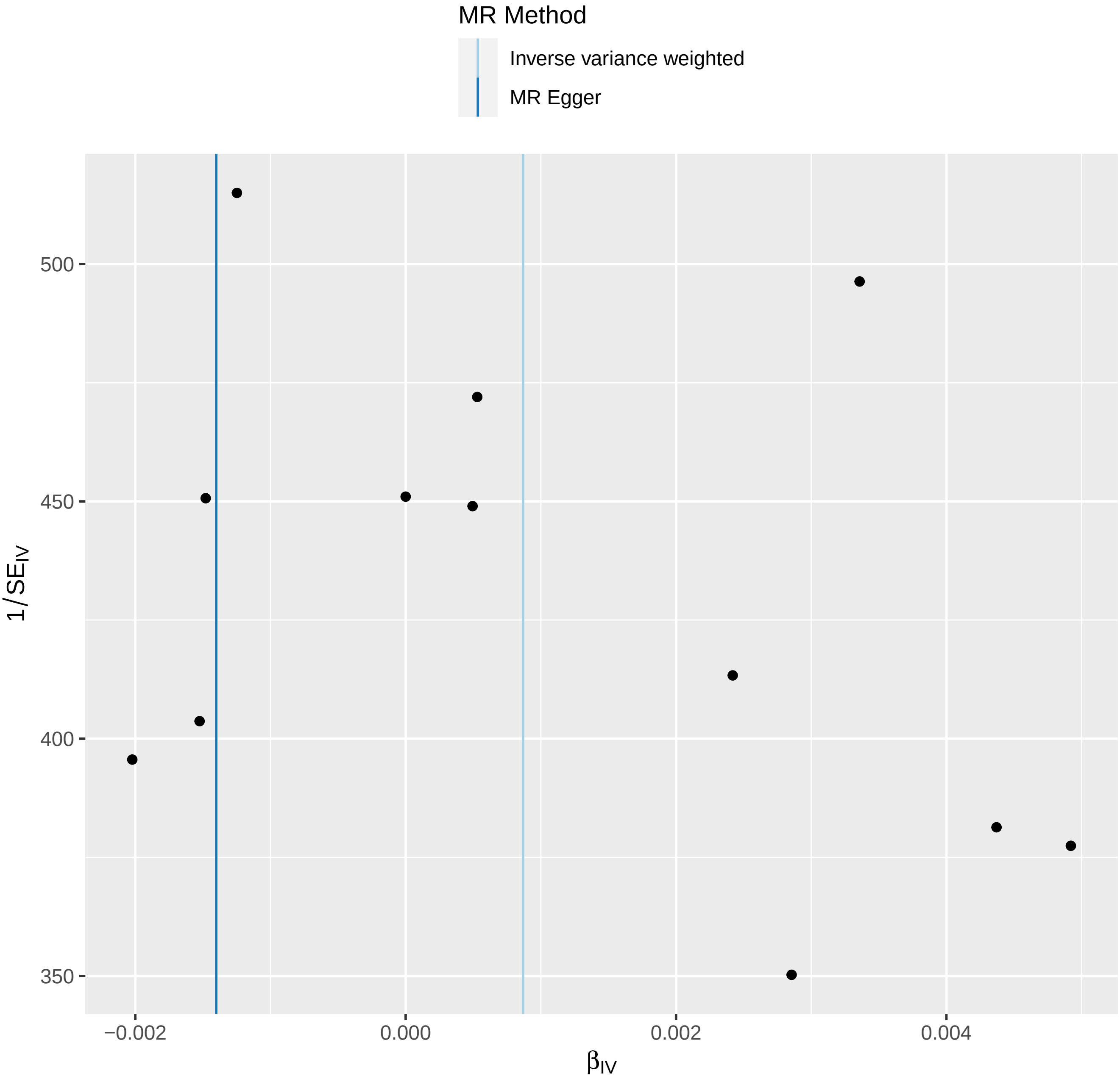

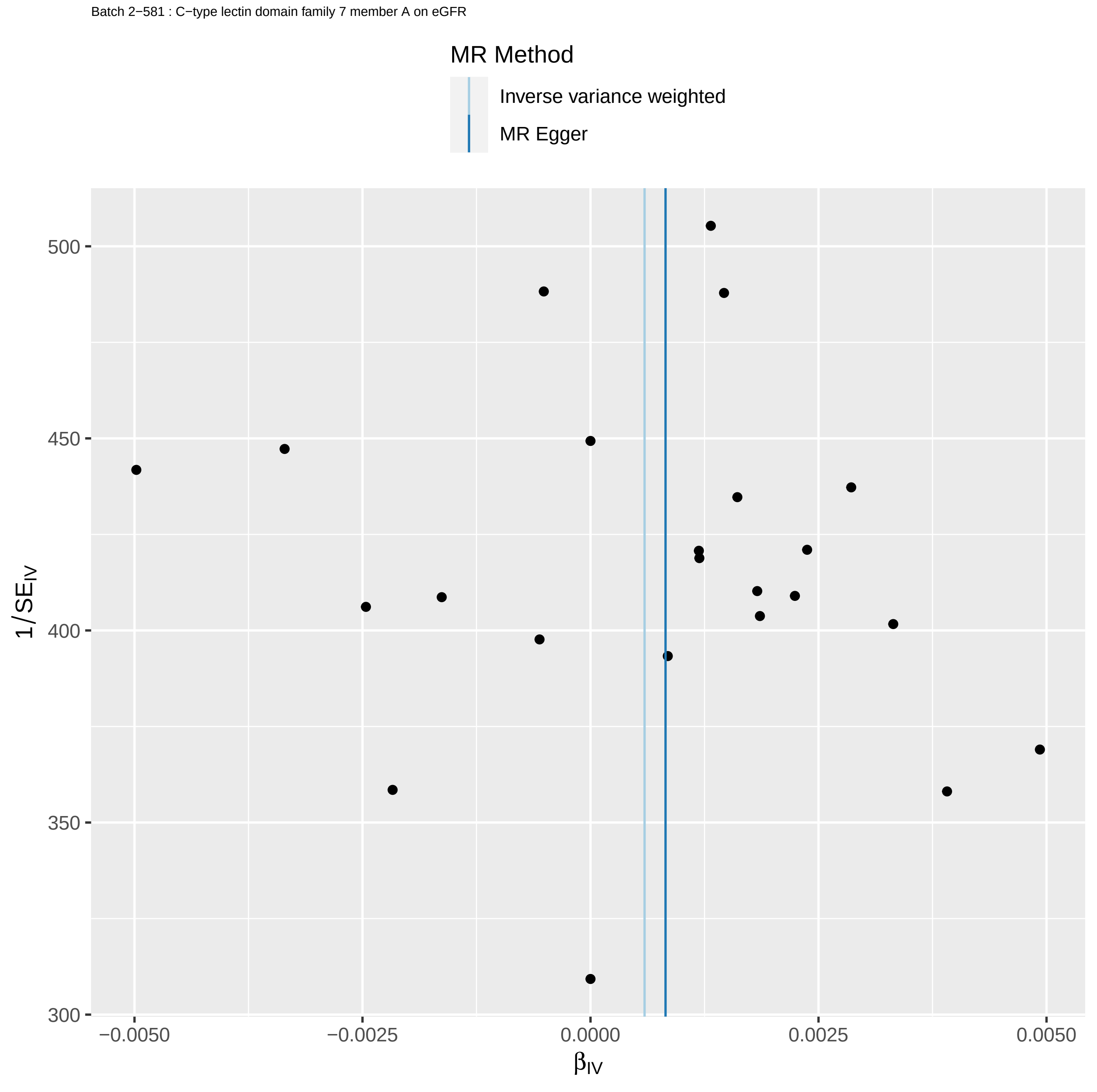
Batch 2−581 : C−type lectin domain family 7 member A on eGFR
MR Method
Inverse variance weighted
MR Egger
1/SE$_{IV}$
500
450
400
350
300
−0.0050
−0.0025
0.0000
0.0025
0.0050
β_{IV}

Batch 2−582 : C−type lectin−like domain family 1 on eGFR

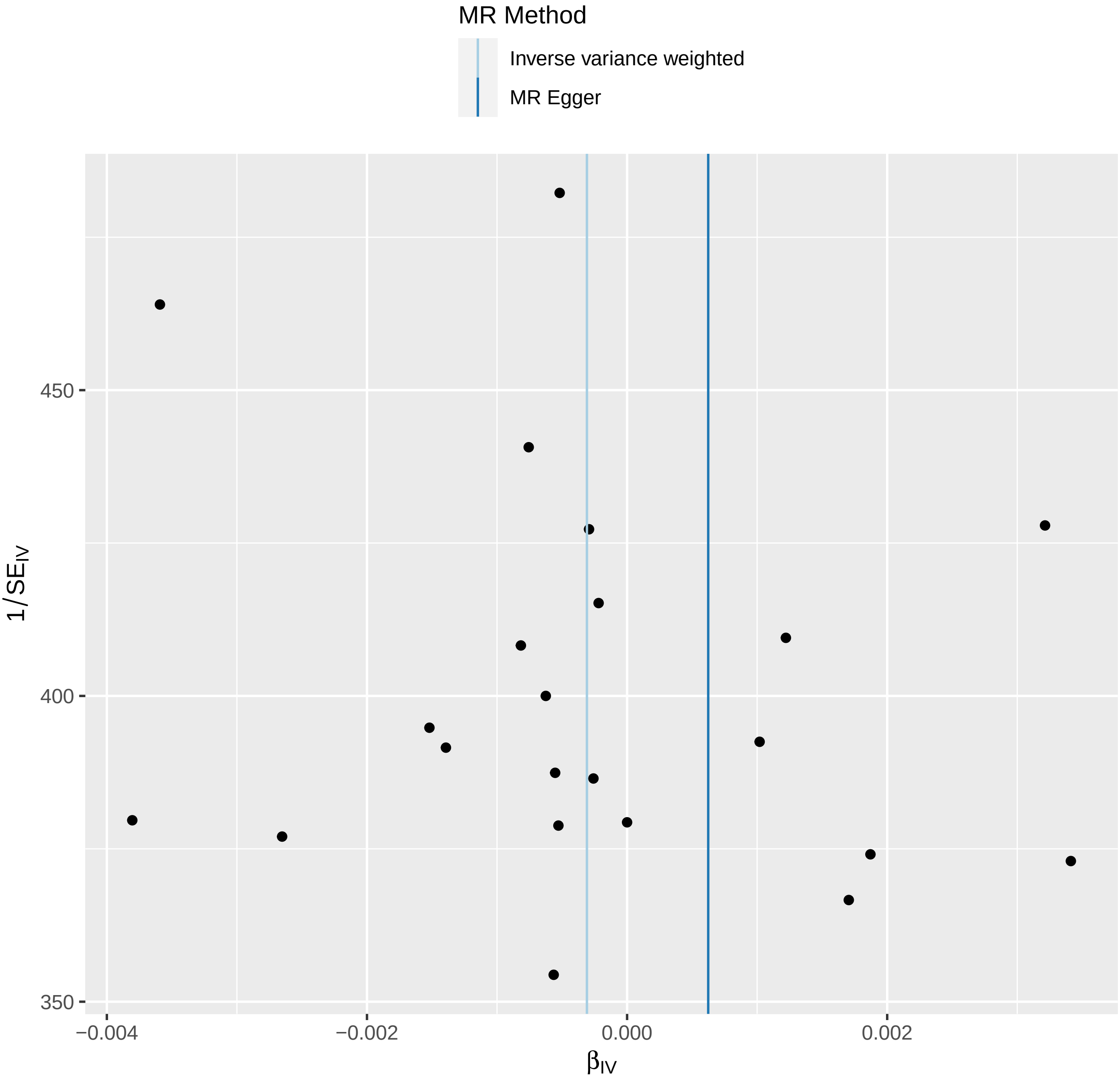

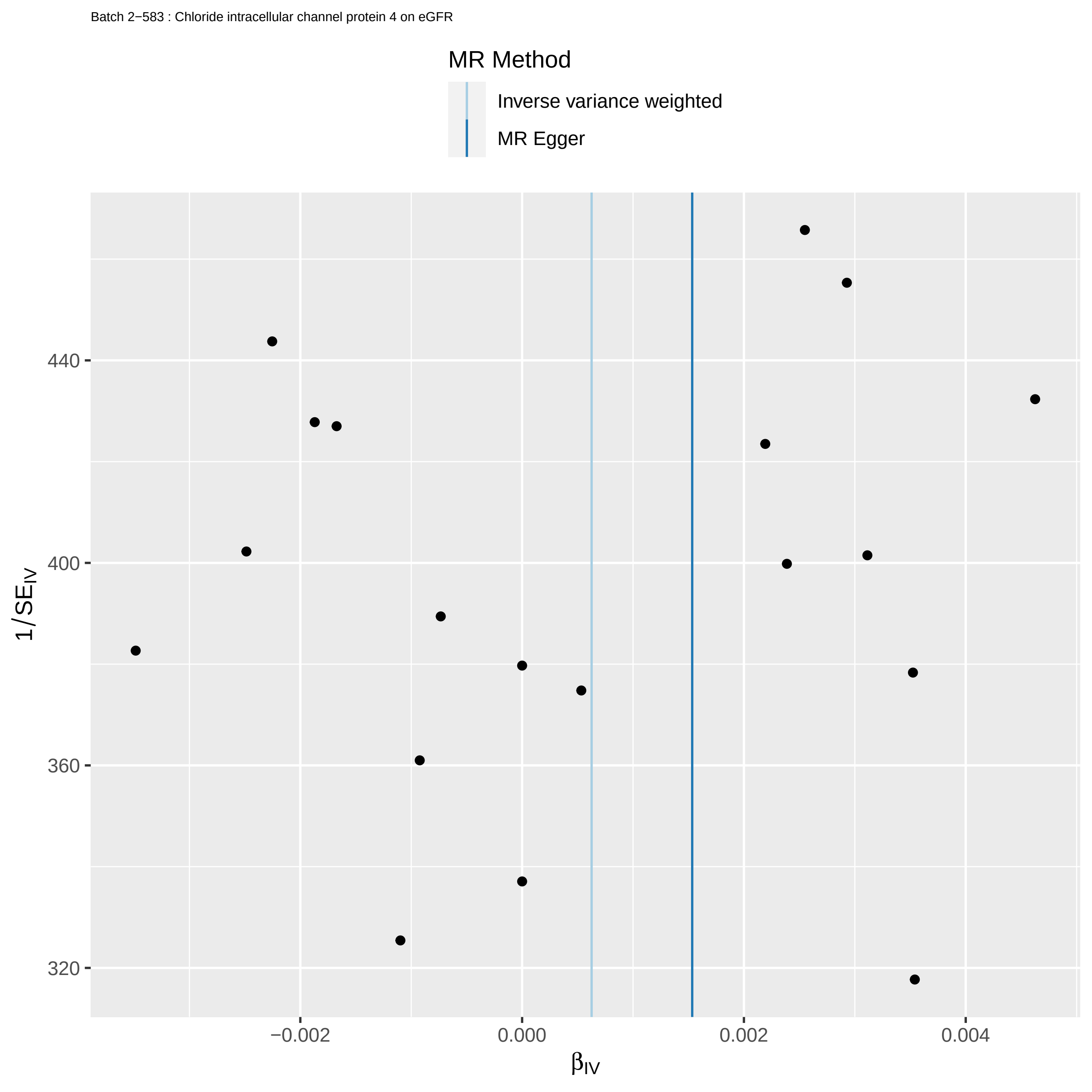

Batch 2−583 : Chloride intracellular channel protein 4 on eGFR
MR Method
Inverse variance weighted
MR Egger
440
400
360
320
1/SE_IV
−0.002
0.000
0.002
0.004
β_IV

Batch 2−584 : Chloride intracellular channel protein 5 on eGFR

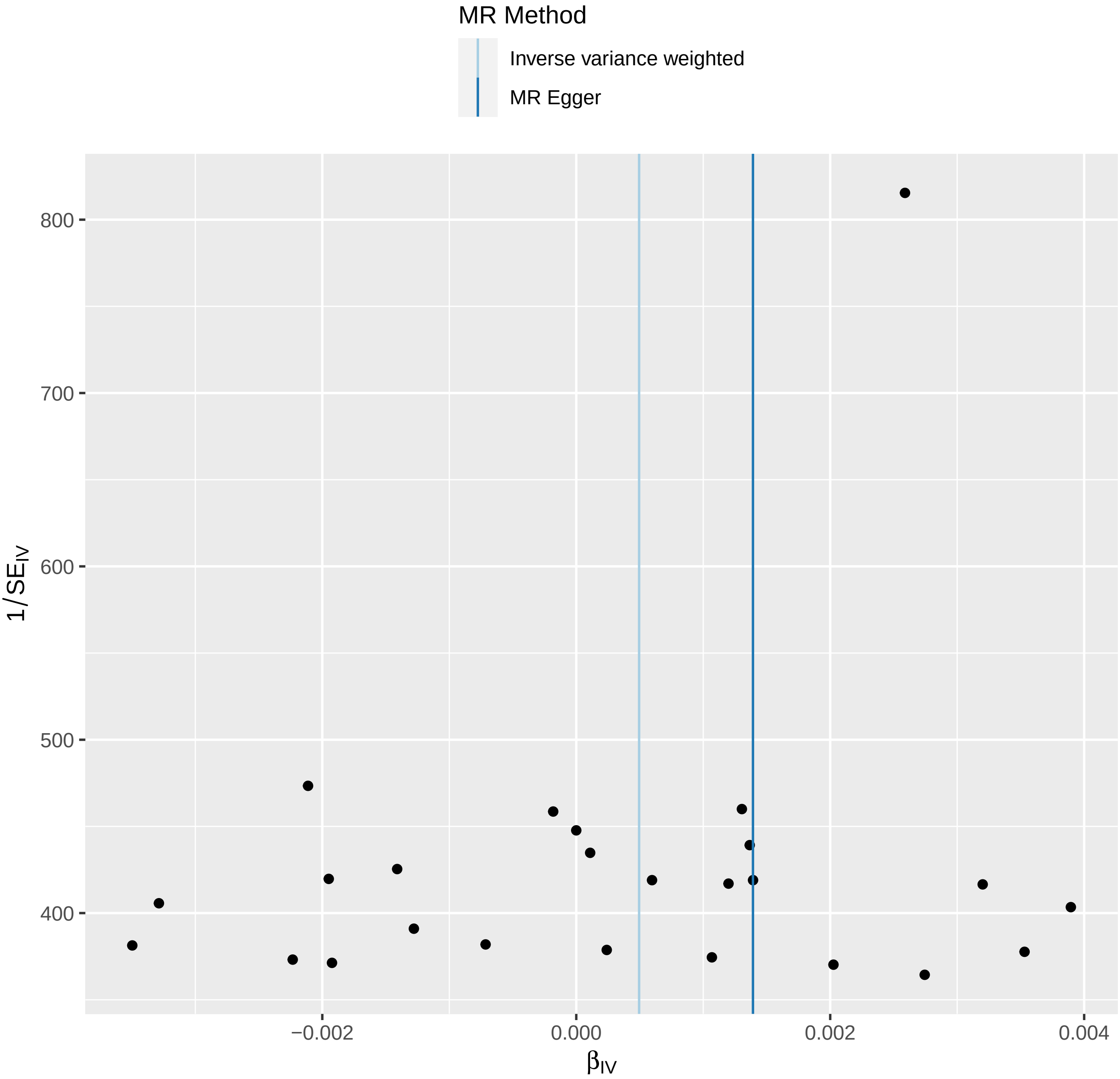

Batch 2−585 : Clathrin interactor 1 on eGFR

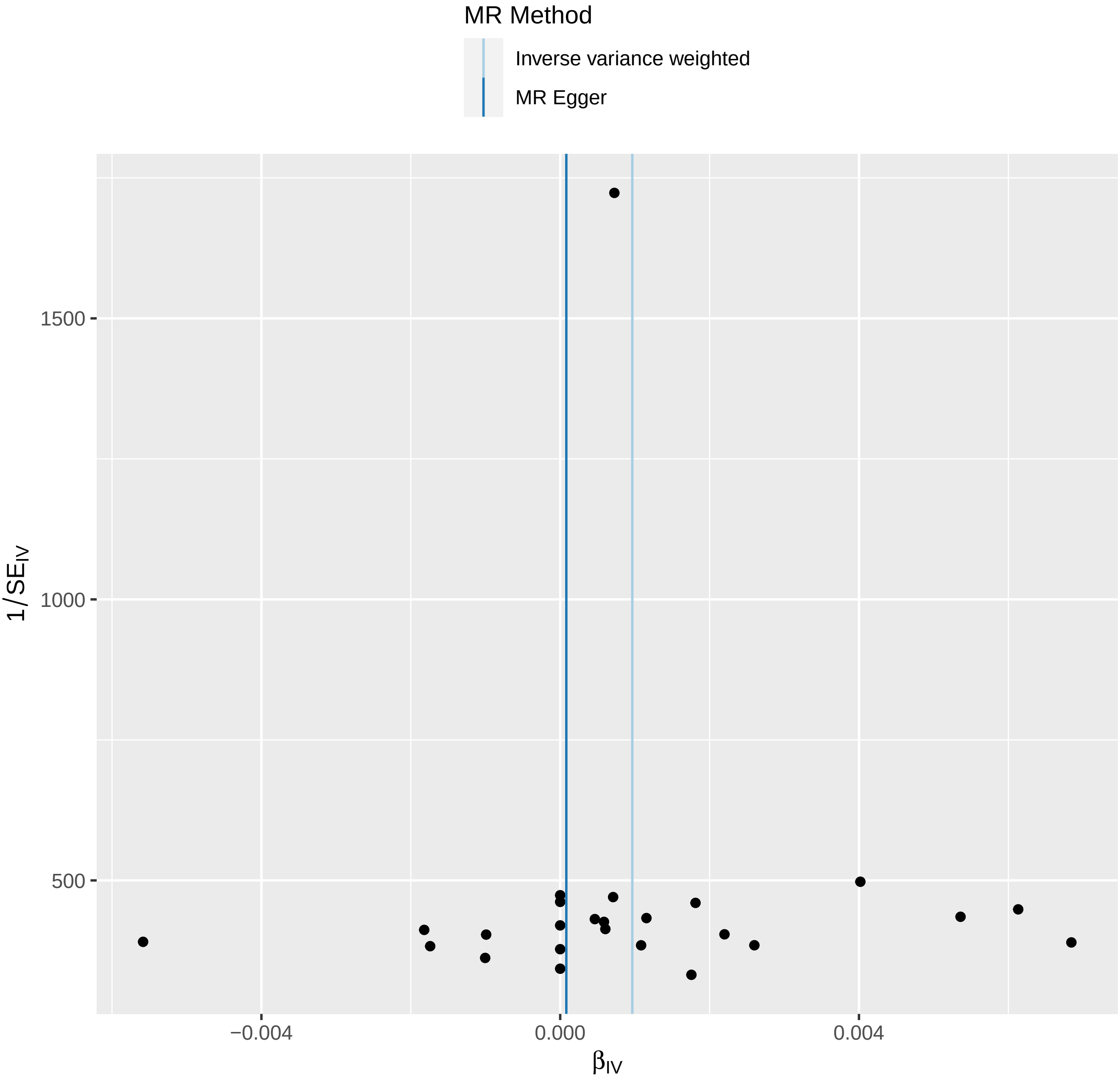

Batch 2−586 : Dual specificity protein kinase CLK2 on eGFR

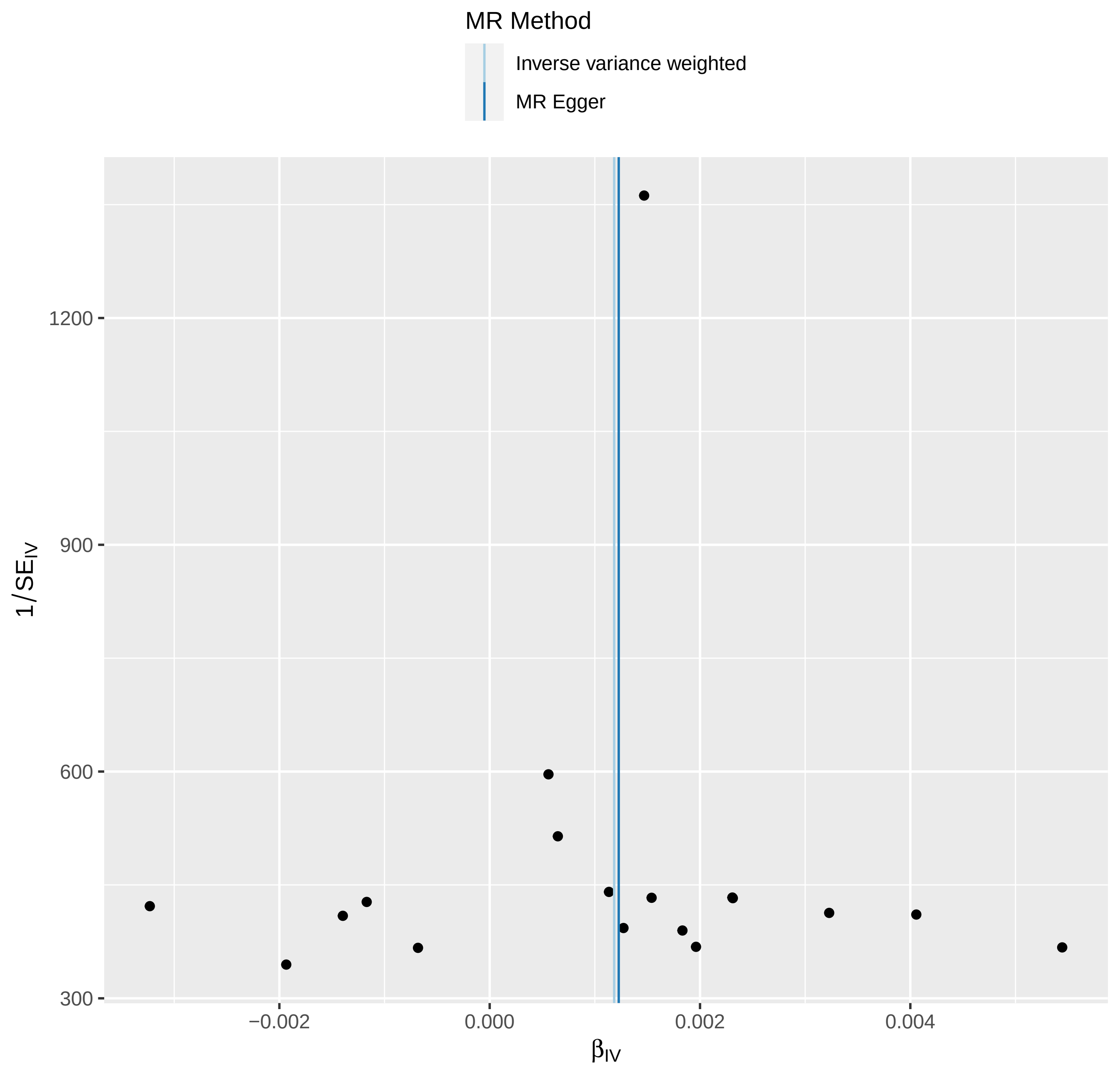

Batch 2−587 : CXADR−like membrane protein on eGFR

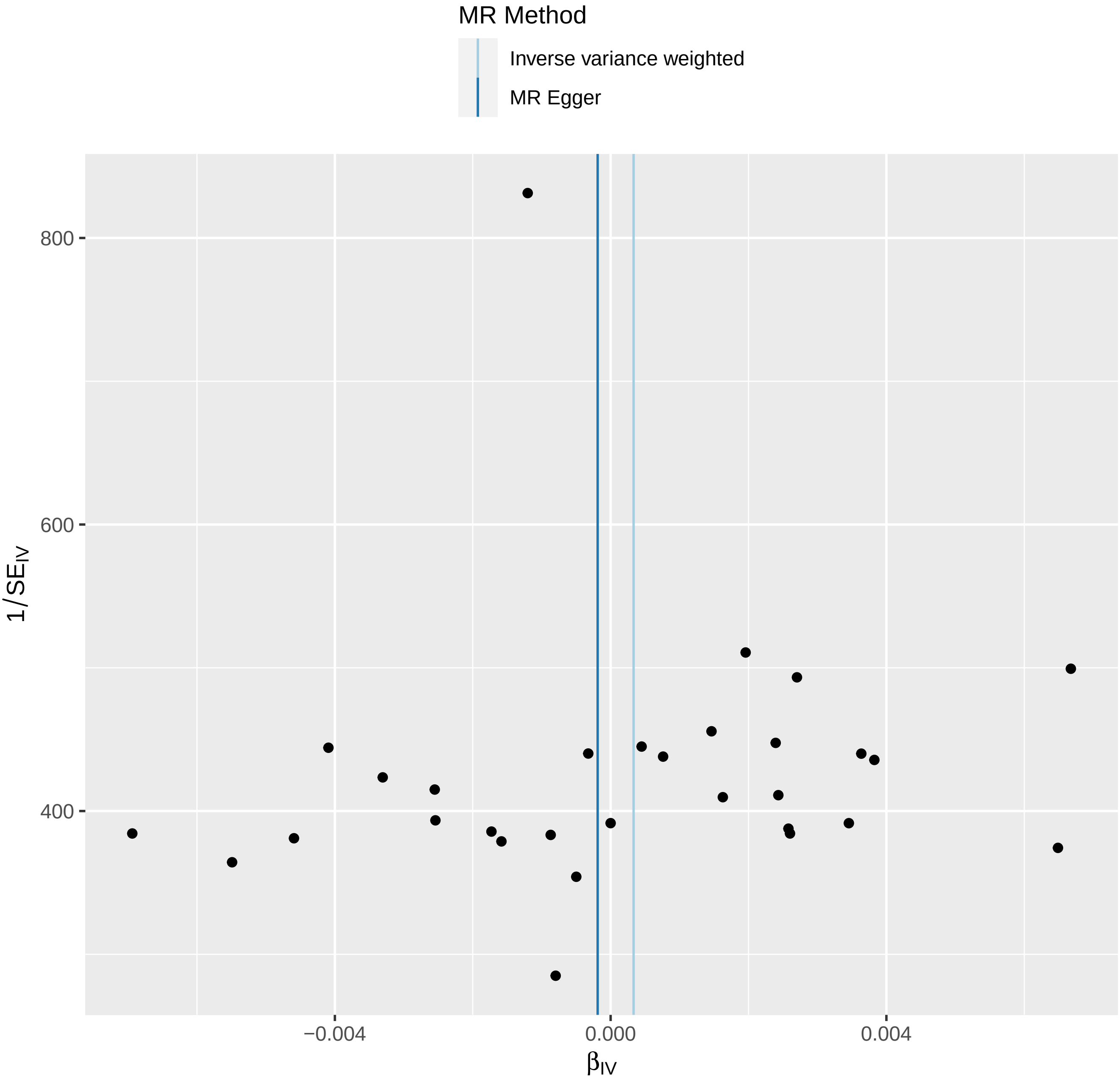

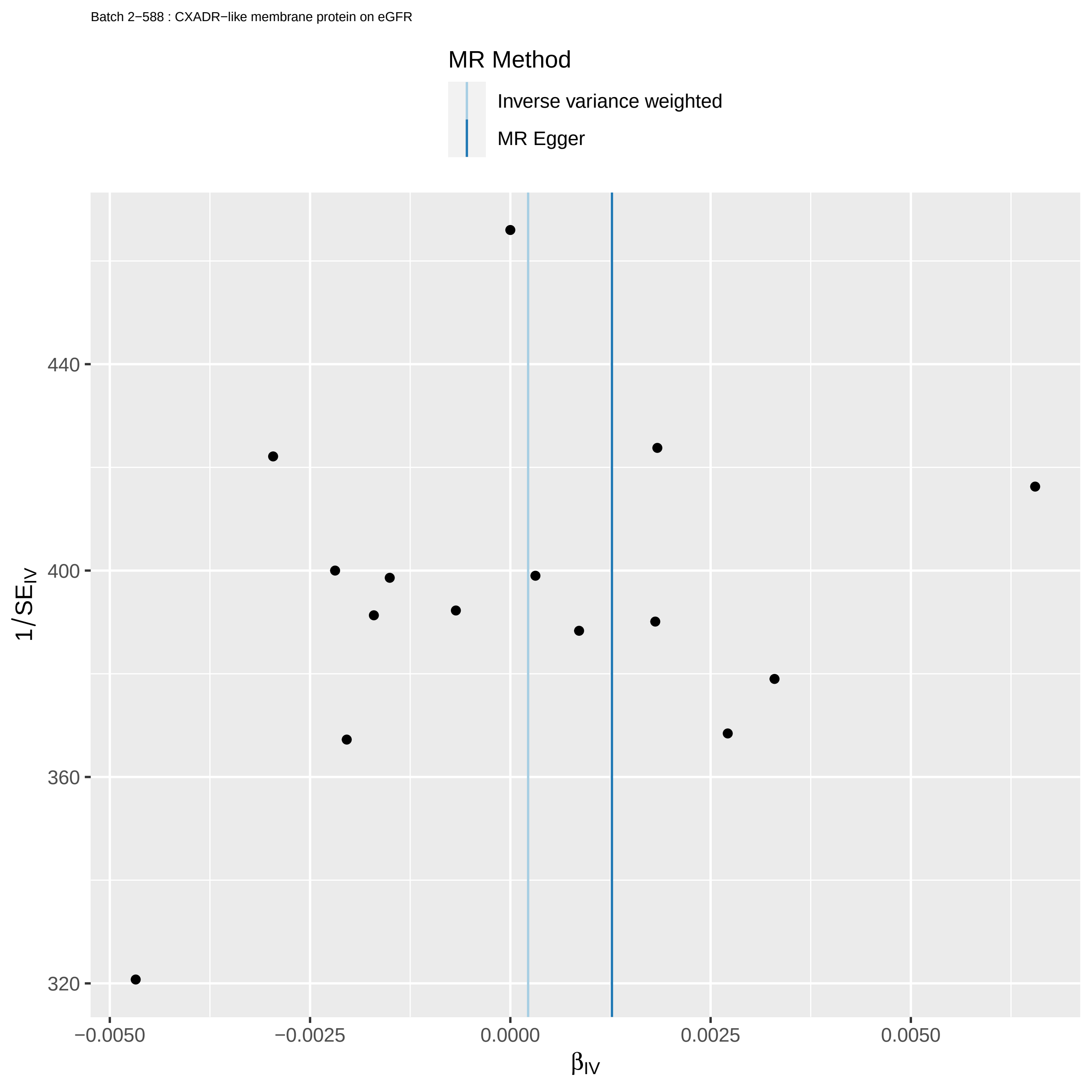

Batch 2−588 : CXADR−like membrane protein on eGFR
MR Method
Inverse variance weighted
MR Egger
440
400
360
320
−0.0050
−0.0025
0.0000
0.0025
0.0050
$1/SE_{IV}$
β_{IV}

Batch 2−589 : Ceroid−lipofuscinosis neuronal protein 5 on eGFR

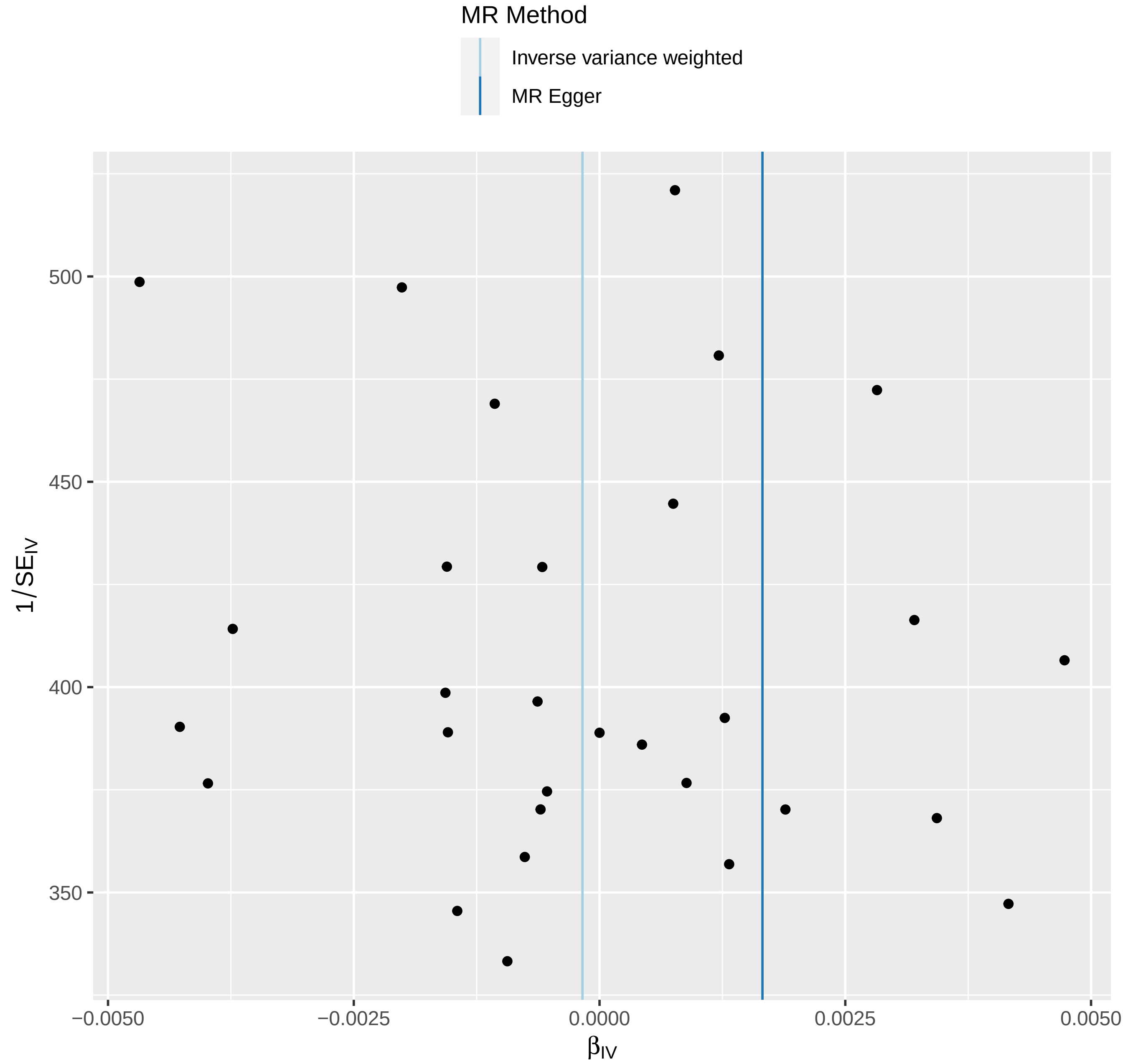

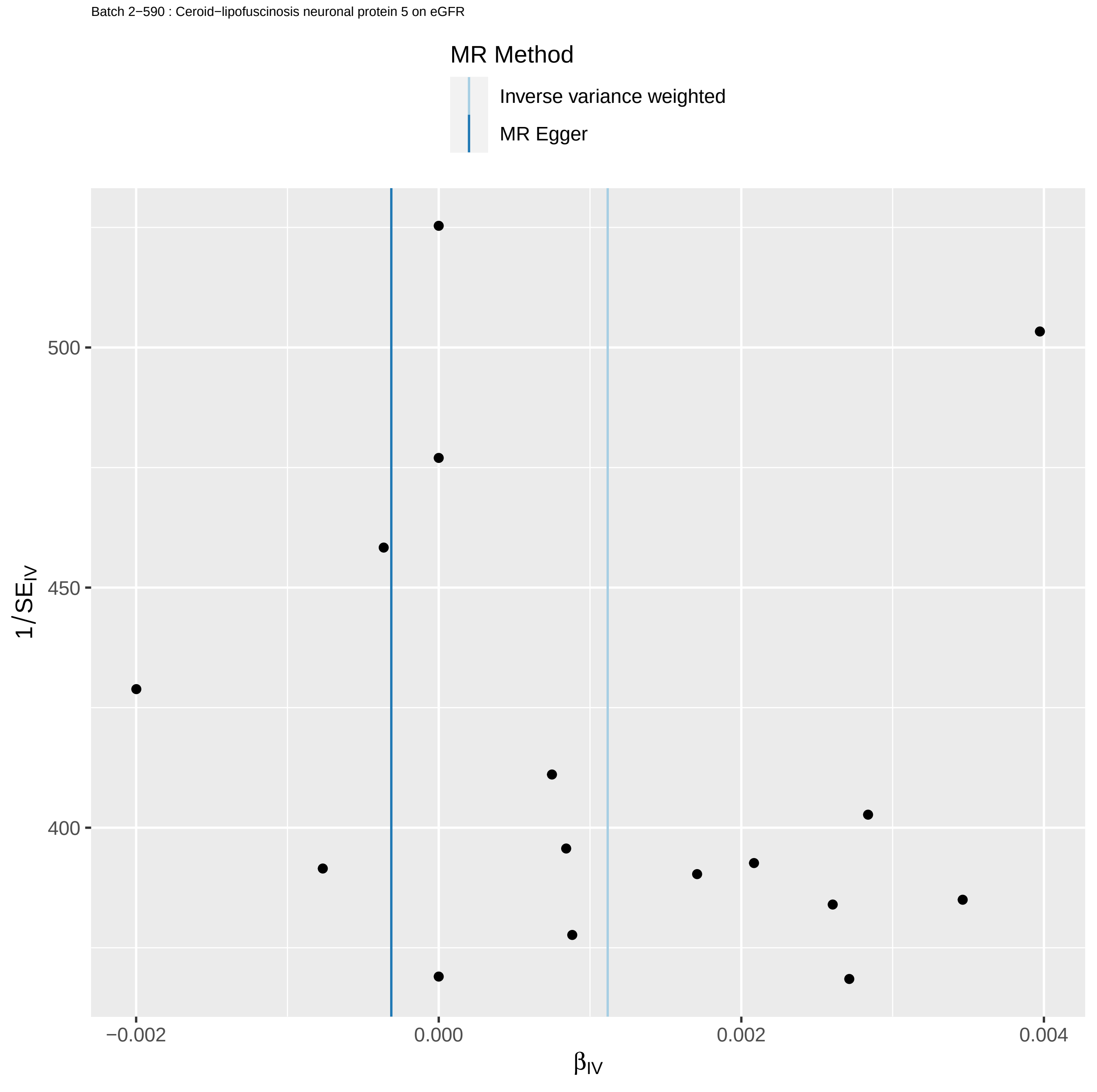
Batch 2−590 : Ceroid−lipofuscinosis neuronal protein 5 on eGFR
MR Method
Inverse variance weighted
MR Egger
500
450
400
1/SE$_{IV}$
−0.002
0.000
0.002
0.004
β_{IV}

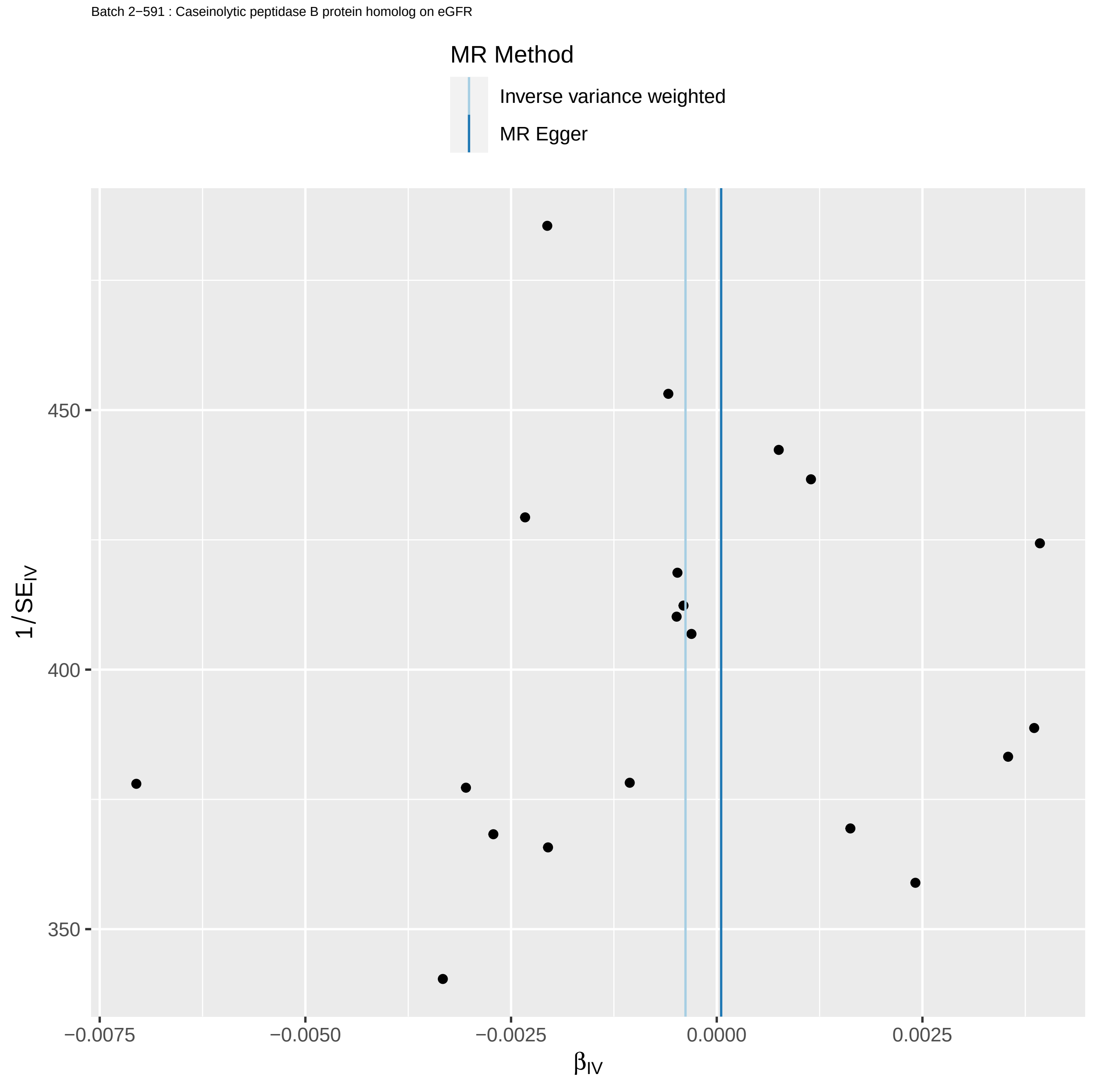

Batch 2−591 : Caseinolytic peptidase B protein homolog on eGFR
MR Method
Inverse variance weighted
MR Egger
450
400
350
1/SE_IV
−0.0075
−0.0050
−0.0025
0.0000
0.0025
β_IV

Batch 2−592 : Colipase on eGFR

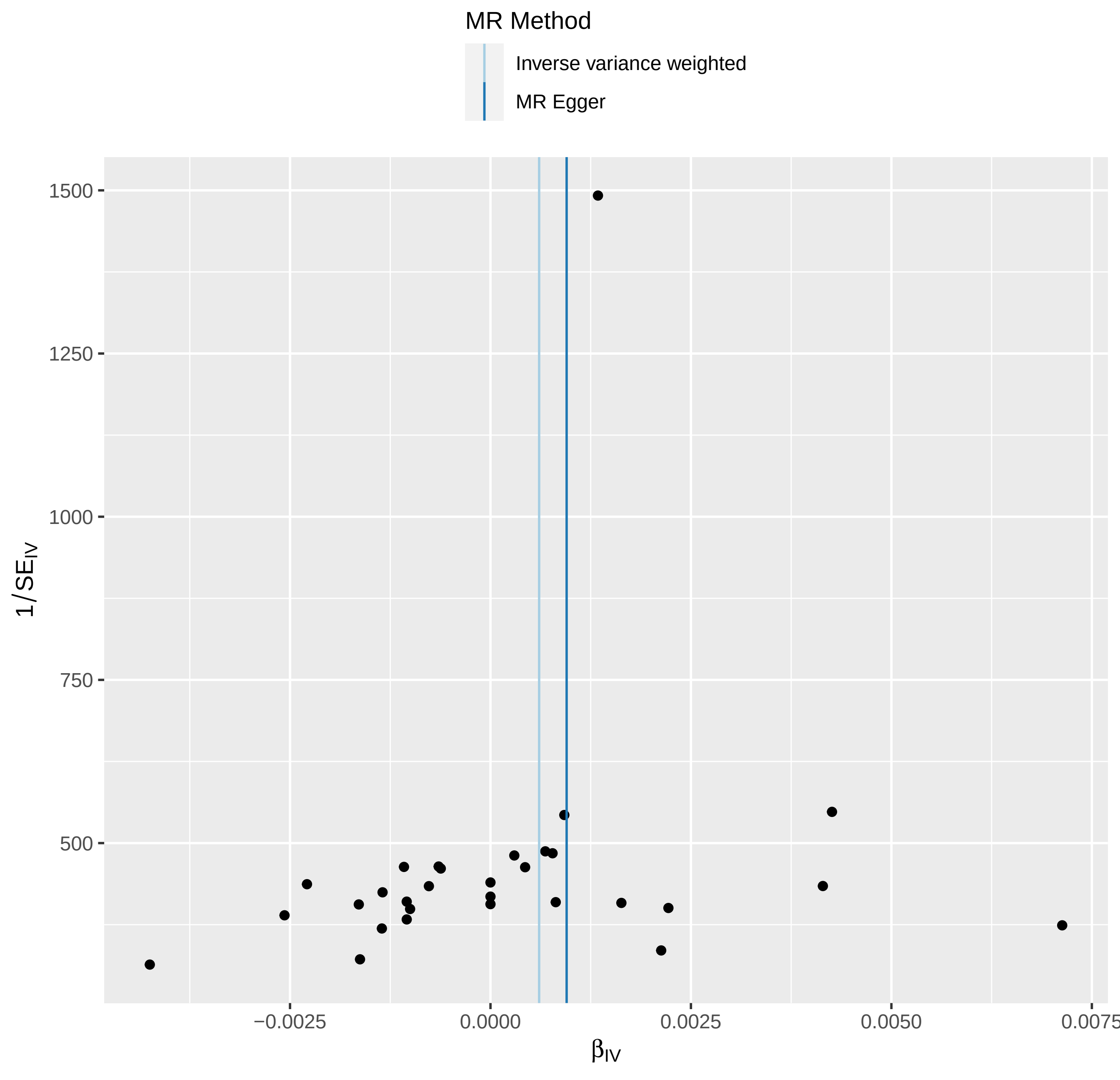

Batch 2−593 : Colipase−like protein 2 on eGFR

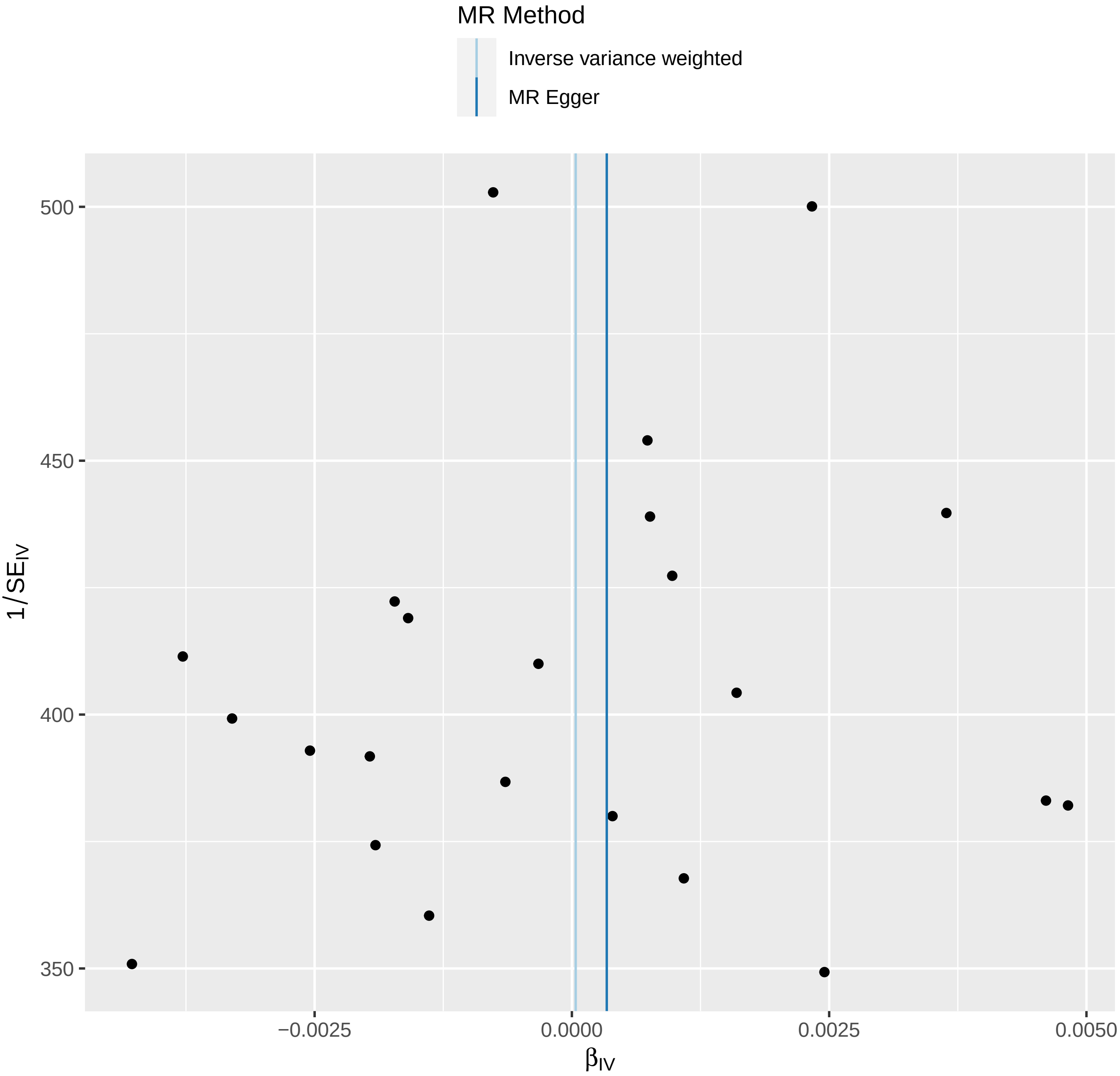

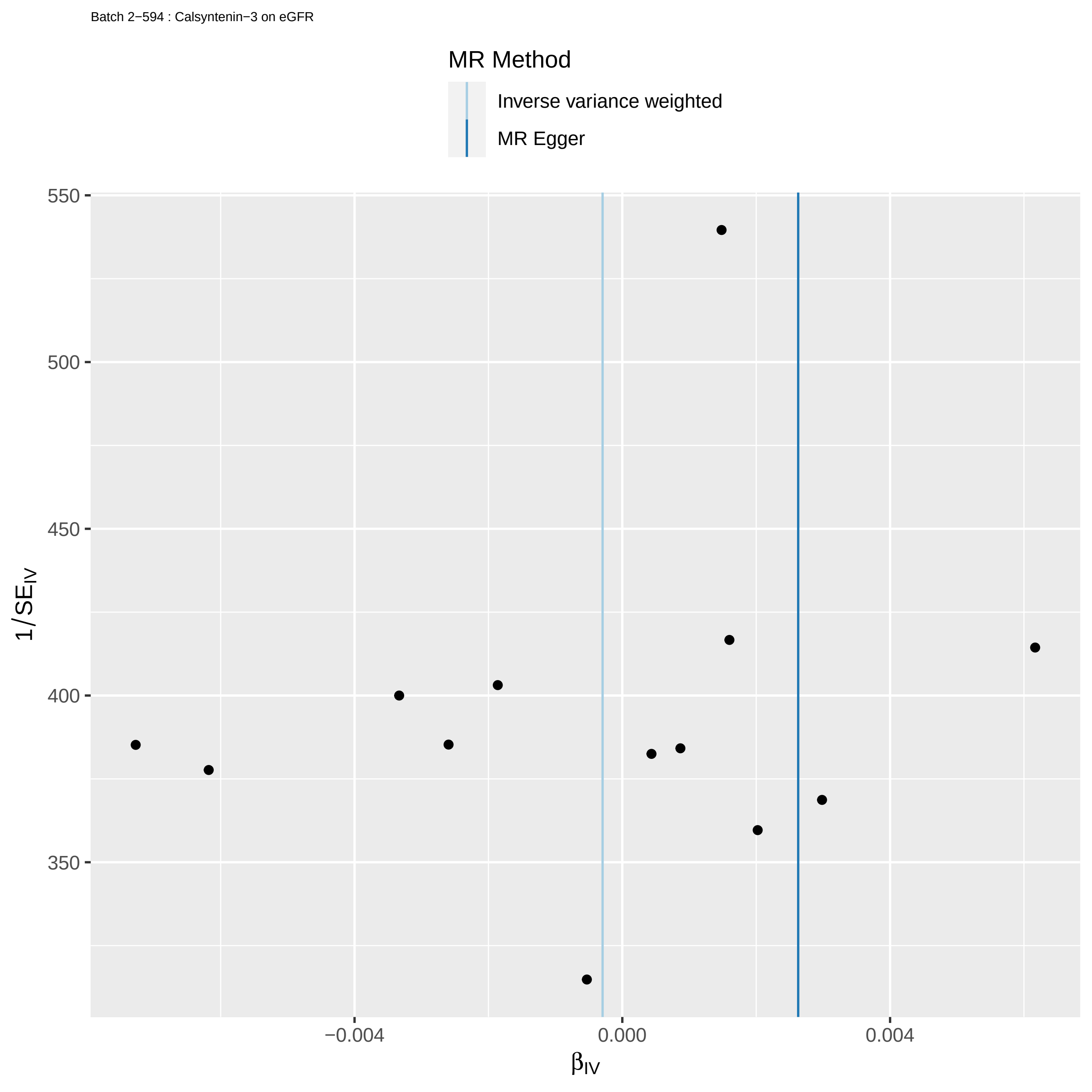

Batch 2−594 : Calsyntenin−3 on eGFR
MR Method
Inverse variance weighted
MR Egger
550
500
450
400
350
1/SE$_{IV}$
−0.004
0.000
0.004
β$_{IV}$

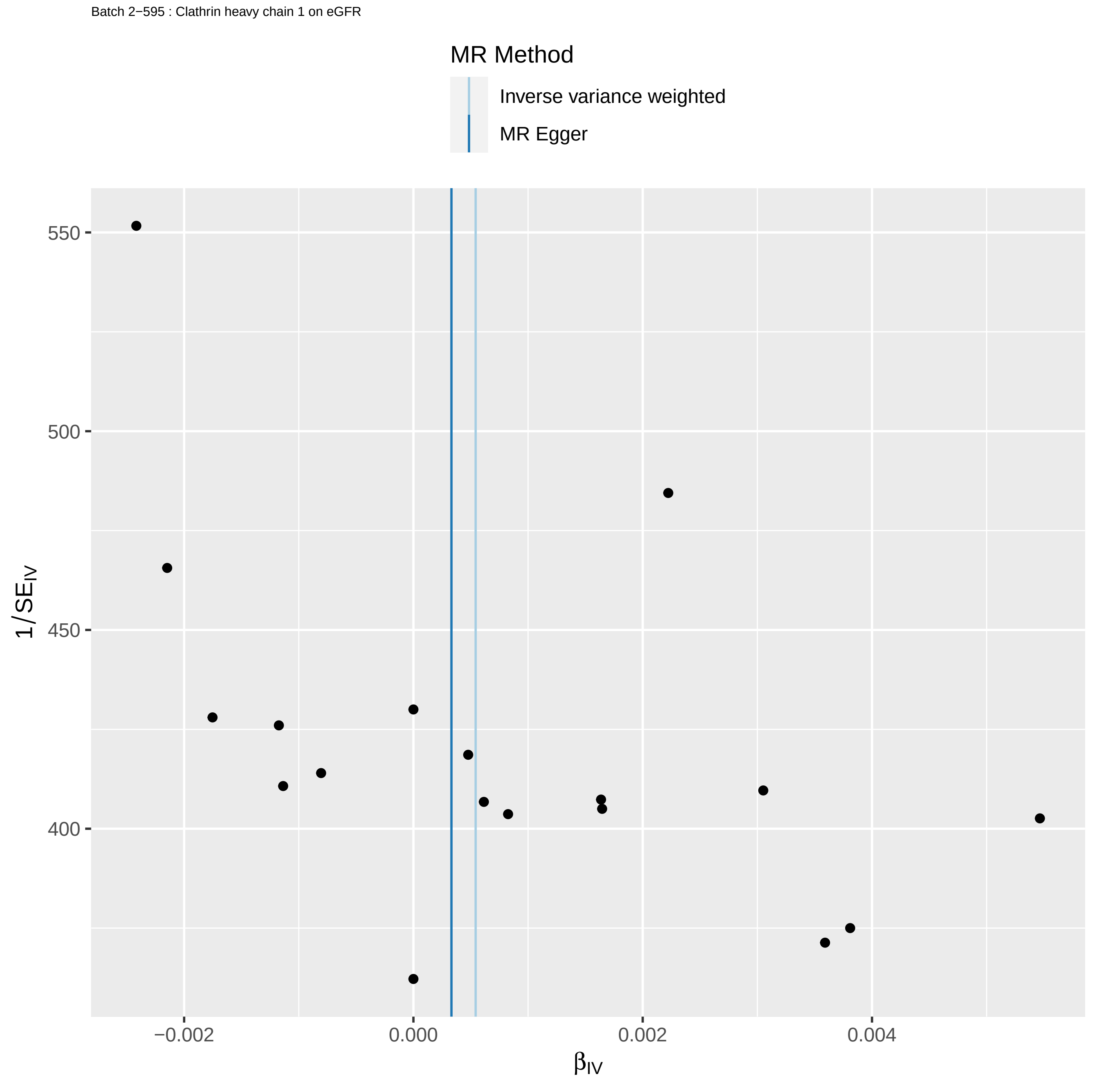
Batch 2−595 : Clathrin heavy chain 1 on eGFR
MR Method
Inverse variance weighted
MR Egger
550
500
450
400
1/SE_IV
−0.002
0.000
0.002
0.004
β_IV

Batch 2−596 : Clusterin on eGFR

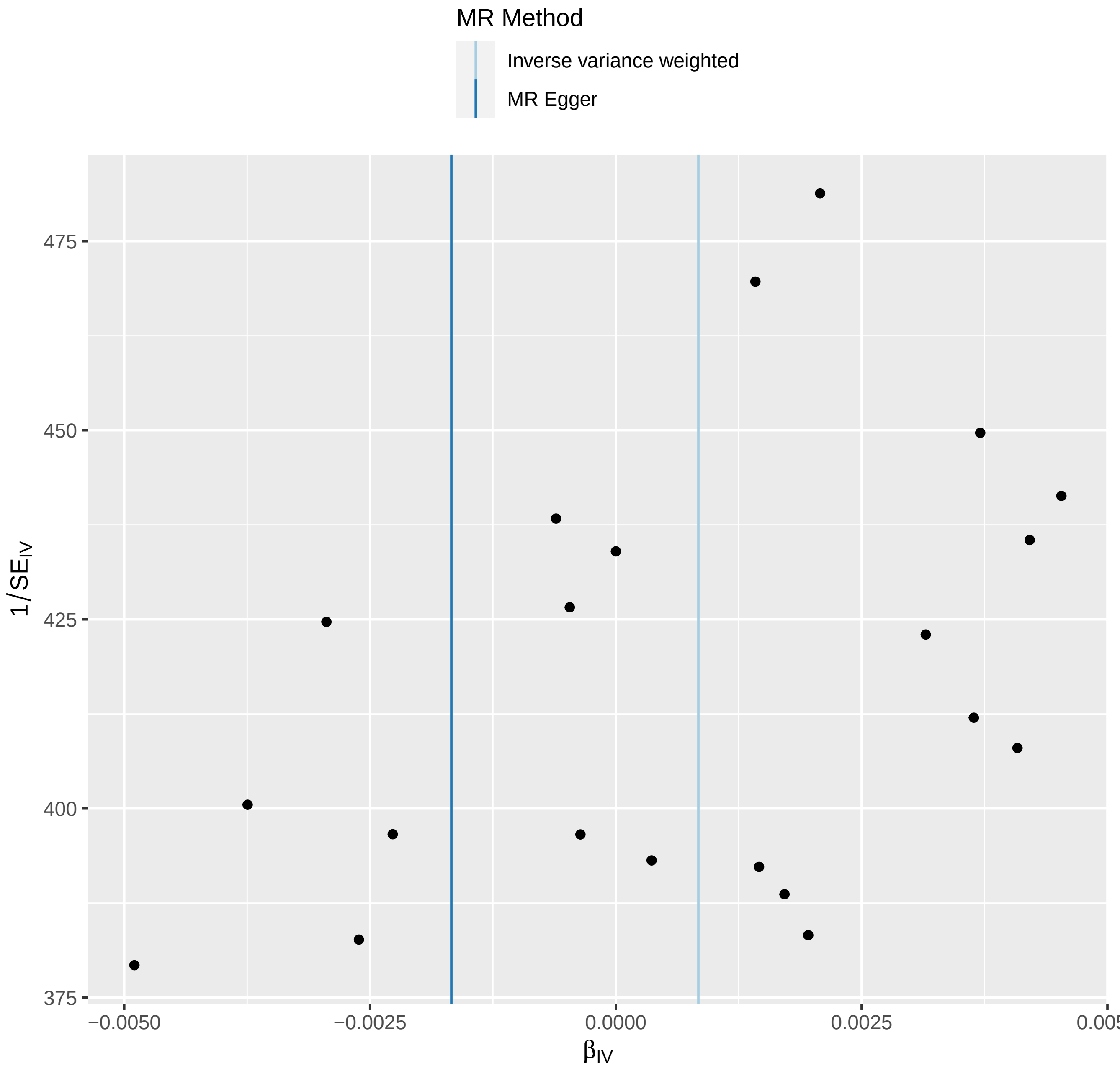

Batch 2−597 : Chymase on eGFR

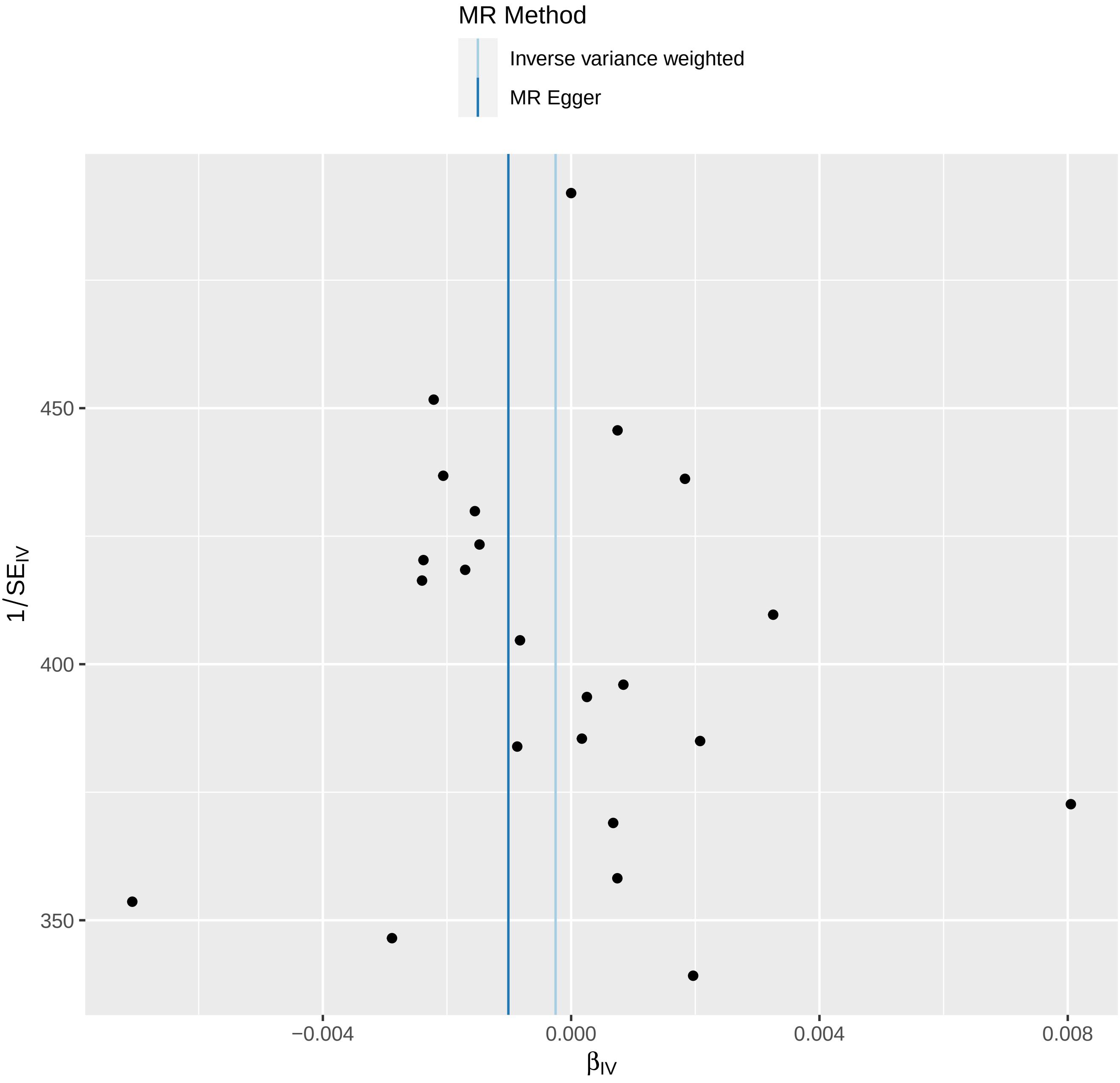

Batch 2−598 : UMP−CMP kinase on eGFR

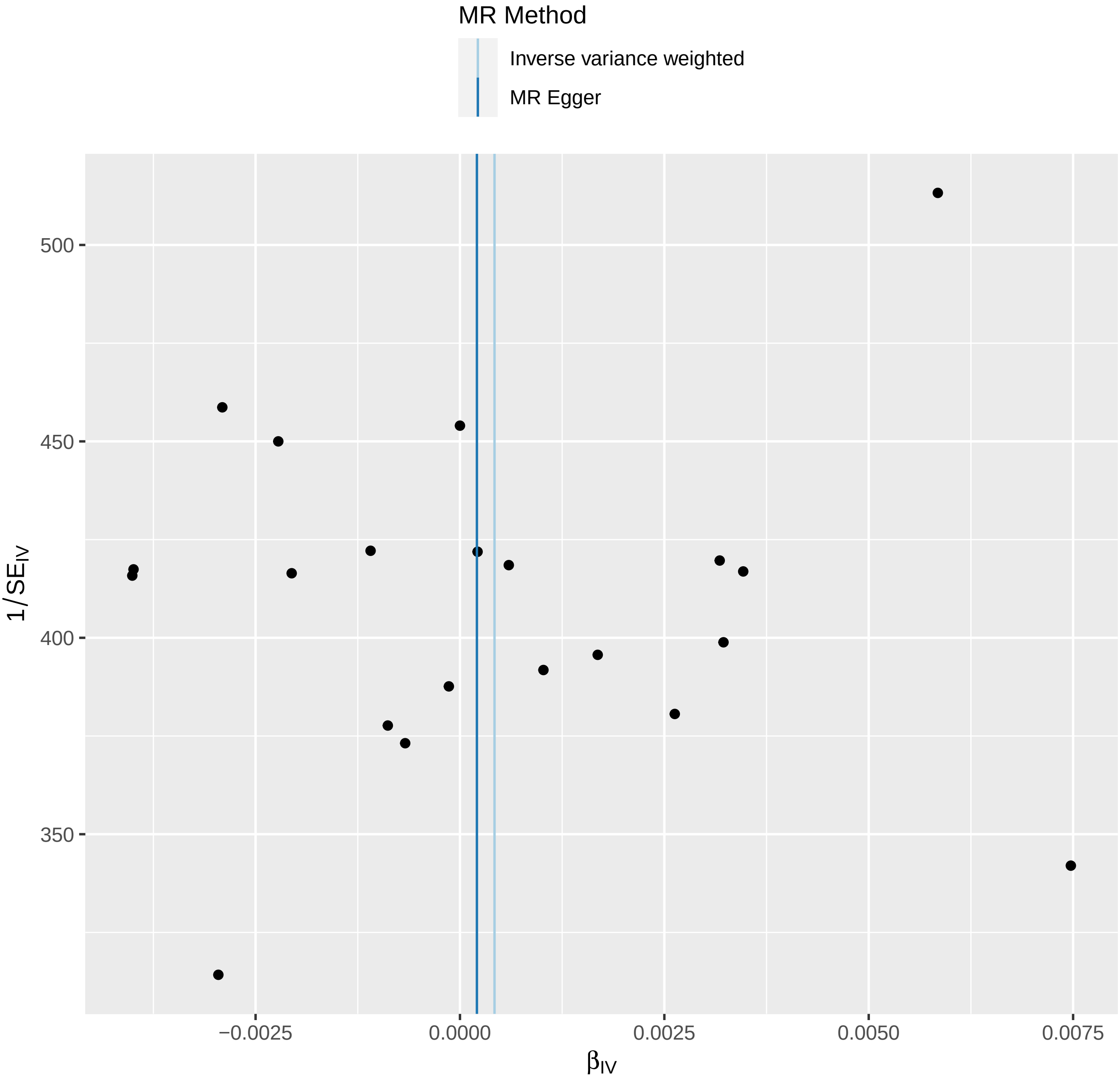

Batch 2−599 : UMP−CMP kinase on eGFR

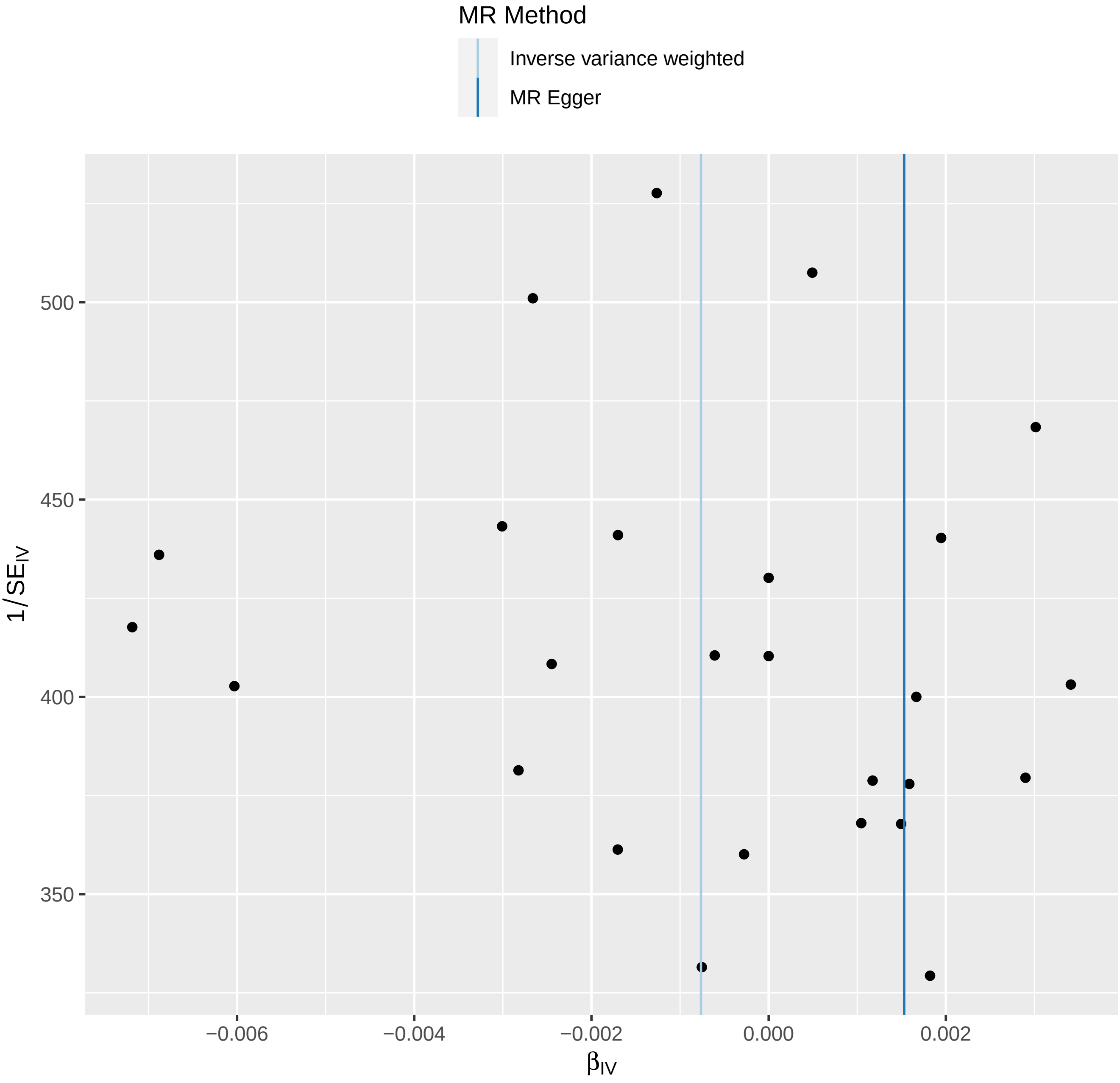

Batch 2−600 : Cytosolic non−specific dipeptidase on eGFR

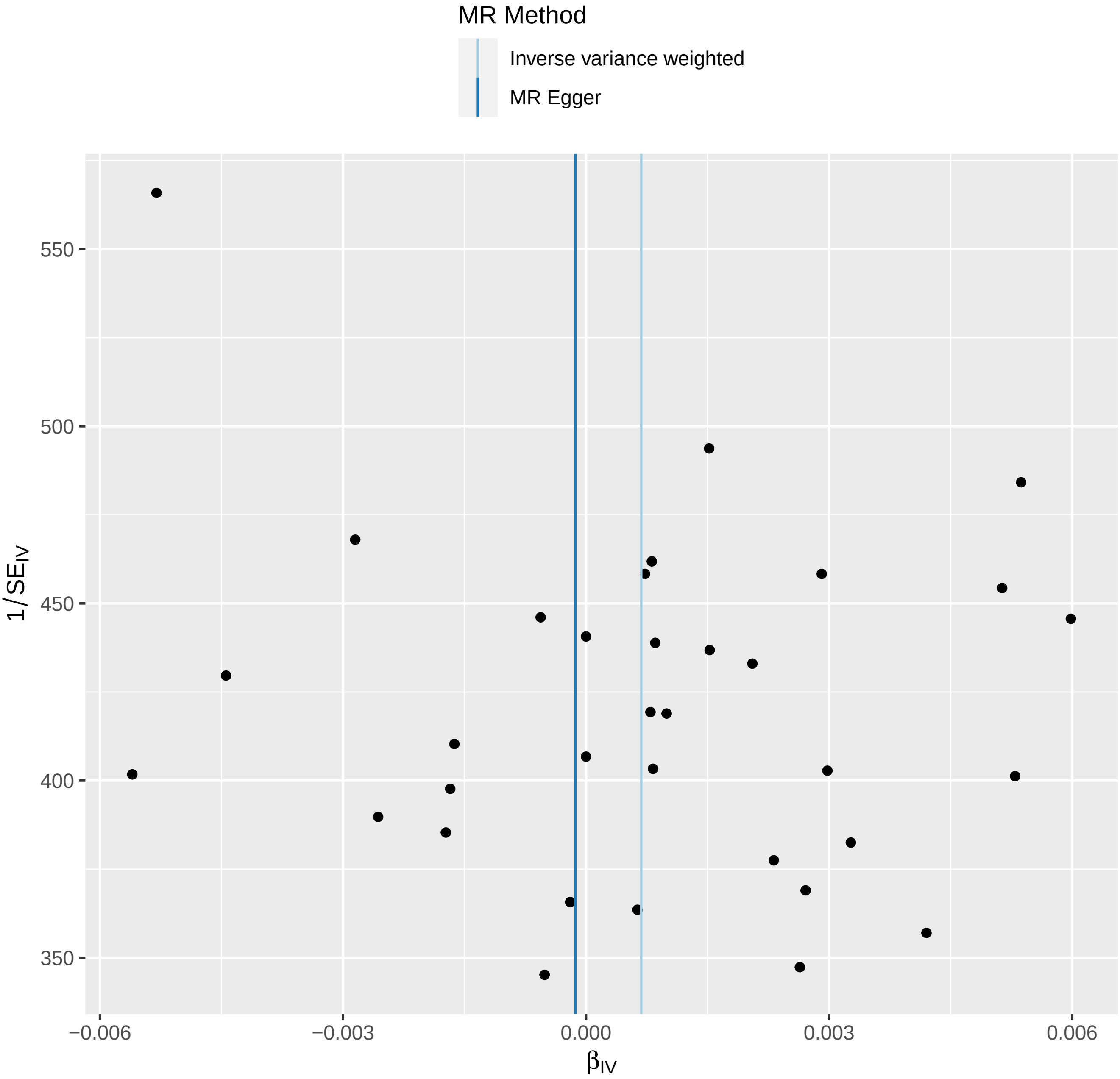

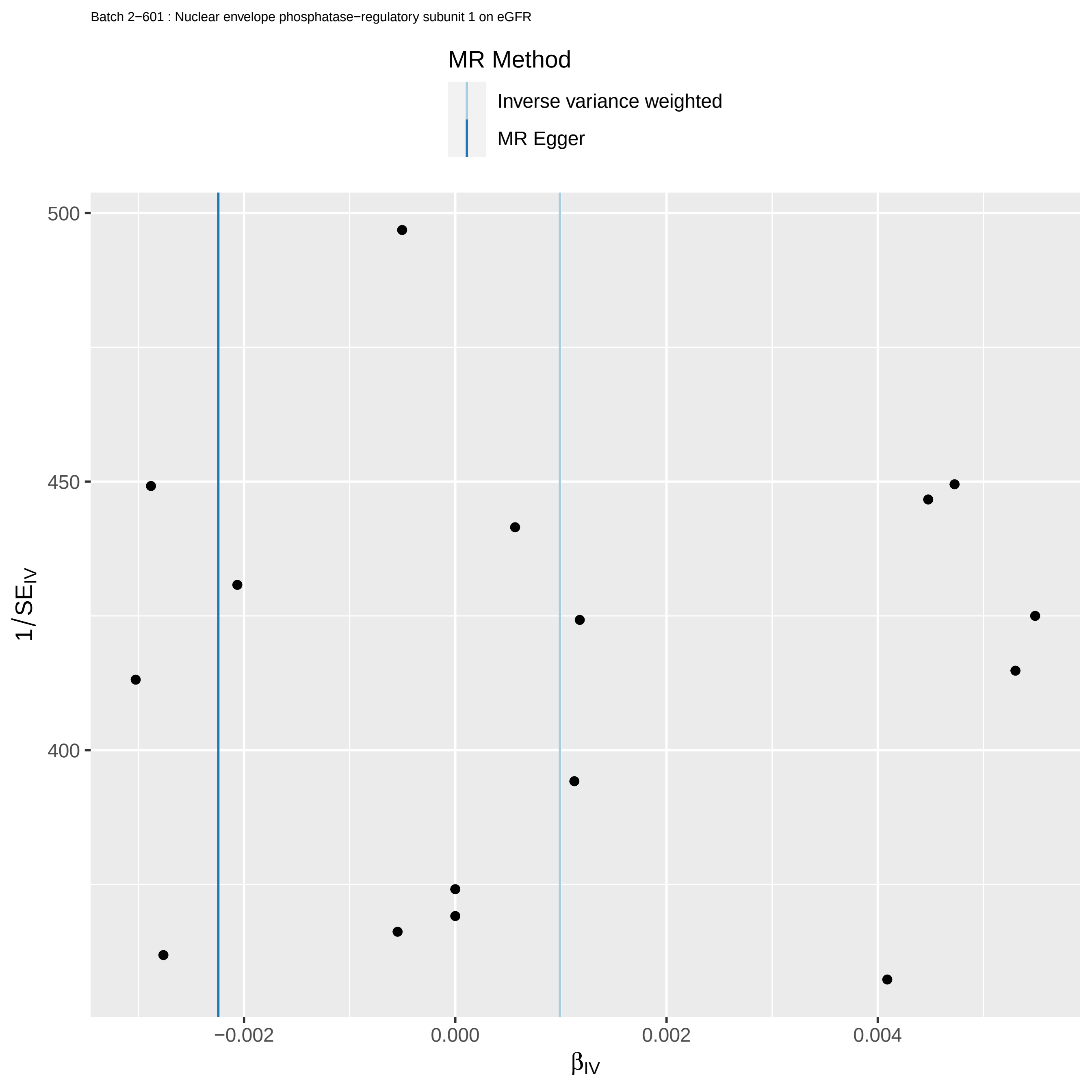
Batch 2−601 : Nuclear envelope phosphatase−regulatory subunit 1 on eGFR
MR Method
Inverse variance weighted
MR Egger
500
450
400
1/SE$_{IV}$
−0.002
0.000
0.002
0.004
β$_{IV}$

Batch 2−602 : CCR4−NOT transcription complex subunit 1 on eGFR

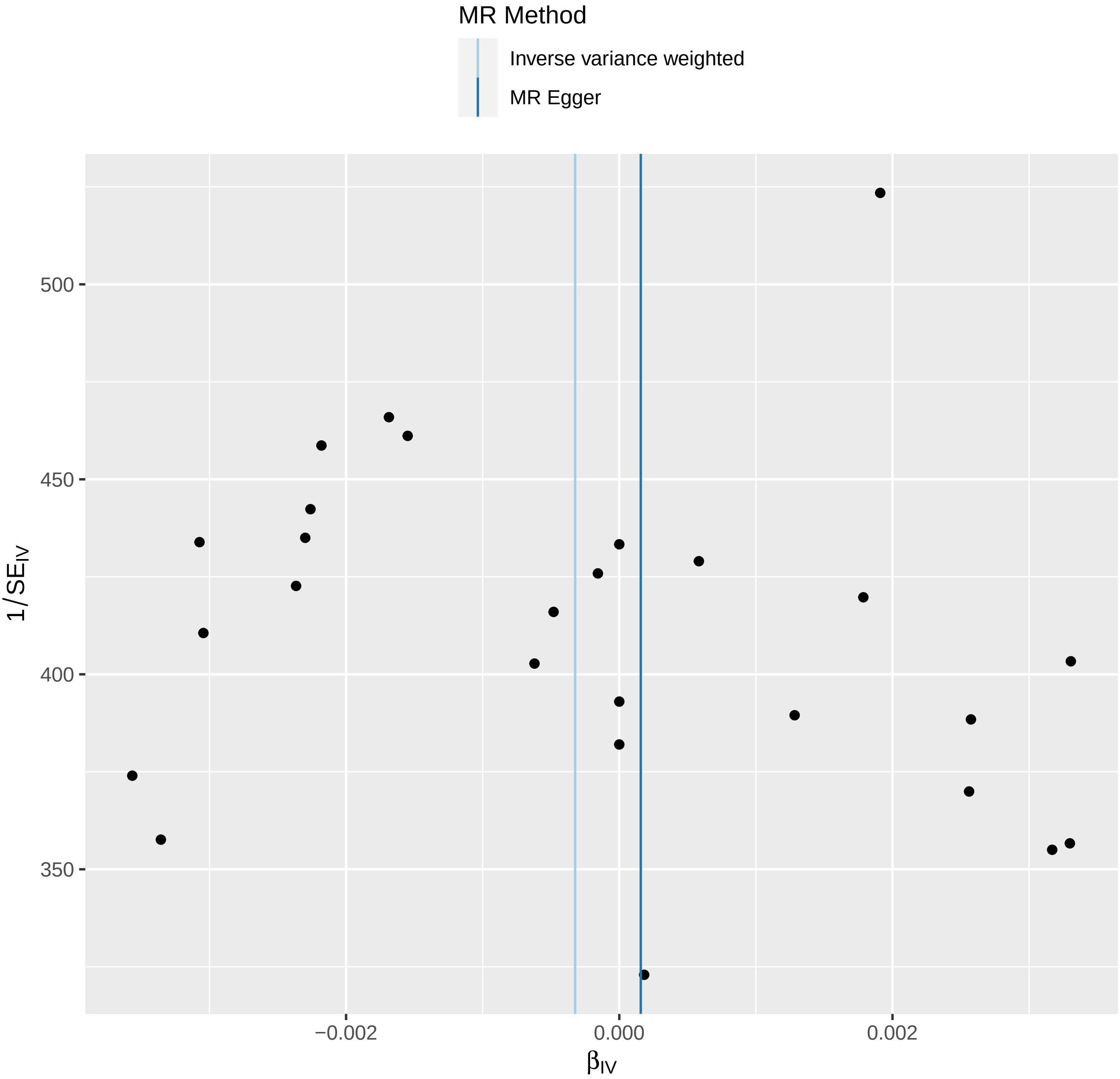

Batch 2−603 : 2',3'−cyclic−nucleotide 3'−phosphodiesterase on eGFR

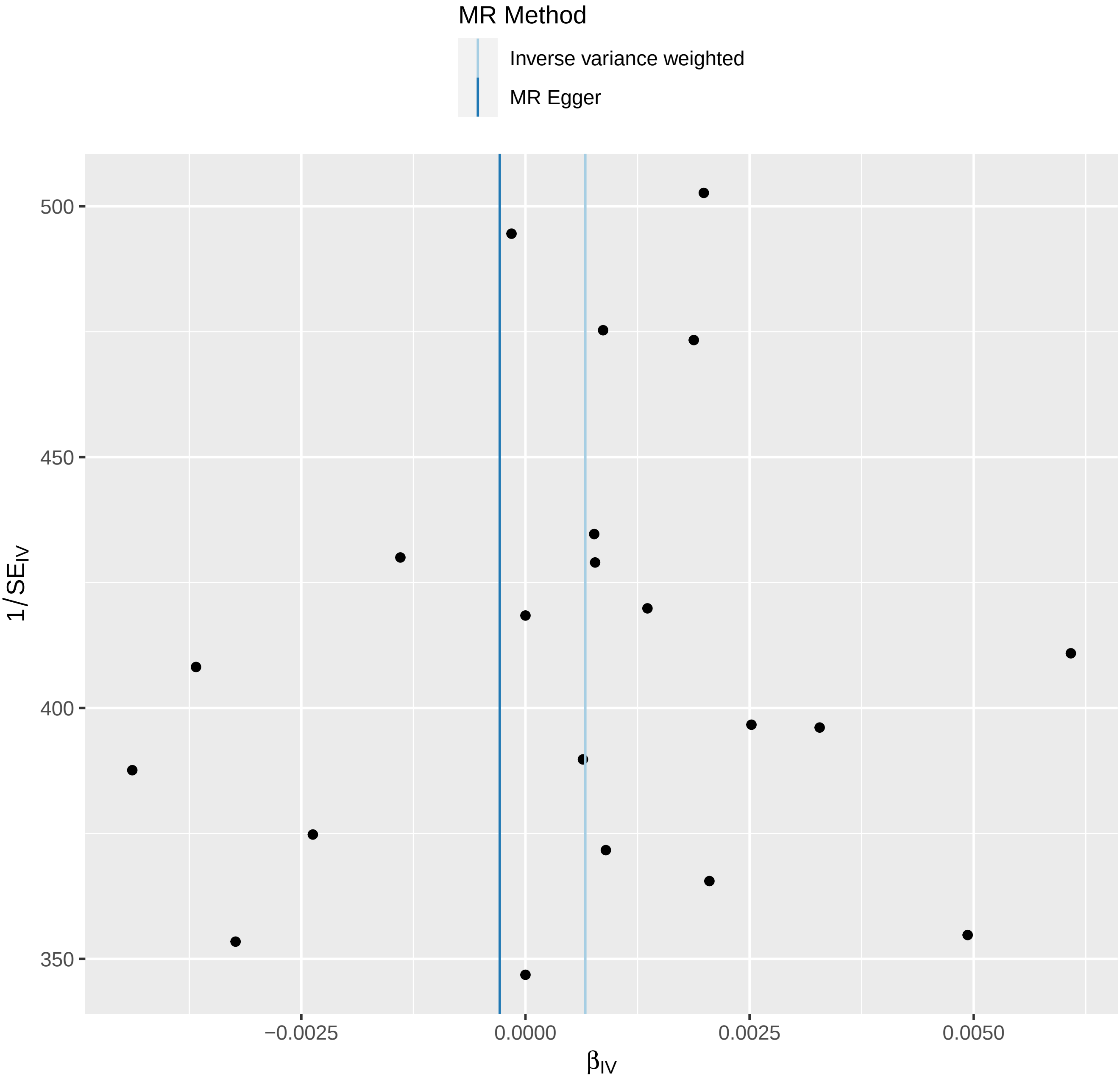

Batch 2−604 : Ciliary neurotrophic factor on eGFR

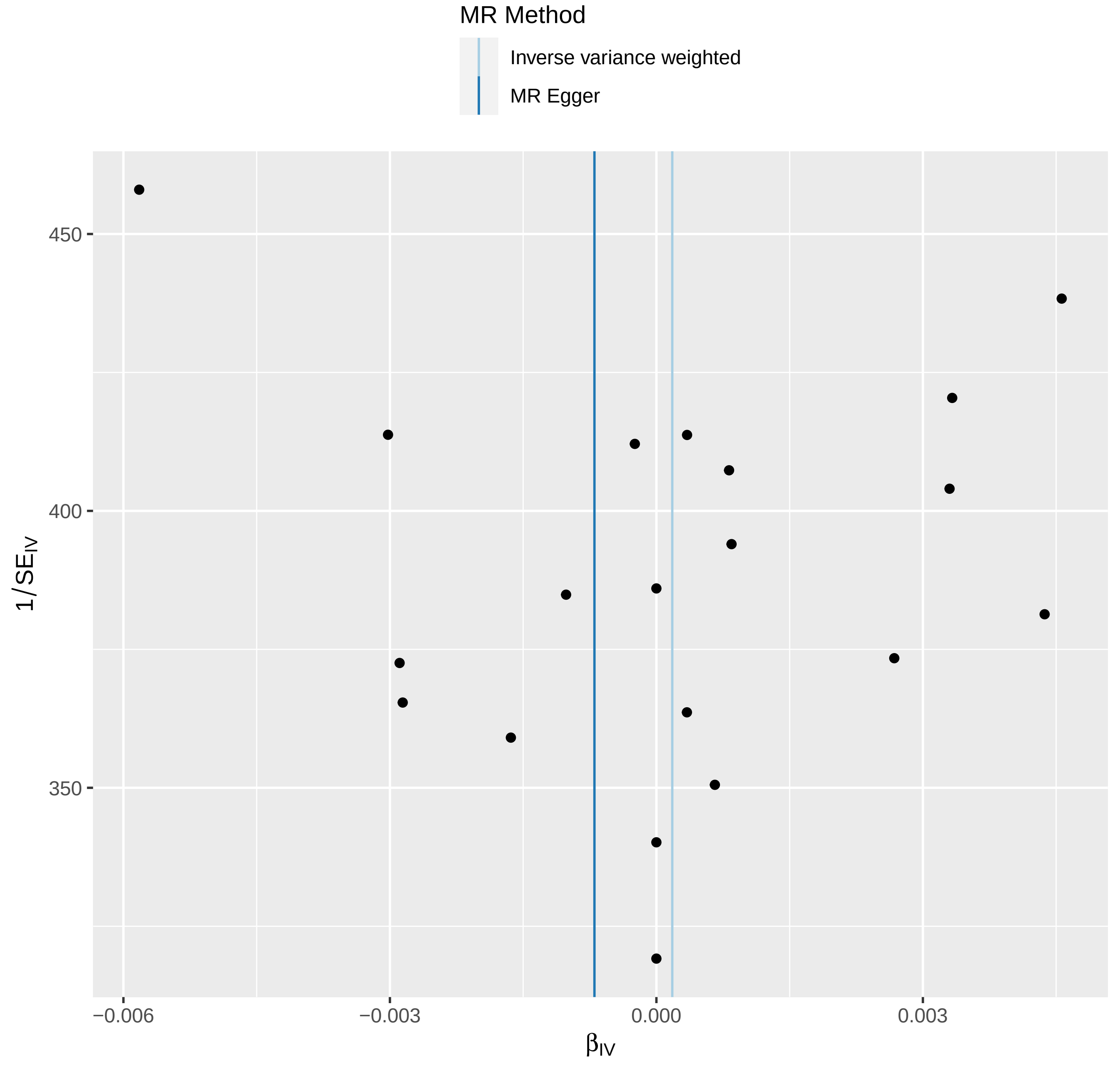

Batch 2−605 : Ciliary neurotrophic factor receptor subunit alpha on eGFR

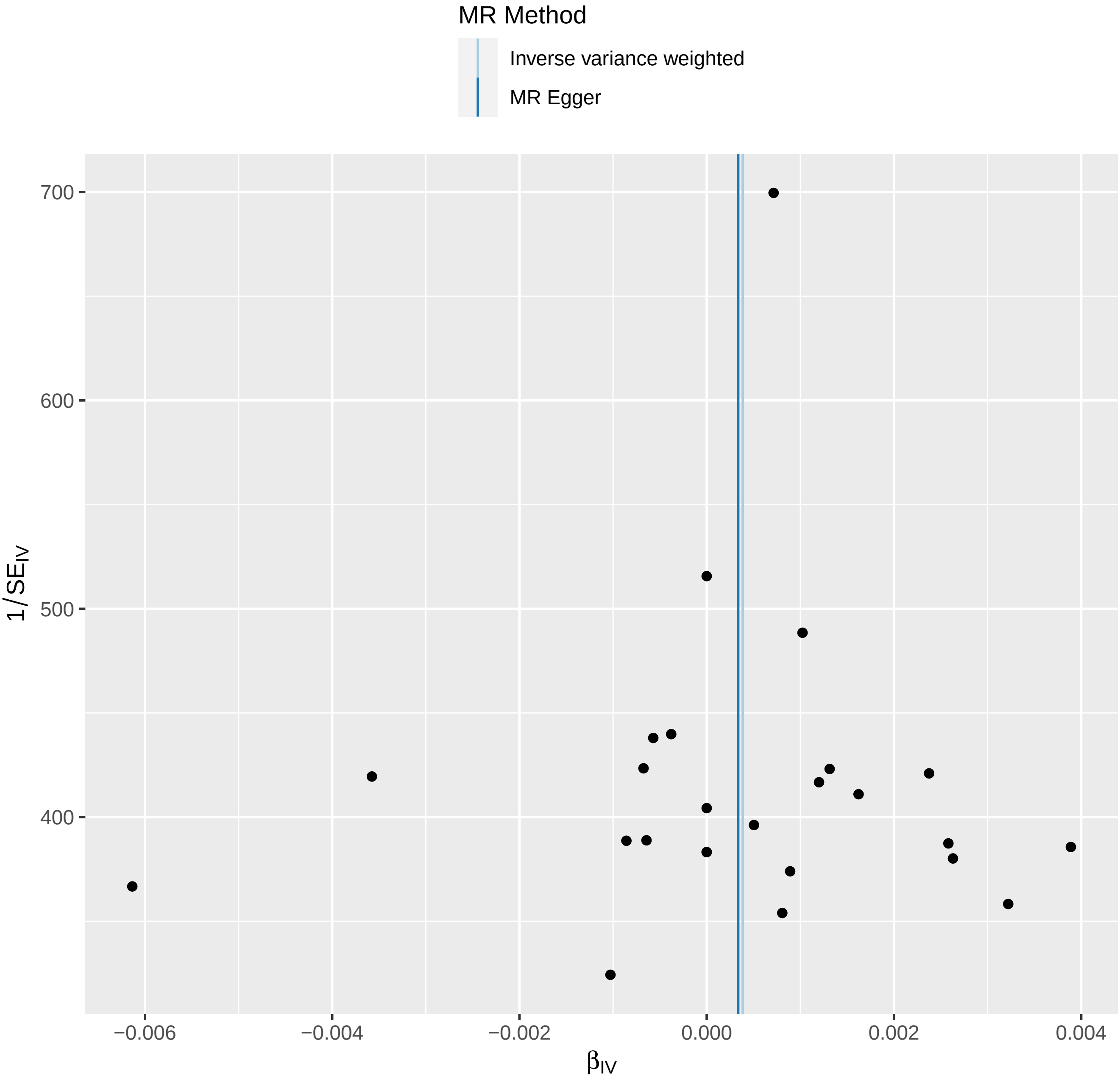

Batch 2−606 : Ciliary neurotrophic factor receptor subunit alpha on eGFR

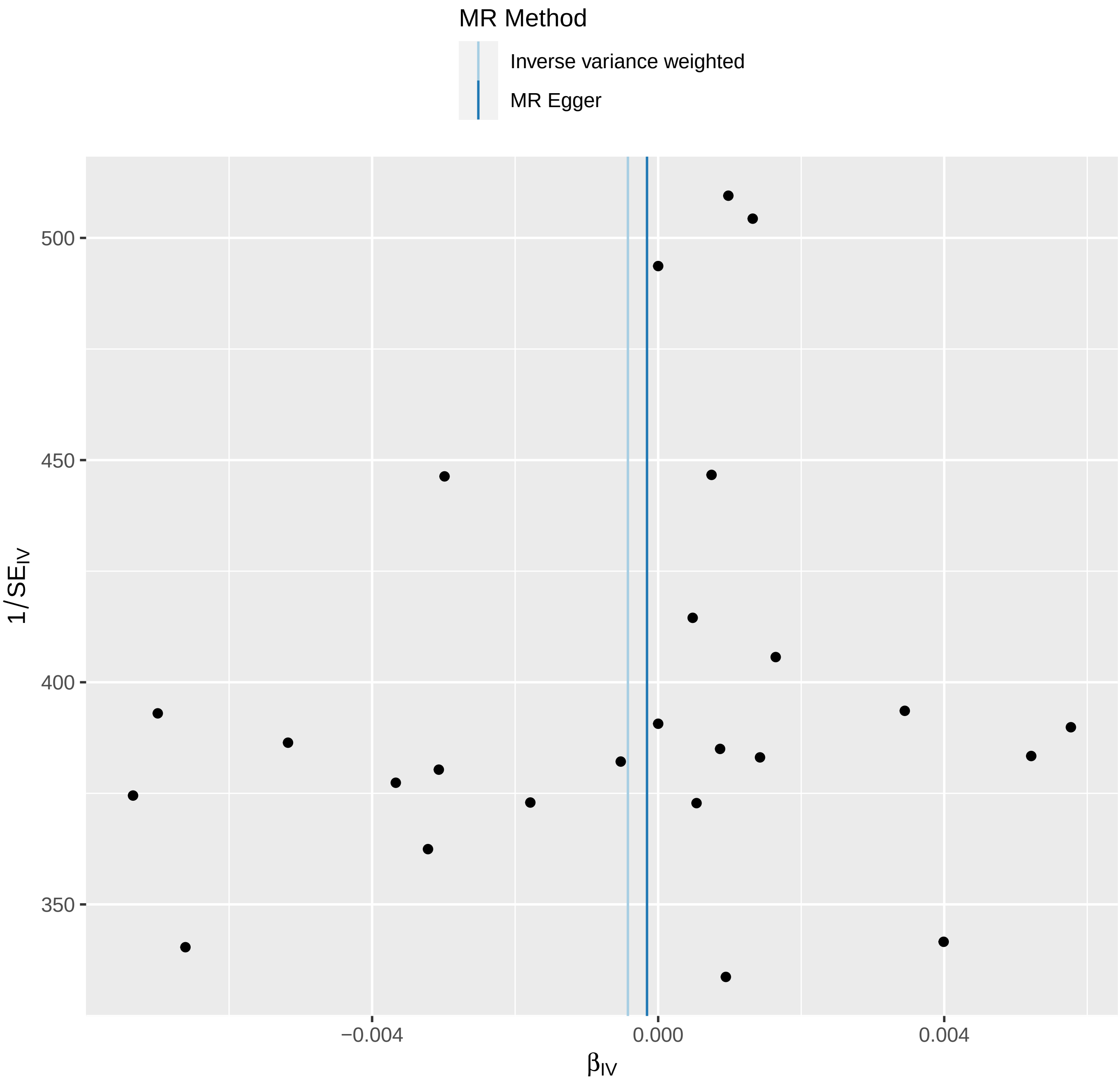

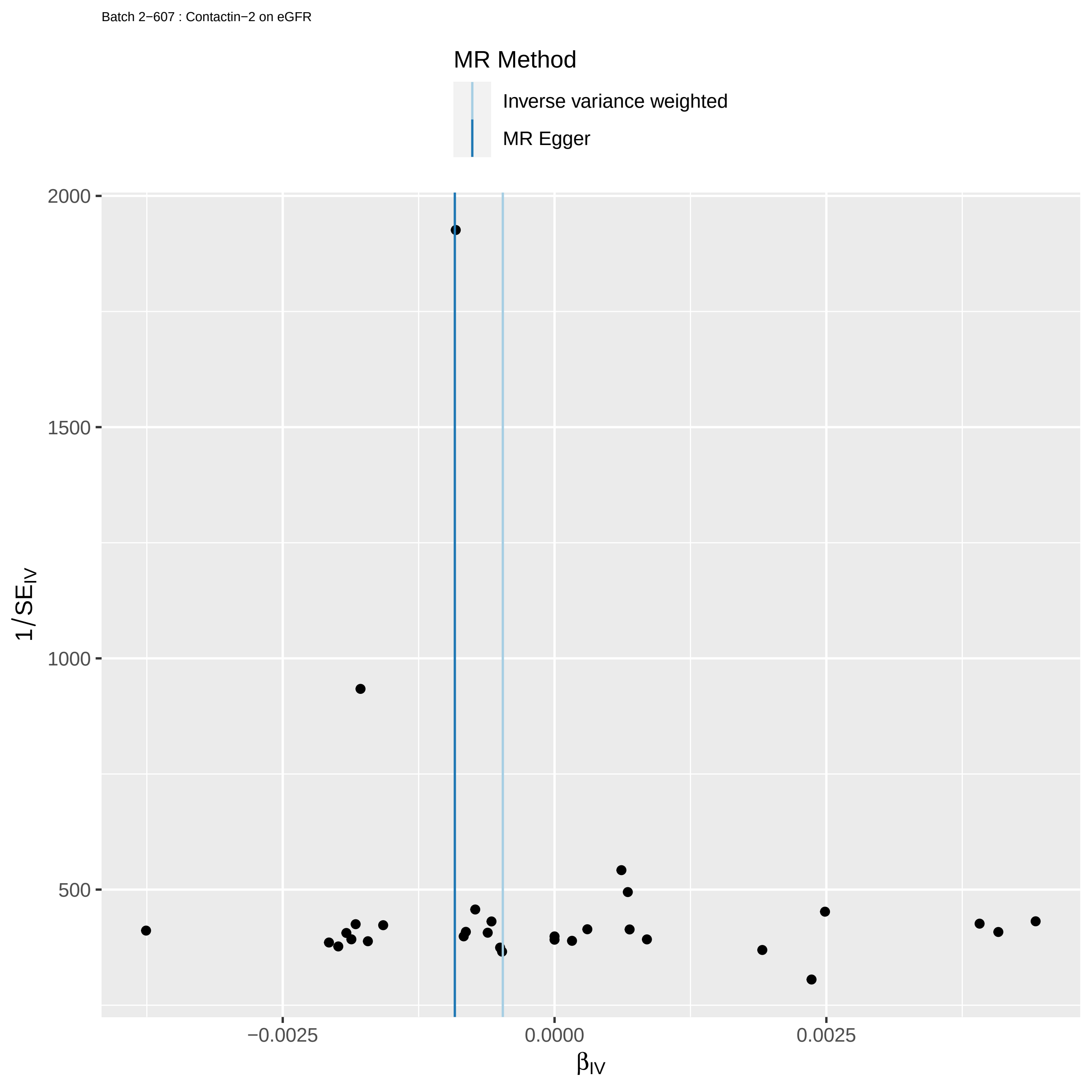

Batch 2−607 : Contactin−2 on eGFR
MR Method
Inverse variance weighted
MR Egger
2000
1500
1000
500
1/SE_IV
−0.0025
0.0000
0.0025
β_IV

Batch 2−608 : Contactin−4 on eGFR

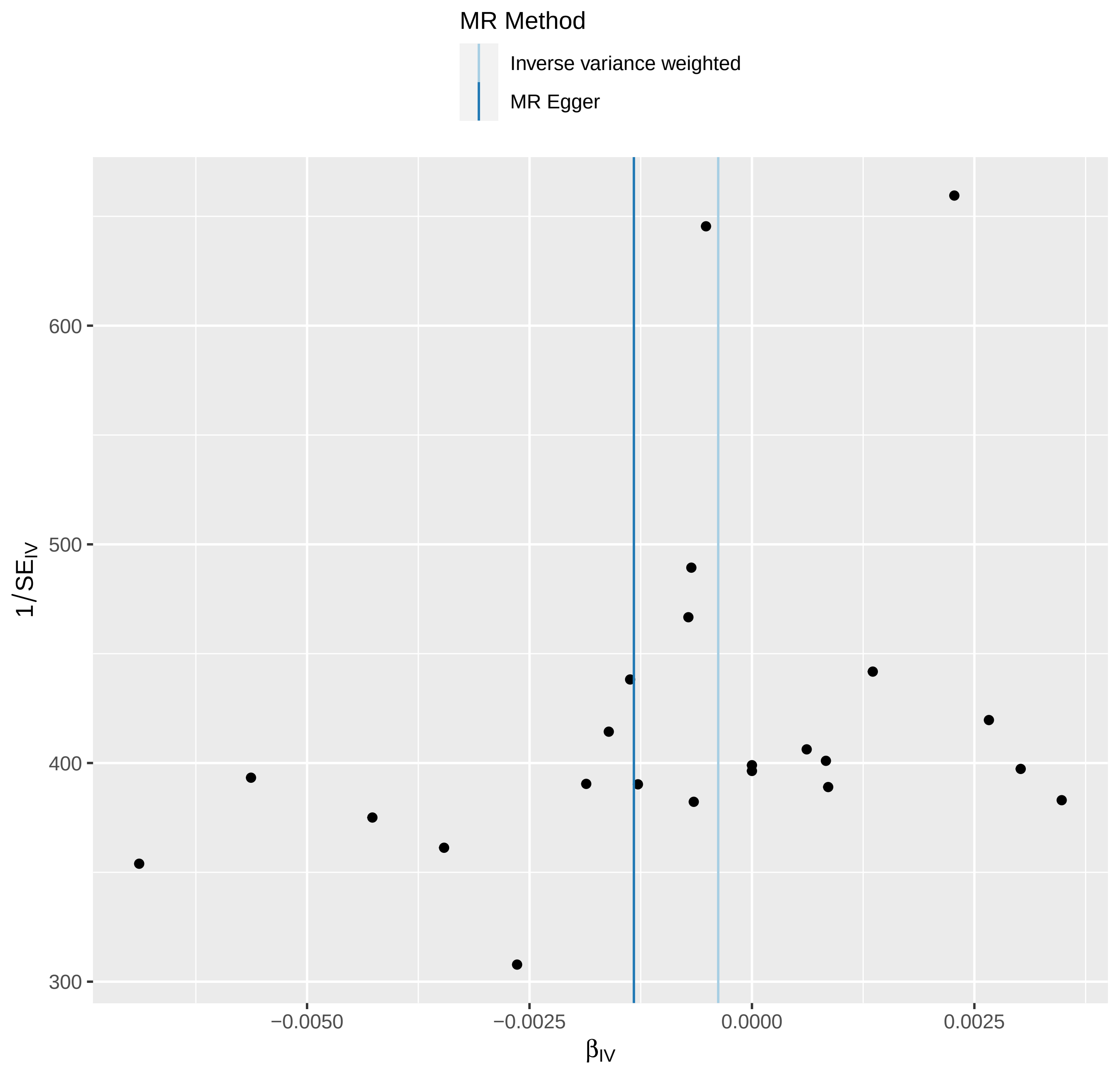

Batch 2−609 : Contactin−5 on eGFR

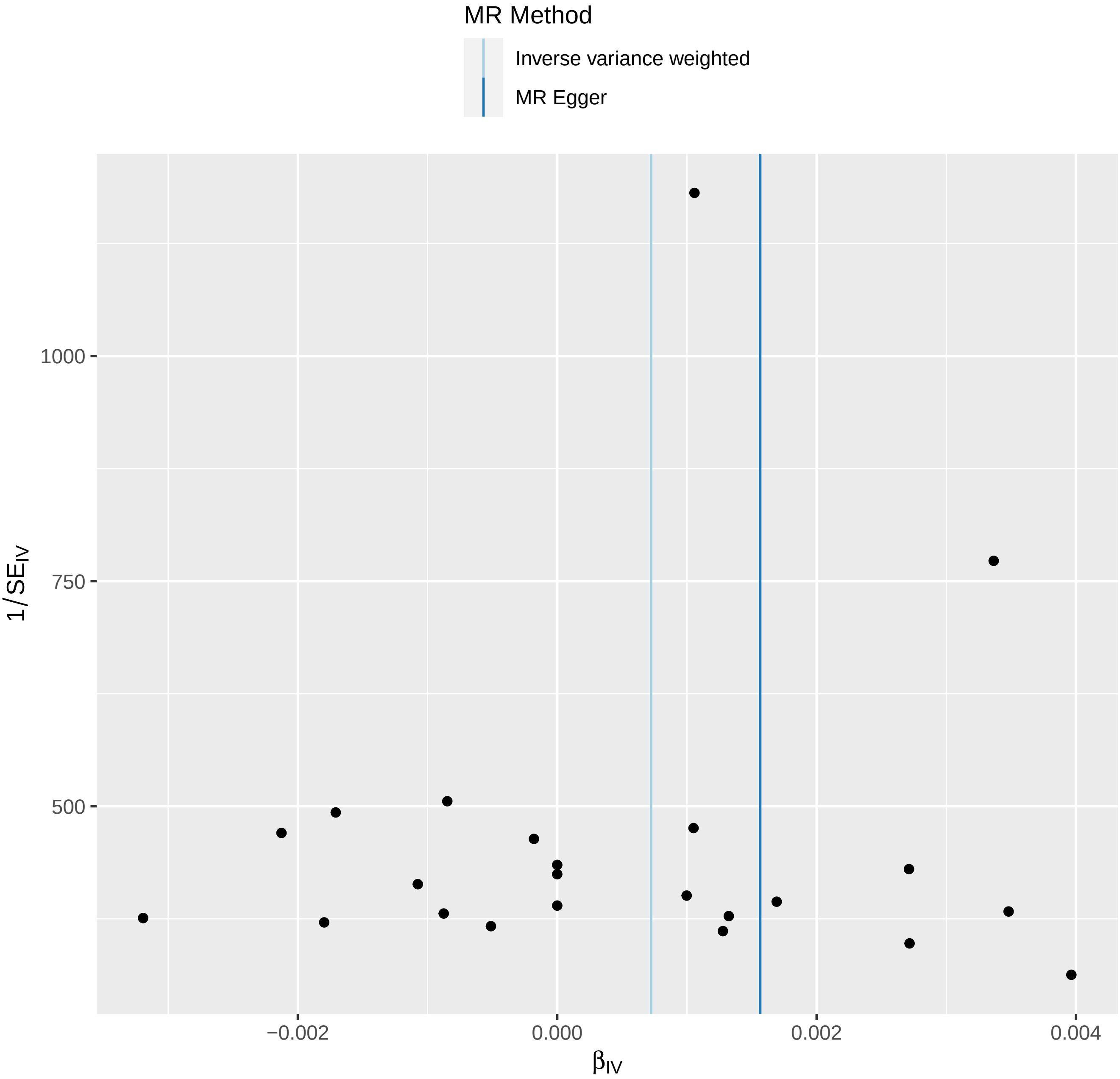

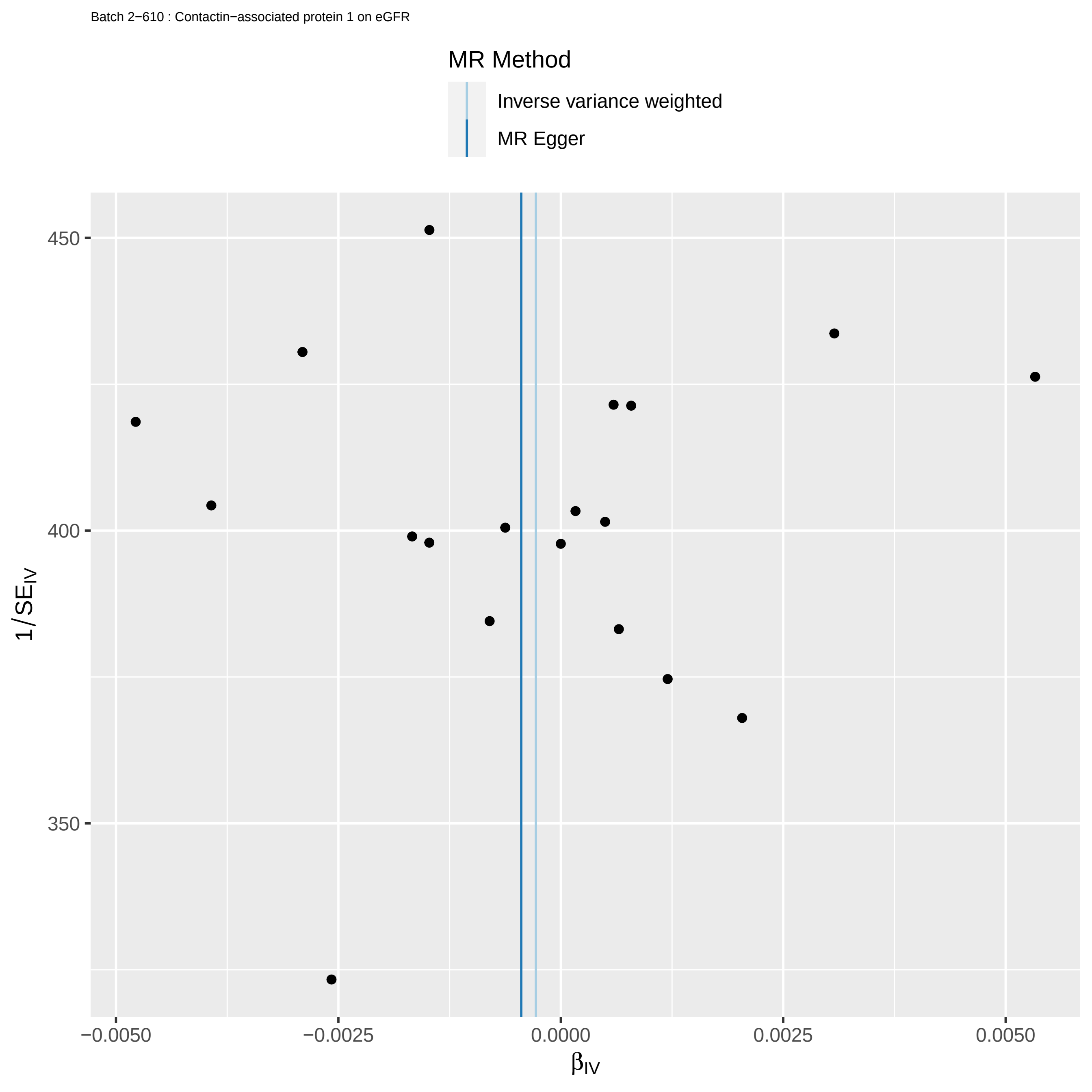

Batch 2−610 : Contactin−associated protein 1 on eGFR
MR Method
Inverse variance weighted
MR Egger
450
400
350
1/SE_IV
−0.0050
−0.0025
0.0000
0.0025
0.0050
β_IV

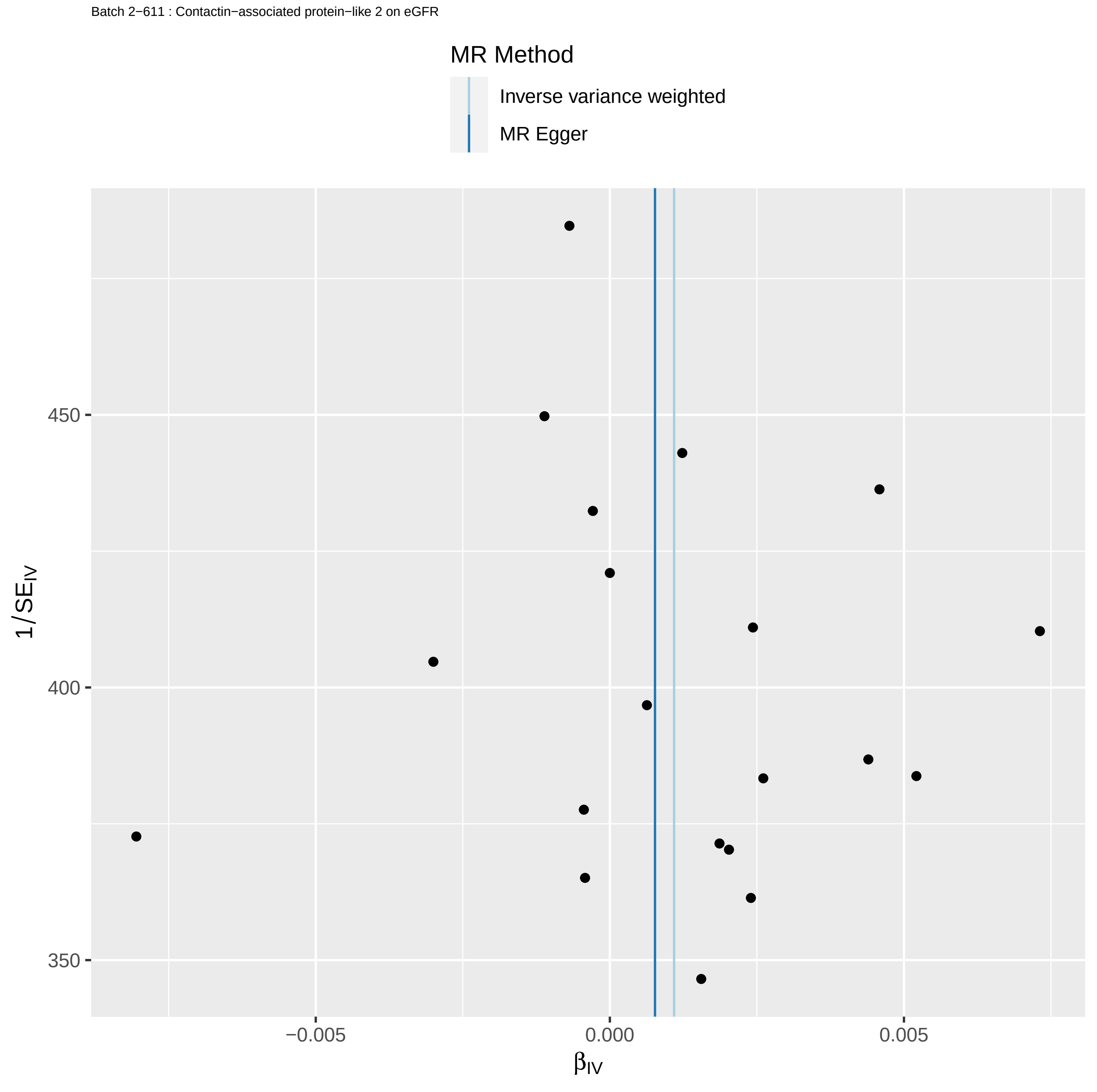
Batch 2−611 : Contactin−associated protein−like 2 on eGFR
MR Method
Inverse variance weighted
MR Egger
450
400
350
1/SE_IV
−0.005
0.000
0.005
β_IV

Batch 2−612 : Cytochrome c oxidase assembly factor 3 homolog, mitochondrial on eGFR

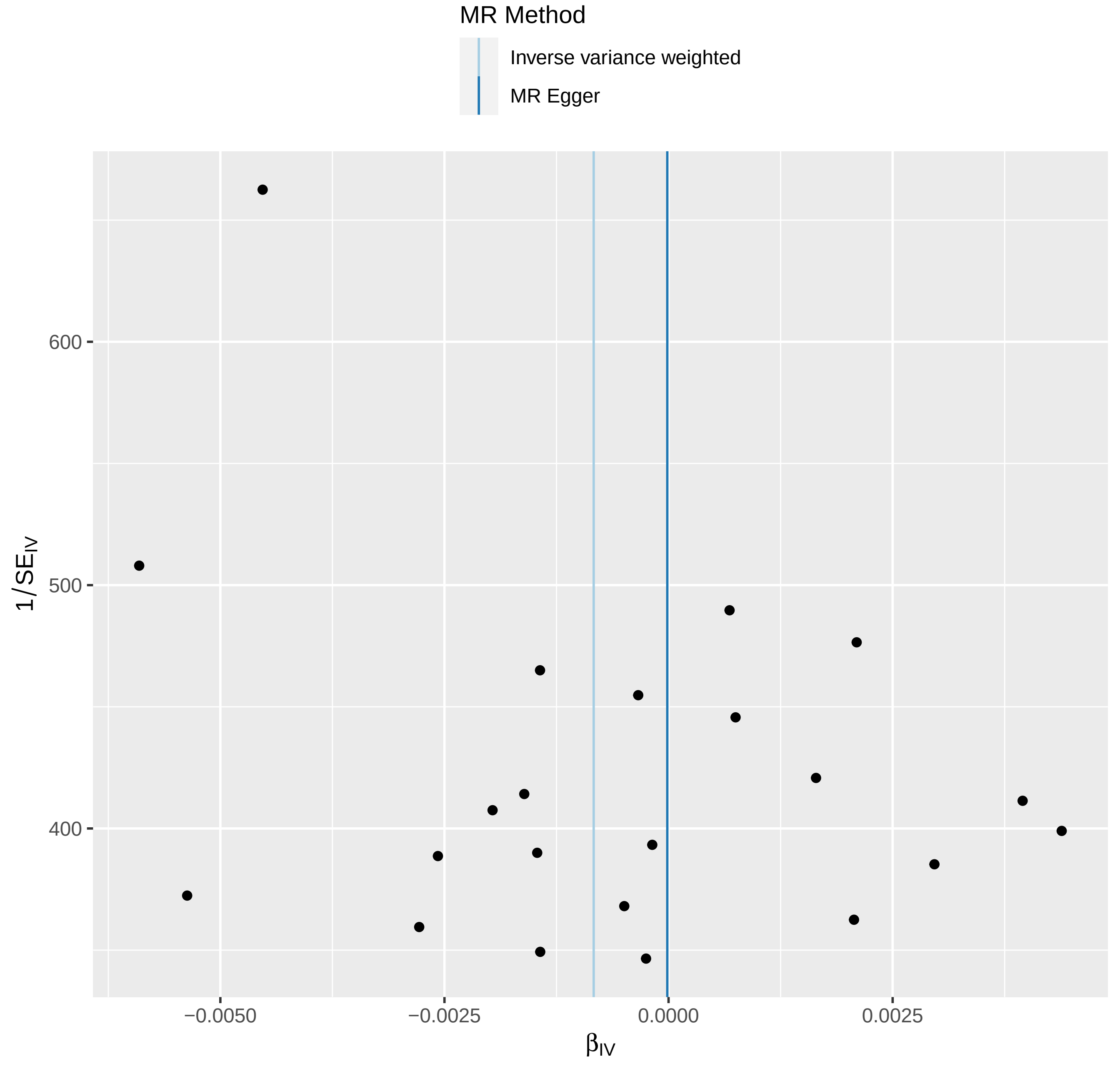

Batch 2−613 : Cochlin on eGFR

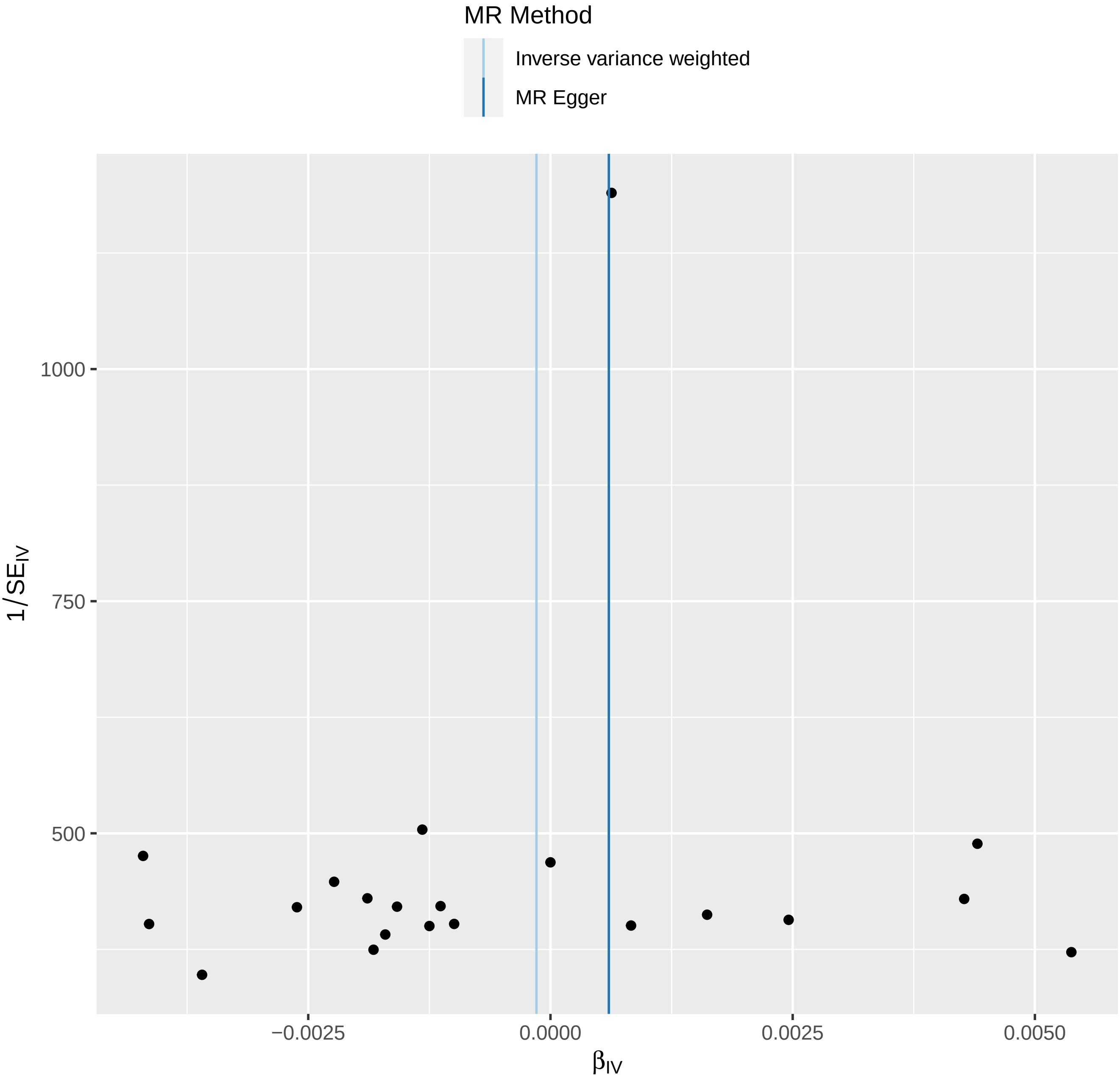

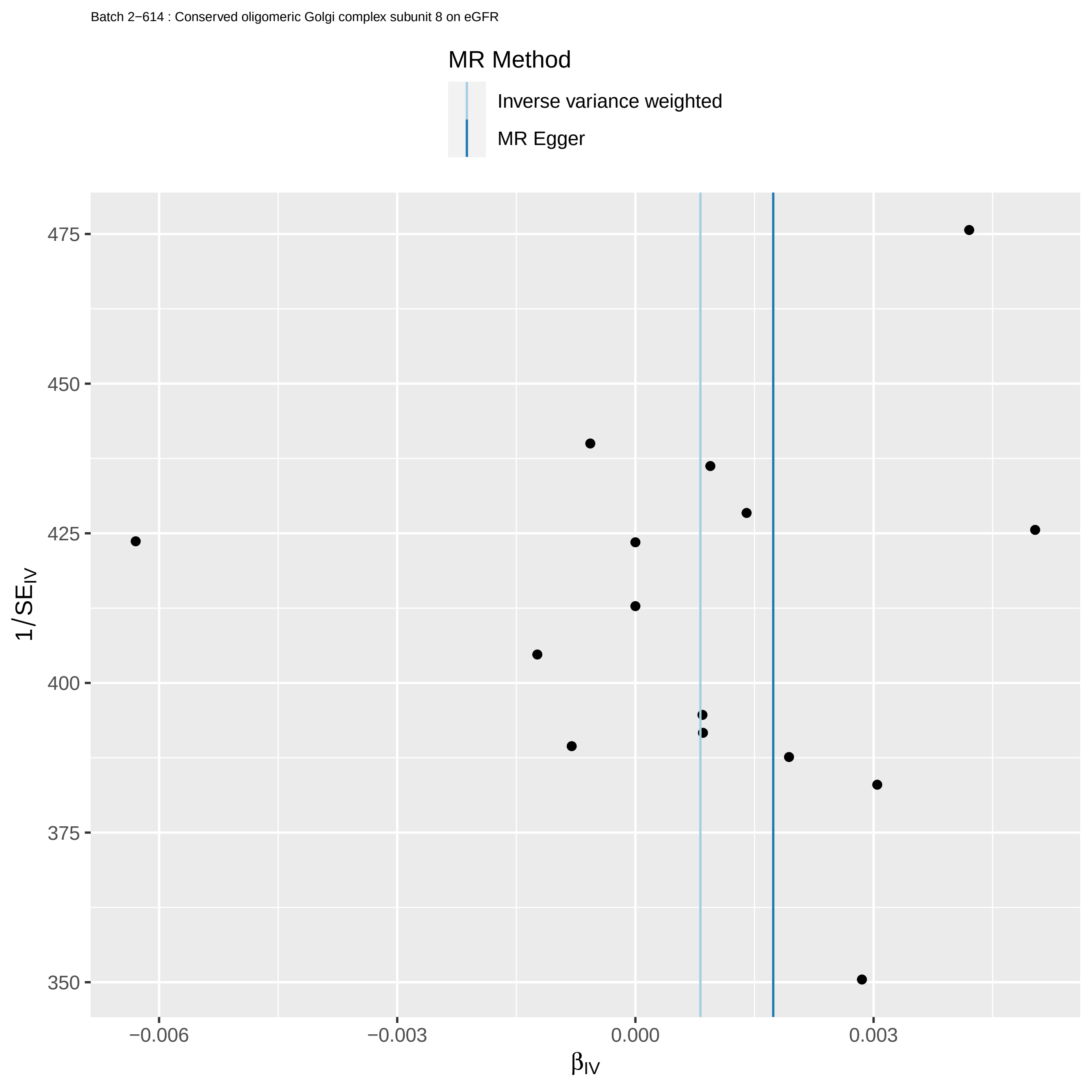
Batch 2−614 : Conserved oligomeric Golgi complex subunit 8 on eGFR
MR Method
Inverse variance weighted
MR Egger
475
450
425
400
375
350
1/SE_IV
−0.006
−0.003
0.000
0.003
β_IV

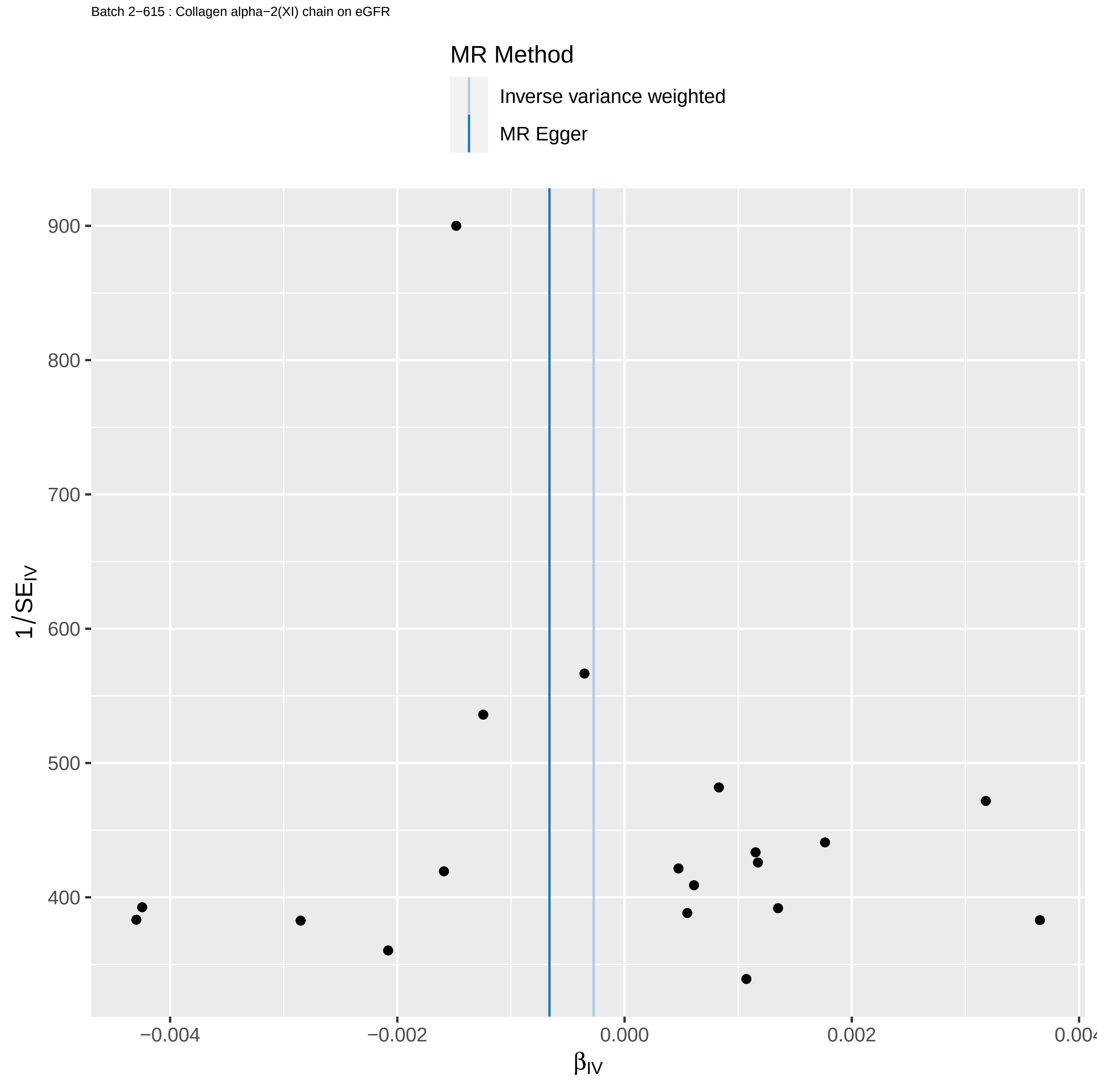
Batch 2−615 : Collagen alpha−2(XI) chain on eGFR
MR Method
Inverse variance weighted
MR Egger
900
800
700
600
500
400
1/SE_IV
−0.004
−0.002
0.000
0.002
0.004
β_IV

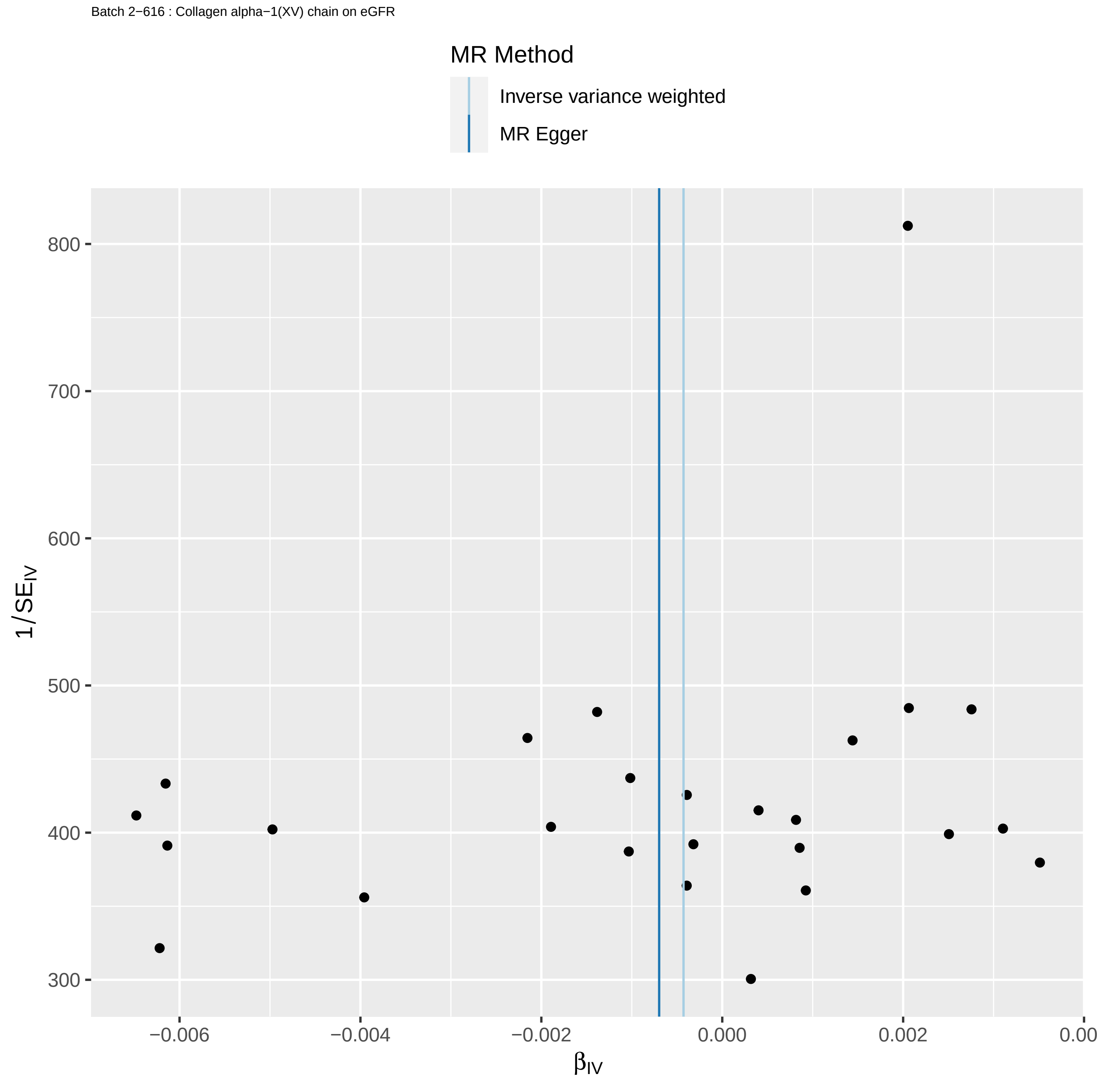
Batch 2−616 : Collagen alpha−1(XV) chain on eGFR
MR Method
Inverse variance weighted
MR Egger
800
700
600
500
400
300
1/SE_IV
−0.006
−0.004
−0.002
0.000
0.002
0.00
β_IV

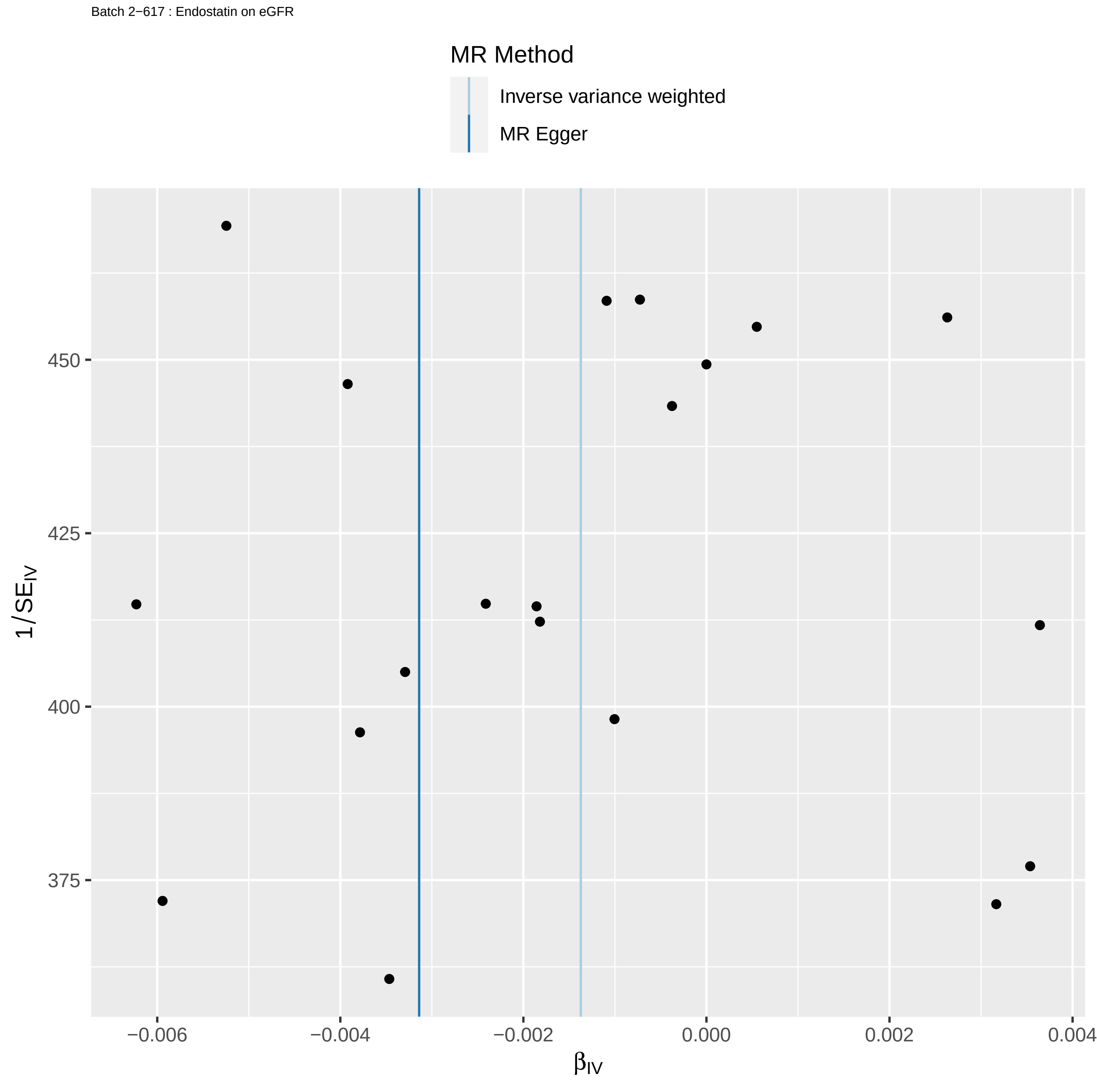

Batch 2−617 : Endostatin on eGFR
MR Method
Inverse variance weighted
MR Egger
450
425
400
375
1/SE_IV
−0.006
−0.004
−0.002
0.000
0.002
0.004
β_IV

Batch 2−618 : Collagen alpha−1(I) chain on eGFR

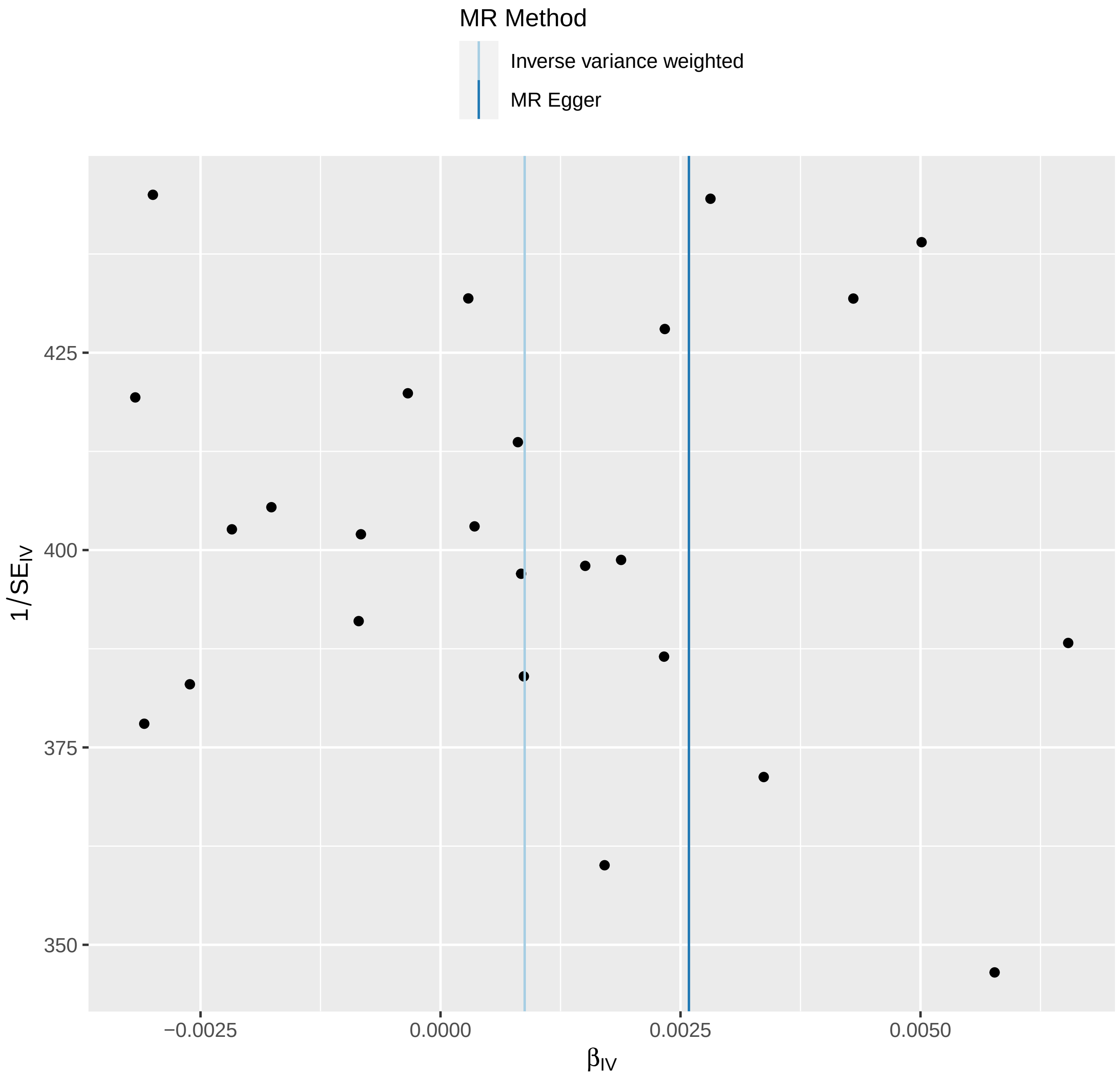

Batch 2−619 : Collagen alpha−1(XX) chain on eGFR

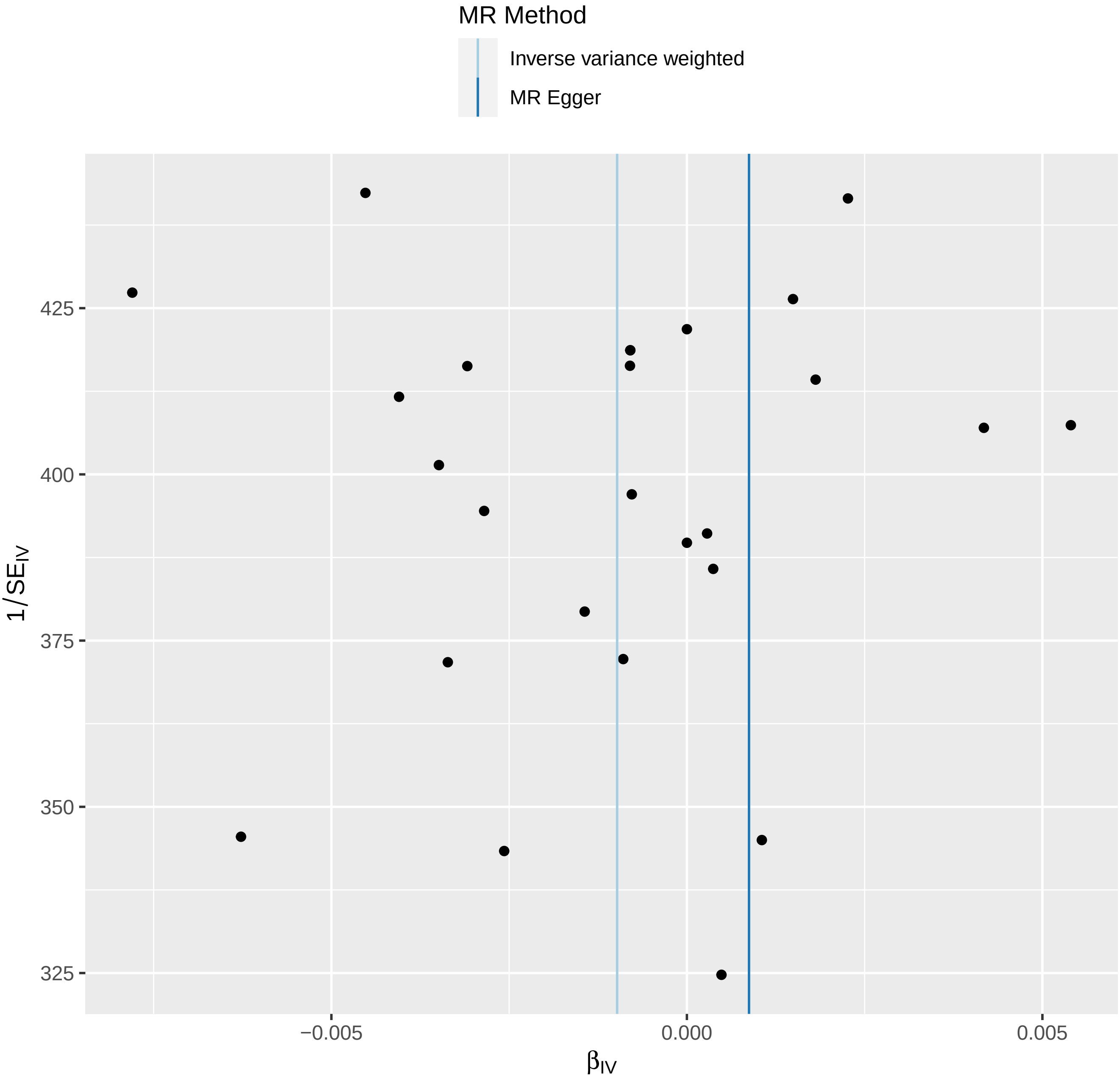

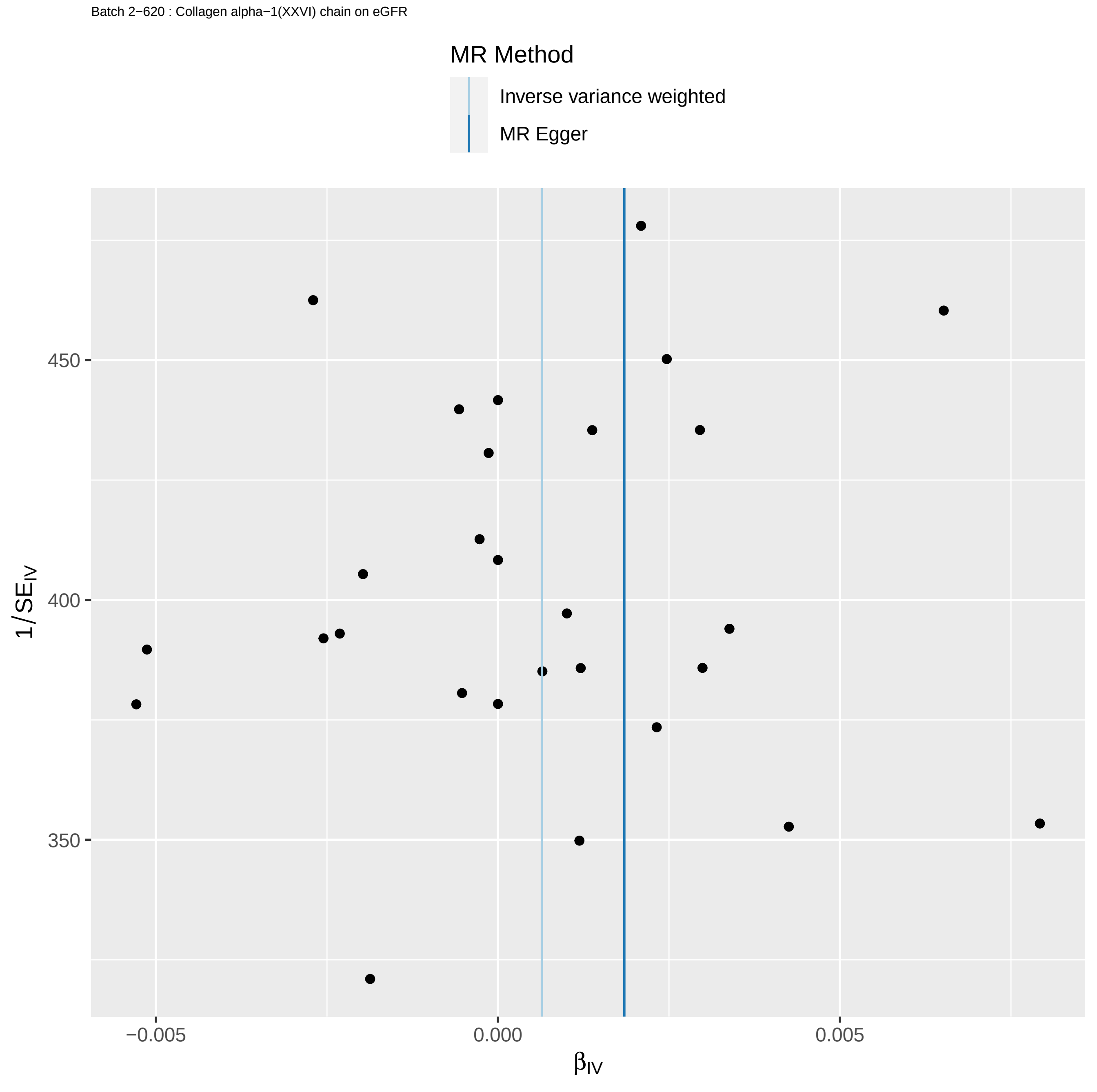
Batch 2−620 : Collagen alpha−1(XXVI) chain on eGFR
MR Method
Inverse variance weighted
MR Egger
450
400
350
1/SE$_{IV}$
−0.005
0.000
0.005
β$_{IV}$

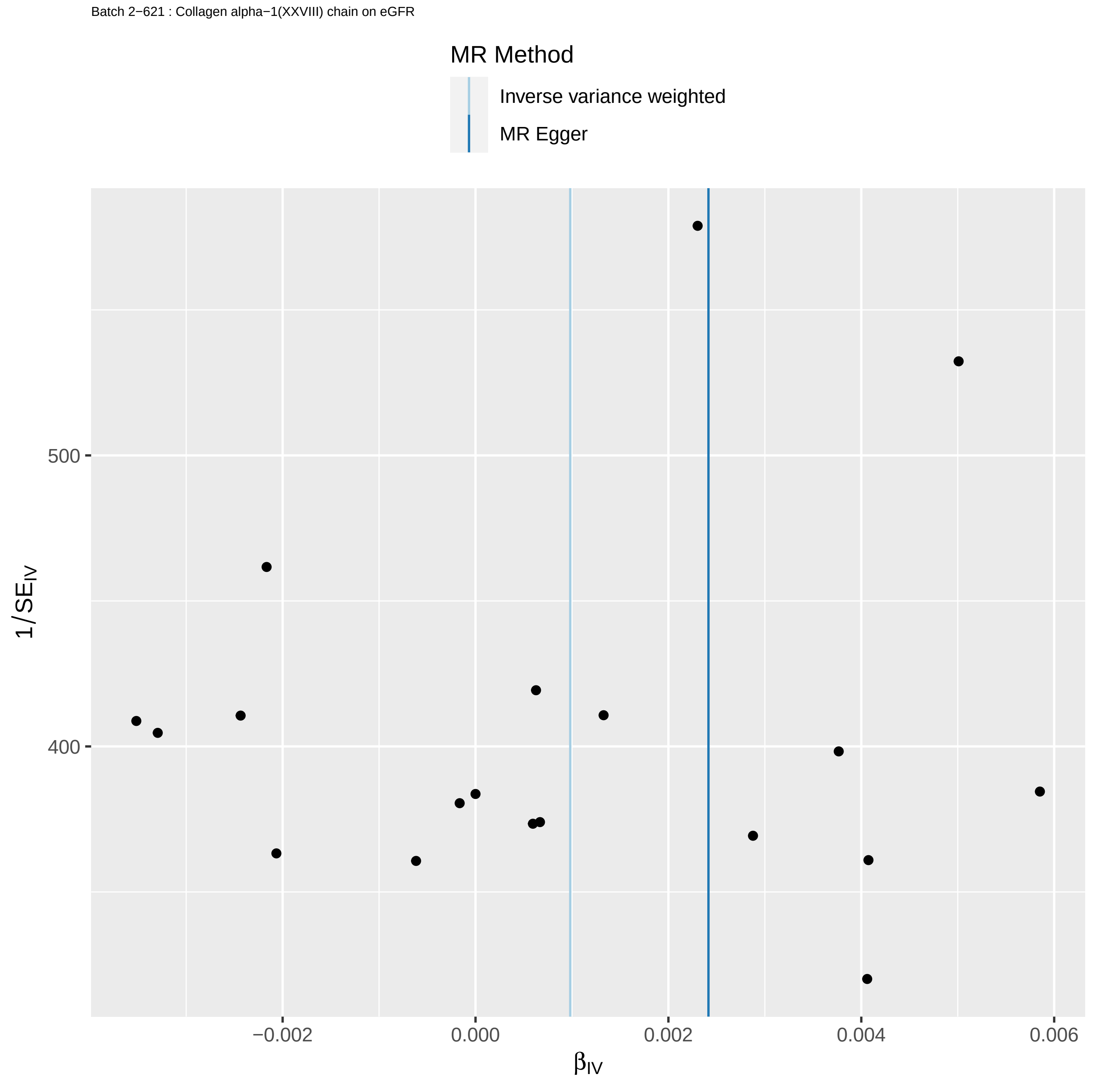
Batch 2−621 : Collagen alpha−1(XXVIII) chain on eGFR
MR Method
Inverse variance weighted
MR Egger
500
400
1/SE_IV
−0.002
0.000
0.002
0.004
0.006
β_IV

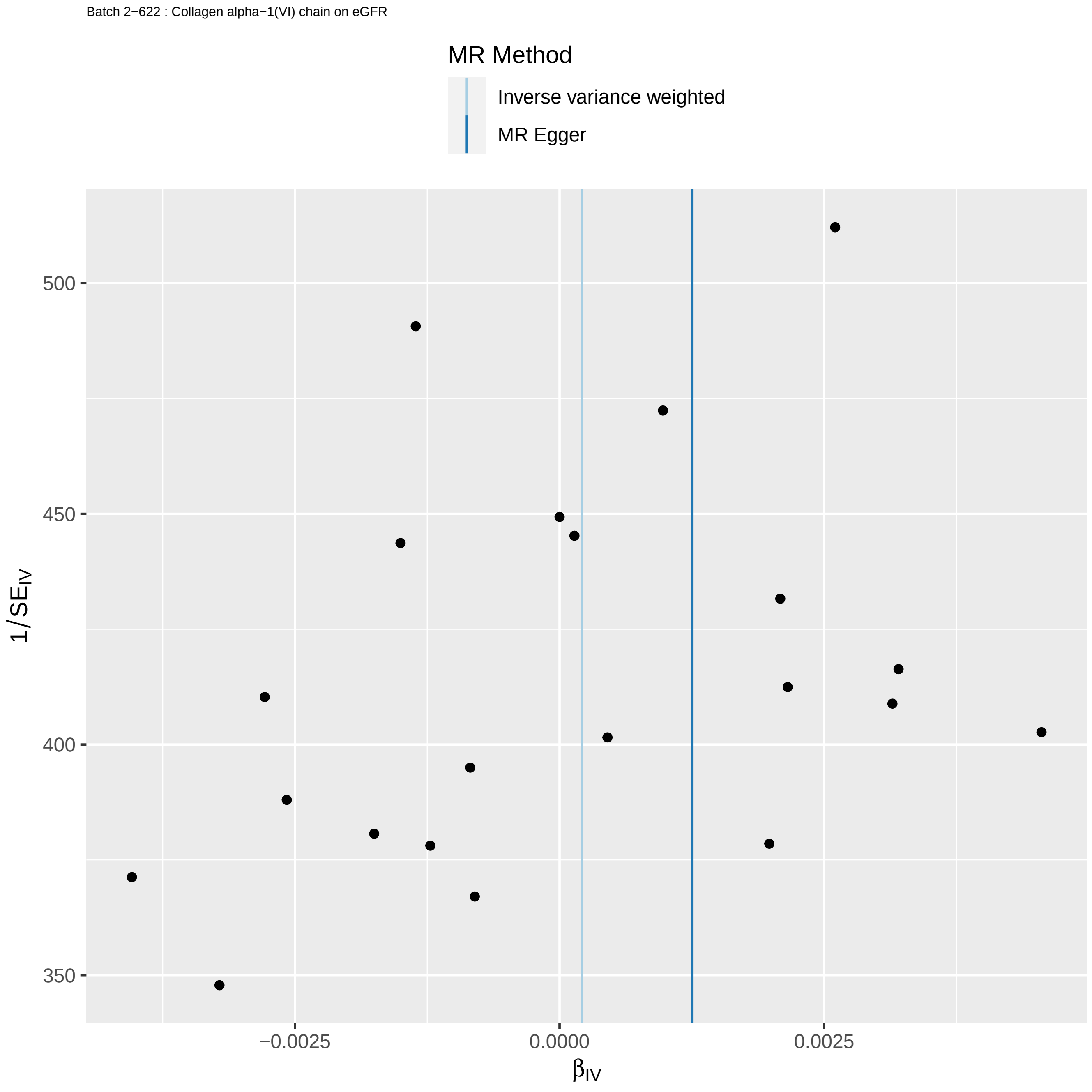

Batch 2−622 : Collagen alpha−1(VI) chain on eGFR
MR Method
Inverse variance weighted
MR Egger
500
450
400
350
1/SE_IV
−0.0025
0.0000
0.0025
β_IV

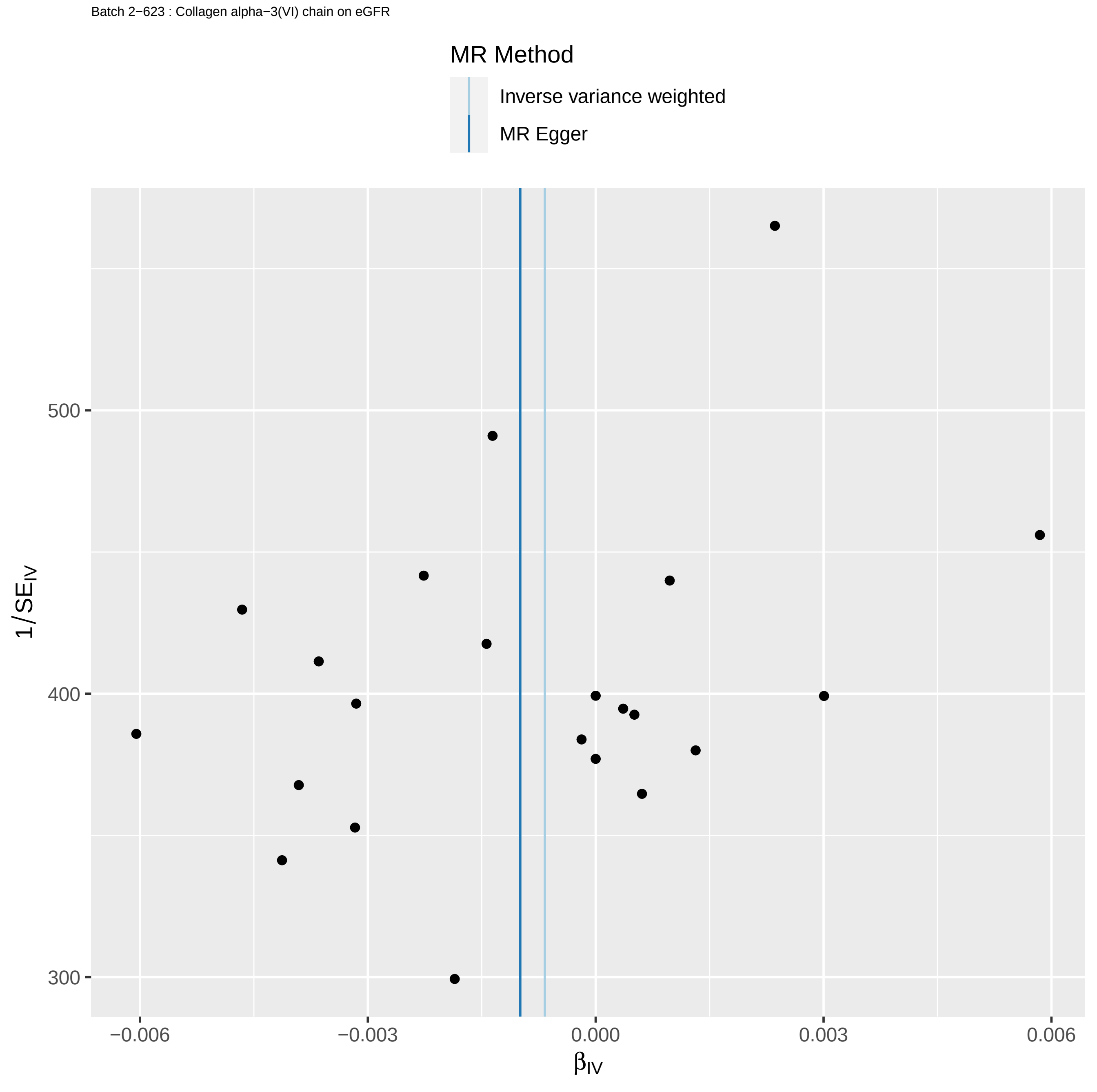

Batch 2−623 : Collagen alpha−3(VI) chain on eGFR
MR Method
Inverse variance weighted
MR Egger
500
400
300
1/SE_IV
−0.006
−0.003
0.000
0.003
0.006
β_IV

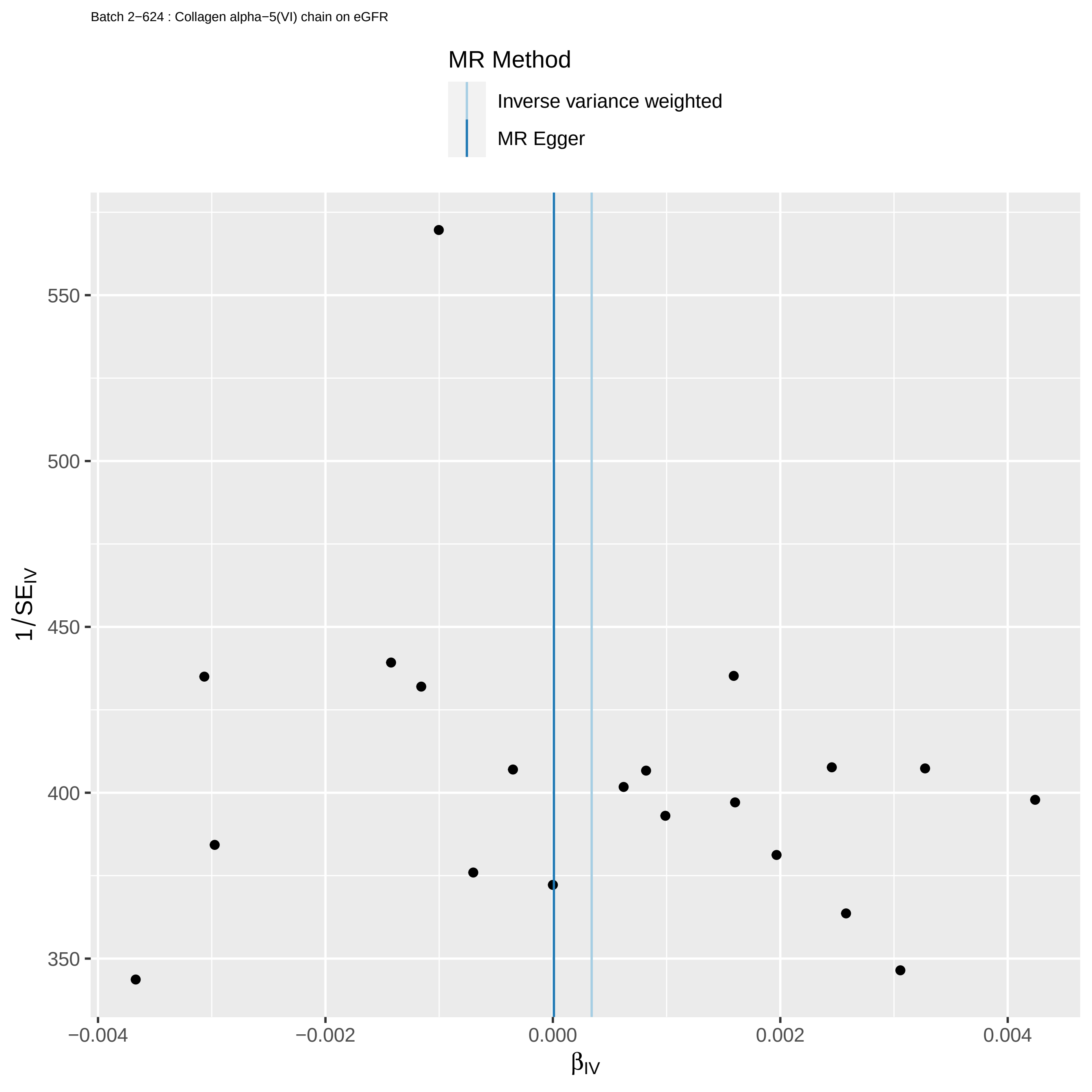

Batch 2−624 : Collagen alpha−5(VI) chain on eGFR
MR Method
Inverse variance weighted
MR Egger
550
500
450
400
350
1/SE$_{IV}$
−0.004
−0.002
0.000
0.002
0.004
β$_{IV}$

Batch 2−625 : Collagen alpha−1(VIII) chain on eGFR

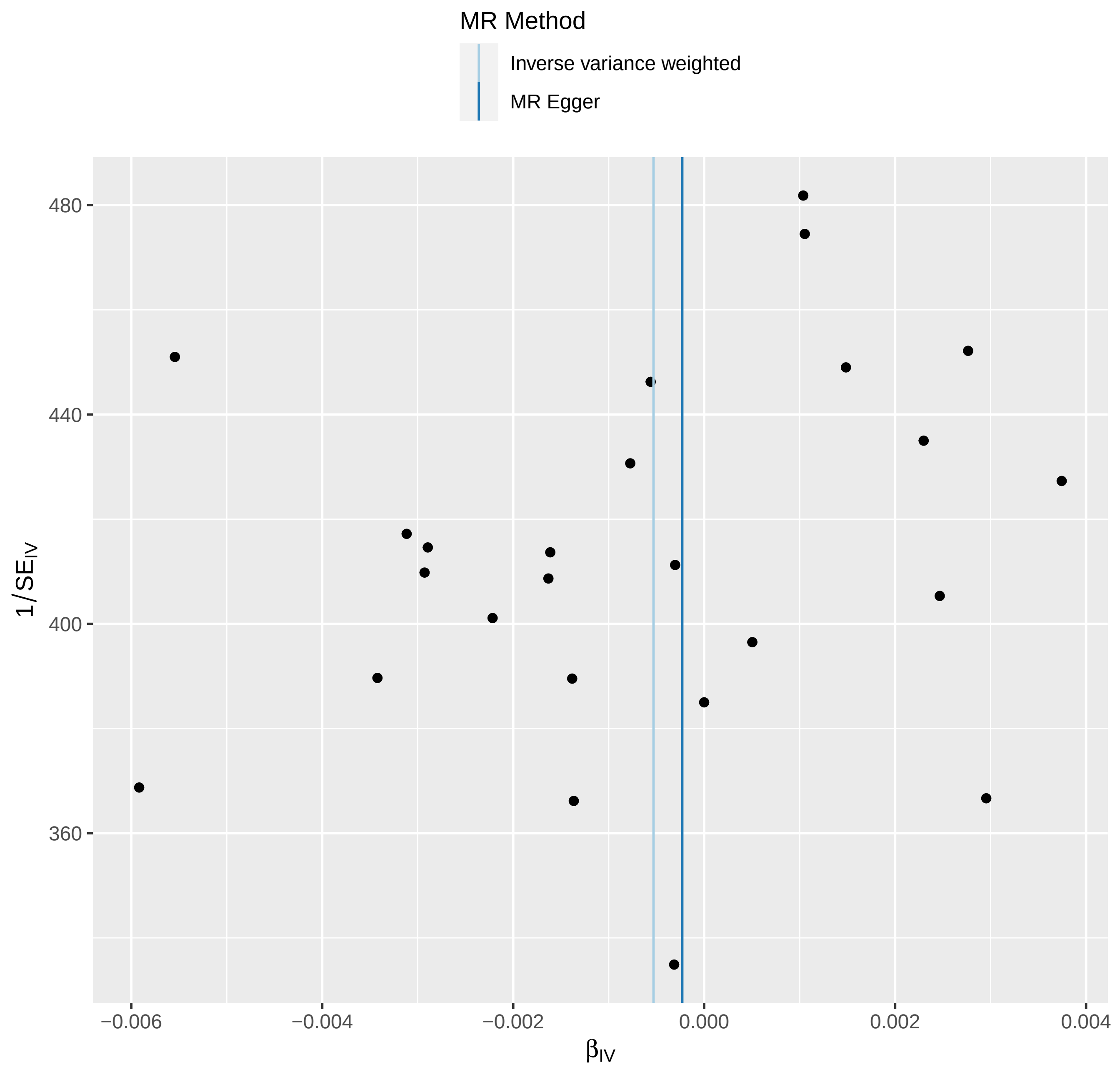

Batch 2−626 : Collagen alpha−2(IX) chain on eGFR

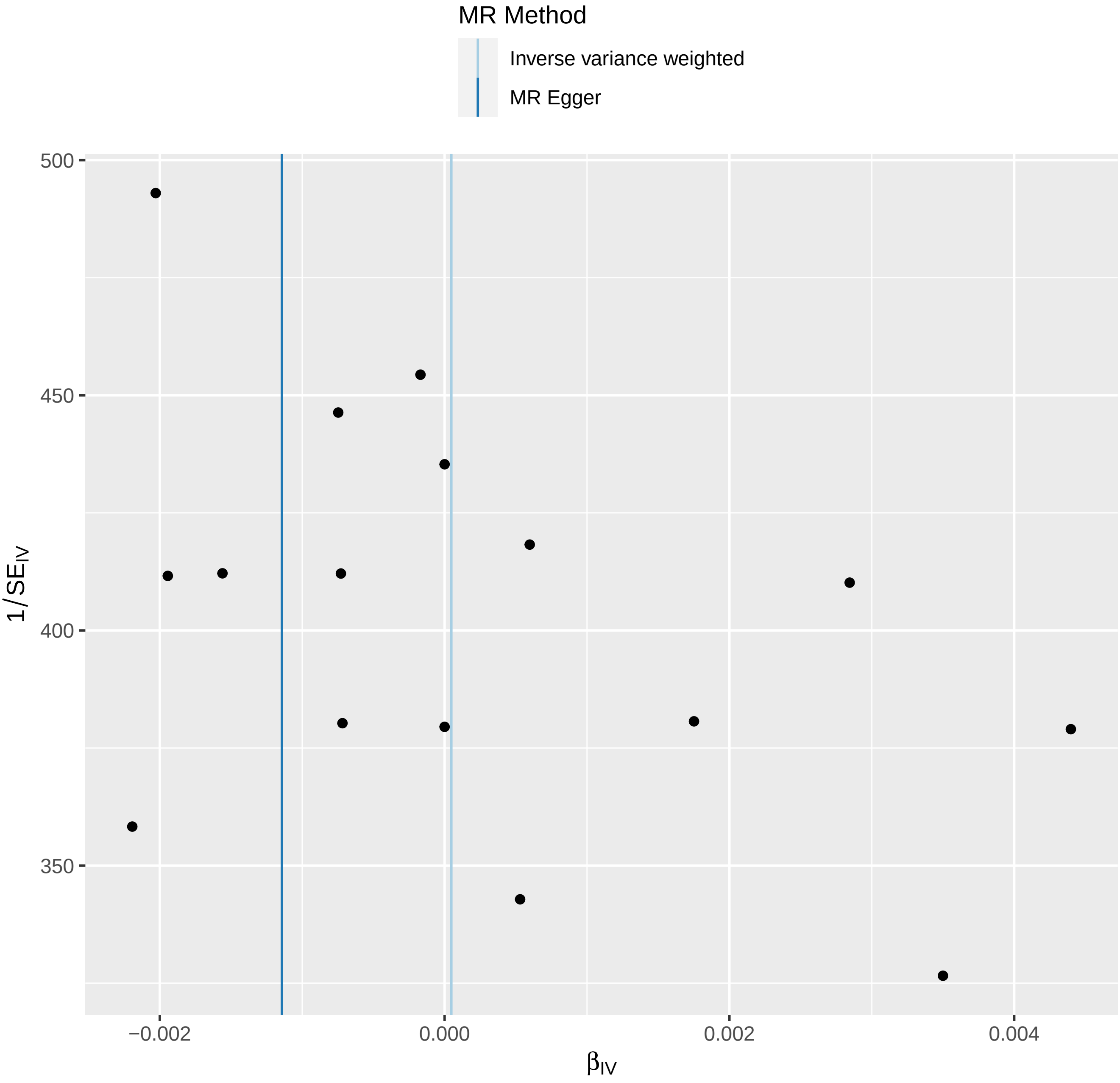

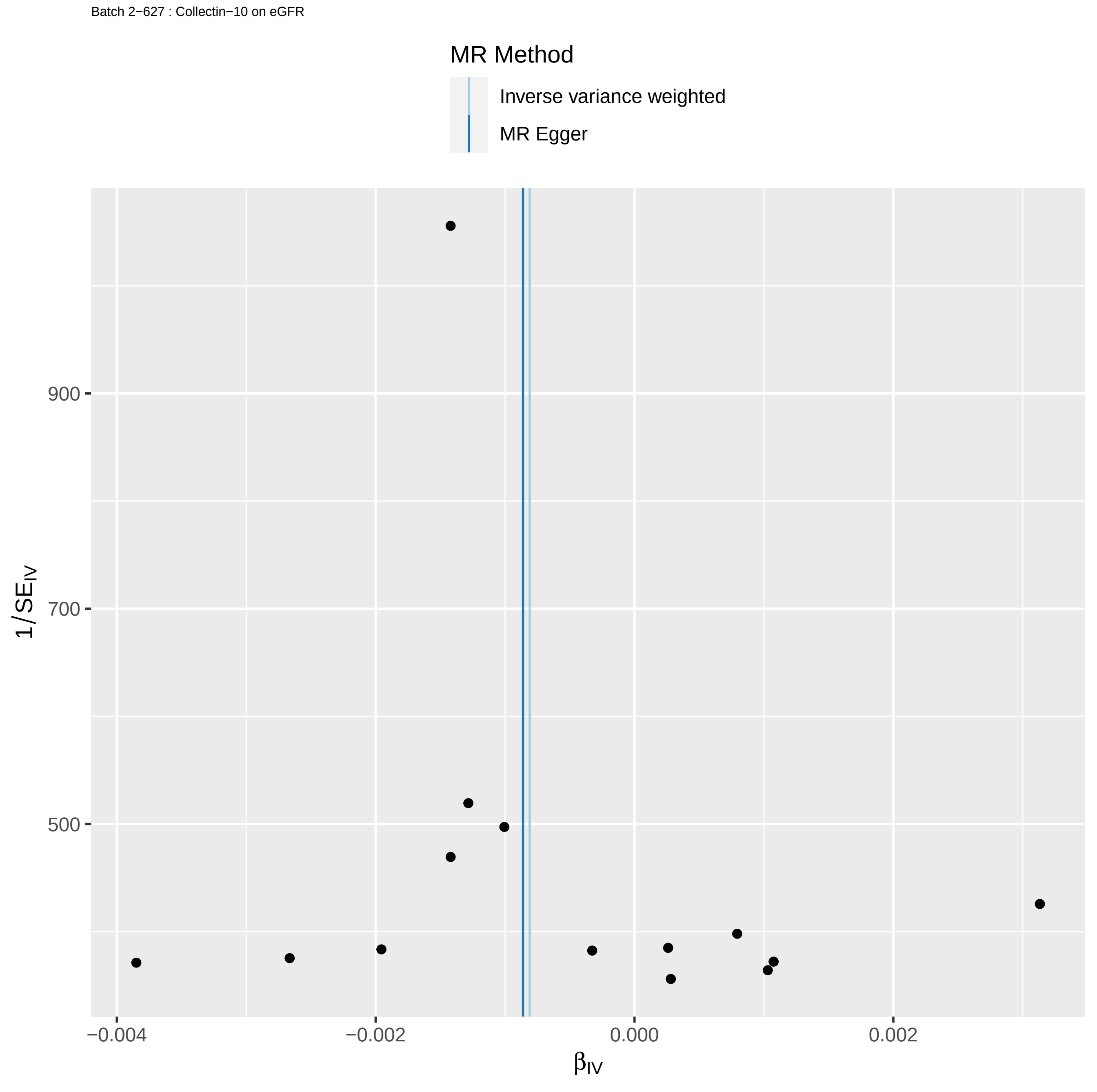

Batch 2−627 : Collectin−10 on eGFR
MR Method
Inverse variance weighted
MR Egger
900
700
500
1/SE_IV
−0.004
−0.002
0.000
0.002
β_IV

Batch 2−628 : Collectin−11 on eGFR

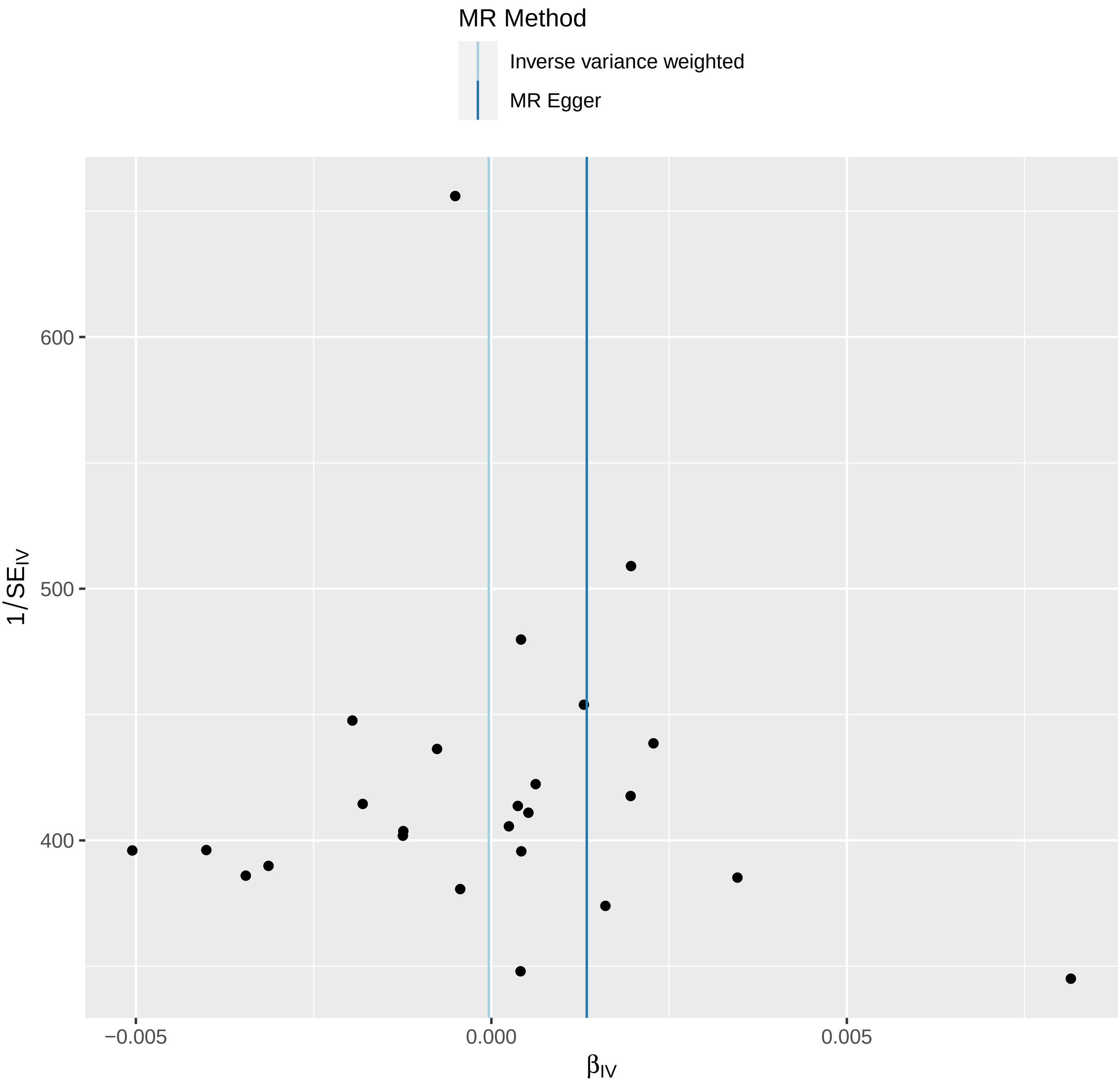

Batch 2−629 : Collectin−12 on eGFR

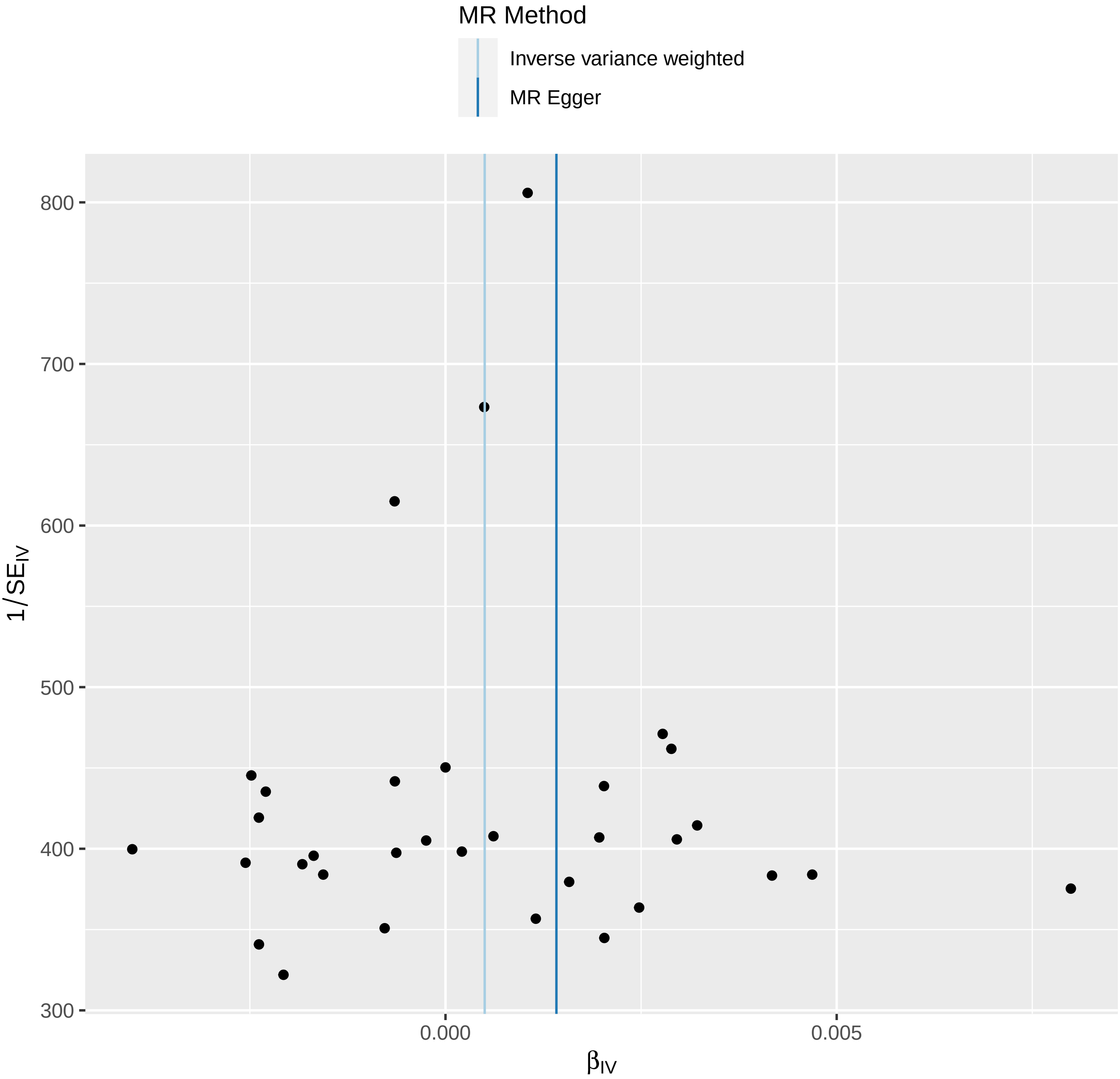

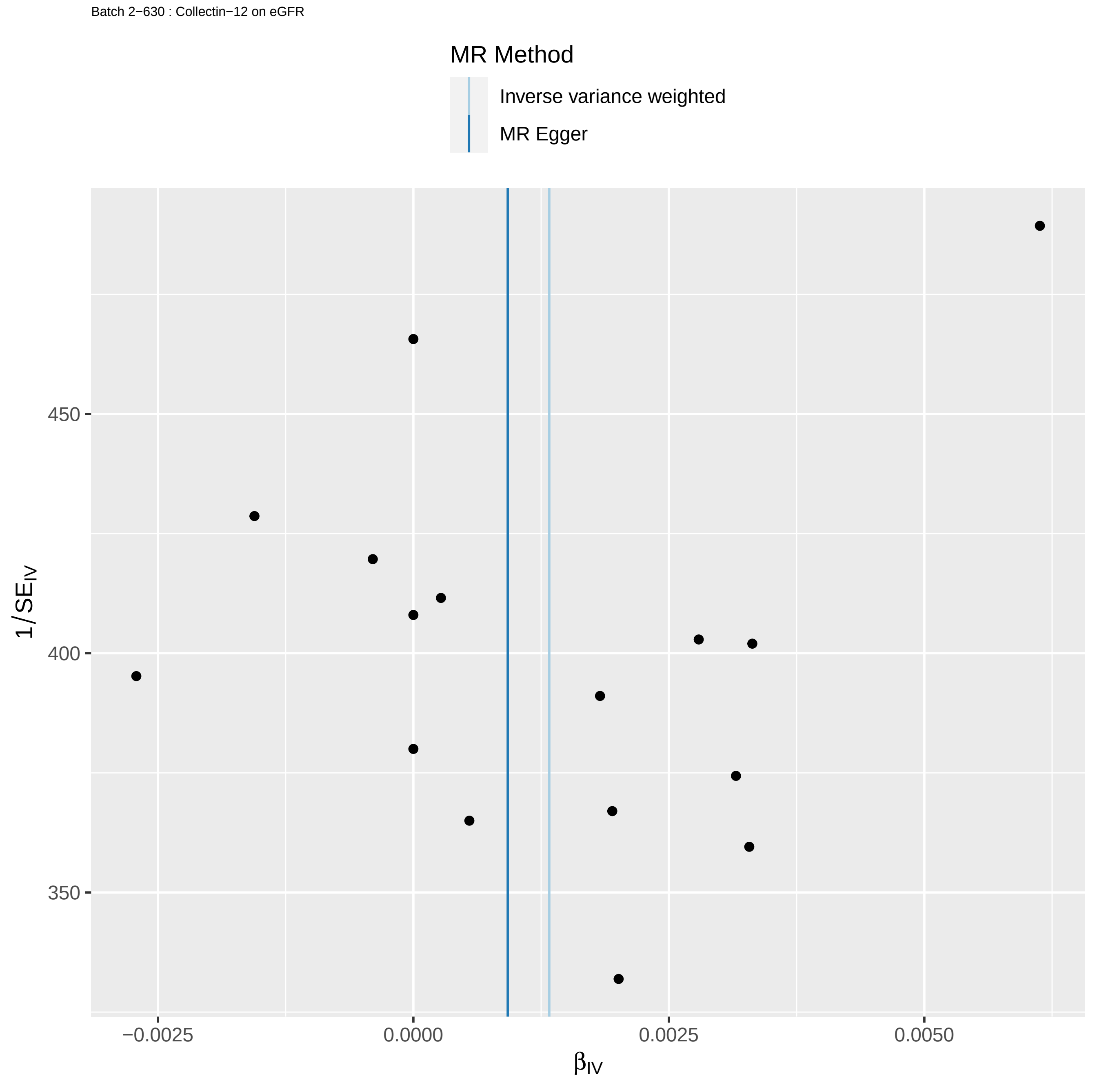

Batch 2−630 : Collectin−12 on eGFR
MR Method
Inverse variance weighted
MR Egger
450
400
350
1/SE_IV
−0.0025
0.0000
0.0025
0.0050
β_IV

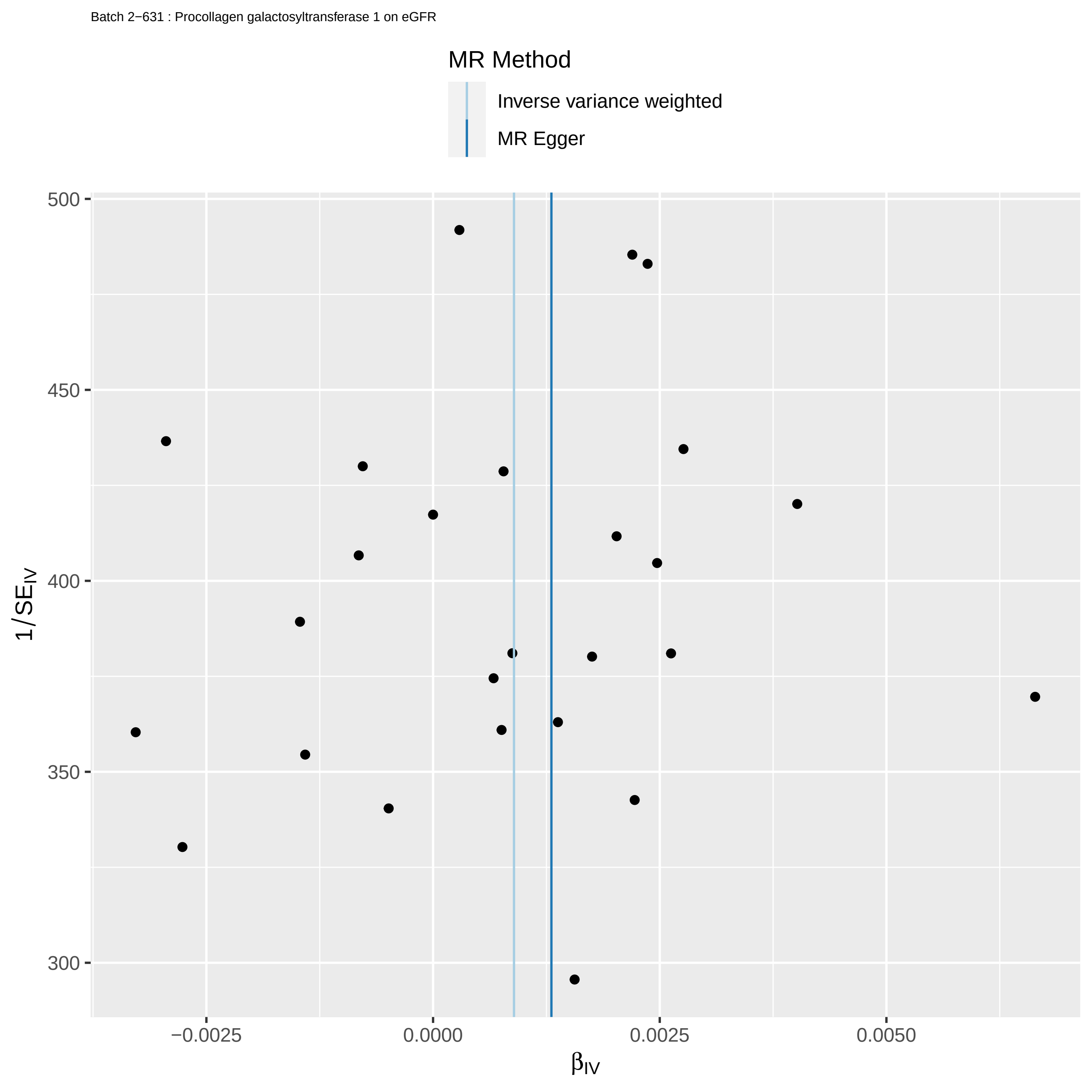

Batch 2−631 : Procollagen galactosyltransferase 1 on eGFR
MR Method
Inverse variance weighted
MR Egger
500
450
400
350
300
1/SE$_{IV}$
−0.0025
0.0000
0.0025
0.0050
β$_{IV}$

Batch 2−632 : Procollagen galactosyltransferase 2 on eGFR

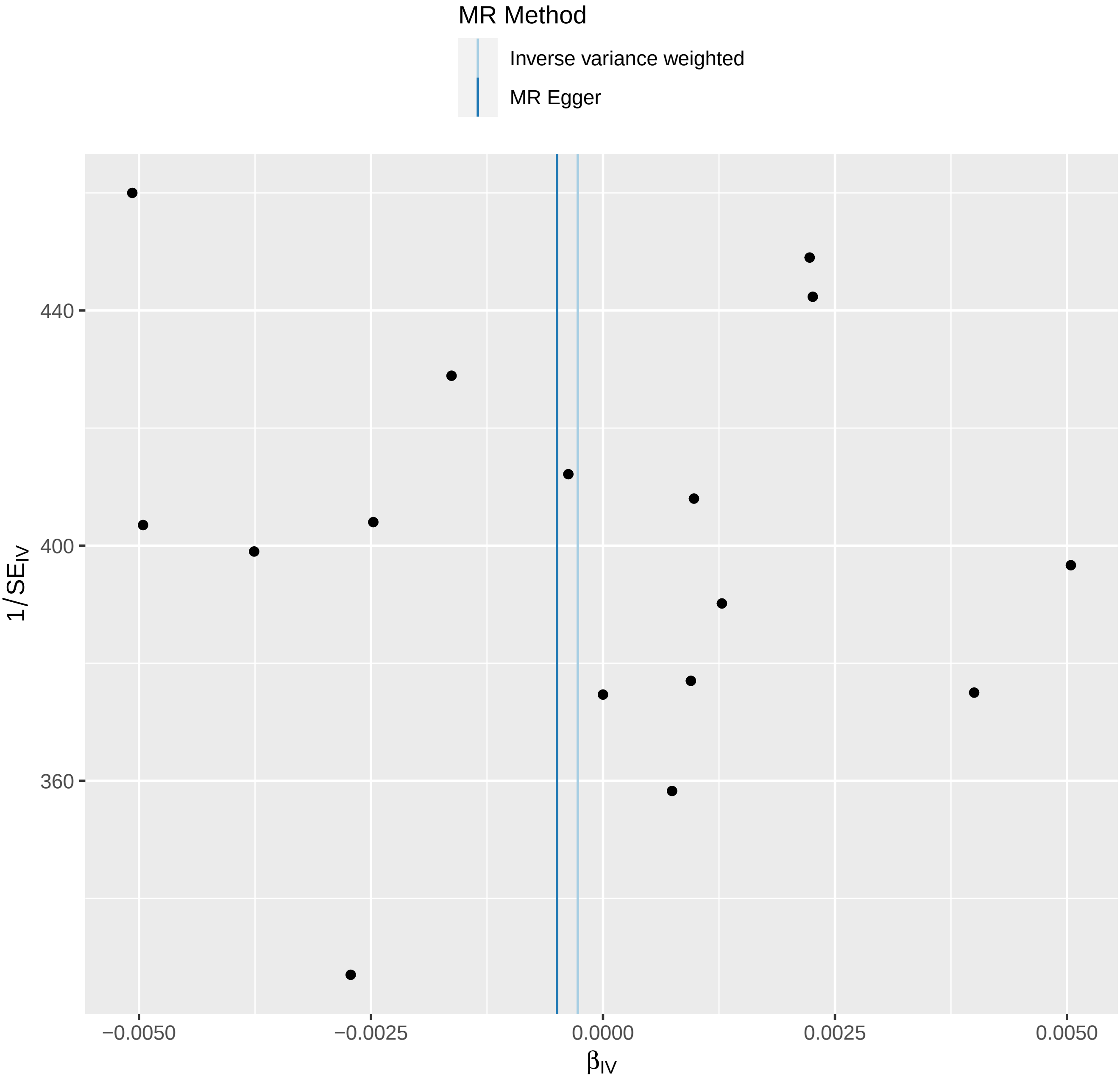

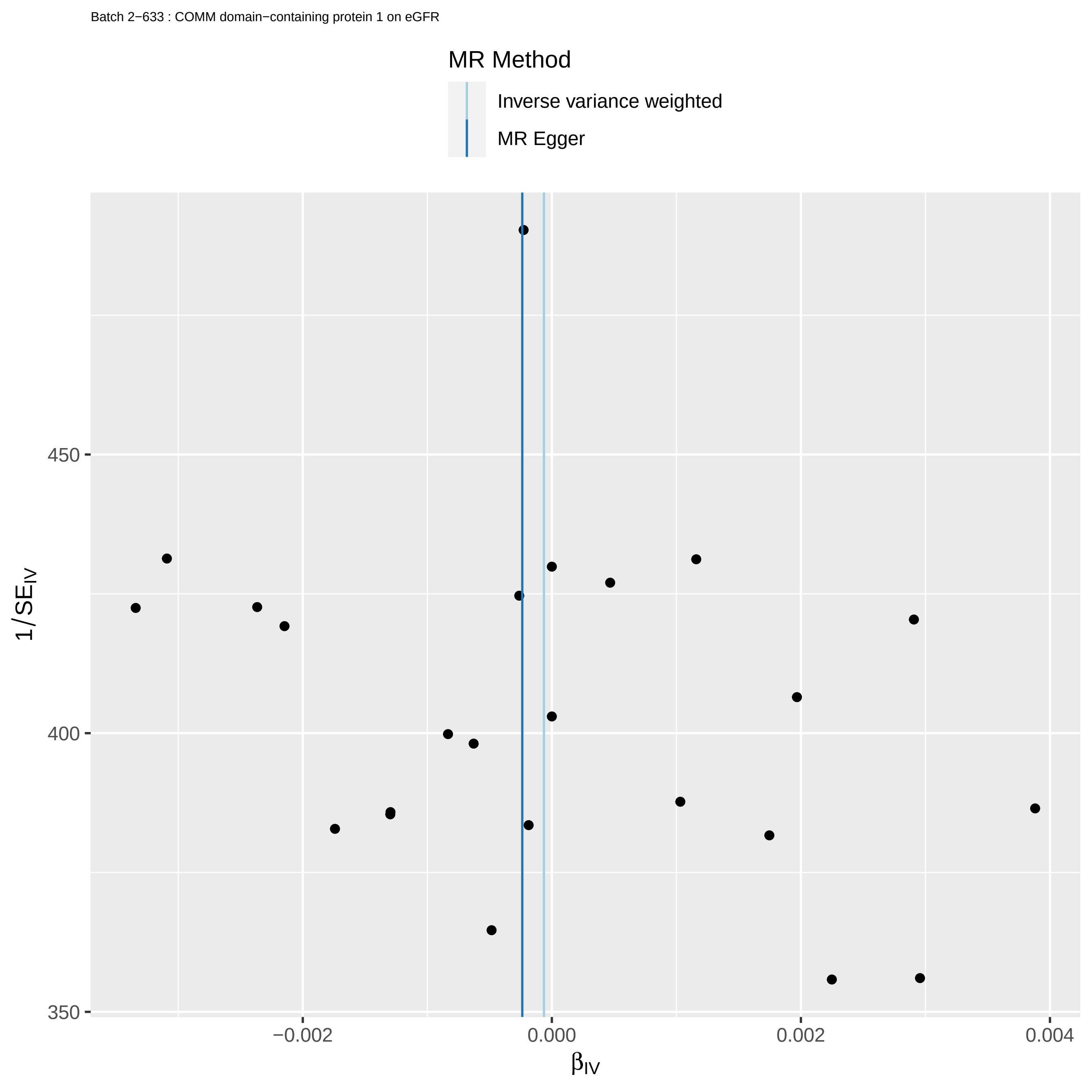

Batch 2−633 : COMM domain−containing protein 1 on eGFR
MR Method
Inverse variance weighted
MR Egger
450
400
350
1/SE_IV
−0.002
0.000
0.002
0.004
β_IV

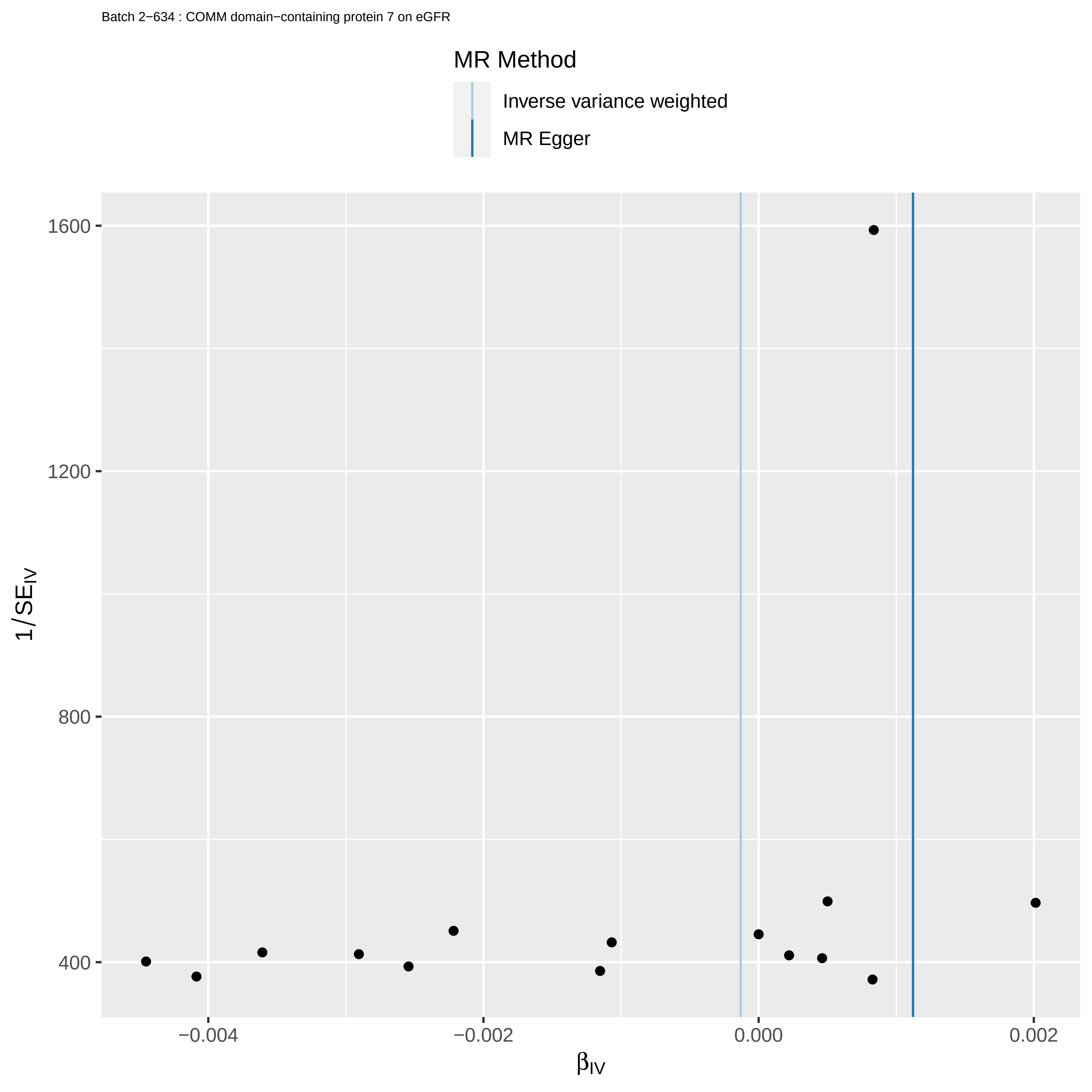

Batch 2−634 : COMM domain−containing protein 7 on eGFR
MR Method
Inverse variance weighted
MR Egger
1600
1200
800
400
1/SE_IV
−0.004
−0.002
0.000
0.002
β_IV

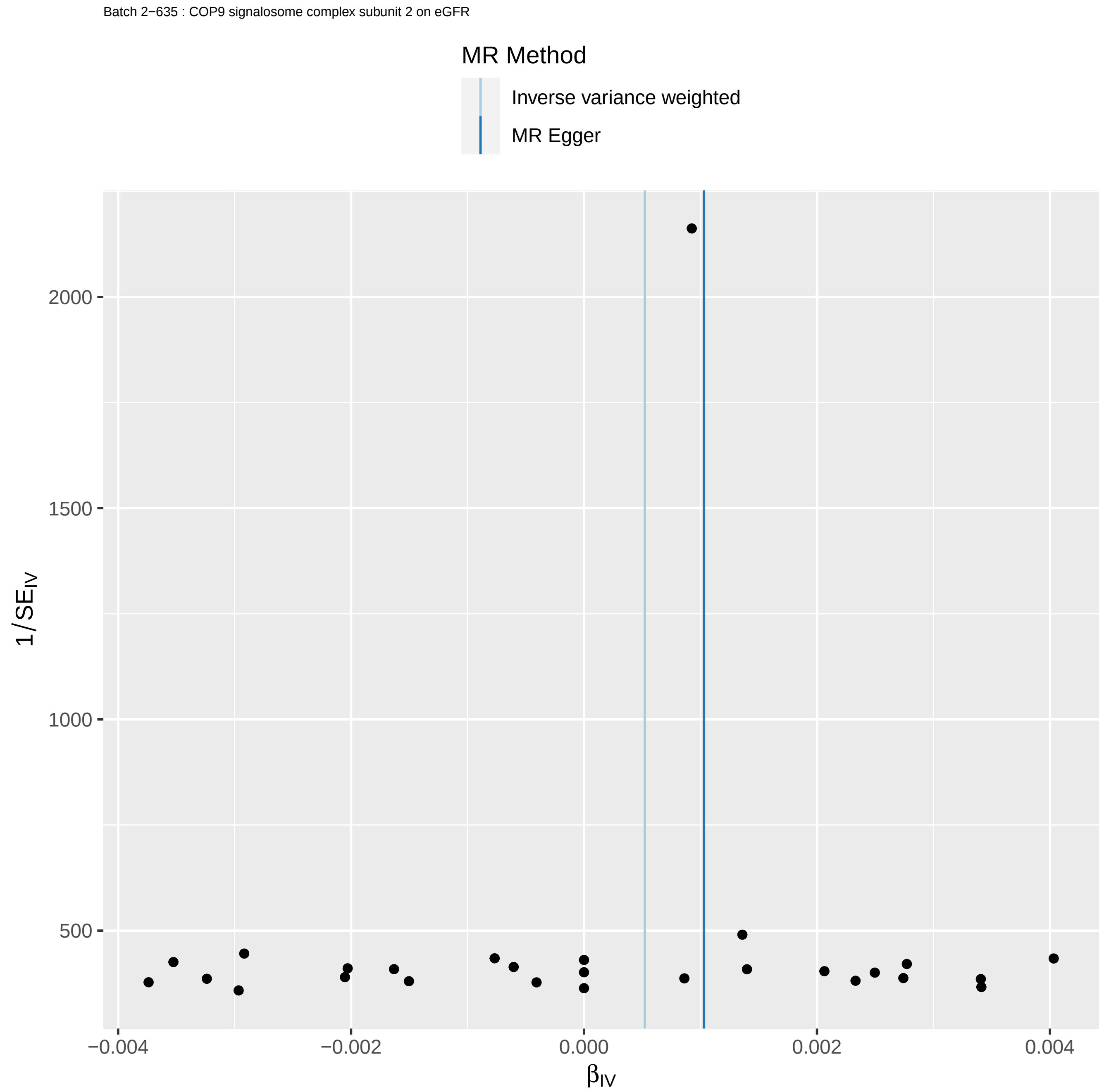
Batch 2−635 : COP9 signalosome complex subunit 2 on eGFR
MR Method
Inverse variance weighted
MR Egger
2000
1500
1000
500
1/SE_IV
−0.004
−0.002
0.000
0.002
0.004
β_IV

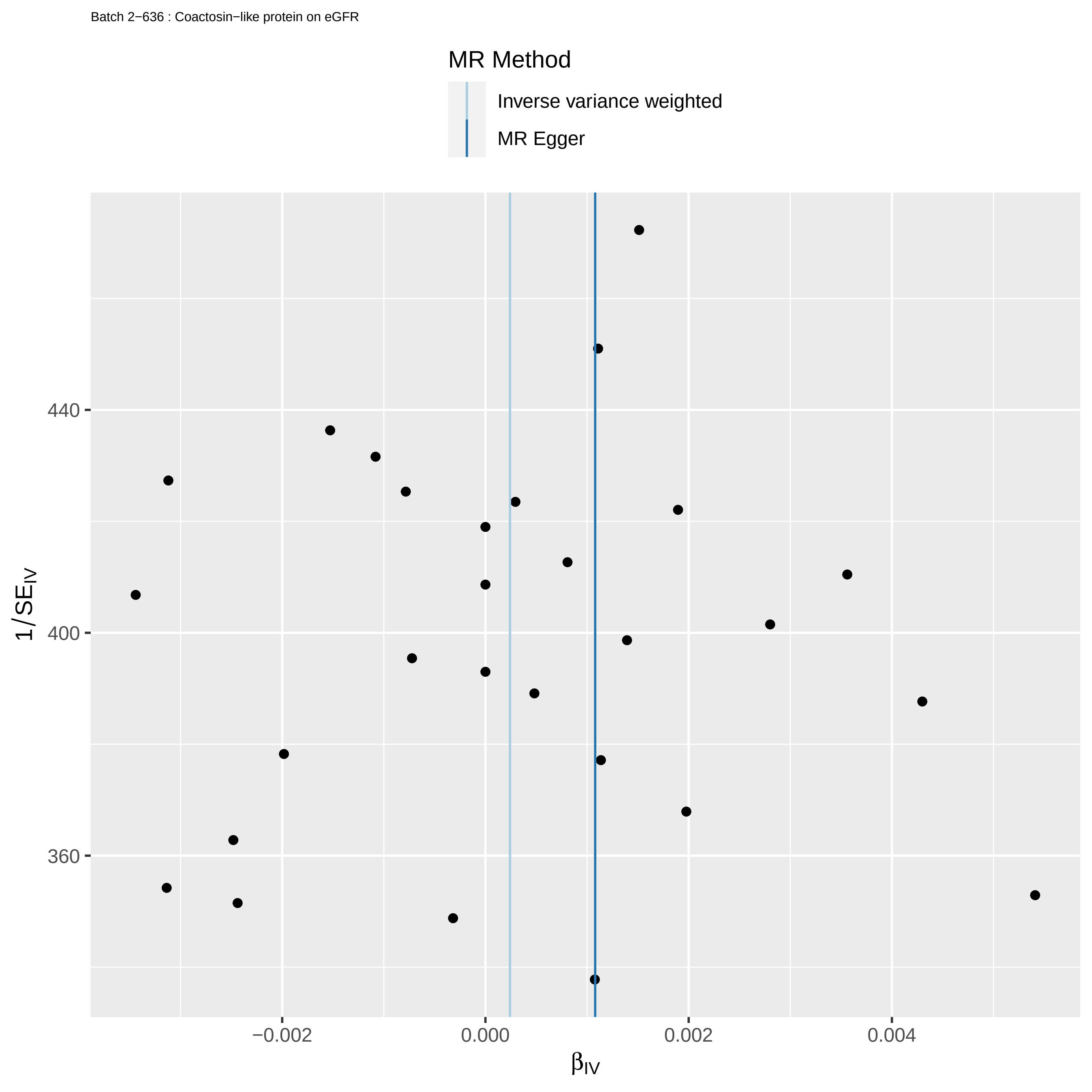
Batch 2−636 : Coactosin−like protein on eGFR
MR Method
Inverse variance weighted
MR Egger
440
400
360
1/SE$_{IV}$
−0.002
0.000
0.002
0.004
β$_{IV}$

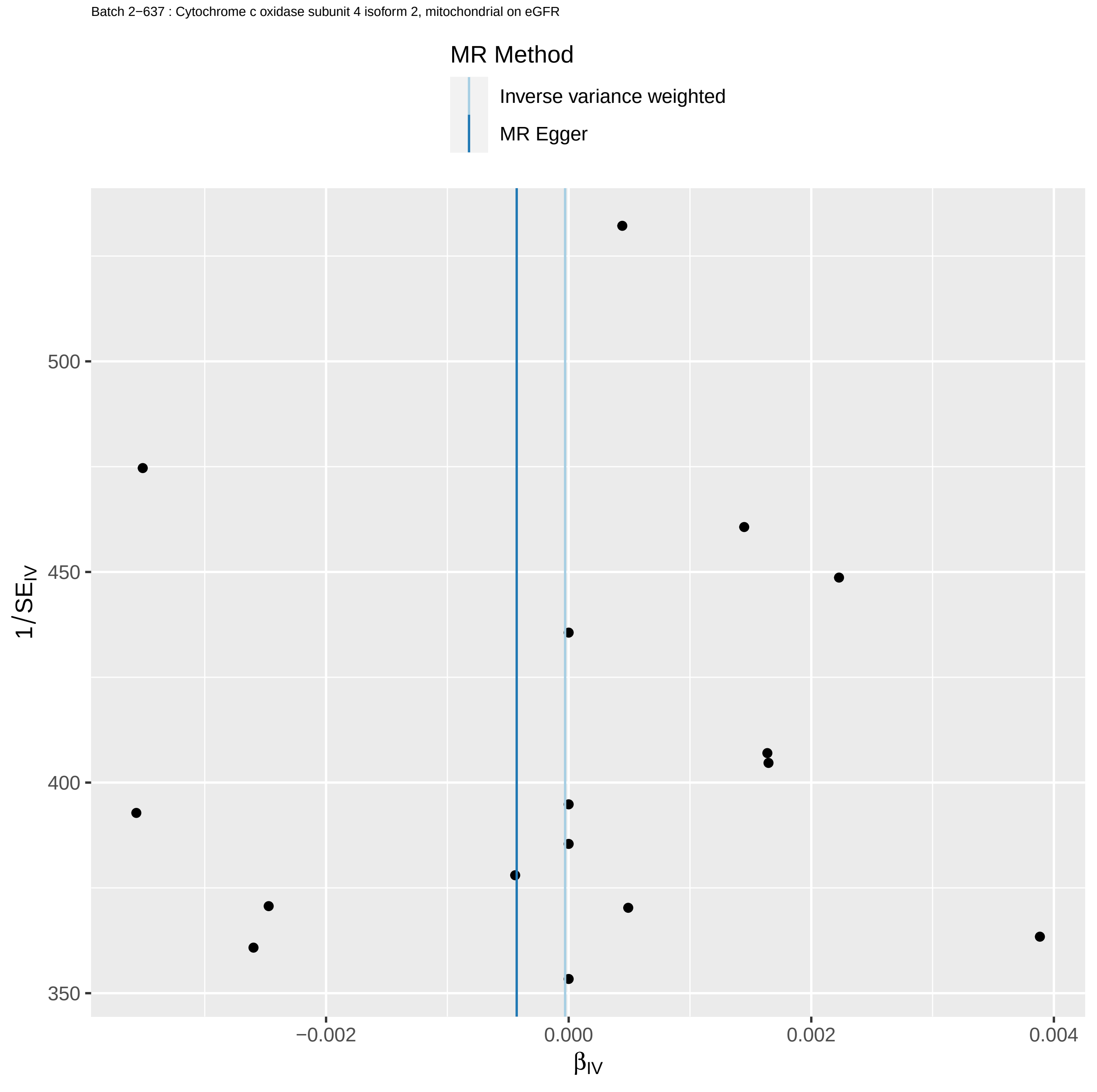

Batch 2−637 : Cytochrome c oxidase subunit 4 isoform 2, mitochondrial on eGFR
MR Method
Inverse variance weighted
MR Egger
500
450
400
350
1/SE$_{IV}$
−0.002
0.000
0.002
0.004
β$_{IV}$

Batch 2−638 : Cytochrome c oxidase subunit 5B, mitochondrial on eGFR

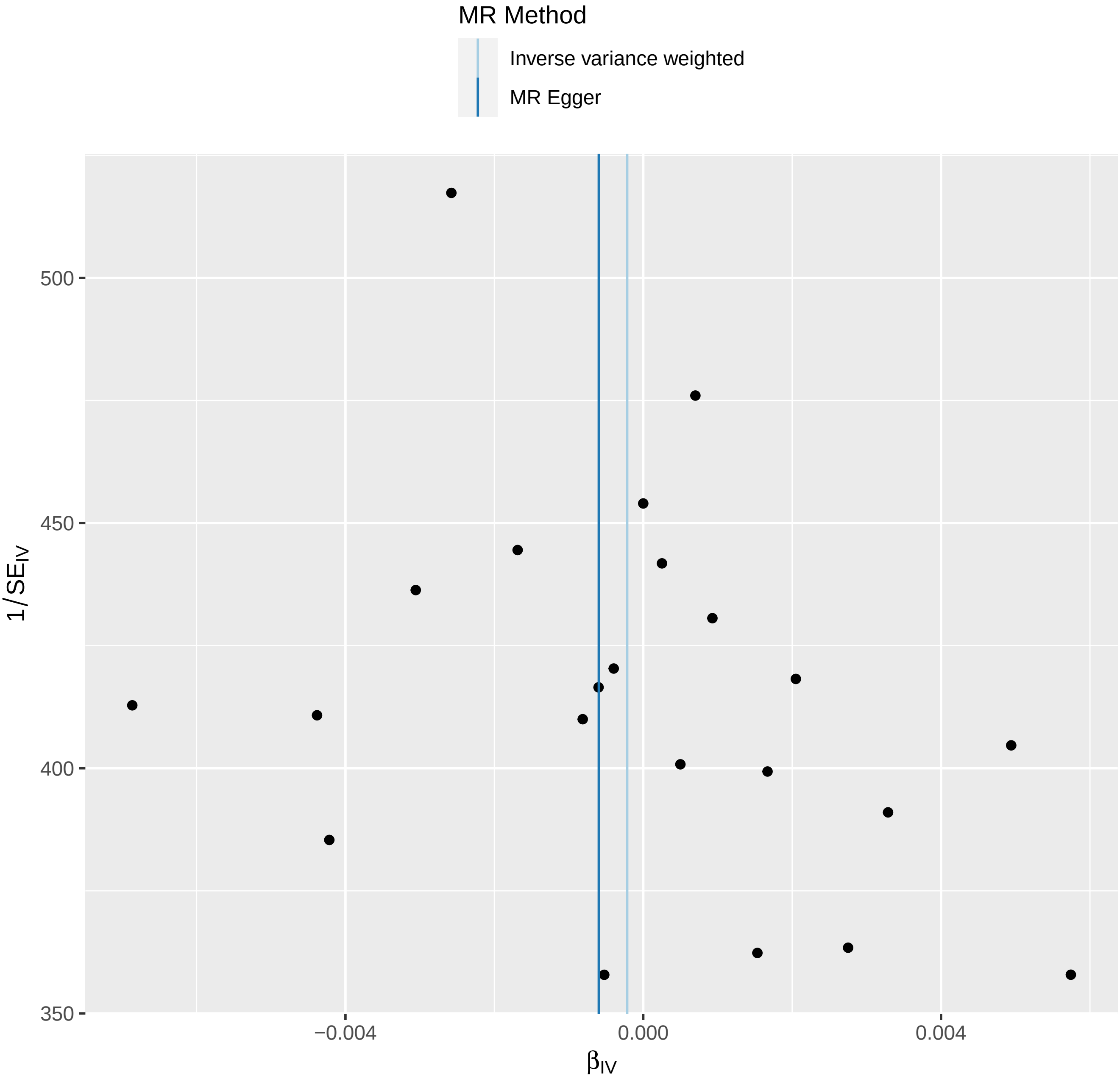

Batch 2−639 : Cytochrome c oxidase subunit 6C on eGFR

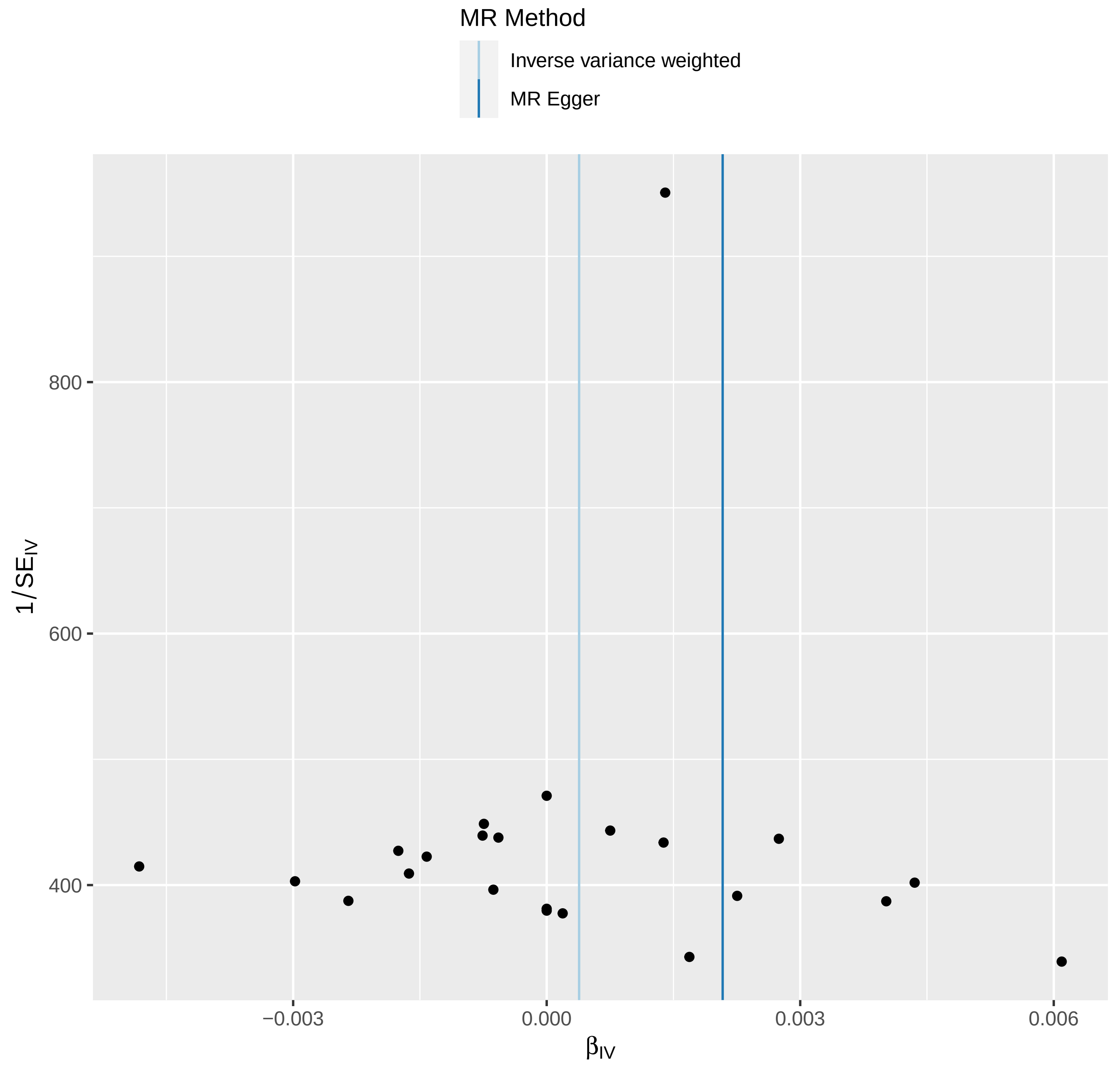

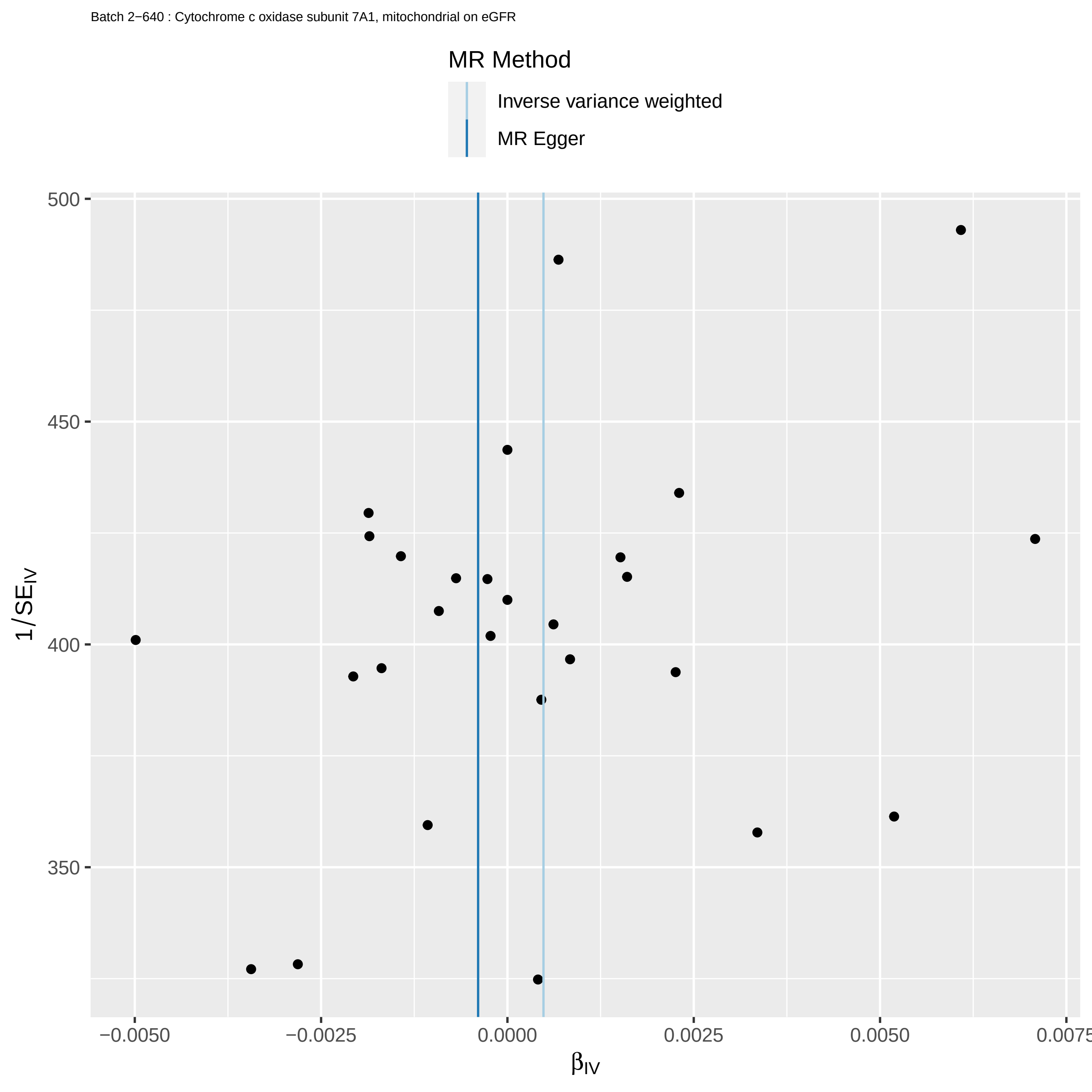

Batch 2−640 : Cytochrome c oxidase subunit 7A1, mitochondrial on eGFR
MR Method
Inverse variance weighted
MR Egger
500
450
400
350
1/SE$_{IV}$
−0.0050
−0.0025
0.0000
0.0025
0.0050
0.0075
β$_{IV}$

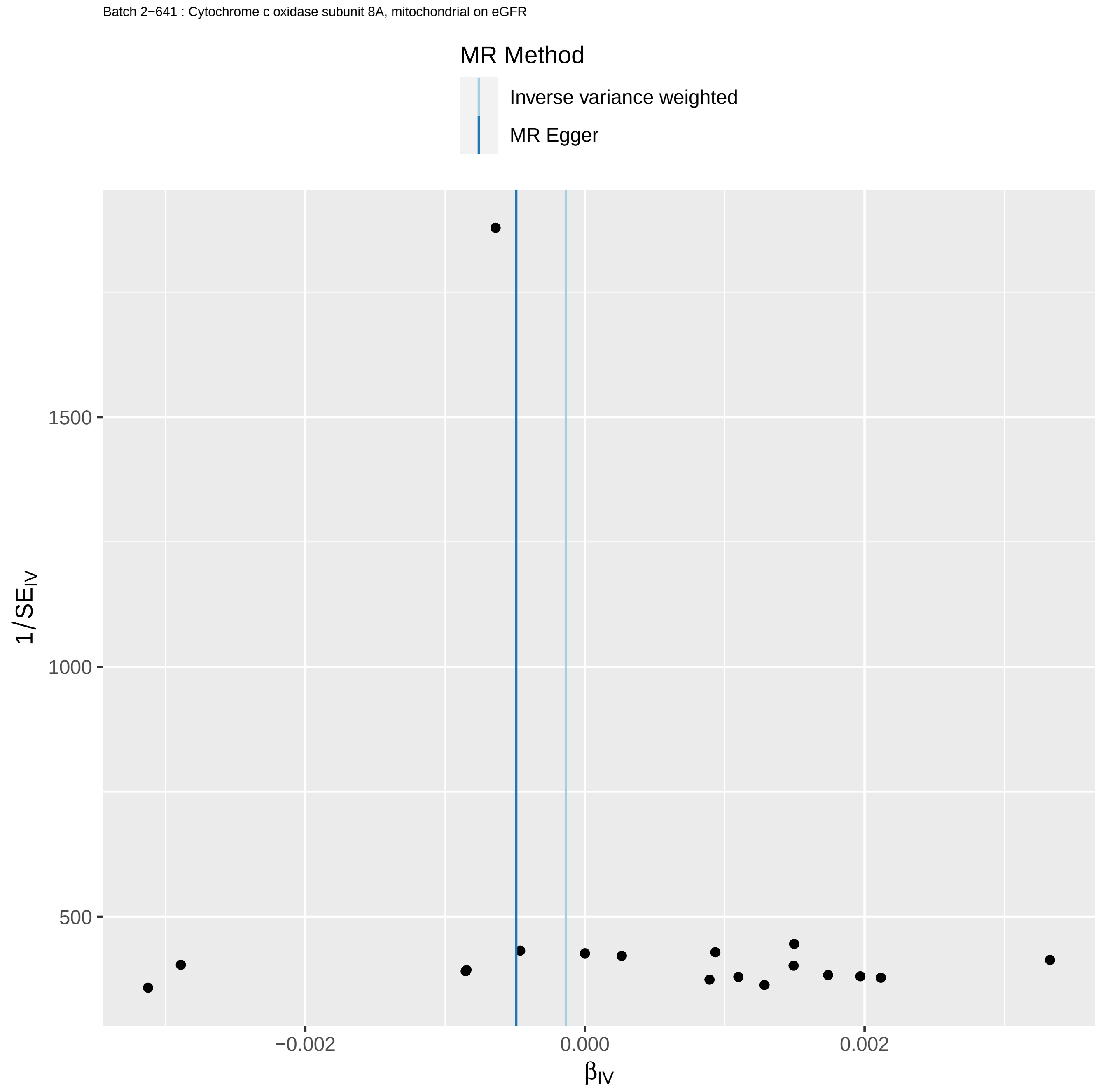

Batch 2−641 : Cytochrome c oxidase subunit 8A, mitochondrial on eGFR
MR Method
Inverse variance weighted
MR Egger
1500
1000
500
1/SE$_{IV}$
−0.002
0.000
0.002
β$_{IV}$

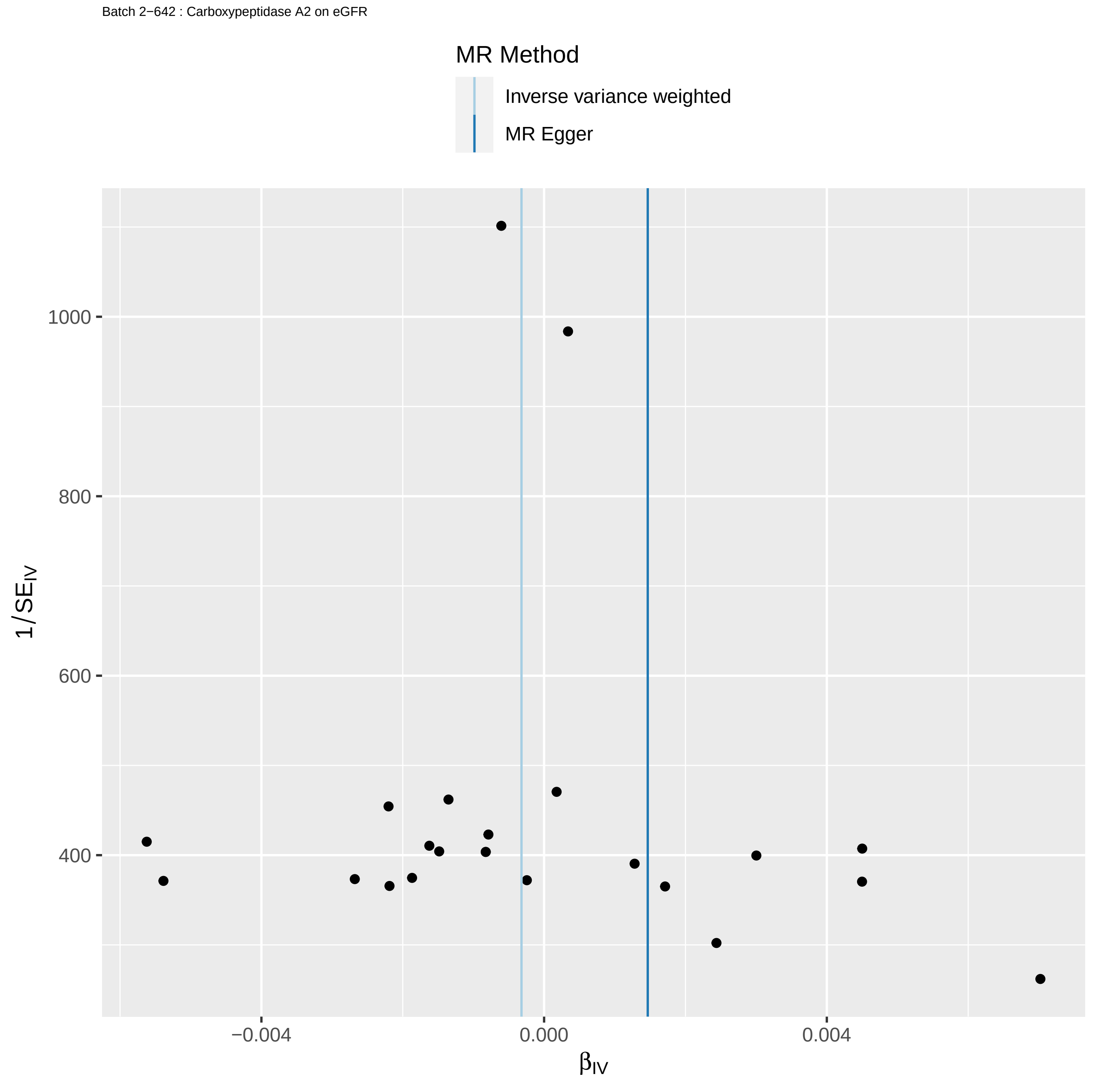

Batch 2−642 : Carboxypeptidase A2 on eGFR
MR Method
Inverse variance weighted
MR Egger
1000
800
600
400
1/SE_IV
−0.004
0.000
0.004
β_IV

Batch 2−643 : Carboxypeptidase A4 on eGFR

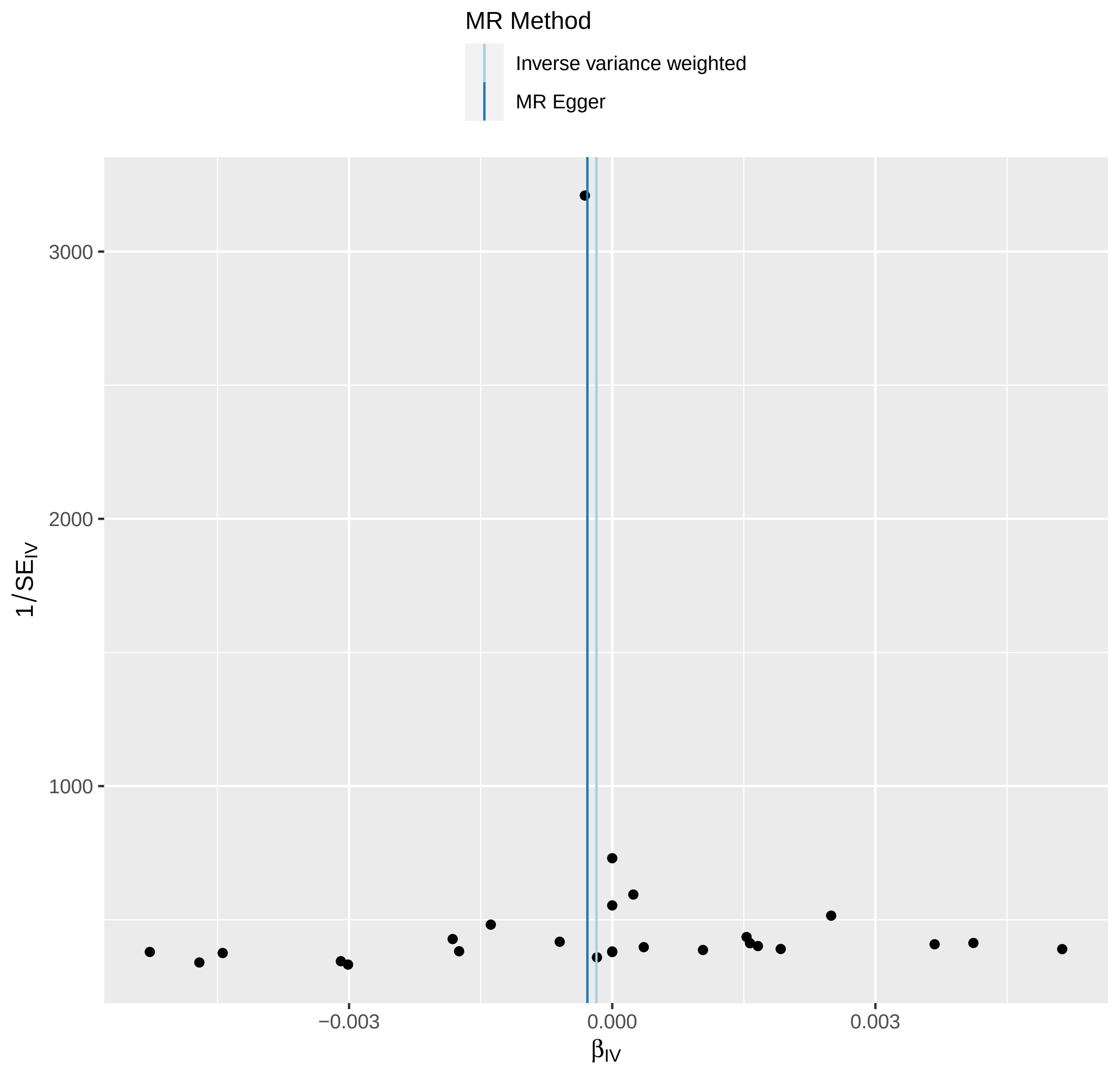

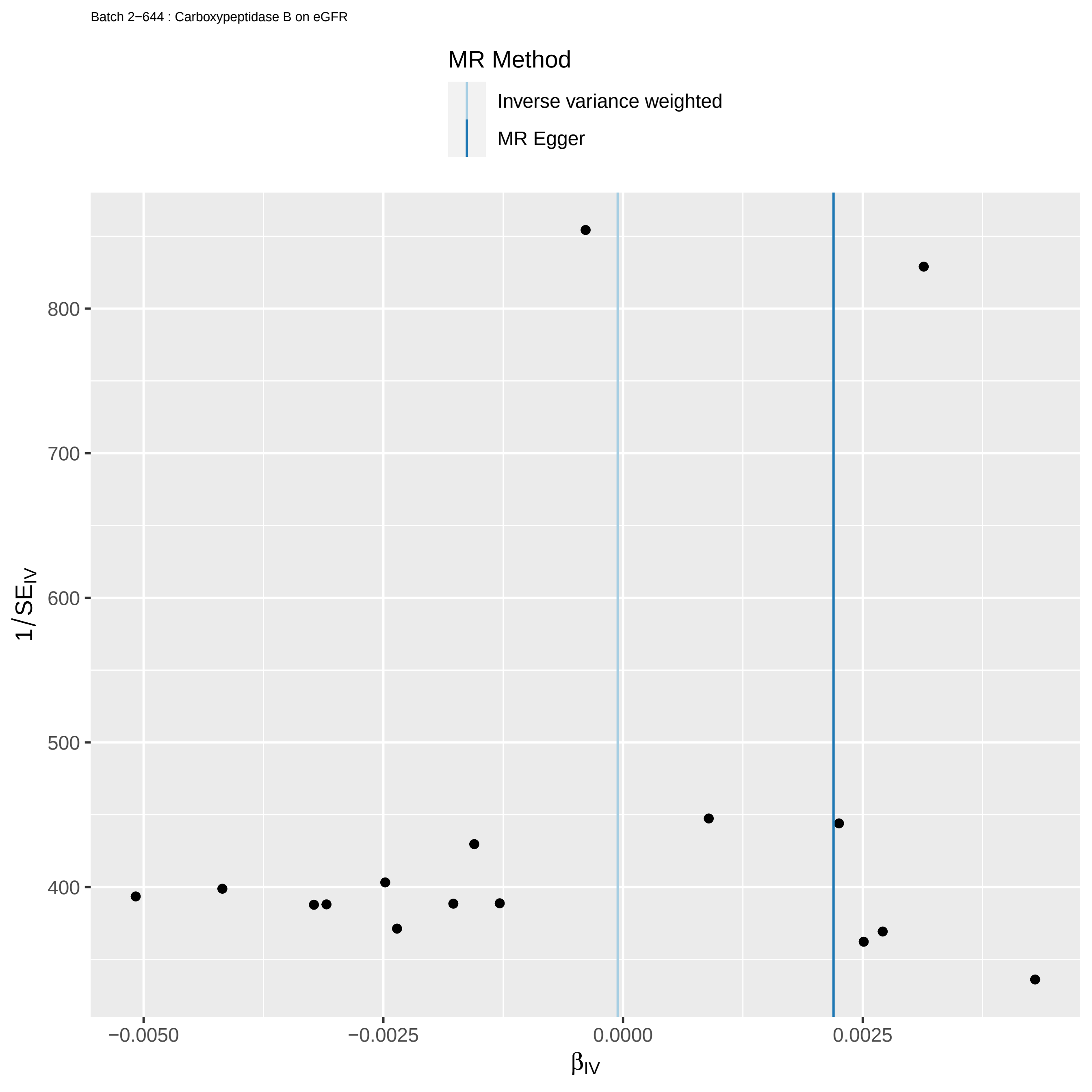
Batch 2−644 : Carboxypeptidase B on eGFR
MR Method
Inverse variance weighted
MR Egger
800
700
600
500
400
1/SE_IV
−0.0050
−0.0025
0.0000
0.0025
β_IV

Batch 2−645 : Carboxypeptidase M on eGFR

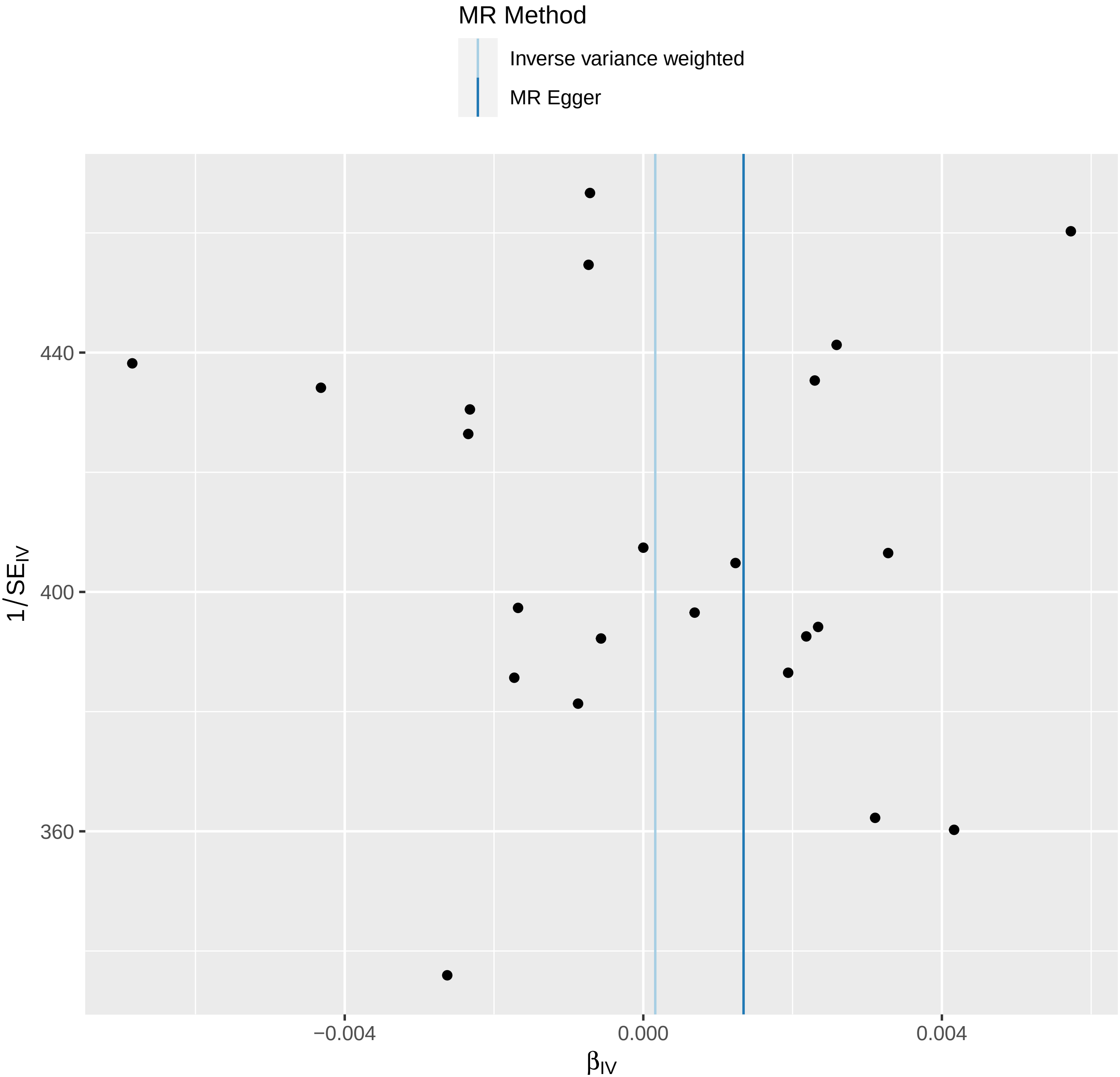

Batch 2−646 : Copine−1 on eGFR

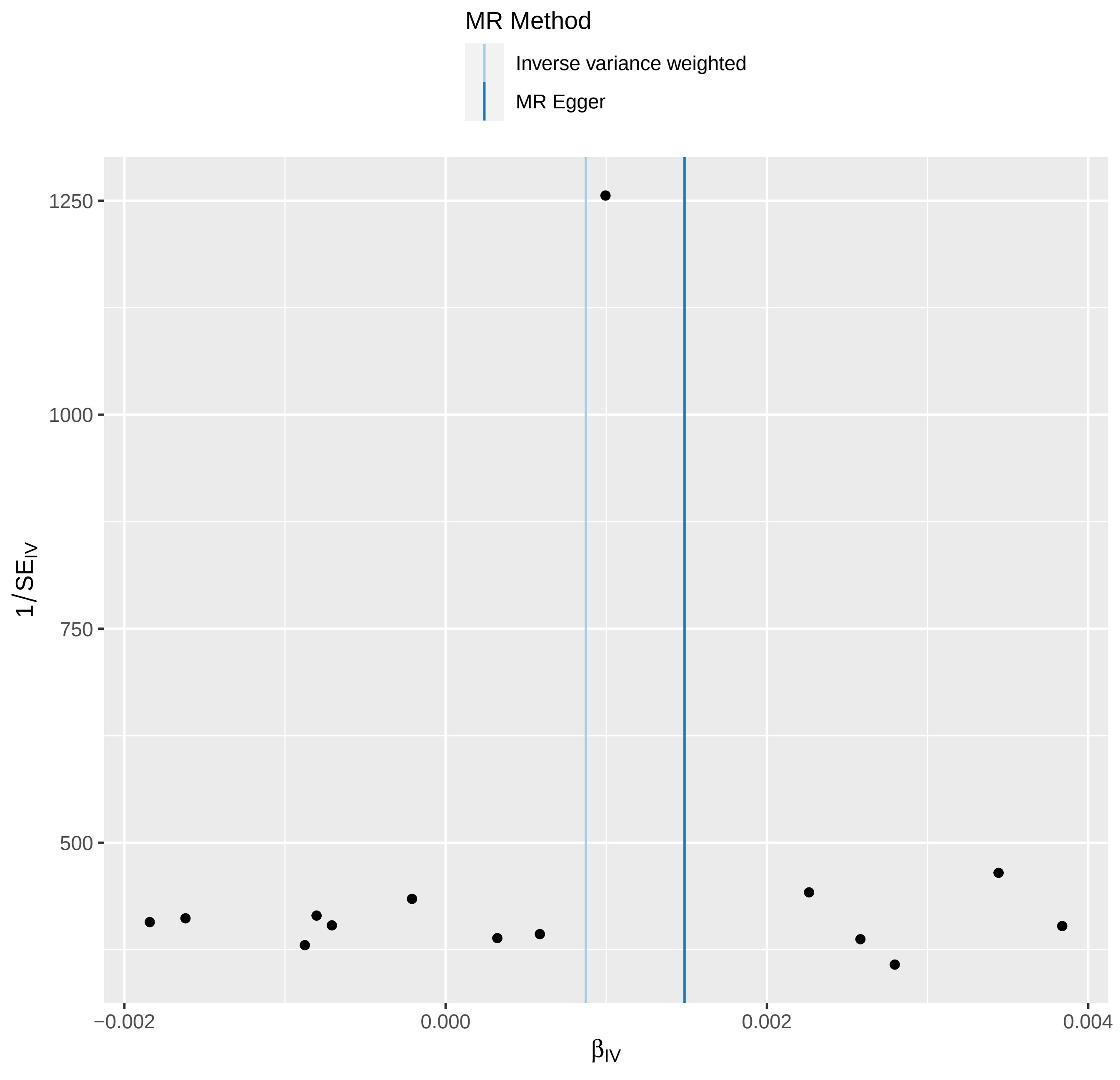

Batch 2−647 : Probable serine carboxypeptidase CPVL on eGFR

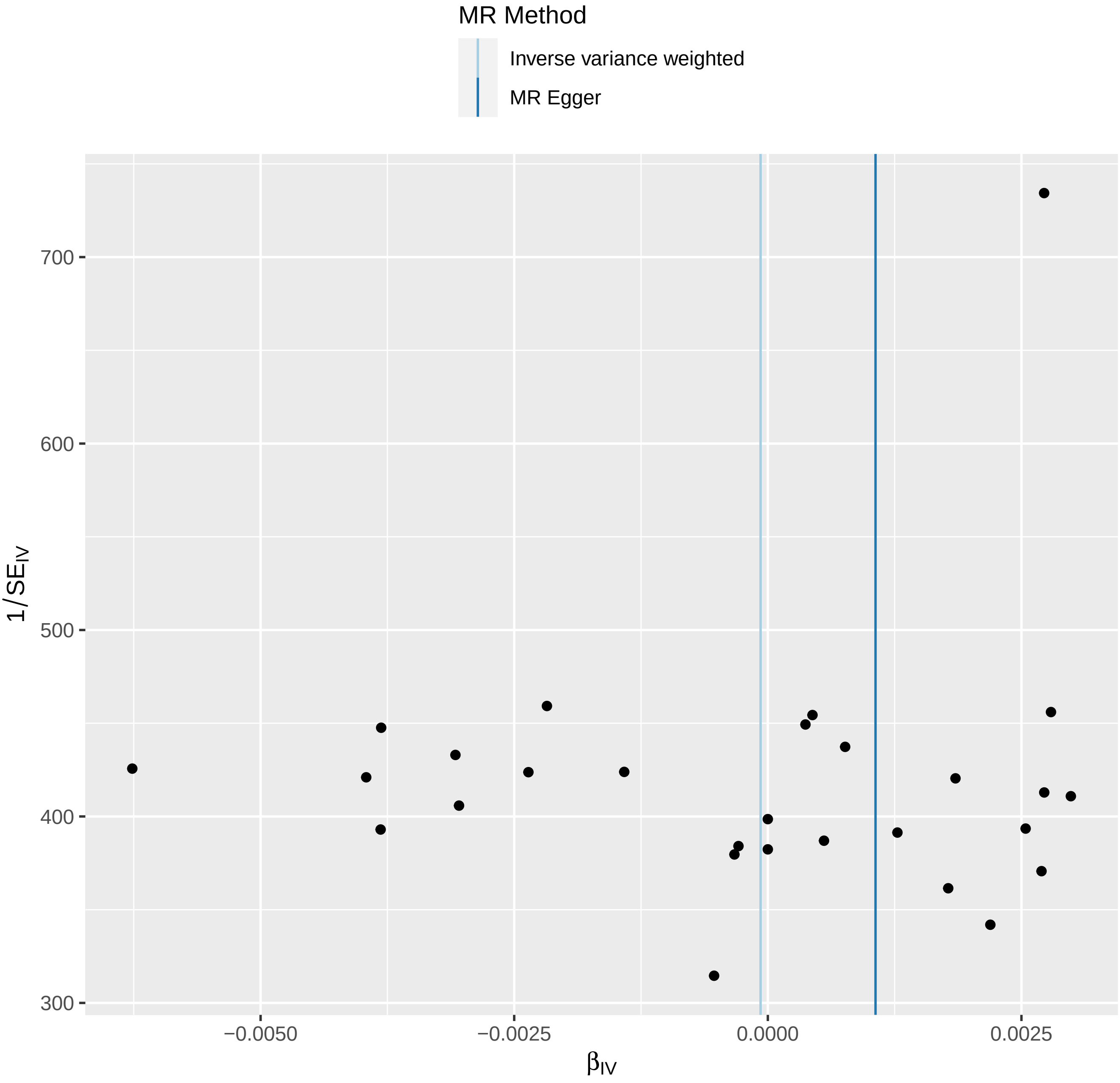

Batch 2−648 : Probable carboxypeptidase X1 on eGFR

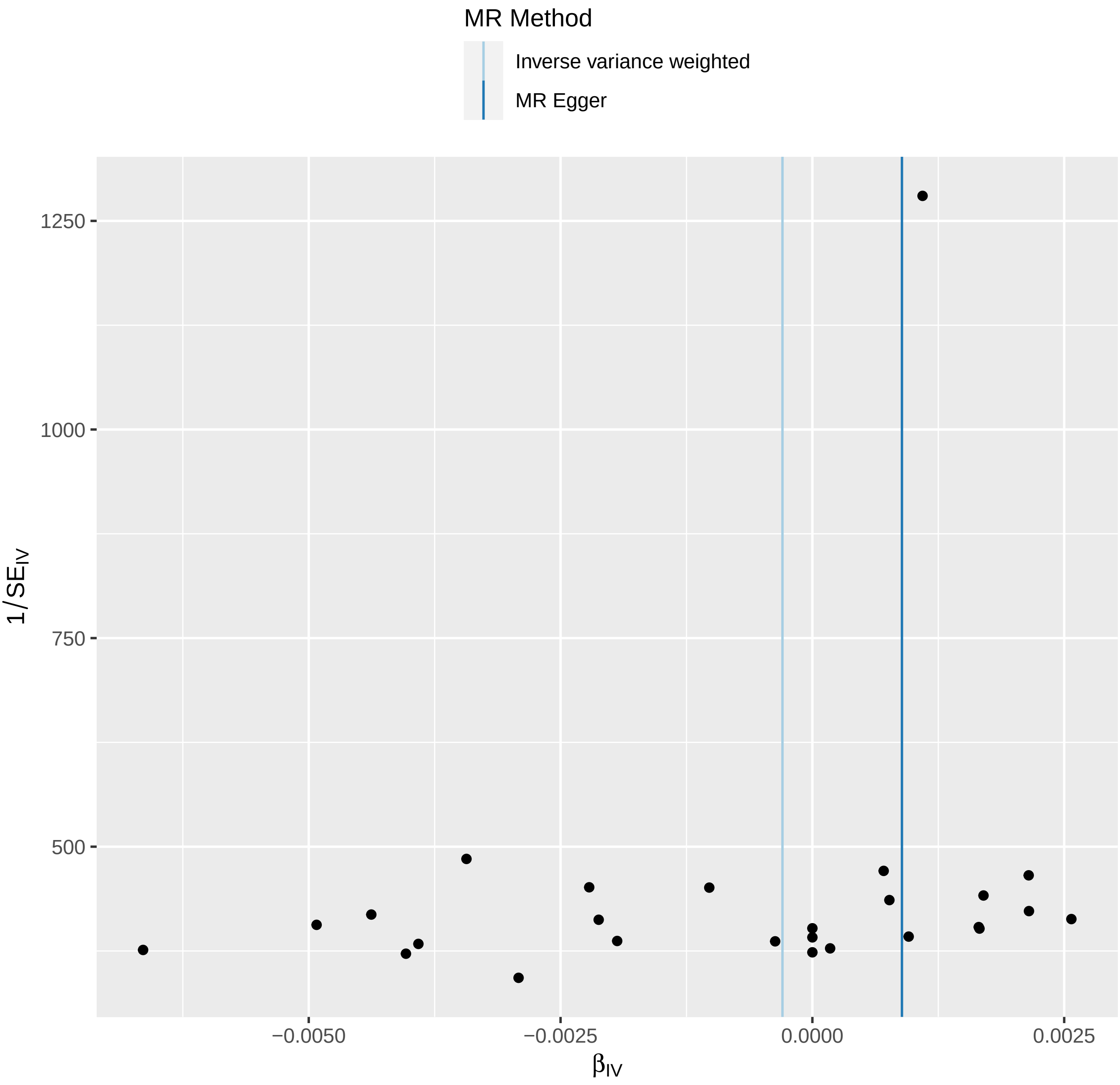

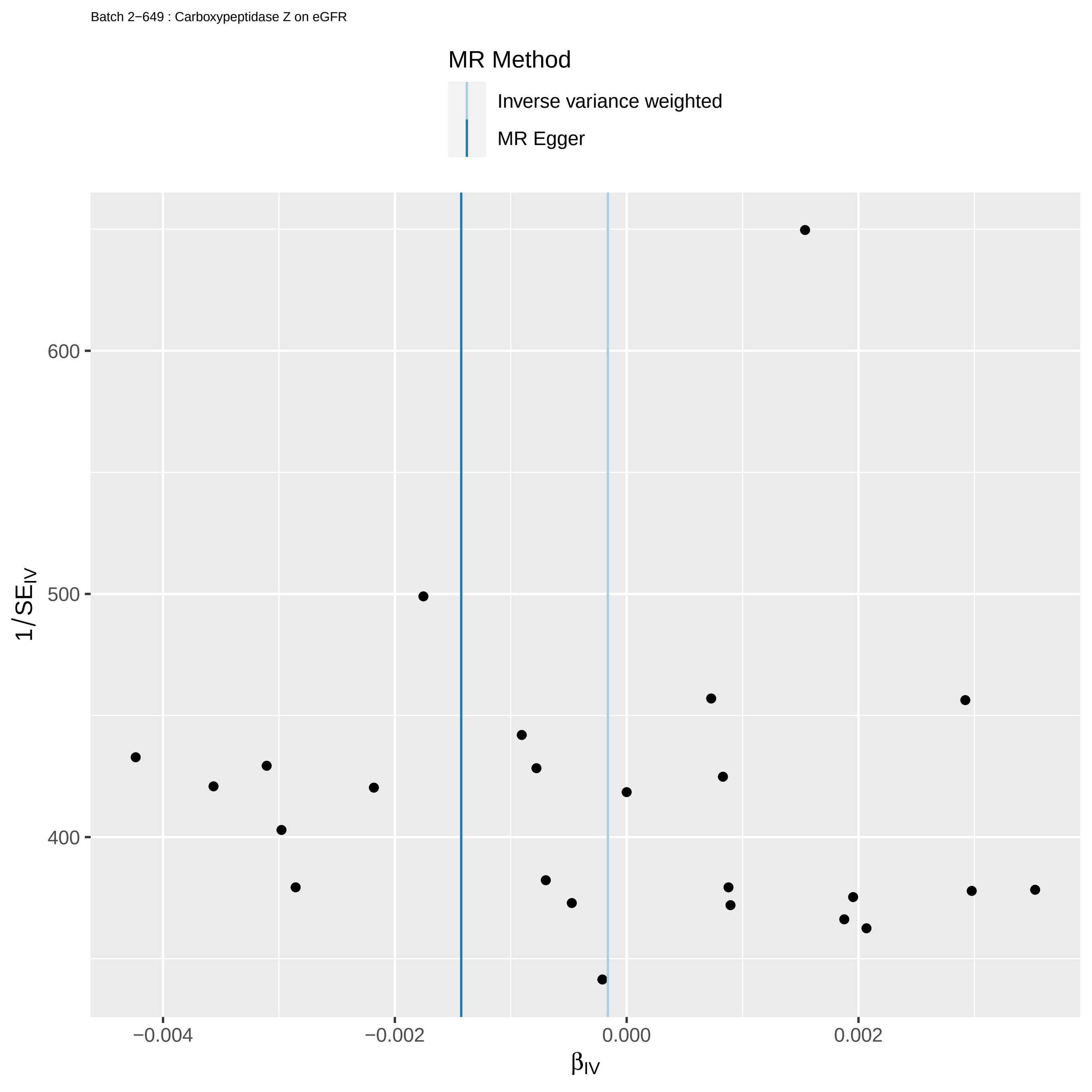

Batch 2−649 : Carboxypeptidase Z on eGFR
MR Method
Inverse variance weighted
MR Egger
600
500
400
1/SE_IV
−0.004
−0.002
0.000
0.002
β_IV

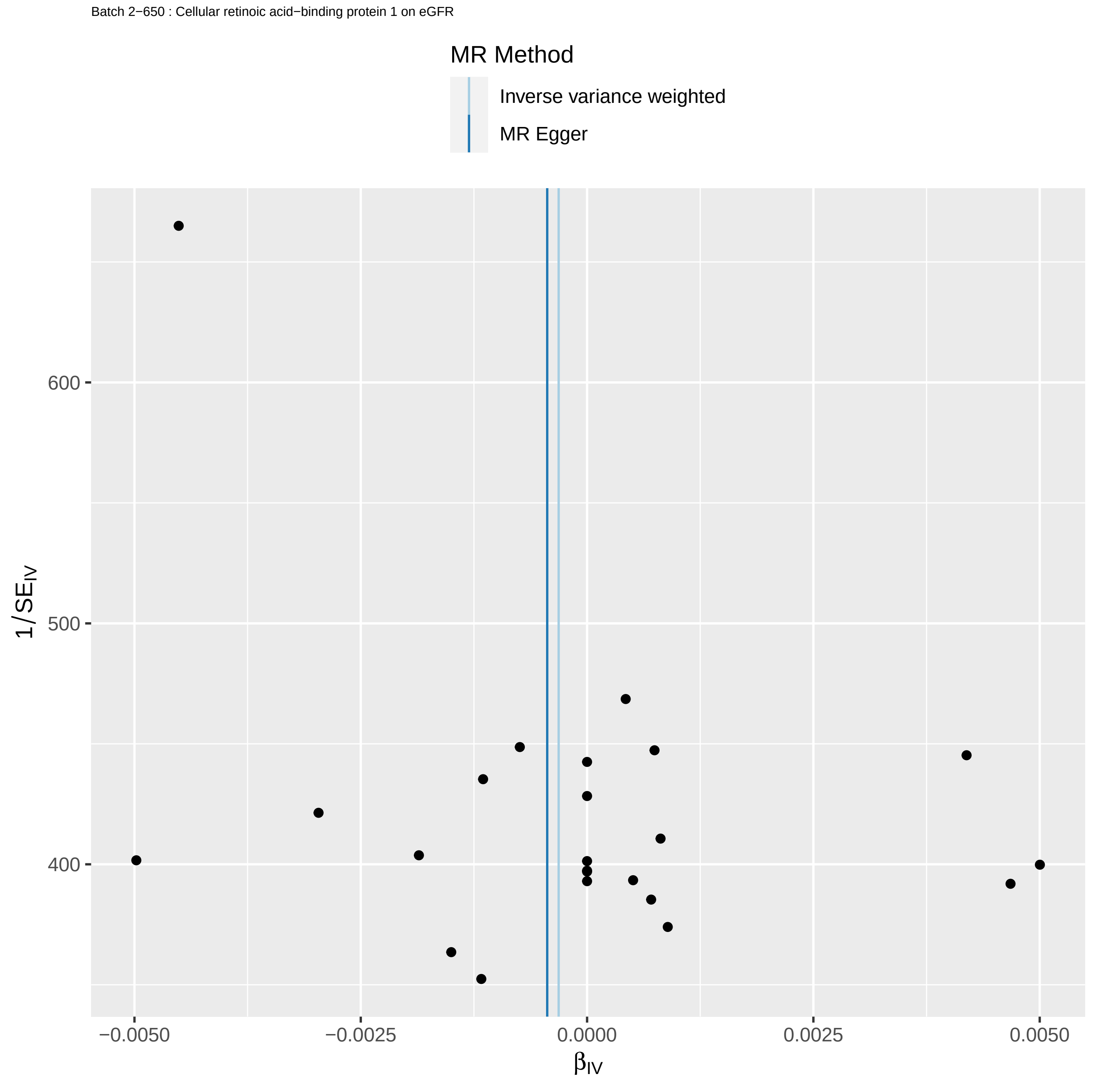
Batch 2−650 : Cellular retinoic acid−binding protein 1 on eGFR
MR Method
Inverse variance weighted
MR Egger
600
500
400
1/SE_IV
−0.0050
−0.0025
0.0000
0.0025
0.0050
β_IV

Batch 2−651 : Cellular retinoic acid−binding protein 2 on eGFR

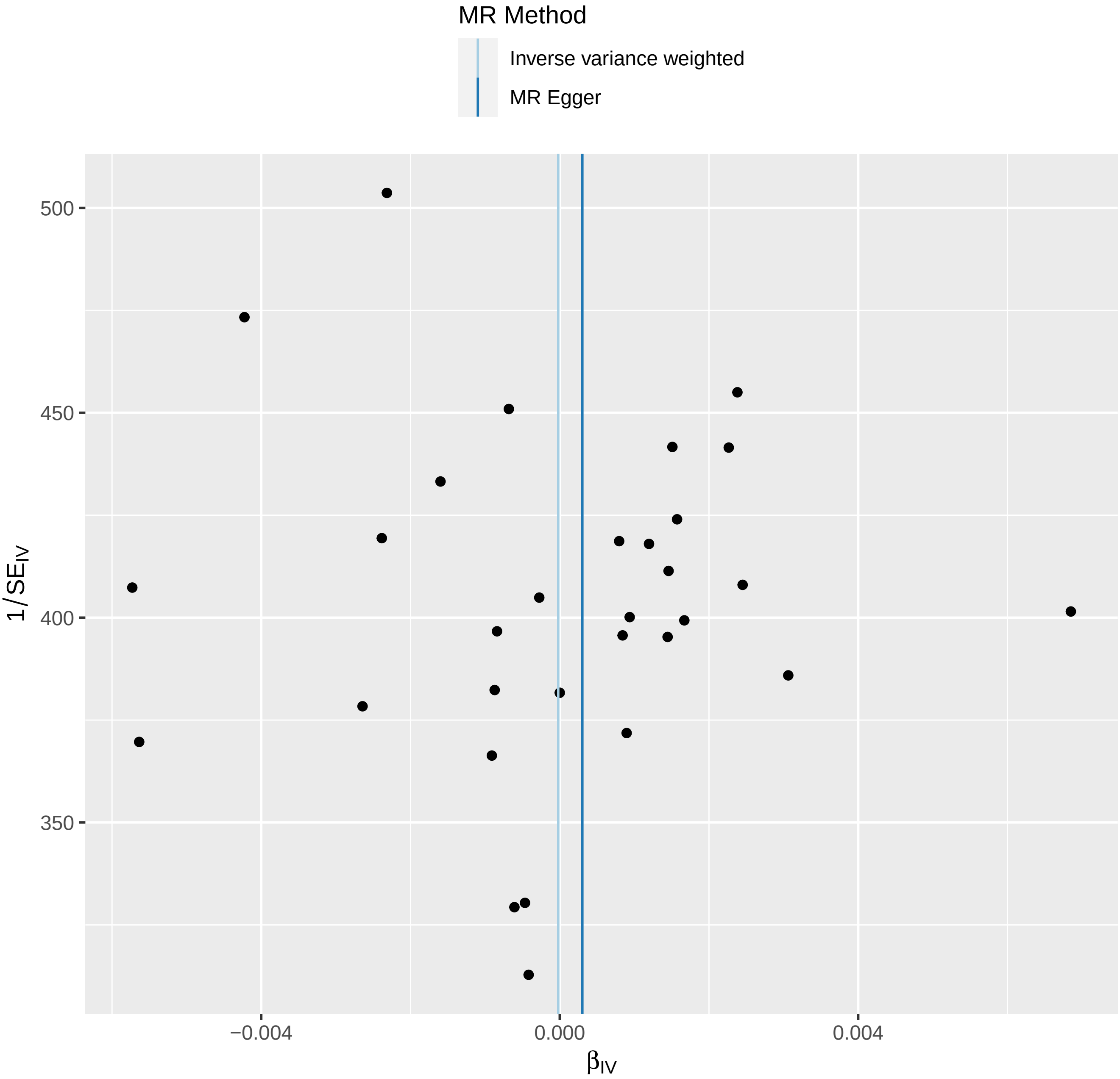

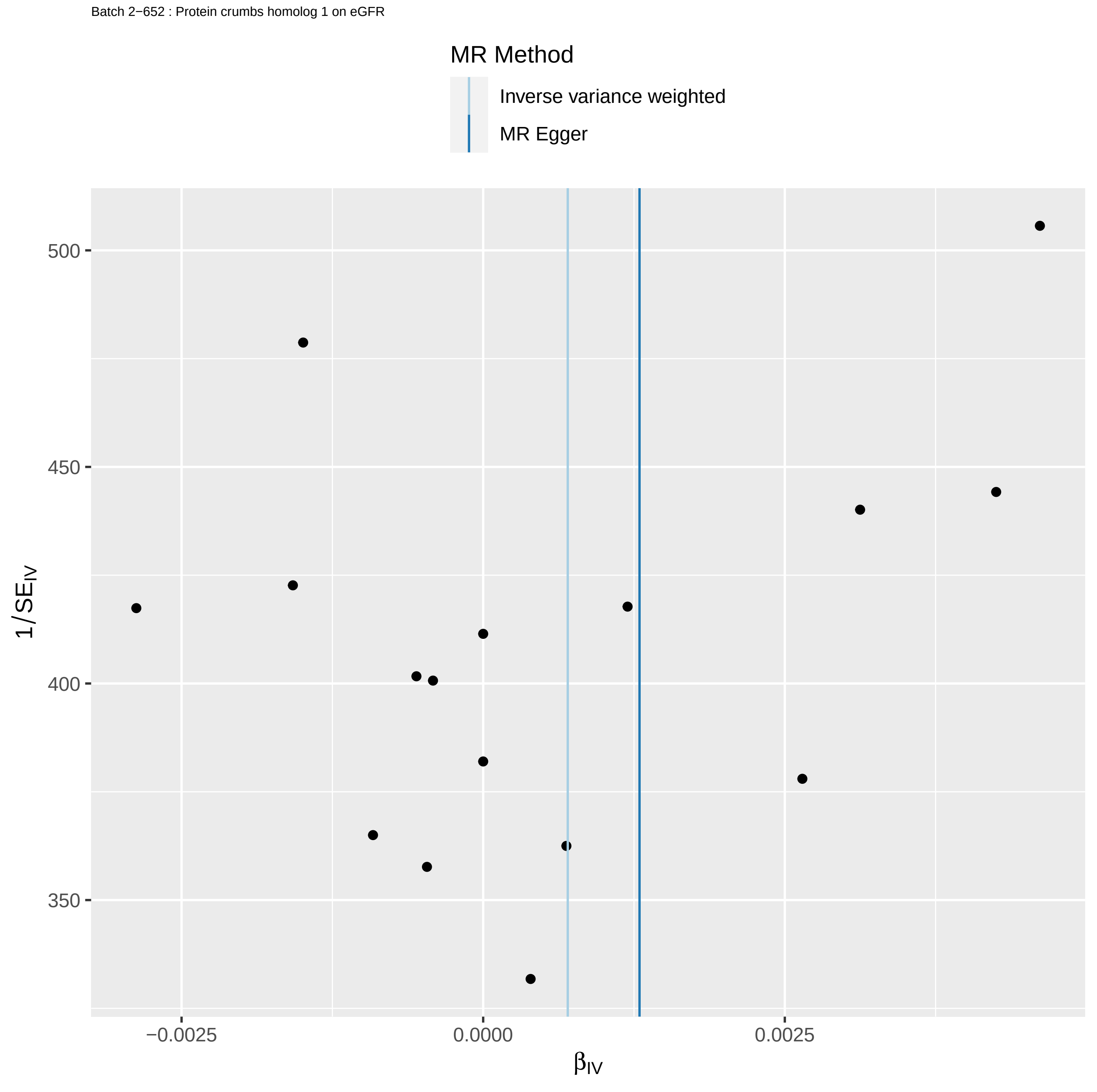
Batch 2−652 : Protein crumbs homolog 1 on eGFR
MR Method
Inverse variance weighted
MR Egger
500
450
400
350
1/SE$_{IV}$
−0.0025
0.0000
0.0025
β$_{IV}$

Batch 2−653 : Cyclic AMP−responsive element−binding protein 3−like protein 1 on eGFR

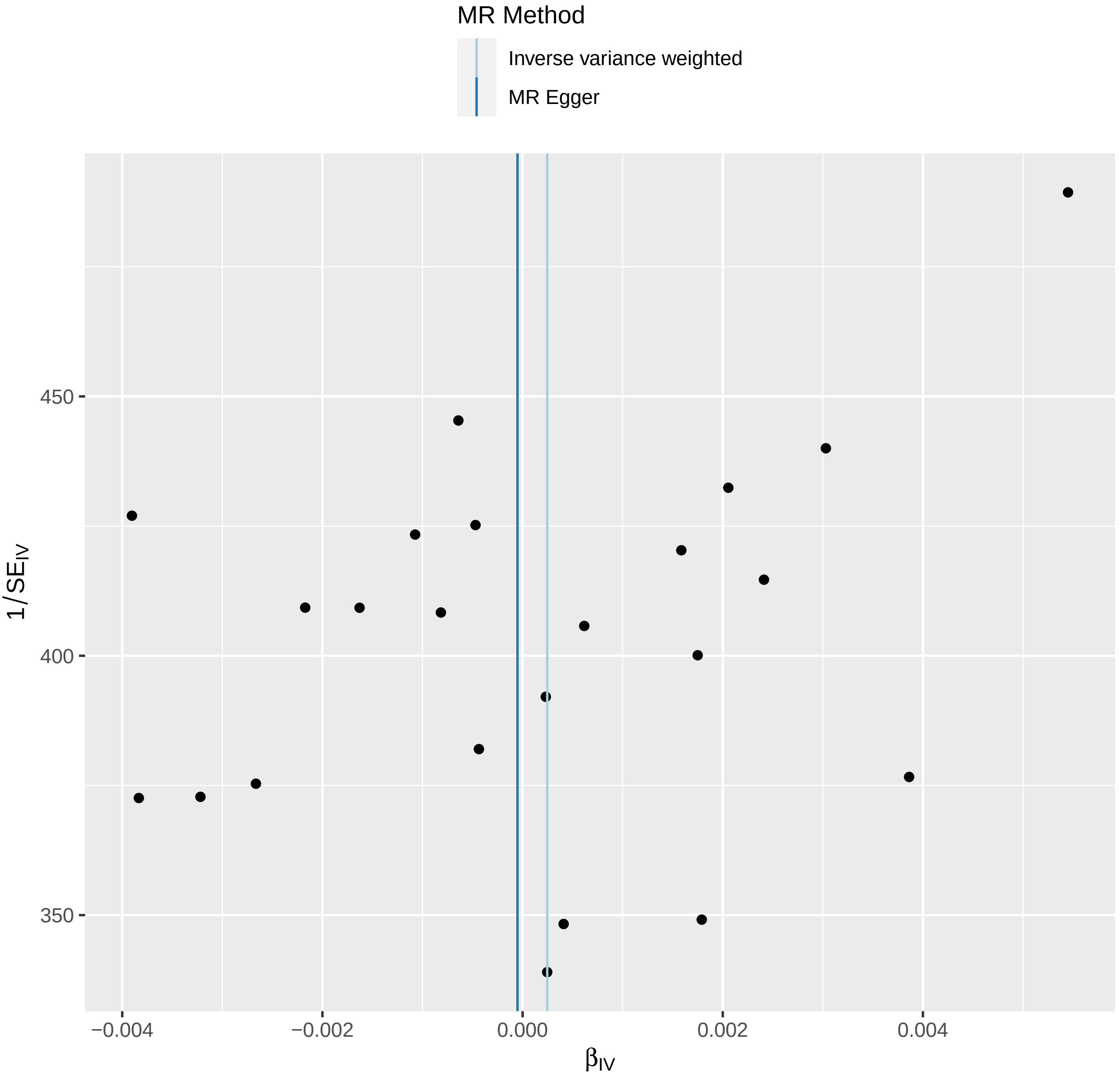

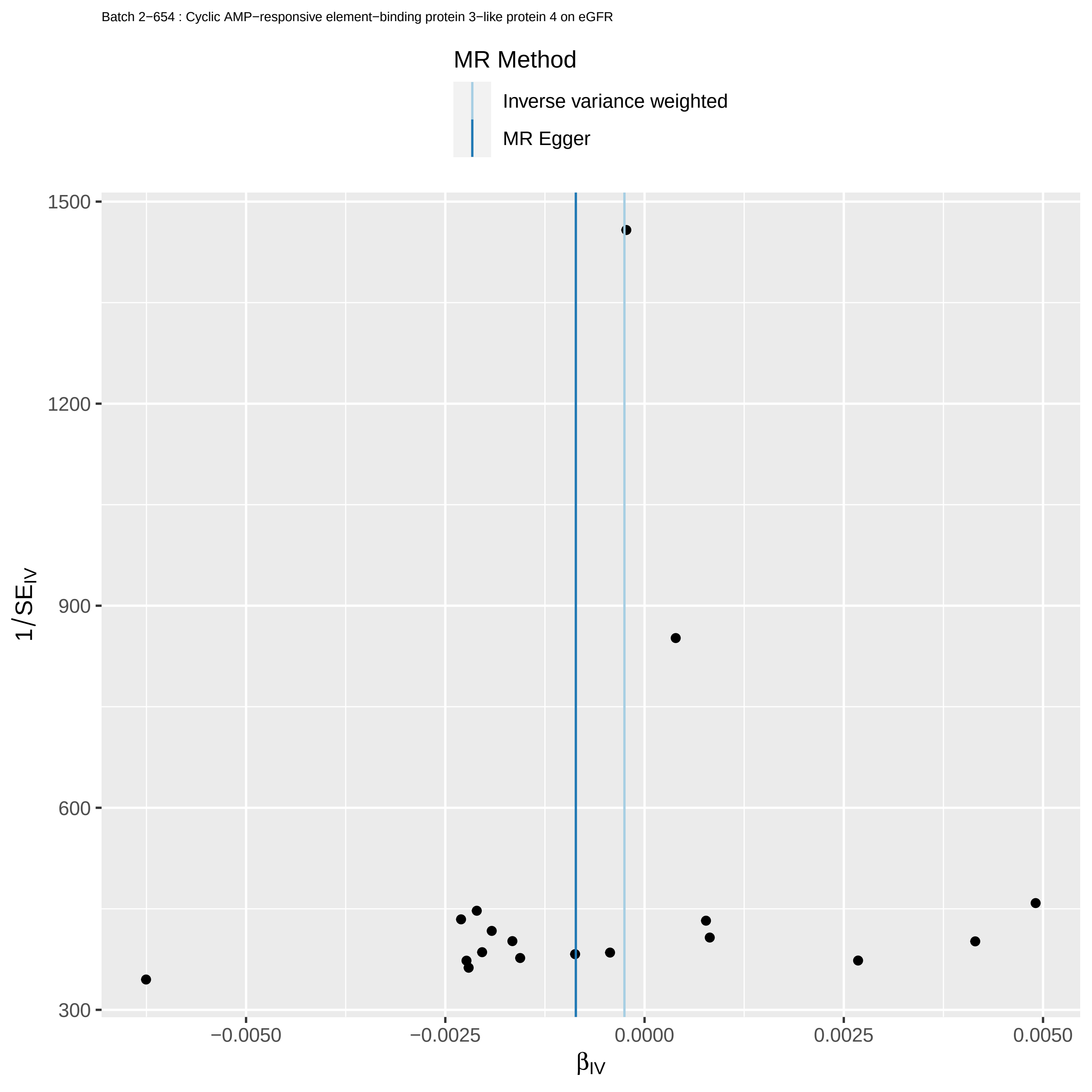

Batch 2−654 : Cyclic AMP−responsive element−binding protein 3−like protein 4 on eGFR
MR Method
Inverse variance weighted
MR Egger
1500
1200
900
600
300
1/SE$_{IV}$
−0.0050
−0.0025
0.0000
0.0025
0.0050
β_{IV}

Batch 2−655 : CREB−binding protein on eGFR

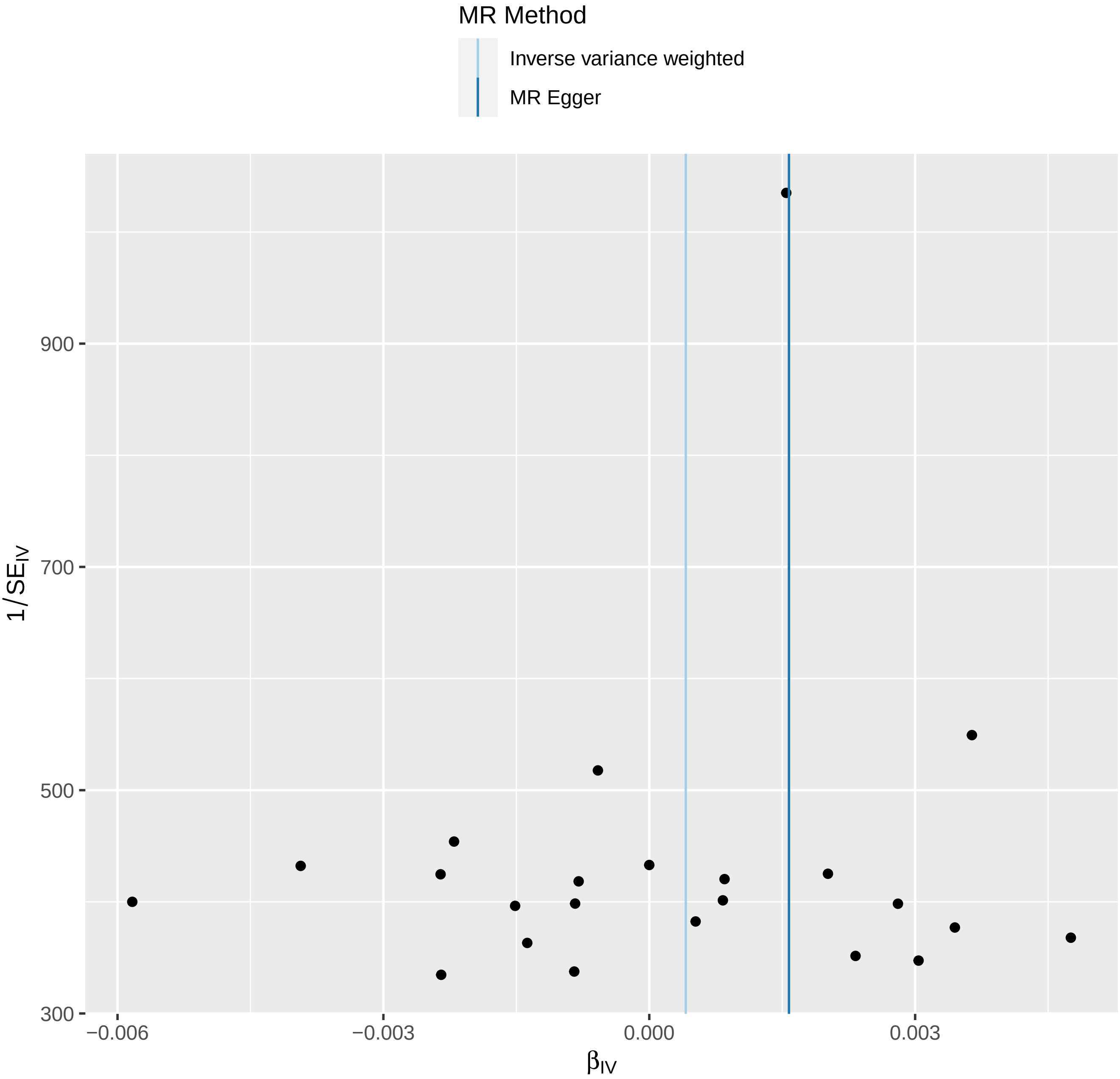

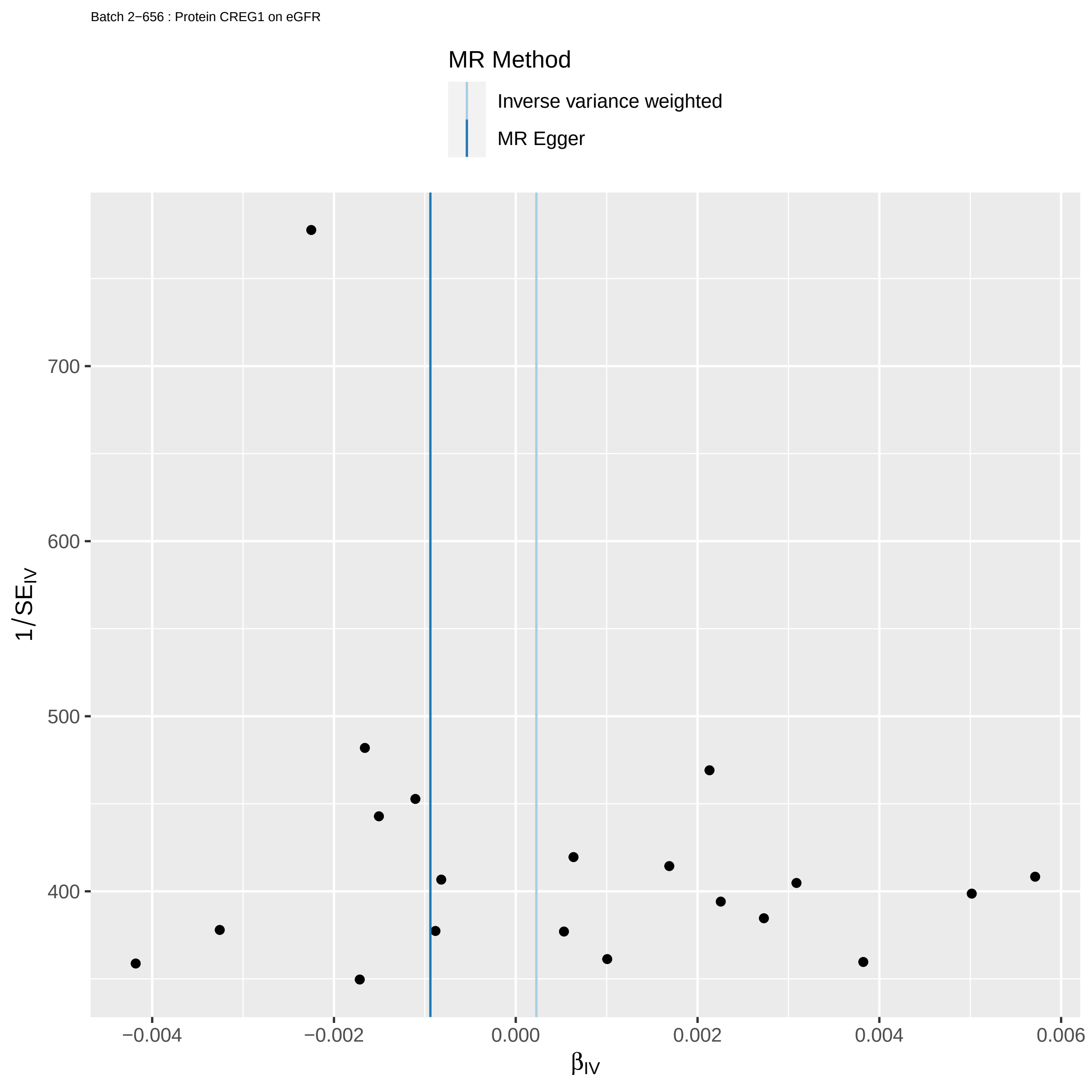

Batch 2−656 : Protein CREG1 on eGFR
MR Method
Inverse variance weighted
MR Egger
1/SE$_{IV}$
700
600
500
400
−0.004
−0.002
0.000
0.002
0.004
0.006
β_{IV}

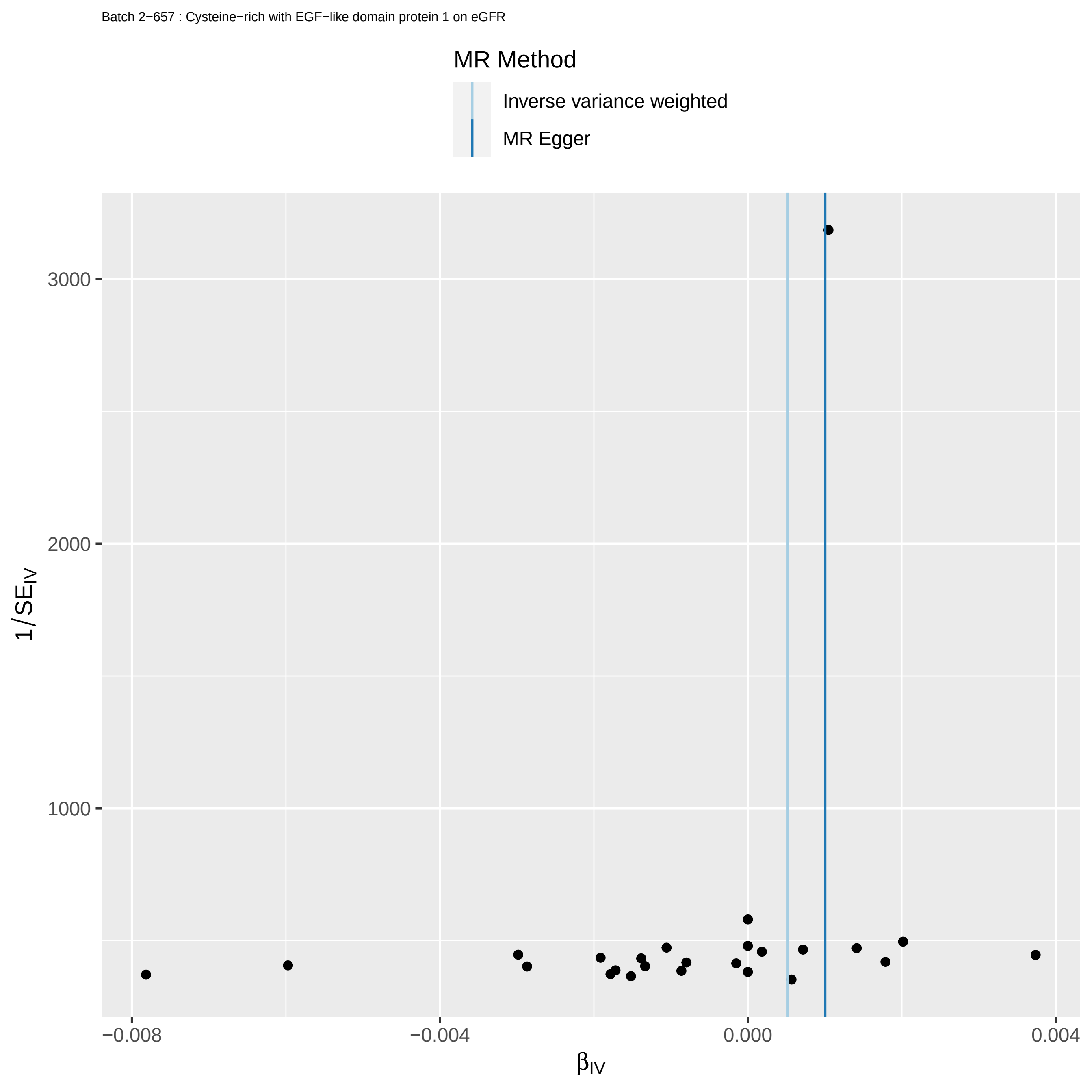

Batch 2−657 : Cysteine−rich with EGF−like domain protein 1 on eGFR
MR Method
Inverse variance weighted
MR Egger
3000
2000
1000
1/SE_IV
−0.008
−0.004
0.000
0.004
β_IV

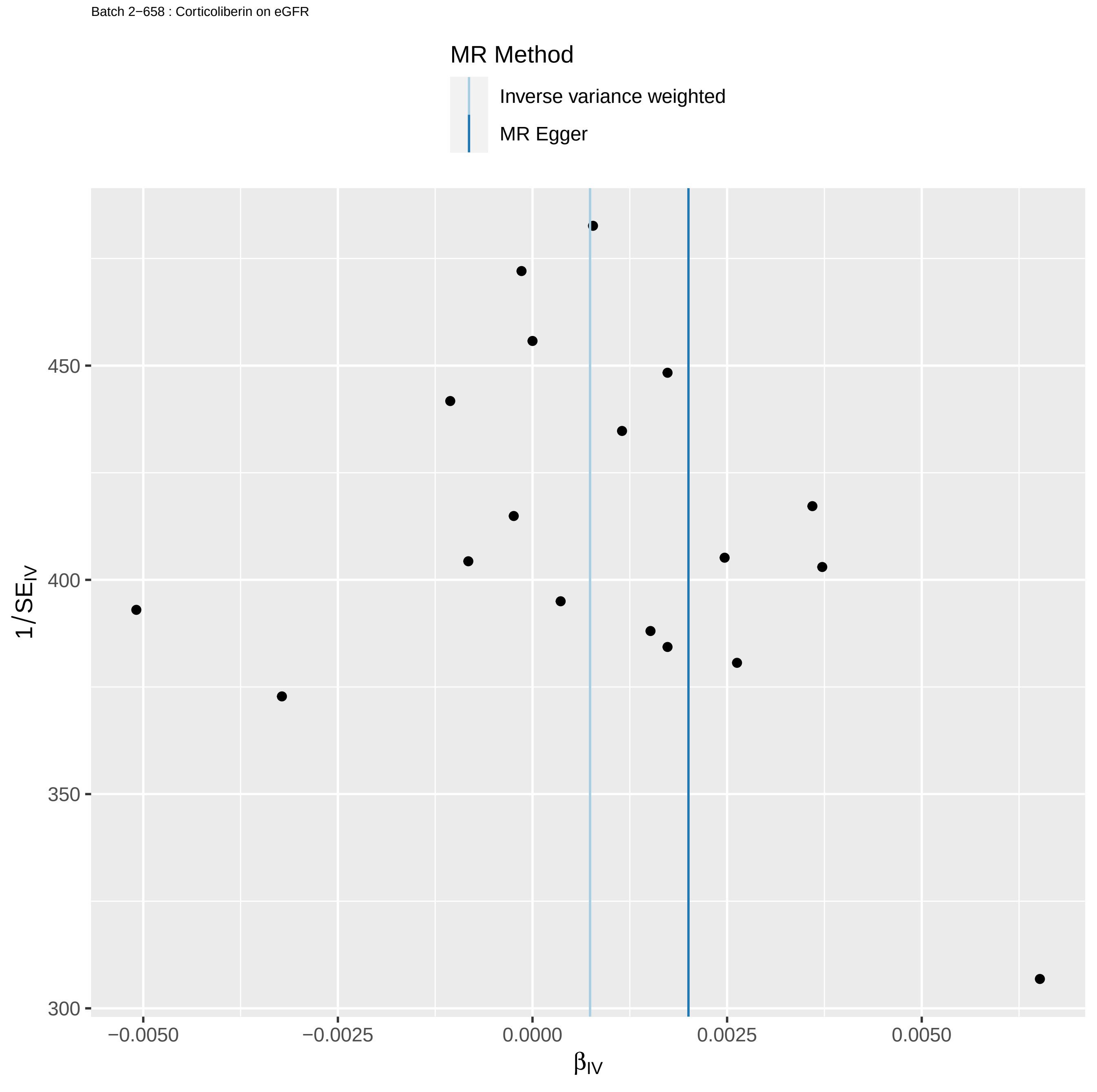
Batch 2−658 : Corticoliberin on eGFR
MR Method
Inverse variance weighted
MR Egger
1/SE$_{IV}$
450
400
350
300
−0.0050
−0.0025
0.0000
0.0025
0.0050
β$_{IV}$

Batch 2−659 : Corticotropin−releasing factor−binding protein on eGFR

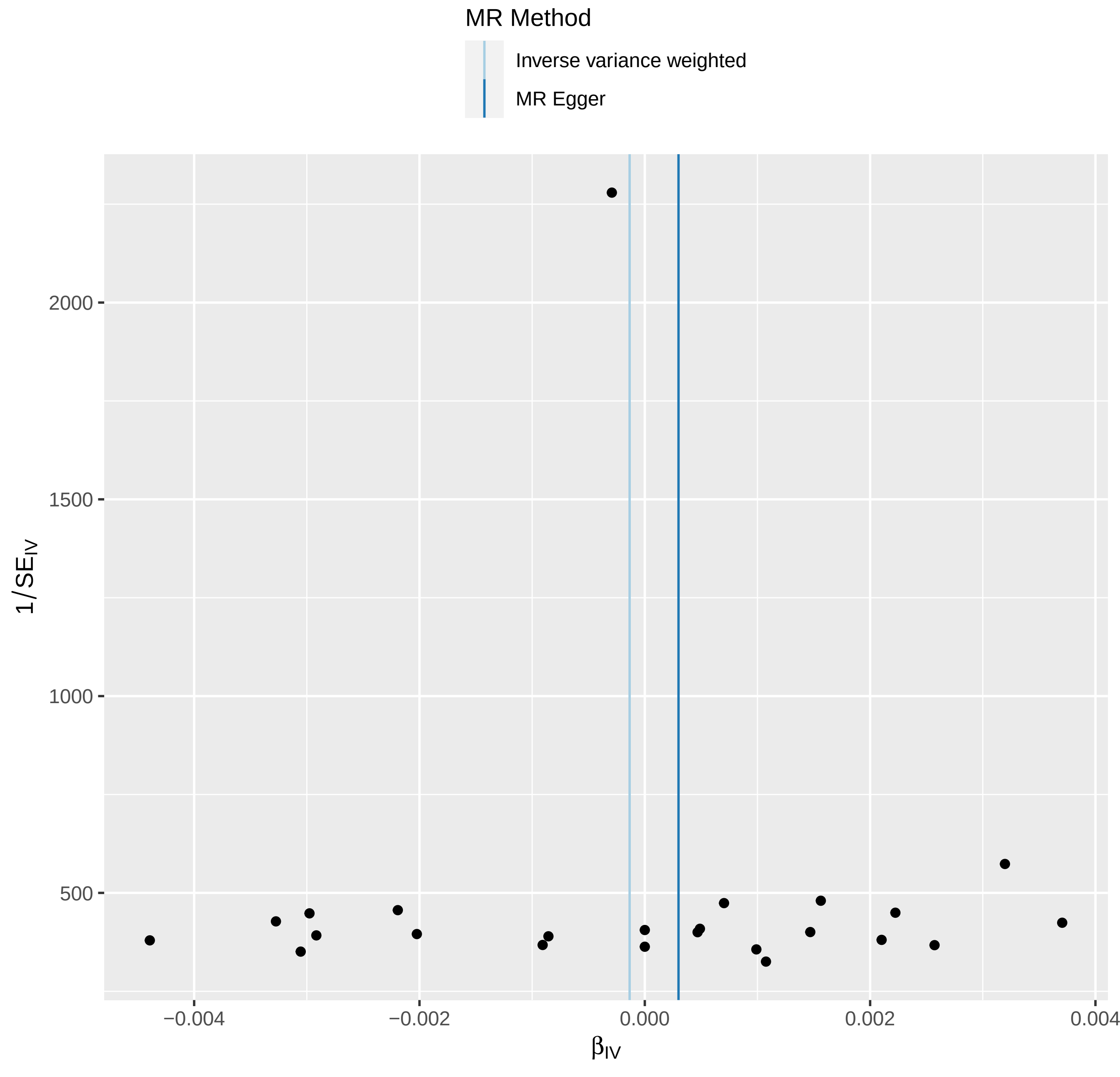

Batch 2−660 : Cysteine−rich motor neuron 1 protein on eGFR

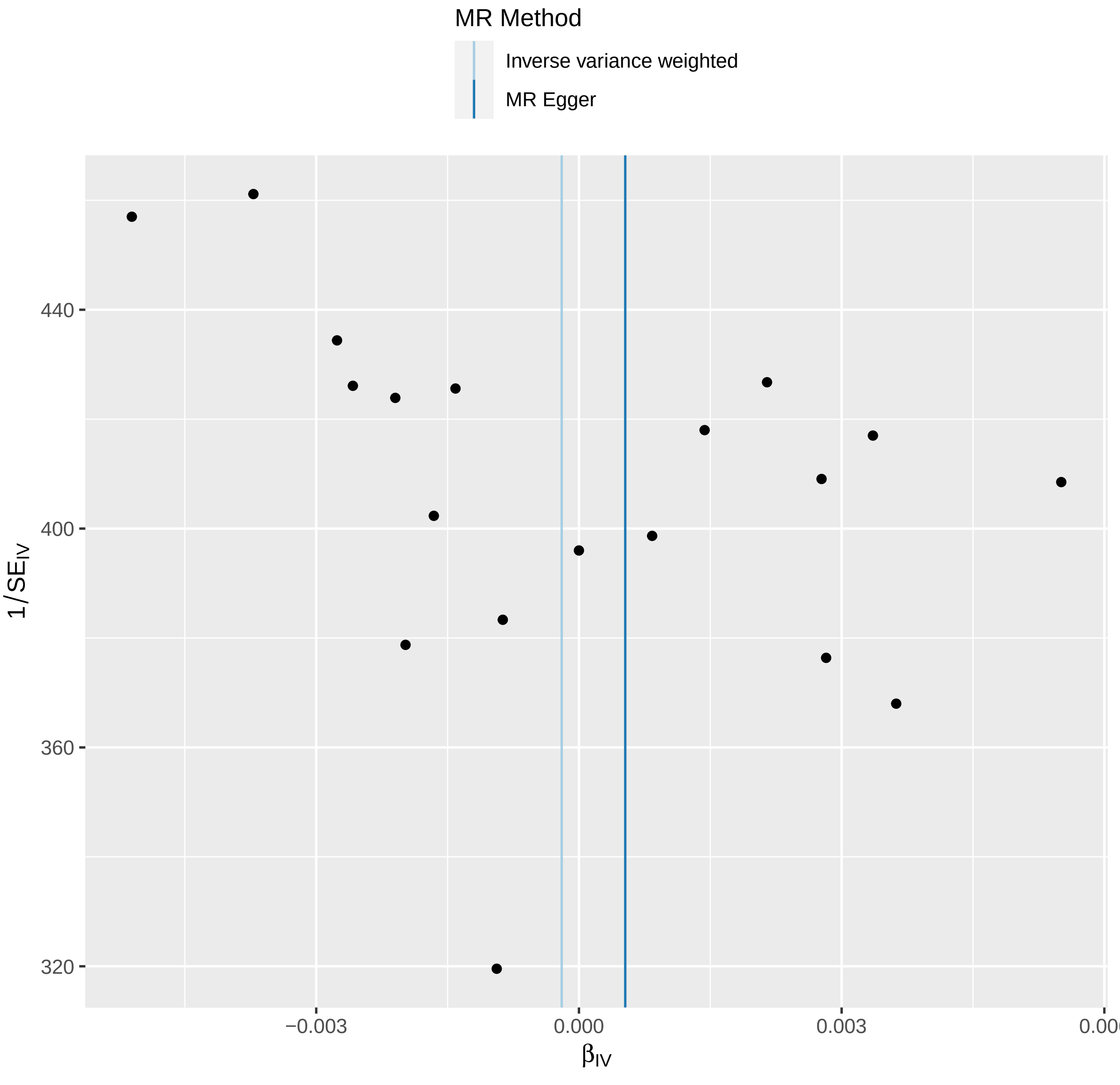

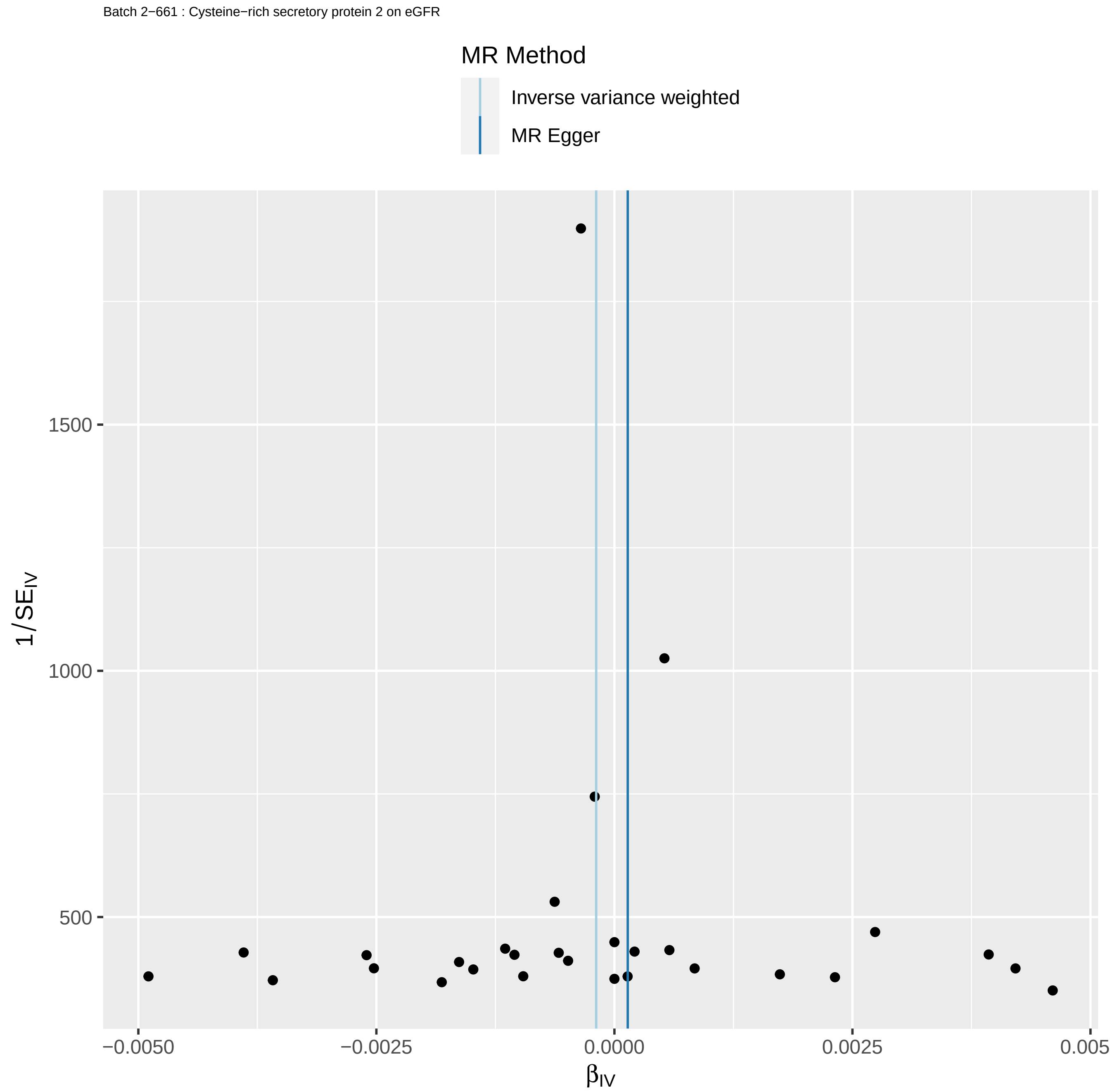

Batch 2−661 : Cysteine−rich secretory protein 2 on eGFR
MR Method
Inverse variance weighted
MR Egger
1500
1000
500
1/SE_IV
−0.0050
−0.0025
0.0000
0.0025
0.005
β_IV

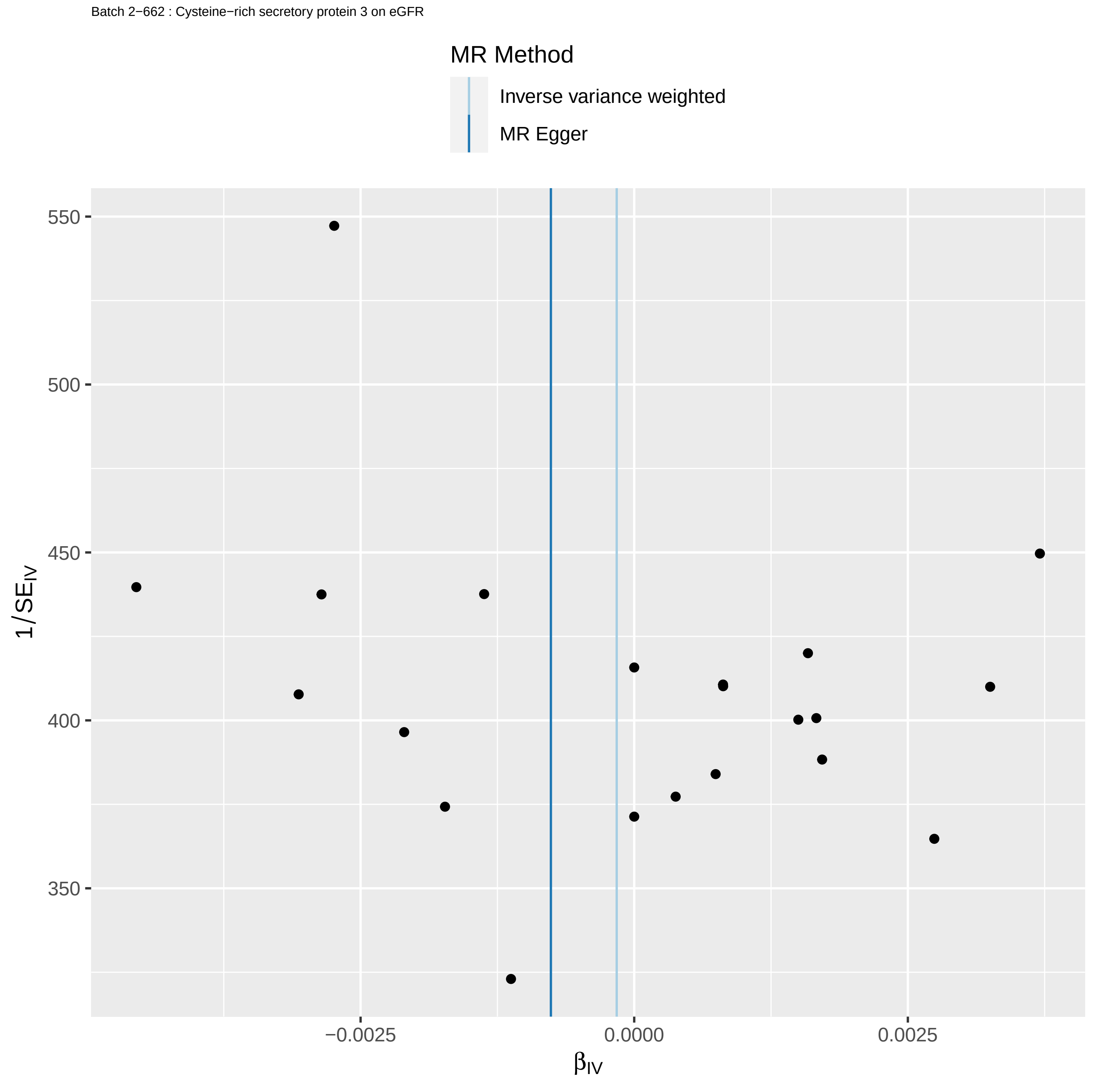

Batch 2−662 : Cysteine−rich secretory protein 3 on eGFR
MR Method
Inverse variance weighted
MR Egger
550
500
450
400
350
1/SE_IV
−0.0025
0.0000
0.0025
β_IV

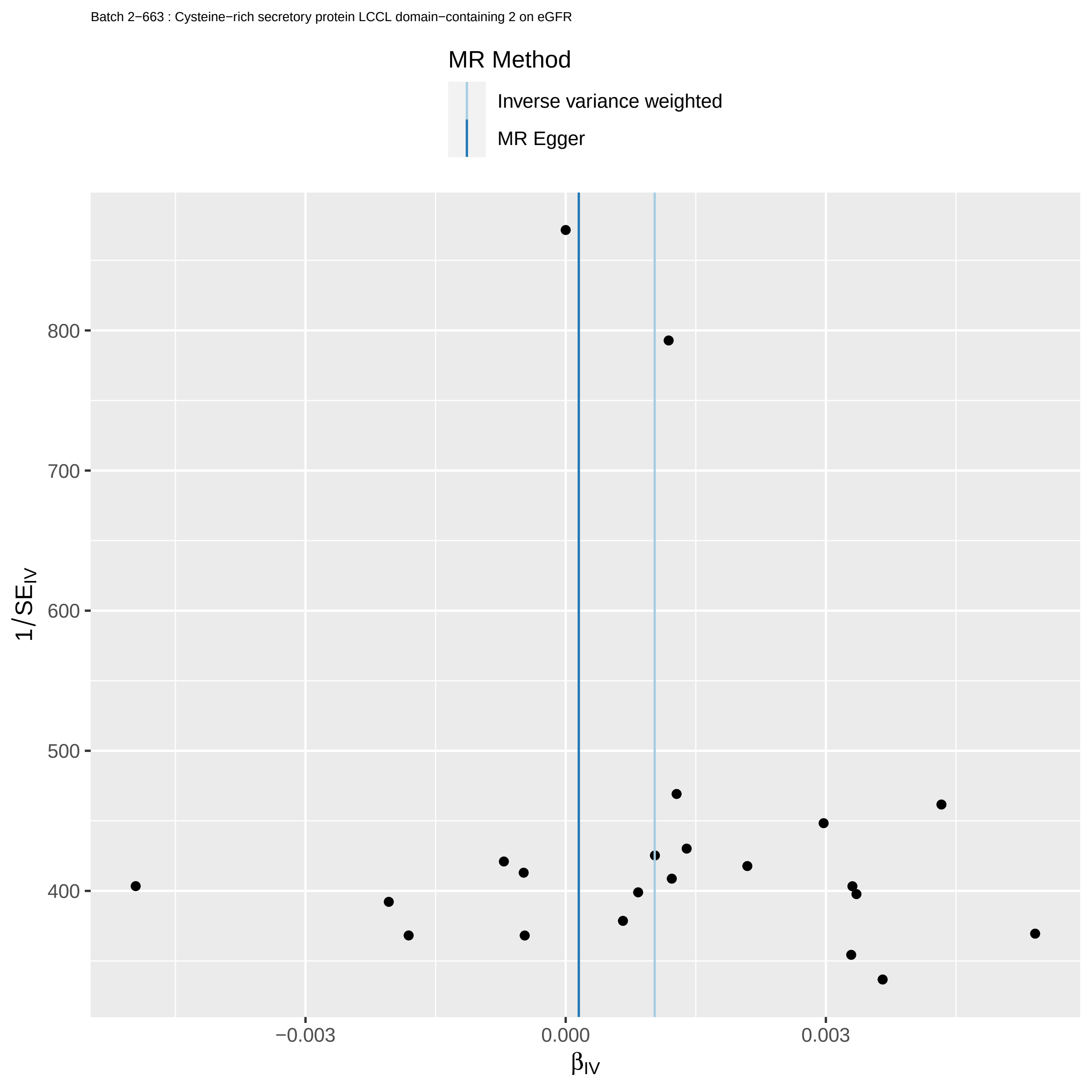
Batch 2−663 : Cysteine−rich secretory protein LCCL domain−containing 2 on eGFR
MR Method
Inverse variance weighted
MR Egger
800
700
600
500
400
1/SE_IV
−0.003
0.000
0.003
β_IV

Batch 2−664 : Adapter molecule crk on eGFR

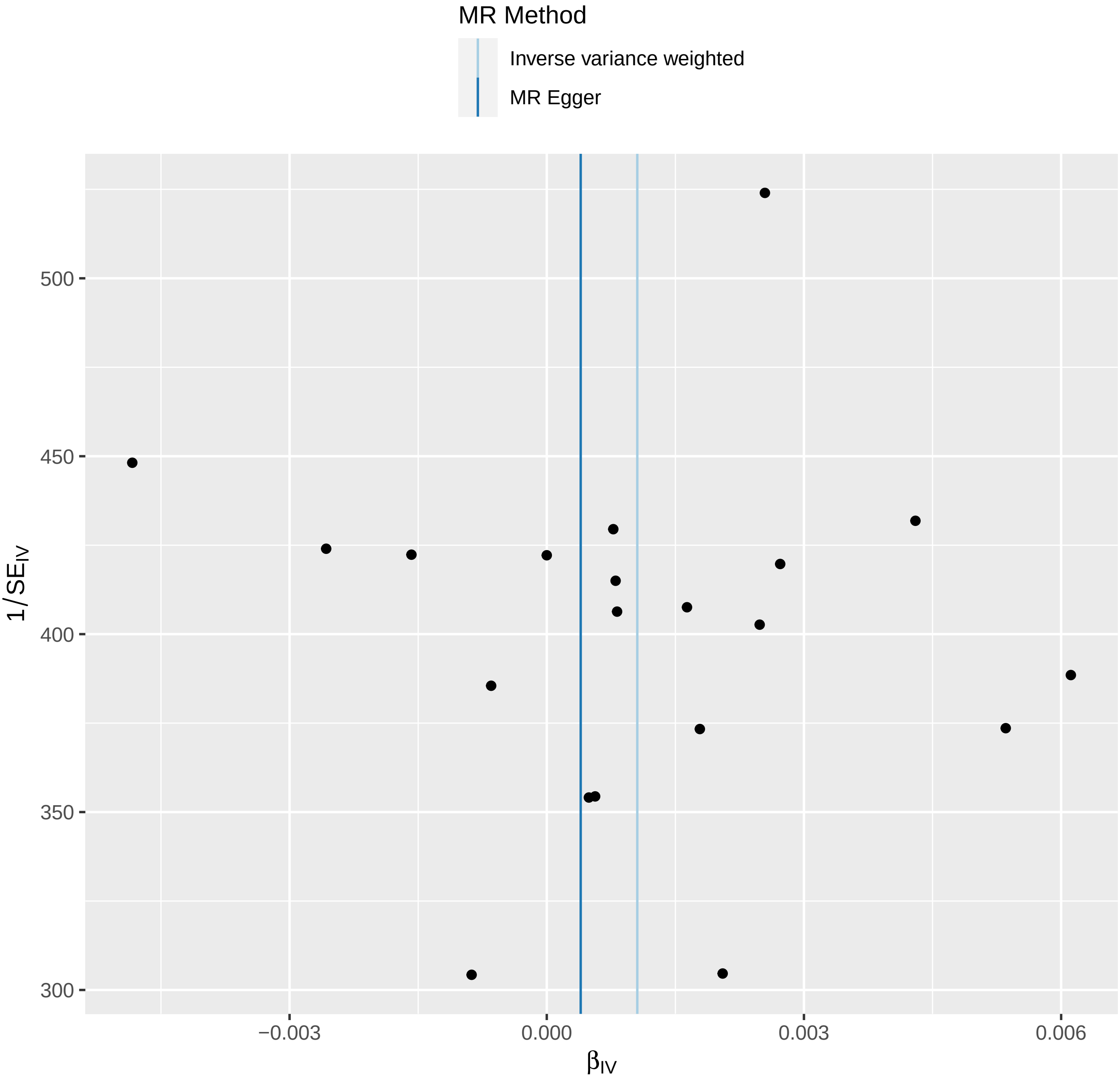

Batch 2−665 : Crk−like protein on eGFR

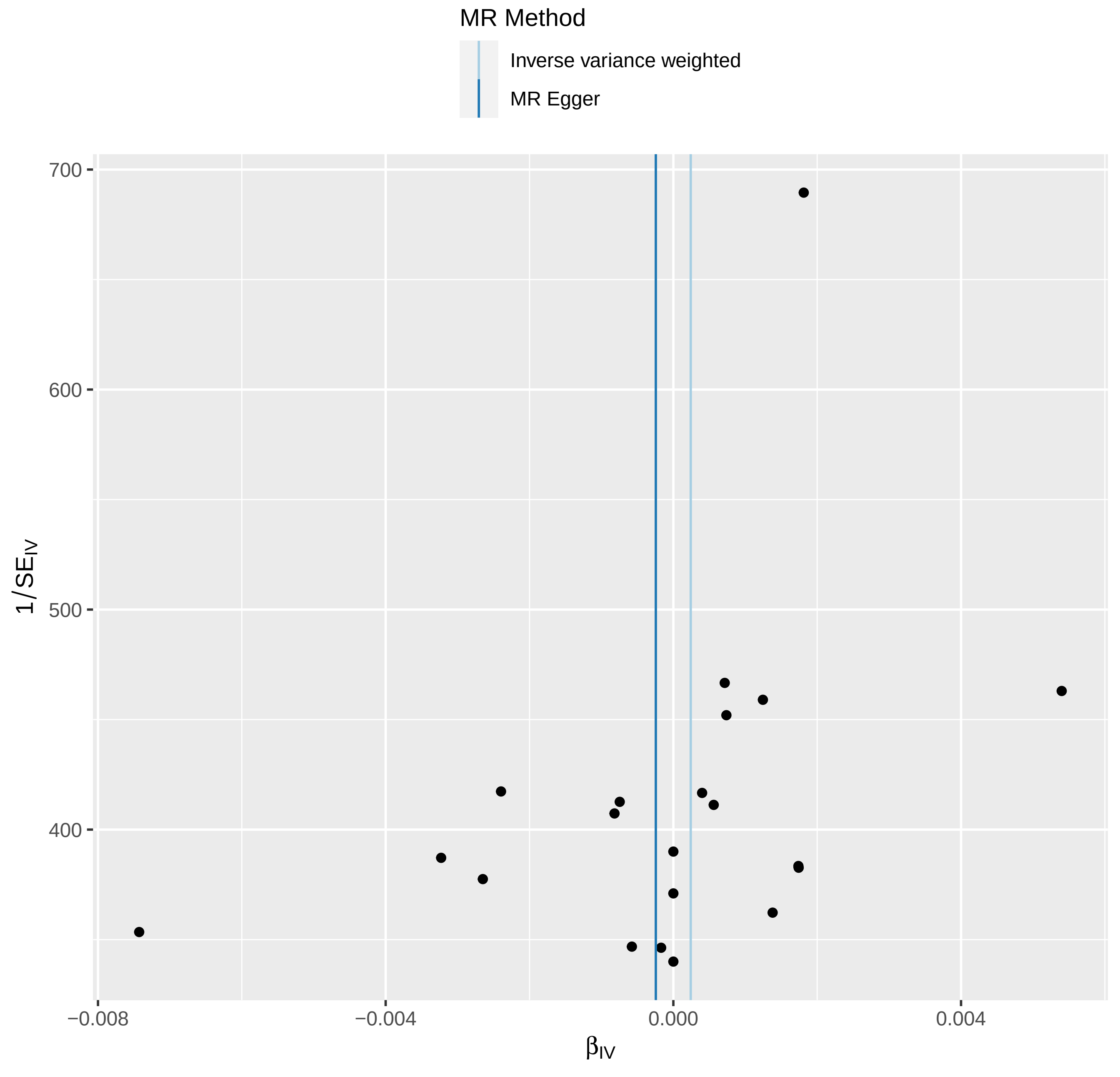

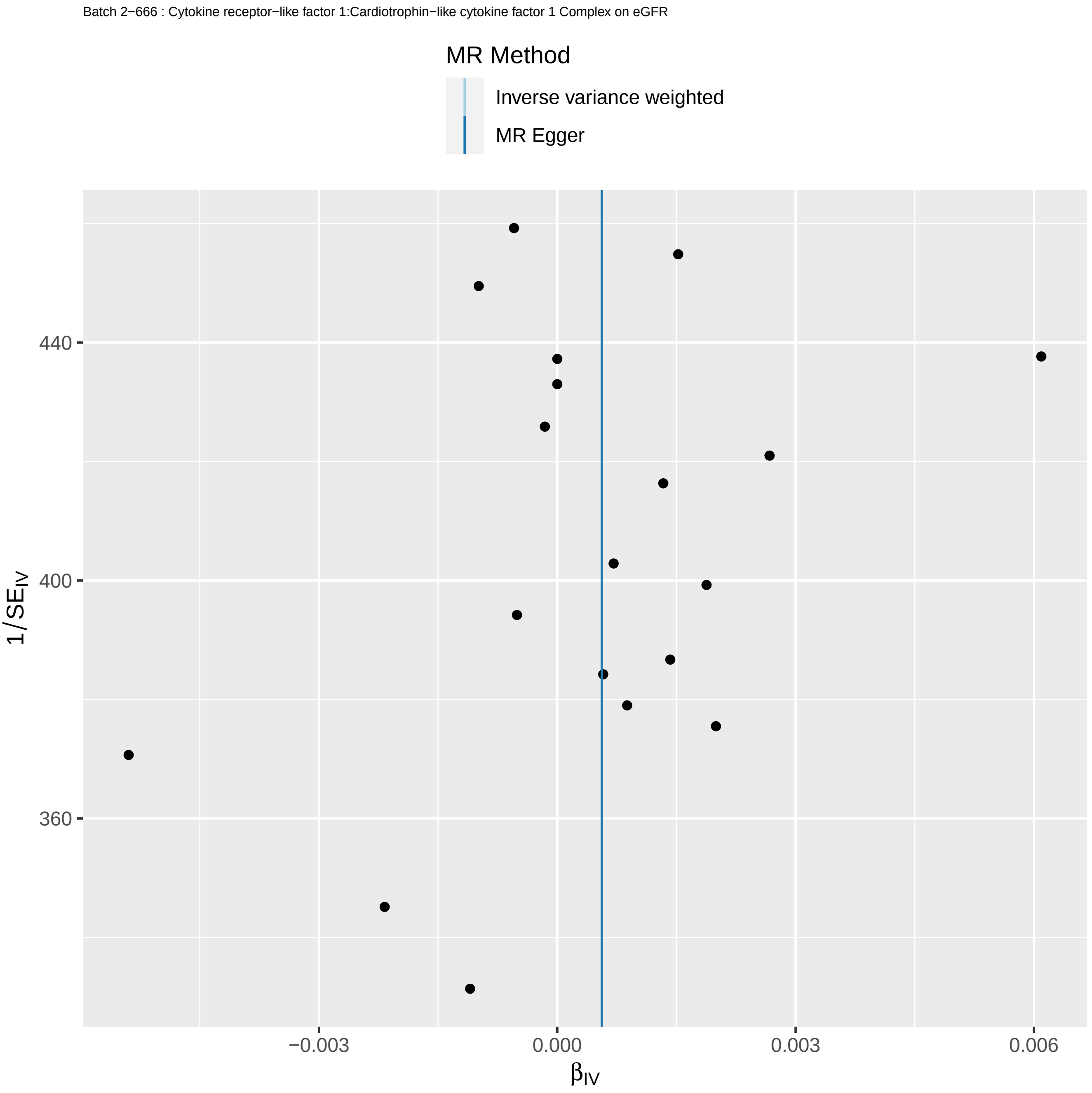

Batch 2−666 : Cytokine receptor−like factor 1:Cardiotrophin−like cytokine factor 1 Complex on eGFR
MR Method
Inverse variance weighted
MR Egger
440
400
360
1/SE_IV
−0.003
0.000
0.003
0.006
β_IV

Batch 2−667 : Cytokine receptor−like factor 2 on eGFR

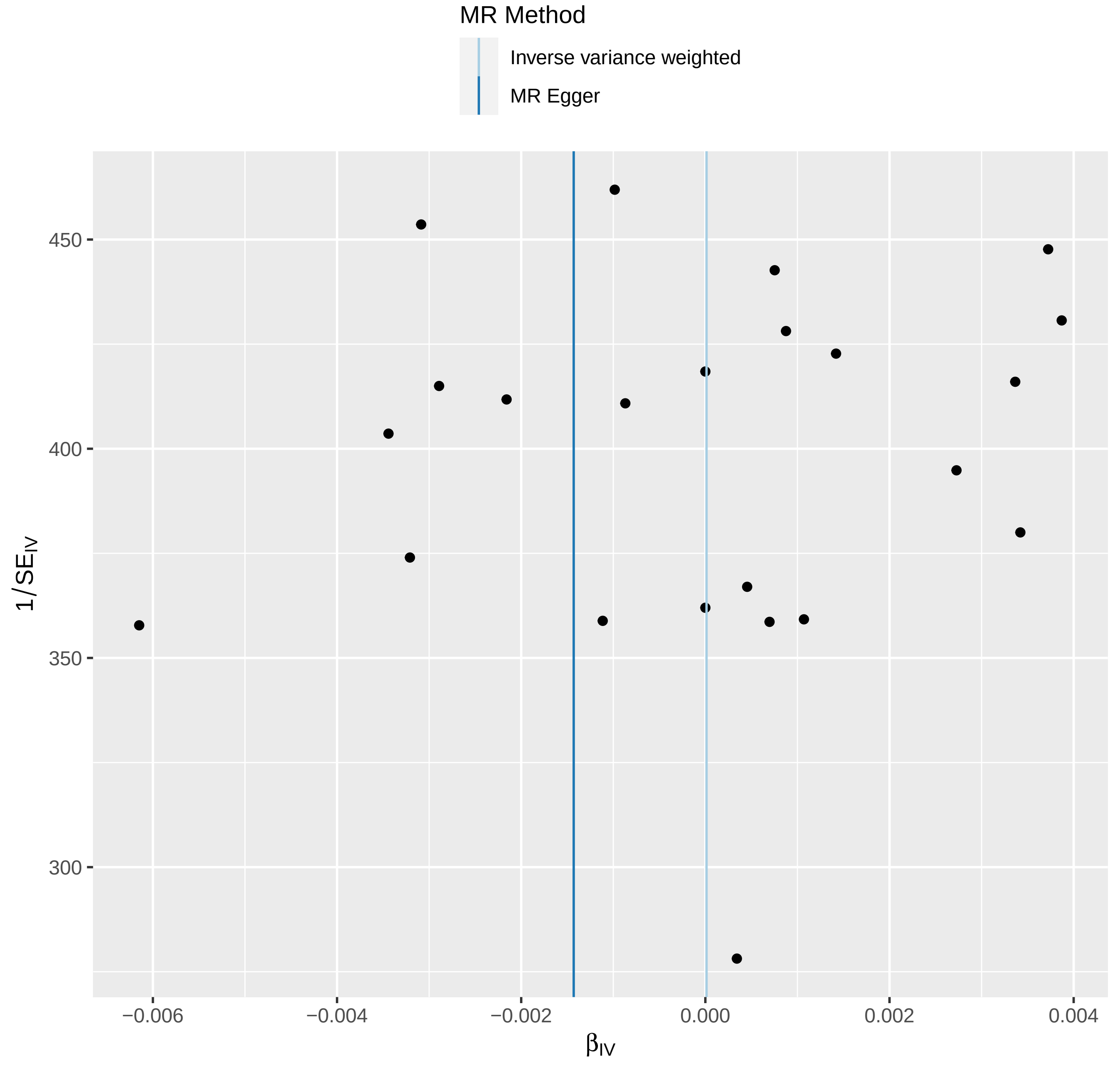

Batch 2−668 : Cytokine receptor−like factor 2 on eGFR

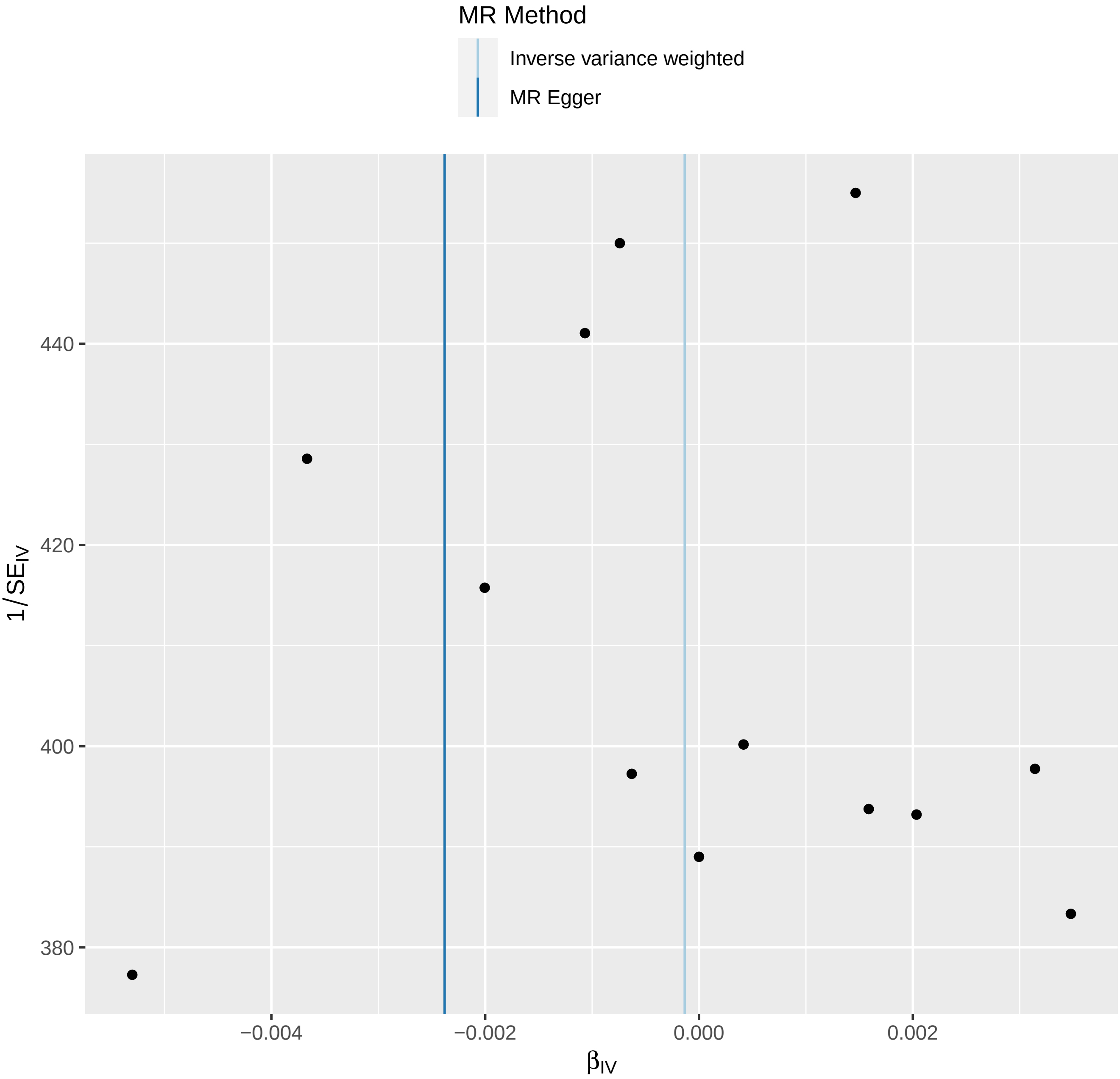

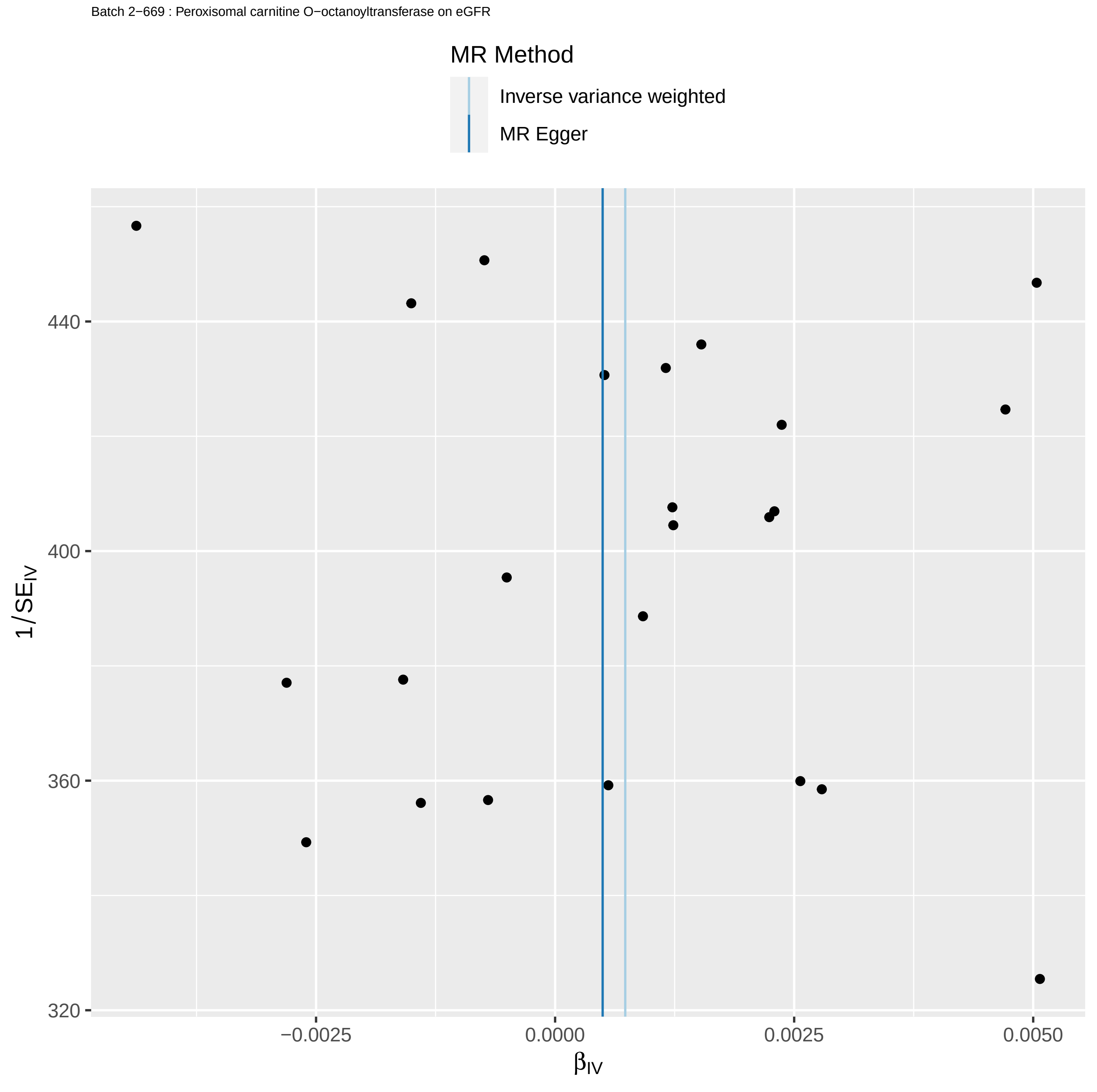

Batch 2−669 : Peroxisomal carnitine O−octanoyltransferase on eGFR
MR Method
Inverse variance weighted
MR Egger
440
400
360
320
1/SE_IV
−0.0025
0.0000
0.0025
0.0050
β_IV

Batch 2−670 : C−reactive protein on eGFR

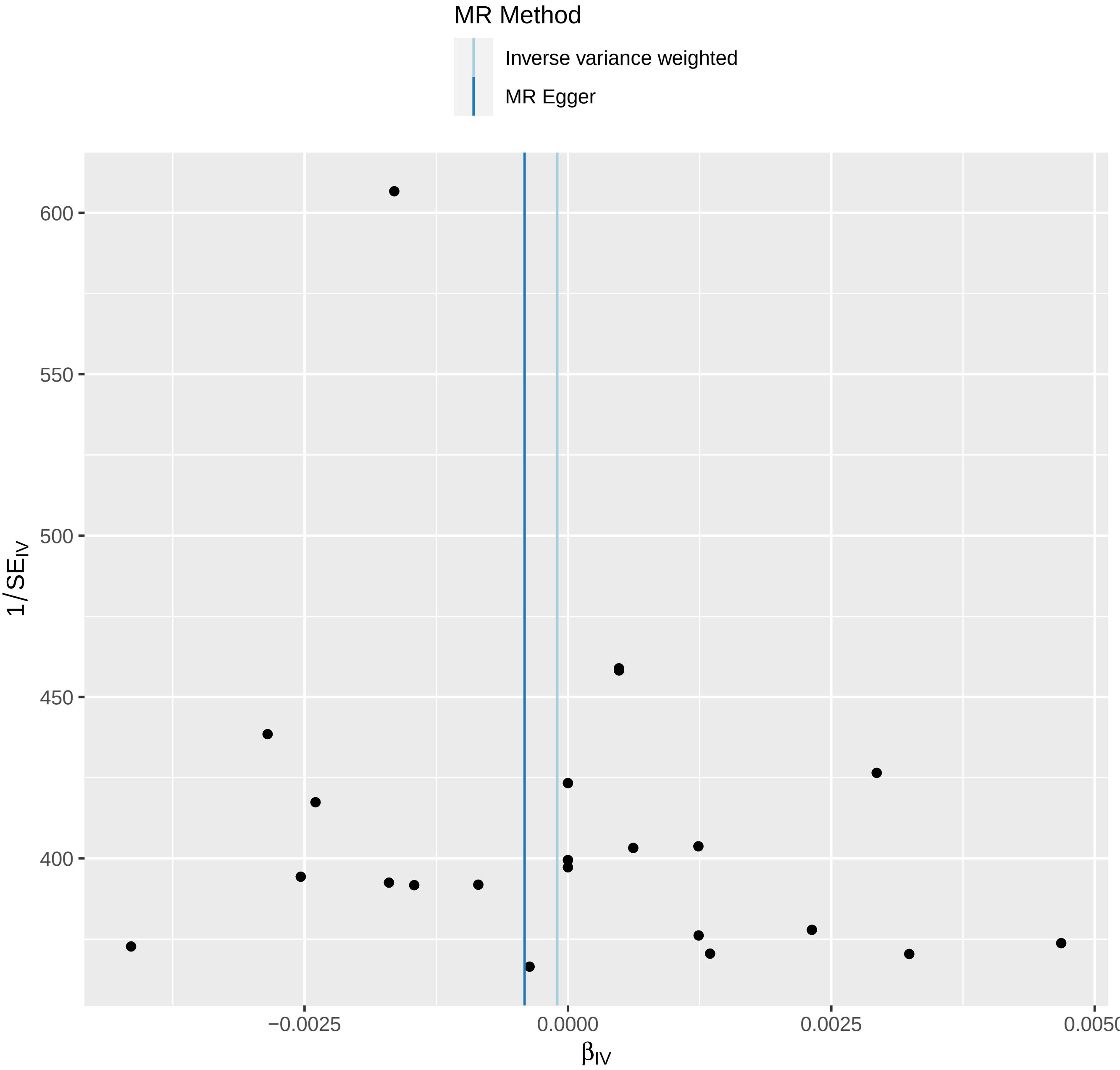

Batch 2−671 : Cytotoxic and regulatory T−cell molecule on eGFR

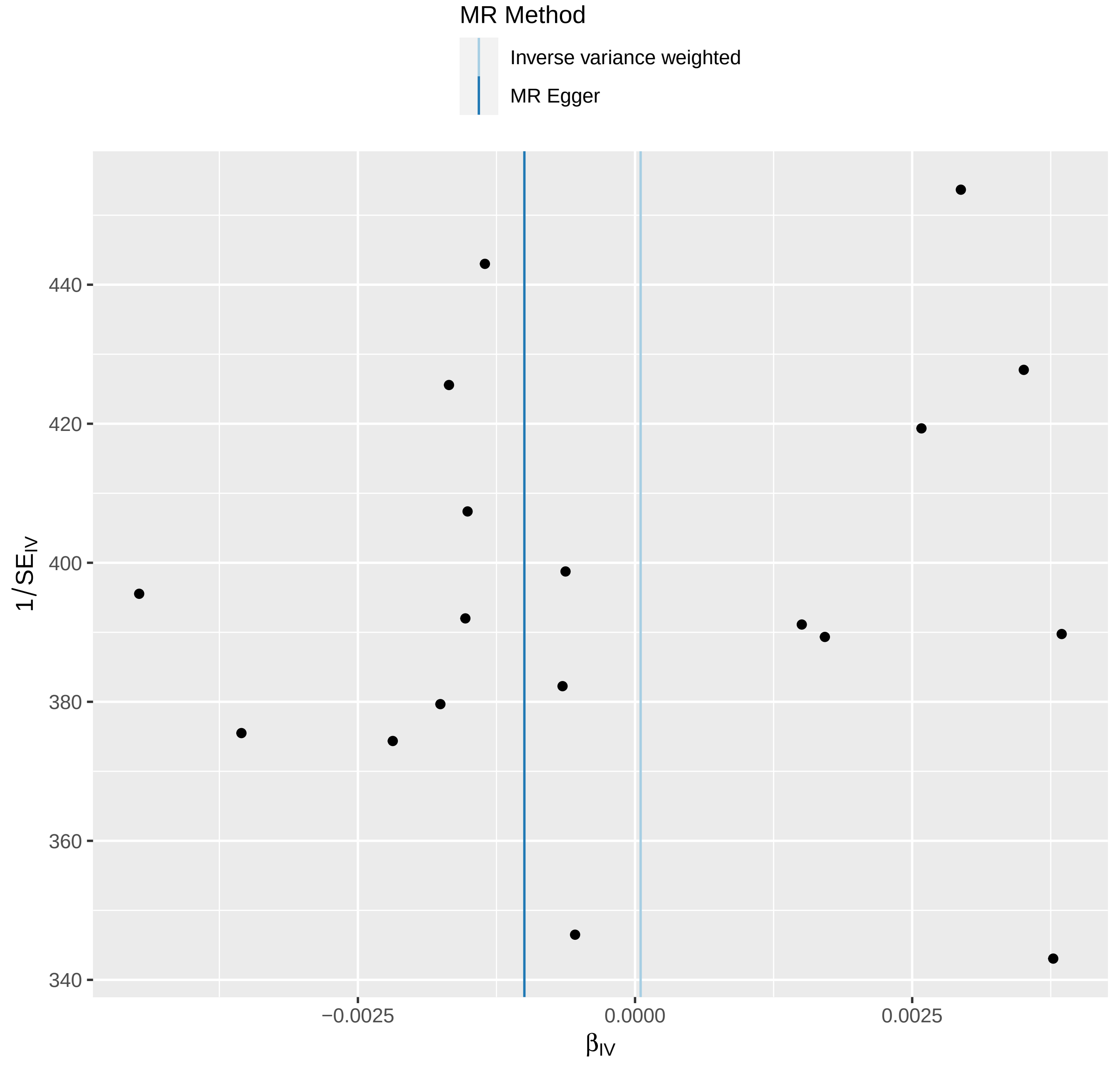

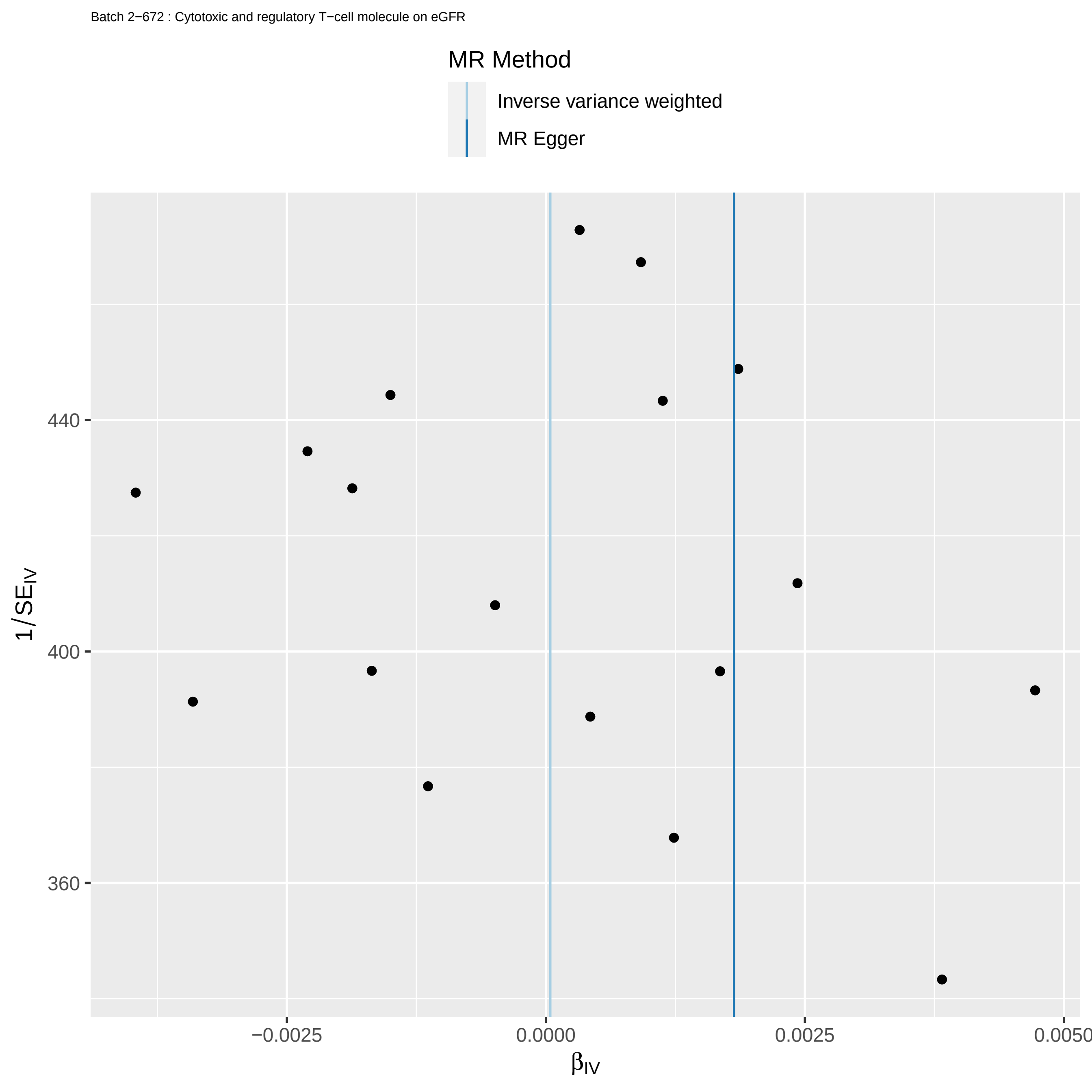

Batch 2−672 : Cytotoxic and regulatory T−cell molecule on eGFR
MR Method
Inverse variance weighted
MR Egger
440
400
360
1/SE$_{IV}$
−0.0025
0.0000
0.0025
0.0050
β$_{IV}$

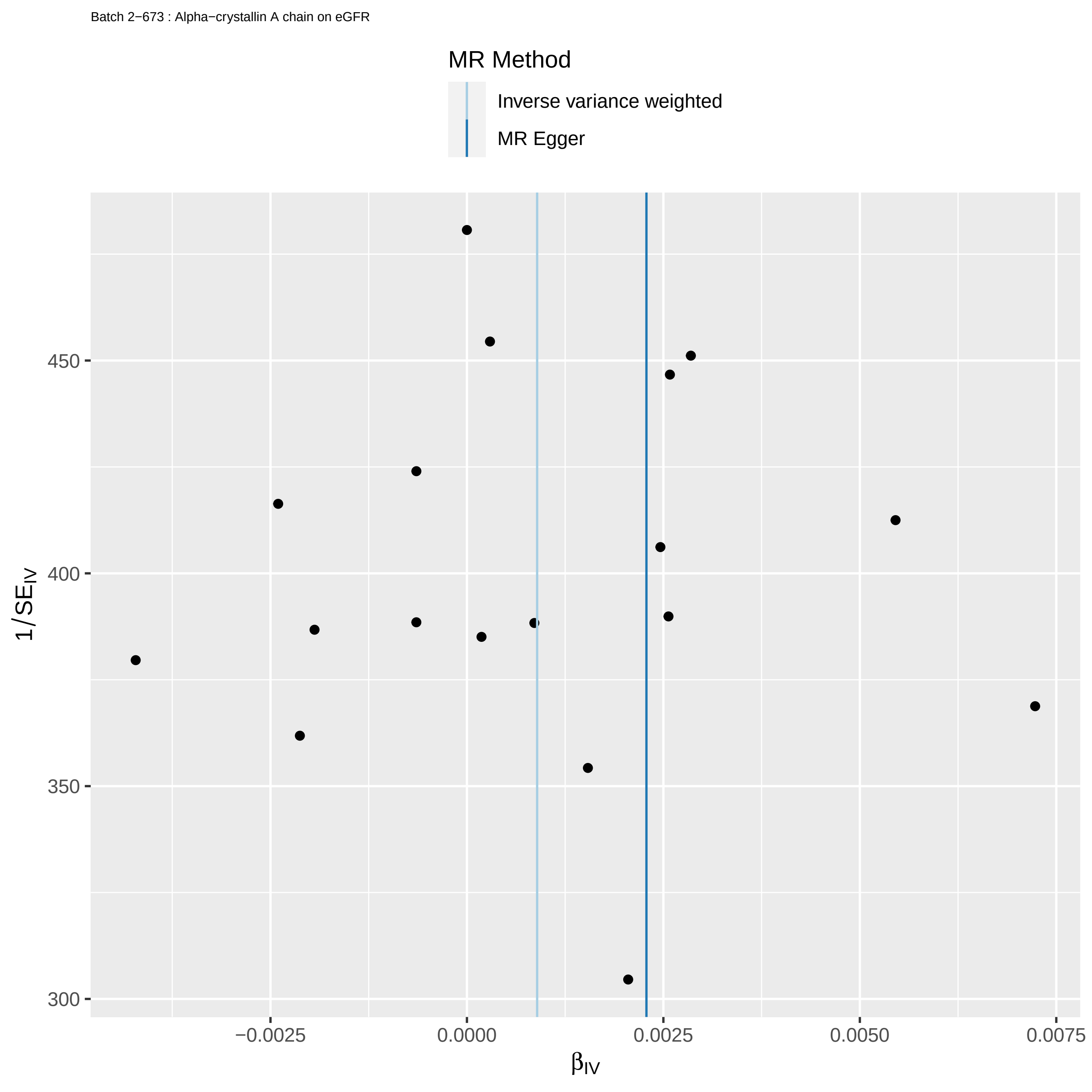

Batch 2−673 : Alpha−crystallin A chain on eGFR
MR Method
Inverse variance weighted
MR Egger
450
400
350
300
1/SE_IV
−0.0025
0.0000
0.0025
0.0050
0.0075
β_IV

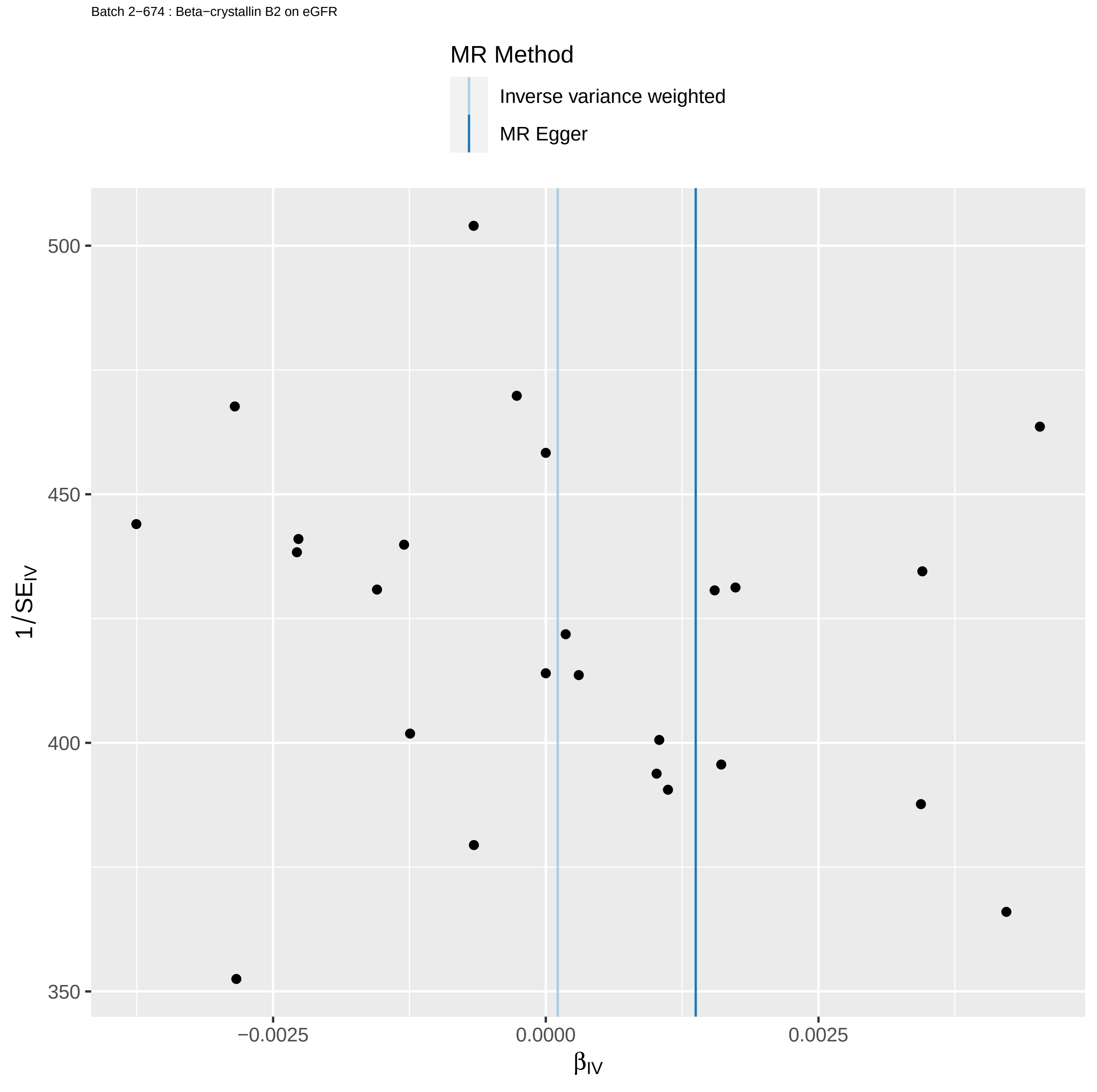

Batch 2−674 : Beta−crystallin B2 on eGFR
MR Method
Inverse variance weighted
MR Egger
500
450
400
350
1/SE_IV
−0.0025
0.0000
0.0025
β_IV

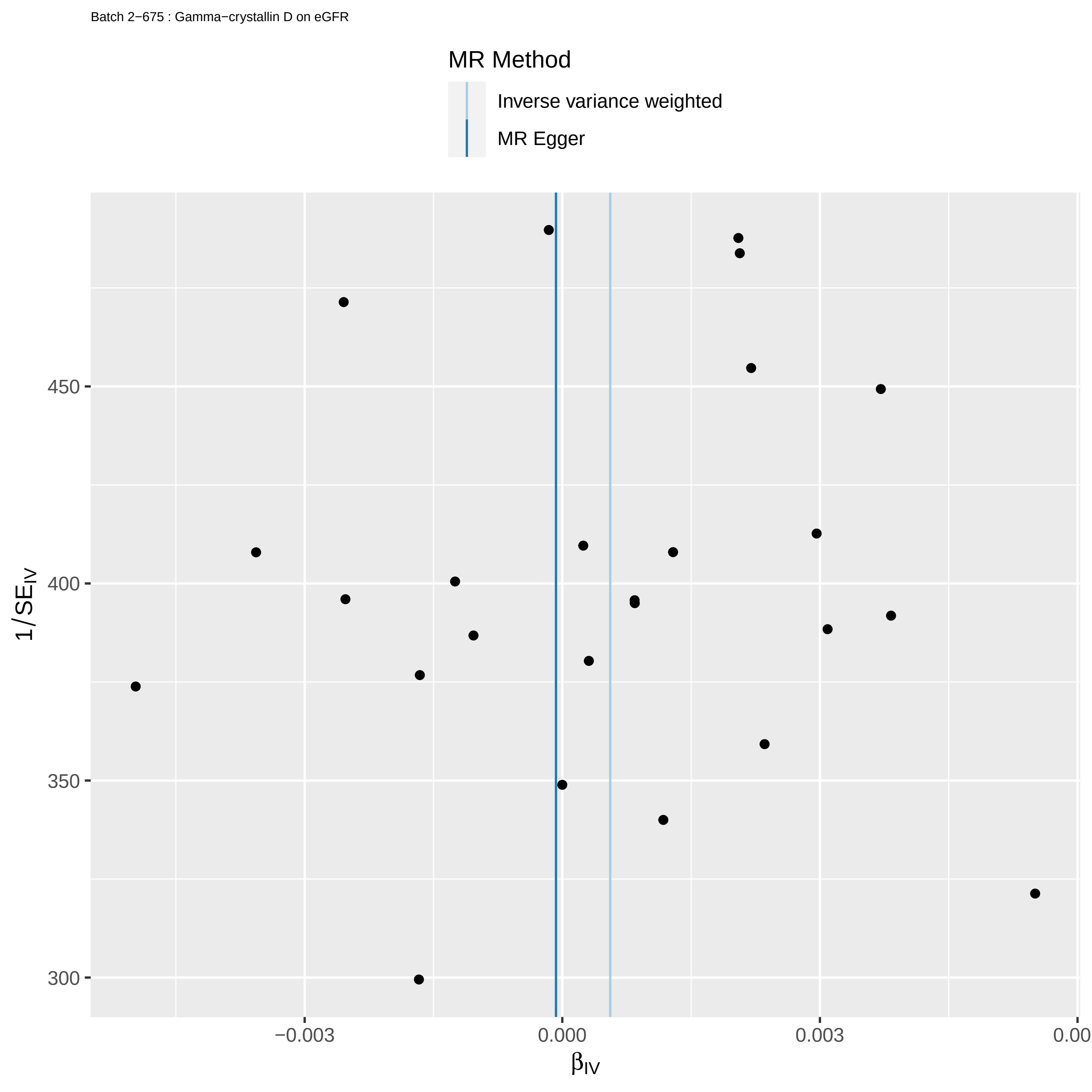

Batch 2−675 : Gamma−crystallin D on eGFR
MR Method
Inverse variance weighted
MR Egger
450
400
350
300
1/SE$_{IV}$
−0.003
0.000
0.003
0.00
β$_{IV}$

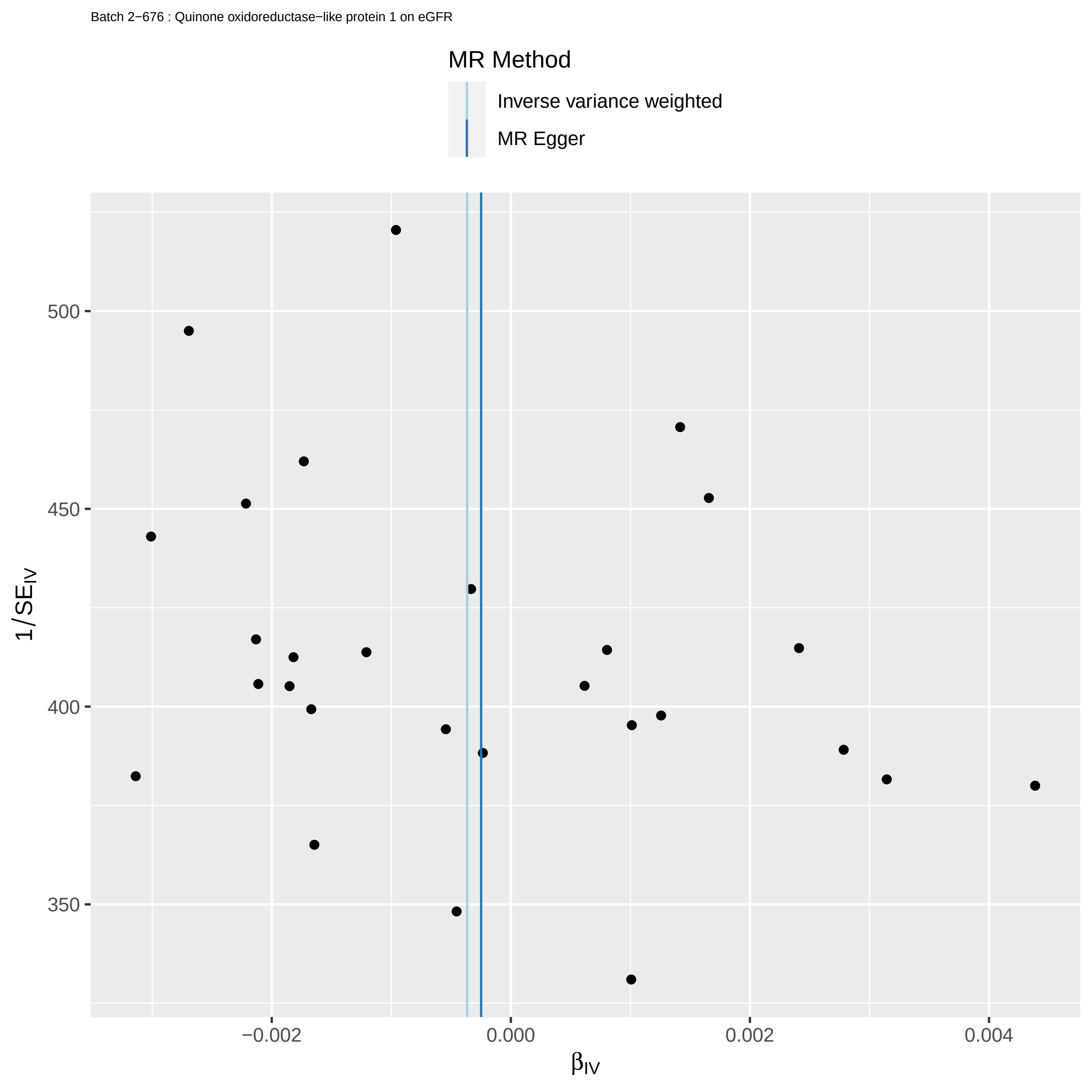

Batch 2−676 : Quinone oxidoreductase−like protein 1 on eGFR
MR Method
Inverse variance weighted
MR Egger
500
450
400
350
1/SE_IV
−0.002
0.000
0.002
0.004
β_IV

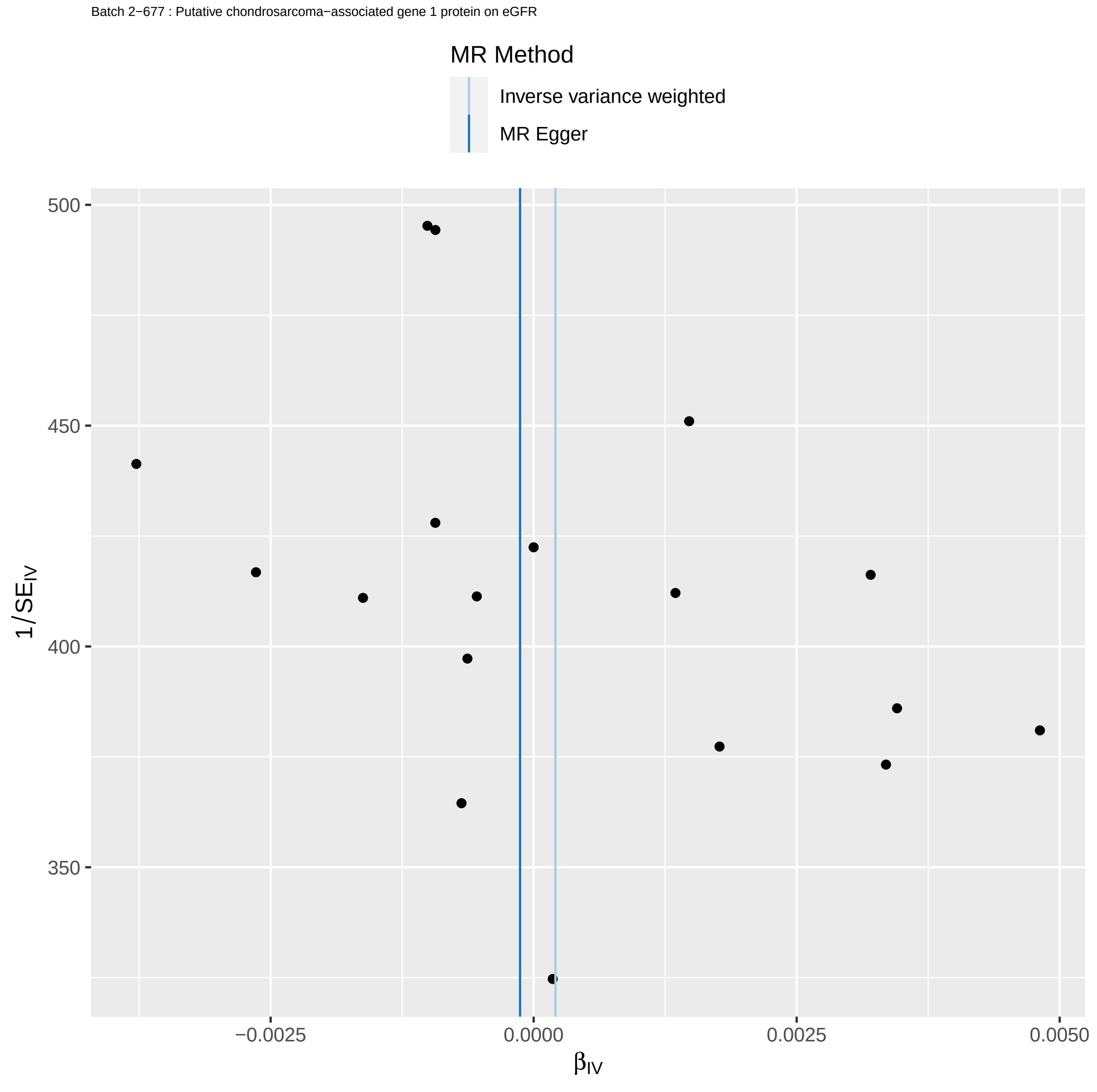
Batch 2−677 : Putative chondrosarcoma−associated gene 1 protein on eGFR
MR Method
Inverse variance weighted
MR Egger
500
450
400
350
1/SE_IV
−0.0025
0.0000
0.0025
0.0050
β_IV

Batch 2−678 : Cold shock domain−containing protein C2 on eGFR

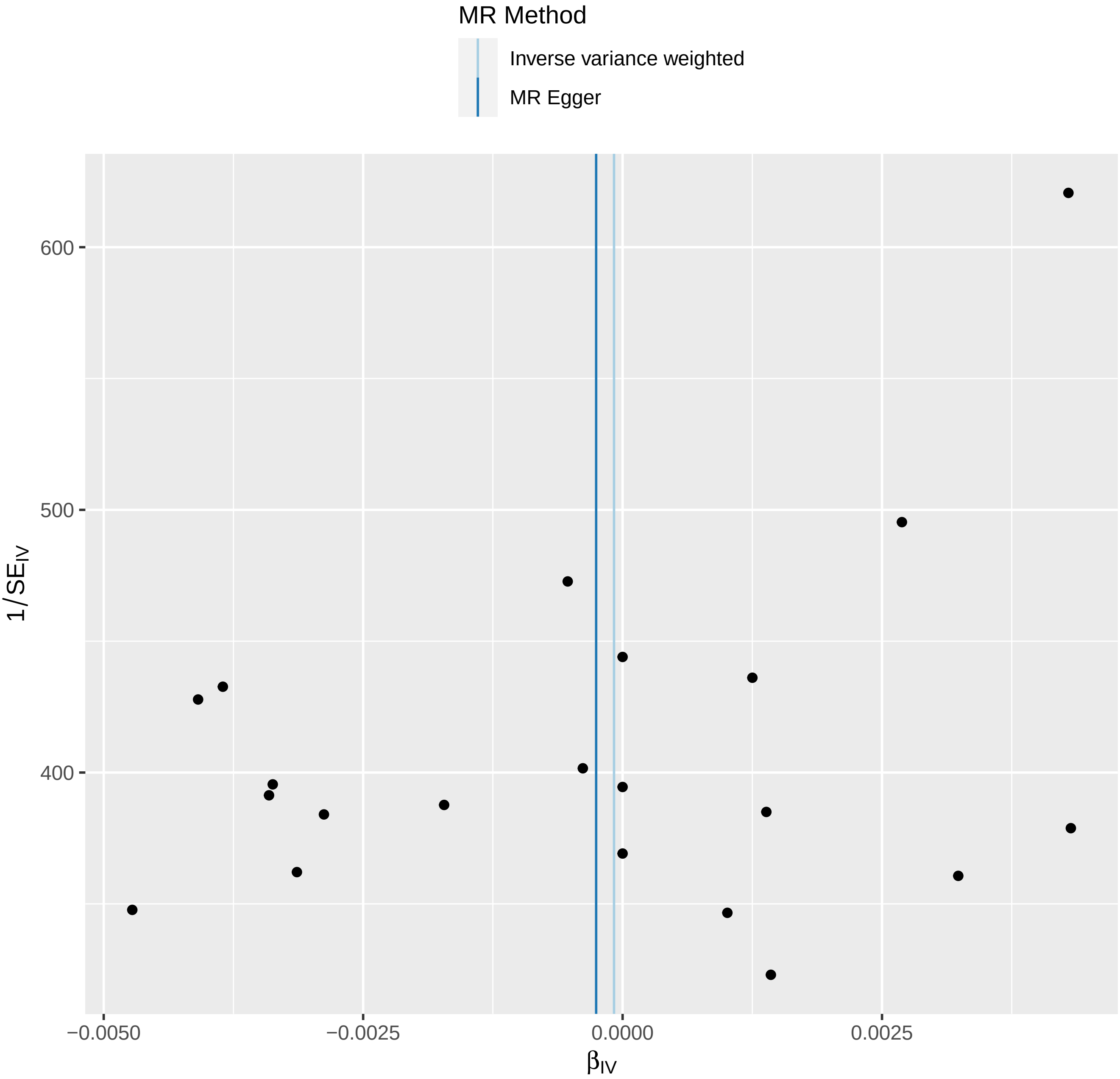

Batch 2−679 : Cold shock domain−containing protein E1 on eGFR

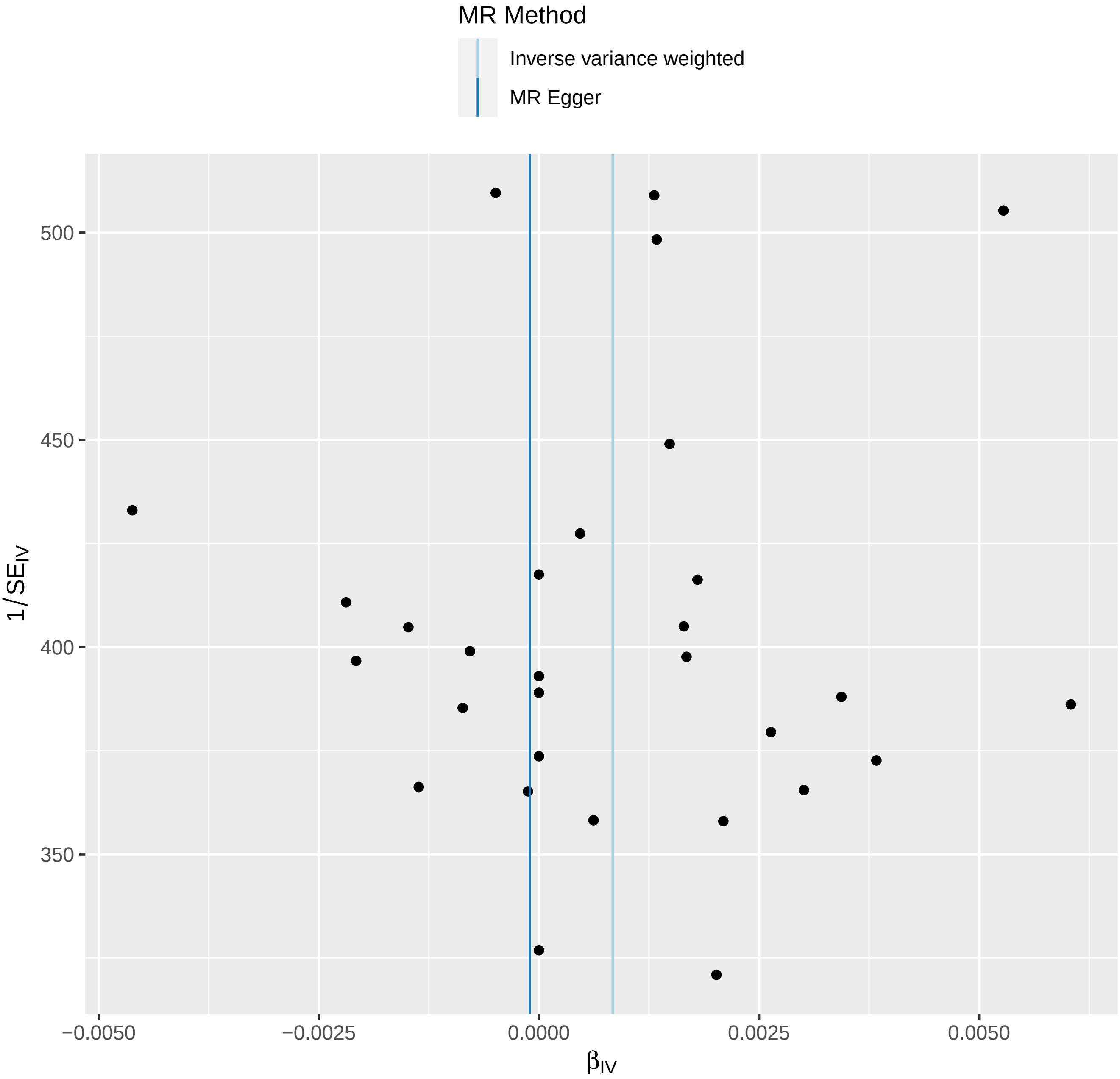

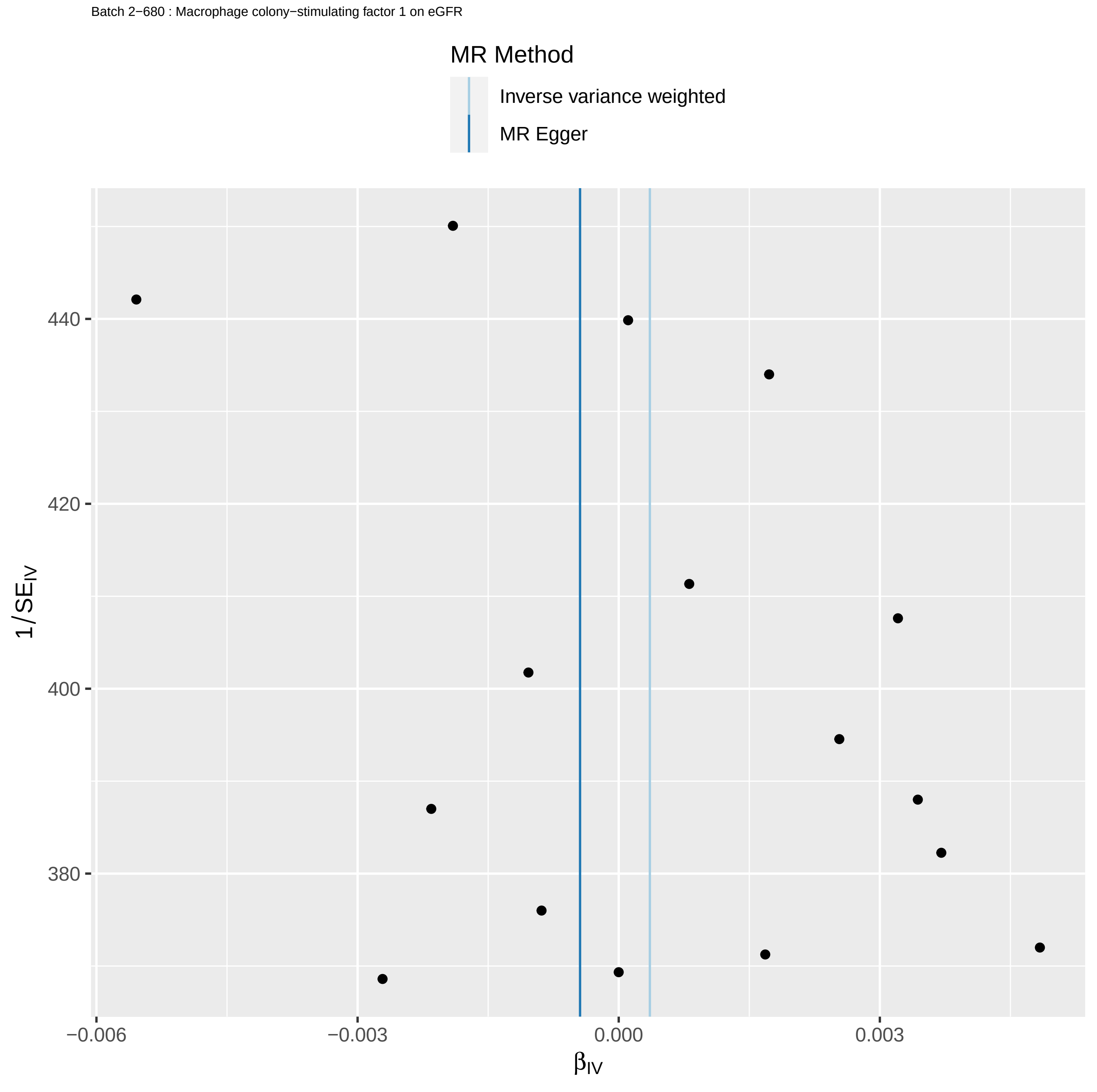
Batch 2−680 : Macrophage colony−stimulating factor 1 on eGFR
MR Method
Inverse variance weighted
MR Egger
440
420
400
380
−0.006
−0.003
0.000
0.003
1/SE_IV
β_IV

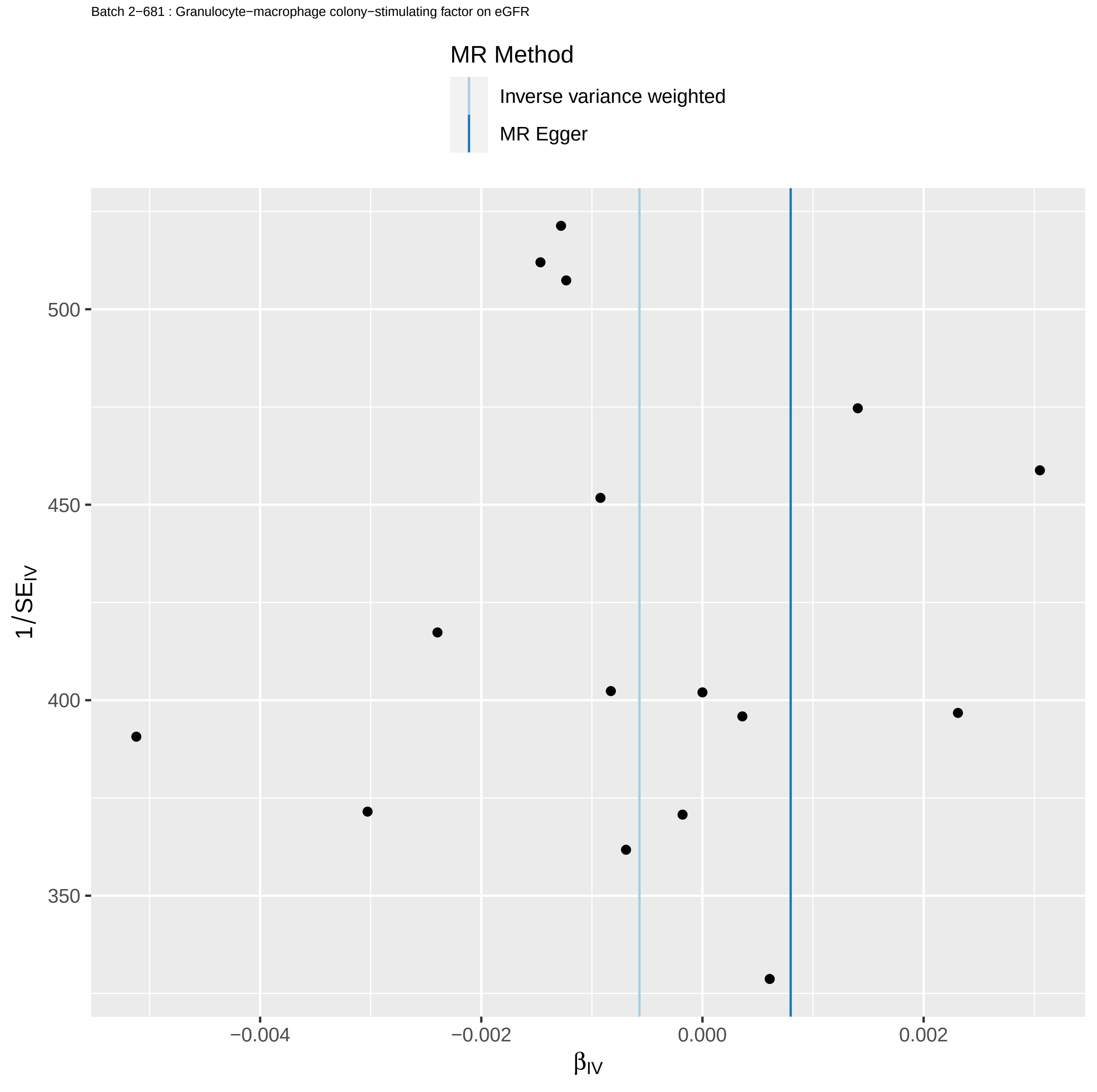
Batch 2−681 : Granulocyte−macrophage colony−stimulating factor on eGFR
MR Method
Inverse variance weighted
MR Egger
500
450
400
350
1/SE_IV
−0.004
−0.002
0.000
0.002
β_IV

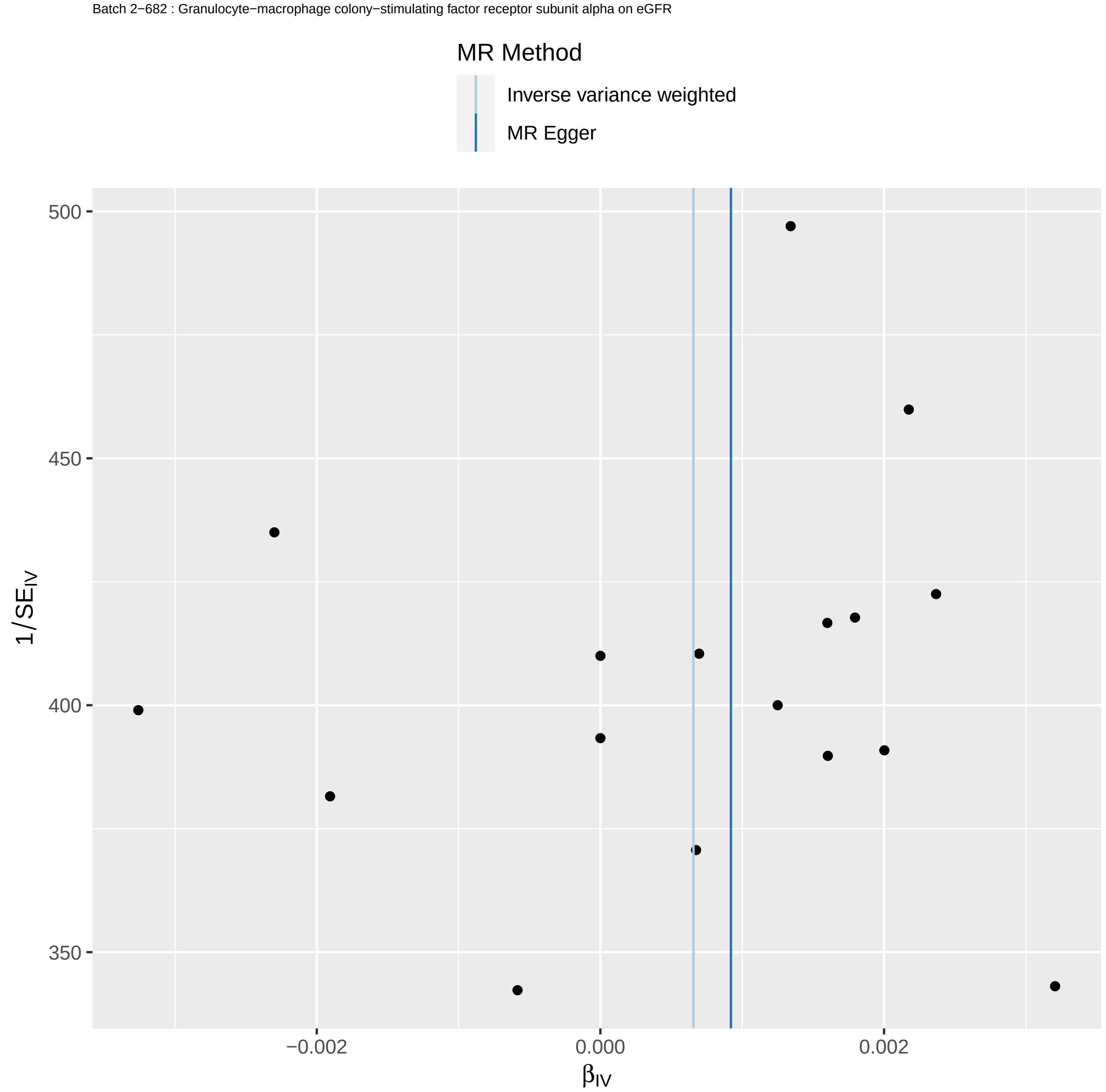
Batch 2−682 : Granulocyte−macrophage colony−stimulating factor receptor subunit alpha on eGFR
MR Method
Inverse variance weighted
MR Egger
500
450
400
350
1/SE_IV
−0.002
0.000
0.002
β_IV

Batch 2−683 : Cytokine receptor common subunit beta on eGFR

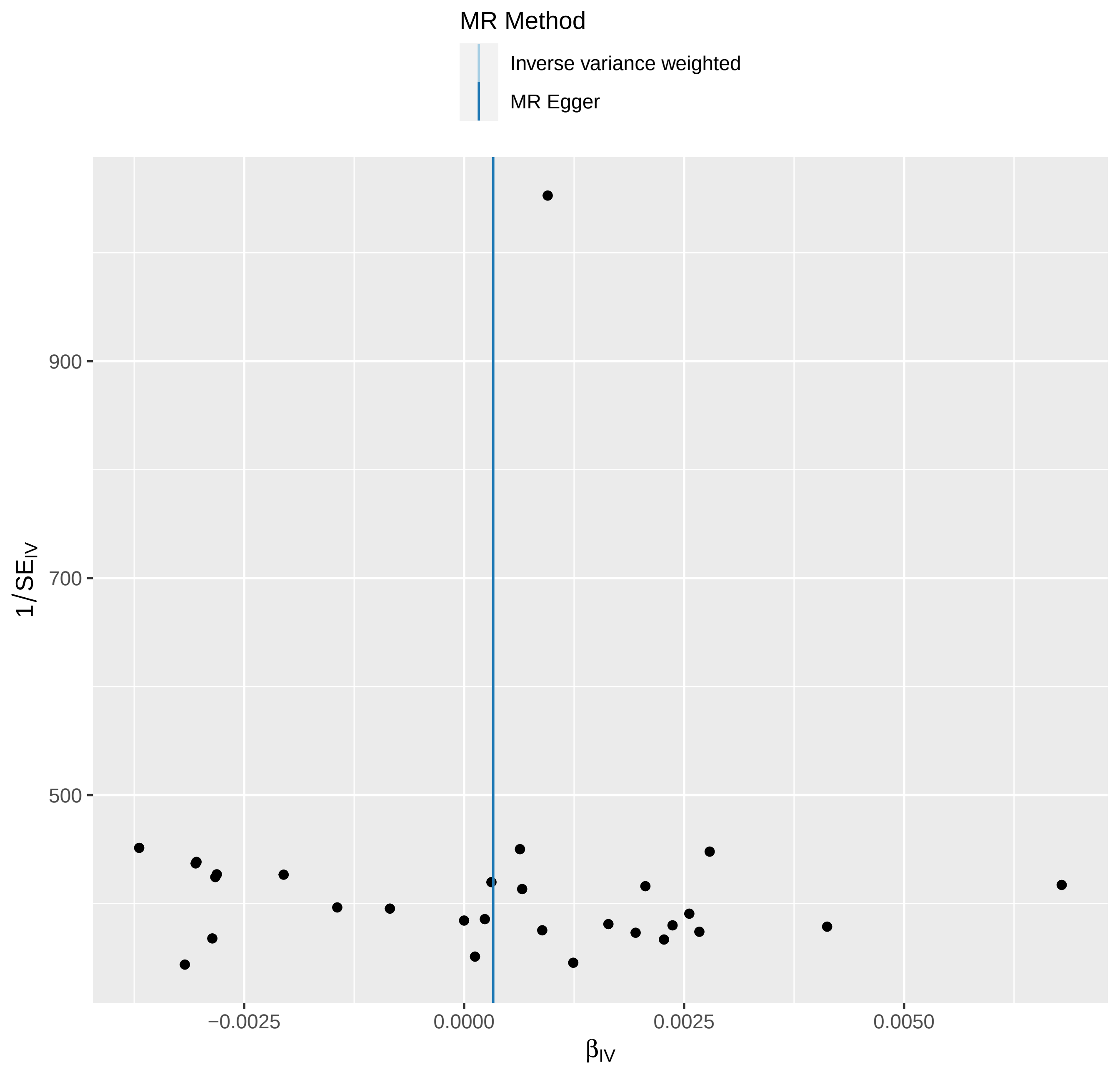

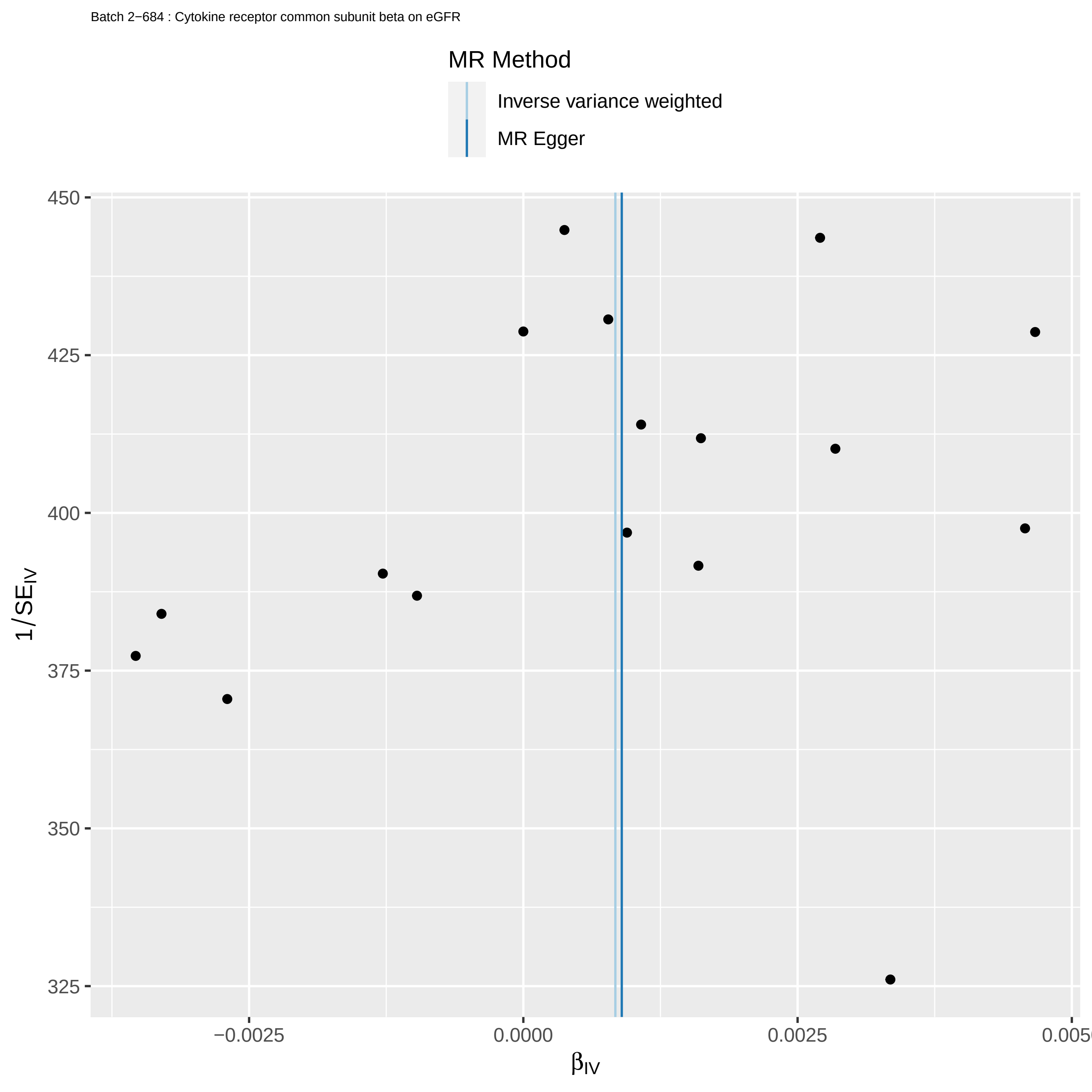

Batch 2−684 : Cytokine receptor common subunit beta on eGFR
MR Method
Inverse variance weighted
MR Egger
450
425
400
375
350
325
1/SE$_{IV}$
−0.0025
0.0000
0.0025
0.005
β$_{IV}$

Batch 2−685 : Granulocyte colony−stimulating factor on eGFR

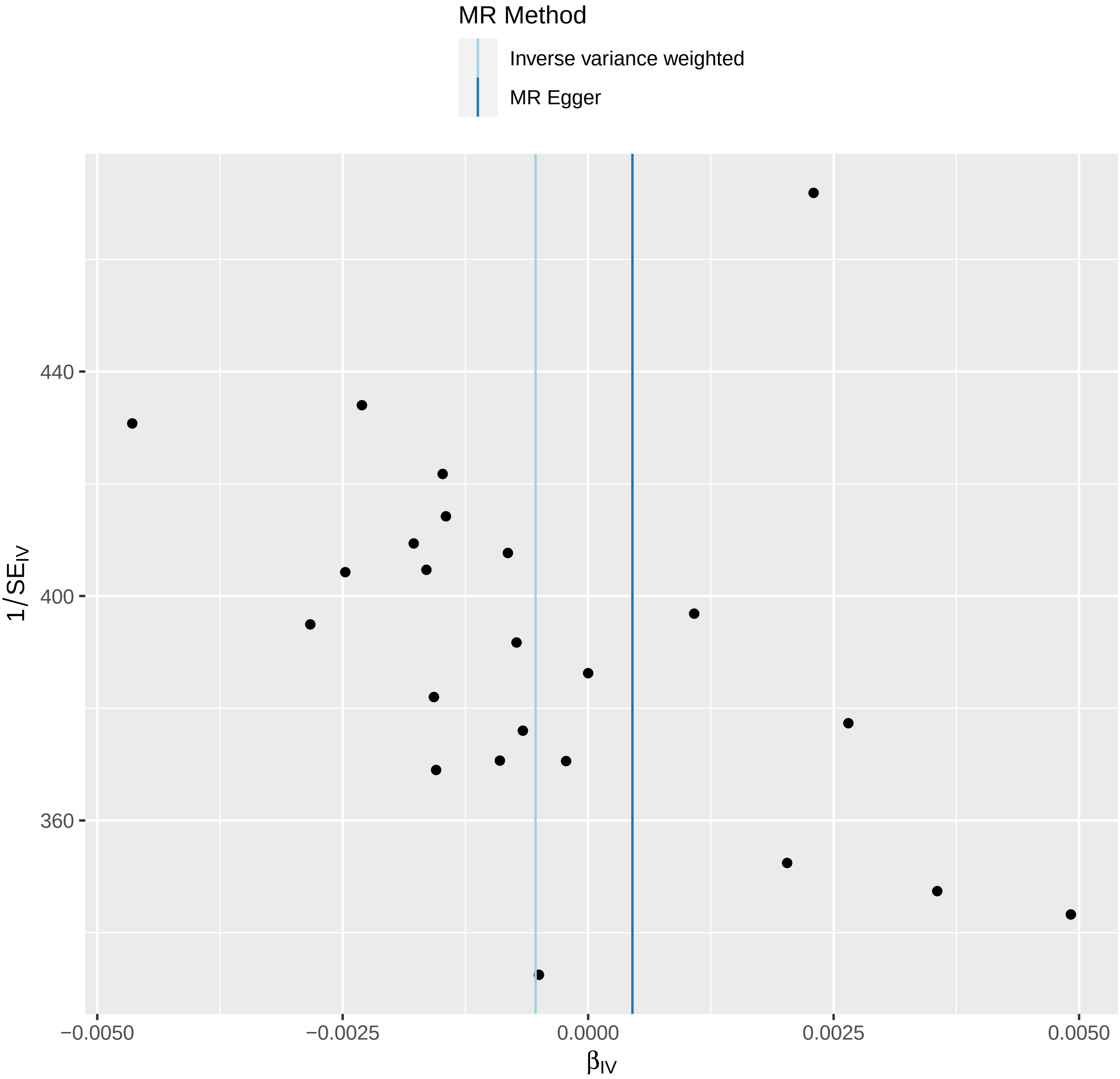

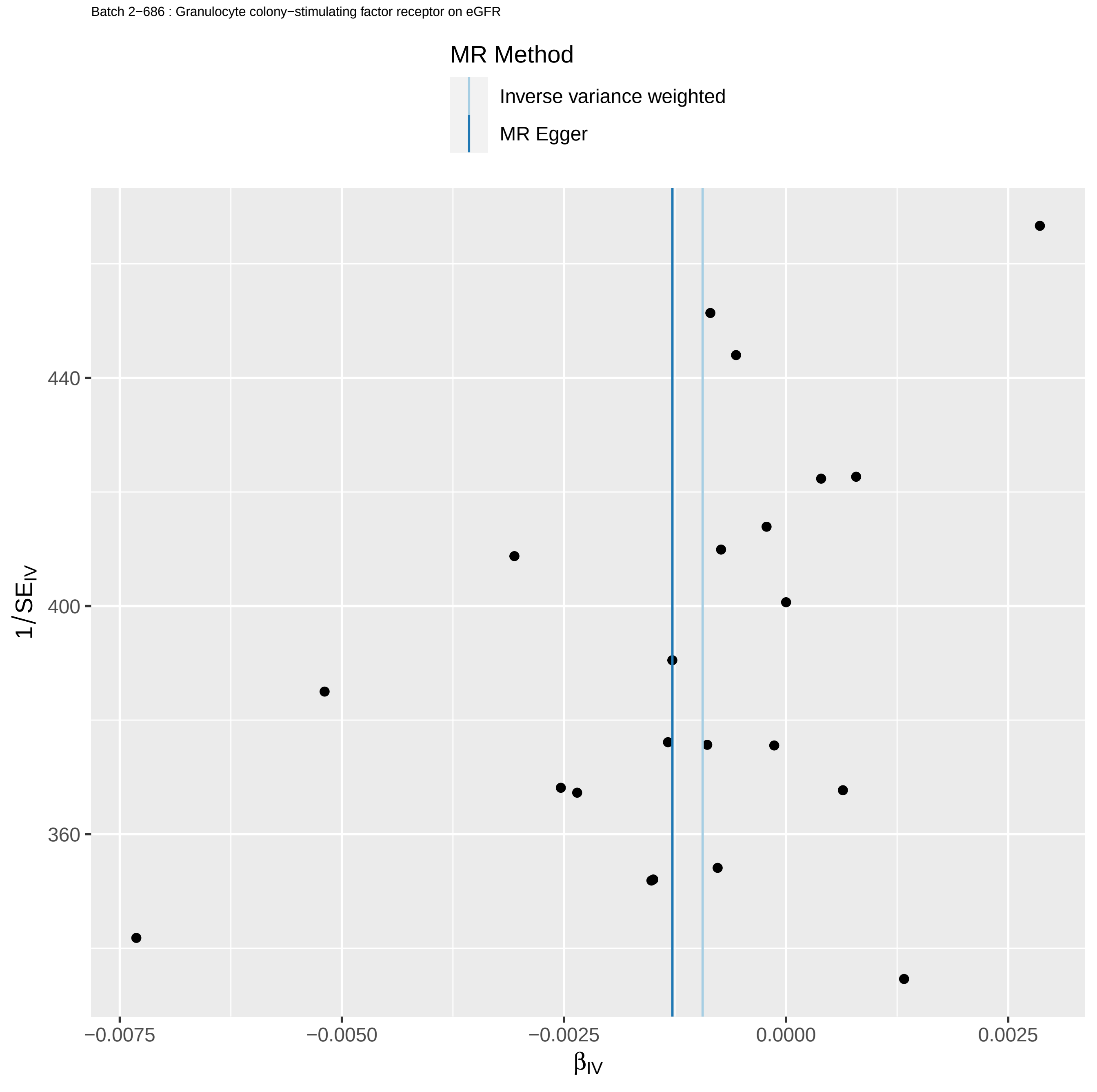

Batch 2−686 : Granulocyte colony−stimulating factor receptor on eGFR
MR Method
Inverse variance weighted
MR Egger
440
400
360
1/SE_IV
−0.0075
−0.0050
−0.0025
0.0000
0.0025
β_IV

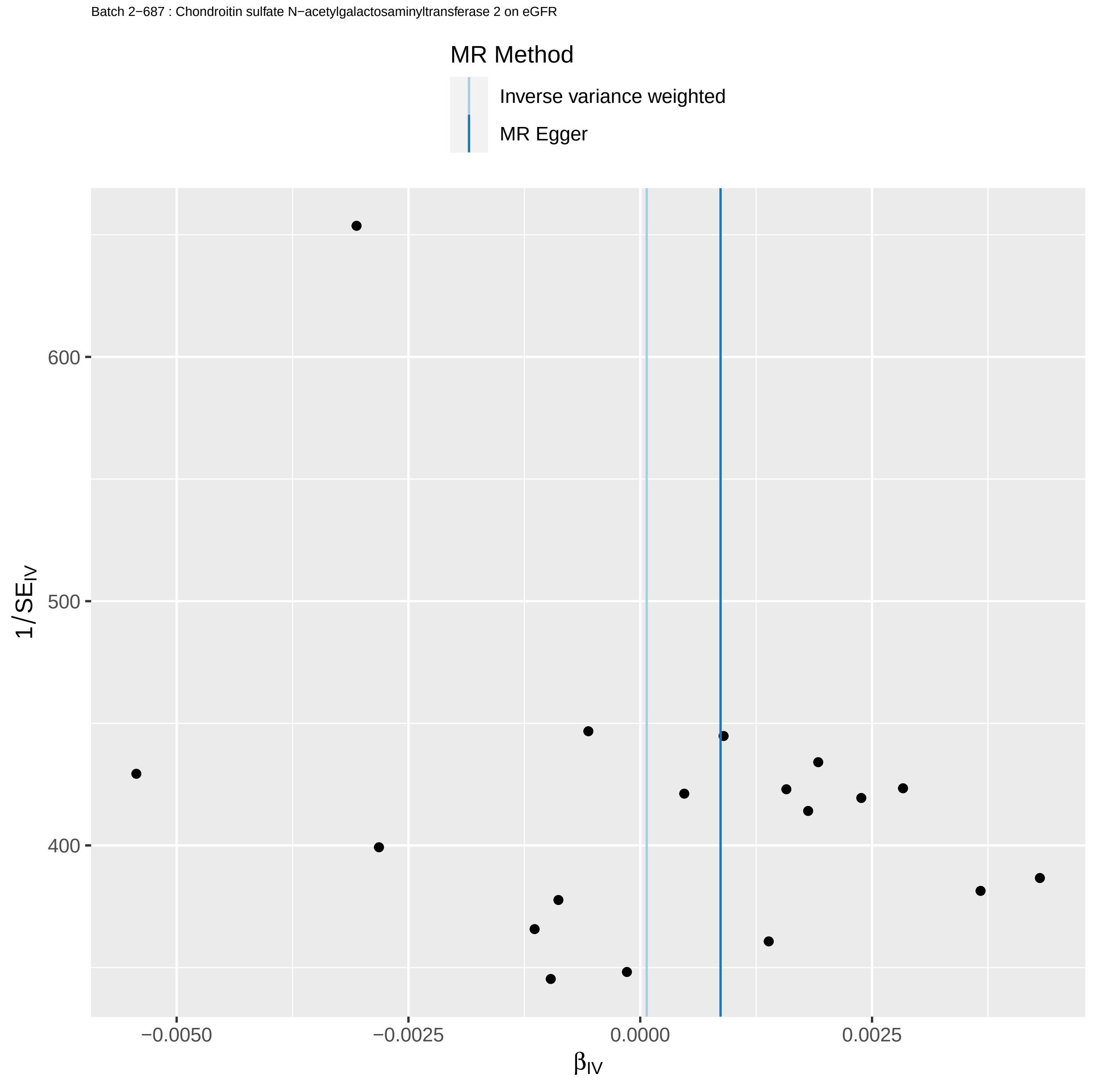

Batch 2−687 : Chondroitin sulfate N−acetylgalactosaminyltransferase 2 on eGFR
MR Method
Inverse variance weighted
MR Egger
600
500
400
1/SE$_{IV}$
−0.0050
−0.0025
0.0000
0.0025
β_{IV}

Batch 2−688 : Chorionic somatomammotropin hormone on eGFR

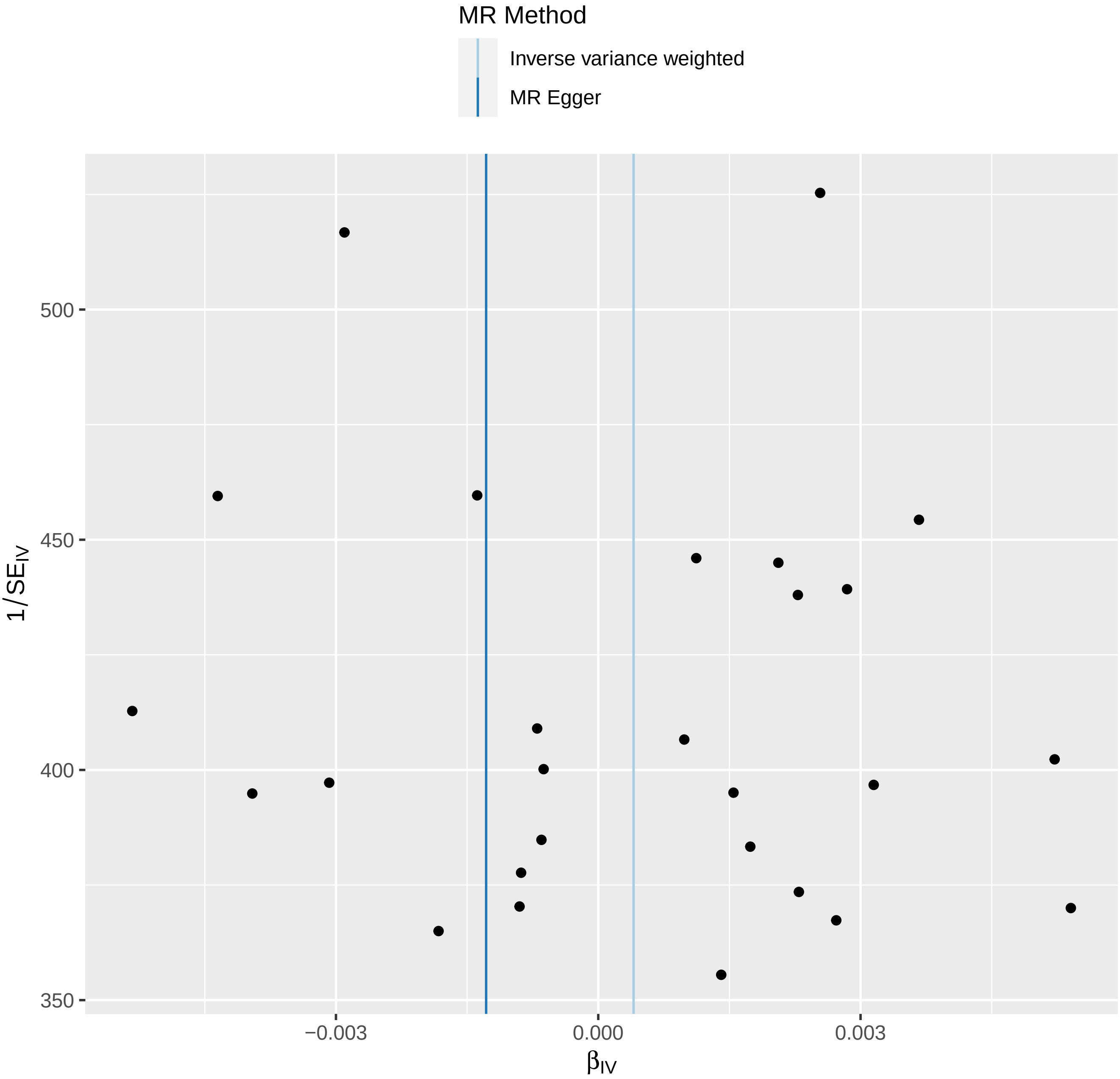

Batch 2−689 : Tyrosine−protein kinase CSK on eGFR

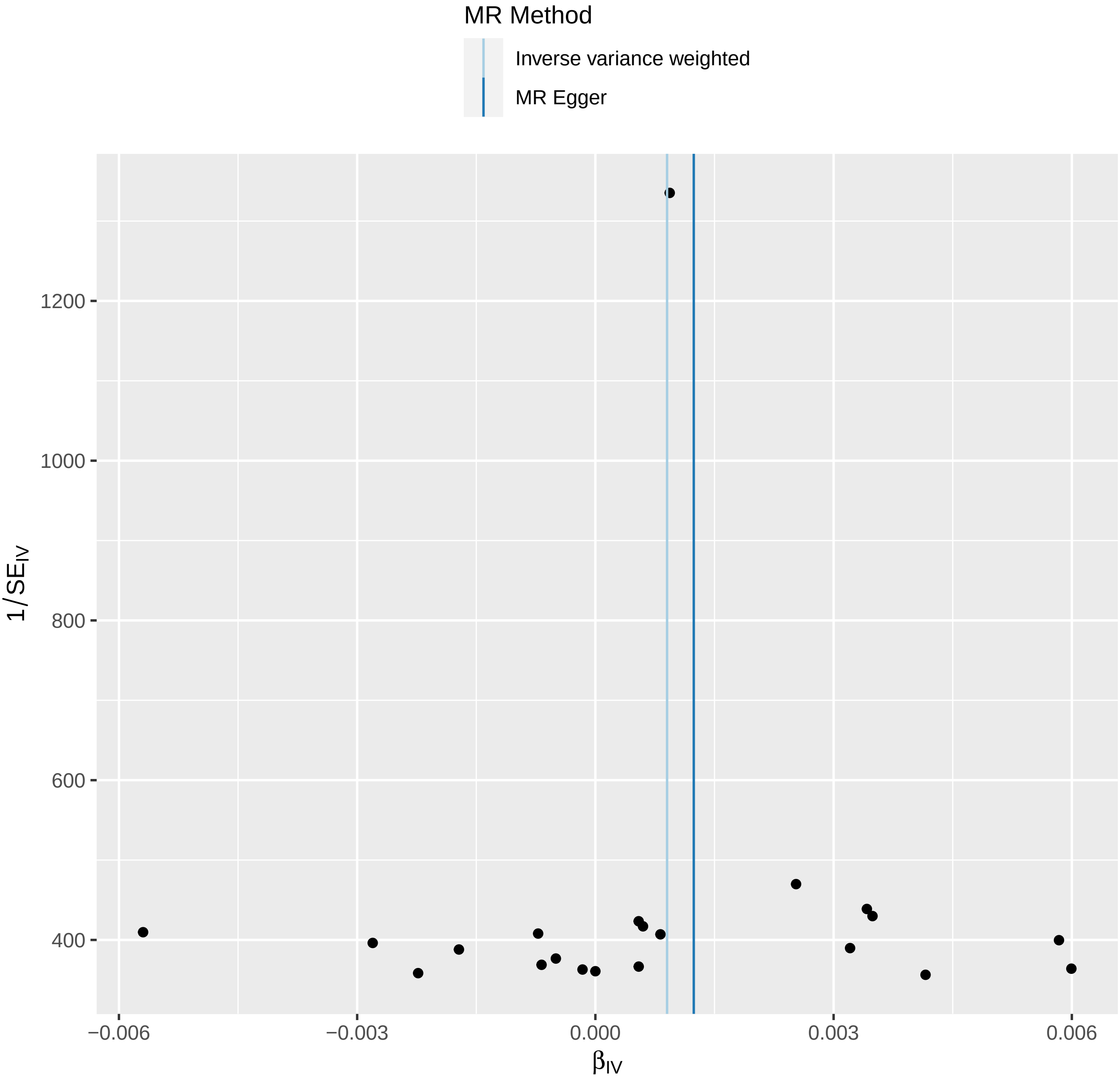

Batch 2−690 : CUB and sushi domain−containing protein 1 on eGFR

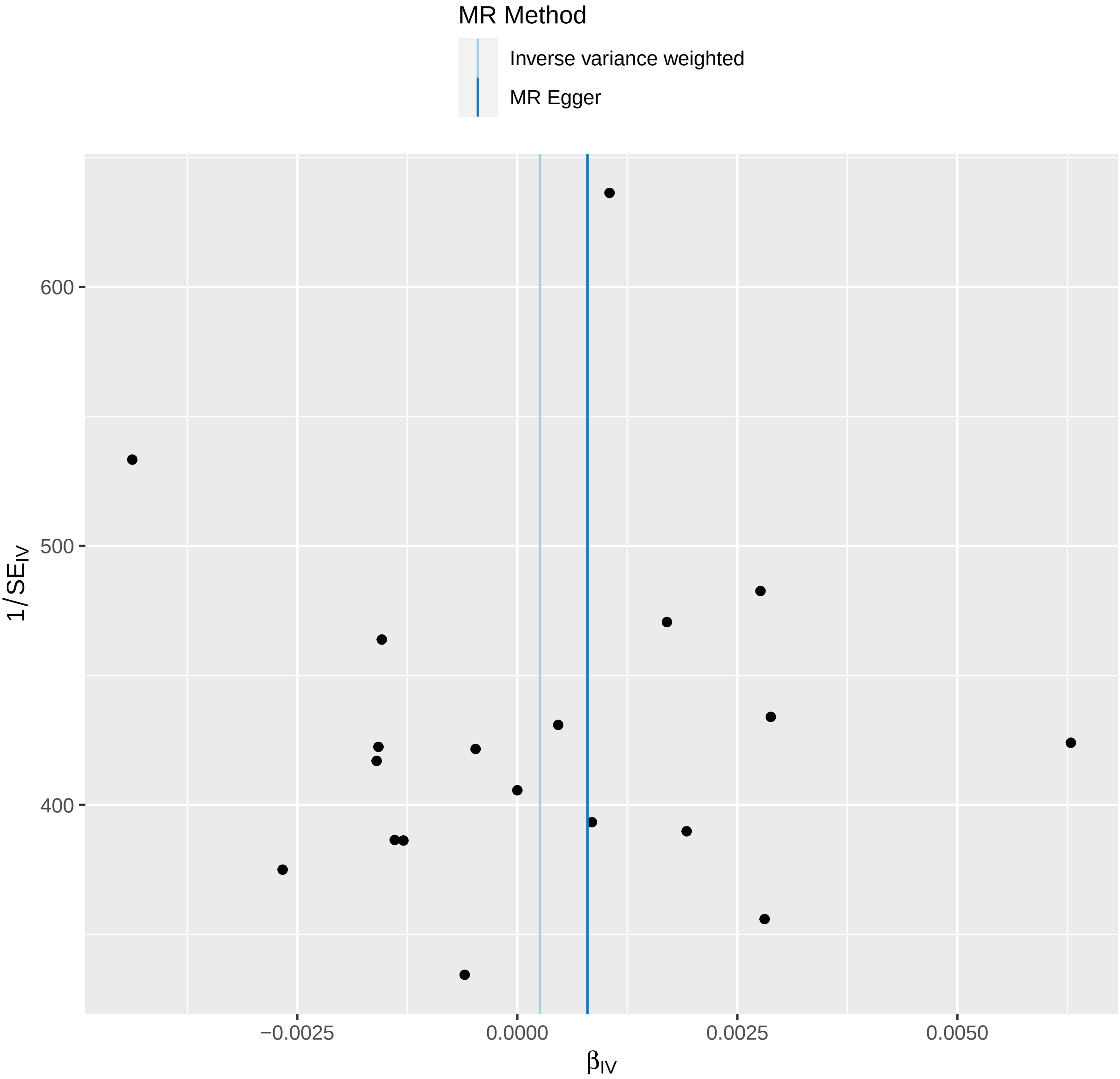

Batch 2−691 : CUB and sushi domain−containing protein 2 on eGFR

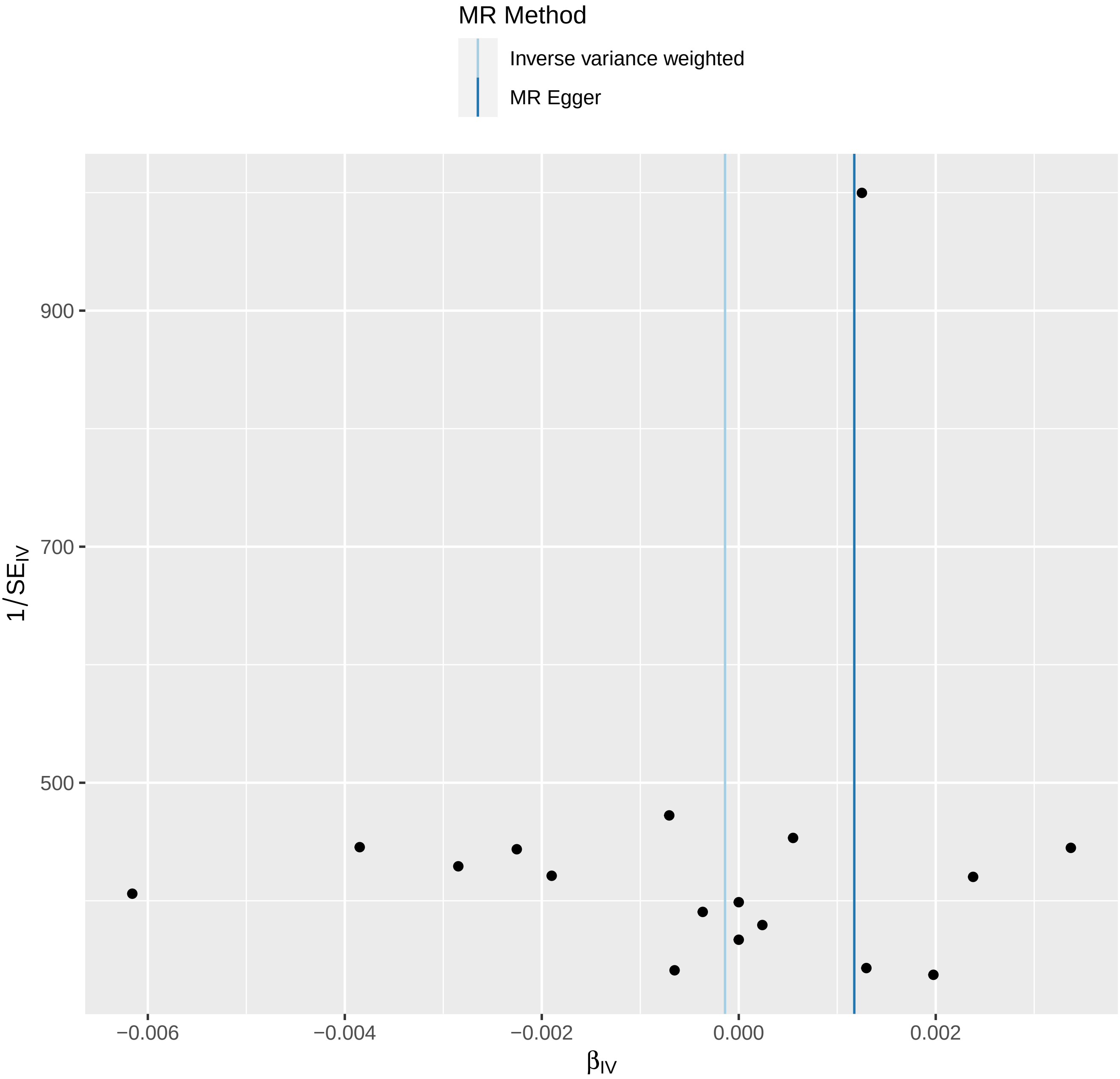

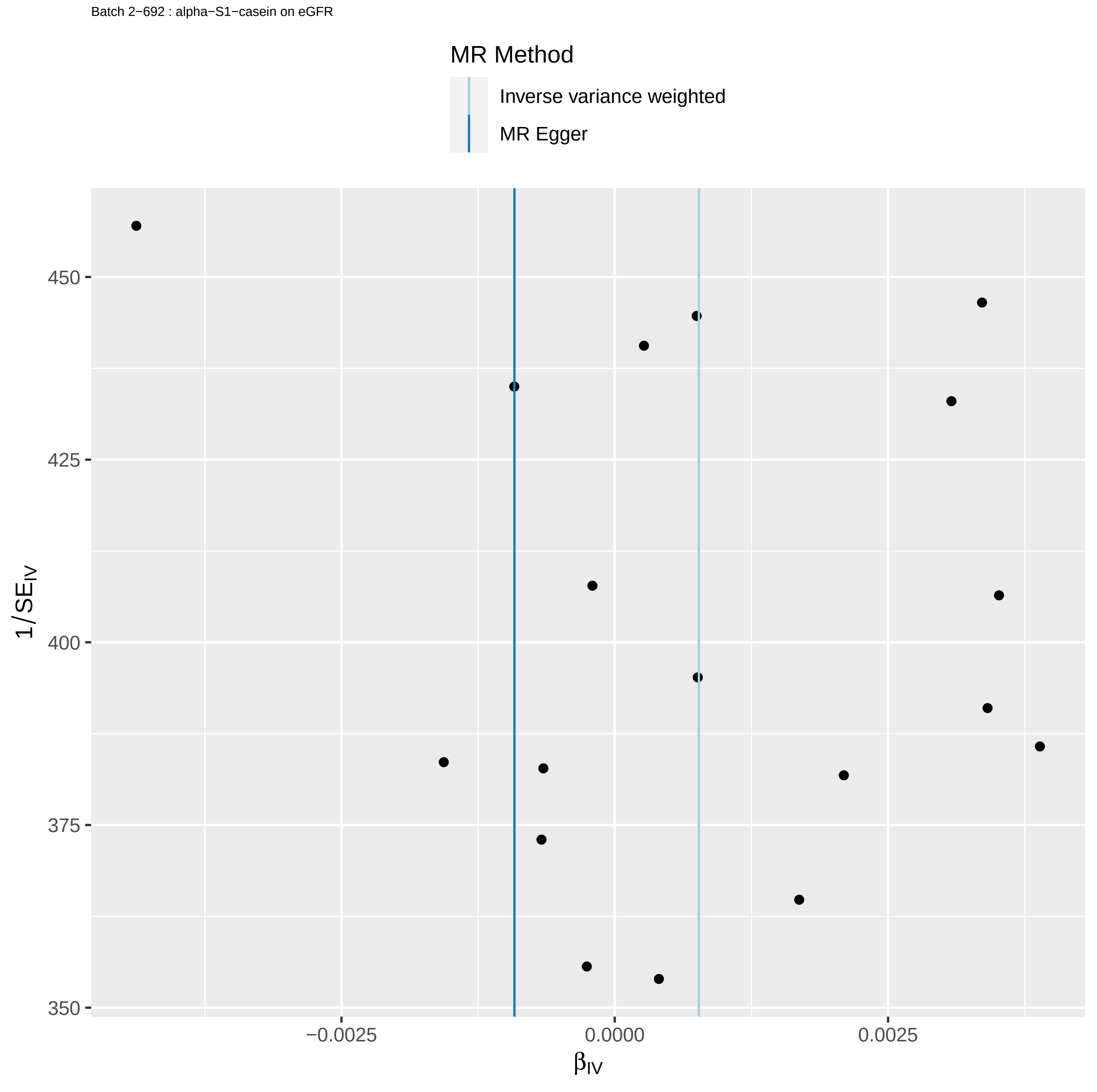

Batch 2−692 : alpha−S1−casein on eGFR
MR Method
Inverse variance weighted
MR Egger
450
425
400
375
350
1/SE$_{IV}$
−0.0025
0.0000
0.0025
β$_{IV}$

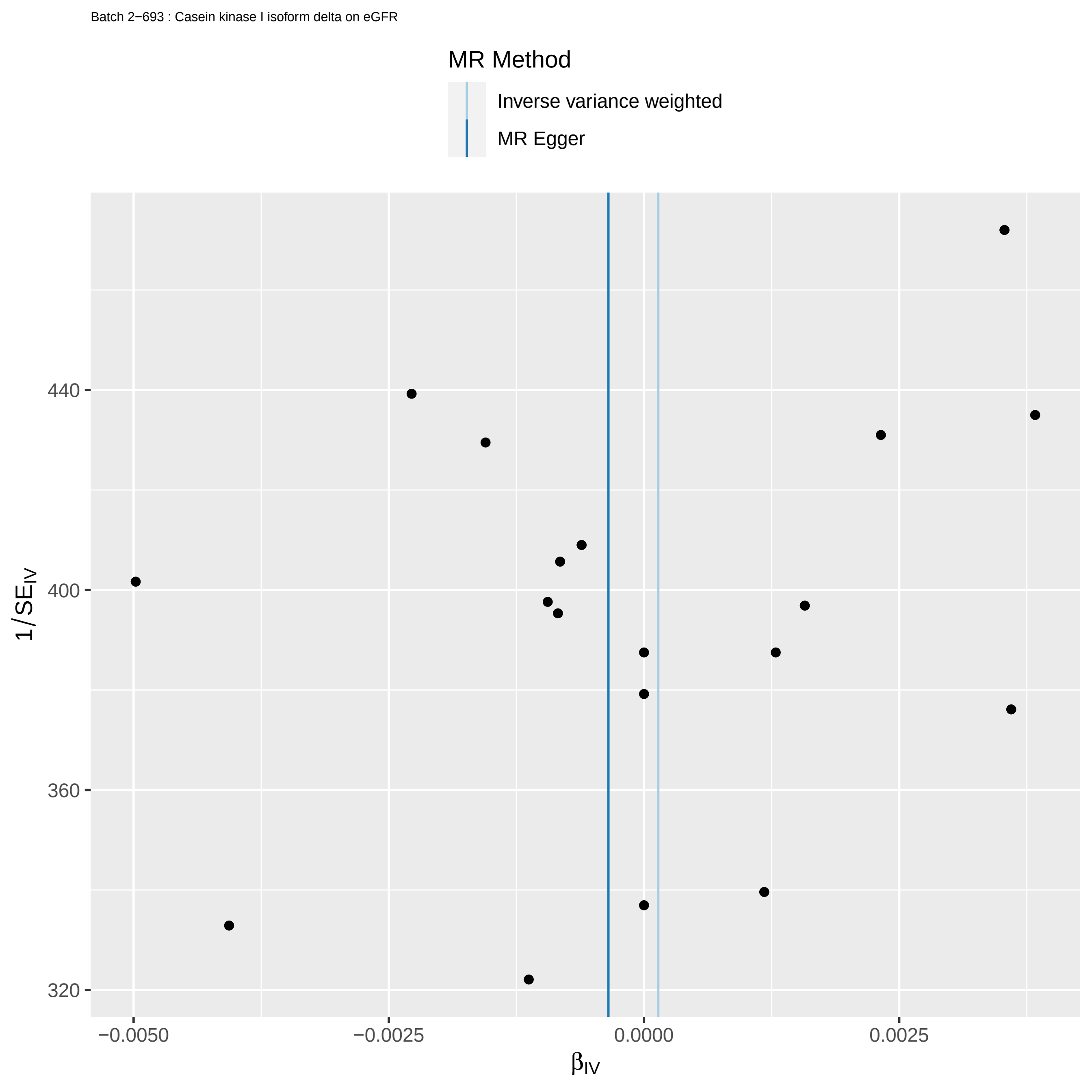

Batch 2−693 : Casein kinase I isoform delta on eGFR
MR Method
Inverse variance weighted
MR Egger
1/SE_IV
440
400
360
320
−0.0050
−0.0025
0.0000
0.0025
β_IV

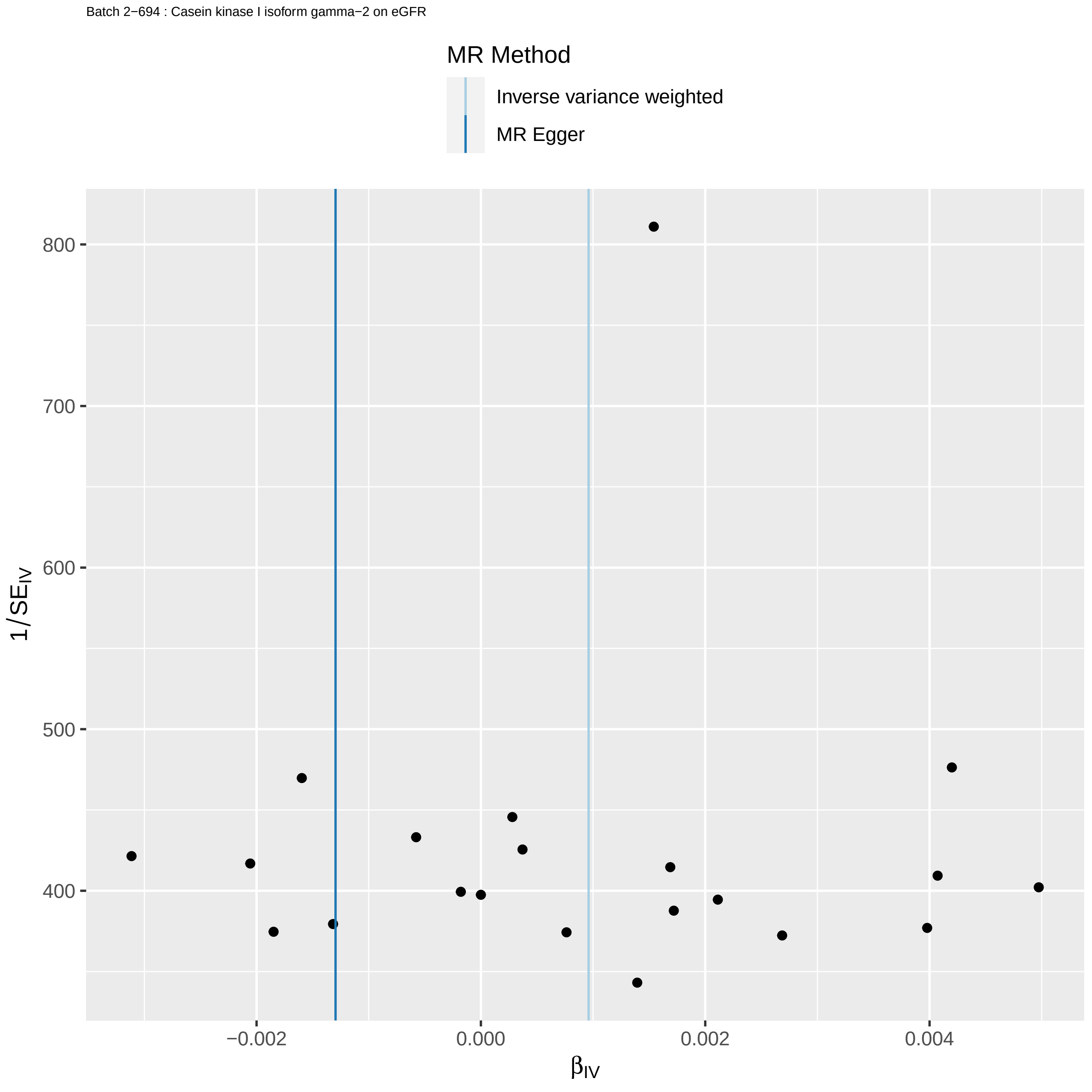
Batch 2−694 : Casein kinase I isoform gamma−2 on eGFR
MR Method
Inverse variance weighted
MR Egger
800
700
600
500
400
1/SE$_{IV}$
−0.002
0.000
0.002
0.004
β$_{IV}$

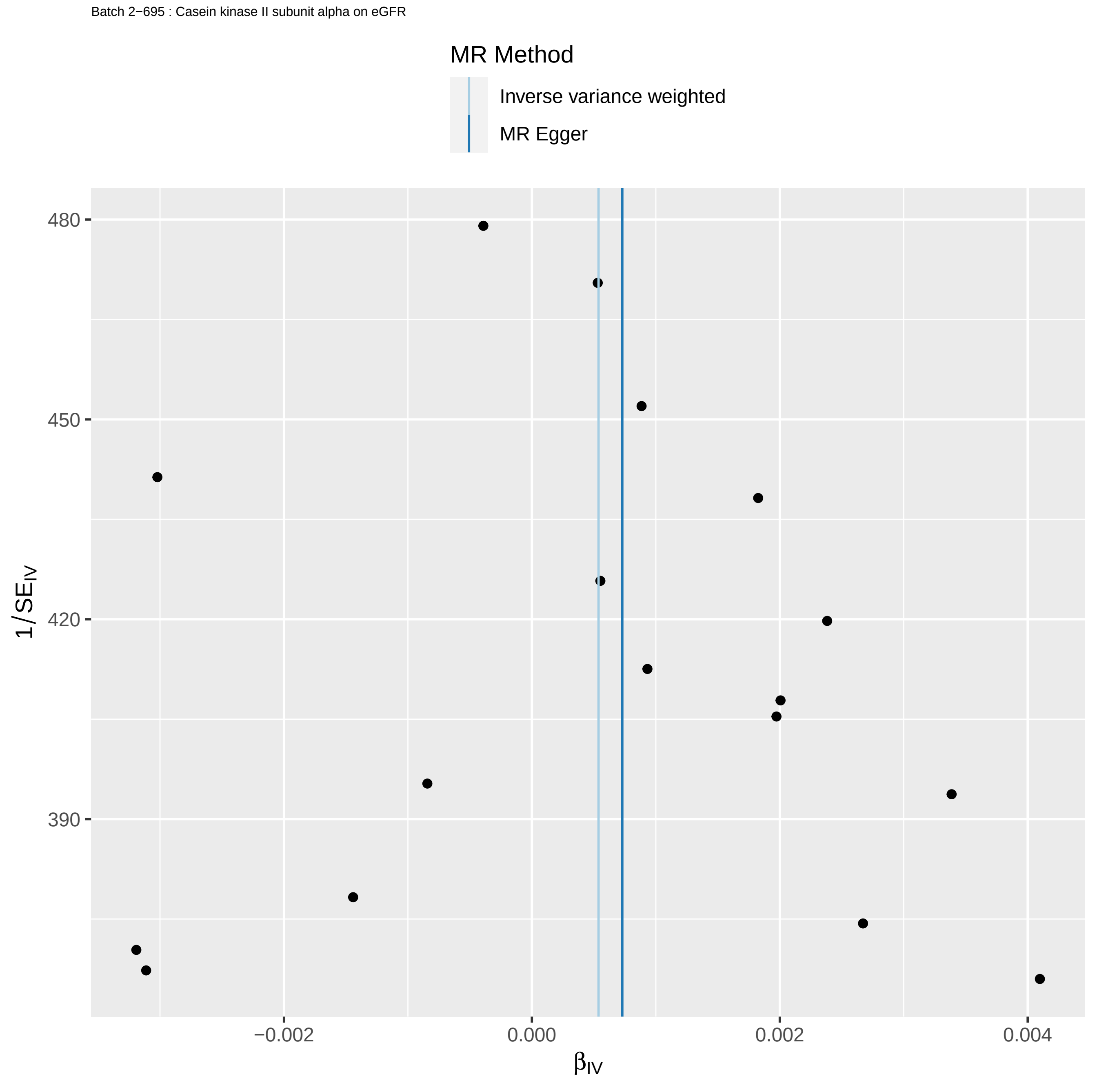
Batch 2−695 : Casein kinase II subunit alpha on eGFR
MR Method
Inverse variance weighted
MR Egger
480
450
420
390
1/SE_IV
−0.002
0.000
0.002
0.004
β_IV

Batch 2−696 : Casein kinase II subunit alpha on eGFR

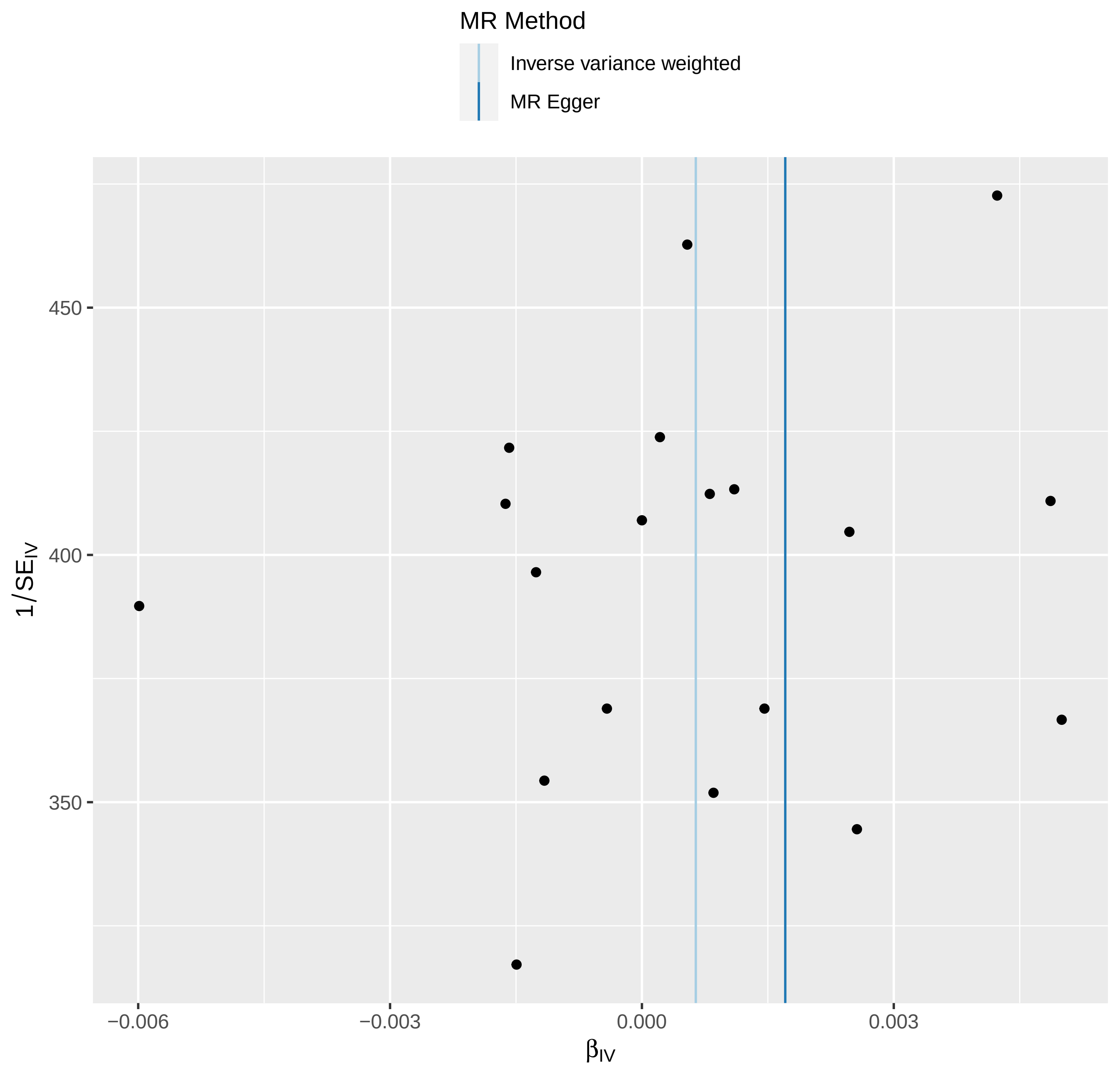

Batch 2−697 : Casein kinase II 2−alpha:2−beta heterotetramer on eGFR

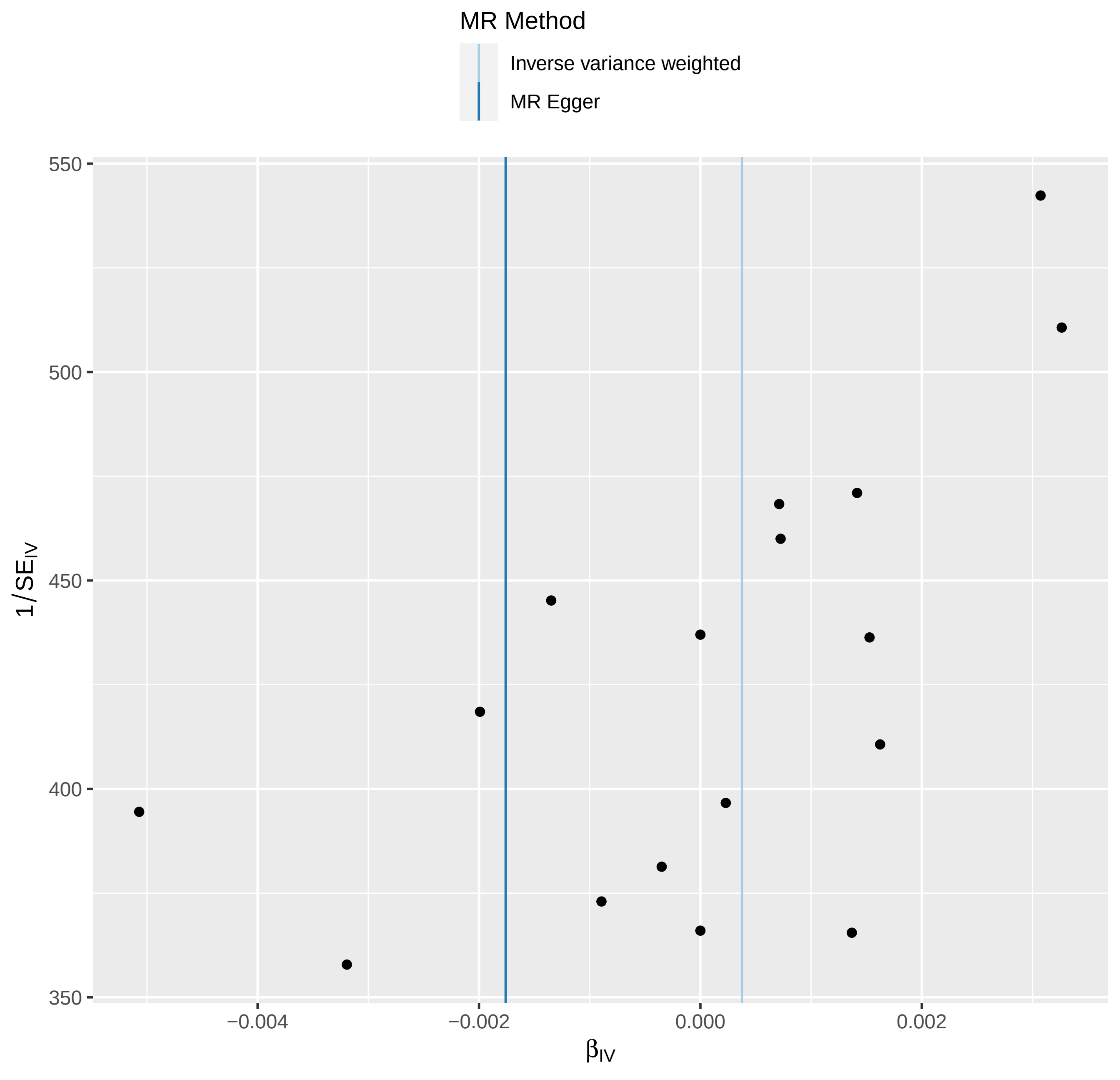

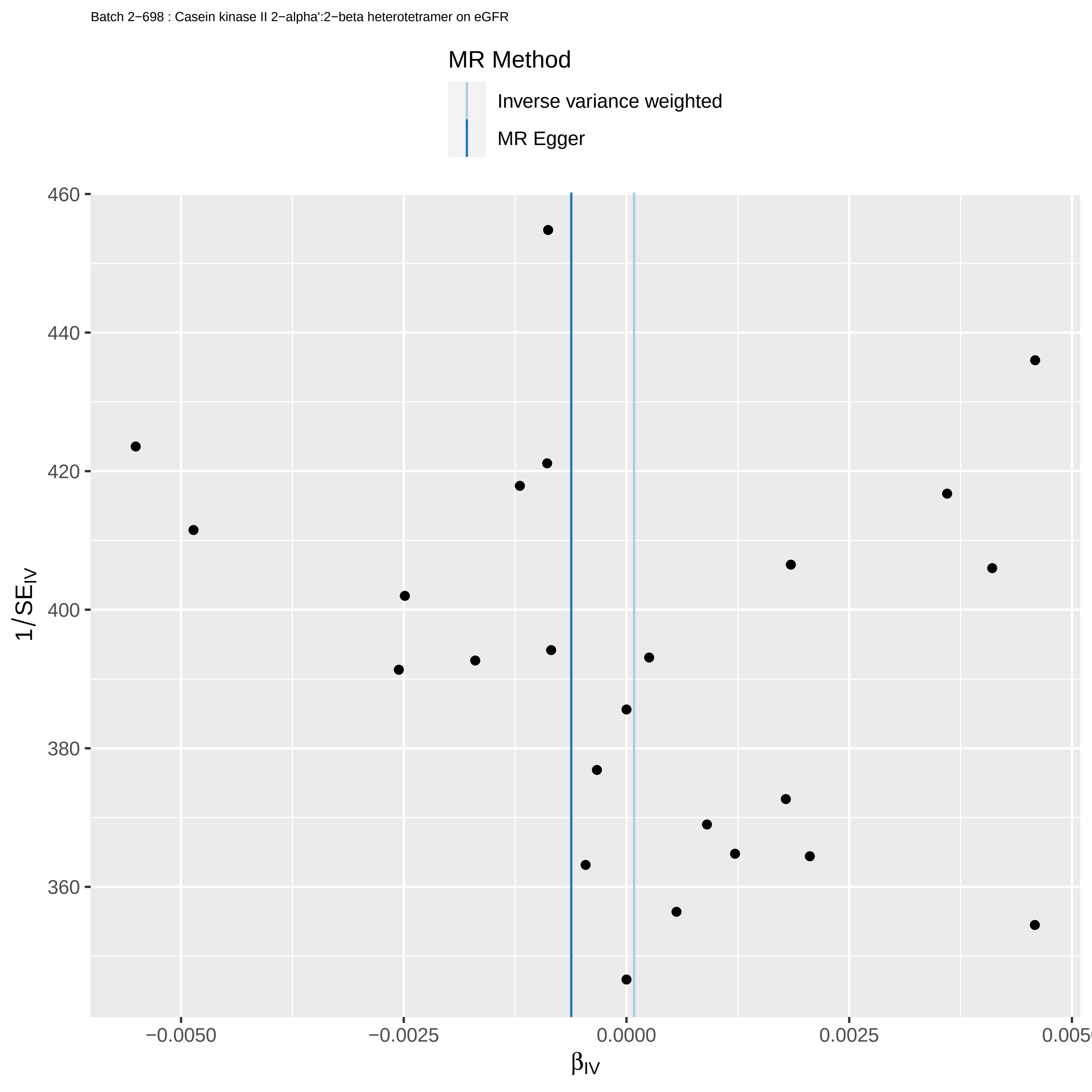
Batch 2−698 : Casein kinase II 2−alpha':2−beta heterotetramer on eGFR
MR Method
Inverse variance weighted
MR Egger
460
440
420
400
380
360
1/SE$_{IV}$
−0.0050
−0.0025
0.0000
0.0025
0.0050
β$_{IV}$

Batch 2−699 : Chondroitin sulfate proteoglycan 4 on eGFR

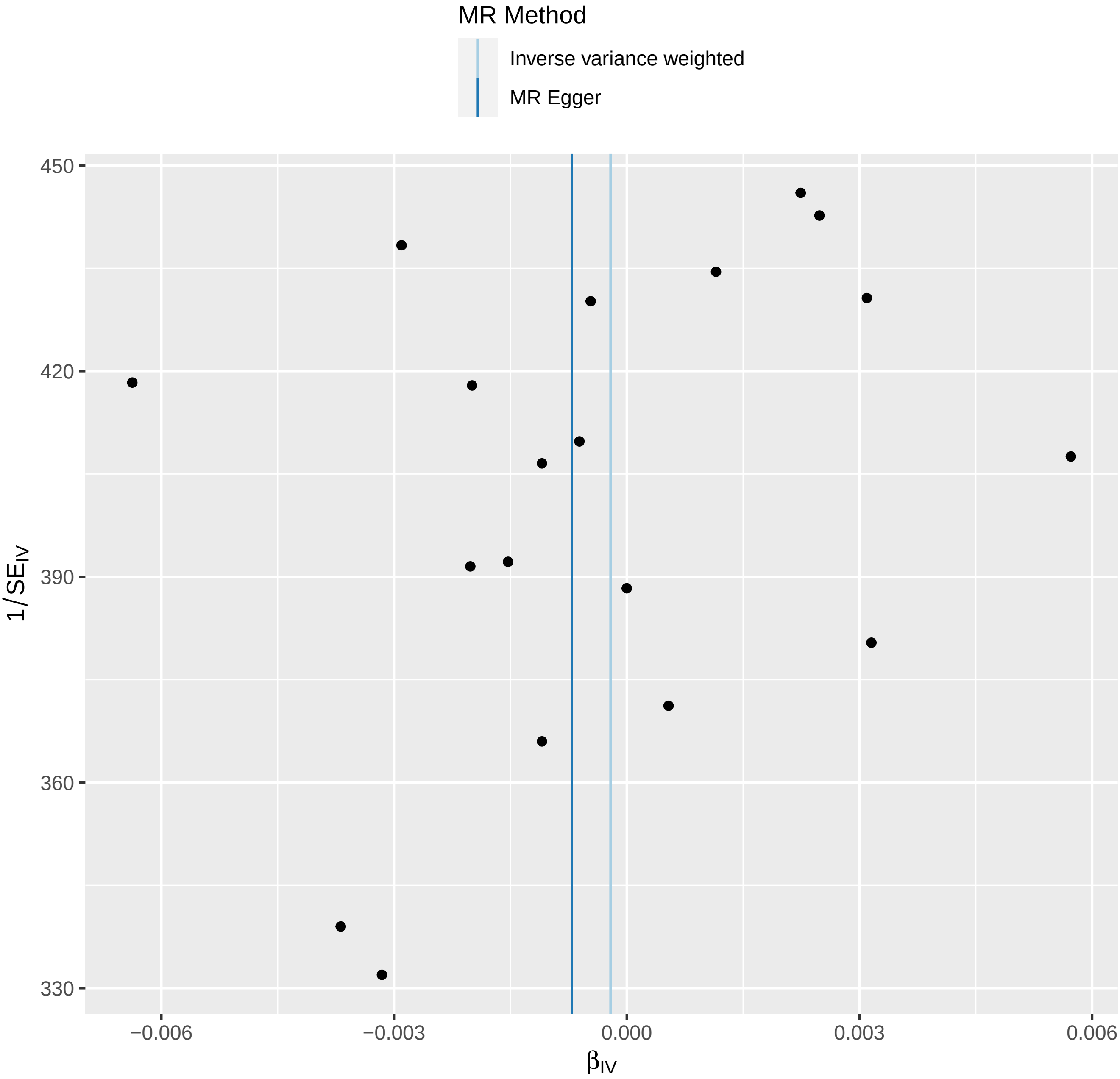

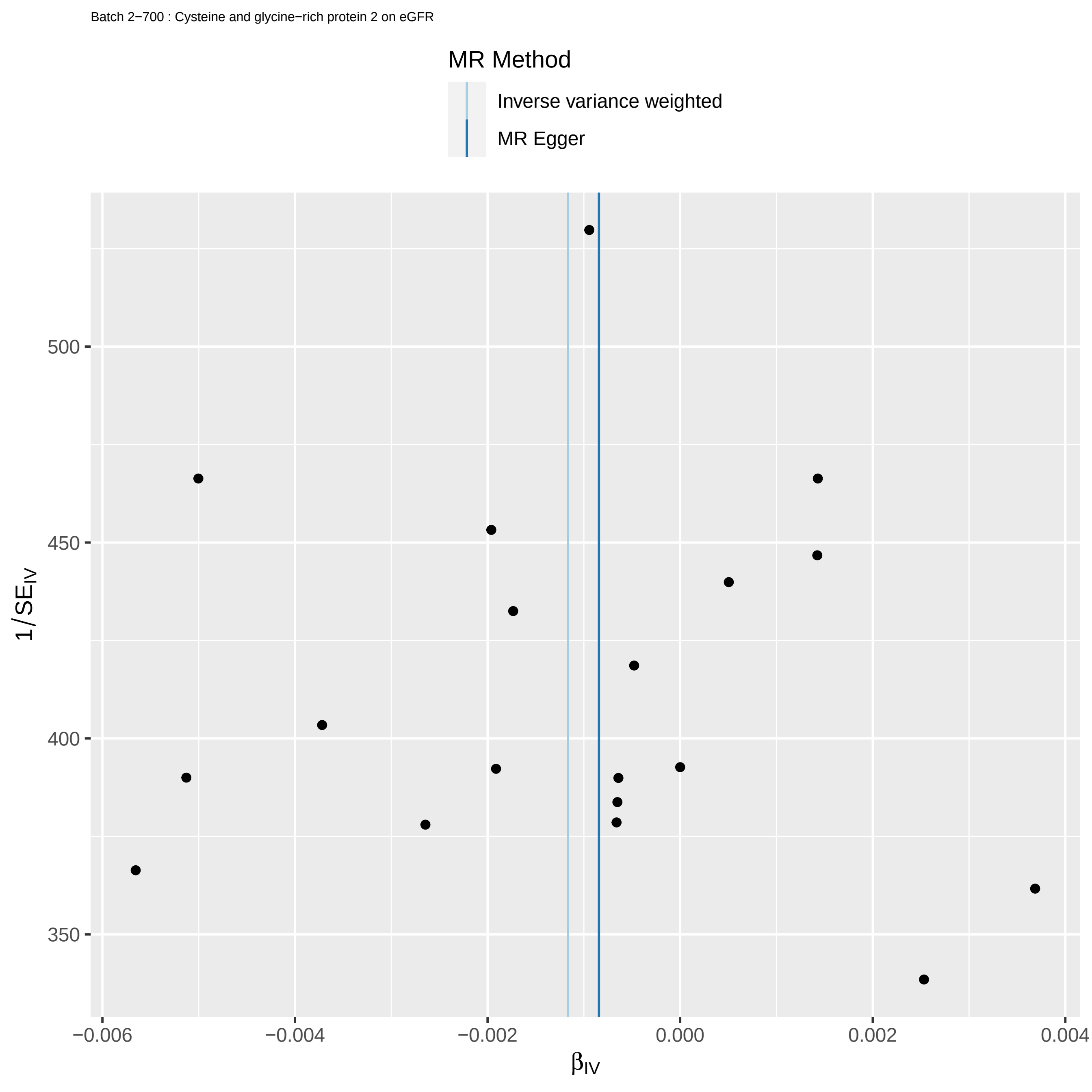

Batch 2−700 : Cysteine and glycine−rich protein 2 on eGFR
MR Method
Inverse variance weighted
MR Egger
500
450
400
350
1/SE_IV
−0.006
−0.004
−0.002
0.000
0.002
0.004
β_IV

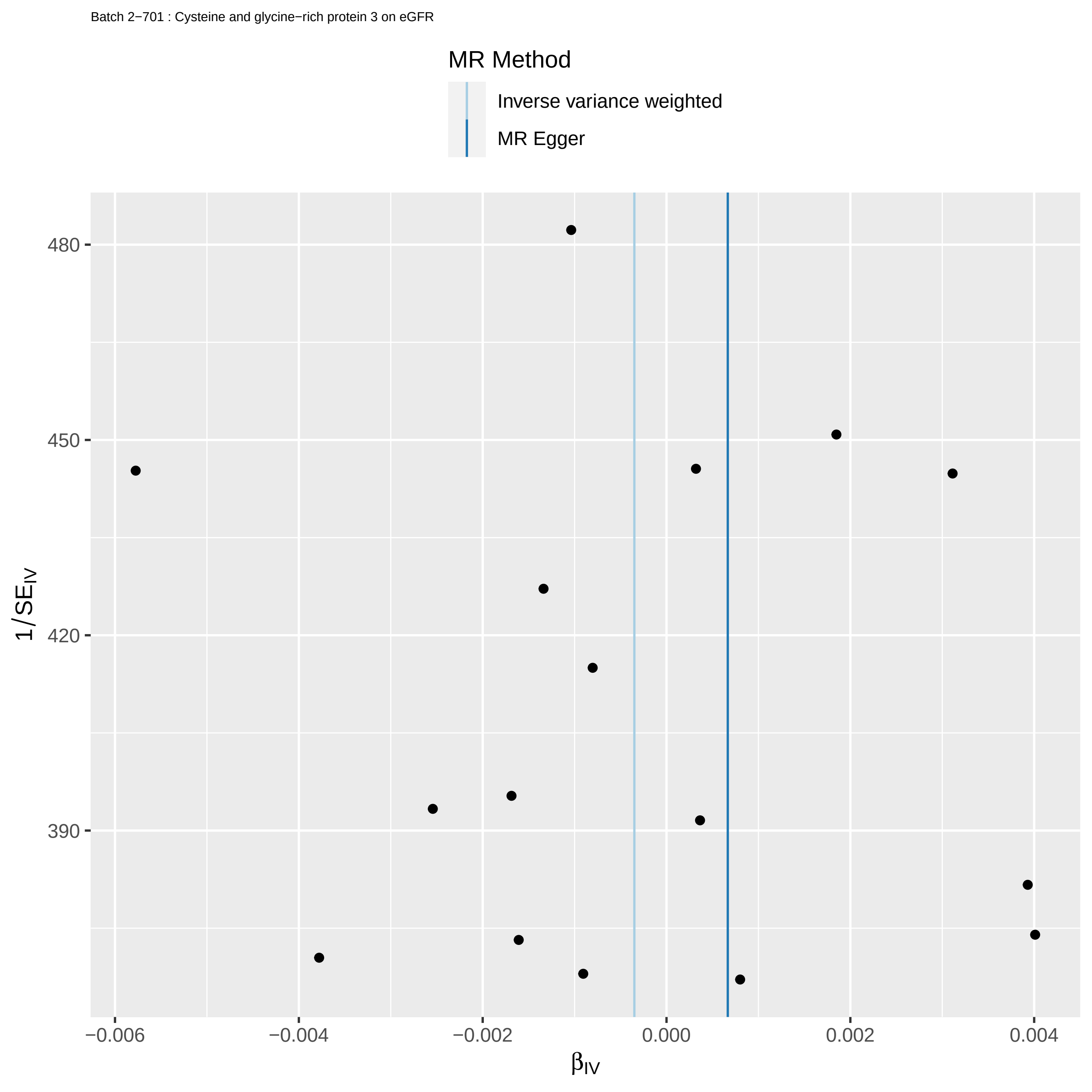

Batch 2−701 : Cysteine and glycine−rich protein 3 on eGFR
MR Method
Inverse variance weighted
MR Egger
480
450
420
390
1/SE_IV
−0.006
−0.004
−0.002
0.000
0.002
0.004
β_IV

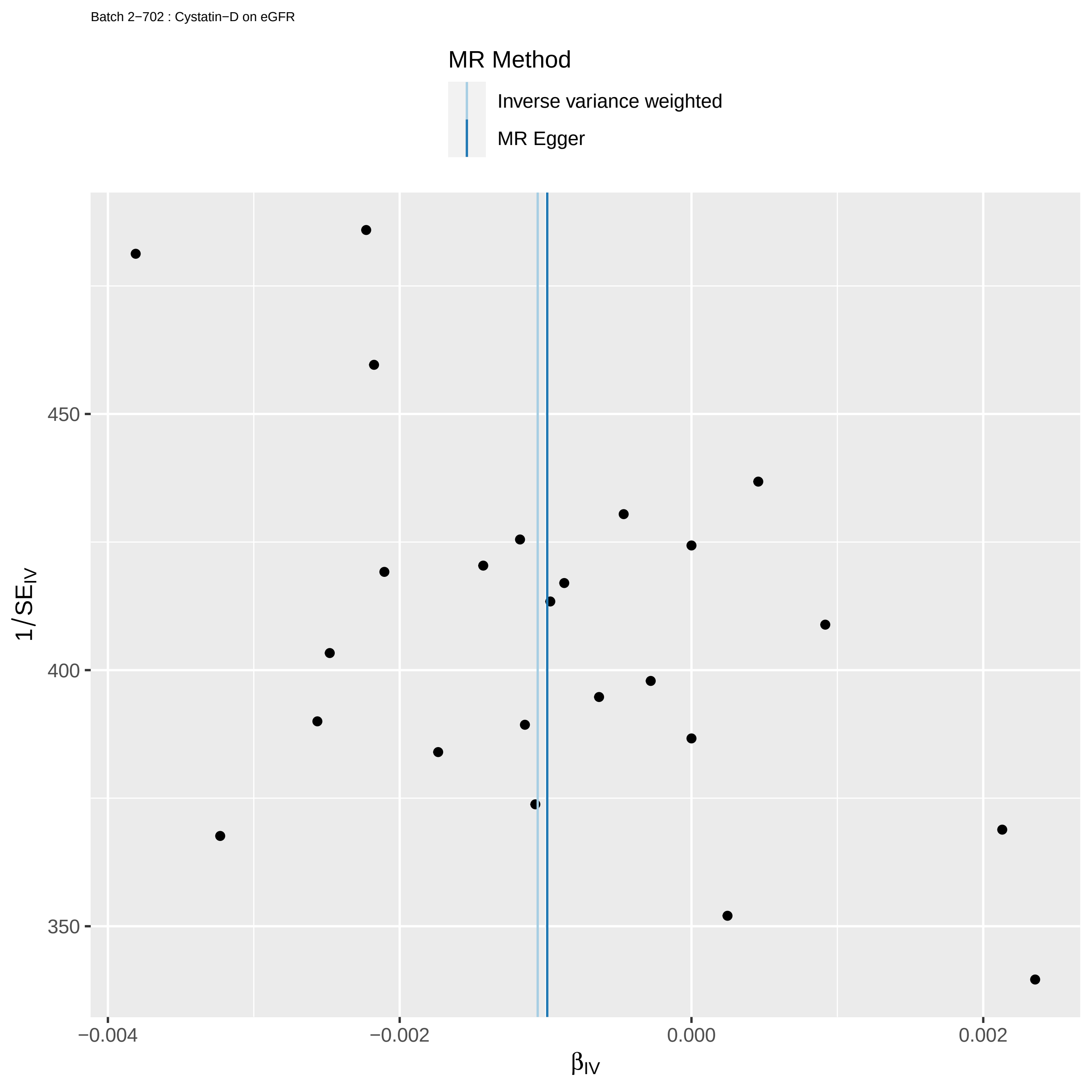

Batch 2−702 : Cystatin−D on eGFR
MR Method
Inverse variance weighted
MR Egger
450
400
350
1/SE$_{IV}$
−0.004
−0.002
0.000
0.002
β$_{IV}$

Batch 2−703 : Cystatin−M on eGFR

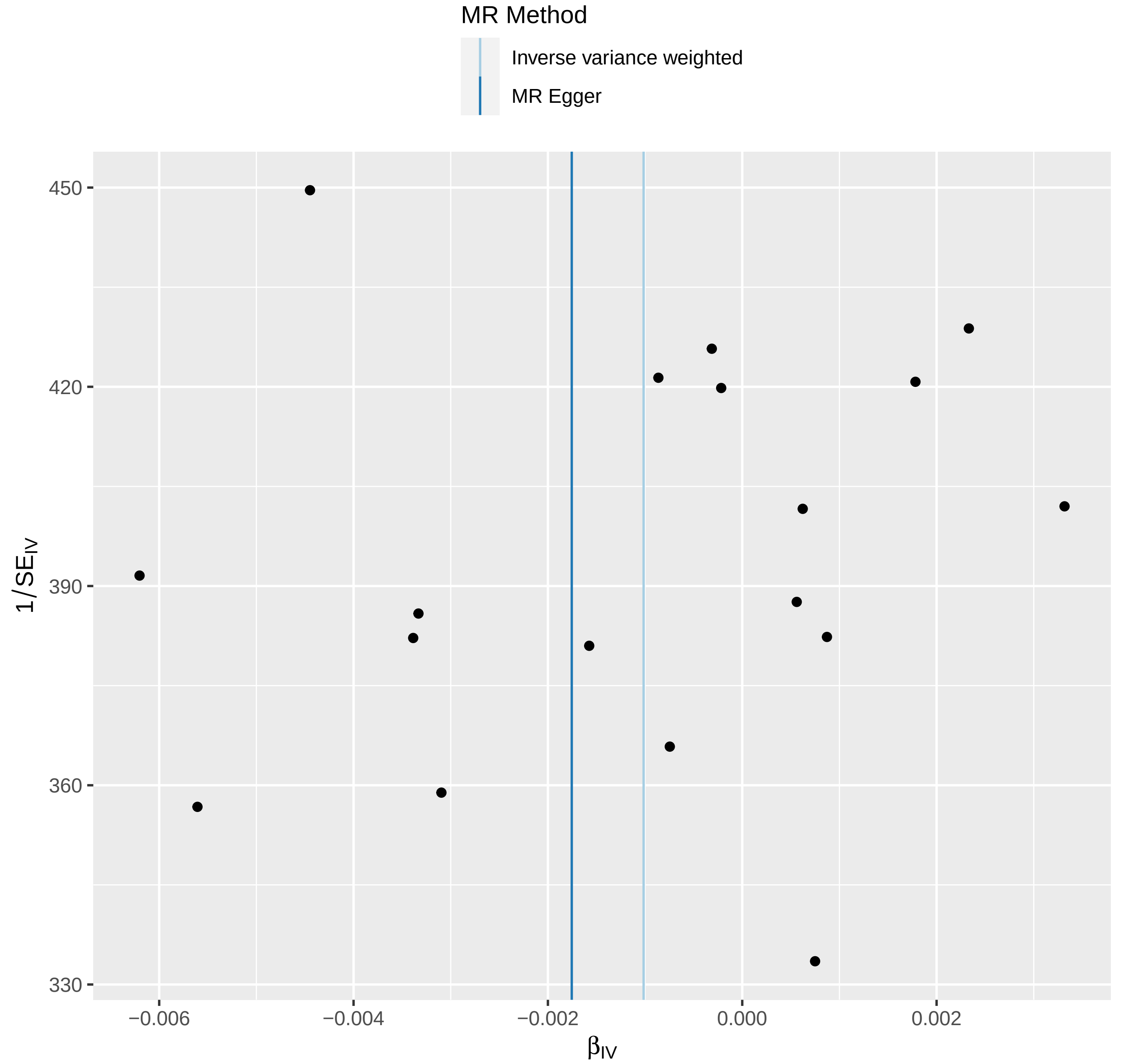

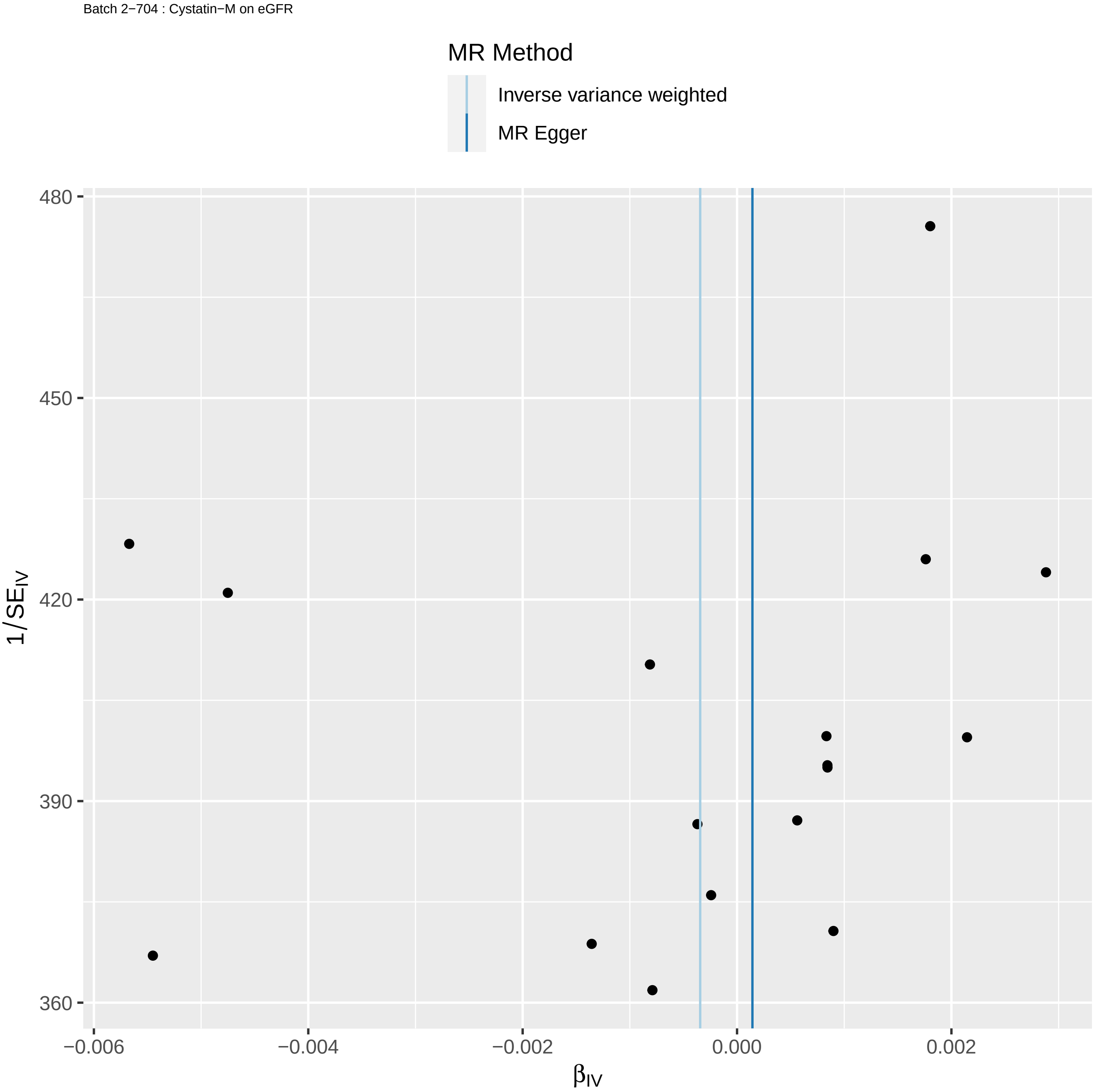
Batch 2−704 : Cystatin−M on eGFR
MR Method
Inverse variance weighted
MR Egger
480
450
420
390
360
1/SE_IV
−0.006
−0.004
−0.002
0.000
0.002
β_IV

Batch 2−705 : Cystatin−F on eGFR

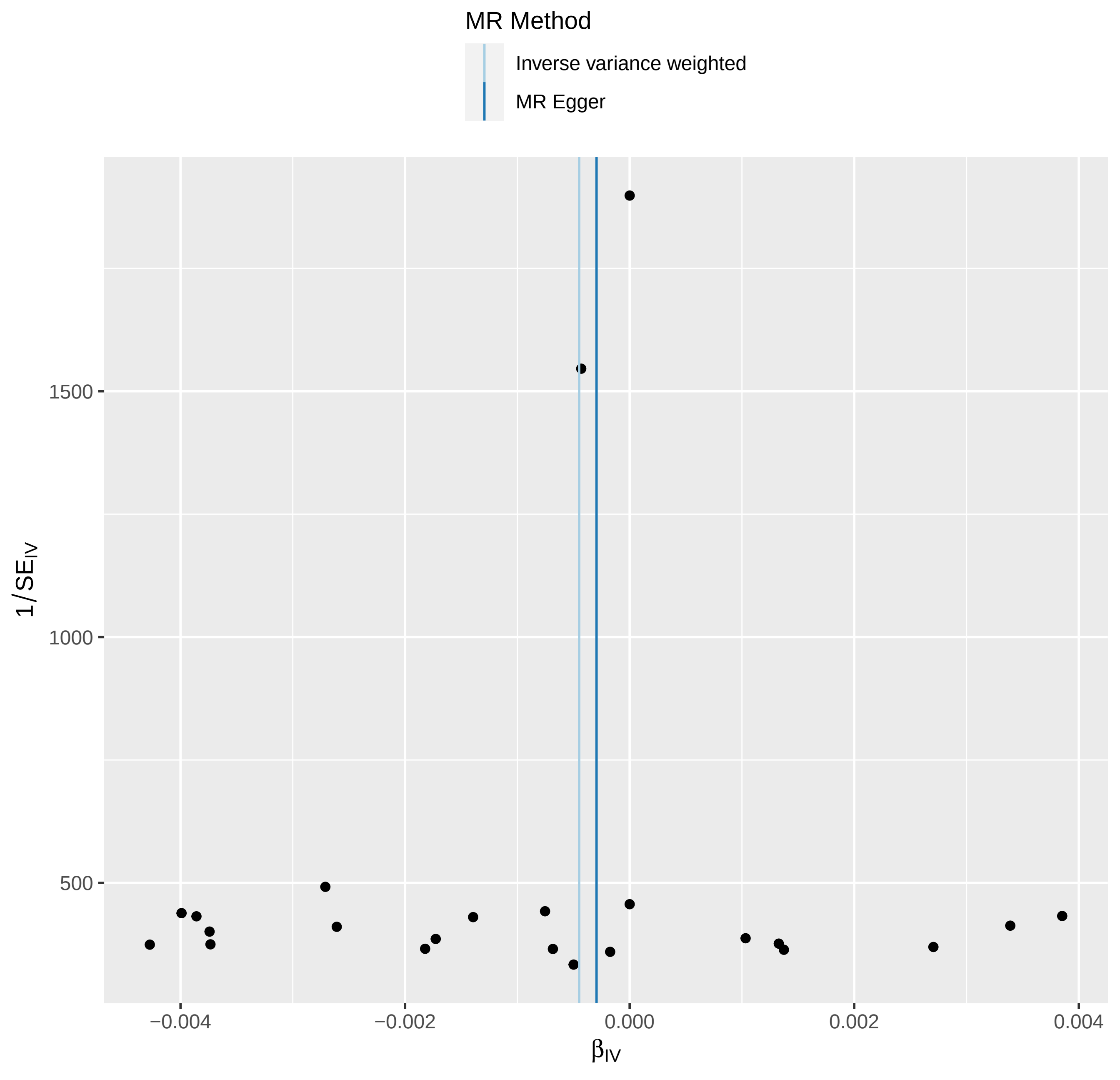

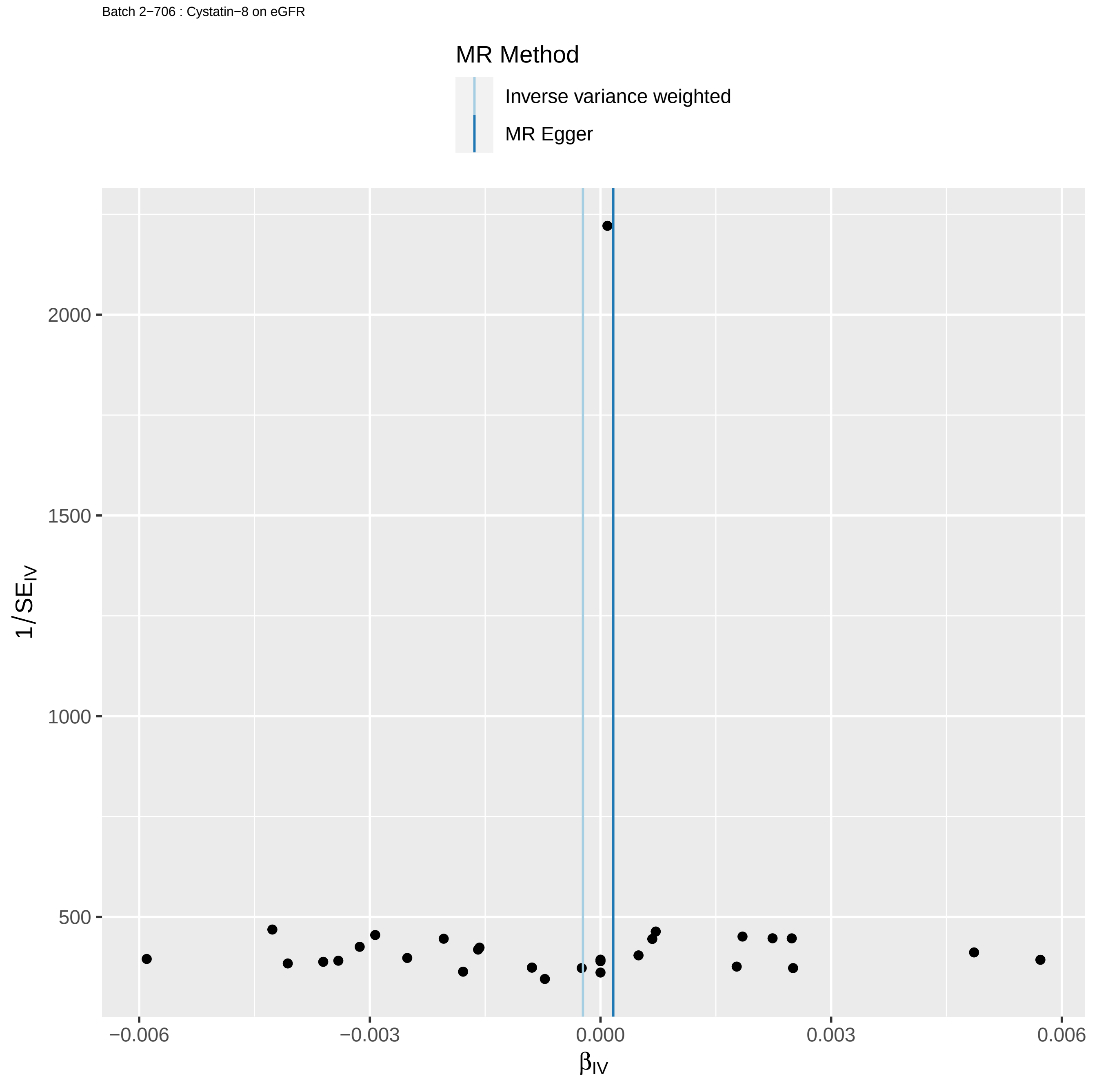

Batch 2−706 : Cystatin−8 on eGFR
MR Method
Inverse variance weighted
MR Egger
2000
1500
1000
500
1/SE_IV
−0.006
−0.003
0.000
0.003
0.006
β_IV

Batch 2−707 : Cancer/testis antigen 55 on eGFR

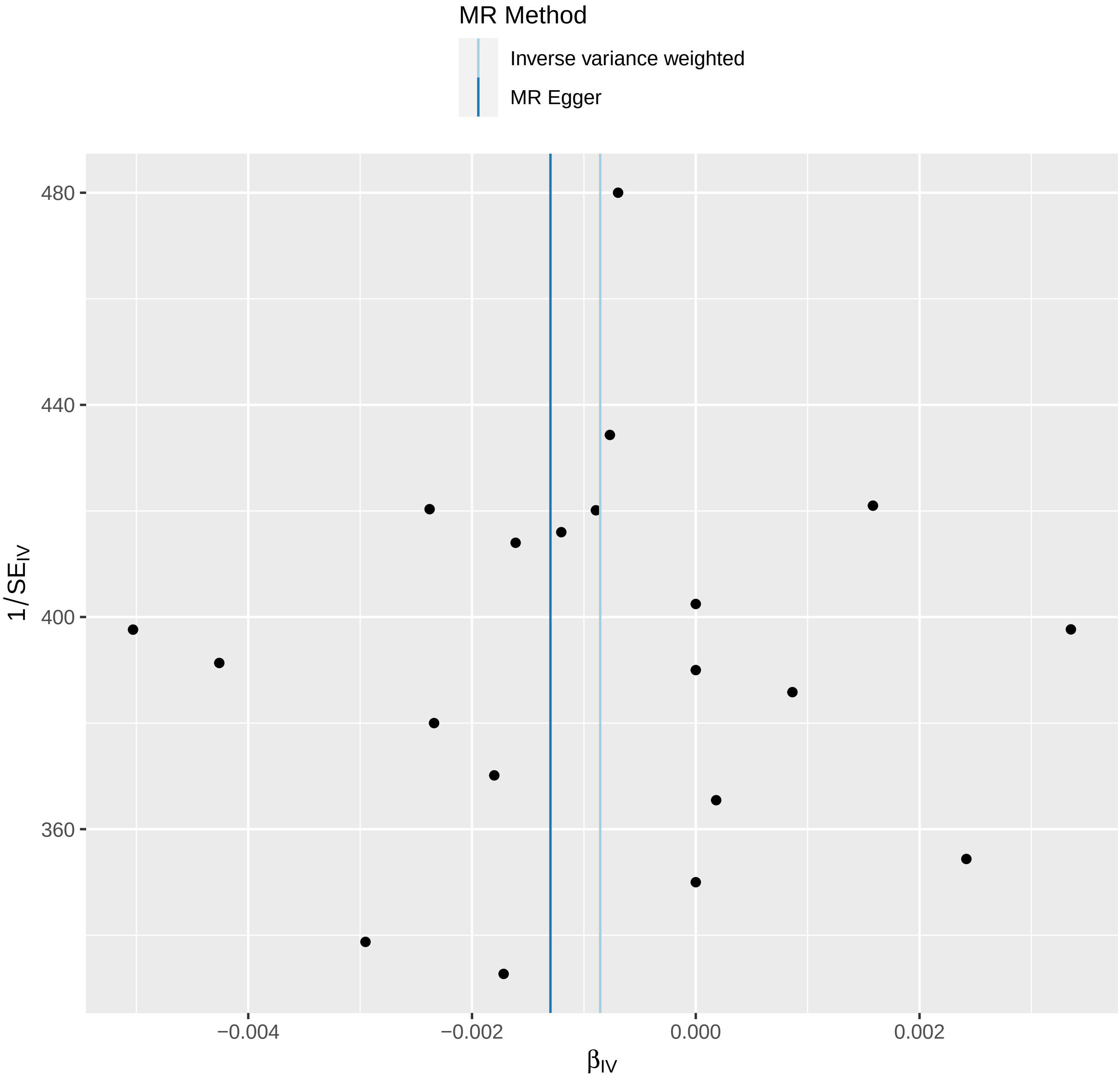

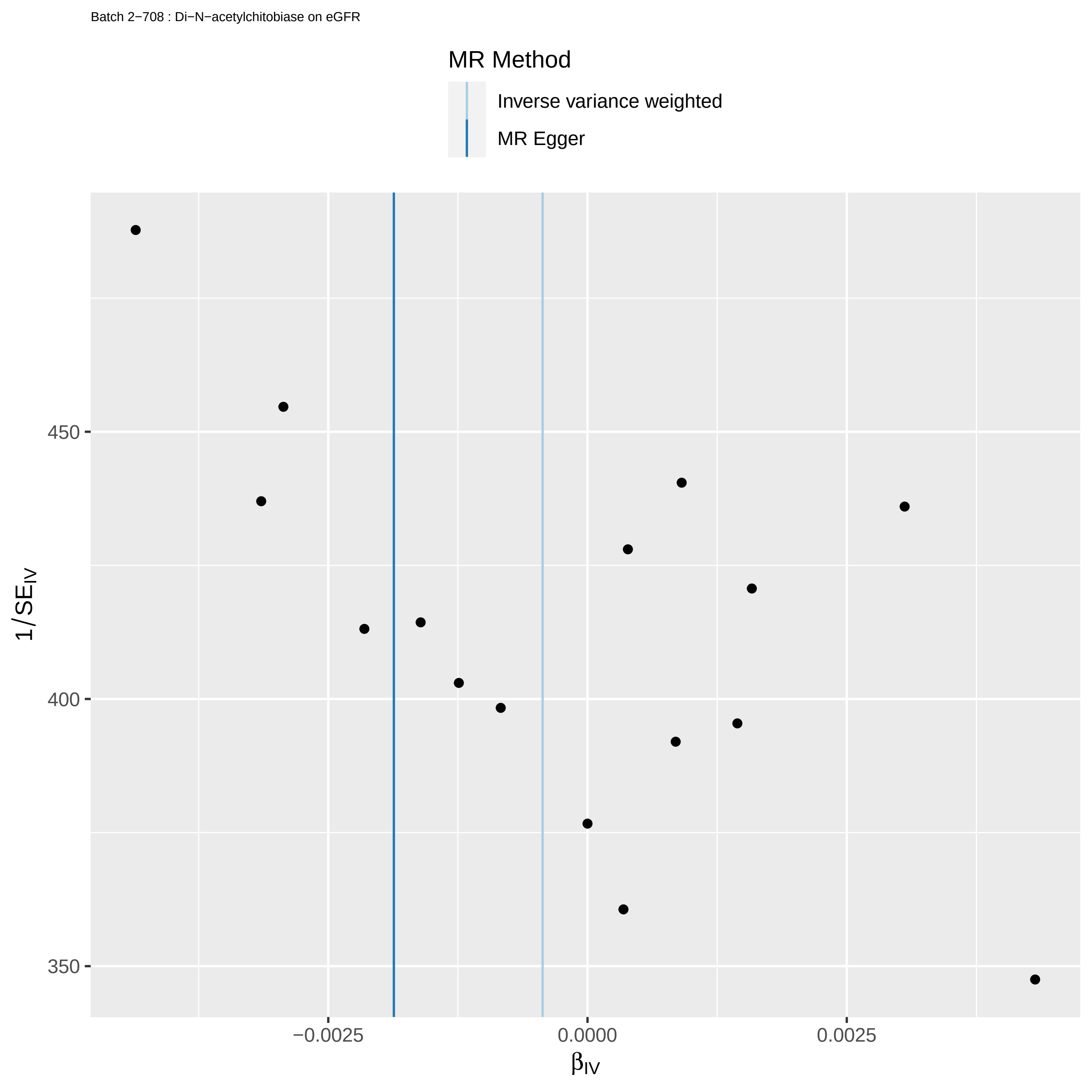
Batch 2−708 : Di−N−acetylchitobiase on eGFR
MR Method
Inverse variance weighted
MR Egger
450
400
350
1/SE_IV
−0.0025
0.0000
0.0025
β_IV

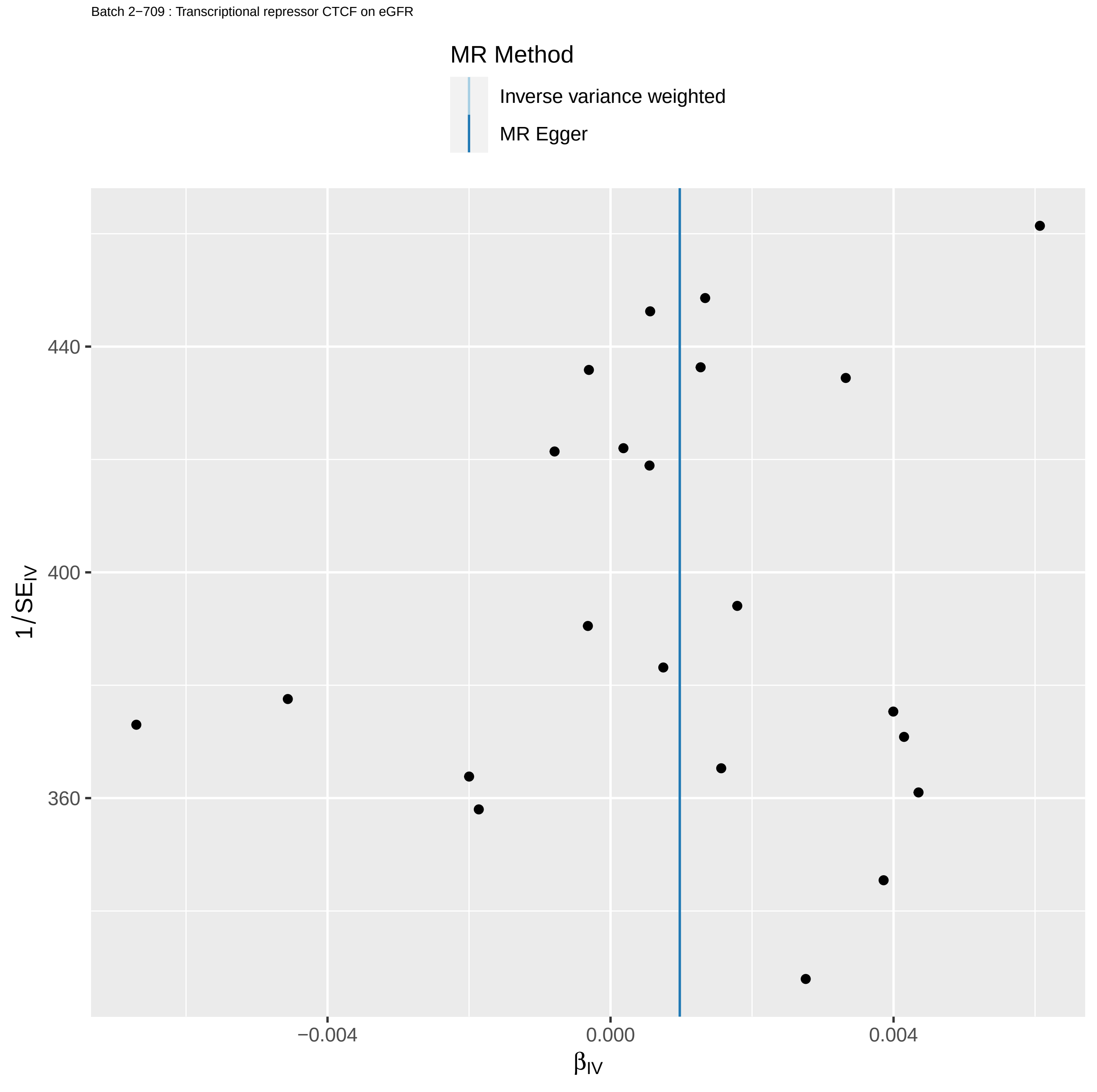

Batch 2−709 : Transcriptional repressor CTCF on eGFR
MR Method
Inverse variance weighted
MR Egger
440
400
360
1/SE$_{IV}$
−0.004
0.000
0.004
β$_{IV}$

Batch 2−710 : Cardiotrophin−1 on eGFR

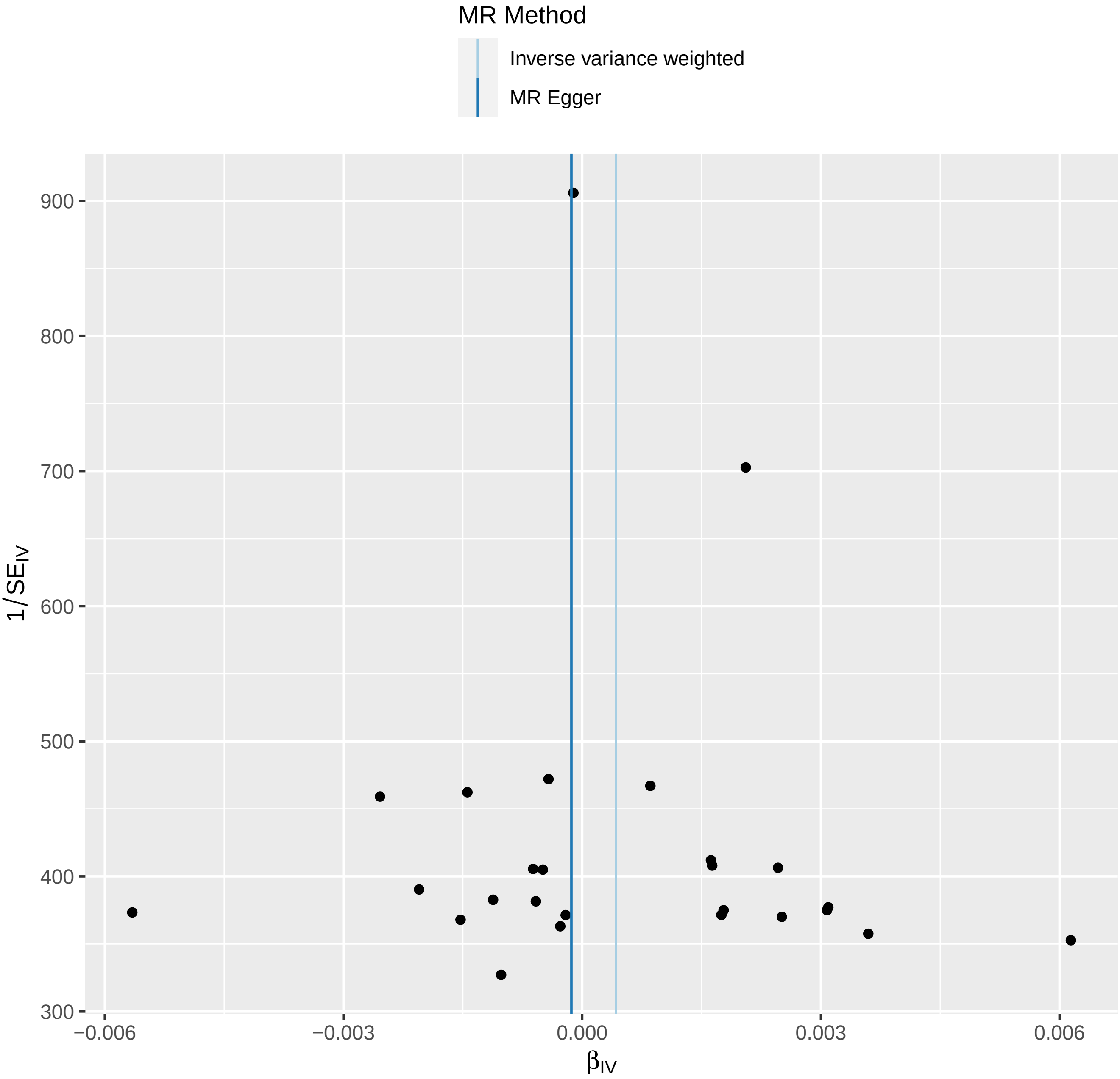

Batch 2−711 : Cardiotrophin−1 on eGFR

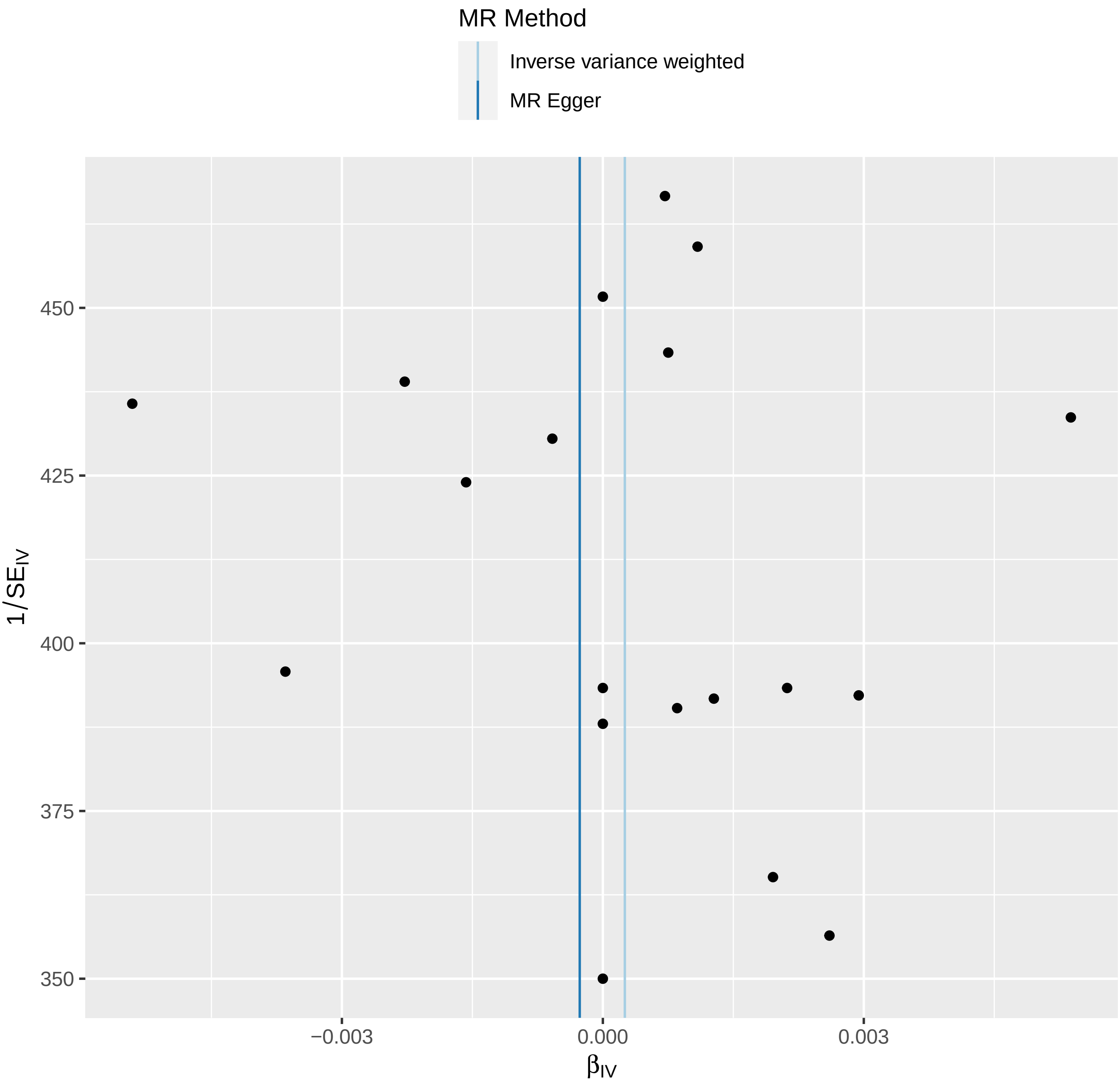

Batch 2−712 : Connective tissue growth factor on eGFR

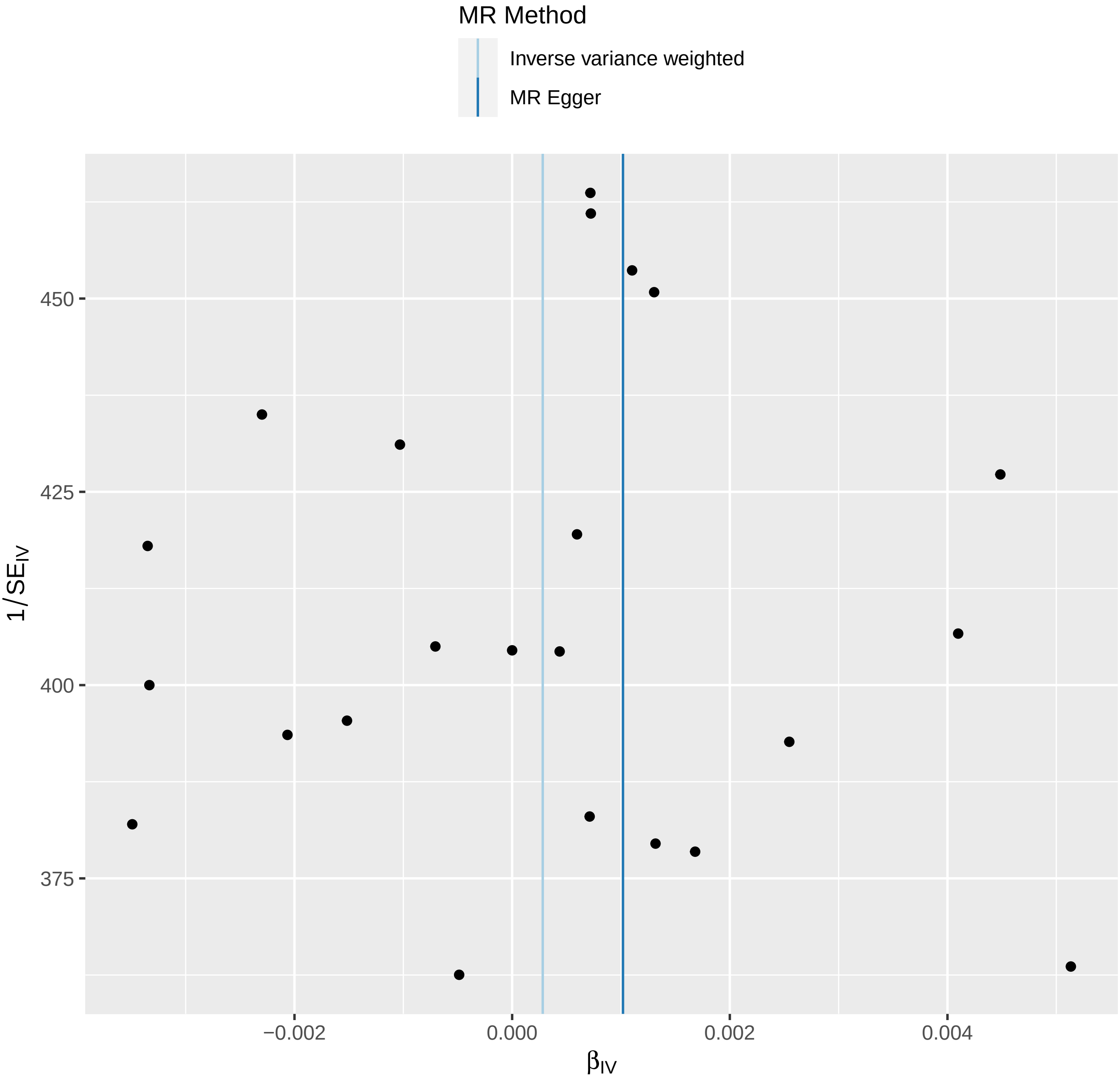

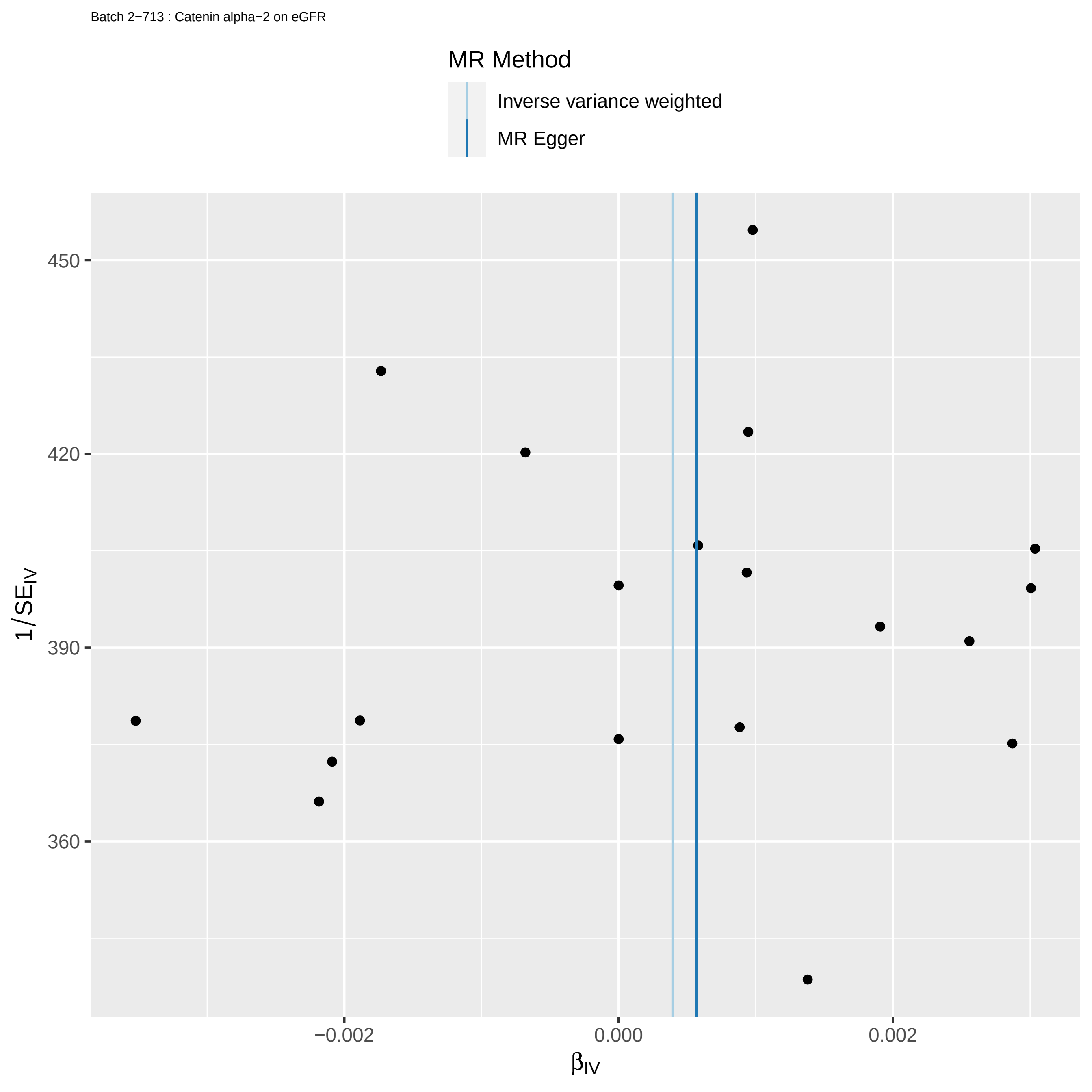

Batch 2−713 : Catenin alpha−2 on eGFR
MR Method
Inverse variance weighted
MR Egger
450
420
390
360
1/SE$_{IV}$
−0.002
0.000
0.002
β$_{IV}$

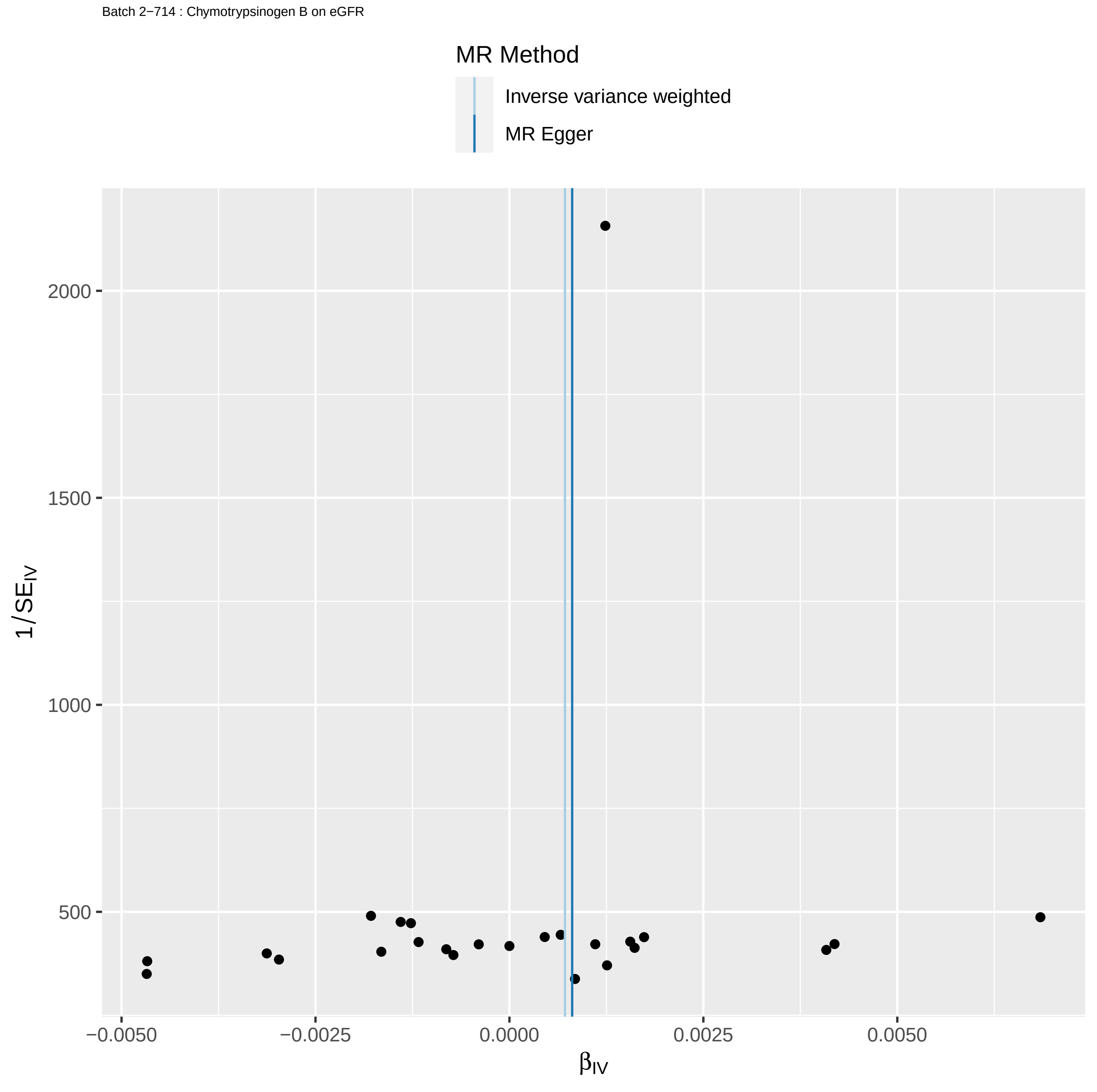

Batch 2−714 : Chymotrypsinogen B on eGFR
MR Method
Inverse variance weighted
MR Egger
2000
1500
1000
500
1/SE_IV
−0.0050
−0.0025
0.0000
0.0025
0.0050
β_IV

Batch 2−715 : Chymotrypsin−C on eGFR

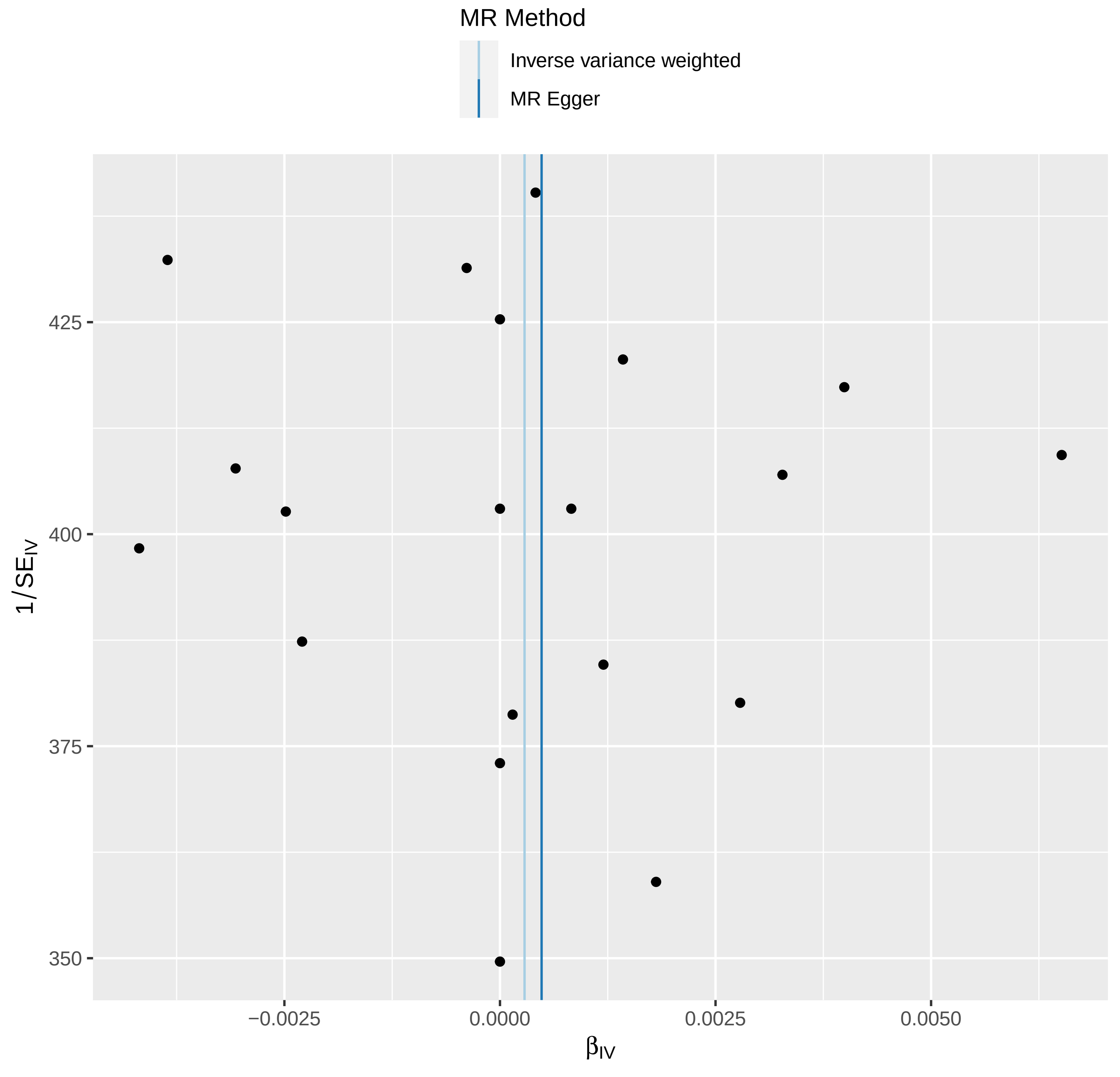

Batch 2−716 : Chymotrypsin−like protease CTRL−1 on eGFR

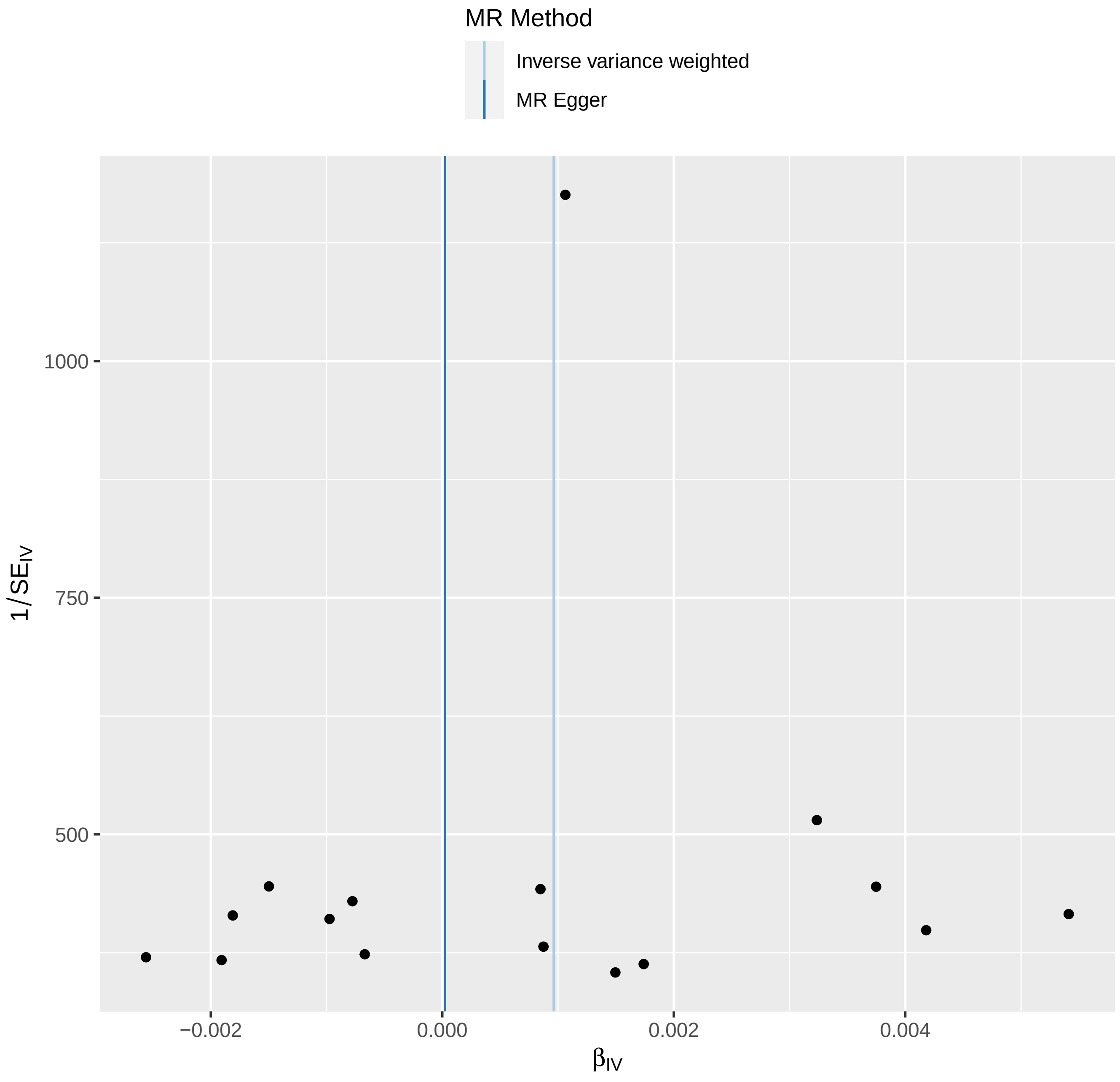

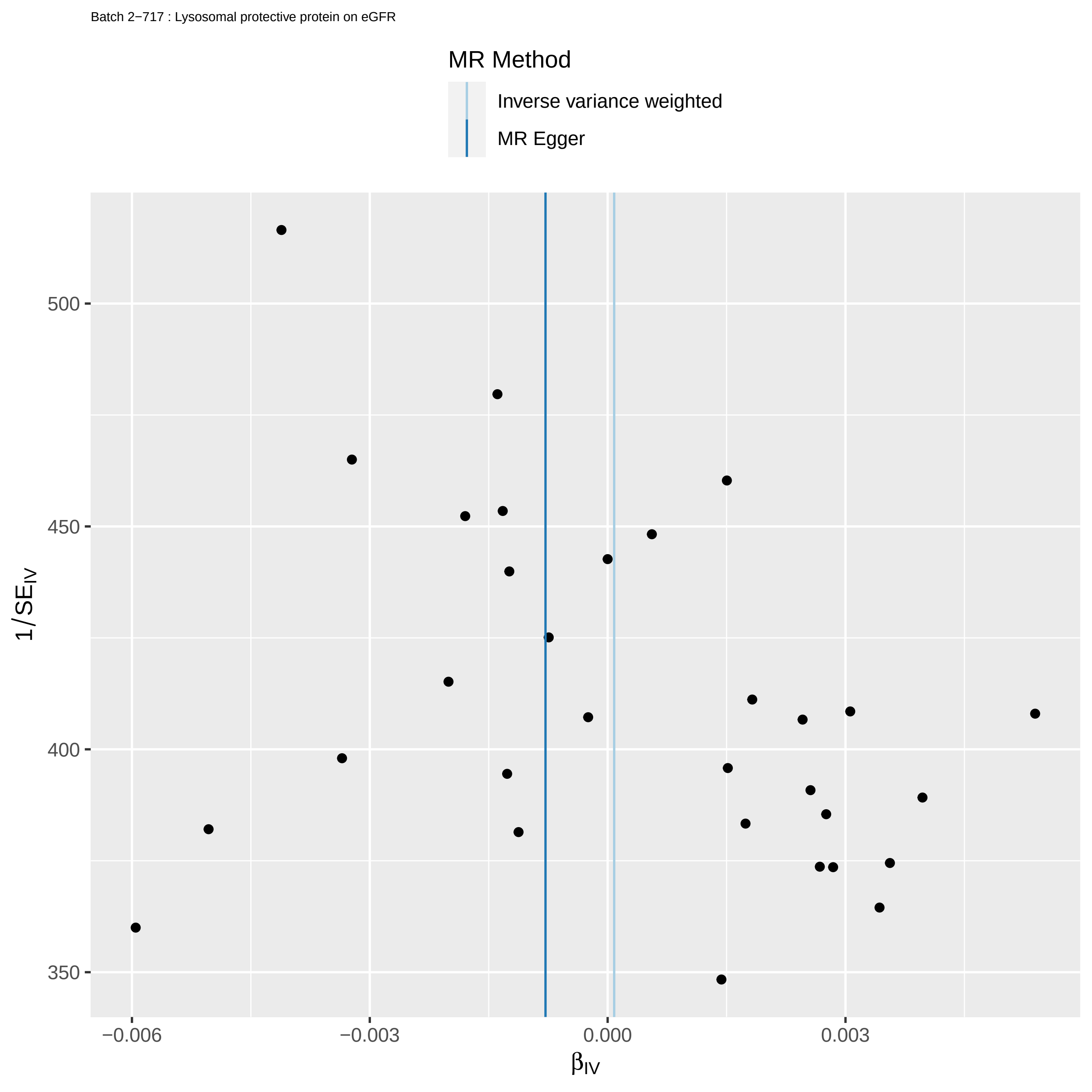

Batch 2−717 : Lysosomal protective protein on eGFR
MR Method
Inverse variance weighted
MR Egger
500
450
400
350
1/SE_IV
−0.006
−0.003
0.000
0.003
β_IV

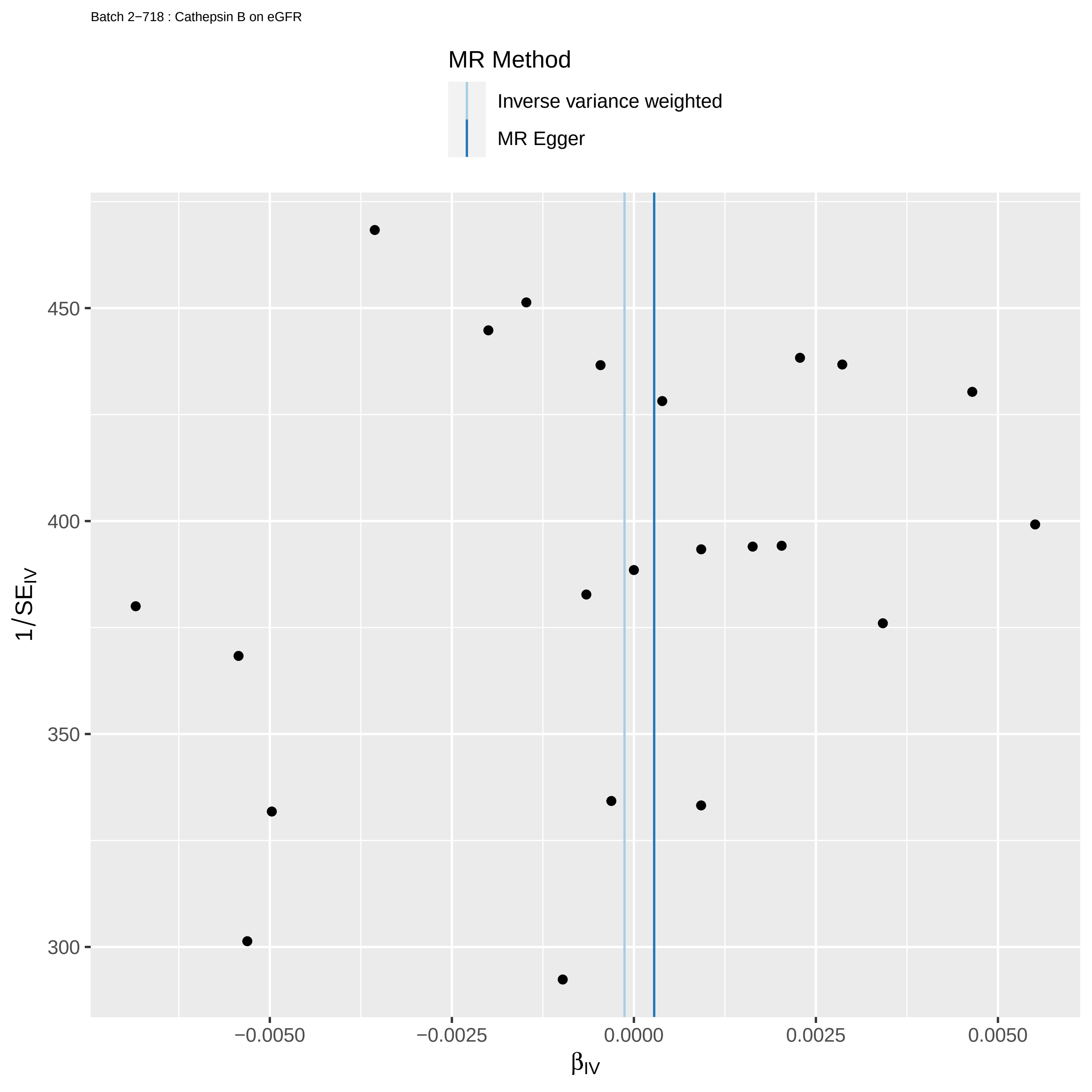

Batch 2−718 : Cathepsin B on eGFR
MR Method
Inverse variance weighted
MR Egger
450
400
350
300
1/SE$_{IV}$
−0.0050
−0.0025
0.0000
0.0025
0.0050
β$_{IV}$

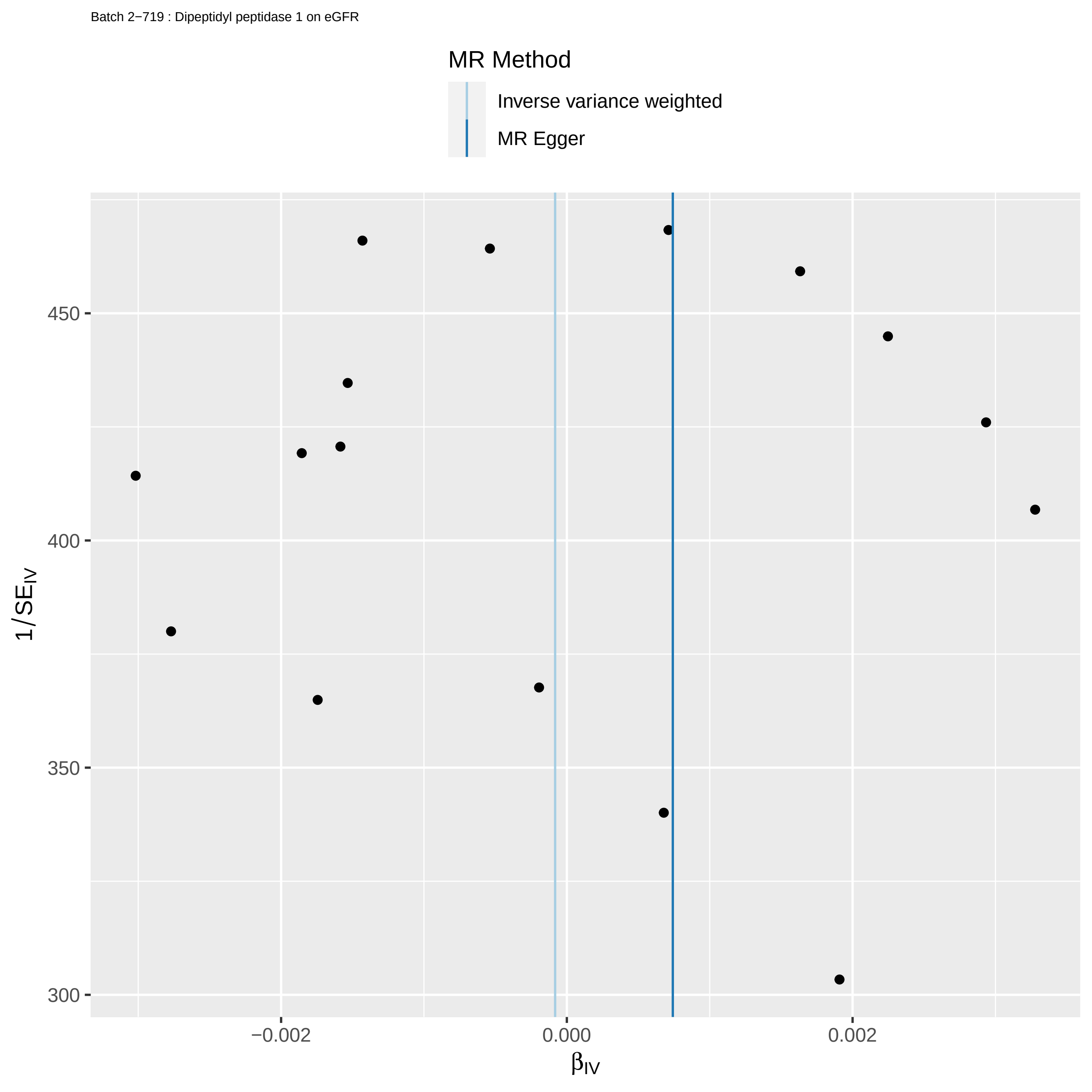

Batch 2−719 : Dipeptidyl peptidase 1 on eGFR
MR Method
Inverse variance weighted
MR Egger
450
400
350
300
1/SE_IV
−0.002
0.000
0.002
β_IV

Batch 2−720 : Cathepsin E on eGFR

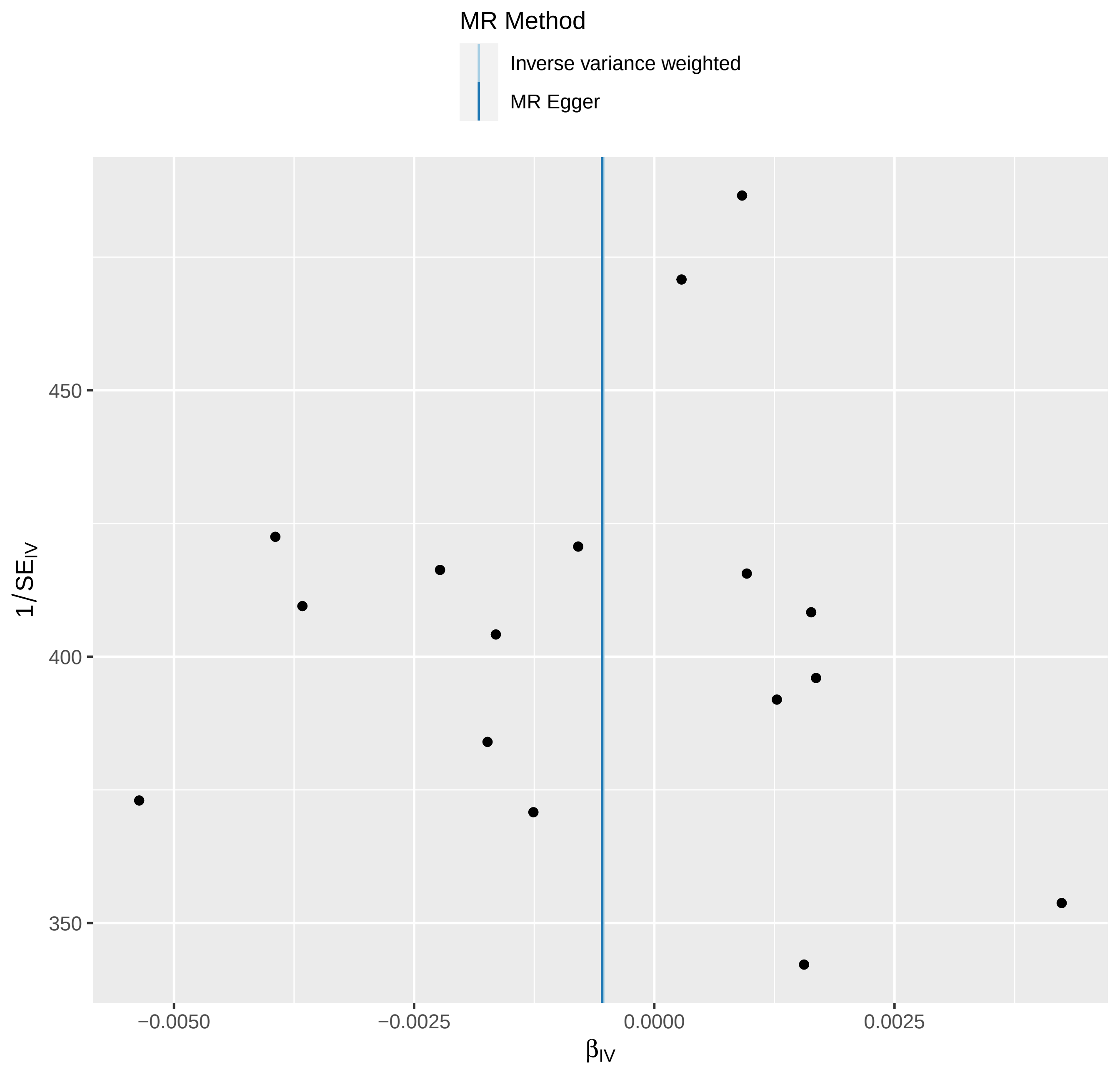

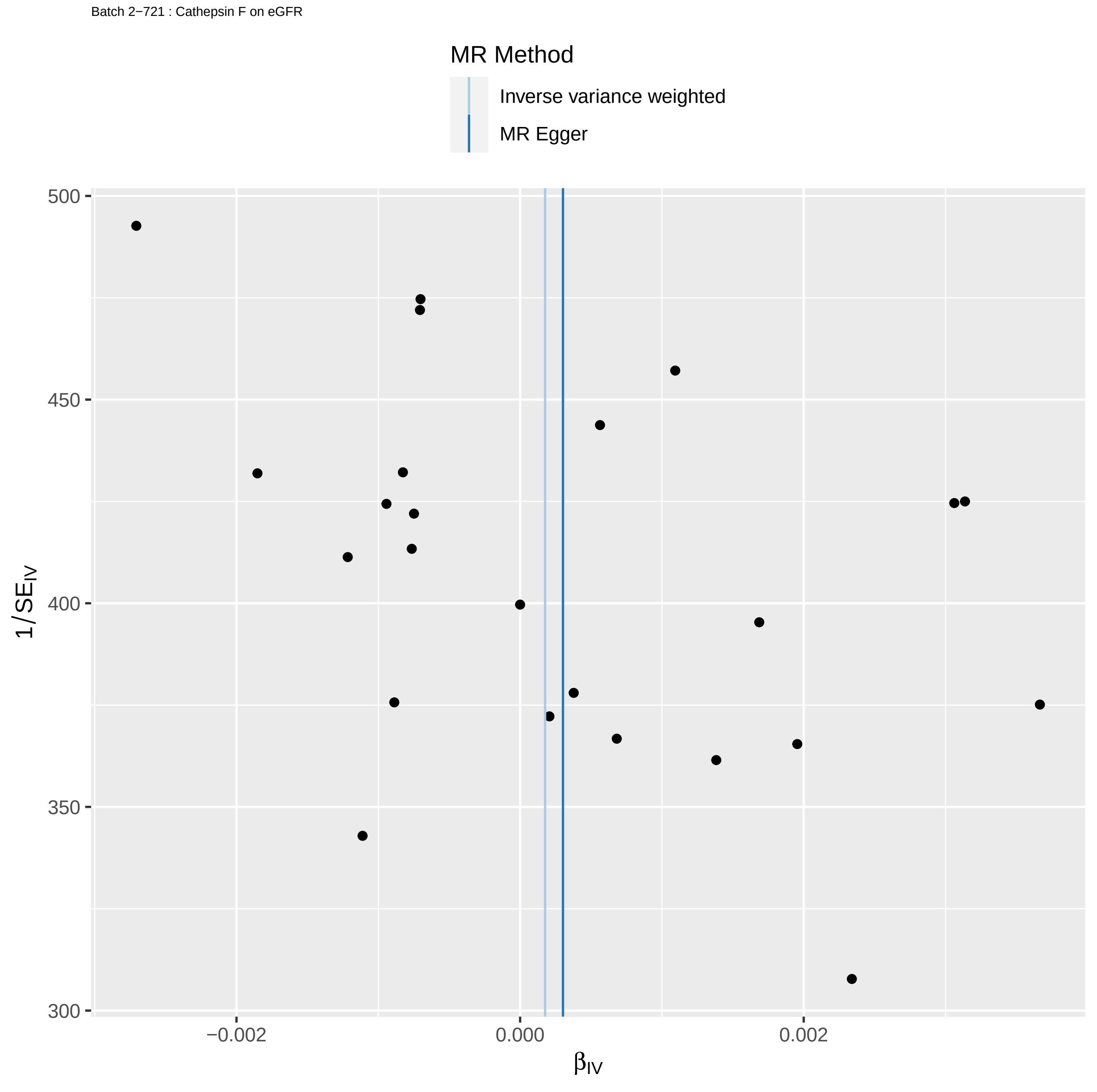

Batch 2−721 : Cathepsin F on eGFR
MR Method
Inverse variance weighted
MR Egger
500
450
400
350
300
1/SE$_{IV}$
−0.002
0.000
0.002
β_{IV}

Batch 2−722 : Cathepsin F on eGFR

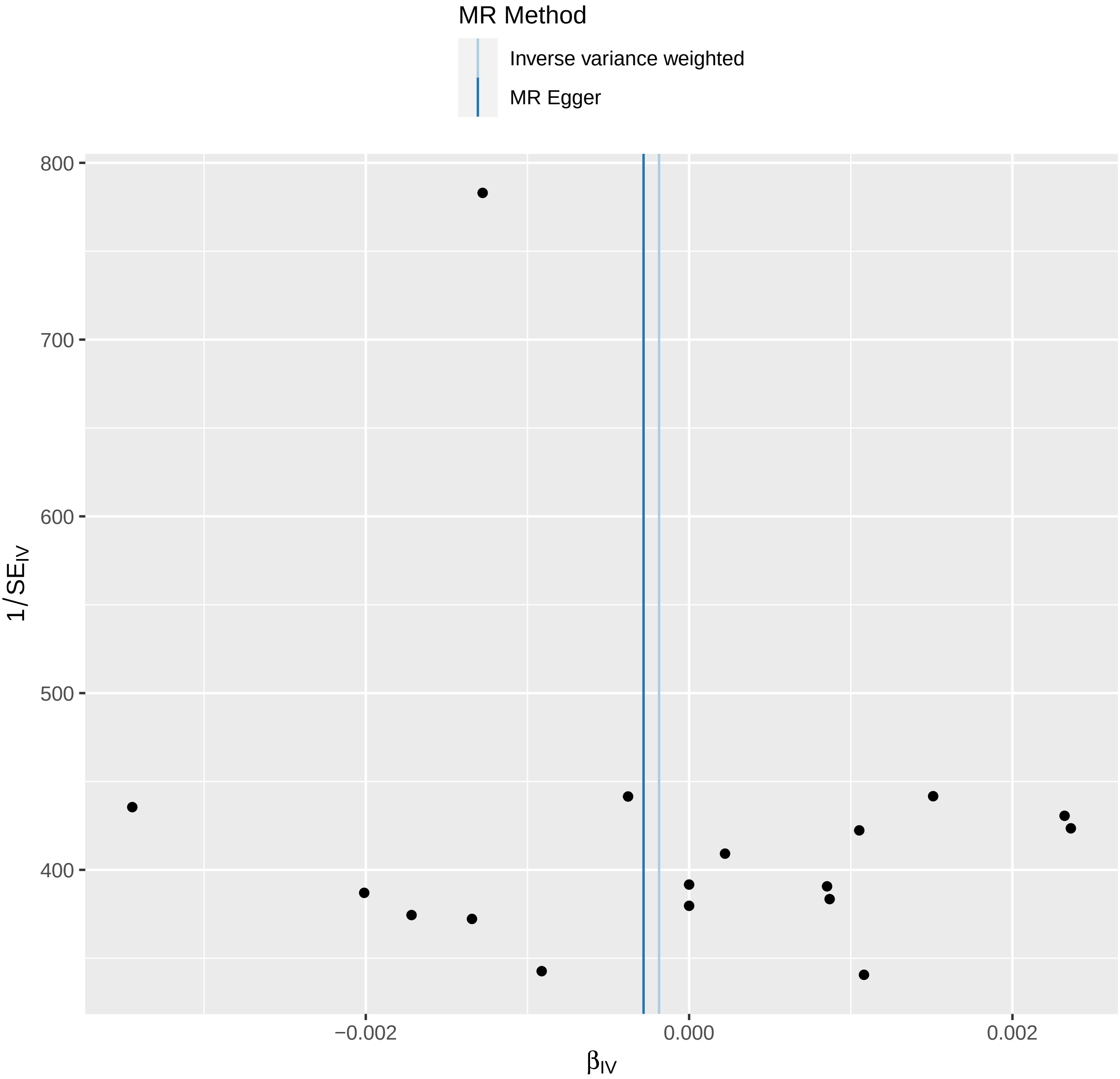

Batch 2−723 : Cathepsin G on eGFR

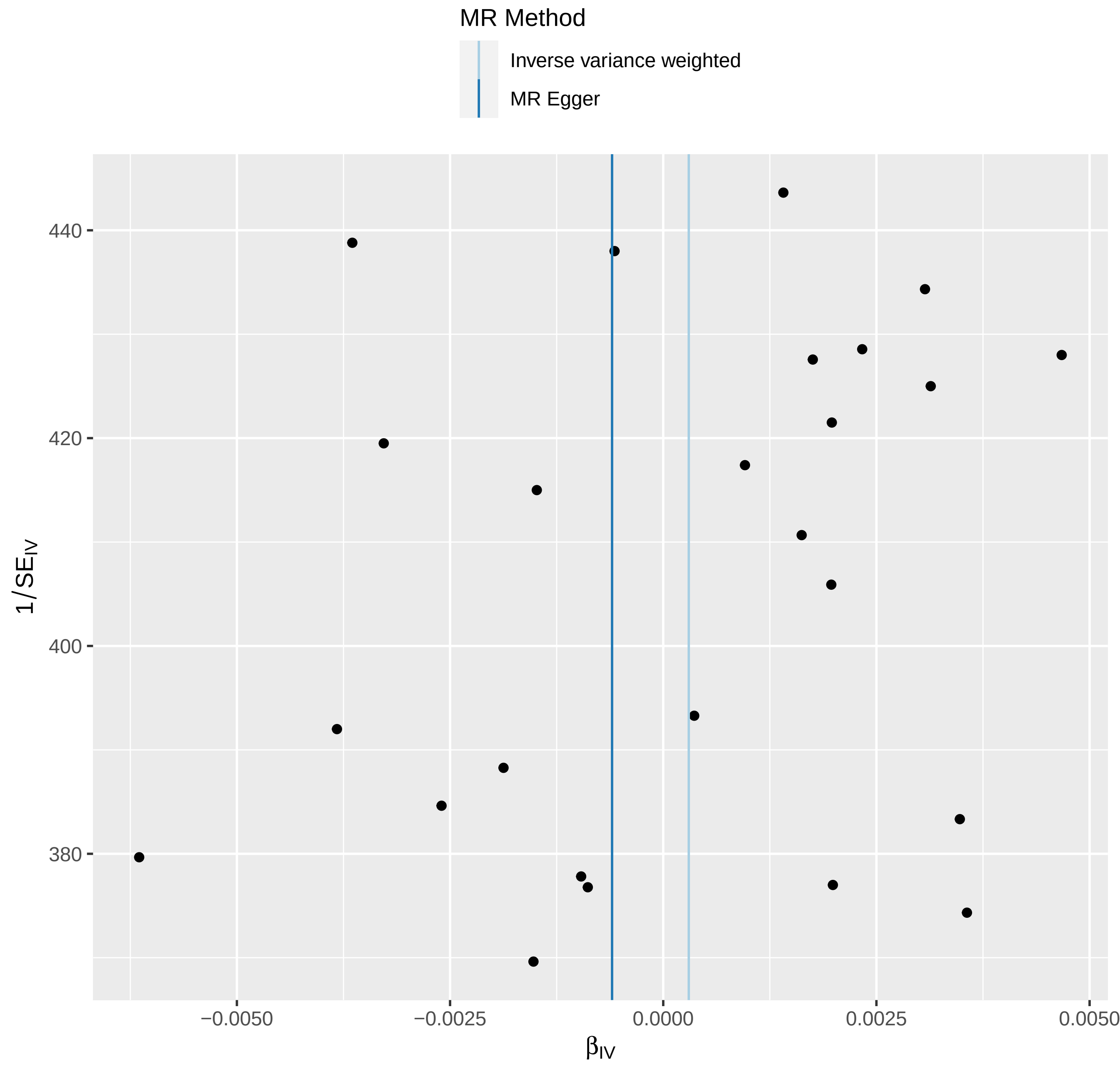

Batch 2−724 : Cathepsin H on eGFR

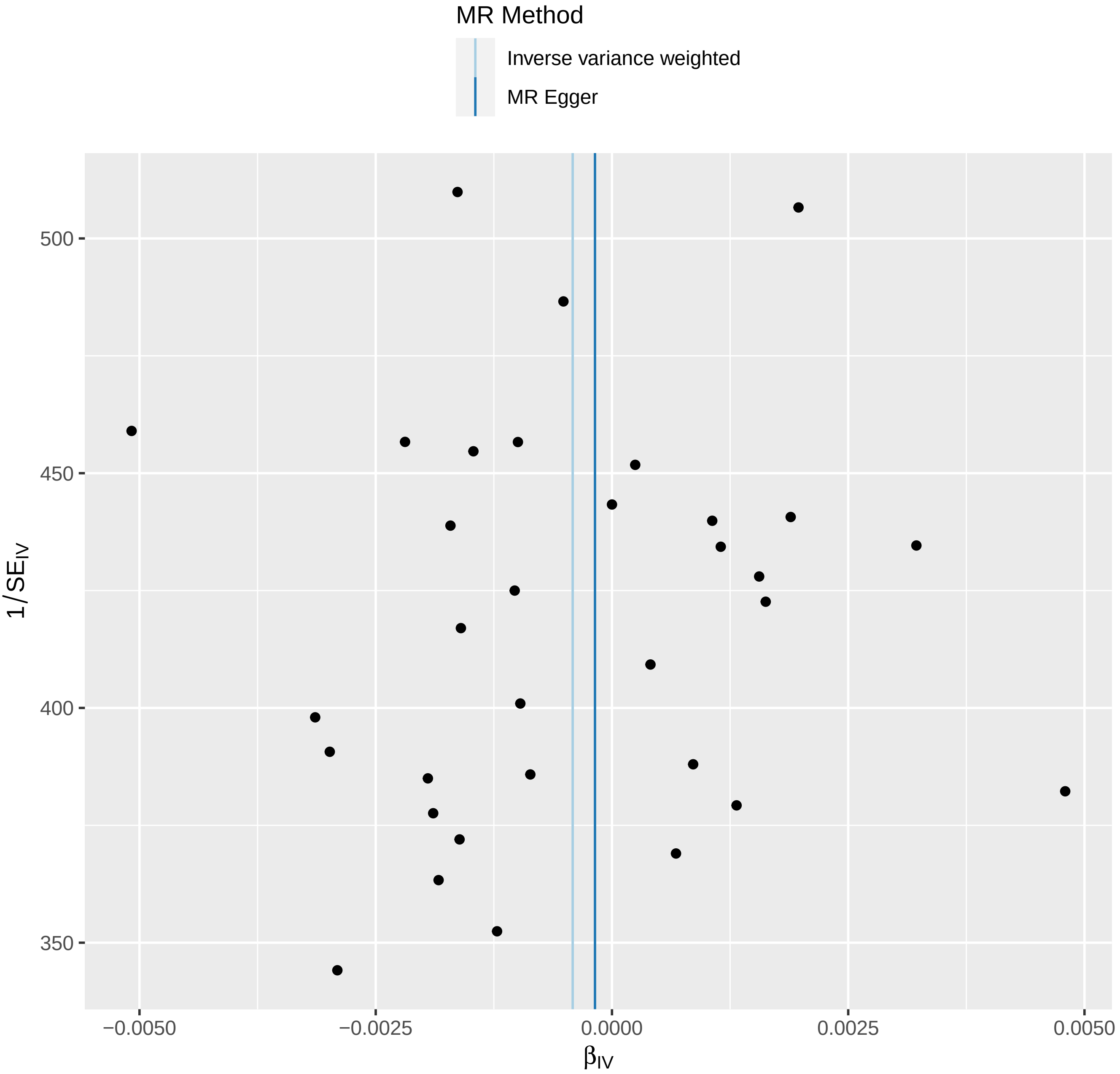

Batch 2−725 : Cathepsin H on eGFR

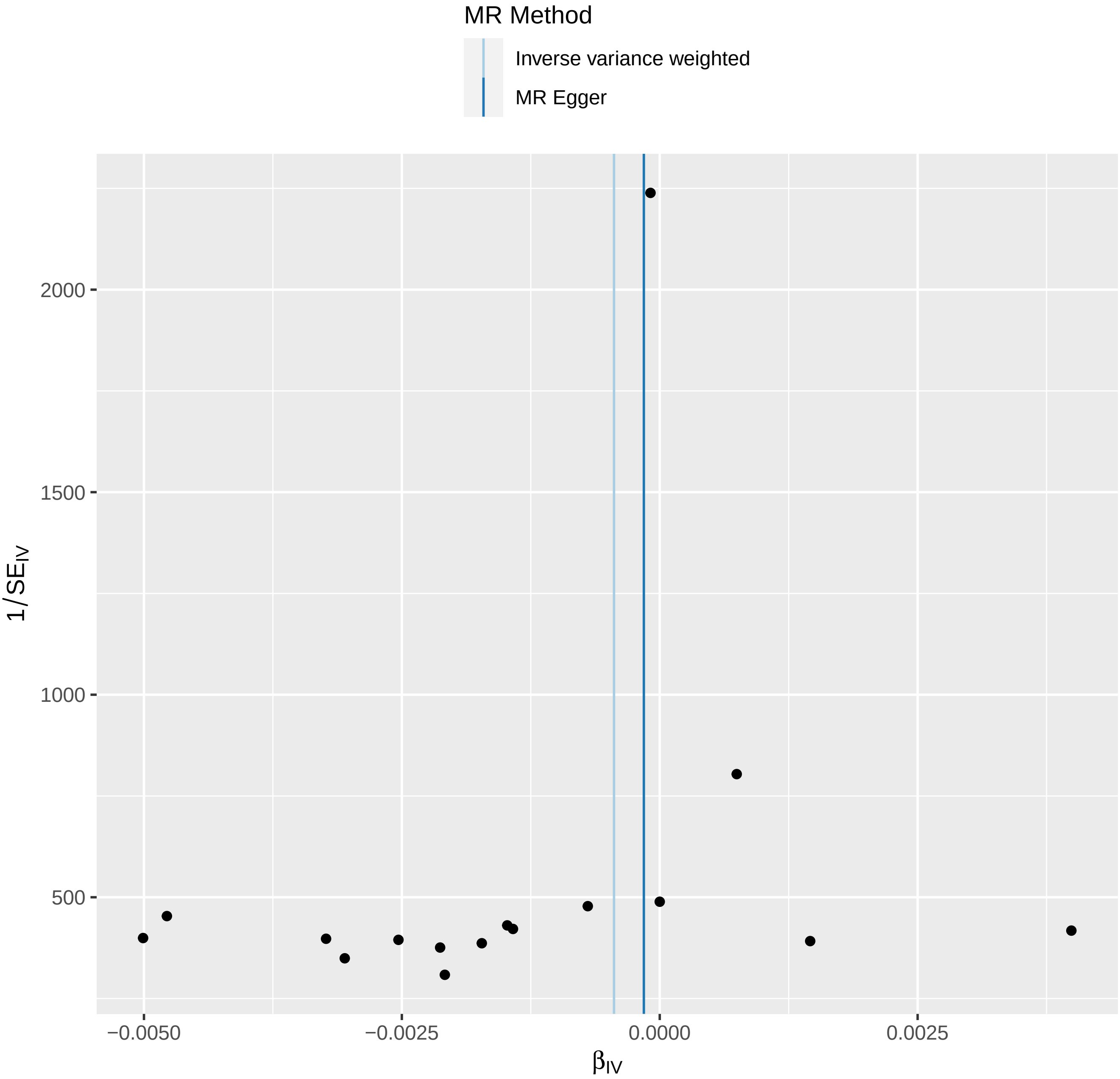

Batch 2−726 : Cathepsin O on eGFR

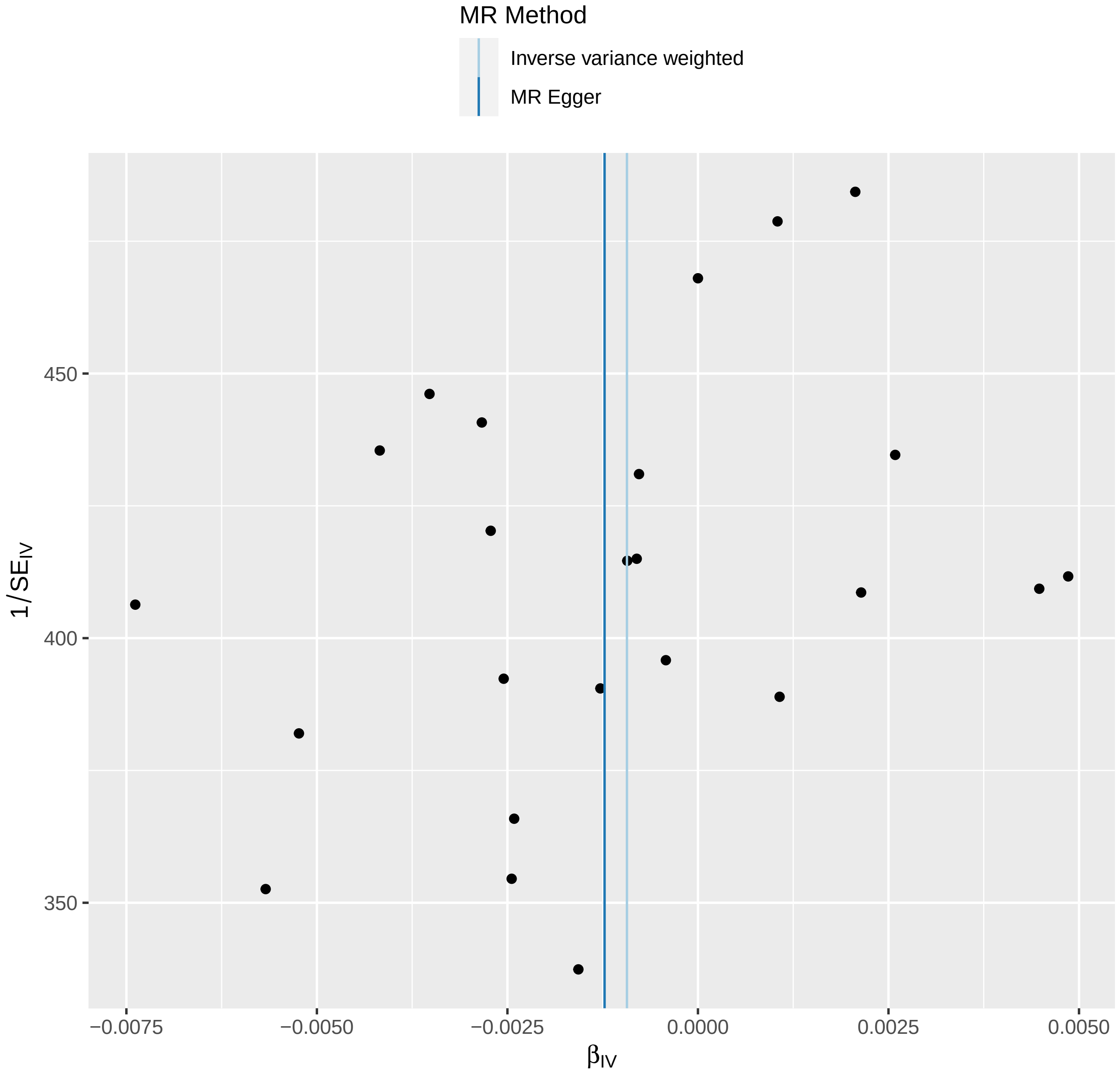

Batch 2−727 : Cathepsin S on eGFR

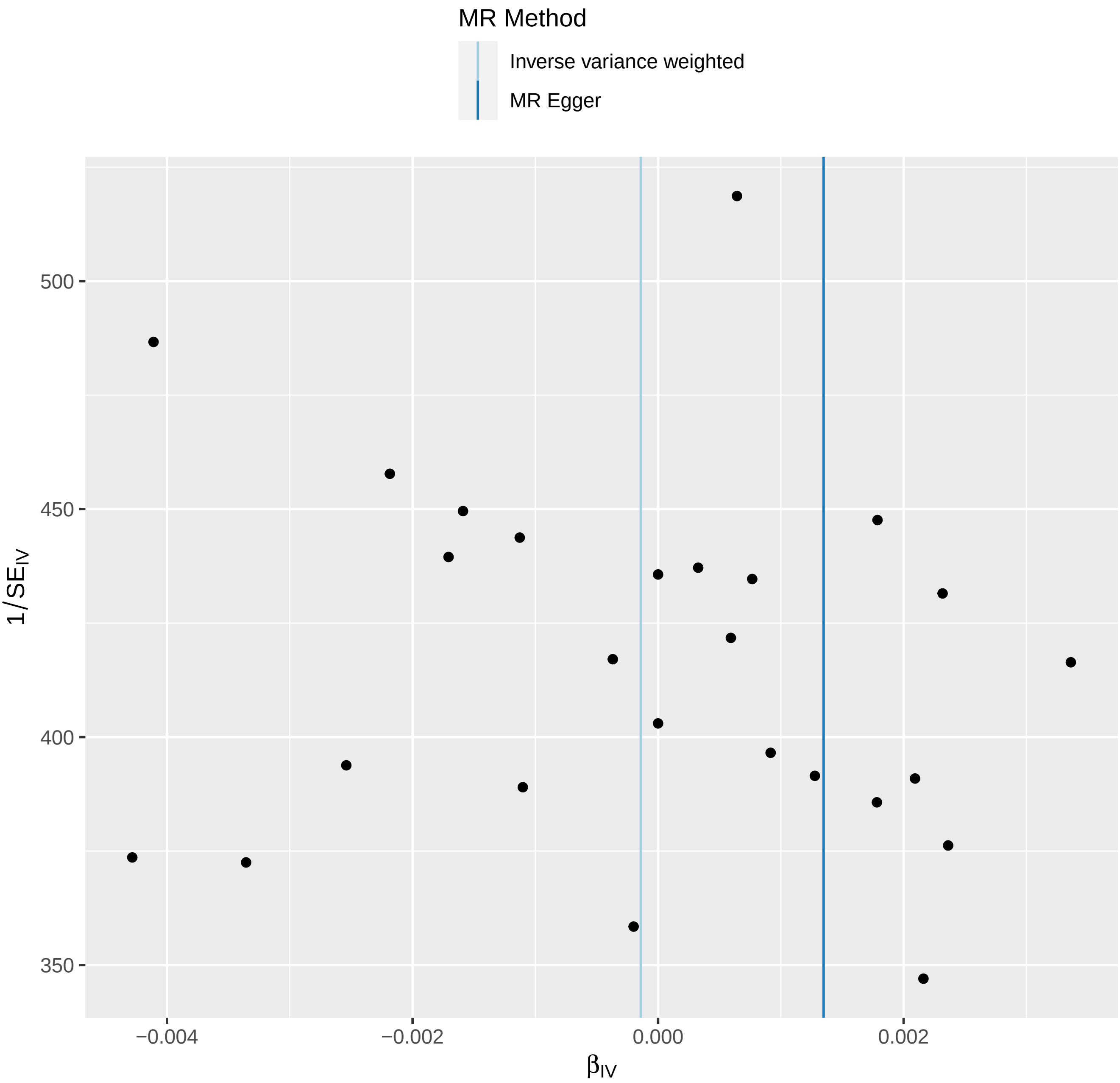

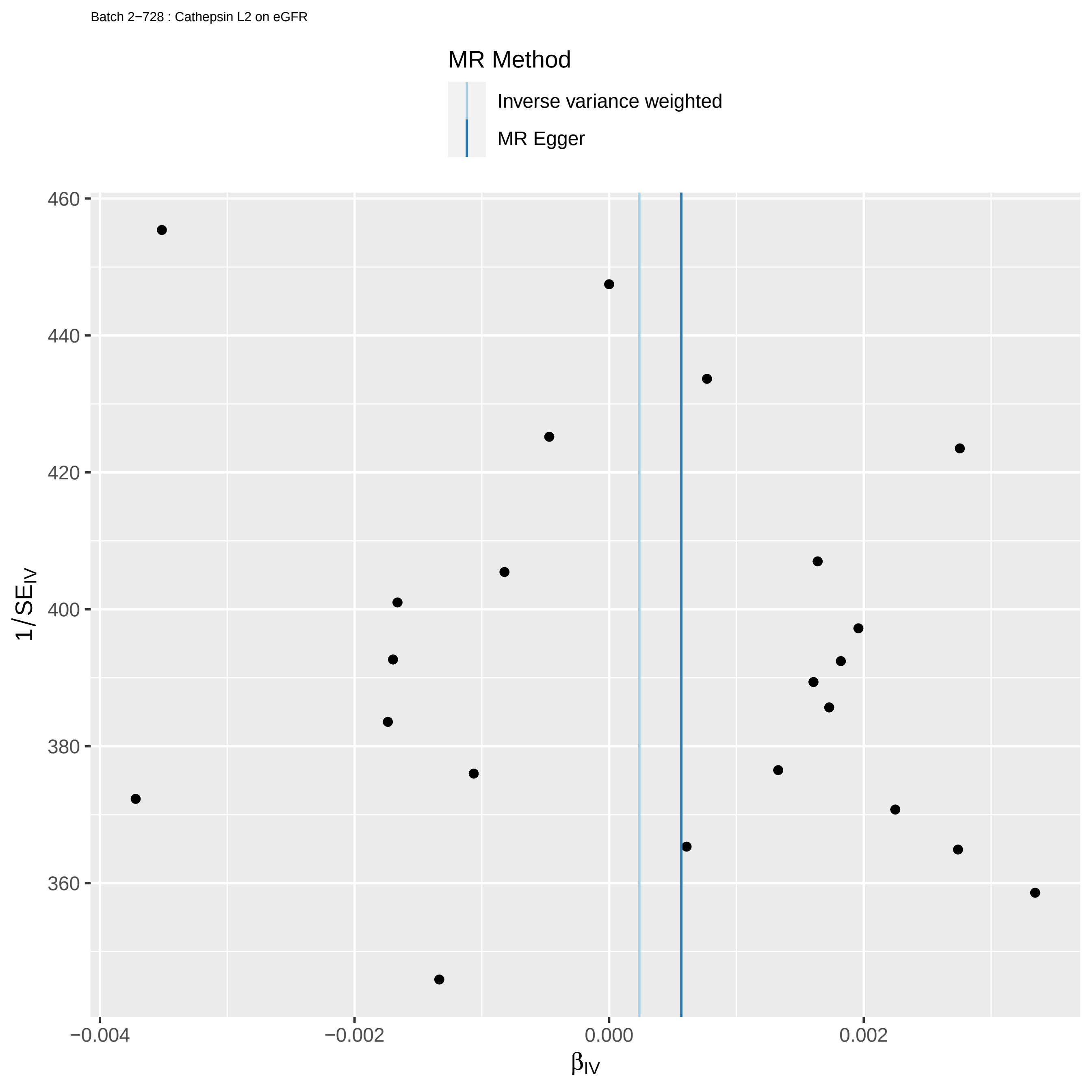

Batch 2−728 : Cathepsin L2 on eGFR
MR Method
Inverse variance weighted
MR Egger
460
440
420
400
380
360
1/SE$_{IV}$
−0.004
−0.002
0.000
0.002
β$_{IV}$

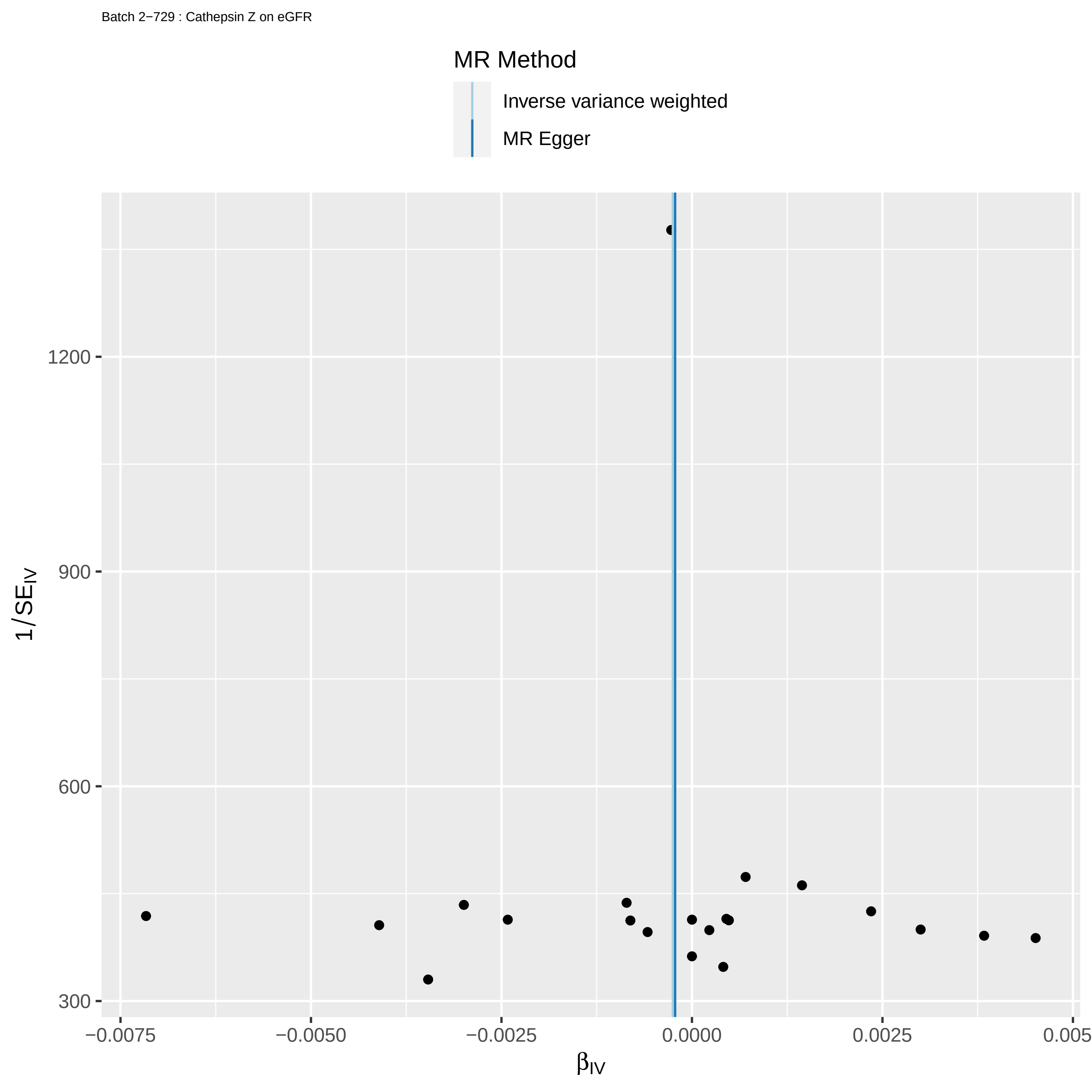

Batch 2−729 : Cathepsin Z on eGFR
MR Method
Inverse variance weighted
MR Egger
1200
900
600
300
1/SE_IV
−0.0075
−0.0050
−0.0025
0.0000
0.0025
0.005
β_IV

Batch 2−730 : Cortexin−3 on eGFR

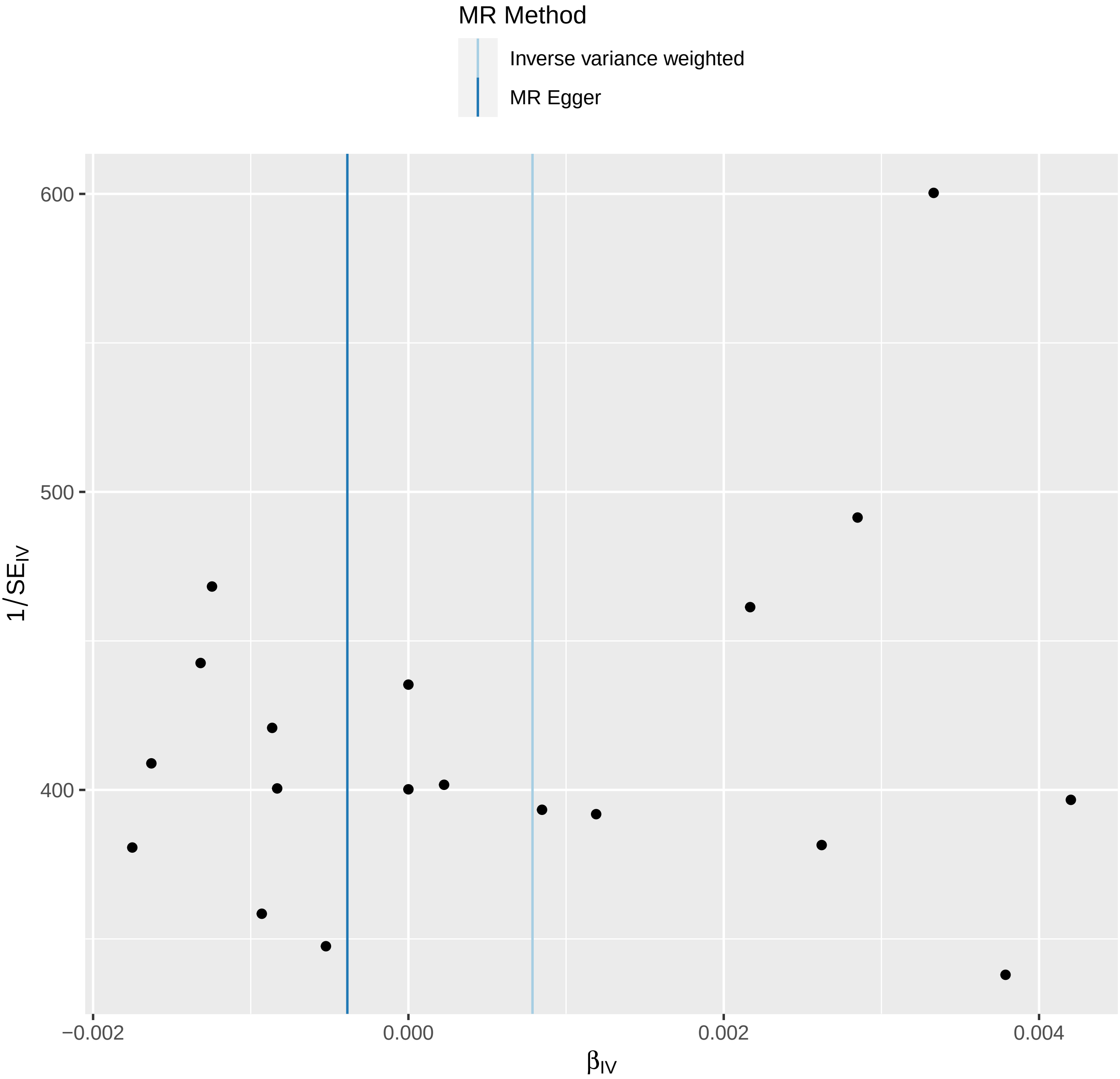

Batch 2−731 : cubilin on eGFR

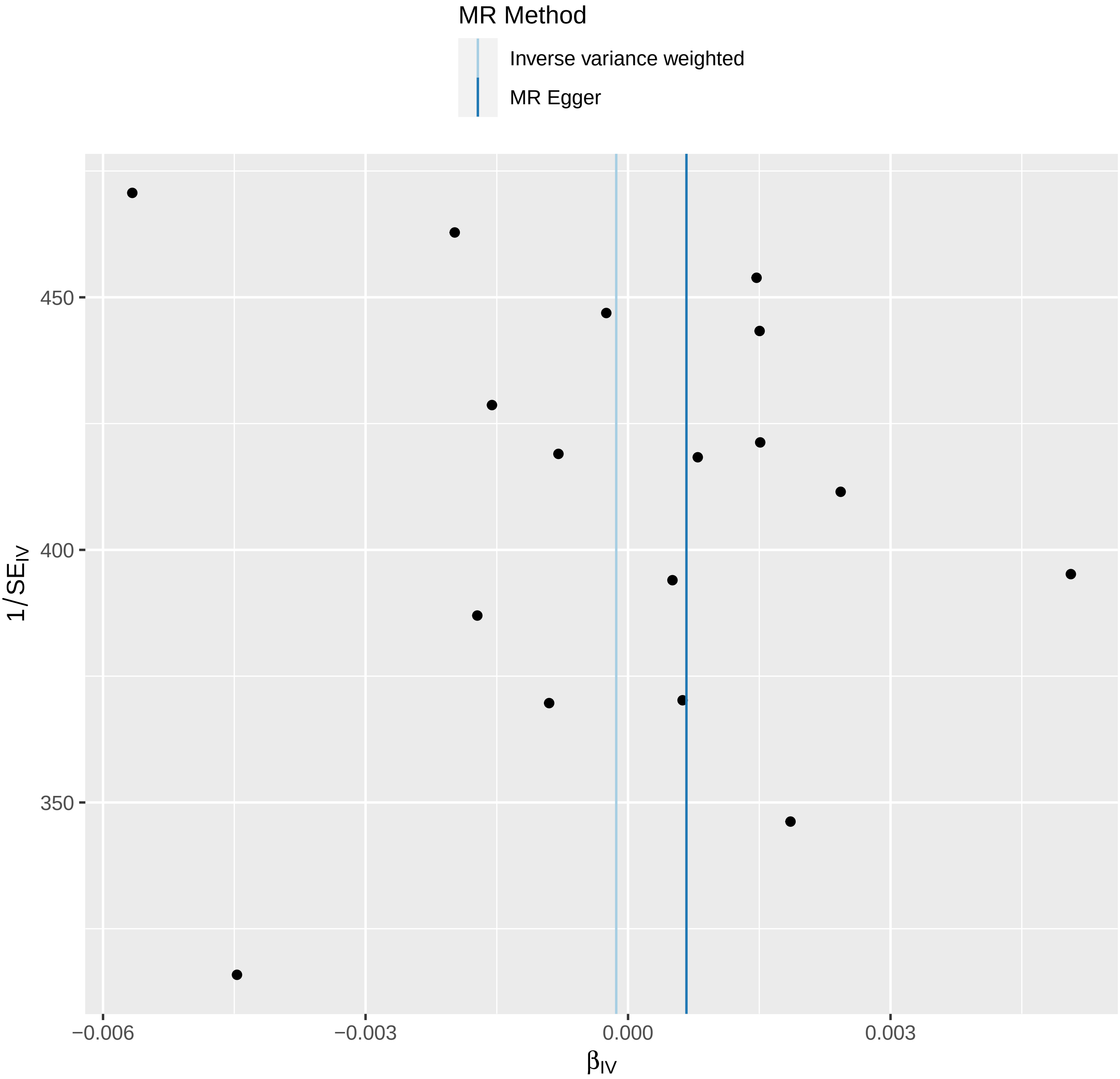

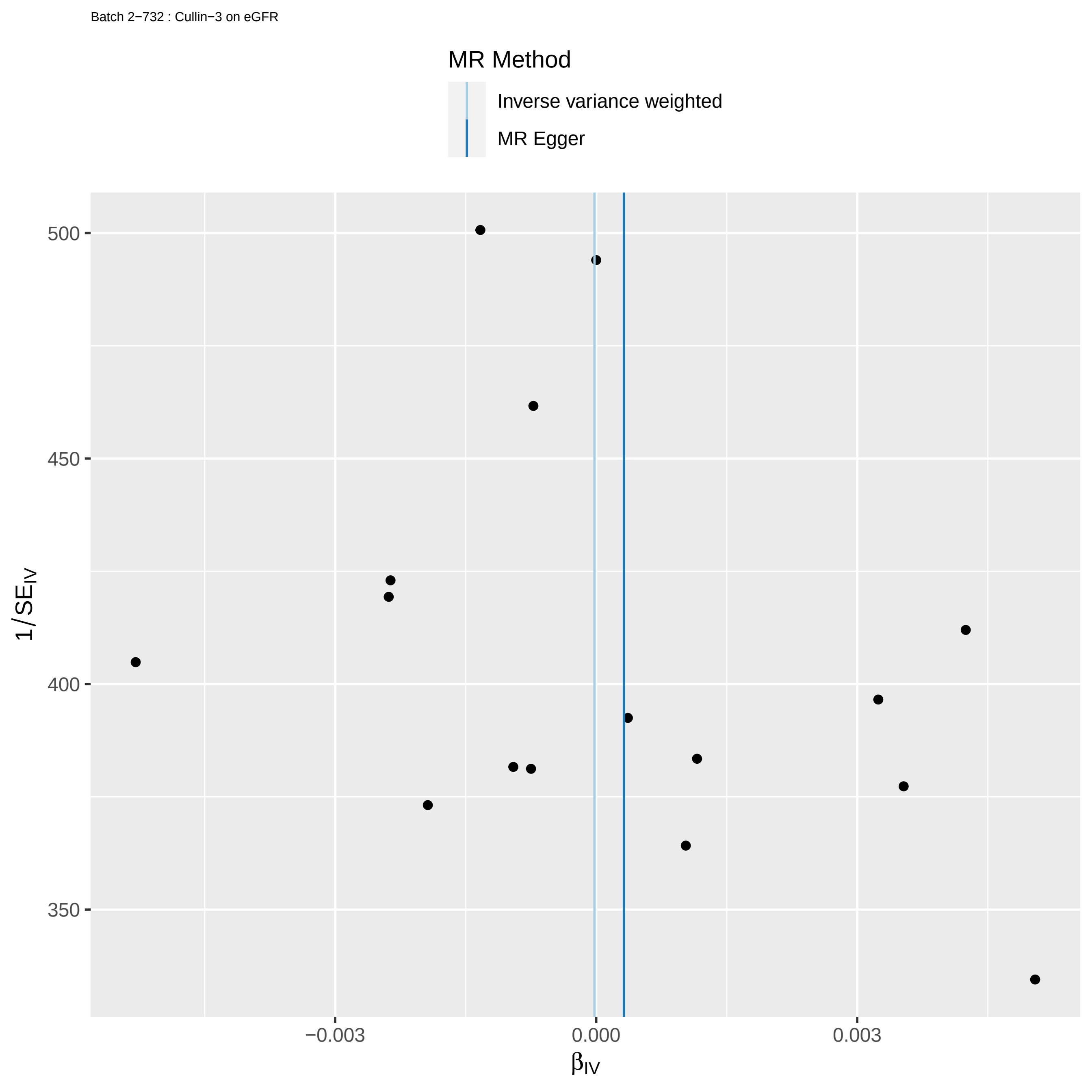

Batch 2−732 : Cullin−3 on eGFR
MR Method
Inverse variance weighted
MR Egger
500
450
400
350
1/SE_IV
−0.003
0.000
0.003
β_IV

Batch 2−733 : Cullin−4B on eGFR

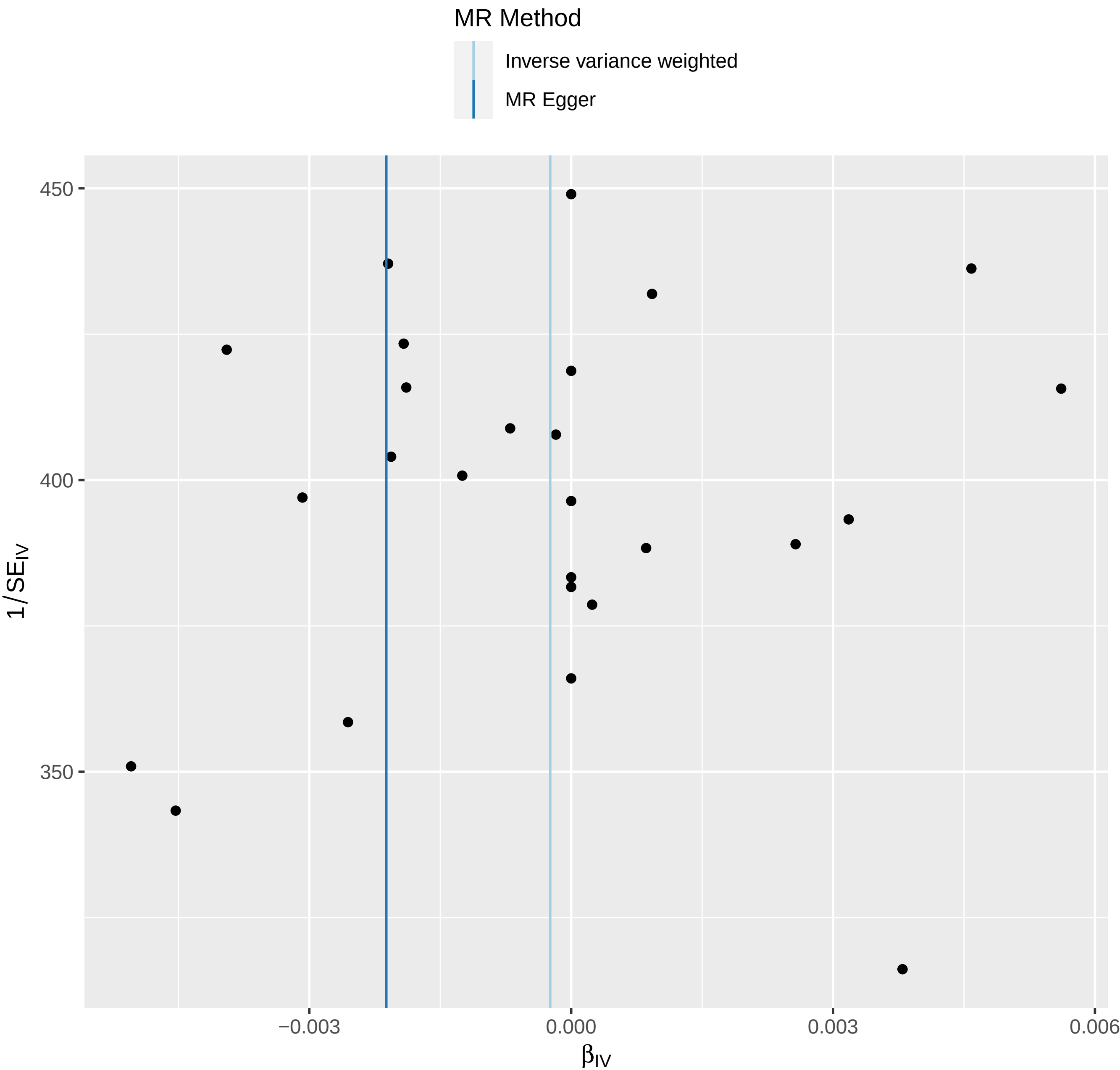

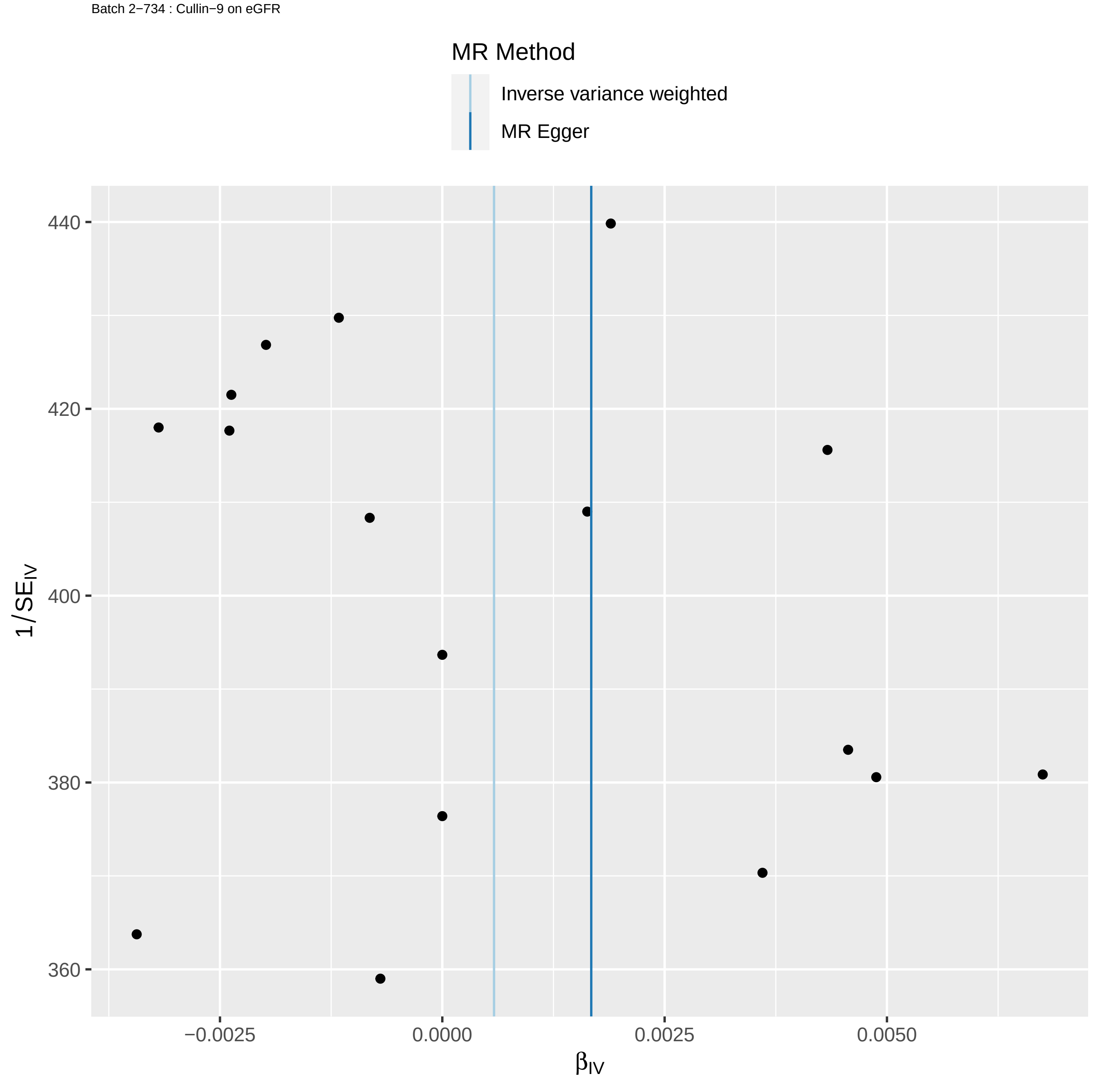
Batch 2−734 : Cullin−9 on eGFR
MR Method
Inverse variance weighted
MR Egger
440
420
400
380
360
1/SE_IV
−0.0025
0.0000
0.0025
0.0050
β_IV

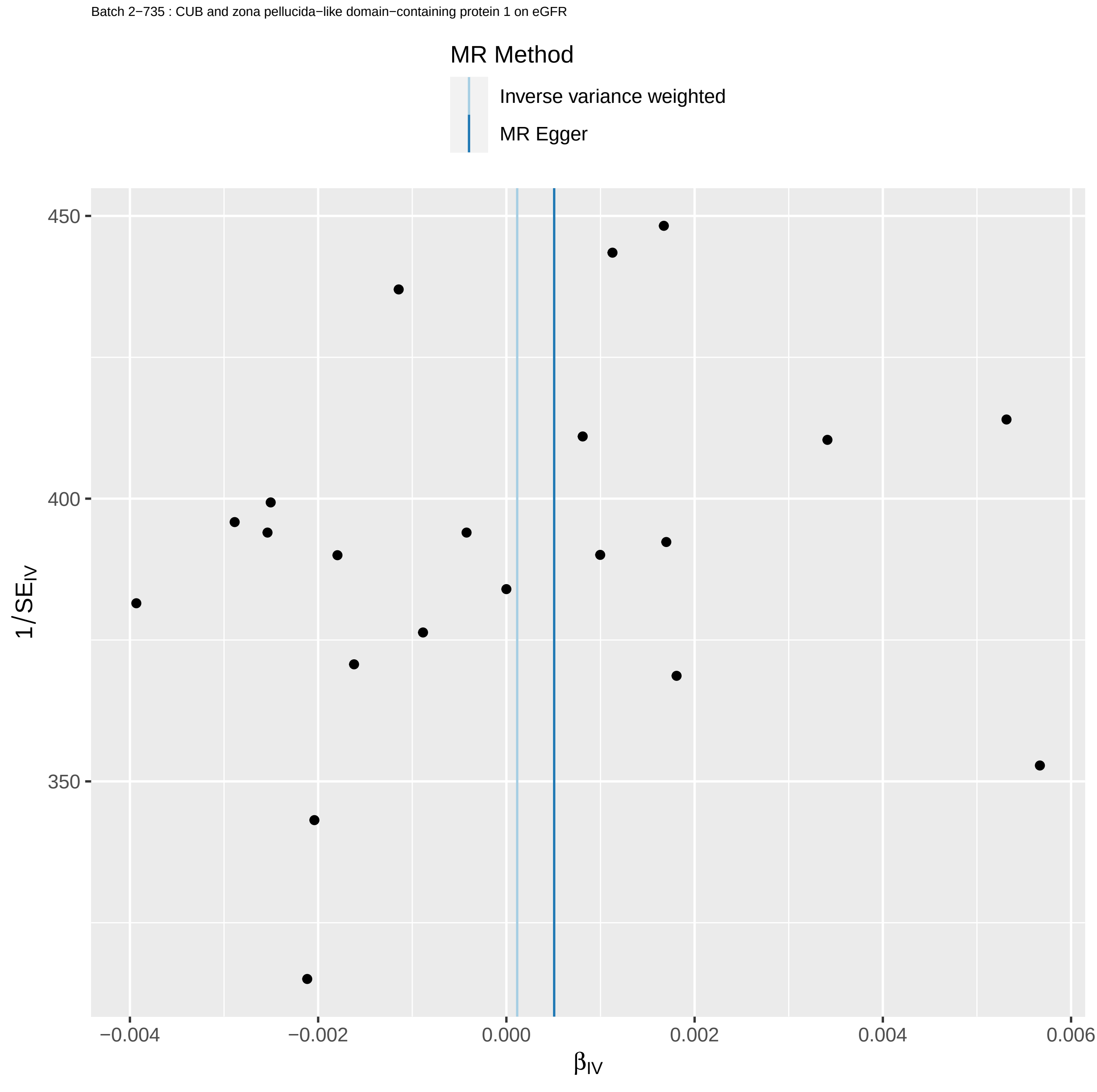

Batch 2−735 : CUB and zona pellucida−like domain−containing protein 1 on eGFR
MR Method
Inverse variance weighted
MR Egger
450
400
350
1/SE$_{IV}$
−0.004
−0.002
0.000
0.002
0.004
0.006
β$_{IV}$

Batch 2−736 : Fractalkine on eGFR

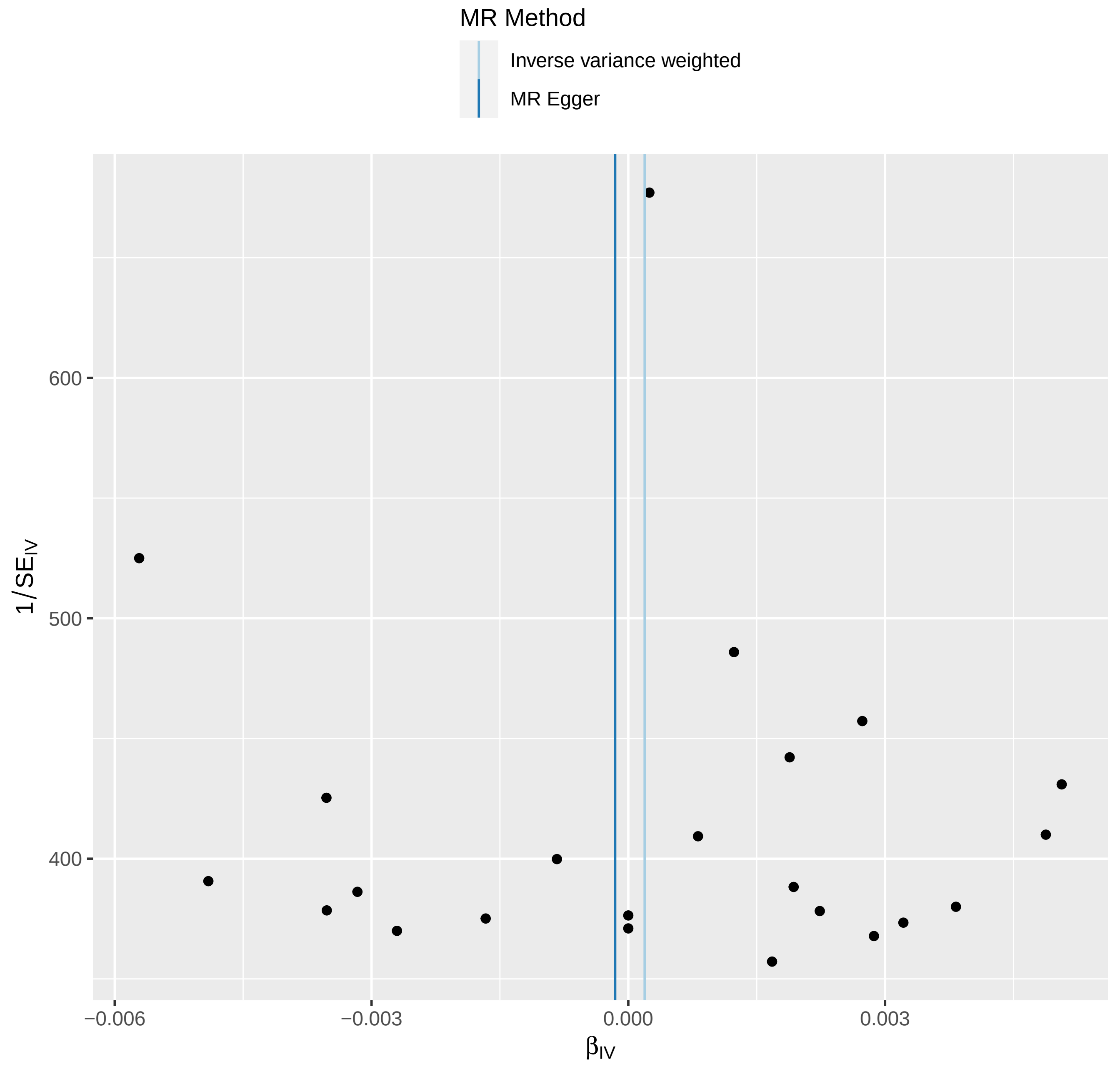

Batch 2−737 : Coxsackievirus and adenovirus receptor on eGFR

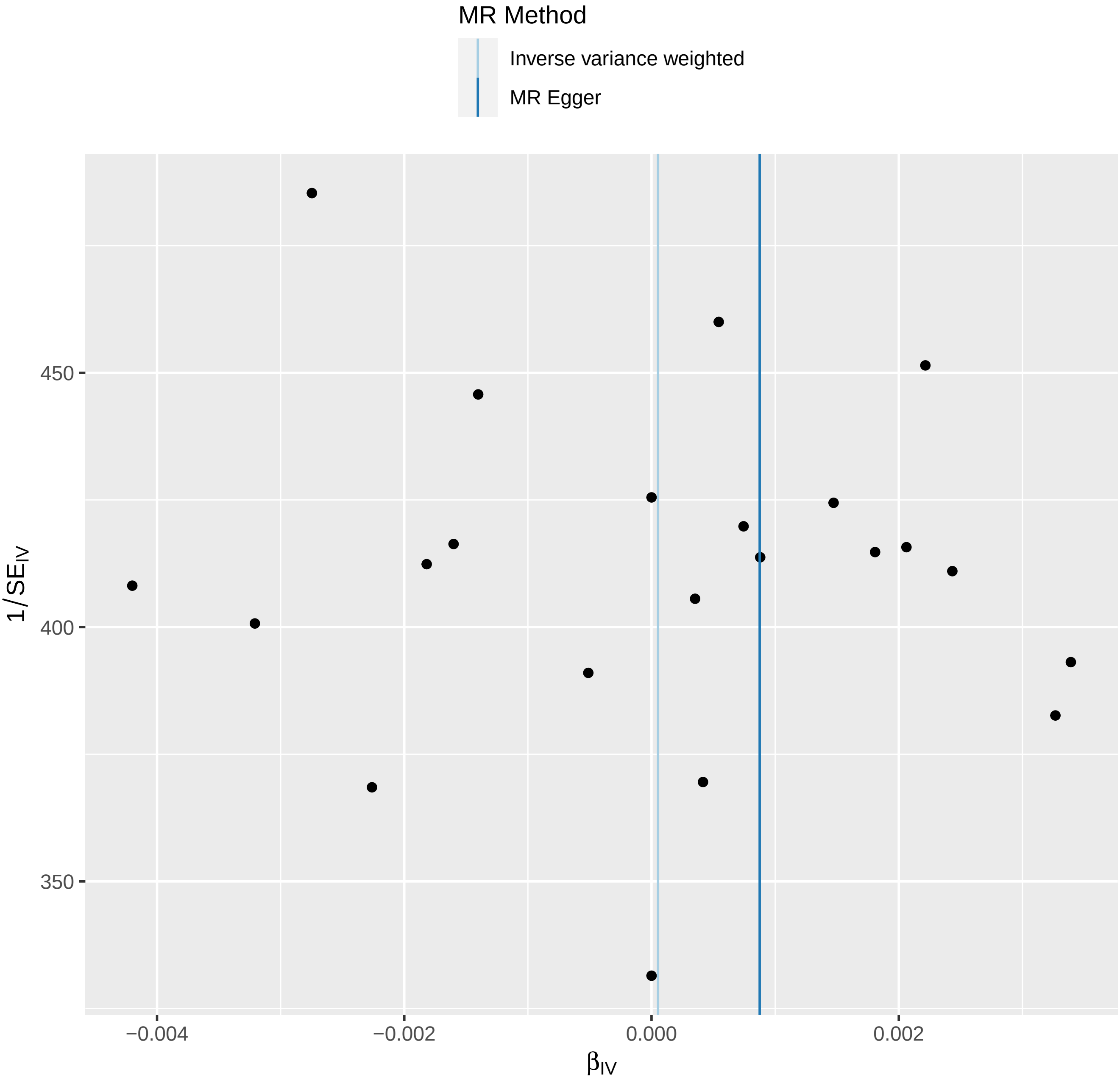

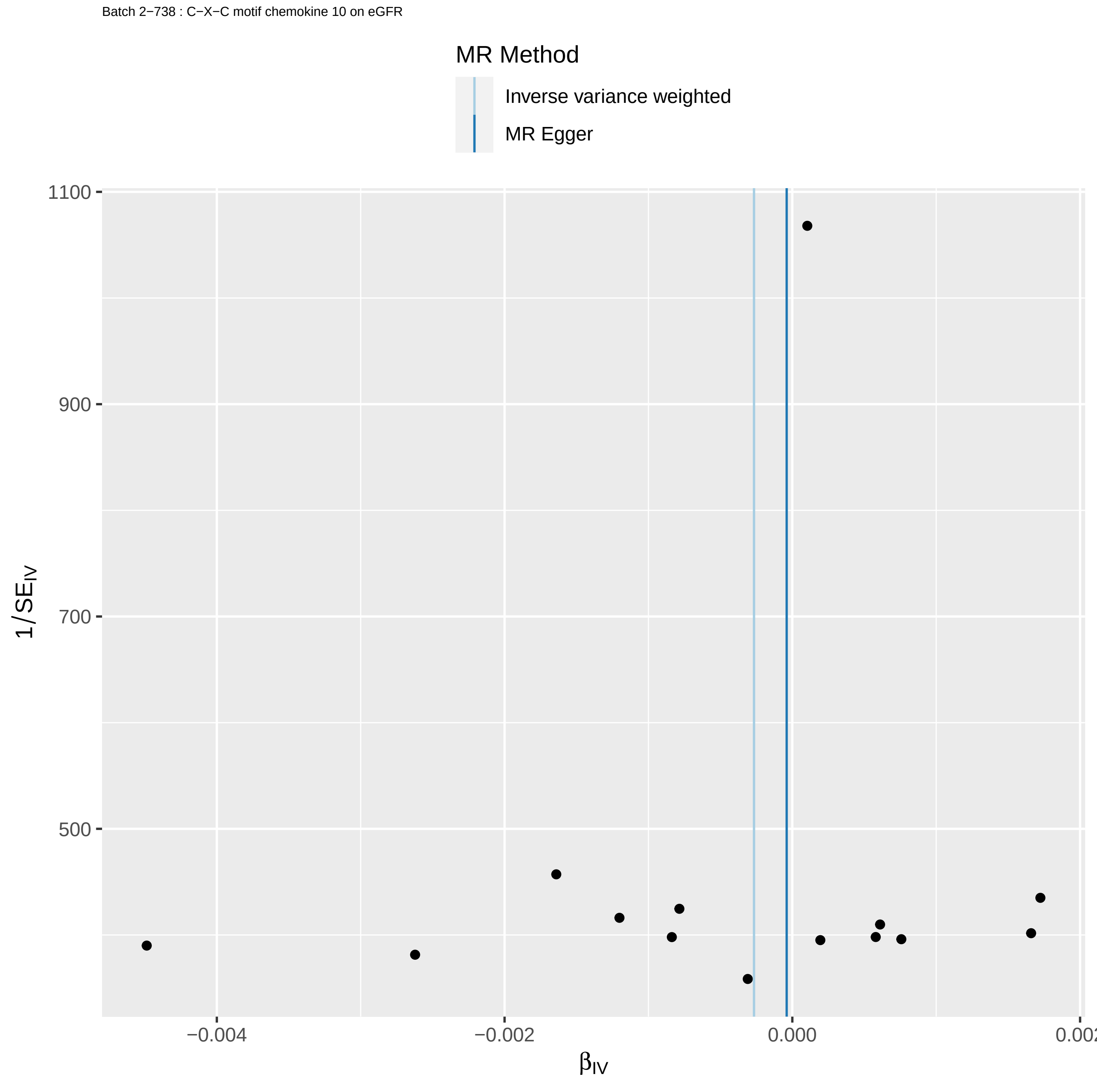
Batch 2−738 : C−X−C motif chemokine 10 on eGFR
MR Method
Inverse variance weighted
MR Egger
1100
900
700
500
1/SE_IV
−0.004
−0.002
0.000
0.002
β_IV

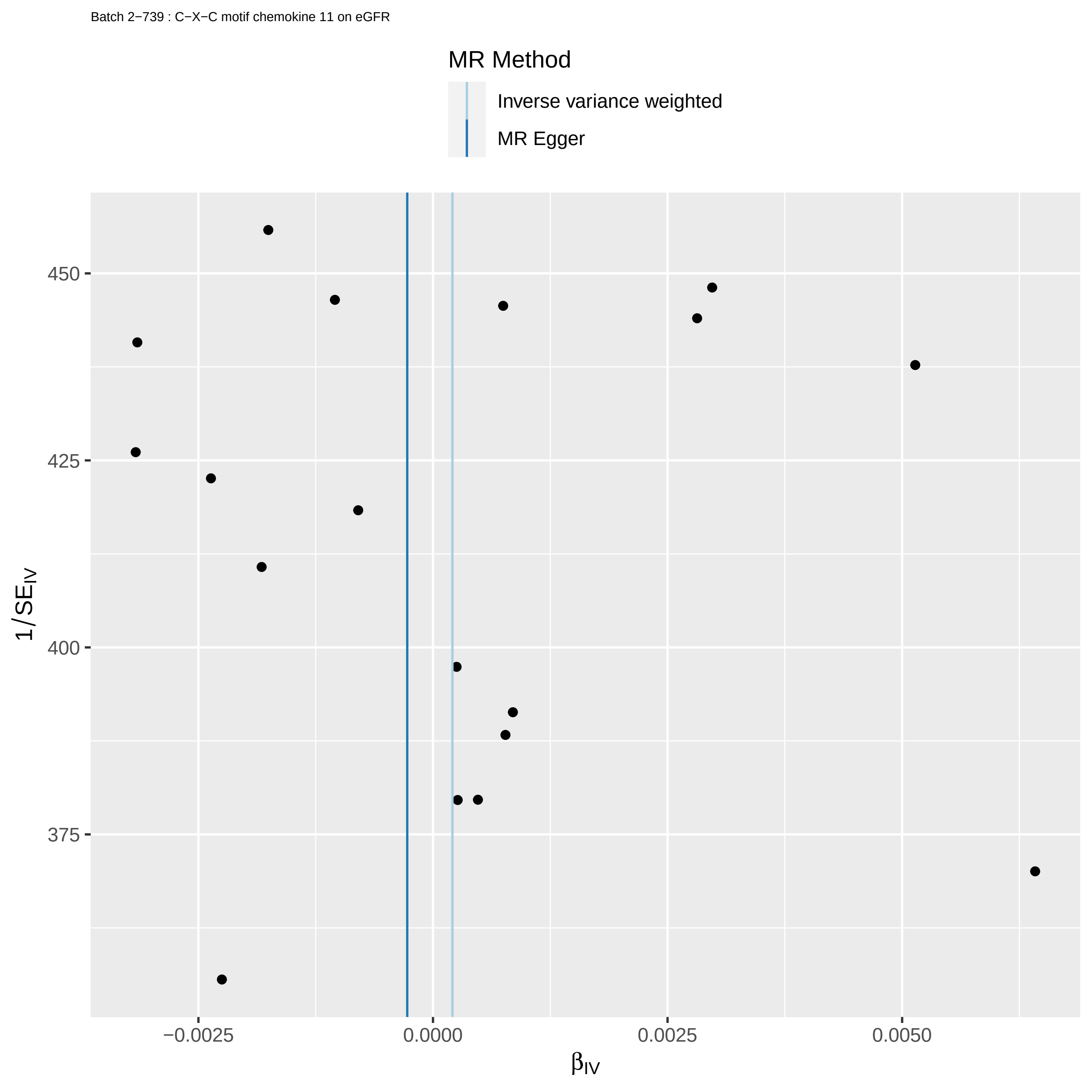
Batch 2−739 : C−X−C motif chemokine 11 on eGFR
MR Method
Inverse variance weighted
MR Egger
450
425
400
375
1/SE$_{IV}$
−0.0025
0.0000
0.0025
0.0050
β$_{IV}$

Batch 2−740 : Stromal cell−derived factor 1 on eGFR

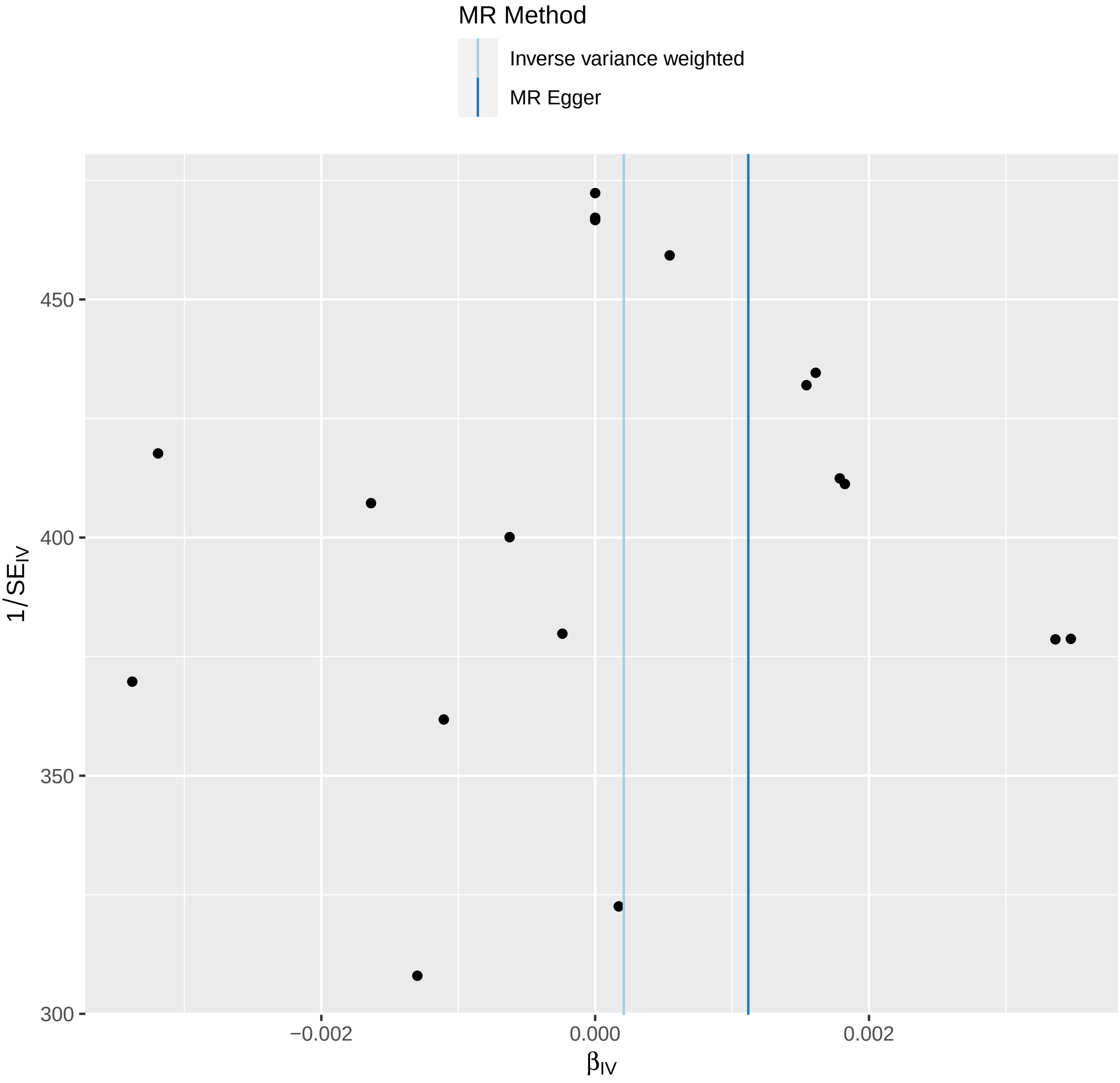

Batch 2−741 : Stromal cell−derived factor 1 on eGFR

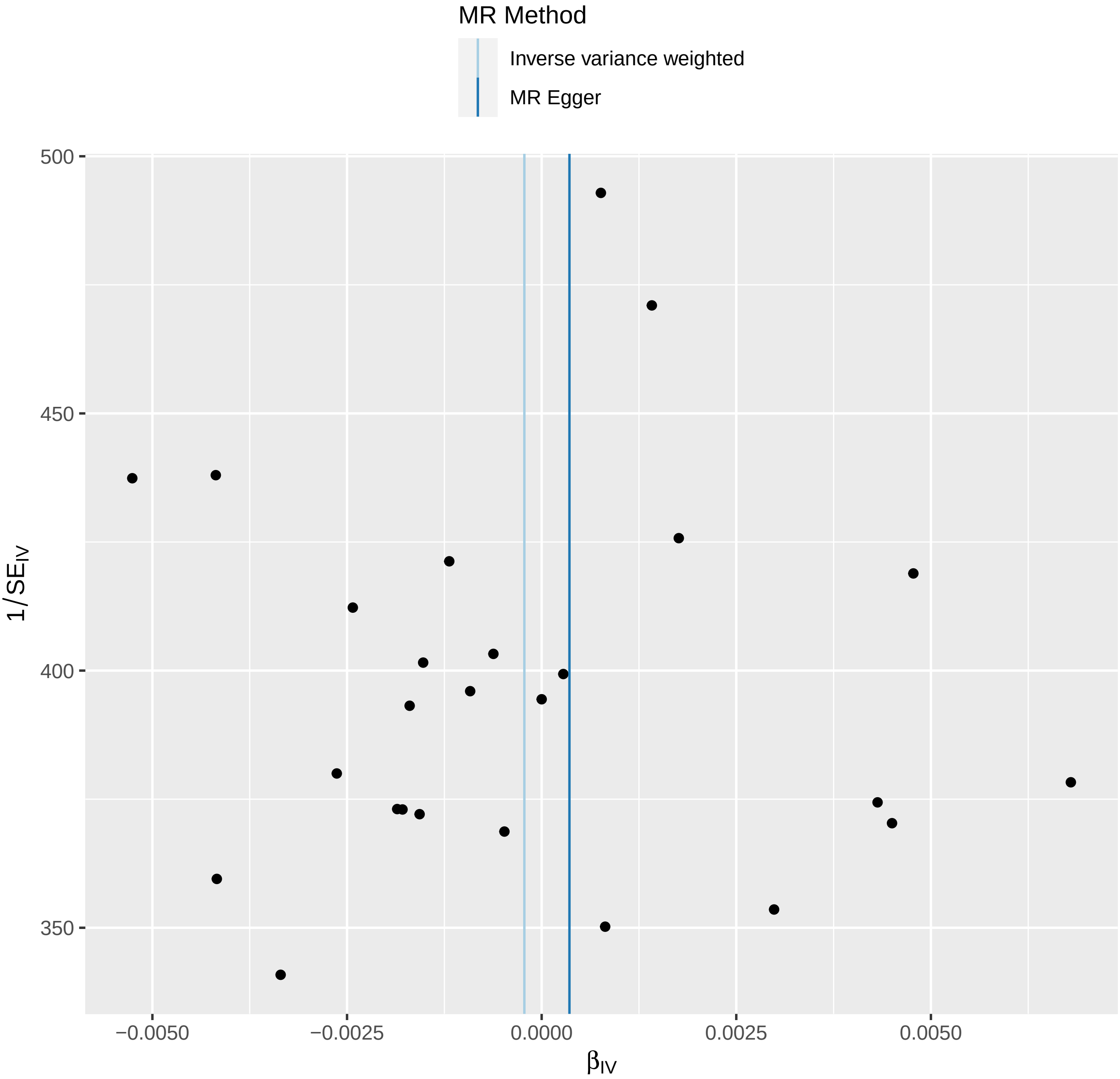

Batch 2−742 : Growth−regulated alpha protein on eGFR

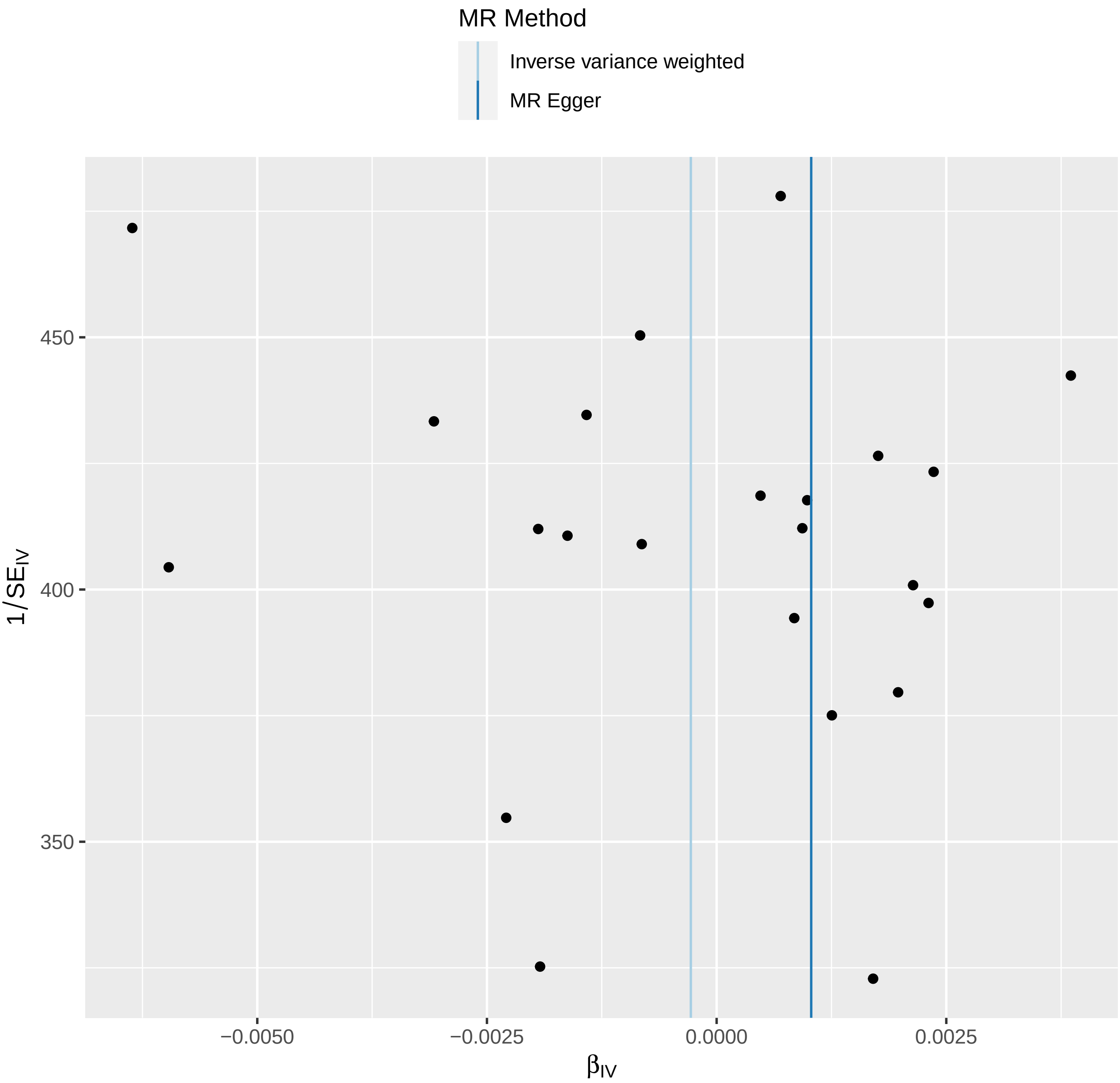

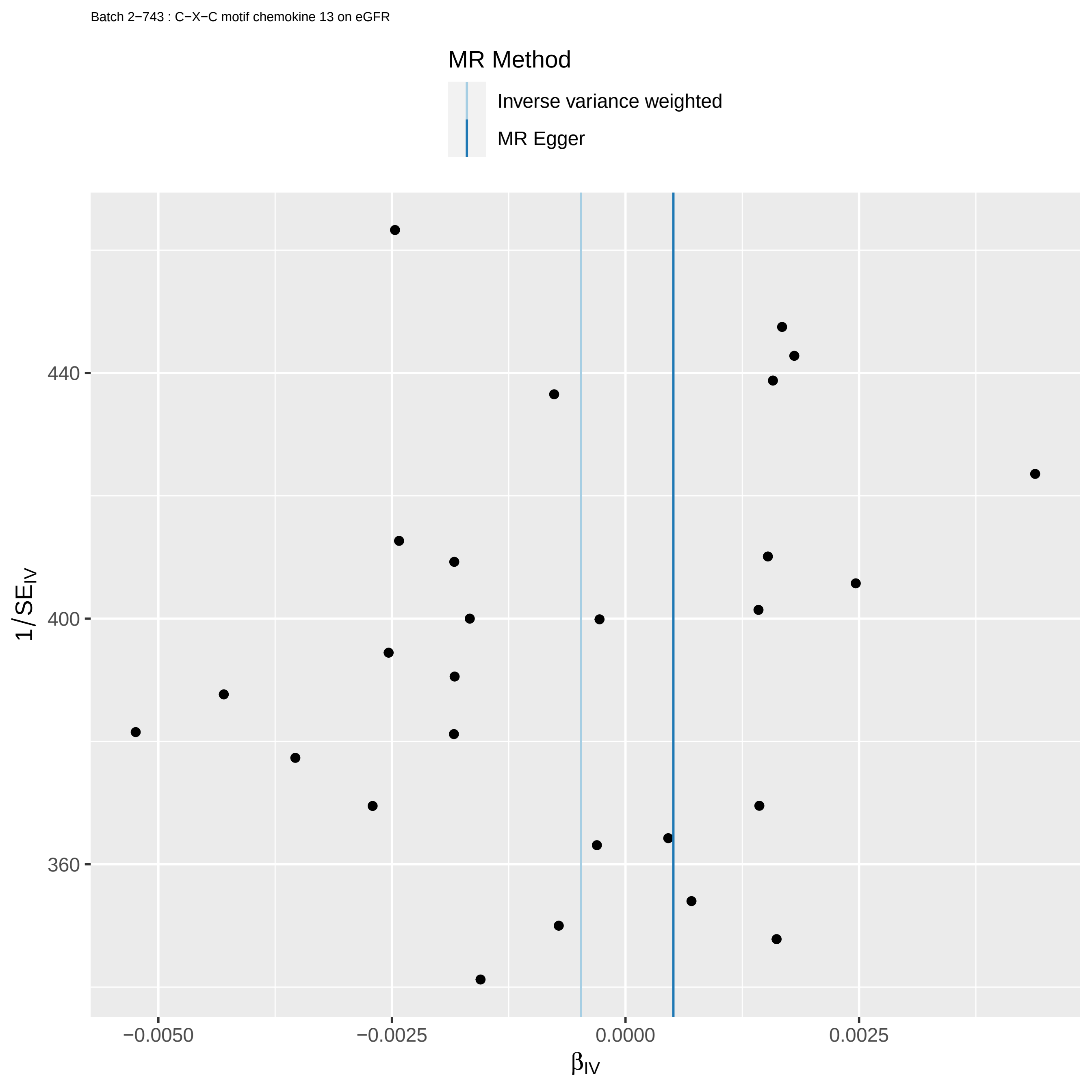

Batch 2−743 : C−X−C motif chemokine 13 on eGFR
MR Method
Inverse variance weighted
MR Egger
440
400
360
1/SE_IV
−0.0050
−0.0025
0.0000
0.0025
β_IV

Batch 2−744 : C−X−C motif chemokine 14 on eGFR

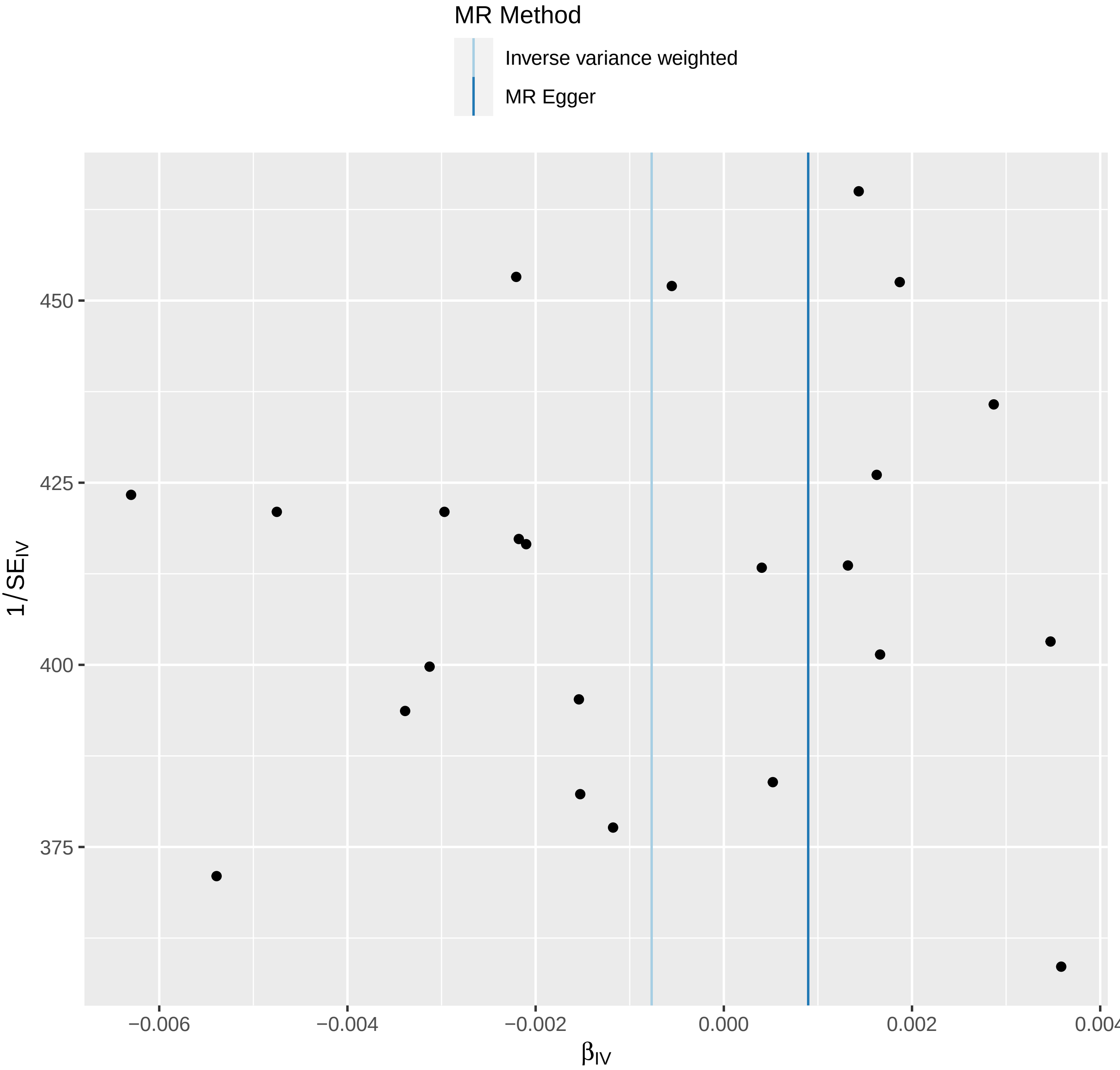

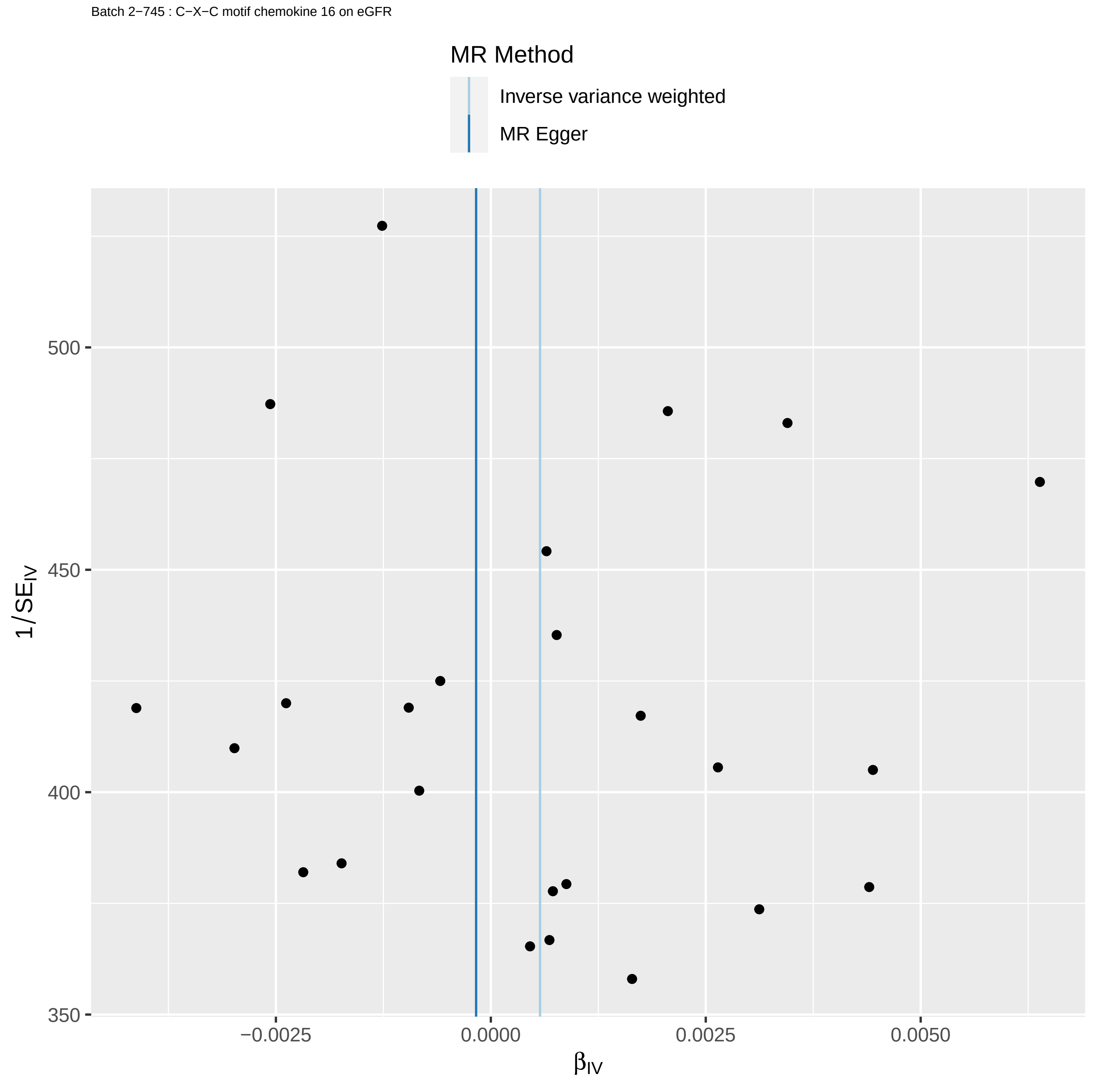
Batch 2−745 : C−X−C motif chemokine 16 on eGFR
MR Method
Inverse variance weighted
MR Egger
500
450
400
350
1/SE_IV
−0.0025
0.0000
0.0025
0.0050
β_IV

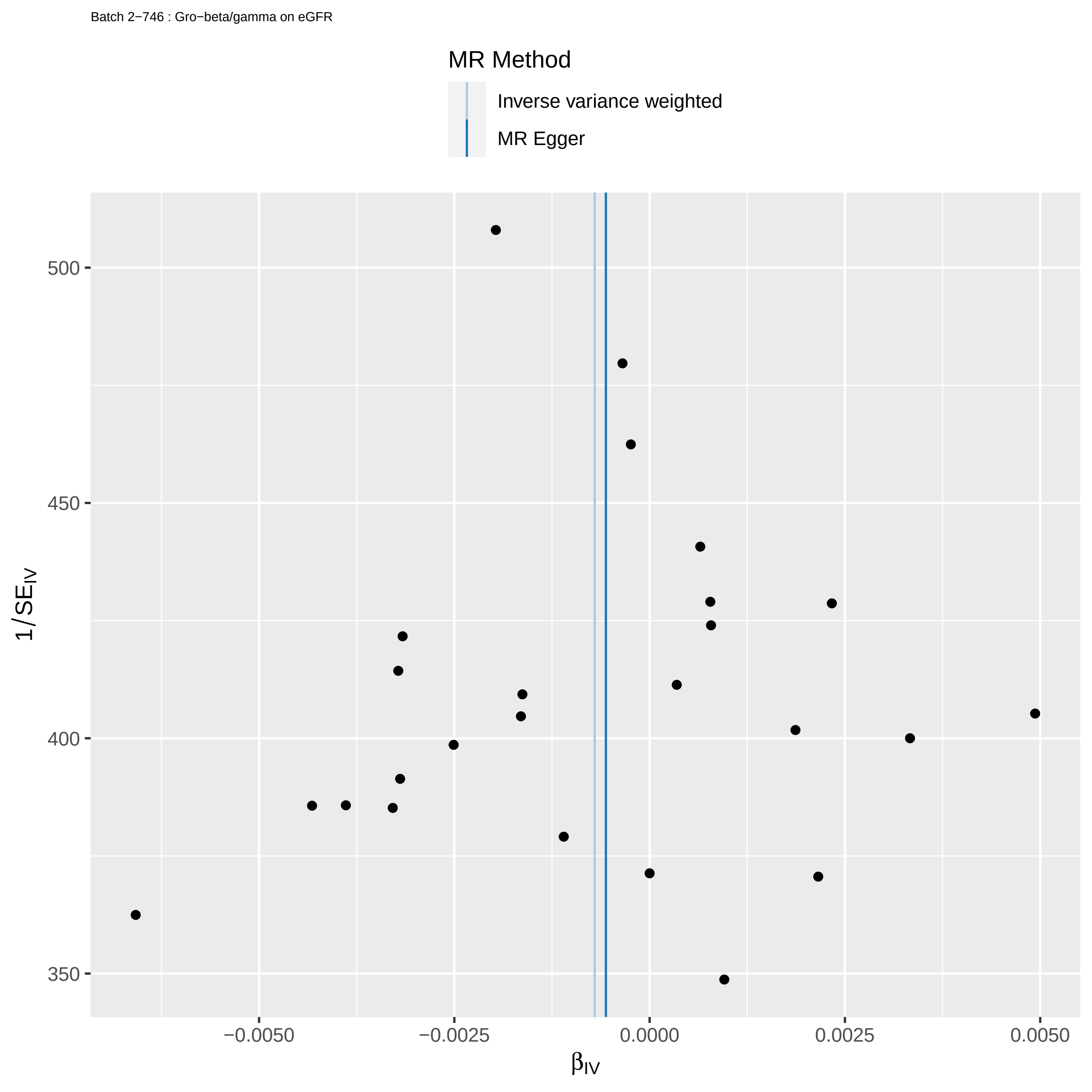

Batch 2−746 : Gro−beta/gamma on eGFR
MR Method
Inverse variance weighted
MR Egger
500
450
400
350
1/SE_IV
−0.0050
−0.0025
0.0000
0.0025
0.0050
β_IV

Batch 2−747 : Gro−beta/gamma on eGFR

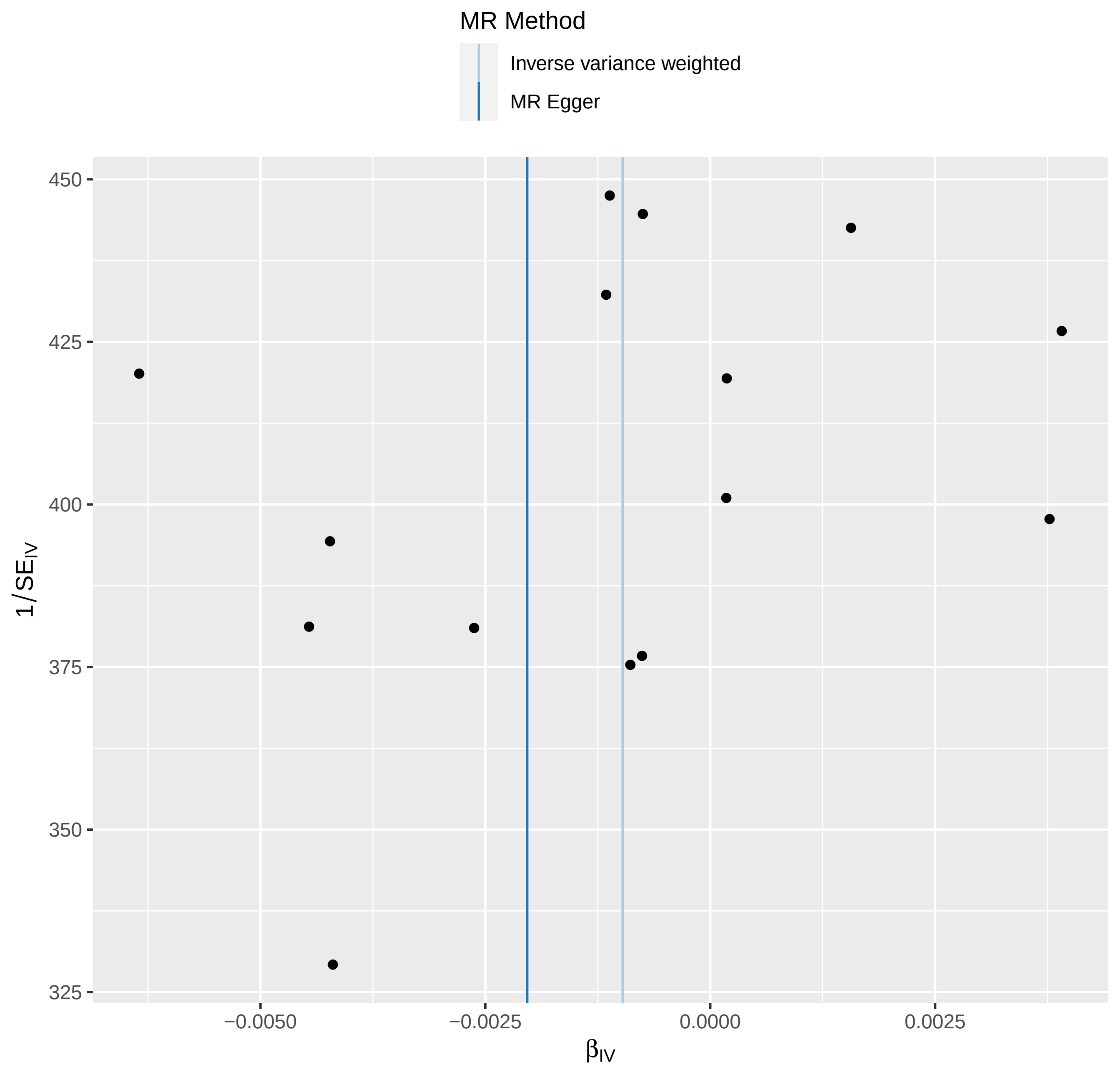

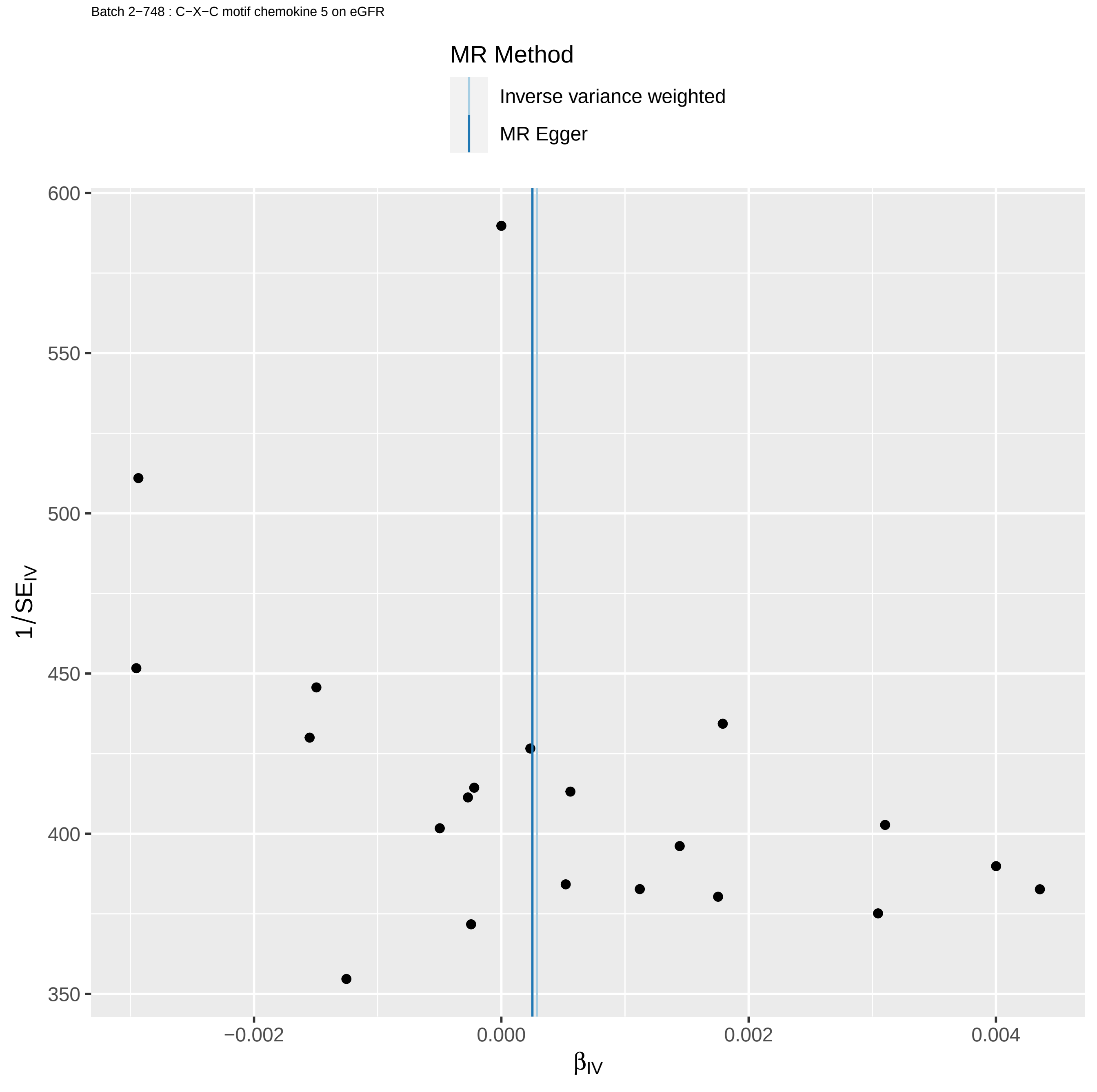

Batch 2−748 : C−X−C motif chemokine 5 on eGFR
MR Method
Inverse variance weighted
MR Egger
600
550
500
450
400
350
1/SE_IV
−0.002
0.000
0.002
0.004
β_IV

Batch 2−749 : Interleukin−8 on eGFR

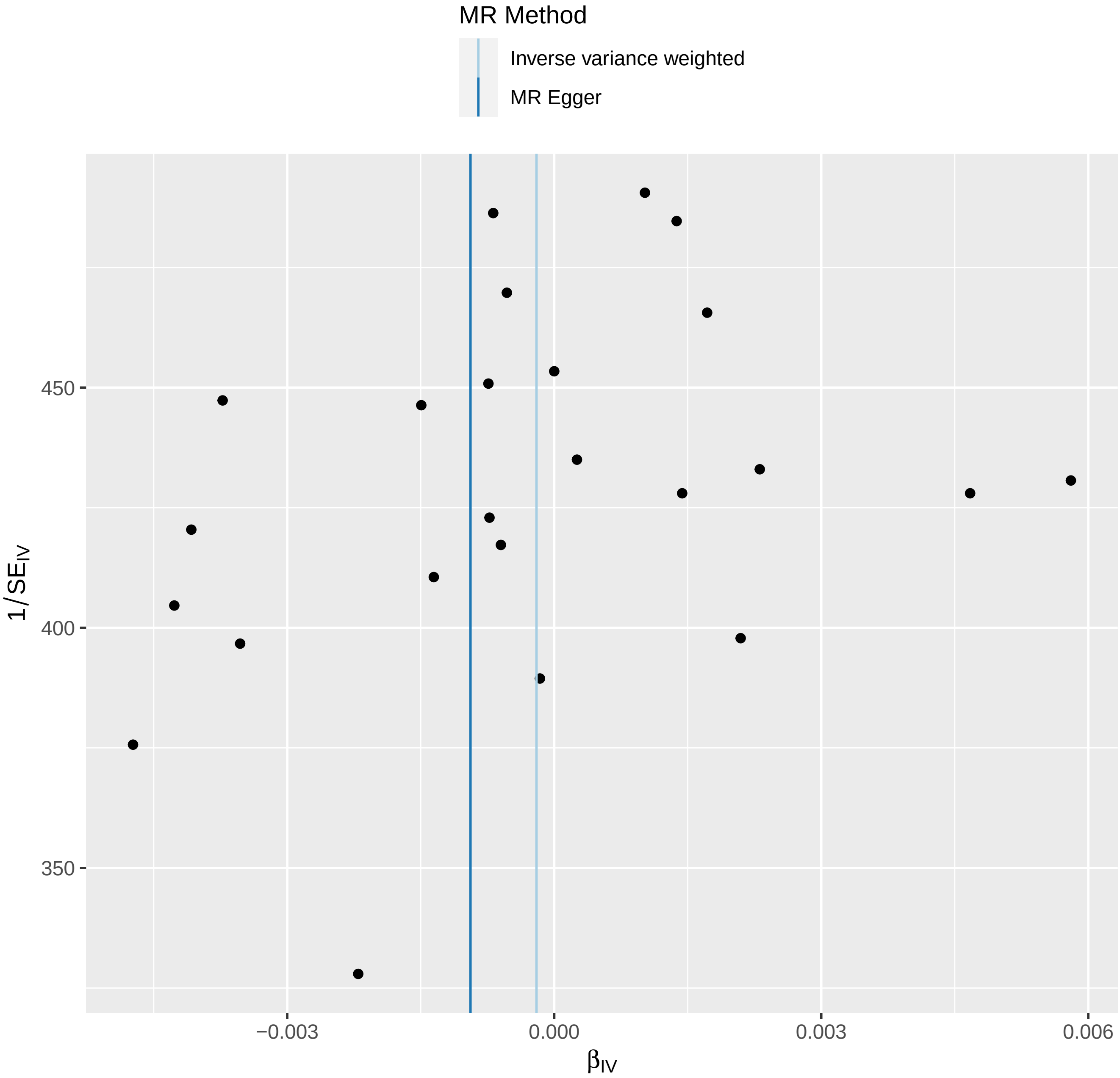

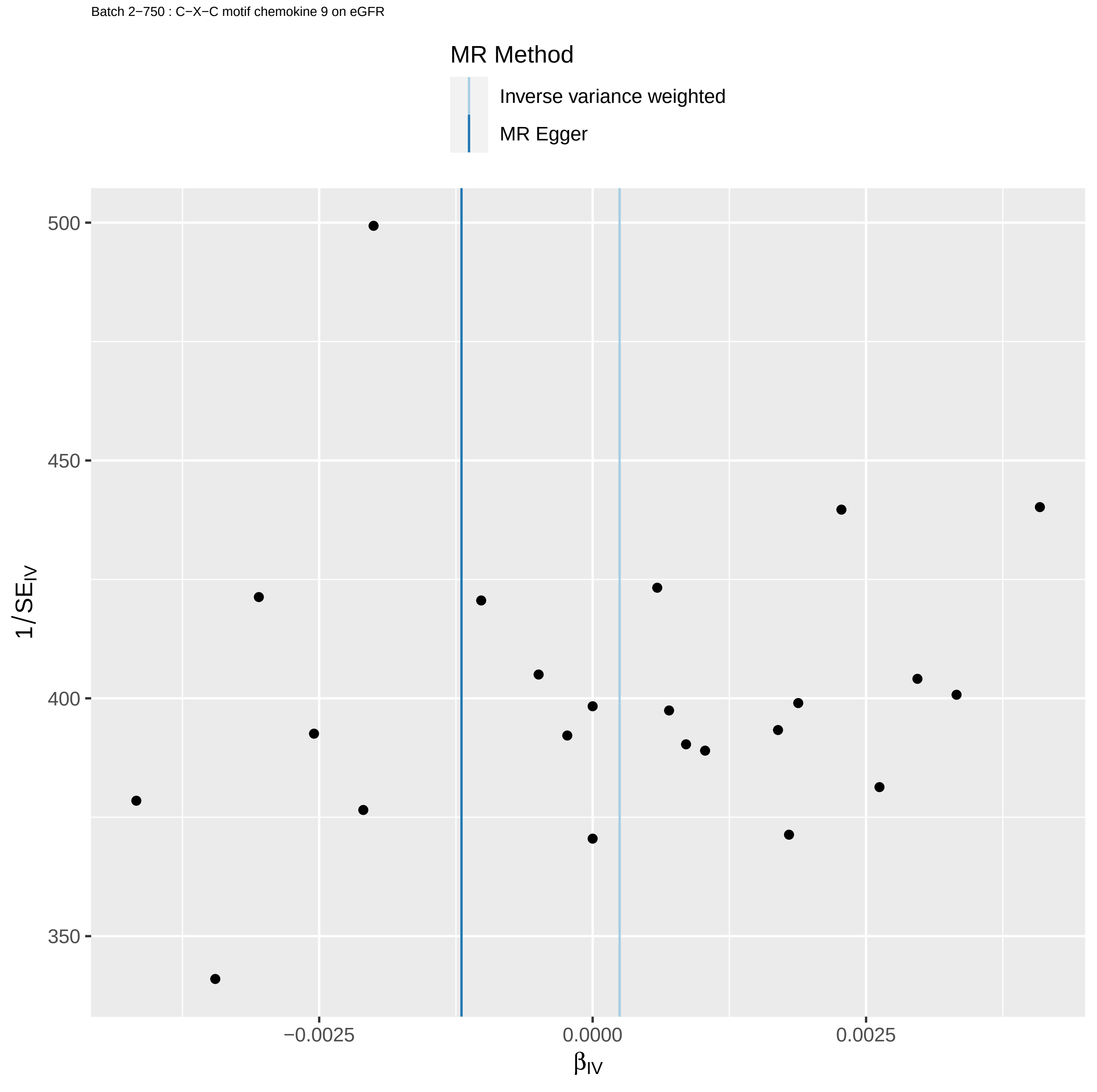
Batch 2−750 : C−X−C motif chemokine 9 on eGFR
MR Method
Inverse variance weighted
MR Egger
500
450
400
350
1/SE_IV
−0.0025
0.0000
0.0025
β_IV

Batch 2−751 : C−X−C motif chemokine 9 on eGFR

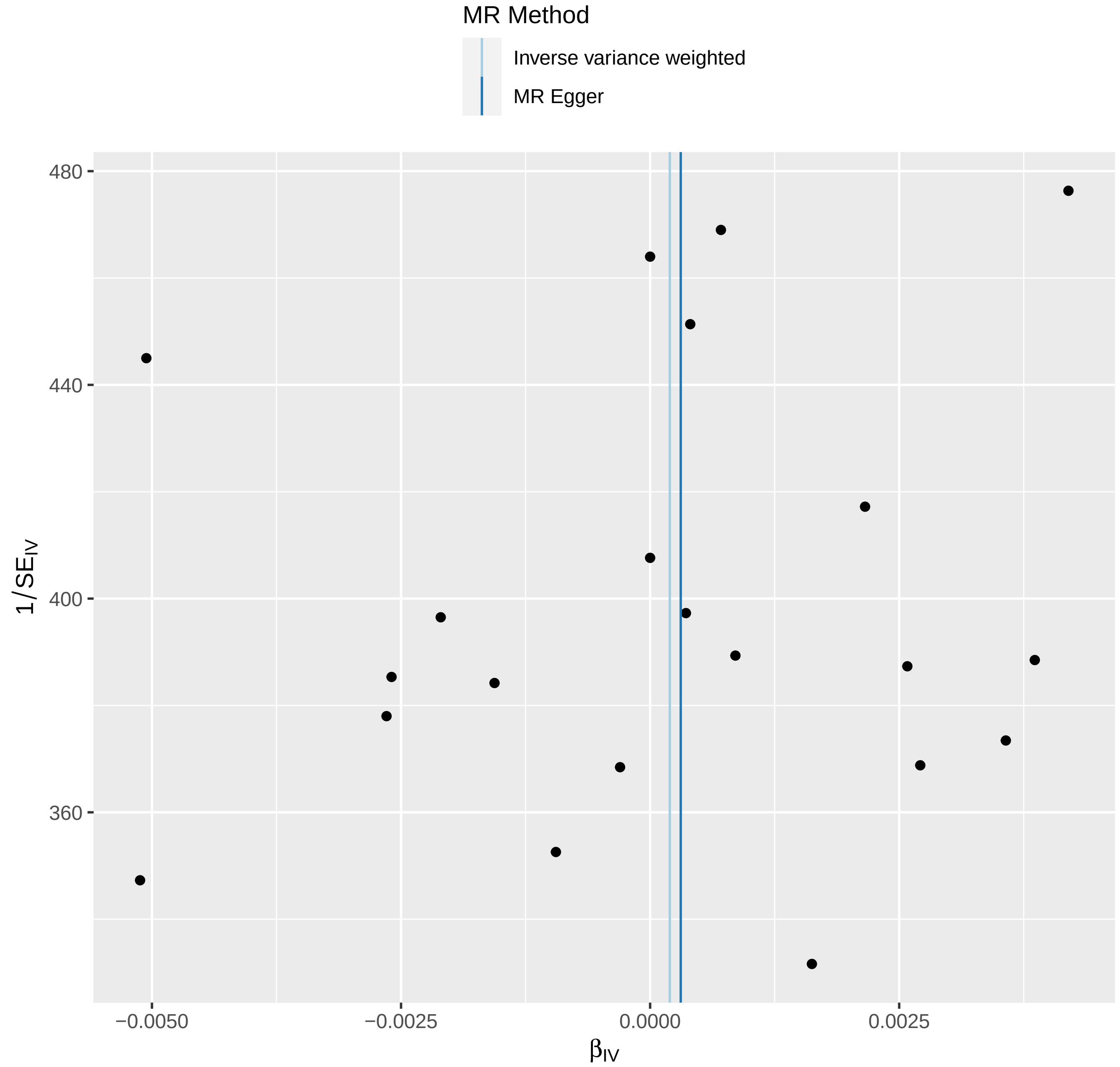

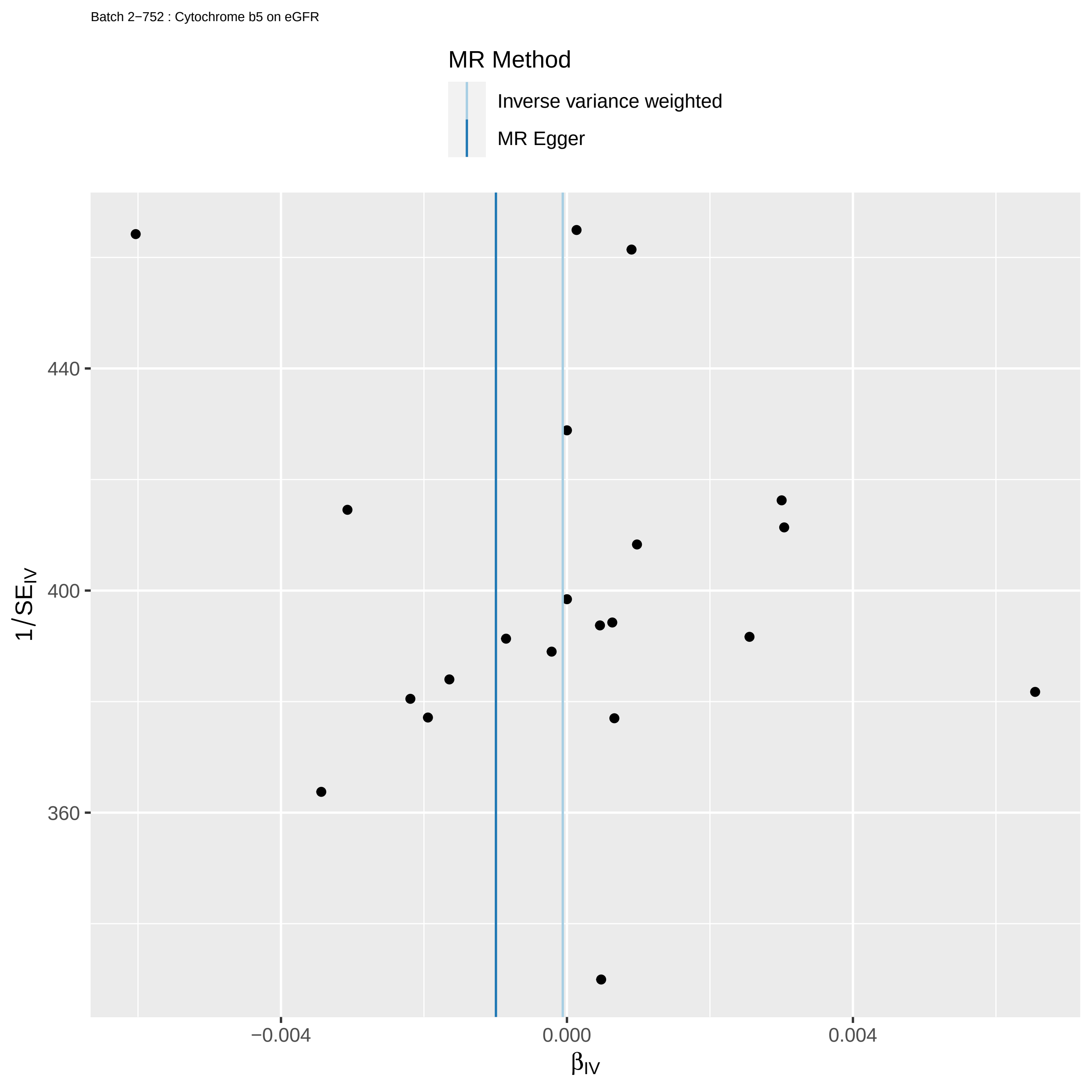
Batch 2−752 : Cytochrome b5 on eGFR
MR Method
Inverse variance weighted
MR Egger
440
400
360
1/SE_IV
−0.004
0.000
0.004
β_IV

Batch 2−753 : NADH−cytochrome b5 reductase 3 on eGFR

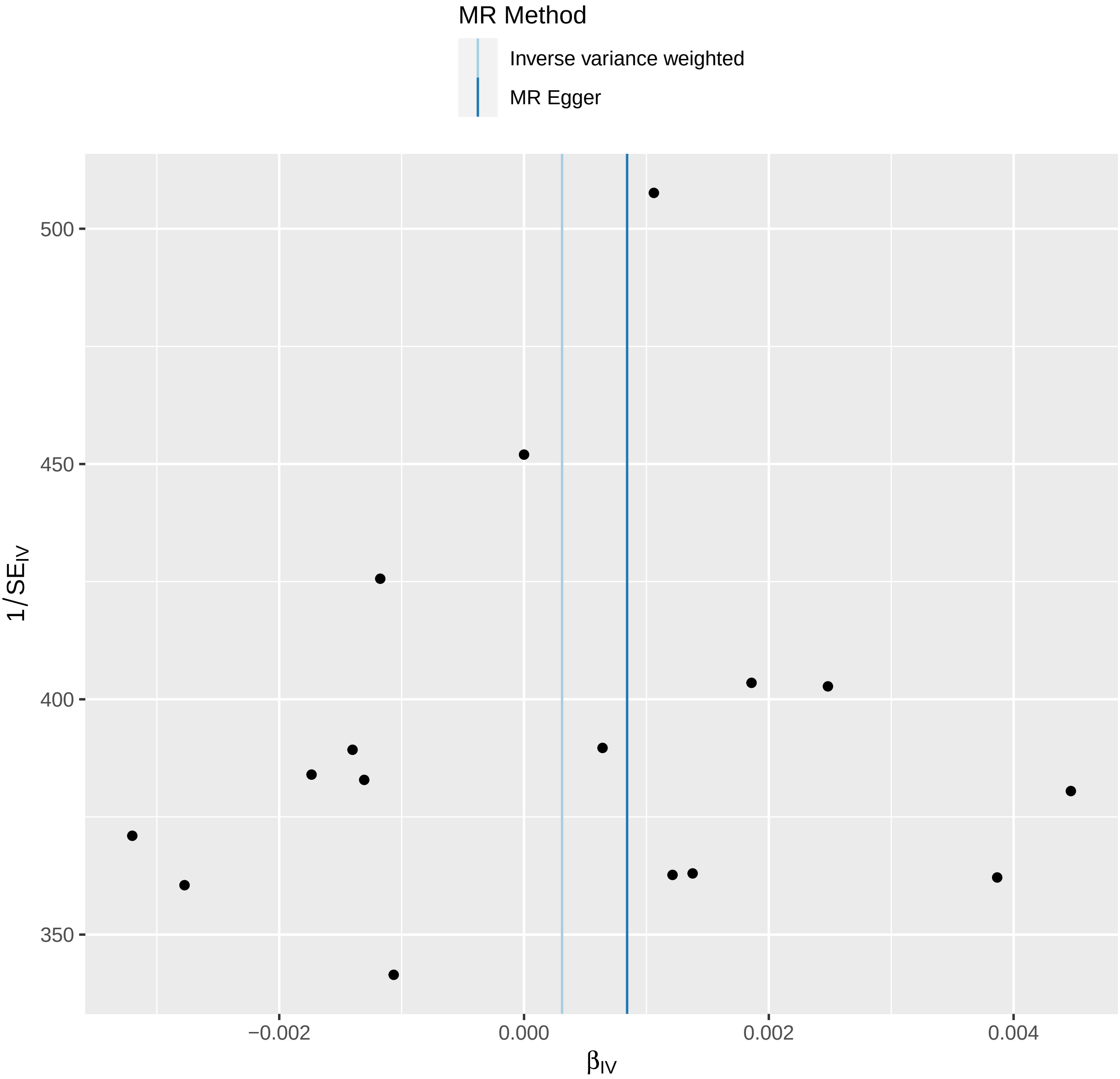

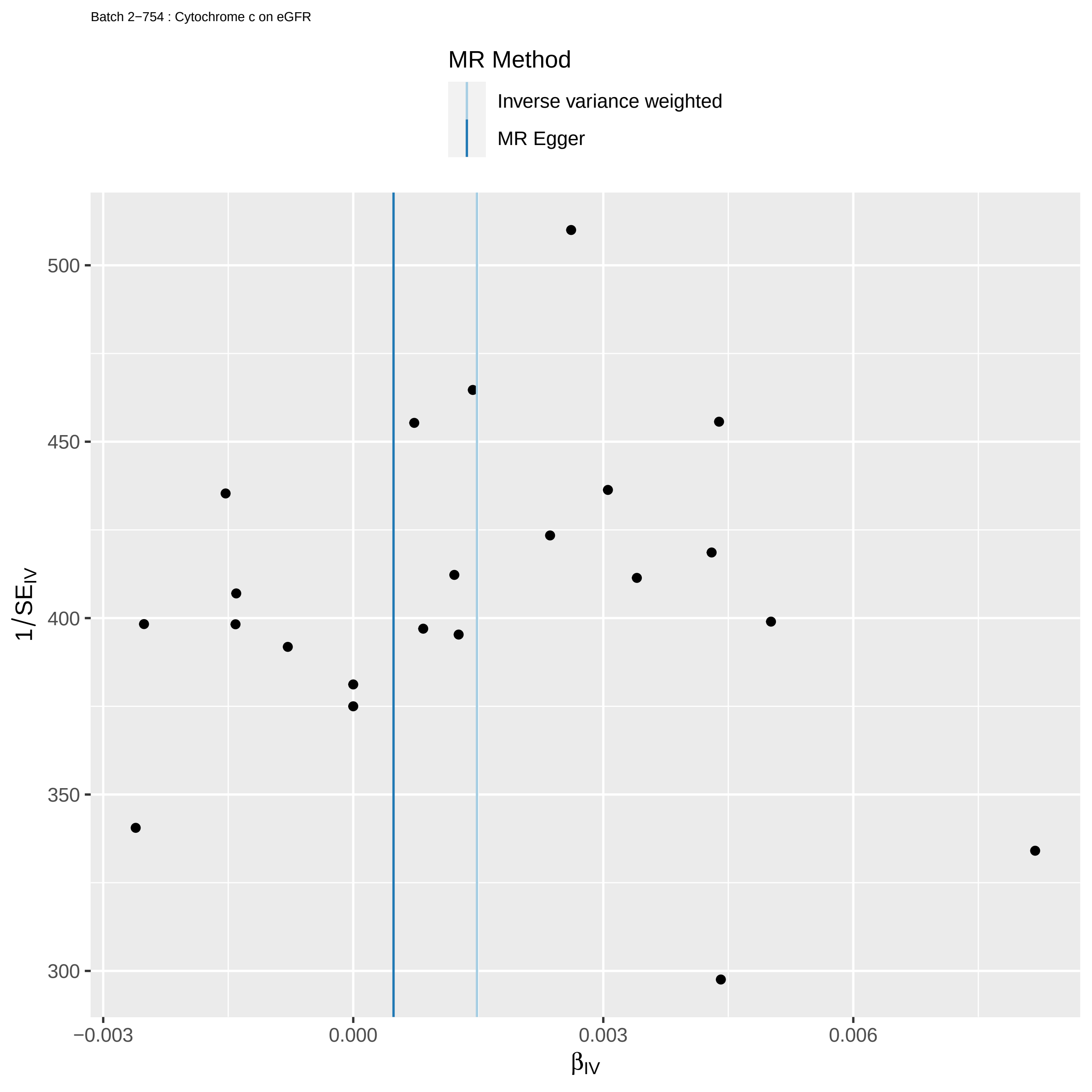
Batch 2−754 : Cytochrome c on eGFR
MR Method
Inverse variance weighted
MR Egger
500
450
400
350
300
1/SE$_{IV}$
−0.003
0.000
0.003
0.006
β$_{IV}$

Batch 2−755 : Cytoglobin on eGFR

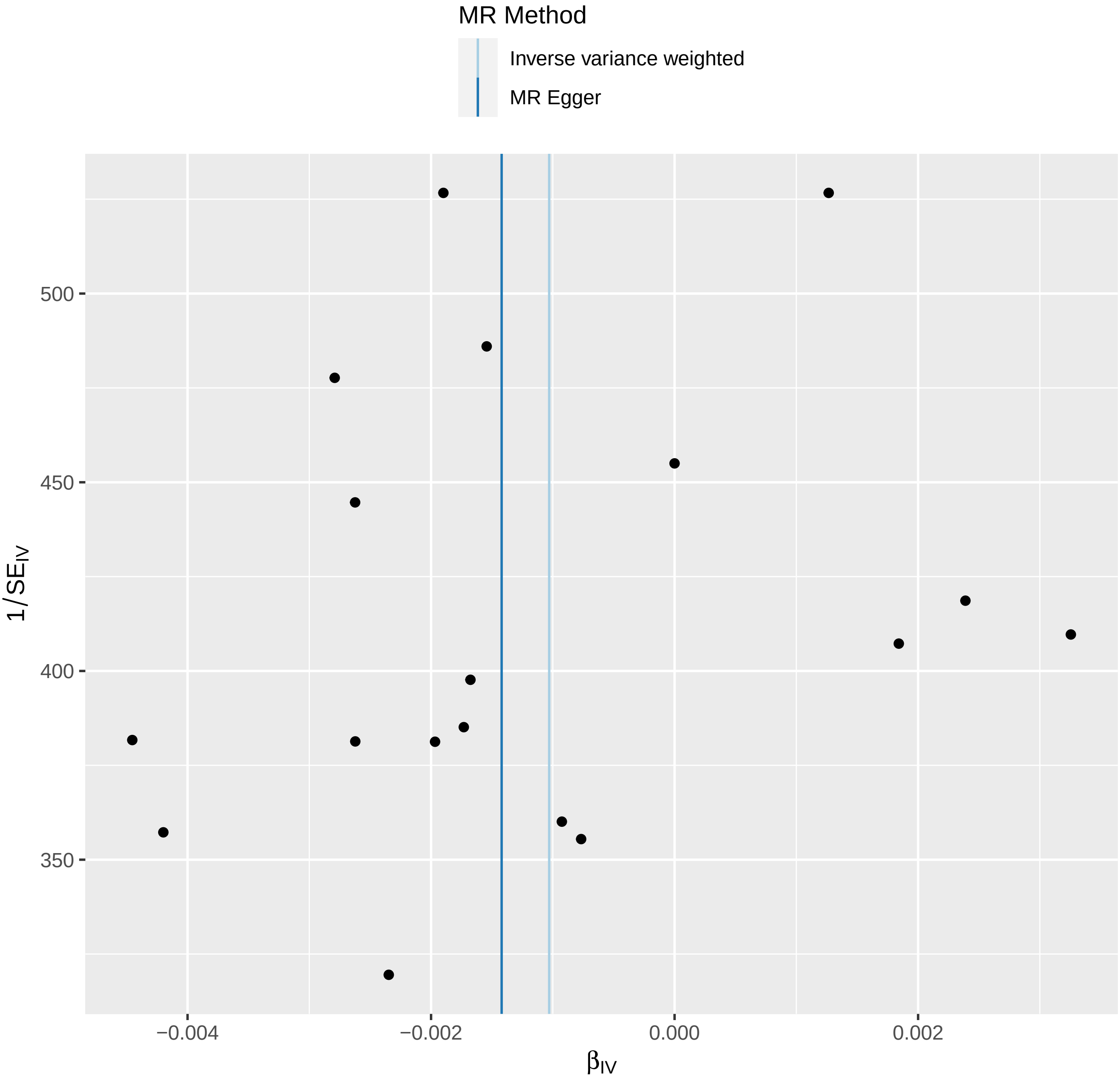

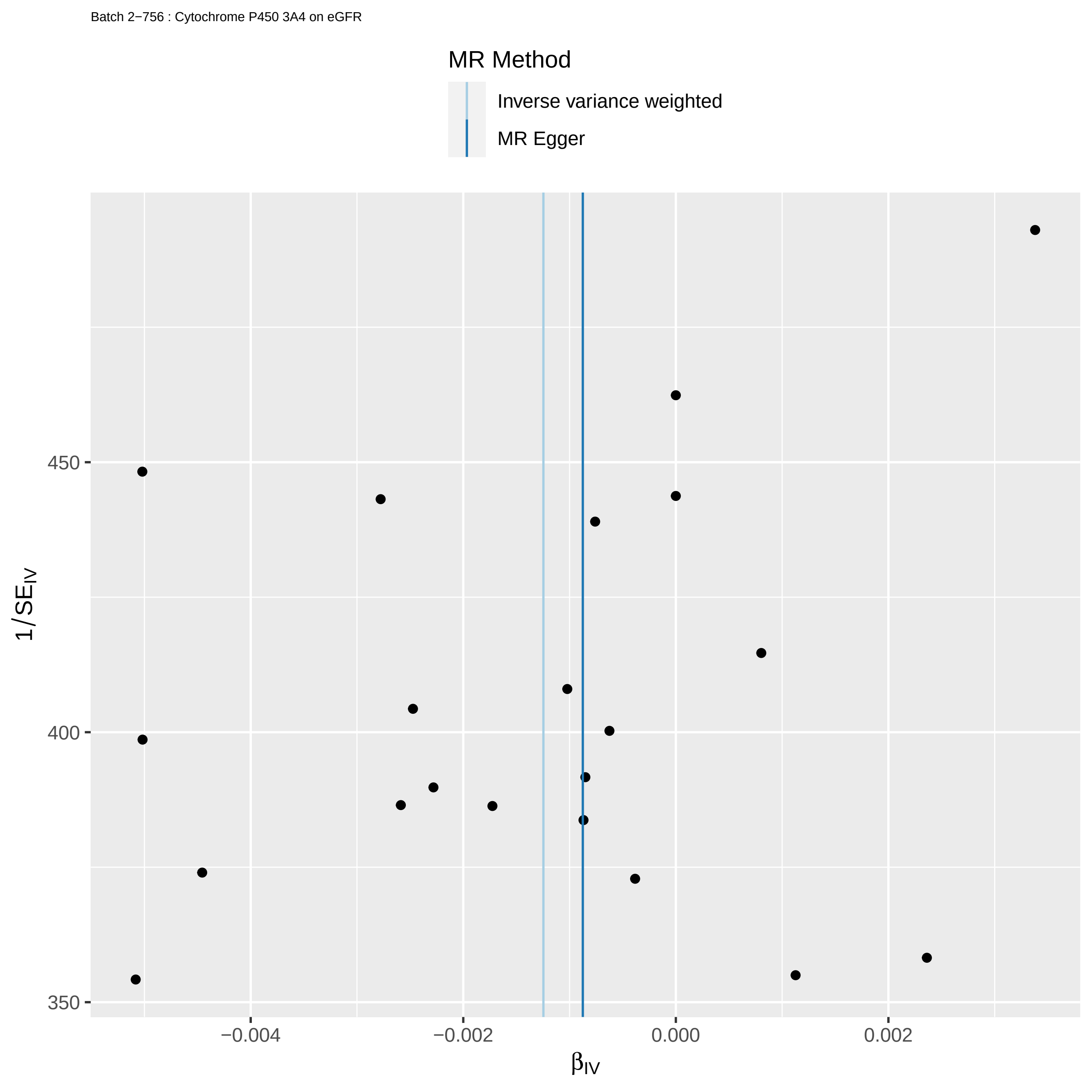

Batch 2−756 : Cytochrome P450 3A4 on eGFR
MR Method
Inverse variance weighted
MR Egger
450
400
350
1/SE_IV
−0.004
−0.002
0.000
0.002
β_IV

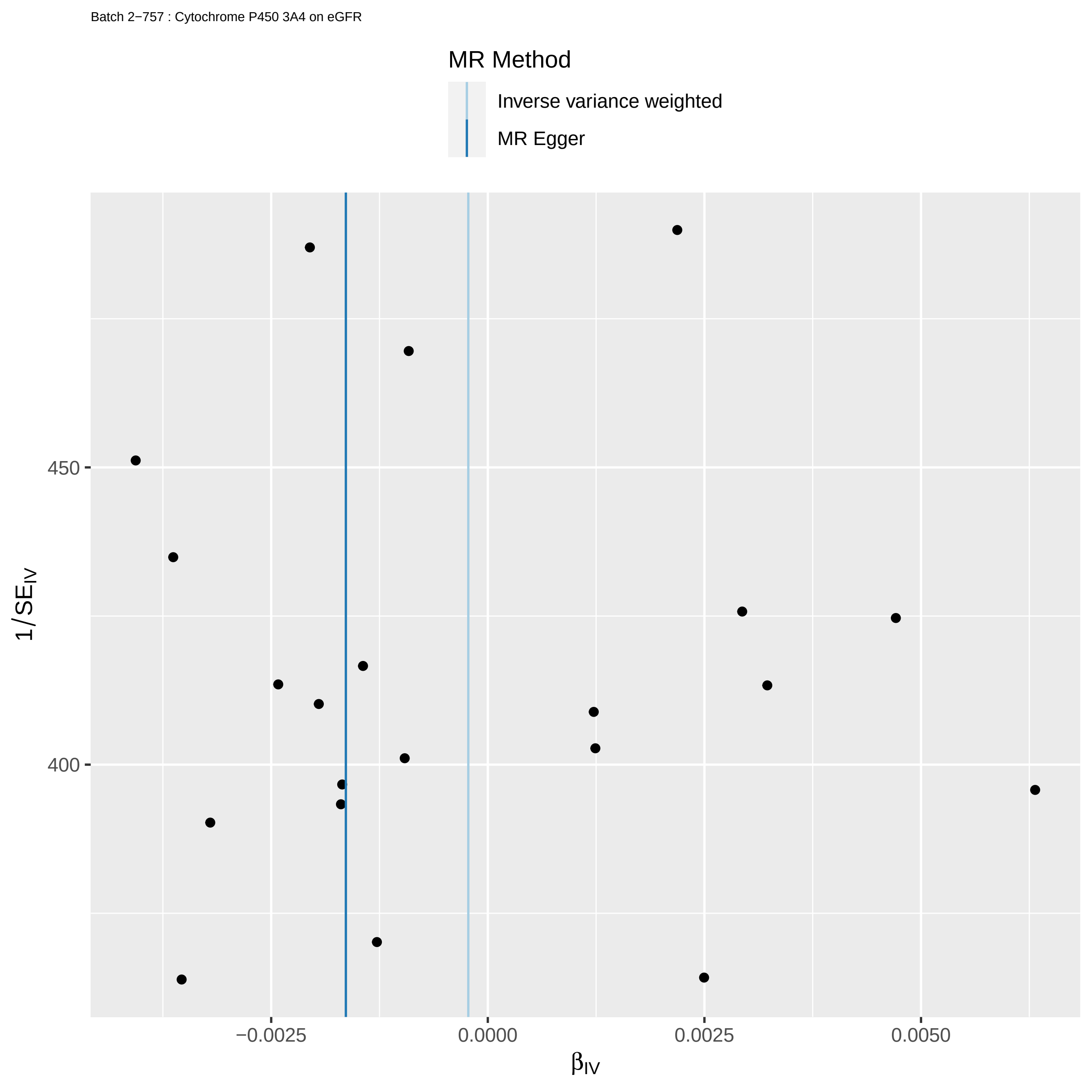
Batch 2−757 : Cytochrome P450 3A4 on eGFR
MR Method
Inverse variance weighted
MR Egger
450
400
1/SE_IV
−0.0025
0.0000
0.0025
0.0050
β_IV

Batch 2−758 : Protein CYR61 on eGFR

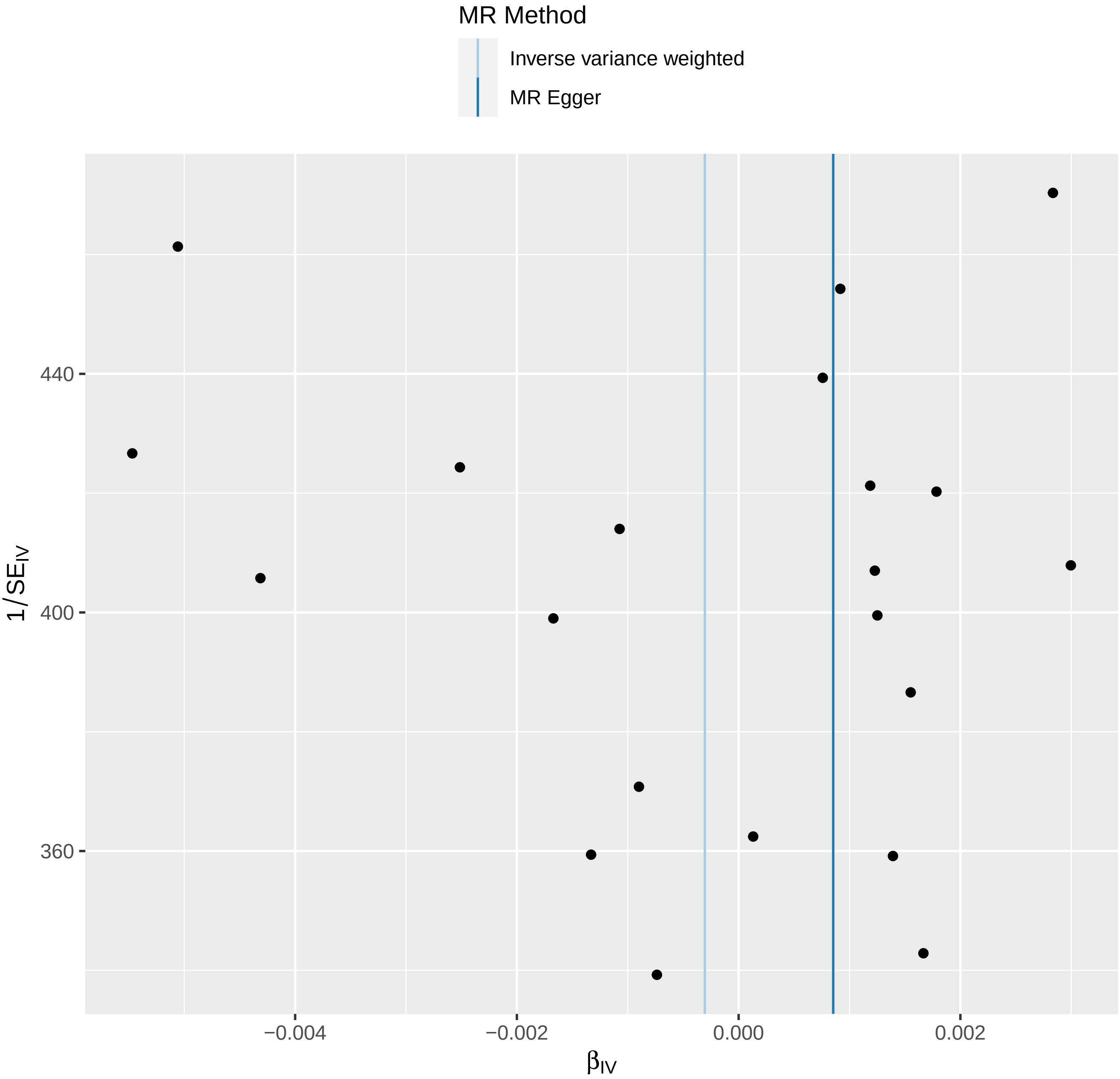

Batch 2−759 : Cytohesin−2 on eGFR

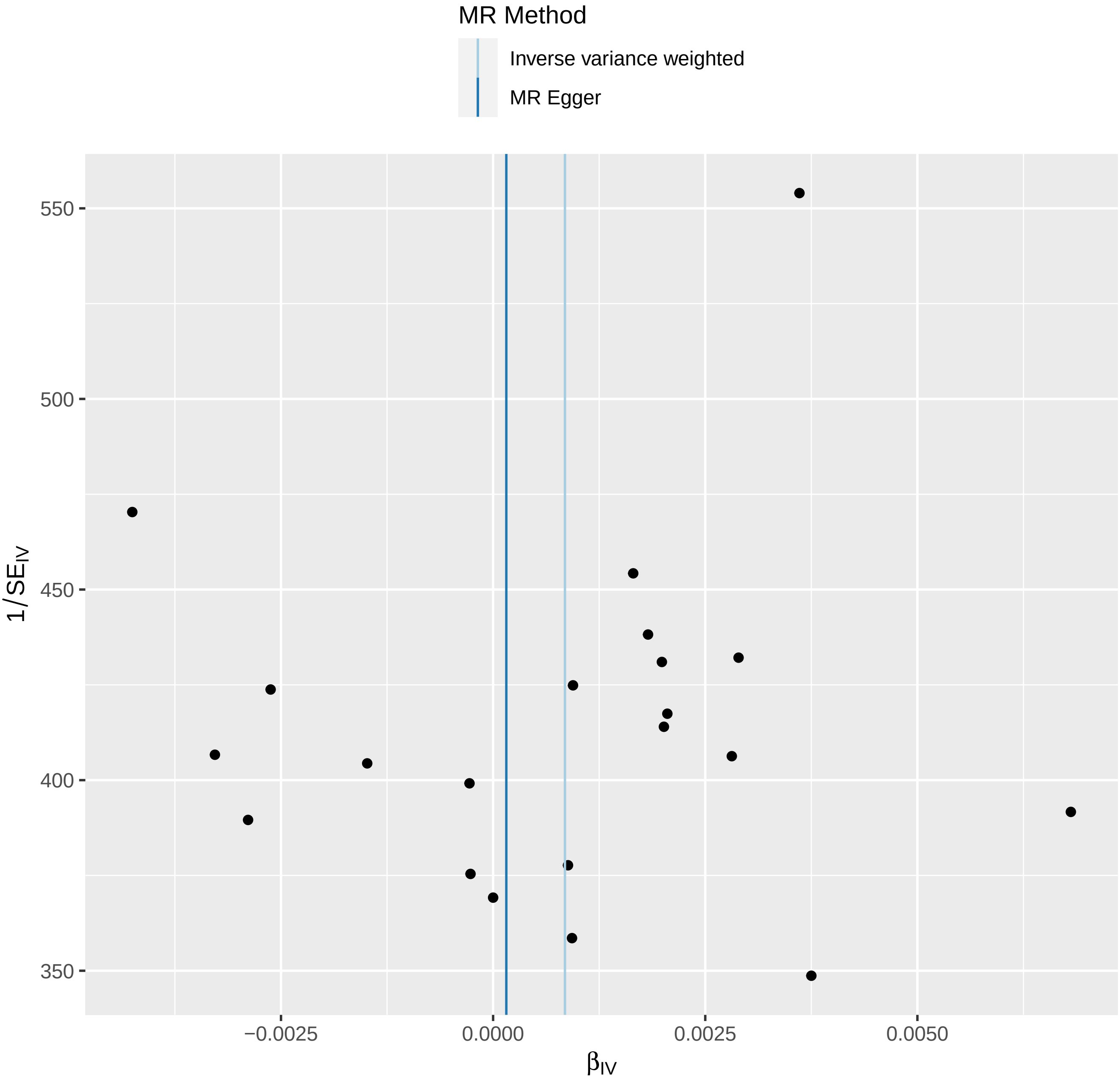

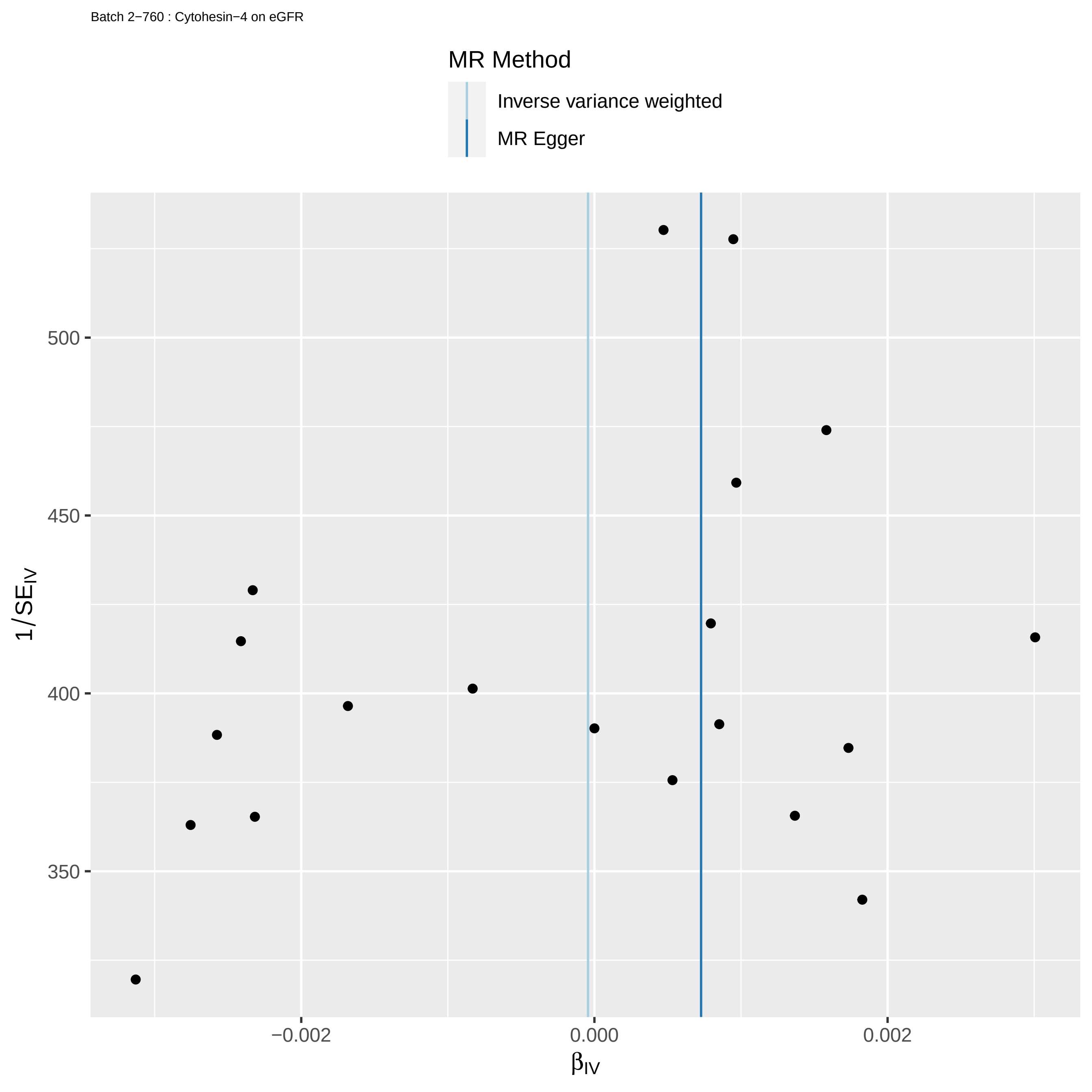
Batch 2−760 : Cytohesin−4 on eGFR
MR Method
Inverse variance weighted
MR Egger
500
450
400
350
1/SE$_{IV}$
−0.002
0.000
0.002
β$_{IV}$

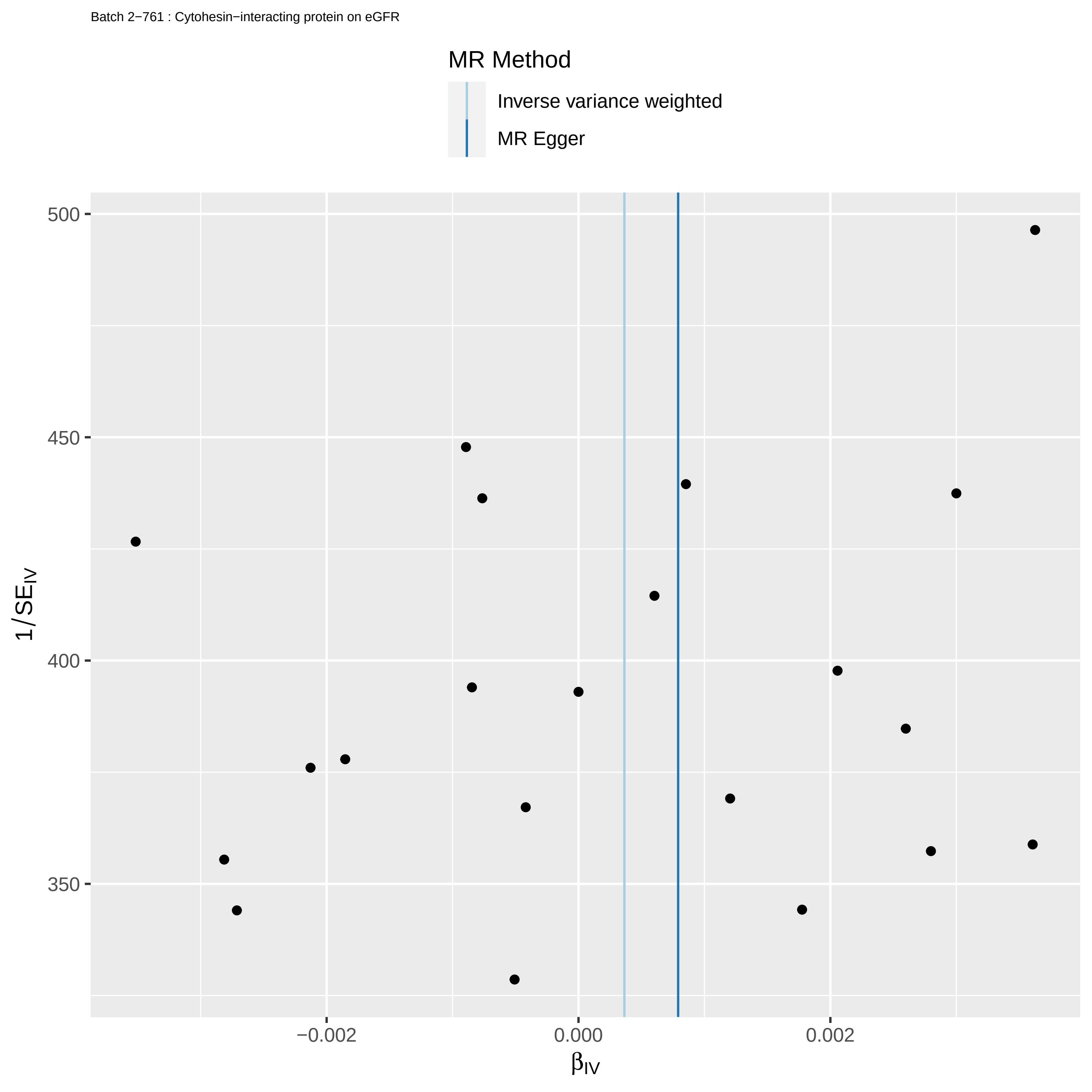

Batch 2−761 : Cytohesin−interacting protein on eGFR
MR Method
Inverse variance weighted
MR Egger
500
450
400
350
1/SE$_{IV}$
−0.002
0.000
0.002
β$_{IV}$

Batch 2−762 : Dystroglycan on eGFR

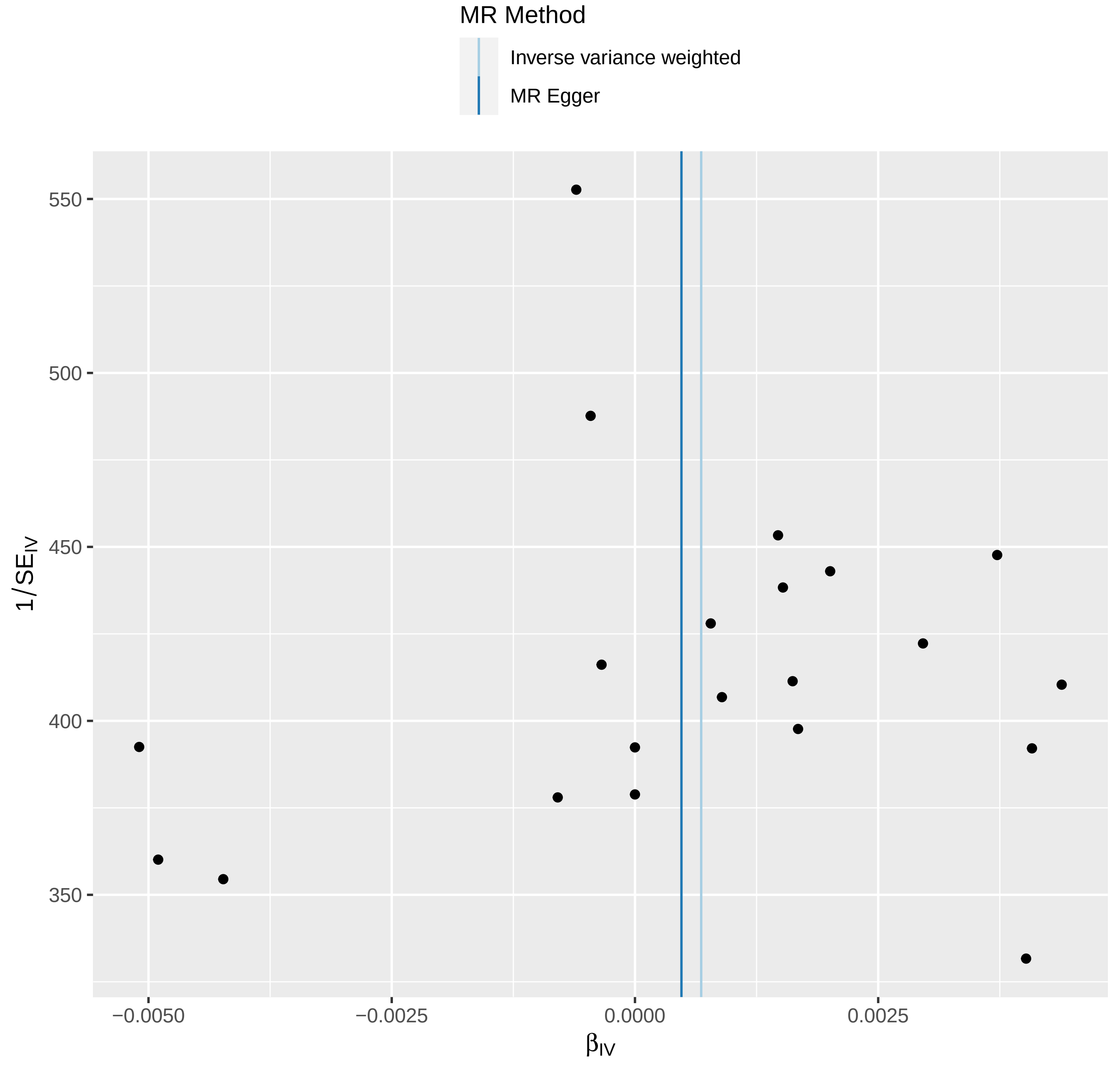

Batch 2−763 : Death−associated protein kinase 2 on eGFR

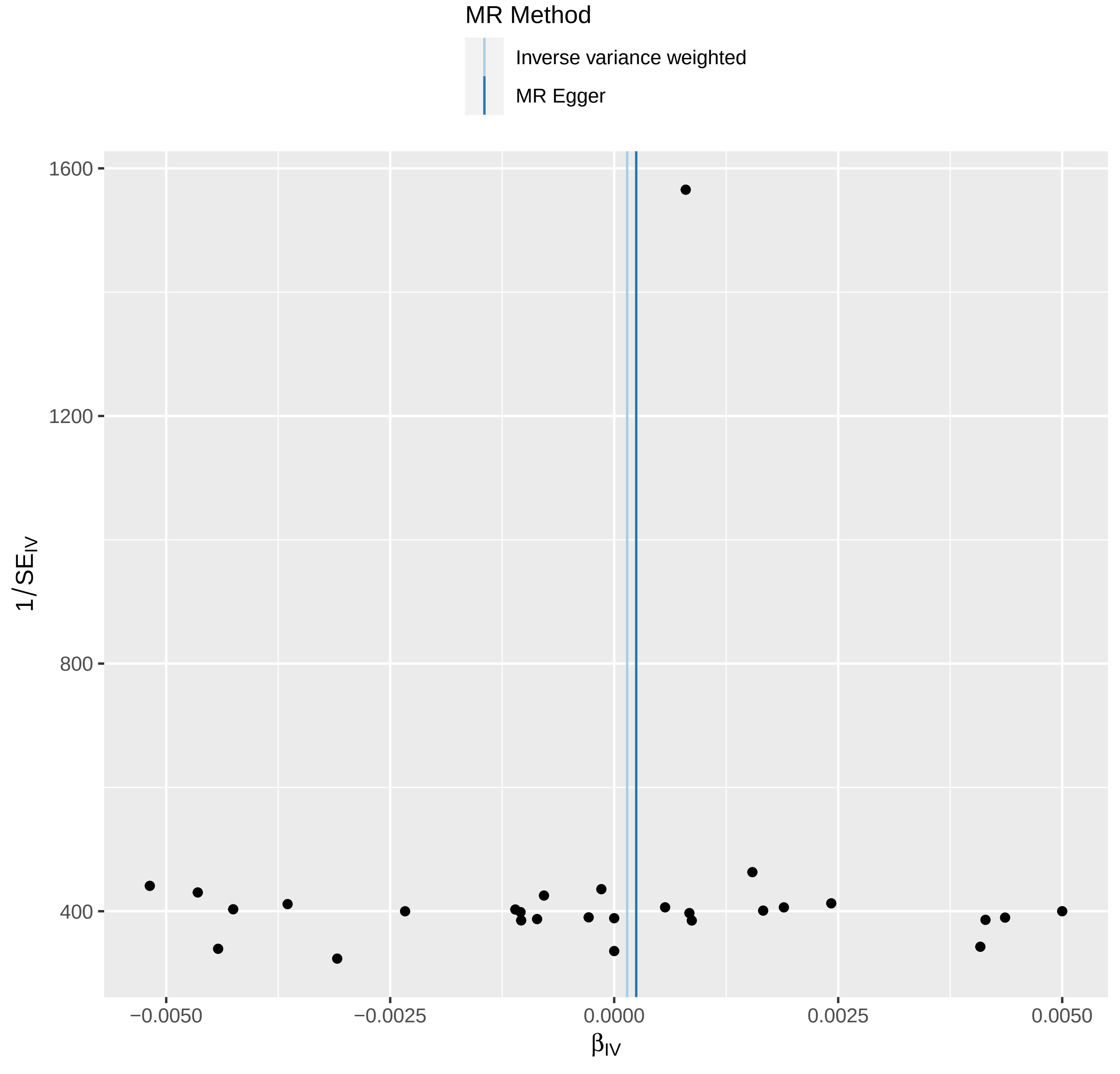

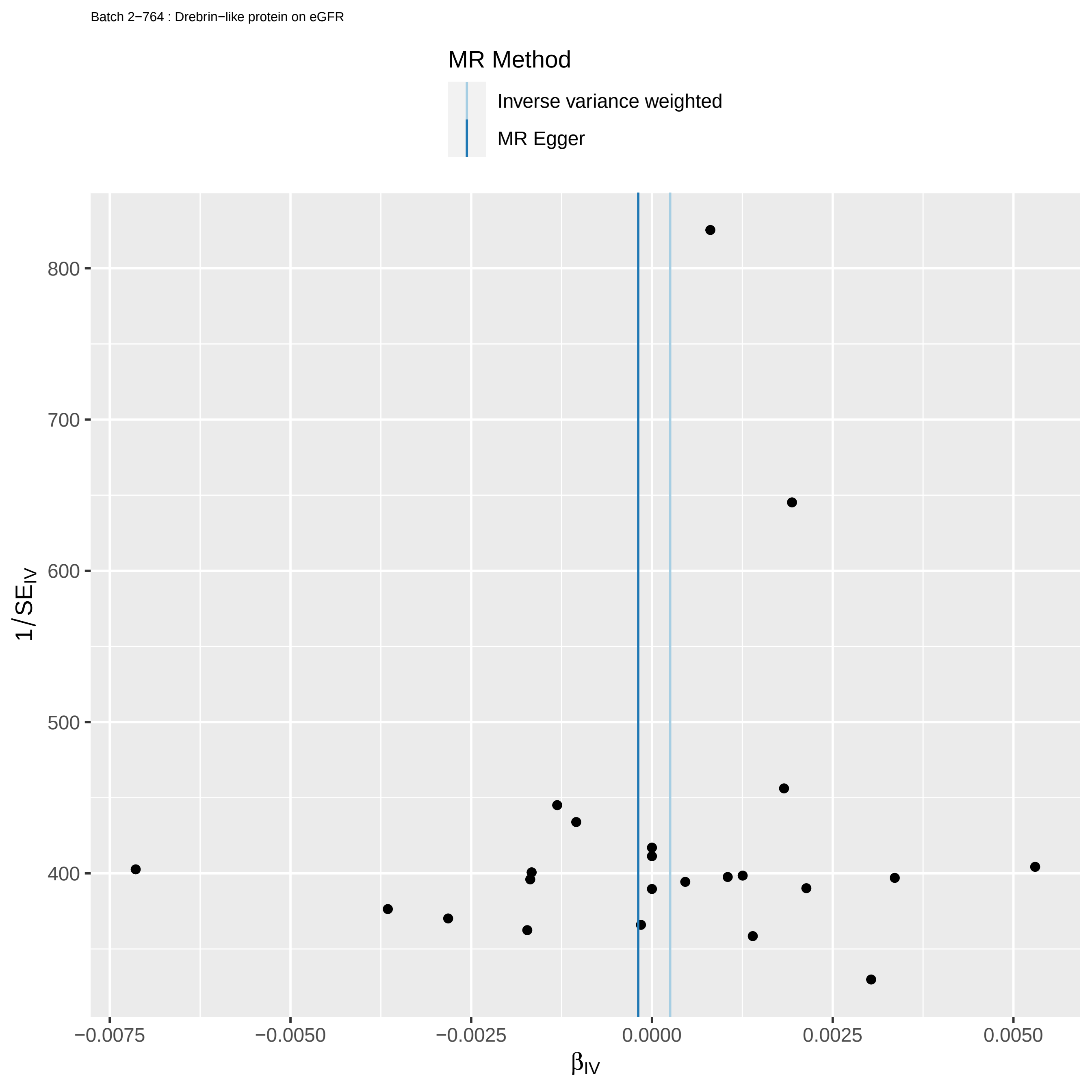

Batch 2−764 : Drebrin−like protein on eGFR
MR Method
Inverse variance weighted
MR Egger
1/SE_IV
800
700
600
500
400
−0.0075
−0.0050
−0.0025
0.0000
0.0025
0.0050
β_IV

Batch 2−765 : DDB1− and CUL4−associated factor 5 on eGFR

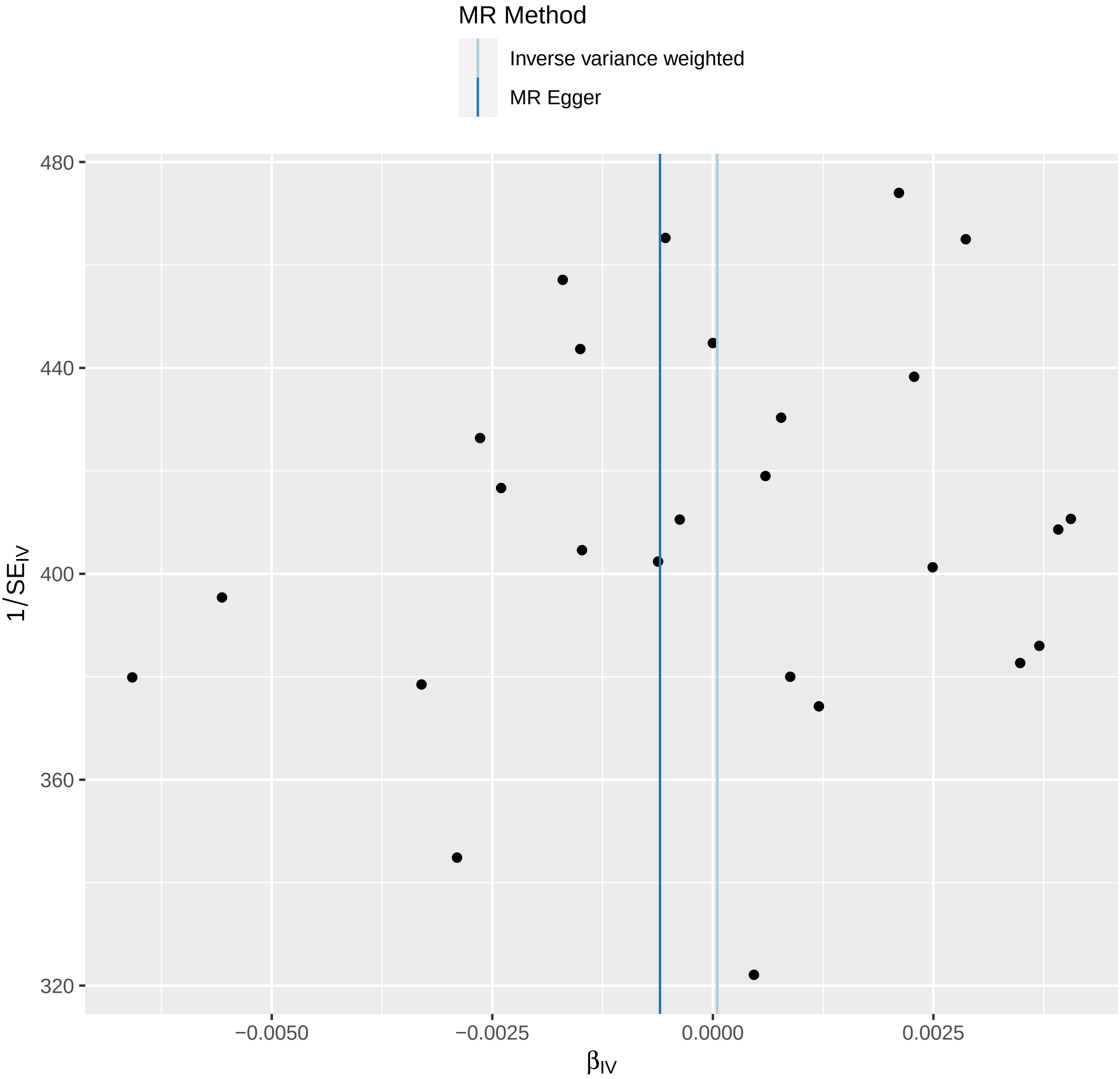

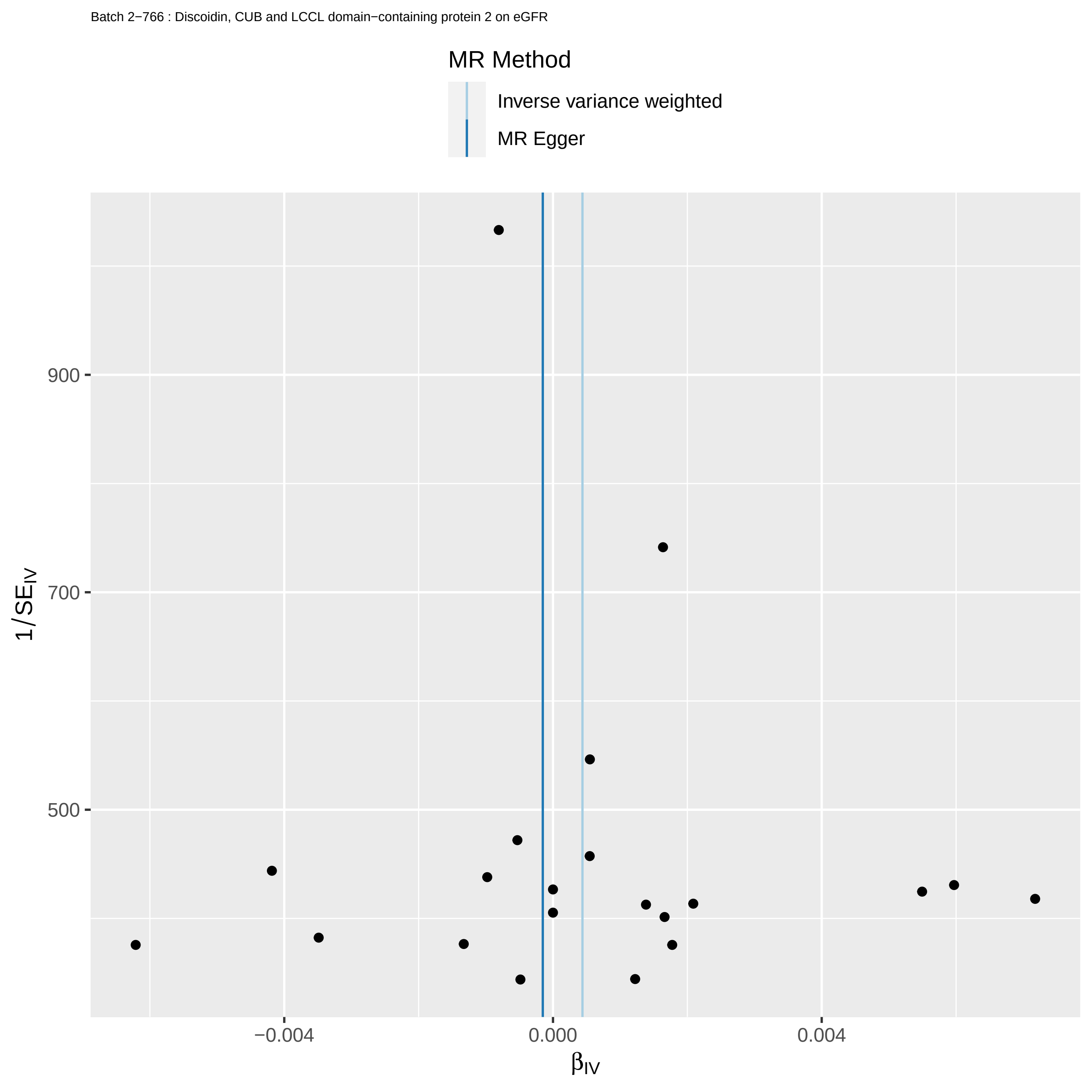

Batch 2−766 : Discoidin, CUB and LCCL domain−containing protein 2 on eGFR
MR Method
Inverse variance weighted
MR Egger
900
700
500
1/SE_IV
−0.004
0.000
0.004
β_IV

Batch 2−767 : Deoxycytidine kinase on eGFR

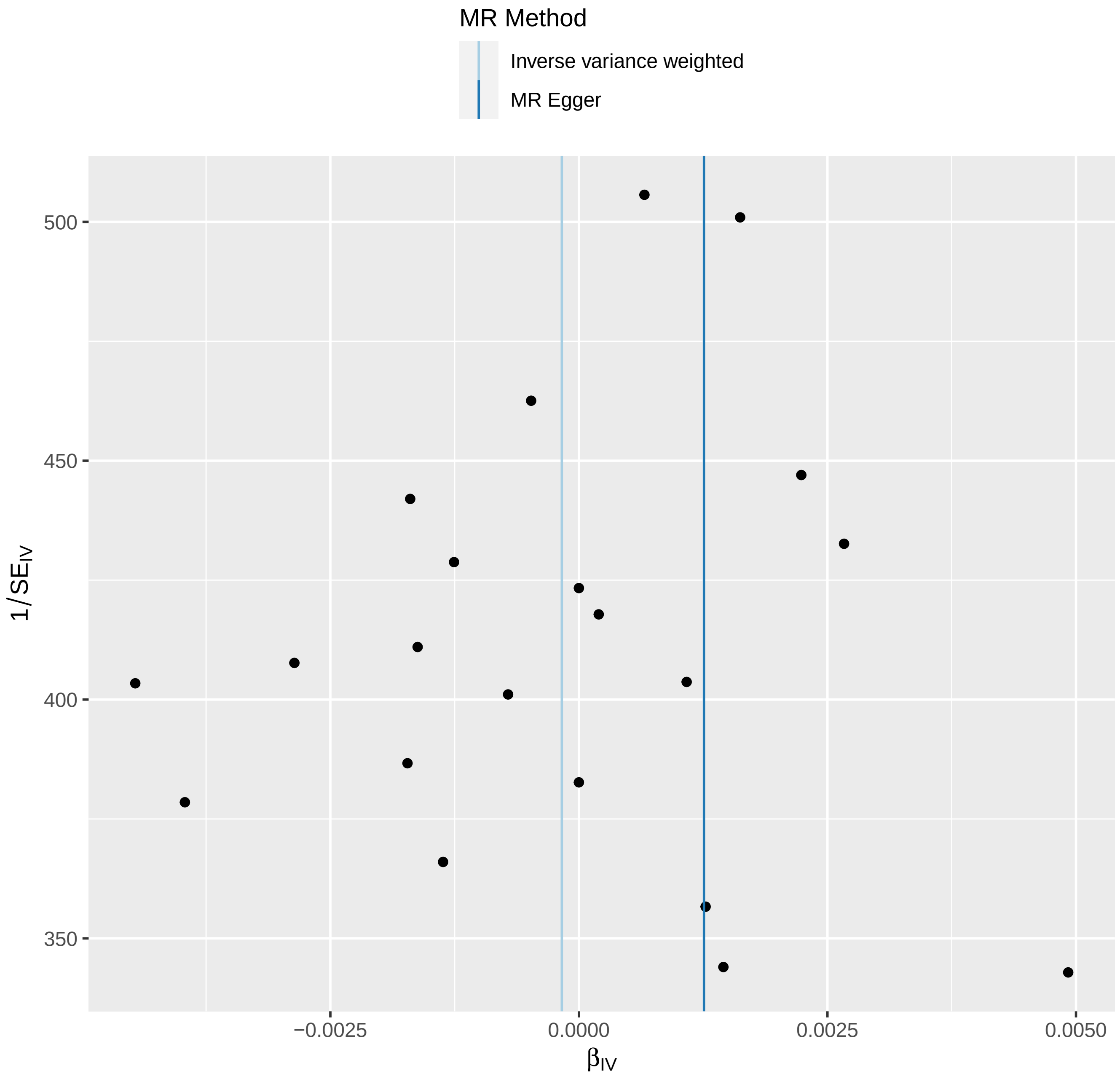

Batch 2−768 : Serine/threonine−protein kinase DCLK3 on eGFR

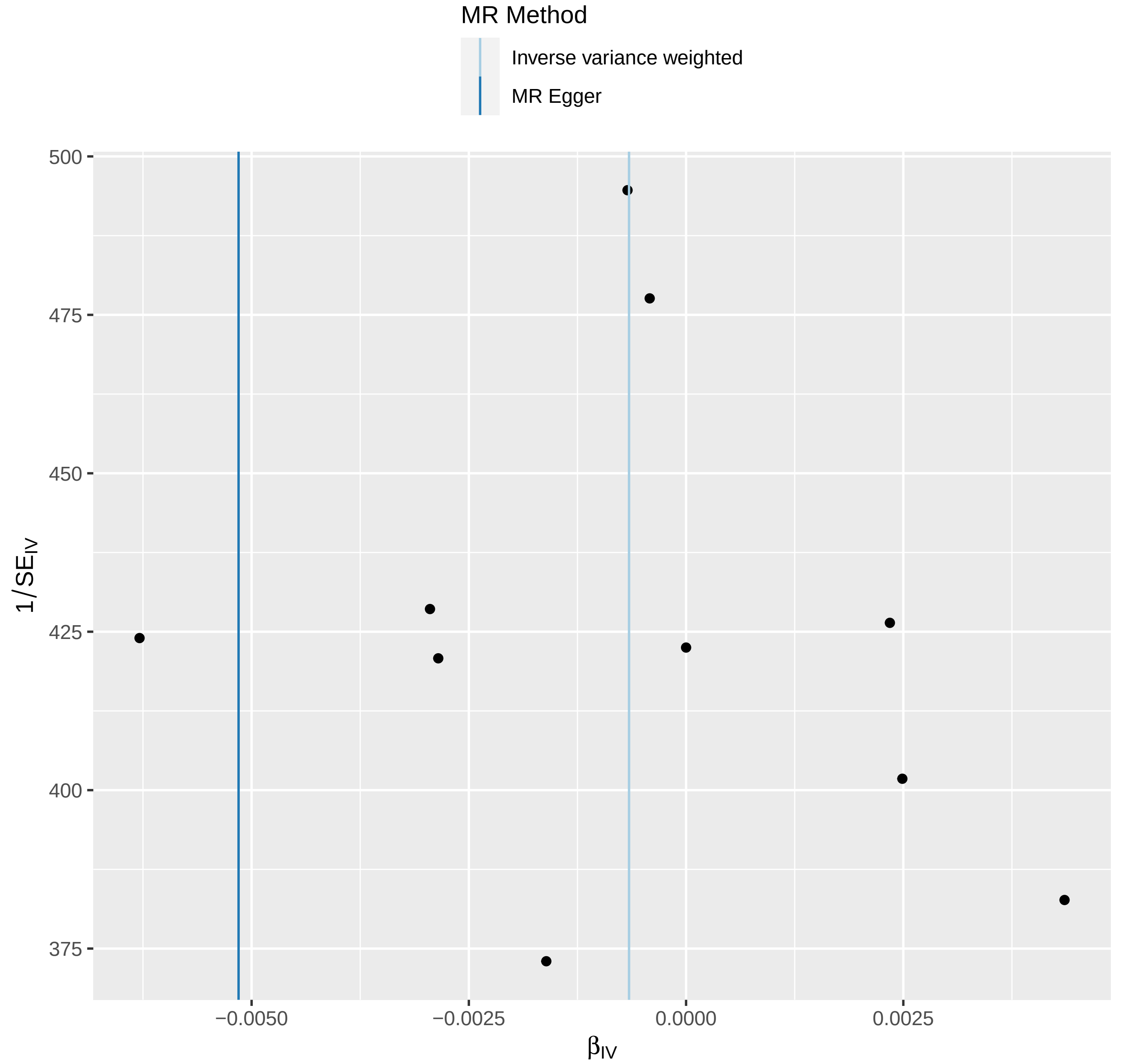

Batch 2−769 : Decorin on eGFR

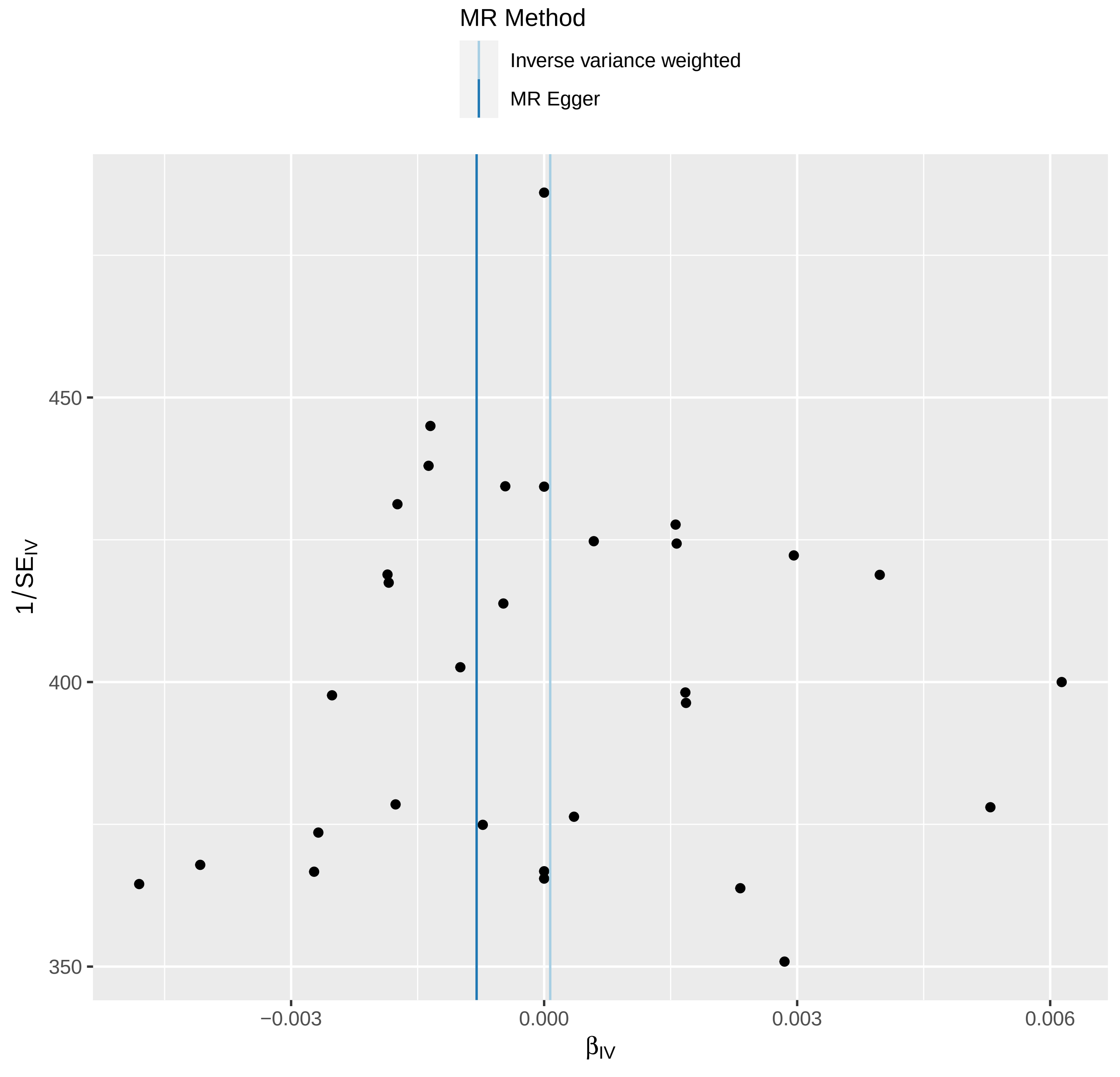

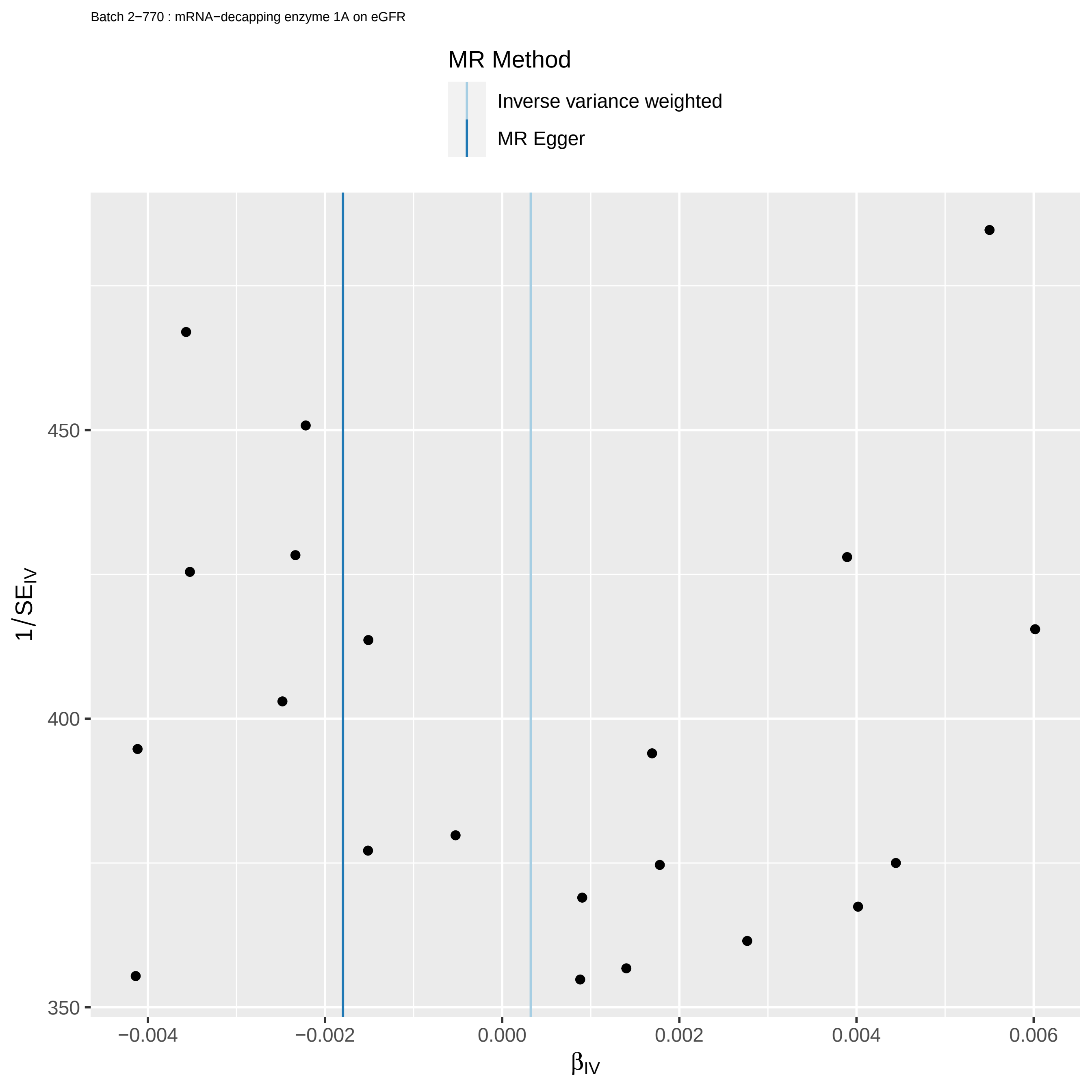
Batch 2−770 : mRNA−decapping enzyme 1A on eGFR
MR Method
Inverse variance weighted
MR Egger
450
400
350
$1/SE_{IV}$
−0.004
−0.002
0.000
0.002
0.004
0.006
β_{IV}

Batch 2−771 : Dynactin subunit 2 on eGFR

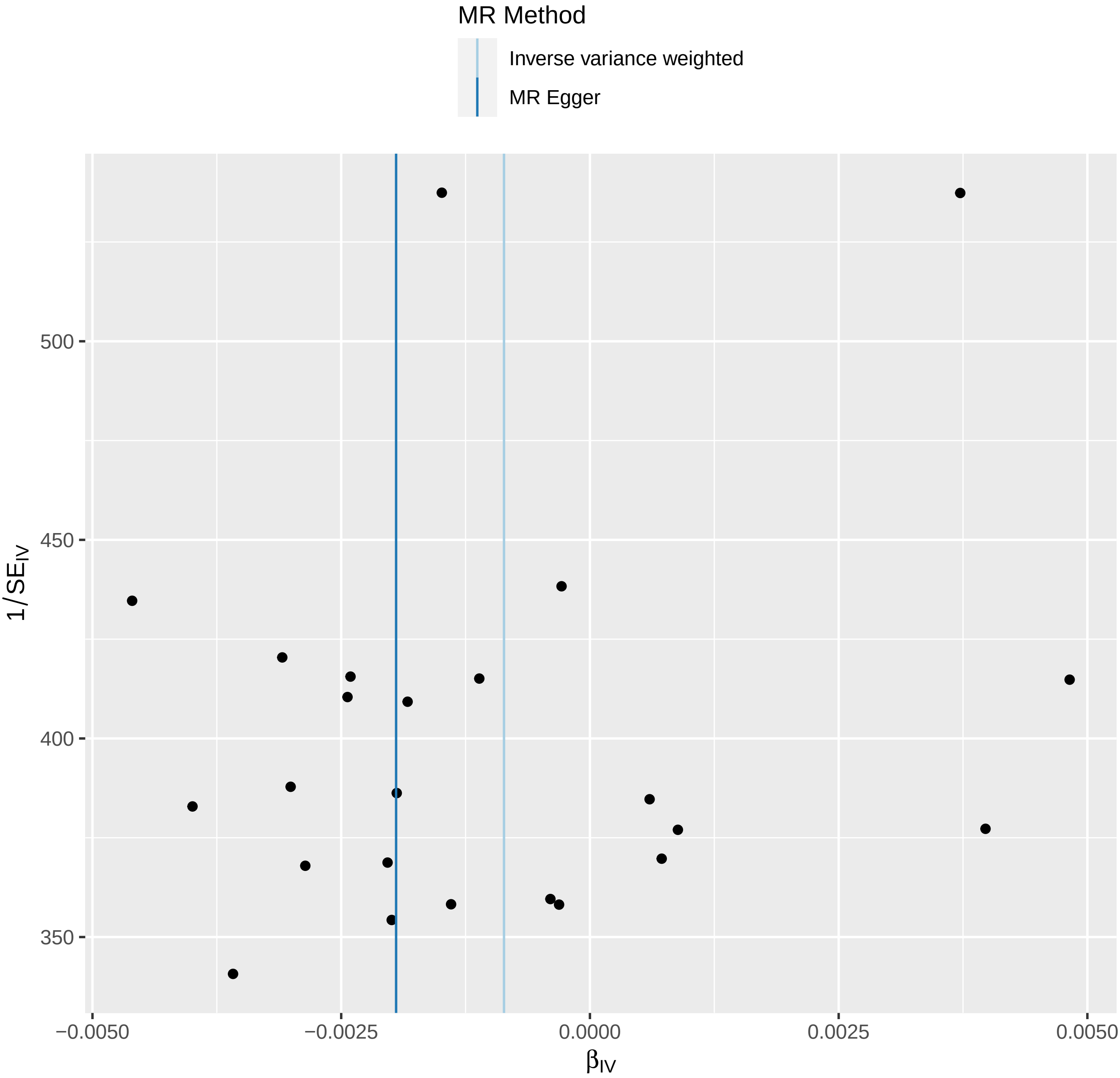

Batch 2−772 : dCTP pyrophosphatase 1 on eGFR

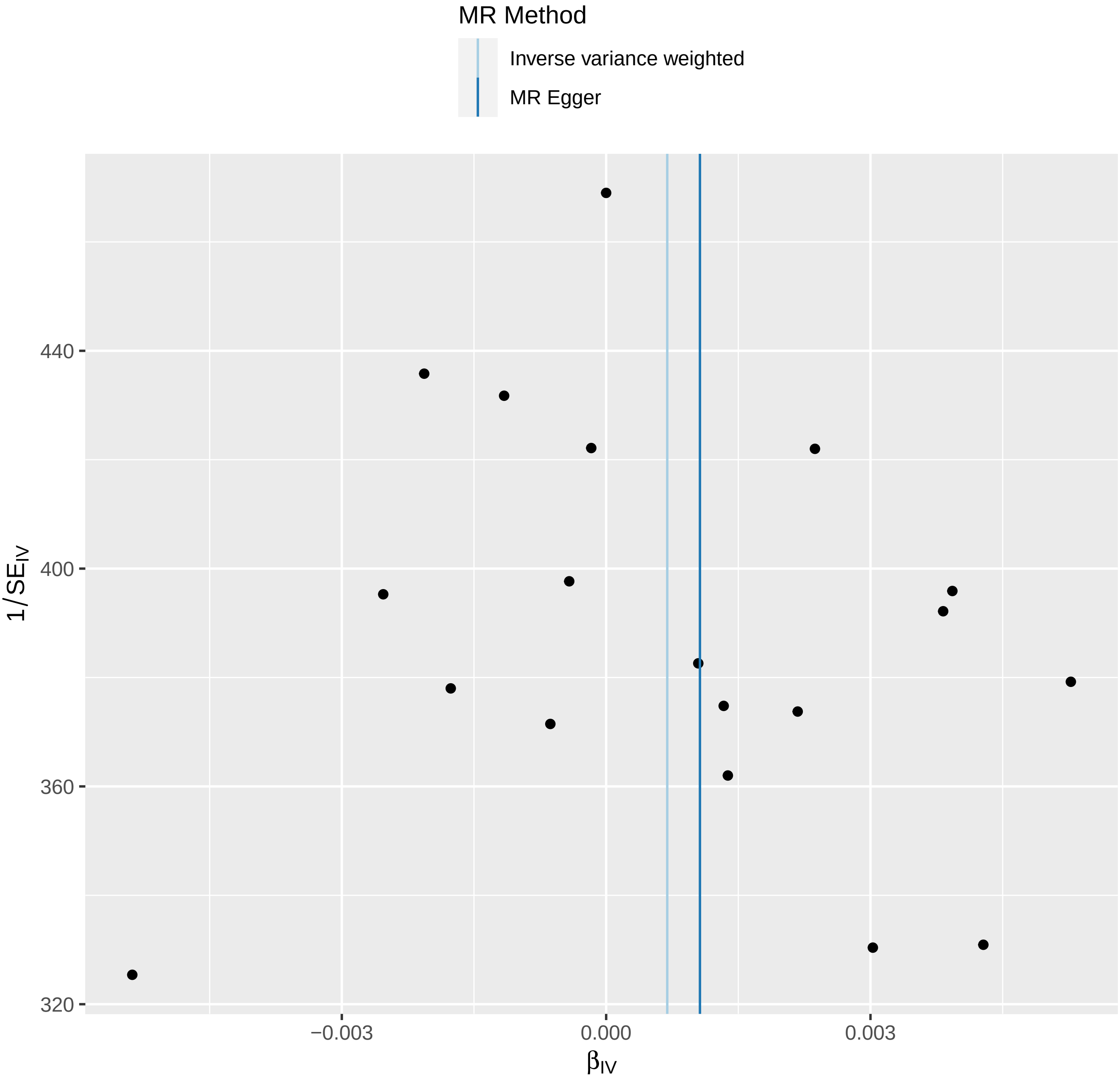

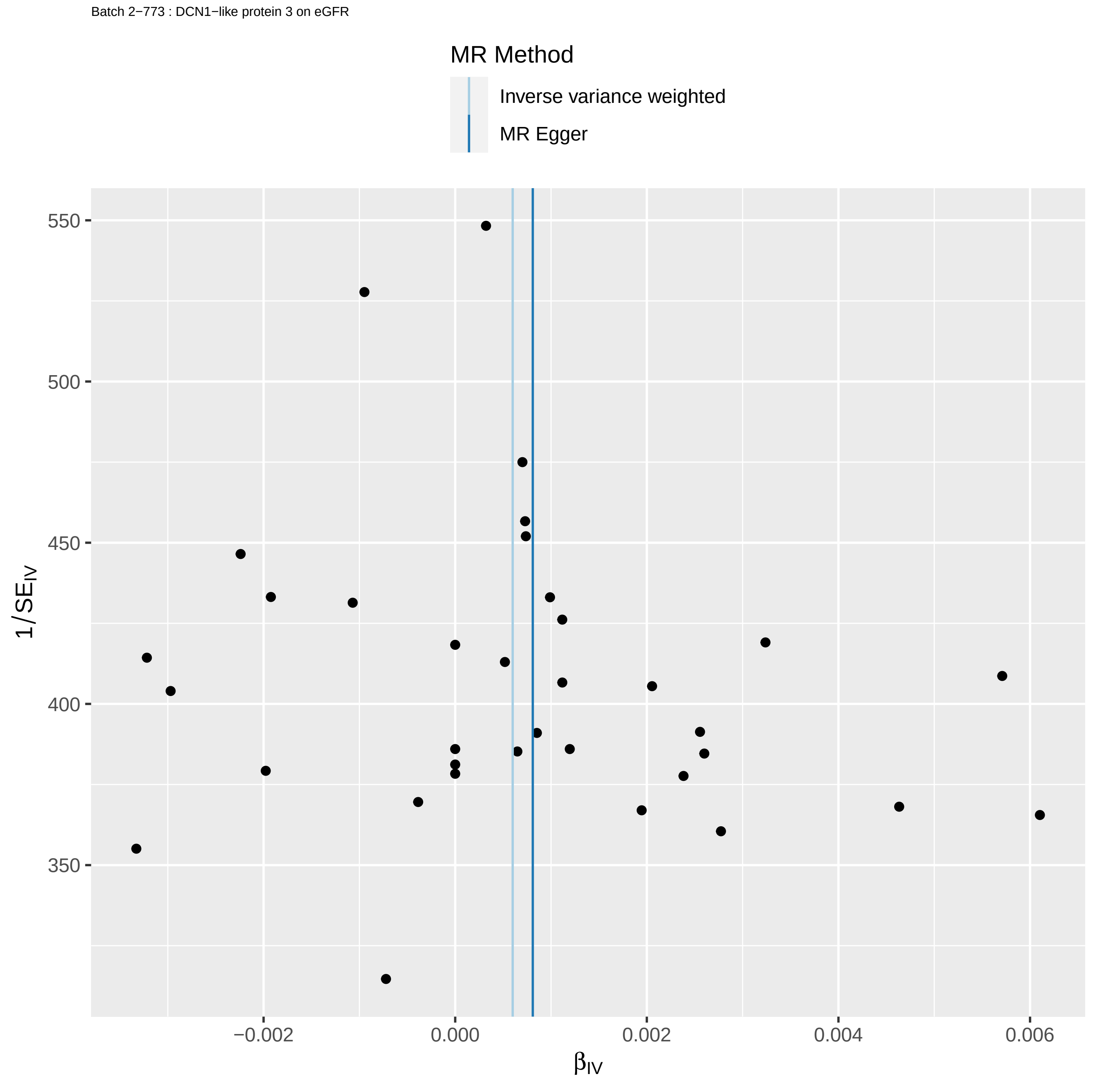

Batch 2−773 : DCN1−like protein 3 on eGFR
MR Method
Inverse variance weighted
MR Egger
550
500
450
400
350
1/SE$_{IV}$
−0.002
0.000
0.002
0.004
0.006
β$_{IV}$

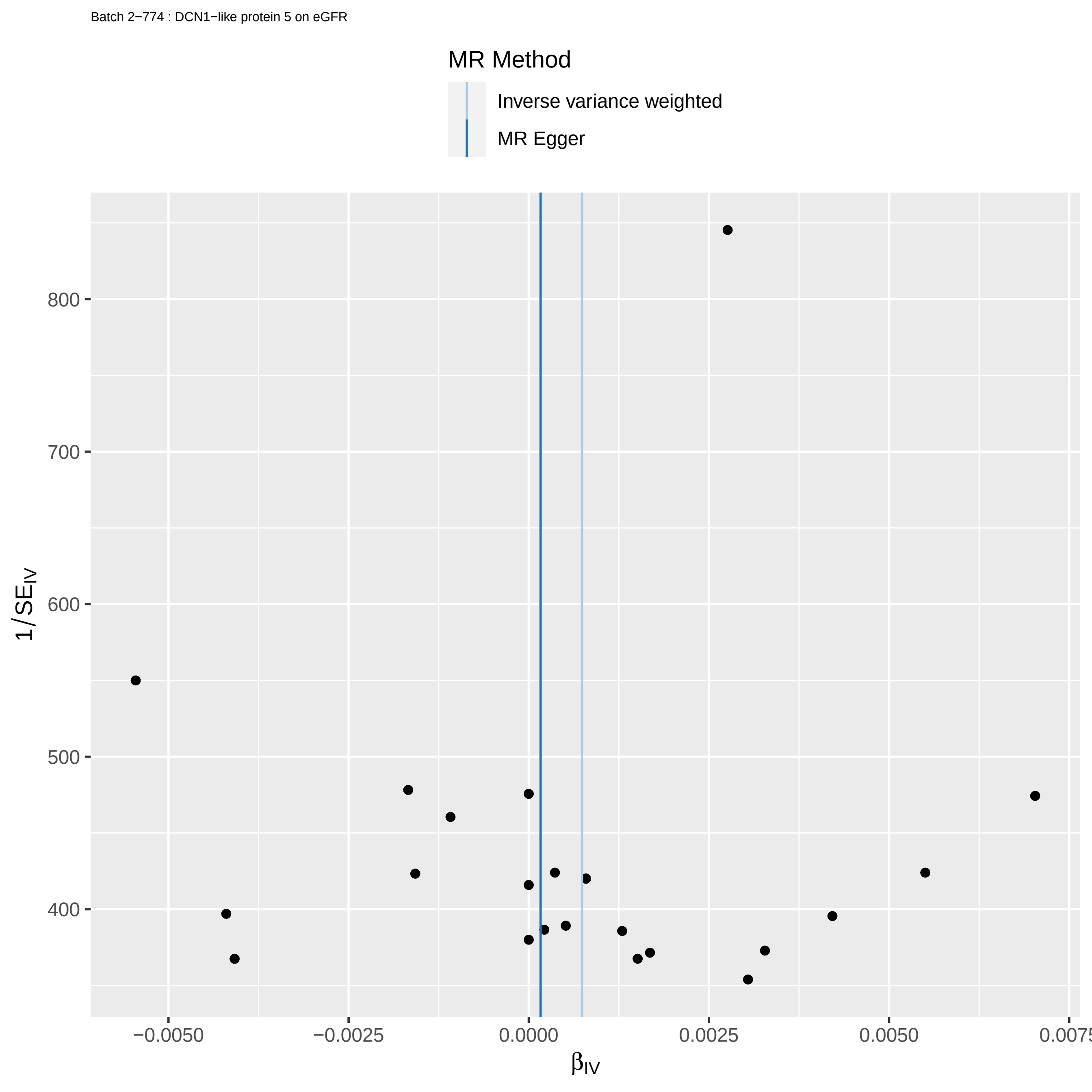
Batch 2−774 : DCN1−like protein 5 on eGFR
MR Method
Inverse variance weighted
MR Egger
800
700
600
500
400
1/SE_IV
−0.0050
−0.0025
0.0000
0.0025
0.0050
0.0075
β_IV

Batch 2−775 : Aromatic−L−amino−acid decarboxylase on eGFR

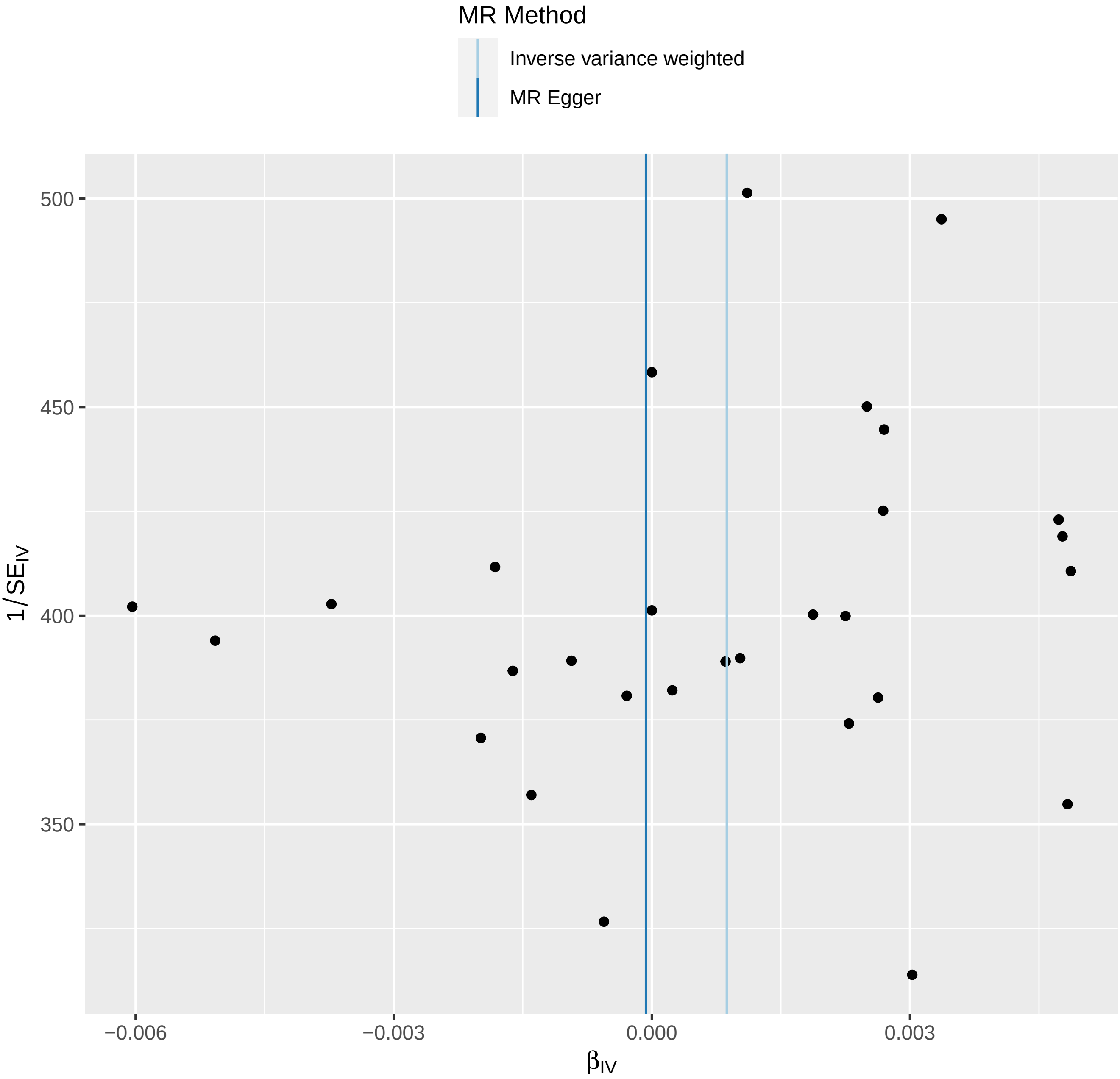

Batch 2−776 : Discoidin domain−containing receptor 2 on eGFR

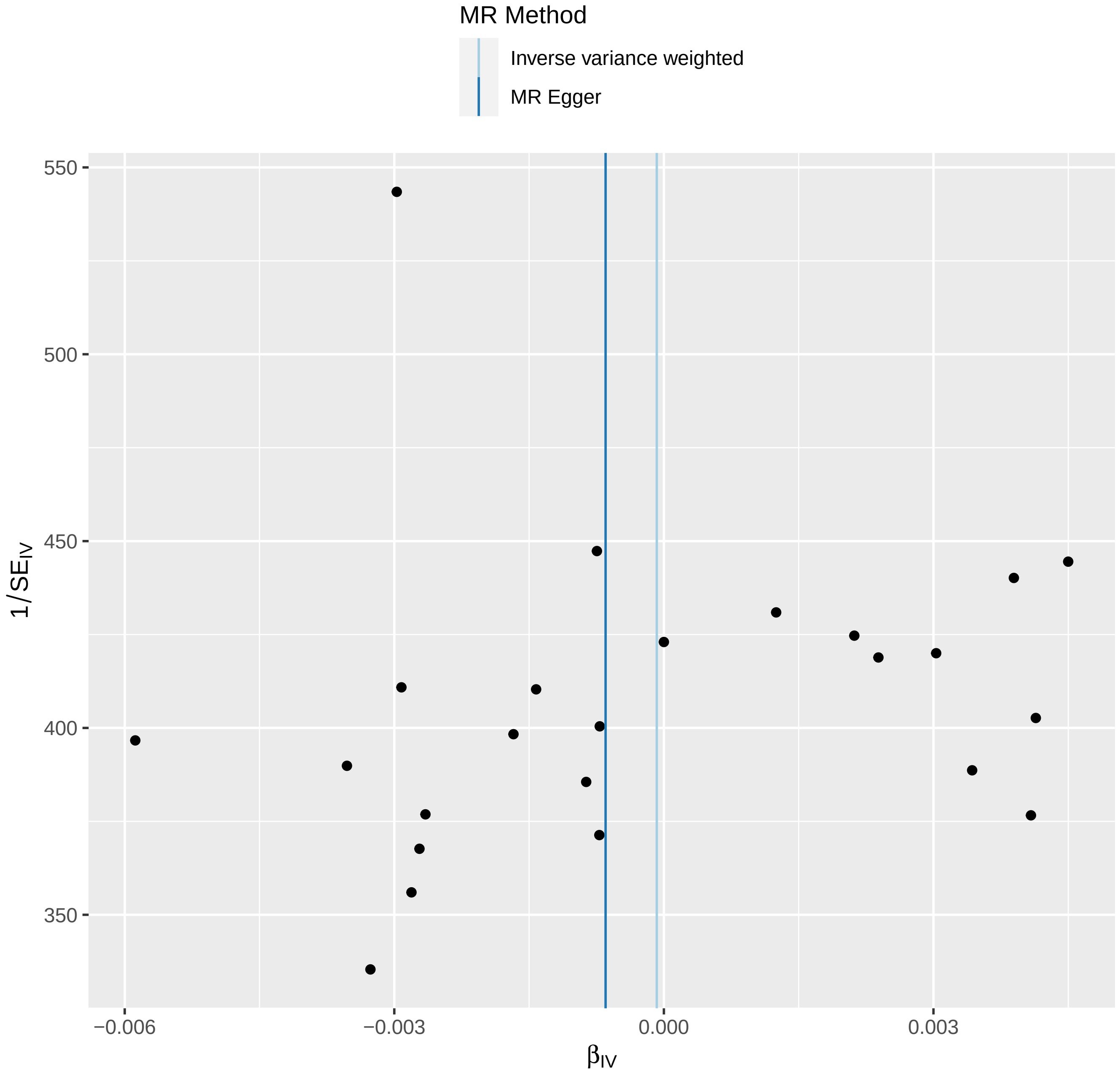

Batch 2−777 : ATP−dependent RNA helicase DDX25 on eGFR

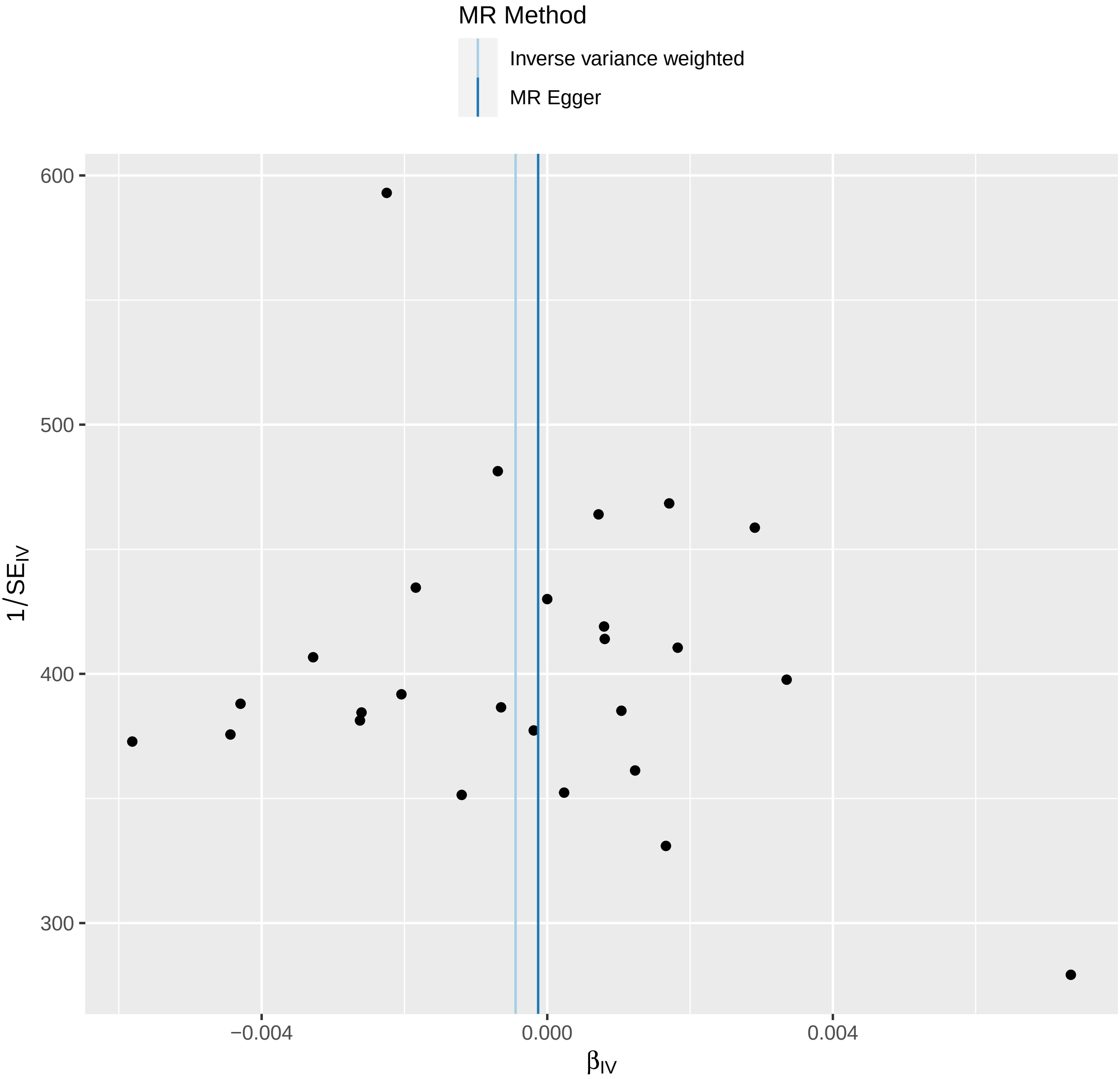

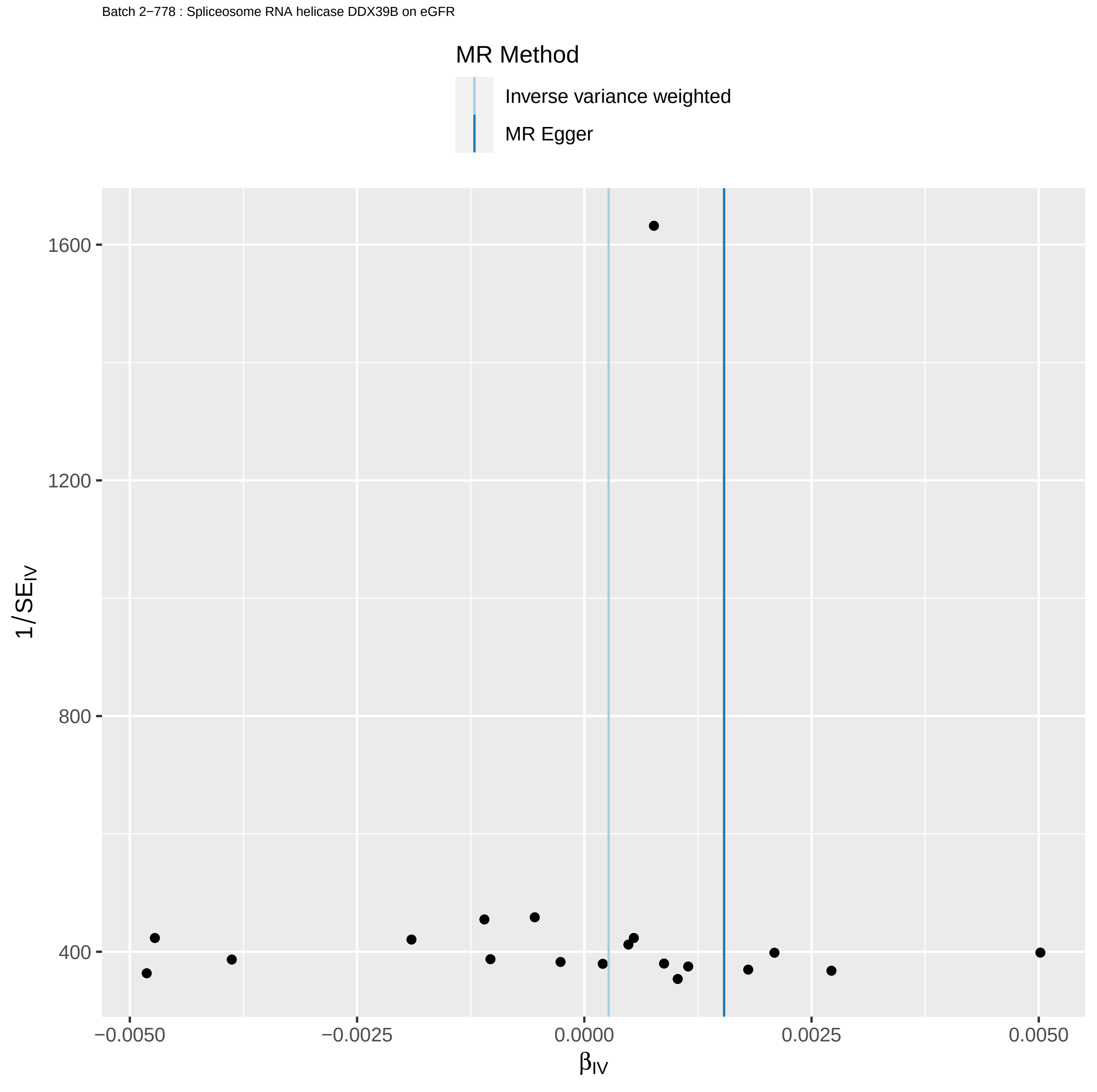
Batch 2−778 : Spliceosome RNA helicase DDX39B on eGFR
MR Method
Inverse variance weighted
MR Egger
1600
1200
800
400
1/SE$_{IV}$
−0.0050
−0.0025
0.0000
0.0025
0.0050
β$_{IV}$

Batch 2−779 : Probable ATP−dependent RNA helicase DDX58 on eGFR

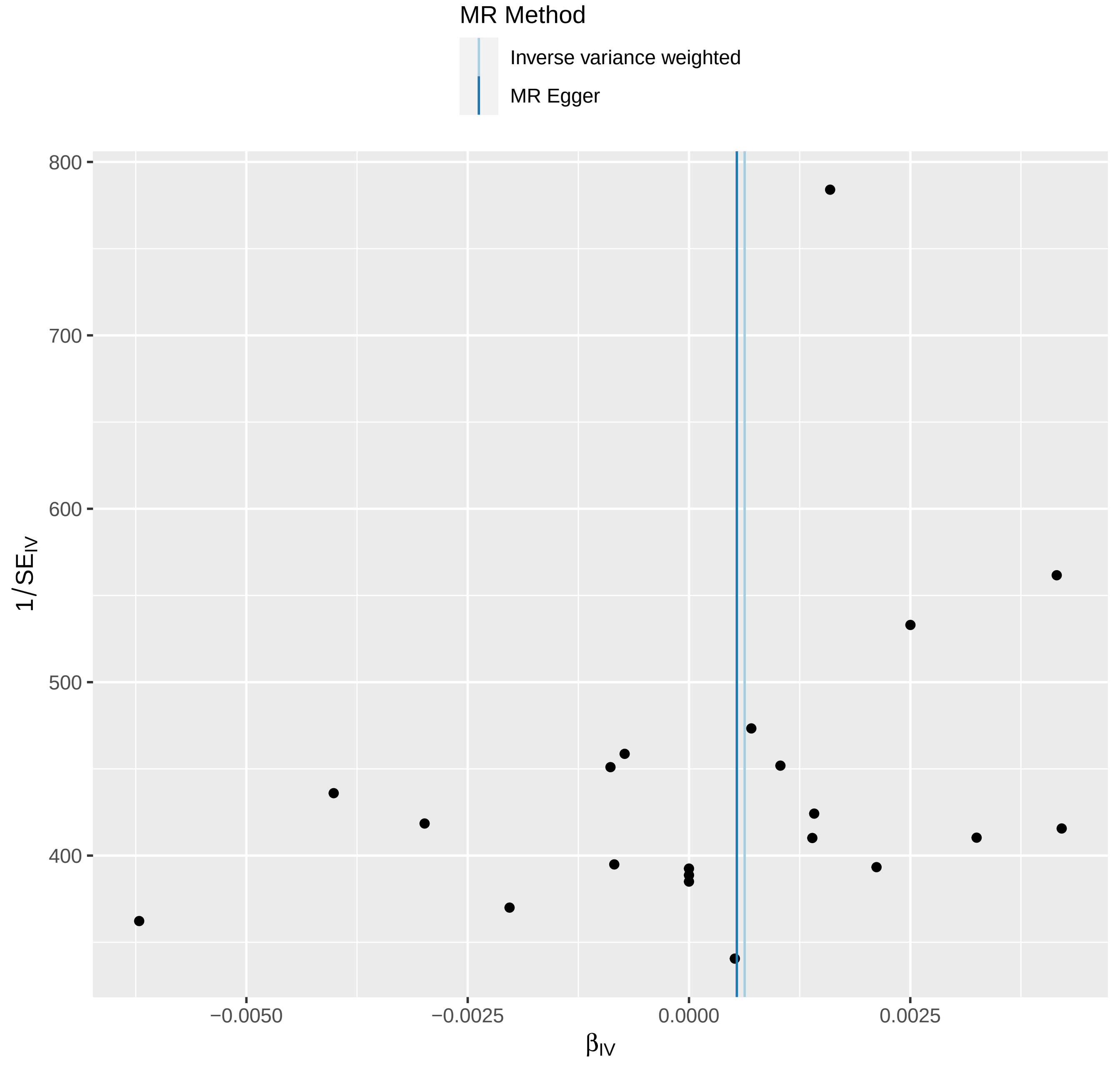

Batch 2−780 : Deformed epidermal autoregulatory factor 1 homolog on eGFR

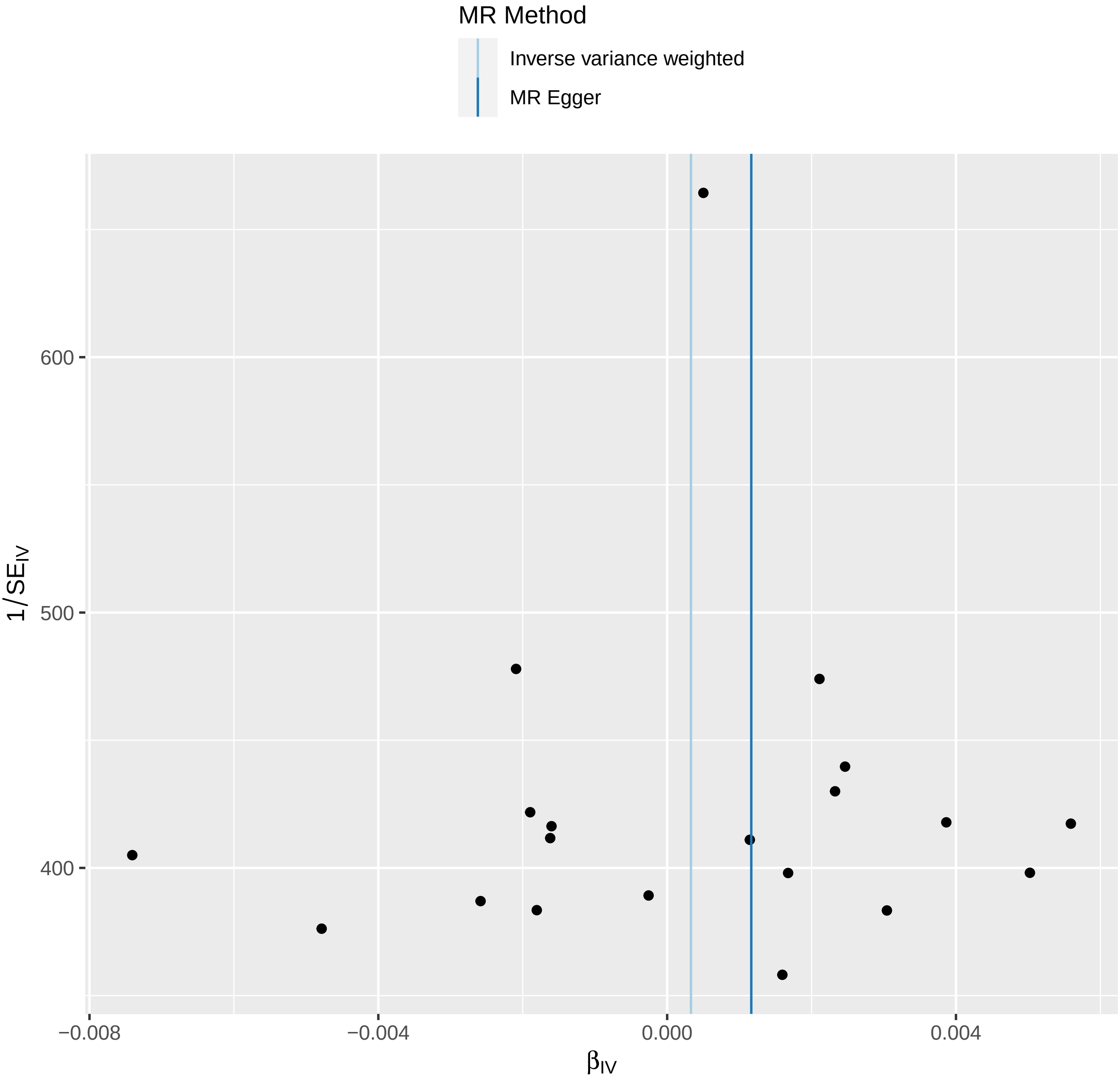

Batch 2−781 : Beta−defensin 103 on eGFR

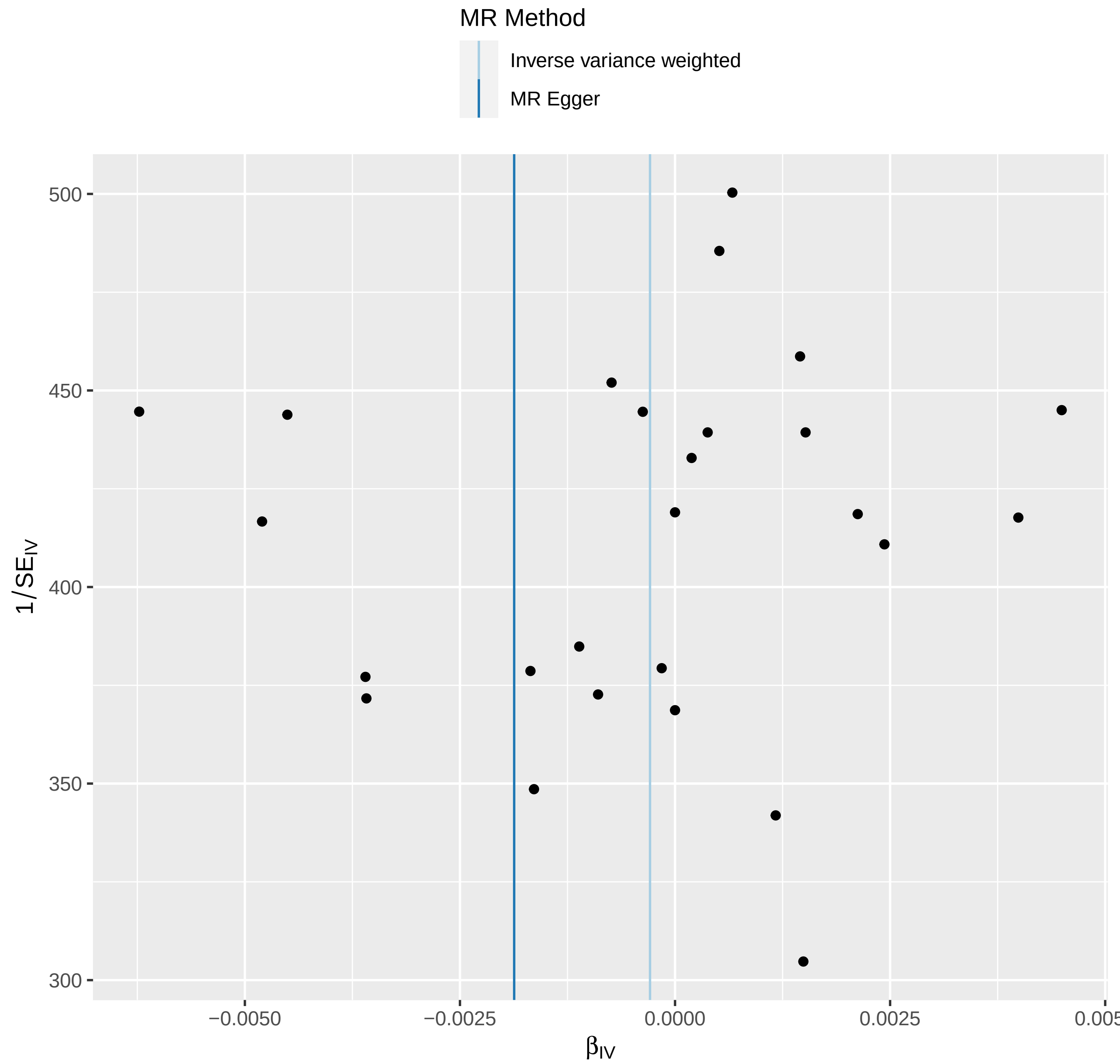

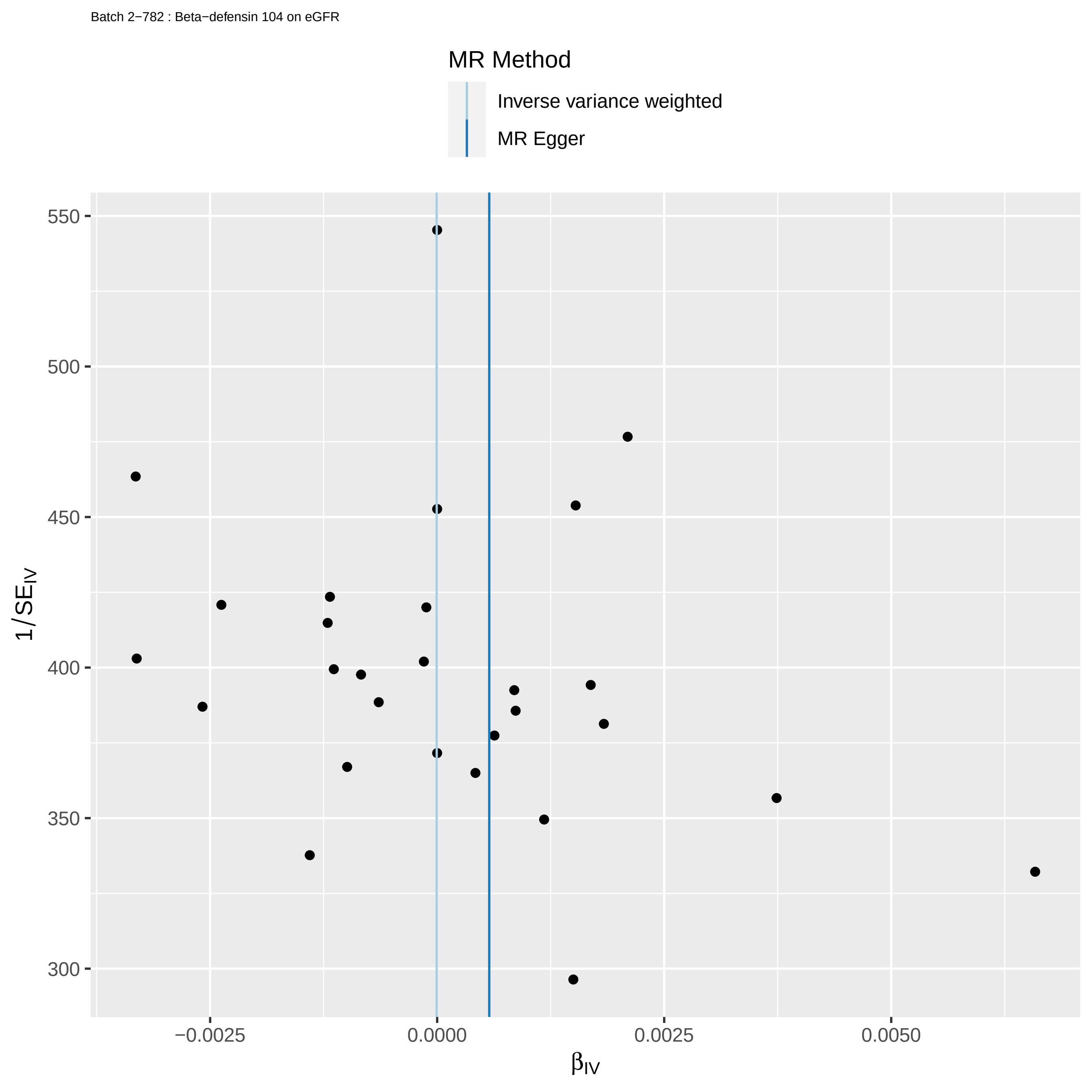

Batch 2−782 : Beta−defensin 104 on eGFR
MR Method
Inverse variance weighted
MR Egger
550
500
450
400
350
300
1/SE_IV
−0.0025
0.0000
0.0025
0.0050
β_IV

Batch 2−783 : Beta−defensin 105 on eGFR

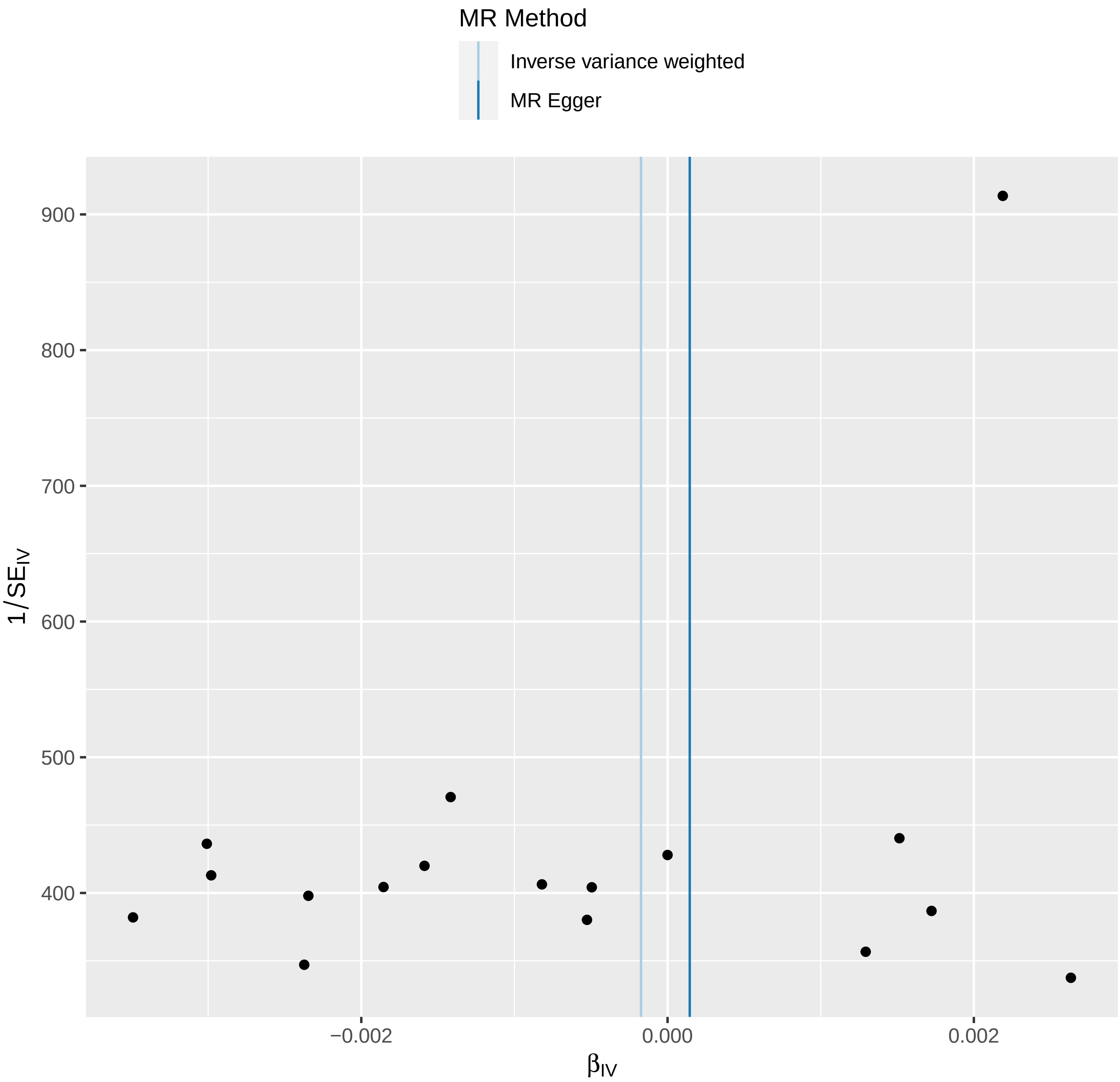

Batch 2−784 : Beta−defensin 106 on eGFR

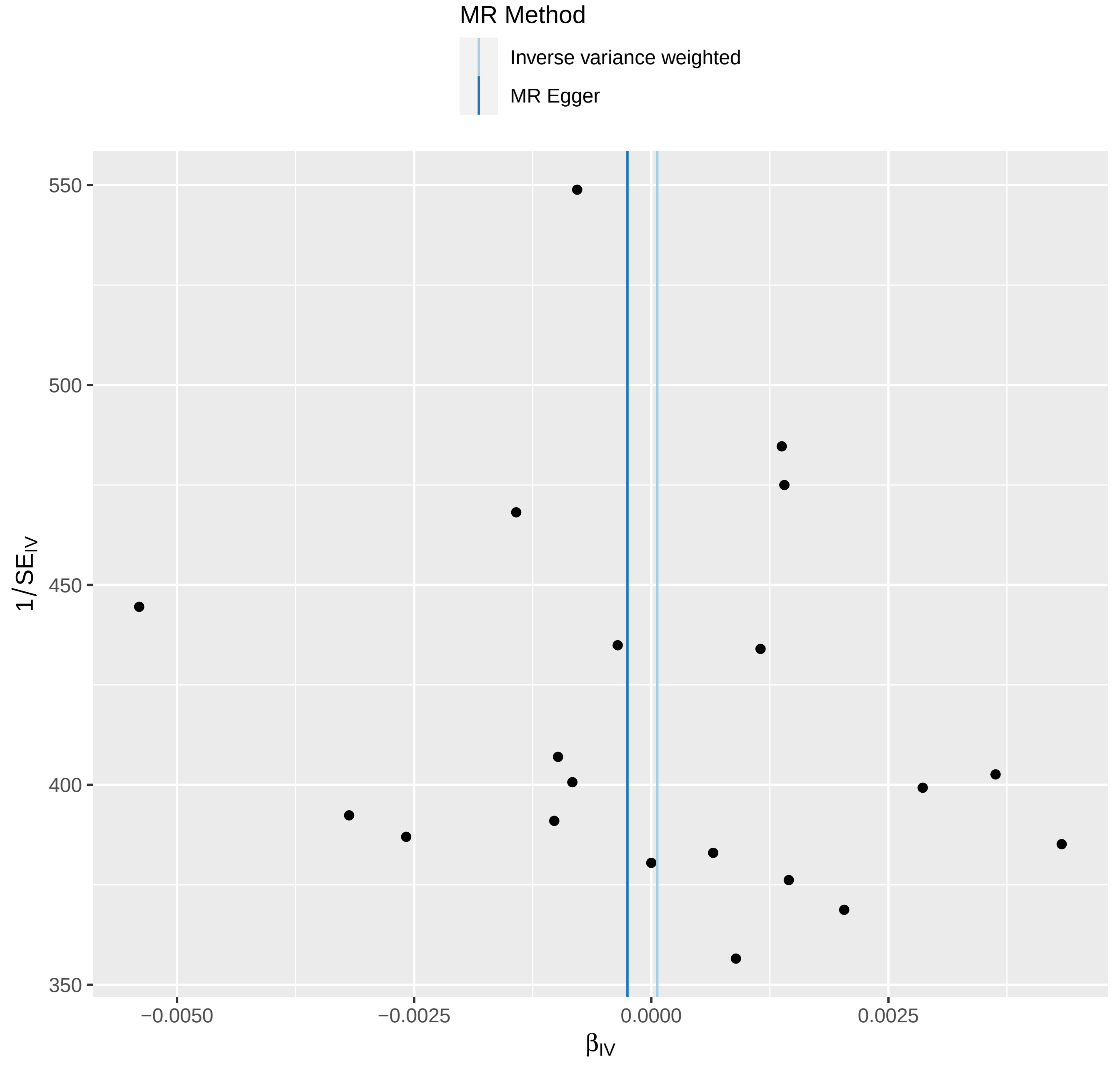

Batch 2−785 : Beta−defensin 107 on eGFR

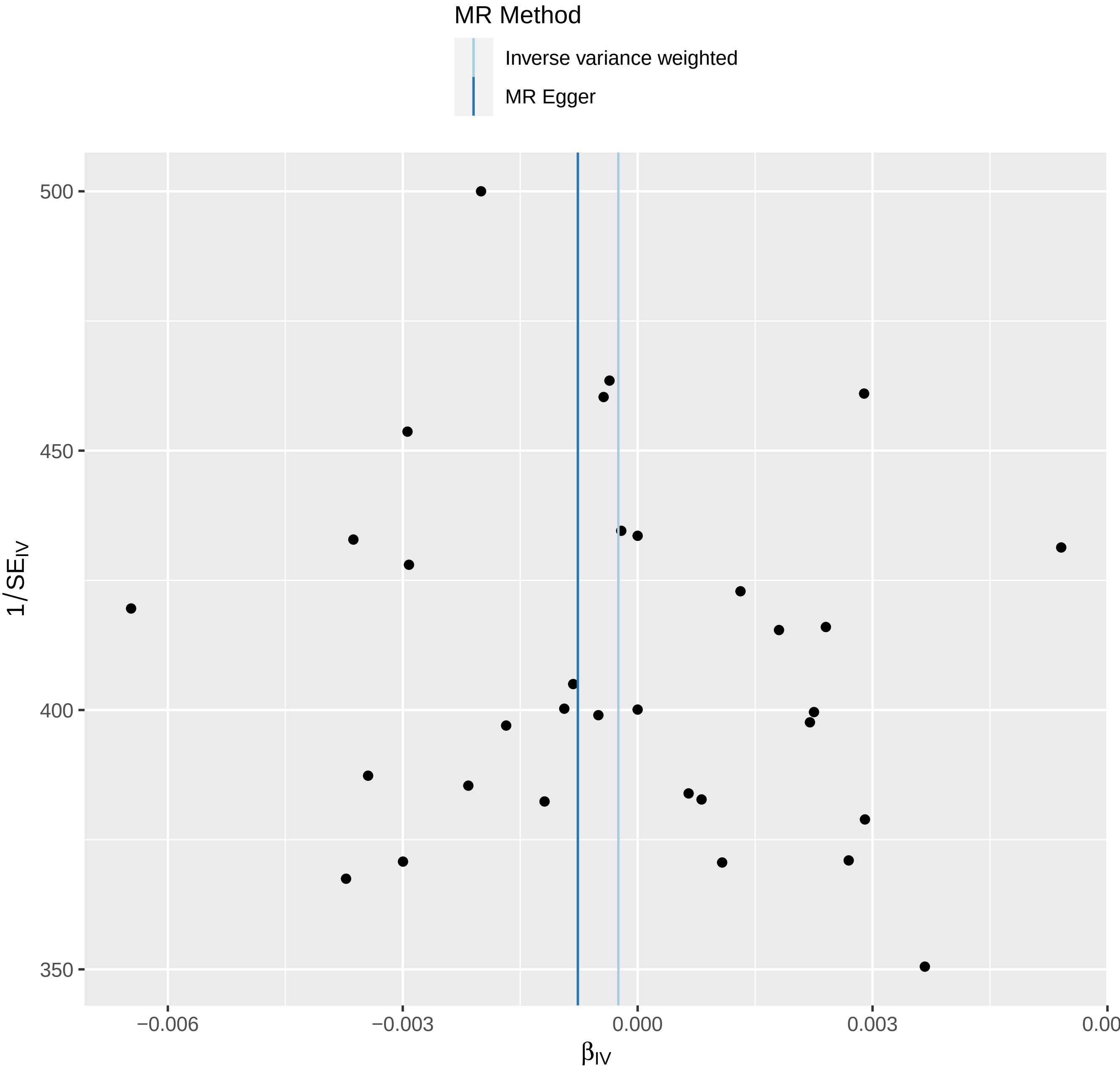

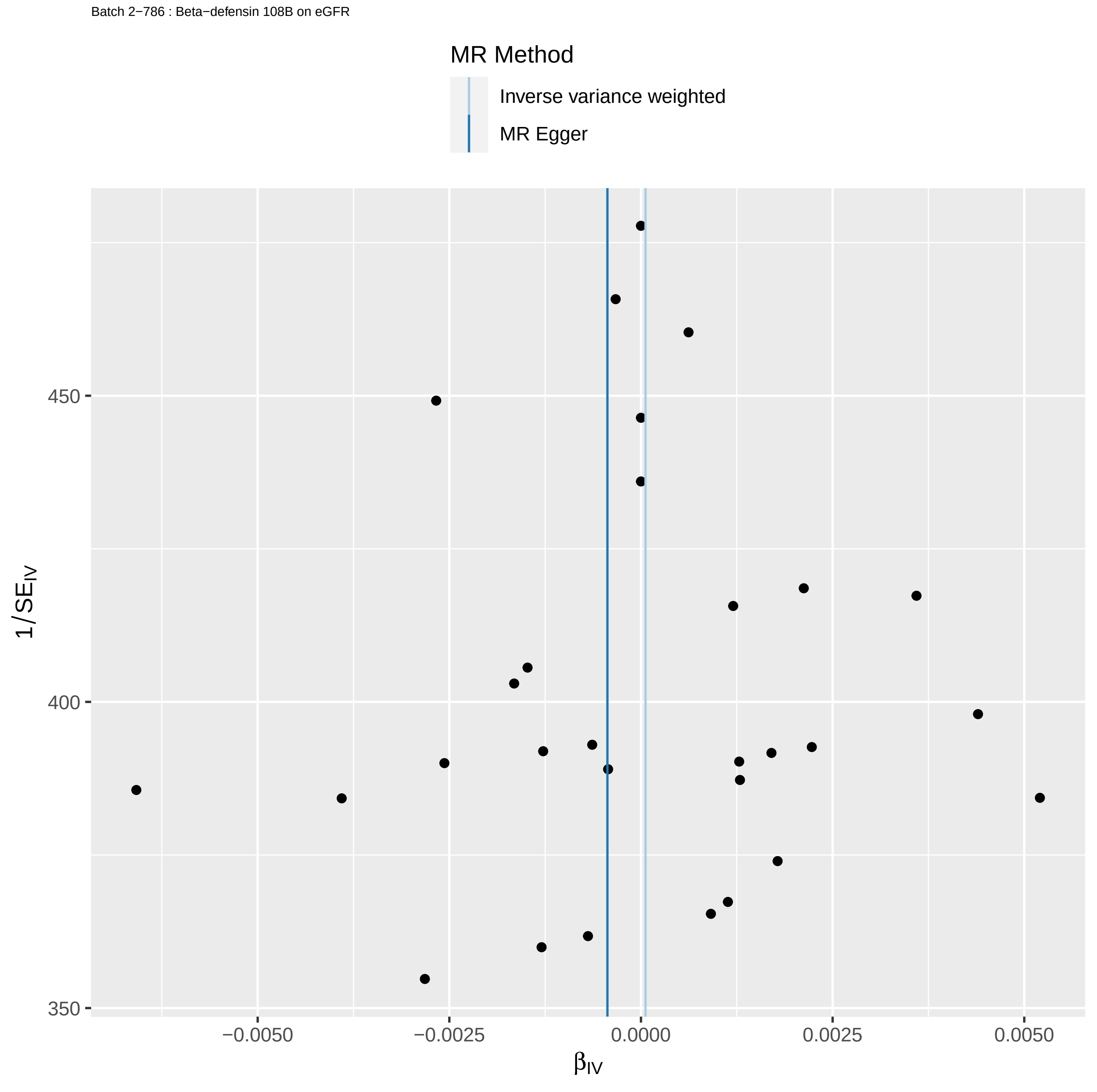
Batch 2−786 : Beta−defensin 108B on eGFR
MR Method
Inverse variance weighted
MR Egger
450
400
350
1/SE_IV
−0.0050
−0.0025
0.0000
0.0025
0.0050
β_IV

Batch 2−787 : Beta−defensin 110 on eGFR

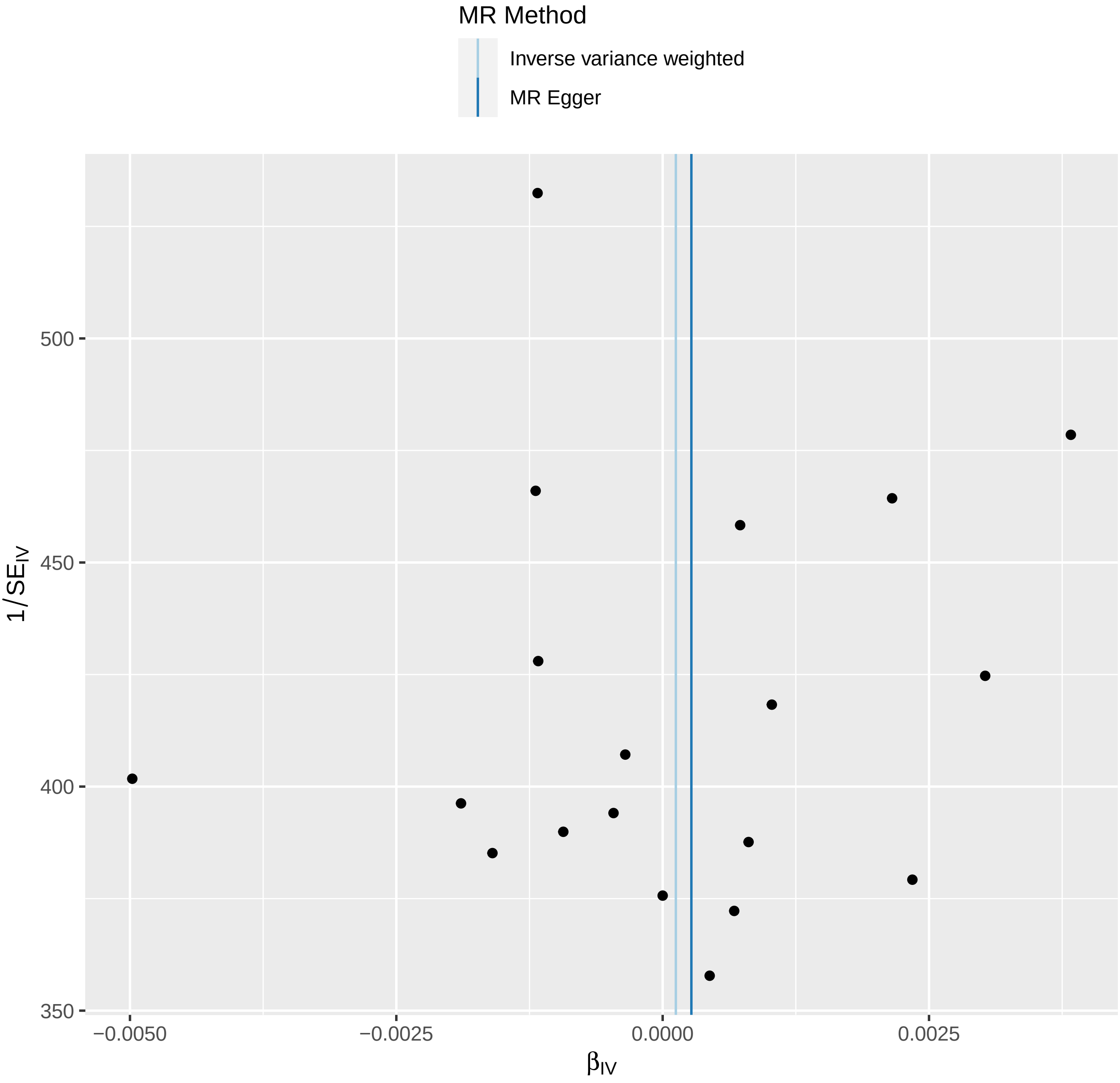

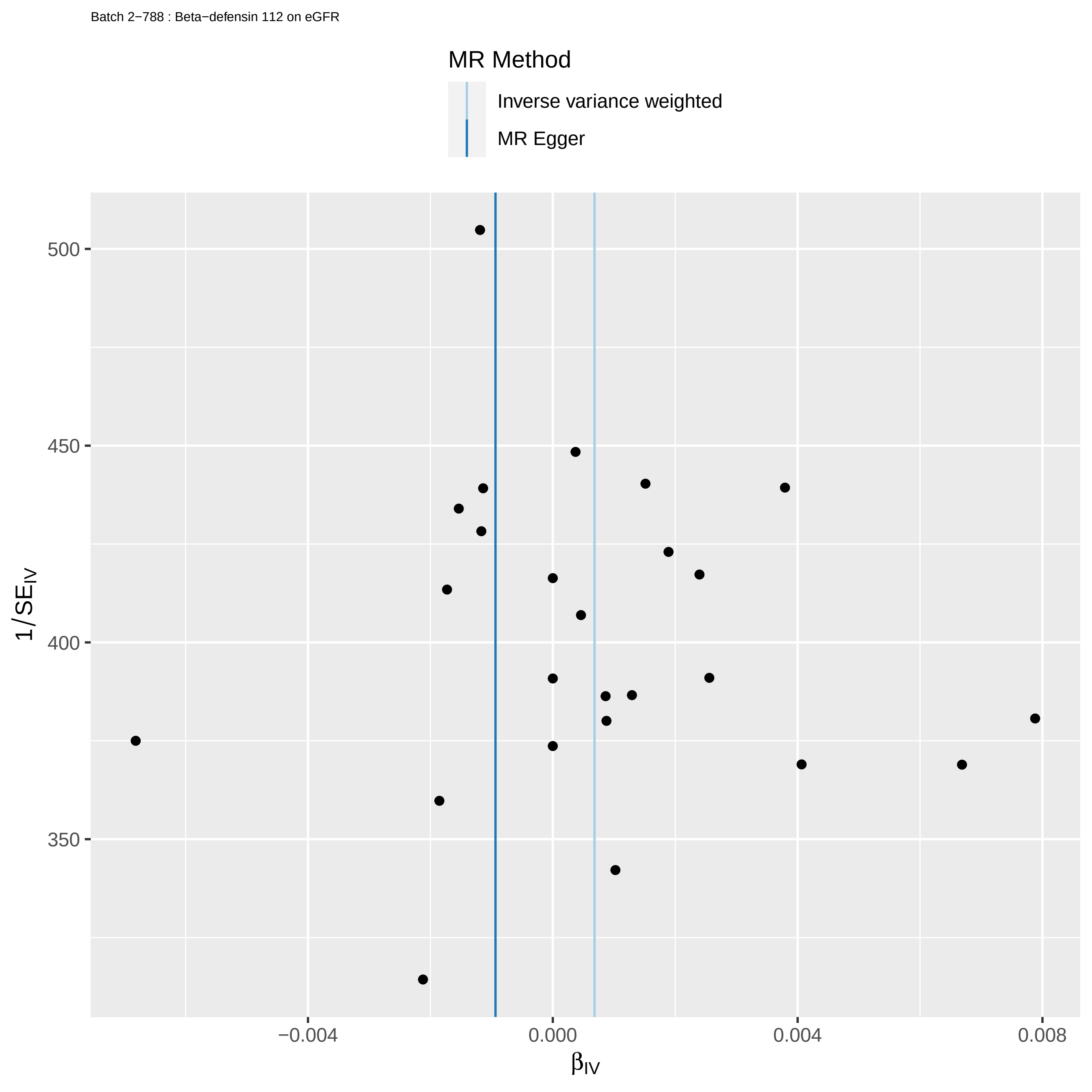

Batch 2−788 : Beta−defensin 112 on eGFR
MR Method
Inverse variance weighted
MR Egger
500
450
400
350
1/SE_IV
−0.004
0.000
0.004
0.008
β_IV

Batch 2−789 : Beta−defensin 113 on eGFR

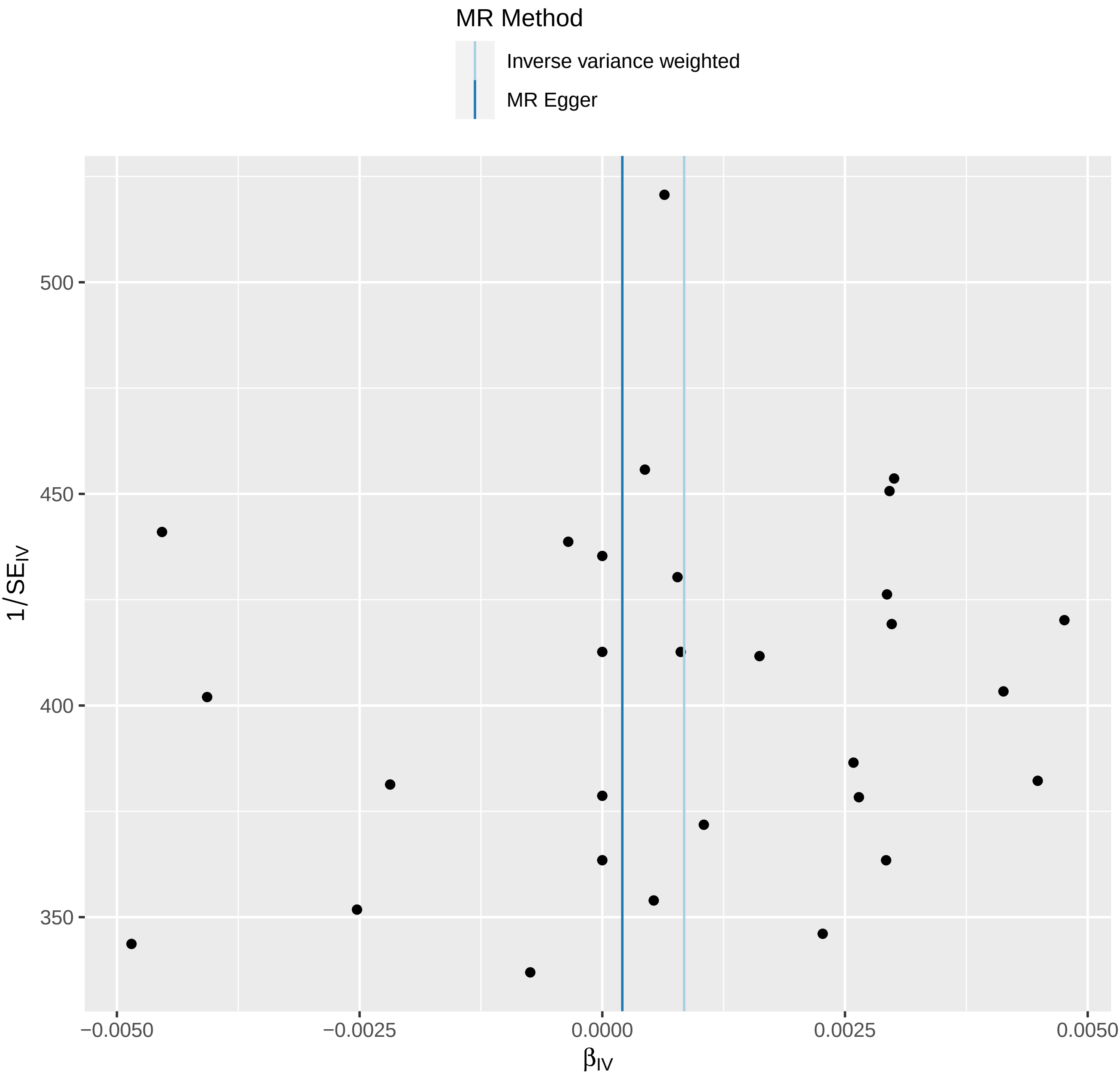

Batch 2−790 : Beta−defensin 115 on eGFR

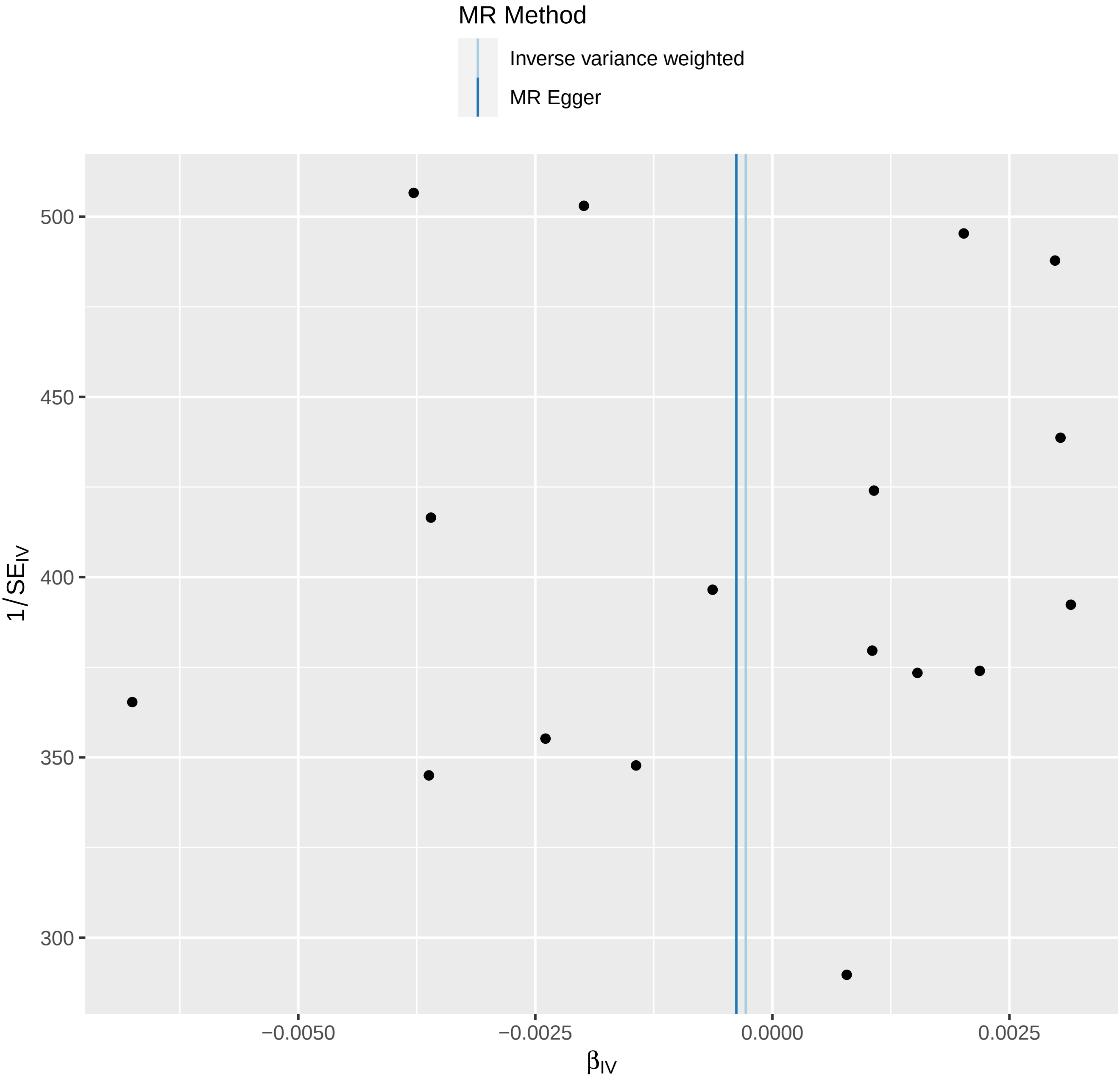

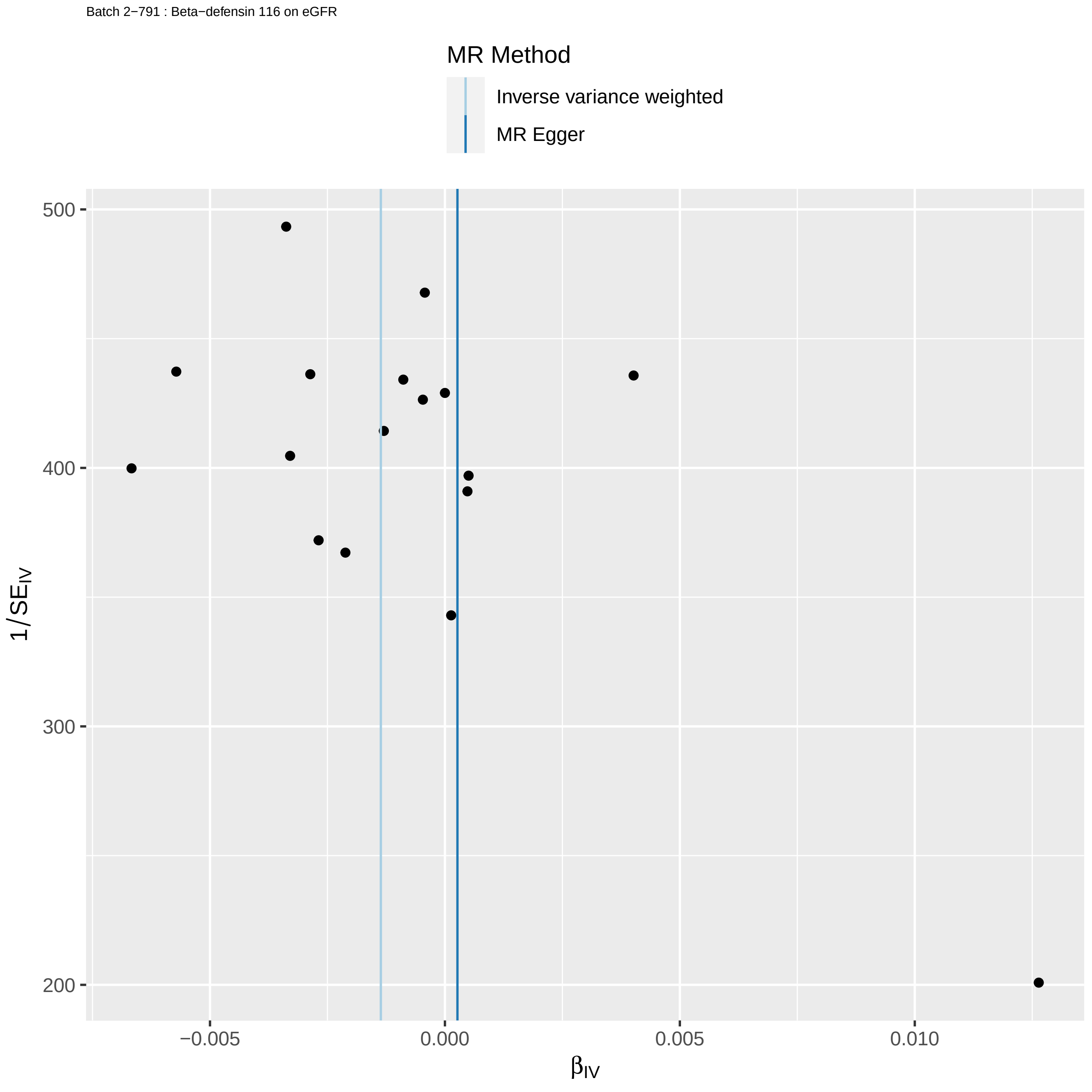

Batch 2−791 : Beta−defensin 116 on eGFR
MR Method
Inverse variance weighted
MR Egger
500
400
300
200
1/SE$_{IV}$
−0.005
0.000
0.005
0.010
β$_{IV}$

Batch 2−792 : Beta−defensin 118 on eGFR

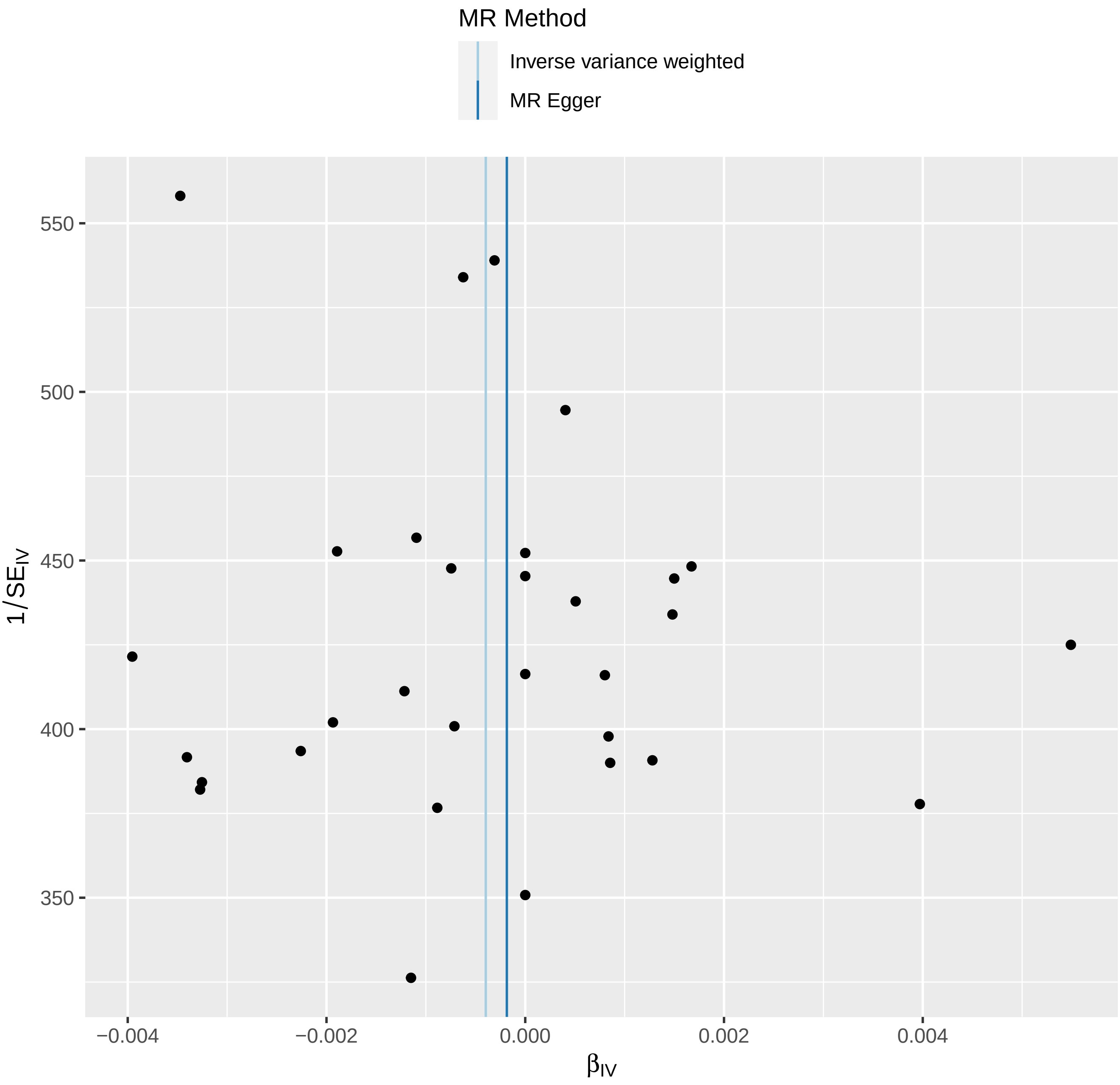

Batch 2−793 : Beta−defensin 119 on eGFR

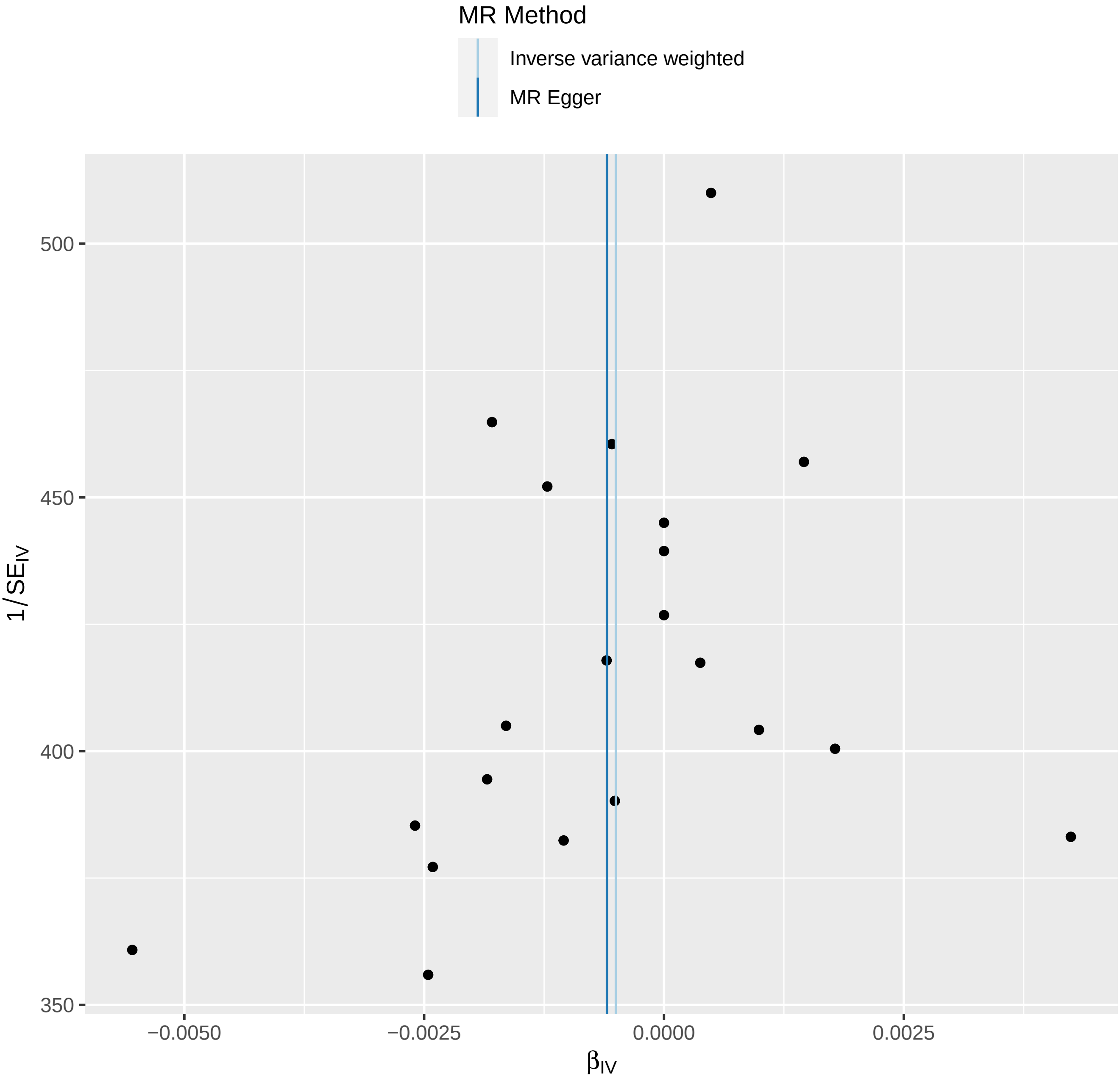

Batch 2−794 : Beta−defensin 119 on eGFR

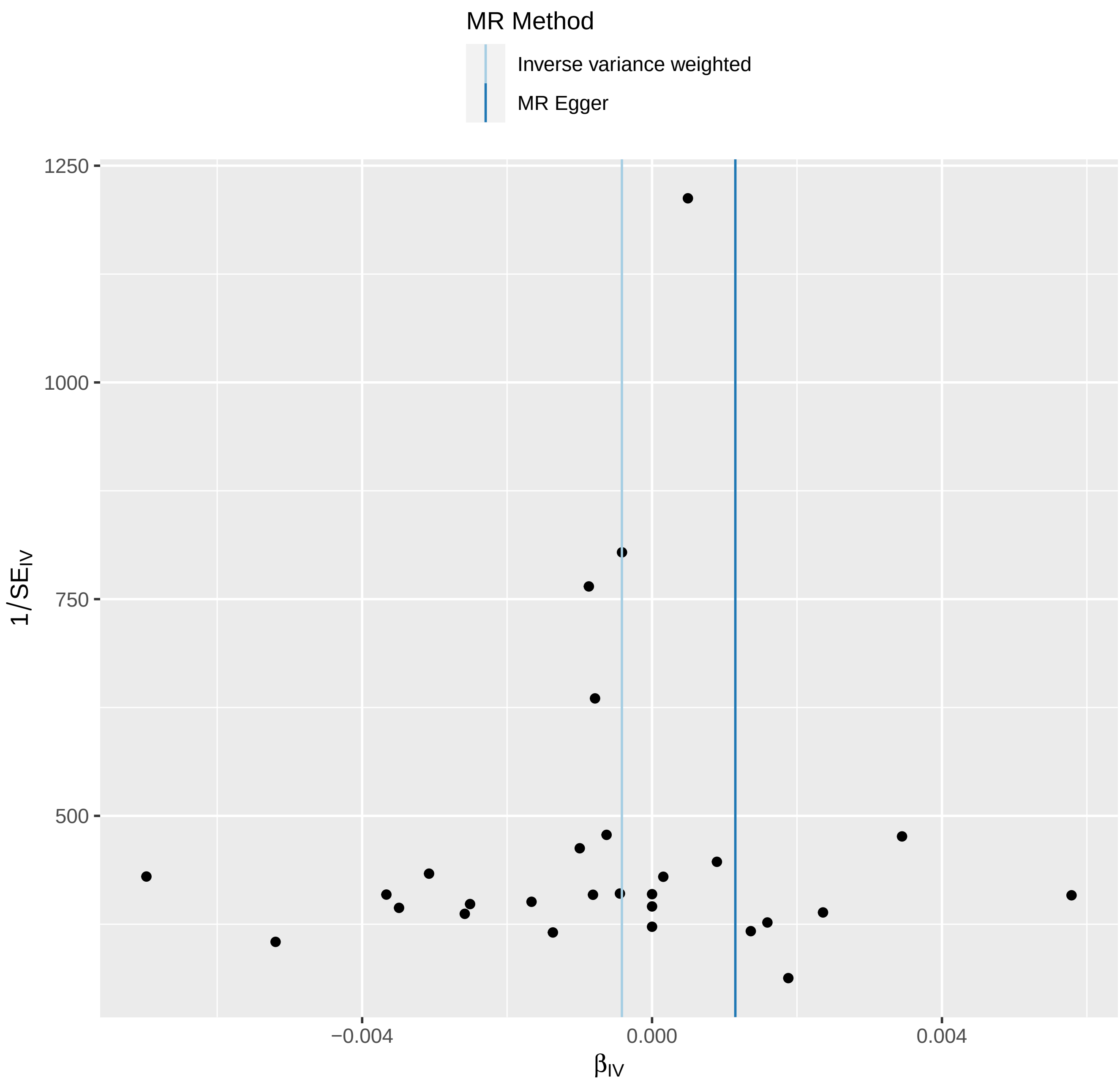

Batch 2−795 : Beta−defensin 119 on eGFR

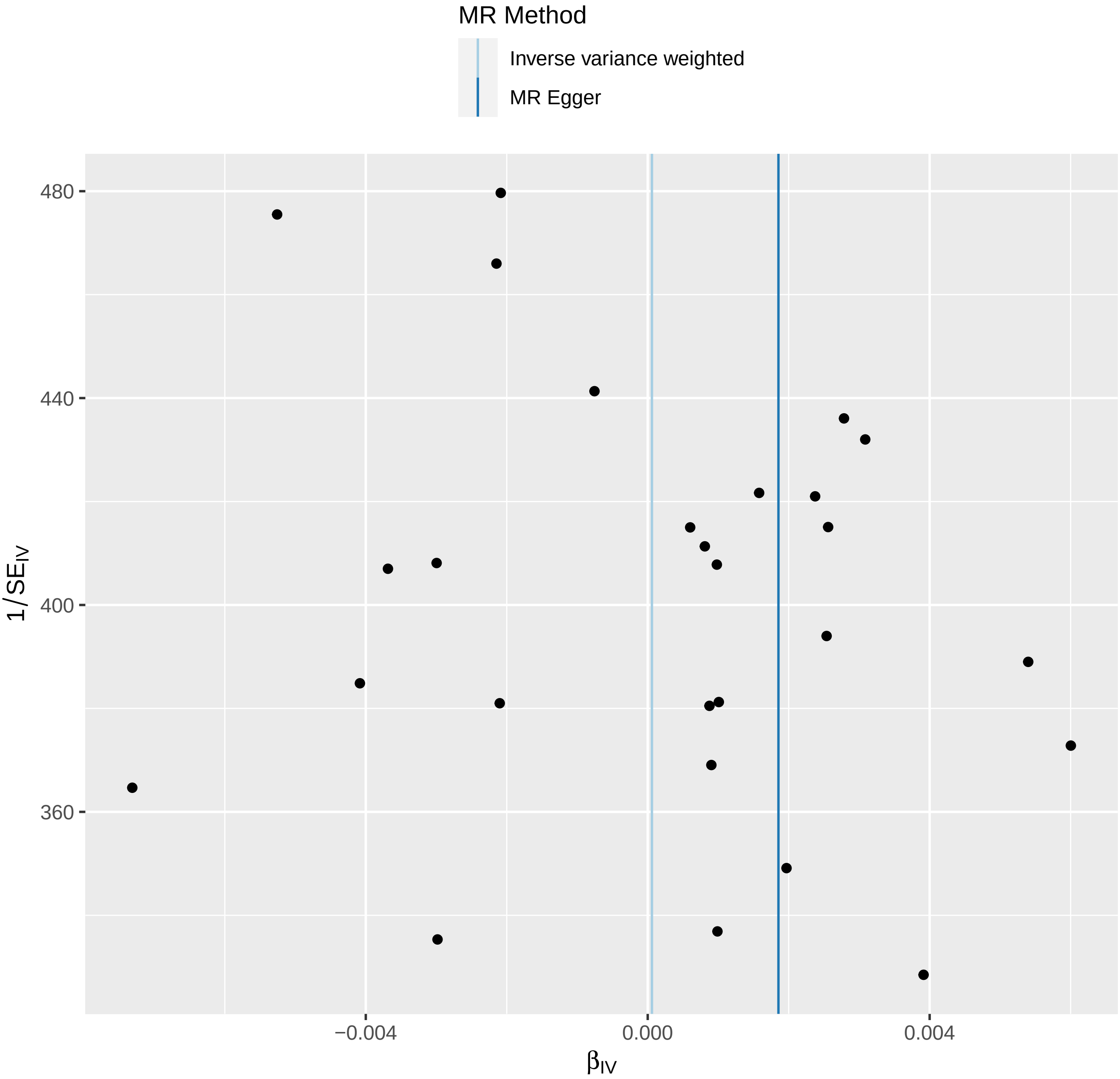

Batch 2−796 : Beta−defensin 119 on eGFR

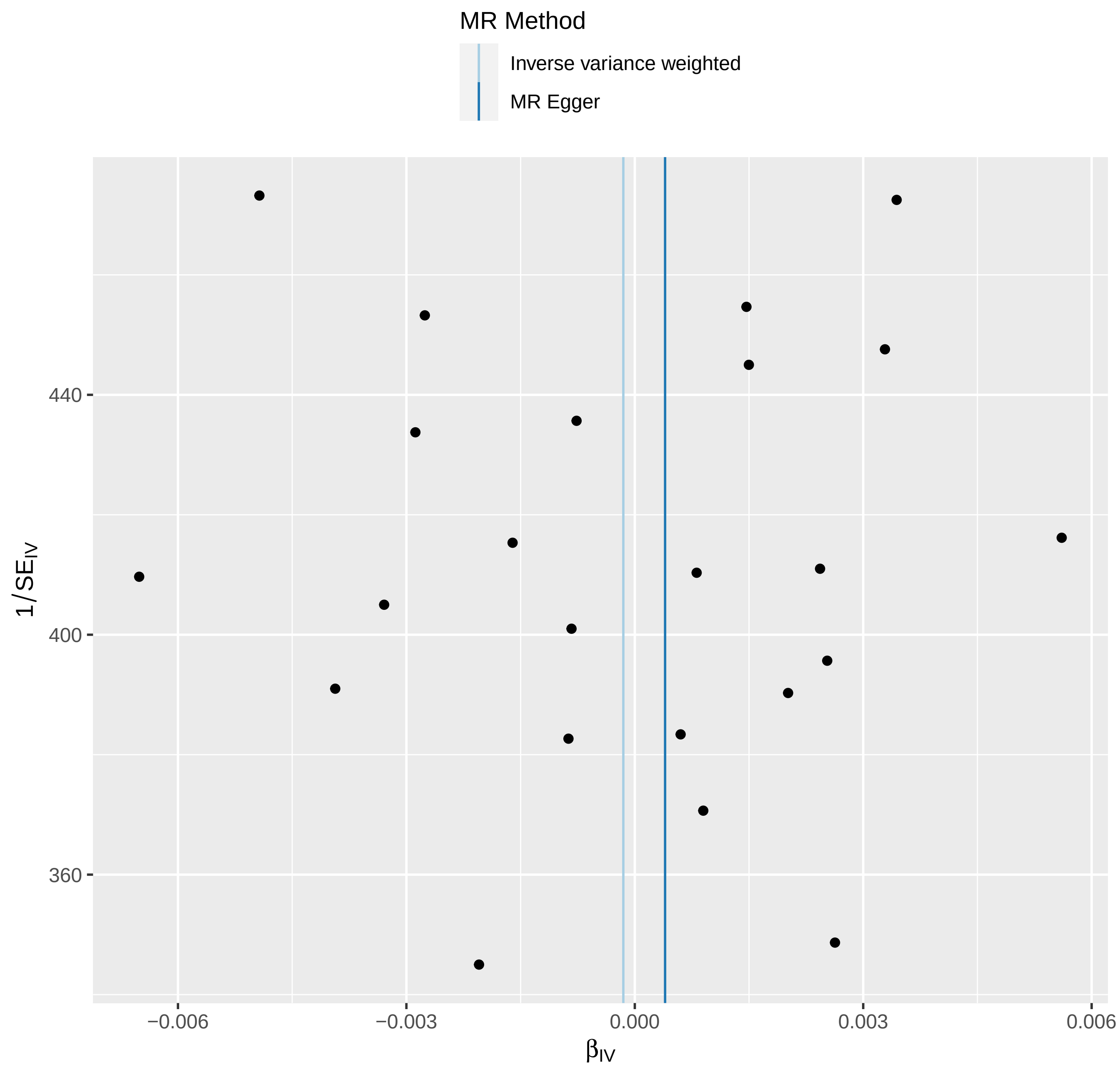

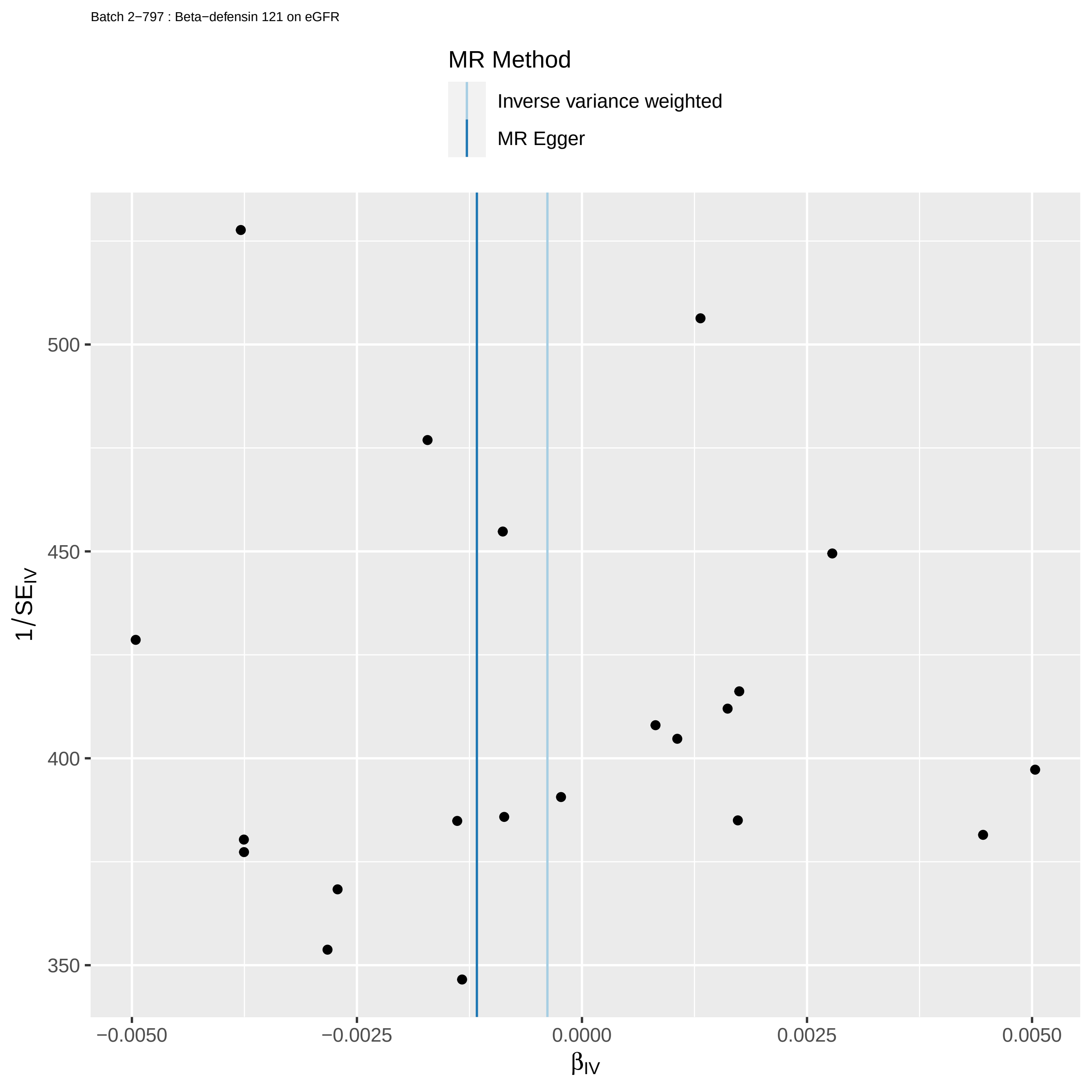
Batch 2−797 : Beta−defensin 121 on eGFR
MR Method
Inverse variance weighted
MR Egger
500
450
400
350
1/SE_IV
−0.0050
−0.0025
0.0000
0.0025
0.0050
β_IV

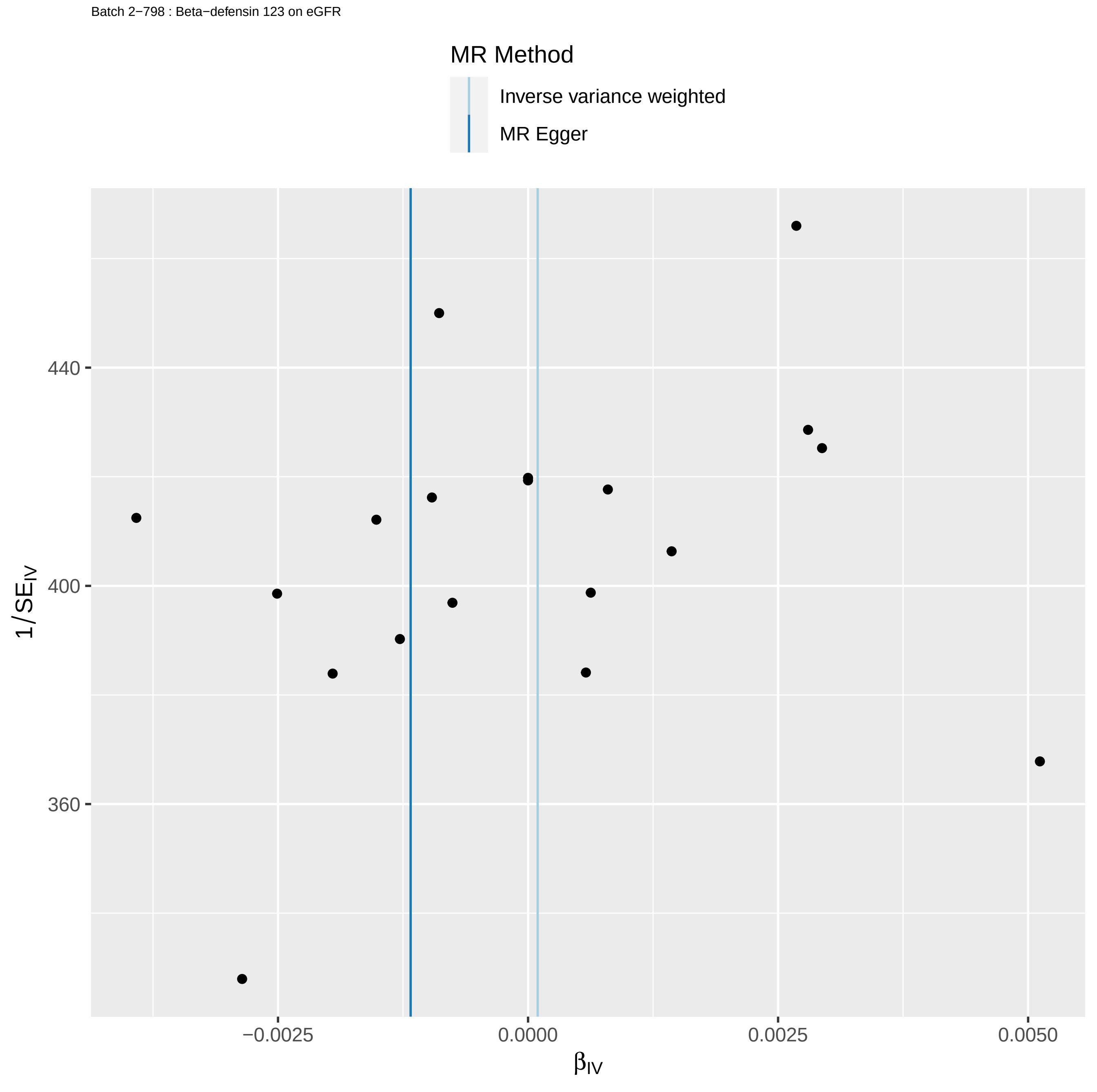

Batch 2−798 : Beta−defensin 123 on eGFR
MR Method
Inverse variance weighted
MR Egger
440
400
360
−0.0025
0.0000
0.0025
0.0050
$1/SE_{IV}$
β_{IV}

Batch 2−799 : Beta−defensin 125 on eGFR

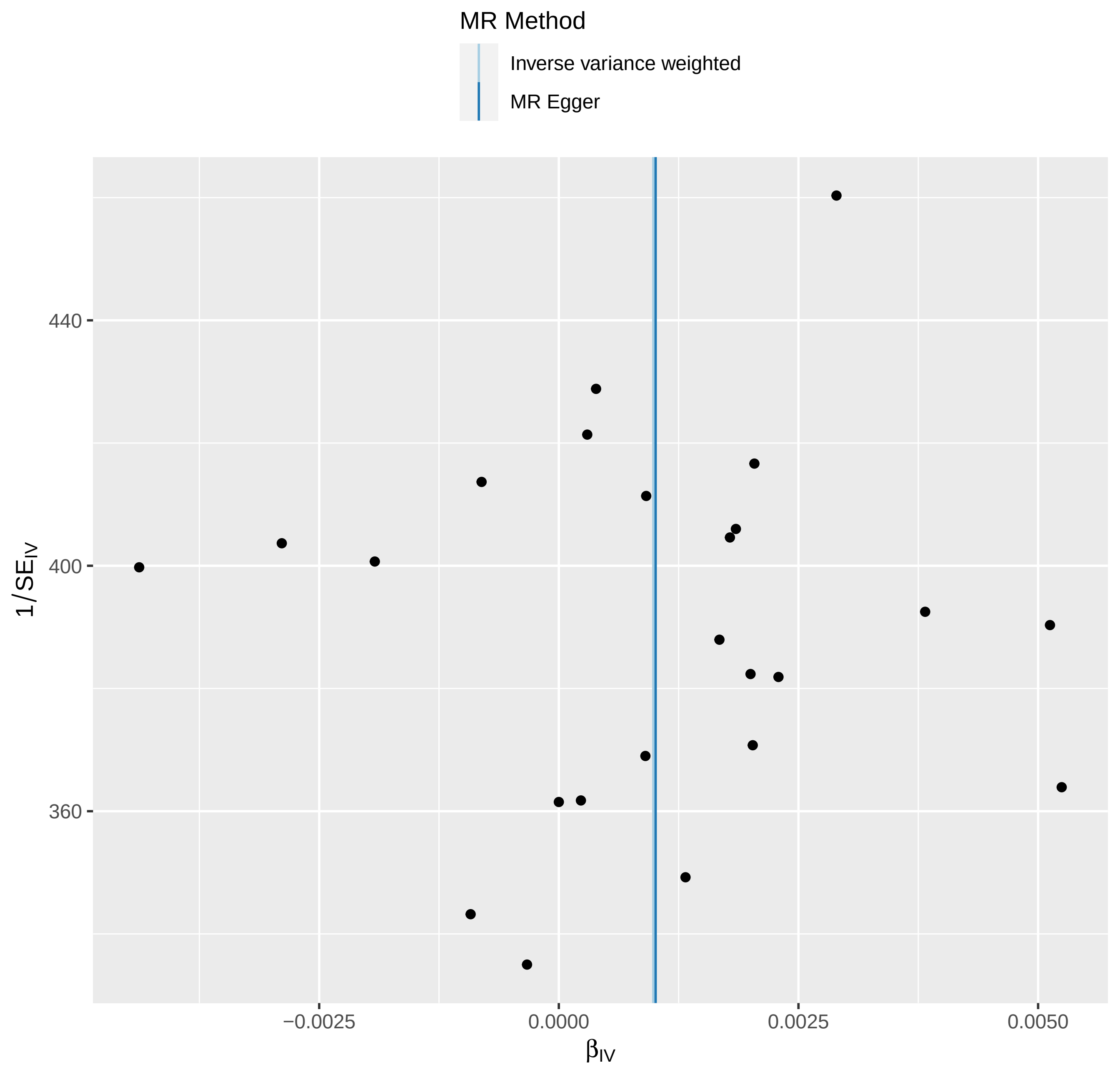

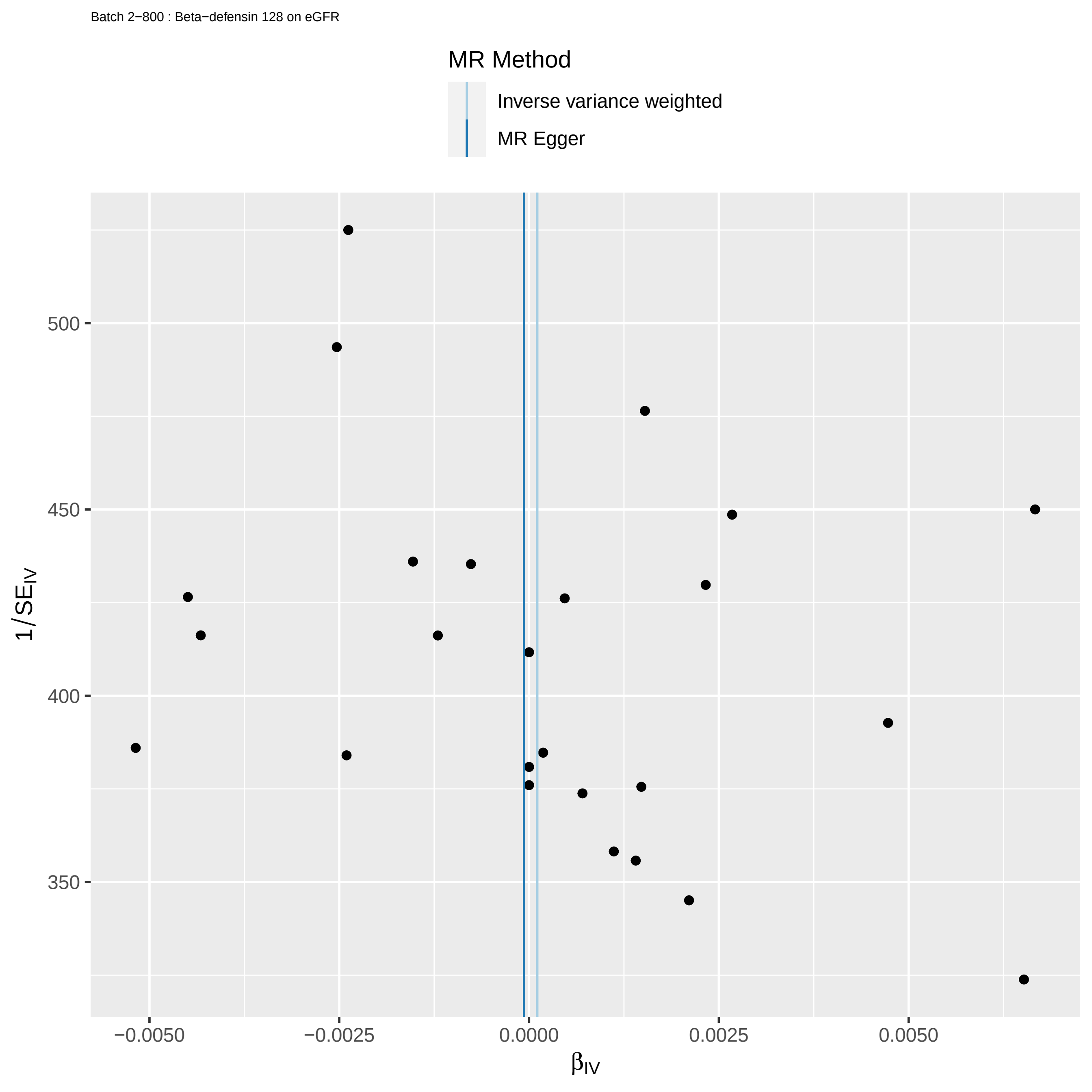
Batch 2−800 : Beta−defensin 128 on eGFR
MR Method
Inverse variance weighted
MR Egger
500
450
400
350
1/SE_IV
−0.0050
−0.0025
0.0000
0.0025
0.0050
β_IV

Batch 2−801 : Beta−defensin 134 on eGFR

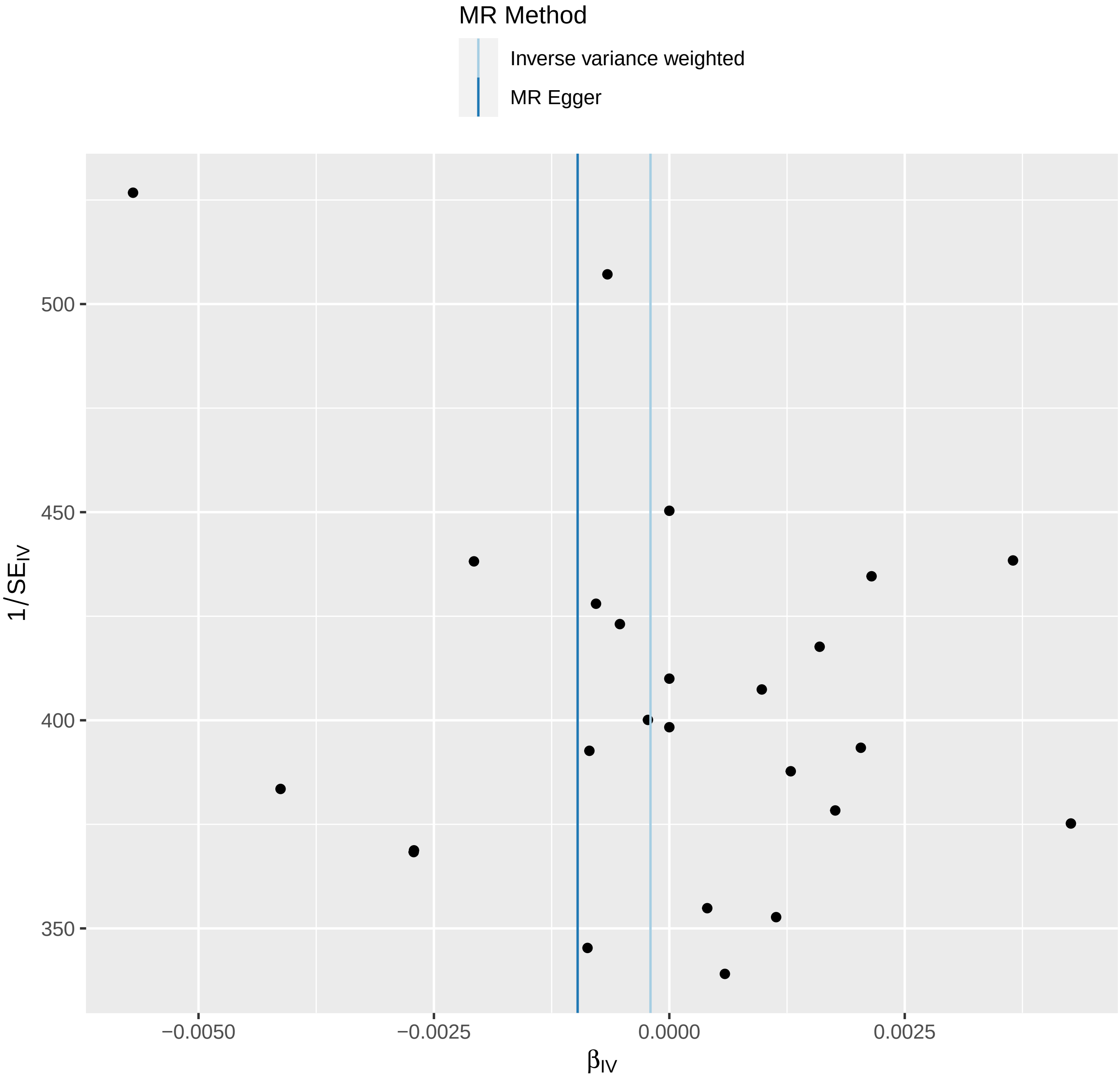

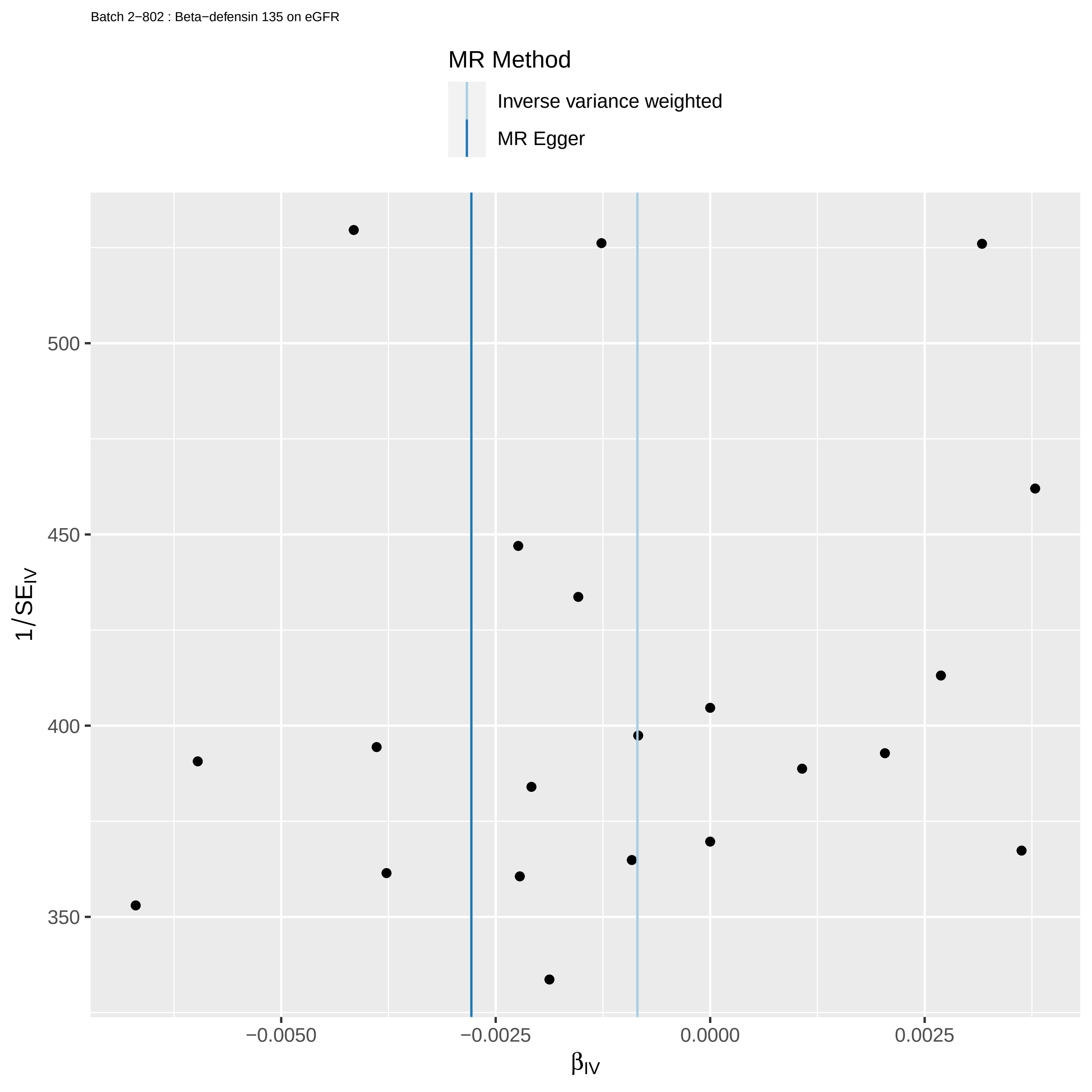

Batch 2−802 : Beta−defensin 135 on eGFR
MR Method
Inverse variance weighted
MR Egger
500
450
400
350
1/SE_IV
−0.0050
−0.0025
0.0000
0.0025
β_IV

Batch 2−803 : Beta−defensin 136 on eGFR

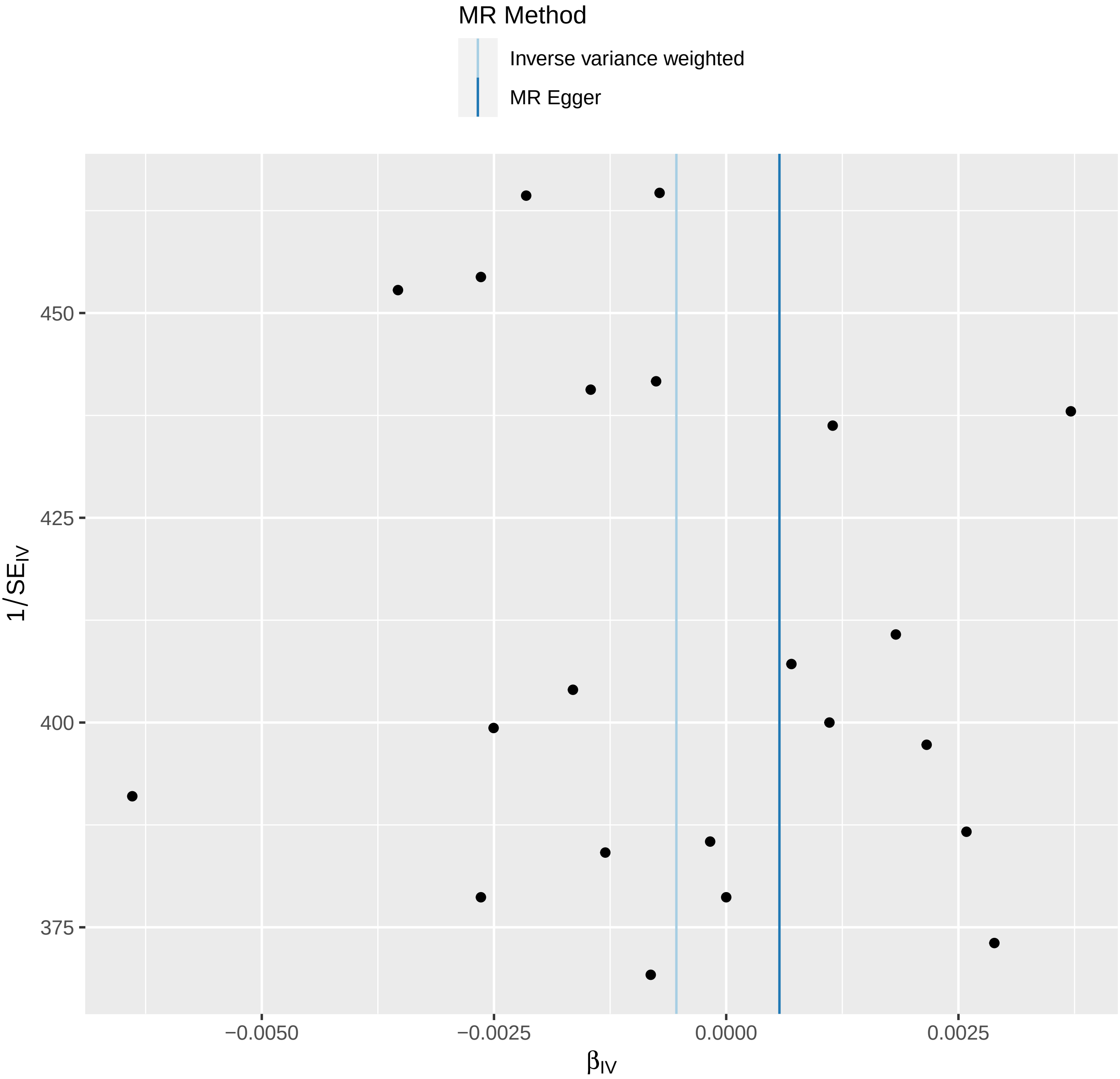

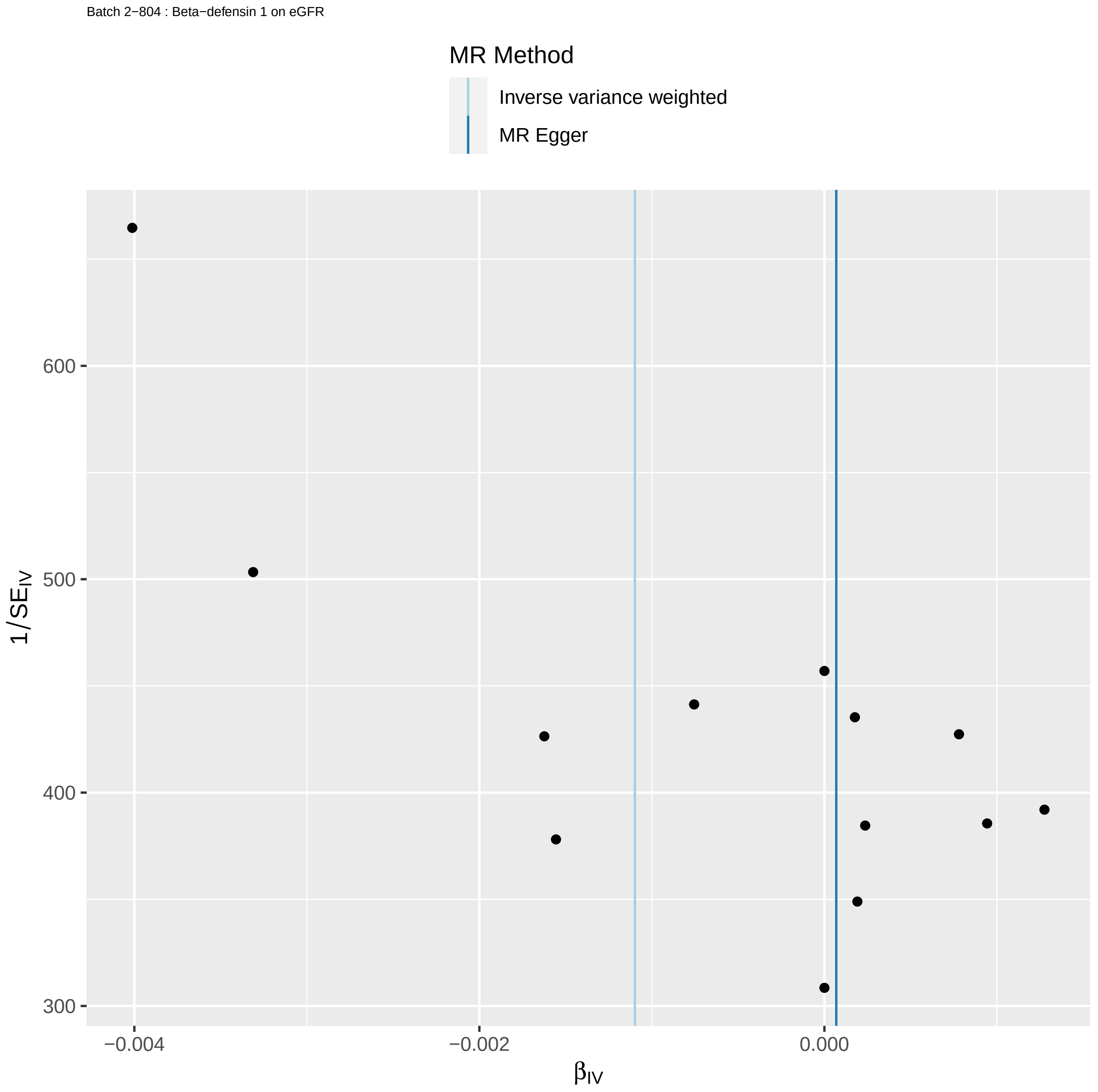

Batch 2−804 : Beta−defensin 1 on eGFR
MR Method
Inverse variance weighted
MR Egger
600
500
400
300
1/SE$_{IV}$
−0.004
−0.002
0.000
β_{IV}

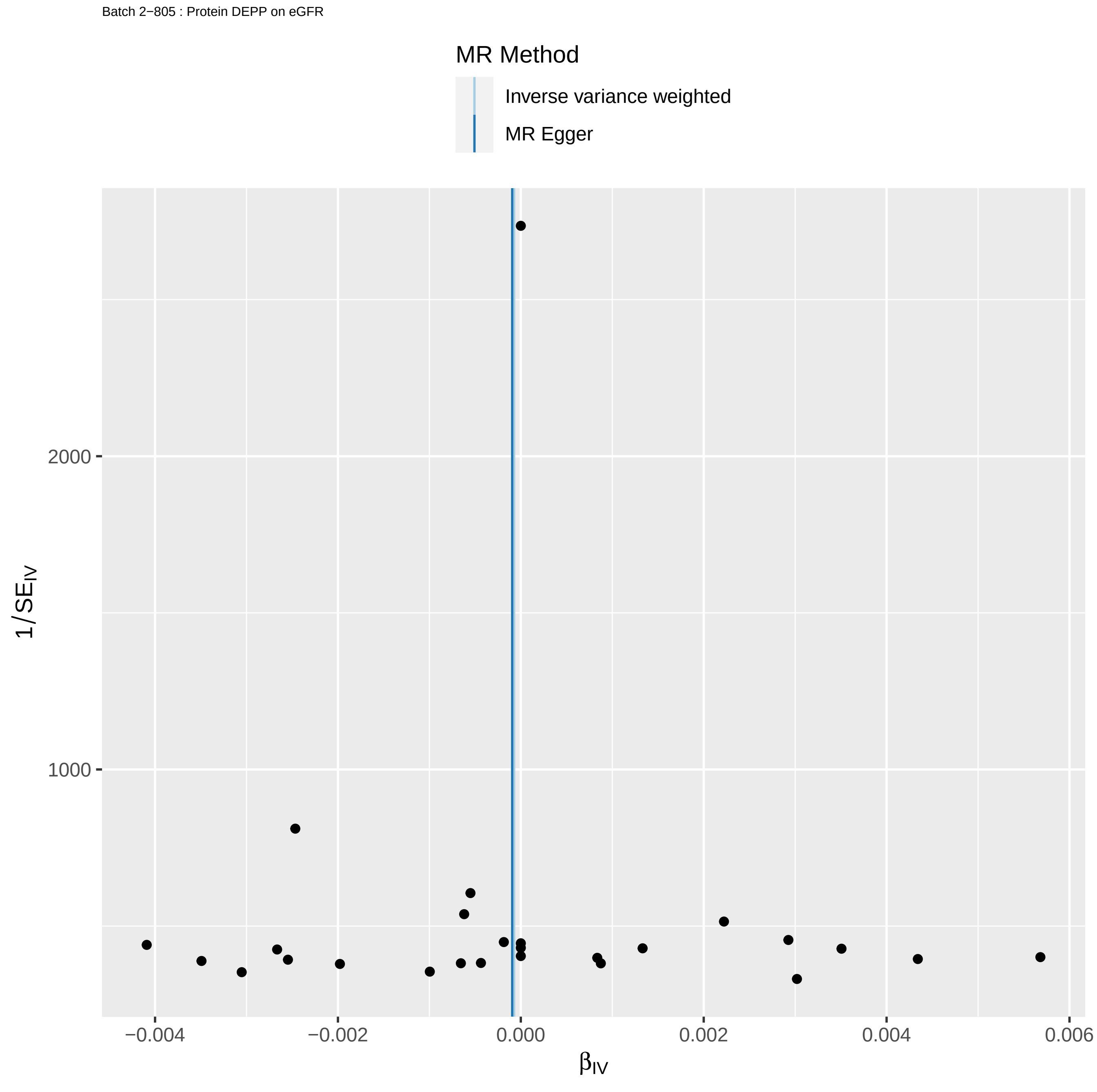

Batch 2−805 : Protein DEPP on eGFR
MR Method
Inverse variance weighted
MR Egger
2000
1000
1/SE_IV
−0.004
−0.002
0.000
0.002
0.004
0.006
β_IV

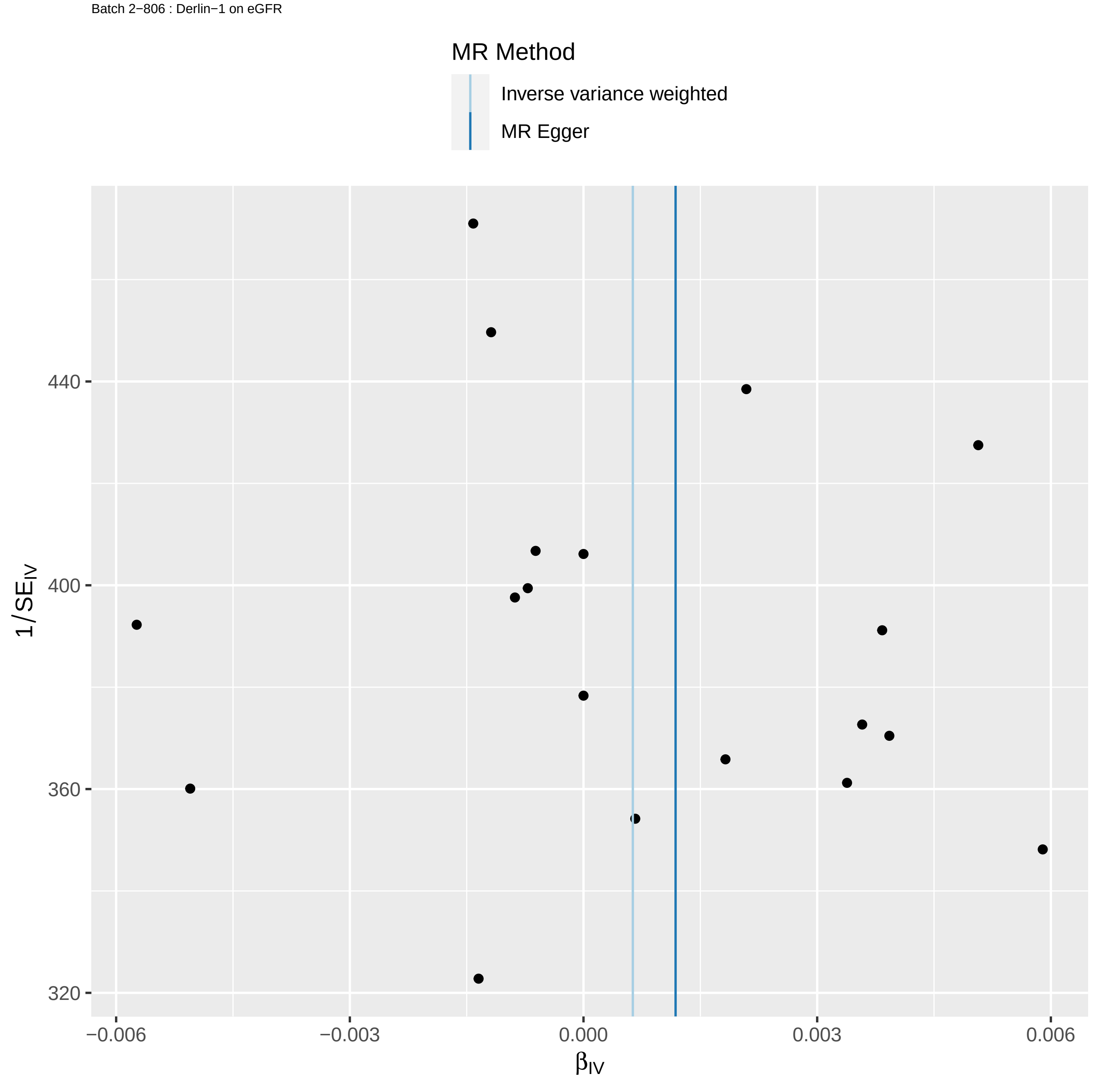

Batch 2−806 : Derlin−1 on eGFR
MR Method
Inverse variance weighted
MR Egger
440
400
360
320
−0.006
−0.003
0.000
0.003
0.006
$1/SE_{IV}$
β_{IV}

Batch 2−807 : Desmin on eGFR

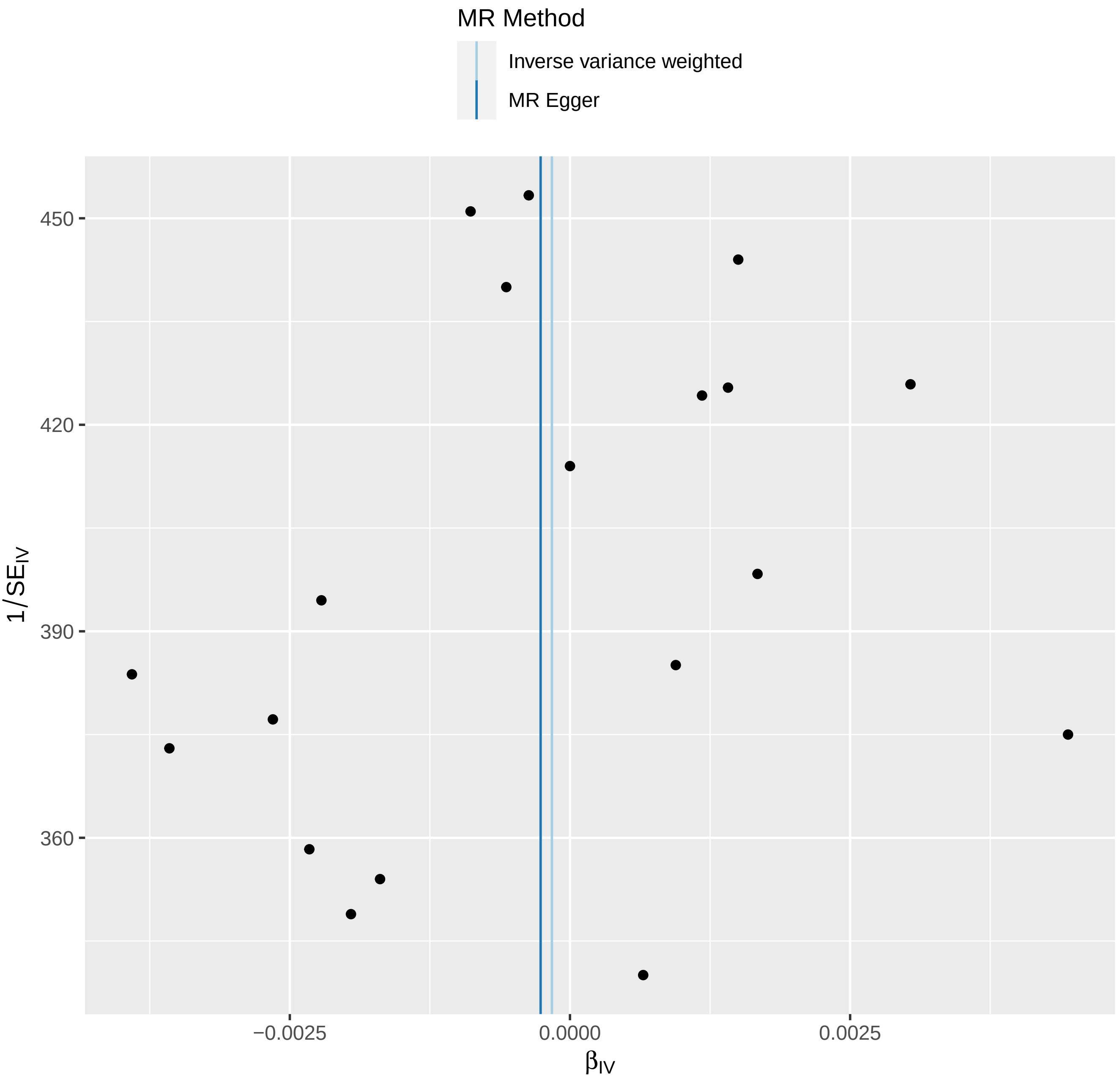

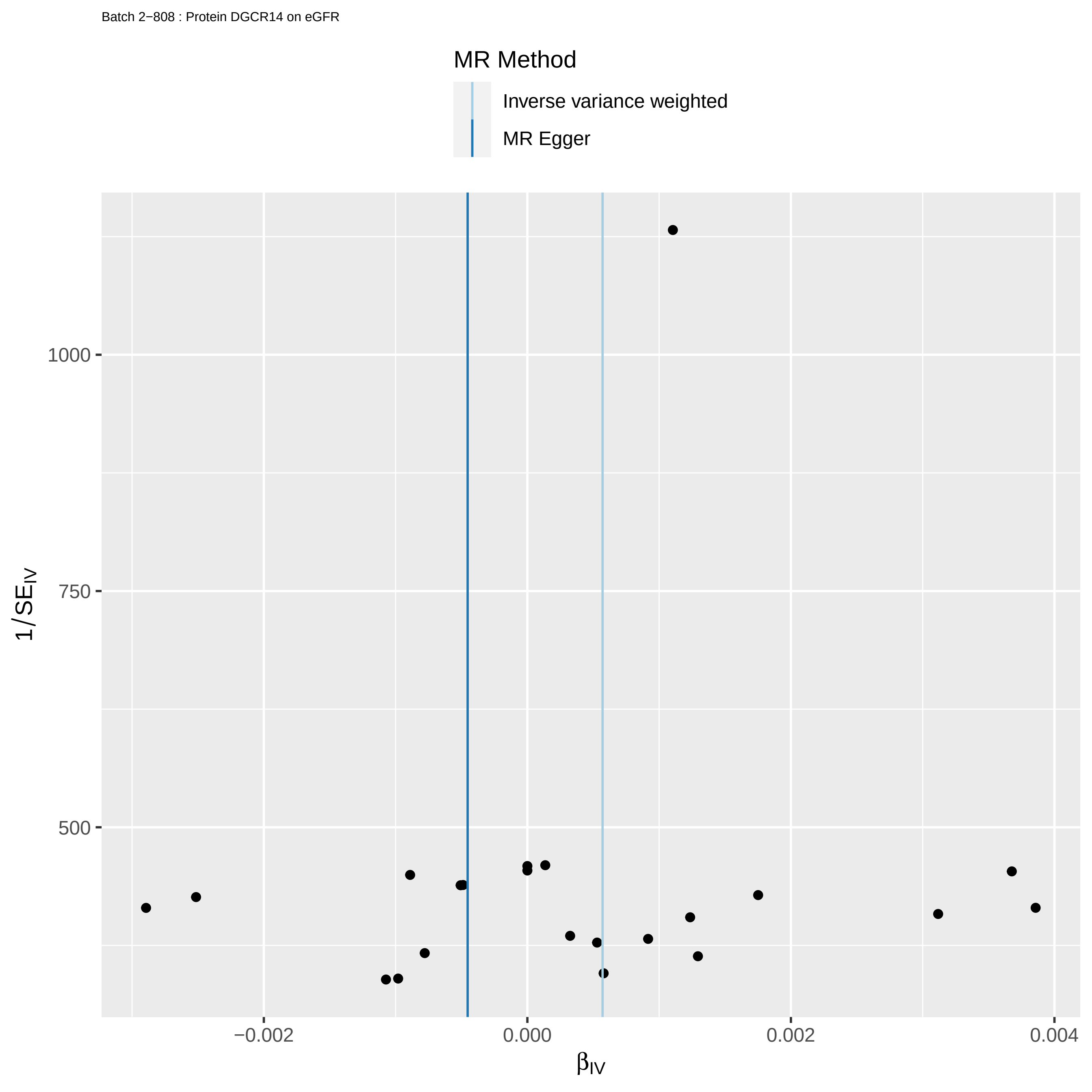
Batch 2−808 : Protein DGCR14 on eGFR
MR Method
Inverse variance weighted
MR Egger
1000
750
500
1/SE_IV
−0.002
0.000
0.002
0.004
β_IV

Batch 2−809 : Integral membrane protein DGCR2/IDD on eGFR

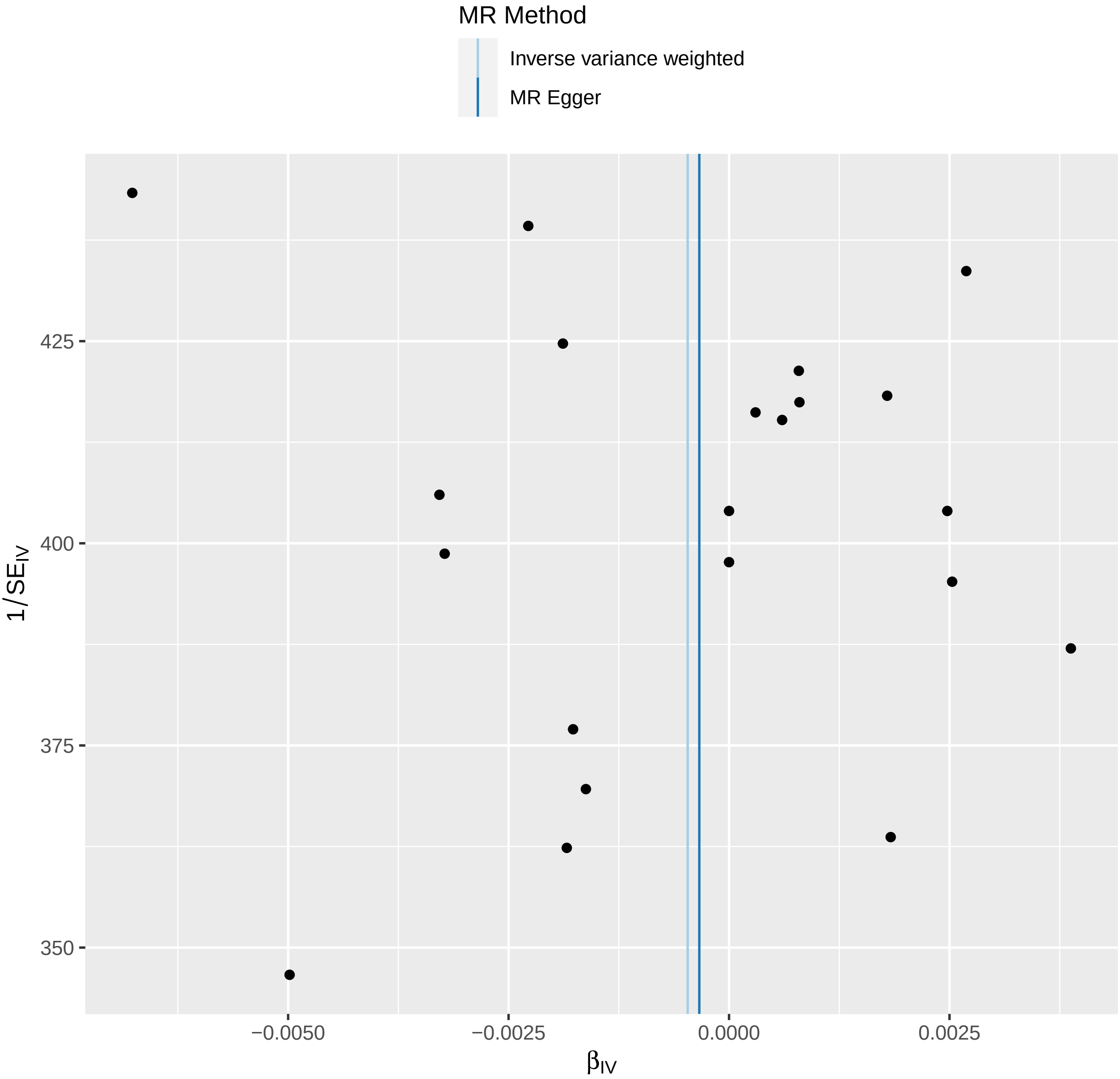

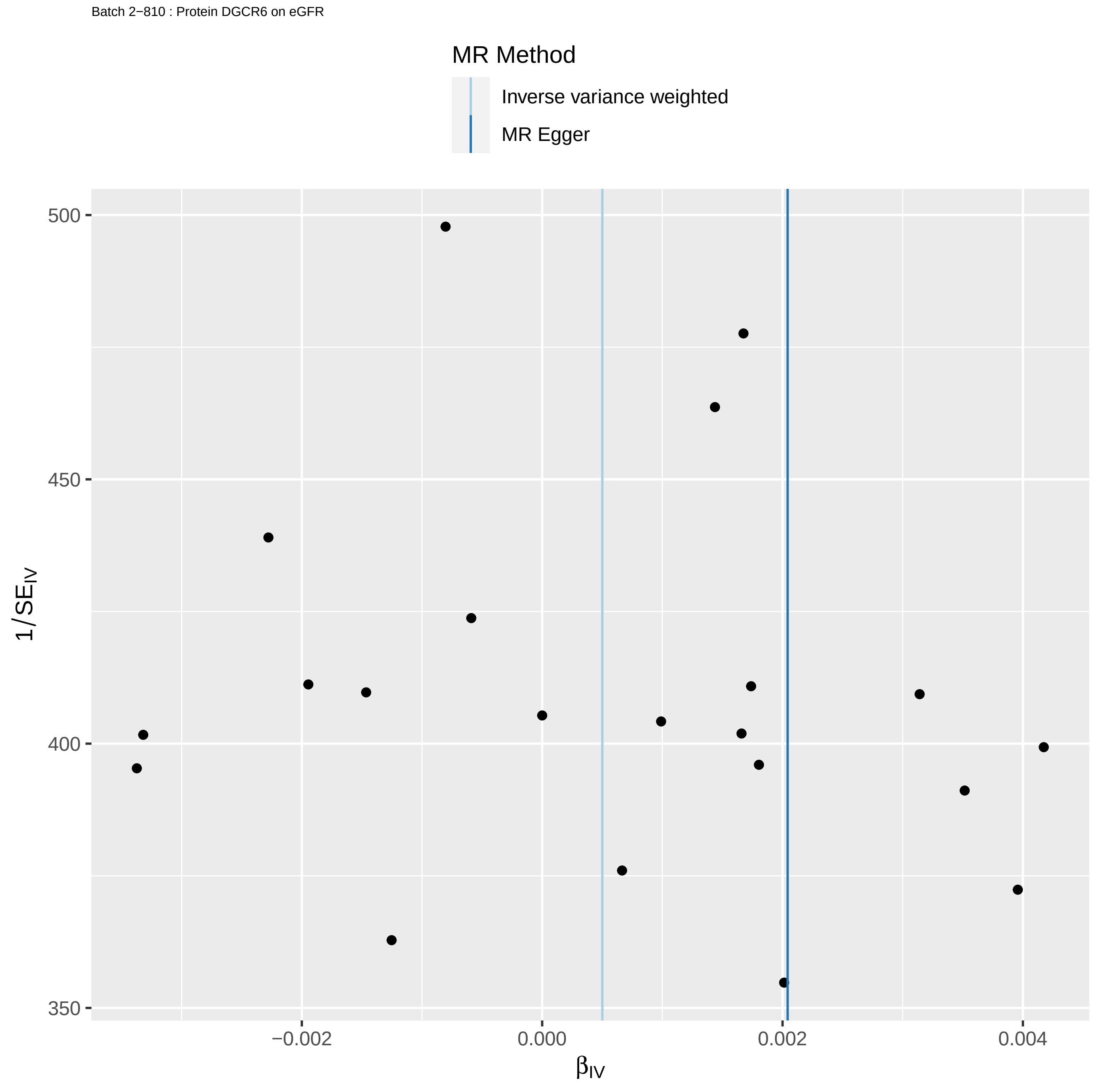
Batch 2−810 : Protein DGCR6 on eGFR
MR Method
Inverse variance weighted
MR Egger
500
450
400
350
−0.002
0.000
0.002
0.004
$1/SE_{IV}$
β_{IV}

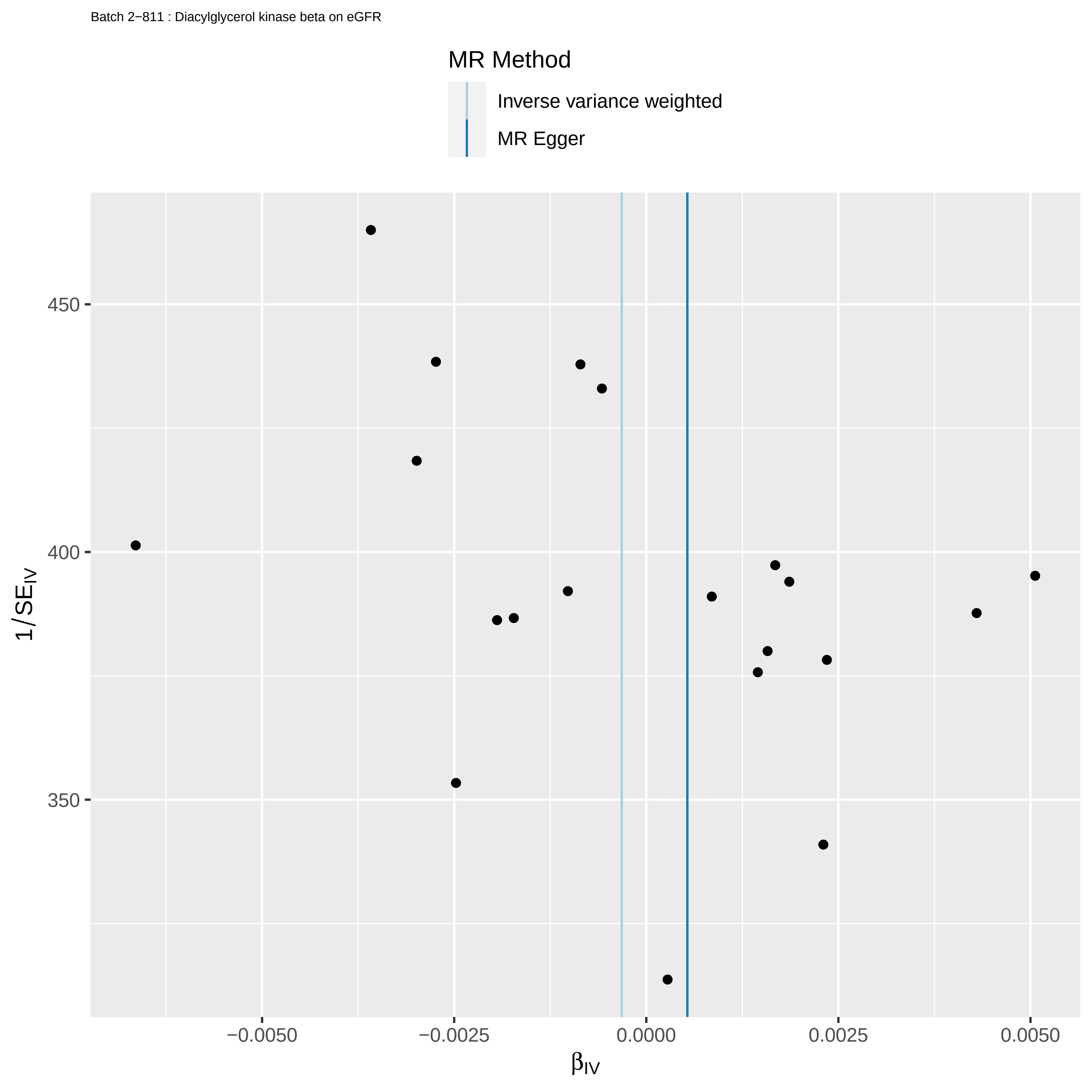

Batch 2−811 : Diacylglycerol kinase beta on eGFR
MR Method
Inverse variance weighted
MR Egger
450
400
350
1/SE_IV
−0.0050
−0.0025
0.0000
0.0025
0.0050
β_IV

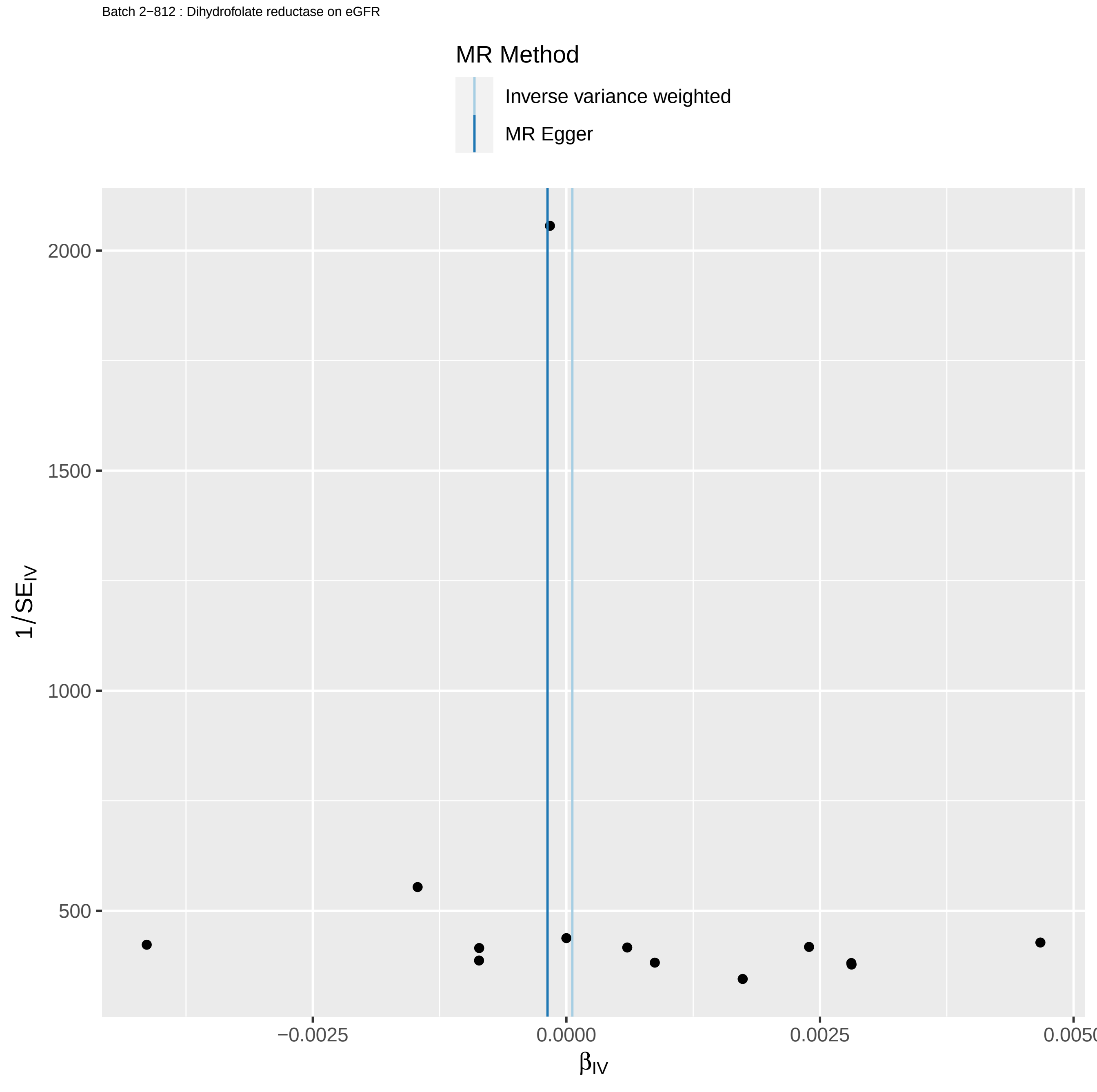

Batch 2−812 : Dihydrofolate reductase on eGFR
MR Method
Inverse variance weighted
MR Egger
2000
1500
1000
500
1/SE_IV
−0.0025
0.0000
0.0025
0.0050
β_IV

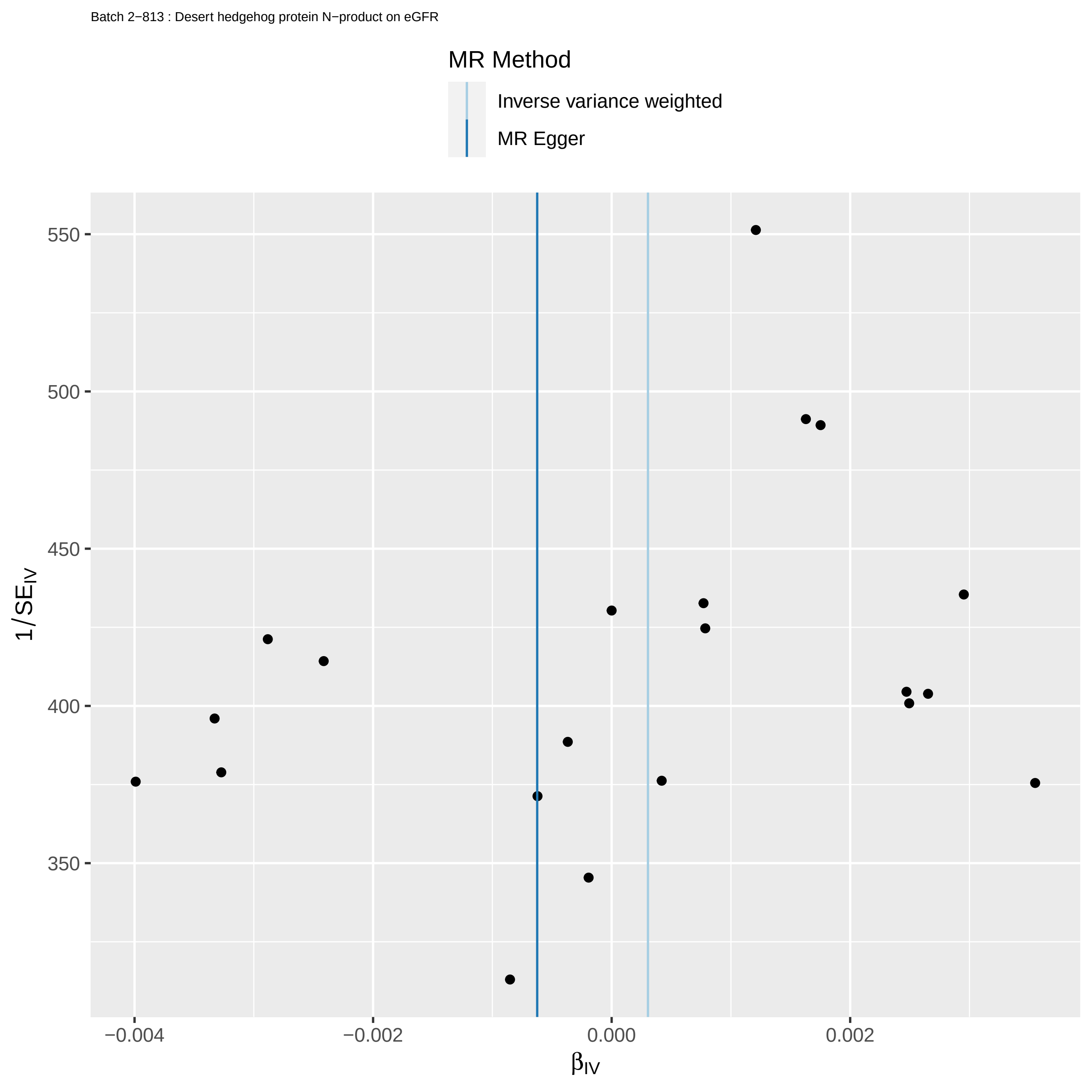
Batch 2−813 : Desert hedgehog protein N−product on eGFR
MR Method
Inverse variance weighted
MR Egger
550
500
450
400
350
1/SE_IV
−0.004
−0.002
0.000
0.002
β_IV

Batch 2−814 : Pre−mRNA−splicing factor ATP−dependent RNA helicase PRP16 on eGFR

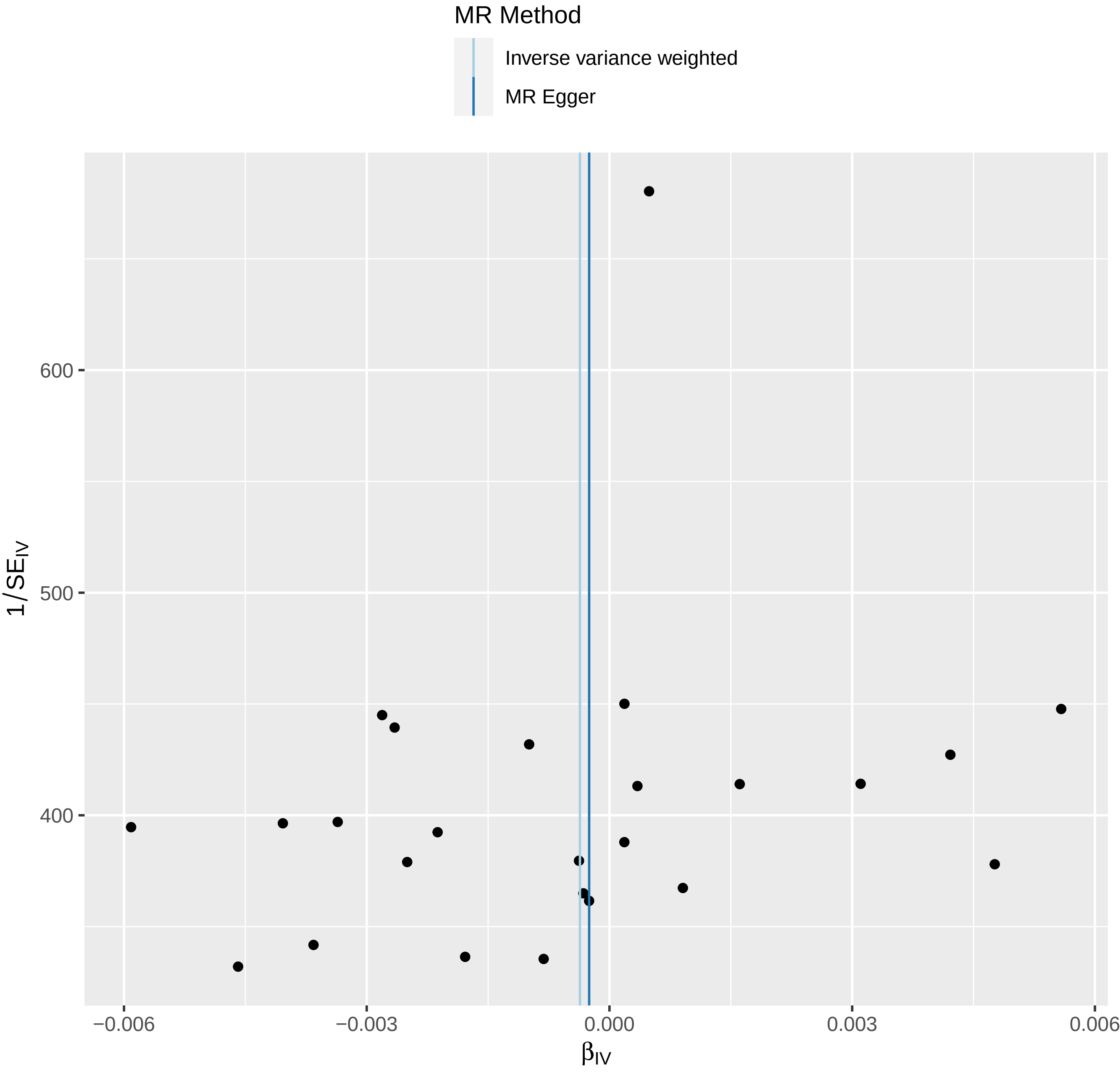

Batch 2−815 : Probable ATP−dependent RNA helicase DHX58 on eGFR

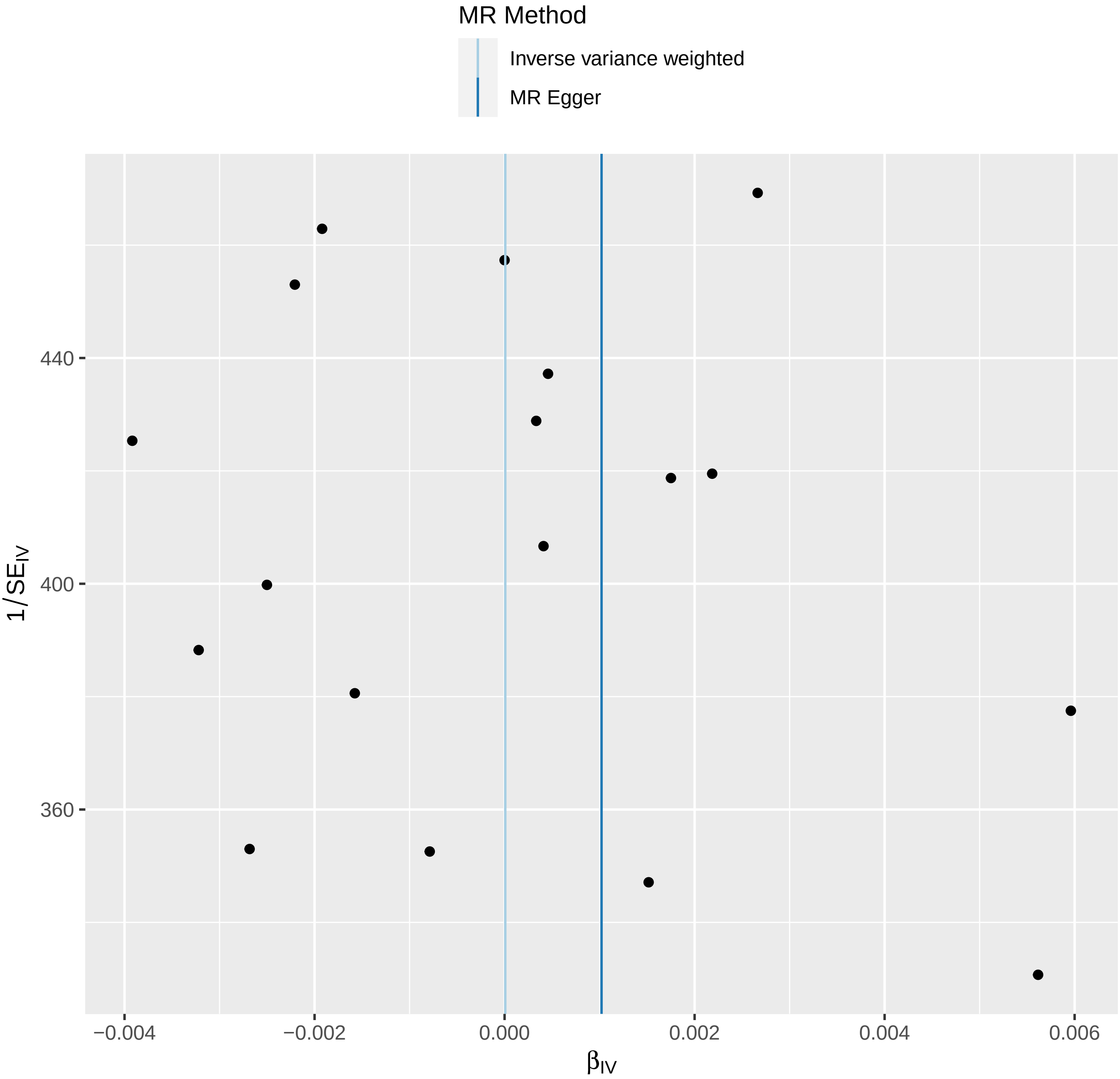

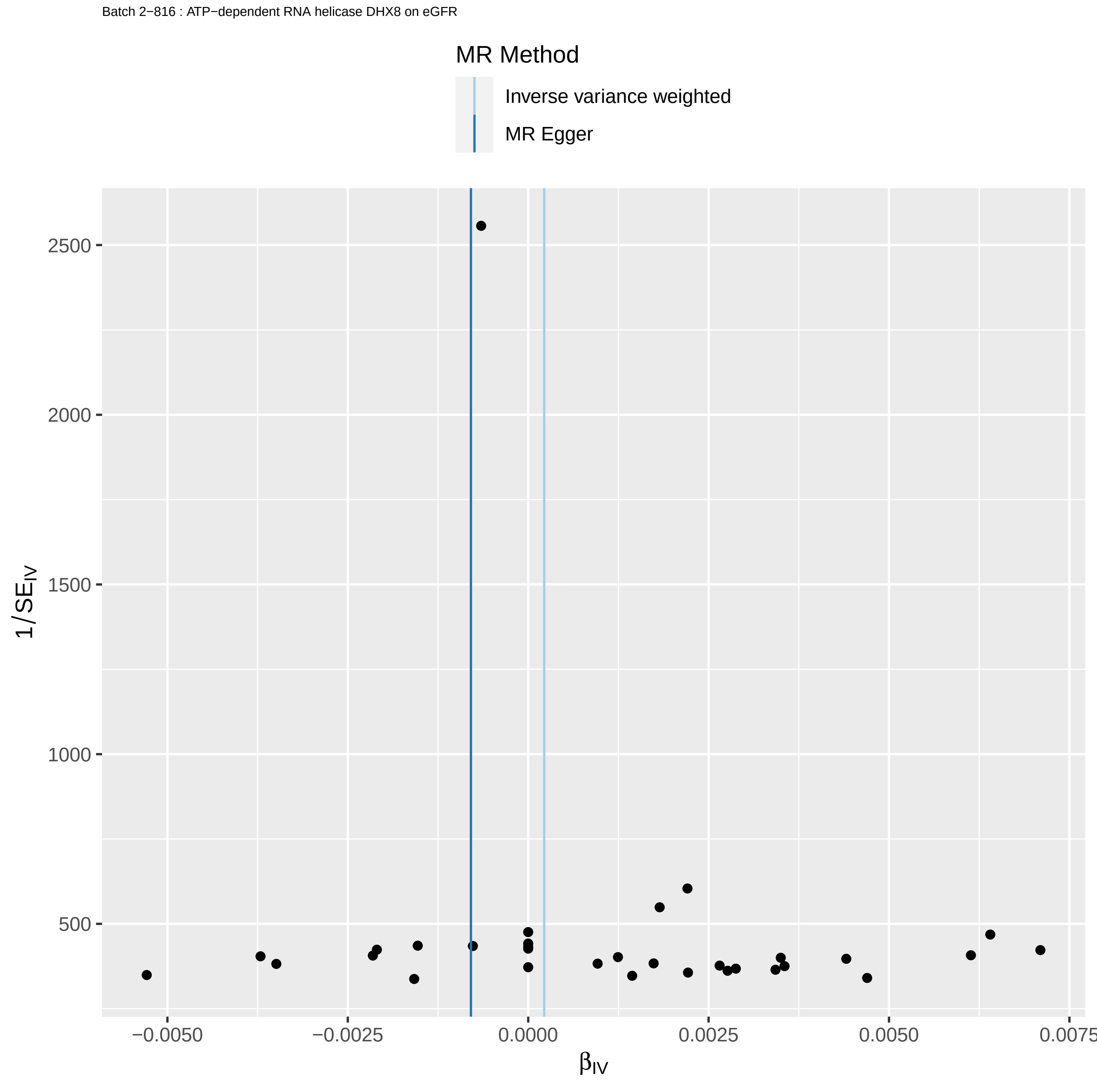
Batch 2−816 : ATP−dependent RNA helicase DHX8 on eGFR
MR Method
Inverse variance weighted
MR Egger
2500
2000
1500
1000
500
1/SE$_{IV}$
−0.0050
−0.0025
0.0000
0.0025
0.0050
0.0075
β$_{IV}$

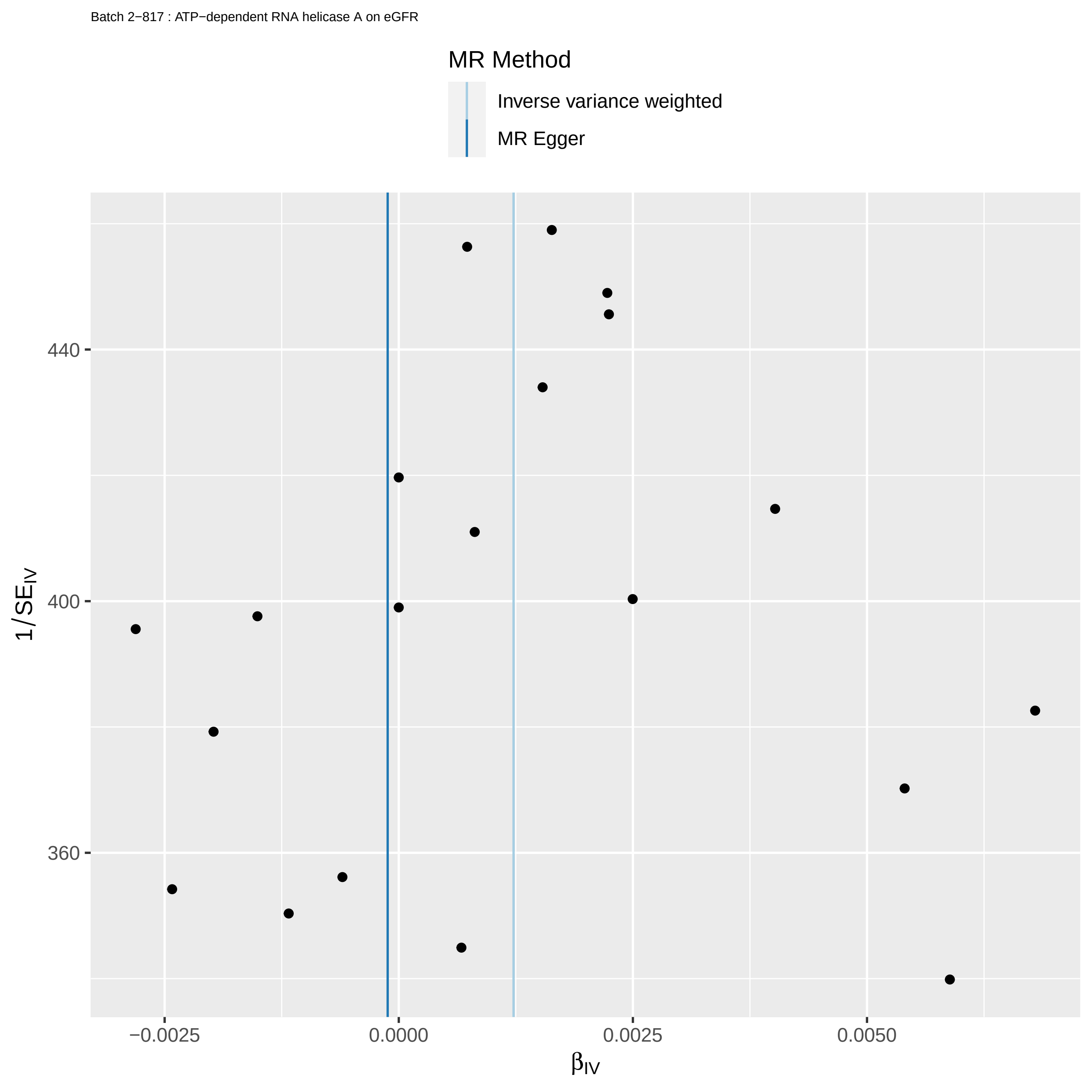
Batch 2−817 : ATP−dependent RNA helicase A on eGFR
MR Method
Inverse variance weighted
MR Egger
440
400
360
1/SE$_{IV}$
−0.0025
0.0000
0.0025
0.0050
β_{IV}

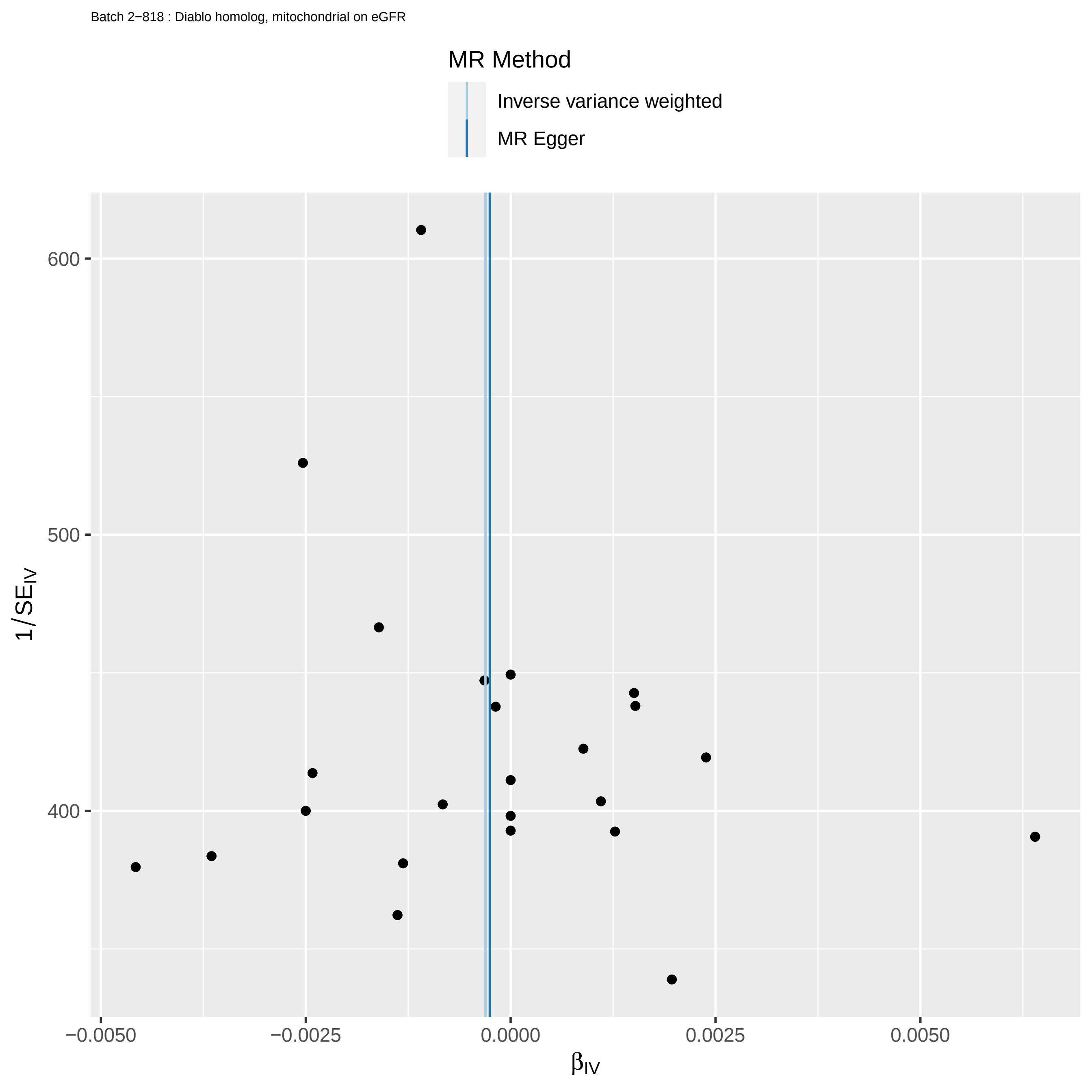

Batch 2−818 : Diablo homolog, mitochondrial on eGFR
MR Method
Inverse variance weighted
MR Egger
600
500
400
1/SE$_{IV}$
−0.0050
−0.0025
0.0000
0.0025
0.0050
β$_{IV}$

Batch 2−819 : Probable dimethyladenosine transferase on eGFR

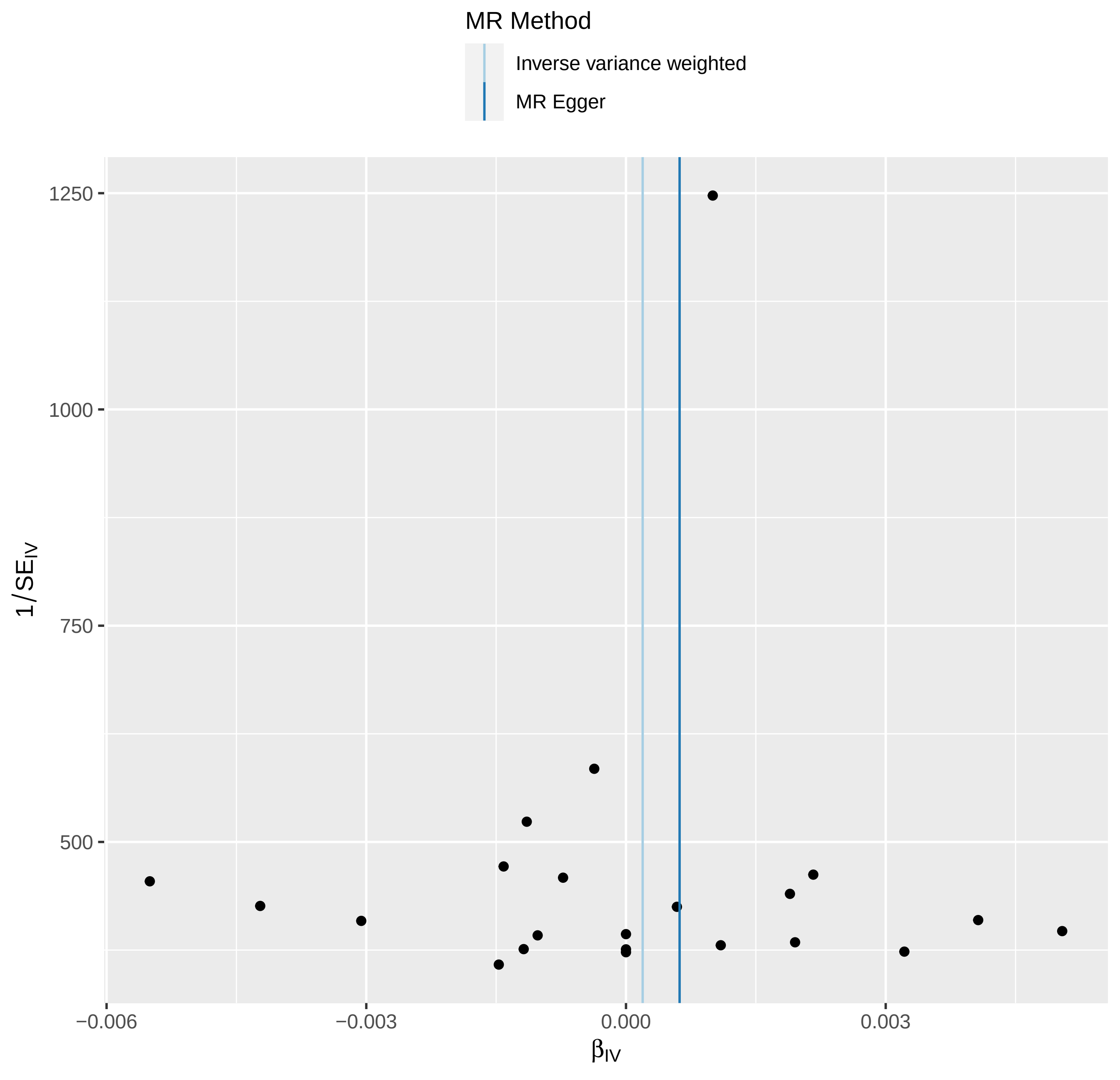

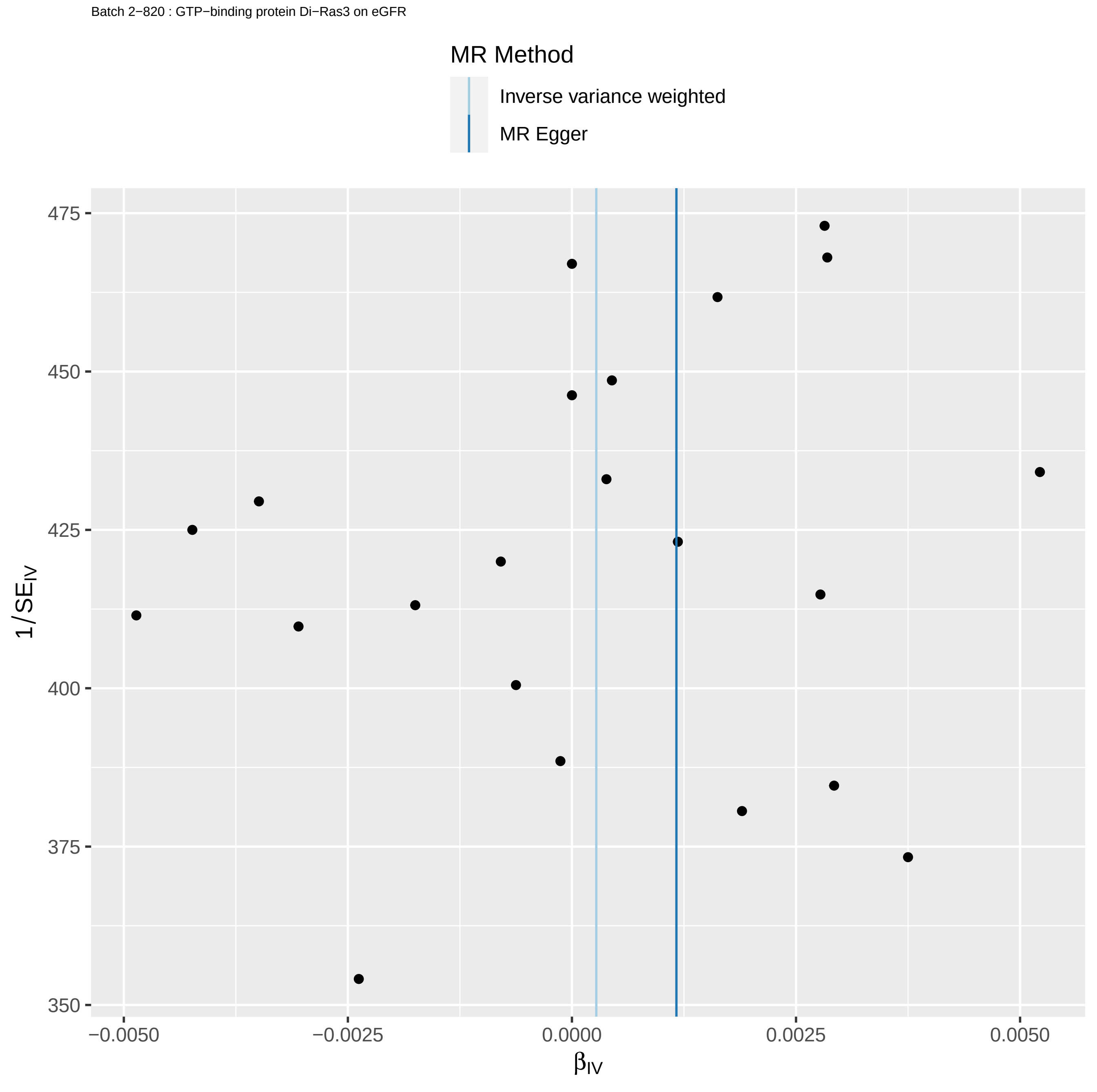
Batch 2−820 : GTP−binding protein Di−Ras3 on eGFR
MR Method
Inverse variance weighted
MR Egger
475
450
425
400
375
350
1/SE$_{IV}$
−0.0050
−0.0025
0.0000
0.0025
0.0050
β$_{IV}$

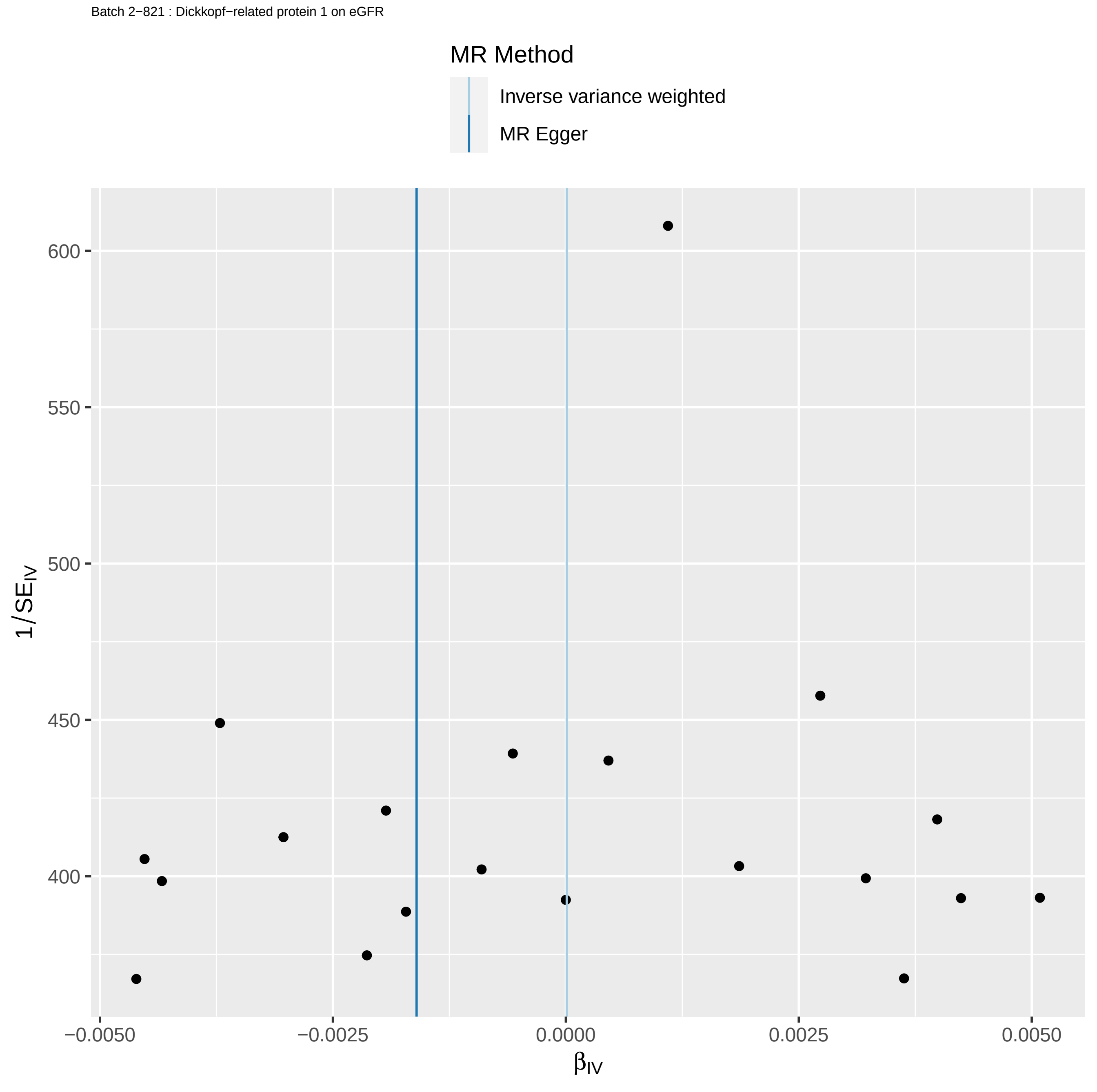

Batch 2−821 : Dickkopf−related protein 1 on eGFR
MR Method
Inverse variance weighted
MR Egger
600
550
500
450
400
1/SE_IV
−0.0050
−0.0025
0.0000
0.0025
0.0050
β_IV

Batch 2−822 : Dickkopf−related protein 3 on eGFR

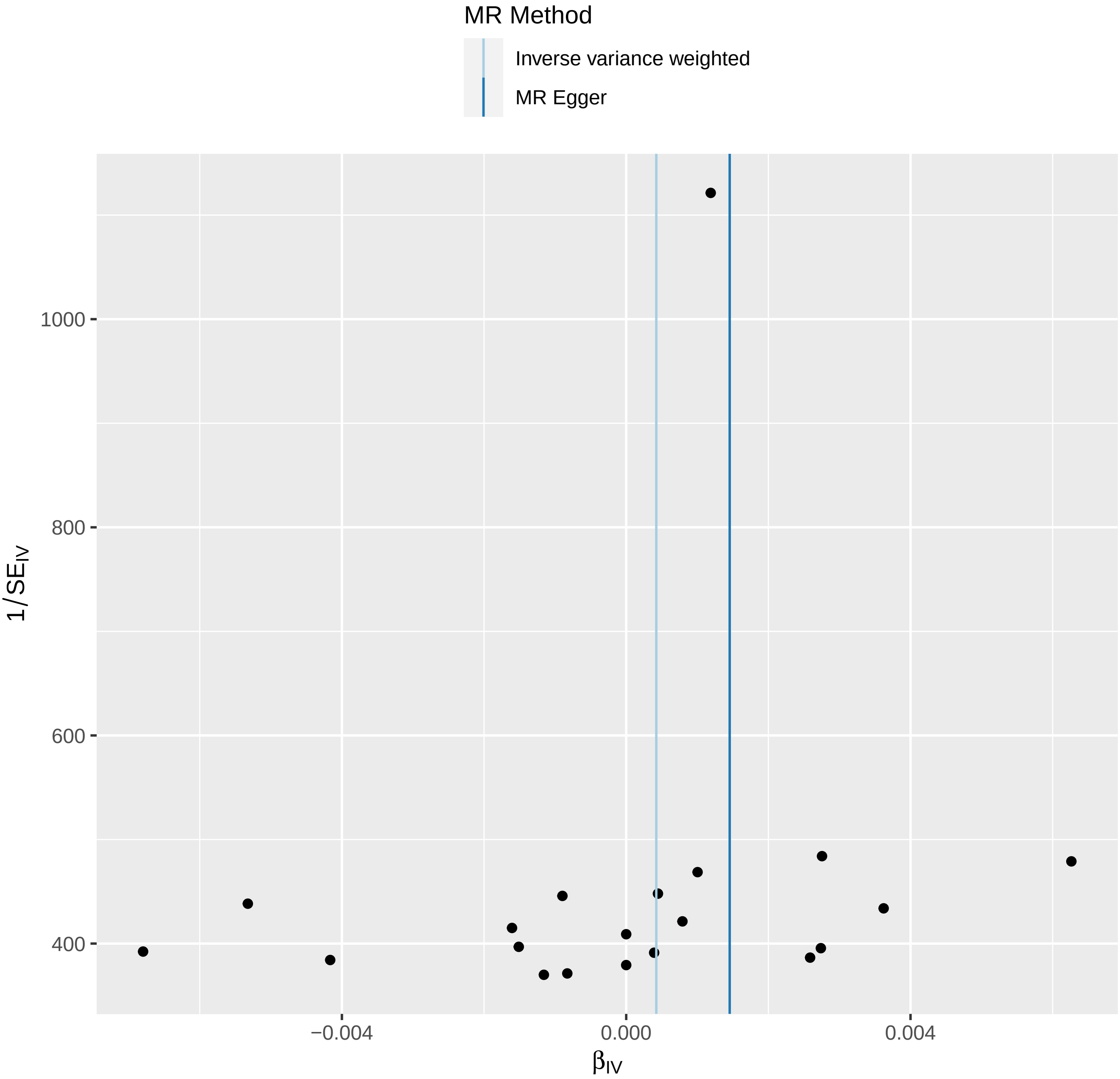

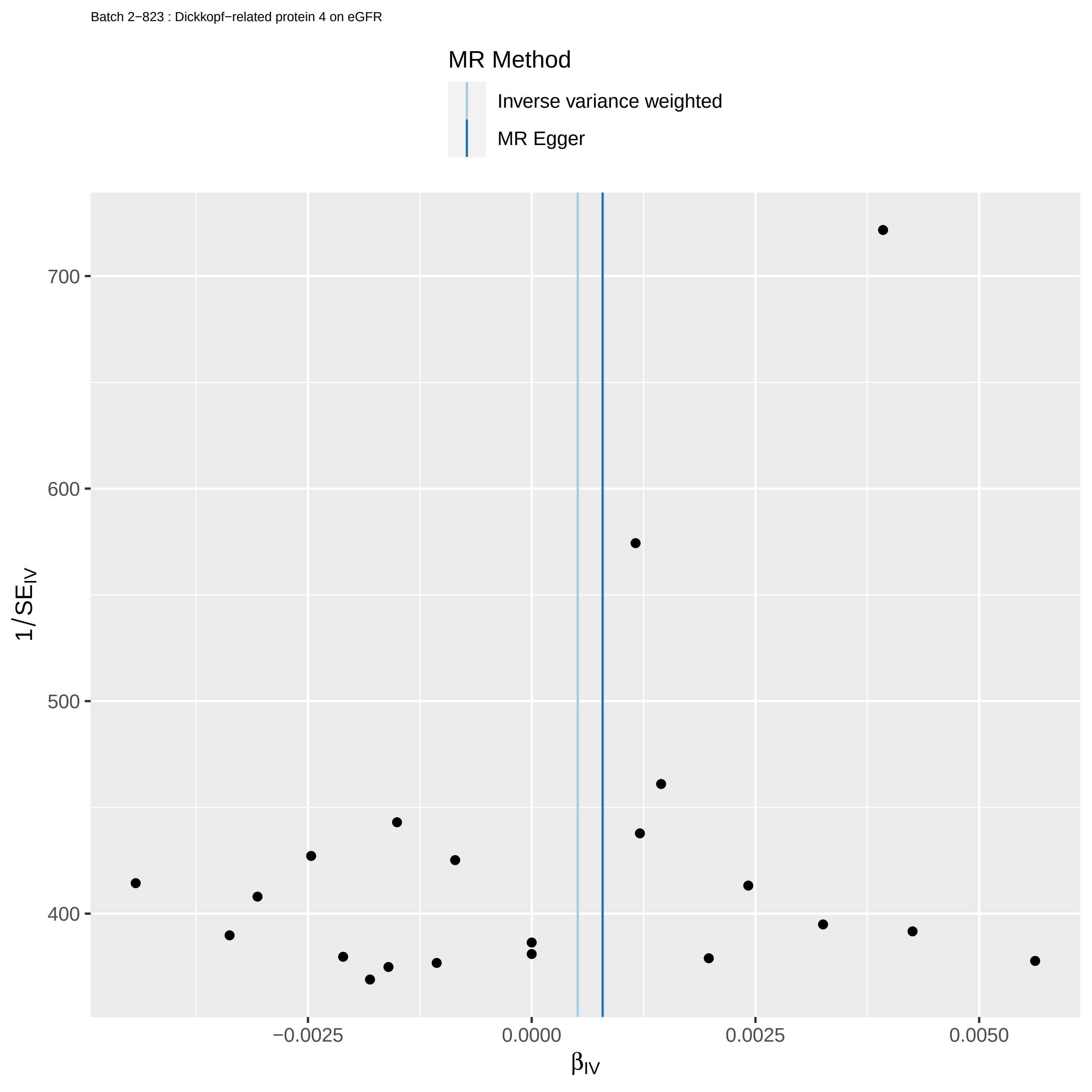

Batch 2−823 : Dickkopf−related protein 4 on eGFR
MR Method
Inverse variance weighted
MR Egger
700
600
500
400
$1/SE_{IV}$
−0.0025
0.0000
0.0025
0.0050
β_{IV}

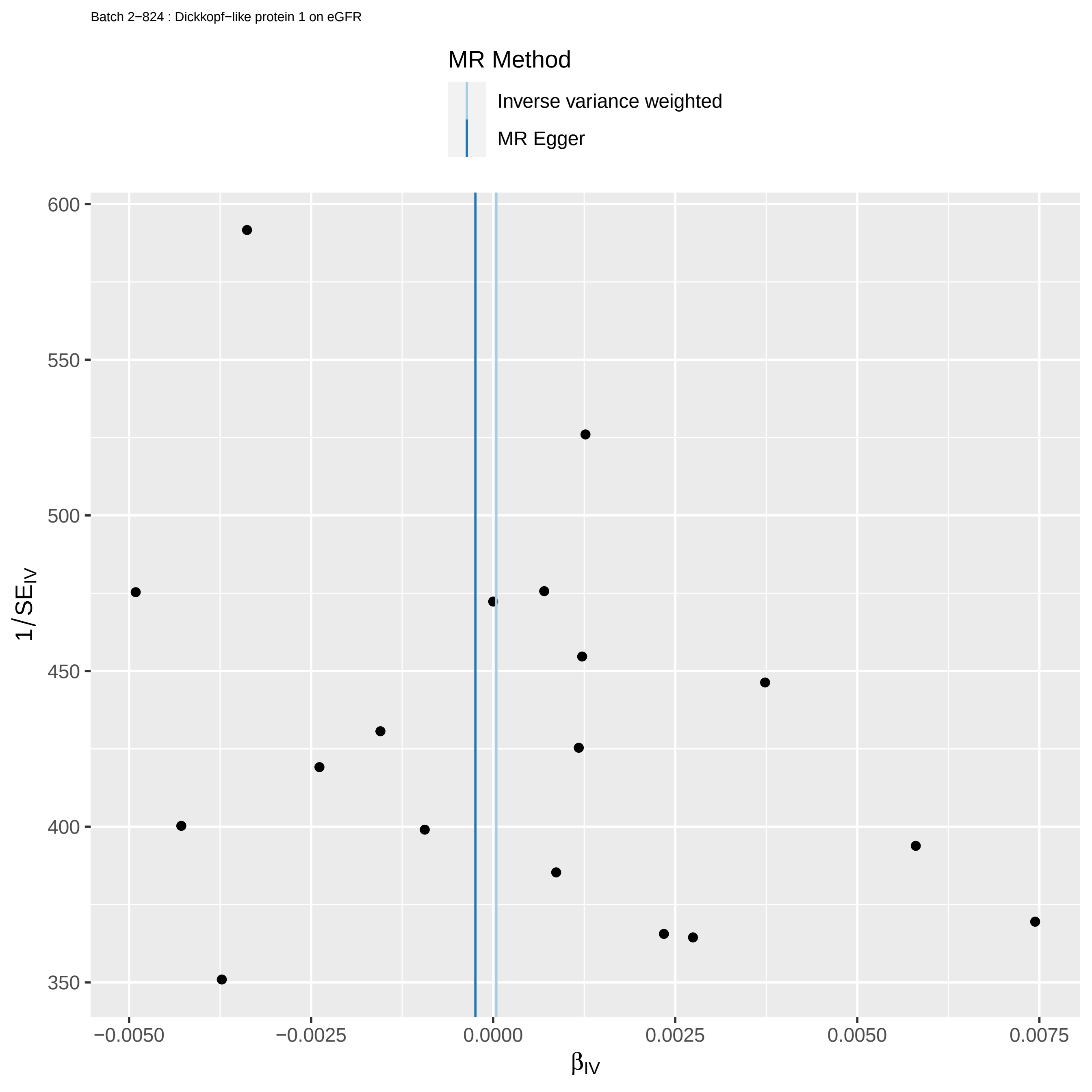

Batch 2−824 : Dickkopf−like protein 1 on eGFR
MR Method
Inverse variance weighted
MR Egger
600
550
500
450
400
350
1/SE_IV
−0.0050
−0.0025
0.0000
0.0025
0.0050
0.0075
β_IV

Batch 2−825 : Dihydrolipoyl dehydrogenase, mitochondrial on eGFR

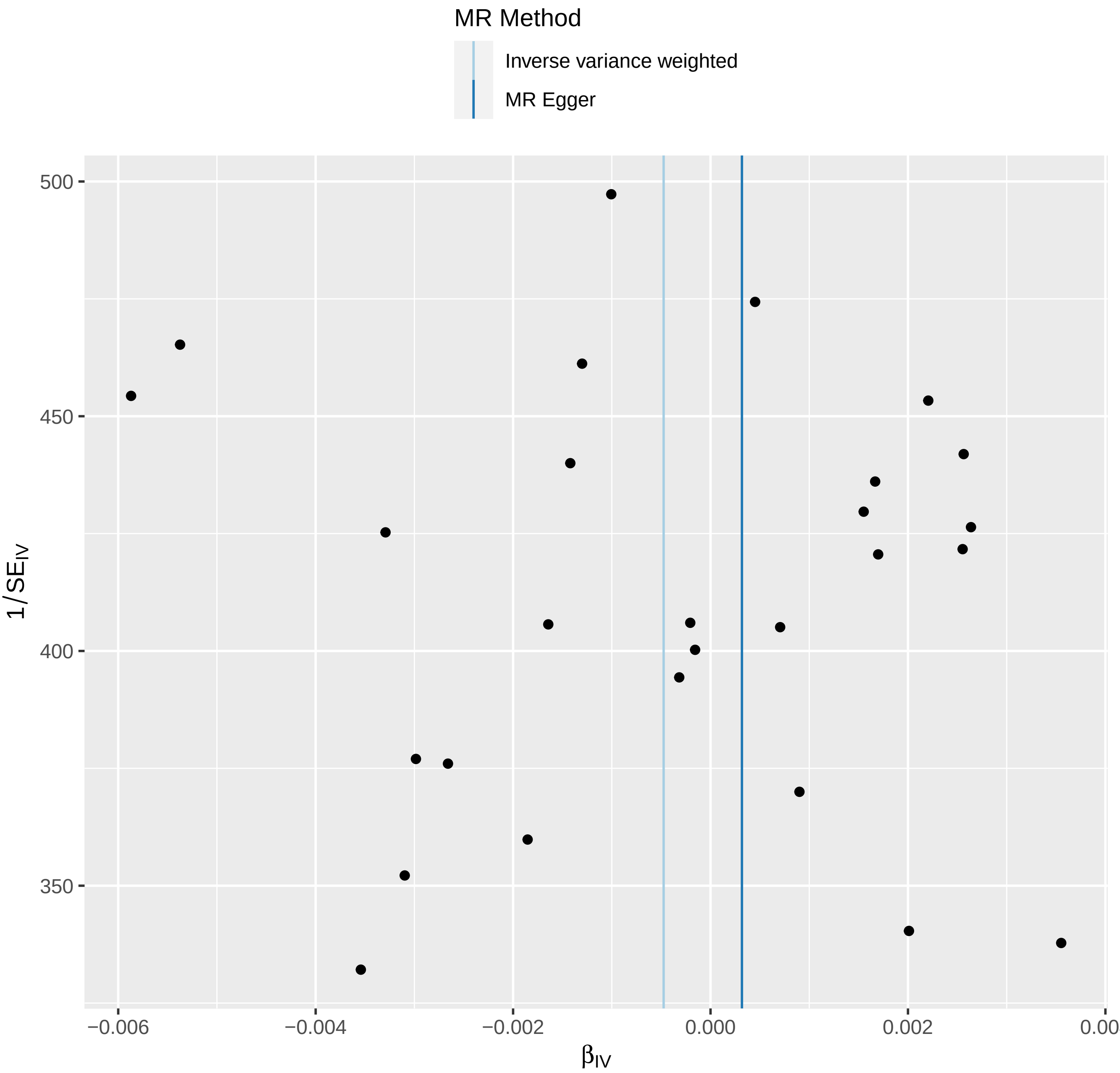

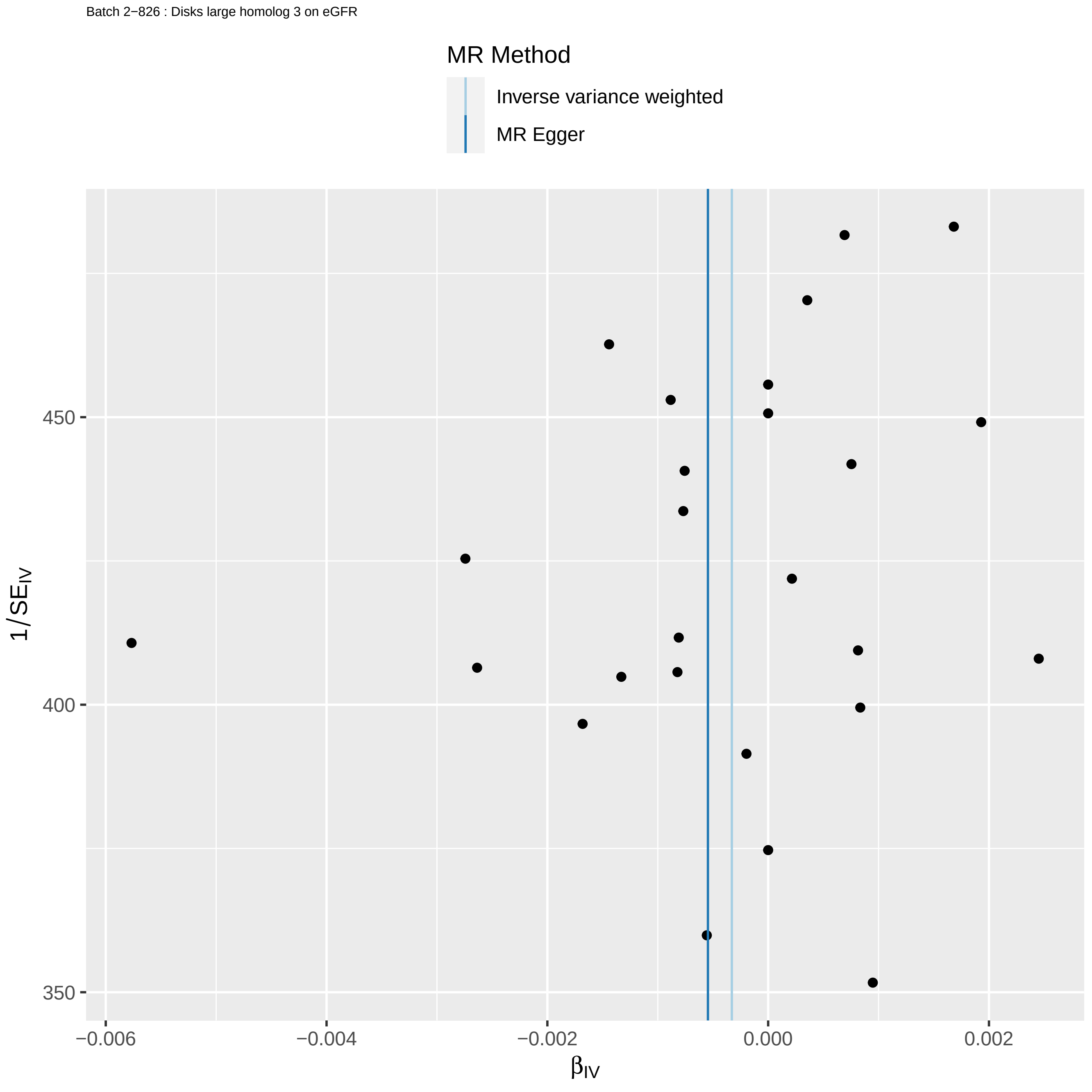

Batch 2−826 : Disks large homolog 3 on eGFR
MR Method
Inverse variance weighted
MR Egger
1/SE$_{IV}$
450
400
350
−0.006
−0.004
−0.002
0.000
0.002
β$_{IV}$

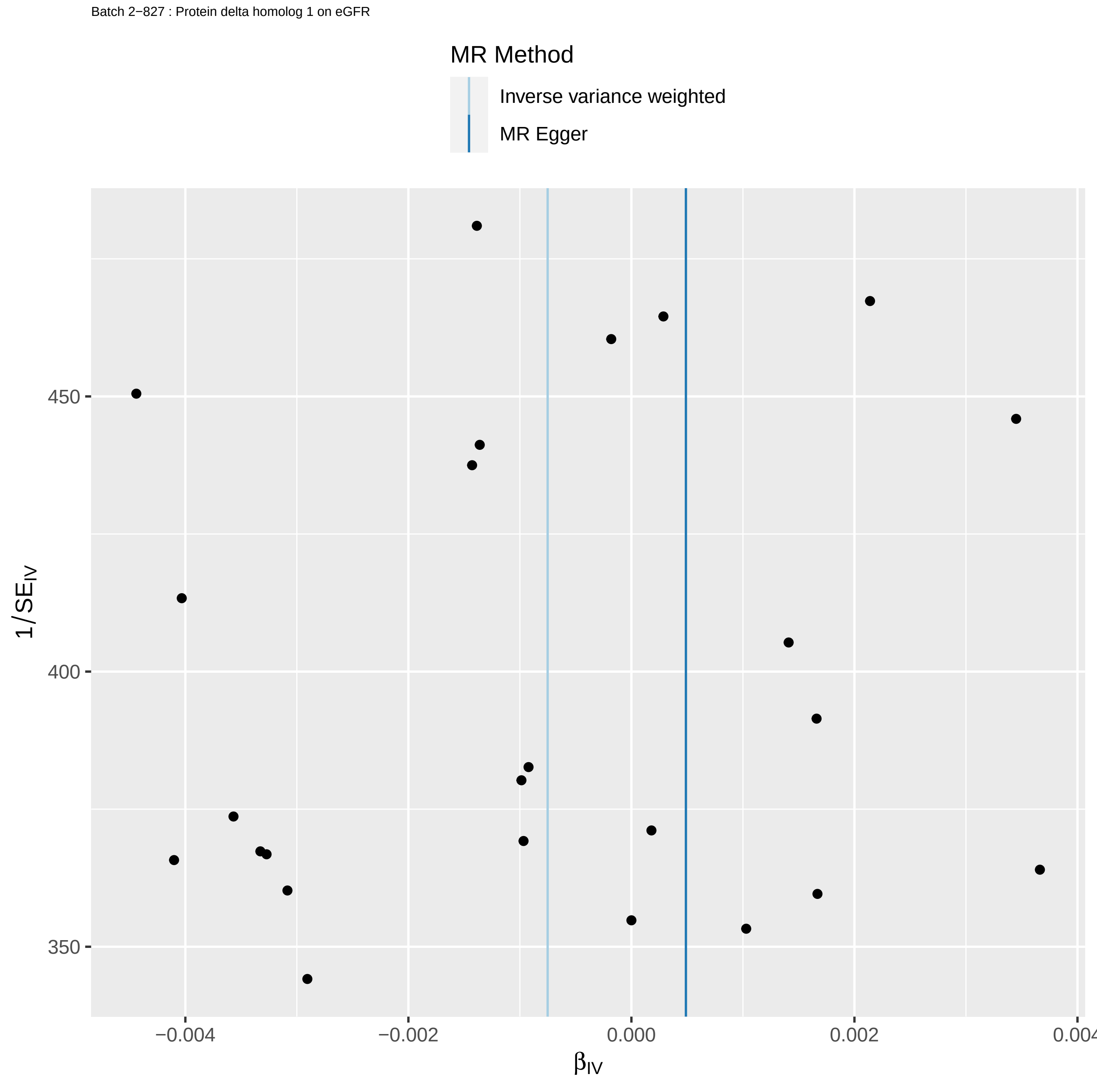

Batch 2−827 : Protein delta homolog 1 on eGFR
MR Method
Inverse variance weighted
MR Egger
450
400
350
1/SE_IV
−0.004
−0.002
0.000
0.002
0.004
β_IV

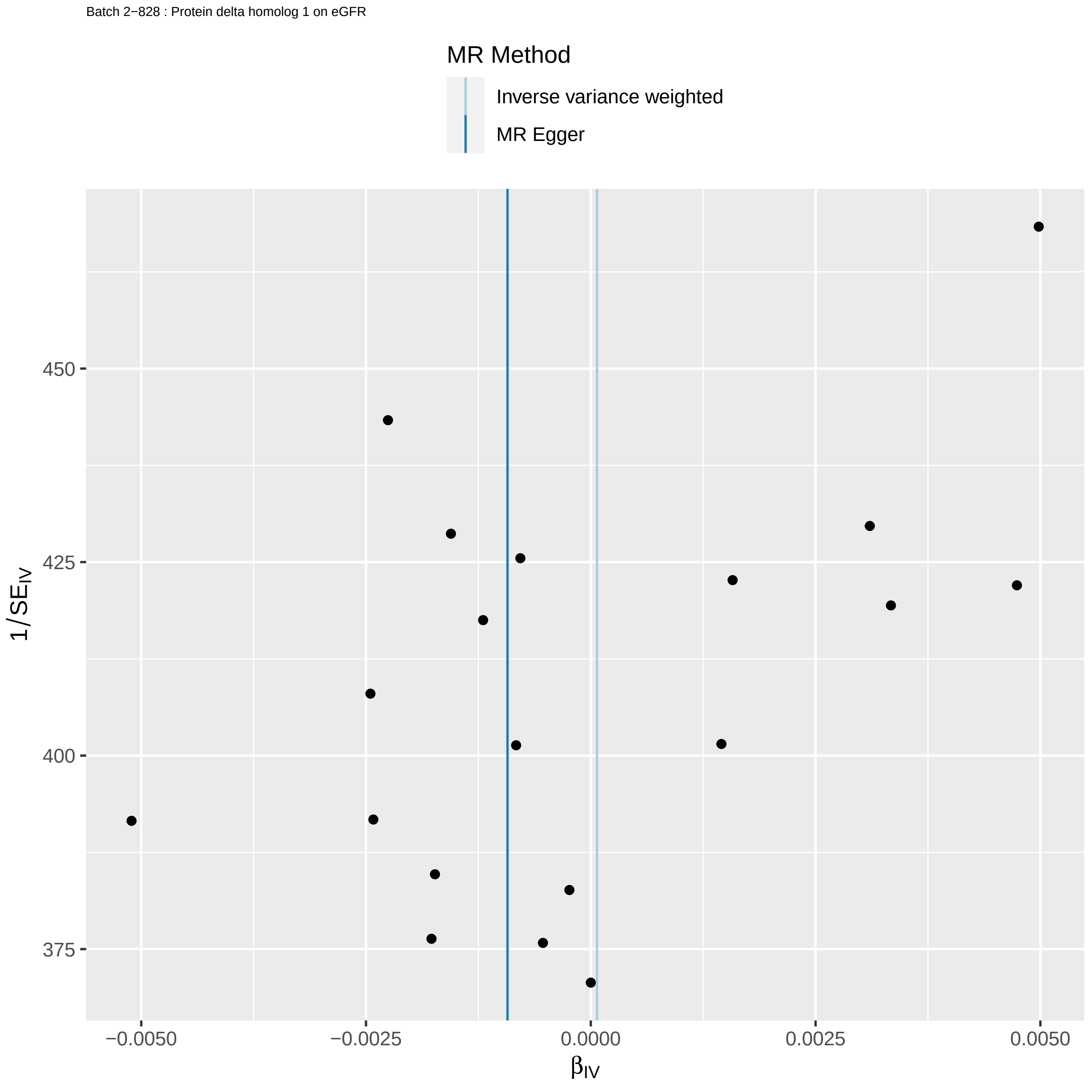

Batch 2−828 : Protein delta homolog 1 on eGFR
MR Method
Inverse variance weighted
MR Egger
450
425
400
375
1/SE_IV
−0.0050
−0.0025
0.0000
0.0025
0.0050
β_IV

Batch 2−829 : Protein delta homolog 2 on eGFR

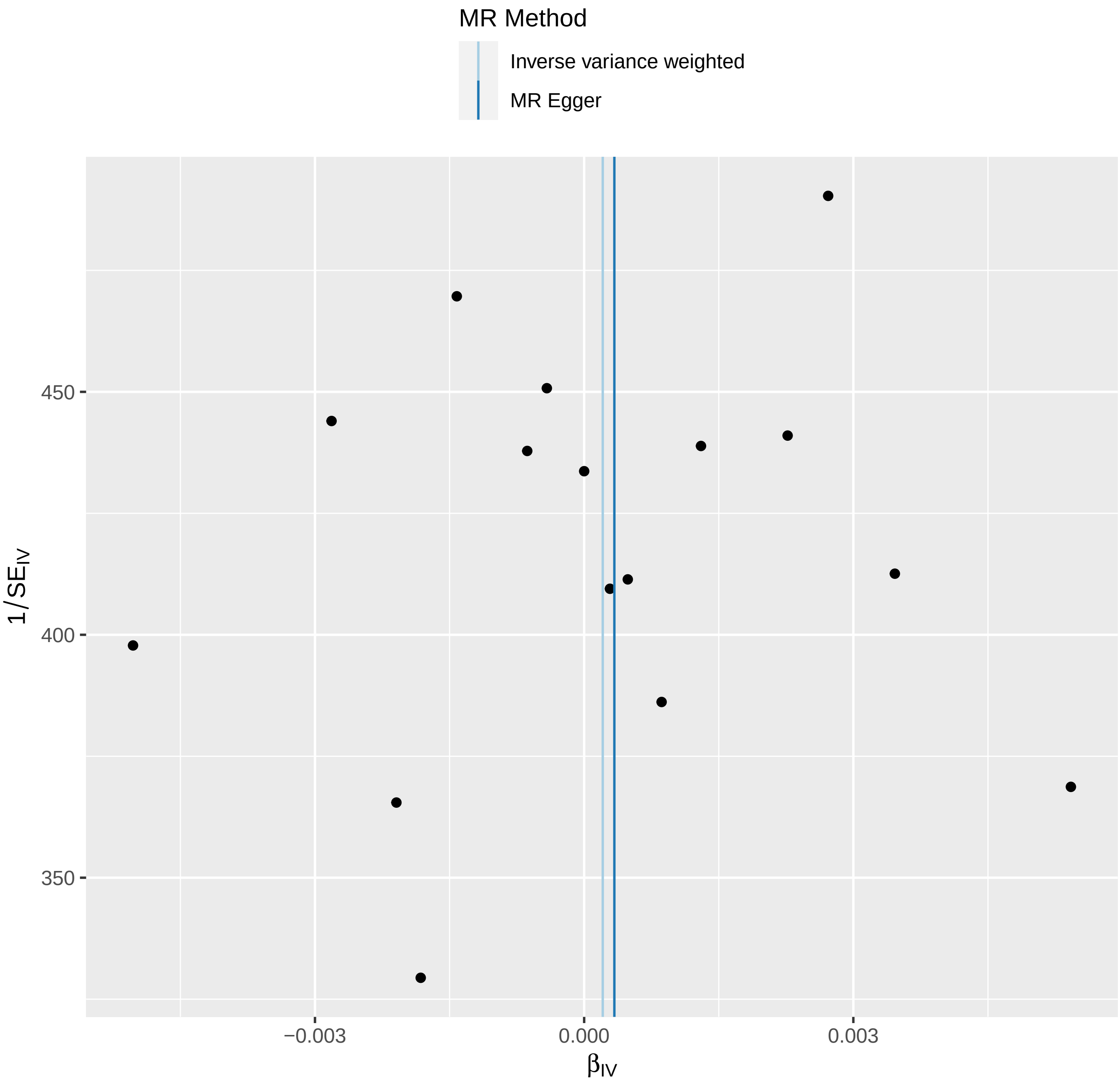

Batch 2−830 : Delta−like protein 1 on eGFR

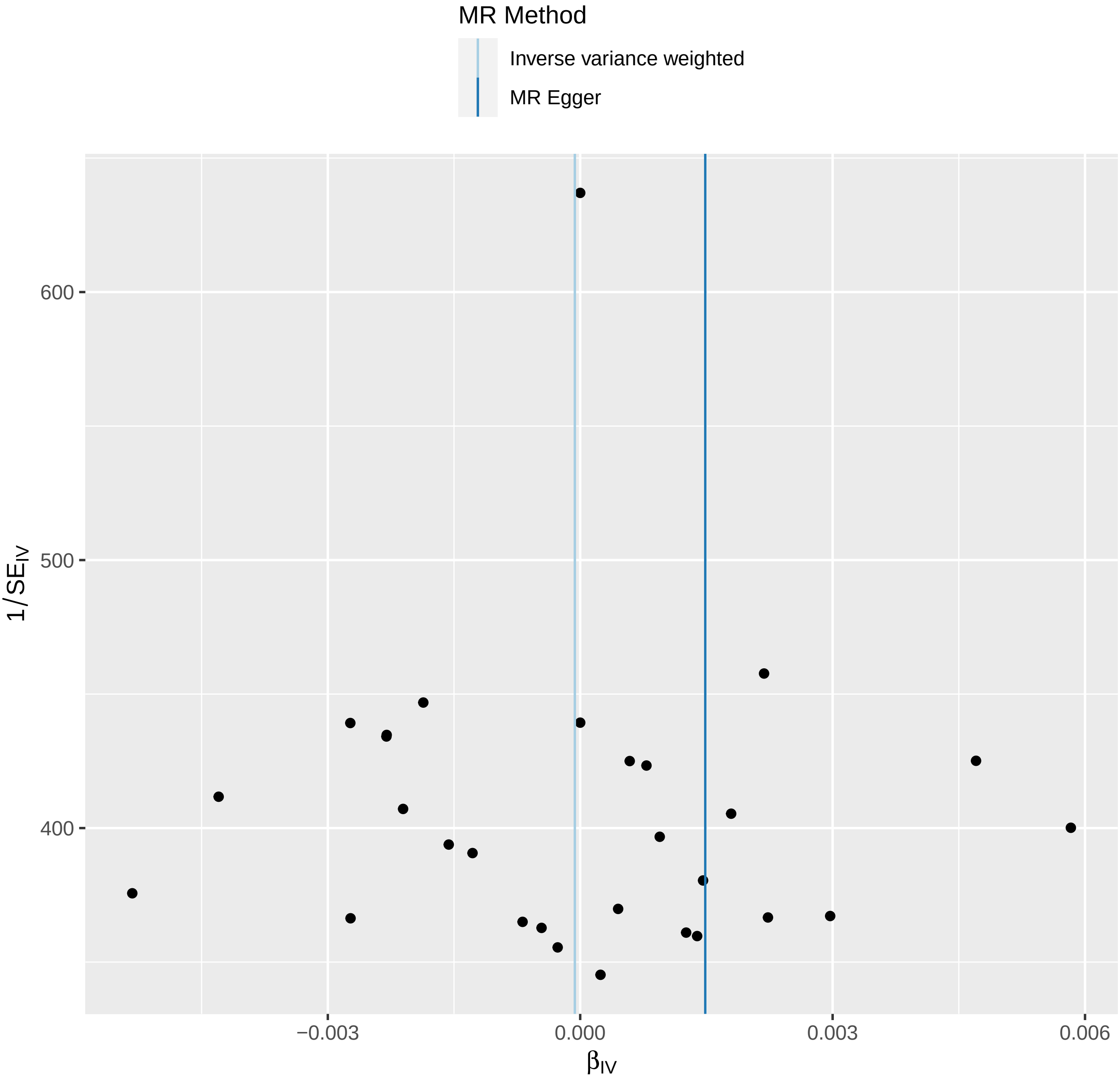

Batch 2−831 : Delta−like protein 1 on eGFR

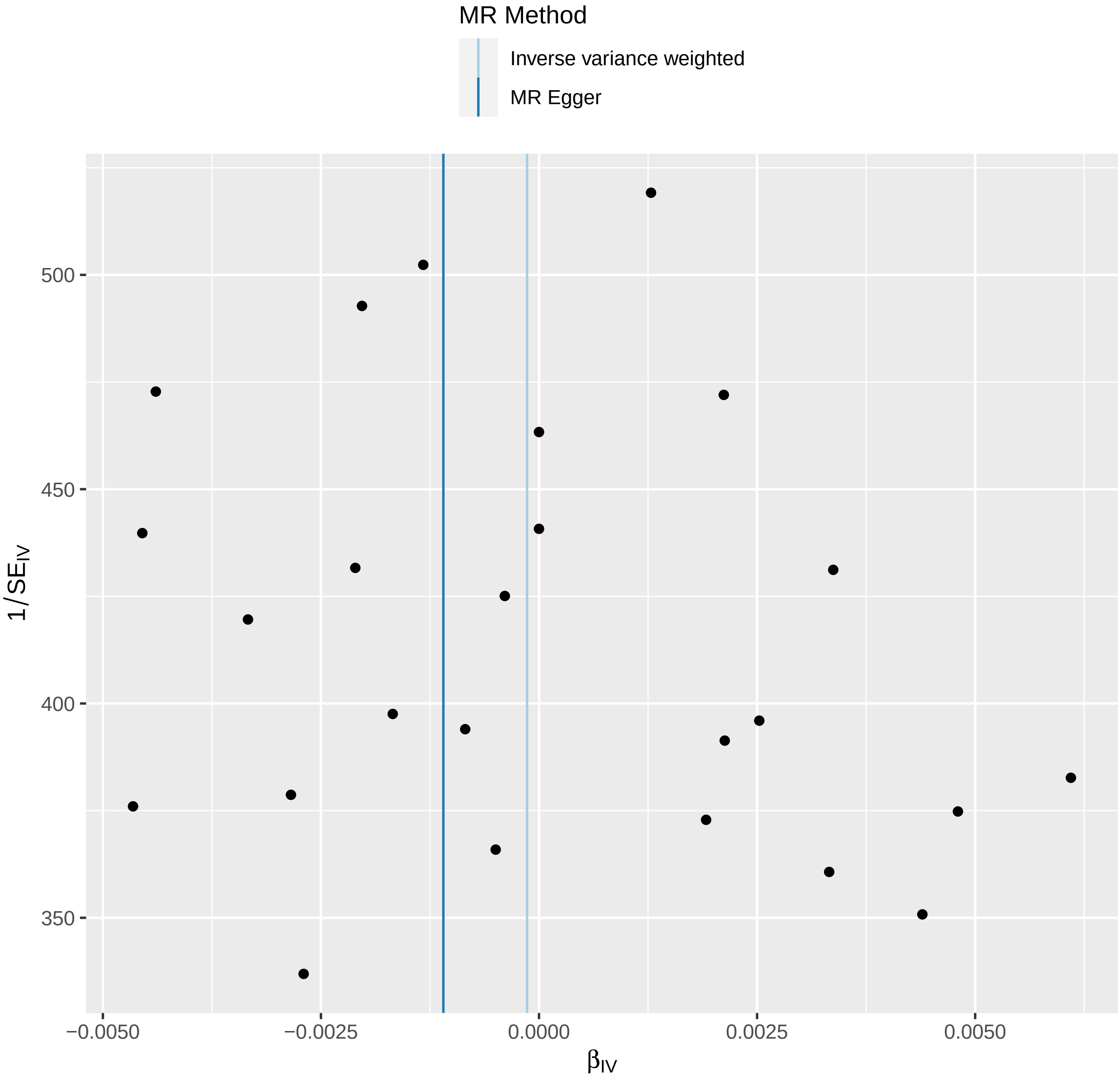

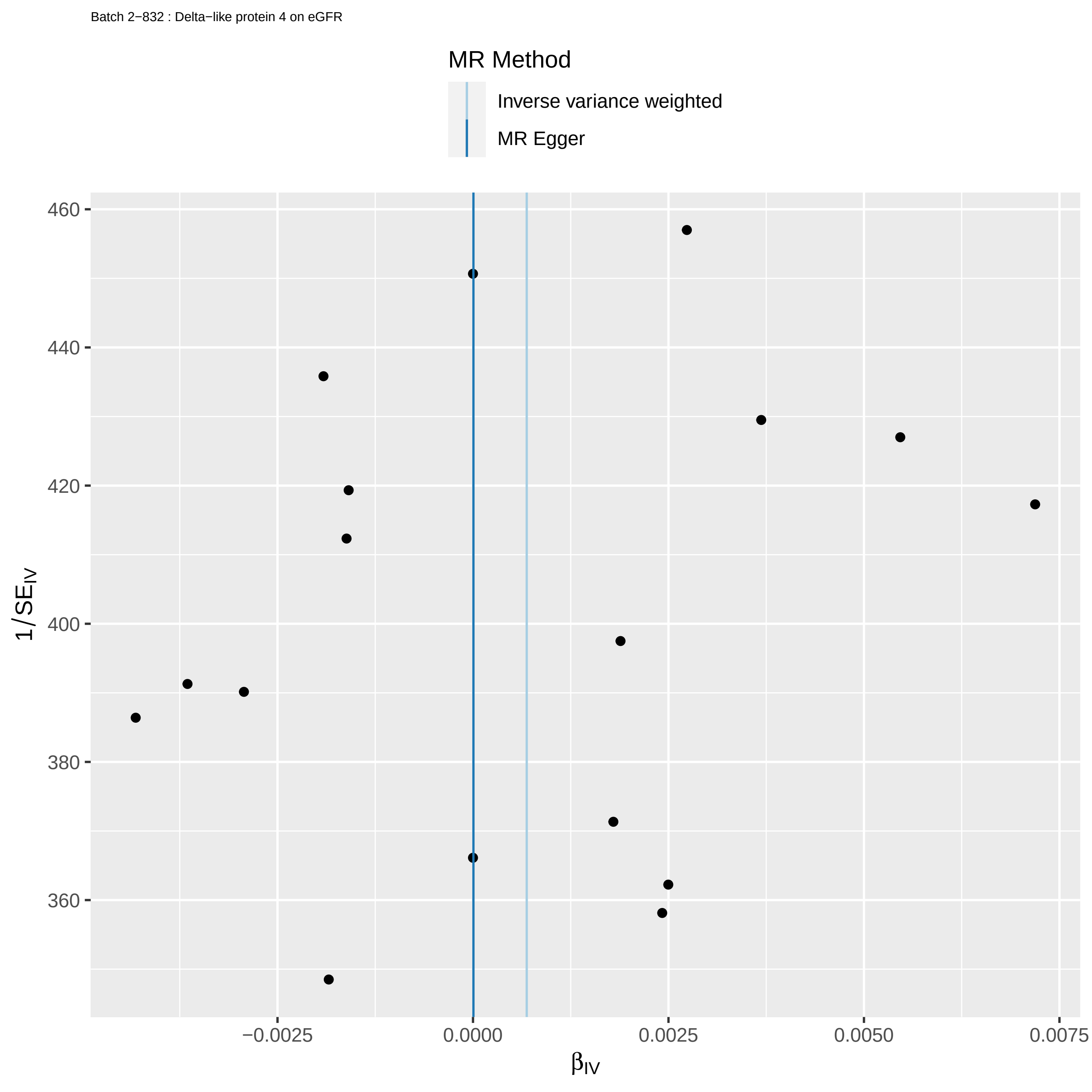

Batch 2−832 : Delta−like protein 4 on eGFR
MR Method
Inverse variance weighted
MR Egger
460
440
420
400
380
360
1/SE$_{IV}$
−0.0025
0.0000
0.0025
0.0050
0.0075
β_{IV}

Batch 2−833 : Dermokine on eGFR

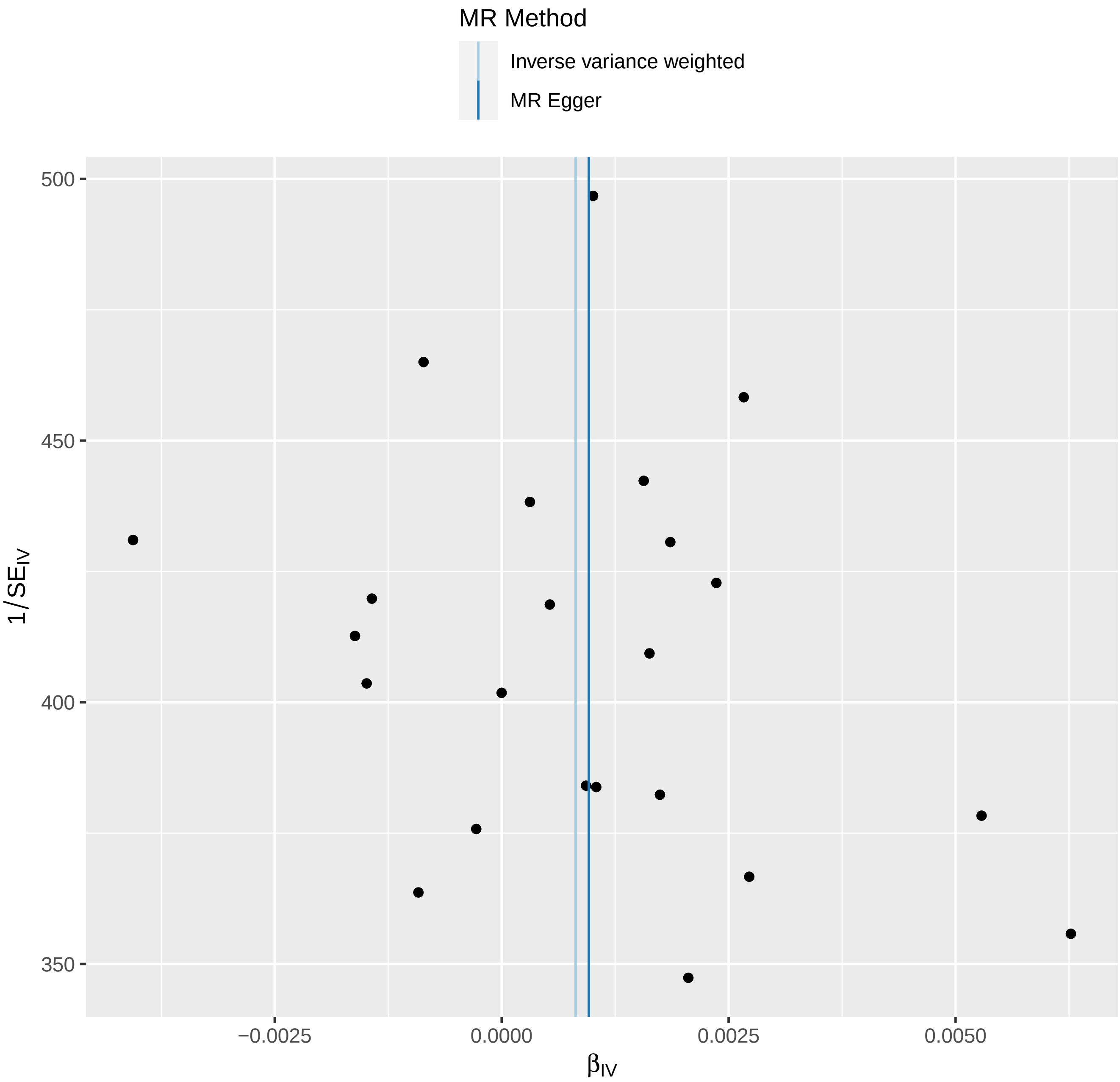

Batch 2−834 : Myotonin−protein kinase on eGFR

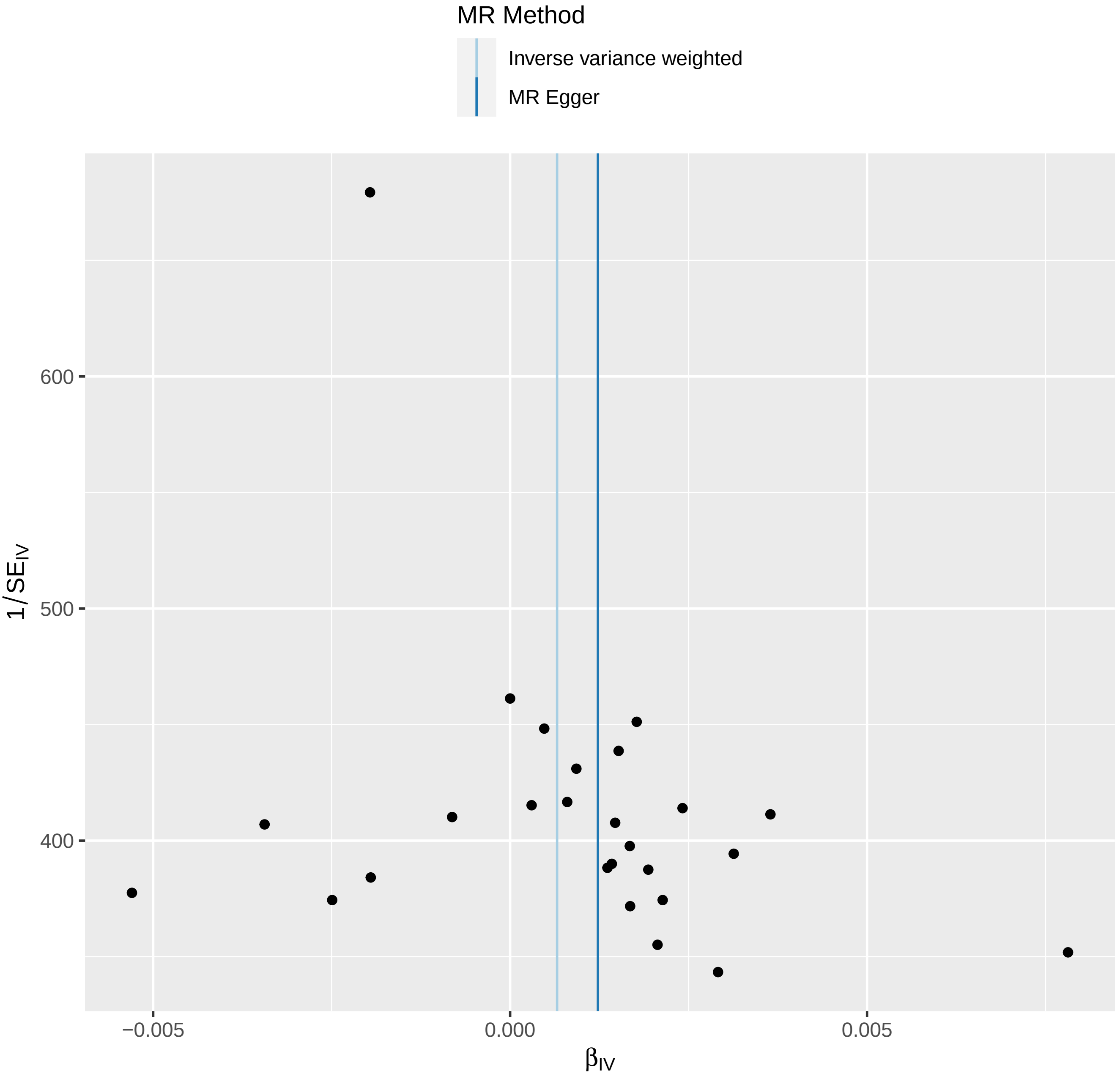

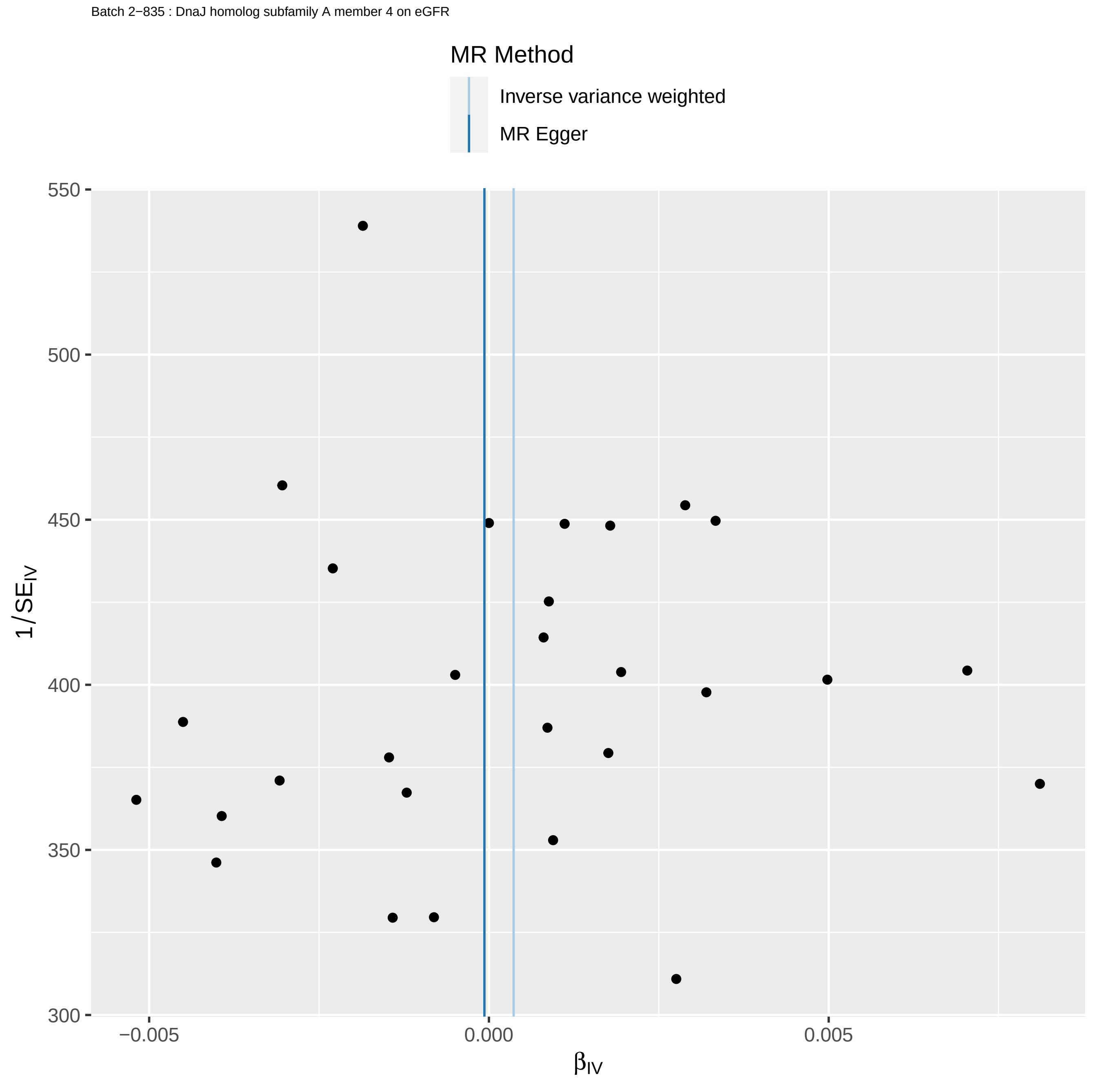

Batch 2−835 : DnaJ homolog subfamily A member 4 on eGFR
MR Method
Inverse variance weighted
MR Egger
550
500
450
400
350
300
1/SE_IV
−0.005
0.000
0.005
β_IV

Batch 2−836 : DnaJ homolog subfamily B member 11 on eGFR

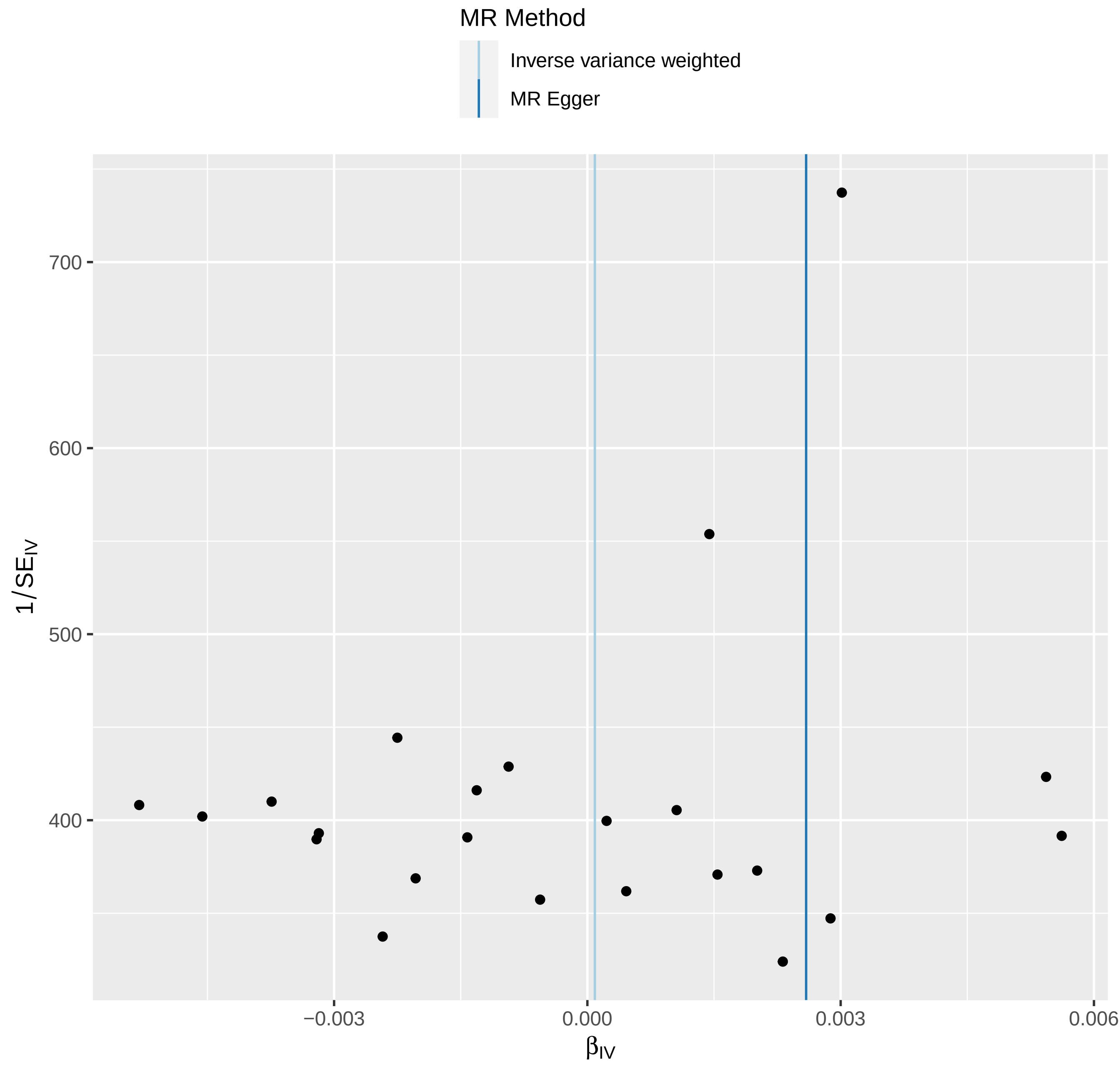

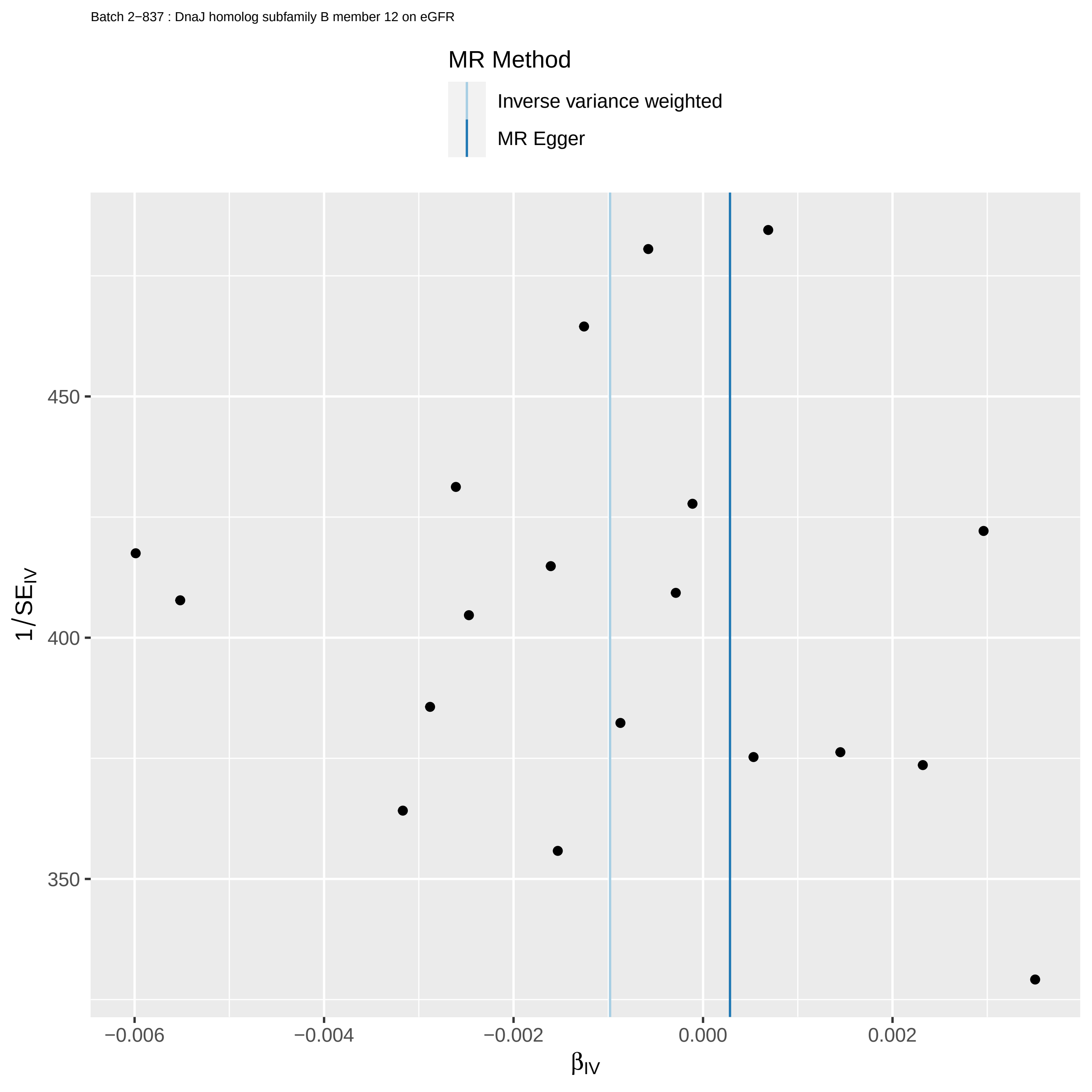

Batch 2−837 : DnaJ homolog subfamily B member 12 on eGFR
MR Method
Inverse variance weighted
MR Egger
450
400
350
1/SE$_{IV}$
−0.006
−0.004
−0.002
0.000
0.002
β$_{IV}$

Batch 2−838 : DnaJ homolog subfamily B member 1 on eGFR

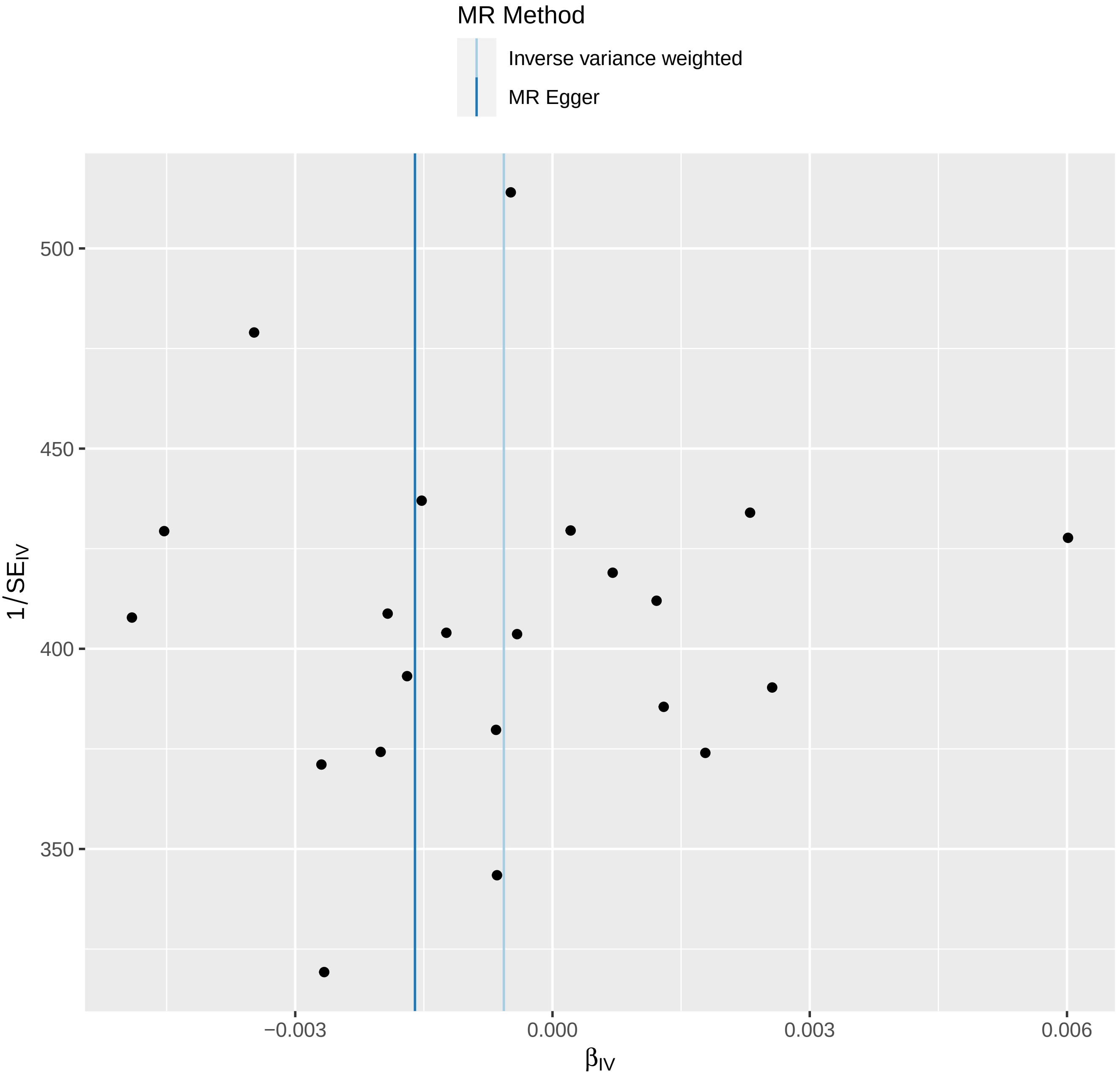

Batch 2−839 : DnaJ homolog subfamily B member 14 on eGFR

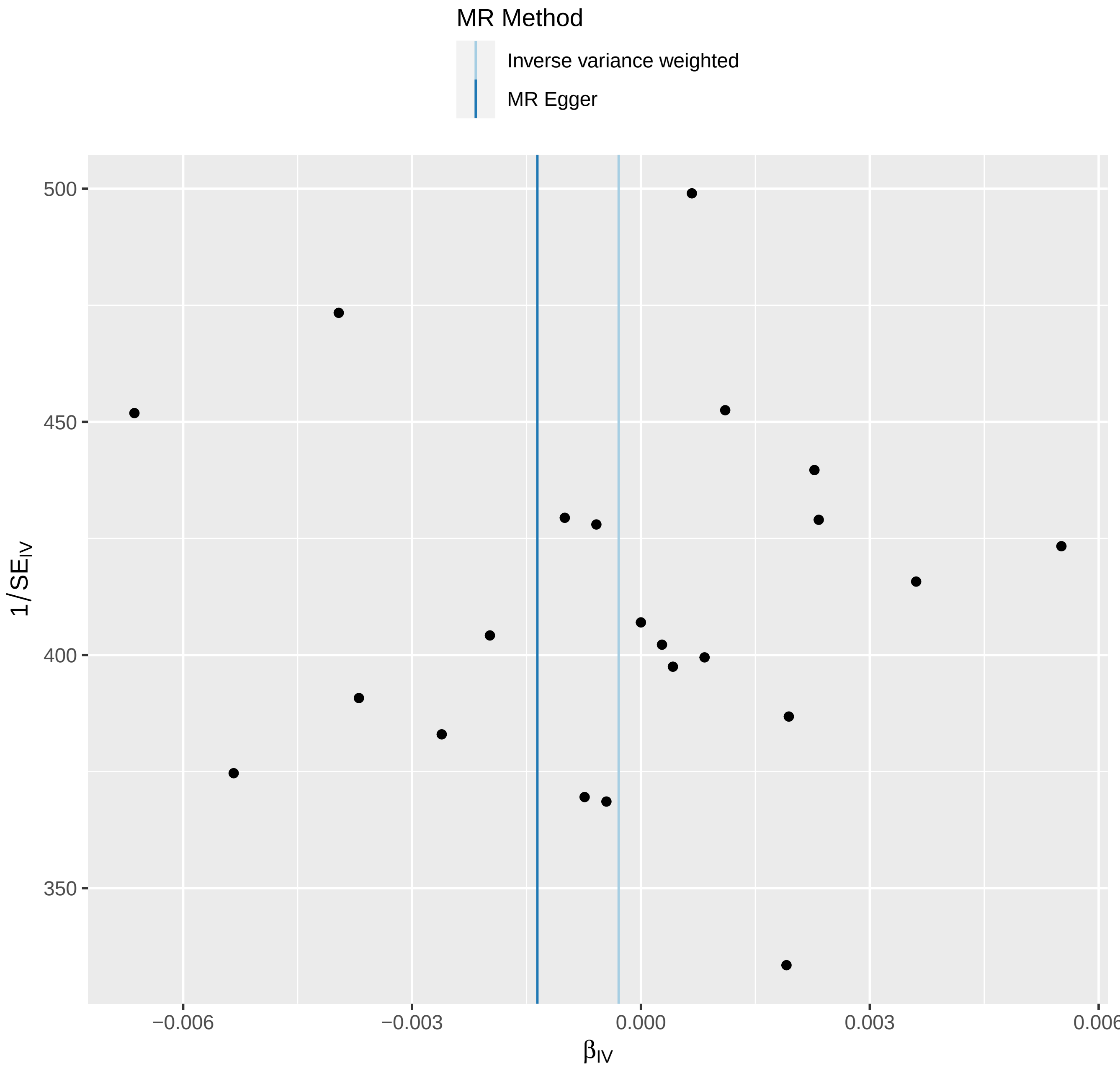

Batch 2−840 : DnaJ homolog subfamily B member 14 on eGFR

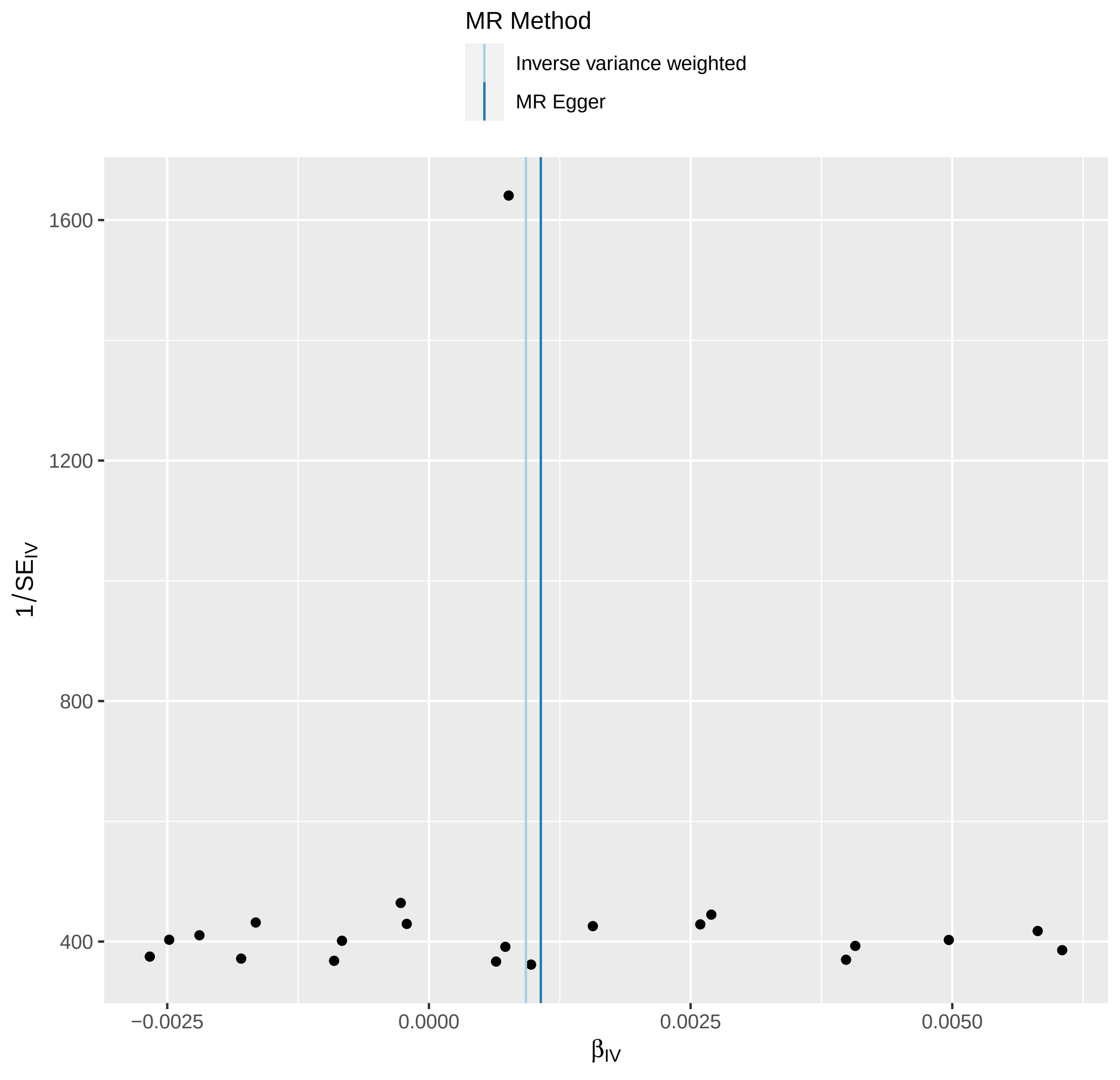

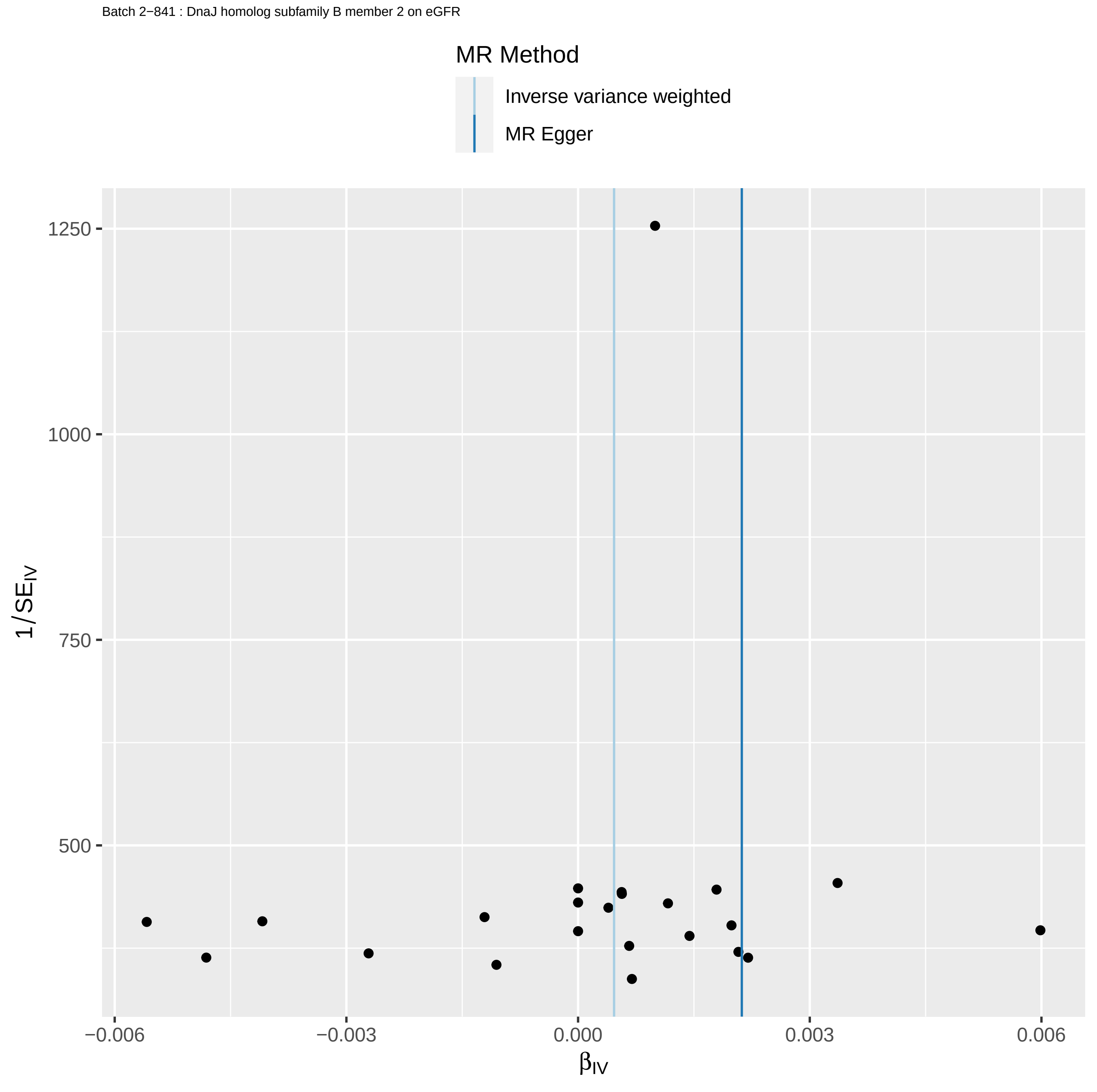
Batch 2−841 : DnaJ homolog subfamily B member 2 on eGFR
MR Method
Inverse variance weighted
MR Egger
1250
1000
750
500
1/SE_IV
−0.006
−0.003
0.000
0.003
0.006
β_IV

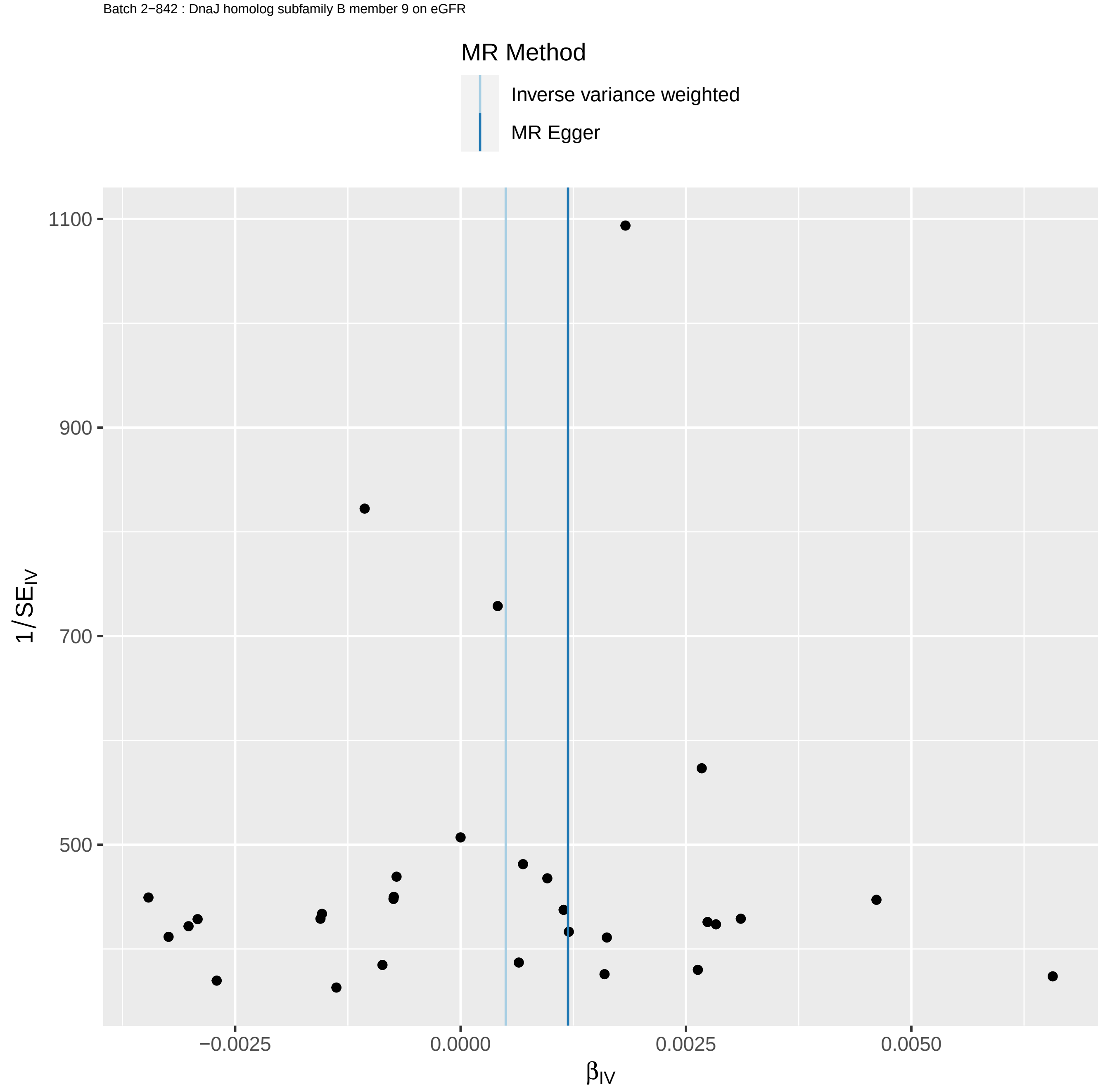

Batch 2−842 : DnaJ homolog subfamily B member 9 on eGFR
MR Method
Inverse variance weighted
MR Egger
1100
900
700
500
1/SE_IV
−0.0025
0.0000
0.0025
0.0050
β_IV

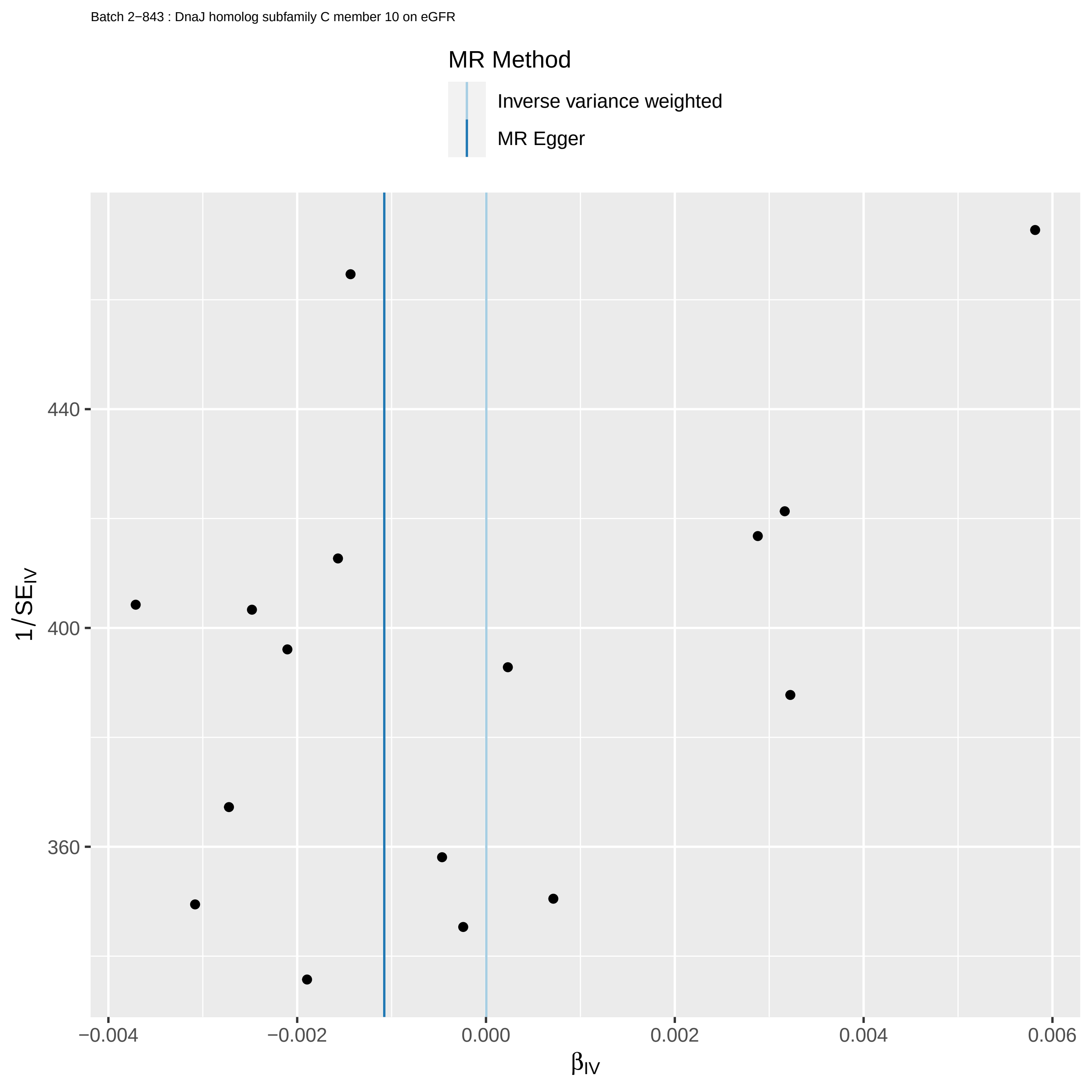

Batch 2−843 : DnaJ homolog subfamily C member 10 on eGFR
MR Method
Inverse variance weighted
MR Egger
440
400
360
$1/SE_{IV}$
−0.004
−0.002
0.000
0.002
0.004
0.006
β_{IV}

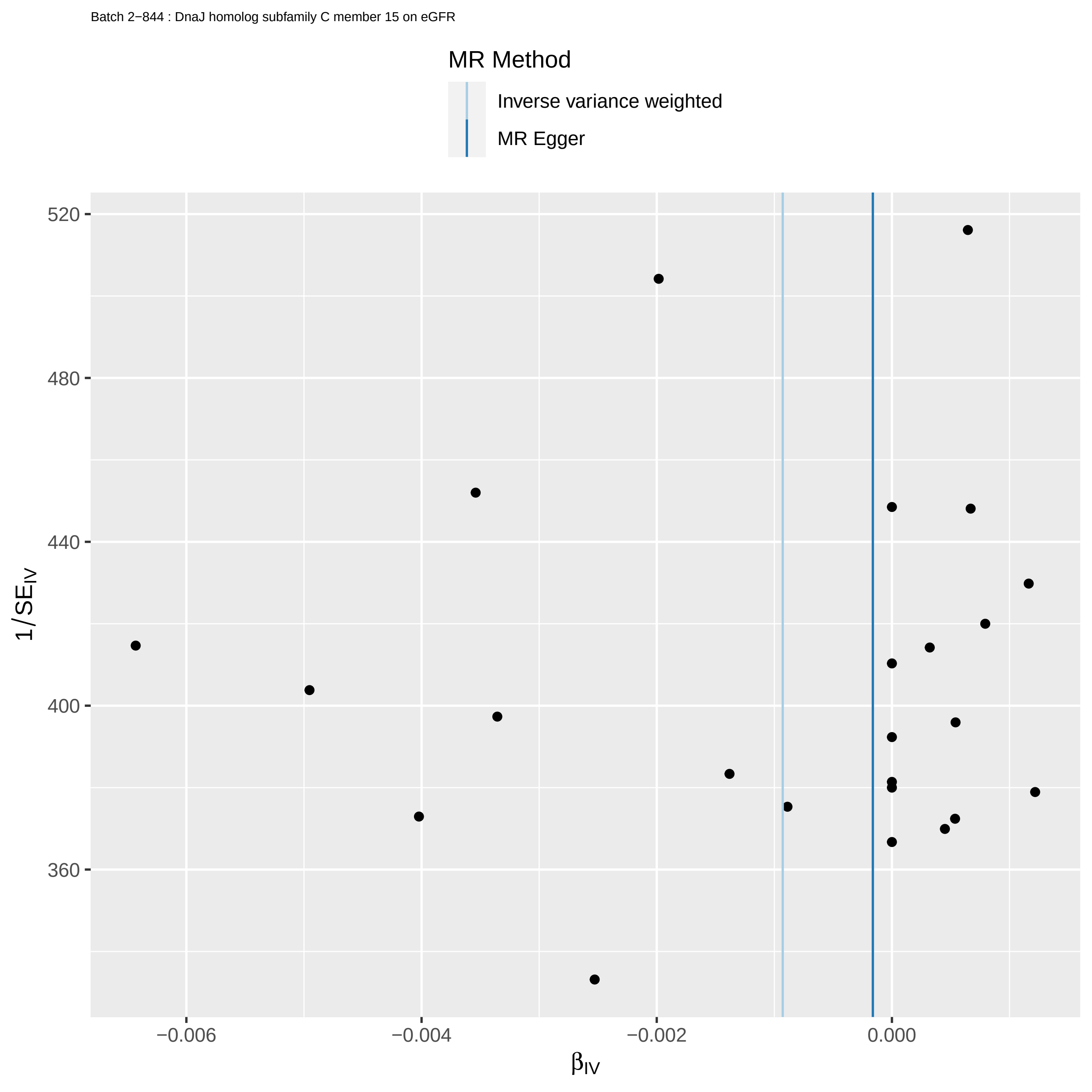

Batch 2−844 : DnaJ homolog subfamily C member 15 on eGFR
MR Method
Inverse variance weighted
MR Egger
520
480
440
400
360
1/SE_IV
−0.006
−0.004
−0.002
0.000
β_IV

Batch 2−845 : DnaJ homolog subfamily C member 17 on eGFR

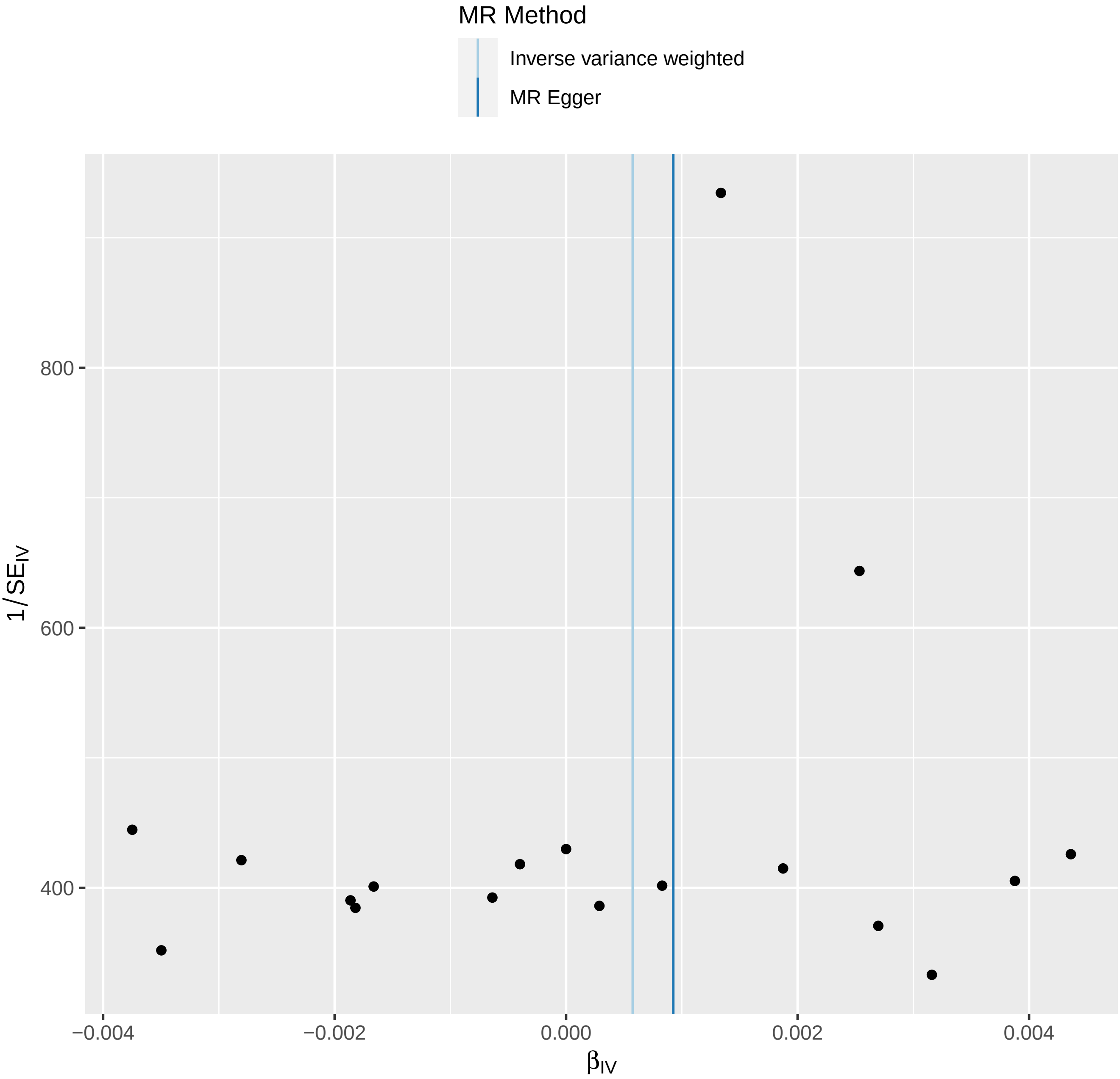

Batch 2−846 : DnaJ homolog subfamily C member 18 on eGFR

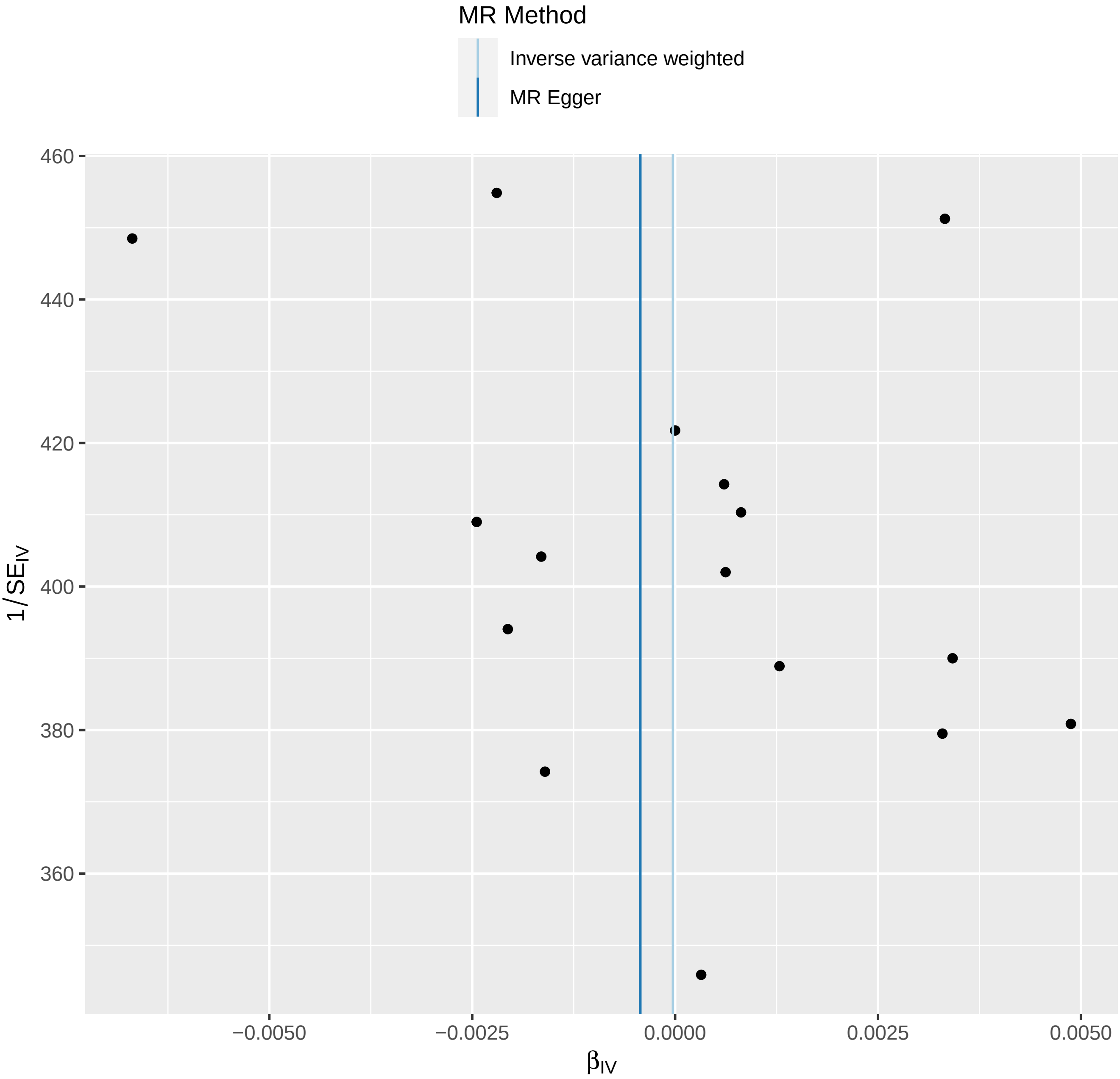

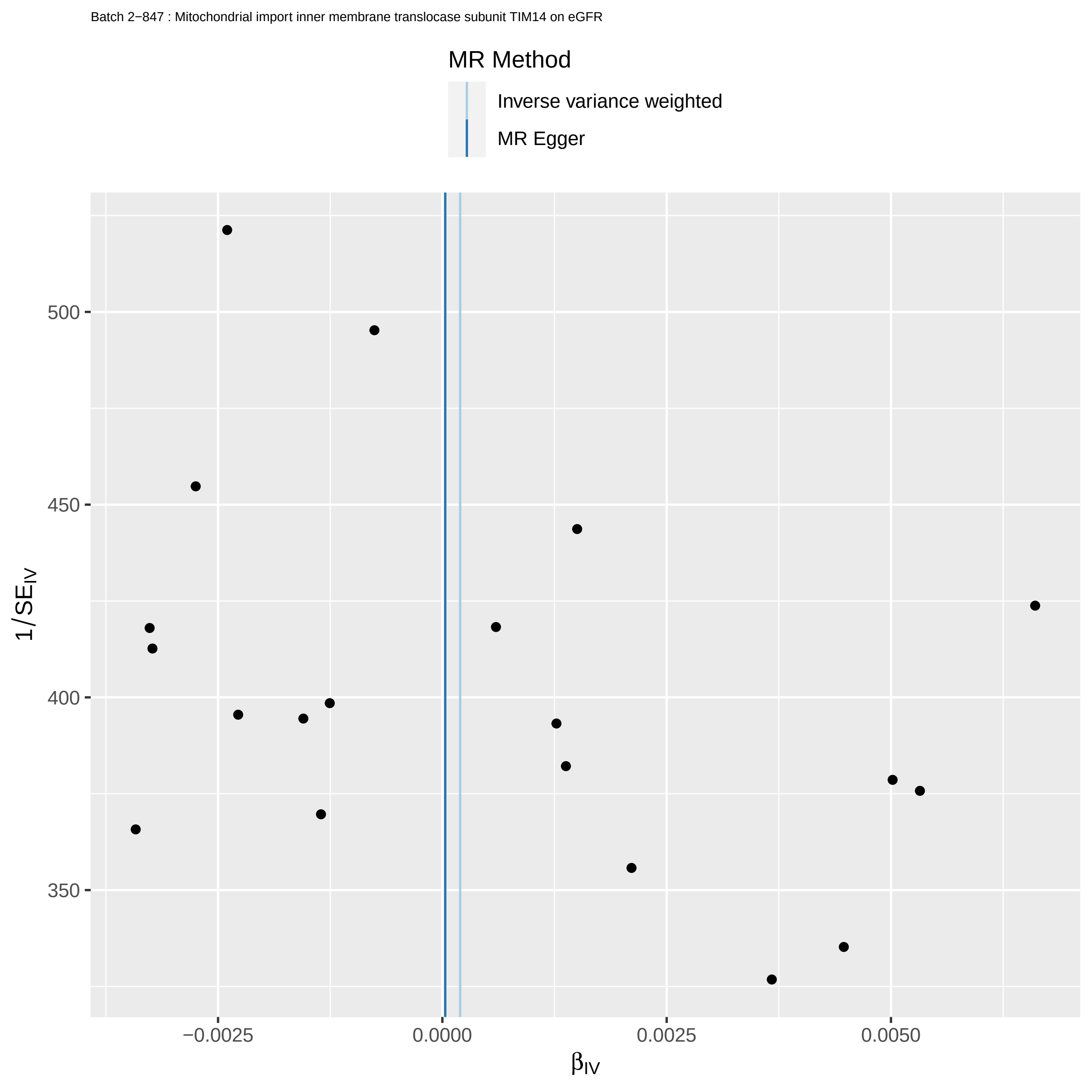
Batch 2−847 : Mitochondrial import inner membrane translocase subunit TIM14 on eGFR
MR Method
Inverse variance weighted
MR Egger
500
450
400
350
1/SE$_{IV}$
−0.0025
0.0000
0.0025
0.0050
β$_{IV}$

Batch 2−848 : DnaJ homolog subfamily C member 27 on eGFR

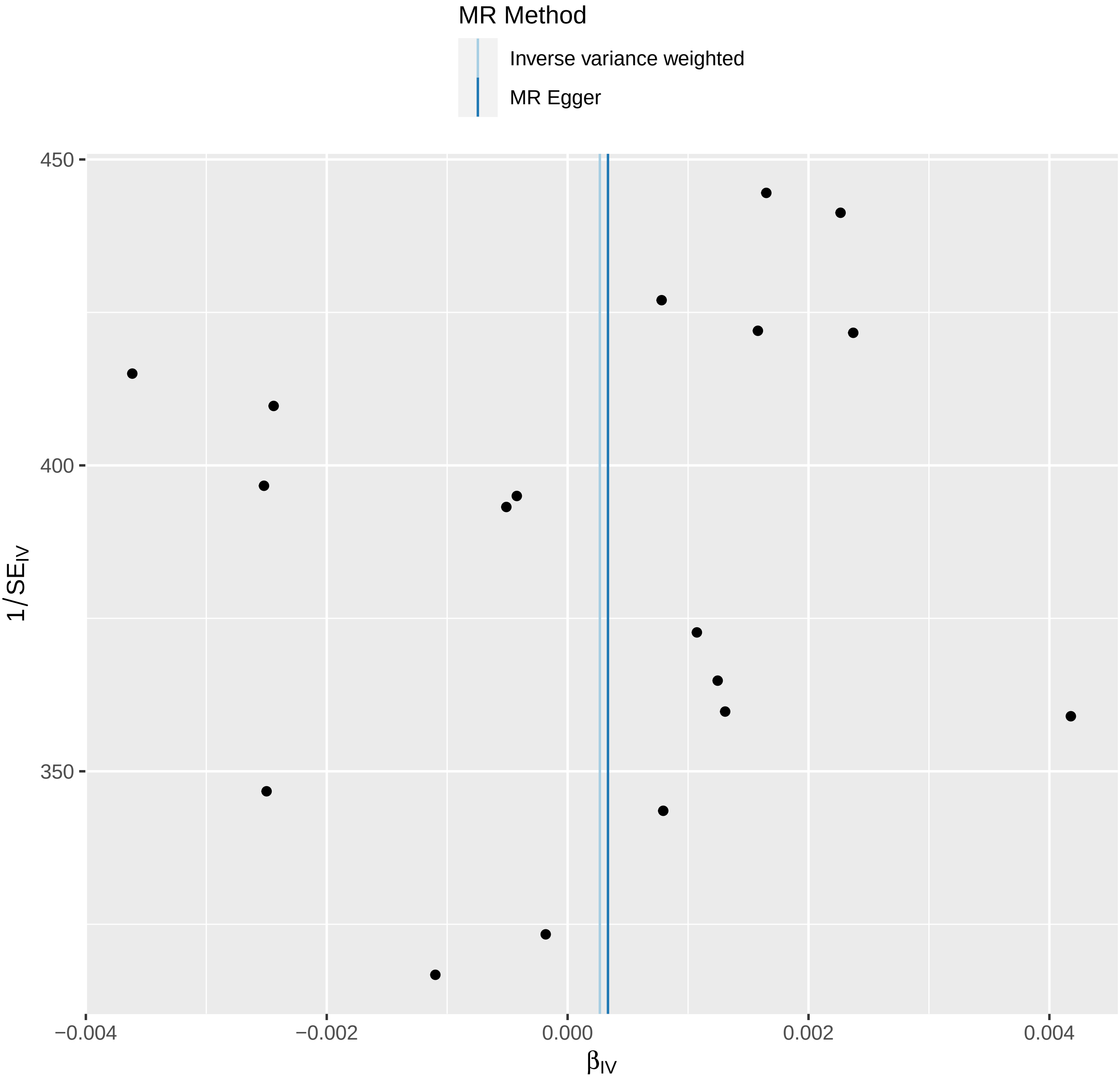

Batch 2−849 : DnaJ homolog subfamily C member 30 on eGFR

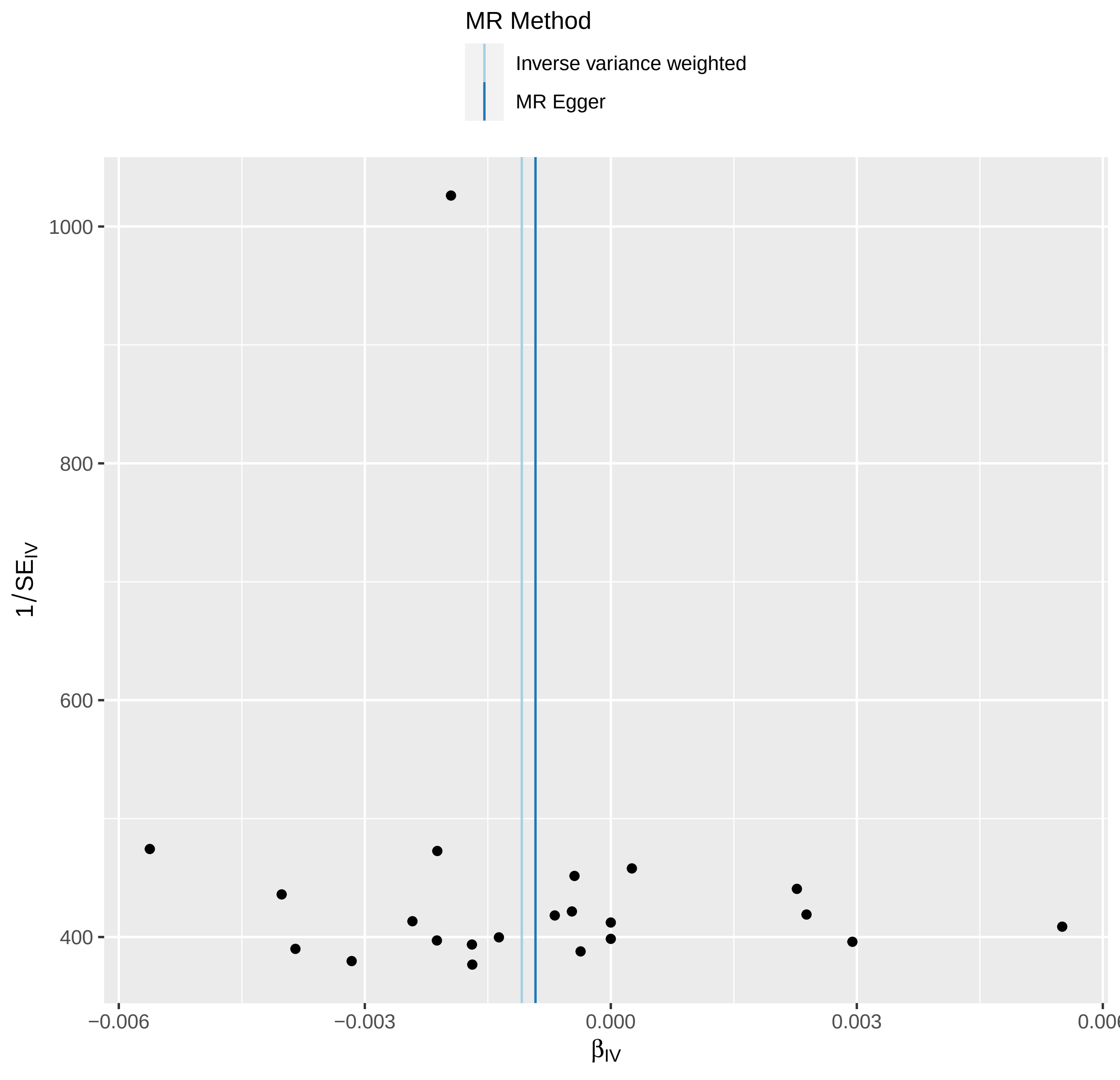

Batch 2−850 : DnaJ homolog subfamily C member 4 on eGFR

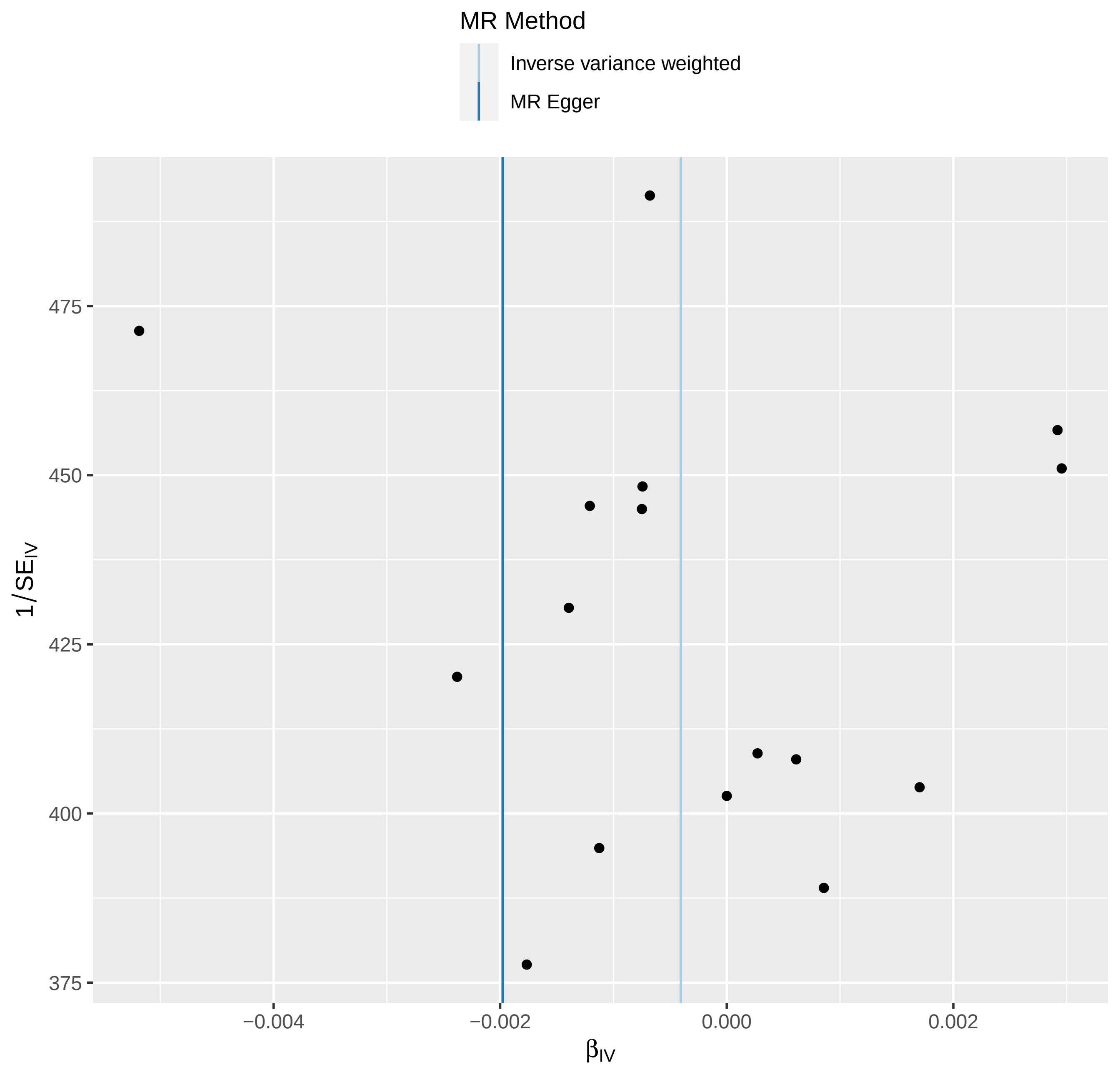

Batch 2−851 : Deoxyribonuclease−2−beta on eGFR

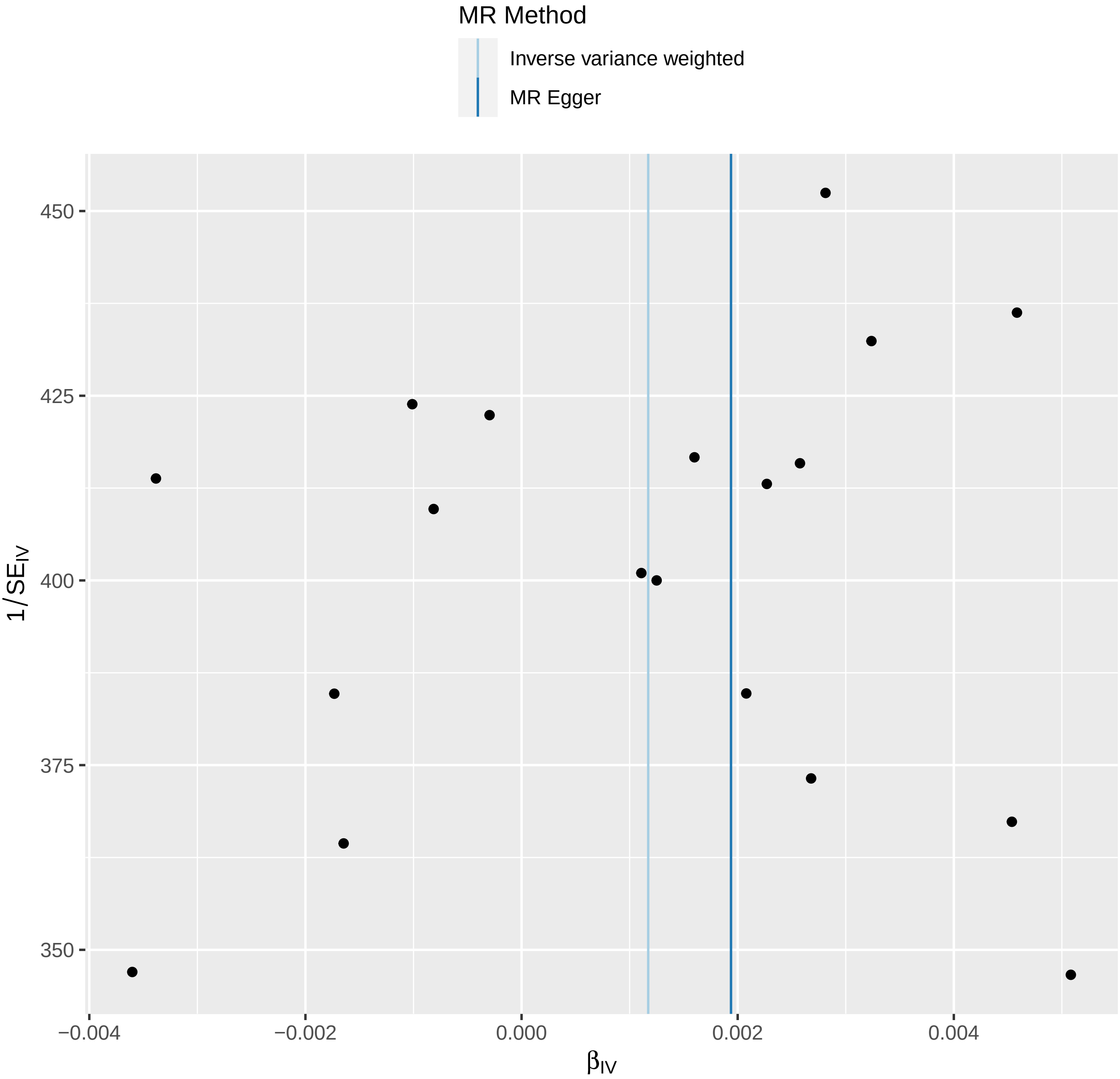

Batch 2−852 : Delta and Notch−like epidermal growth factor−related receptor on eGFR

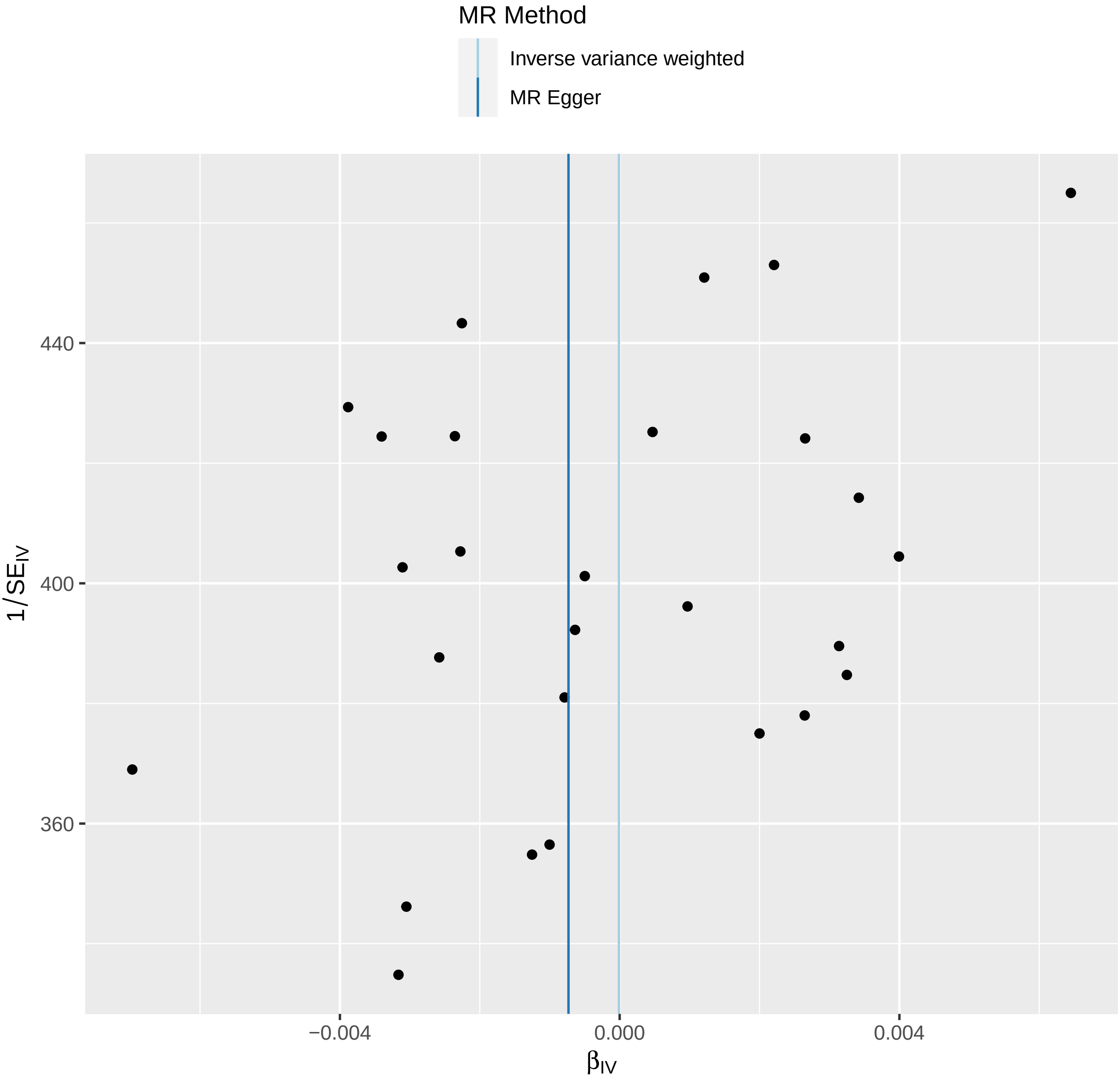

Batch 2−853 : Delta and Notch−like epidermal growth factor−related receptor on eGFR

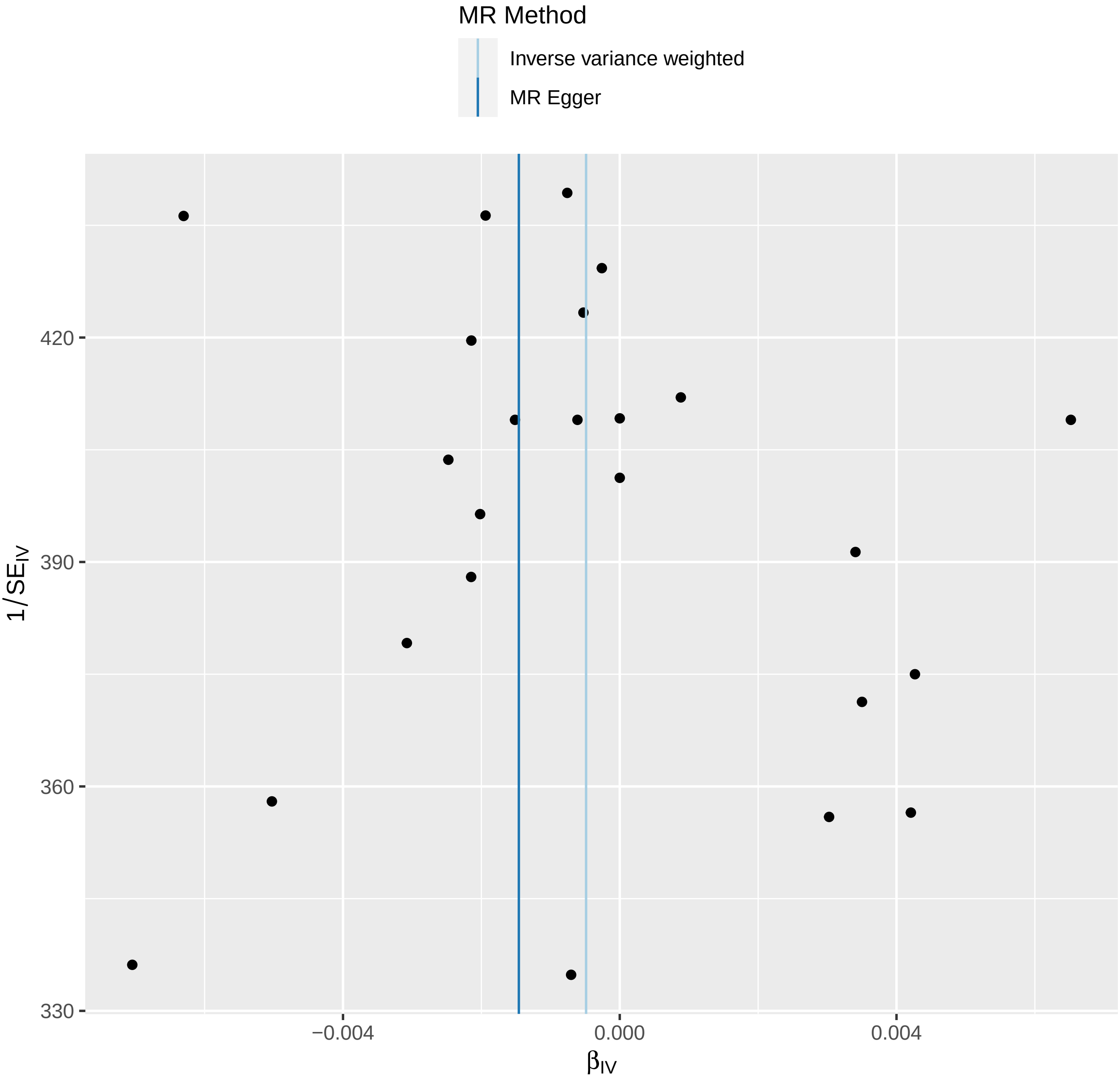

Batch 2−854 : Dedicator of cytokinesis protein 9 on eGFR

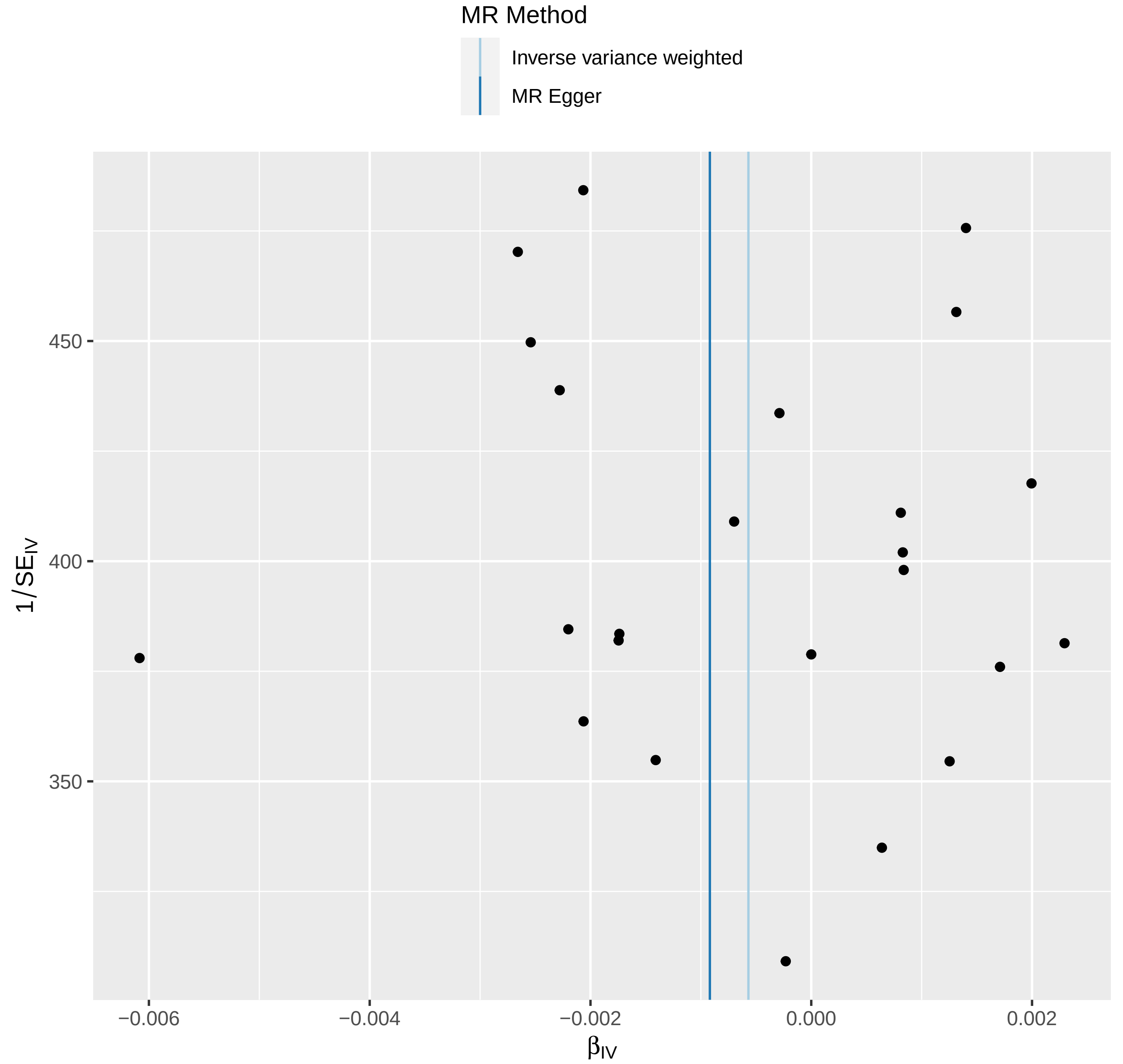

Batch 2−855 : Docking protein 2 on eGFR

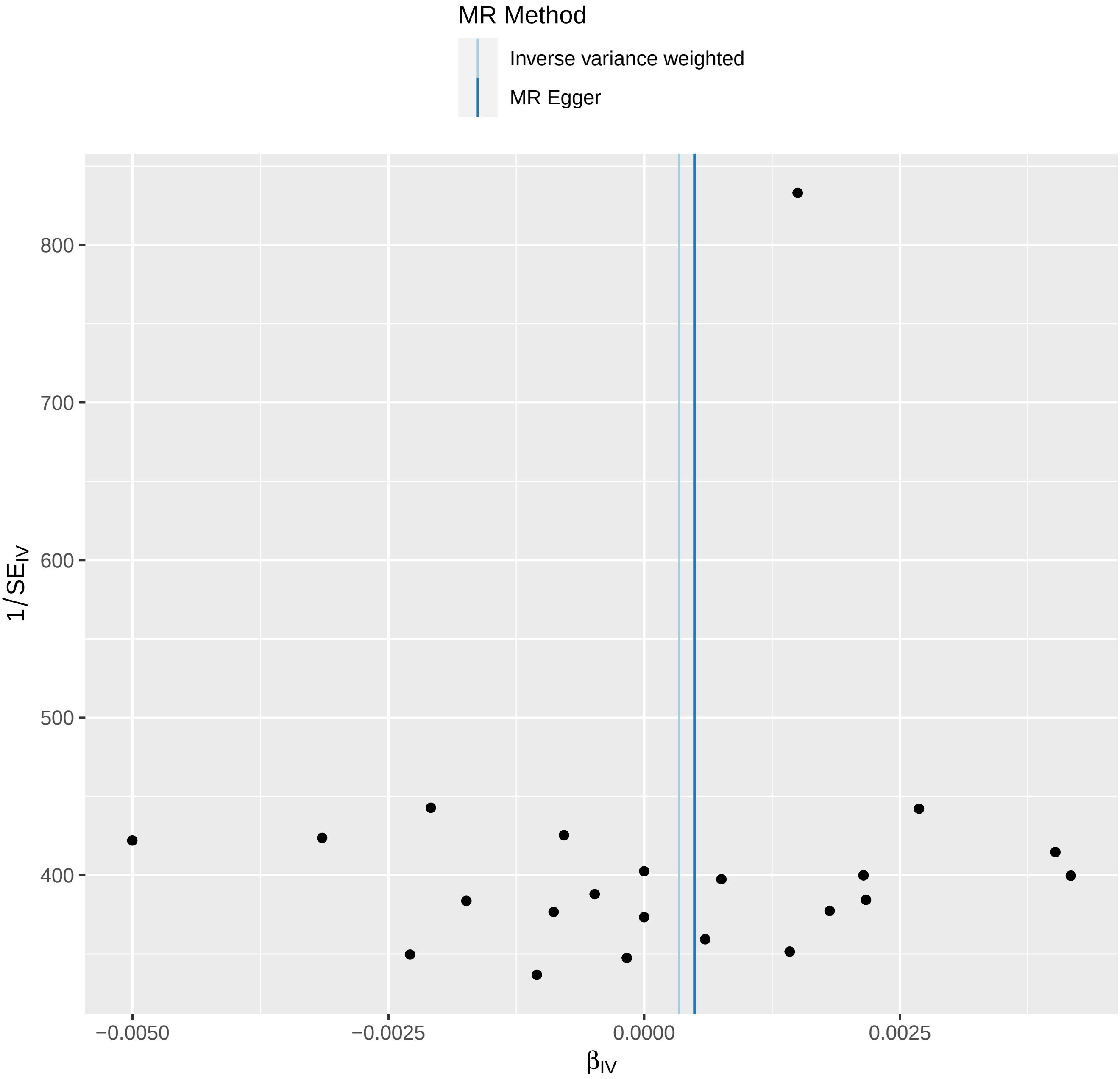

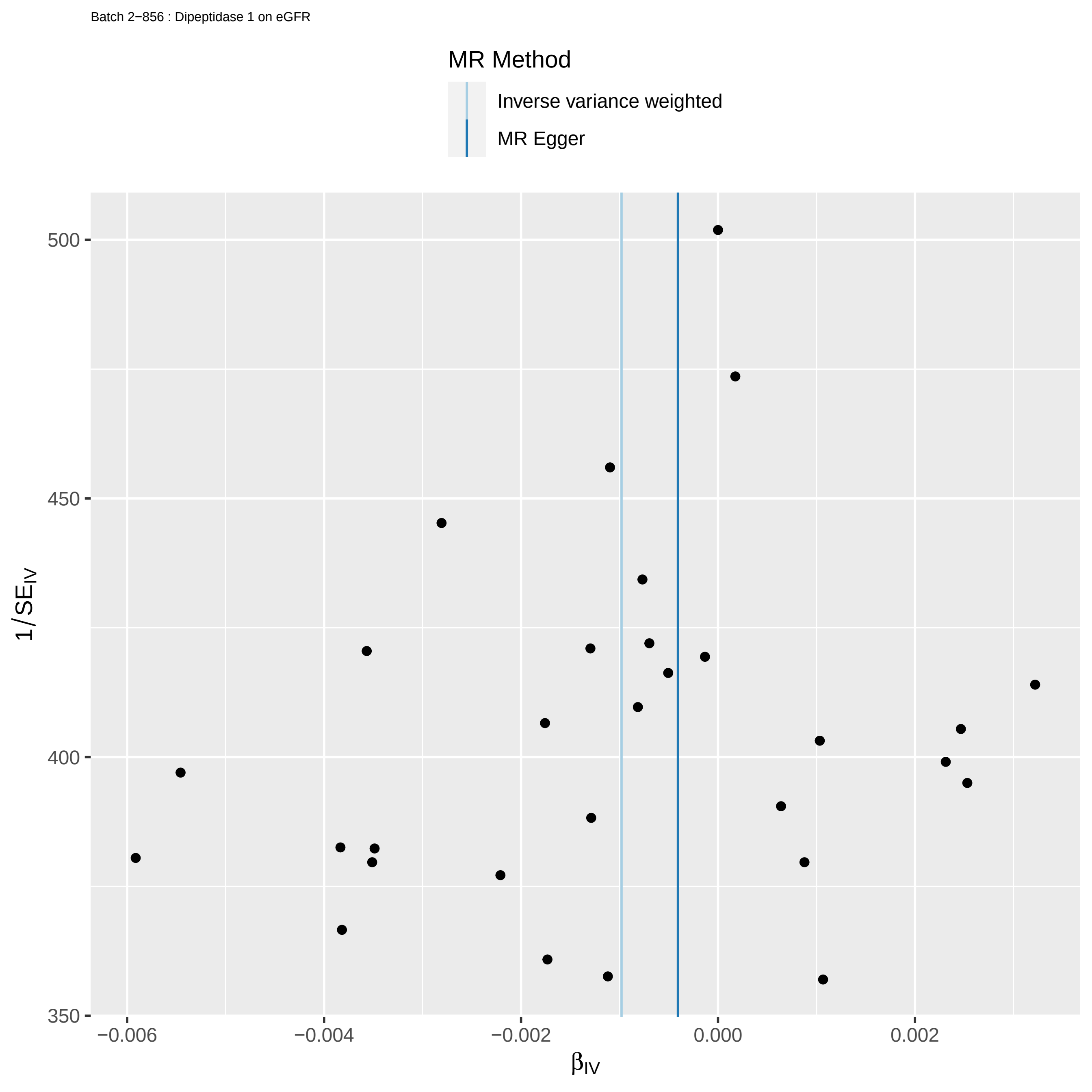
Batch 2−856 : Dipeptidase 1 on eGFR
MR Method
Inverse variance weighted
MR Egger
500
450
400
350
1/SE$_{IV}$
−0.006
−0.004
−0.002
0.000
0.002
β$_{IV}$

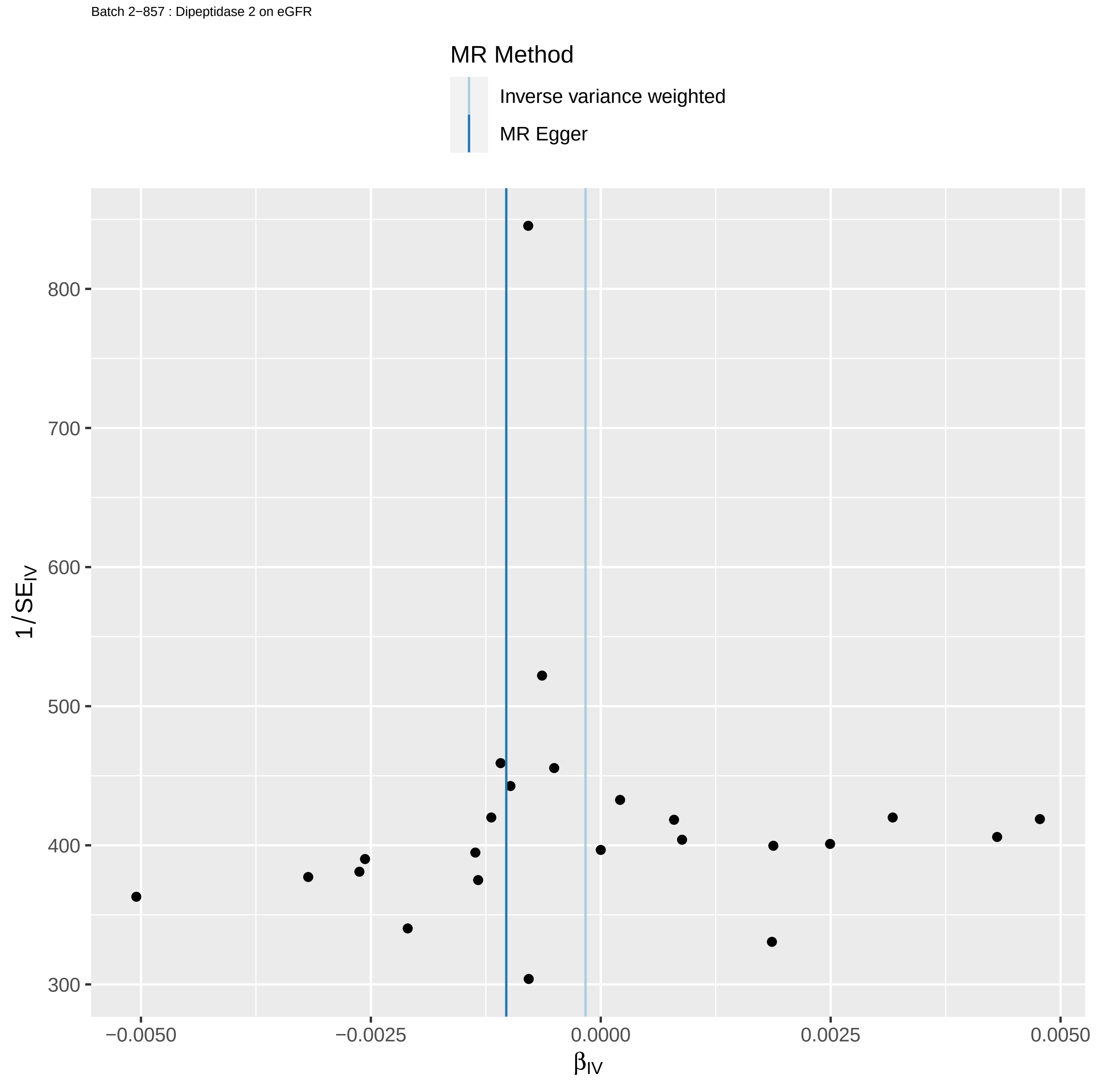

Batch 2−857 : Dipeptidase 2 on eGFR
MR Method
Inverse variance weighted
MR Egger
800
700
600
500
400
300
1/SE$_{IV}$
−0.0050
−0.0025
0.0000
0.0025
0.0050
β$_{IV}$

Batch 2−858 : Inactive dipeptidyl peptidase 10 on eGFR

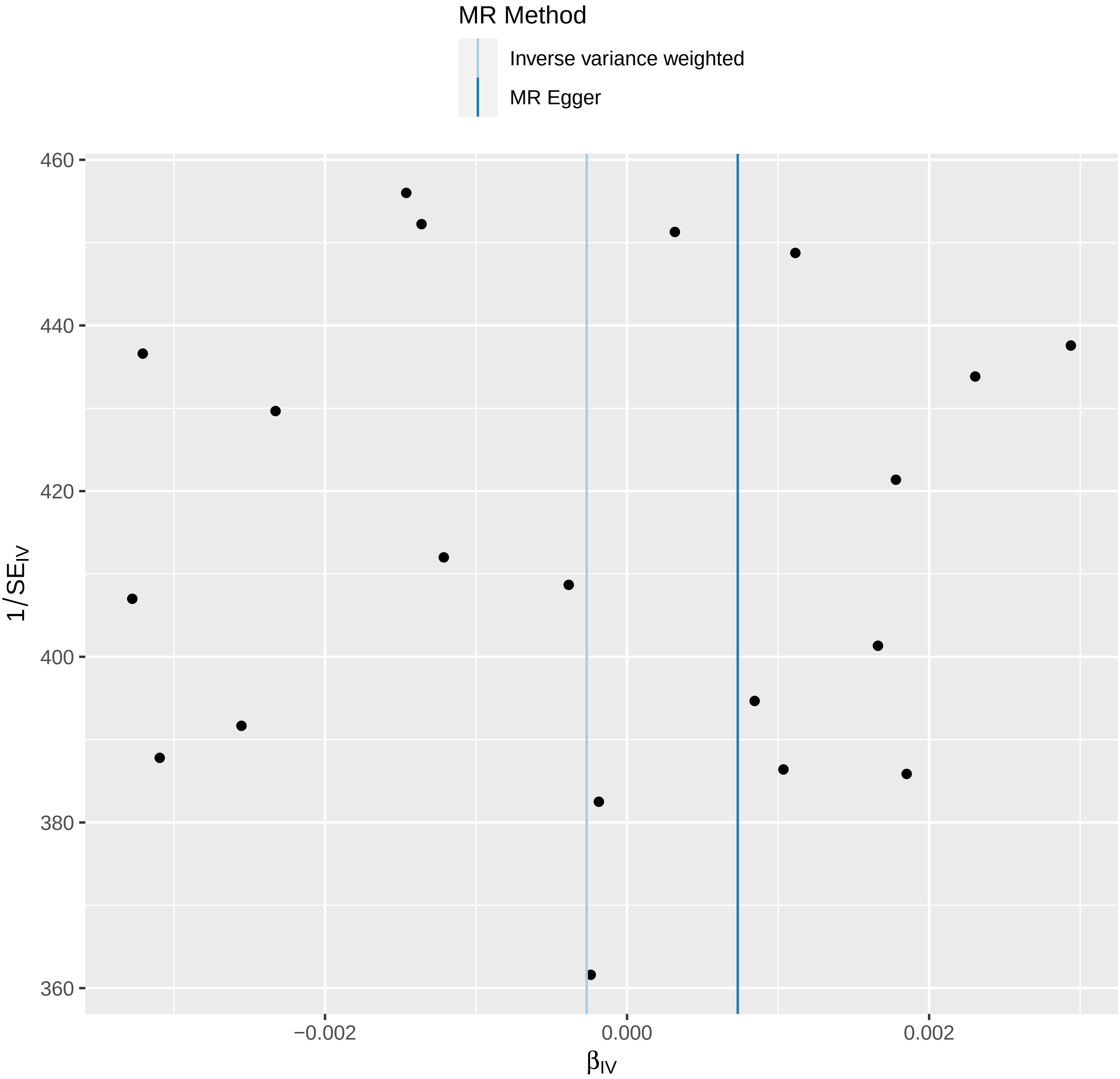

Batch 2−859 : Dipeptidyl peptidase 2 on eGFR

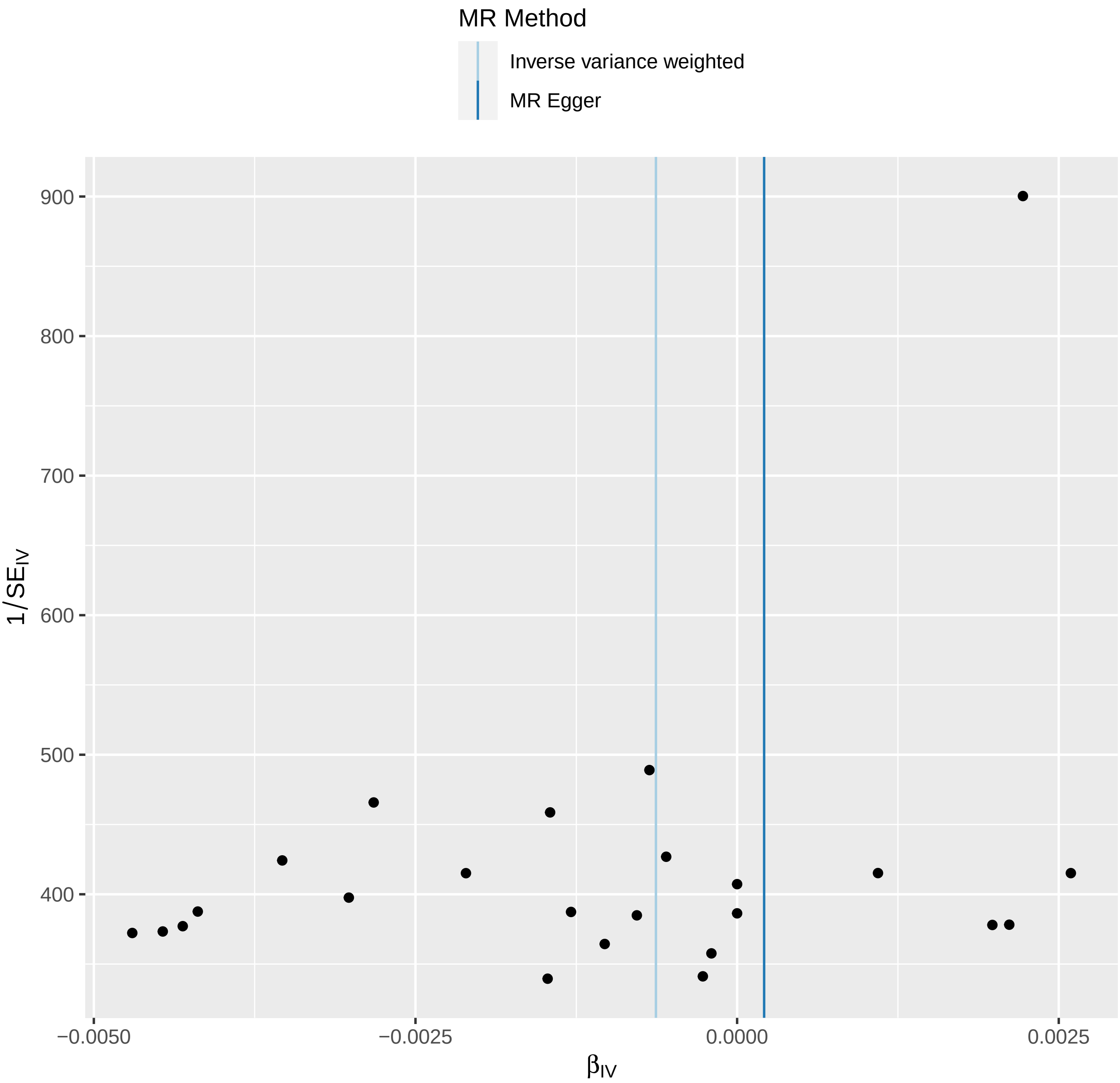

Batch 2−860 : Dipeptidyl peptidase 2 on eGFR

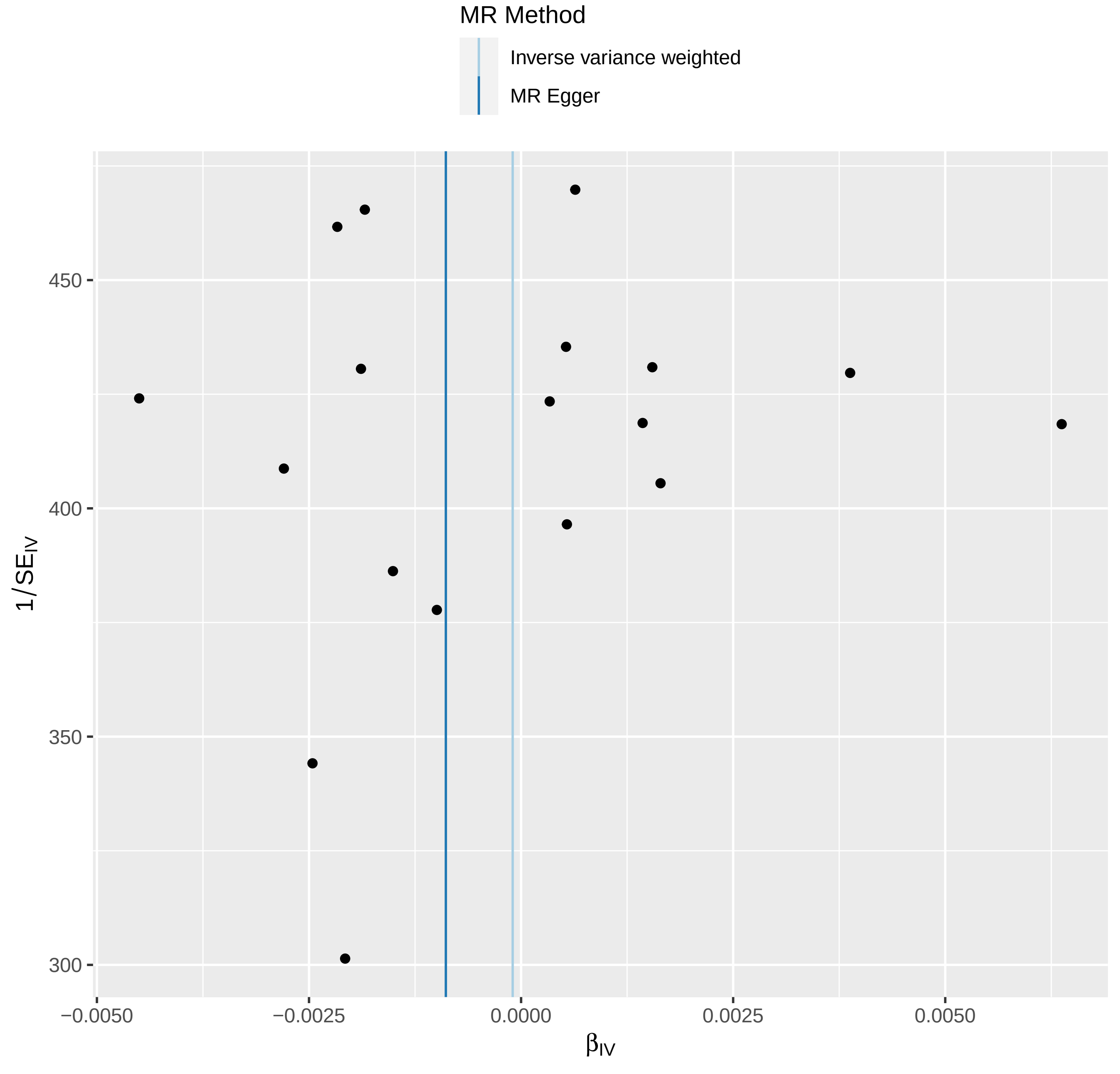

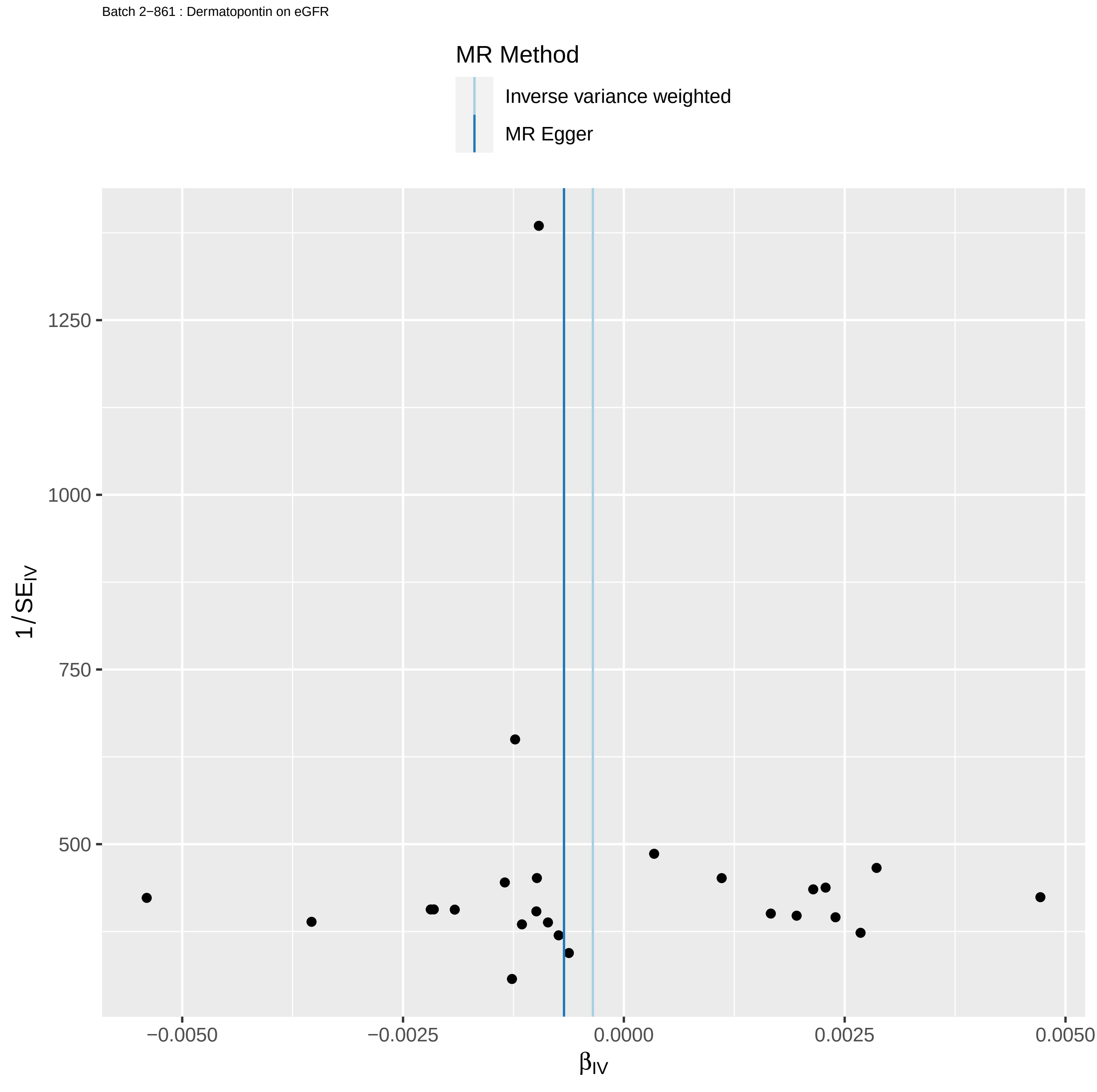
Batch 2−861 : Dermatopontin on eGFR
MR Method
Inverse variance weighted
MR Egger
1250
1000
750
500
1/SE_IV
−0.0050
−0.0025
0.0000
0.0025
0.0050
β_IV

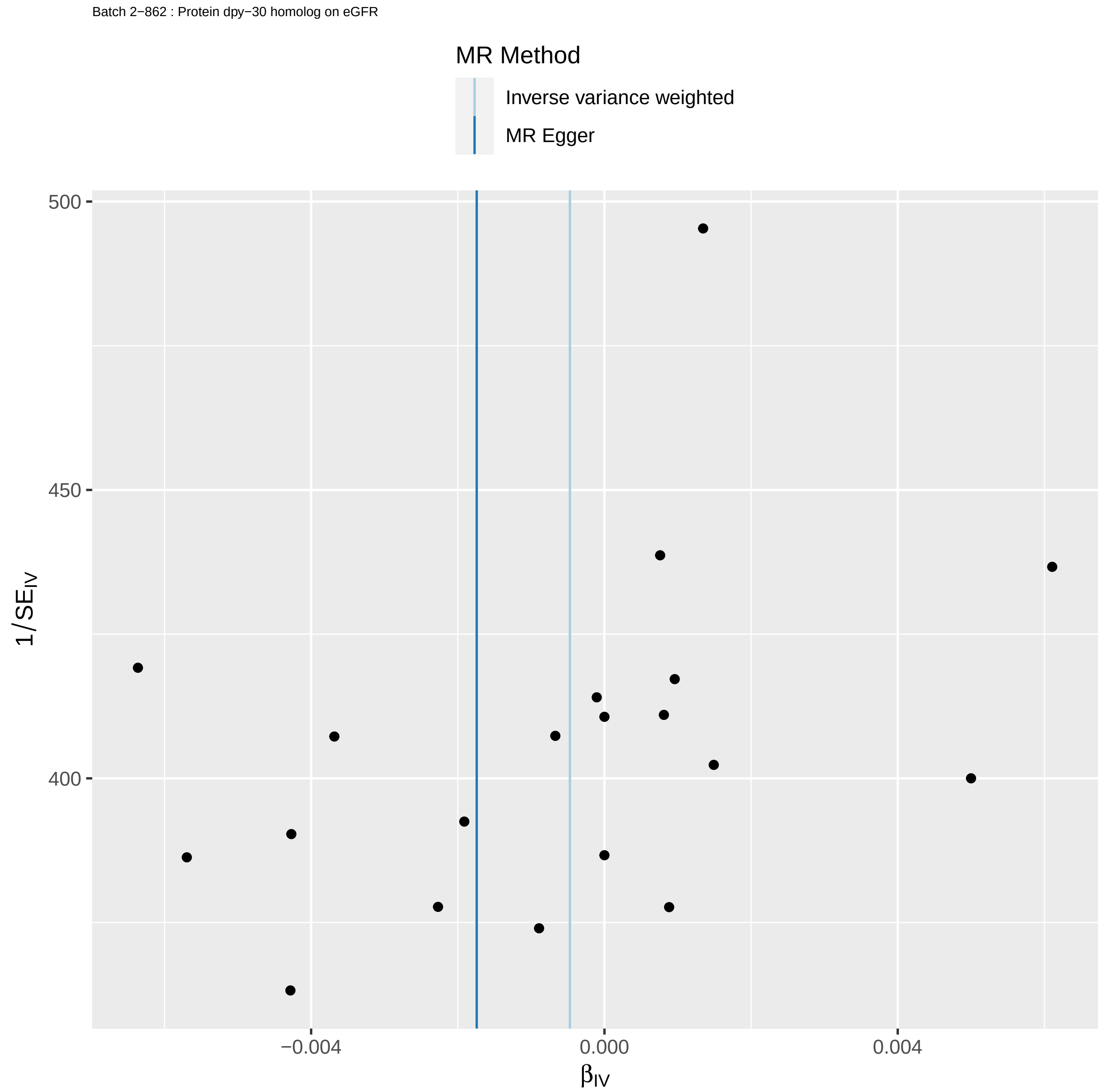

Batch 2−862 : Protein dpy−30 homolog on eGFR
MR Method
Inverse variance weighted
MR Egger
500
450
400
1/SE$_{IV}$
−0.004
0.000
0.004
β$_{IV}$

Batch 2−863 : Dihydropyrimidinase−related protein 3 on eGFR

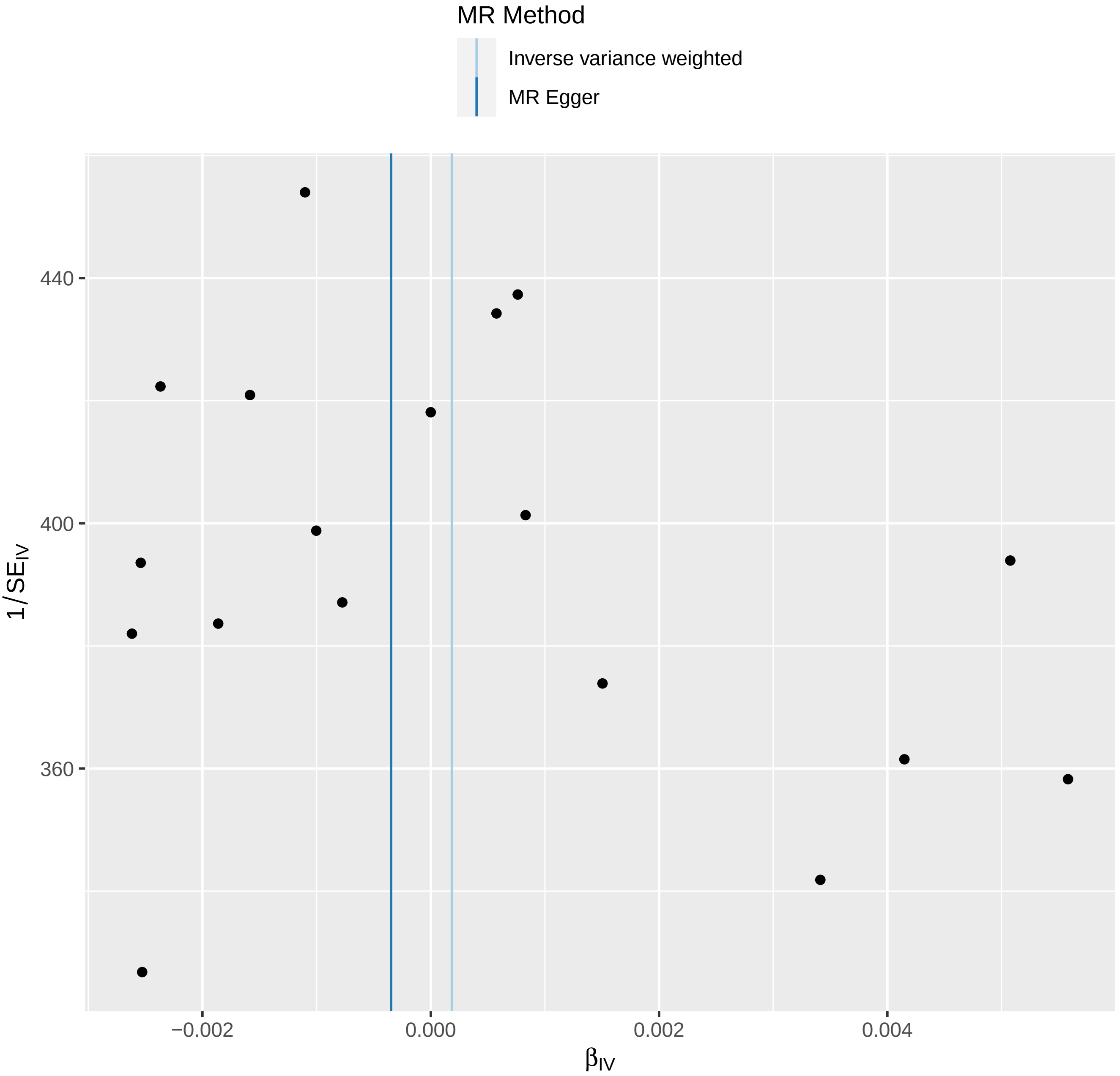

Batch 2−864 : Dihydropyrimidinase−related protein 5 on eGFR

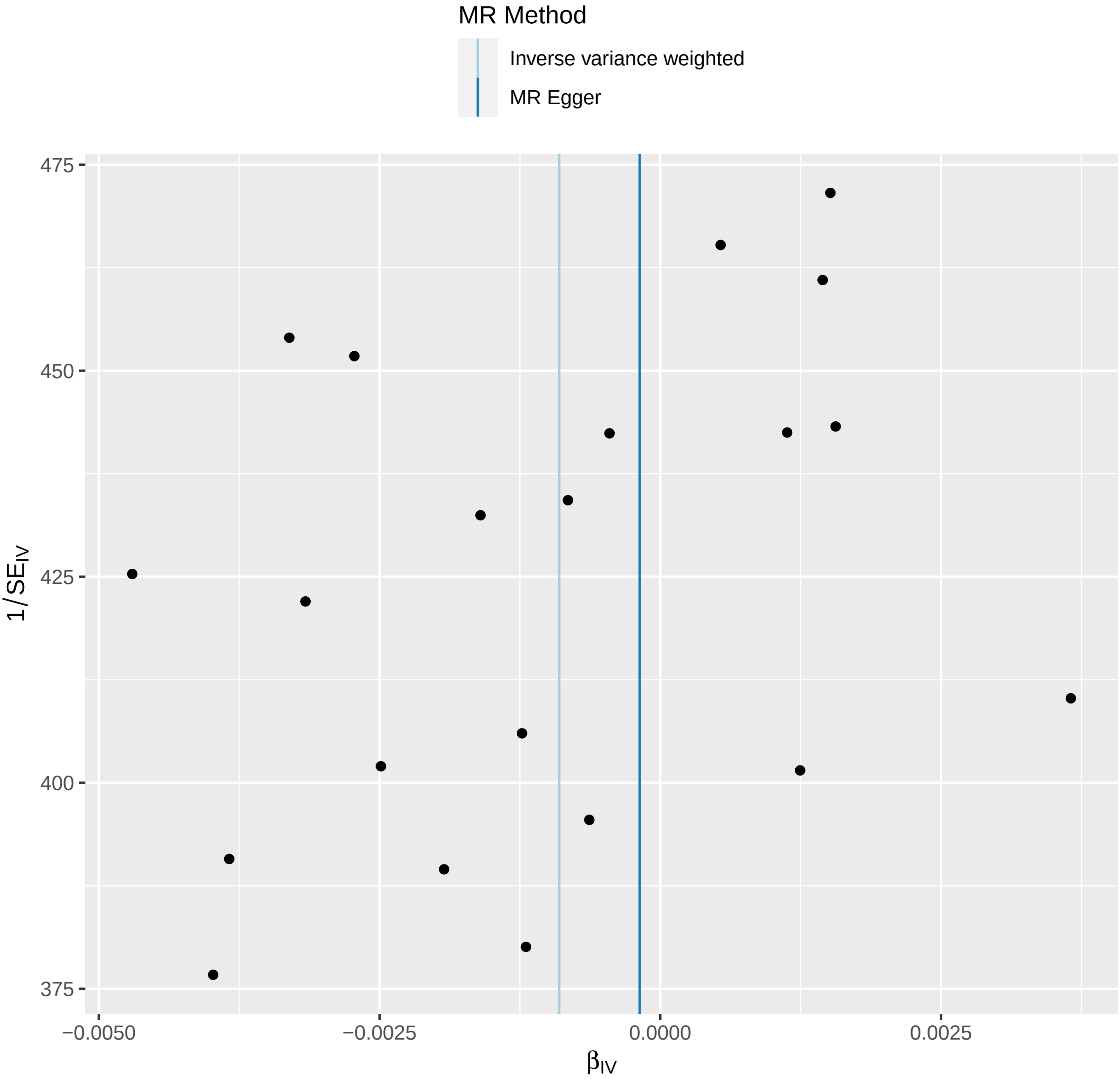

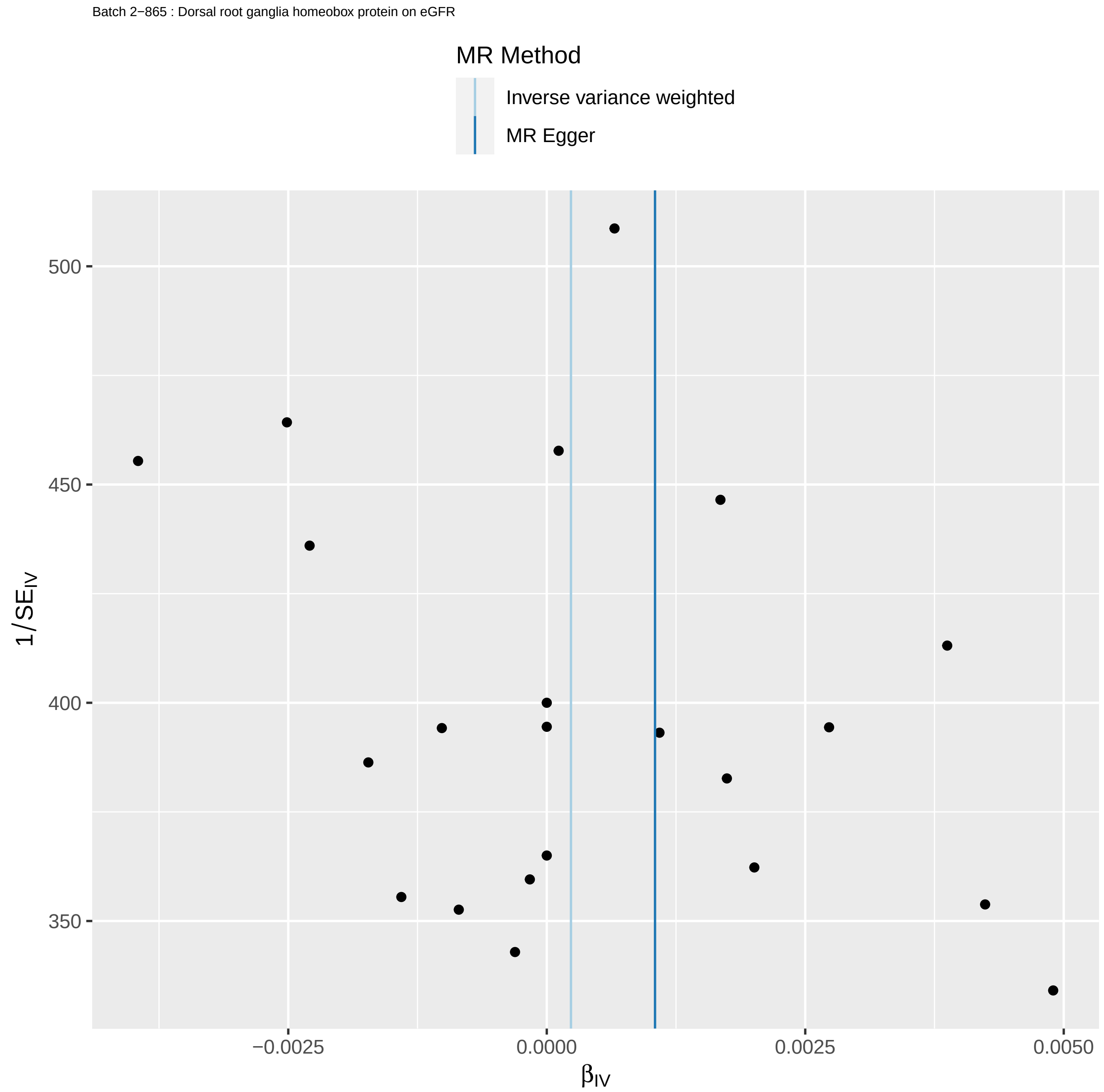
Batch 2−865 : Dorsal root ganglia homeobox protein on eGFR
MR Method
Inverse variance weighted
MR Egger
500
450
400
350
1/SE_IV
−0.0025
0.0000
0.0025
0.0050
β_IV

Batch 2−866 : Desmocollin−2 on eGFR

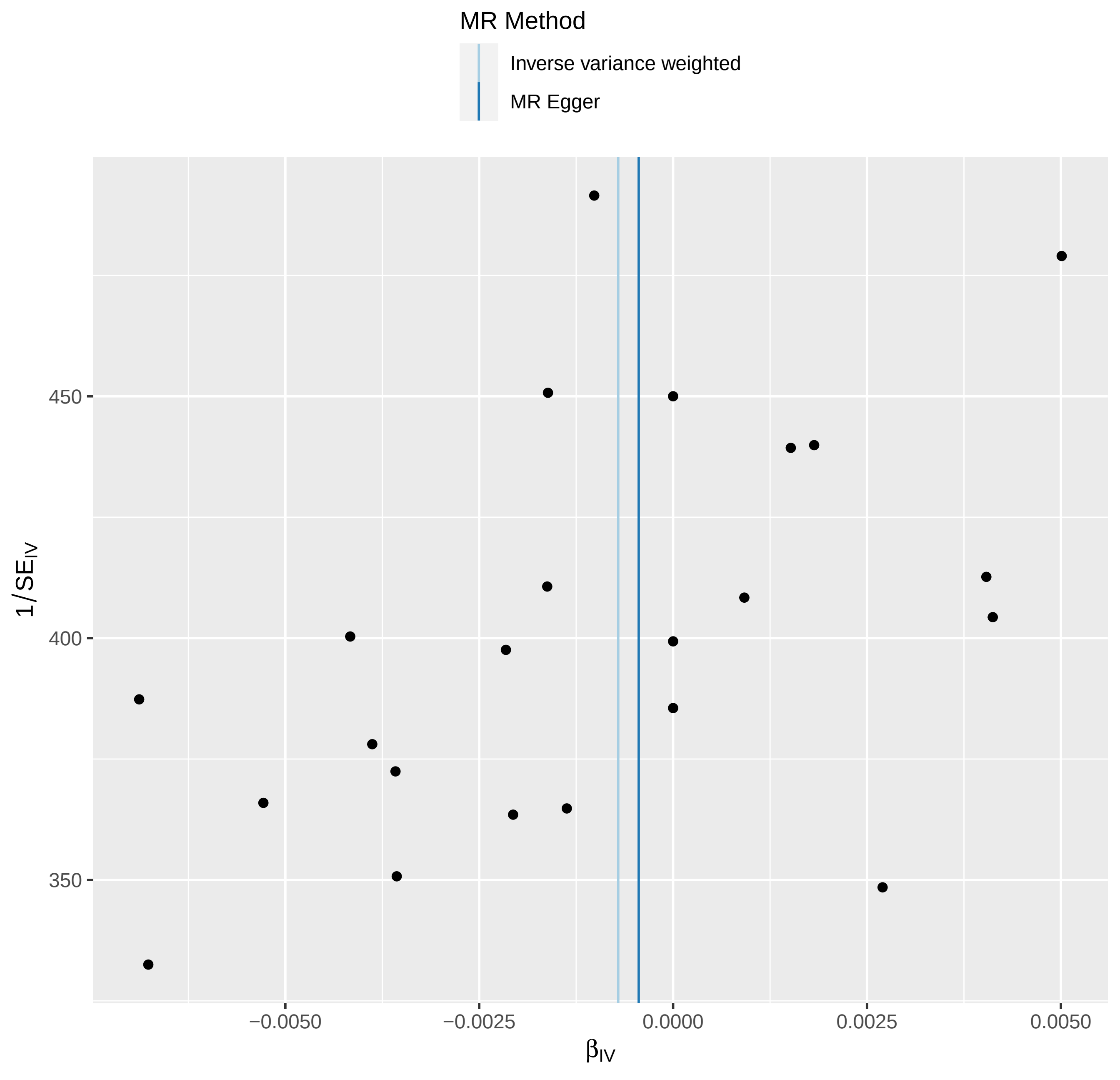

Batch 2−867 : Desmocollin−3 on eGFR

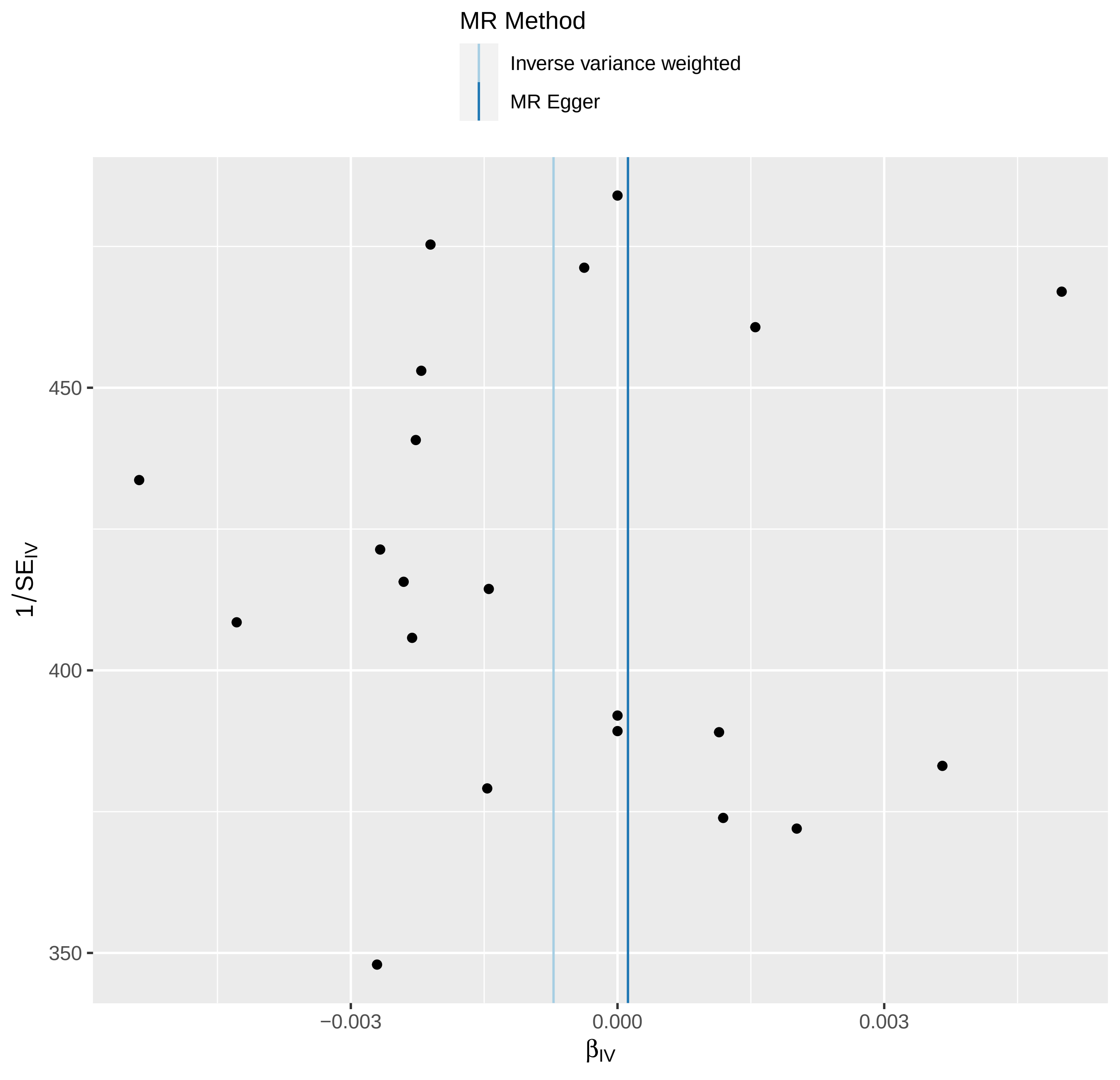

Batch 2−868 : Down syndrome cell adhesion molecule on eGFR

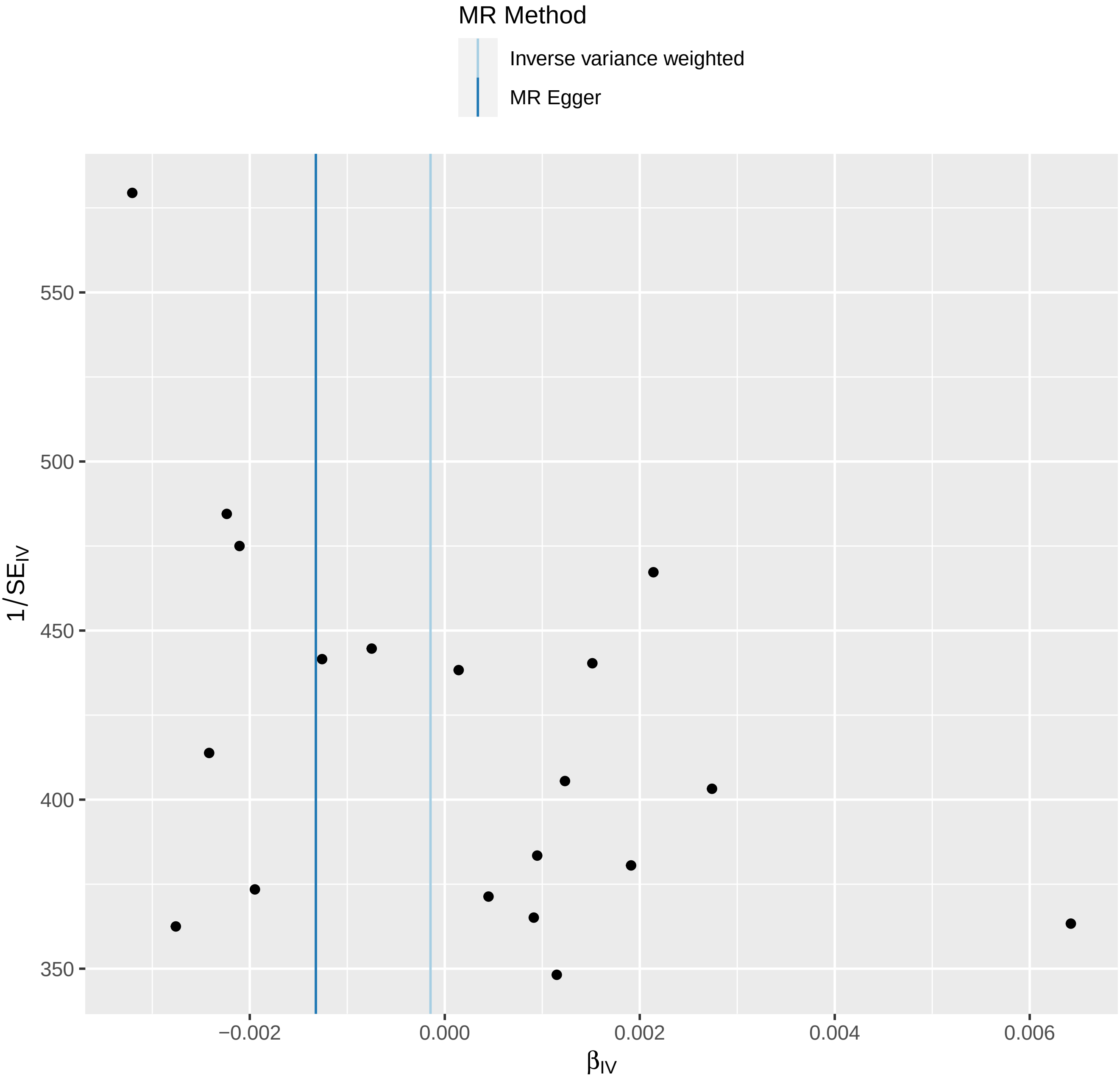

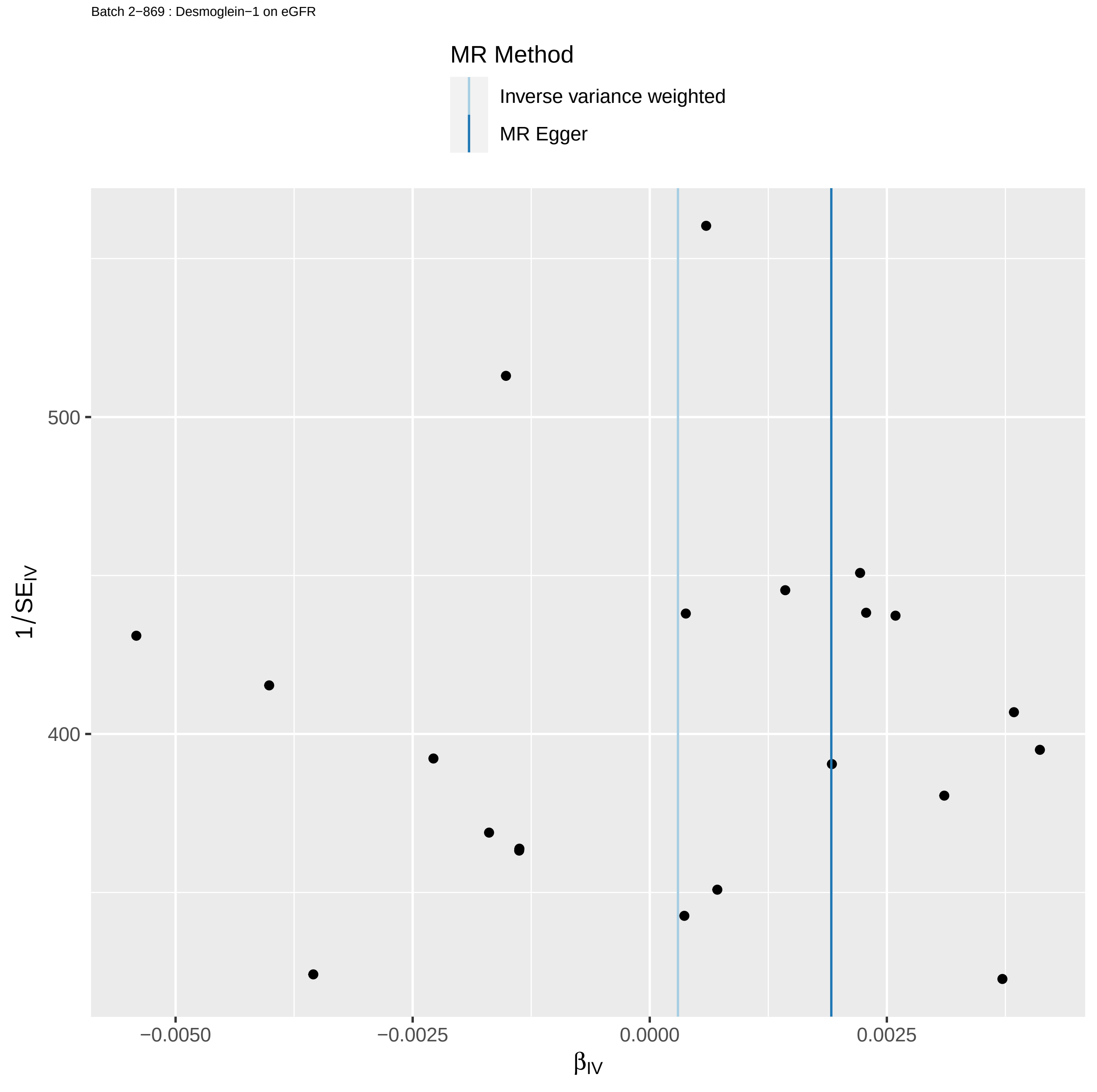

Batch 2−869 : Desmoglein−1 on eGFR
MR Method
Inverse variance weighted
MR Egger
500
400
1/SE_IV
−0.0050
−0.0025
0.0000
0.0025
β_IV

Batch 2−870 : Desmoglein−2 on eGFR

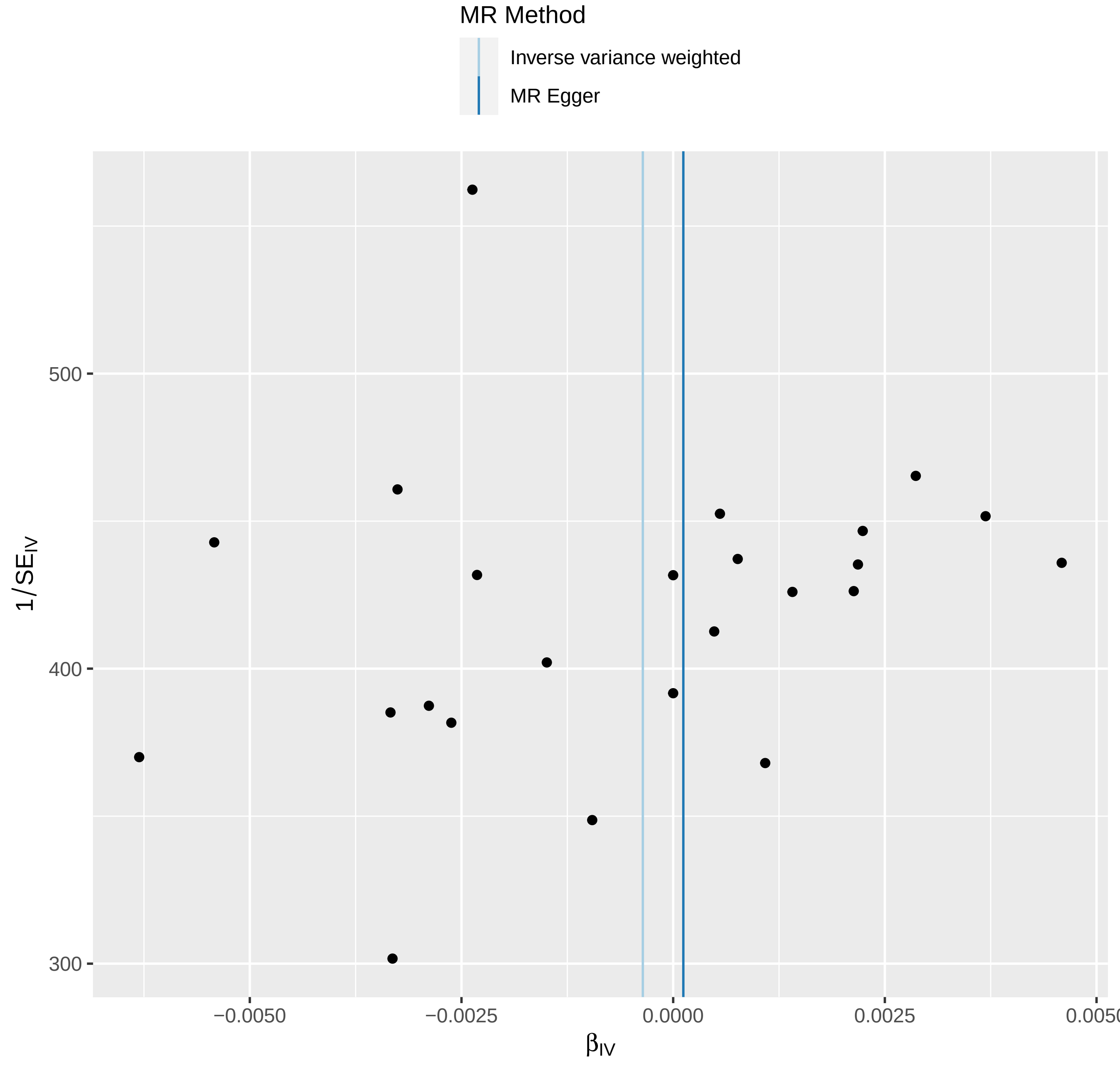

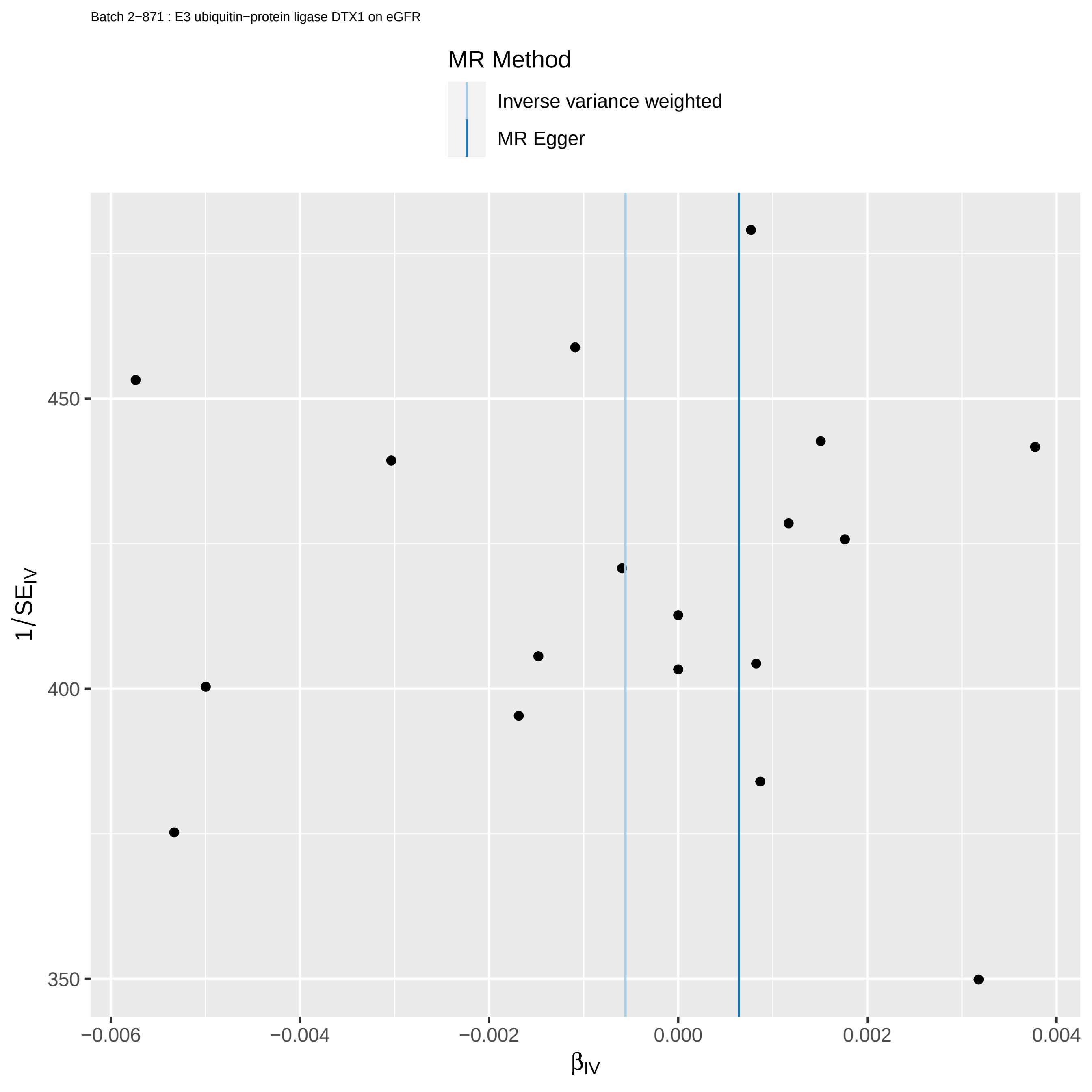
Batch 2−871 : E3 ubiquitin−protein ligase DTX1 on eGFR
MR Method
Inverse variance weighted
MR Egger
450
400
350
−0.006
−0.004
−0.002
0.000
0.002
0.004
$1/SE_{IV}$
β_{IV}

Batch 2−872 : E3 ubiquitin−protein ligase DTX3L on eGFR

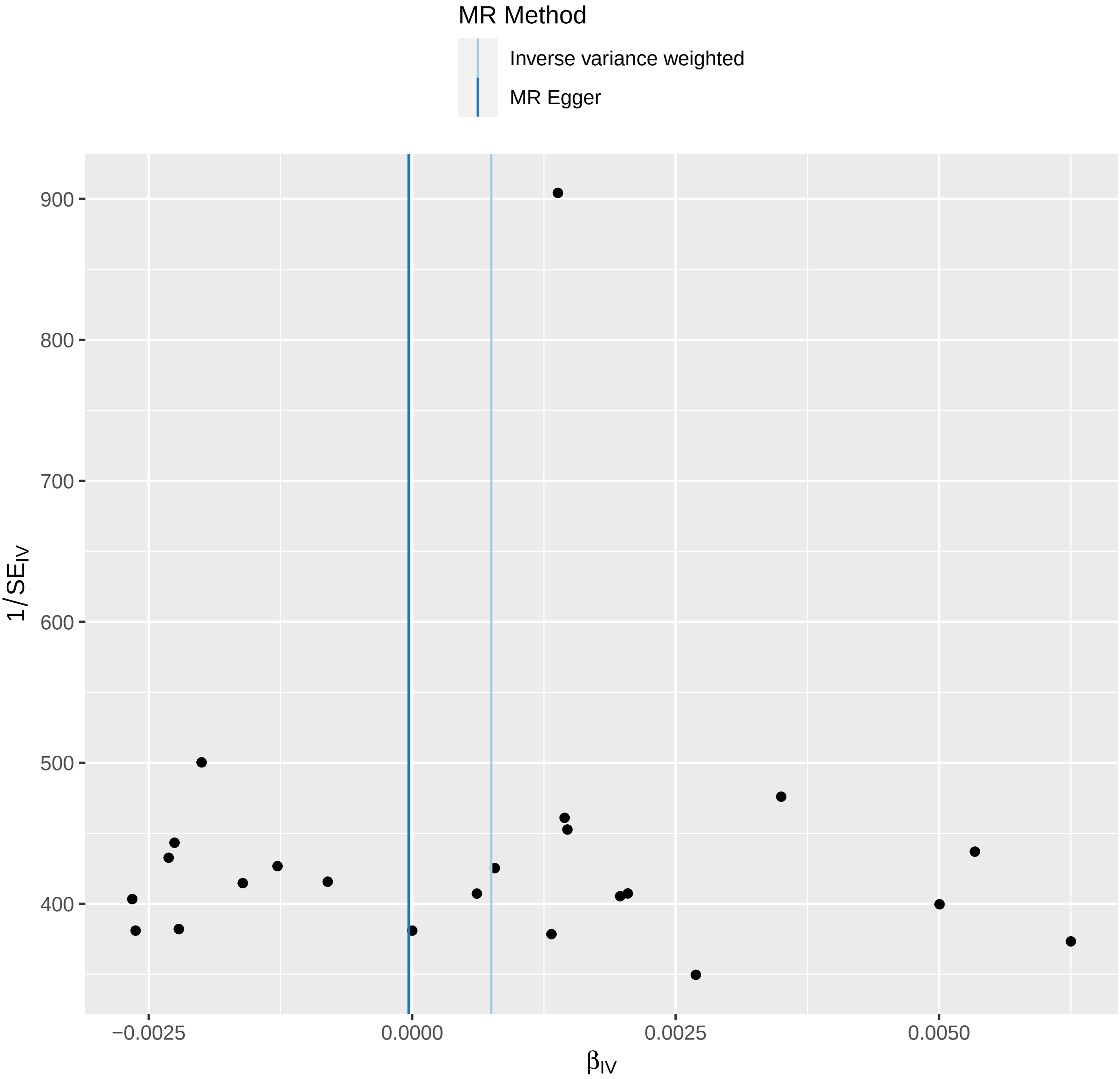

Batch 2−873 : Dual specificity protein phosphatase 13 isoform A on eGFR

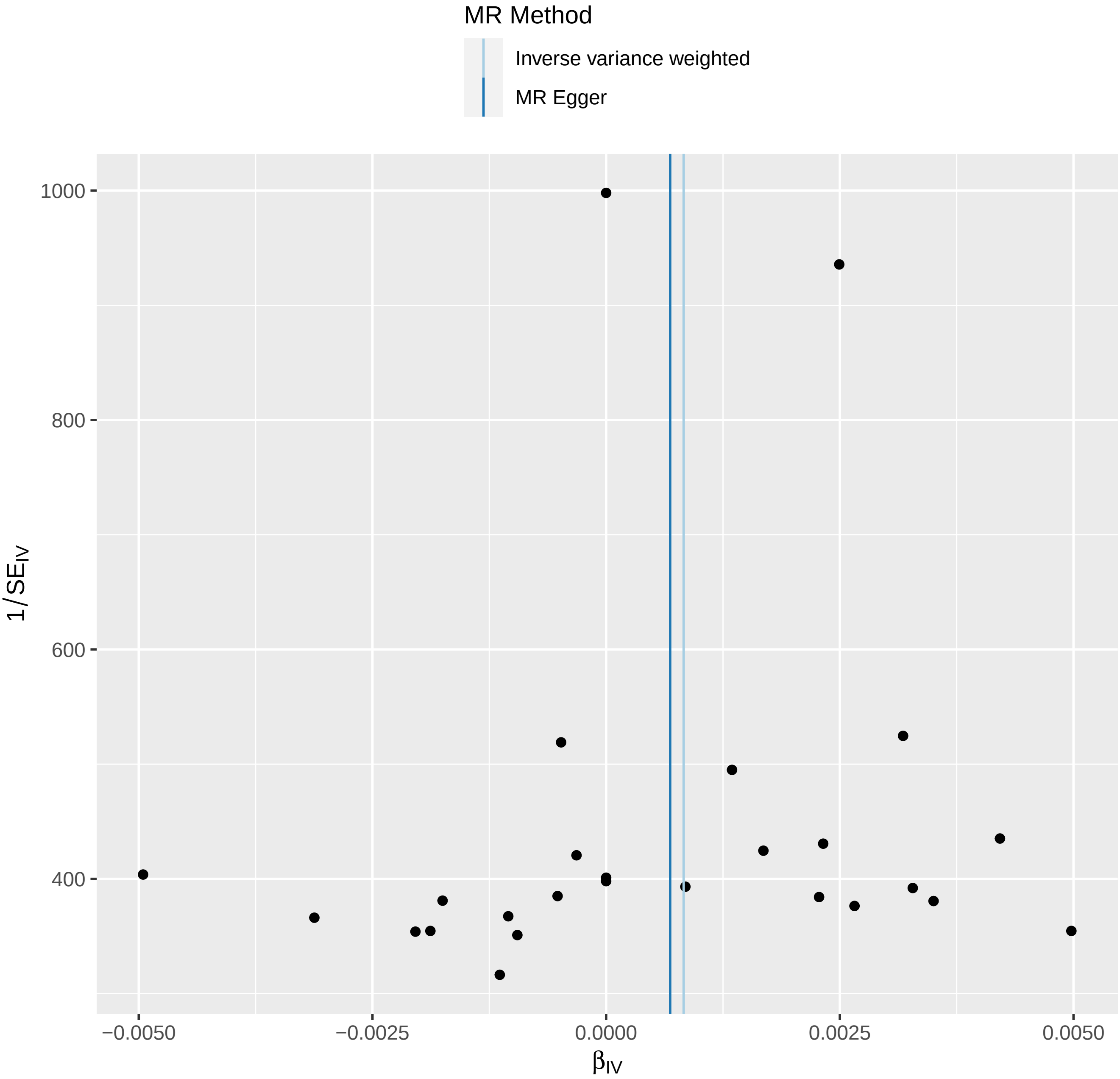

Batch 2−874 : Dual specificity protein phosphatase 15 on eGFR

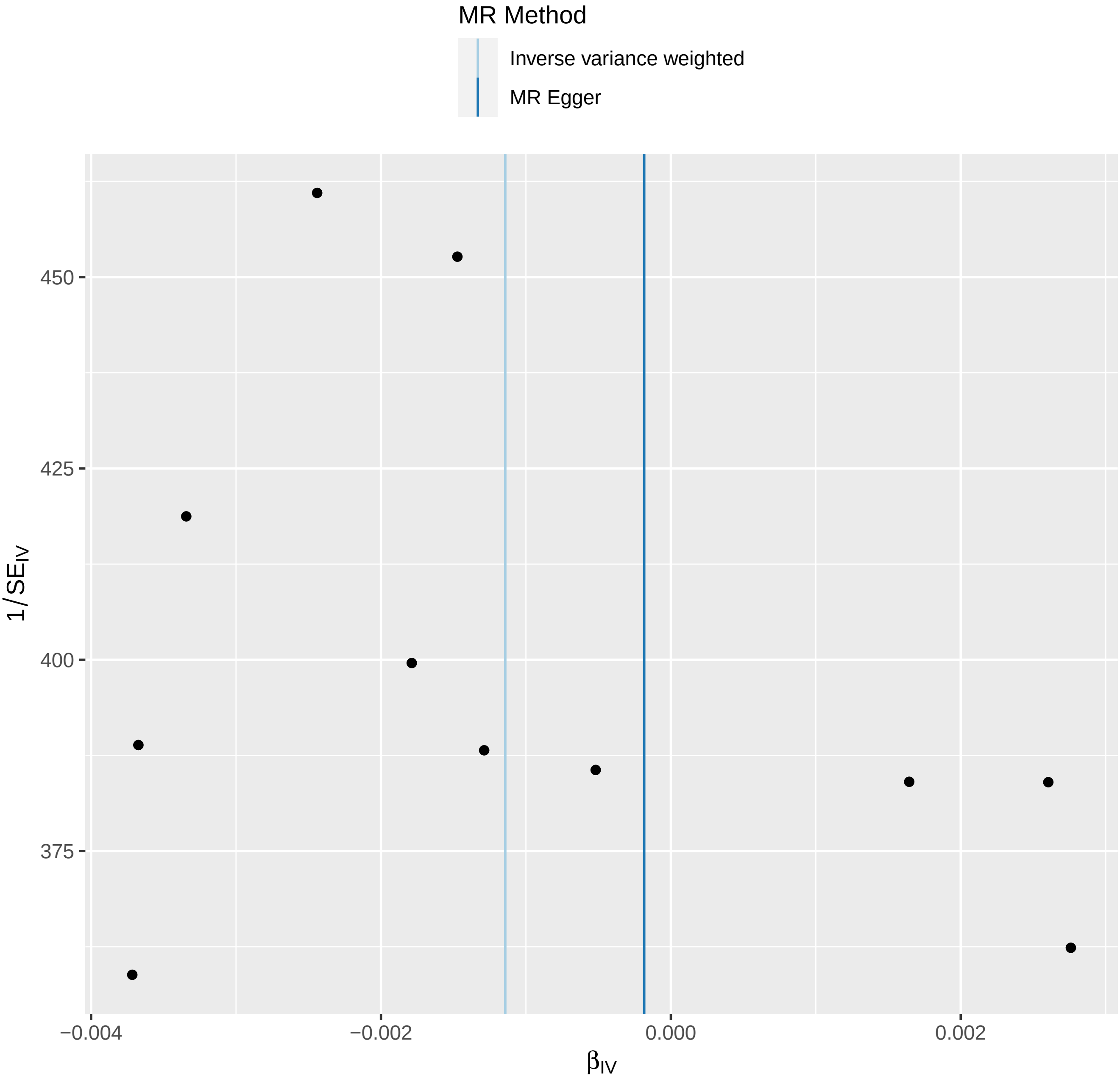

Batch 2−875 : Dual specificity protein phosphatase 16 on eGFR

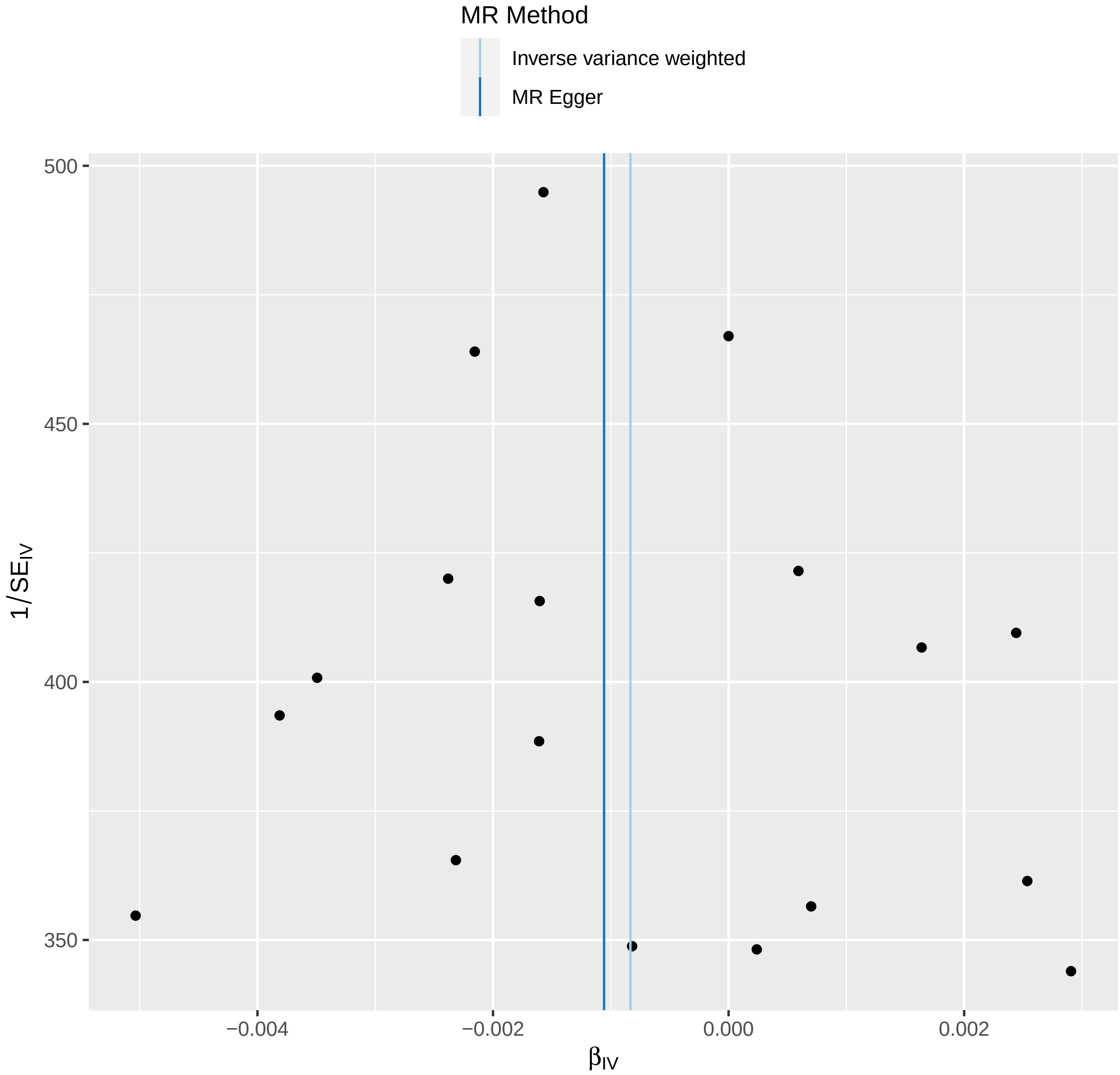

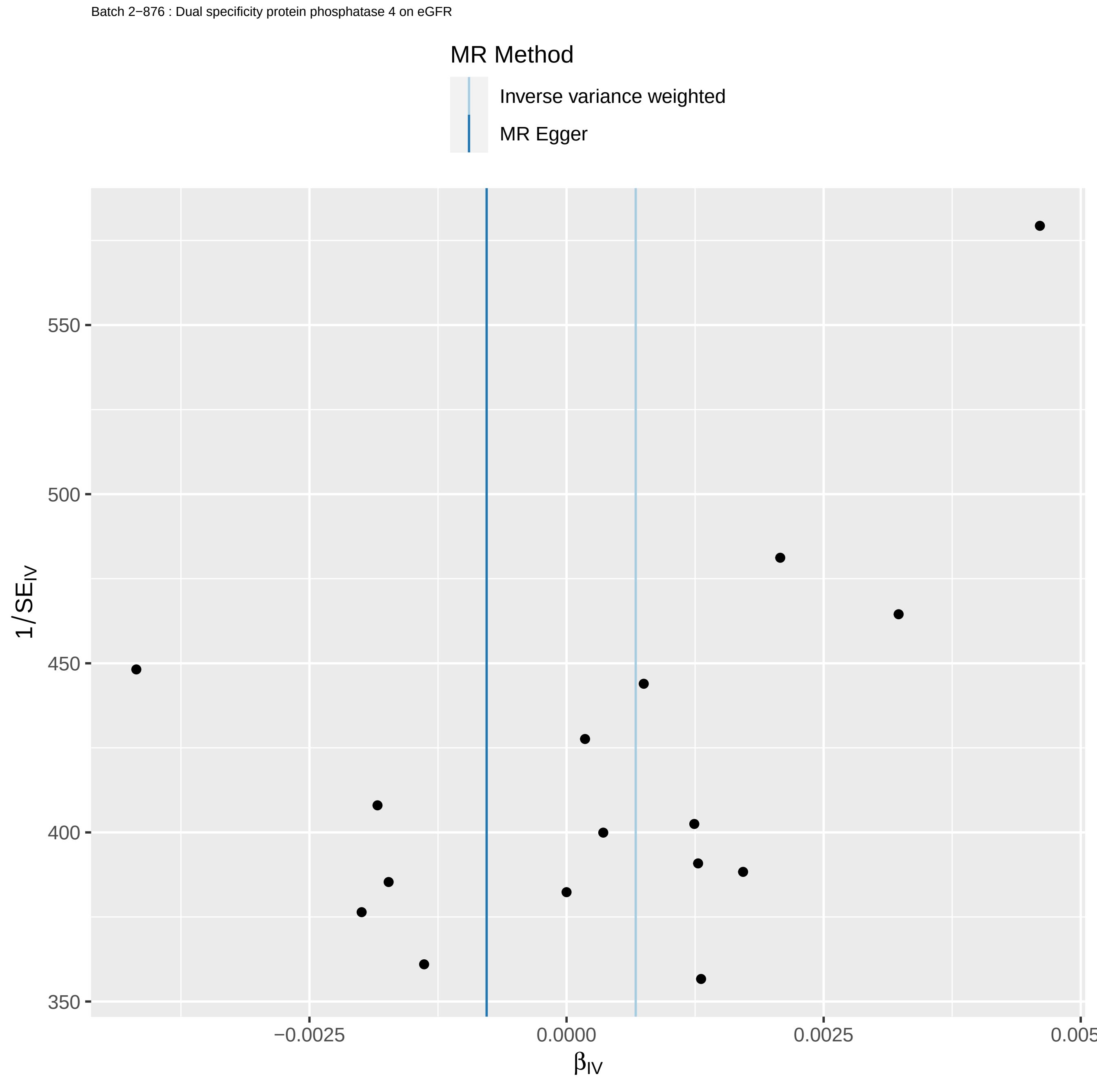
Batch 2−876 : Dual specificity protein phosphatase 4 on eGFR
MR Method
Inverse variance weighted
MR Egger
550
500
450
400
350
1/SE$_{IV}$
−0.0025
0.0000
0.0025
0.005
β$_{IV}$

Batch 2−877 : Dual specificity protein phosphatase 6 on eGFR

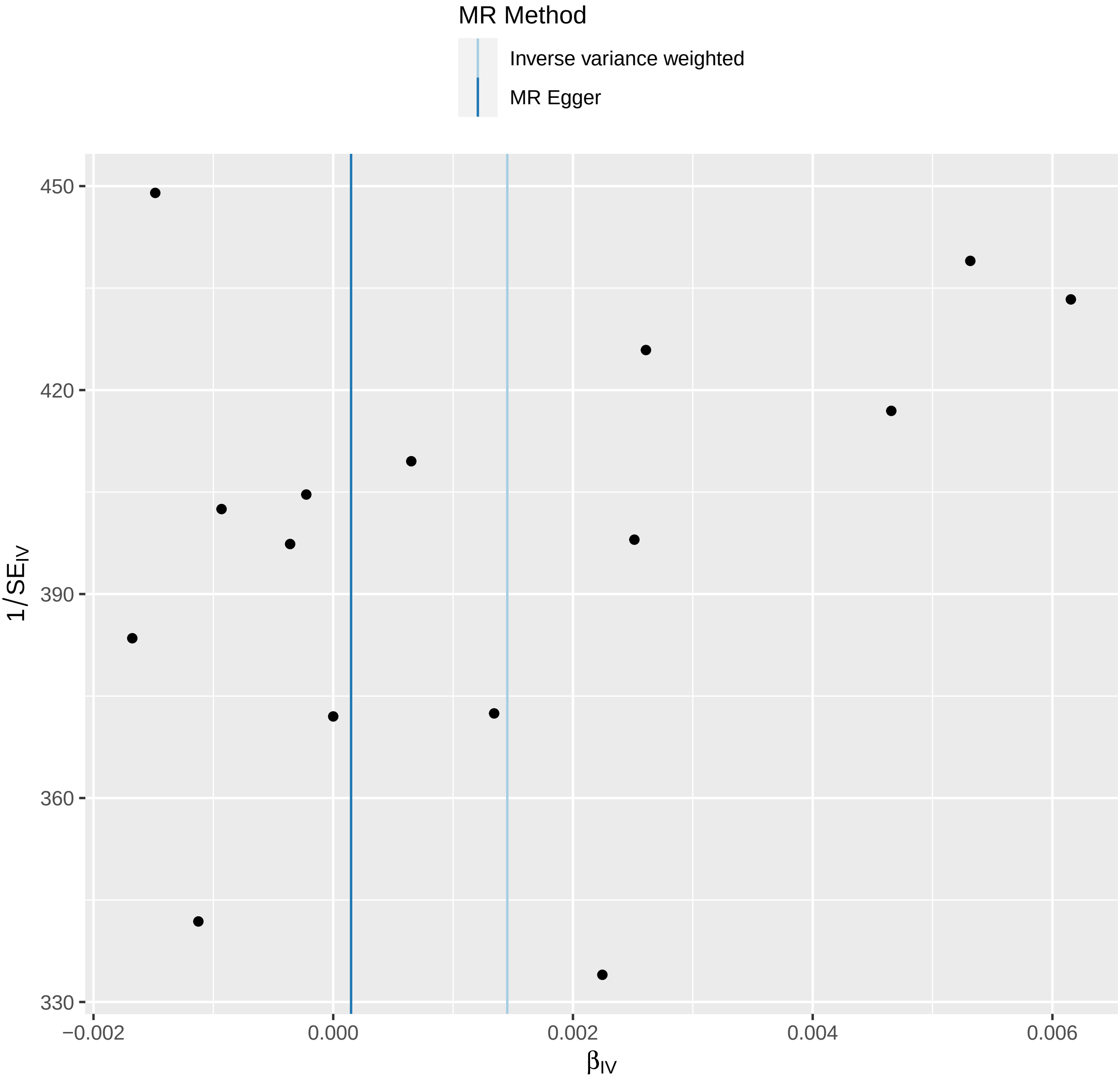

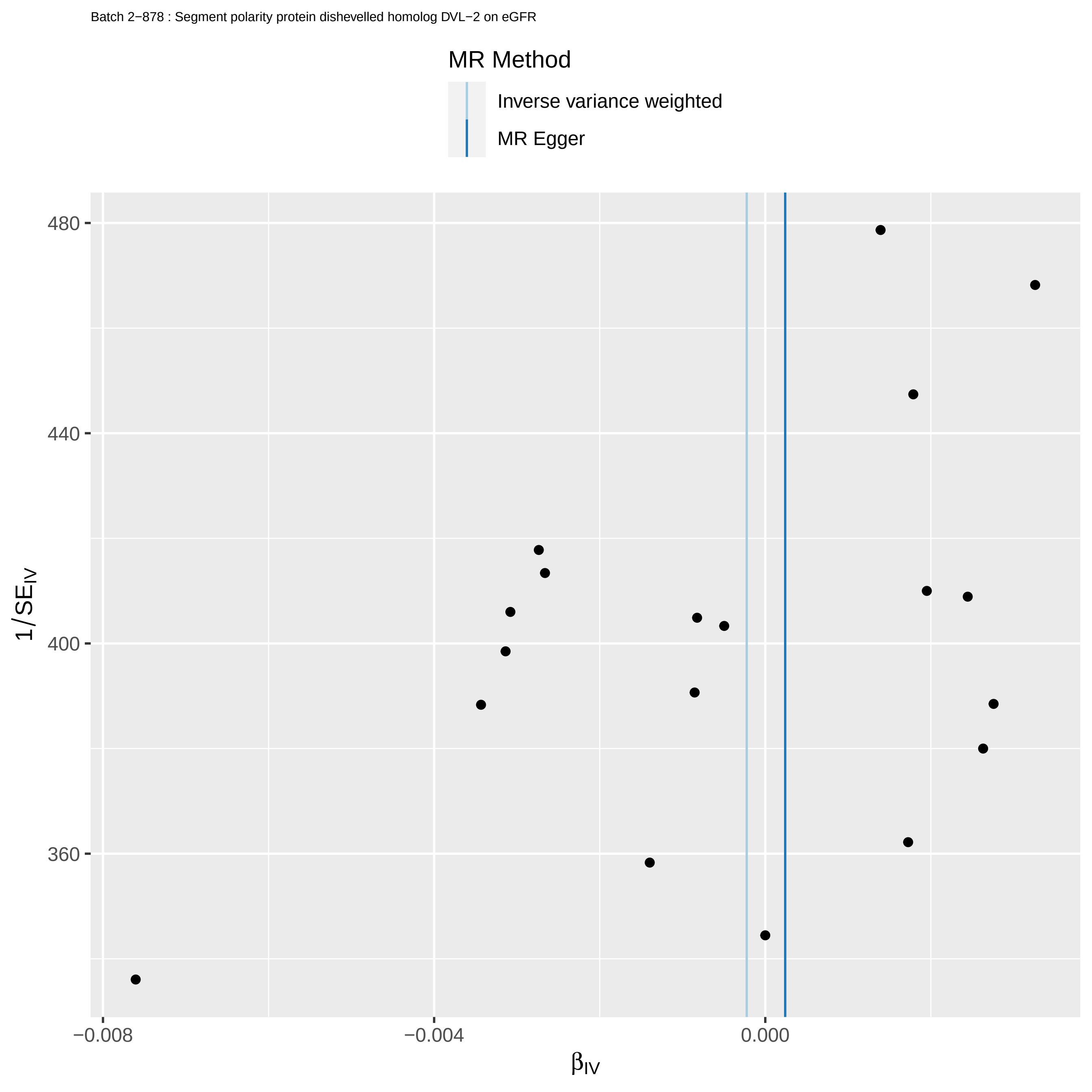

Batch 2−878 : Segment polarity protein dishevelled homolog DVL−2 on eGFR
MR Method
Inverse variance weighted
MR Egger
480
440
400
360
1/SE_IV
−0.008
−0.004
0.000
β_IV

Batch 2−879 : Dynactin−associated protein on eGFR

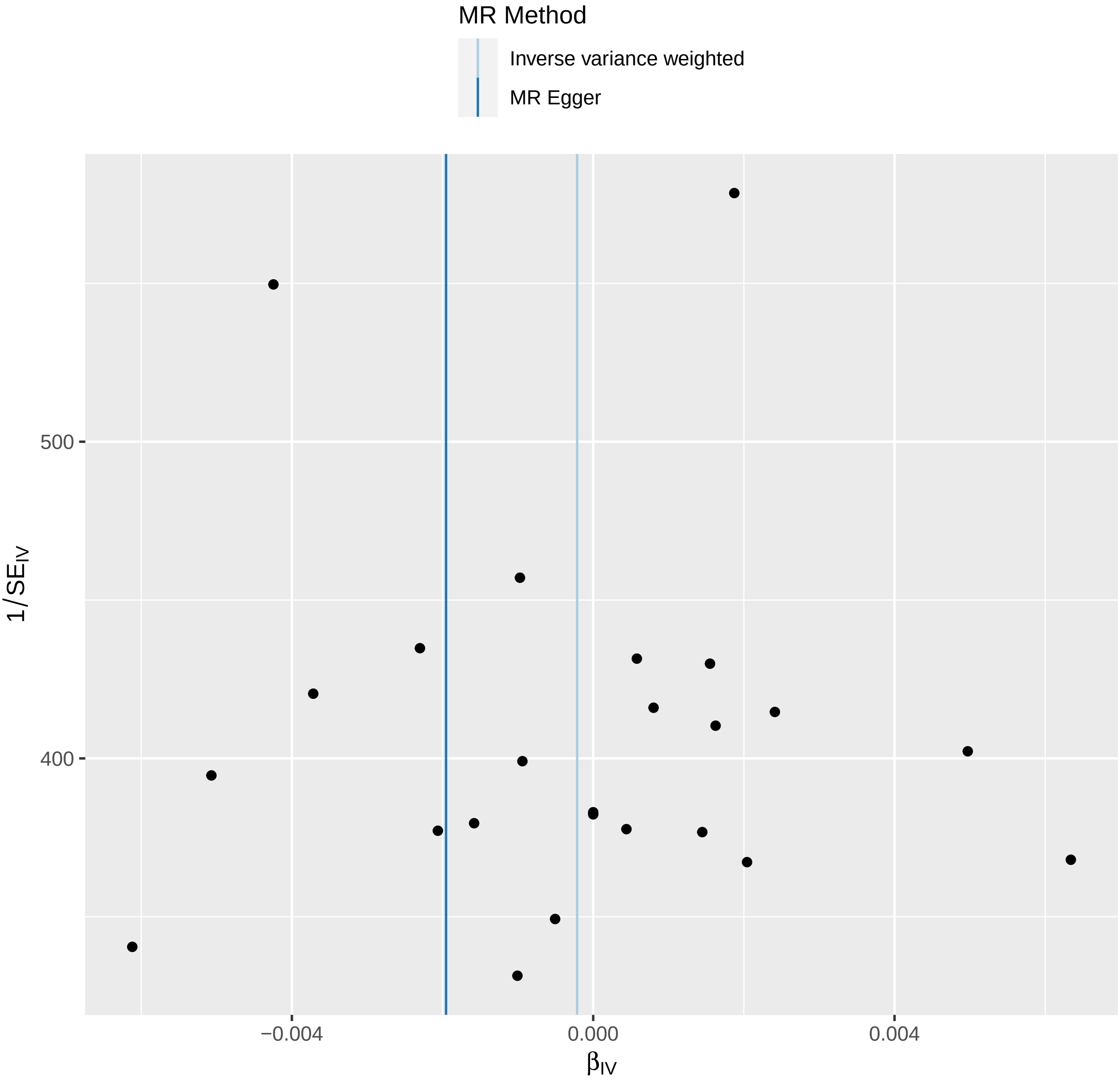

Batch 2−880 : Dynein light chain 1, cytoplasmic on eGFR

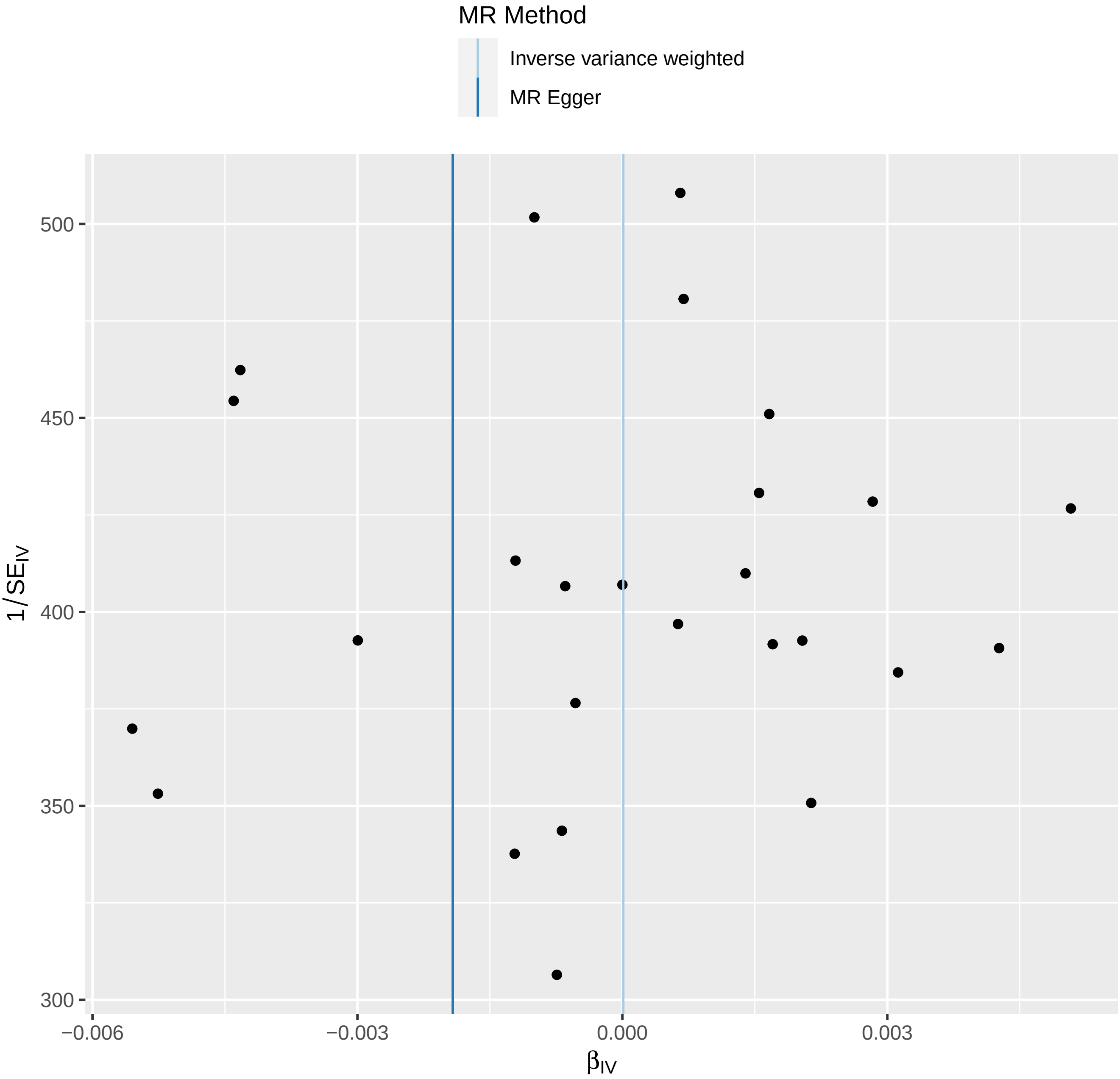

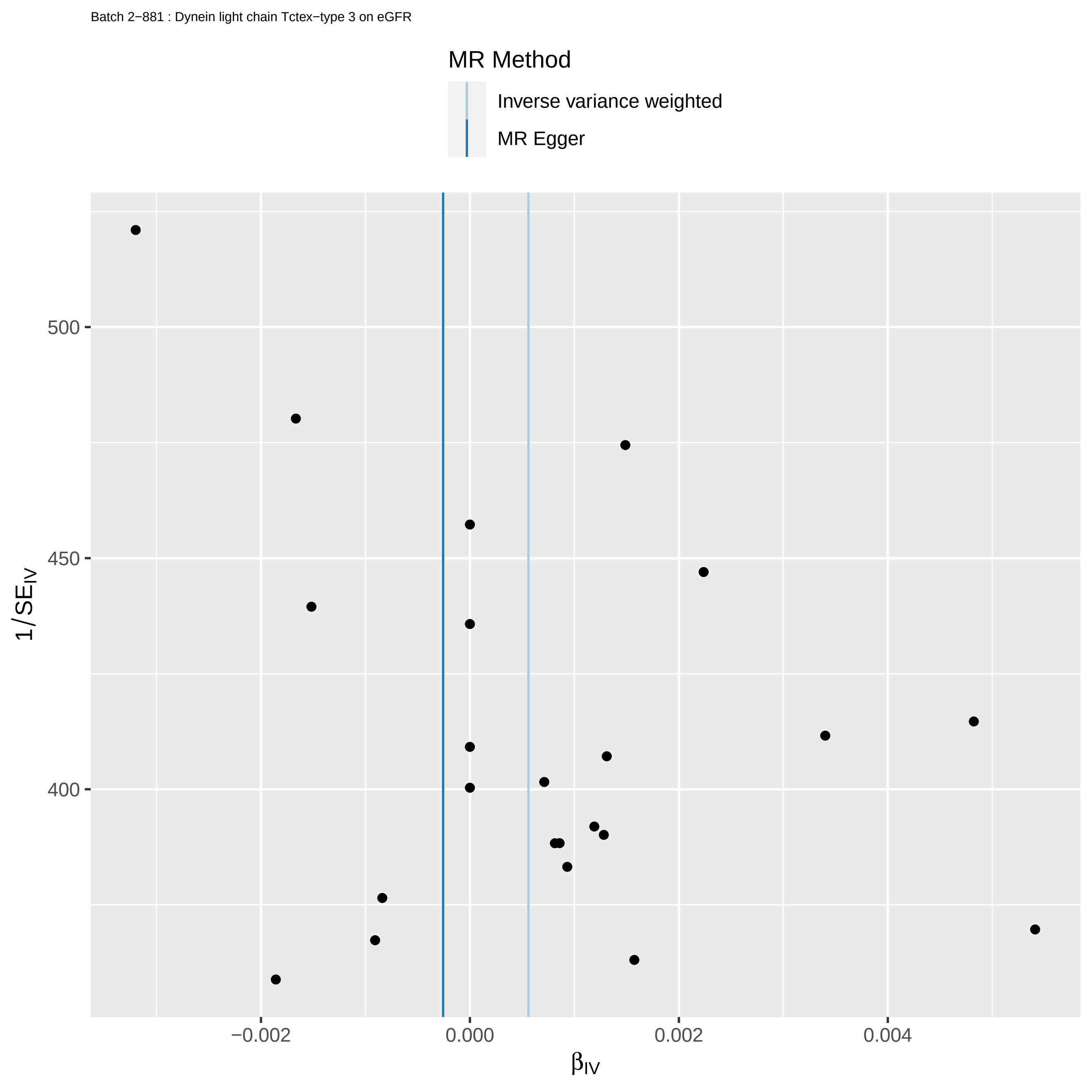
Batch 2−881 : Dynein light chain Tctex−type 3 on eGFR
MR Method
Inverse variance weighted
MR Egger
500
450
400
1/SE_IV
−0.002
0.000
0.002
0.004
β_IV

Batch 2−882 : Dual specificity tyrosine−phosphorylation−regulated kinase 3 on eGFR

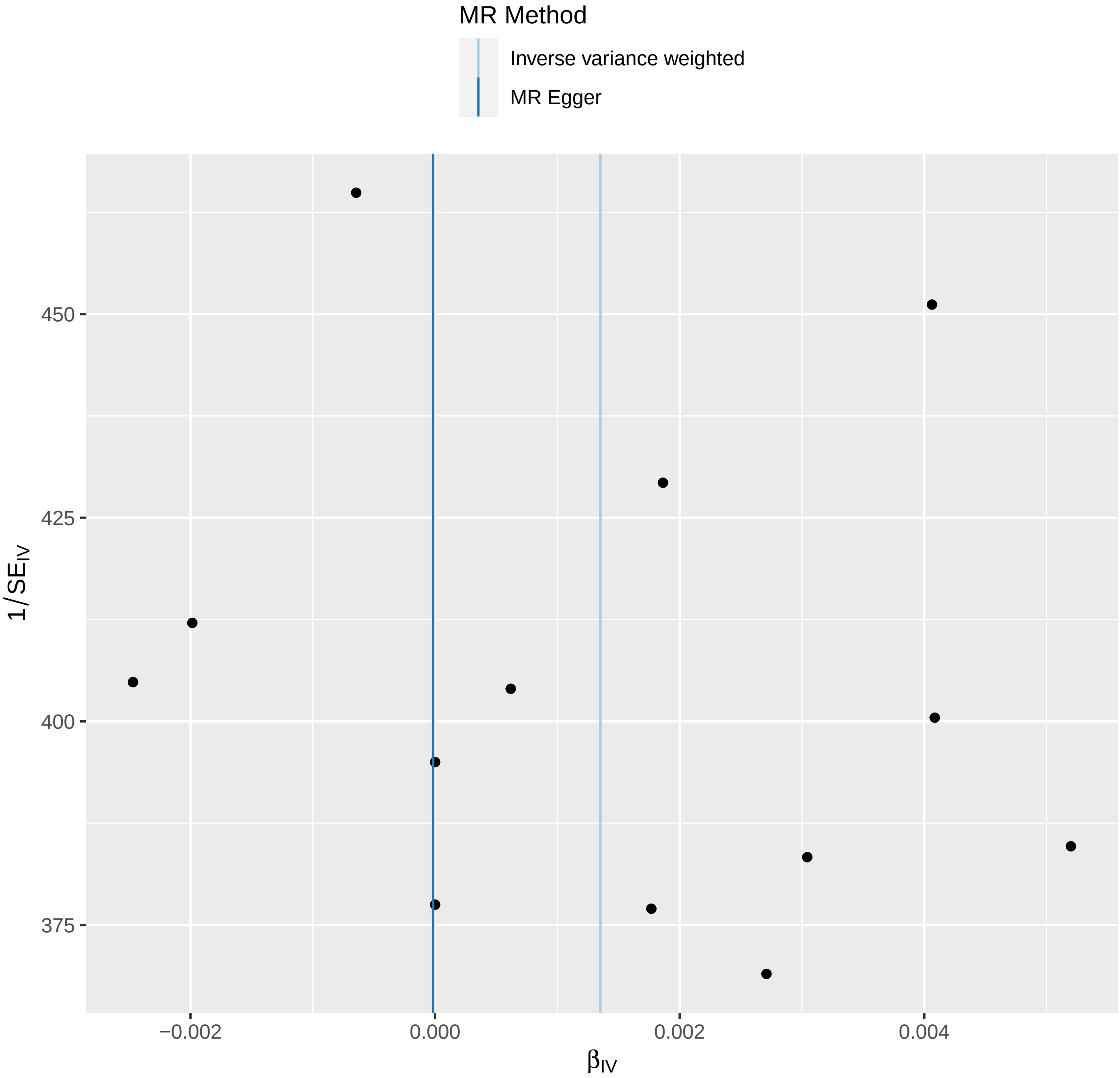

Batch 2−883 : Endothelin−converting enzyme 1 on eGFR

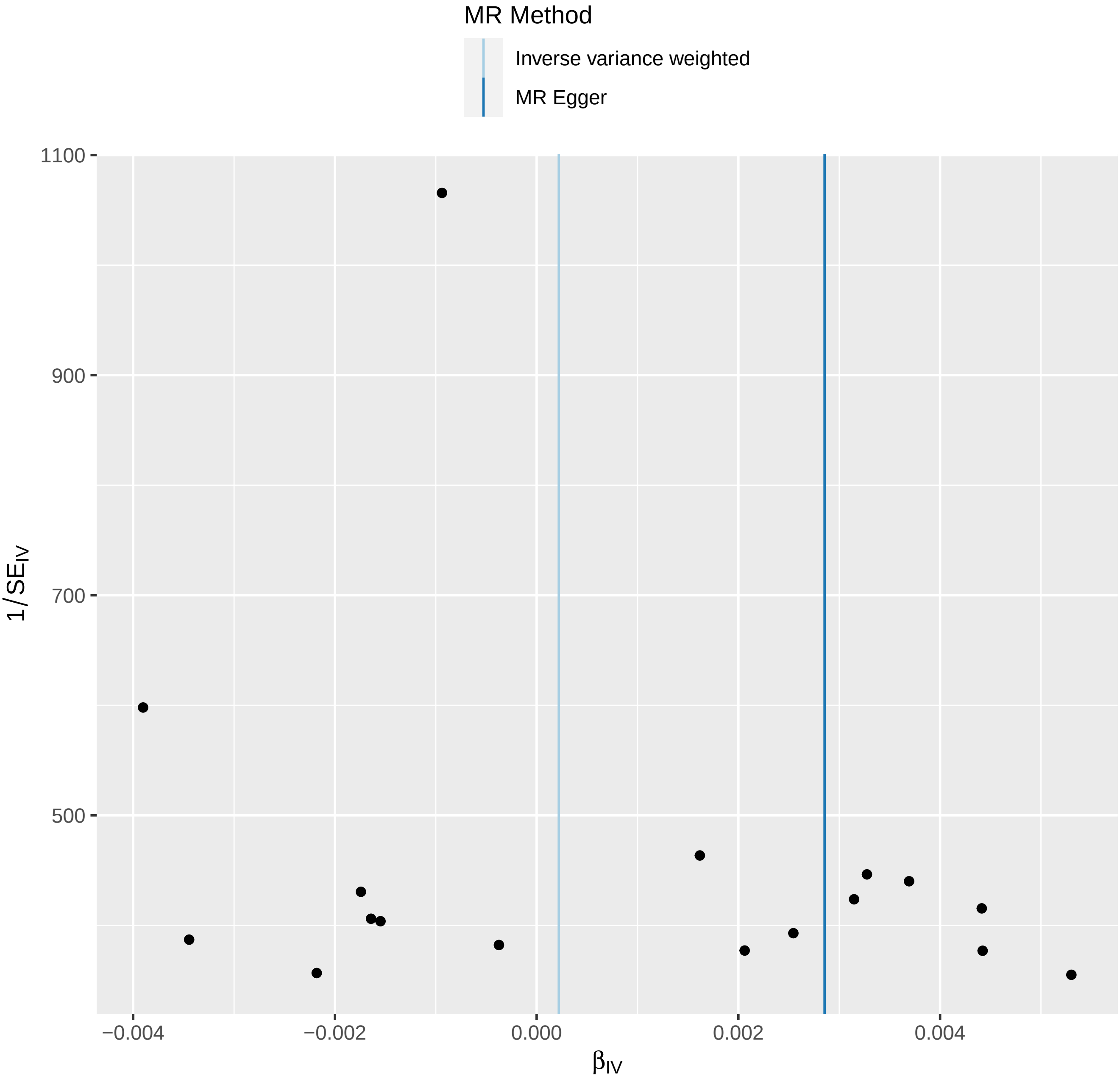

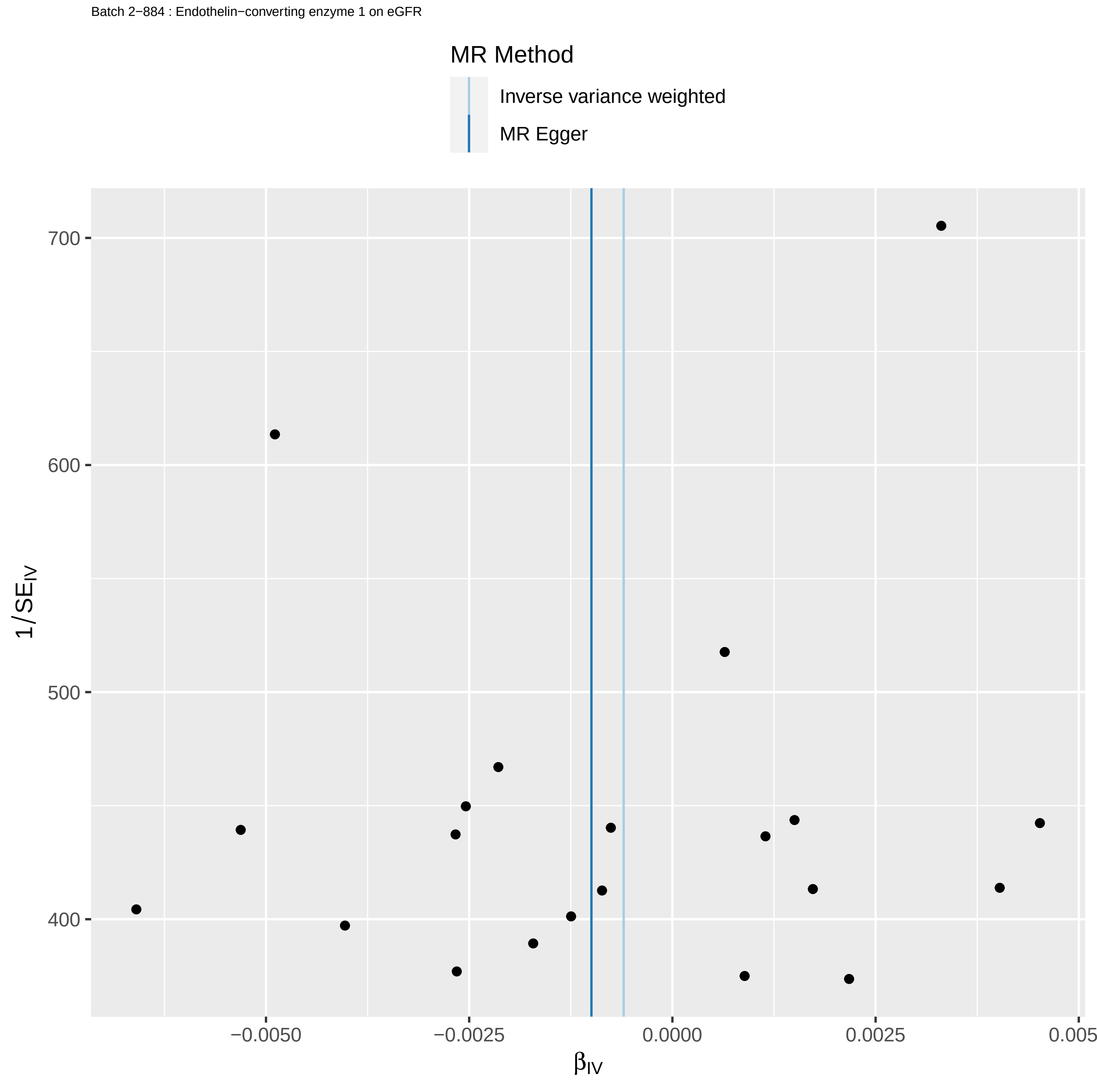

Batch 2−884 : Endothelin−converting enzyme 1 on eGFR
MR Method
Inverse variance weighted
MR Egger
700
600
500
400
1/SE_IV
−0.0050
−0.0025
0.0000
0.0025
0.005
β_IV

Batch 2−885 : Ectodysplasin−A, secreted form on eGFR

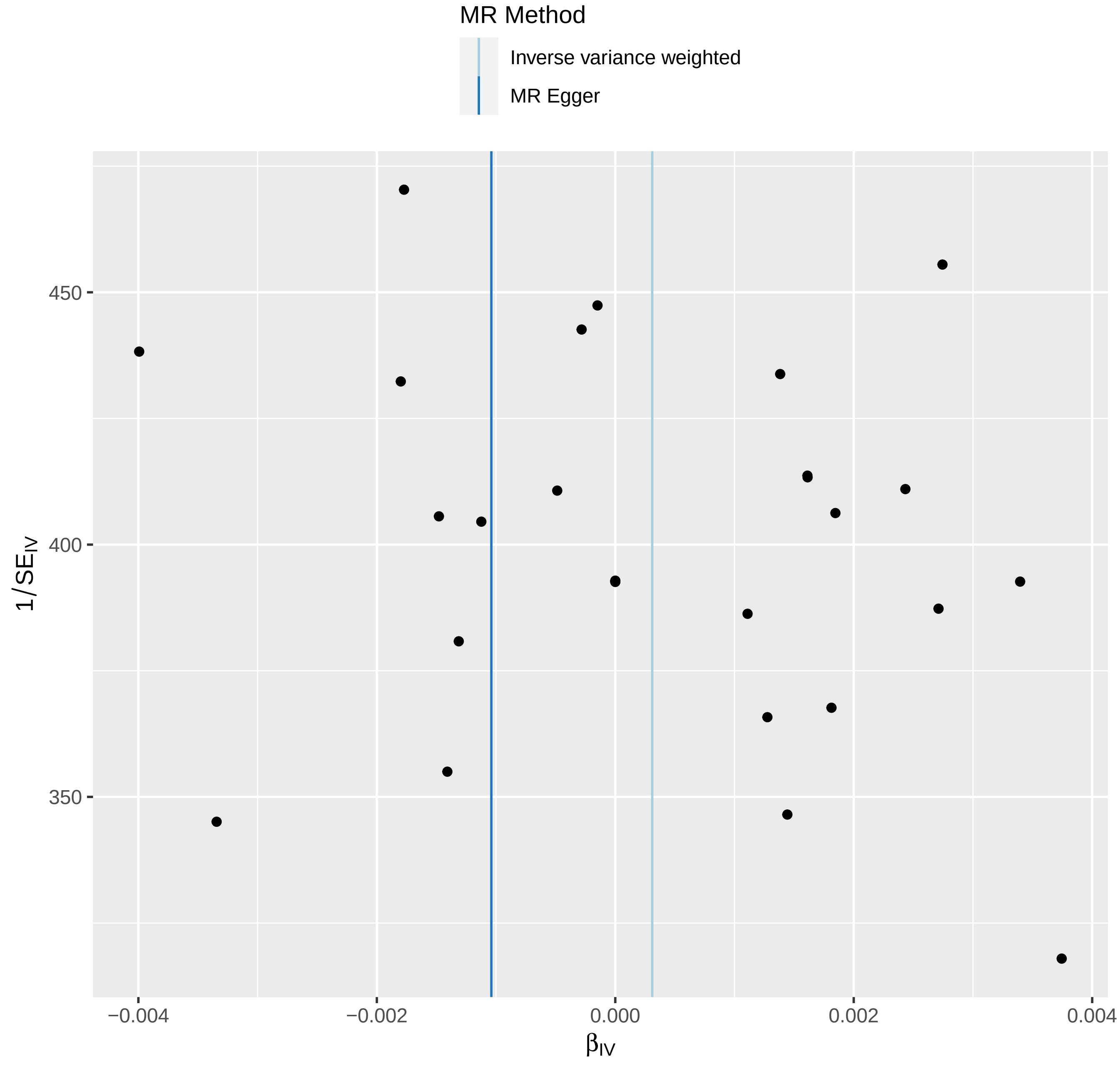

Batch 2−886 : Ectodysplasin−A, secreted form on eGFR

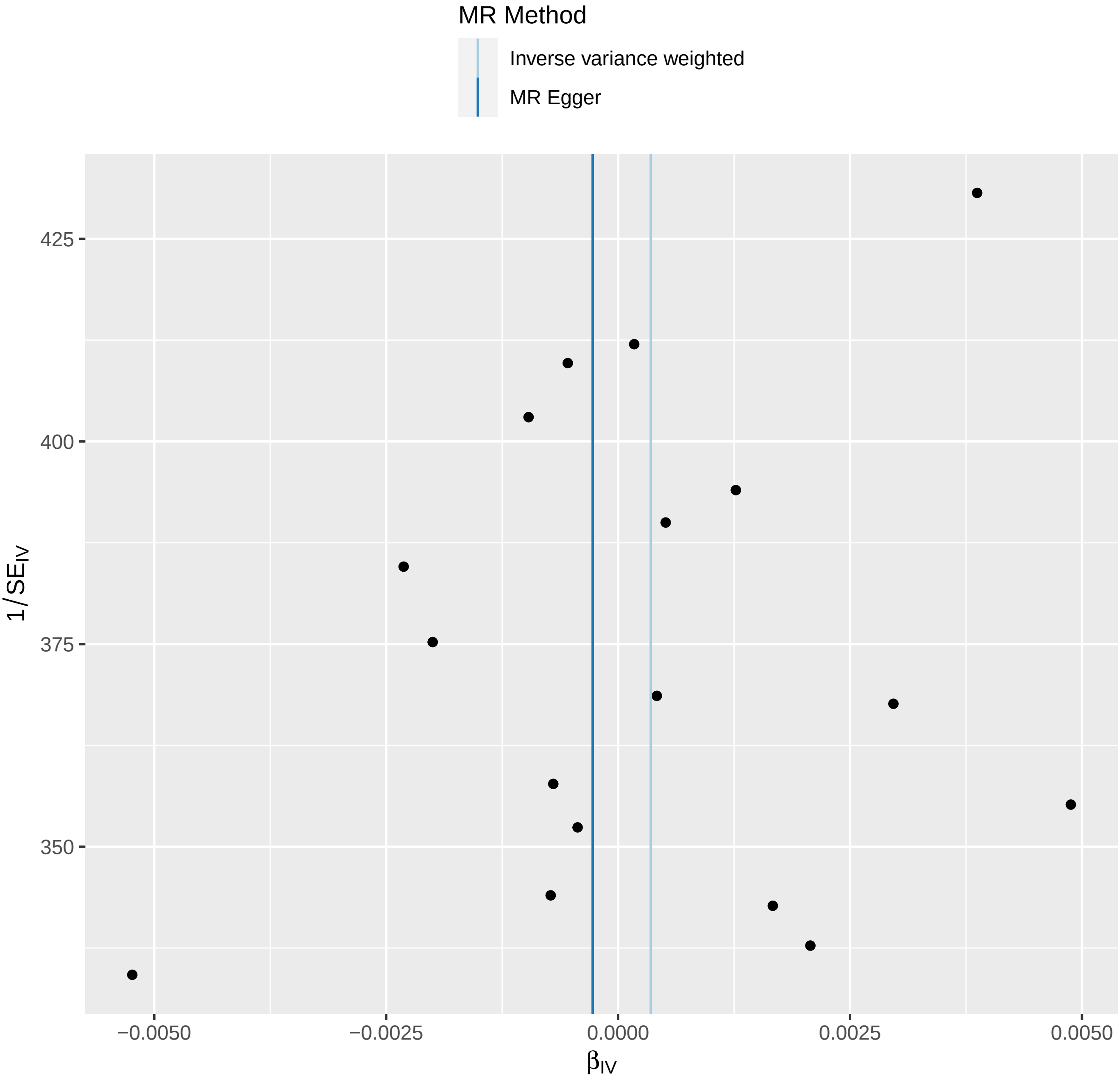

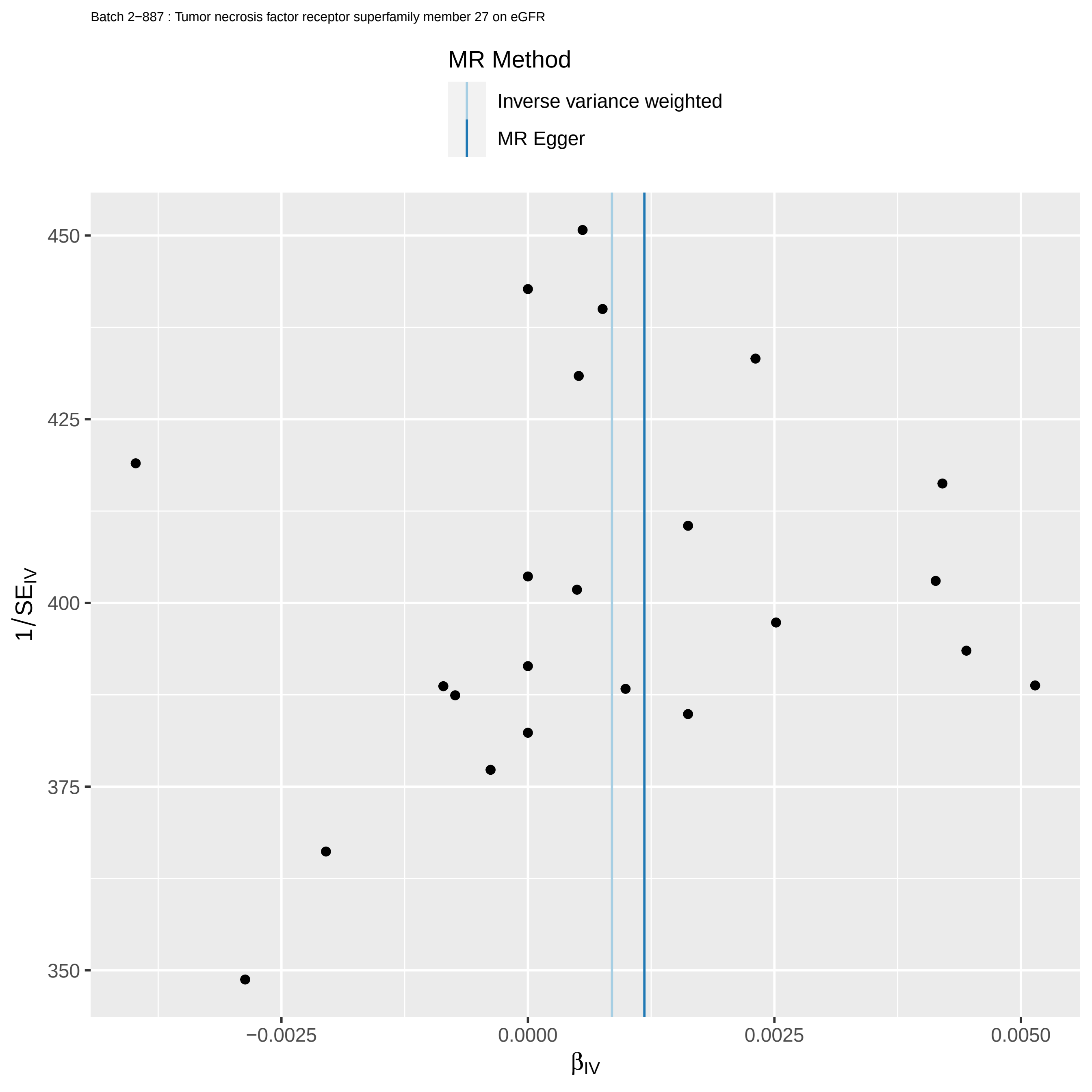

Batch 2−887 : Tumor necrosis factor receptor superfamily member 27 on eGFR
MR Method
Inverse variance weighted
MR Egger
450
425
400
375
350
1/SE_IV
−0.0025
0.0000
0.0025
0.0050
β_IV

Batch 2−888 : Tumor necrosis factor receptor superfamily member EDAR on eGFR

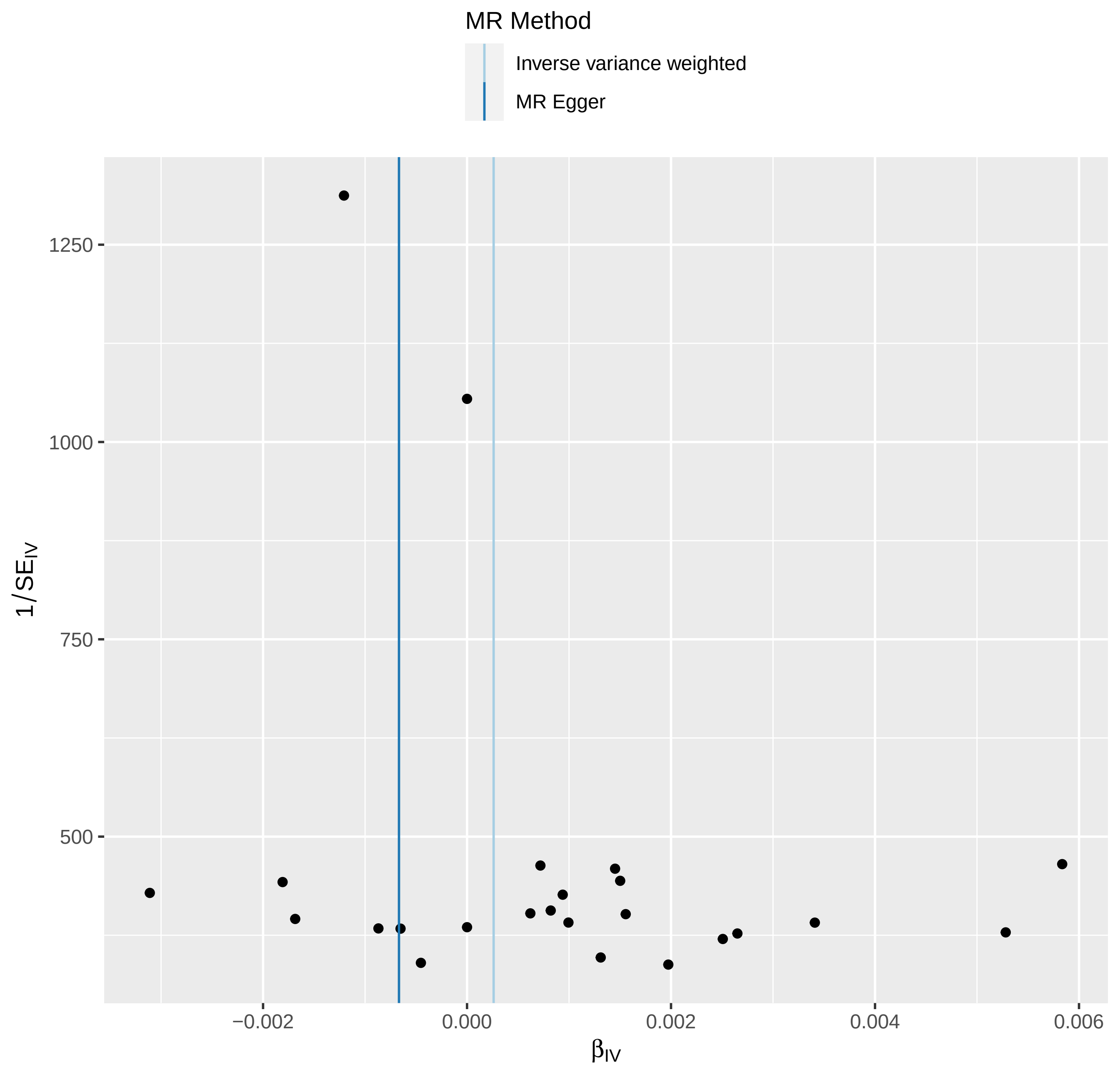

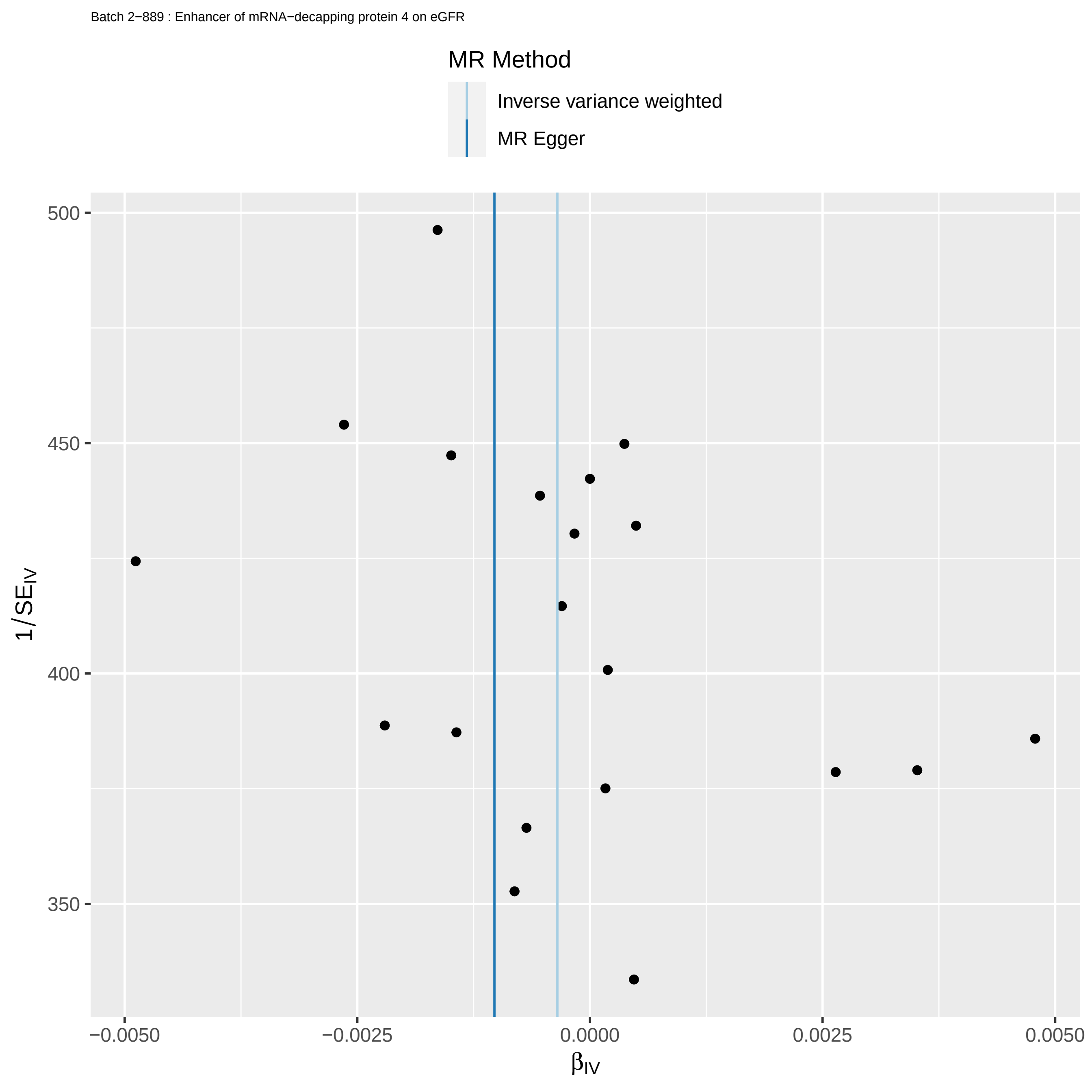
Batch 2−889 : Enhancer of mRNA−decapping protein 4 on eGFR
MR Method
Inverse variance weighted
MR Egger
500
450
400
350
1/SE_IV
−0.0050
−0.0025
0.0000
0.0025
0.0050
β_IV

Batch 2−890 : Epididymal secretory protein E3−beta on eGFR

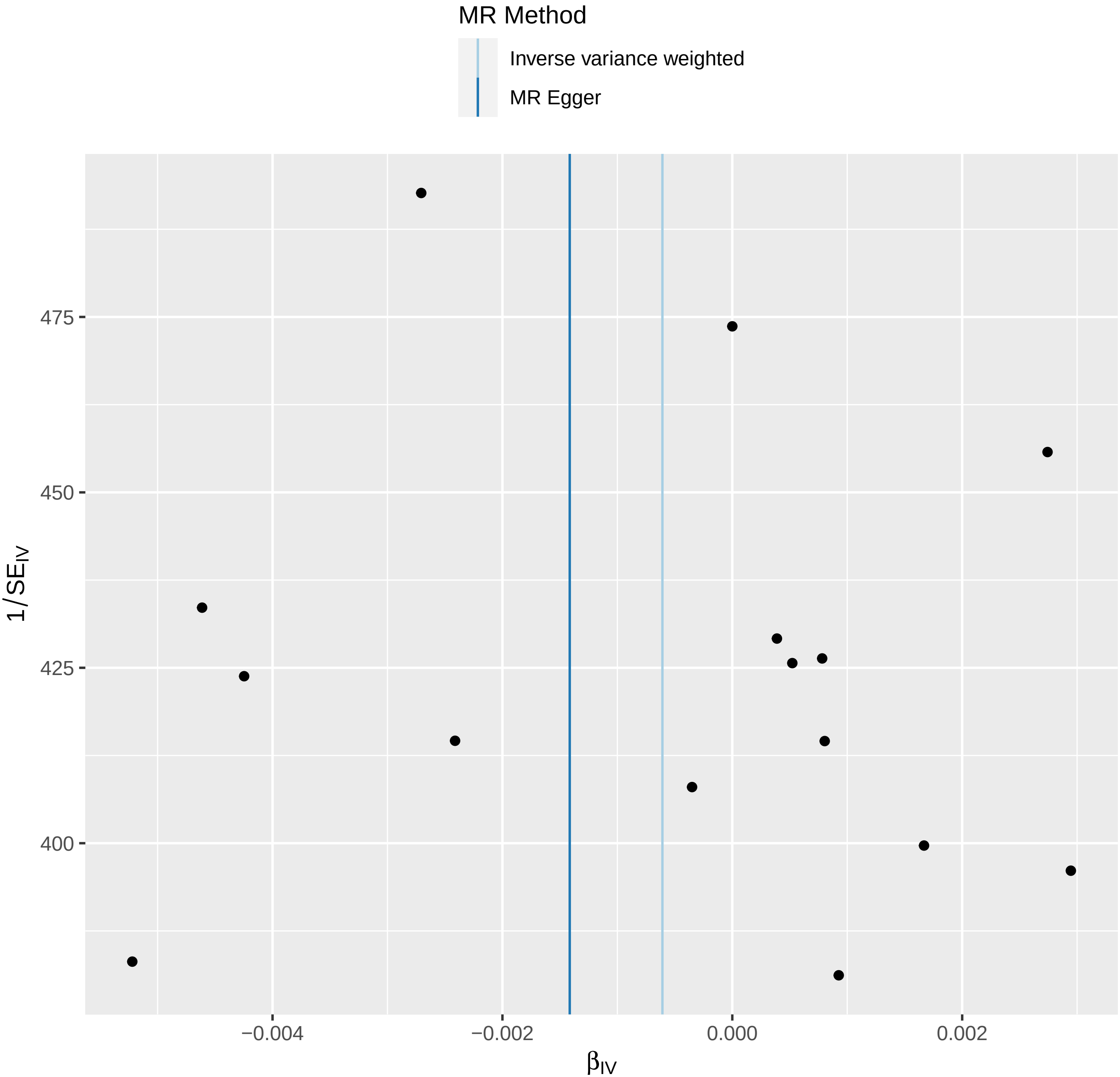

Batch 2−891 : Endothelial differentiation−related factor 1 on eGFR

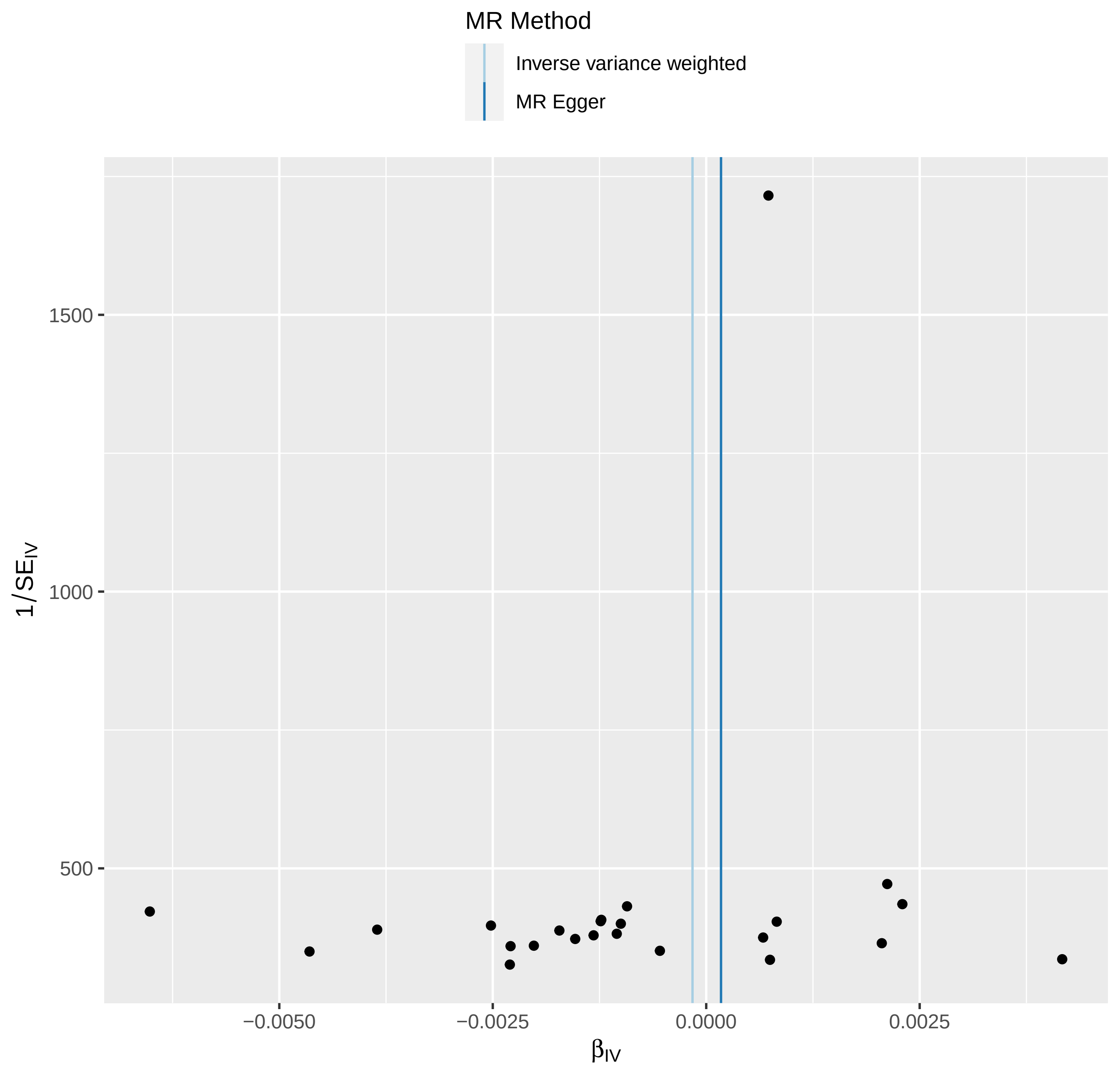

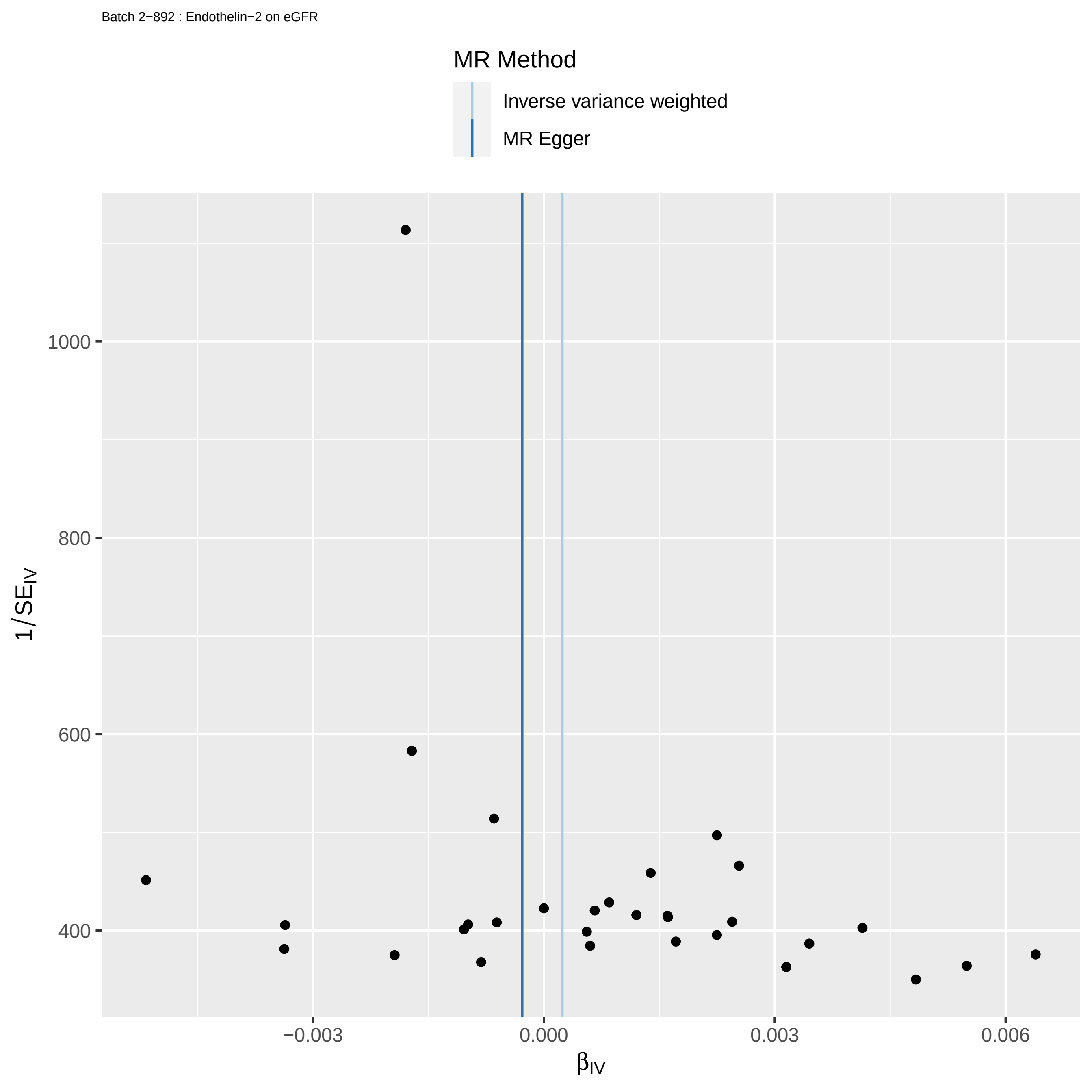

Batch 2−892 : Endothelin−2 on eGFR
MR Method
Inverse variance weighted
MR Egger
1000
800
600
400
1/SE_IV
−0.003
0.000
0.003
0.006
β_IV

Batch 2−893 : Early endosome antigen 1 on eGFR

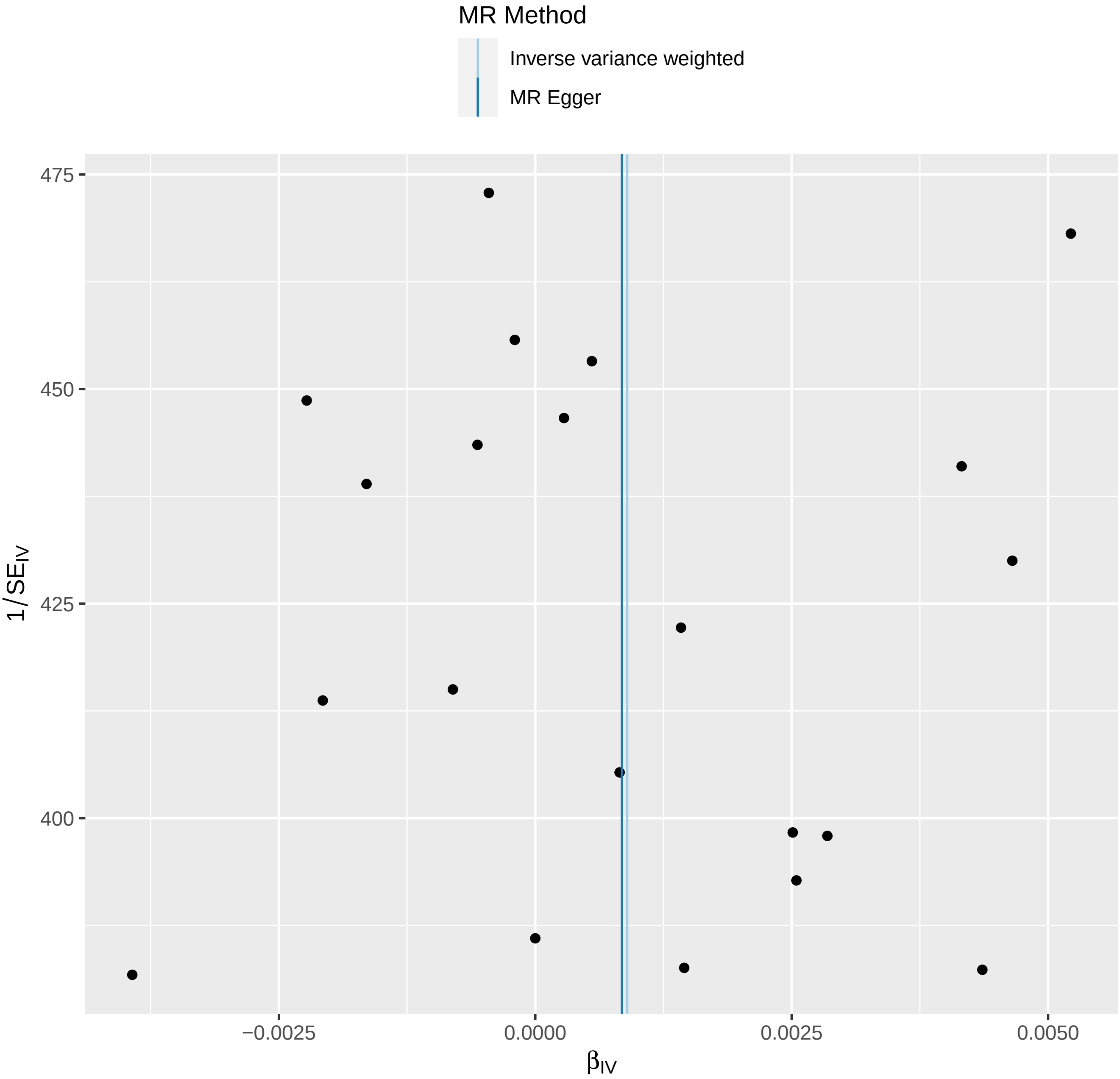

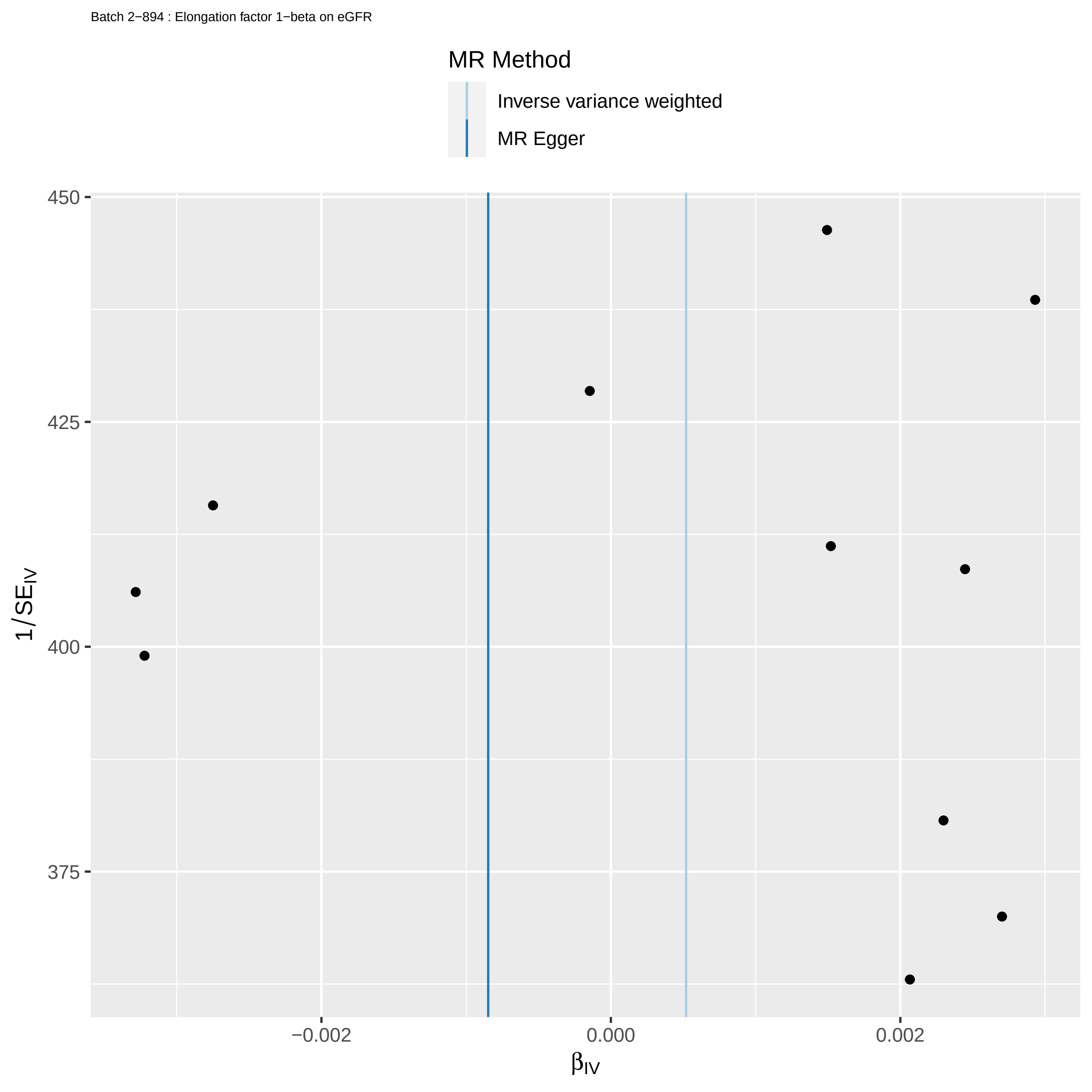

Batch 2−894 : Elongation factor 1−beta on eGFR
MR Method
Inverse variance weighted
MR Egger
450
425
400
375
1/SE_IV
−0.002
0.000
0.002
β_IV

Batch 2−895 : EF−hand calcium−binding domain−containing protein 14 on eGFR

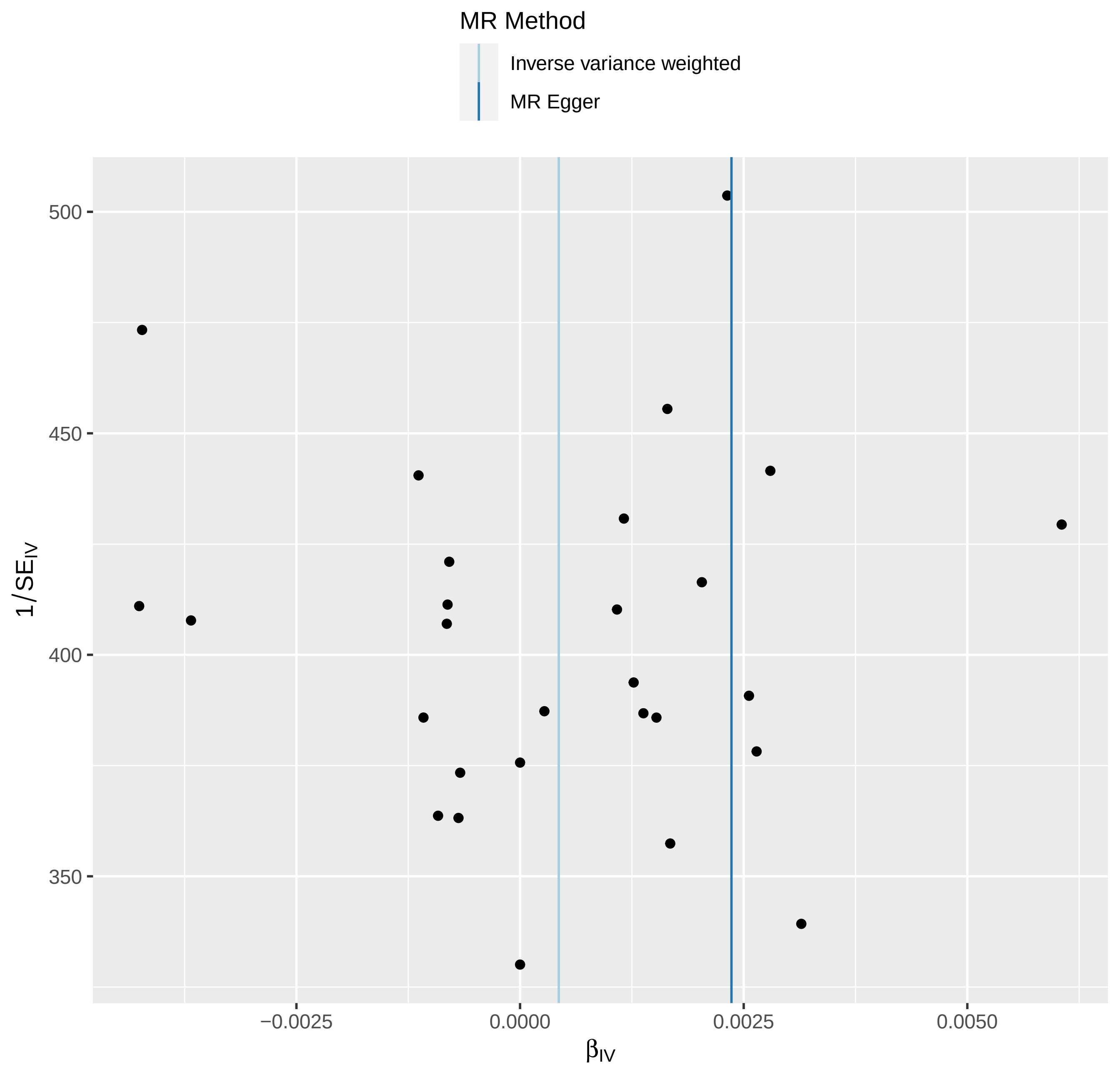

Batch 2−896 : Calcium uptake protein 3, mitochondrial on eGFR

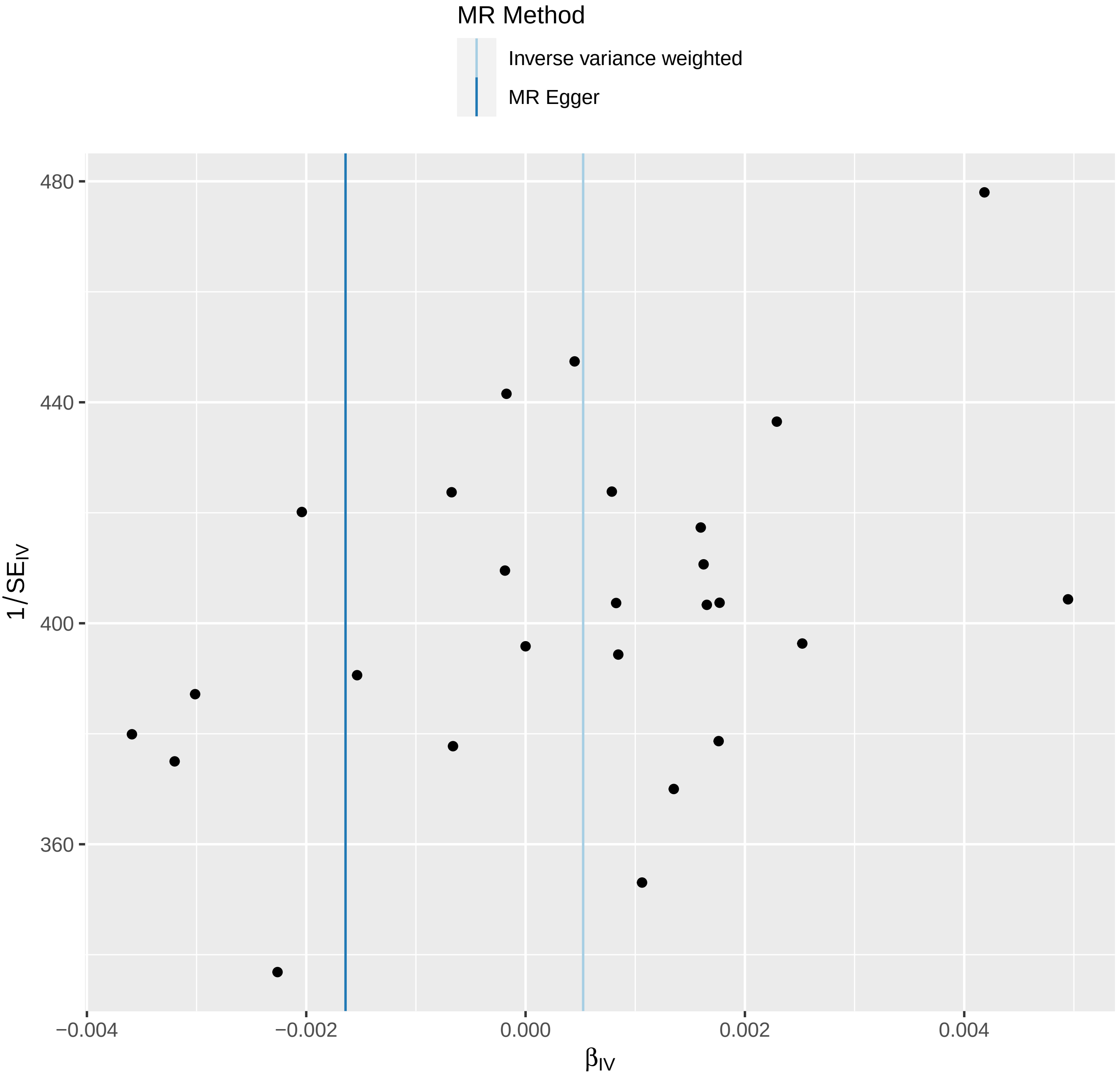

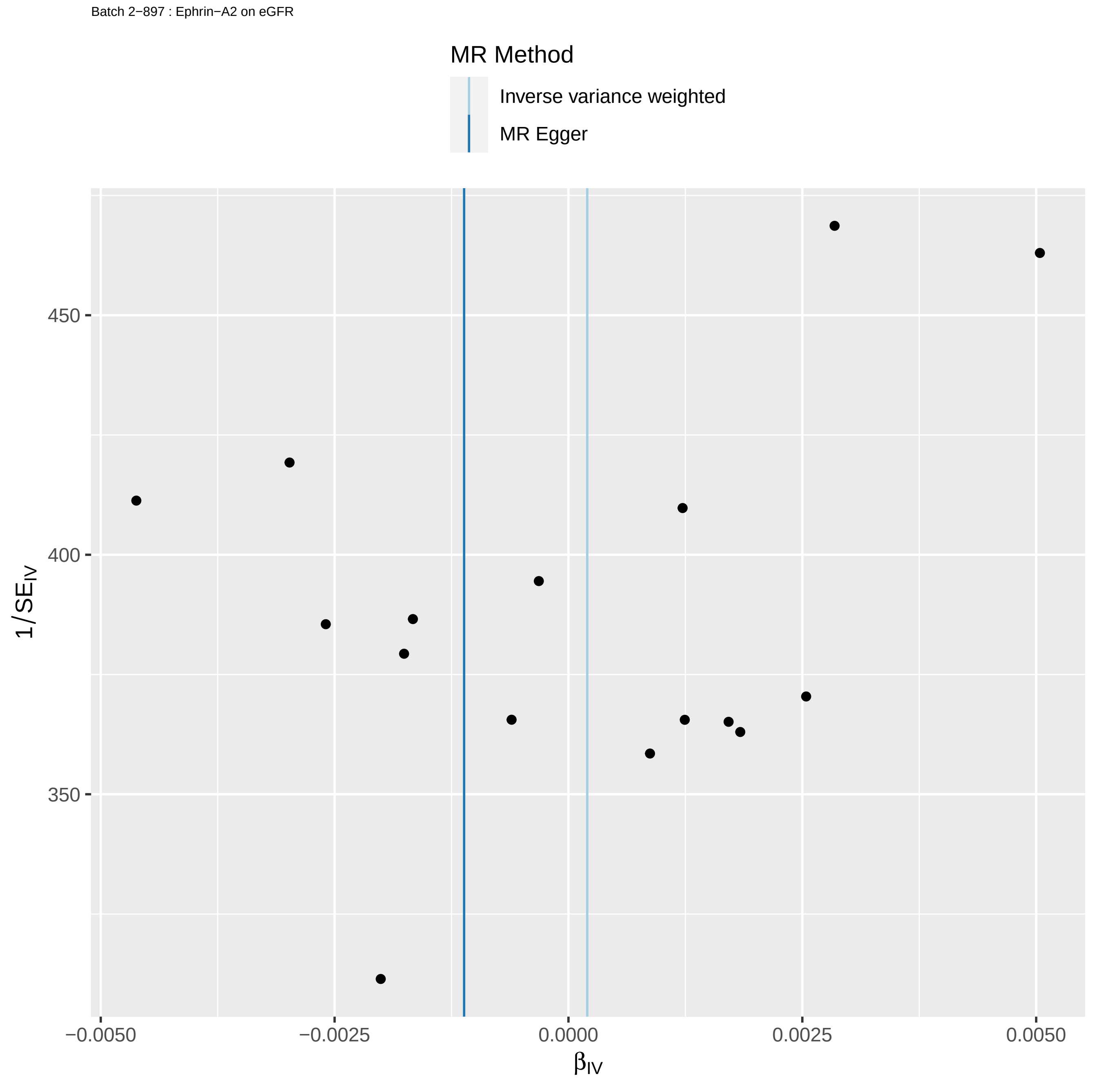

Batch 2−897 : Ephrin−A2 on eGFR
MR Method
Inverse variance weighted
MR Egger
450
400
350
1/SE_IV
−0.0050
−0.0025
0.0000
0.0025
0.0050
β_IV

Batch 2−898 : Ephrin−A3 on eGFR

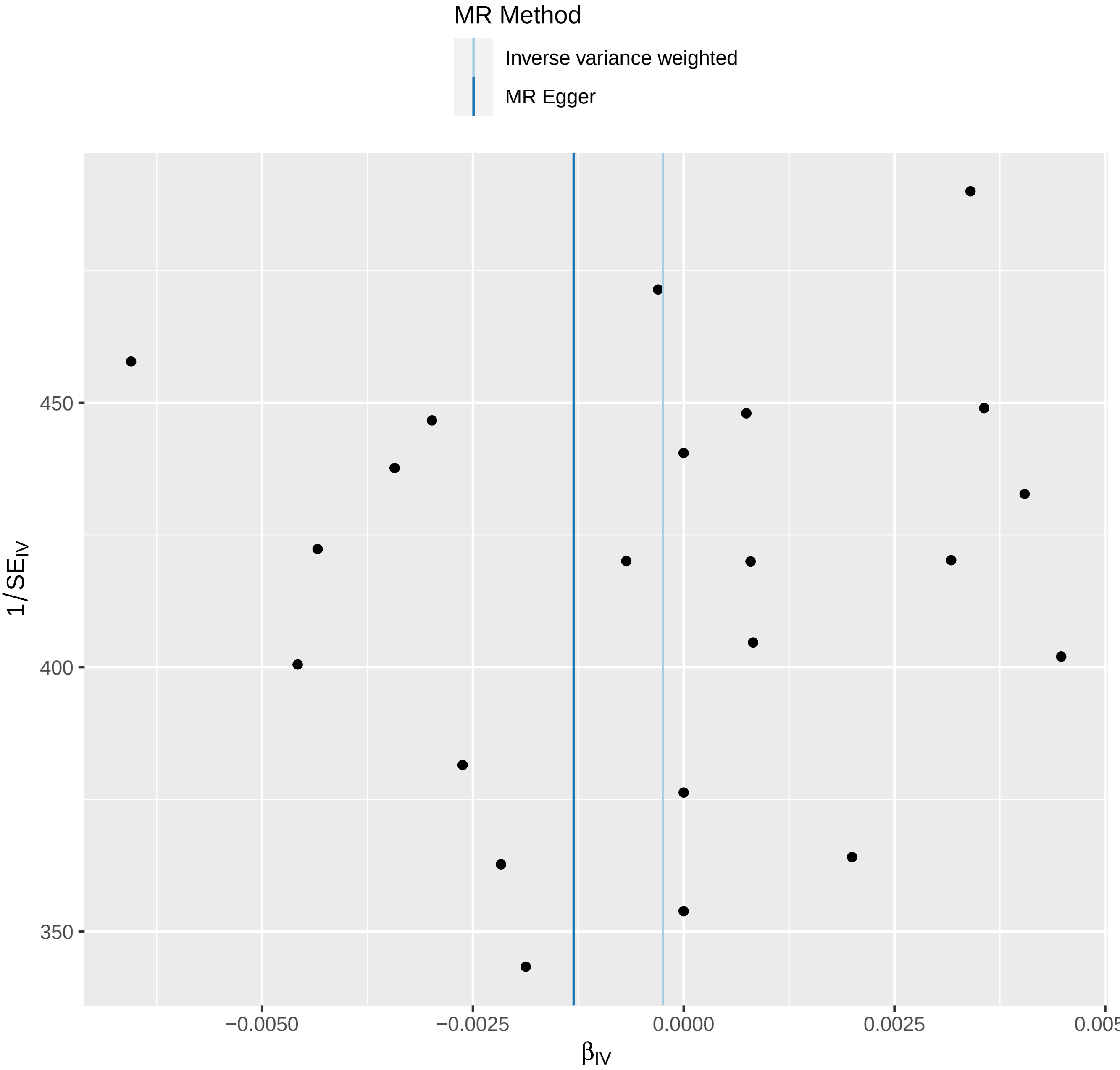

Batch 2−899 : Ephrin−A3 on eGFR

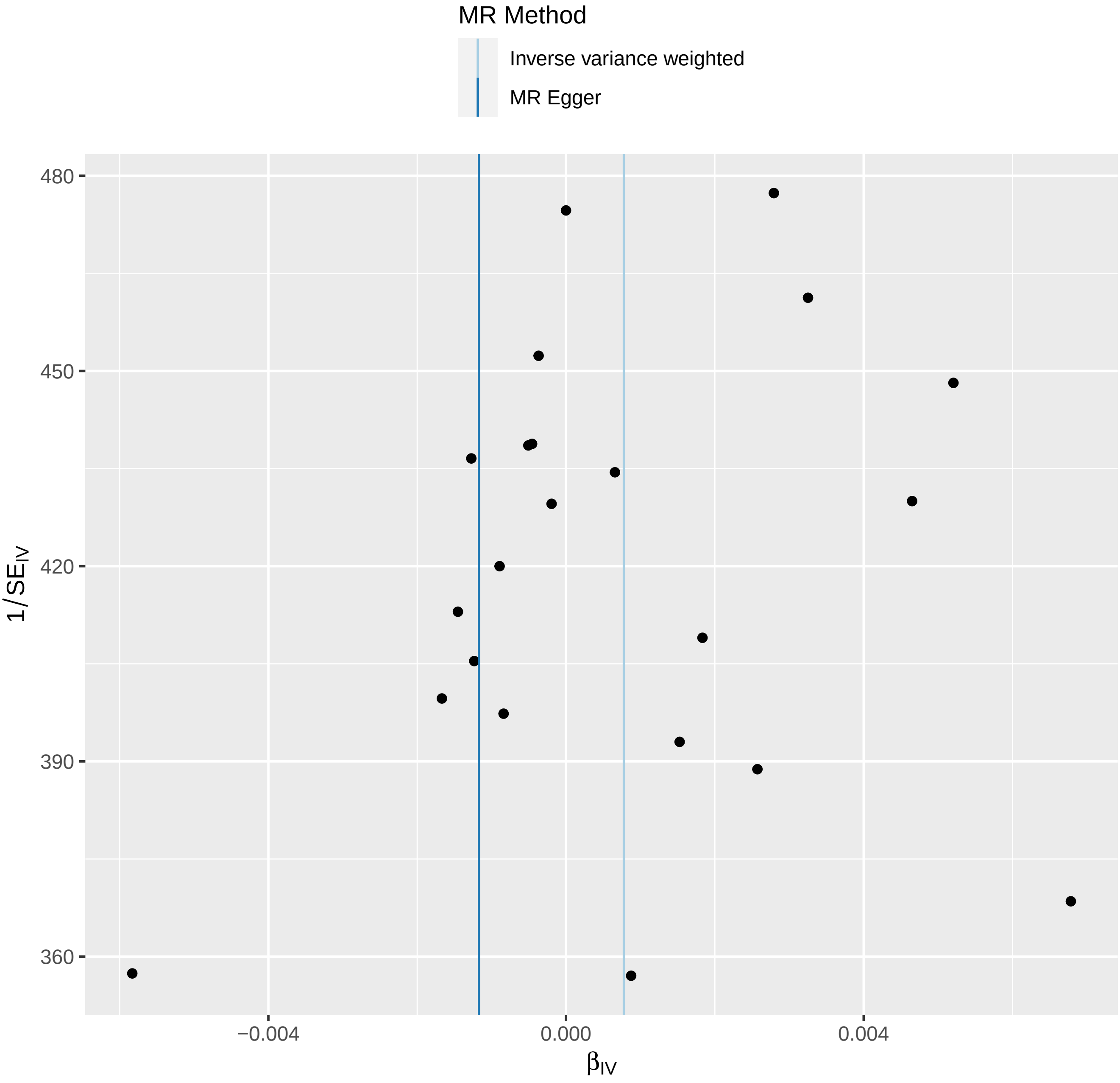

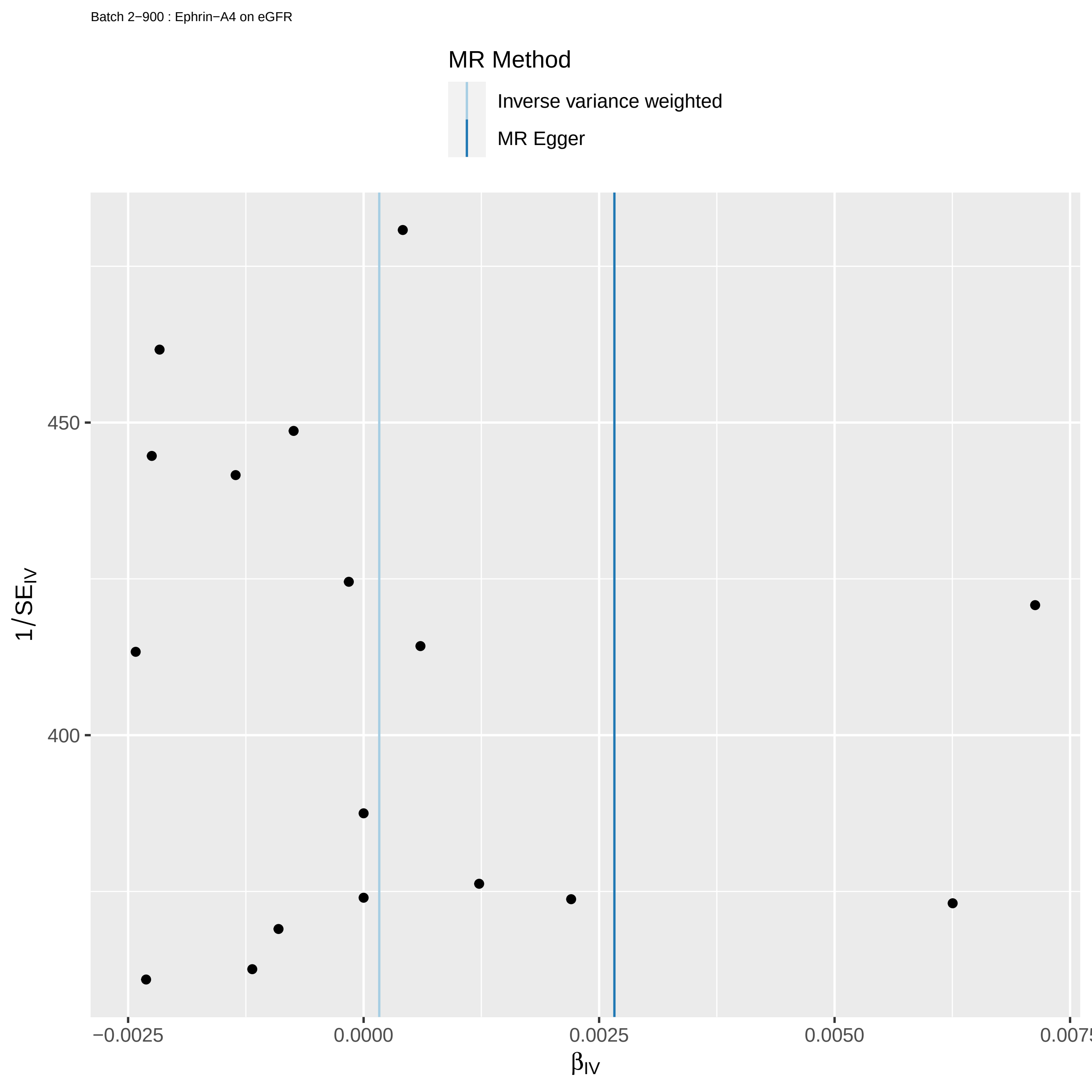

Batch 2−900 : Ephrin−A4 on eGFR
MR Method
Inverse variance weighted
MR Egger
450
400
1/SE_IV
−0.0025
0.0000
0.0025
0.0050
0.0075
β_IV

Batch 2−901 : Ephrin−A5 on eGFR

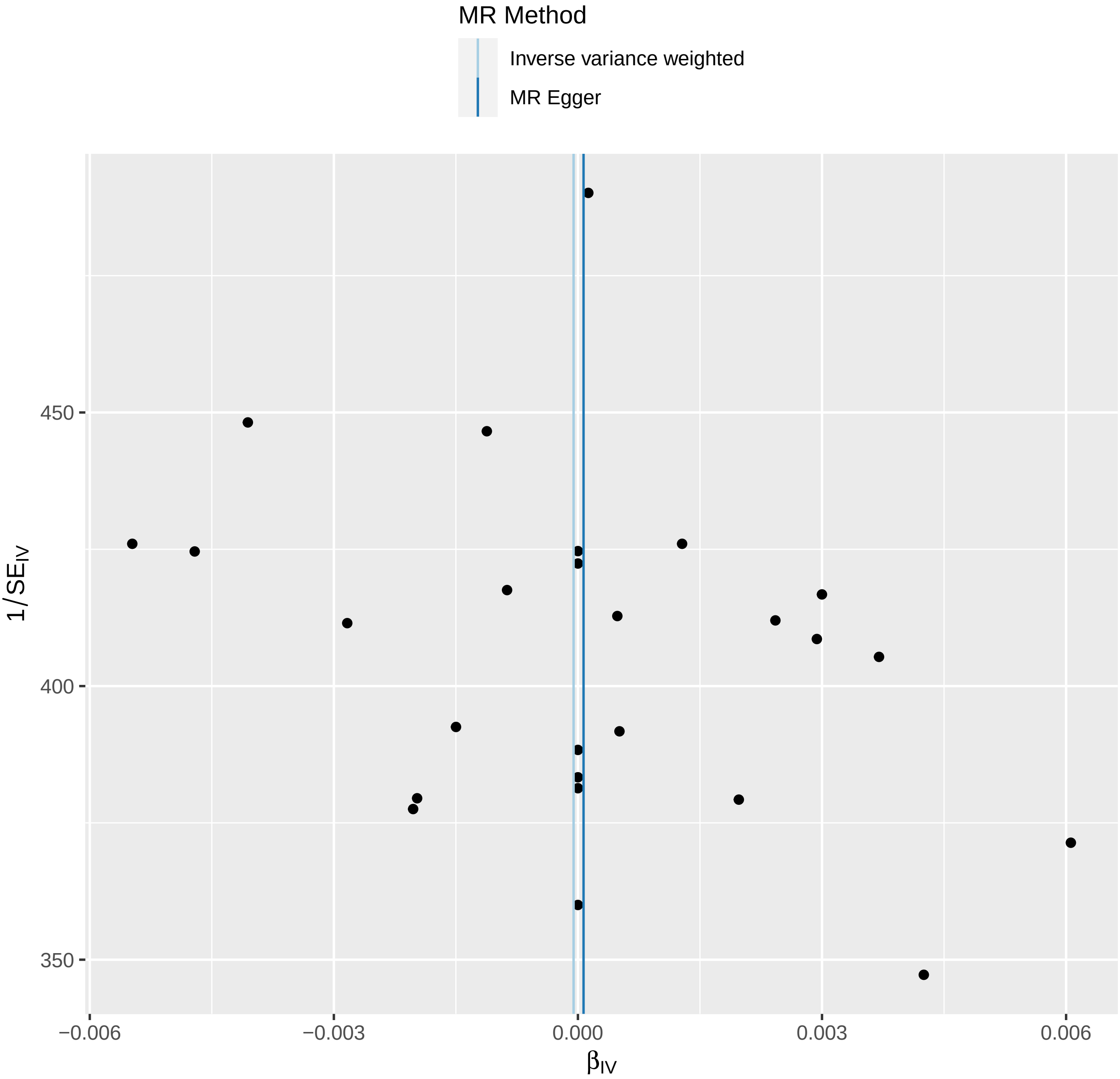

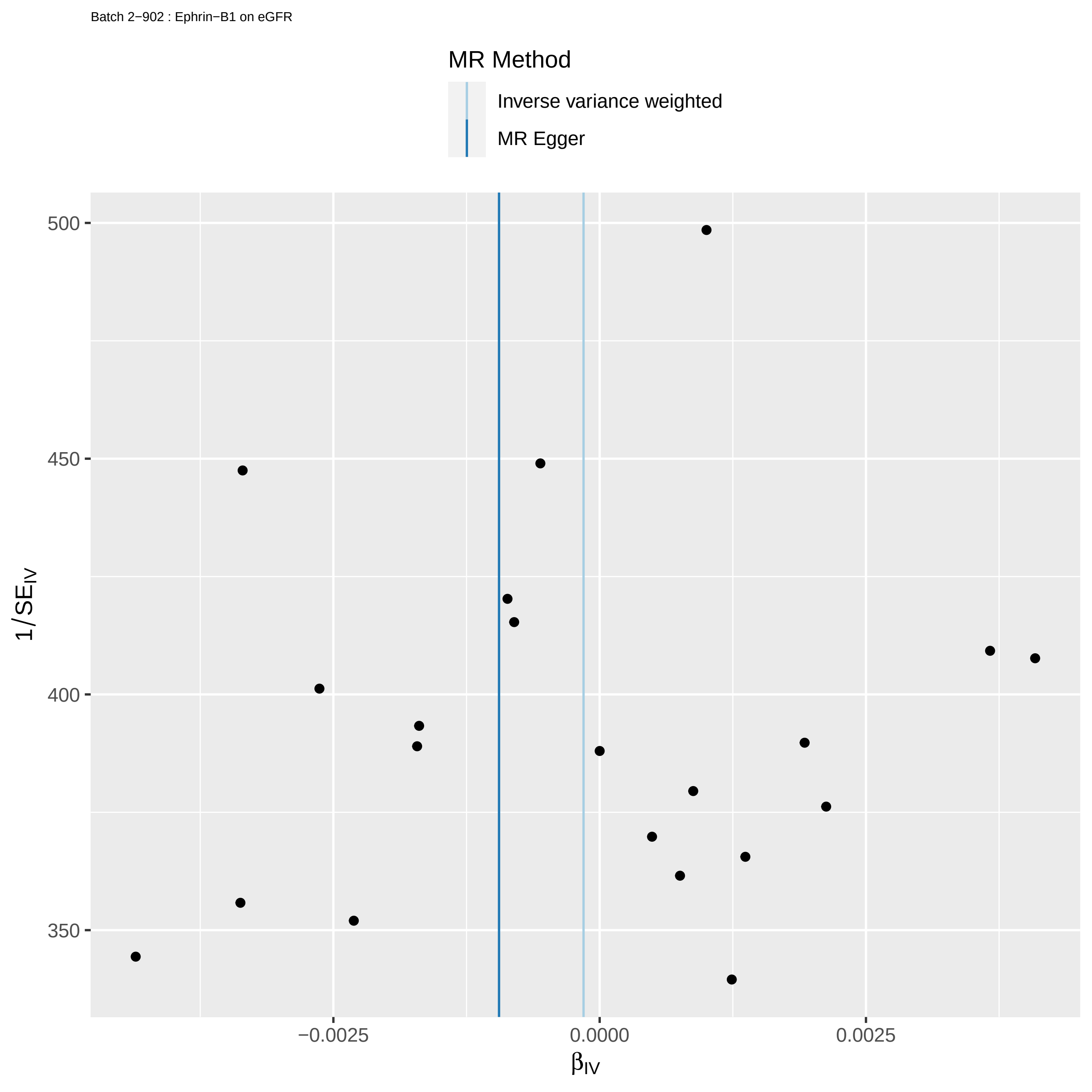

Batch 2−902 : Ephrin−B1 on eGFR
MR Method
Inverse variance weighted
MR Egger
500
450
400
350
1/SE$_{IV}$
−0.0025
0.0000
0.0025
β$_{IV}$

Batch 2−903 : Ephrin−B1 on eGFR

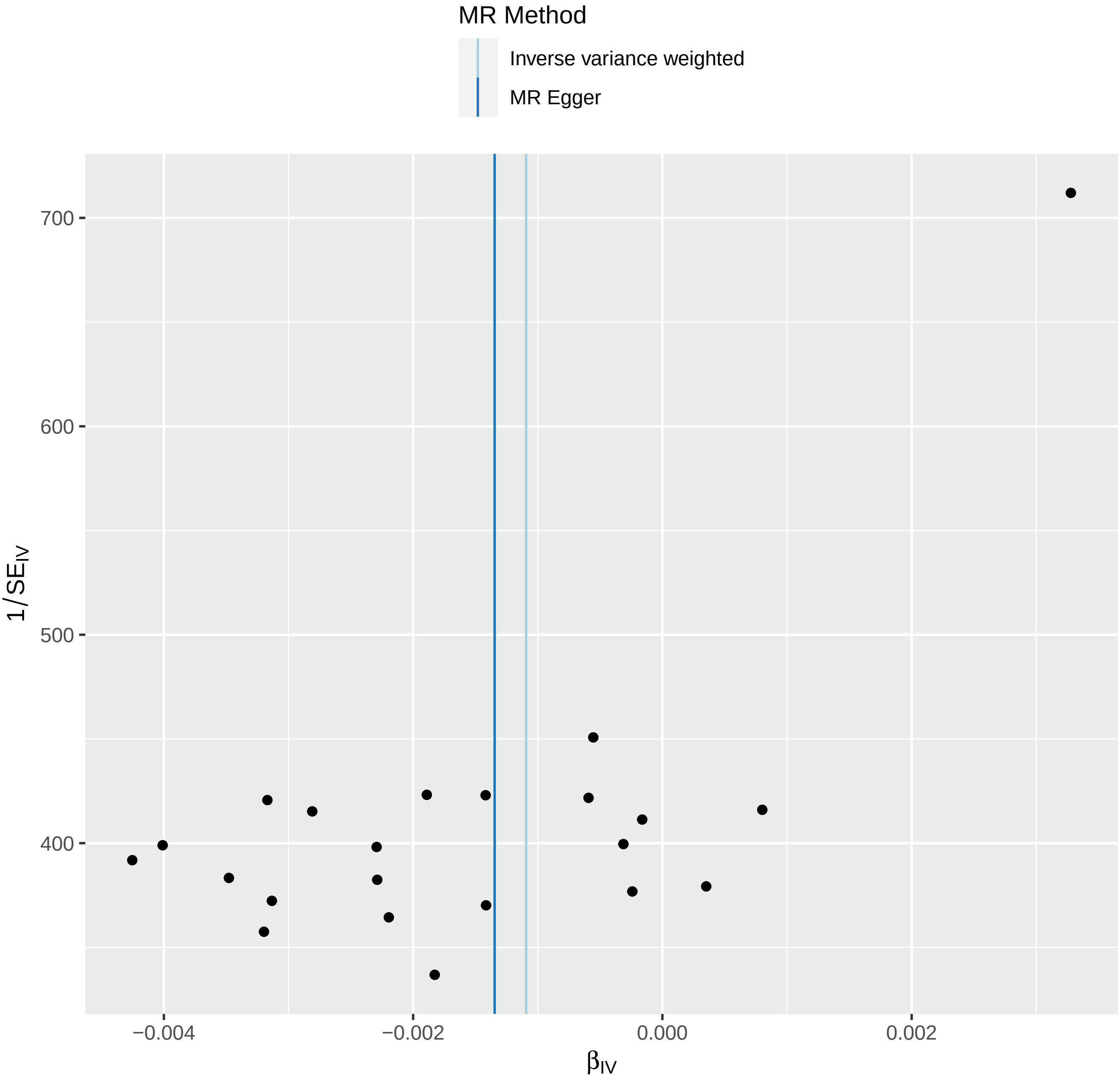

Batch 2−904 : Ephrin−B2 on eGFR

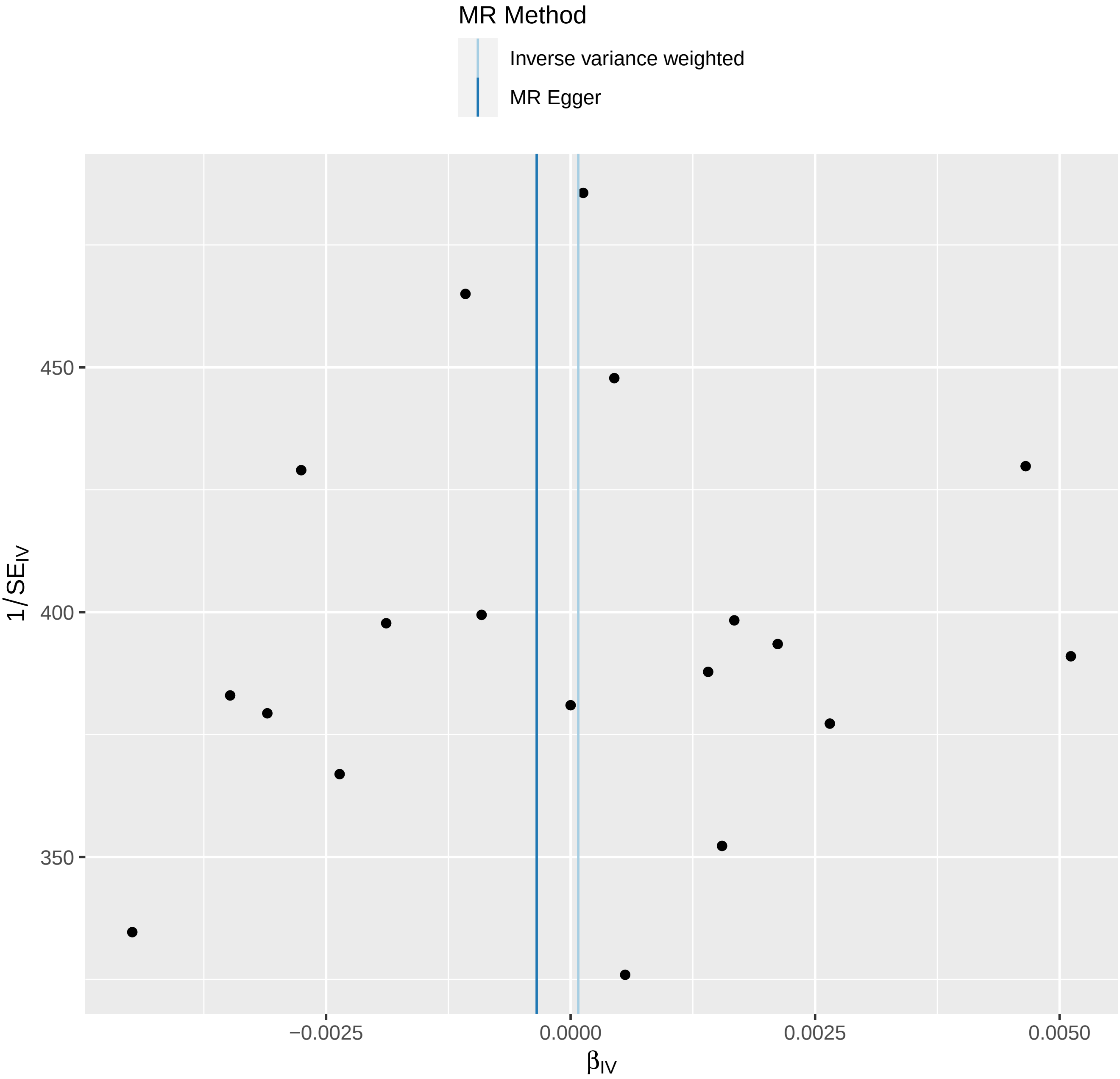

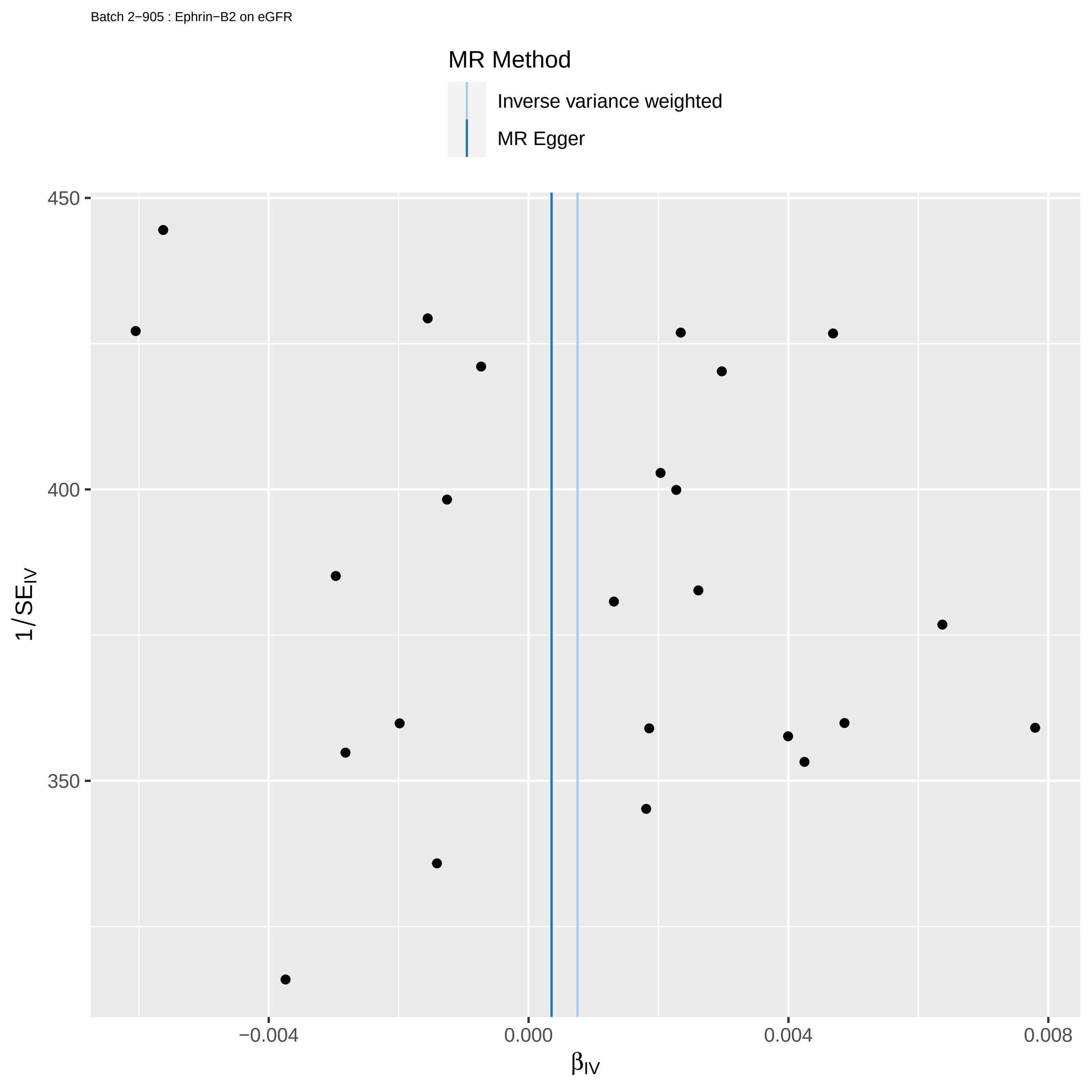

Batch 2−905 : Ephrin−B2 on eGFR
MR Method
Inverse variance weighted
MR Egger
450
400
350
1/SE_IV
−0.004
0.000
0.004
0.008
β_IV

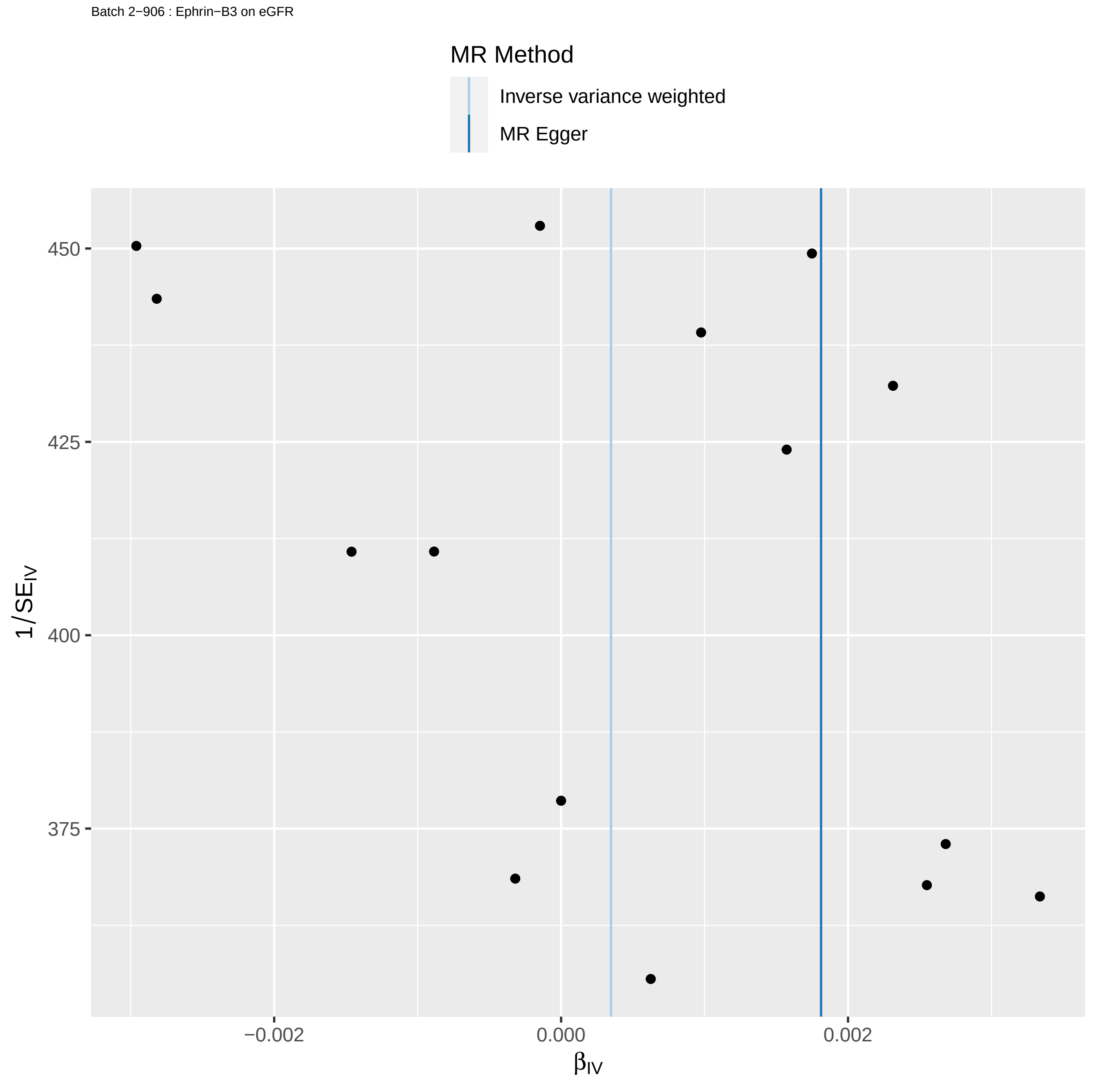

Batch 2−906 : Ephrin−B3 on eGFR
MR Method
Inverse variance weighted
MR Egger
450
425
400
375
1/SE_IV
−0.002
0.000
0.002
β_IV

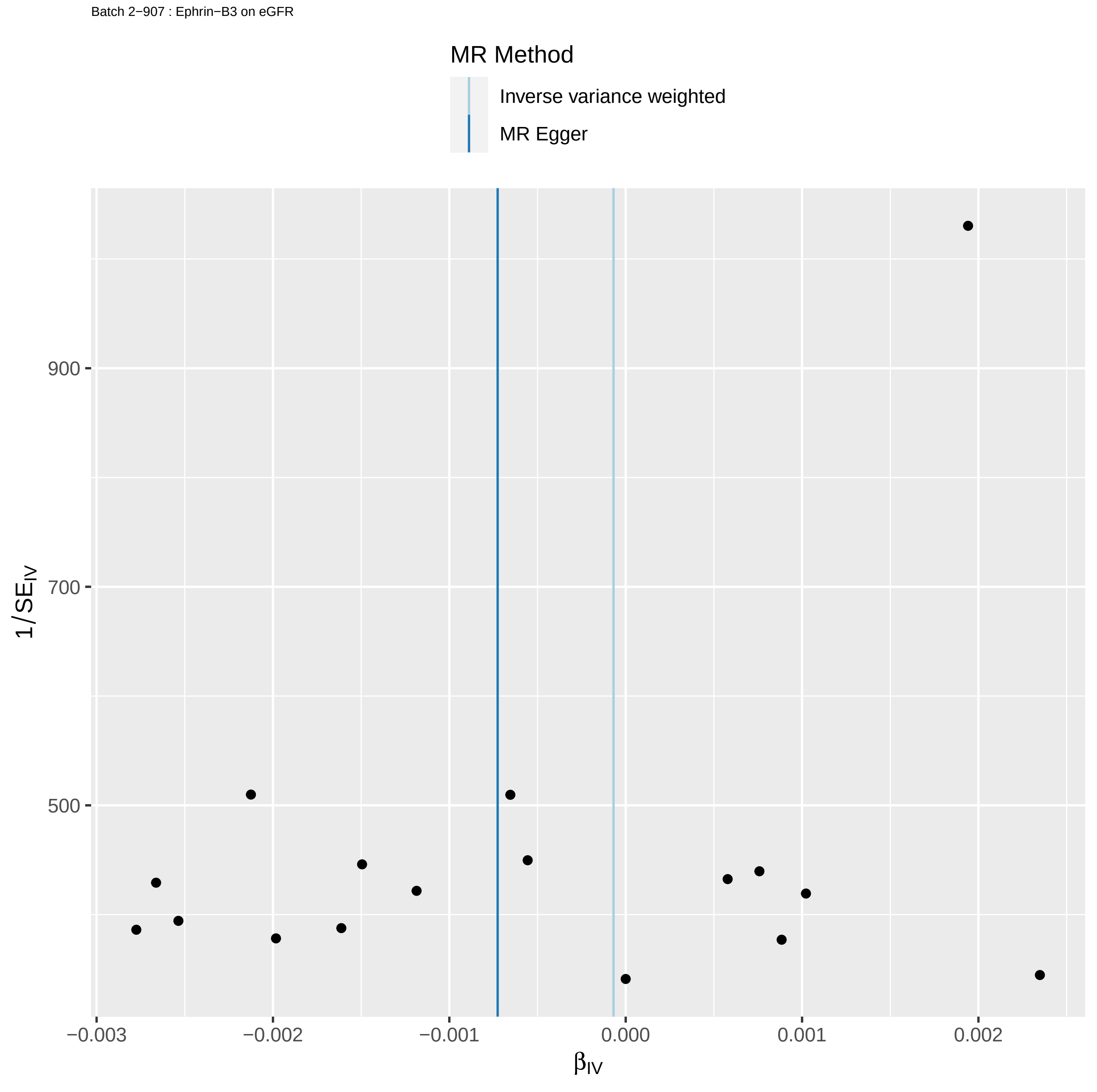
Batch 2−907 : Ephrin−B3 on eGFR
MR Method
Inverse variance weighted
MR Egger
900
700
500
1/SE_IV
−0.003
−0.002
−0.001
0.000
0.001
0.002
β_IV

Batch 2−908 : Epidermal growth factor on eGFR

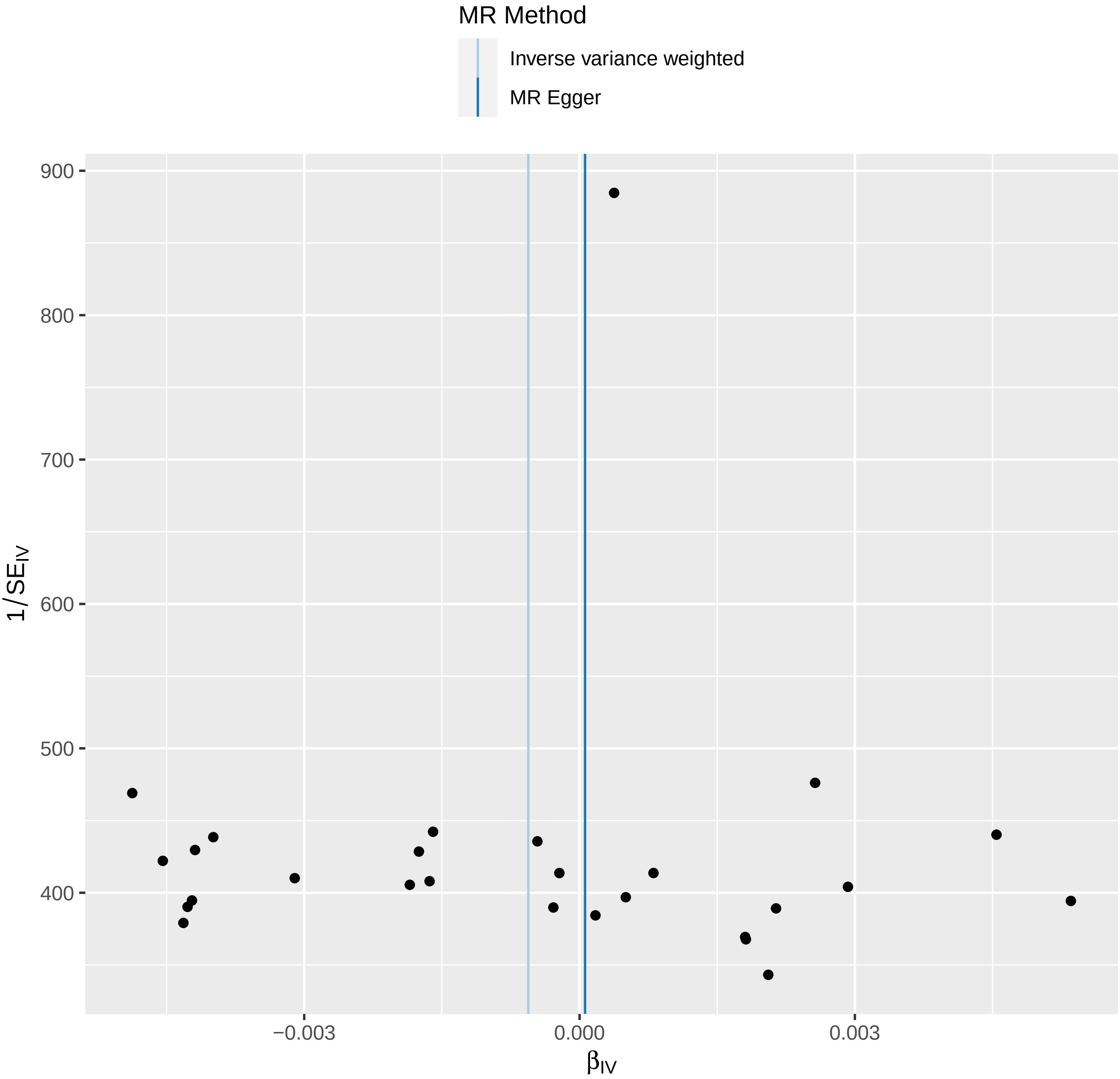

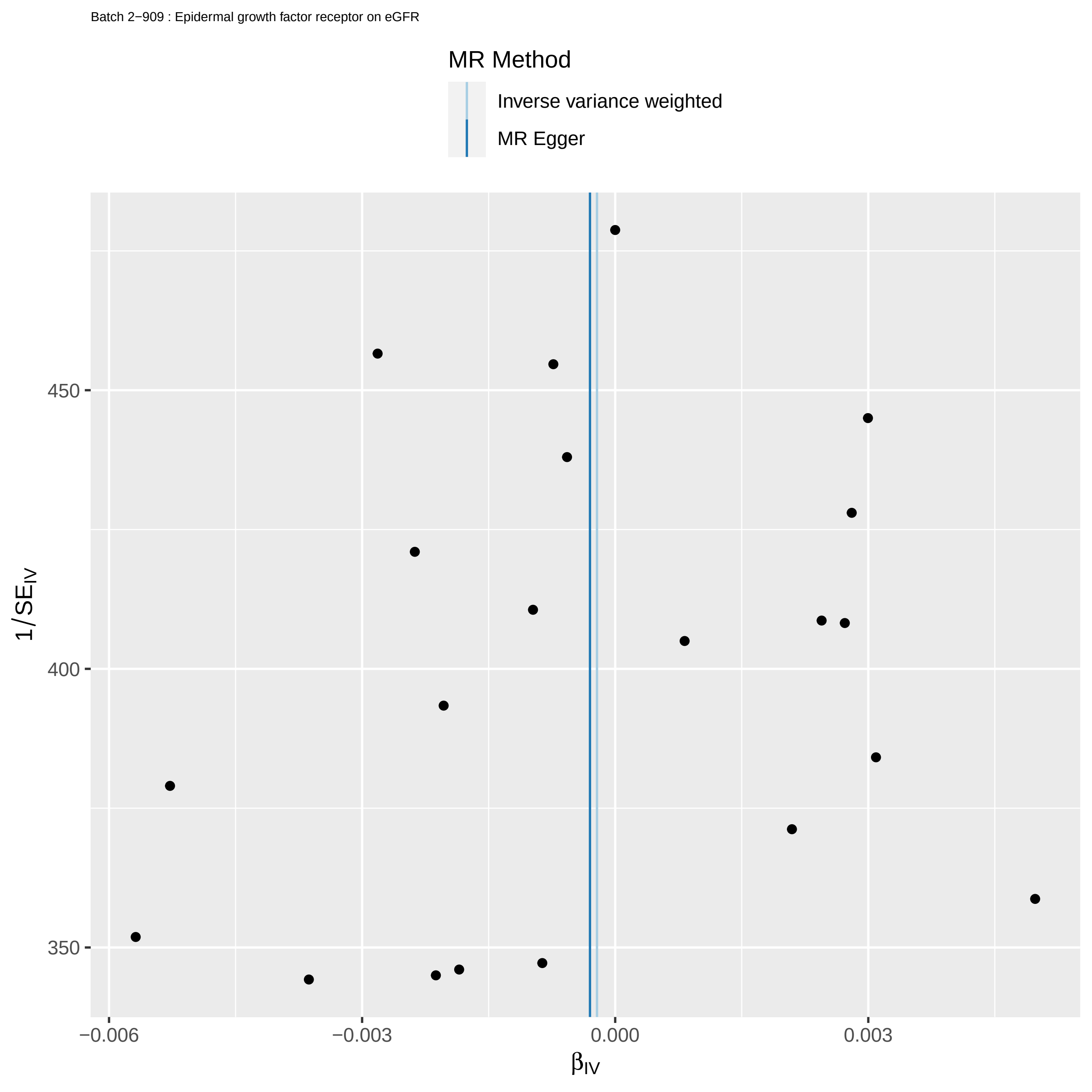

Batch 2−909 : Epidermal growth factor receptor on eGFR
MR Method
Inverse variance weighted
MR Egger
450
400
350
1/SE$_{IV}$
−0.006
−0.003
0.000
0.003
β$_{IV}$

Batch 2−910 : Epidermal growth factor receptor variant III on eGFR

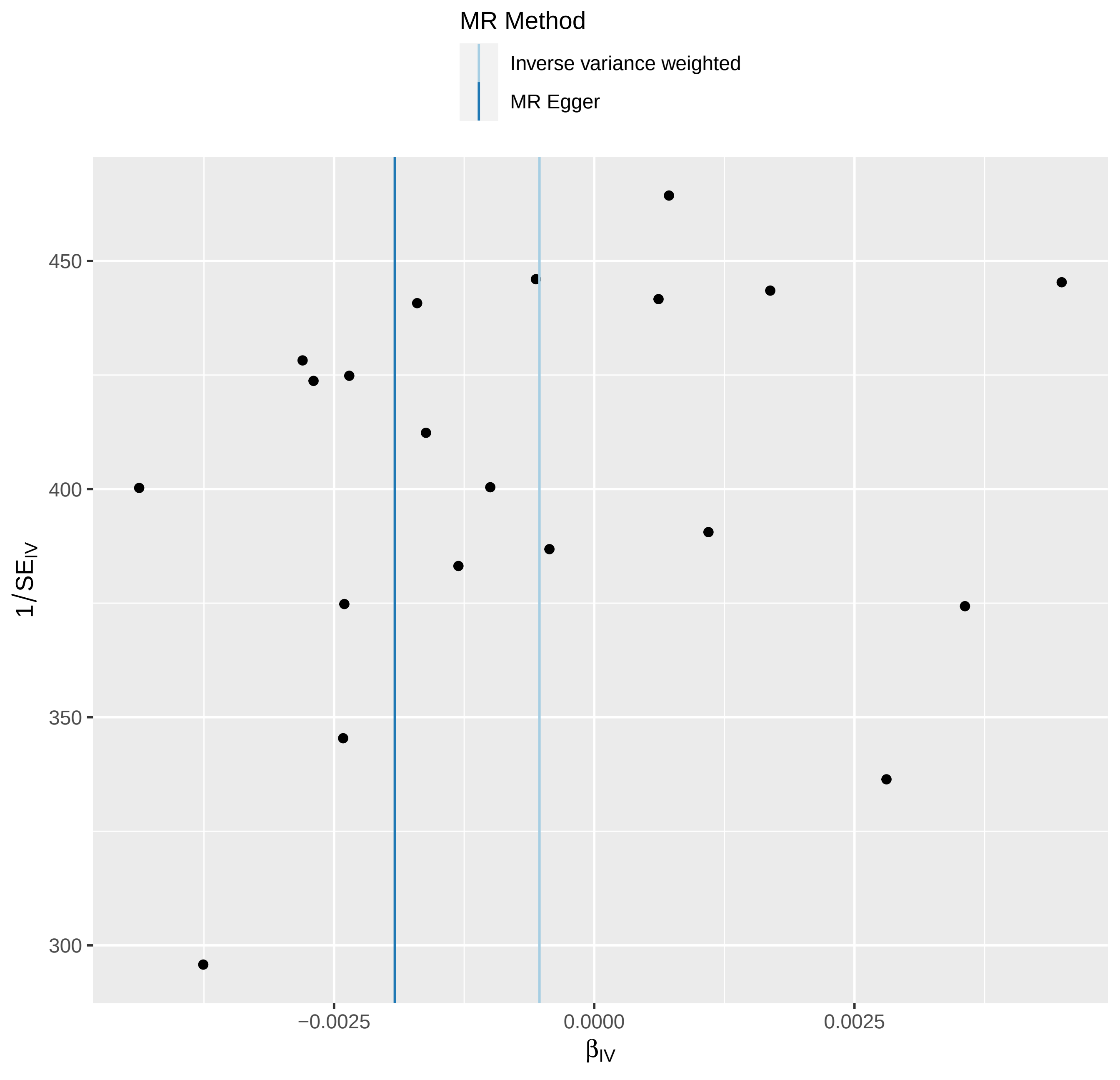

Batch 2−911 : EH domain−binding protein 1 on eGFR

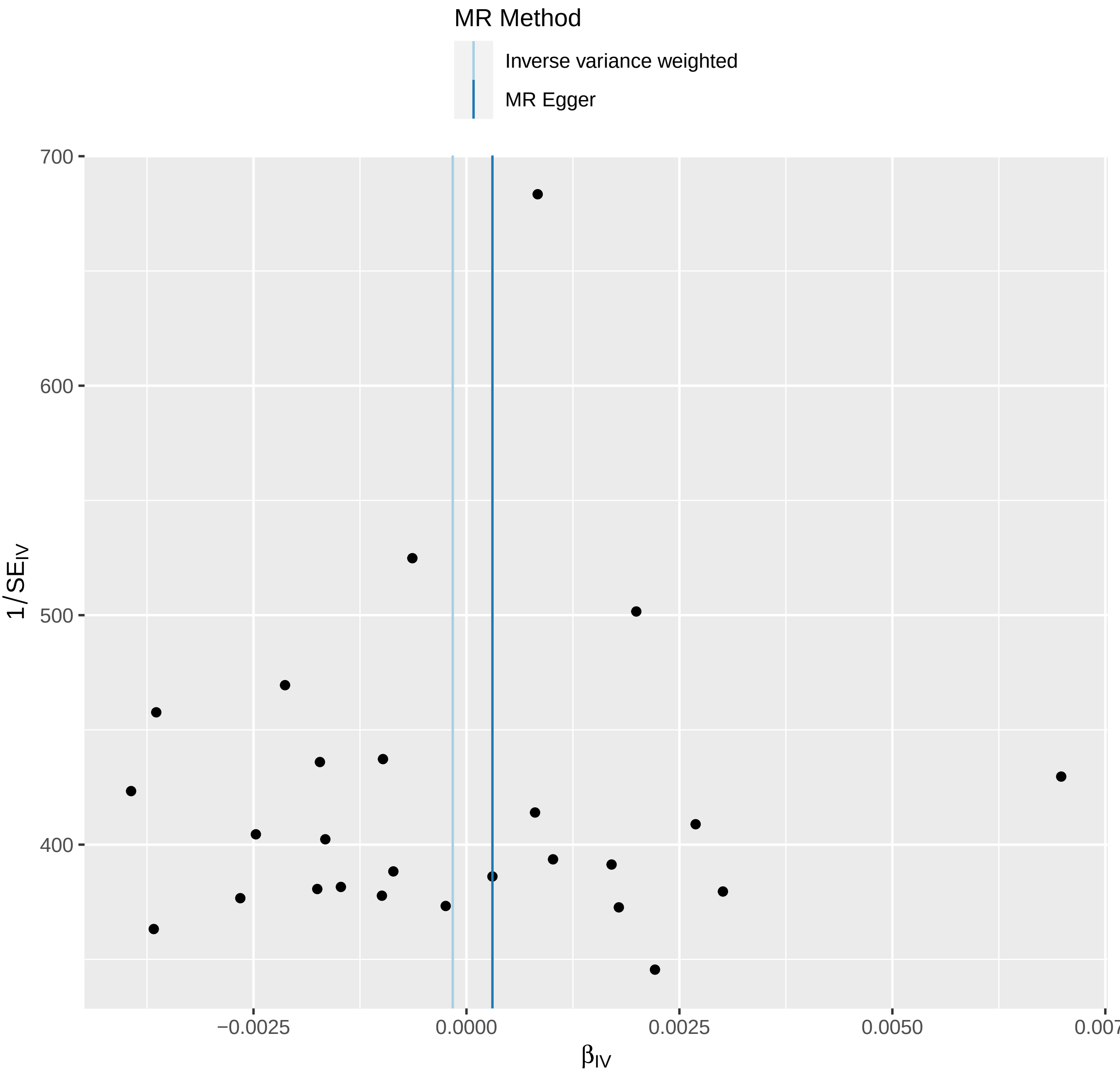

Batch 2−912 : EH domain−containing protein 4 on eGFR

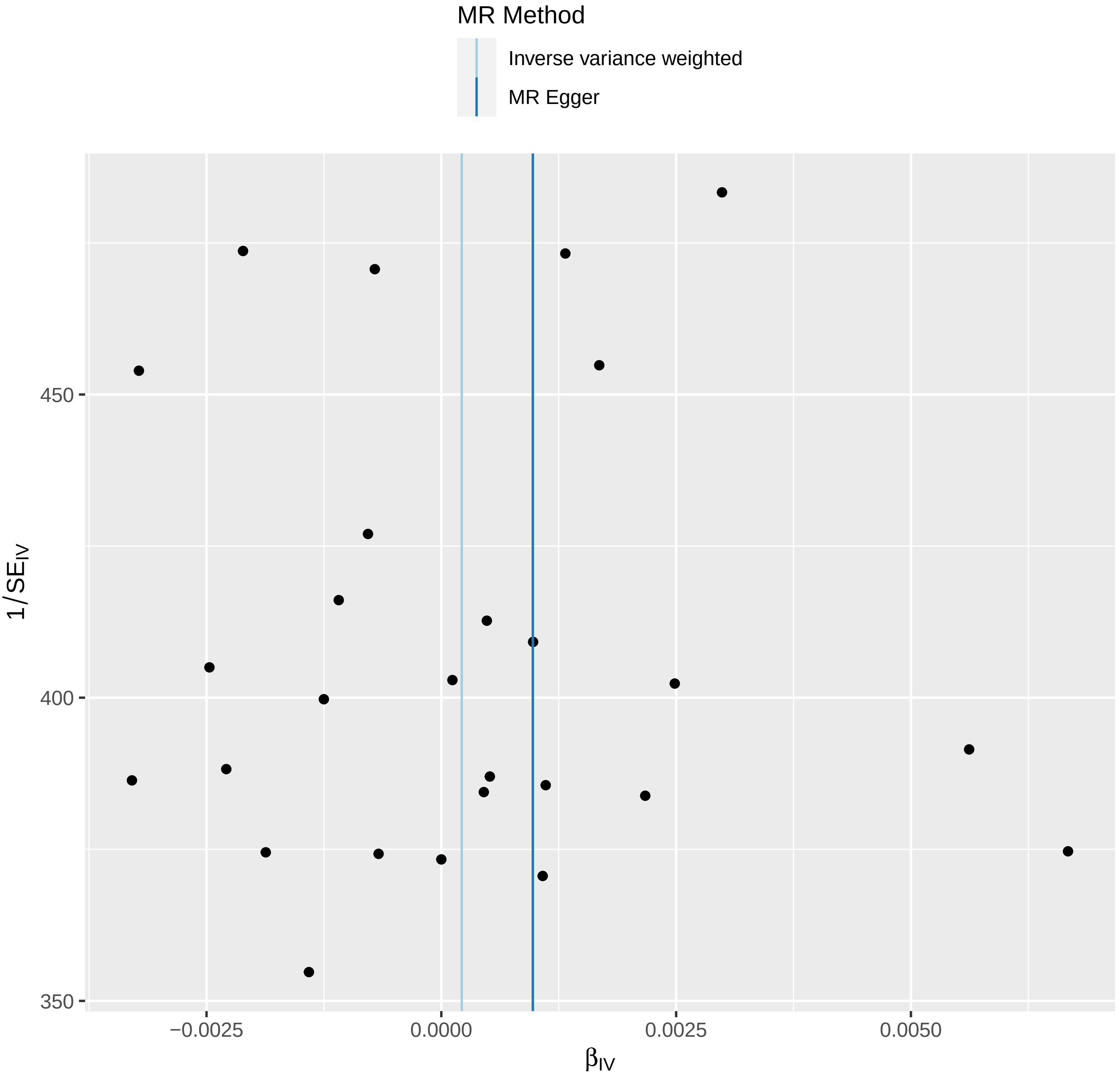

Batch 2−913 : ETS homologous factor on eGFR

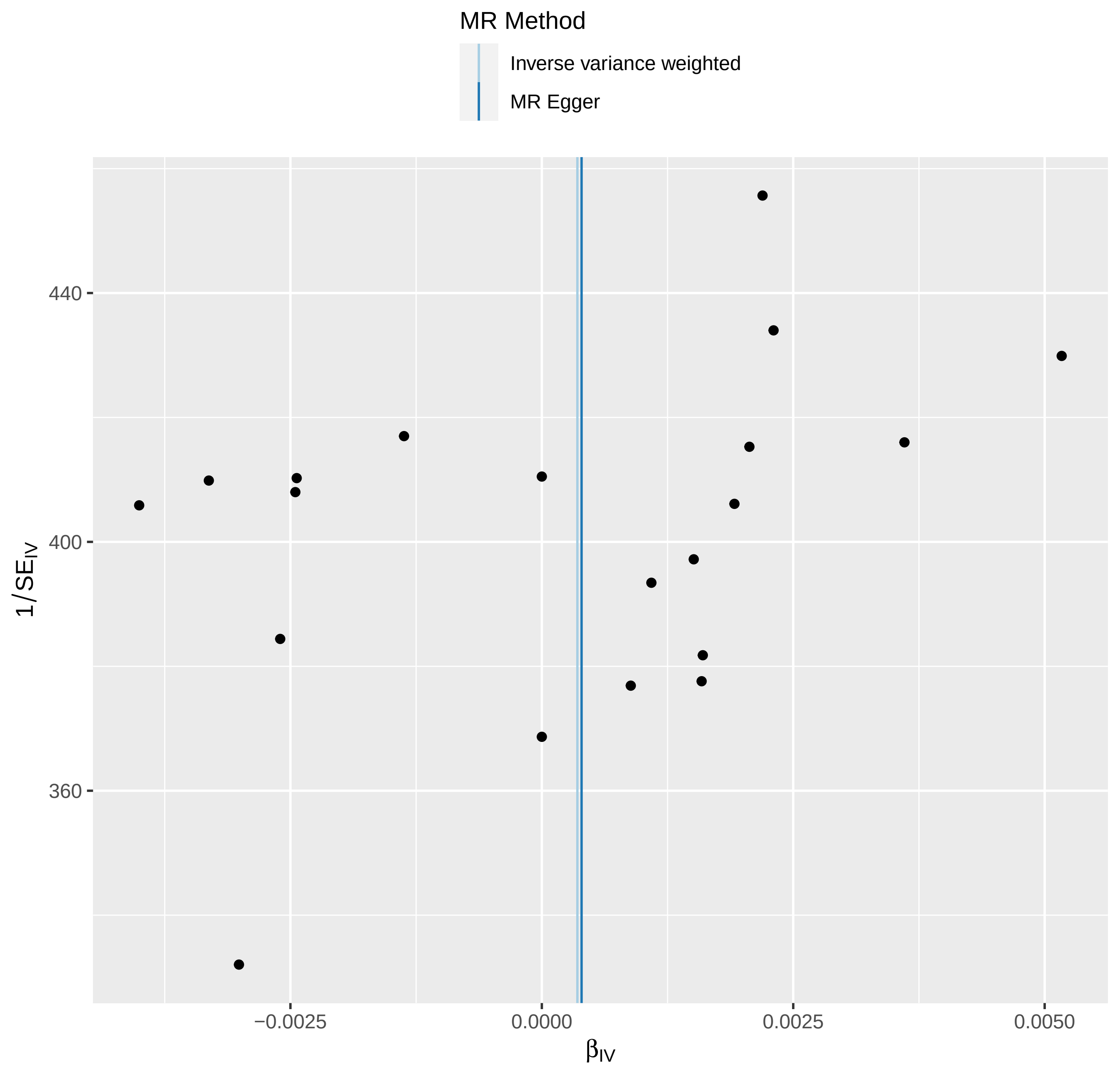

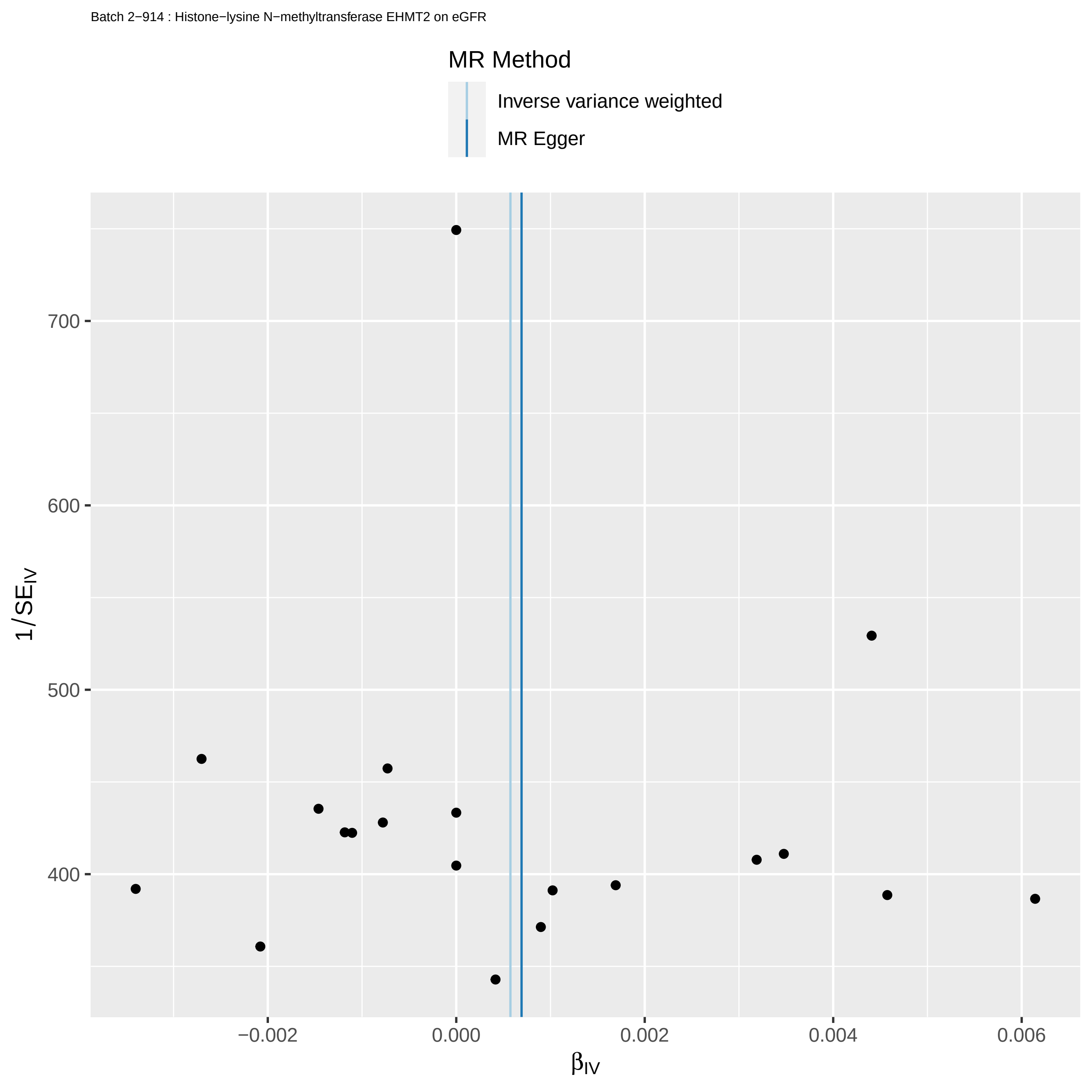

Batch 2−914 : Histone−lysine N−methyltransferase EHMT2 on eGFR
MR Method
Inverse variance weighted
MR Egger
700
600
500
400
−0.002
0.000
0.002
0.004
0.006
1/SE_IV
β_IV

Batch 2−915 : EP300−interacting inhibitor of differentiation 3 on eGFR

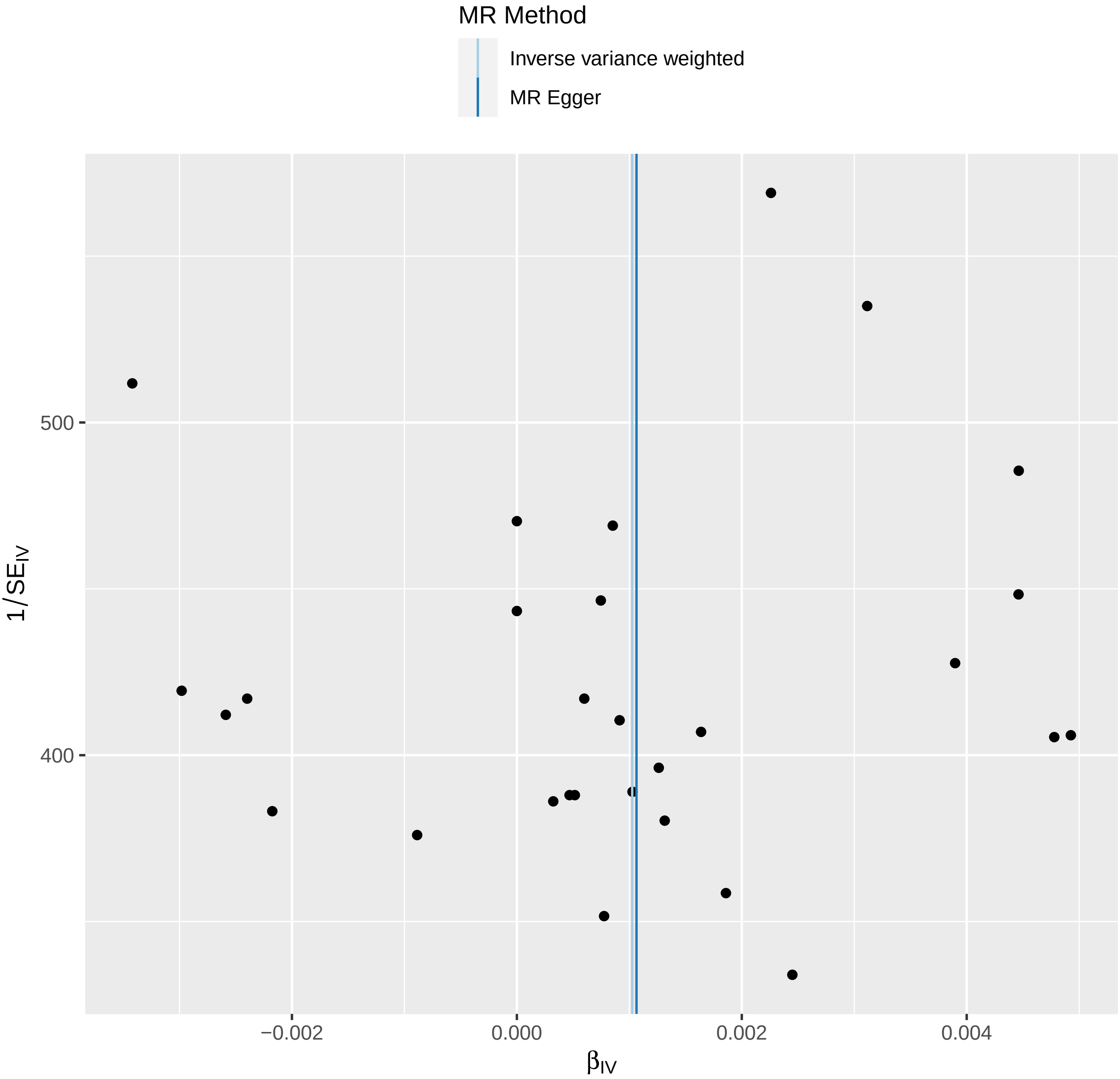

Batch 2−916 : Probable RNA−binding protein EIF1AD on eGFR

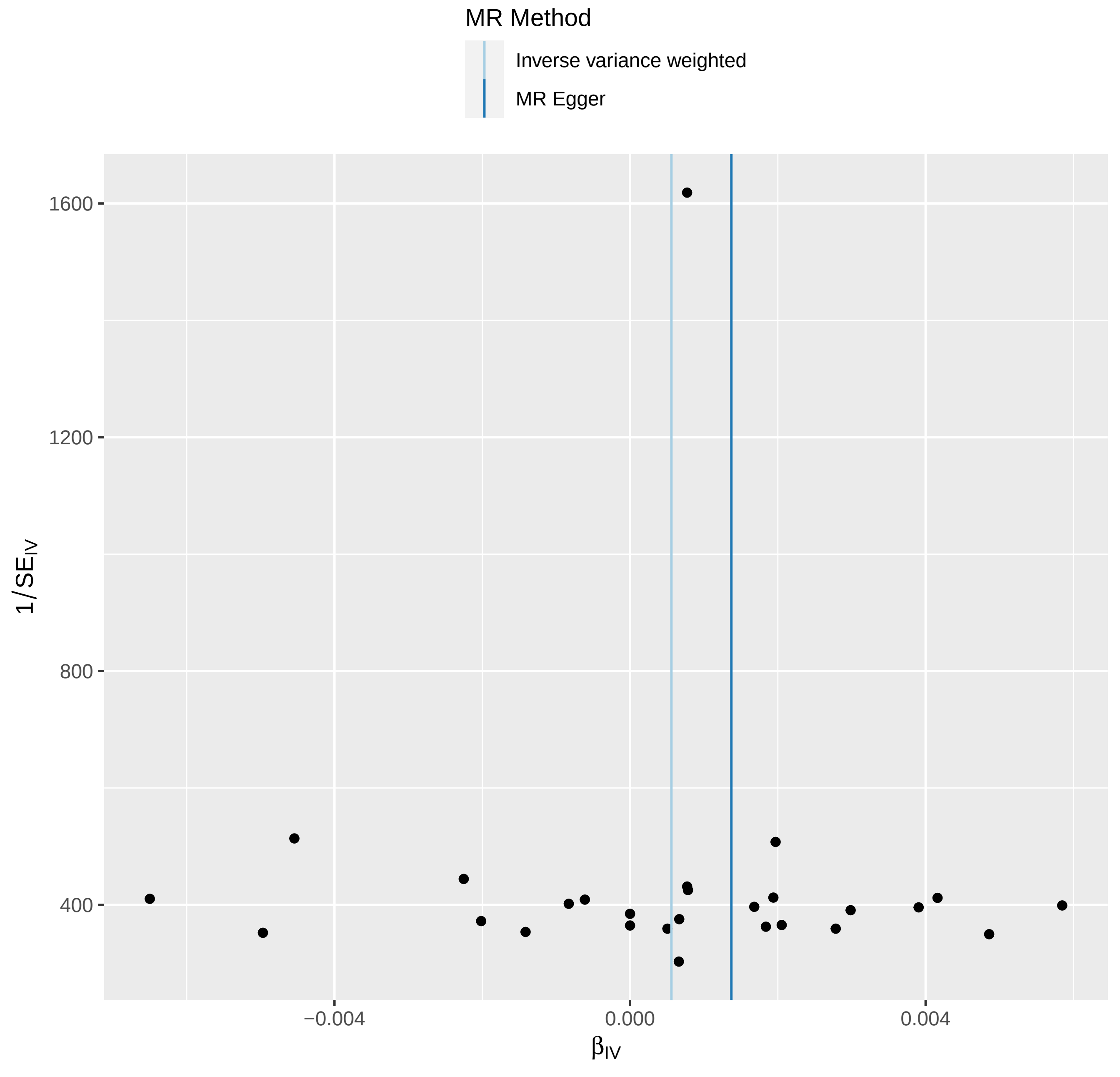

Batch 2−917 : Eukaryotic translation initiation factor 1A, X−chromosomal on eGFR

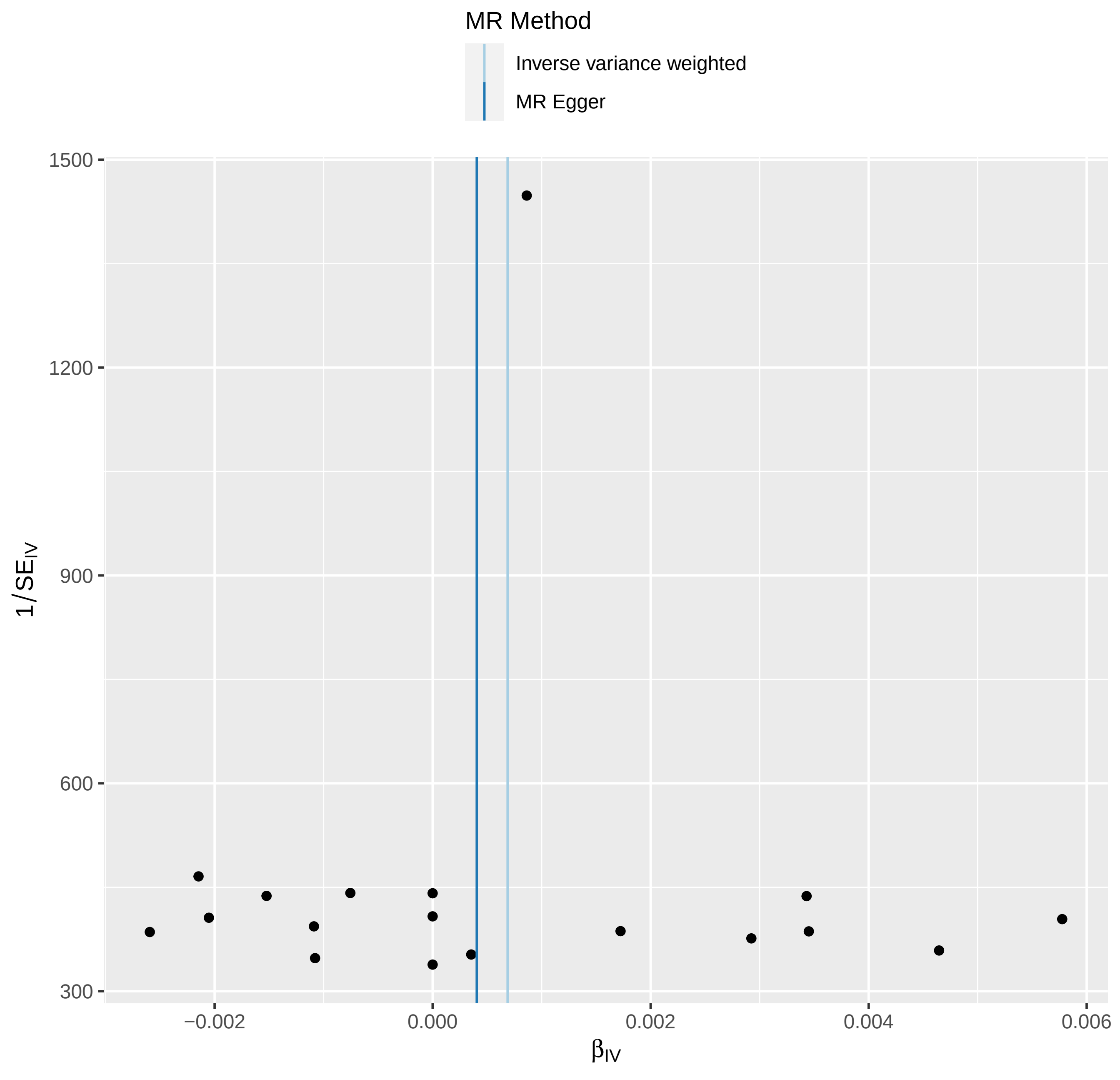

Batch 2−918 : Translation initiation factor eIF−2B subunit alpha on eGFR

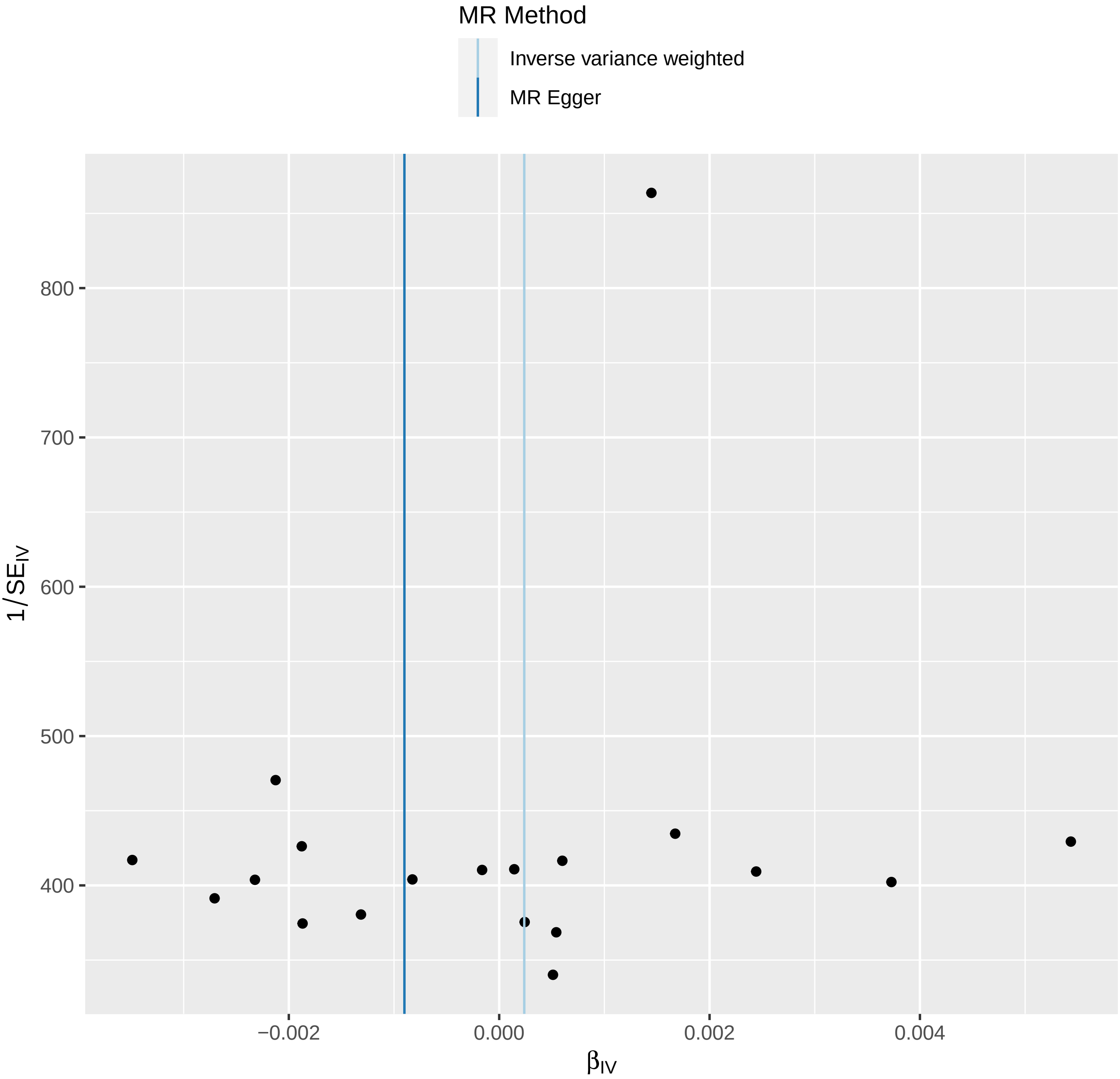

Batch 2−919 : Eukaryotic translation initiation factor 3 subunit G on eGFR

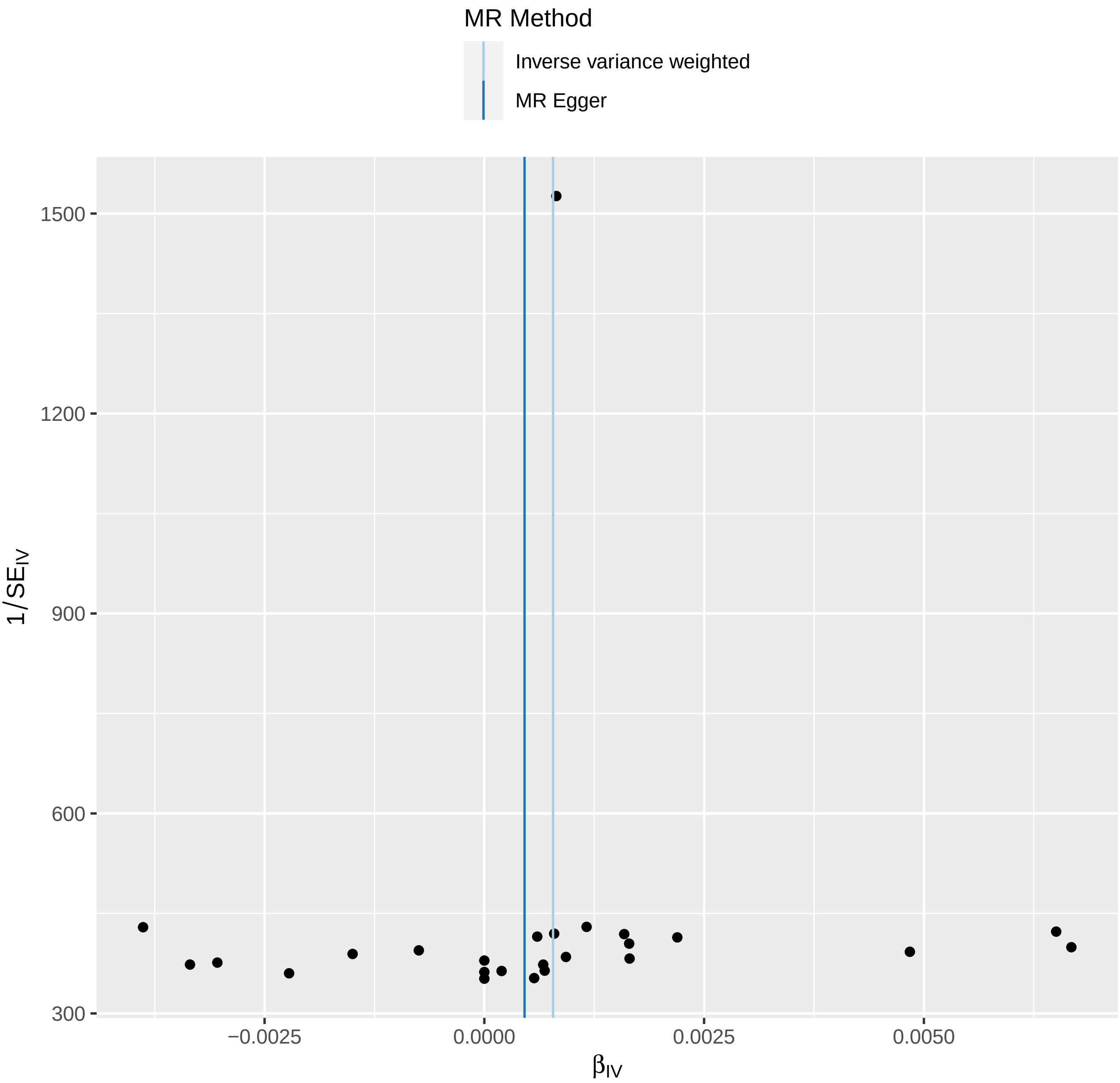

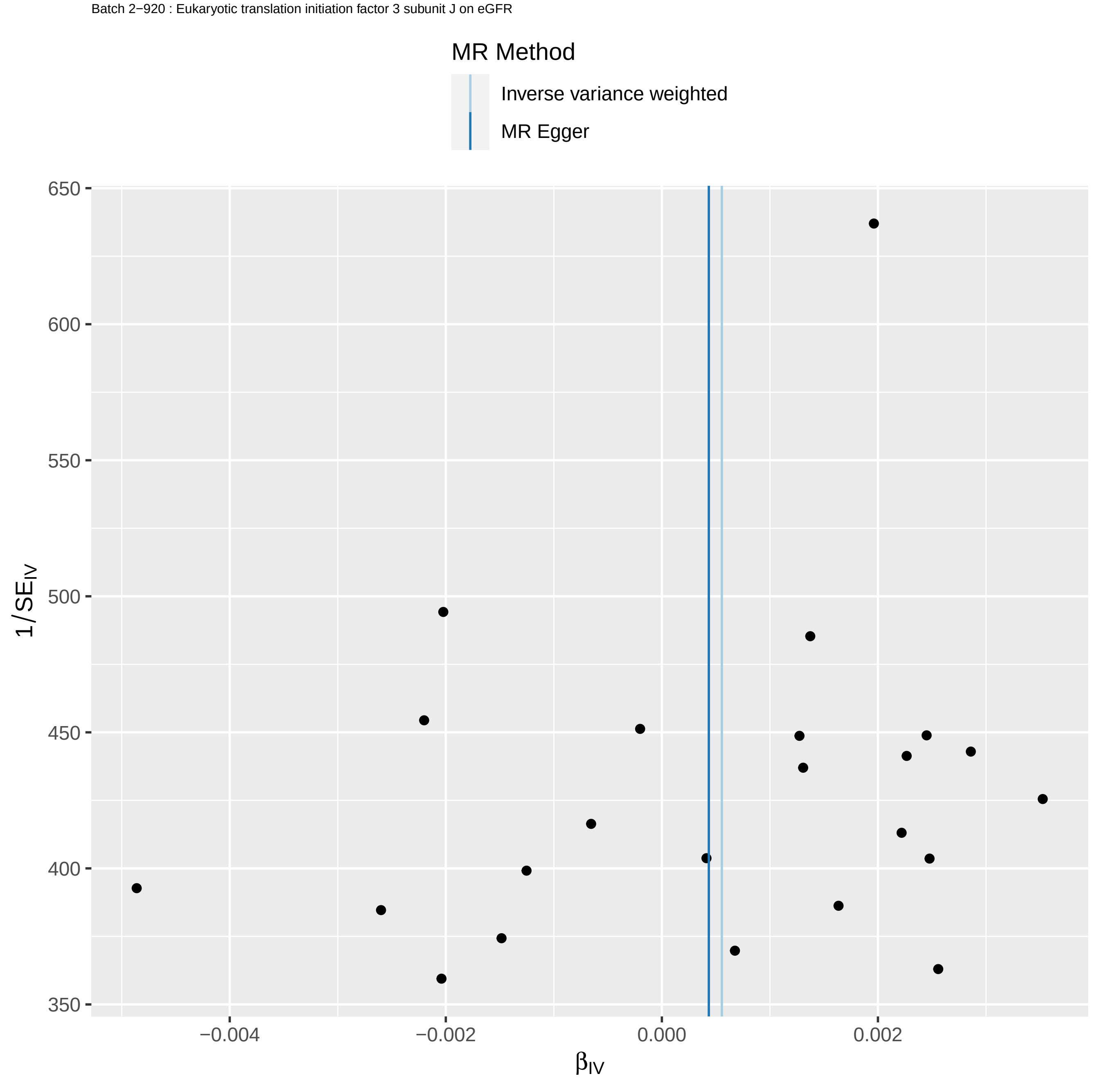

Batch 2−920 : Eukaryotic translation initiation factor 3 subunit J on eGFR
MR Method
Inverse variance weighted
MR Egger
650
600
550
500
450
400
350
1/SE$_{IV}$
−0.004
−0.002
0.000
0.002
β_{IV}

Batch 2−921 : Eukaryotic initiation factor 4A−III on eGFR

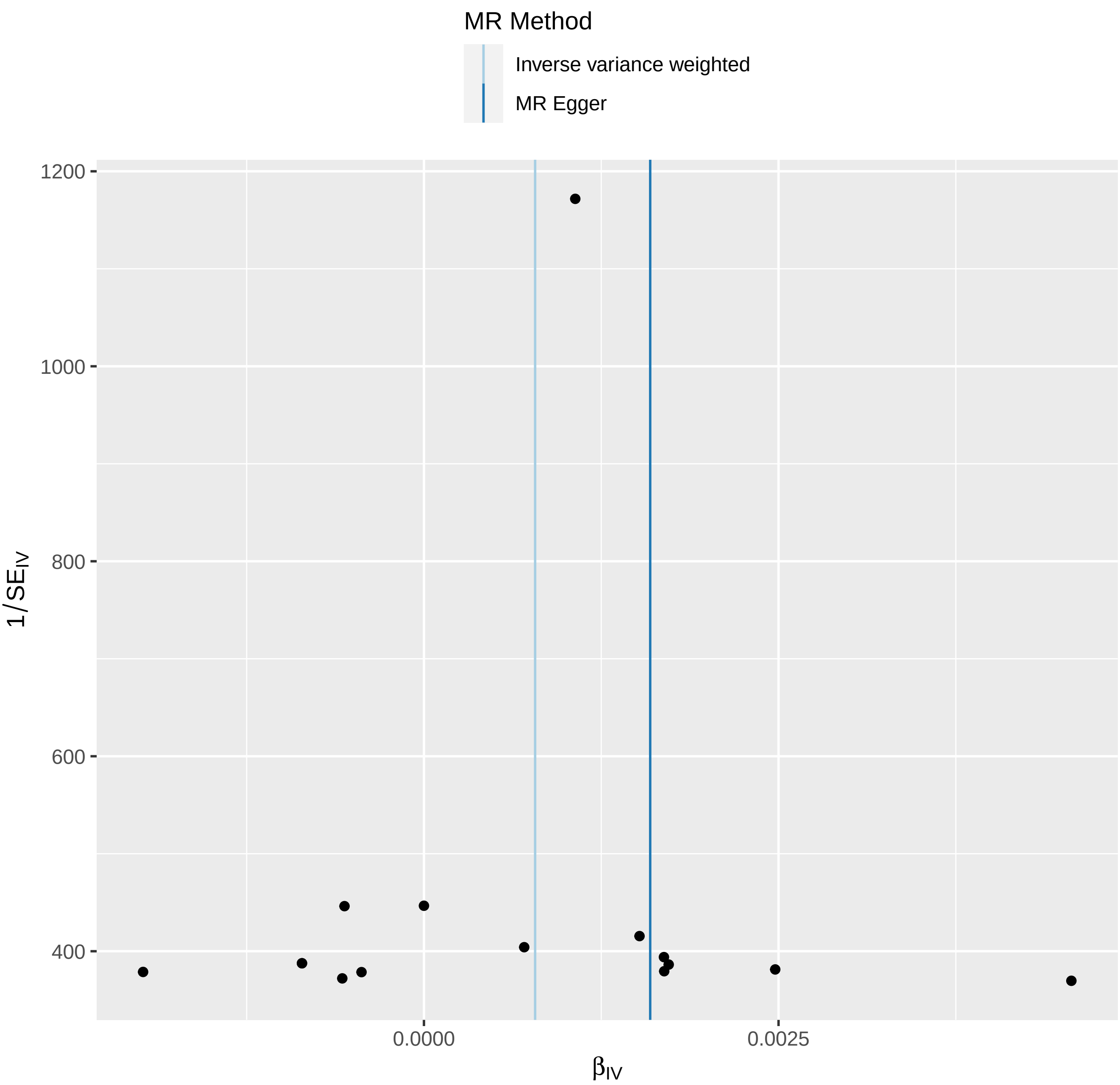

Batch 2−922 : Eukaryotic translation initiation factor 4B on eGFR

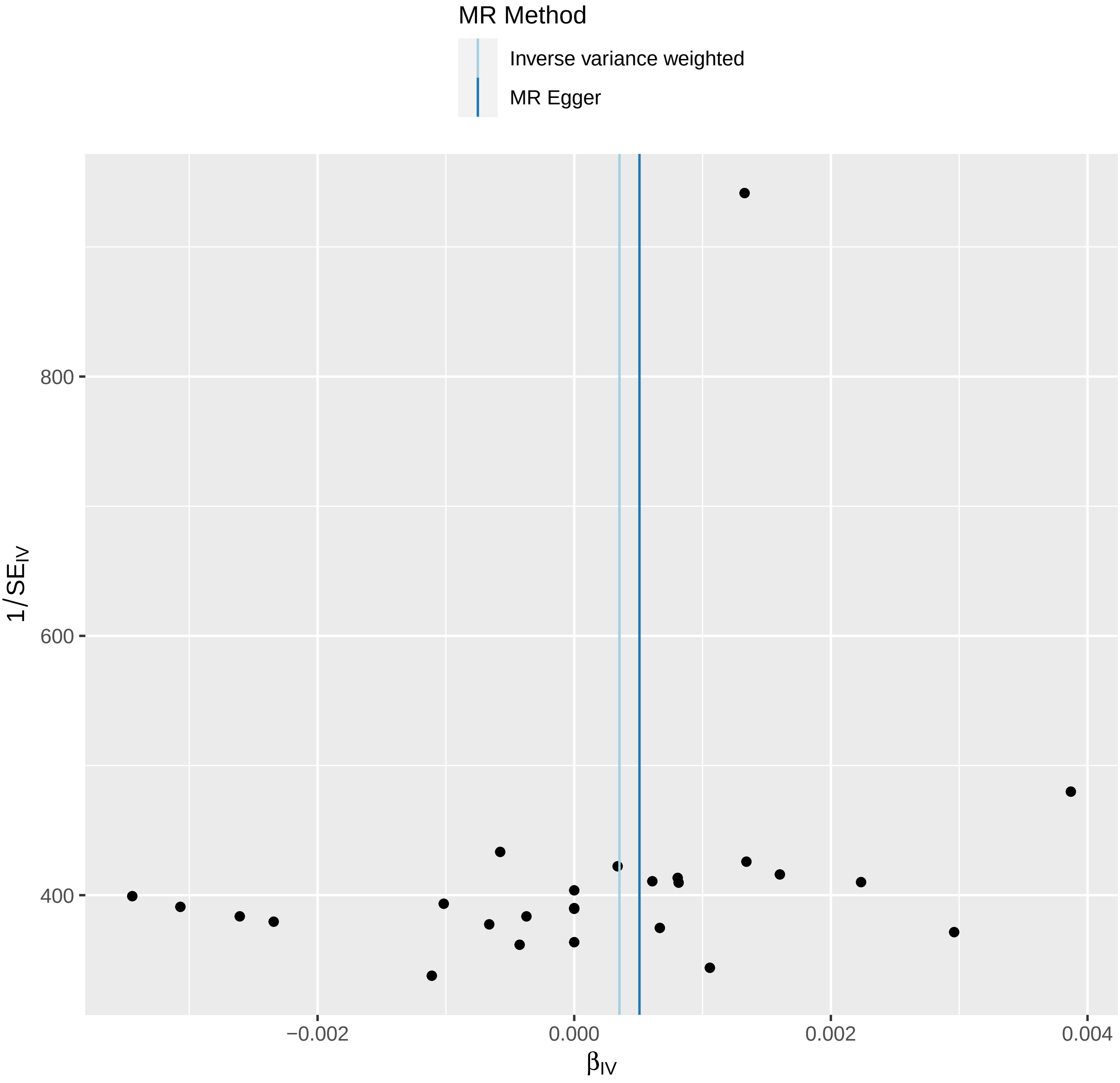

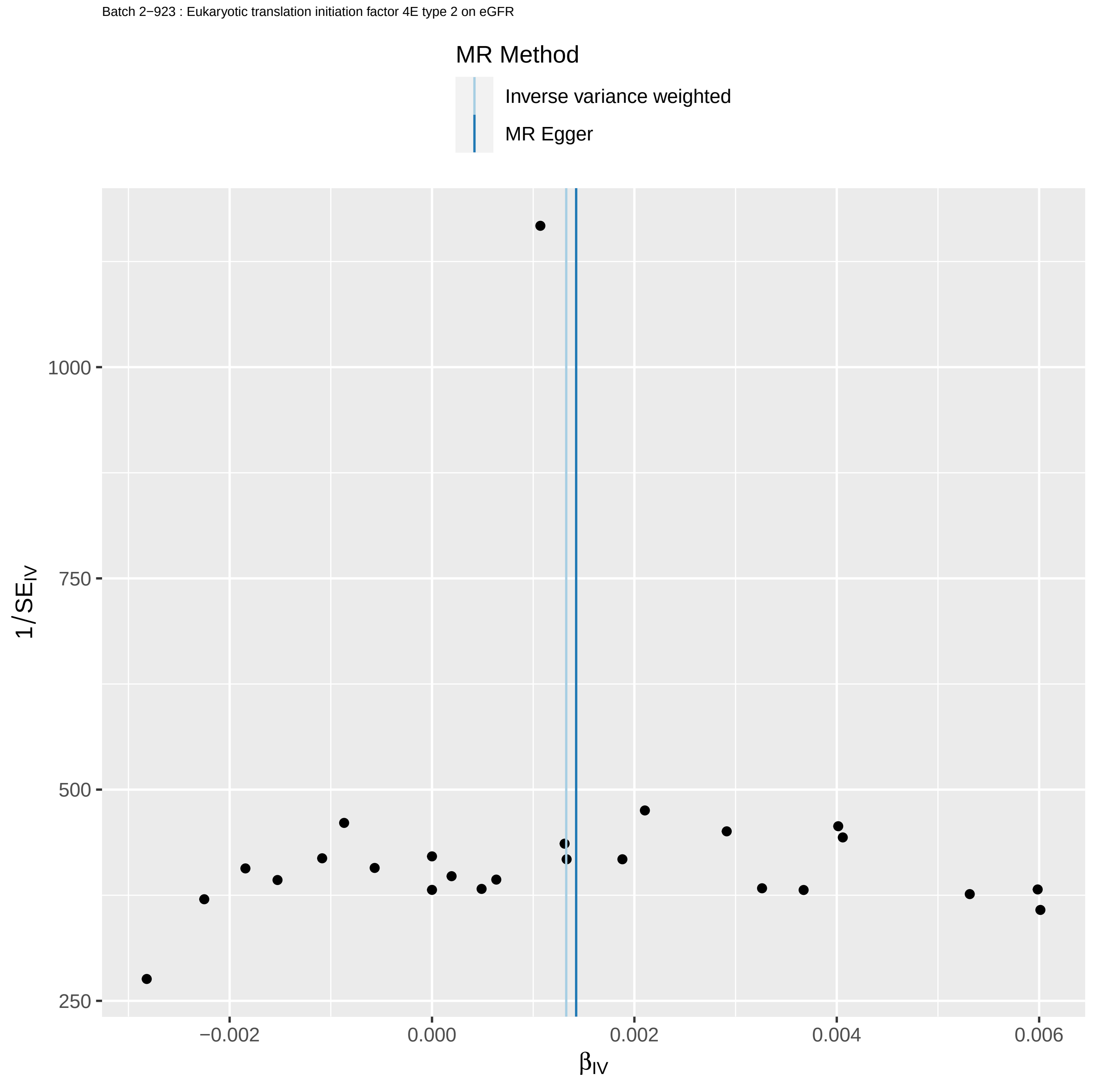
Batch 2−923 : Eukaryotic translation initiation factor 4E type 2 on eGFR
MR Method
Inverse variance weighted
MR Egger
1/SE_IV
1000
750
500
250
−0.002
0.000
0.002
0.004
0.006
β_IV

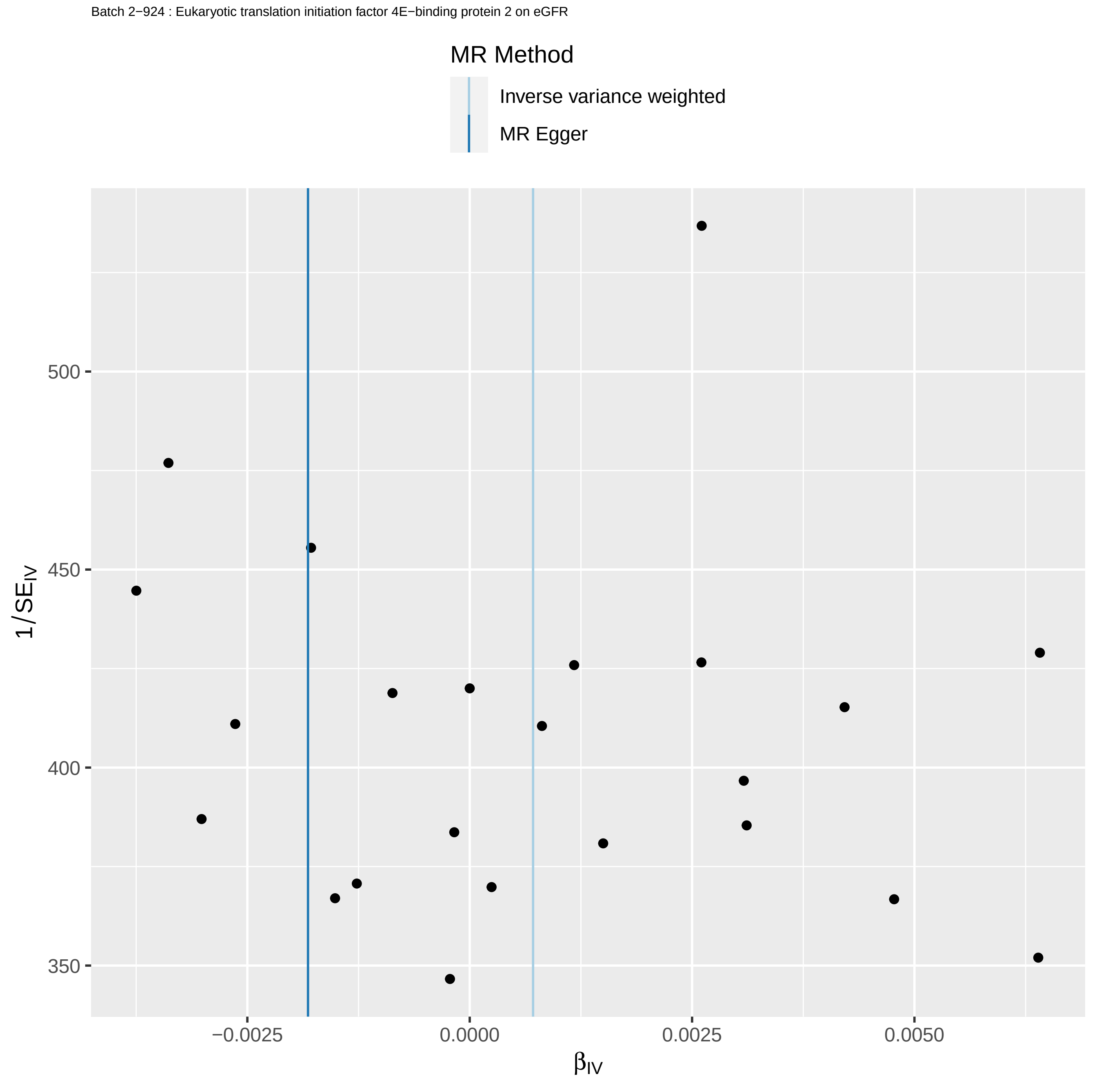

Batch 2−924 : Eukaryotic translation initiation factor 4E−binding protein 2 on eGFR
MR Method
Inverse variance weighted
MR Egger
500
450
400
350
$1/SE_{IV}$
−0.0025
0.0000
0.0025
0.0050
β_{IV}

Batch 2−925 : Eukaryotic translation initiation factor 4 gamma 3 on eGFR

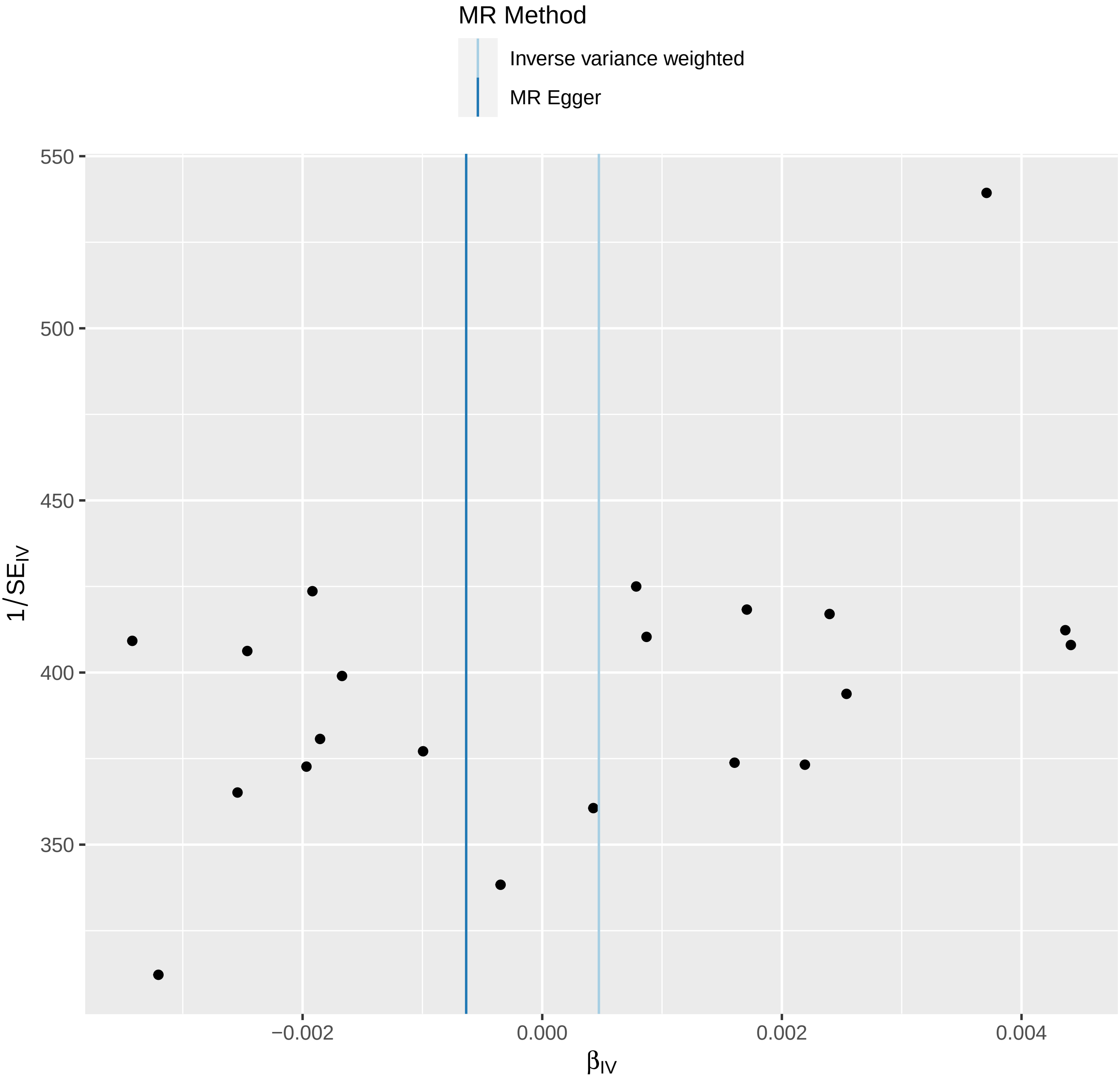

Batch 2−926 : Eukaryotic translation initiation factor 5 on eGFR

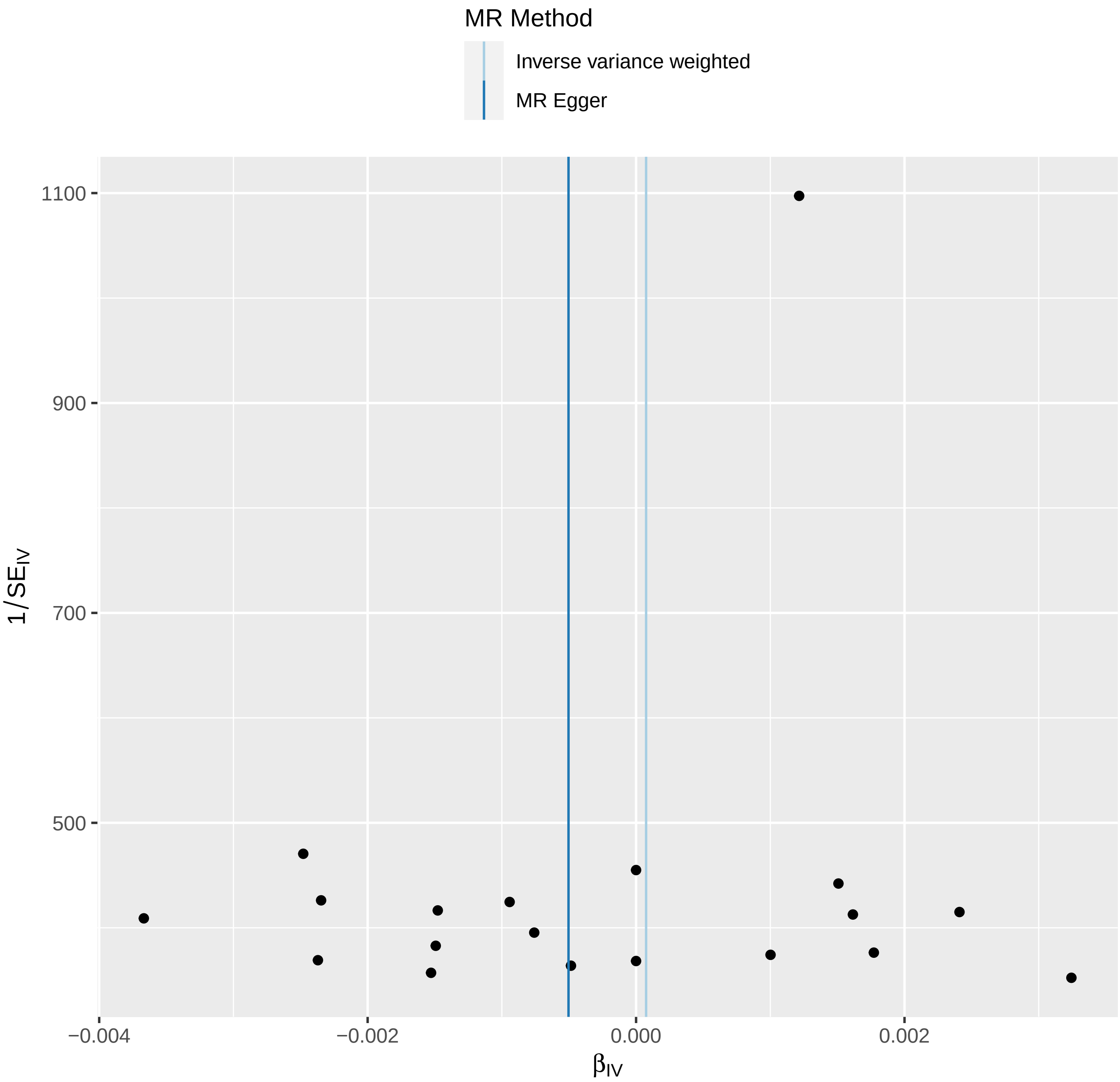

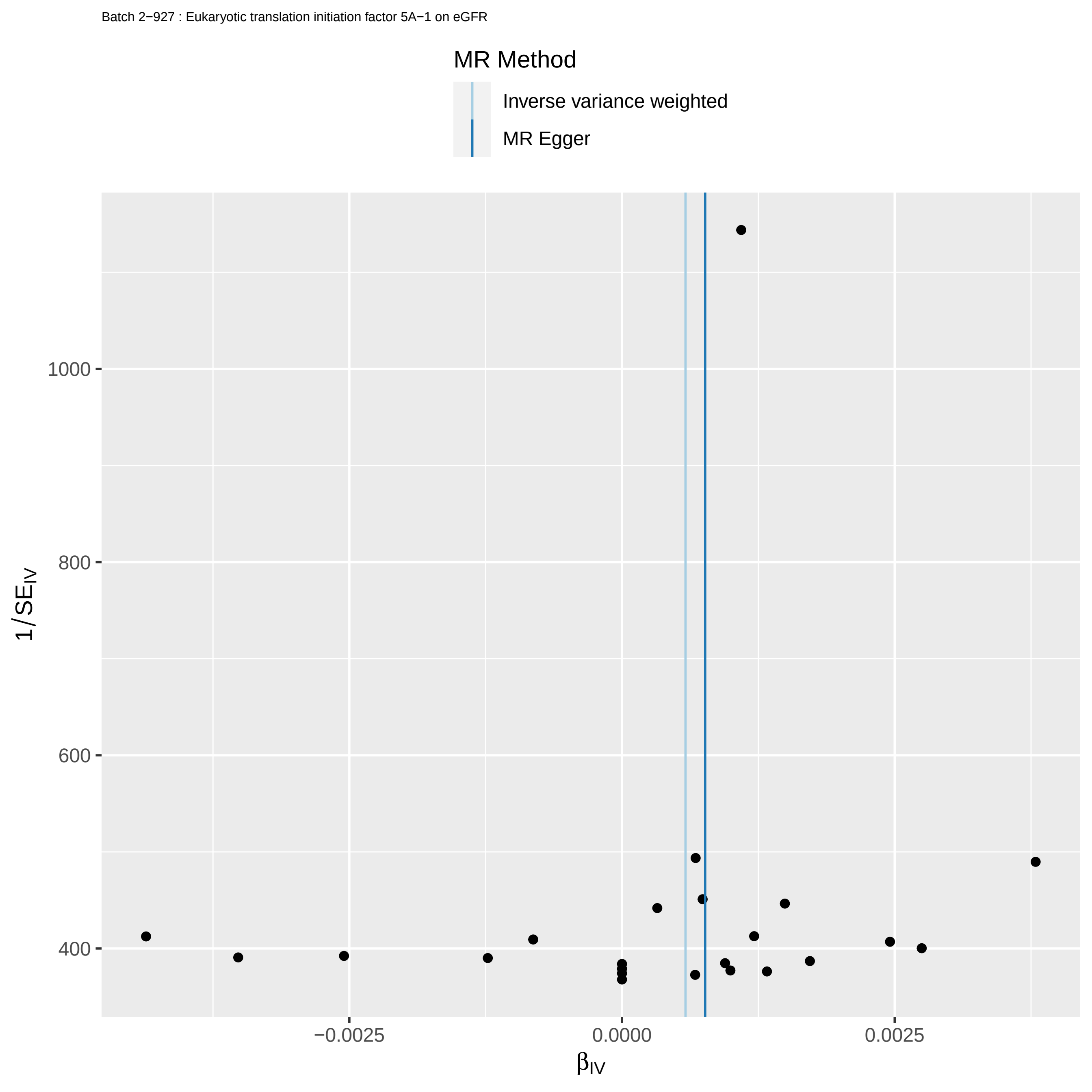
Batch 2−927 : Eukaryotic translation initiation factor 5A−1 on eGFR
MR Method
Inverse variance weighted
MR Egger
1000
800
600
400
1/SE_IV
−0.0025
0.0000
0.0025
β_IV

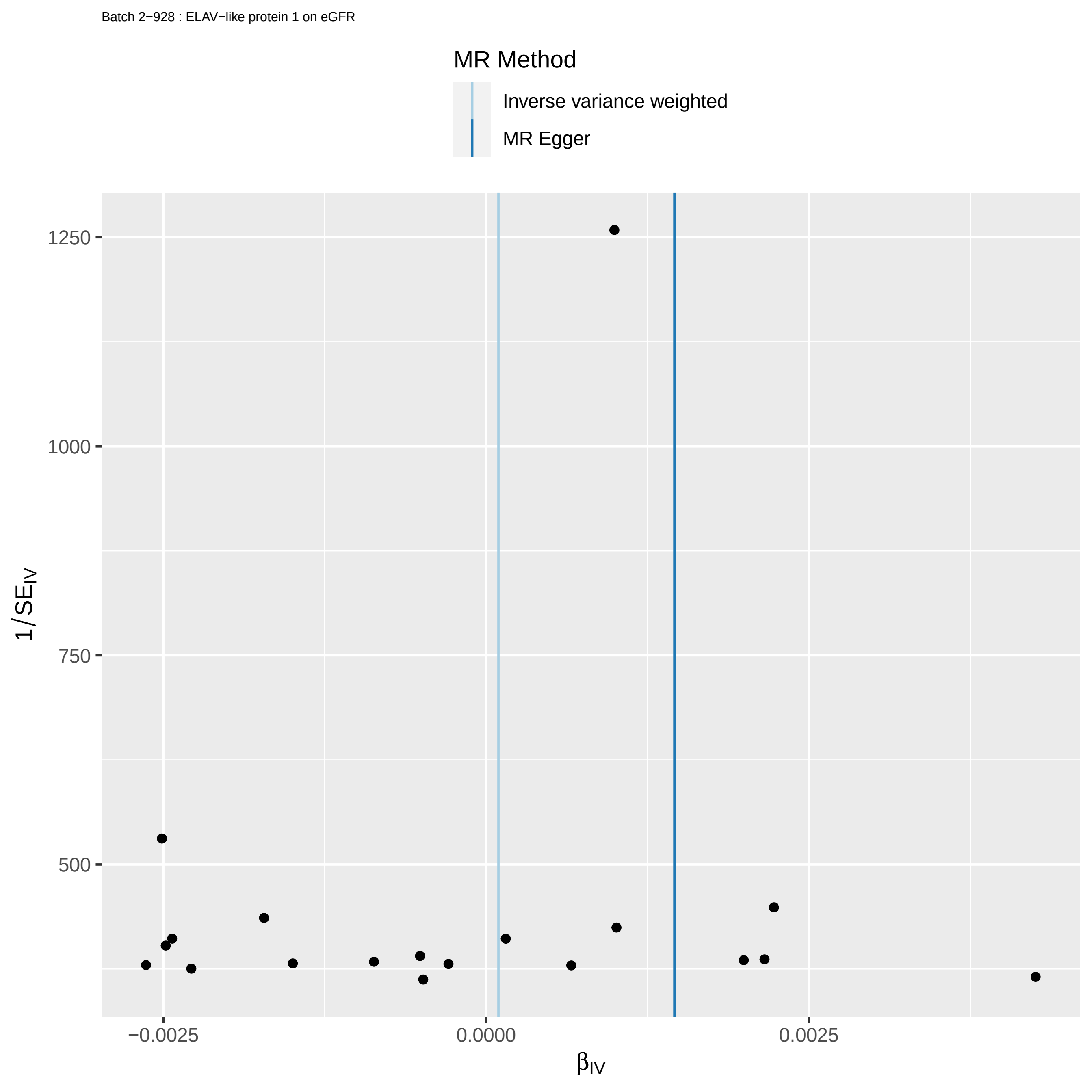

Batch 2−928 : ELAV−like protein 1 on eGFR
MR Method
Inverse variance weighted
MR Egger
1250
1000
750
500
1/SE_IV
−0.0025
0.0000
0.0025
β_IV

Batch 2−929 : ETS−related transcription factor Elf−5 on eGFR

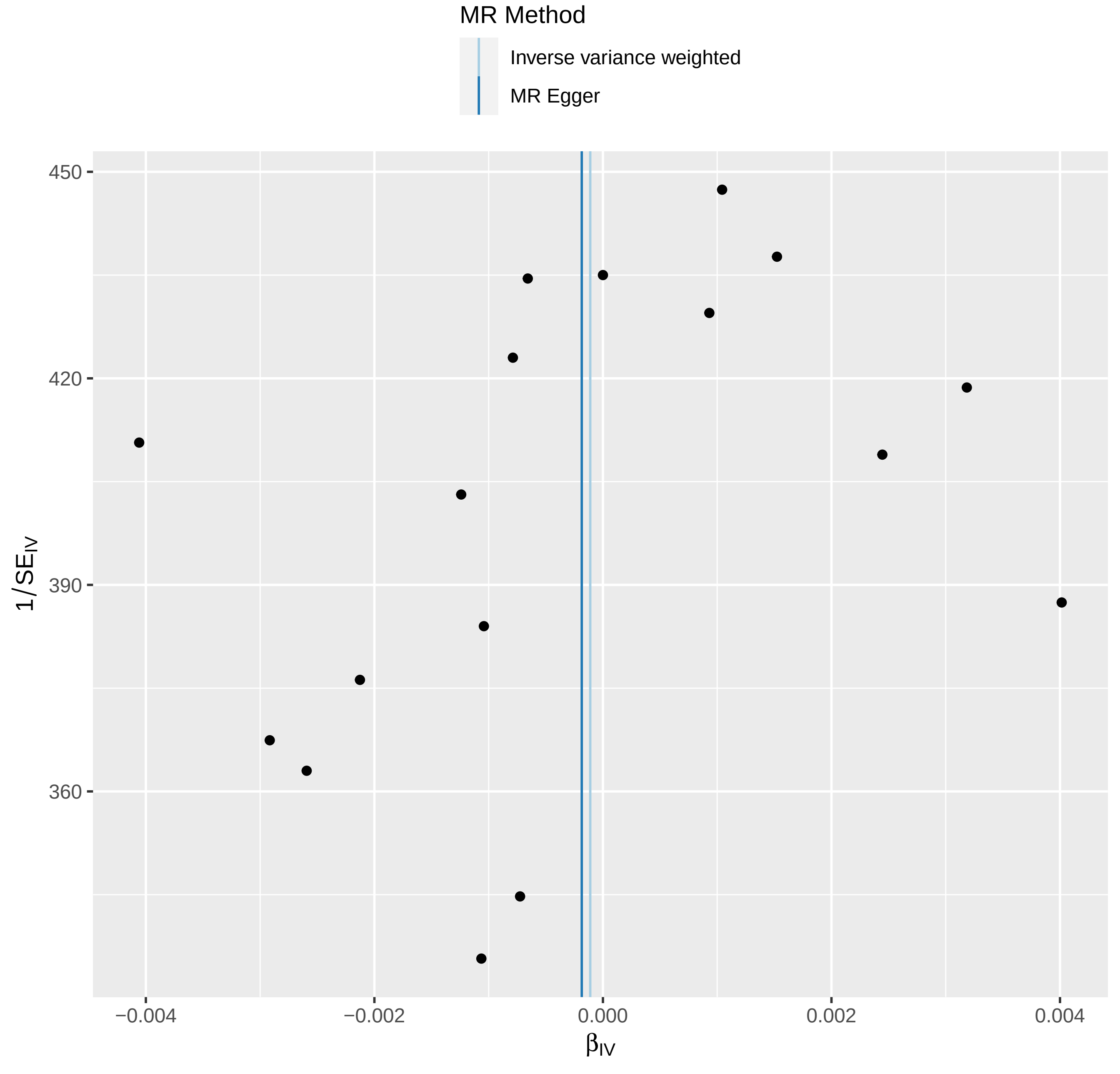

Batch 2−930 : ETS domain−containing protein Elk−1 on eGFR

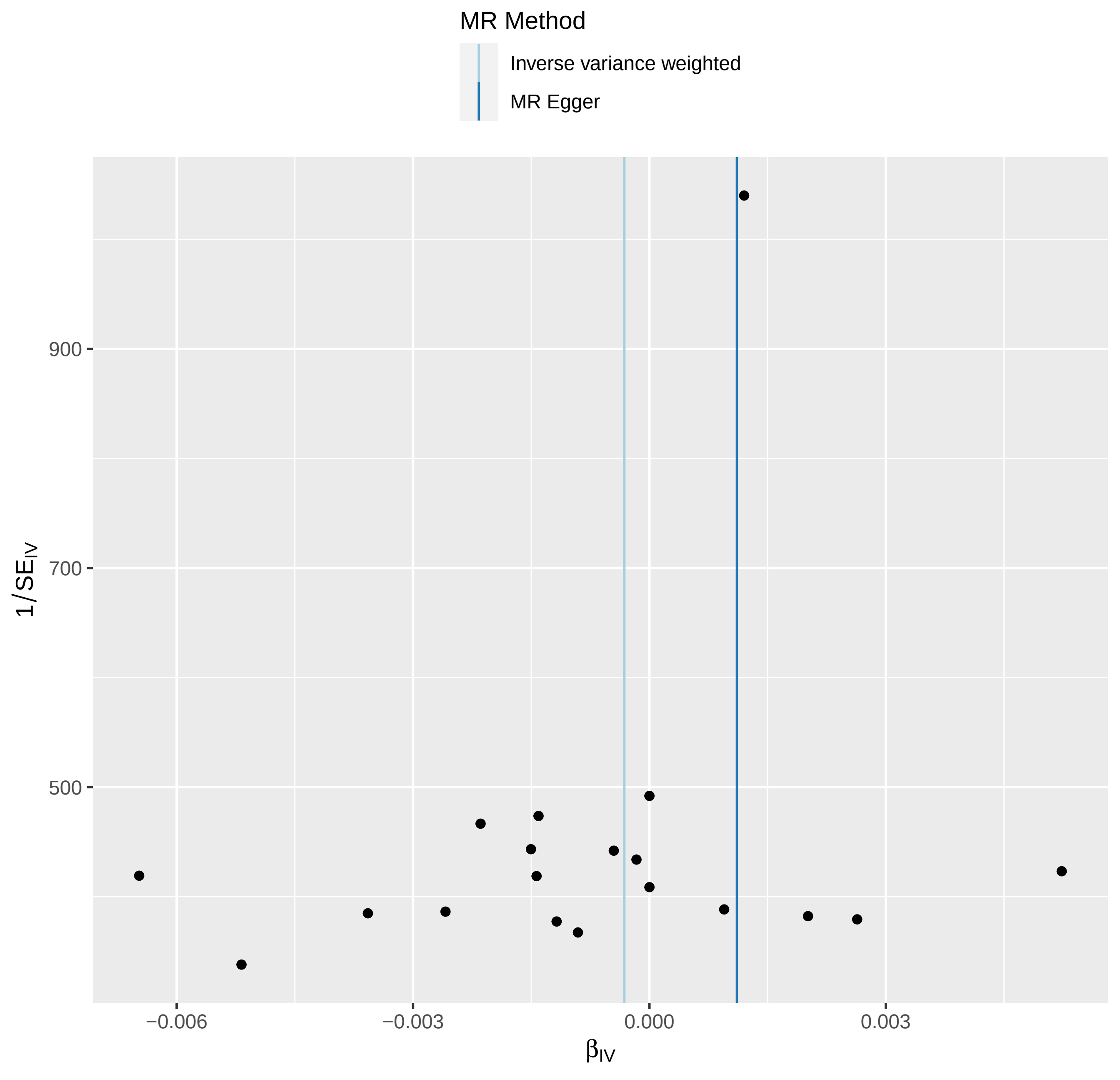

Batch 2−931 : ETS domain−containing protein Elk−3 on eGFR

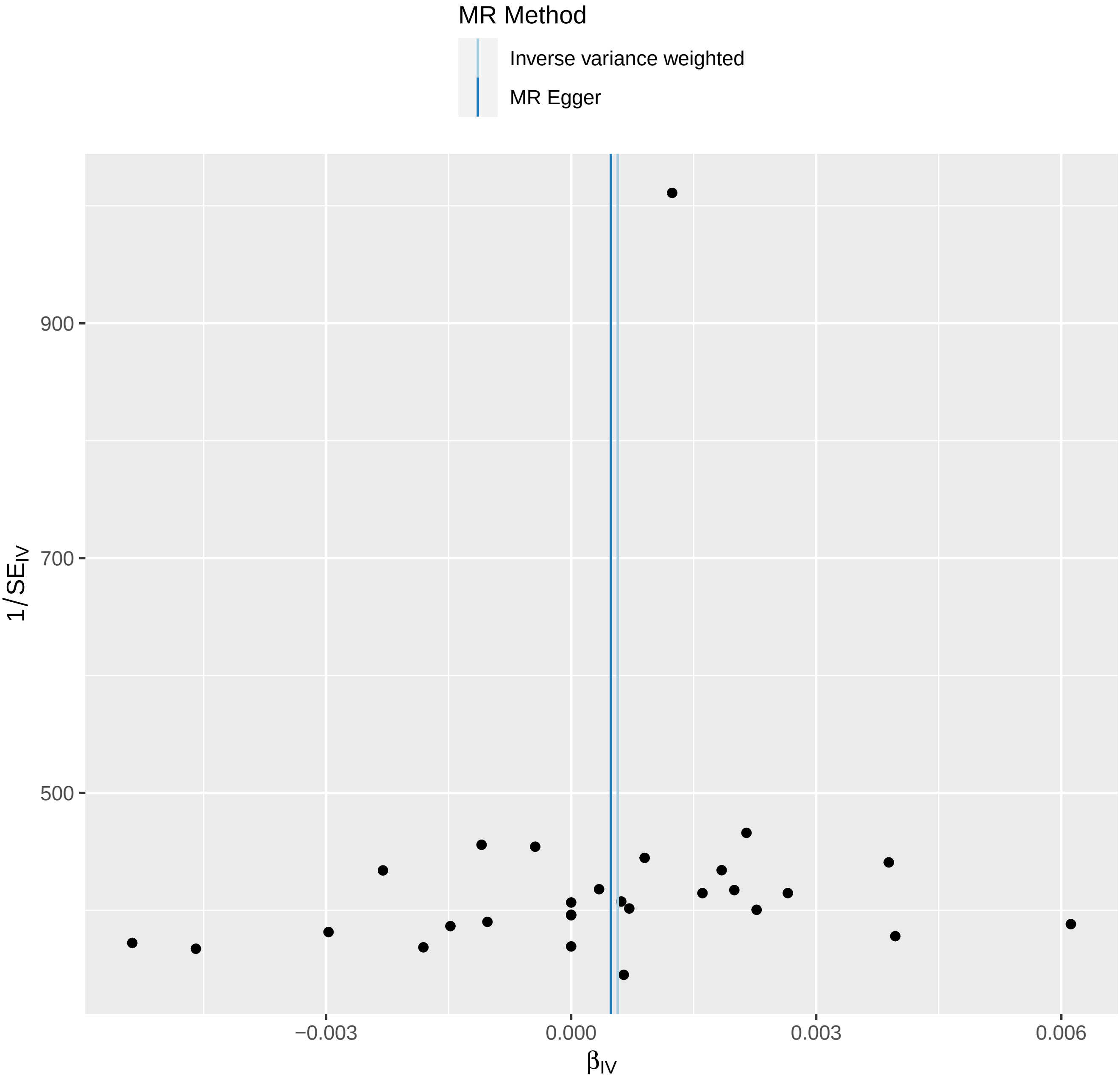

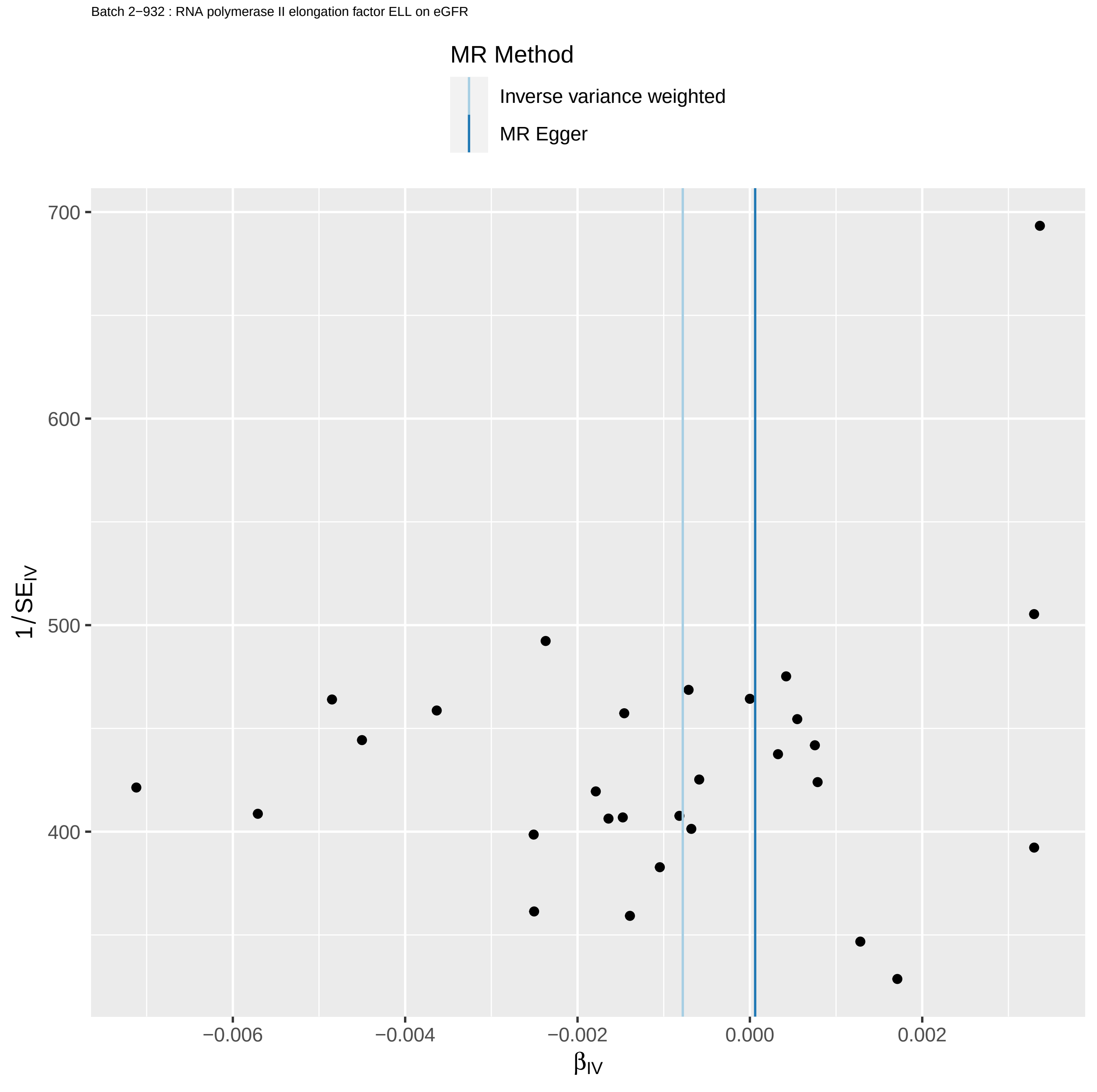
Batch 2−932 : RNA polymerase II elongation factor ELL on eGFR
MR Method
Inverse variance weighted
MR Egger
700
600
500
400
1/SE_IV
−0.006
−0.004
−0.002
0.000
0.002
β_IV

Batch 2−933 : RNA polymerase II elongation factor ELL2 on eGFR

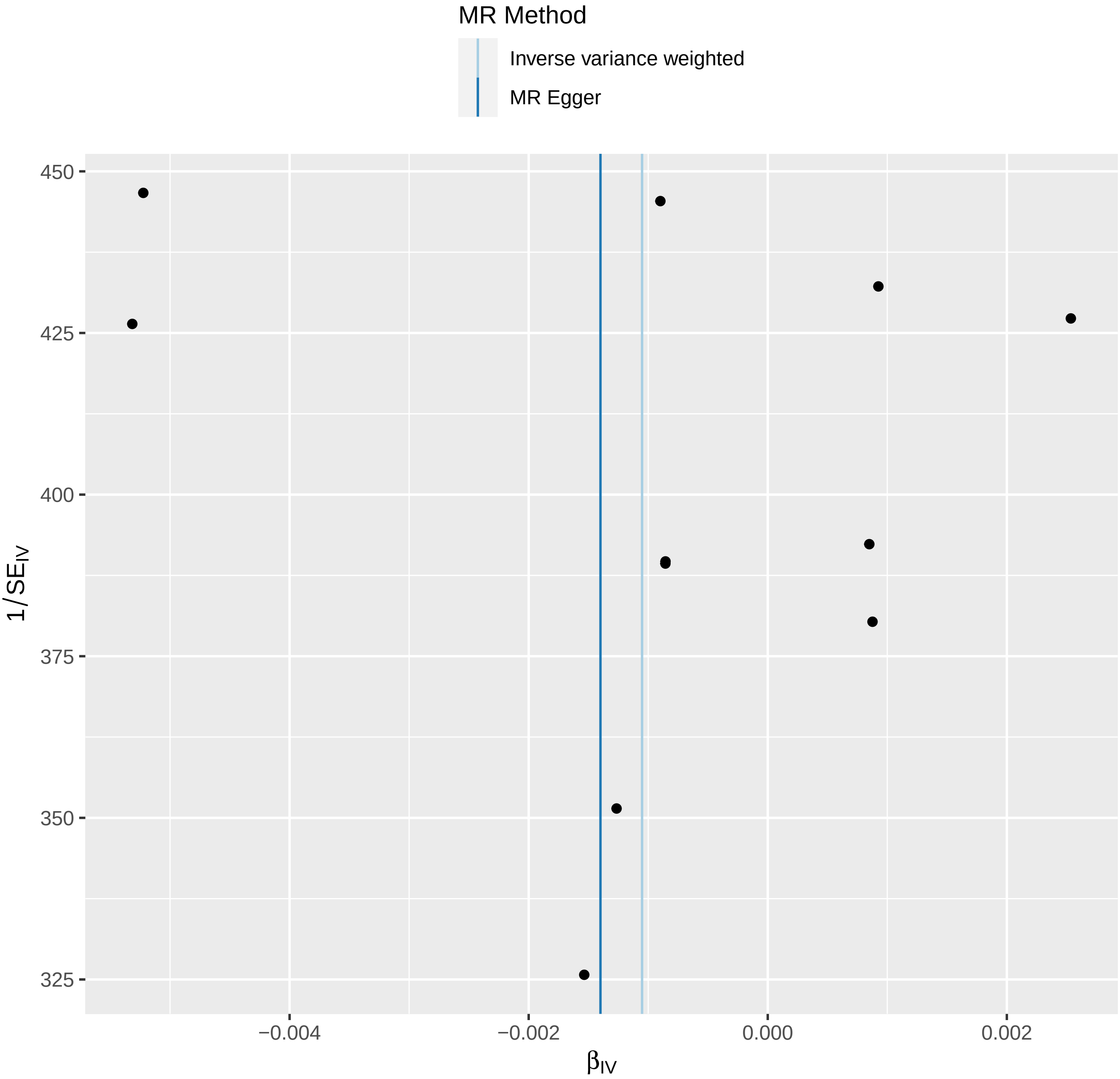

Batch 2−934 : Engulfment and cell motility protein 1 on eGFR

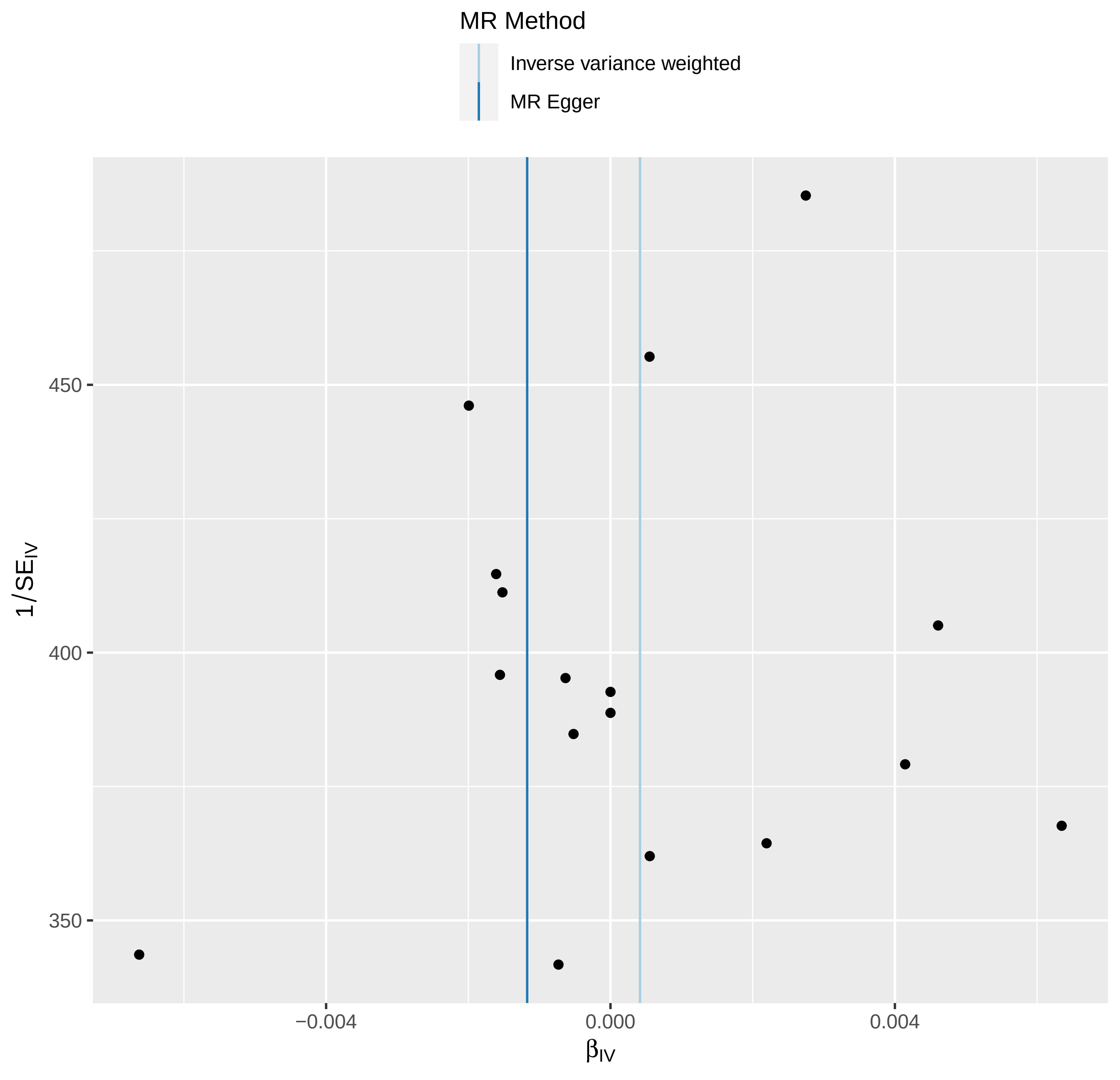

Batch 2−935 : ER membrane protein complex subunit 1 on eGFR

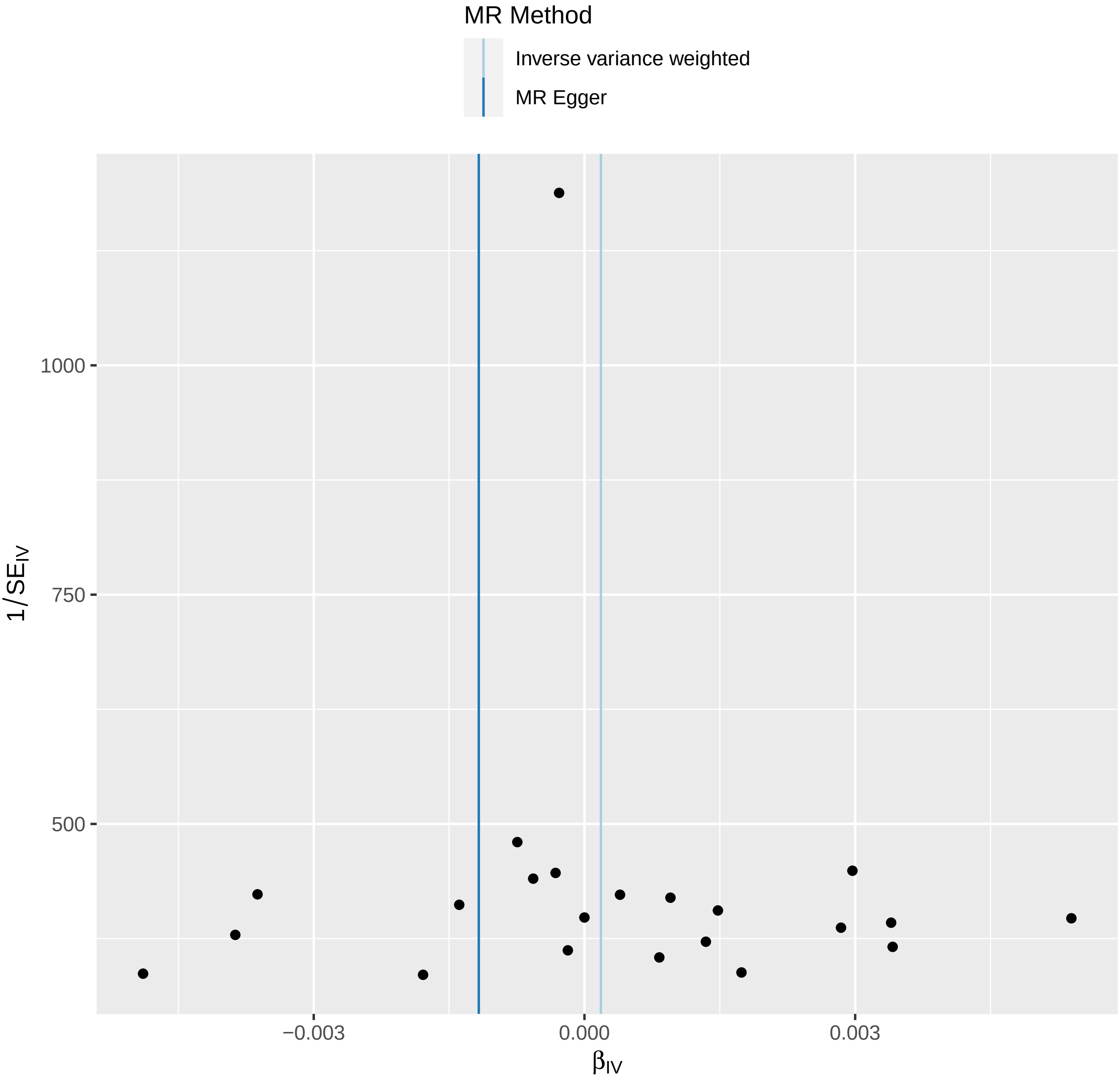

Batch 2−936 : ER membrane protein complex subunit 4 on eGFR

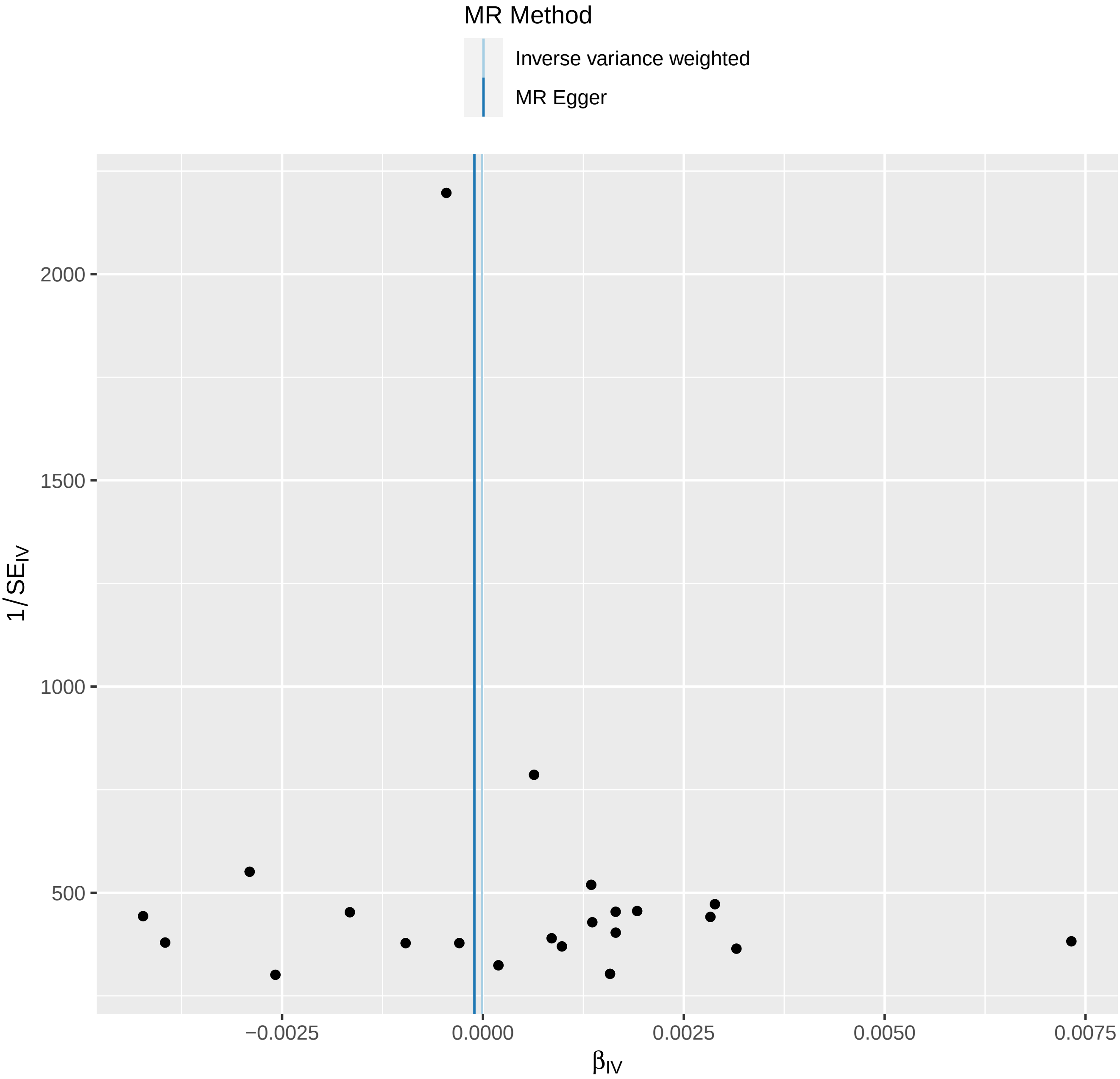

Batch 2−937 : EMI domain−containing protein 1 on eGFR

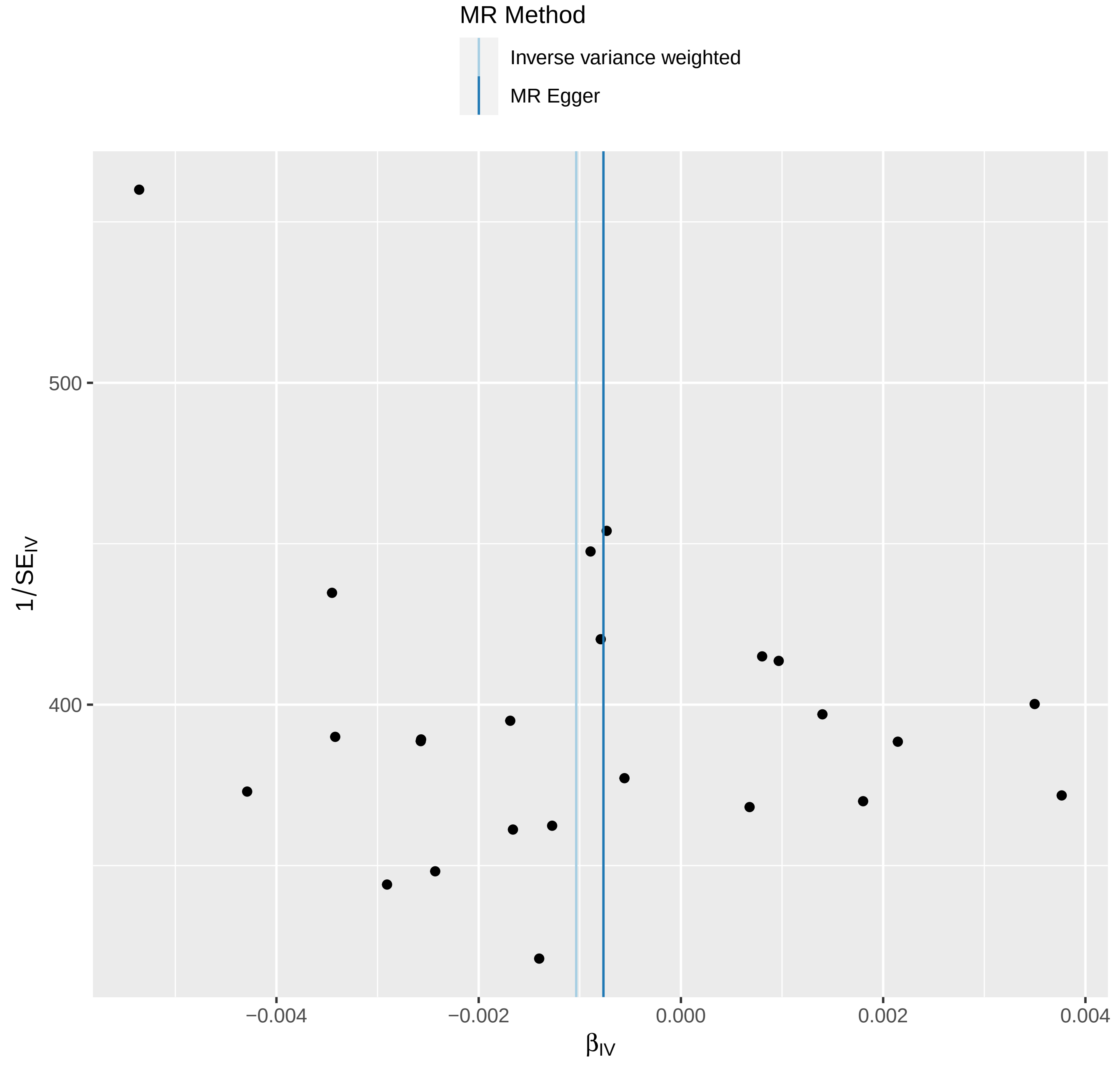

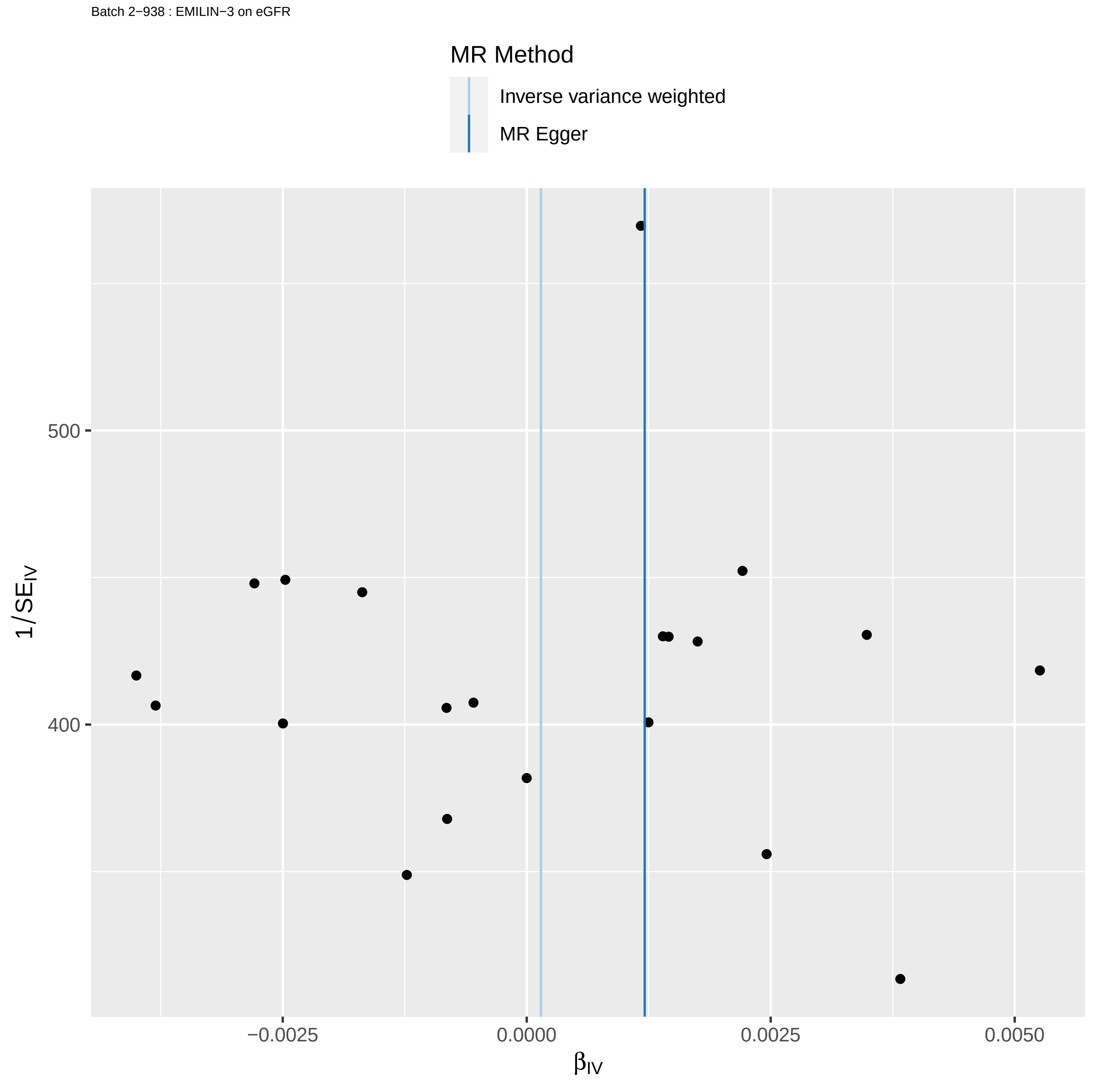
Batch 2−938 : EMILIN−3 on eGFR
MR Method
Inverse variance weighted
MR Egger
500
400
1/SE_IV
−0.0025
0.0000
0.0025
0.0050
β_IV

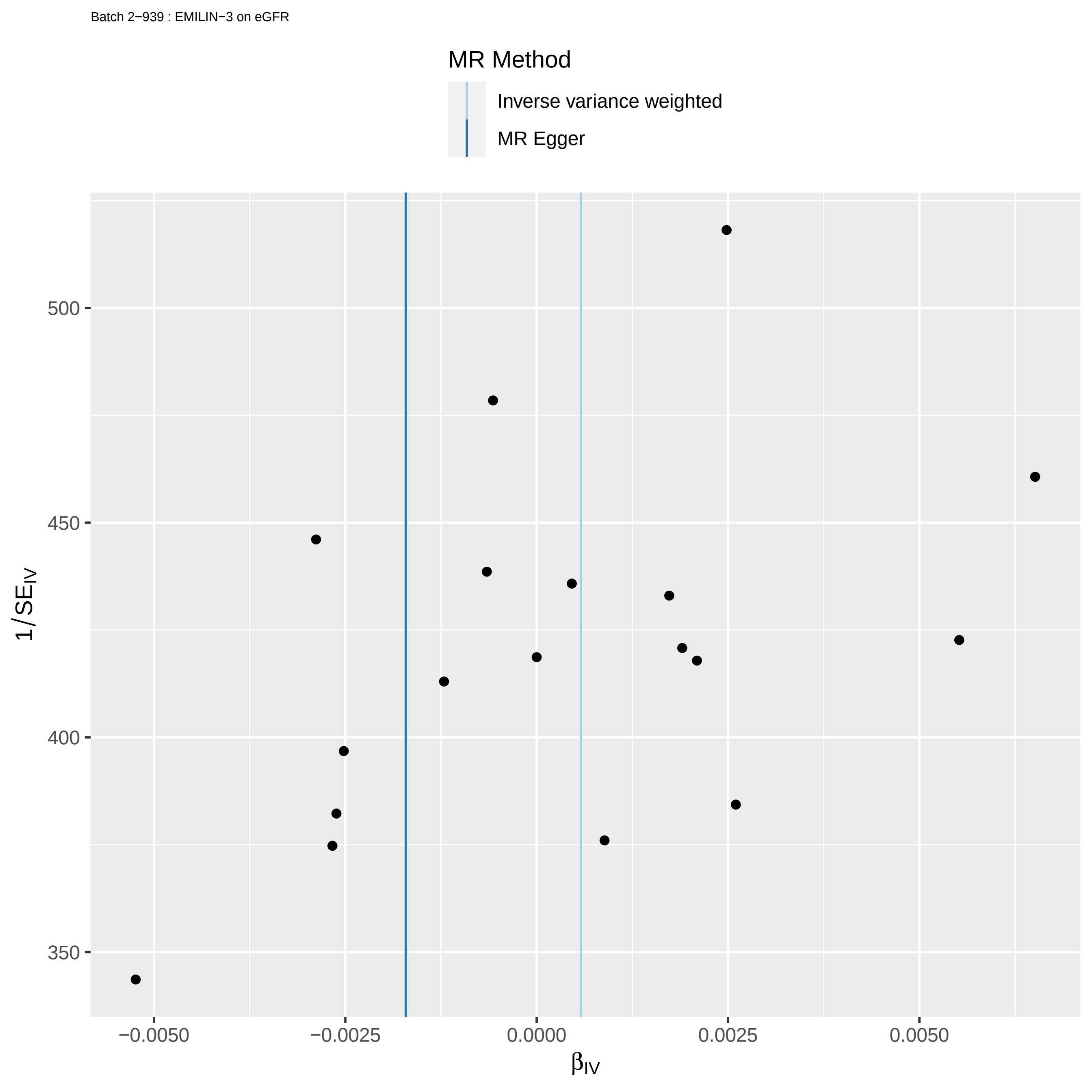

Batch 2−939 : EMILIN−3 on eGFR
MR Method
Inverse variance weighted
MR Egger
500
450
400
350
1/SE_IV
−0.0050
−0.0025
0.0000
0.0025
0.0050
β_IV

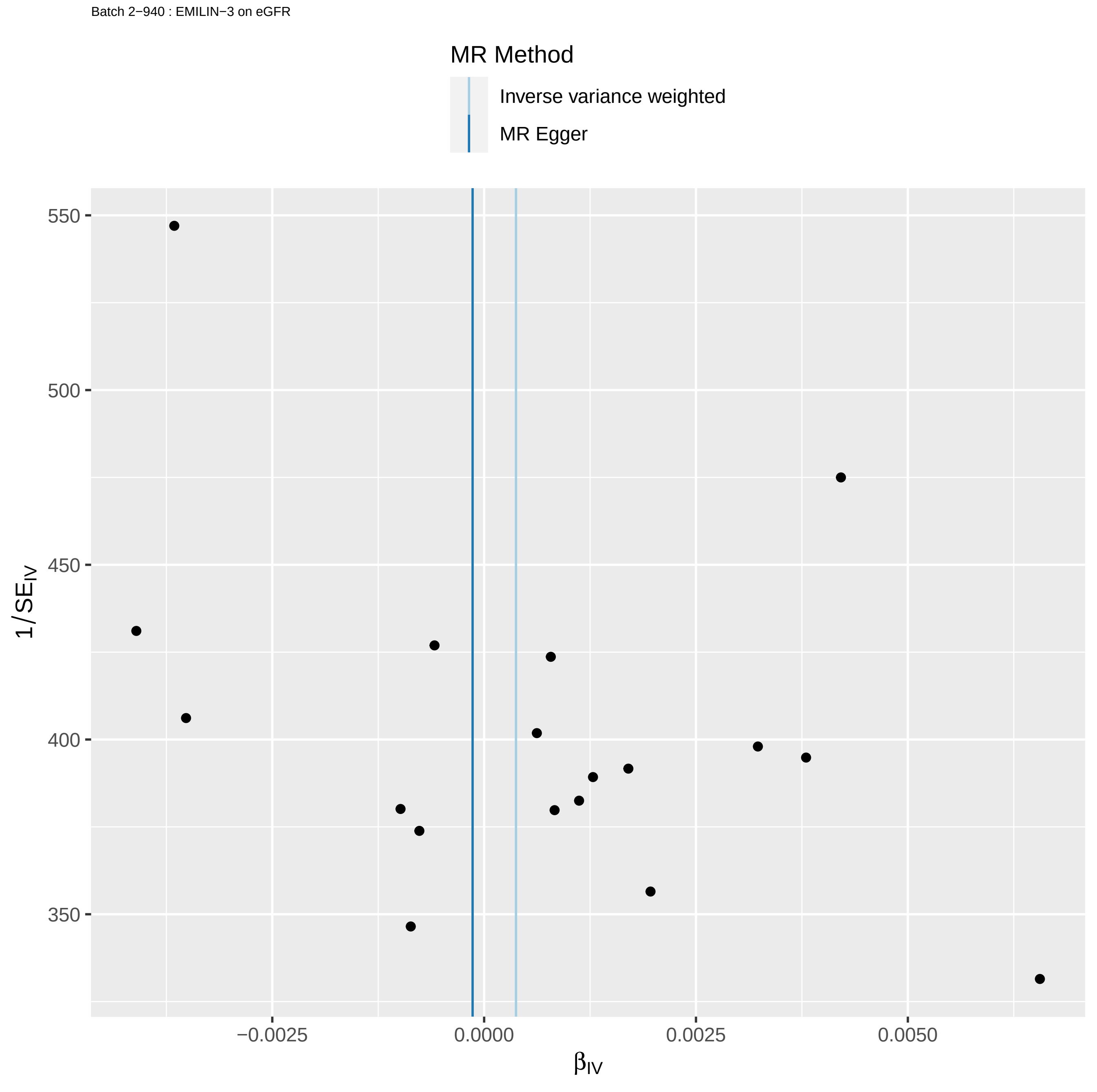

Batch 2−940 : EMILIN−3 on eGFR
MR Method
Inverse variance weighted
MR Egger
550
500
450
400
350
1/SE_IV
−0.0025
0.0000
0.0025
0.0050
β_IV

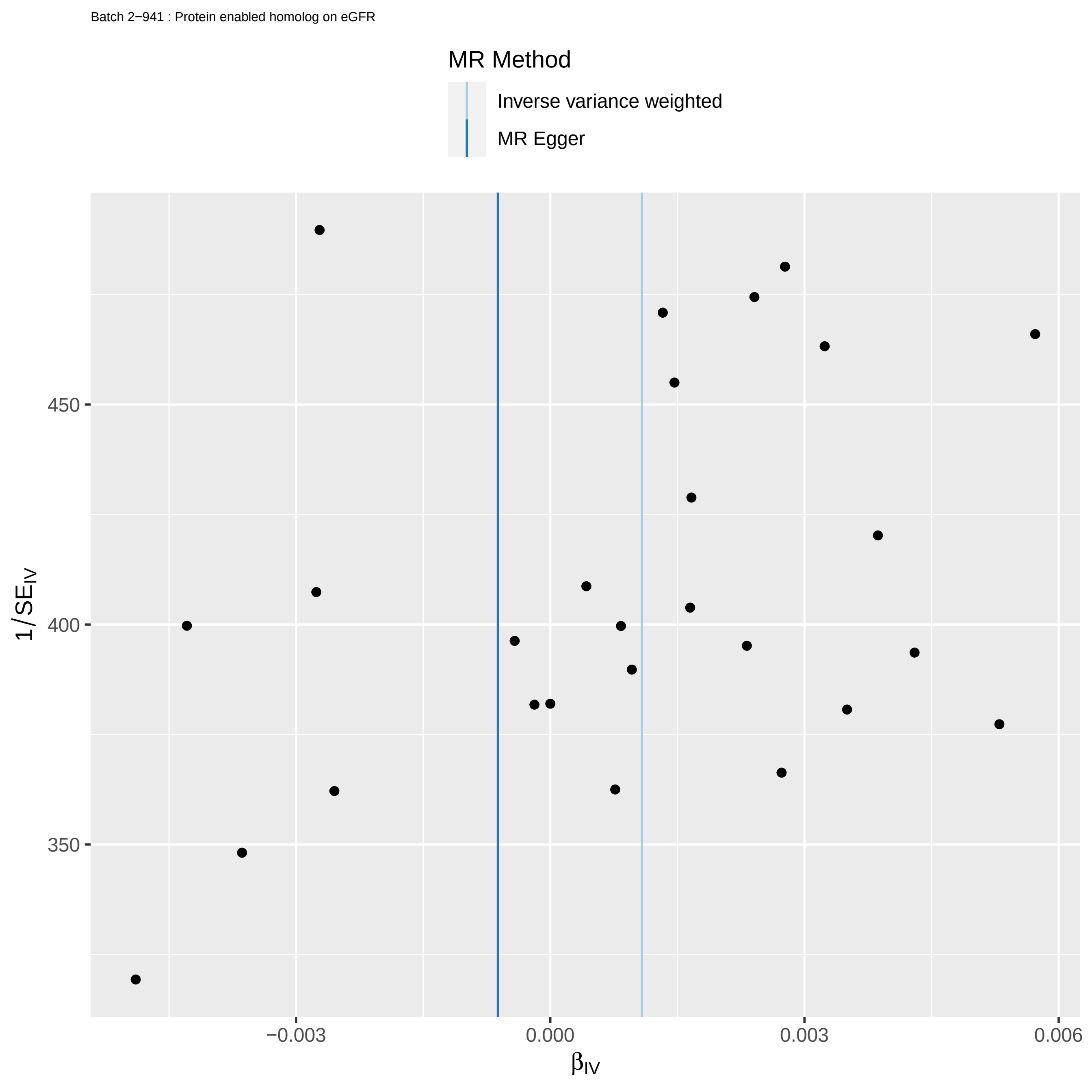

Batch 2−941 : Protein enabled homolog on eGFR
MR Method
Inverse variance weighted
MR Egger
450
400
350
1/SE_IV
−0.003
0.000
0.003
0.006
β_IV

Batch 2−942 : Poly(U)−specific endoribonuclease on eGFR

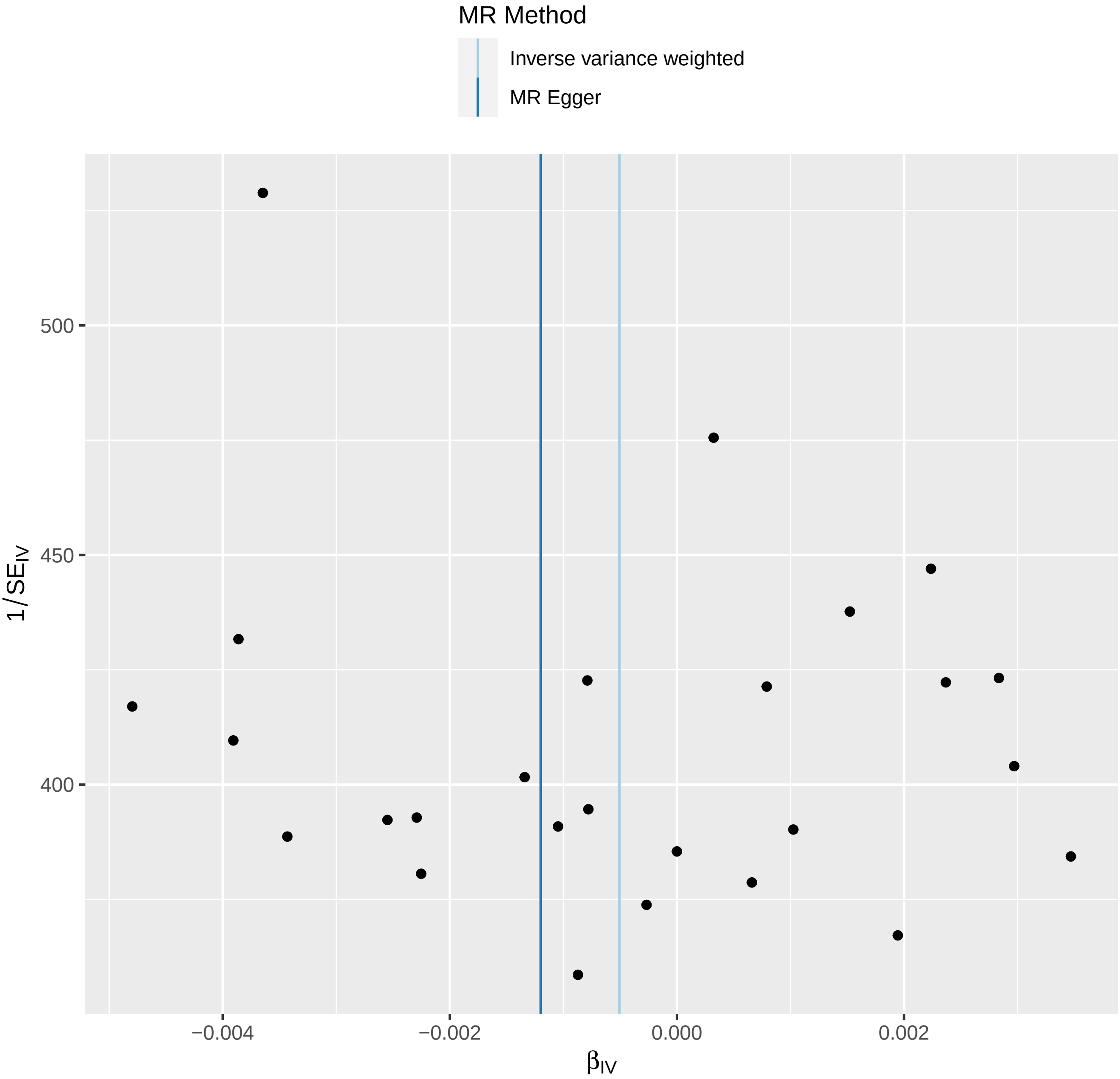

Batch 2−943 : Endoglin on eGFR

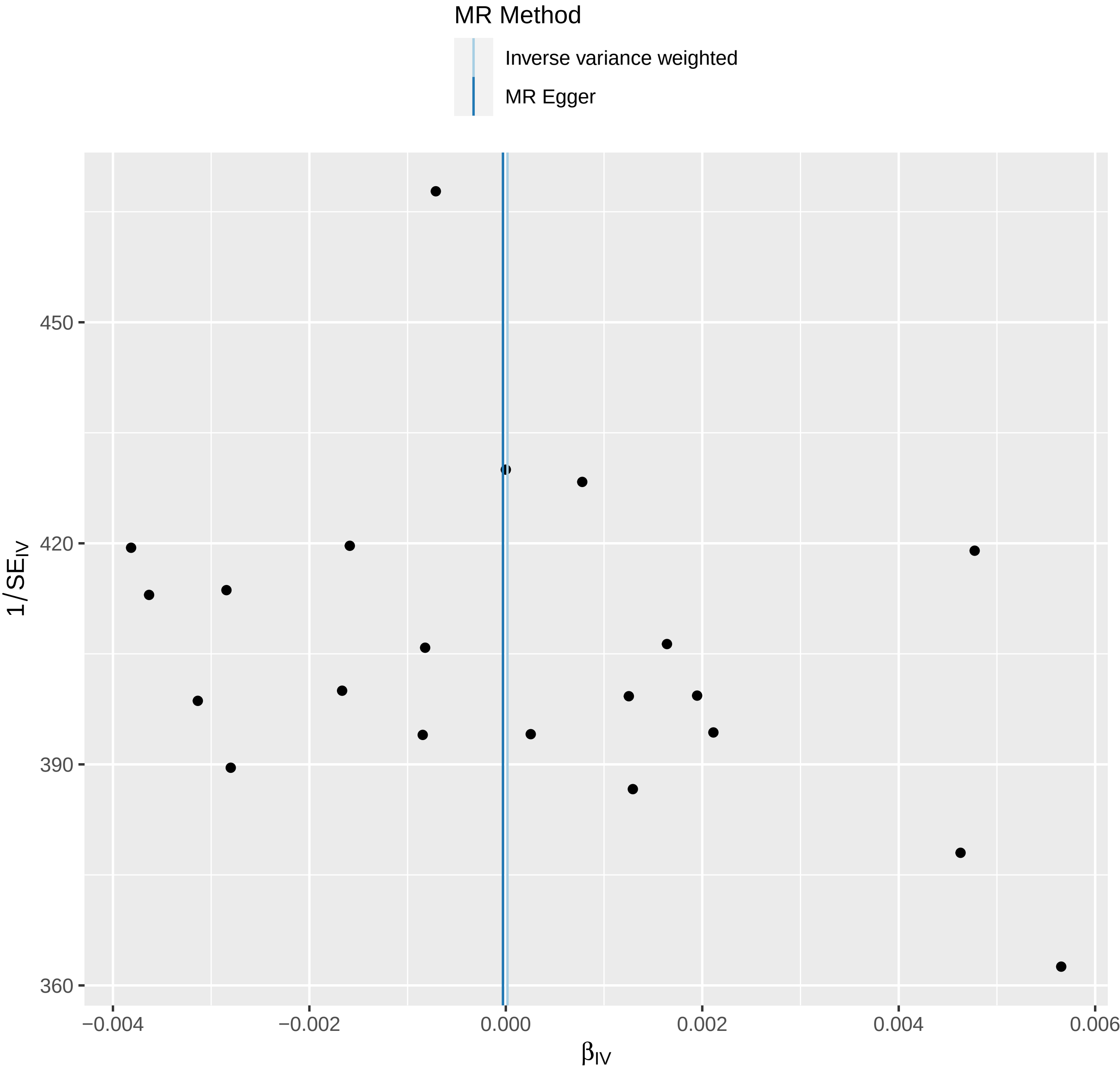

Batch 2−944 : Gamma−enolase on eGFR

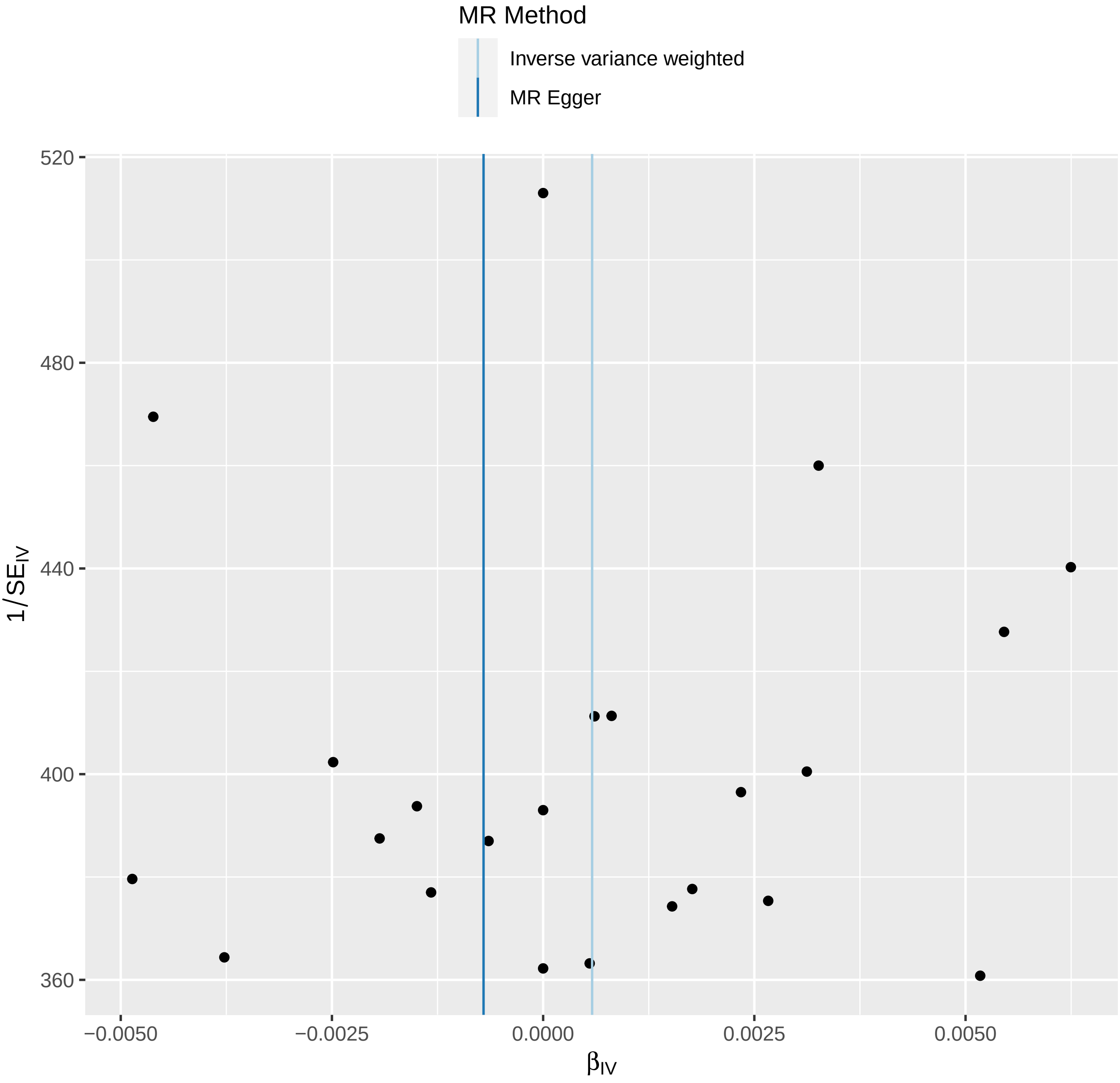

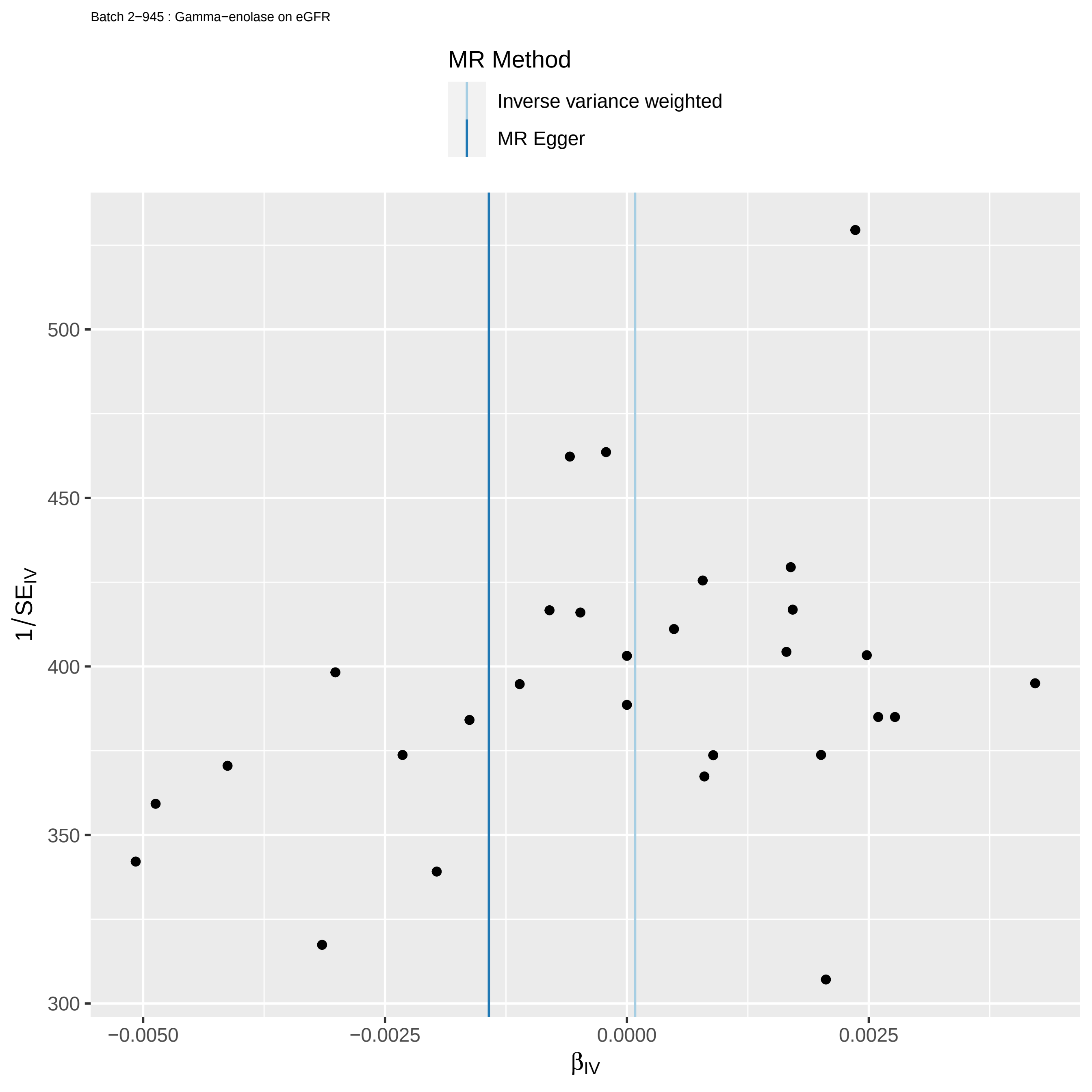

Batch 2−945 : Gamma−enolase on eGFR
MR Method
Inverse variance weighted
MR Egger
500
450
400
350
300
1/SE$_{IV}$
−0.0050
−0.0025
0.0000
0.0025
β$_{IV}$

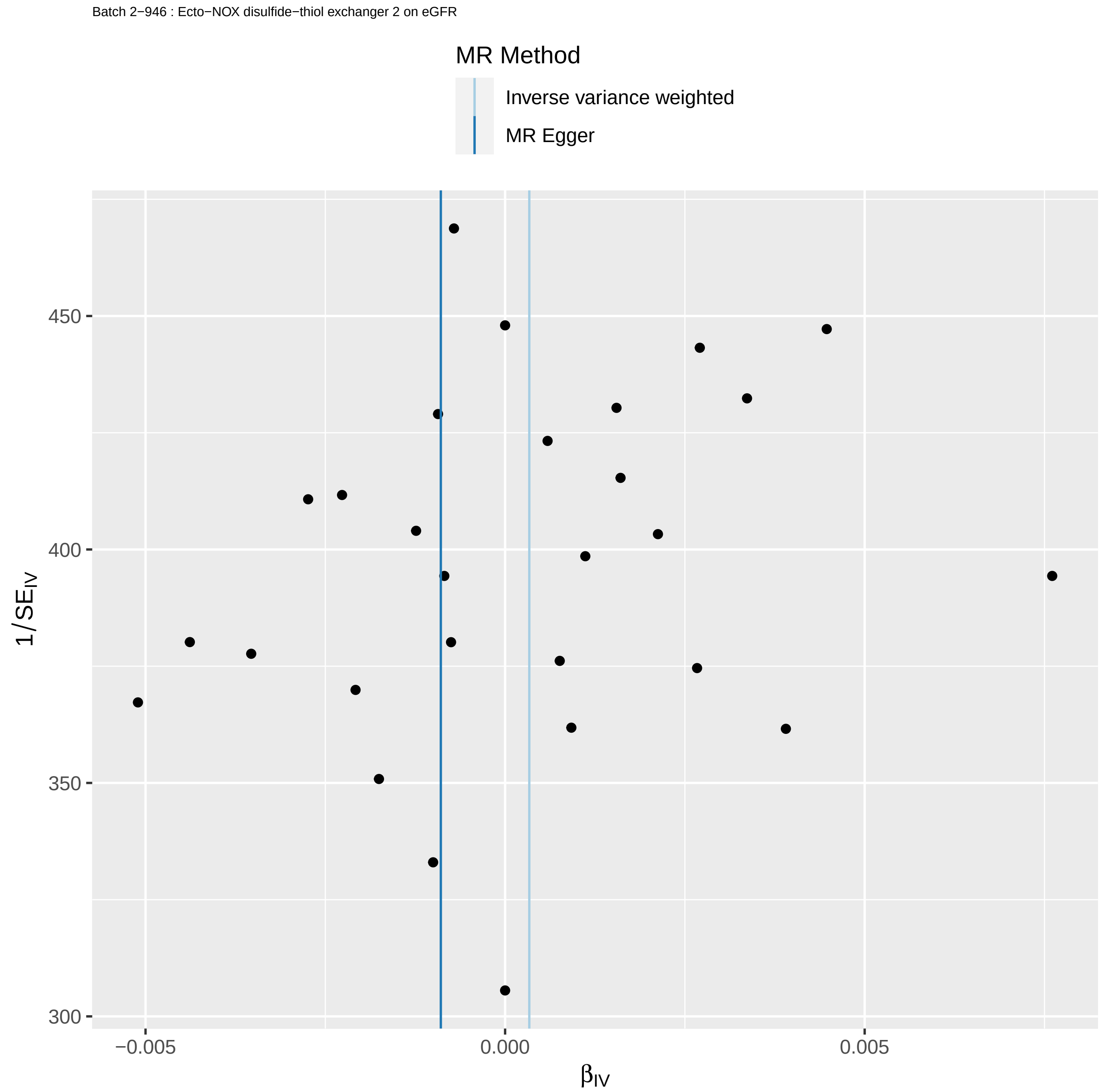
Batch 2−946 : Ecto−NOX disulfide−thiol exchanger 2 on eGFR
MR Method
Inverse variance weighted
MR Egger
450
400
350
300
1/SE_IV
−0.005
0.000
0.005
β_IV

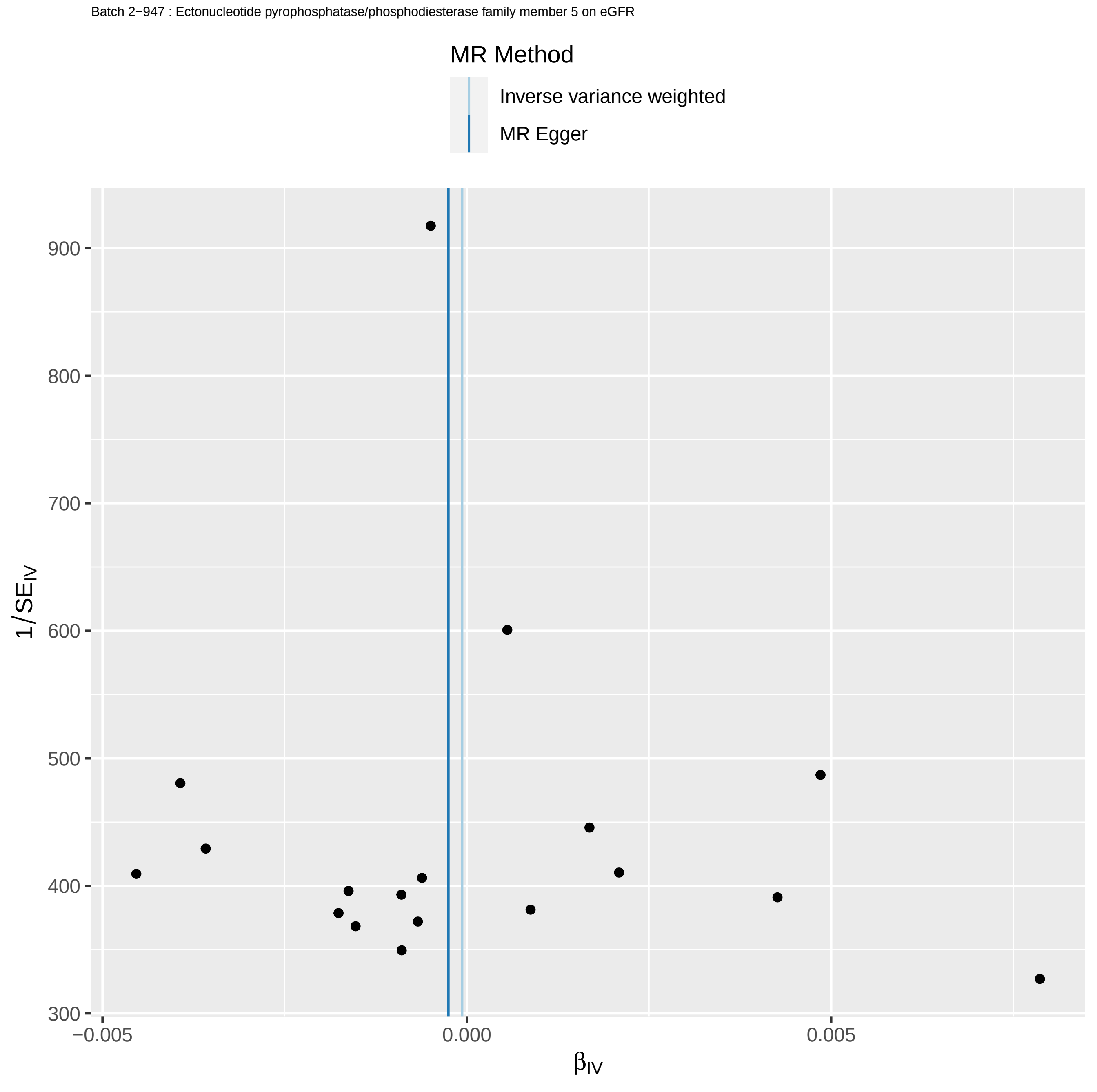

Batch 2−947 : Ectonucleotide pyrophosphatase/phosphodiesterase family member 5 on eGFR
MR Method
Inverse variance weighted
MR Egger
900
800
700
600
500
400
300
1/SE$_{IV}$
−0.005
0.000
0.005
β$_{IV}$

Batch 2−948 : Ectonucleotide pyrophosphatase/phosphodiesterase family member 7 on eGFR

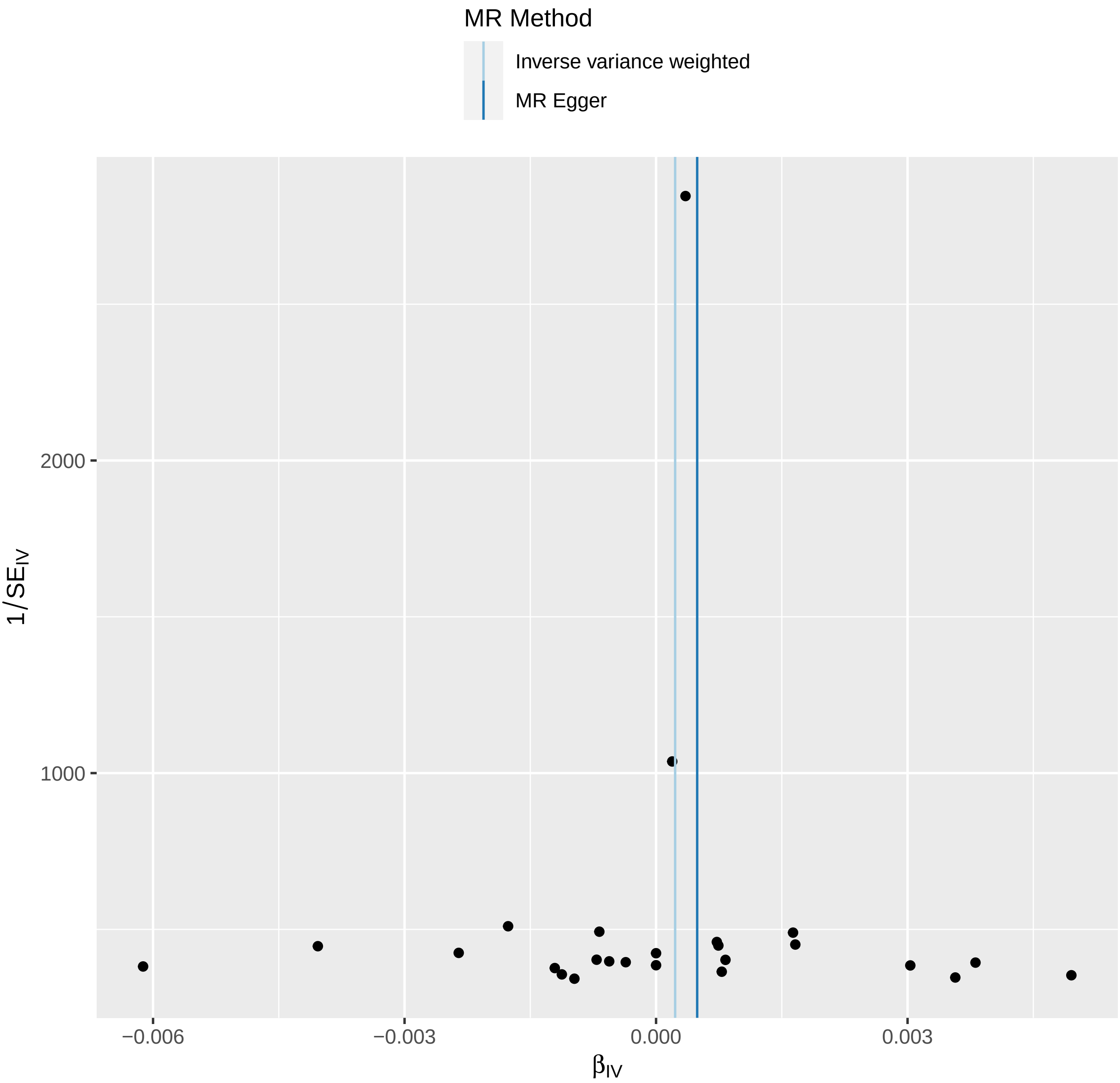

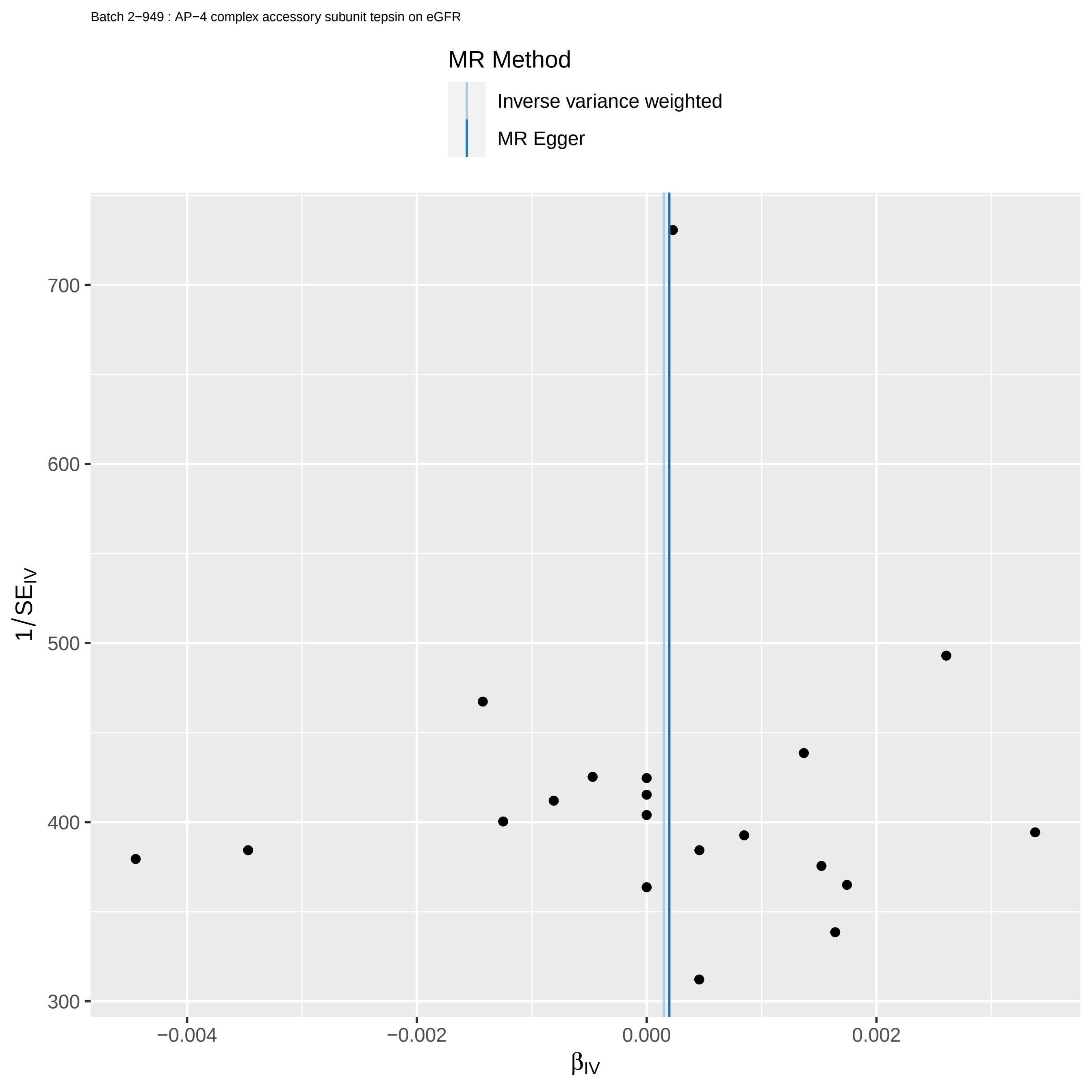

Batch 2−949 : AP−4 complex accessory subunit tepsin on eGFR
MR Method
Inverse variance weighted
MR Egger
700
600
500
400
300
1/SE_IV
−0.004
−0.002
0.000
0.002
β_IV

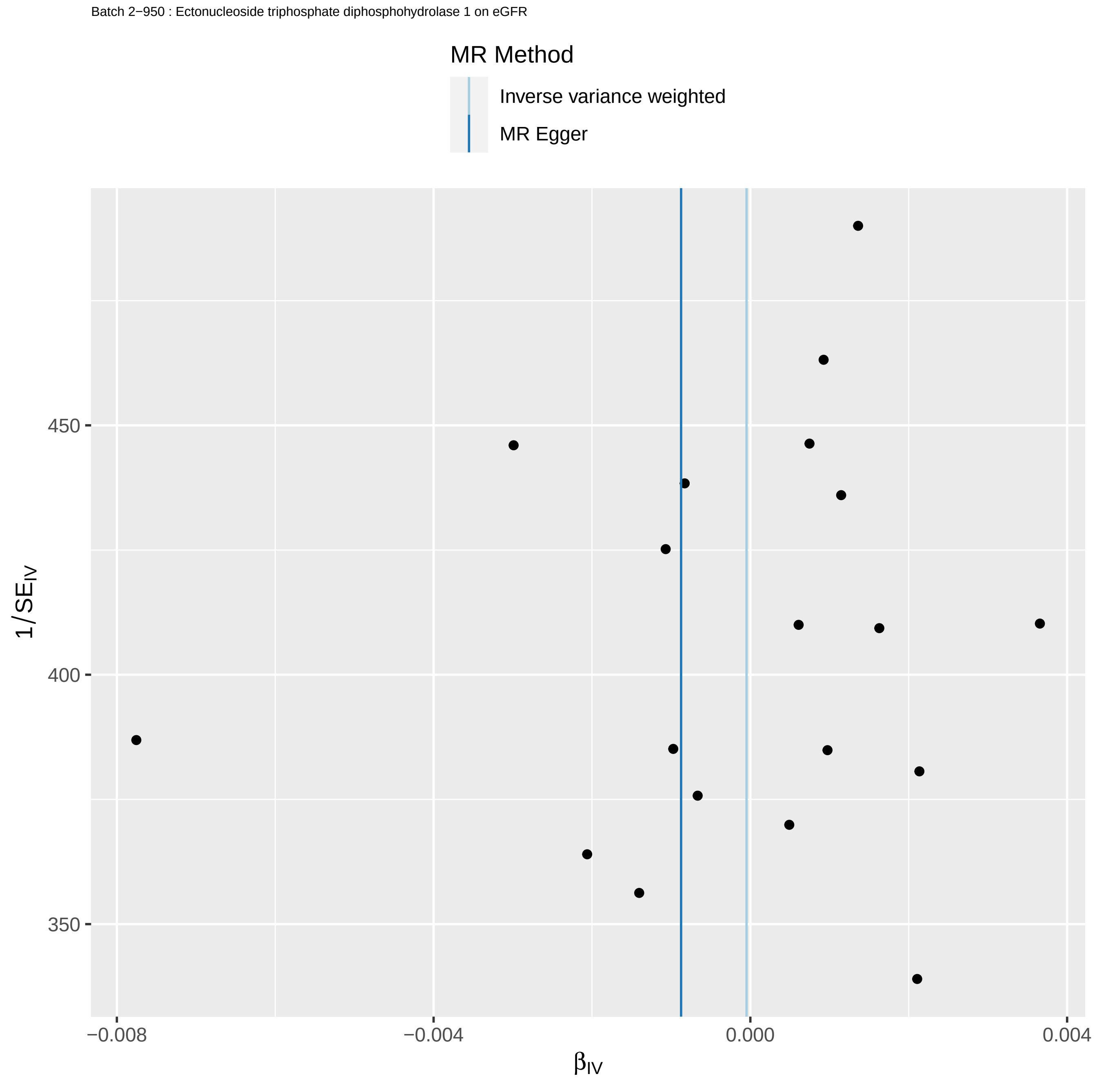
Batch 2−950 : Ectonucleoside triphosphate diphosphohydrolase 1 on eGFR
MR Method
Inverse variance weighted
MR Egger
450
400
350
1/SE_IV
−0.008
−0.004
0.000
0.004
β_IV

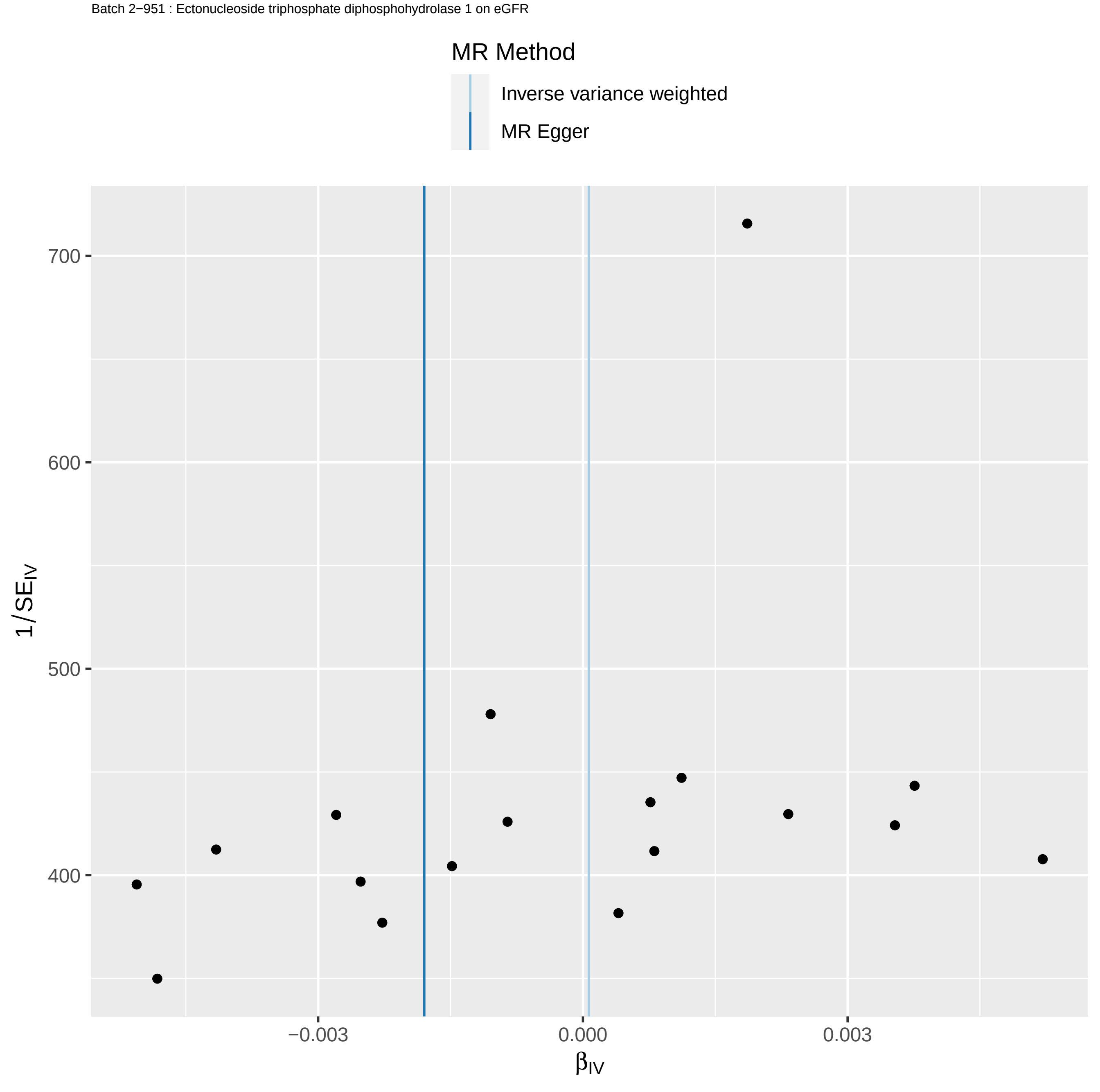
Batch 2−951 : Ectonucleoside triphosphate diphosphohydrolase 1 on eGFR
MR Method
Inverse variance weighted
MR Egger
700
600
500
400
1/SE_IV
−0.003
0.000
0.003
β_IV

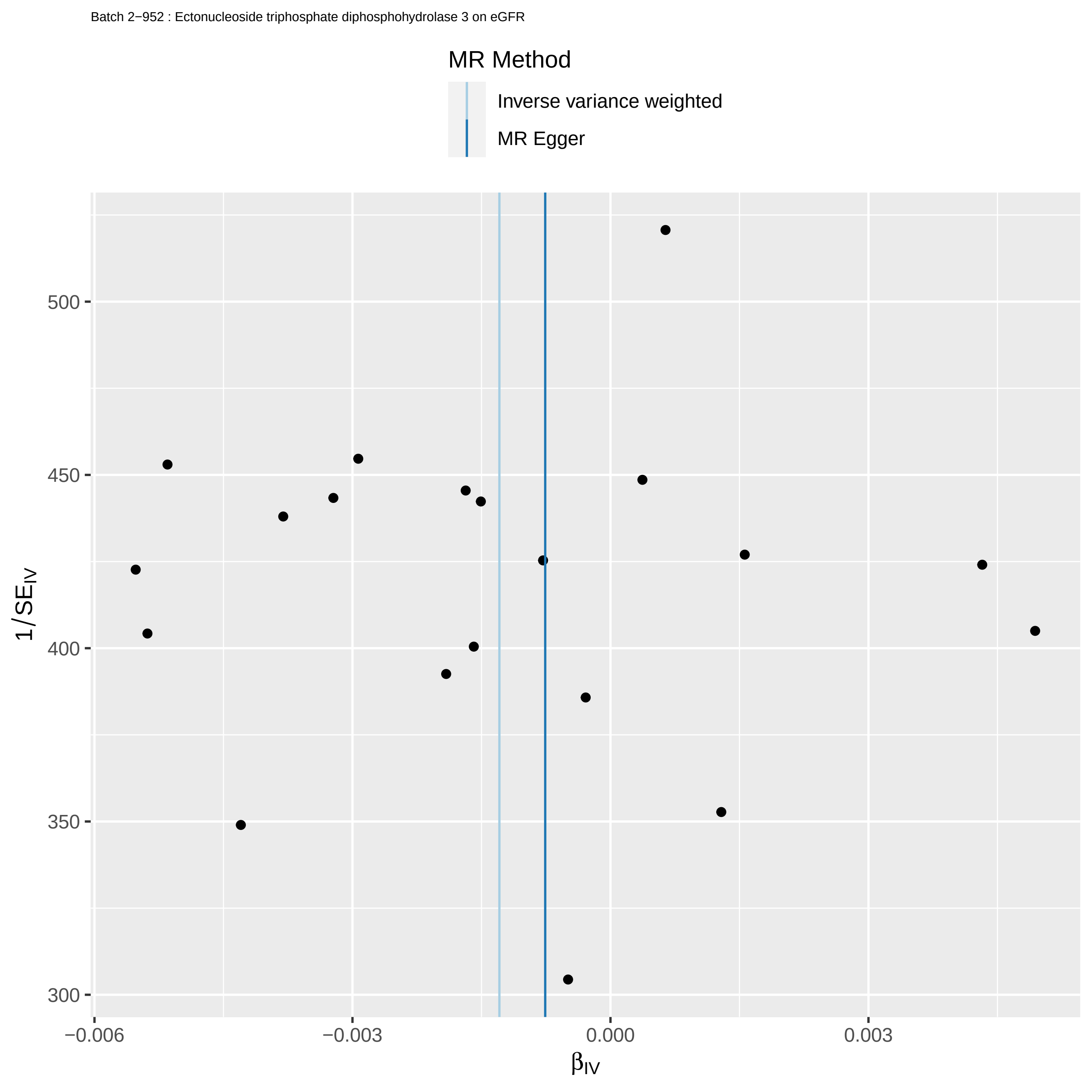

Batch 2−952 : Ectonucleoside triphosphate diphosphohydrolase 3 on eGFR
MR Method
Inverse variance weighted
MR Egger
500
450
400
350
300
1/SE_IV
−0.006
−0.003
0.000
0.003
β_IV

Batch 2−953 : Ectonucleoside triphosphate diphosphohydrolase 5 on eGFR

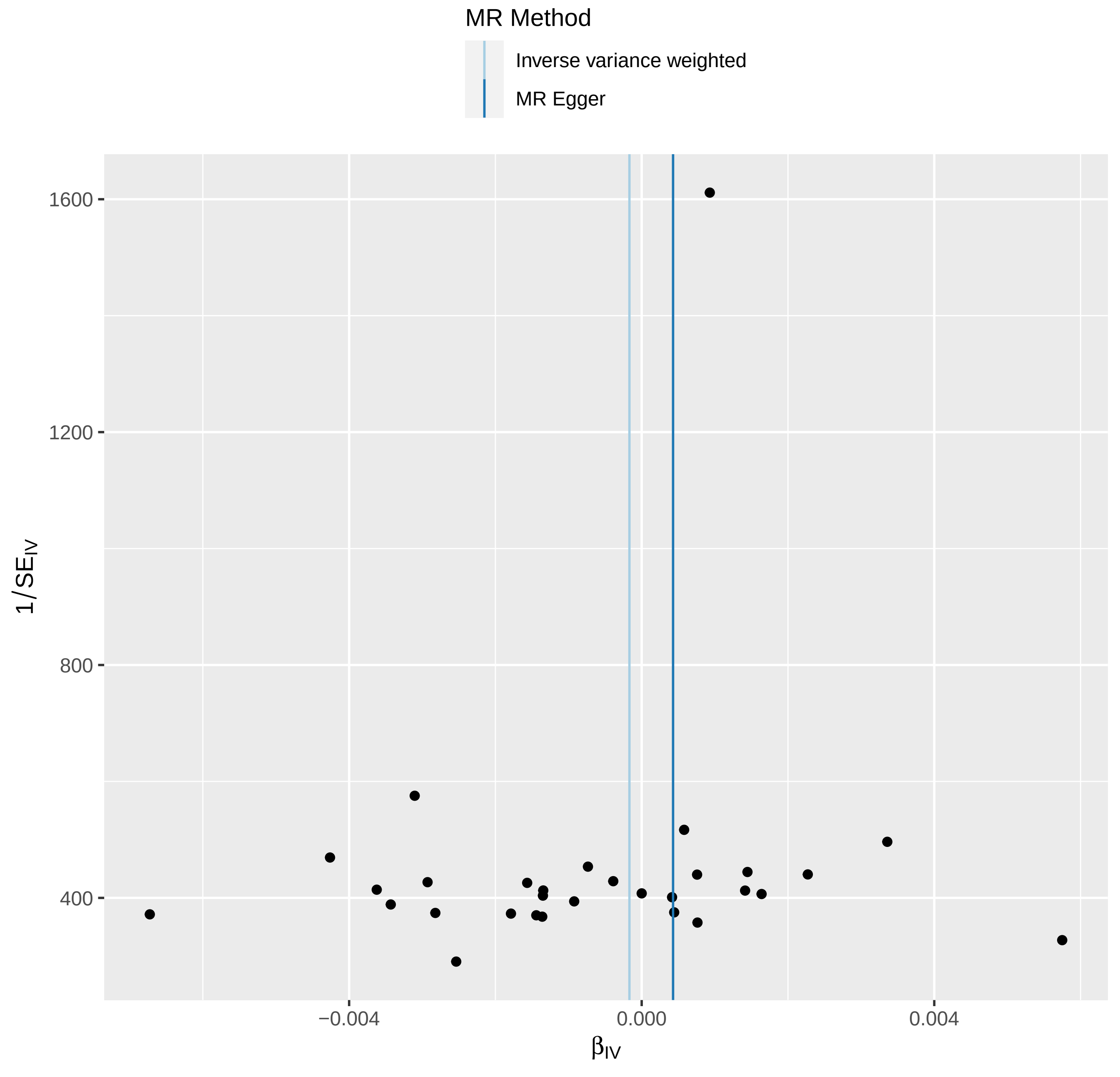

Batch 2−954 : Ectonucleoside triphosphate diphosphohydrolase 6 on eGFR

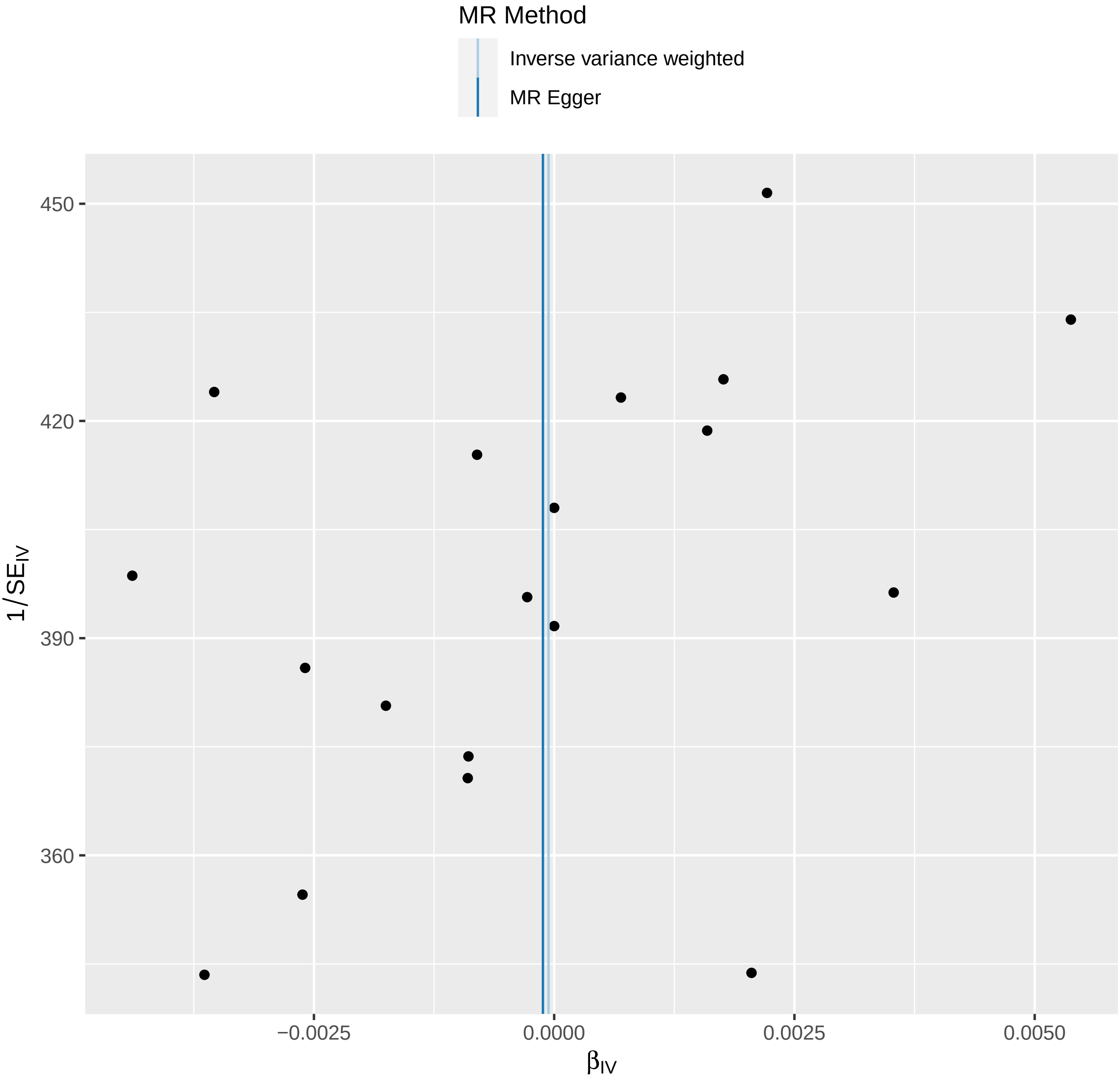

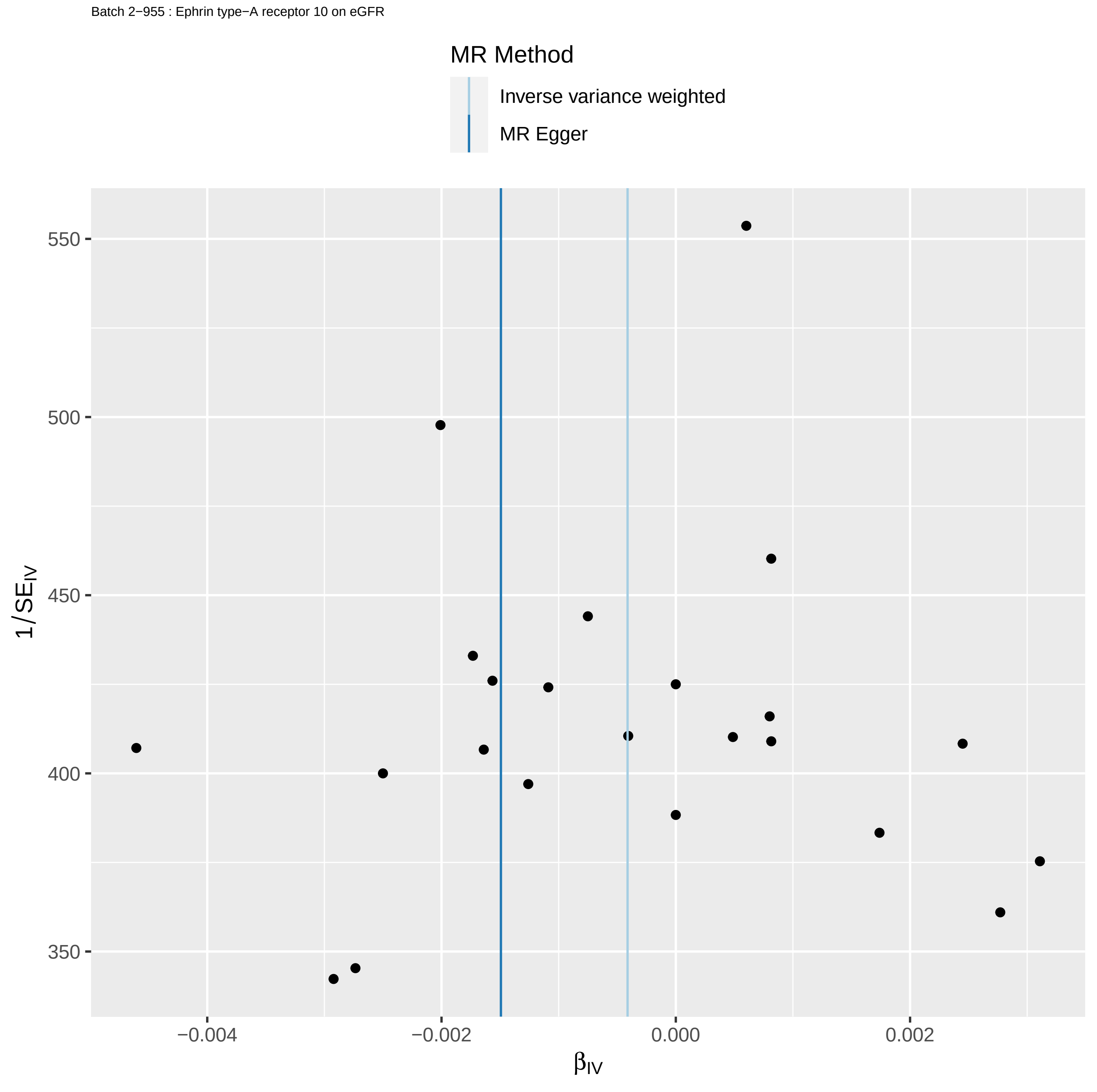

Batch 2−955 : Ephrin type−A receptor 10 on eGFR
MR Method
Inverse variance weighted
MR Egger
550
500
450
400
350
1/SE_IV
−0.004
−0.002
0.000
0.002
β_IV

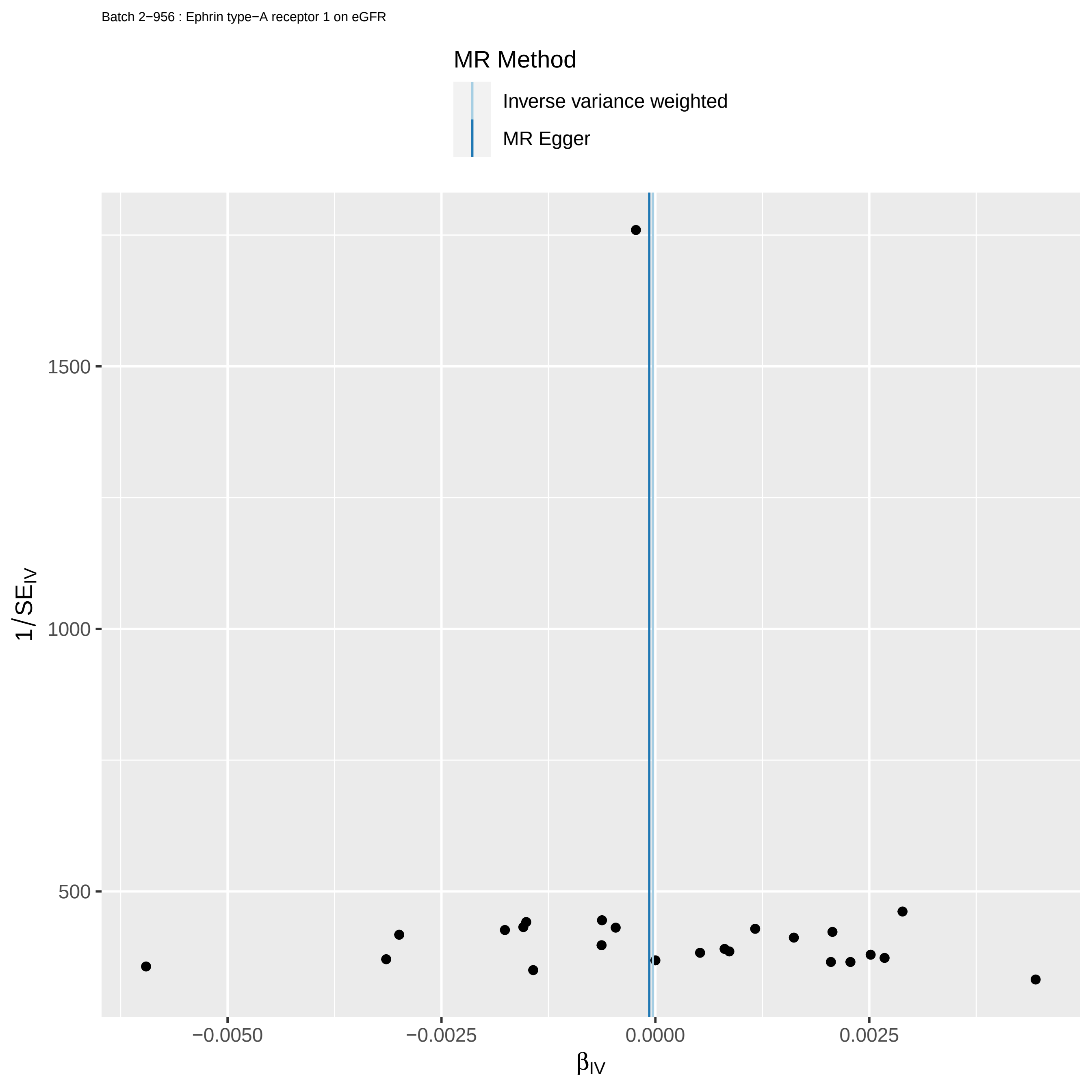

Batch 2−956 : Ephrin type−A receptor 1 on eGFR
MR Method
Inverse variance weighted
MR Egger
1/SE_IV
1500
1000
500
−0.0050
−0.0025
0.0000
0.0025
β_IV

Batch 2−957 : Ephrin type−A receptor 2 on eGFR

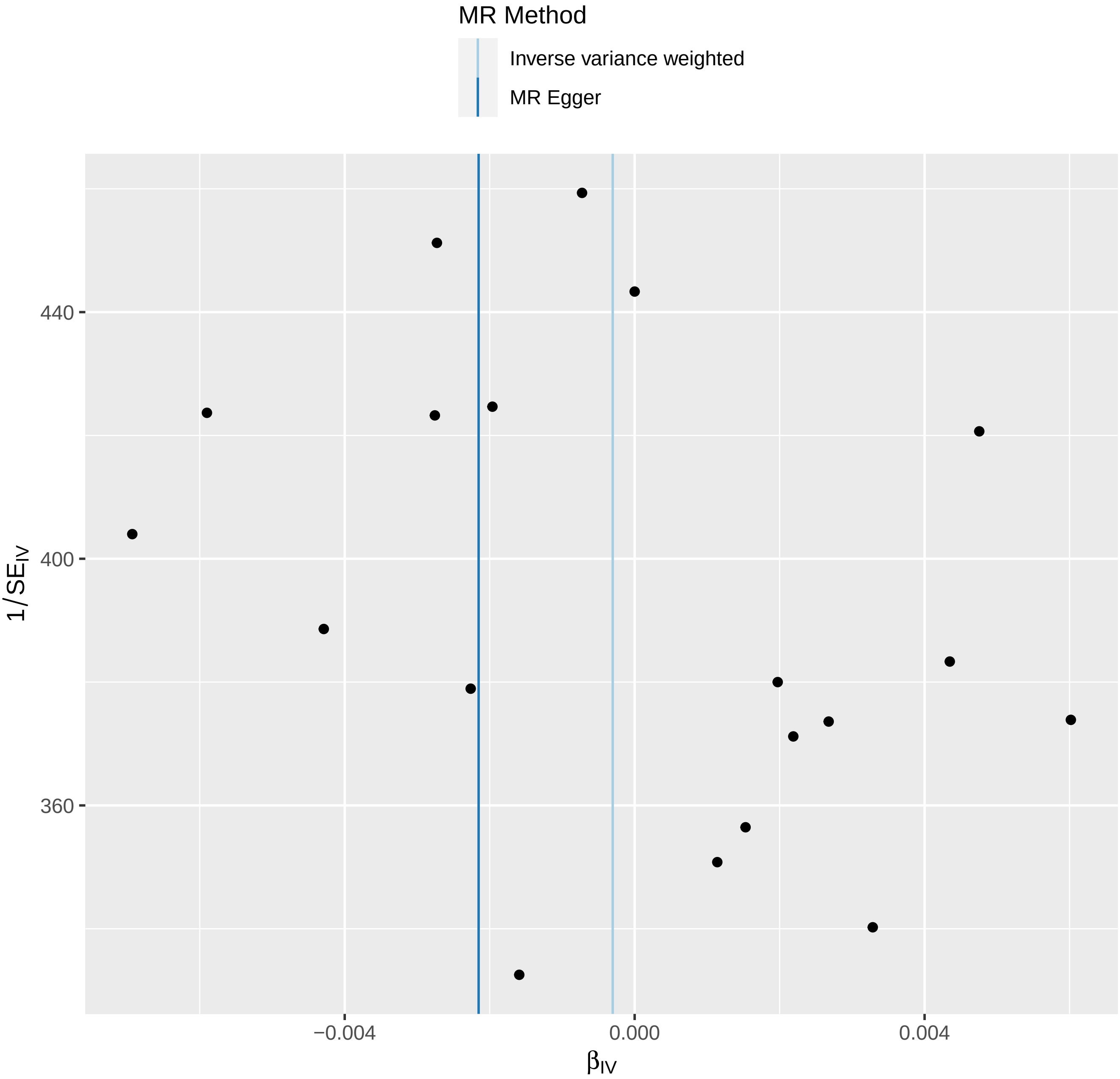

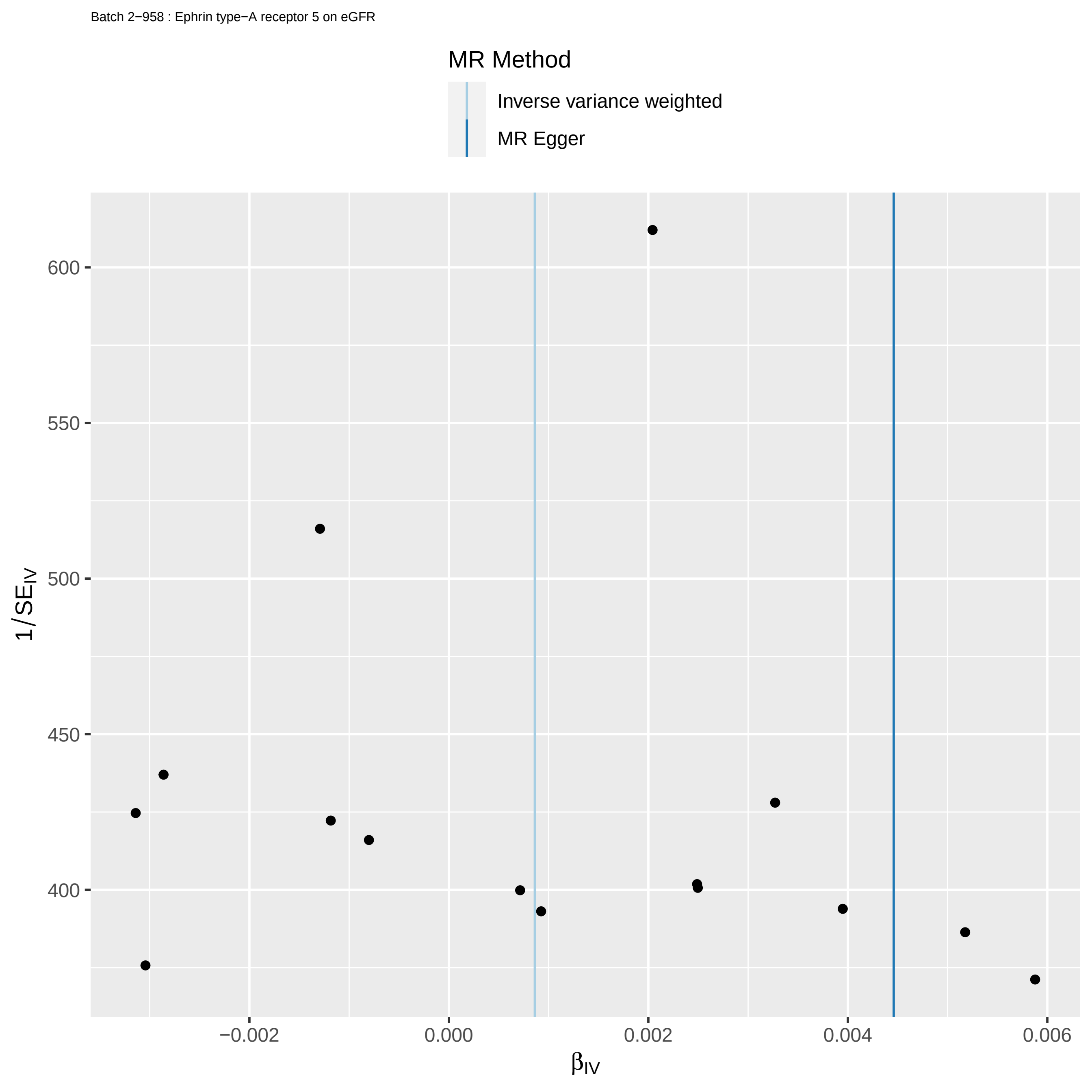

Batch 2−958 : Ephrin type−A receptor 5 on eGFR
MR Method
Inverse variance weighted
MR Egger
600
550
500
450
400
1/SE$_{IV}$
−0.002
0.000
0.002
0.004
0.006
β$_{IV}$

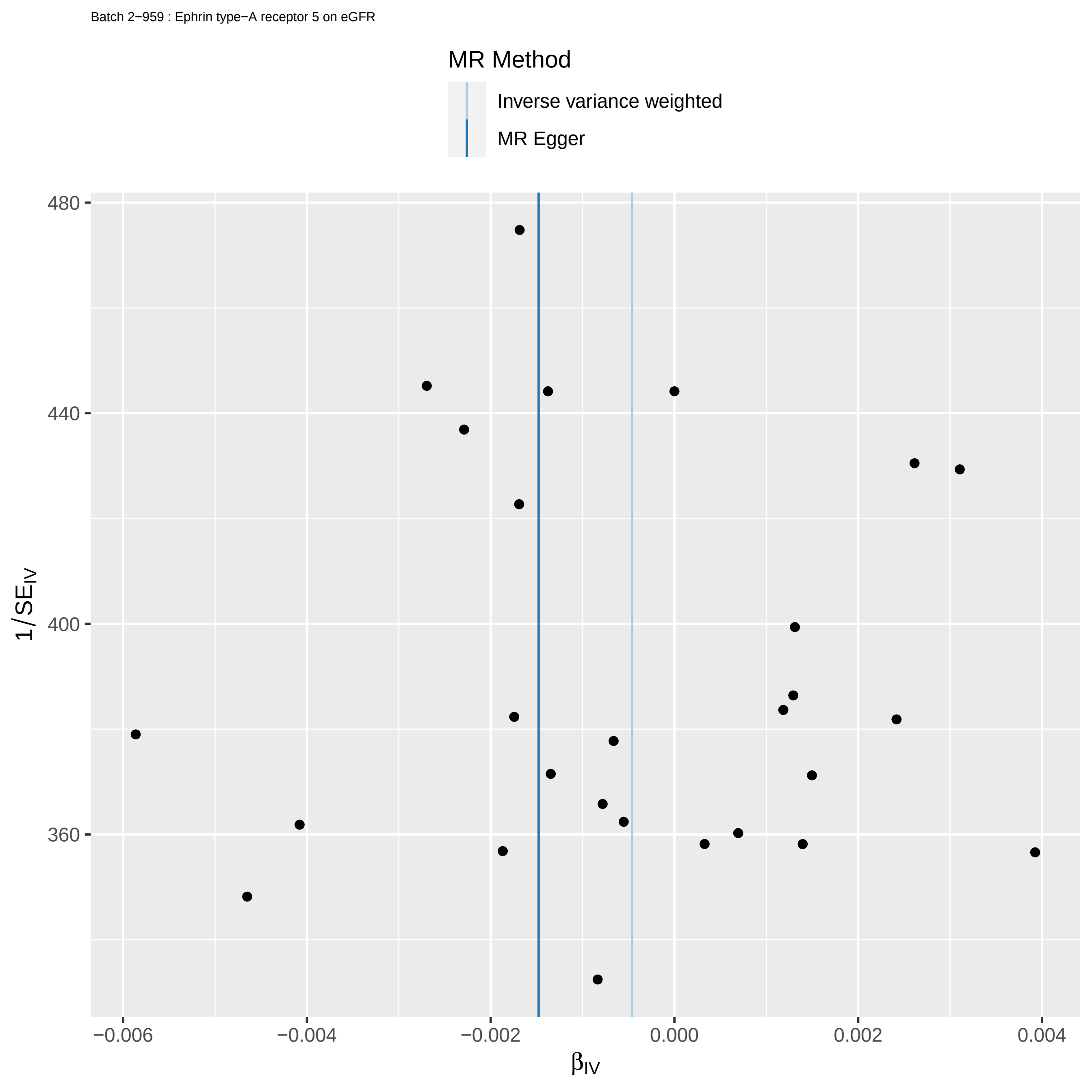
Batch 2−959 : Ephrin type−A receptor 5 on eGFR
MR Method
Inverse variance weighted
MR Egger
480
440
400
360
$1/SE_{IV}$
−0.006
−0.004
−0.002
0.000
0.002
0.004
β_{IV}

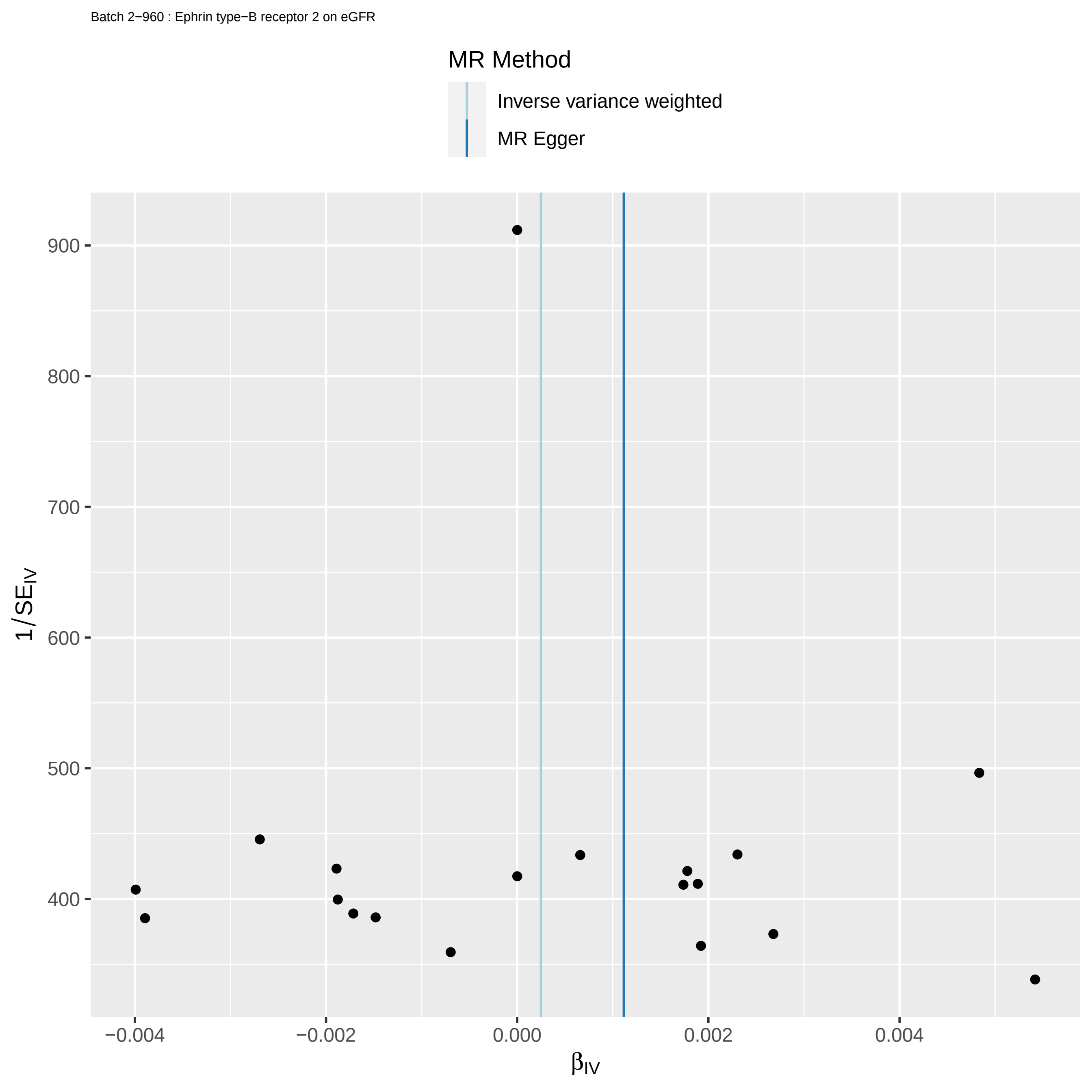

Batch 2−960 : Ephrin type−B receptor 2 on eGFR
MR Method
Inverse variance weighted
MR Egger
900
800
700
600
500
400
1/SE$_{IV}$
−0.004
−0.002
0.000
0.002
0.004
β_{IV}

Batch 2−961 : Ephrin type−B receptor 2 on eGFR

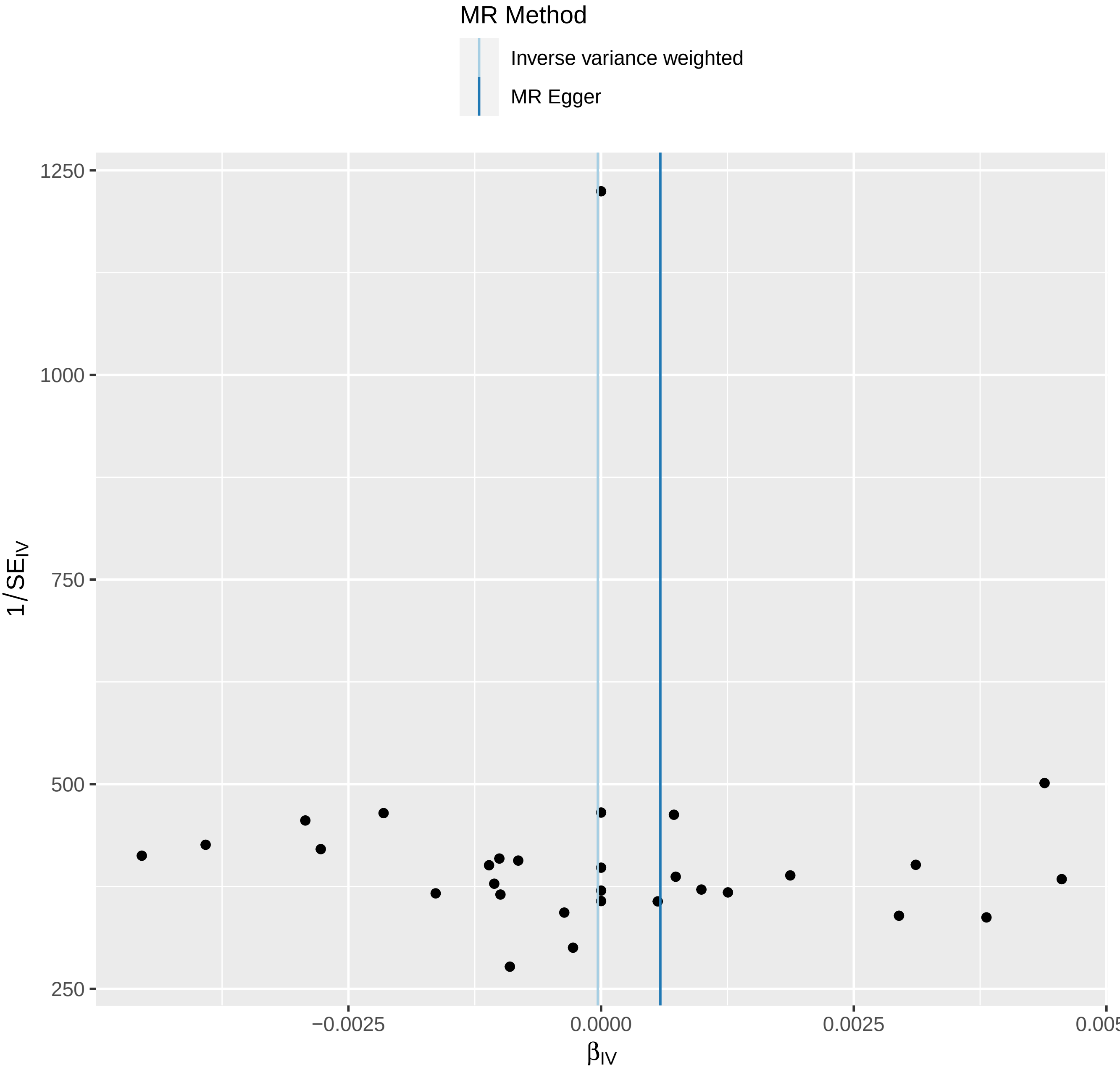

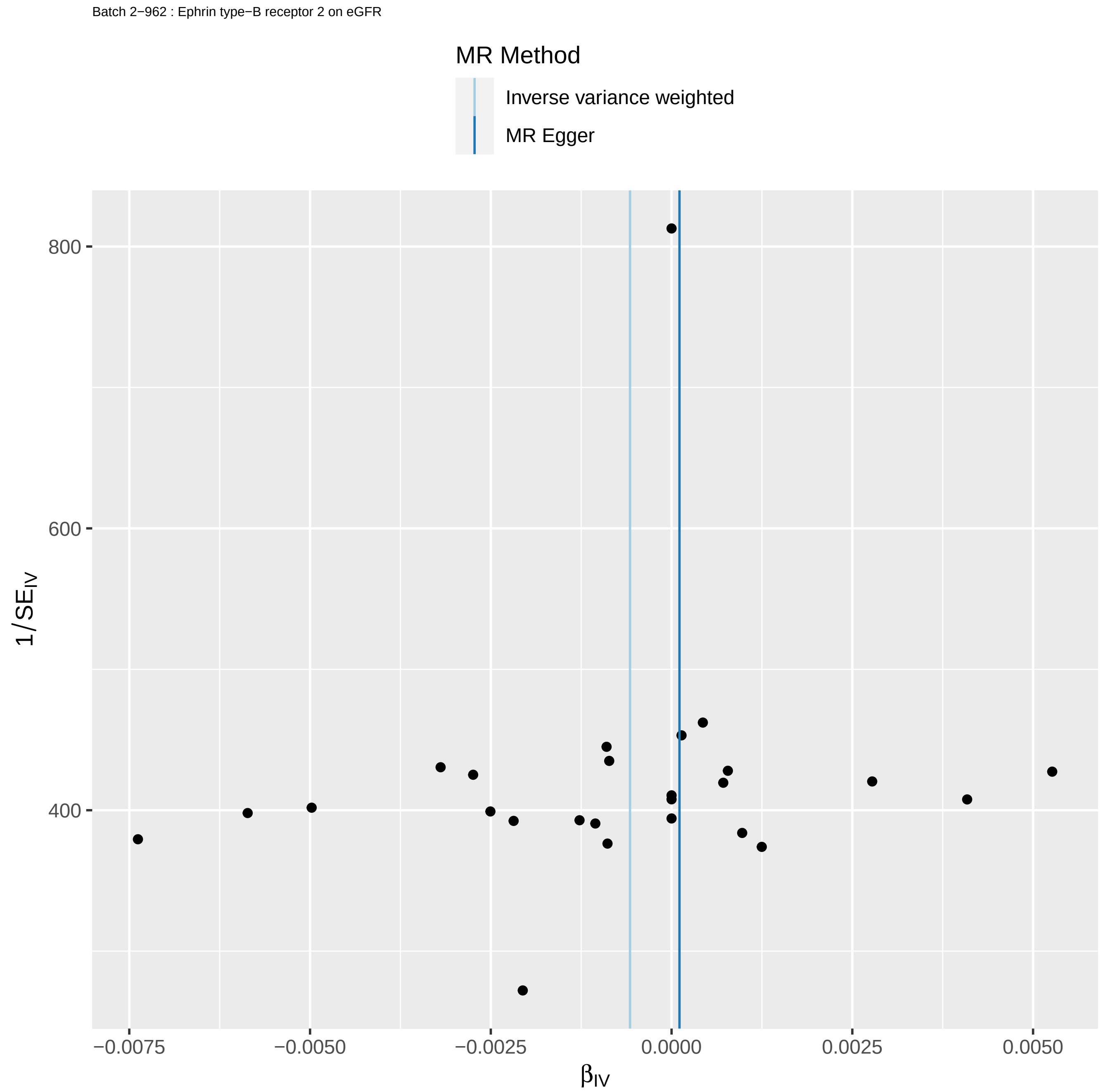

Batch 2−962 : Ephrin type−B receptor 2 on eGFR
MR Method
Inverse variance weighted
MR Egger
800
600
400
1/SE$_{IV}$
−0.0075
−0.0050
−0.0025
0.0000
0.0025
0.0050
β$_{IV}$

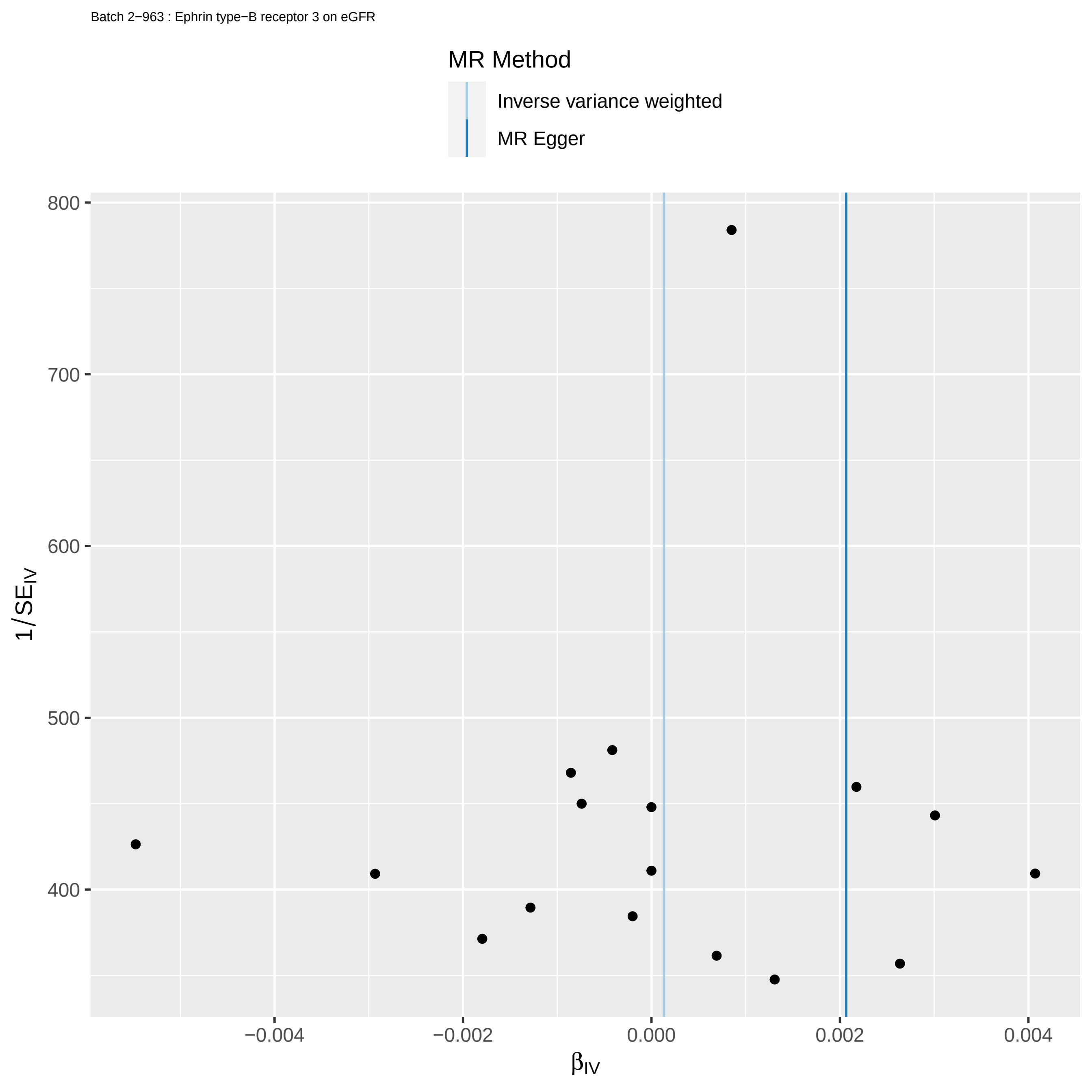

Batch 2−963 : Ephrin type−B receptor 3 on eGFR
MR Method
Inverse variance weighted
MR Egger
800
700
600
500
400
$1/SE_{IV}$
−0.004
−0.002
0.000
0.002
0.004
β_{IV}

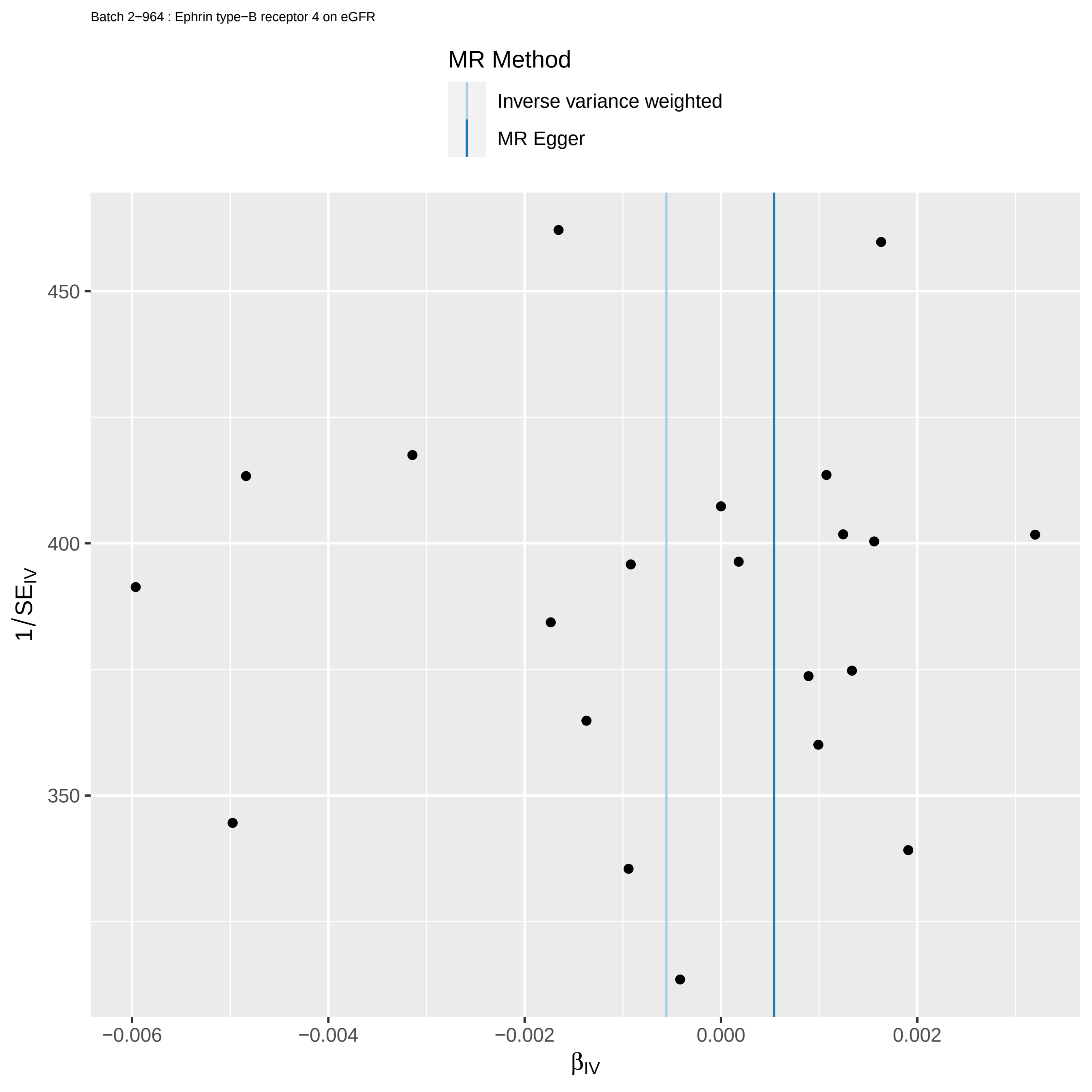

Batch 2−964 : Ephrin type−B receptor 4 on eGFR
MR Method
Inverse variance weighted
MR Egger
450
400
350
1/SE_IV
−0.006
−0.004
−0.002
0.000
0.002
β_IV

Batch 2−965 : Ephrin type−B receptor 6 on eGFR

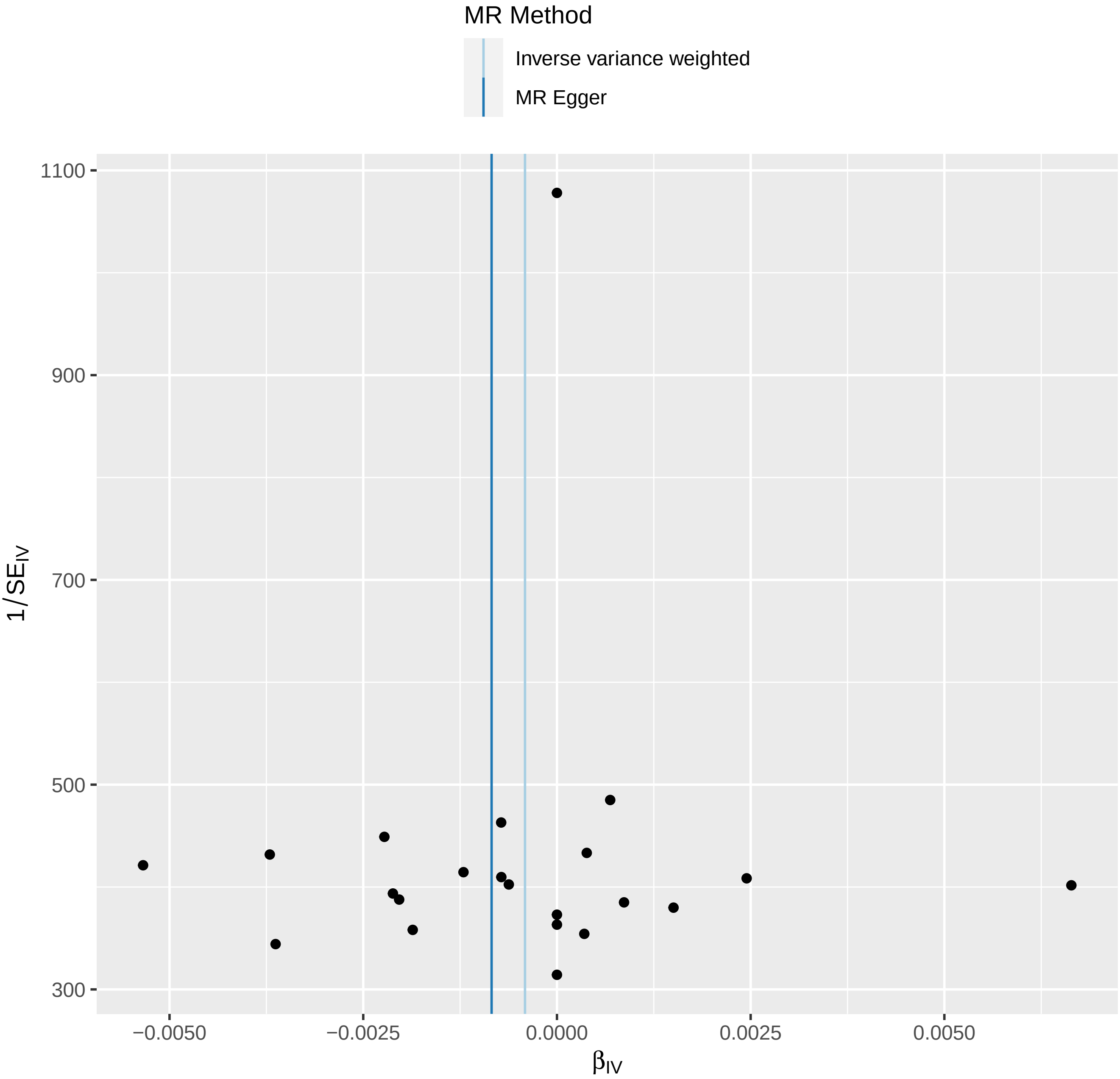

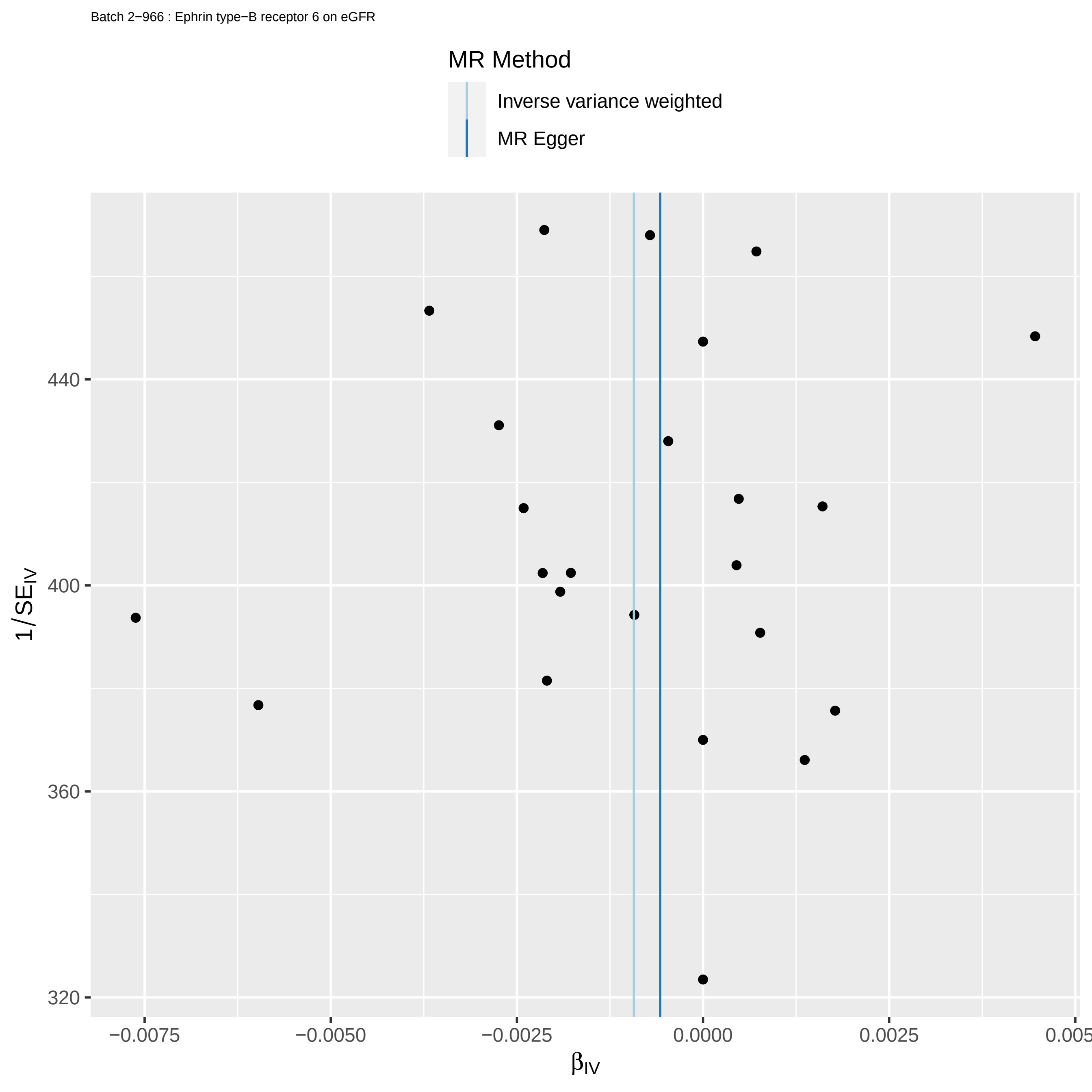

Batch 2−966 : Ephrin type−B receptor 6 on eGFR
MR Method
Inverse variance weighted
MR Egger
440
400
360
320
1/SE_IV
−0.0075
−0.0050
−0.0025
0.0000
0.0025
0.005
β_IV

Batch 2−967 : Erythropoietin on eGFR

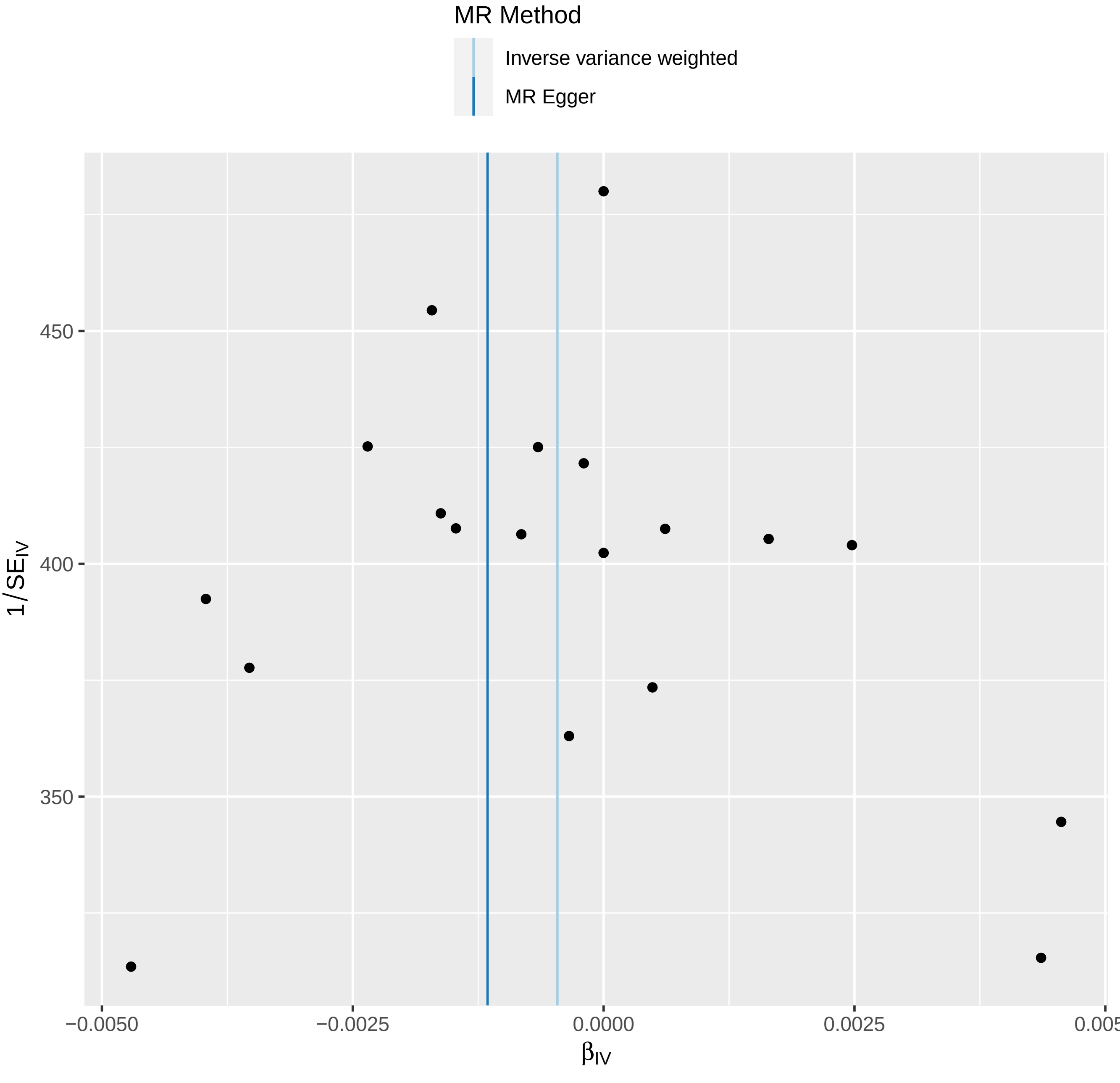

Batch 2−968 : Erythropoietin receptor on eGFR

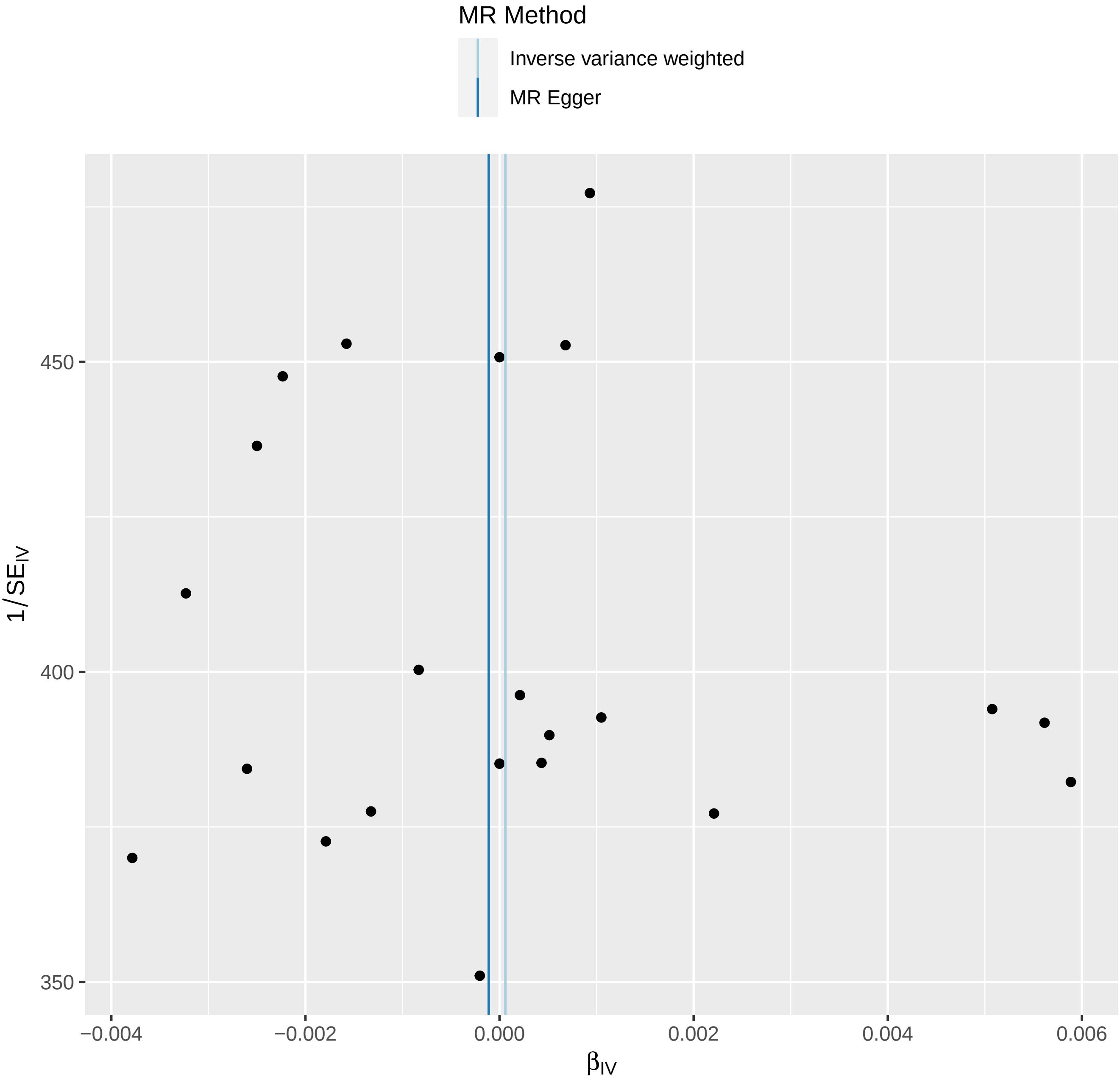

Batch 2−969 : Epidermal growth factor receptor substrate 15−like 1 on eGFR

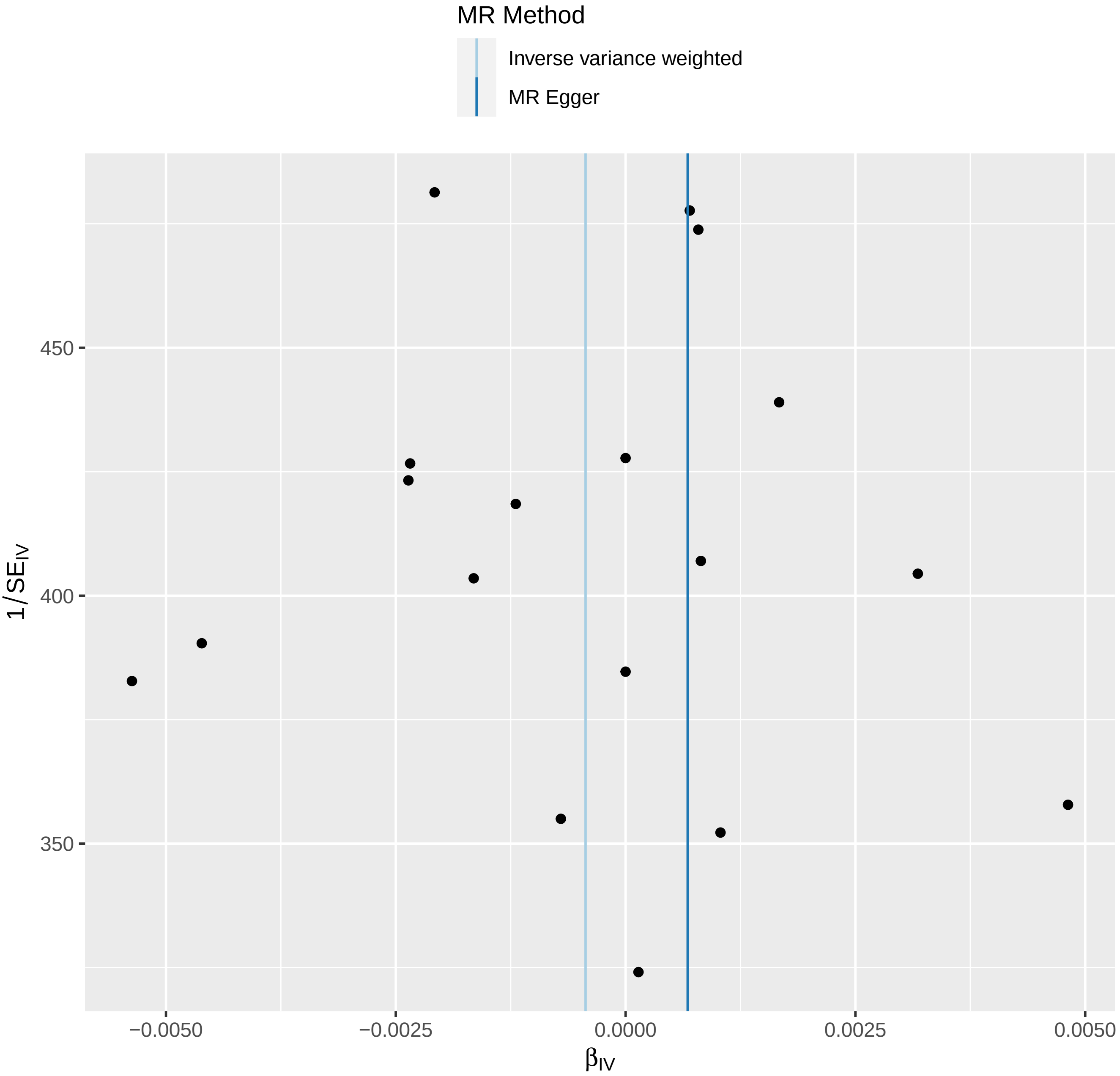

Batch 2−970 : Epiphycan on eGFR

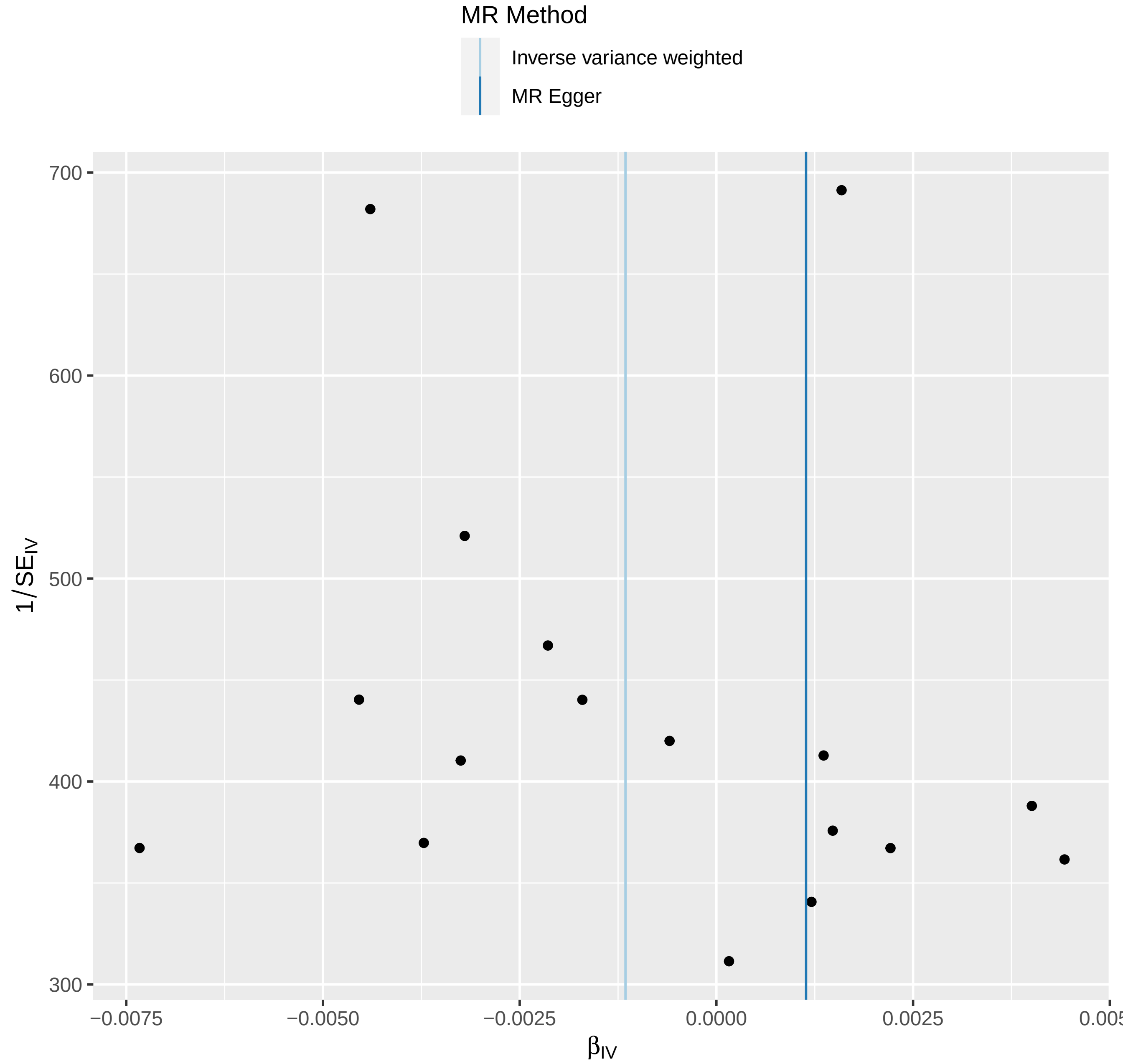

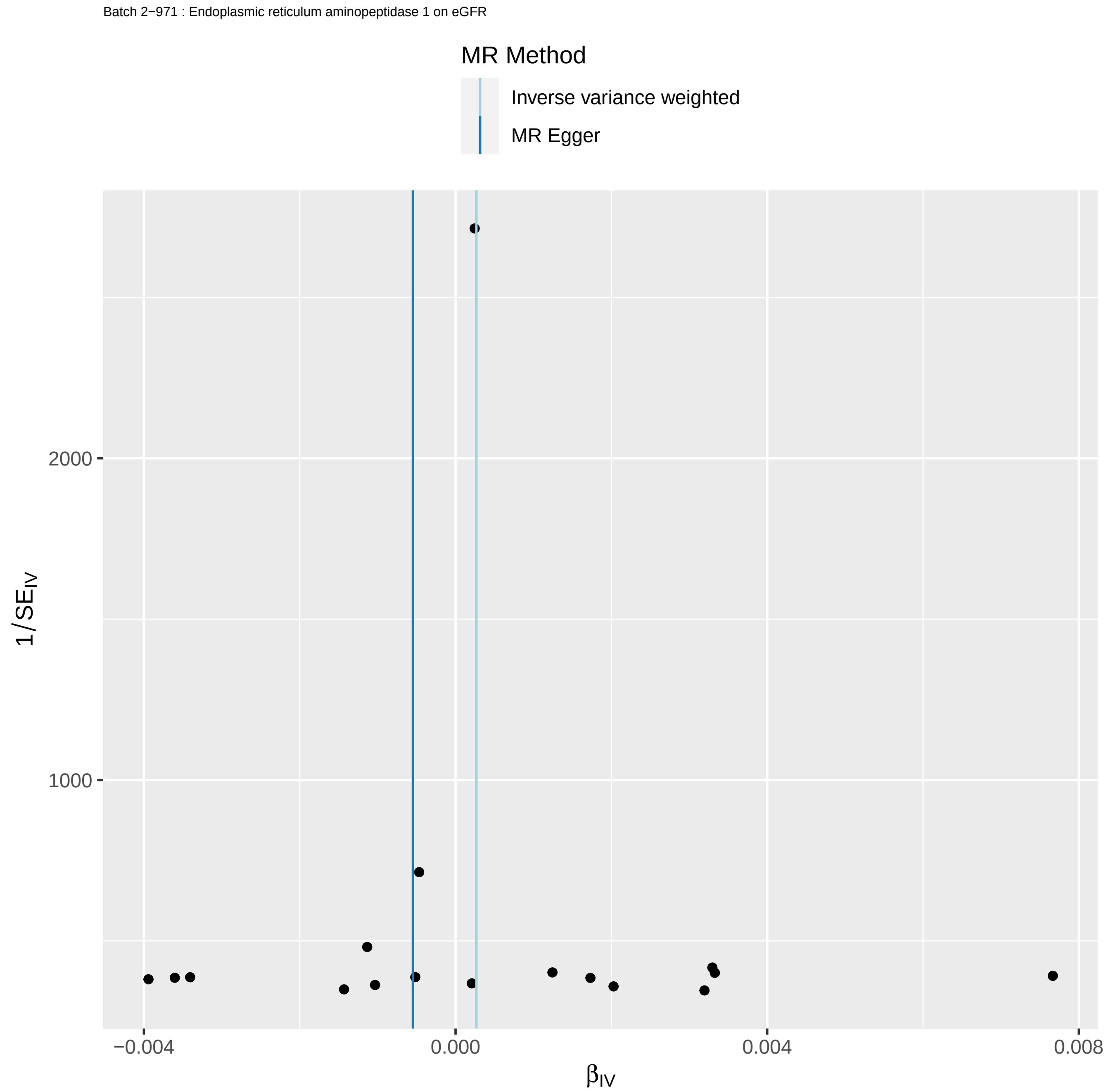
Batch 2−971 : Endoplasmic reticulum aminopeptidase 1 on eGFR
MR Method
Inverse variance weighted
MR Egger
2000
1000
1/SE_IV
−0.004
0.000
0.004
0.008
β_IV

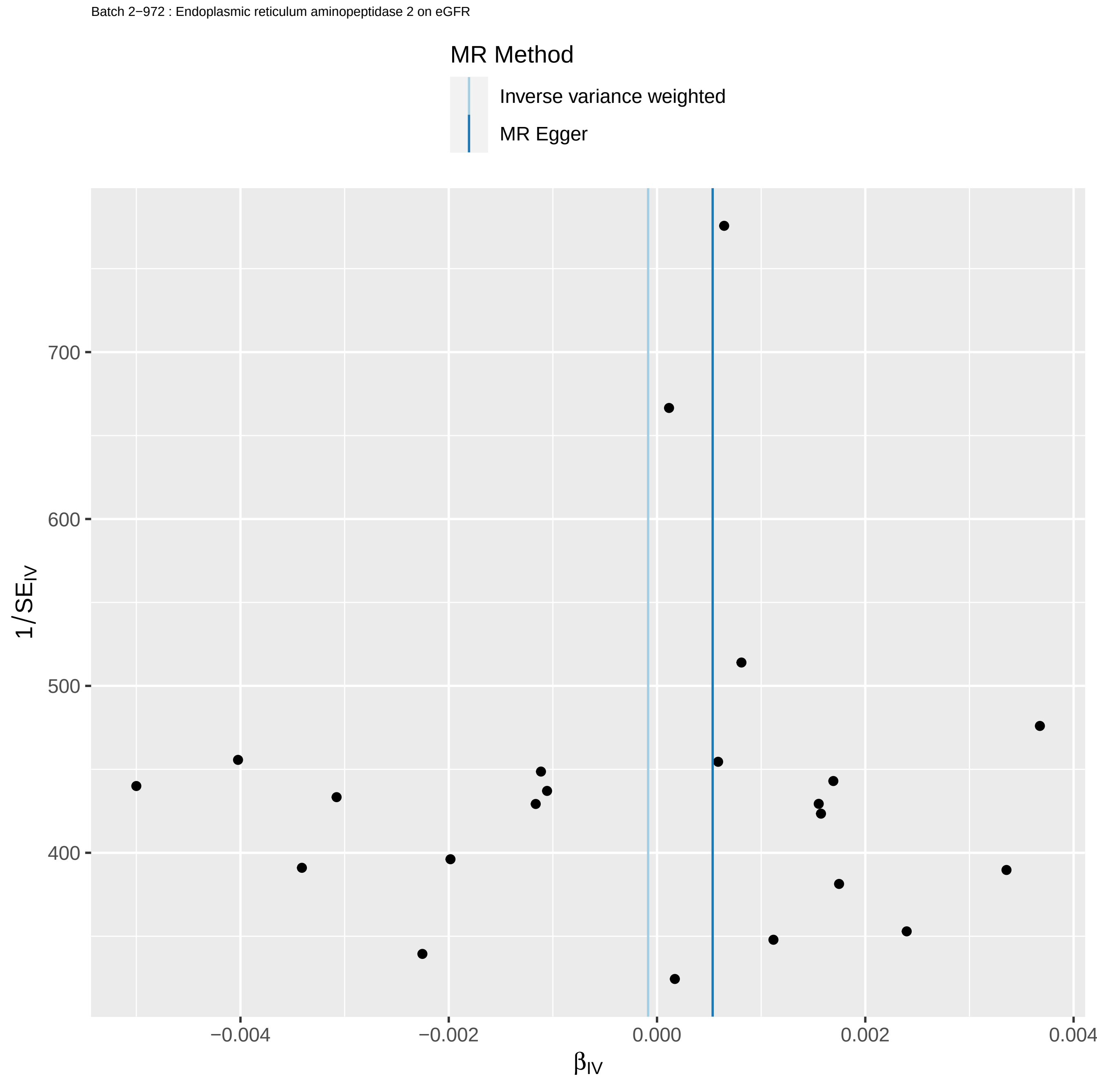

Batch 2−972 : Endoplasmic reticulum aminopeptidase 2 on eGFR
MR Method
Inverse variance weighted
MR Egger
700
600
500
400
1/SE_IV
−0.004
−0.002
0.000
0.002
0.004
β_IV

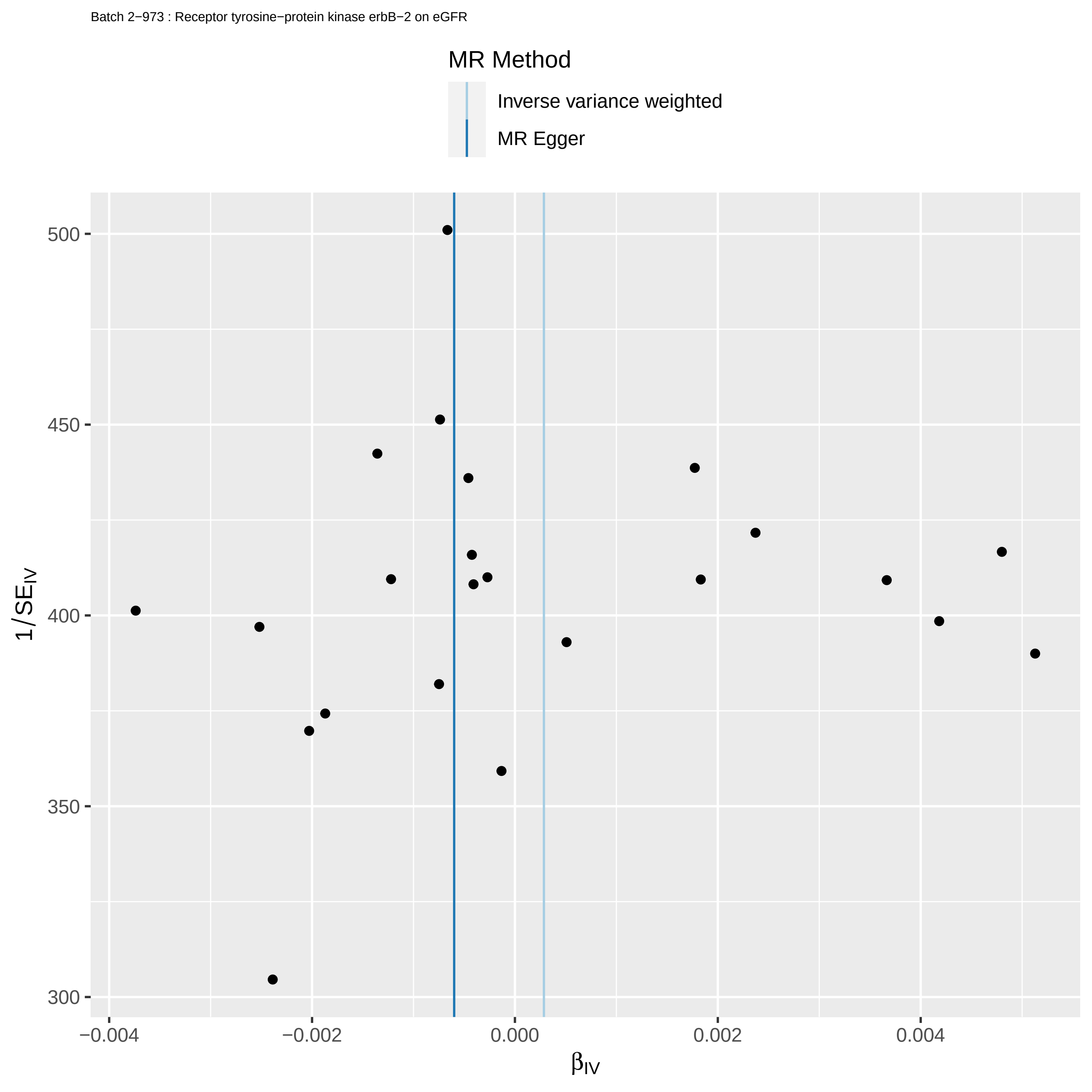

Batch 2−973 : Receptor tyrosine−protein kinase erbB−2 on eGFR
MR Method
Inverse variance weighted
MR Egger
1/SE_IV
β_IV
500
450
400
350
300
−0.004
−0.002
0.000
0.002
0.004

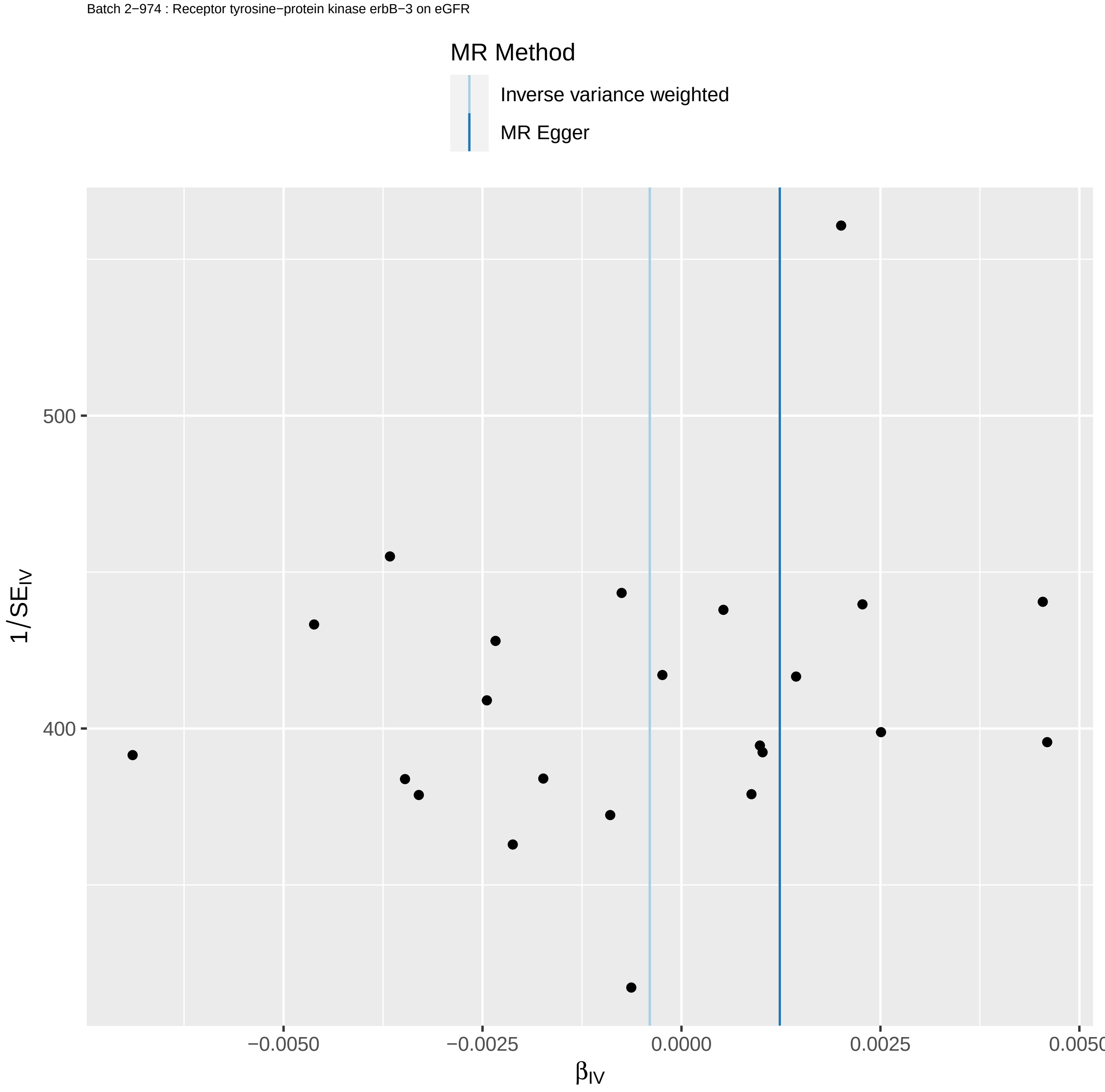

Batch 2−974 : Receptor tyrosine−protein kinase erbB−3 on eGFR
MR Method
Inverse variance weighted
MR Egger
500
400
1/SE$_{IV}$
−0.0050
−0.0025
0.0000
0.0025
0.0050
β$_{IV}$

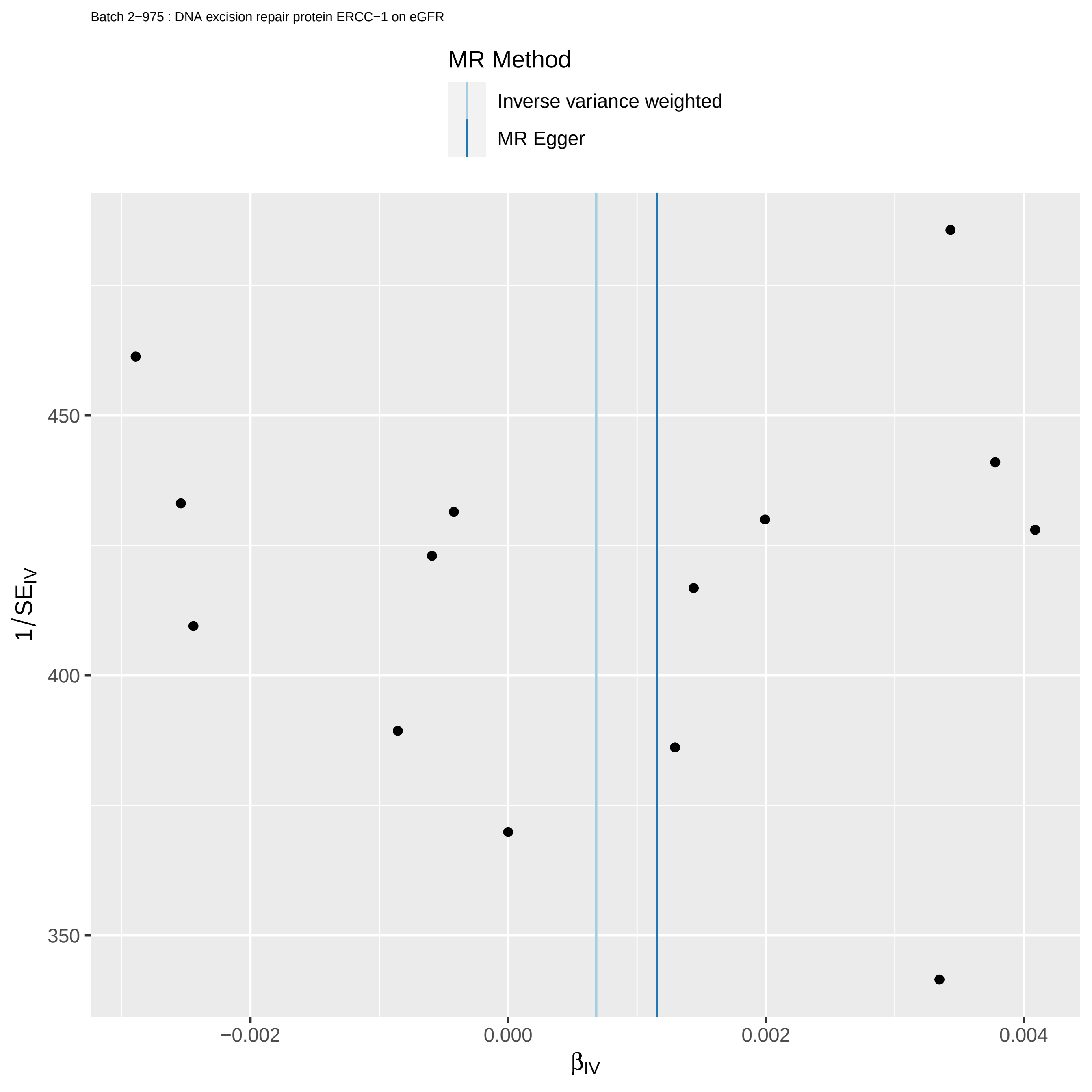

Batch 2−975 : DNA excision repair protein ERCC−1 on eGFR
MR Method
Inverse variance weighted
MR Egger
450
400
350
1/SE_IV
−0.002
0.000
0.002
0.004
β_IV

Batch 2−976 : DNA repair endonuclease XPF on eGFR

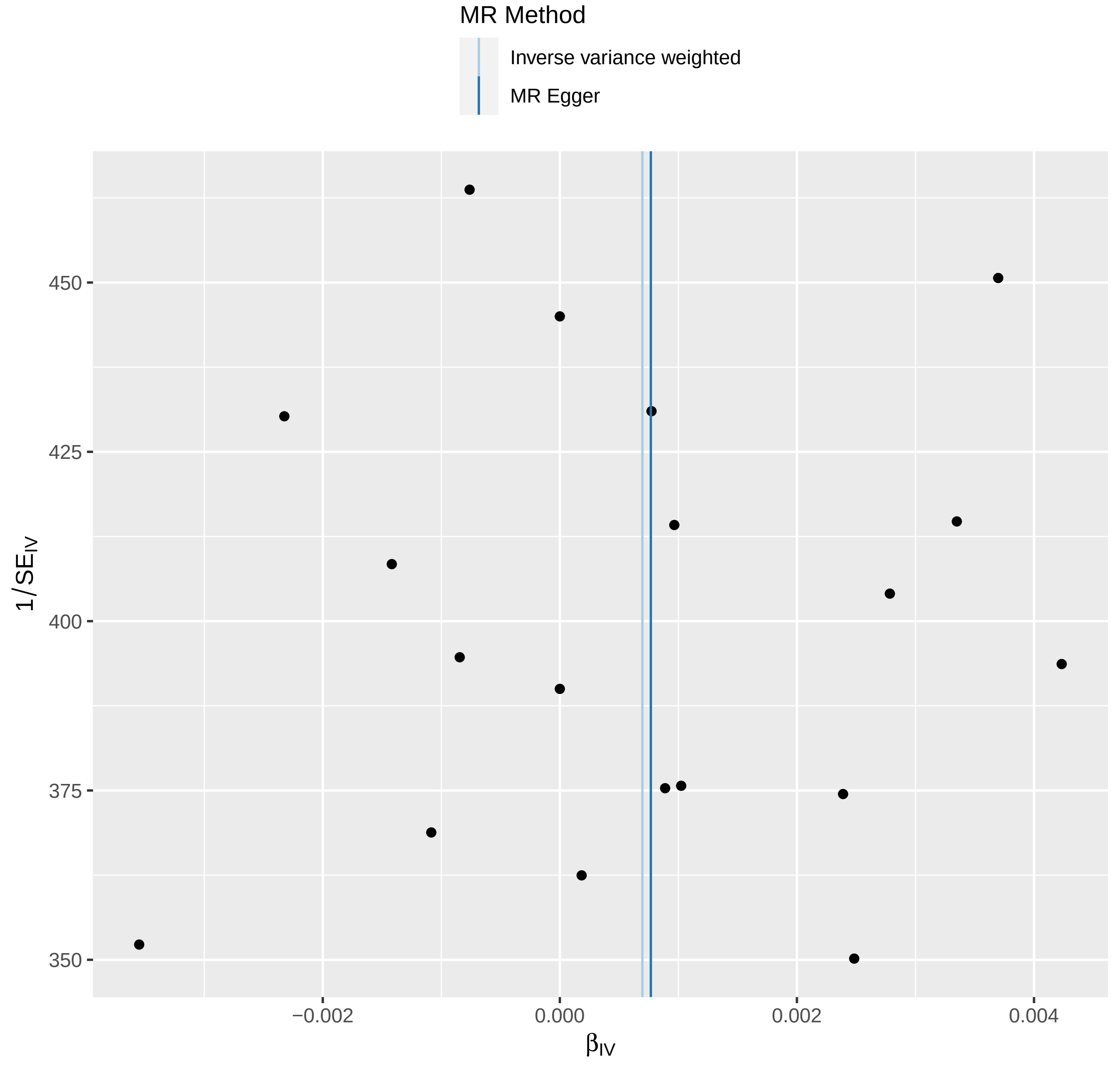

Batch 2−977 : Epiregulin on eGFR

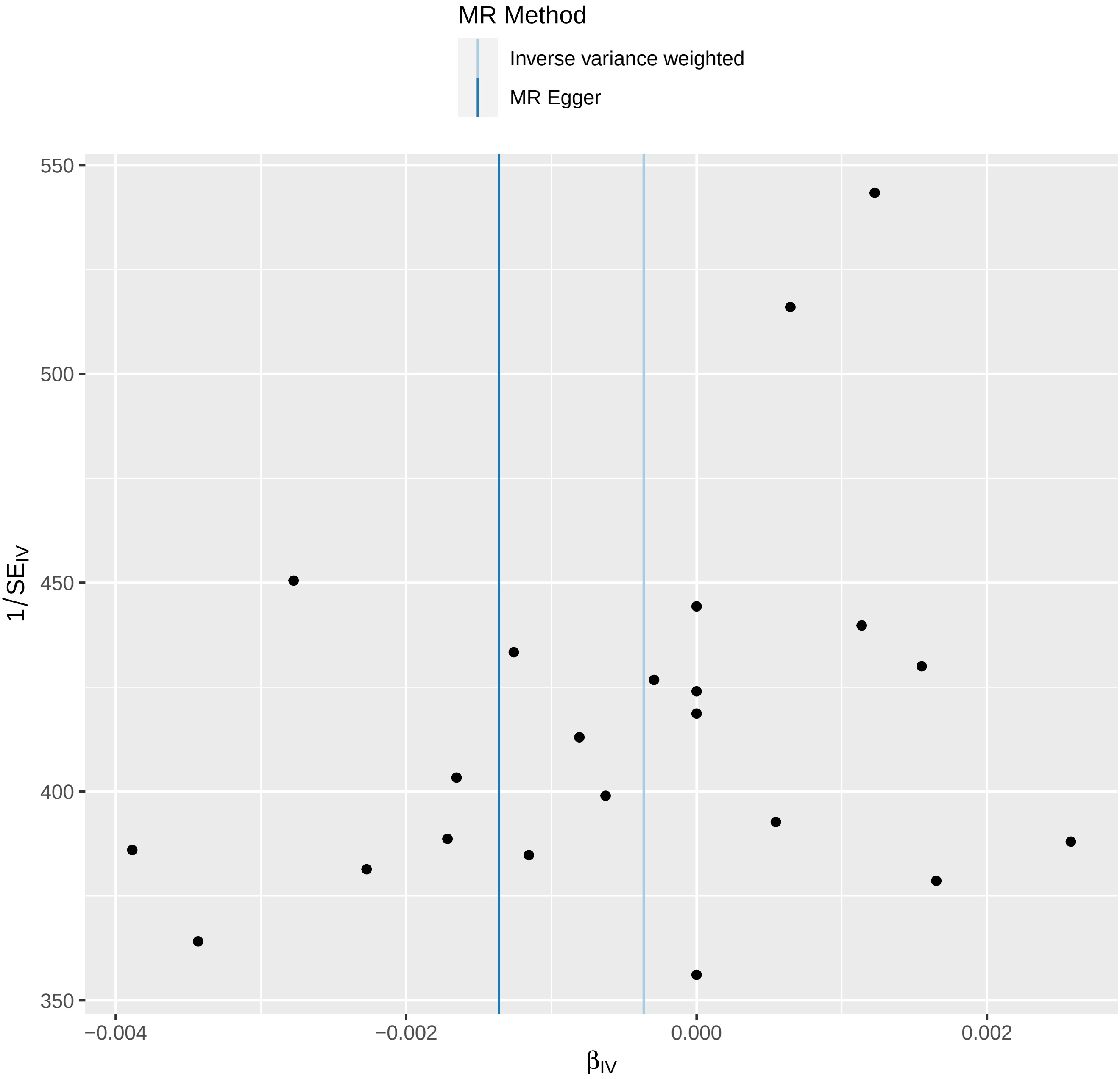

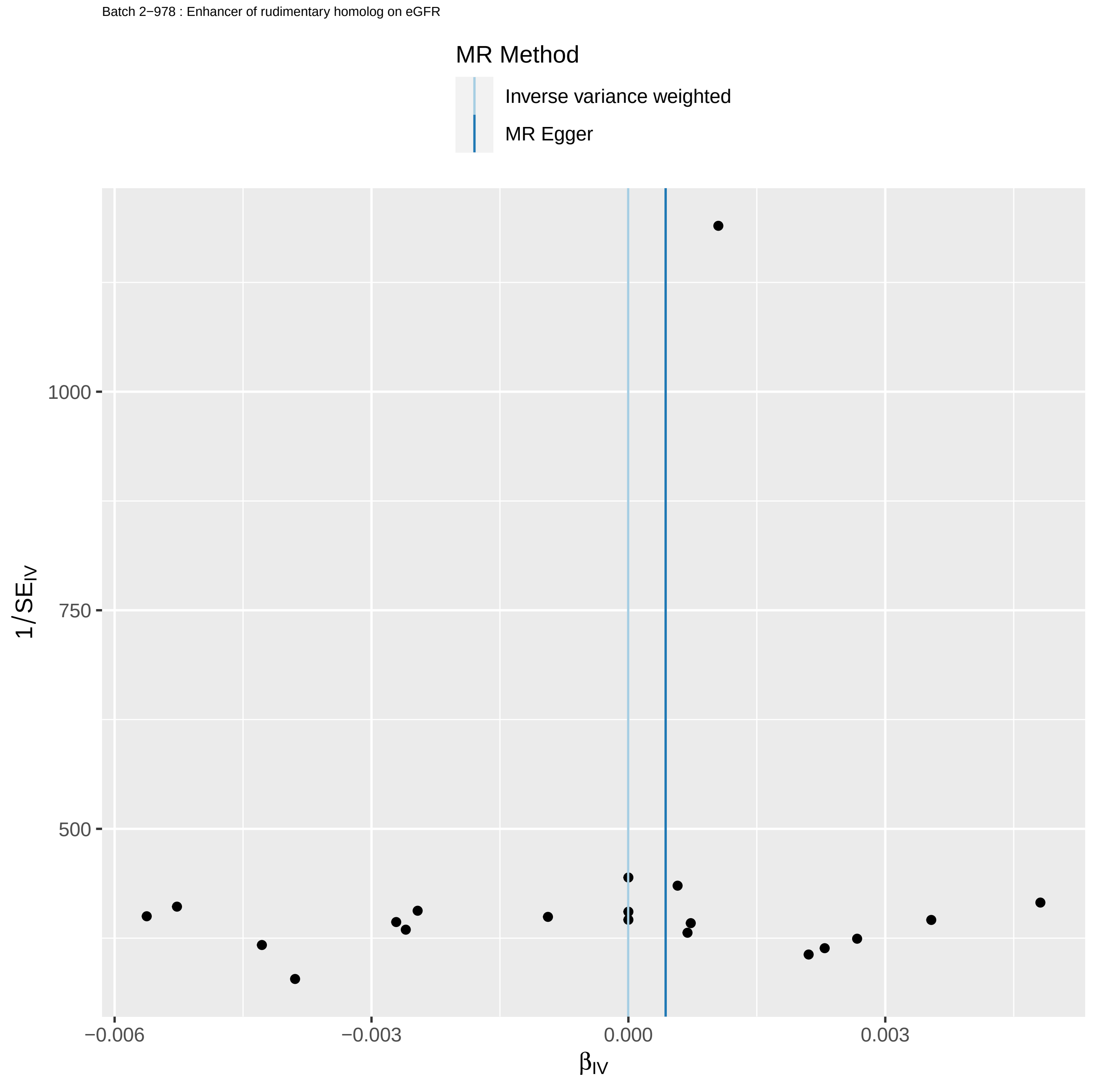
Batch 2−978 : Enhancer of rudimentary homolog on eGFR
MR Method
Inverse variance weighted
MR Egger
1000
750
500
1/SE$_{IV}$
−0.006
−0.003
0.000
0.003
β$_{IV}$

Batch 2−979 : Endoplasmic reticulum lectin 1 on eGFR

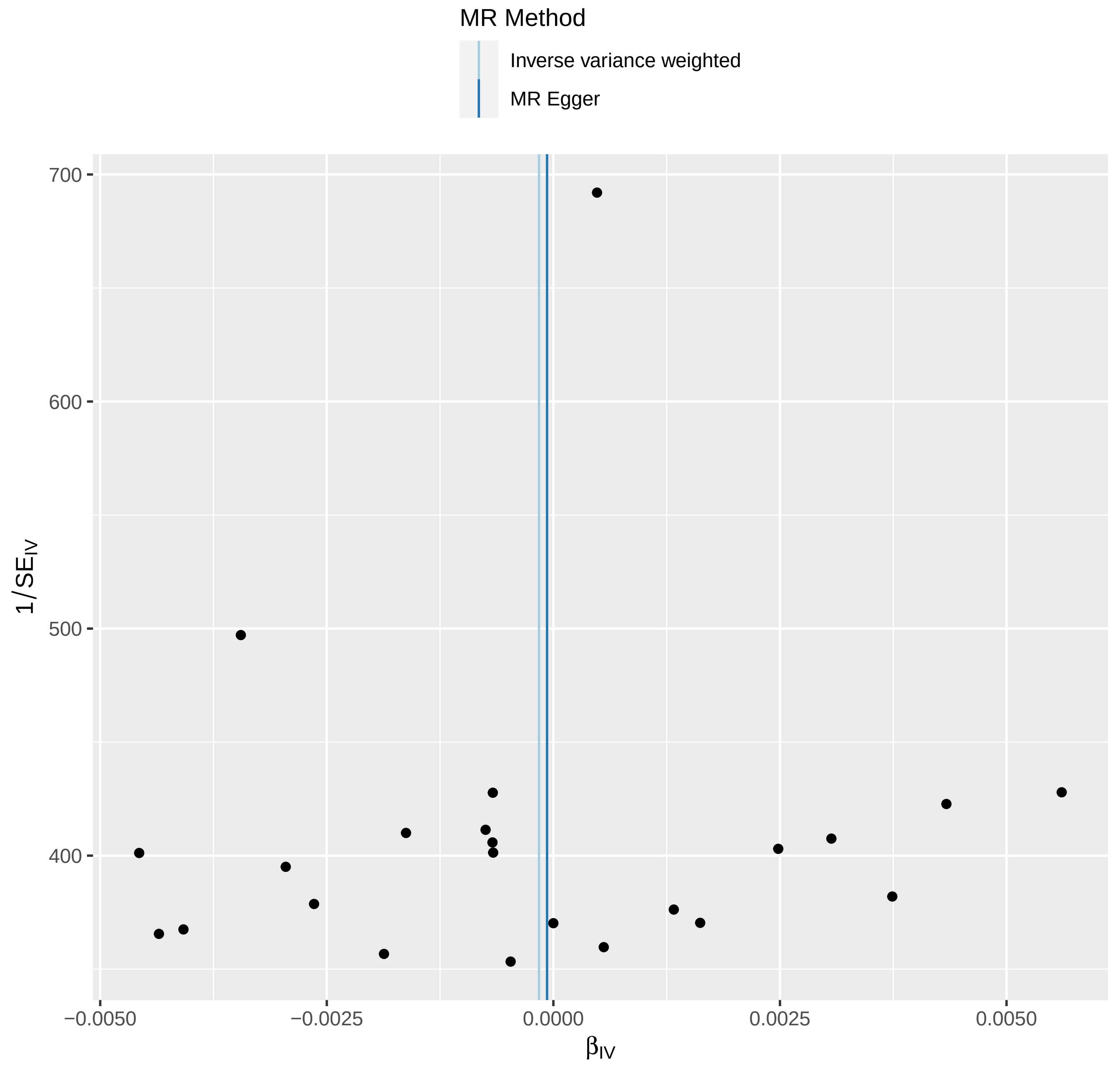

Batch 2−980 : Erlin−1 on eGFR

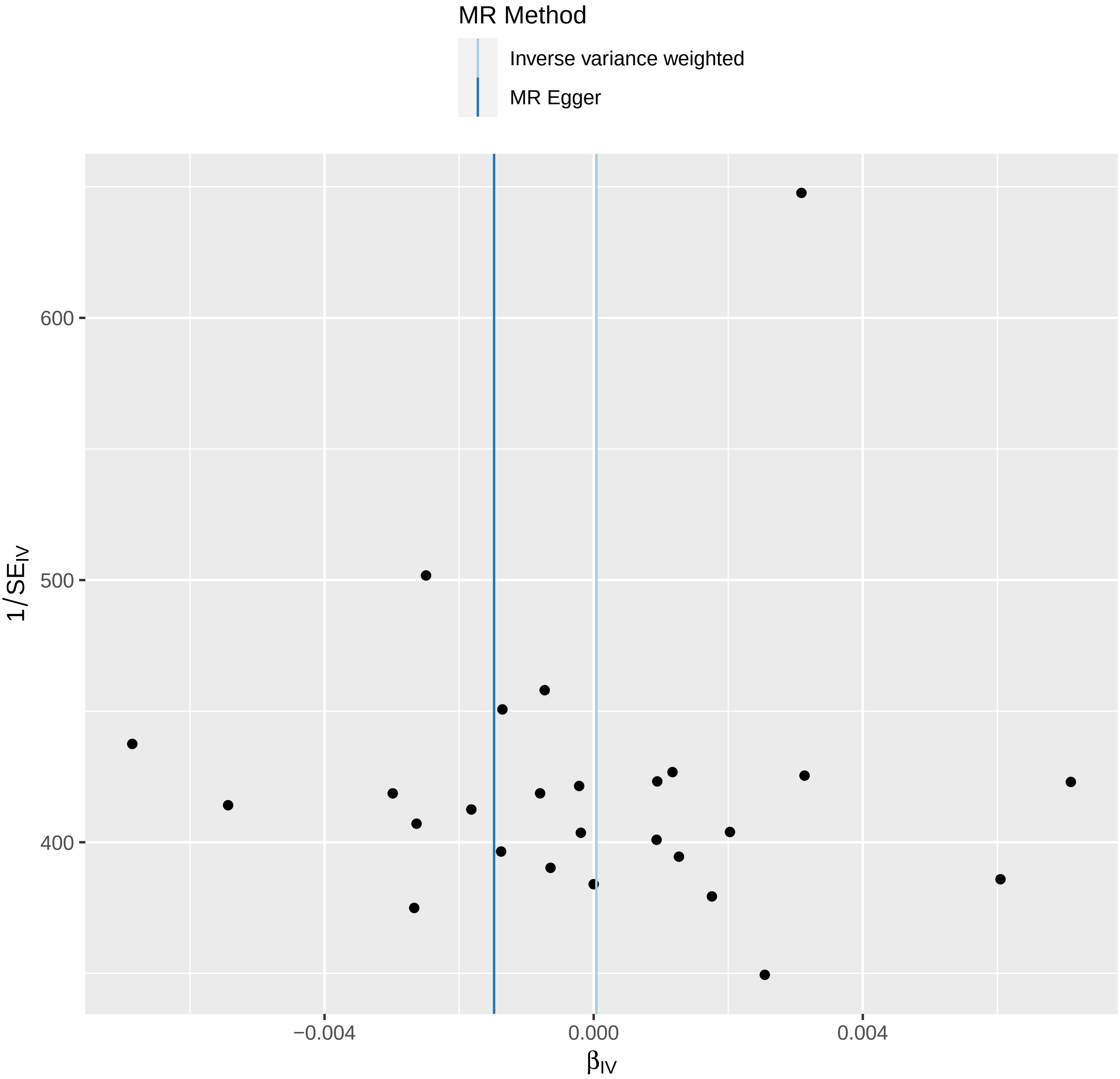

Batch 2−981 : Erythroid membrane−associated protein on eGFR

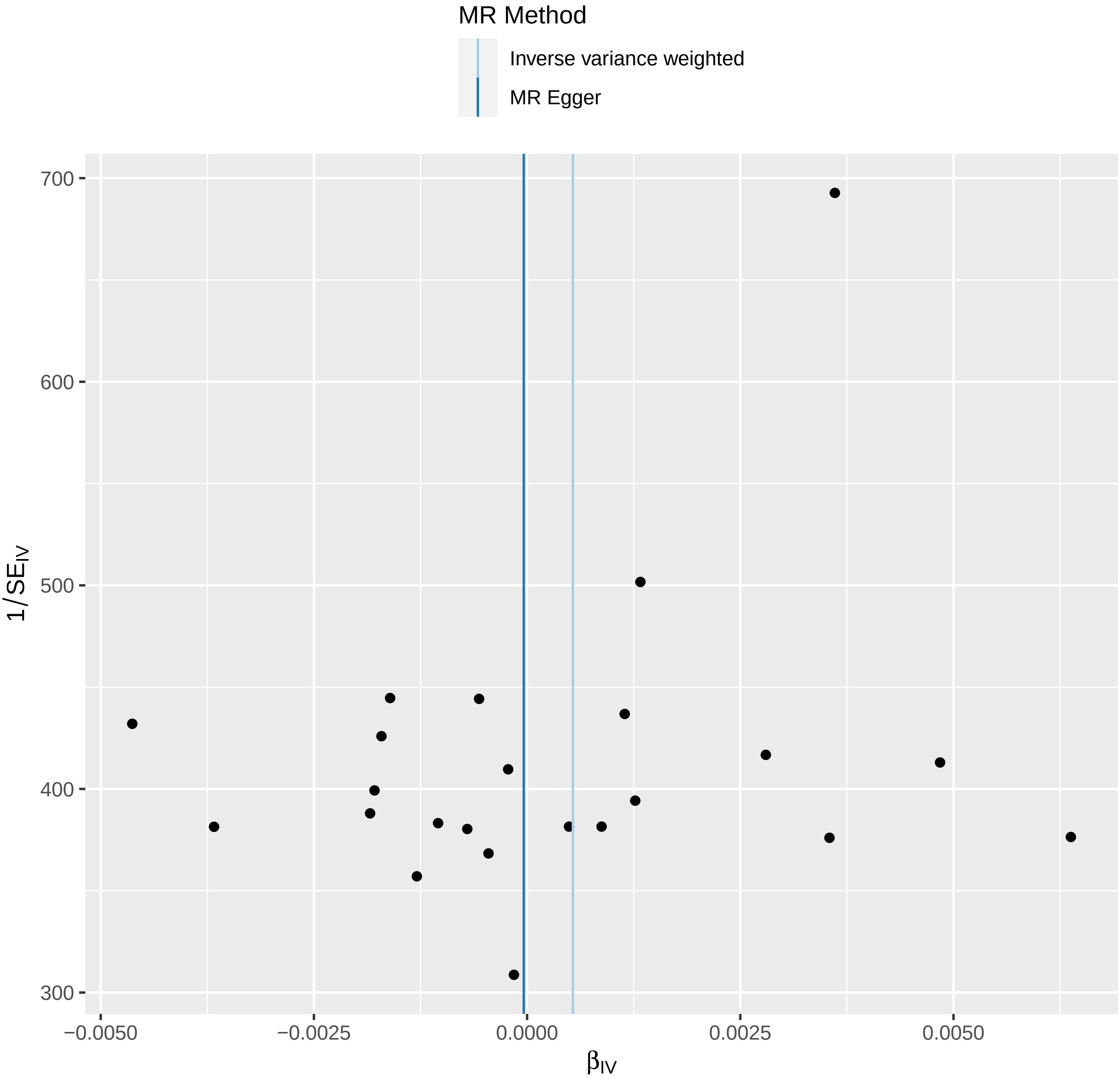

Batch 2−982 : ERO1−like protein alpha on eGFR

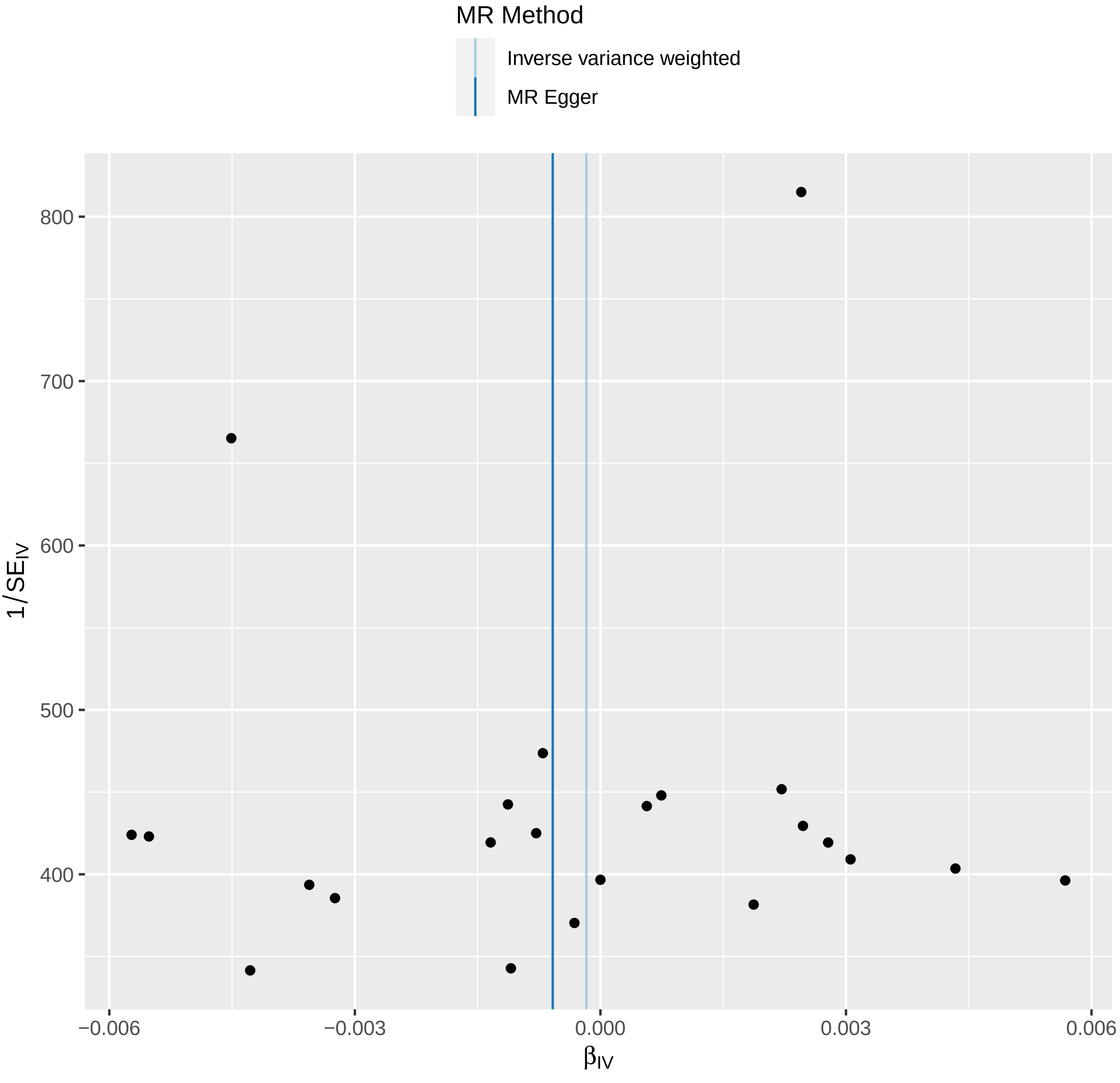

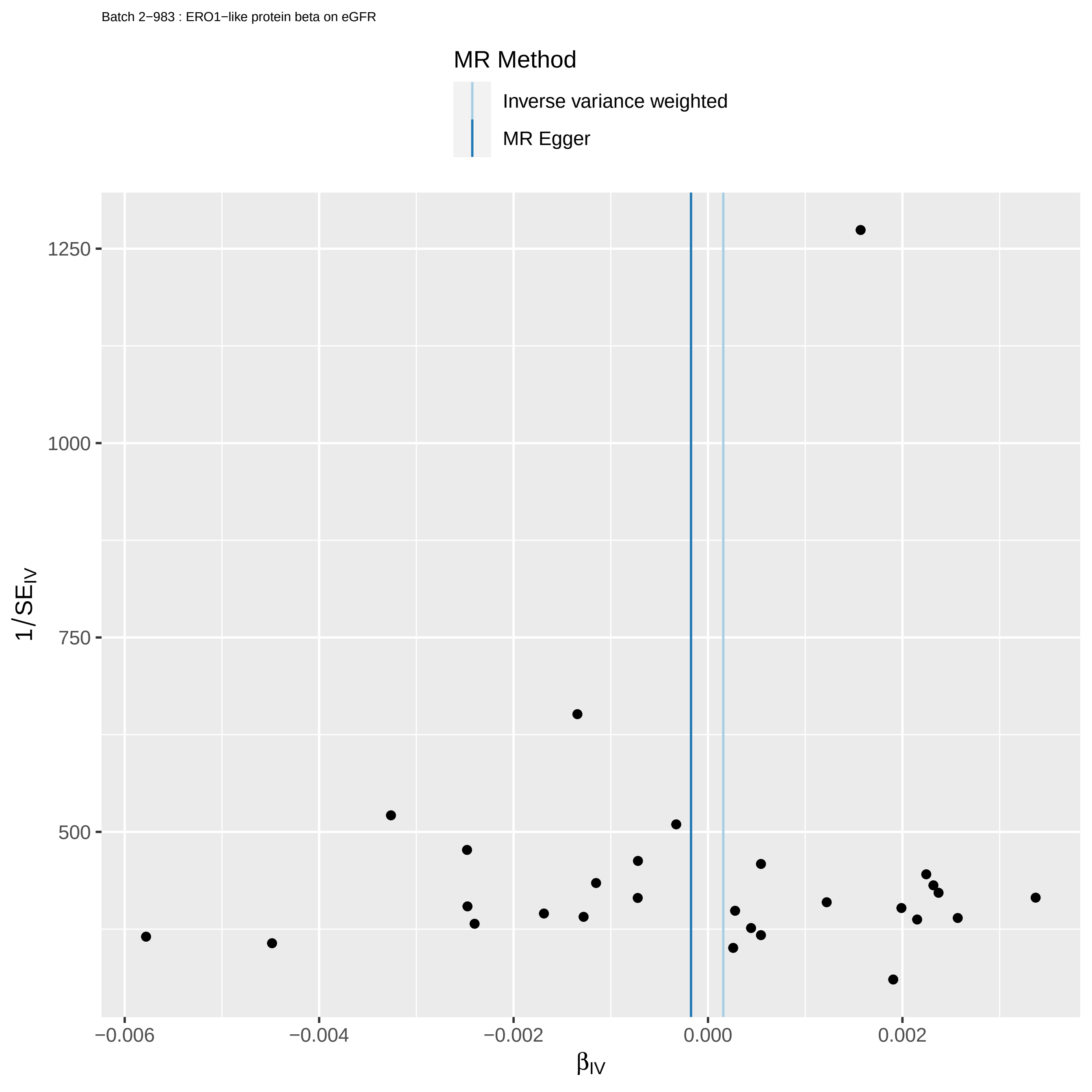
Batch 2−983 : ERO1−like protein beta on eGFR
MR Method
Inverse variance weighted
MR Egger
1/SE_IV
1250
1000
750
500
−0.006
−0.004
−0.002
0.000
0.002
β_IV

Batch 2−984 : Endoplasmic reticulum resident protein 29 on eGFR

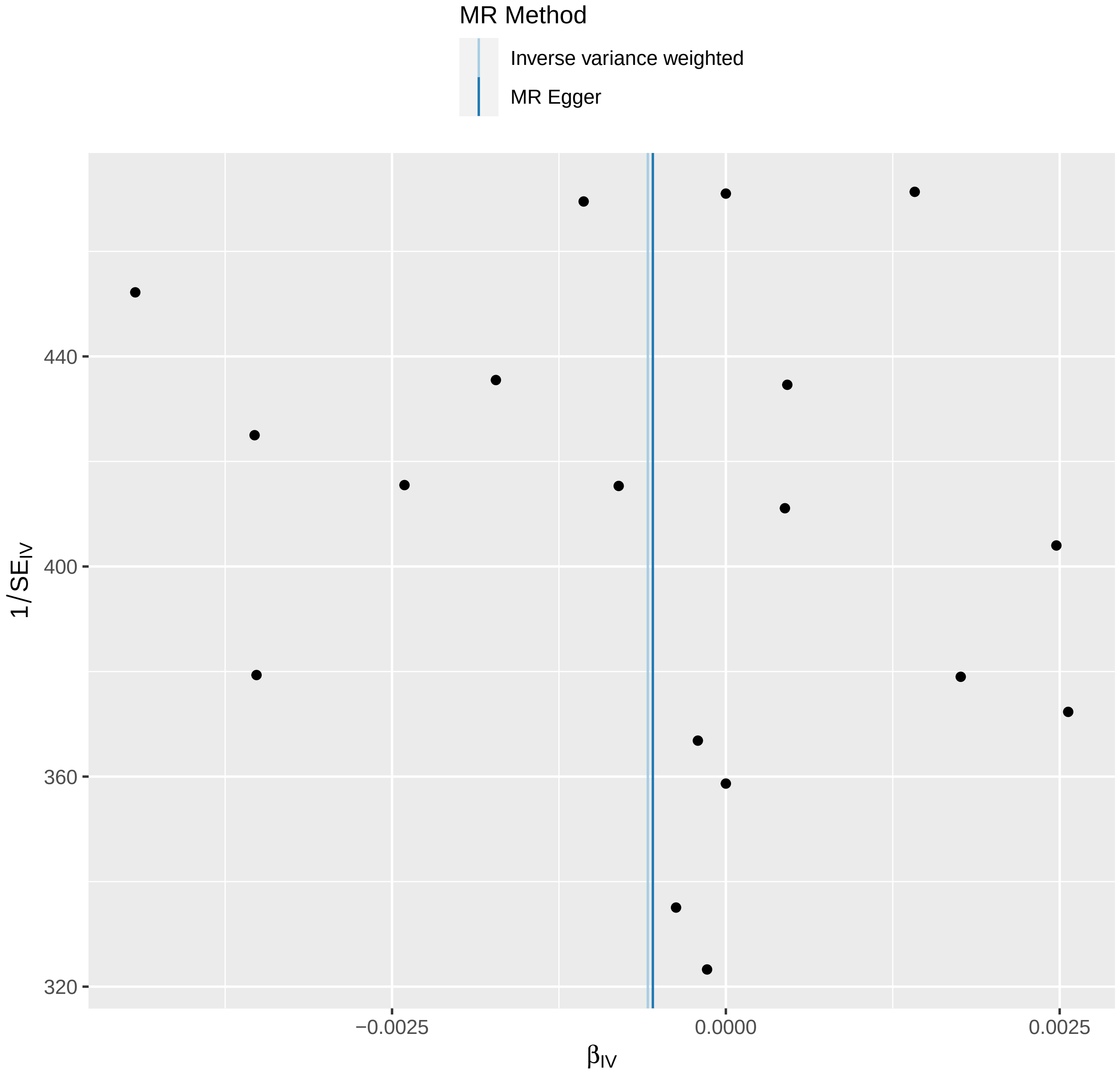

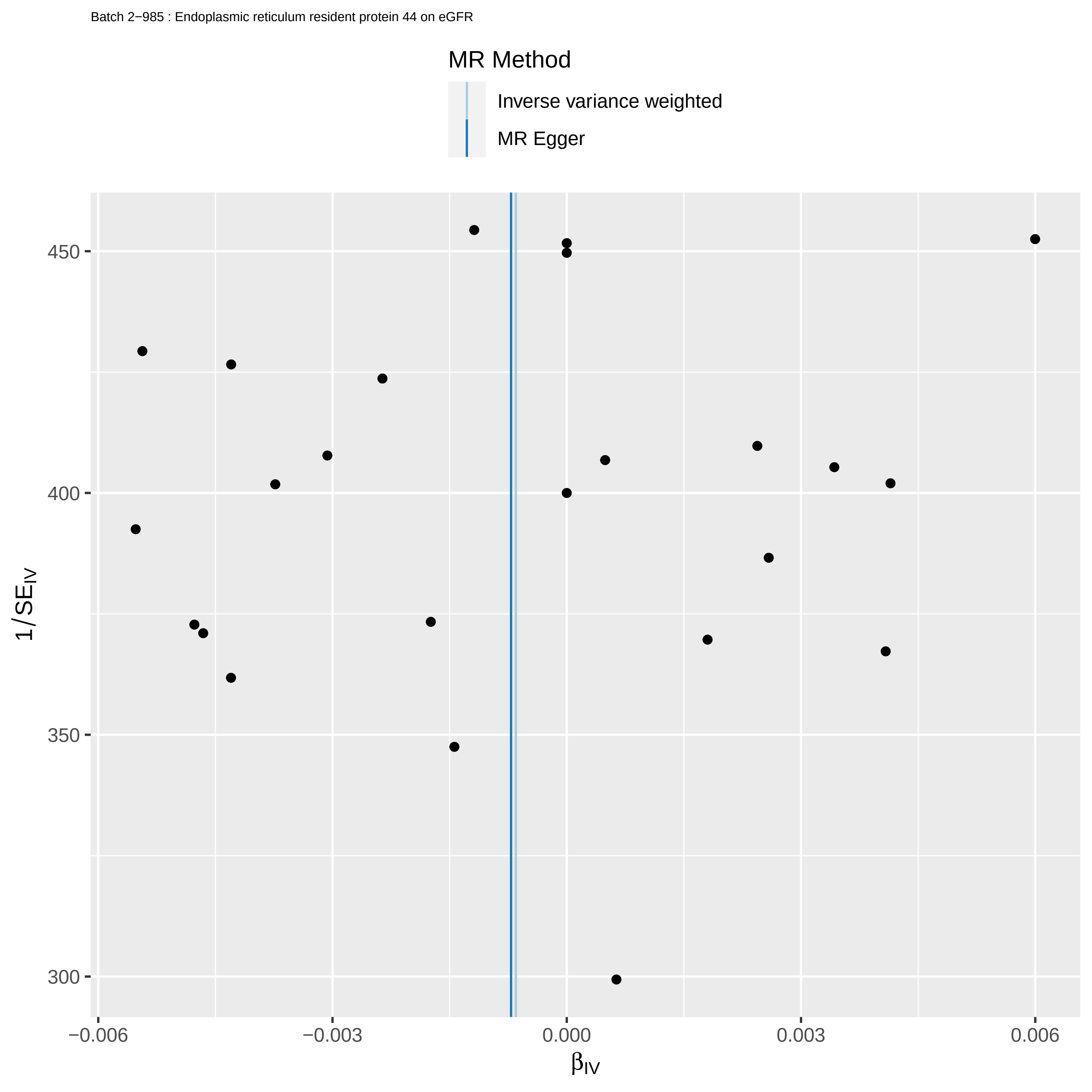

Batch 2−985 : Endoplasmic reticulum resident protein 44 on eGFR
MR Method
Inverse variance weighted
MR Egger
450
400
350
300
1/SE_IV
−0.006
−0.003
0.000
0.003
0.006
β_IV

Batch 2−986 : Endogenous retrovirus group V member 1 Env polyprotein on eGFR

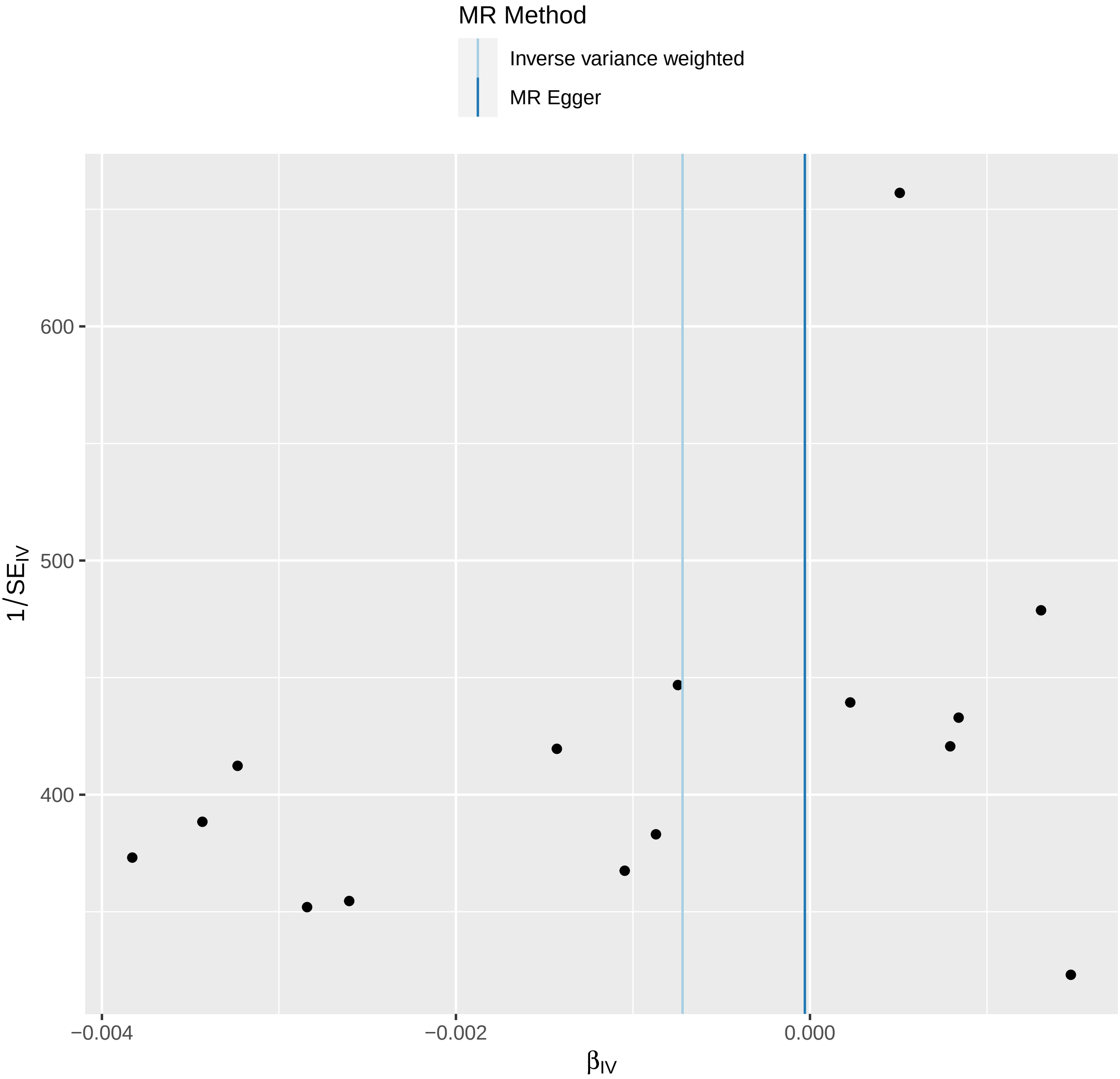

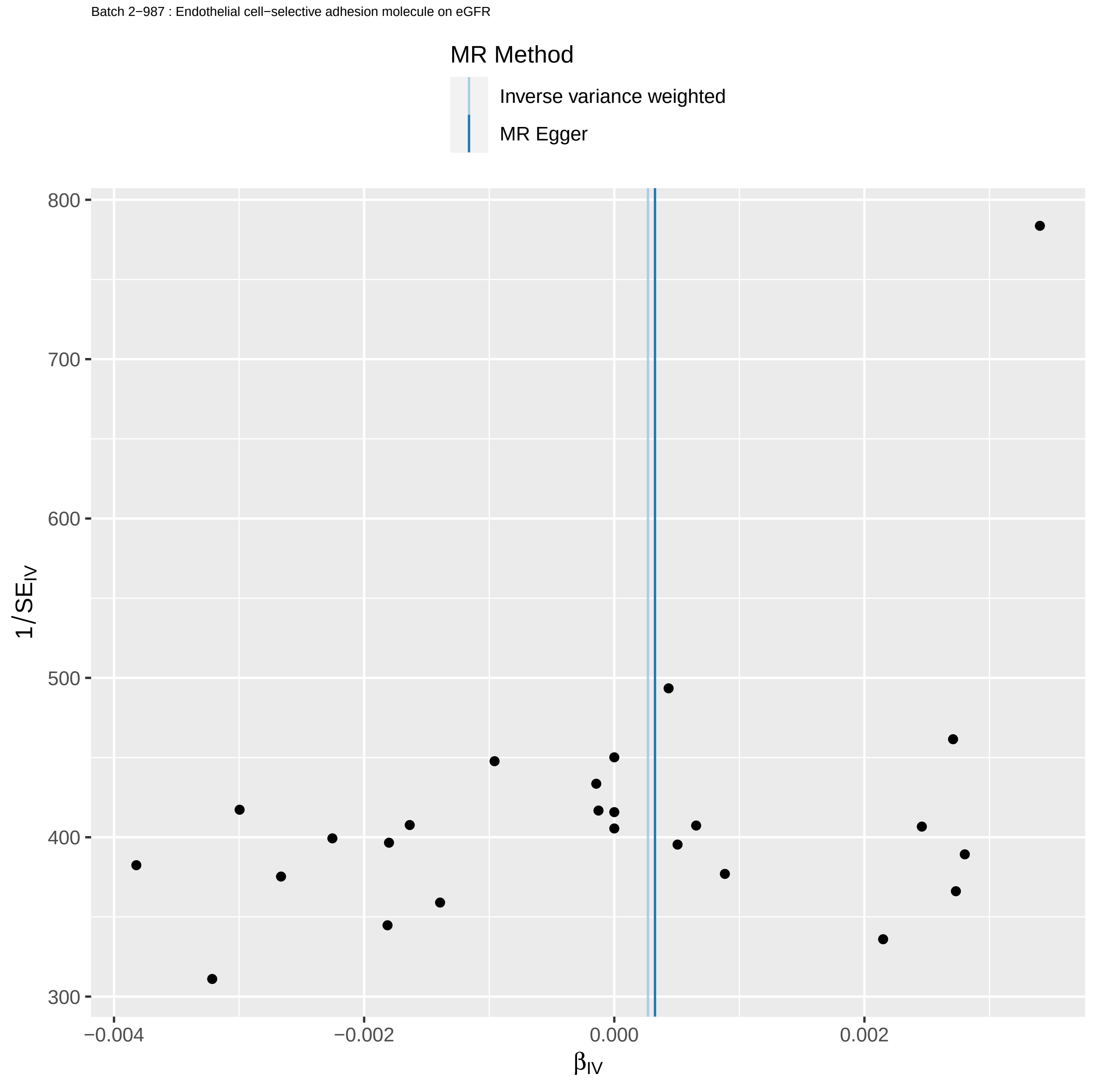
Batch 2−987 : Endothelial cell−selective adhesion molecule on eGFR
MR Method
Inverse variance weighted
MR Egger
800
700
600
500
400
300
1/SE_IV
−0.004
−0.002
0.000
0.002
β_IV

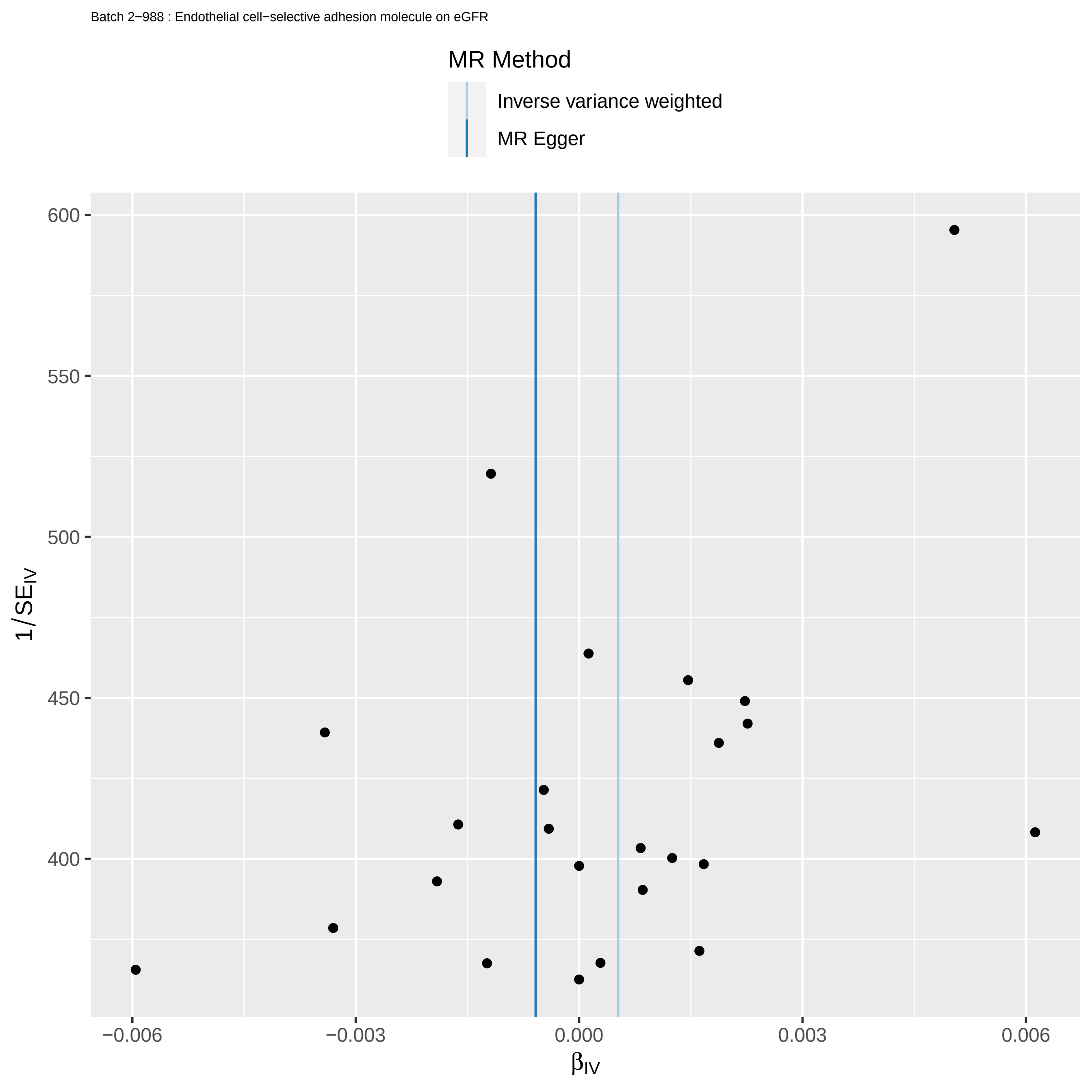

Batch 2−988 : Endothelial cell−selective adhesion molecule on eGFR
MR Method
Inverse variance weighted
MR Egger
600
550
500
450
400
1/SE_IV
−0.006
−0.003
0.000
0.003
0.006
β_IV

Batch 2−989 : S−formylglutathione hydrolase on eGFR

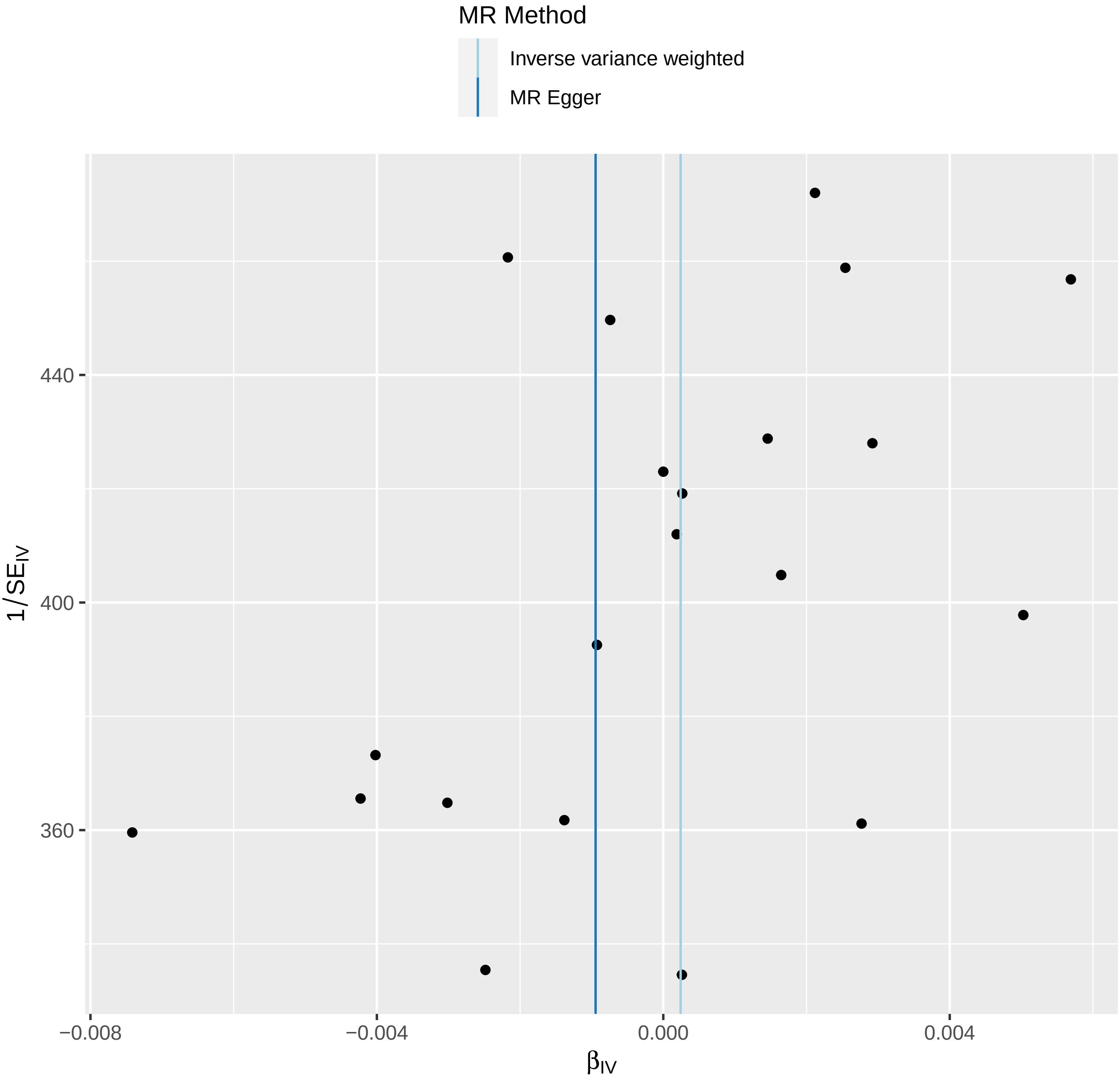

Batch 2−990 : Endothelial cell−specific molecule 1 on eGFR

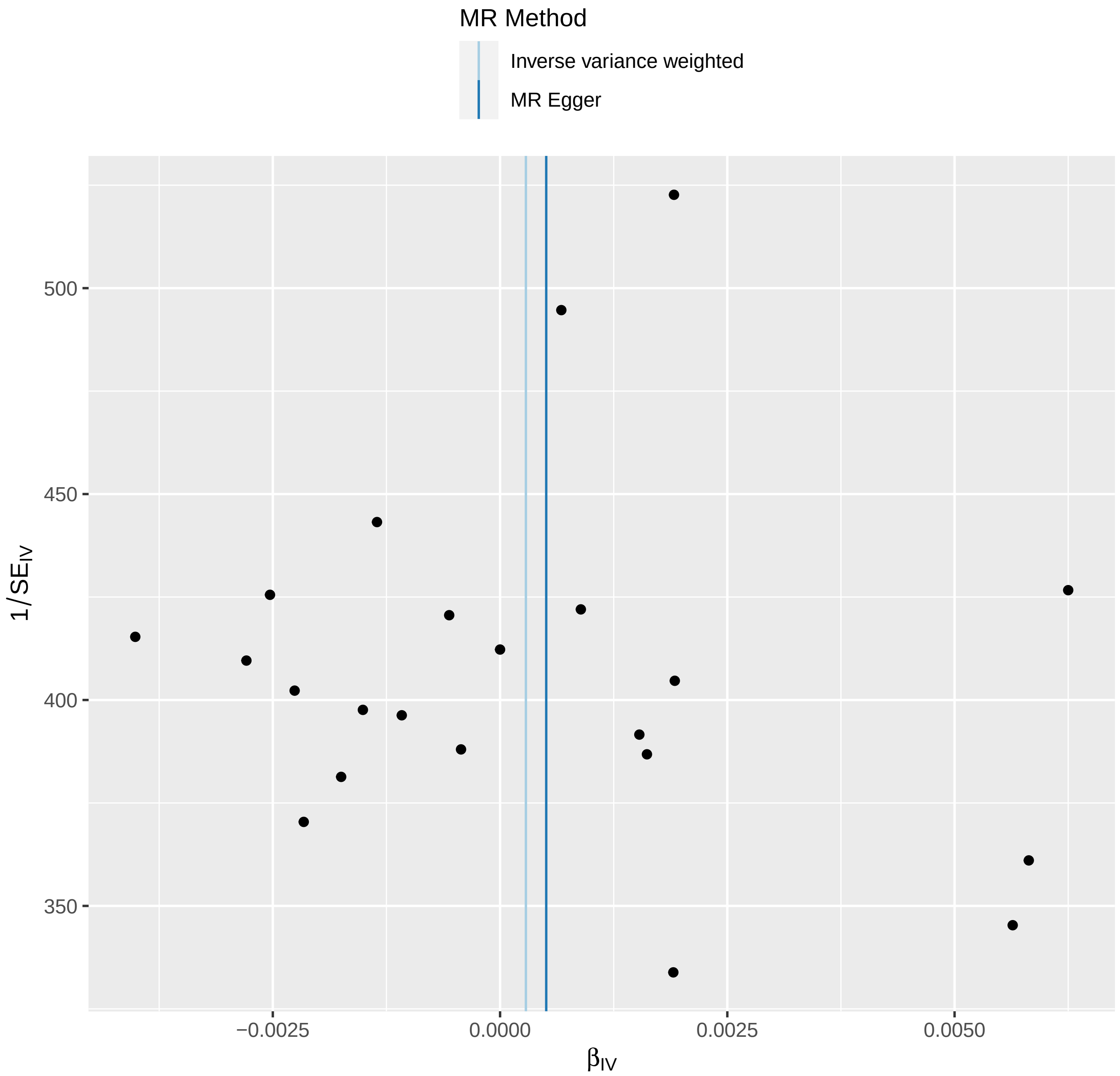

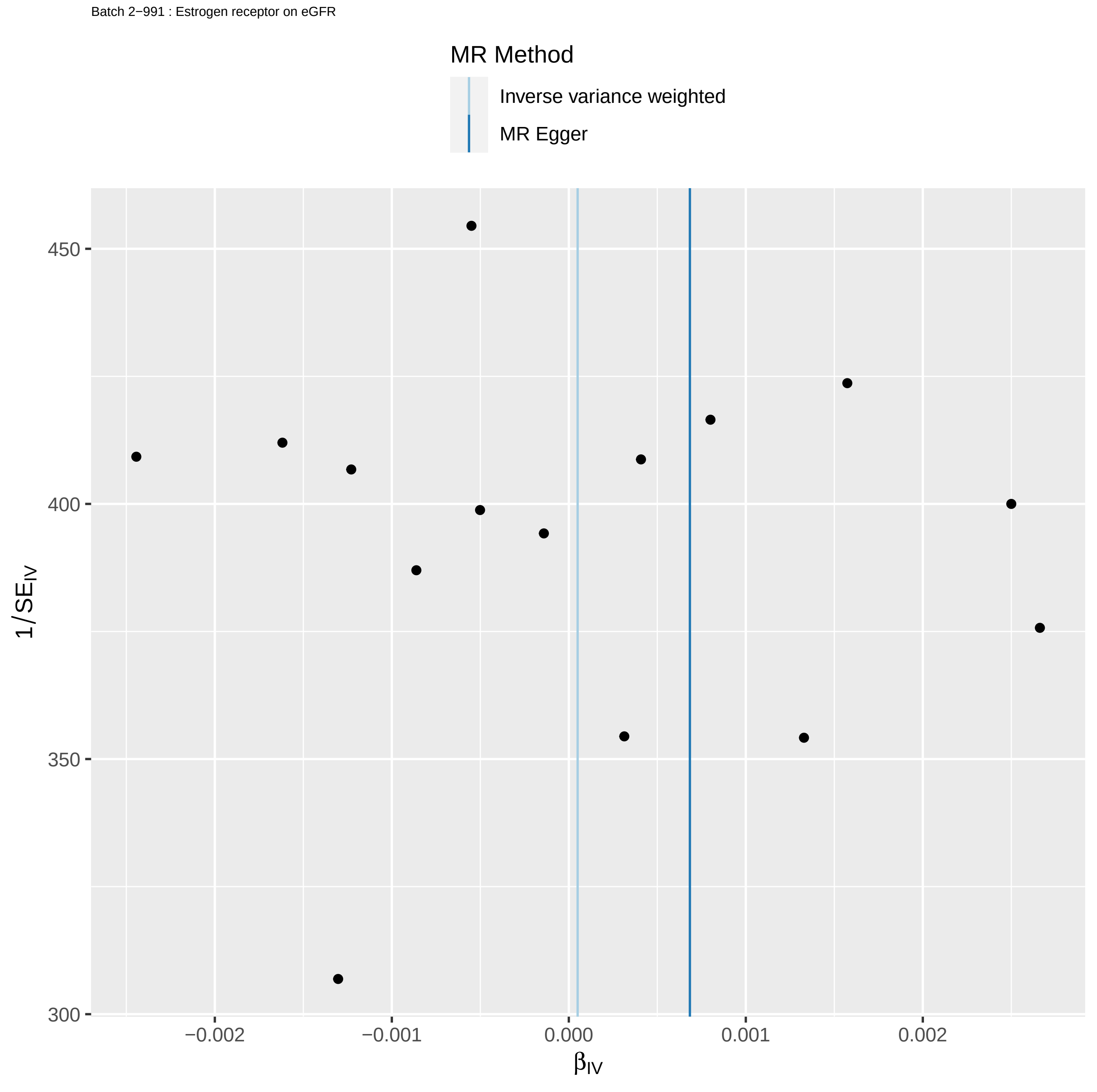
Batch 2−991 : Estrogen receptor on eGFR
MR Method
Inverse variance weighted
MR Egger
450
400
350
300
1/SE_IV
−0.002
−0.001
0.000
0.001
0.002
β_IV

Batch 2−992 : Persulfide dioxygenase ETHE1, mitochondrial on eGFR

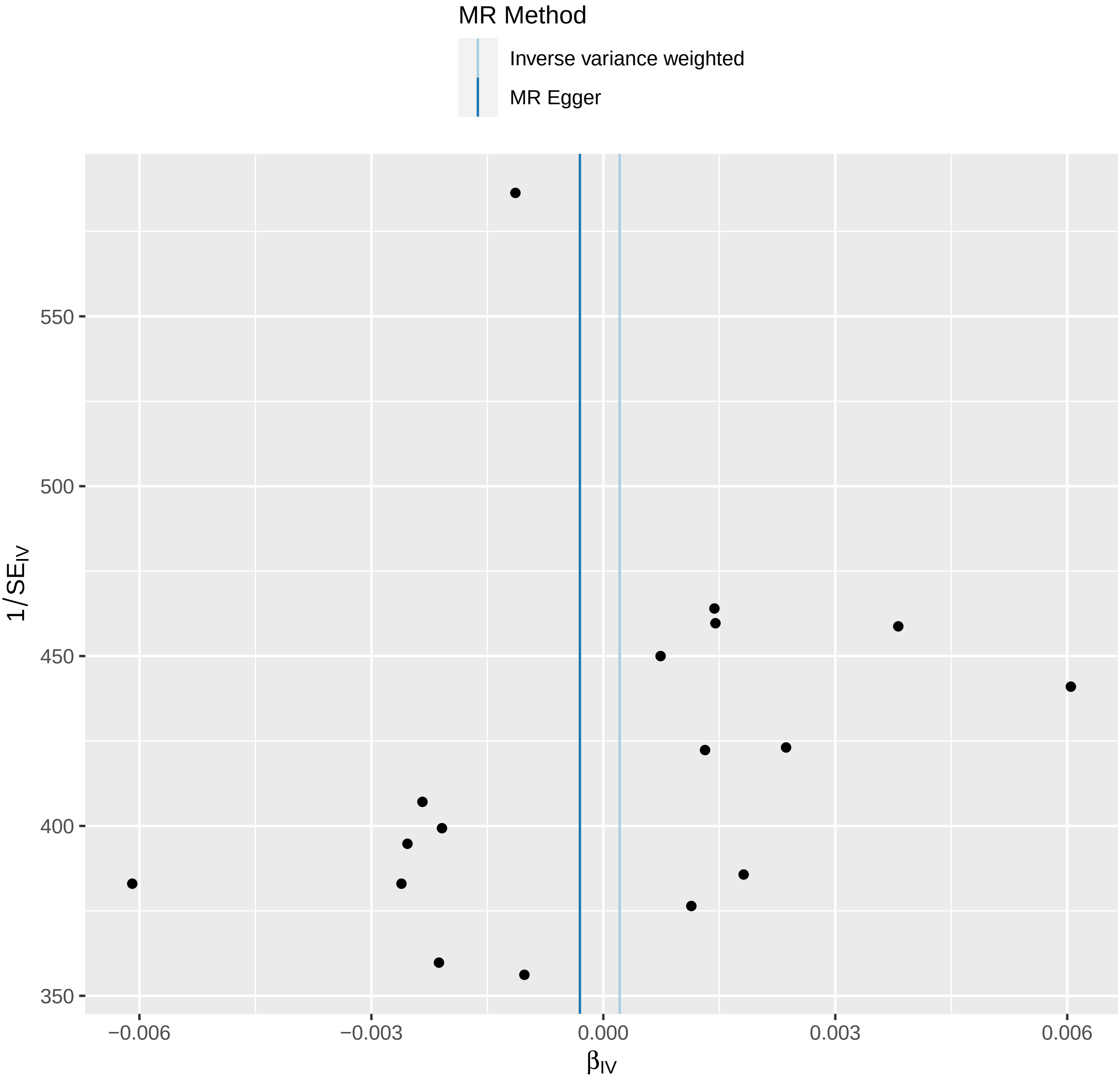

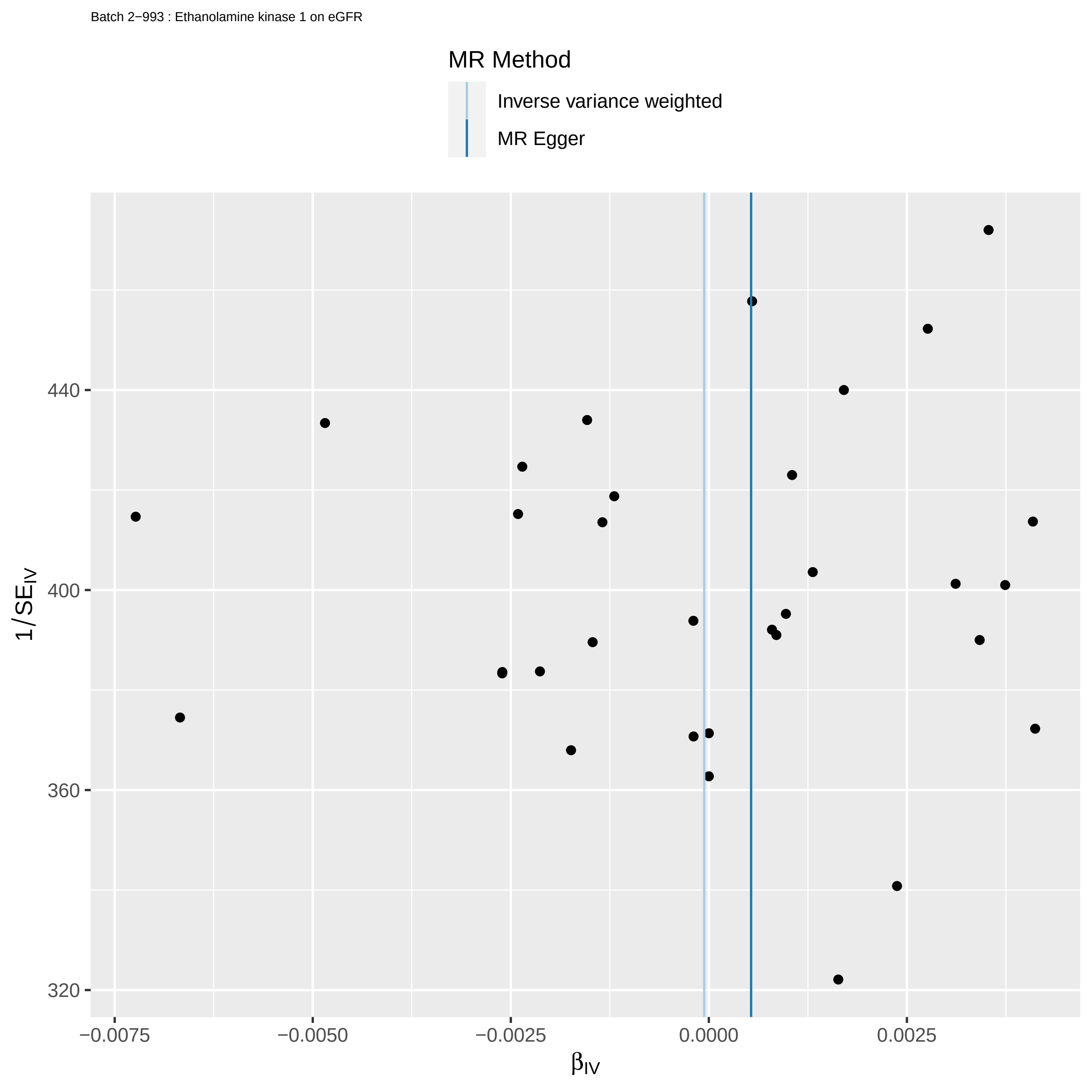

Batch 2−993 : Ethanolamine kinase 1 on eGFR
MR Method
Inverse variance weighted
MR Egger
1/SE$_{IV}$
440
400
360
320
−0.0075
−0.0050
−0.0025
0.0000
0.0025
β$_{IV}$

Batch 2−994 : Protein C−ets−2 on eGFR

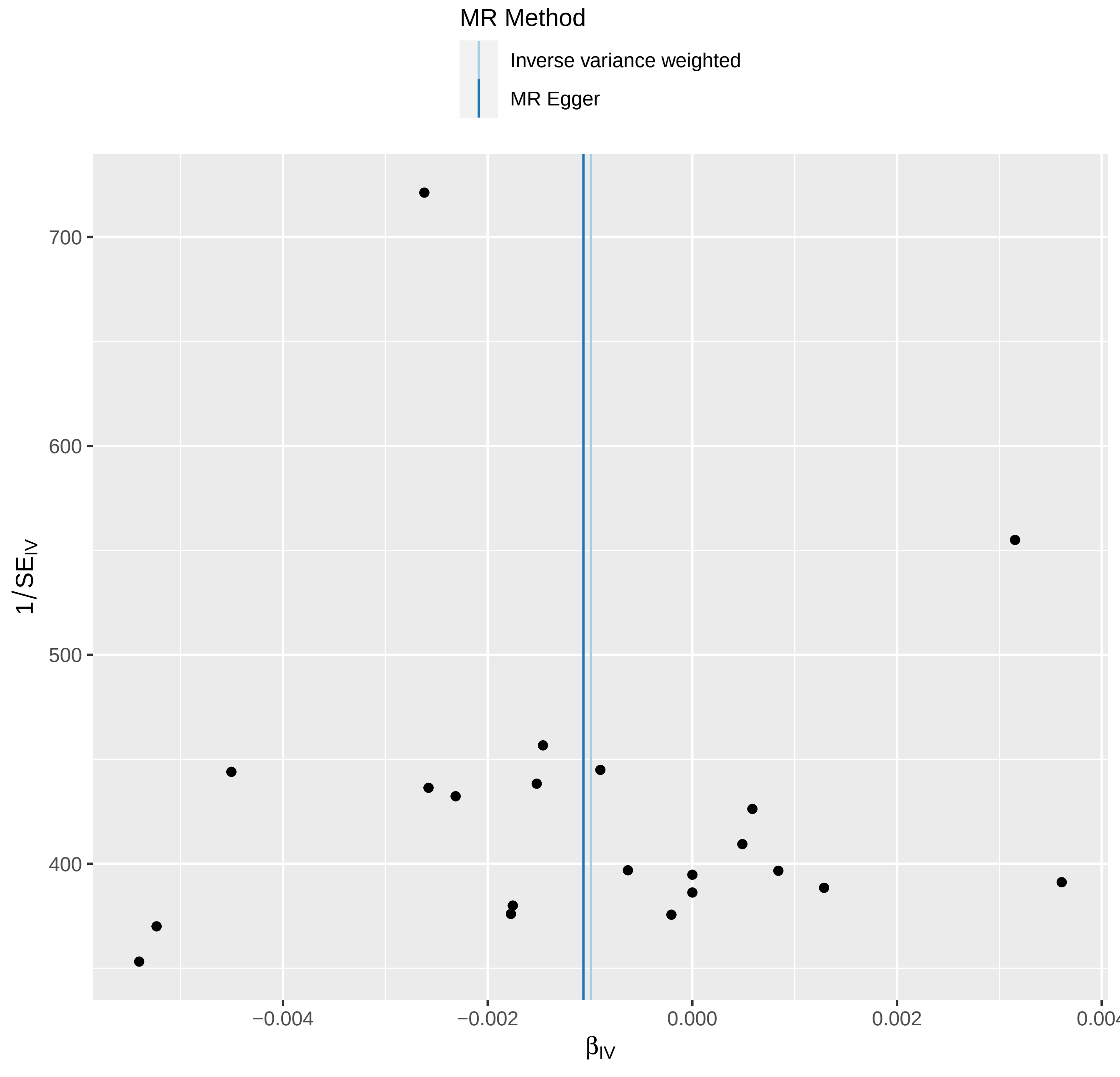

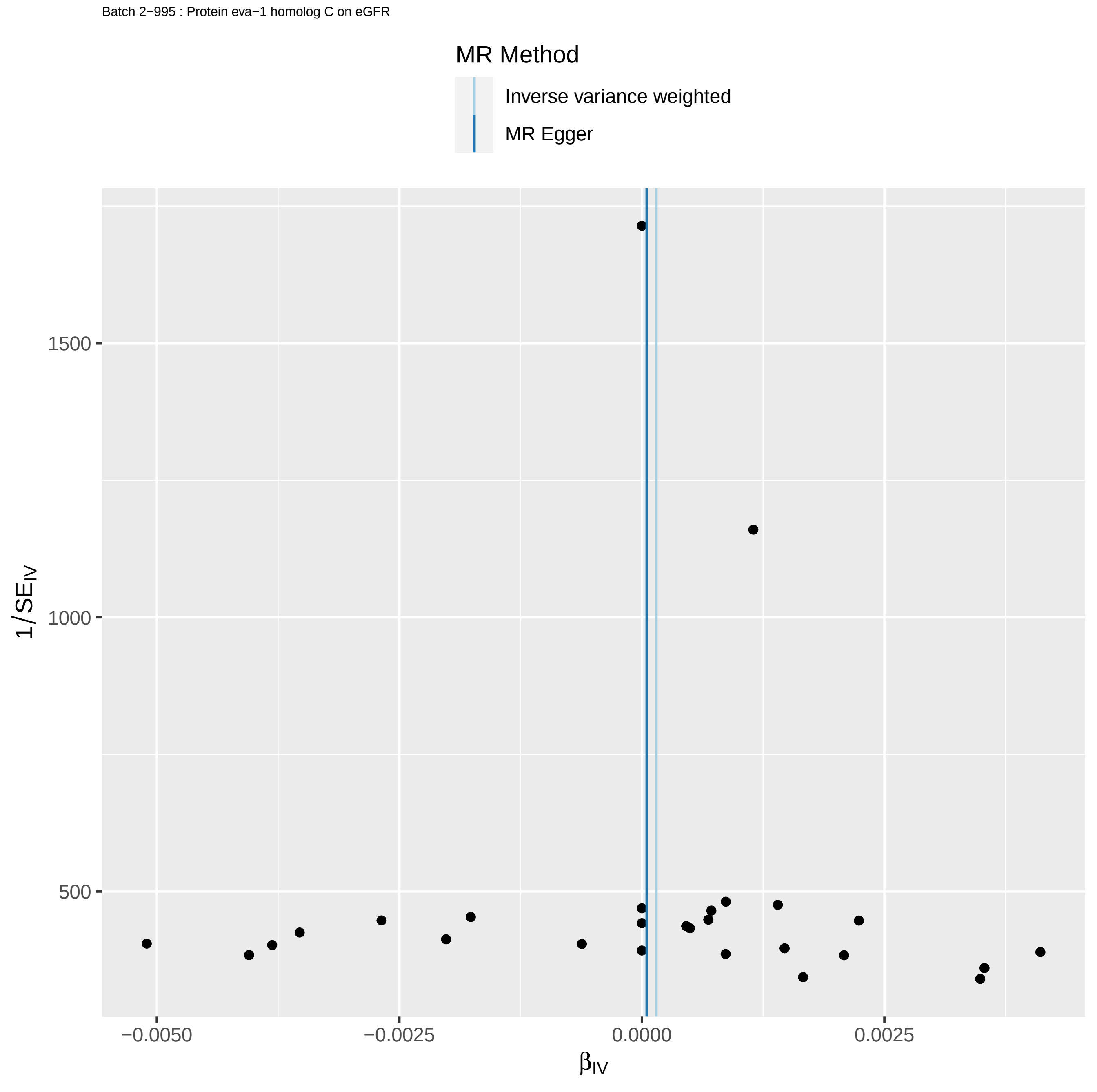

Batch 2−995 : Protein eva−1 homolog C on eGFR
MR Method
Inverse variance weighted
MR Egger
1500
1000
500
1/SE$_{IV}$
−0.0050
−0.0025
0.0000
0.0025
β$_{IV}$

Batch 2−996 : Protein eva−1 homolog C on eGFR

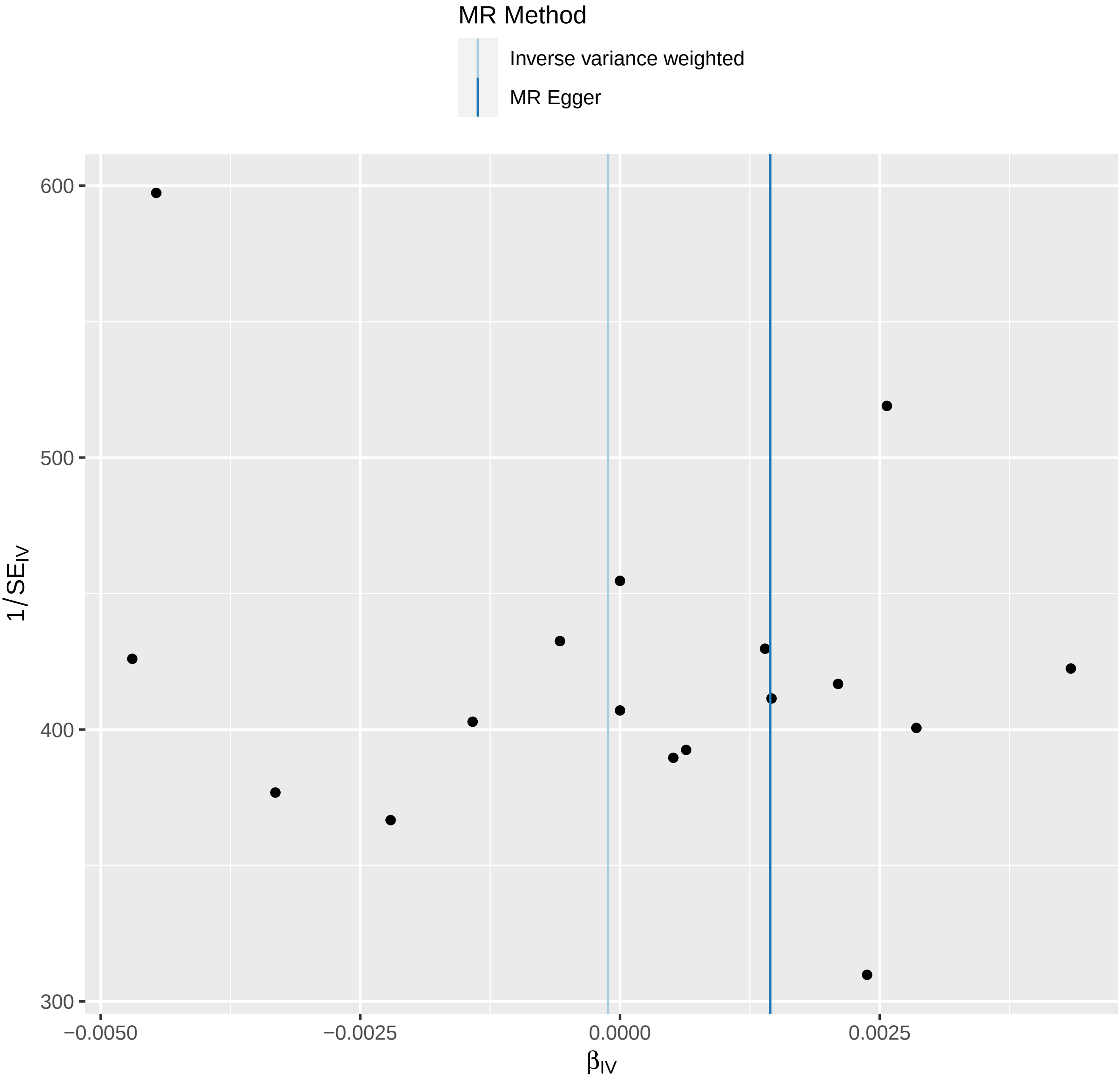

Batch 2−997 : Protein EVI2B on eGFR

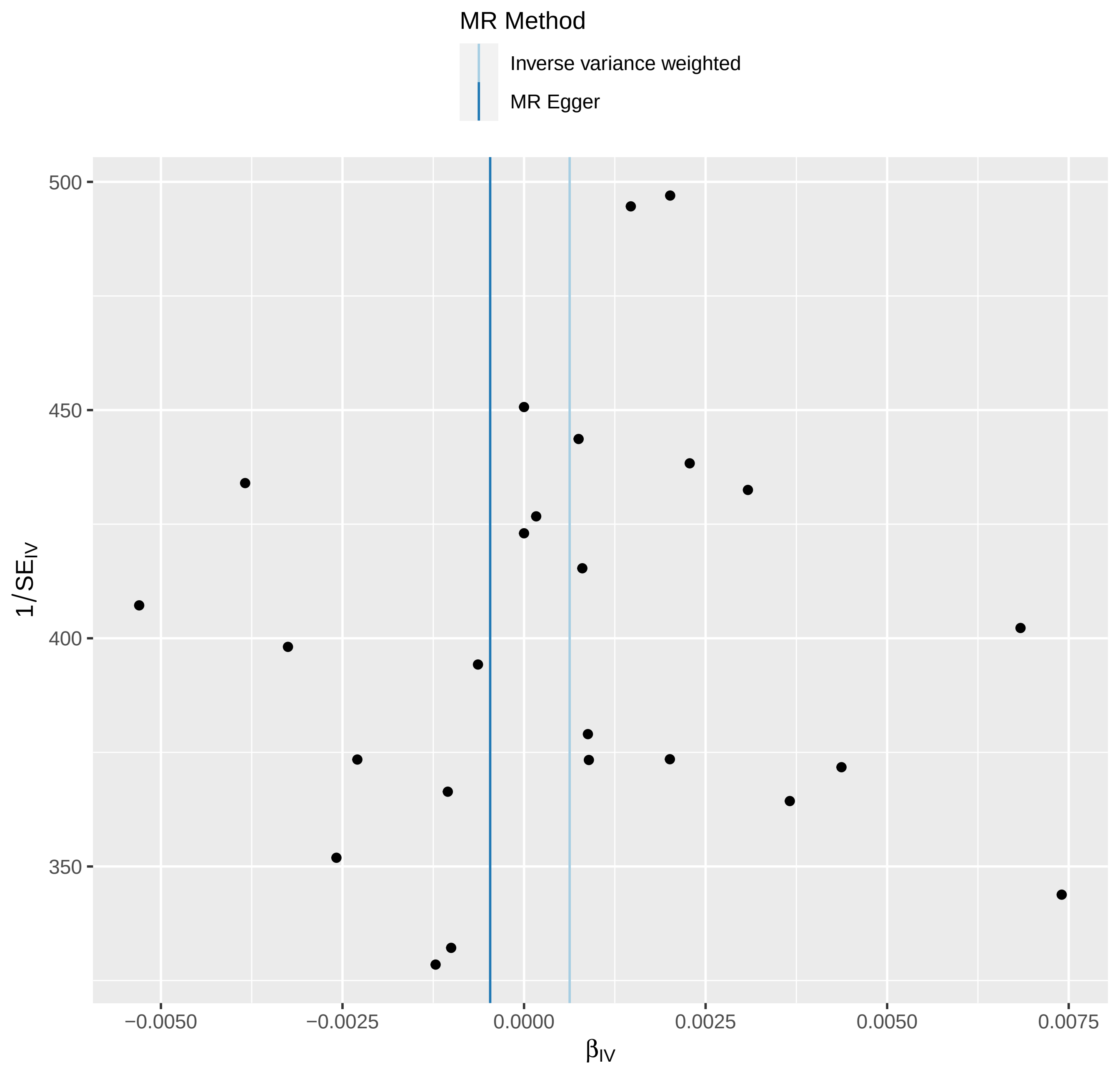

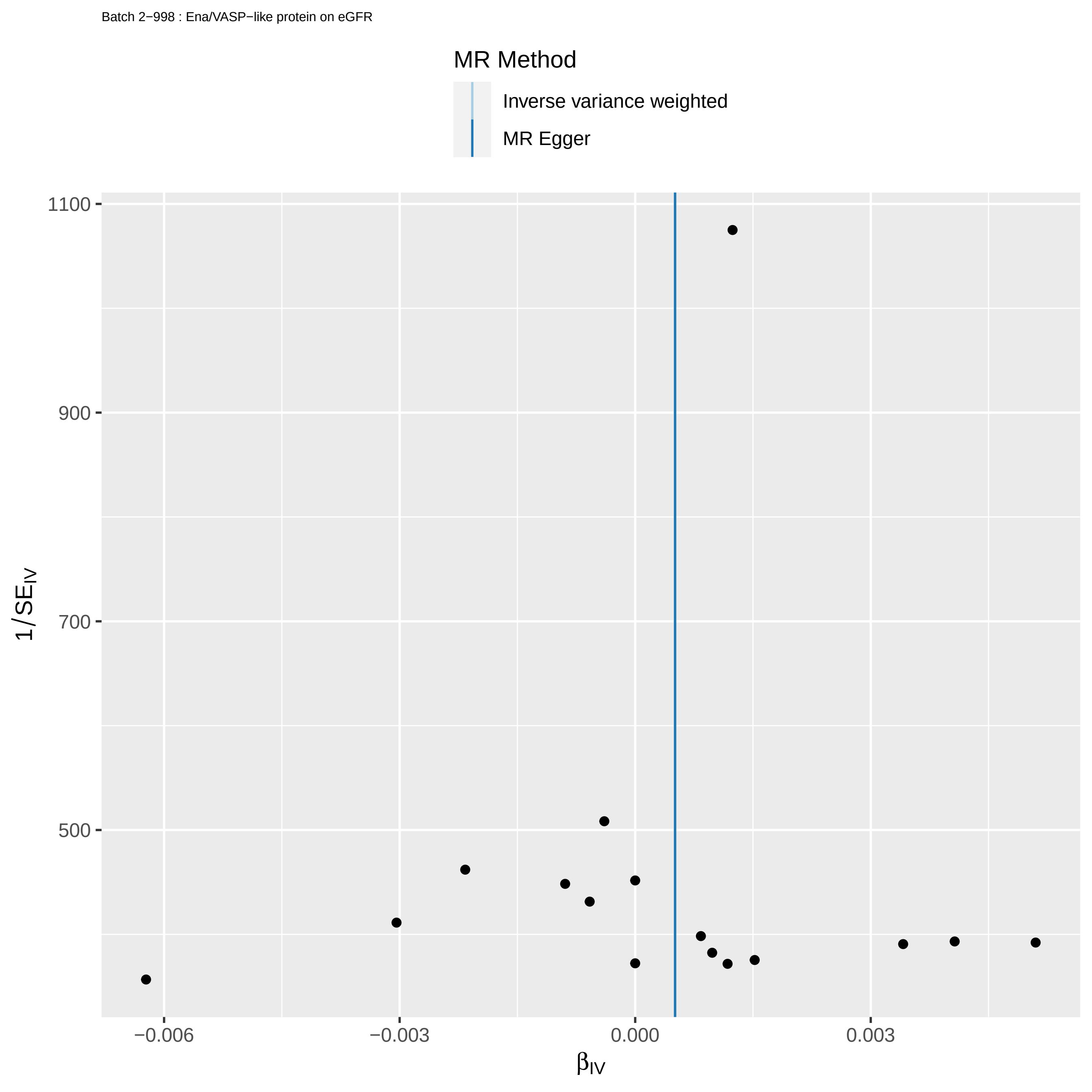

Batch 2−998 : Ena/VASP−like protein on eGFR
MR Method
Inverse variance weighted
MR Egger
1100
900
700
500
1/SE$_{IV}$
−0.006
−0.003
0.000
0.003
β$_{IV}$

Batch 2−999 : Envoplakin on eGFR

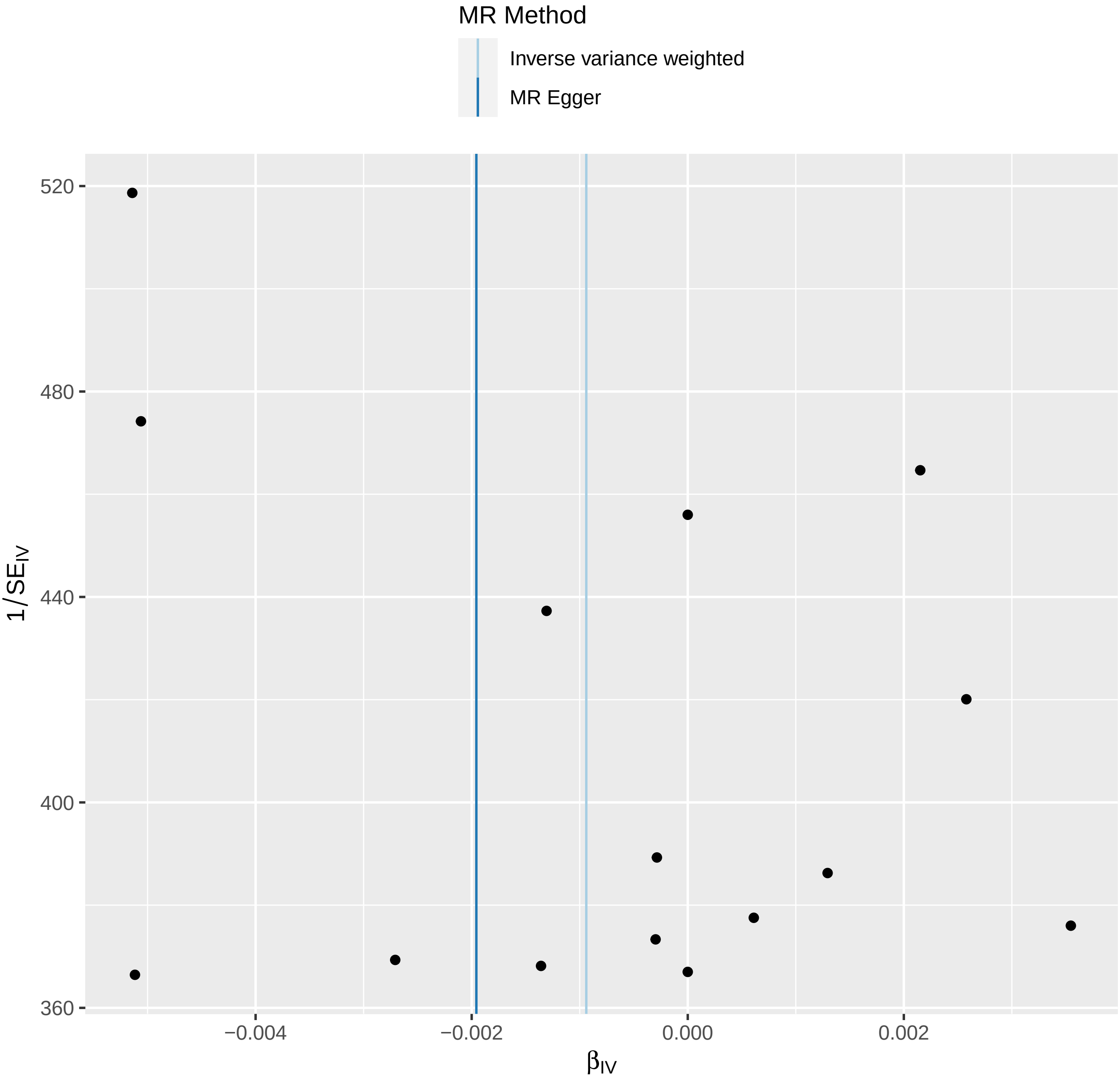

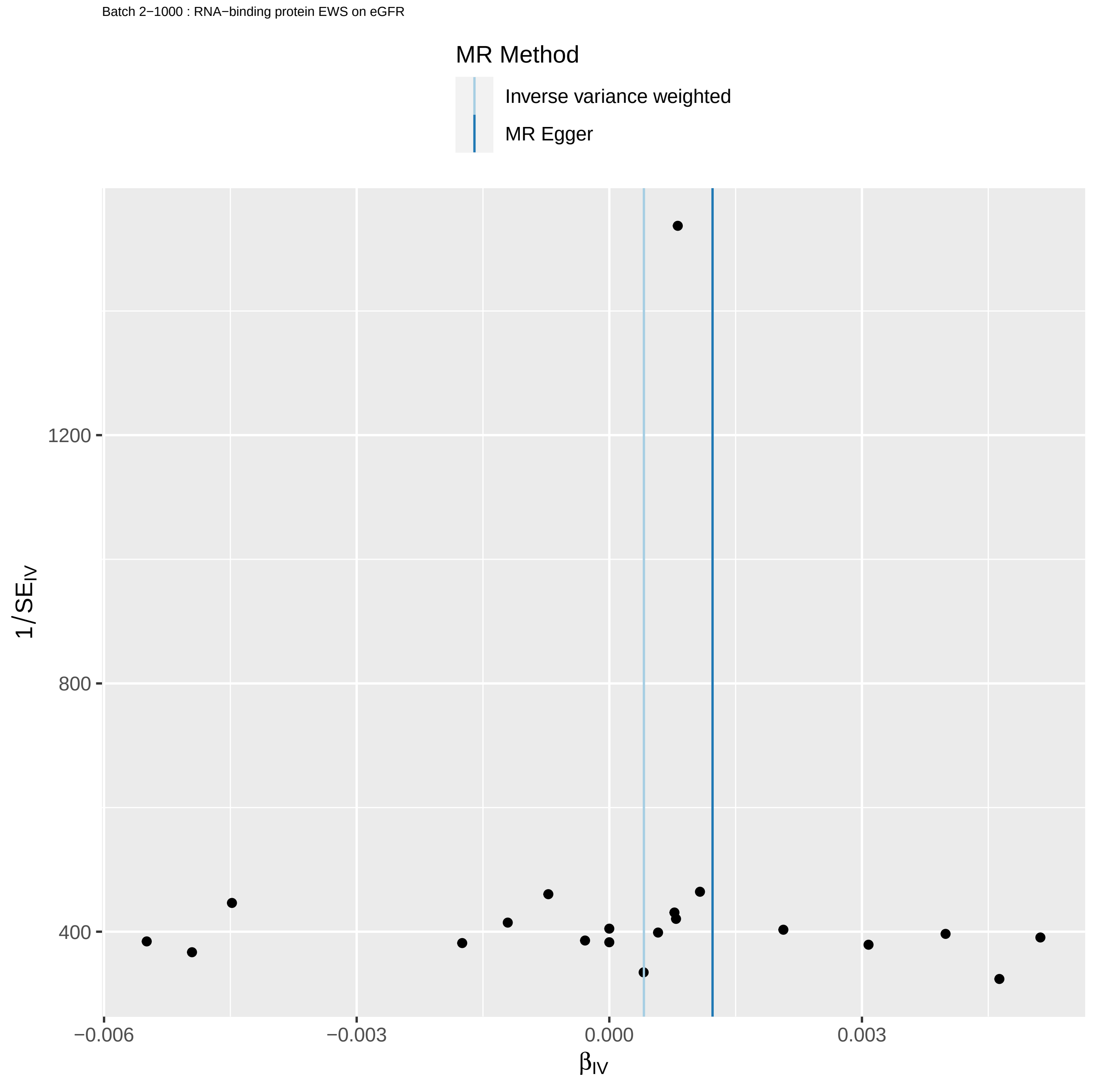
Batch 2−1000 : RNA−binding protein EWS on eGFR
MR Method
Inverse variance weighted
MR Egger
1200
800
400
1/SE_IV
−0.006
−0.003
0.000
0.003
β_IV

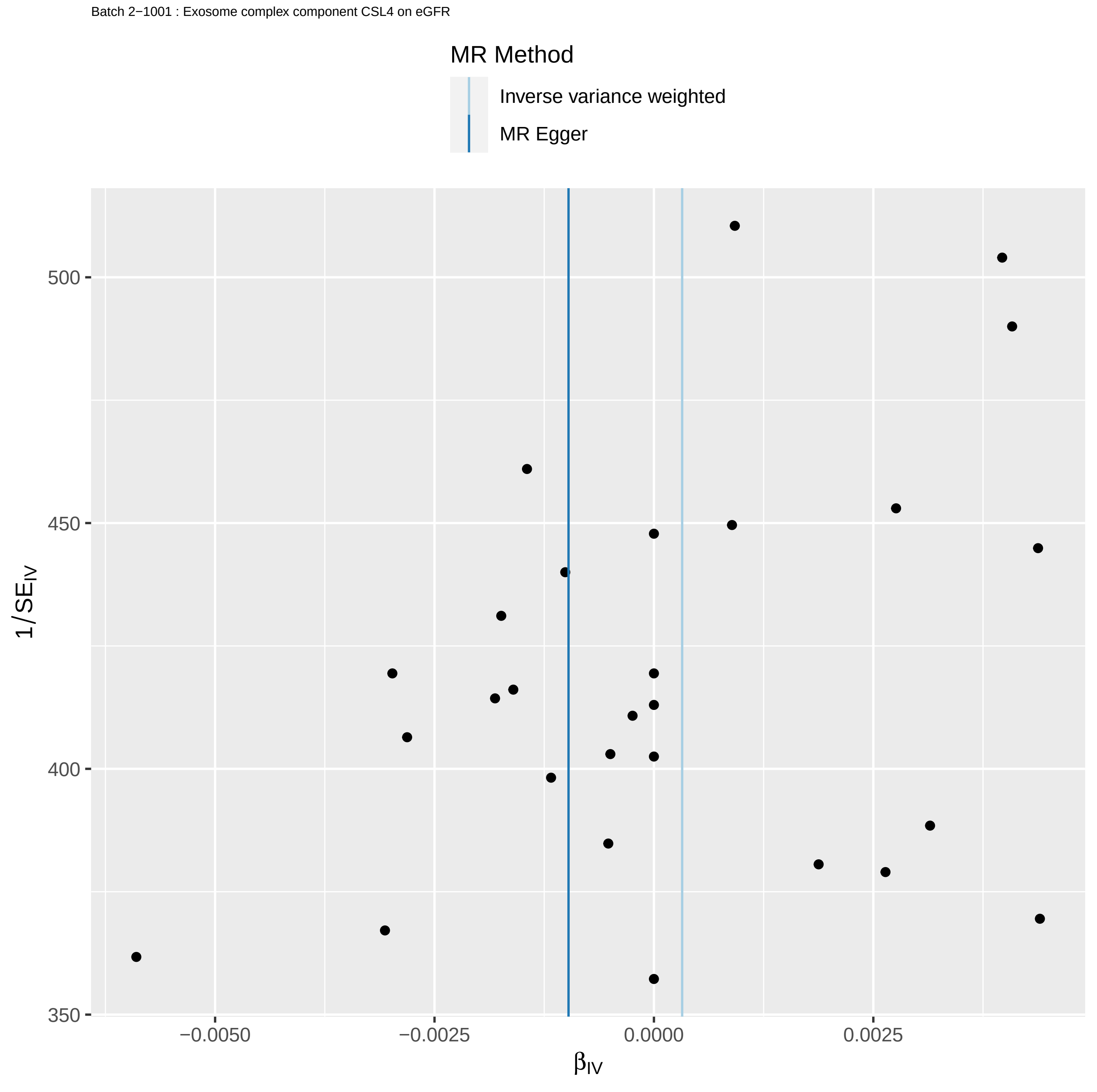
Batch 2−1001 : Exosome complex component CSL4 on eGFR
MR Method
Inverse variance weighted
MR Egger
500
450
400
350
1/SE$_{IV}$
−0.0050
−0.0025
0.0000
0.0025
β$_{IV}$

Batch 2−1002 : Exosome complex component RRP40 on eGFR

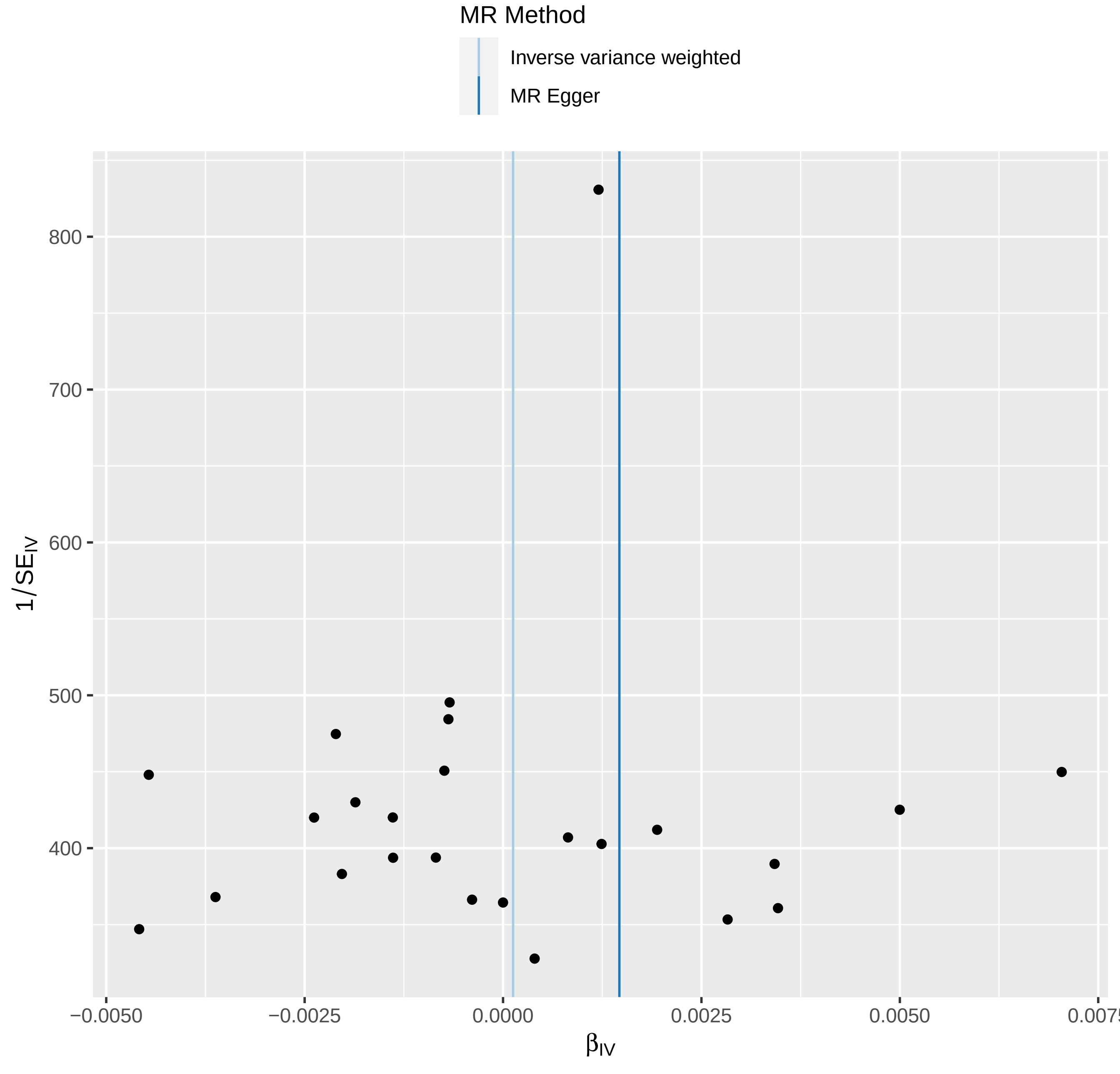

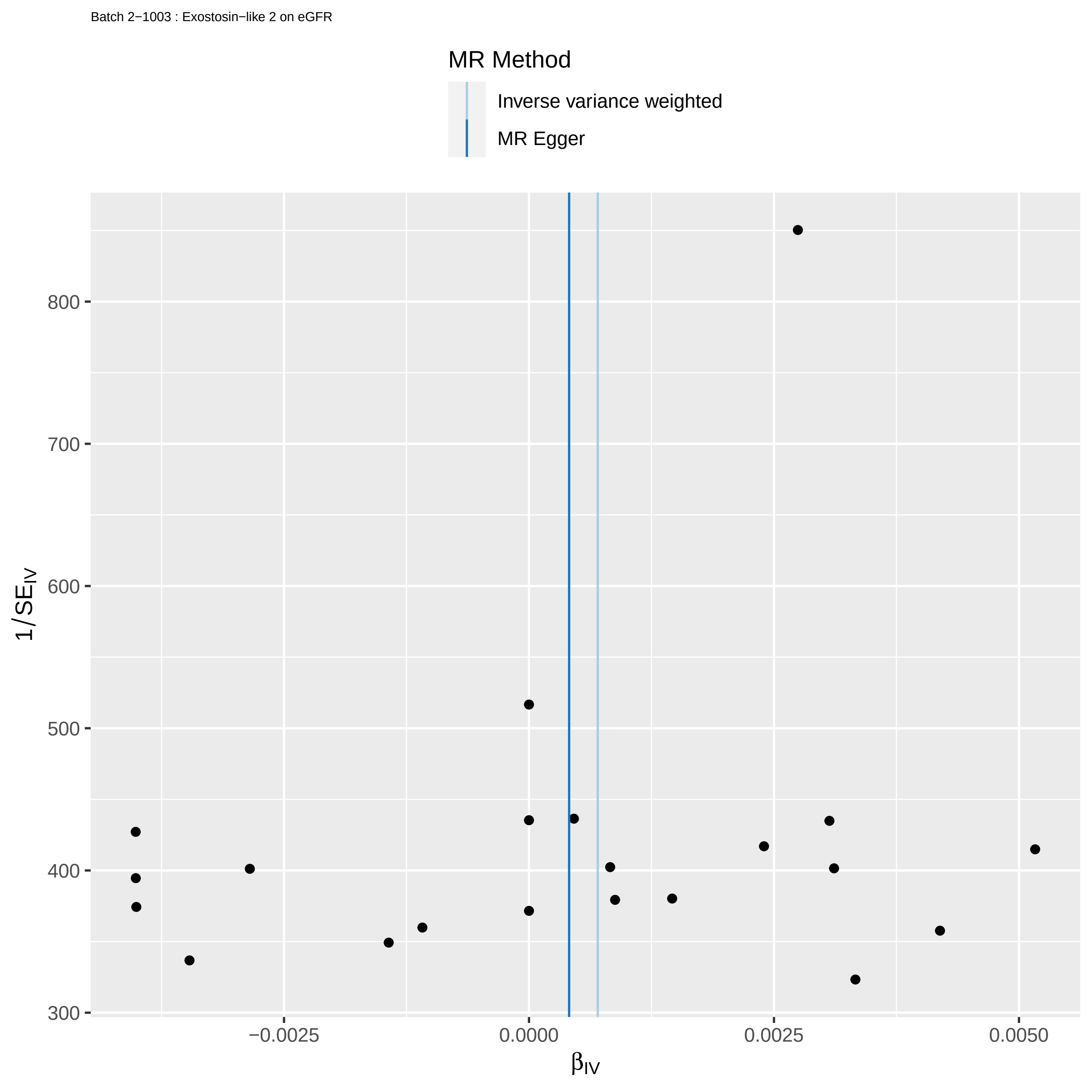
Batch 2−1003 : Exostosin−like 2 on eGFR
MR Method
Inverse variance weighted
MR Egger
800
700
600
500
400
300
1/SE_IV
−0.0025
0.0000
0.0025
0.0050
β_IV

Batch 2−1004 : Ezrin on eGFR

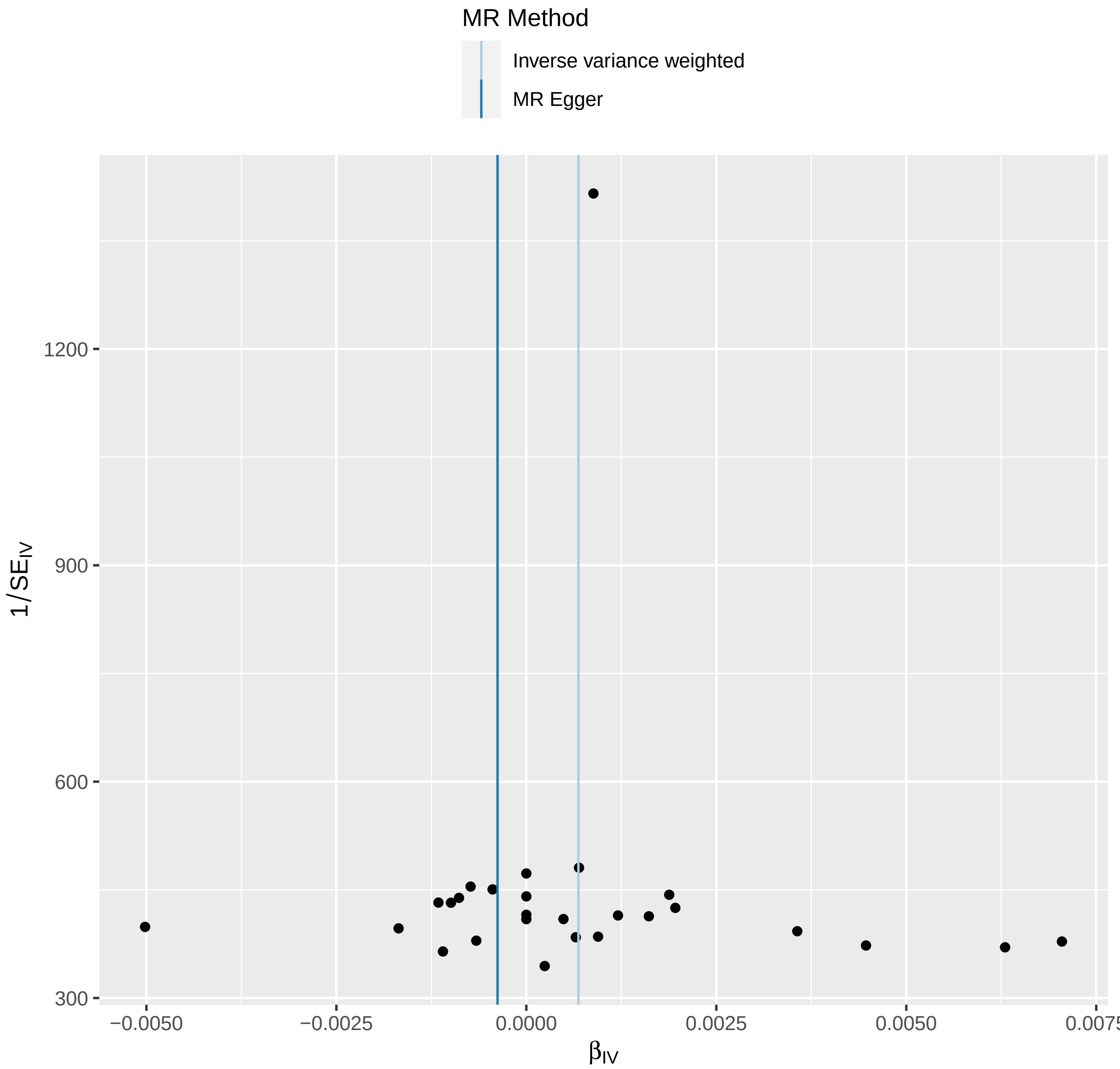

Batch 2−1005 : Coagulation factor Xa on eGFR

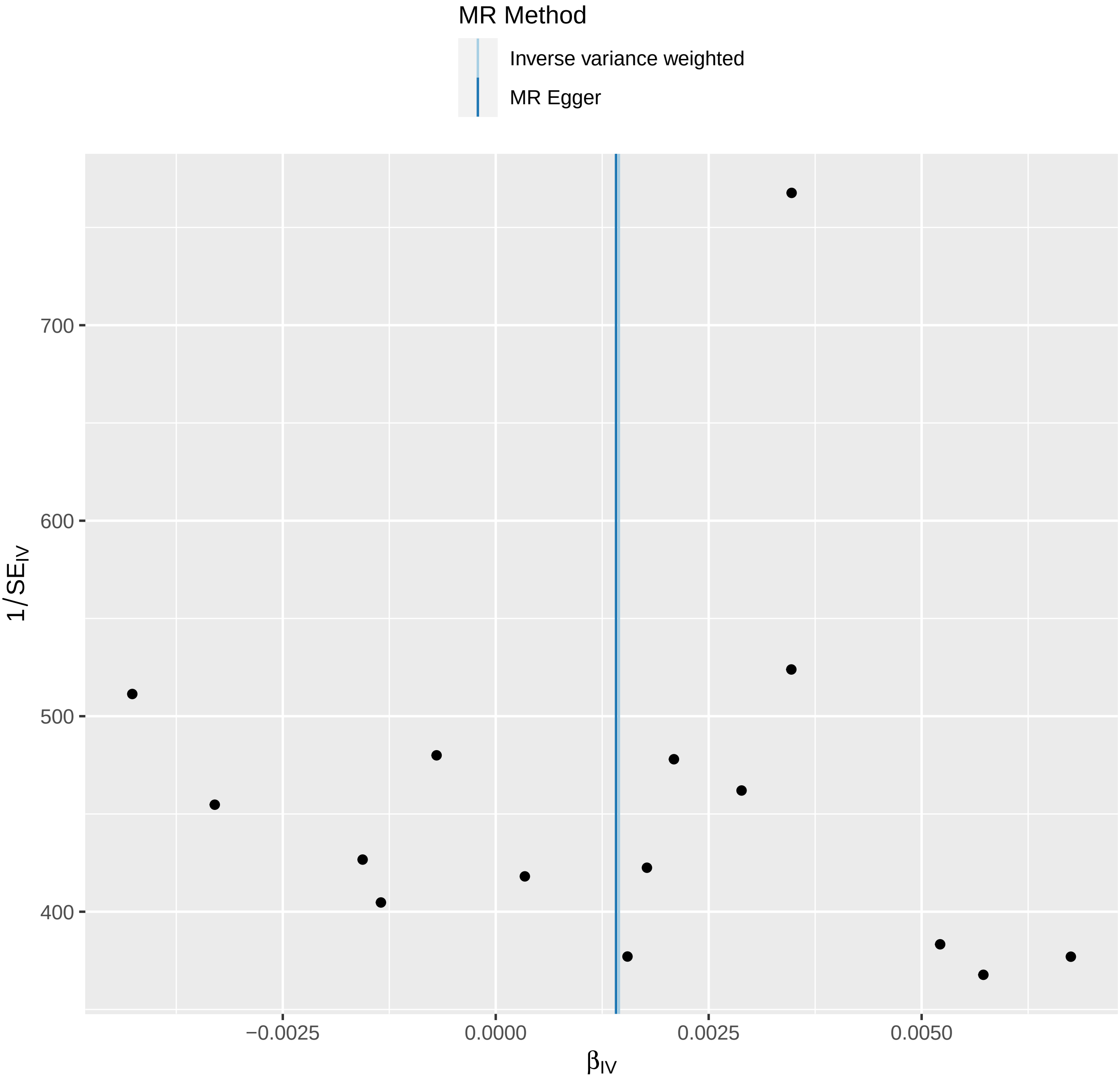

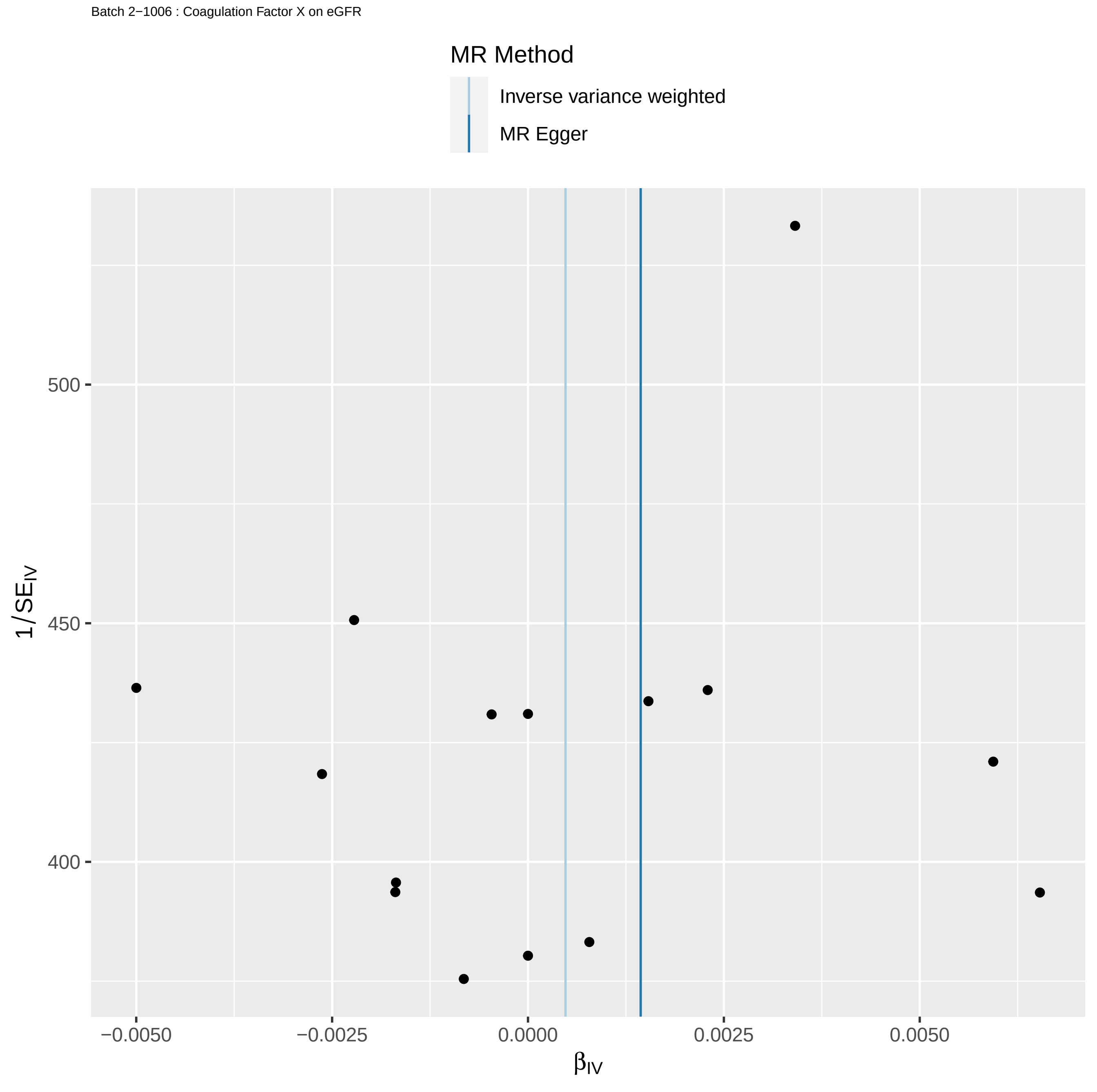

Batch 2−1006 : Coagulation Factor X on eGFR
MR Method
Inverse variance weighted
MR Egger
500
450
400
1/SE_IV
−0.0050
−0.0025
0.0000
0.0025
0.0050
β_IV

Batch 2−1007 : Prothrombin on eGFR

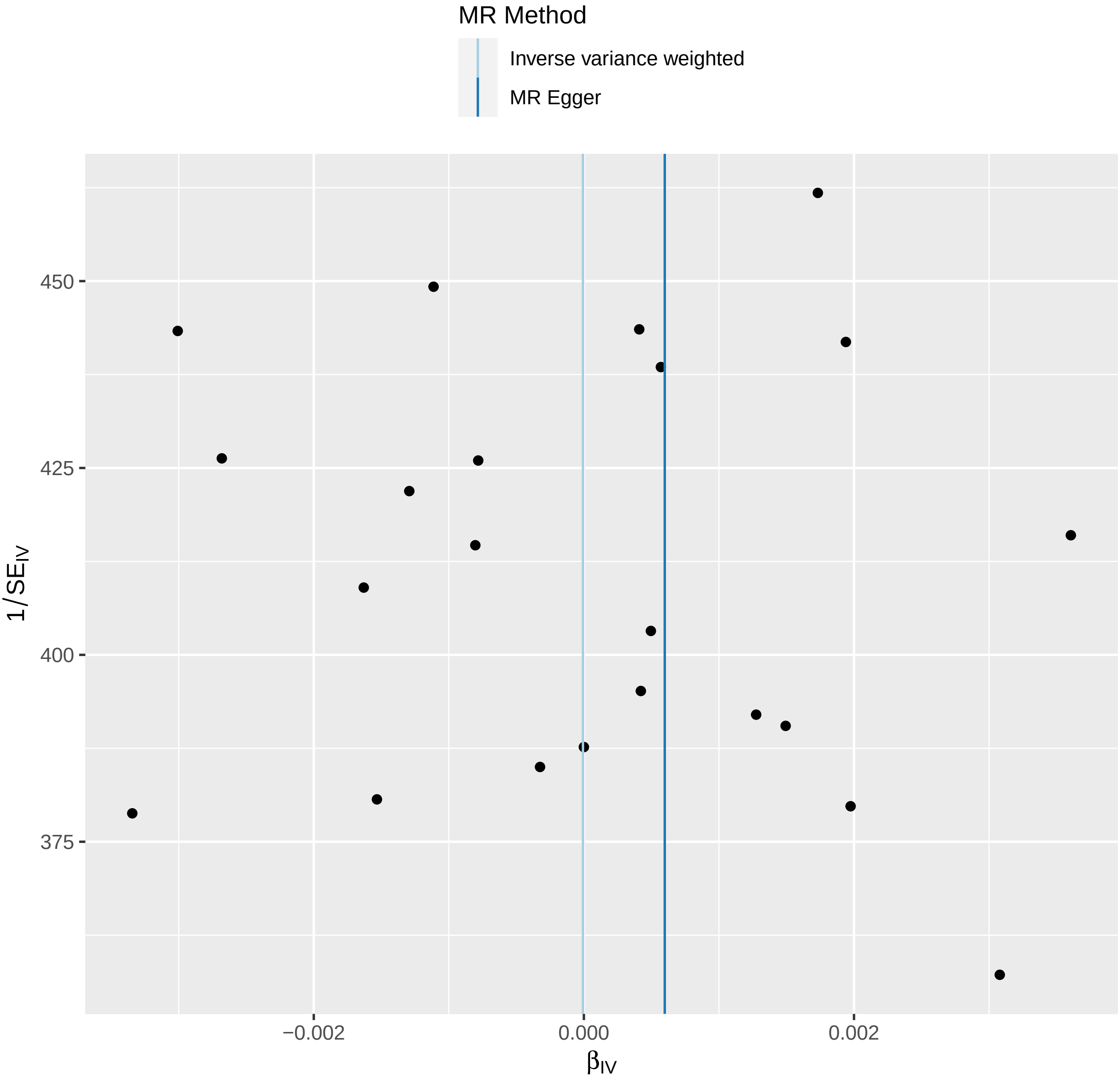

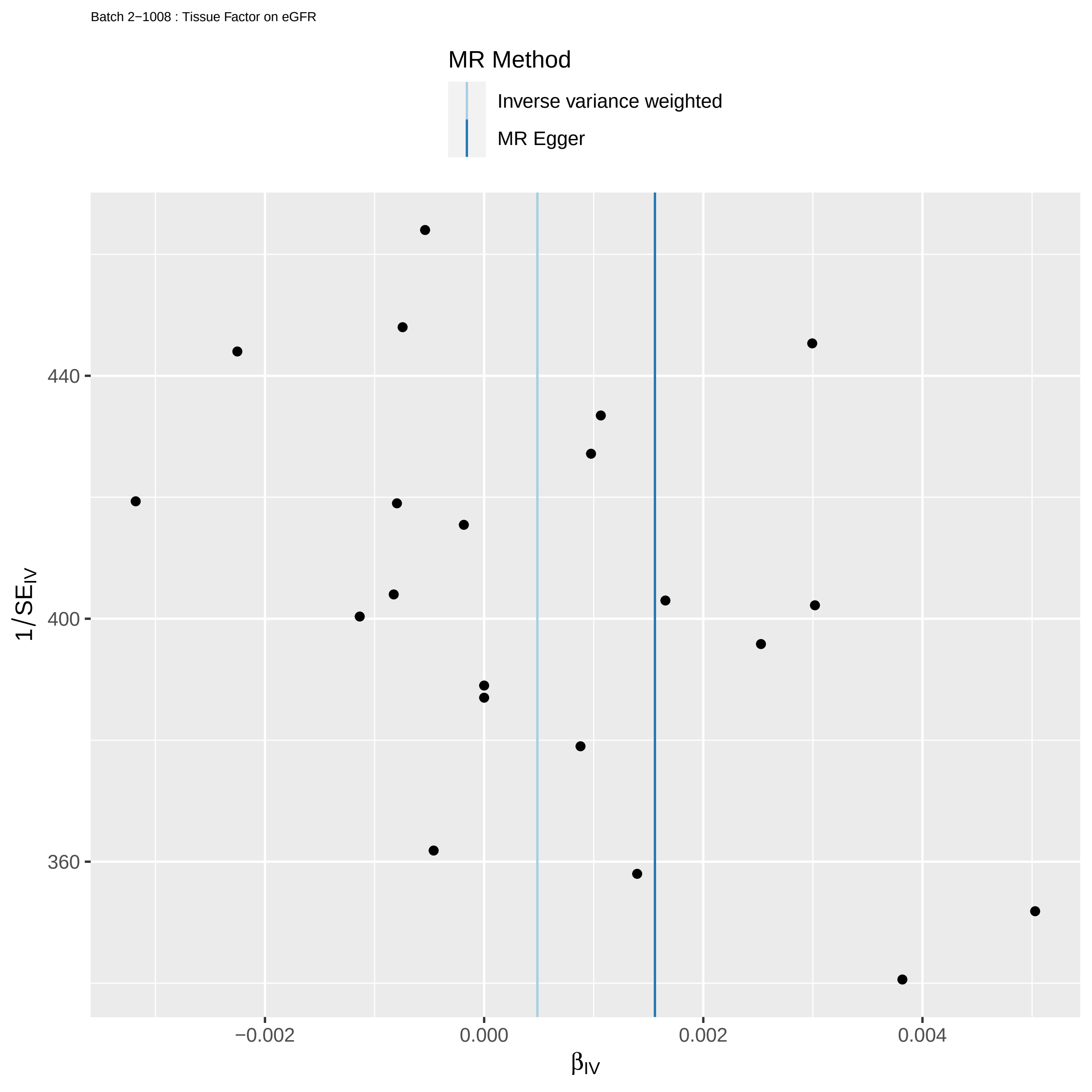
Batch 2−1008 : Tissue Factor on eGFR
MR Method
Inverse variance weighted
MR Egger
440
400
360
1/SE_IV
−0.002
0.000
0.002
0.004
β_IV

Batch 2−1009 : Coagulation Factor VIII on eGFR

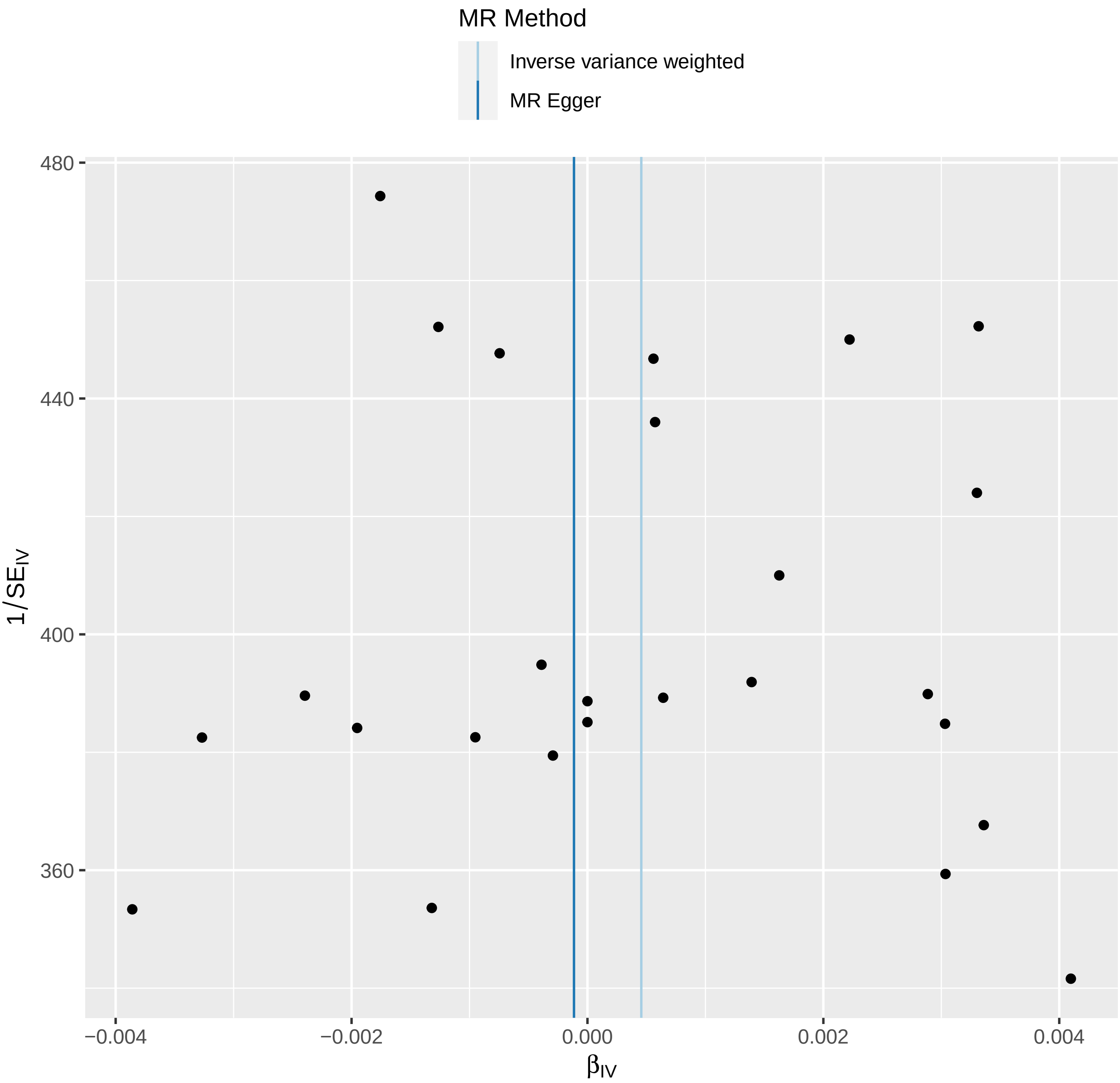

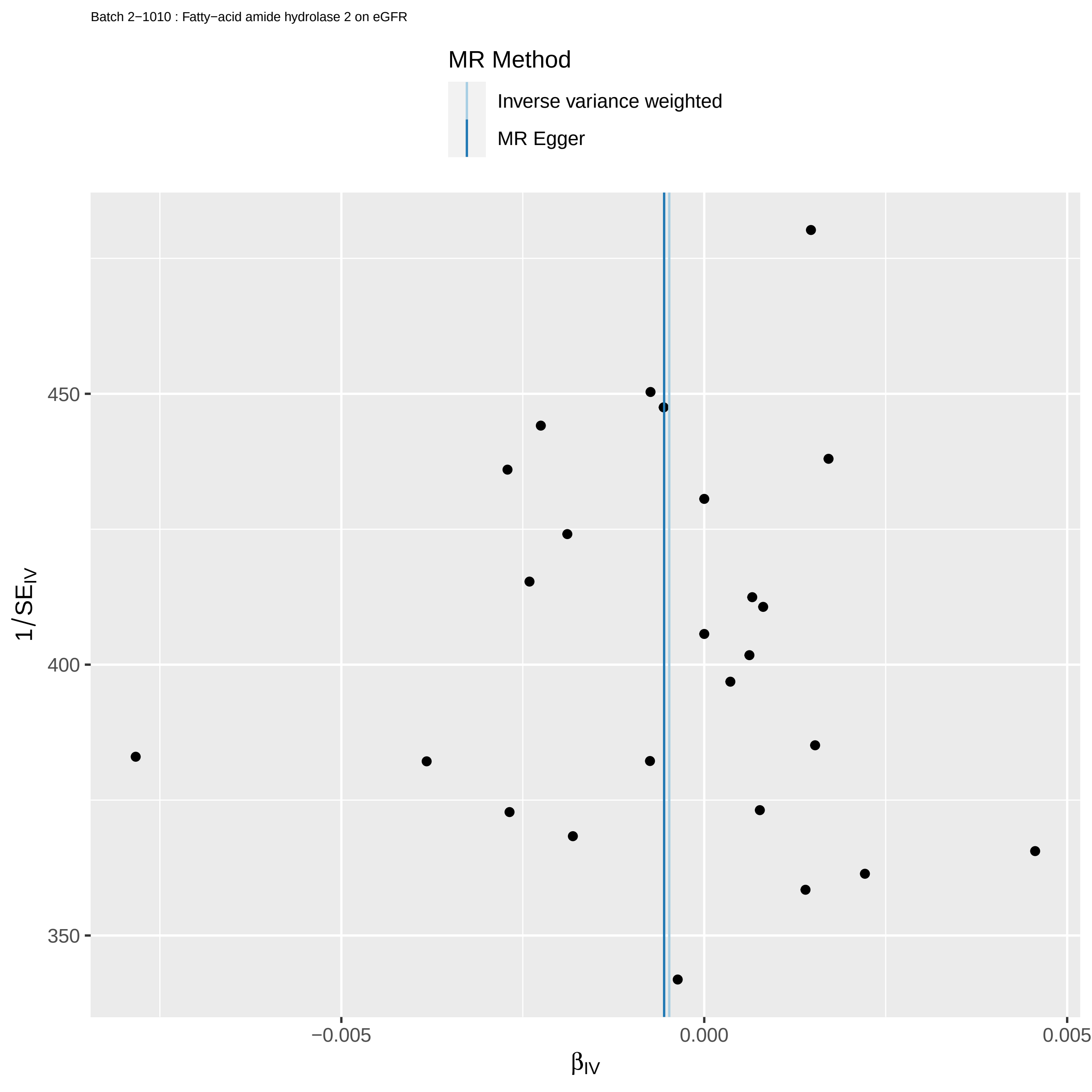

Batch 2−1010 : Fatty−acid amide hydrolase 2 on eGFR
MR Method
Inverse variance weighted
MR Egger
450
400
350
−0.005
0.000
0.005
1/SE_IV
β_IV

Batch 2−1011 : Fatty acid−binding protein, liver on eGFR

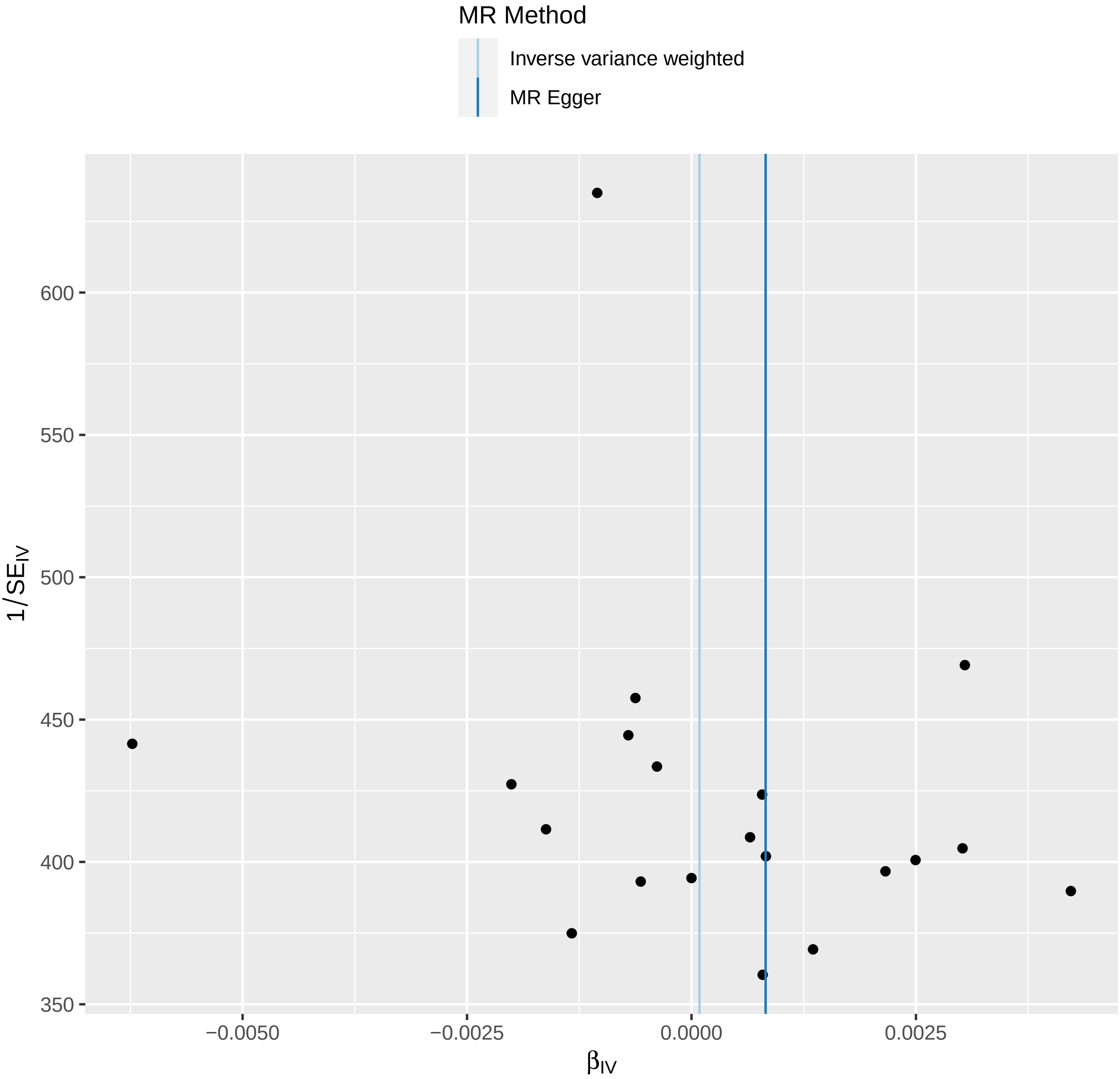

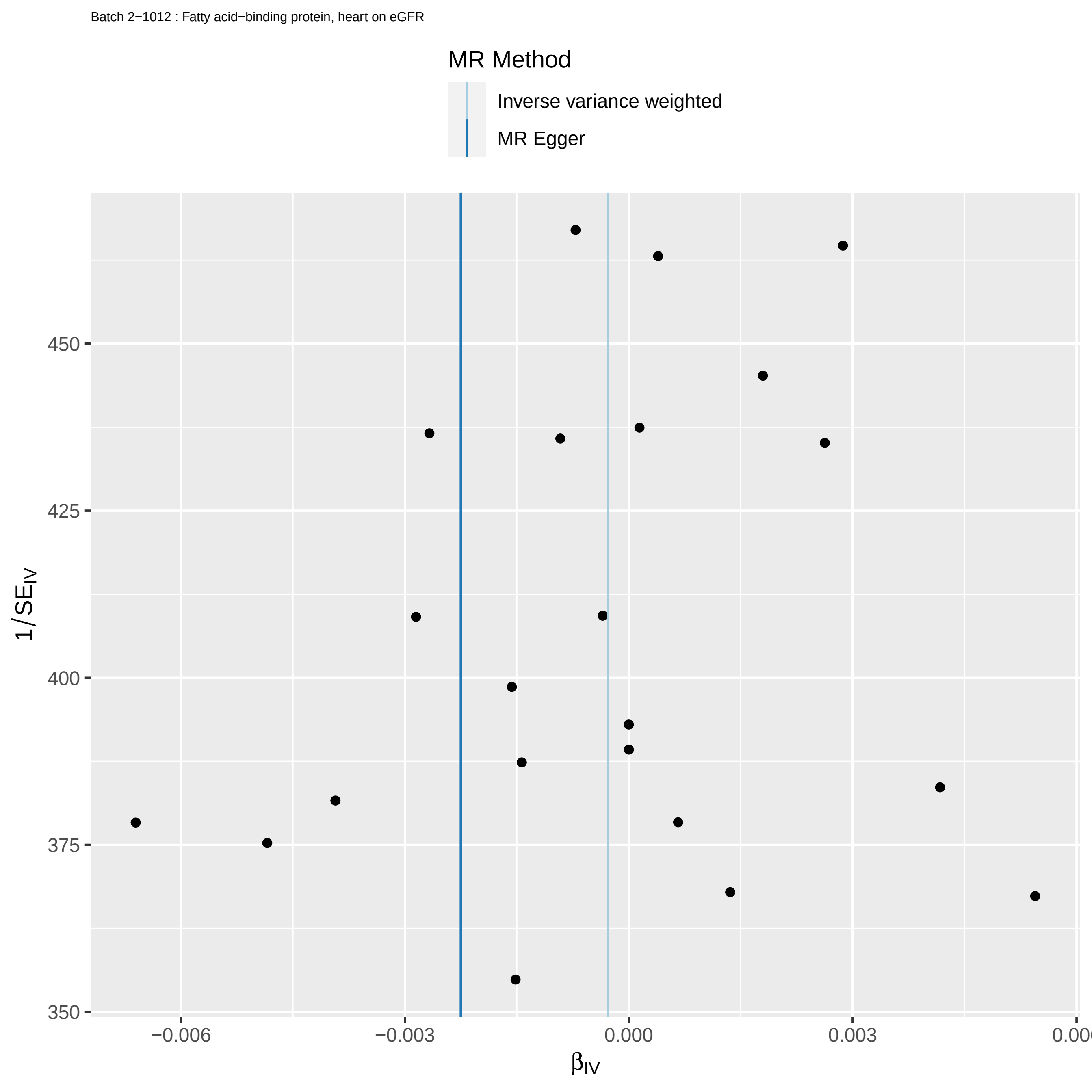
Batch 2−1012 : Fatty acid−binding protein, heart on eGFR
MR Method
Inverse variance weighted
MR Egger
1/SE$_{IV}$
450
425
400
375
350
−0.006
−0.003
0.000
0.003
β$_{IV}$

Batch 2−1013 : Fatty acid−binding protein, adipocyte on eGFR

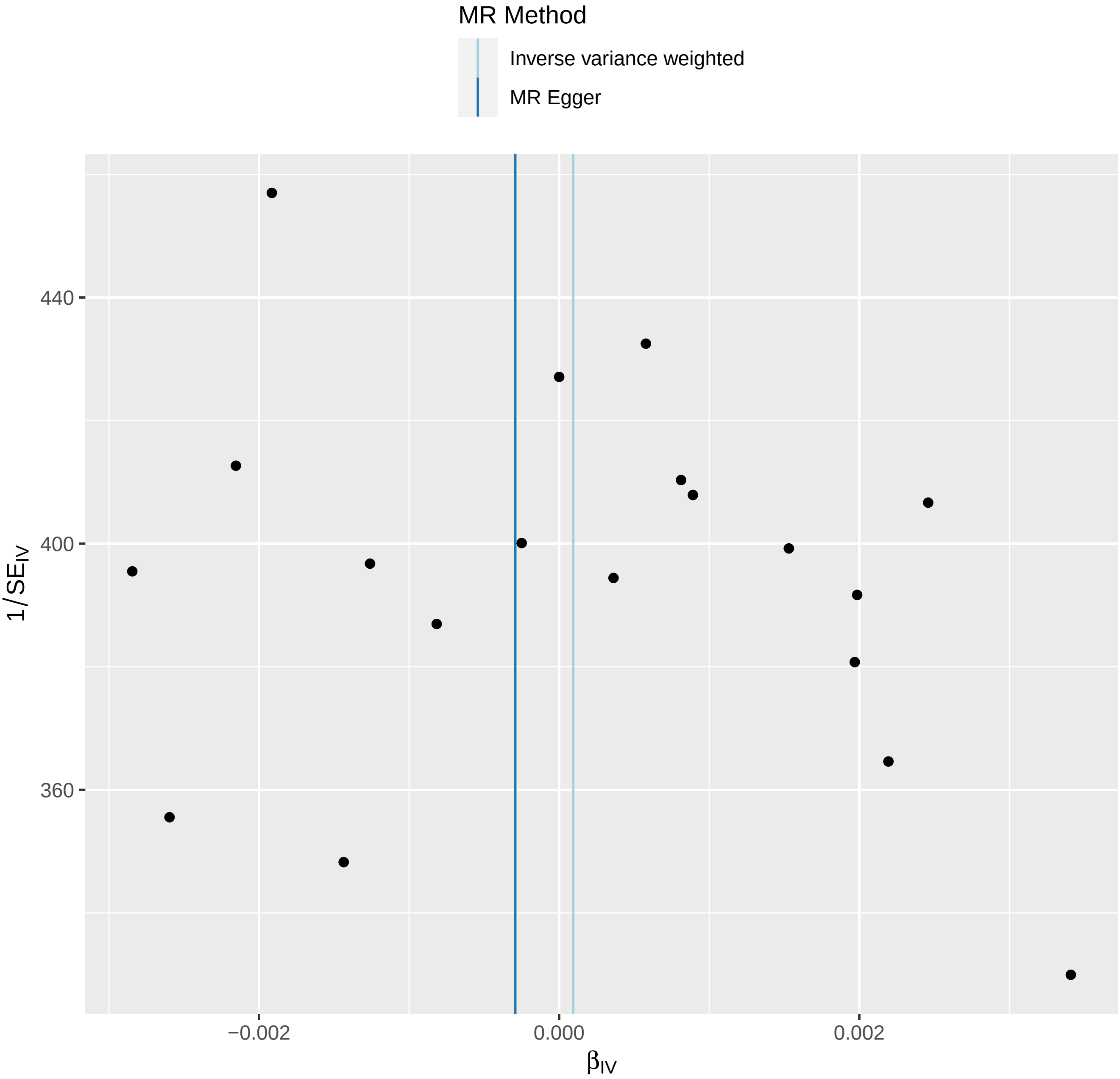

Batch 2−1014 : Fatty acid−binding protein, epidermal on eGFR

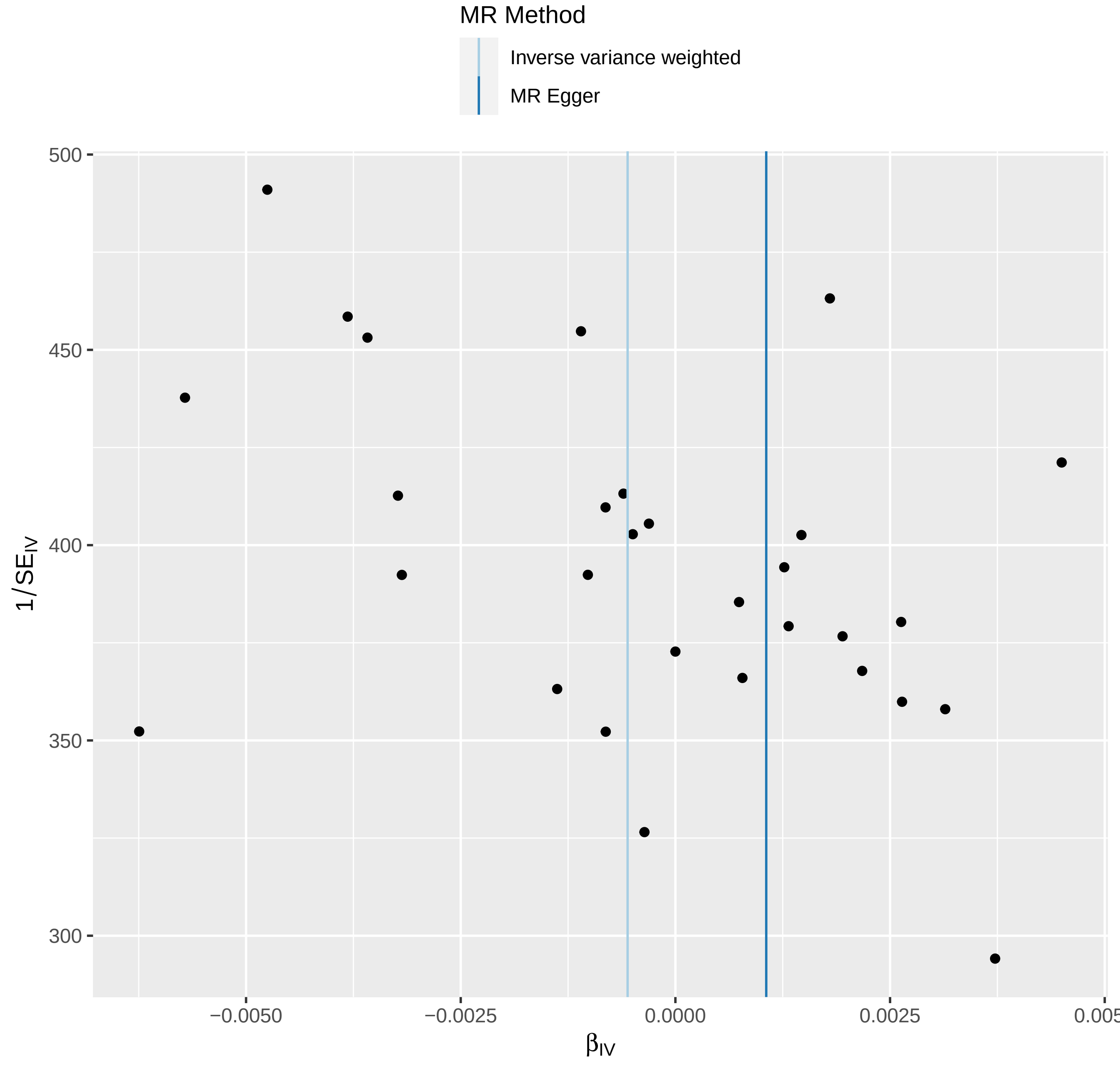

Batch 2−1015 : FAS−associated factor 2 on eGFR

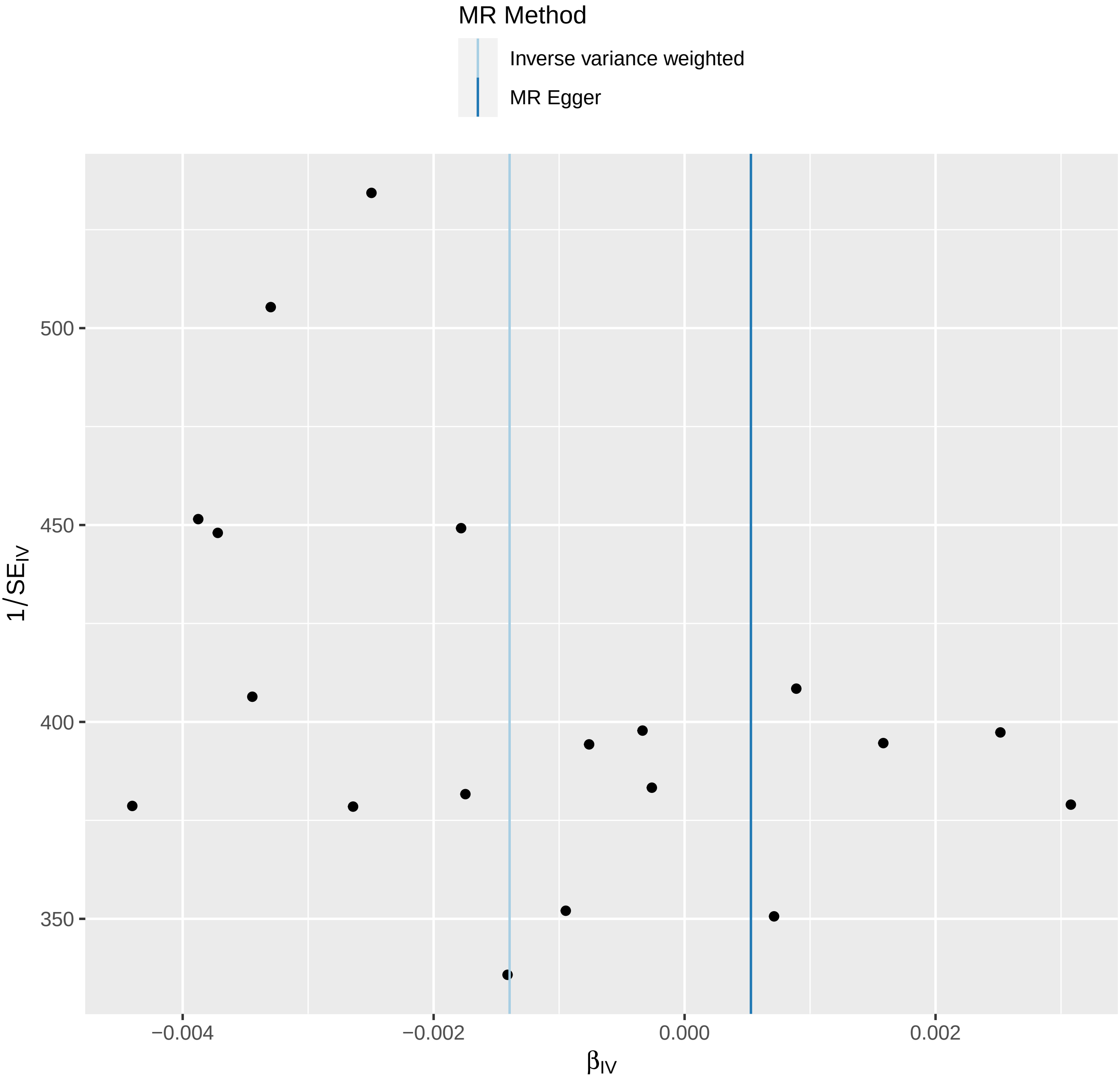

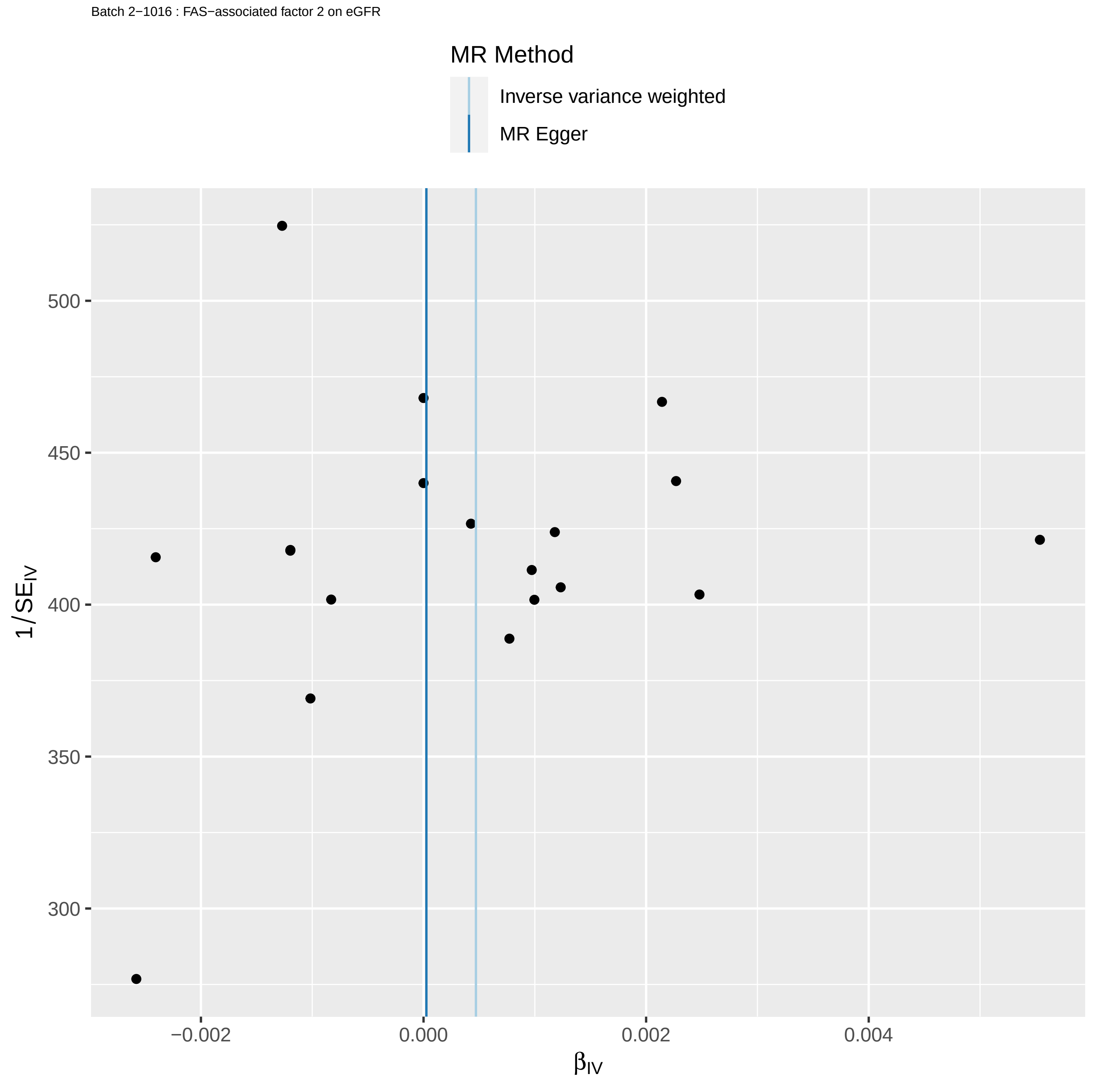
Batch 2−1016 : FAS−associated factor 2 on eGFR
MR Method
Inverse variance weighted
MR Egger
500
450
400
350
300
1/SE_IV
−0.002
0.000
0.002
0.004
β_IV

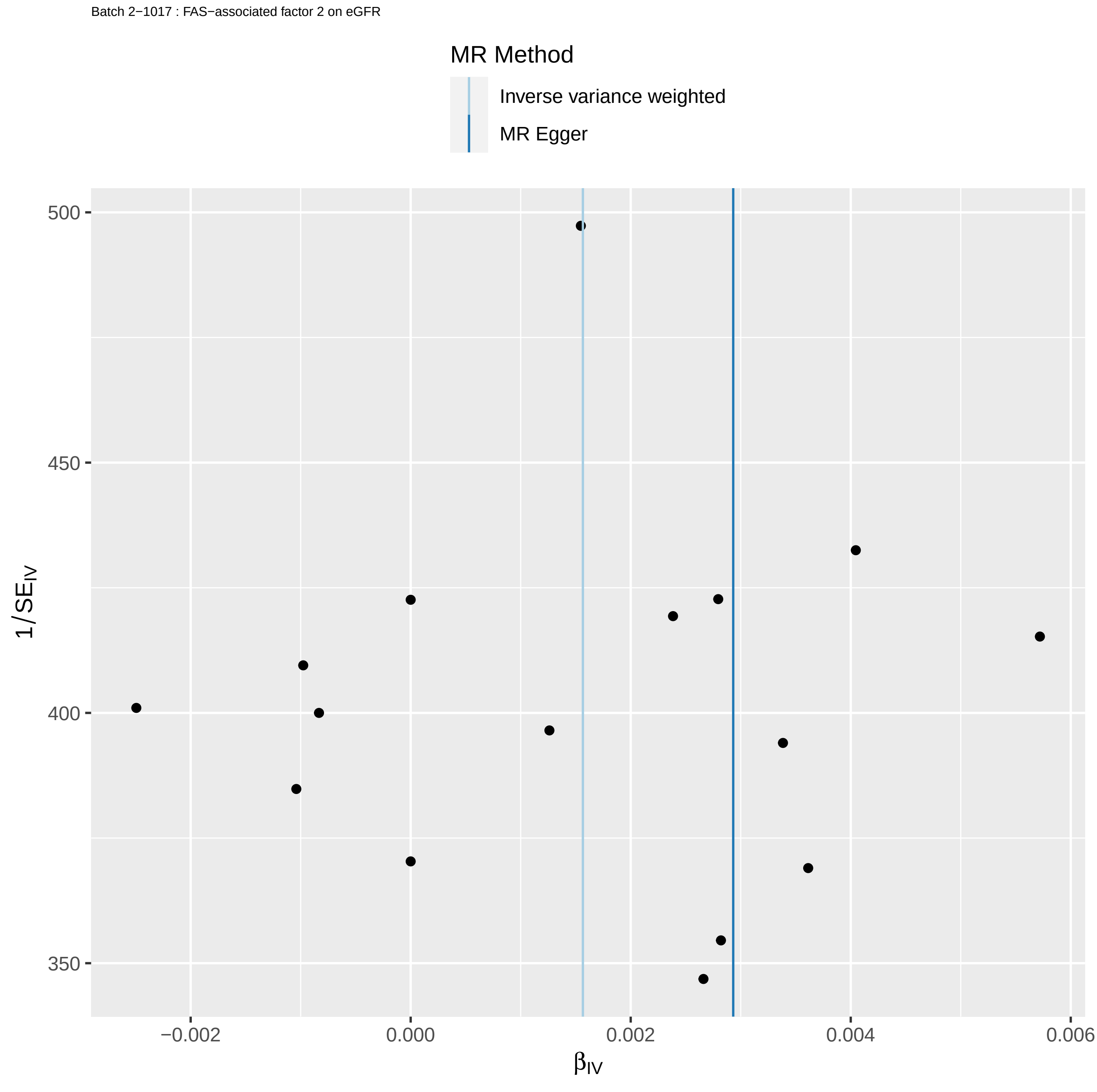

Batch 2−1017 : FAS−associated factor 2 on eGFR
MR Method
Inverse variance weighted
MR Egger
500
450
400
350
1/SE_IV
−0.002
0.000
0.002
0.004
0.006
β_IV

Batch 2−1018 : Fumarylacetoacetase on eGFR

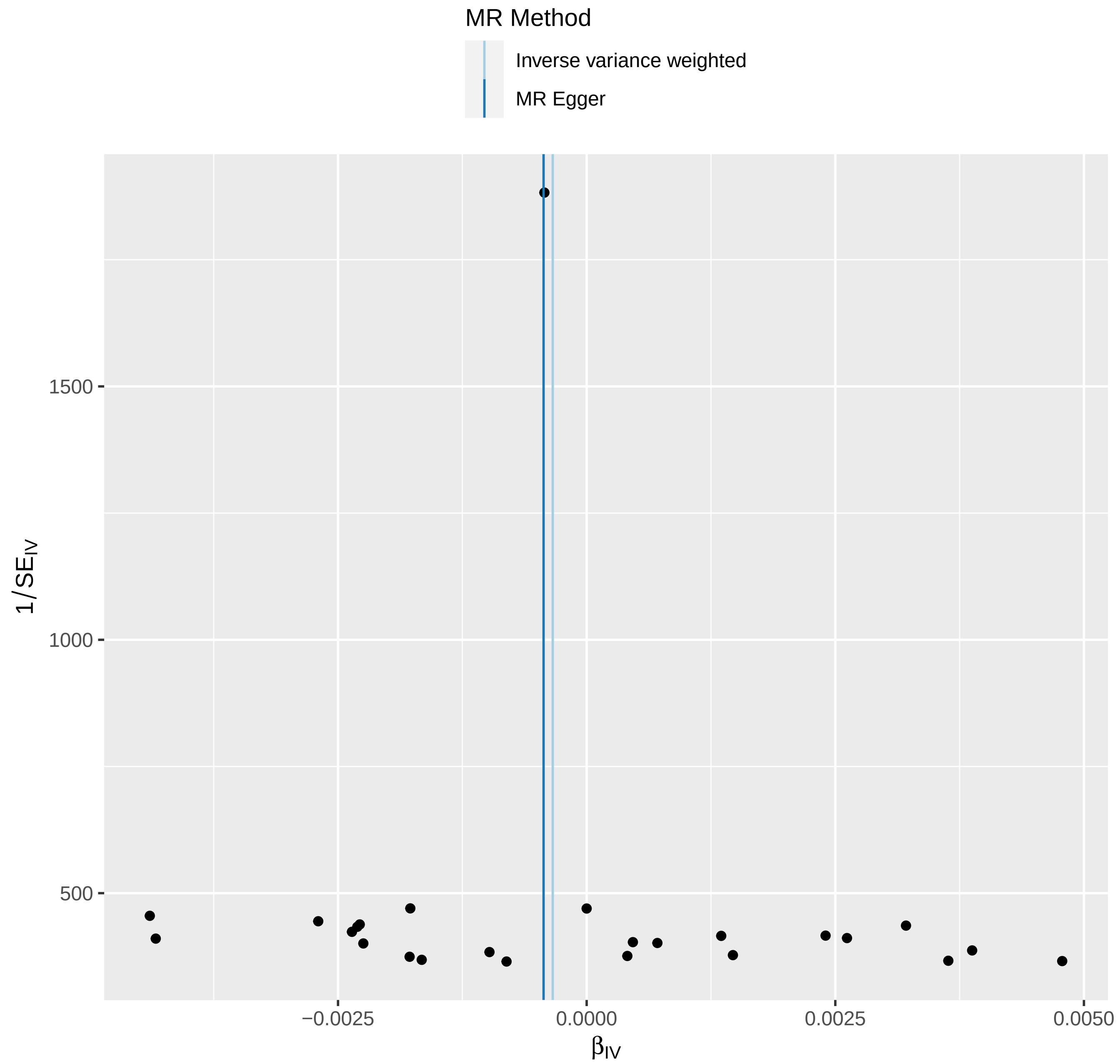

Batch 2−1019 : Fas apoptotic inhibitory molecule 3 on eGFR

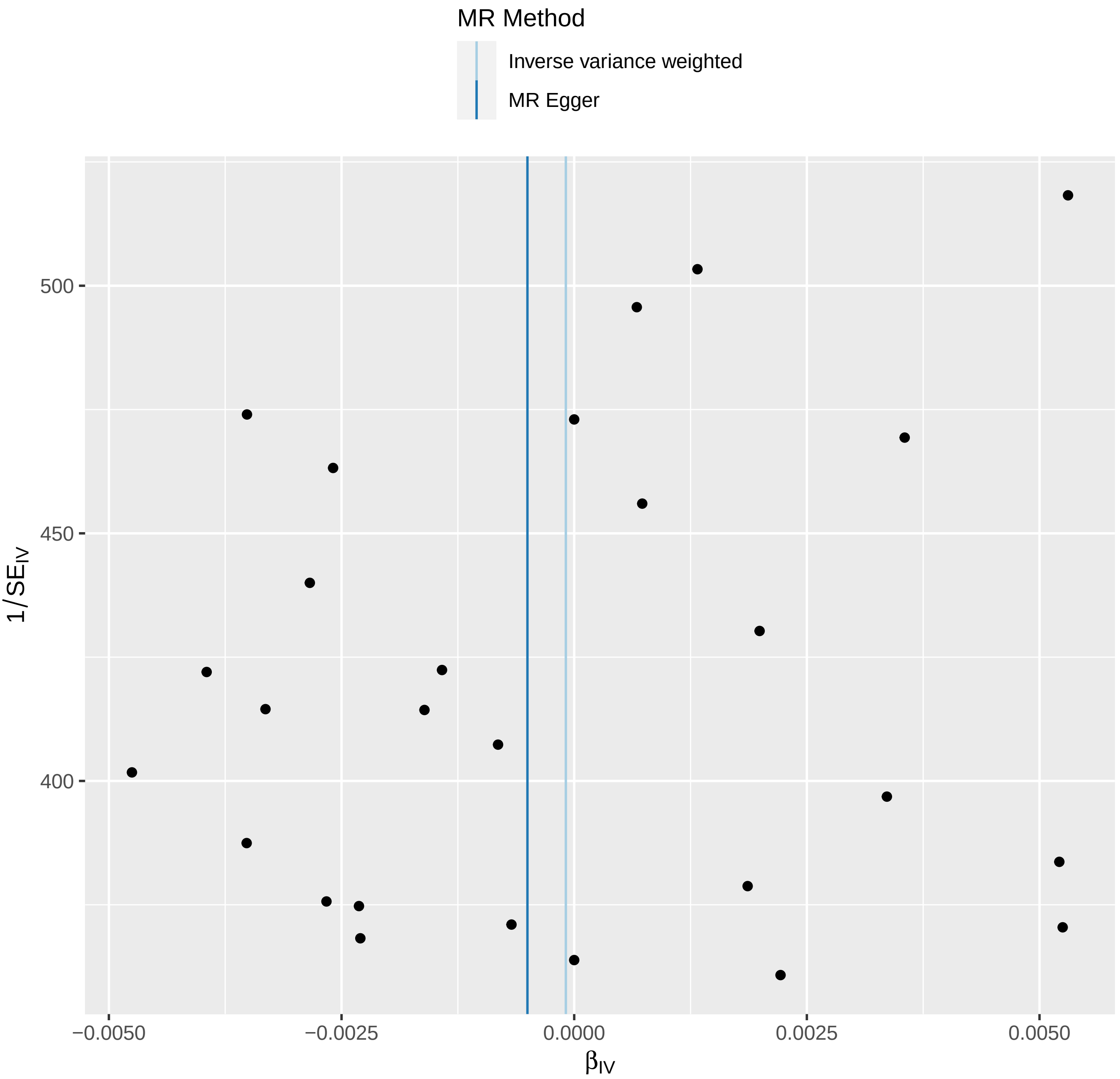

Batch 2−1020 : Protein FAM107A on eGFR

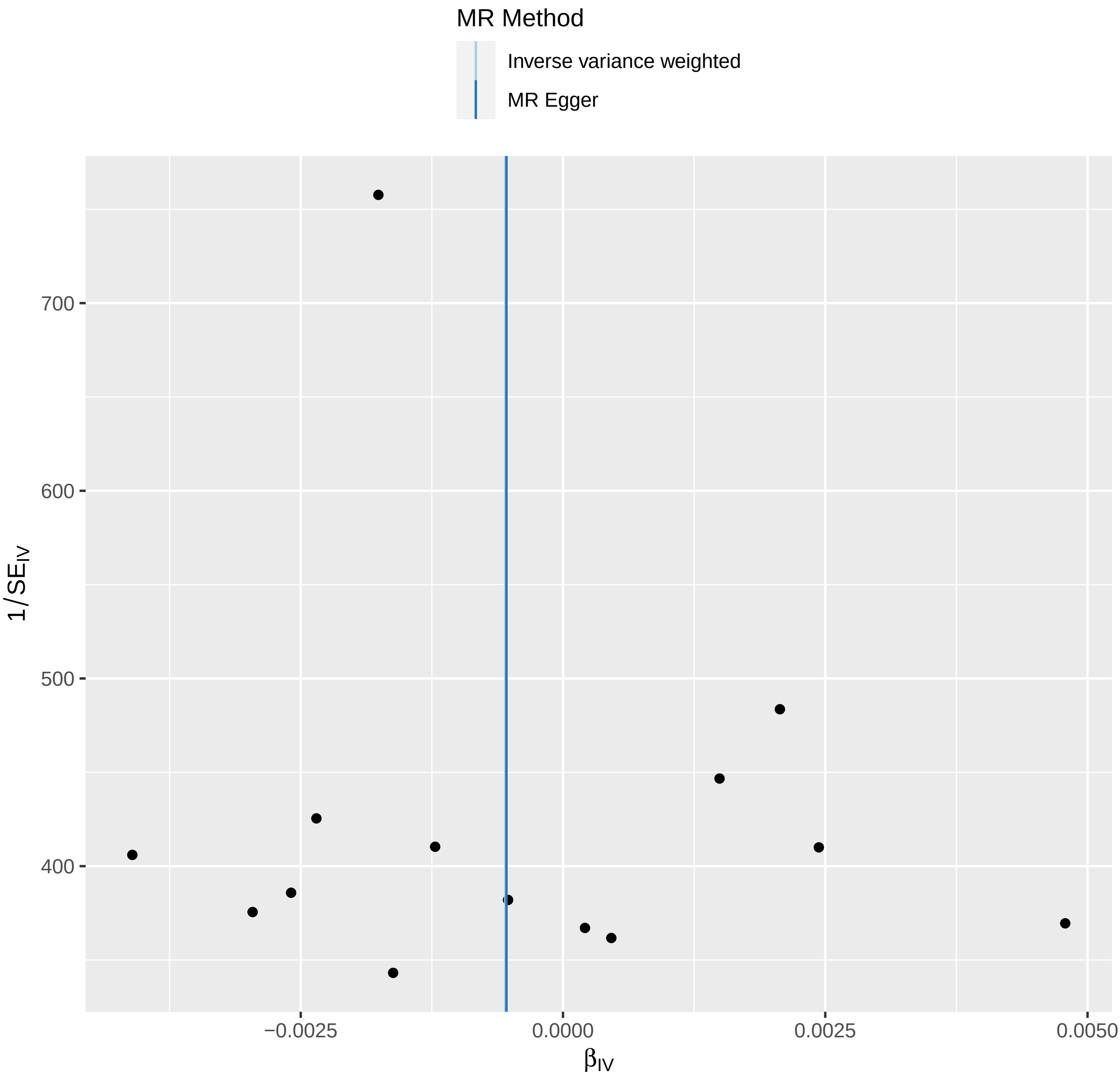

Batch 2−1021 : Protein FAM107B on eGFR

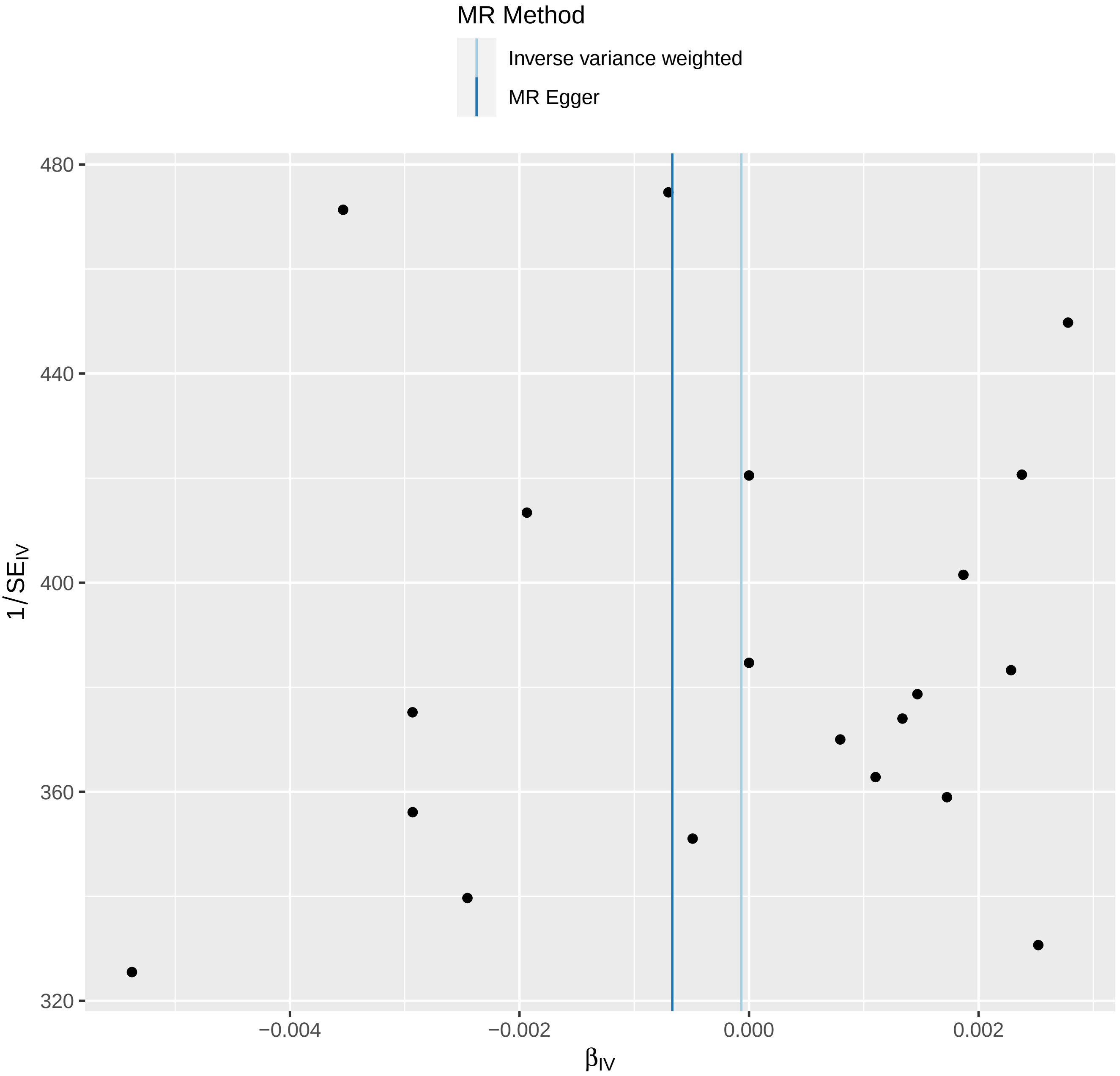

Batch 2−1022 : Protein FAM107B on eGFR

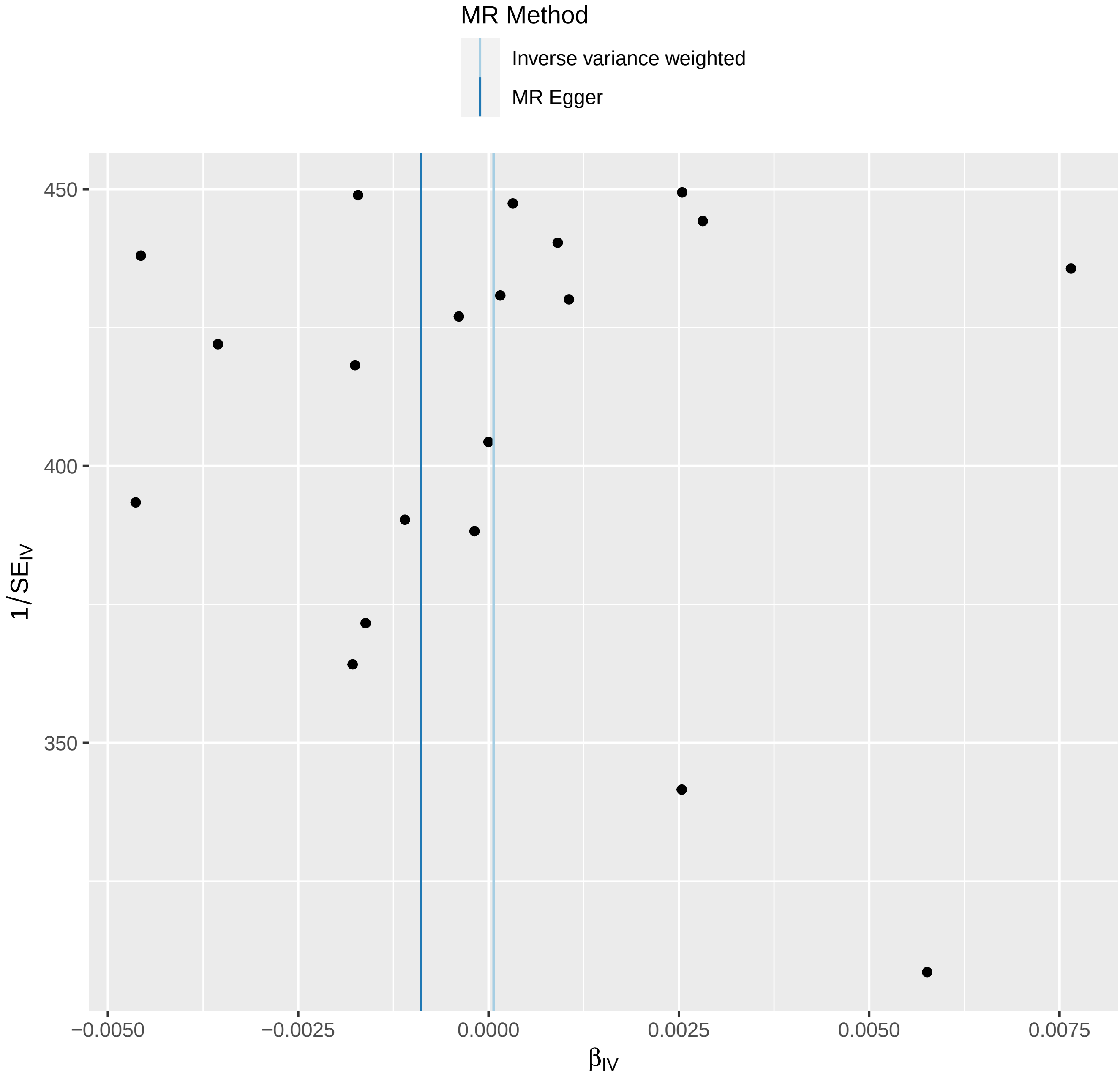

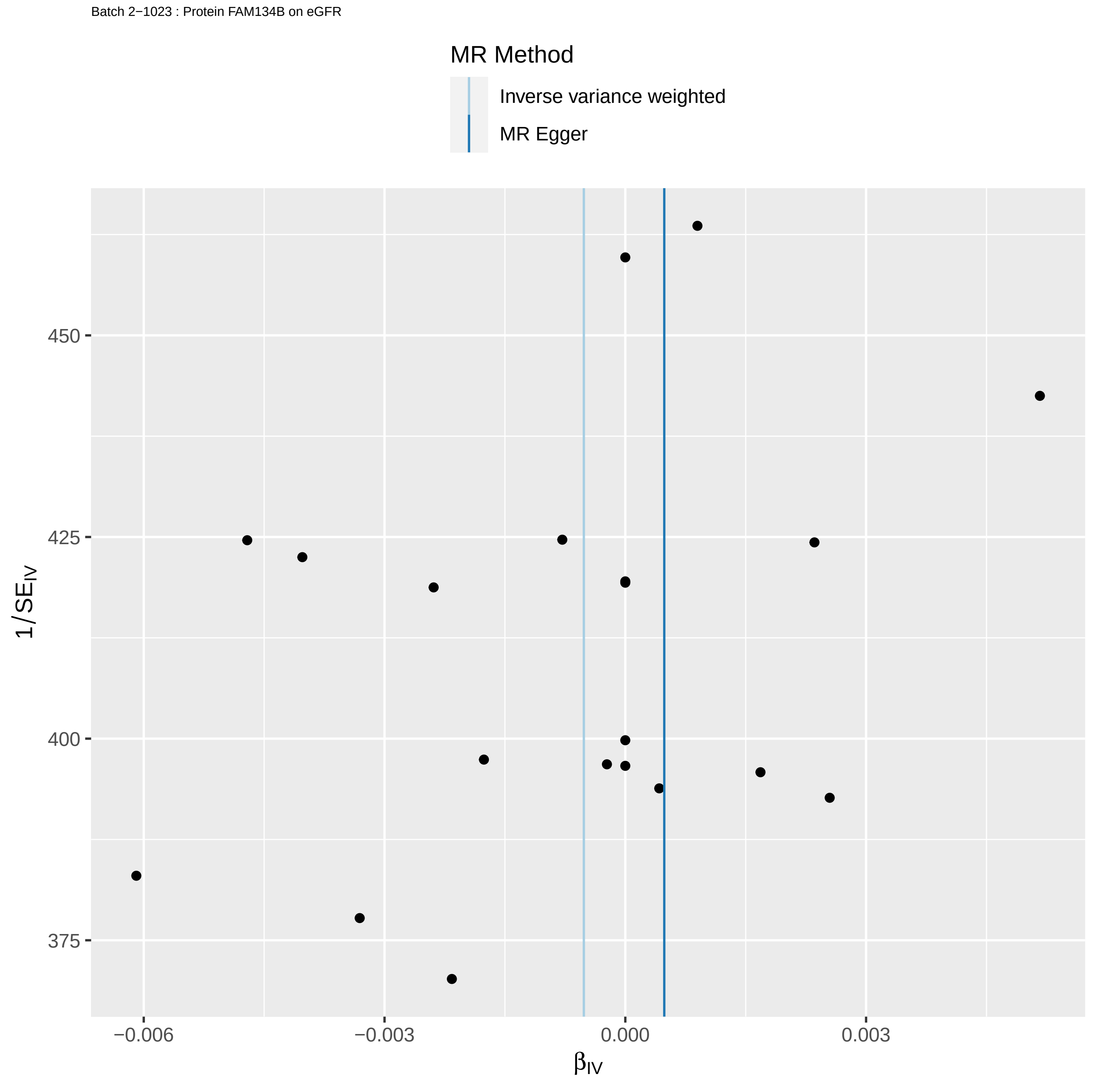
Batch 2−1023 : Protein FAM134B on eGFR
MR Method
Inverse variance weighted
MR Egger
450
425
400
375
1/SE_IV
−0.006
−0.003
0.000
0.003
β_IV

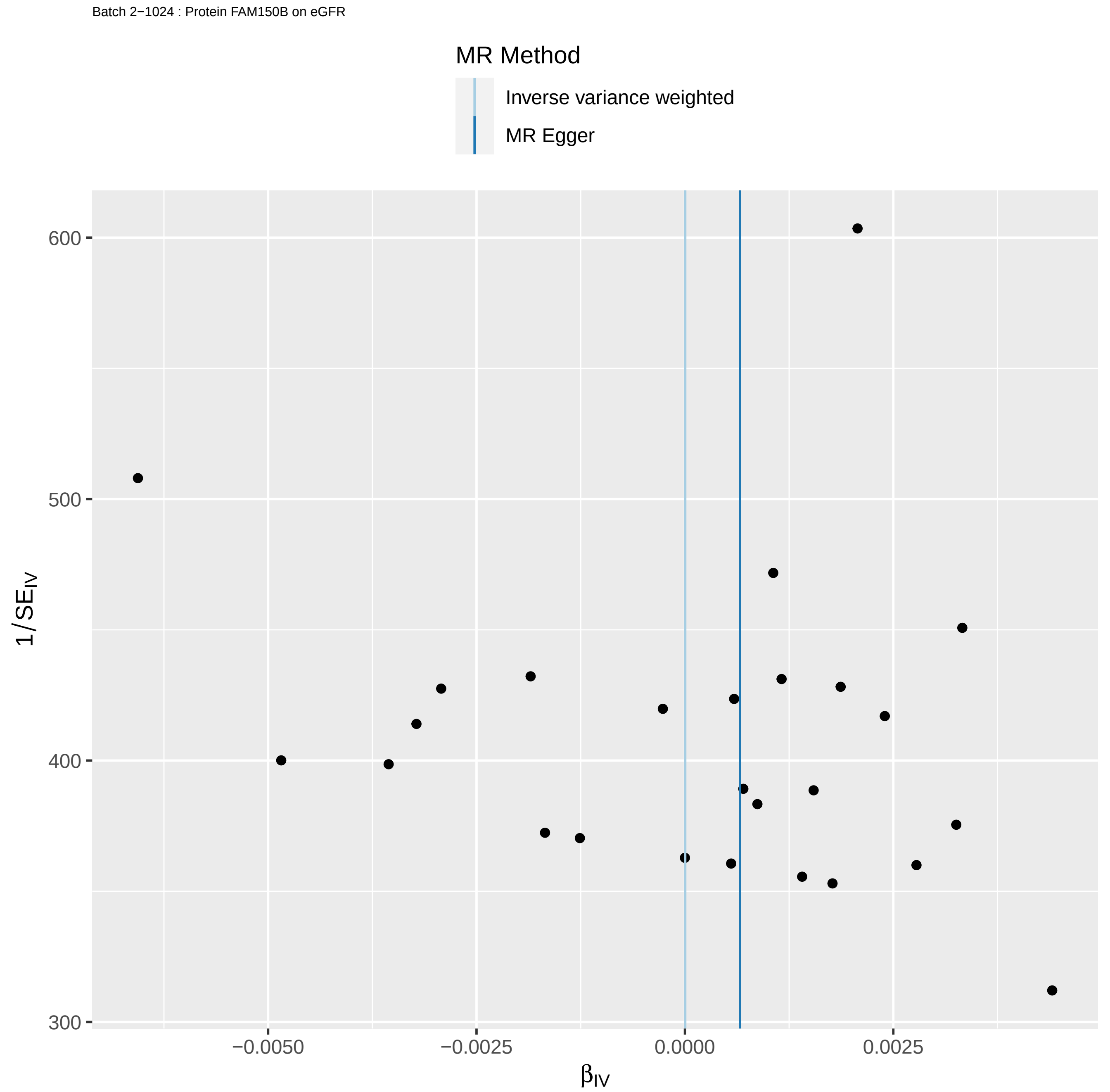

Batch 2−1024 : Protein FAM150B on eGFR
MR Method
Inverse variance weighted
MR Egger
600
500
400
300
1/SE$_{IV}$
−0.0050
−0.0025
0.0000
0.0025
β$_{IV}$

Batch 2−1025 : Protein FAM151A on eGFR

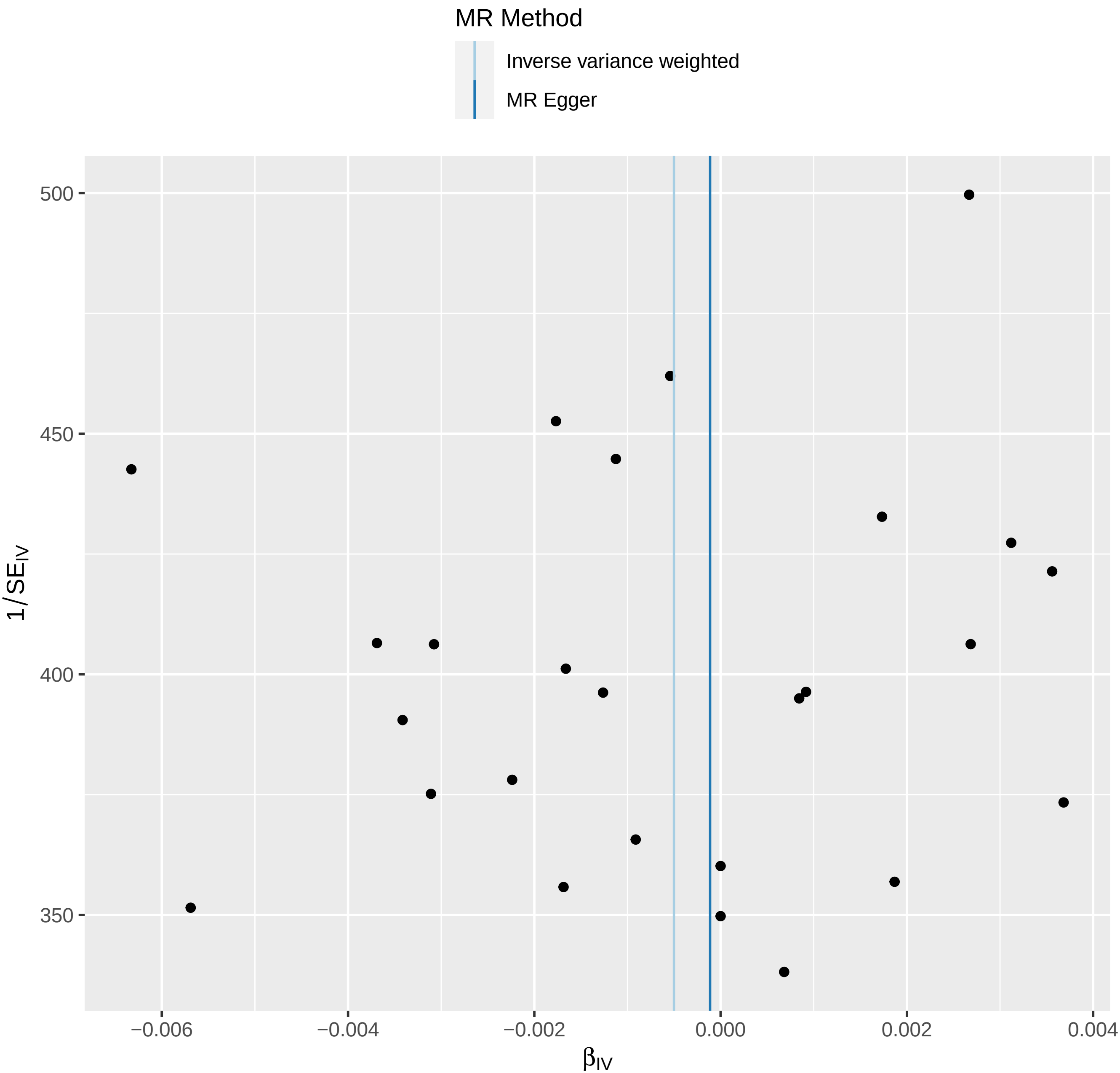

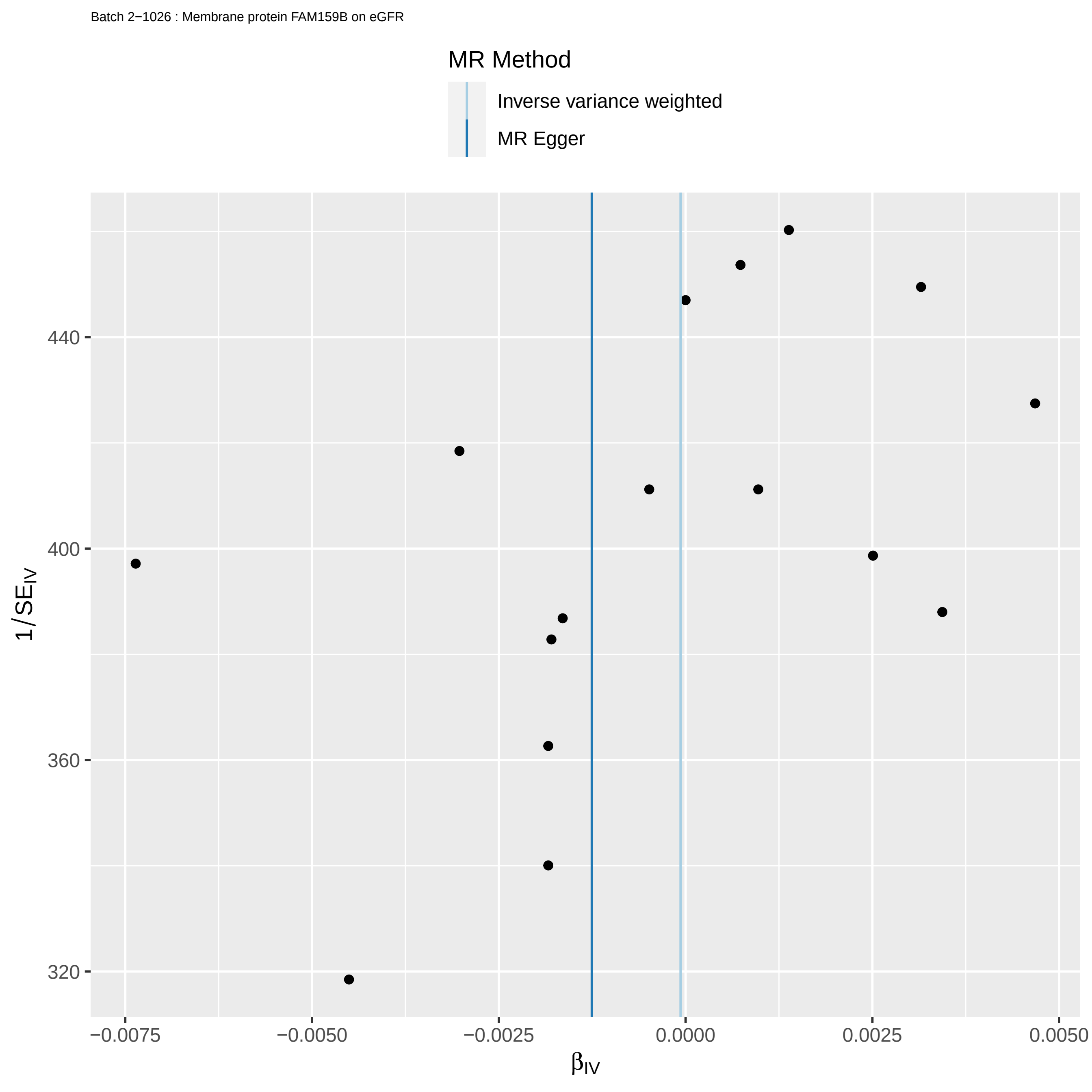
Batch 2−1026 : Membrane protein FAM159B on eGFR
MR Method
Inverse variance weighted
MR Egger
440
400
360
320
1/SE_IV
−0.0075
−0.0050
−0.0025
0.0000
0.0025
0.0050
β_IV

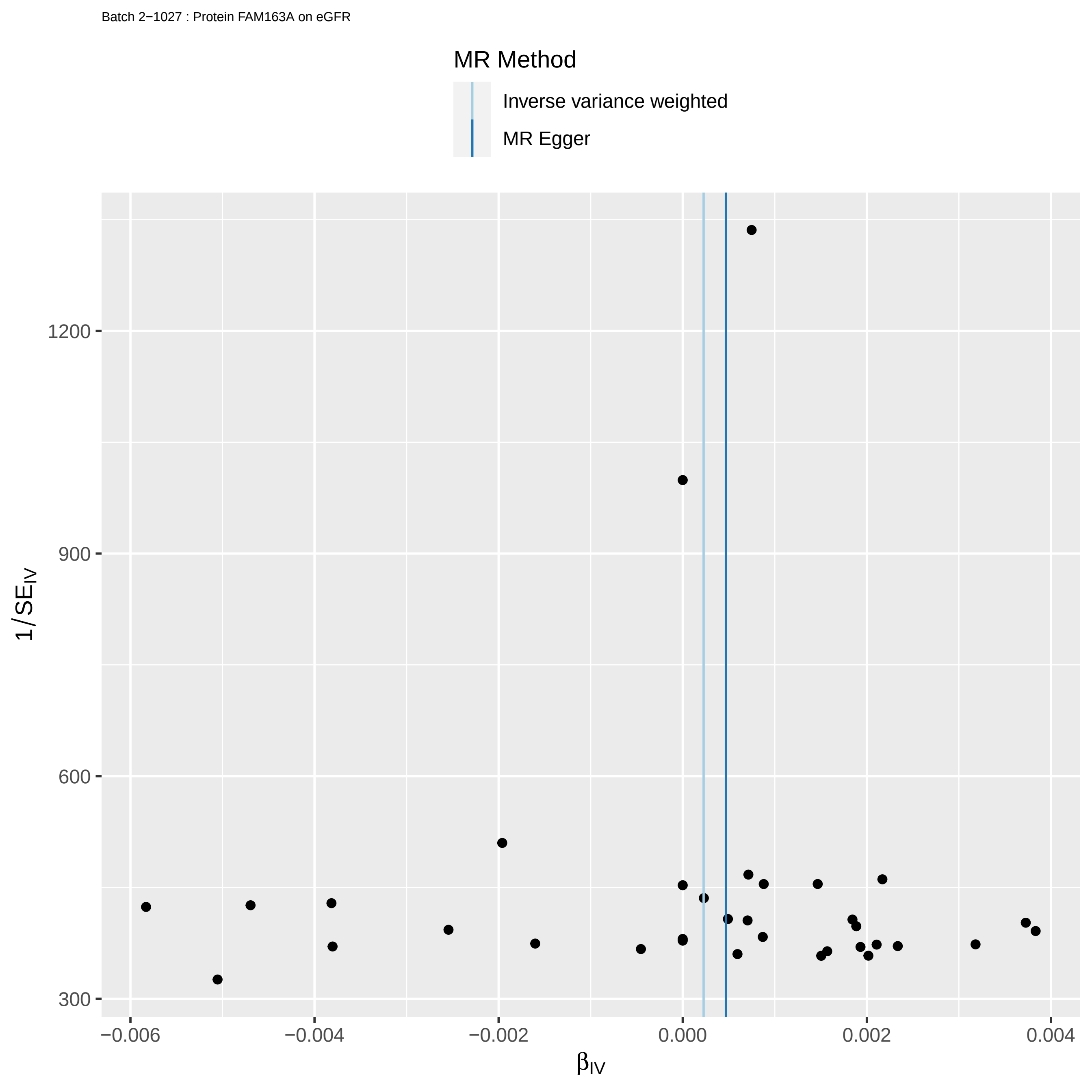

Batch 2−1027 : Protein FAM163A on eGFR
MR Method
Inverse variance weighted
MR Egger
1200
900
600
300
1/SE$_{IV}$
−0.006
−0.004
−0.002
0.000
0.002
0.004
β$_{IV}$

Batch 2−1028 : Protein FAM163B on eGFR

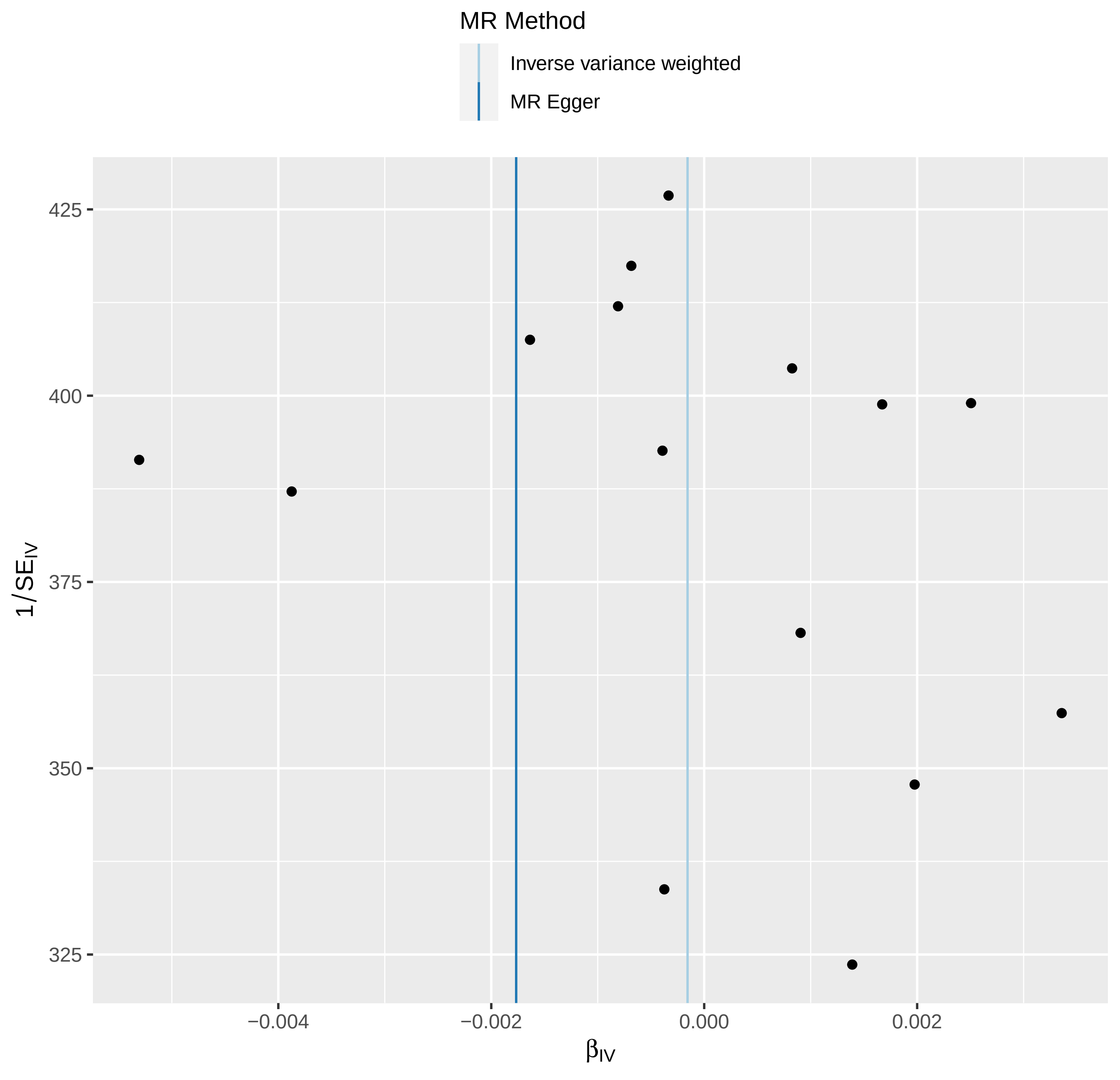

Batch 2−1029 : Protein FAM171B on eGFR

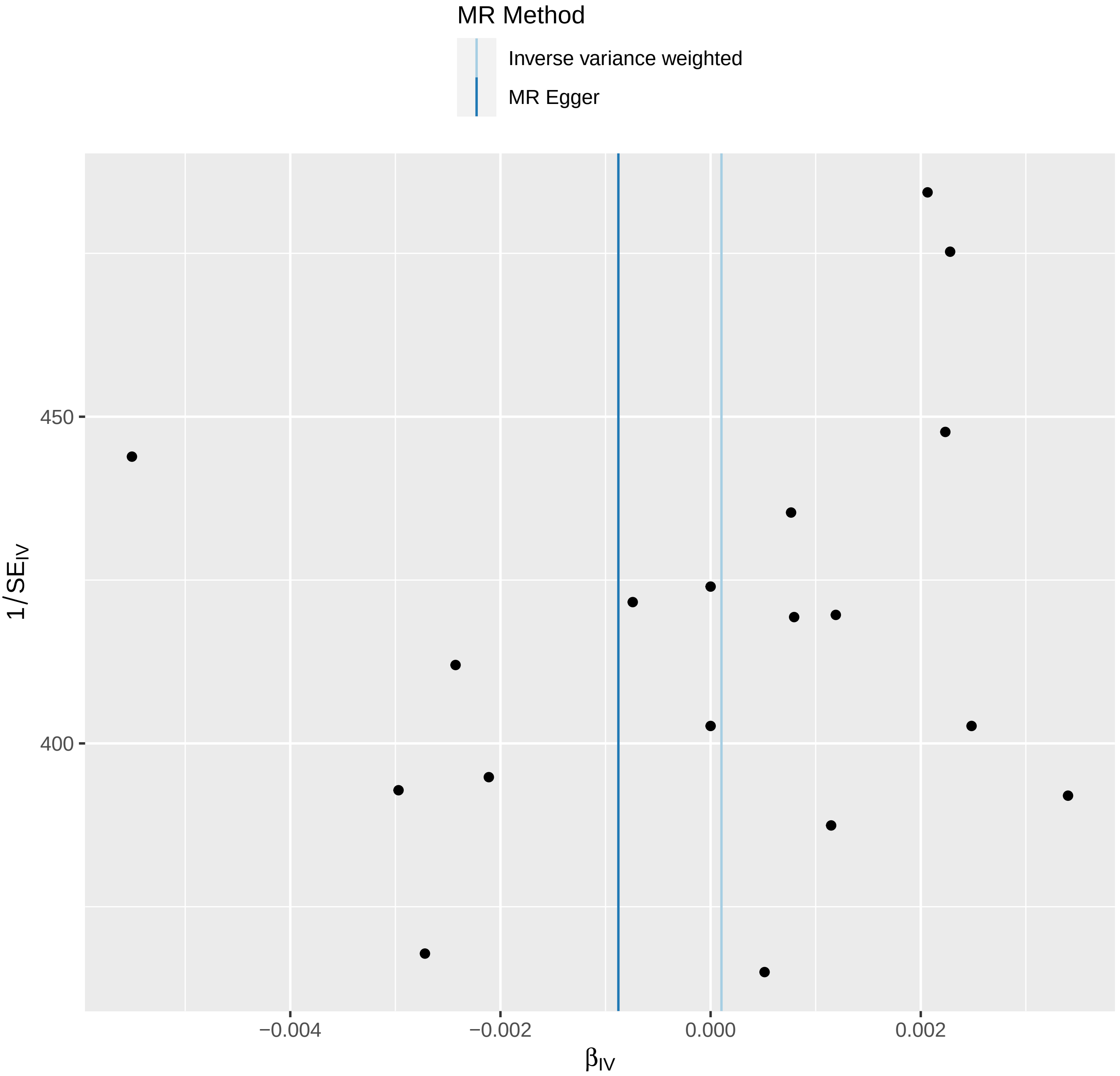

Batch 2−1030 : Protein FAM171B on eGFR

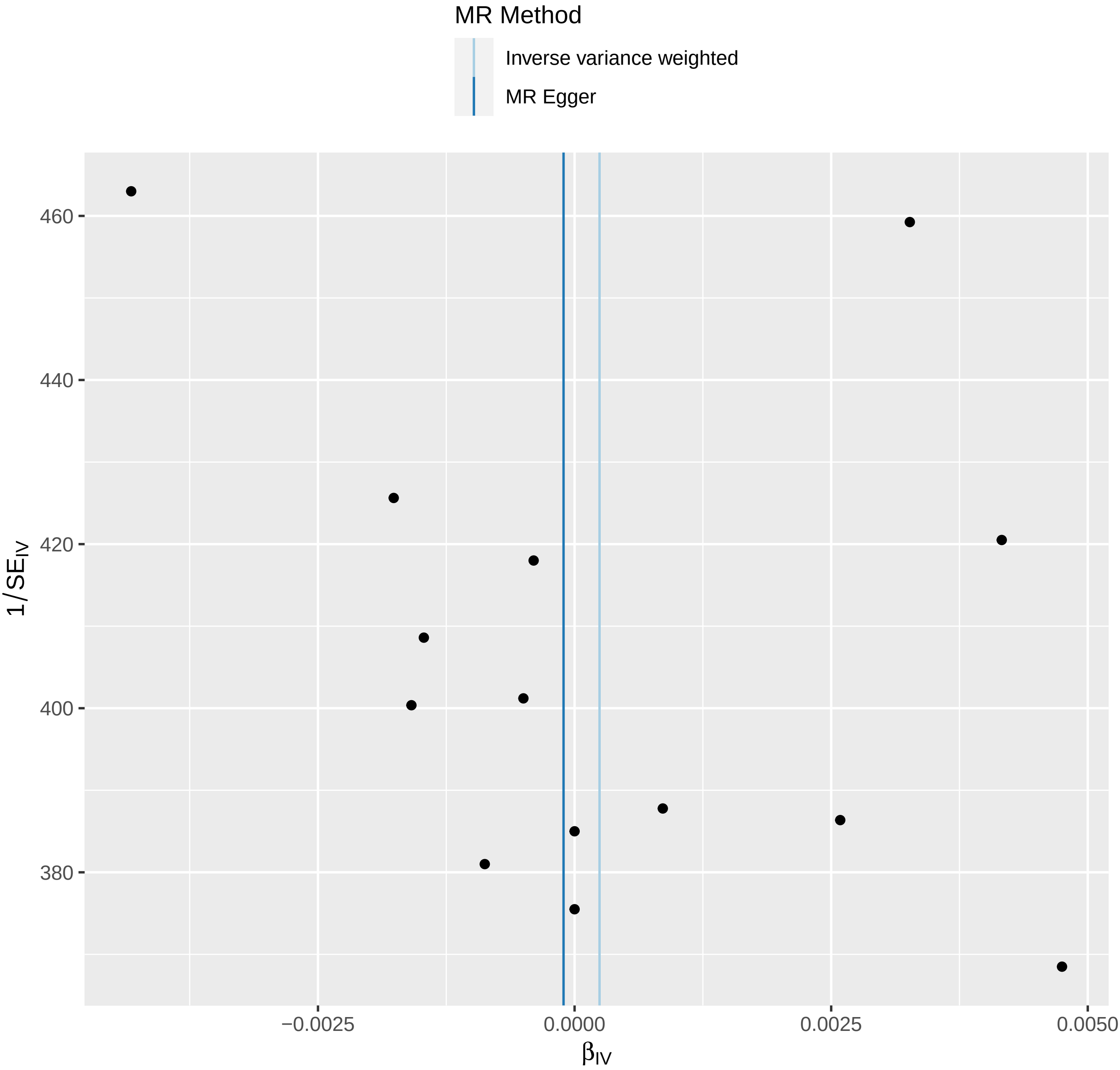

Batch 2−1031 : Protein FAM171B on eGFR

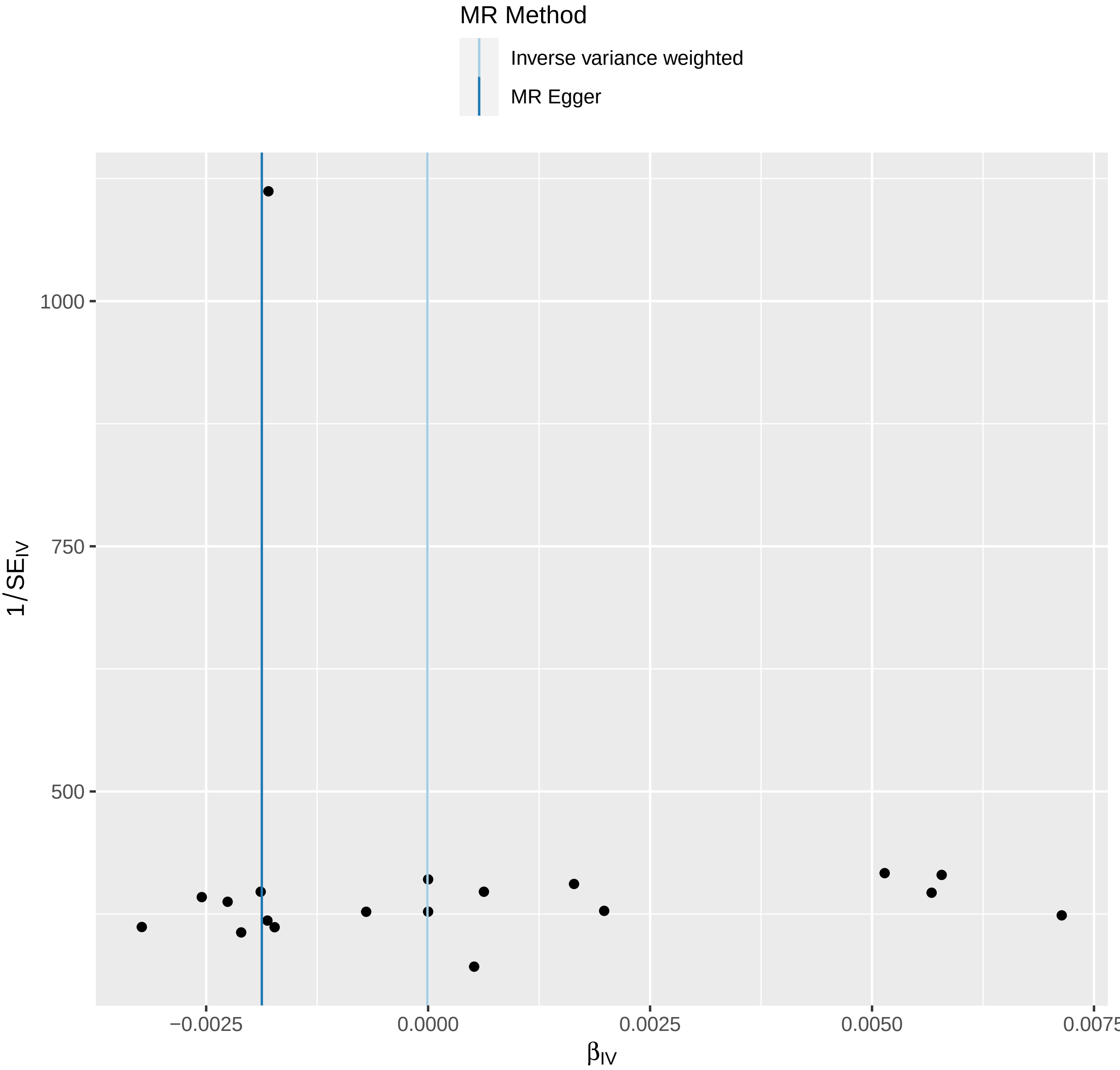

Batch 2−1032 : Protein FAM172A on eGFR

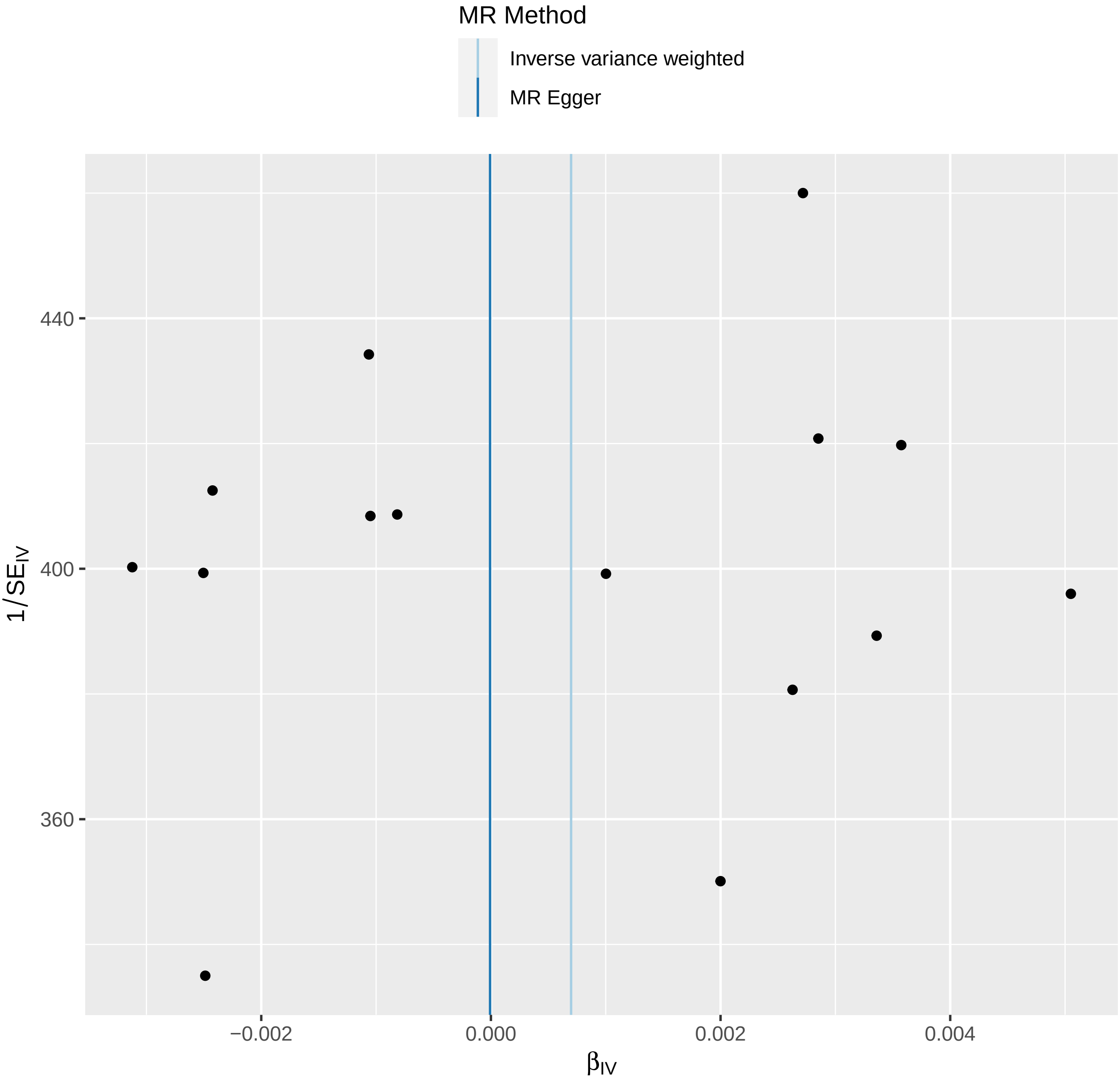

Batch 2−1033 : Protein FAM173A on eGFR

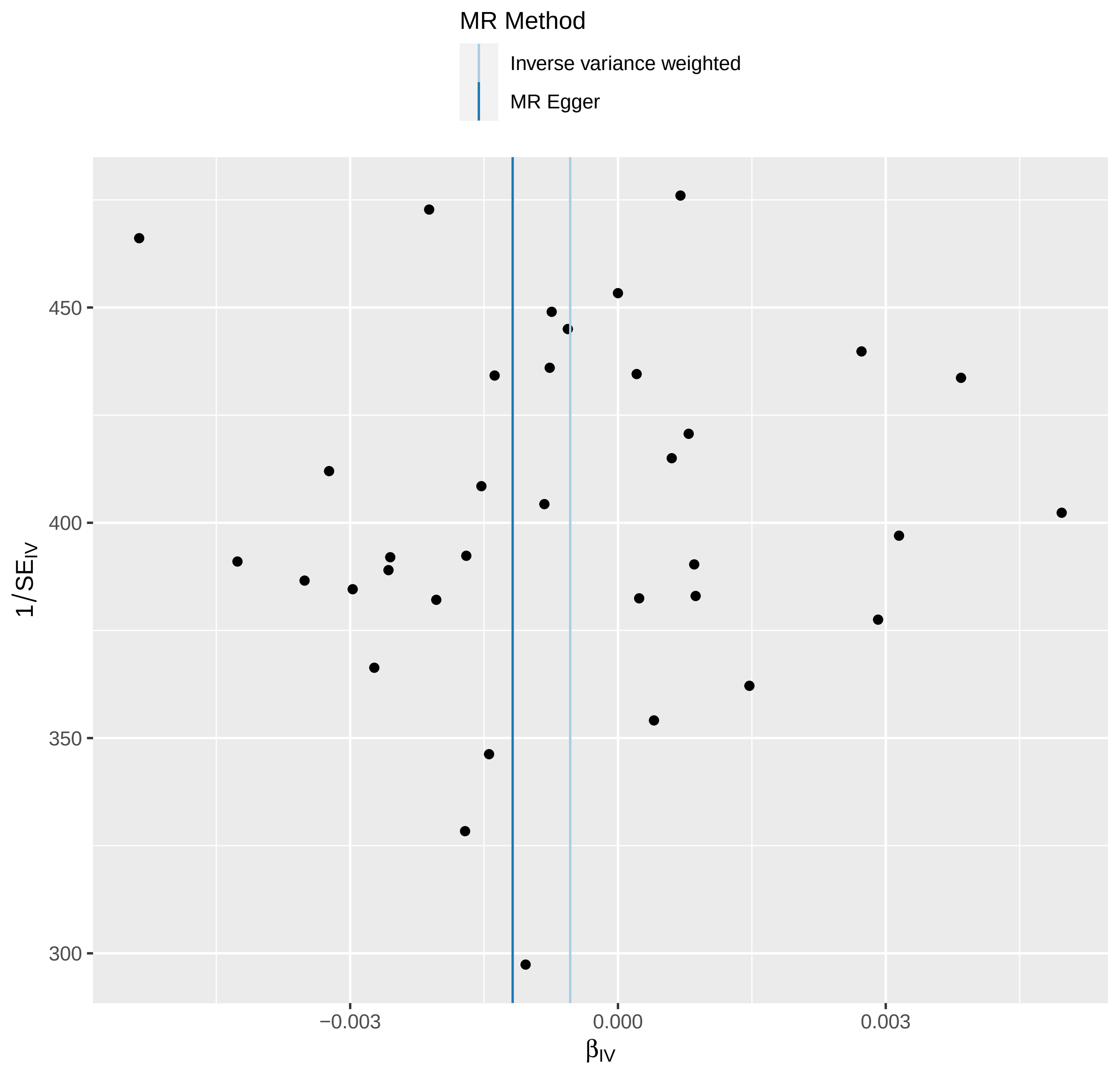

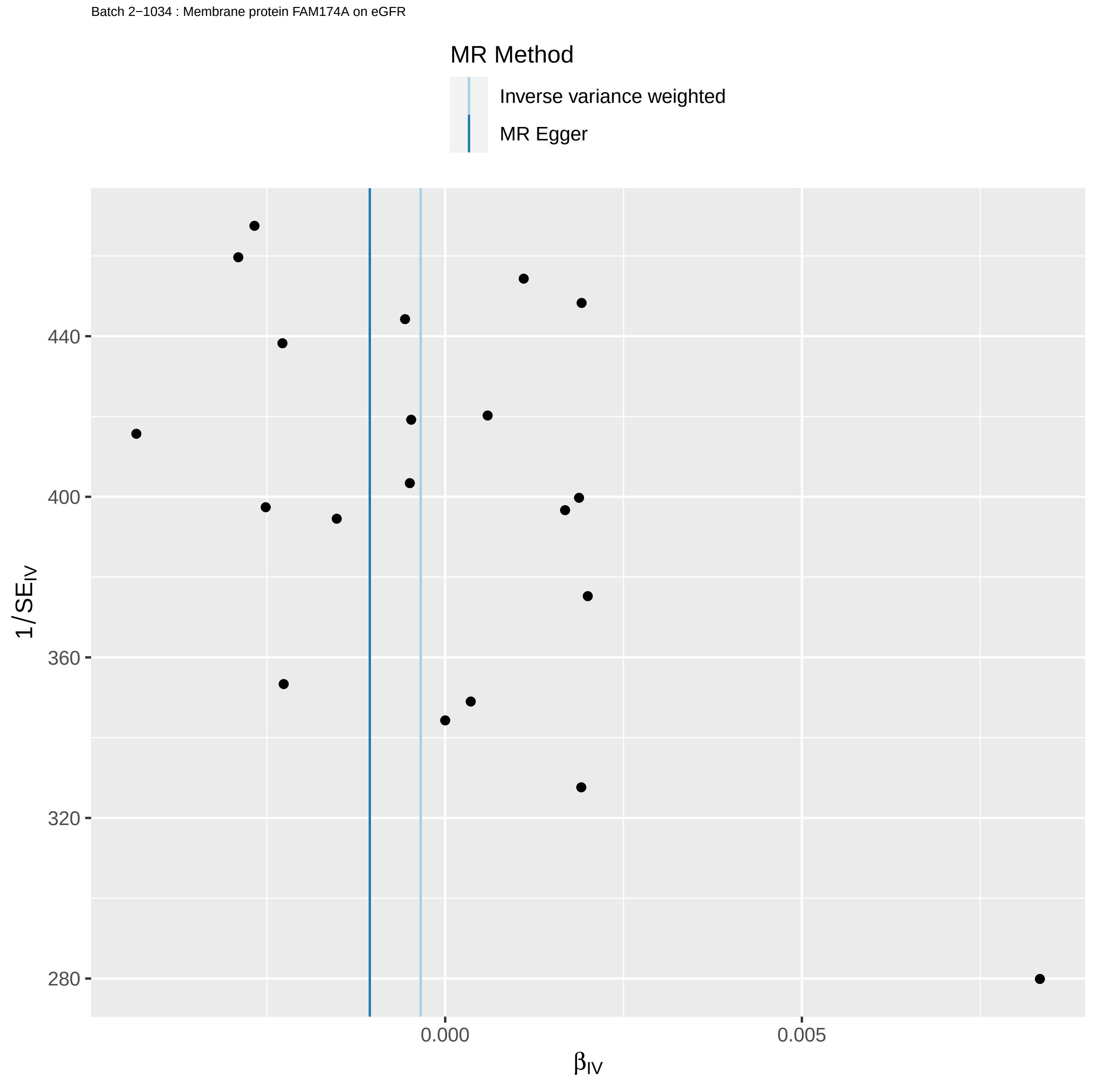

Batch 2−1034 : Membrane protein FAM174A on eGFR
MR Method
Inverse variance weighted
MR Egger
440
400
360
320
280
1/SE_IV
0.000
0.005
β_IV

Batch 2−1035 : Membrane protein FAM174B on eGFR

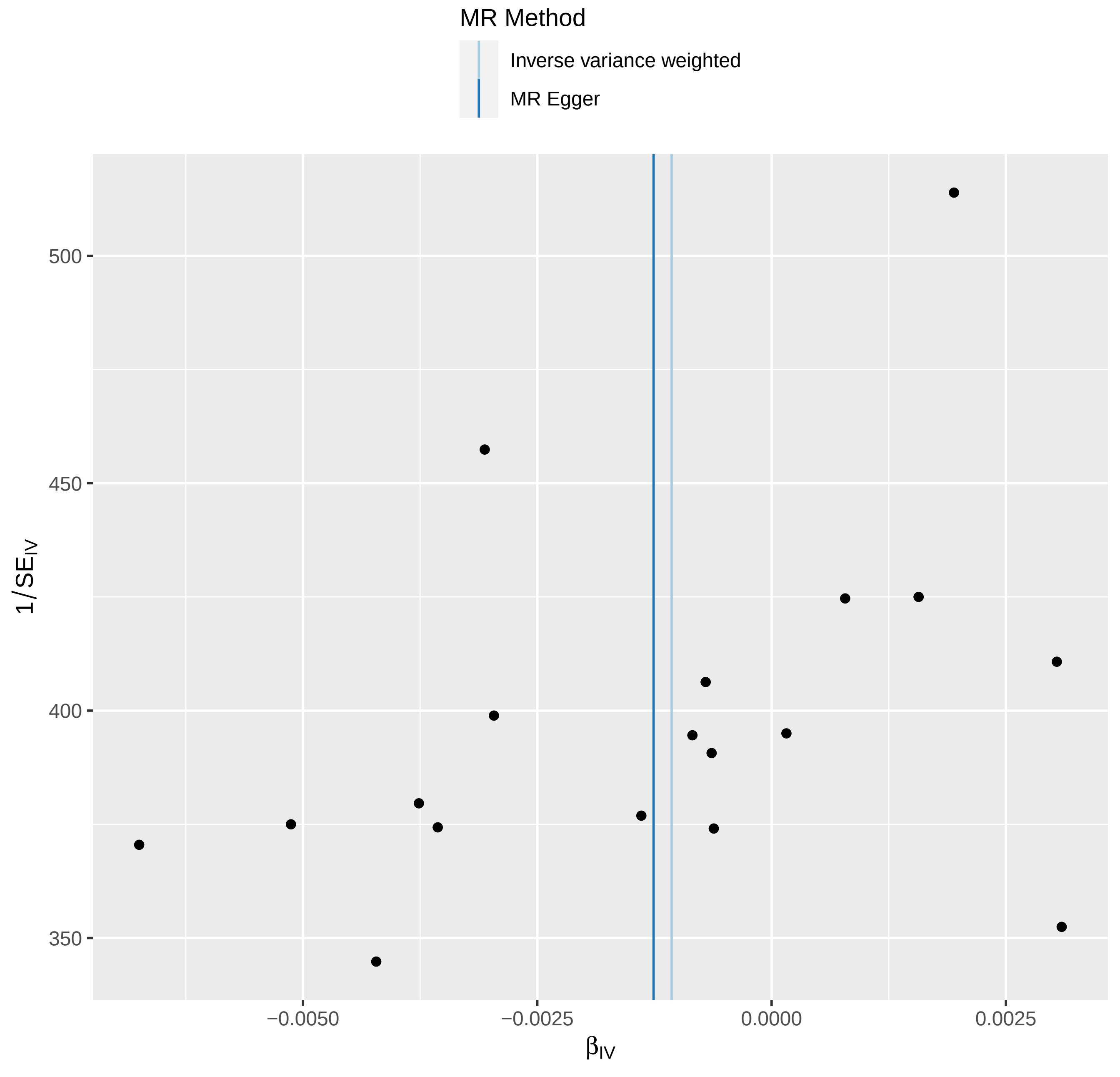

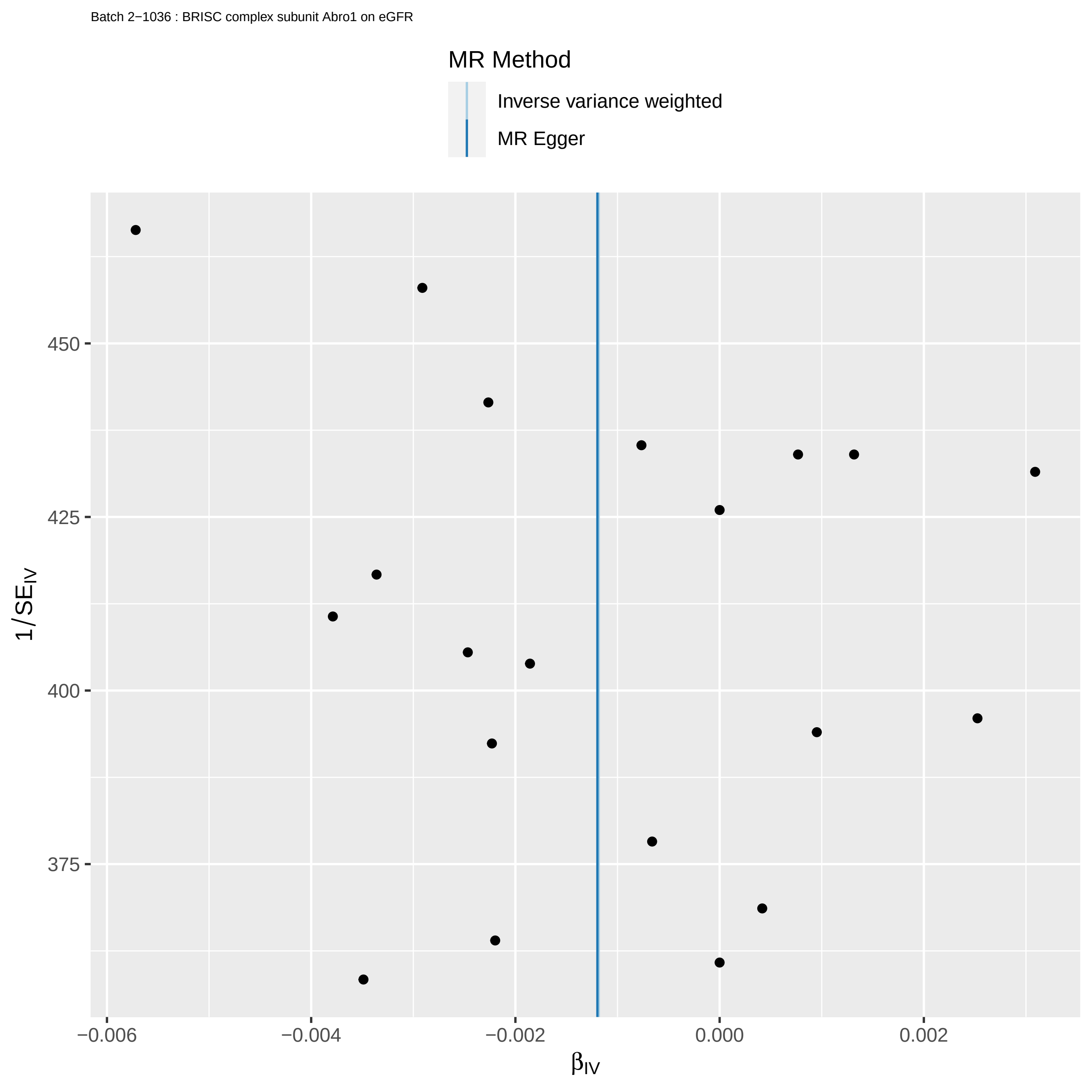
Batch 2−1036 : BRISC complex subunit Abro1 on eGFR
MR Method
Inverse variance weighted
MR Egger
450
425
400
375
1/SE$_{IV}$
−0.006
−0.004
−0.002
0.000
0.002
β$_{IV}$

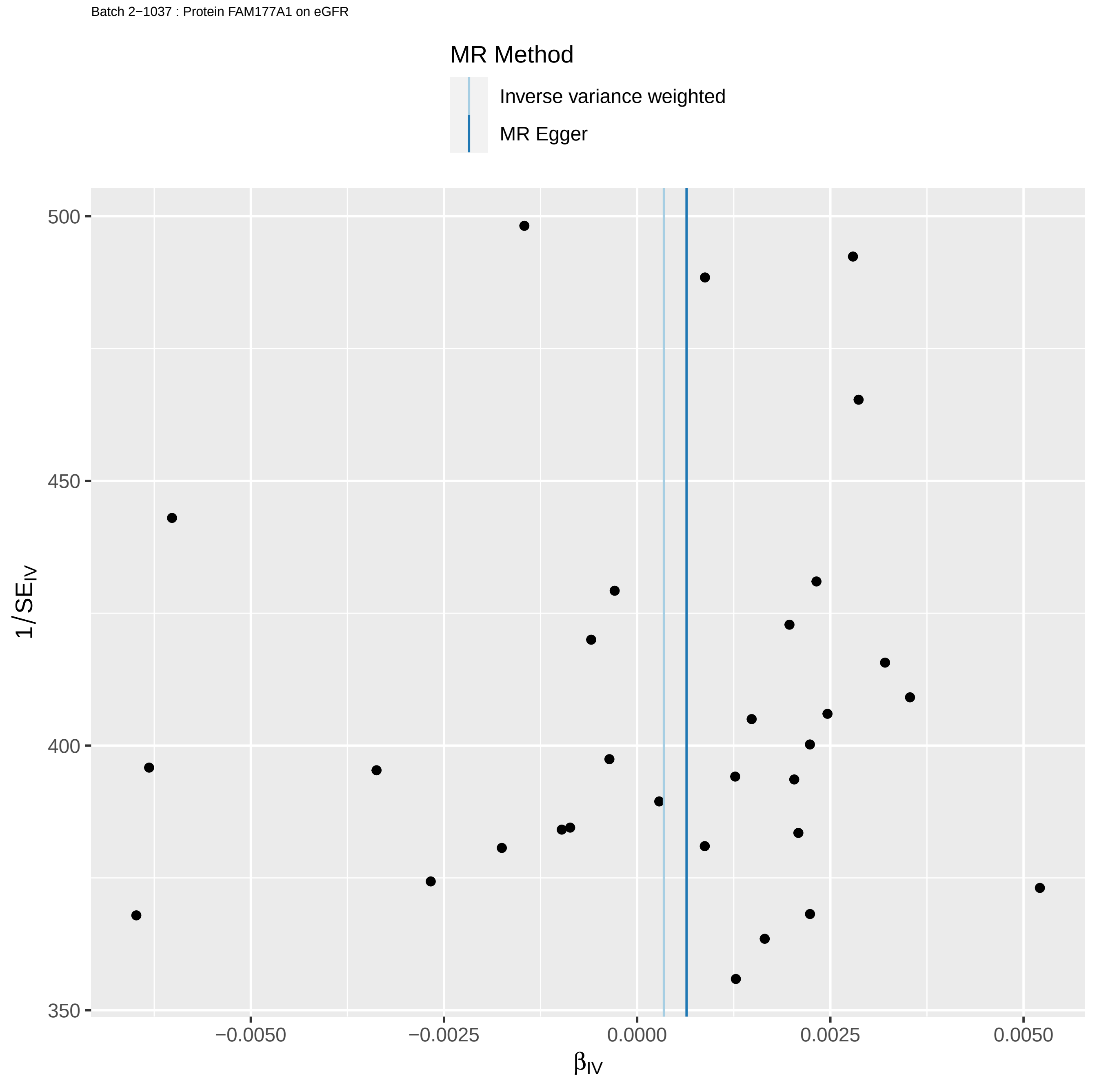

Batch 2−1037 : Protein FAM177A1 on eGFR
MR Method
Inverse variance weighted
MR Egger
500
450
400
350
1/SE_IV
−0.0050
−0.0025
0.0000
0.0025
0.0050
β_IV

Batch 2−1038 : Protein FAM189A2 on eGFR

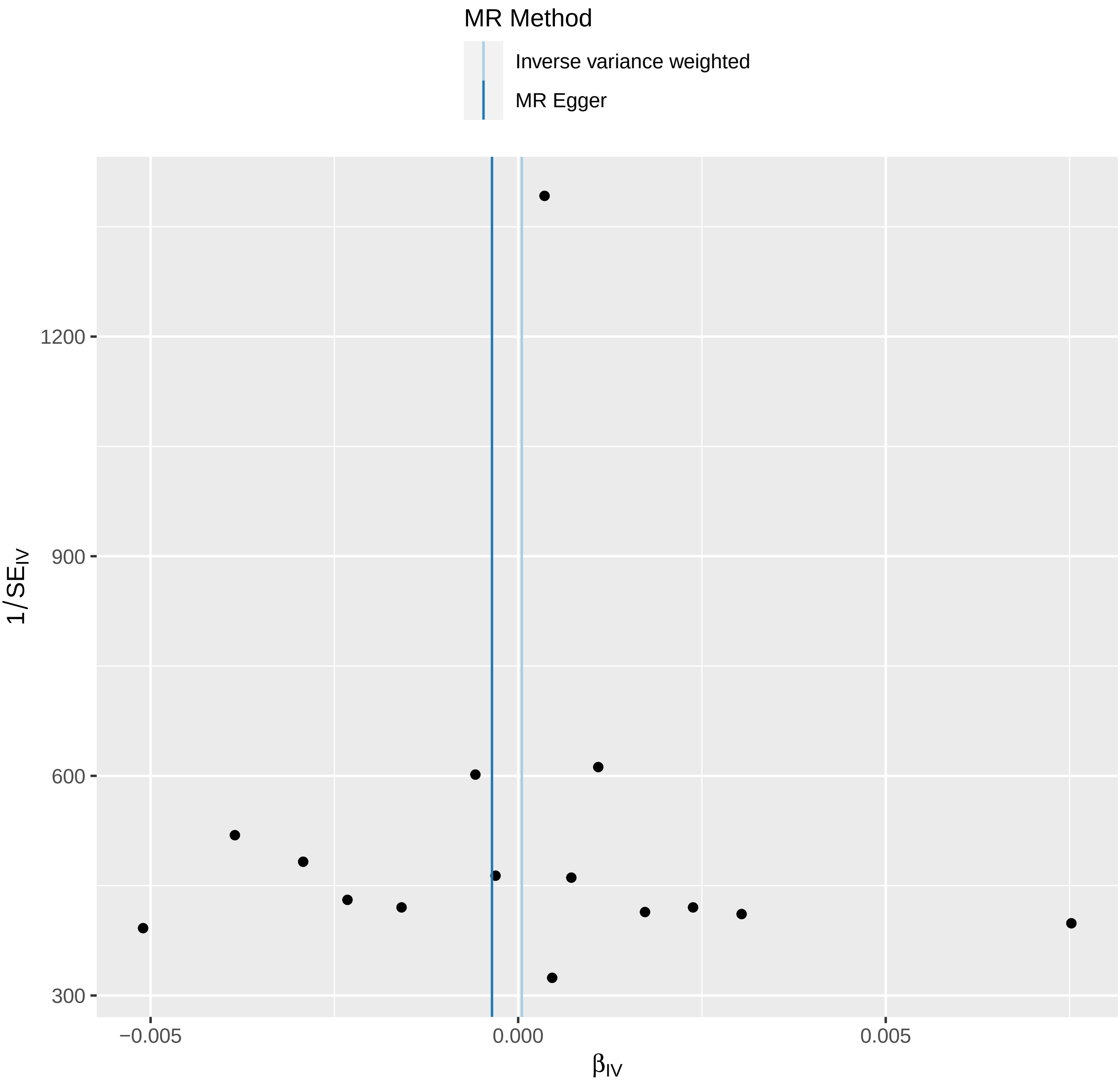

Batch 2−1039 : Protein FAM189A2 on eGFR

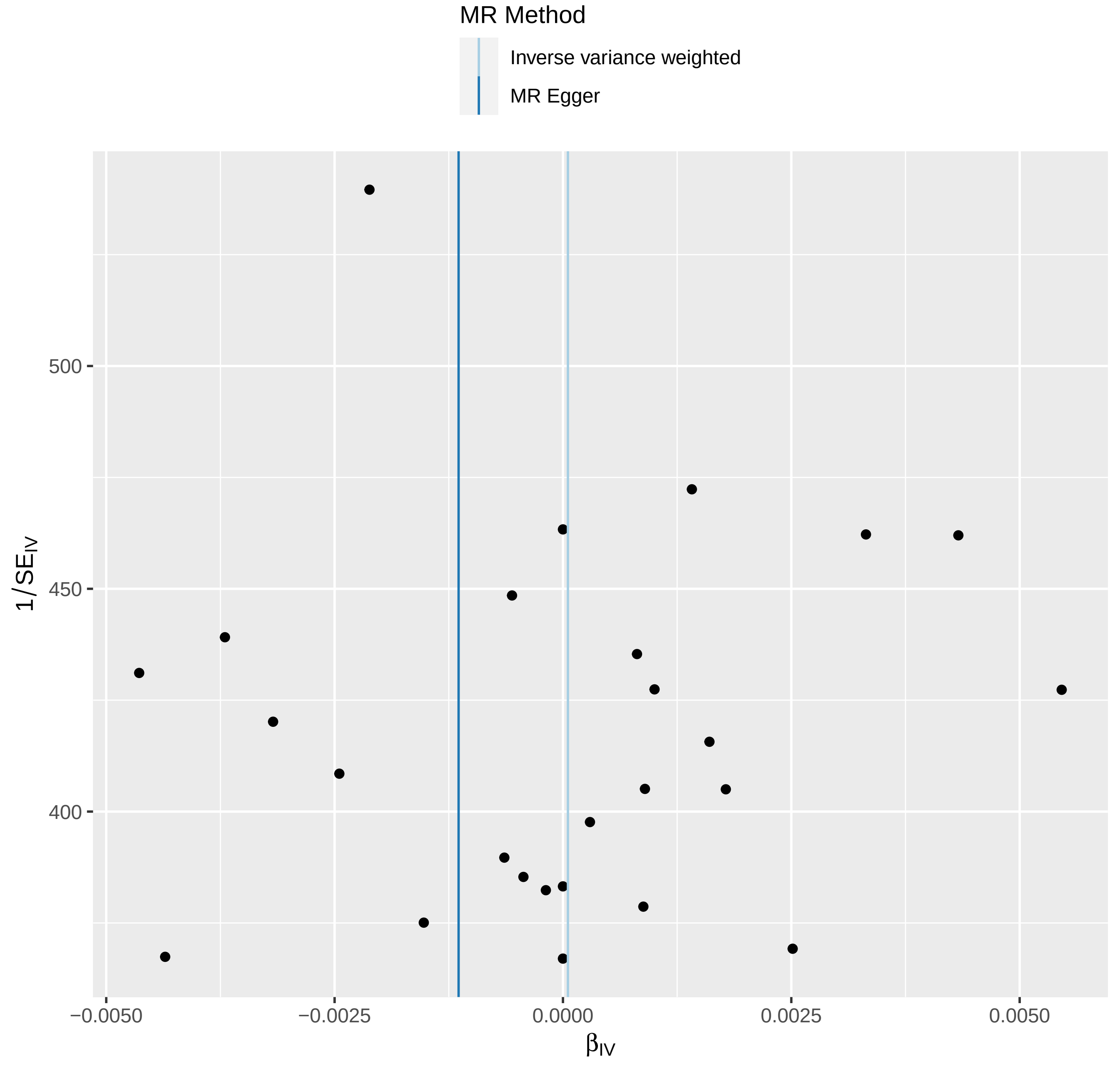

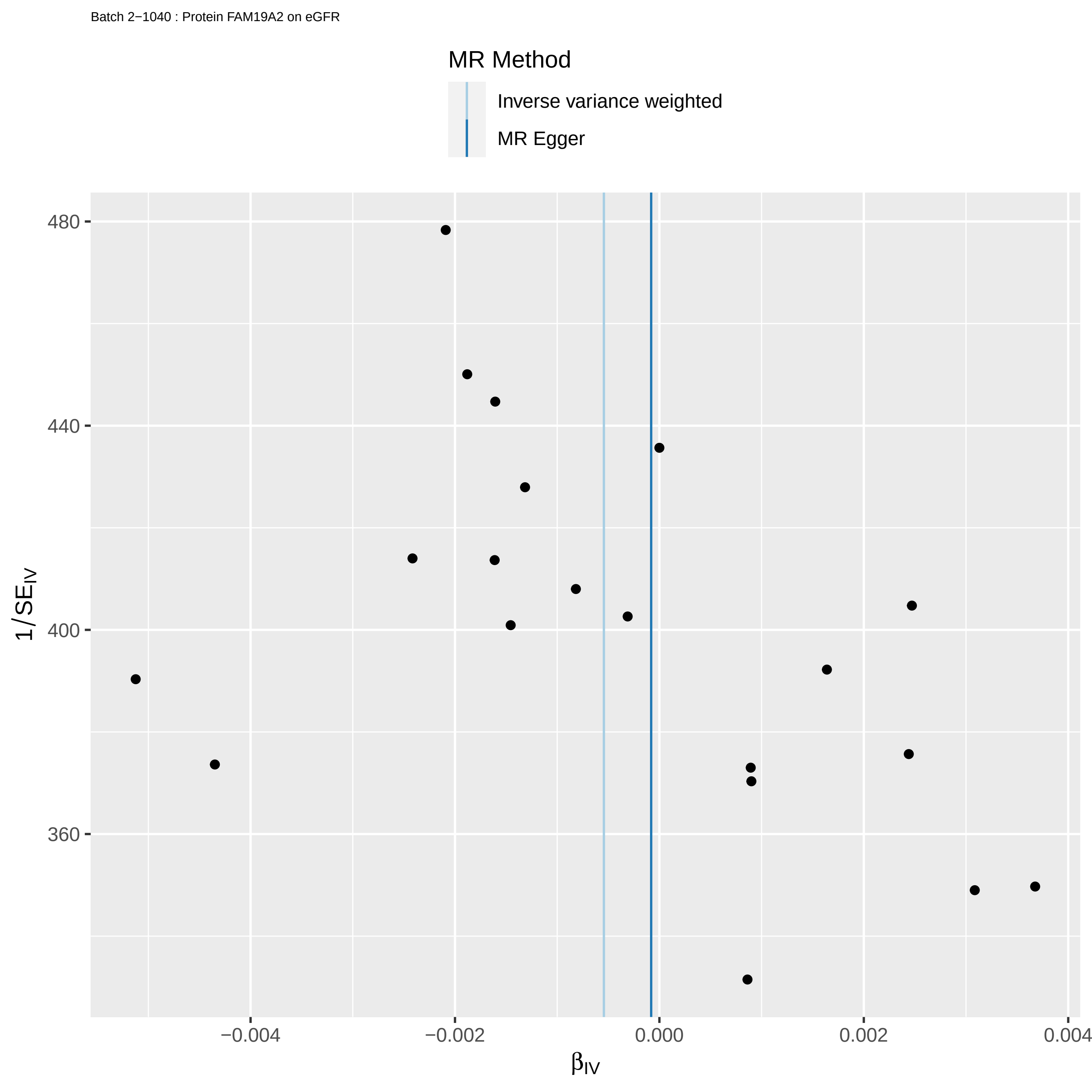
Batch 2−1040 : Protein FAM19A2 on eGFR
MR Method
Inverse variance weighted
MR Egger
480
440
400
360
$1/SE_{IV}$
−0.004
−0.002
0.000
0.002
0.004
β_{IV}

Batch 2−1041 : Protein FAM19A3 on eGFR

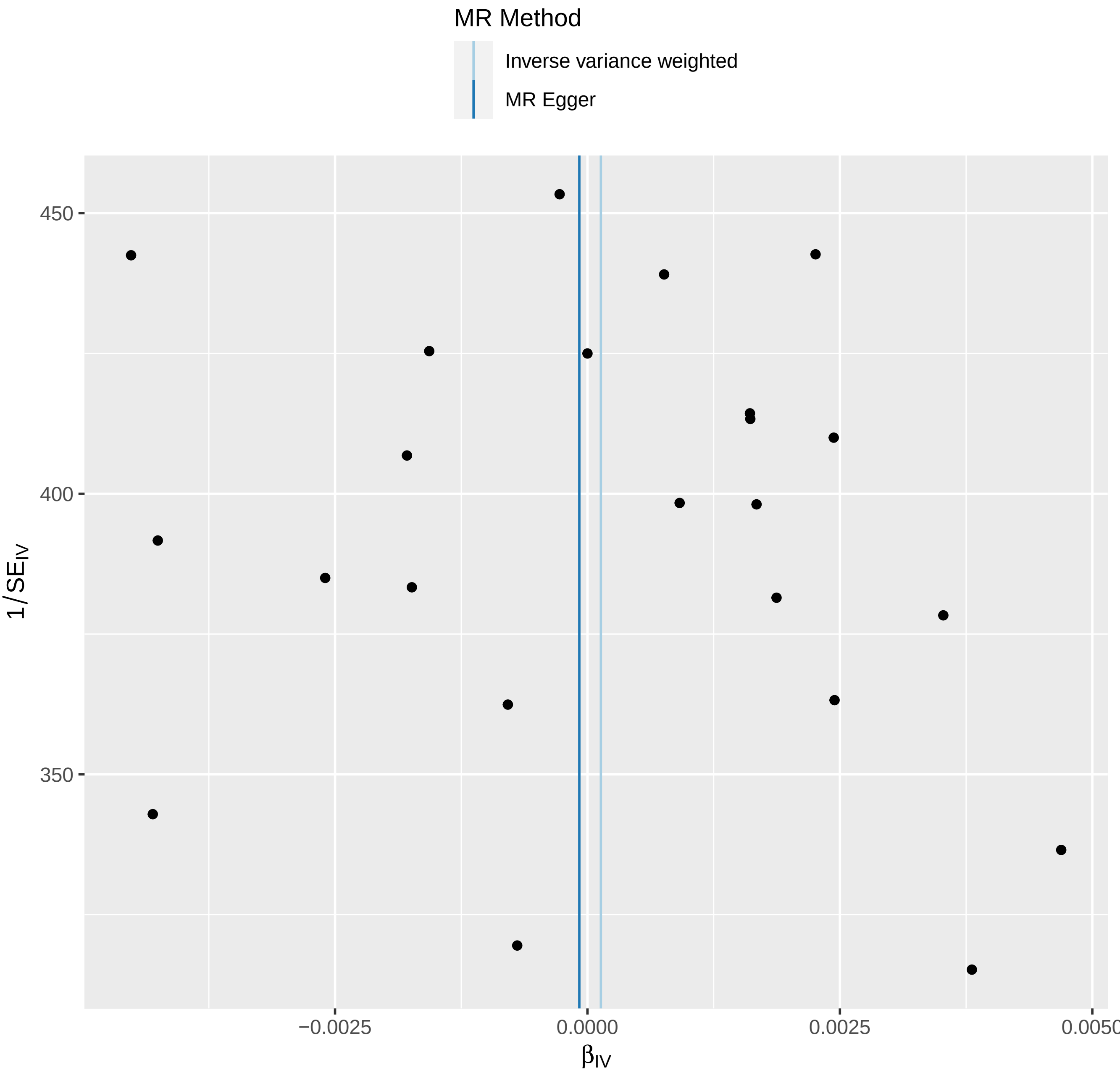

Batch 2−1042 : Protein FAM19A4 on eGFR

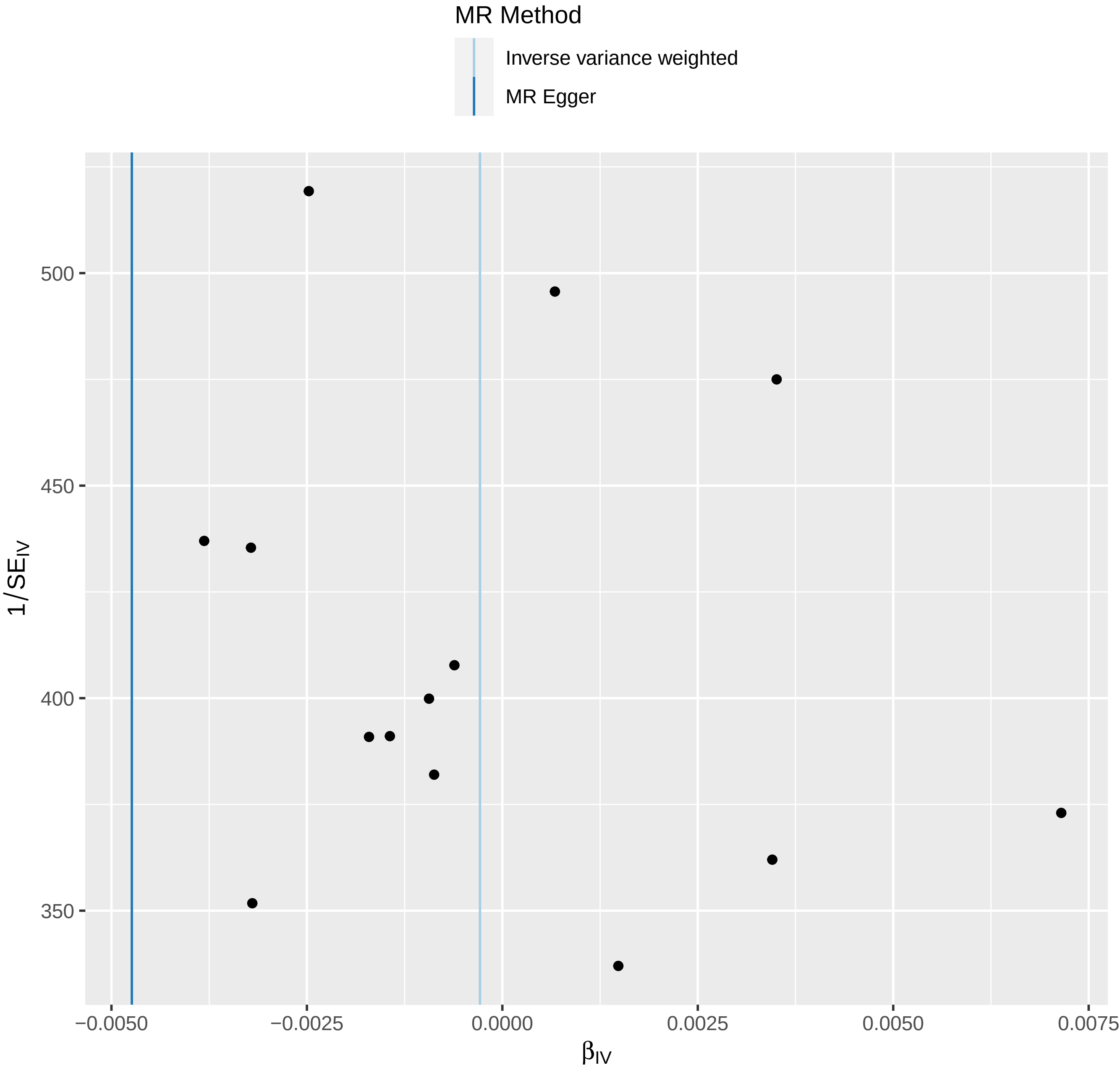

Batch 2−1043 : Protein FAM19A5 on eGFR

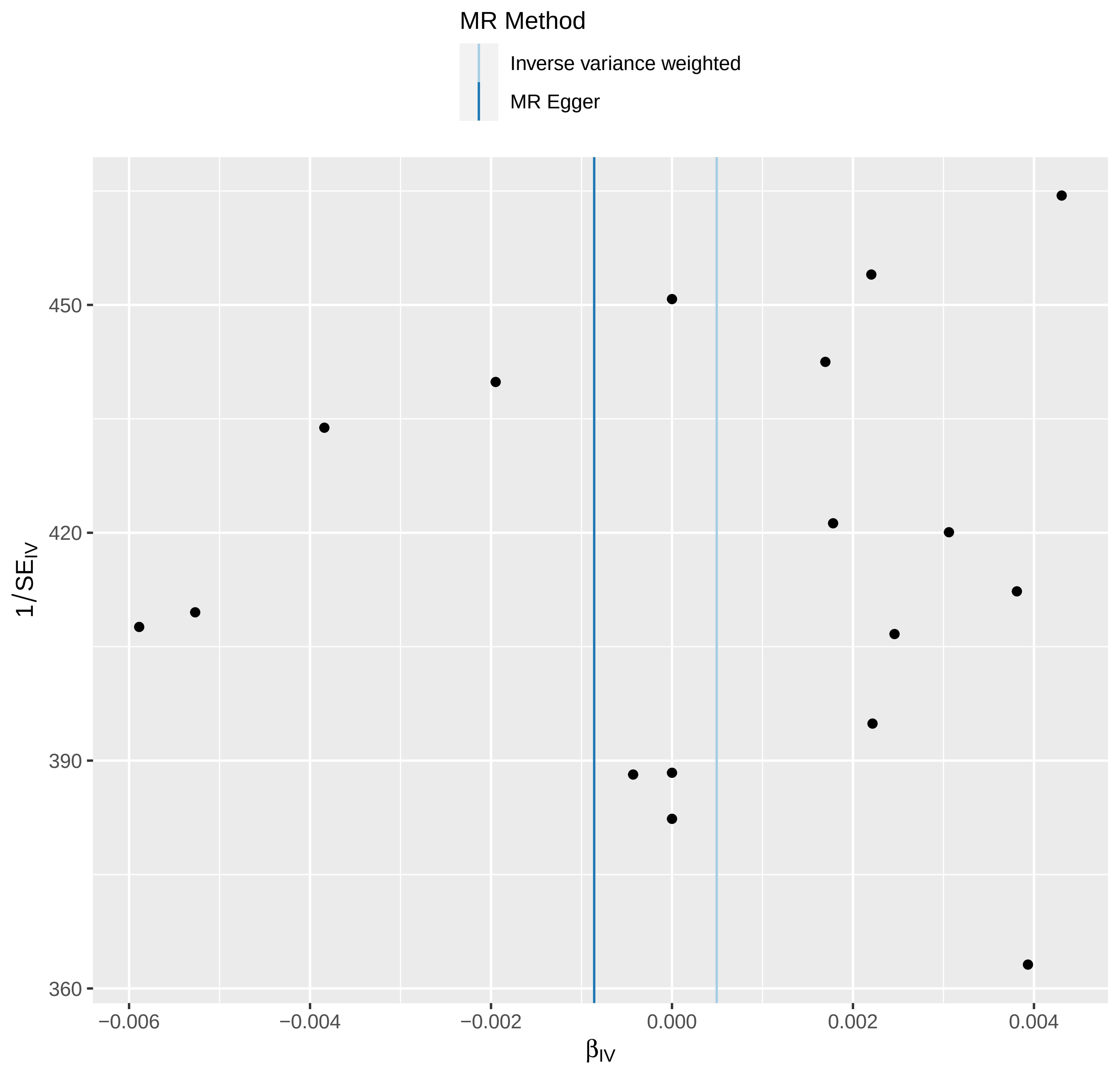

Batch 2−1044 : Protein FAM209B on eGFR

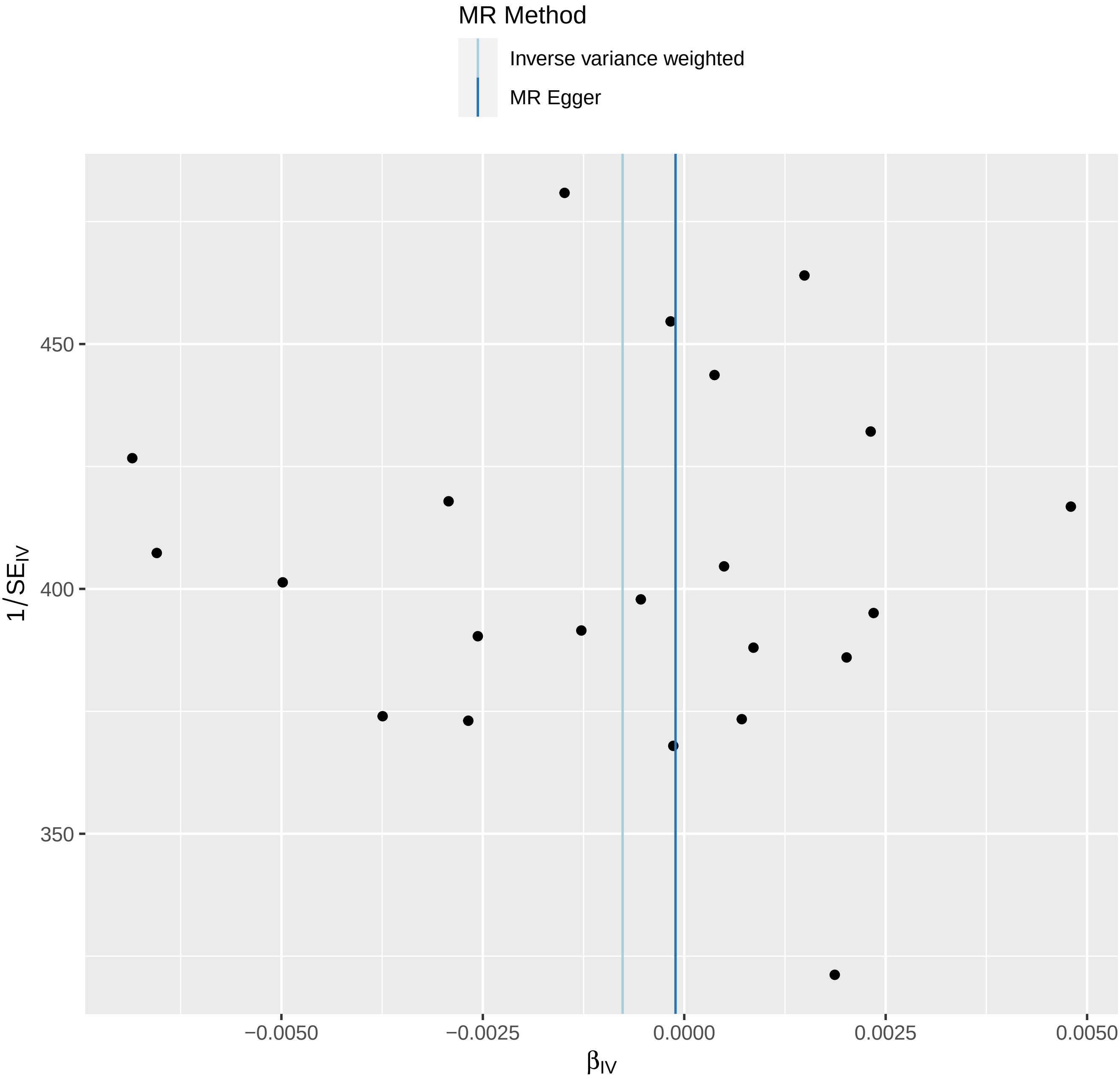

Batch 2−1045 : Pseudokinase FAM20A on eGFR

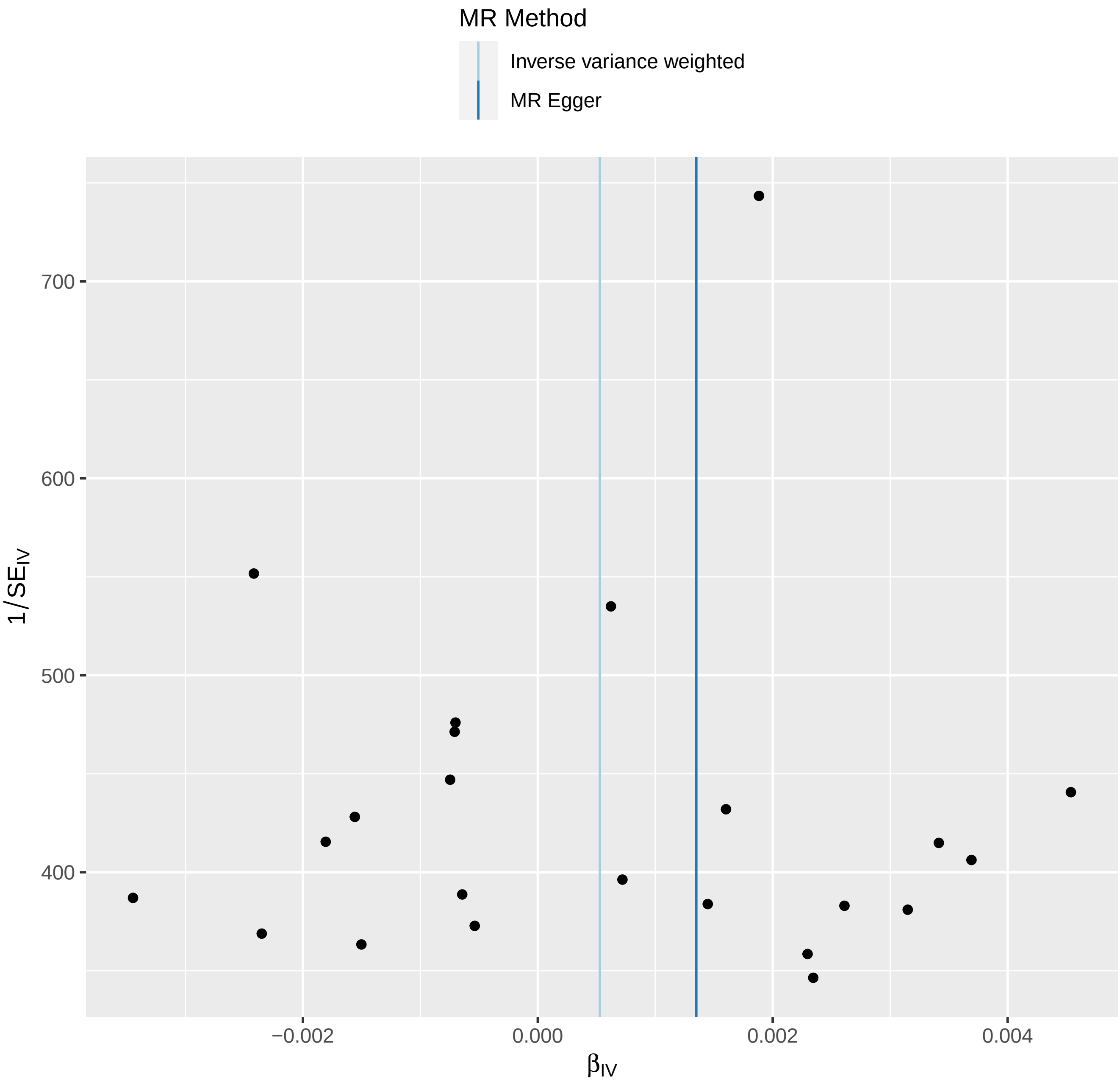

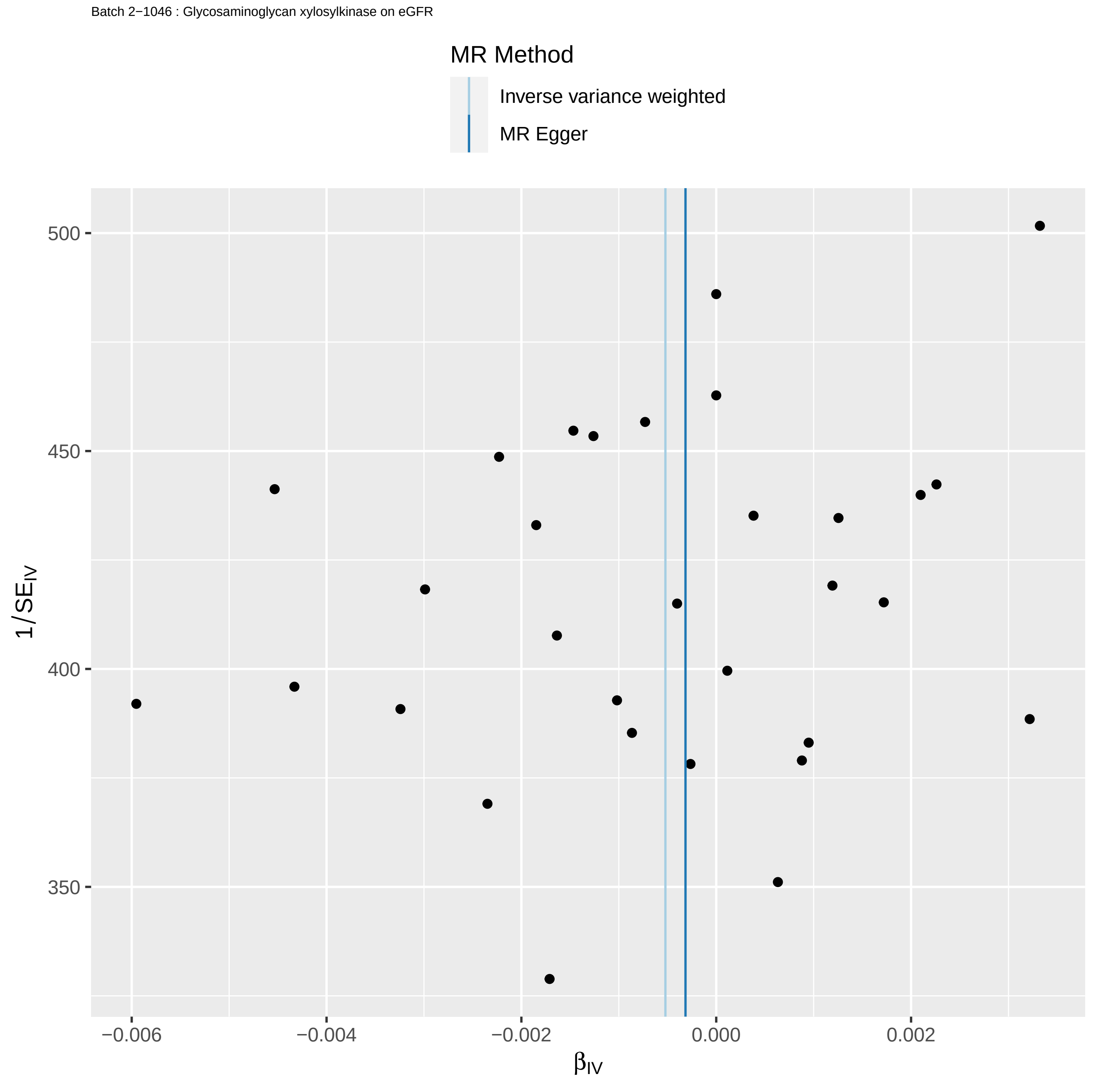

Batch 2−1046 : Glycosaminoglycan xylosylkinase on eGFR
MR Method
Inverse variance weighted
MR Egger
500
450
400
350
1/SE_IV
−0.006
−0.004
−0.002
0.000
0.002
β_IV

Batch 2−1047 : Redox−regulatory protein FAM213A on eGFR

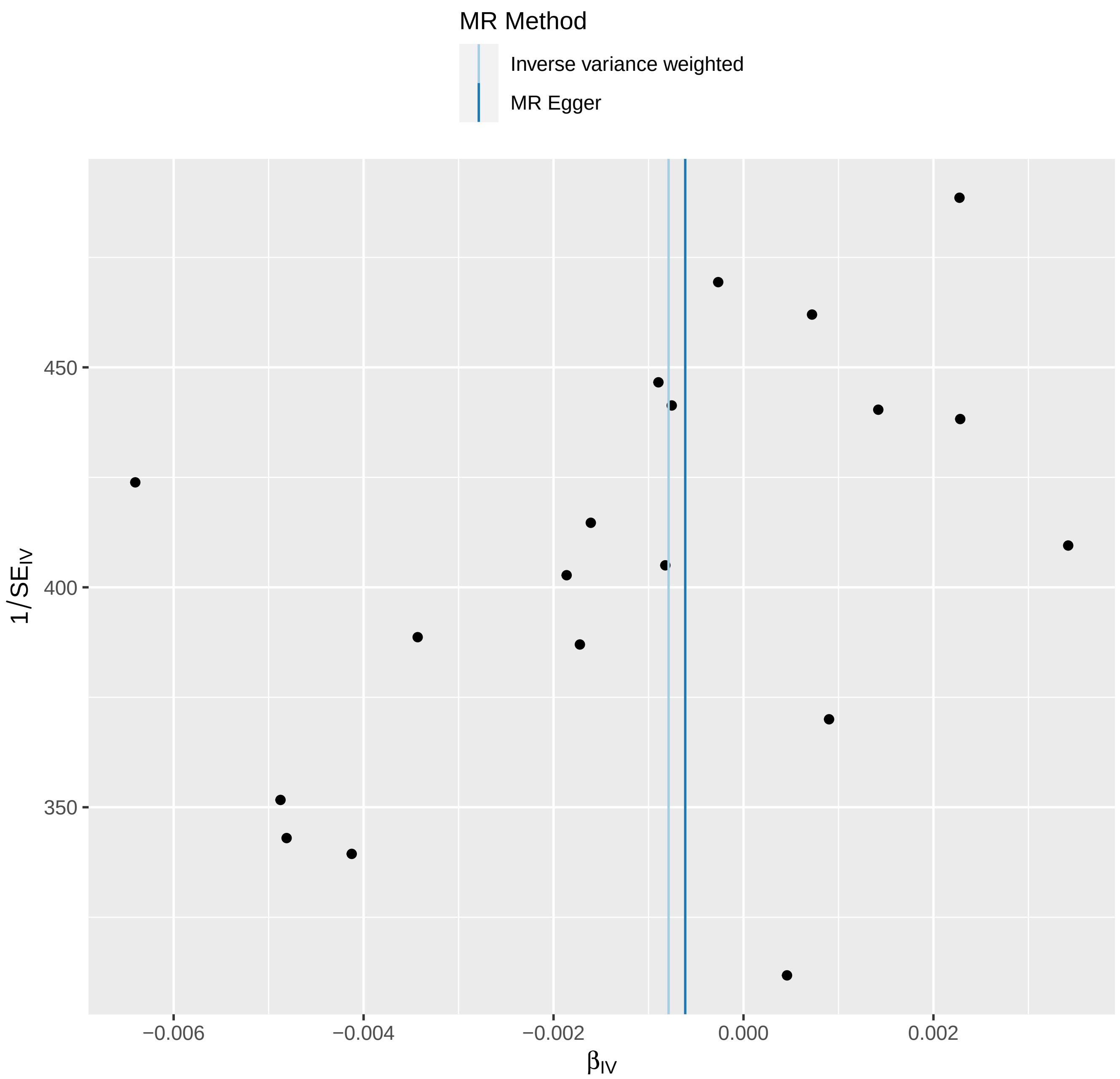

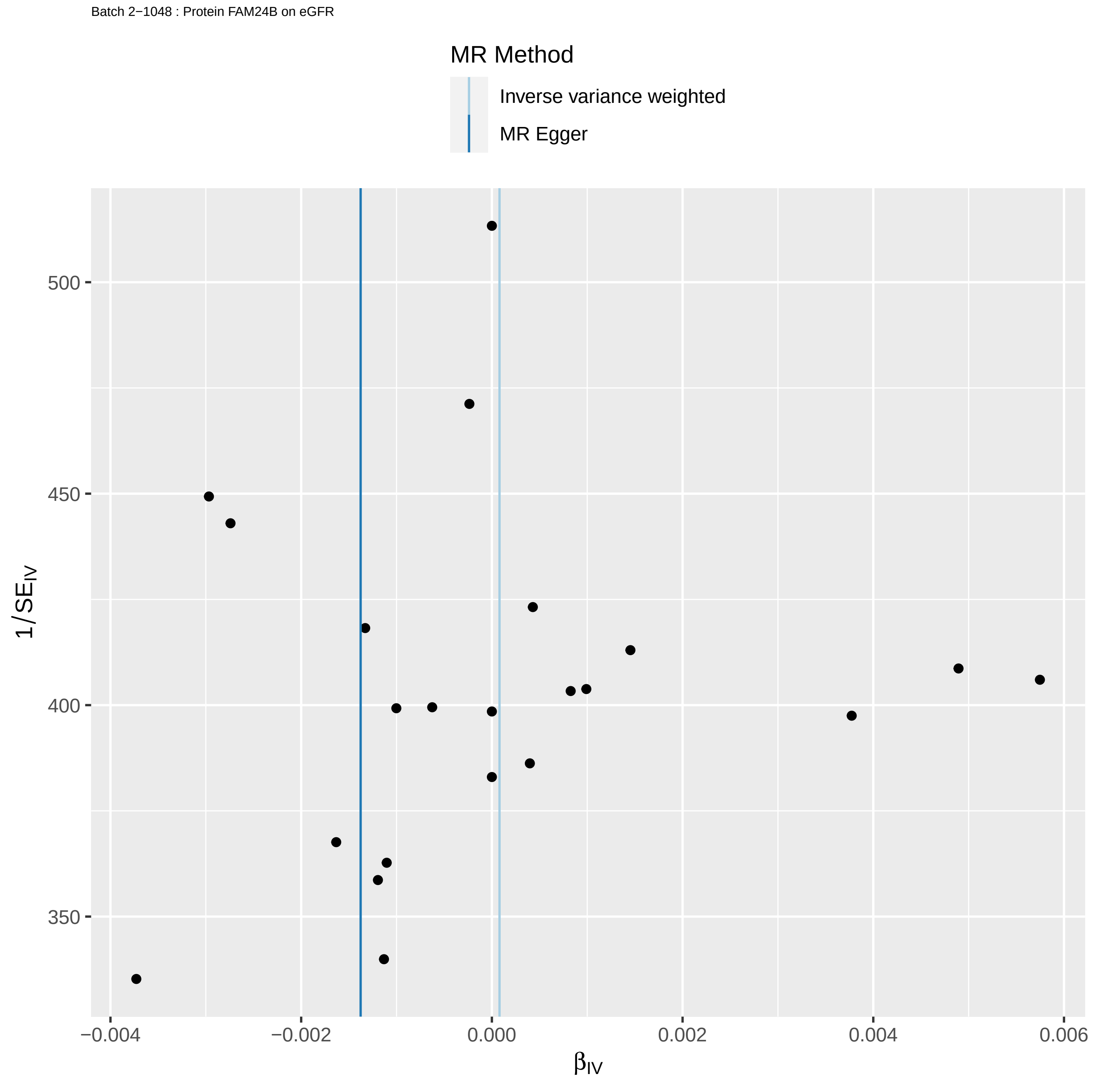
Batch 2−1048 : Protein FAM24B on eGFR
MR Method
Inverse variance weighted
MR Egger
500
450
400
350
1/SE$_{IV}$
−0.004
−0.002
0.000
0.002
0.004
0.006
β$_{IV}$

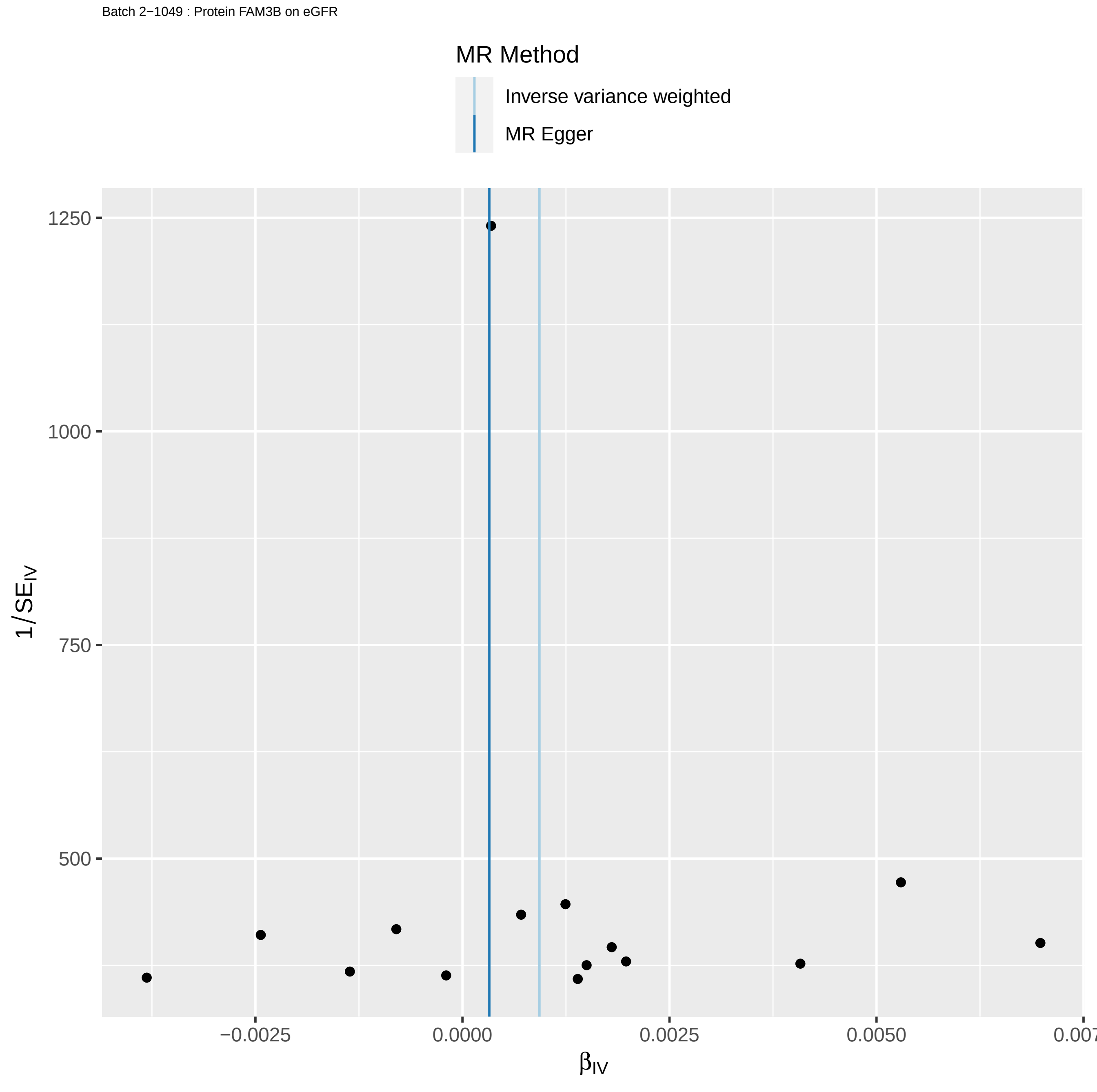
Batch 2−1049 : Protein FAM3B on eGFR
MR Method
Inverse variance weighted
MR Egger
1250
1000
750
500
1/SE_IV
−0.0025
0.0000
0.0025
0.0050
0.007
β_IV

Batch 2−1050 : Protein FAM3B on eGFR

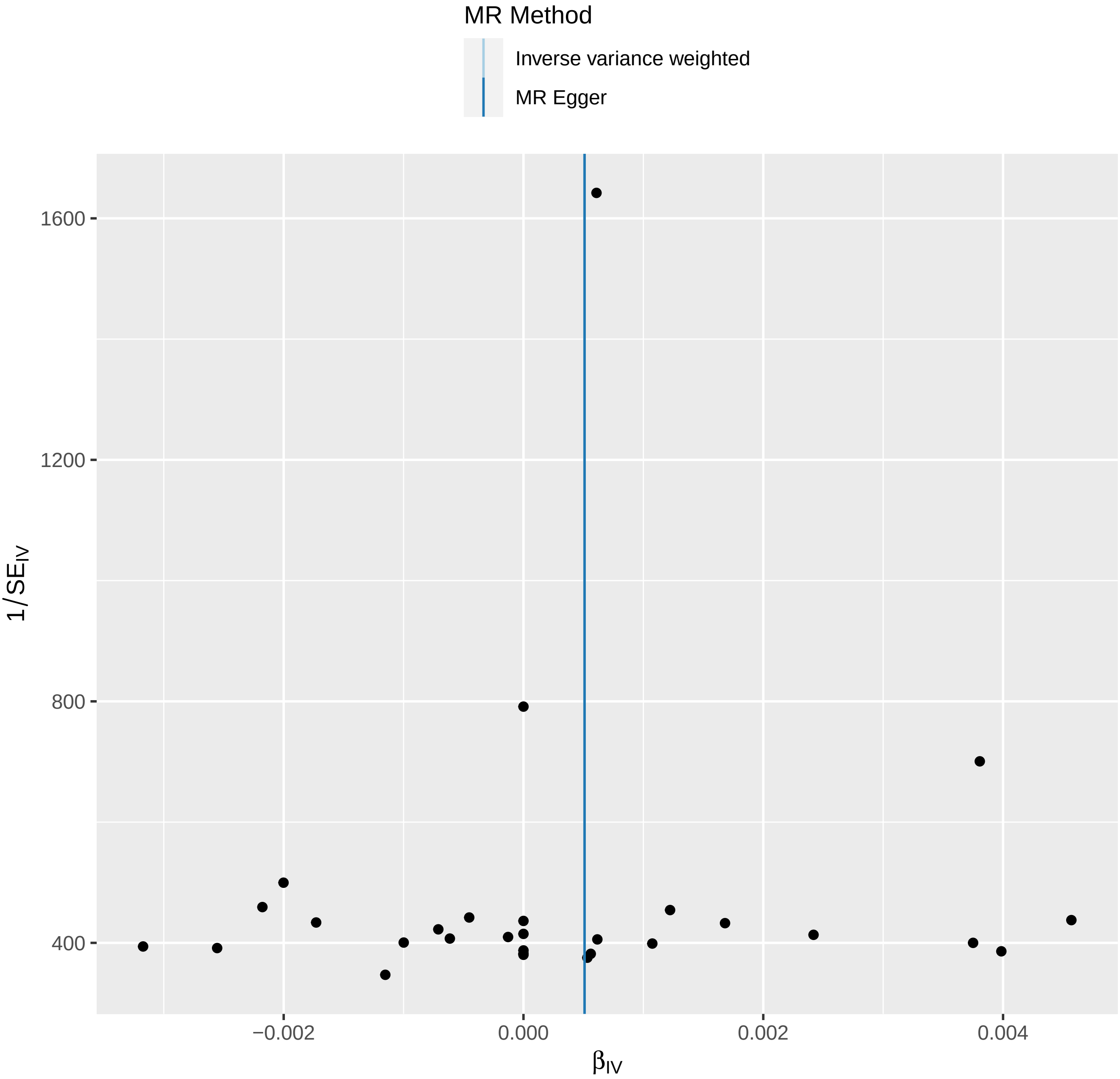

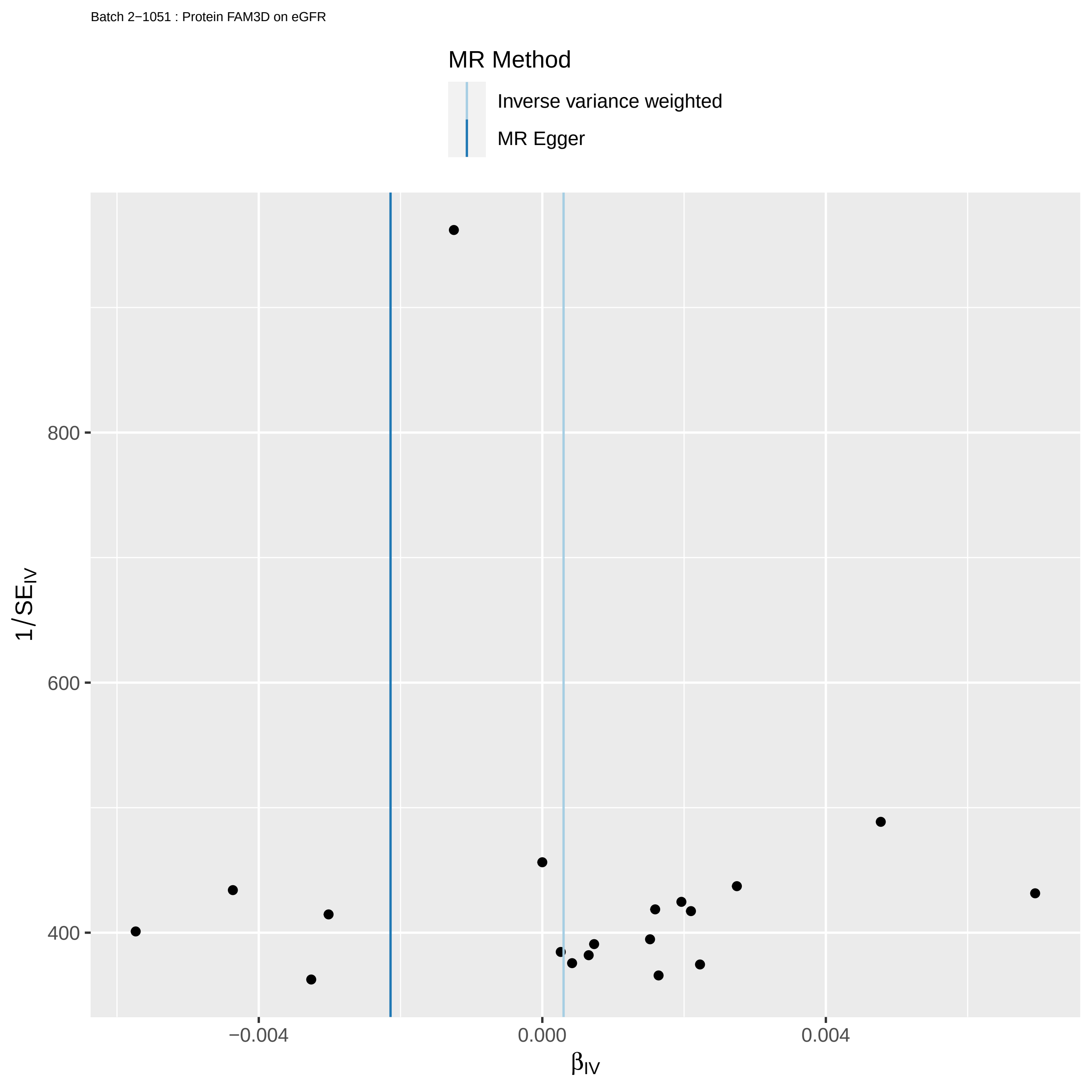
Batch 2−1051 : Protein FAM3D on eGFR
MR Method
Inverse variance weighted
MR Egger
1/SE_IV
800
600
400
−0.004
0.000
0.004
β_IV

Batch 2−1052 : MIP18 family protein FAM96A on eGFR

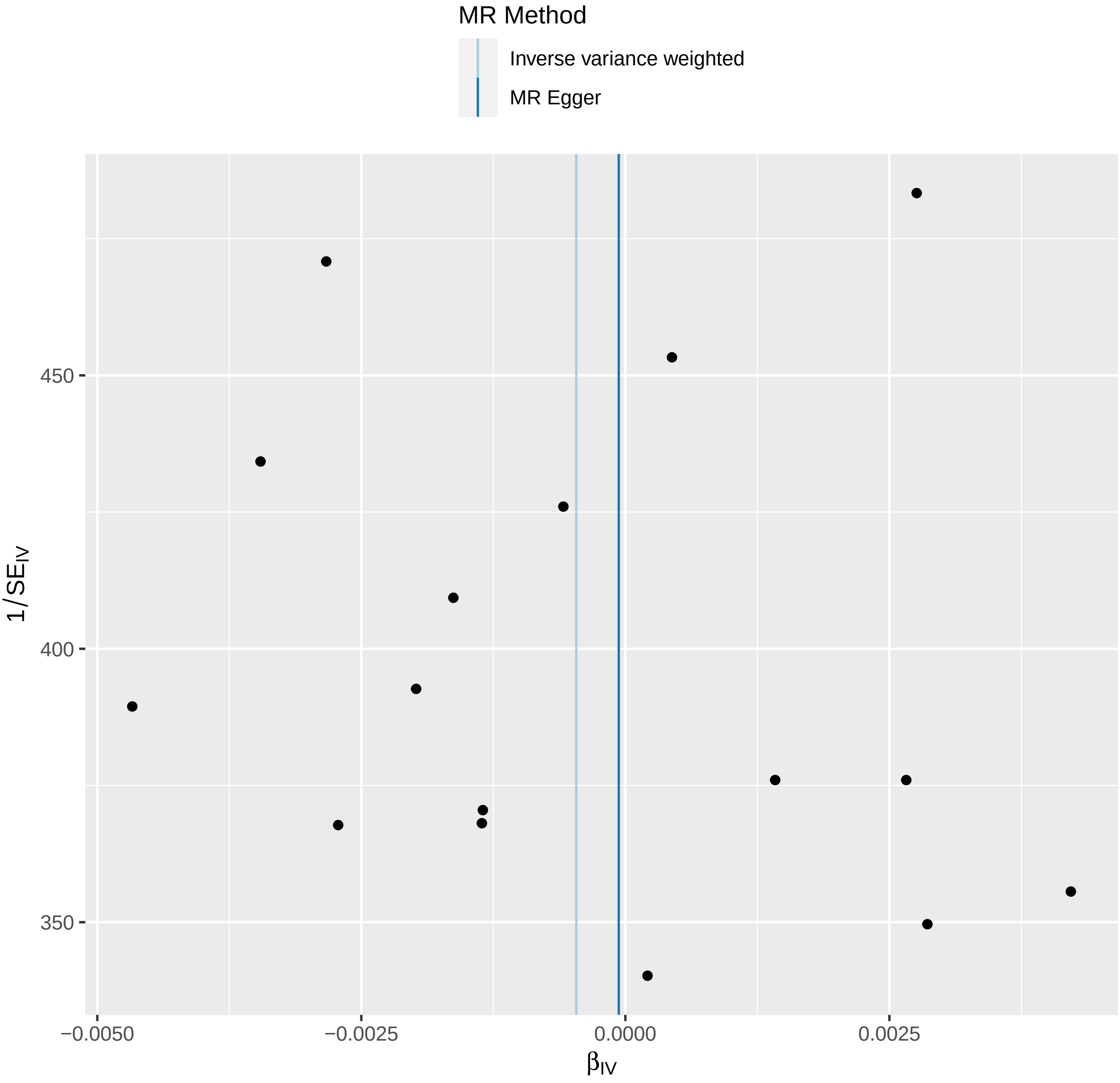

Batch 2−1053 : E3 ubiquitin−protein ligase FANCL on eGFR

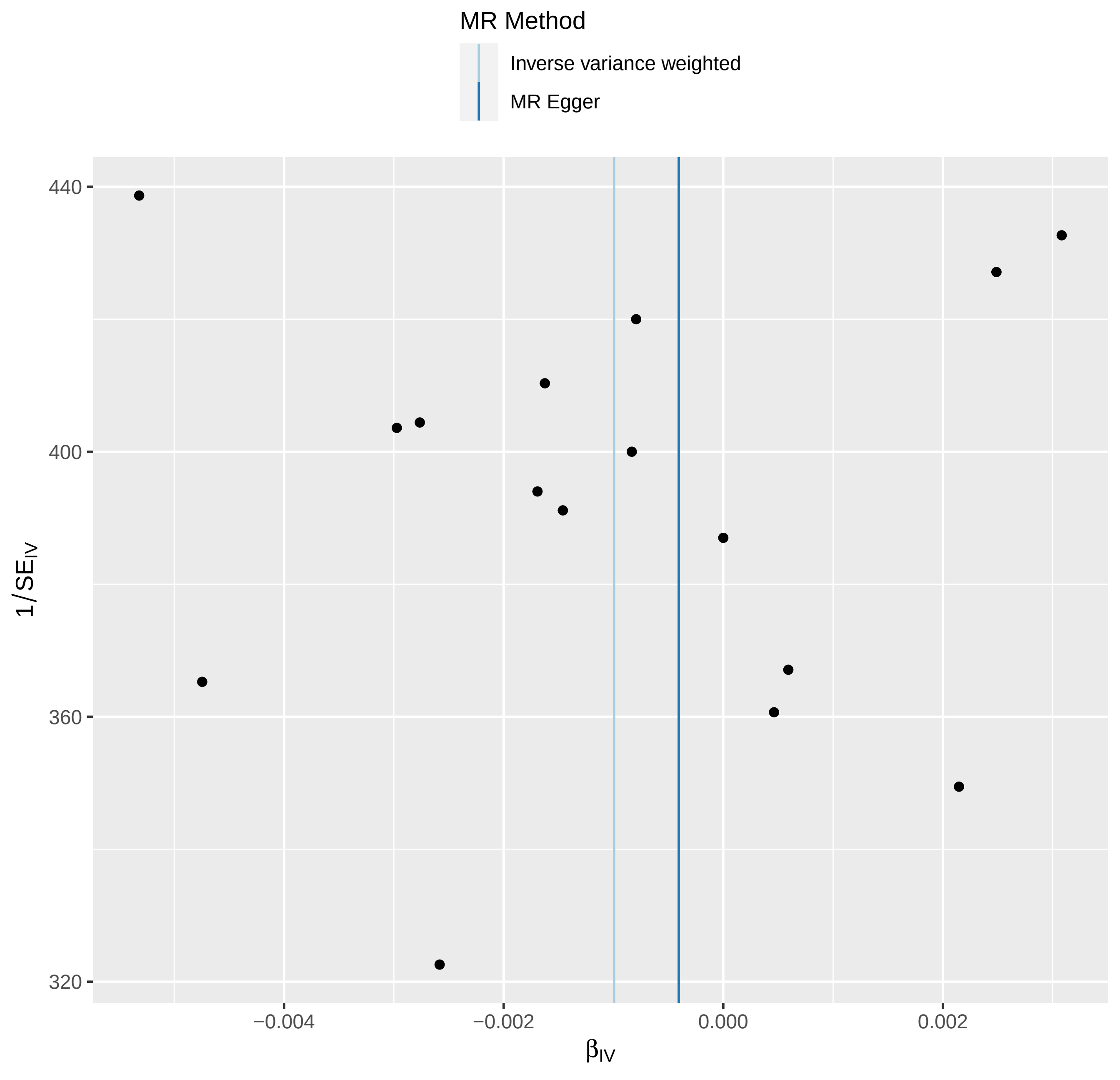

Batch 2−1054 : Prolyl endopeptidase FAP on eGFR

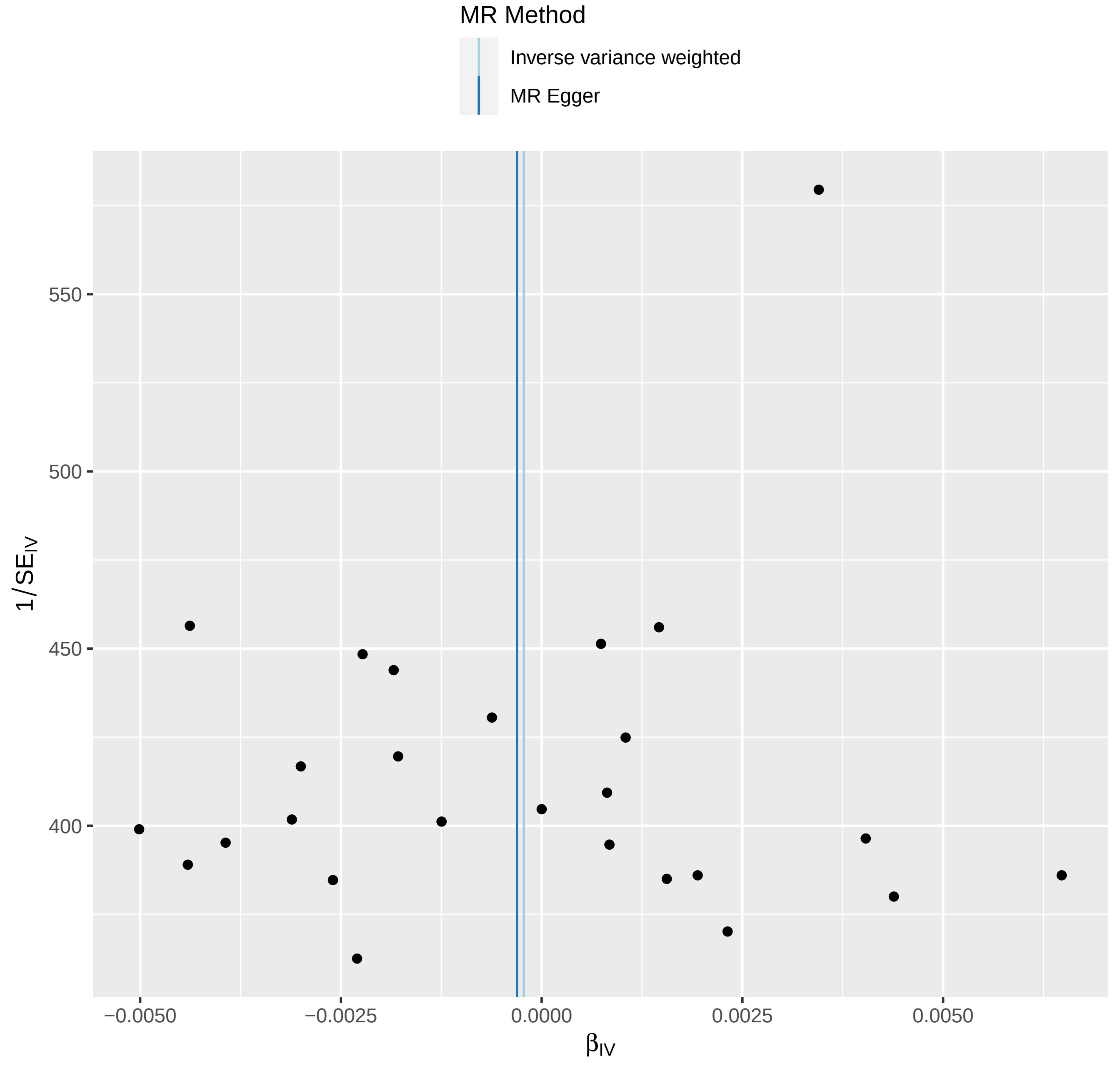

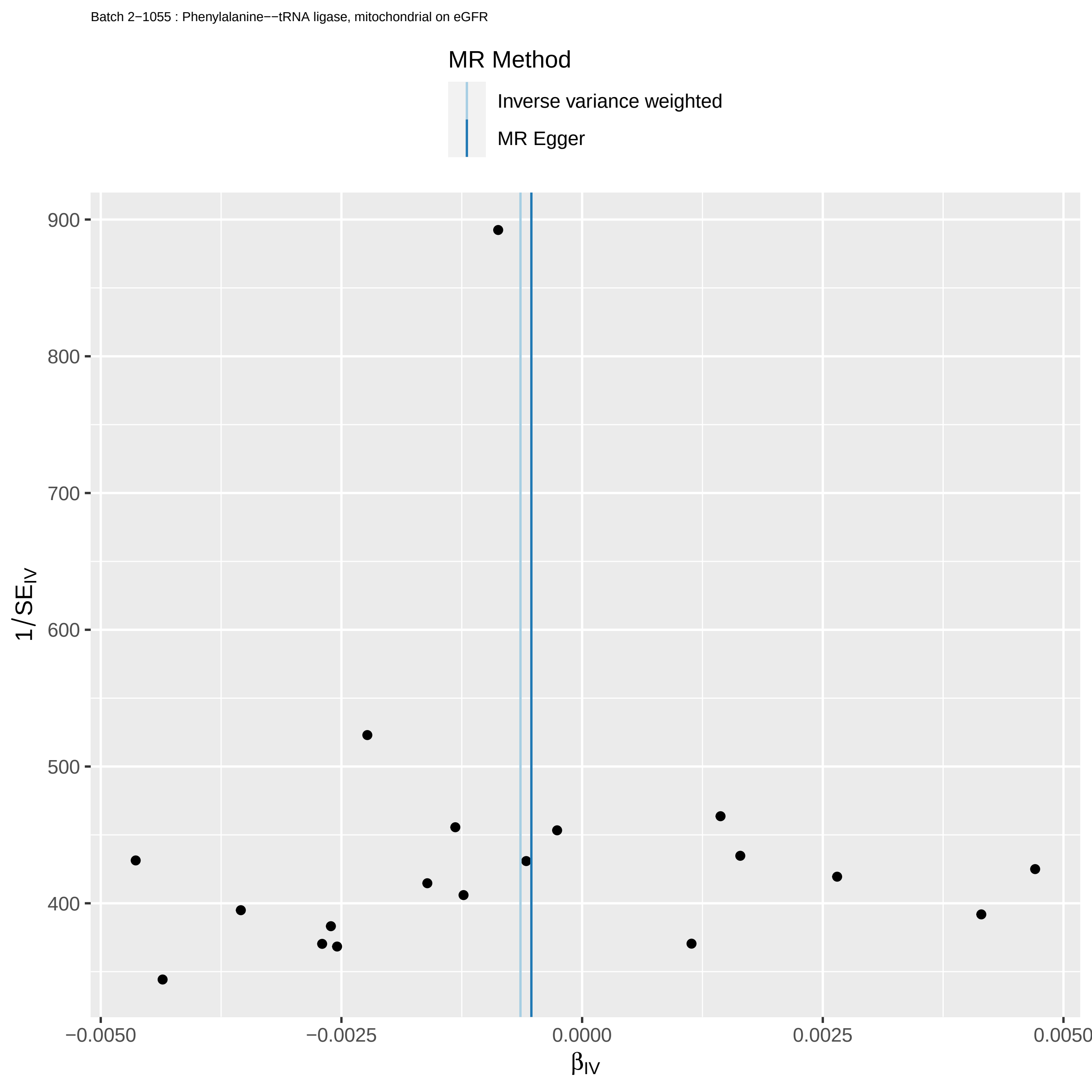

Batch 2−1055 : Phenylalanine−−tRNA ligase, mitochondrial on eGFR
MR Method
Inverse variance weighted
MR Egger
900
800
700
600
500
400
1/SE$_{IV}$
−0.0050
−0.0025
0.0000
0.0025
0.0050
β$_{IV}$

Batch 2−1056 : Tumor necrosis factor receptor superfamily member 6 on eGFR

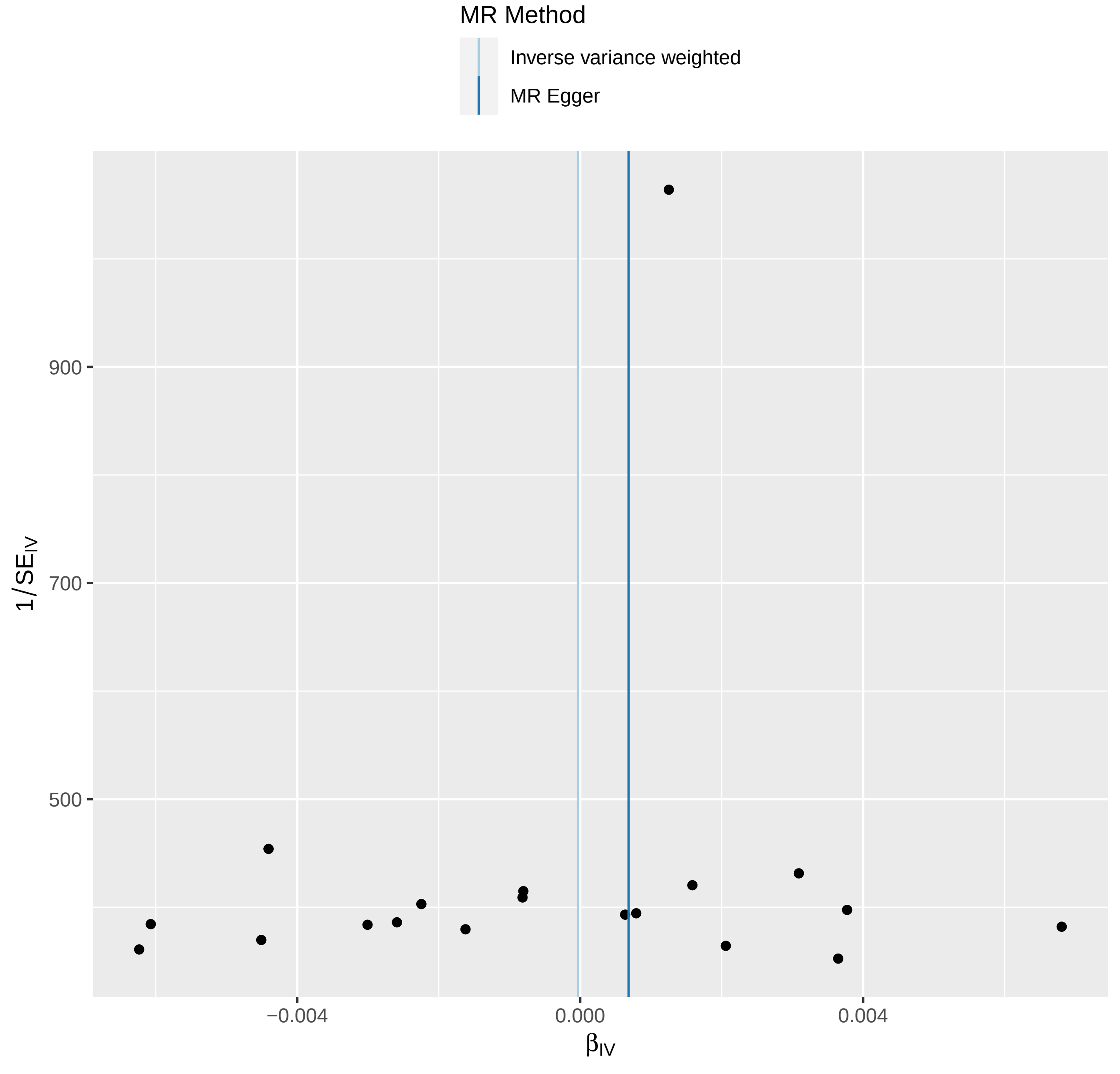

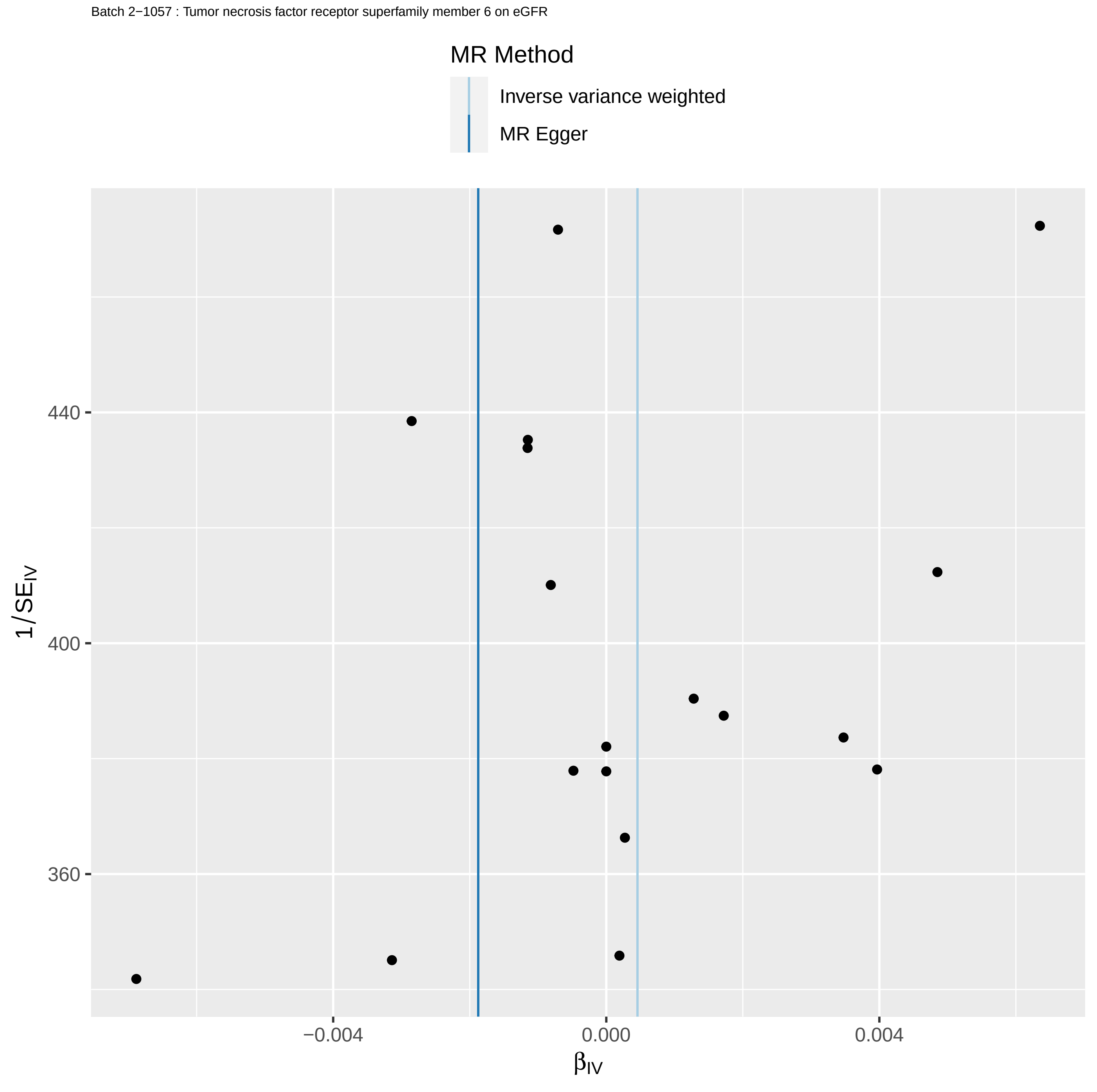
Batch 2−1057 : Tumor necrosis factor receptor superfamily member 6 on eGFR
MR Method
Inverse variance weighted
MR Egger
440
400
360
1/SE_IV
−0.004
0.000
0.004
β_IV

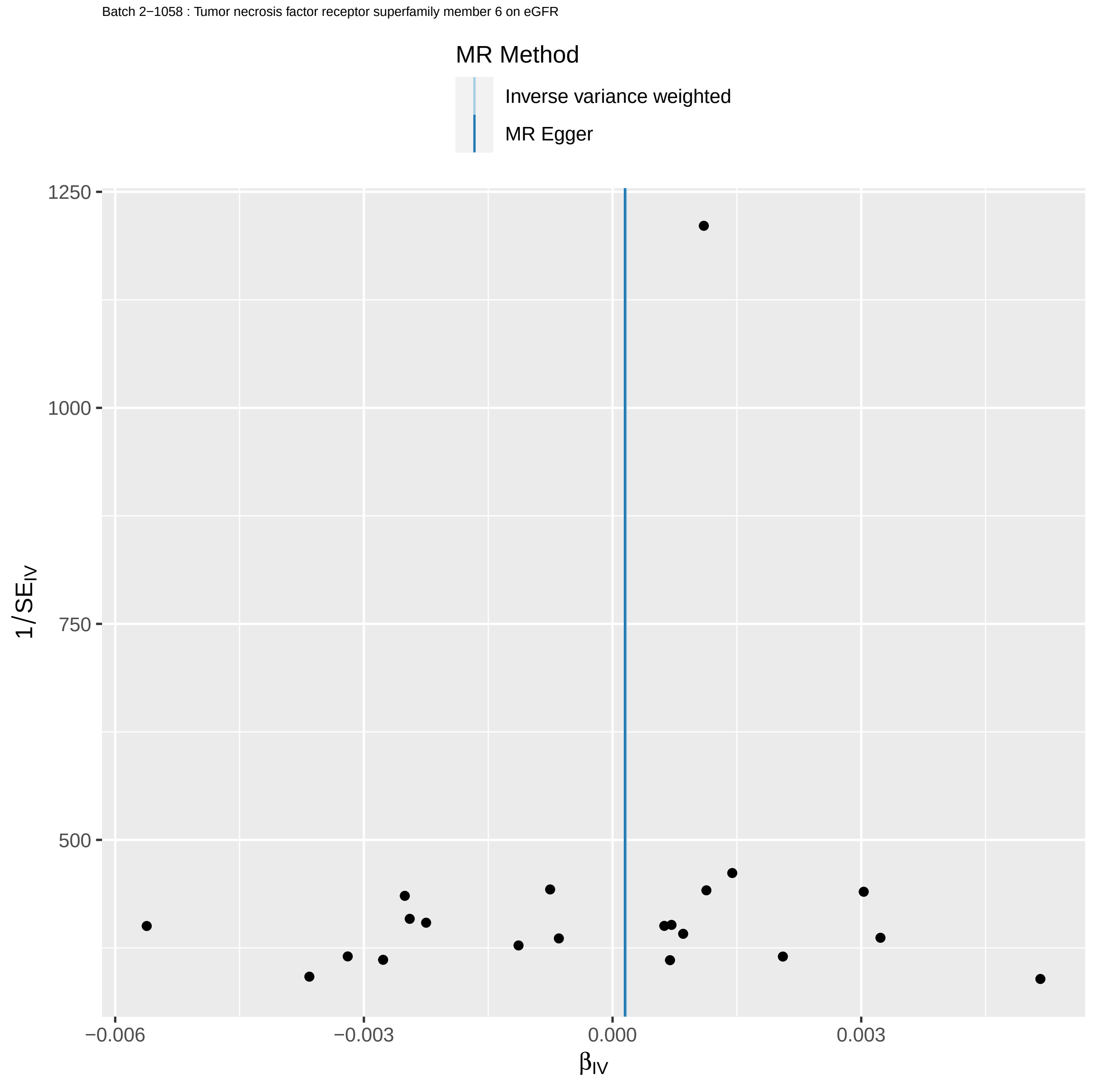
Batch 2−1058 : Tumor necrosis factor receptor superfamily member 6 on eGFR
MR Method
Inverse variance weighted
MR Egger
1250
1000
750
500
1/SE$_{IV}$
−0.006
−0.003
0.000
0.003
β$_{IV}$

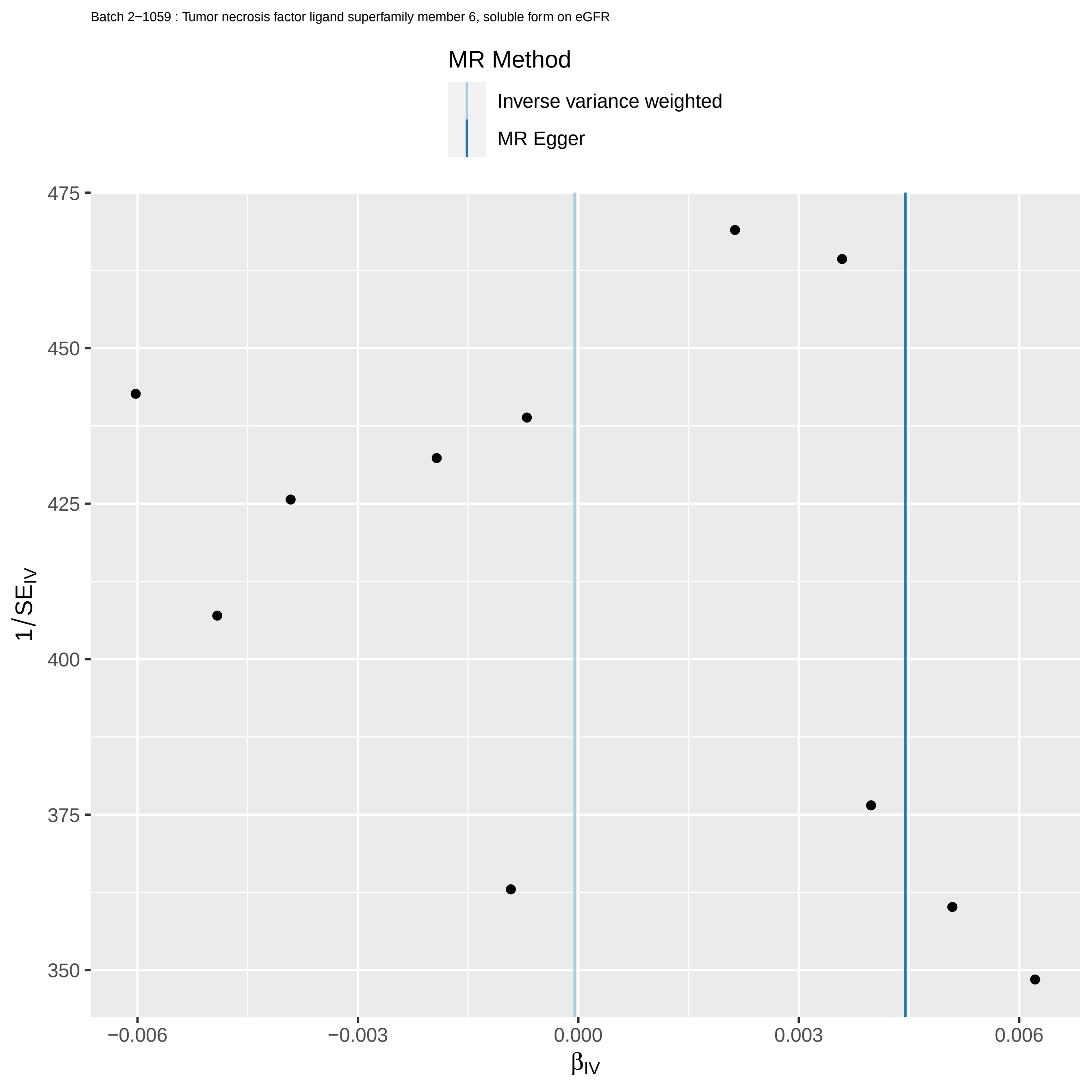
Batch 2−1059 : Tumor necrosis factor ligand superfamily member 6, soluble form on eGFR
MR Method
Inverse variance weighted
MR Egger
475
450
425
400
375
350
1/SE_IV
−0.006
−0.003
0.000
0.003
0.006
β_IV

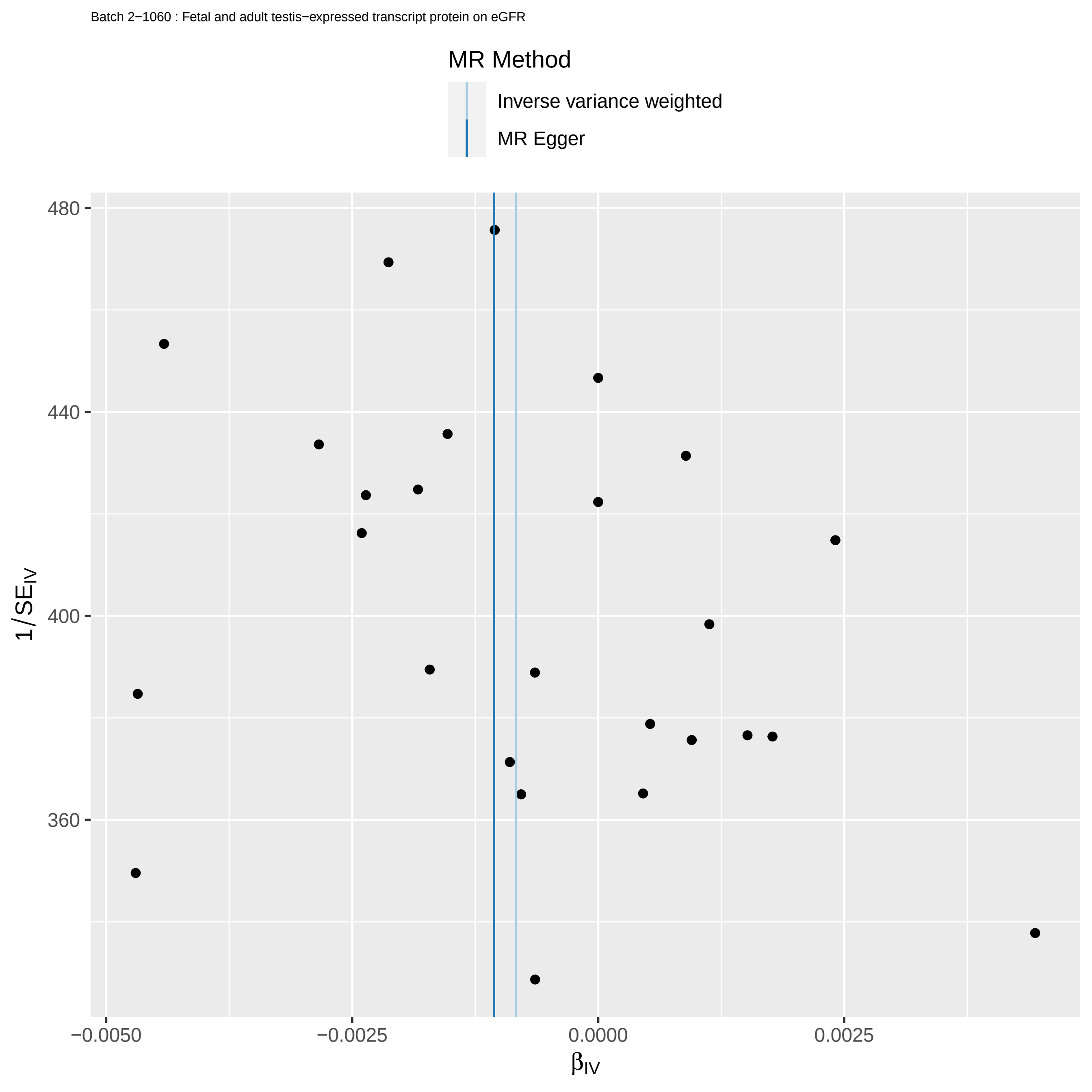

Batch 2−1060 : Fetal and adult testis−expressed transcript protein on eGFR
MR Method
Inverse variance weighted
MR Egger
480
440
400
360
1/SE$_{IV}$
−0.0050
−0.0025
0.0000
0.0025
β_{IV}

Batch 2−1061 : Fatty acid hydroxylase domain−containing protein 2 on eGFR

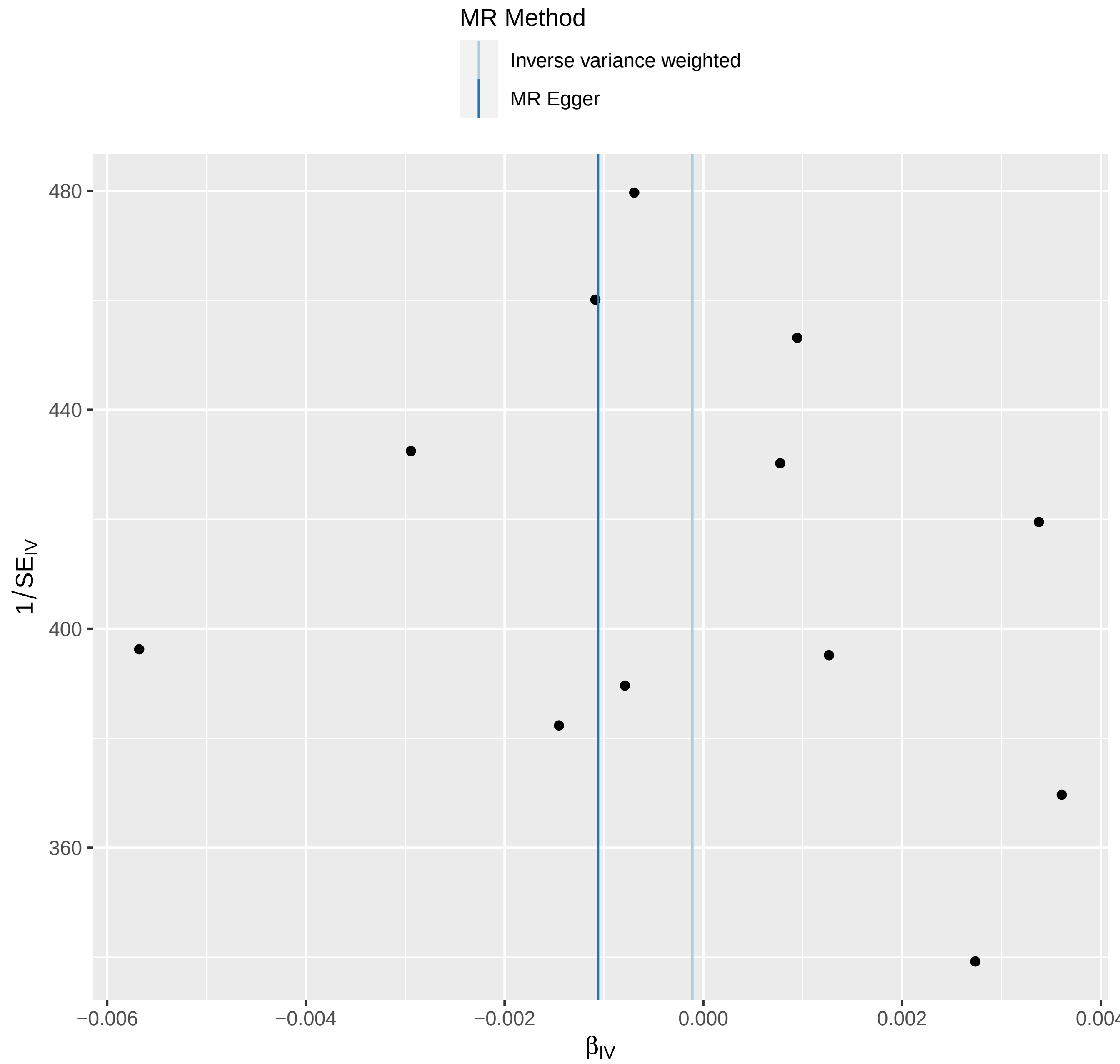

Batch 2−1062 : Fibulin−1 on eGFR

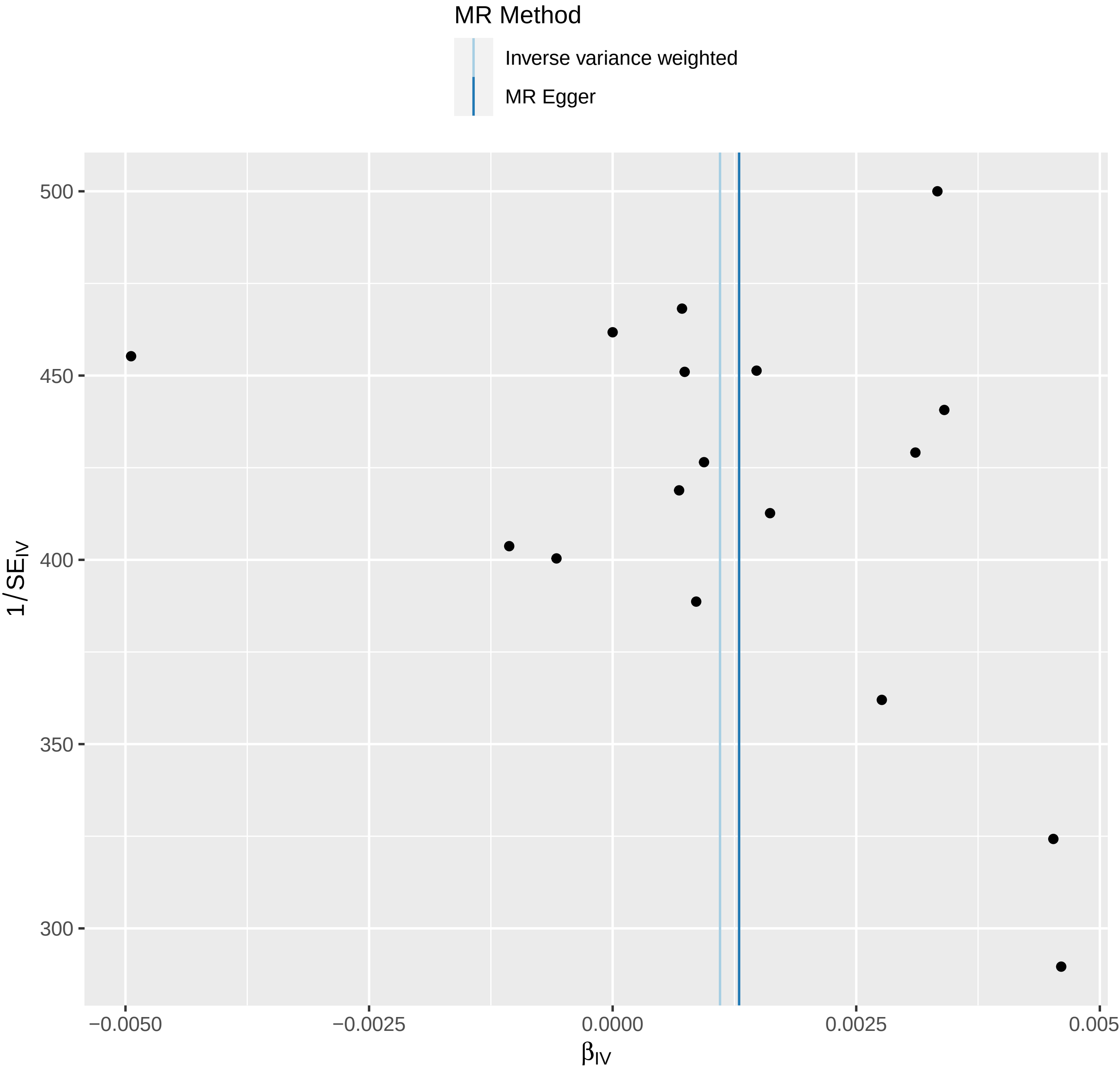

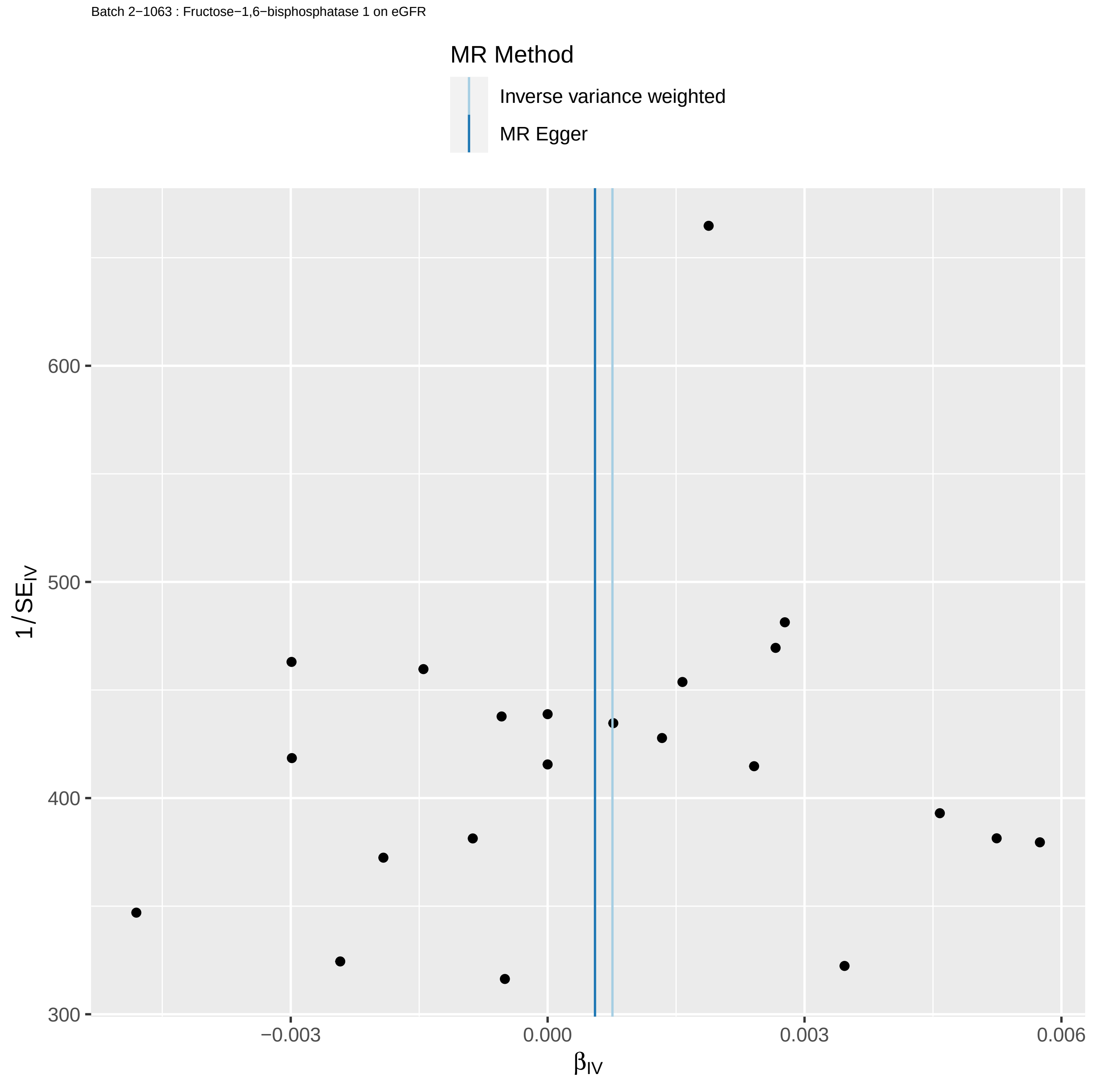

Batch 2−1063 : Fructose−1,6−bisphosphatase 1 on eGFR
MR Method
Inverse variance weighted
MR Egger
600
500
400
300
1/SE_IV
−0.003
0.000
0.003
0.006
β_IV

Batch 2−1064 : Fructose−1,6−bisphosphatase isozyme 2 on eGFR

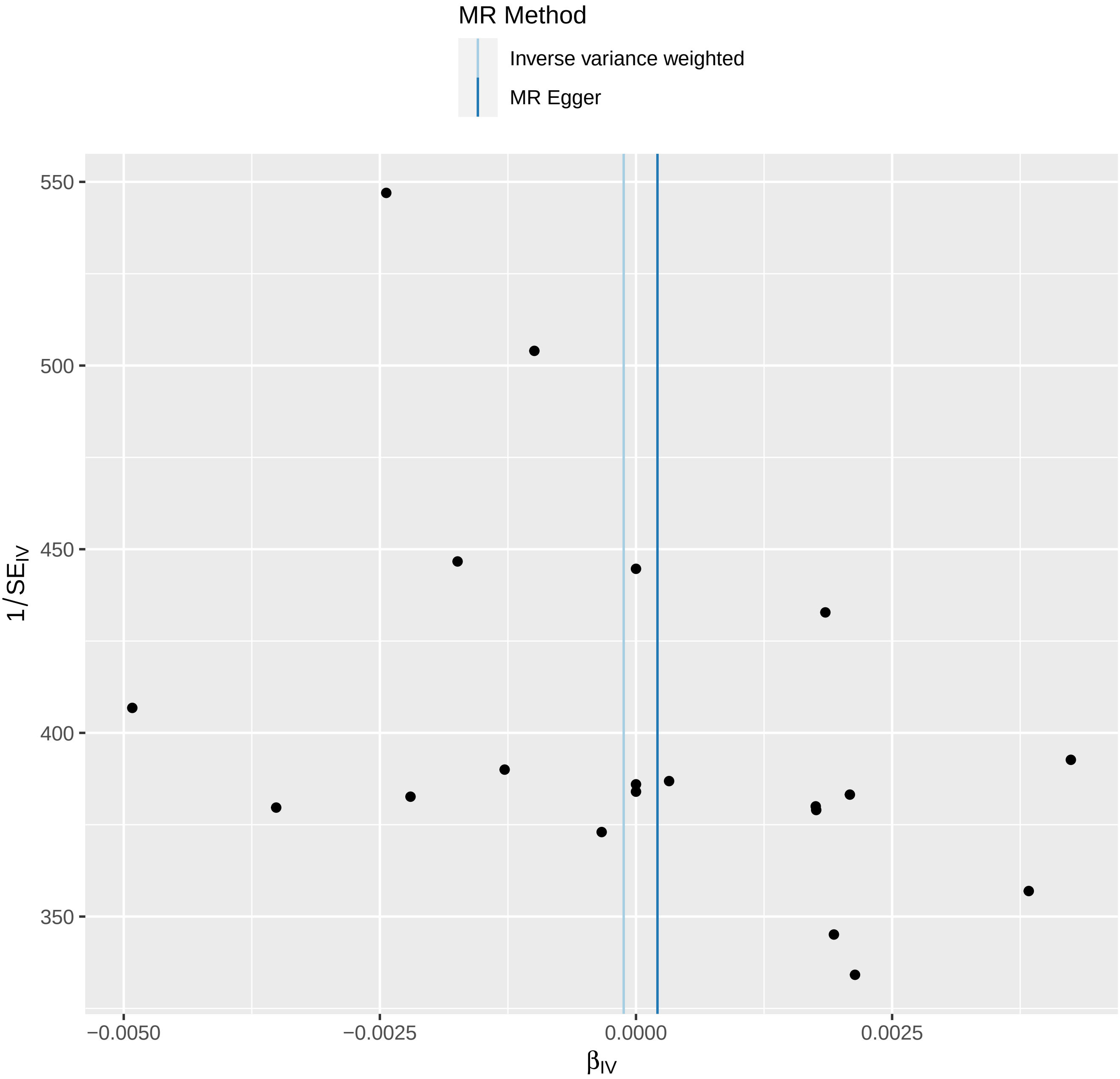

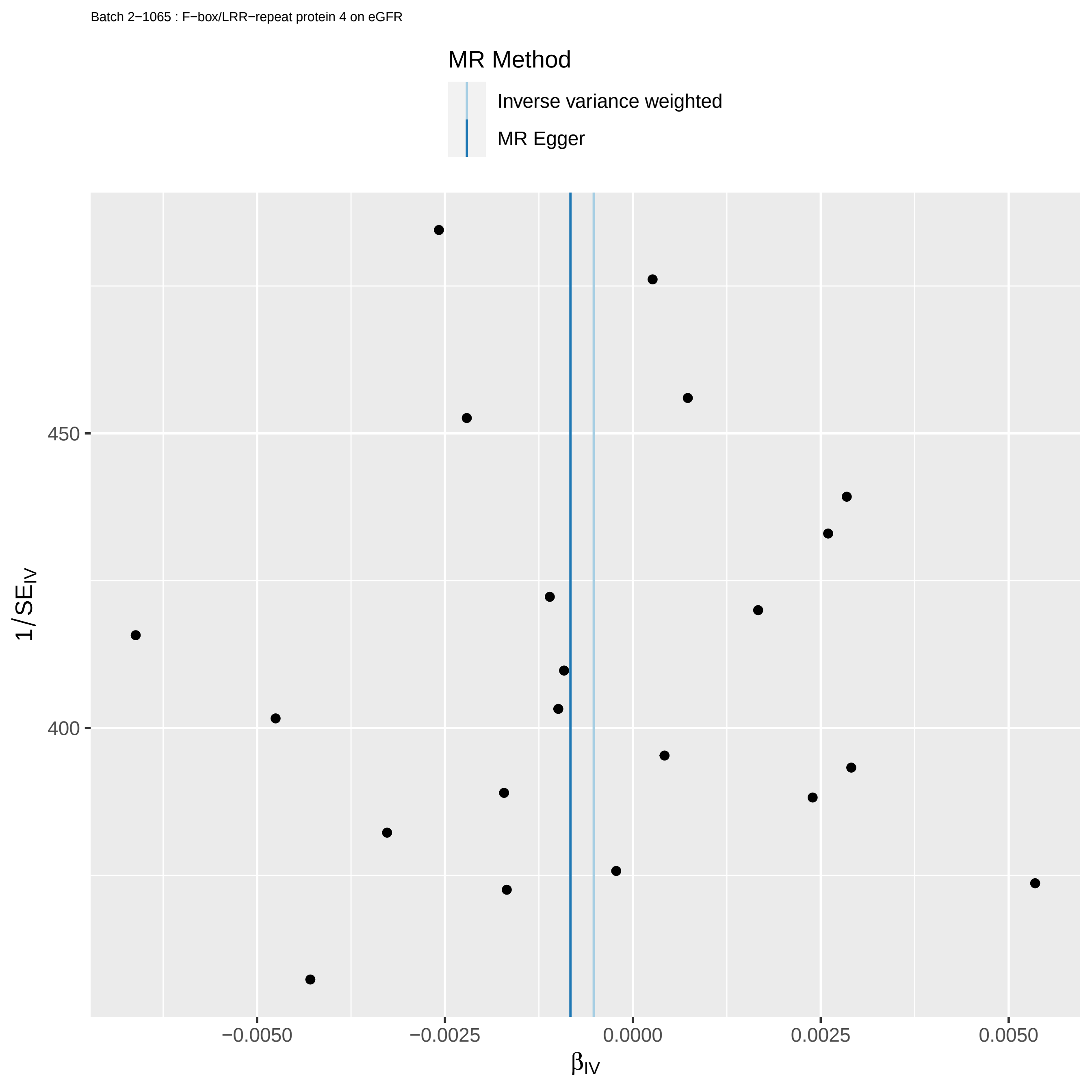
Batch 2−1065 : F−box/LRR−repeat protein 4 on eGFR
MR Method
Inverse variance weighted
MR Egger
450
400
1/SE$_{IV}$
−0.0050
−0.0025
0.0000
0.0025
0.0050
β_{IV}

Batch 2−1066 : F−box/LRR−repeat protein 4 on eGFR

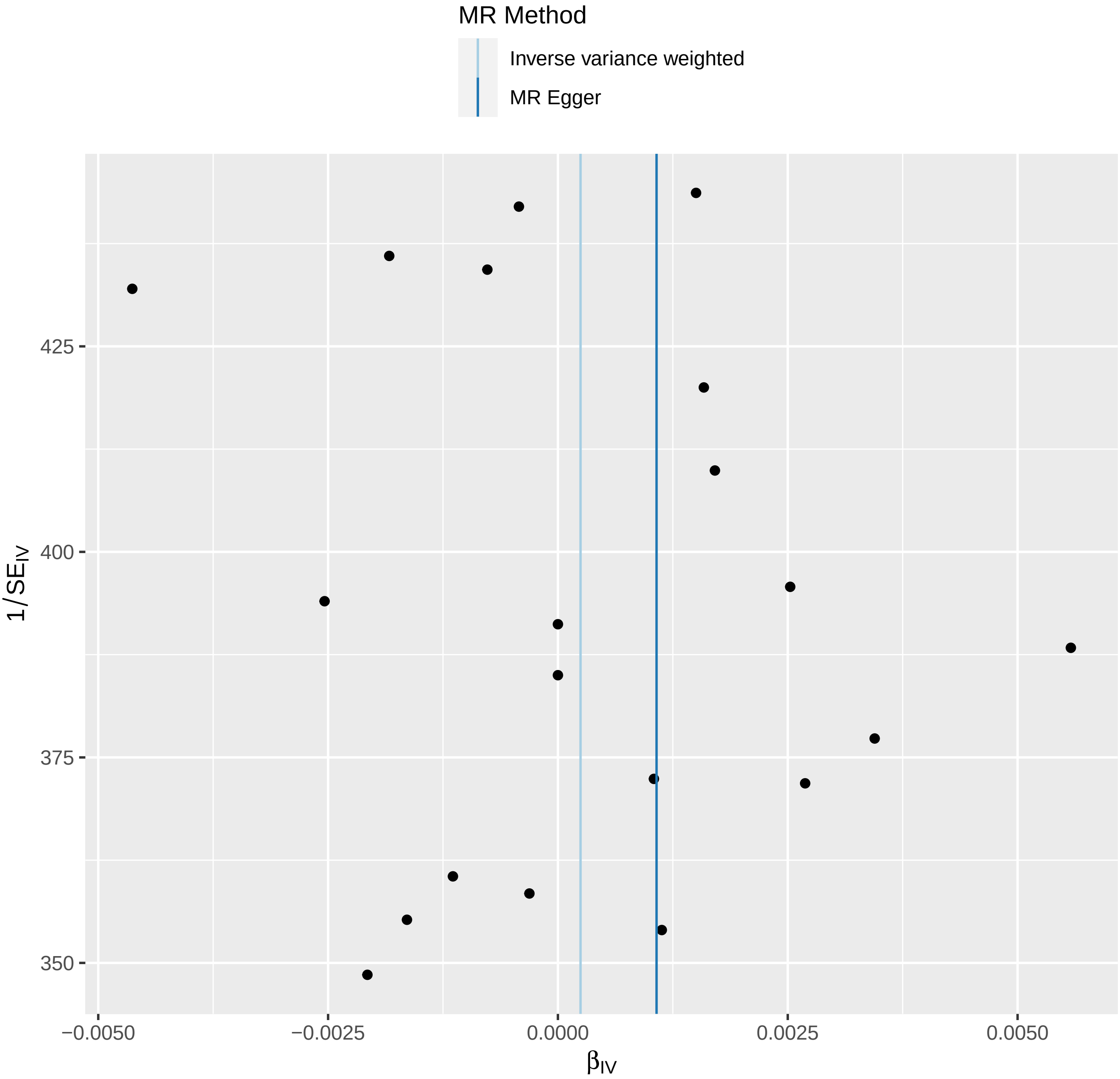

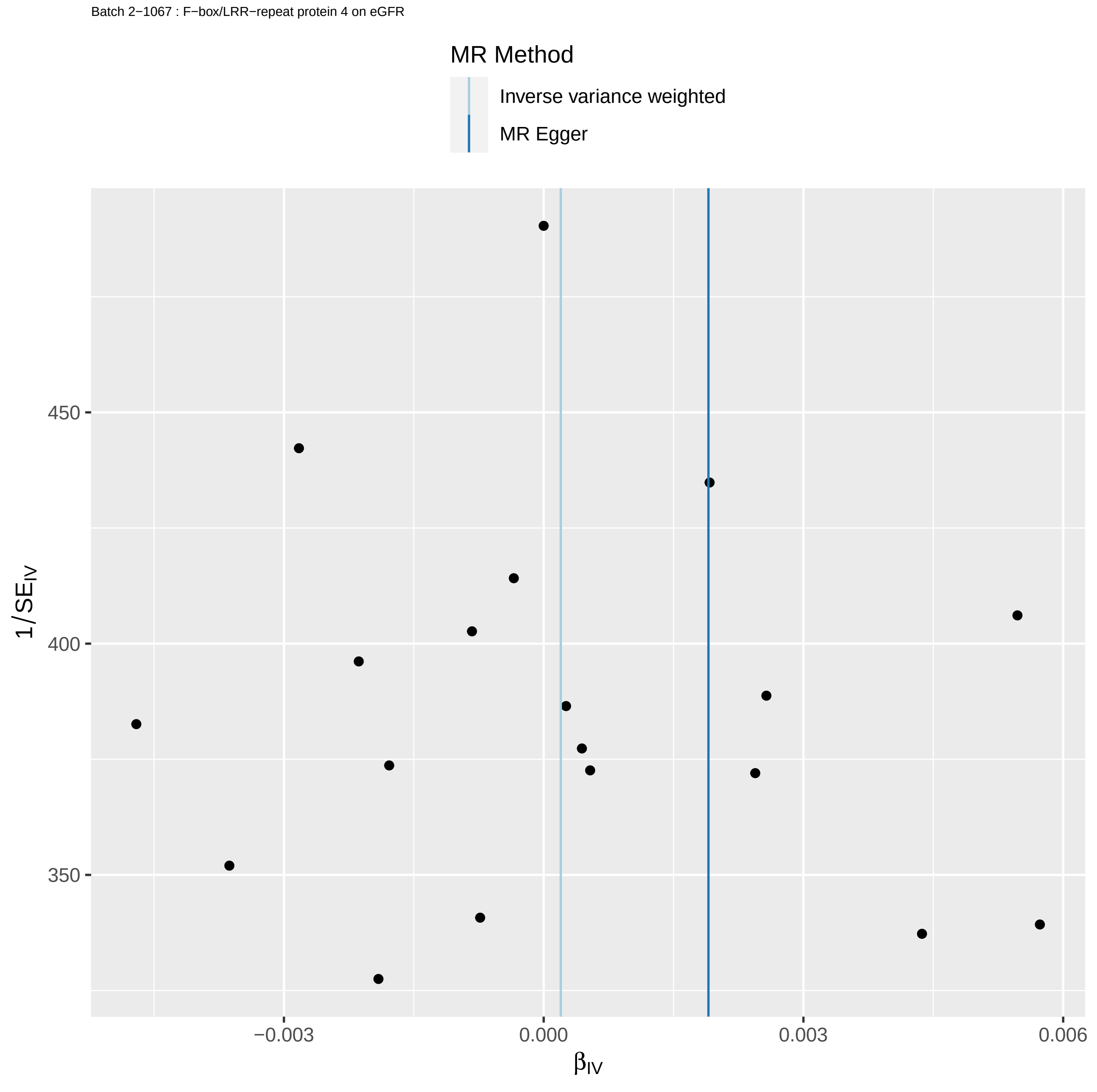

Batch 2−1067 : F−box/LRR−repeat protein 4 on eGFR
MR Method
Inverse variance weighted
MR Egger
450
400
350
1/SE_IV
−0.003
0.000
0.003
0.006
β_IV

Batch 2−1068 : F−box/LRR−repeat protein 5 on eGFR

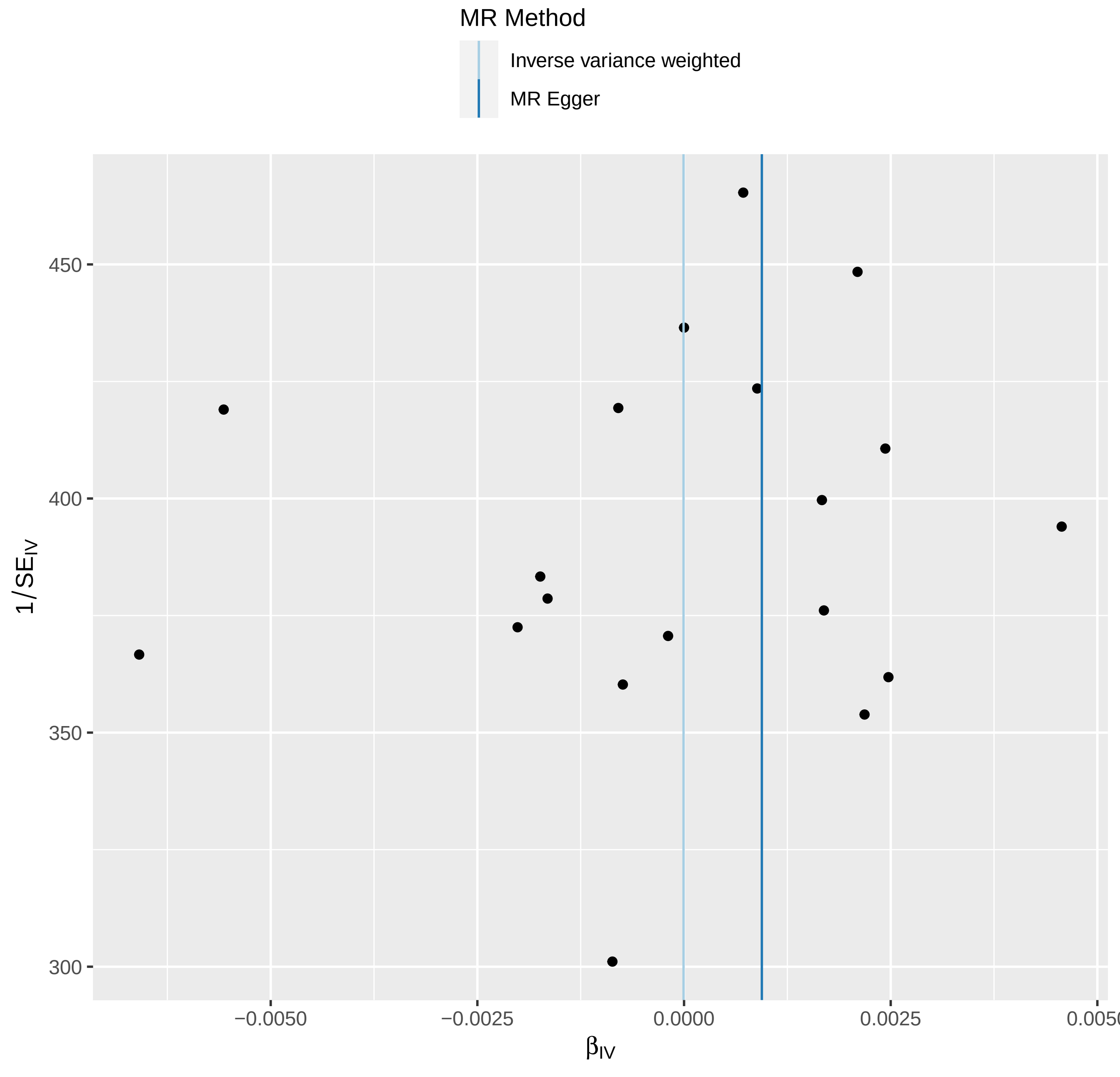

Batch 2−1069 : F−box only protein 3 on eGFR

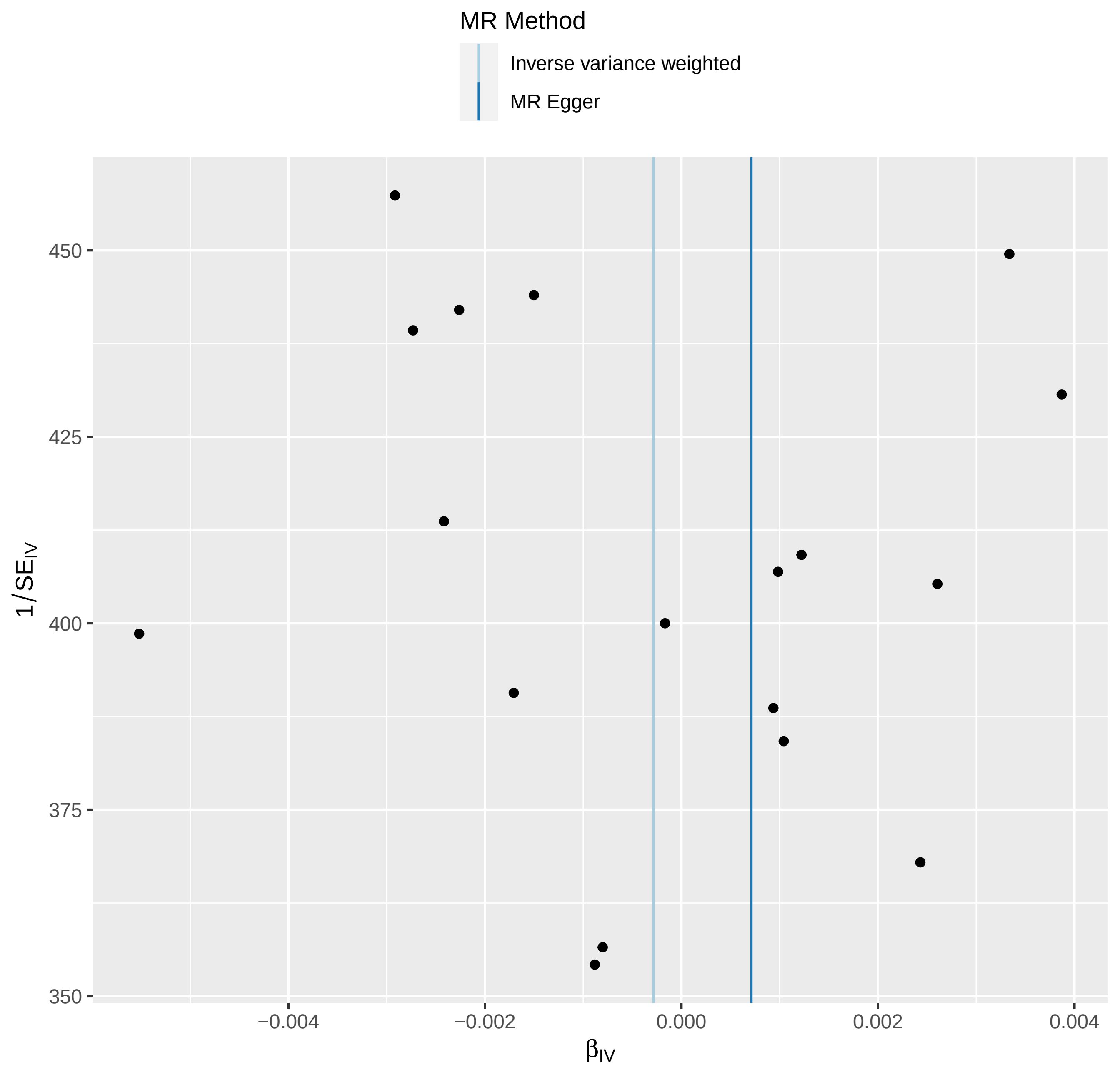

Batch 2−1070 : High affinity immunoglobulin alpha and immunoglobulin mu Fc receptor on eGFR

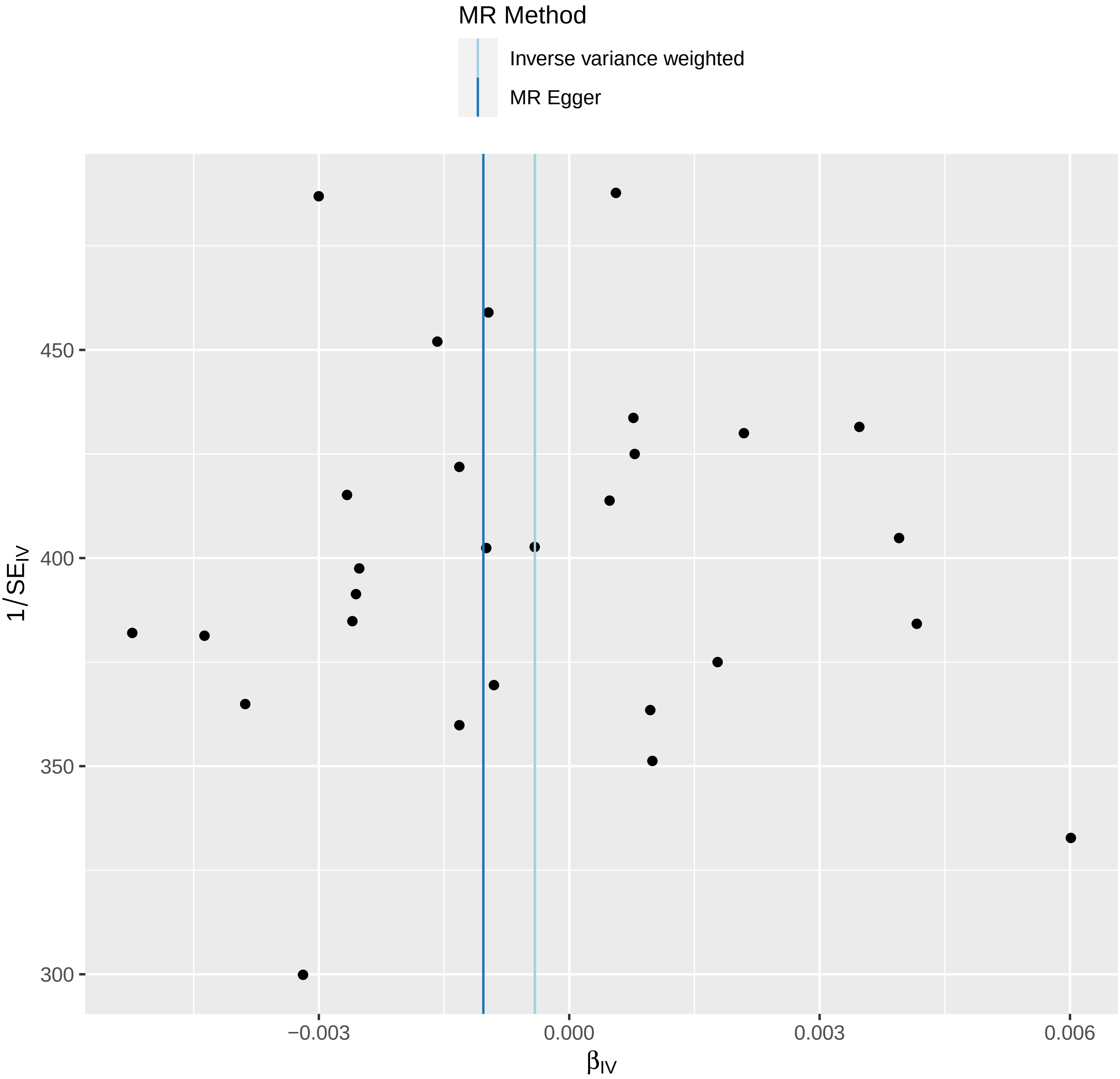

Batch 2−1071 : Immunoglobulin alpha Fc receptor on eGFR

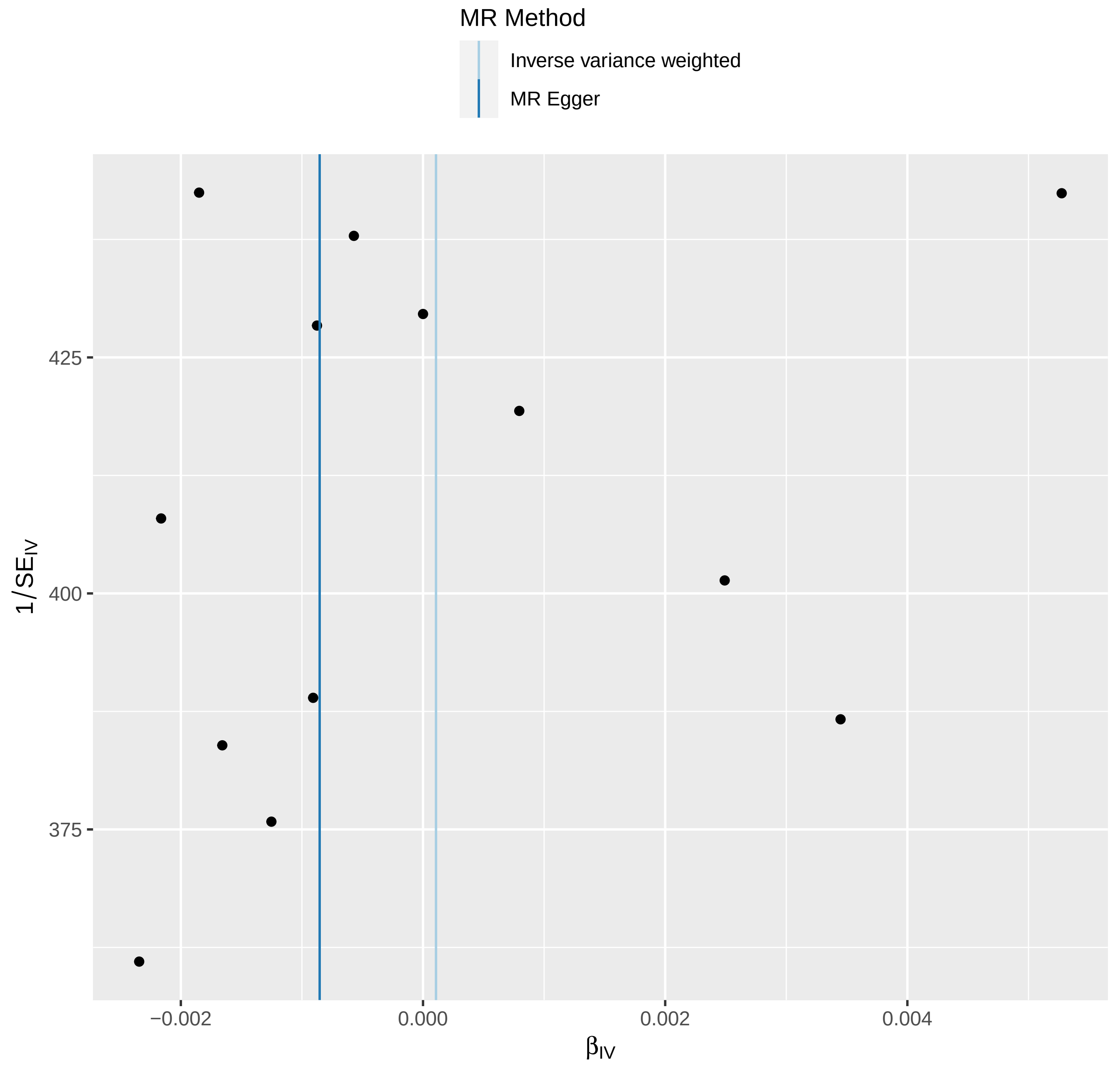

Batch 2−1072 : Low affinity immunoglobulin epsilon Fc receptor on eGFR

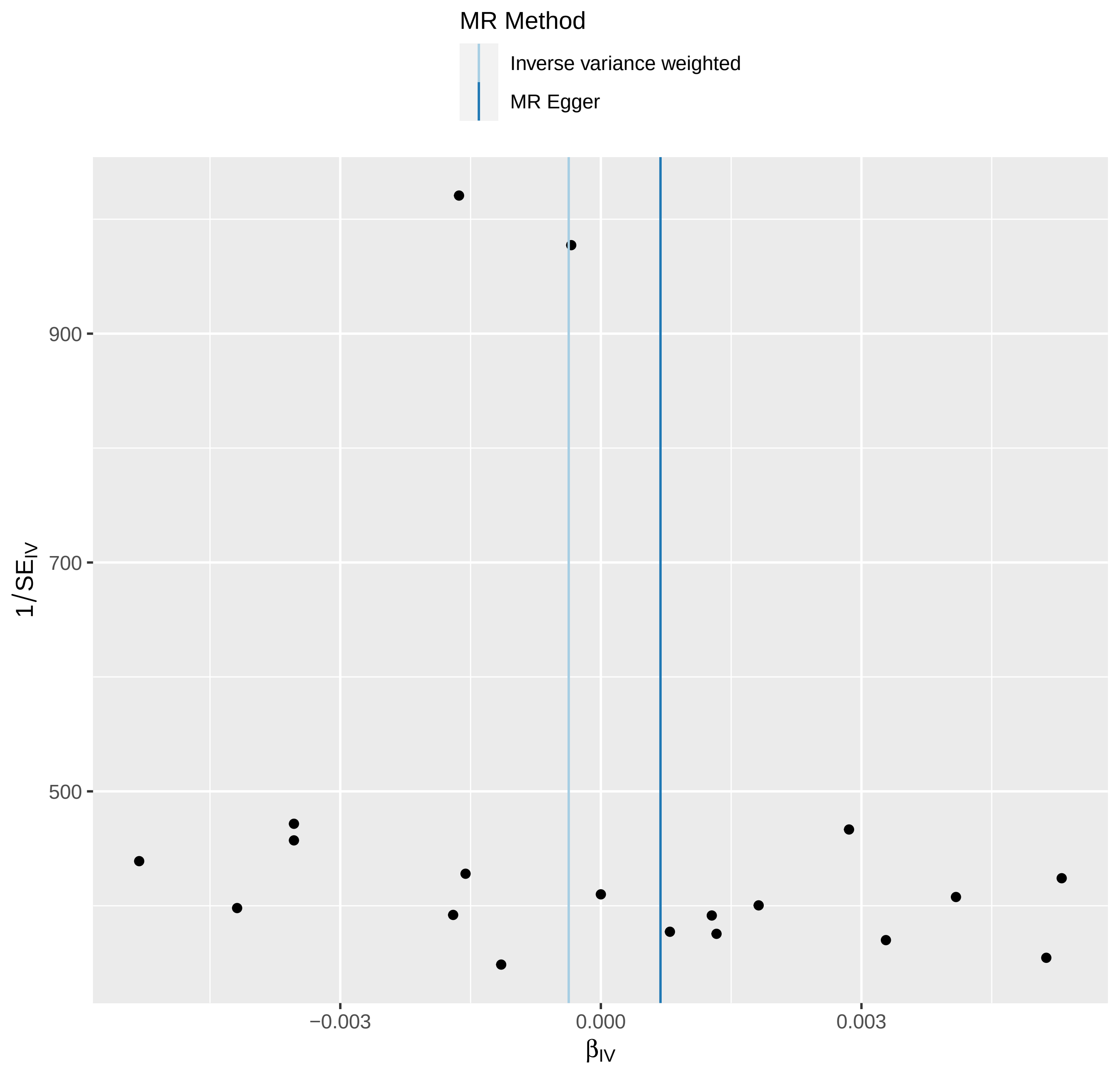

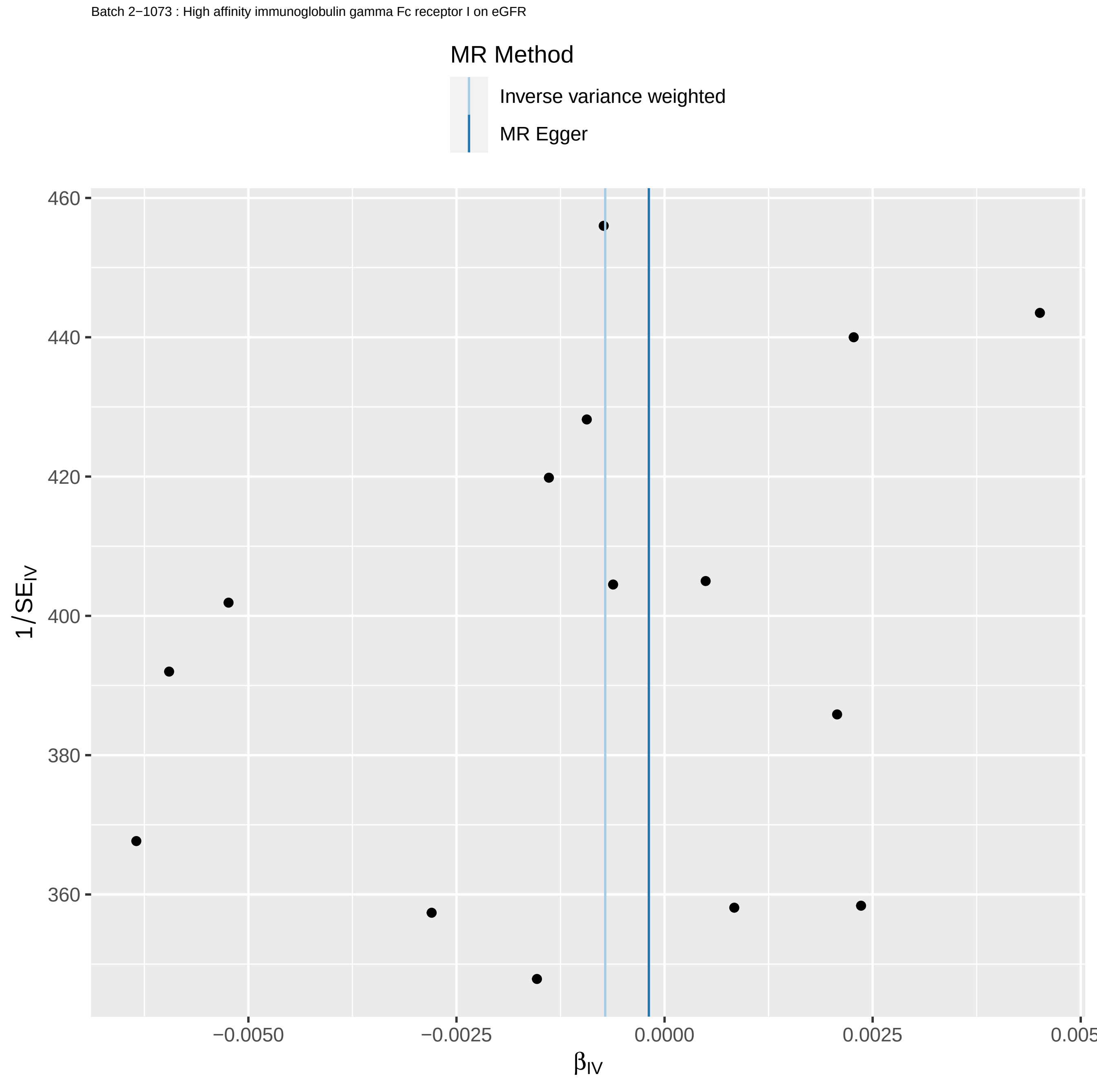

Batch 2−1073 : High affinity immunoglobulin gamma Fc receptor I on eGFR
MR Method
Inverse variance weighted
MR Egger
460
440
420
400
380
360
1/SE_IV
−0.0050
−0.0025
0.0000
0.0025
0.005
β_IV

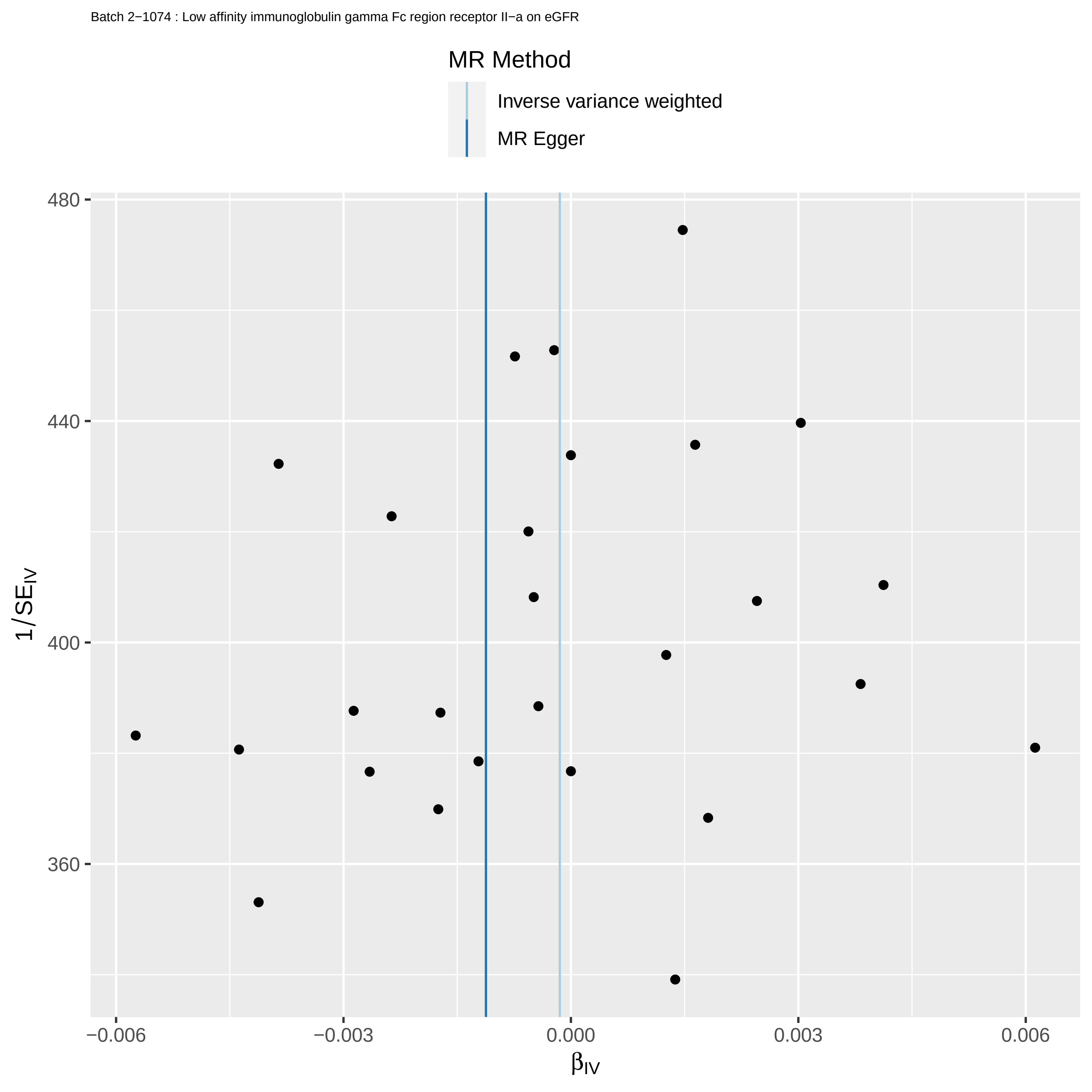

Batch 2−1074 : Low affinity immunoglobulin gamma Fc region receptor II−a on eGFR
MR Method
Inverse variance weighted
MR Egger
480
440
400
360
1/SE$_{IV}$
−0.006
−0.003
0.000
0.003
0.006
β$_{IV}$

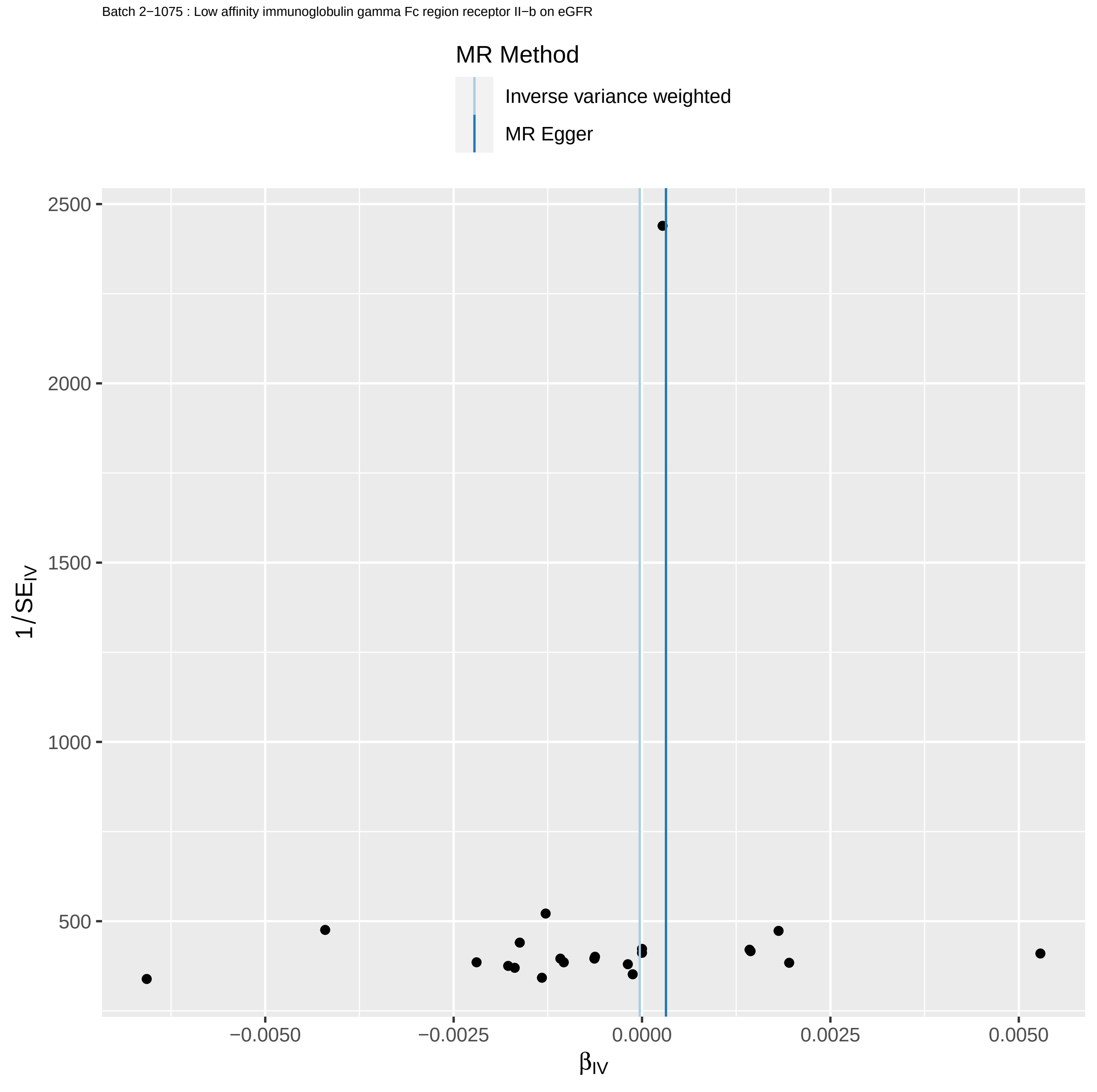

Batch 2−1075 : Low affinity immunoglobulin gamma Fc region receptor II−b on eGFR
MR Method
Inverse variance weighted
MR Egger
2500
2000
1500
1000
500
1/SE$_{IV}$
−0.0050
−0.0025
0.0000
0.0025
0.0050
β$_{IV}$

Batch 2−1076 : Low affinity immunoglobulin gamma Fc region receptor III−B on eGFR

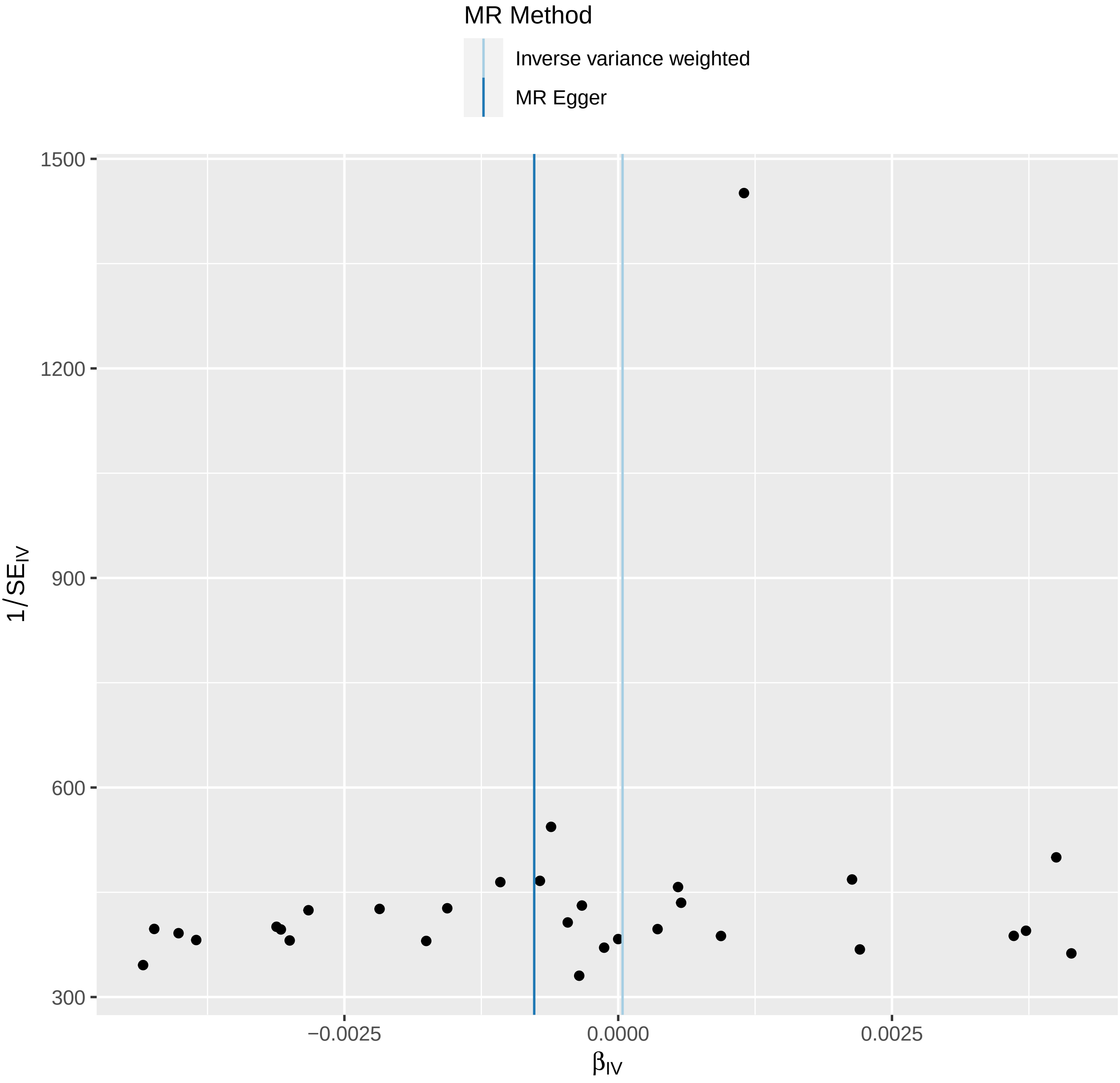

Batch 2−1077 : Ficolin−1 on eGFR

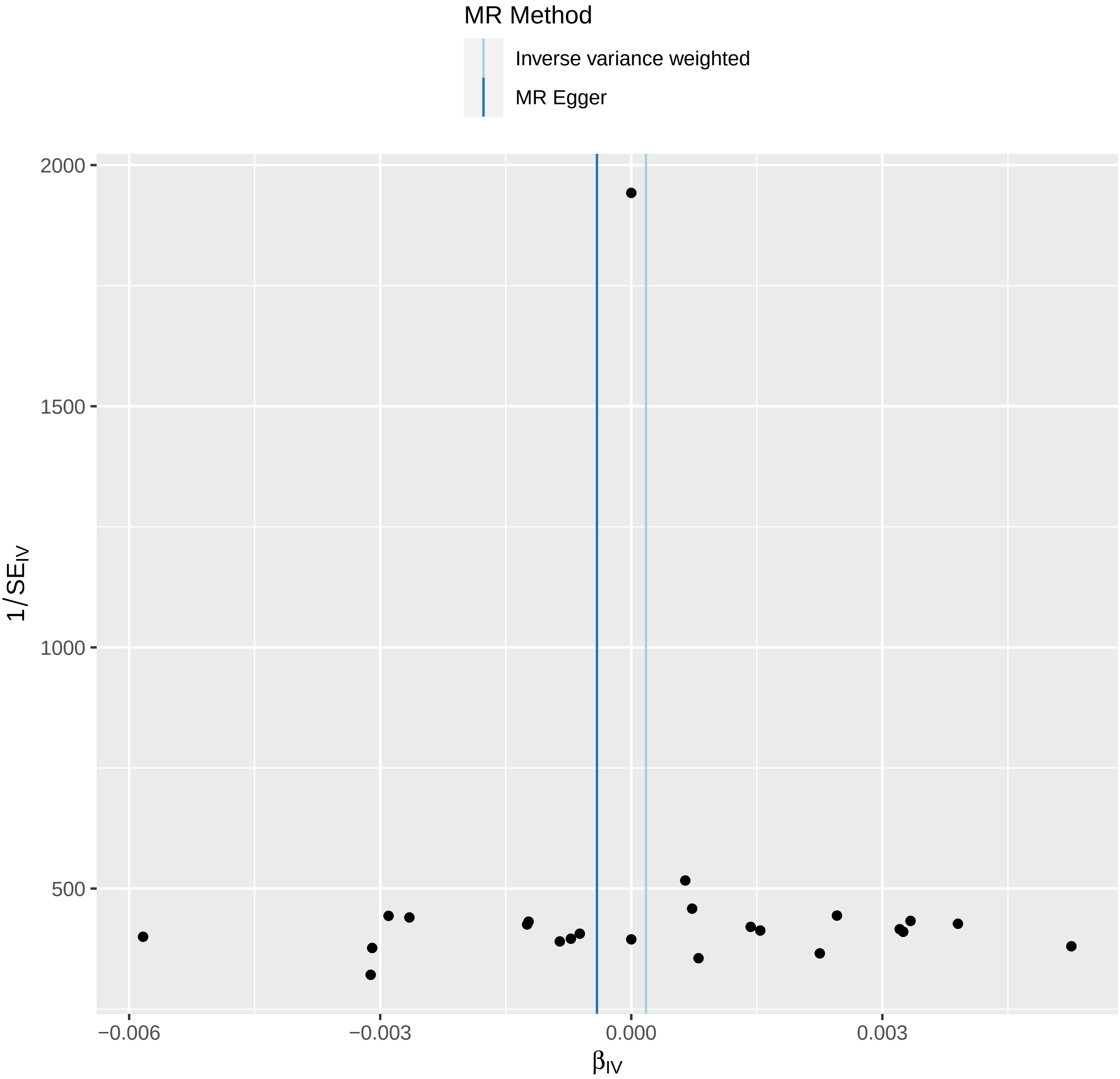

Batch 2−1078 : Ficolin−2 on eGFR

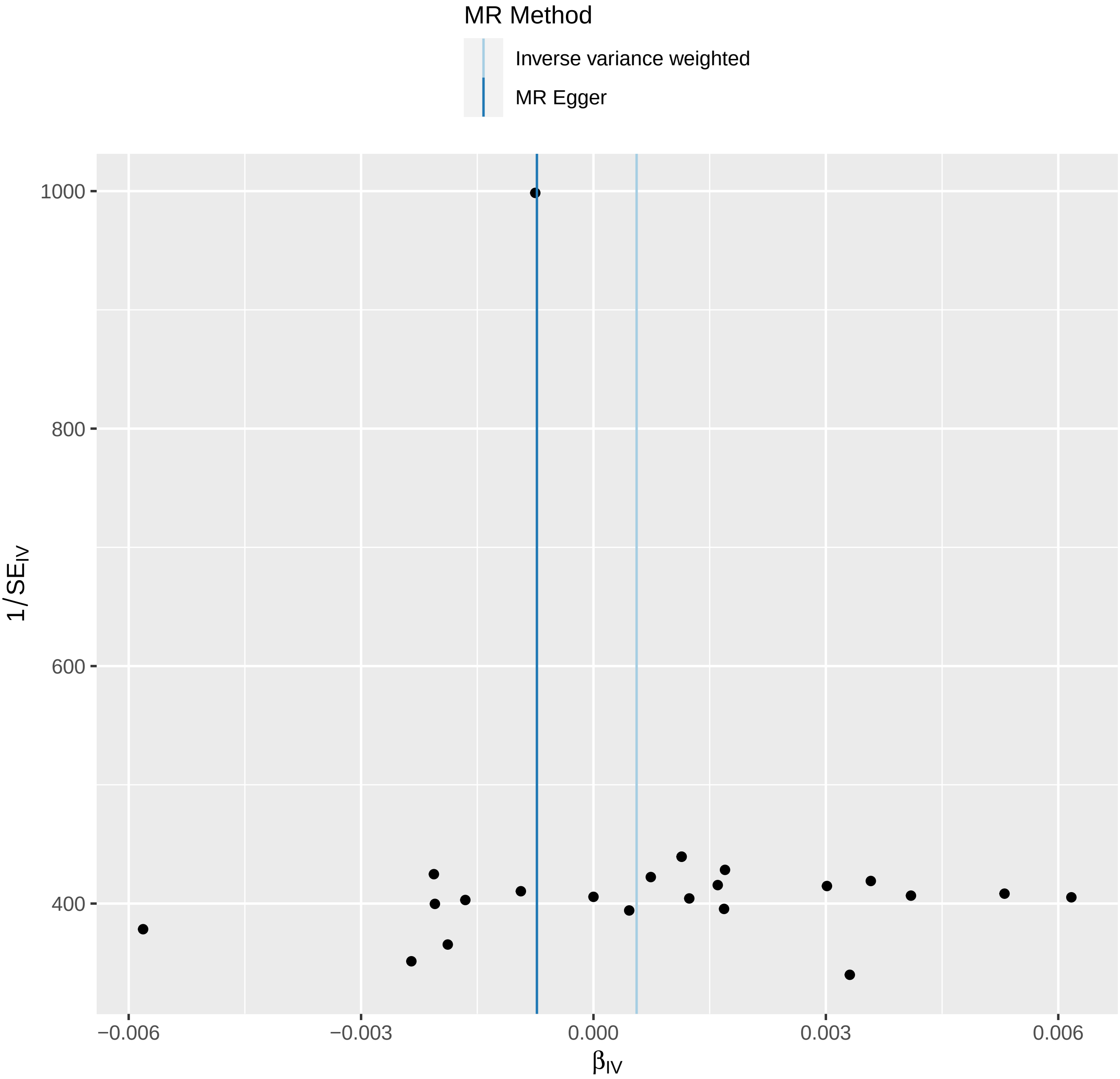

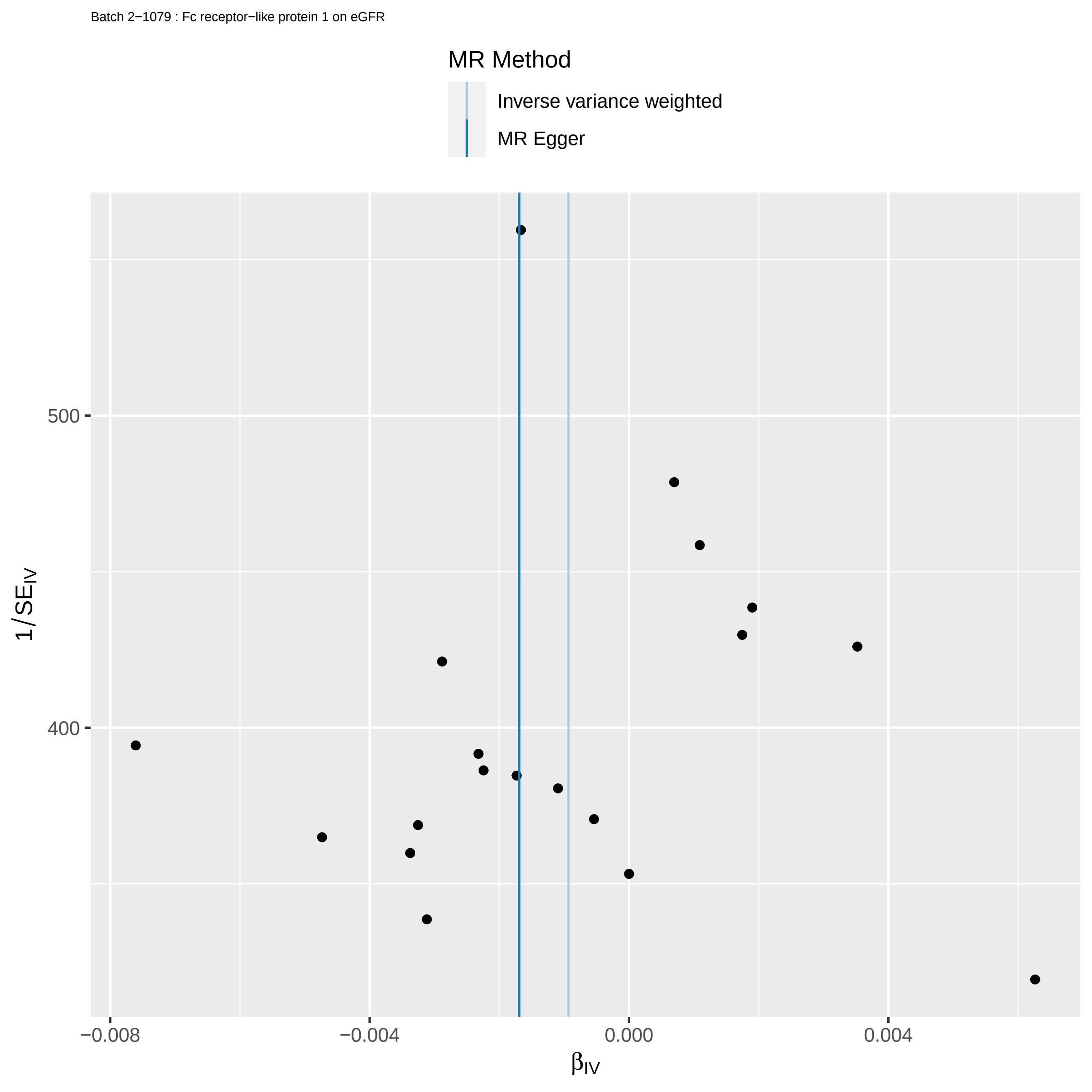

Batch 2−1079 : Fc receptor−like protein 1 on eGFR
MR Method
Inverse variance weighted
MR Egger
500
400
1/SE_IV
−0.008
−0.004
0.000
0.004
β_IV

Batch 2−1080 : Fc receptor−like protein 2 on eGFR

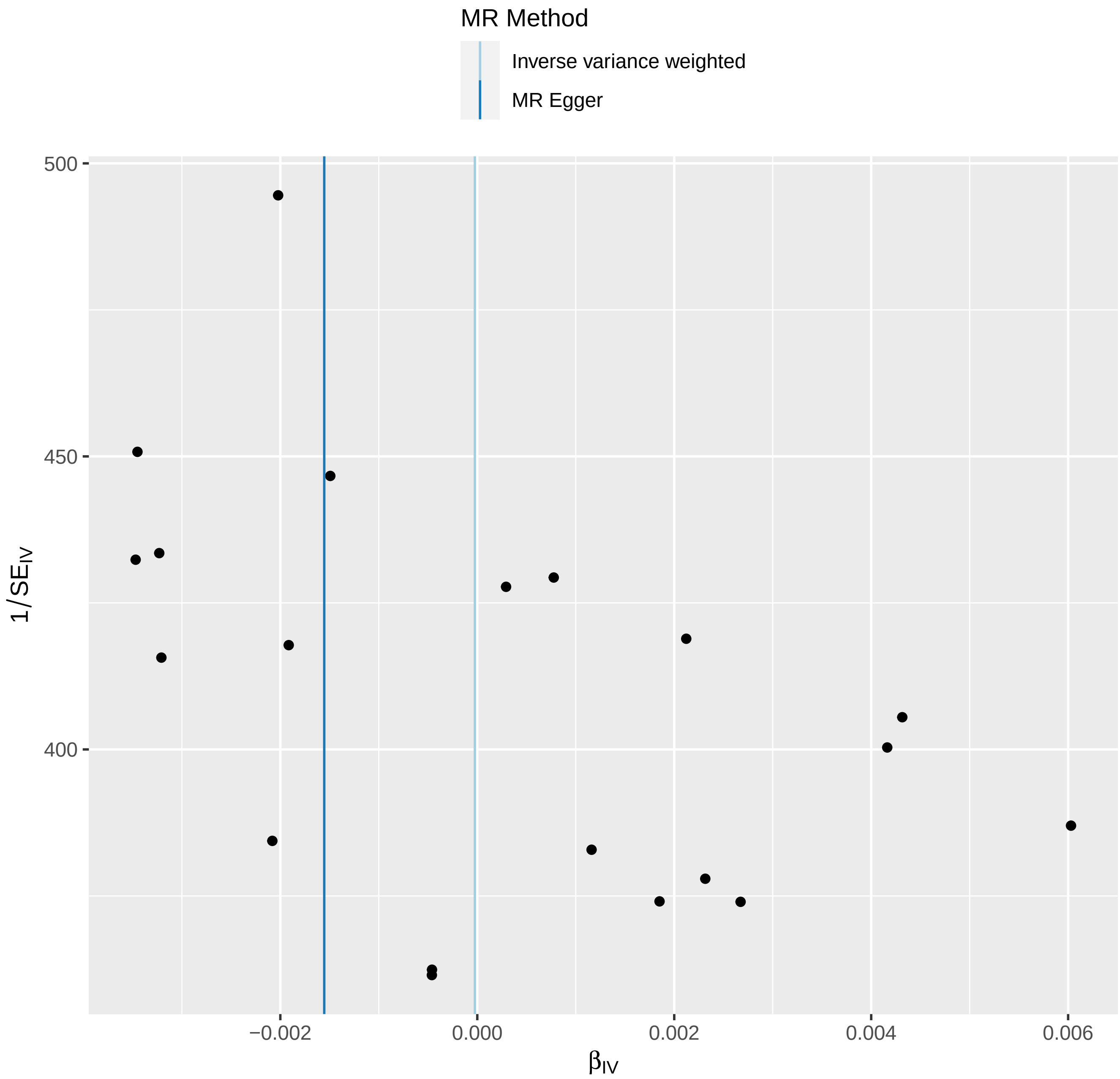

Batch 2−1081 : Fc receptor−like protein 3 on eGFR

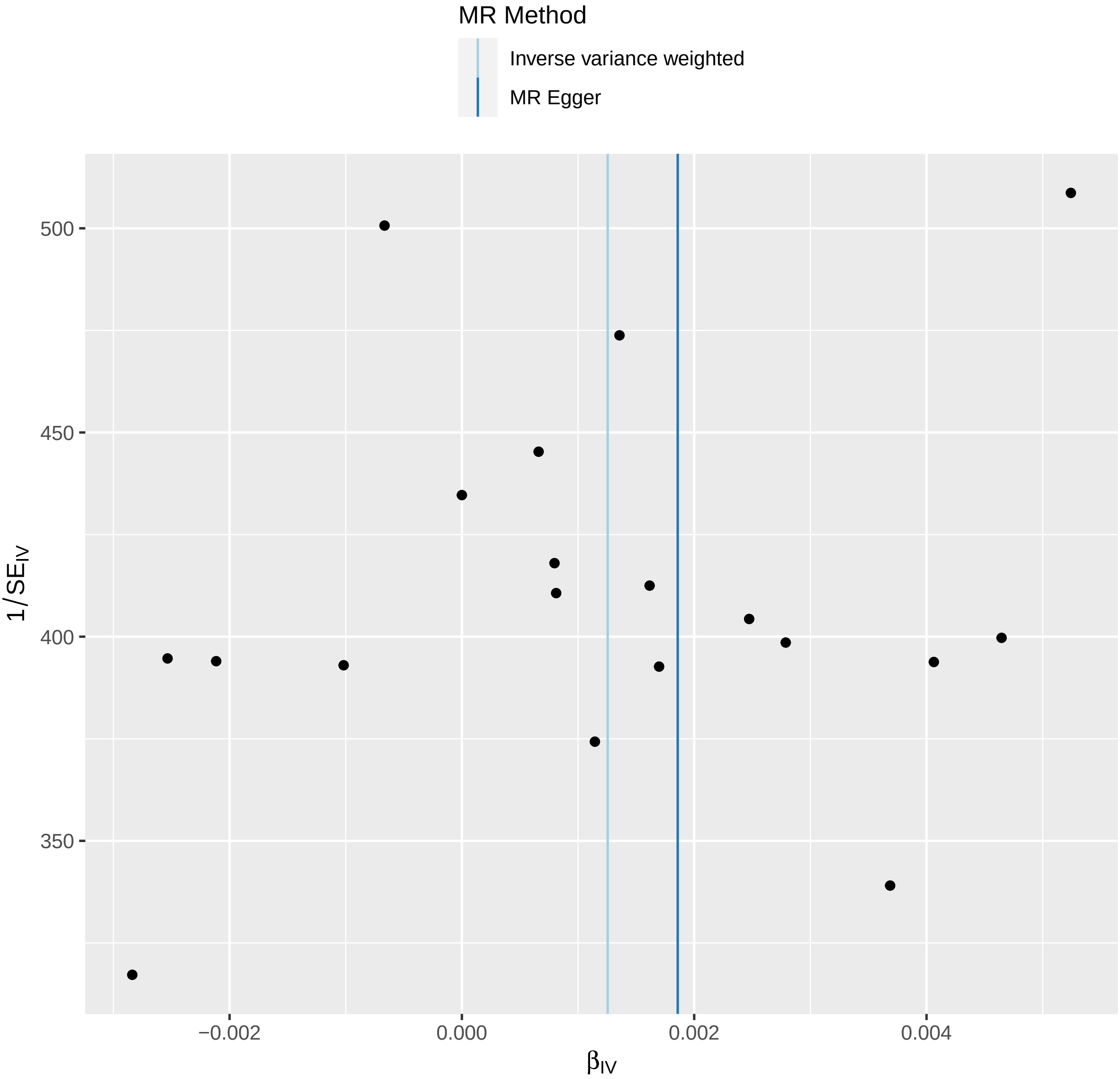

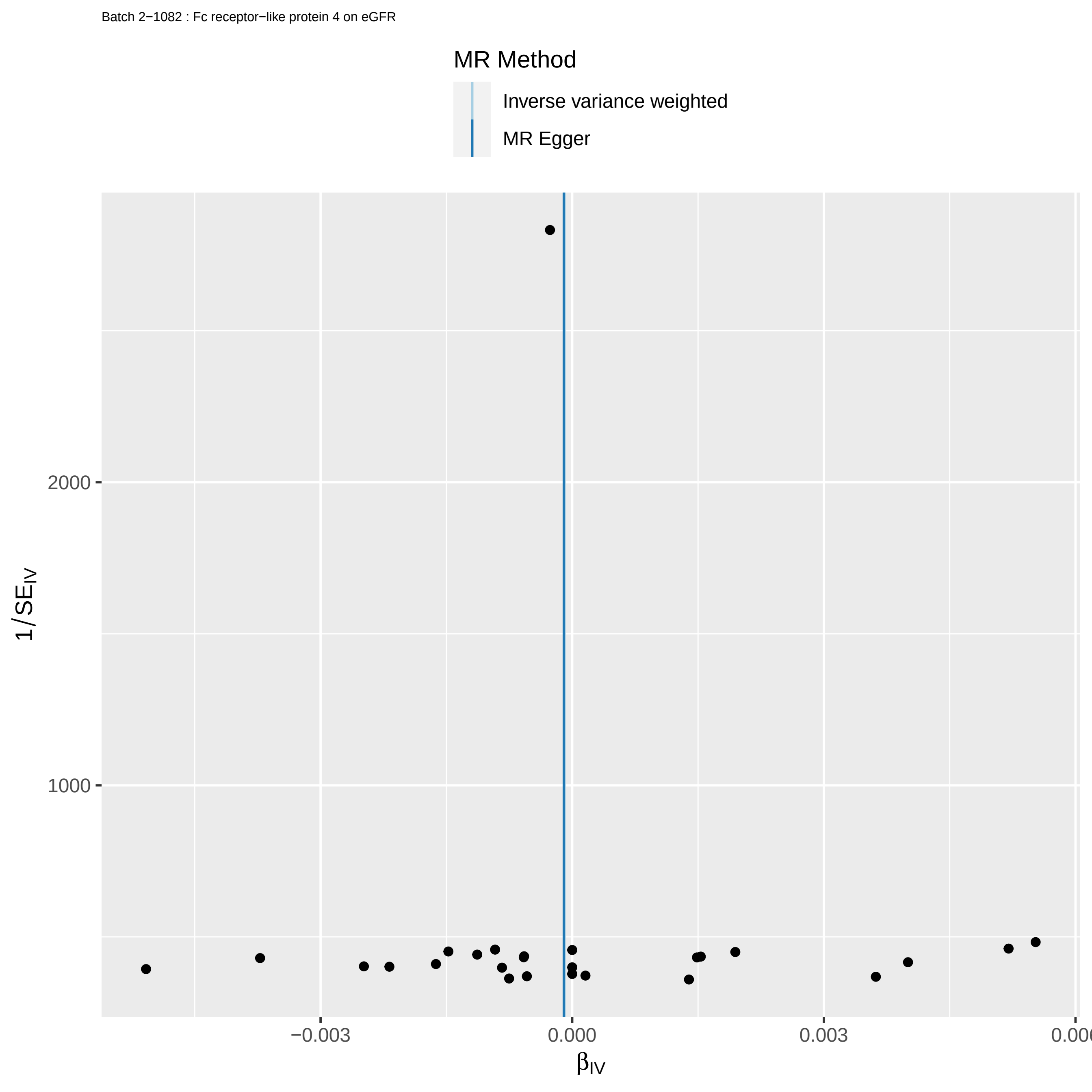
Batch 2−1082 : Fc receptor−like protein 4 on eGFR
MR Method
Inverse variance weighted
MR Egger
2000
1000
1/SE$_{IV}$
−0.003
0.000
0.003
β$_{IV}$

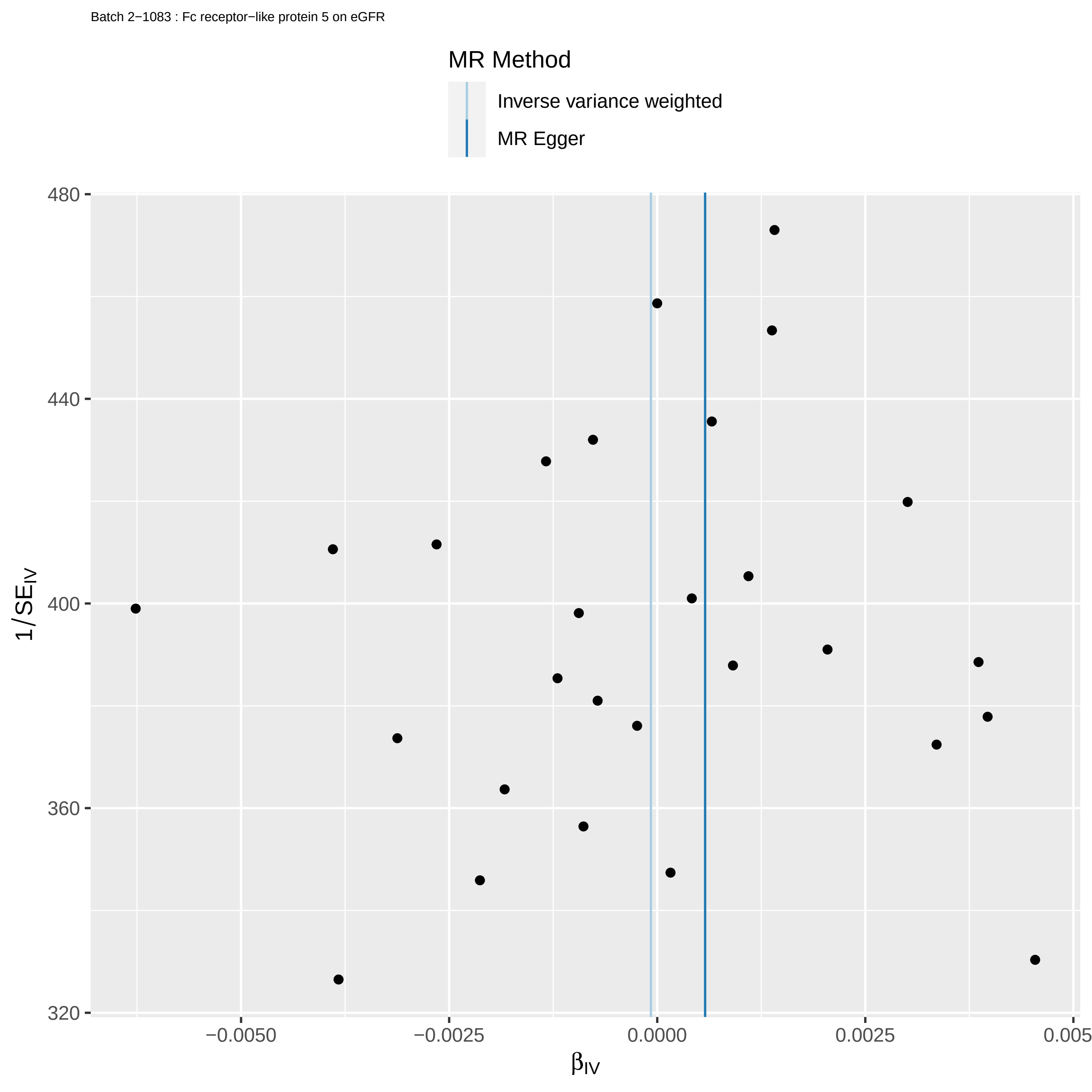
Batch 2−1083 : Fc receptor−like protein 5 on eGFR
MR Method
Inverse variance weighted
MR Egger
480
440
400
360
320
1/SE$_{IV}$
−0.0050
−0.0025
0.0000
0.0025
0.005
β_{IV}

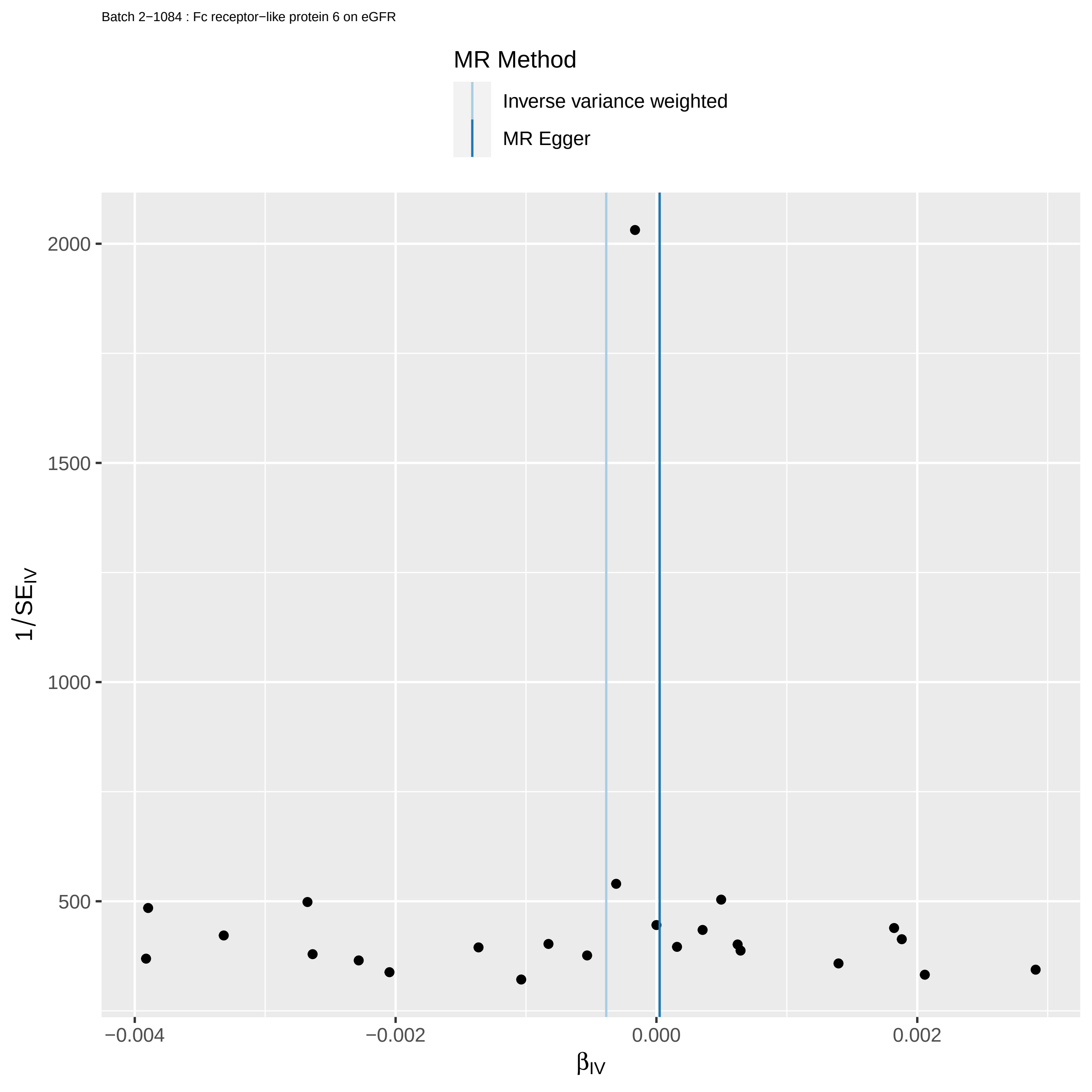
Batch 2−1084 : Fc receptor−like protein 6 on eGFR
MR Method
Inverse variance weighted
MR Egger
2000
1500
1000
500
1/SE_IV
−0.004
−0.002
0.000
0.002
β_IV

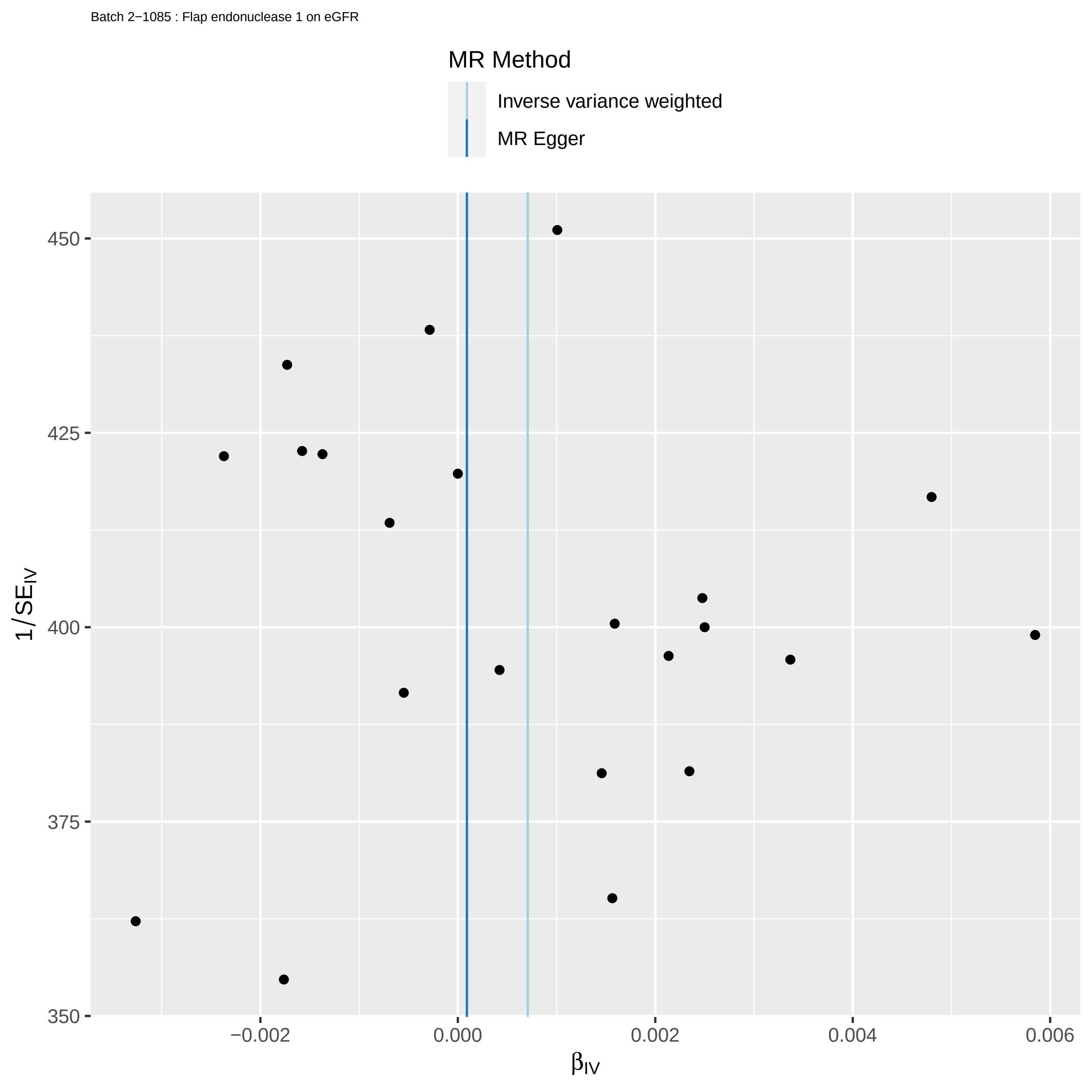
Batch 2−1085 : Flap endonuclease 1 on eGFR
MR Method
Inverse variance weighted
MR Egger
1/SE_IV
450
425
400
375
350
−0.002
0.000
0.002
0.004
0.006
β_IV

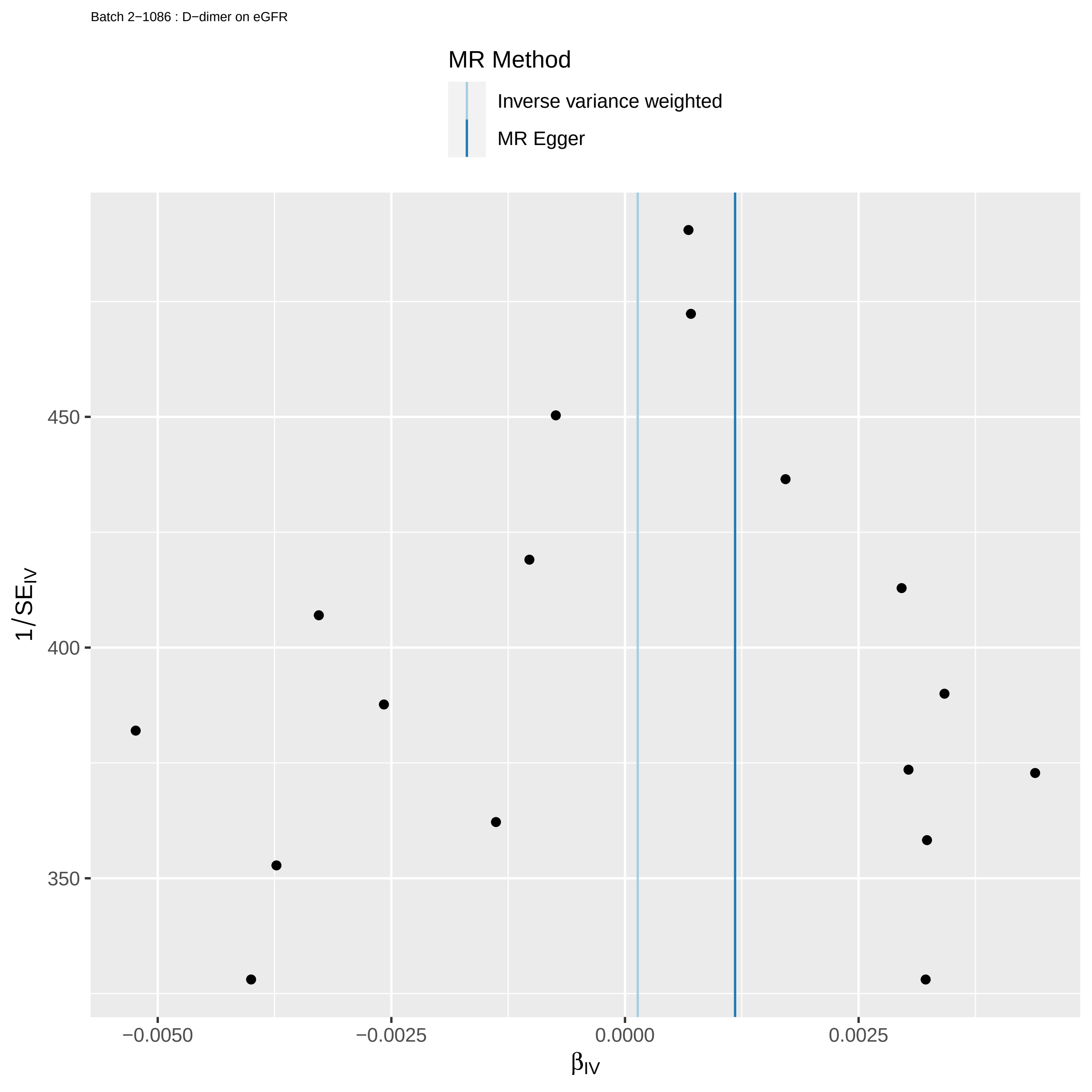
Batch 2−1086 : D−dimer on eGFR
MR Method
Inverse variance weighted
MR Egger
450
400
350
1/SE$_{IV}$
−0.0050
−0.0025
0.0000
0.0025
β$_{IV}$

Batch 2−1087 : Fibroblast growth factor 10 on eGFR

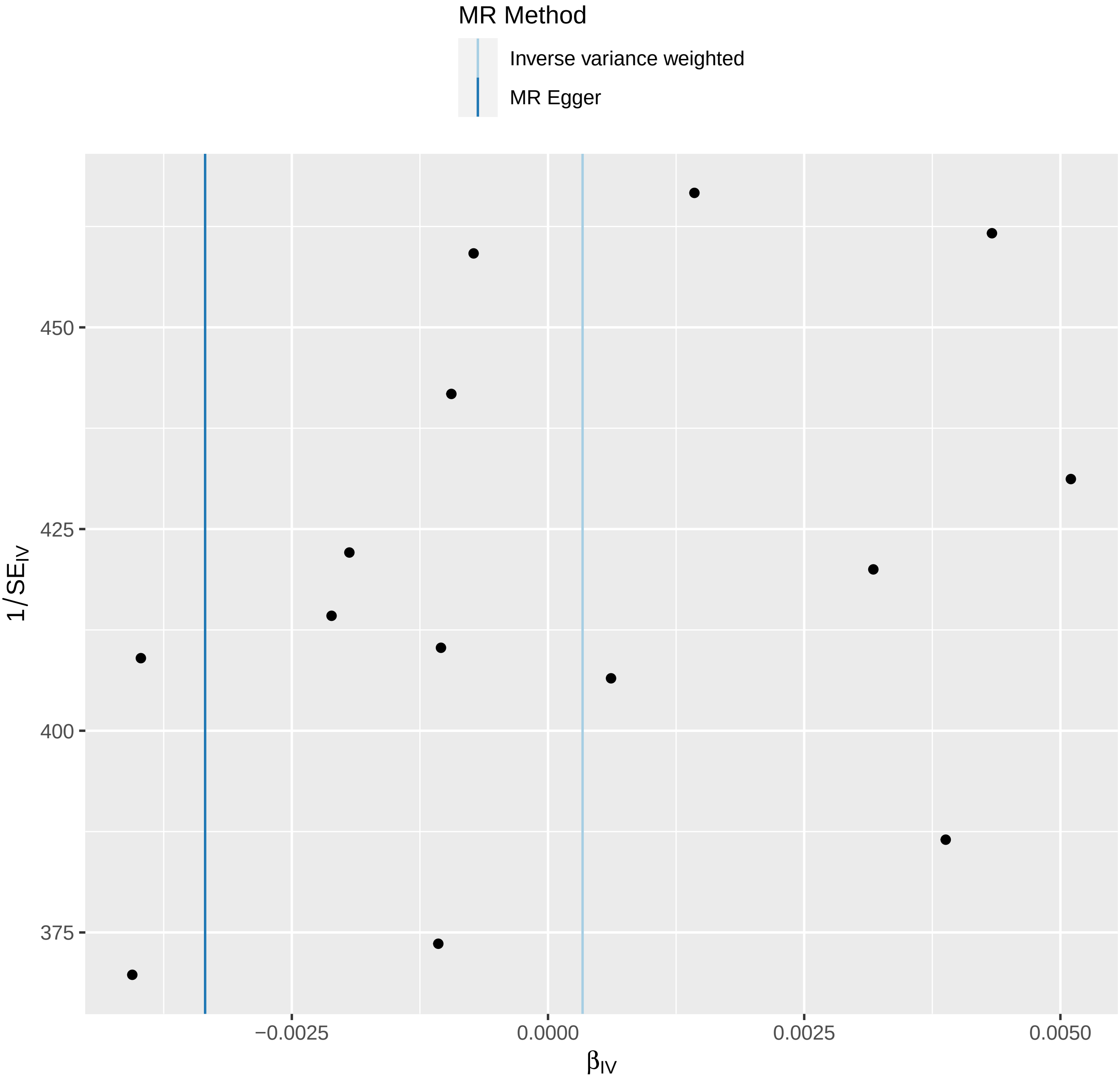

Batch 2−1088 : Fibroblast growth factor 1 on eGFR

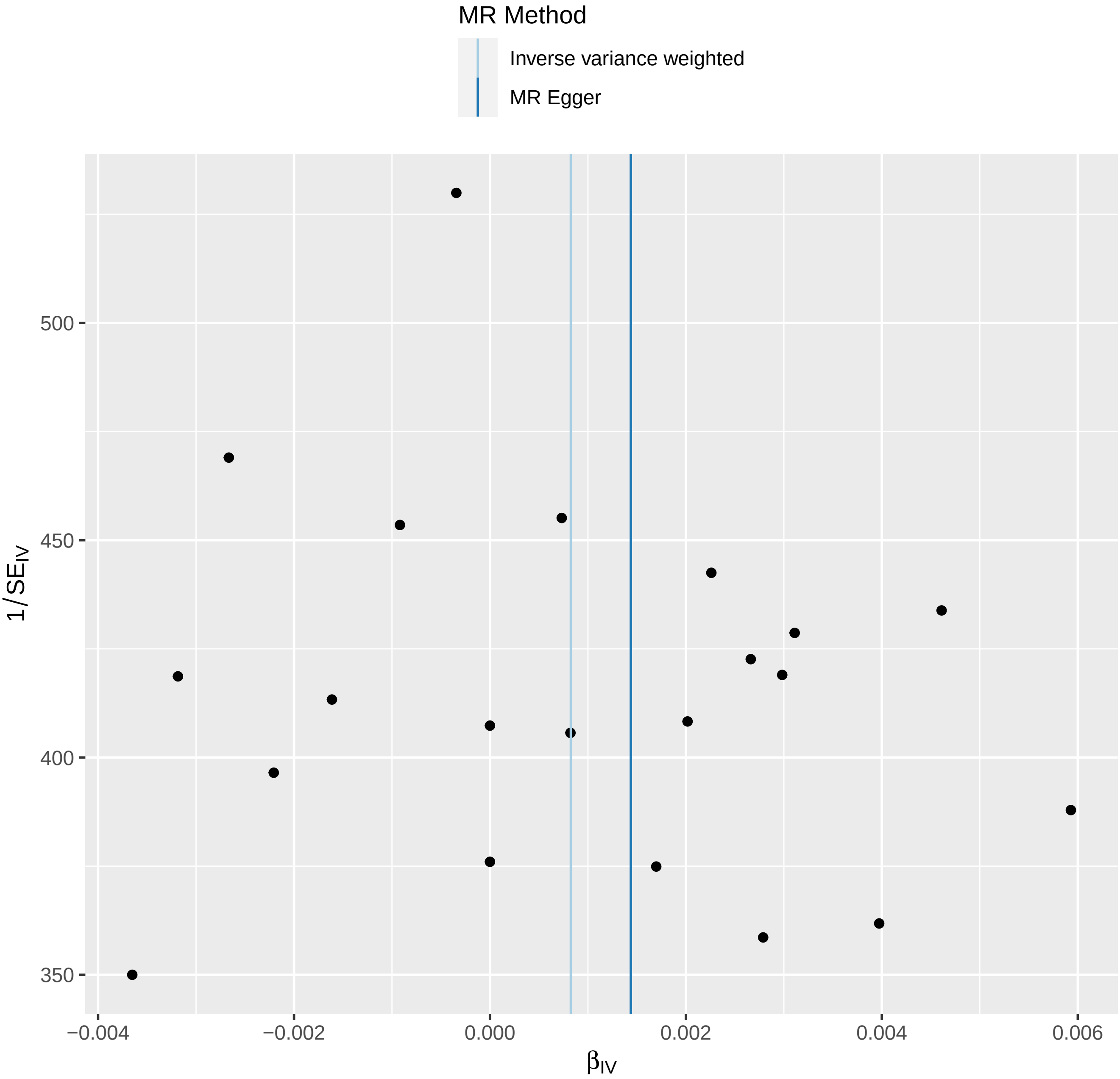

Batch 2−1089 : Fibroblast growth factor 16 on eGFR

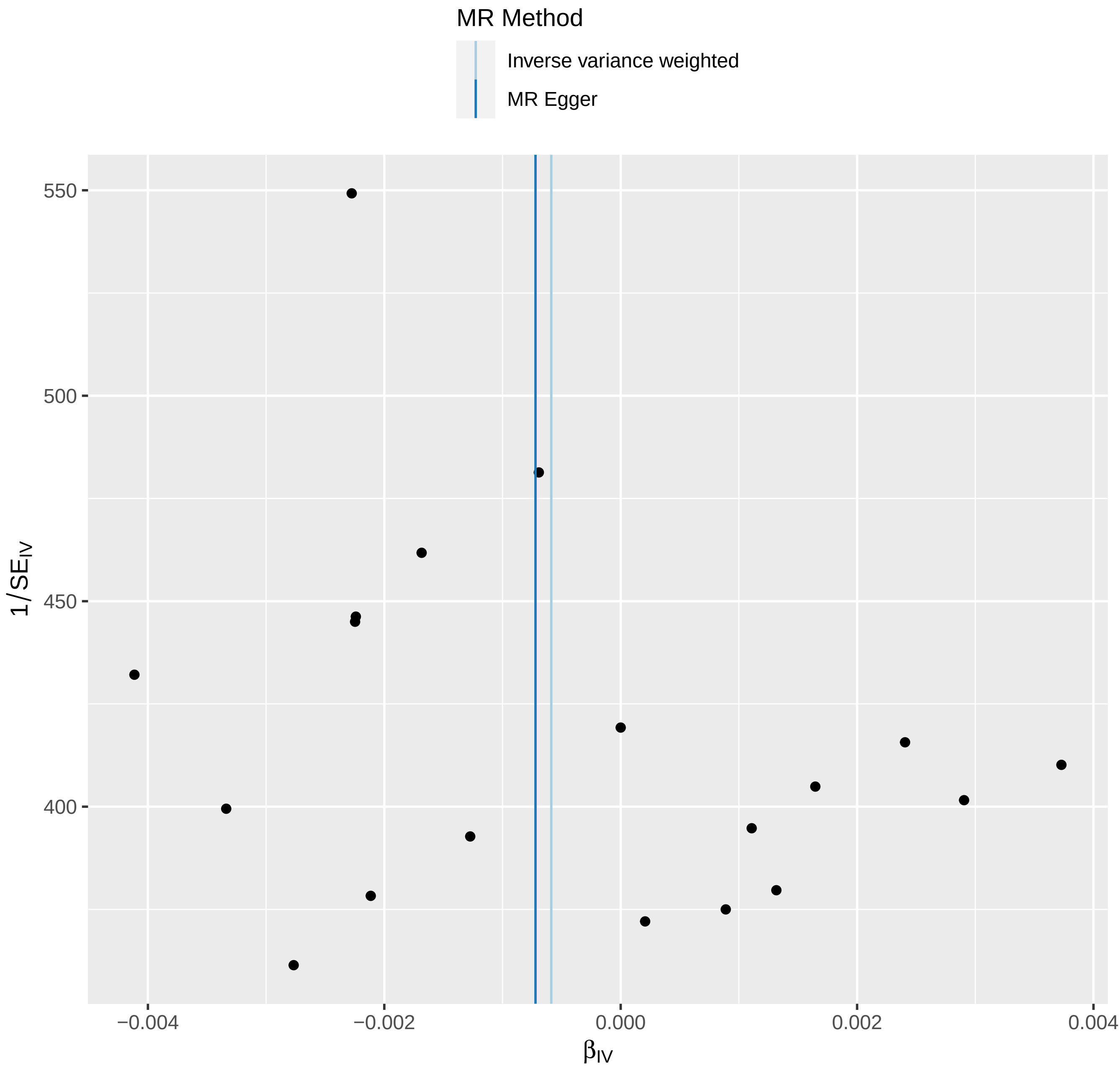

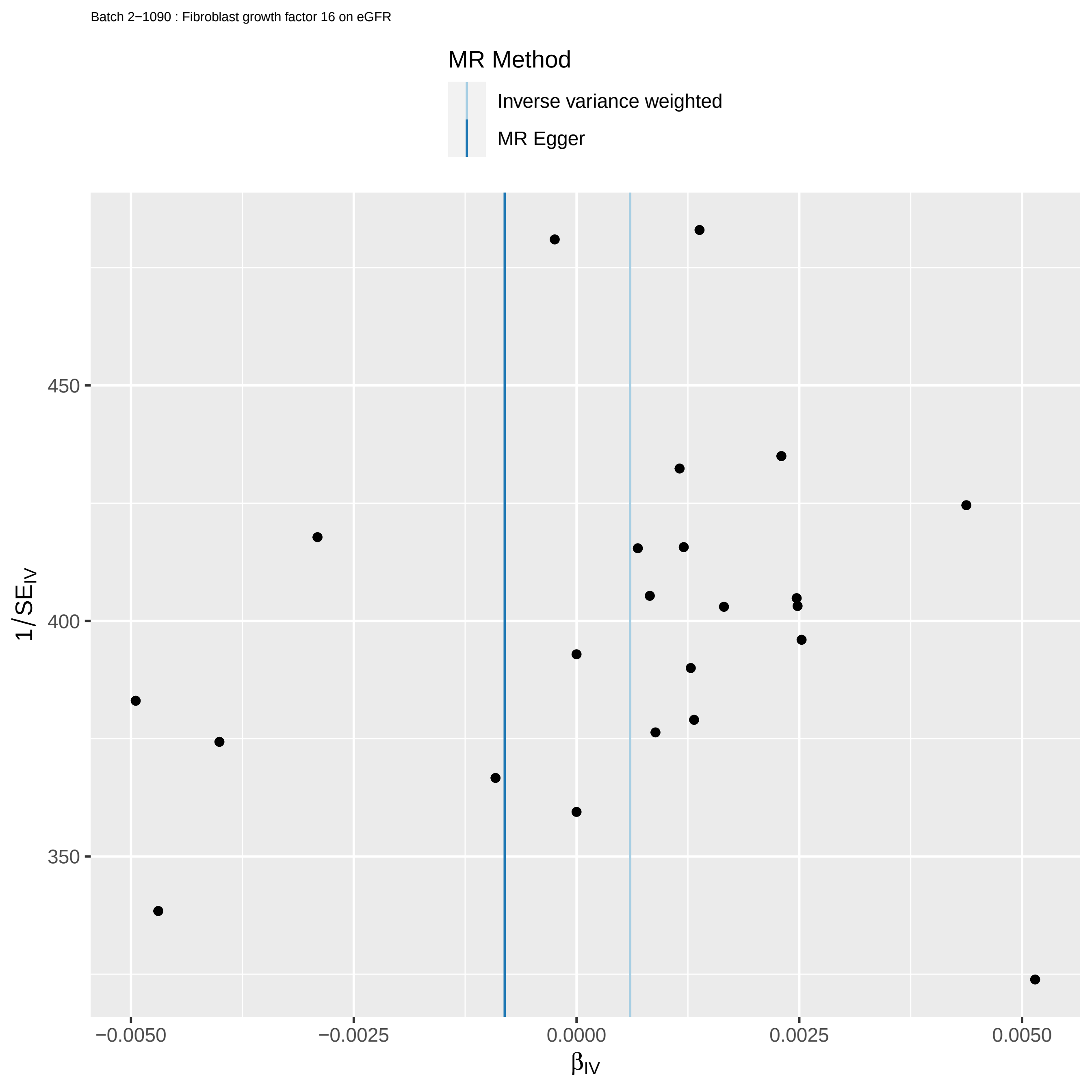
Batch 2−1090 : Fibroblast growth factor 16 on eGFR
MR Method
Inverse variance weighted
MR Egger
450
400
350
1/SE_IV
−0.0050
−0.0025
0.0000
0.0025
0.0050
β_IV

Batch 2−1091 : Fibroblast growth factor 19 on eGFR

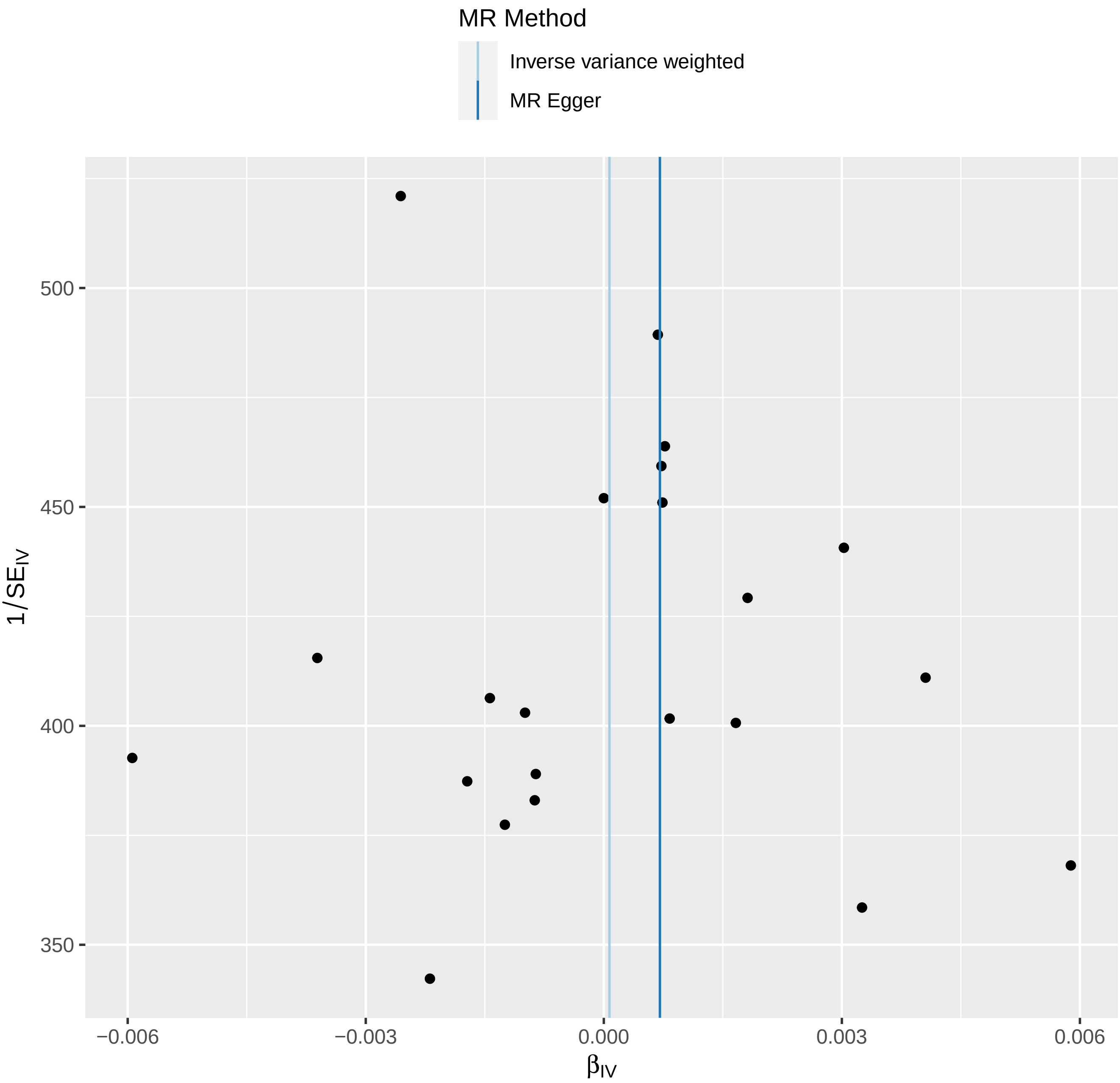

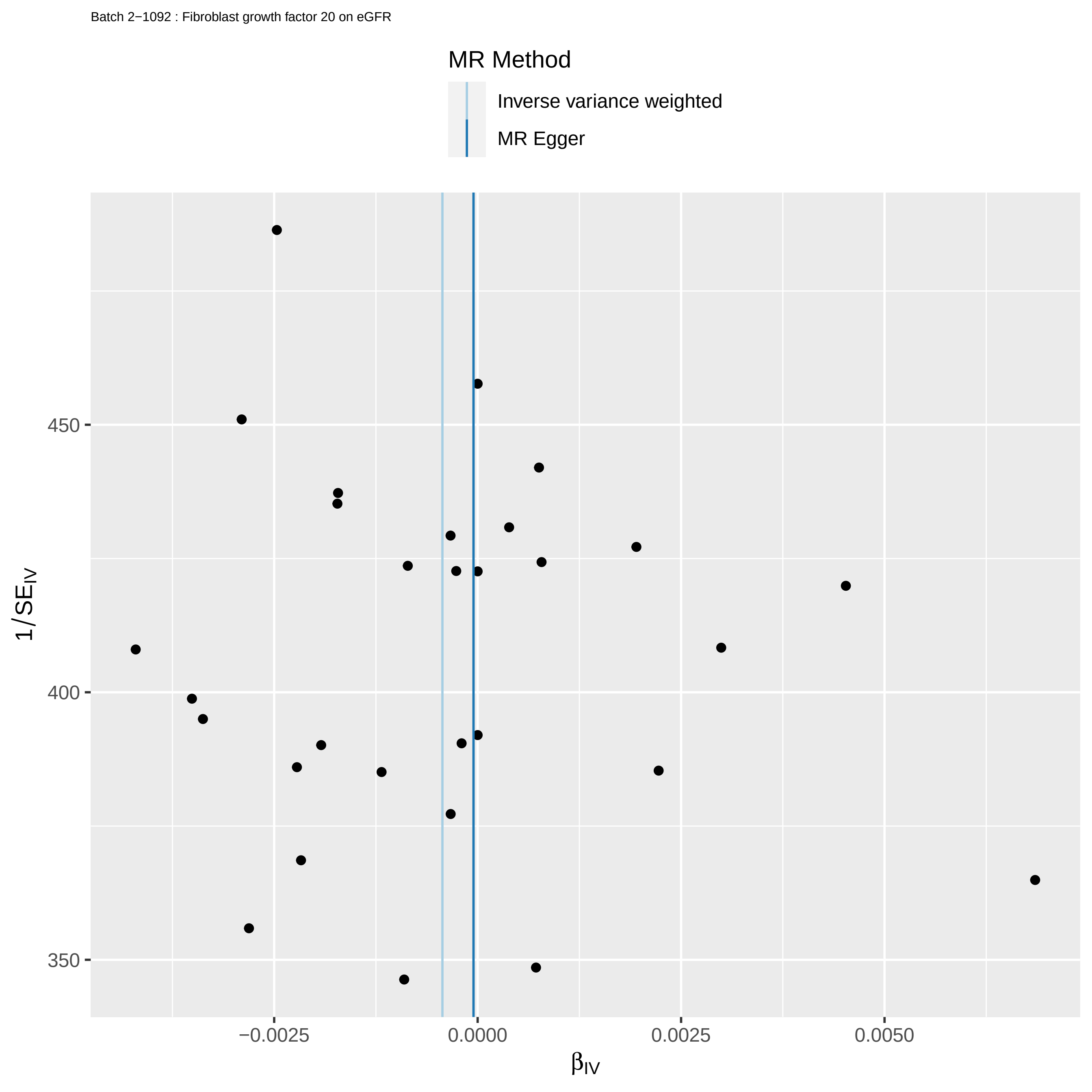

Batch 2−1092 : Fibroblast growth factor 20 on eGFR
MR Method
Inverse variance weighted
MR Egger
450
400
350
1/SE$_{IV}$
−0.0025
0.0000
0.0025
0.0050
β$_{IV}$

Batch 2−1093 : Fibroblast growth factor 23 on eGFR

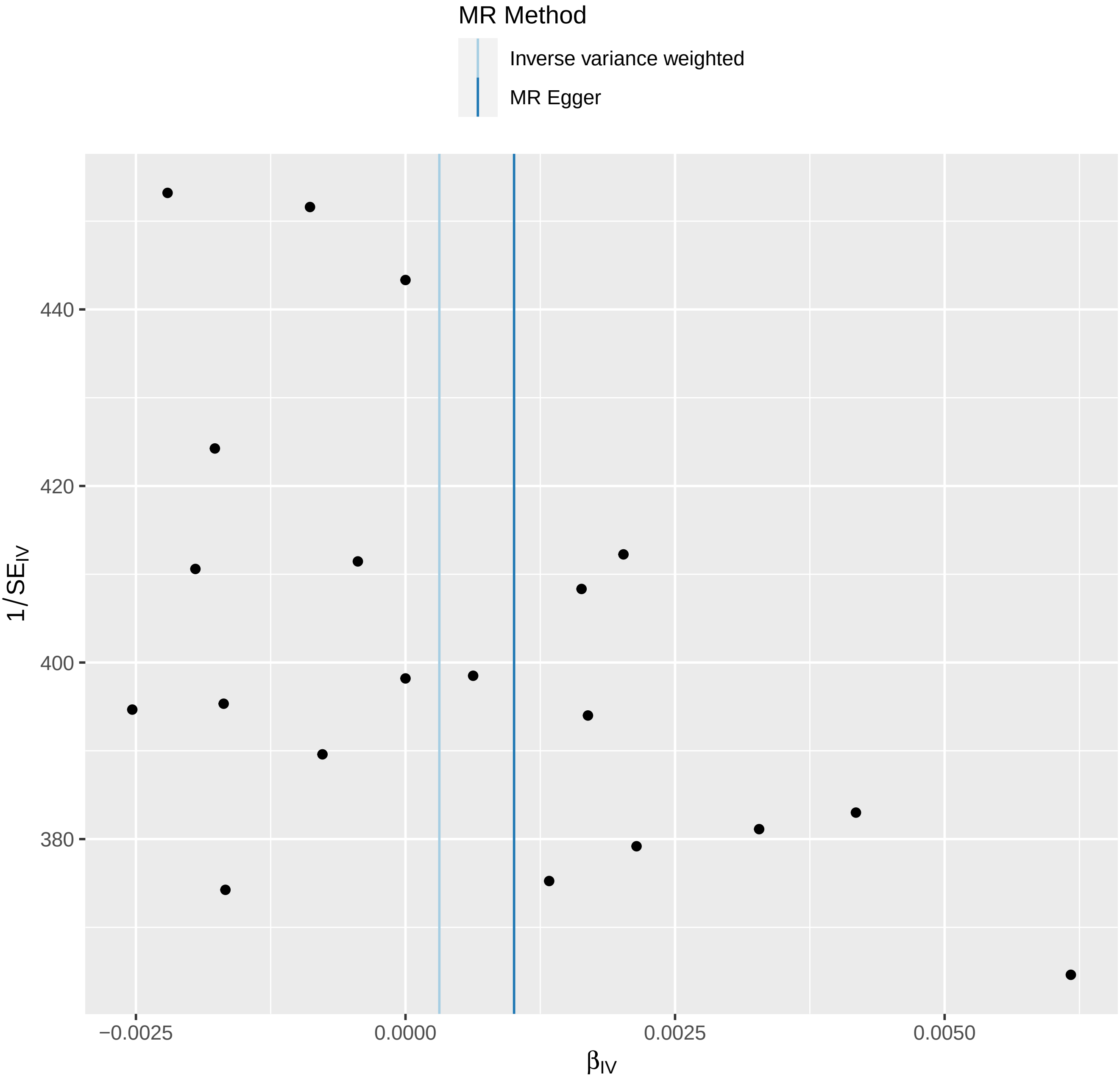

Batch 2−1094 : Fibroblast growth factor 4 on eGFR

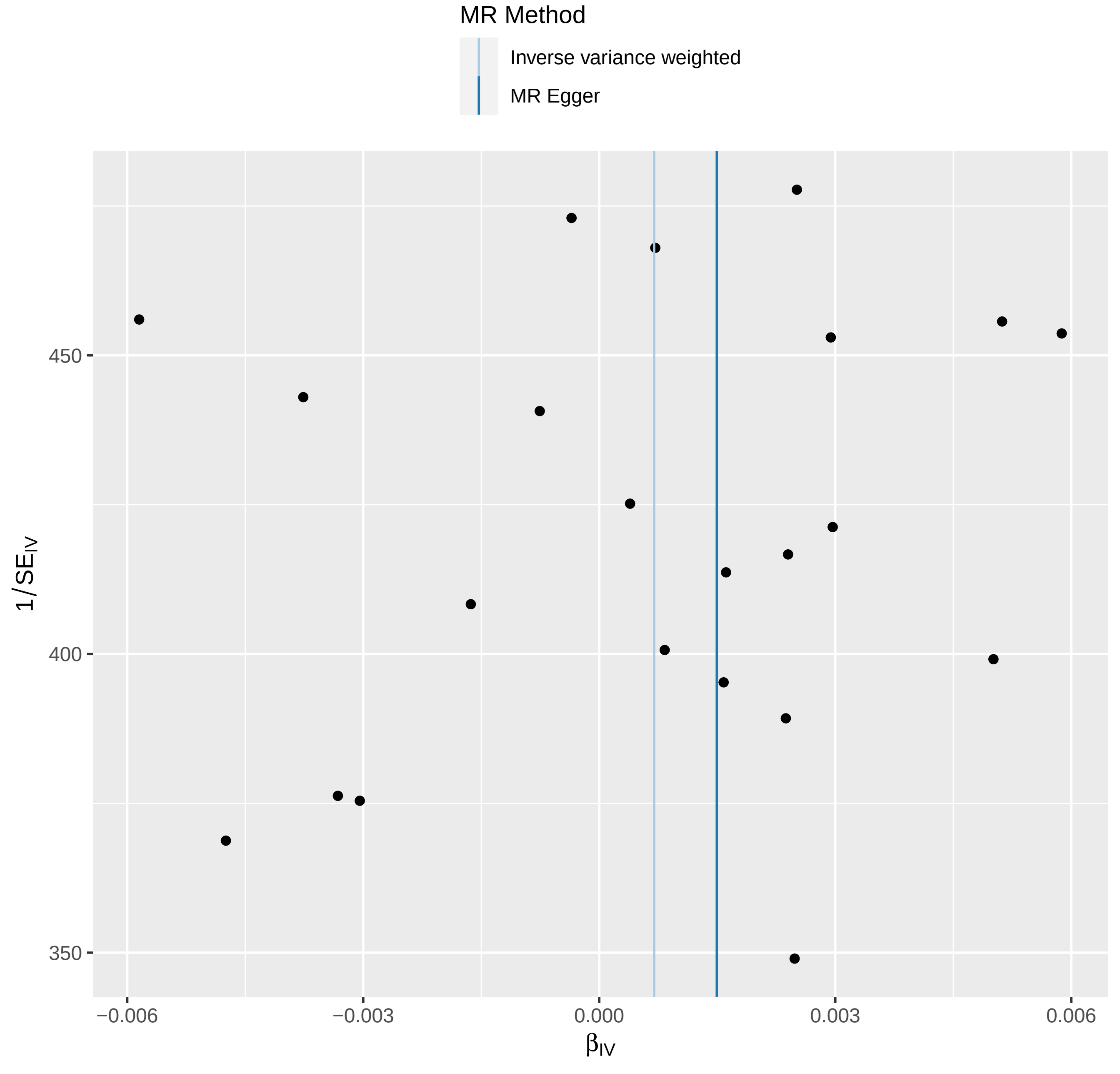

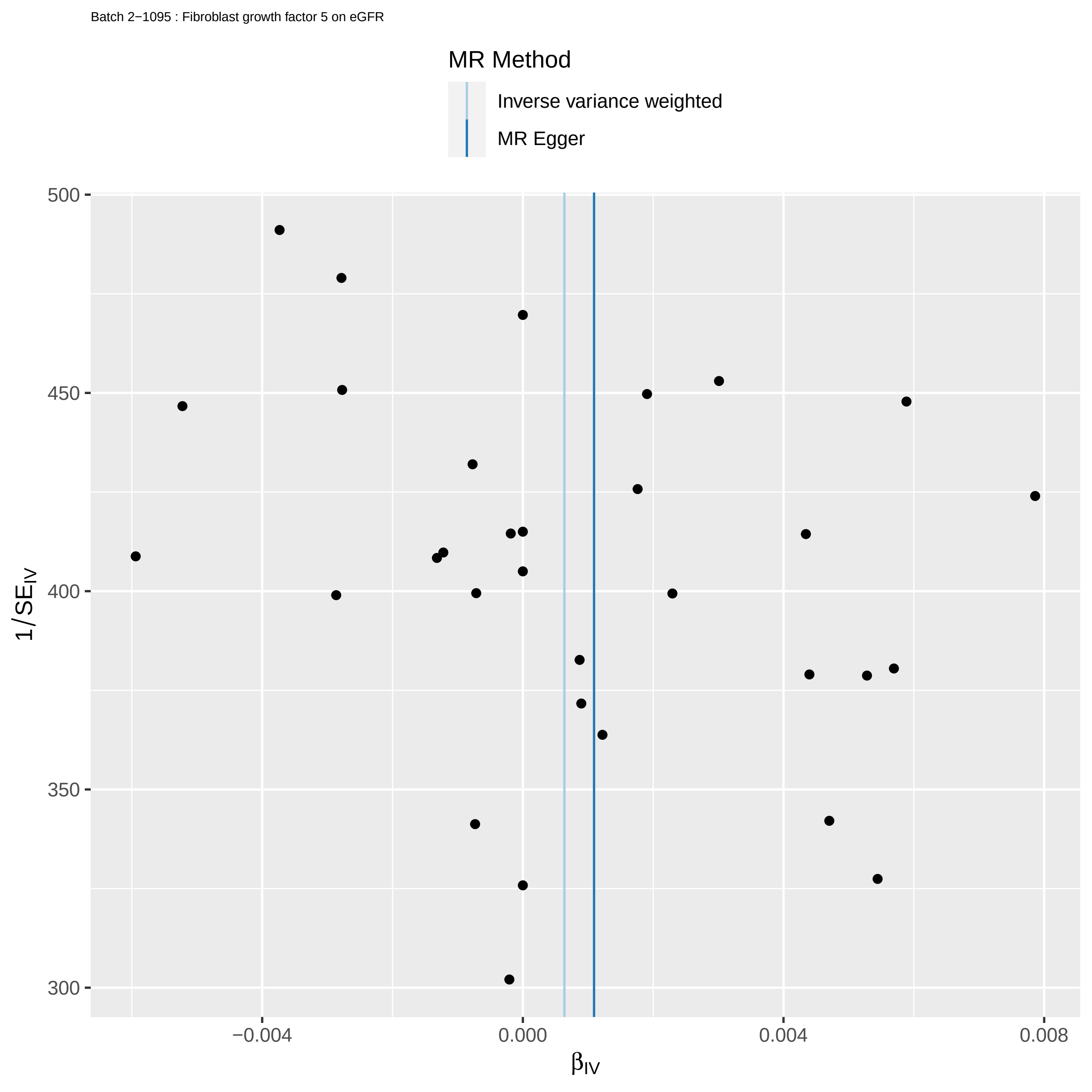

Batch 2−1095 : Fibroblast growth factor 5 on eGFR
MR Method
Inverse variance weighted
MR Egger
500
450
400
350
300
1/SE$_{IV}$
−0.004
0.000
0.004
0.008
β$_{IV}$

Batch 2−1096 : Fibroblast growth factor 6 on eGFR

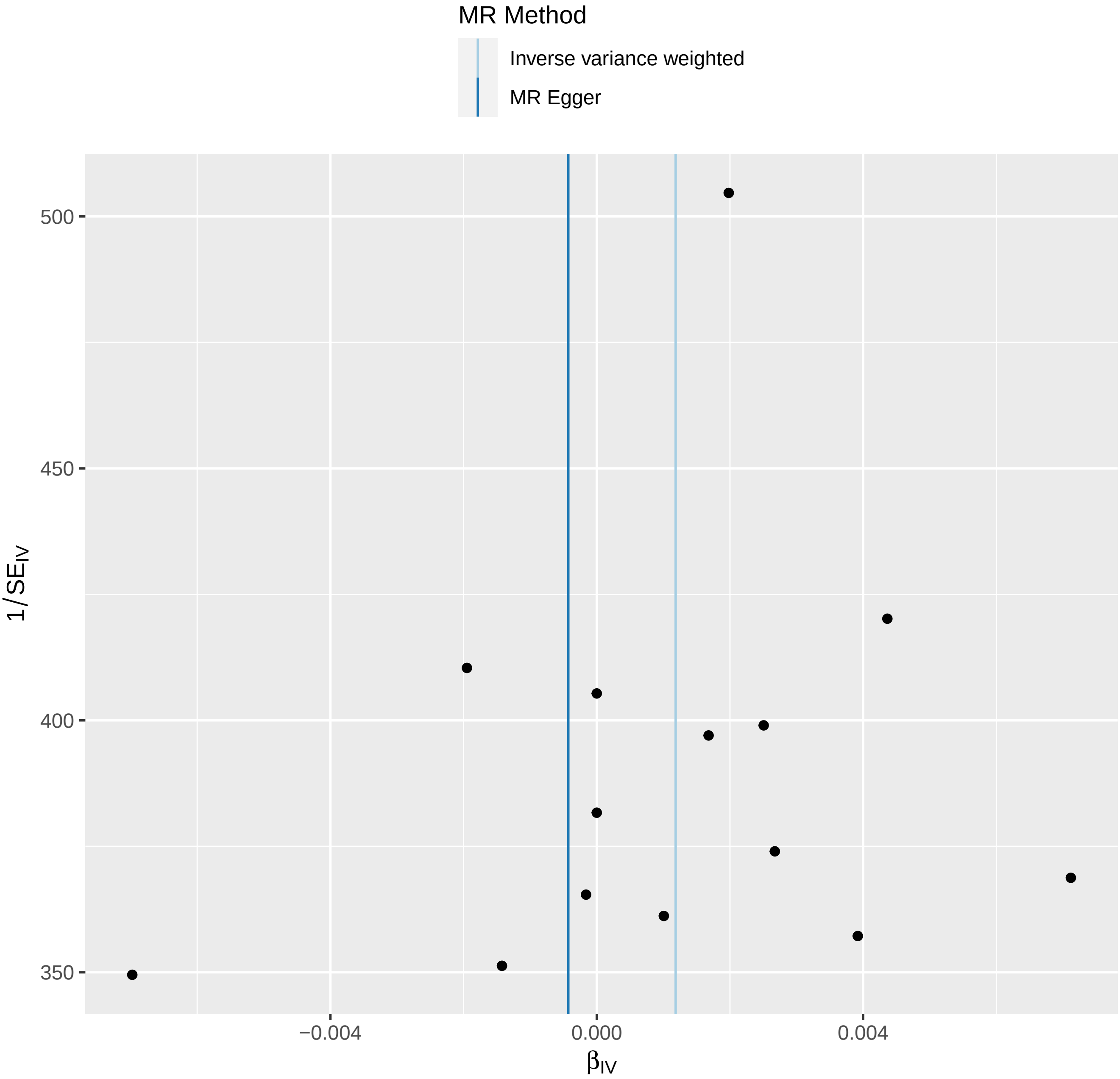

Batch 2−1097 : Fibroblast growth factor 7 on eGFR

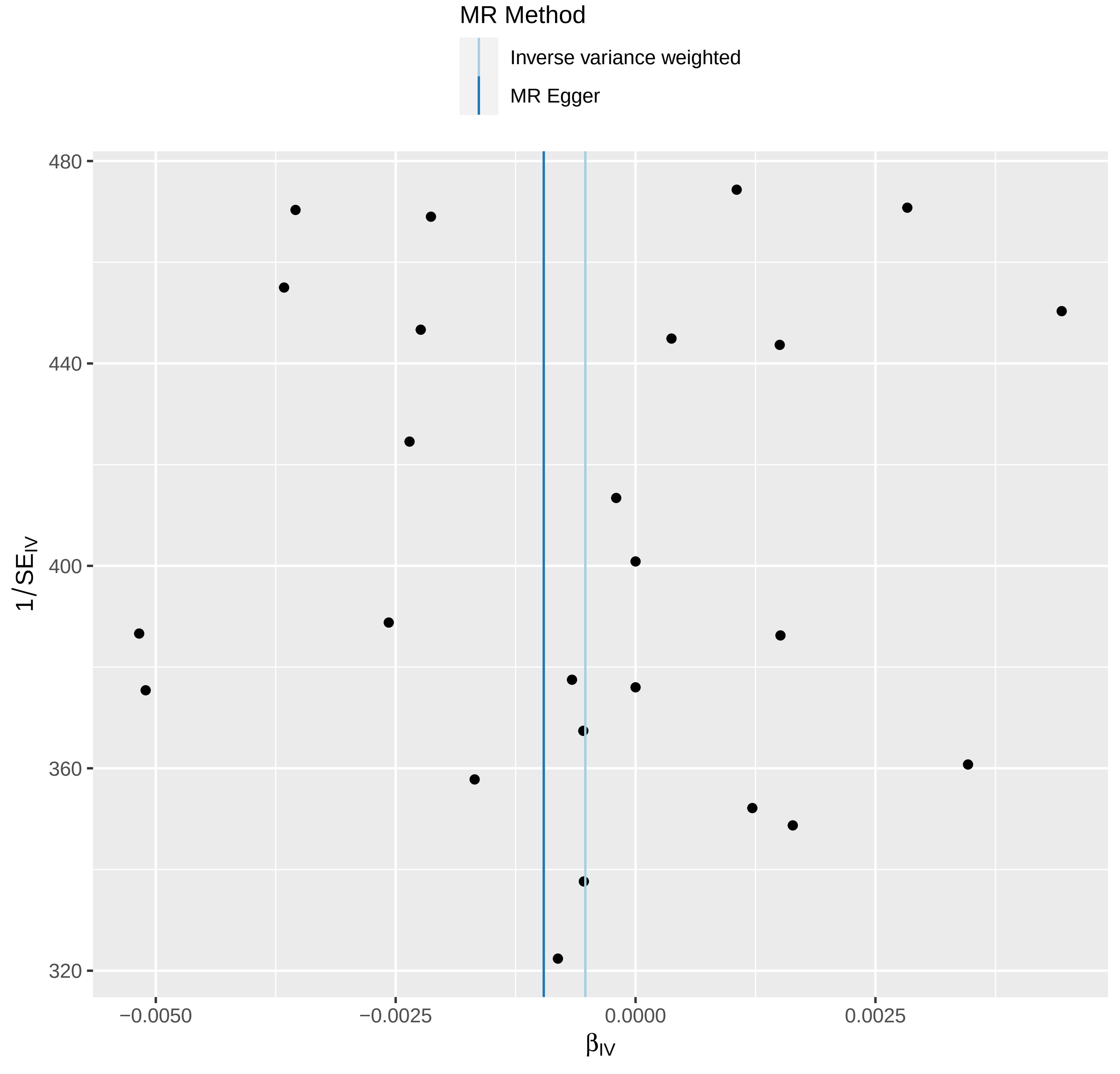

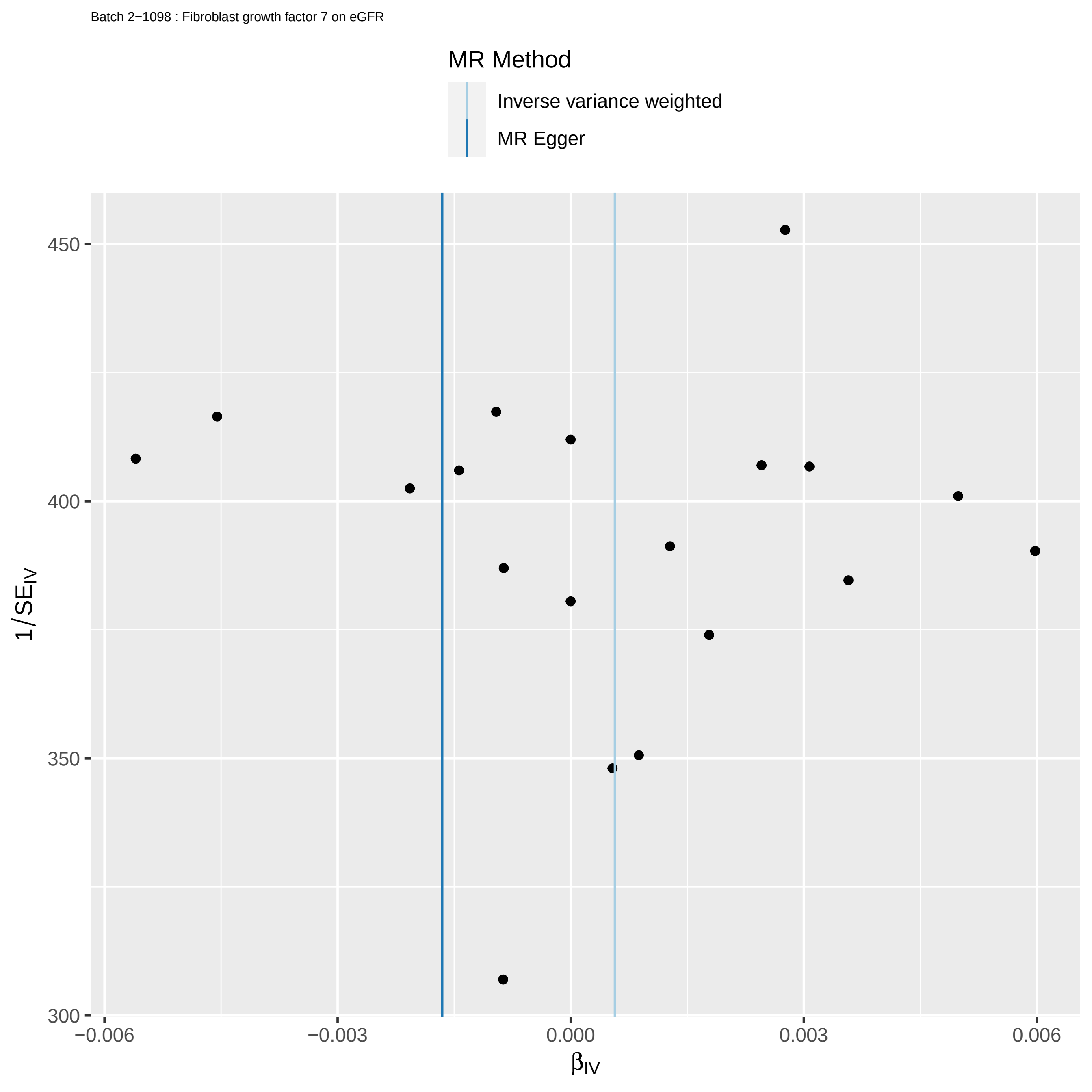

Batch 2−1098 : Fibroblast growth factor 7 on eGFR
MR Method
Inverse variance weighted
MR Egger
450
400
350
300
1/SE_IV
−0.006
−0.003
0.000
0.003
0.006
β_IV

Batch 2−1099 : Fibroblast growth factor 8 isoform B on eGFR

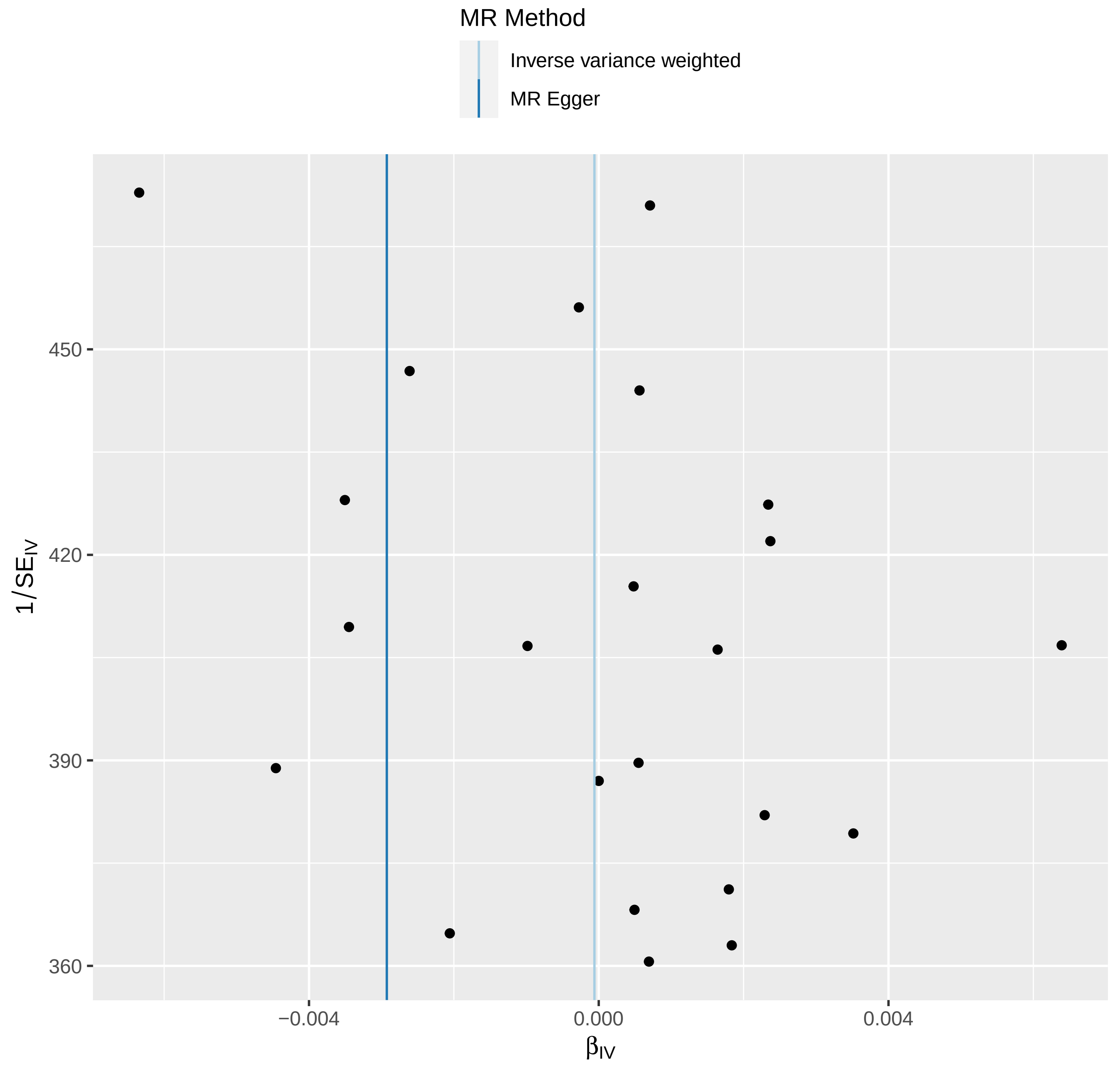

Batch 2−1100 : Fibroblast growth factor 8 isoform A on eGFR

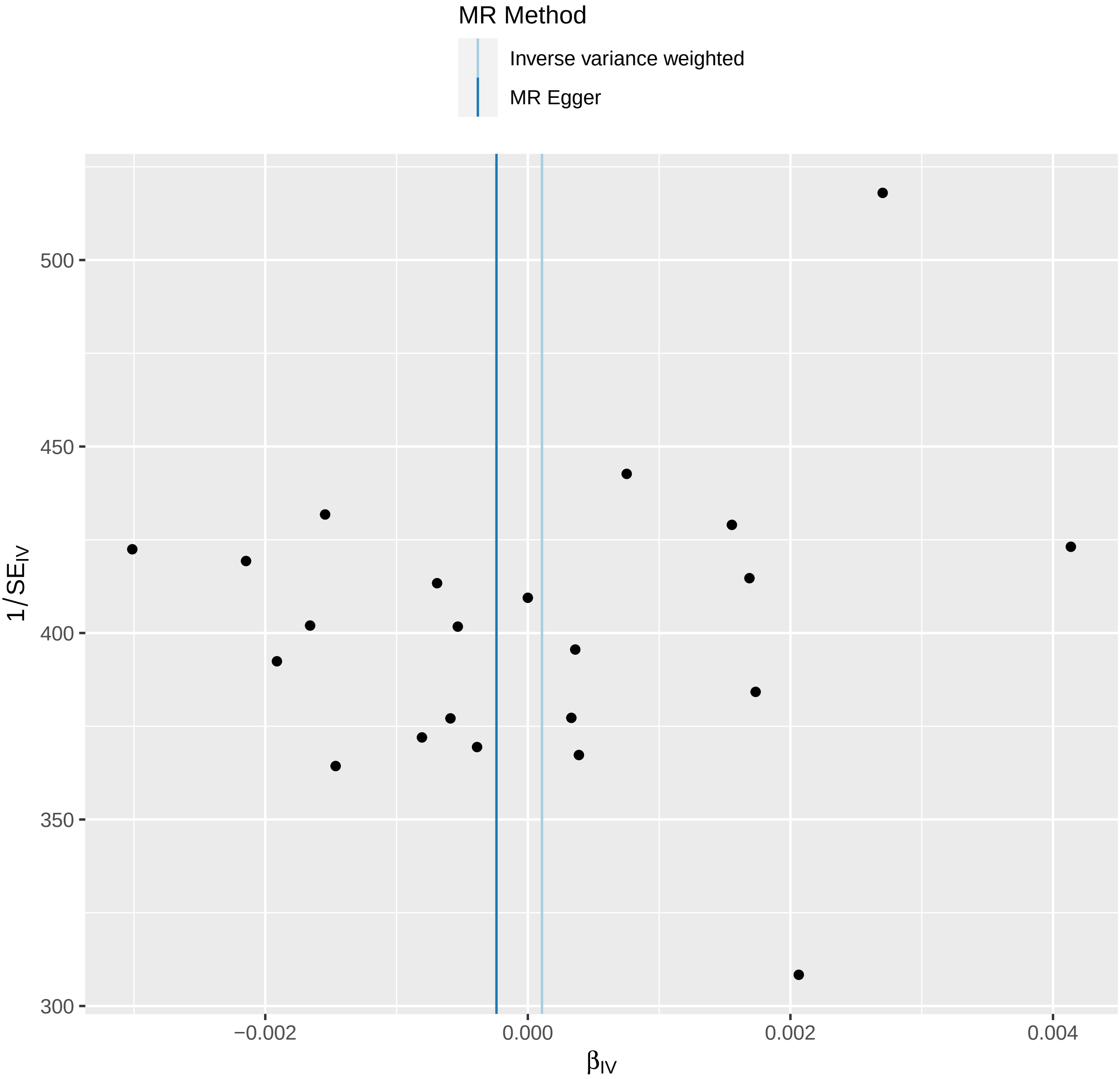

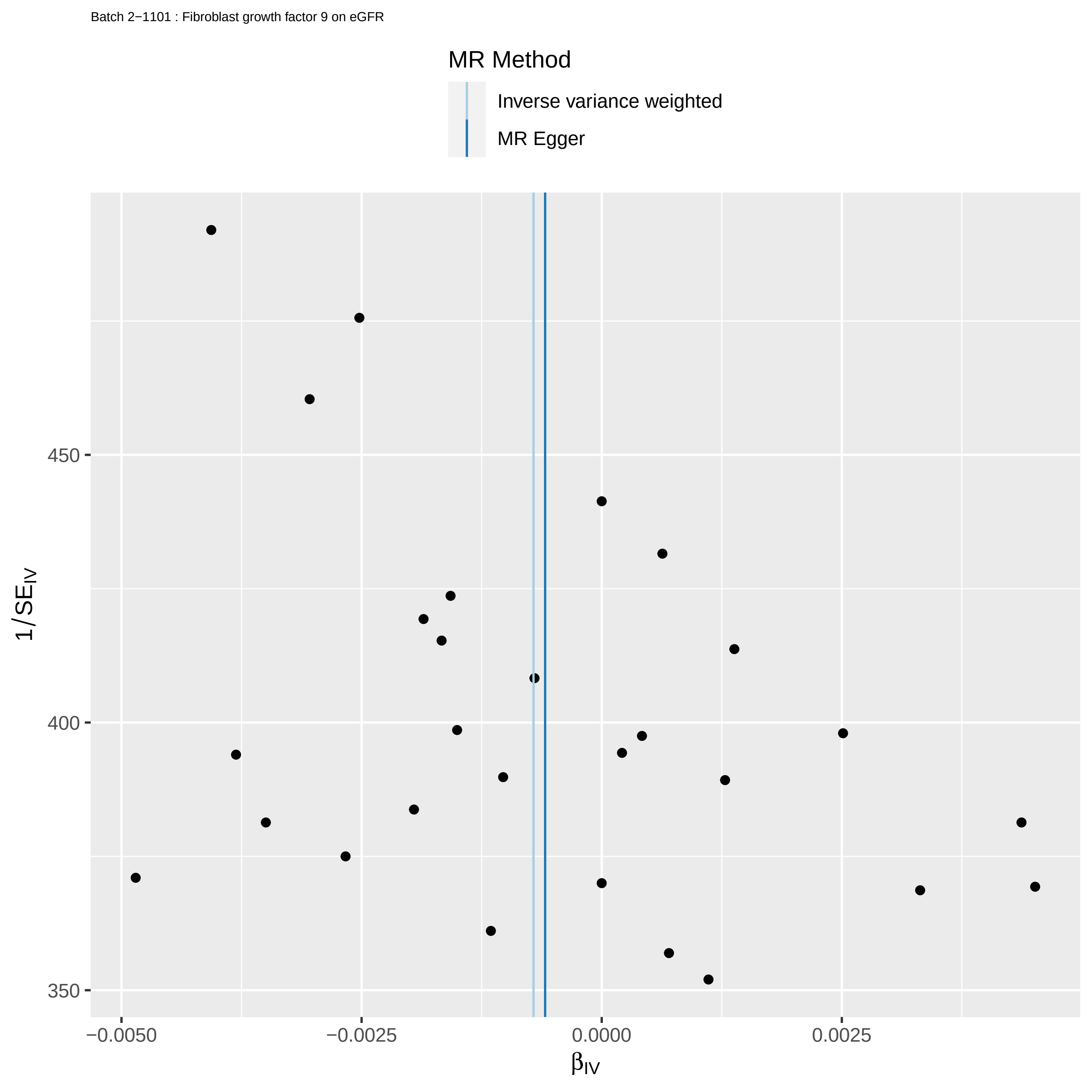

Batch 2−1101 : Fibroblast growth factor 9 on eGFR
MR Method
Inverse variance weighted
MR Egger
450
400
350
1/SE_IV
−0.0050
−0.0025
0.0000
0.0025
β_IV

Batch 2−1102 : Fibroblast growth factor receptor 2 on eGFR

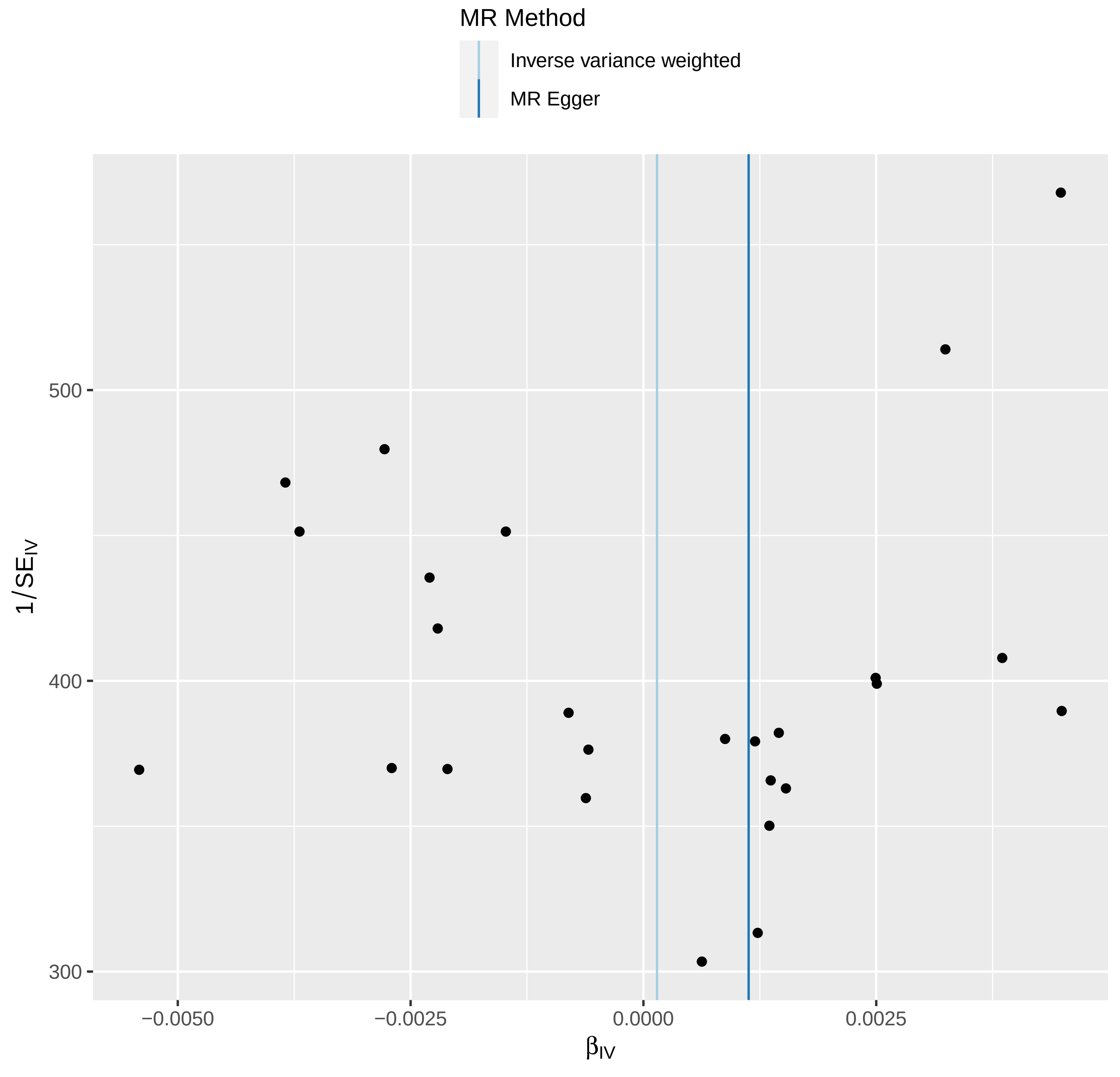

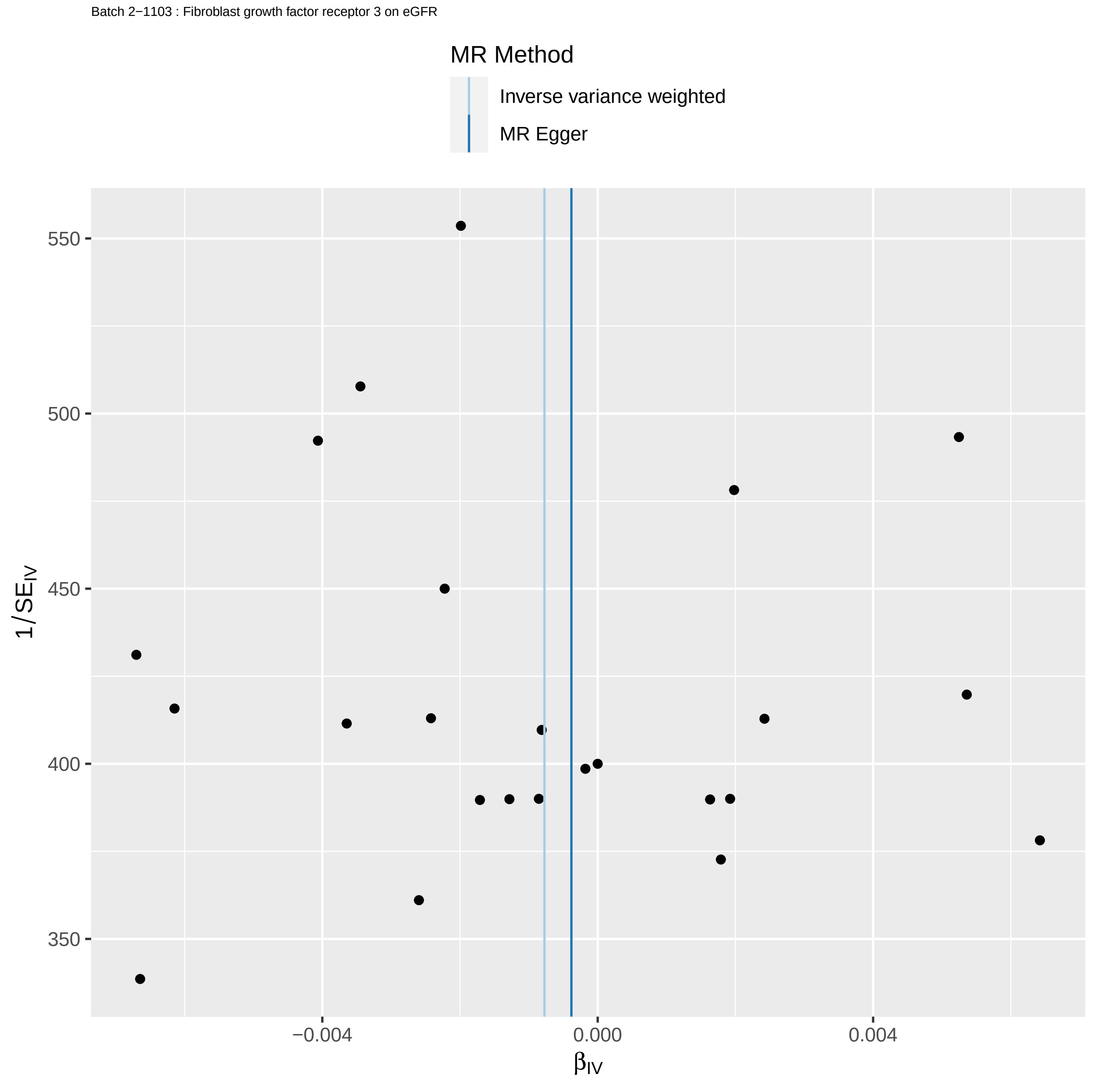
Batch 2−1103 : Fibroblast growth factor receptor 3 on eGFR
MR Method
Inverse variance weighted
MR Egger
550
500
450
400
350
1/SE_IV
−0.004
0.000
0.004
β_IV

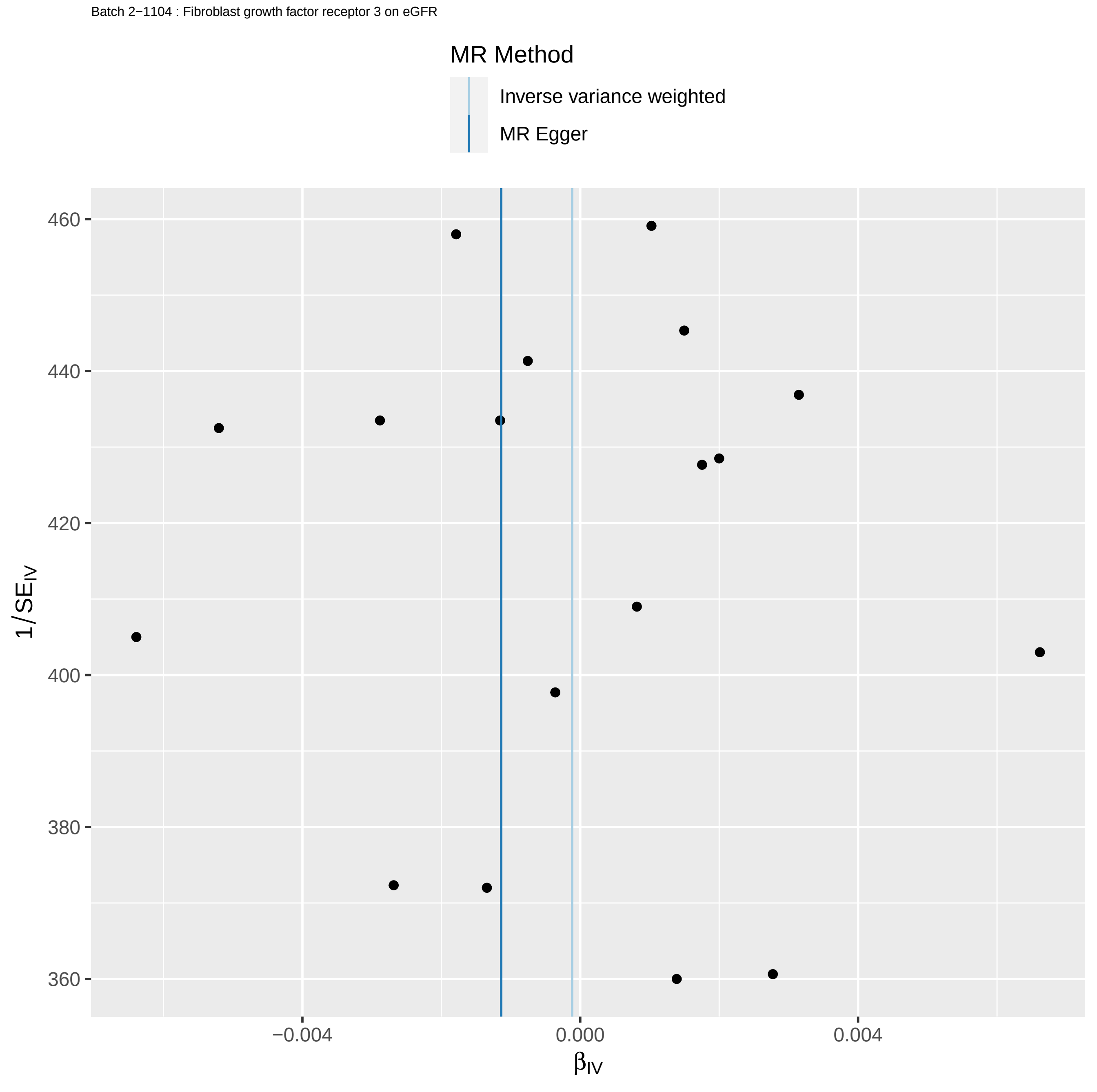
Batch 2−1104 : Fibroblast growth factor receptor 3 on eGFR
MR Method
Inverse variance weighted
MR Egger
460
440
420
400
380
360
1/SE$_{IV}$
−0.004
0.000
0.004
β_{IV}

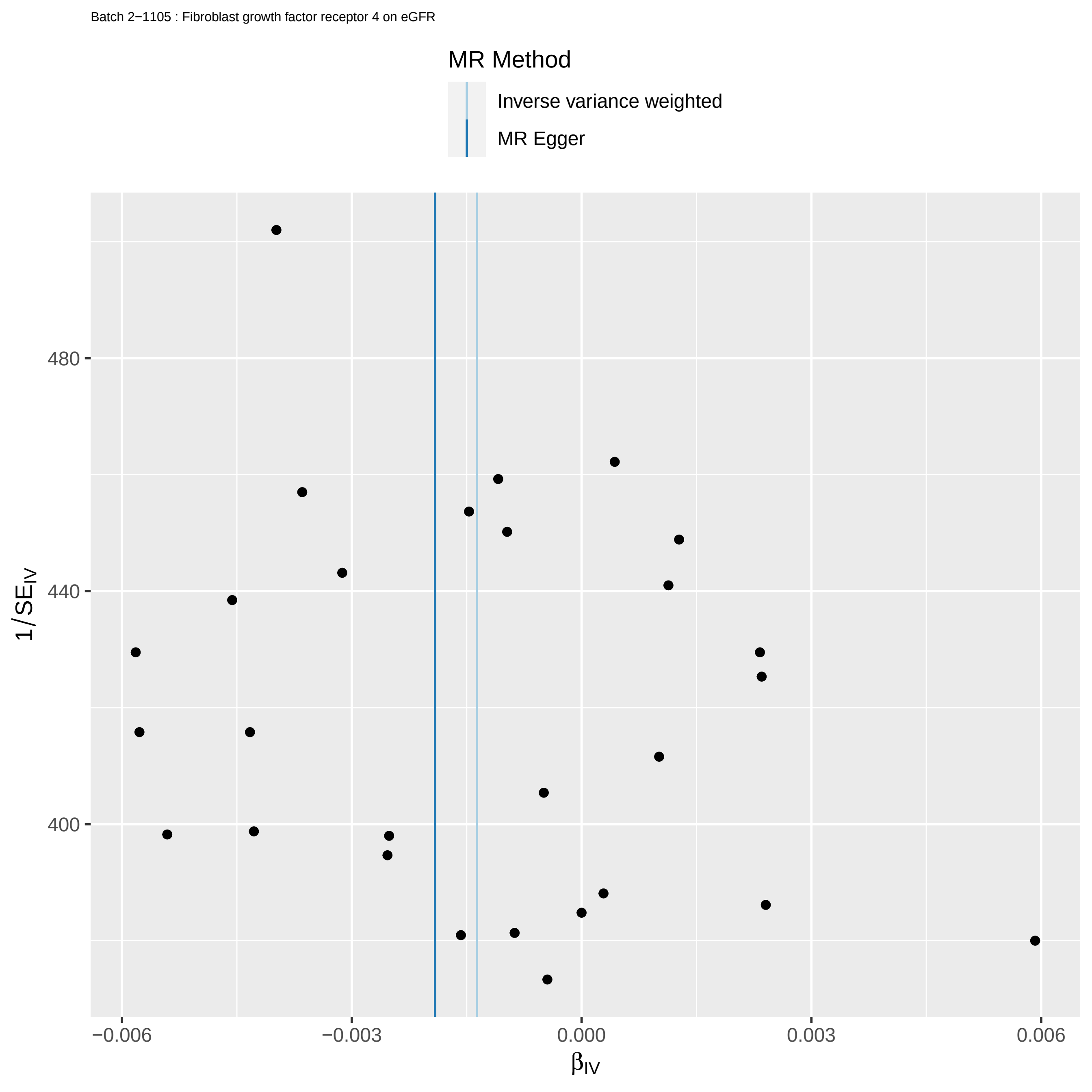
Batch 2−1105 : Fibroblast growth factor receptor 4 on eGFR
MR Method
Inverse variance weighted
MR Egger
480
440
400
1/SE_IV
−0.006
−0.003
0.000
0.003
0.006
β_IV

Batch 2−1106 : Tyrosine−protein kinase Fgr on eGFR

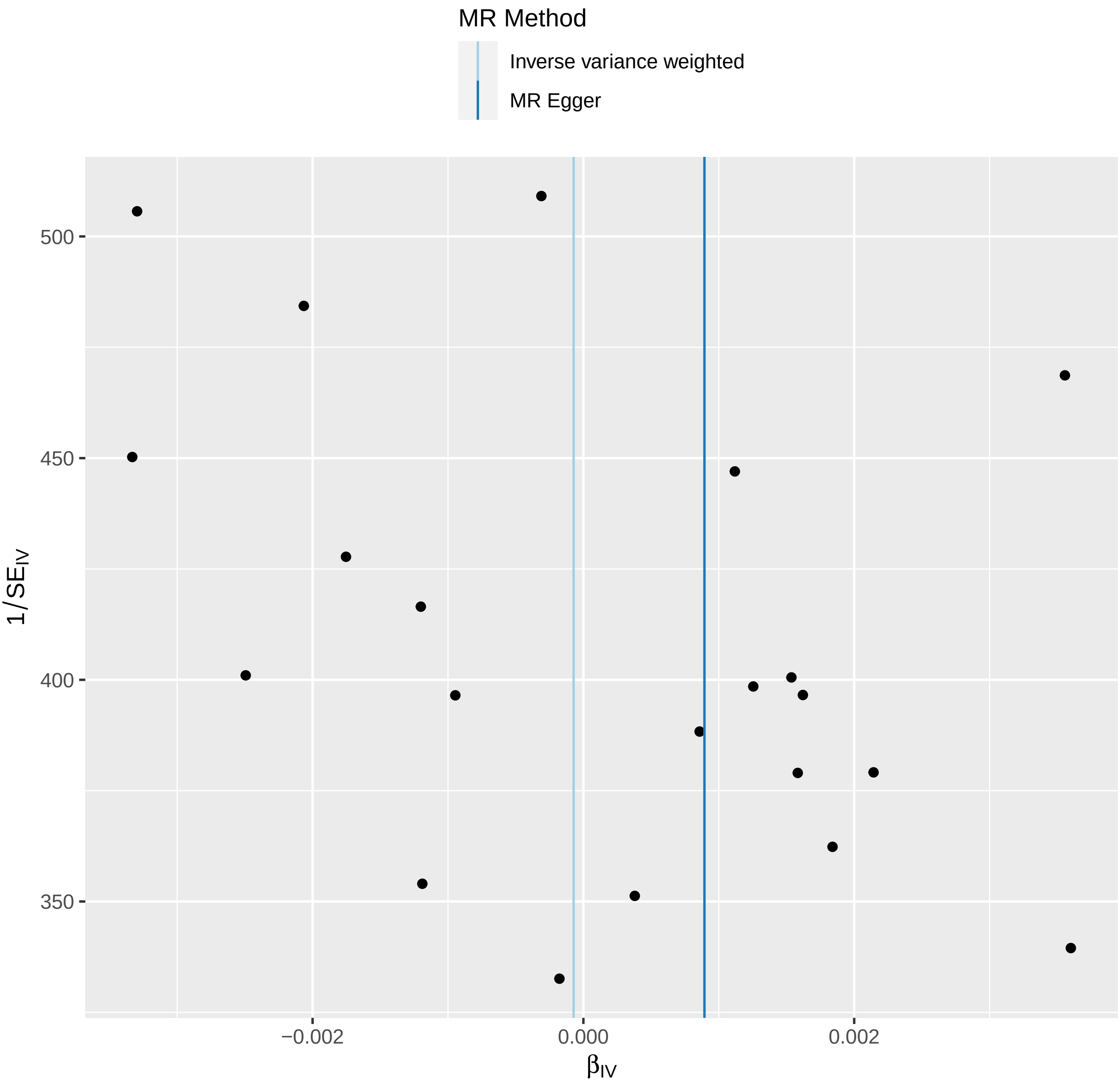

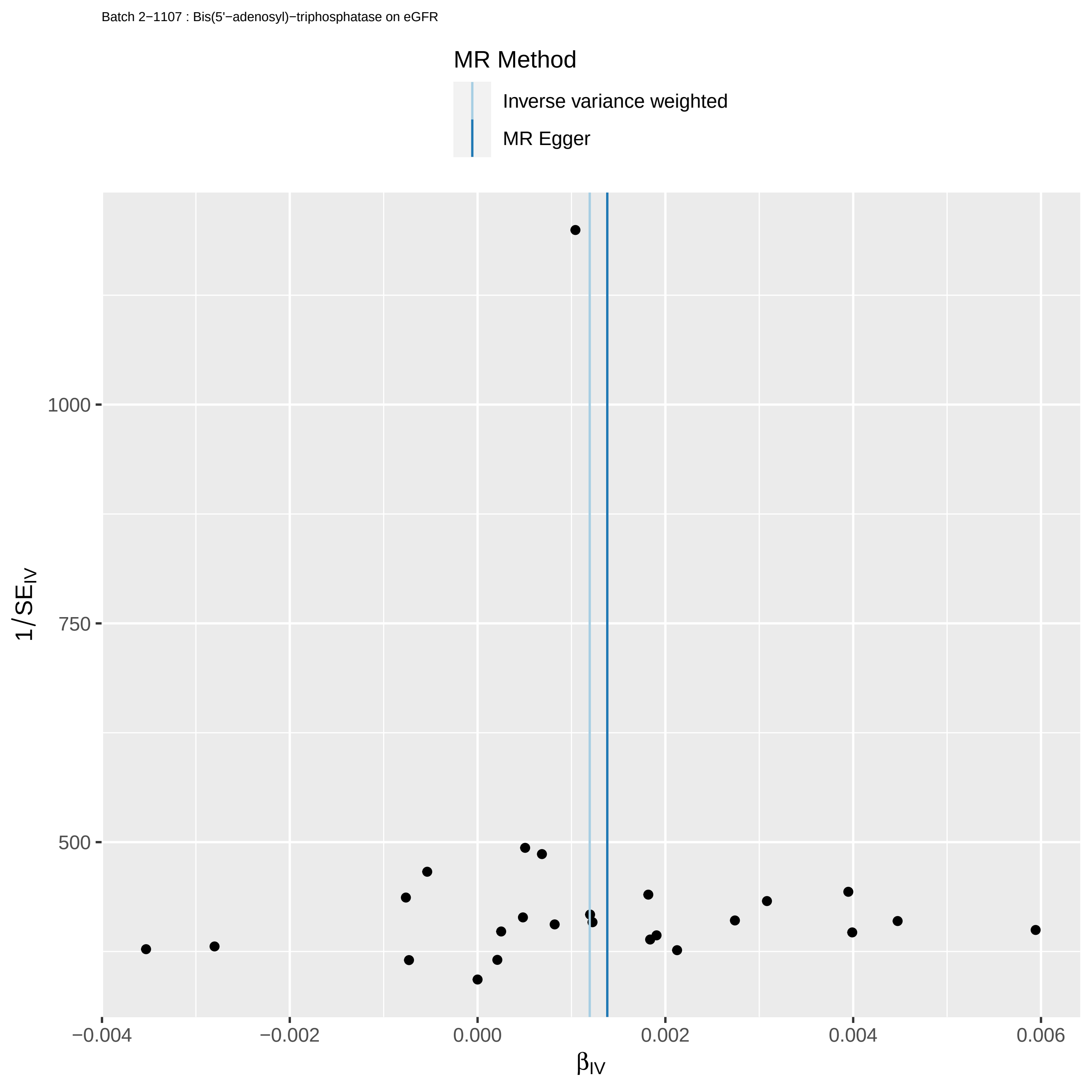
Batch 2−1107 : Bis(5'−adenosyl)−triphosphatase on eGFR
MR Method
Inverse variance weighted
MR Egger
1000
750
500
1/SE_IV
−0.004
−0.002
0.000
0.002
0.004
0.006
β_IV

Batch 2−1108 : Fibrinogen C domain−containing protein 1 on eGFR

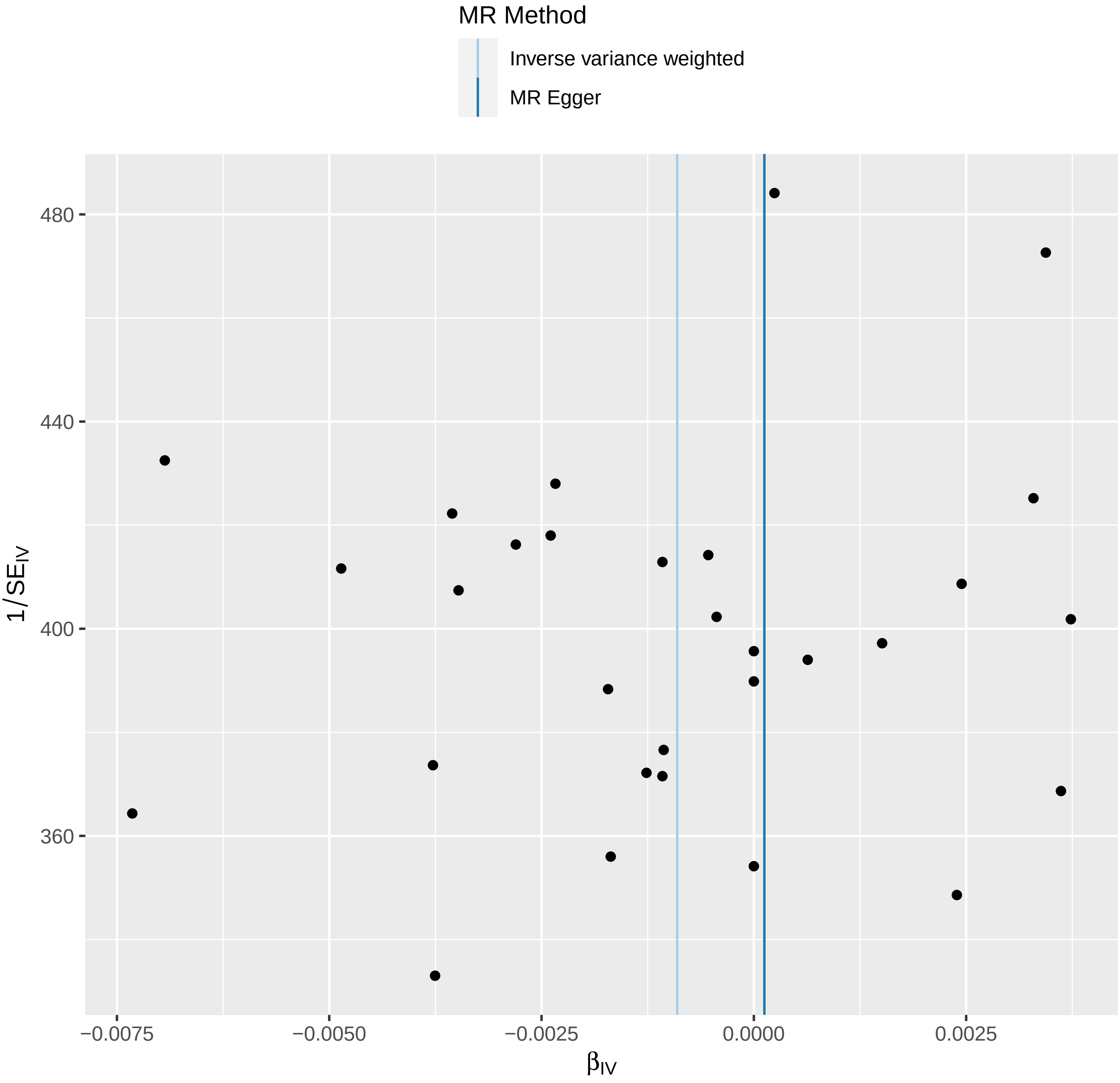

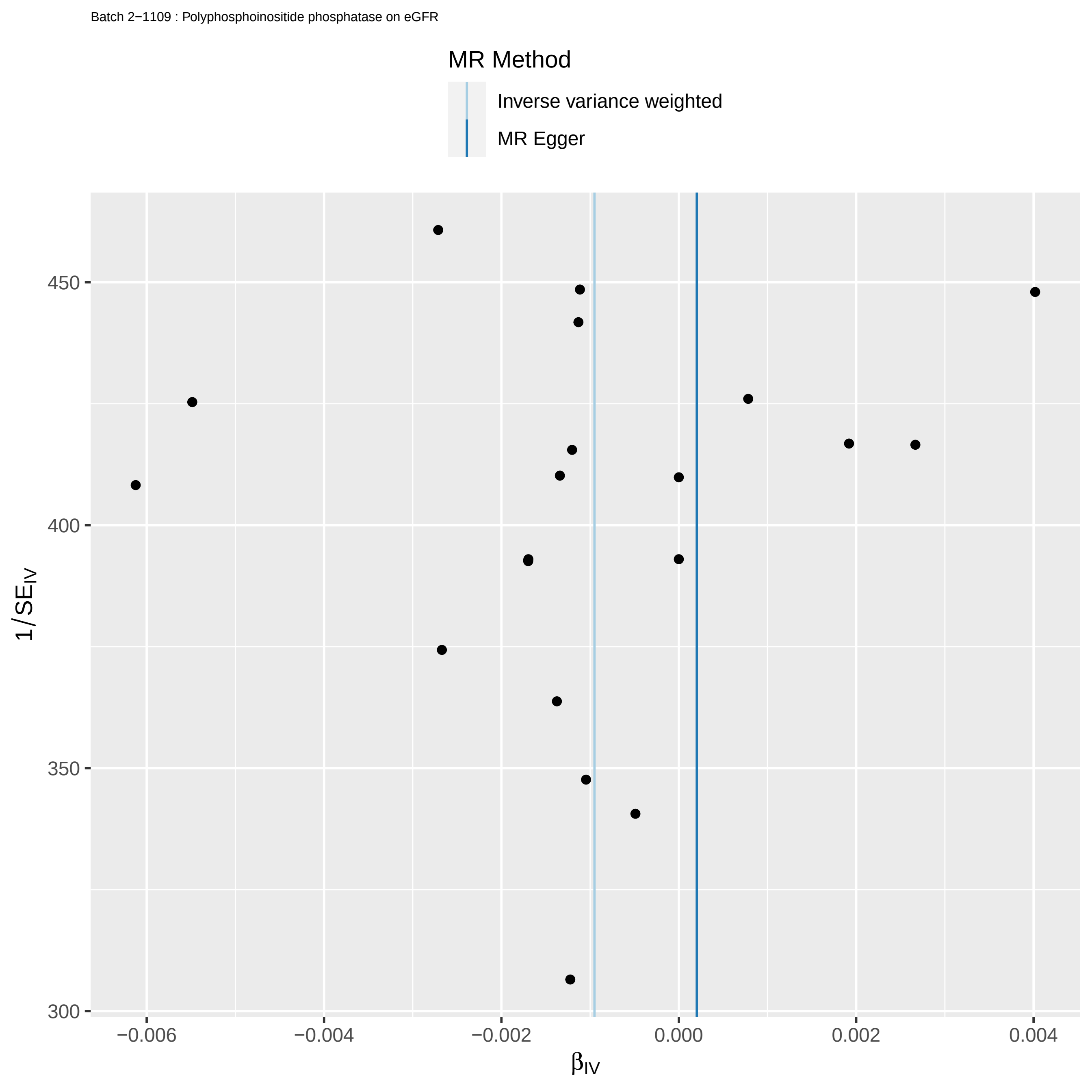
Batch 2−1109 : Polyphosphoinositide phosphatase on eGFR
MR Method
Inverse variance weighted
MR Egger
450
400
350
300
1/SE$_{IV}$
−0.006
−0.004
−0.002
0.000
0.002
0.004
β$_{IV}$

Batch 2−1110 : Vascular endothelial growth factor D on eGFR

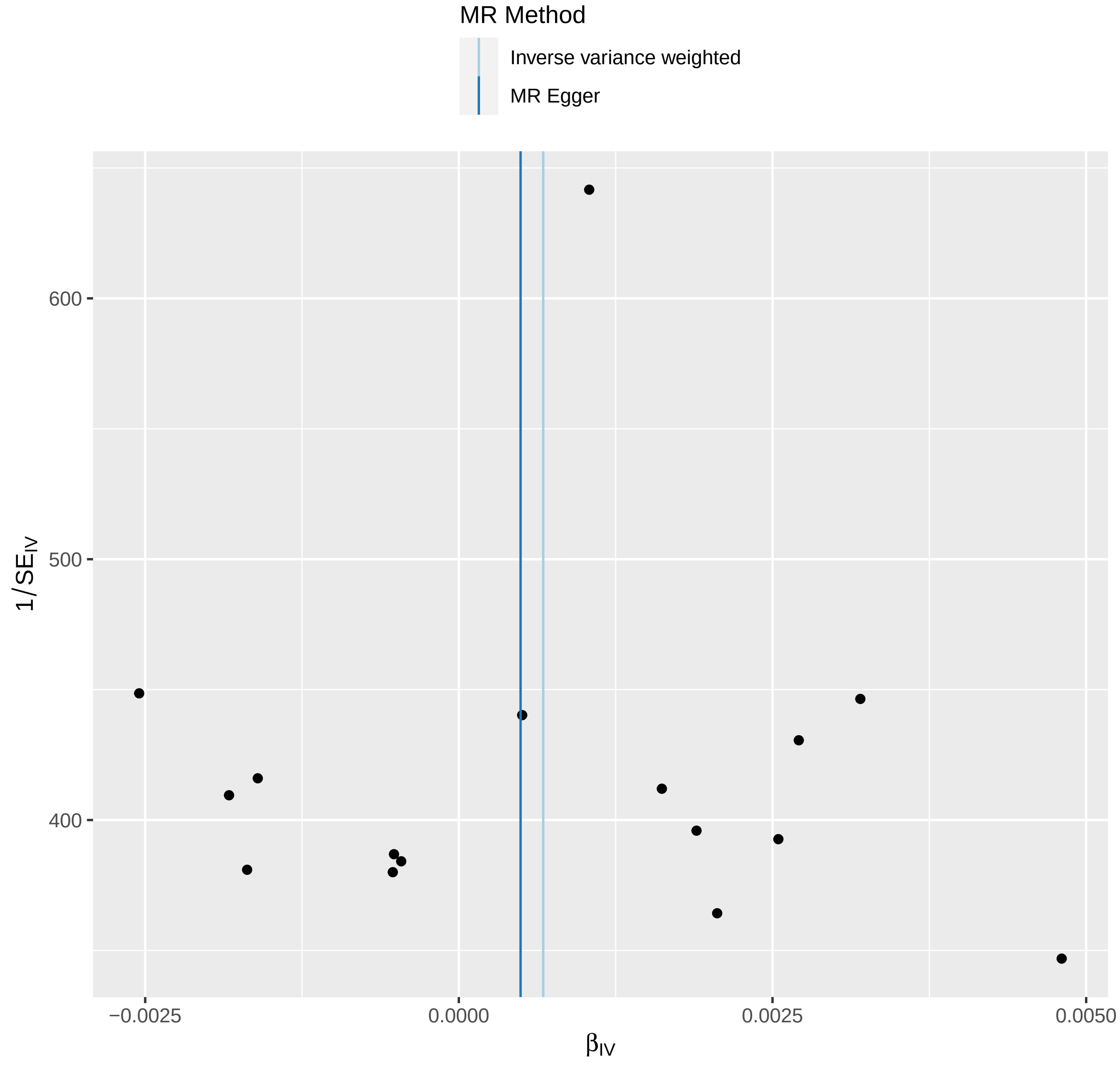

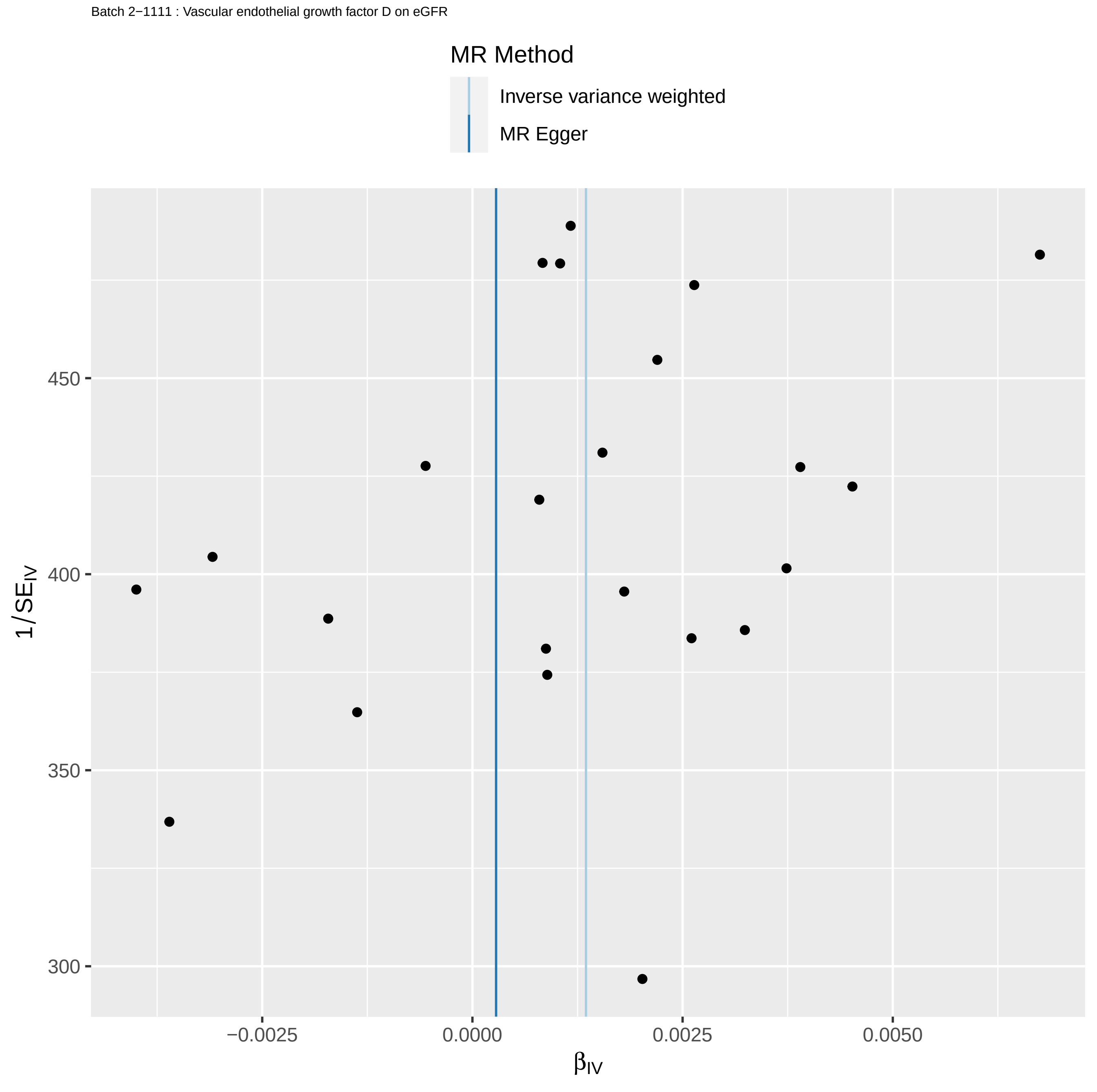
Batch 2−1111 : Vascular endothelial growth factor D on eGFR
MR Method
Inverse variance weighted
MR Egger
450
400
350
300
1/SE_IV
−0.0025
0.0000
0.0025
0.0050
β_IV

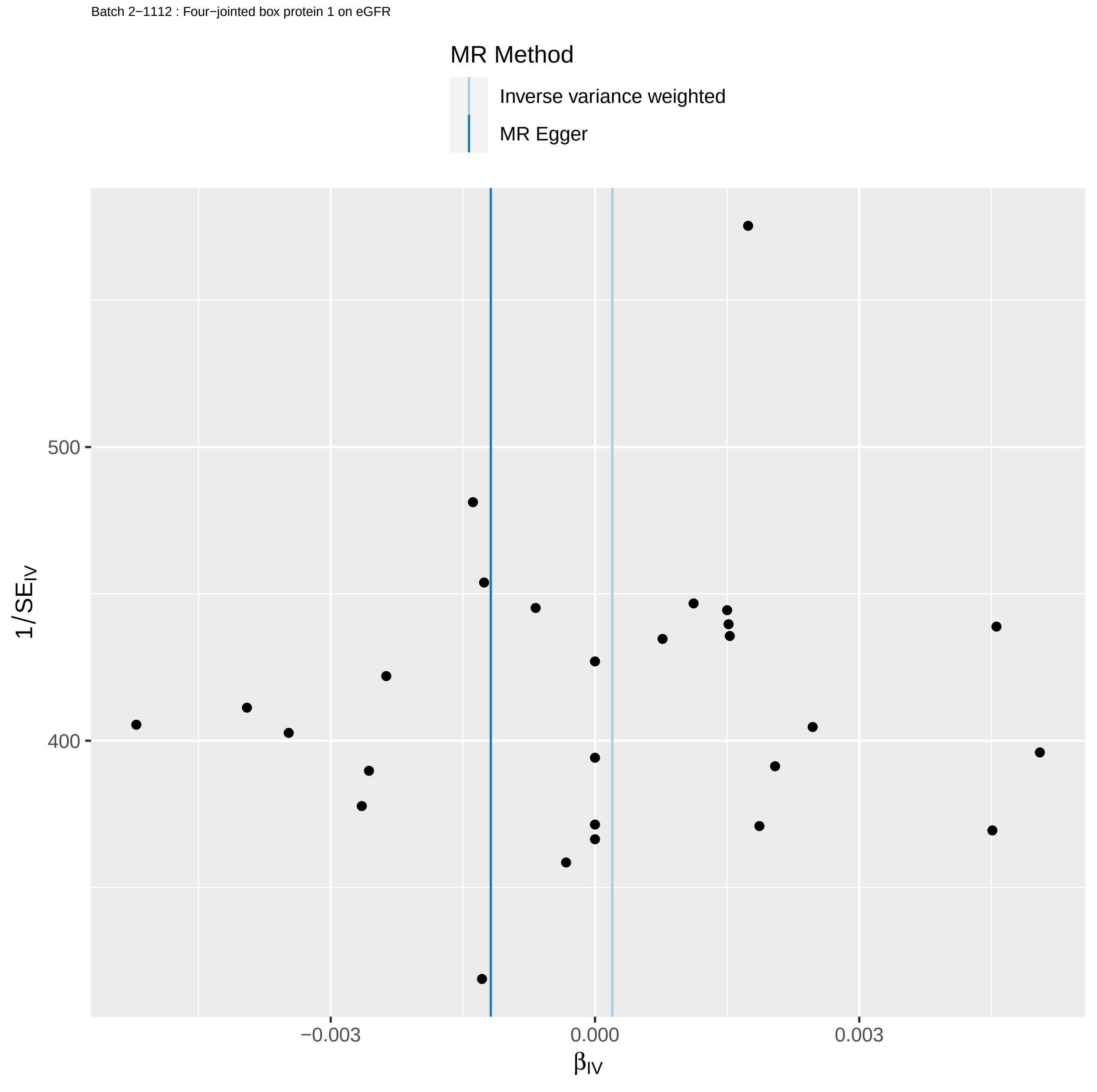

Batch 2−1112 : Four−jointed box protein 1 on eGFR
MR Method
Inverse variance weighted
MR Egger
500
400
1/SE_IV
−0.003
0.000
0.003
β_IV

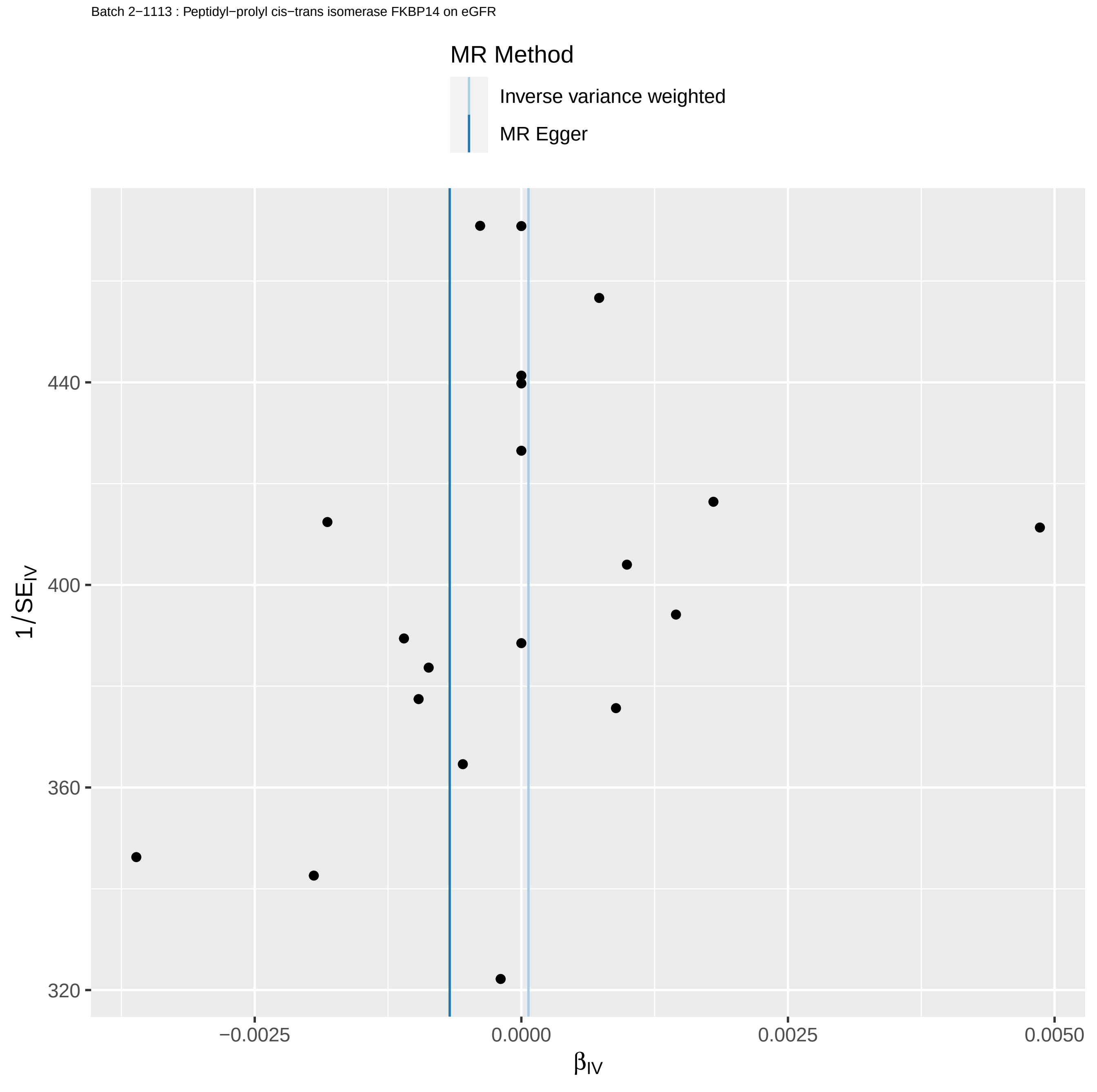

Batch 2−1113 : Peptidyl−prolyl cis−trans isomerase FKBP14 on eGFR
MR Method
Inverse variance weighted
MR Egger
440
400
360
320
1/SE_IV
−0.0025
0.0000
0.0025
0.0050
β_IV

Batch 2−1114 : Peptidyl−prolyl cis−trans isomerase FKBP2 on eGFR

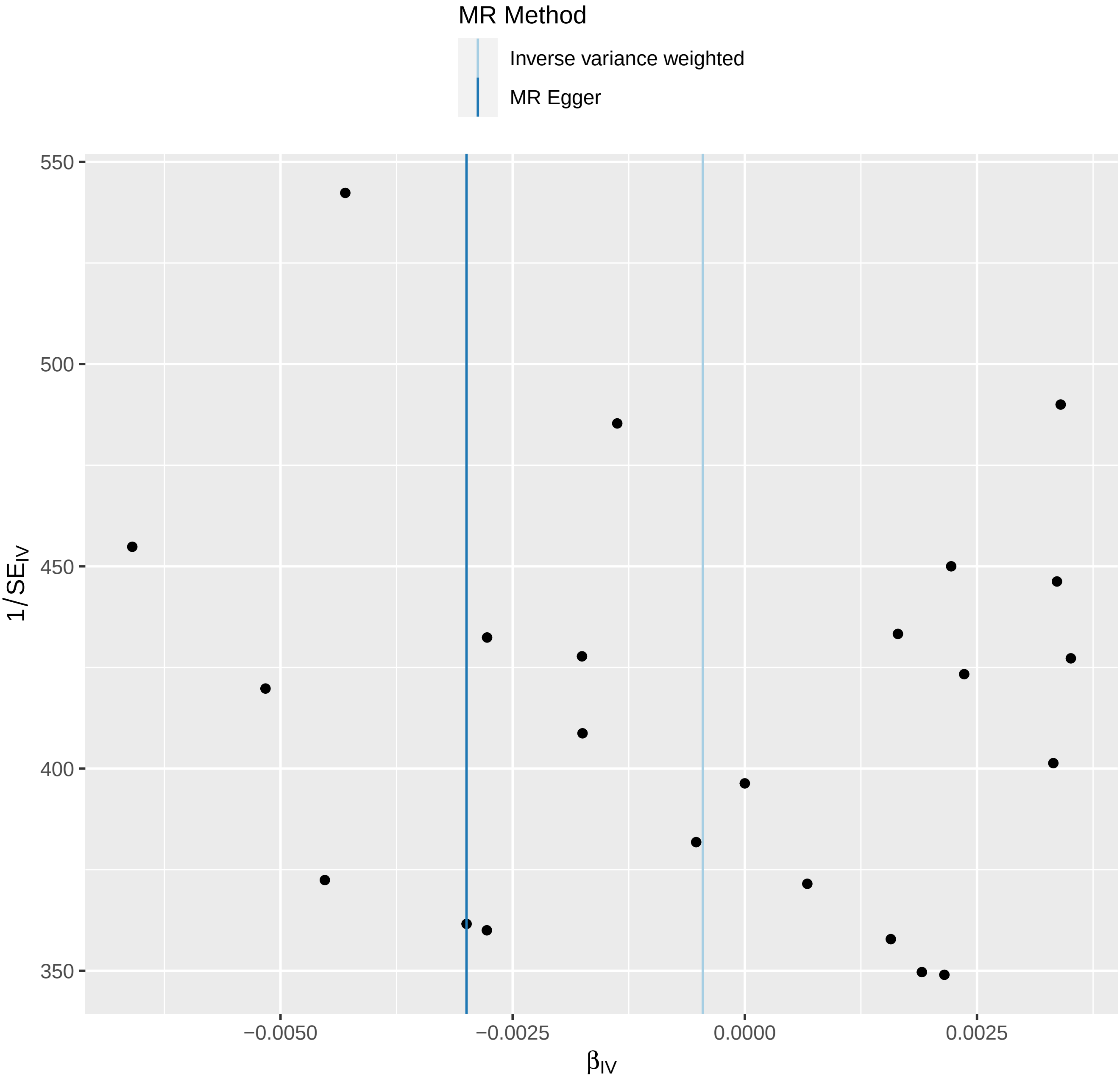

Batch 2−1115 : Inactive peptidyl−prolyl cis−trans isomerase FKBP6 on eGFR

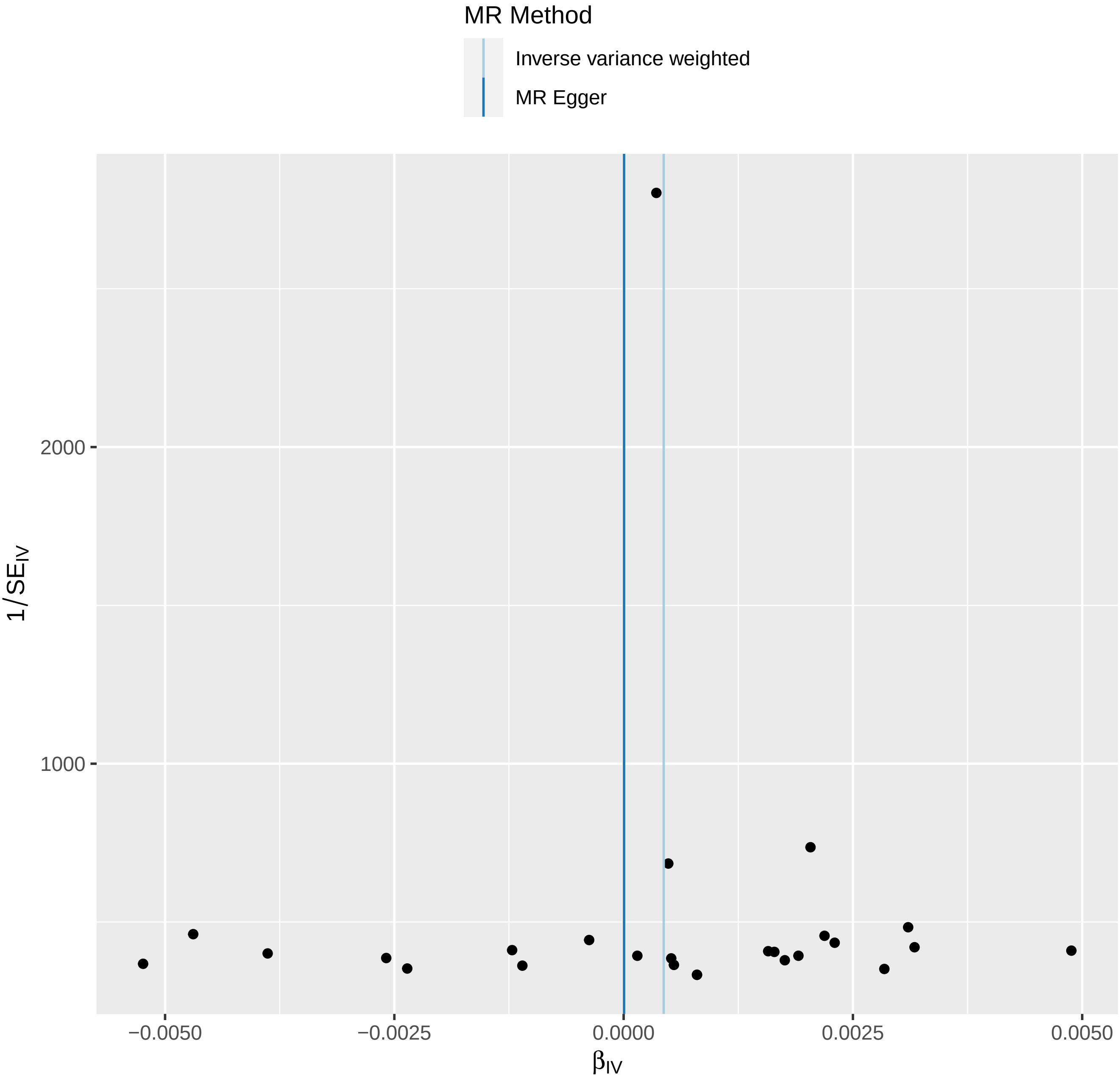

Batch 2−1116 : Peptidyl−prolyl cis−trans isomerase FKBP7 on eGFR

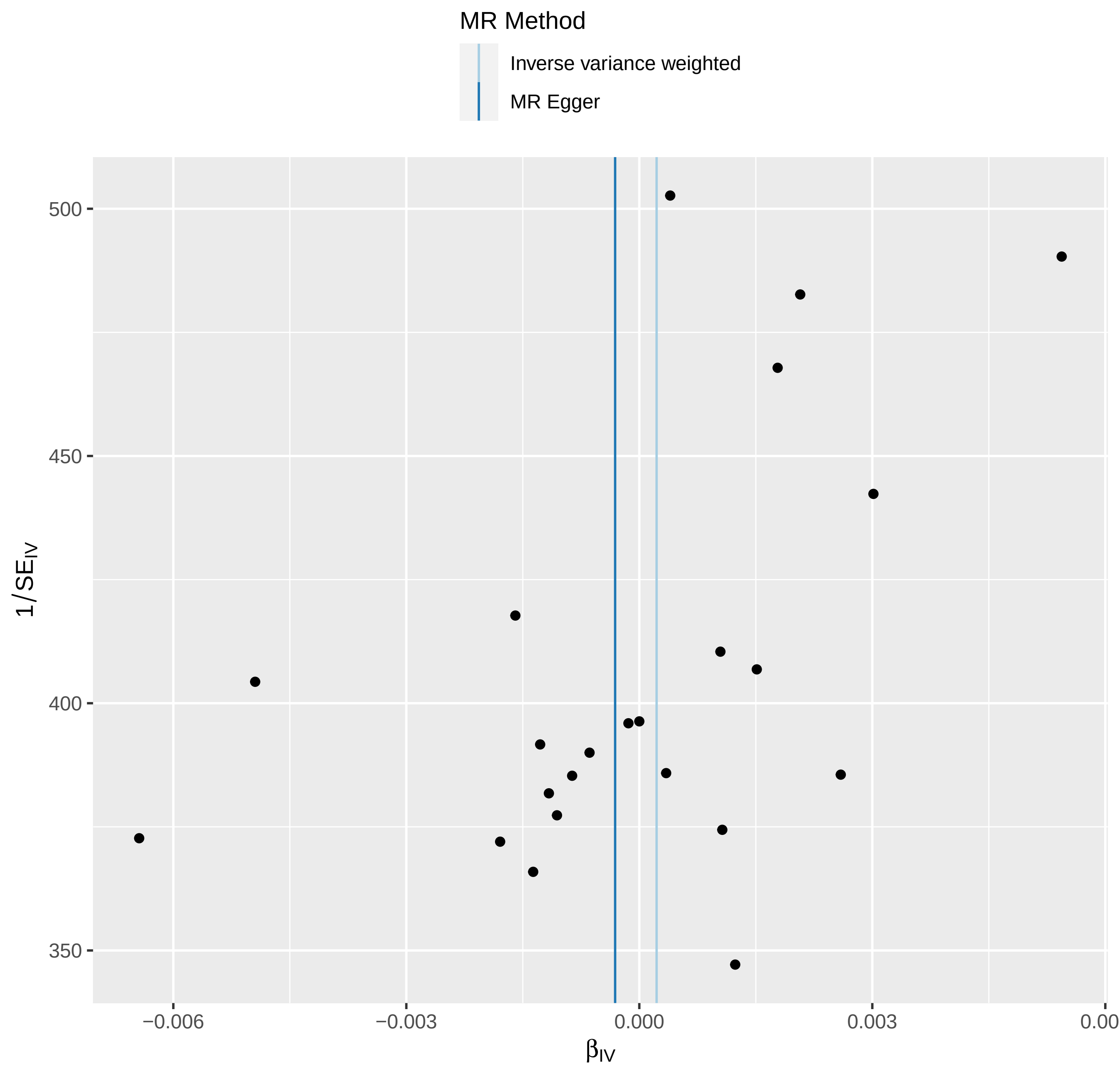

Batch 2−1117 : Filamin−A on eGFR

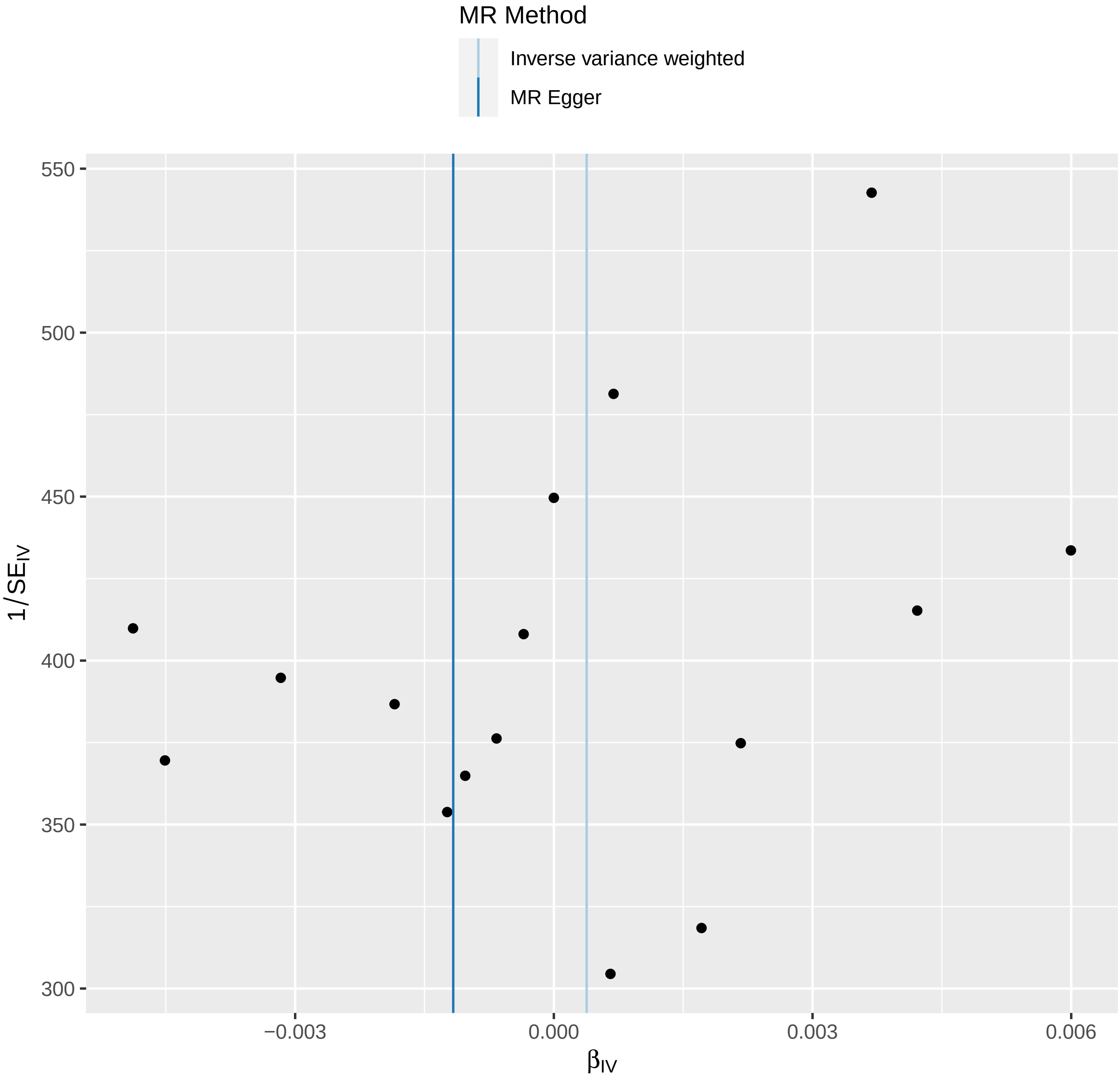

Batch 2−1118 : Filamin−A on eGFR

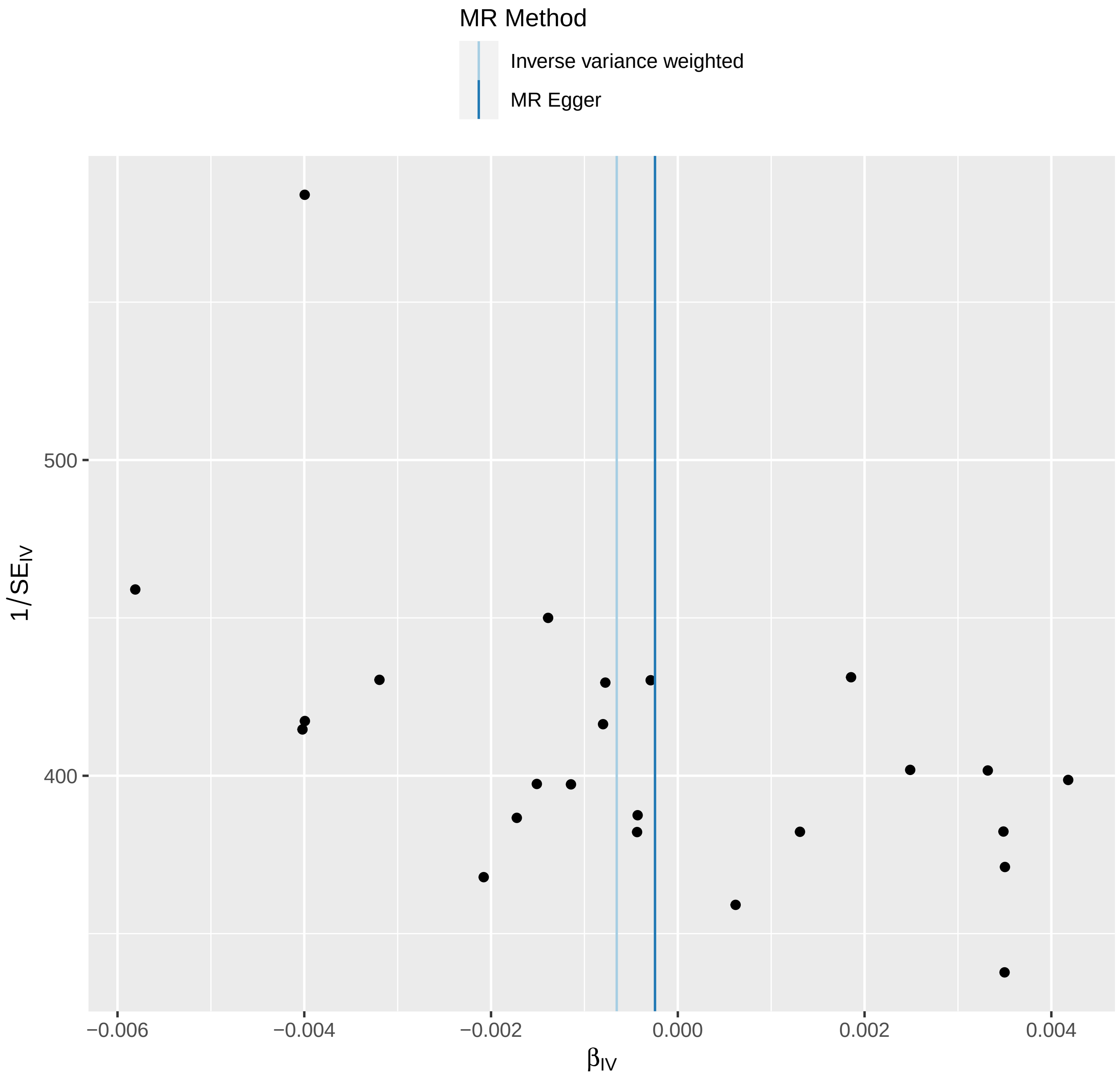

Batch 2−1119 : Filamin−A on eGFR

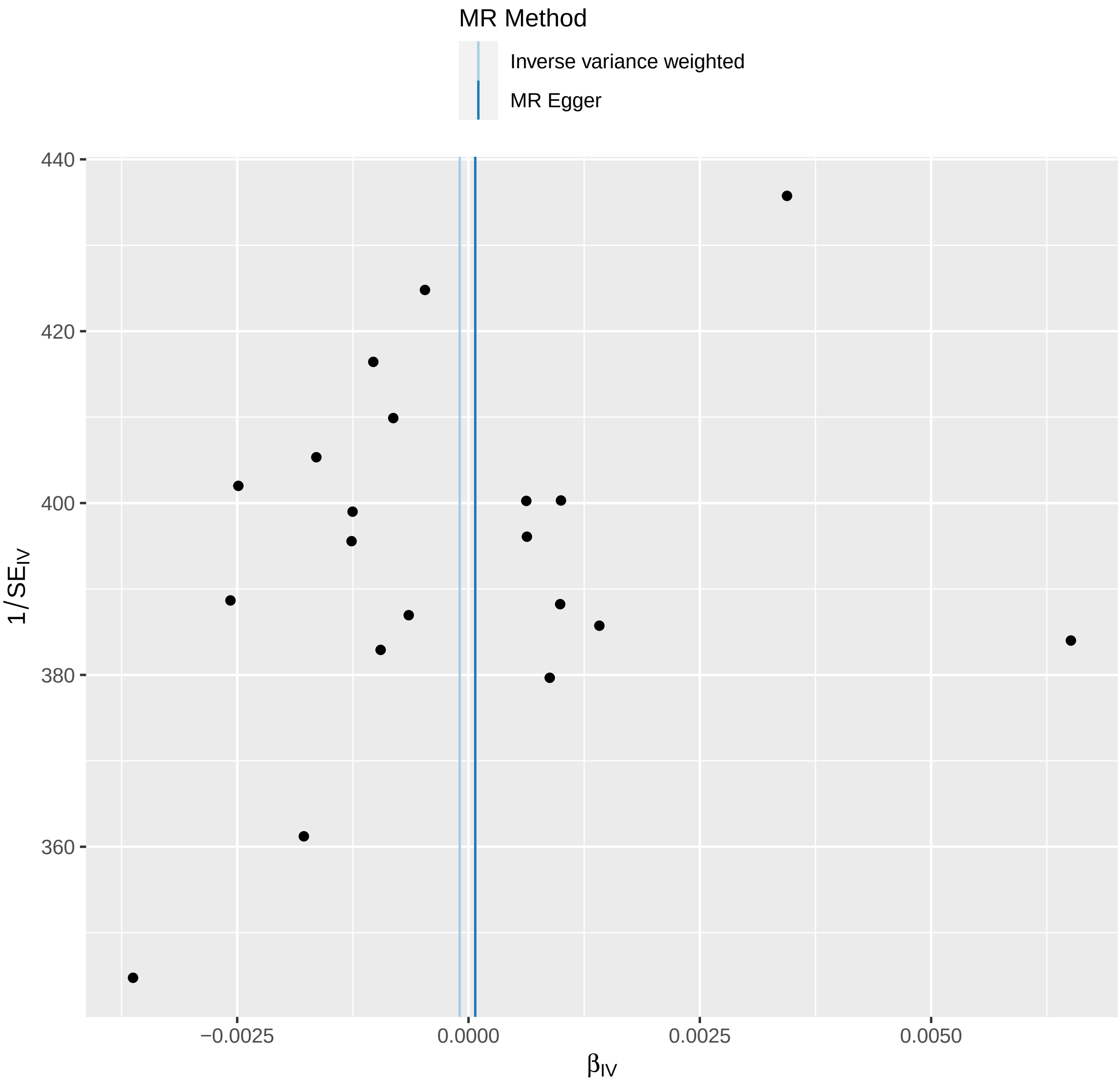

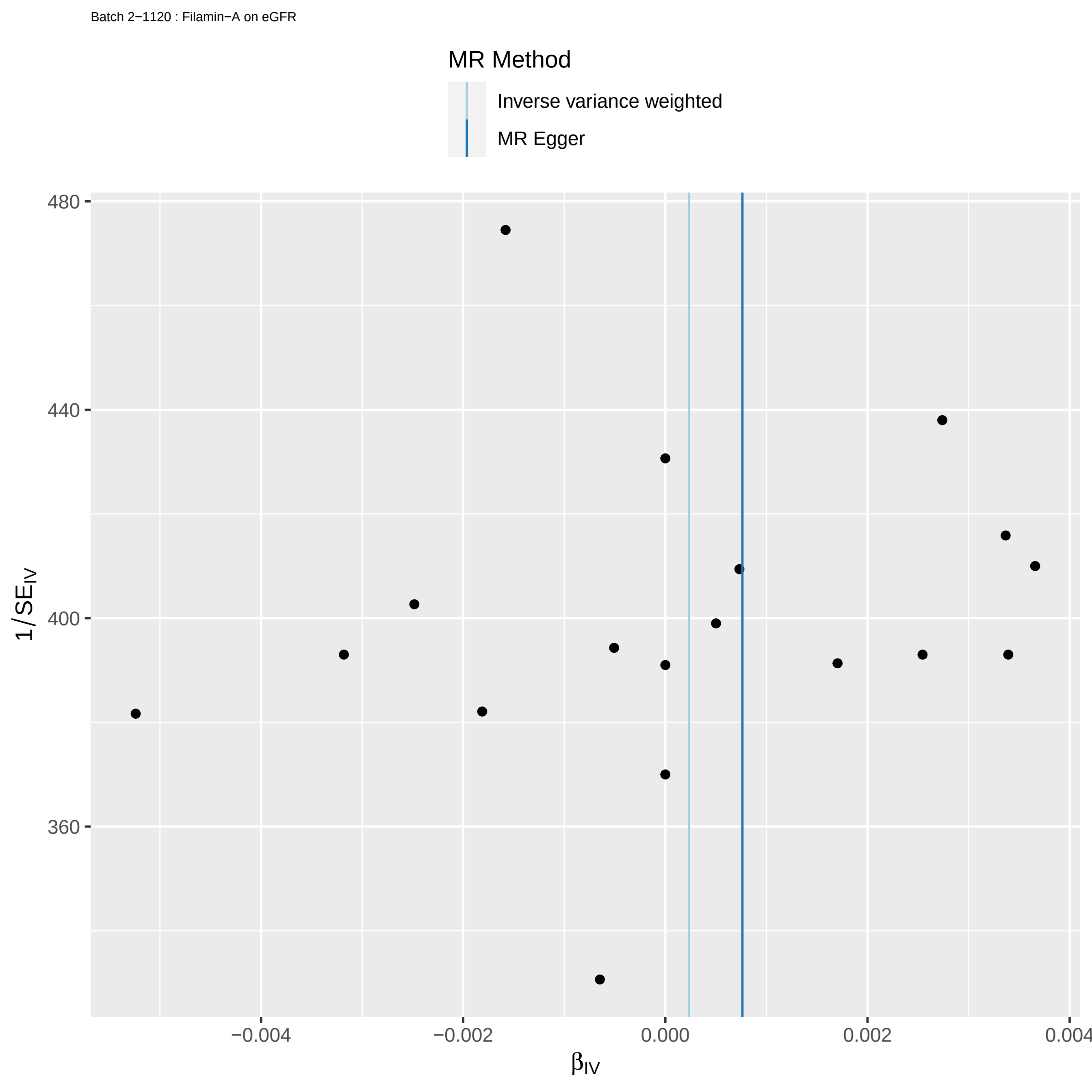

Batch 2−1120 : Filamin−A on eGFR
MR Method
Inverse variance weighted
MR Egger
480
440
400
360
1/SE_IV
−0.004
−0.002
0.000
0.002
0.004
β_IV

Batch 2−1121 : Leucine−rich repeat transmembrane protein FLRT1 on eGFR

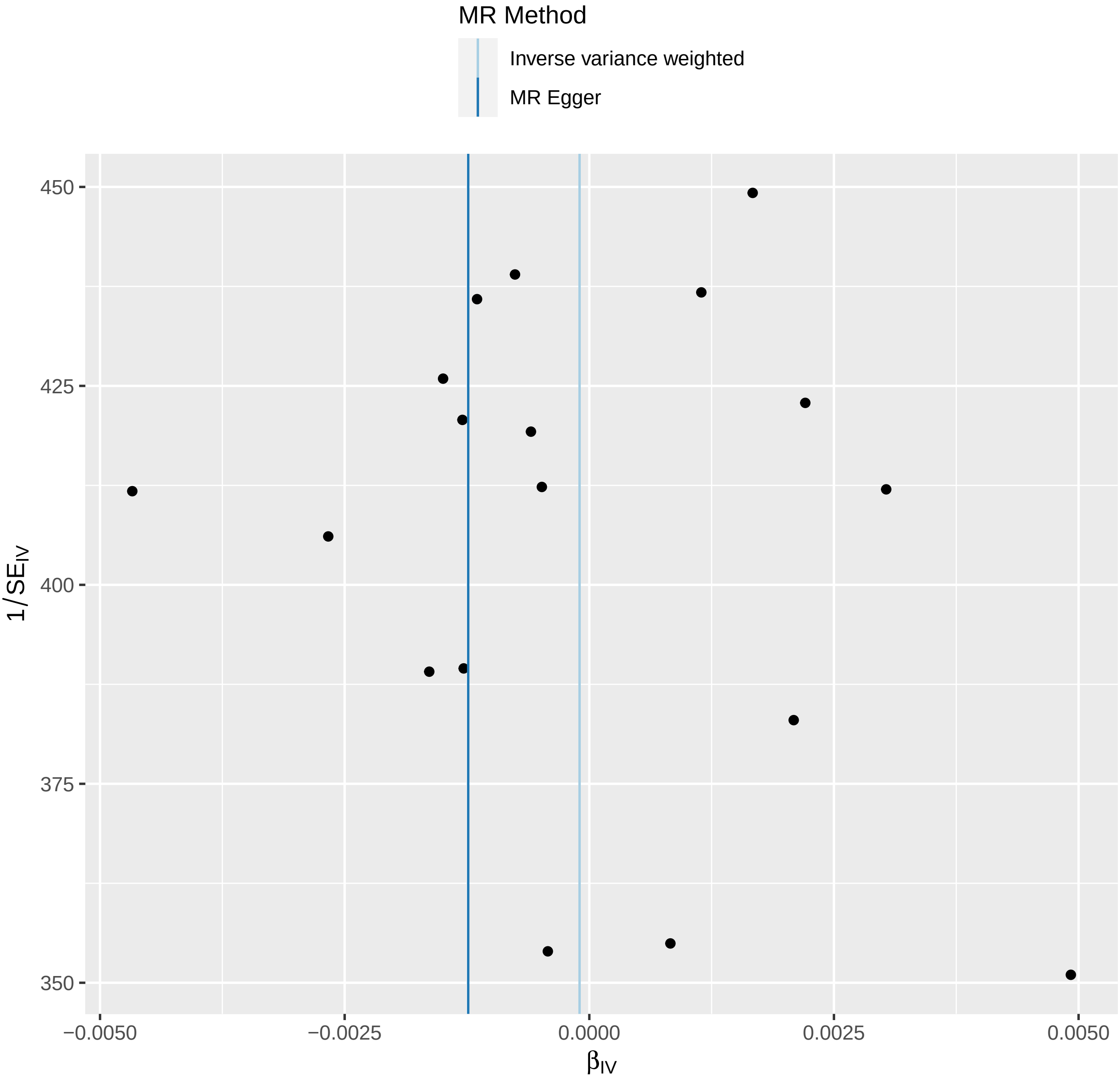

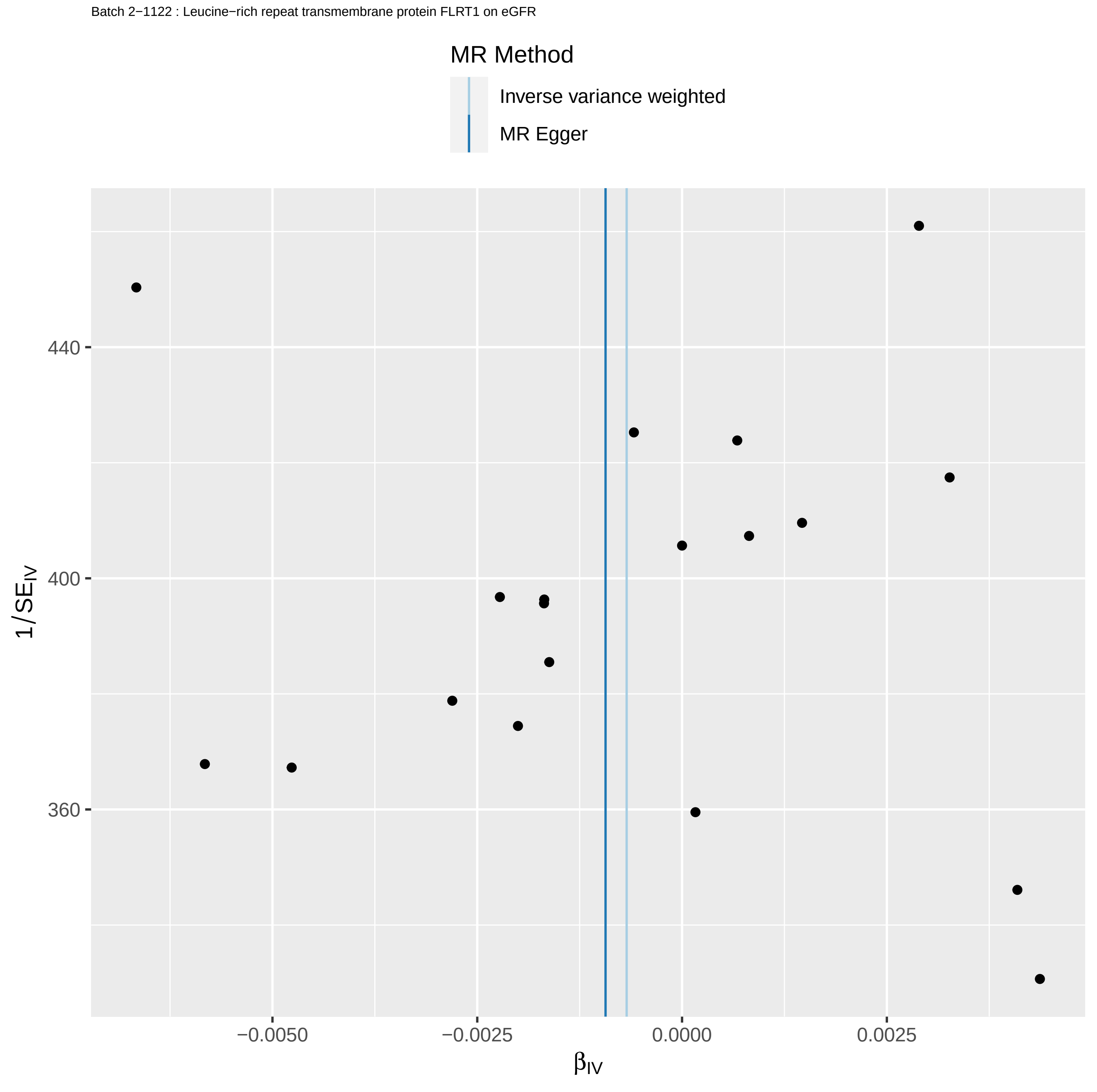
Batch 2−1122 : Leucine−rich repeat transmembrane protein FLRT1 on eGFR
MR Method
Inverse variance weighted
MR Egger
440
400
360
1/SE_IV
−0.0050
−0.0025
0.0000
0.0025
β_IV

Batch 2−1123 : Leucine−rich repeat transmembrane protein FLRT2 on eGFR

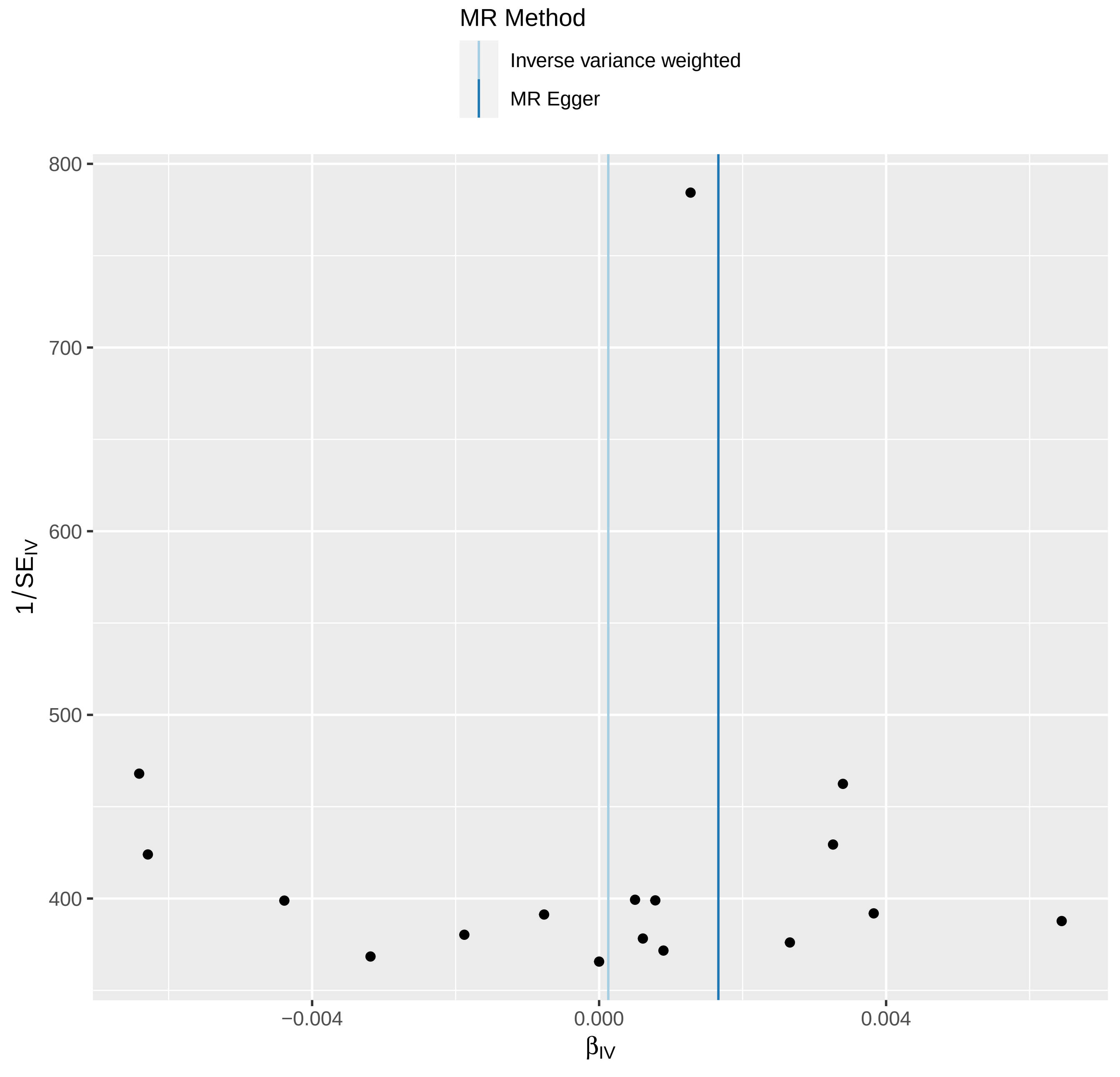

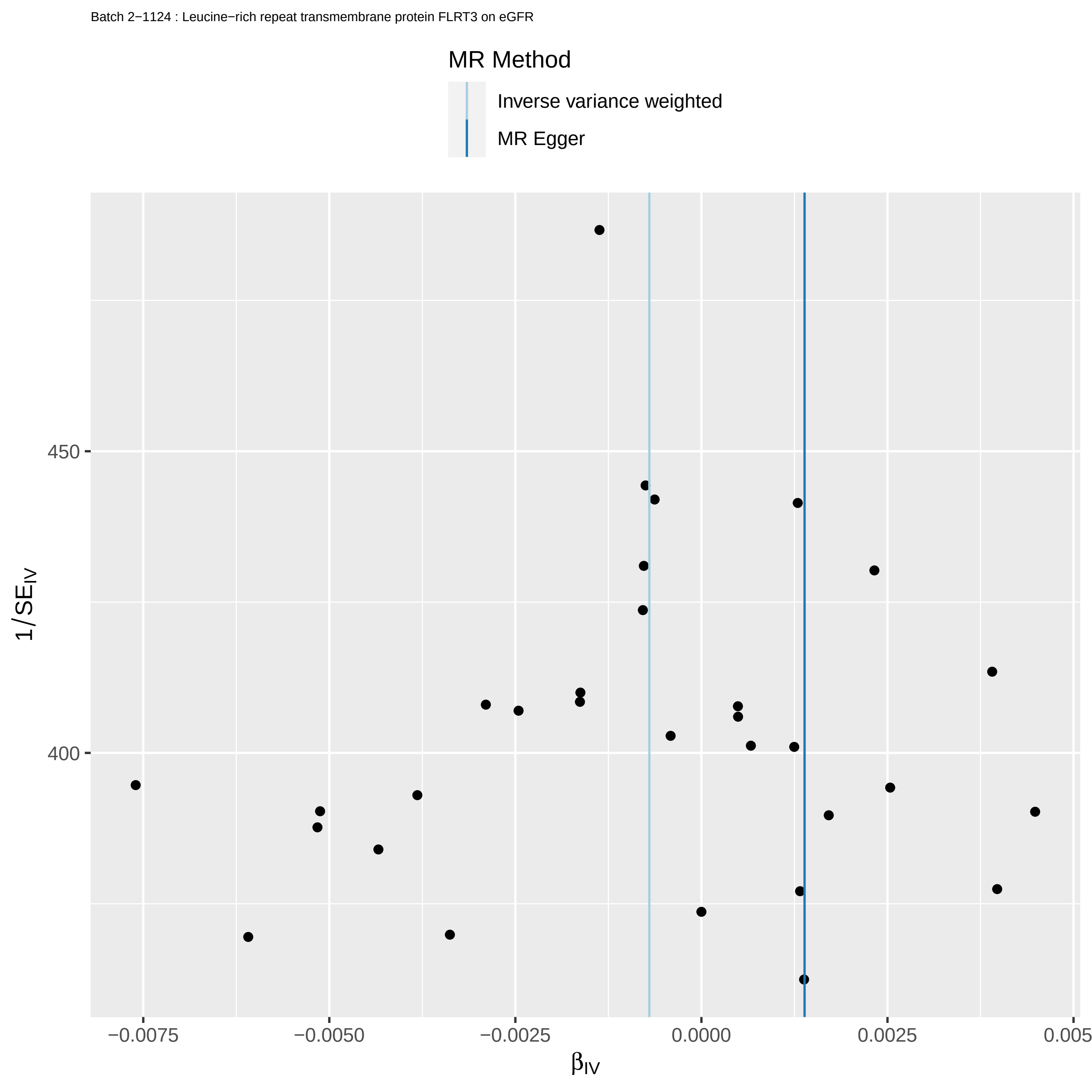

Batch 2−1124 : Leucine−rich repeat transmembrane protein FLRT3 on eGFR
MR Method
Inverse variance weighted
MR Egger
450
400
1/SE$_{IV}$
−0.0075
−0.0050
−0.0025
0.0000
0.0025
0.005
β_{IV}

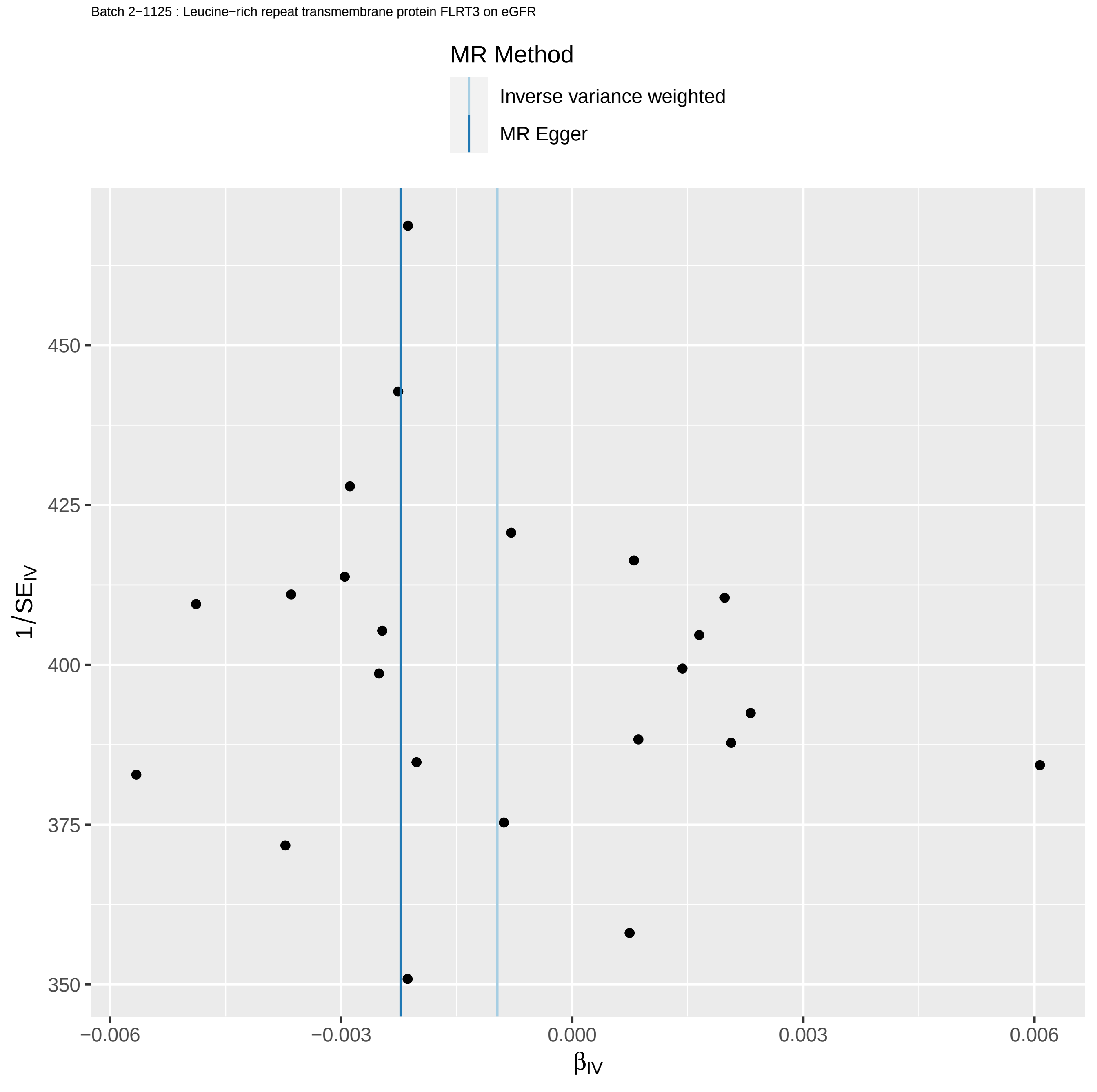
Batch 2−1125 : Leucine−rich repeat transmembrane protein FLRT3 on eGFR
MR Method
Inverse variance weighted
MR Egger
1/SE$_{IV}$
450
425
400
375
350
−0.006
−0.003
0.000
0.003
0.006
β$_{IV}$

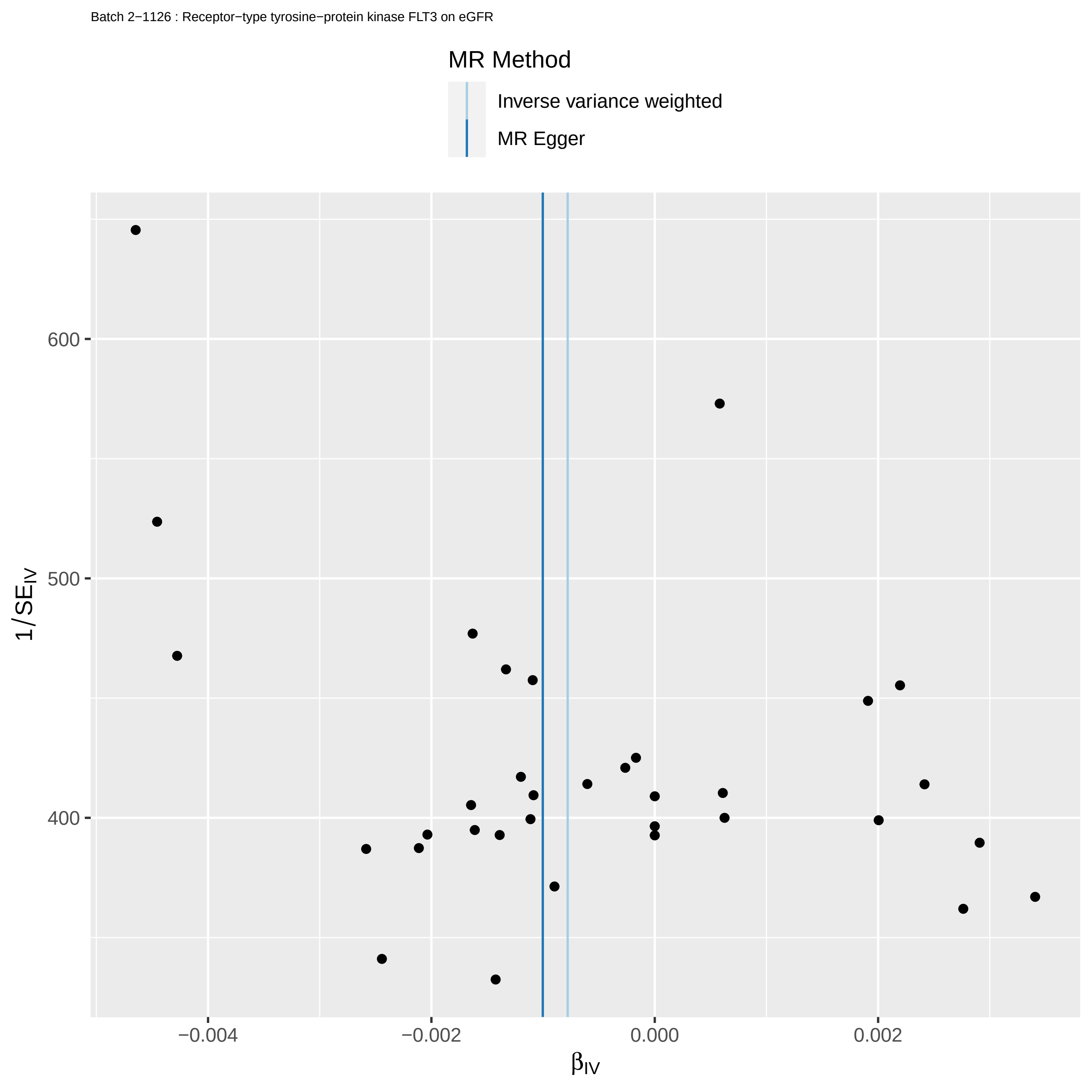

Batch 2−1126 : Receptor−type tyrosine−protein kinase FLT3 on eGFR
MR Method
Inverse variance weighted
MR Egger
600
500
400
1/SE$_{IV}$
−0.004
−0.002
0.000
0.002
β_{IV}

Batch 2−1127 : Fms−related tyrosine kinase 3 ligand on eGFR

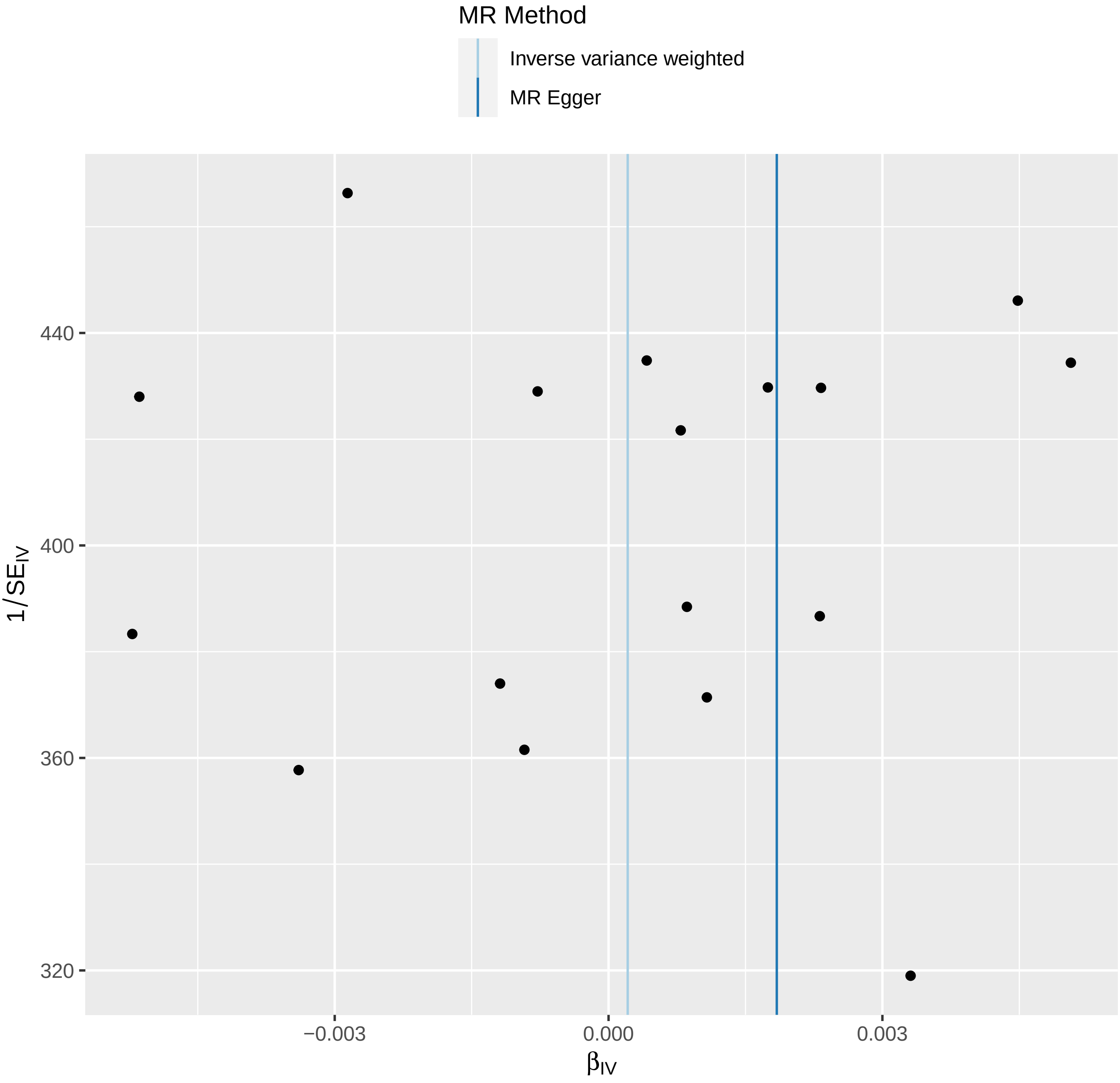

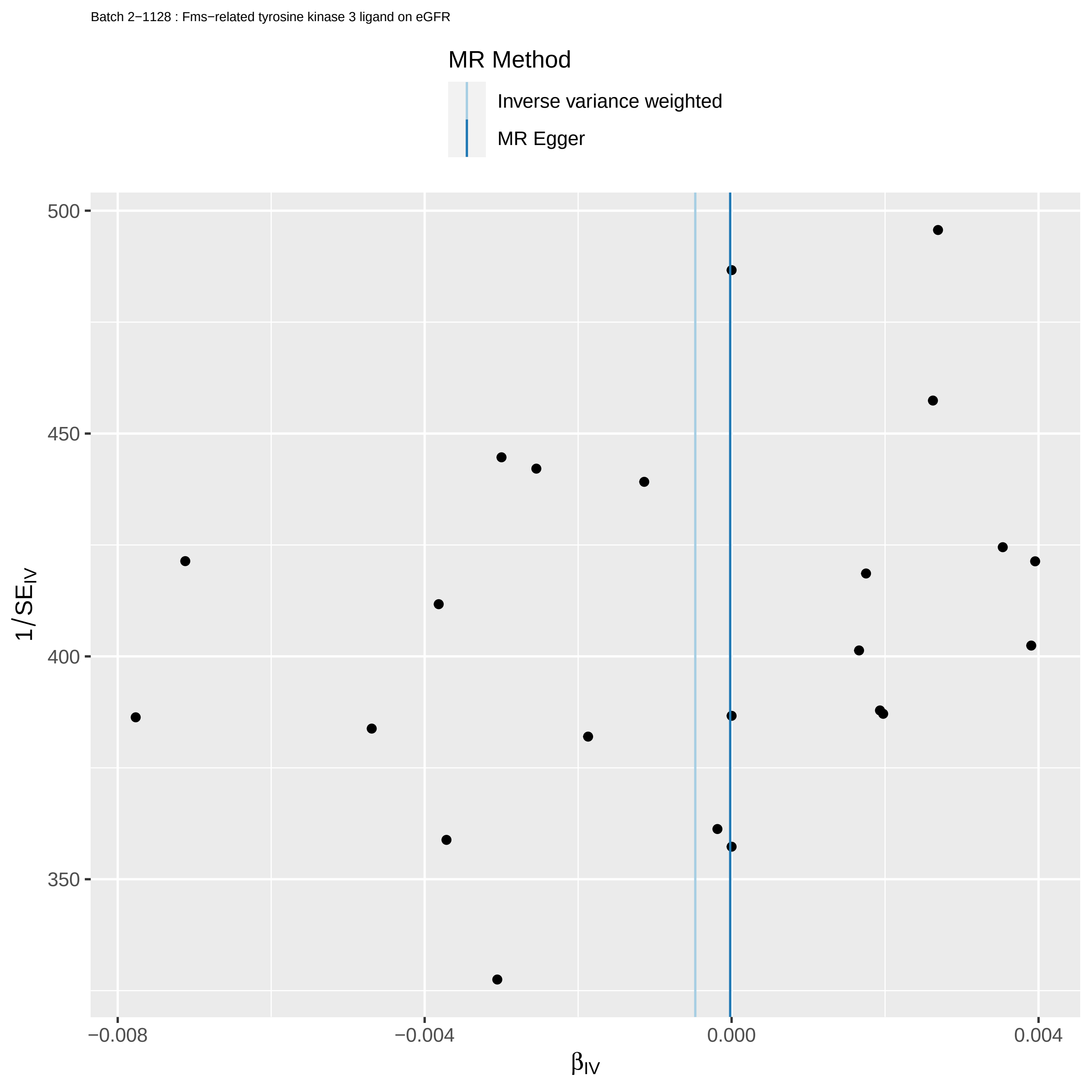

Batch 2−1128 : Fms−related tyrosine kinase 3 ligand on eGFR
MR Method
Inverse variance weighted
MR Egger
500
450
400
350
1/SE$_{IV}$
−0.008
−0.004
0.000
0.004
β$_{IV}$

Batch 2−1129 : Vascular endothelial growth factor receptor 3 on eGFR

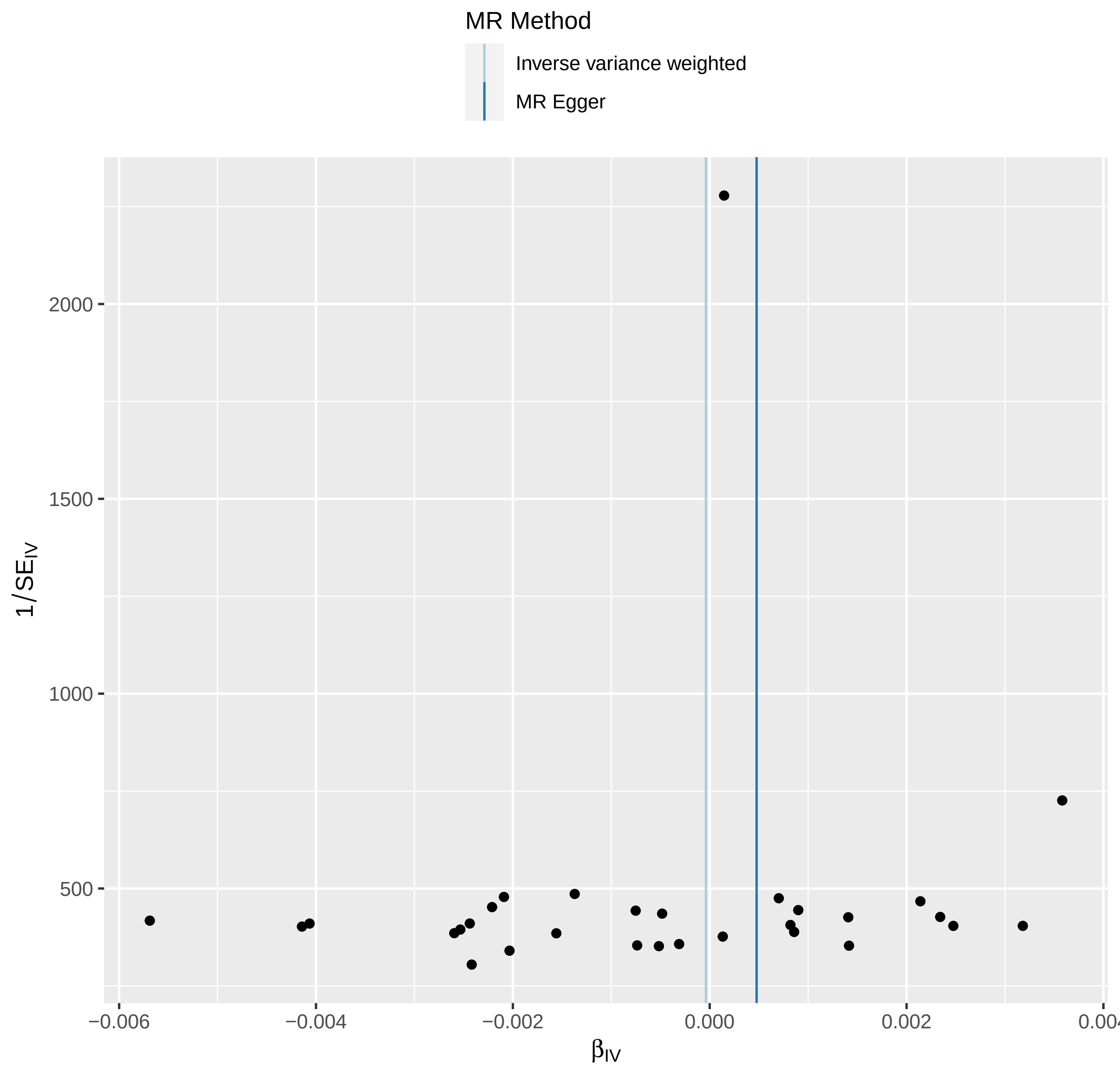

Batch 2−1130 : Feline leukemia virus subgroup C receptor−related protein 1 on eGFR

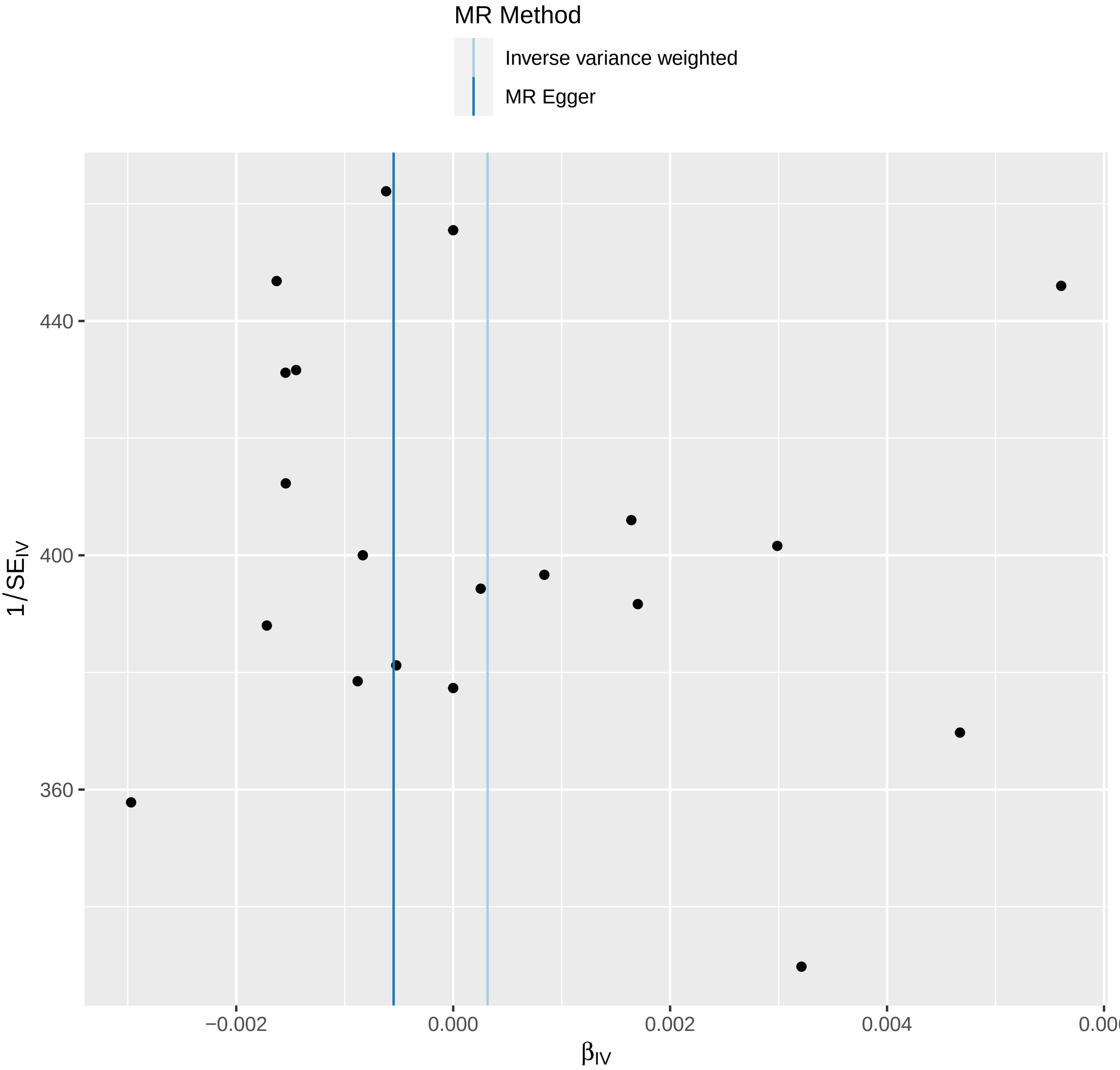

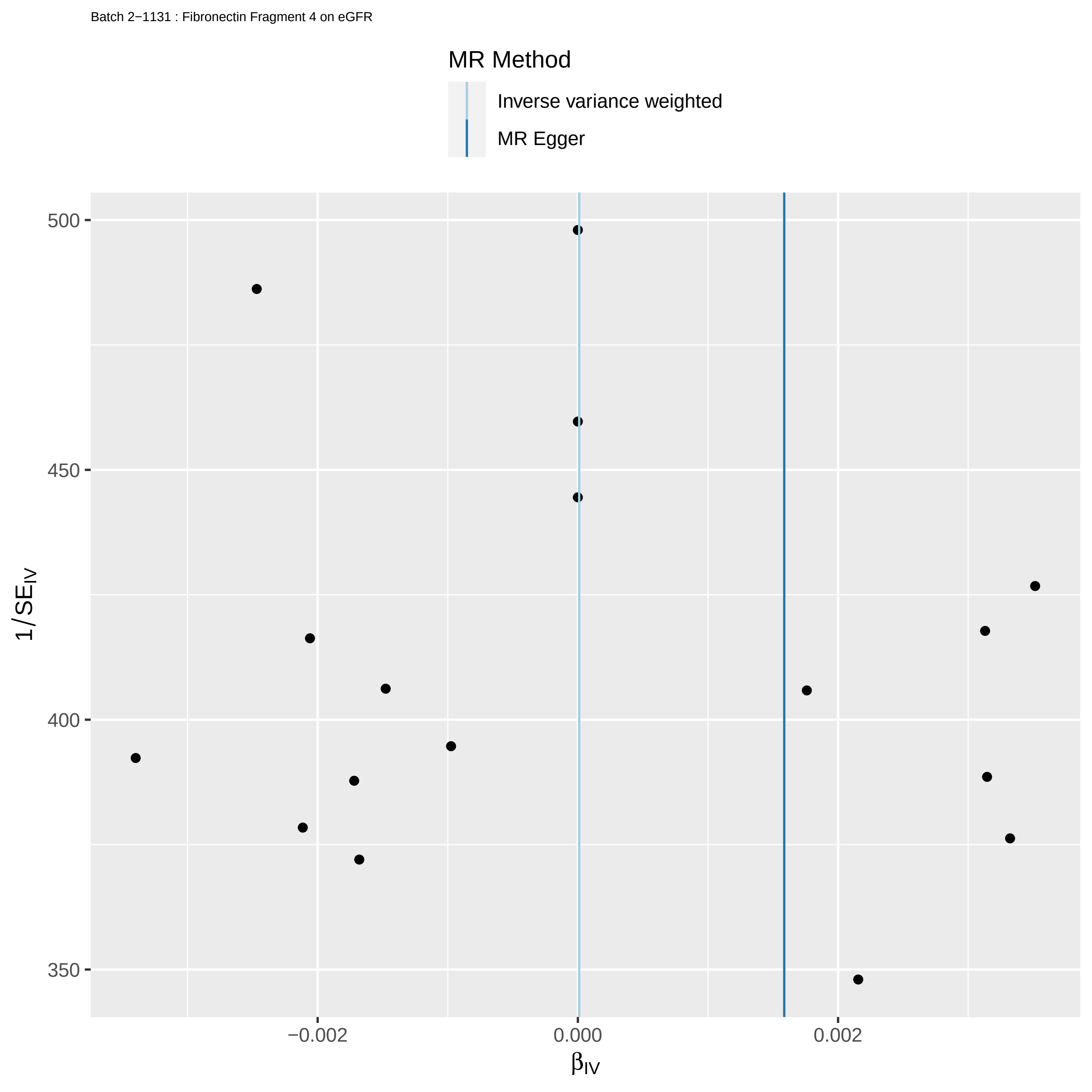

Batch 2−1131 : Fibronectin Fragment 4 on eGFR
MR Method
Inverse variance weighted
MR Egger
500
450
400
350
1/SE$_{IV}$
−0.002
0.000
0.002
β$_{IV}$

Batch 2−1132 : Fibronectin type III domain−containing protein 4 on eGFR

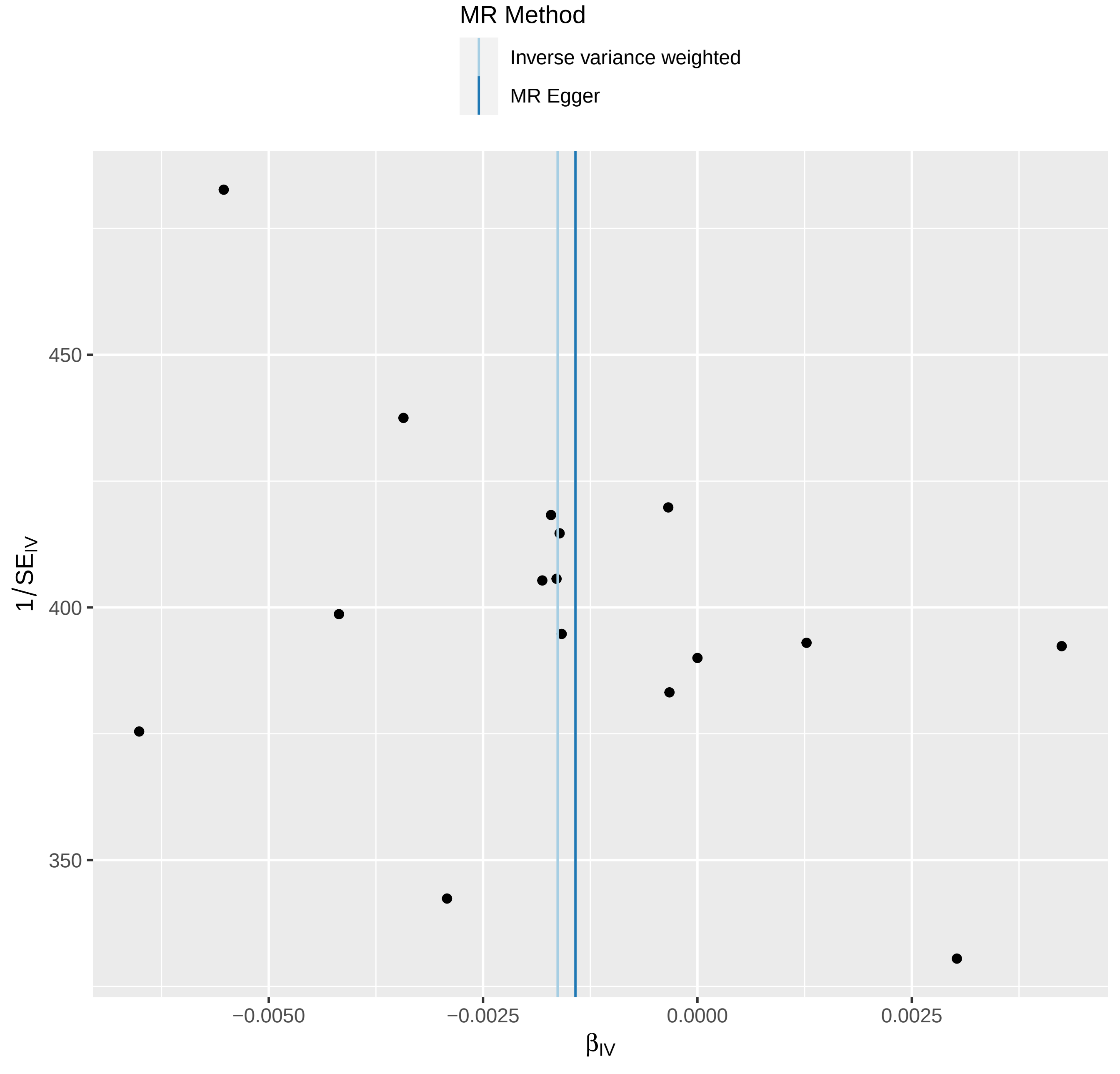

Batch 2−1133 : Fibronectin type III domain−containing protein 5 on eGFR

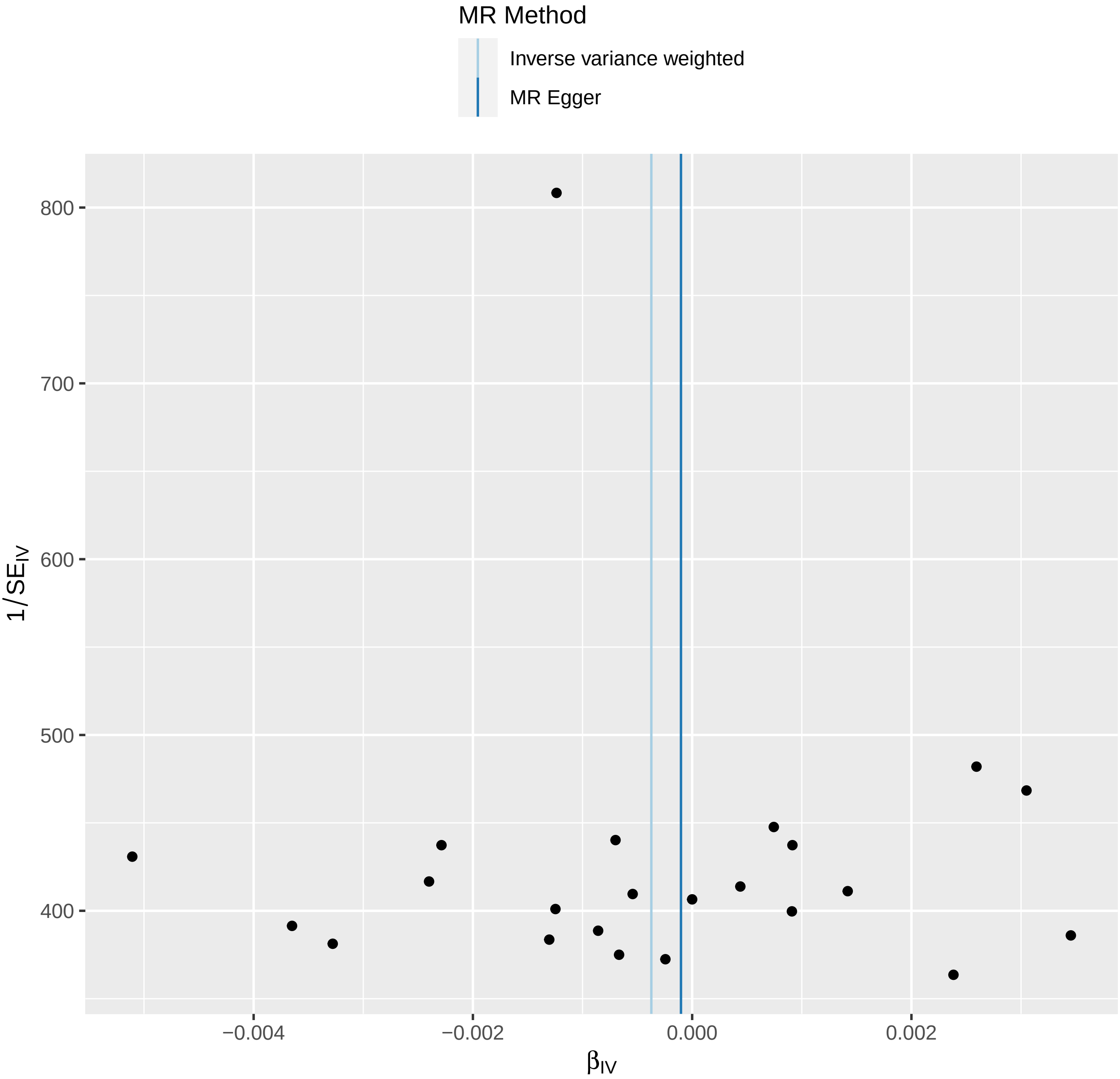

Batch 2−1134 : Glutamate carboxypeptidase 2 on eGFR

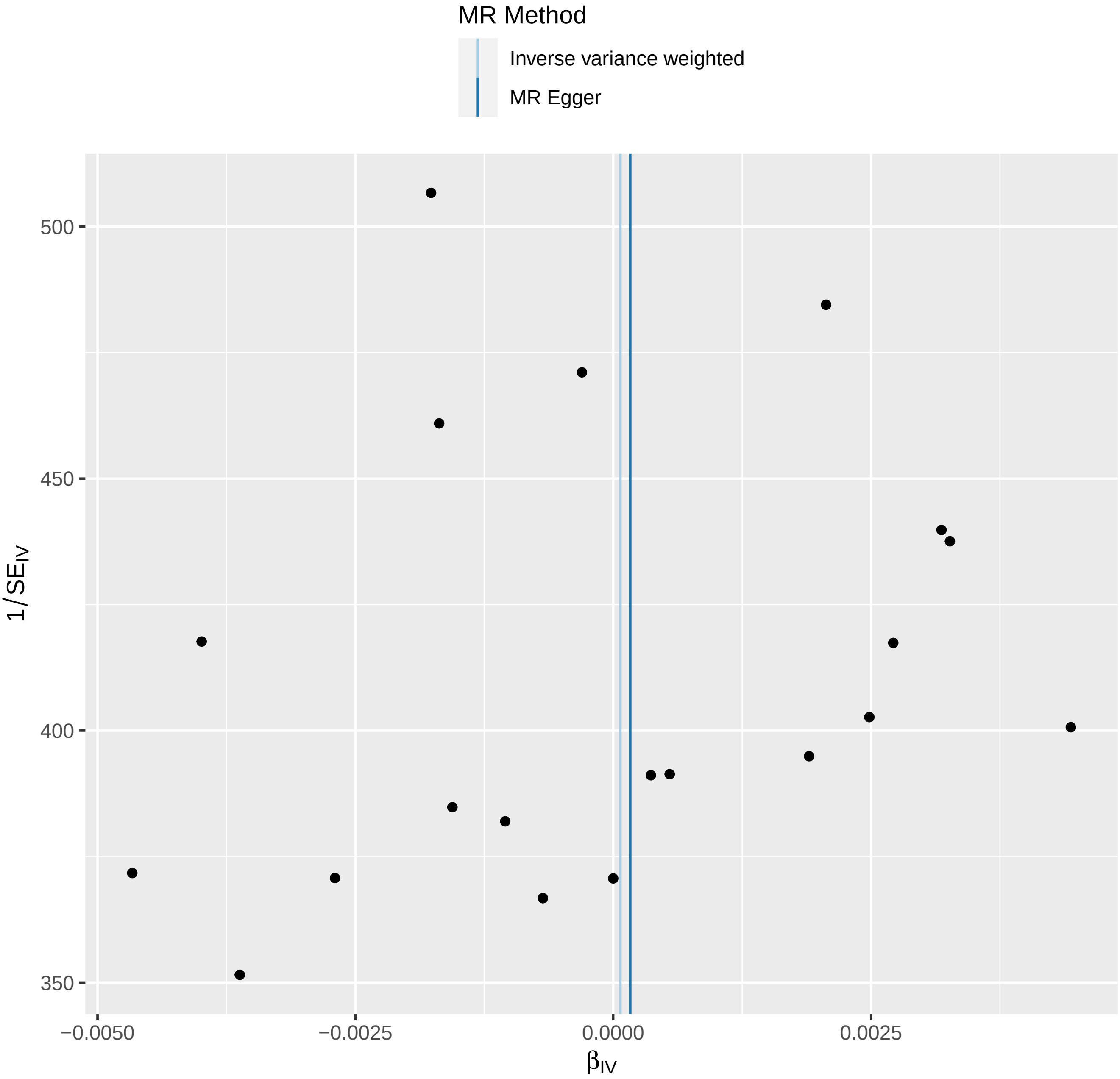

Batch 2−1135 : Glutamate carboxypeptidase 2 on eGFR

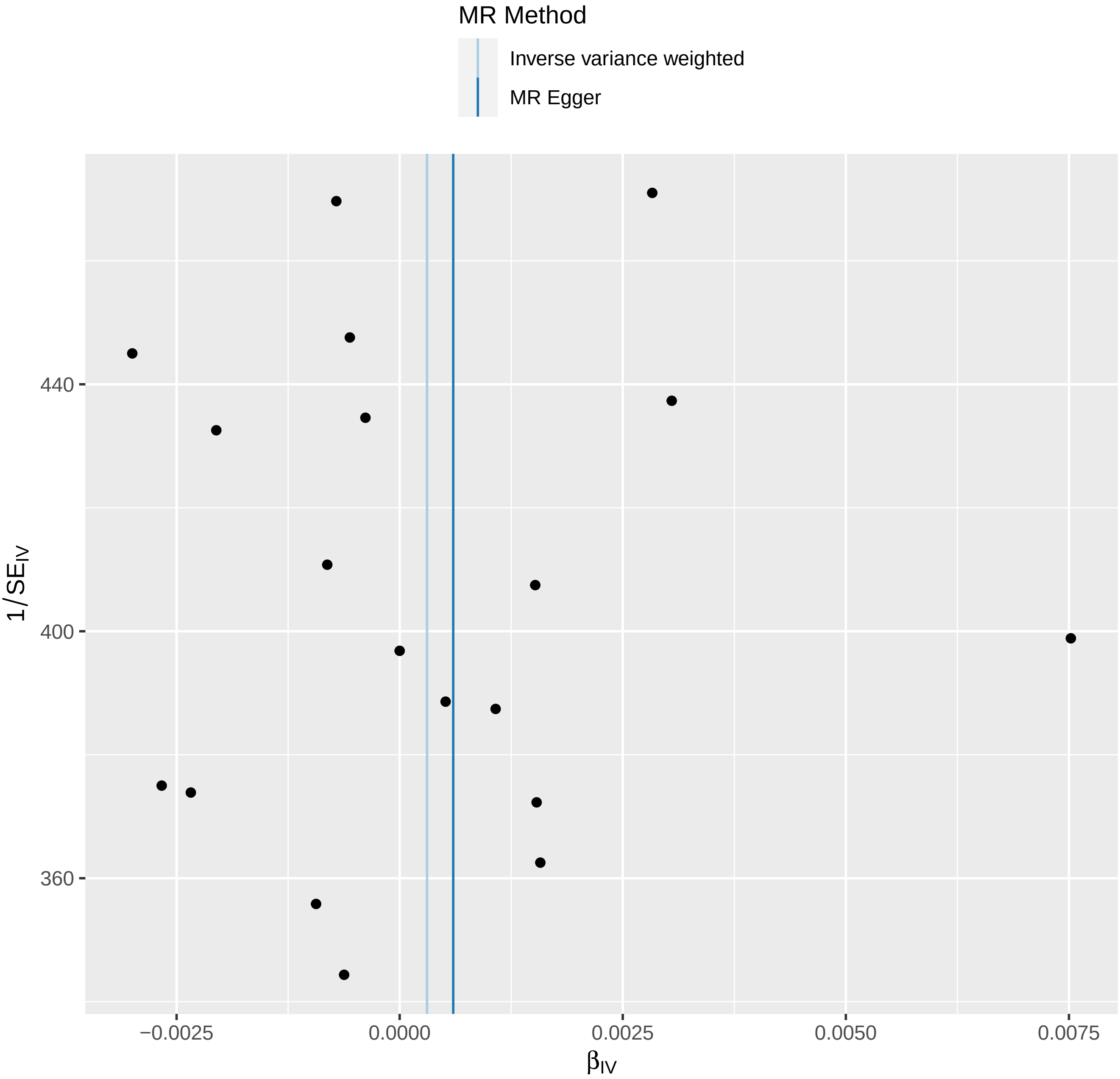

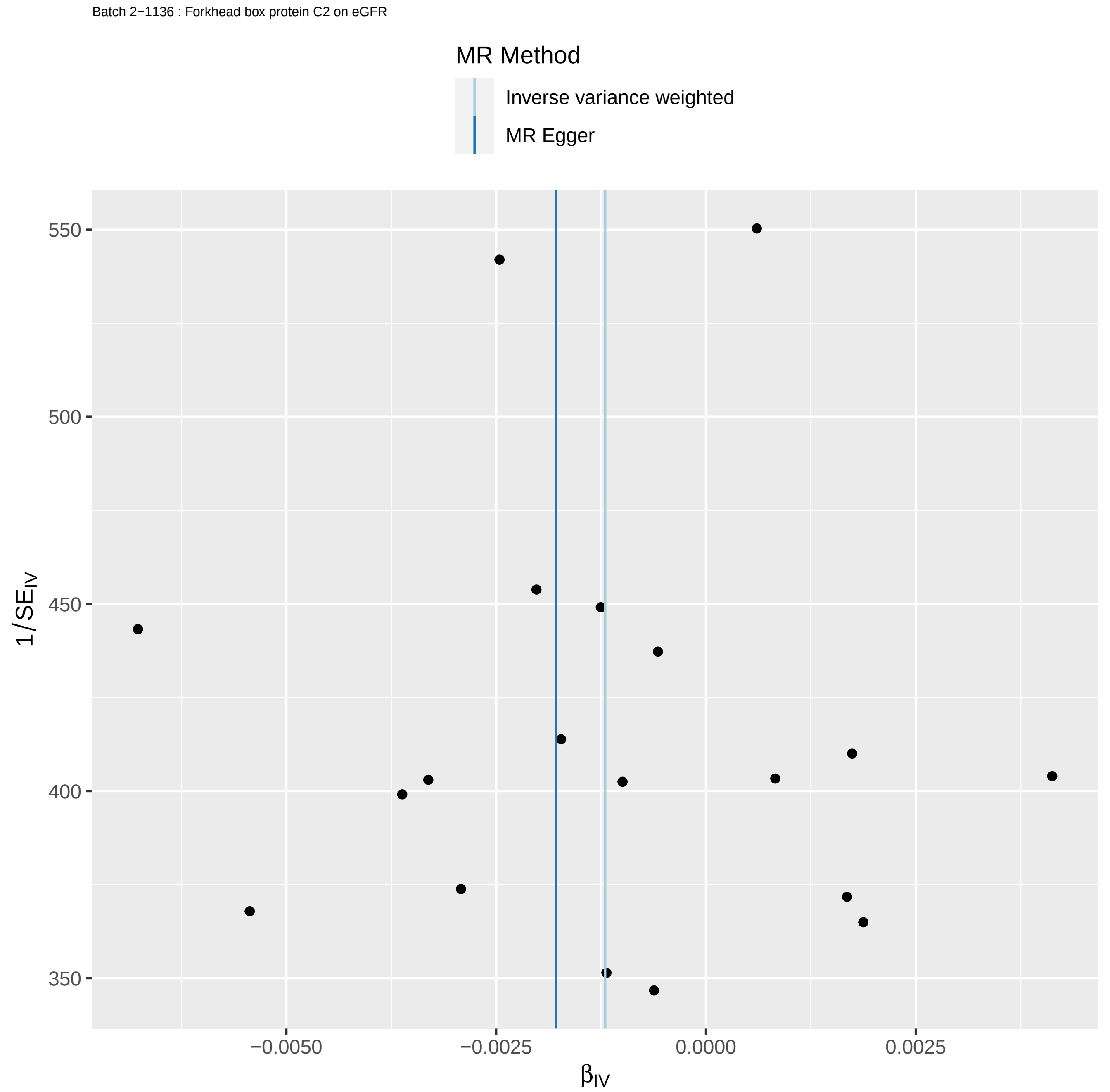
Batch 2−1136 : Forkhead box protein C2 on eGFR
MR Method
Inverse variance weighted
MR Egger
550
500
450
400
350
1/SE_IV
−0.0050
−0.0025
0.0000
0.0025
β_IV

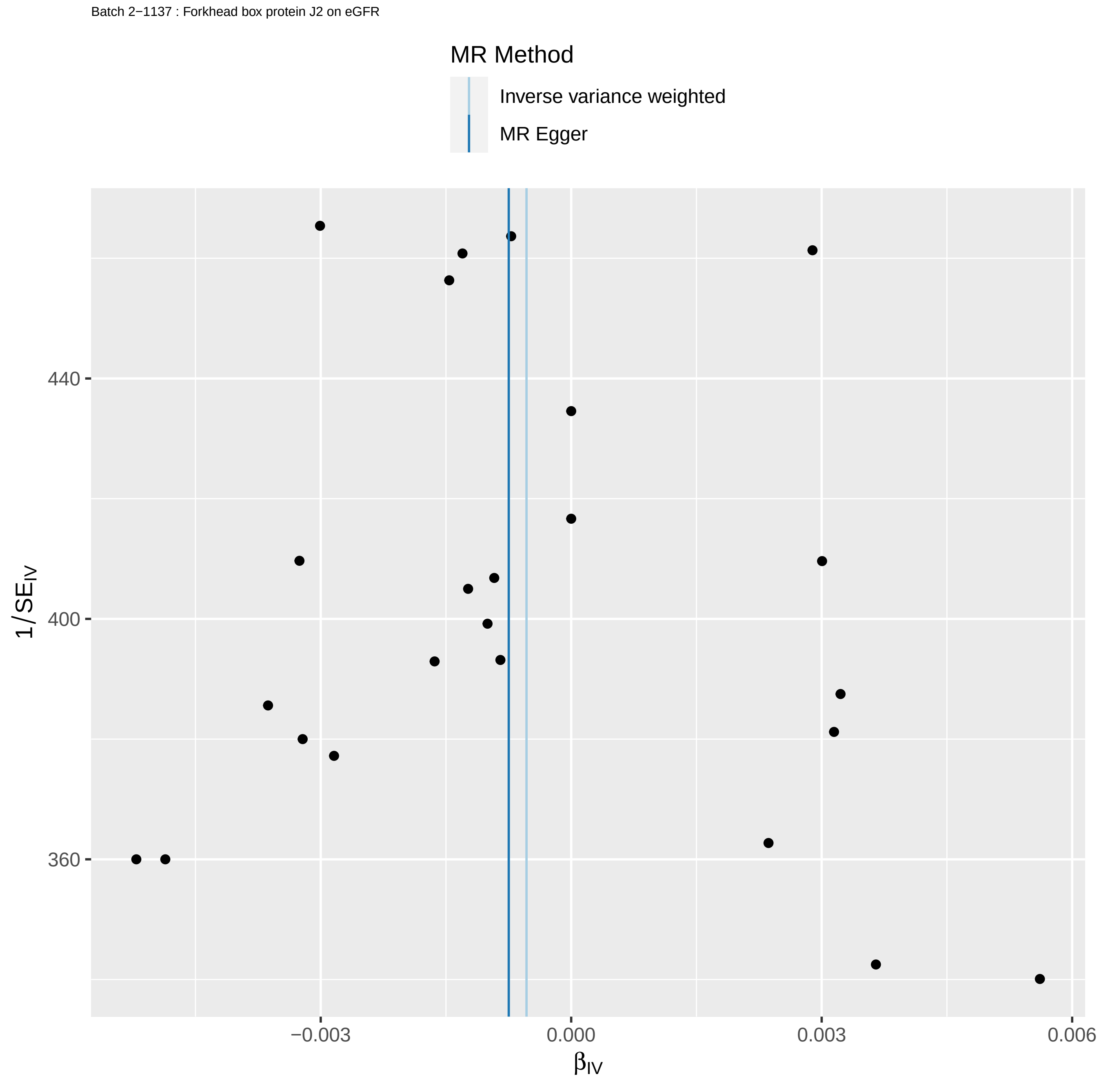
Batch 2−1137 : Forkhead box protein J2 on eGFR
MR Method
Inverse variance weighted
MR Egger
440
400
360
1/SE_IV
−0.003
0.000
0.003
0.006
β_IV

Batch 2−1138 : Forkhead box protein L2 on eGFR

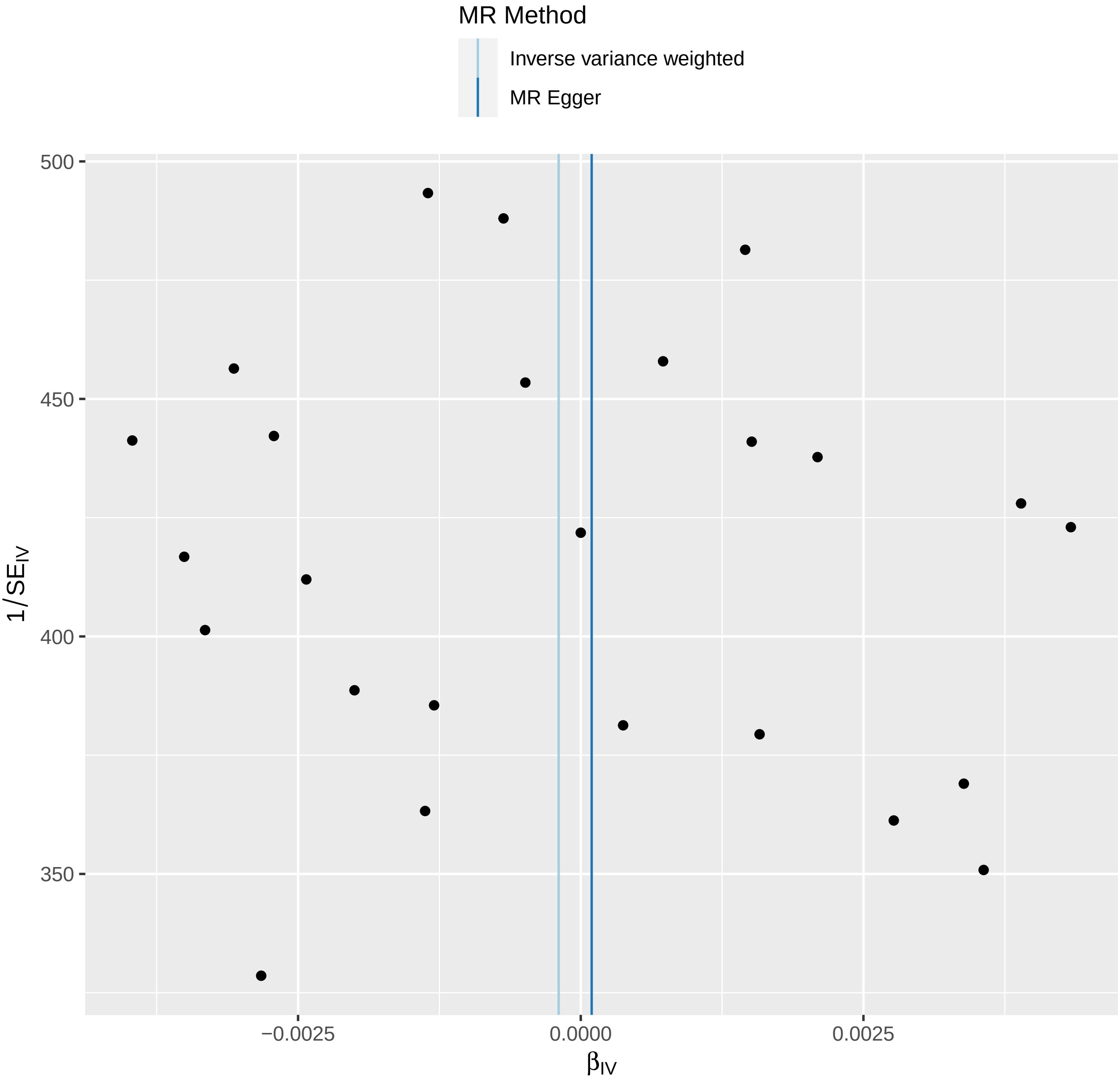

Batch 2−1139 : Tyrosine−protein kinase FRK on eGFR

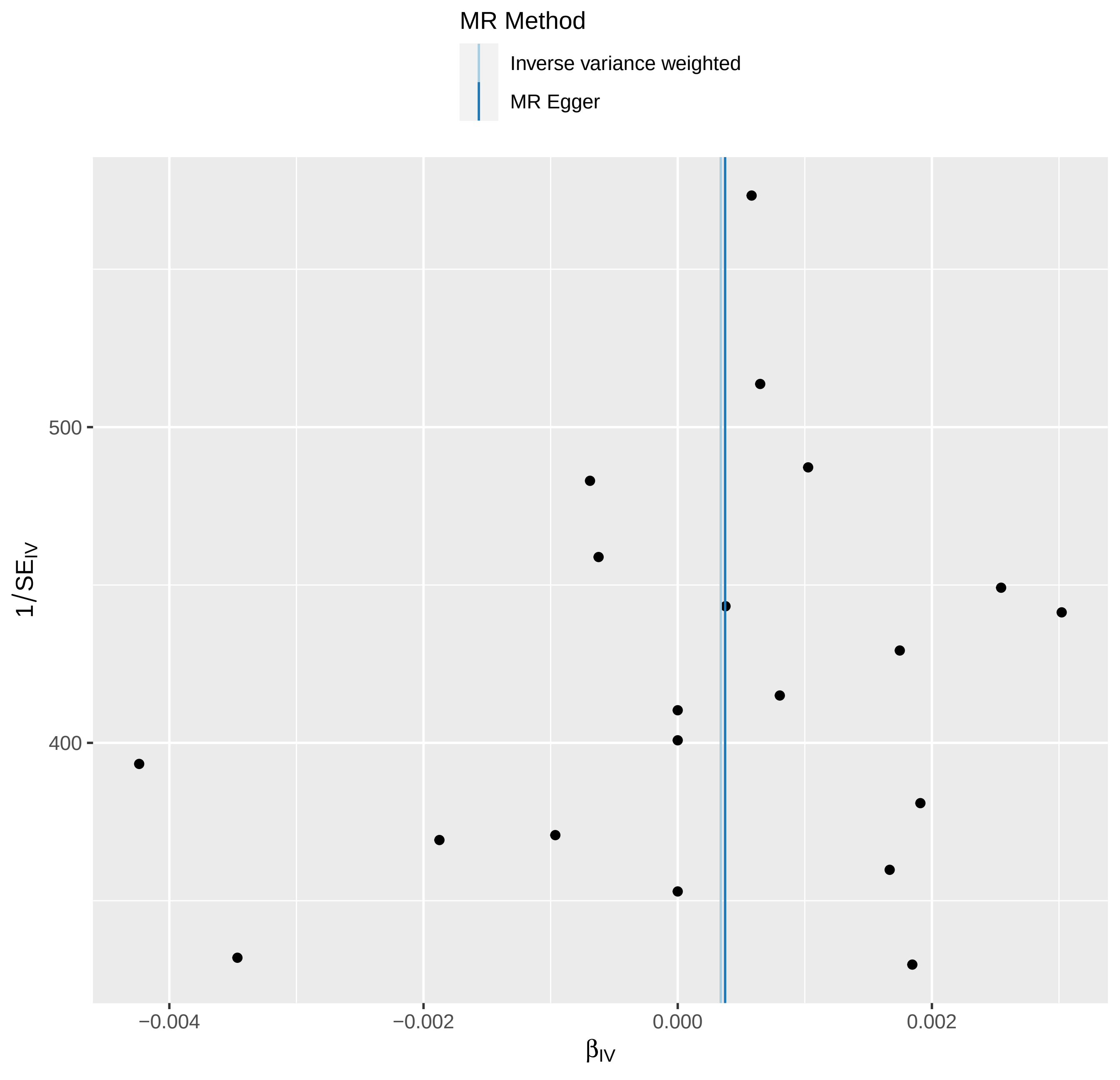

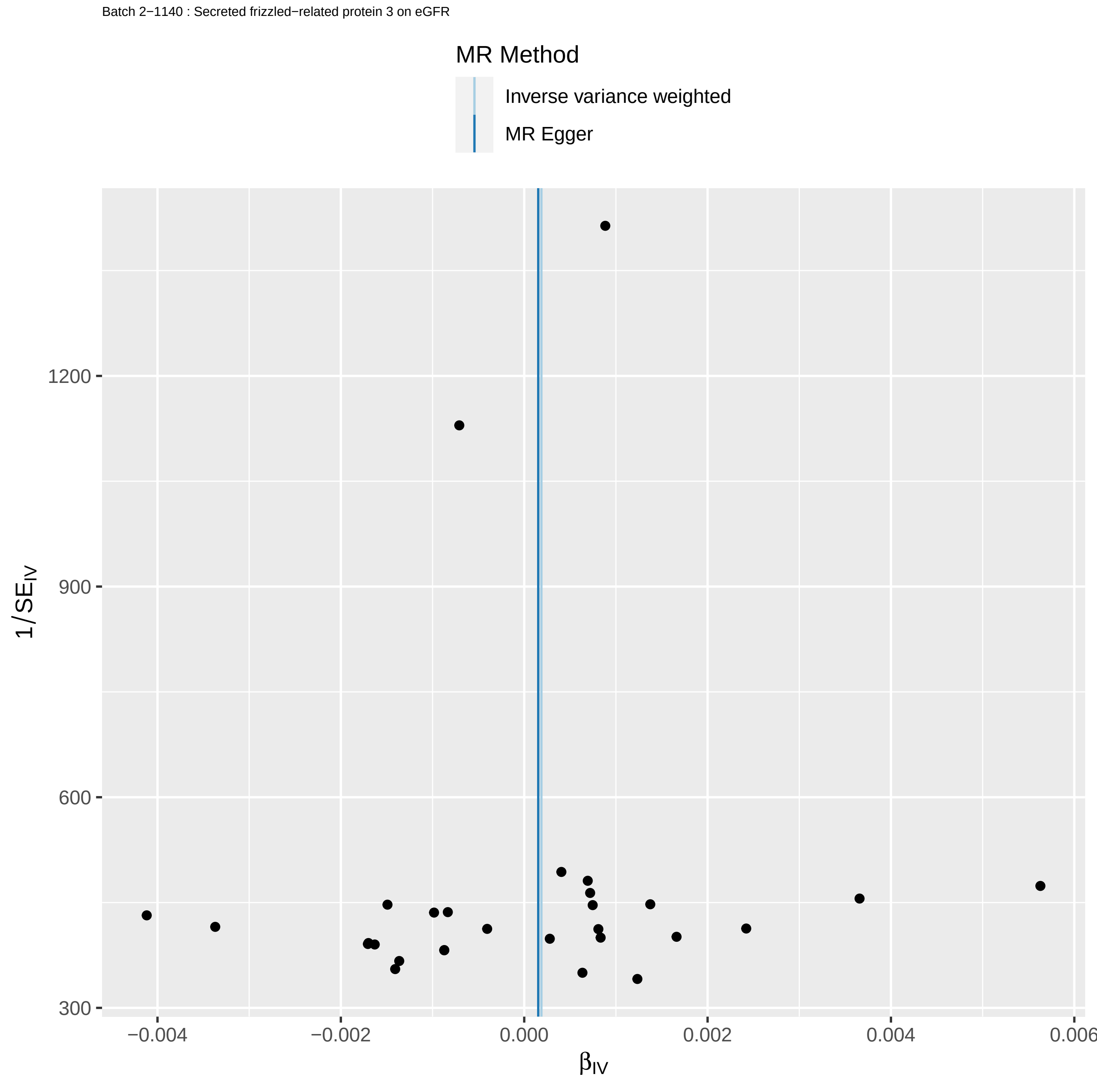

Batch 2−1140 : Secreted frizzled−related protein 3 on eGFR
MR Method
Inverse variance weighted
MR Egger
1200
900
600
300
1/SE_IV
−0.004
−0.002
0.000
0.002
0.004
0.006
β_IV

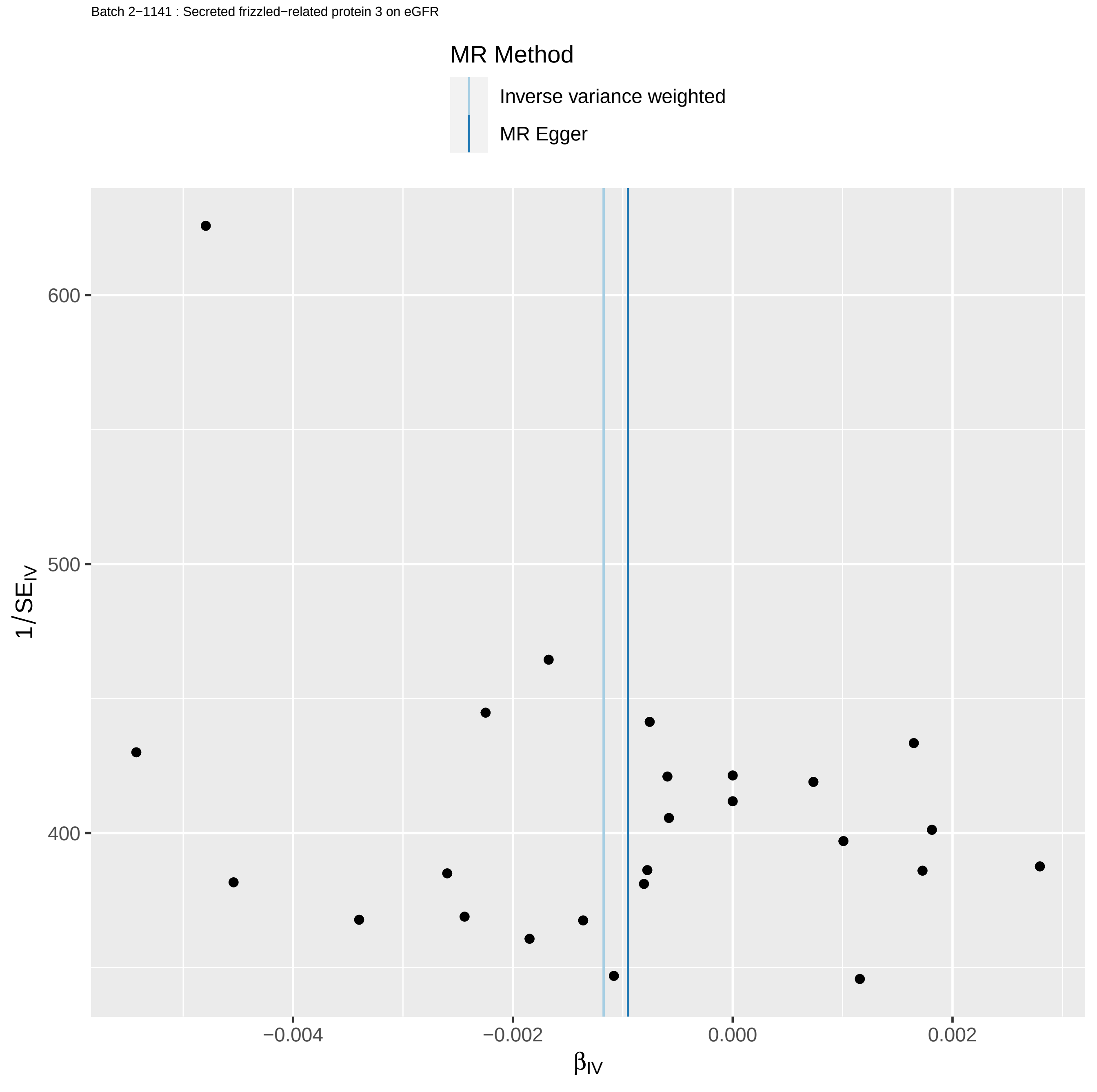
Batch 2−1141 : Secreted frizzled−related protein 3 on eGFR
MR Method
Inverse variance weighted
MR Egger
600
500
400
1/SE$_{IV}$
−0.004
−0.002
0.000
0.002
β$_{IV}$

Batch 2−1142 : Follistatin on eGFR

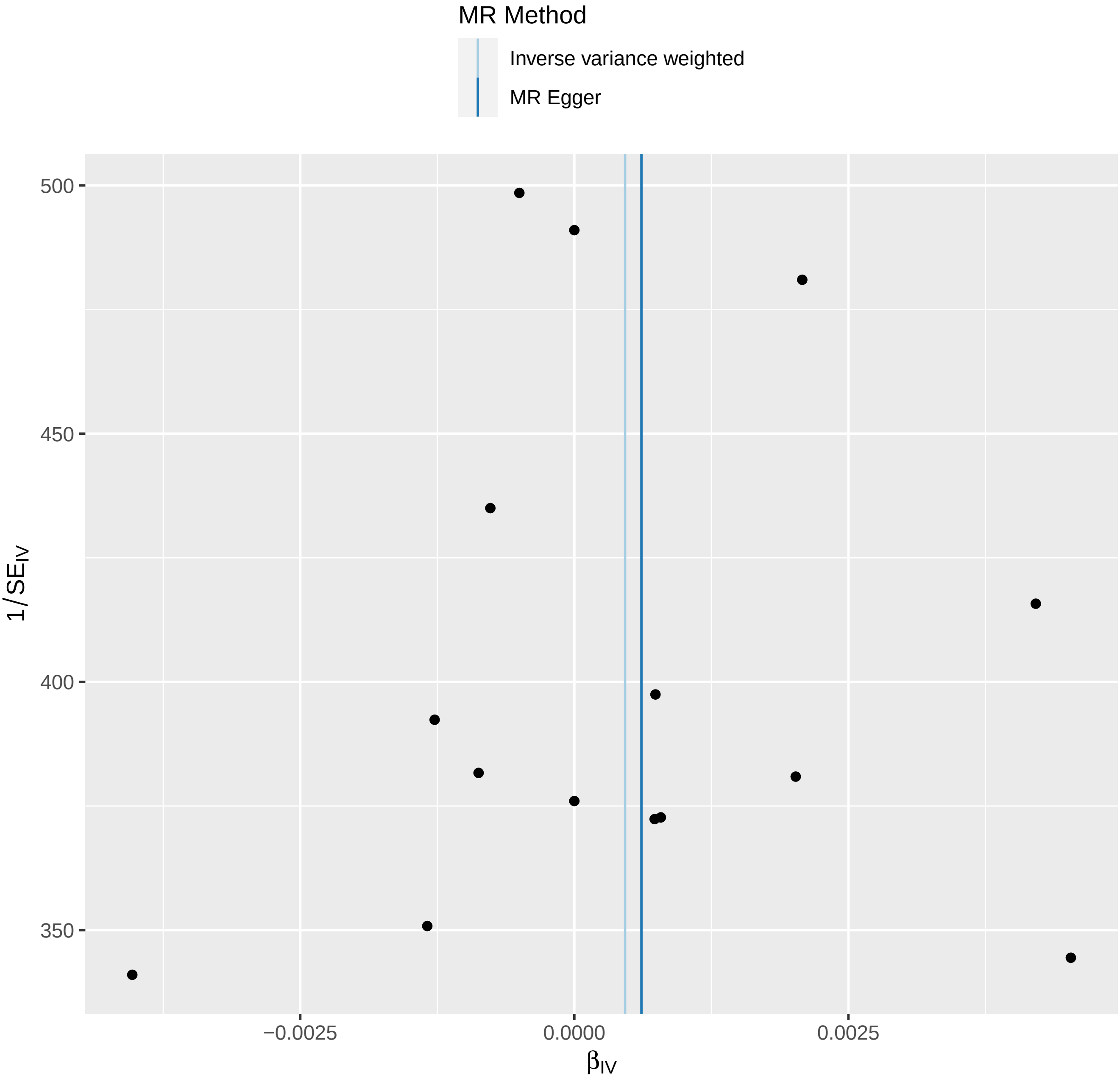

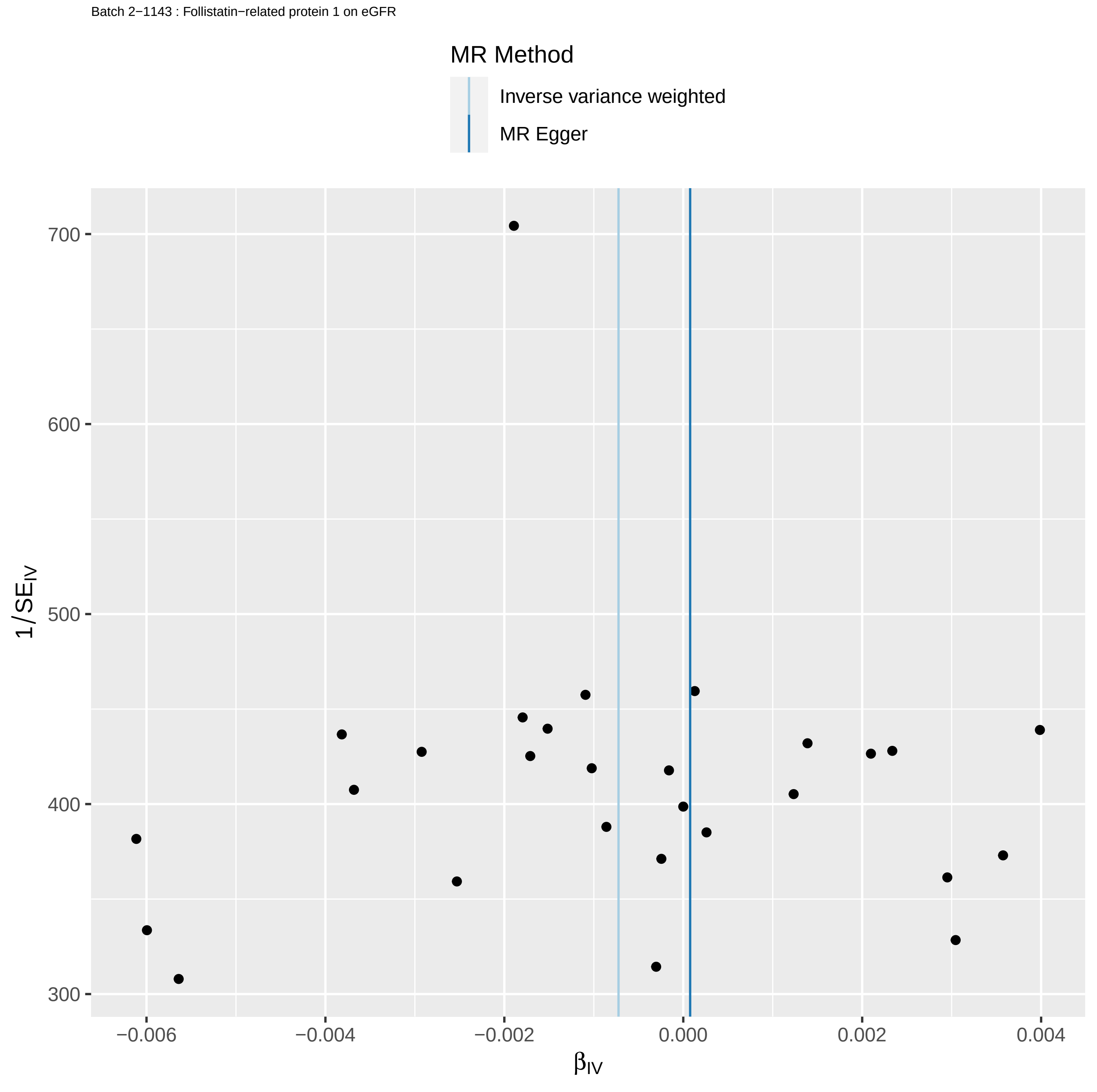

Batch 2−1143 : Follistatin−related protein 1 on eGFR
MR Method
Inverse variance weighted
MR Egger
700
600
500
400
300
1/SE$_{IV}$
−0.006
−0.004
−0.002
0.000
0.002
0.004
β$_{IV}$

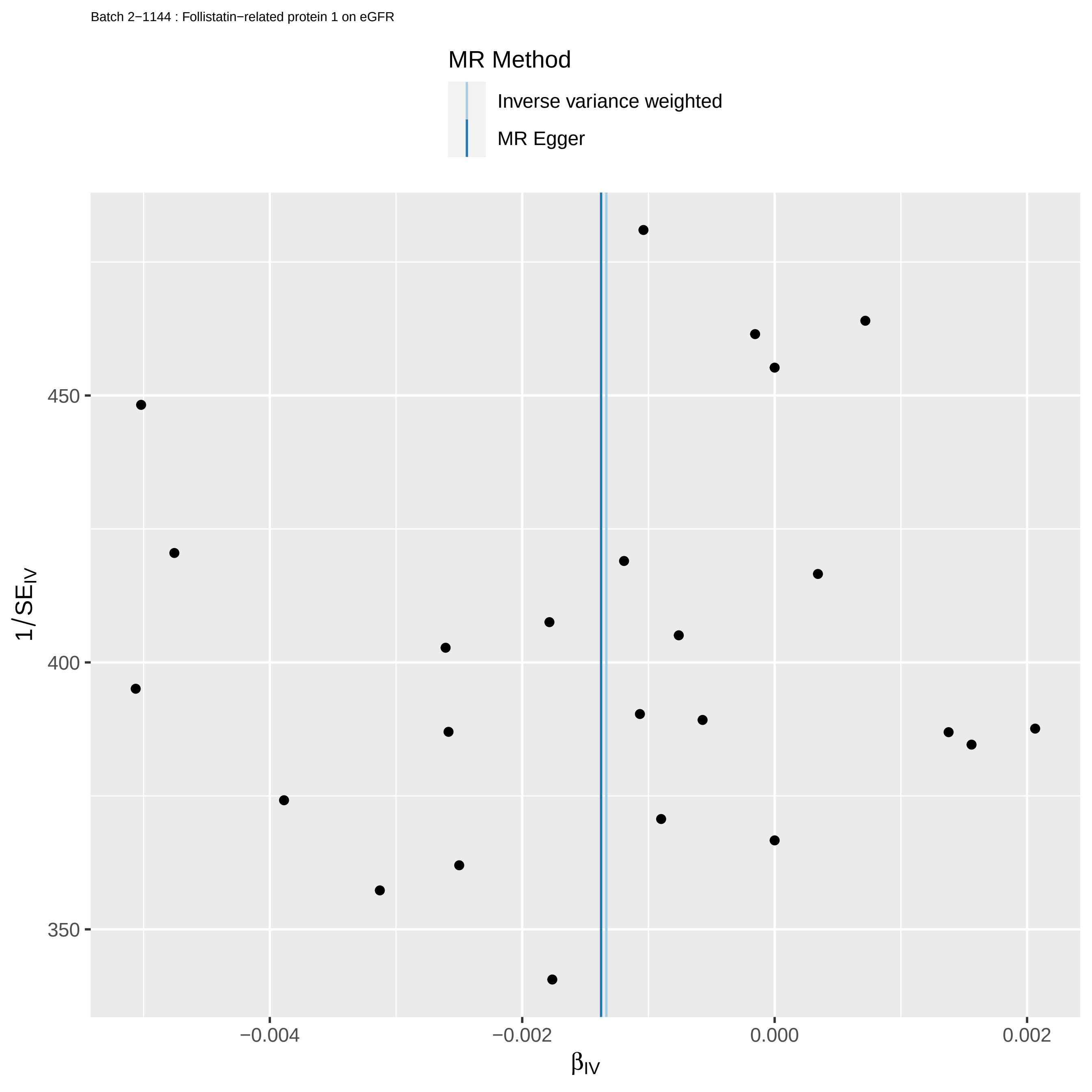
Batch 2−1144 : Follistatin−related protein 1 on eGFR
MR Method
Inverse variance weighted
MR Egger
450
400
350
1/SE_IV
−0.004
−0.002
0.000
0.002
β_IV

Batch 2−1145 : Follistatin−related protein 3 on eGFR

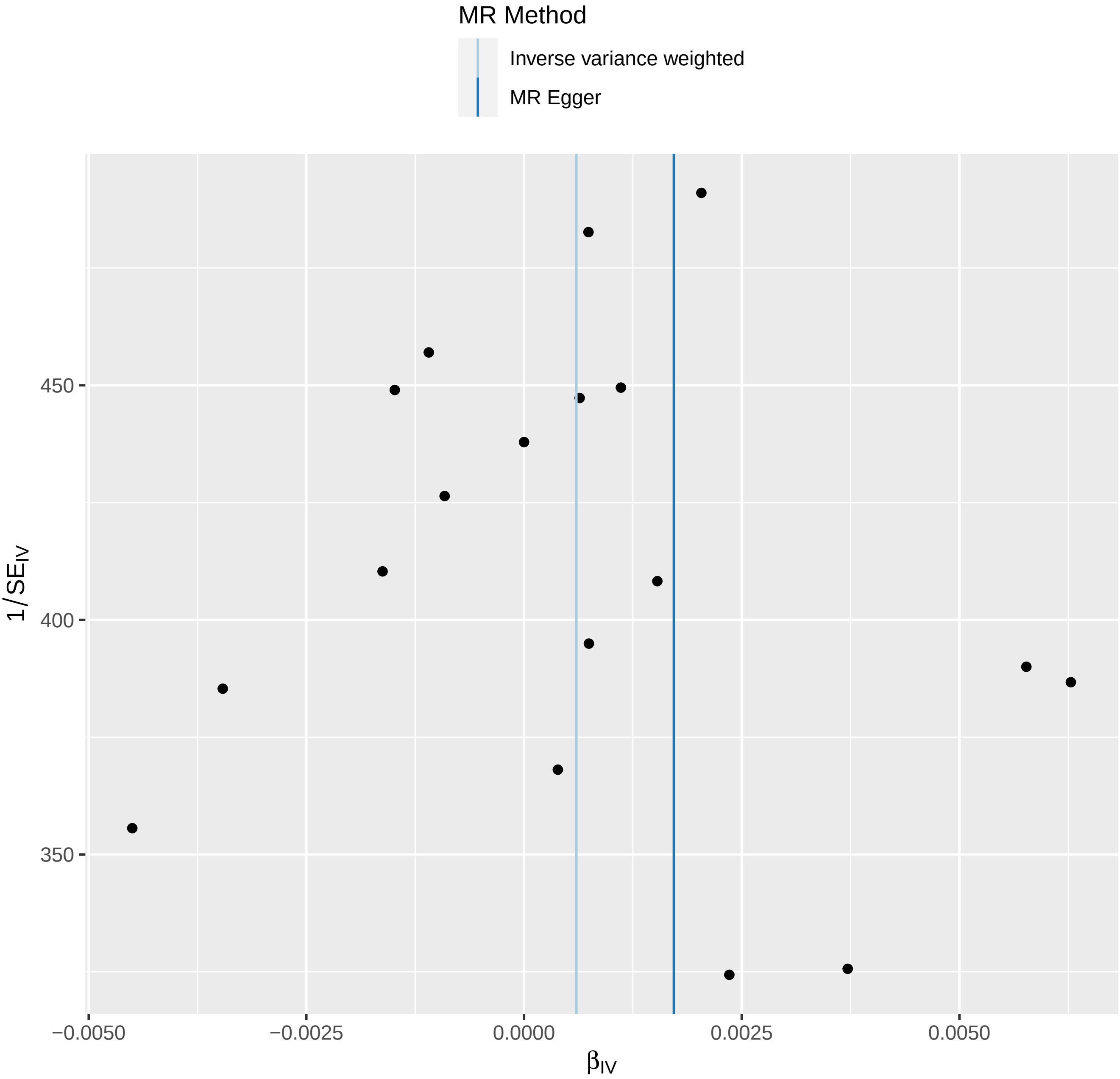

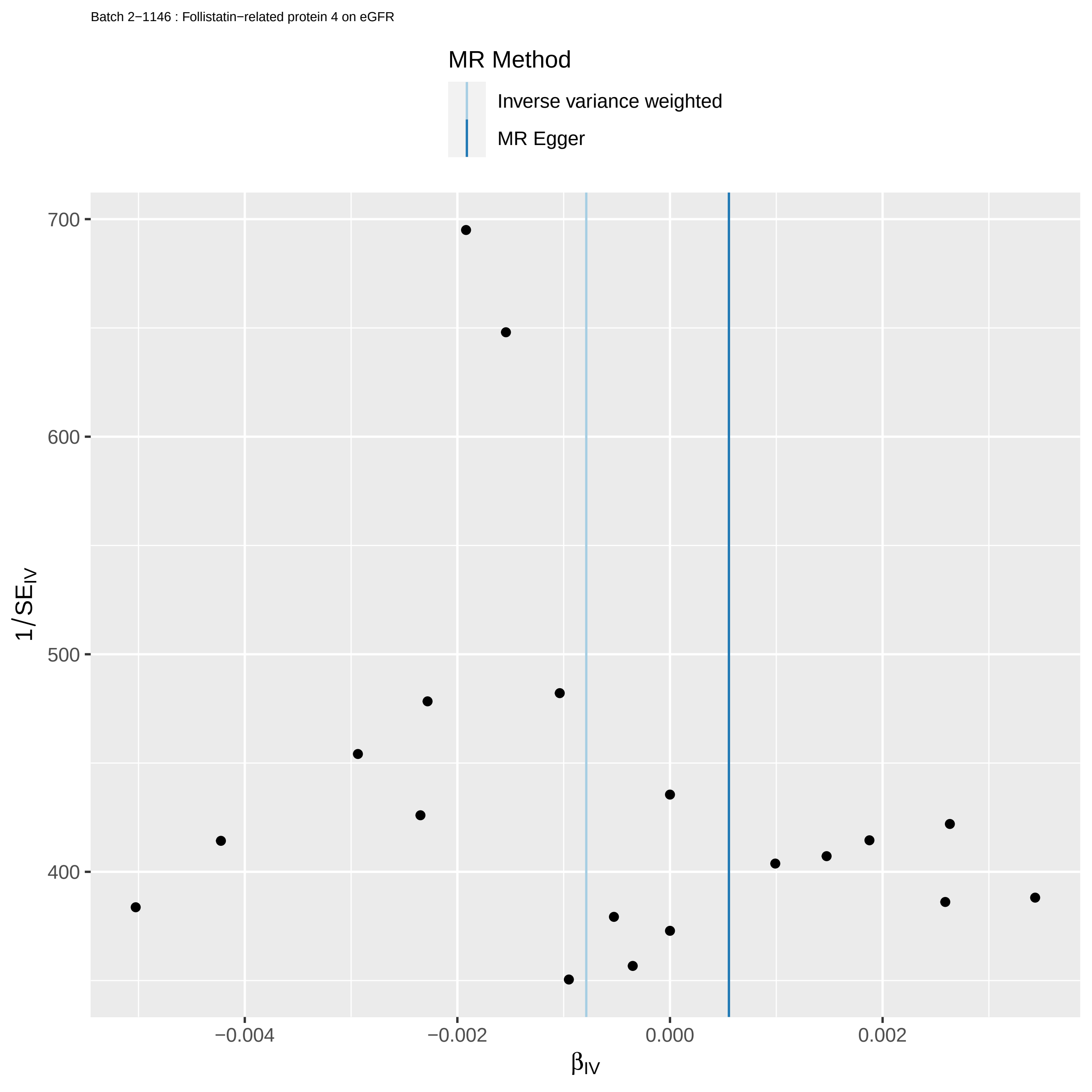

Batch 2−1146 : Follistatin−related protein 4 on eGFR
MR Method
Inverse variance weighted
MR Egger
700
600
500
400
1/SE$_{IV}$
−0.004
−0.002
0.000
0.002
β$_{IV}$

Batch 2−1147 : Follistatin−related protein 5 on eGFR

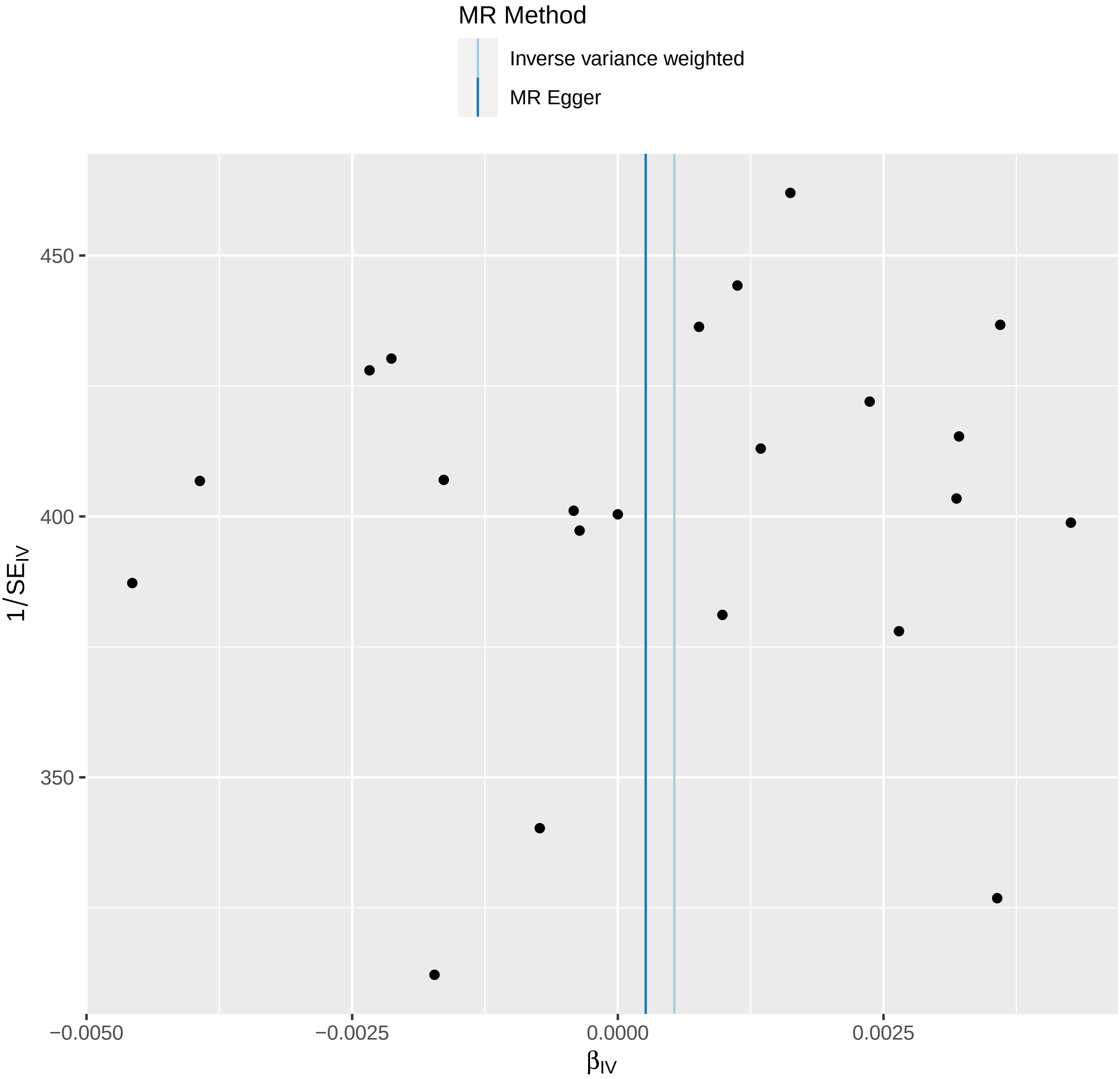

Batch 2−1148 : Ferritin on eGFR

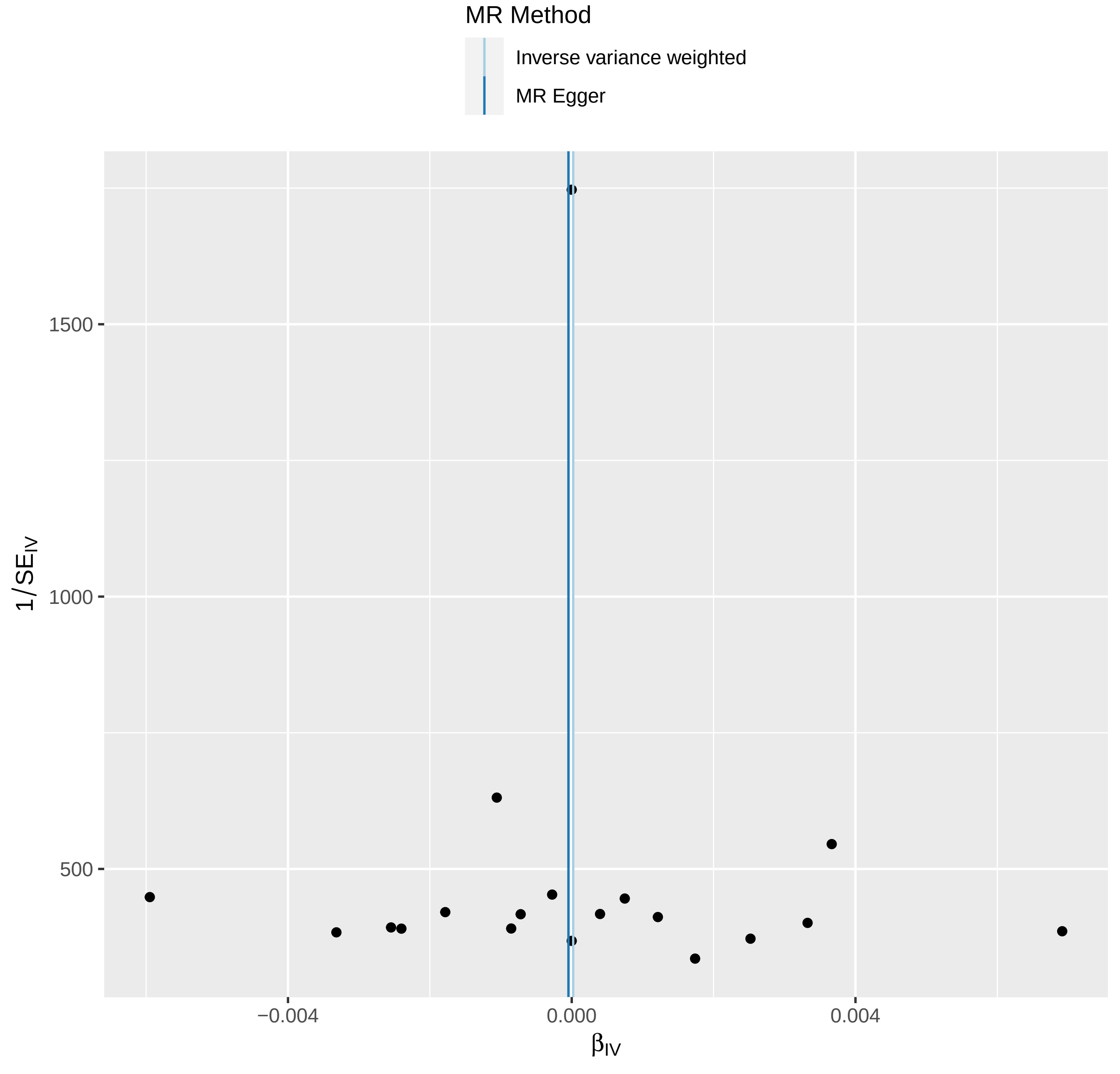

Batch 2−1149 : Ferritin on eGFR

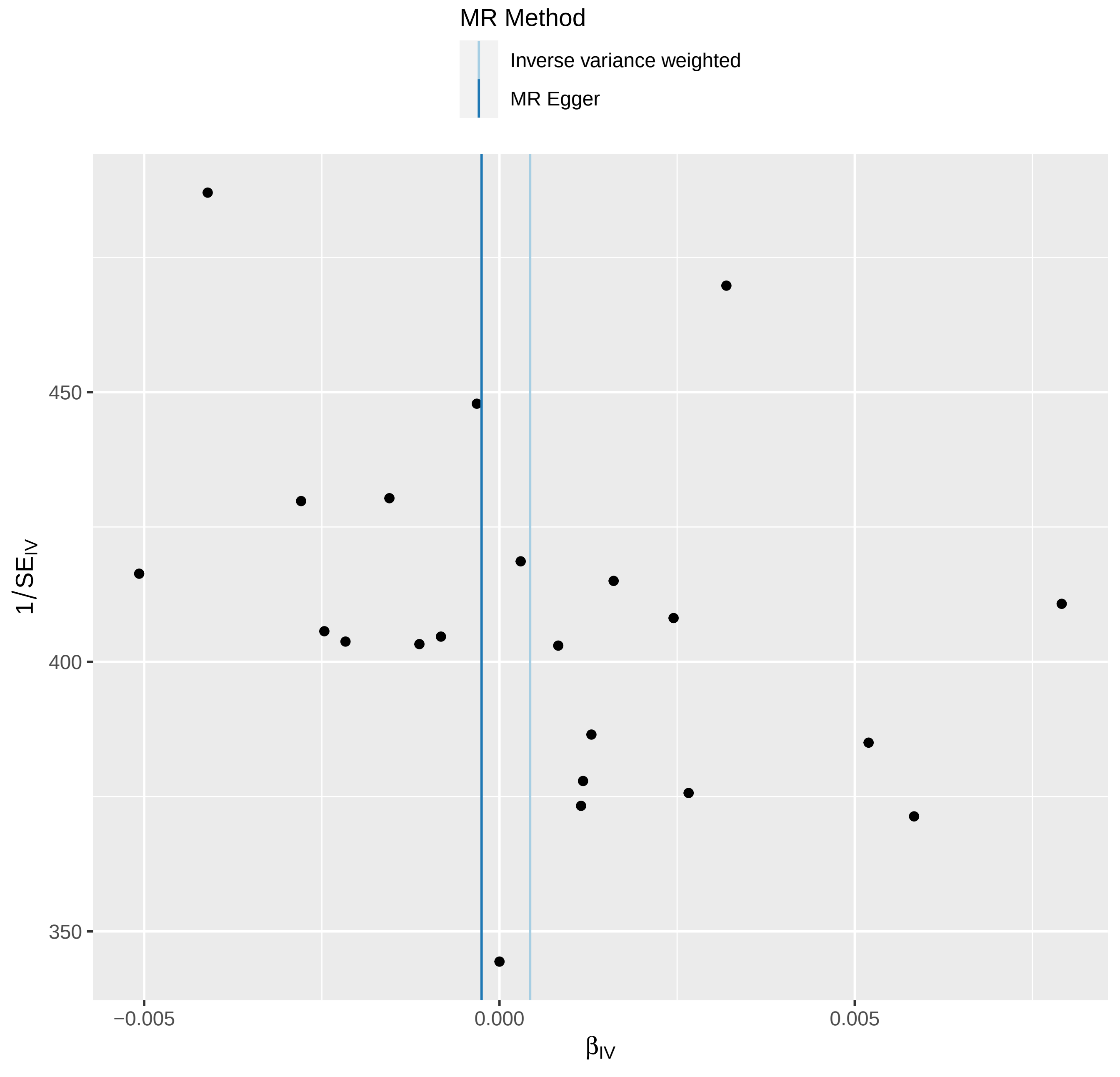

Batch 2−1150 : Furin on eGFR

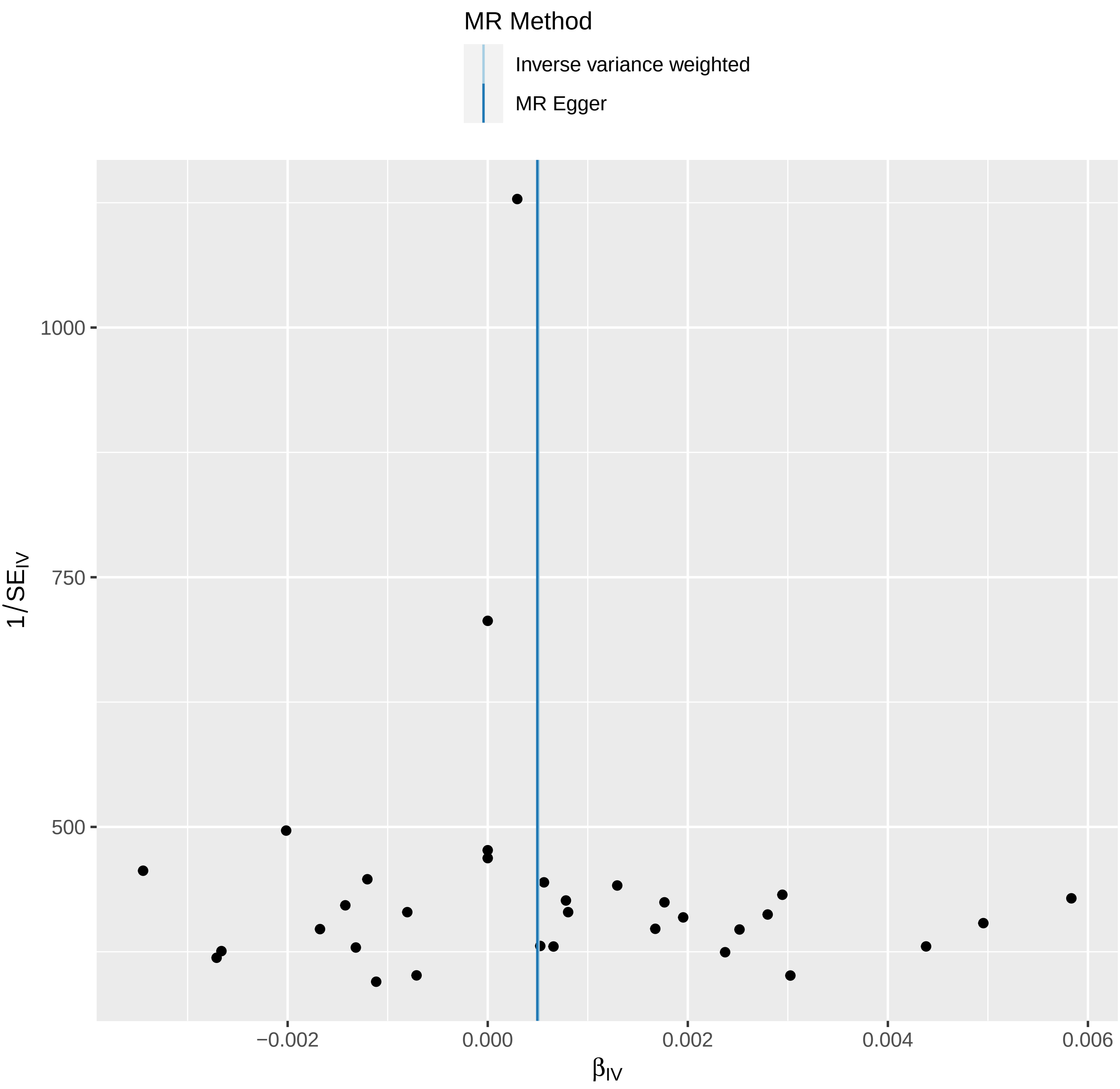

Batch 2−1151 : Alpha−(1,3)−fucosyltransferase 10 on eGFR

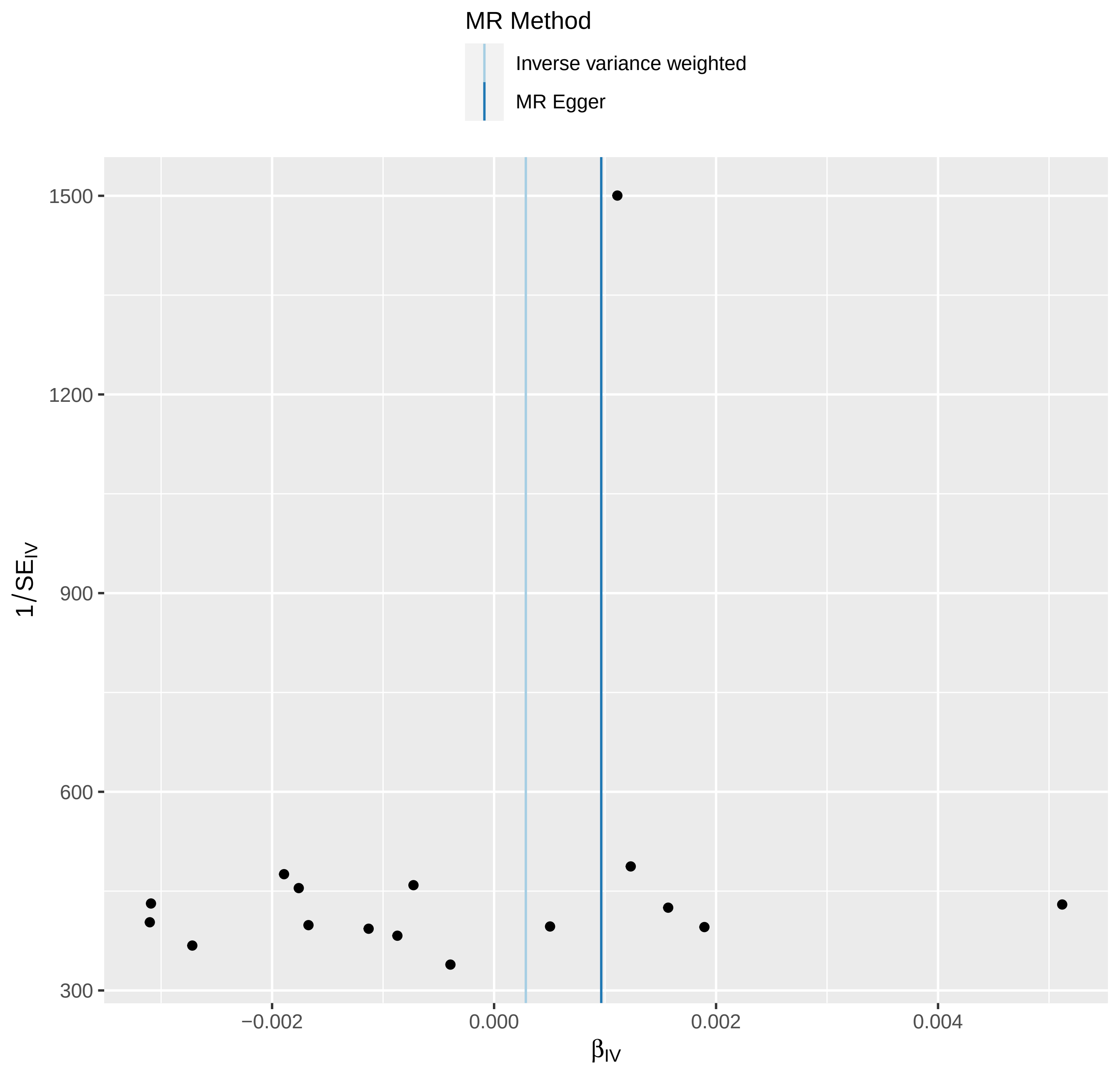

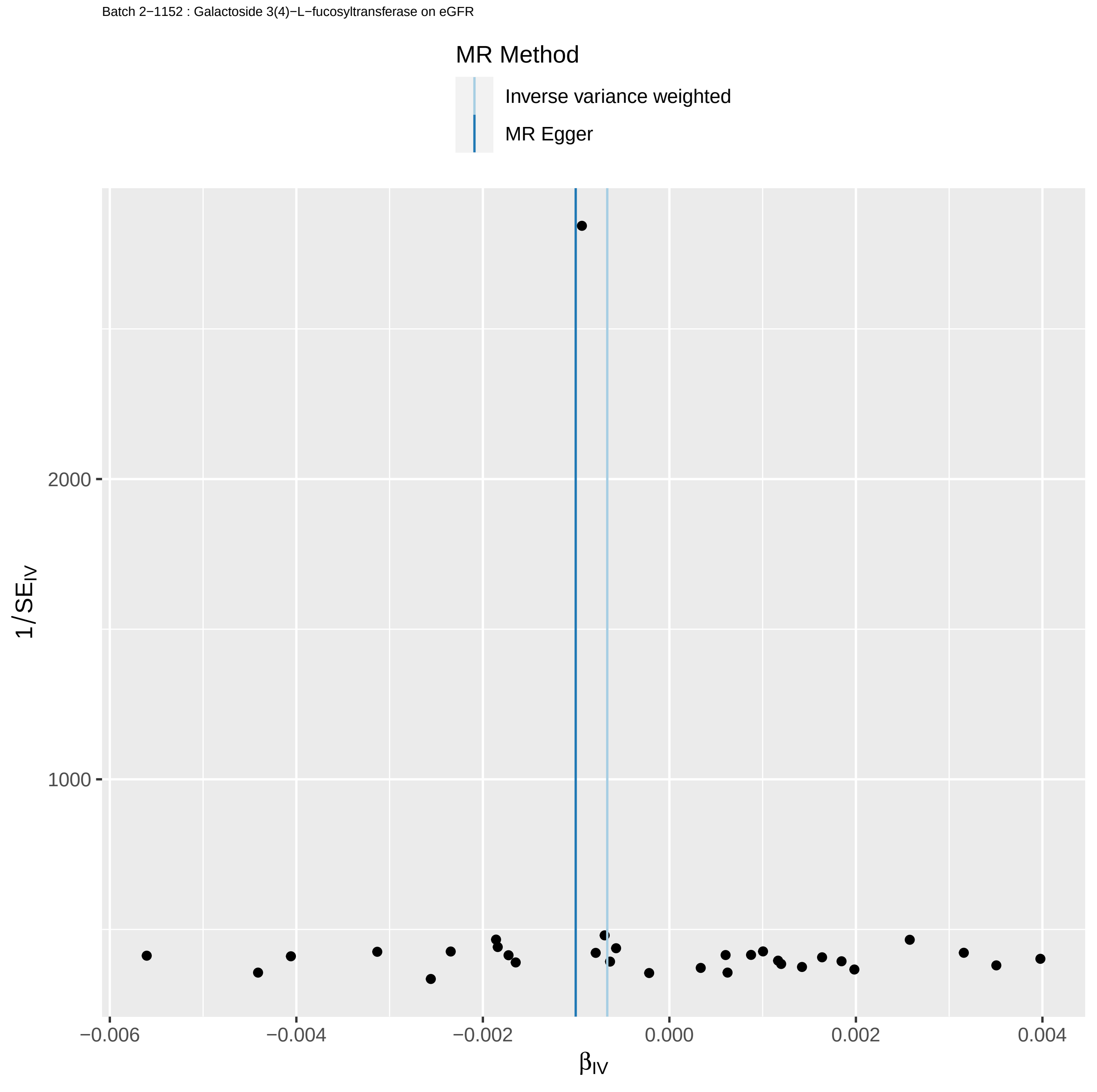

Batch 2−1152 : Galactoside 3(4)−L−fucosyltransferase on eGFR
MR Method
Inverse variance weighted
MR Egger
2000
1000
1/SE$_{IV}$
−0.006
−0.004
−0.002
0.000
0.002
0.004
β$_{IV}$

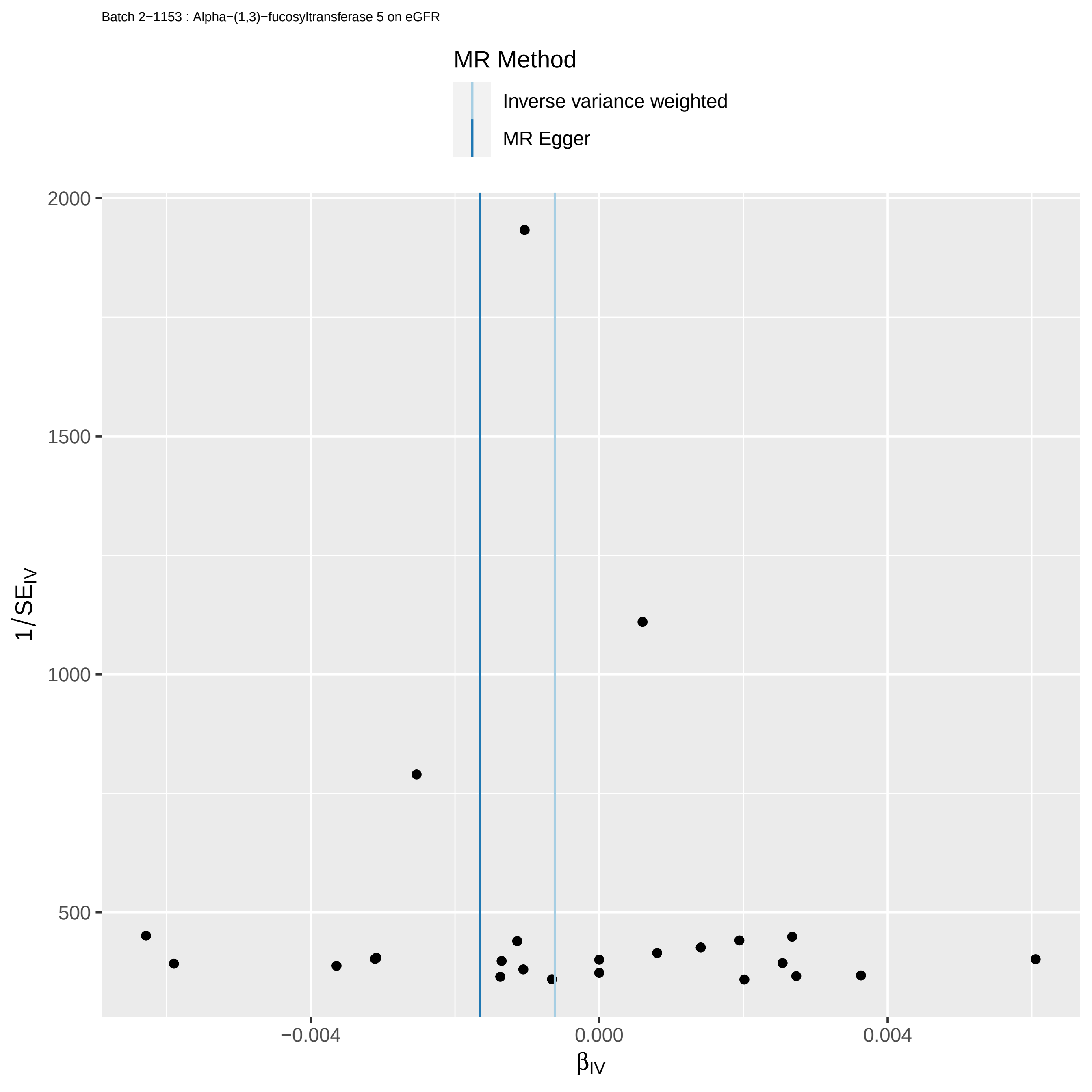
Batch 2−1153 : Alpha−(1,3)−fucosyltransferase 5 on eGFR
MR Method
Inverse variance weighted
MR Egger
2000
1500
1000
500
1/SE_IV
−0.004
0.000
0.004
β_IV

Batch 2−1154 : Alpha−(1,6)−fucosyltransferase on eGFR

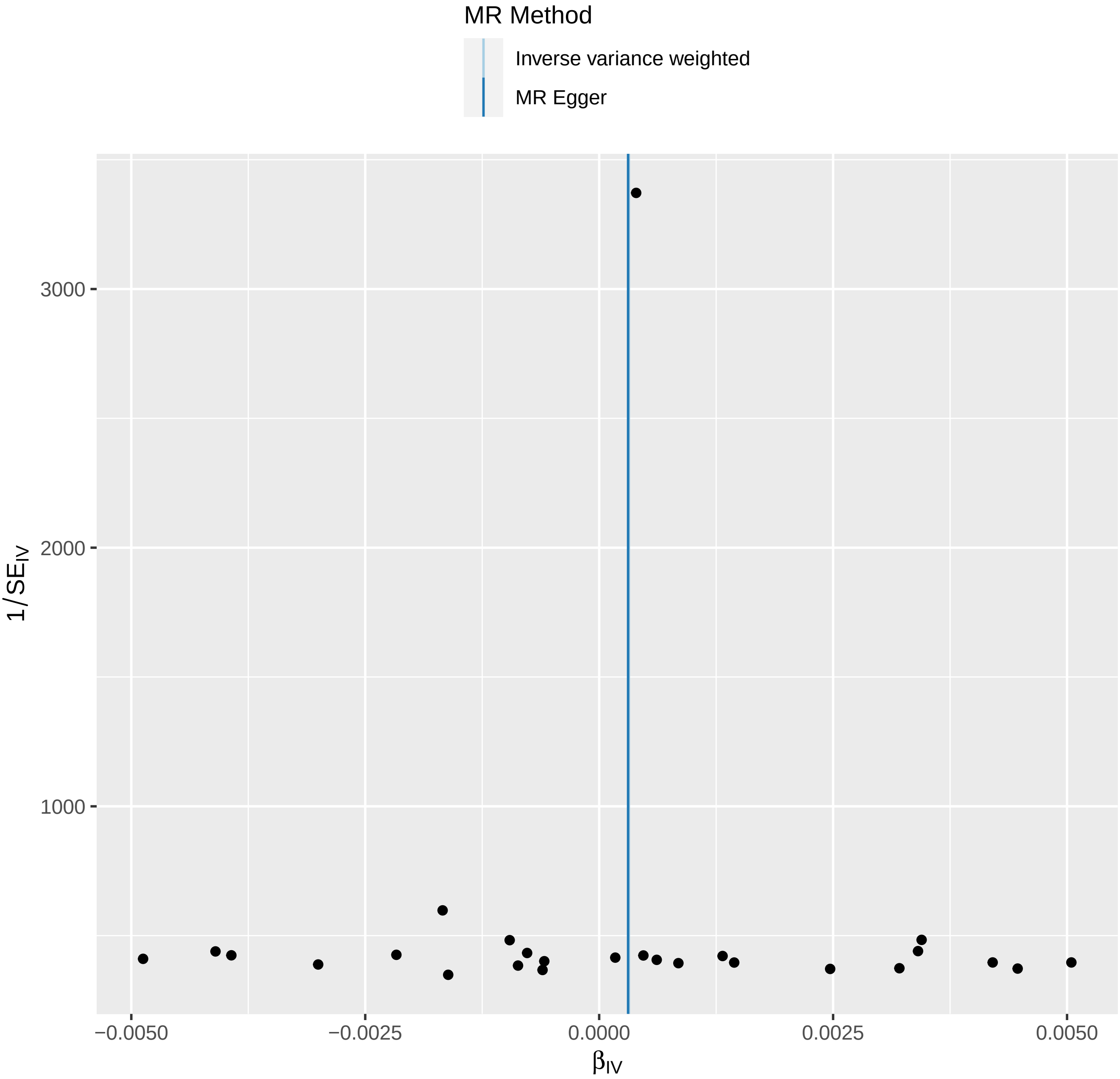

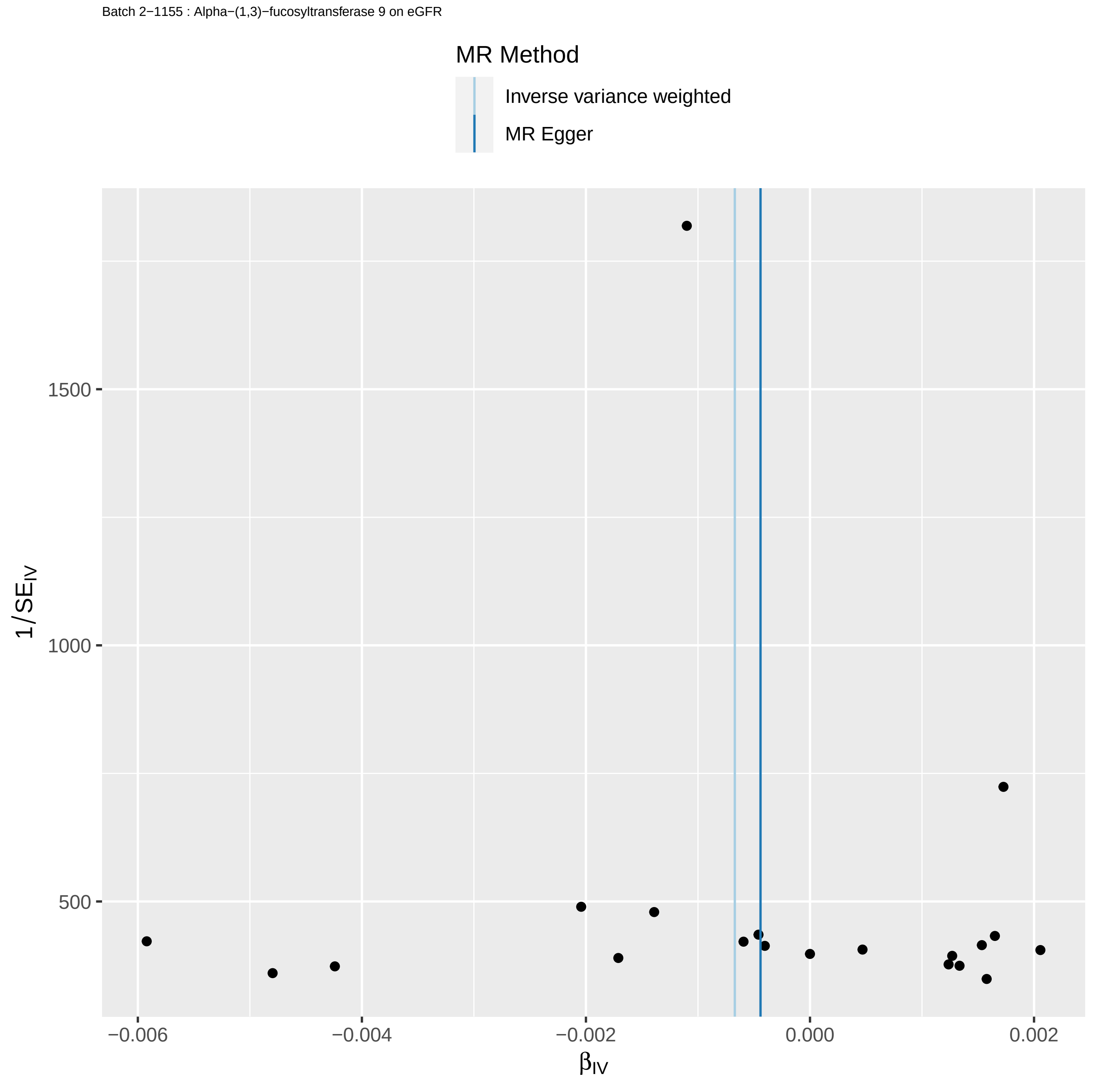
Batch 2−1155 : Alpha−(1,3)−fucosyltransferase 9 on eGFR
MR Method
Inverse variance weighted
MR Egger
1500
1000
500
1/SE$_{IV}$
−0.006
−0.004
−0.002
0.000
0.002
β$_{IV}$

Batch 2−1156 : Fragile X mental retardation syndrome−related protein 1 on eGFR

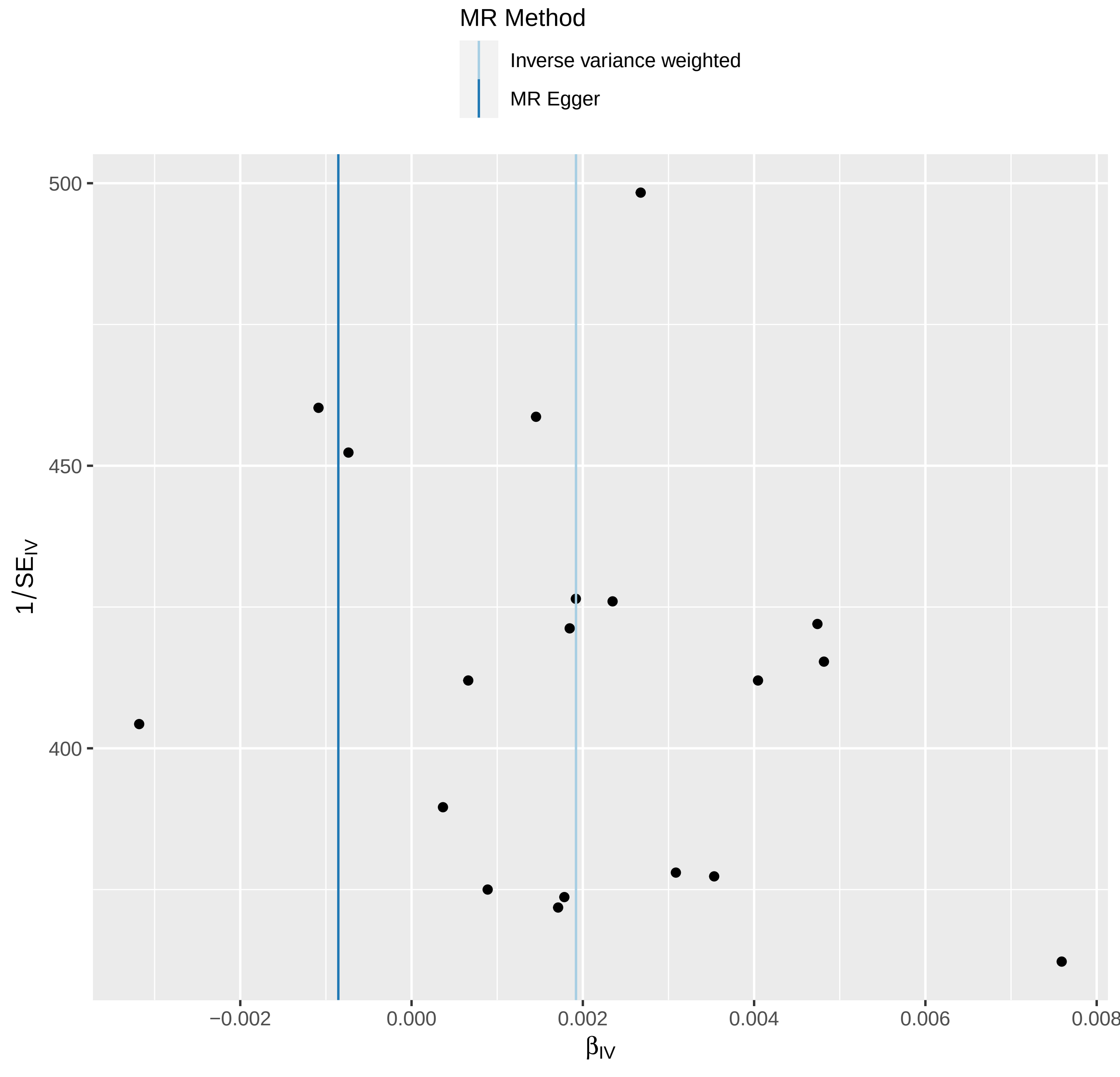

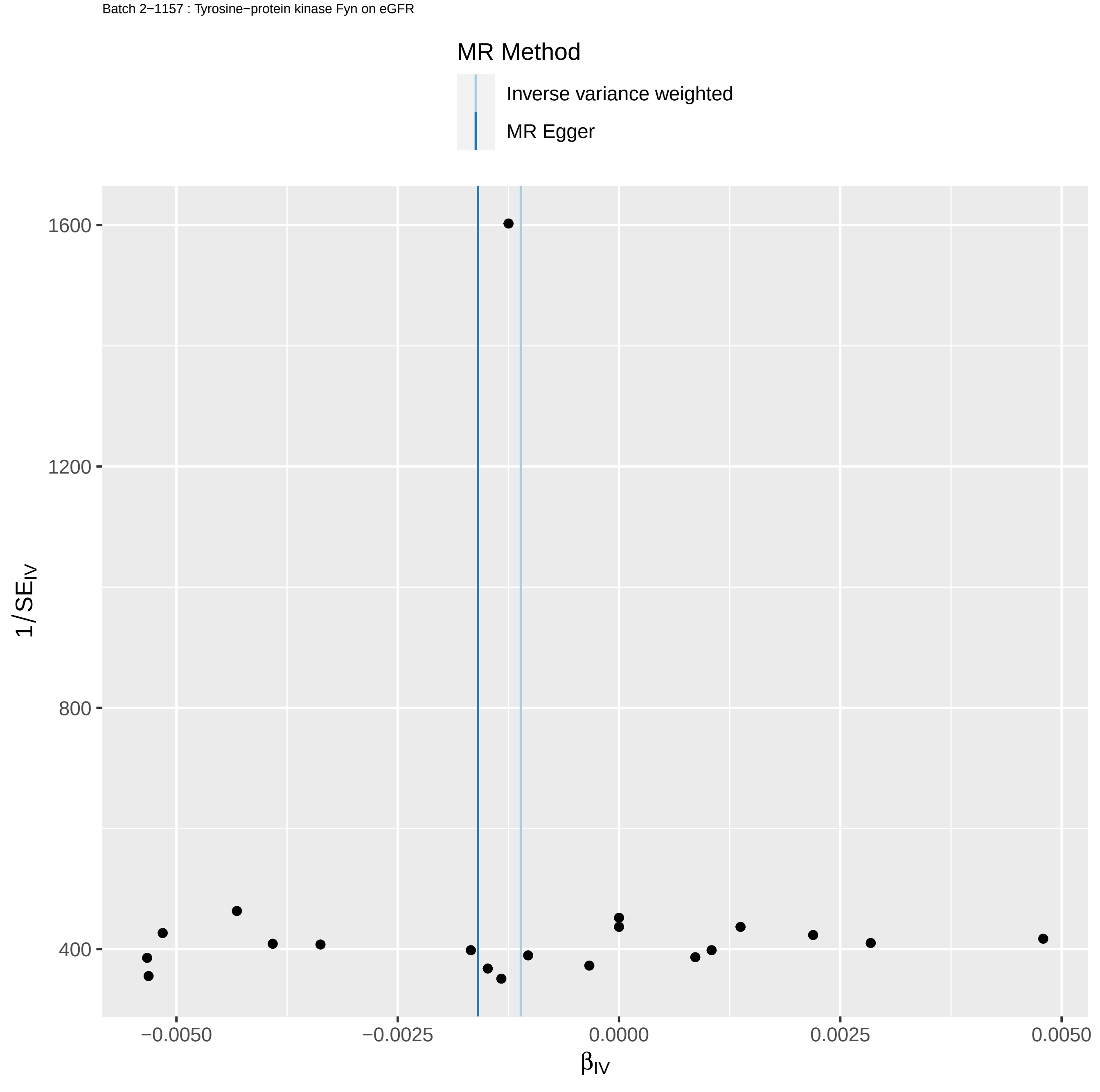

Batch 2−1157 : Tyrosine−protein kinase Fyn on eGFR
MR Method
Inverse variance weighted
MR Egger
1600
1200
800
400
1/SE$_{IV}$
−0.0050
−0.0025
0.0000
0.0025
0.0050
β_{IV}

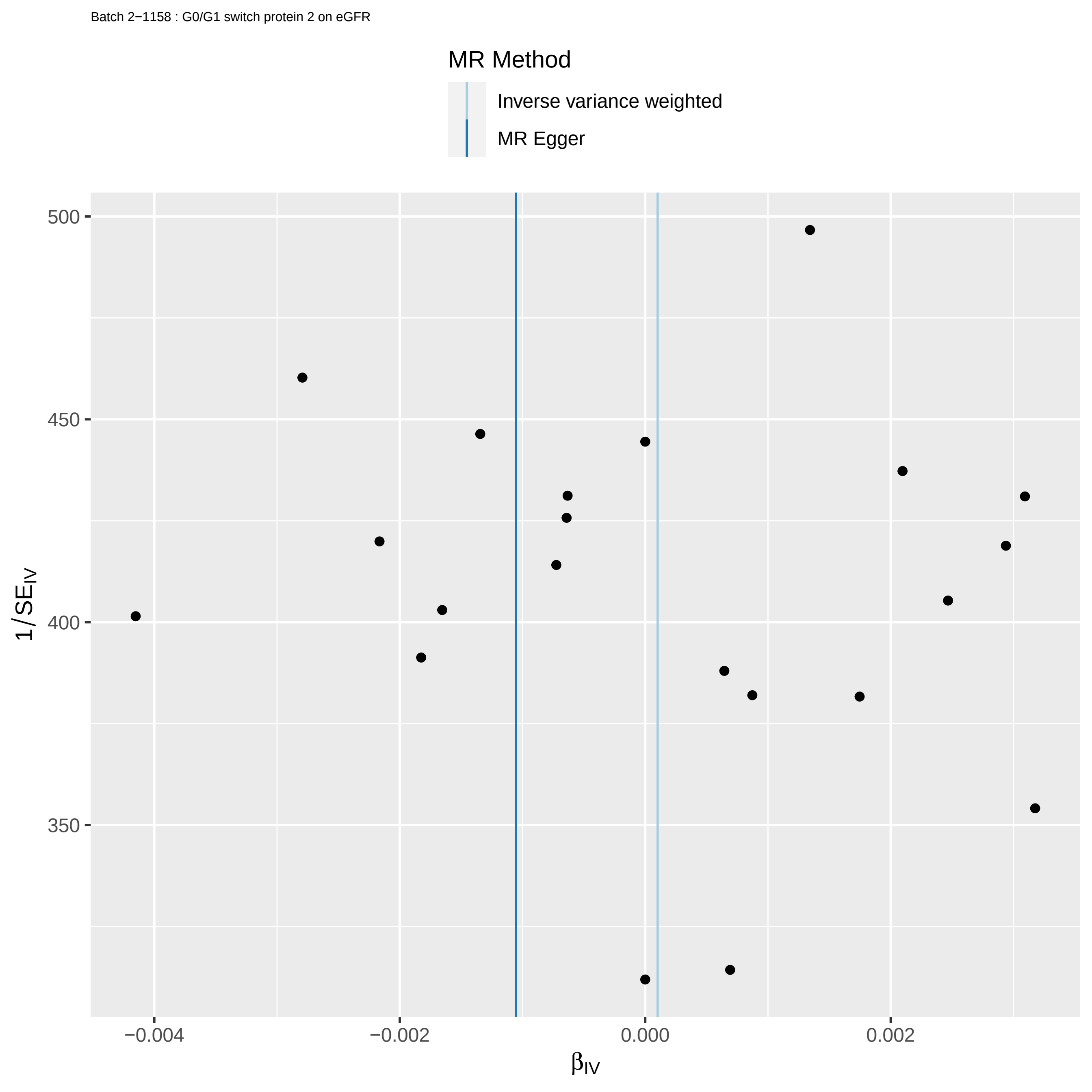
Batch 2−1158 : G0/G1 switch protein 2 on eGFR
MR Method
Inverse variance weighted
MR Egger
500
450
400
350
1/SE_IV
−0.004
−0.002
0.000
0.002
β_IV

Batch 2−1159 : Protein G6b on eGFR

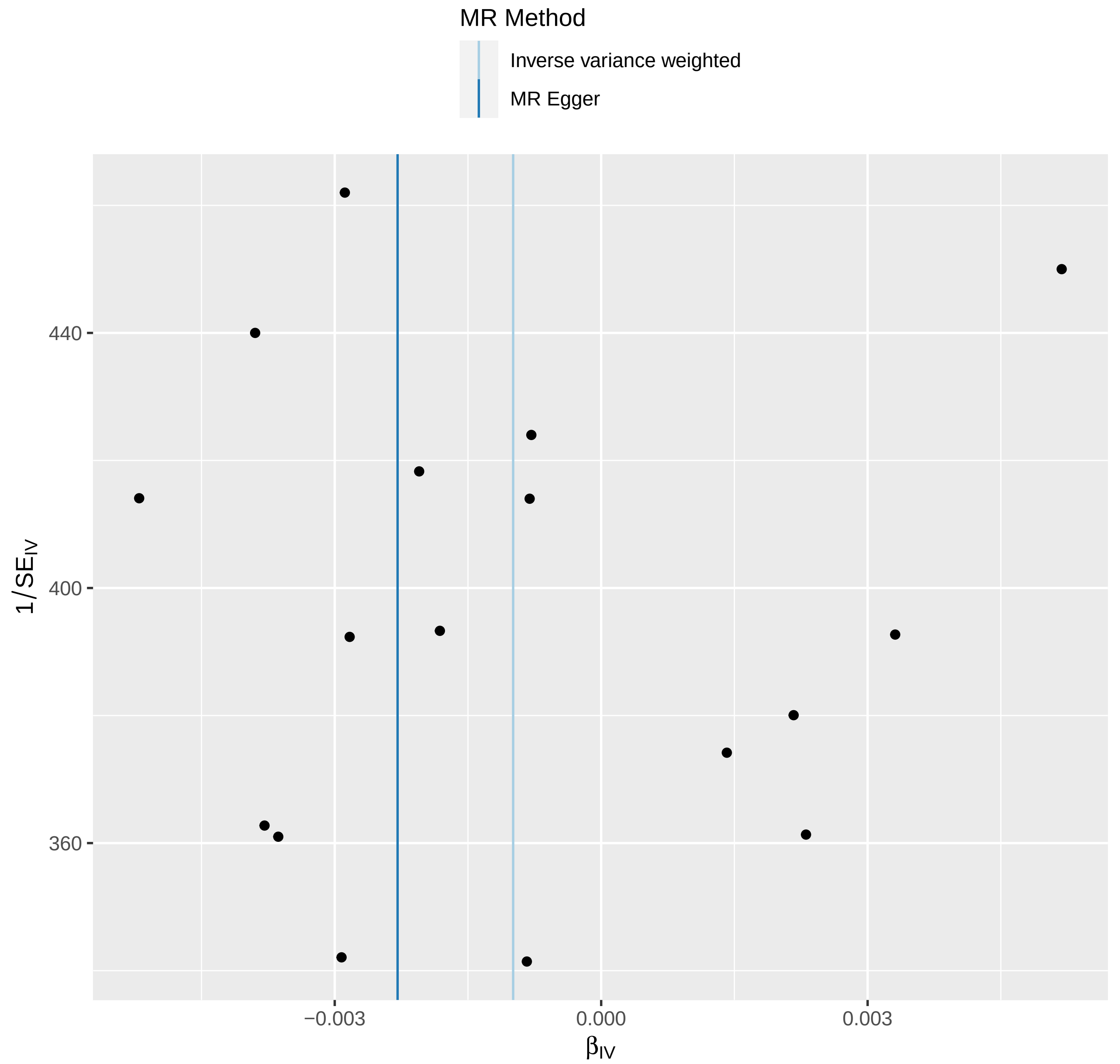

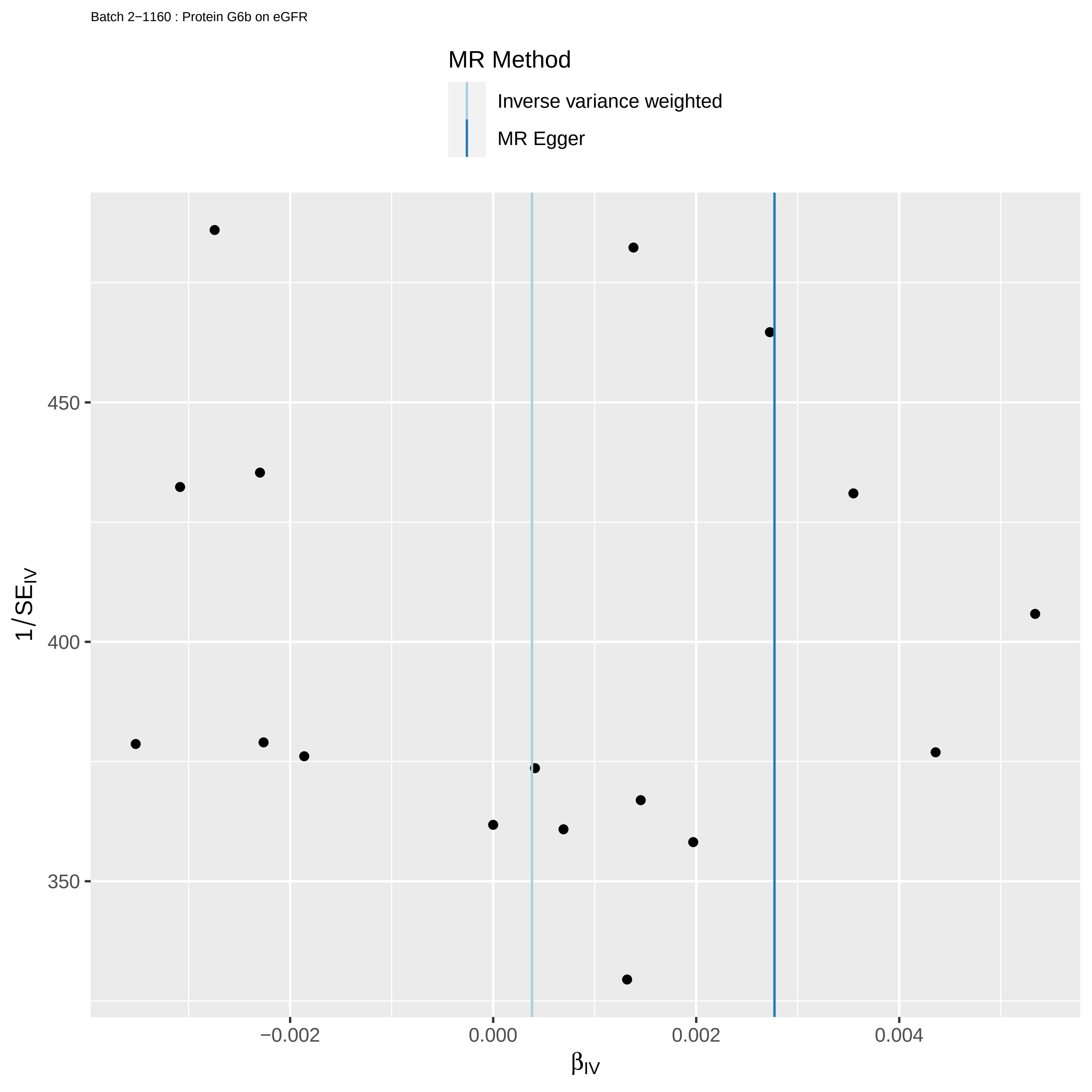
Batch 2−1160 : Protein G6b on eGFR
MR Method
Inverse variance weighted
MR Egger
450
400
350
1/SE$_{IV}$
−0.002
0.000
0.002
0.004
β$_{IV}$

Batch 2−1161 : Gamma−aminobutyric acid receptor−associated protein−like 1 on eGFR

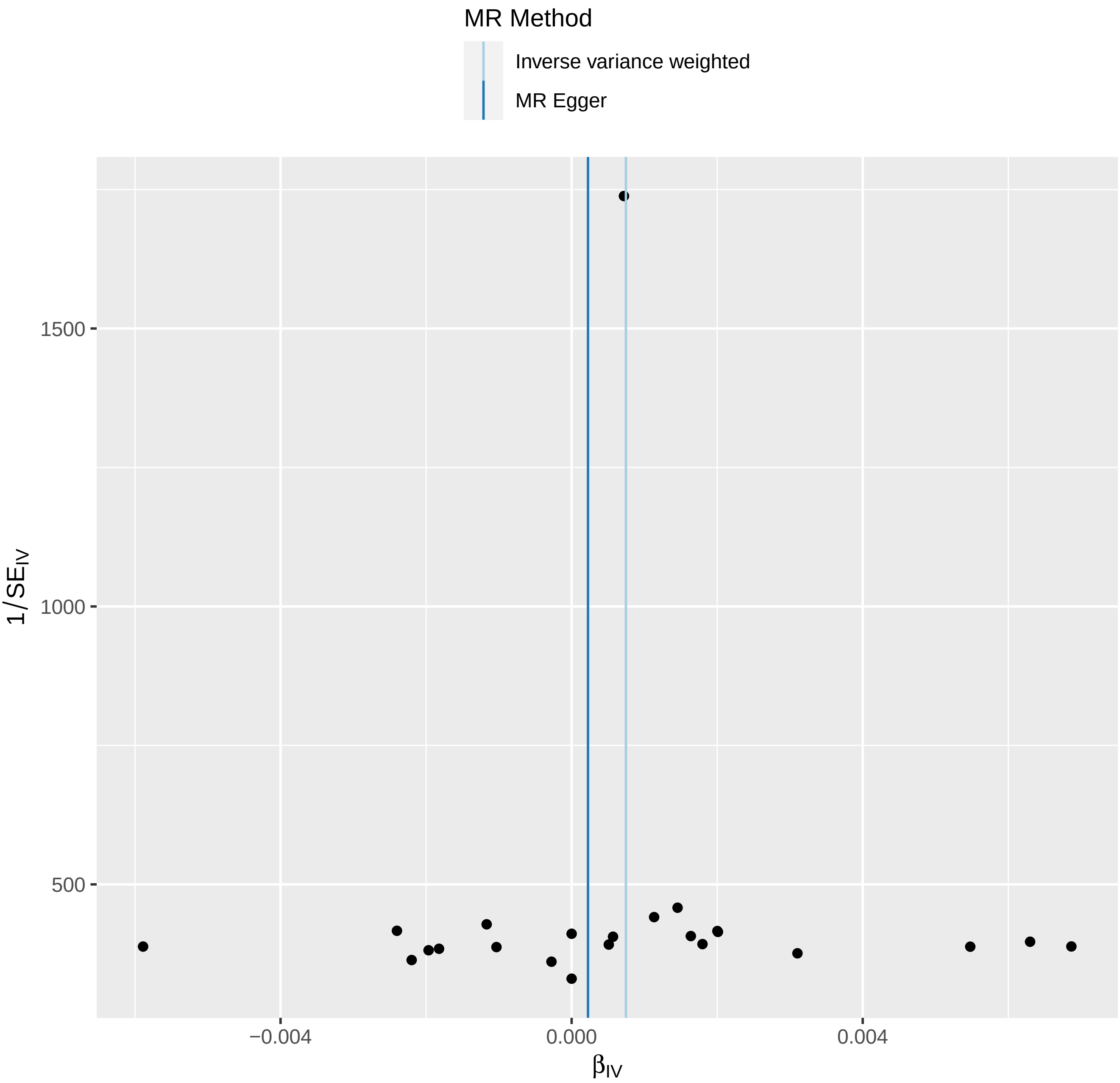

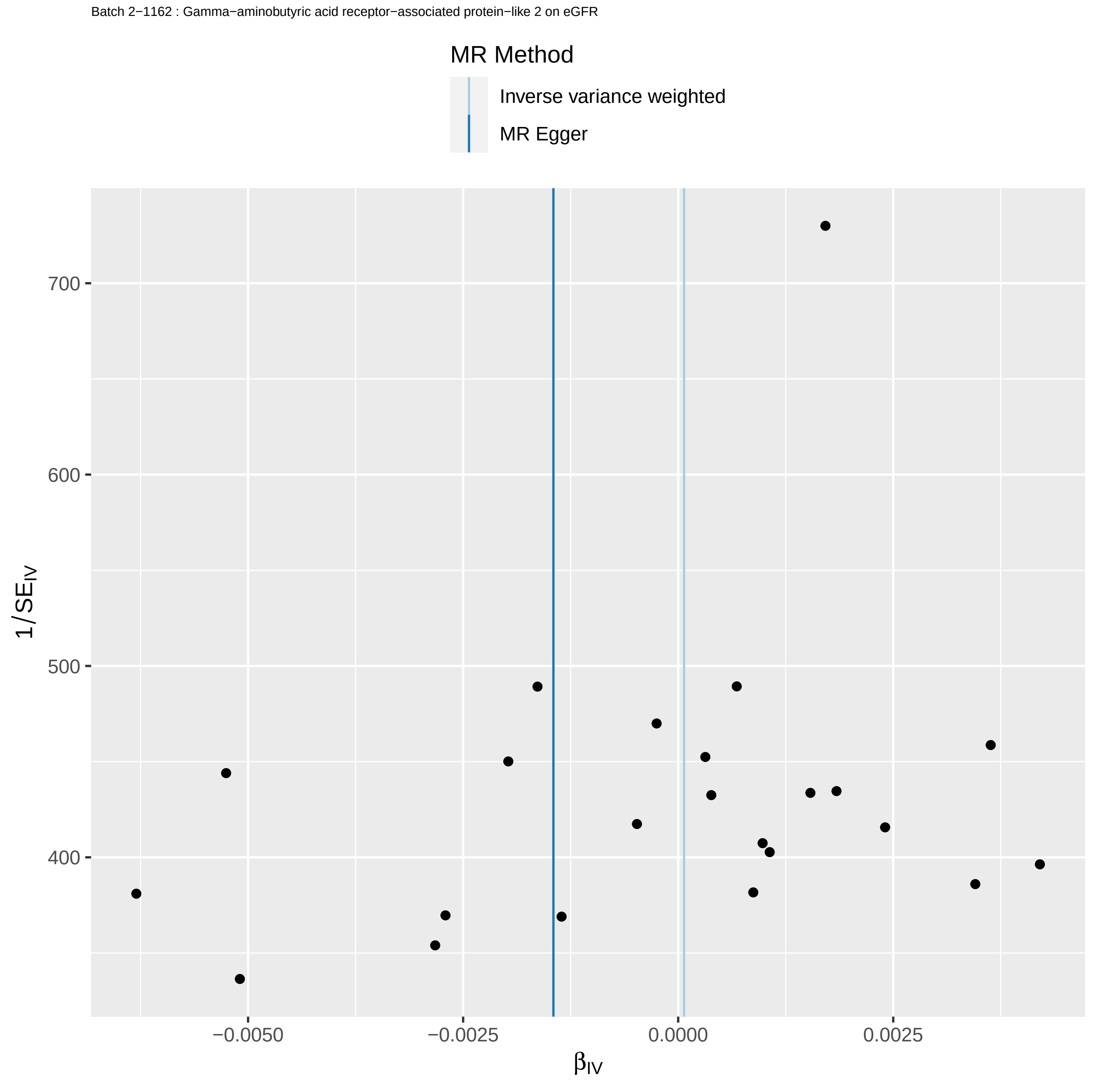

Batch 2−1162 : Gamma−aminobutyric acid receptor−associated protein−like 2 on eGFR
MR Method
Inverse variance weighted
MR Egger
700
600
500
400
1/SE$_{IV}$
−0.0050
−0.0025
0.0000
0.0025
β$_{IV}$

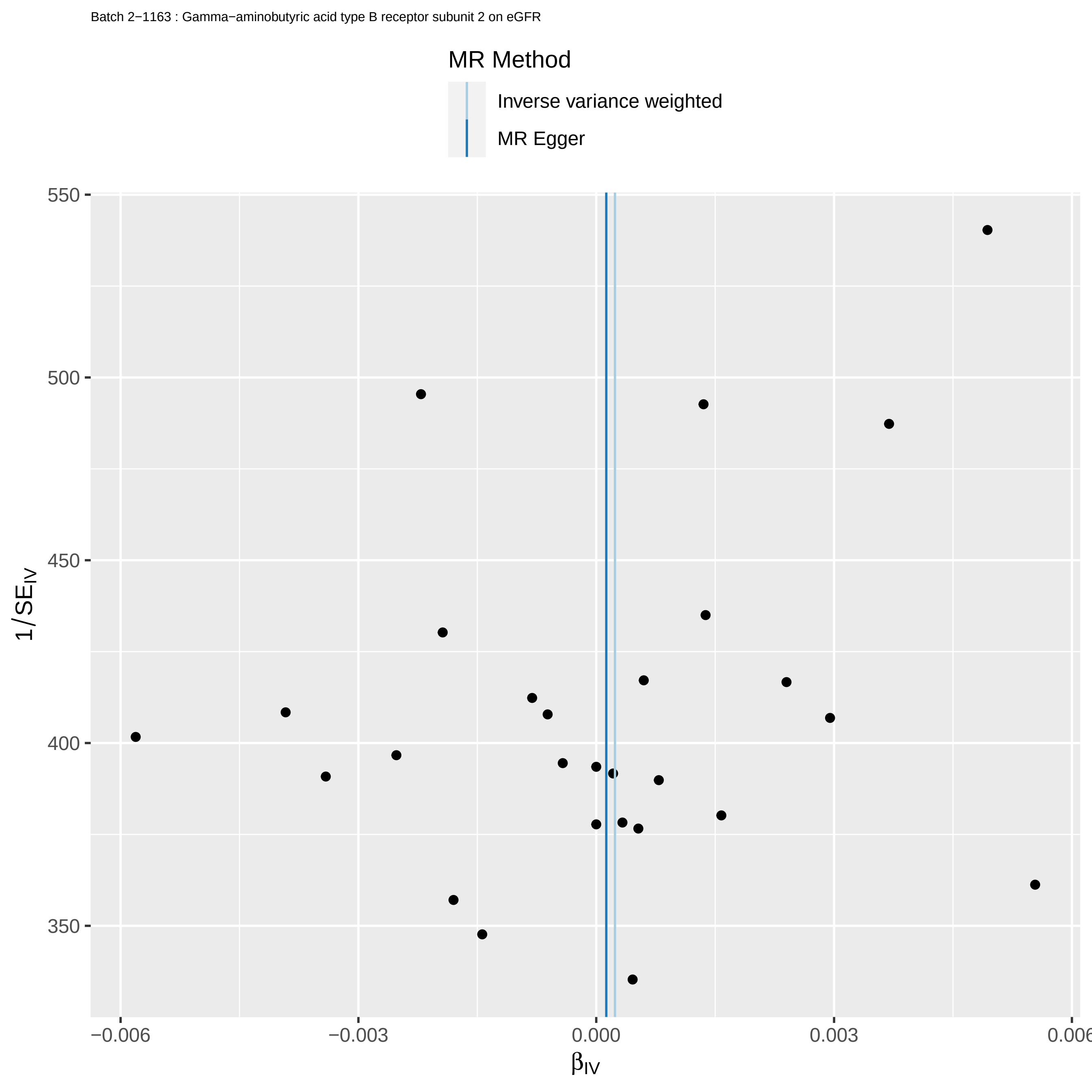

Batch 2−1163 : Gamma−aminobutyric acid type B receptor subunit 2 on eGFR
MR Method
Inverse variance weighted
MR Egger
550
500
450
400
350
1/SE$_{IV}$
−0.006
−0.003
0.000
0.003
0.006
β$_{IV}$

Batch 2−1164 : Gamma−aminobutyric acid type B receptor subunit 2 on eGFR

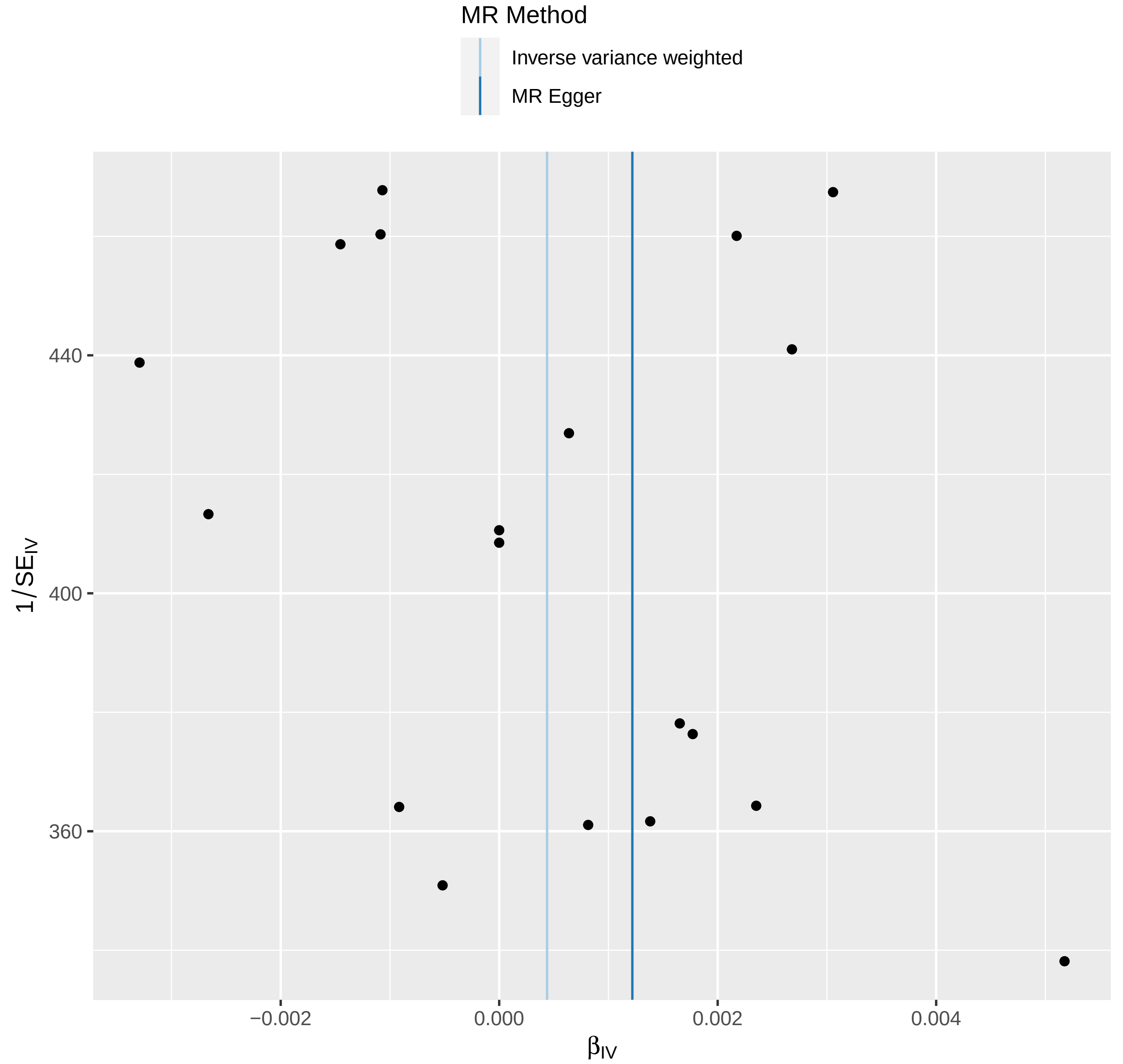

Batch 2−1165 : Growth arrest and DNA damage−inducible proteins−interacting protein 1 on eGFR

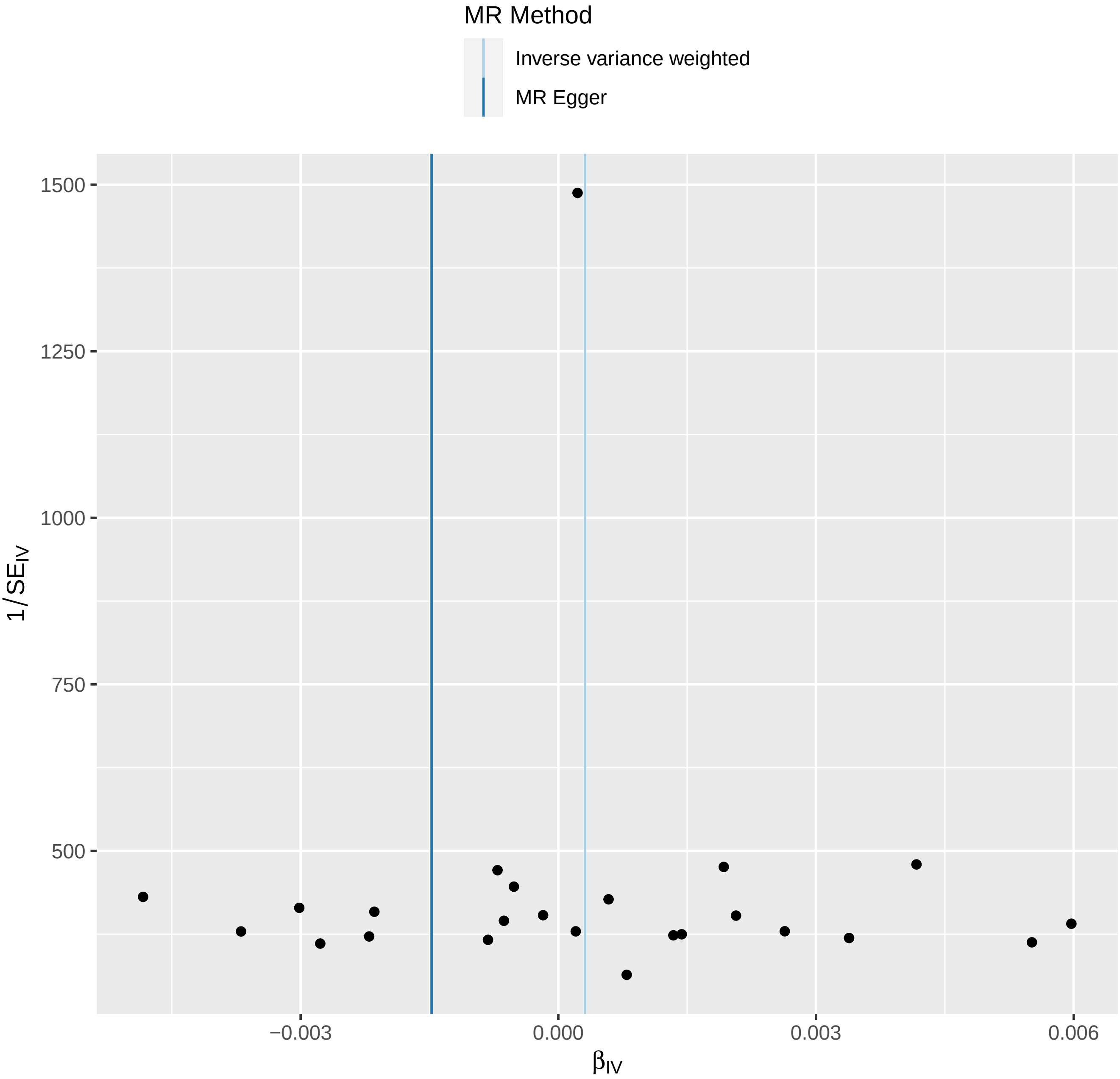

Batch 2−1166 : Galanin peptides on eGFR

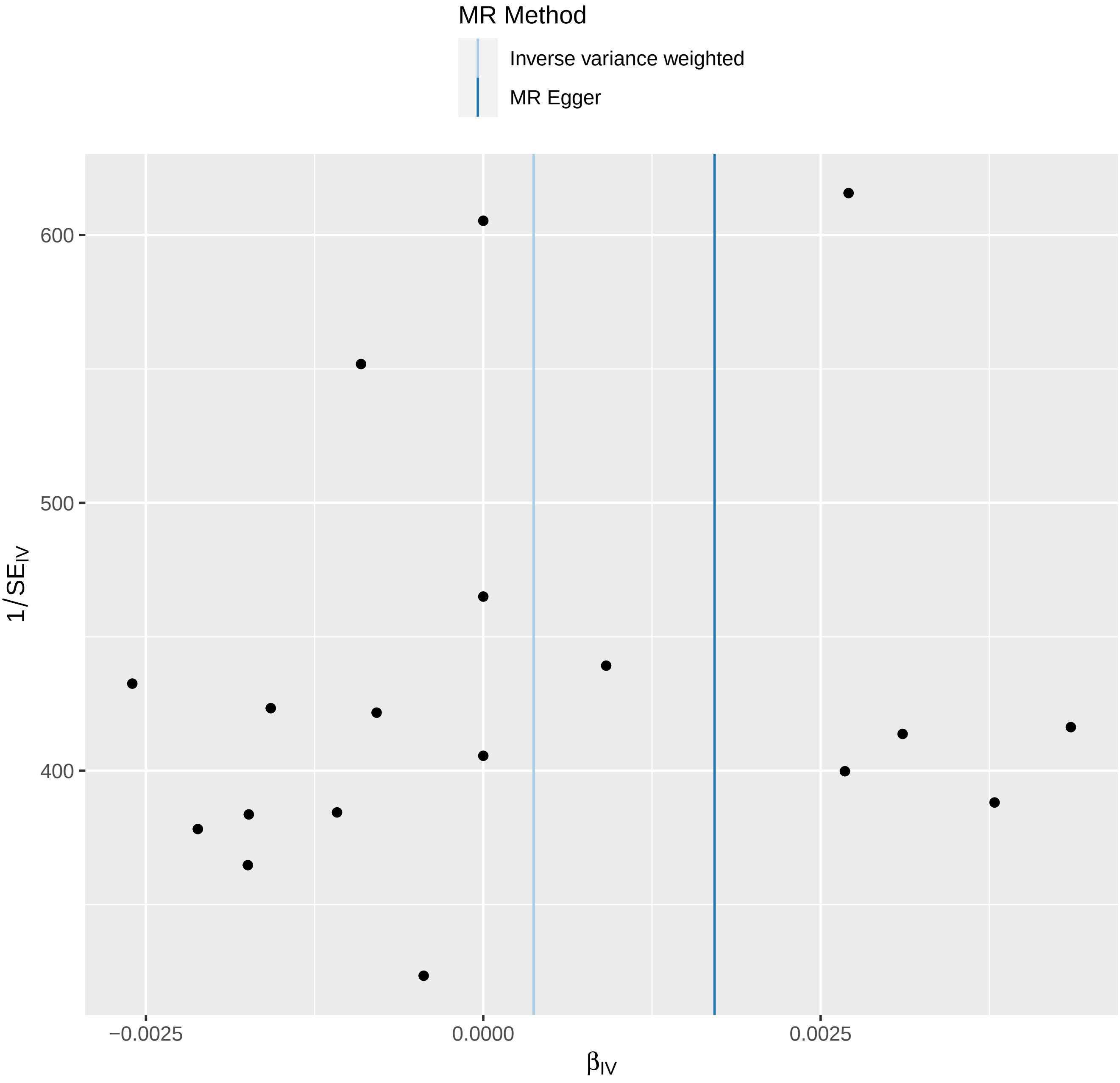

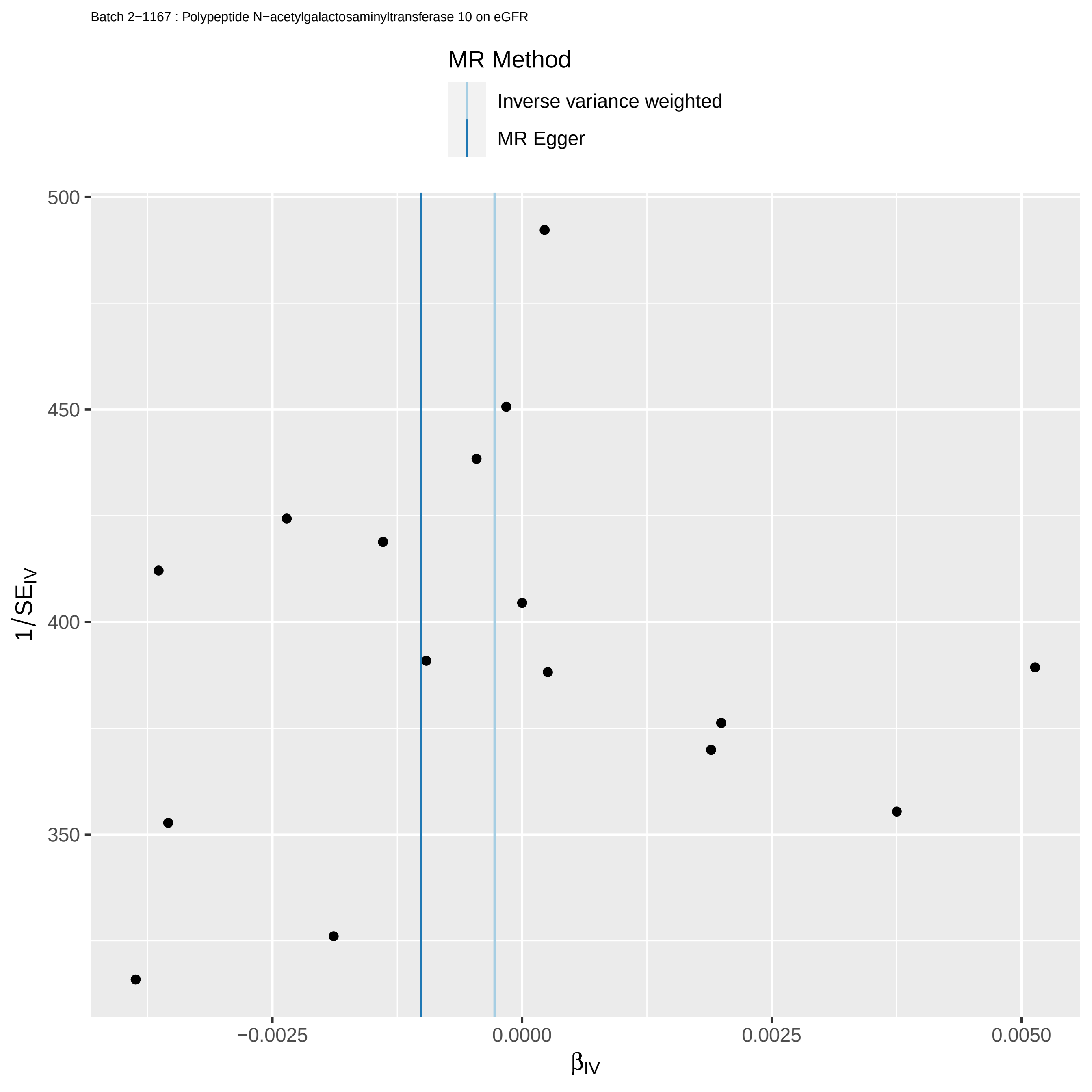

Batch 2−1167 : Polypeptide N−acetylgalactosaminyltransferase 10 on eGFR
MR Method
Inverse variance weighted
MR Egger
500
450
400
350
1/SE$_{IV}$
−0.0025
0.0000
0.0025
0.0050
β$_{IV}$

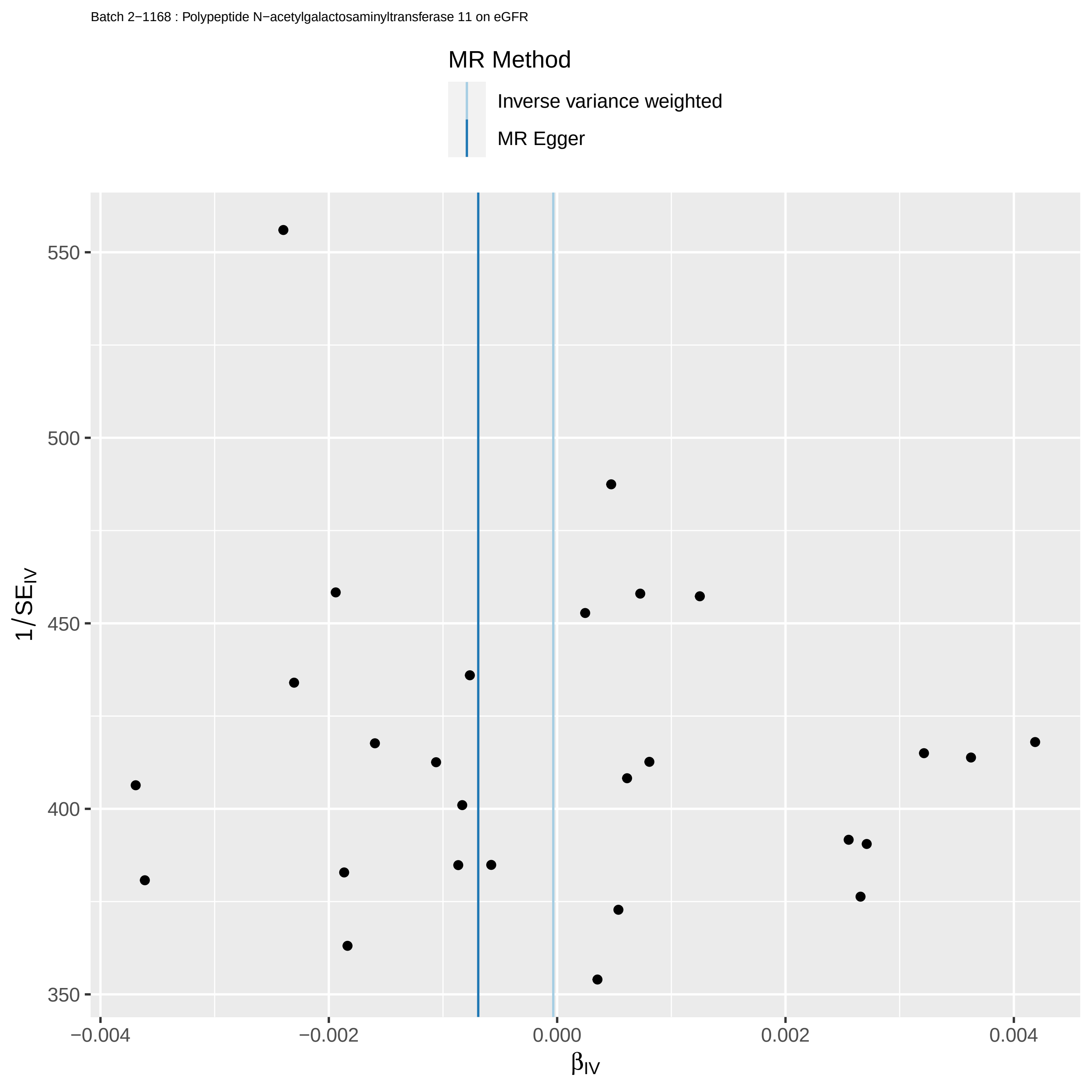
Batch 2−1168 : Polypeptide N−acetylgalactosaminyltransferase 11 on eGFR
MR Method
Inverse variance weighted
MR Egger
550
500
450
400
350
1/SE_IV
−0.004
−0.002
0.000
0.002
0.004
β_IV

Batch 2−1169 : Polypeptide N−acetylgalactosaminyltransferase 16 on eGFR

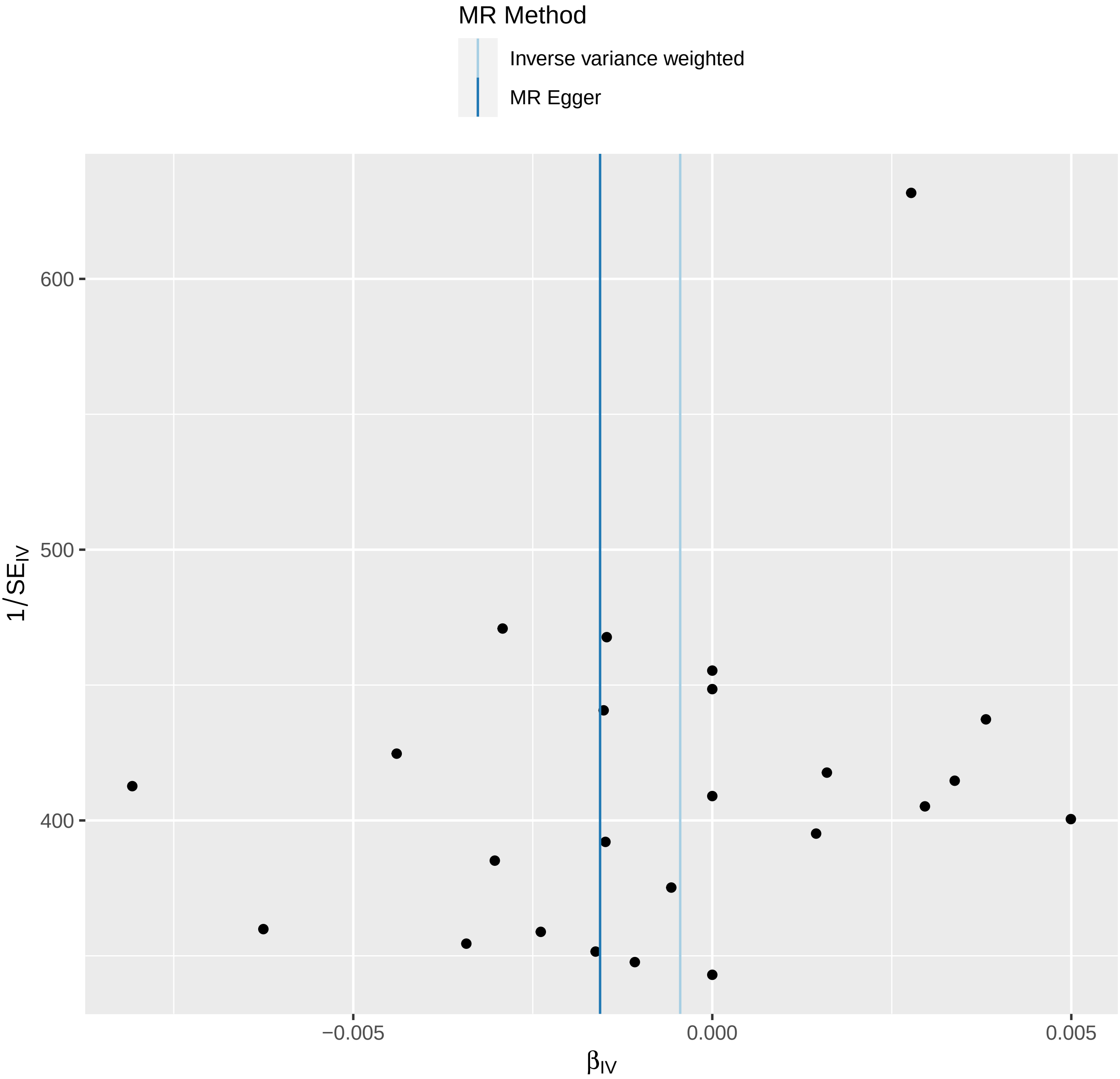

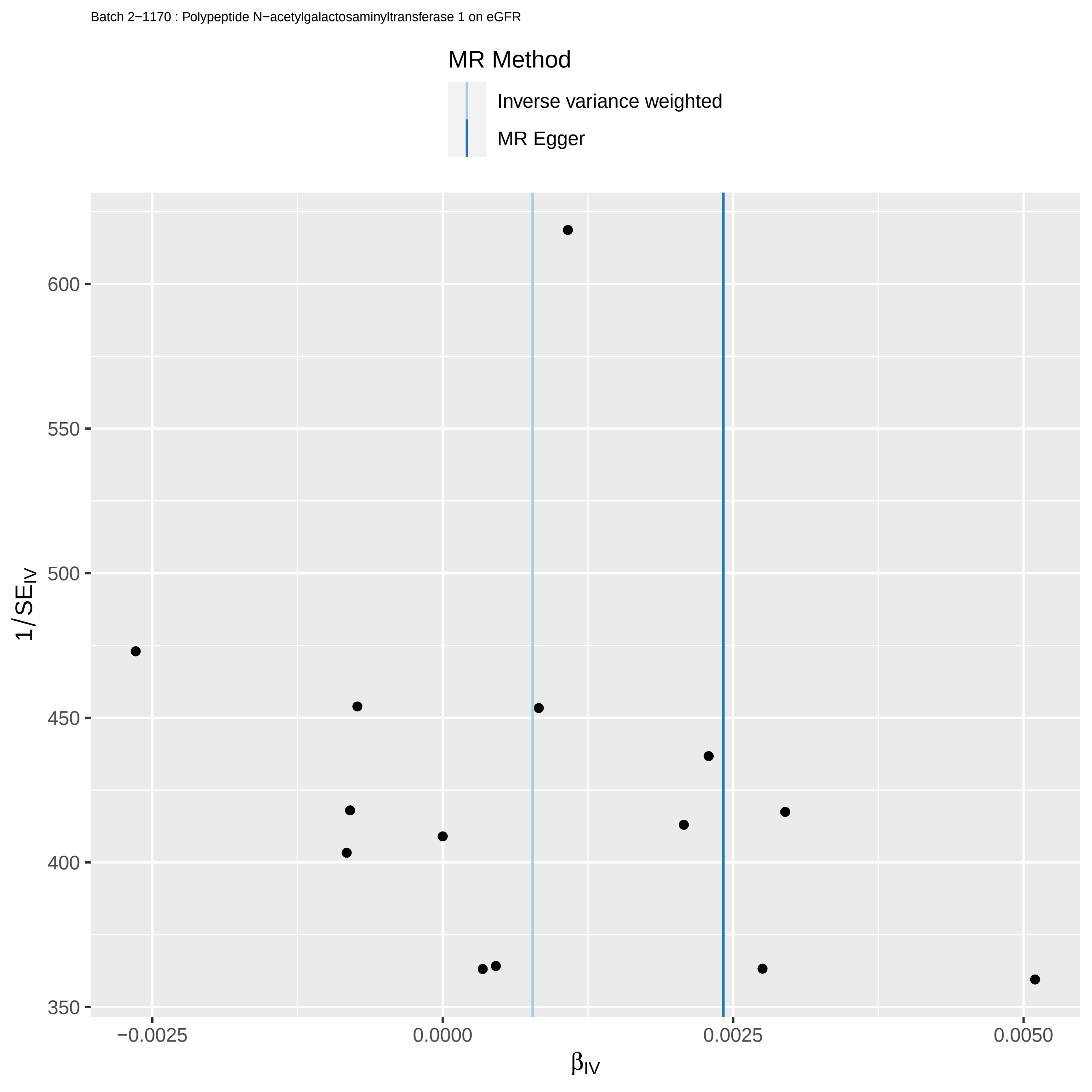

Batch 2−1170 : Polypeptide N−acetylgalactosaminyltransferase 1 on eGFR
MR Method
Inverse variance weighted
MR Egger
600
550
500
450
400
350
1/SE$_{IV}$
−0.0025
0.0000
0.0025
0.0050
β$_{IV}$

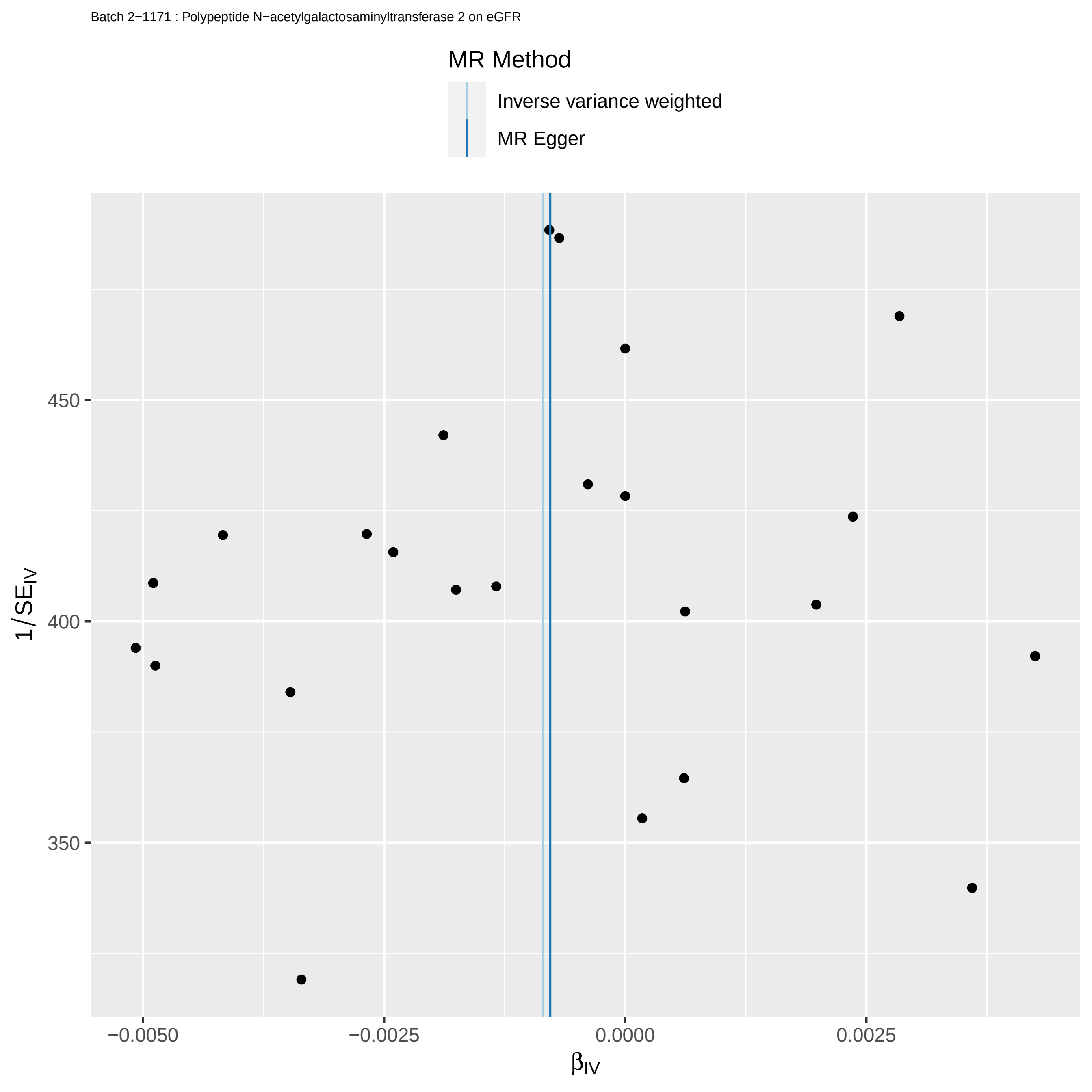
Batch 2−1171 : Polypeptide N−acetylgalactosaminyltransferase 2 on eGFR
MR Method
Inverse variance weighted
MR Egger
450
400
350
1/SE$_{IV}$
−0.0050
−0.0025
0.0000
0.0025
β$_{IV}$

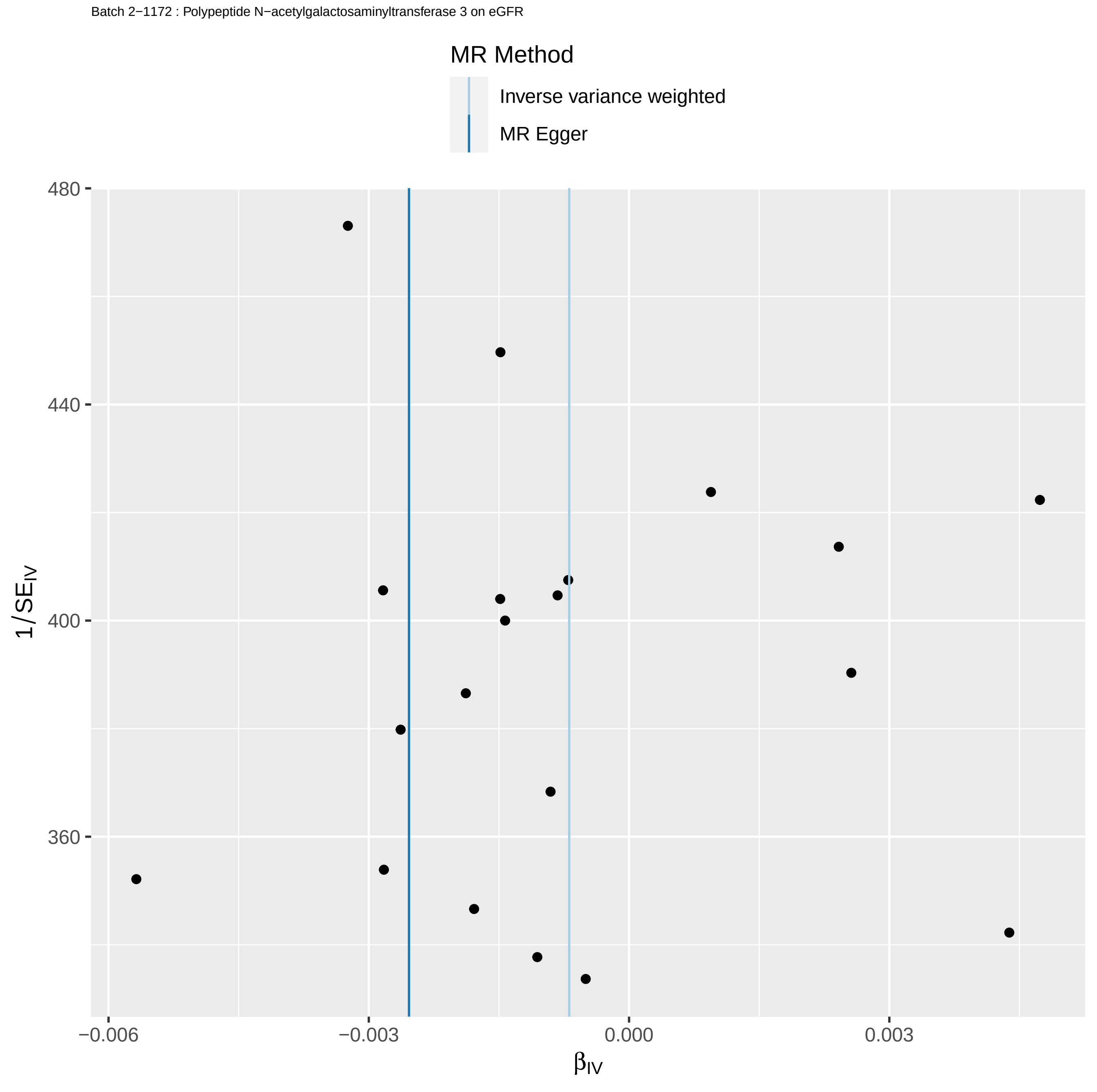

Batch 2−1172 : Polypeptide N−acetylgalactosaminyltransferase 3 on eGFR
MR Method
Inverse variance weighted
MR Egger
480
440
400
360
1/SE_IV
−0.006
−0.003
0.000
0.003
β_IV

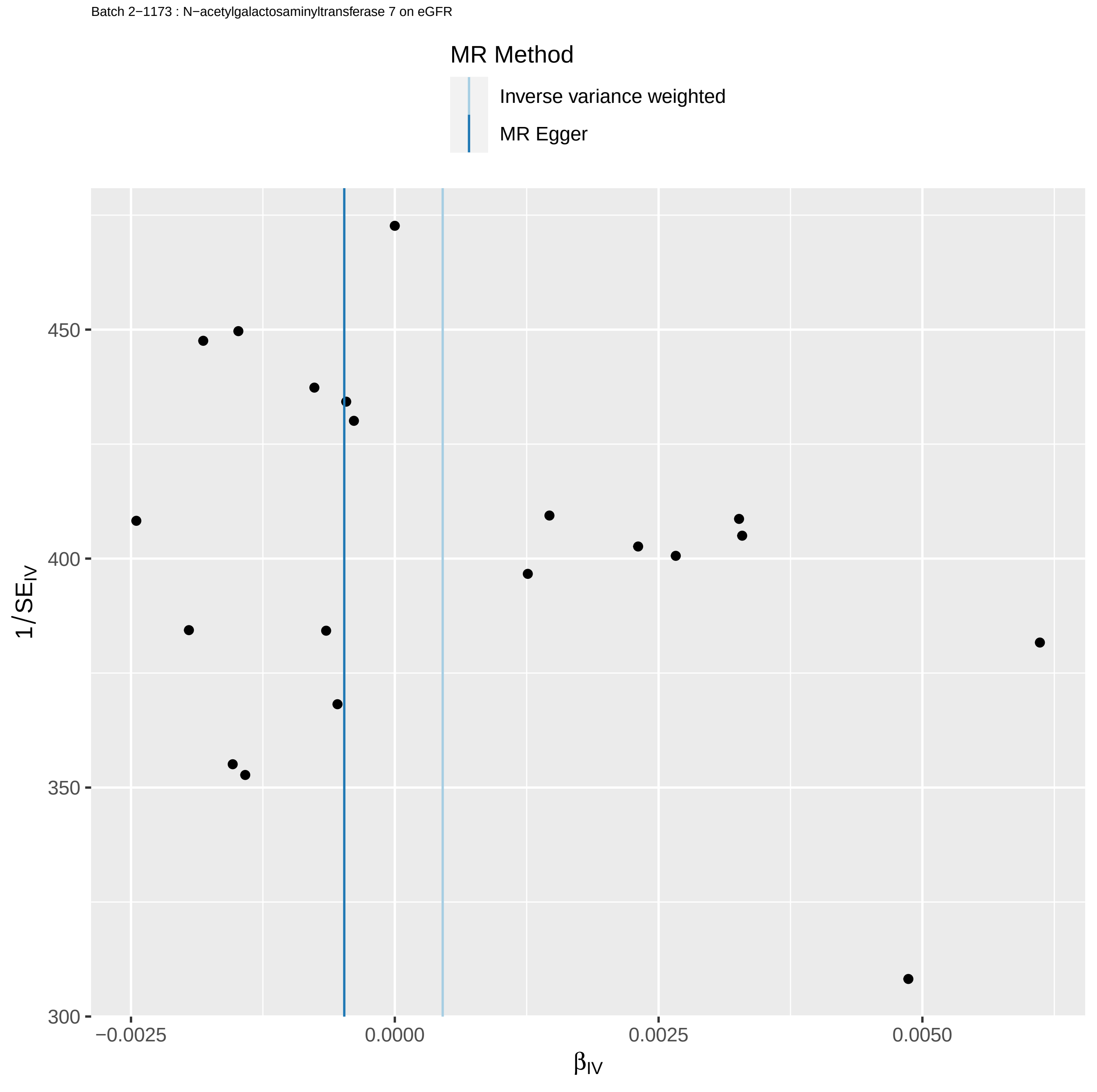

Batch 2−1173 : N−acetylgalactosaminyltransferase 7 on eGFR
MR Method
Inverse variance weighted
MR Egger
450
400
350
300
1/SE_IV
−0.0025
0.0000
0.0025
0.0050
β_IV

Batch 2−1174 : Galanin−like peptide on eGFR

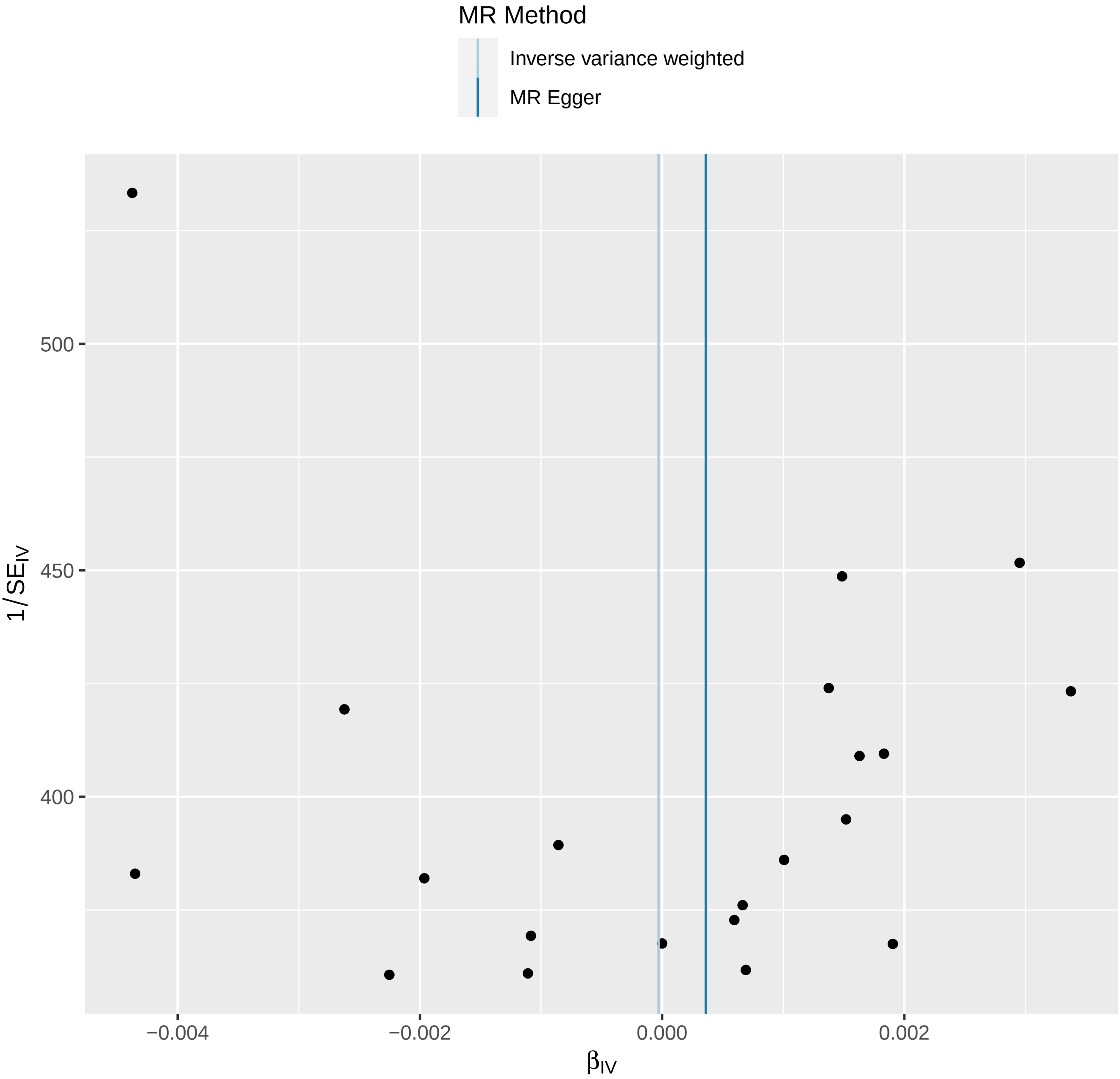

Batch 2−1175 : Gigaxonin on eGFR

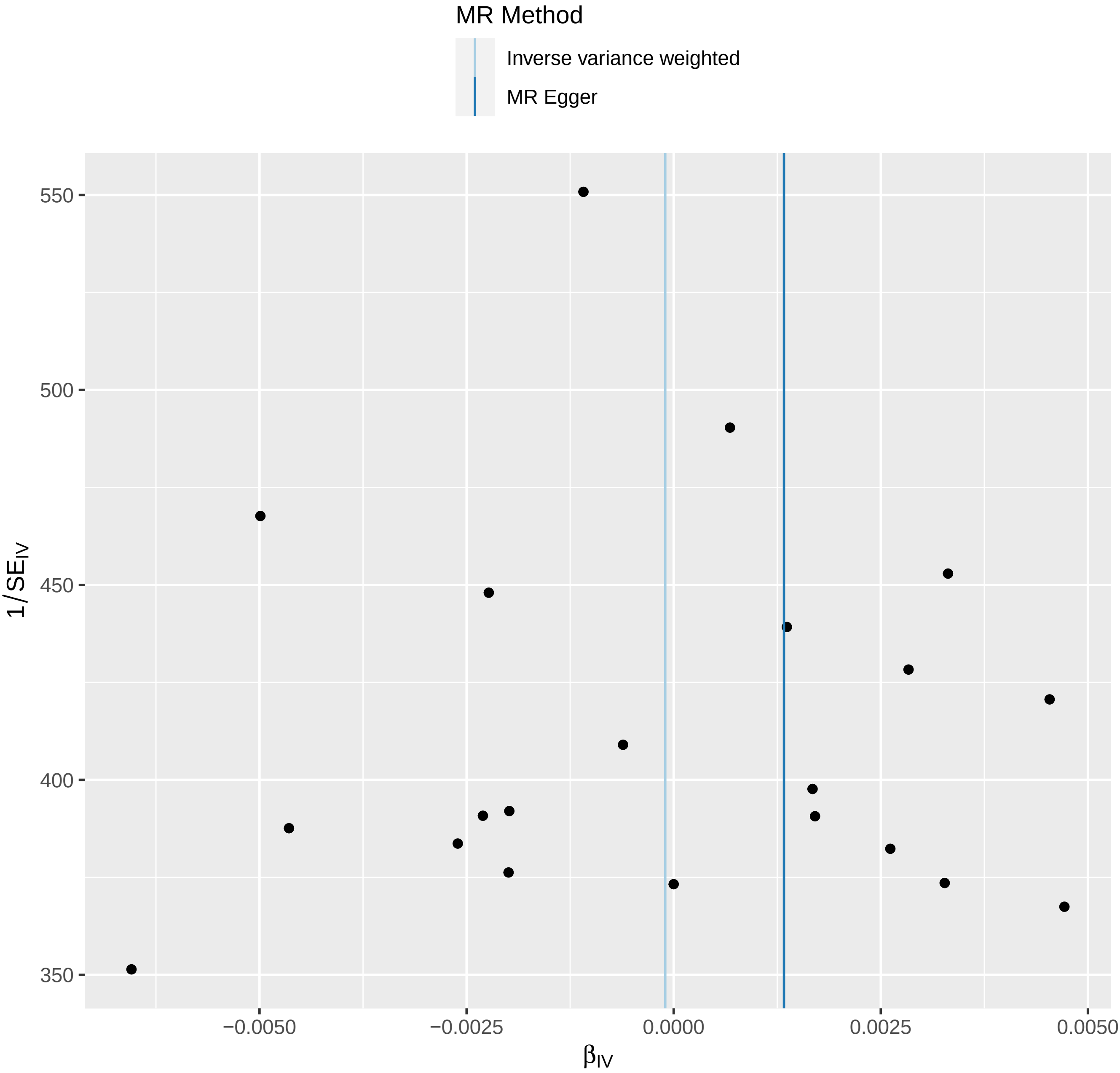

Batch 2−1176 : Glyceraldehyde−3−phosphate dehydrogenase, testis−specific on eGFR

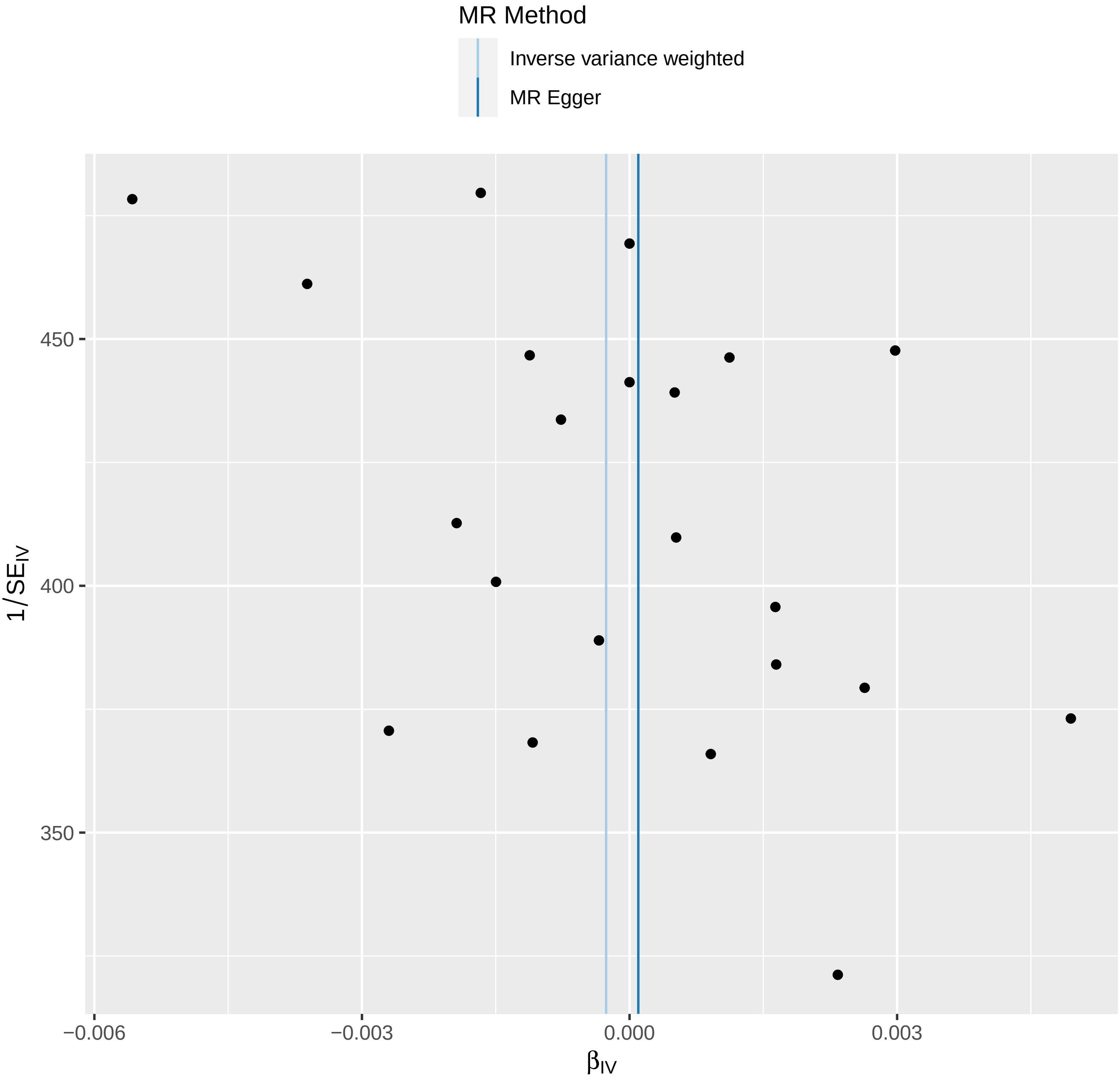

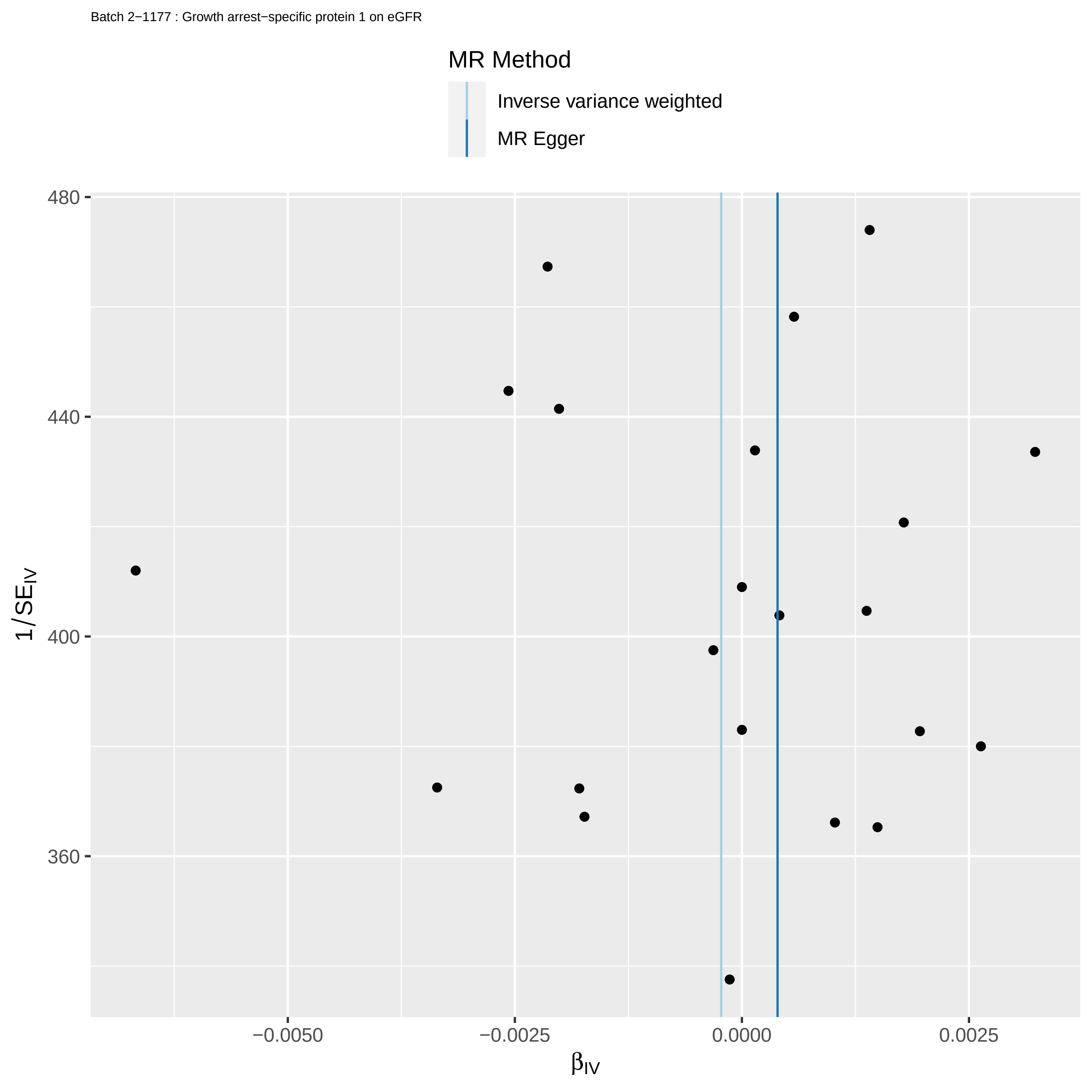

Batch 2−1177 : Growth arrest−specific protein 1 on eGFR
MR Method
Inverse variance weighted
MR Egger
480
440
400
360
1/SE_IV
−0.0050
−0.0025
0.0000
0.0025
β_IV

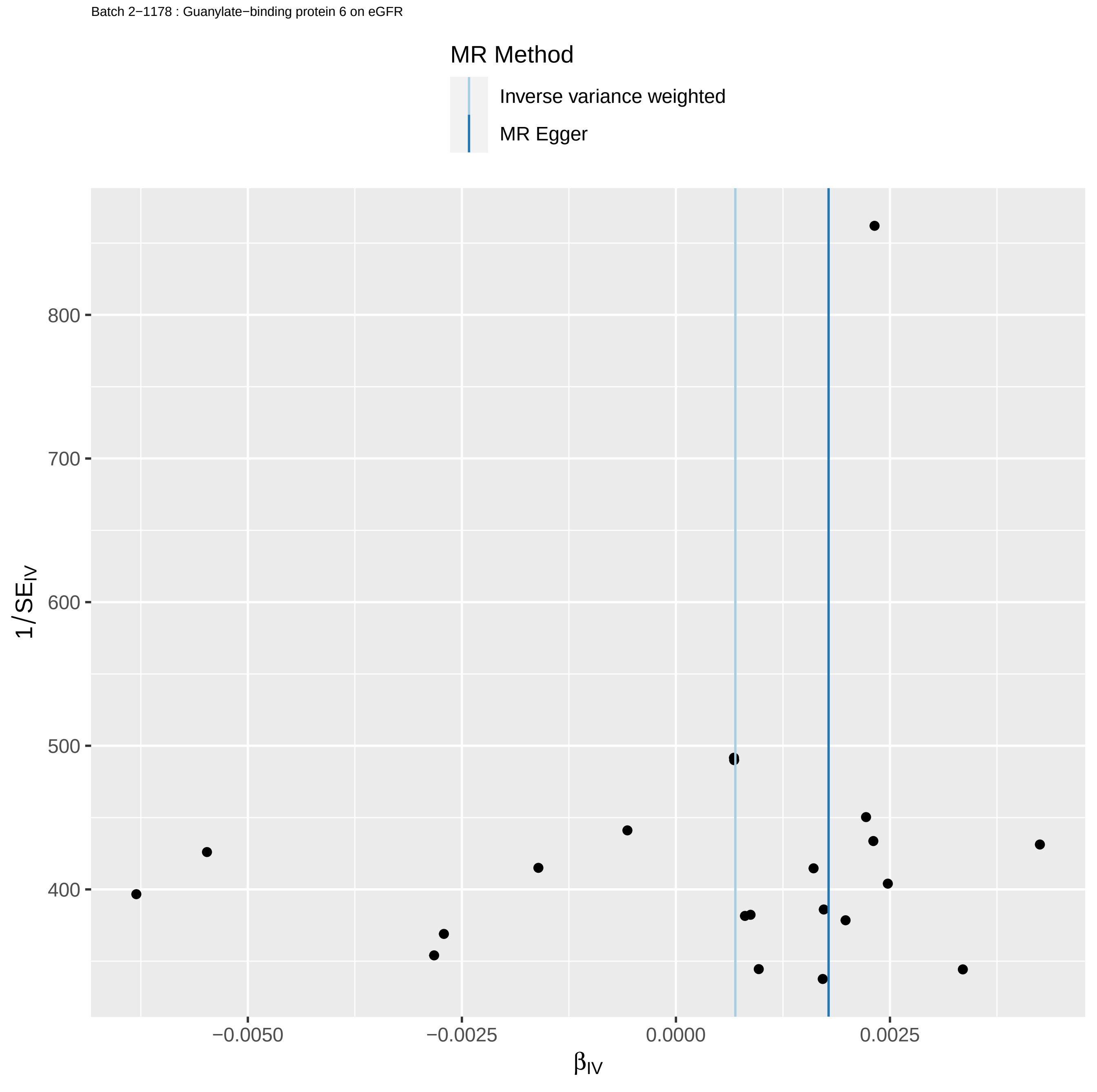

Batch 2−1178 : Guanylate−binding protein 6 on eGFR
MR Method
Inverse variance weighted
MR Egger
800
700
600
500
400
1/SE$_{IV}$
−0.0050
−0.0025
0.0000
0.0025
β_{IV}

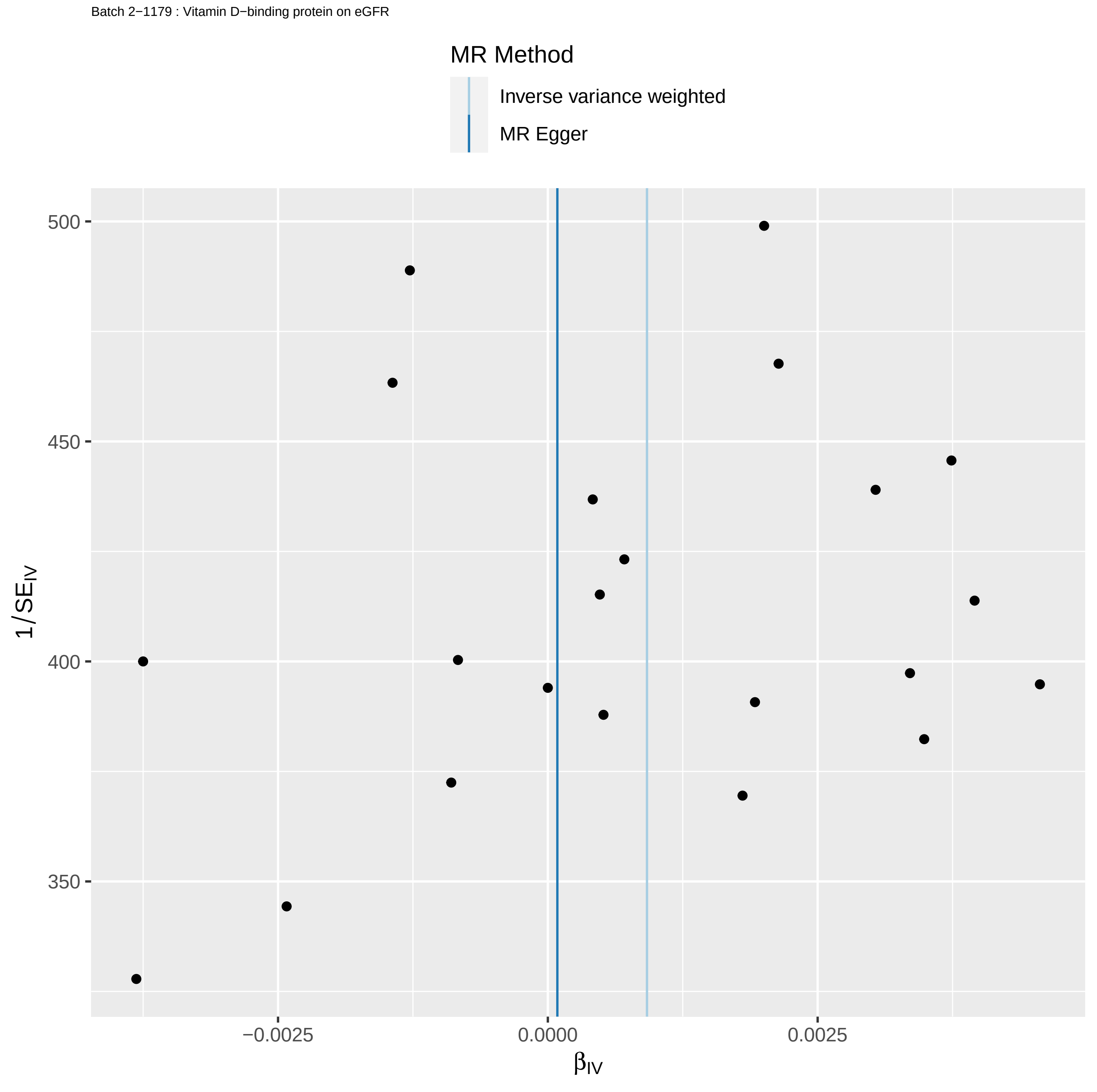

Batch 2−1179 : Vitamin D−binding protein on eGFR
MR Method
Inverse variance weighted
MR Egger
500
450
400
350
1/SE_IV
−0.0025
0.0000
0.0025
β_IV

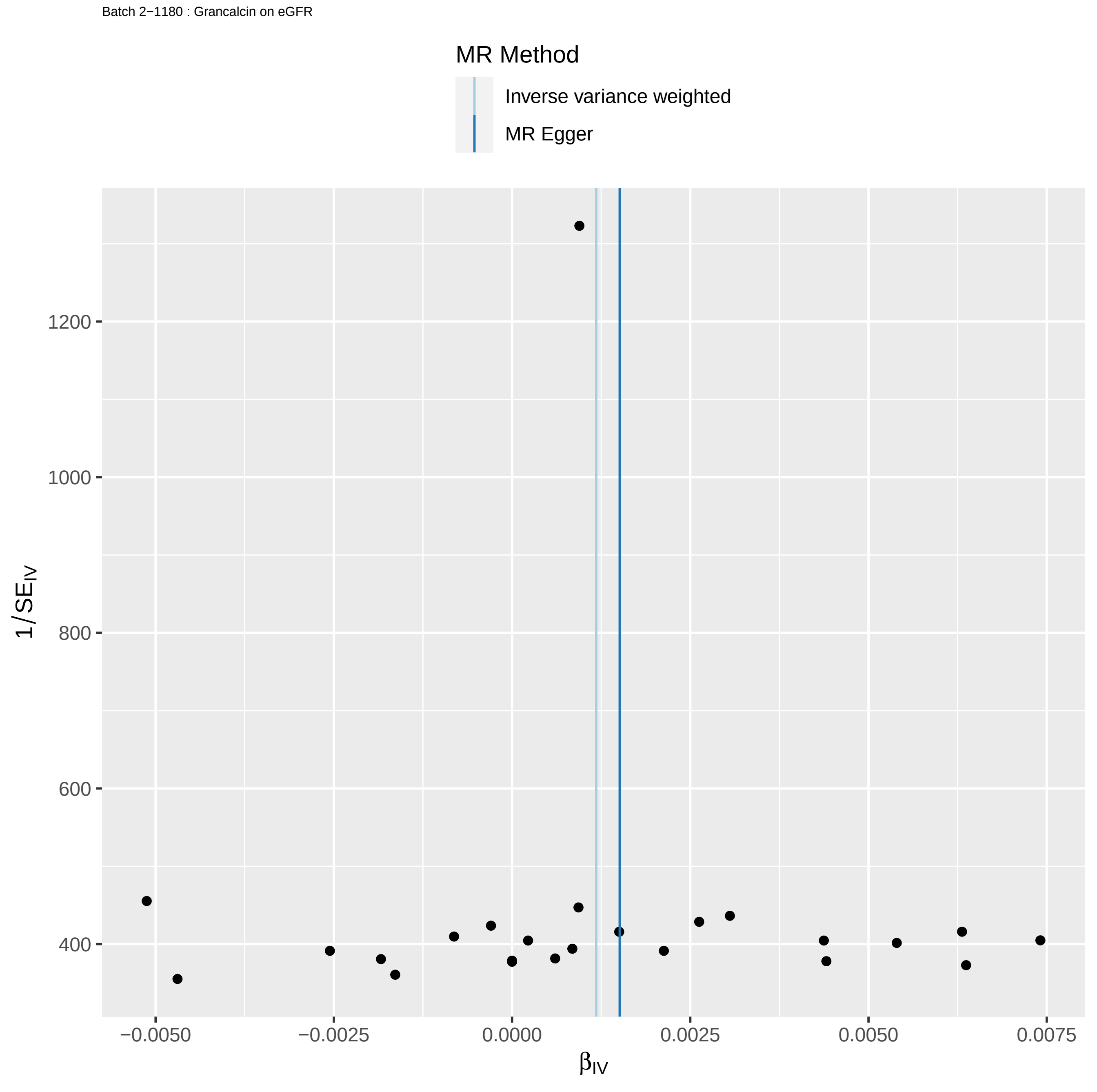

Batch 2−1180 : Grancalcin on eGFR
MR Method
Inverse variance weighted
MR Egger
1200
1000
800
600
400
1/SE$_{IV}$
−0.0050
−0.0025
0.0000
0.0025
0.0050
0.0075
β$_{IV}$

Batch 2−1181 : Glucagon on eGFR

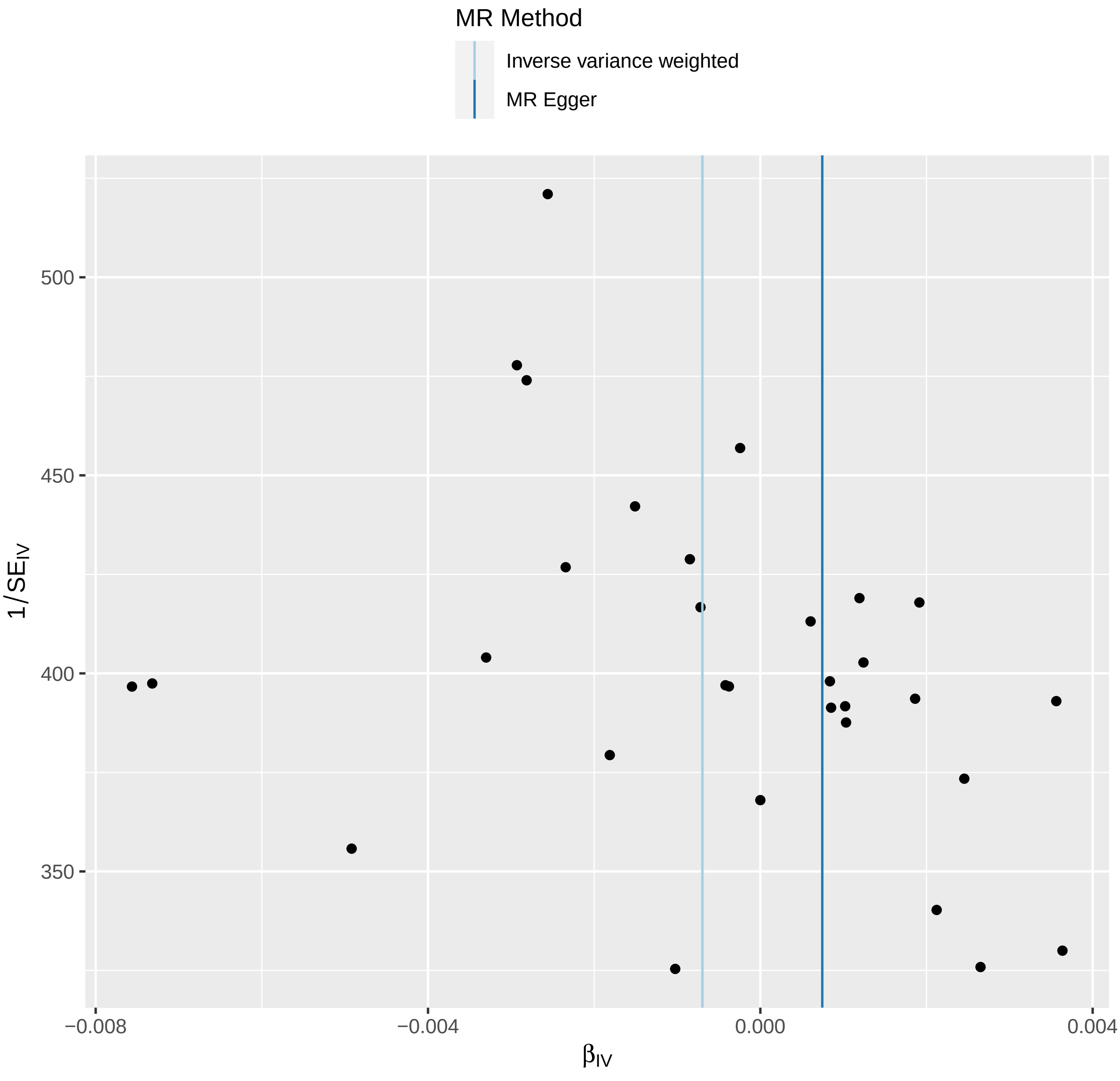

Batch 2−1182 : GTP cyclohydrolase 1 on eGFR

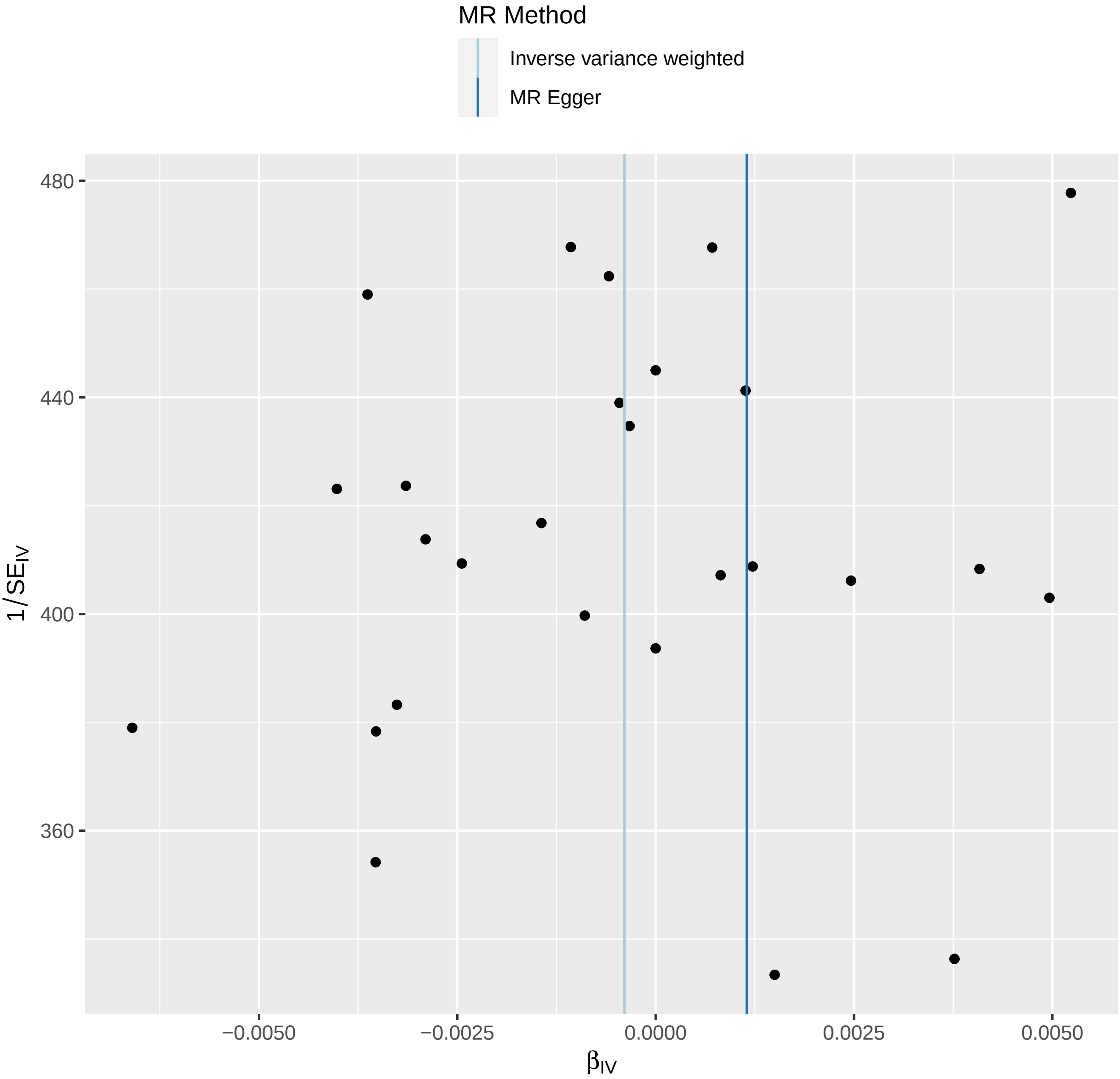

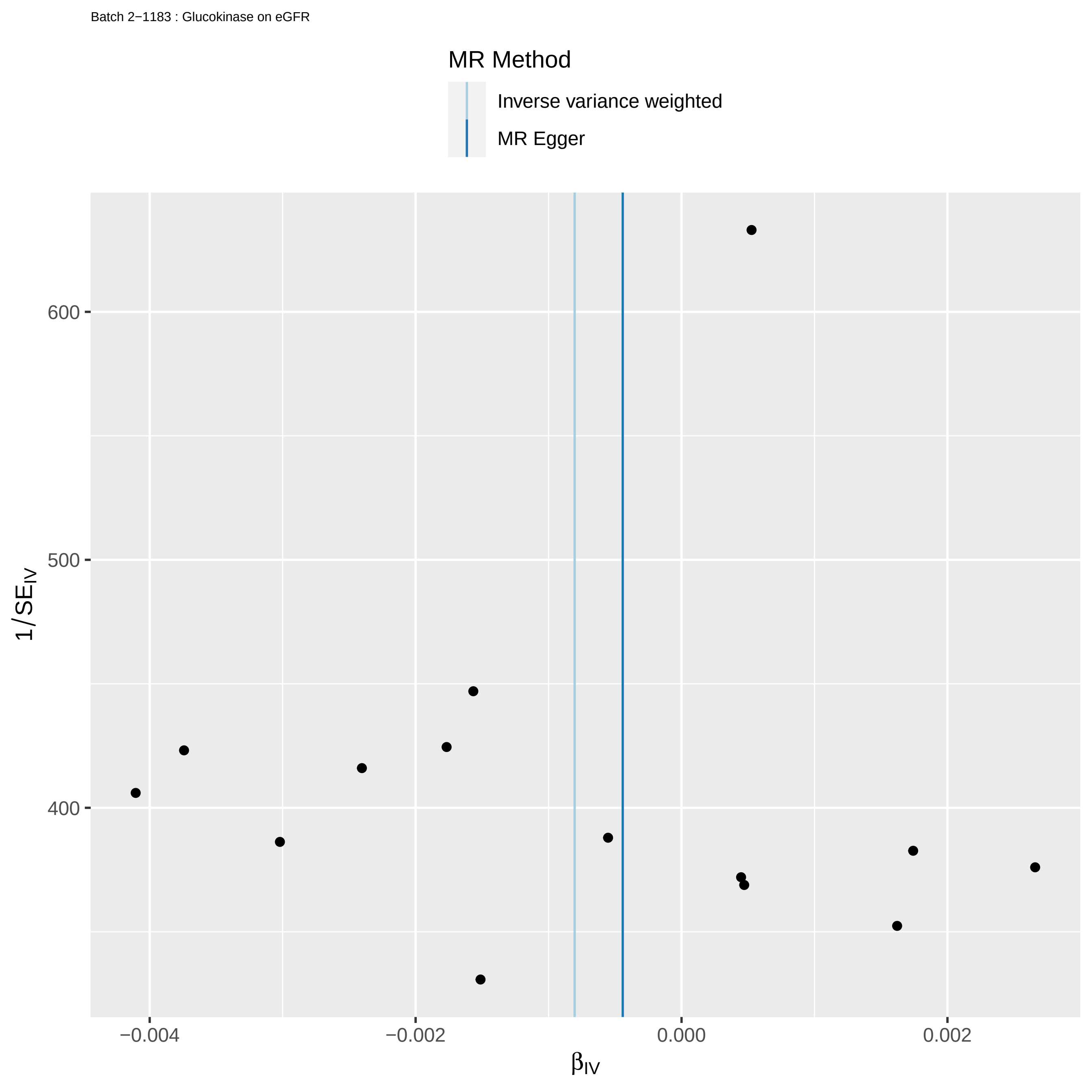
Batch 2−1183 : Glucokinase on eGFR
MR Method
Inverse variance weighted
MR Egger
600
500
400
1/SE$_{IV}$
−0.004
−0.002
0.000
0.002
β$_{IV}$

Batch 2−1184 : Glucokinase regulatory protein on eGFR

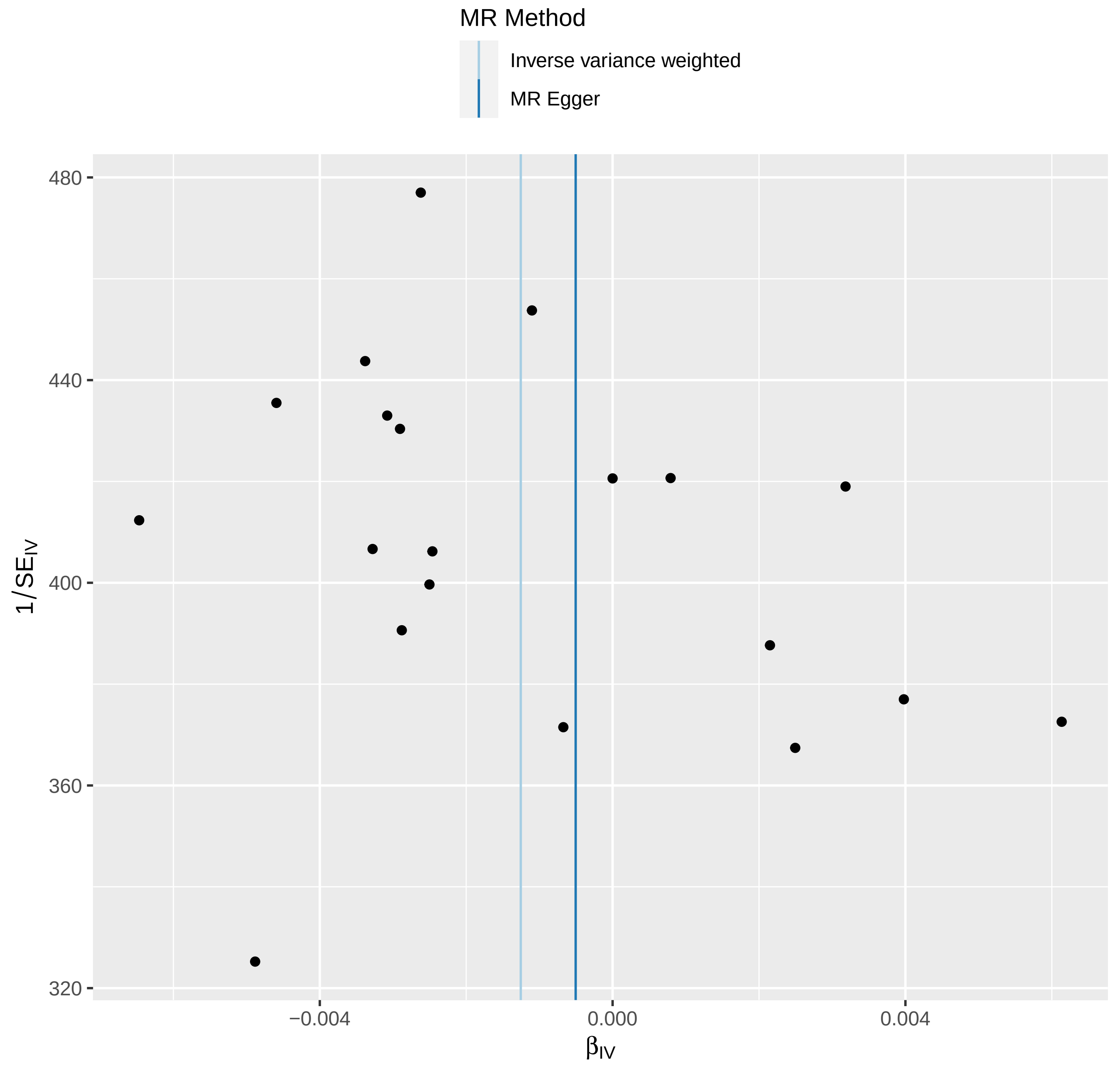

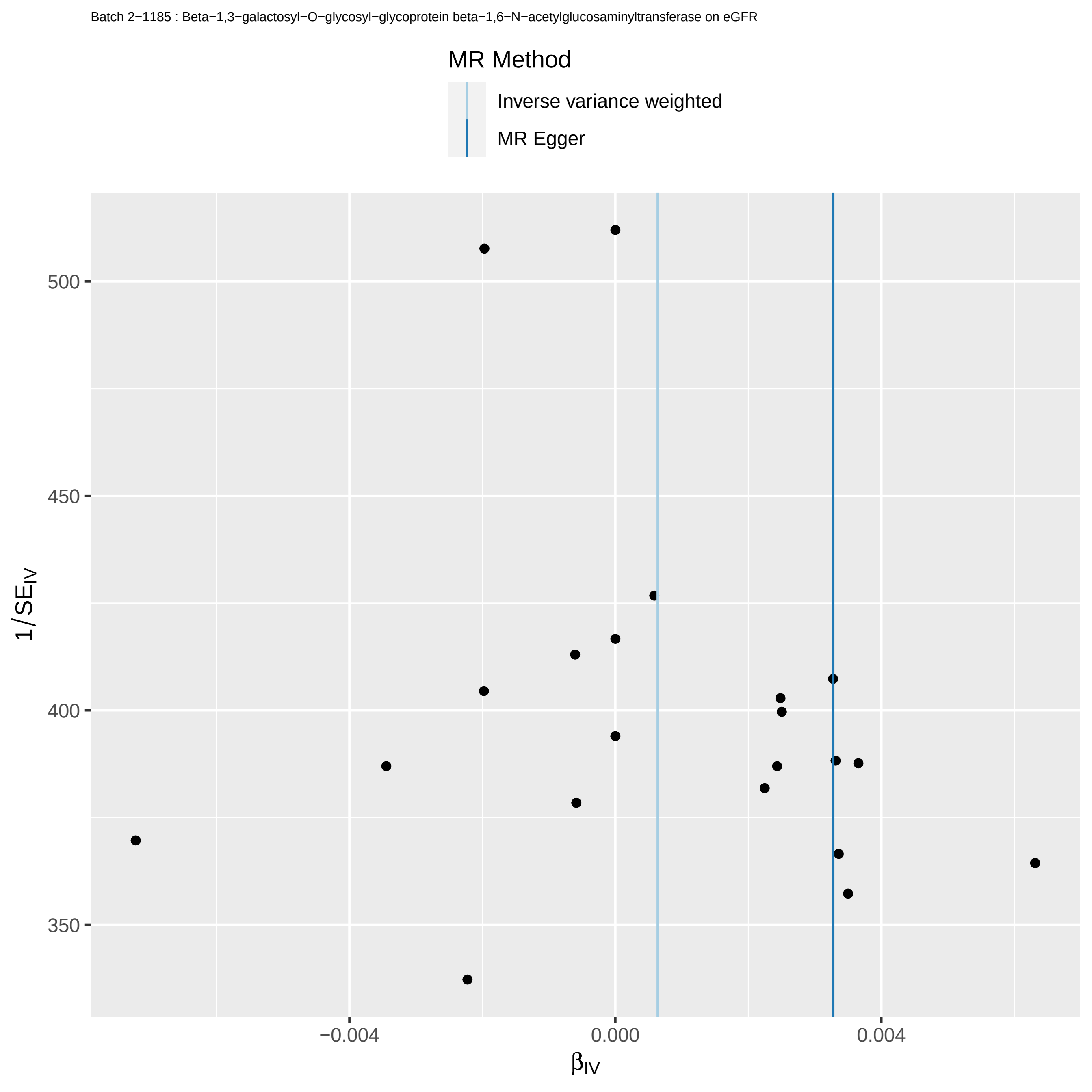

Batch 2−1185 : Beta−1,3−galactosyl−O−glycosyl−glycoprotein beta−1,6−N−acetylglucosaminyltransferase on eGFR
MR Method
Inverse variance weighted
MR Egger
500
450
400
350
1/SE_IV
−0.004
0.000
0.004
β_IV

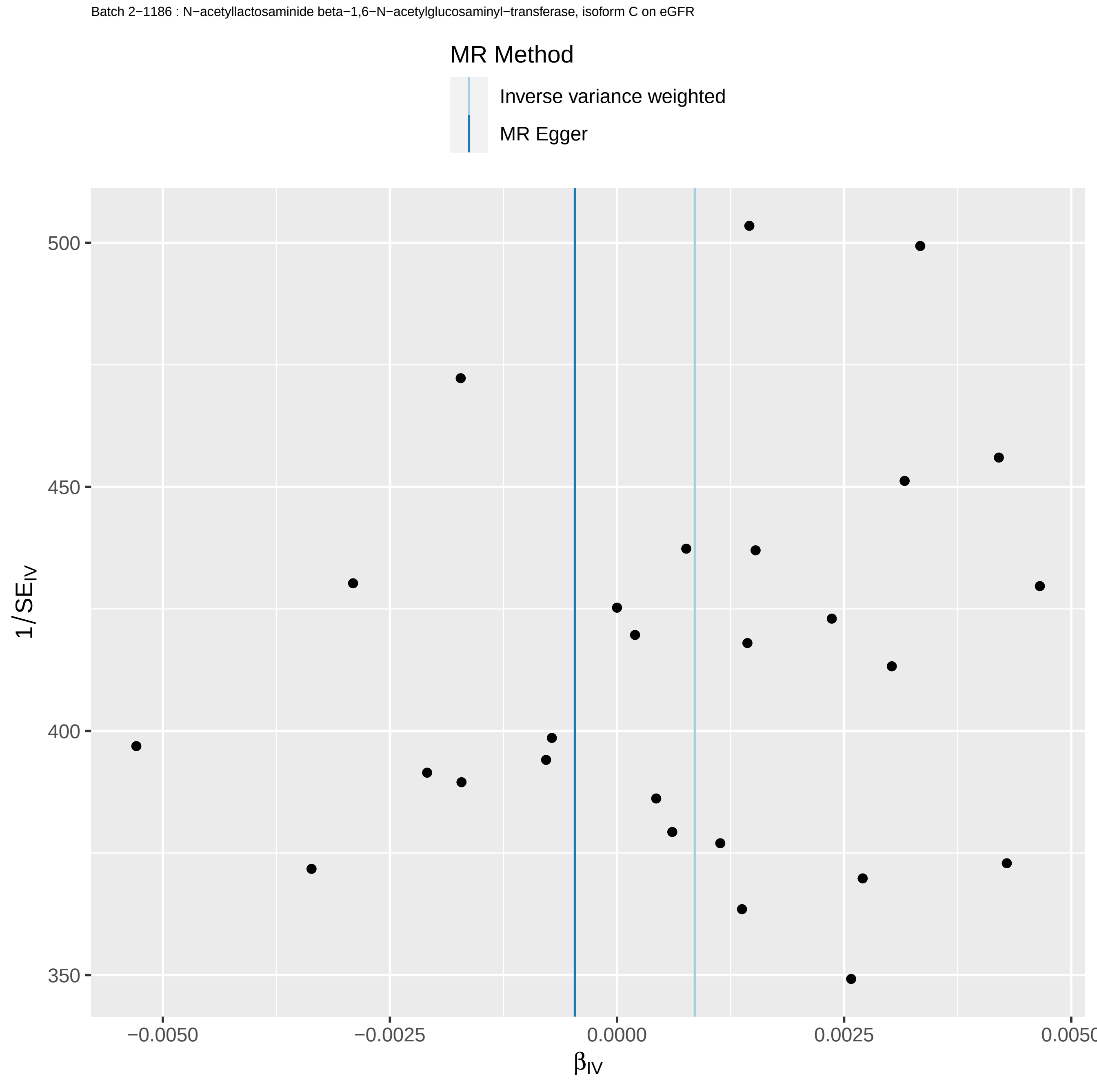

Batch 2−1186 : N−acetyllactosaminide beta−1,6−N−acetylglucosaminyl−transferase, isoform C on eGFR
MR Method
Inverse variance weighted
MR Egger
500
450
400
350
1/SE$_{IV}$
−0.0050
−0.0025
0.0000
0.0025
0.0050
β$_{IV}$

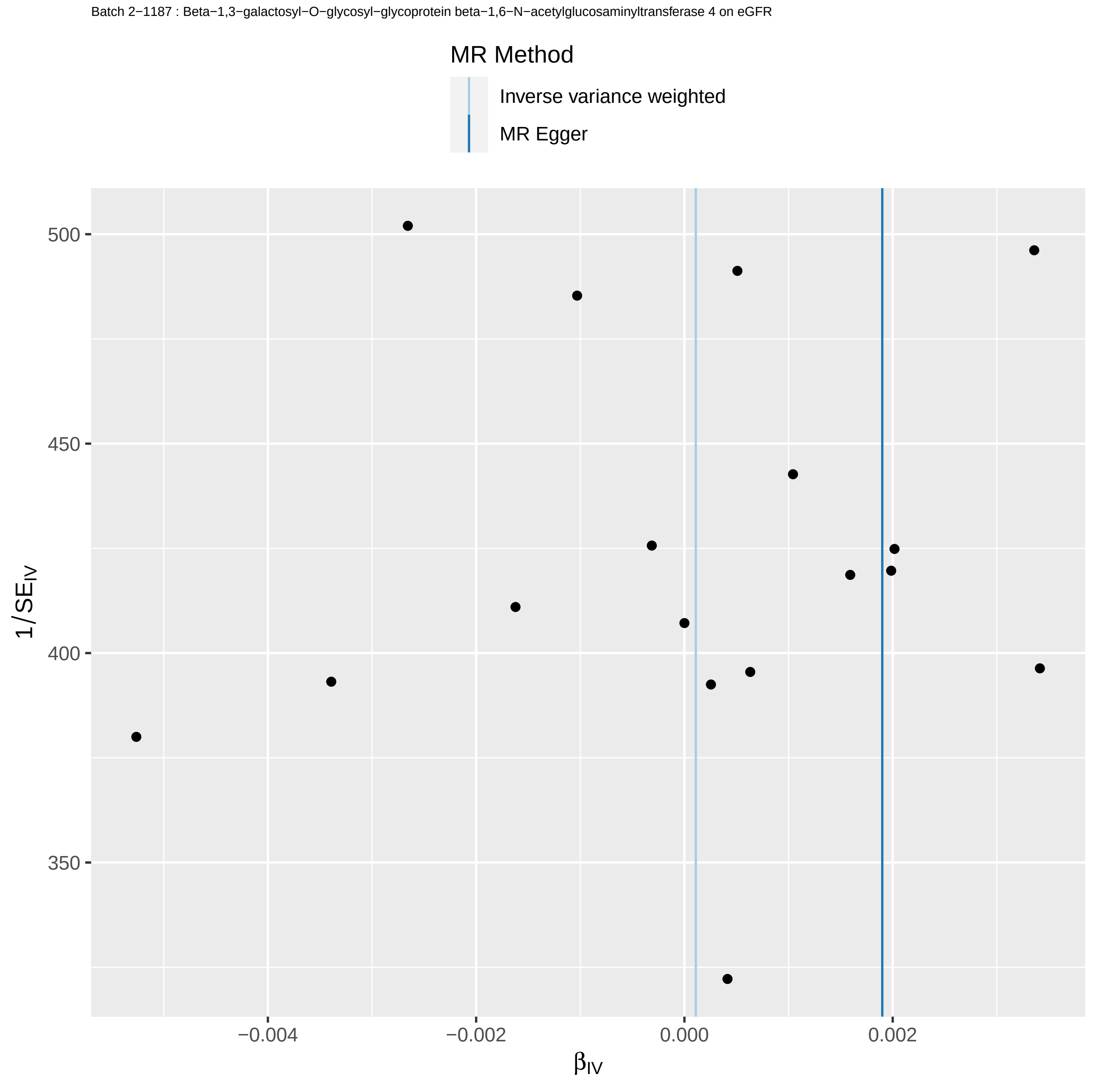
Batch 2−1187 : Beta−1,3−galactosyl−O−glycosyl−glycoprotein beta−1,6−N−acetylglucosaminyltransferase 4 on eGFR
MR Method
Inverse variance weighted
MR Egger
500
450
400
350
1/SE_IV
−0.004
−0.002
0.000
0.002
β_IV

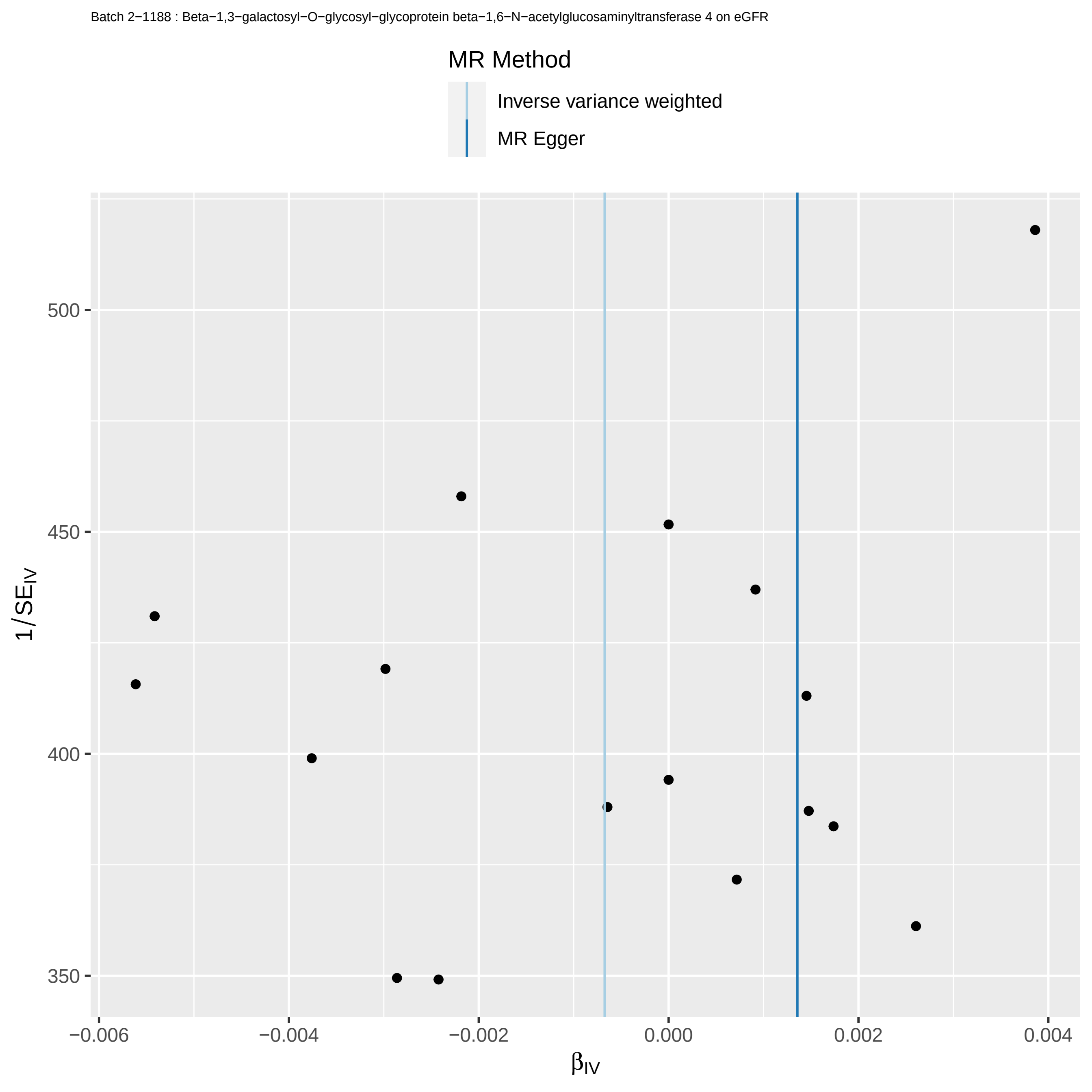

Batch 2−1188 : Beta−1,3−galactosyl−O−glycosyl−glycoprotein beta−1,6−N−acetylglucosaminyltransferase 4 on eGFR
MR Method
Inverse variance weighted
MR Egger
500
450
400
350
1/SE_IV
−0.006
−0.004
−0.002
0.000
0.002
0.004
β_IV

Batch 2−1189 : Guanine deaminase on eGFR

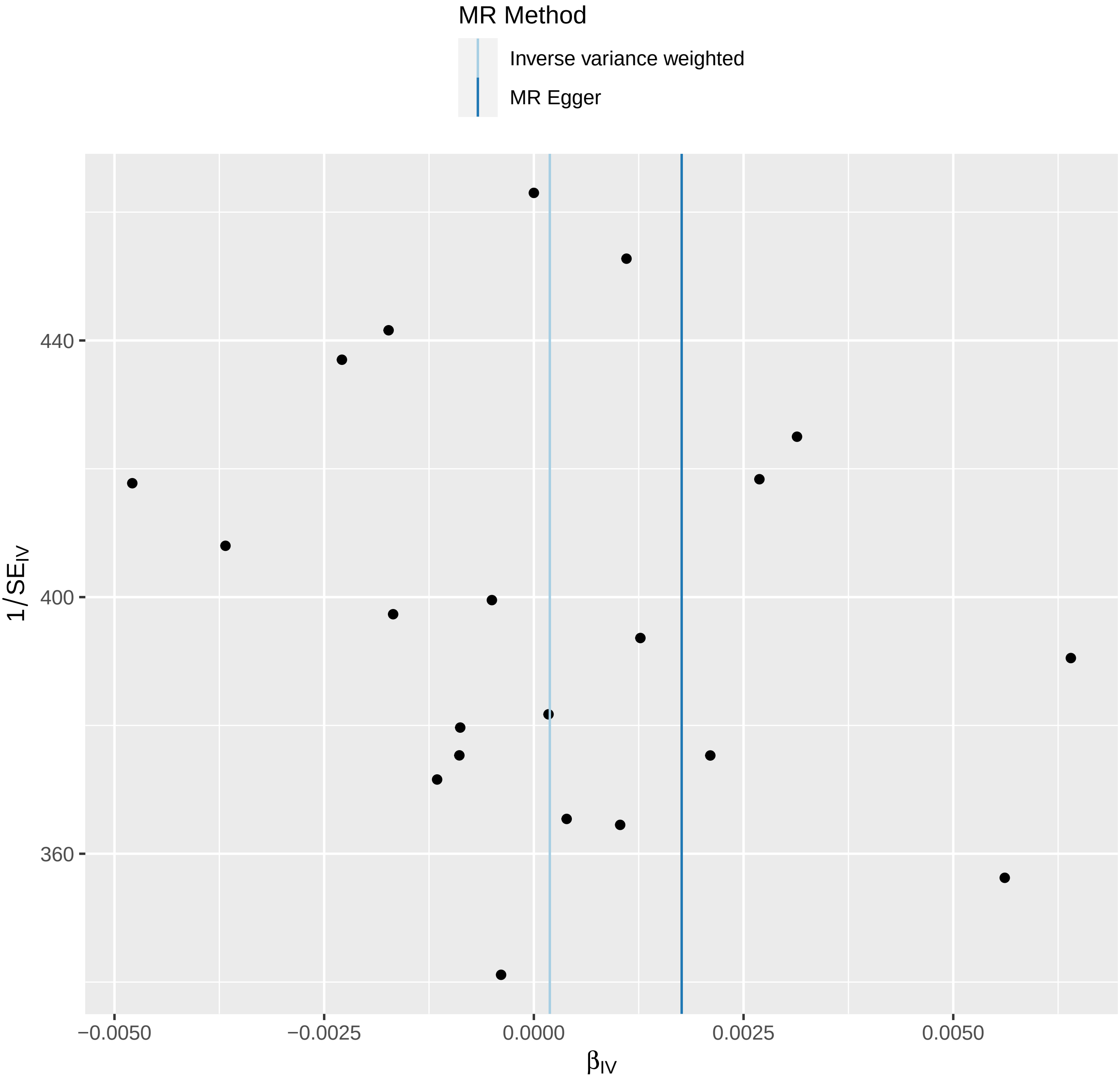

Batch 2−1190 : Growth/differentiation factor 11 on eGFR

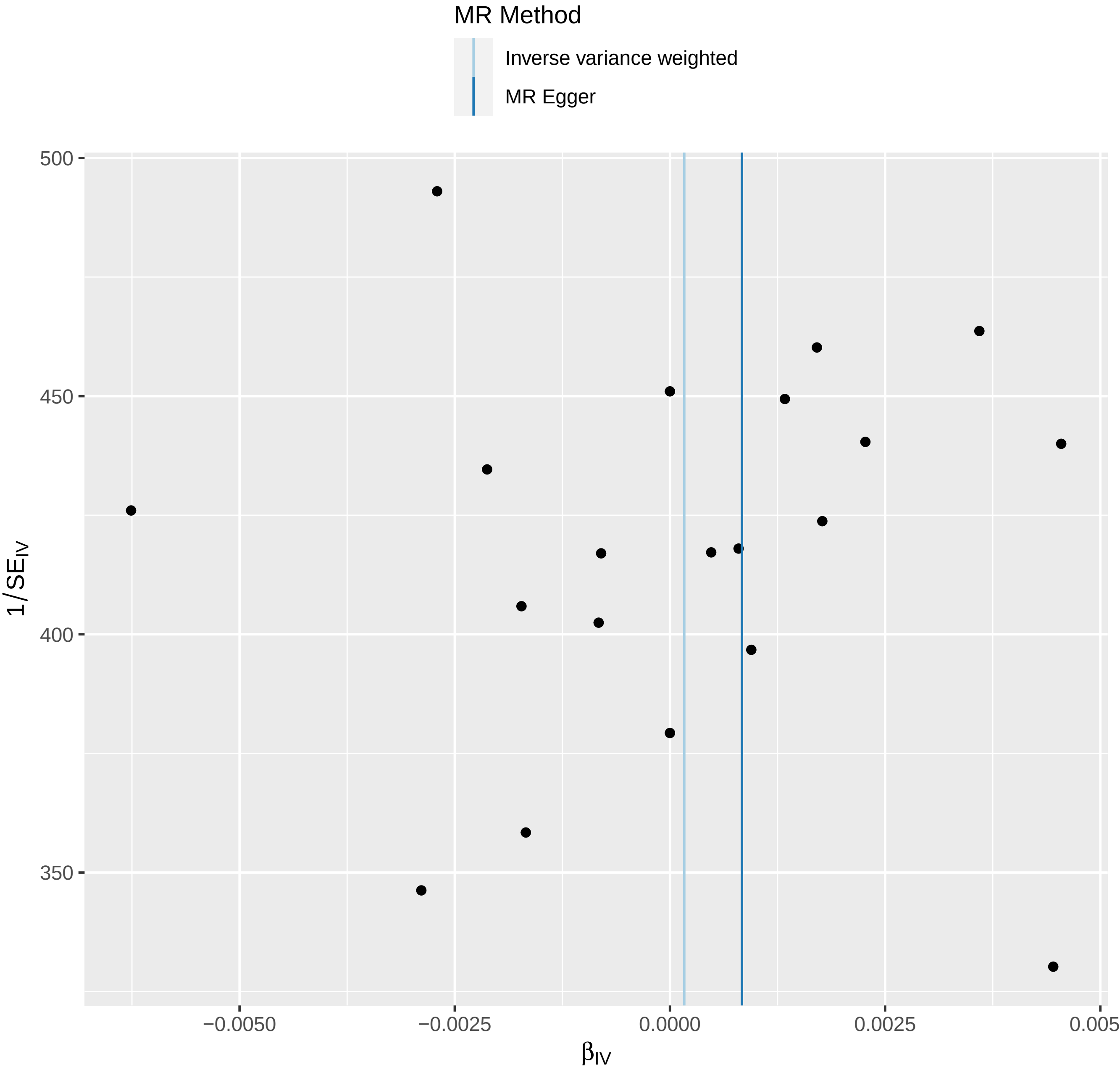

Batch 2−1191 : Growth/differentiation factor 11 on eGFR

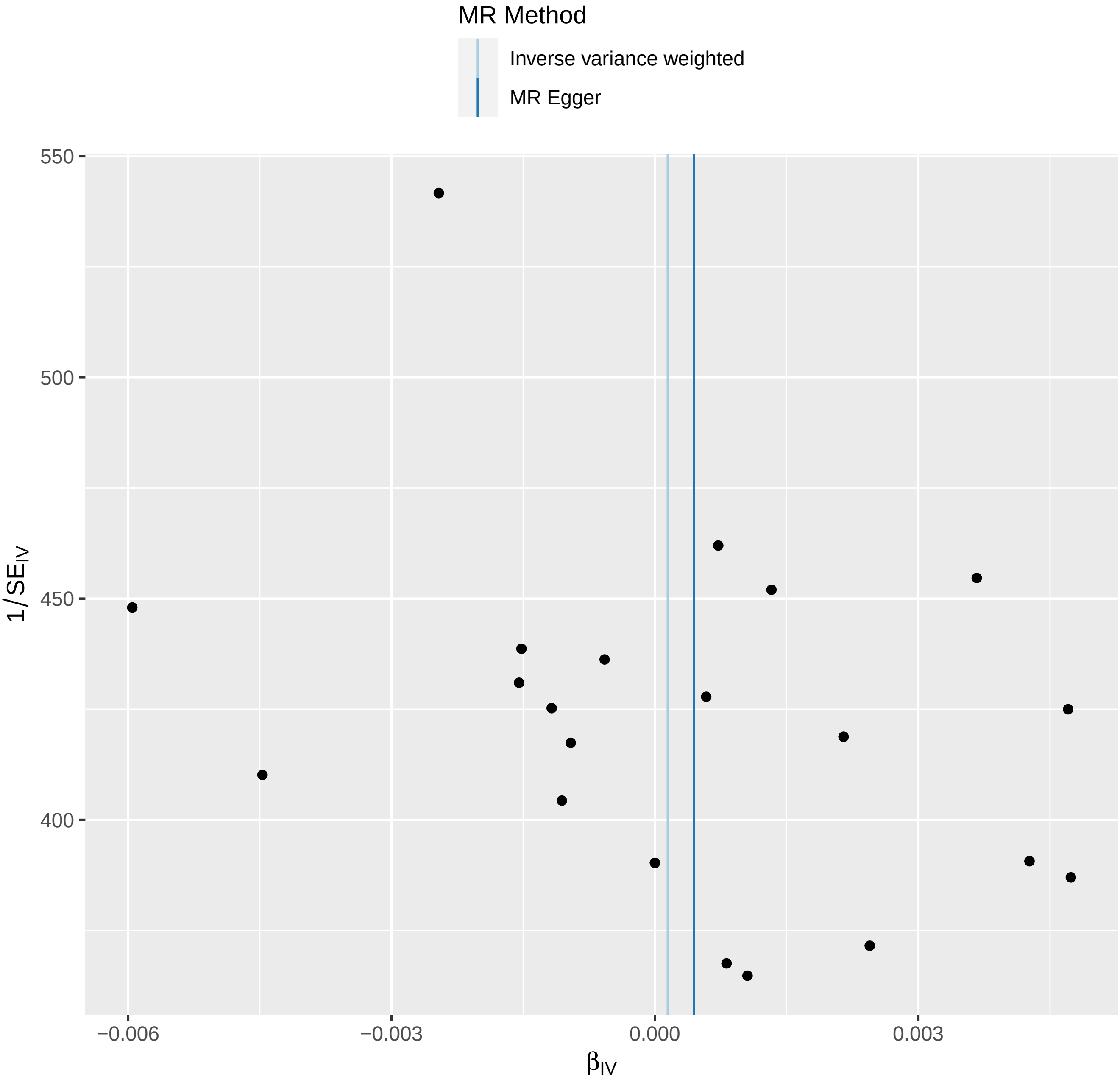

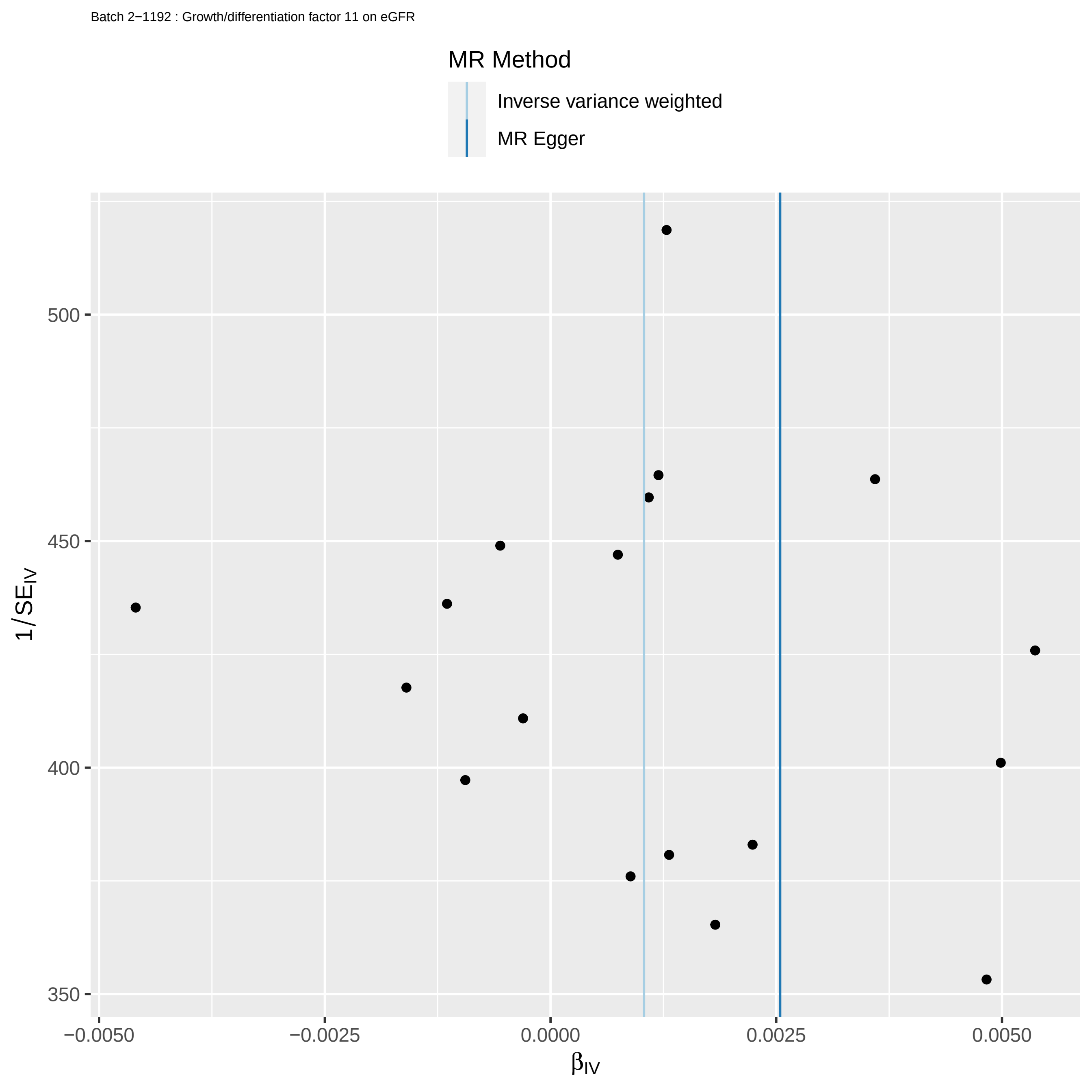

Batch 2−1192 : Growth/differentiation factor 11 on eGFR
MR Method
Inverse variance weighted
MR Egger
500
450
400
350
1/SE_IV
−0.0050
−0.0025
0.0000
0.0025
0.0050
β_IV

Batch 2−1193 : Growth/differentiation factor 11 on eGFR

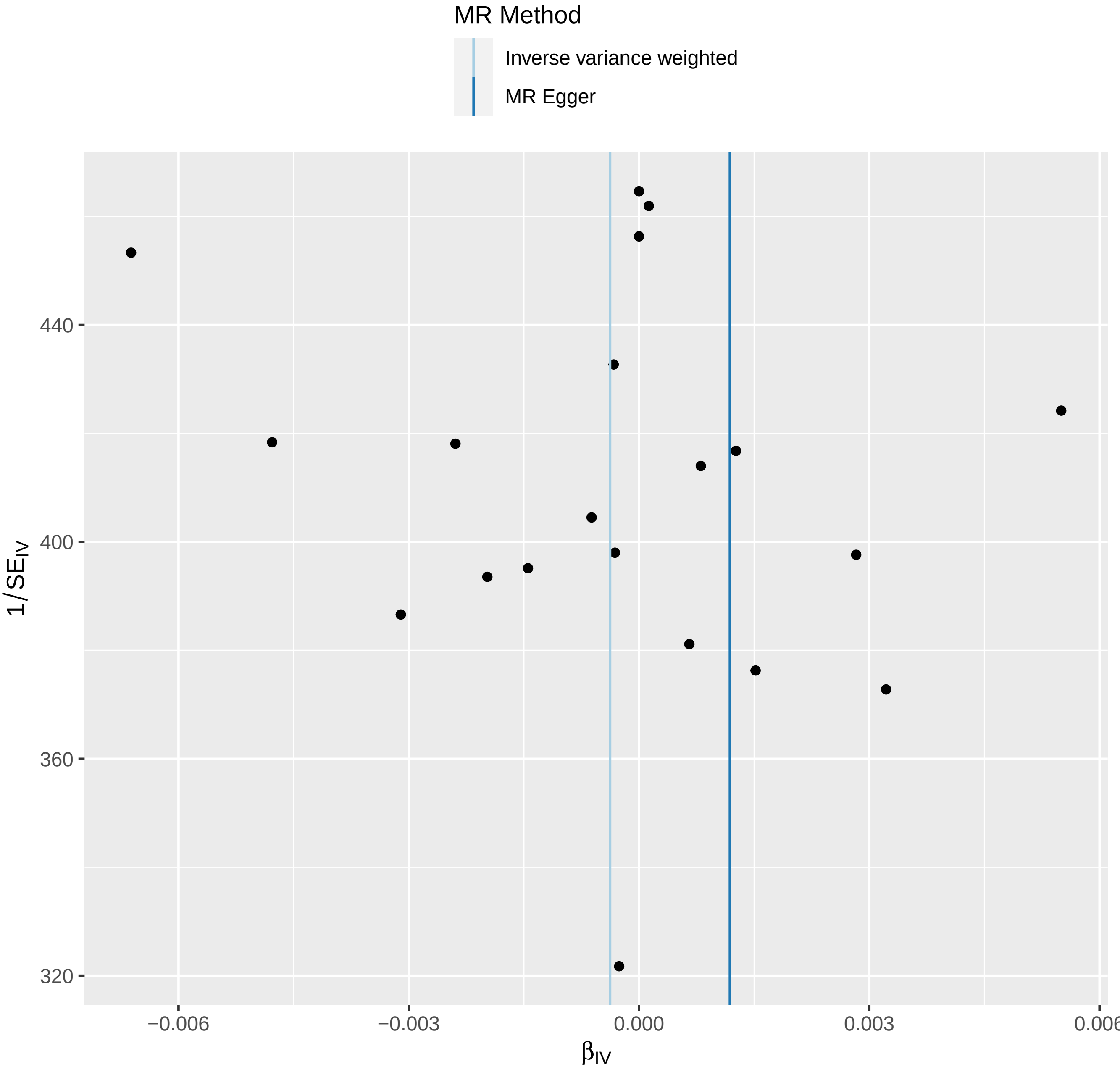

Batch 2−1194 : Growth/differentiation factor 11/8 on eGFR

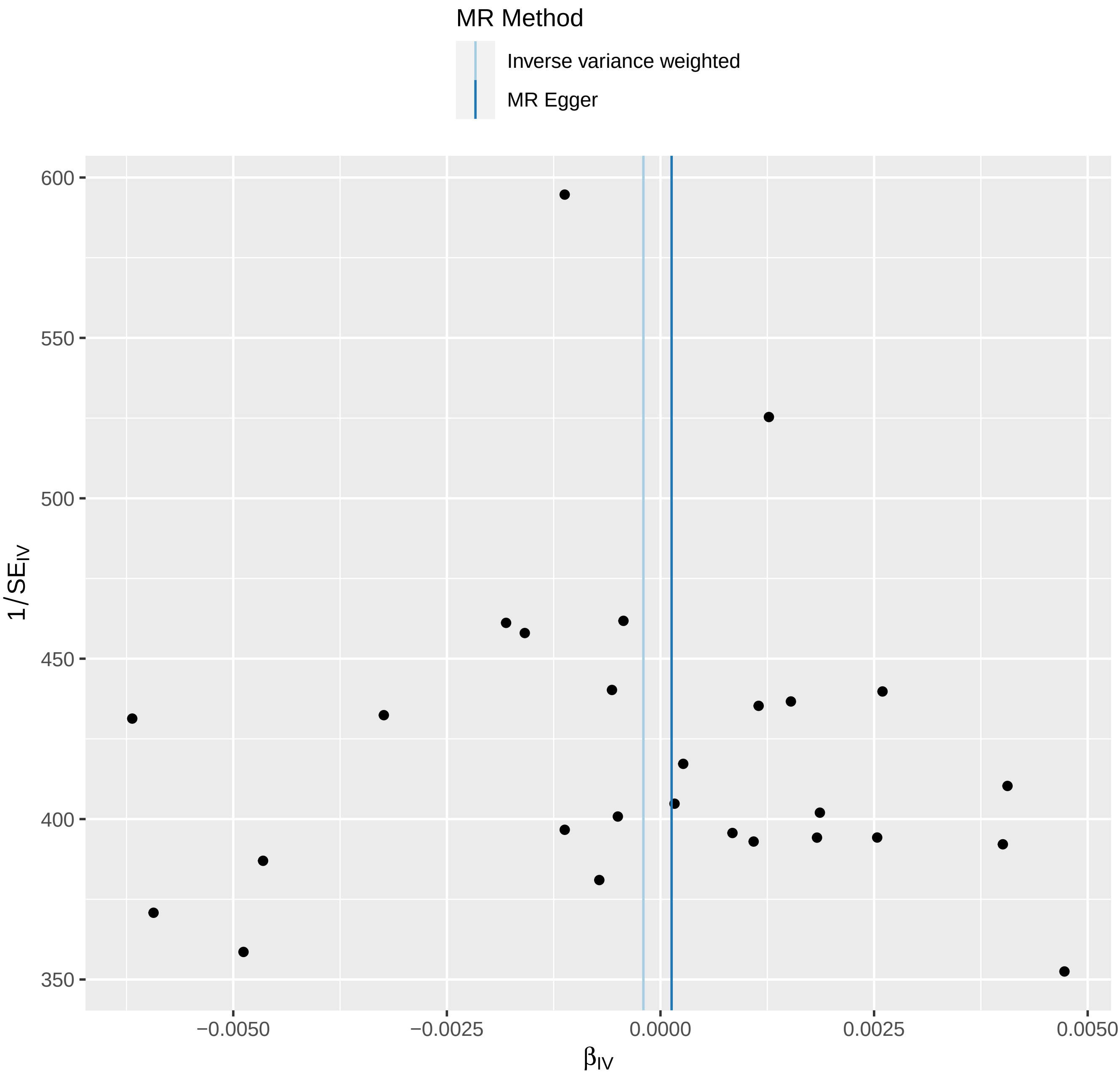

Batch 2−1195 : Growth/differentiation factor 15 on eGFR

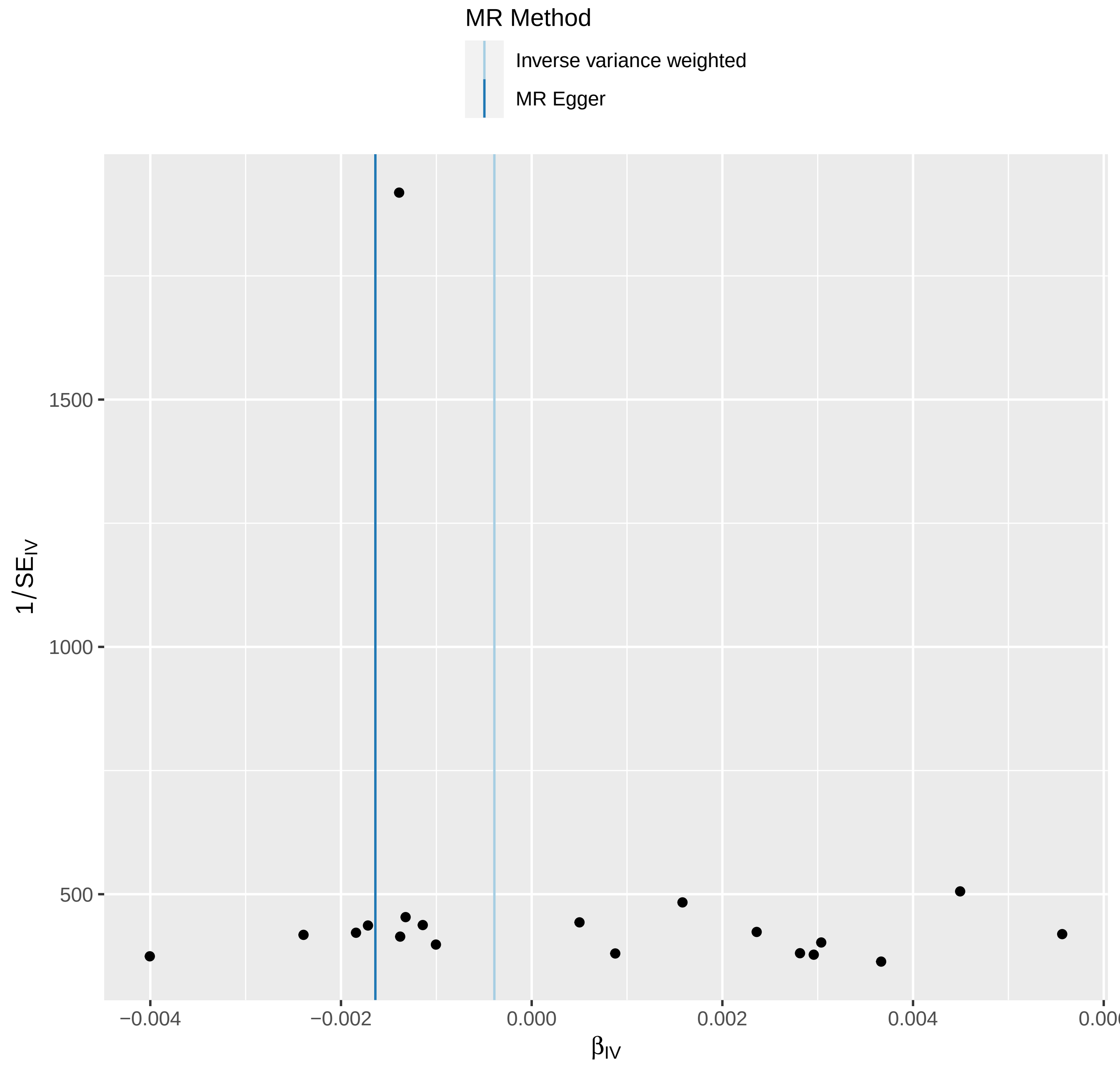

Batch 2−1196 : Growth/differentiation factor 5 on eGFR

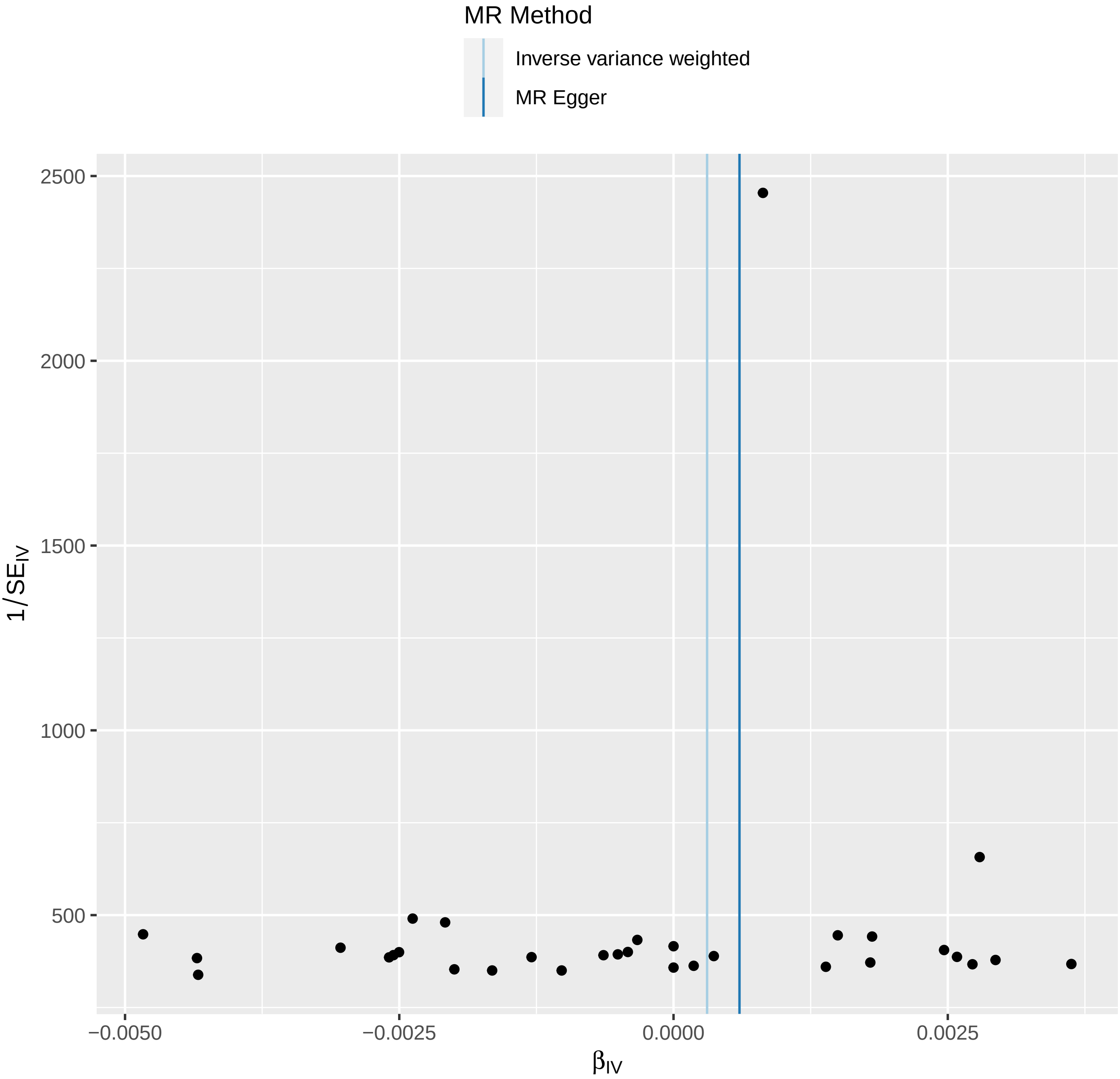

Batch 2−1197 : Growth/differentiation factor 9 on eGFR

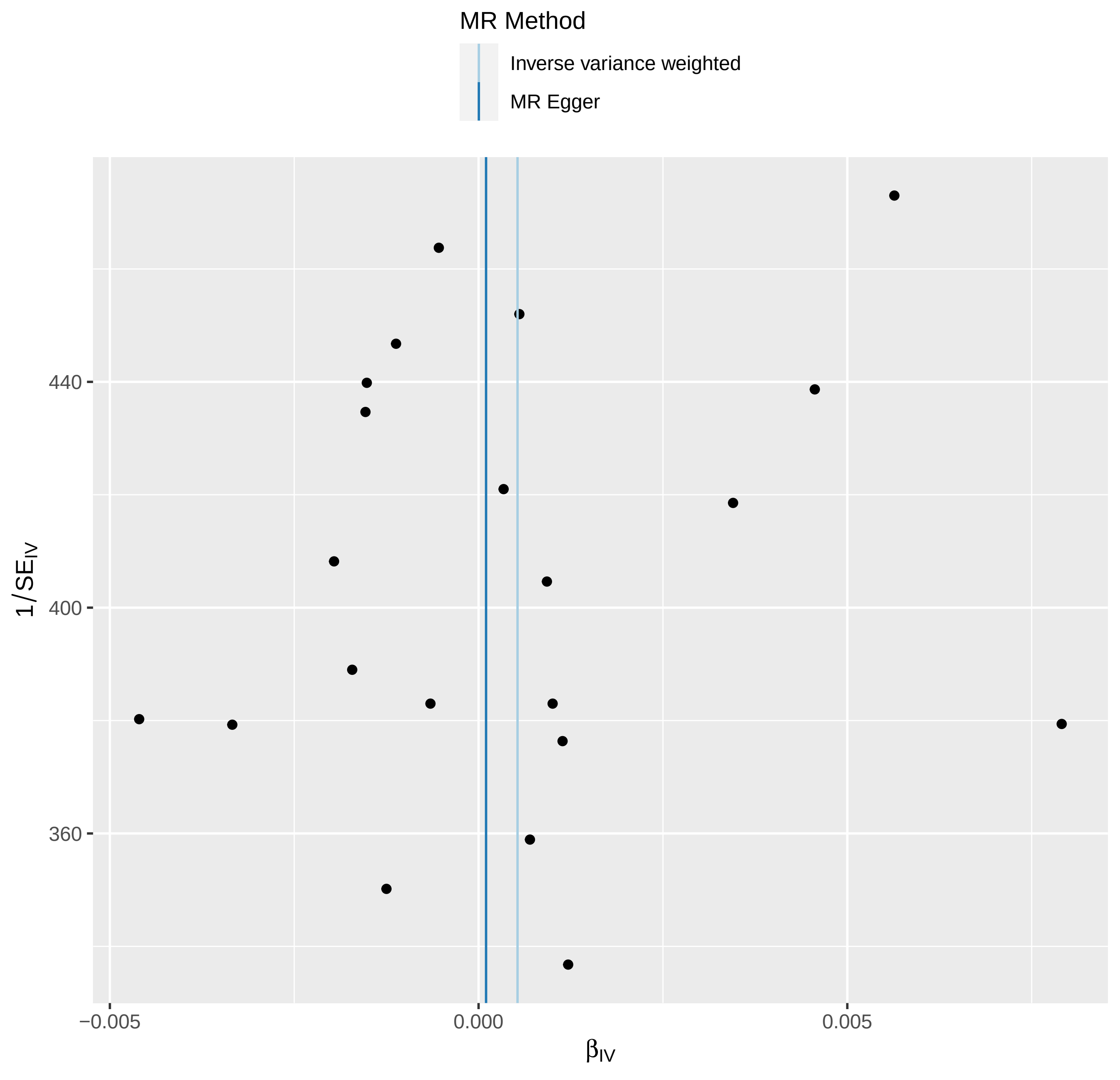

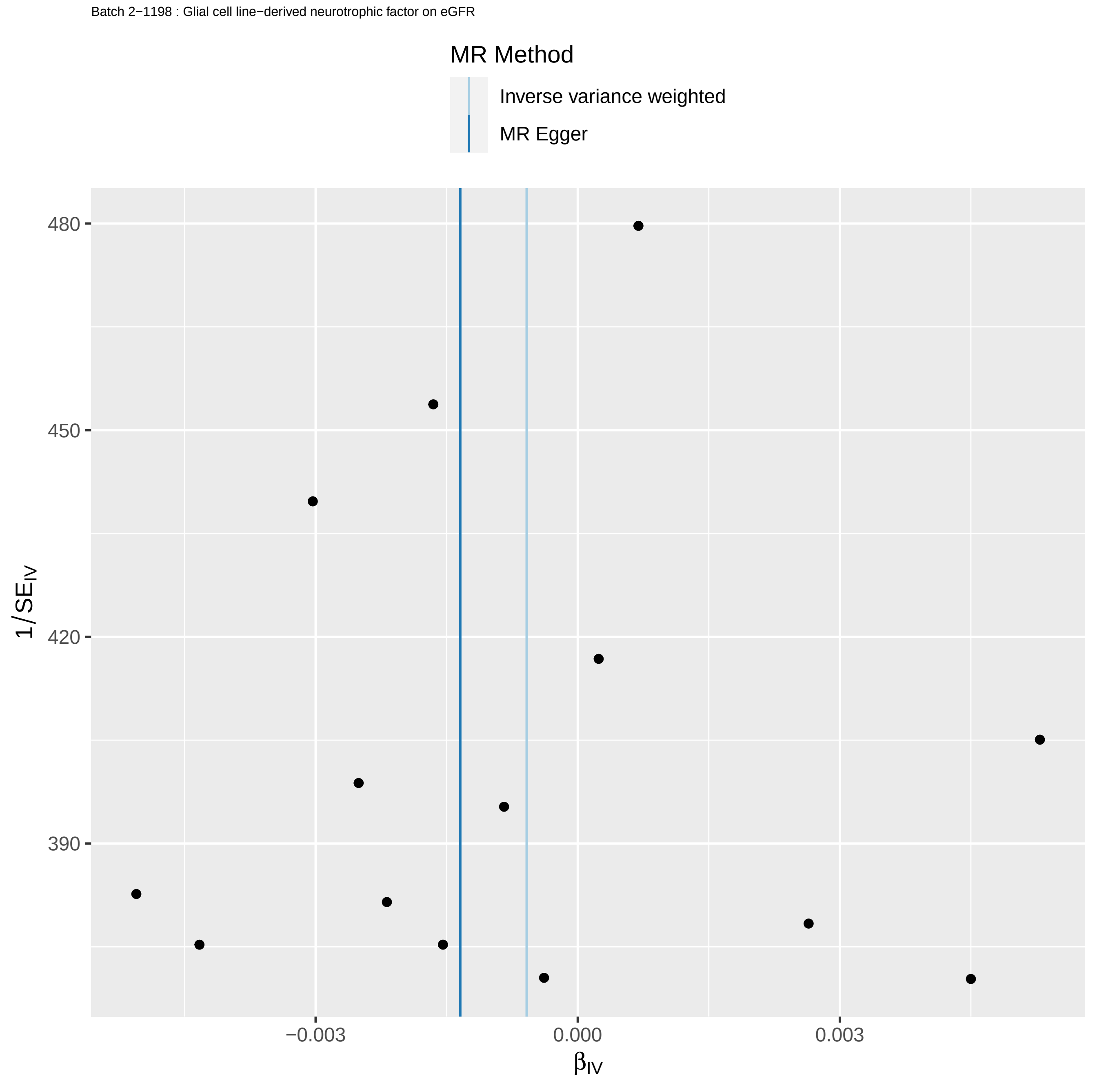

Batch 2−1198 : Glial cell line−derived neurotrophic factor on eGFR
MR Method
Inverse variance weighted
MR Egger
480
450
420
390
1/SE_IV
−0.003
0.000
0.003
β_IV

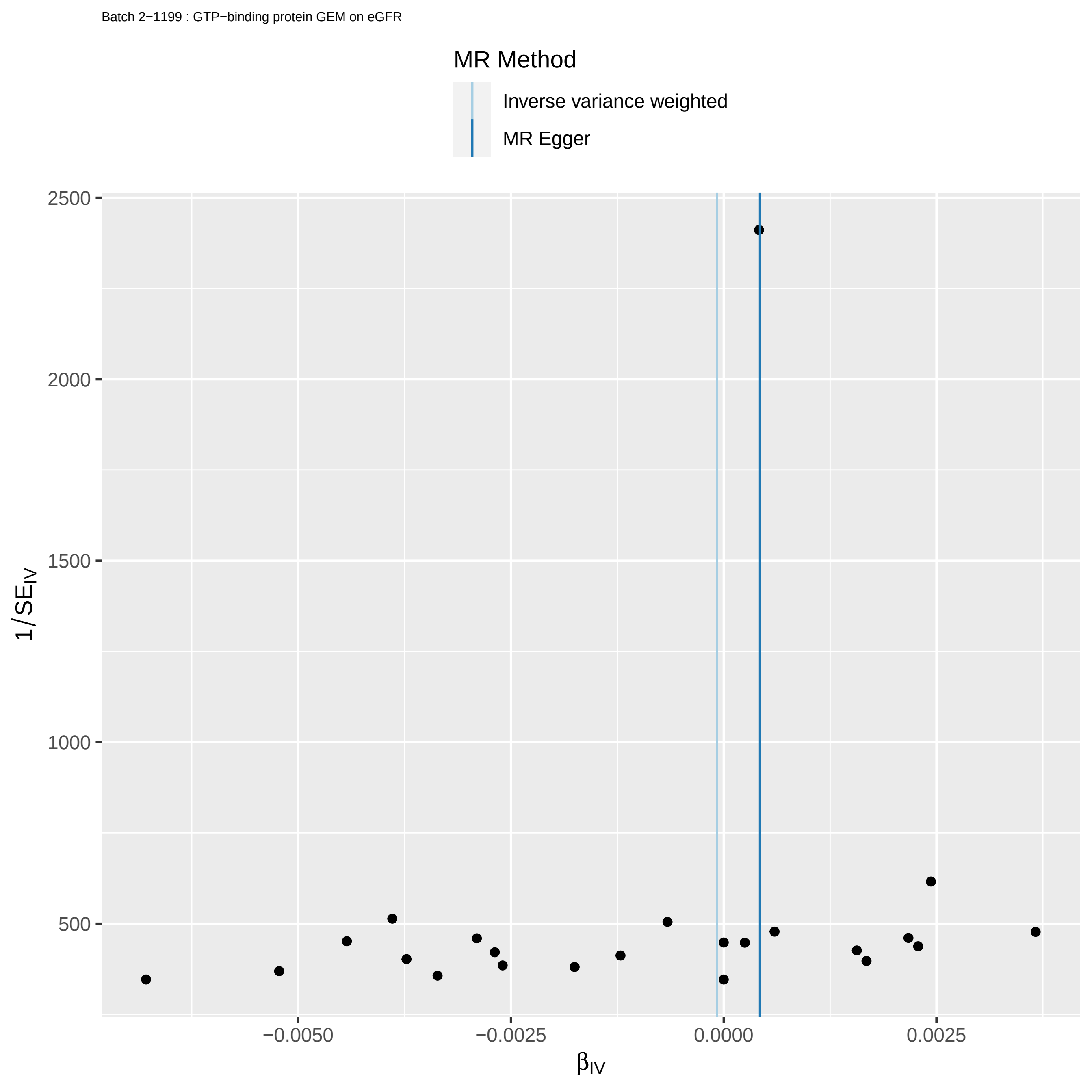

Batch 2−1199 : GTP−binding protein GEM on eGFR
MR Method
Inverse variance weighted
MR Egger
2500
2000
1500
1000
500
1/SE_IV
−0.0050
−0.0025
0.0000
0.0025
β_IV

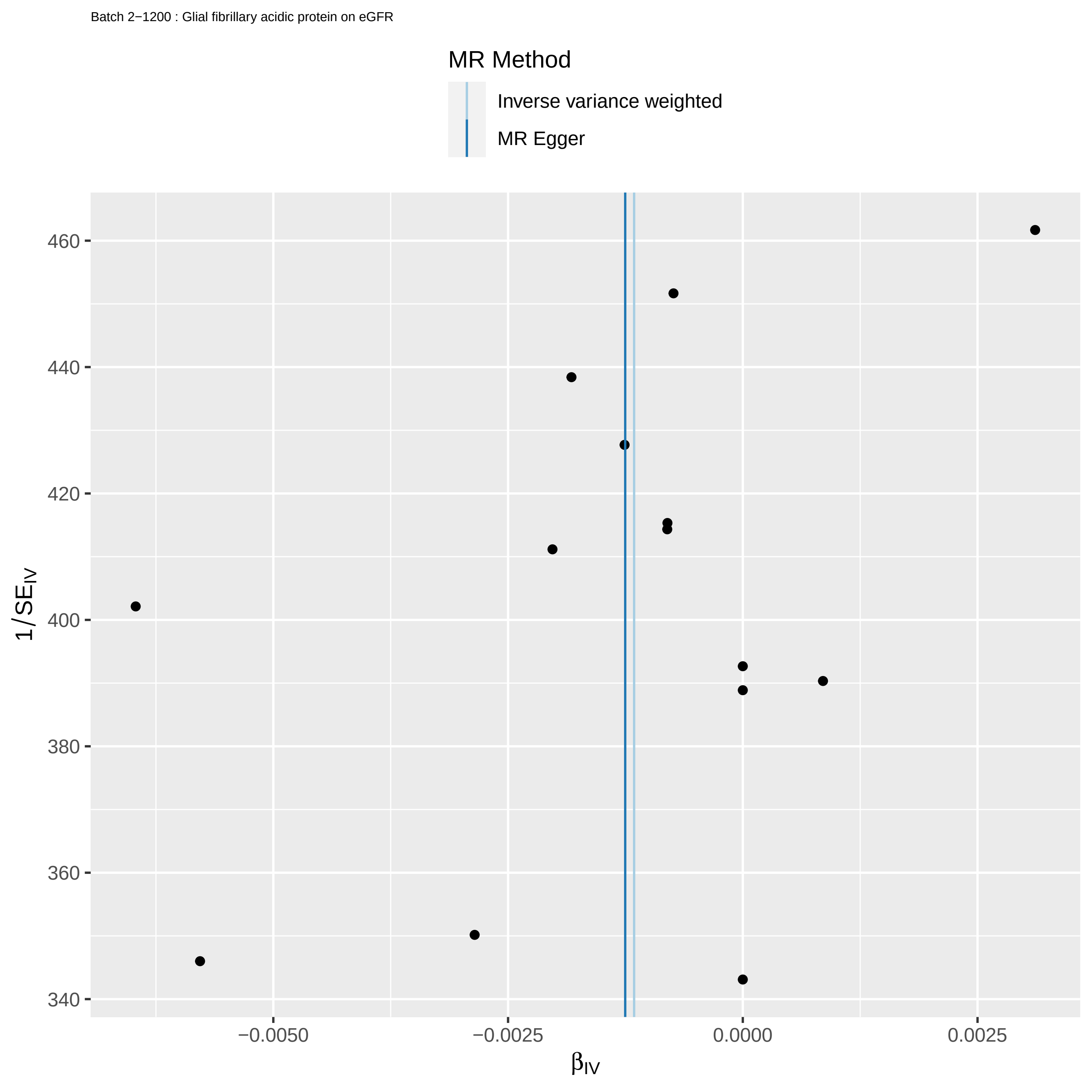

Batch 2−1200 : Glial fibrillary acidic protein on eGFR
MR Method
Inverse variance weighted
MR Egger
460
440
420
400
380
360
340
1/SE_IV
−0.0050
−0.0025
0.0000
0.0025
β_IV

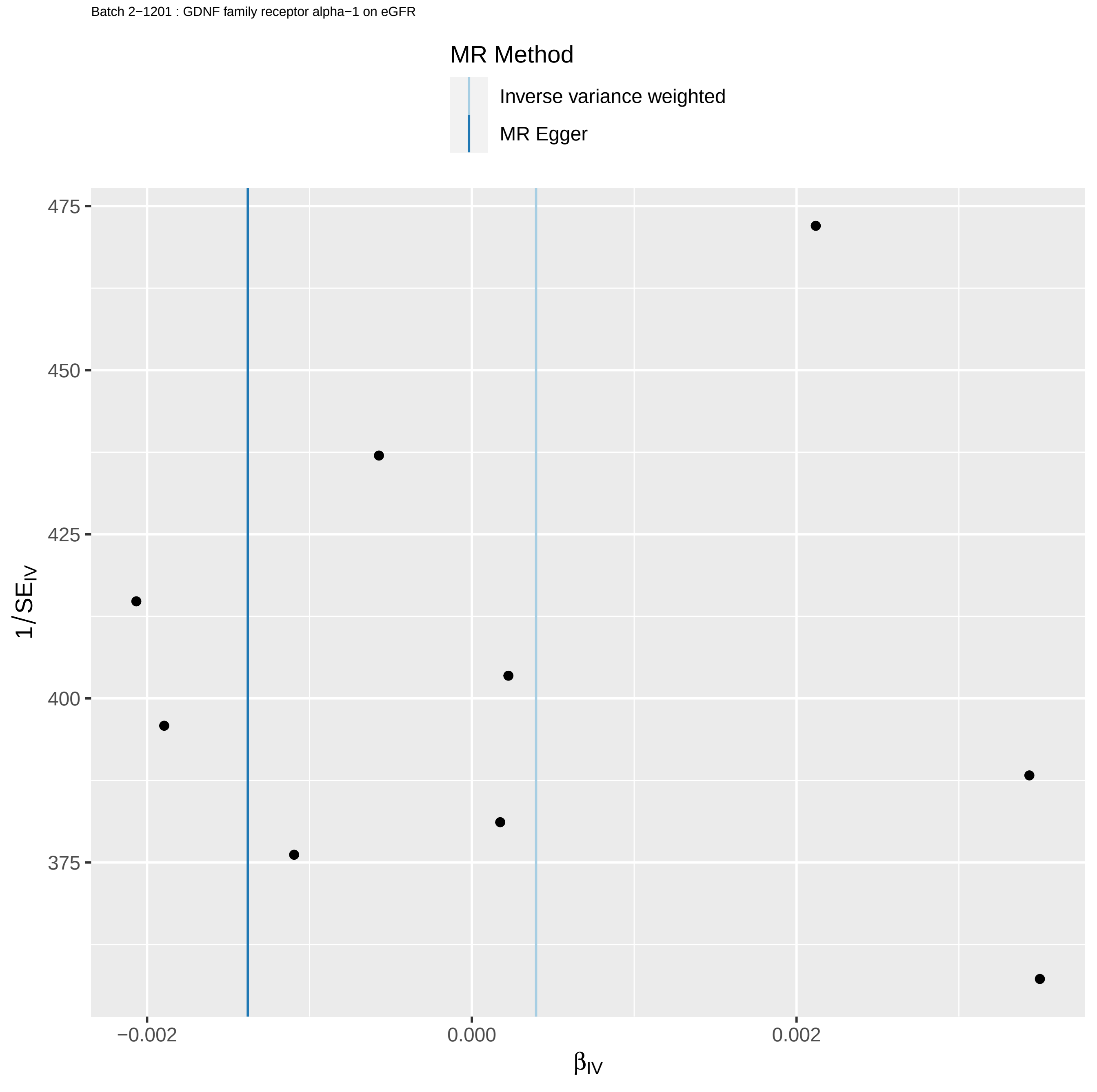
Batch 2−1201 : GDNF family receptor alpha−1 on eGFR
MR Method
Inverse variance weighted
MR Egger
475
450
425
400
375
1/SE_IV
−0.002
0.000
0.002
β_IV

Batch 2−1202 : GDNF family receptor alpha−2 on eGFR

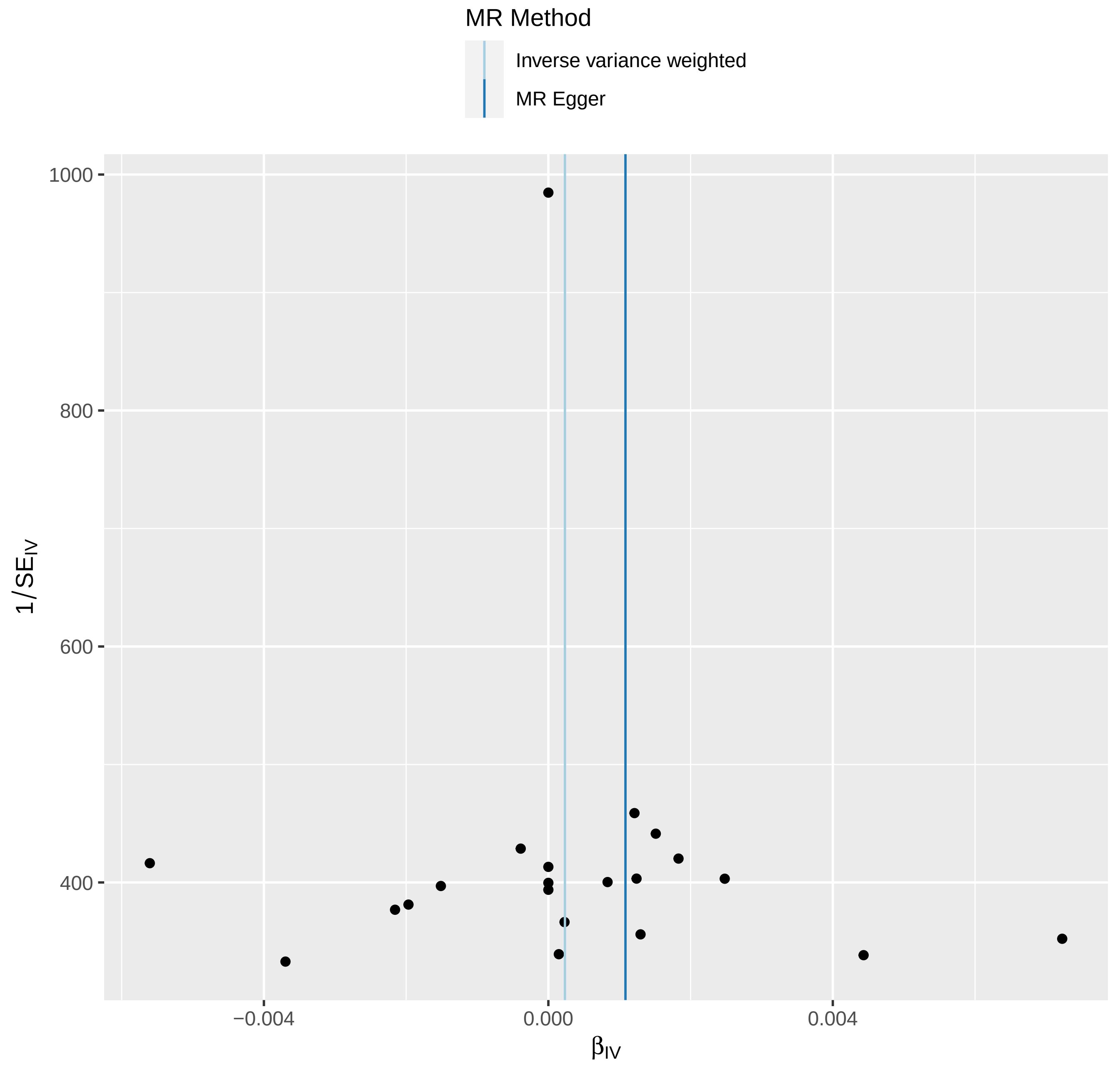

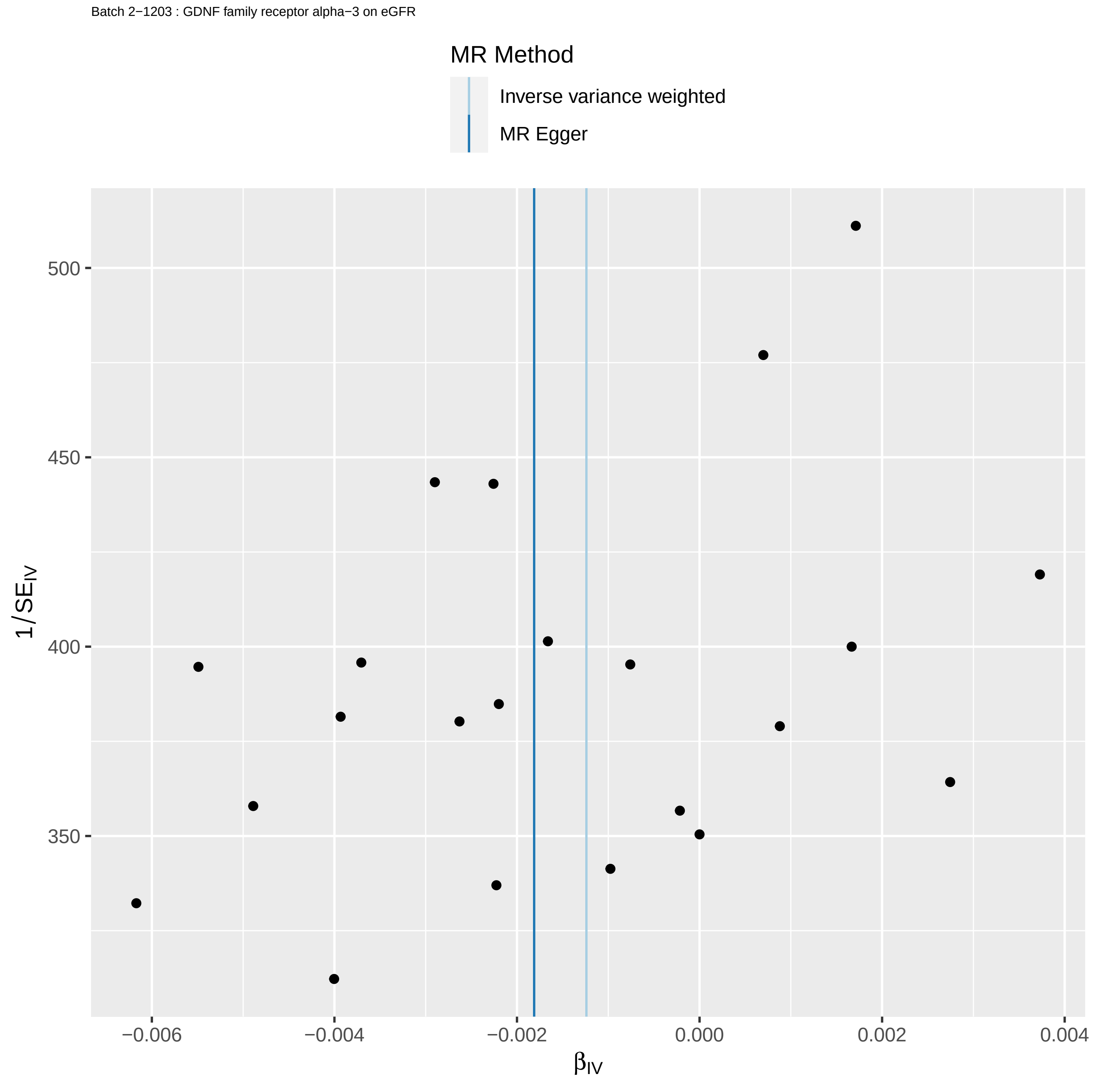
Batch 2−1203 : GDNF family receptor alpha−3 on eGFR
MR Method
Inverse variance weighted
MR Egger
500
450
400
350
1/SE$_{IV}$
−0.006
−0.004
−0.002
0.000
0.002
0.004
β$_{IV}$

Batch 2−1204 : GDNF family receptor alpha−like on eGFR

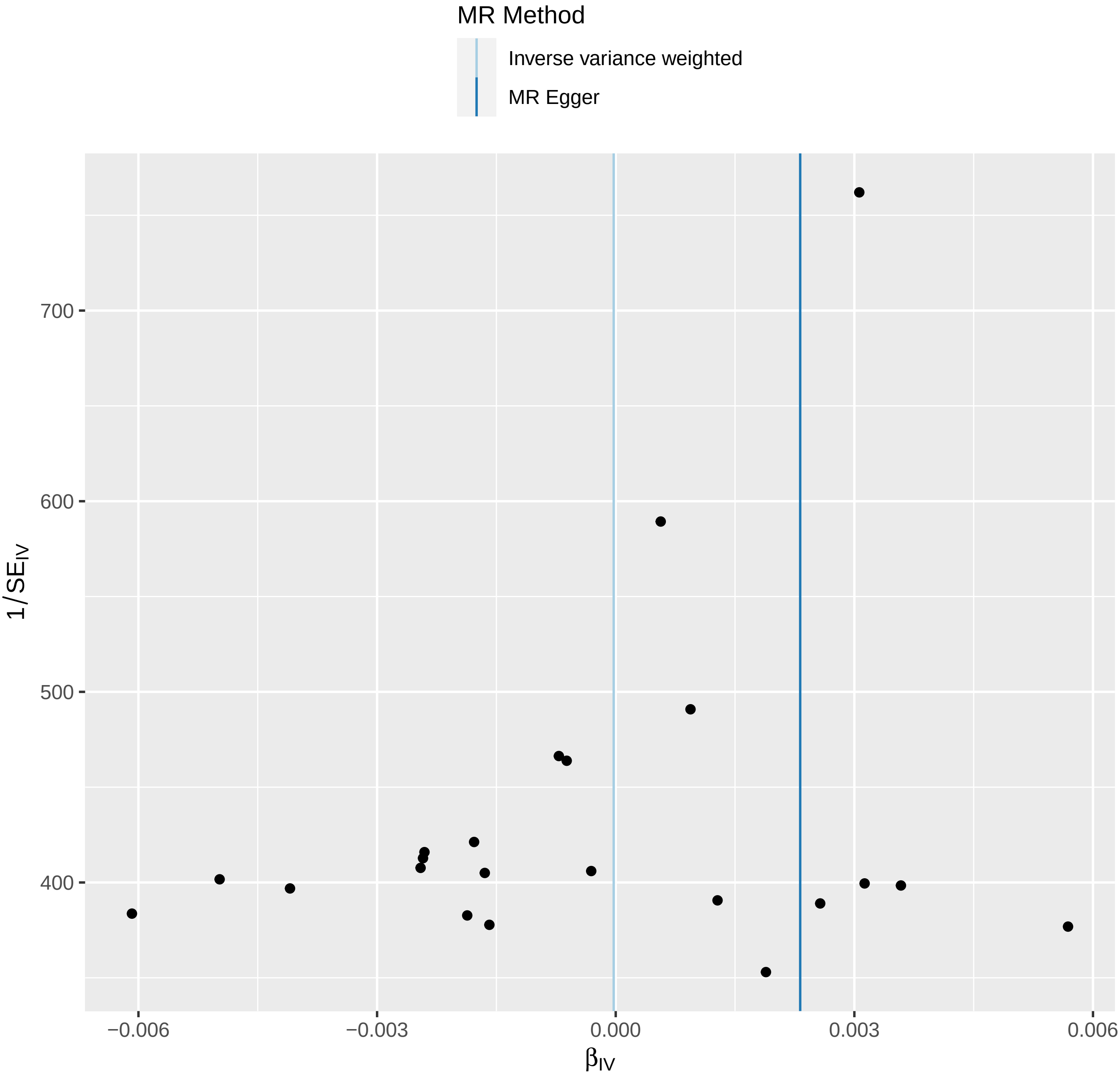

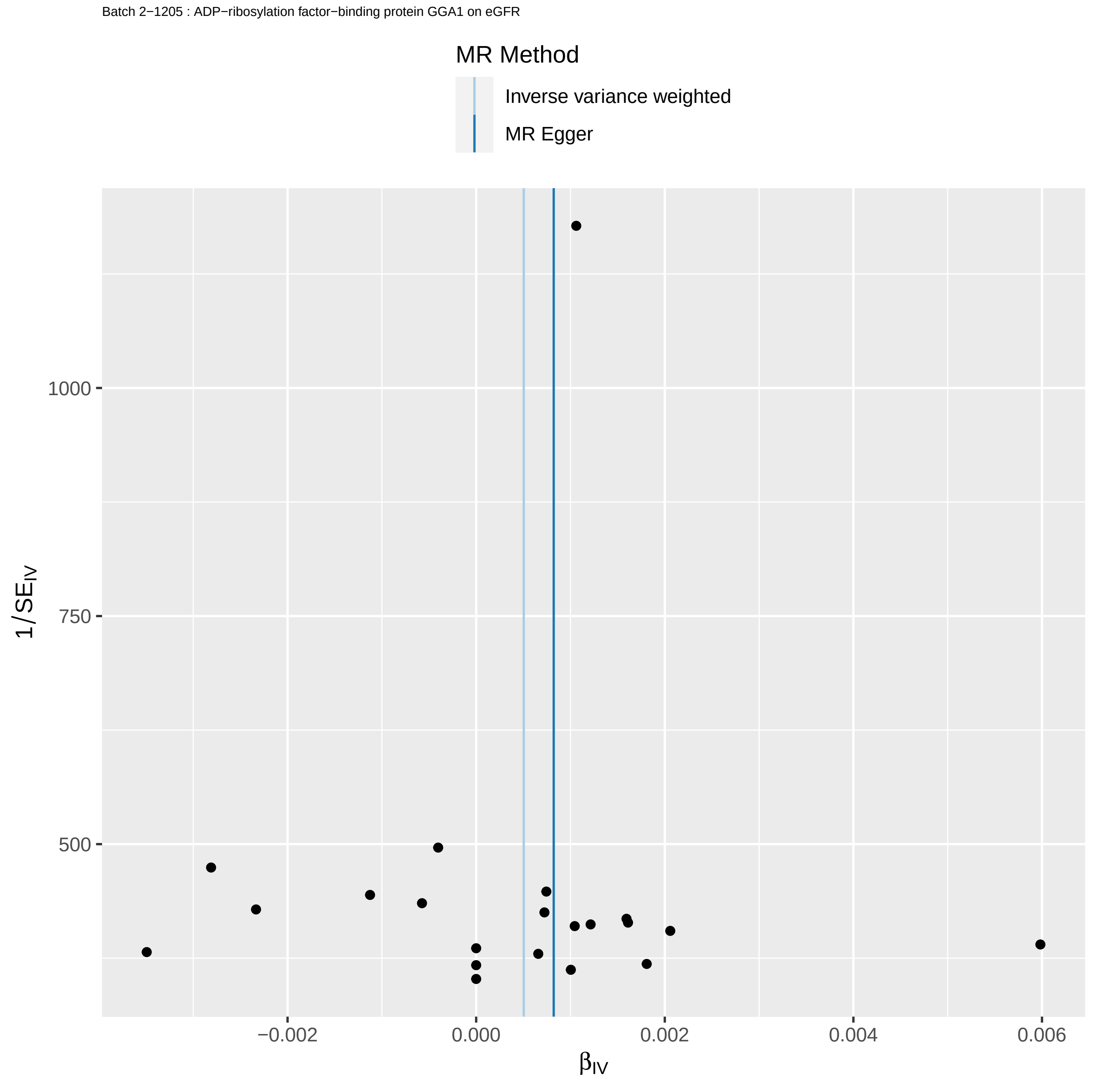

Batch 2−1205 : ADP−ribosylation factor−binding protein GGA1 on eGFR
MR Method
Inverse variance weighted
MR Egger
1000
750
500
1/SE_IV
−0.002
0.000
0.002
0.004
0.006
β_IV

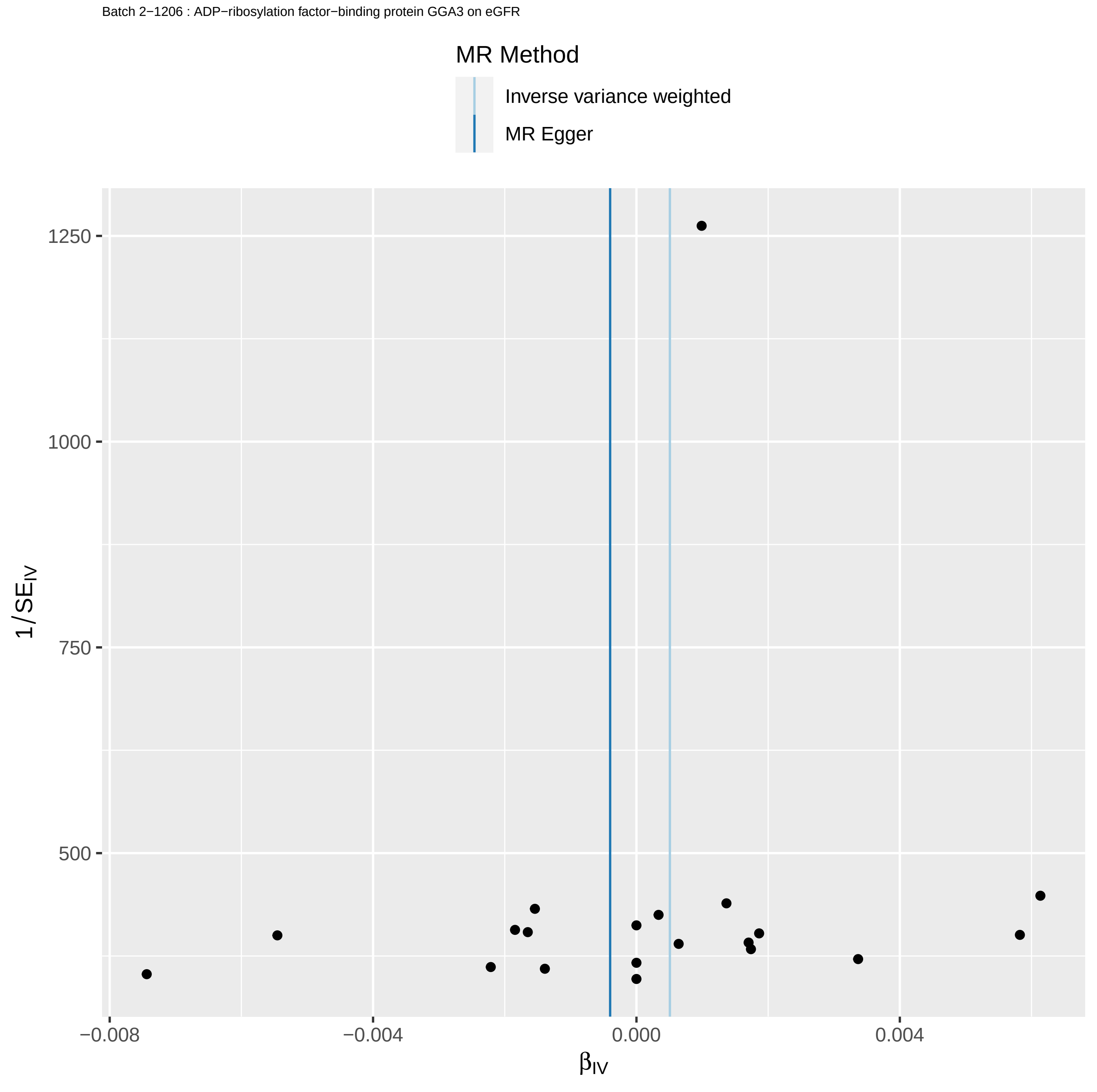
Batch 2−1206 : ADP−ribosylation factor−binding protein GGA3 on eGFR
MR Method
Inverse variance weighted
MR Egger
1250
1000
750
500
1/SE_IV
−0.008
−0.004
0.000
0.004
β_IV

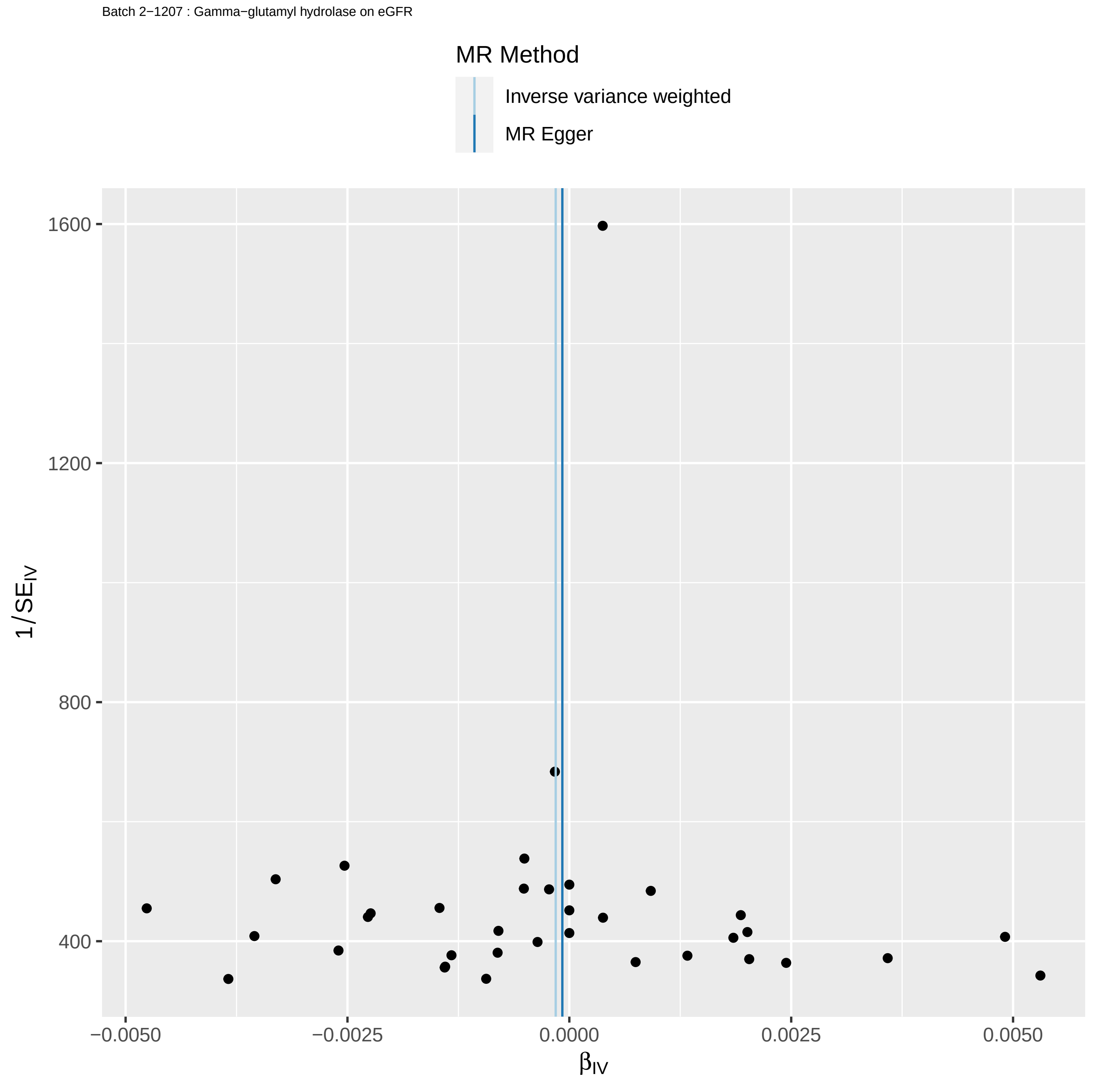

Batch 2−1207 : Gamma−glutamyl hydrolase on eGFR
MR Method
Inverse variance weighted
MR Egger
1600
1200
800
400
1/SE_IV
−0.0050
−0.0025
0.0000
0.0025
0.0050
β_IV

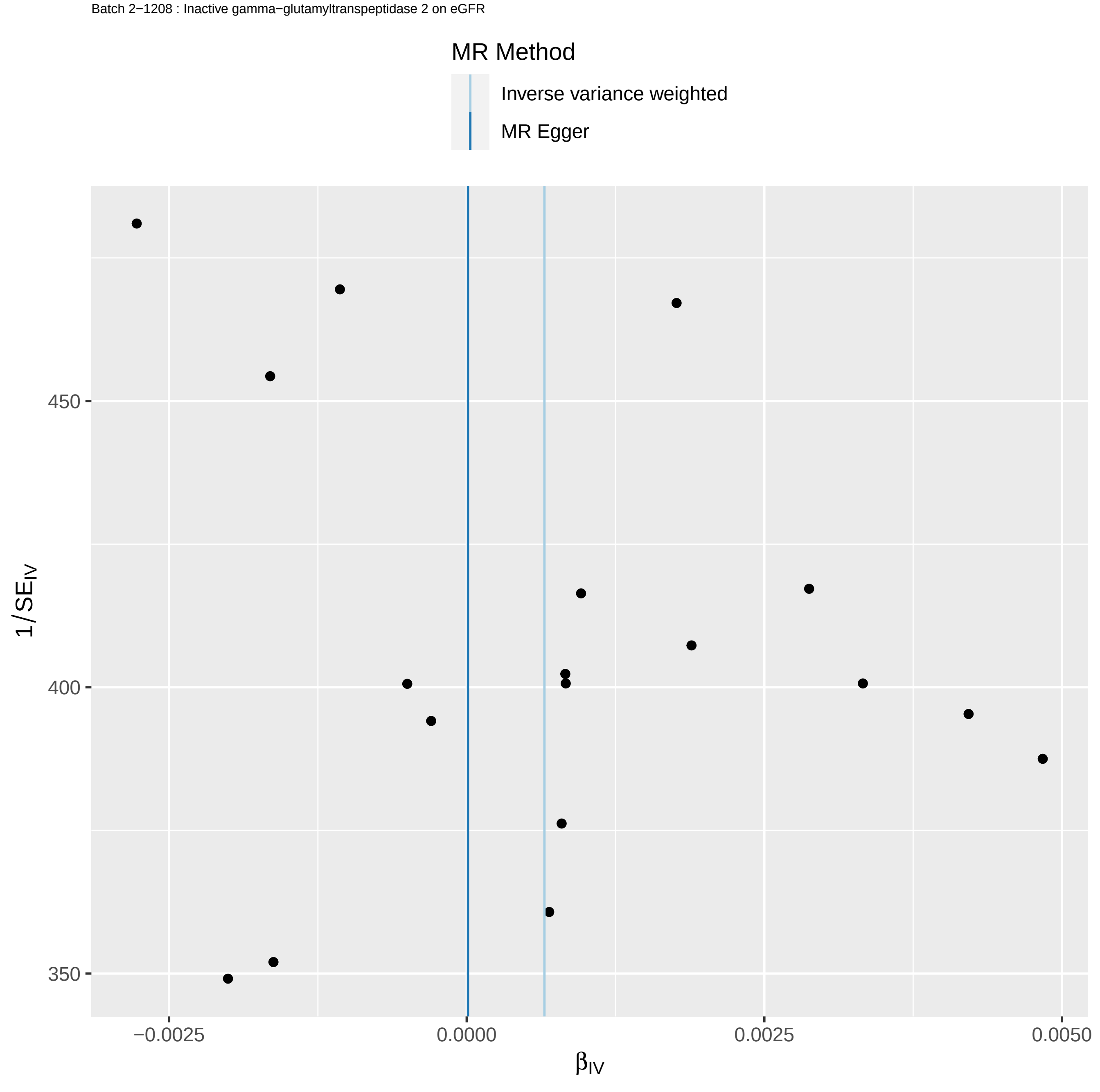

Batch 2−1208 : Inactive gamma−glutamyltranspeptidase 2 on eGFR
MR Method
Inverse variance weighted
MR Egger
450
400
350
1/SE$_{IV}$
−0.0025
0.0000
0.0025
0.0050
β$_{IV}$

Batch 2−1209 : Somatotropin on eGFR

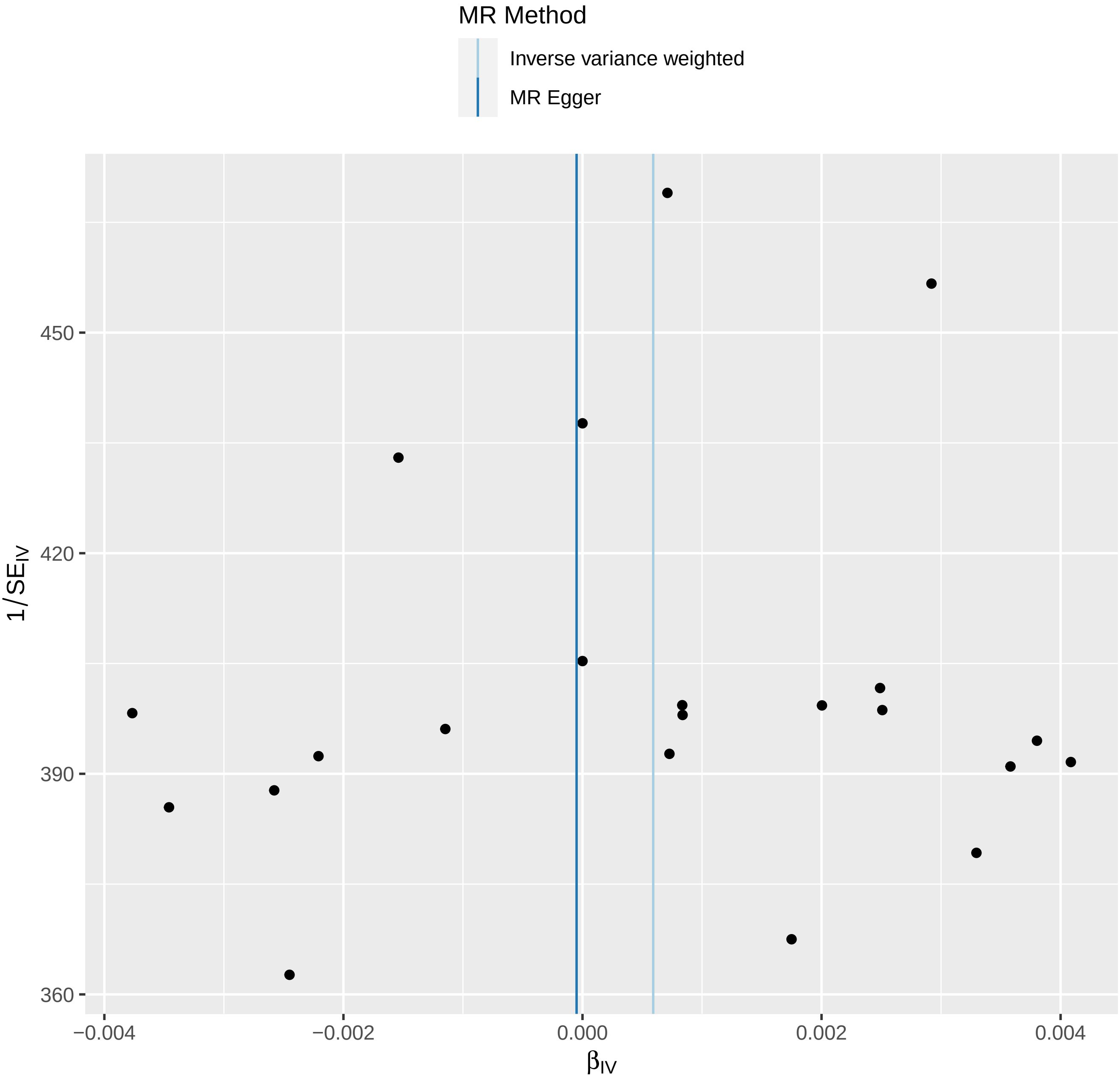

Batch 2−1210 : GH3 domain−containing protein on eGFR

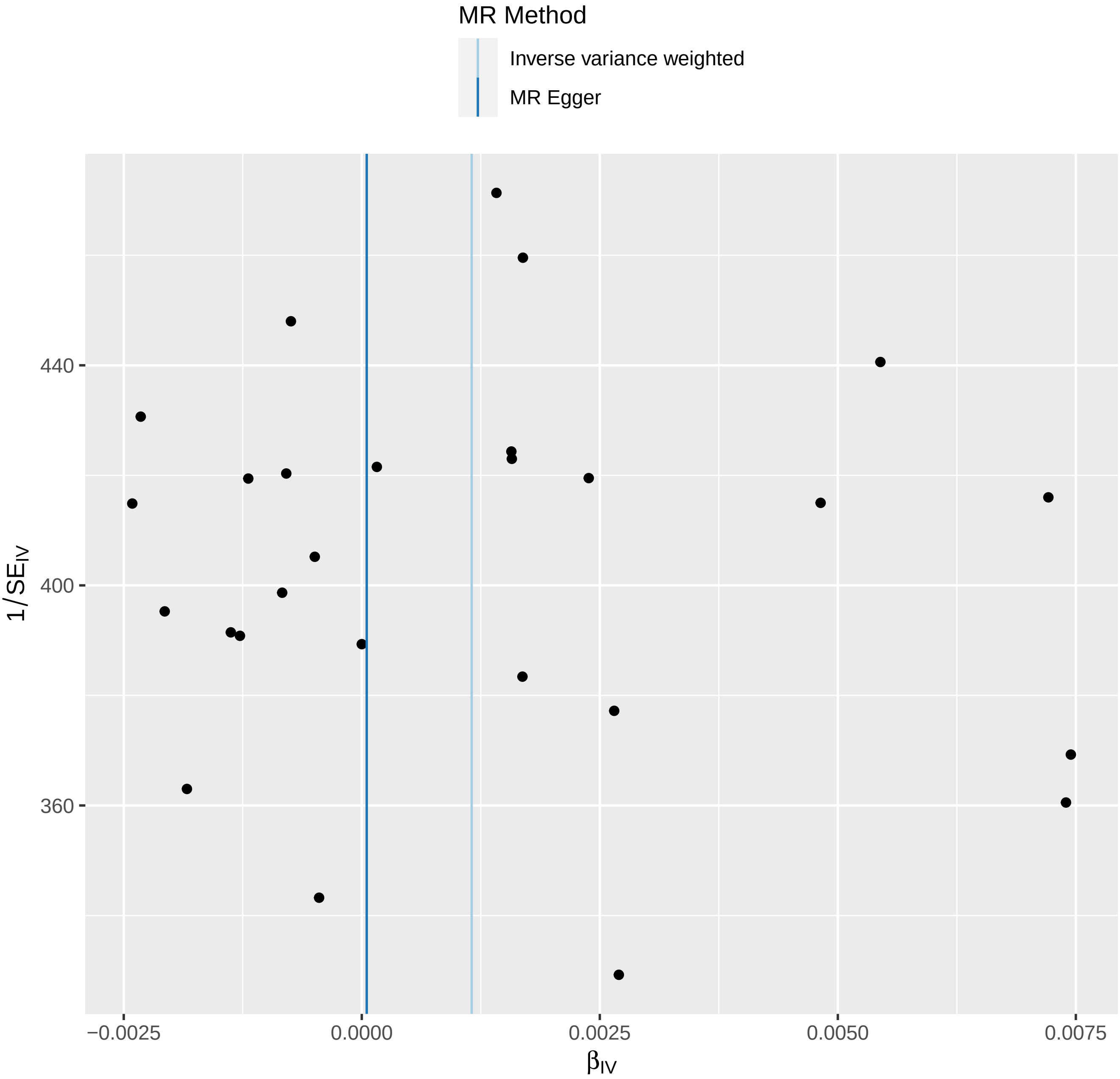

Batch 2−1211 : Growth hormone receptor on eGFR

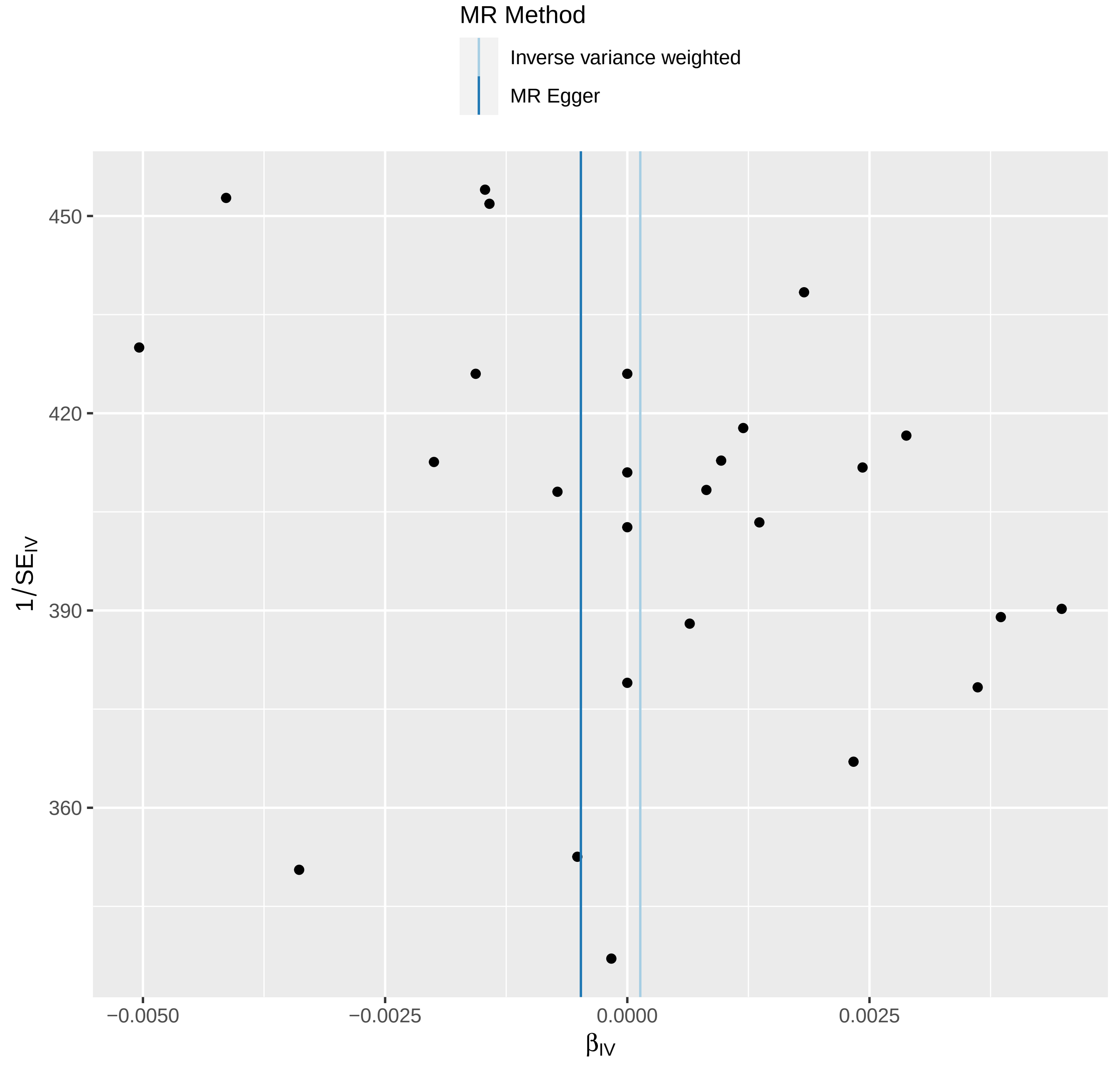

Batch 2−1212 : Appetite−regulating hormone on eGFR

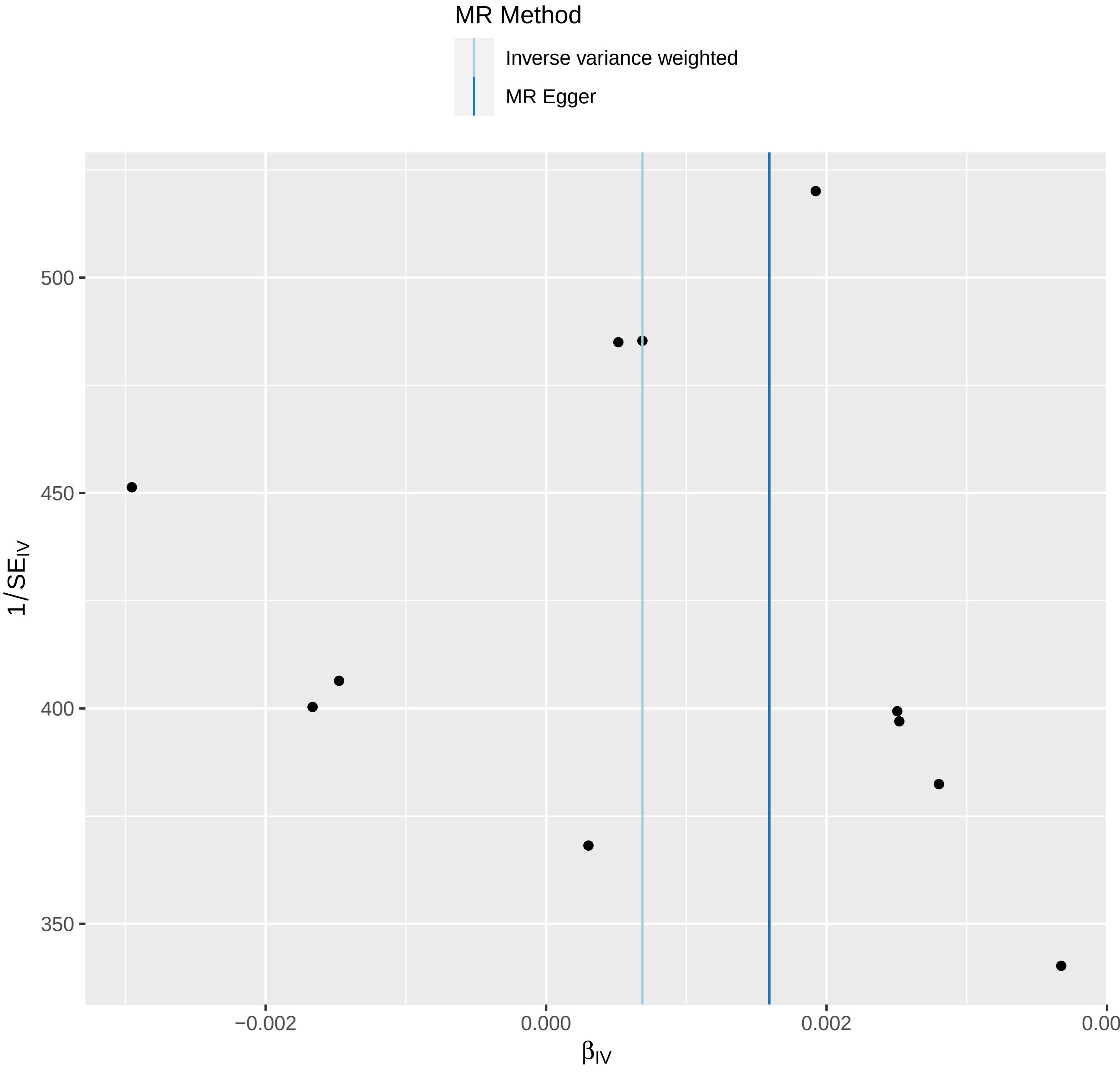

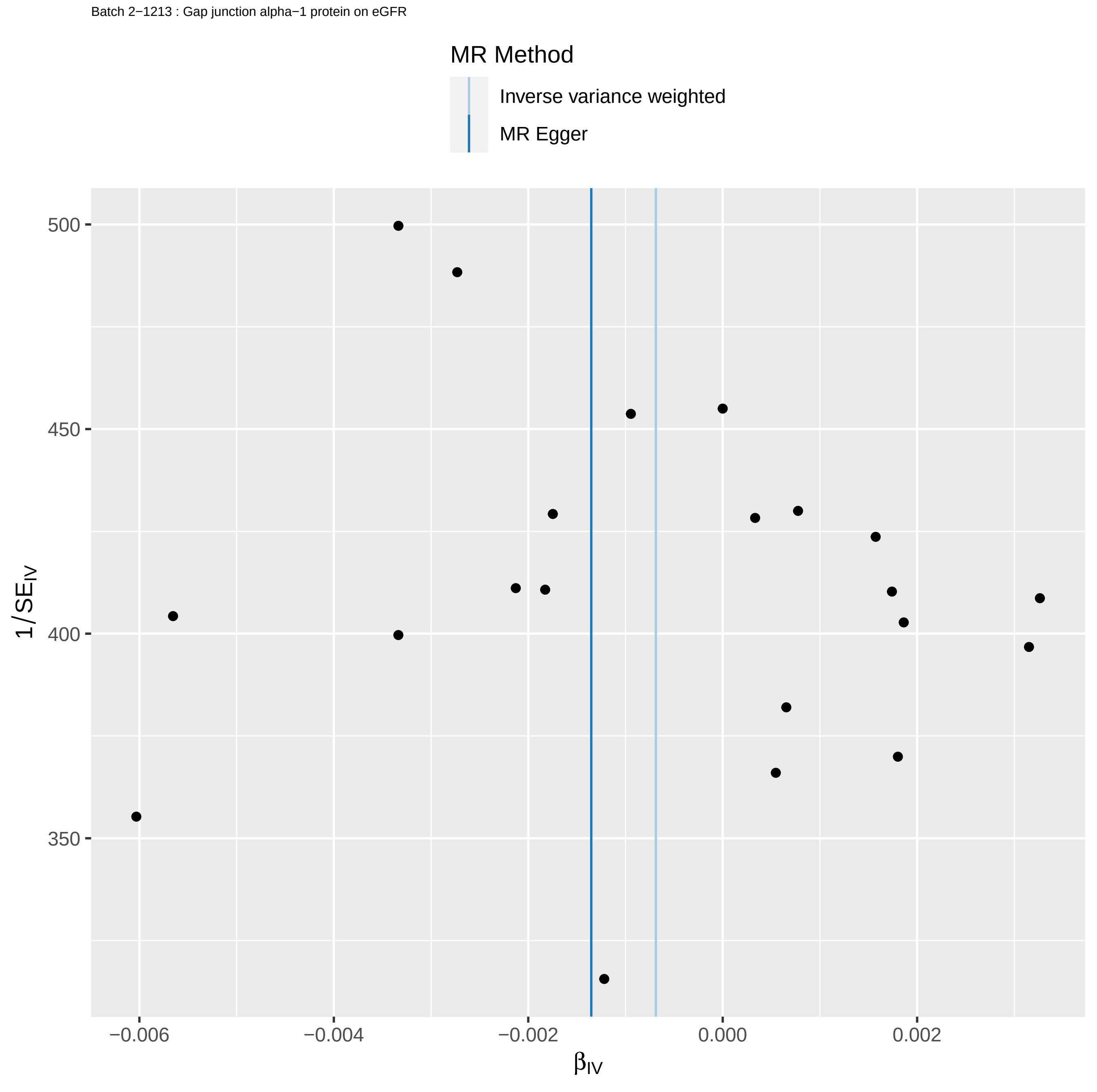
Batch 2−1213 : Gap junction alpha−1 protein on eGFR
MR Method
Inverse variance weighted
MR Egger
500
450
400
350
1/SE_IV
−0.006
−0.004
−0.002
0.000
0.002
β_IV

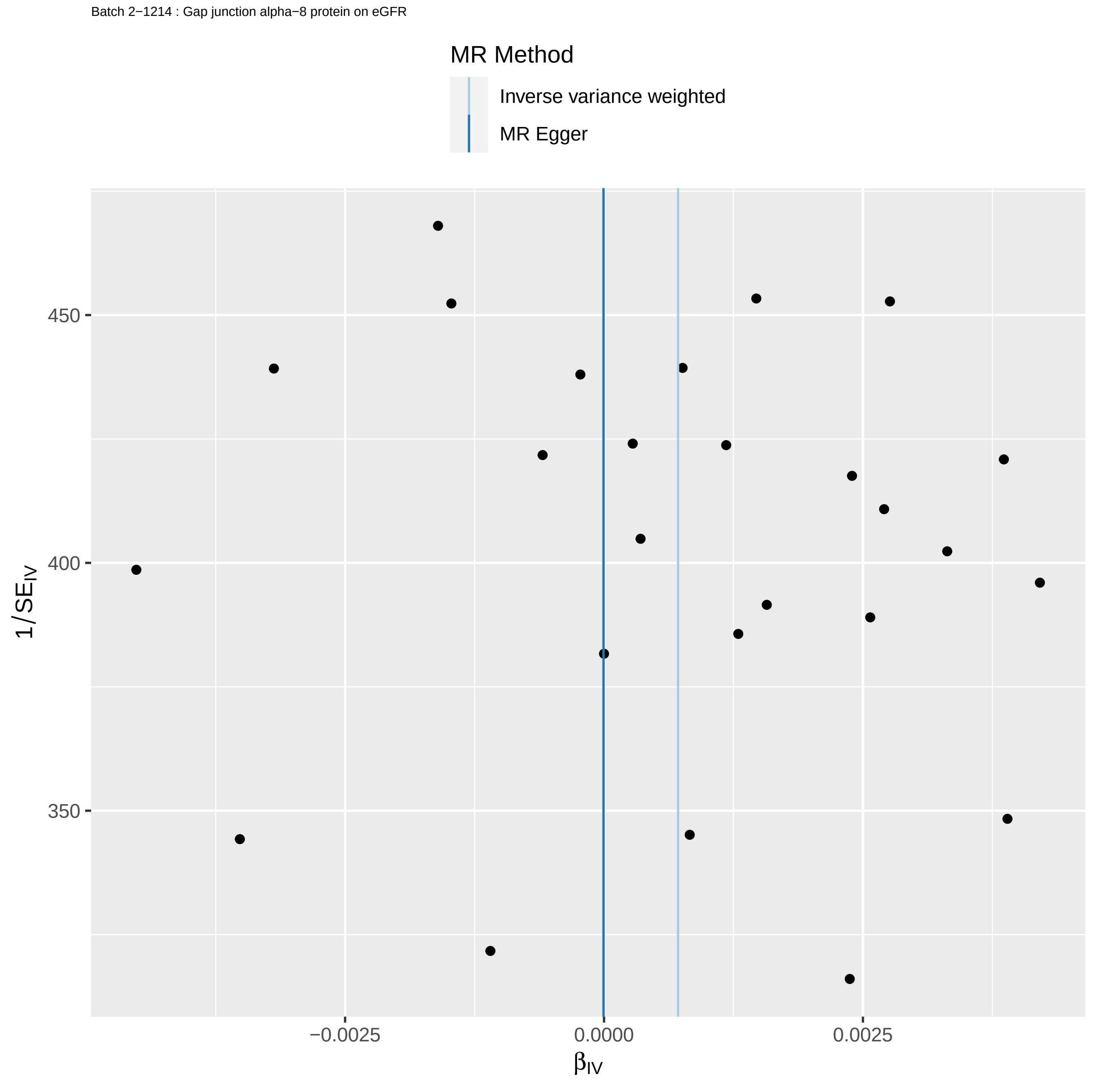

Batch 2−1214 : Gap junction alpha−8 protein on eGFR
MR Method
Inverse variance weighted
MR Egger
450
400
350
1/SE_IV
−0.0025
0.0000
0.0025
β_IV

Batch 2−1215 : Gap junction delta−2 protein on eGFR

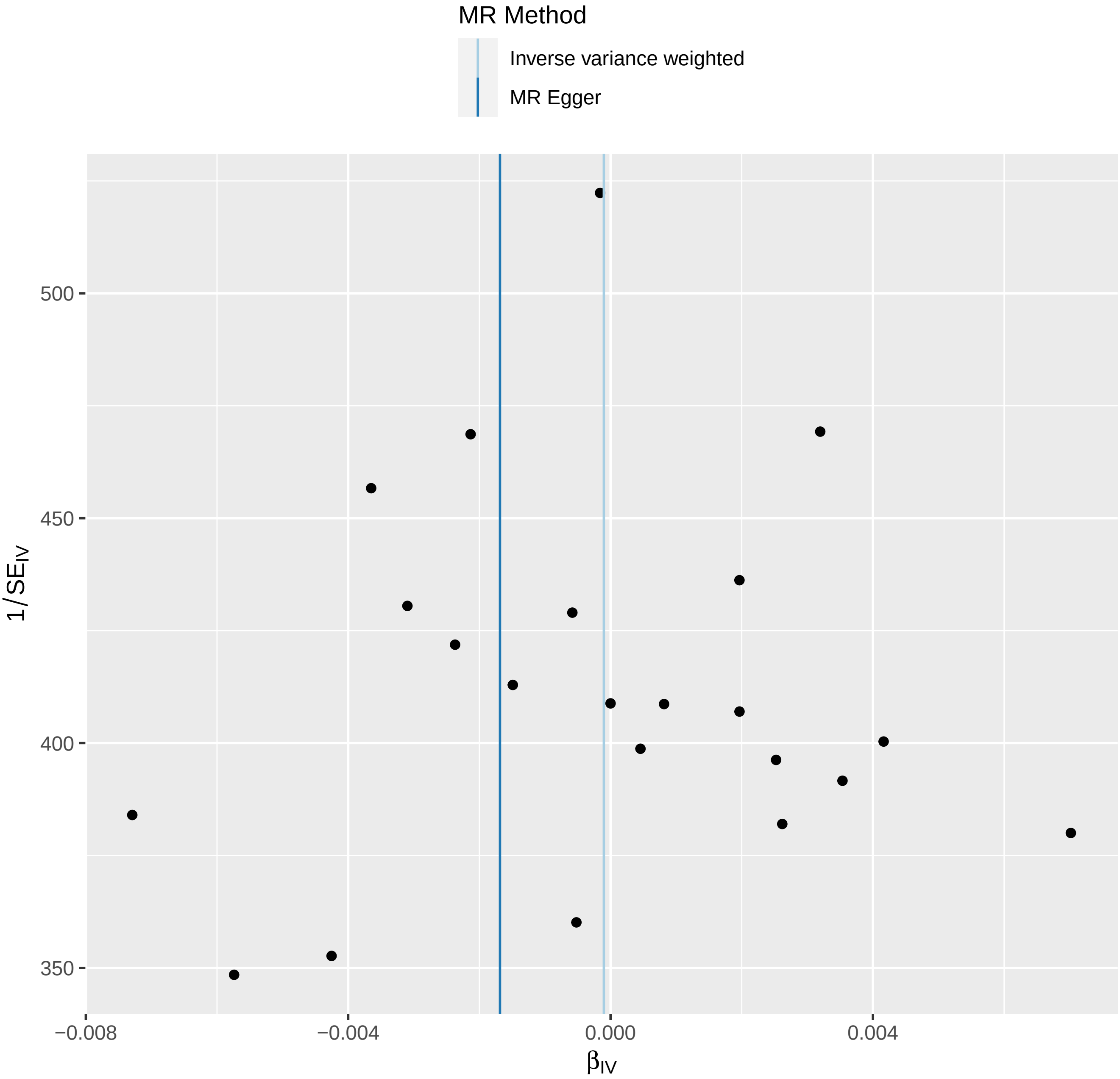

Batch 2−1216 : D−glucuronyl C5−epimerase on eGFR

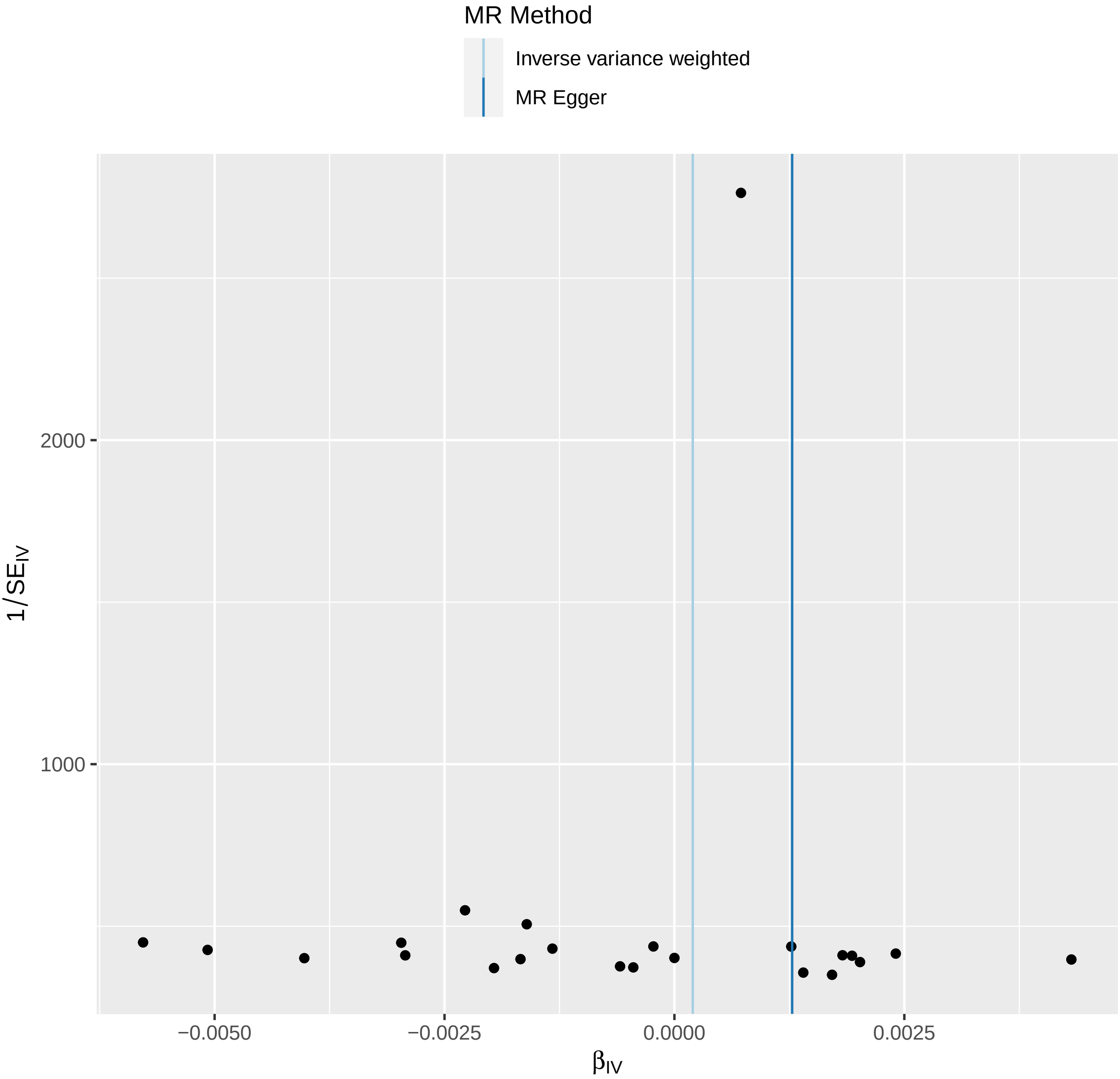

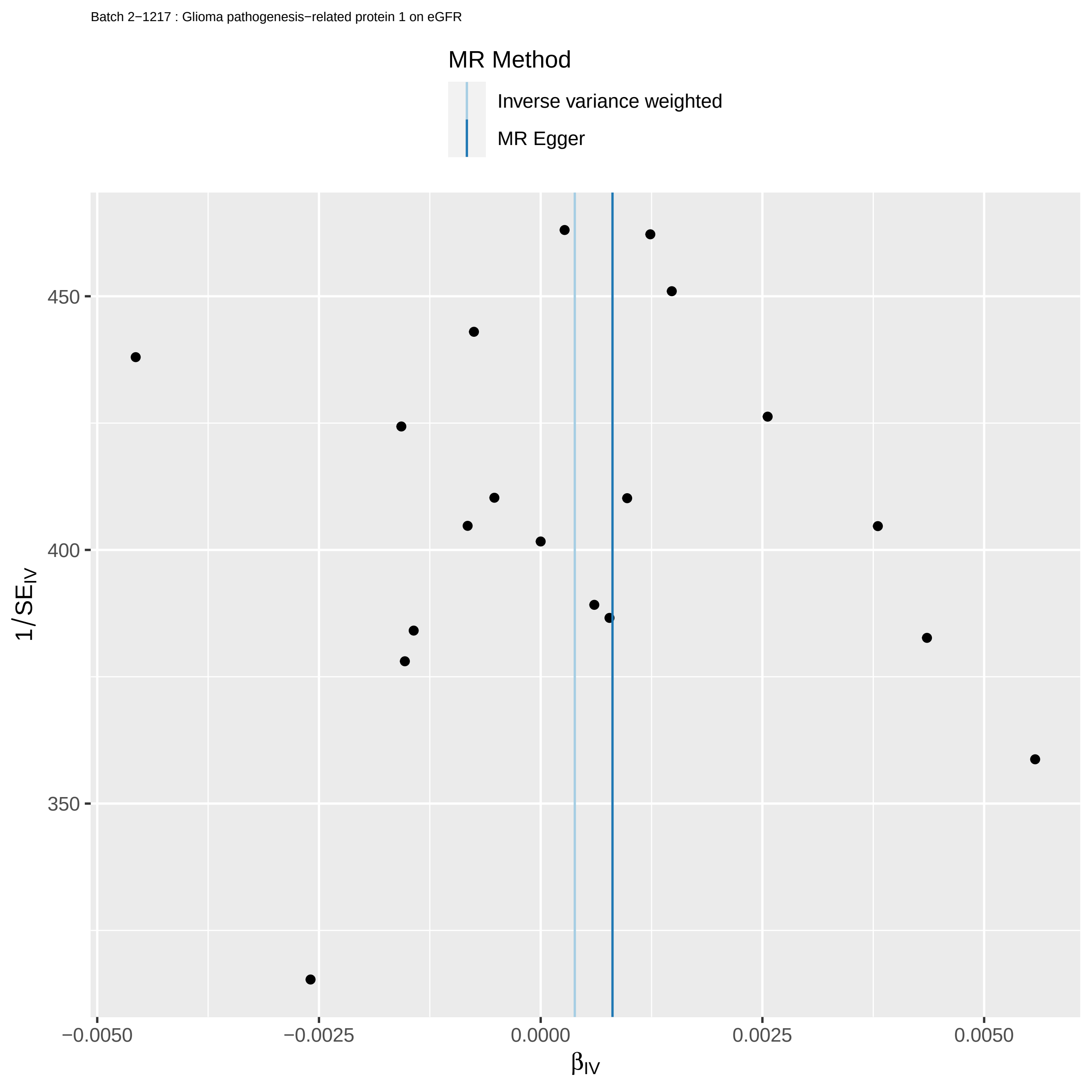

Batch 2−1217 : Glioma pathogenesis−related protein 1 on eGFR
MR Method
Inverse variance weighted
MR Egger
450
400
350
1/SE_IV
−0.0050
−0.0025
0.0000
0.0025
0.0050
β_IV

Batch 2−1218 : Lactoylglutathione lyase on eGFR

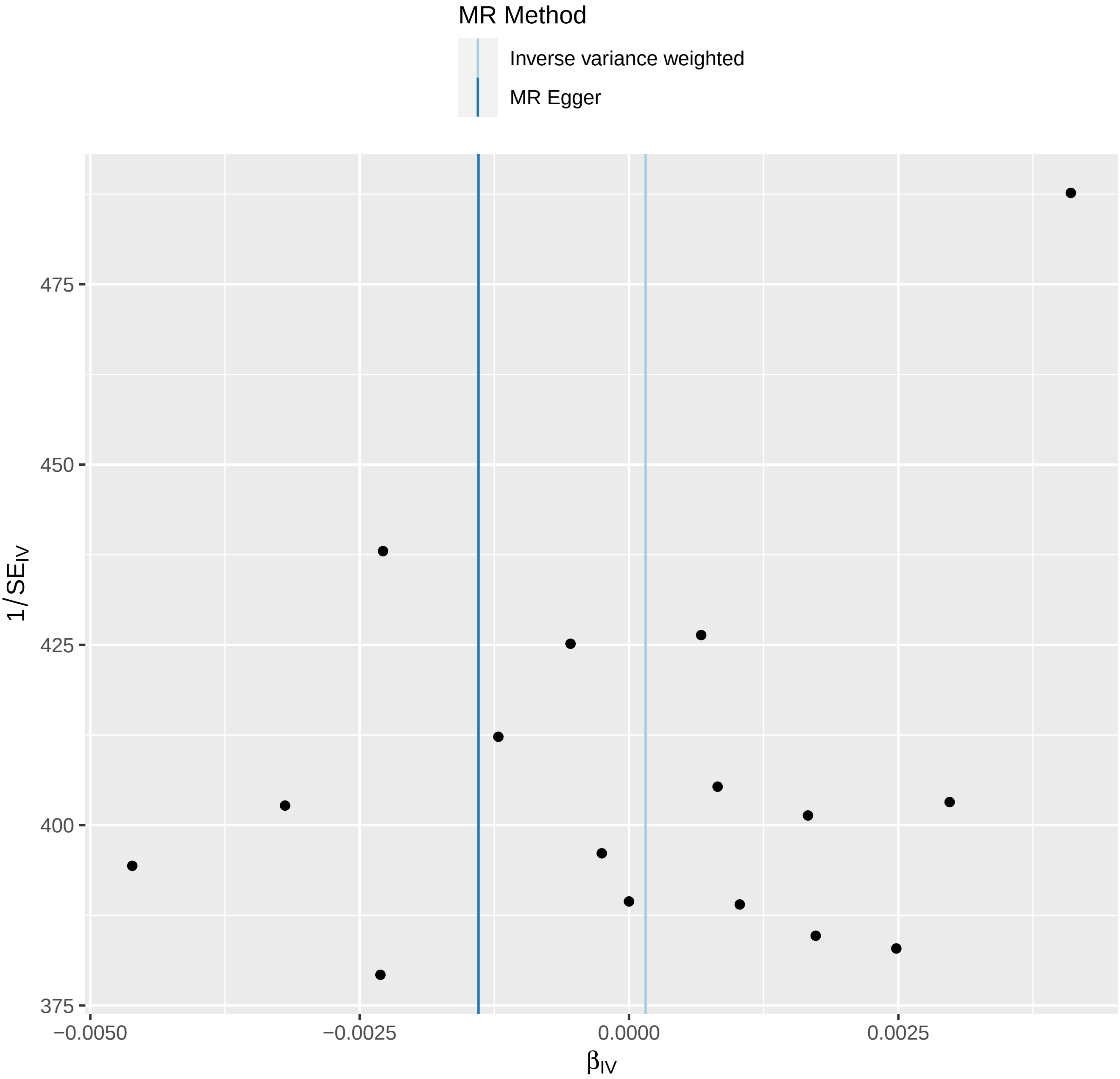

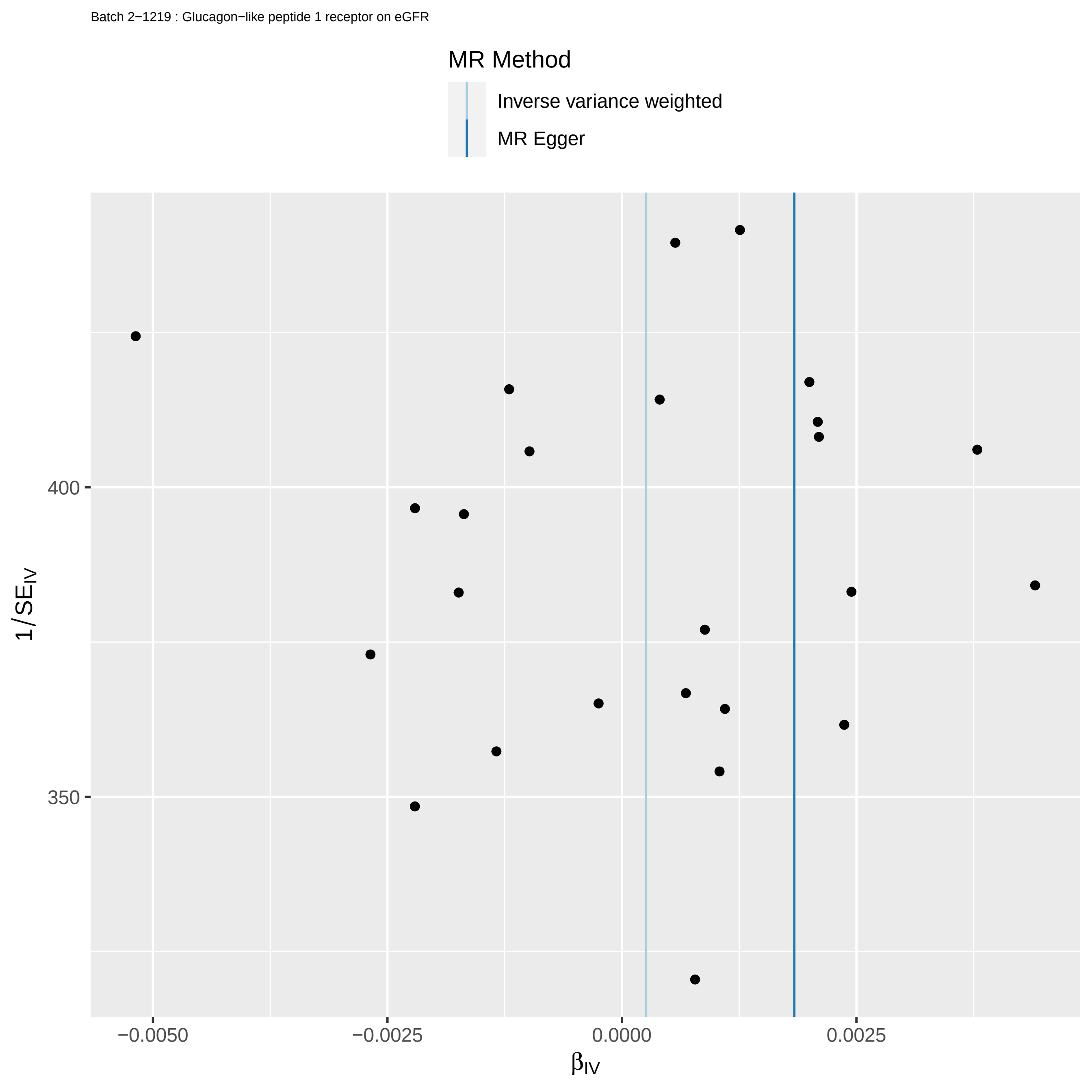
Batch 2−1219 : Glucagon−like peptide 1 receptor on eGFR
MR Method
Inverse variance weighted
MR Egger
400
350
1/SE$_{IV}$
−0.0050
−0.0025
0.0000
0.0025
β$_{IV}$

Batch 2−1220 : Glutaredoxin−2, mitochondrial on eGFR

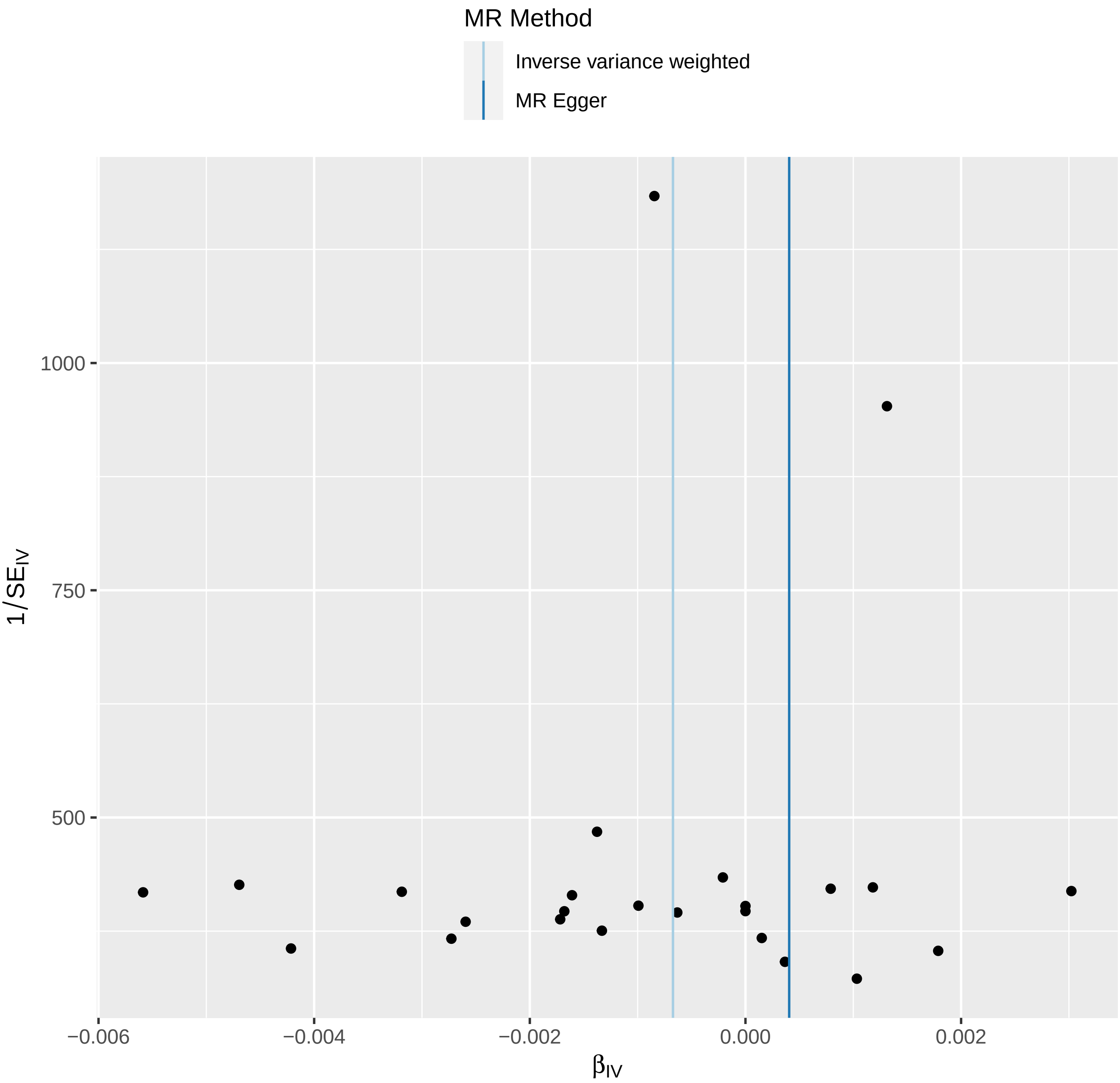

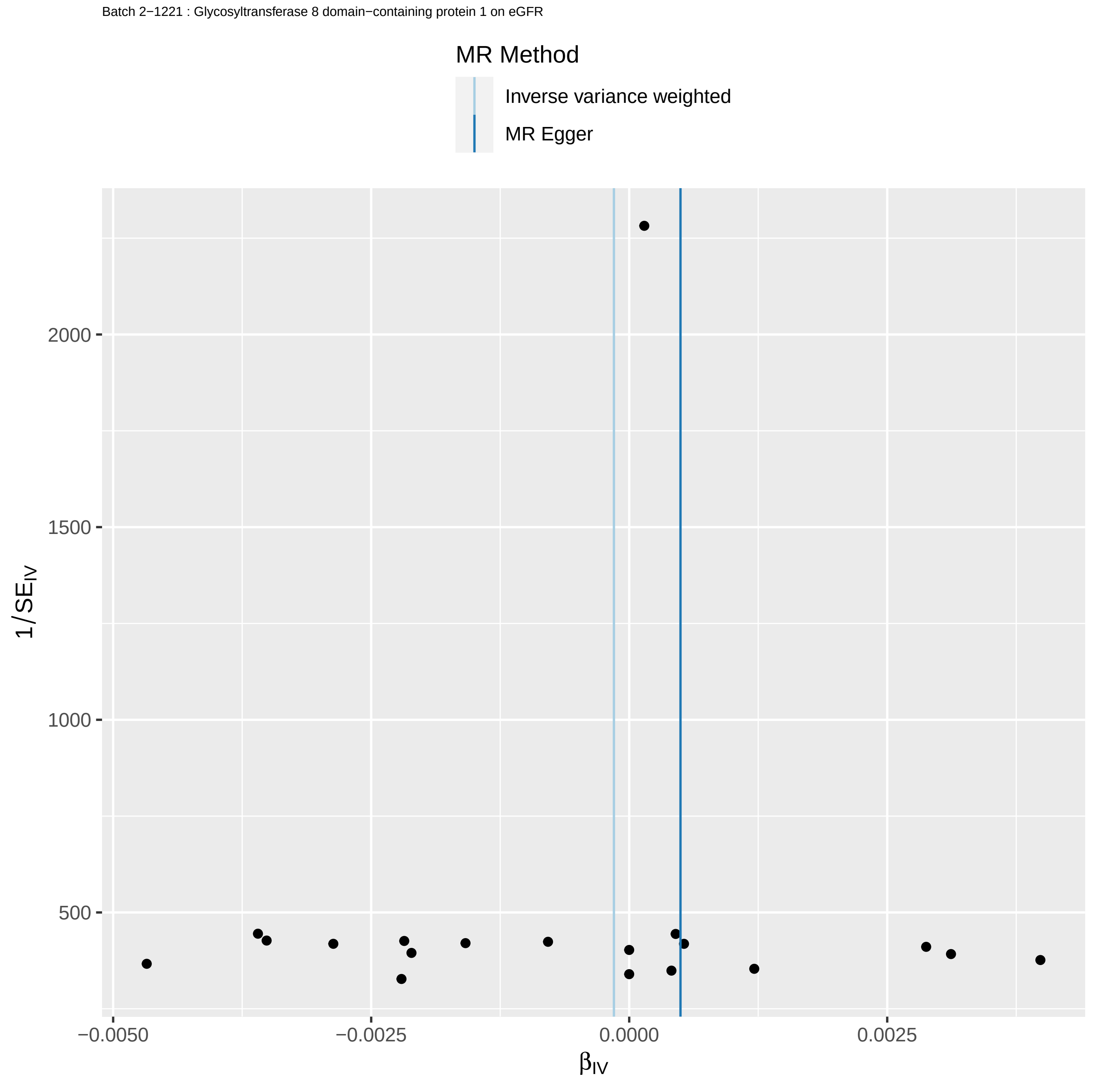

Batch 2−1221 : Glycosyltransferase 8 domain−containing protein 1 on eGFR
MR Method
Inverse variance weighted
MR Egger
2000
1500
1000
500
1/SE$_{IV}$
−0.0050
−0.0025
0.0000
0.0025
β$_{IV}$

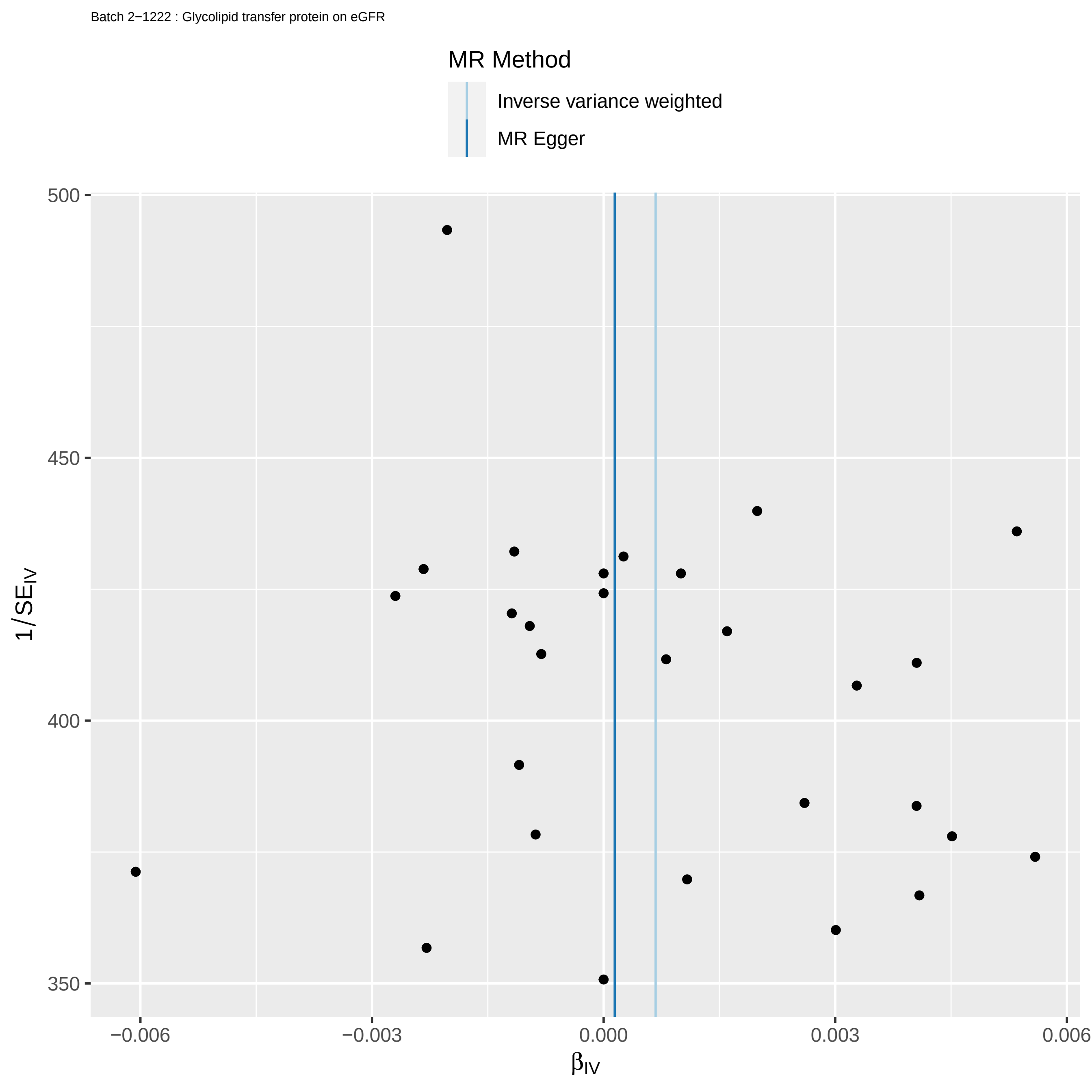
Batch 2−1222 : Glycolipid transfer protein on eGFR
MR Method
Inverse variance weighted
MR Egger
500
450
400
350
1/SE$_{IV}$
−0.006
−0.003
0.000
0.003
0.006
β$_{IV}$

Batch 2−1223 : Glycolipid transfer protein domain−containing protein 2 on eGFR

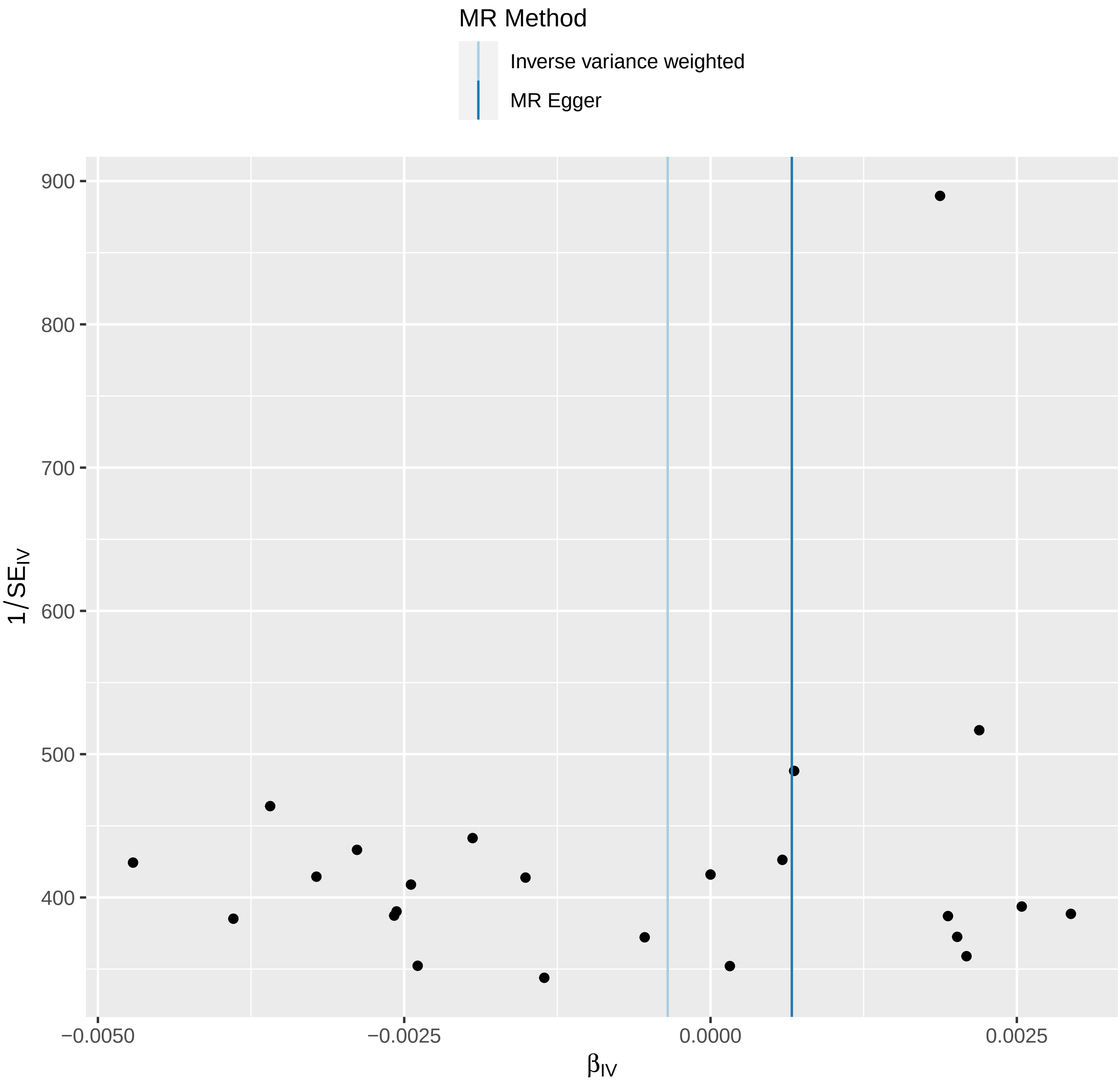

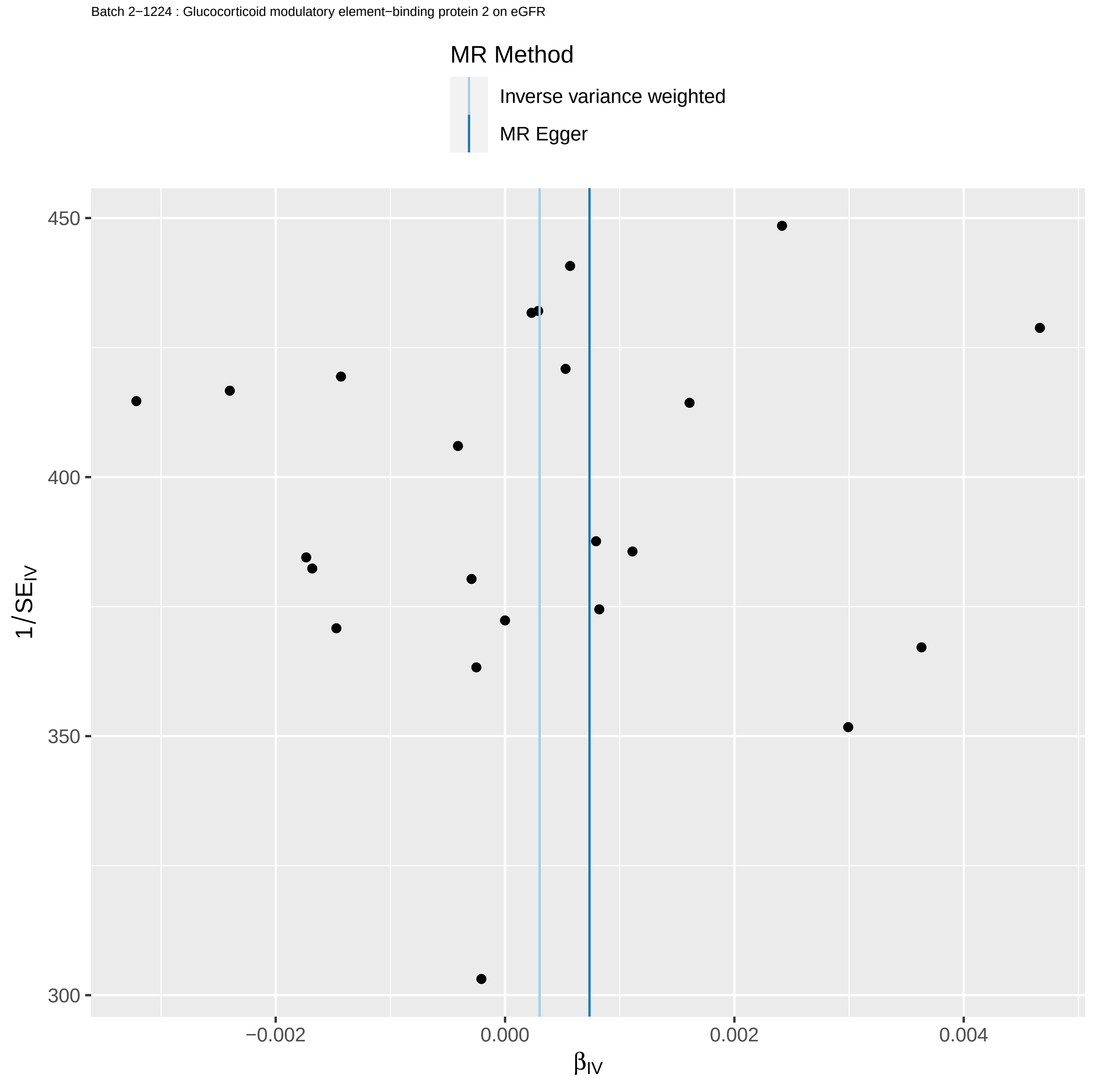

Batch 2−1224 : Glucocorticoid modulatory element−binding protein 2 on eGFR
MR Method
Inverse variance weighted
MR Egger
450
400
350
300
1/SE$_{IV}$
−0.002
0.000
0.002
0.004
β$_{IV}$

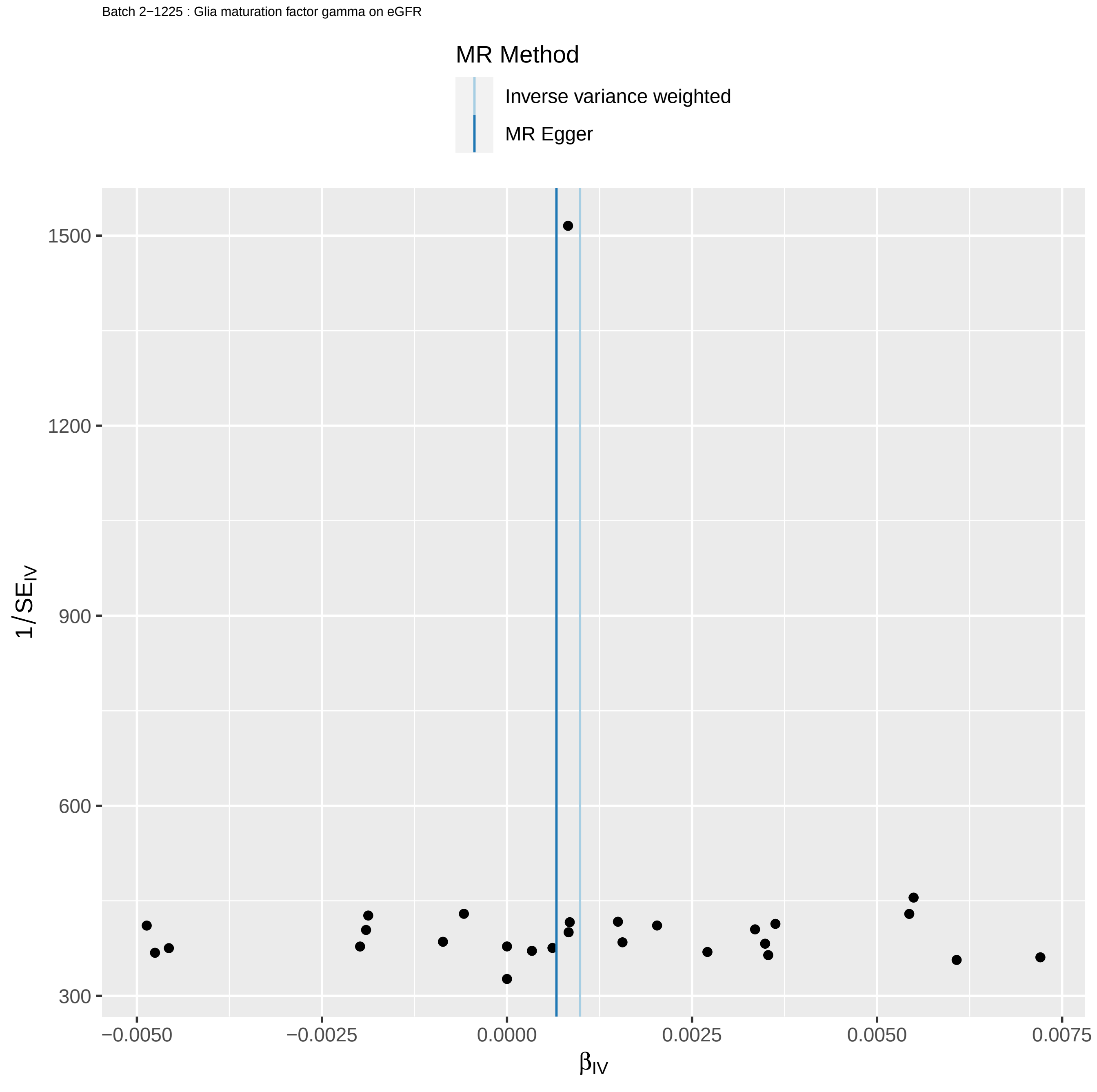

Batch 2−1225 : Glia maturation factor gamma on eGFR
MR Method
Inverse variance weighted
MR Egger
1500
1200
900
600
300
1/SE_IV
−0.0050
−0.0025
0.0000
0.0025
0.0050
0.0075
β_IV

Batch 2−1226 : Guanine nucleotide−binding protein G(k) subunit alpha on eGFR

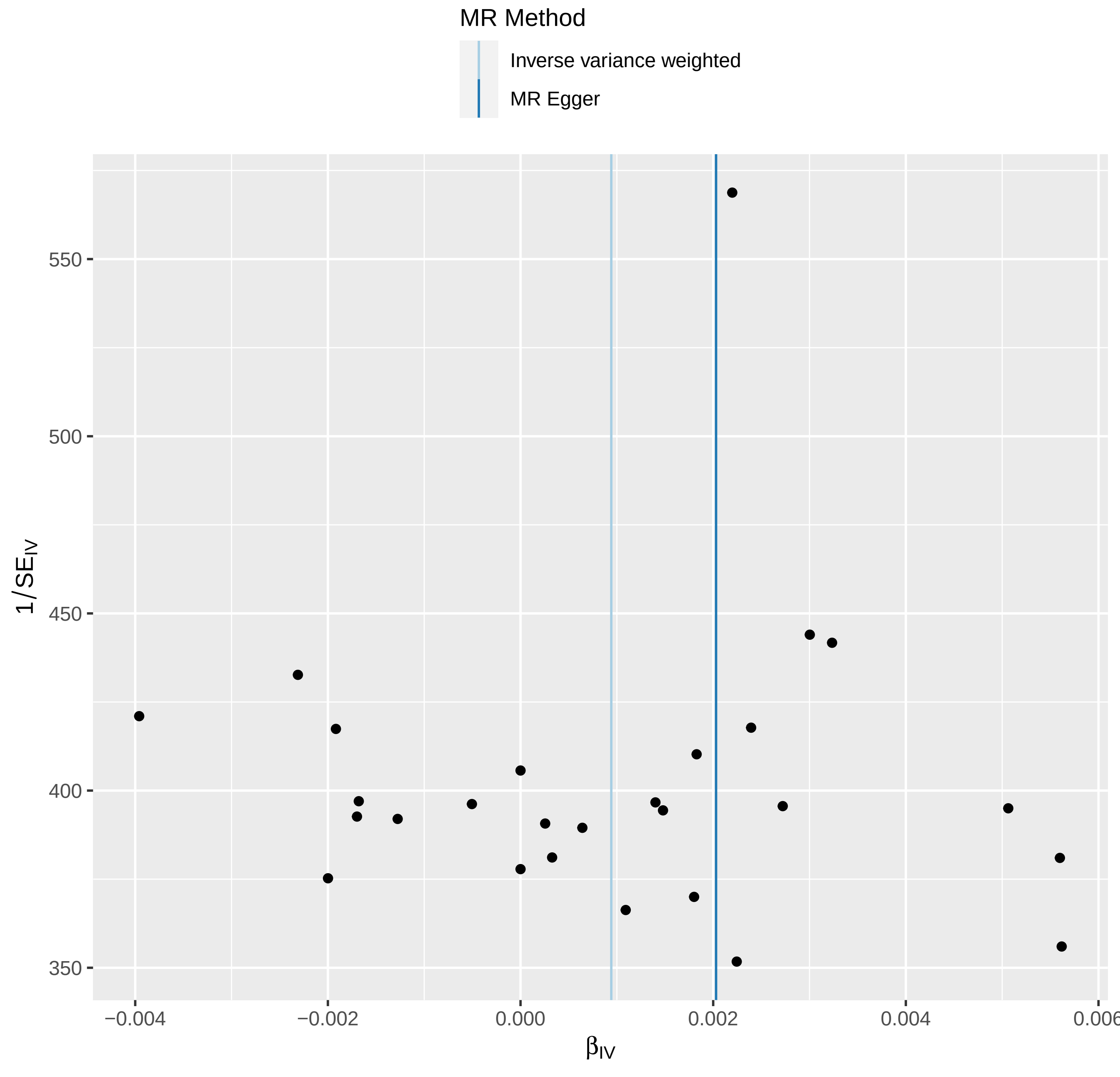

Batch 2−1227 : Guanine nucleotide−binding protein G(I)/G(S)/G(O) subunit gamma−T2 on eGFR

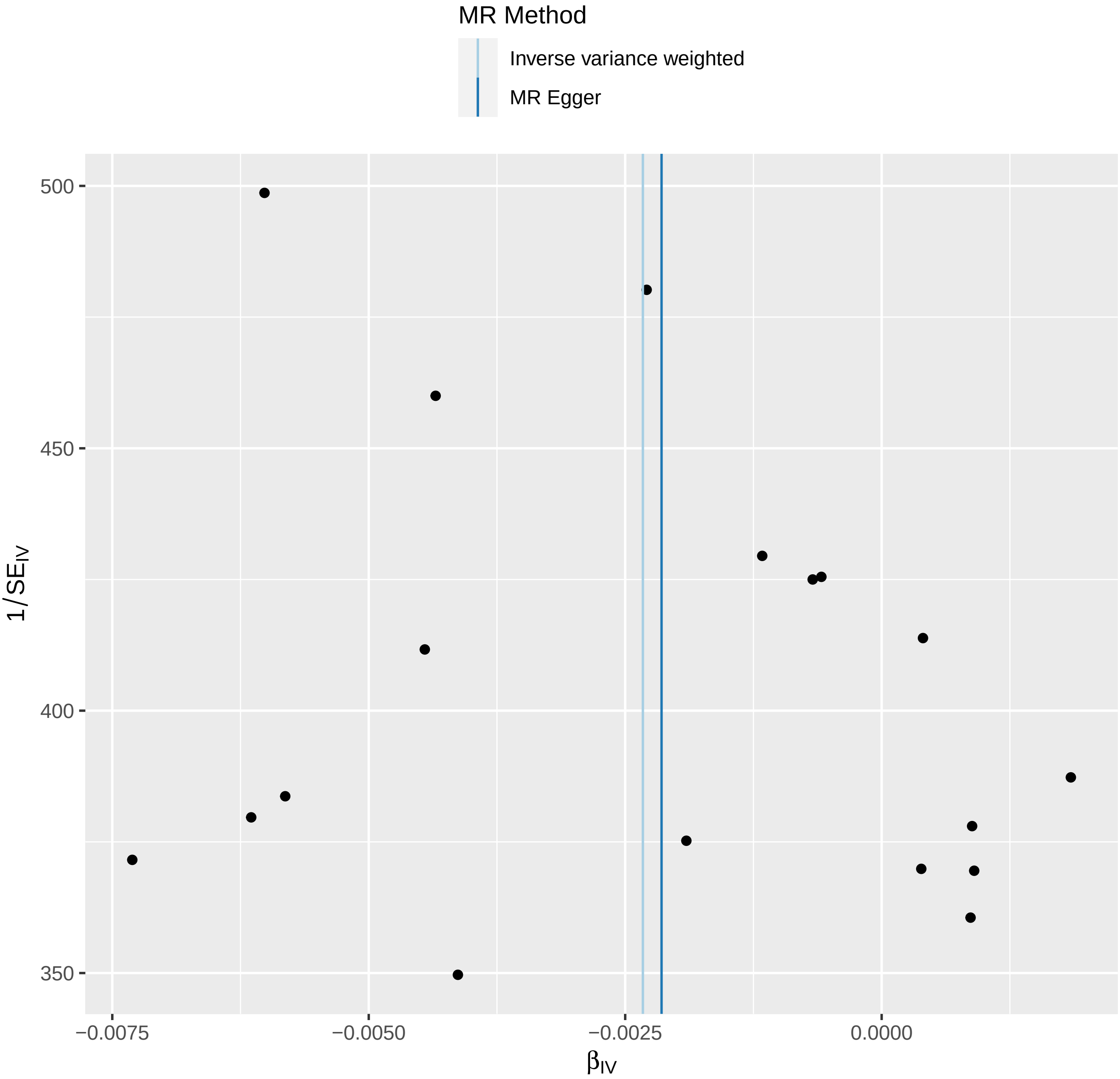

Batch 2−1228 : Granulysin on eGFR

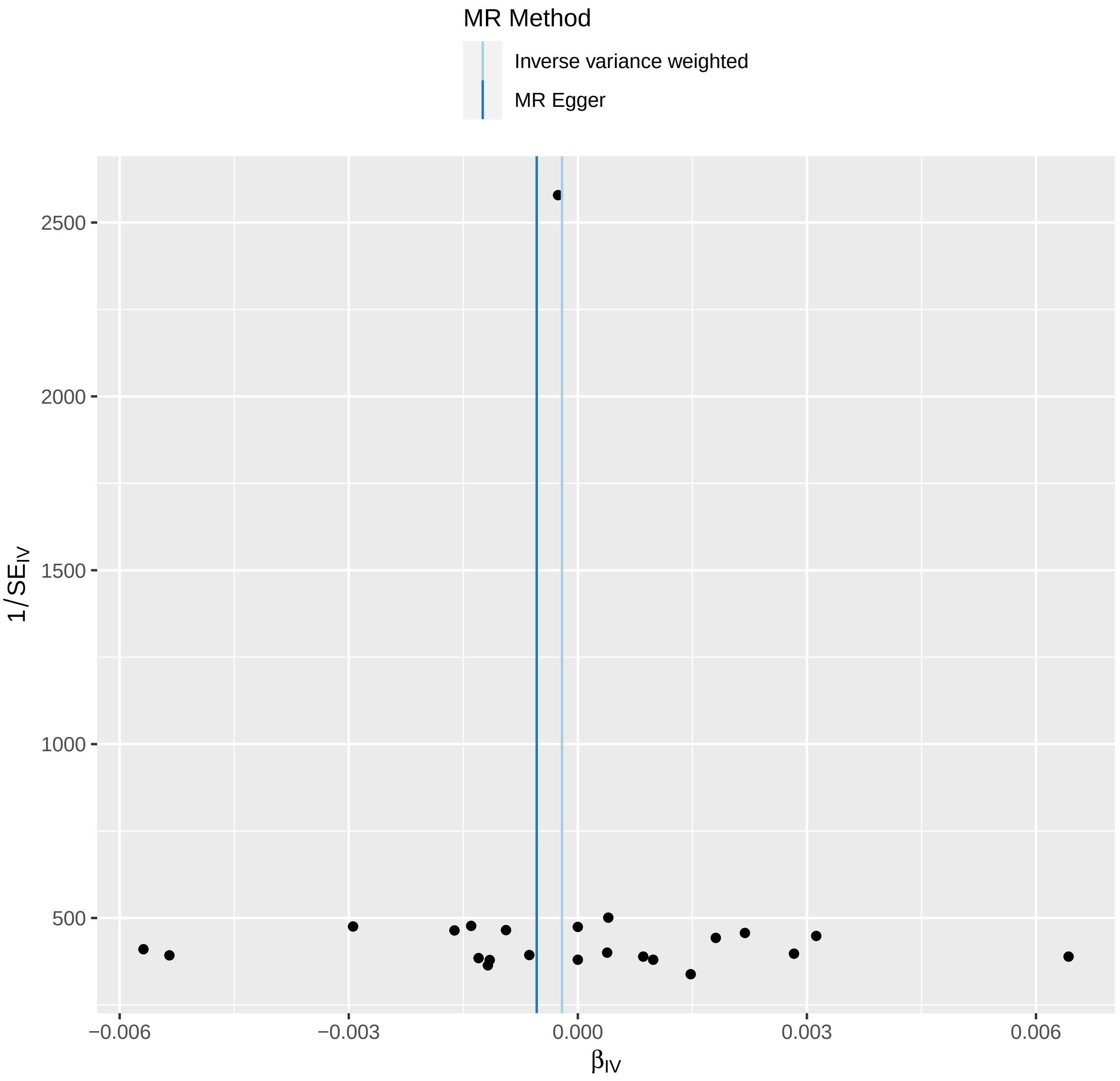

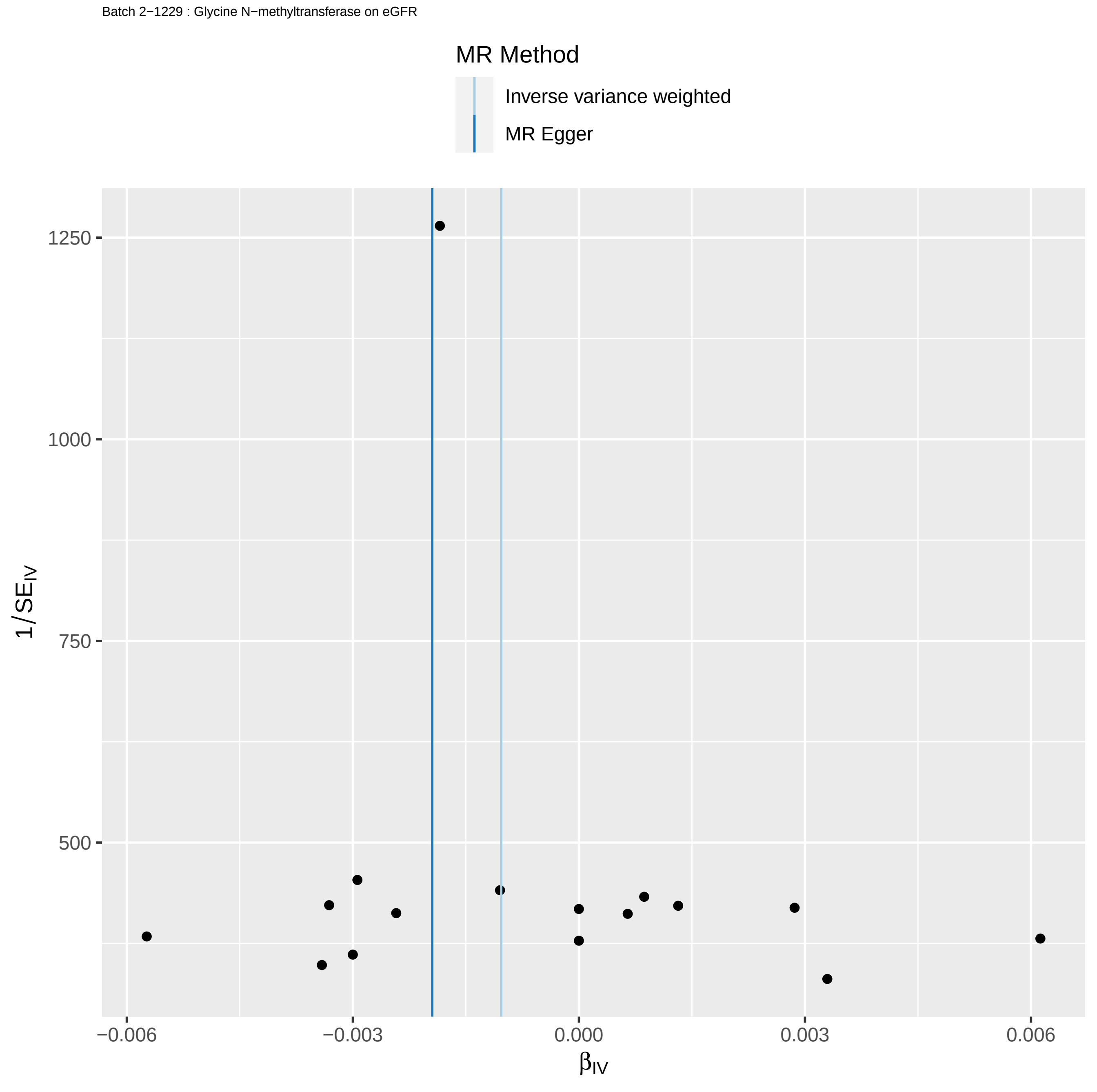
Batch 2−1229 : Glycine N−methyltransferase on eGFR
MR Method
Inverse variance weighted
MR Egger
1250
1000
750
500
1/SE_IV
−0.006
−0.003
0.000
0.003
0.006
β_IV

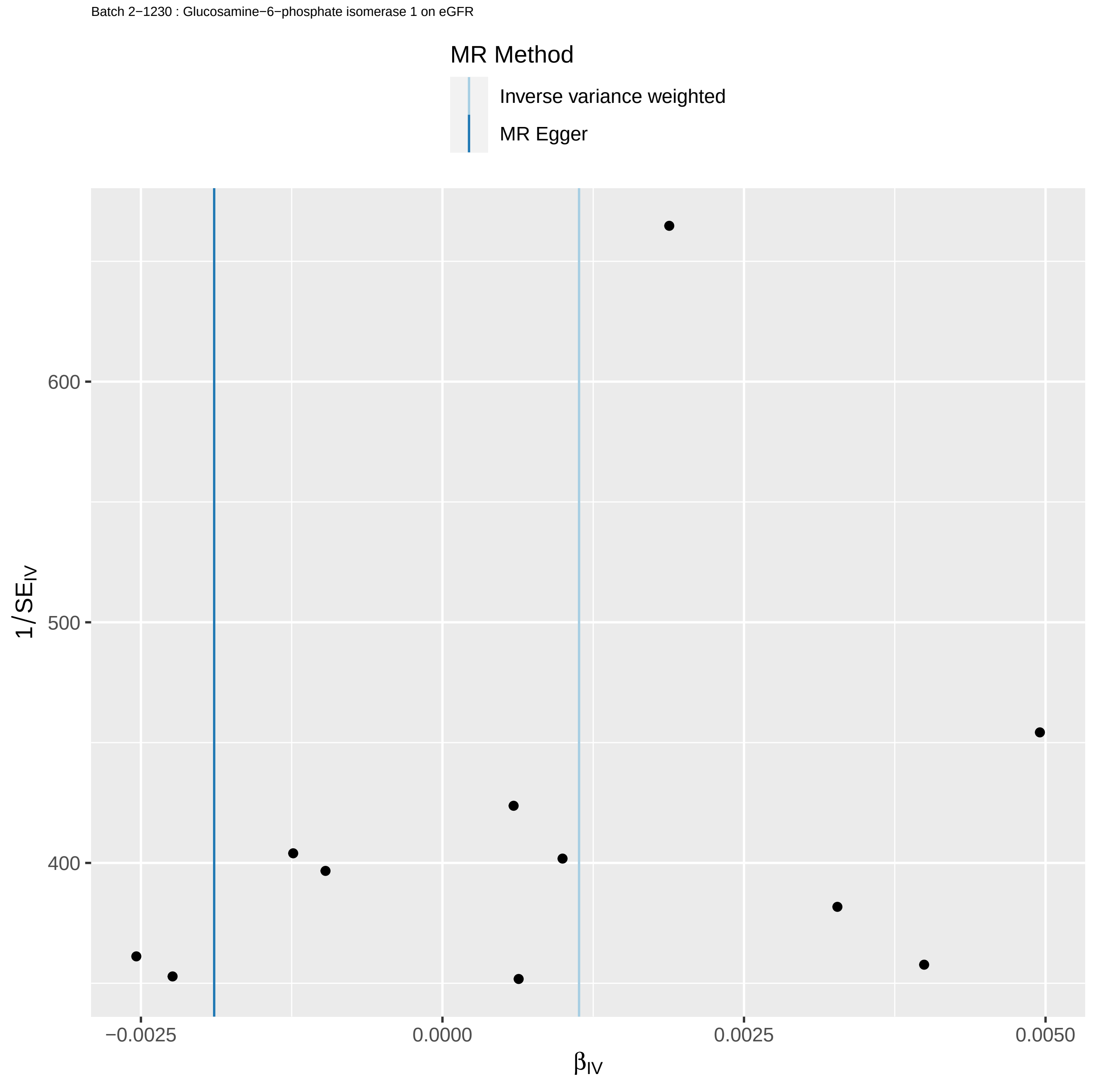

Batch 2−1230 : Glucosamine−6−phosphate isomerase 1 on eGFR
MR Method
Inverse variance weighted
MR Egger
600
500
400
1/SE_IV
−0.0025
0.0000
0.0025
0.0050
β_IV

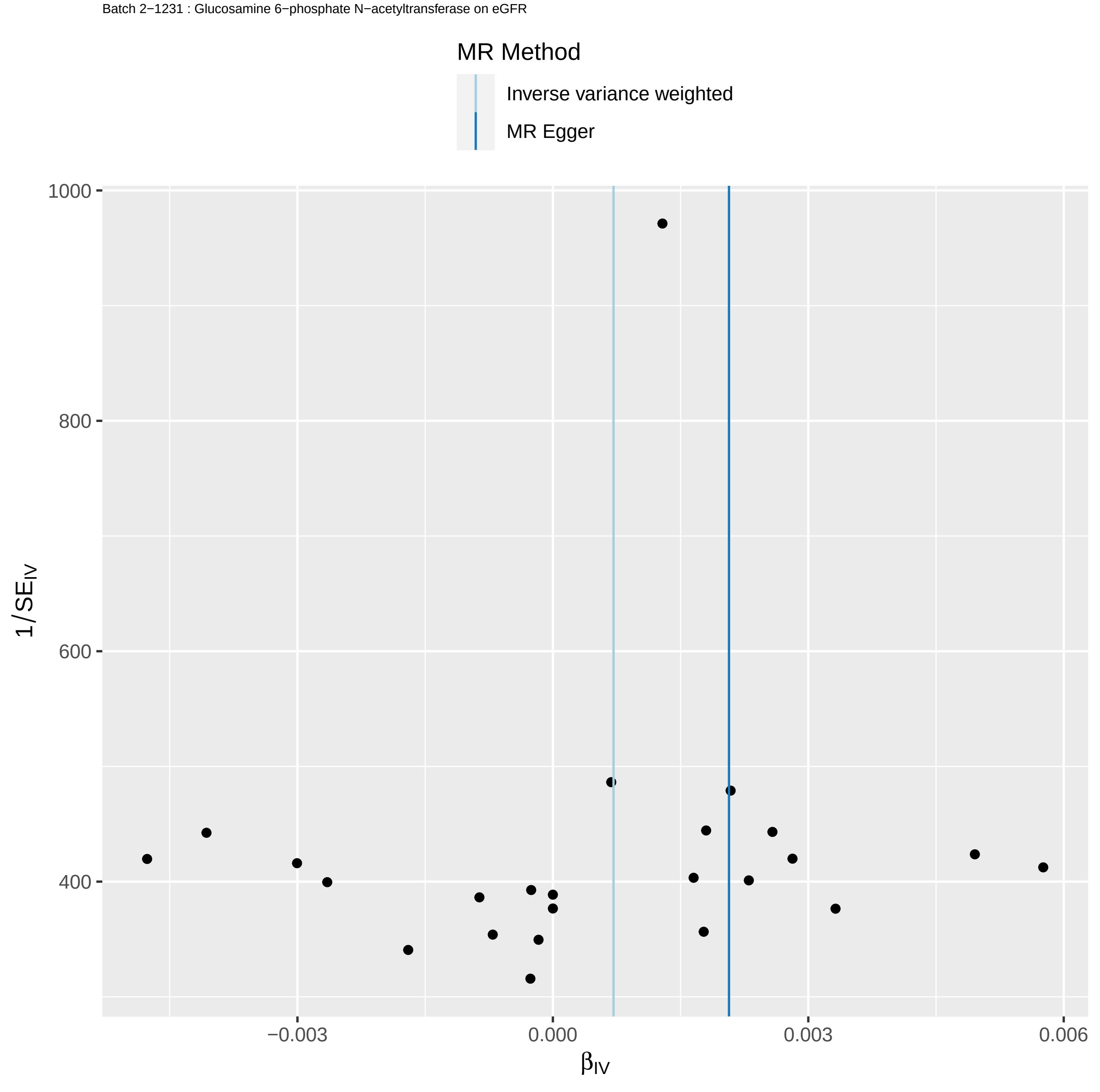
Batch 2−1231 : Glucosamine 6−phosphate N−acetyltransferase on eGFR
MR Method
Inverse variance weighted
MR Egger
1000
800
600
400
1/SE_IV
−0.003
0.000
0.003
0.006
β_IV

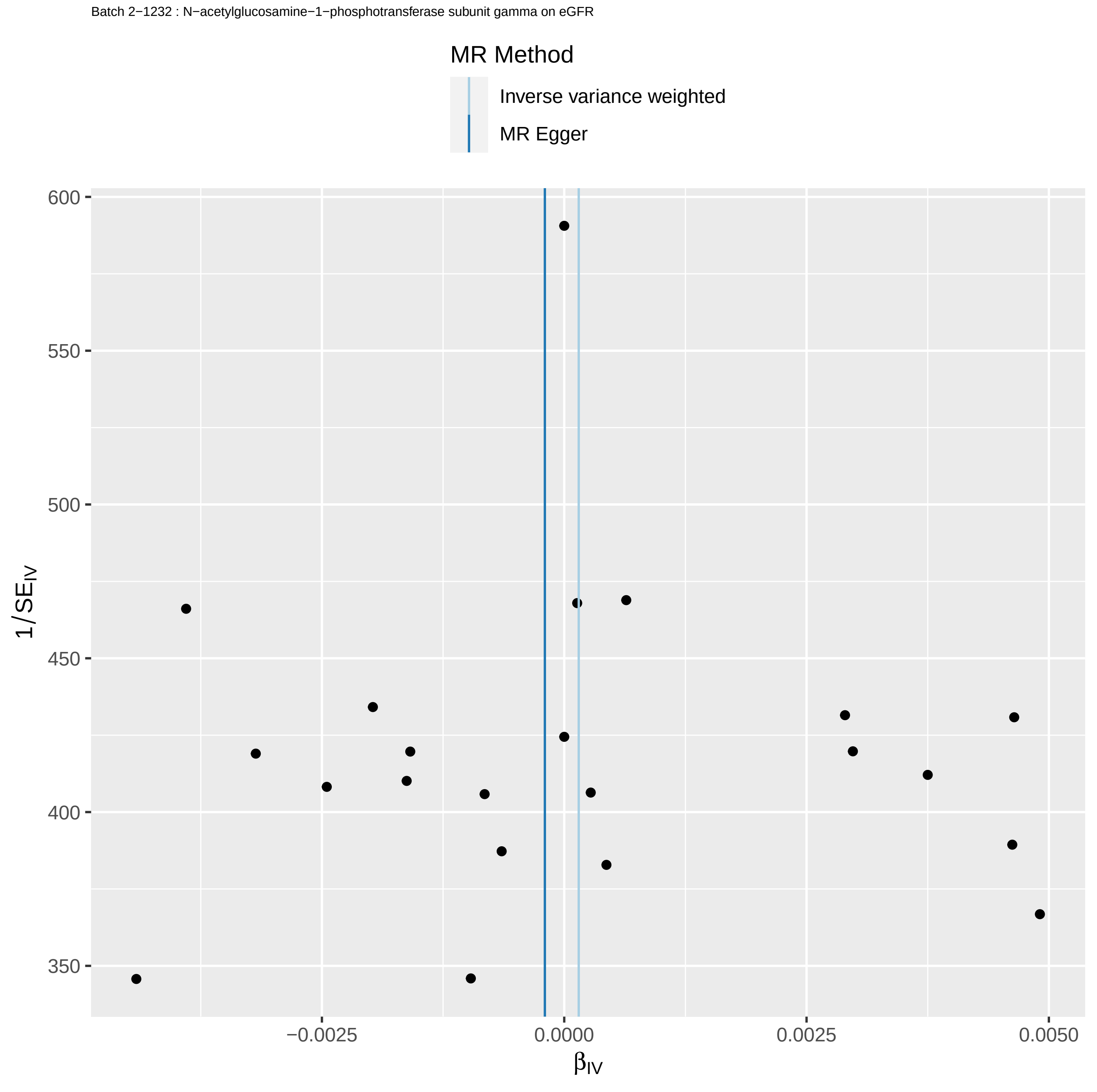
Batch 2−1232 : N−acetylglucosamine−1−phosphotransferase subunit gamma on eGFR
MR Method
Inverse variance weighted
MR Egger
600
550
500
450
400
350
1/SE_IV
−0.0025
0.0000
0.0025
0.0050
β_IV

Batch 2−1233 : Progonadoliberin−1 on eGFR

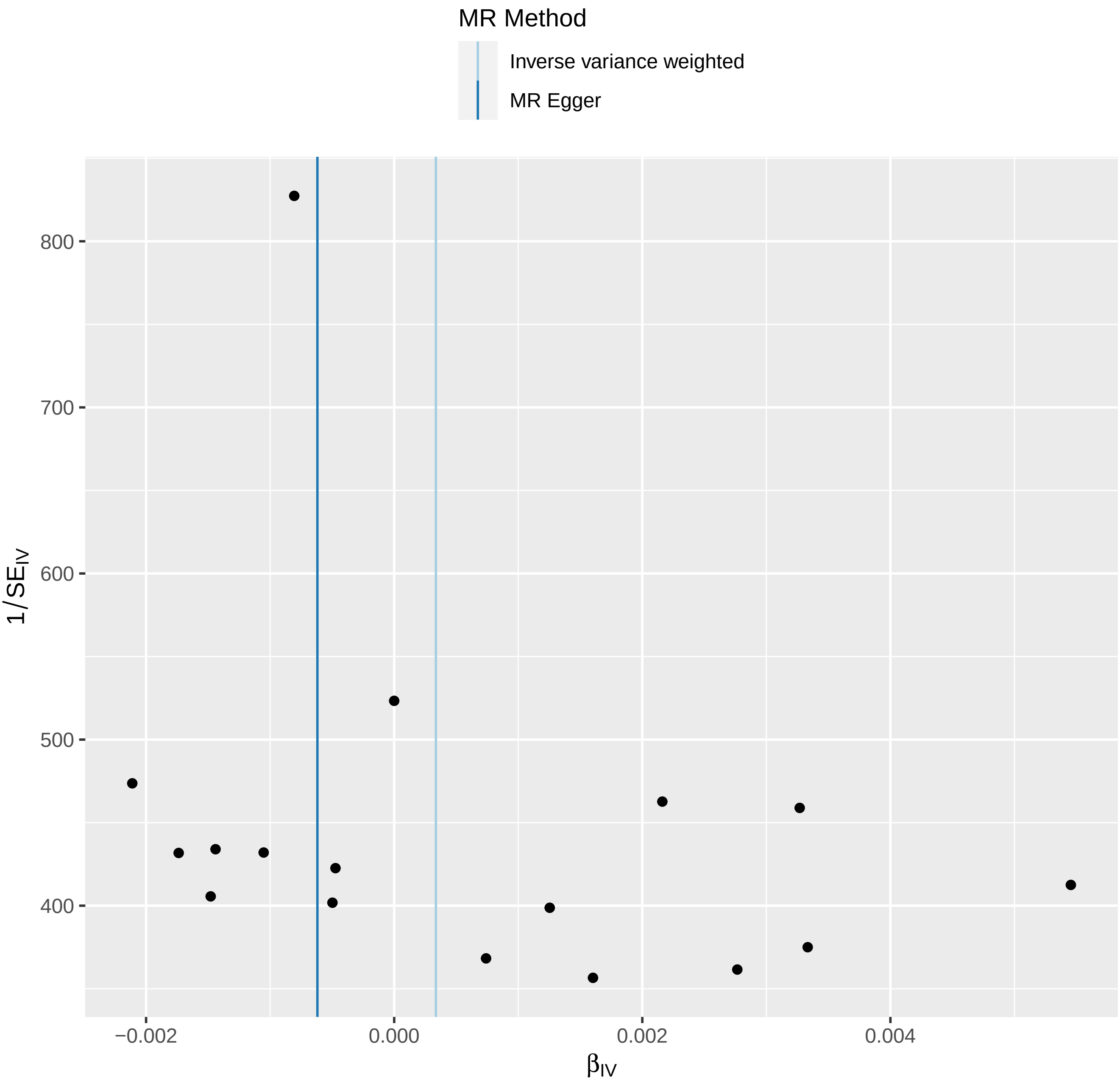

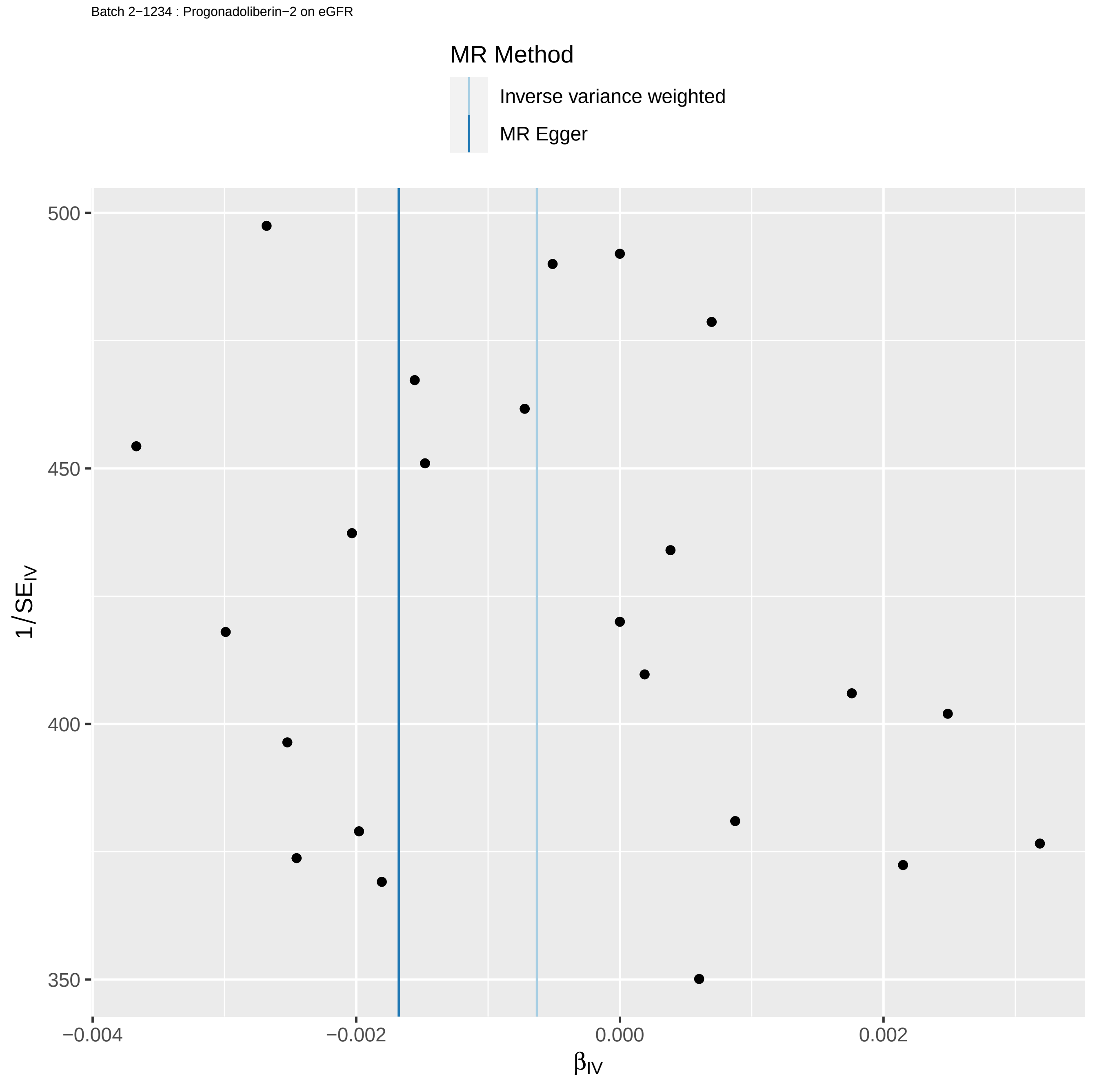
Batch 2−1234 : Progonadoliberin−2 on eGFR
MR Method
Inverse variance weighted
MR Egger
500
450
400
350
1/SE$_{IV}$
−0.004
−0.002
0.000
0.002
β$_{IV}$

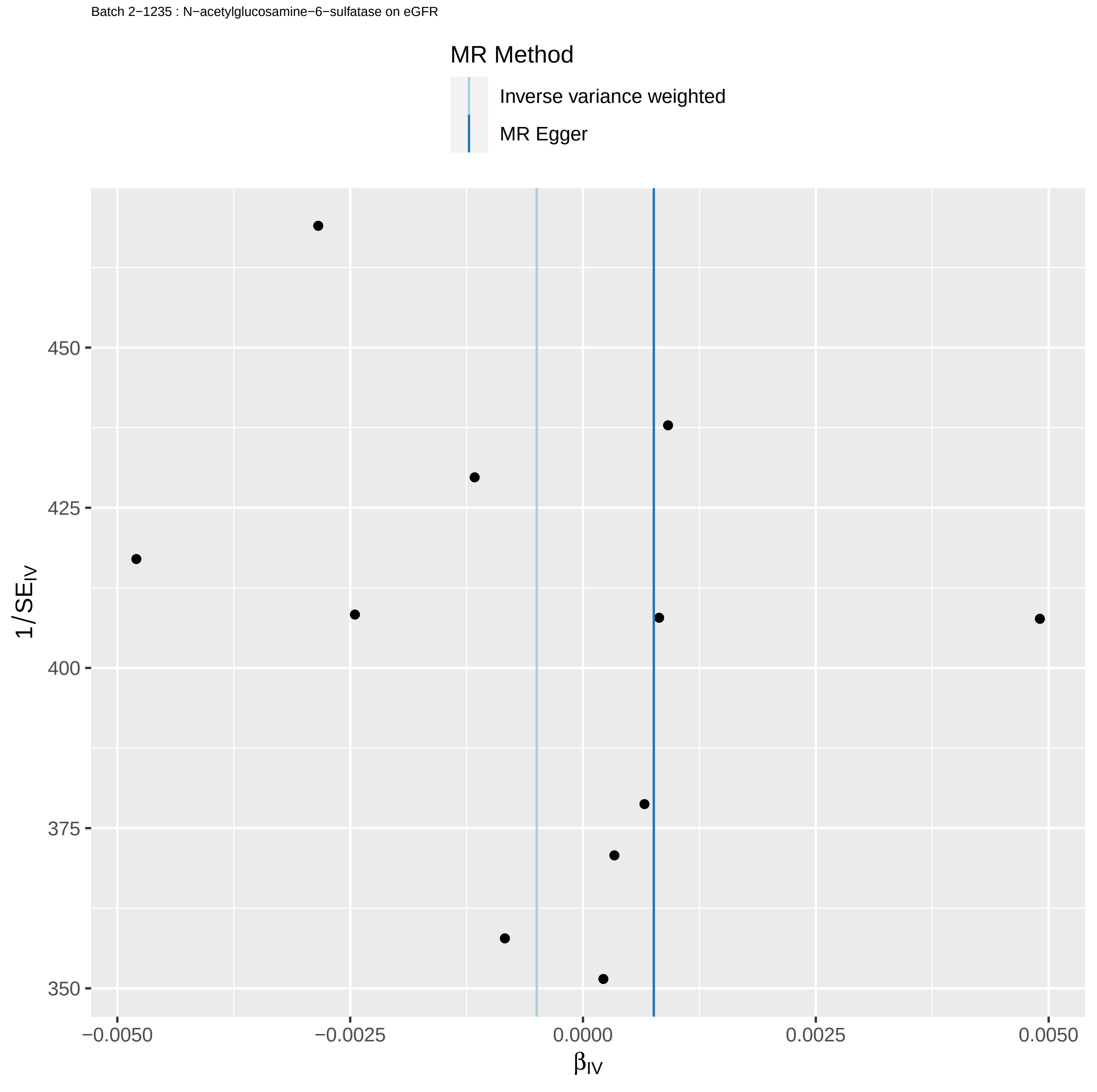

Batch 2−1235 : N−acetylglucosamine−6−sulfatase on eGFR
MR Method
Inverse variance weighted
MR Egger
450
425
400
375
350
$1/SE_{IV}$
−0.0050
−0.0025
0.0000
0.0025
0.0050
β_{IV}

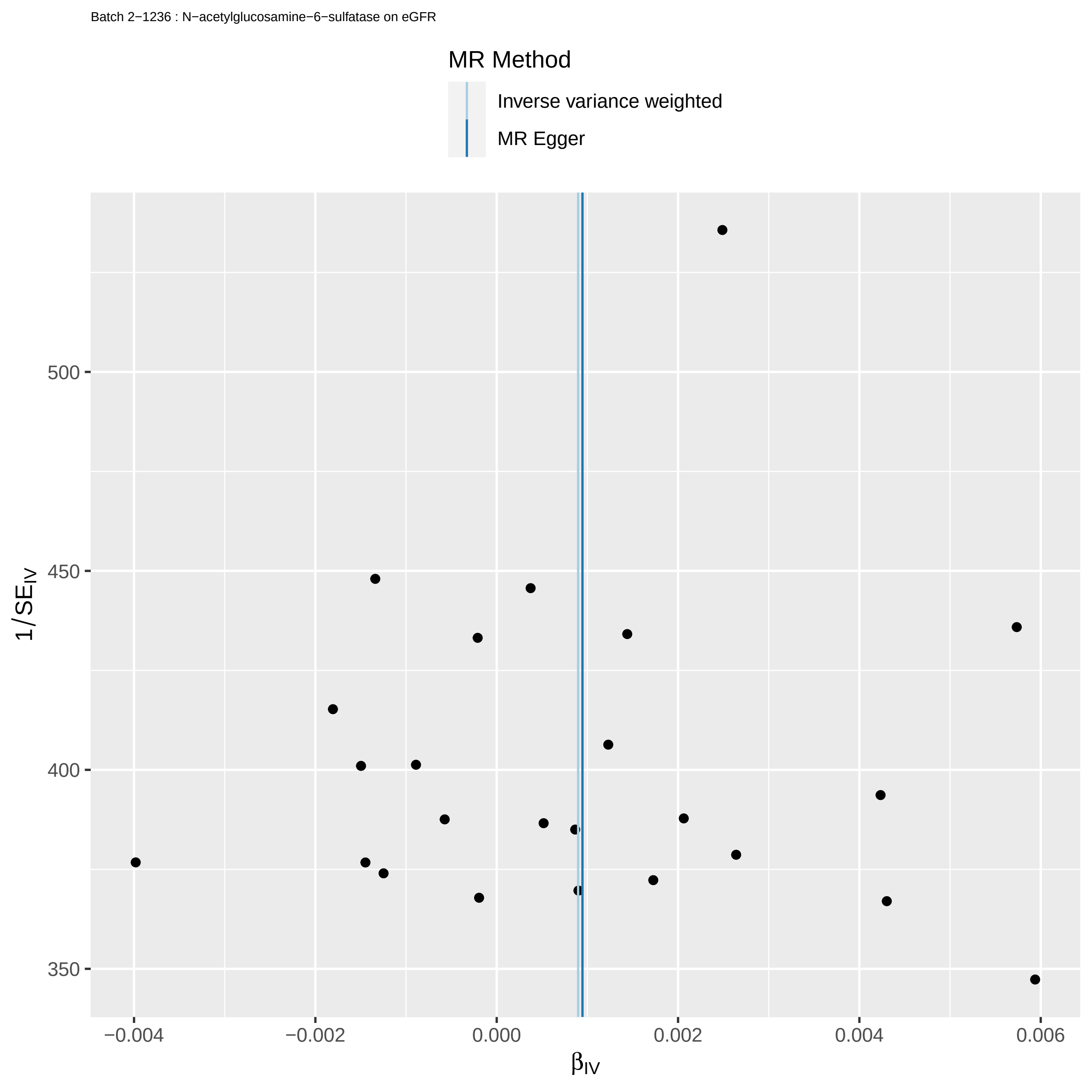

Batch 2−1236 : N−acetylglucosamine−6−sulfatase on eGFR
MR Method
Inverse variance weighted
MR Egger
500
450
400
350
1/SE$_{IV}$
−0.004
−0.002
0.000
0.002
0.004
0.006
β$_{IV}$

Batch 2−1237 : Golgi membrane protein 1 on eGFR

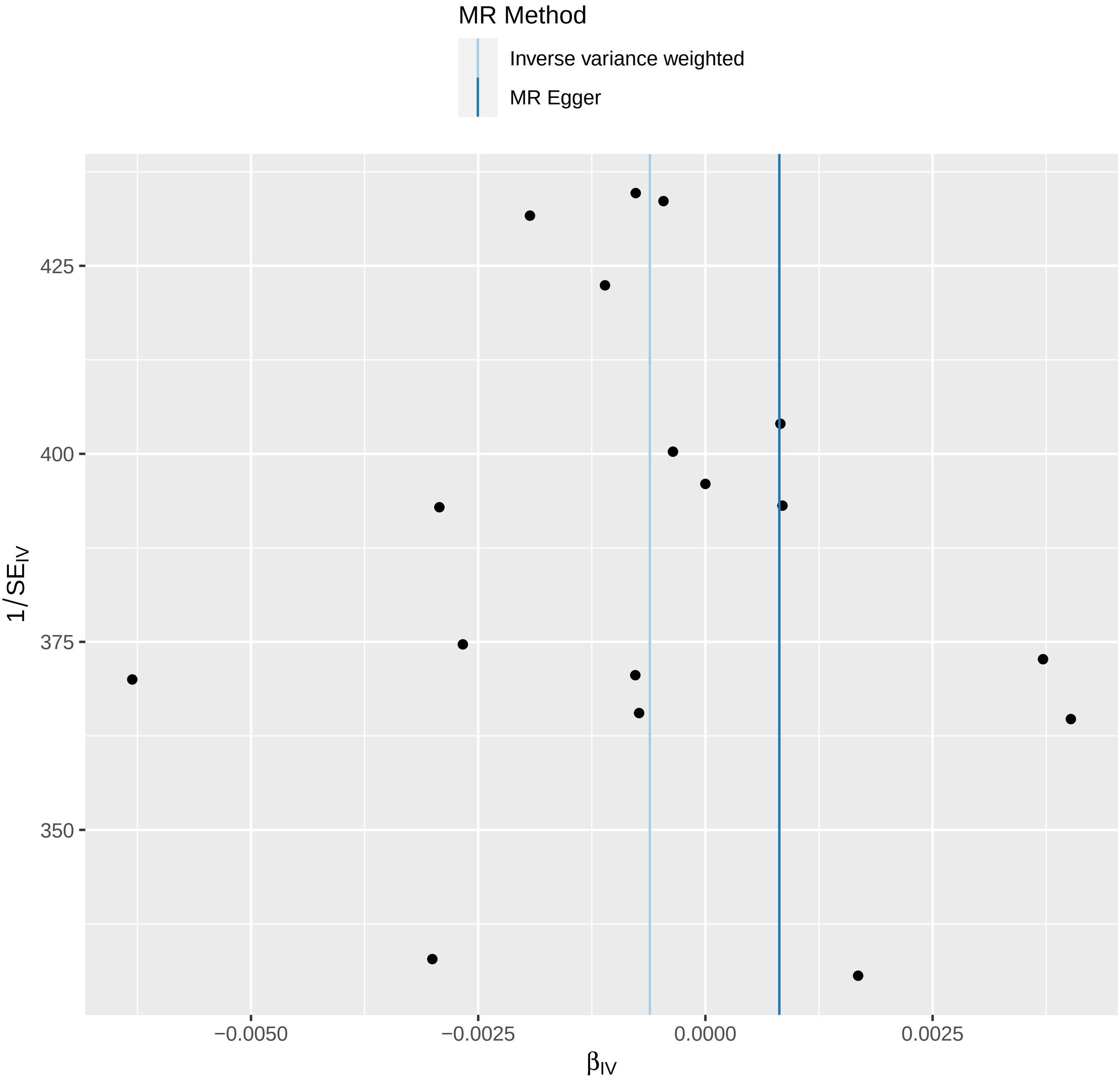

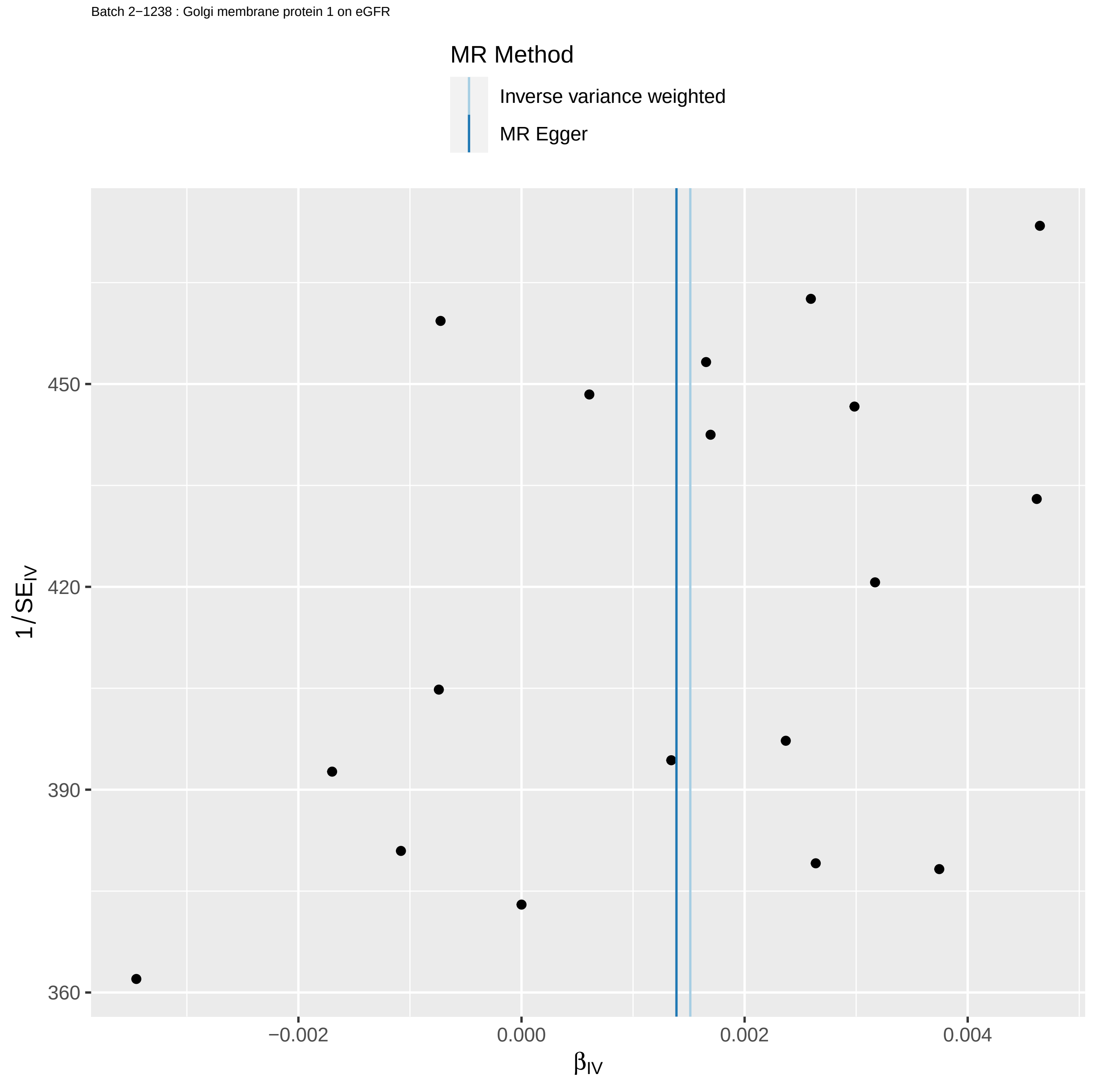

Batch 2−1238 : Golgi membrane protein 1 on eGFR
MR Method
Inverse variance weighted
MR Egger
1/SE$_{IV}$
450
420
390
360
−0.002
0.000
0.002
0.004
β$_{IV}$

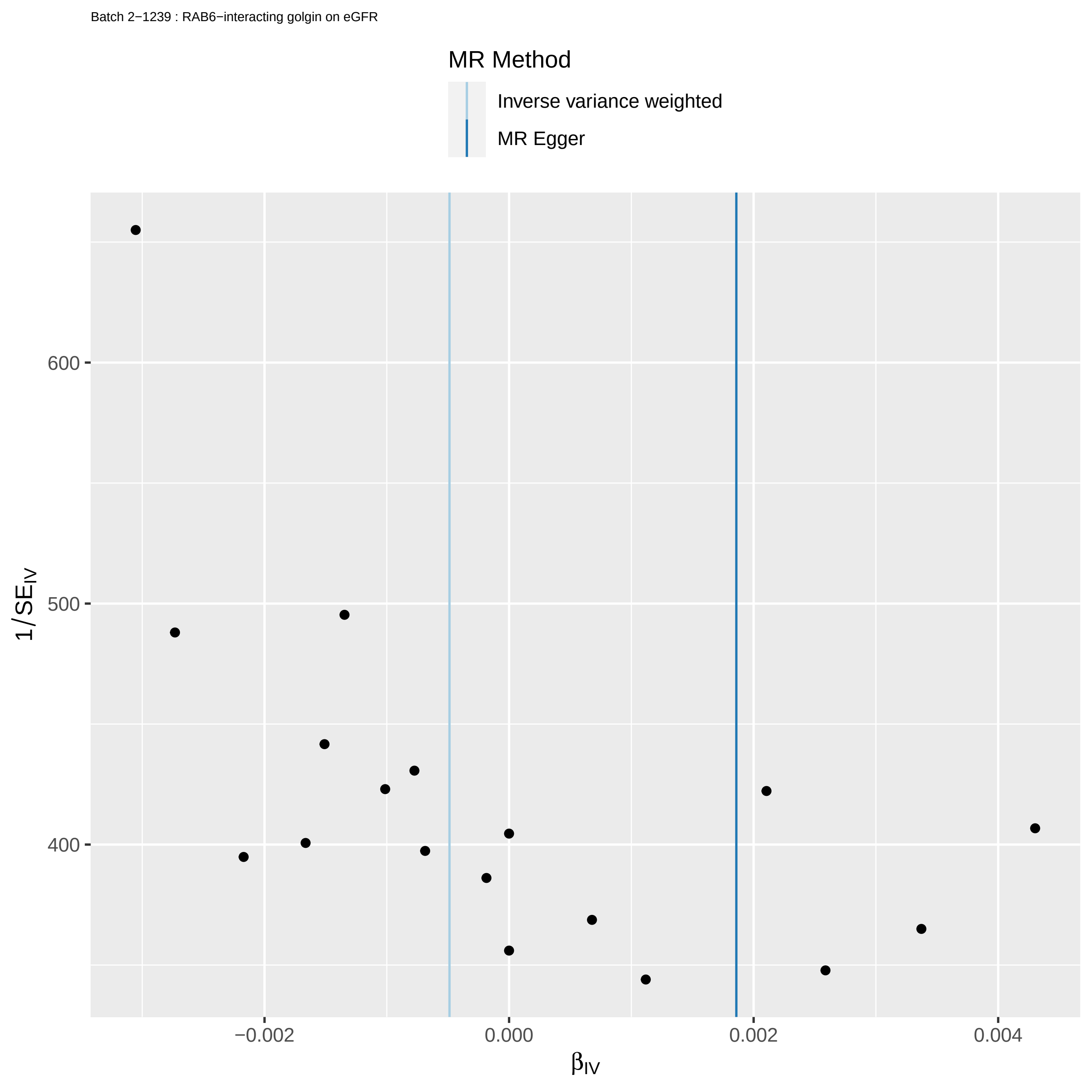
Batch 2−1239 : RAB6−interacting golgin on eGFR
MR Method
Inverse variance weighted
MR Egger
600
500
400
1/SE$_{IV}$
−0.002
0.000
0.002
0.004
β$_{IV}$

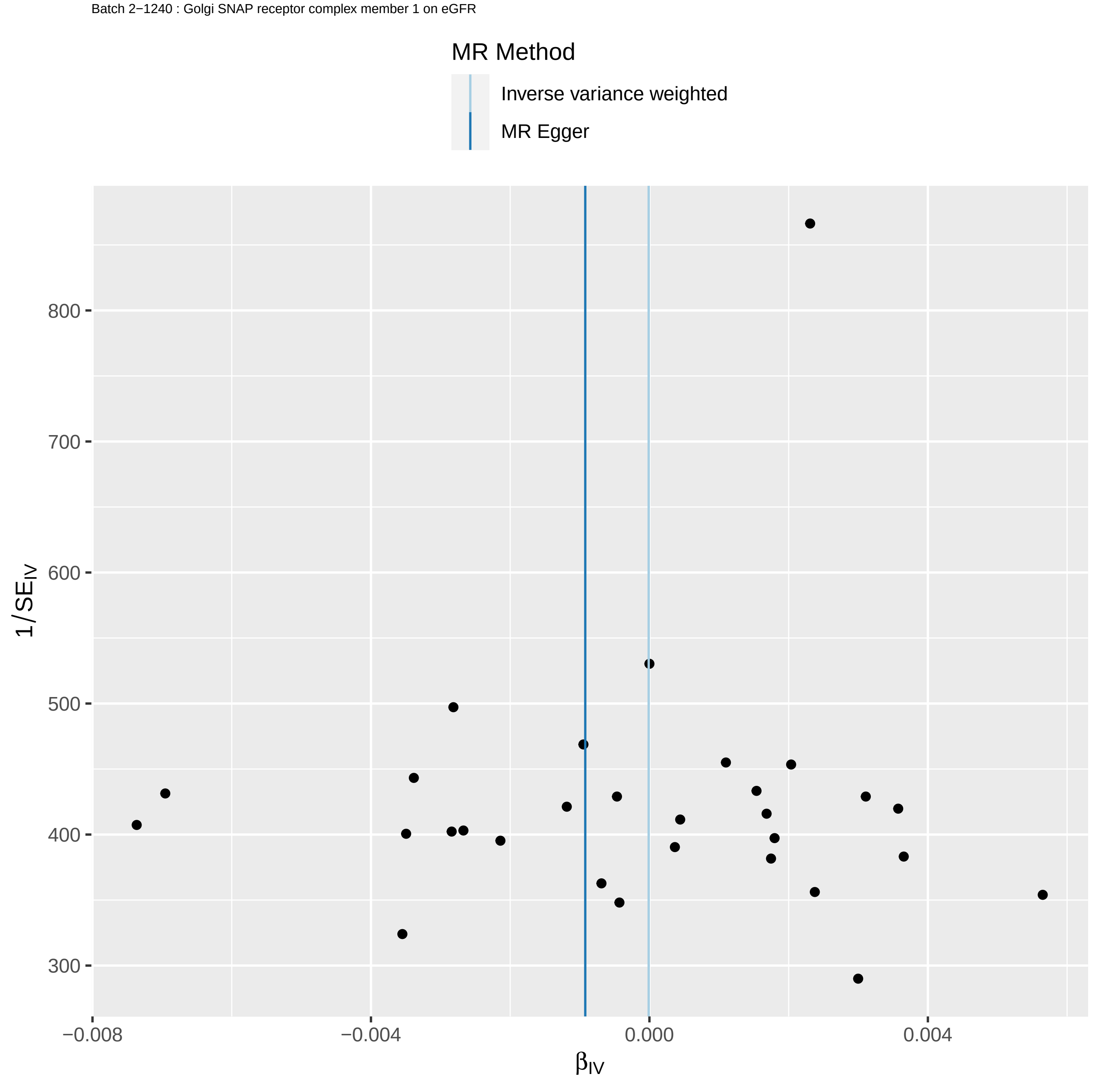

Batch 2−1240 : Golgi SNAP receptor complex member 1 on eGFR
MR Method
Inverse variance weighted
MR Egger
800
700
600
500
400
300
1/SE$_{IV}$
−0.008
−0.004
0.000
0.004
β$_{IV}$

Batch 2−1241 : Aspartate aminotransferase, cytoplasmic on eGFR

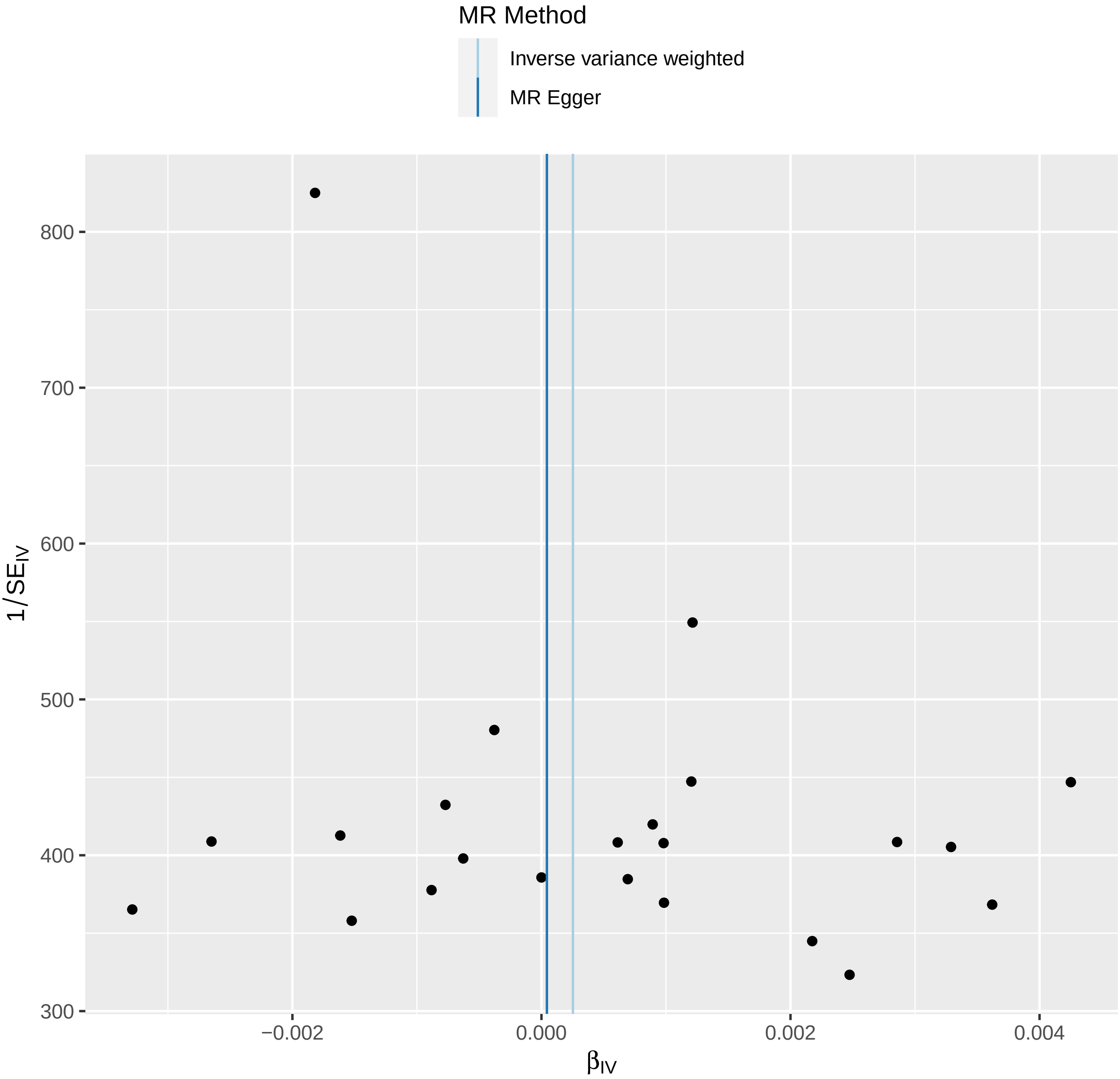

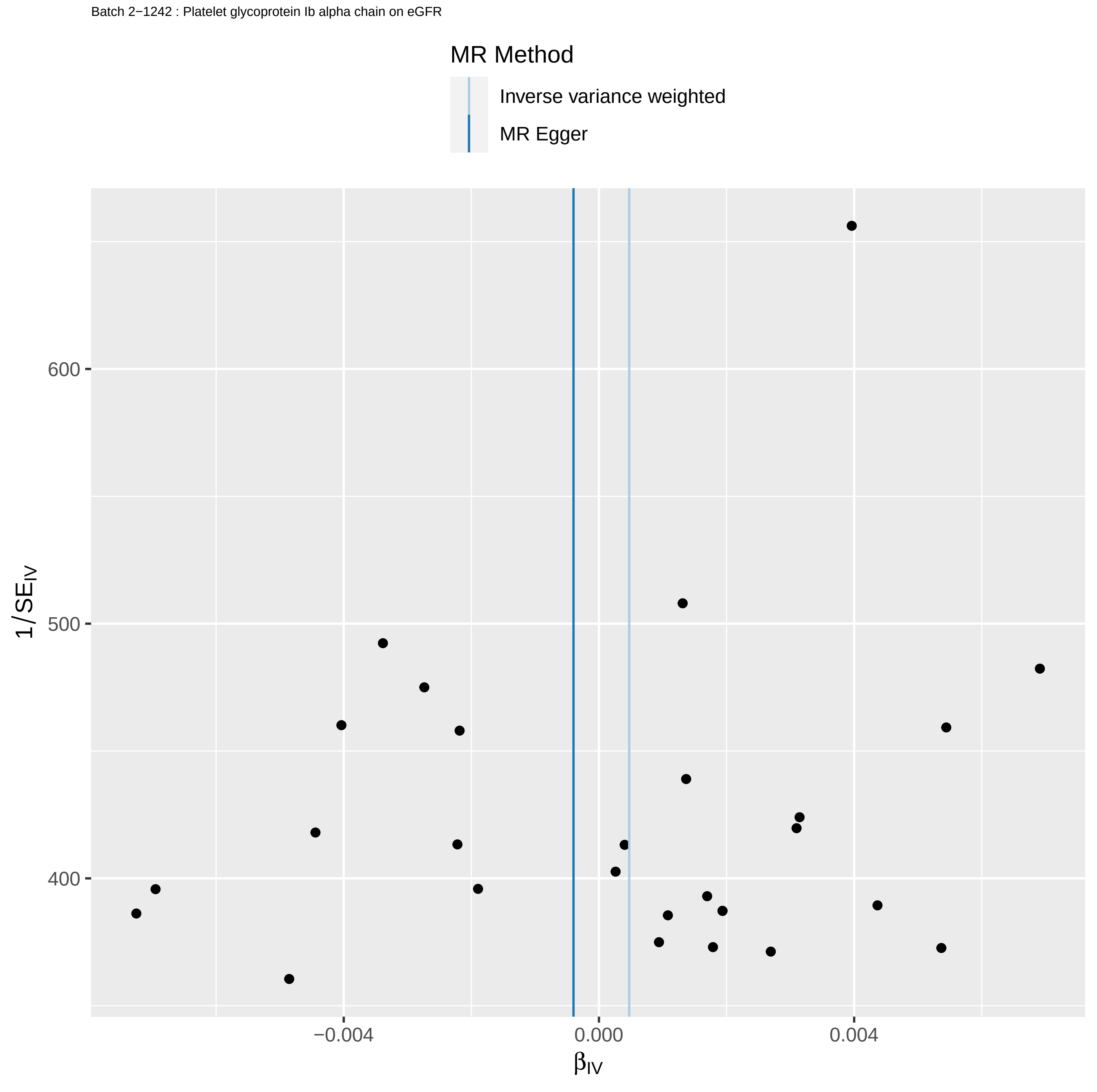
Batch 2−1242 : Platelet glycoprotein Ib alpha chain on eGFR
MR Method
Inverse variance weighted
MR Egger
600
500
400
$1/SE_{IV}$
−0.004
0.000
0.004
β_{IV}

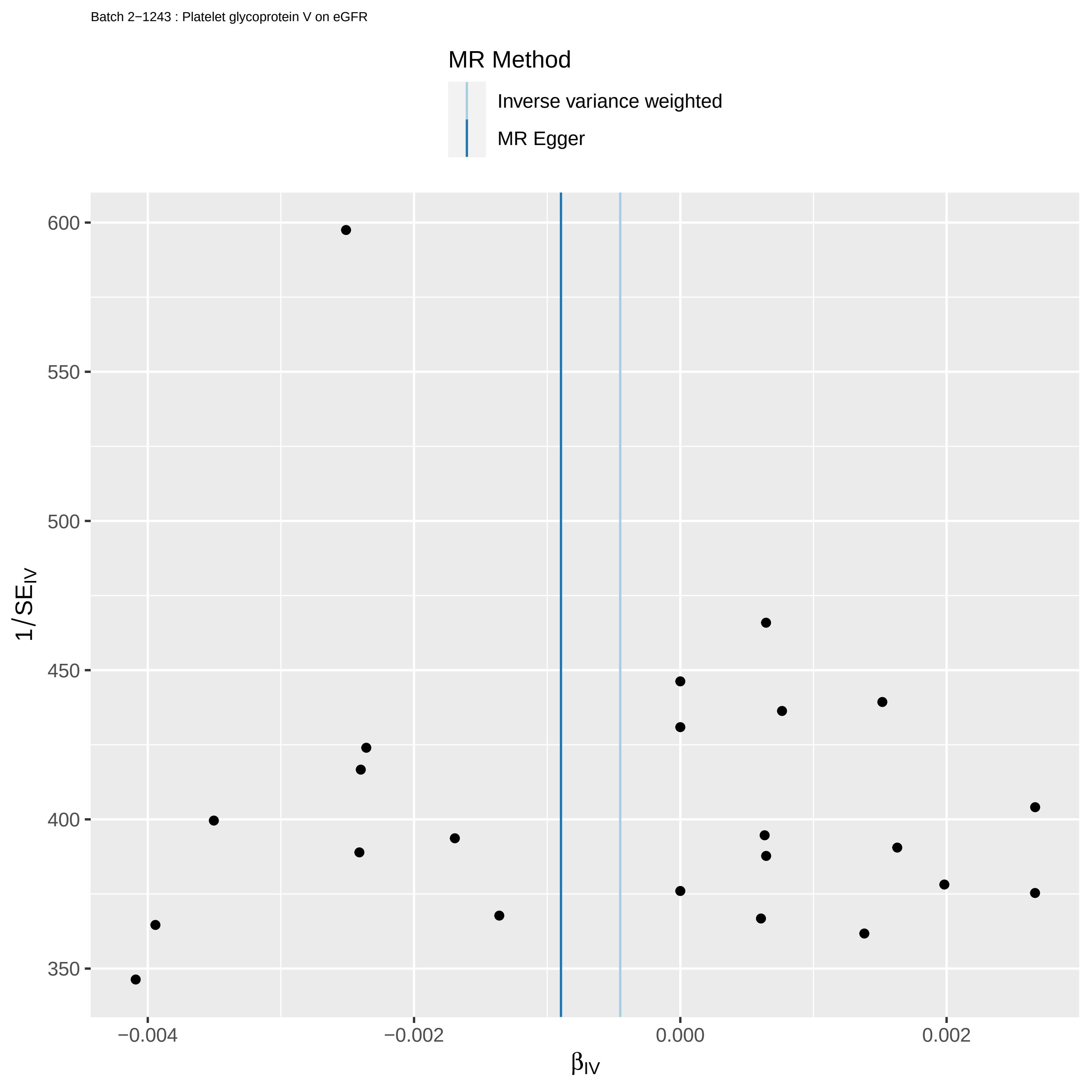
Batch 2−1243 : Platelet glycoprotein V on eGFR
MR Method
Inverse variance weighted
MR Egger
600
550
500
450
400
350
1/SE_IV
−0.004
−0.002
0.000
0.002
β_IV

Batch 2−1244 : Cell surface A33 antigen on eGFR

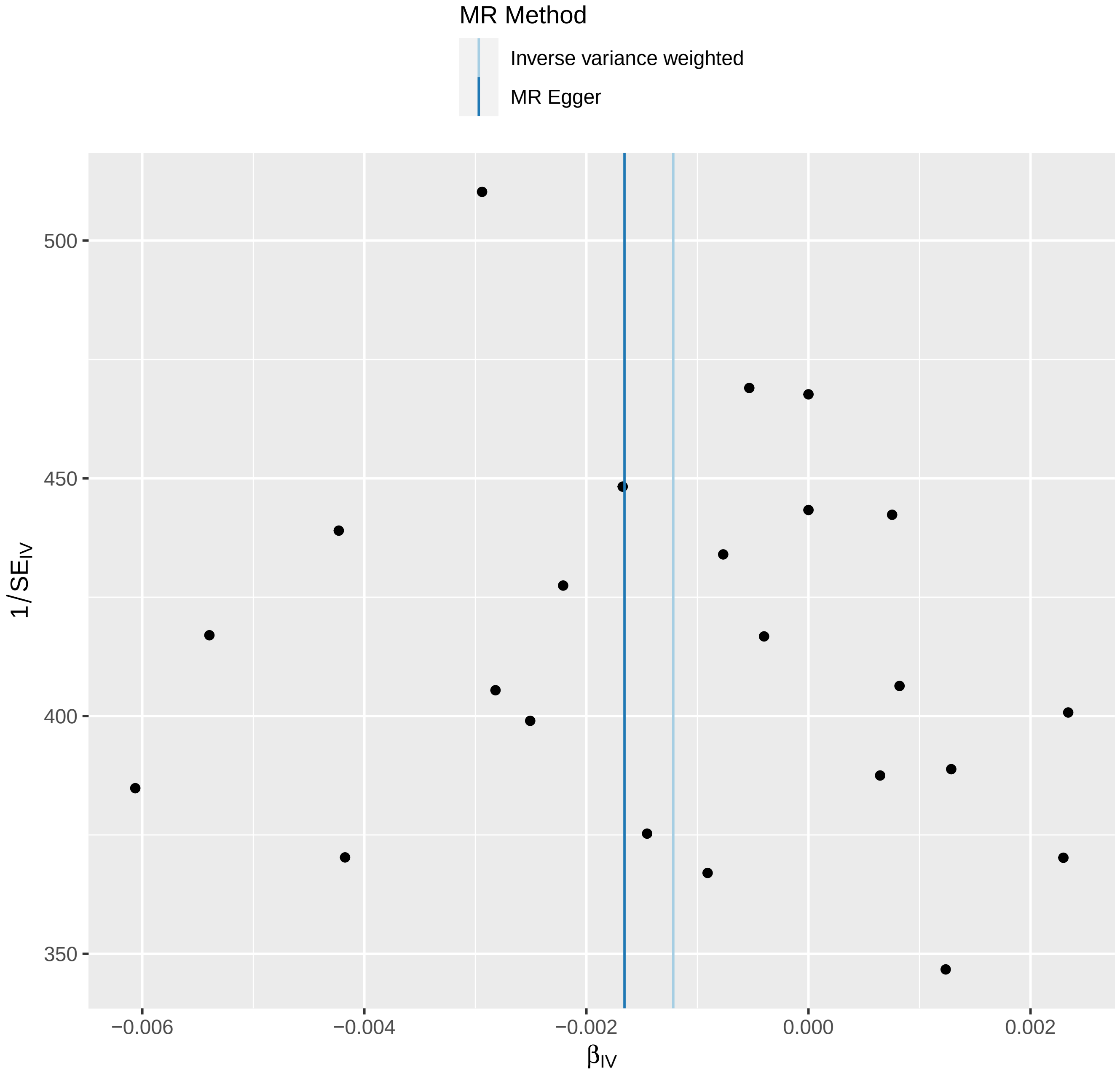

Batch 2−1245 : Glypican−1 on eGFR

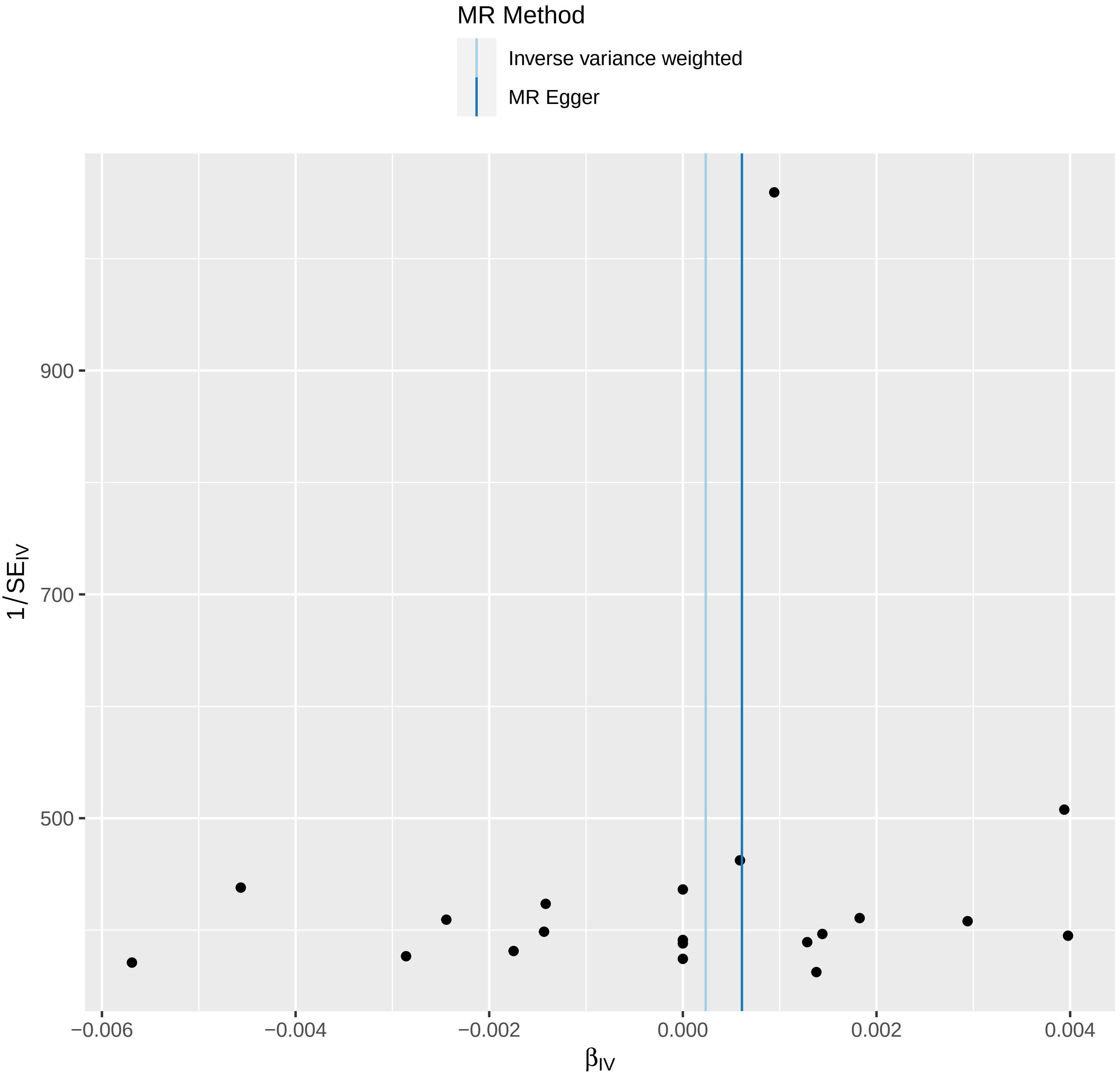

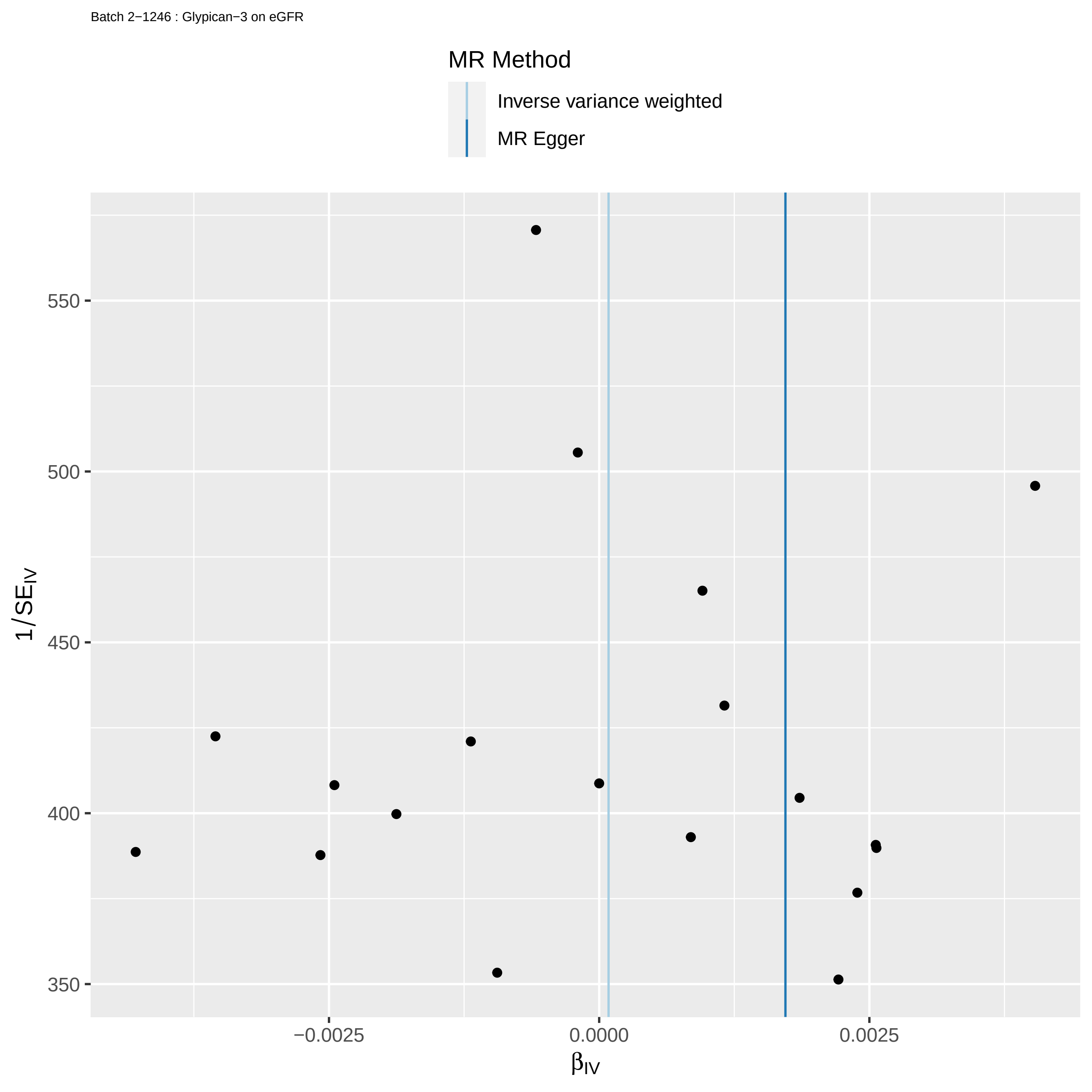
Batch 2−1246 : Glypican−3 on eGFR
MR Method
Inverse variance weighted
MR Egger
550
500
450
400
350
1/SE_IV
−0.0025
0.0000
0.0025
β_IV

Batch 2−1247 : Glypican−5 on eGFR

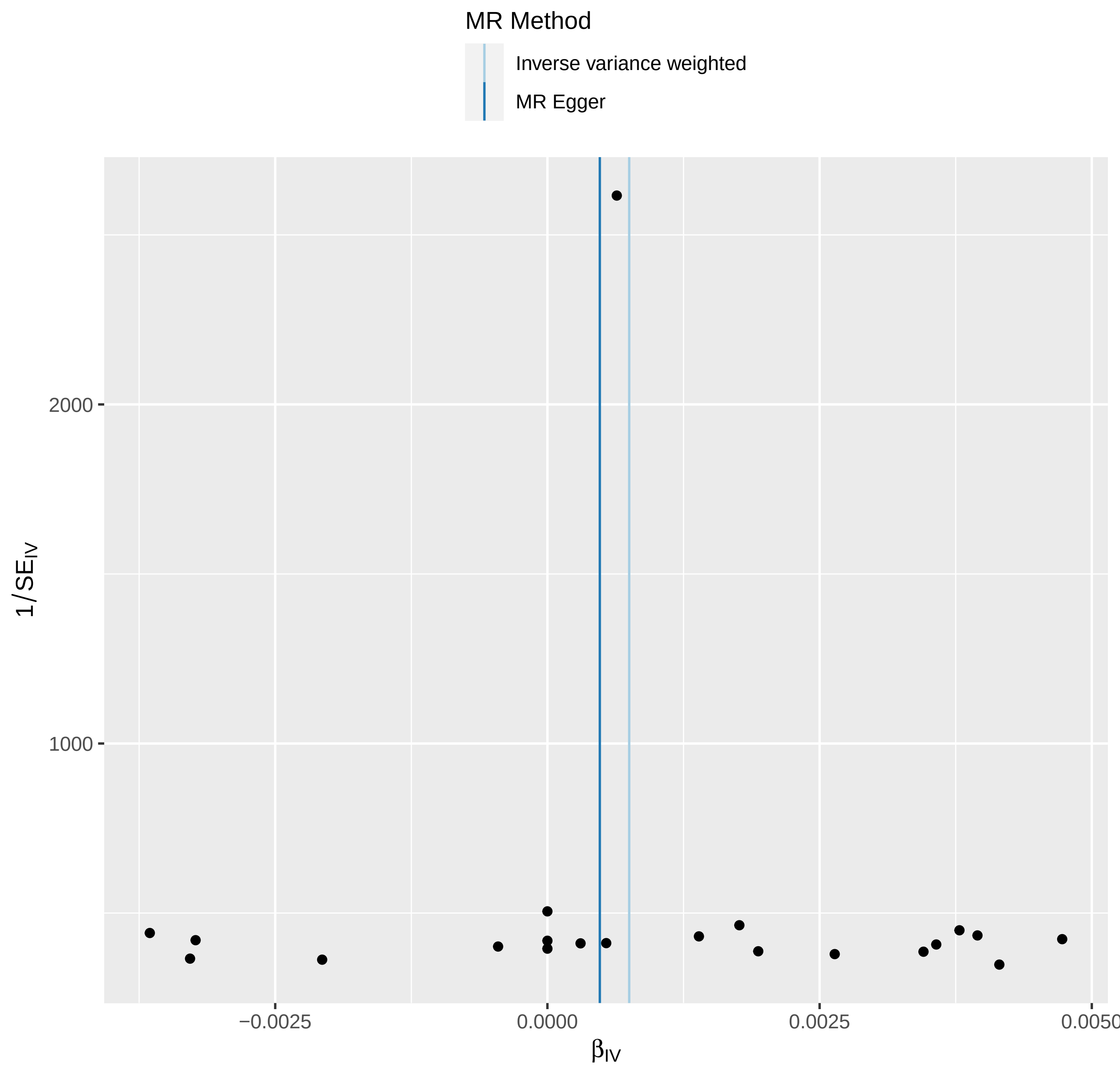

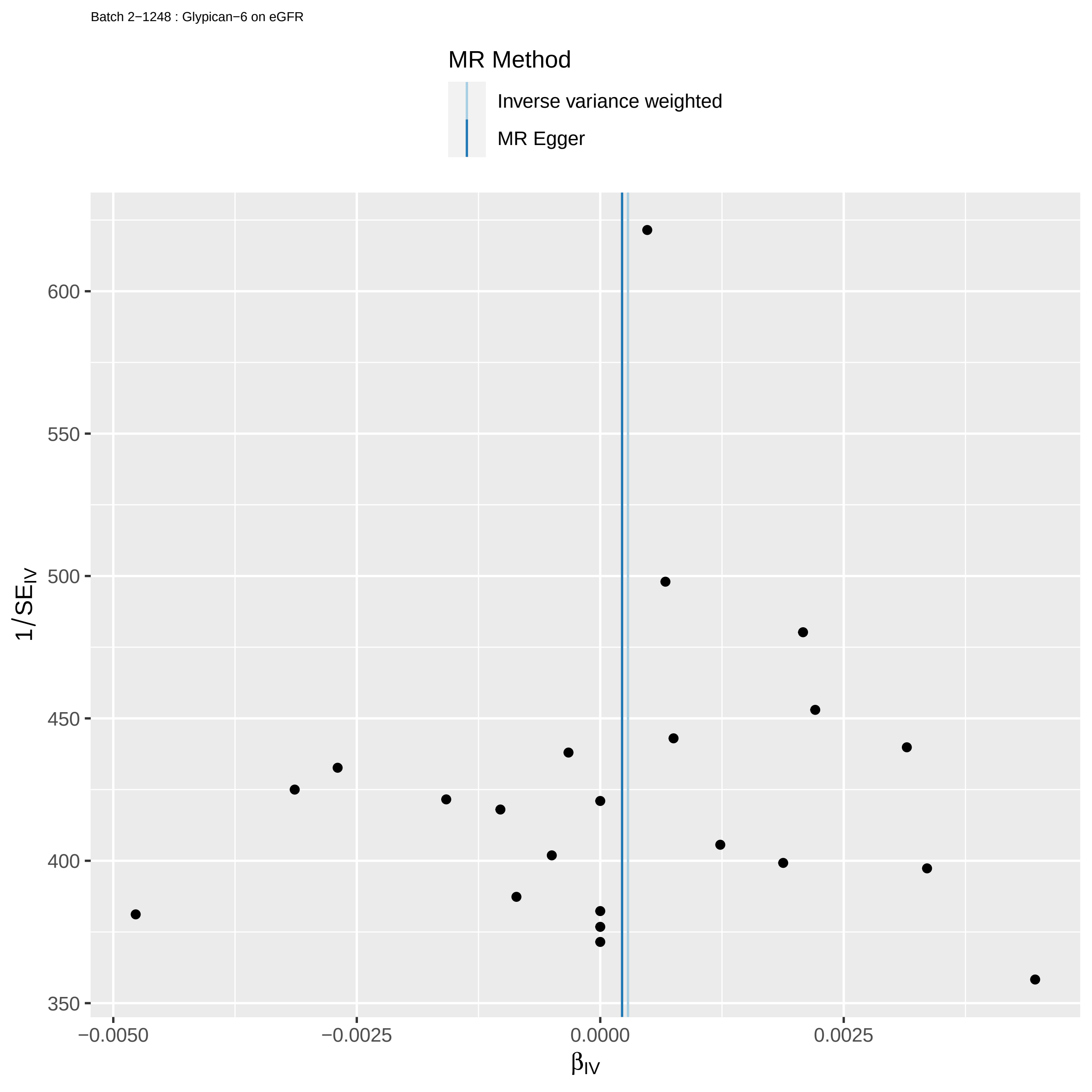
Batch 2−1248 : Glypican−6 on eGFR
MR Method
Inverse variance weighted
MR Egger
600
550
500
450
400
350
1/SE_IV
−0.0050
−0.0025
0.0000
0.0025
β_IV

Batch 2−1249 : Glycerophosphocholine phosphodiesterase GPCPD1 on eGFR

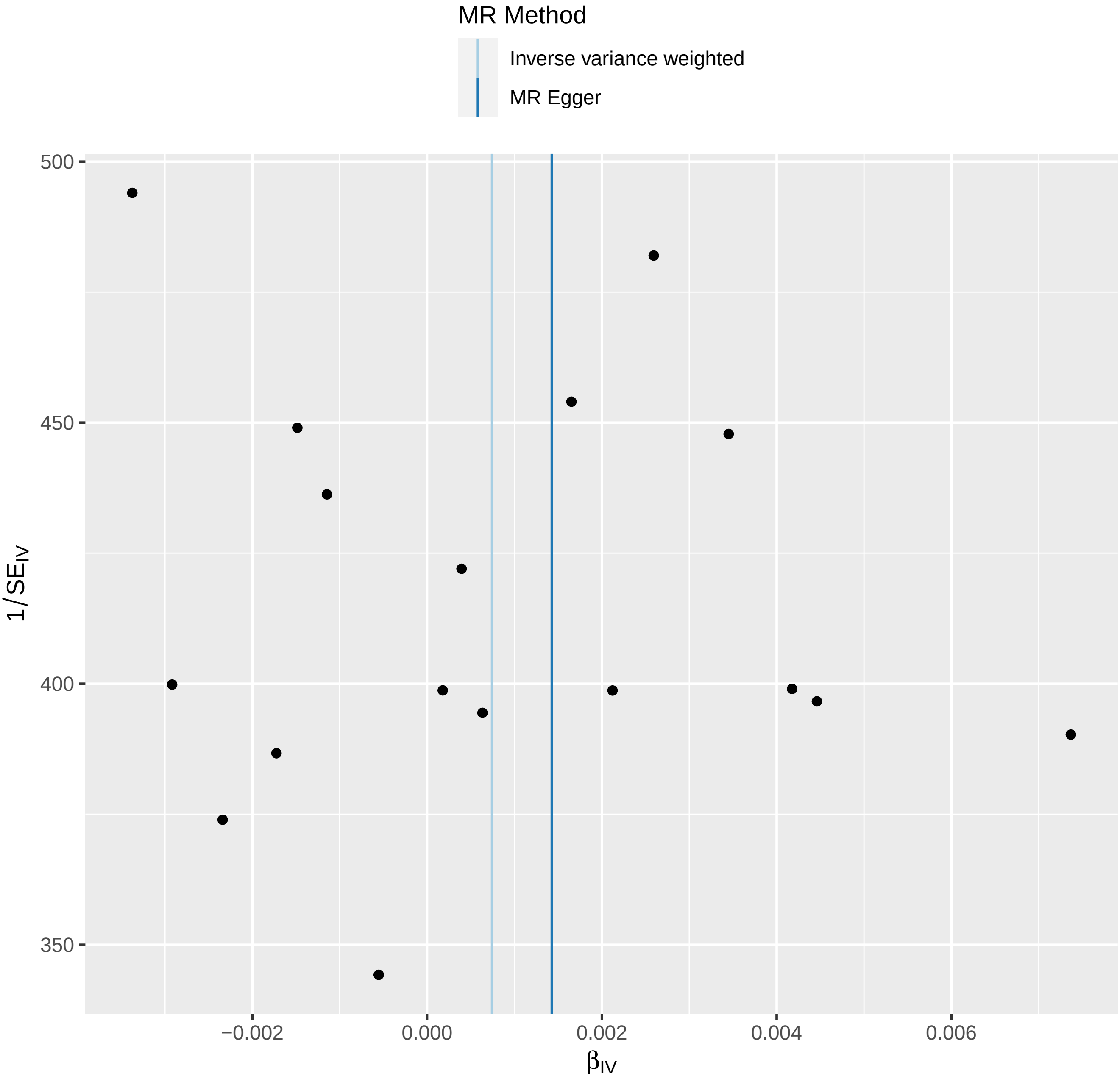

Batch 2−1250 : Glycerol−3−phosphate dehydrogenase 1−like protein on eGFR

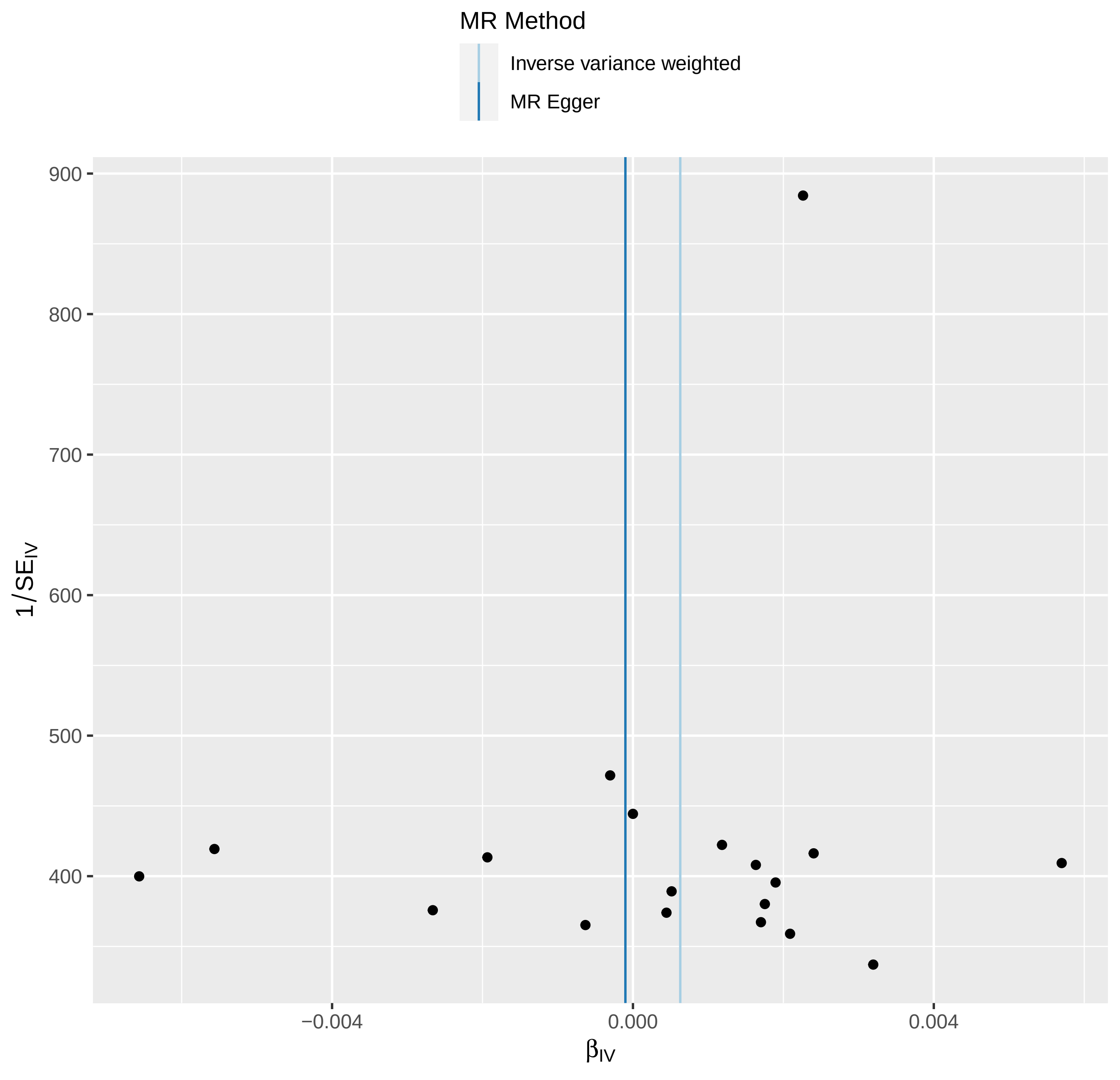

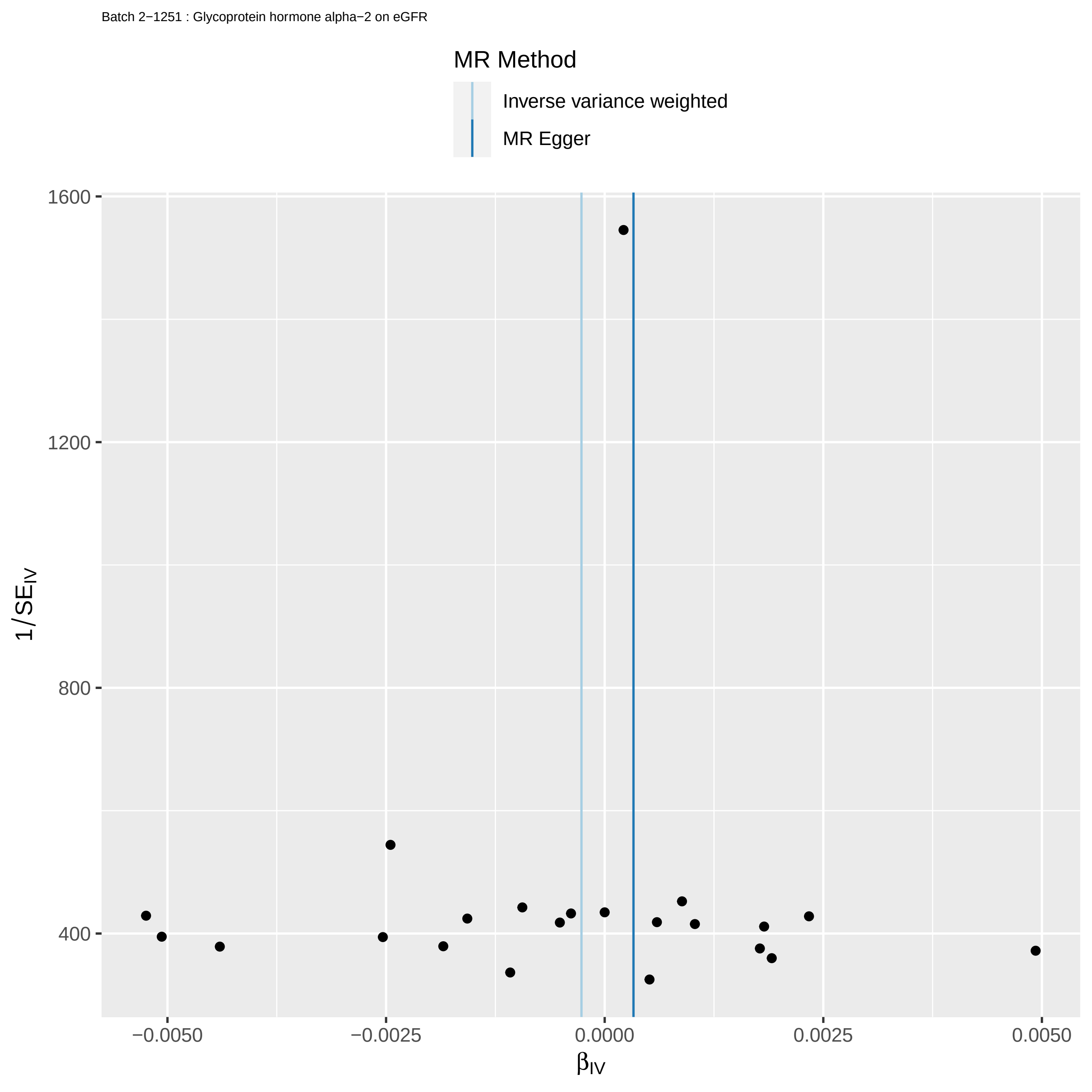
Batch 2−1251 : Glycoprotein hormone alpha−2 on eGFR
MR Method
Inverse variance weighted
MR Egger
1600
1200
800
400
1/SE_IV
−0.0050
−0.0025
0.0000
0.0025
0.0050
β_IV

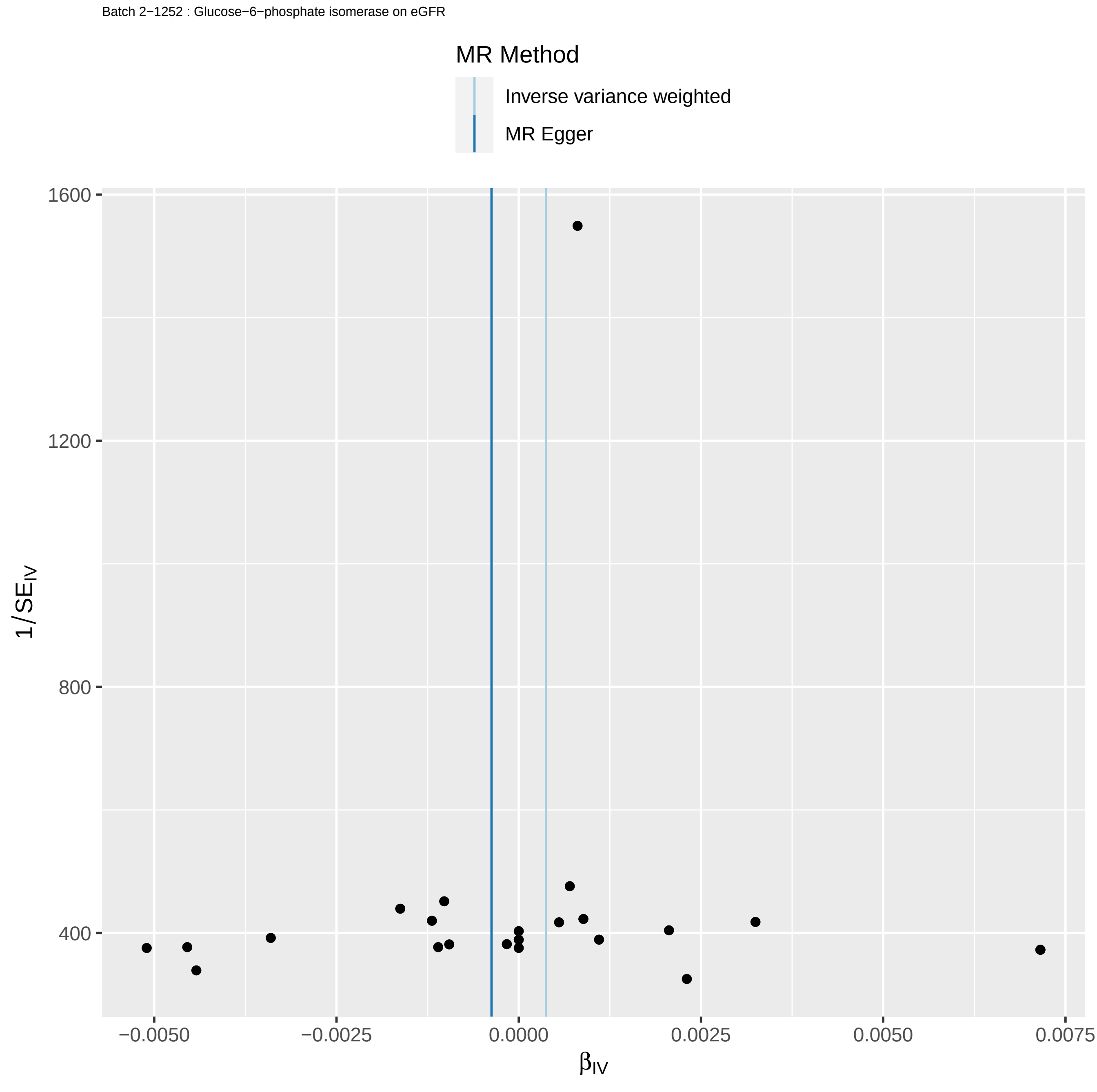

Batch 2−1252 : Glucose−6−phosphate isomerase on eGFR
MR Method
Inverse variance weighted
MR Egger
1600
1200
800
400
1/SE_IV
−0.0050
−0.0025
0.0000
0.0025
0.0050
0.0075
β_IV

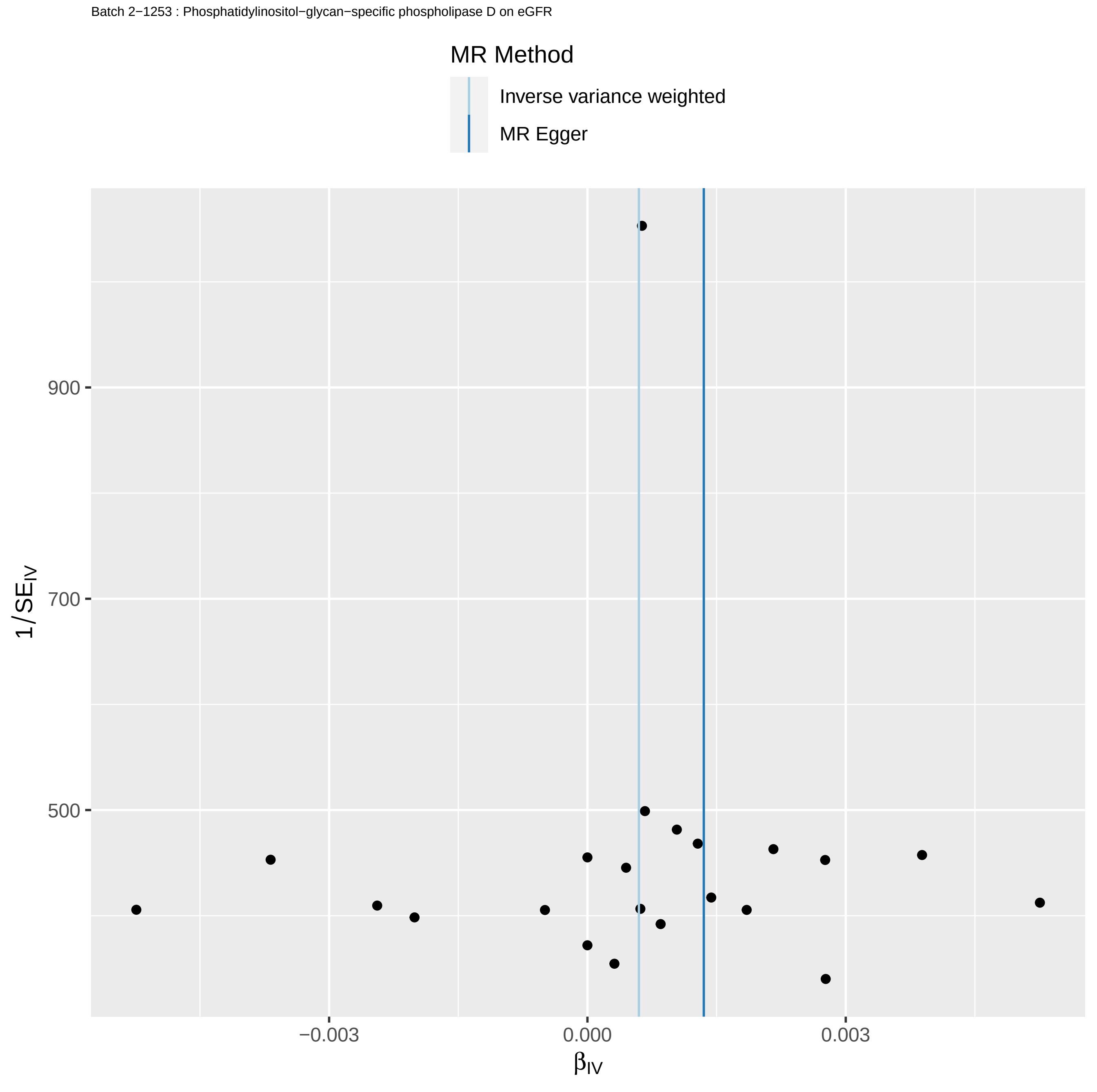

Batch 2−1253 : Phosphatidylinositol−glycan−specific phospholipase D on eGFR
MR Method
Inverse variance weighted
MR Egger
900
700
500
1/SE_IV
−0.003
0.000
0.003
β_IV

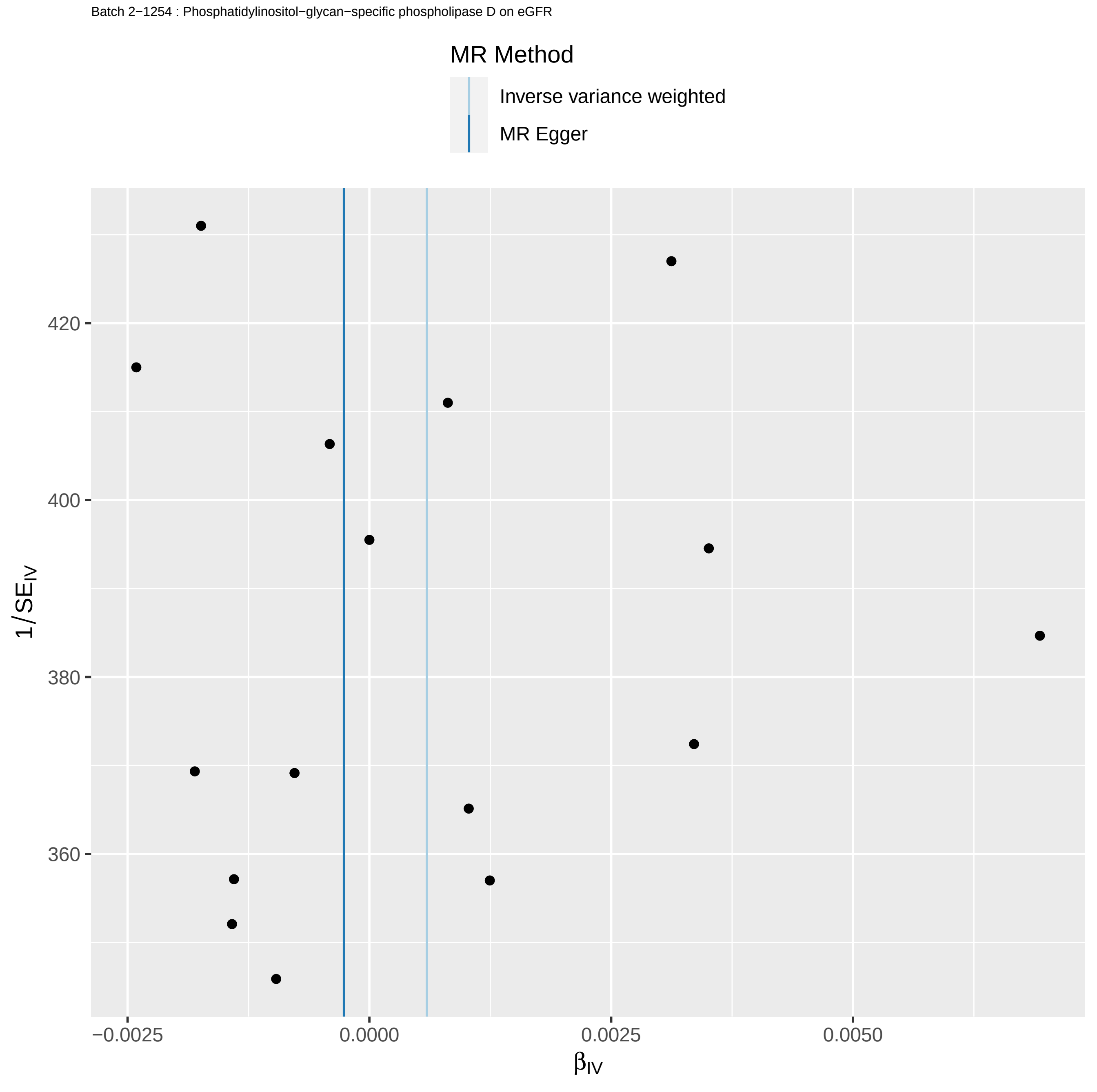

Batch 2−1254 : Phosphatidylinositol−glycan−specific phospholipase D on eGFR
MR Method
Inverse variance weighted
MR Egger
420
400
380
360
1/SE$_{IV}$
−0.0025
0.0000
0.0025
0.0050
β$_{IV}$

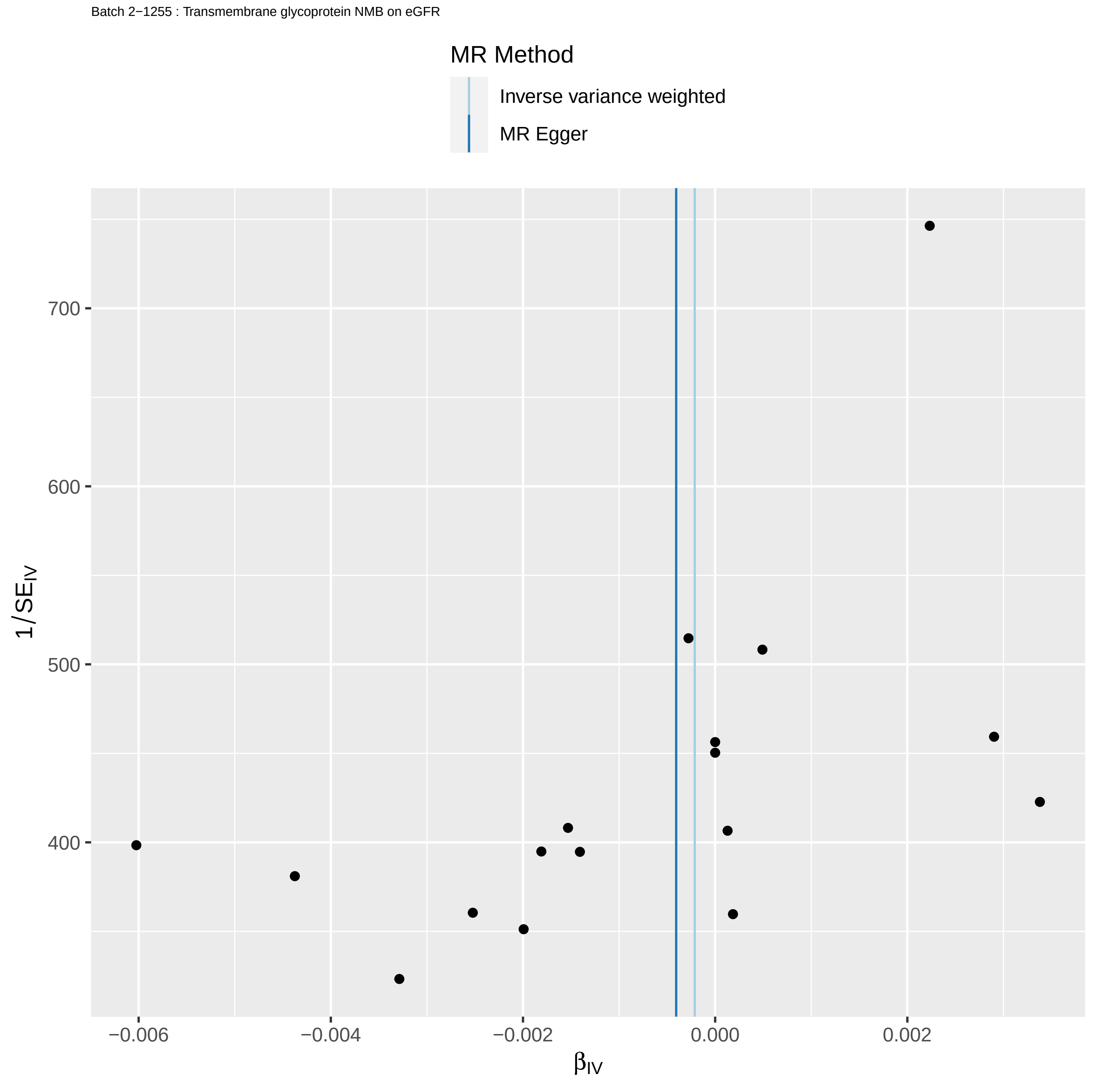

Batch 2−1255 : Transmembrane glycoprotein NMB on eGFR
MR Method
Inverse variance weighted
MR Egger
700
600
500
400
1/SE_IV
−0.006
−0.004
−0.002
0.000
0.002
β_IV

Batch 2−1256 : Transmembrane glycoprotein NMB on eGFR

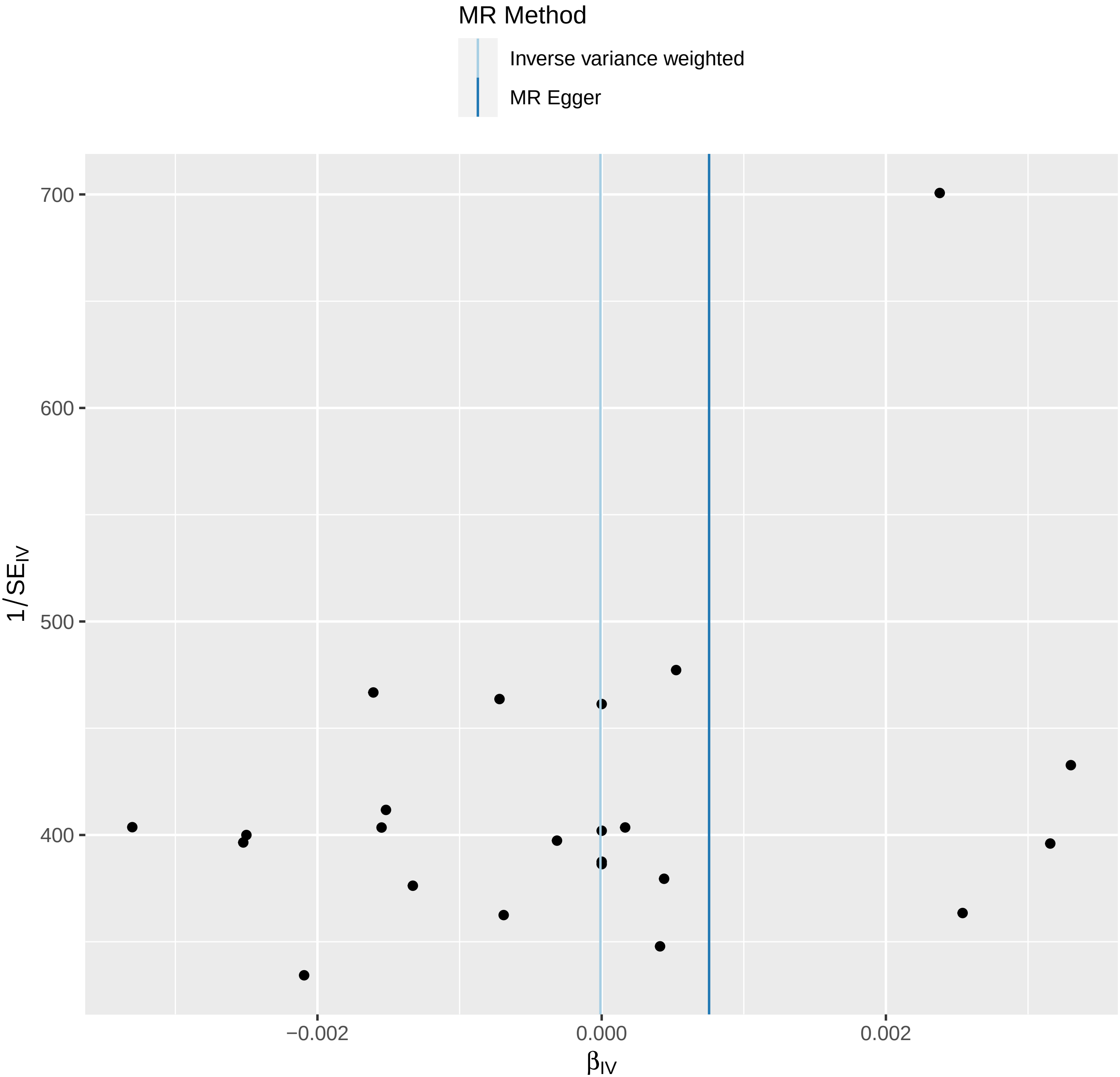

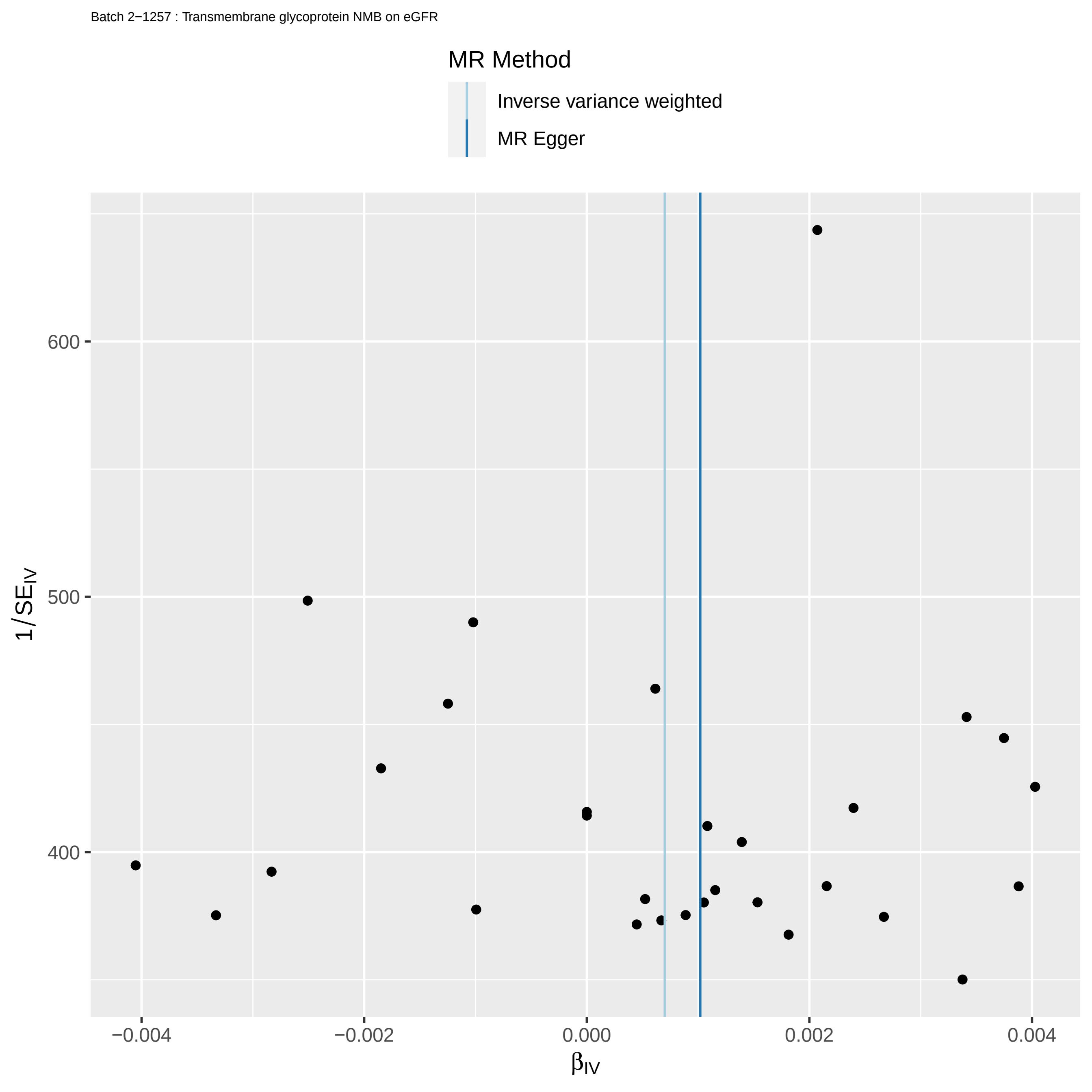
Batch 2−1257 : Transmembrane glycoprotein NMB on eGFR
MR Method
Inverse variance weighted
MR Egger
600
500
400
1/SE$_{IV}$
−0.004
−0.002
0.000
0.002
0.004
β_{IV}

Batch 2−1258 : Transmembrane glycoprotein NMB on eGFR

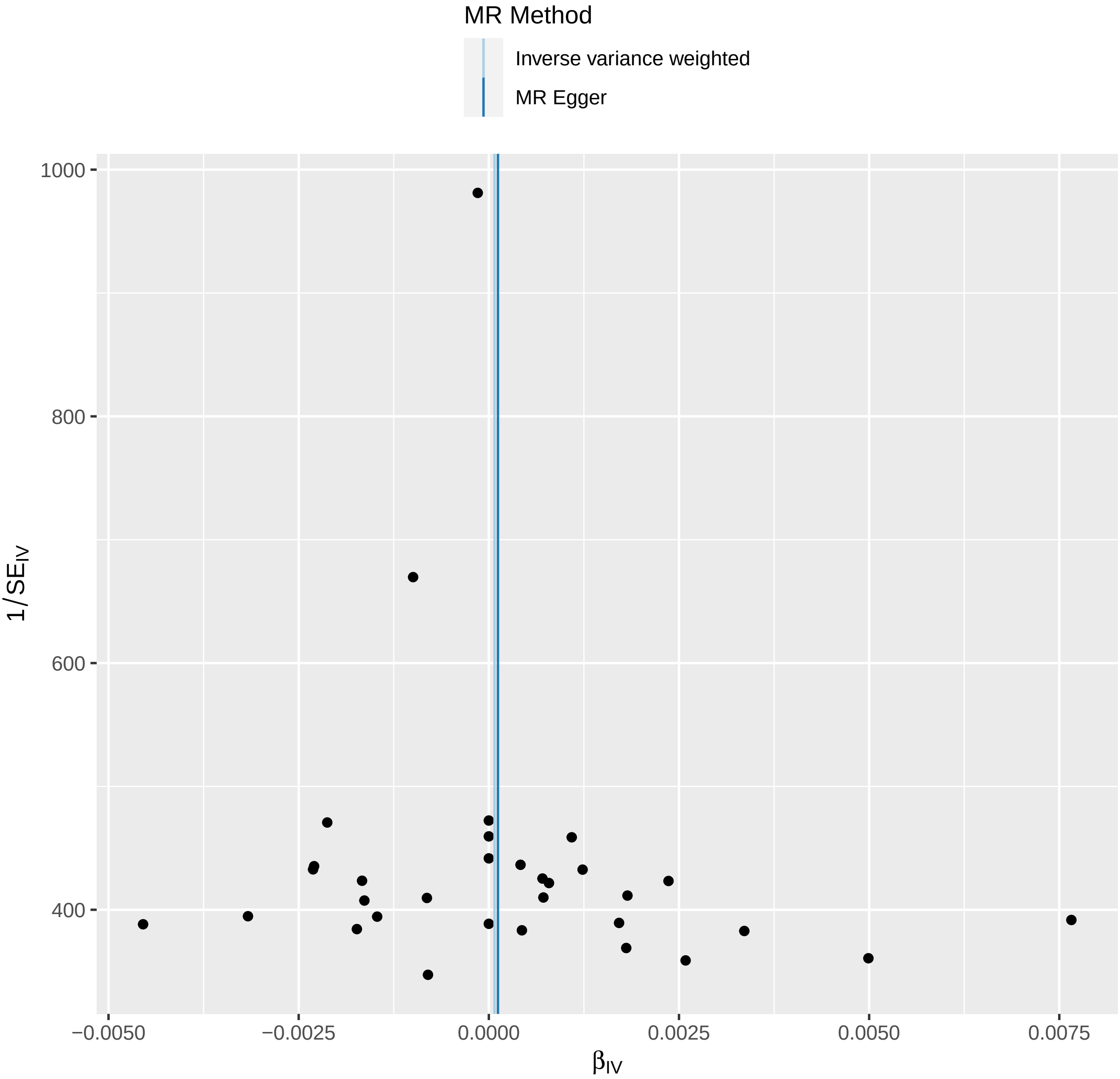

Batch 2−1259 : Probable G−protein coupled receptor 101 on eGFR

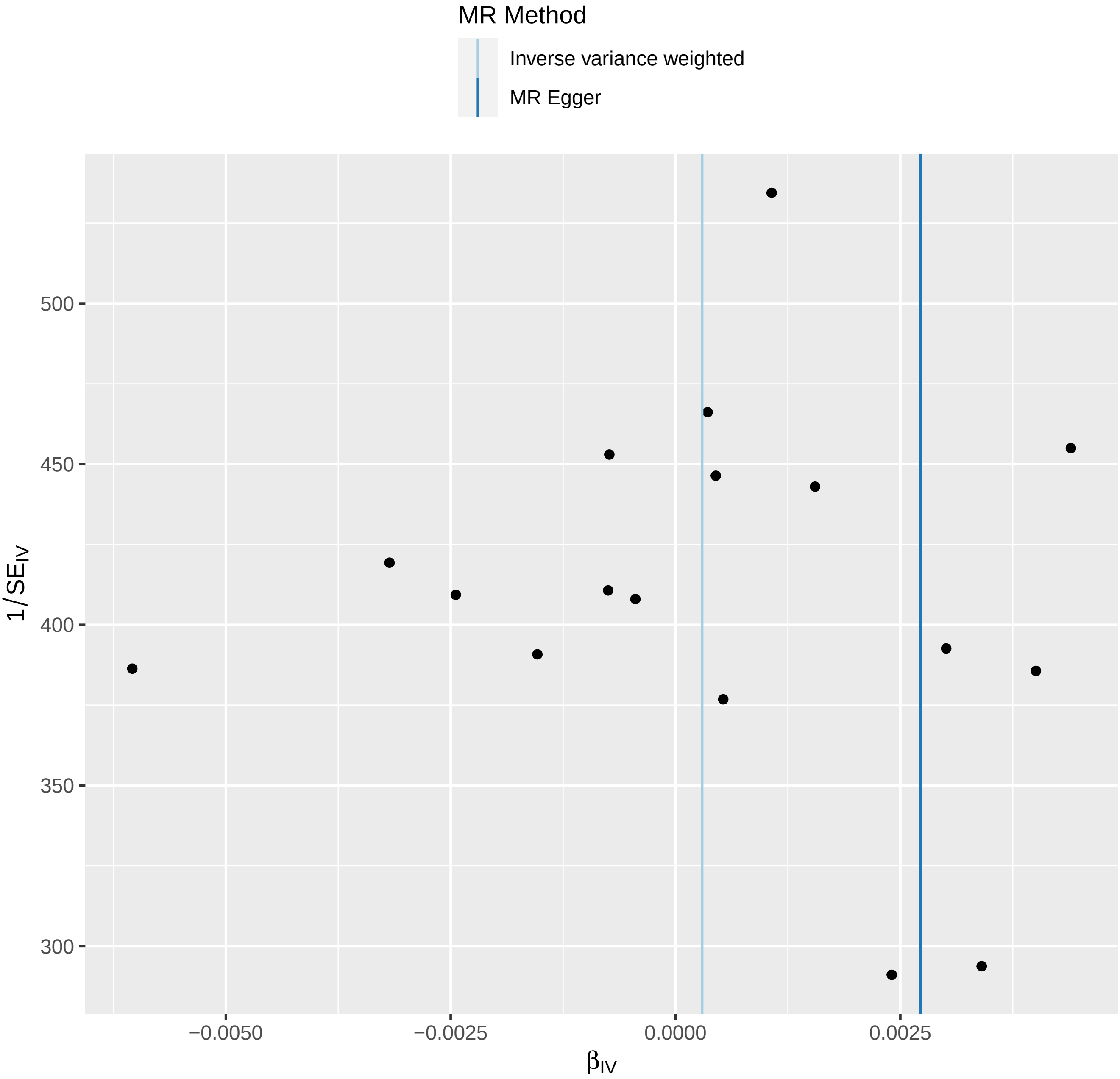

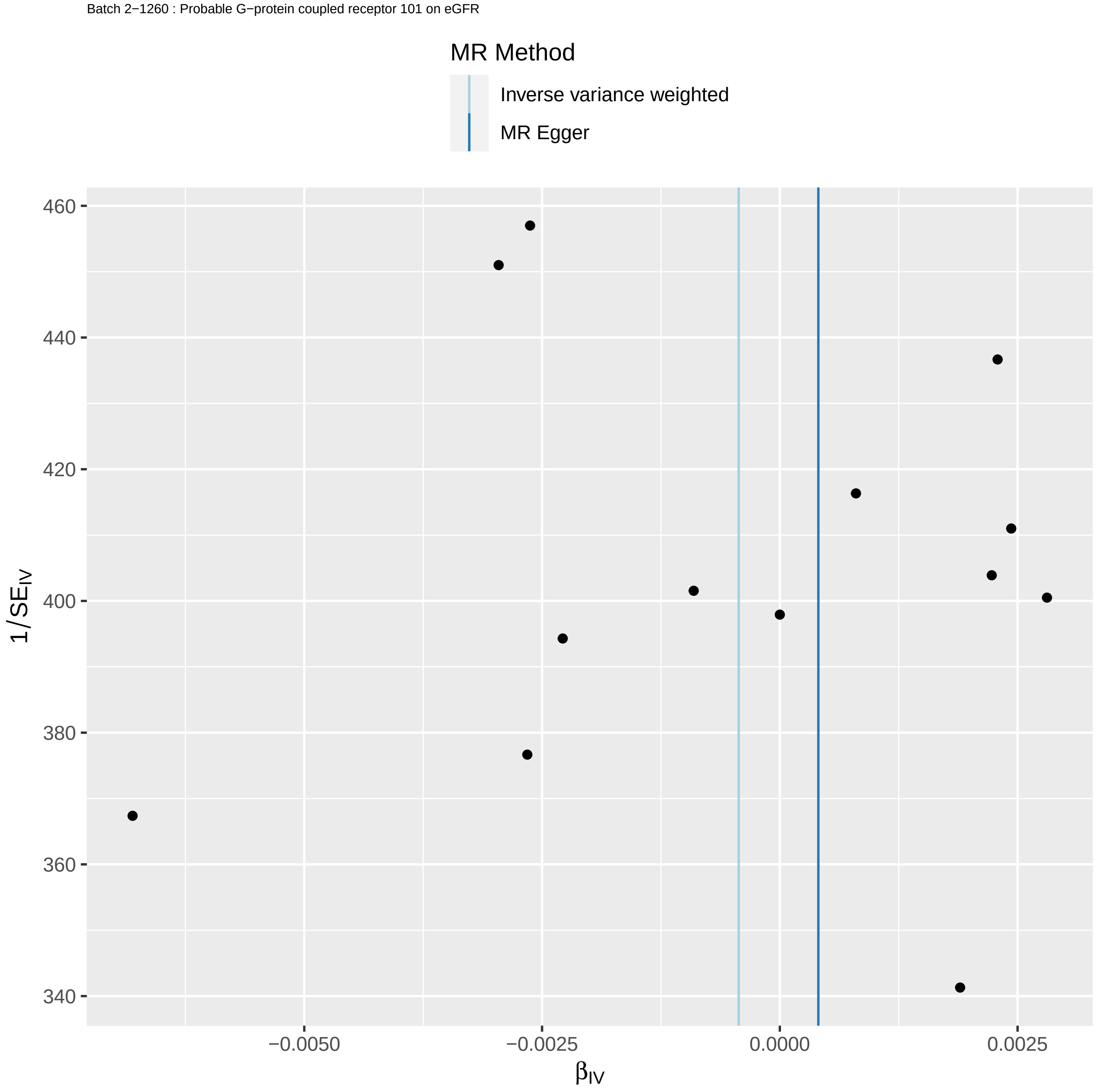
Batch 2−1260 : Probable G−protein coupled receptor 101 on eGFR
MR Method
Inverse variance weighted
MR Egger
460
440
420
400
380
360
340
1/SE$_{IV}$
−0.0050
−0.0025
0.0000
0.0025
β$_{IV}$

Batch 2−1261 : Protein GPR107 on eGFR

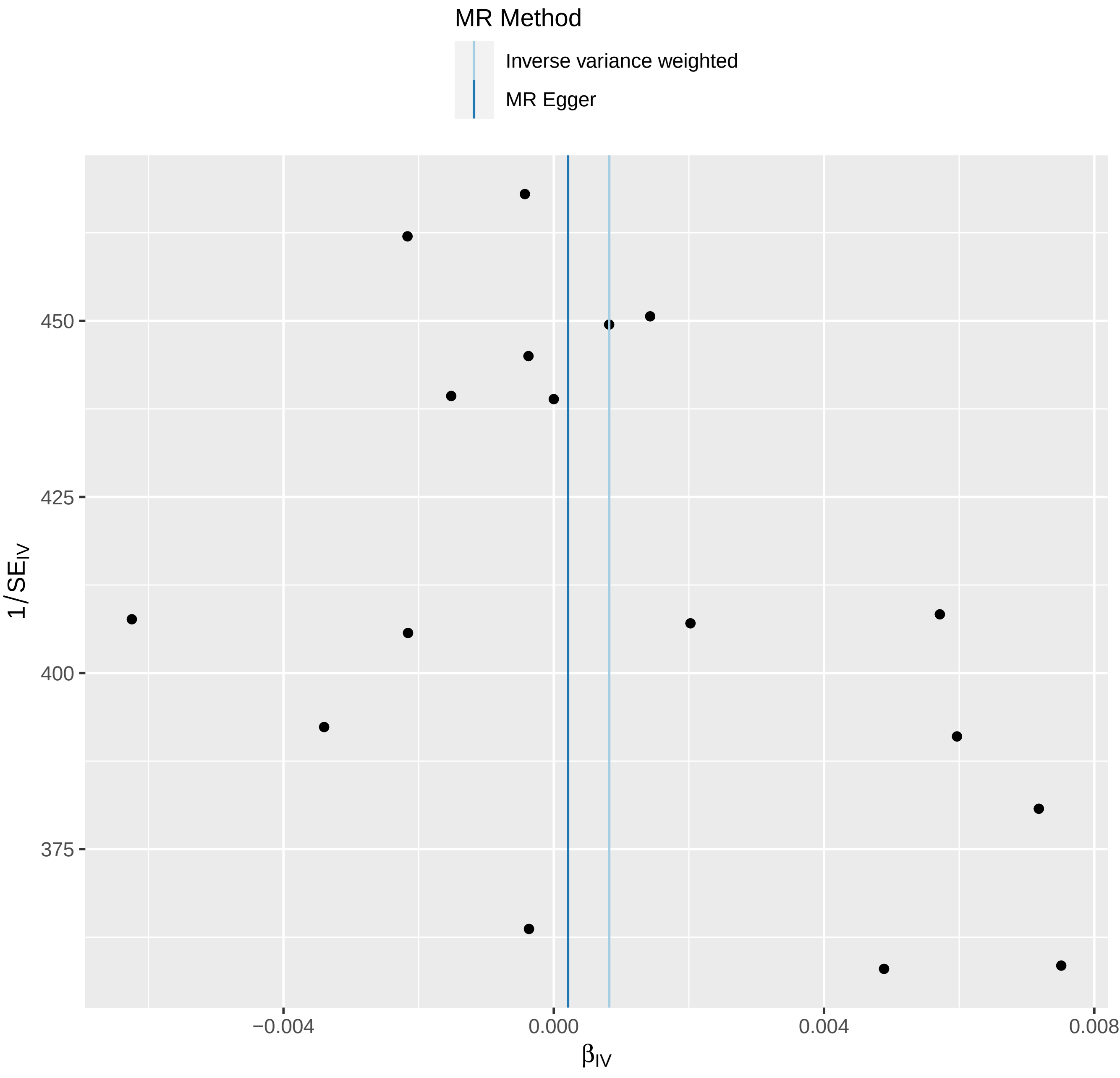

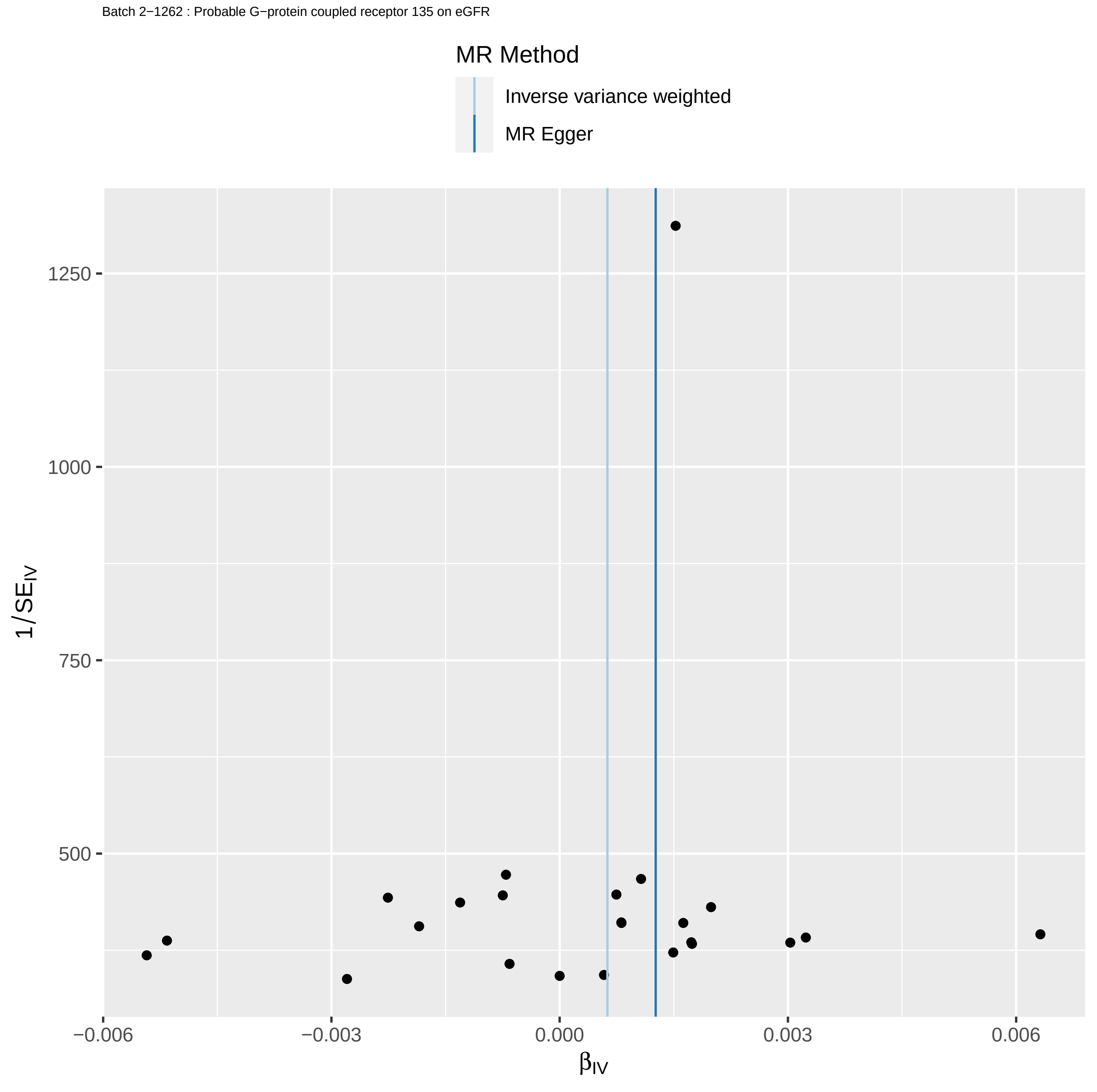

Batch 2−1262 : Probable G−protein coupled receptor 135 on eGFR
MR Method
Inverse variance weighted
MR Egger
1250
1000
750
500
1/SE_IV
−0.006
−0.003
0.000
0.003
0.006
β_IV

Batch 2−1263 : G−protein coupled receptor 26 on eGFR

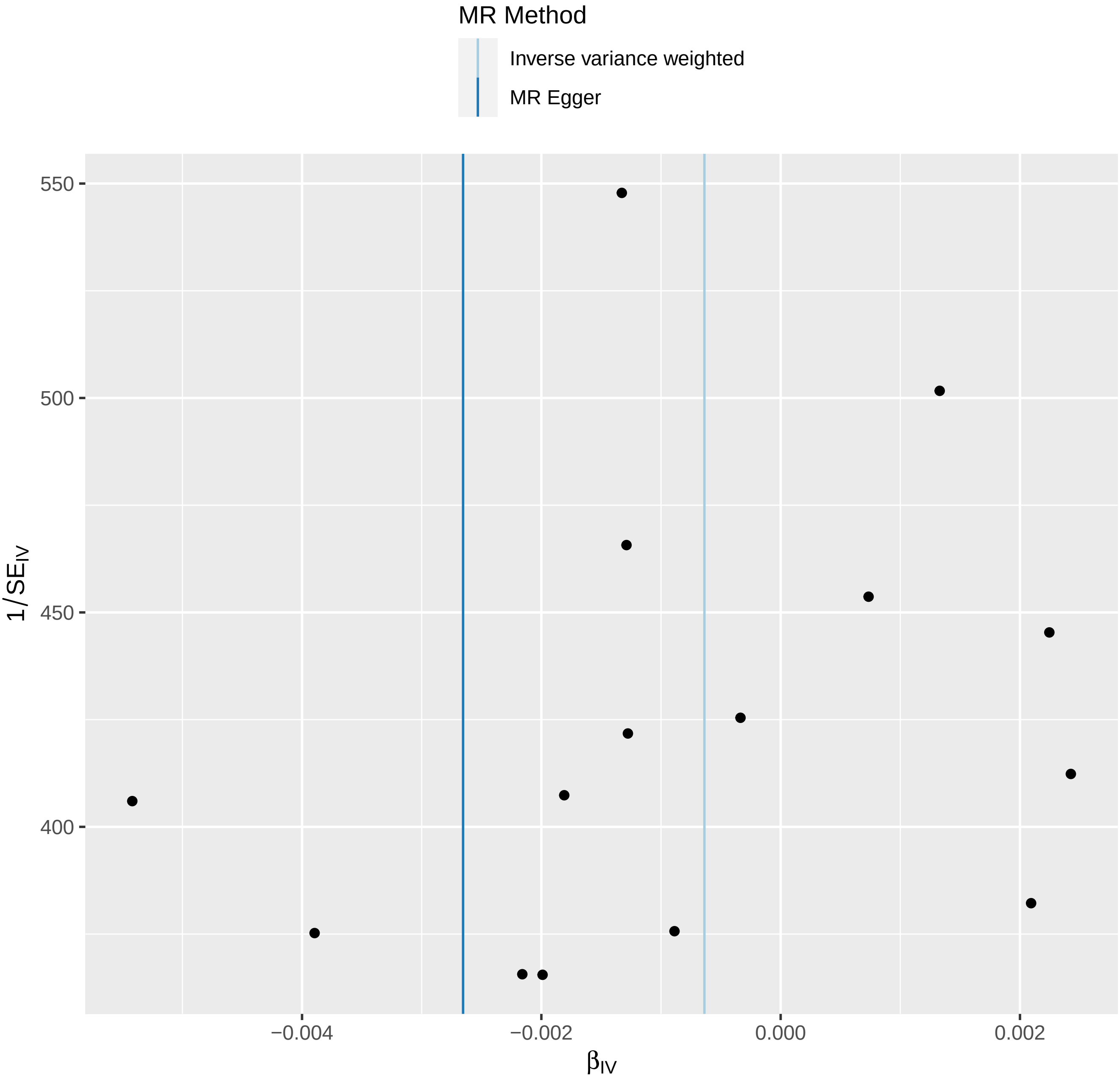

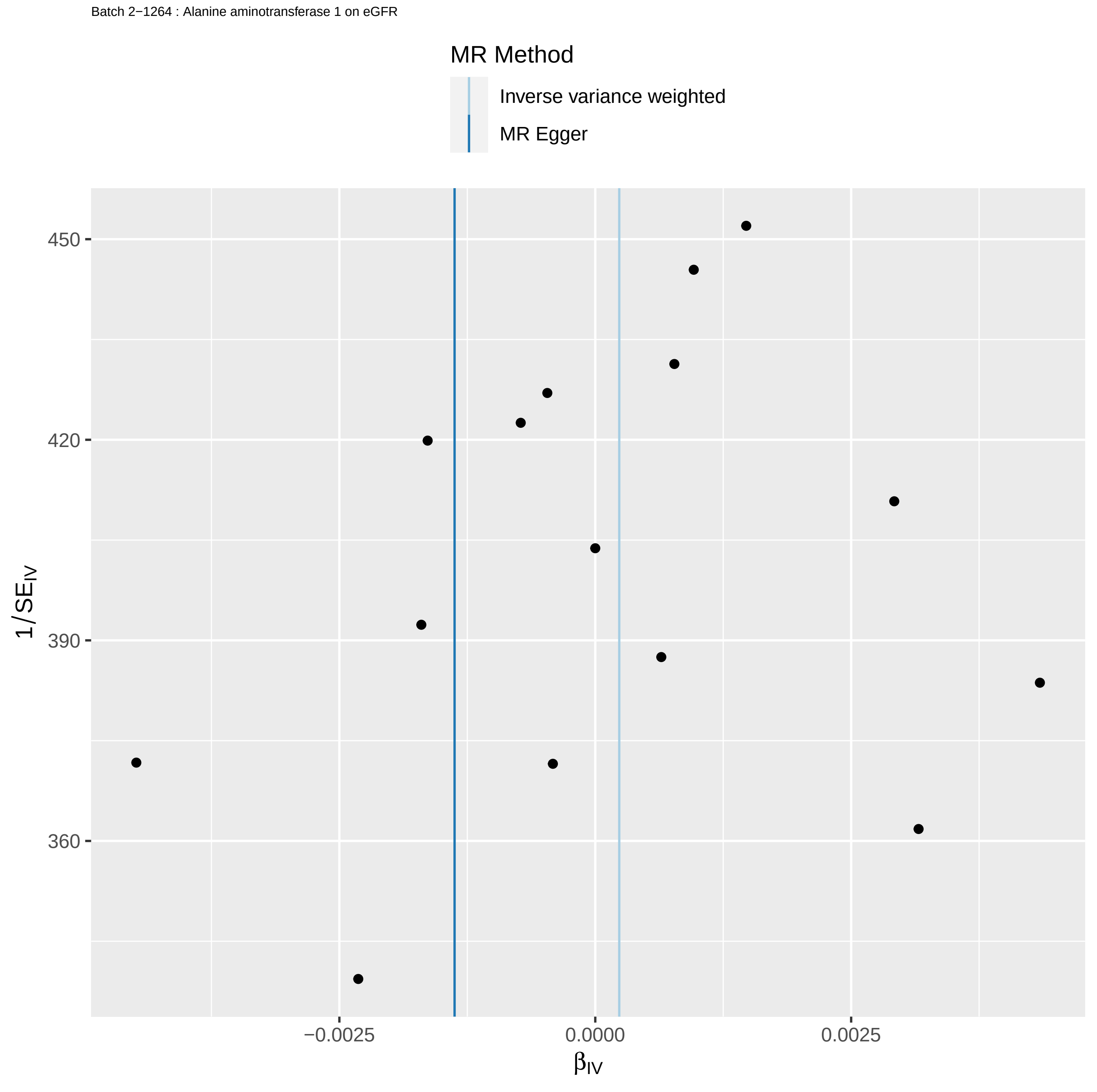

Batch 2−1264 : Alanine aminotransferase 1 on eGFR
MR Method
Inverse variance weighted
MR Egger
450
420
390
360
1/SE$_{IV}$
−0.0025
0.0000
0.0025
β$_{IV}$

Batch 2−1265 : Glutathione peroxidase 7 on eGFR

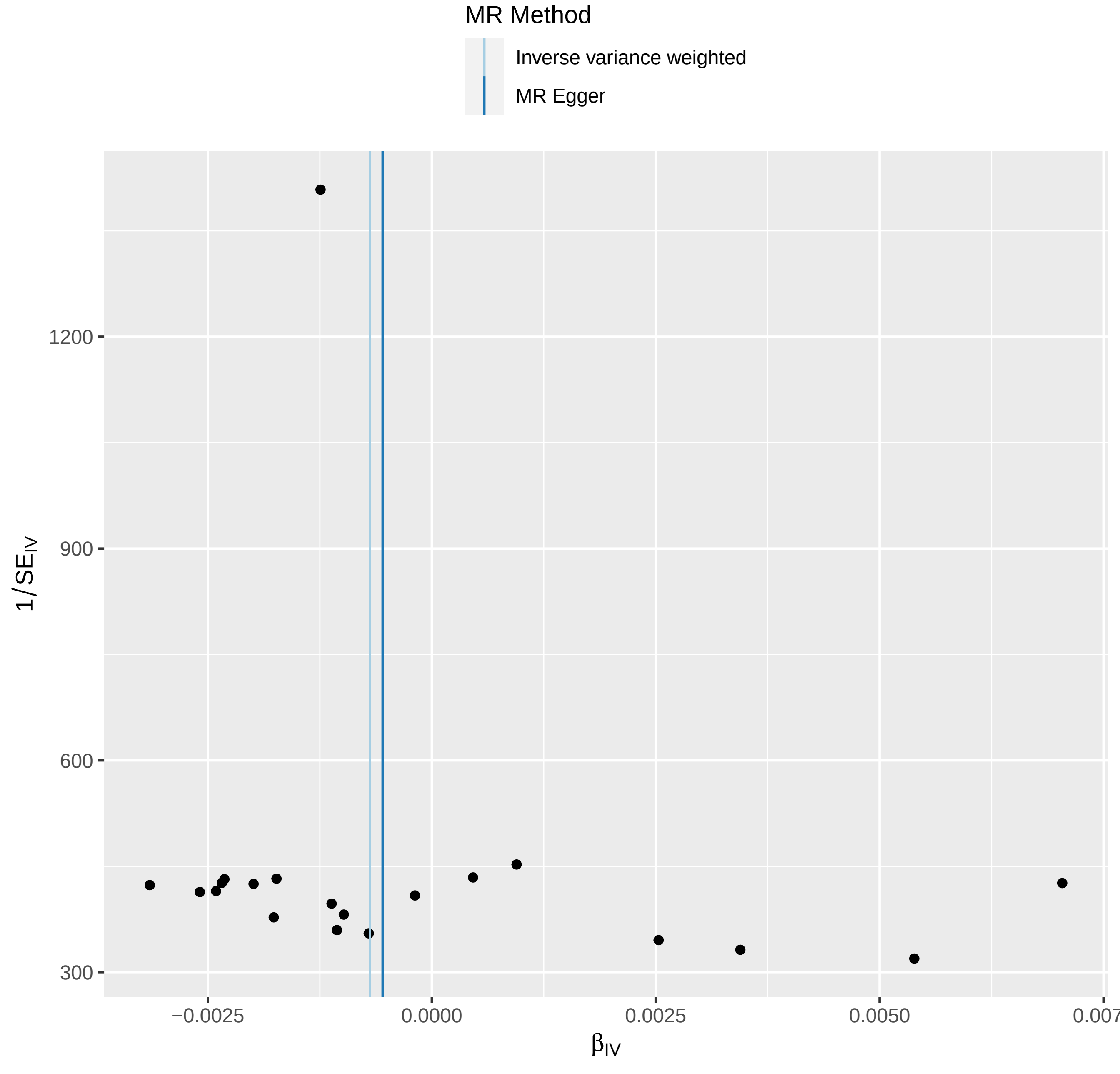

Batch 2−1266 : GRAM domain−containing protein 1C on eGFR

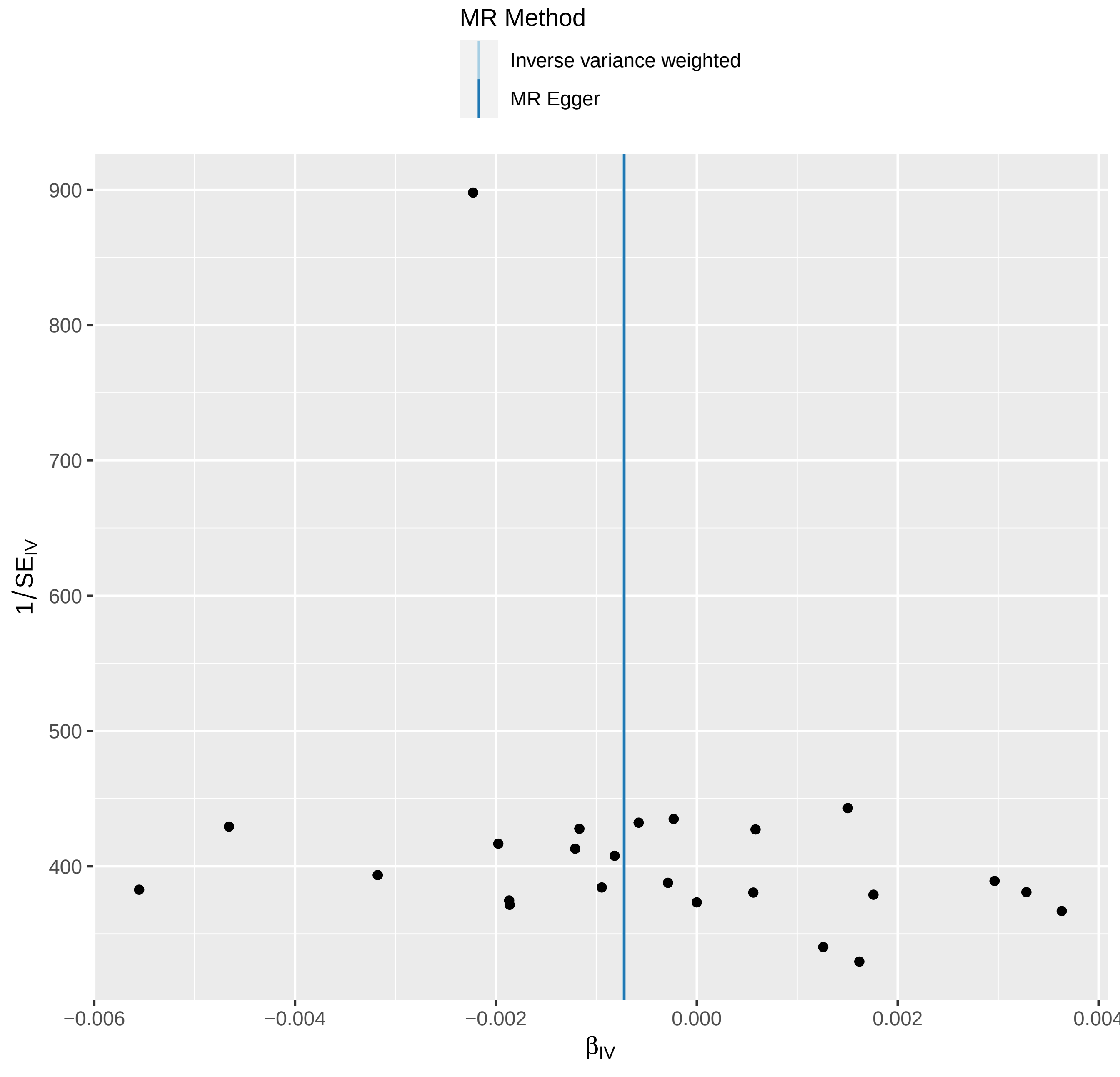

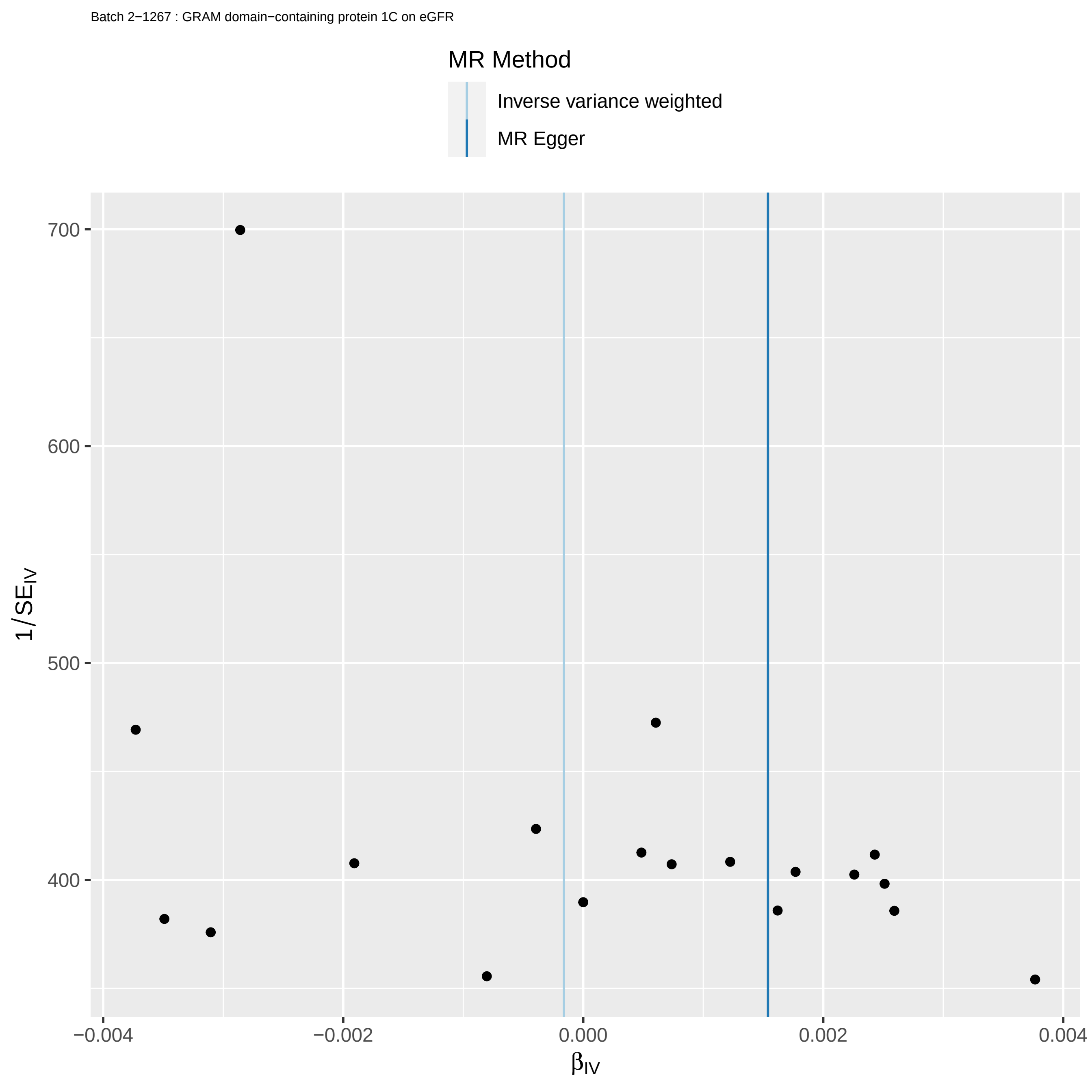
Batch 2−1267 : GRAM domain−containing protein 1C on eGFR
MR Method
Inverse variance weighted
MR Egger
700
600
500
400
1/SE_IV
−0.004
−0.002
0.000
0.002
0.004
β_IV

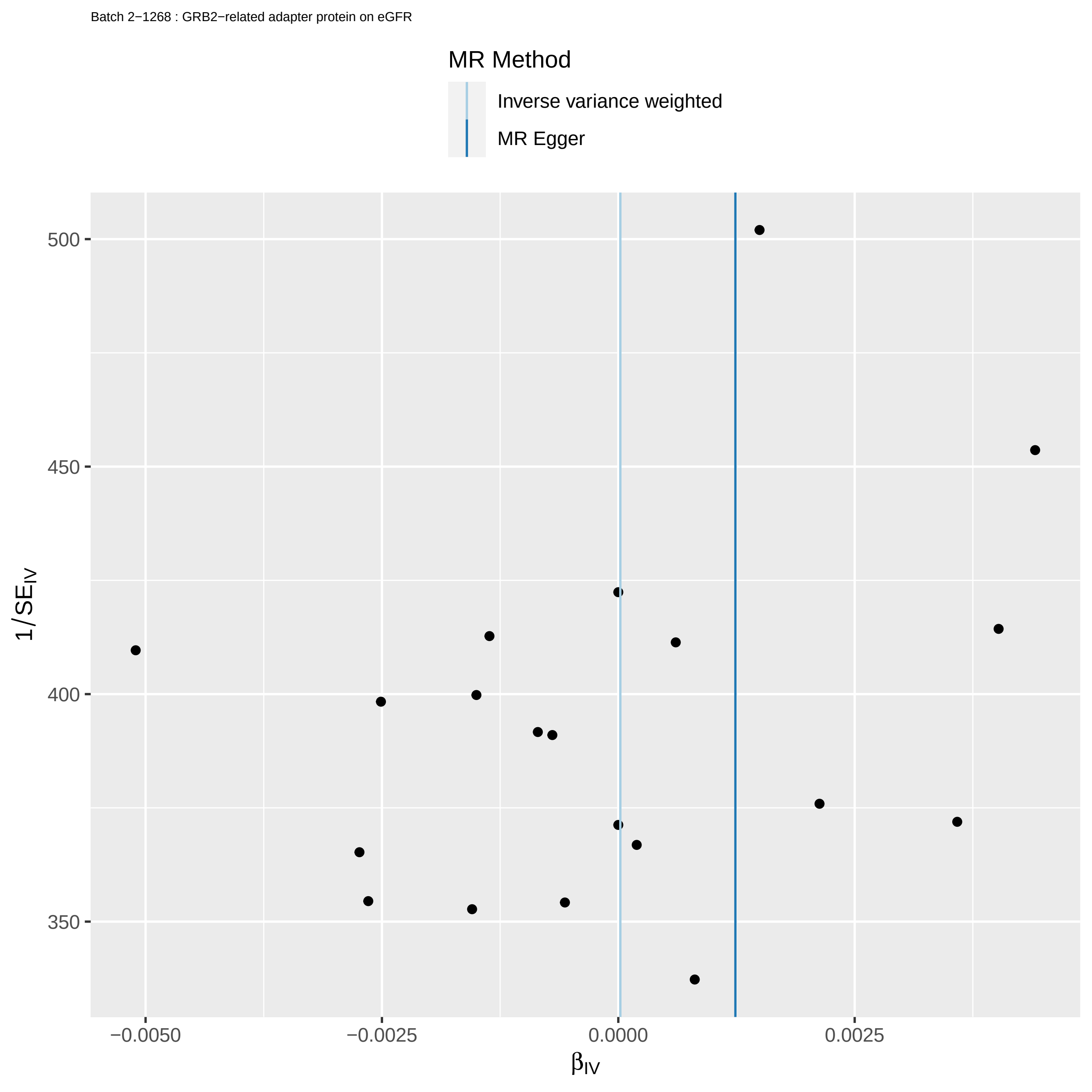
Batch 2−1268 : GRB2−related adapter protein on eGFR
MR Method
Inverse variance weighted
MR Egger
500
450
400
350
1/SE_IV
−0.0050
−0.0025
0.0000
0.0025
β_IV

Batch 2−1269 : GRB2−related adapter protein 2 on eGFR

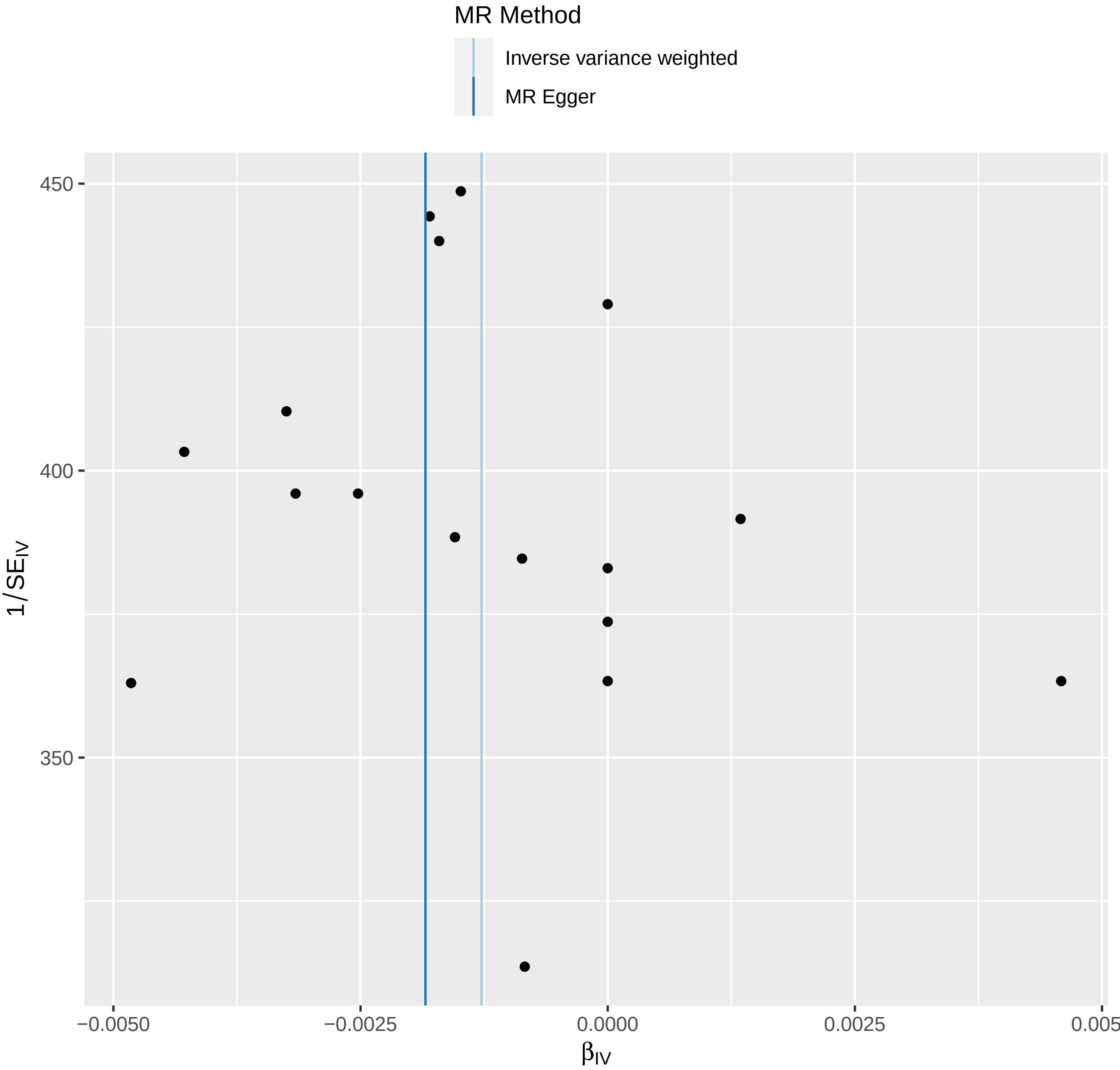

Batch 2−1270 : Growth factor receptor−bound protein 14 on eGFR

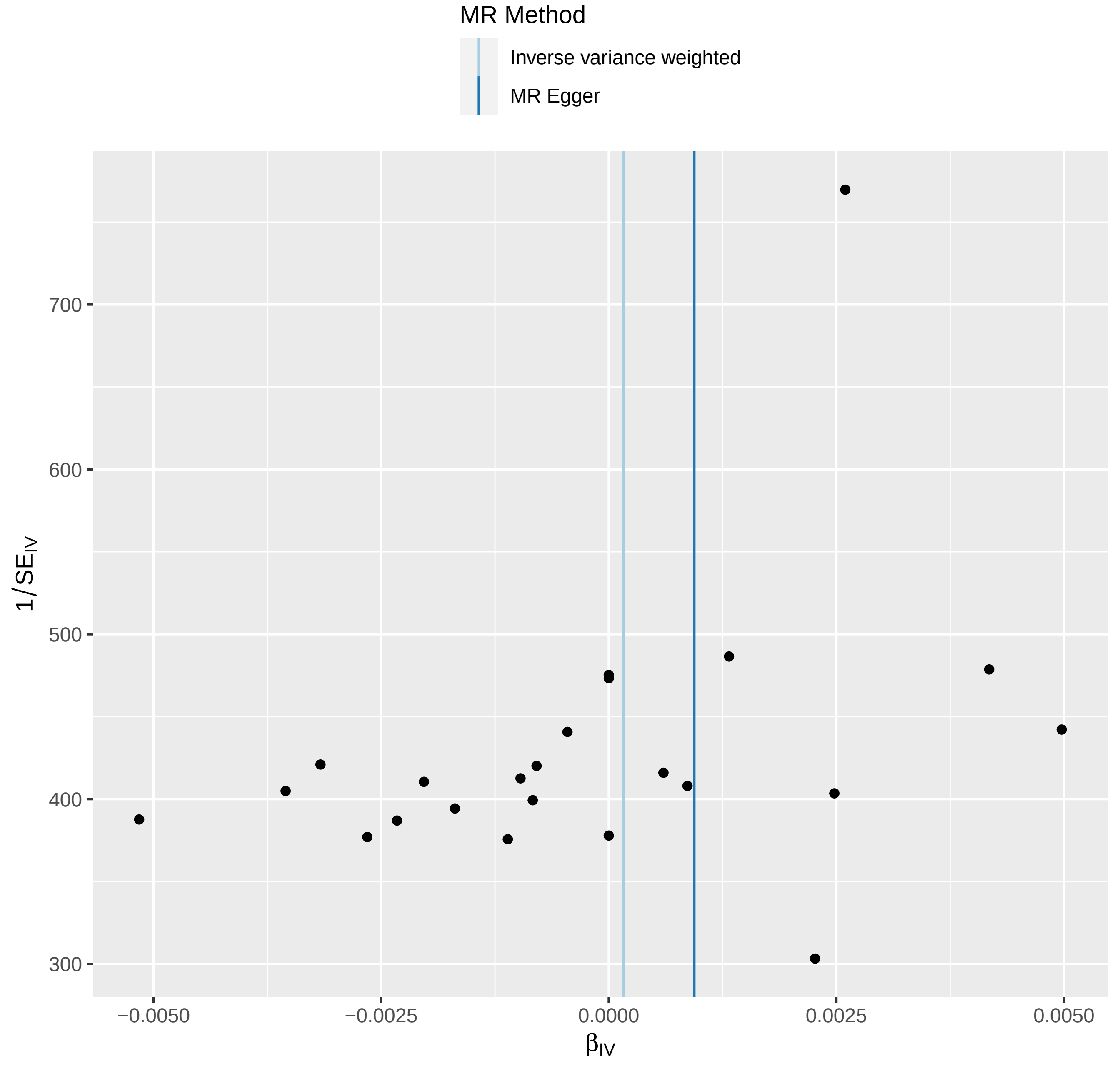

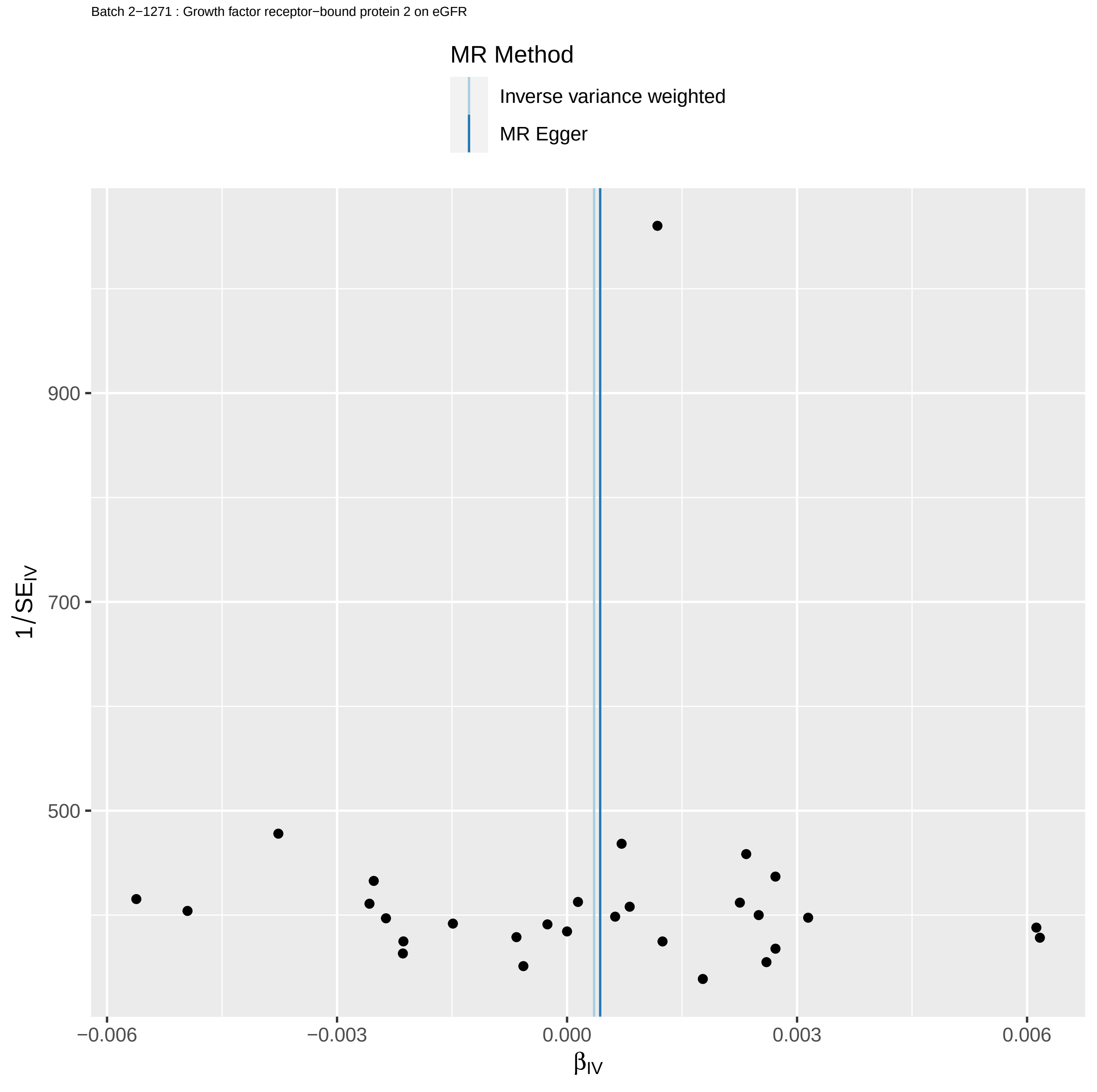
Batch 2−1271 : Growth factor receptor−bound protein 2 on eGFR
MR Method
Inverse variance weighted
MR Egger
900
700
500
1/SE_IV
−0.006
−0.003
0.000
0.003
0.006
β_IV

Batch 2−1272 : Growth factor receptor−bound protein 7 on eGFR

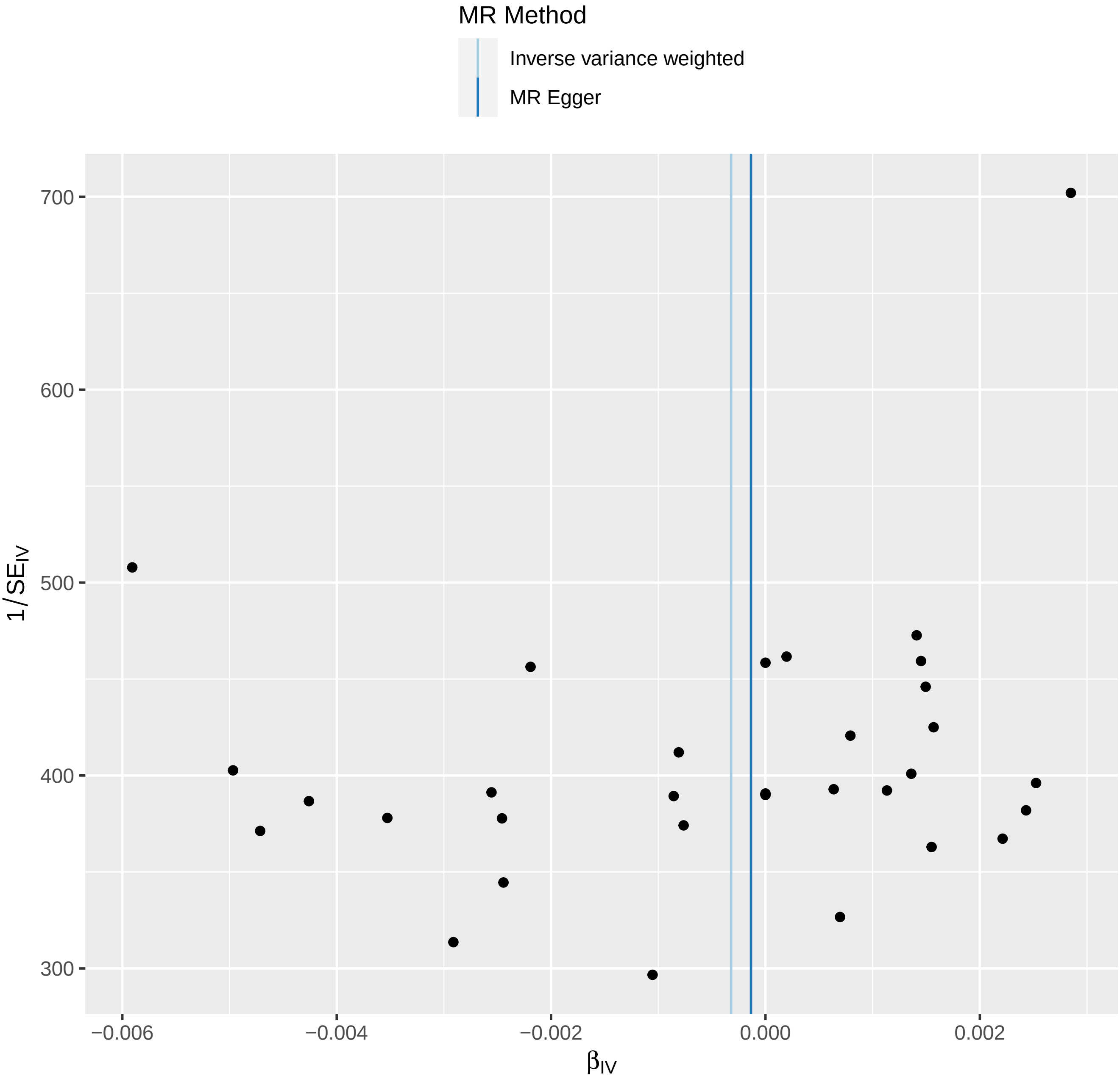

Batch 2−1273 : Gremlin−1 on eGFR

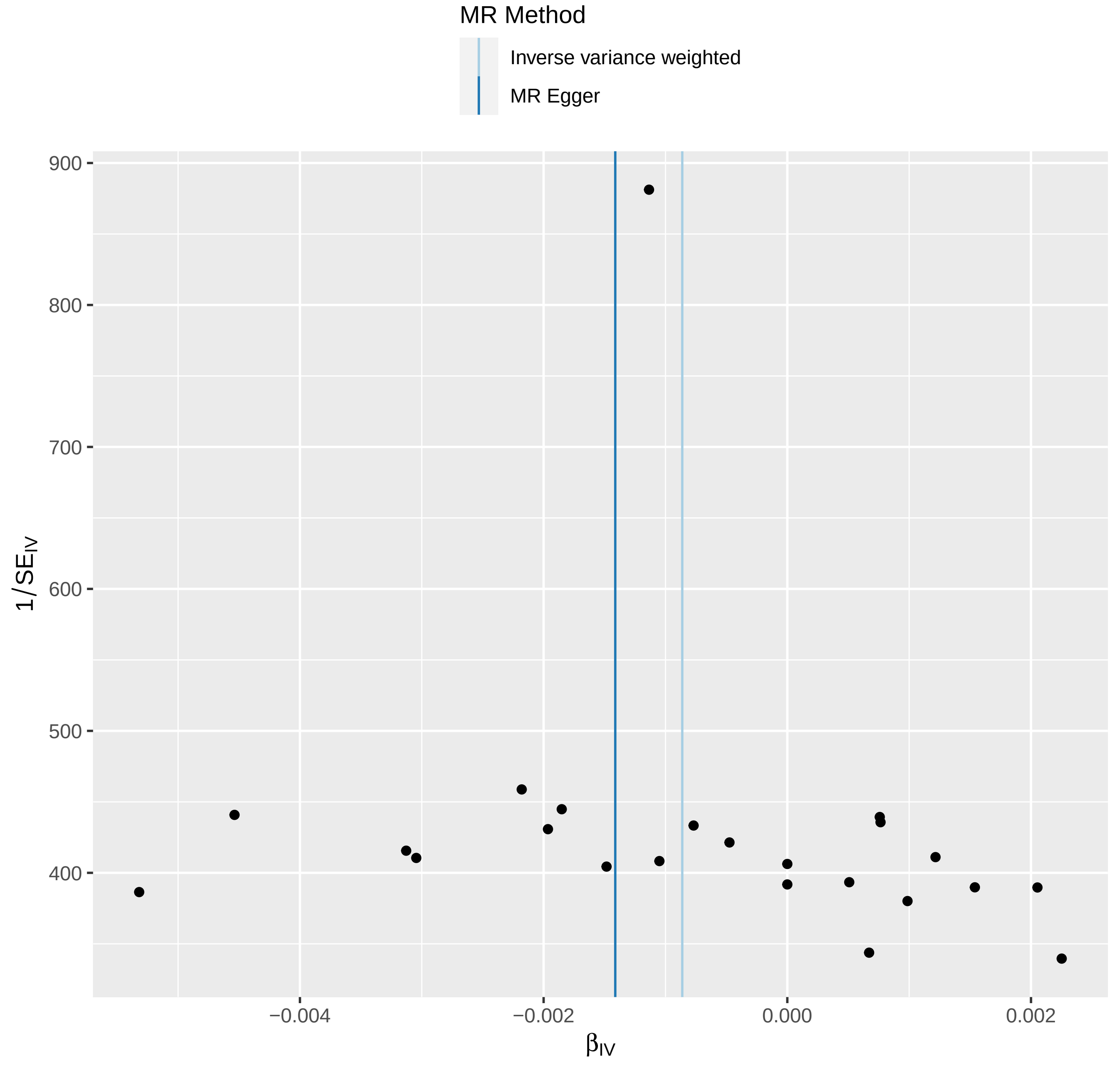

Batch 2−1274 : Gremlin−2 on eGFR

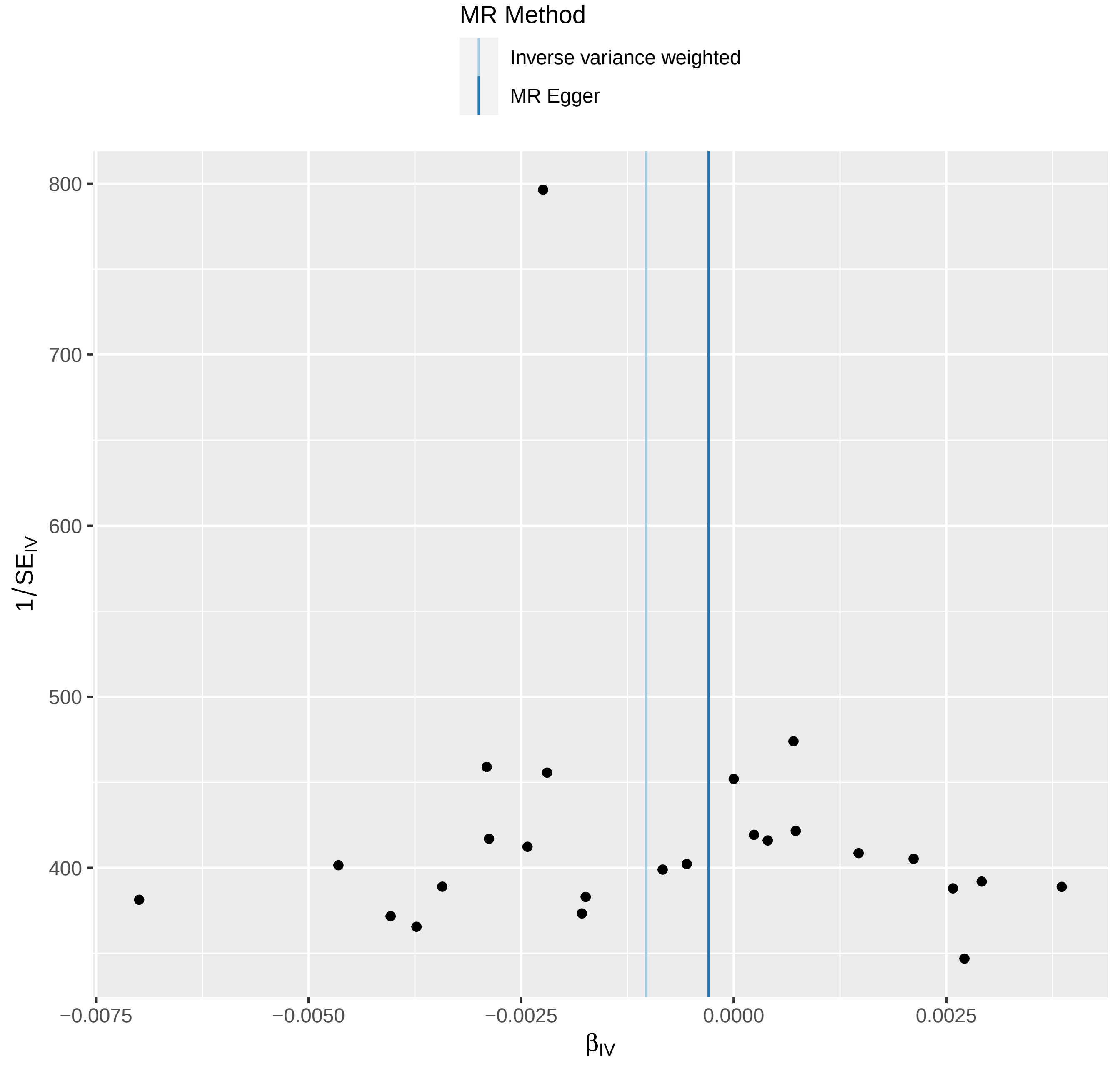

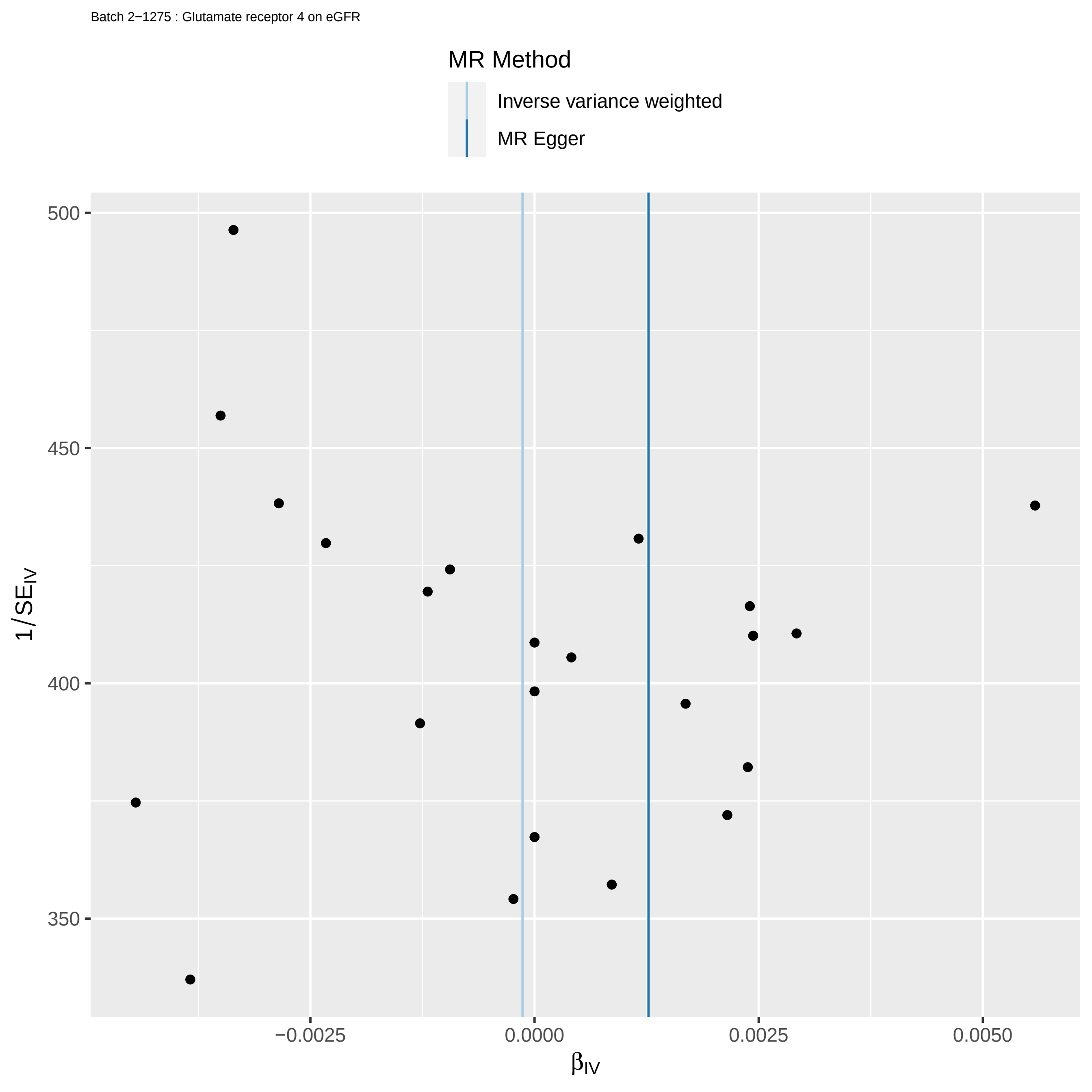

Batch 2−1275 : Glutamate receptor 4 on eGFR
MR Method
Inverse variance weighted
MR Egger
500
450
400
350
1/SE_IV
−0.0025
0.0000
0.0025
0.0050
β_IV

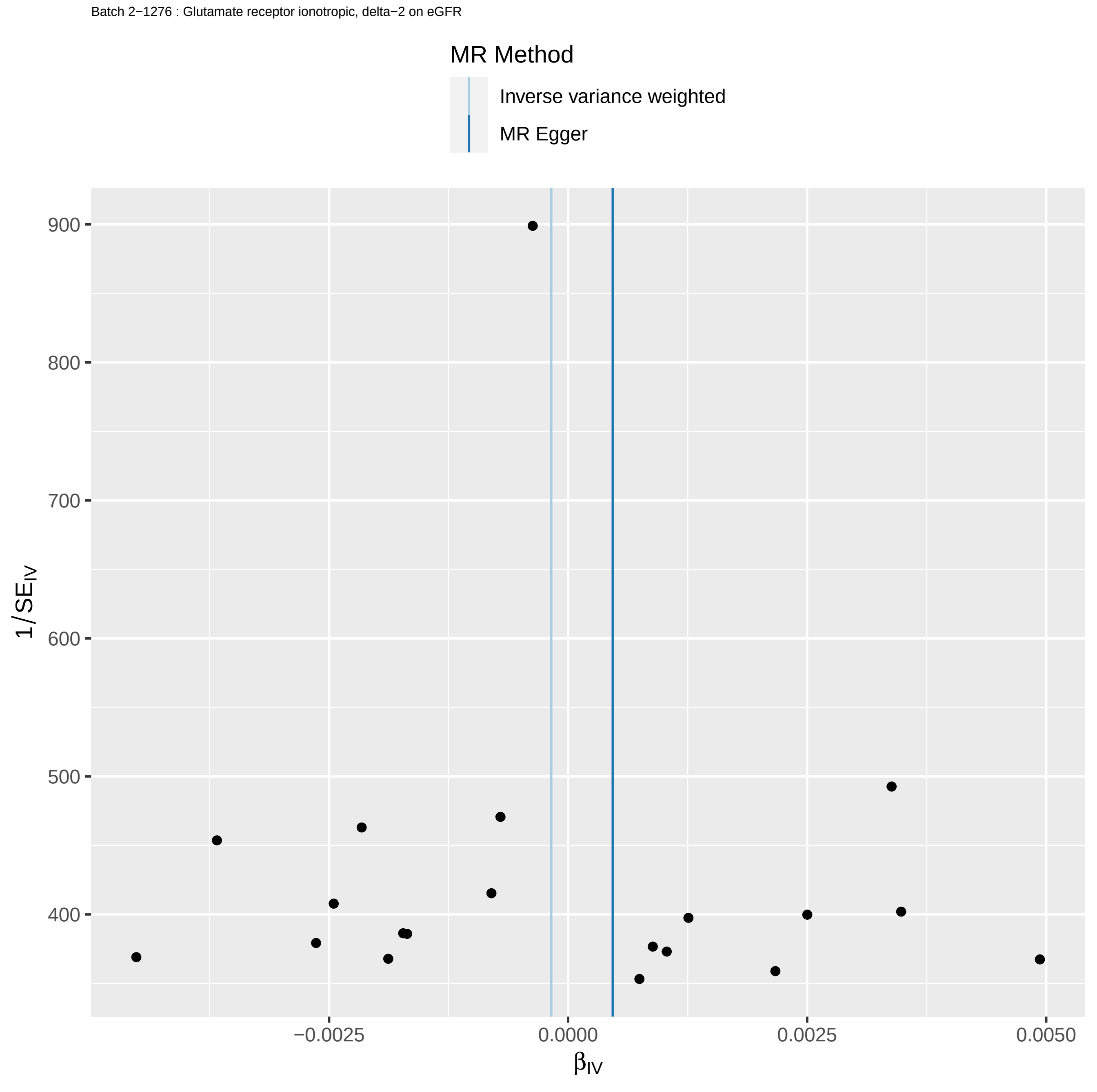
Batch 2−1276 : Glutamate receptor ionotropic, delta−2 on eGFR
MR Method
Inverse variance weighted
MR Egger
900
800
700
600
500
400
1/SE$_{IV}$
−0.0025
0.0000
0.0025
0.0050
β_{IV}

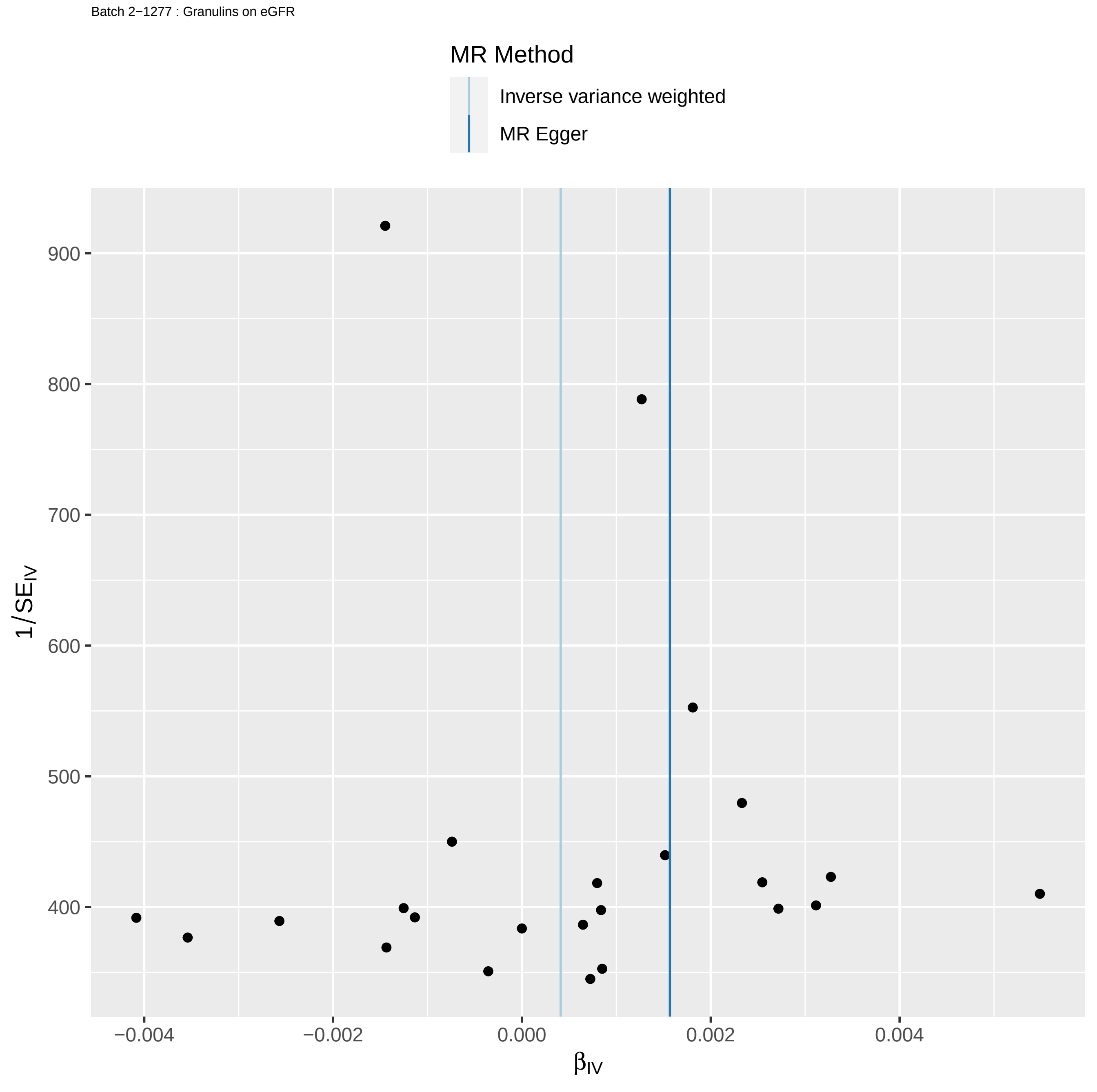
Batch 2−1277 : Granulins on eGFR
MR Method
Inverse variance weighted
MR Egger
900
800
700
600
500
400
1/SE_IV
−0.004
−0.002
0.000
0.002
0.004
β_IV

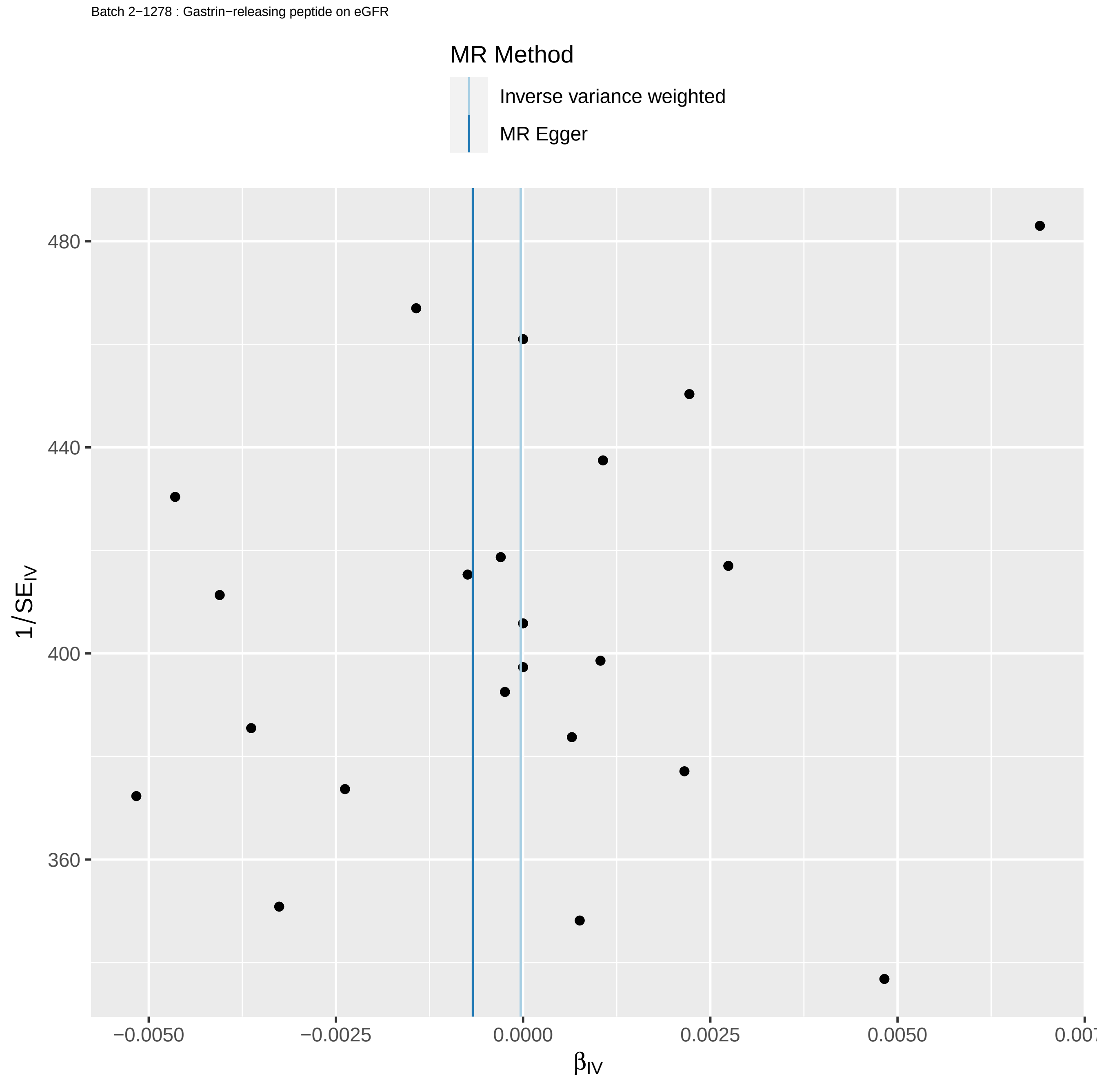
Batch 2−1278 : Gastrin−releasing peptide on eGFR
MR Method
Inverse variance weighted
MR Egger
480
440
400
360
1/SE_IV
−0.0050
−0.0025
0.0000
0.0025
0.0050
0.007
β_IV

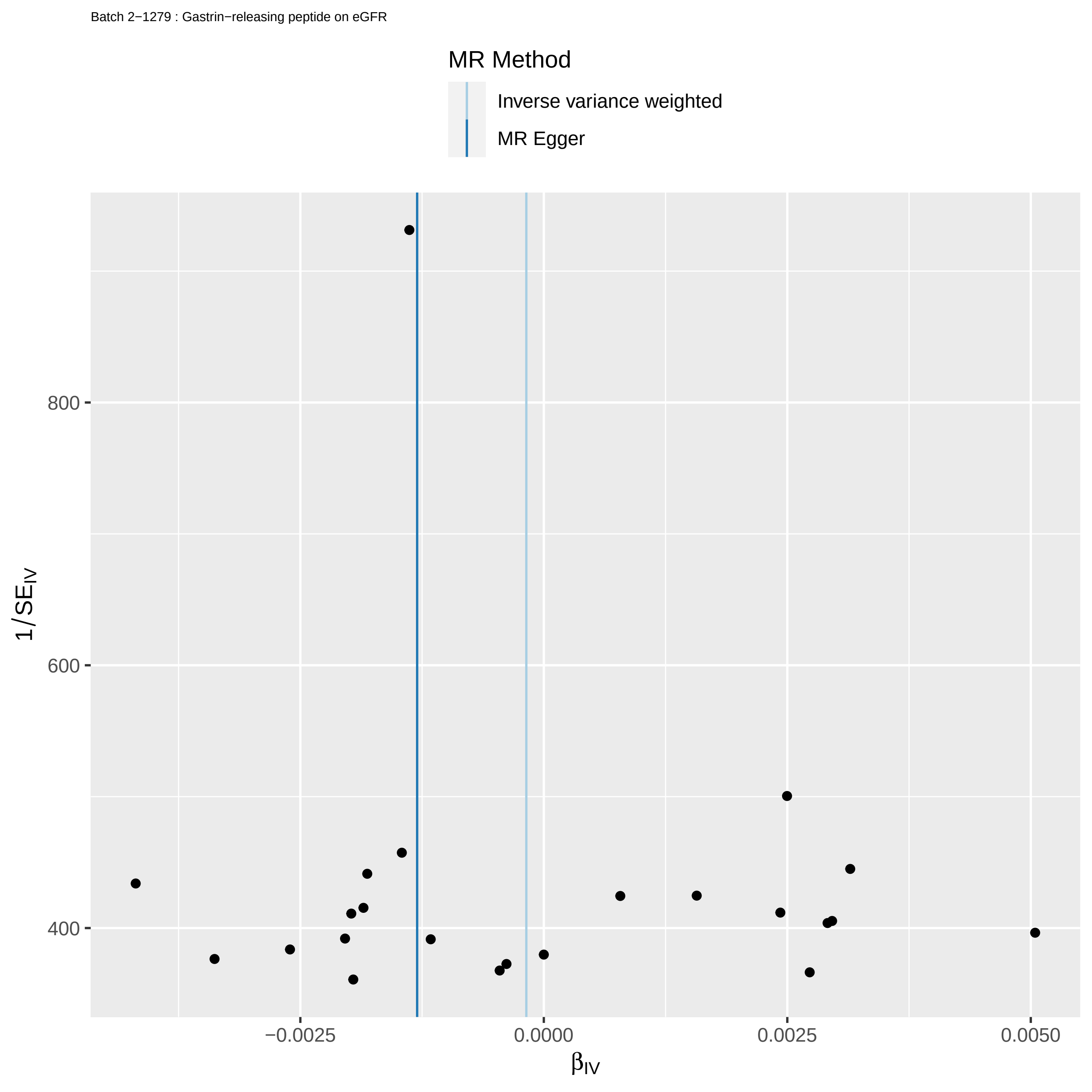

Batch 2−1279 : Gastrin−releasing peptide on eGFR
MR Method
Inverse variance weighted
MR Egger
800
600
400
1/SE_IV
−0.0025
0.0000
0.0025
0.0050
β_IV

Batch 2−1280 : Gastrin−releasing peptide on eGFR

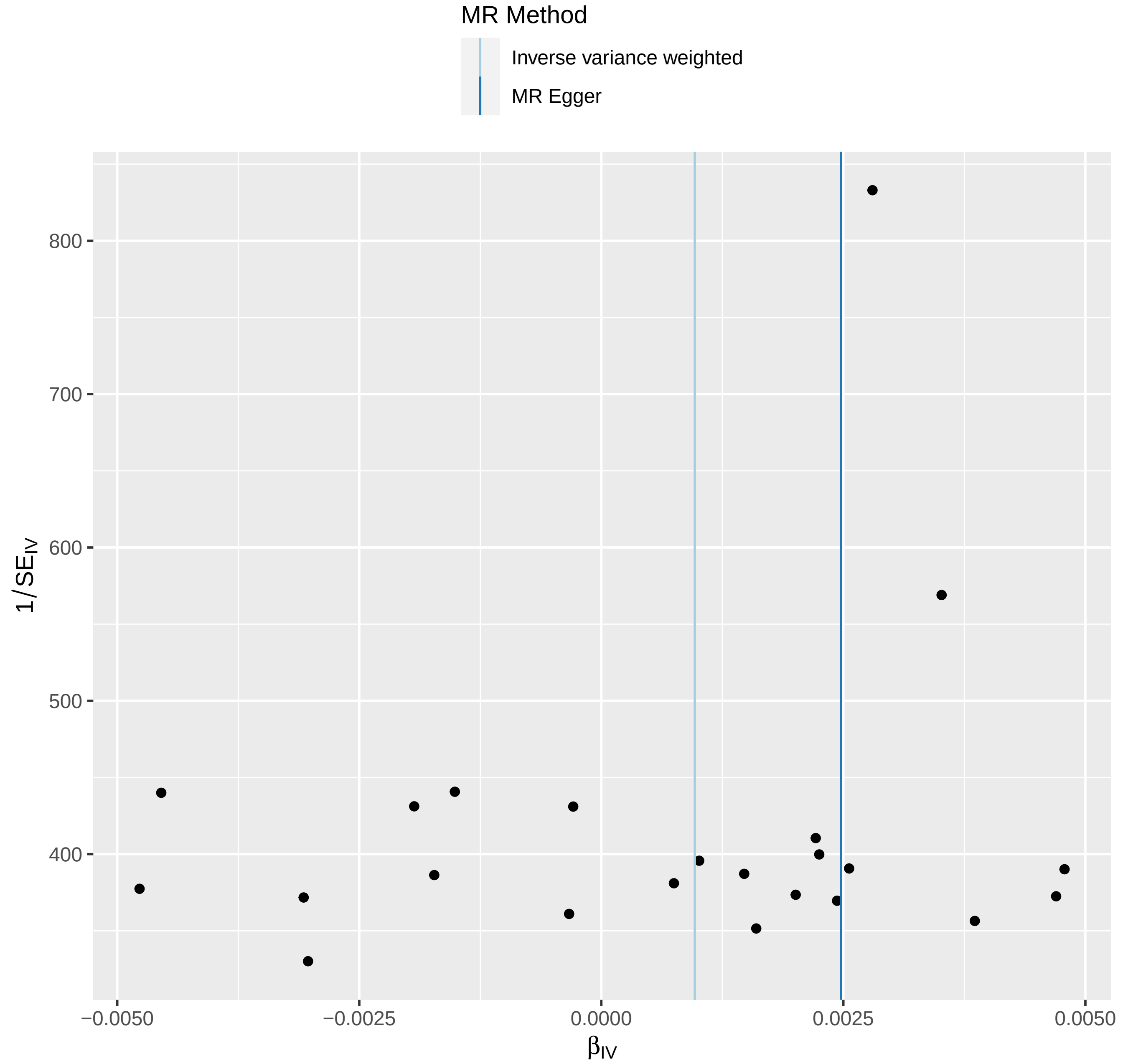

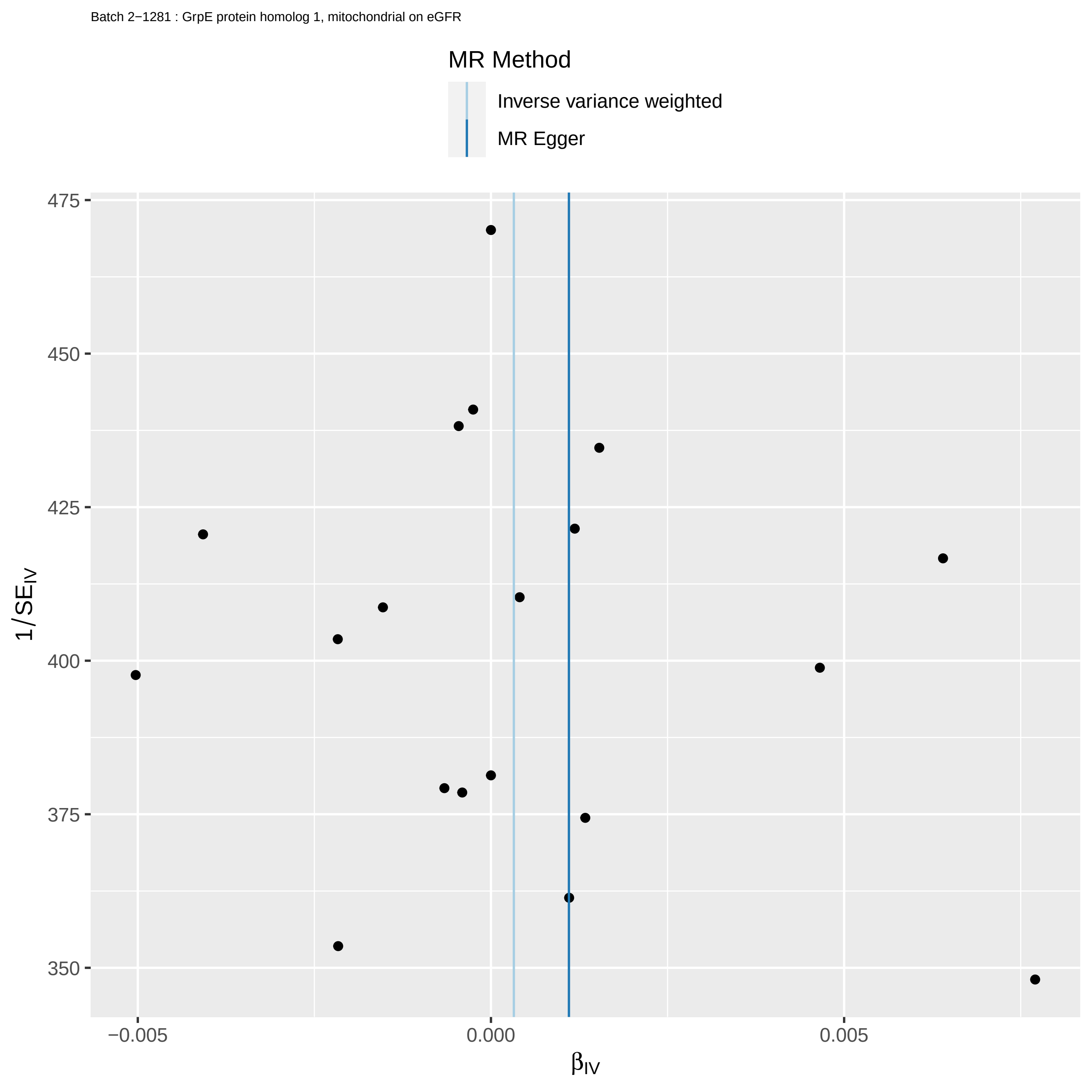

Batch 2−1281 : GrpE protein homolog 1, mitochondrial on eGFR
MR Method
Inverse variance weighted
MR Egger
475
450
425
400
375
350
1/SE_IV
−0.005
0.000
0.005
β_IV

Batch 2−1282 : GSK3−beta interaction protein on eGFR

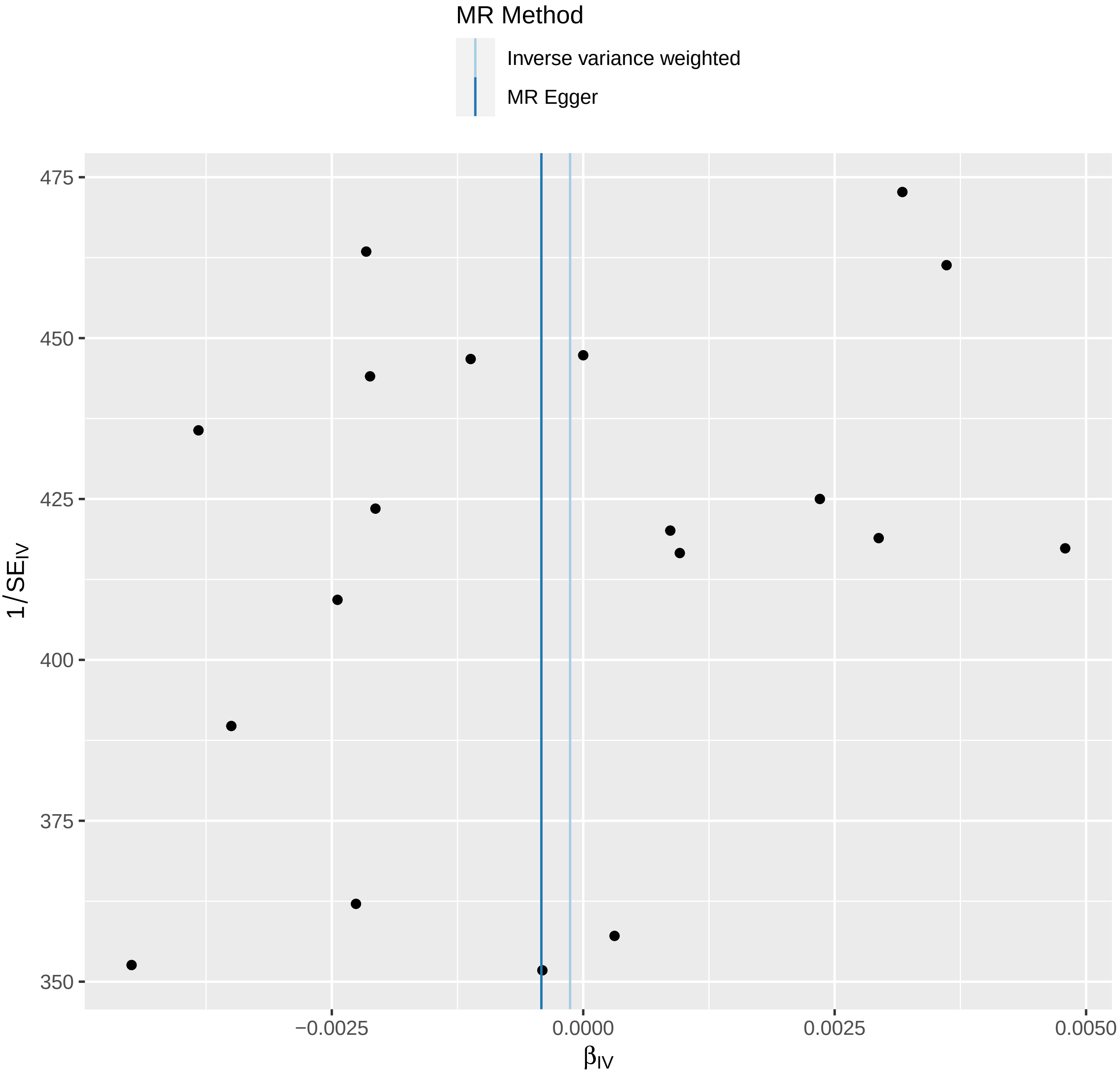

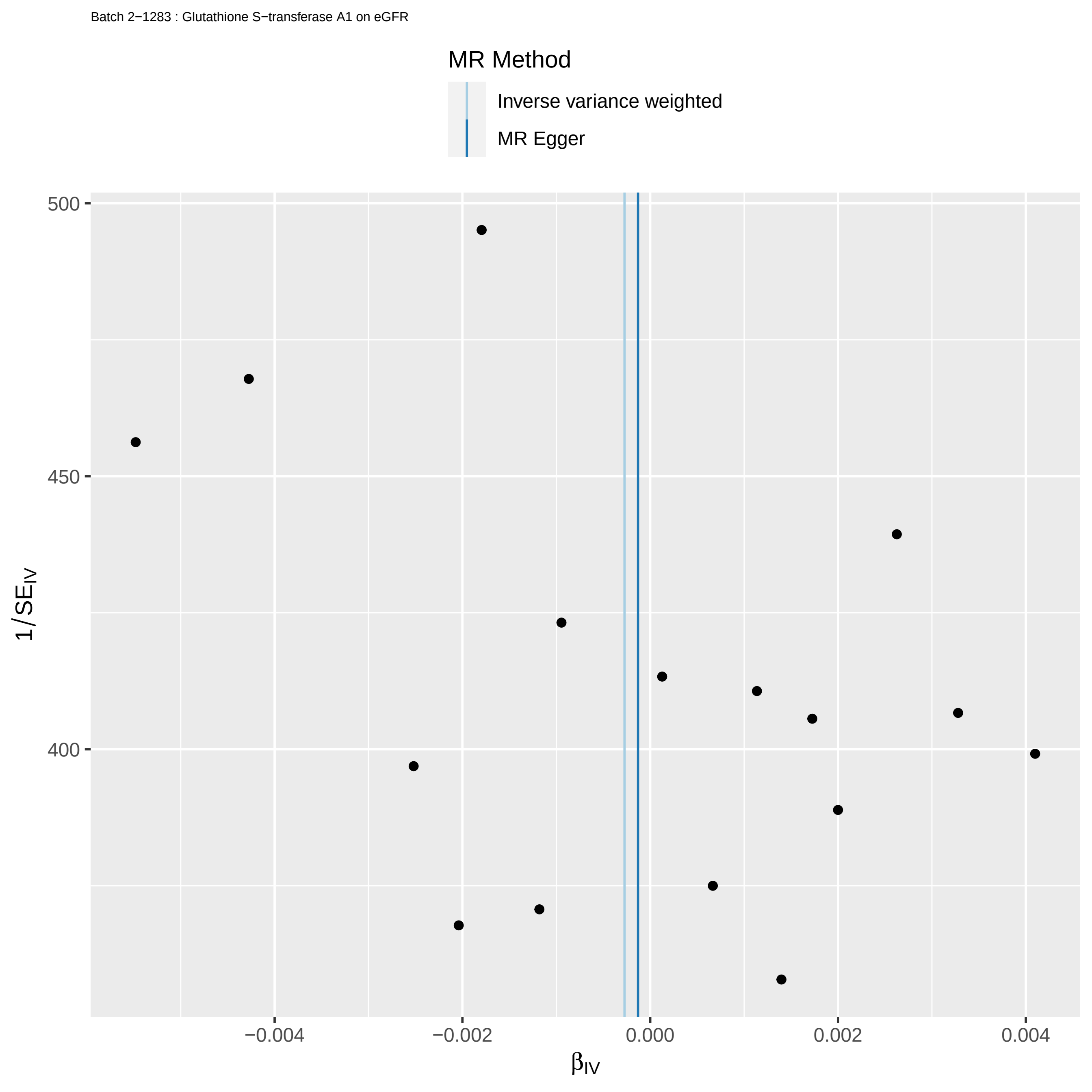
Batch 2−1283 : Glutathione S−transferase A1 on eGFR
MR Method
Inverse variance weighted
MR Egger
500
450
400
1/SE_IV
−0.004
−0.002
0.000
0.002
0.004
β_IV

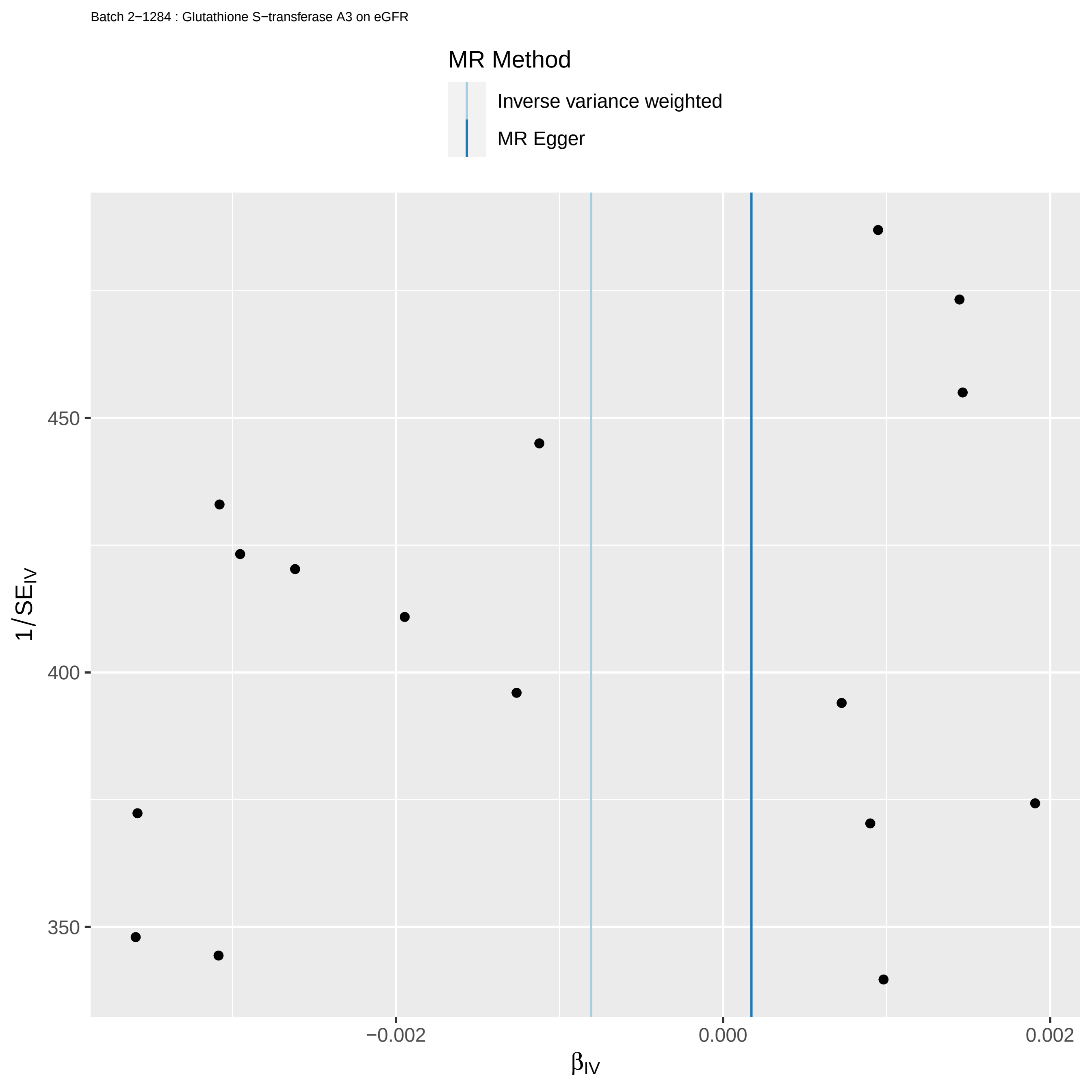

Batch 2−1284 : Glutathione S−transferase A3 on eGFR
MR Method
Inverse variance weighted
MR Egger
450
400
350
1/SE_IV
−0.002
0.000
0.002
β_IV

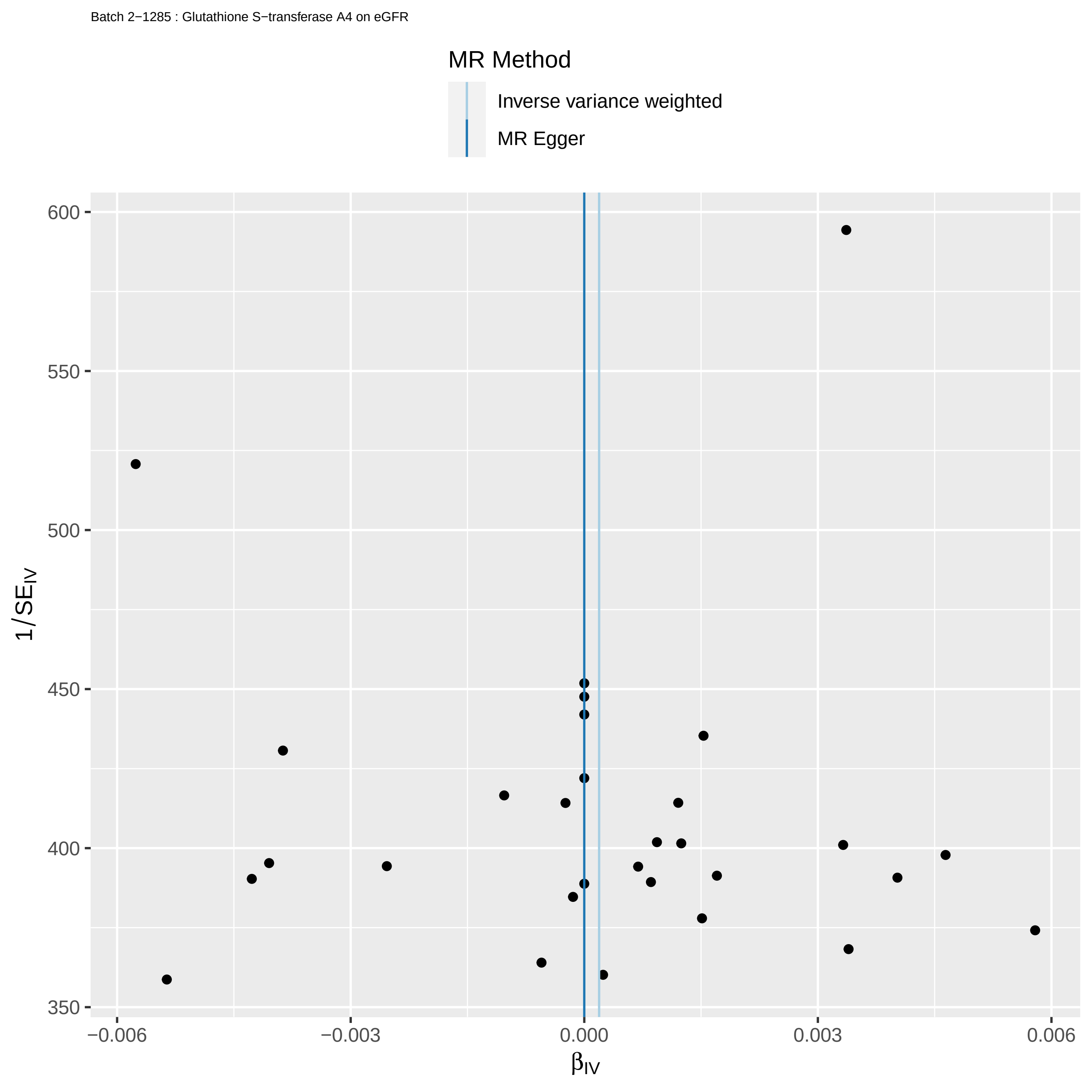
Batch 2−1285 : Glutathione S−transferase A4 on eGFR
MR Method
Inverse variance weighted
MR Egger
600
550
500
450
400
350
1/SE$_{IV}$
−0.006
−0.003
0.000
0.003
0.006
β$_{IV}$

Batch 2−1286 : Glutathione S−transferase kappa 1 on eGFR

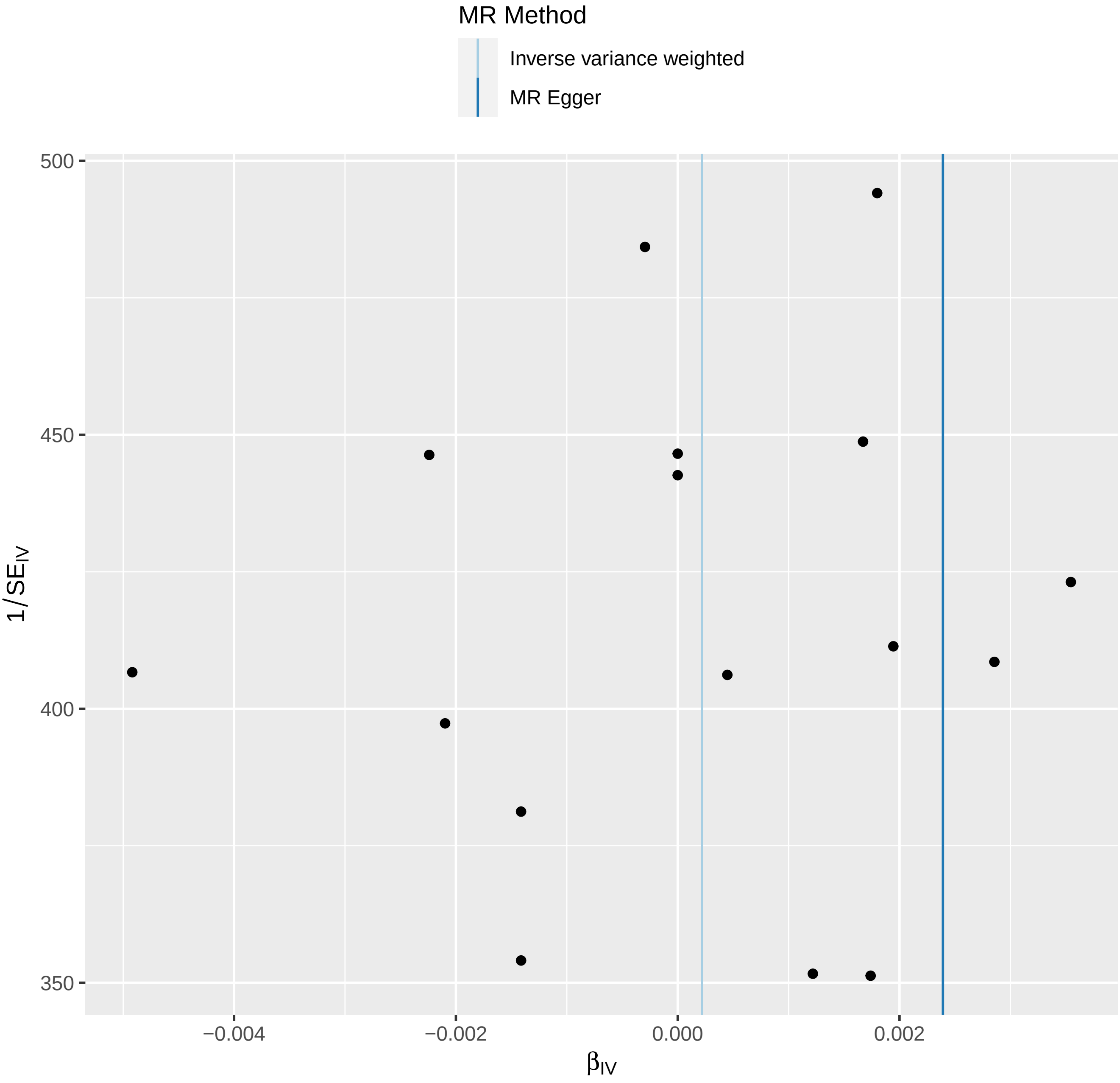

Batch 2−1287 : Glutathione S−transferase Mu 1 on eGFR

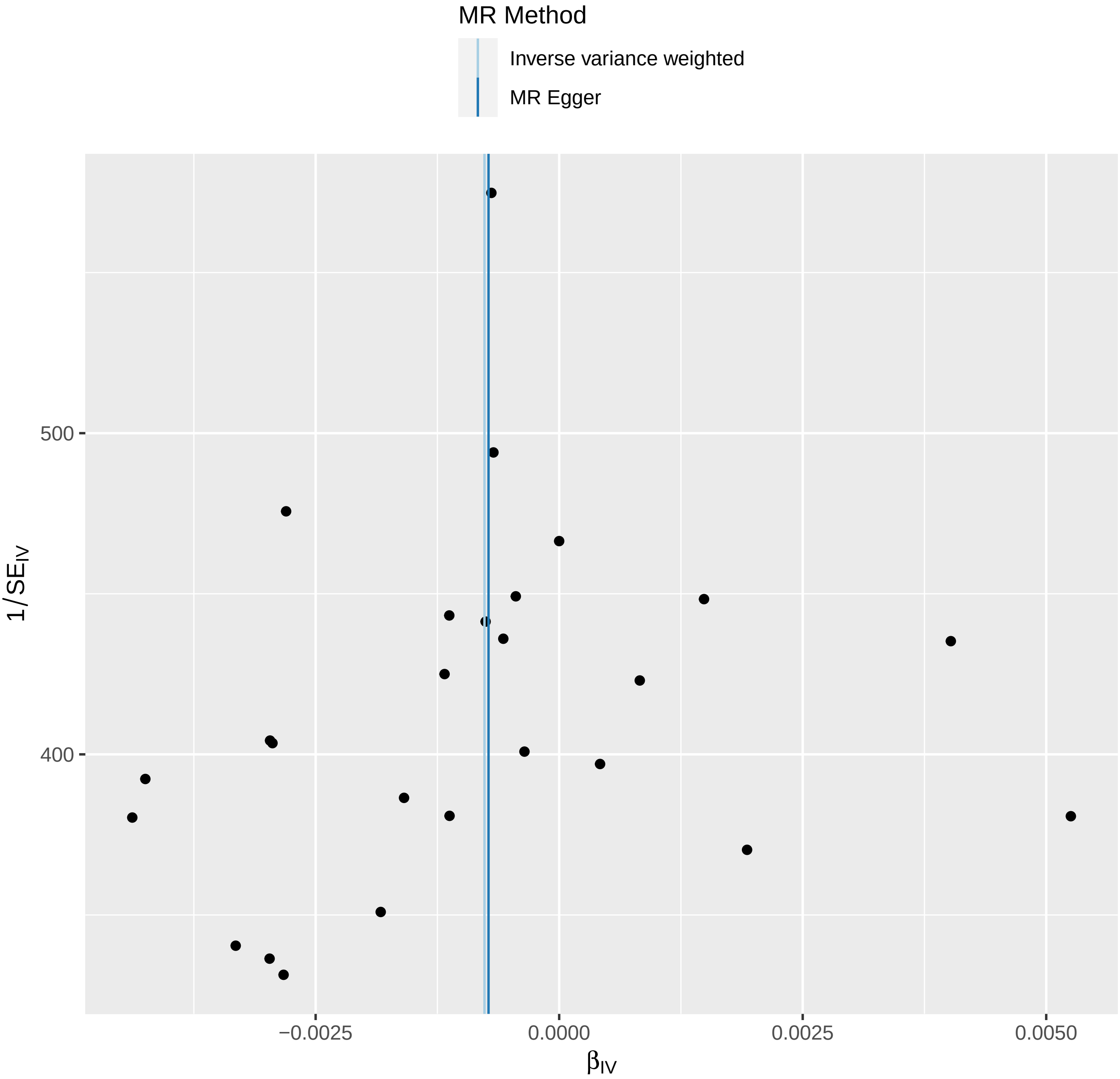

Batch 2−1288 : Glutathione S−transferase omega−1 on eGFR

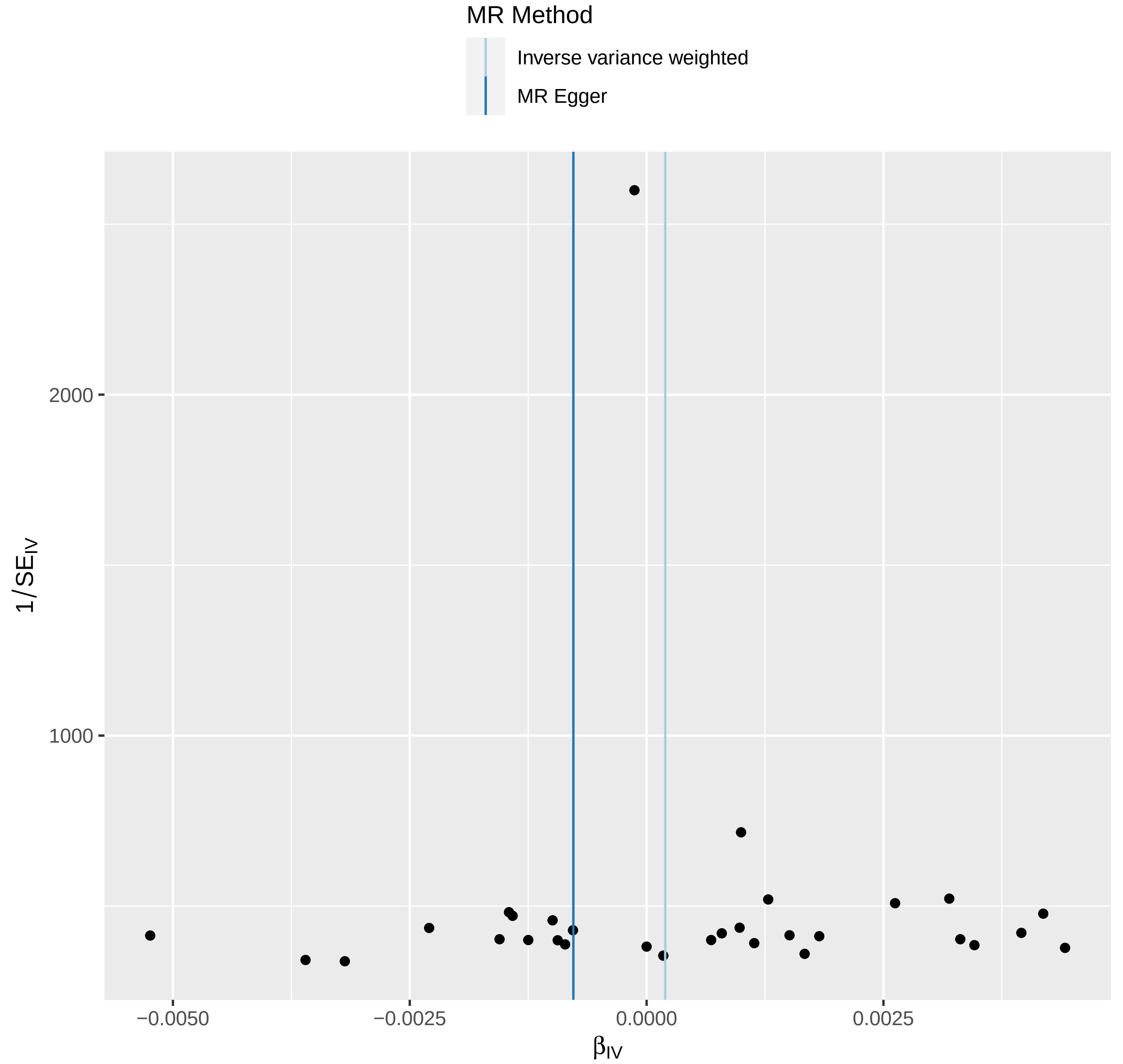

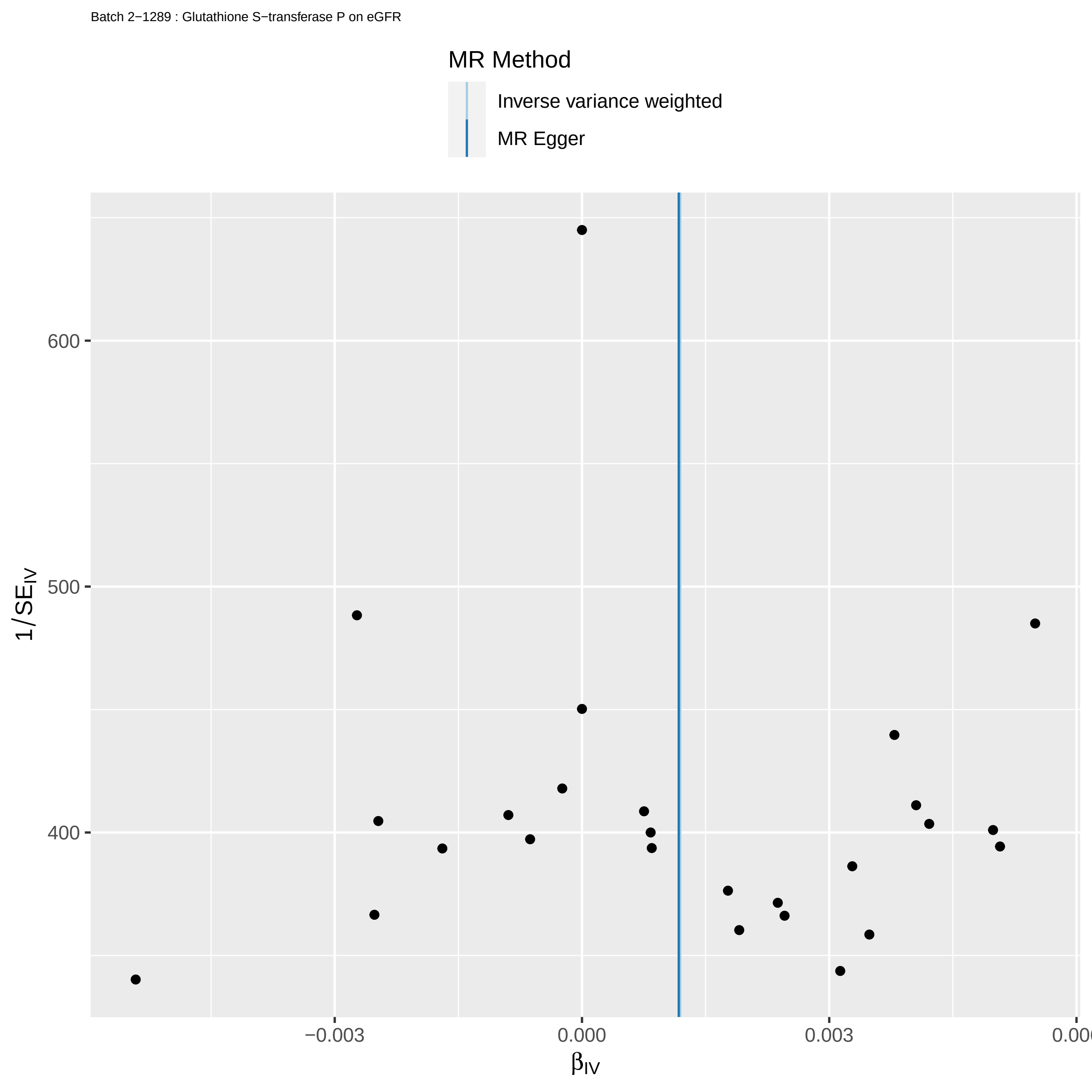

Batch 2−1289 : Glutathione S−transferase P on eGFR
MR Method
Inverse variance weighted
MR Egger
600
500
400
1/SE_IV
−0.003
0.000
0.003
β_IV

Batch 2−1290 : Glutathione S−transferase P on eGFR

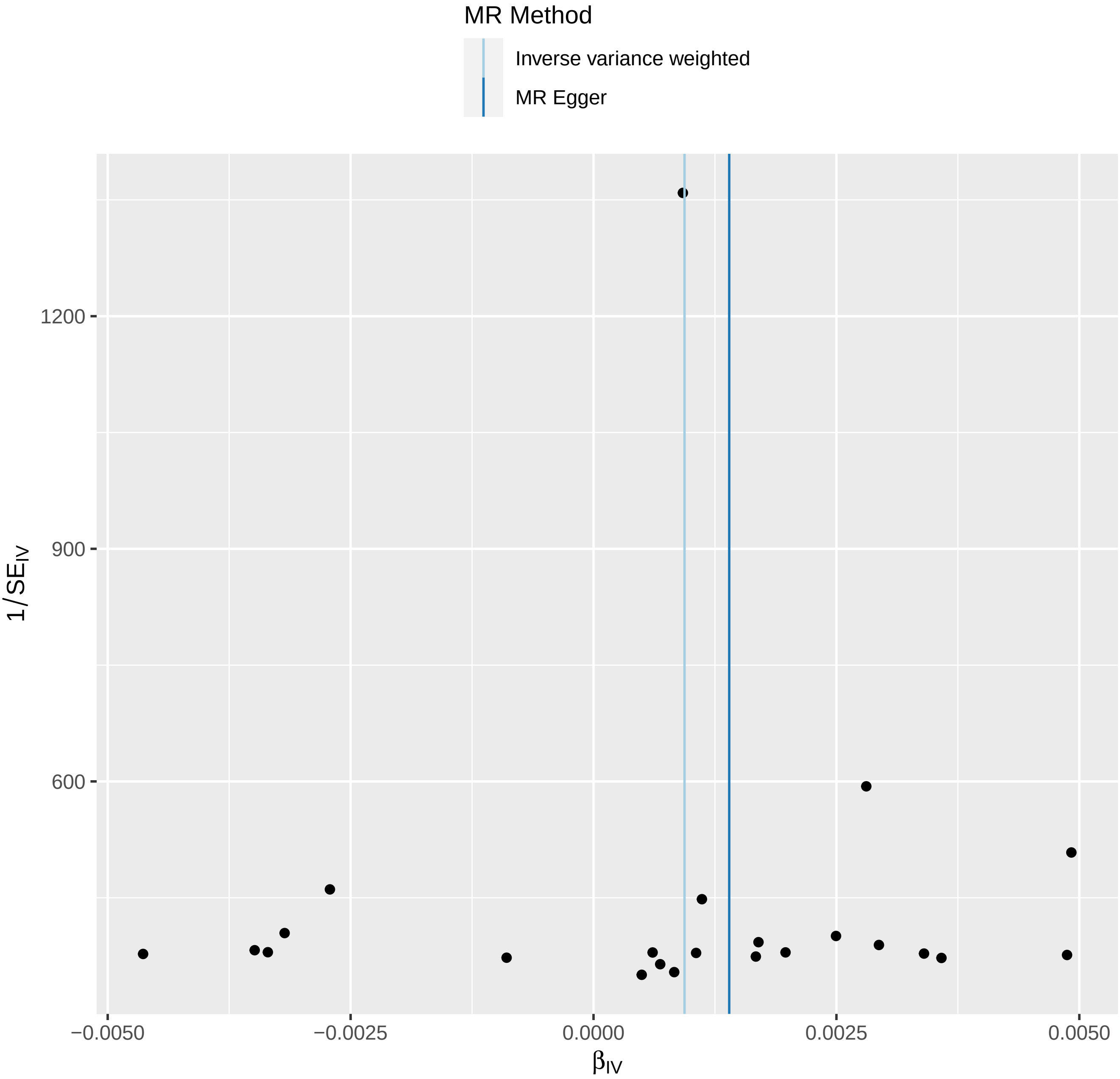

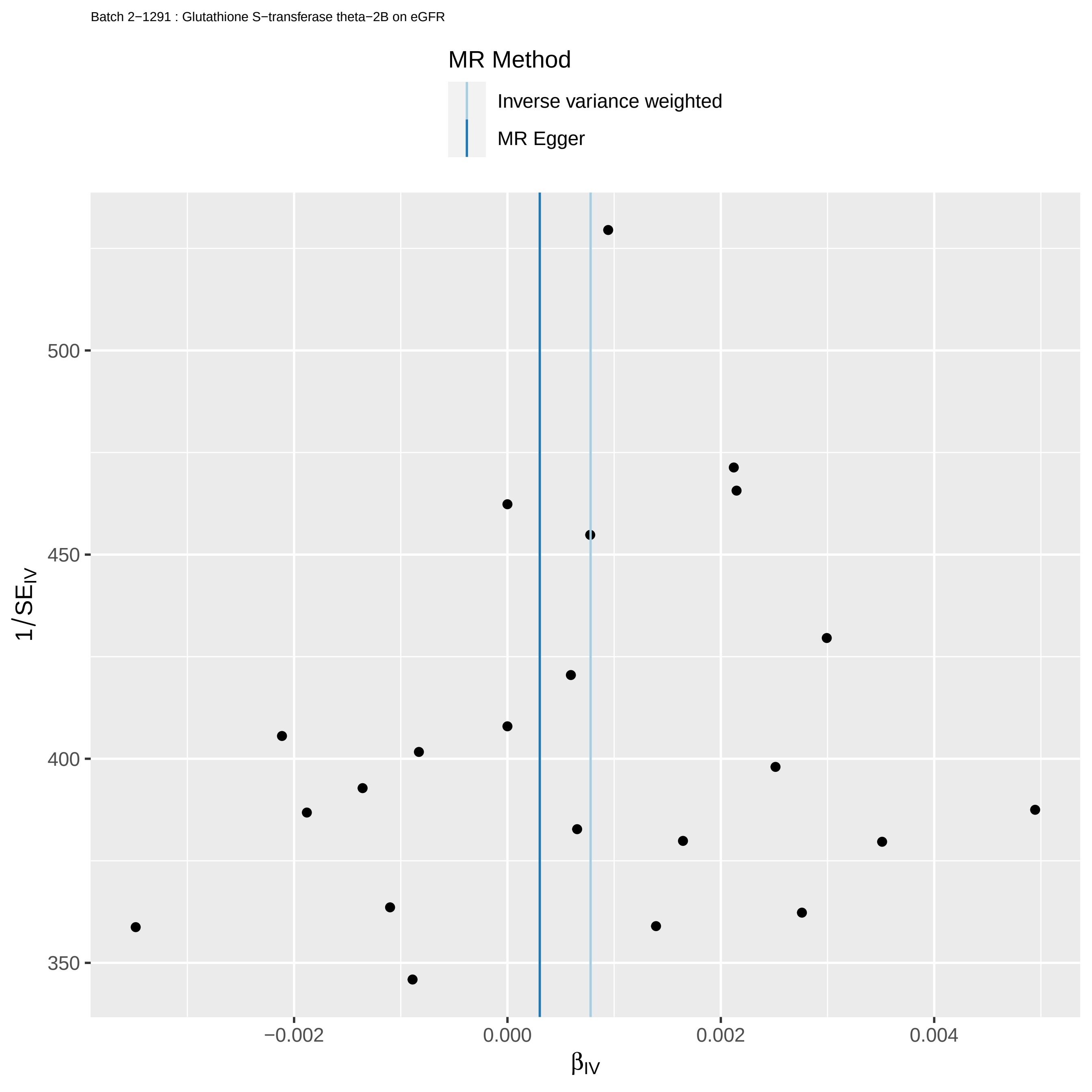
Batch 2−1291 : Glutathione S−transferase theta−2B on eGFR
MR Method
Inverse variance weighted
MR Egger
500
450
400
350
1/SE$_{IV}$
−0.002
0.000
0.002
0.004
β$_{IV}$

Batch 2−1292 : General transcription factor II−I on eGFR

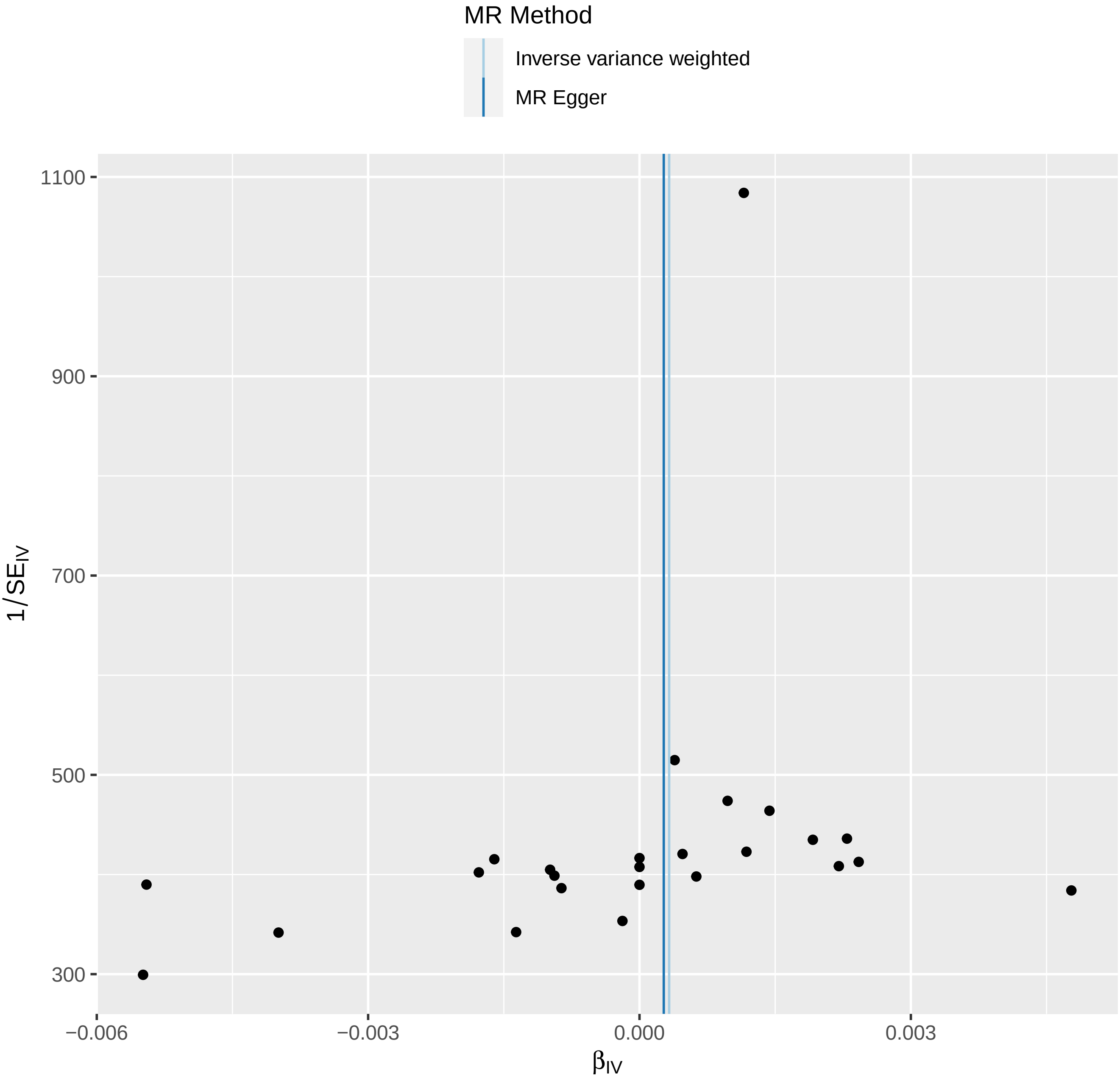

Batch 2−1293 : Guanylyl cyclase−activating protein 1 on eGFR

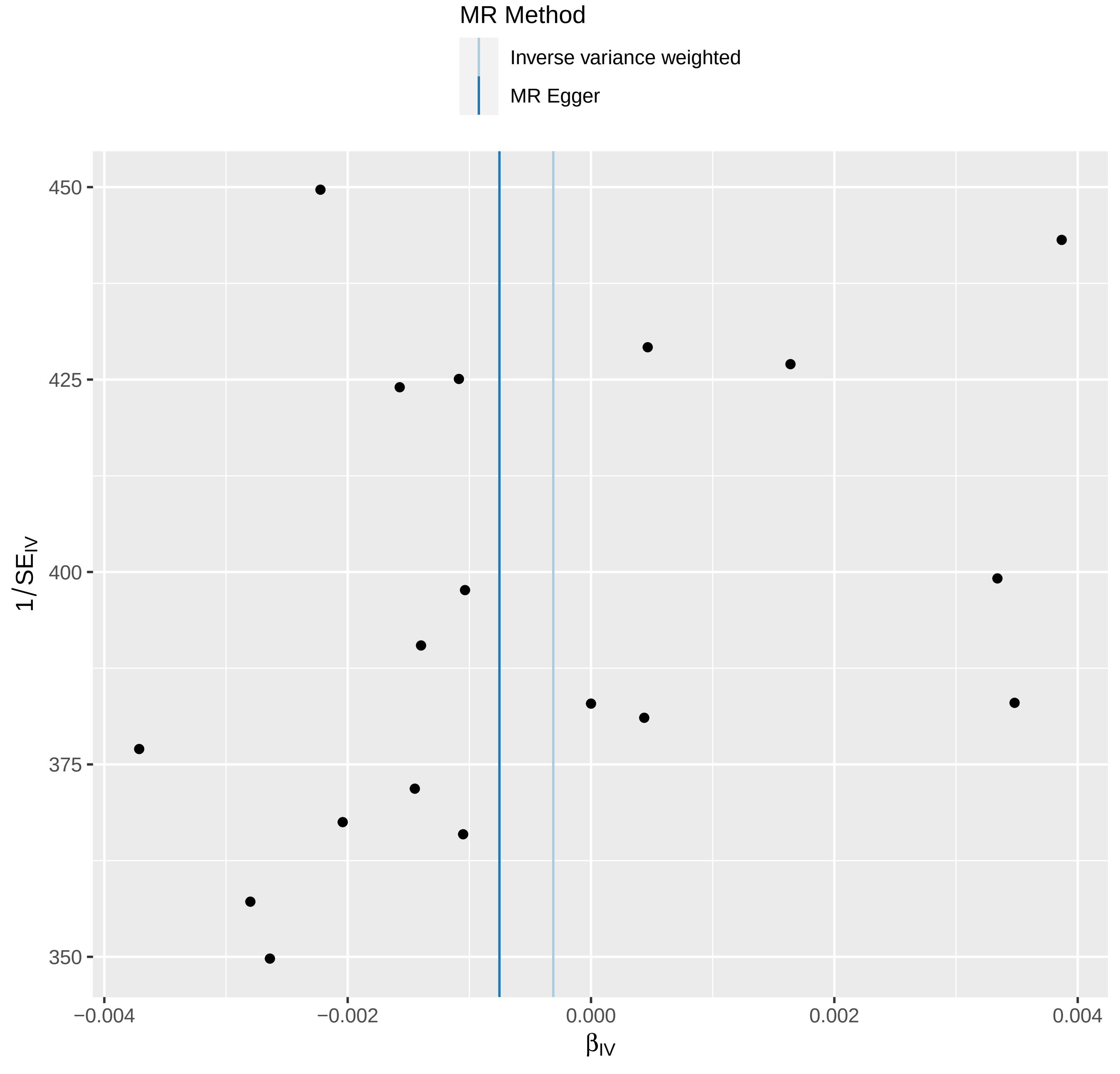

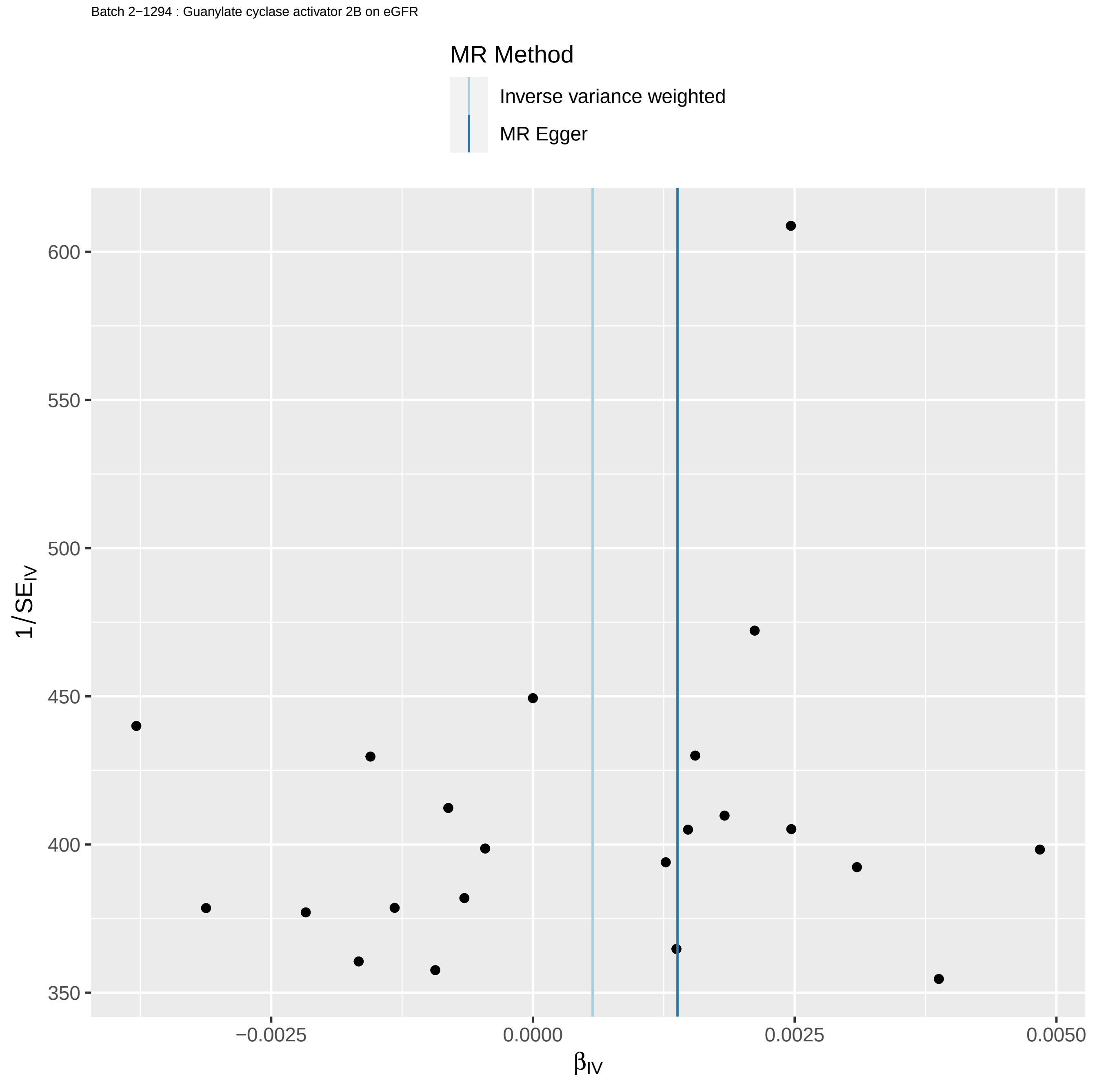

Batch 2−1294 : Guanylate cyclase activator 2B on eGFR
MR Method
Inverse variance weighted
MR Egger
600
550
500
450
400
350
1/SE_IV
−0.0025
0.0000
0.0025
0.0050
β_IV

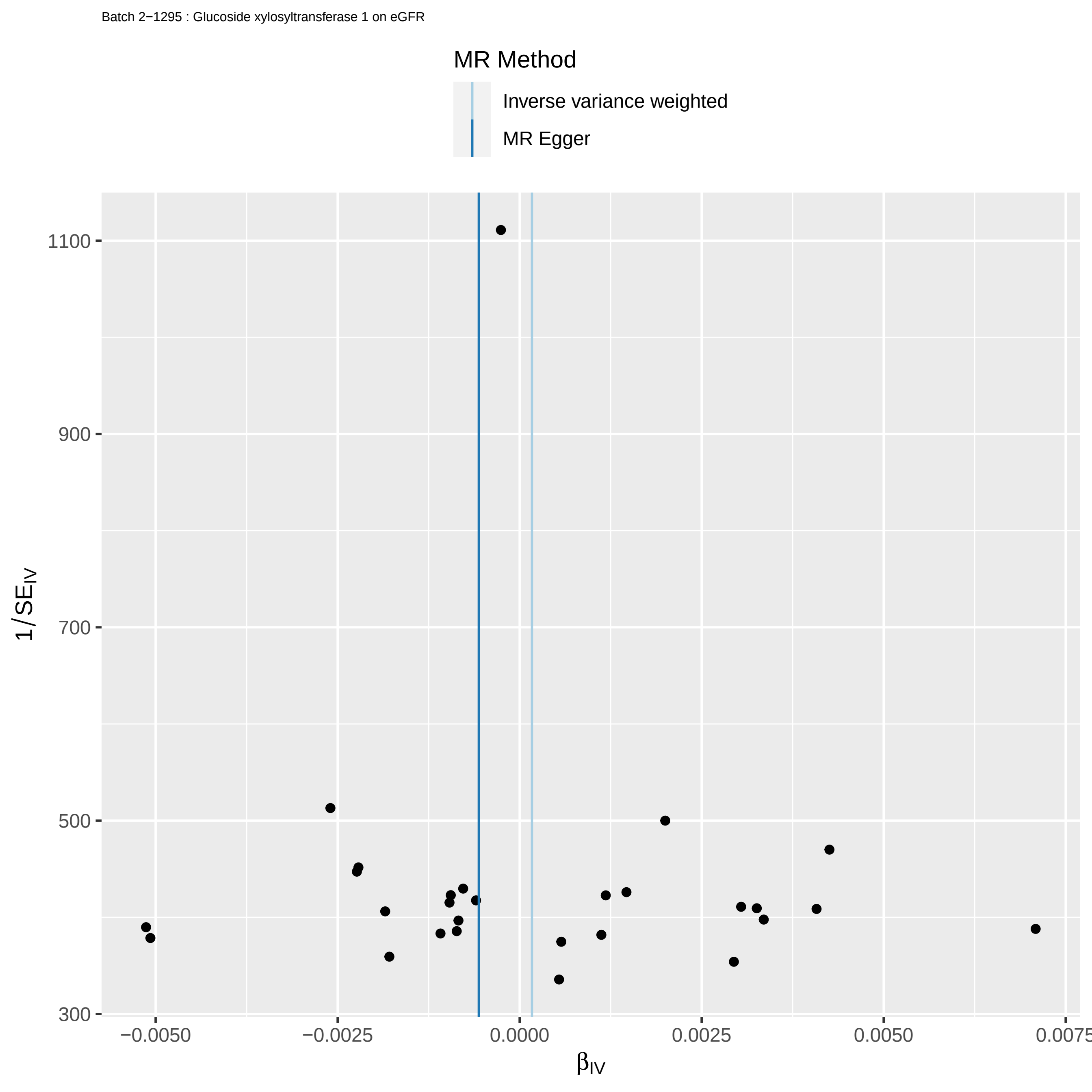
Batch 2−1295 : Glucoside xylosyltransferase 1 on eGFR
MR Method
Inverse variance weighted
MR Egger
1100
900
700
500
300
1/SE_IV
−0.0050
−0.0025
0.0000
0.0025
0.0050
0.0075
β_IV

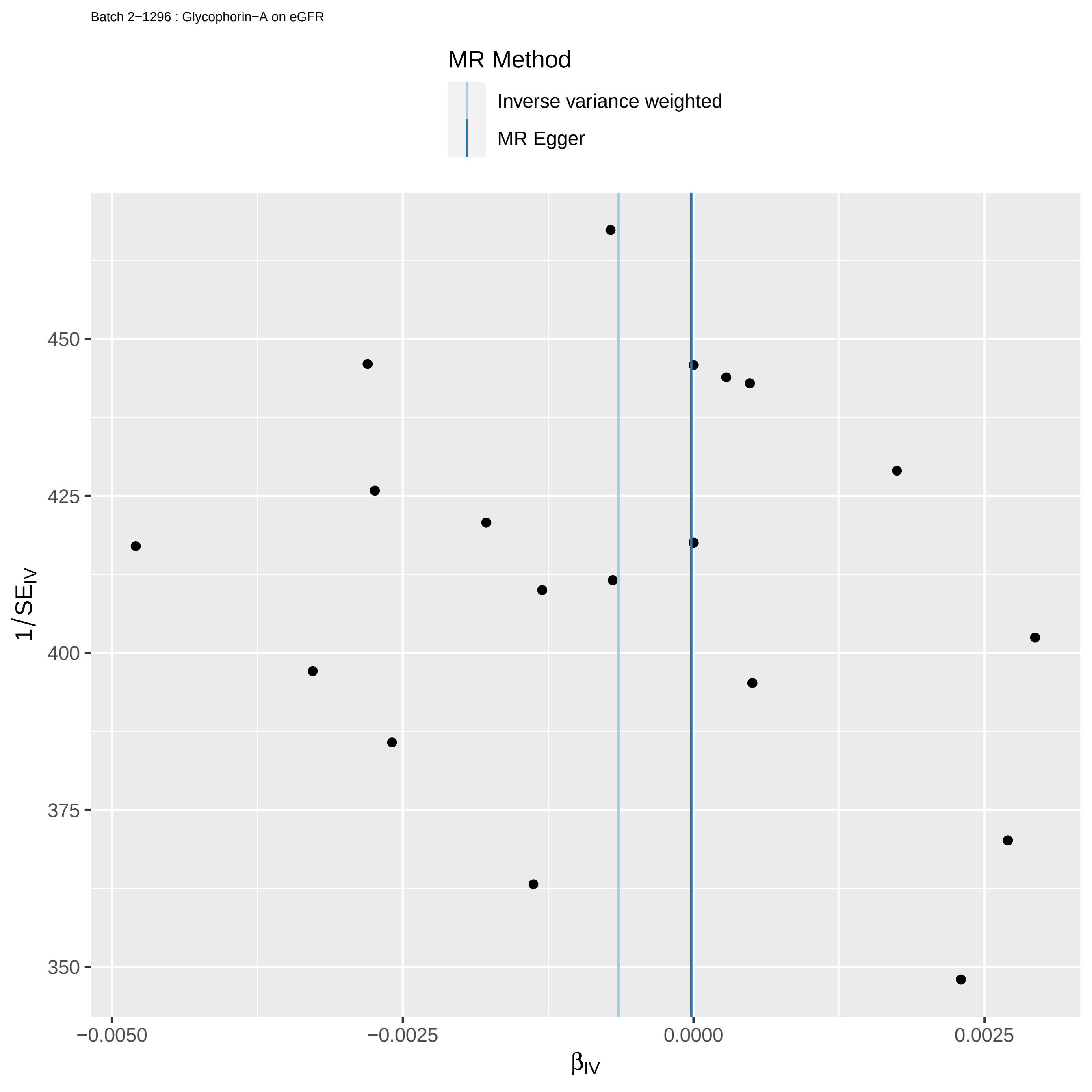

Batch 2−1296 : Glycophorin−A on eGFR
MR Method
Inverse variance weighted
MR Egger
450
425
400
375
350
1/SE_IV
−0.0050
−0.0025
0.0000
0.0025
β_IV

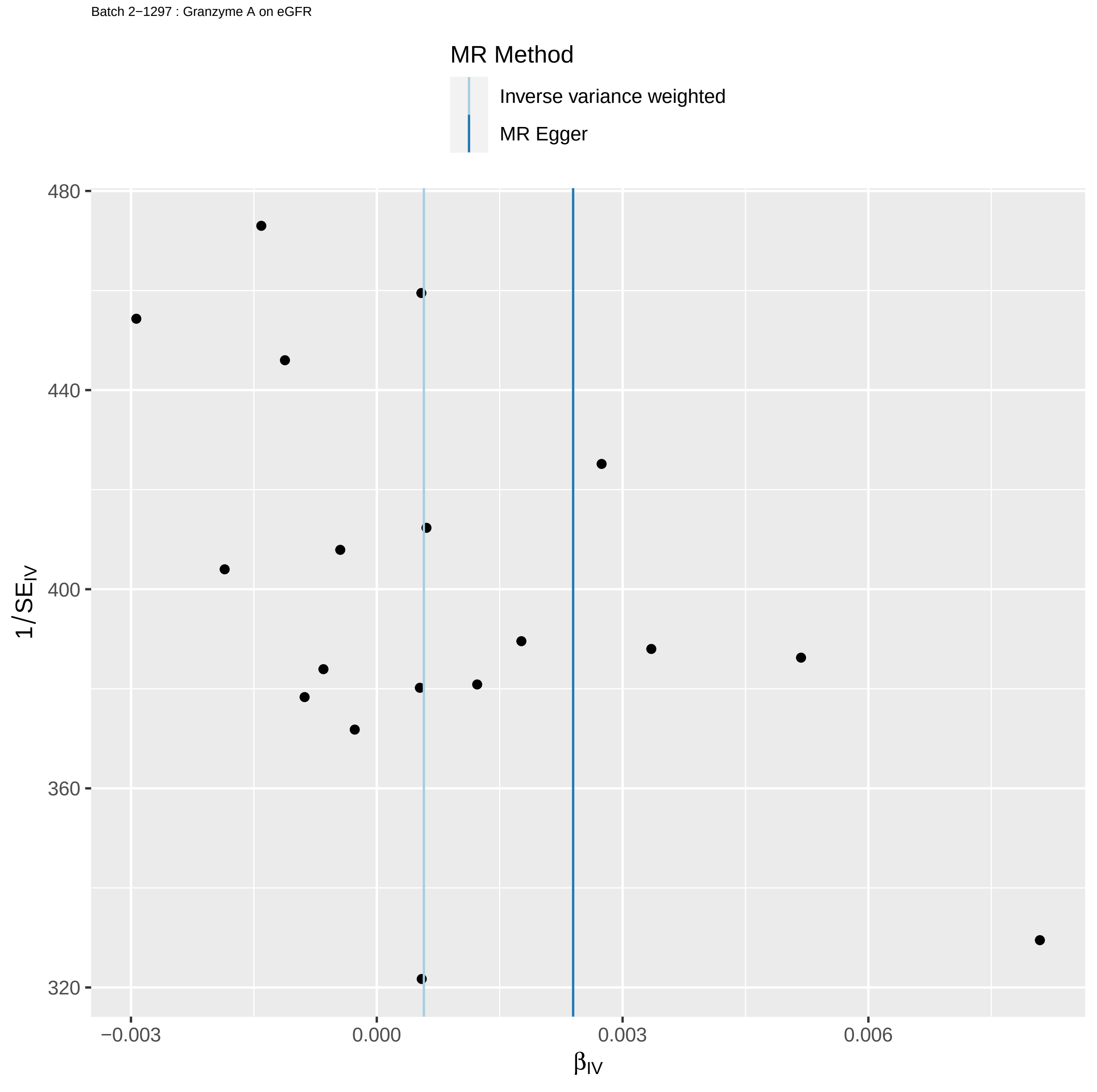

Batch 2−1297 : Granzyme A on eGFR
MR Method
Inverse variance weighted
MR Egger
480
440
400
360
320
1/SE_IV
−0.003
0.000
0.003
0.006
β_IV

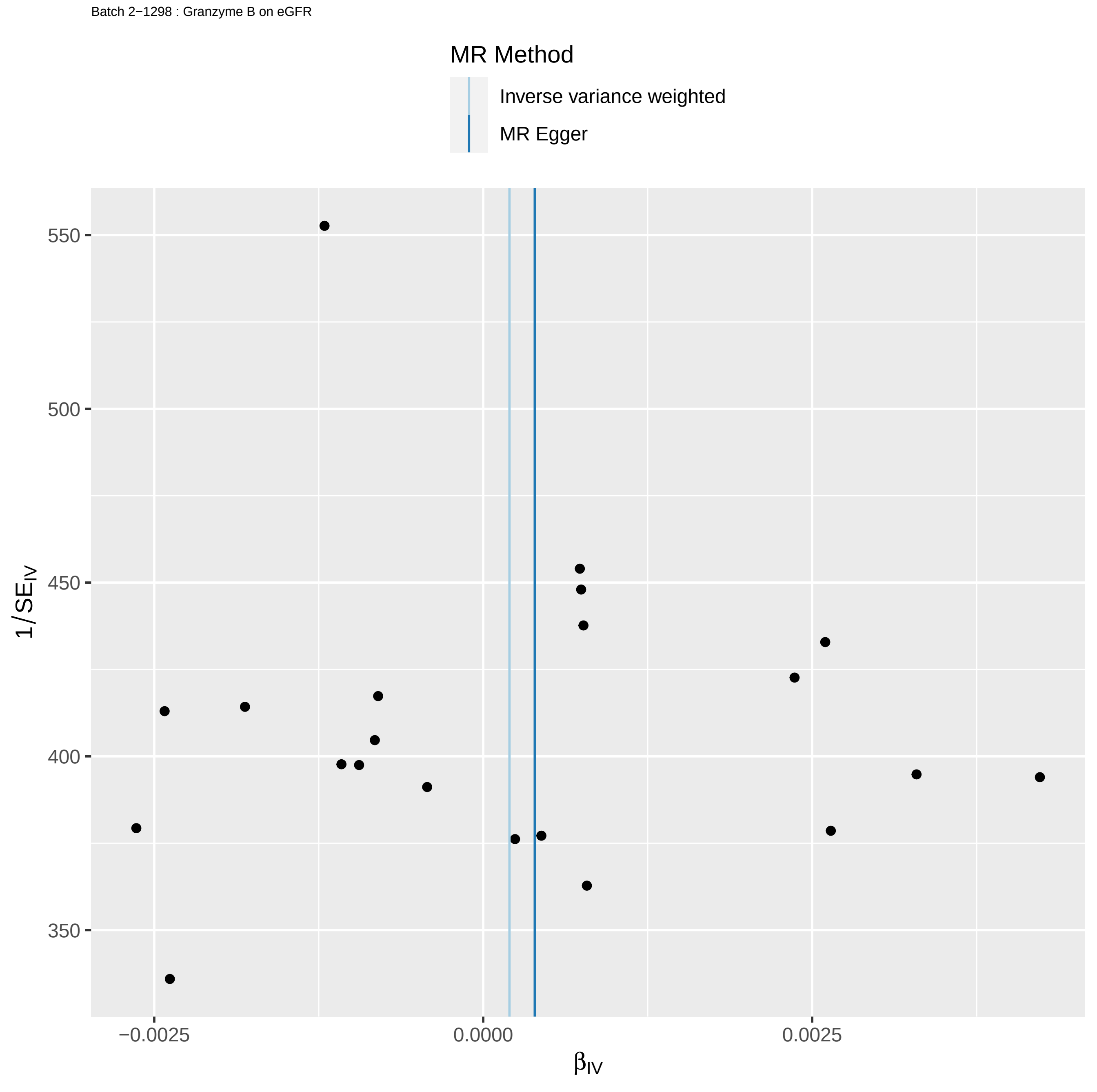

Batch 2−1298 : Granzyme B on eGFR
MR Method
Inverse variance weighted
MR Egger
550
500
450
400
350
1/SE$_{IV}$
−0.0025
0.0000
0.0025
β$_{IV}$

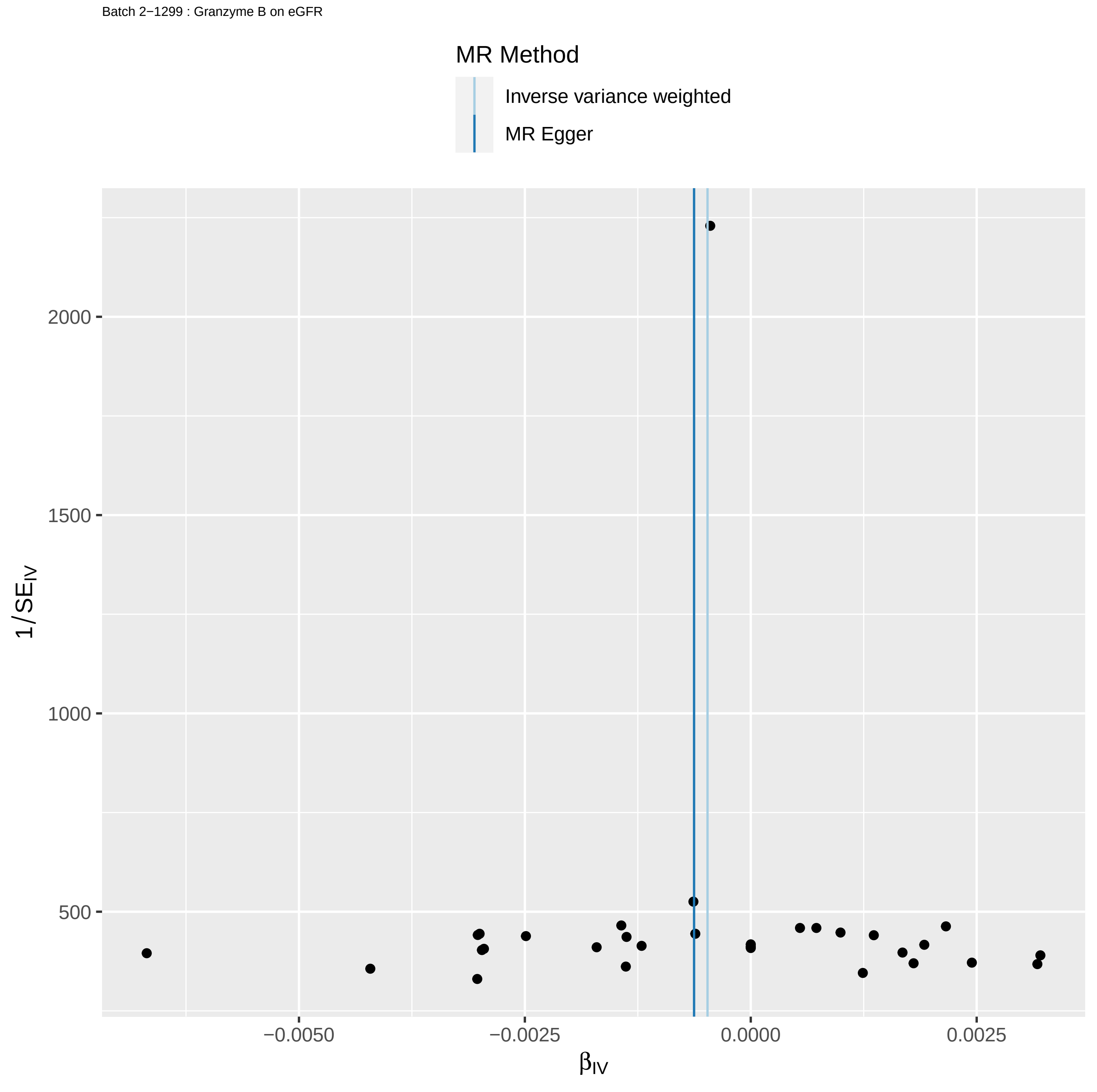

Batch 2−1299 : Granzyme B on eGFR
MR Method
Inverse variance weighted
MR Egger
2000
1500
1000
500
1/SE_IV
−0.0050
−0.0025
0.0000
0.0025
β_IV

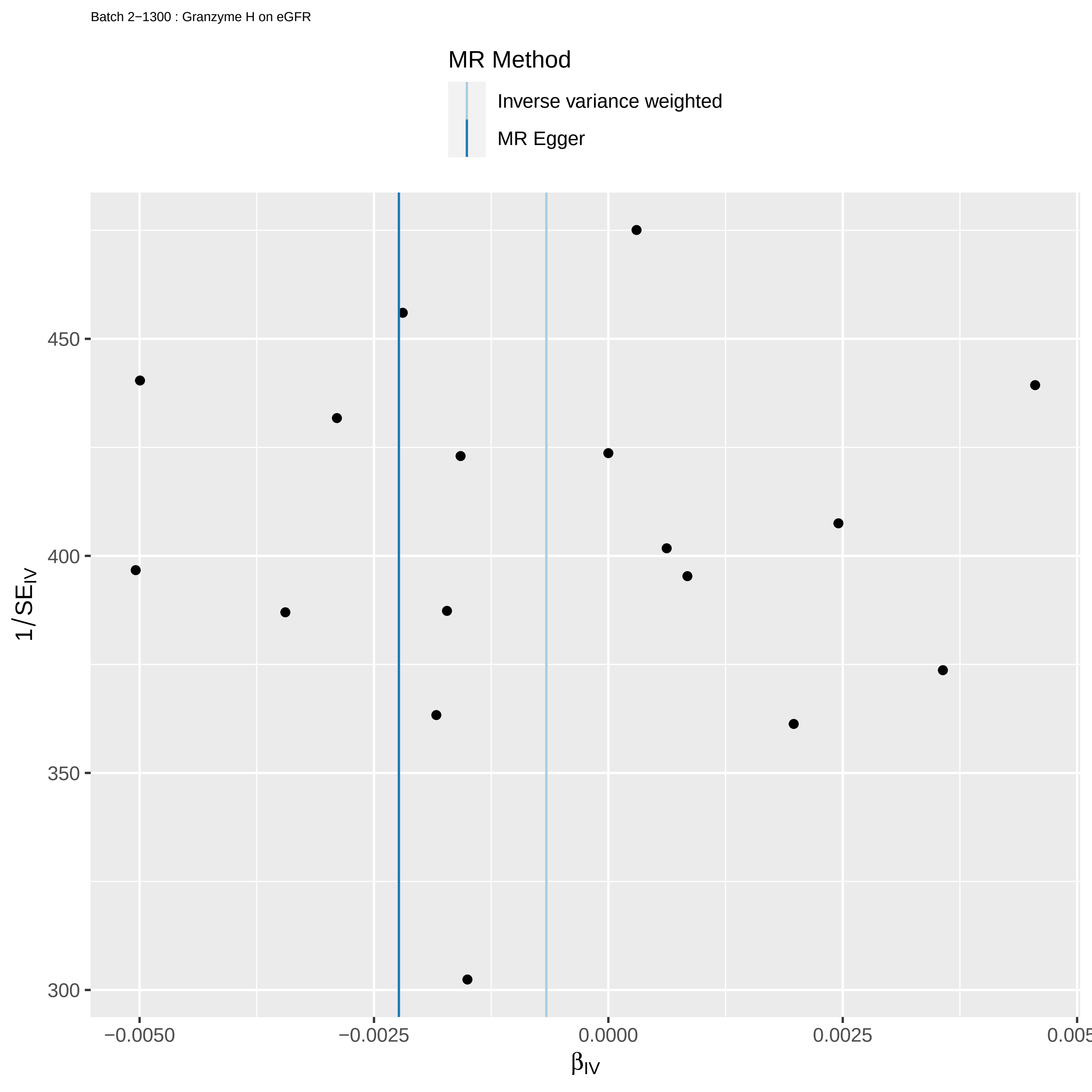

Batch 2−1300 : Granzyme H on eGFR
MR Method
Inverse variance weighted
MR Egger
450
400
350
300
1/SE_IV
−0.0050
−0.0025
0.0000
0.0025
0.005
β_IV

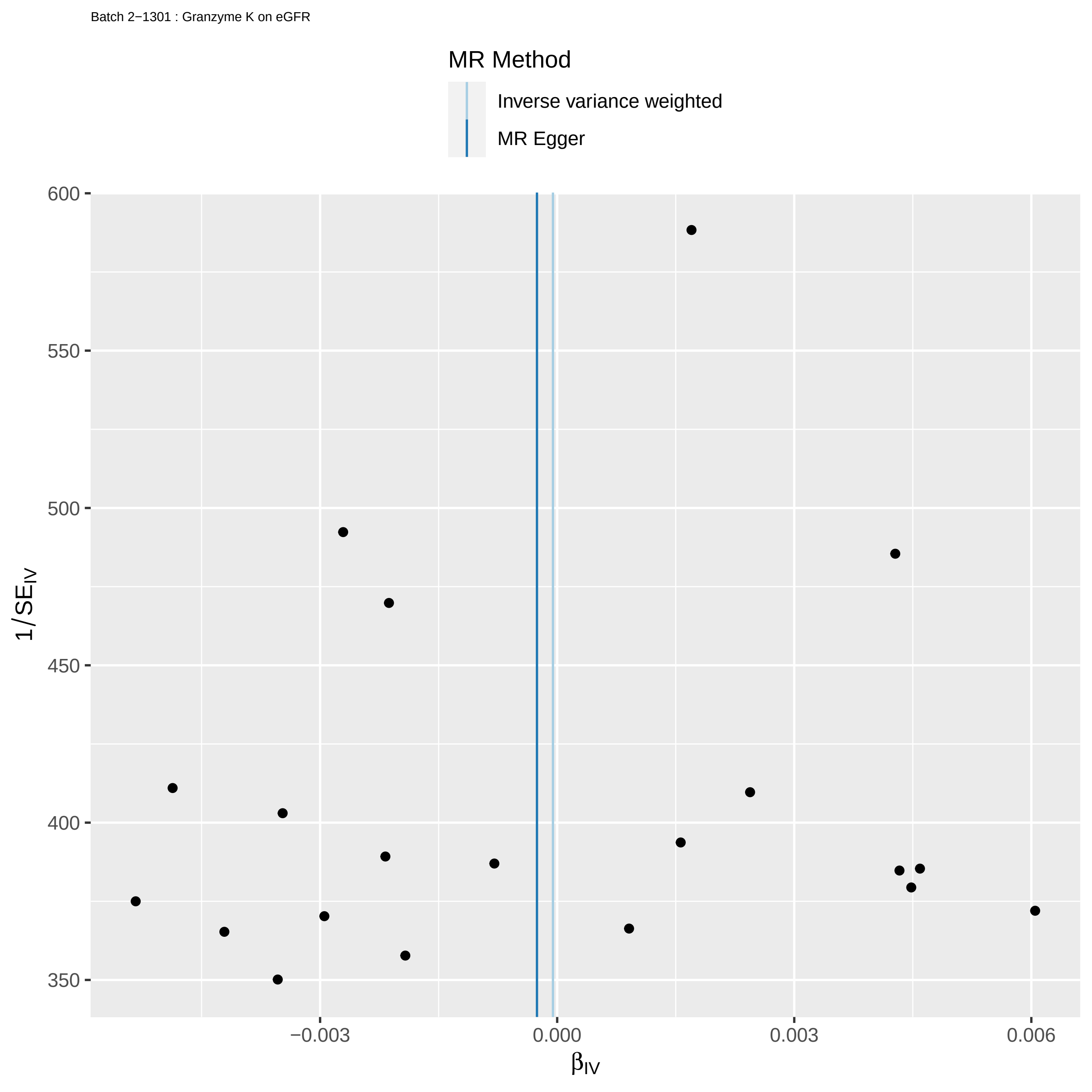

Batch 2−1301 : Granzyme K on eGFR
MR Method
Inverse variance weighted
MR Egger
600
550
500
450
400
350
$1/SE_{IV}$
−0.003
0.000
0.003
0.006
β_{IV}

Batch 2−1302 : Granzyme M on eGFR

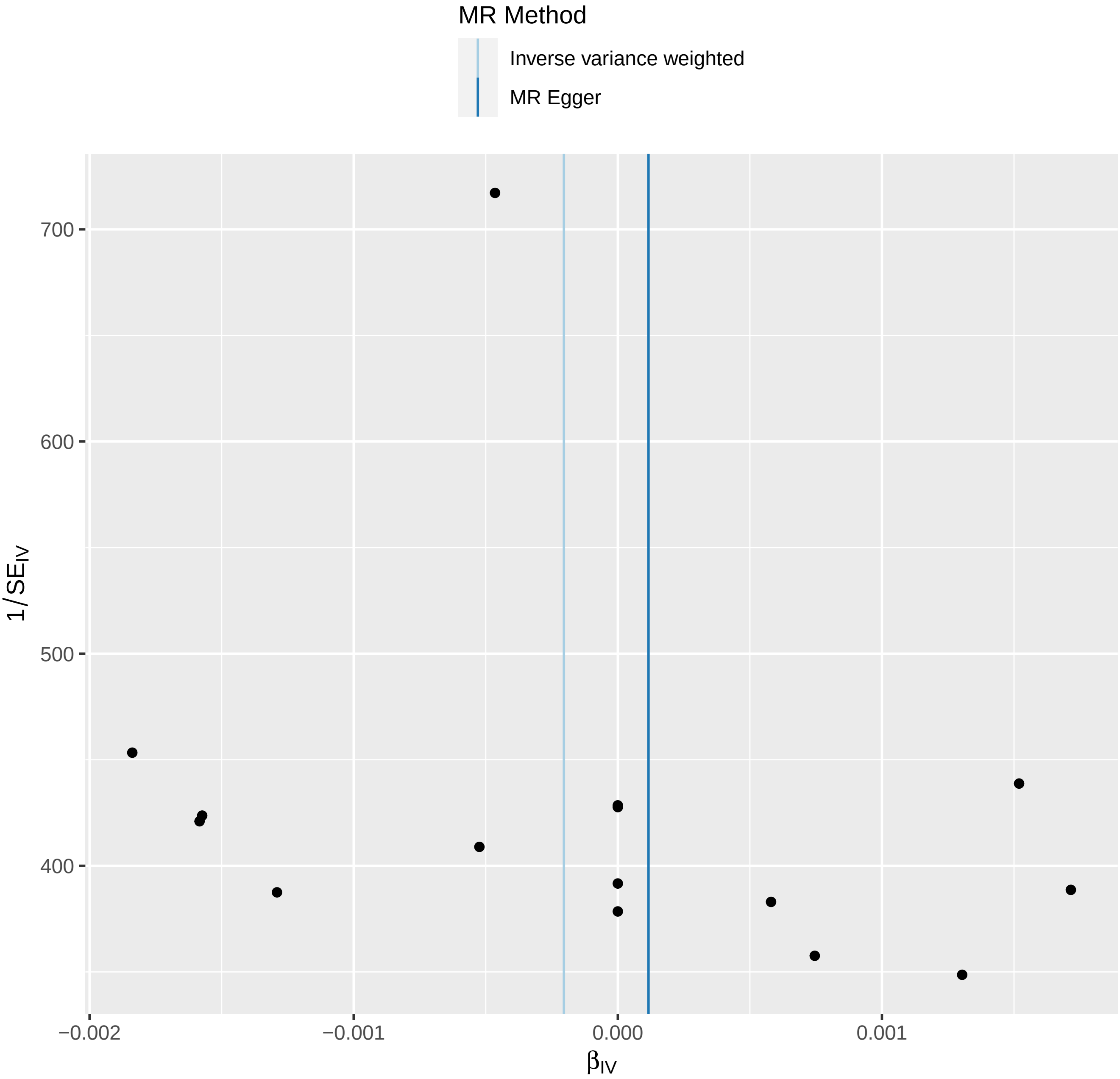

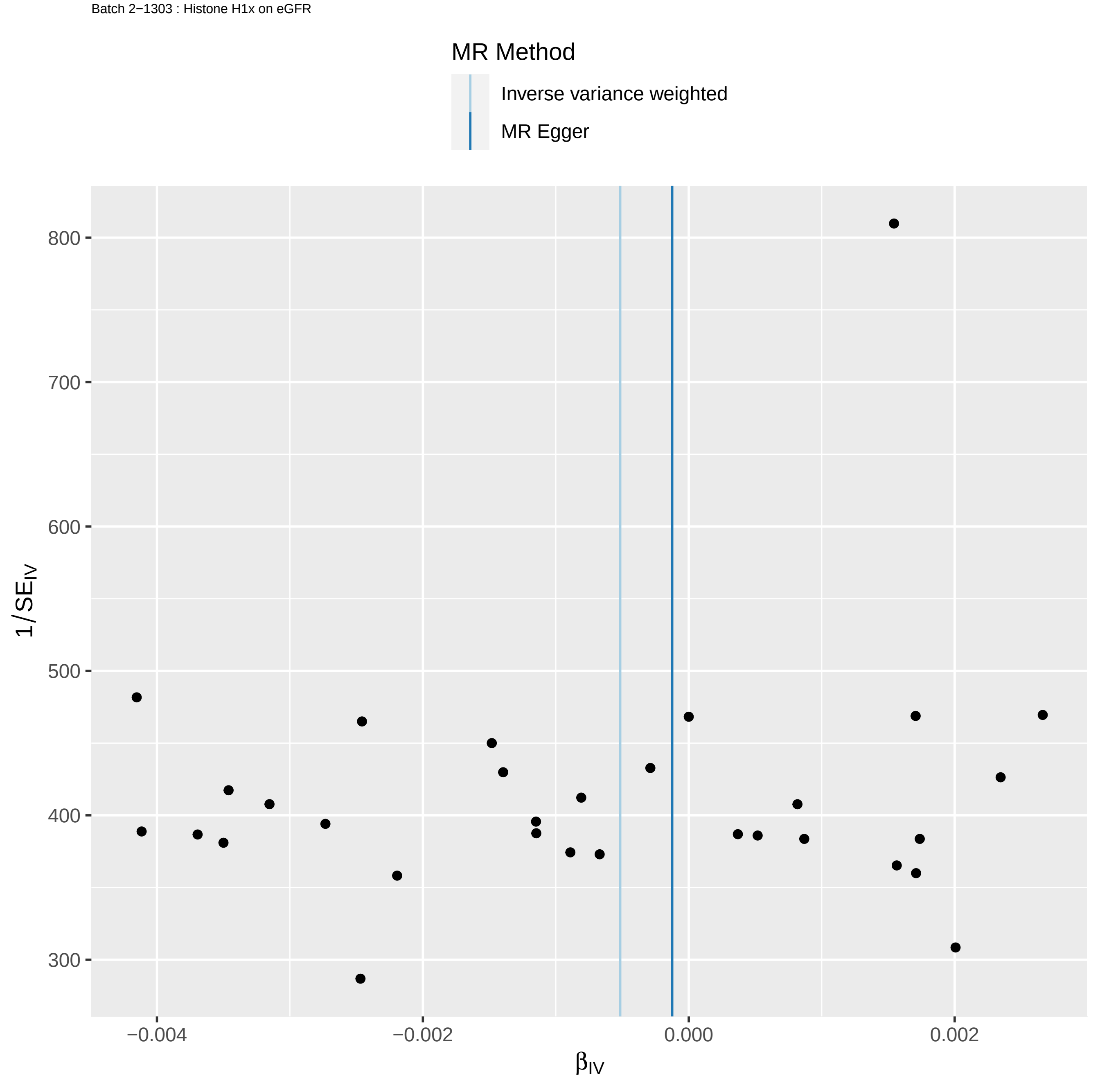

Batch 2−1303 : Histone H1x on eGFR
MR Method
Inverse variance weighted
MR Egger
800
700
600
500
400
300
1/SE_IV
−0.004
−0.002
0.000
0.002
β_IV

Batch 2−1304 : Histone H2A.z on eGFR

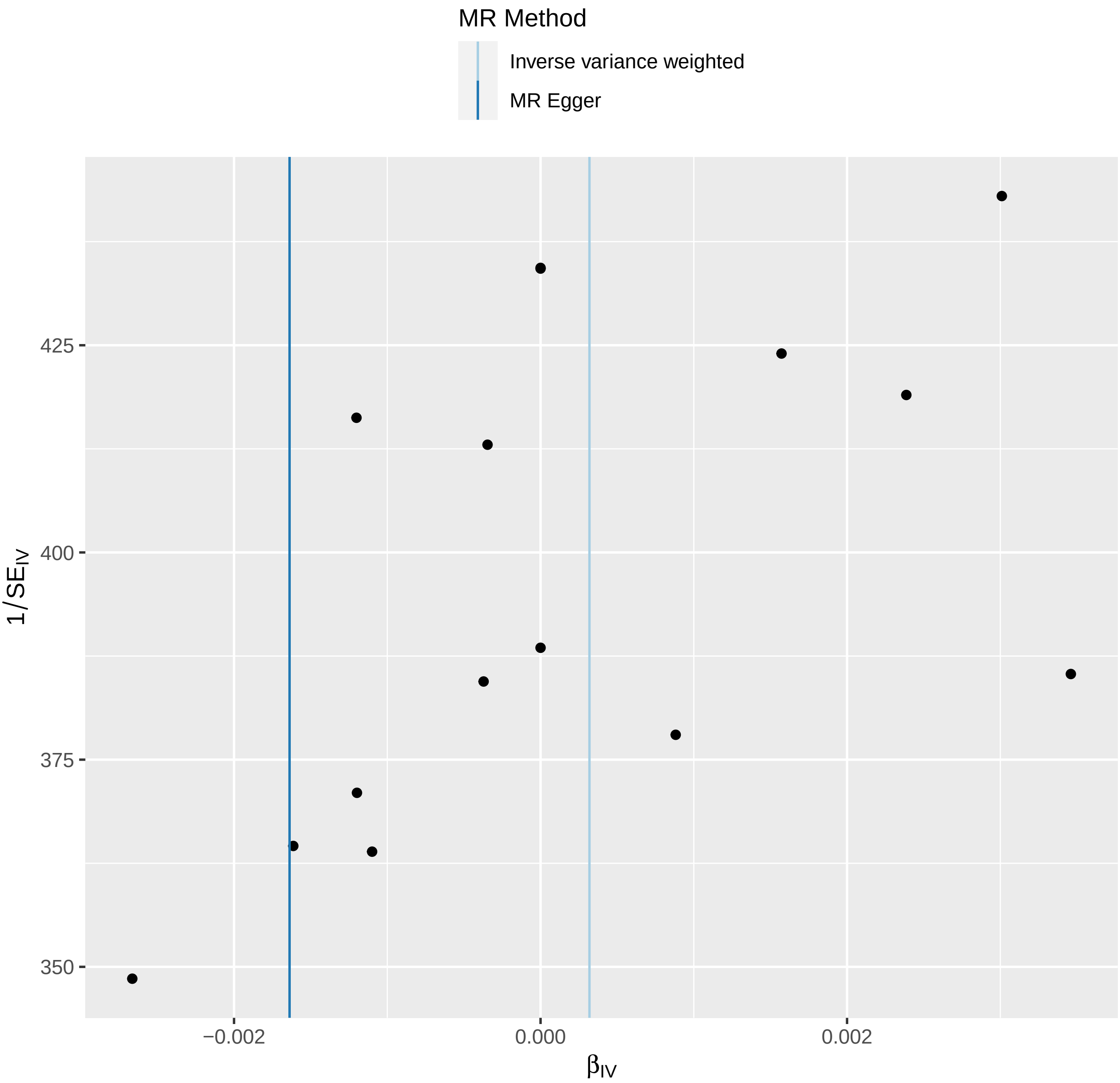

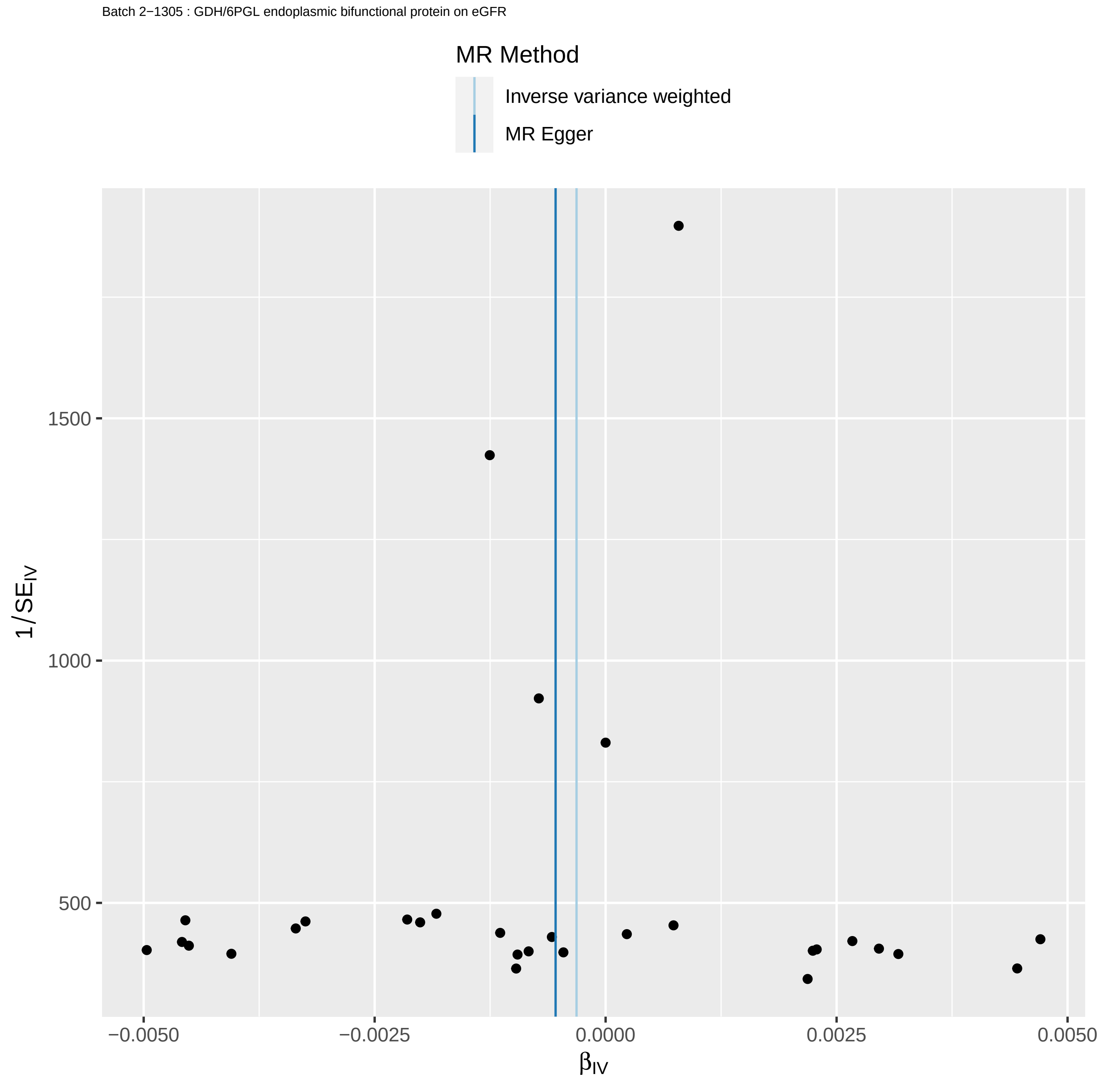
Batch 2−1305 : GDH/6PGL endoplasmic bifunctional protein on eGFR
MR Method
Inverse variance weighted
MR Egger
1500
1000
500
1/SE_IV
−0.0050
−0.0025
0.0000
0.0025
0.0050
β_IV

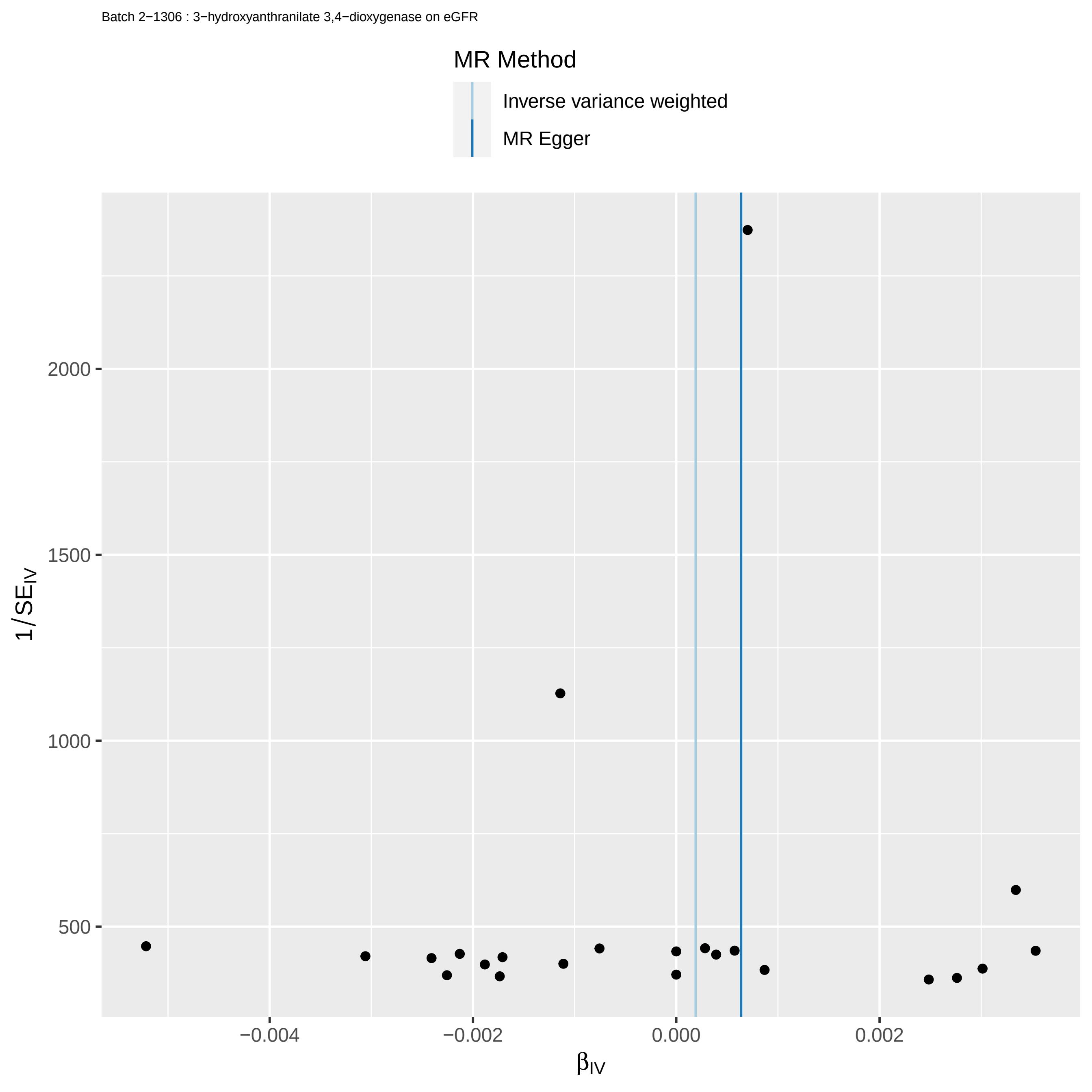
Batch 2−1306 : 3−hydroxyanthranilate 3,4−dioxygenase on eGFR
MR Method
Inverse variance weighted
MR Egger
2000
1500
1000
500
1/SE_IV
−0.004
−0.002
0.000
0.002
β_IV

Batch 2−1307 : Hepcidin on eGFR

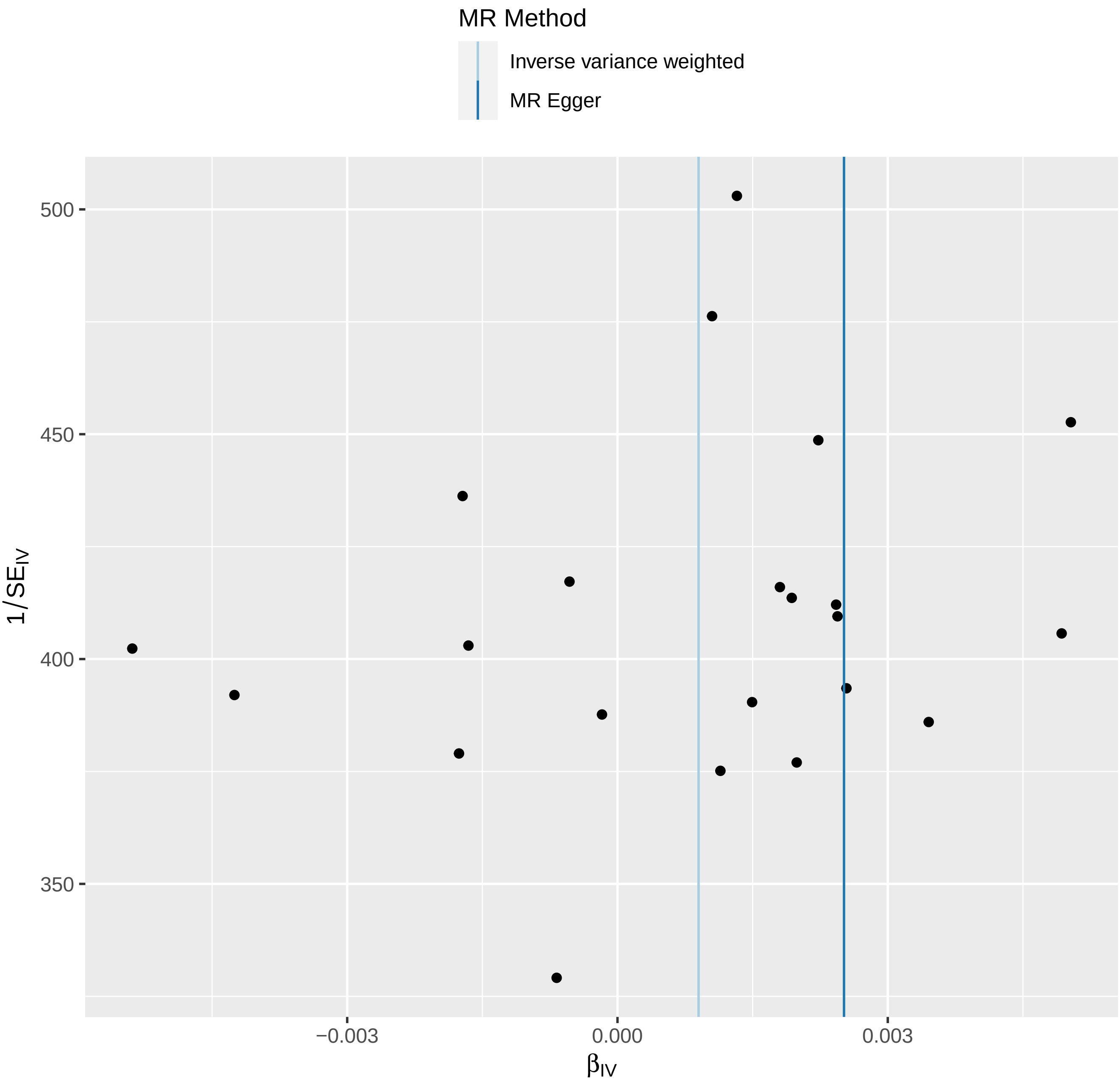

Batch 2−1308 : Hyaluronan and proteoglycan link protein 1 on eGFR

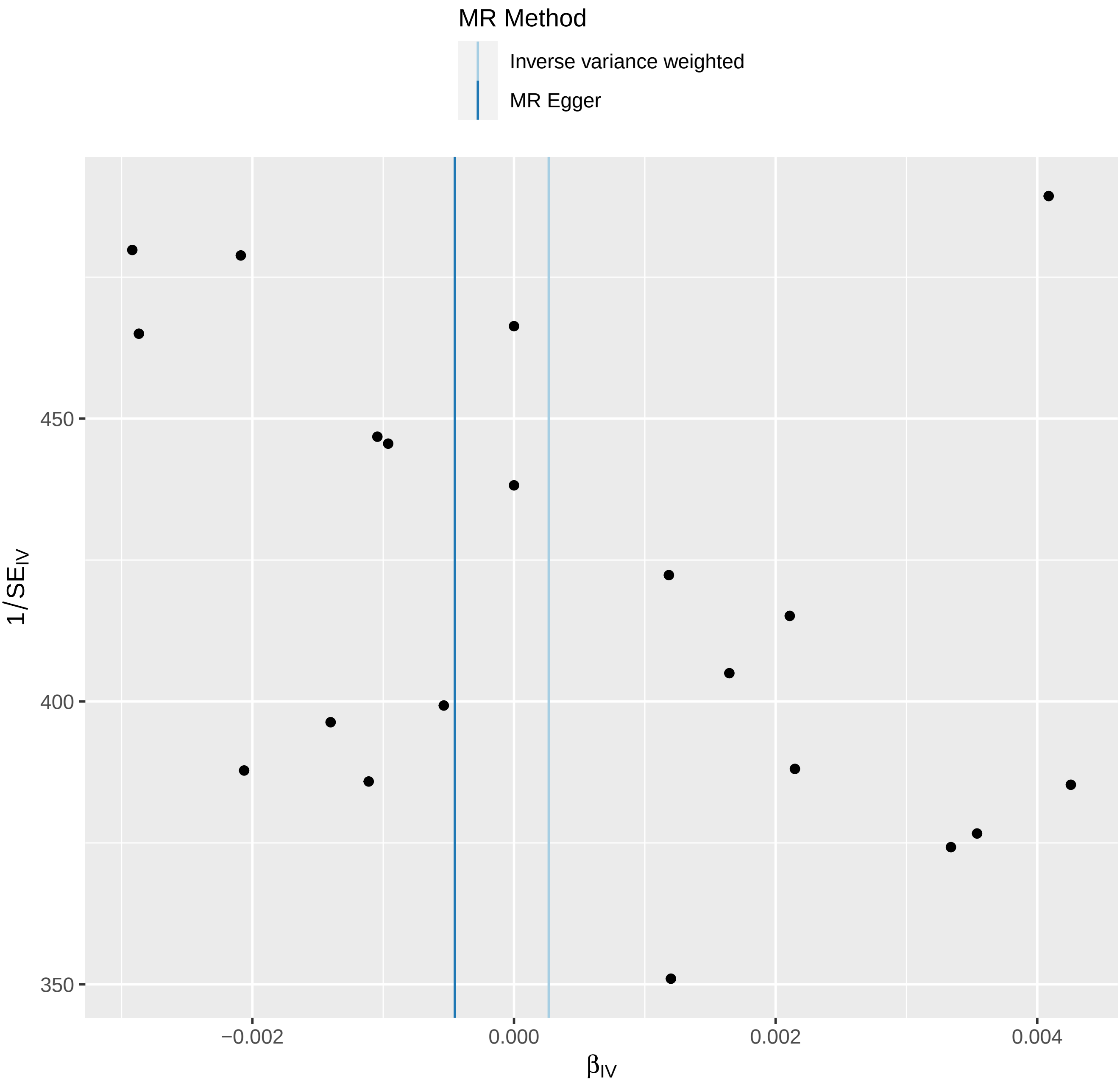

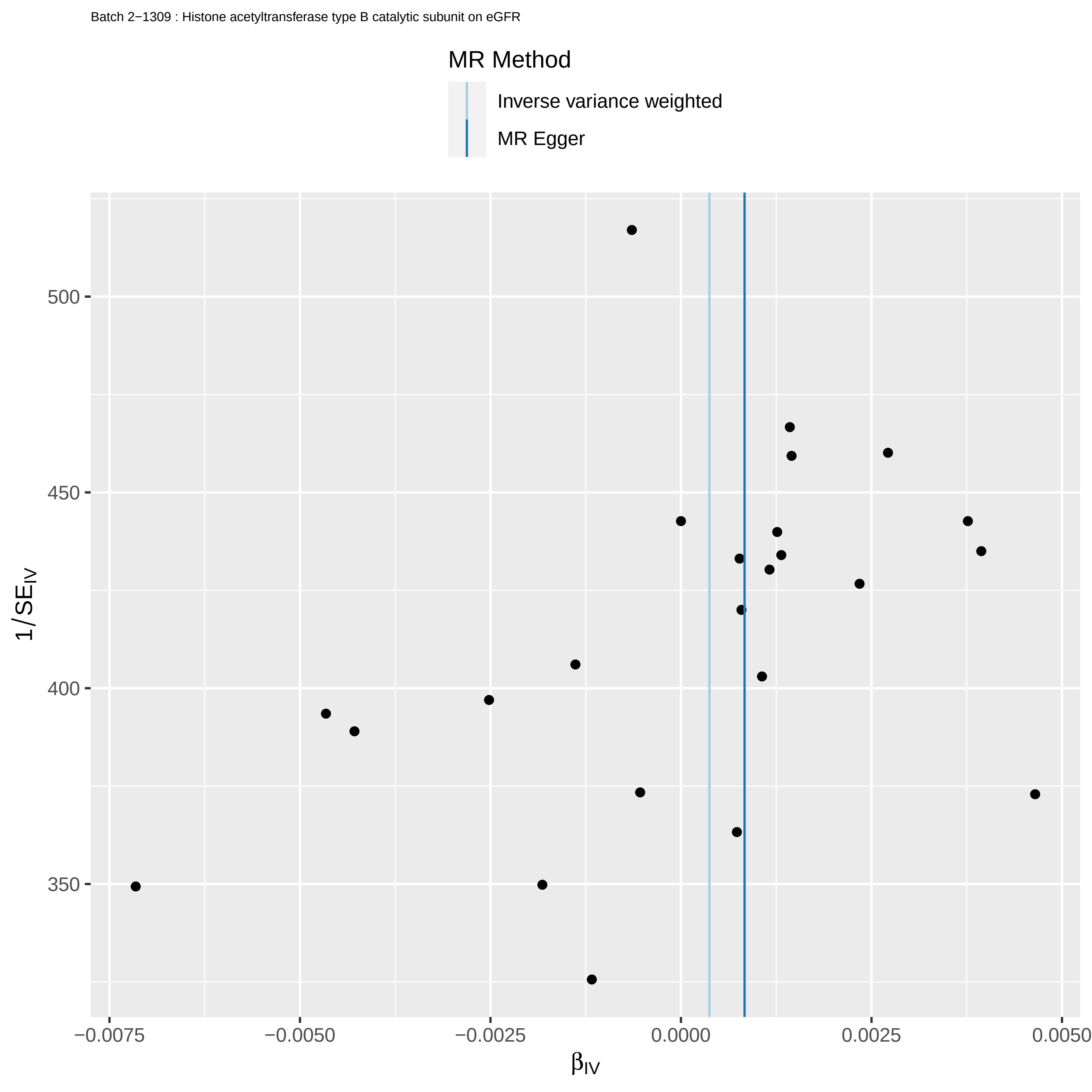
Batch 2−1309 : Histone acetyltransferase type B catalytic subunit on eGFR
MR Method
Inverse variance weighted
MR Egger
500
450
400
350
1/SE_IV
−0.0075
−0.0050
−0.0025
0.0000
0.0025
0.0050
β_IV

Batch 2−1310 : Hepatitis A virus cellular receptor 1 on eGFR

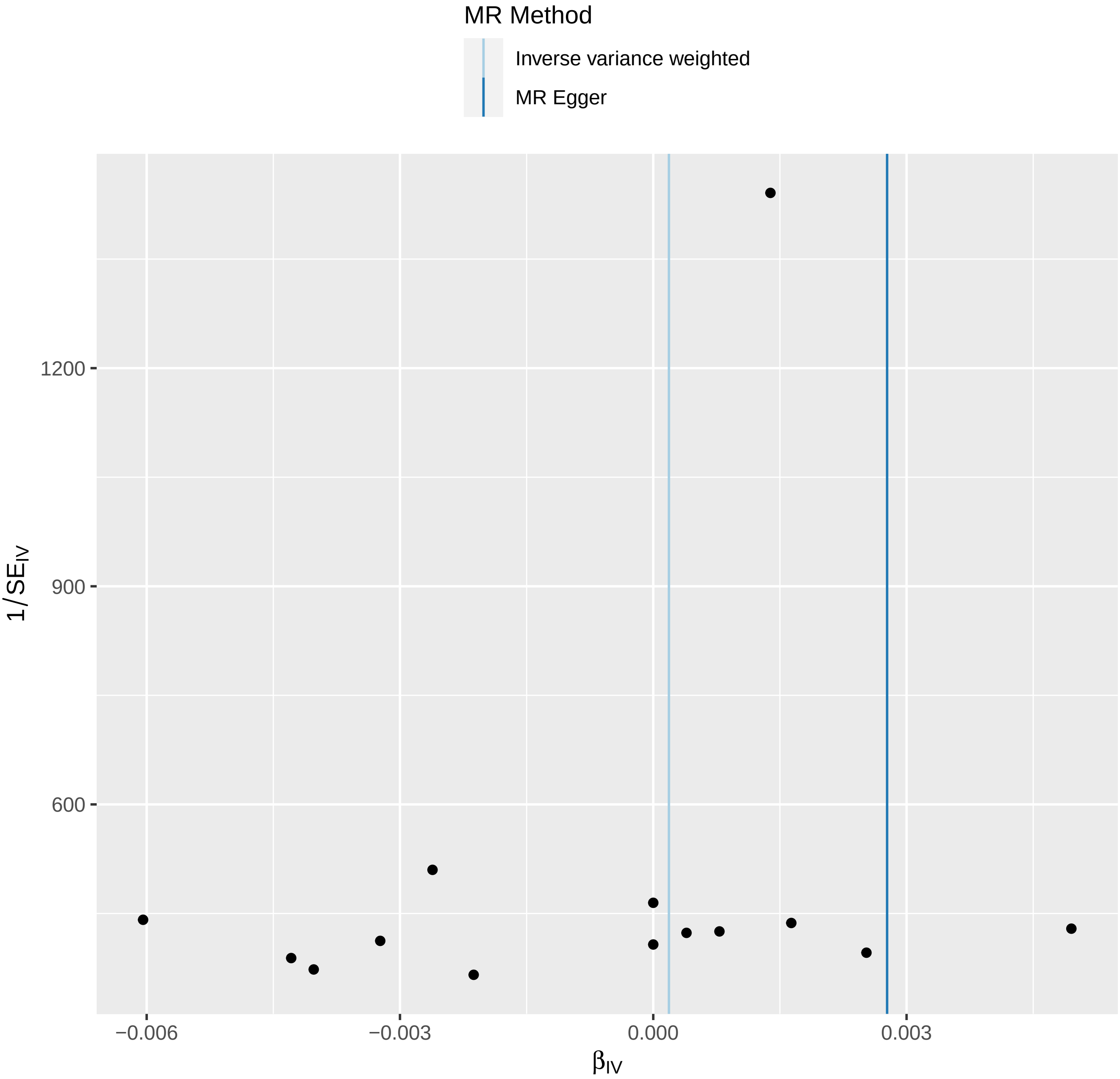

Batch 2−1311 : Hepatitis A virus cellular receptor 2 on eGFR

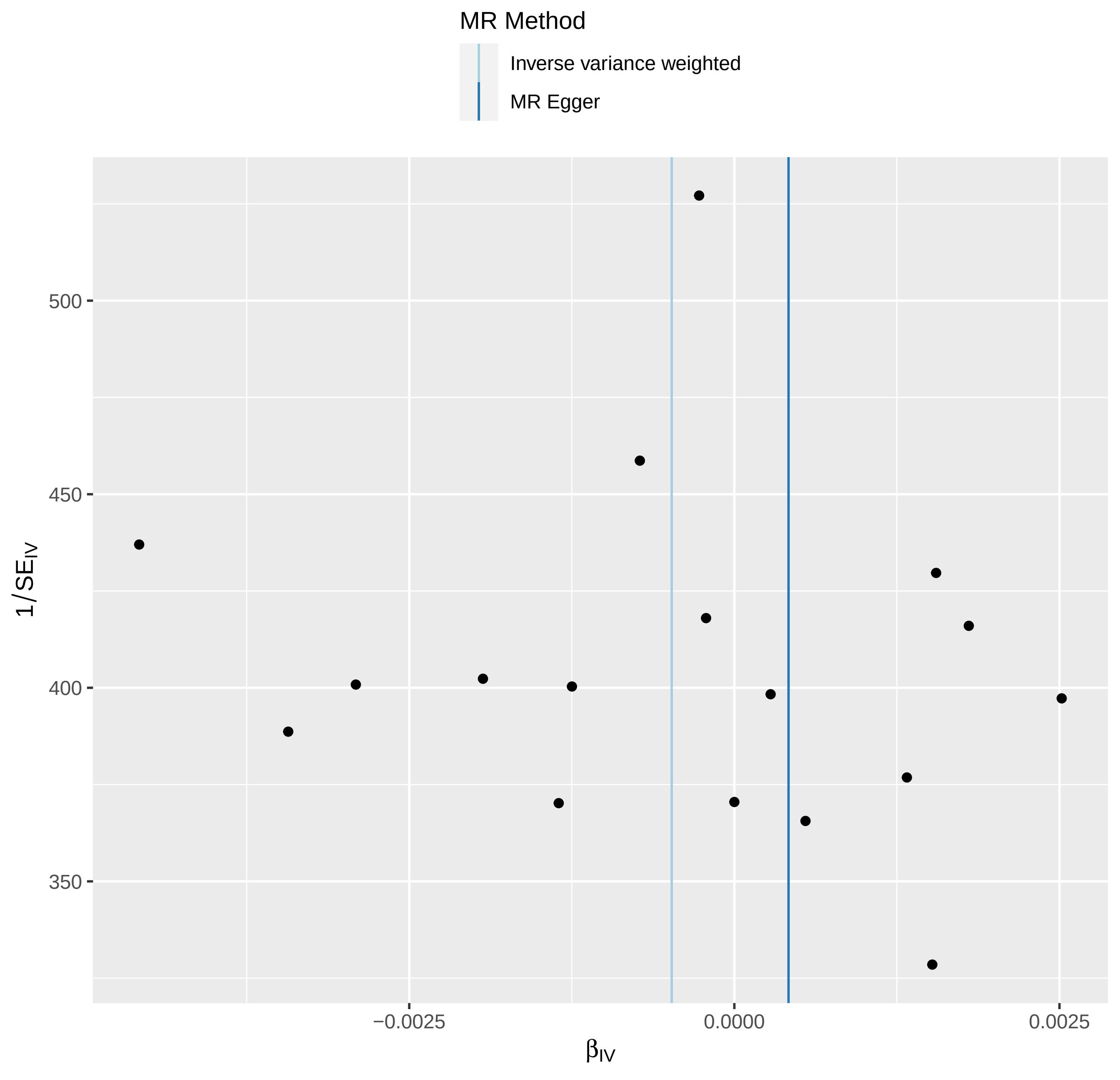

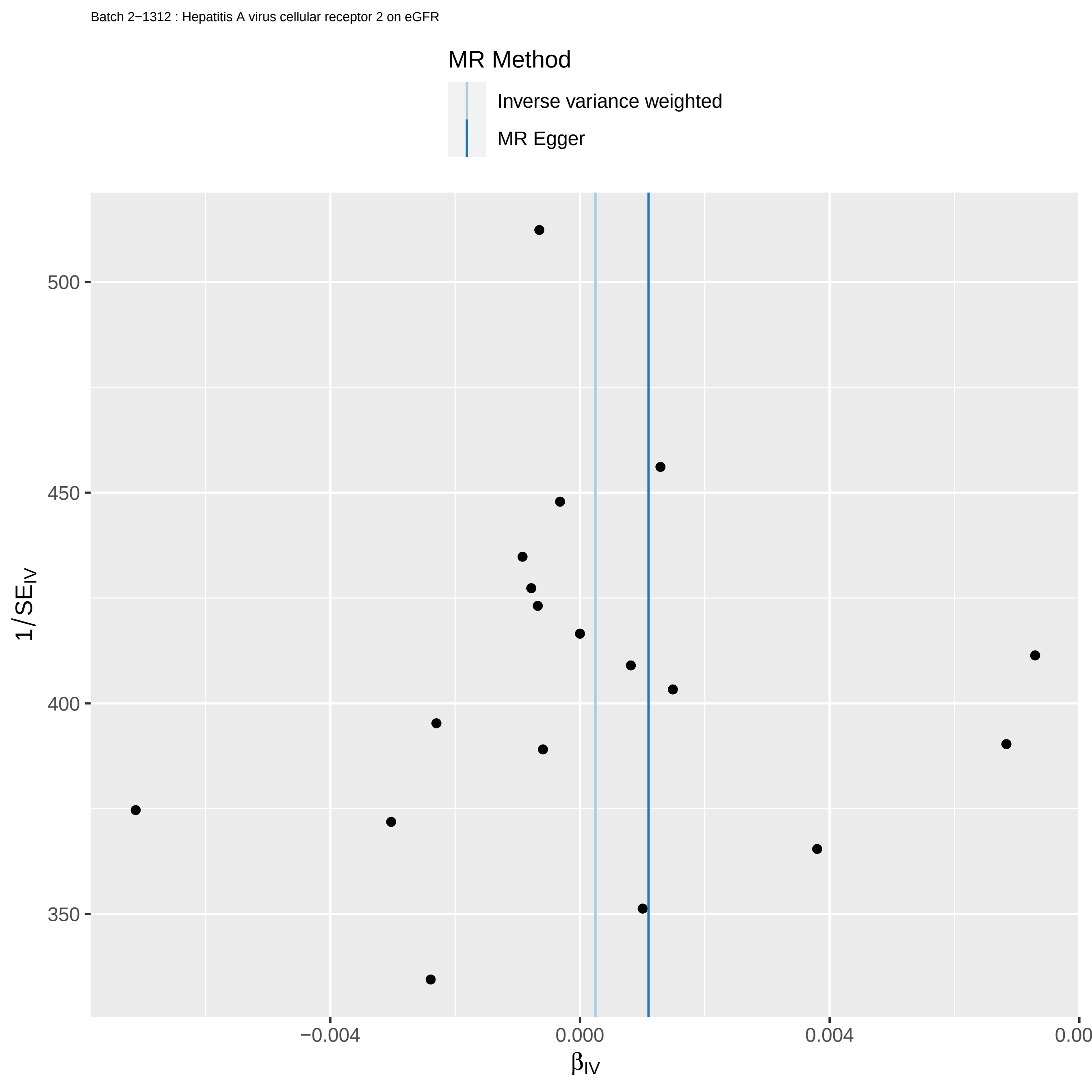

Batch 2−1312 : Hepatitis A virus cellular receptor 2 on eGFR
MR Method
Inverse variance weighted
MR Egger
500
450
400
350
1/SE_IV
−0.004
0.000
0.004
0.00
β_IV

Batch 2−1313 : Heparin−binding EGF−like growth factor on eGFR

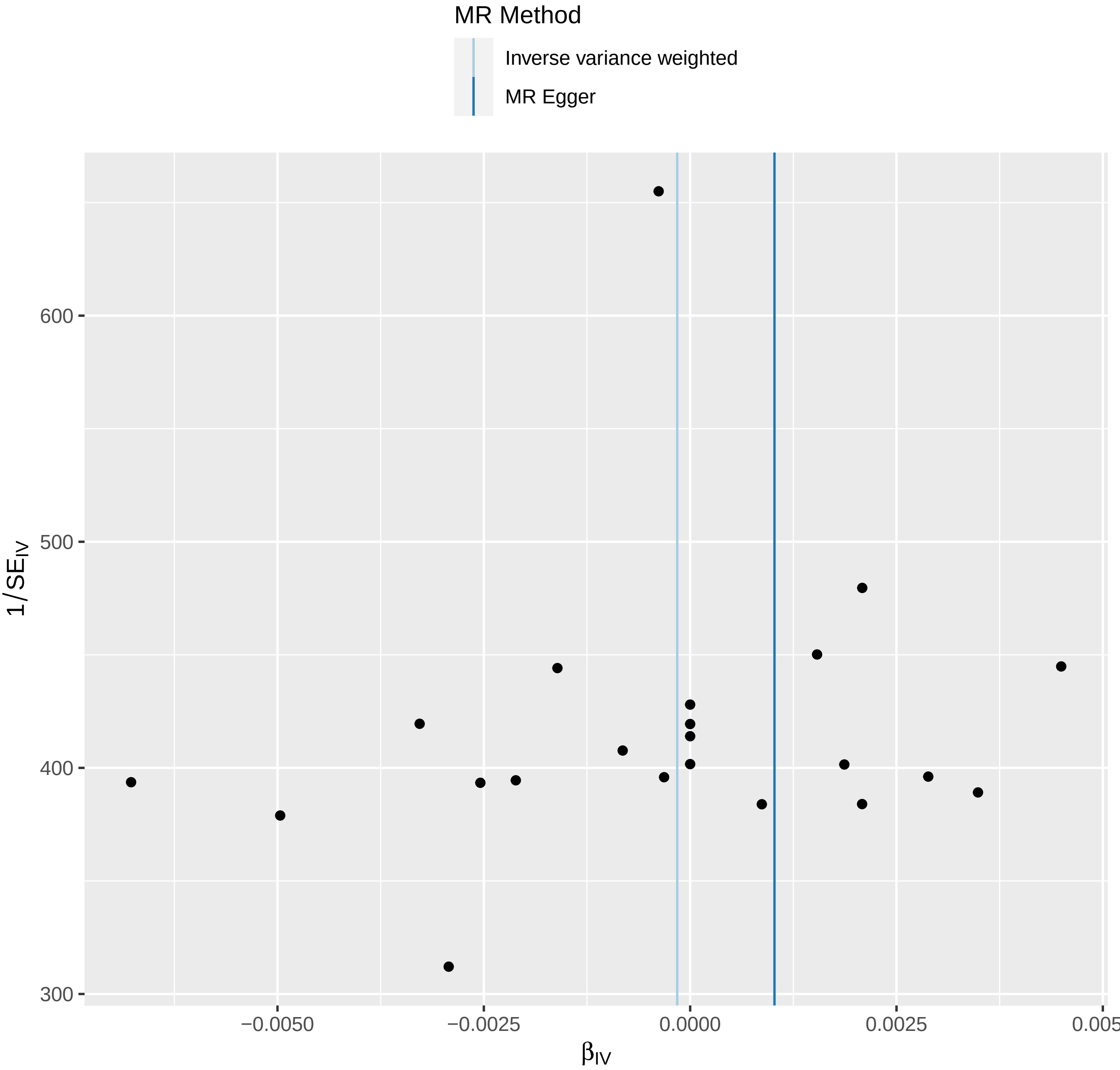

Batch 2−1314 : Heparin−binding EGF−like growth factor on eGFR

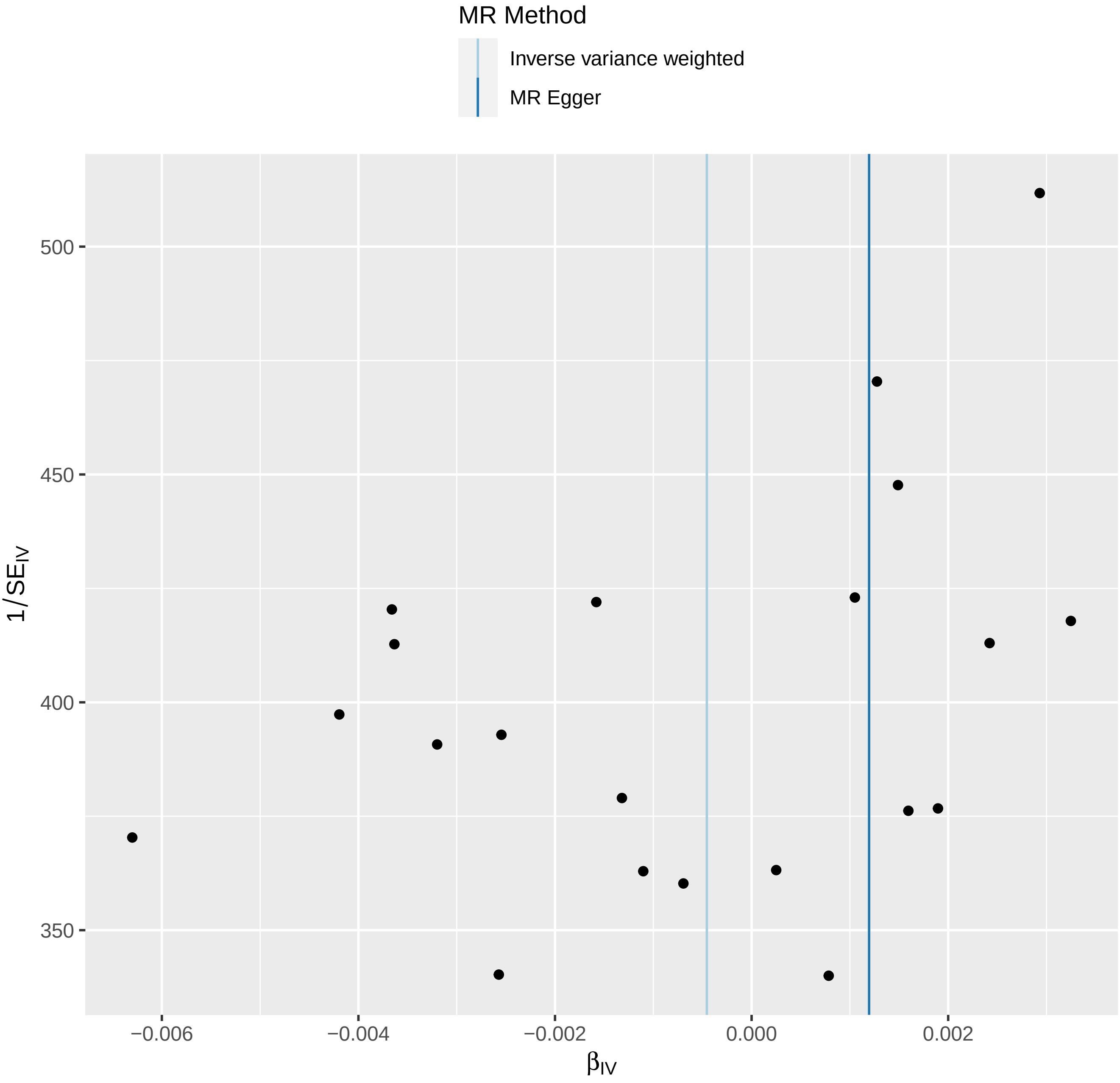

Batch 2−1315 : Hemoglobin subunit gamma−1 on eGFR

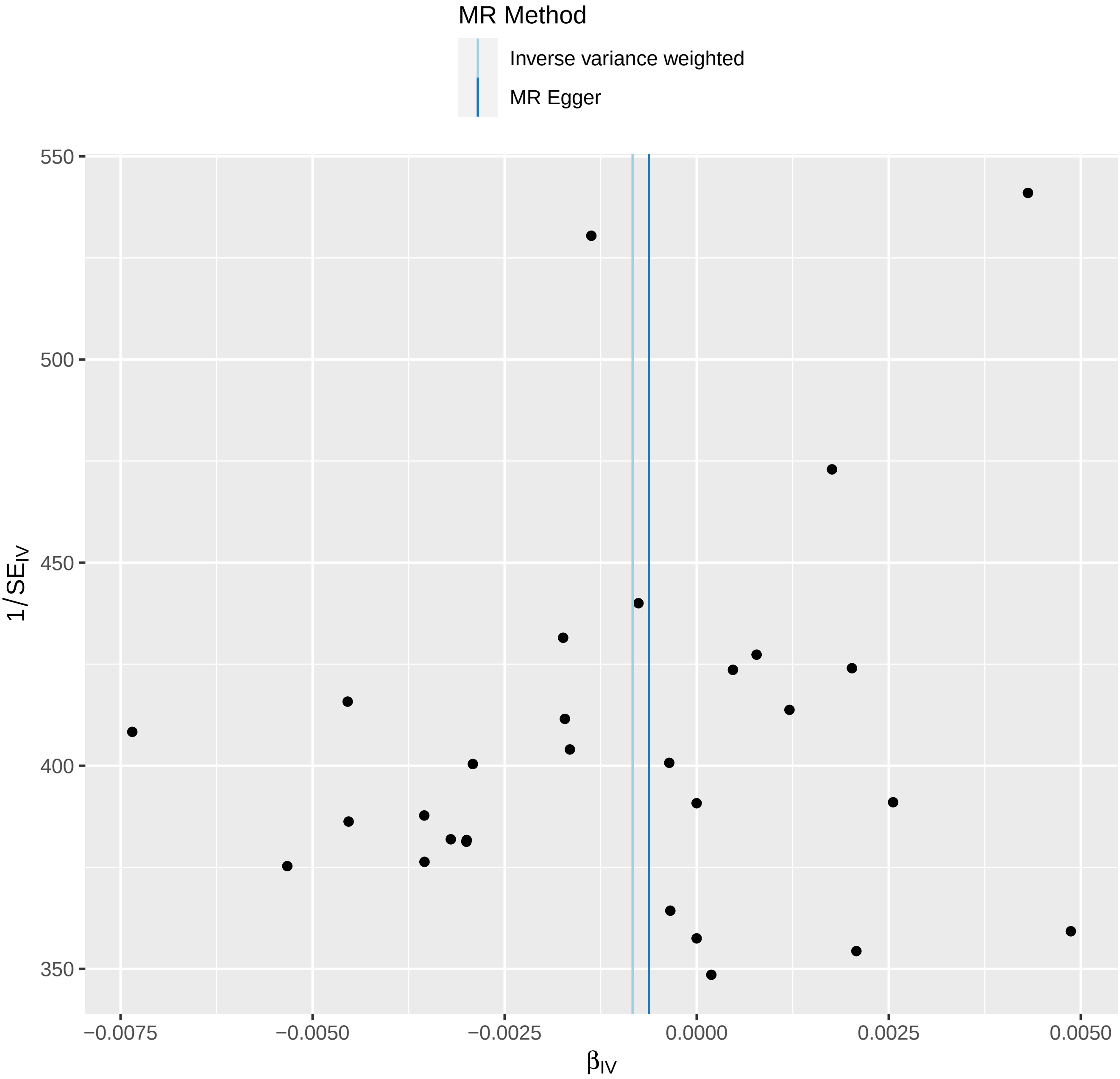

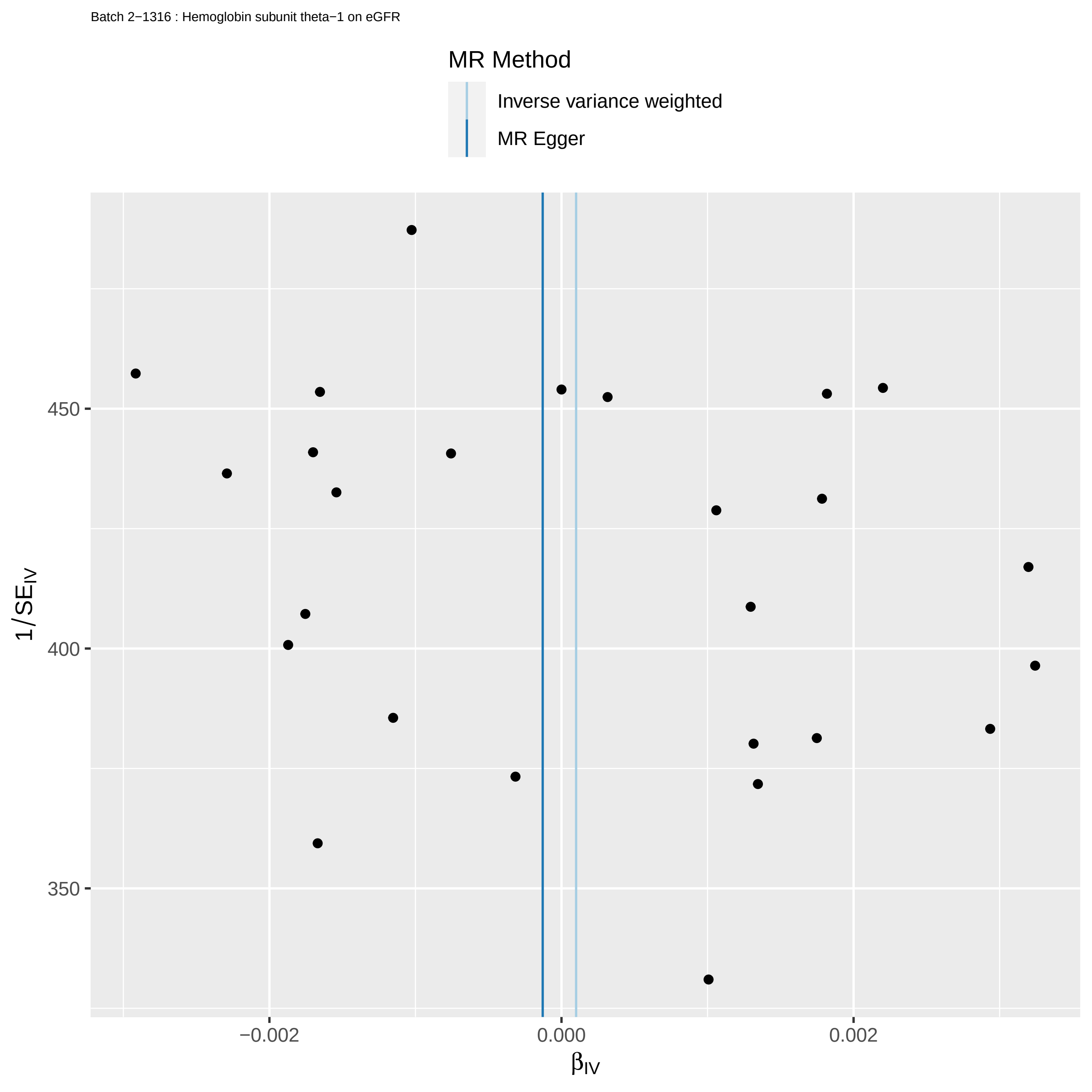

Batch 2−1316 : Hemoglobin subunit theta−1 on eGFR
MR Method
Inverse variance weighted
MR Egger
450
400
350
1/SE_IV
−0.002
0.000
0.002
β_IV

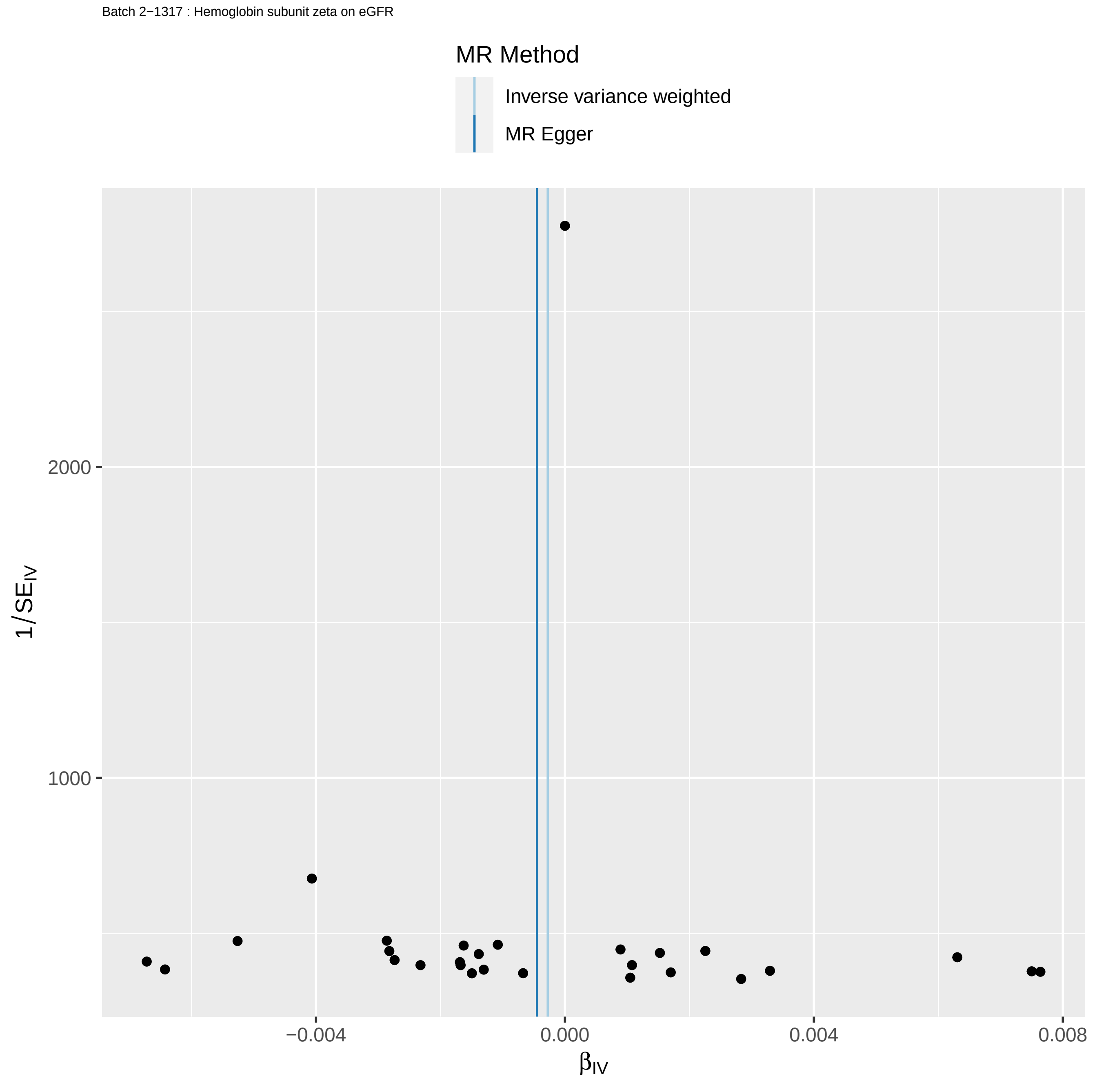

Batch 2−1317 : Hemoglobin subunit zeta on eGFR
MR Method
Inverse variance weighted
MR Egger
2000
1000
1/SE_IV
−0.004
0.000
0.004
0.008
β_IV

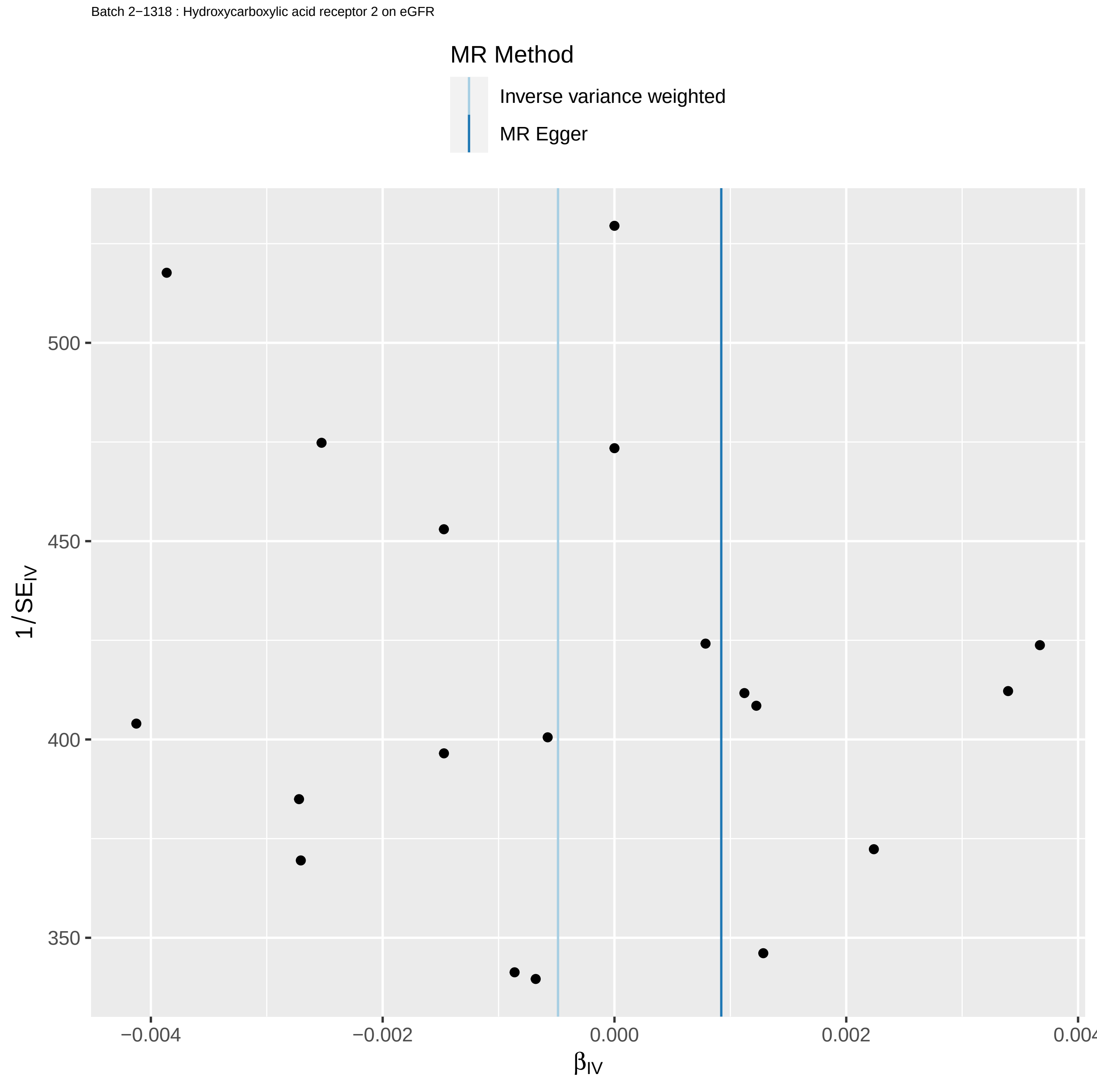
Batch 2−1318 : Hydroxycarboxylic acid receptor 2 on eGFR
MR Method
Inverse variance weighted
MR Egger
500
450
400
350
1/SE_IV
−0.004
−0.002
0.000
0.002
0.004
β_IV

Batch 2−1319 : Tyrosine−protein kinase HCK on eGFR

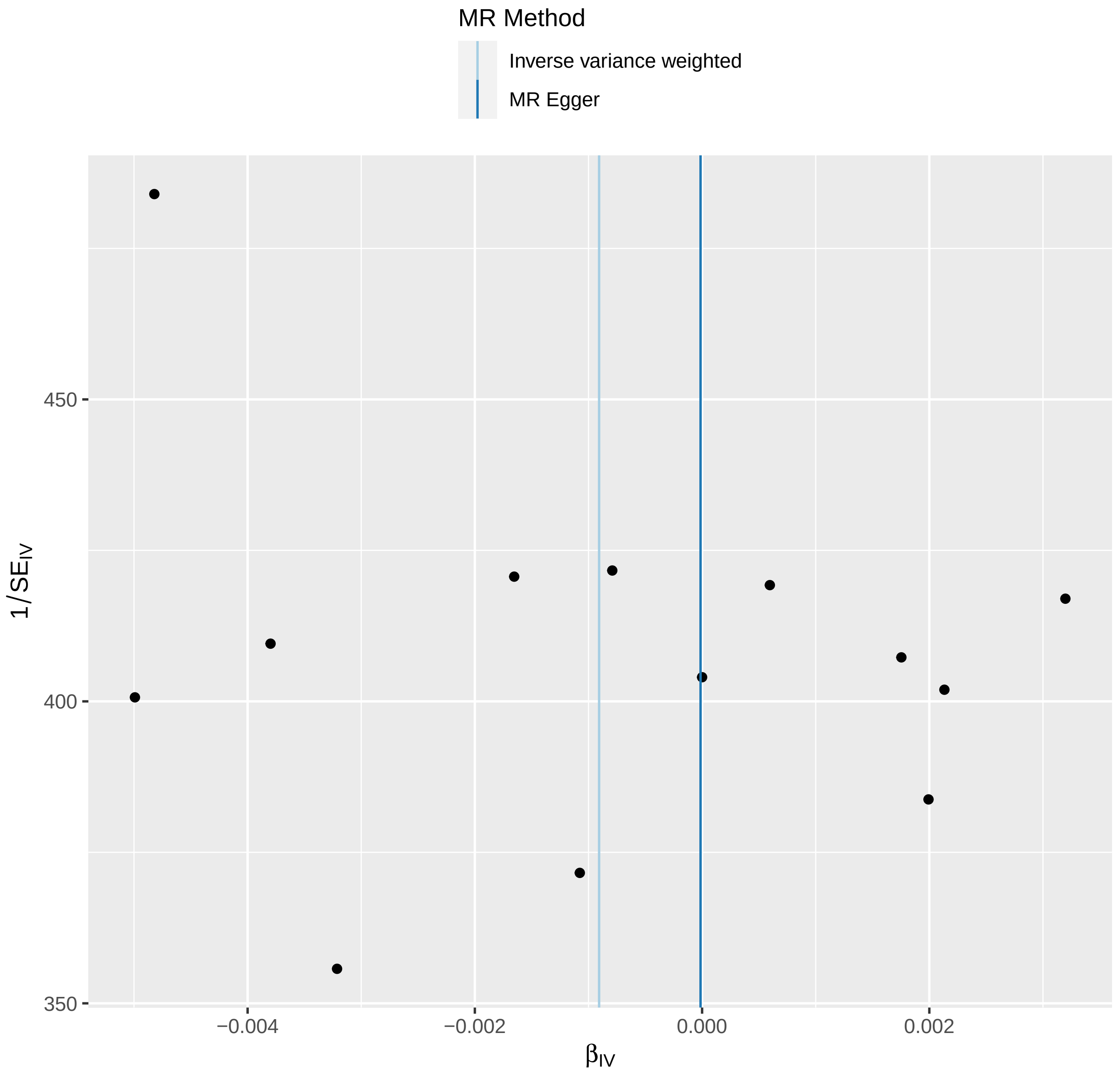

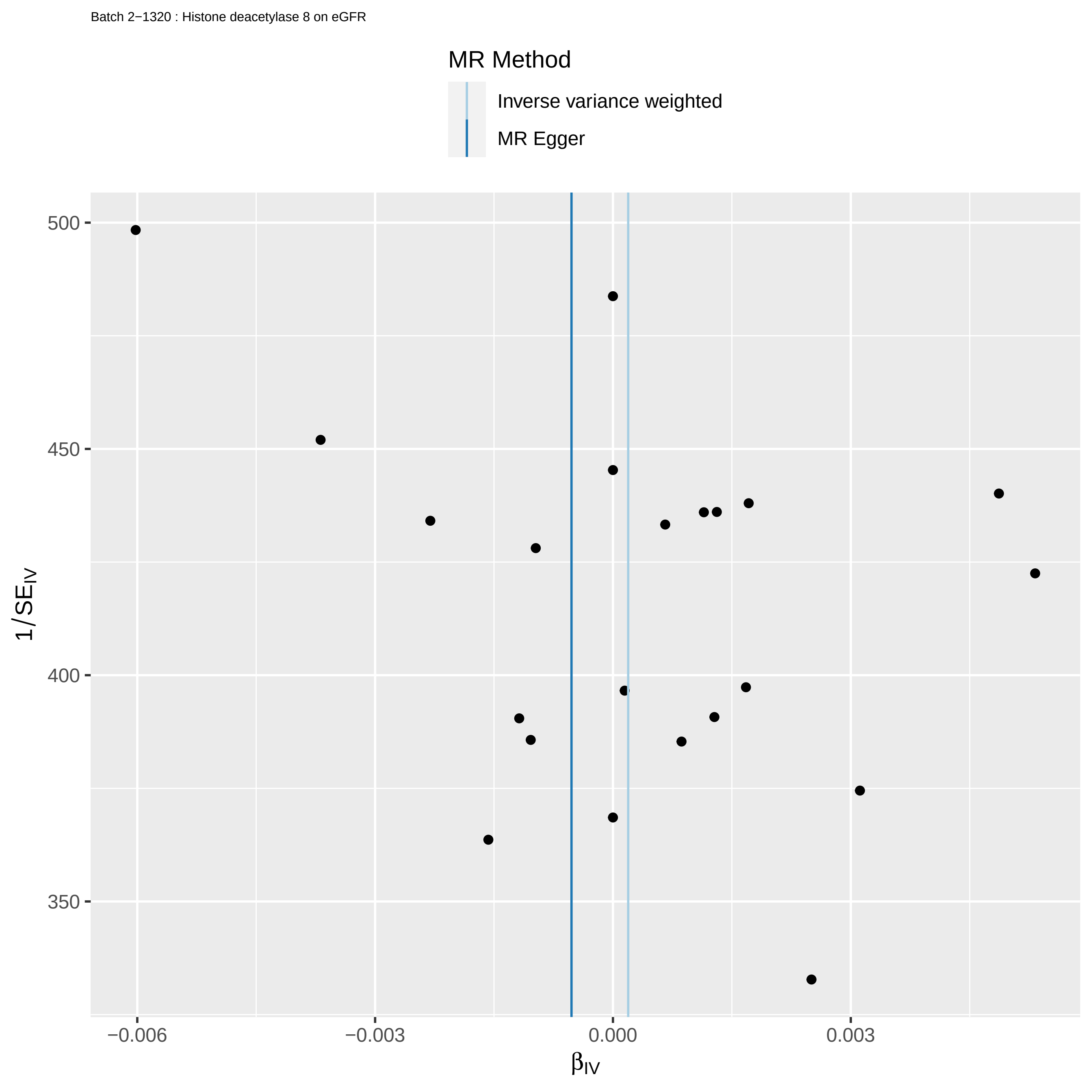

Batch 2−1320 : Histone deacetylase 8 on eGFR
MR Method
Inverse variance weighted
MR Egger
500
450
400
350
1/SE$_{IV}$
−0.006
−0.003
0.000
0.003
β$_{IV}$

Batch 2−1321 : Hepatoma−derived growth factor−related protein 2 on eGFR

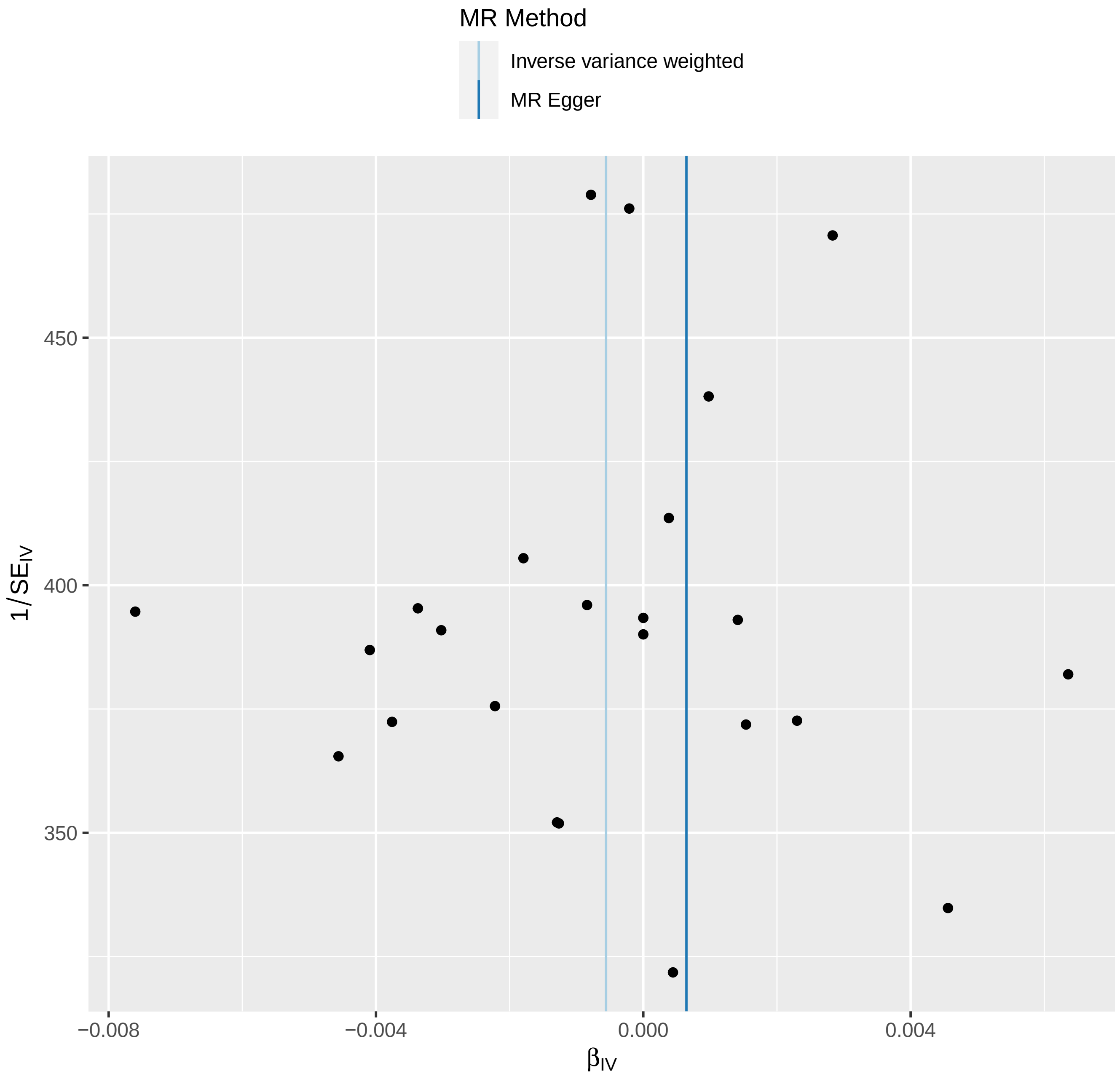

Batch 2−1322 : Haloacid dehalogenase−like hydrolase domain−containing protein 2 on eGFR

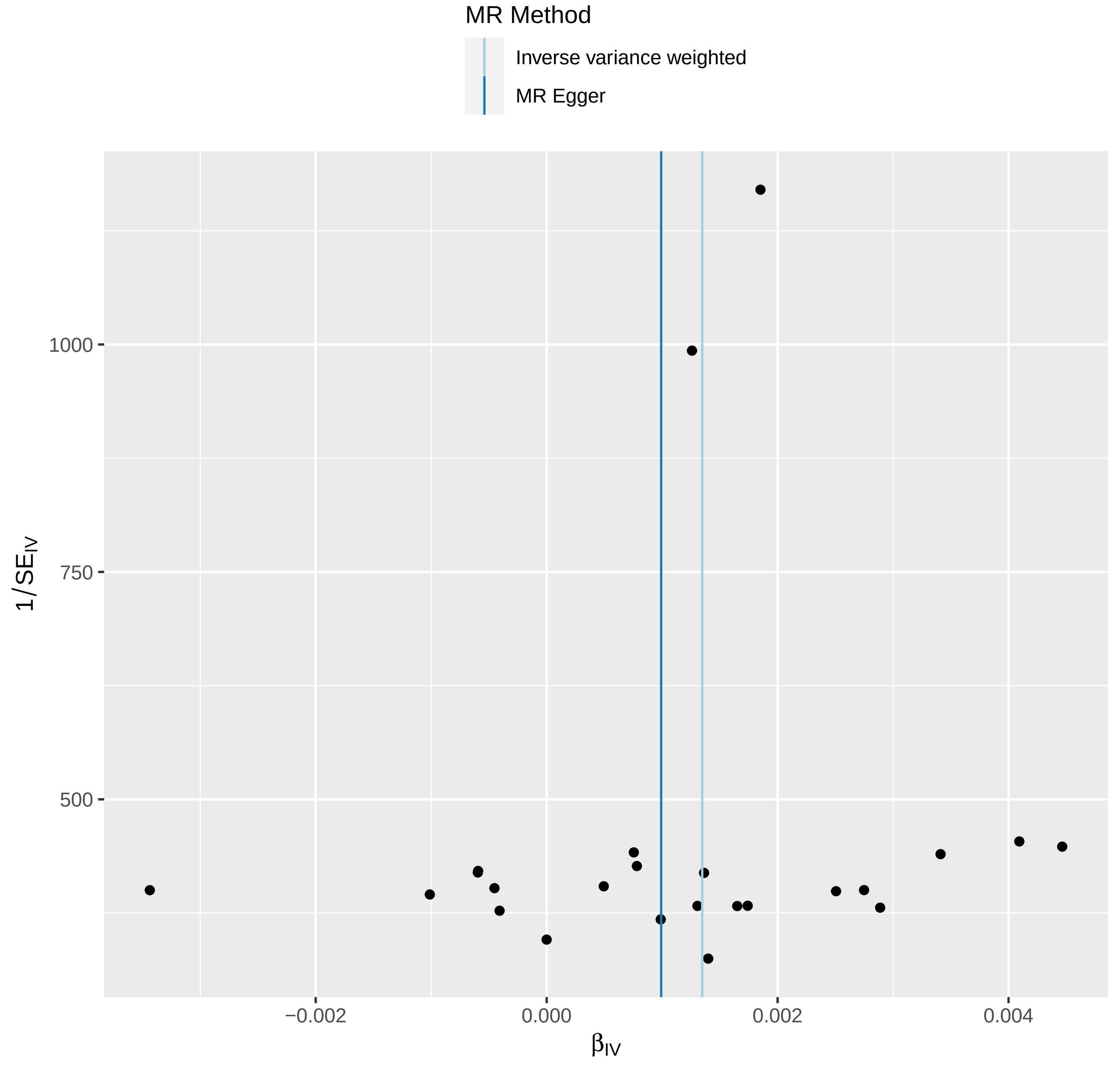

Batch 2−1323 : Vigilin on eGFR

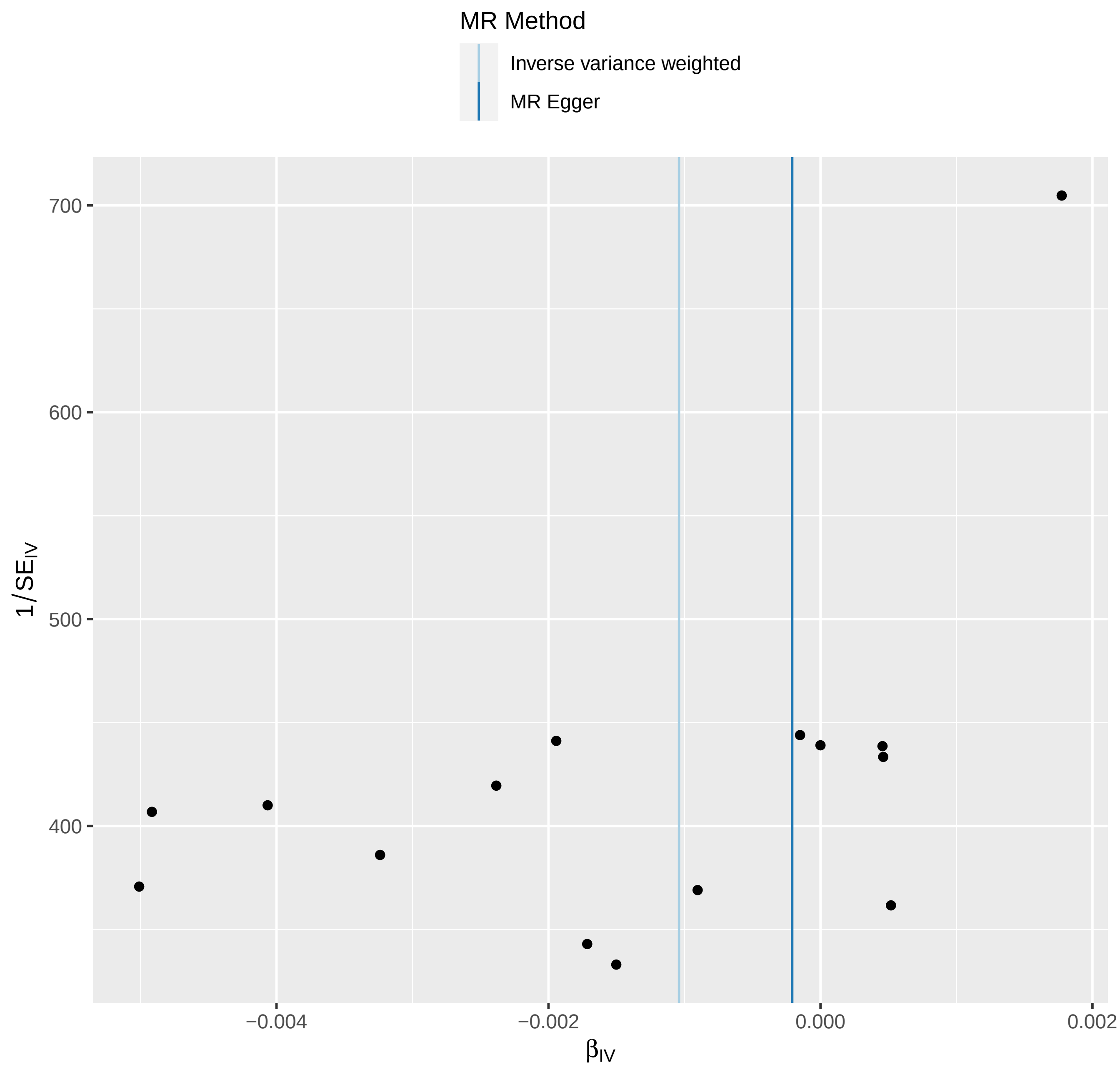

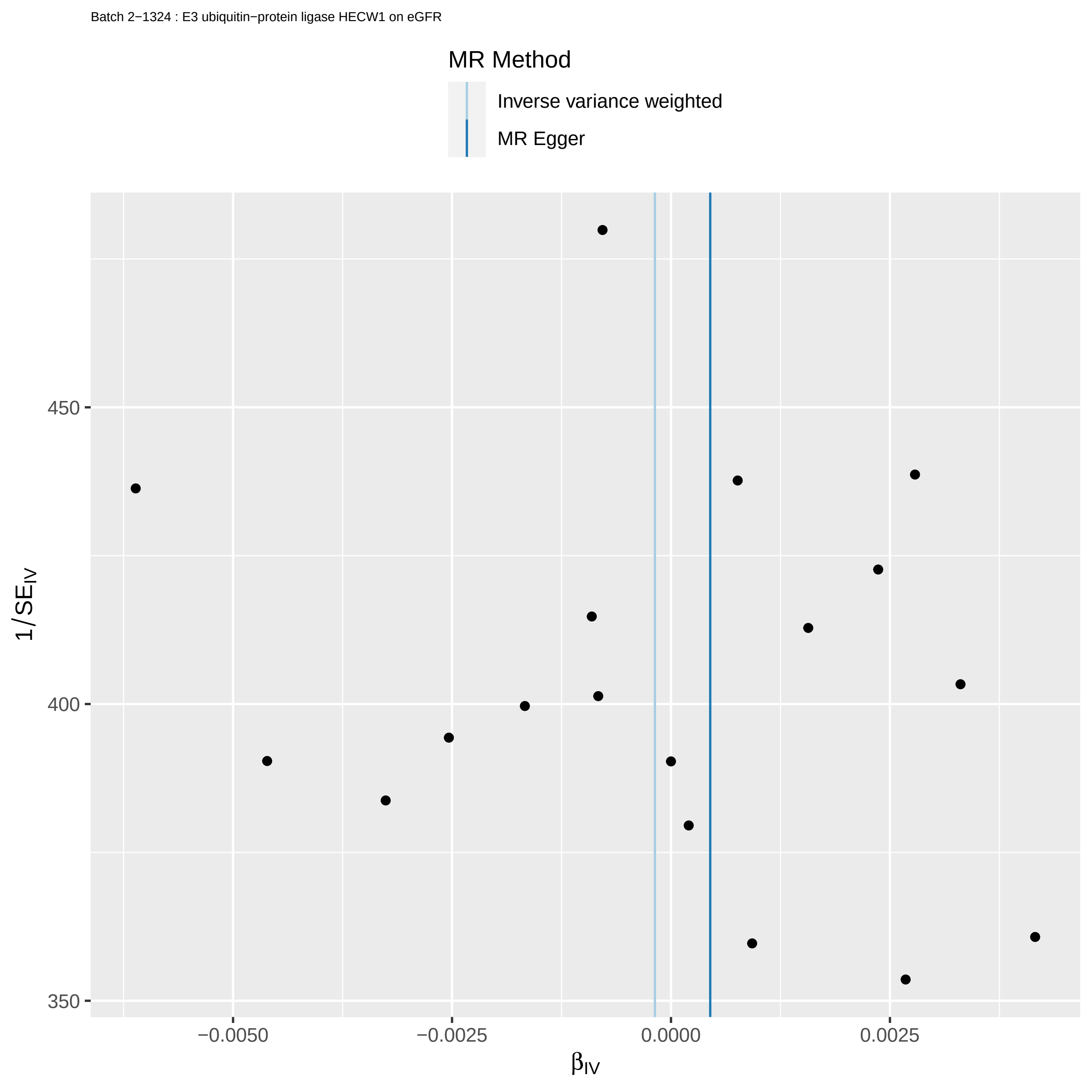

Batch 2−1324 : E3 ubiquitin−protein ligase HECW1 on eGFR
MR Method
Inverse variance weighted
MR Egger
450
400
350
1/SE_IV
−0.0050
−0.0025
0.0000
0.0025
β_IV

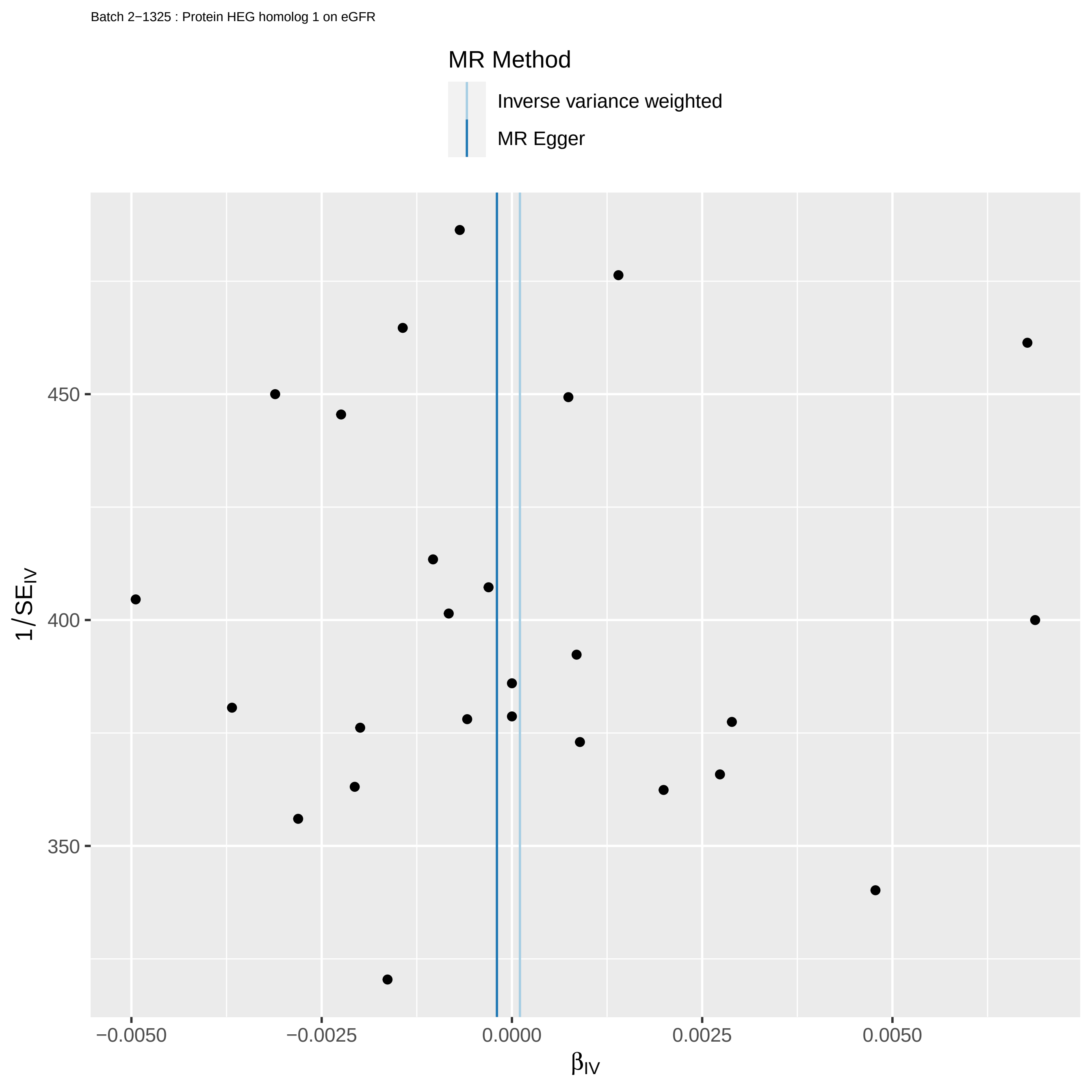

Batch 2−1325 : Protein HEG homolog 1 on eGFR
MR Method
Inverse variance weighted
MR Egger
450
400
350
1/SE$_{IV}$
−0.0050
−0.0025
0.0000
0.0025
0.0050
β$_{IV}$

Batch 2−1326 : HEPACAM family member 2 on eGFR

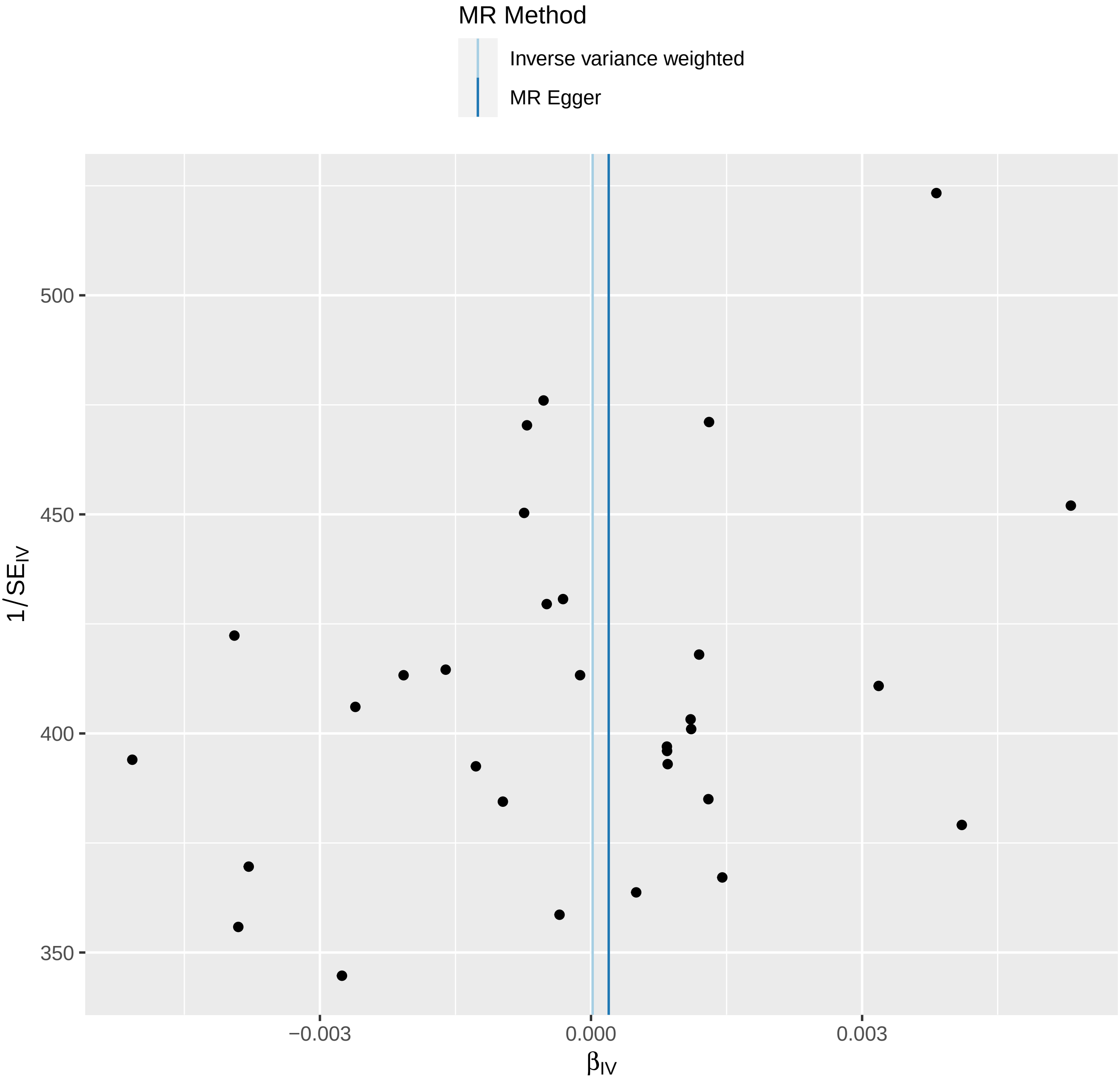

Batch 2−1327 : HEPACAM family member 2 on eGFR

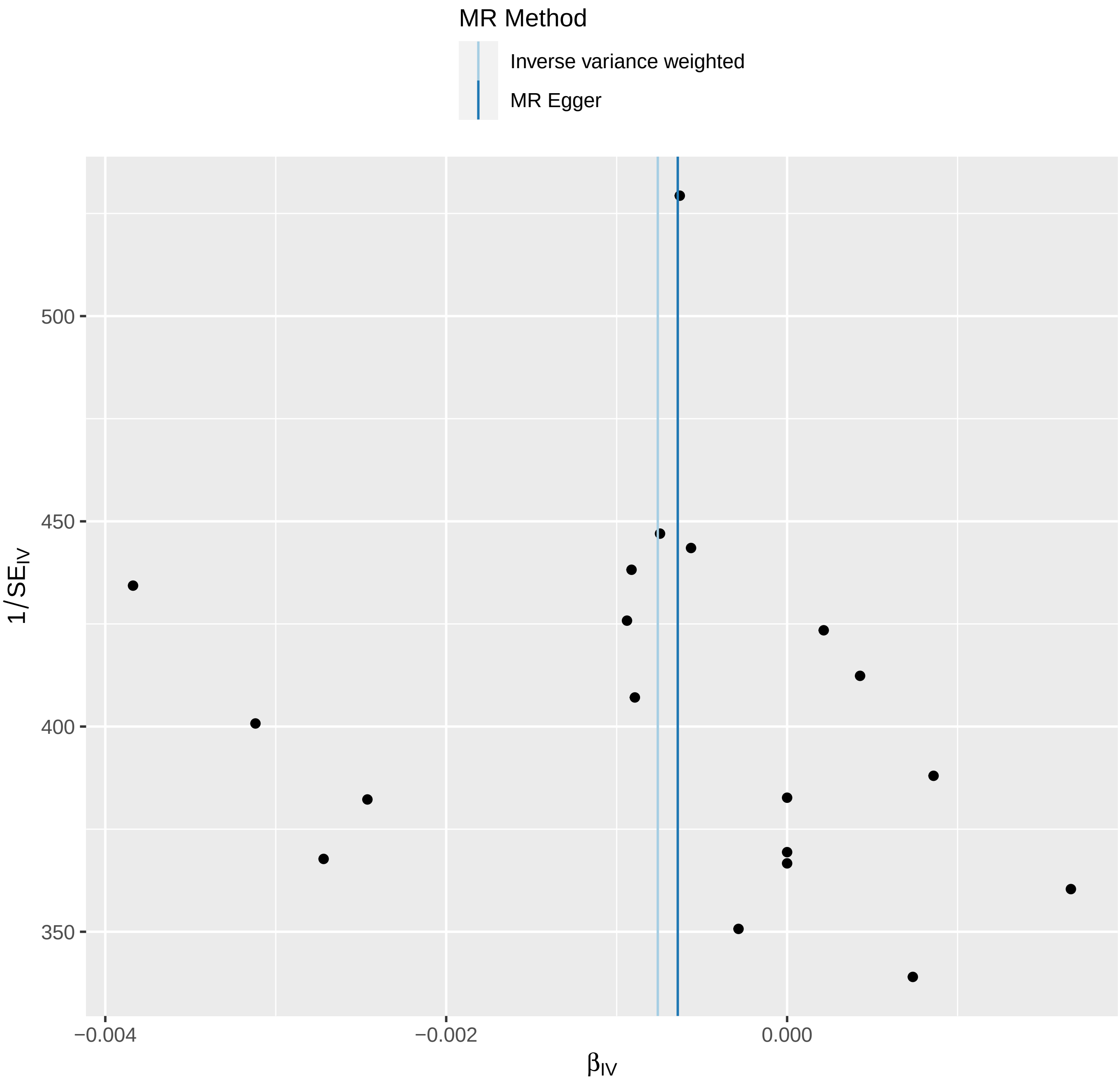

Batch 2−1328 : Hephaestin−like protein 1 on eGFR

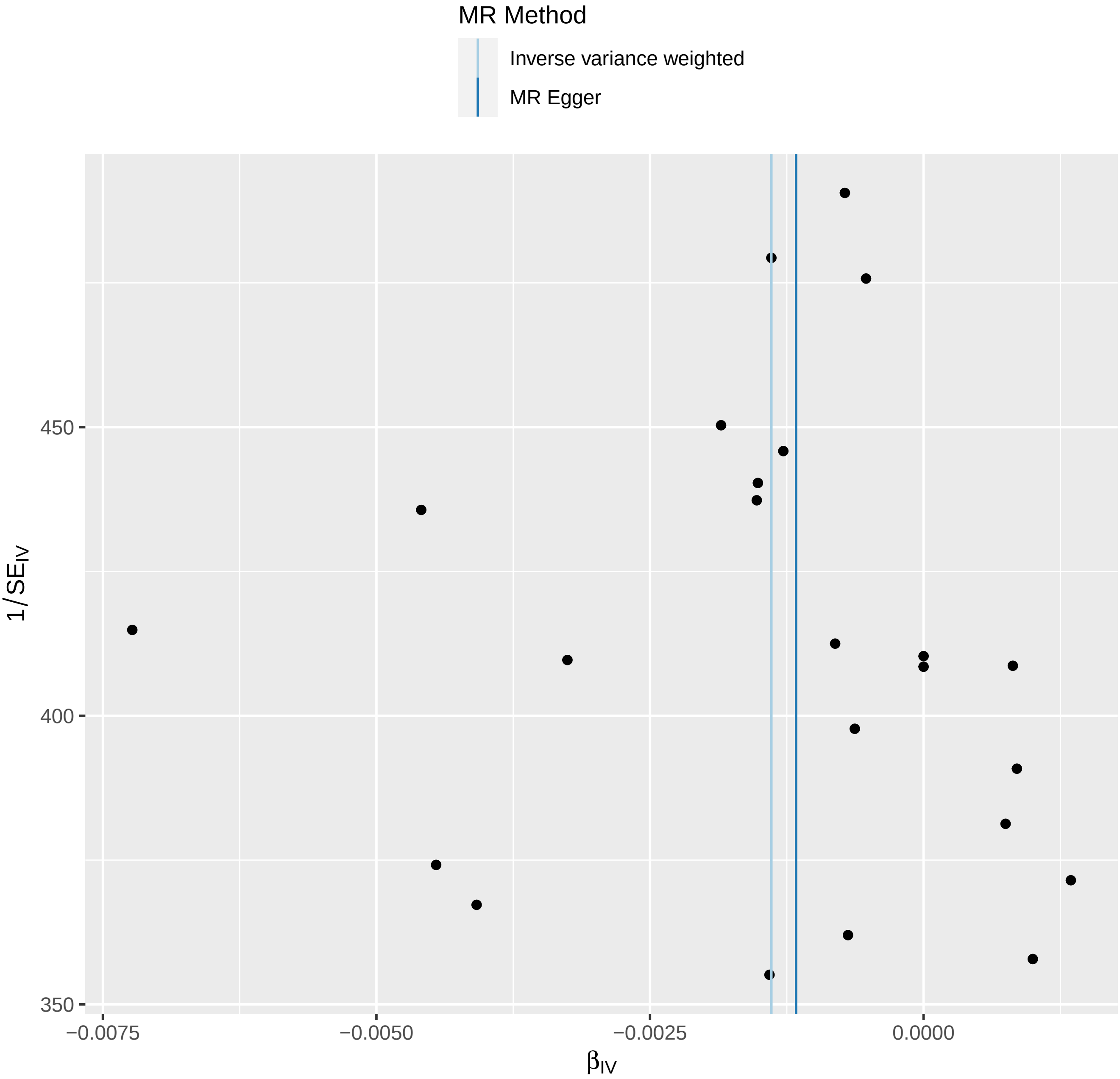

Batch 2−1329 : Probable E3 ubiquitin−protein ligase HERC1 on eGFR

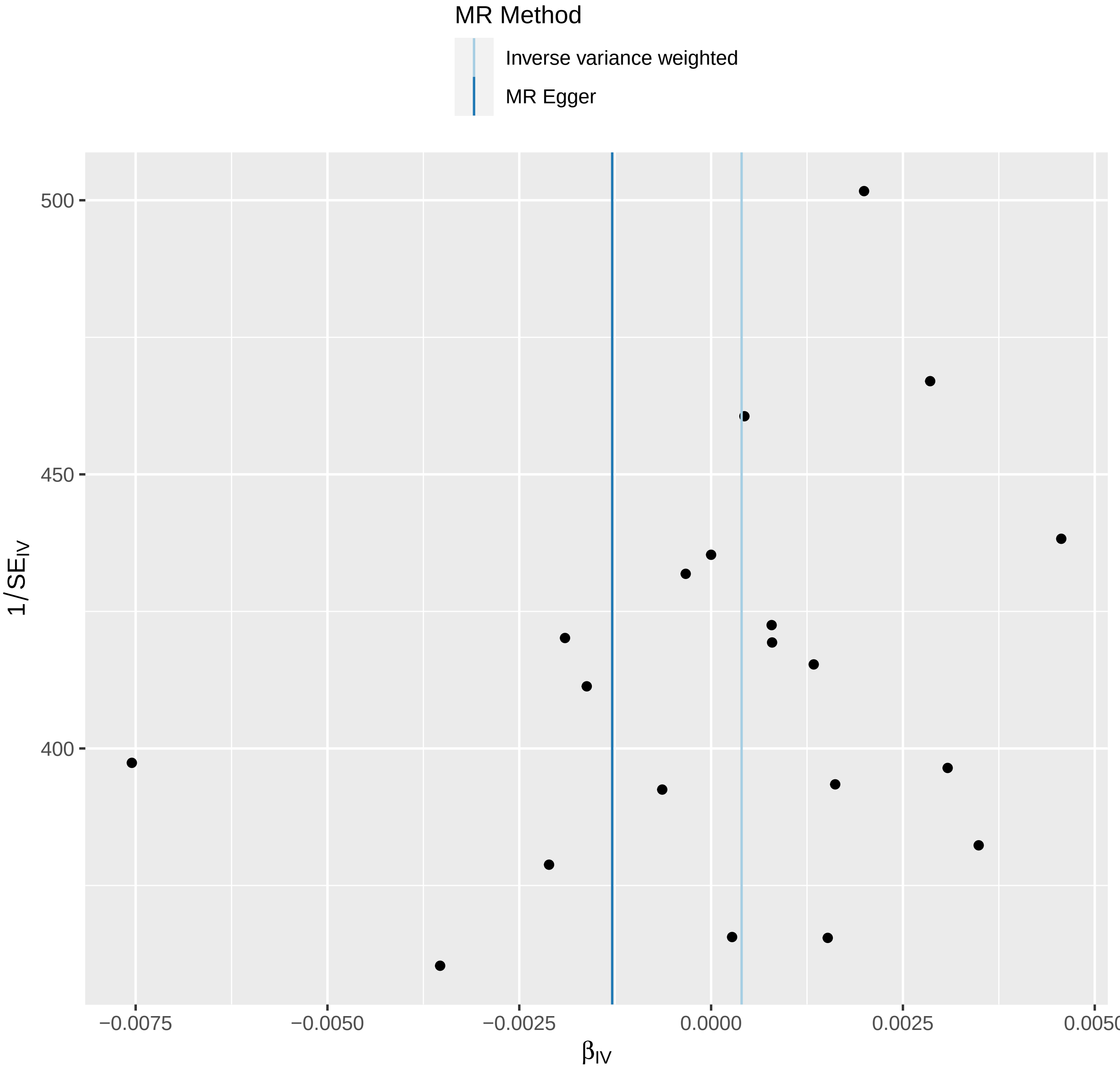

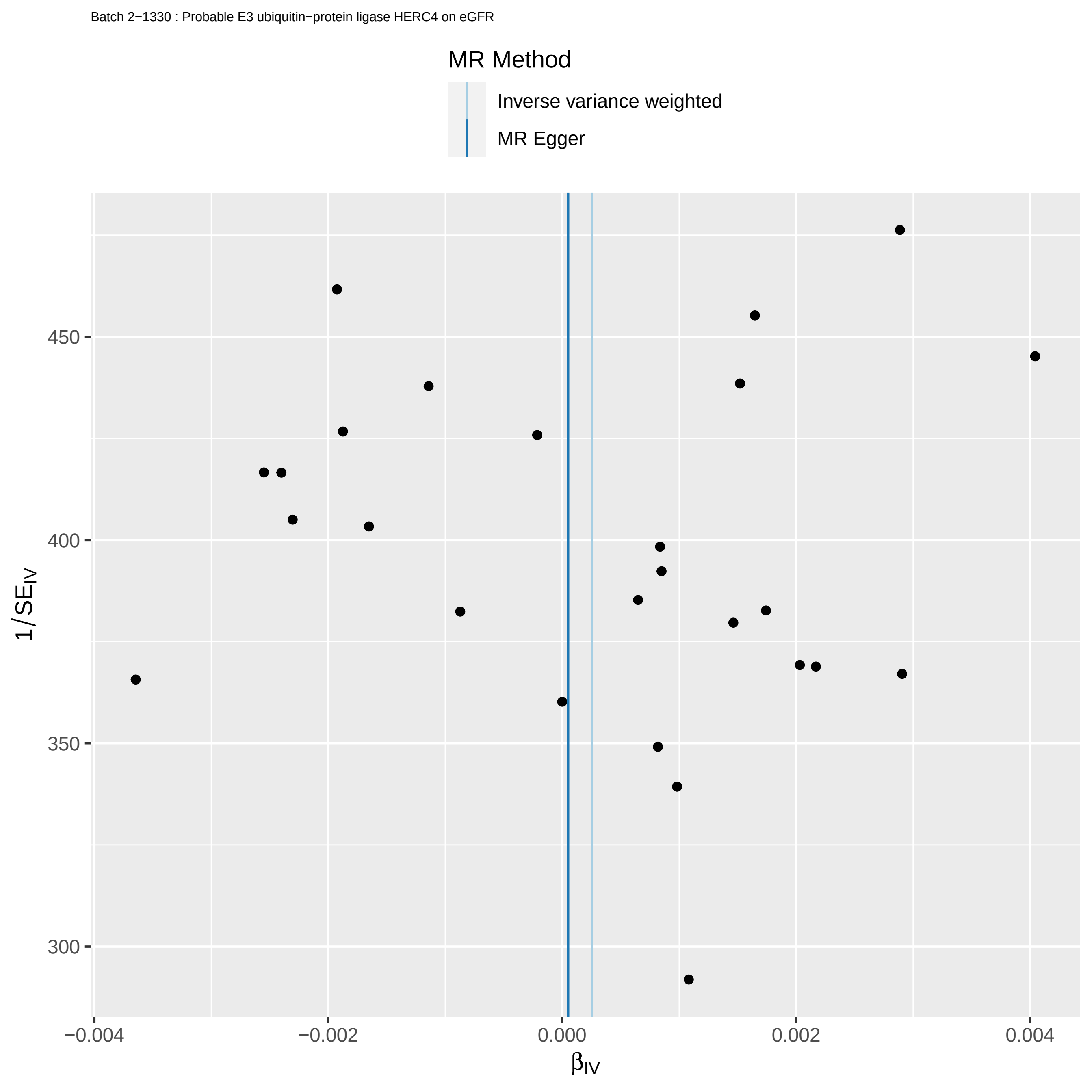

Batch 2−1330 : Probable E3 ubiquitin−protein ligase HERC4 on eGFR
MR Method
Inverse variance weighted
MR Egger
450
400
350
300
1/SE$_{IV}$
−0.004
−0.002
0.000
0.002
0.004
β$_{IV}$

Batch 2−1331 : E3 ISG15−−protein ligase HERC5 on eGFR

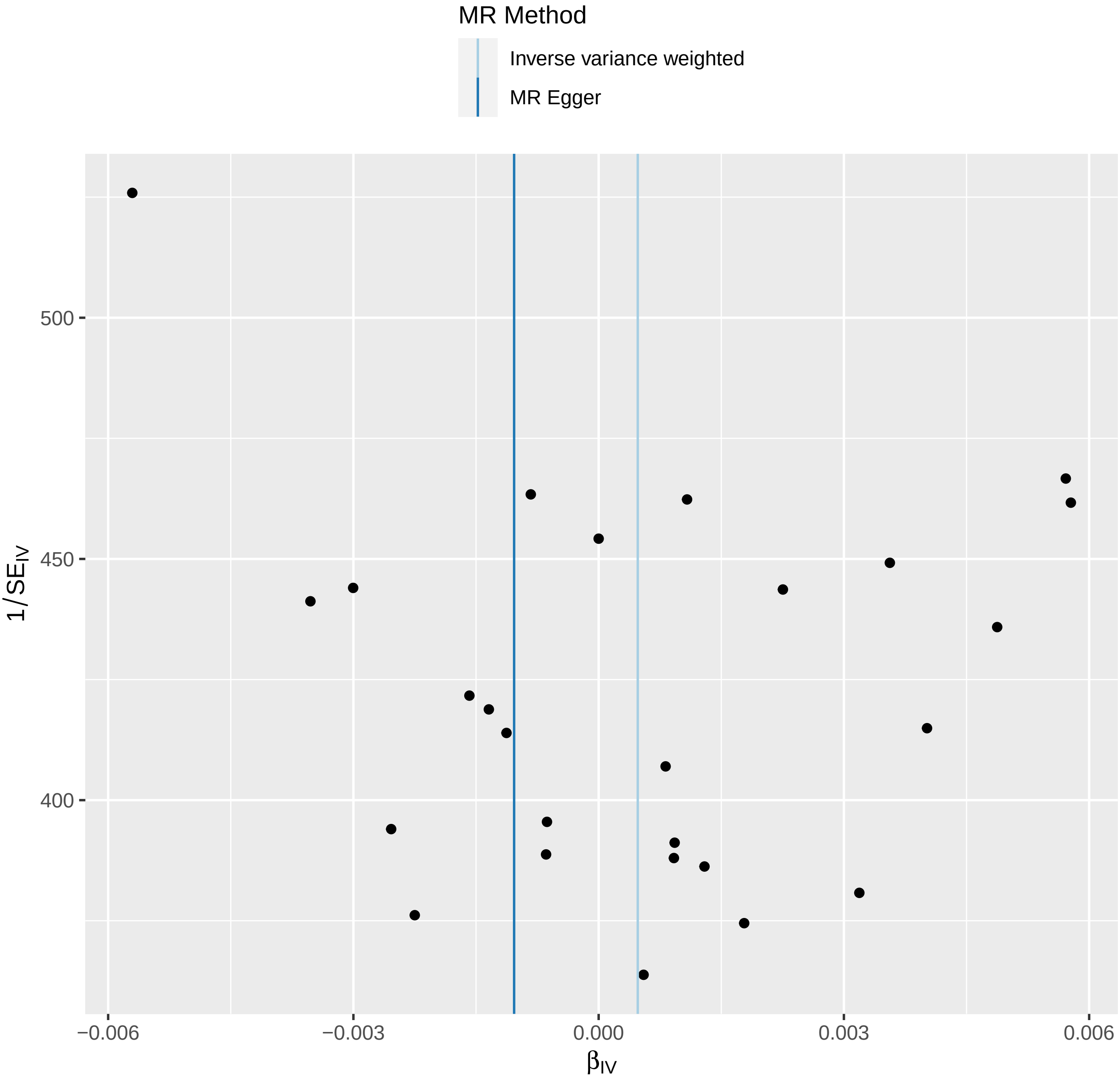

Batch 2−1332 : Hemojuvelin on eGFR

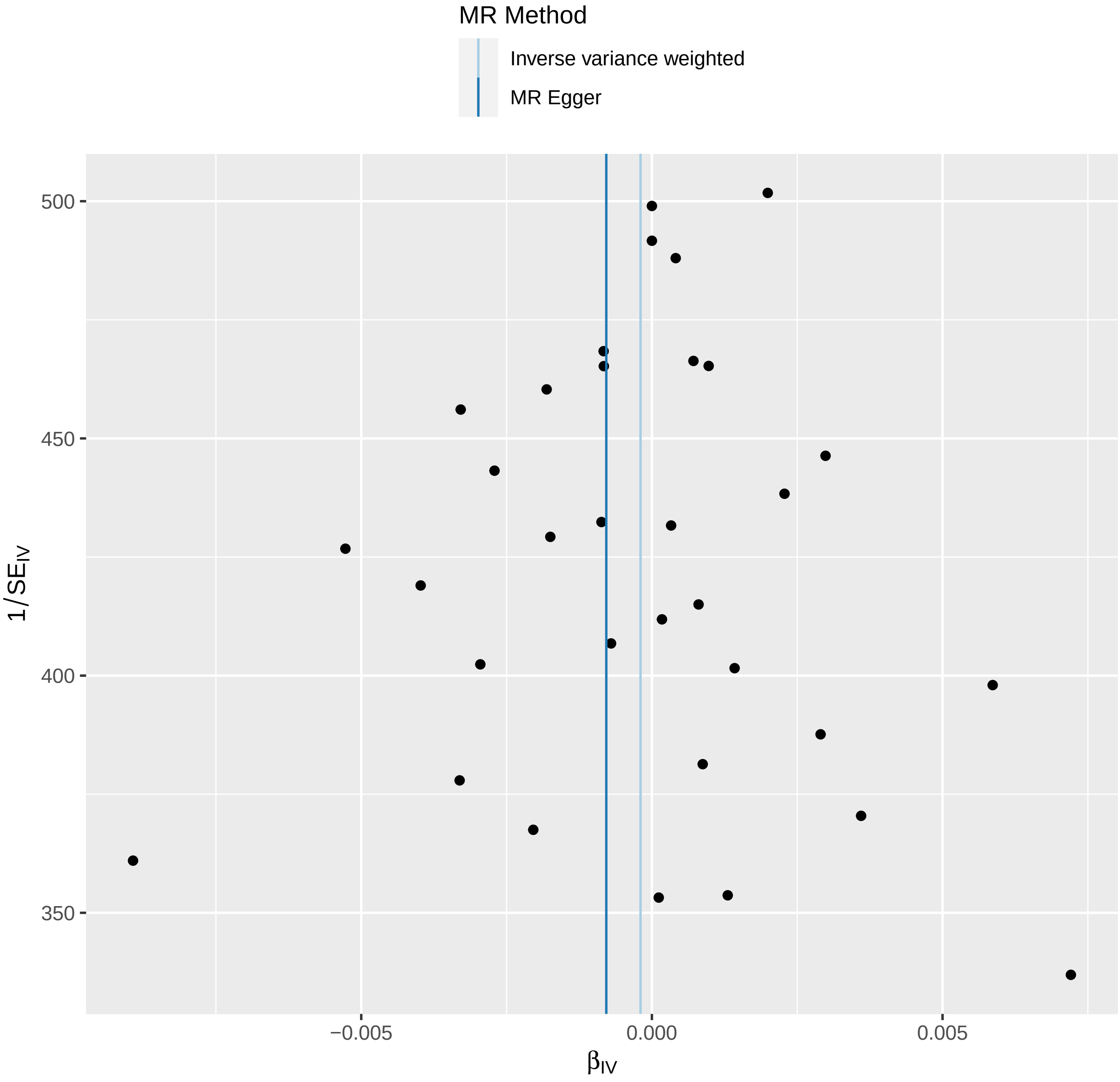

Batch 2−1333 : Homogentisate 1,2−dioxygenase on eGFR

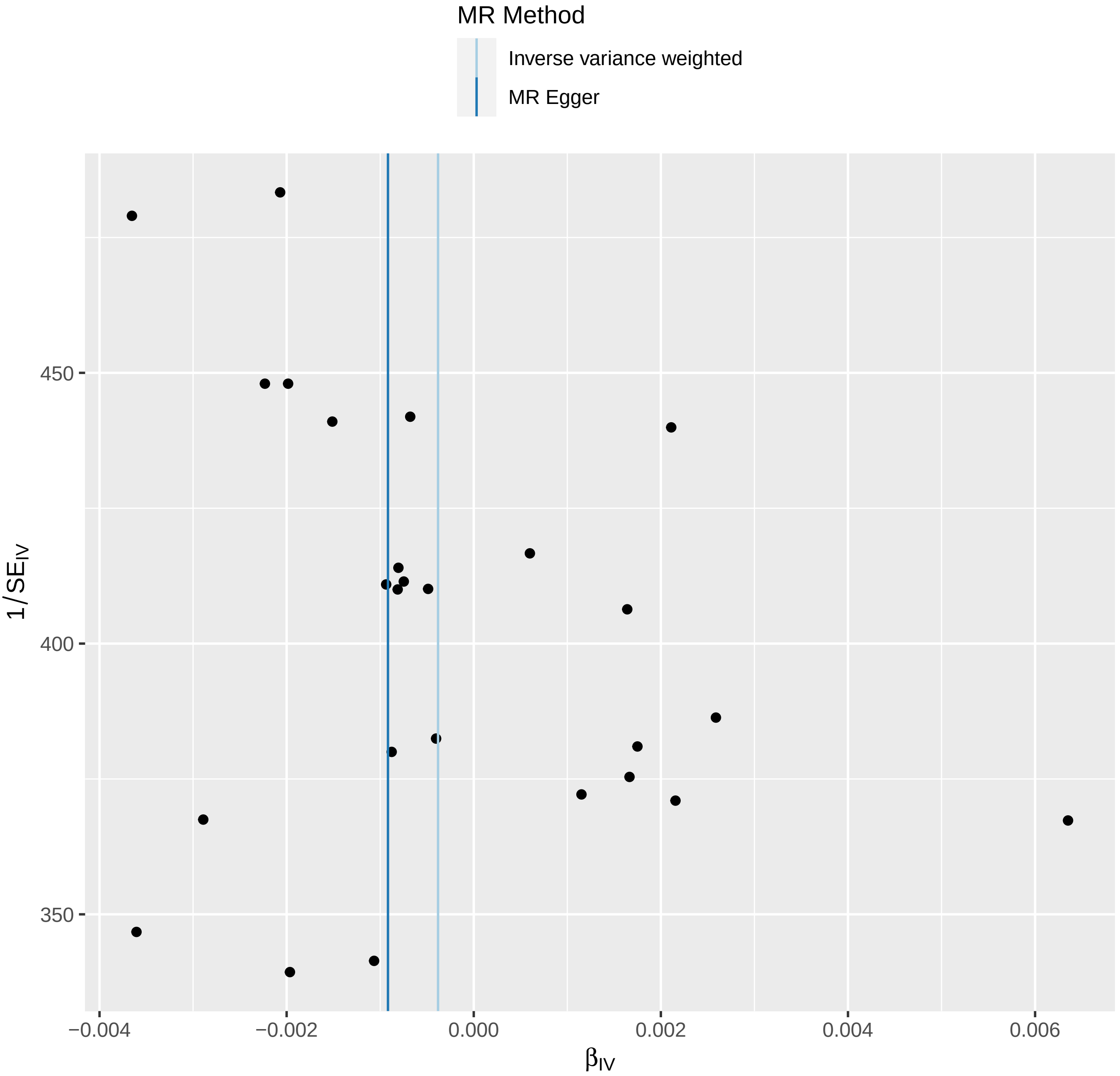

Batch 2−1334 : Hepatocyte growth factor on eGFR

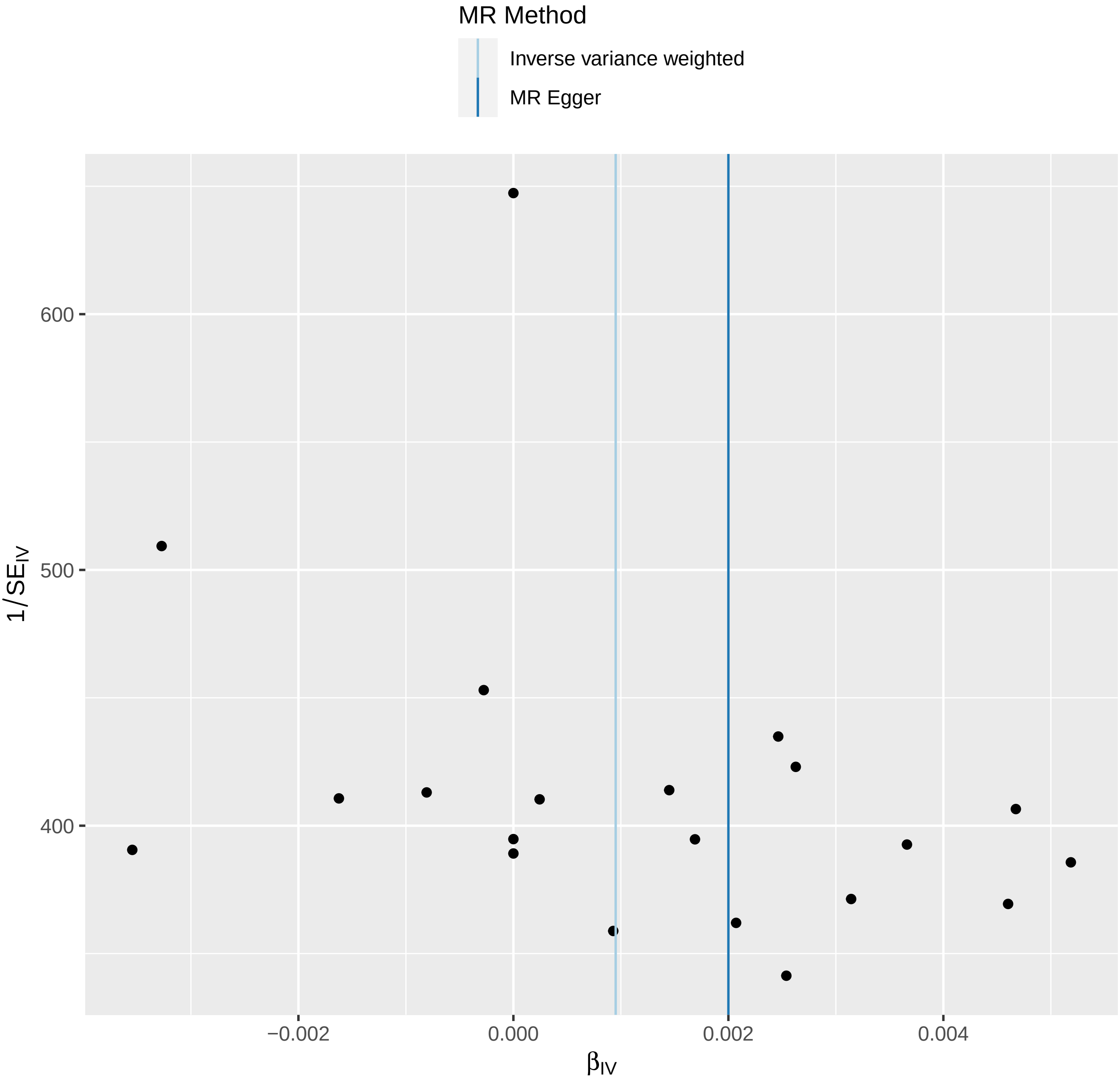

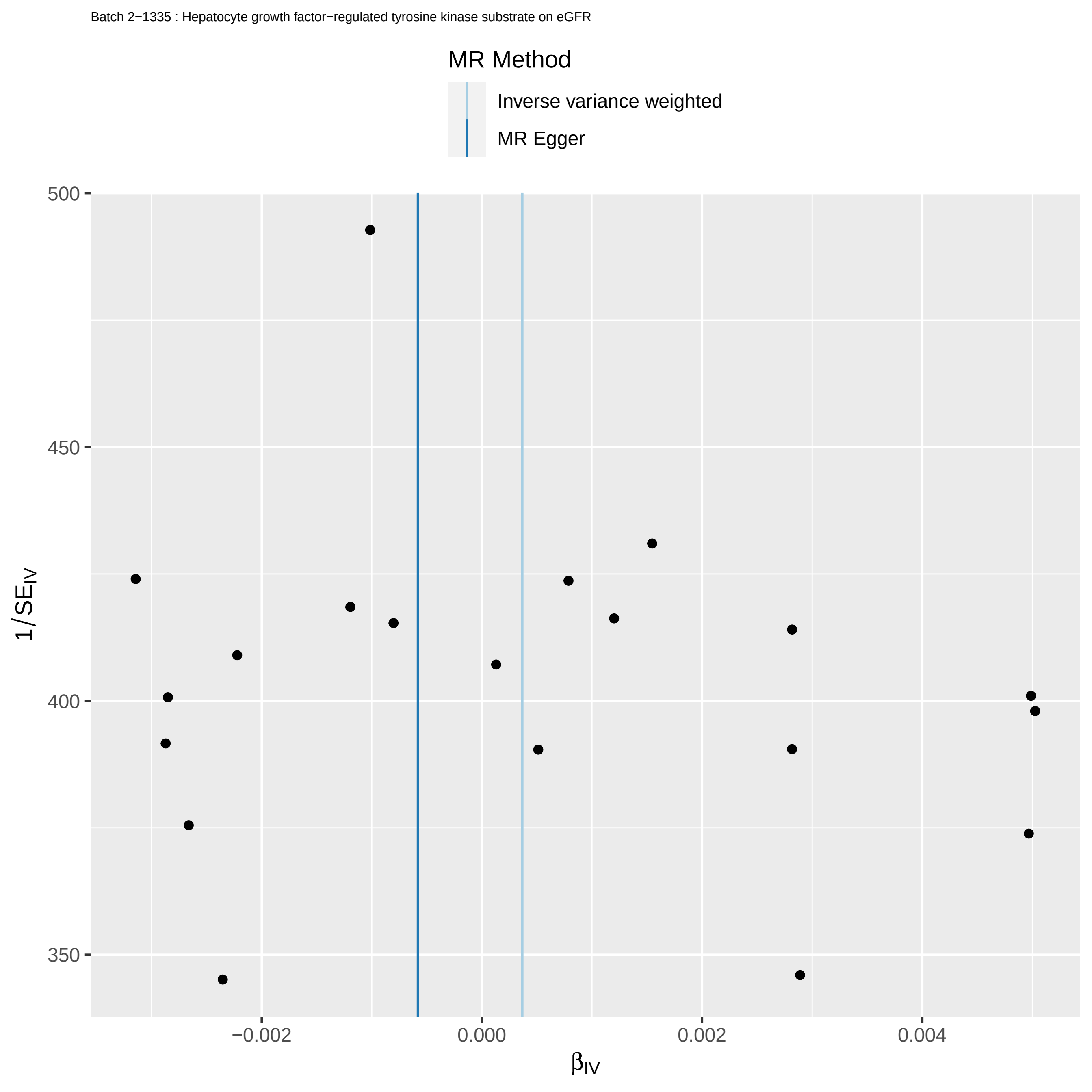

Batch 2−1335 : Hepatocyte growth factor−regulated tyrosine kinase substrate on eGFR
MR Method
Inverse variance weighted
MR Egger
500
450
400
350
1/SE$_{IV}$
−0.002
0.000
0.002
0.004
β$_{IV}$

Batch 2−1336 : HERV−H LTR−associating protein 2 on eGFR

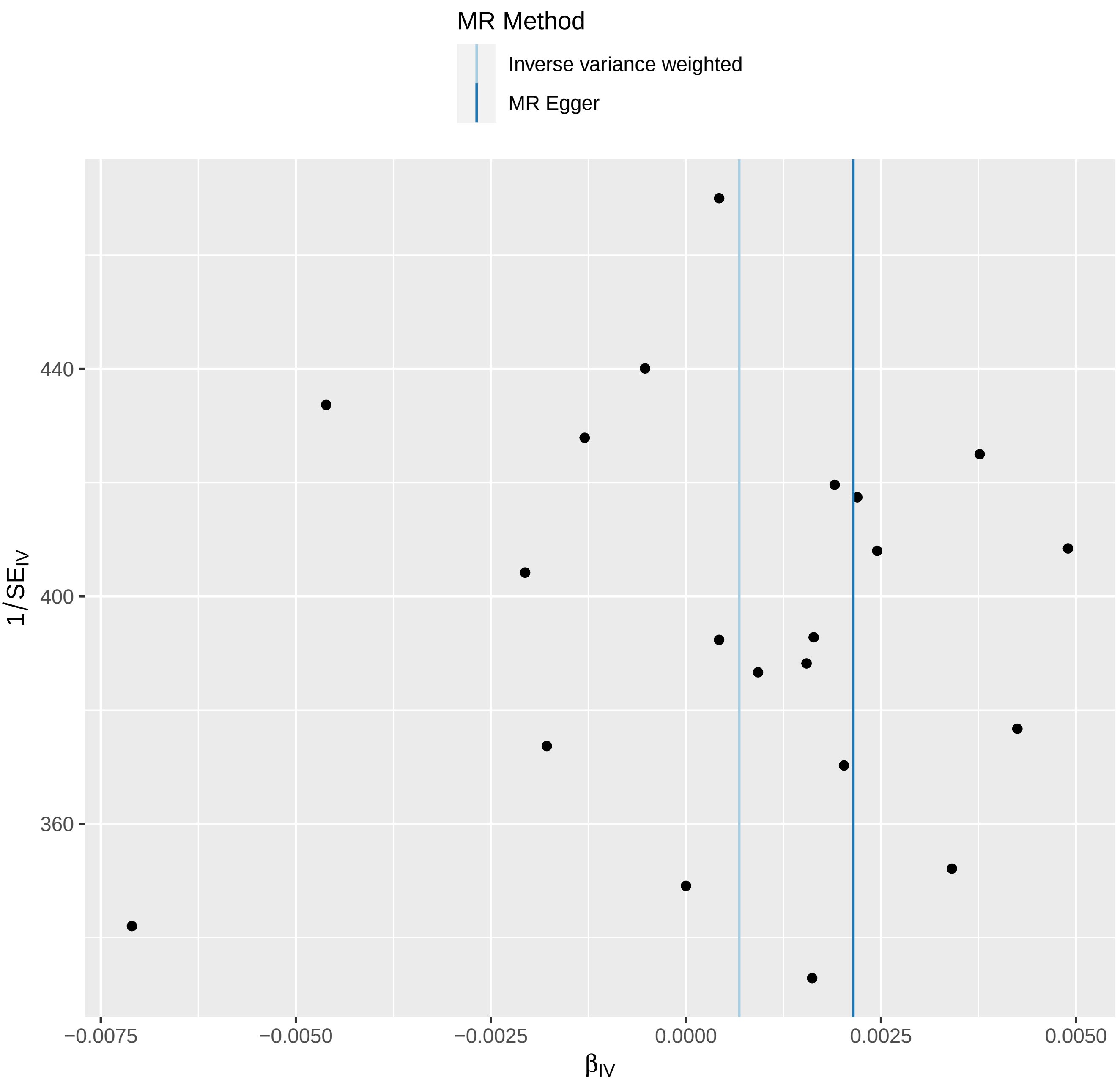

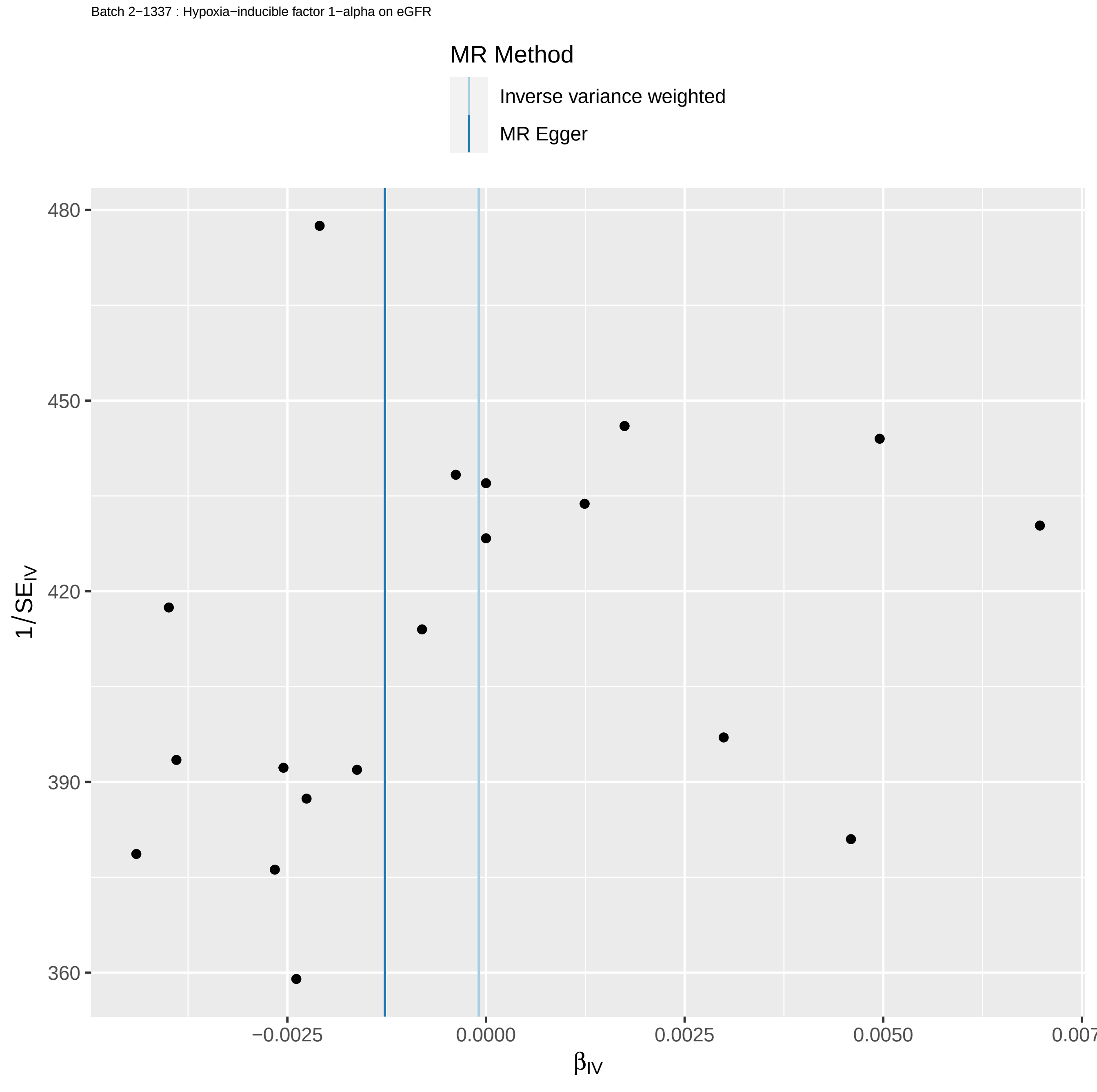
Batch 2−1337 : Hypoxia−inducible factor 1−alpha on eGFR
MR Method
Inverse variance weighted
MR Egger
480
450
420
390
360
1/SE_IV
−0.0025
0.0000
0.0025
0.0050
0.007
β_IV

Batch 2−1338 : Histidine triad nucleotide−binding protein 1 on eGFR

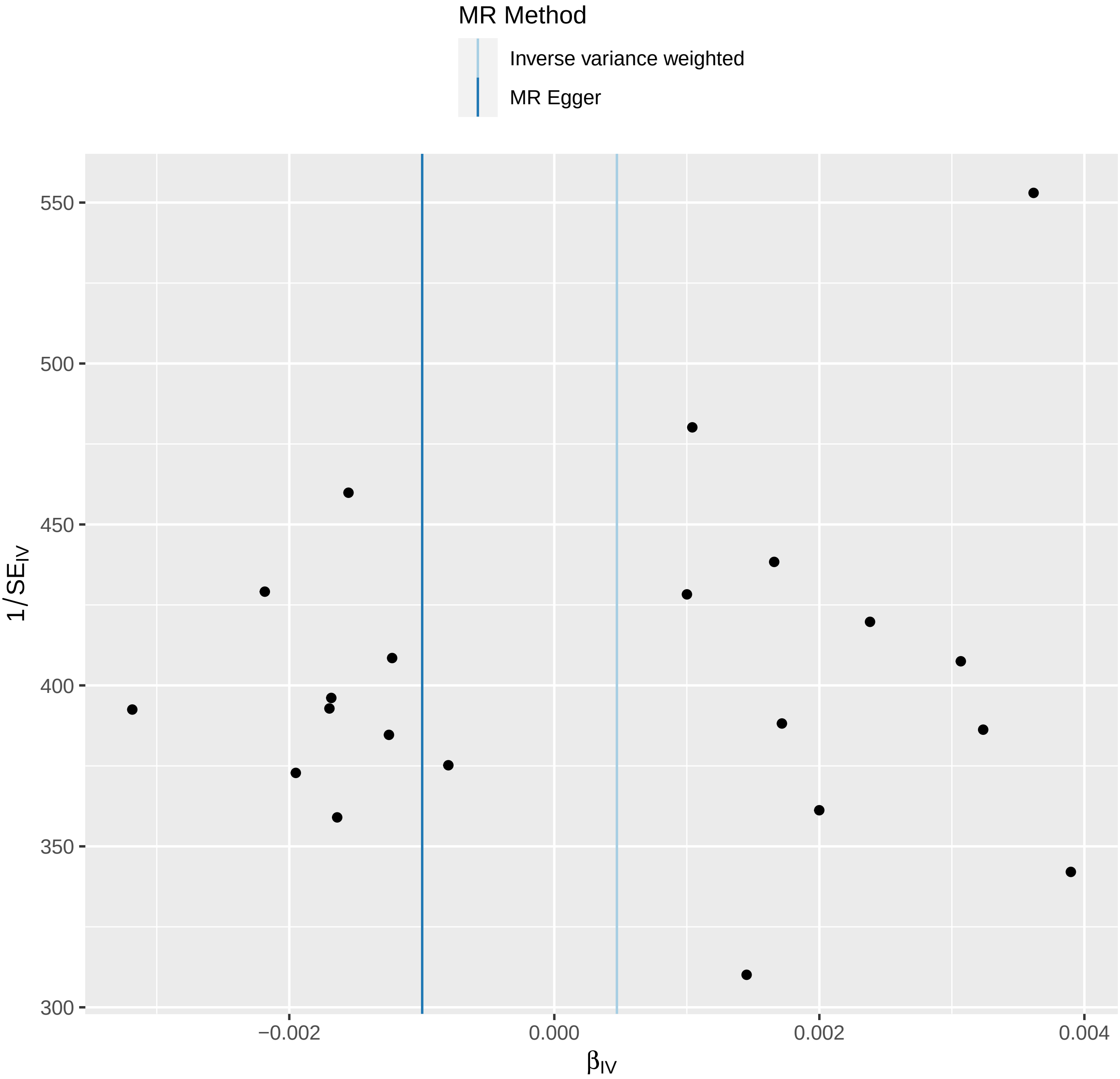

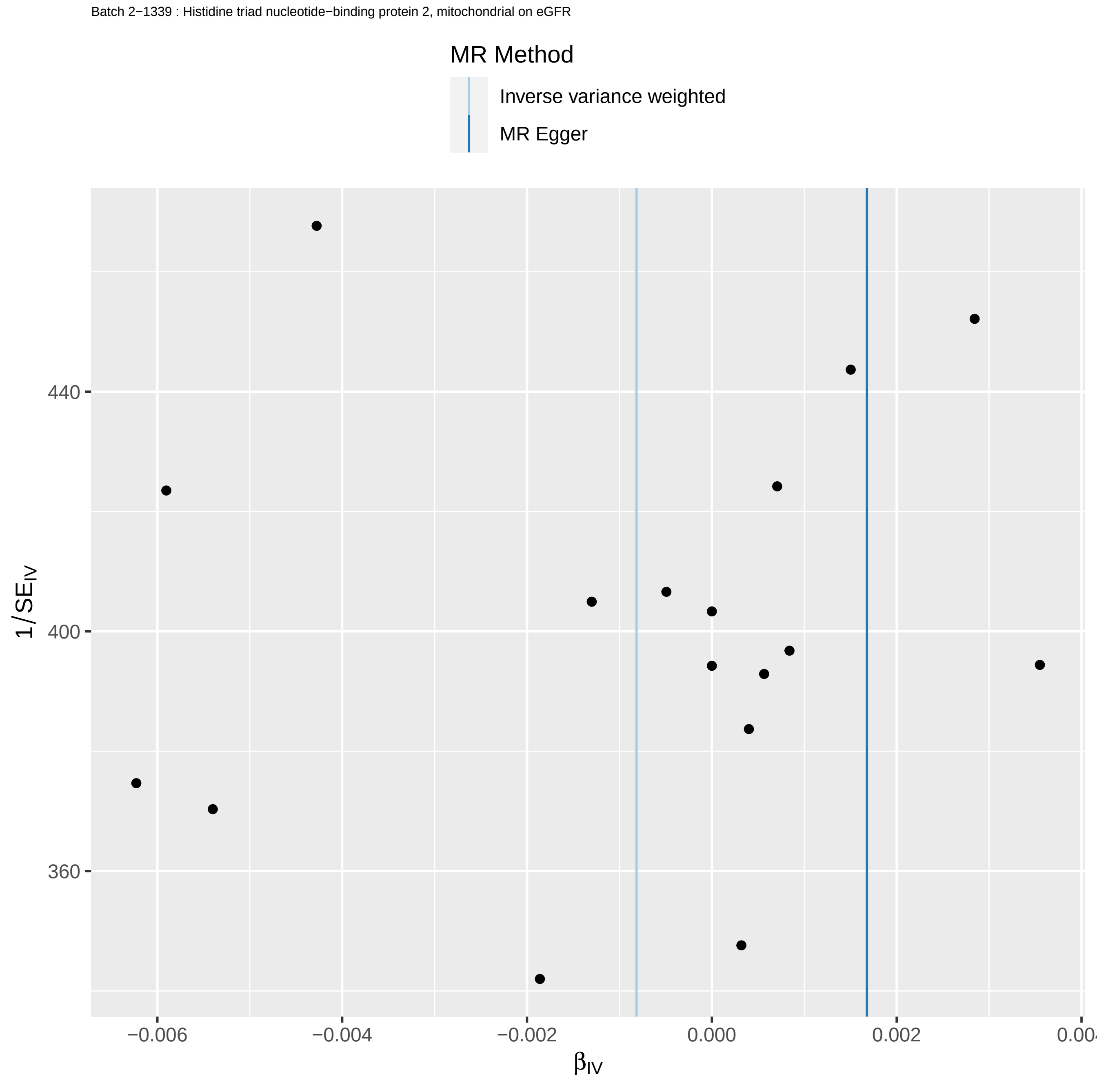

Batch 2−1339 : Histidine triad nucleotide−binding protein 2, mitochondrial on eGFR
MR Method
Inverse variance weighted
MR Egger
440
400
360
1/SE_IV
−0.006
−0.004
−0.002
0.000
0.002
β_IV

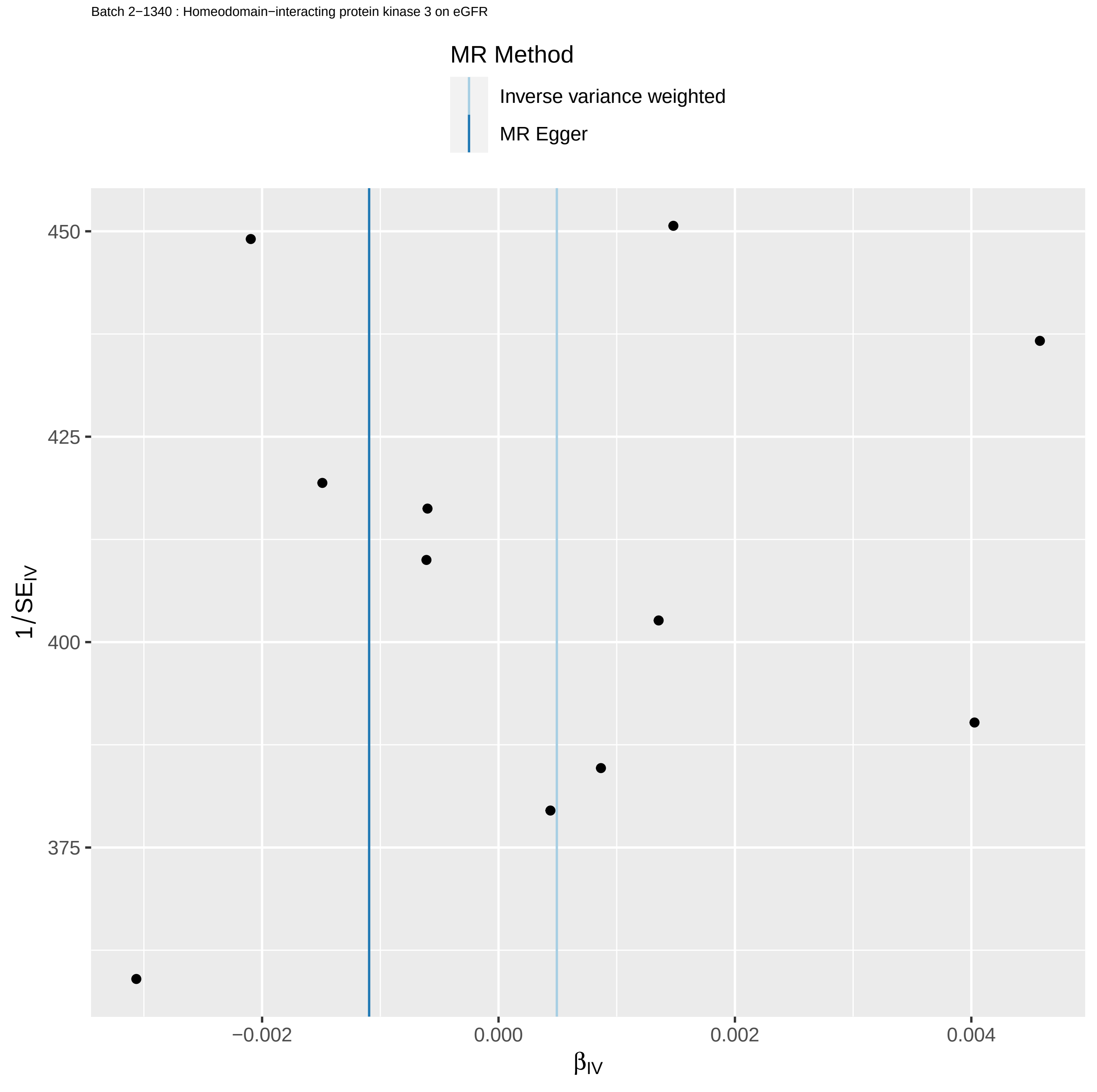
Batch 2−1340 : Homeodomain−interacting protein kinase 3 on eGFR
MR Method
Inverse variance weighted
MR Egger
450
425
400
375
1/SE$_{IV}$
−0.002
0.000
0.002
0.004
β$_{IV}$

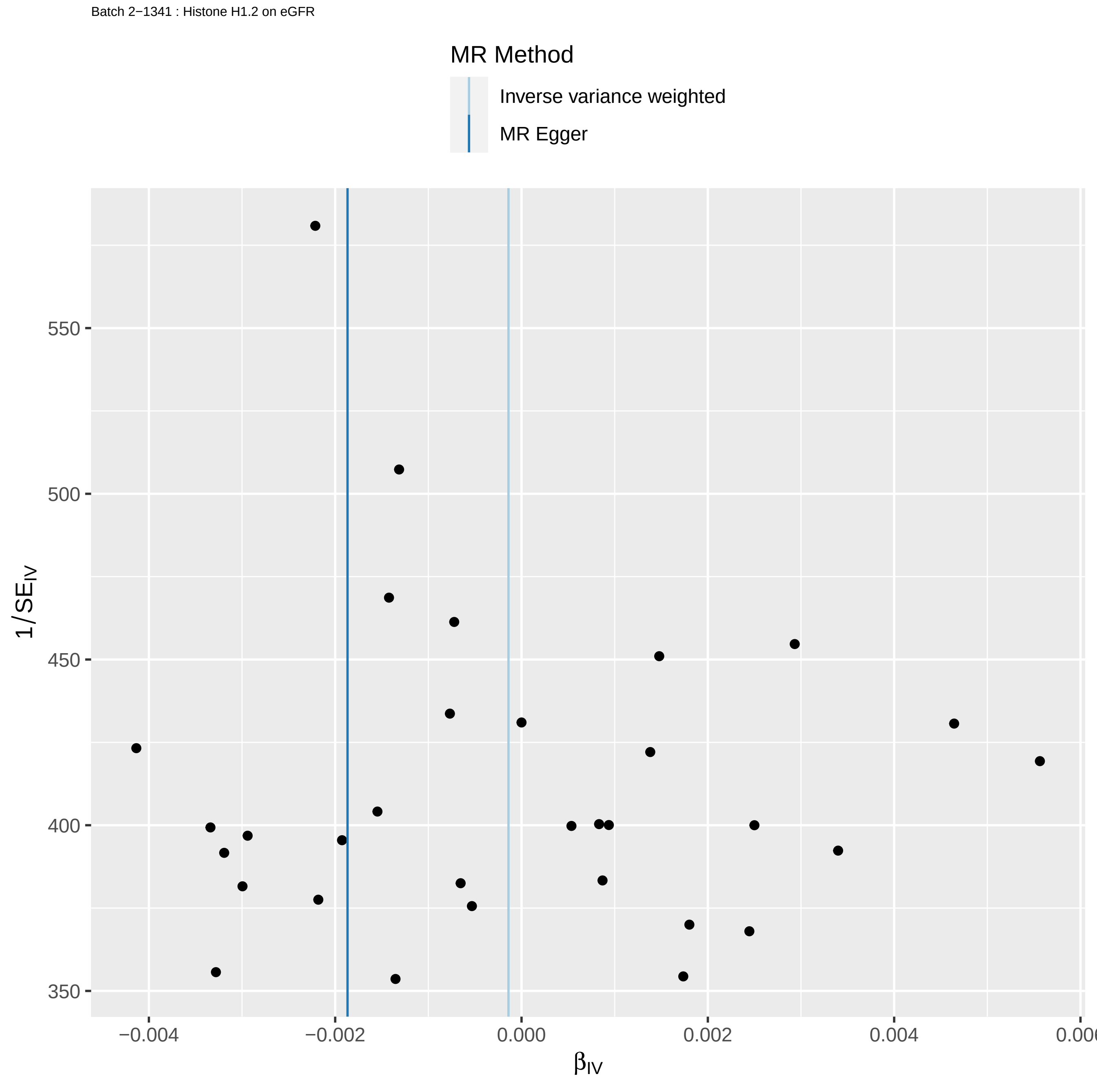

Batch 2−1341 : Histone H1.2 on eGFR
MR Method
Inverse variance weighted
MR Egger
550
500
450
400
350
1/SE$_{IV}$
−0.004
−0.002
0.000
0.002
0.004
β$_{IV}$

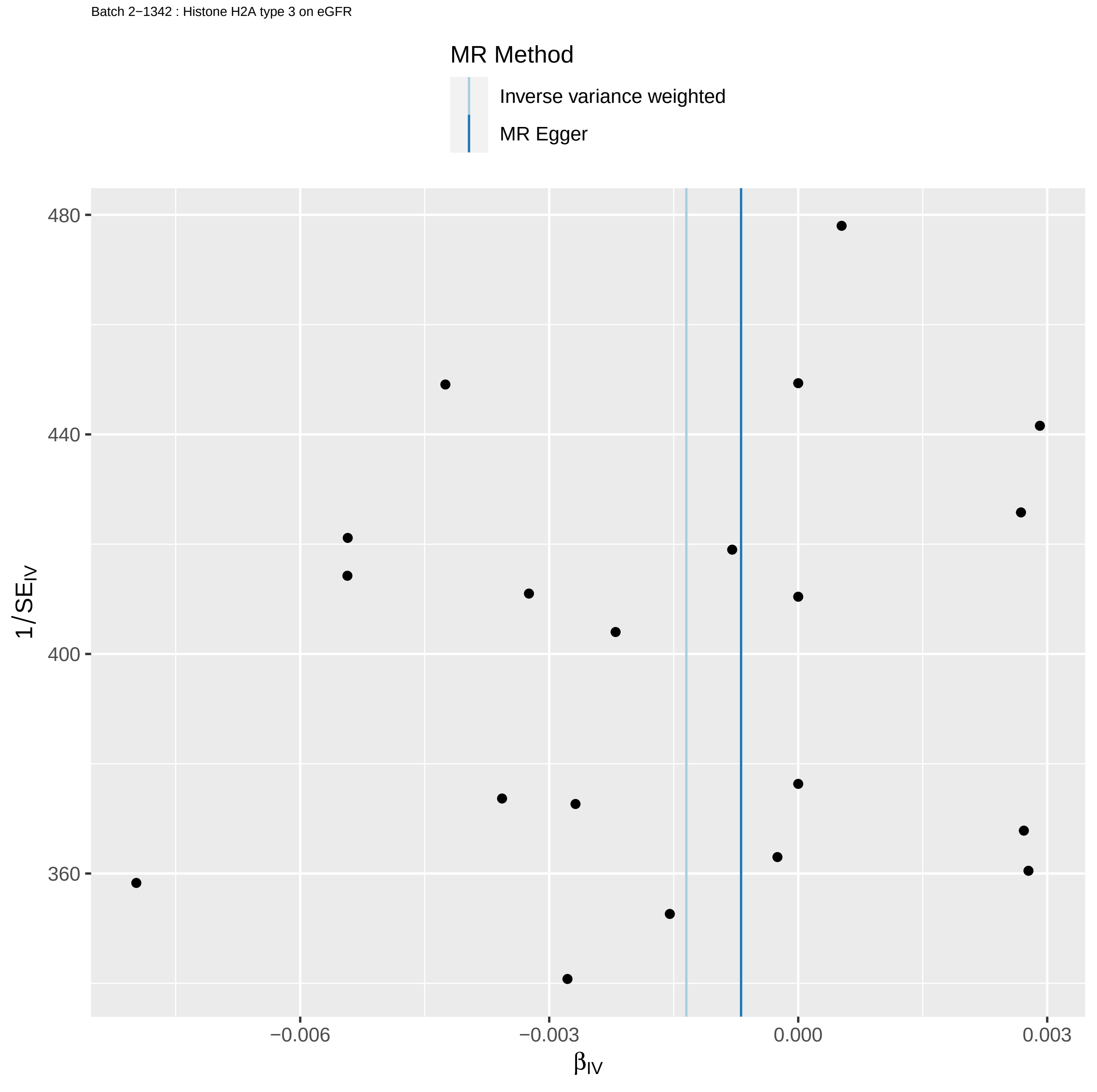
Batch 2−1342 : Histone H2A type 3 on eGFR
MR Method
Inverse variance weighted
MR Egger
480
440
400
360
1/SE_IV
−0.006
−0.003
0.000
0.003
β_IV

Batch 2−1343 : Hexokinase−1 on eGFR

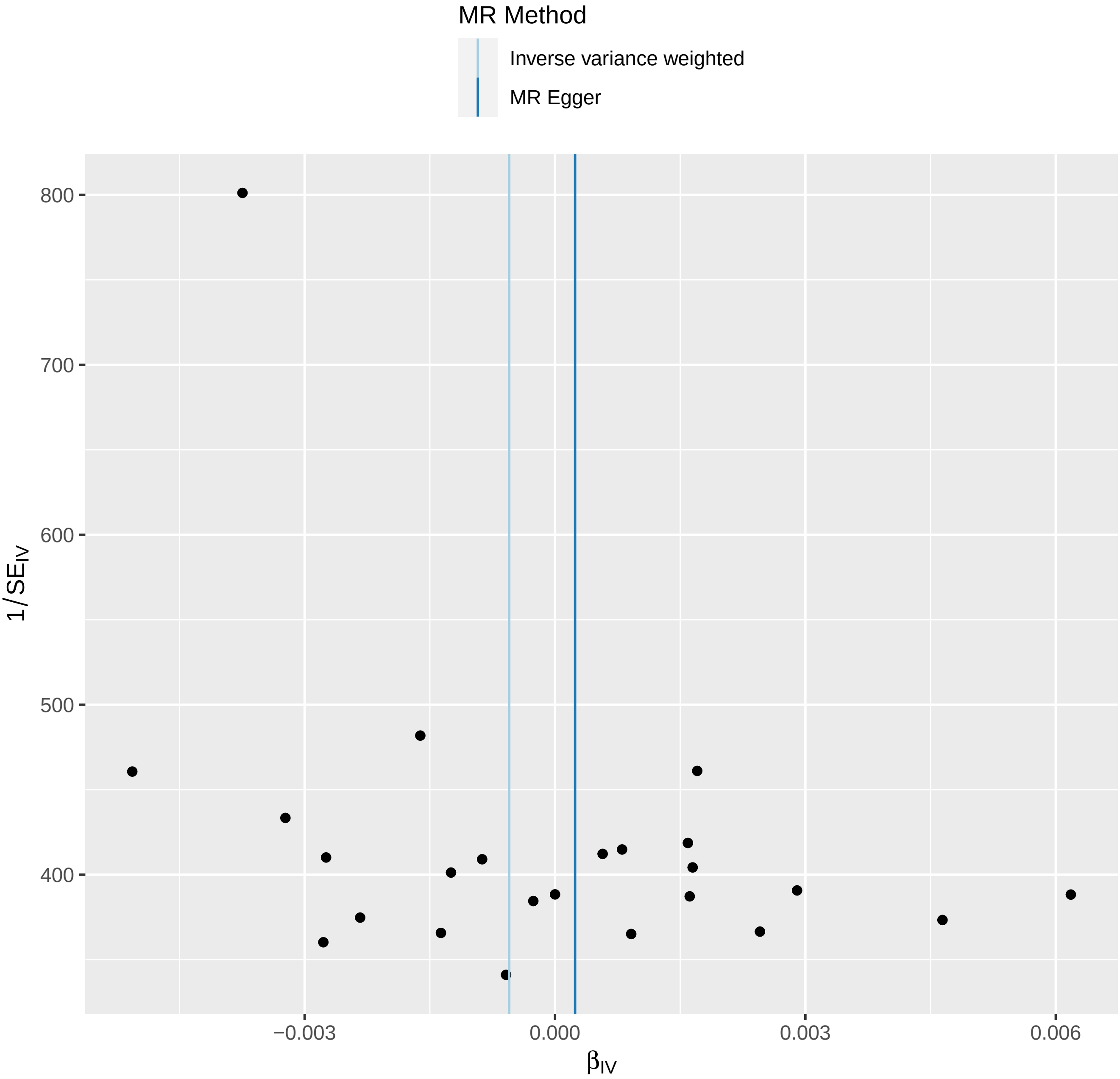

Batch 2−1344 : Hexokinase−2 on eGFR

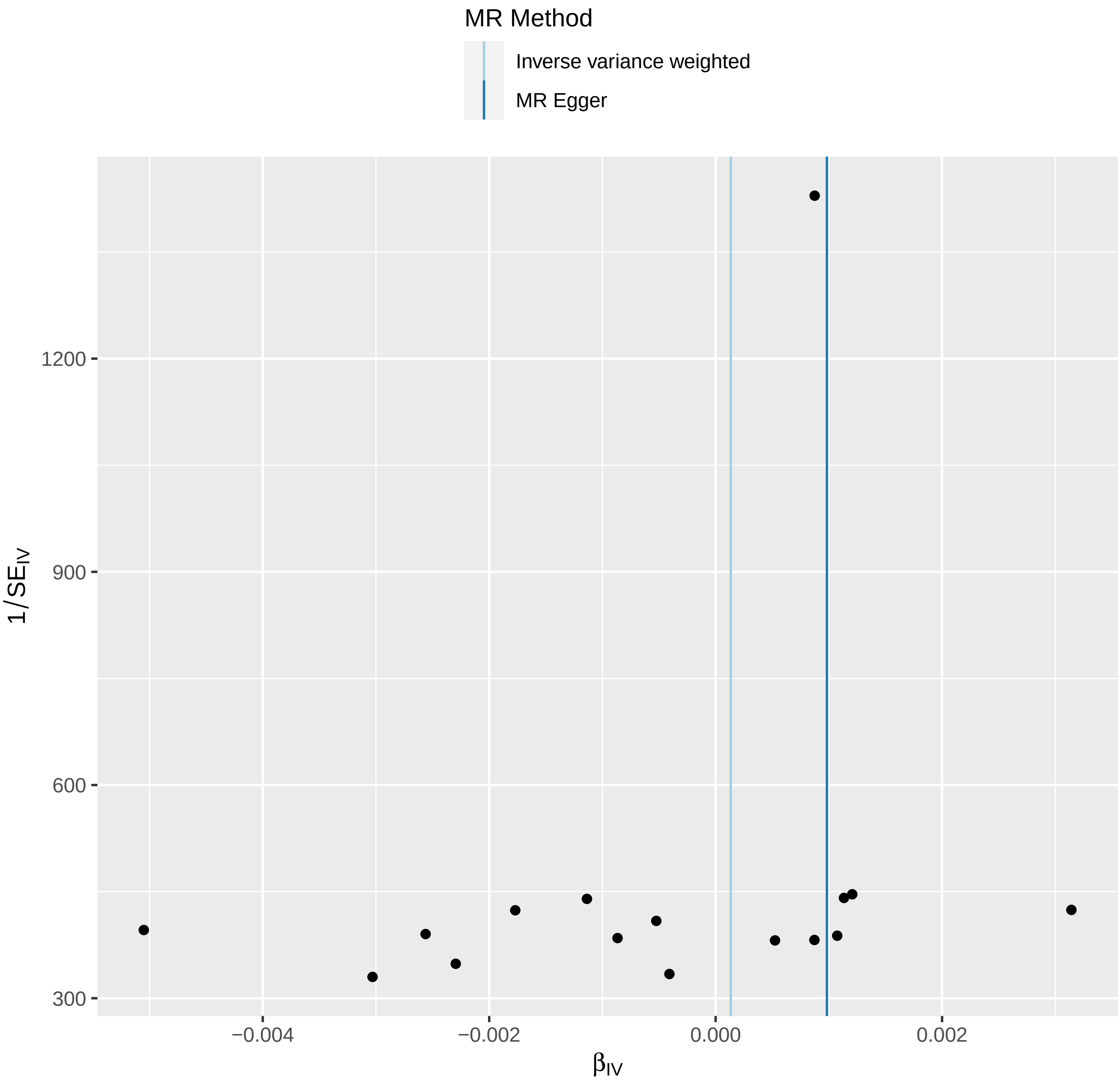

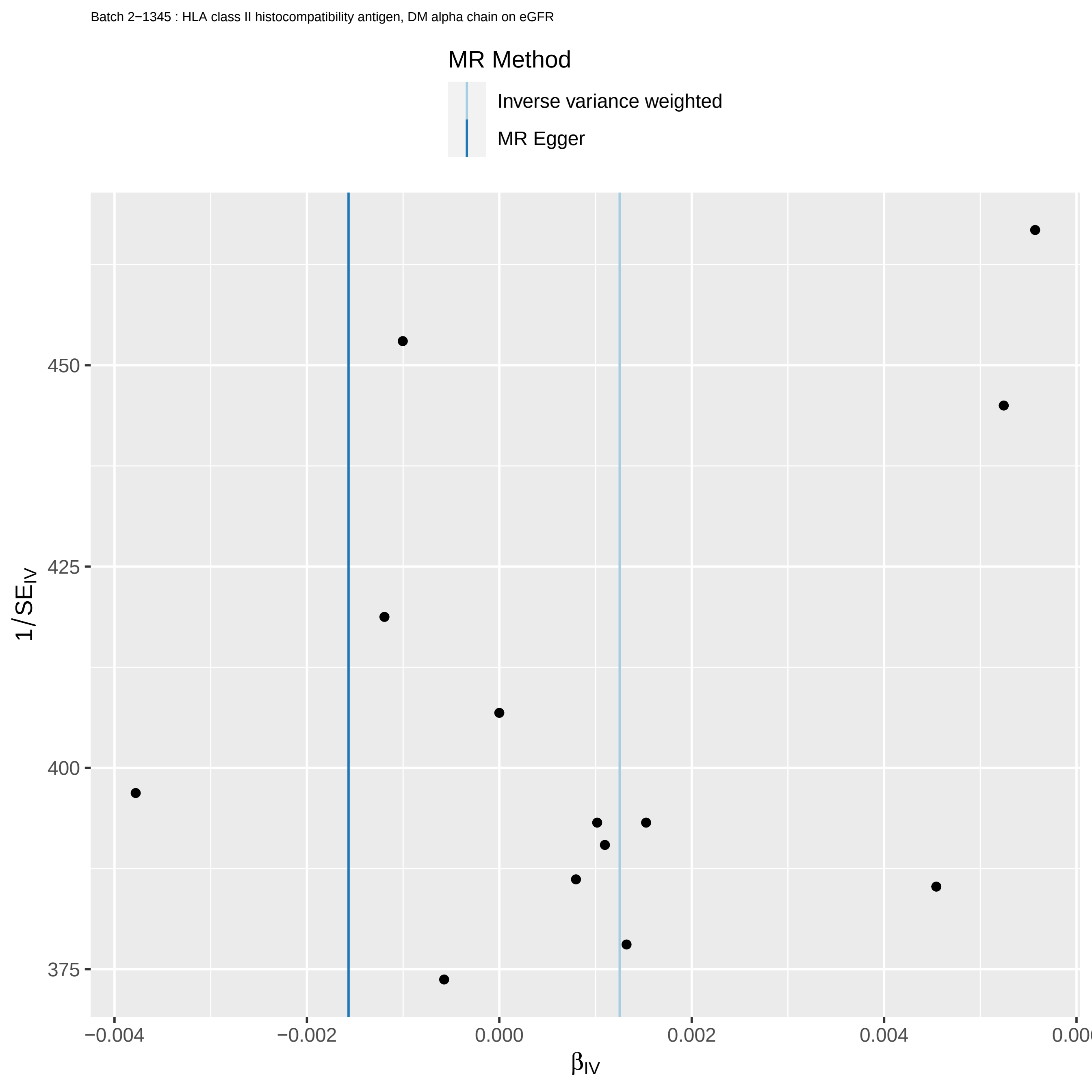

Batch 2−1345 : HLA class II histocompatibility antigen, DM alpha chain on eGFR
MR Method
Inverse variance weighted
MR Egger
450
425
400
375
1/SE_IV
−0.004
−0.002
0.000
0.002
0.004
β_IV

Batch 2−1346 : HLA class II histocompatibility antigen, DP beta 1 chain on eGFR

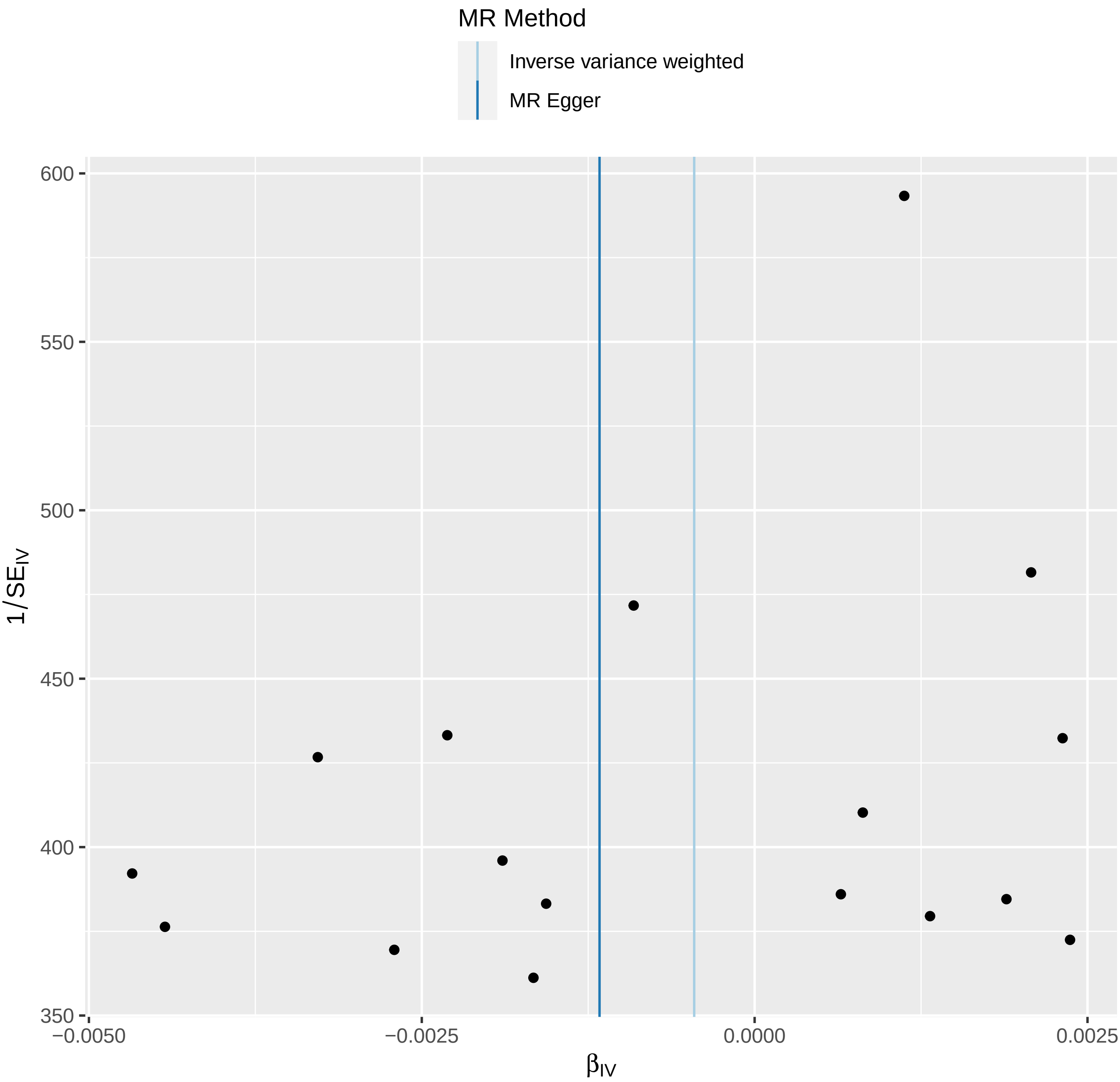

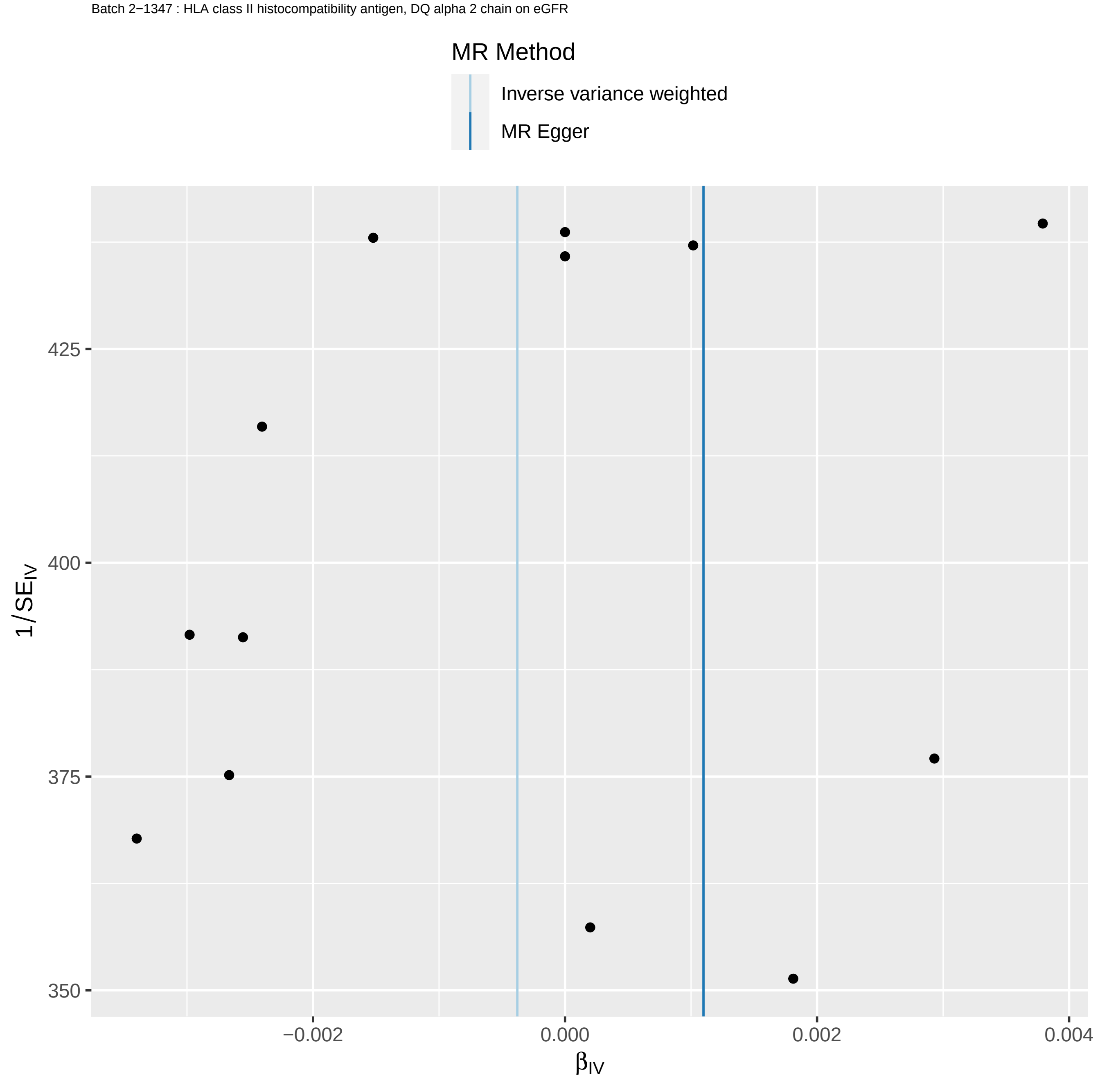
Batch 2−1347 : HLA class II histocompatibility antigen, DQ alpha 2 chain on eGFR
MR Method
Inverse variance weighted
MR Egger
425
400
375
350
1/SE_IV
−0.002
0.000
0.002
0.004
β_IV

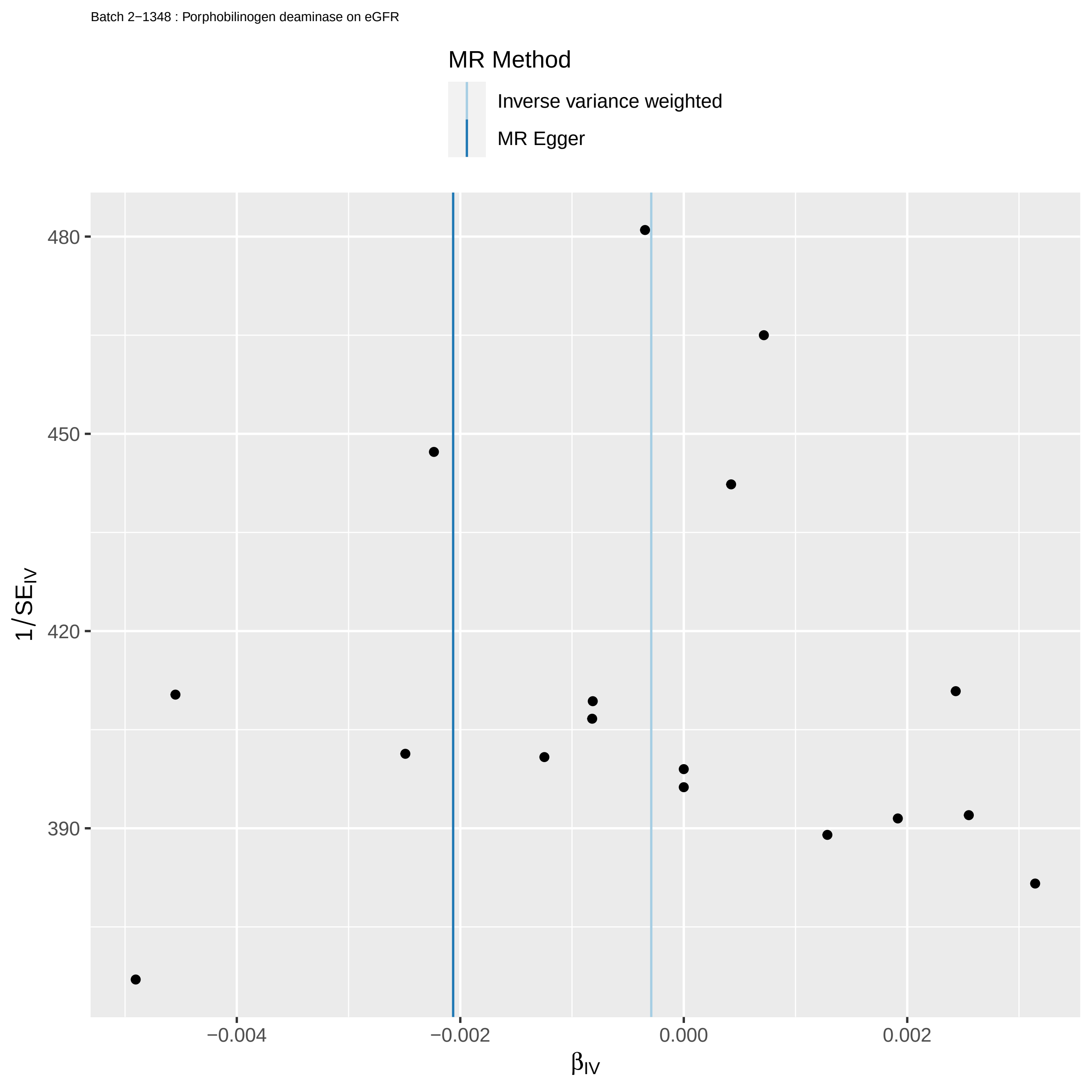

Batch 2−1348 : Porphobilinogen deaminase on eGFR
MR Method
Inverse variance weighted
MR Egger
480
450
420
390
1/SE$_{IV}$
−0.004
−0.002
0.000
0.002
β$_{IV}$

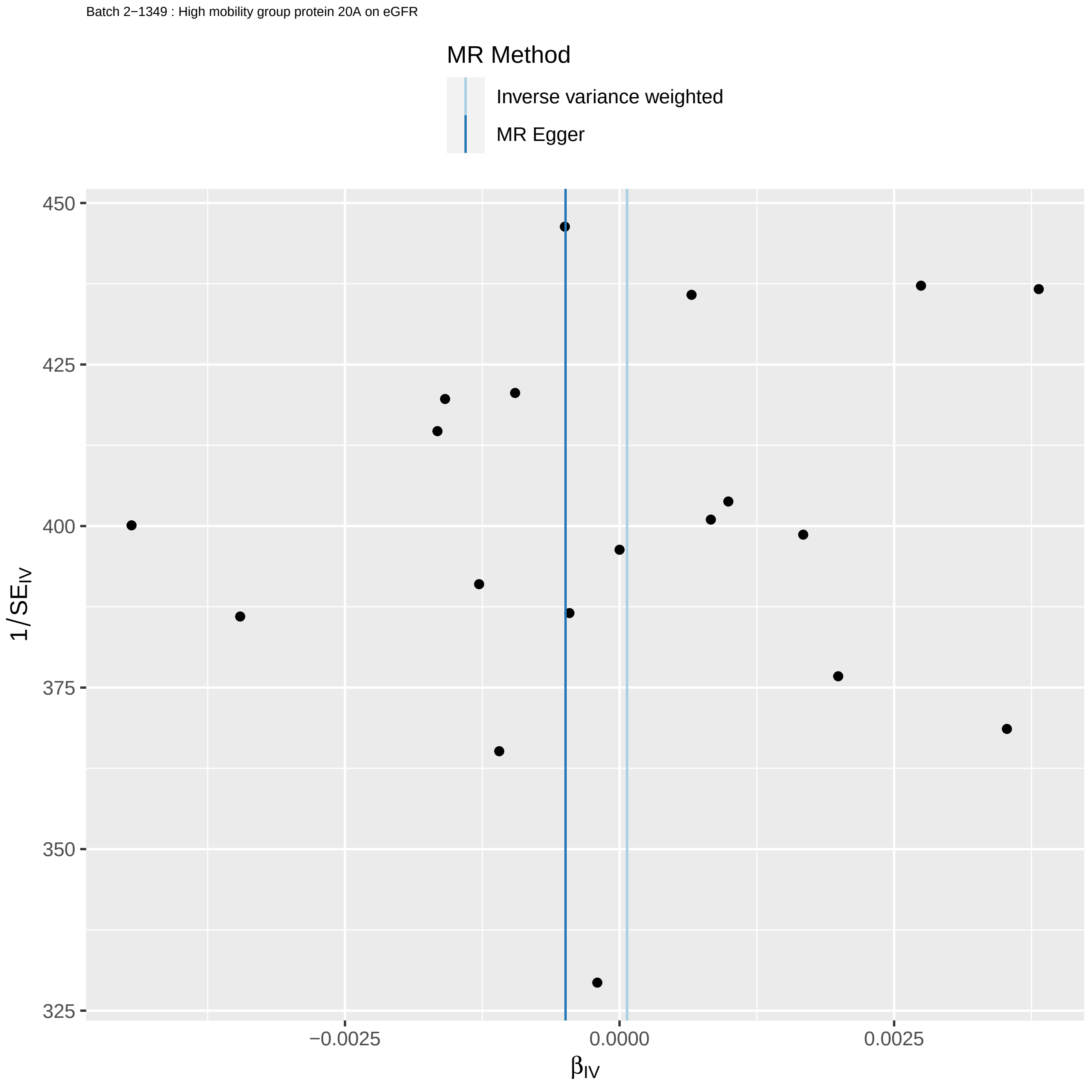

Batch 2−1349 : High mobility group protein 20A on eGFR
MR Method
Inverse variance weighted
MR Egger
450
425
400
375
350
325
1/SE_IV
−0.0025
0.0000
0.0025
β_IV

Batch 2−1350 : SWI/SNF−related matrix−associated actin−dependent regulator of chromatin subfamily E member 1−related on eGFR

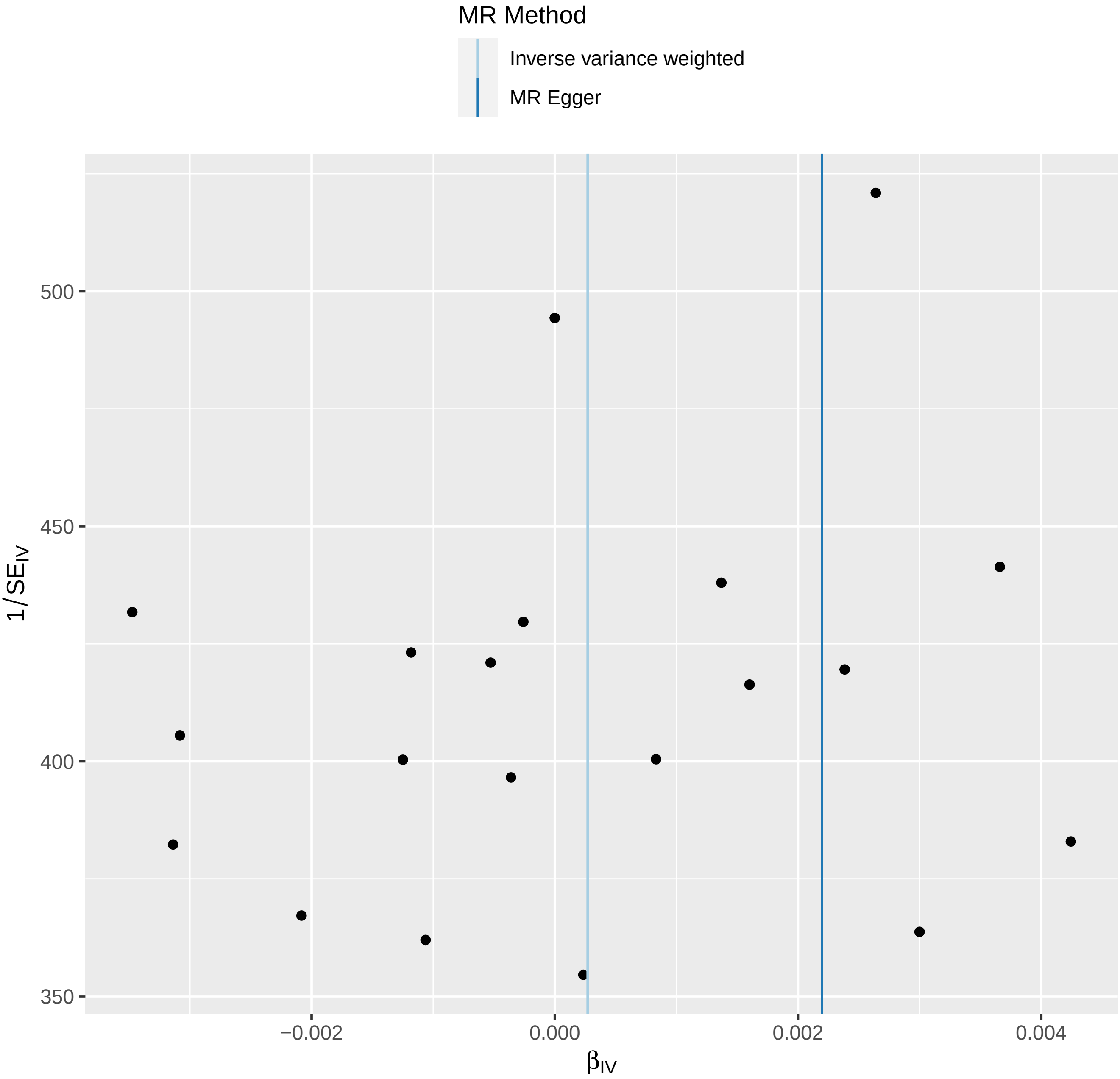

Batch 2−1351 : High mobility group protein B1 on eGFR

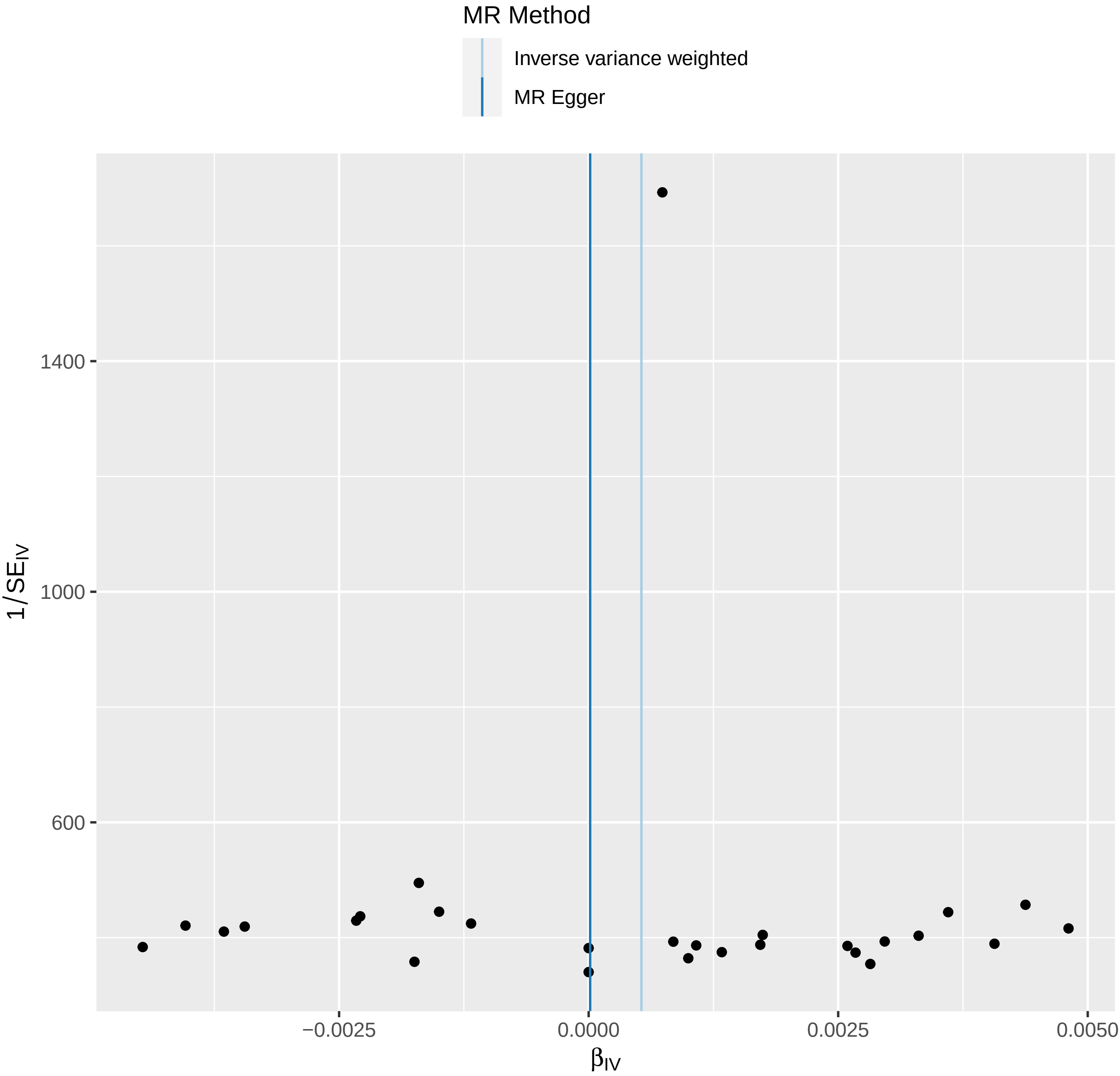

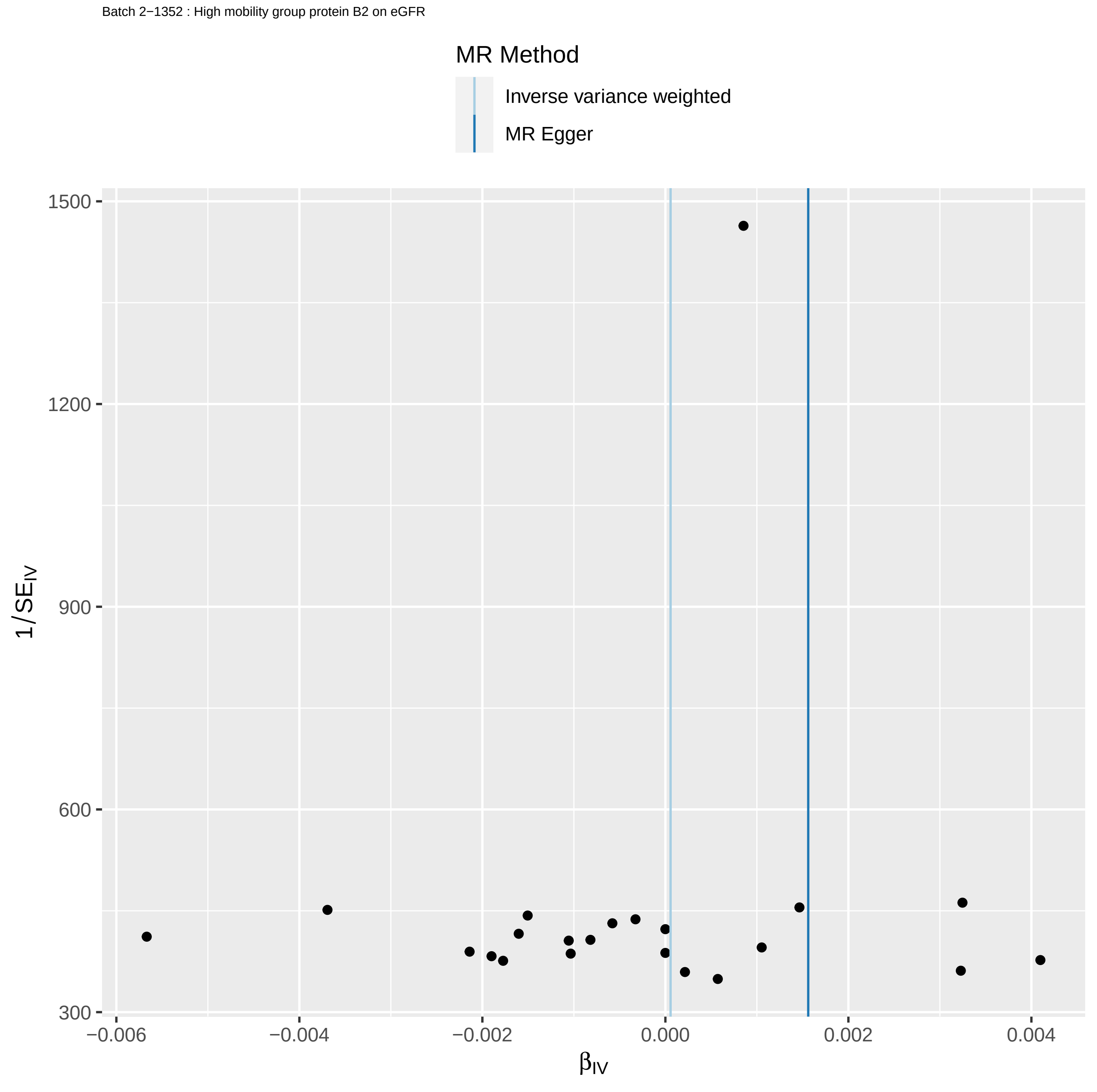

Batch 2−1352 : High mobility group protein B2 on eGFR
MR Method
Inverse variance weighted
MR Egger
1500
1200
900
600
300
1/SE_IV
−0.006
−0.004
−0.002
0.000
0.002
0.004
β_IV

Batch 2−1353 : High mobility group protein B3 on eGFR

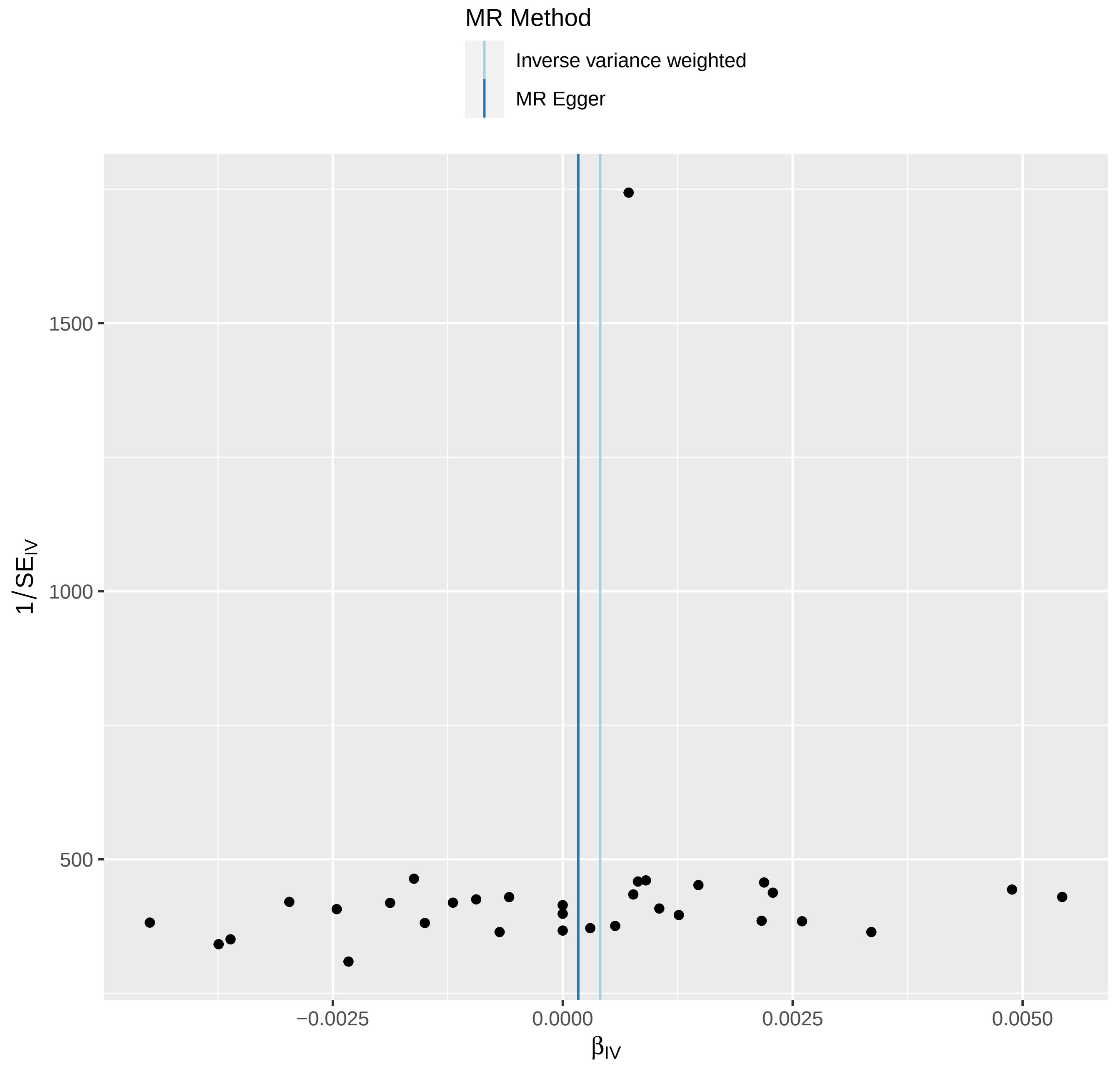

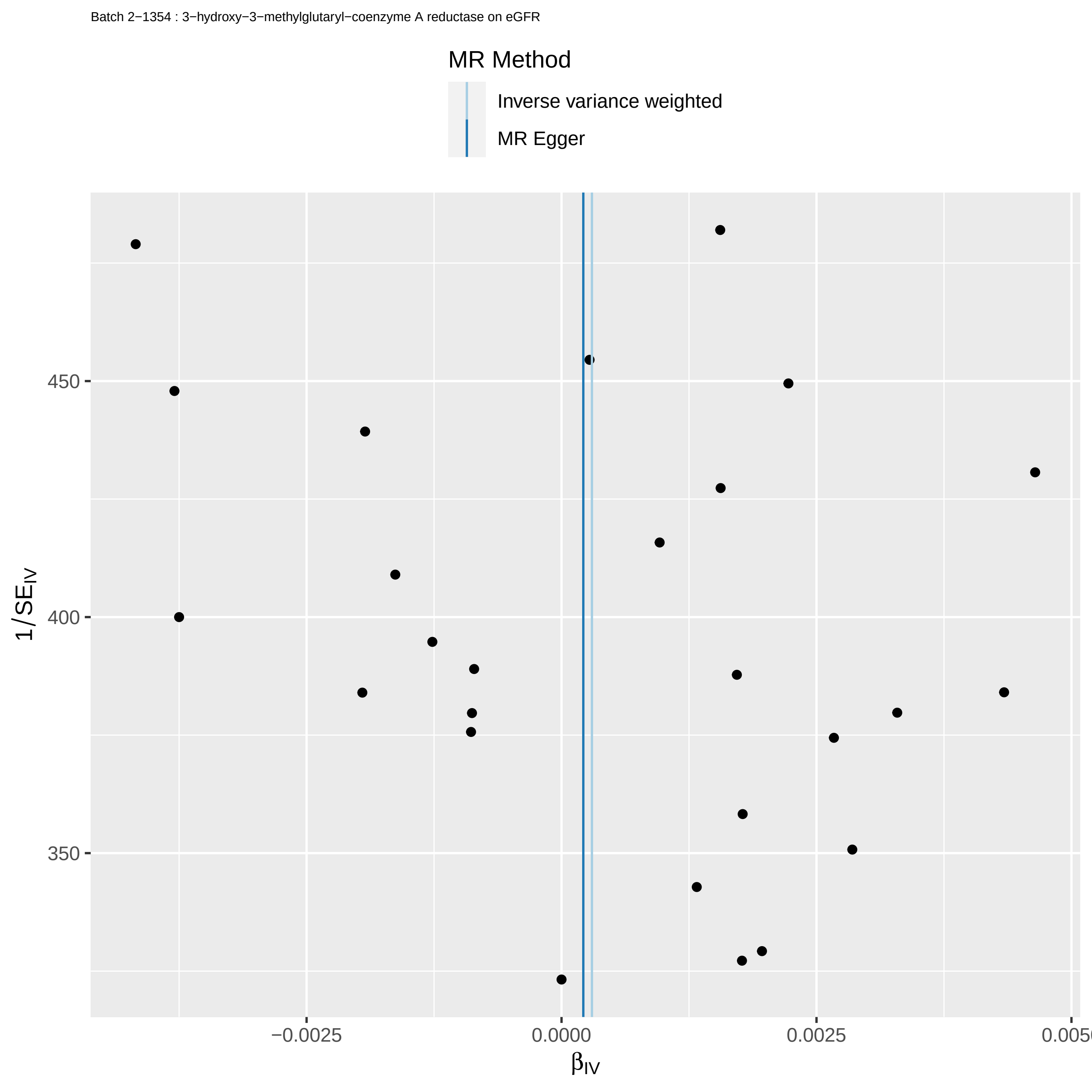
Batch 2−1354 : 3−hydroxy−3−methylglutaryl−coenzyme A reductase on eGFR
MR Method
Inverse variance weighted
MR Egger
450
400
350
1/SE_IV
−0.0025
0.0000
0.0025
0.0050
β_IV

Batch 2−1355 : Hydroxymethylglutaryl−CoA synthase, cytoplasmic on eGFR

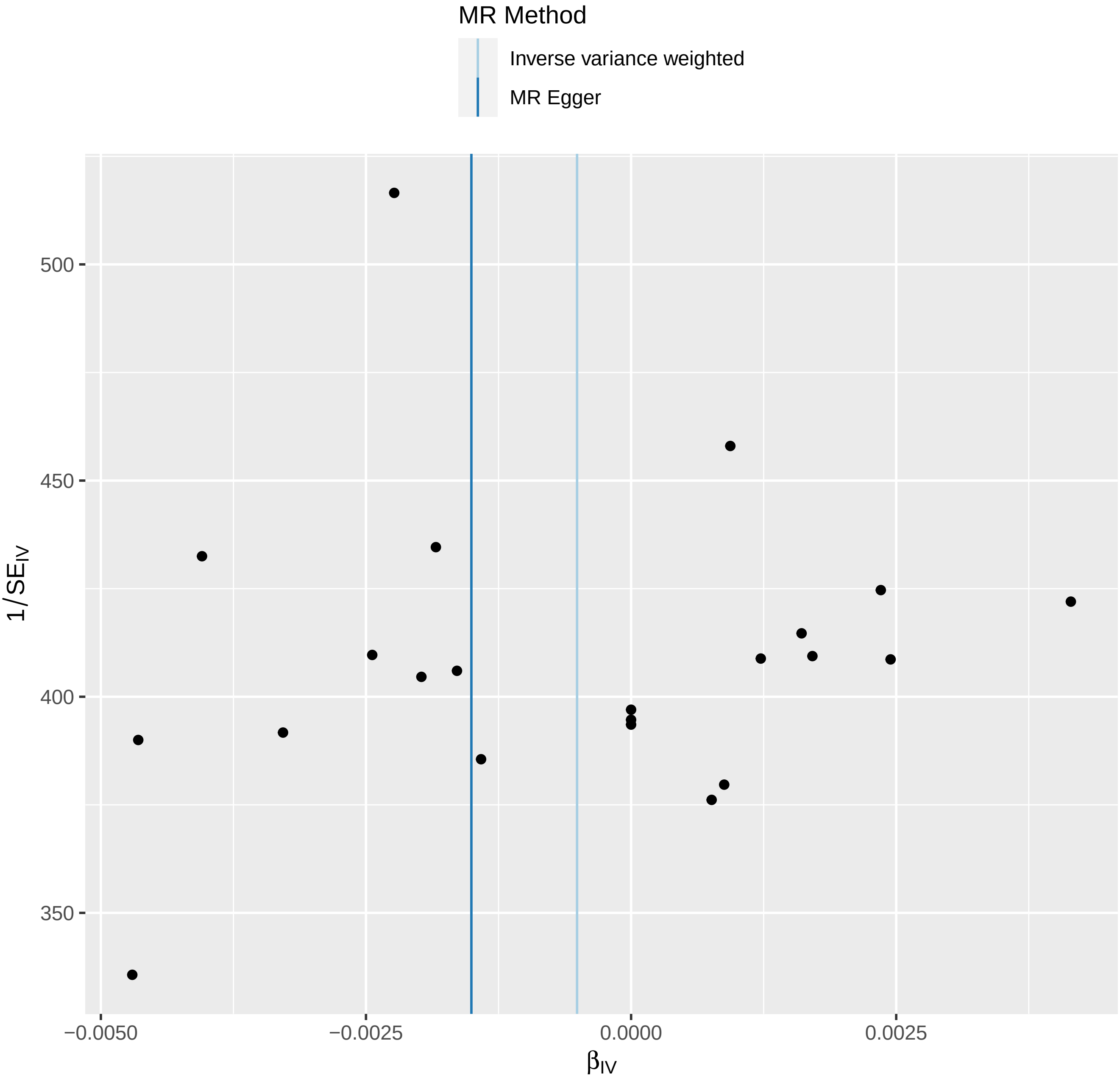

Batch 2−1356 : Hydroxymethylglutaryl−CoA synthase, mitochondrial on eGFR

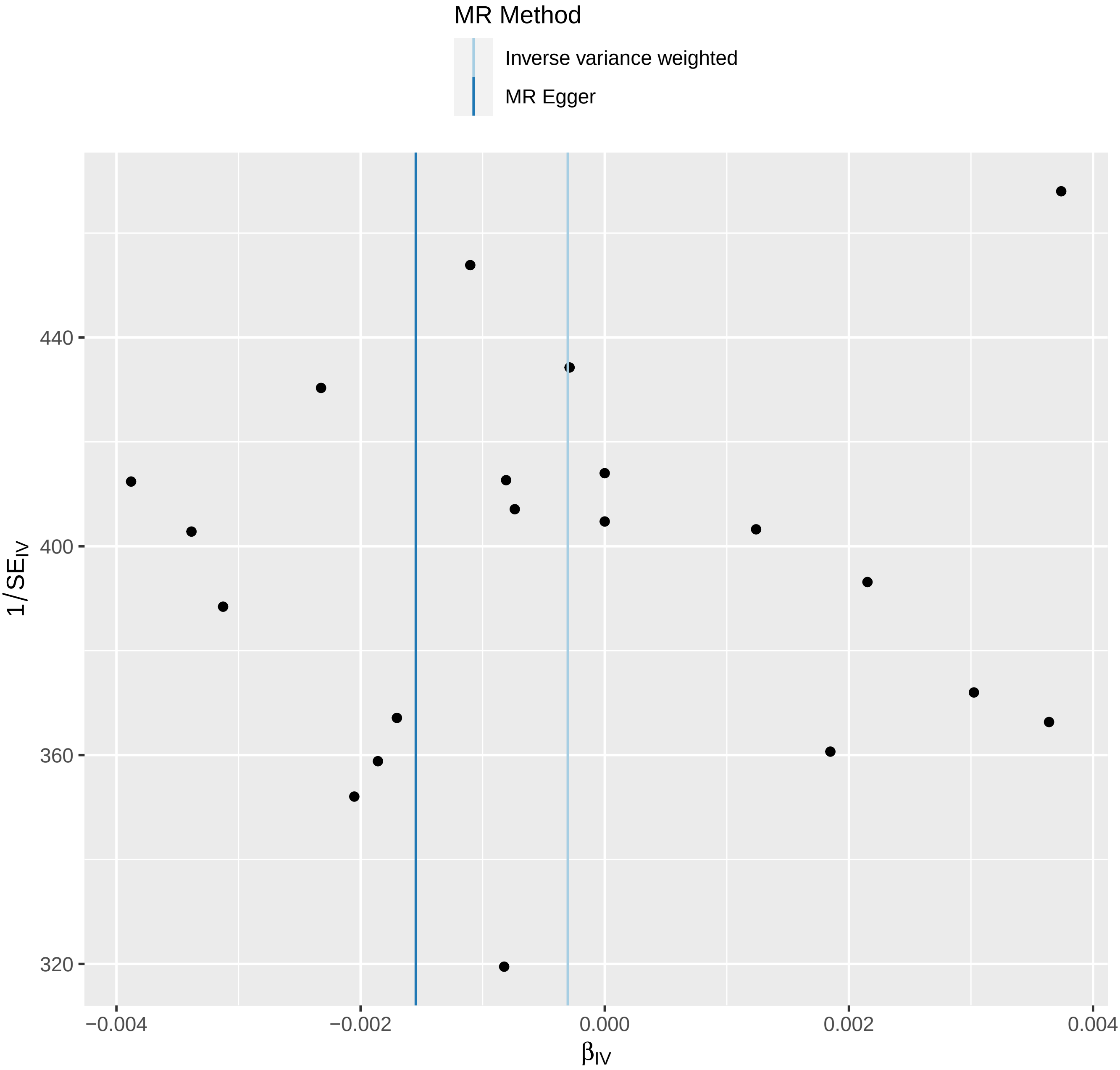

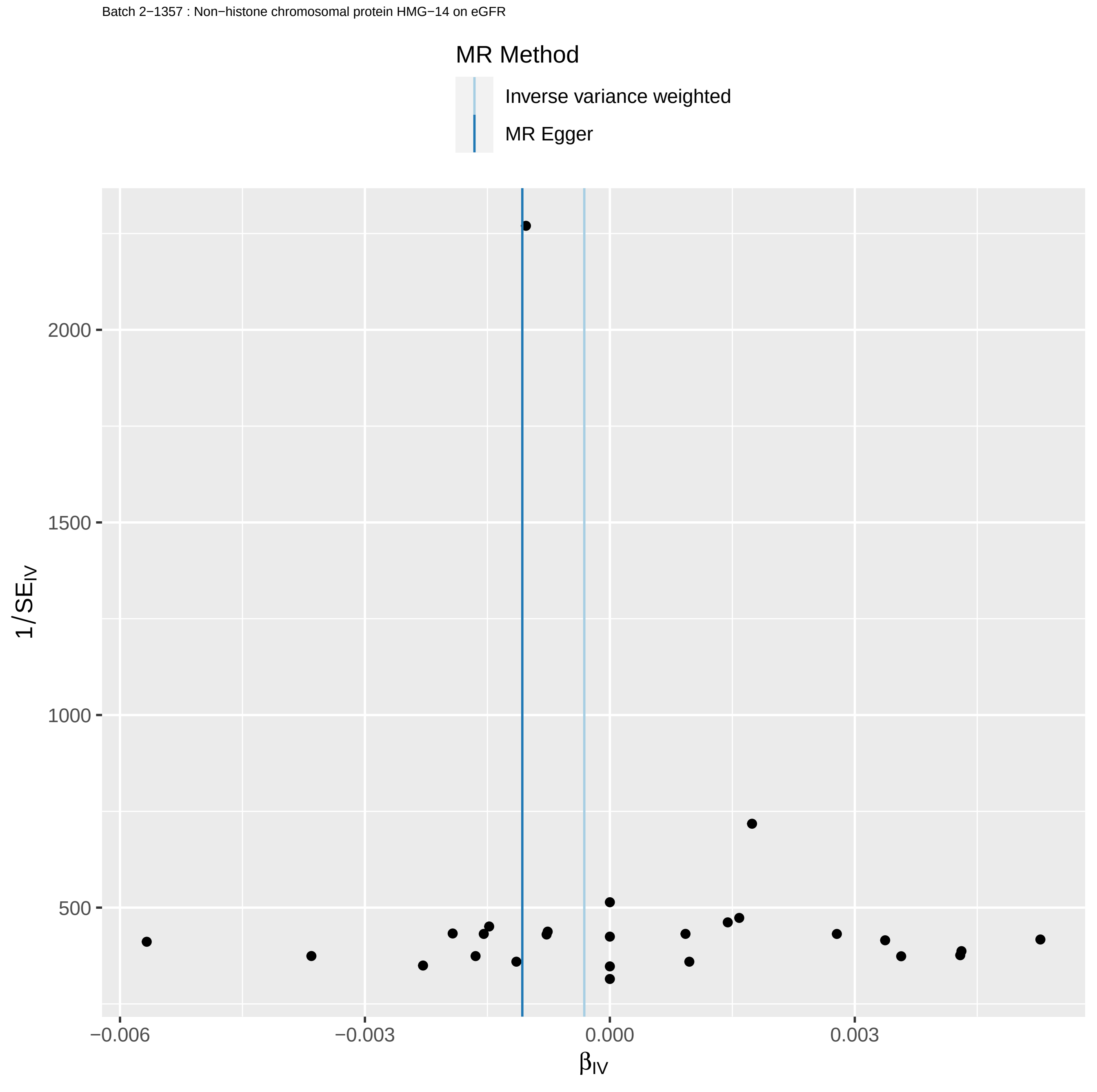
Batch 2−1357 : Non−histone chromosomal protein HMG−14 on eGFR
MR Method
Inverse variance weighted
MR Egger
2000
1500
1000
500
1/SE$_{IV}$
−0.006
−0.003
0.000
0.003
β$_{IV}$

Batch 2−1358 : Heme oxygenase 2 on eGFR

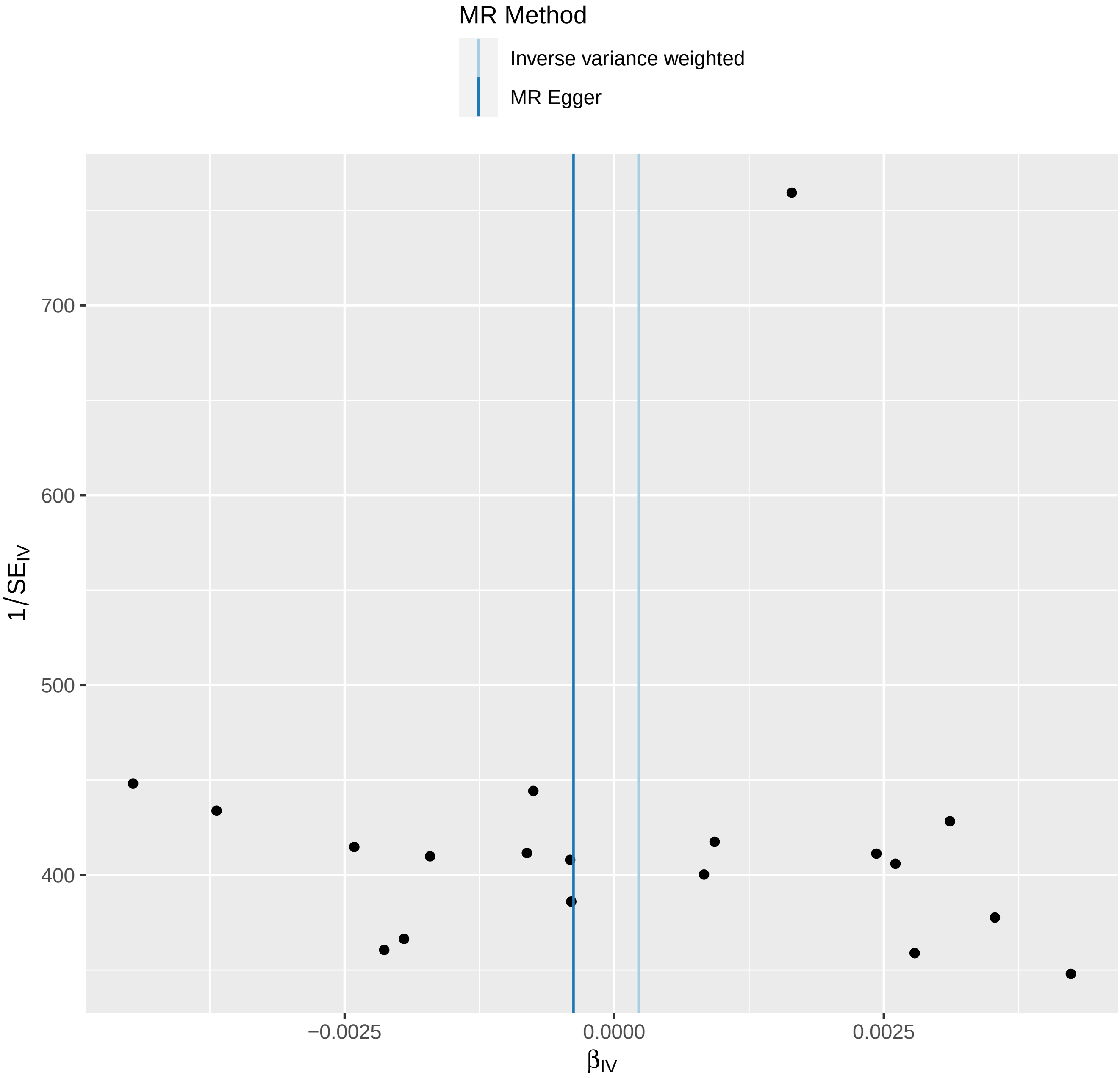

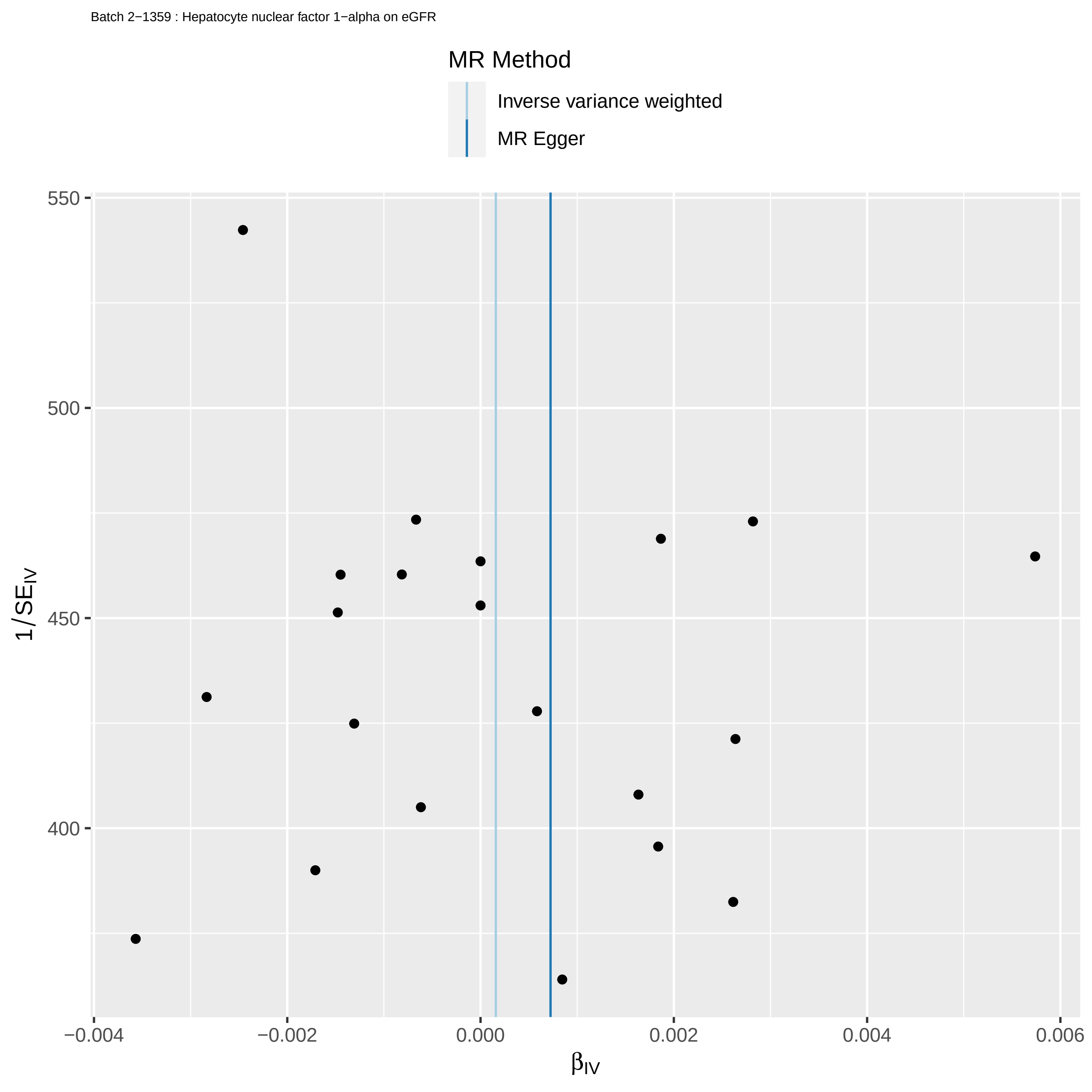

Batch 2−1359 : Hepatocyte nuclear factor 1−alpha on eGFR
MR Method
Inverse variance weighted
MR Egger
550
500
450
400
1/SE_IV
−0.004
−0.002
0.000
0.002
0.004
0.006
β_IV

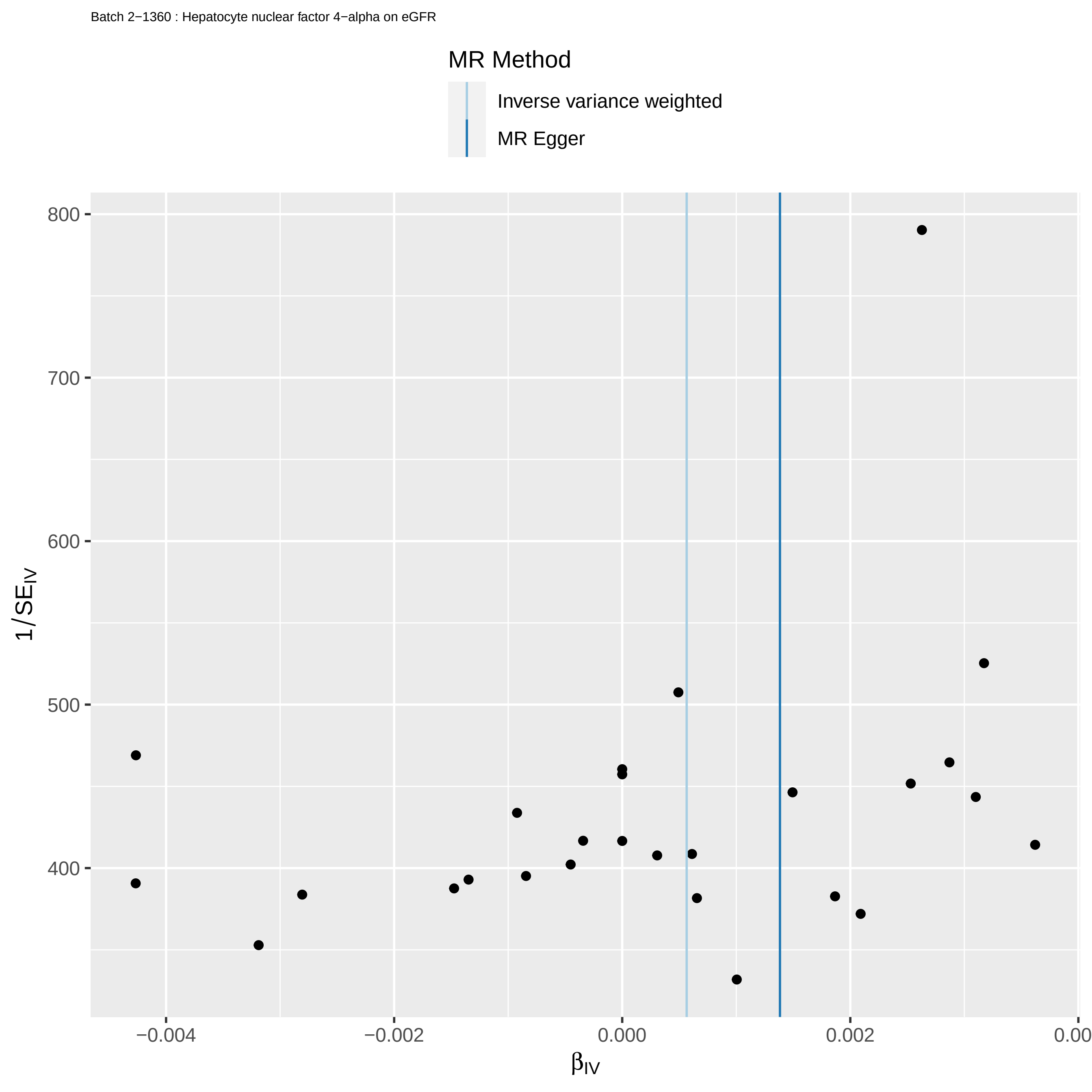
Batch 2−1360 : Hepatocyte nuclear factor 4−alpha on eGFR
MR Method
Inverse variance weighted
MR Egger
800
700
600
500
400
1/SE$_{IV}$
−0.004
−0.002
0.000
0.002
0.00
β$_{IV}$

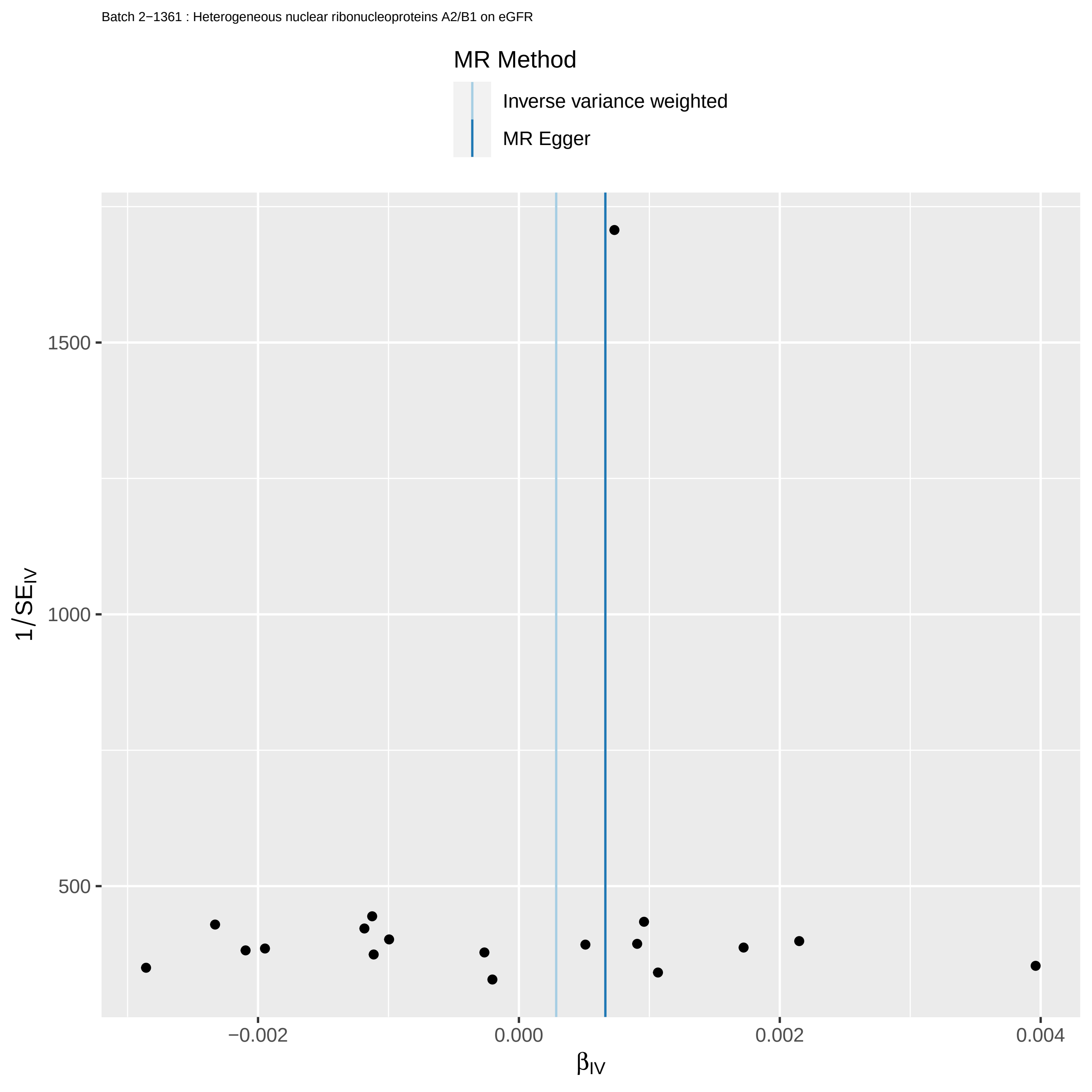
Batch 2−1361 : Heterogeneous nuclear ribonucleoproteins A2/B1 on eGFR
MR Method
Inverse variance weighted
MR Egger
1500
1000
500
1/SE_IV
−0.002
0.000
0.002
0.004
β_IV

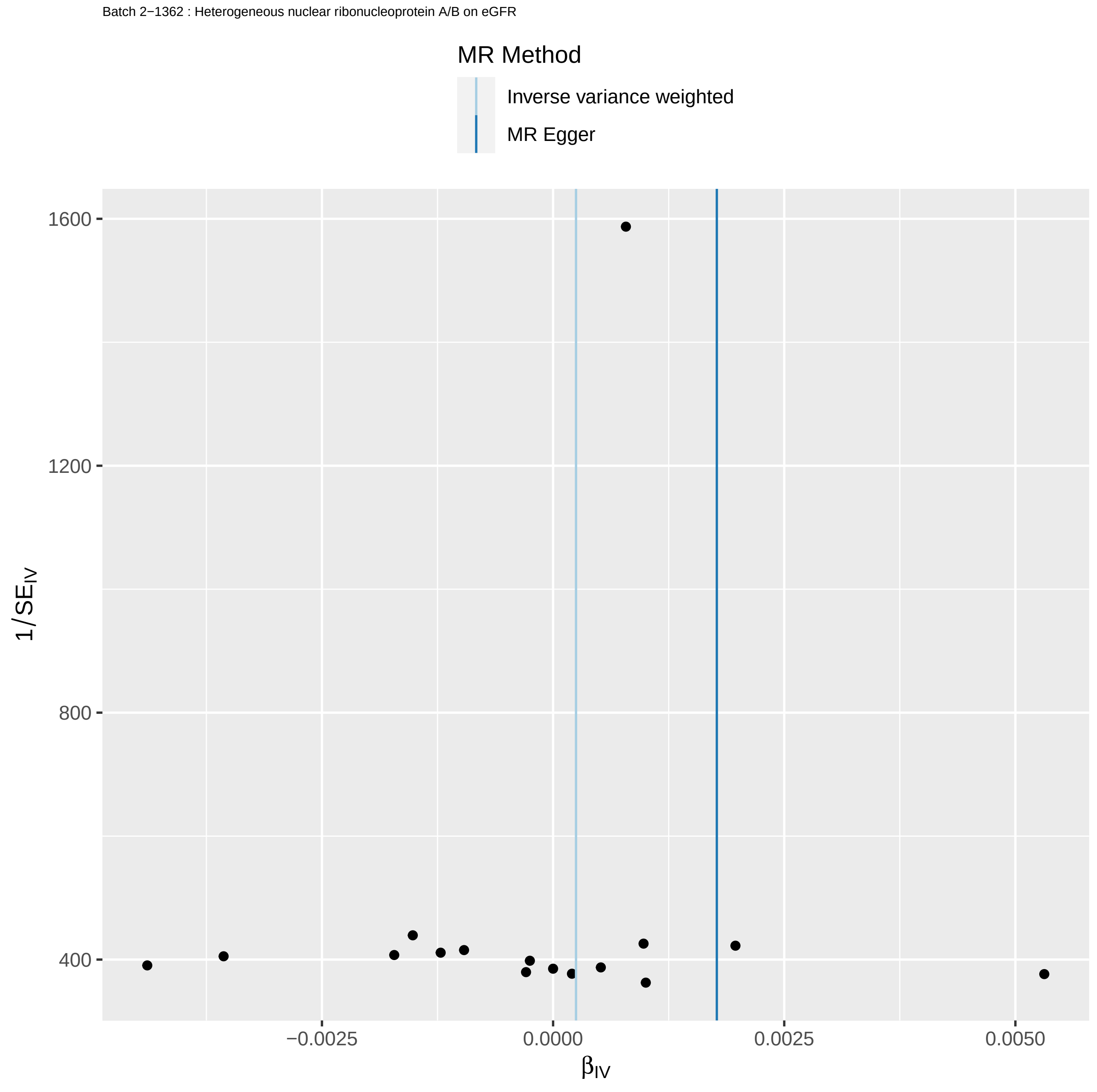

Batch 2−1362 : Heterogeneous nuclear ribonucleoprotein A/B on eGFR
MR Method
Inverse variance weighted
MR Egger
1600
1200
800
400
1/SE_IV
−0.0025
0.0000
0.0025
0.0050
β_IV

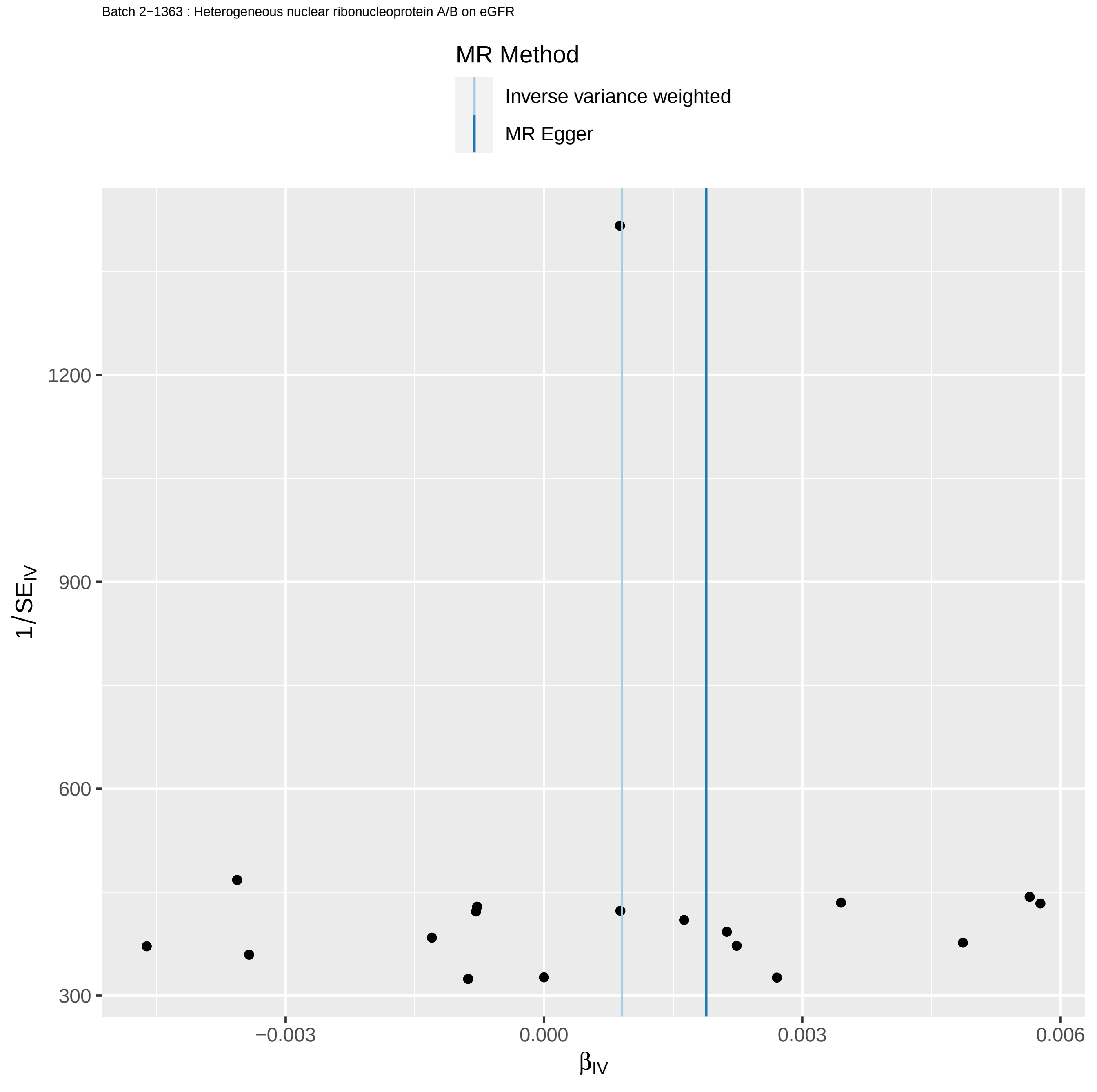
Batch 2−1363 : Heterogeneous nuclear ribonucleoprotein A/B on eGFR
MR Method
Inverse variance weighted
MR Egger
1200
900
600
300
−0.003
0.000
0.003
0.006
1/SE_IV
β_IV

Batch 2−1364 : Heterogeneous nuclear ribonucleoproteins C1/C2 on eGFR

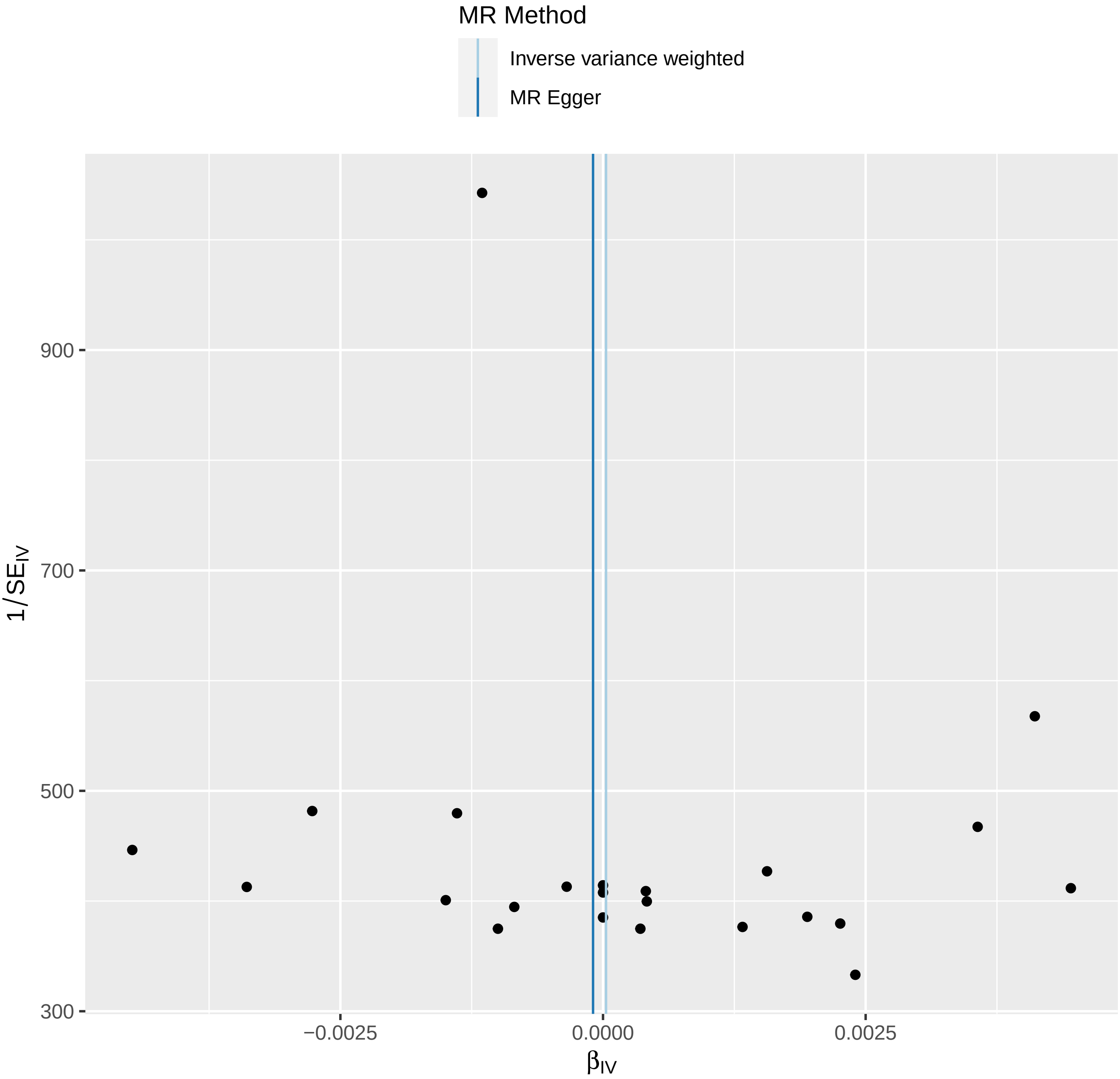

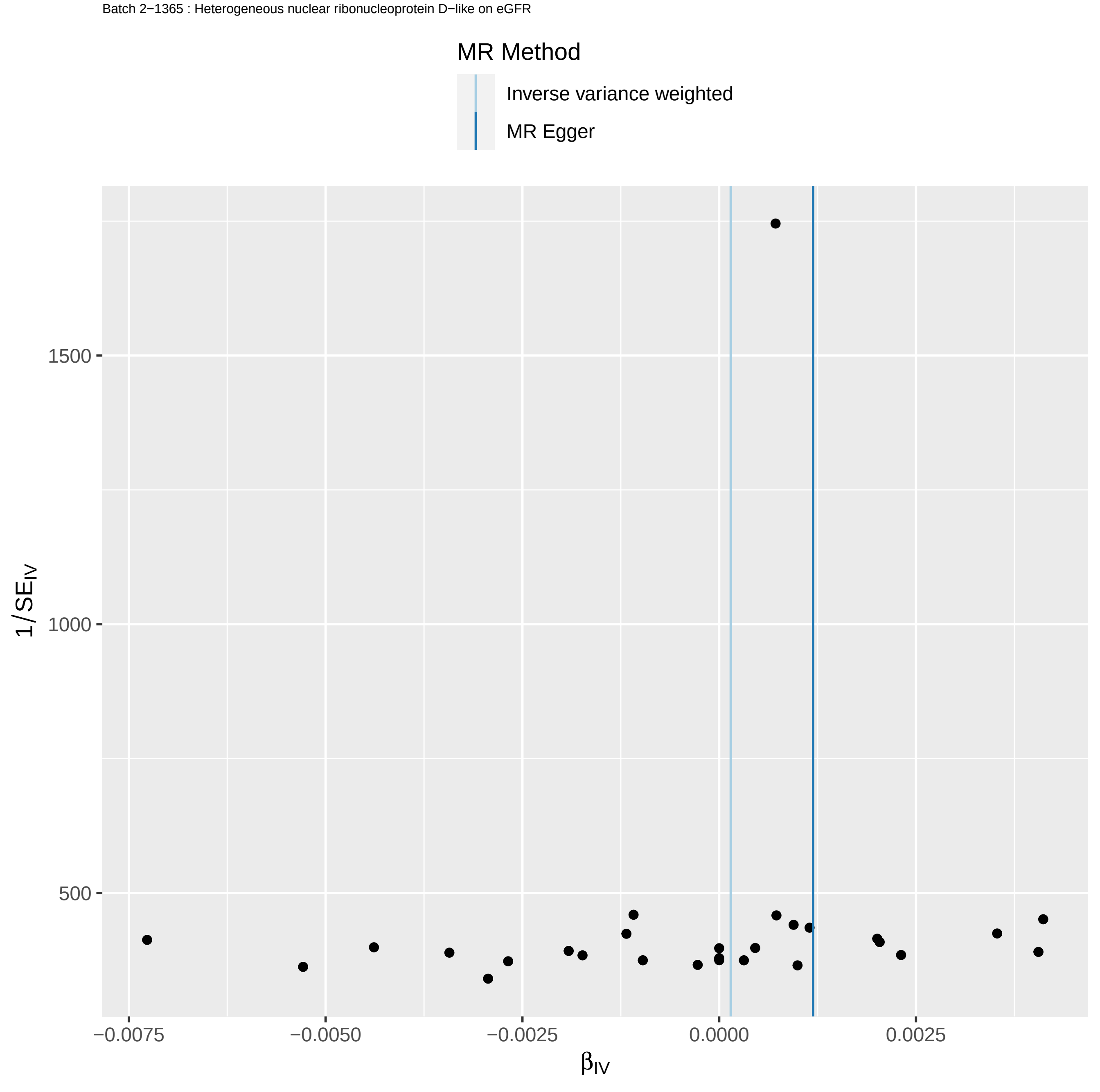

Batch 2−1365 : Heterogeneous nuclear ribonucleoprotein D−like on eGFR
MR Method
Inverse variance weighted
MR Egger
1500
1000
500
1/SE$_{IV}$
−0.0075
−0.0050
−0.0025
0.0000
0.0025
β$_{IV}$

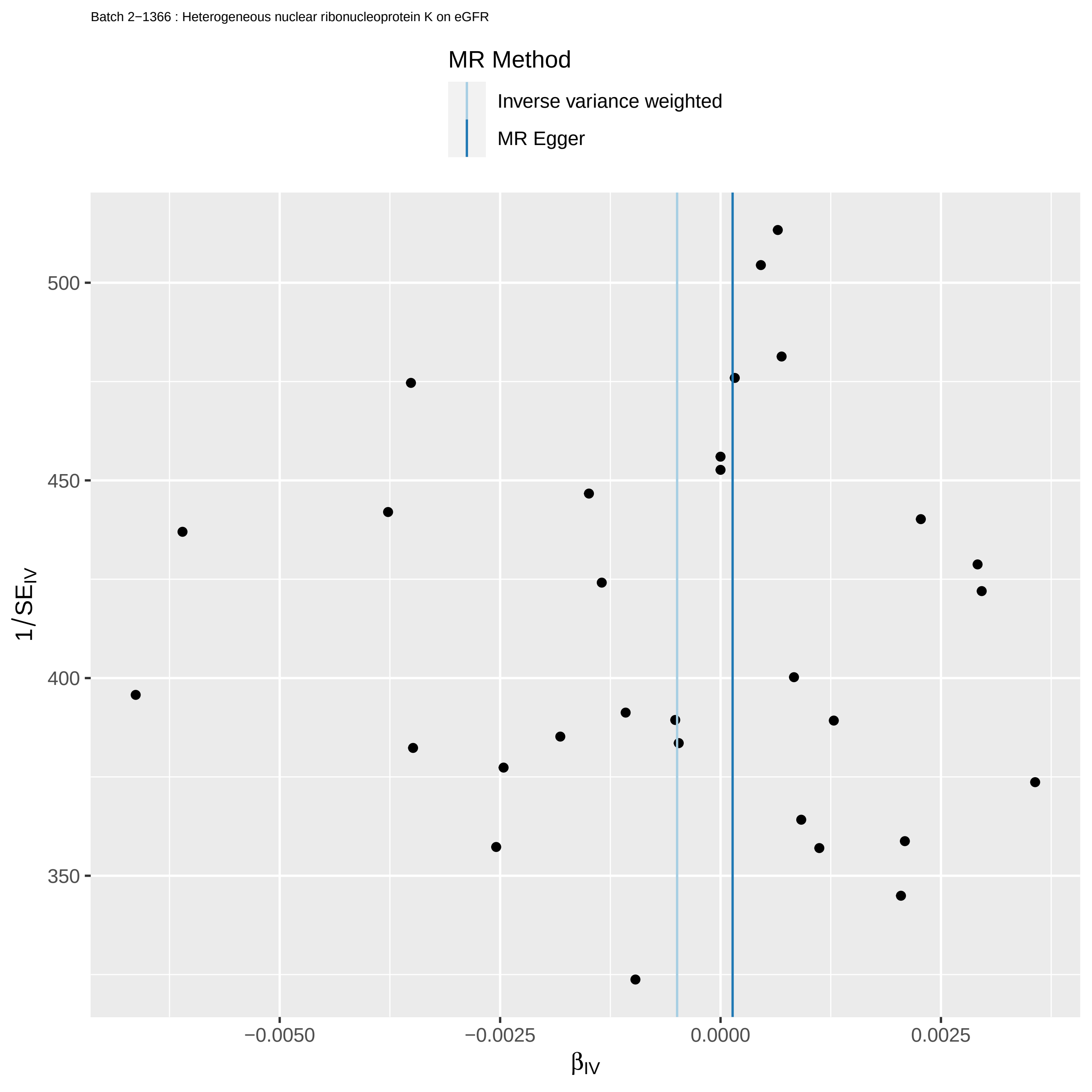

Batch 2−1366 : Heterogeneous nuclear ribonucleoprotein K on eGFR
MR Method
Inverse variance weighted
MR Egger
500
450
400
350
1/SE_IV
−0.0050
−0.0025
0.0000
0.0025
β_IV

Batch 2−1367 : Heterogeneous nuclear ribonucleoprotein M on eGFR

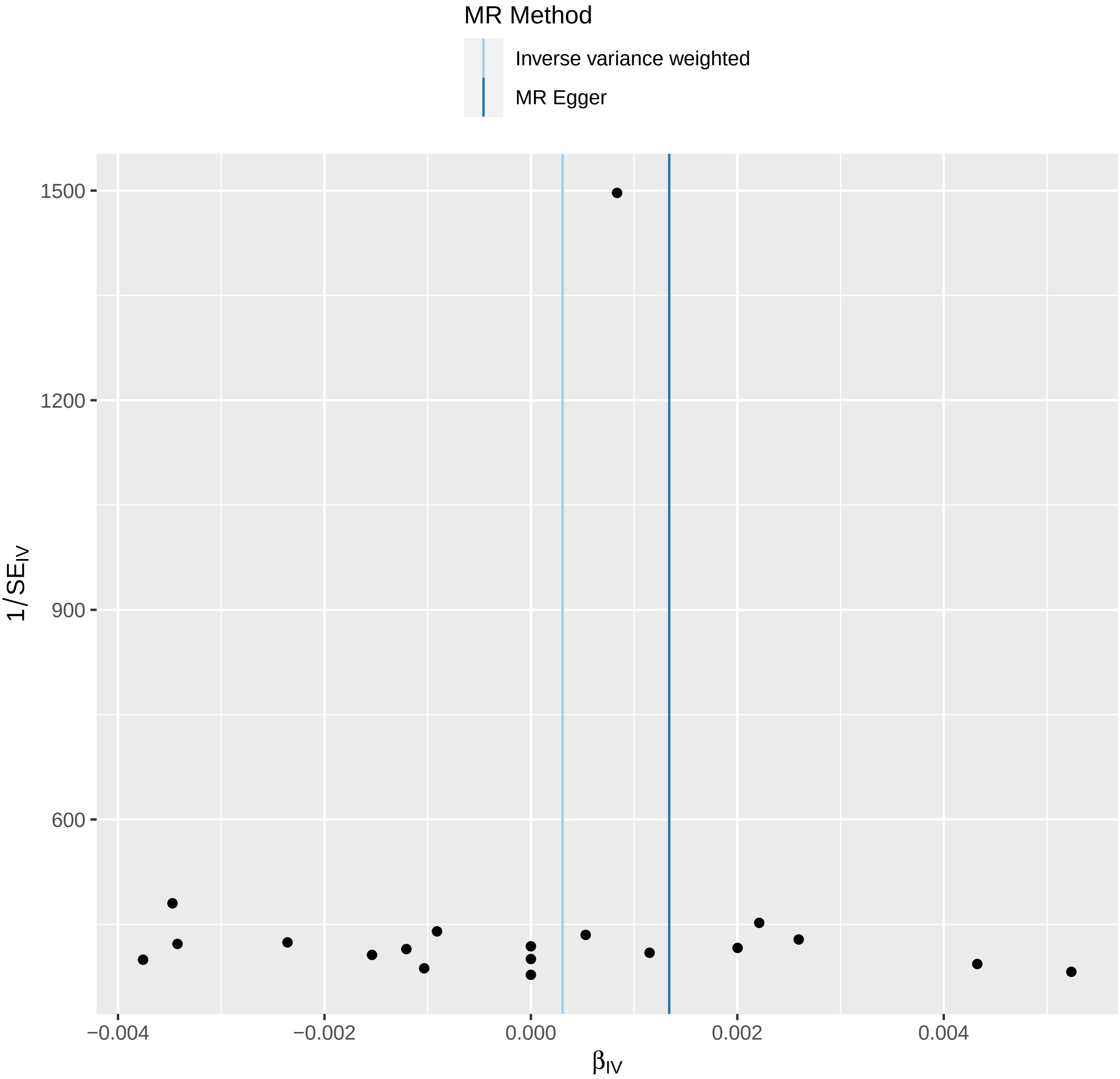

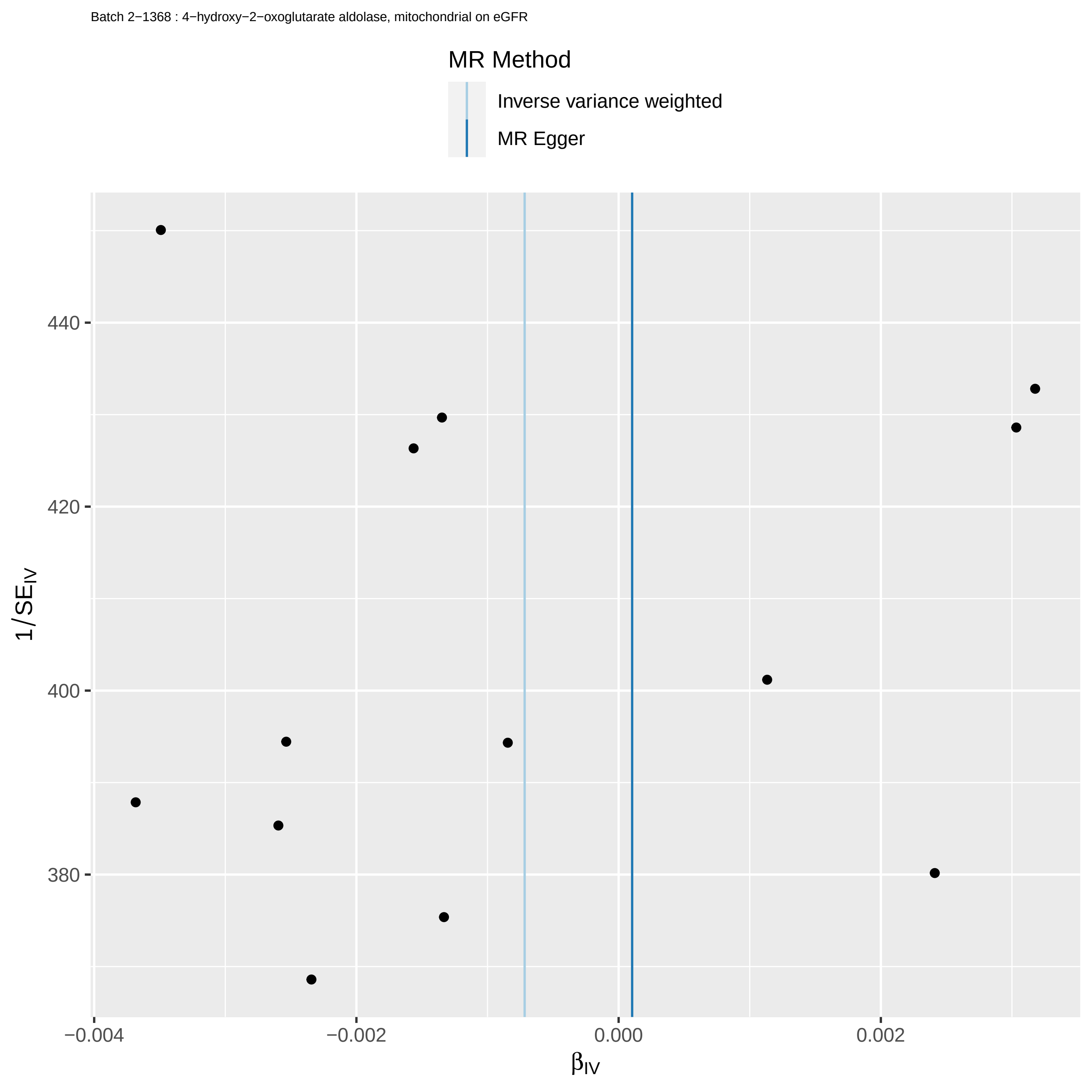

Batch 2−1368 : 4−hydroxy−2−oxoglutarate aldolase, mitochondrial on eGFR
MR Method
Inverse variance weighted
MR Egger
440
420
400
380
1/SE_IV
−0.004
−0.002
0.000
0.002
β_IV

Batch 2−1369 : Haptoglobin on eGFR

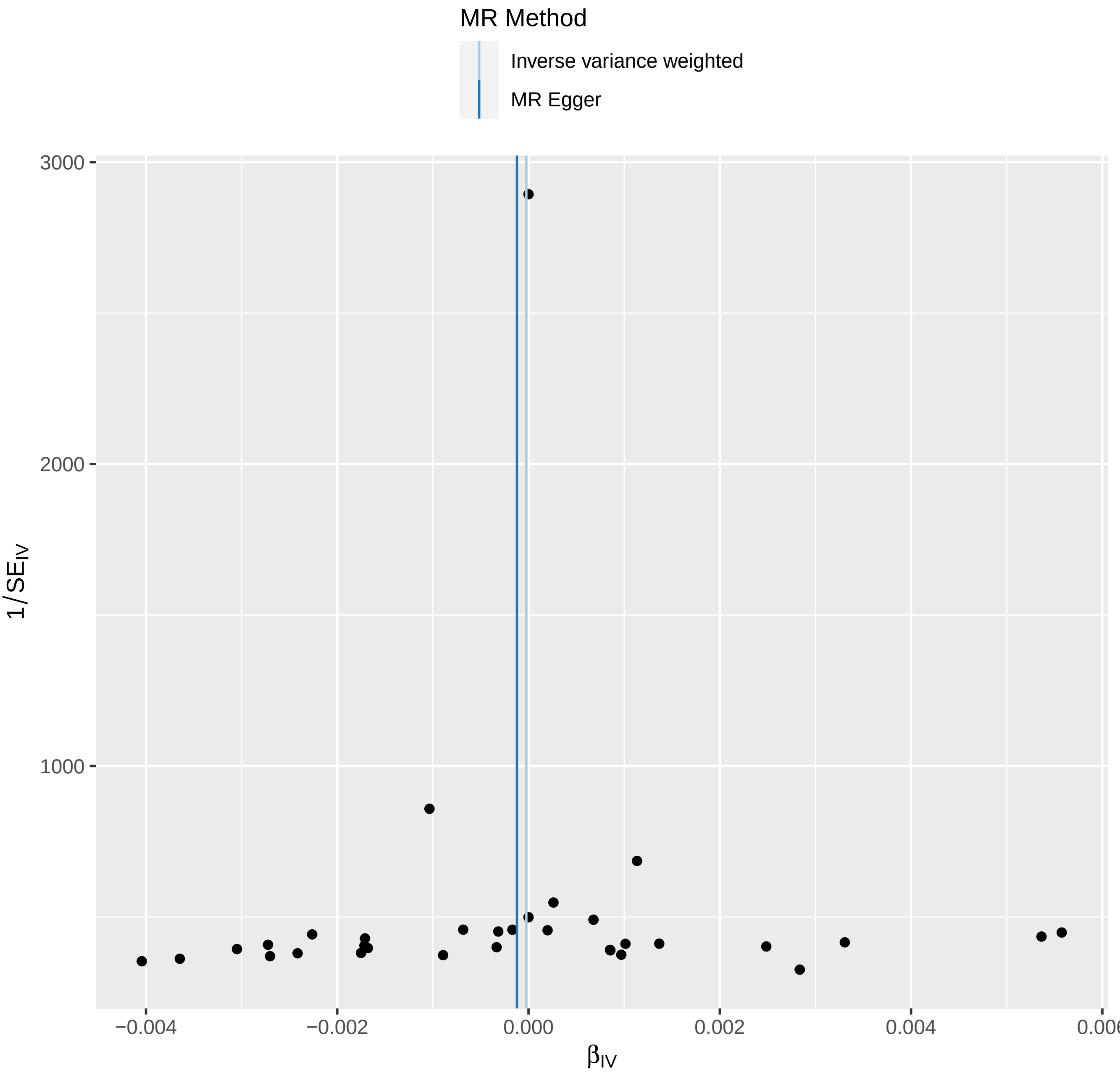

Batch 2−1370 : 15−hydroxyprostaglandin dehydrogenase [NAD(+)] on eGFR

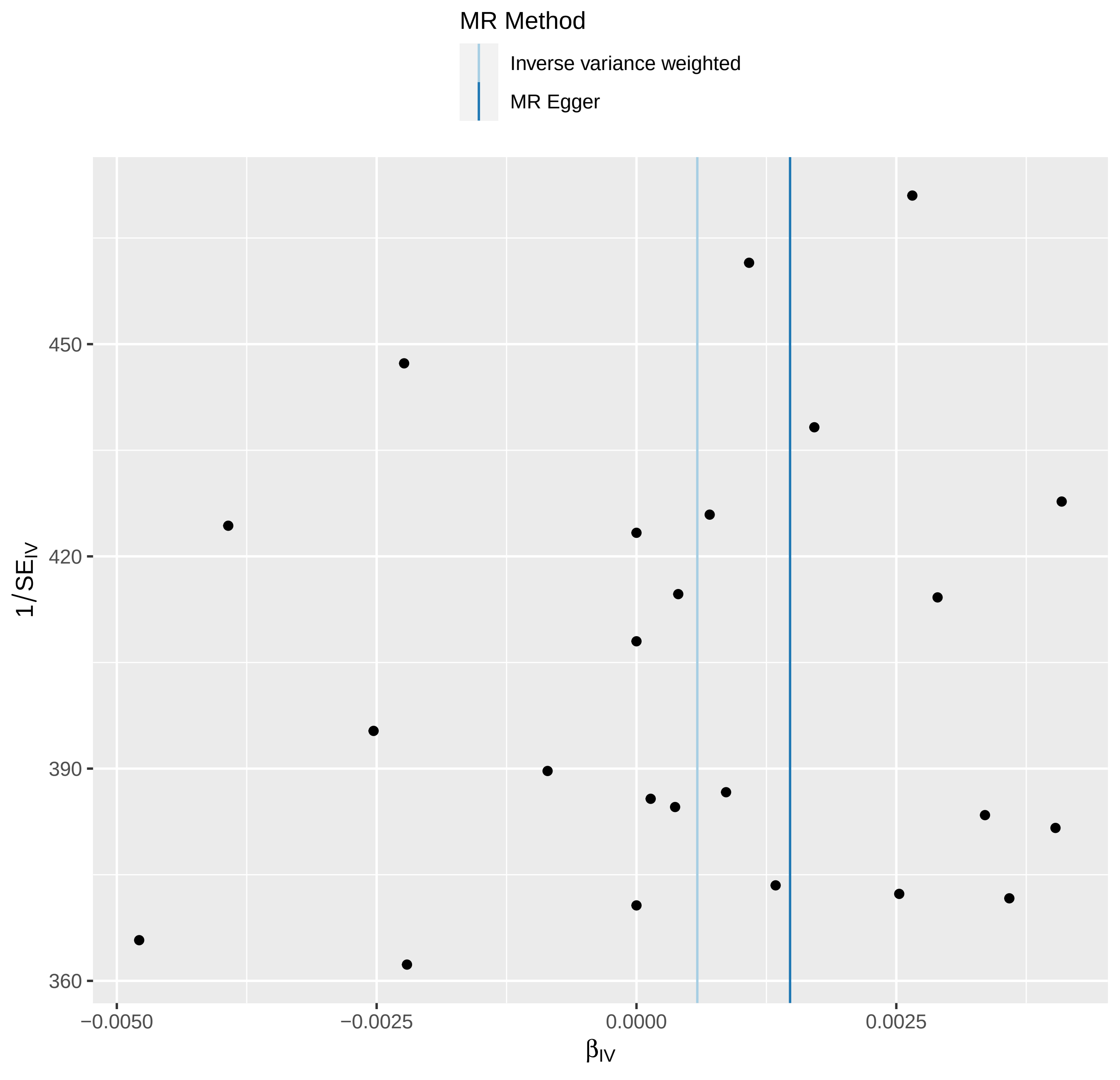

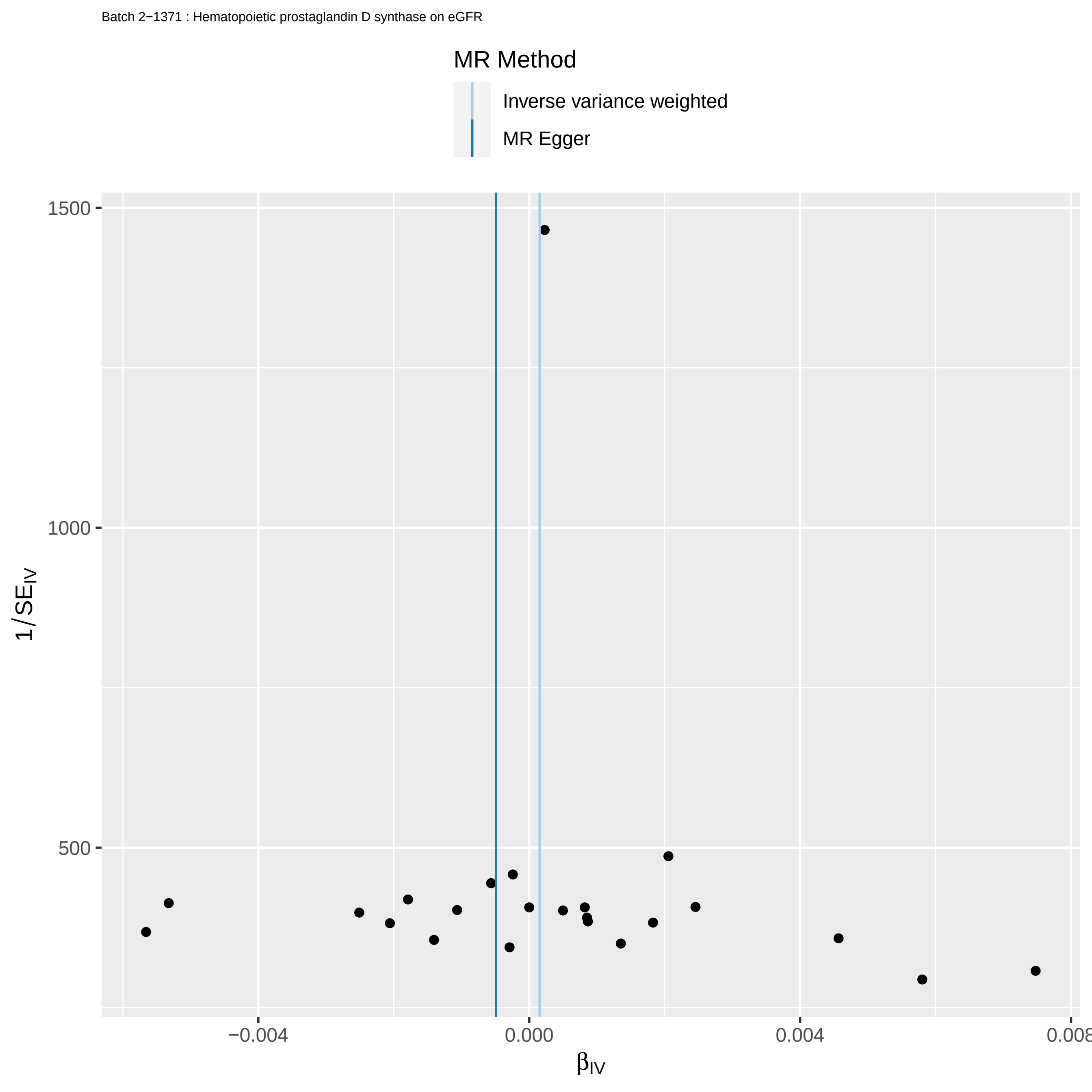

Batch 2−1371 : Hematopoietic prostaglandin D synthase on eGFR
MR Method
Inverse variance weighted
MR Egger
1500
1000
500
1/SE_IV
−0.004
0.000
0.004
0.008
β_IV

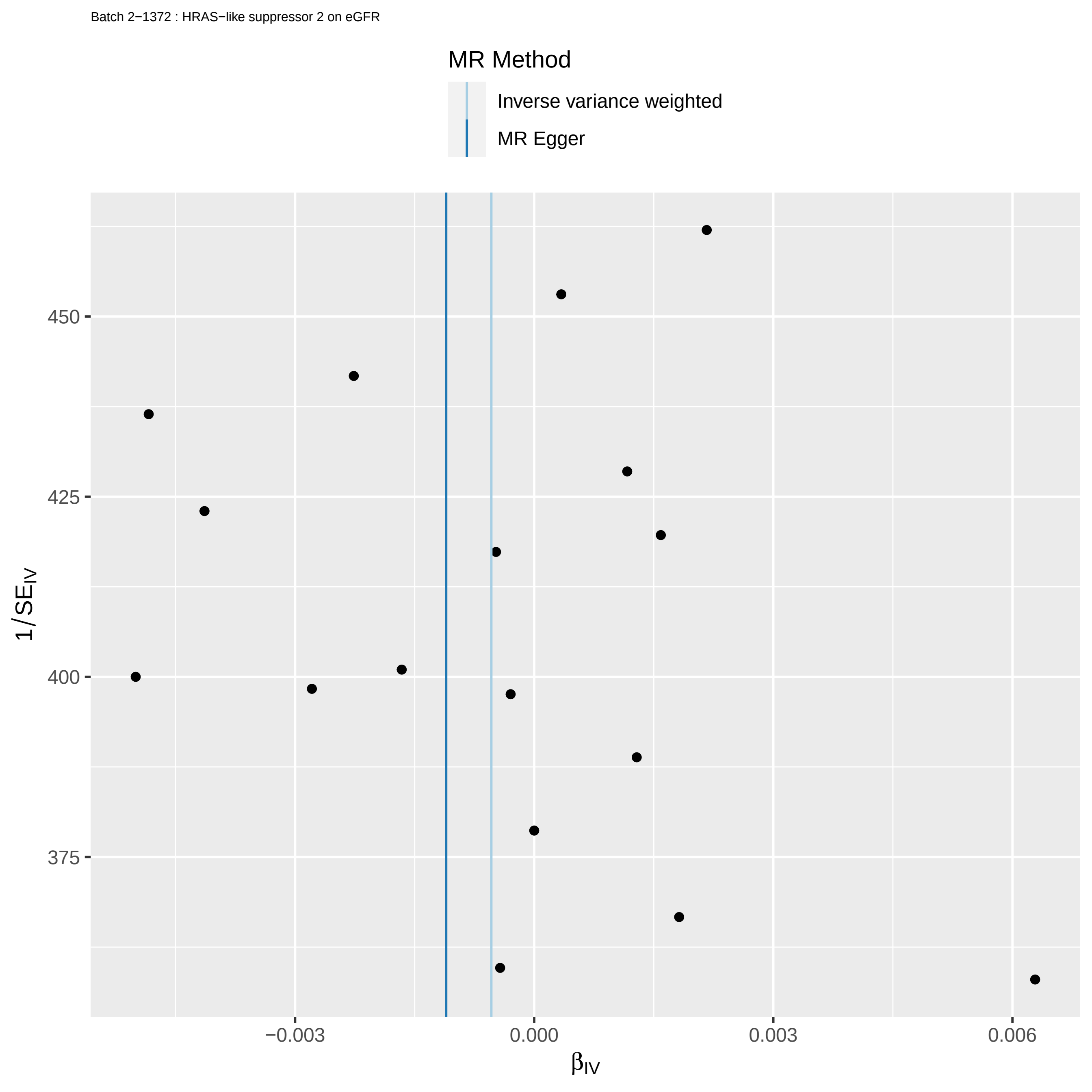

Batch 2−1372 : HRAS−like suppressor 2 on eGFR
MR Method
Inverse variance weighted
MR Egger
450
425
400
375
1/SE_IV
−0.003
0.000
0.003
0.006
β_IV

Batch 2−1373 : Activator of apoptosis harakiri on eGFR

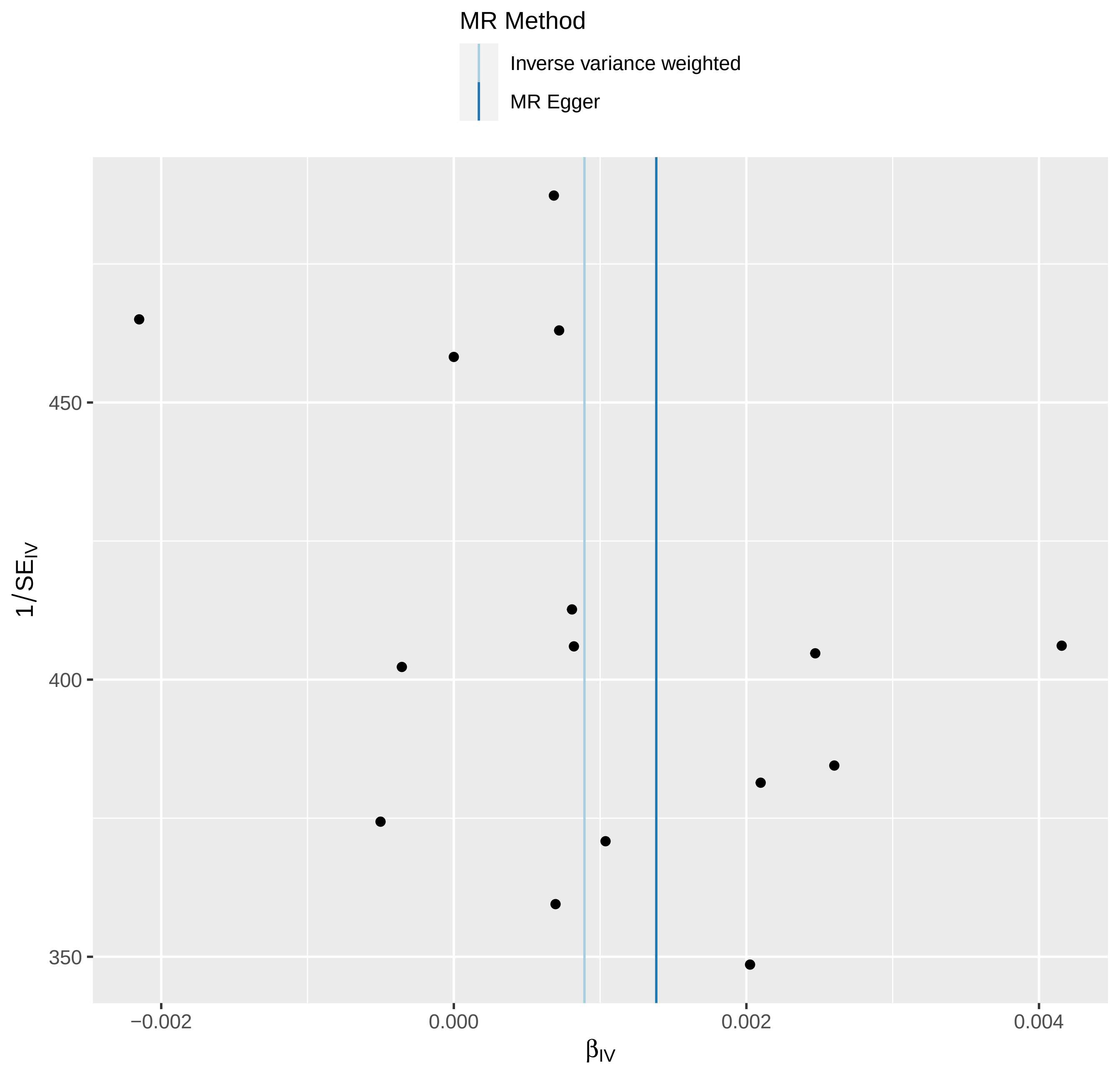

Batch 2−1374 : Ribonuclease UK114 on eGFR

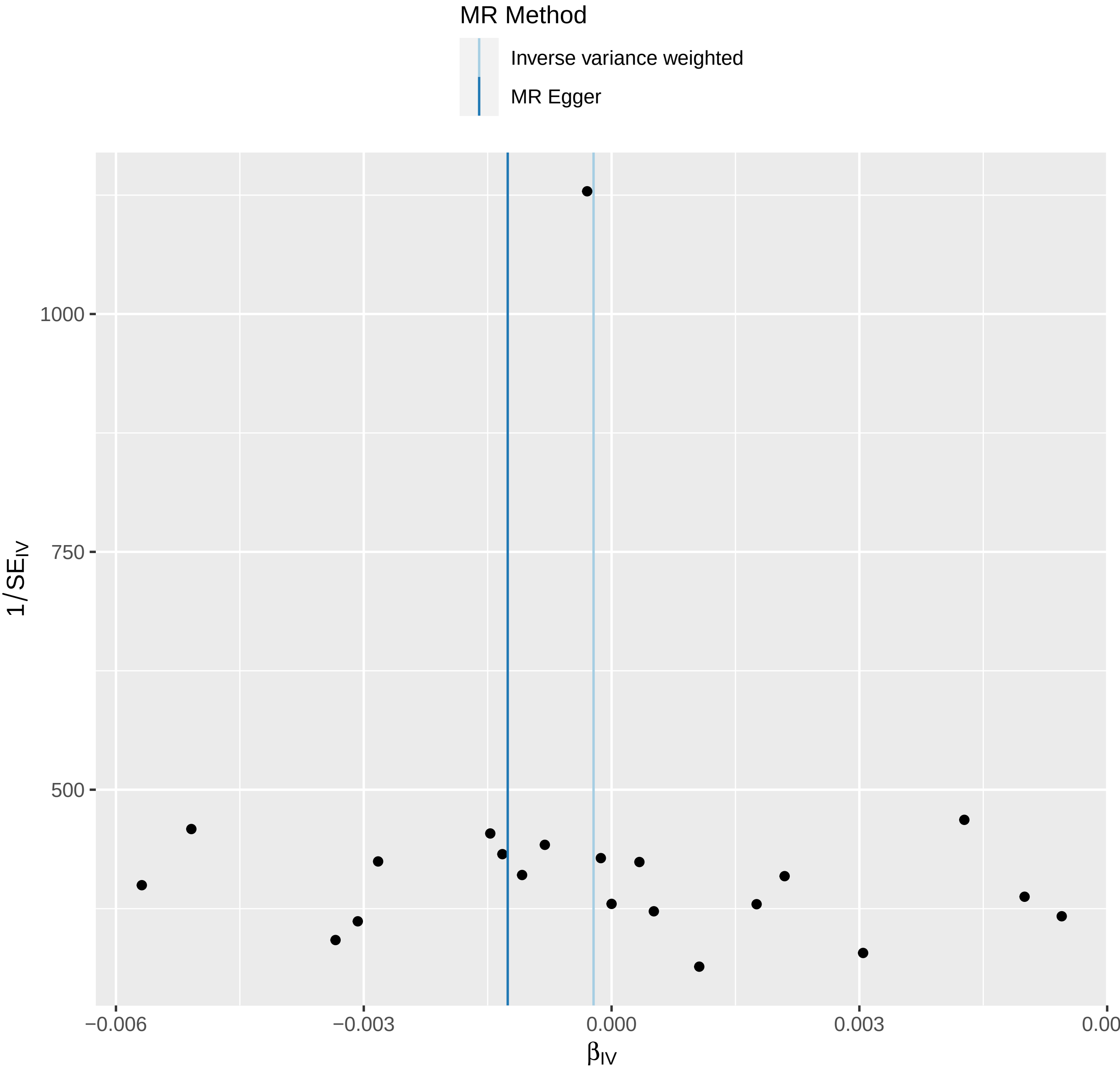

Batch 2−1375 : Heparan sulfate glucosamine 3−O−sulfotransferase 3A1 on eGFR

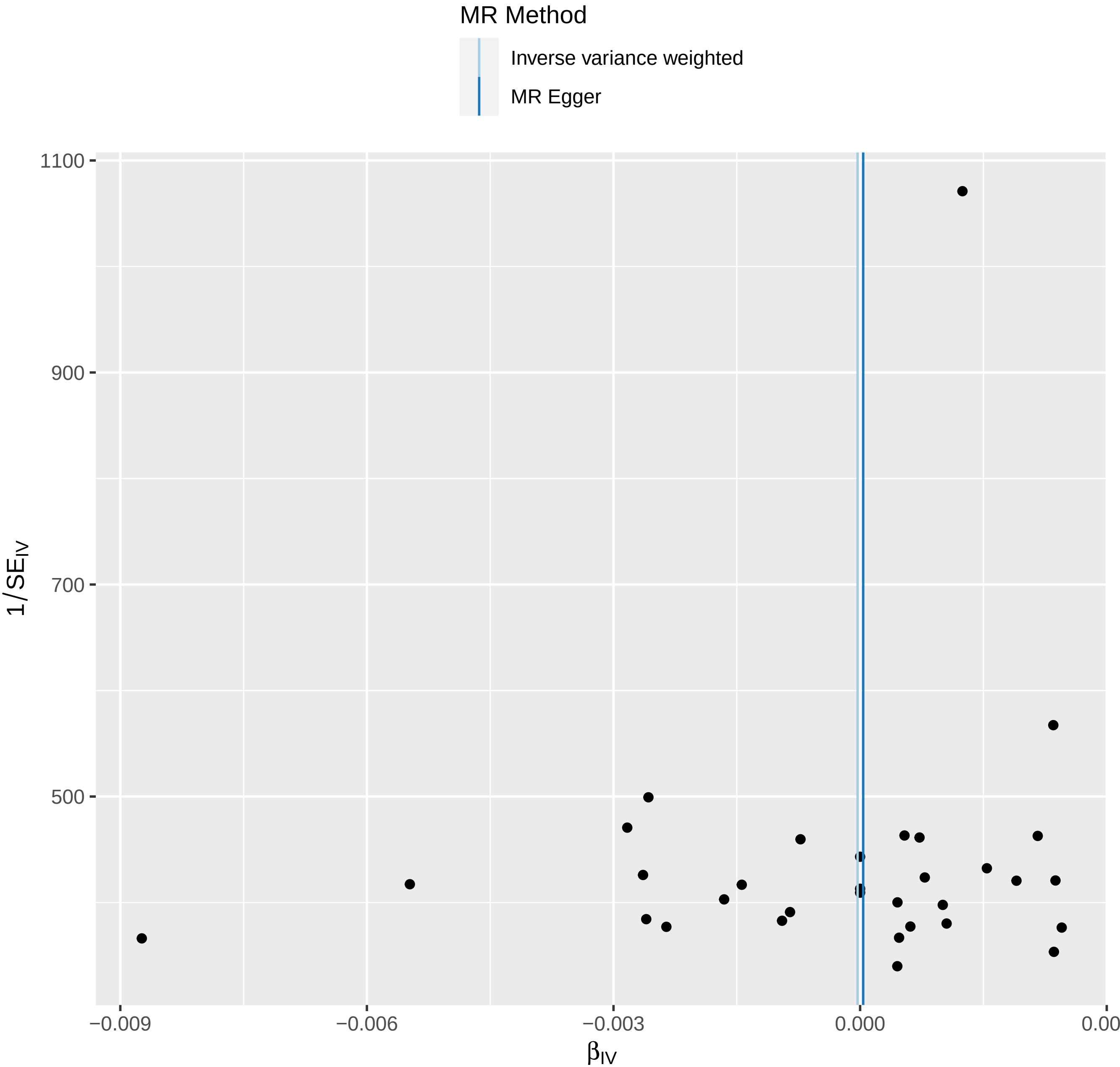

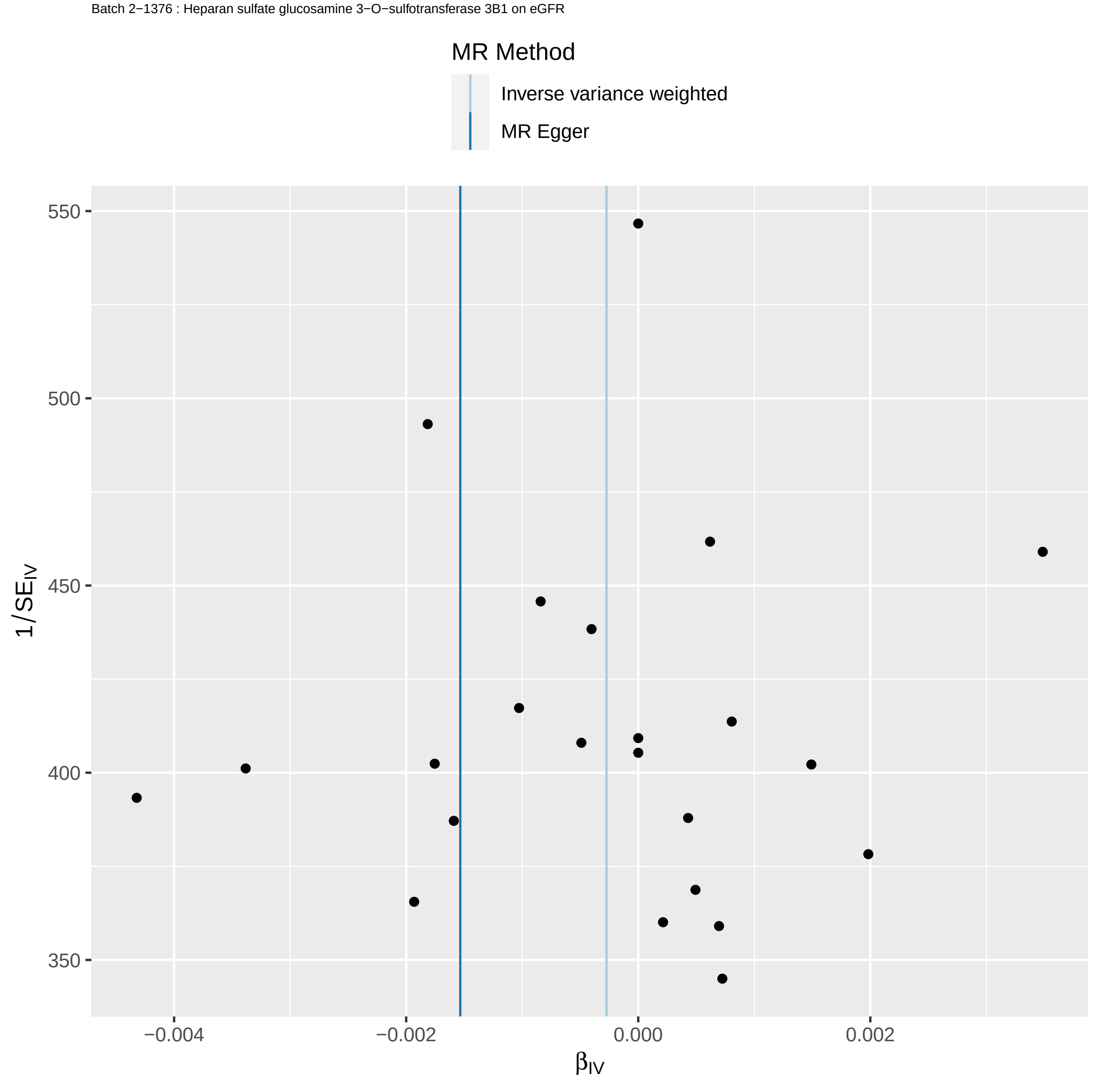
Batch 2−1376 : Heparan sulfate glucosamine 3−O−sulfotransferase 3B1 on eGFR
MR Method
Inverse variance weighted
MR Egger
550
500
450
400
350
1/SE$_{IV}$
−0.004
−0.002
0.000
0.002
β_{IV}

Batch 2−1377 : Heparan sulfate glucosamine 3−O−sulfotransferase 4 on eGFR

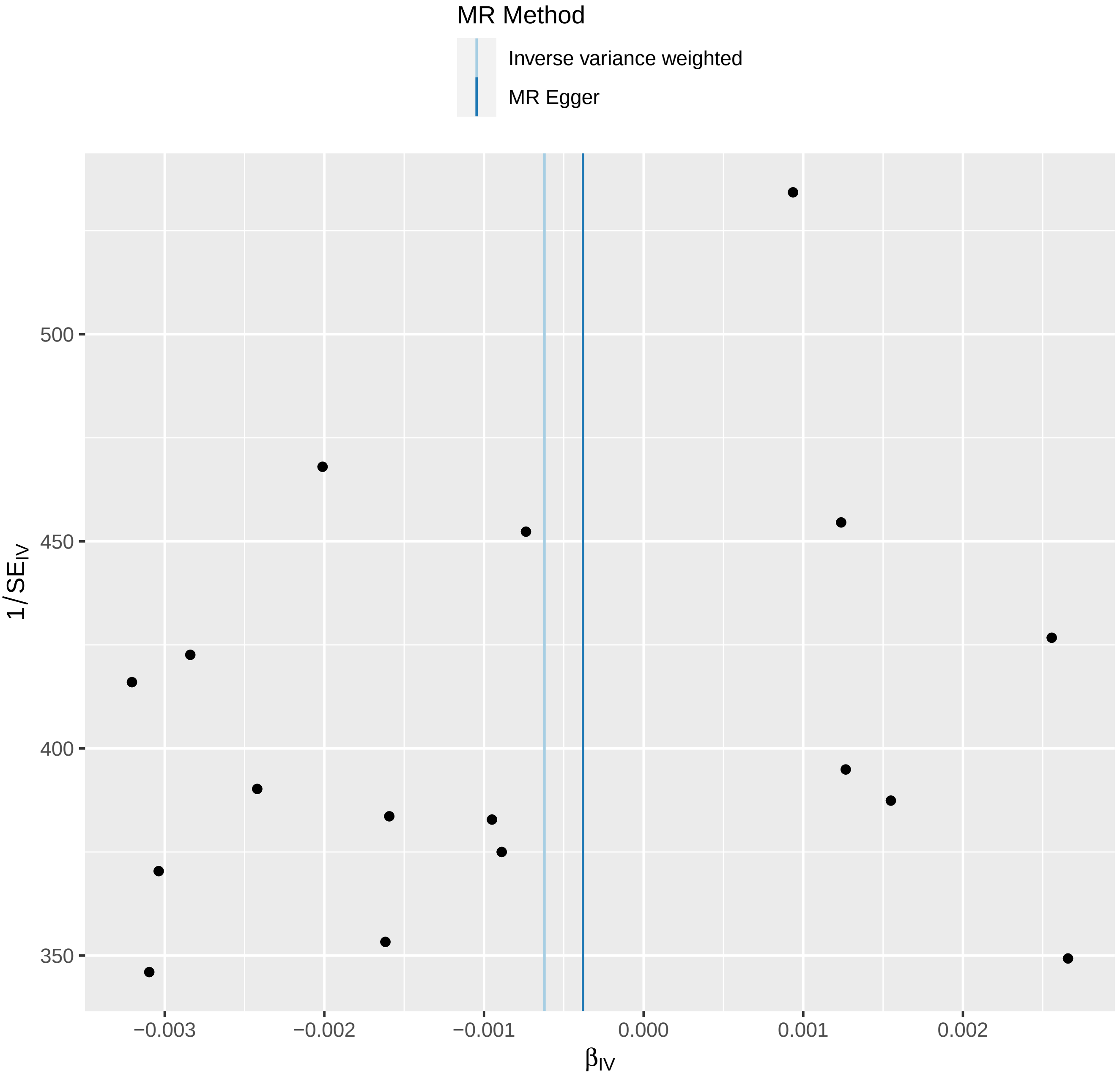

Batch 2−1378 : Heparan−sulfate 6−O−sulfotransferase 1 on eGFR

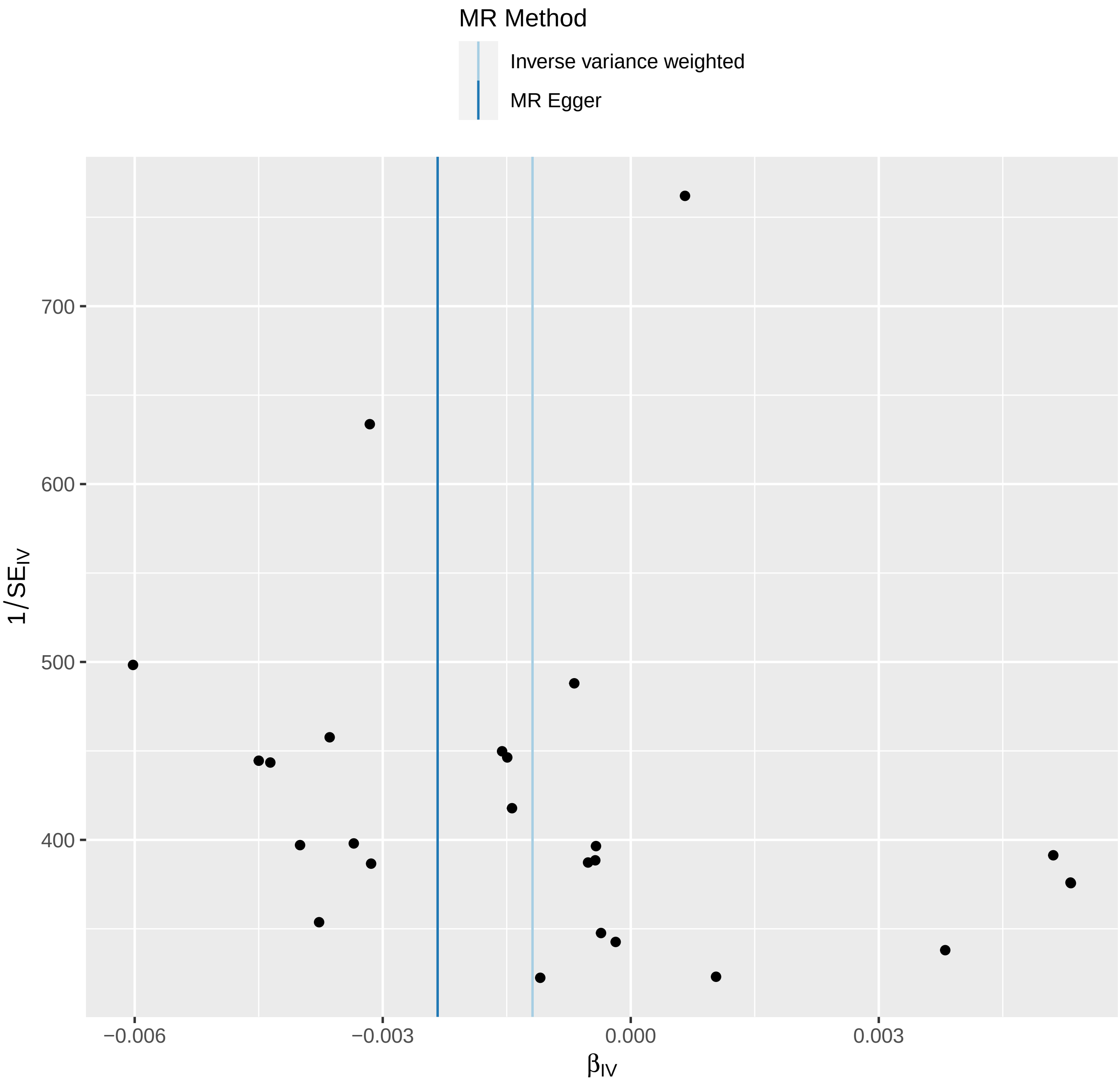

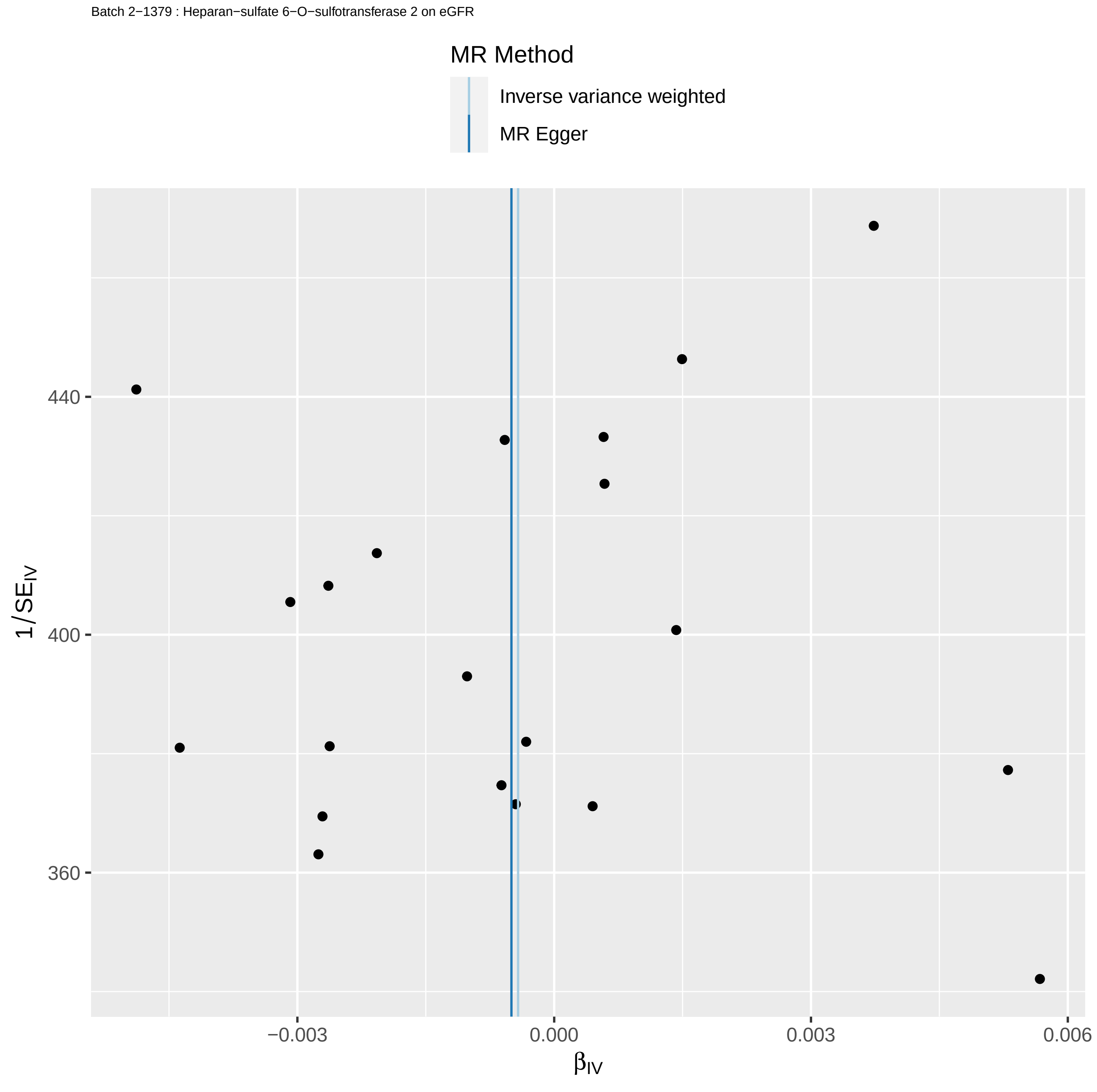

Batch 2−1379 : Heparan−sulfate 6−O−sulfotransferase 2 on eGFR
MR Method
Inverse variance weighted
MR Egger
440
400
360
1/SE_IV
−0.003
0.000
0.003
0.006
β_IV

Batch 2−1380 : 3−hydroxyacyl−CoA dehydrogenase type−2 on eGFR

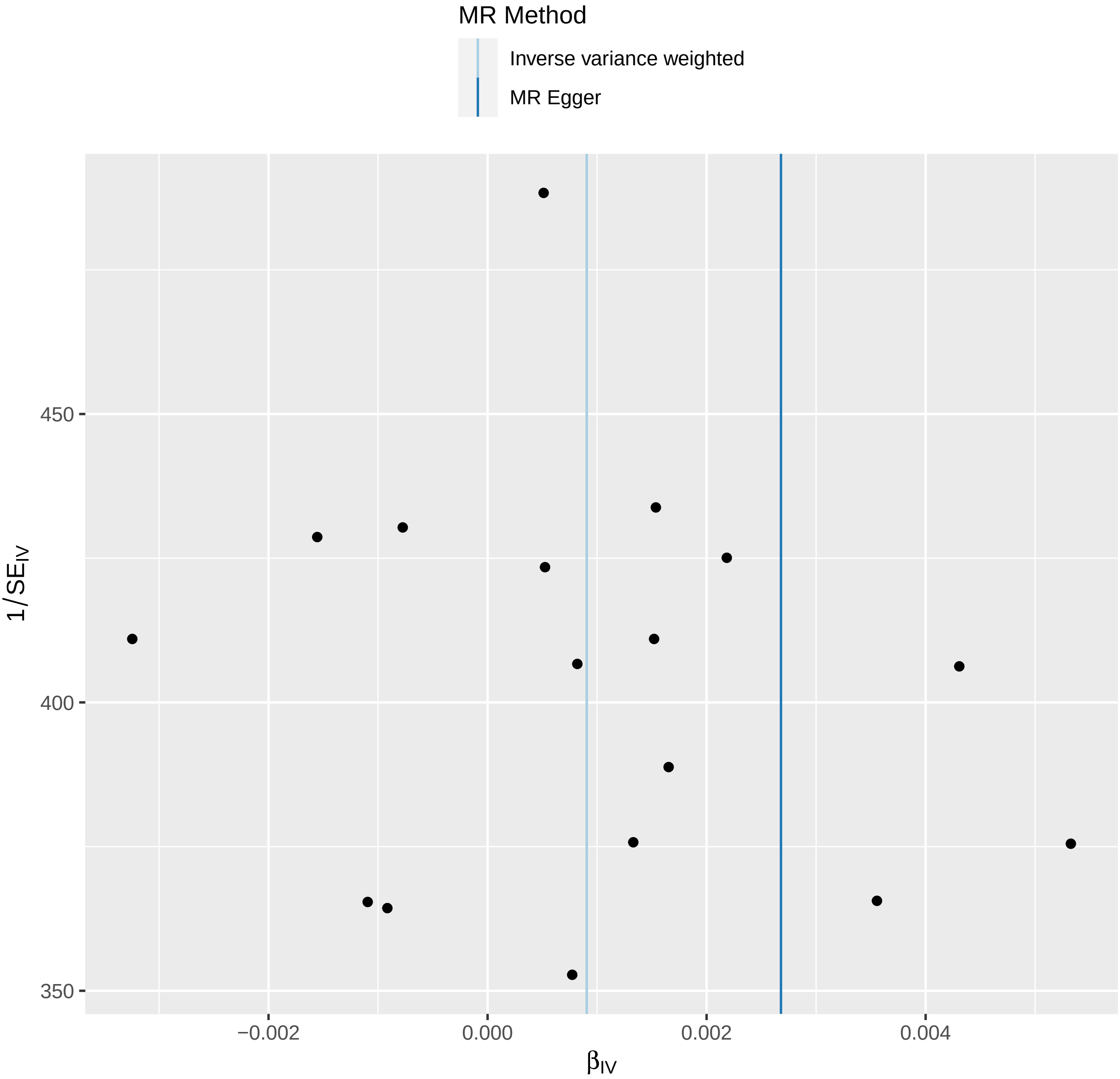

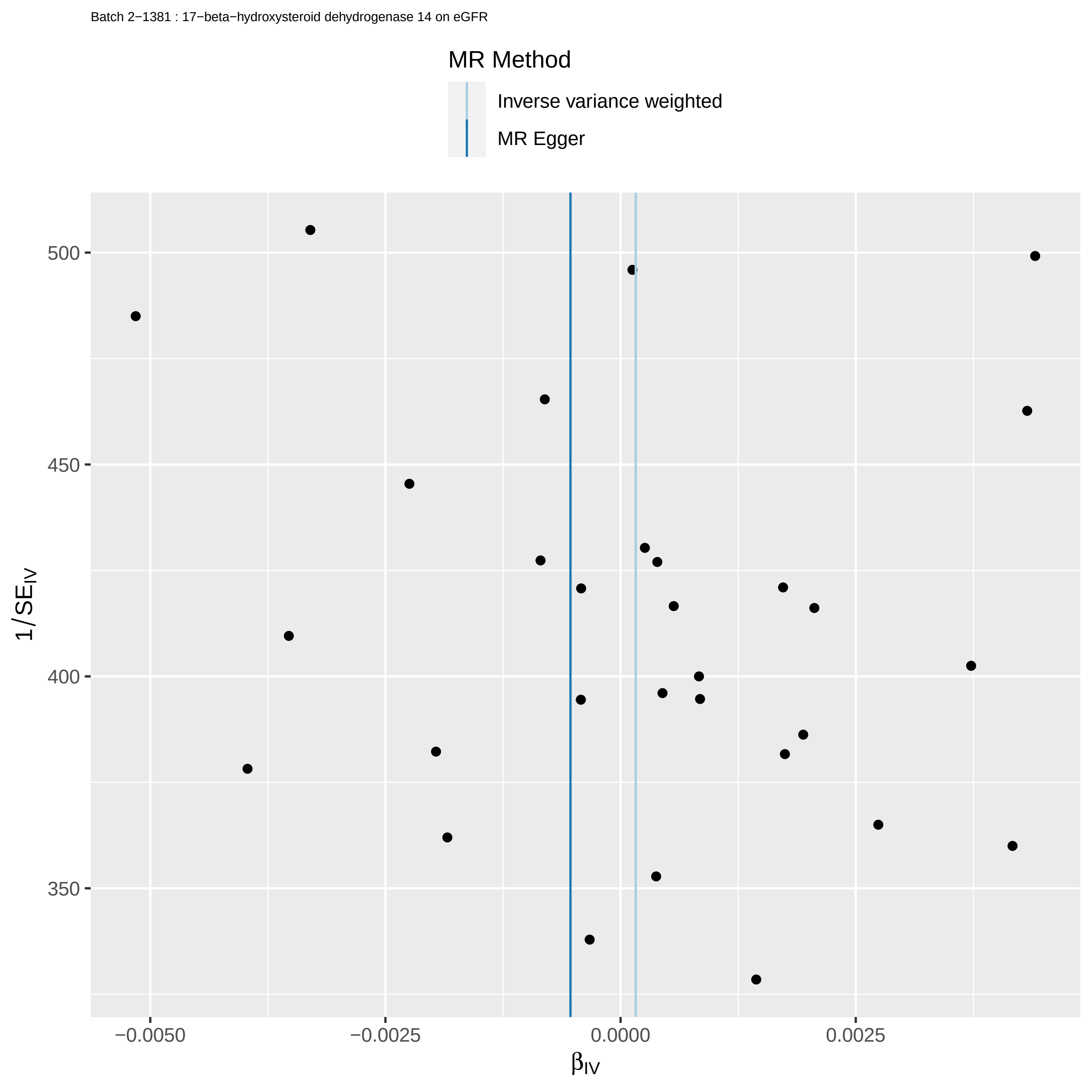

Batch 2−1381 : 17−beta−hydroxysteroid dehydrogenase 14 on eGFR
MR Method
Inverse variance weighted
MR Egger
500
450
400
350
1/SE_IV
−0.0050
−0.0025
0.0000
0.0025
β_IV

Batch 2−1382 : Estradiol 17−beta−dehydrogenase 1 on eGFR

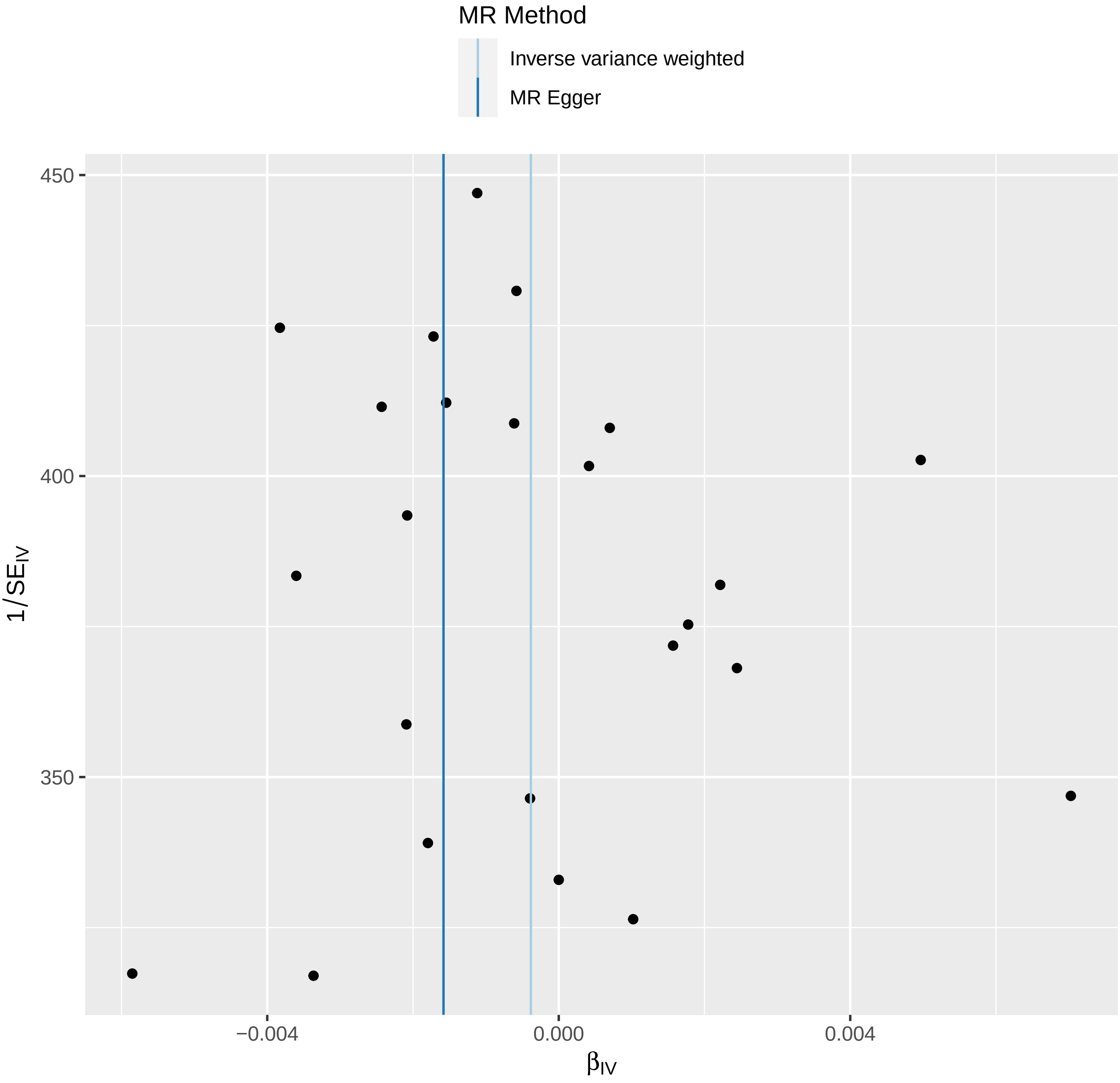

Batch 2−1383 : Estradiol 17−beta−dehydrogenase 2 on eGFR

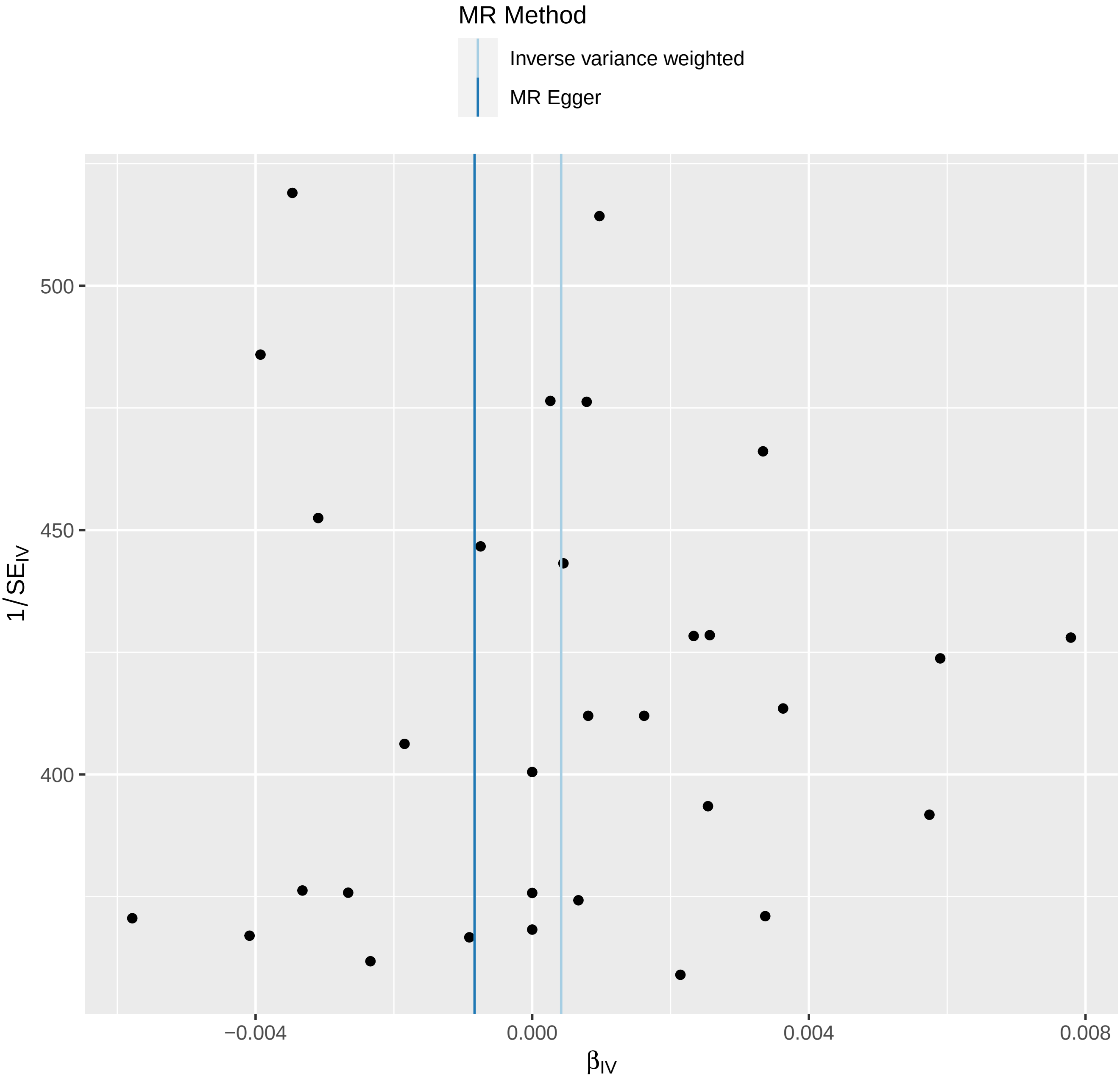

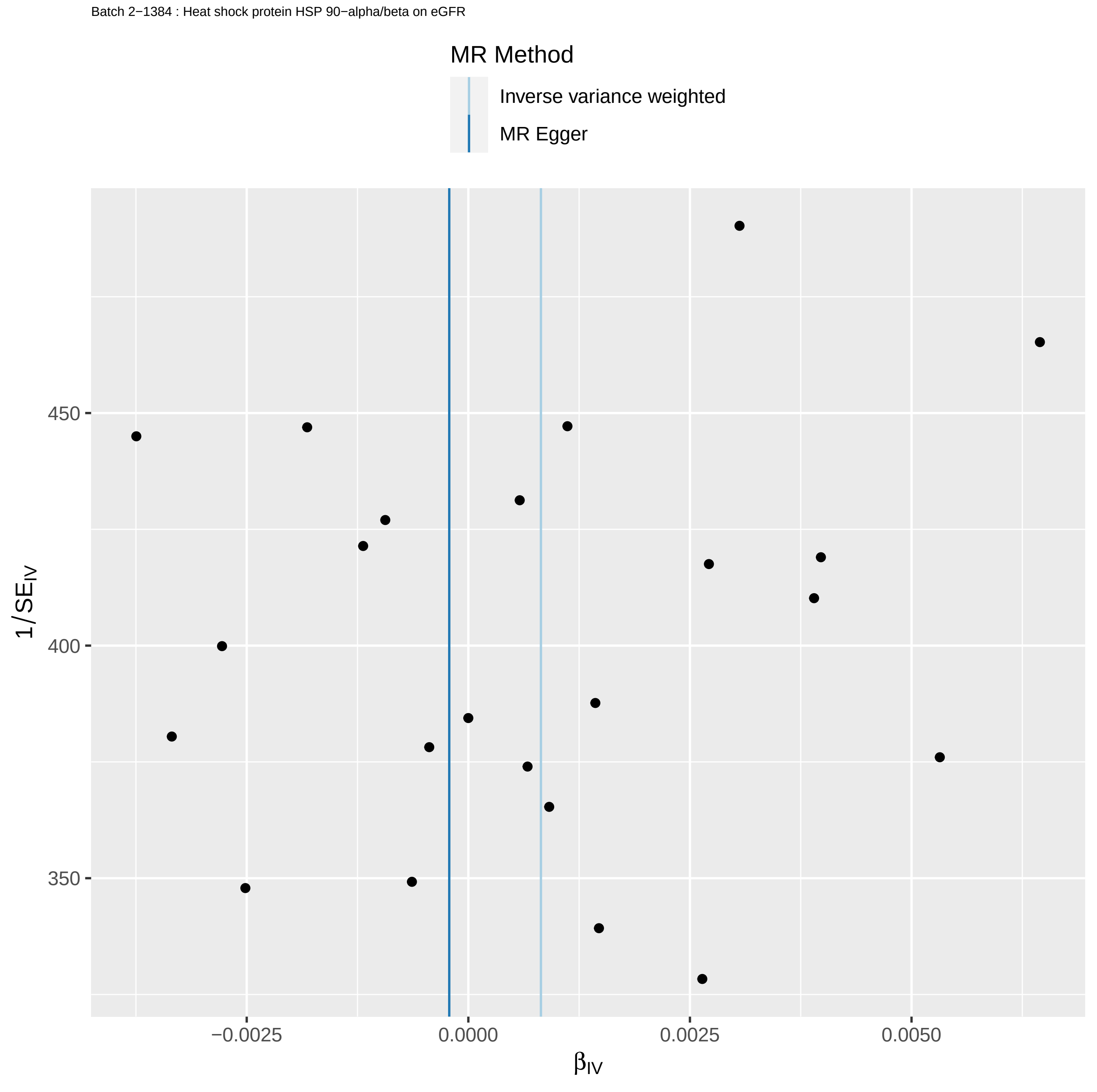
Batch 2−1384 : Heat shock protein HSP 90−alpha/beta on eGFR
MR Method
Inverse variance weighted
MR Egger
450
400
350
1/SE_IV
−0.0025
0.0000
0.0025
0.0050
β_IV

Batch 2−1385 : Heat shock protein HSP 90−beta on eGFR

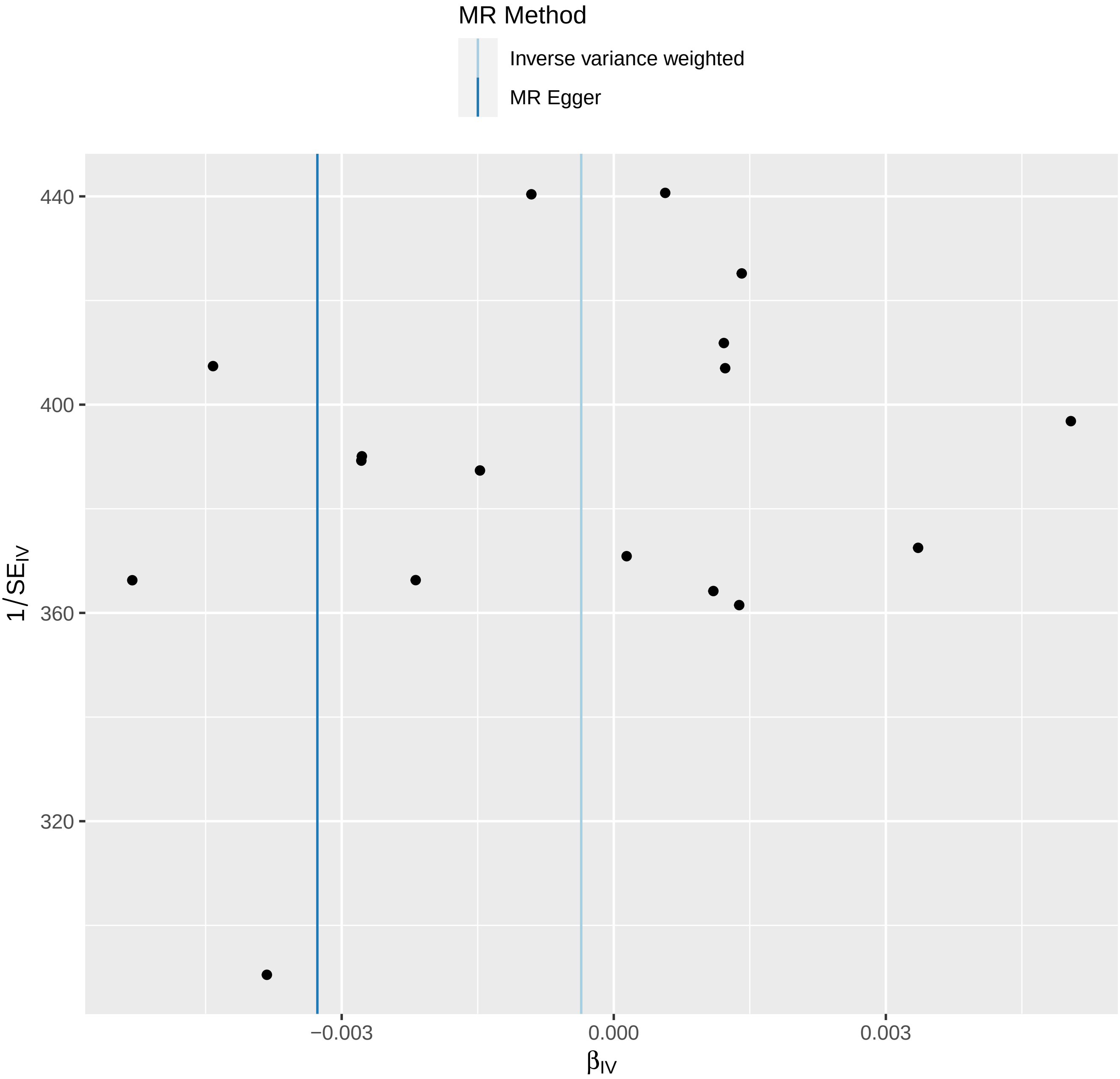

Batch 2−1386 : Endoplasmin on eGFR

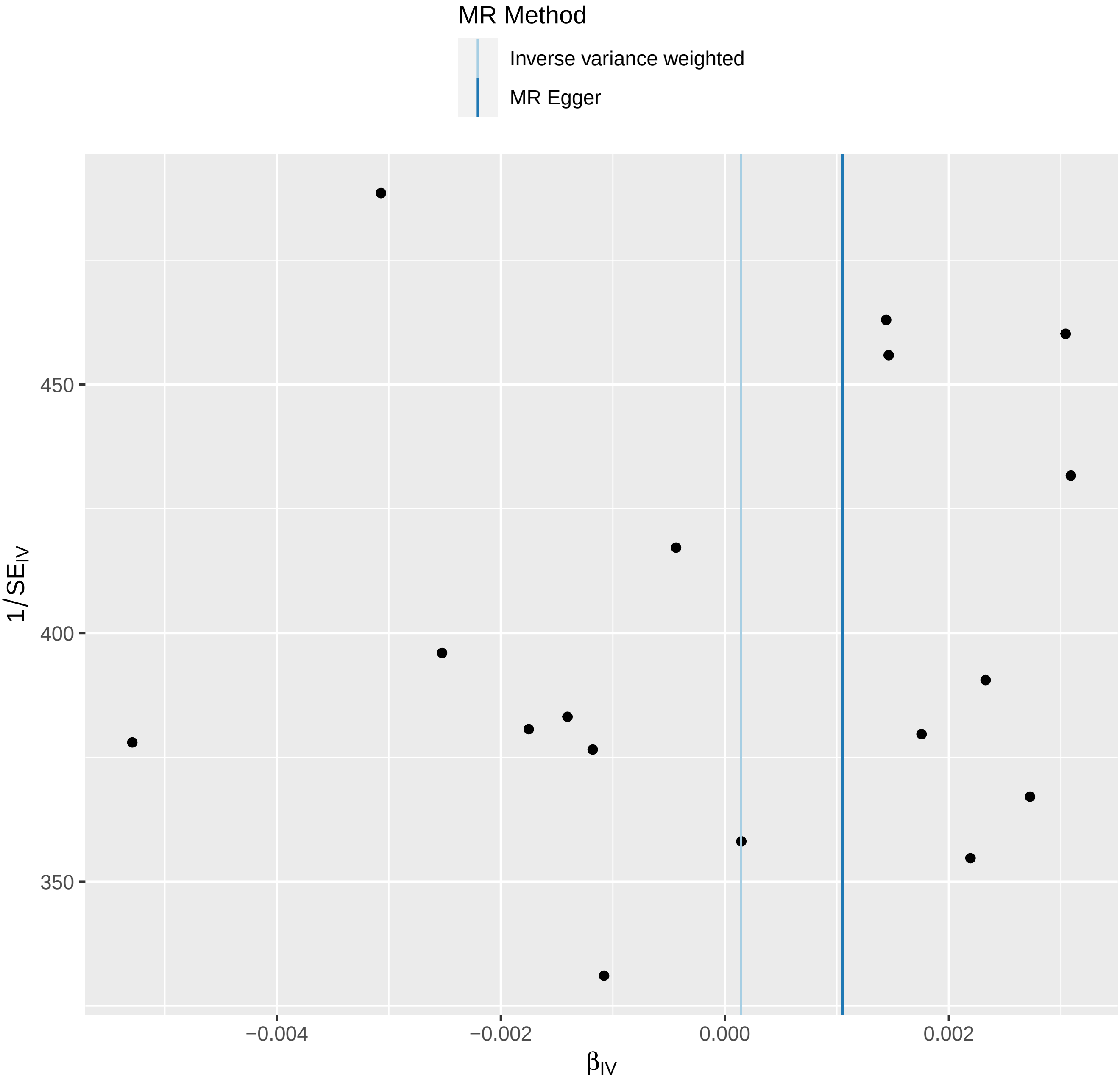

Batch 2−1387 : Heat shock 70 kDa protein 1−like on eGFR

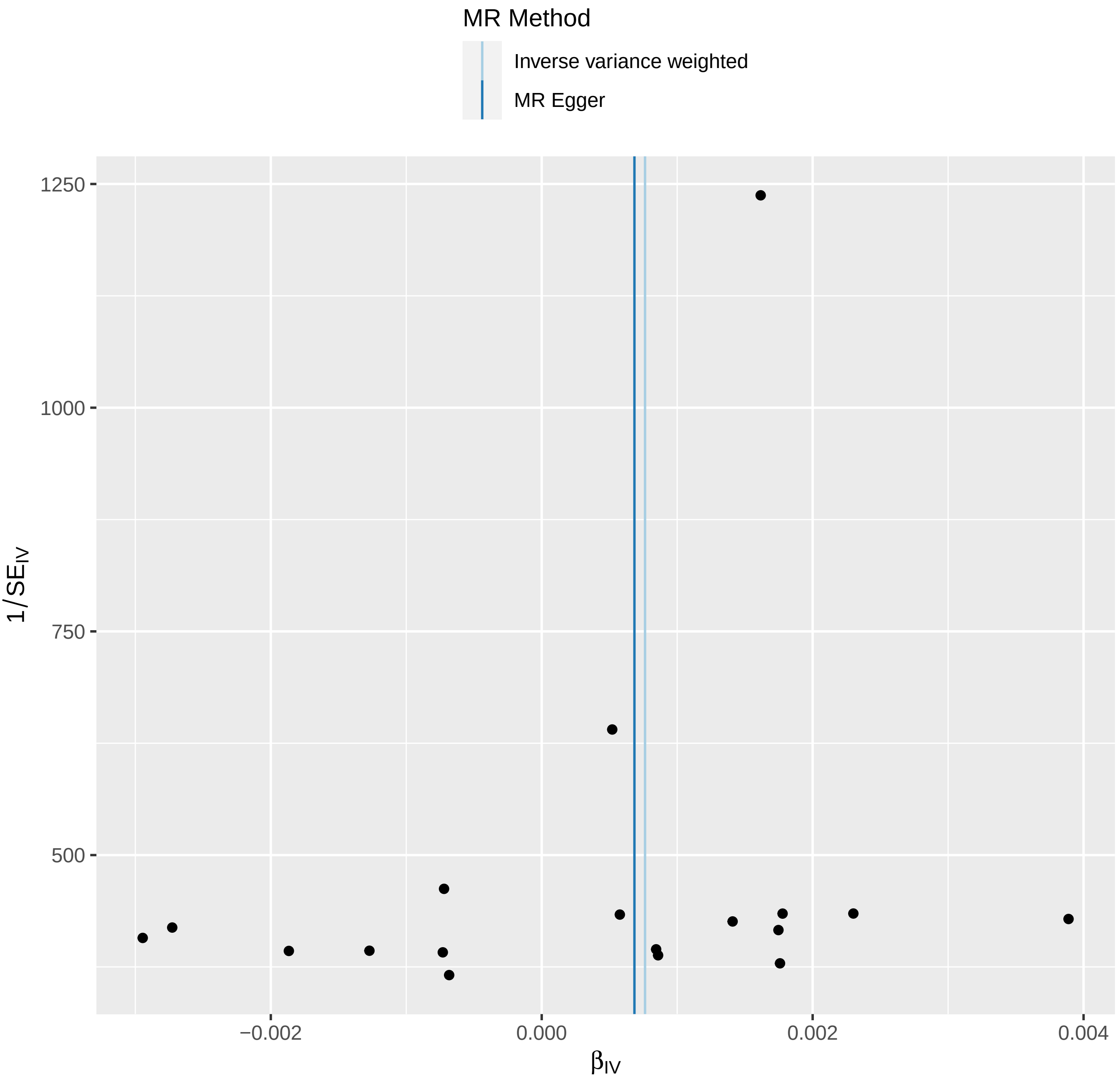

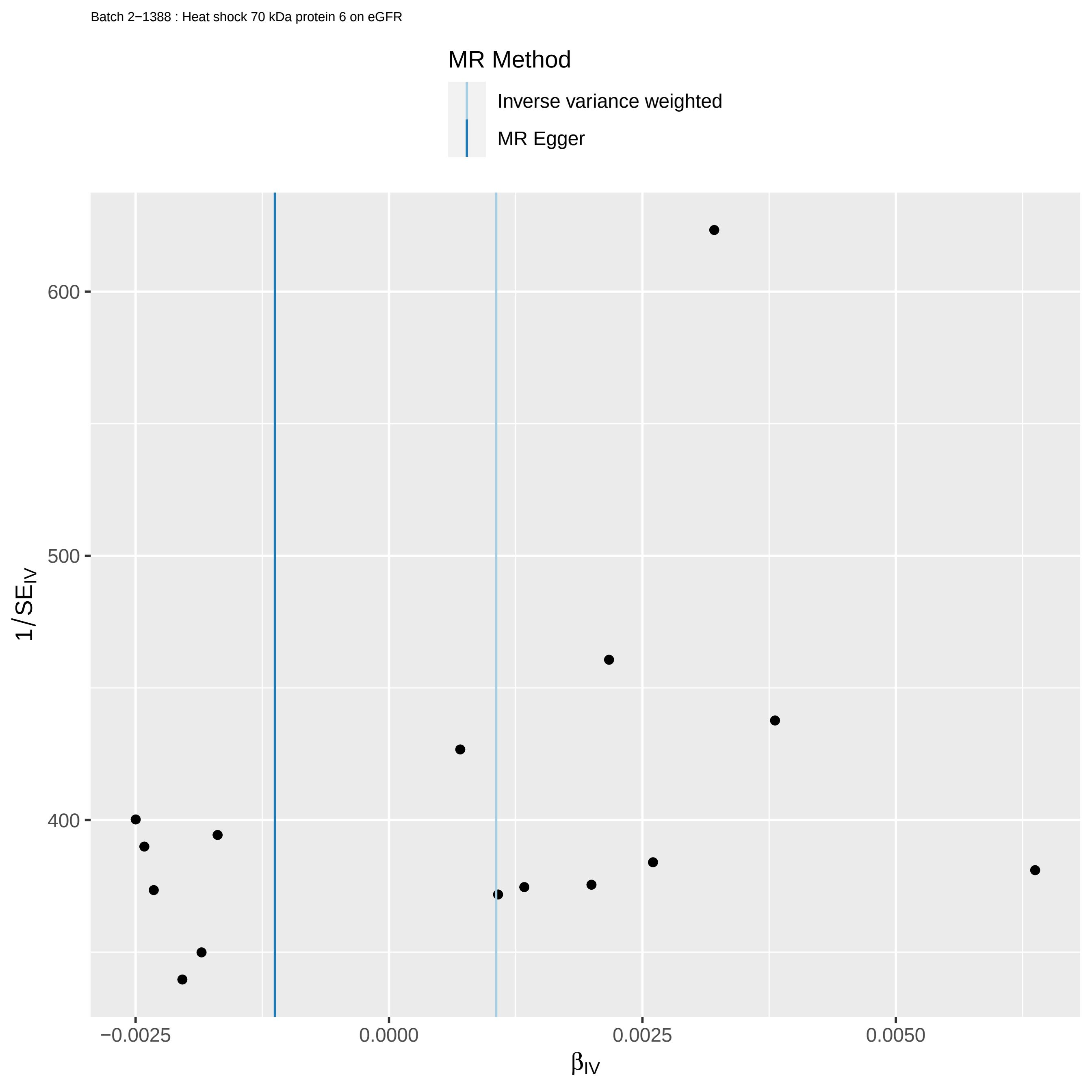

Batch 2−1388 : Heat shock 70 kDa protein 6 on eGFR
MR Method
Inverse variance weighted
MR Egger
600
500
400
1/SE_IV
−0.0025
0.0000
0.0025
0.0050
β_IV

Batch 2−1389 : Oxidoreductase HTATIP2 on eGFR

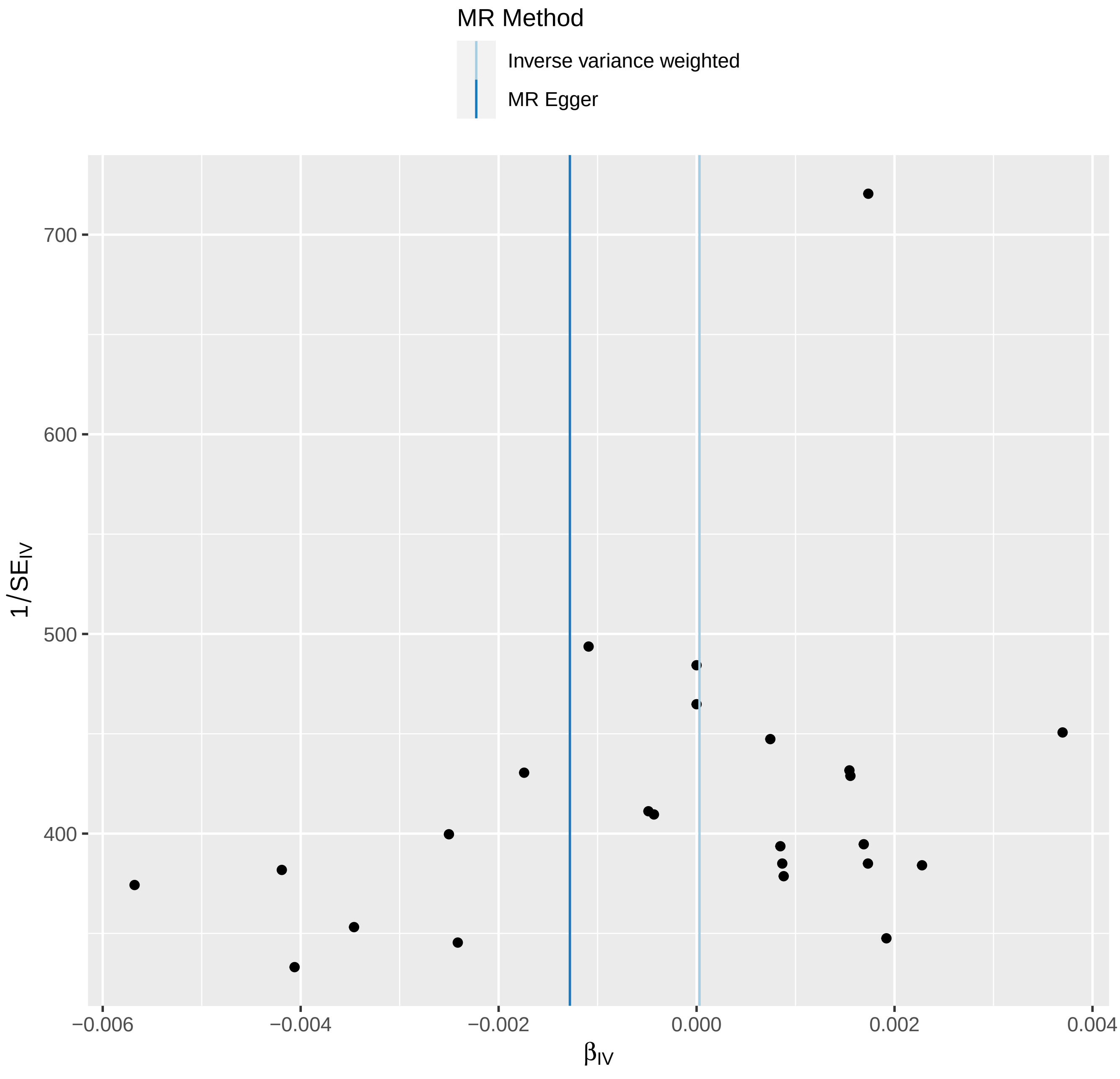

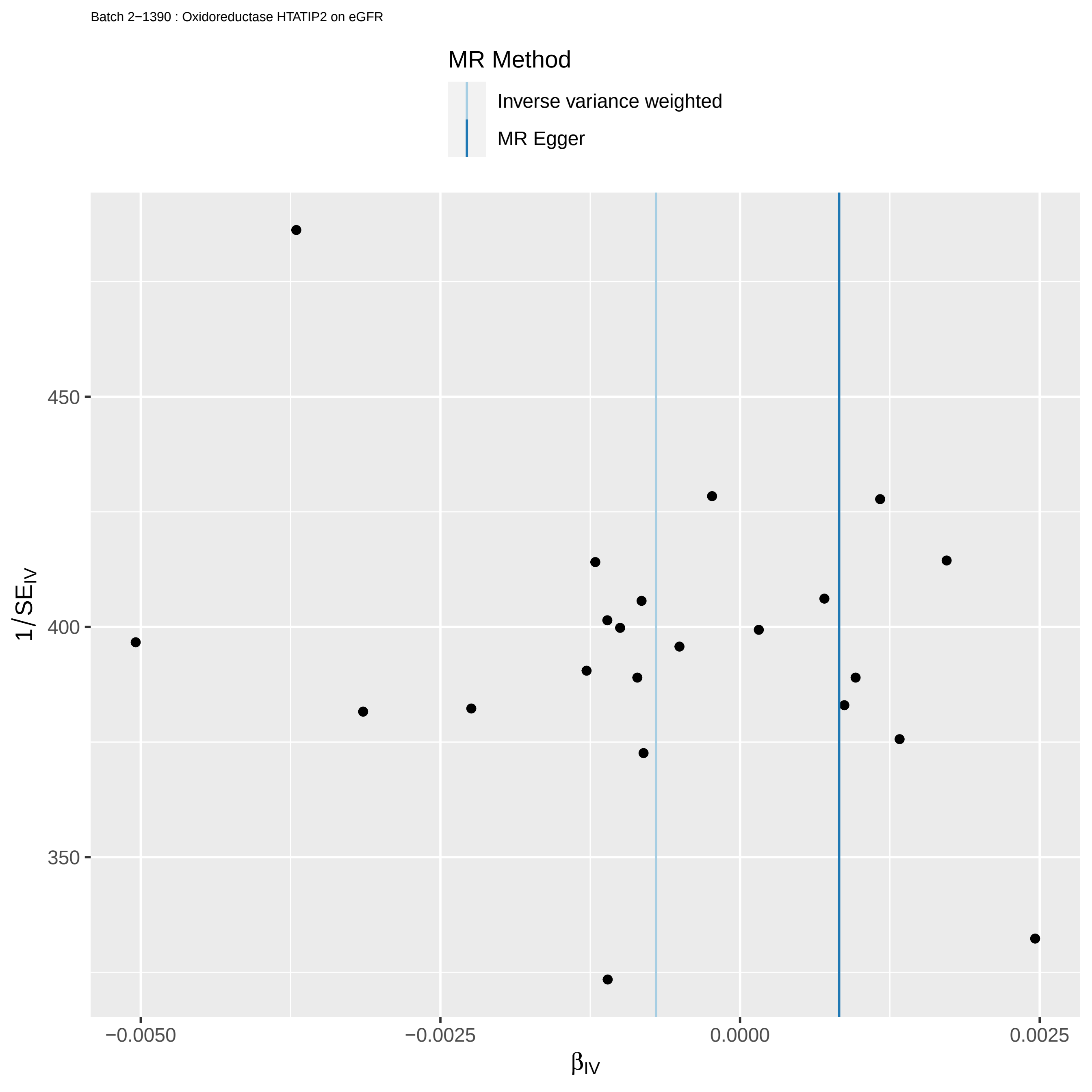

Batch 2−1390 : Oxidoreductase HTATIP2 on eGFR
MR Method
Inverse variance weighted
MR Egger
450
400
350
−0.0050
−0.0025
0.0000
0.0025
β_{IV}
$1/SE_{IV}$

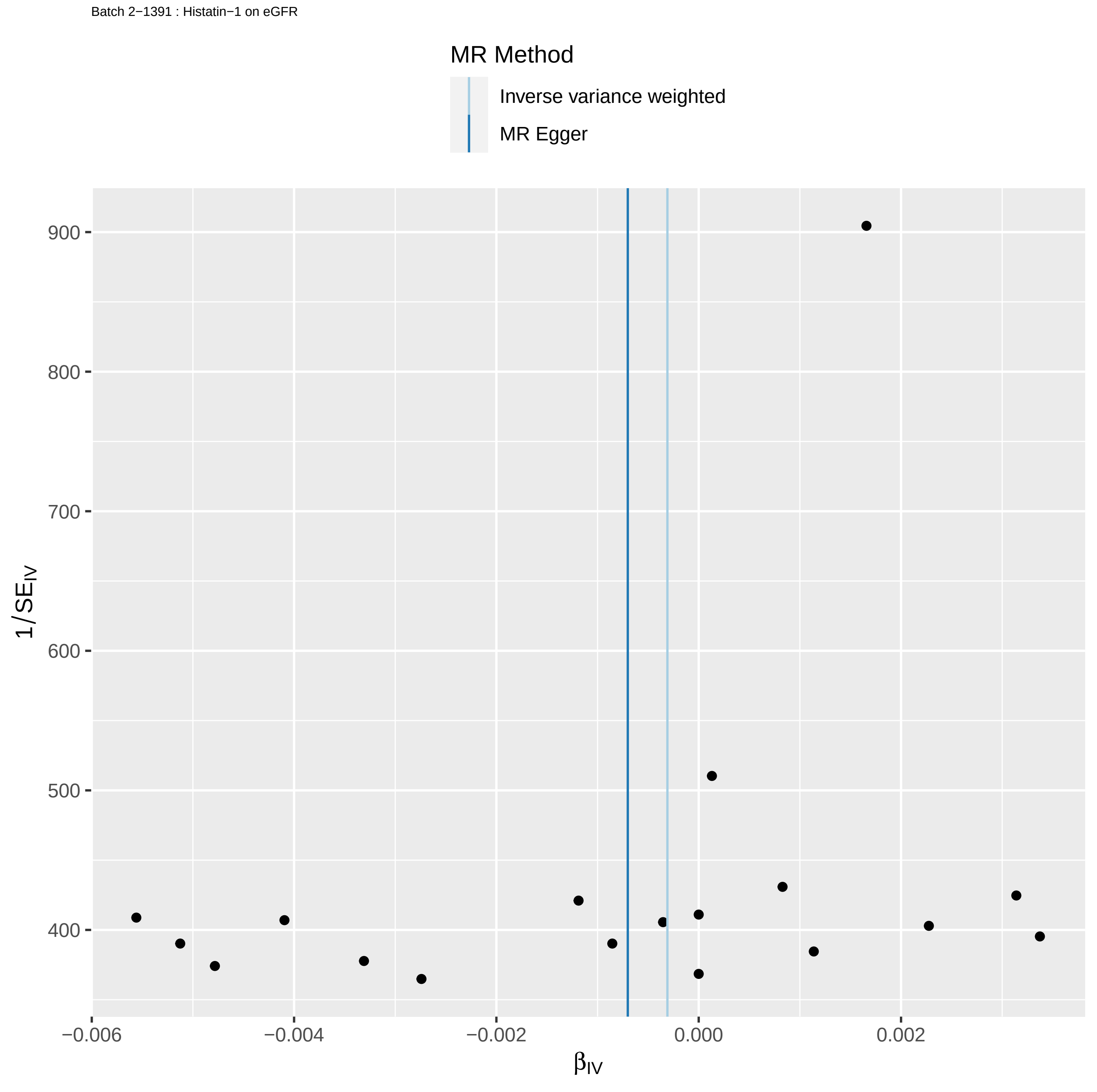

Batch 2−1391 : Histatin−1 on eGFR
MR Method
Inverse variance weighted
MR Egger
900
800
700
600
500
400
1/SE_IV
−0.006
−0.004
−0.002
0.000
0.002
β_IV

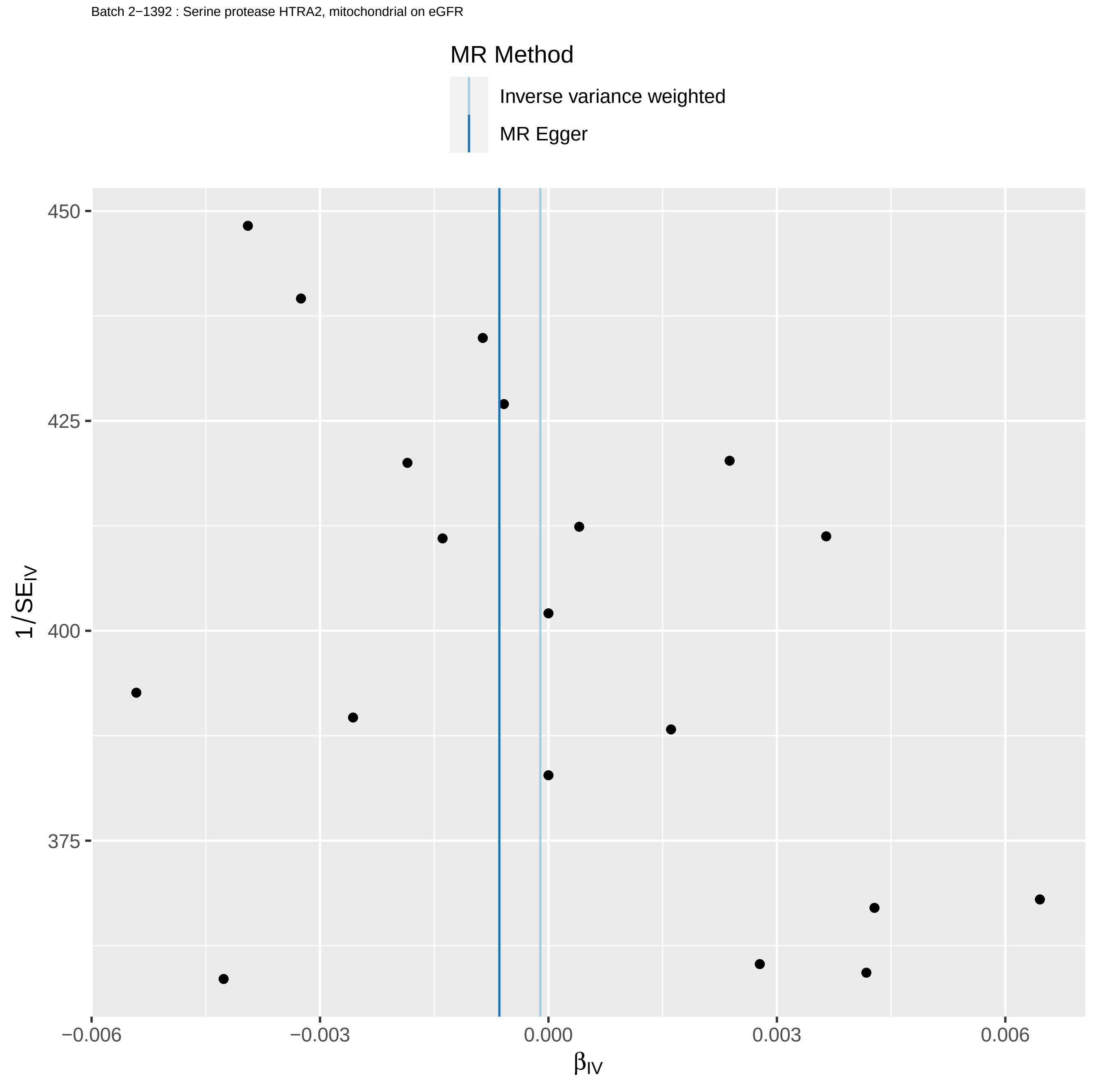
Batch 2−1392 : Serine protease HTRA2, mitochondrial on eGFR
MR Method
Inverse variance weighted
MR Egger
450
425
400
375
1/SE_IV
−0.006
−0.003
0.000
0.003
0.006
β_IV

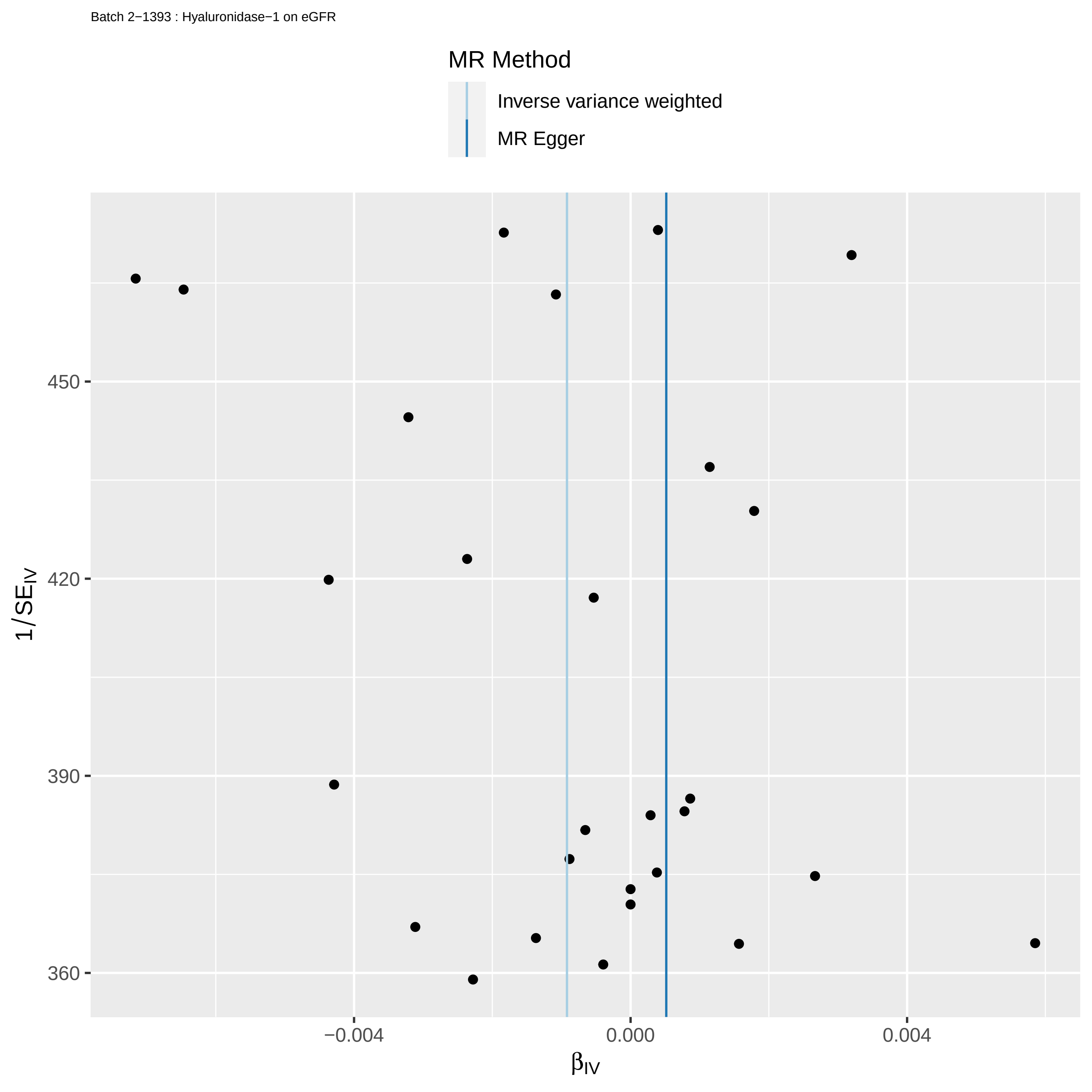
Batch 2−1393 : Hyaluronidase−1 on eGFR
MR Method
Inverse variance weighted
MR Egger
450
420
390
360
1/SE_IV
−0.004
0.000
0.004
β_IV

Batch 2−1394 : Isoleucine−−tRNA ligase, cytoplasmic on eGFR

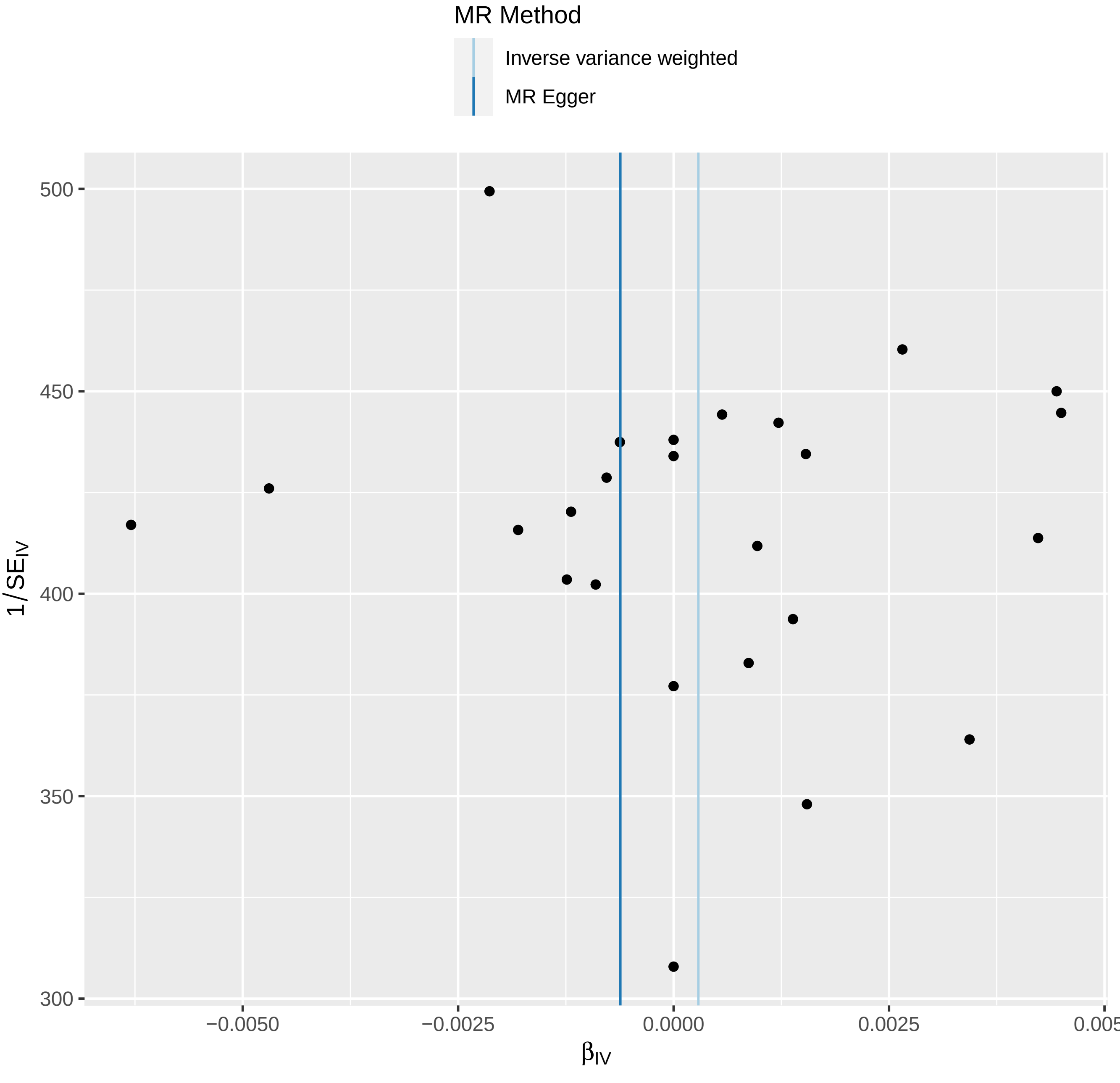

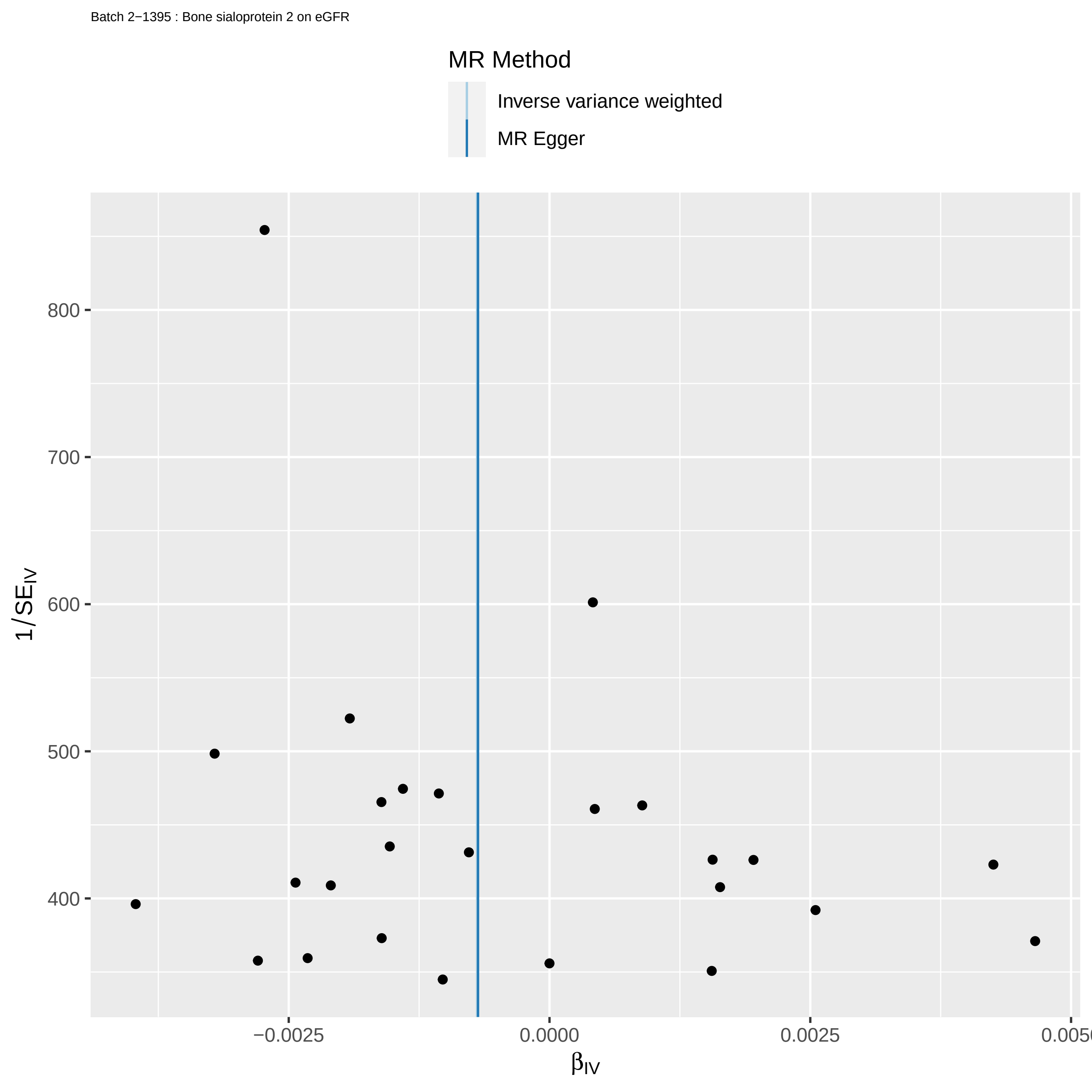

Batch 2−1395 : Bone sialoprotein 2 on eGFR
MR Method
Inverse variance weighted
MR Egger
800
700
600
500
400
1/SE_IV
−0.0025
0.0000
0.0025
0.0050
β_IV

Batch 2−1396 : Bone sialoprotein 2 on eGFR

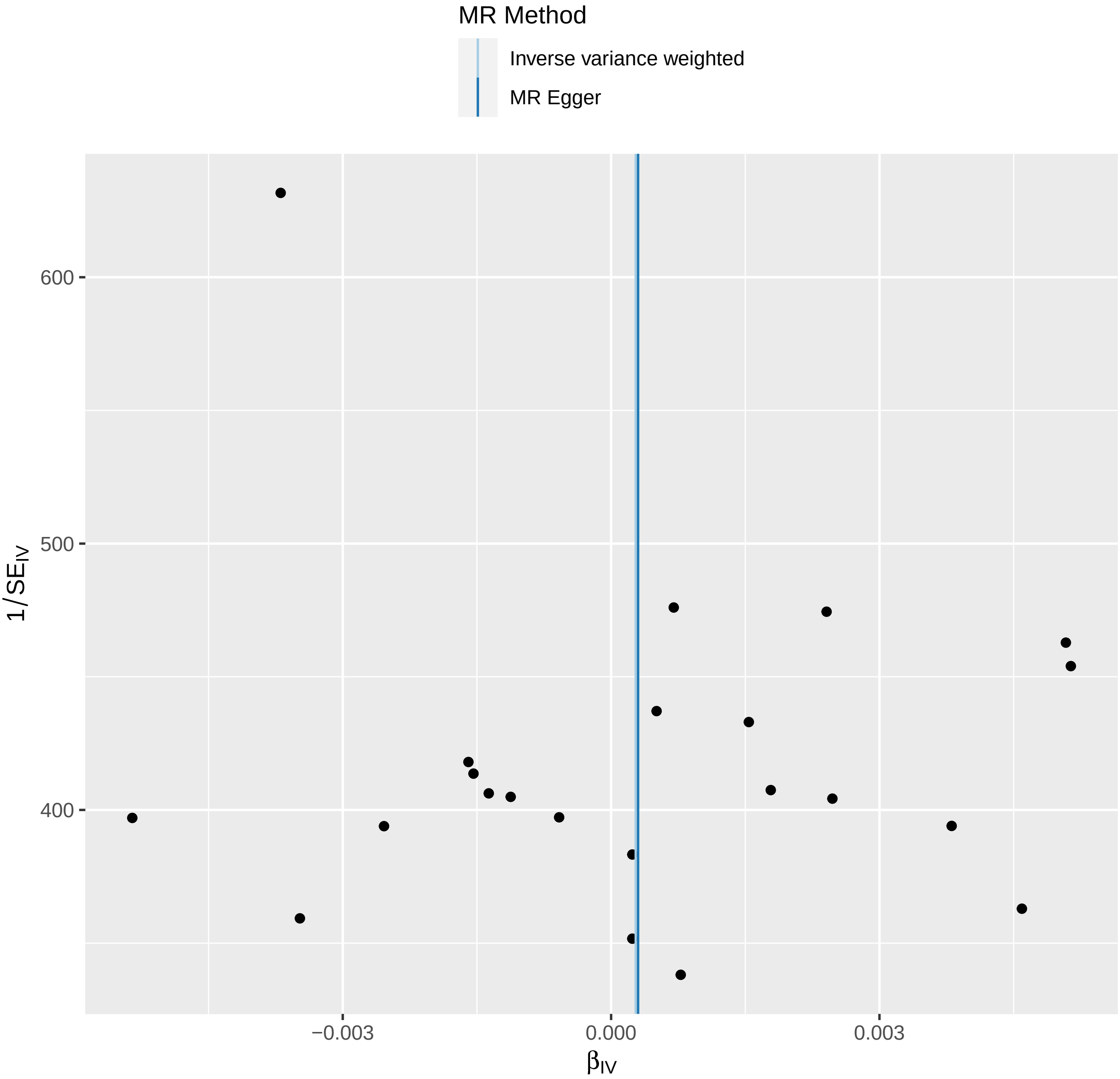

Batch 2−1397 : Intercellular adhesion molecule 1 on eGFR

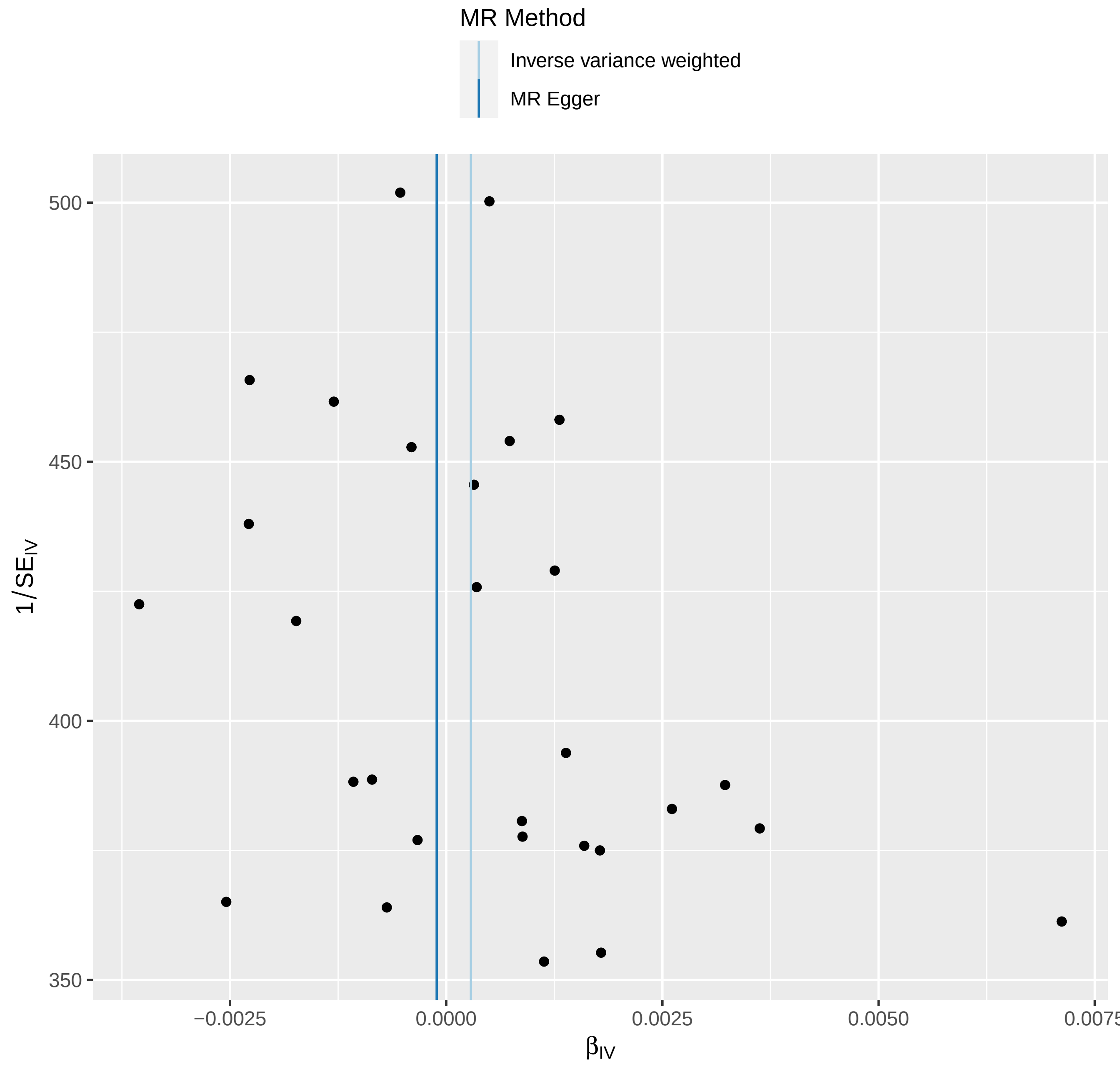

Batch 2−1398 : Intercellular adhesion molecule 2 on eGFR

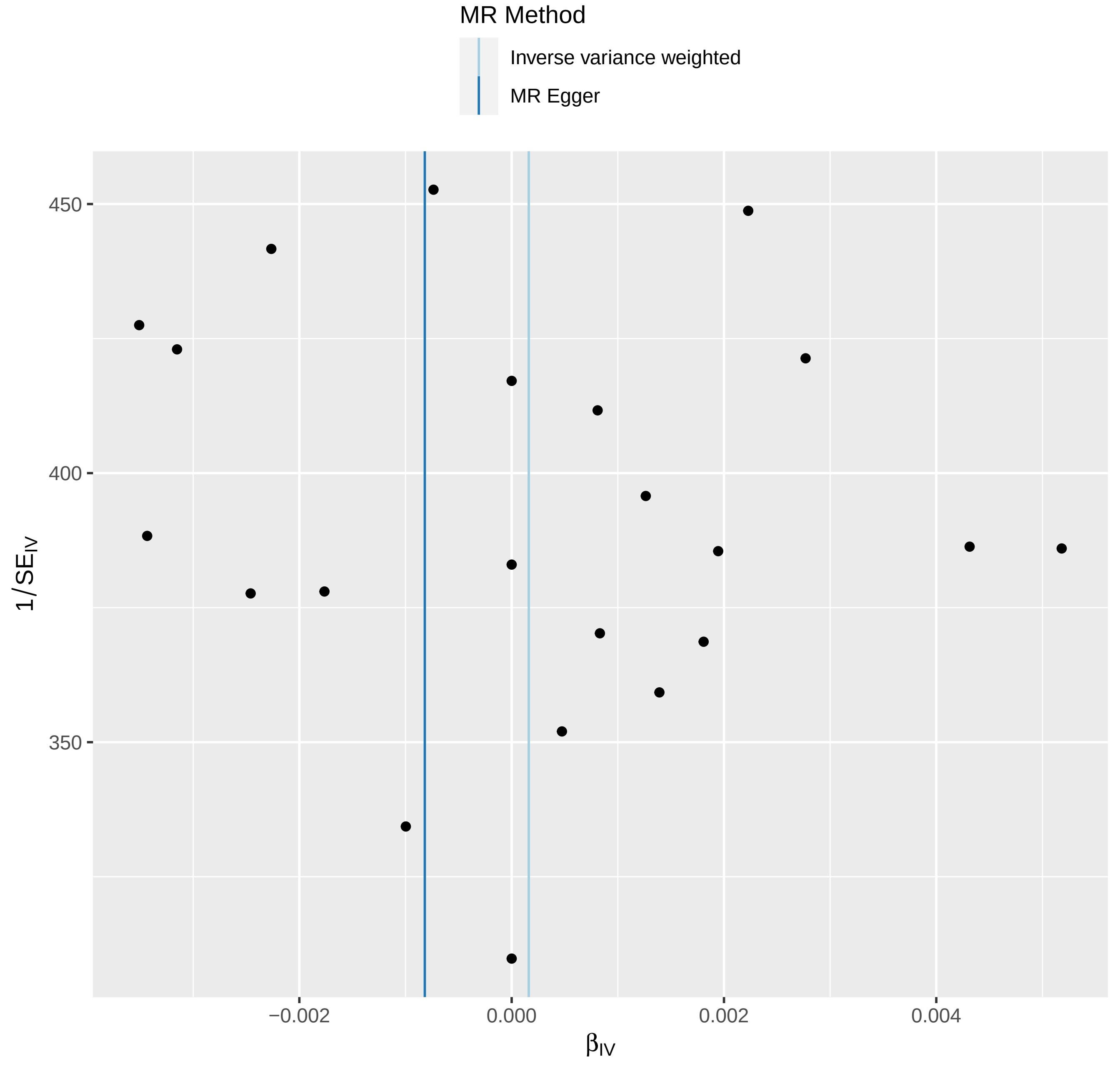

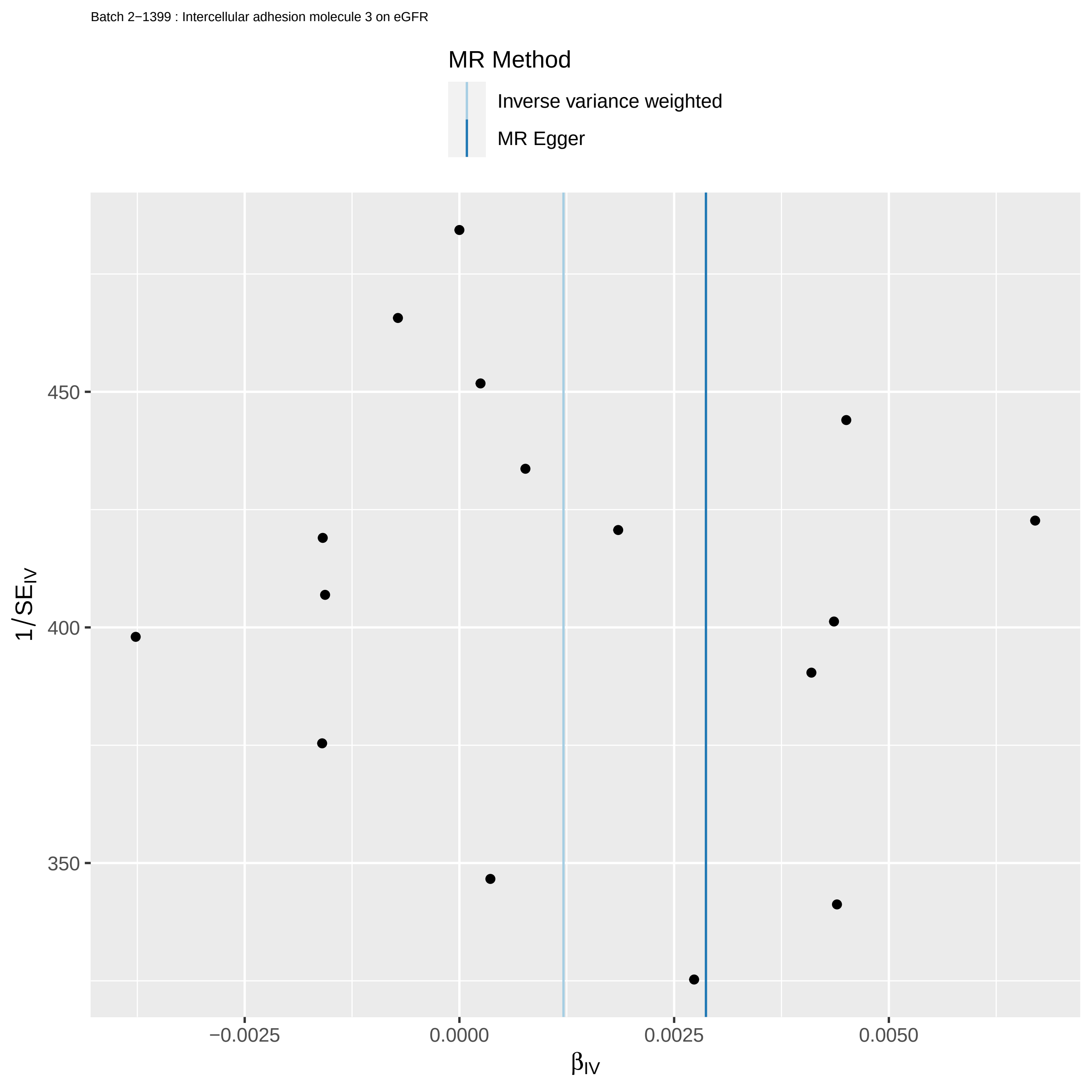

Batch 2−1399 : Intercellular adhesion molecule 3 on eGFR
MR Method
Inverse variance weighted
MR Egger
450
400
350
1/SE_IV
−0.0025
0.0000
0.0025
0.0050
β_IV

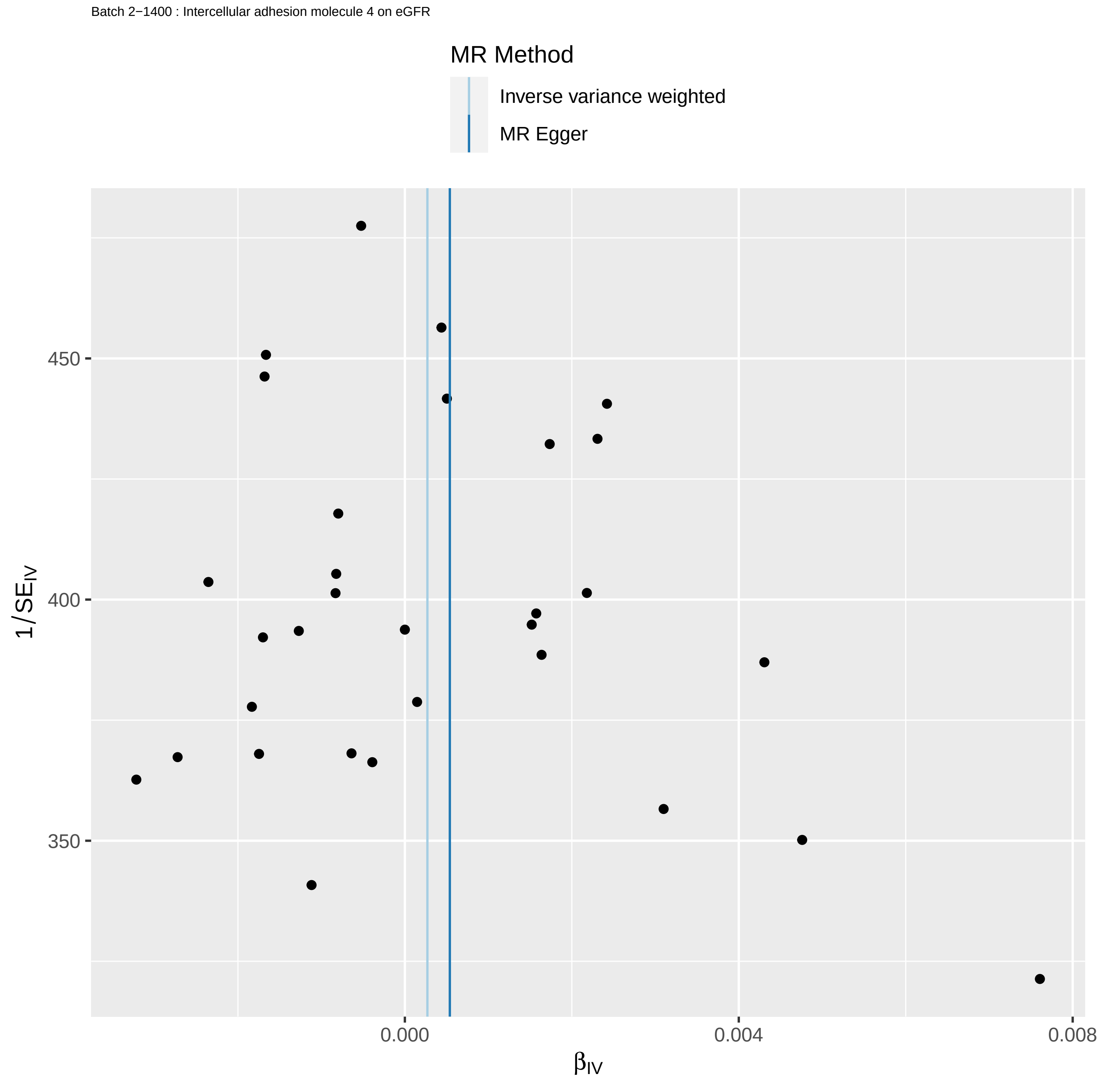

Batch 2−1400 : Intercellular adhesion molecule 4 on eGFR
MR Method
Inverse variance weighted
MR Egger
450
400
350
1/SE_IV
0.000
0.004
0.008
β_IV

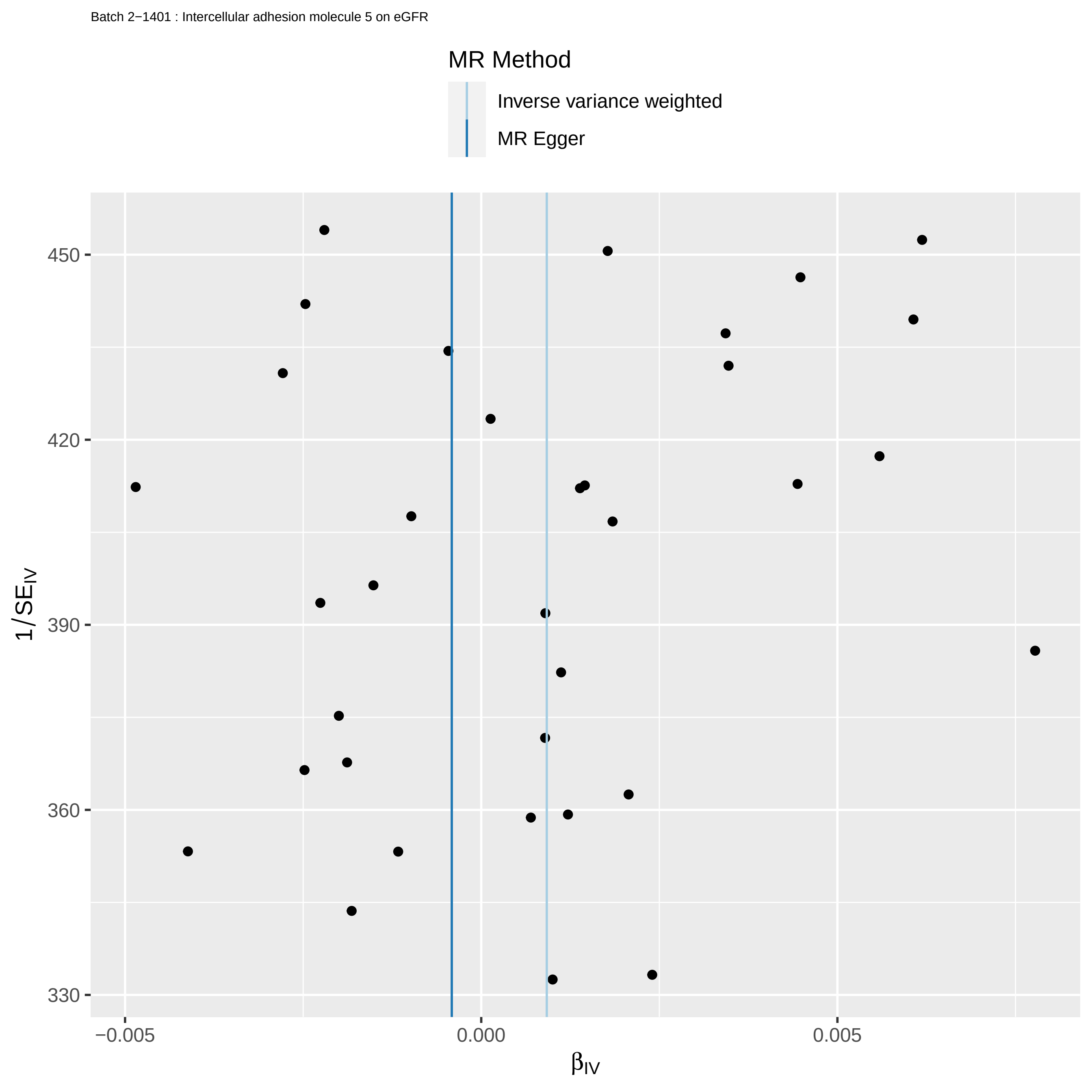

Batch 2−1401 : Intercellular adhesion molecule 5 on eGFR
MR Method
Inverse variance weighted
MR Egger
450
420
390
360
330
1/SE_IV
−0.005
0.000
0.005
β_IV

Batch 2−1402 : Intercellular adhesion molecule 5 on eGFR

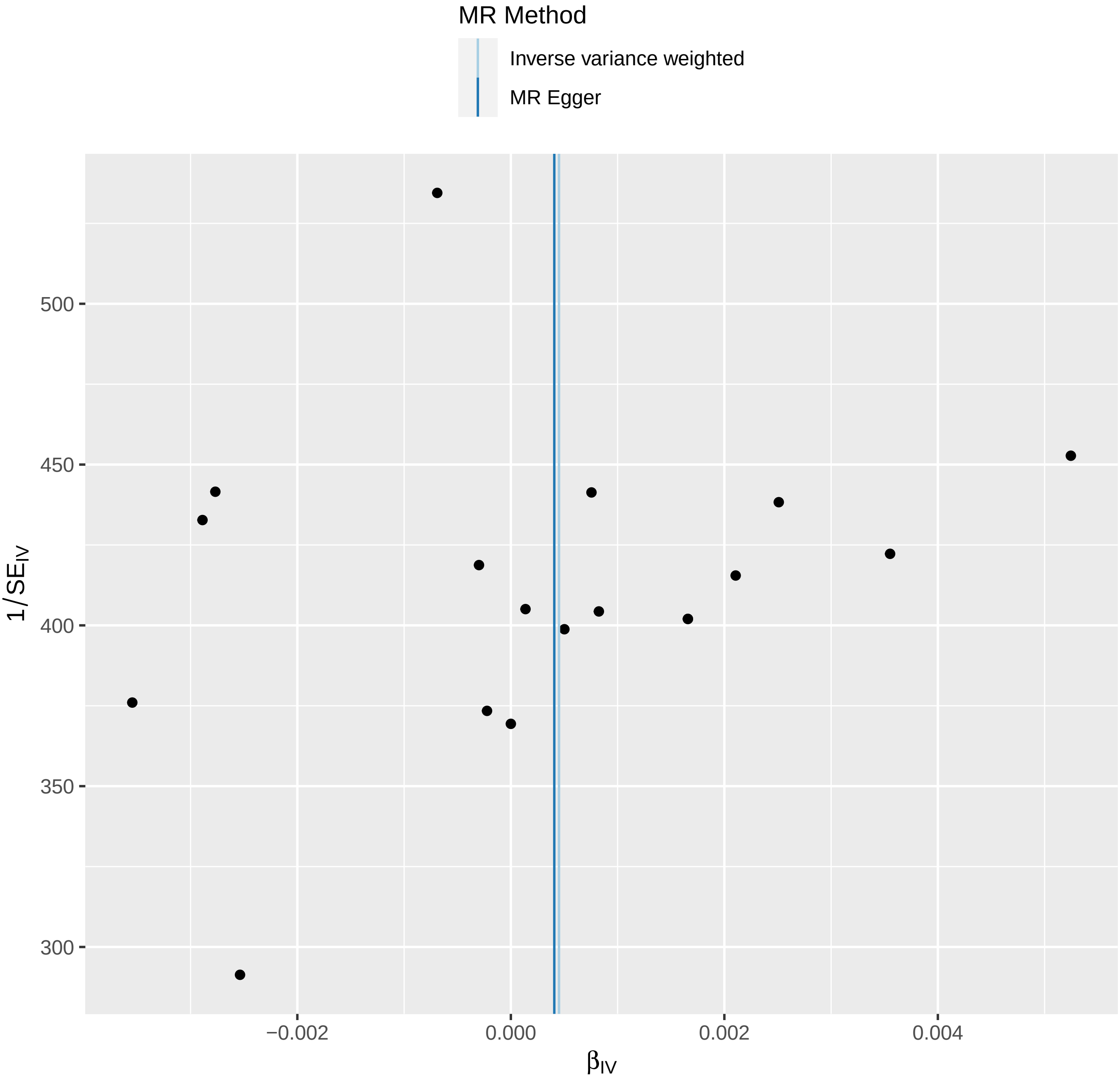

Batch 2−1403 : Inducible T−cell costimulator on eGFR

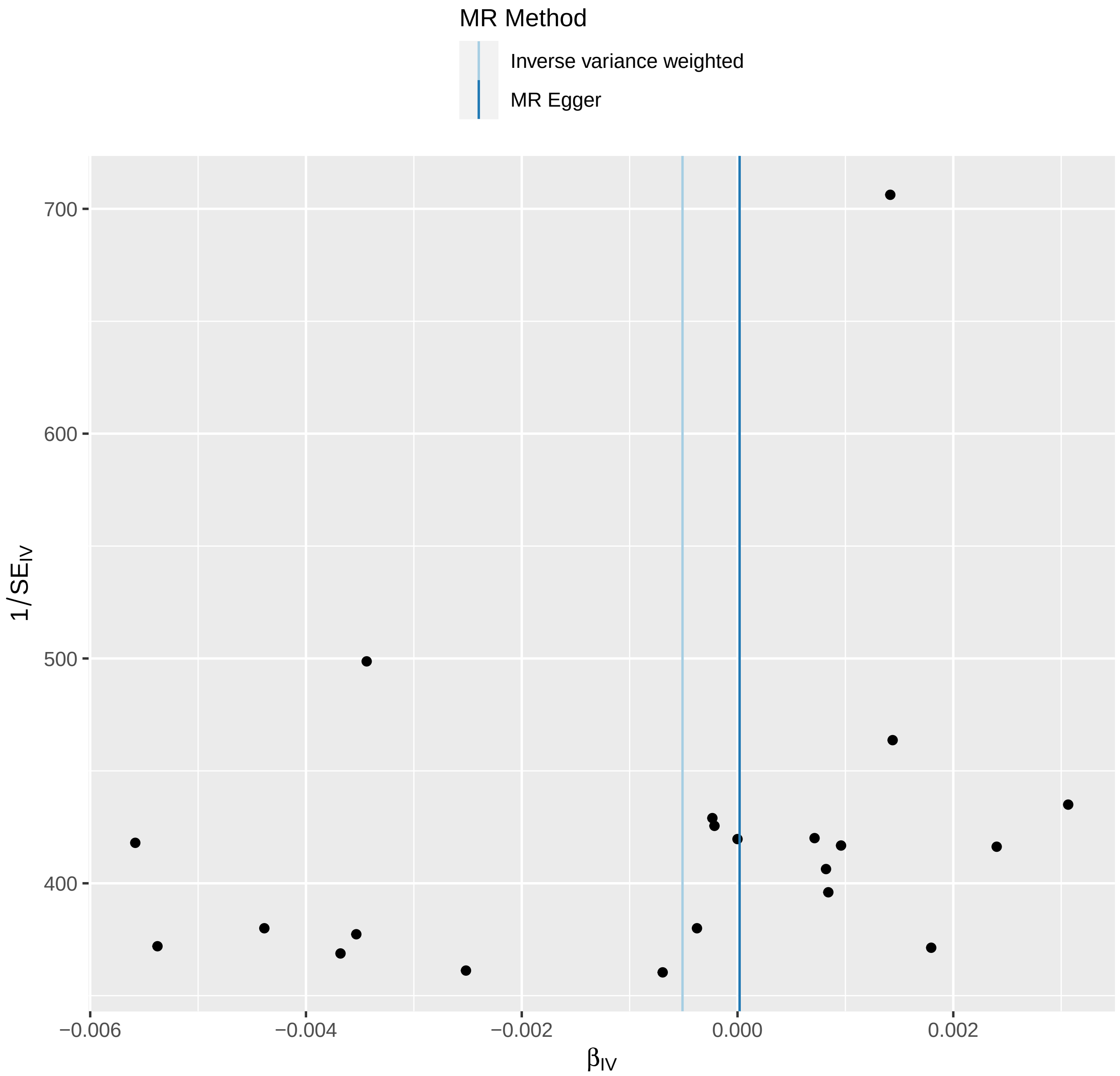

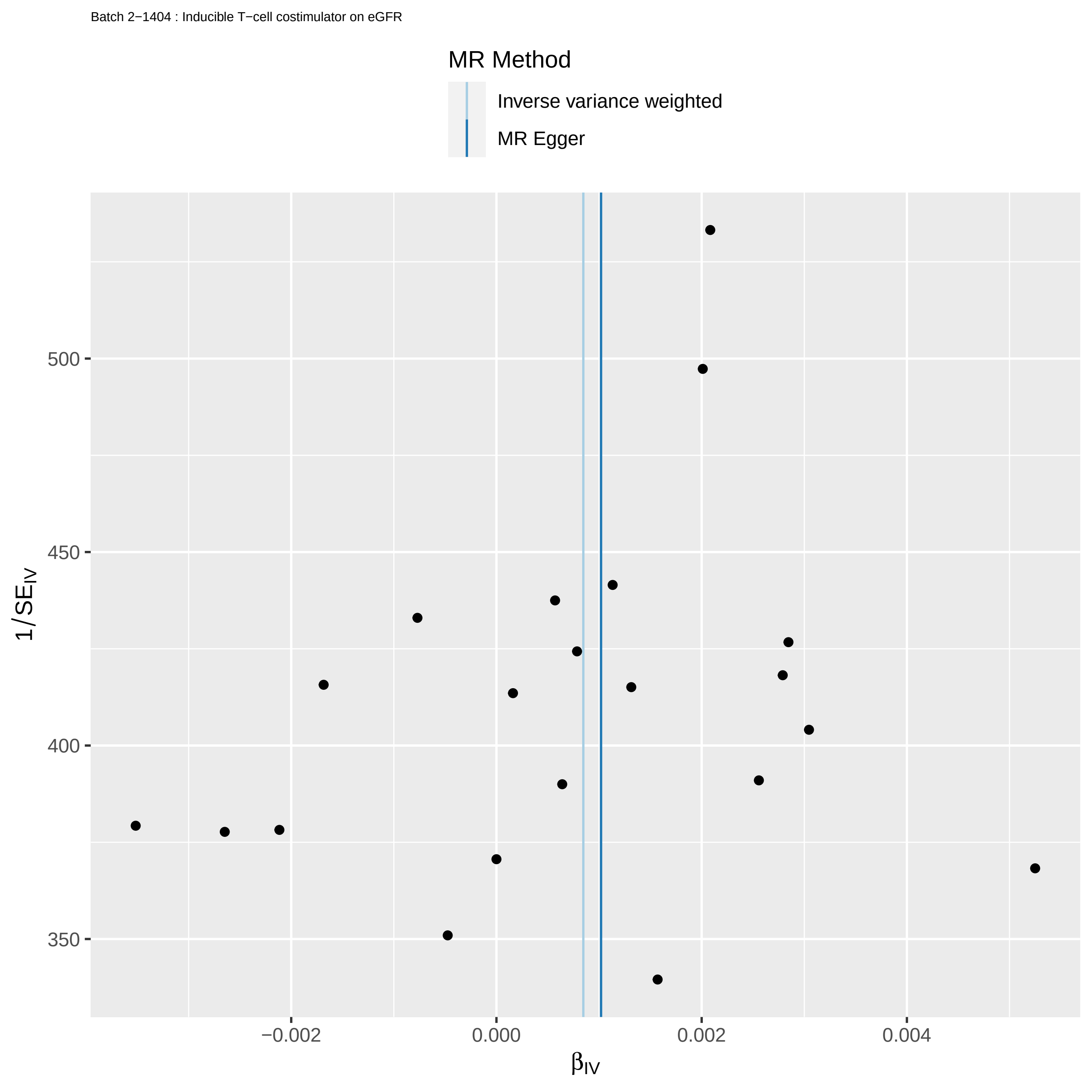
Batch 2−1404 : Inducible T−cell costimulator on eGFR
MR Method
Inverse variance weighted
MR Egger
500
450
400
350
1/SE_IV
−0.002
0.000
0.002
0.004
β_IV

Batch 2−1405 : ICOS ligand on eGFR

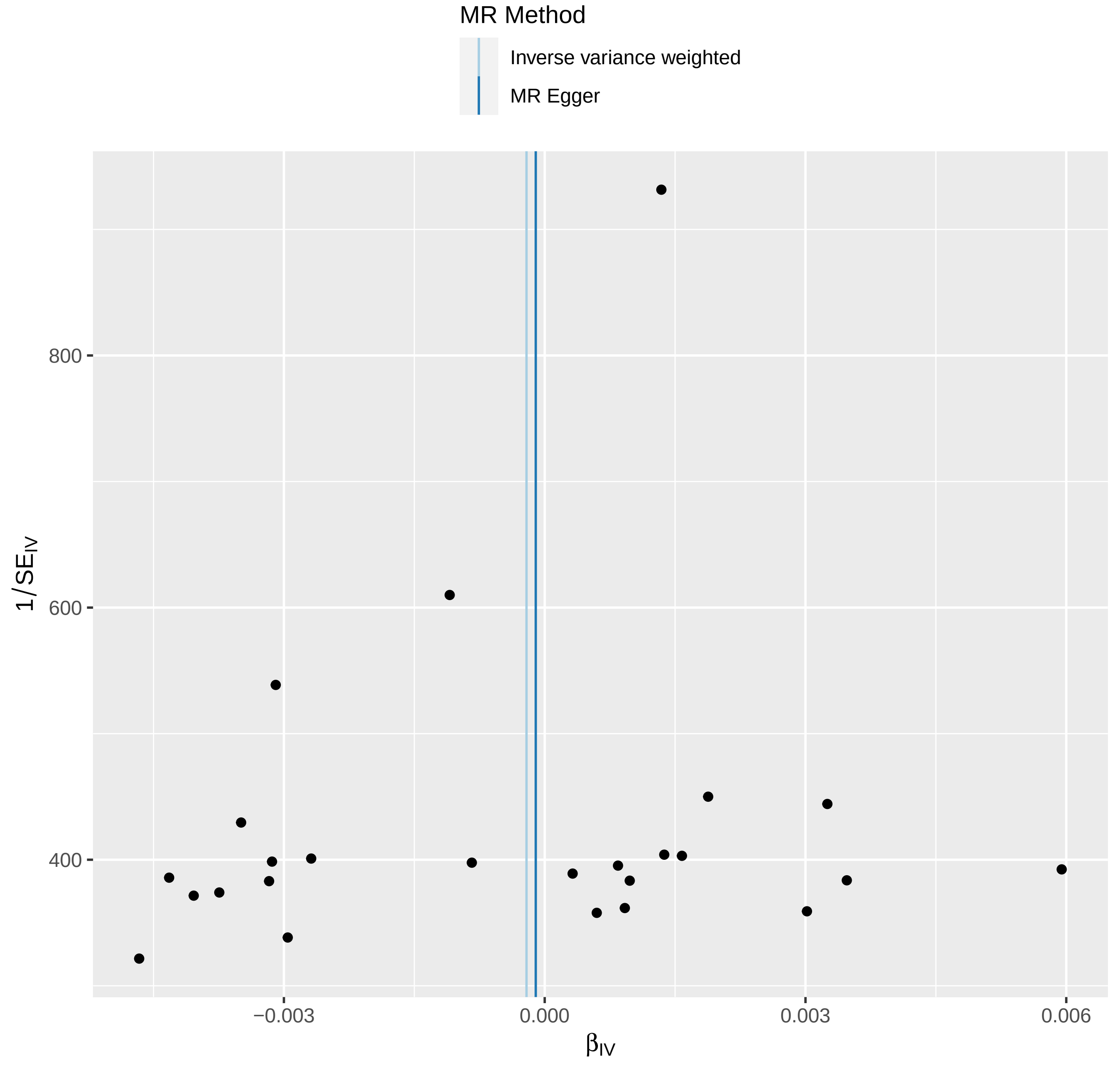

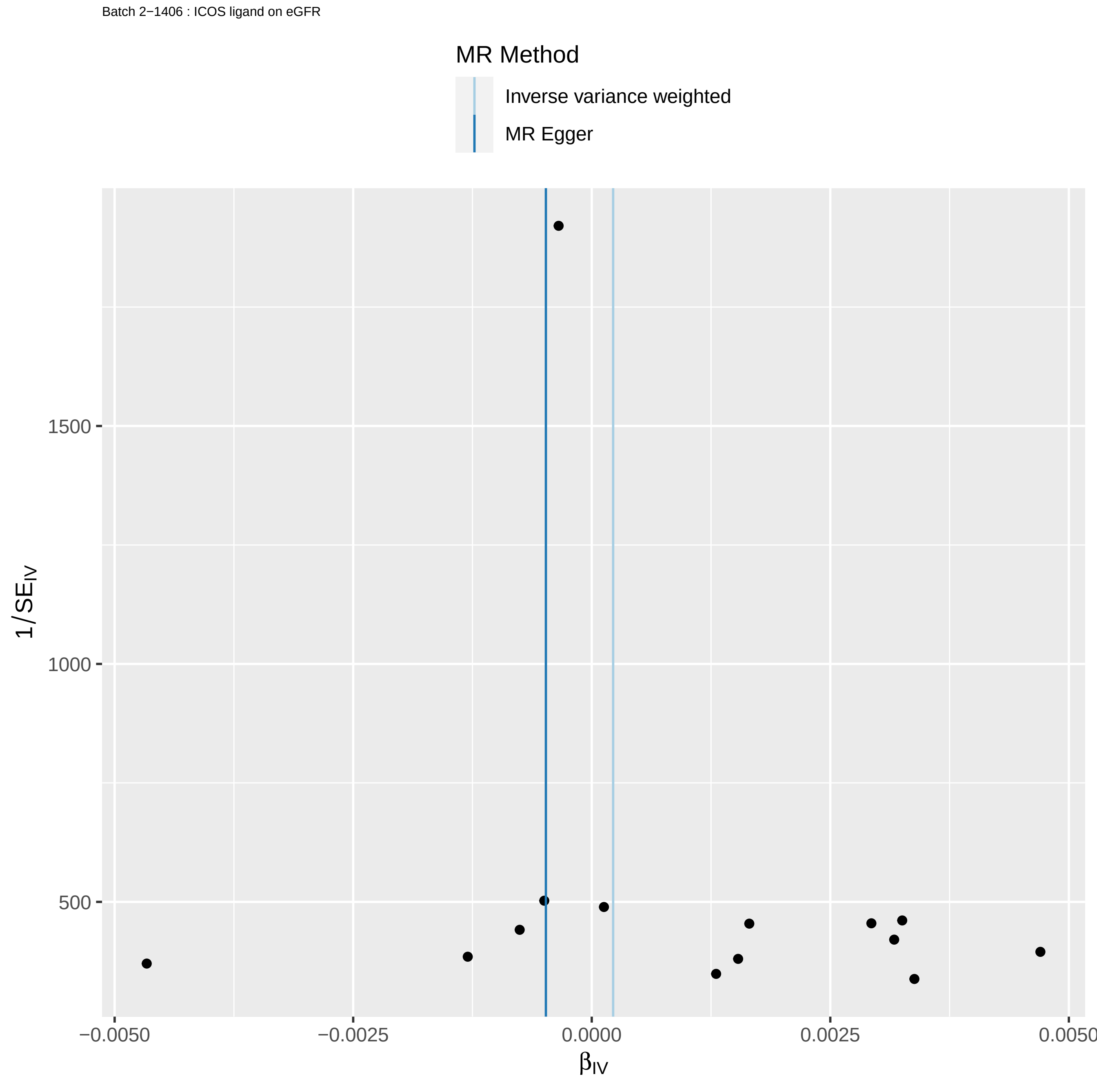
Batch 2−1406 : ICOS ligand on eGFR
MR Method
Inverse variance weighted
MR Egger
1500
1000
500
1/SE$_{IV}$
−0.0050
−0.0025
0.0000
0.0025
0.0050
β$_{IV}$

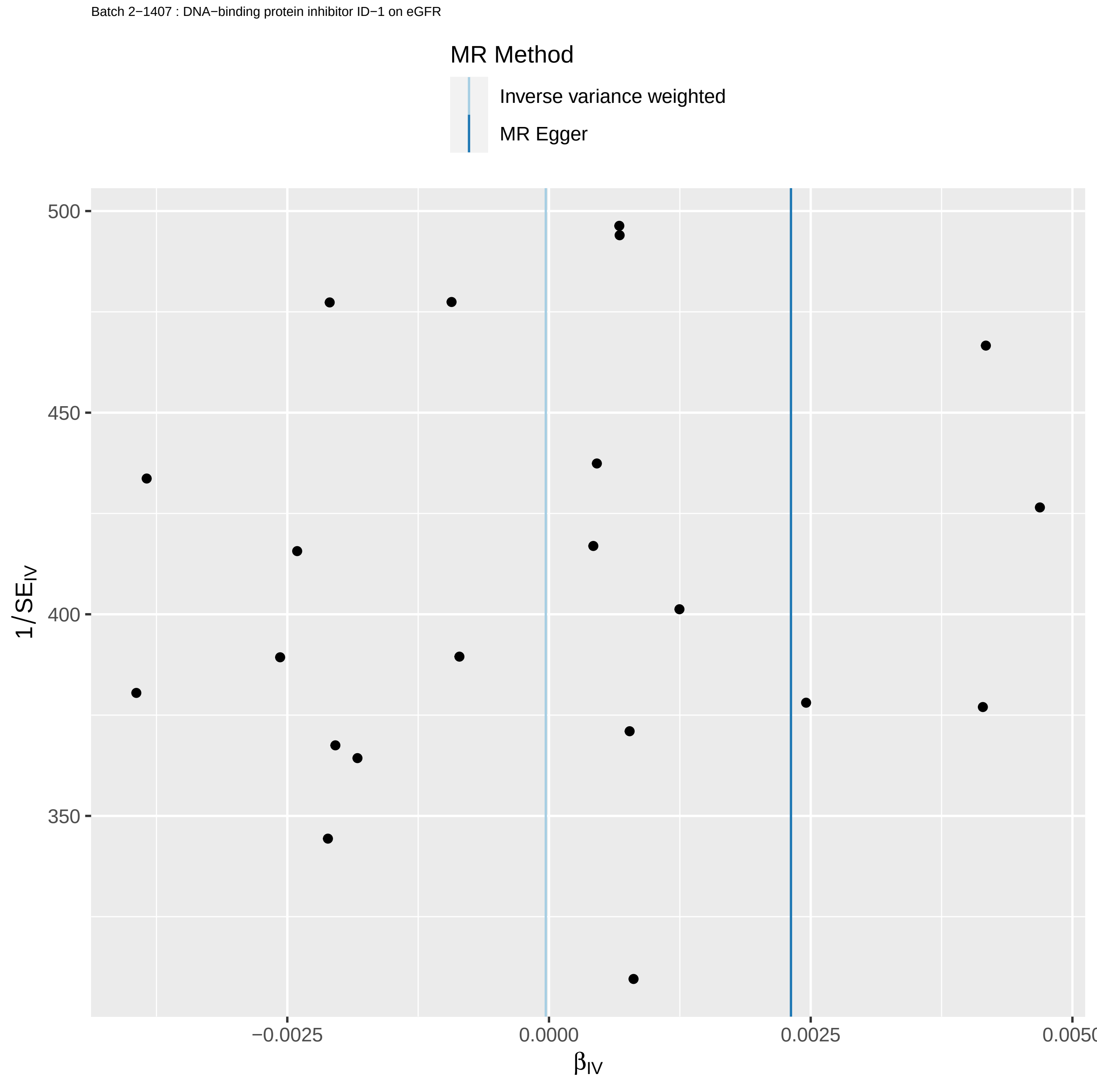

Batch 2−1407 : DNA−binding protein inhibitor ID−1 on eGFR
MR Method
Inverse variance weighted
MR Egger
500
450
400
350
1/SE$_{IV}$
−0.0025
0.0000
0.0025
0.0050
β$_{IV}$

Batch 2−1408 : DNA−binding protein inhibitor ID−2 on eGFR

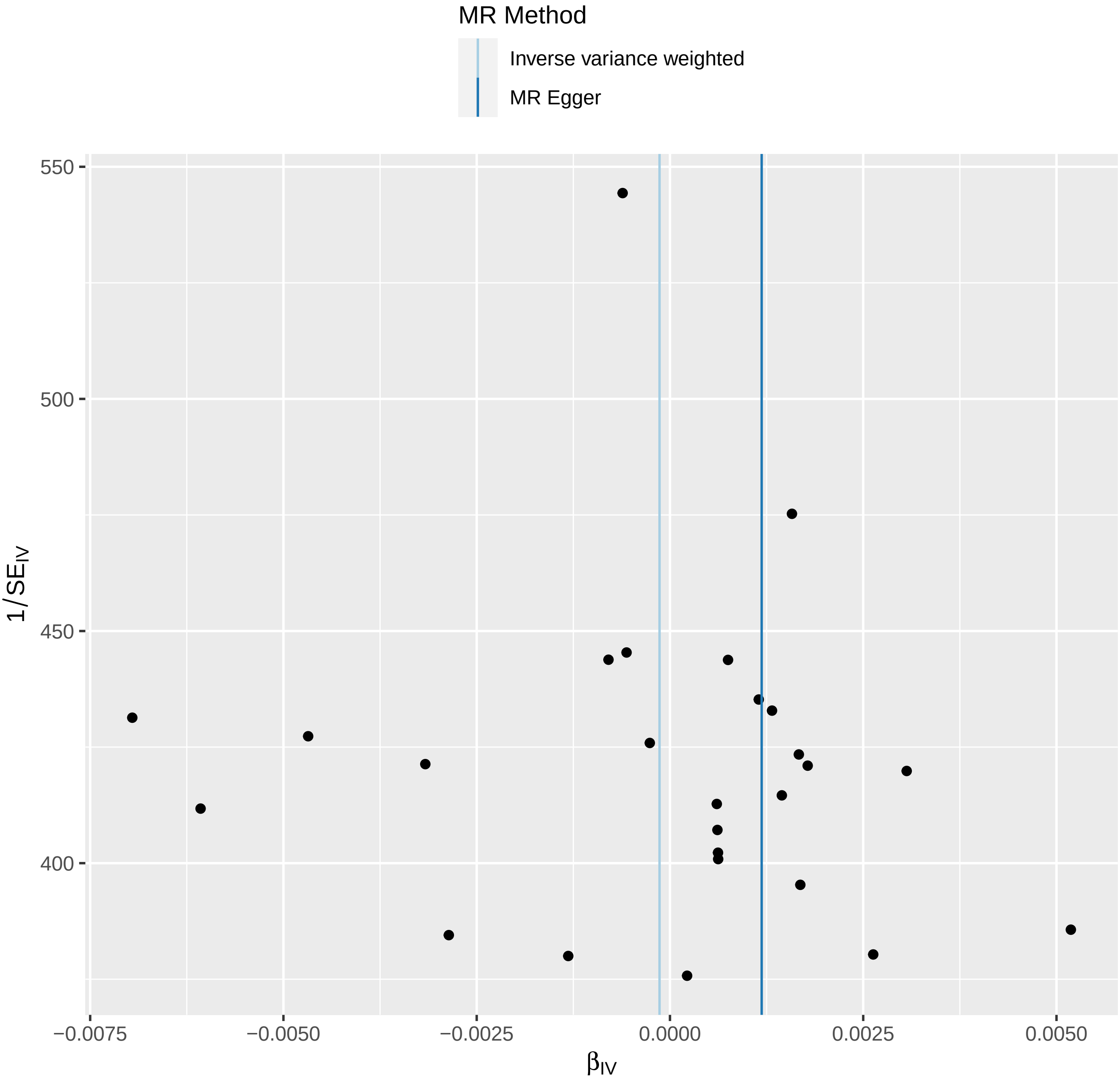

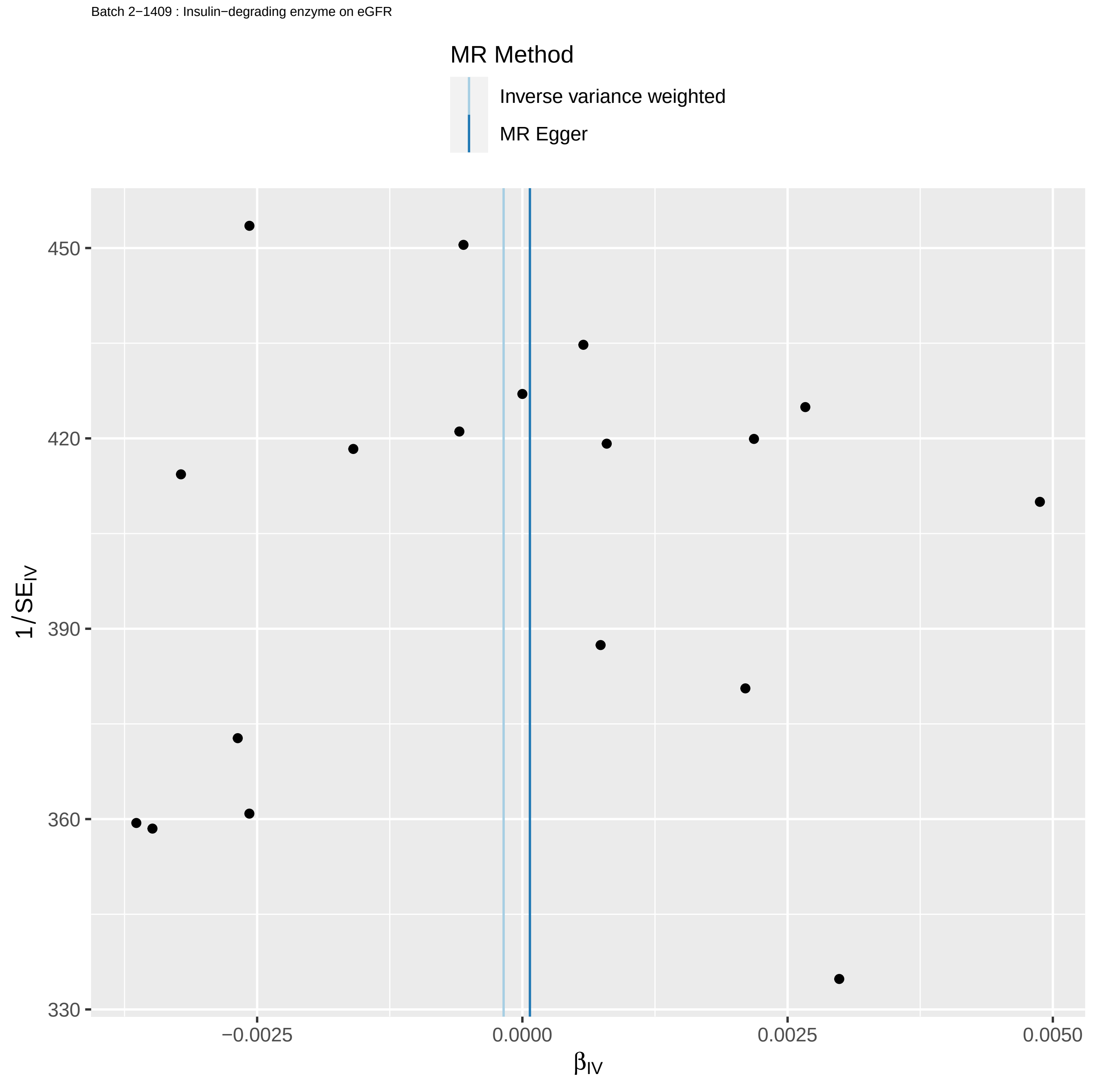

Batch 2−1409 : Insulin−degrading enzyme on eGFR
MR Method
Inverse variance weighted
MR Egger
1/SE_IV
450
420
390
360
330
−0.0025
0.0000
0.0025
0.0050
β_IV

Batch 2−1410 : Indoleamine 2,3−dioxygenase 1 on eGFR

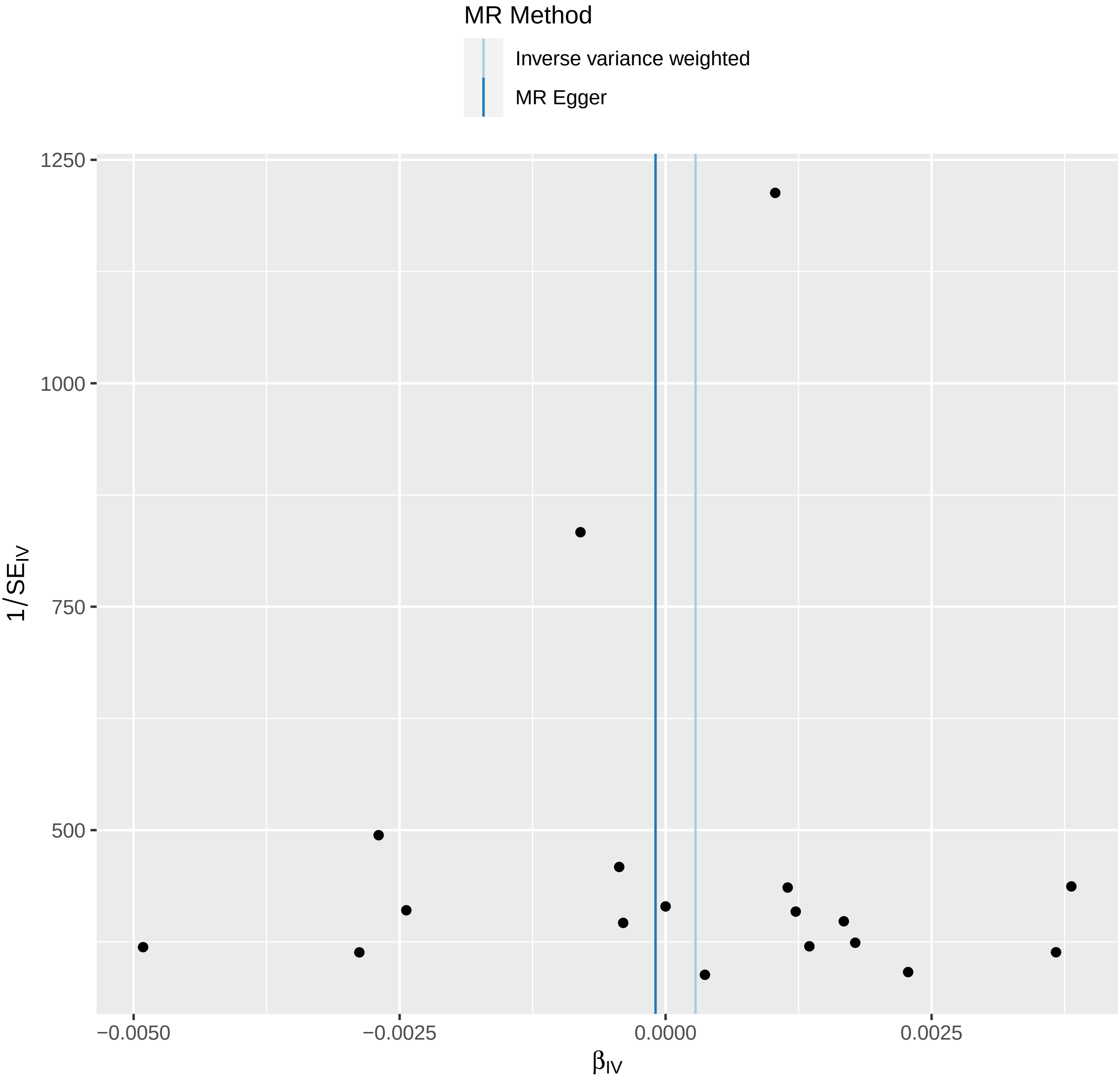

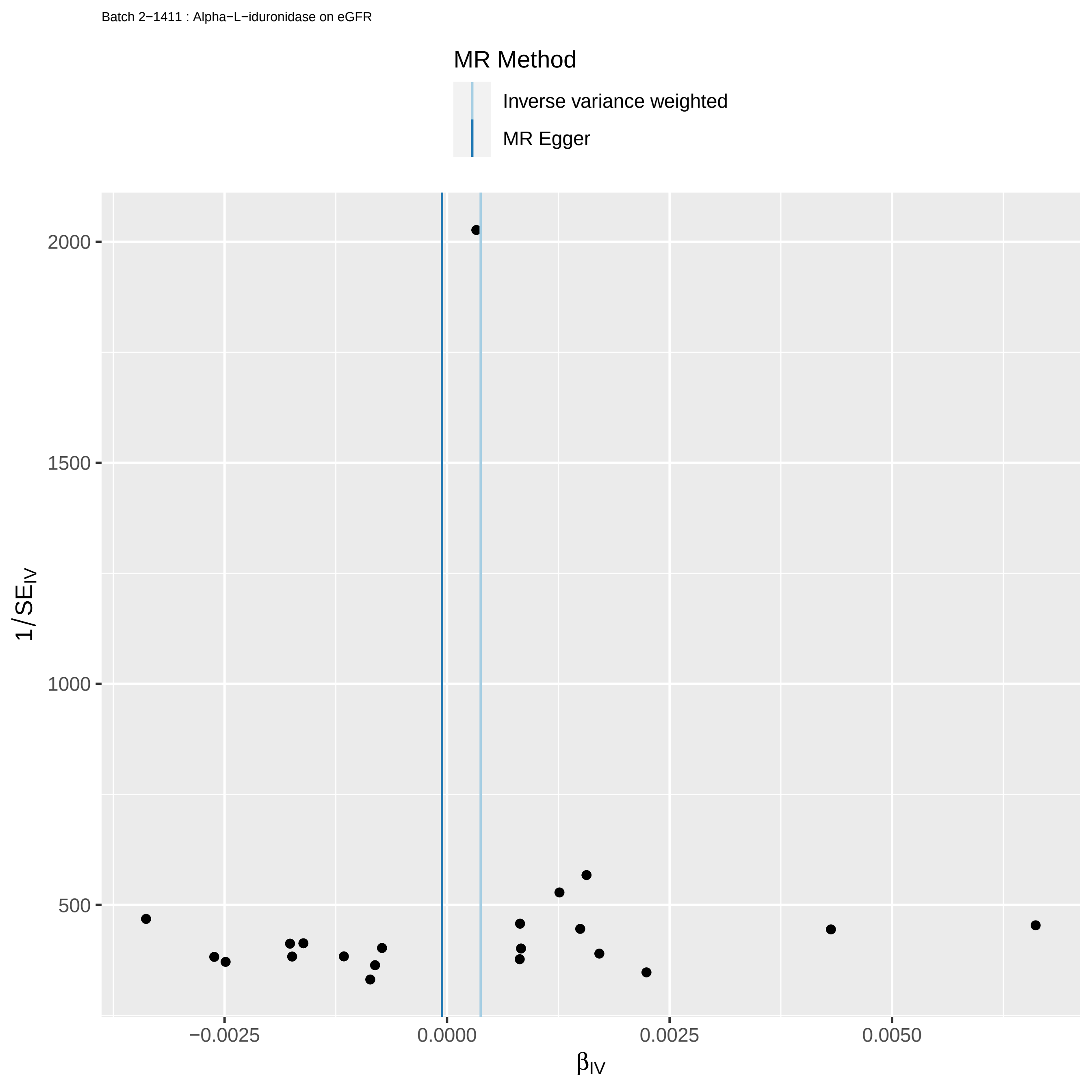

Batch 2−1411 : Alpha−L−iduronidase on eGFR
MR Method
Inverse variance weighted
MR Egger
2000
1500
1000
500
1/SE_IV
−0.0025
0.0000
0.0025
0.0050
β_IV

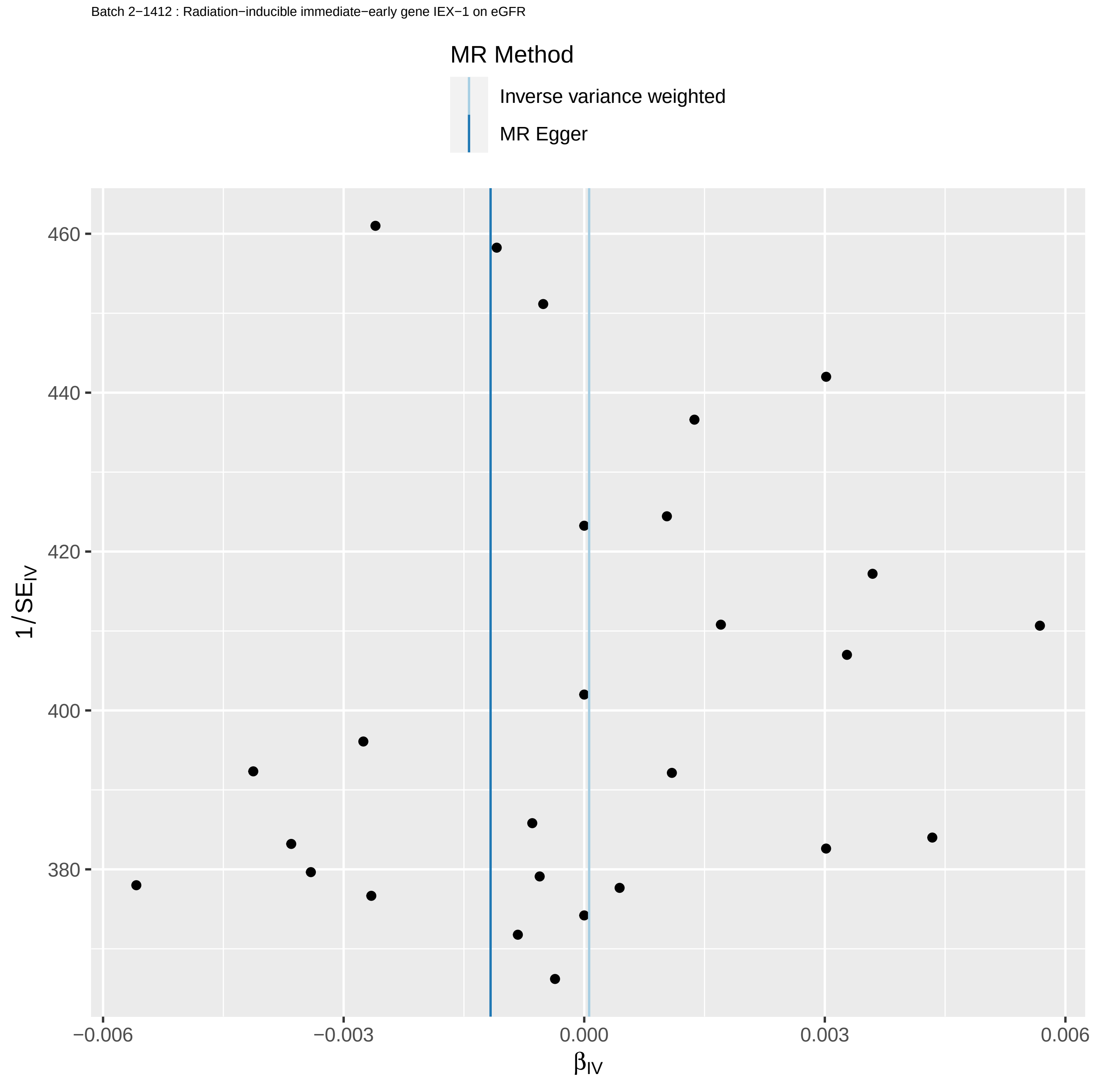

Batch 2−1412 : Radiation−inducible immediate−early gene IEX−1 on eGFR
MR Method
Inverse variance weighted
MR Egger
460
440
420
400
380
1/SE$_{IV}$
−0.006
−0.003
0.000
0.003
0.006
β$_{IV}$

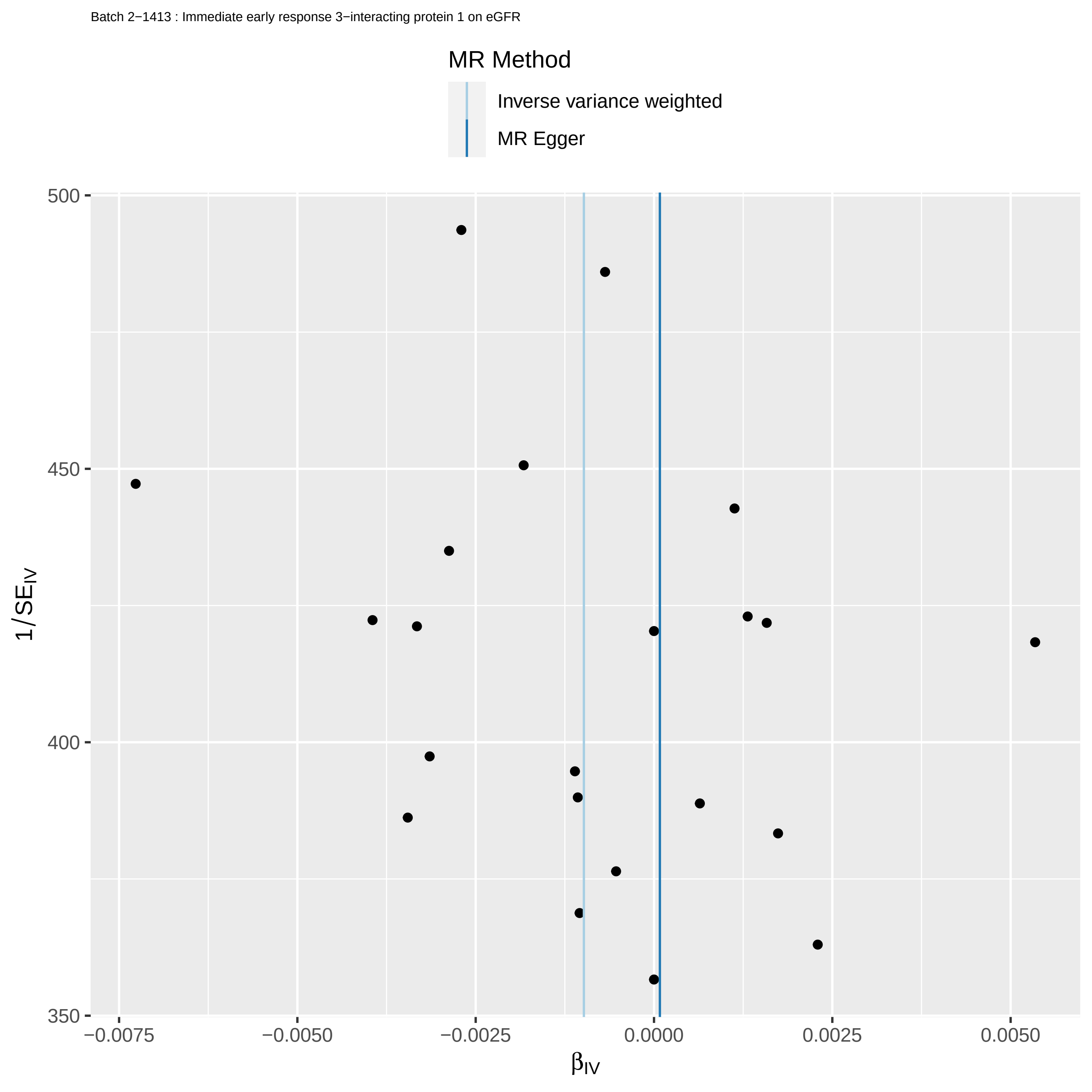
Batch 2−1413 : Immediate early response 3−interacting protein 1 on eGFR
MR Method
Inverse variance weighted
MR Egger
500
450
400
350
1/SE$_{IV}$
−0.0075
−0.0050
−0.0025
0.0000
0.0025
0.0050
β$_{IV}$

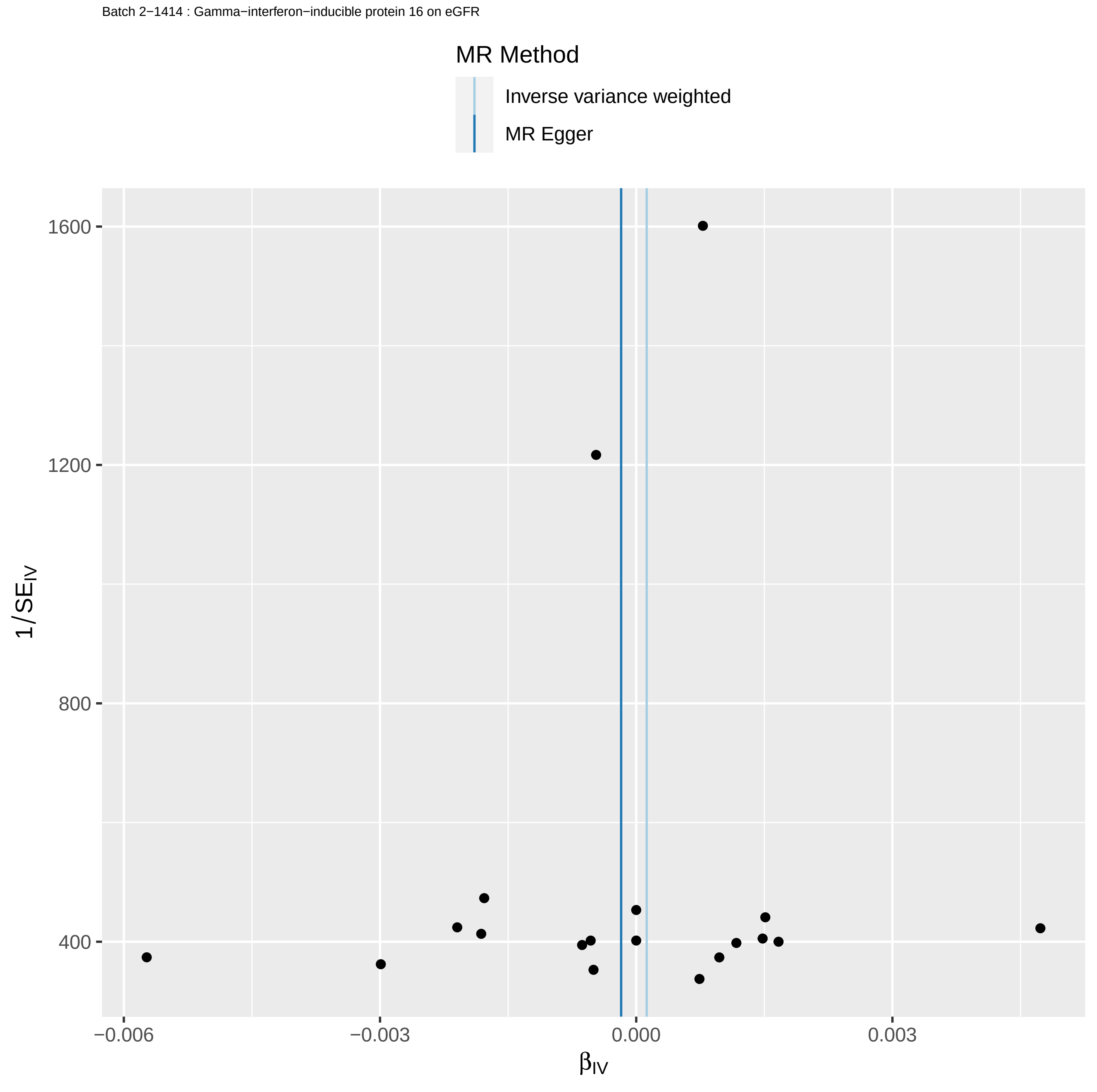
Batch 2−1414 : Gamma−interferon−inducible protein 16 on eGFR
MR Method
Inverse variance weighted
MR Egger
1600
1200
800
400
1/SE$_{IV}$
−0.006
−0.003
0.000
0.003
β$_{IV}$

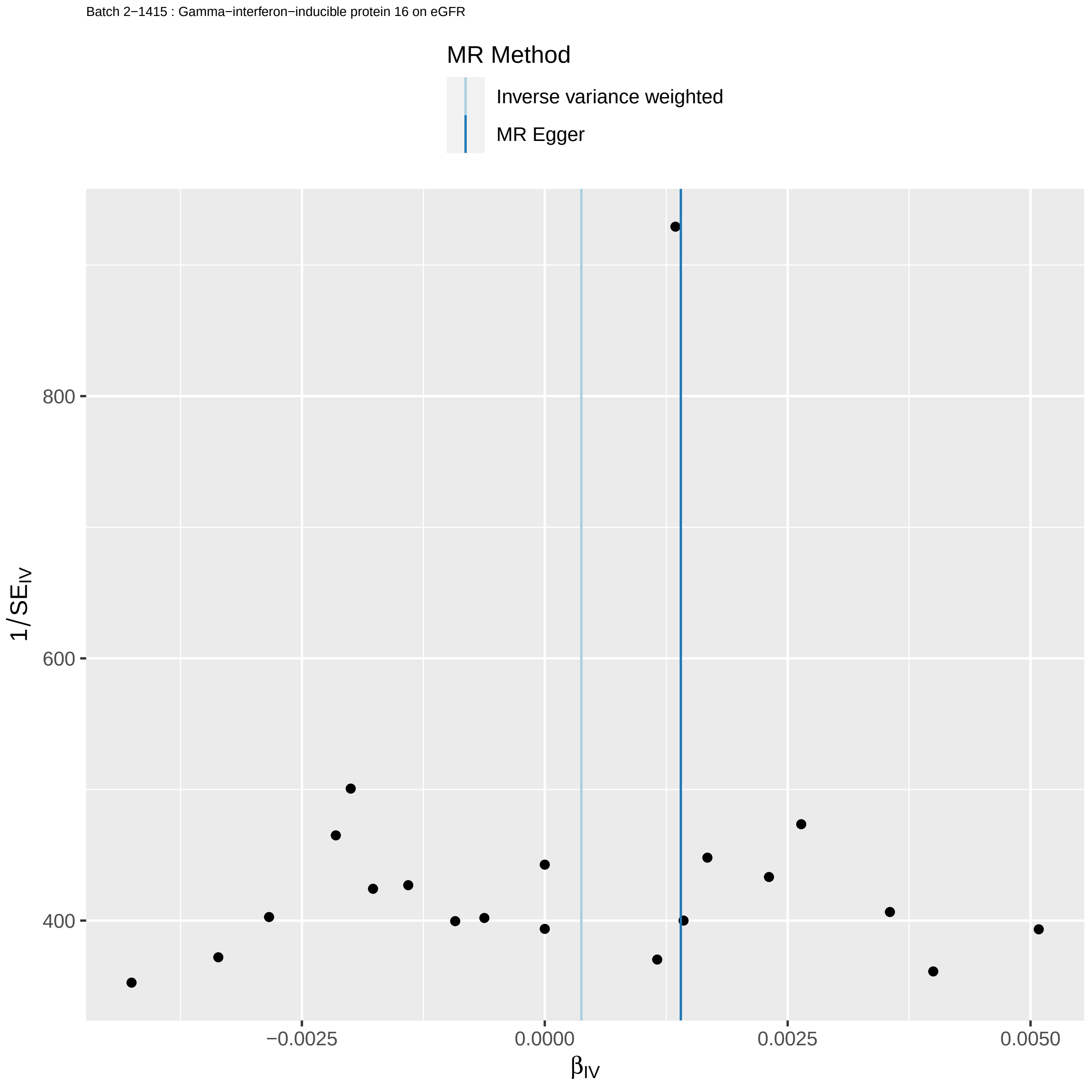

Batch 2−1415 : Gamma−interferon−inducible protein 16 on eGFR
MR Method
Inverse variance weighted
MR Egger
800
600
400
1/SE$_{IV}$
−0.0025
0.0000
0.0025
0.0050
β$_{IV}$

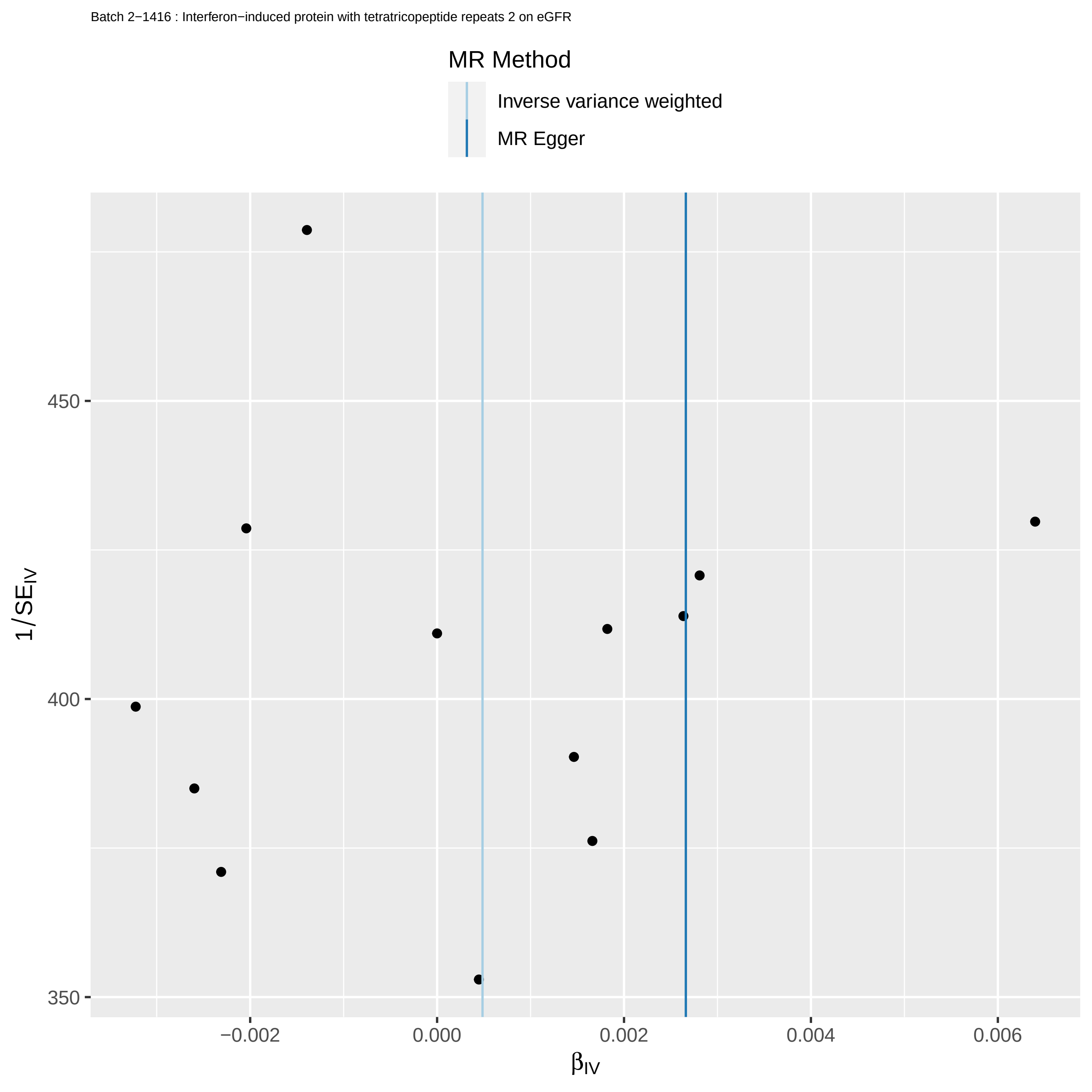

Batch 2−1416 : Interferon−induced protein with tetratricopeptide repeats 2 on eGFR
MR Method
Inverse variance weighted
MR Egger
450
400
350
1/SE$_{IV}$
−0.002
0.000
0.002
0.004
0.006
β$_{IV}$

Batch 2−1417 : Interferon−induced protein with tetratricopeptide repeats 3 on eGFR

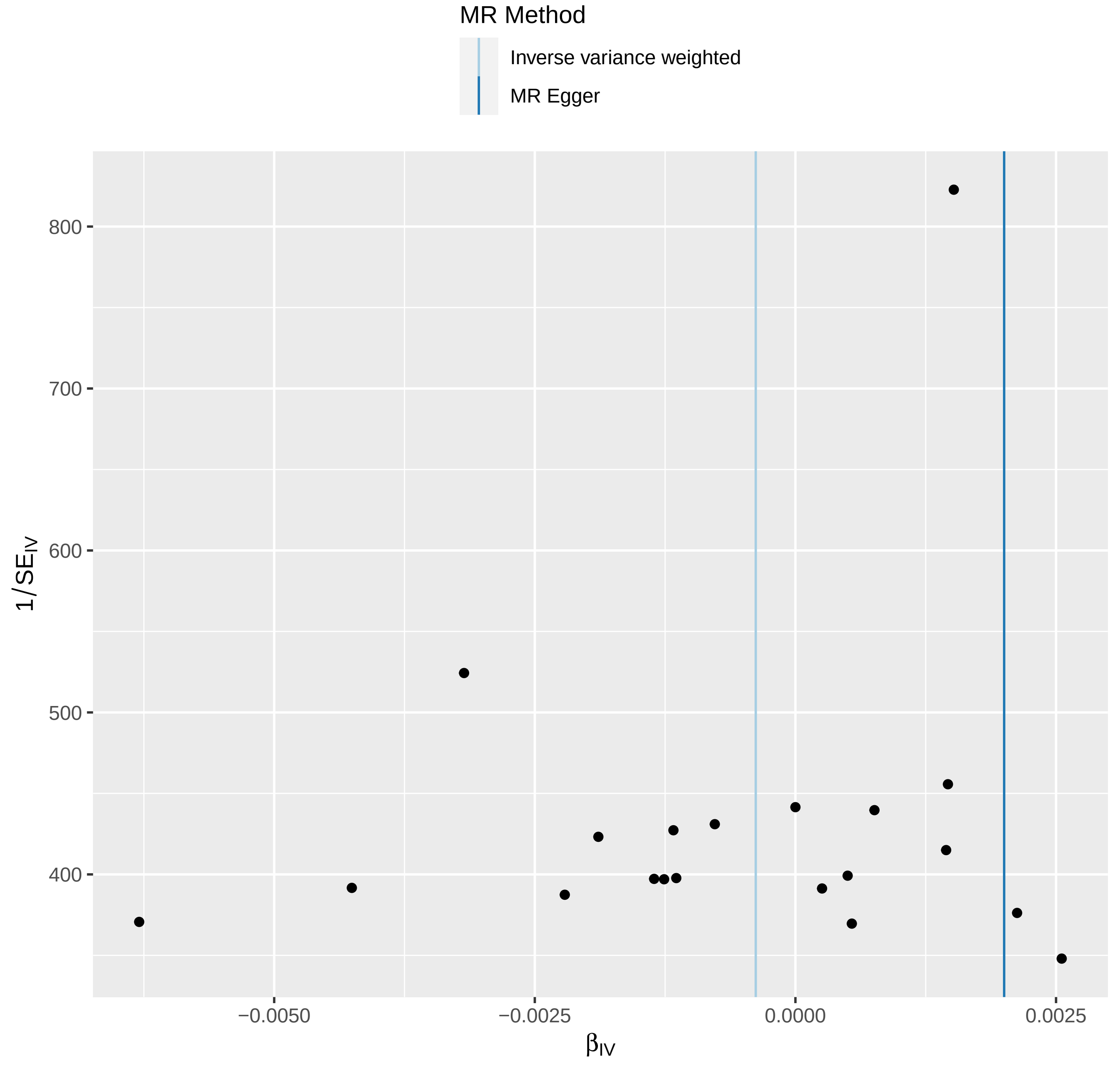

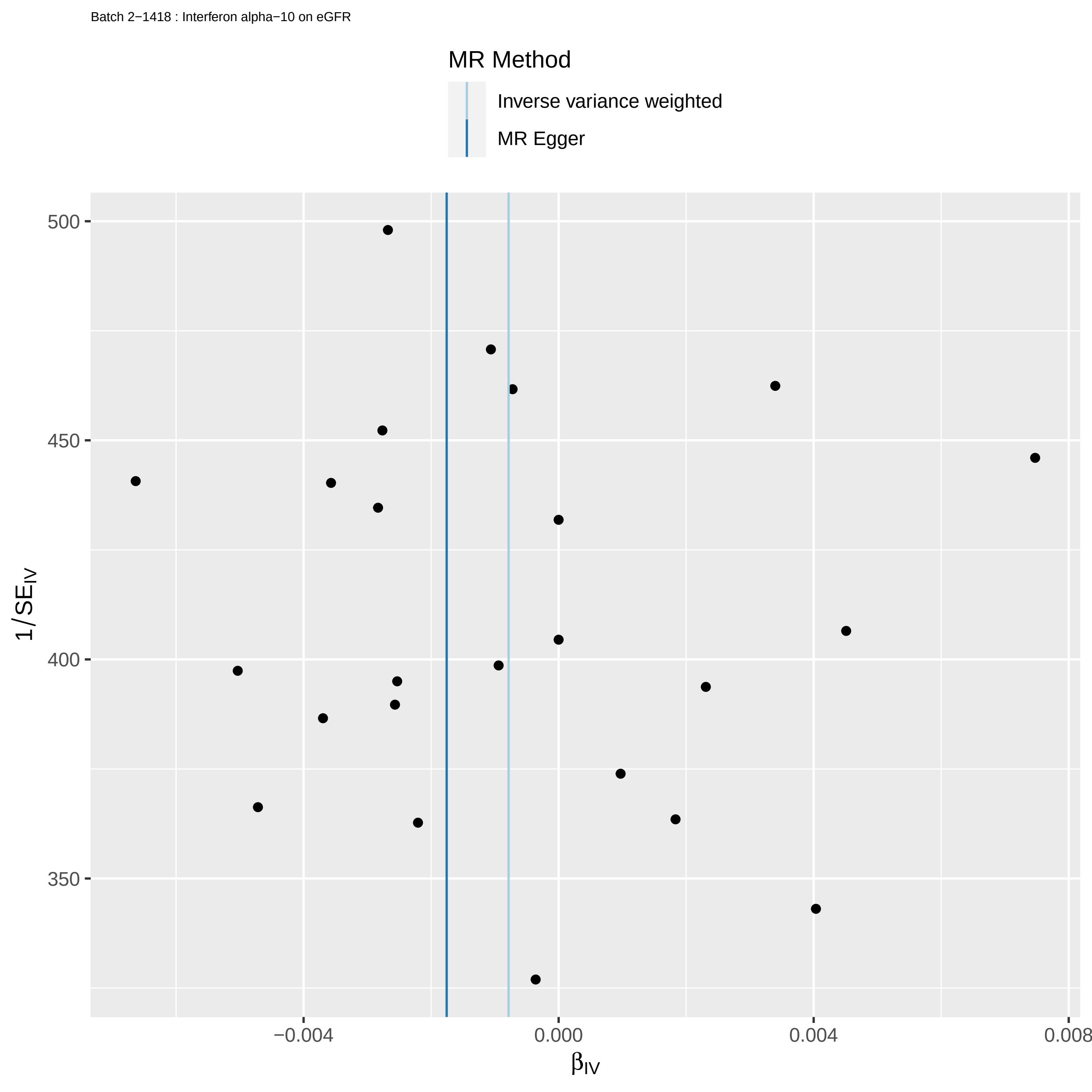

Batch 2−1418 : Interferon alpha−10 on eGFR
MR Method
Inverse variance weighted
MR Egger
500
450
400
350
1/SE_IV
−0.004
0.000
0.004
0.008
β_IV

Batch 2−1419 : Interferon alpha−10 on eGFR

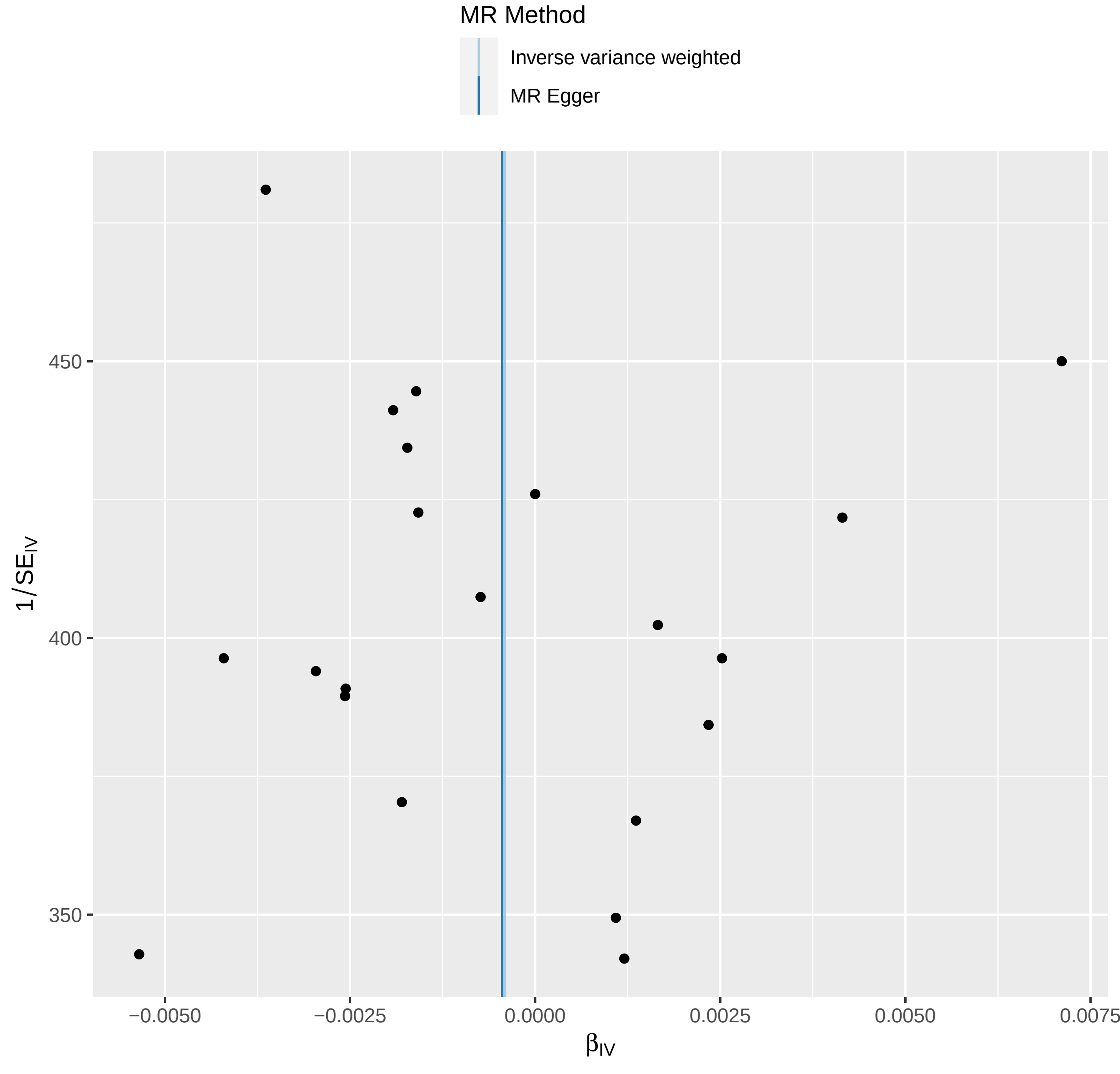

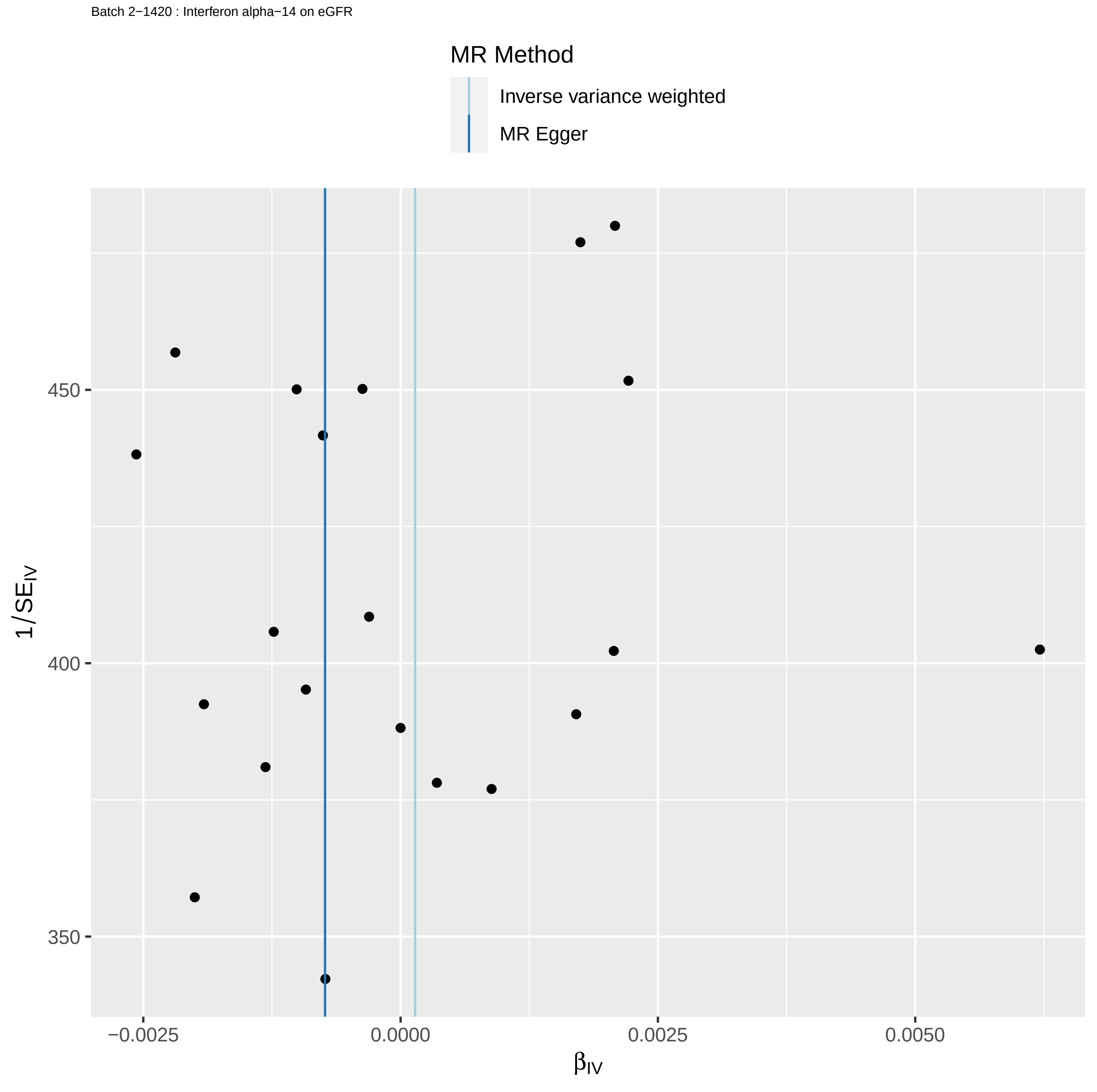
Batch 2−1420 : Interferon alpha−14 on eGFR
MR Method
Inverse variance weighted
MR Egger
450
400
350
1/SE_IV
−0.0025
0.0000
0.0025
0.0050
β_IV

Batch 2−1421 : Interferon alpha−4 on eGFR

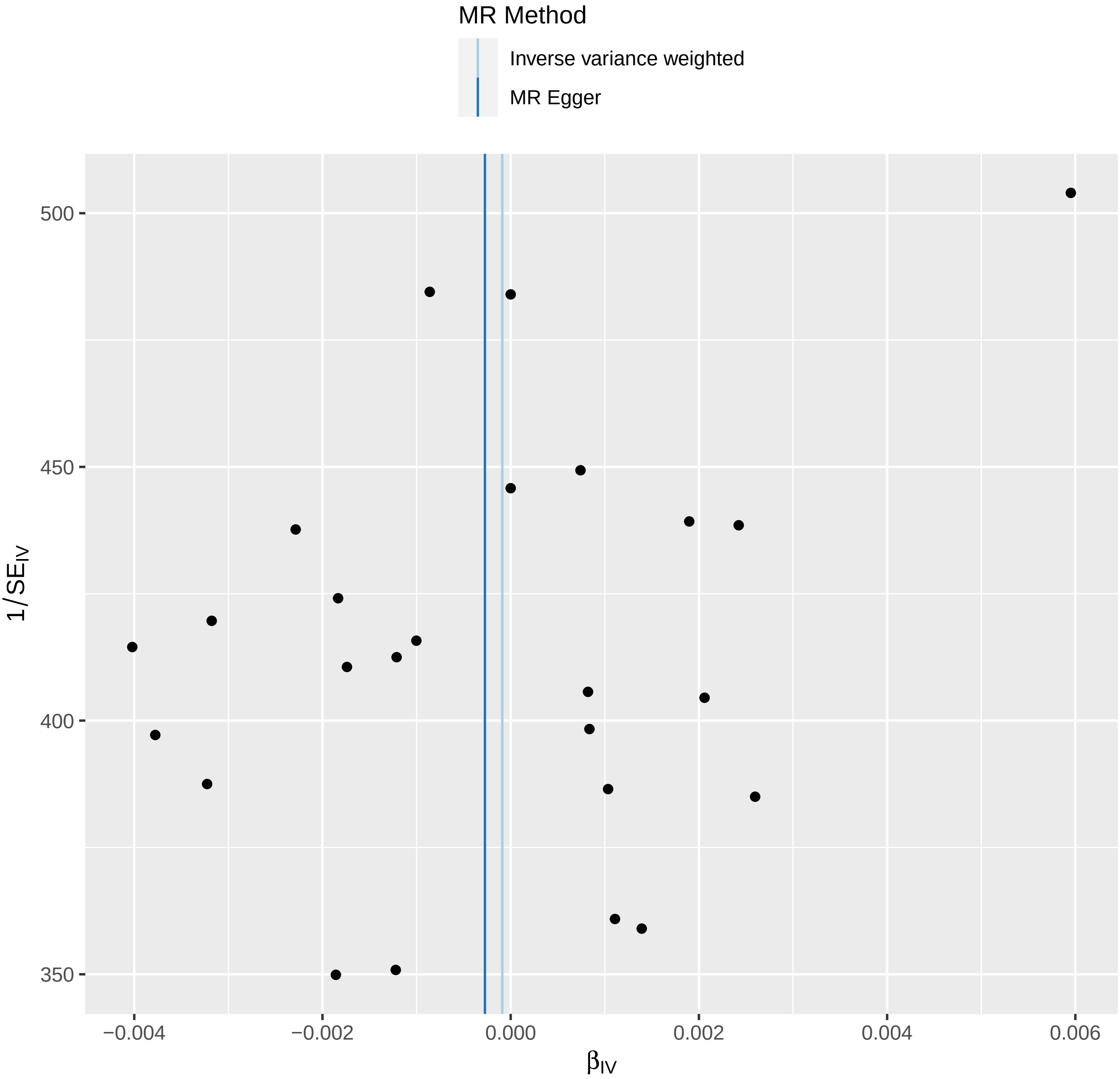

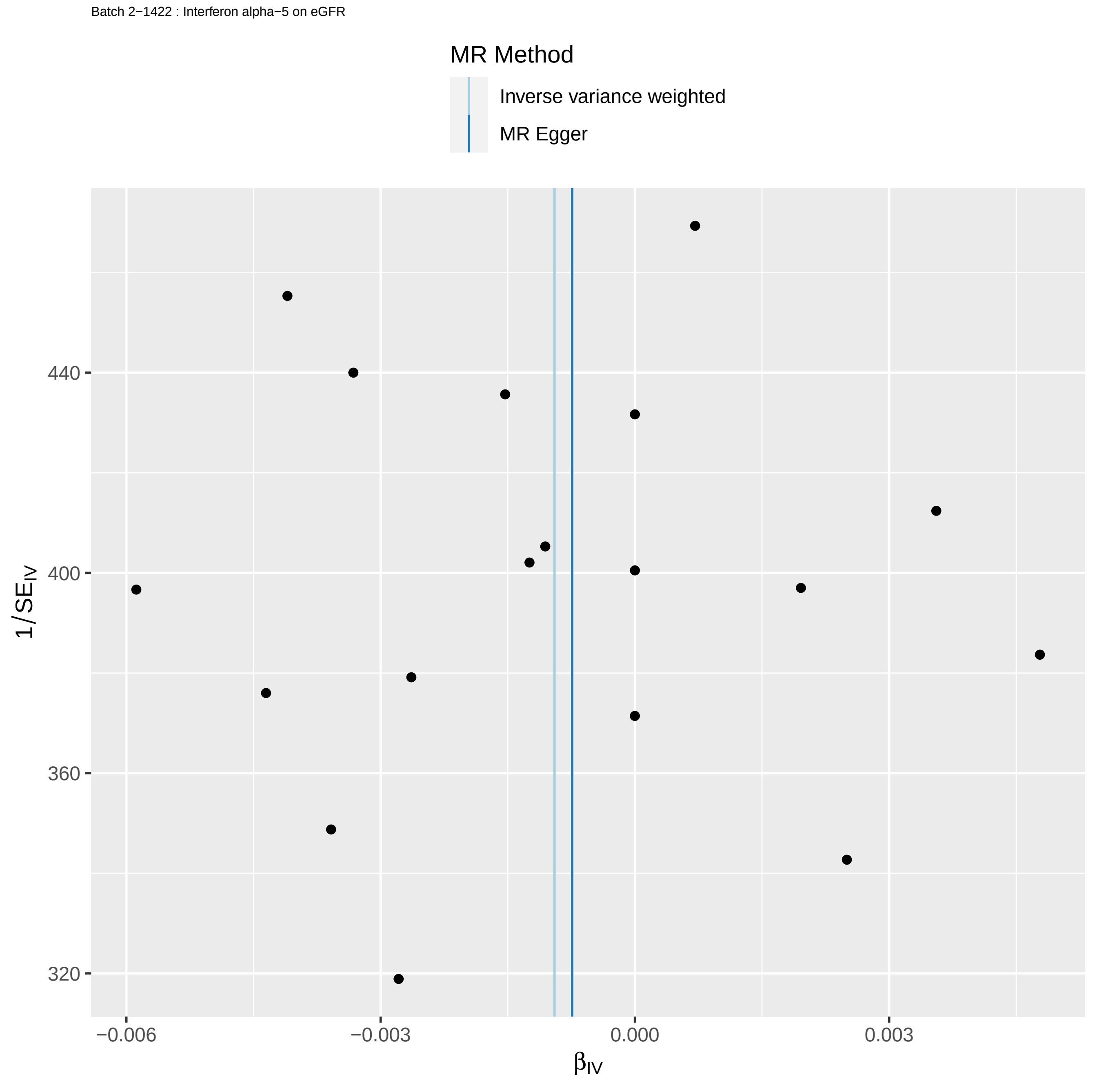
Batch 2−1422 : Interferon alpha−5 on eGFR
MR Method
Inverse variance weighted
MR Egger
440
400
360
320
1/SE_IV
−0.006
−0.003
0.000
0.003
β_IV

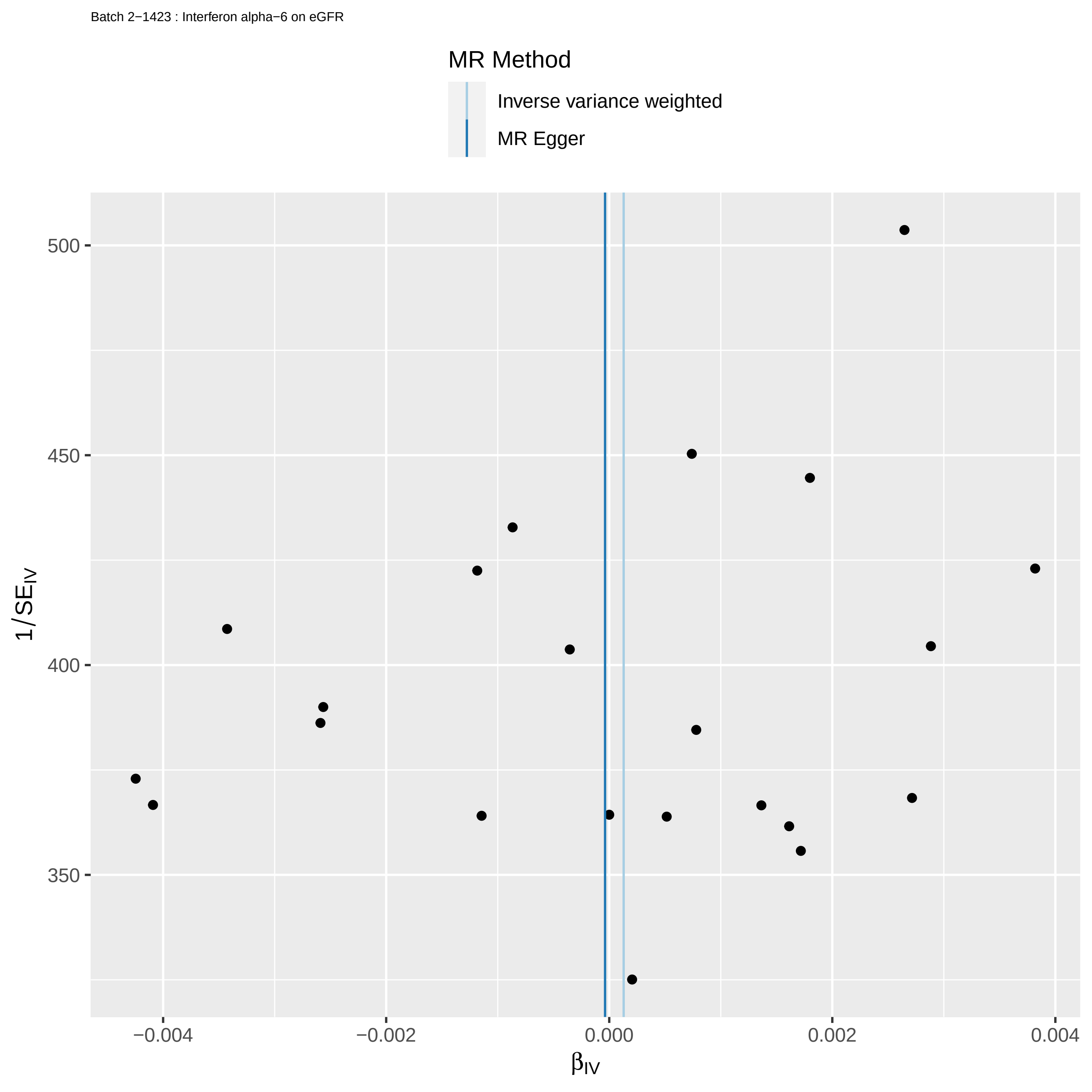

Batch 2−1423 : Interferon alpha−6 on eGFR
MR Method
Inverse variance weighted
MR Egger
500
450
400
350
1/SE$_{IV}$
−0.004
−0.002
0.000
0.002
0.004
β_{IV}

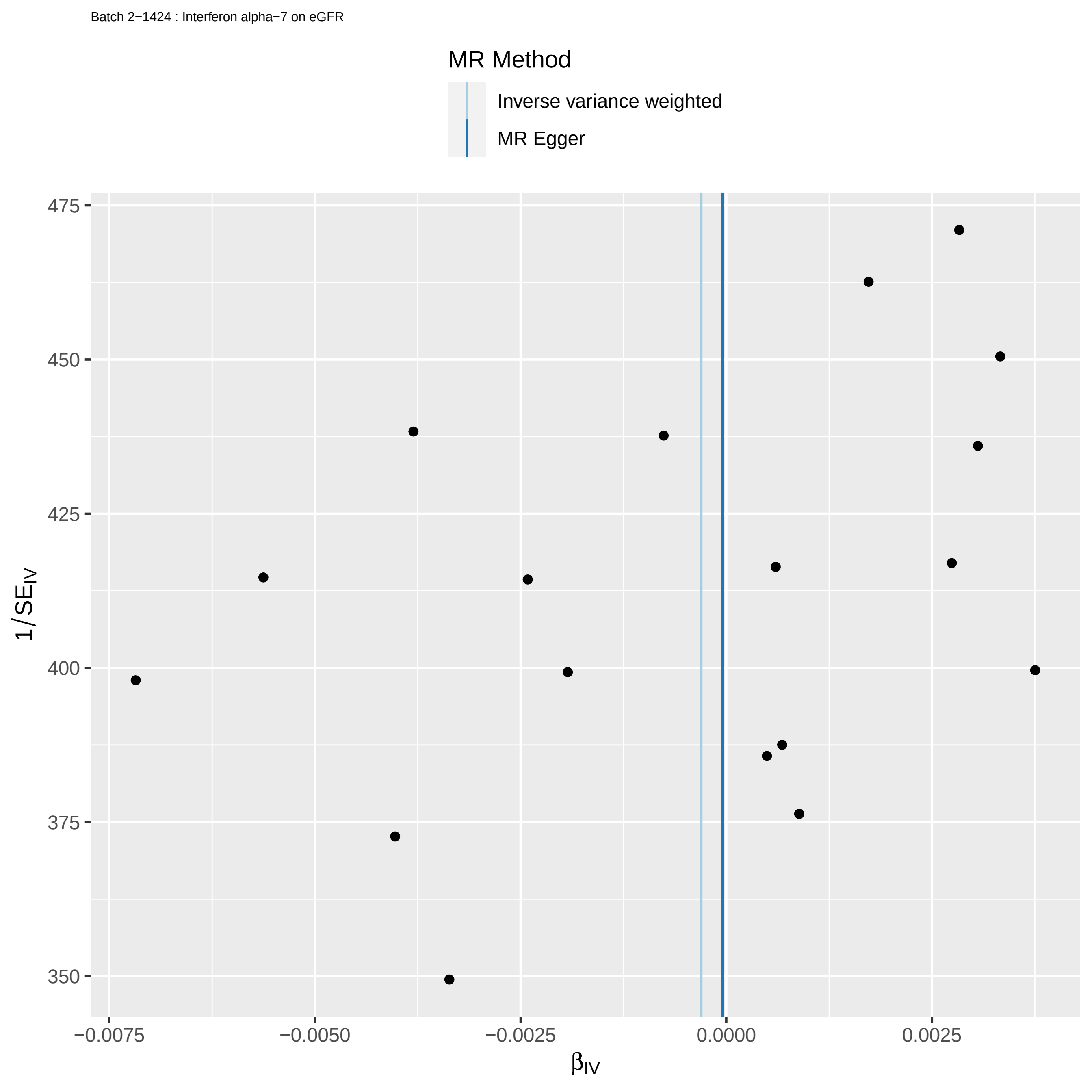

Batch 2−1424 : Interferon alpha−7 on eGFR
MR Method
Inverse variance weighted
MR Egger
475
450
425
400
375
350
1/SE$_{IV}$
−0.0075
−0.0050
−0.0025
0.0000
0.0025
β$_{IV}$

Batch 2−1425 : Interferon alpha−8 on eGFR

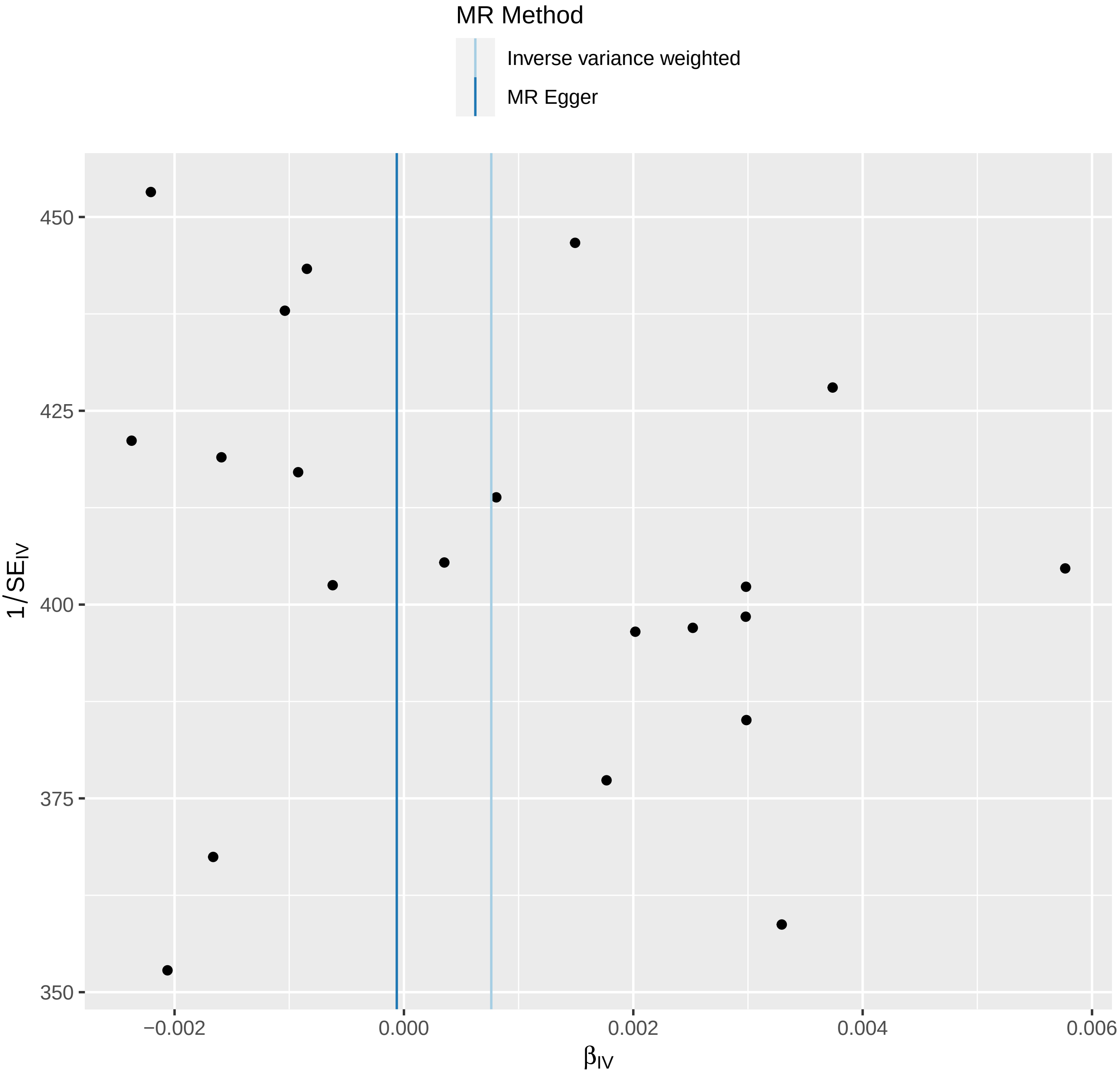

Batch 2−1426 : Interferon alpha/beta receptor 1 on eGFR

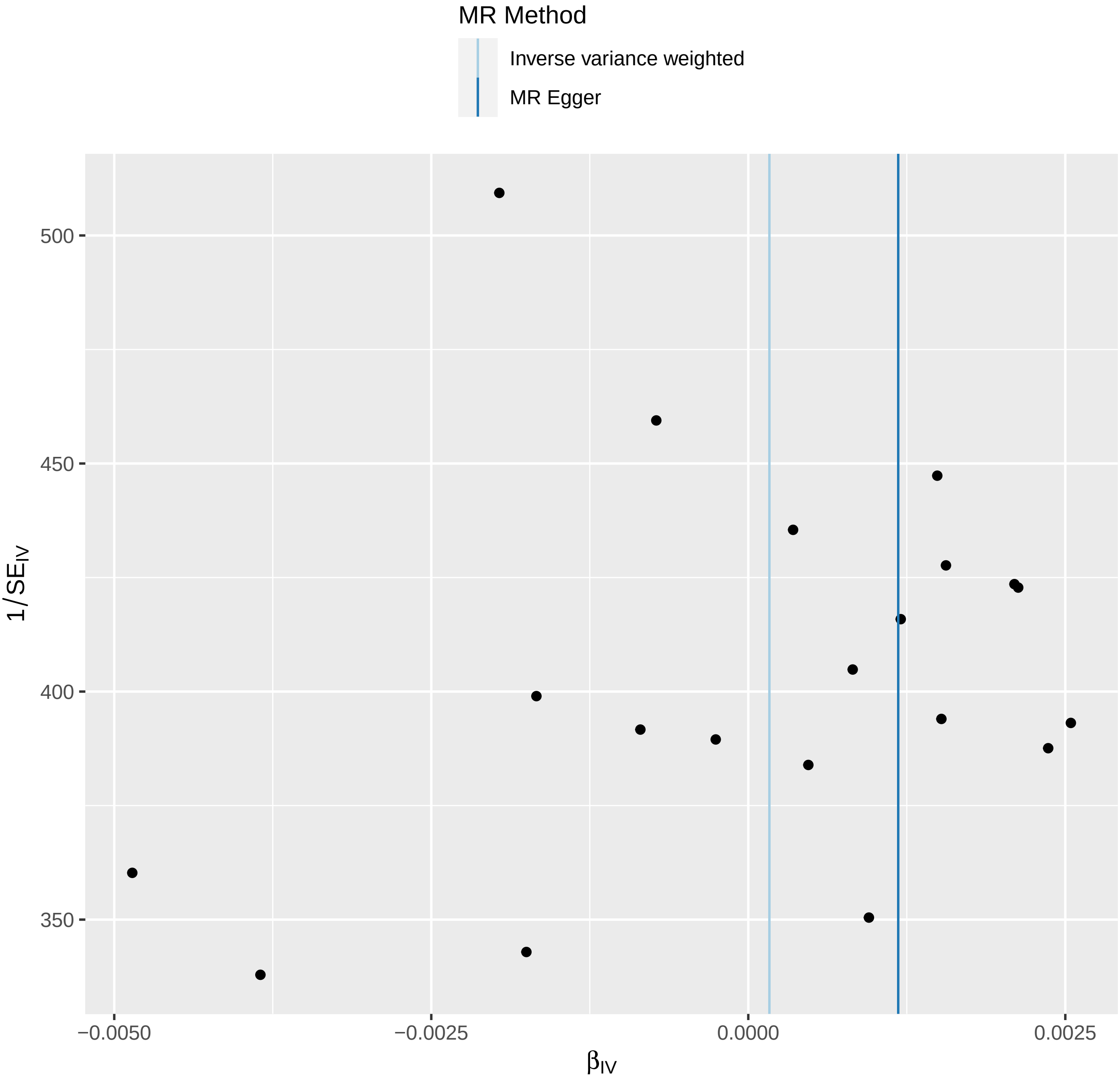

Batch 2−1427 : Interferon beta on eGFR

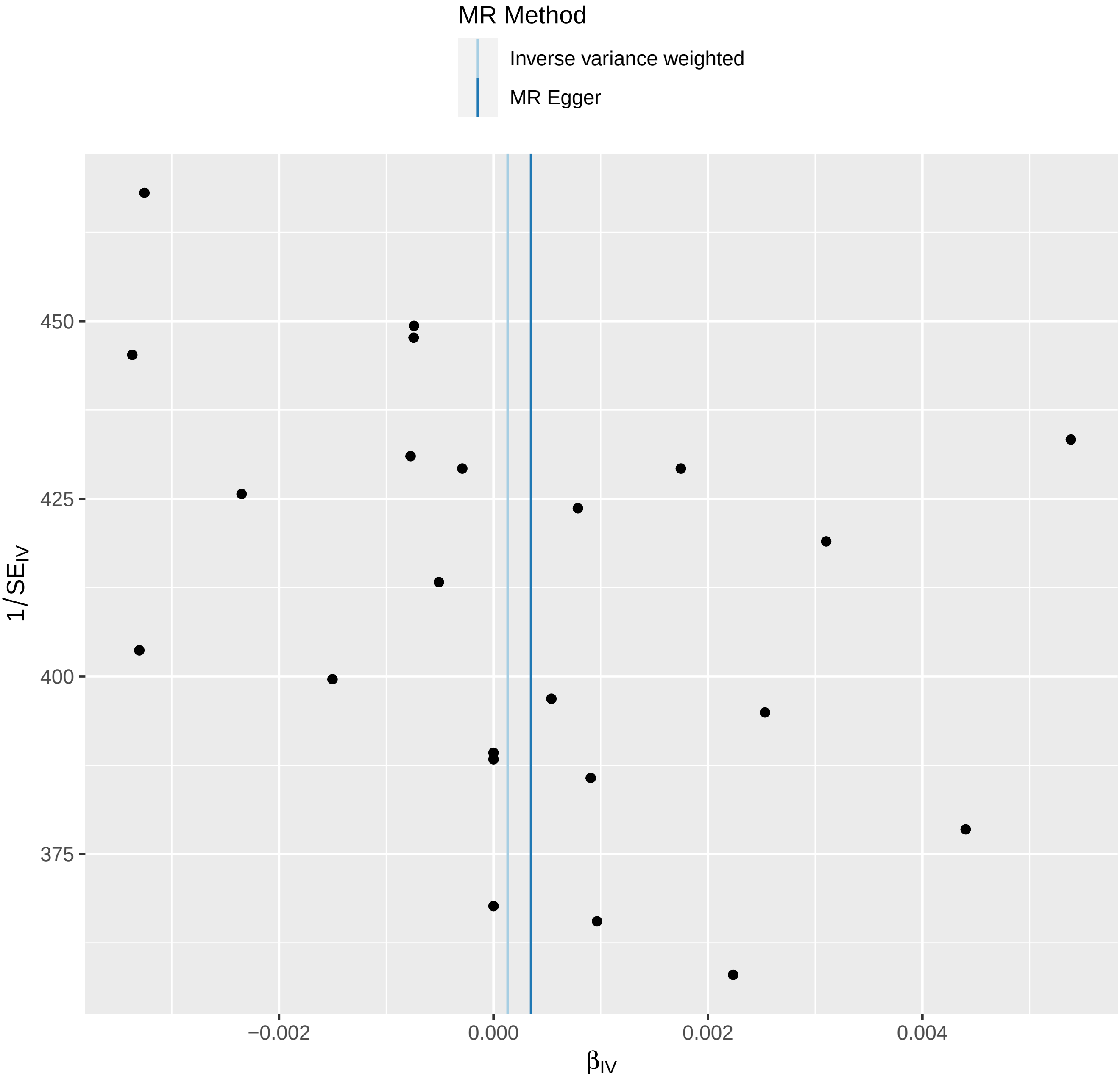

Batch 2−1428 : Interferon gamma on eGFR

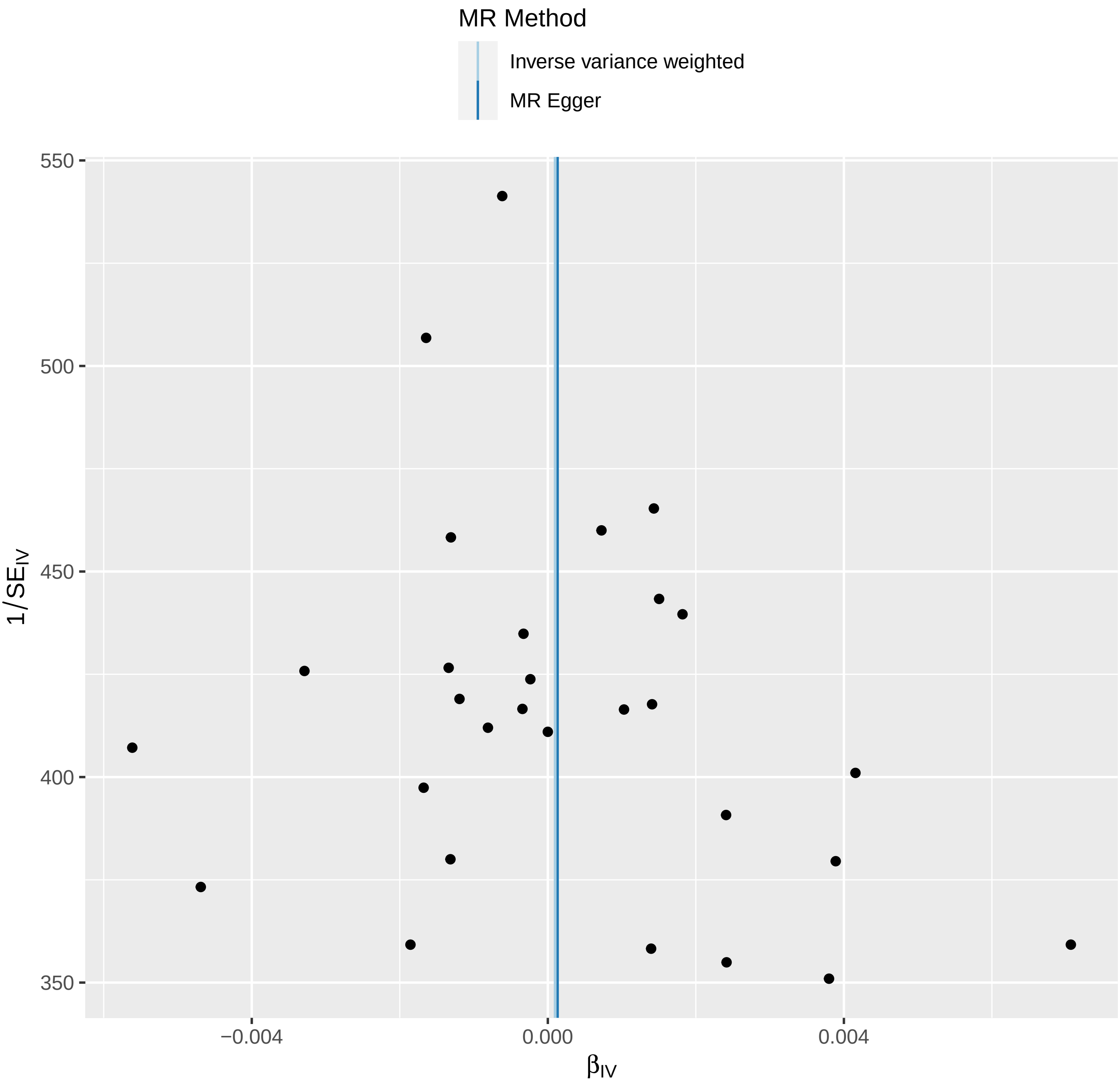

Batch 2−1429 : Interferon gamma on eGFR

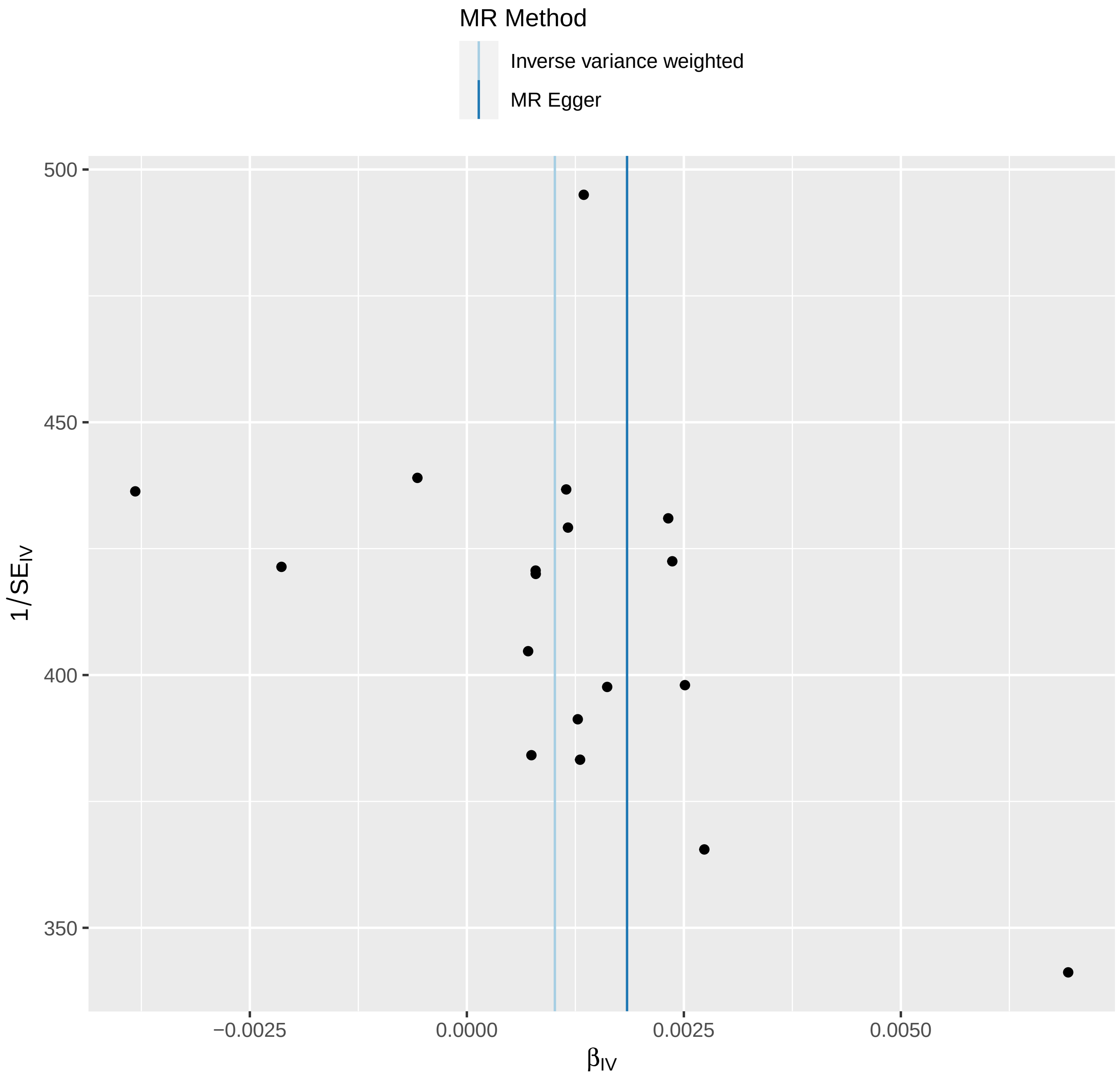

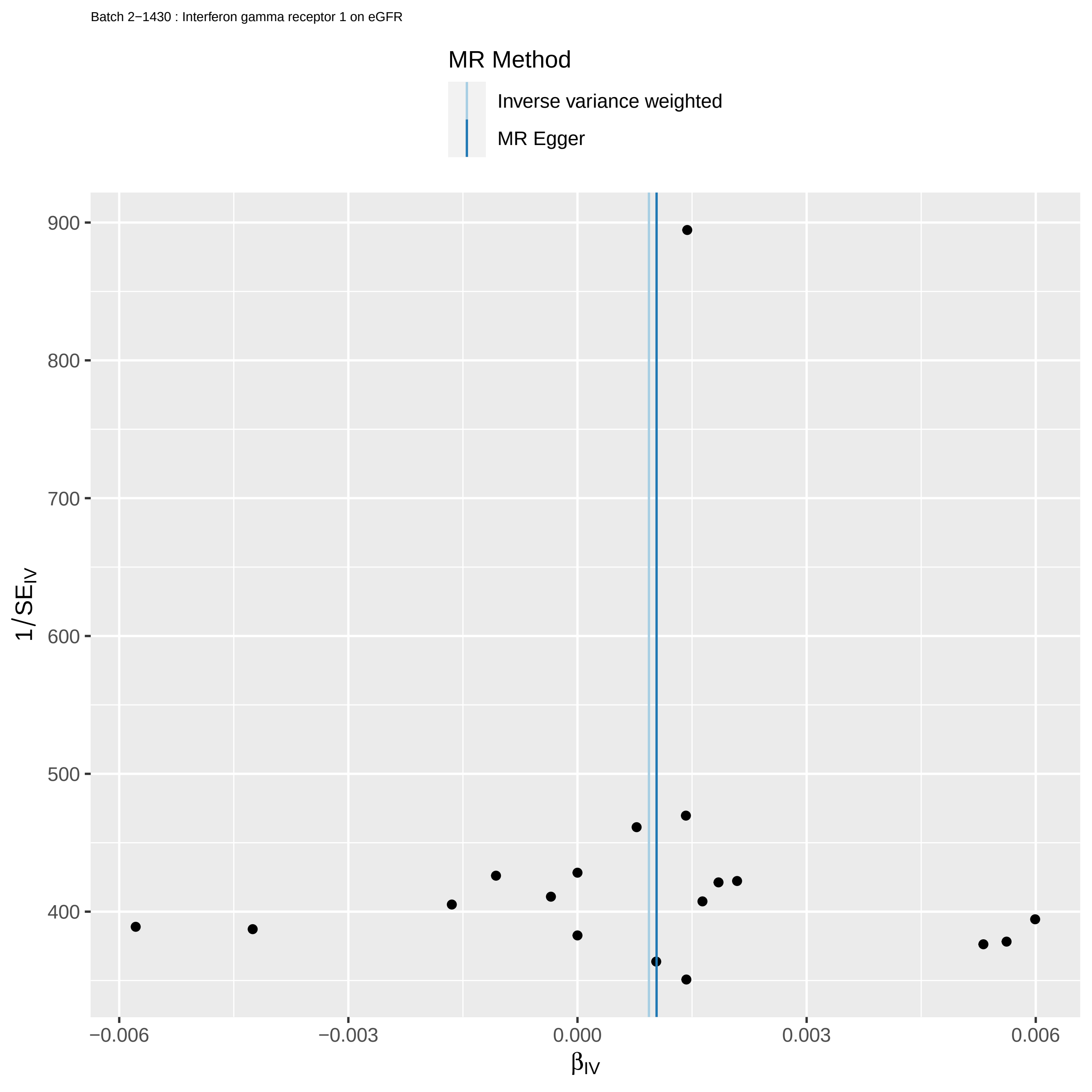

Batch 2−1430 : Interferon gamma receptor 1 on eGFR
MR Method
Inverse variance weighted
MR Egger
900
800
700
600
500
400
1/SE$_{IV}$
−0.006
−0.003
0.000
0.003
0.006
β$_{IV}$

Batch 2−1431 : Interferon gamma receptor 2 on eGFR

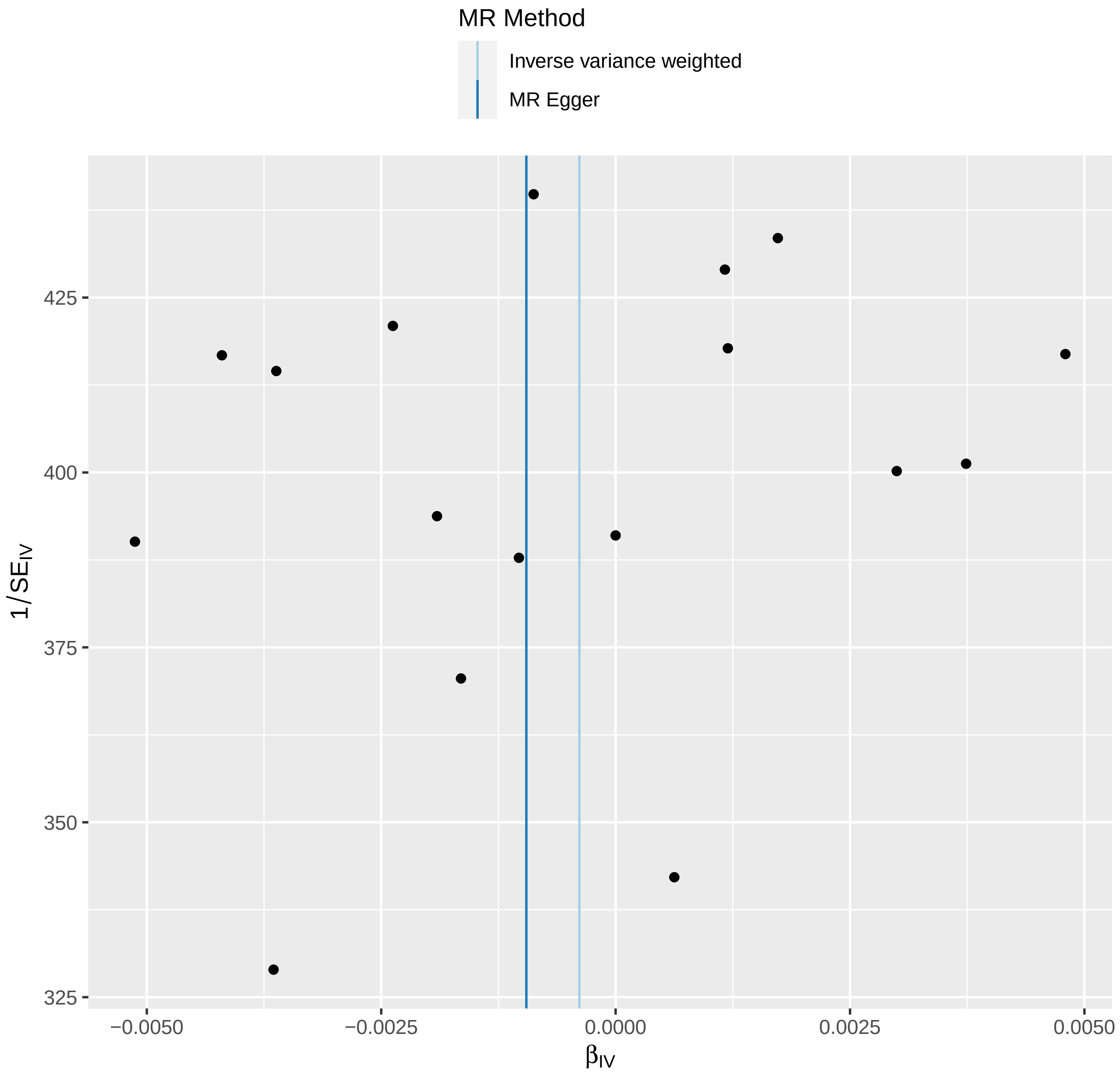

Batch 2−1432 : Interferon gamma receptor 2 on eGFR

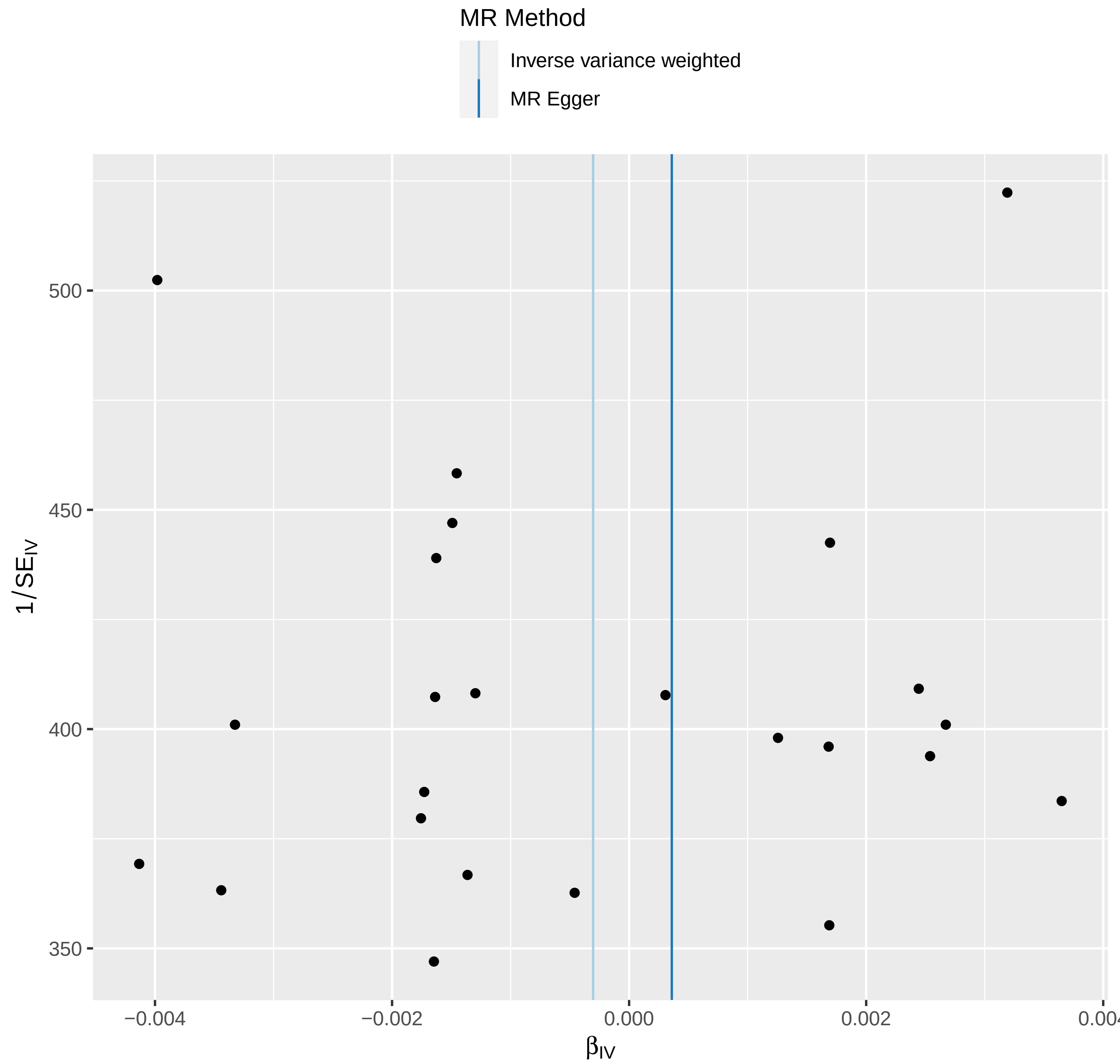

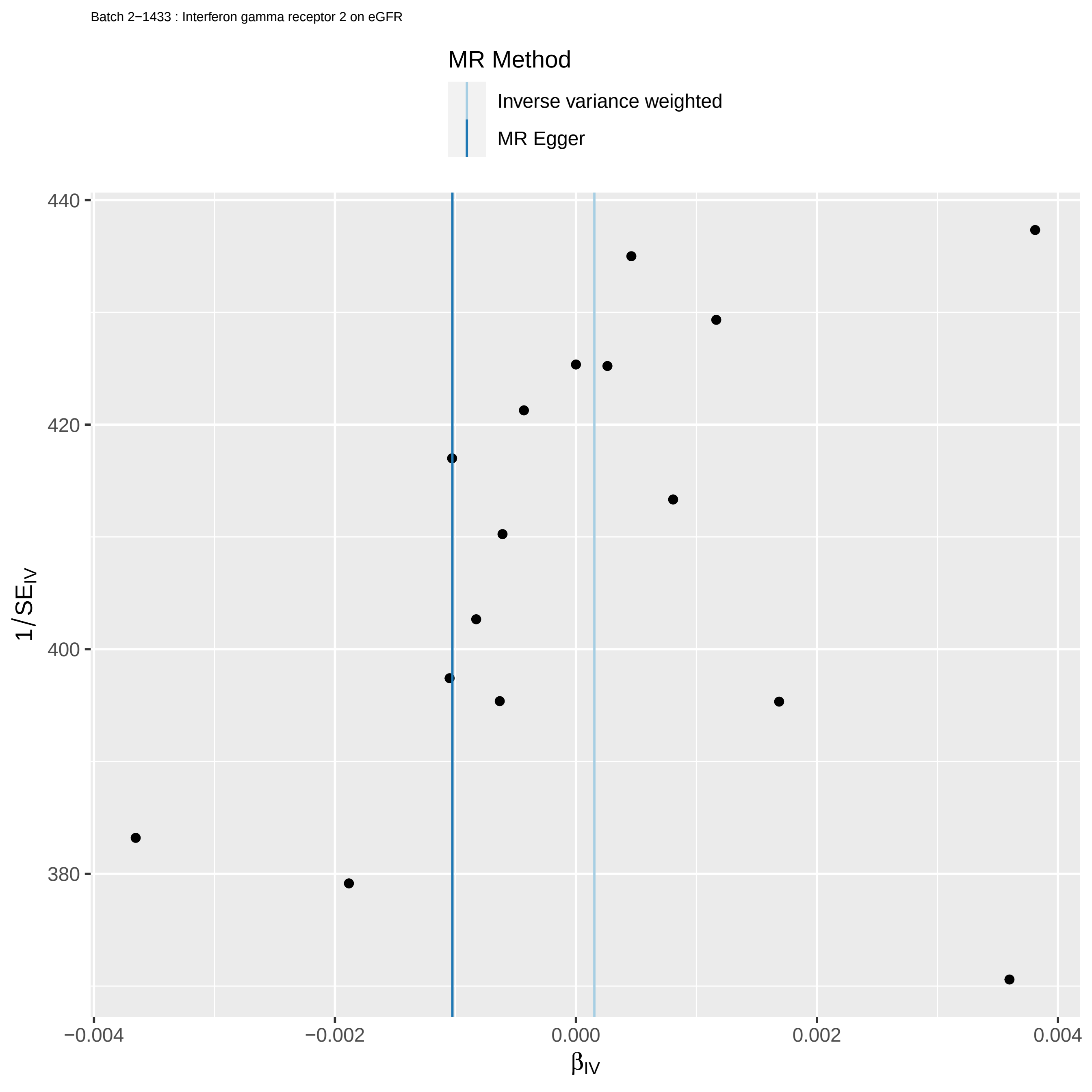

Batch 2−1433 : Interferon gamma receptor 2 on eGFR
MR Method
Inverse variance weighted
MR Egger
440
420
400
380
1/SE$_{IV}$
−0.004
−0.002
0.000
0.002
0.004
β$_{IV}$

Batch 2−1434 : Interferon lambda−1 on eGFR

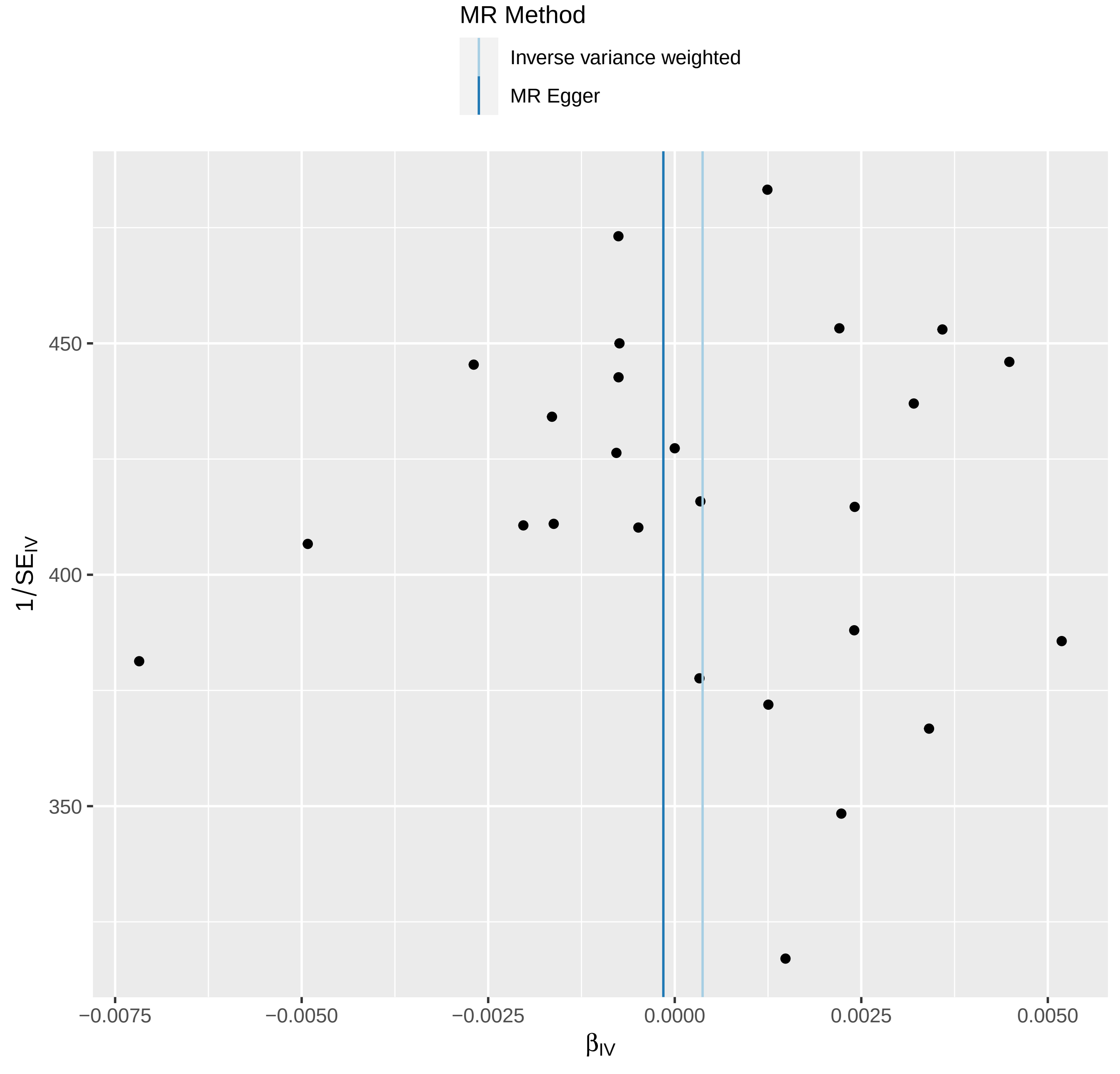

Batch 2−1435 : Interferon lambda−1 on eGFR

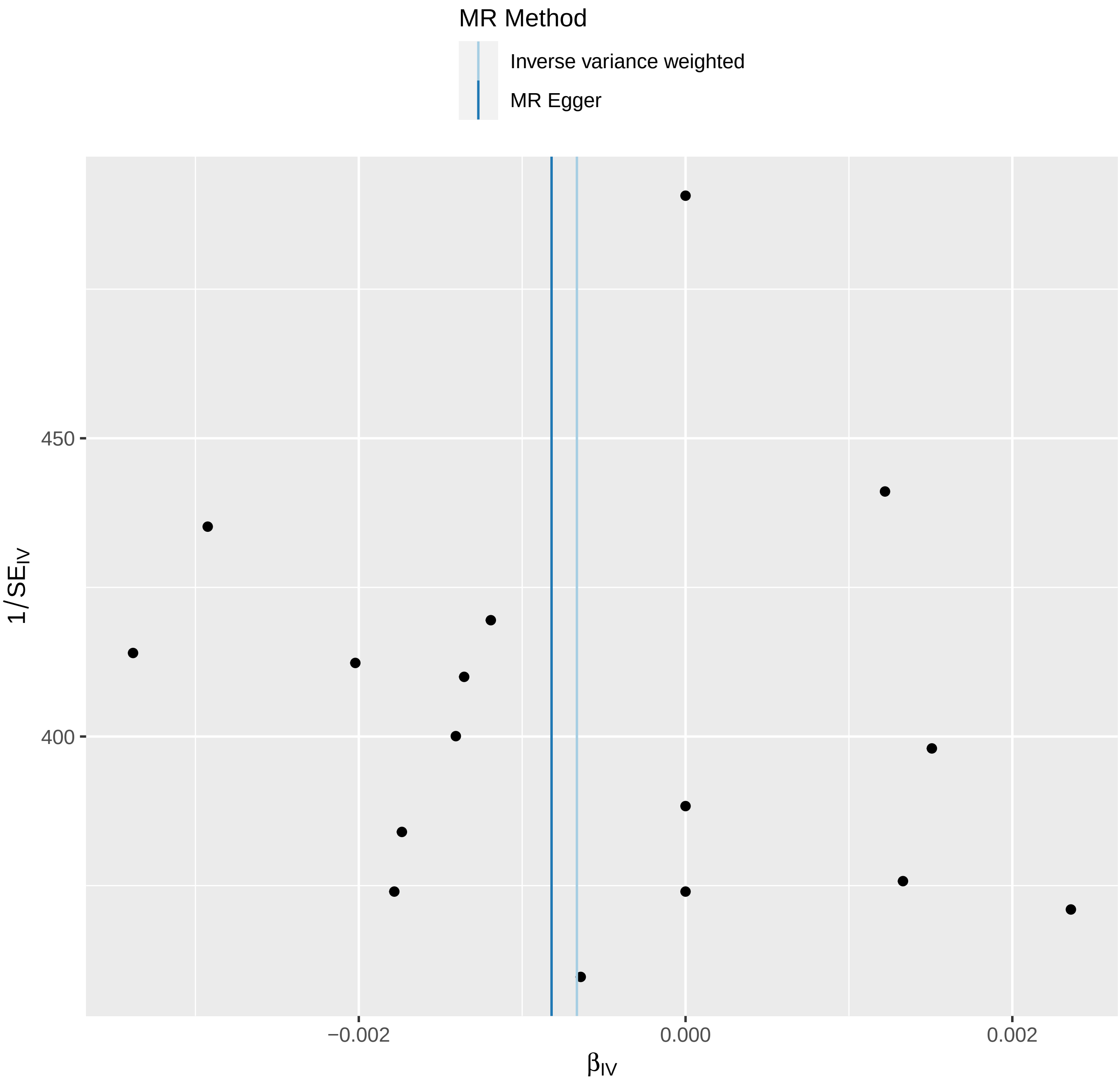

Batch 2−1436 : Interferon lambda−2 on eGFR

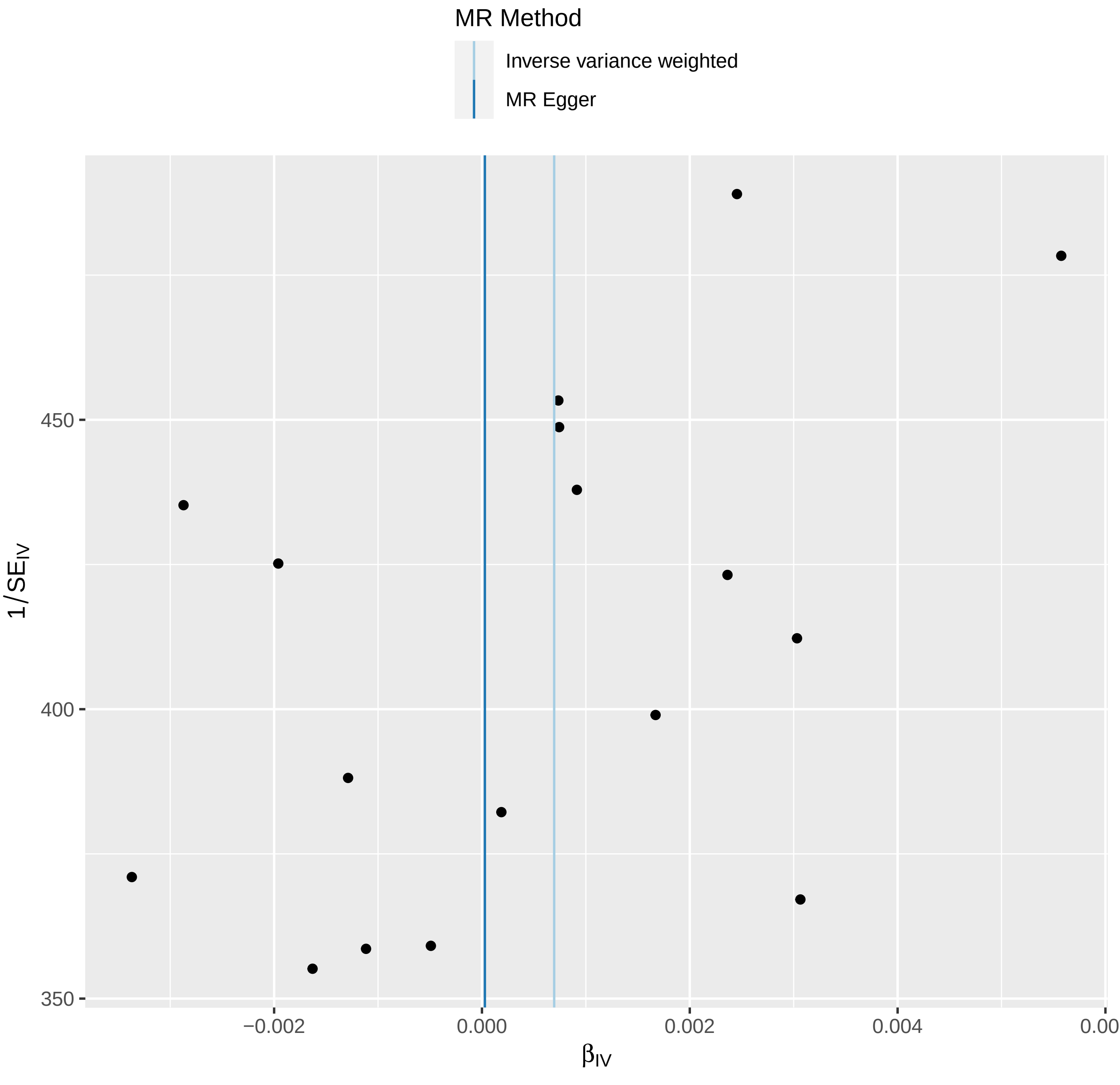

Batch 2−1437 : Interferon lambda receptor 1 on eGFR

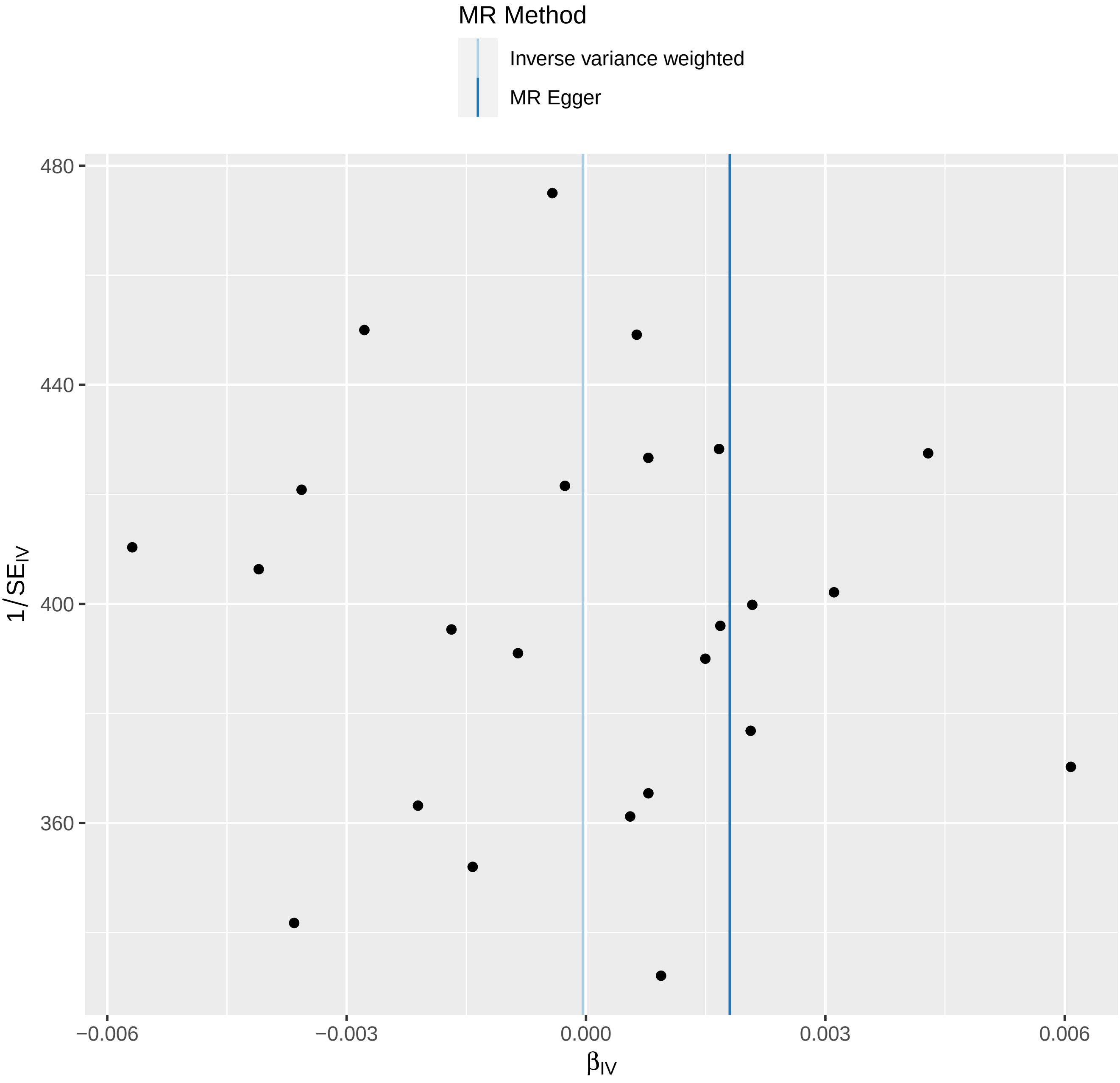

Batch 2−1438 : Interferon omega−1 on eGFR

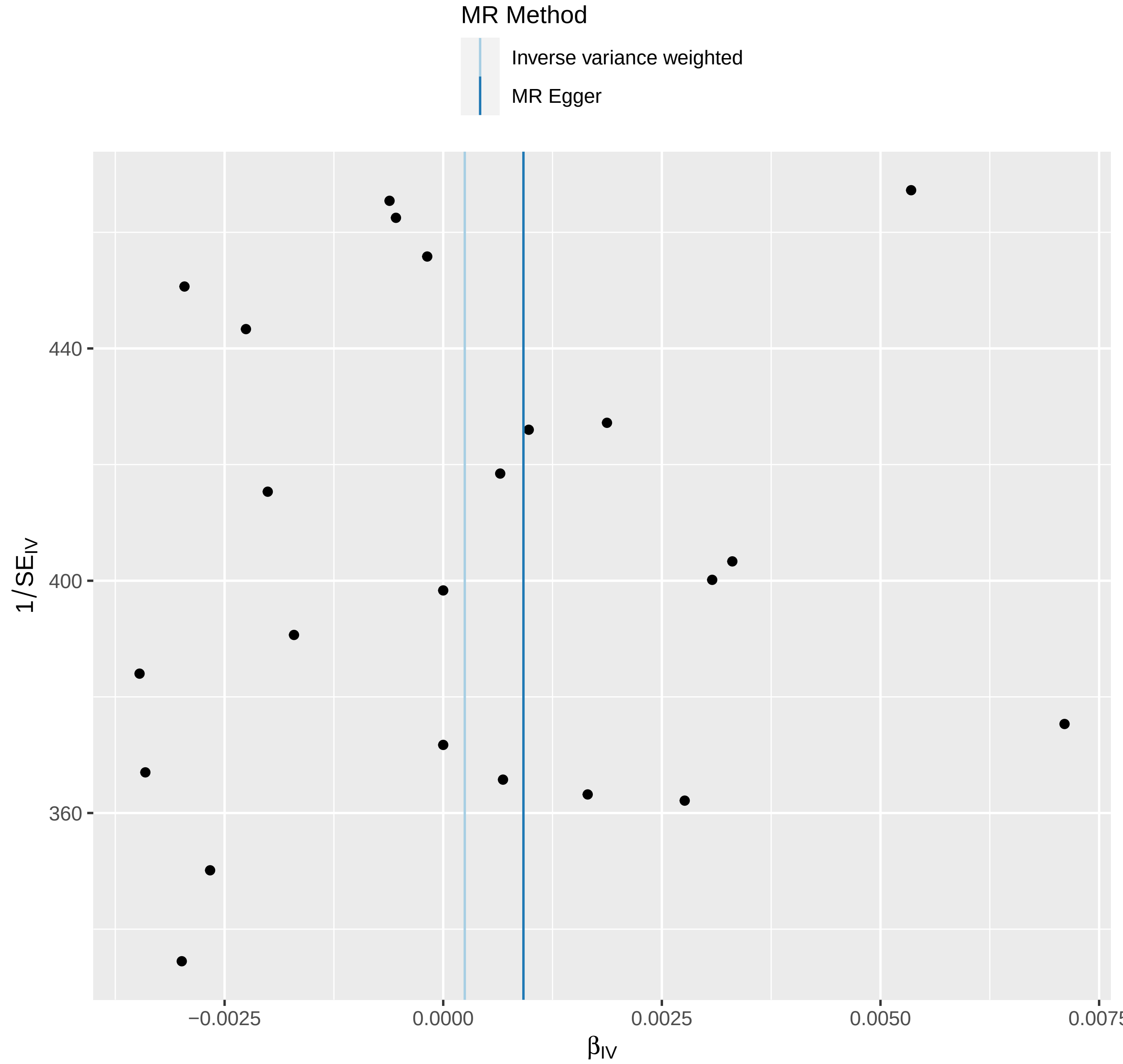

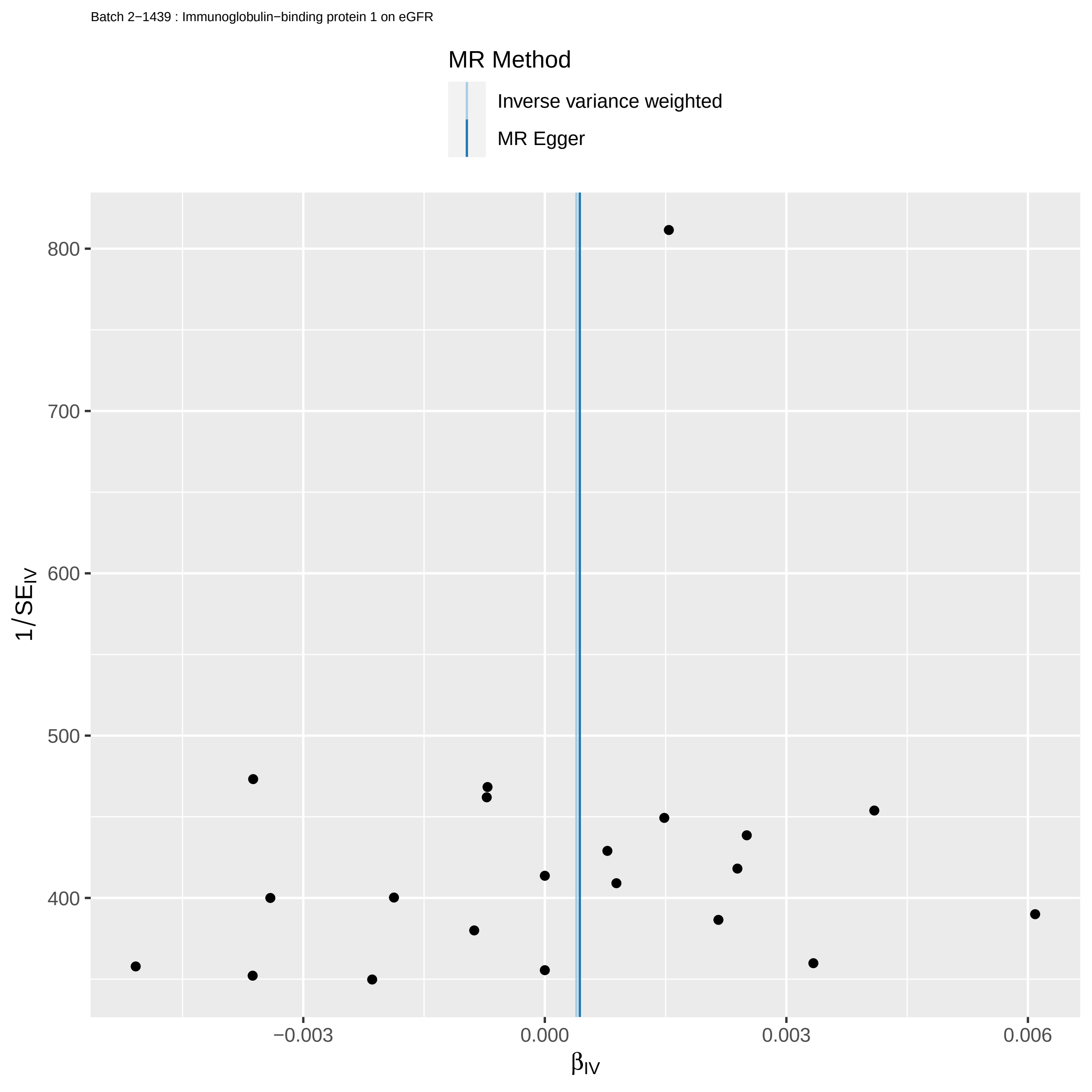
Batch 2−1439 : Immunoglobulin−binding protein 1 on eGFR
MR Method
Inverse variance weighted
MR Egger
800
700
600
500
400
1/SE_IV
−0.003
0.000
0.003
0.006
β_IV

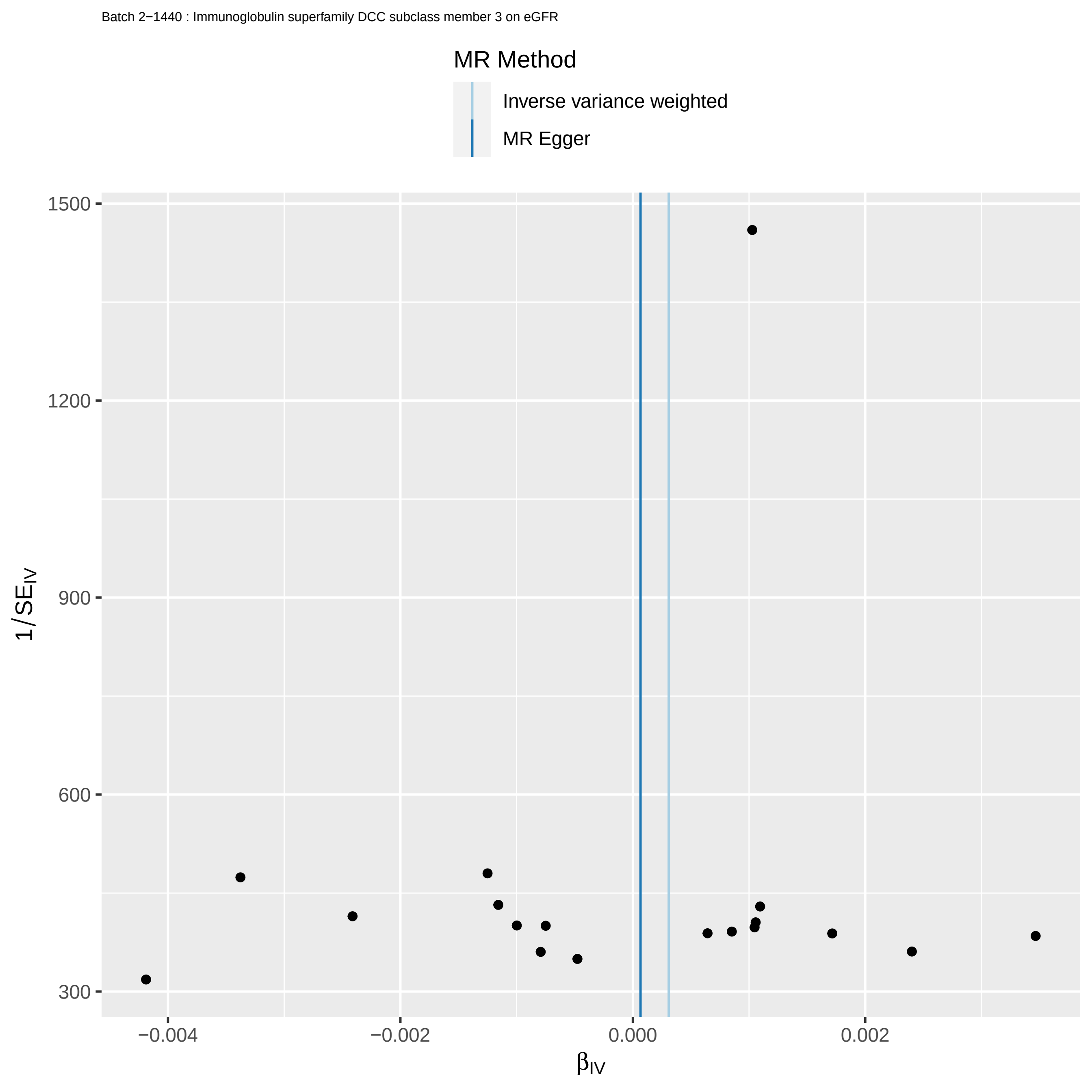
Batch 2−1440 : Immunoglobulin superfamily DCC subclass member 3 on eGFR
MR Method
Inverse variance weighted
MR Egger
1500
1200
900
600
300
1/SE_IV
−0.004
−0.002
0.000
0.002
β_IV

Batch 2−1441 : Immunoglobulin superfamily DCC subclass member 3 on eGFR

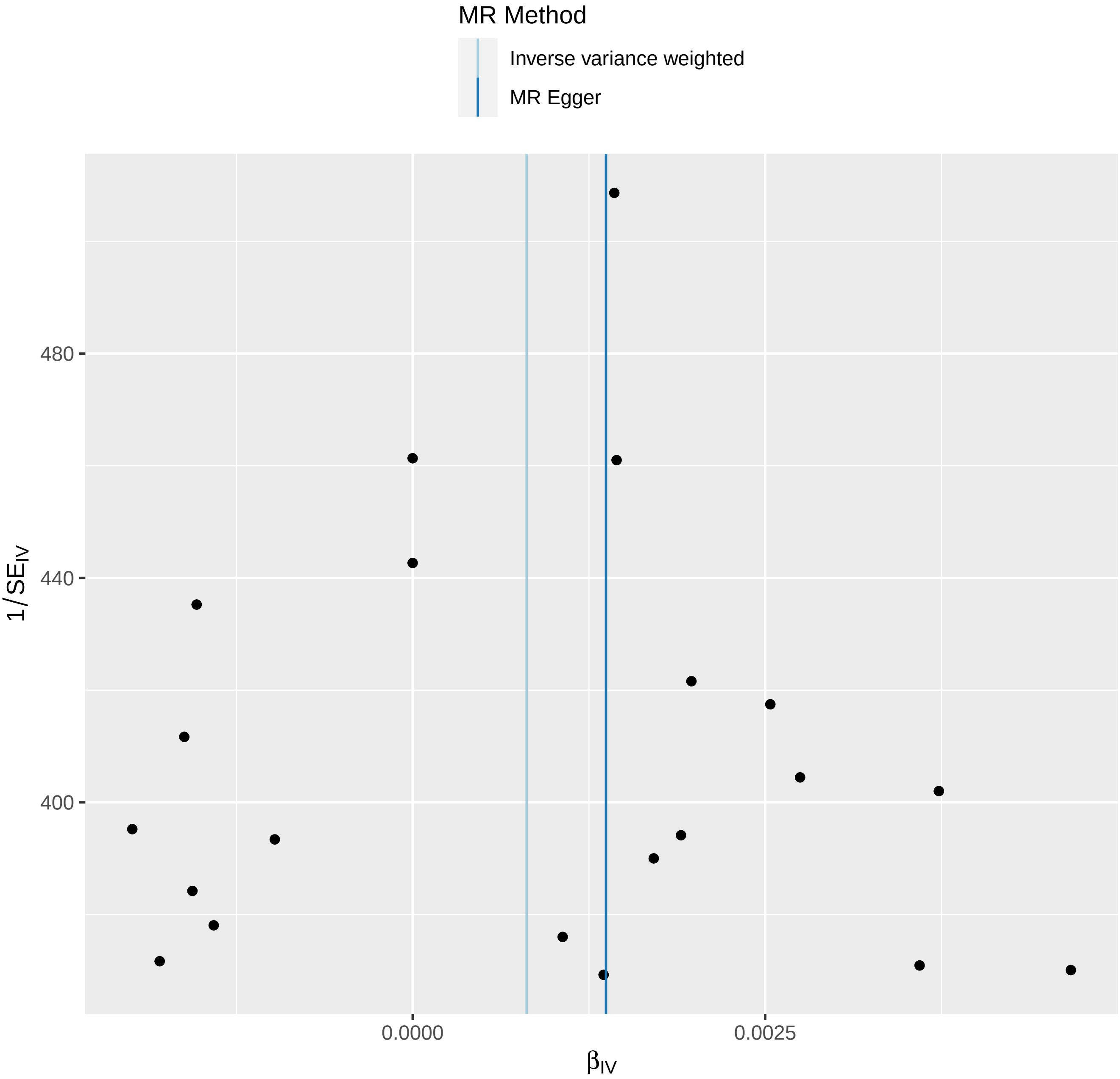

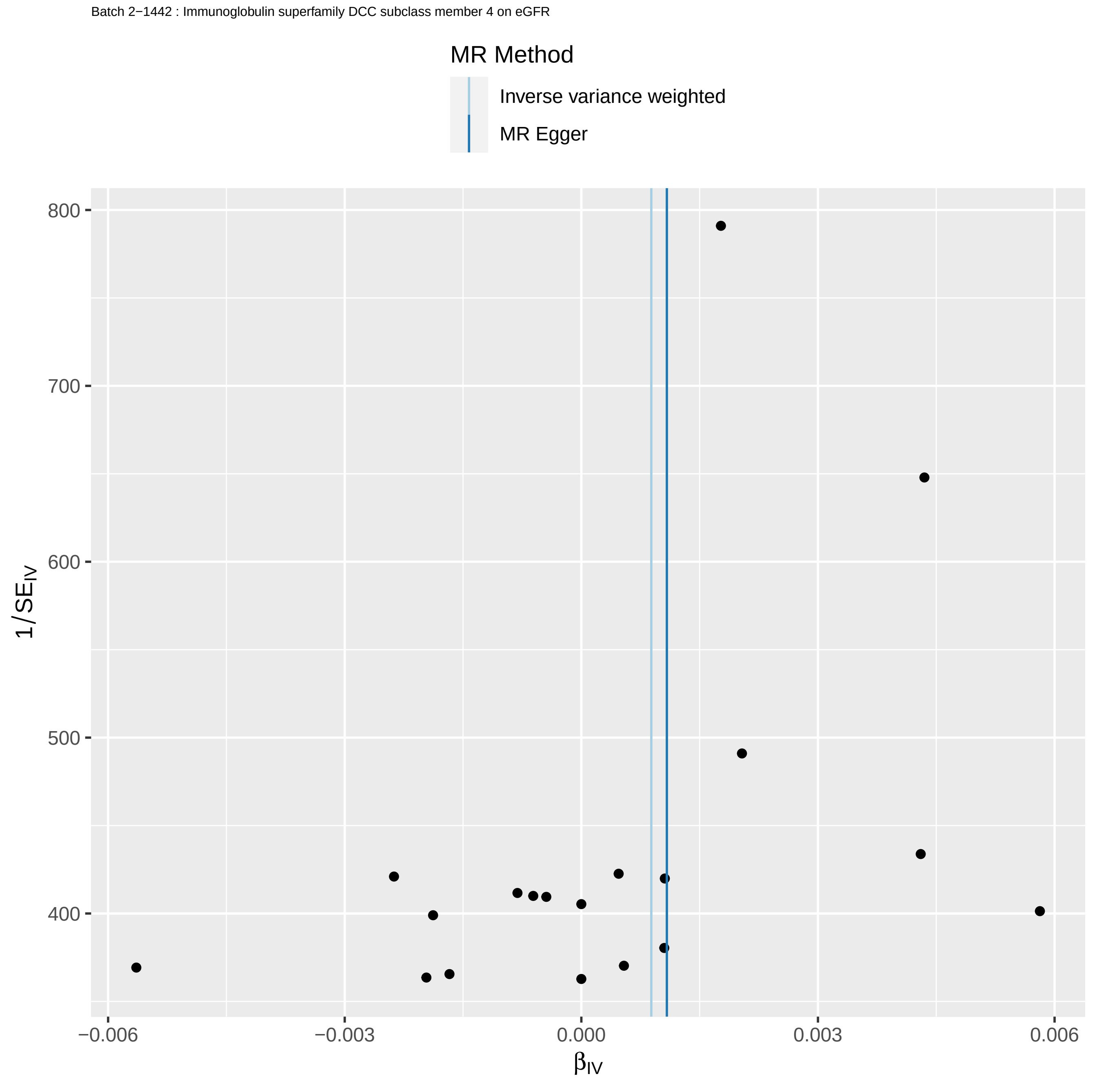
Batch 2−1442 : Immunoglobulin superfamily DCC subclass member 4 on eGFR
MR Method
Inverse variance weighted
MR Egger
800
700
600
500
400
1/SE_IV
−0.006
−0.003
0.000
0.003
0.006
β_IV

Batch 2−1443 : Insulin−like growth factor I on eGFR

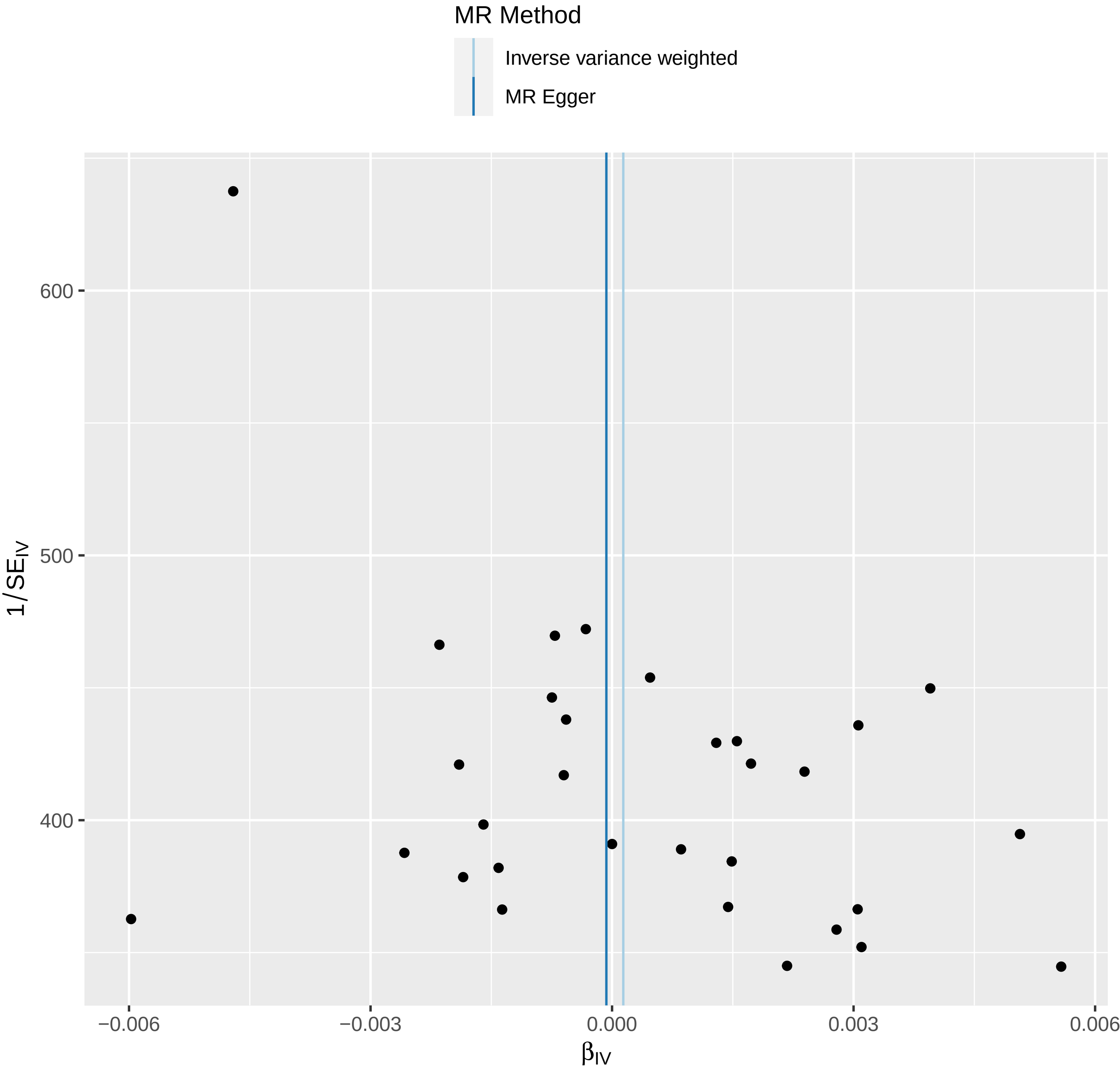

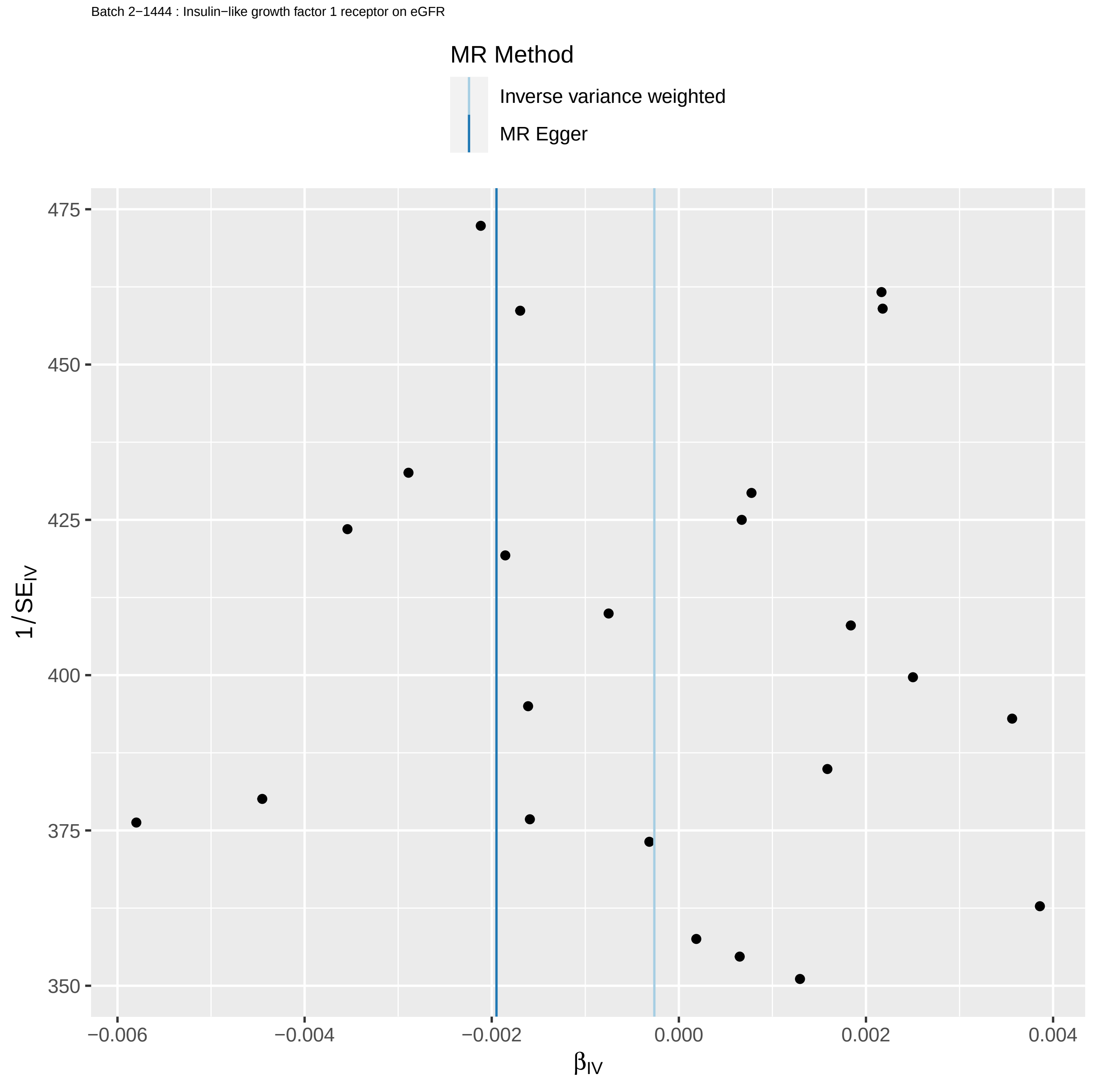

Batch 2−1444 : Insulin−like growth factor 1 receptor on eGFR
MR Method
Inverse variance weighted
MR Egger
475
450
425
400
375
350
1/SE$_{IV}$
−0.006
−0.004
−0.002
0.000
0.002
0.004
β$_{IV}$

Batch 2−1445 : Cation−independent mannose−6−phosphate receptor on eGFR

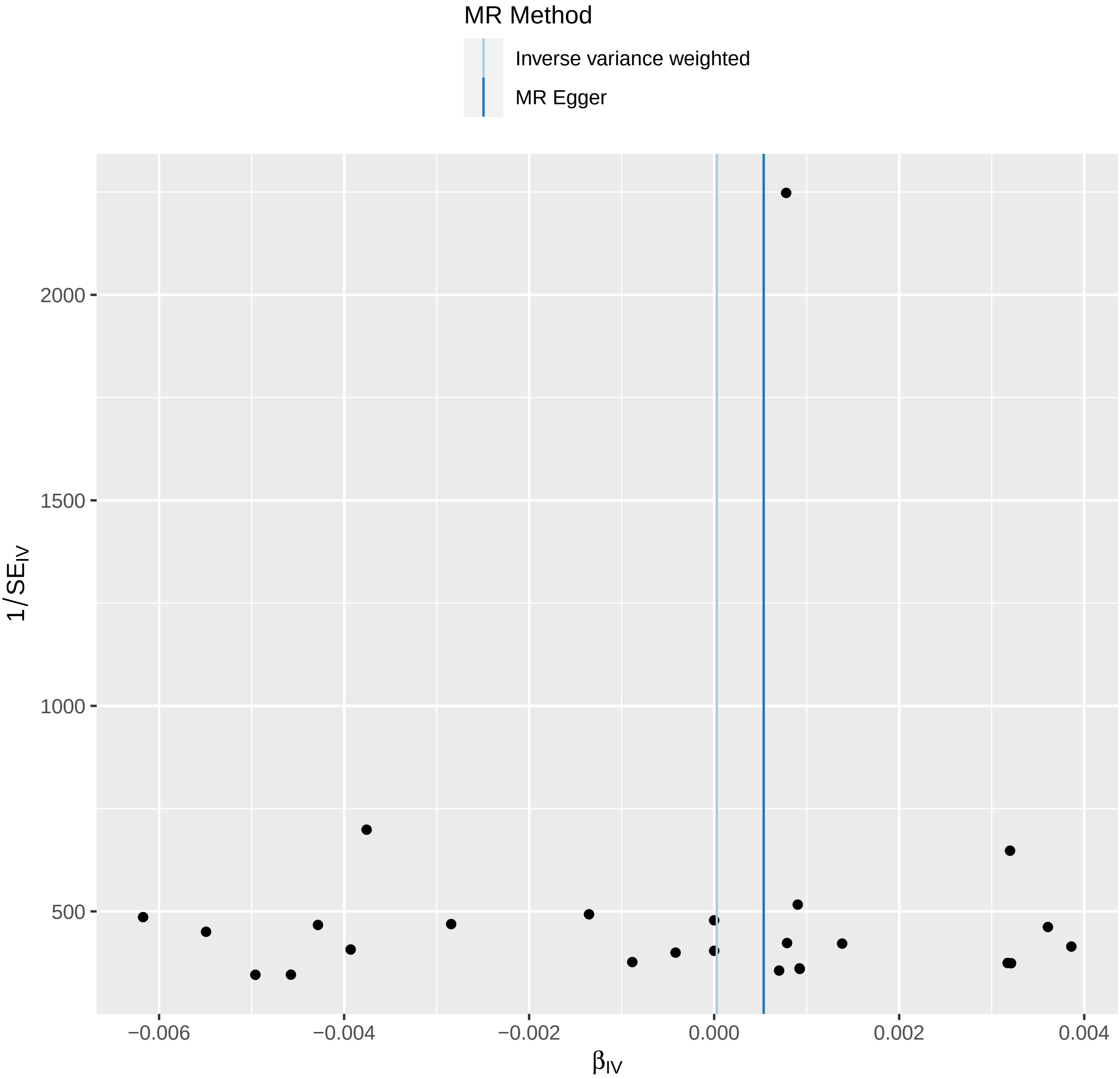

Batch 2−1446 : Insulin−like growth factor−binding protein 1 on eGFR

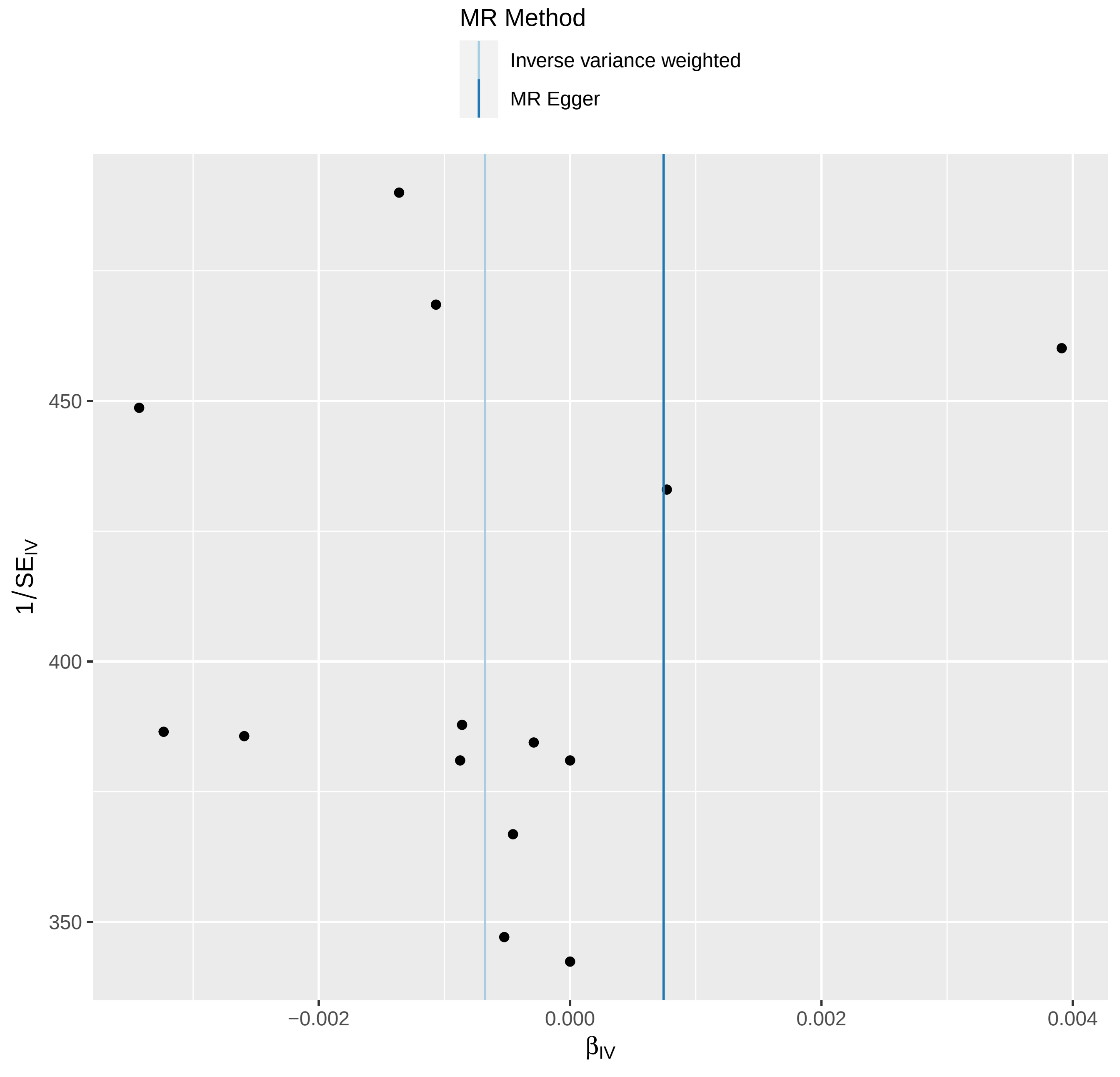

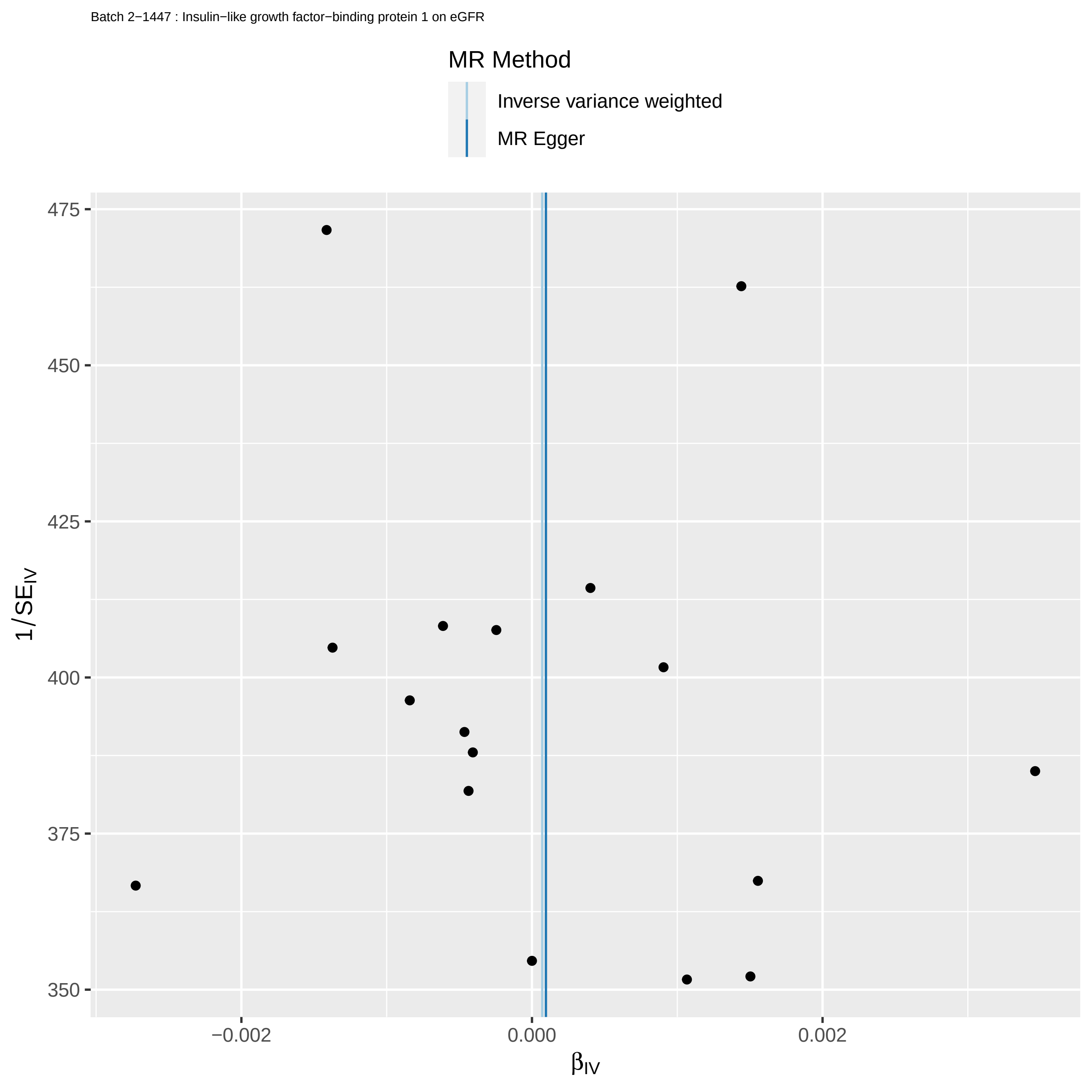

Batch 2−1447 : Insulin−like growth factor−binding protein 1 on eGFR
MR Method
Inverse variance weighted
MR Egger
475
450
425
400
375
350
1/SE_IV
−0.002
0.000
0.002
β_IV

Batch 2−1448 : Insulin−like growth factor−binding protein 2 on eGFR

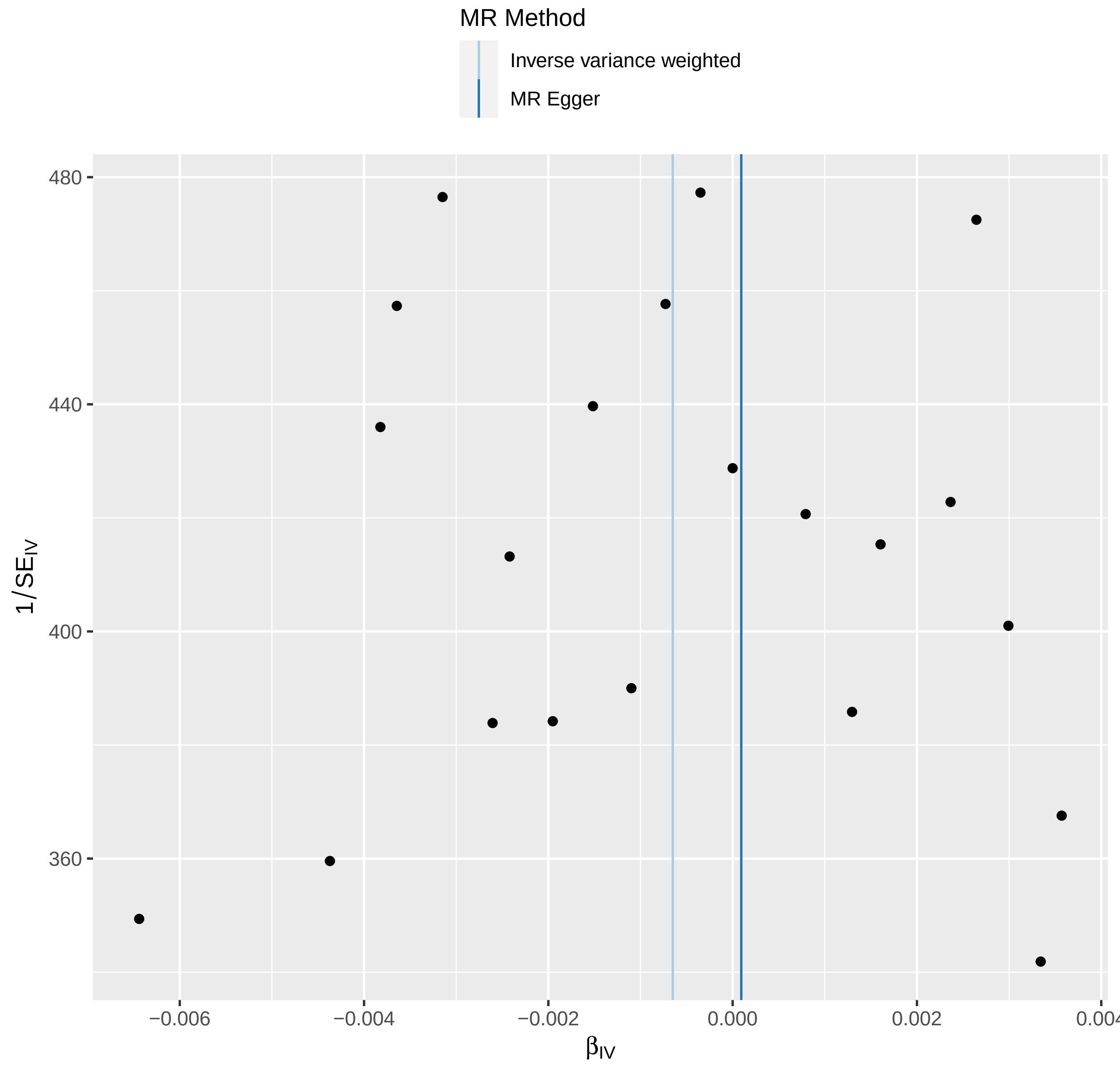

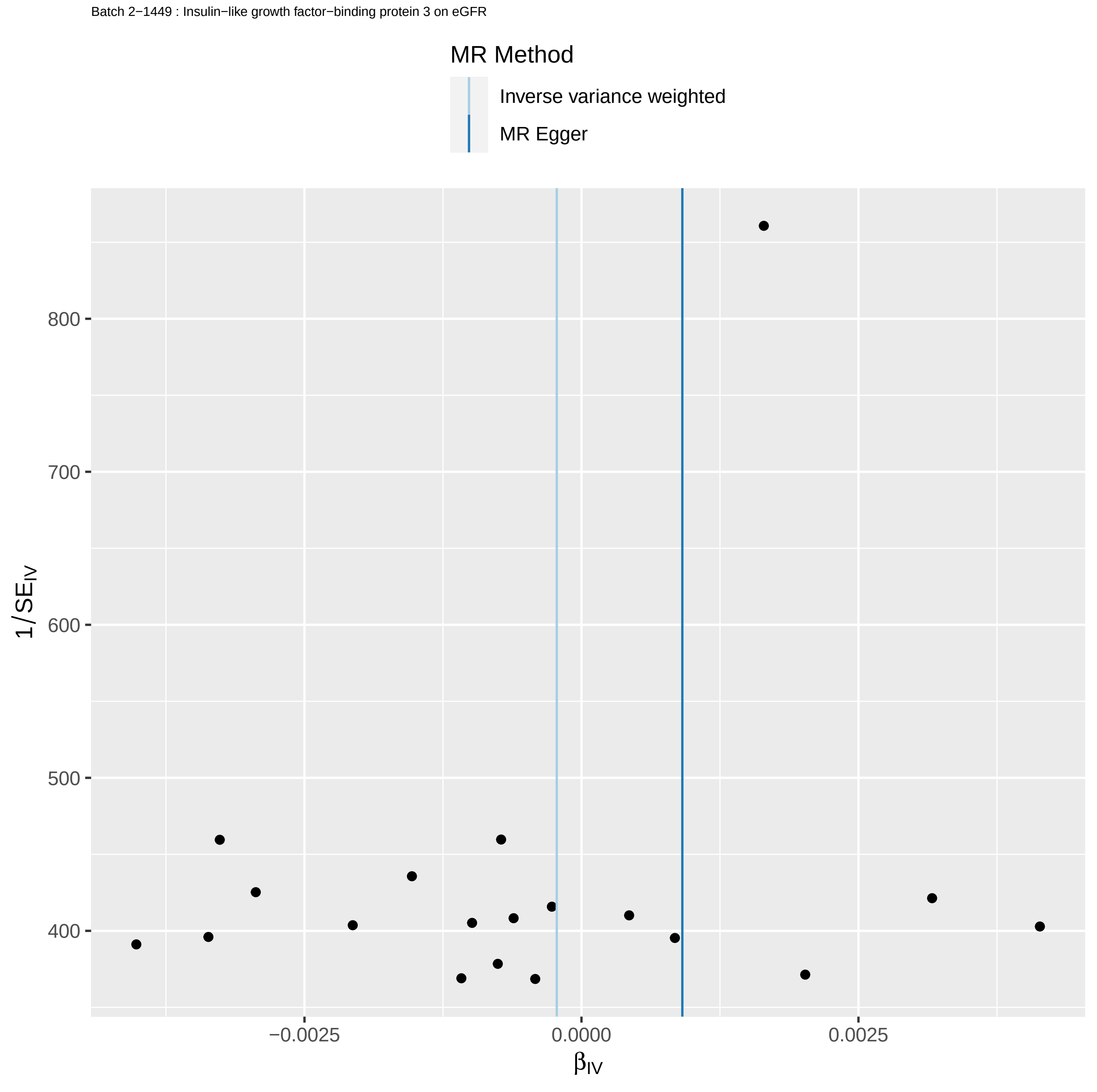
Batch 2−1449 : Insulin−like growth factor−binding protein 3 on eGFR
MR Method
Inverse variance weighted
MR Egger
800
700
600
500
400
1/SE_IV
−0.0025
0.0000
0.0025
β_IV

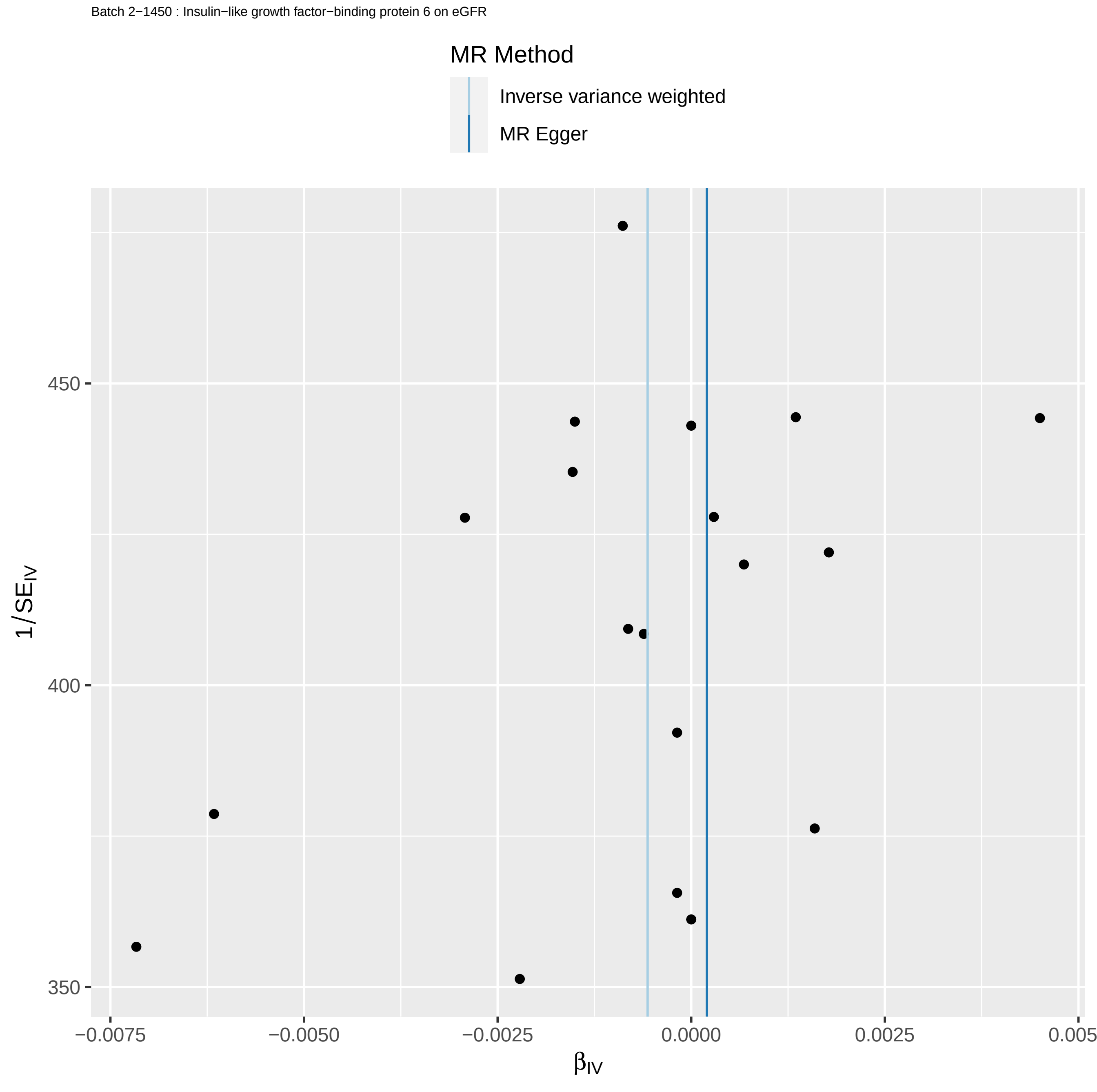
Batch 2−1450 : Insulin−like growth factor−binding protein 6 on eGFR
MR Method
Inverse variance weighted
MR Egger
450
400
350
1/SE_IV
−0.0075
−0.0050
−0.0025
0.0000
0.0025
0.005
β_IV

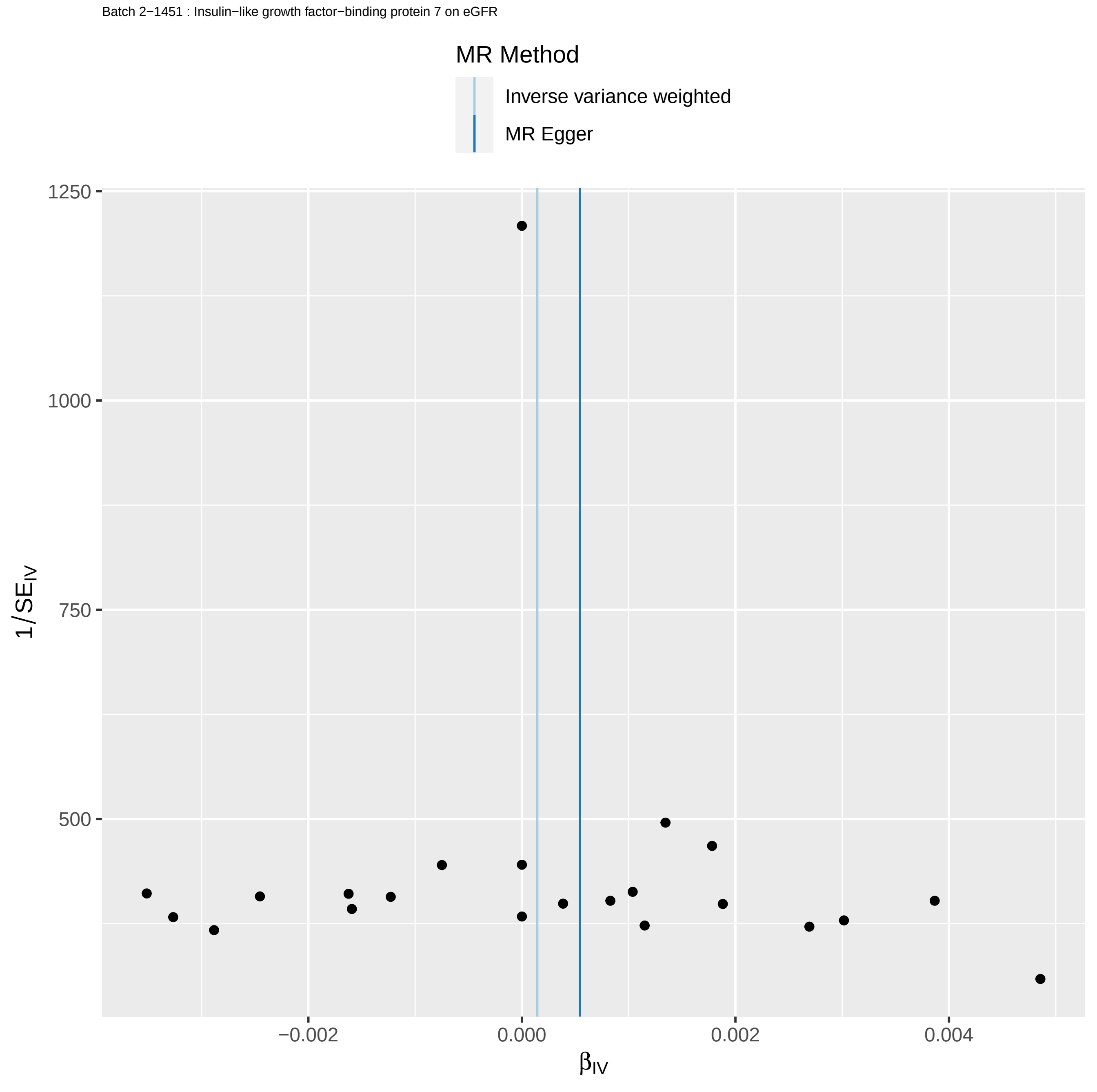

Batch 2−1451 : Insulin−like growth factor−binding protein 7 on eGFR
MR Method
Inverse variance weighted
MR Egger
1250
1000
750
500
1/SE$_{IV}$
−0.002
0.000
0.002
0.004
β$_{IV}$

Batch 2−1452 : Insulin−like growth factor−binding protein−like 1 on eGFR

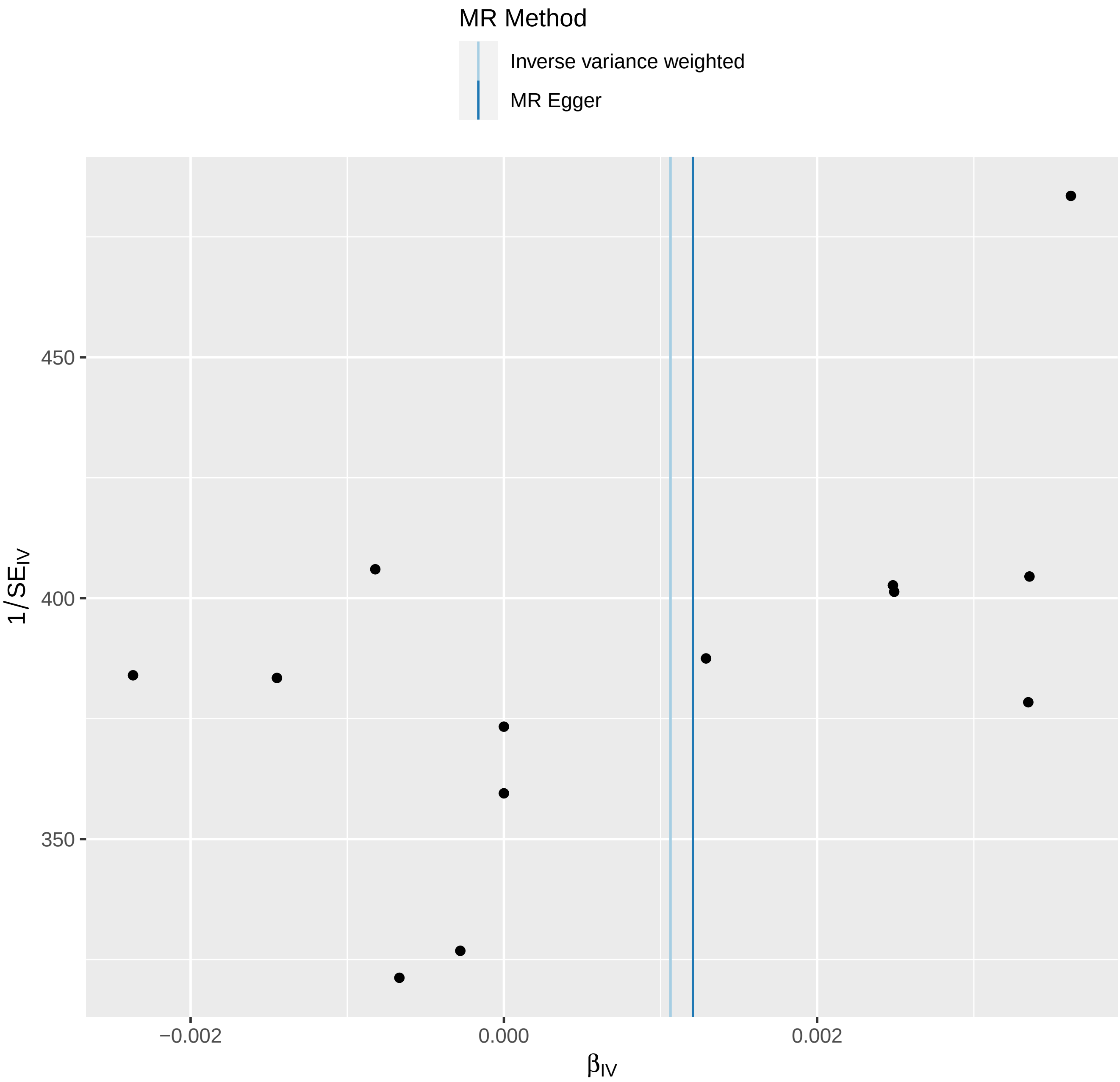

Batch 2−1453 : Insulin growth factor−like family member 3 on eGFR

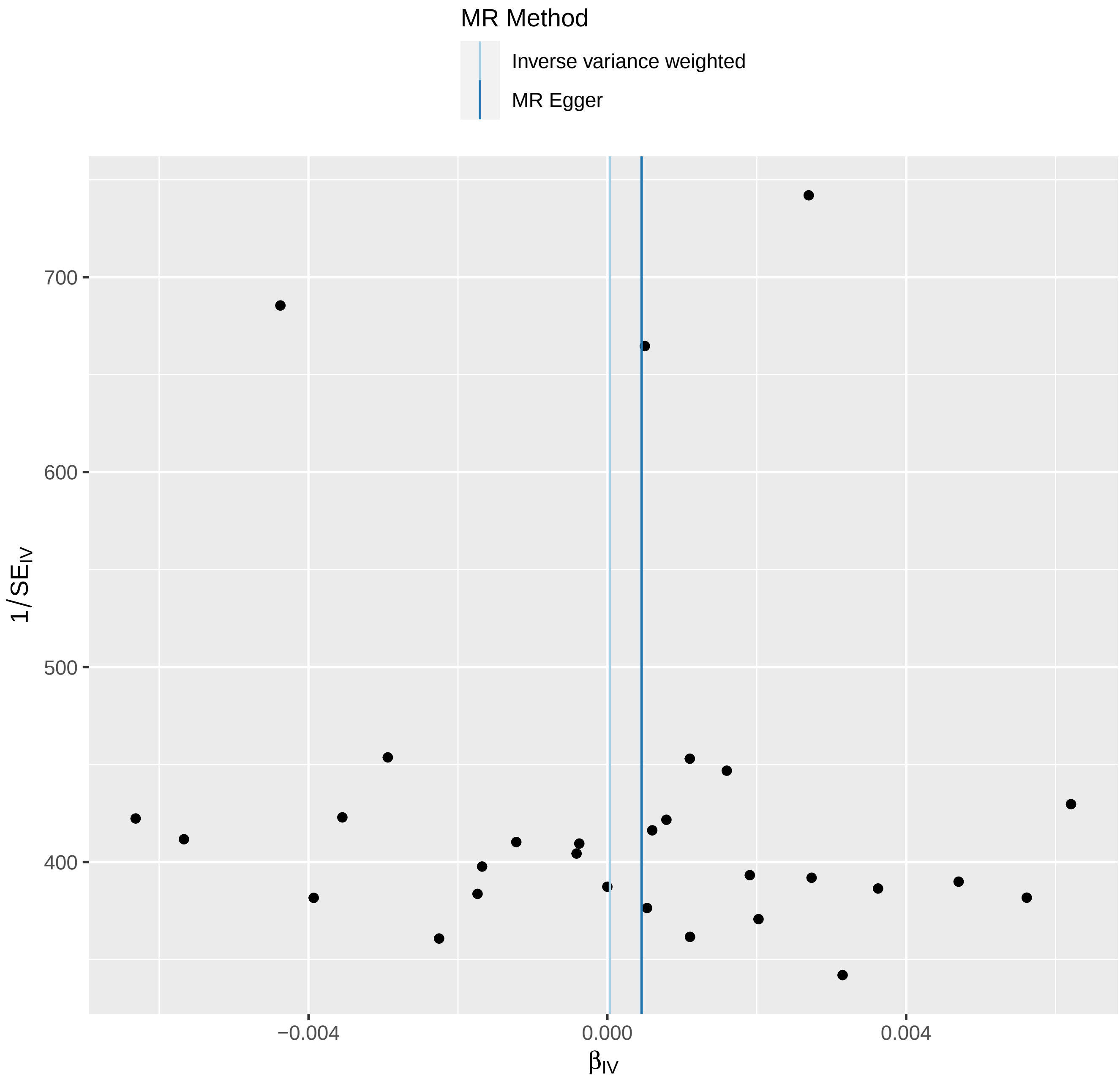

Batch 2−1454 : Insulin growth factor−like family member 4 on eGFR

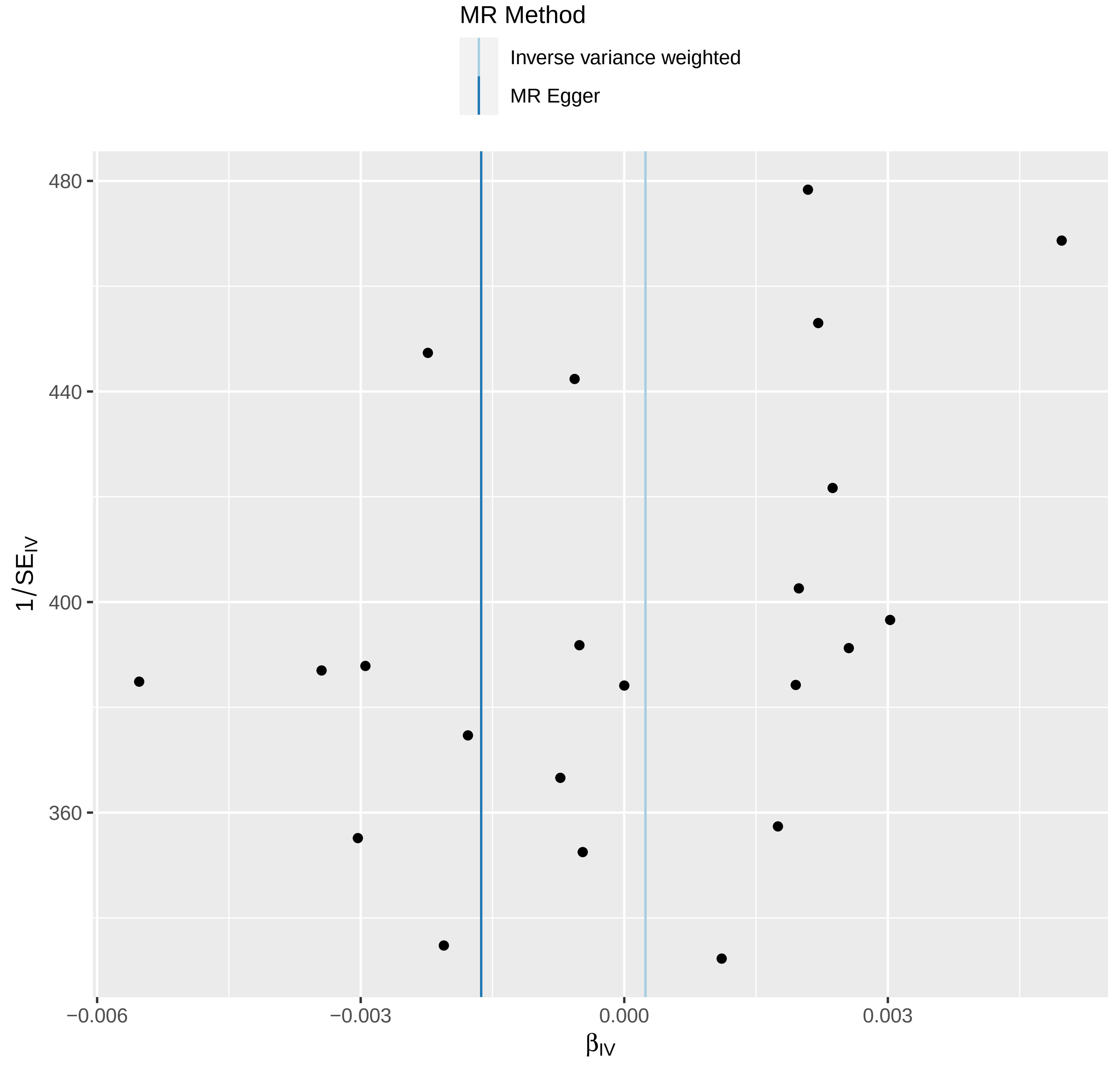

Batch 2−1455 : IGF−like family receptor 1 on eGFR

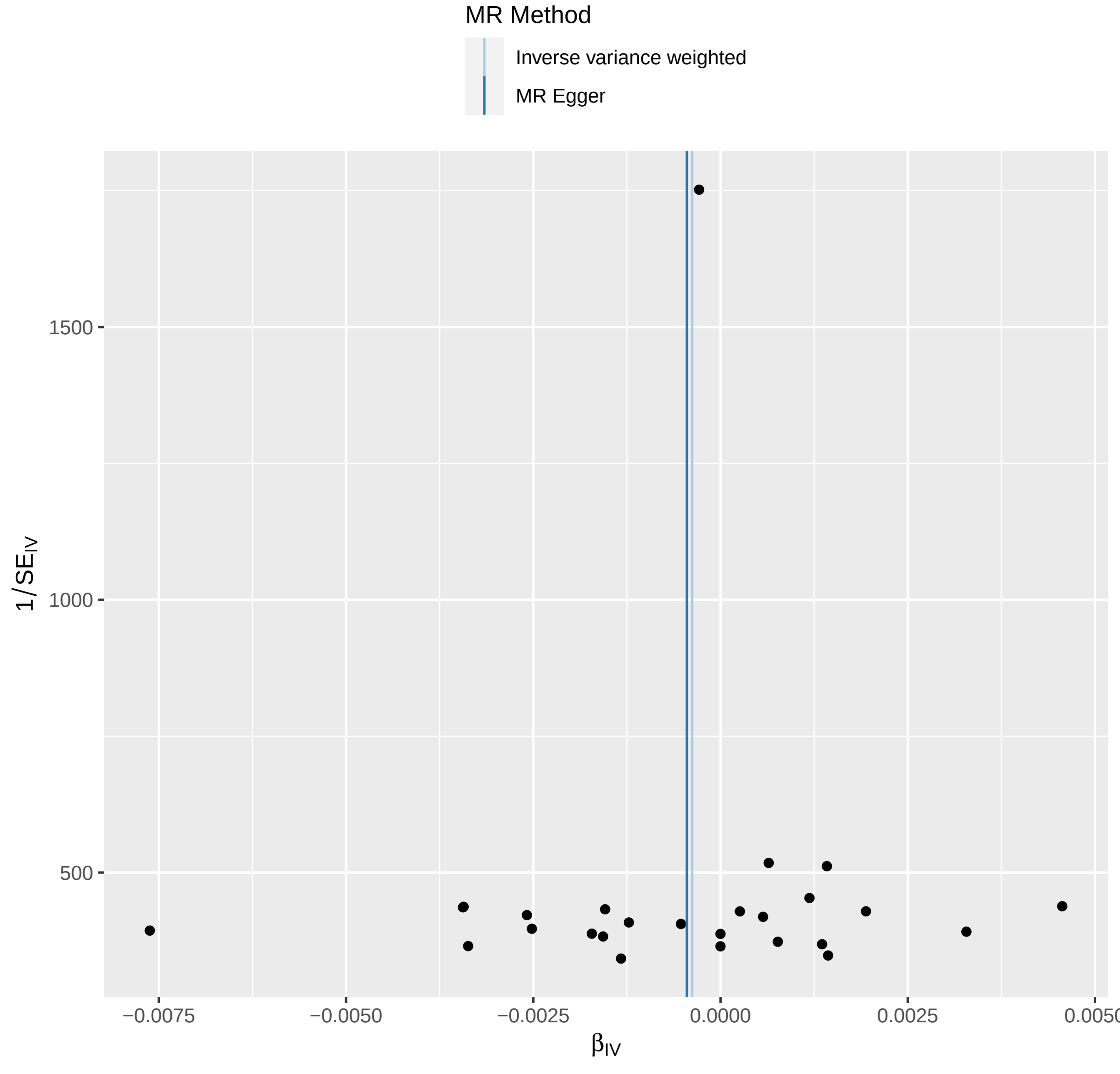

Batch 2−1456 : Immunoglobulin E on eGFR

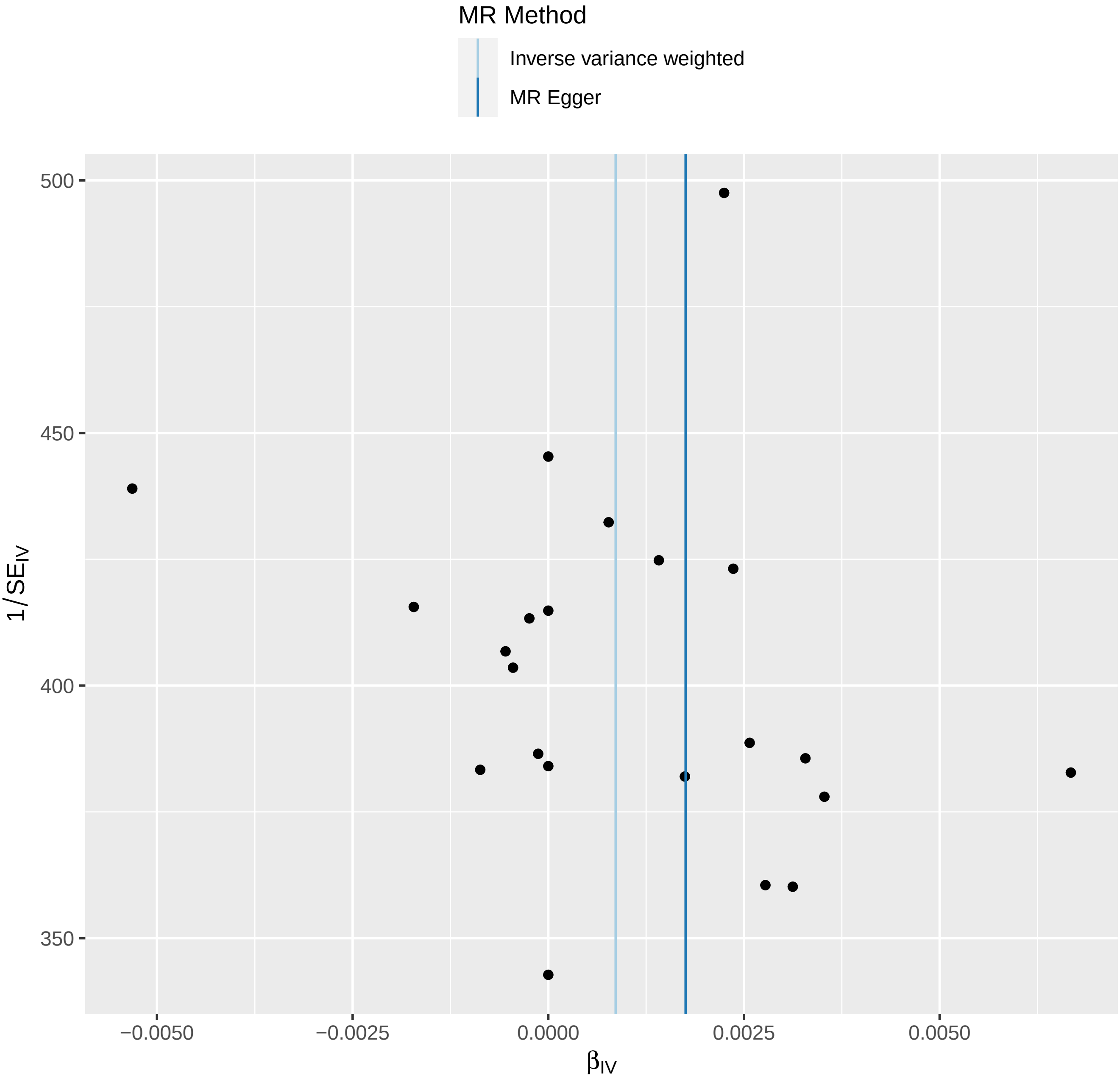

Batch 2−1457 : Immunoglobulin J chain on eGFR

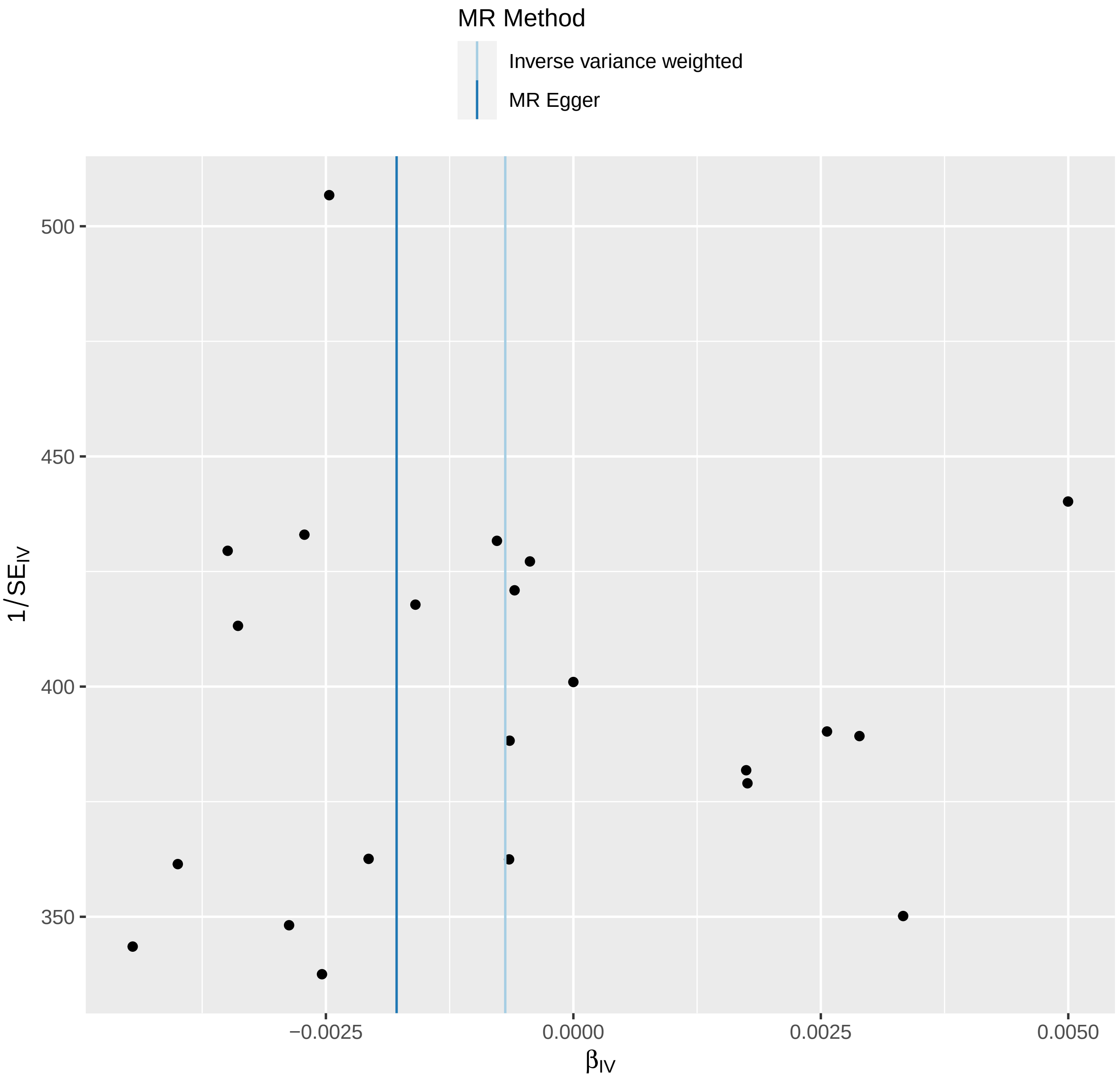

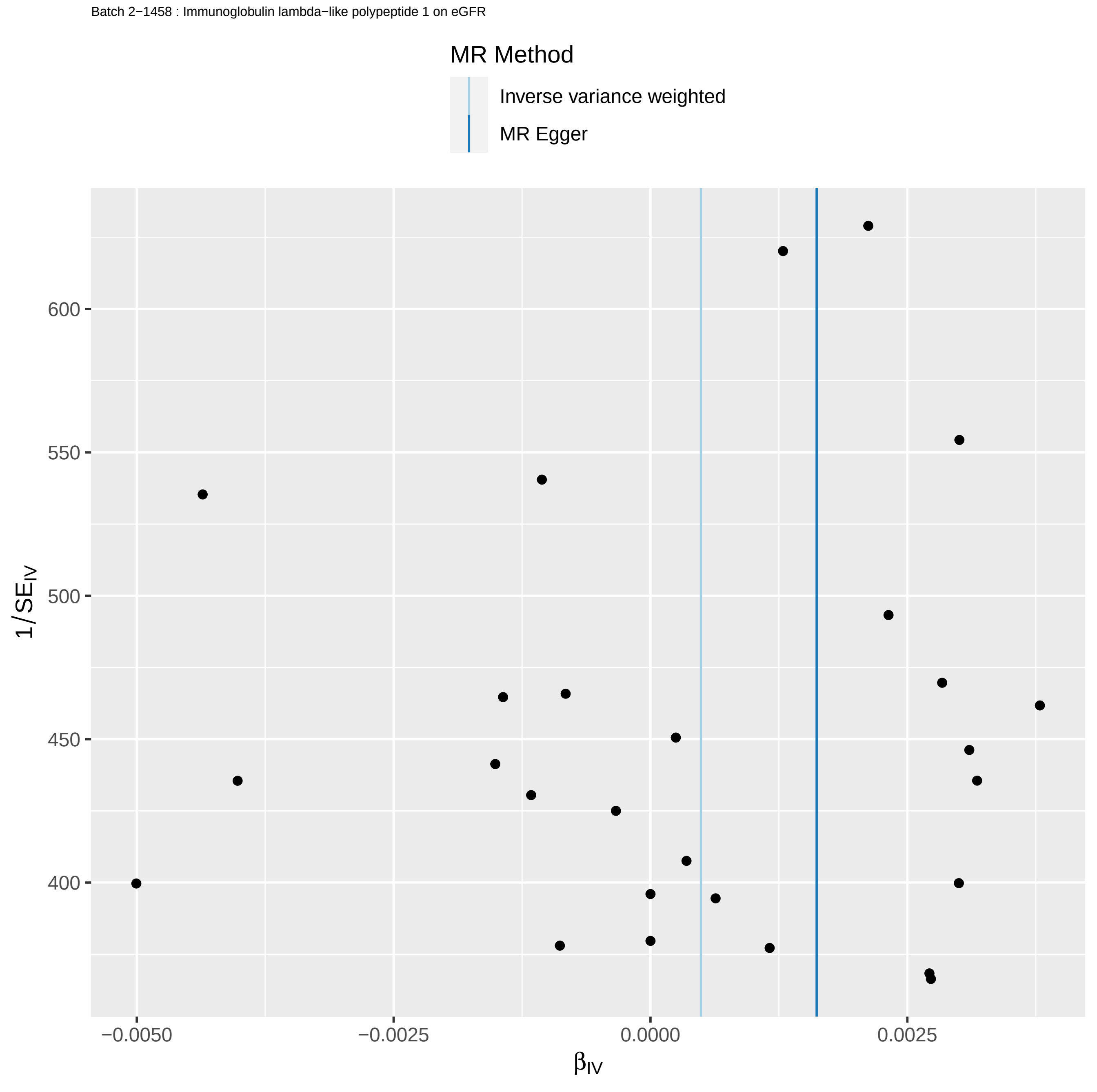

Batch 2−1458 : Immunoglobulin lambda−like polypeptide 1 on eGFR
MR Method
Inverse variance weighted
MR Egger
600
550
500
450
400
1/SE_IV
−0.0050
−0.0025
0.0000
0.0025
β_IV

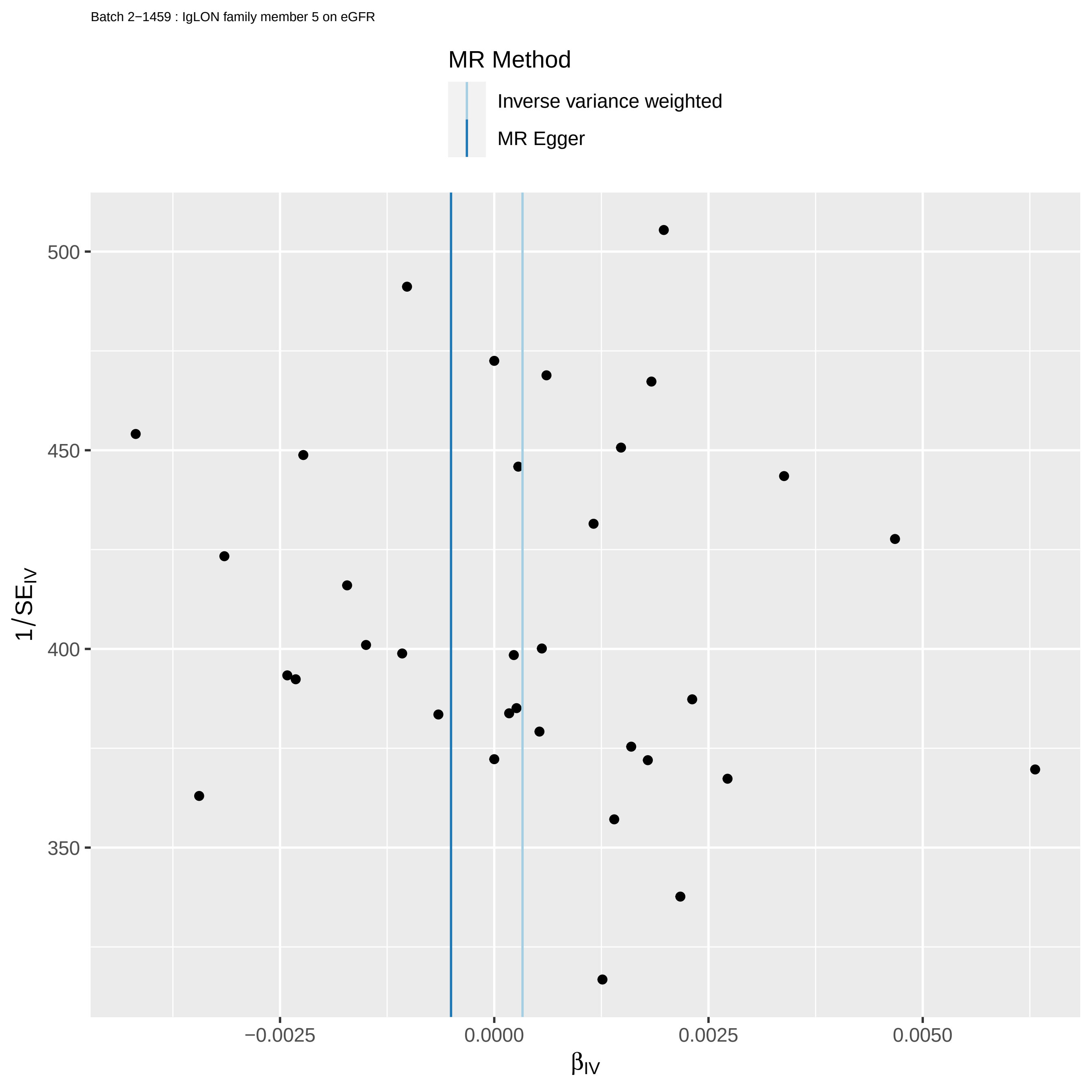

Batch 2−1459 : IgLON family member 5 on eGFR
MR Method
Inverse variance weighted
MR Egger
500
450
400
350
1/SE$_{IV}$
−0.0025
0.0000
0.0025
0.0050
β_{IV}

Batch 2−1460 : Immunoglobulin superfamily member 11 on eGFR

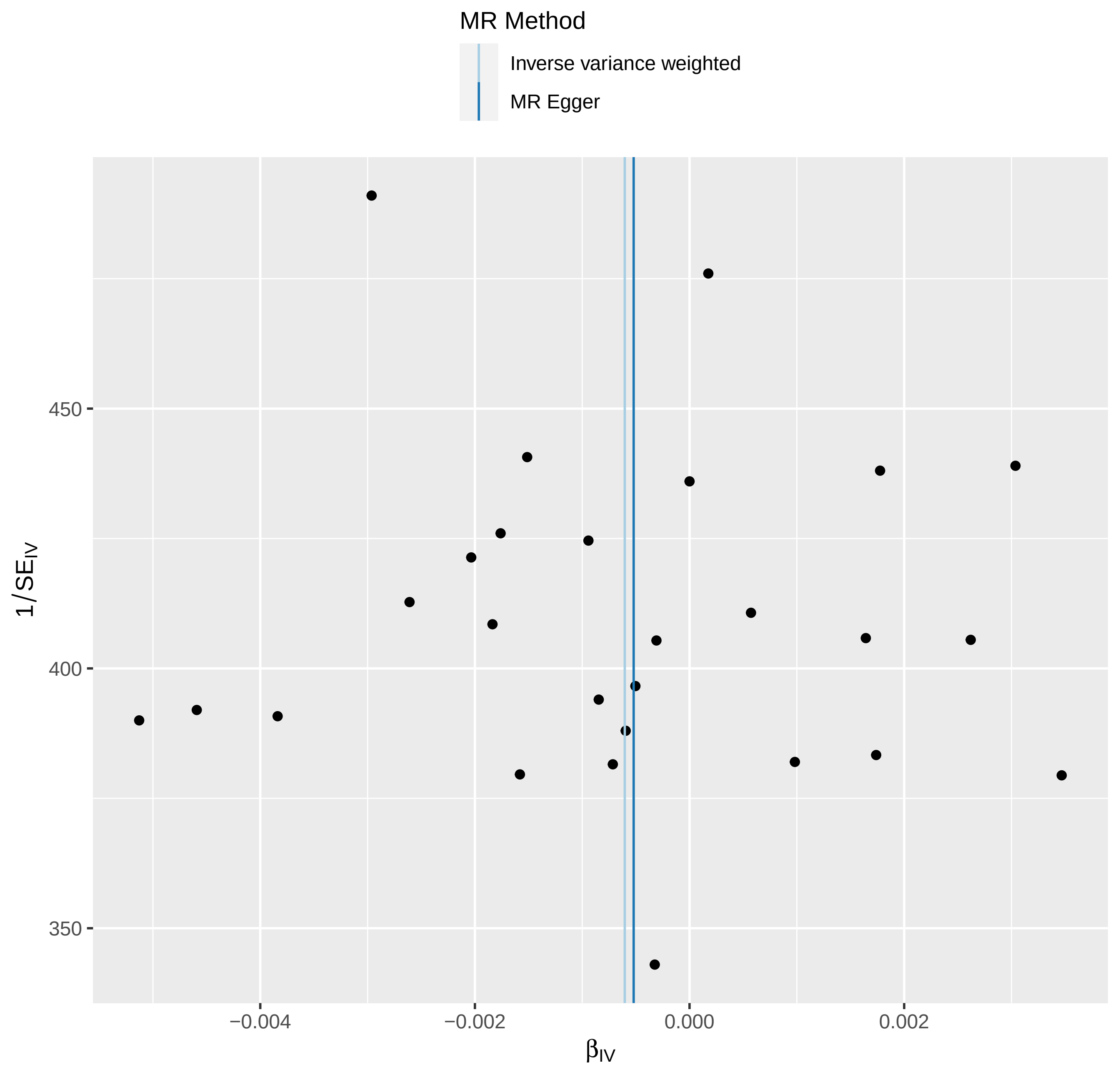

Batch 2−1461 : Immunoglobulin superfamily member 11 on eGFR

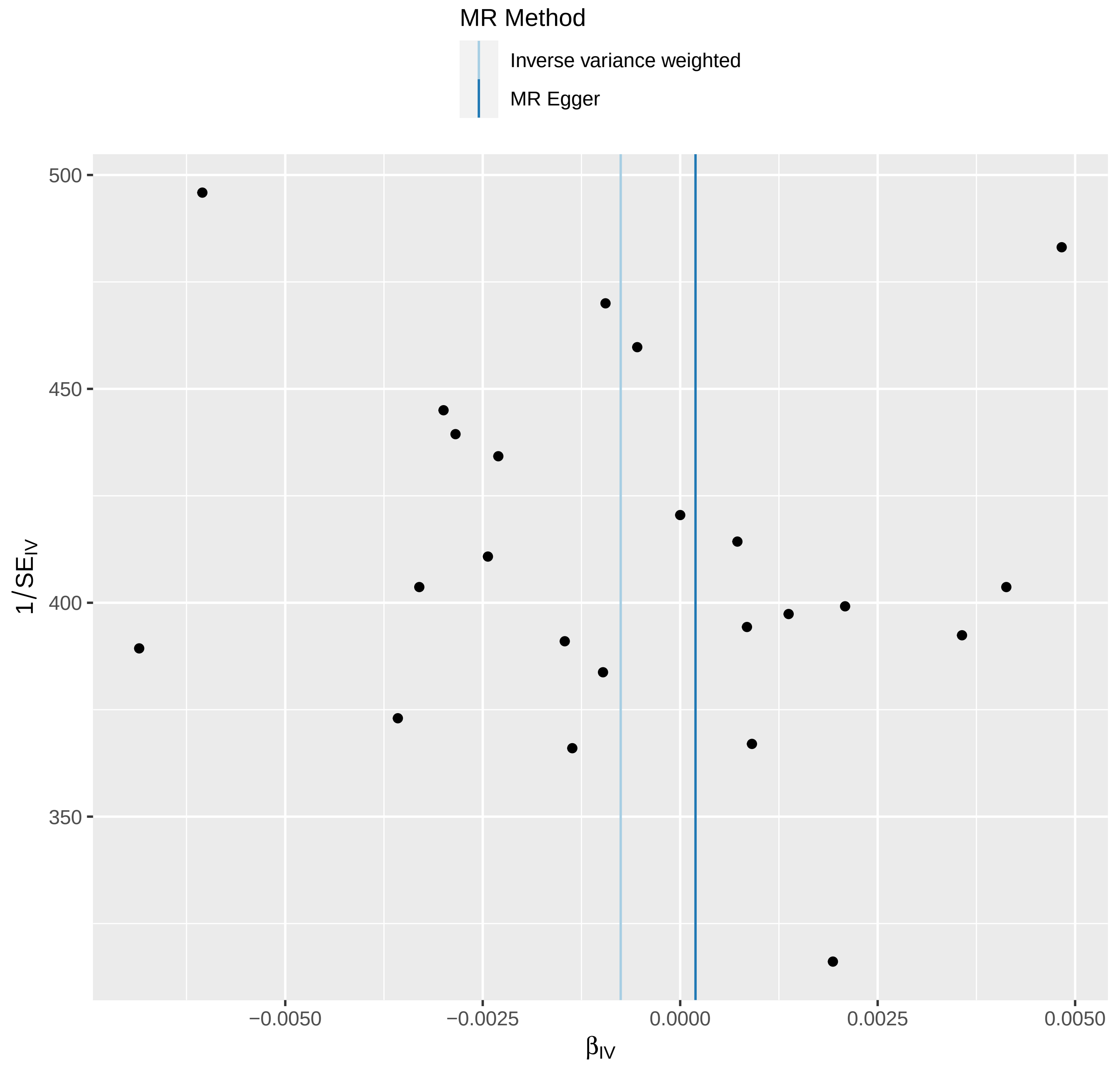

Batch 2−1462 : Immunoglobulin superfamily member 8 on eGFR

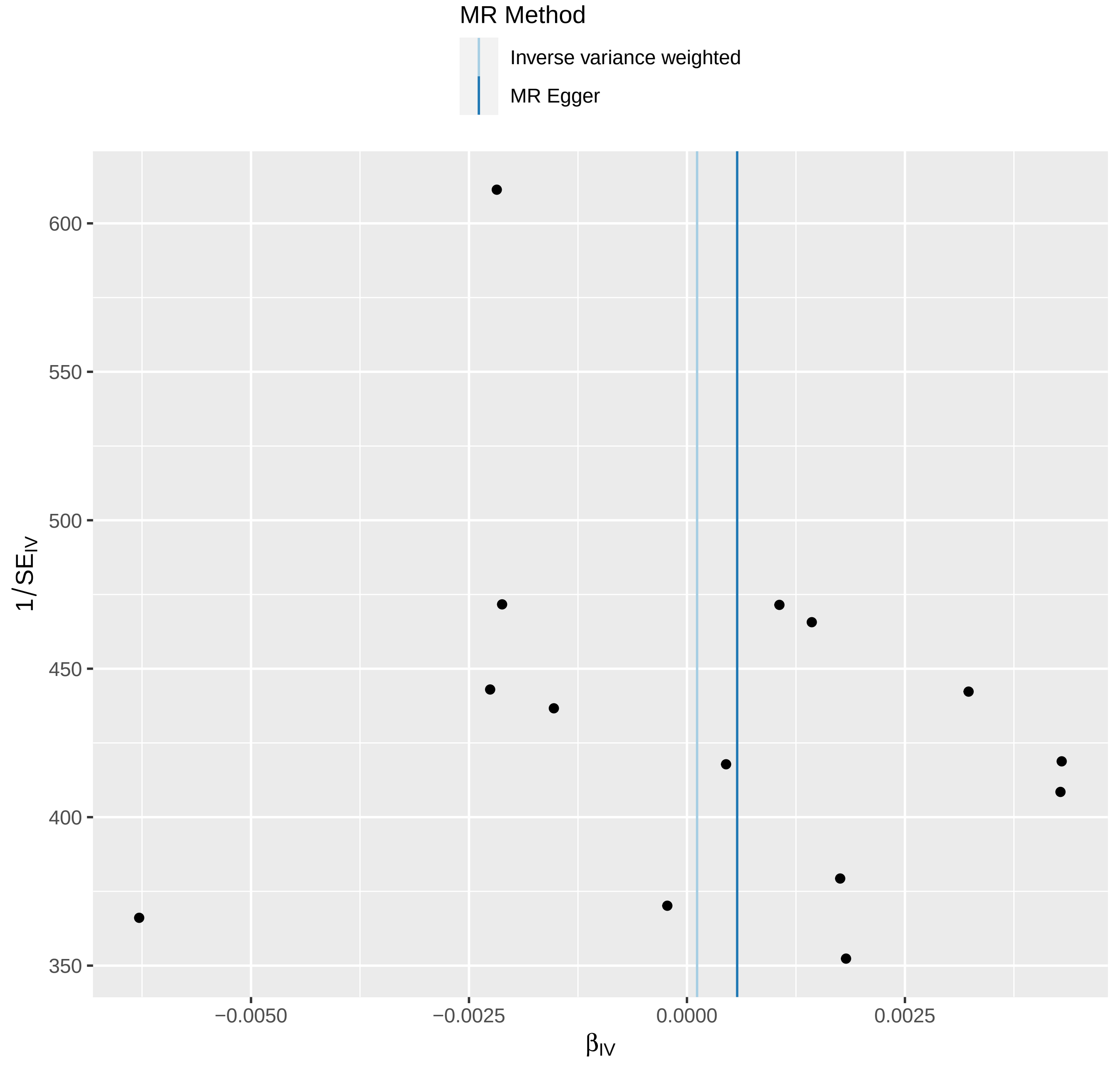

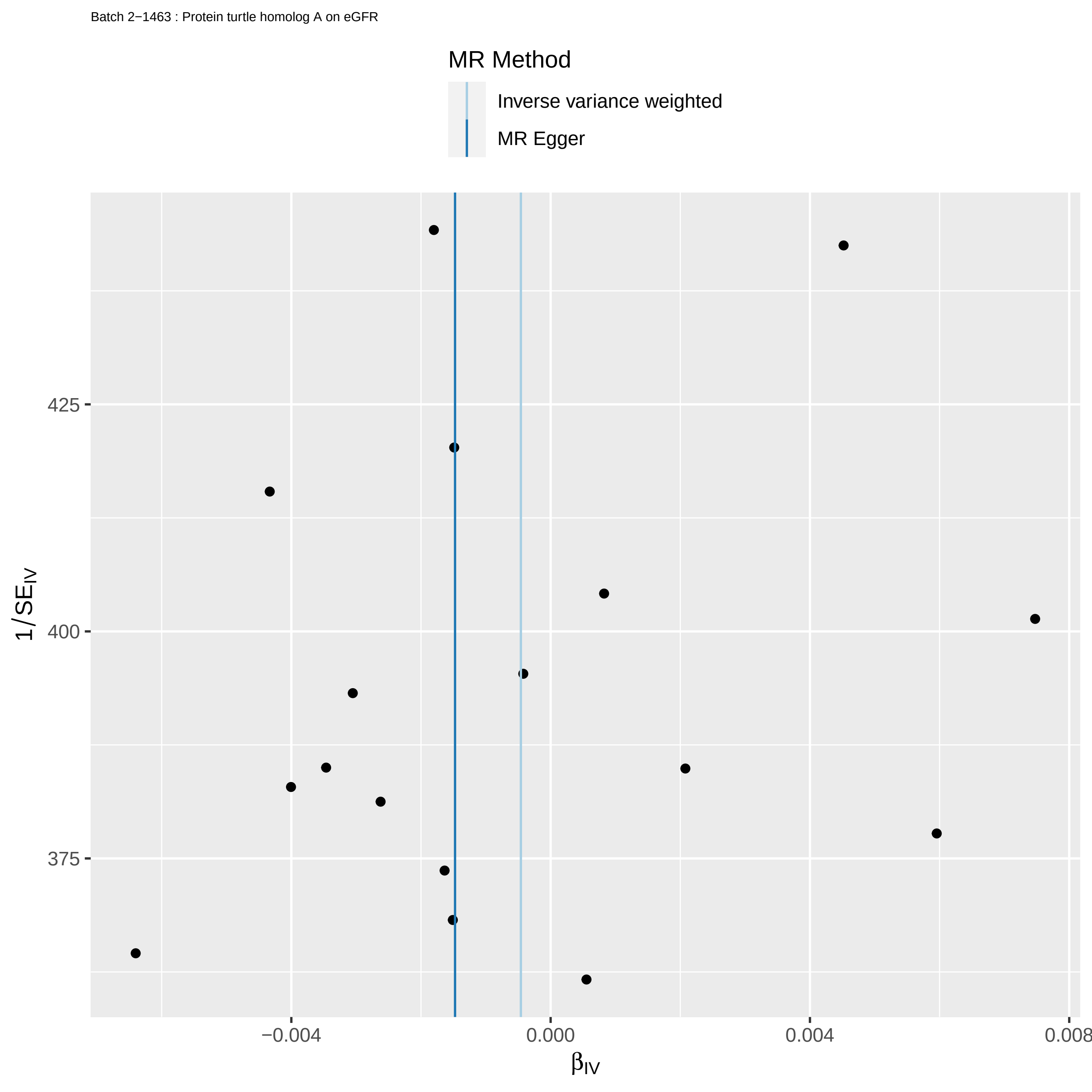

Batch 2−1463 : Protein turtle homolog A on eGFR
MR Method
Inverse variance weighted
MR Egger
425
400
375
1/SE_IV
−0.004
0.000
0.004
0.008
β_IV

Batch 2−1464 : Interleukin−10 on eGFR

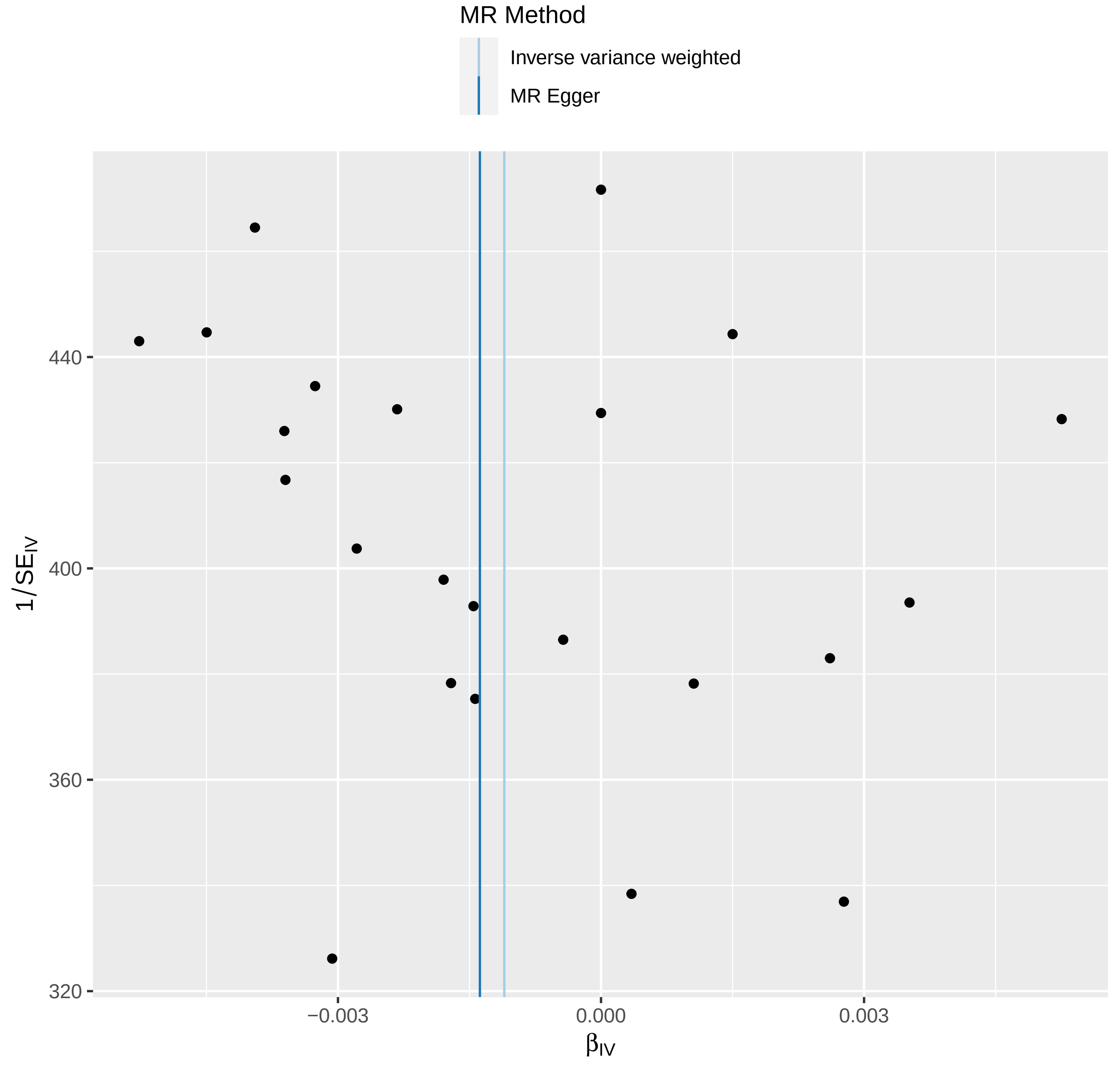

Batch 2−1465 : Interleukin−10 receptor subunit beta on eGFR

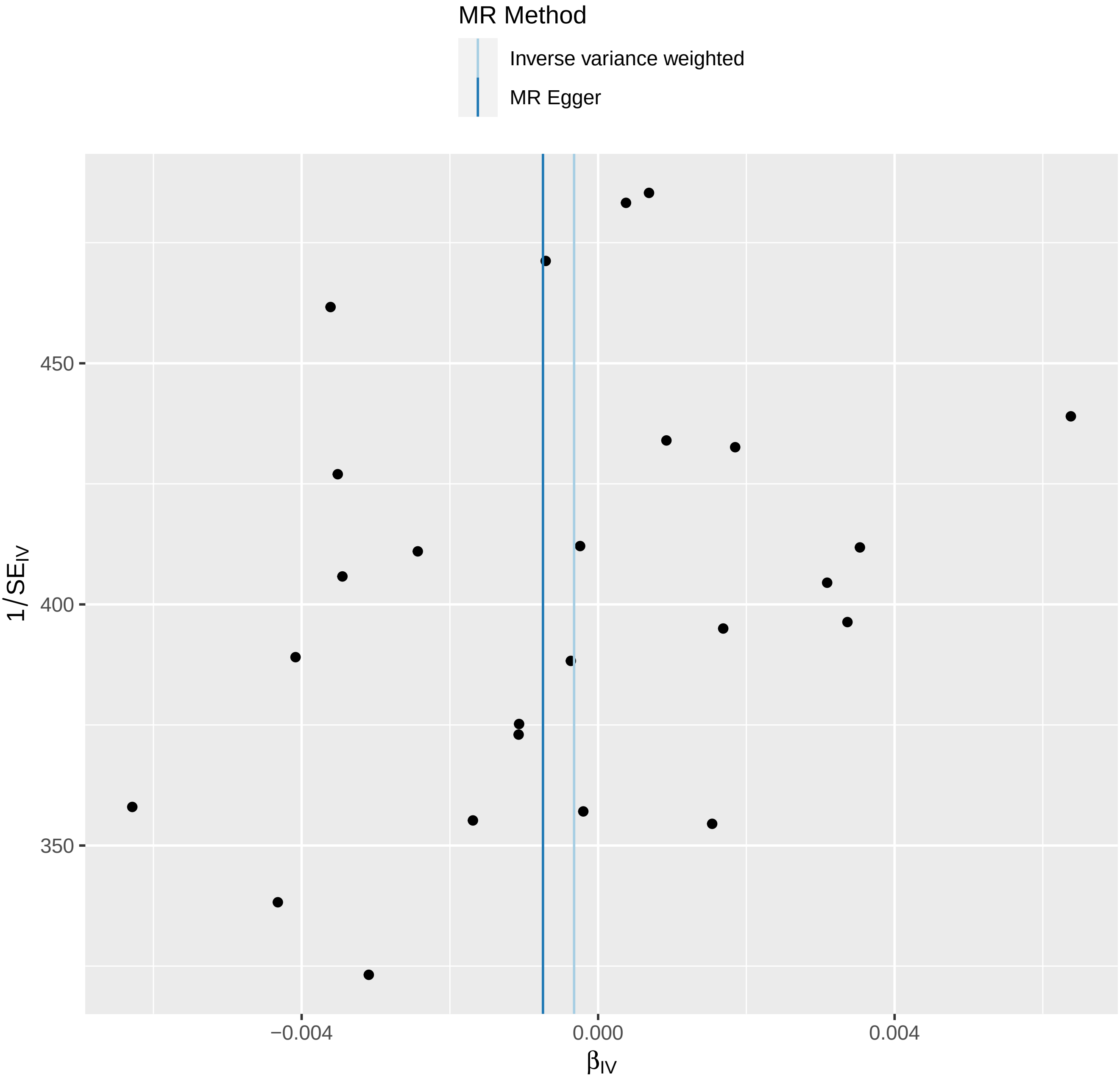

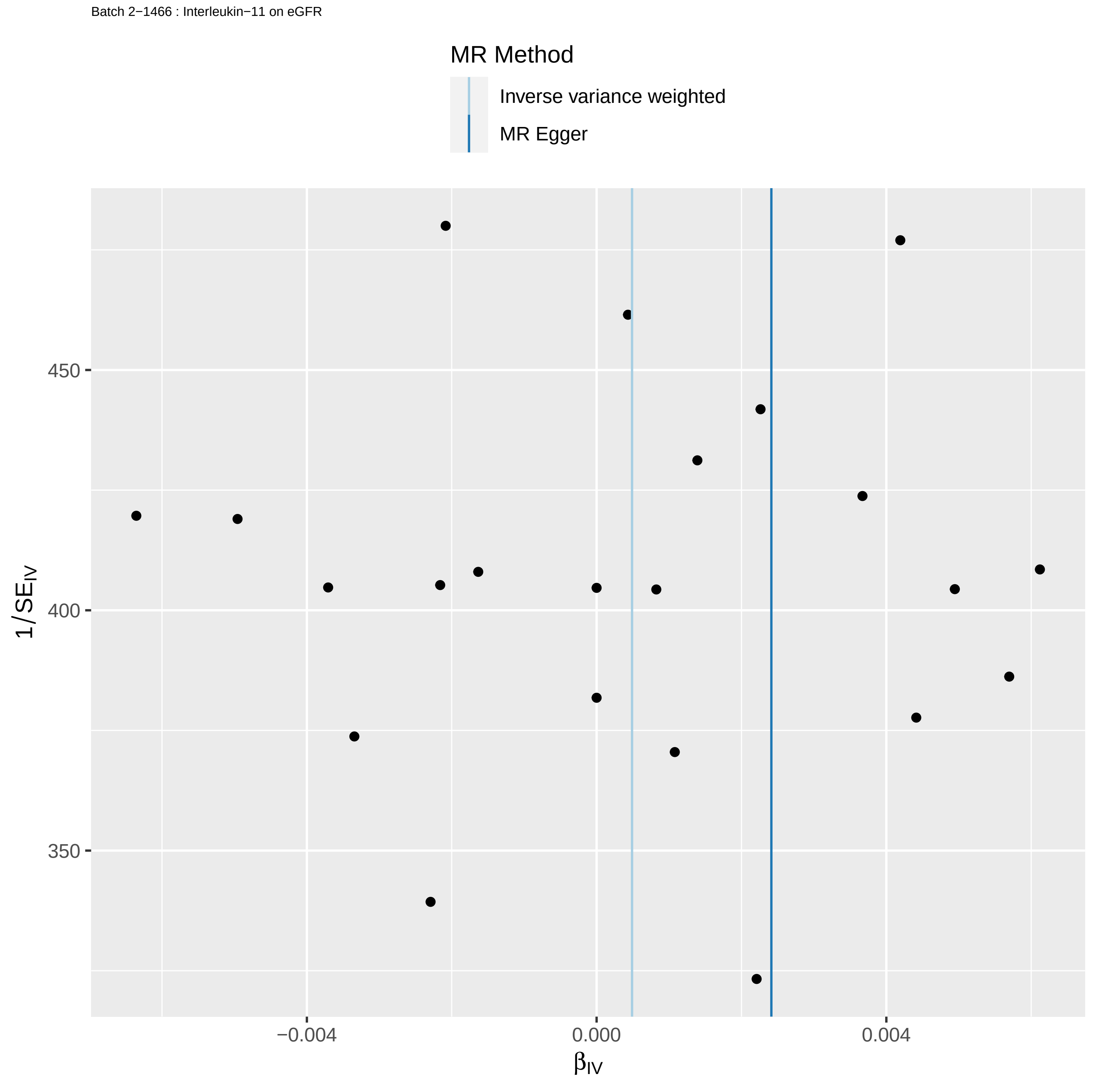
Batch 2−1466 : Interleukin−11 on eGFR
MR Method
Inverse variance weighted
MR Egger
450
400
350
1/SE$_{IV}$
−0.004
0.000
0.004
β$_{IV}$

Batch 2−1467 : Interleukin−11 receptor subunit alpha on eGFR

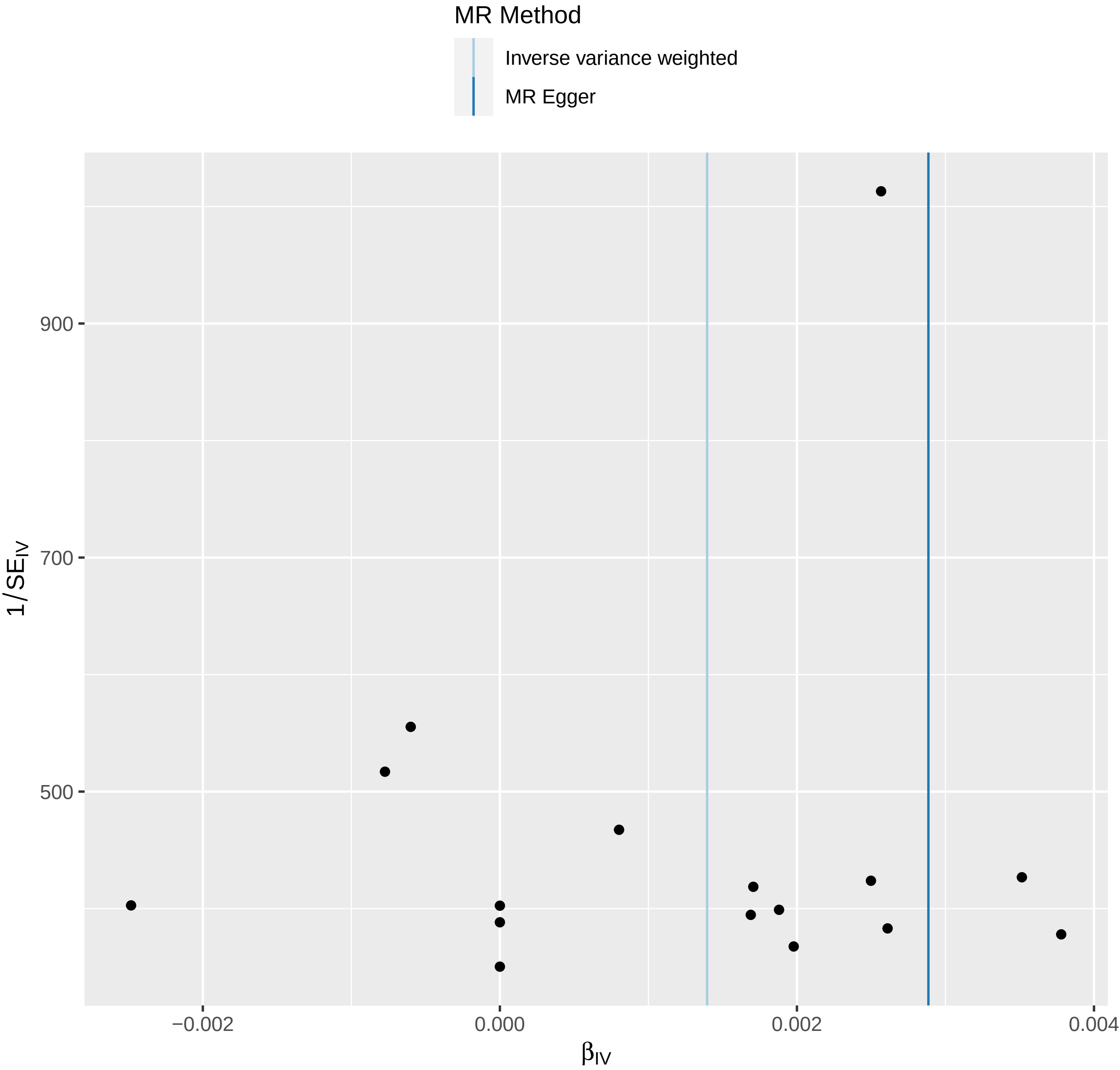

Batch 2−1468 : Interleukin−12 on eGFR

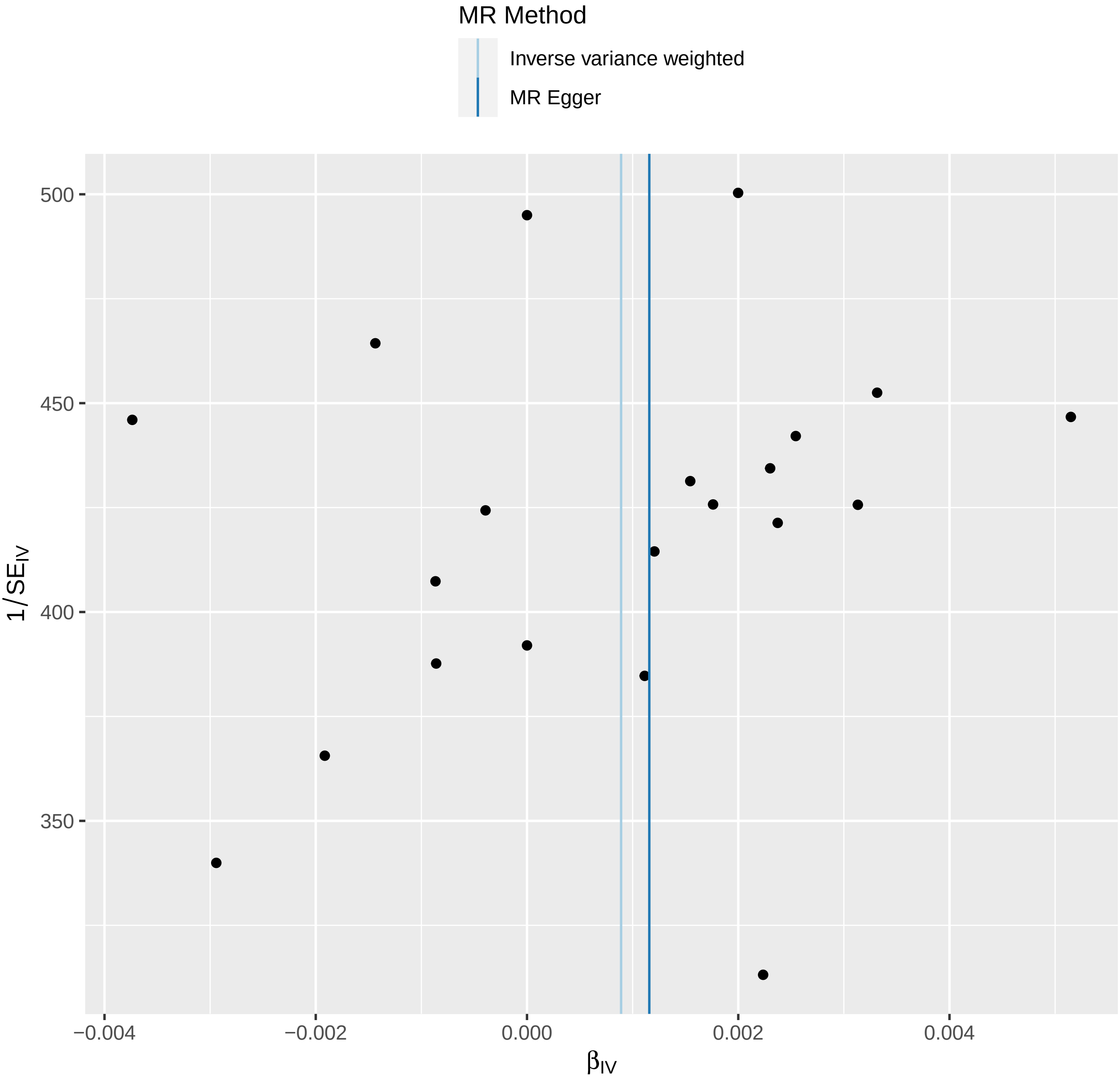

Batch 2−1469 : Interleukin−12 on eGFR

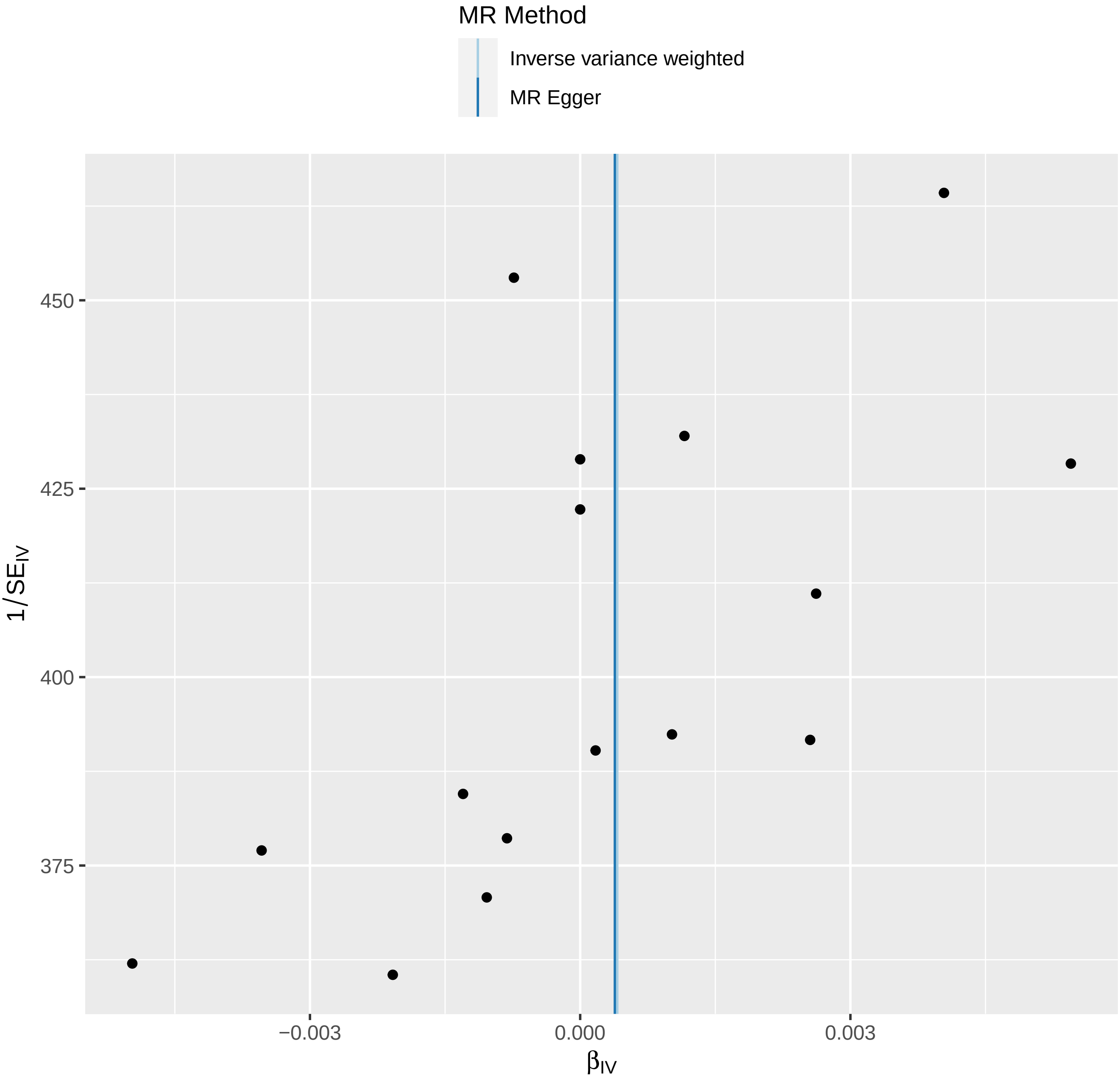

Batch 2−1470 : Interleukin−12 on eGFR

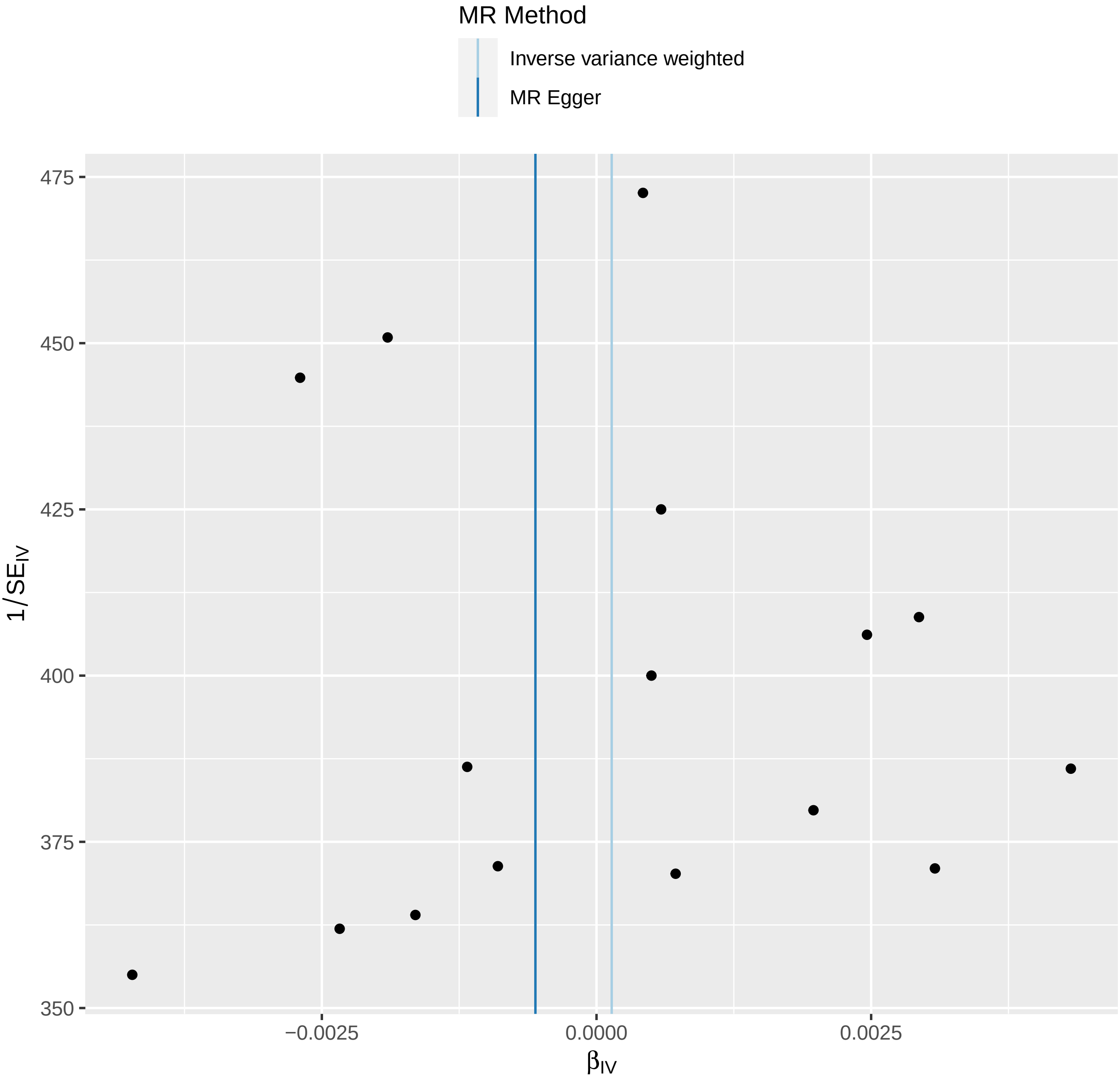

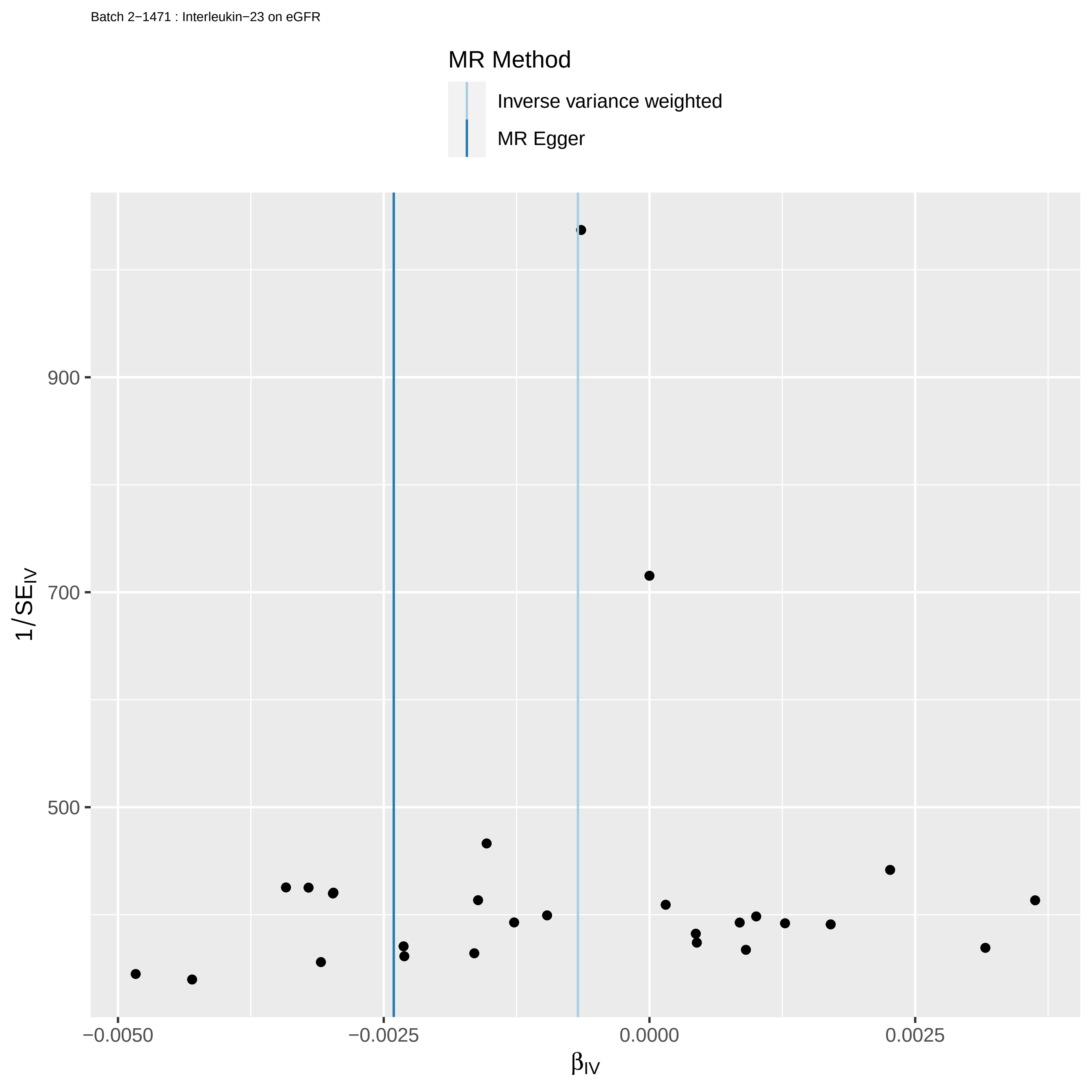
Batch 2−1471 : Interleukin−23 on eGFR
MR Method
Inverse variance weighted
MR Egger
900
700
500
1/SE$_{IV}$
−0.0050
−0.0025
0.0000
0.0025
β$_{IV}$

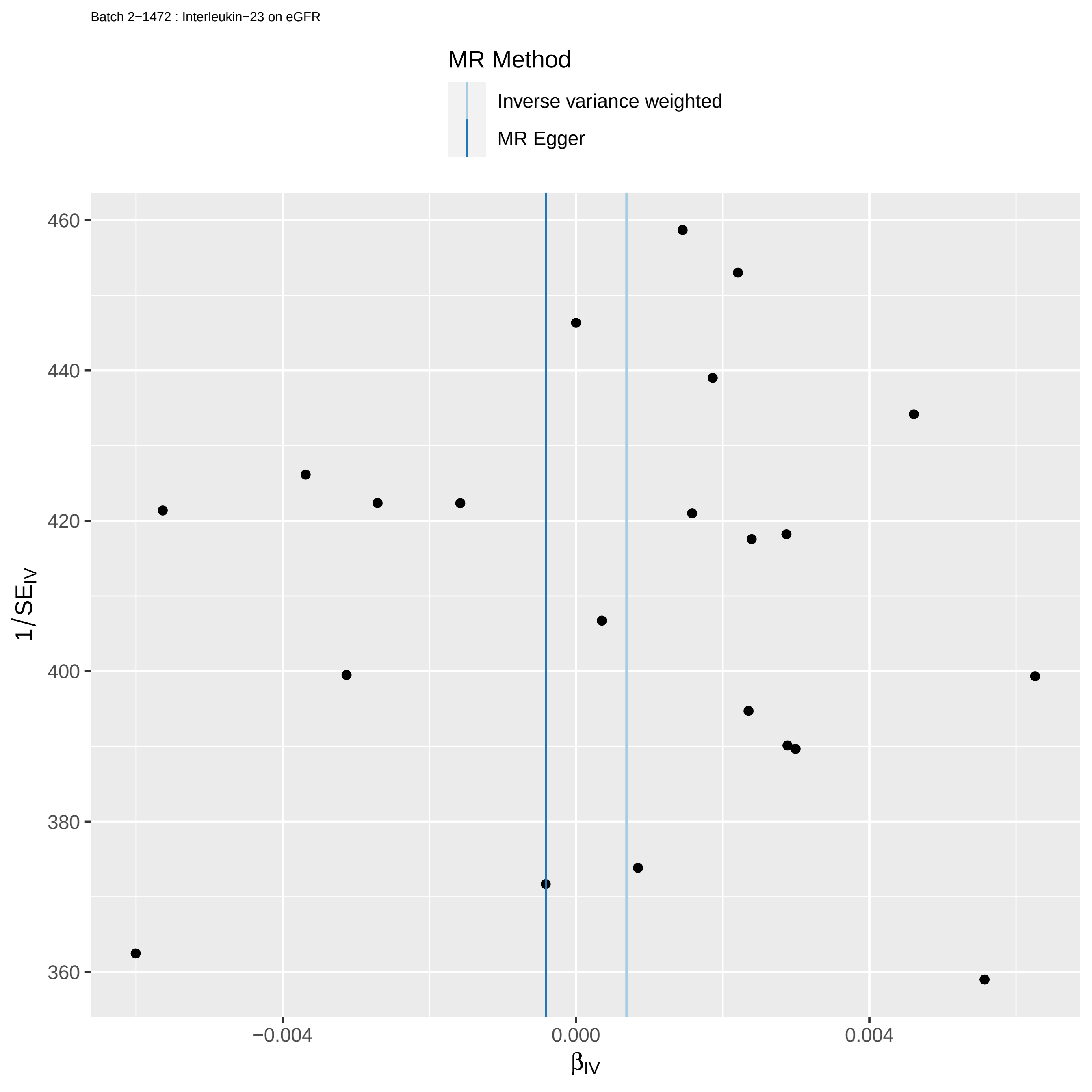

Batch 2−1472 : Interleukin−23 on eGFR
MR Method
Inverse variance weighted
MR Egger
460
440
420
400
380
360
1/SE_IV
−0.004
0.000
0.004
β_IV

Batch 2−1473 : Interleukin−12 receptor subunit beta−1 on eGFR

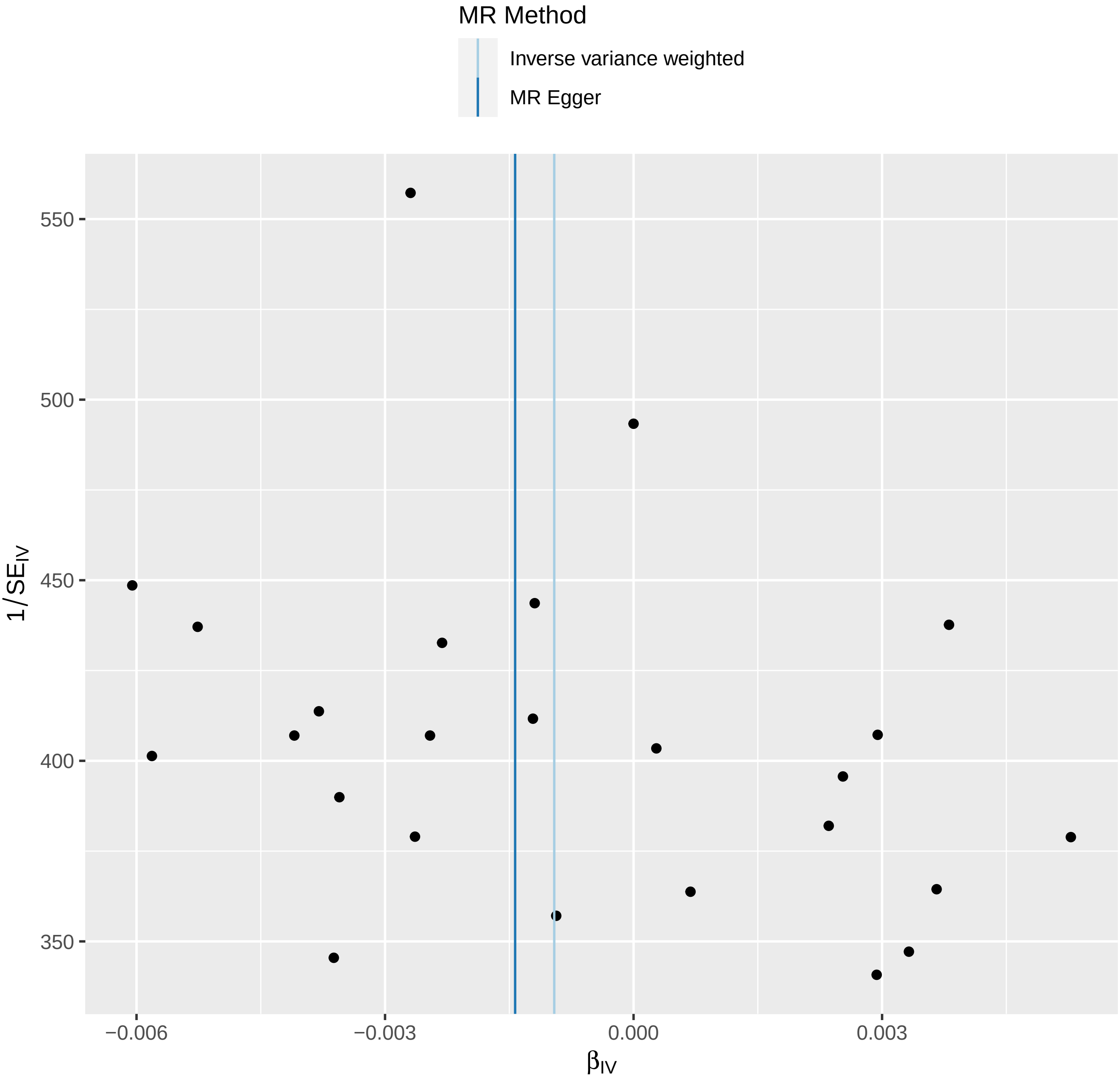

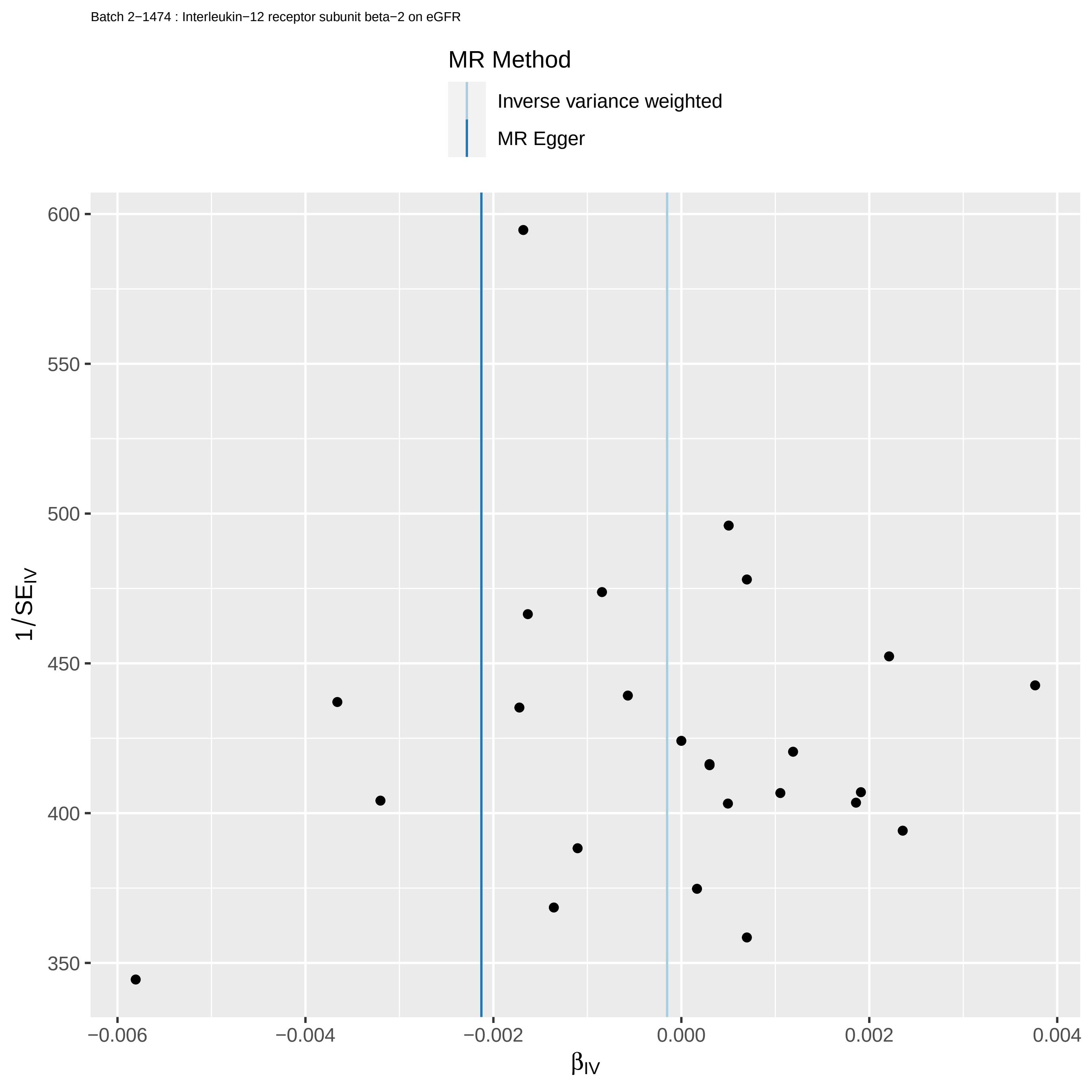

Batch 2−1474 : Interleukin−12 receptor subunit beta−2 on eGFR
MR Method
Inverse variance weighted
MR Egger
600
550
500
450
400
350
1/SE_IV
−0.006
−0.004
−0.002
0.000
0.002
0.004
β_IV

Batch 2−1475 : Interleukin−13 on eGFR

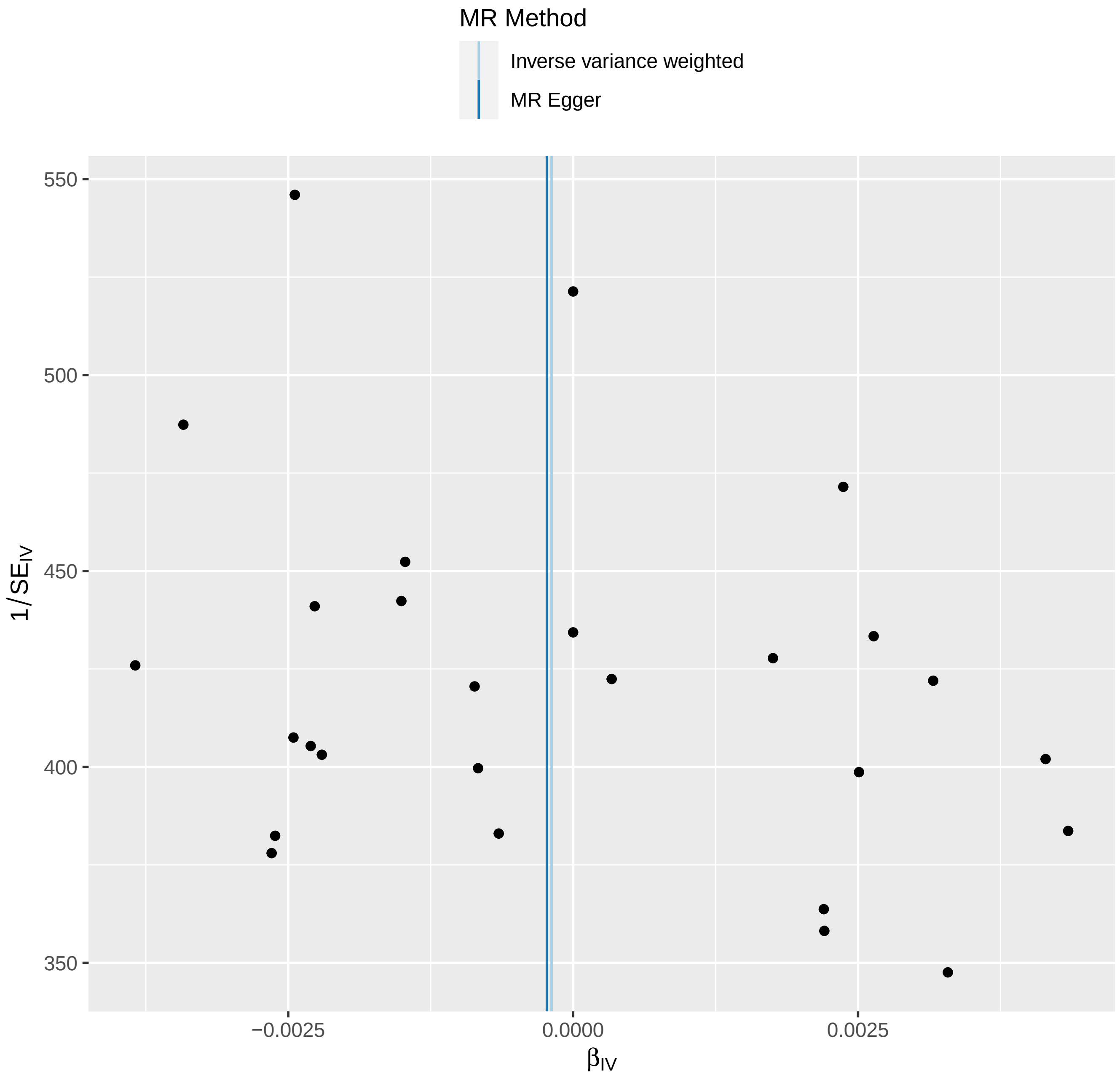

Batch 2−1476 : Interleukin−13 receptor subunit alpha−1 on eGFR

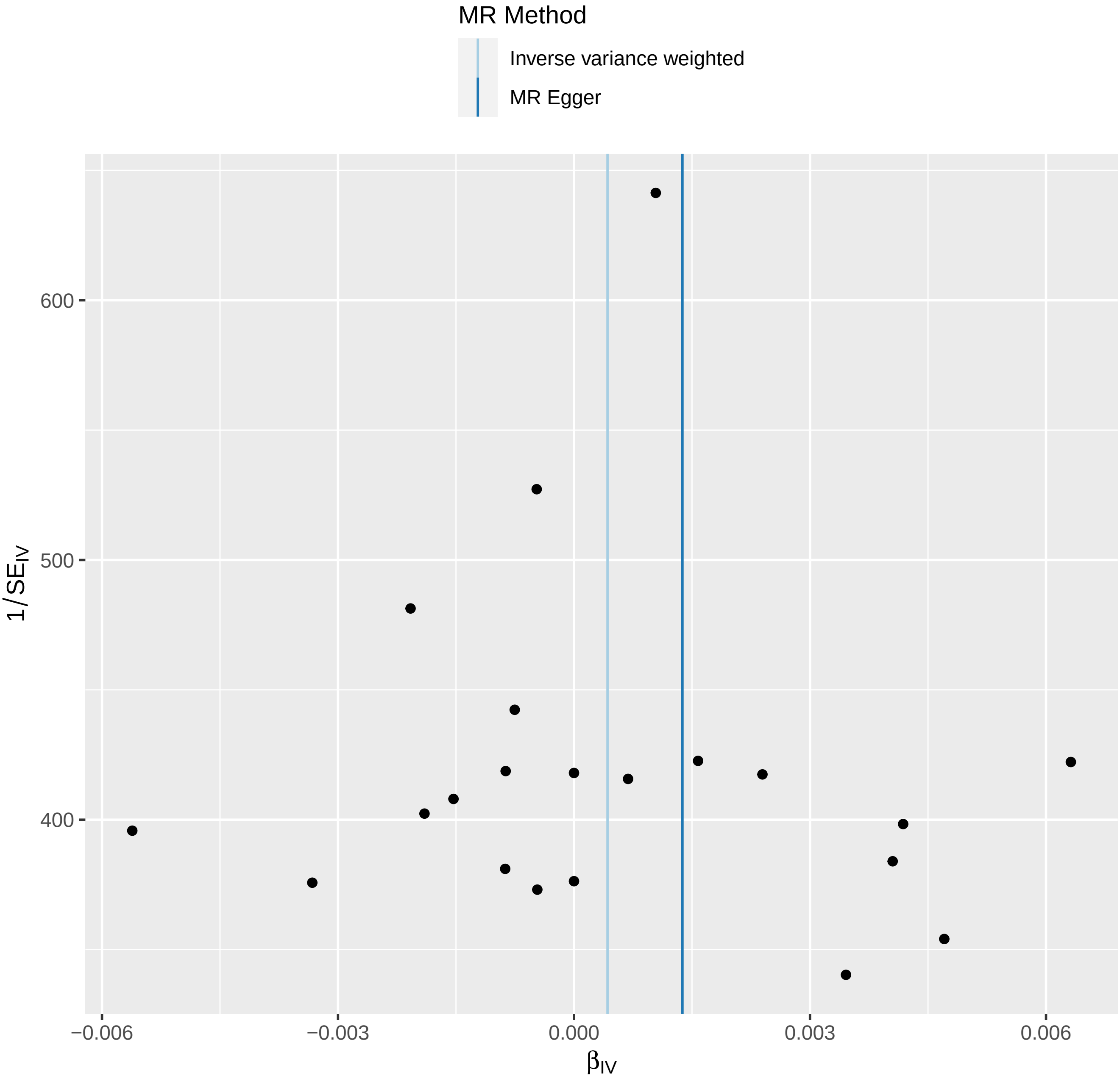

Batch 2−1477 : Interleukin−15 receptor subunit alpha on eGFR

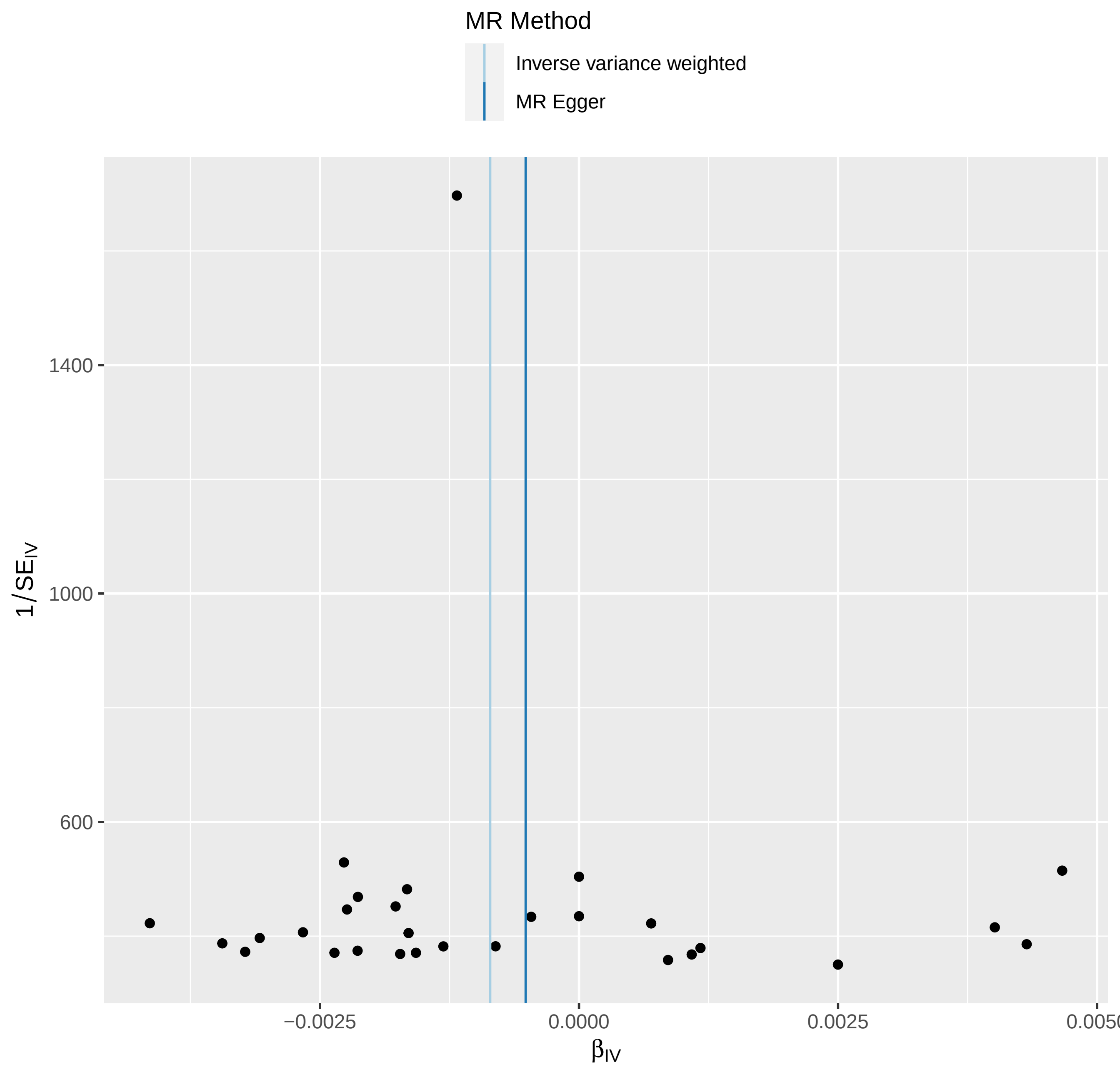

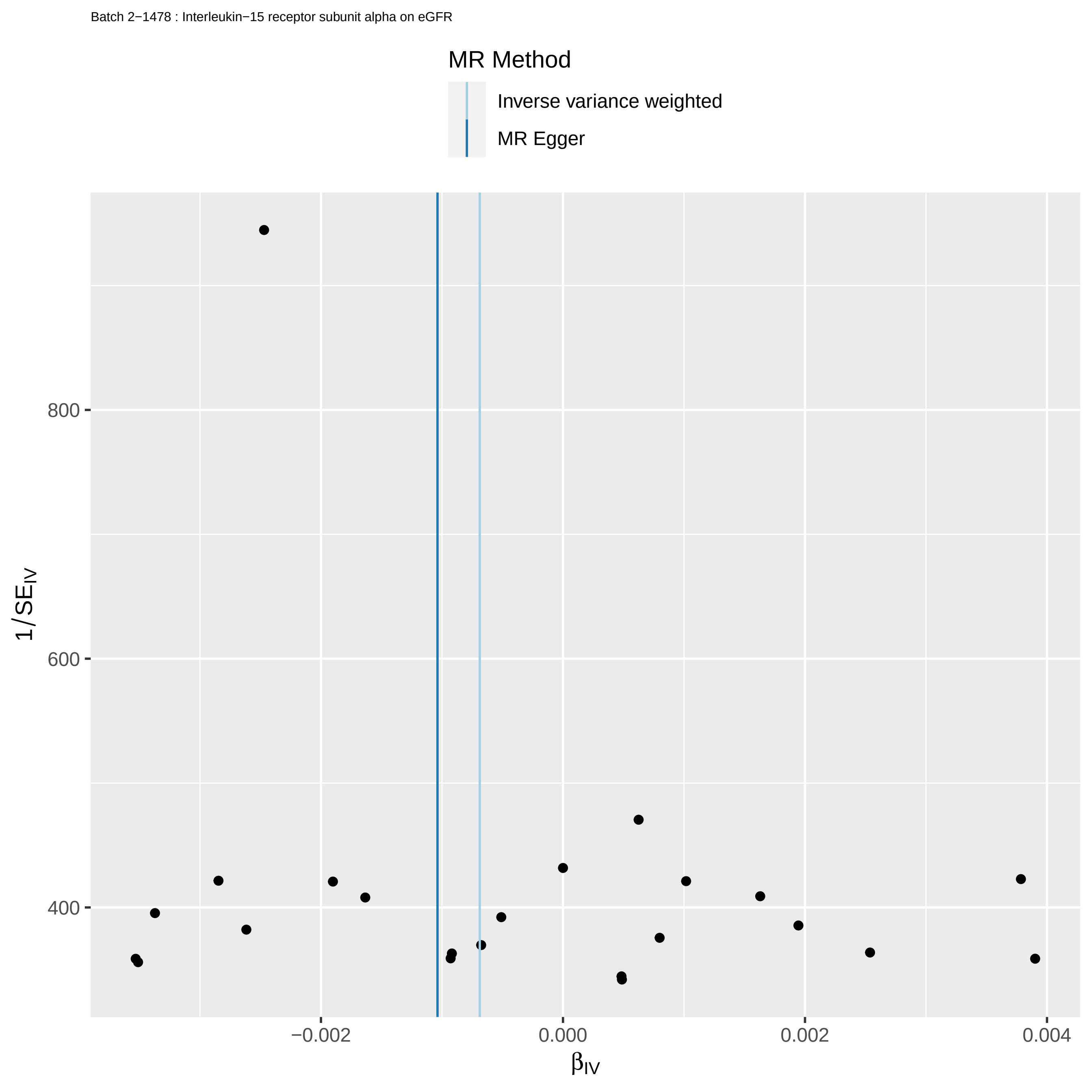
Batch 2−1478 : Interleukin−15 receptor subunit alpha on eGFR
MR Method
Inverse variance weighted
MR Egger
800
600
400
1/SE_IV
−0.002
0.000
0.002
0.004
β_IV

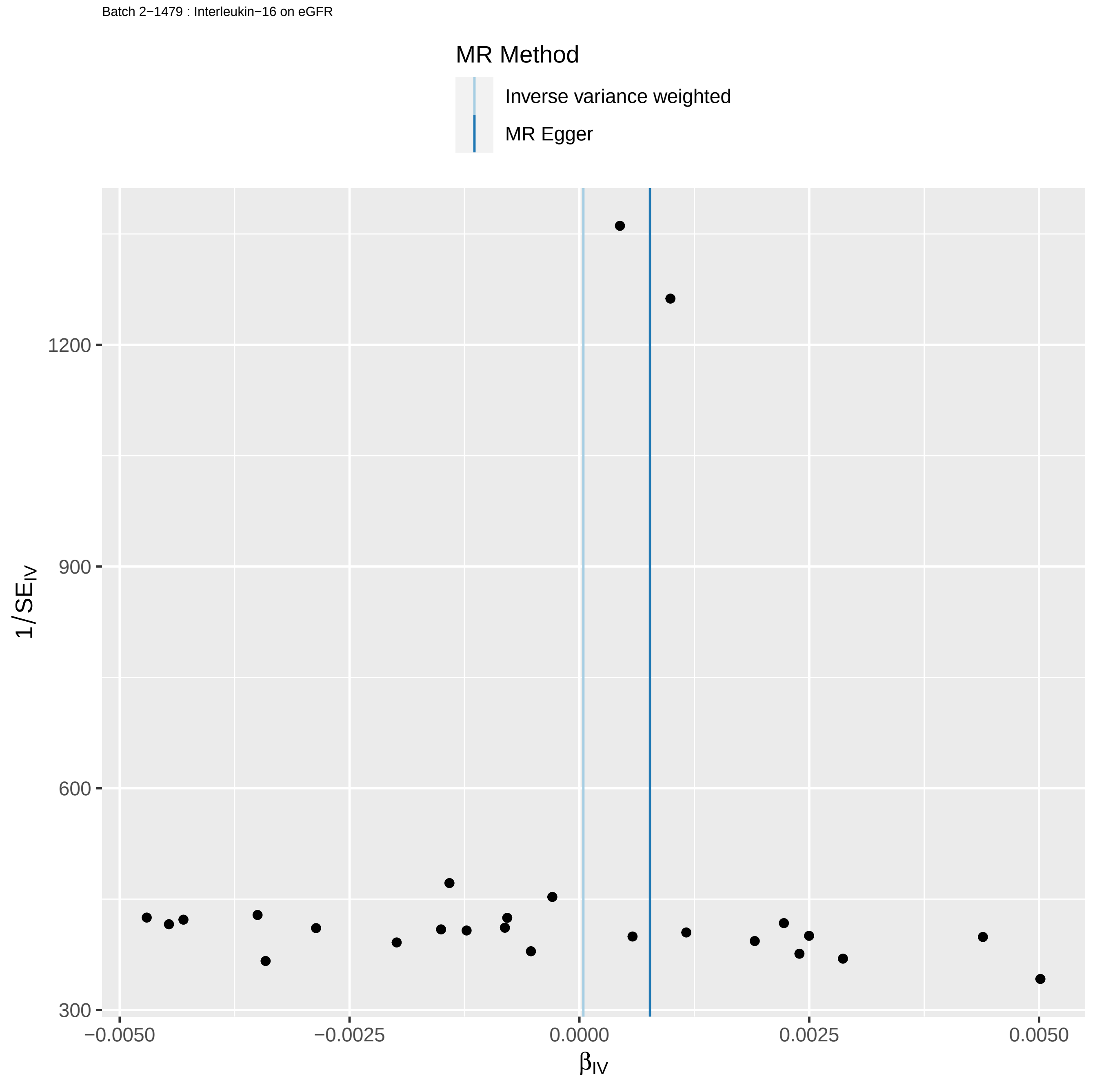
Batch 2−1479 : Interleukin−16 on eGFR
MR Method
Inverse variance weighted
MR Egger
1200
900
600
300
−0.0050
−0.0025
0.0000
0.0025
0.0050
β_{IV}
$1/SE_{IV}$

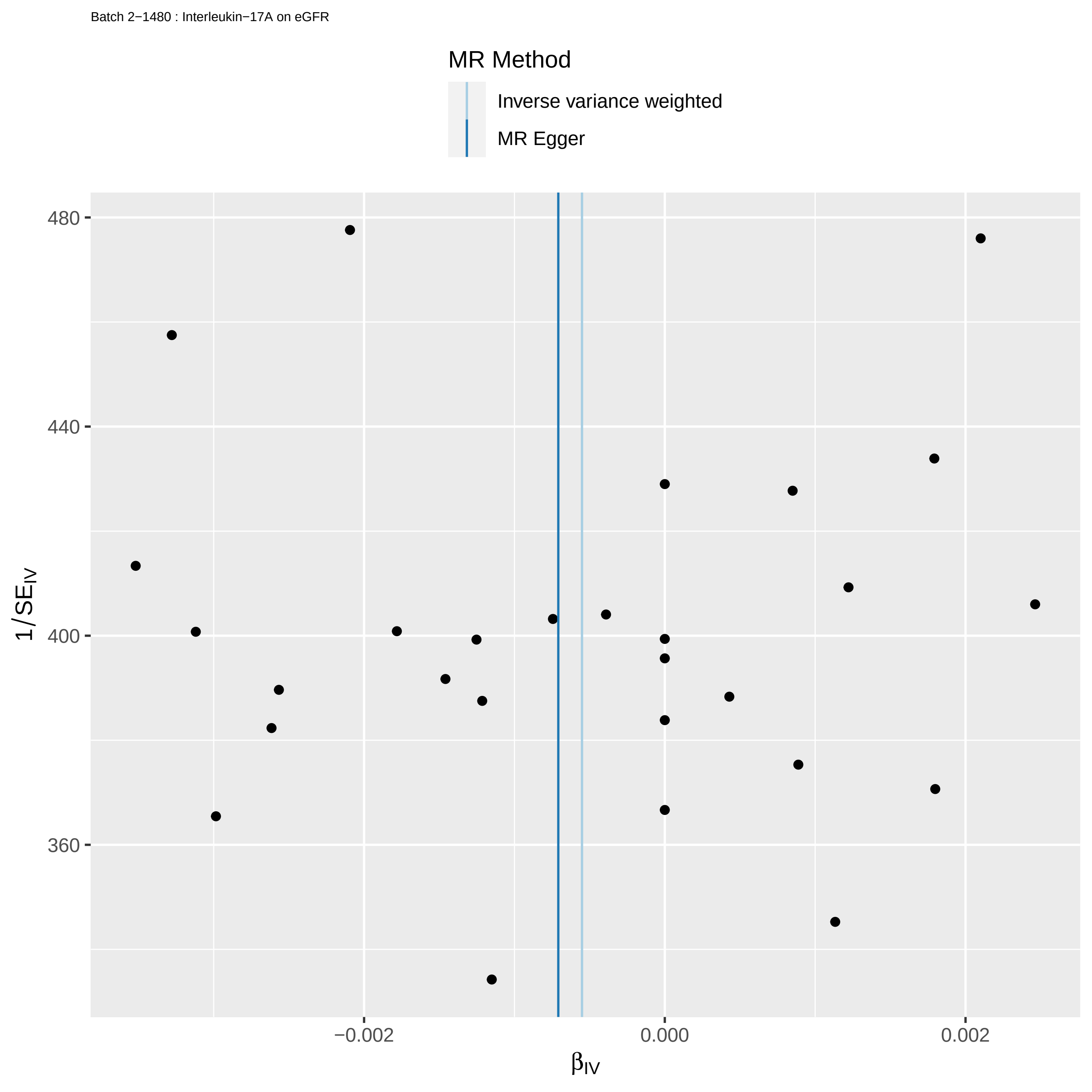

Batch 2−1480 : Interleukin−17A on eGFR
MR Method
Inverse variance weighted
MR Egger
480
440
400
360
1/SE_IV
−0.002
0.000
0.002
β_IV

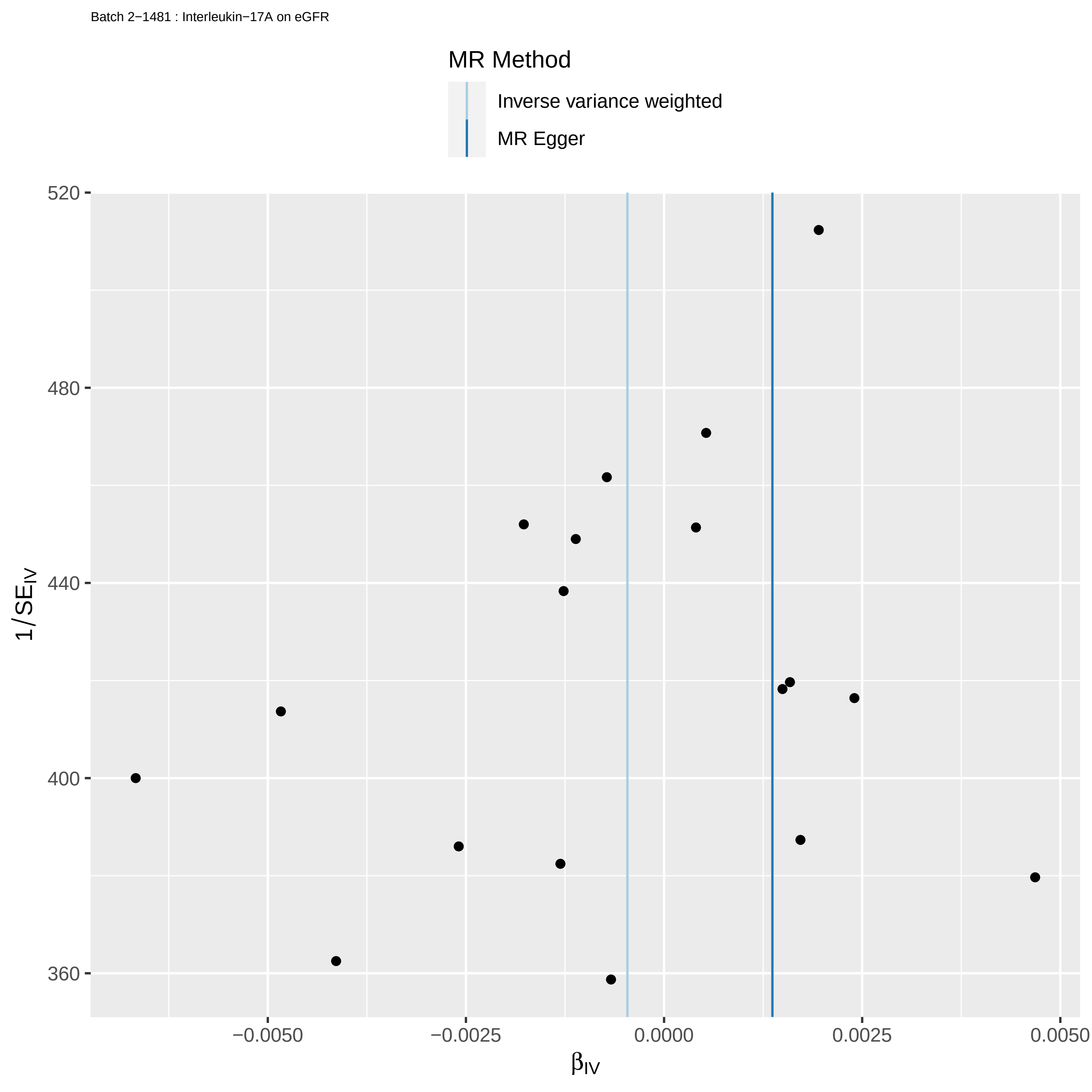

Batch 2−1481 : Interleukin−17A on eGFR
MR Method
Inverse variance weighted
MR Egger
520
480
440
400
360
1/SE_IV
−0.0050
−0.0025
0.0000
0.0025
0.0050
β_IV

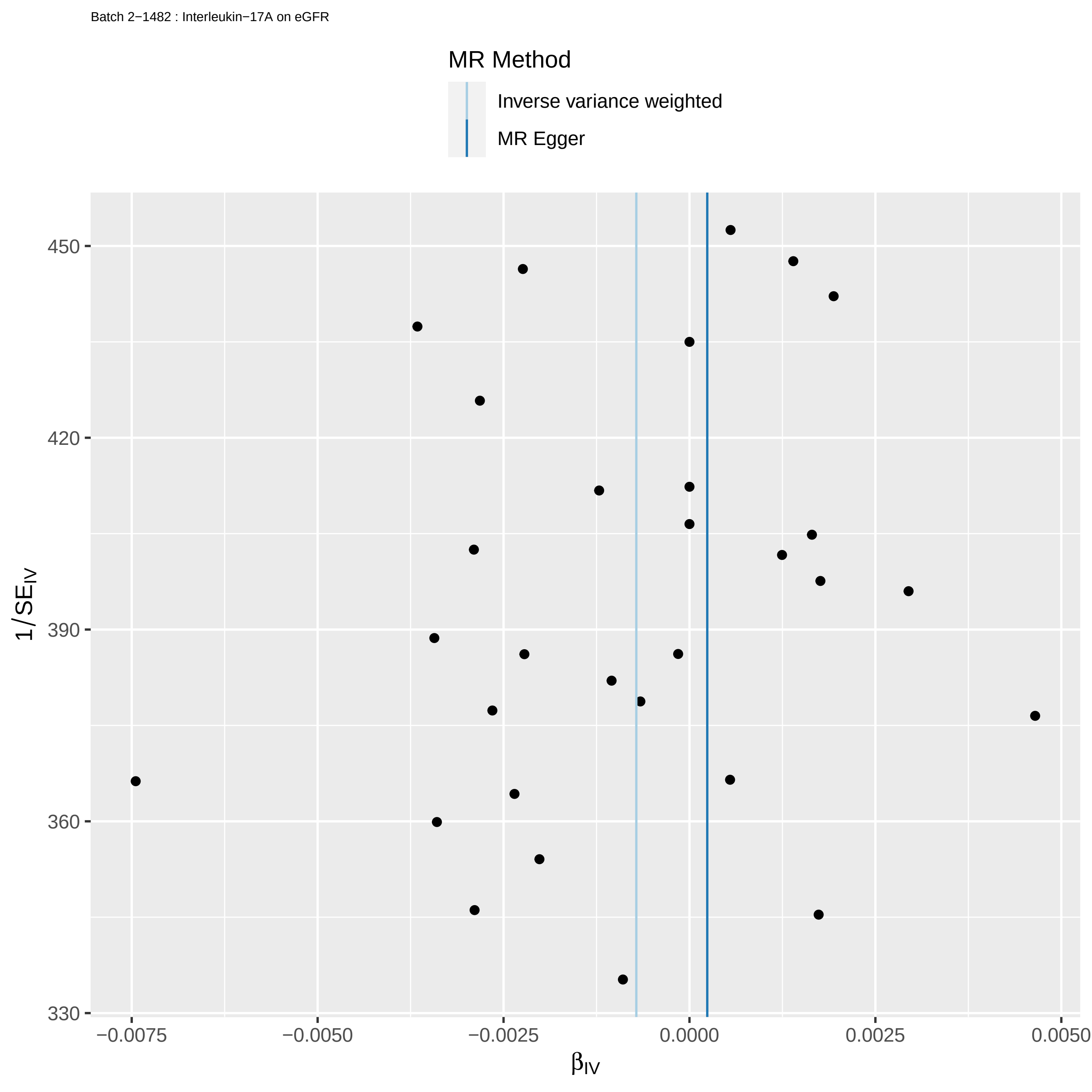

Batch 2−1482 : Interleukin−17A on eGFR
MR Method
Inverse variance weighted
MR Egger
450
420
390
360
330
1/SE_IV
−0.0075
−0.0050
−0.0025
0.0000
0.0025
0.0050
β_IV

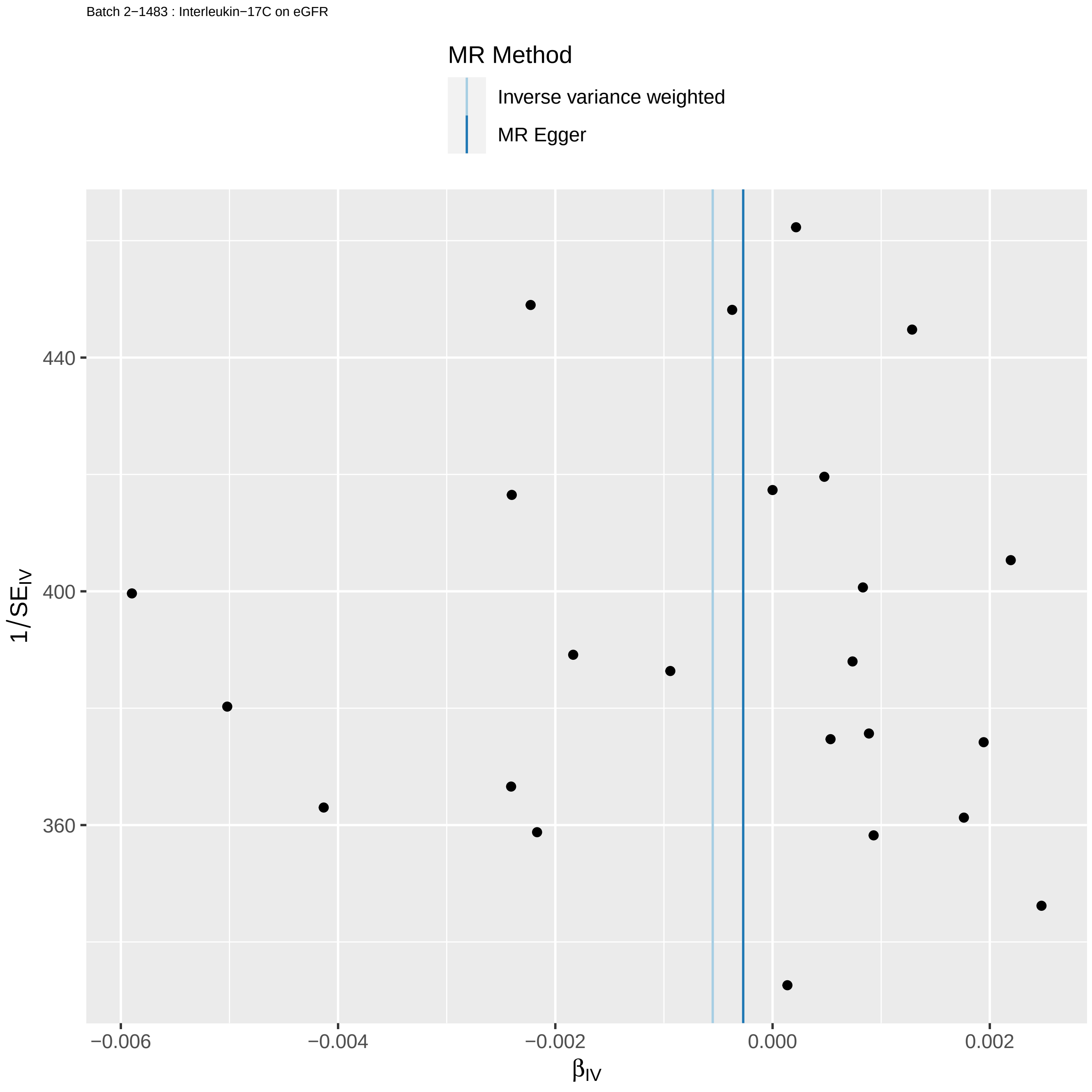
Batch 2−1483 : Interleukin−17C on eGFR
MR Method
Inverse variance weighted
MR Egger
440
400
360
1/SE_IV
−0.006
−0.004
−0.002
0.000
0.002
β_IV

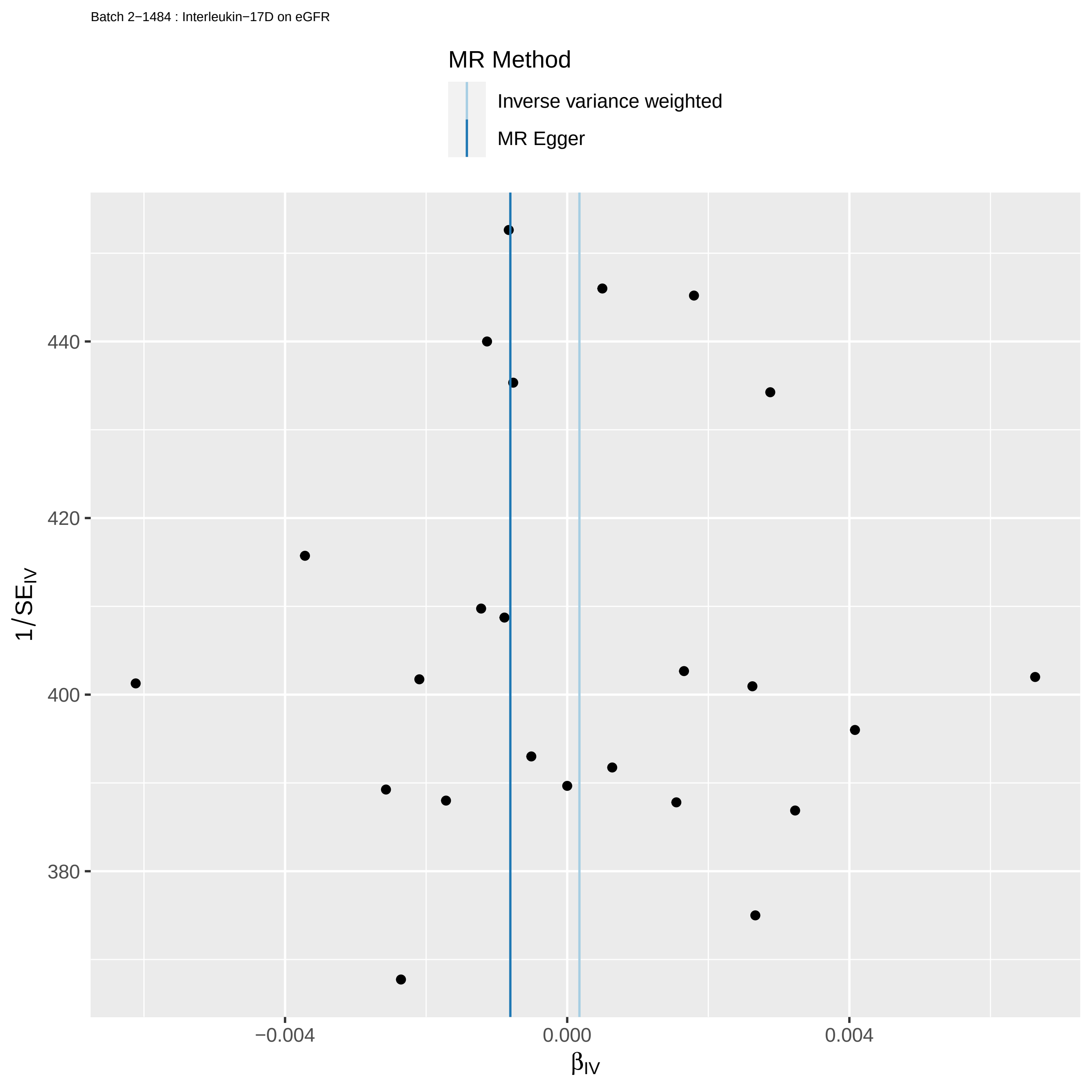

Batch 2−1484 : Interleukin−17D on eGFR
MR Method
Inverse variance weighted
MR Egger
440
420
400
380
$1/SE_{IV}$
−0.004
0.000
0.004
β_{IV}

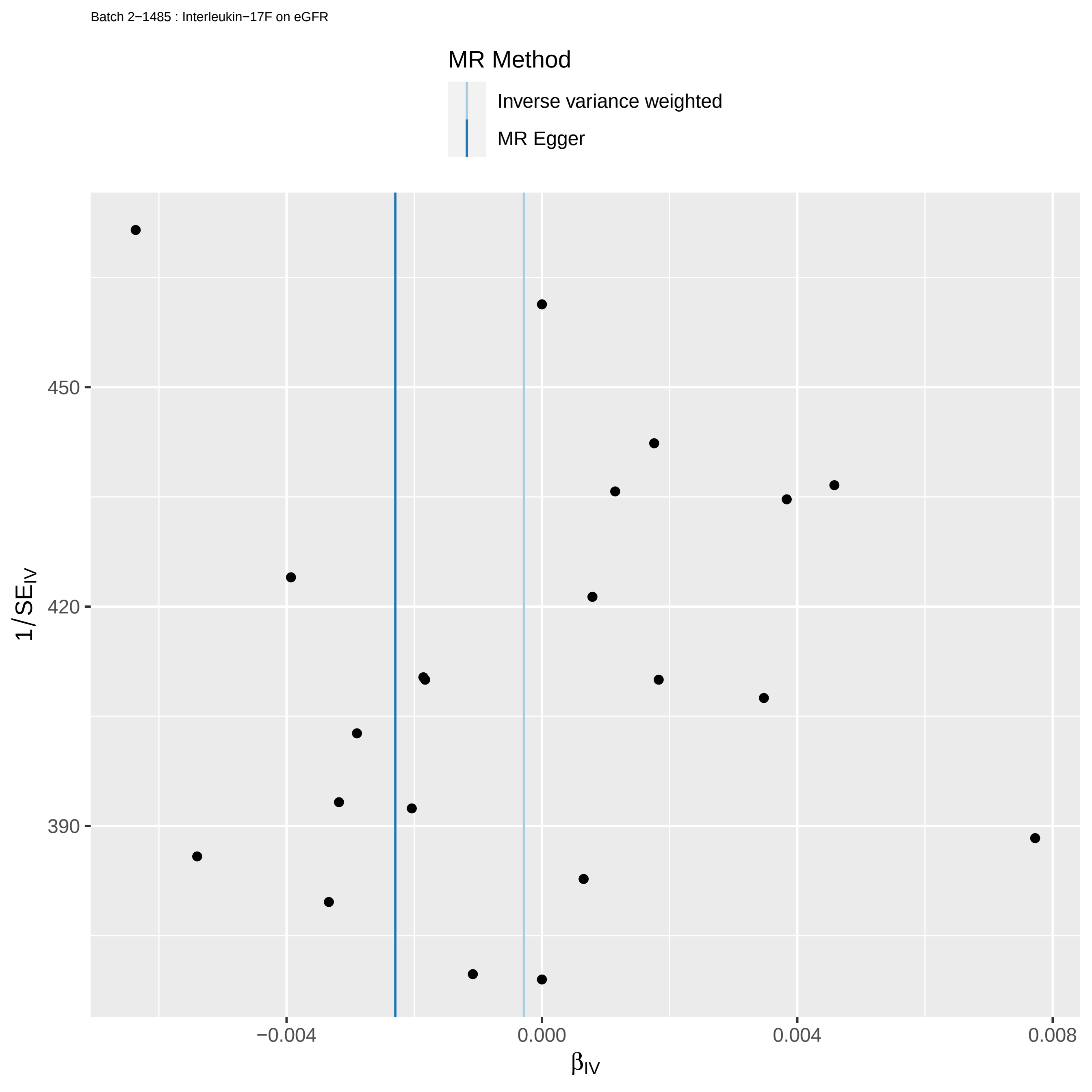

Batch 2−1485 : Interleukin−17F on eGFR
MR Method
Inverse variance weighted
MR Egger
450
420
390
1/SE$_{IV}$
−0.004
0.000
0.004
0.008
β$_{IV}$

Batch 2−1486 : Interleukin−17 receptor A on eGFR

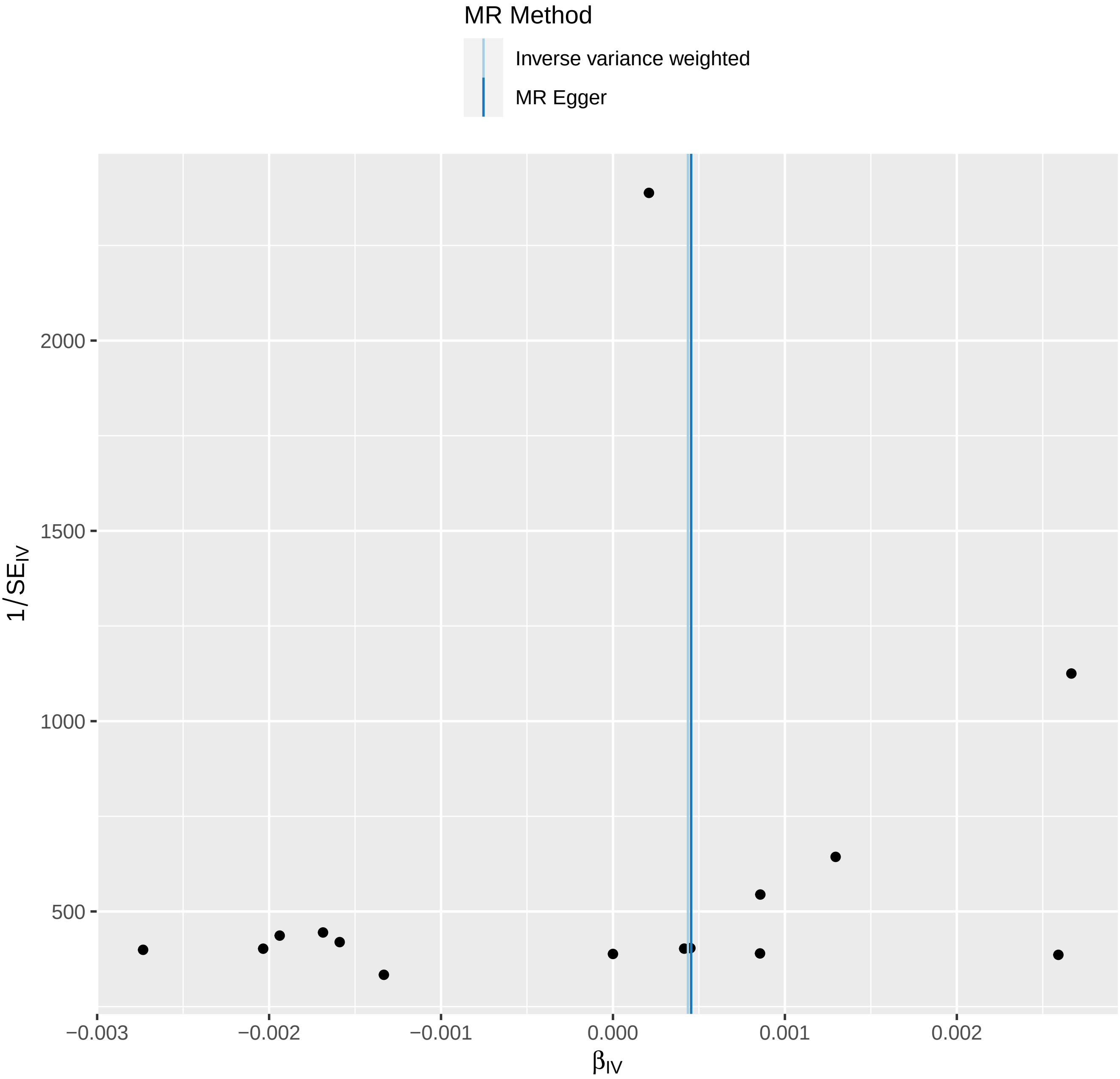

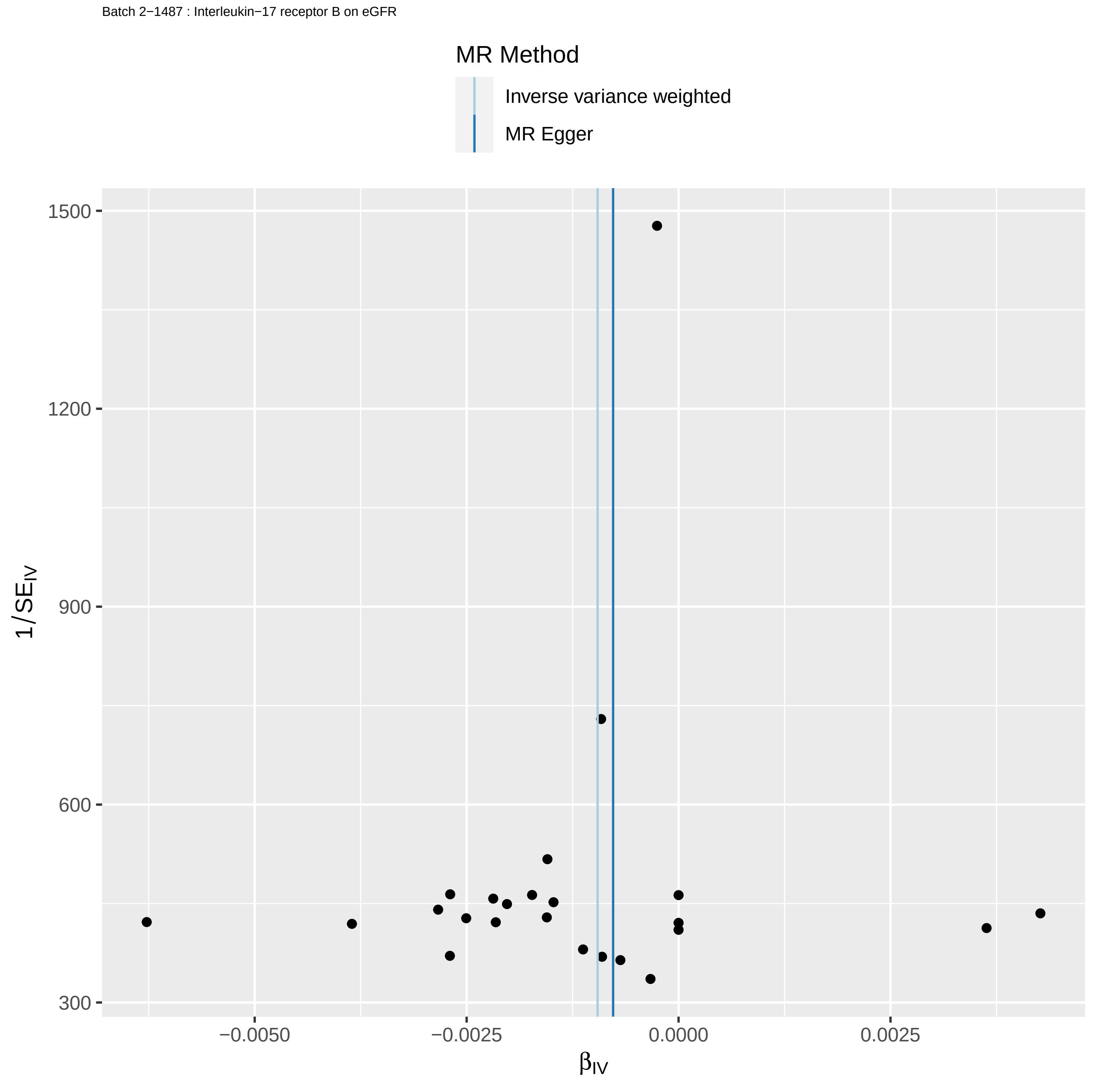

Batch 2−1487 : Interleukin−17 receptor B on eGFR
MR Method
Inverse variance weighted
MR Egger
1500
1200
900
600
300
1/SE$_{IV}$
−0.0050
−0.0025
0.0000
0.0025
β$_{IV}$

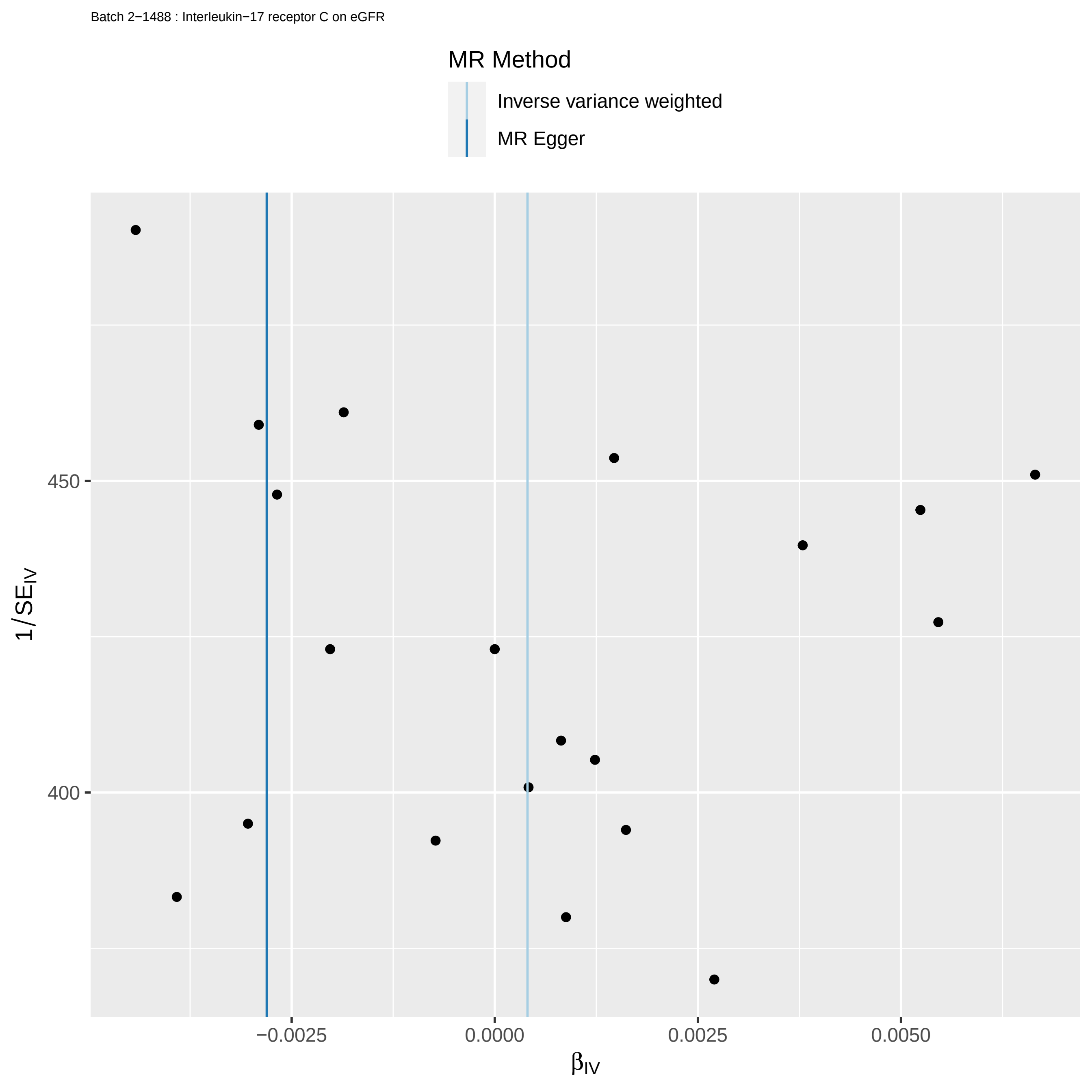

Batch 2−1488 : Interleukin−17 receptor C on eGFR
MR Method
Inverse variance weighted
MR Egger
450
400
1/SE$_{IV}$
−0.0025
0.0000
0.0025
0.0050
β$_{IV}$

Batch 2−1489 : Interleukin−17 receptor D on eGFR

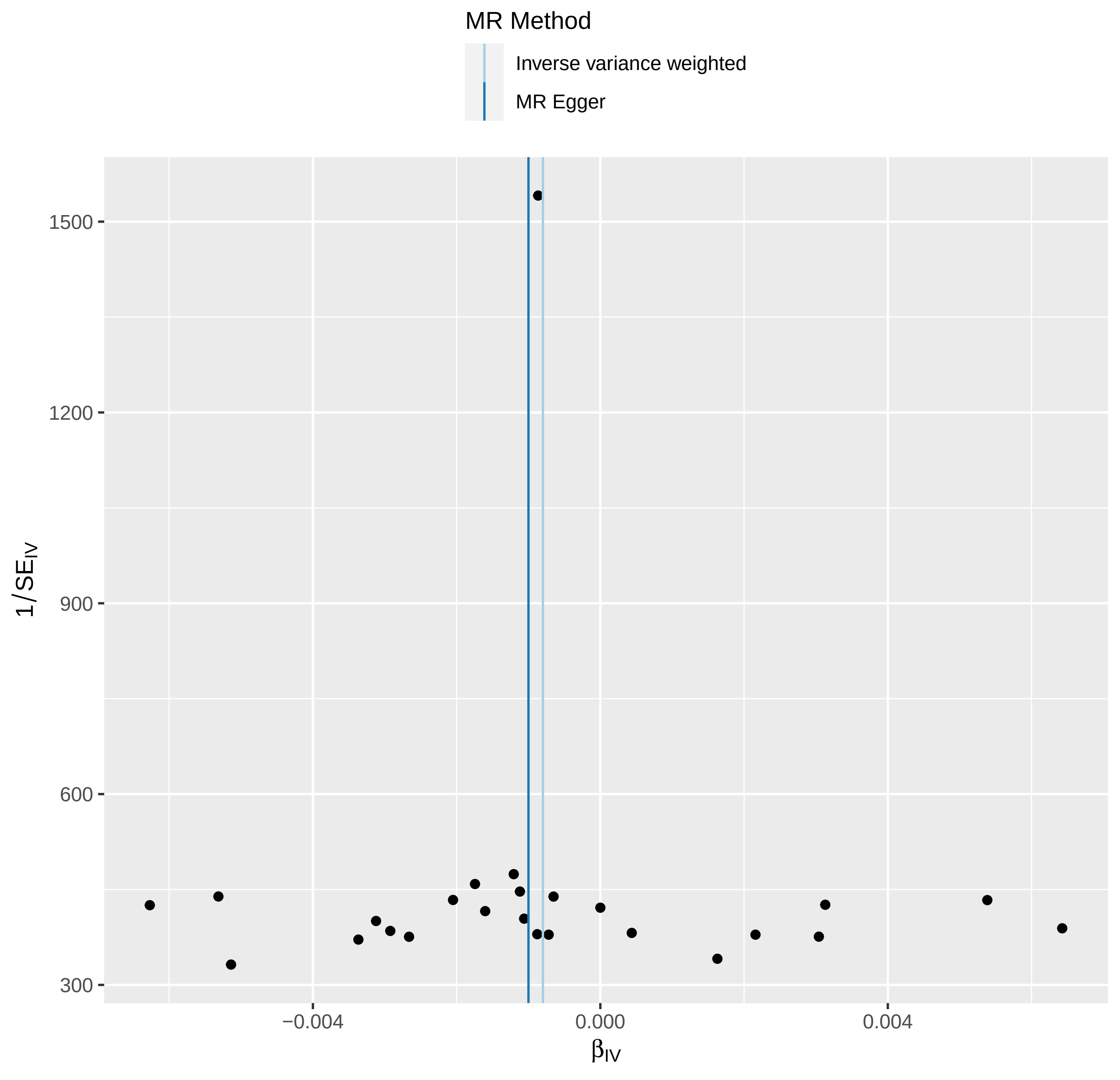

Batch 2−1490 : Interleukin−18−binding protein on eGFR

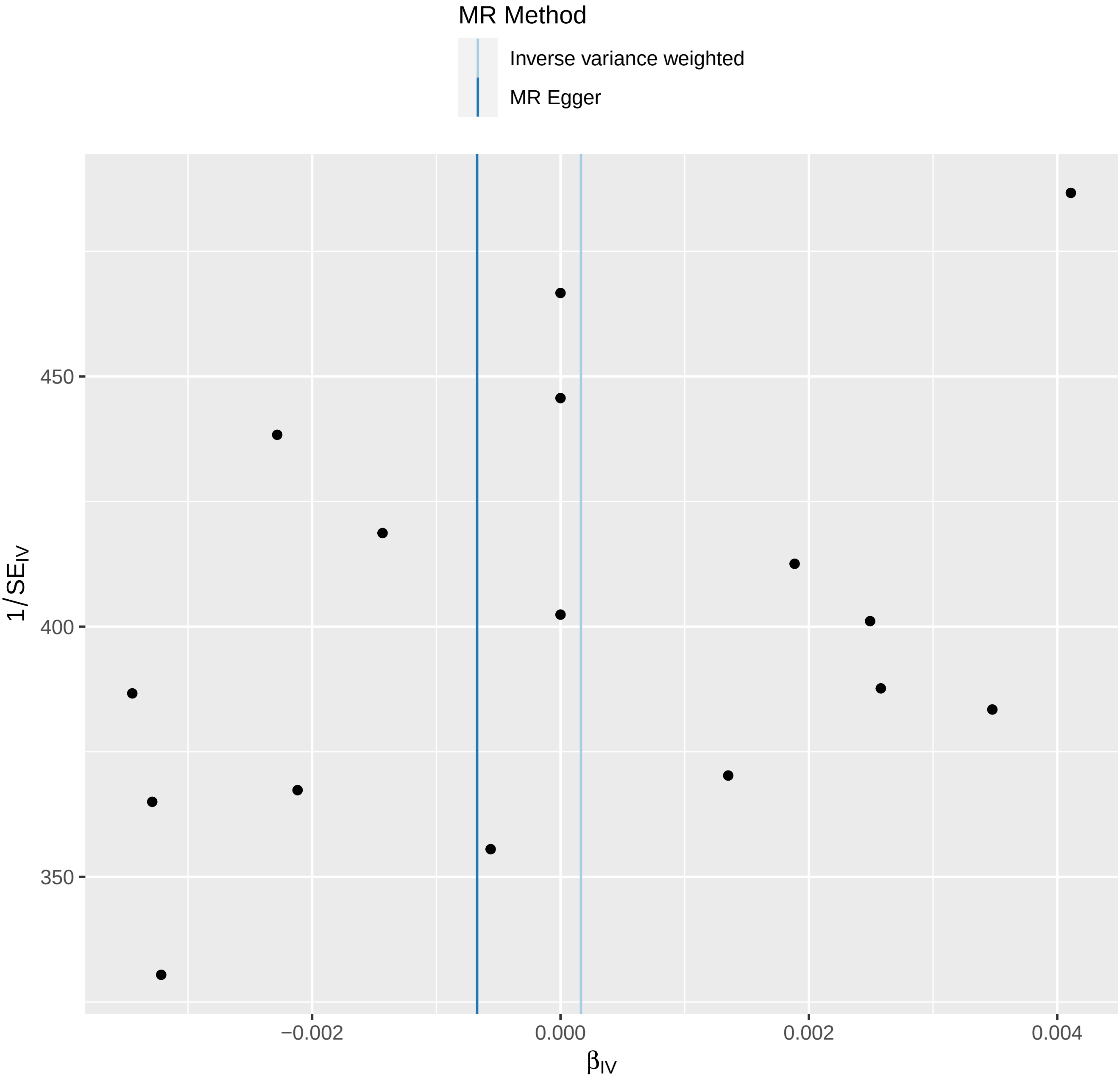

Batch 2−1491 : Interleukin−18 receptor 1 on eGFR

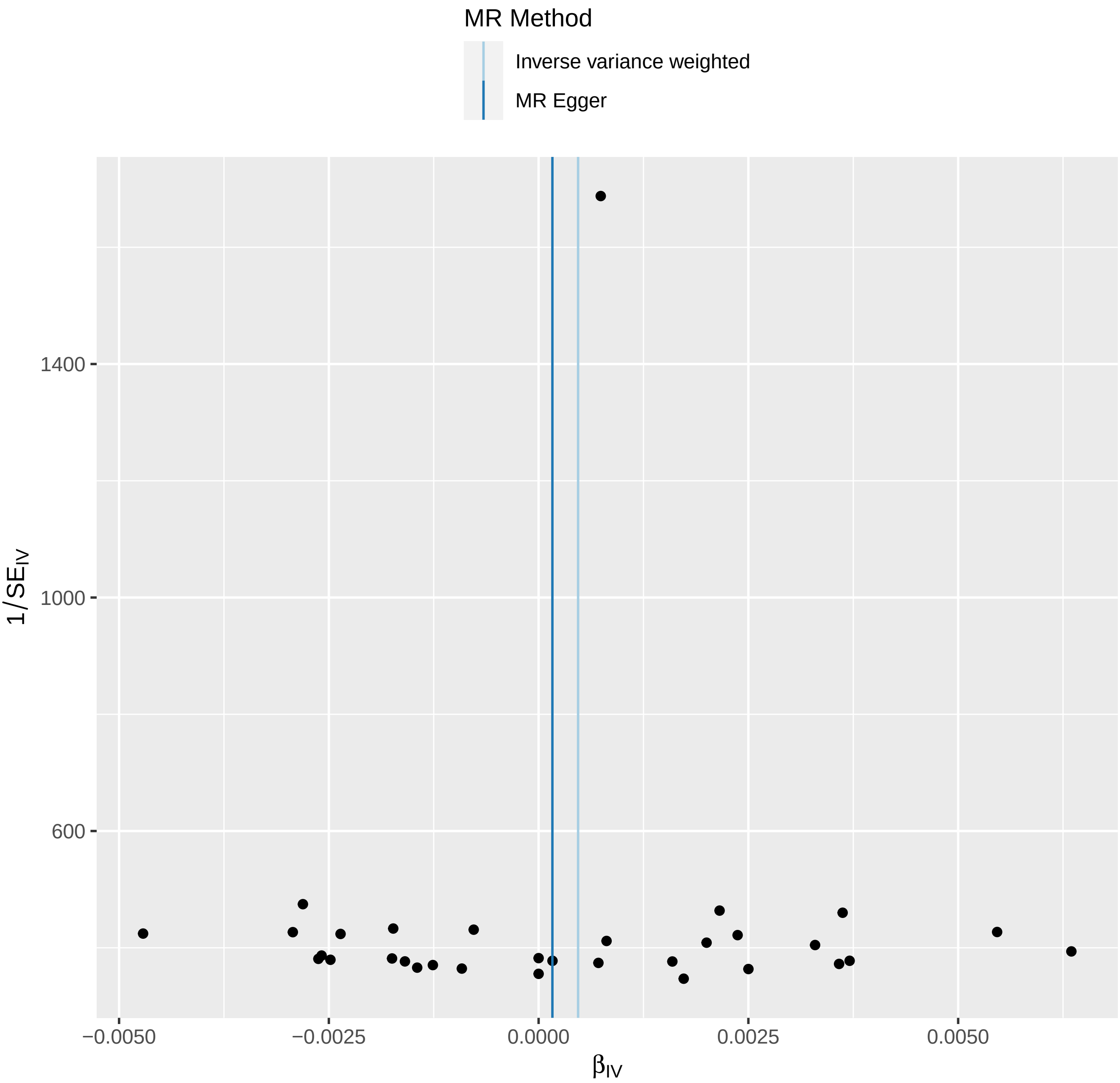

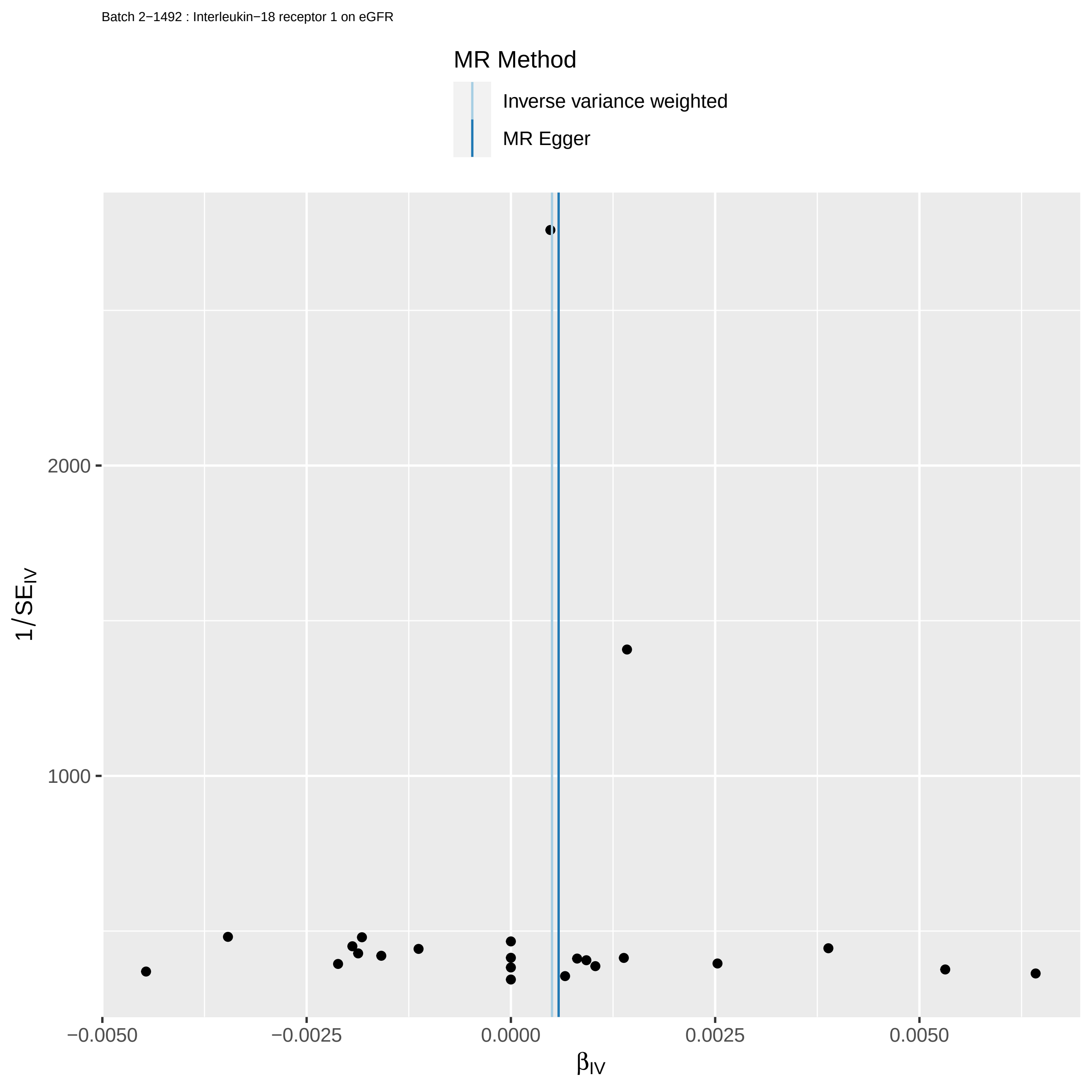
Batch 2−1492 : Interleukin−18 receptor 1 on eGFR
MR Method
Inverse variance weighted
MR Egger
2000
1000
1/SE_IV
−0.0050
−0.0025
0.0000
0.0025
0.0050
β_IV

Batch 2−1493 : Interleukin−19 on eGFR

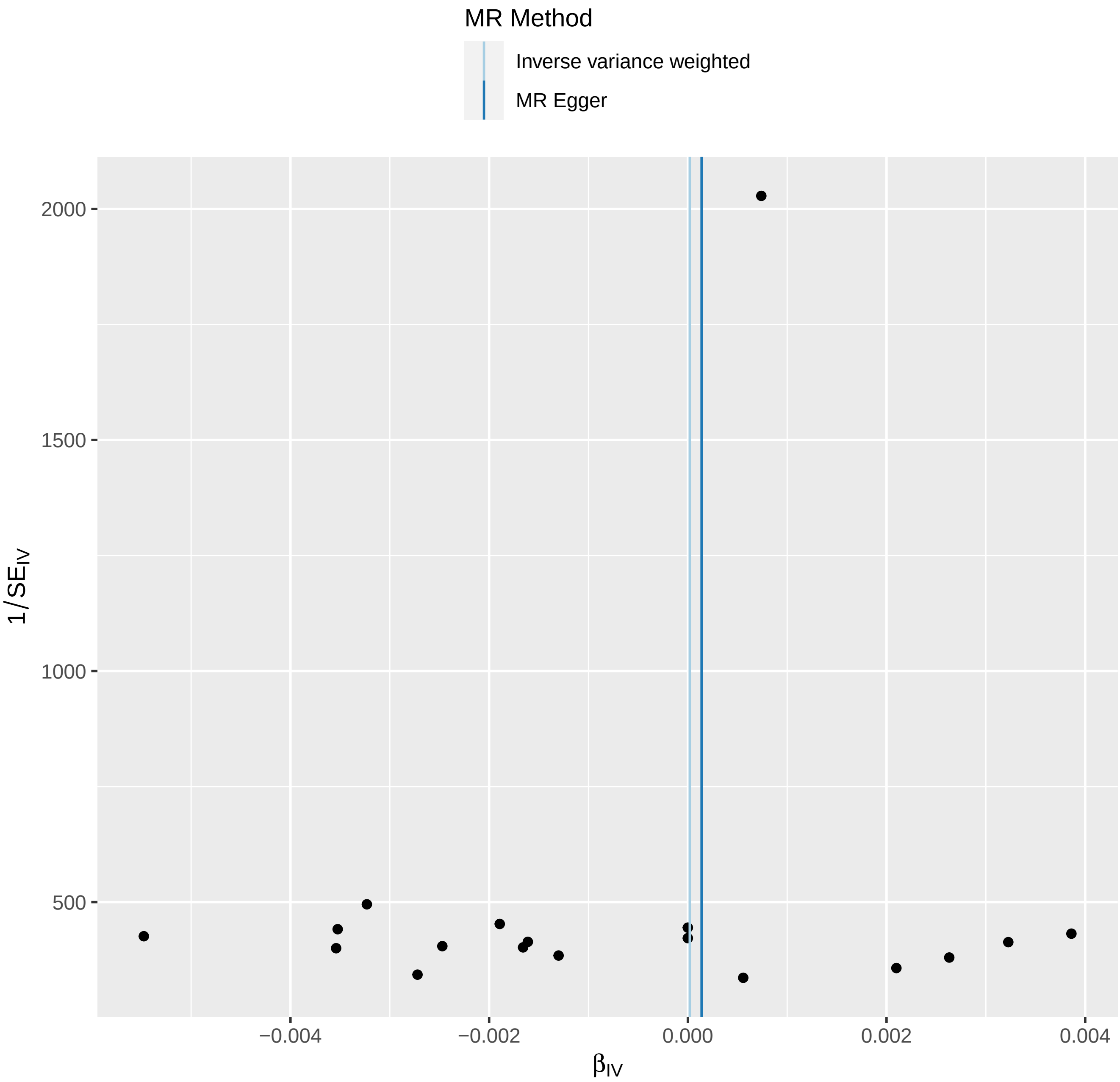

Batch 2−1494 : Interleukin−1 alpha on eGFR

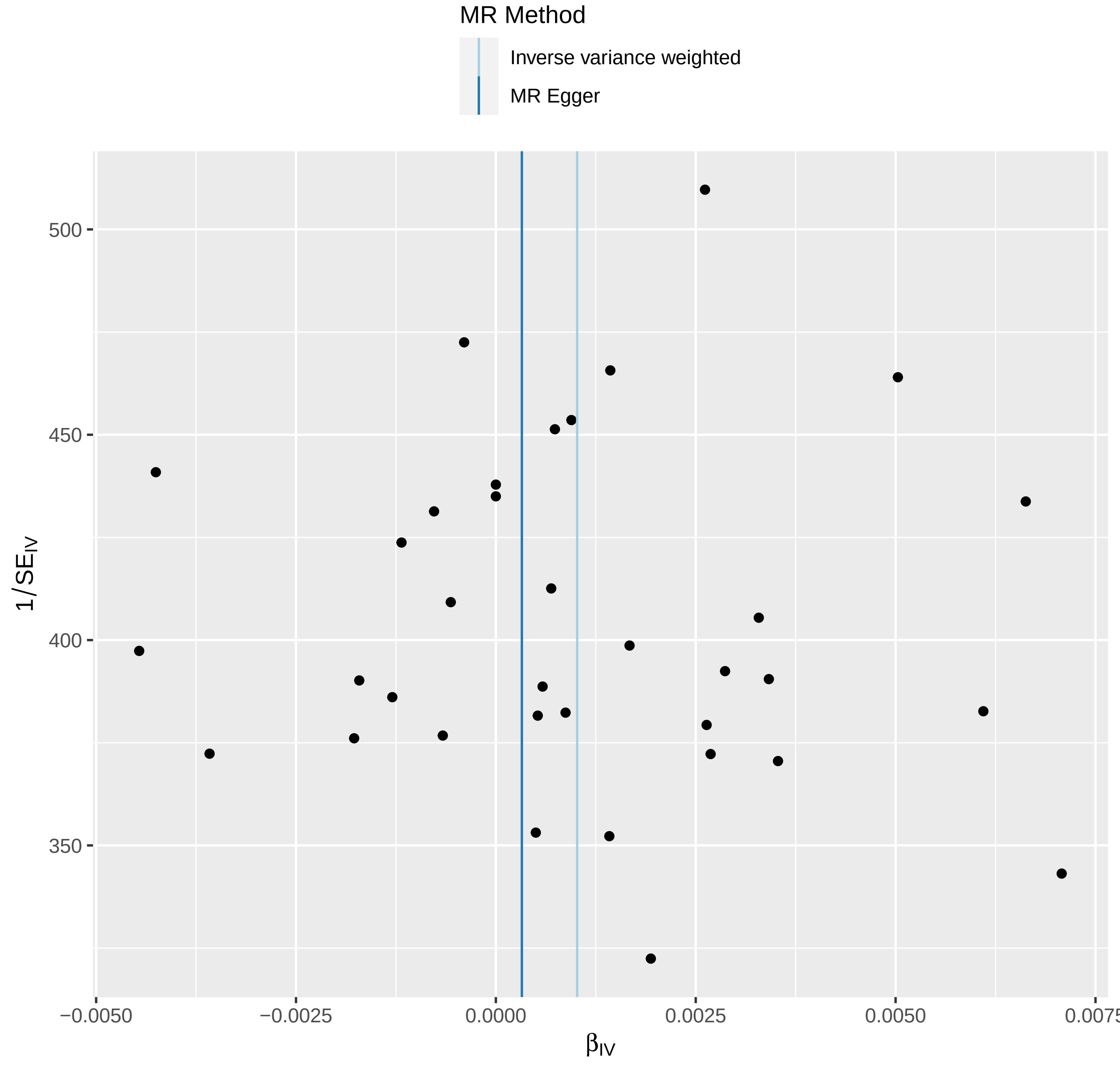

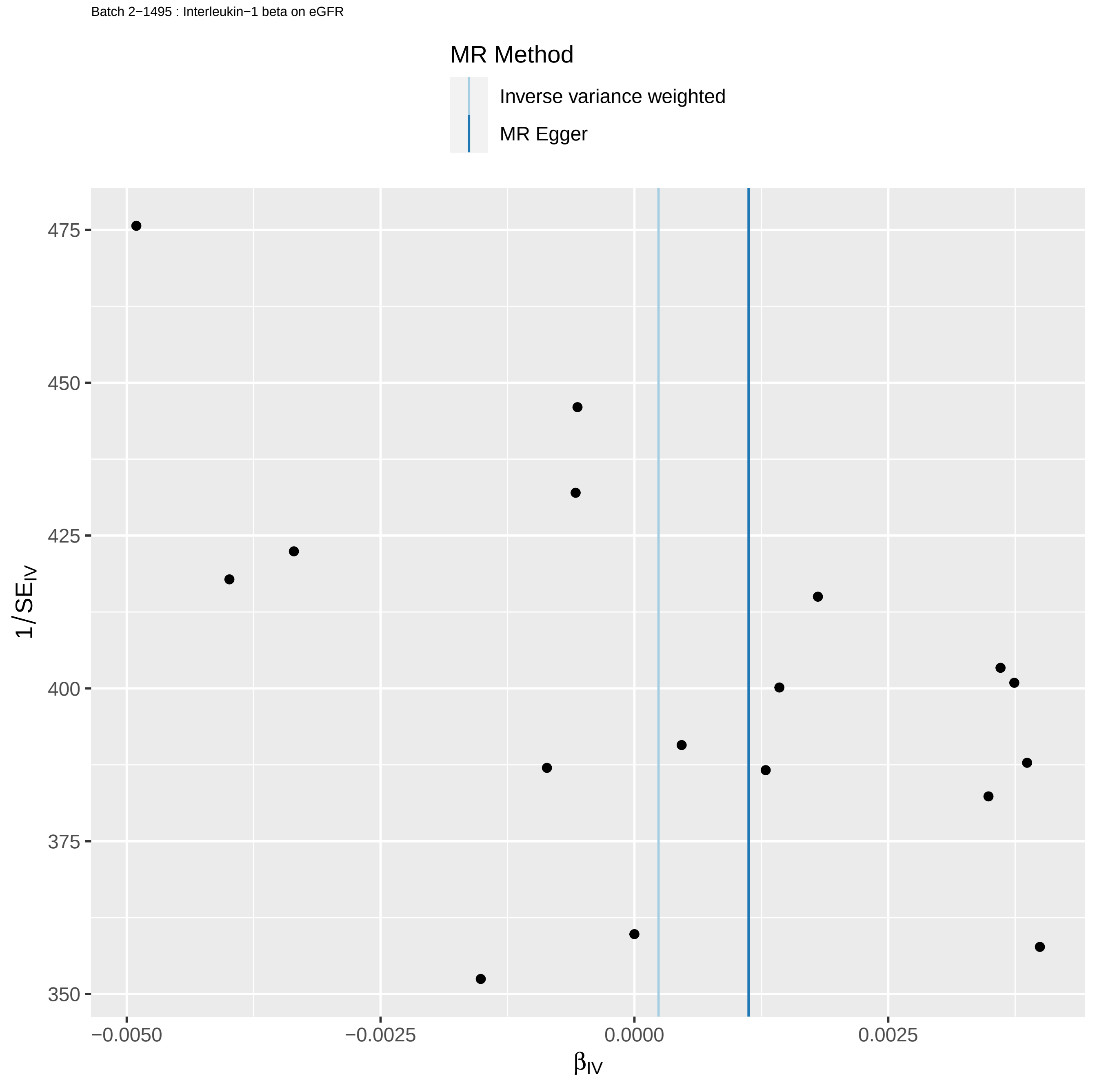
Batch 2−1495 : Interleukin−1 beta on eGFR
MR Method
Inverse variance weighted
MR Egger
475
450
425
400
375
350
1/SE$_{IV}$
−0.0050
−0.0025
0.0000
0.0025
β$_{IV}$

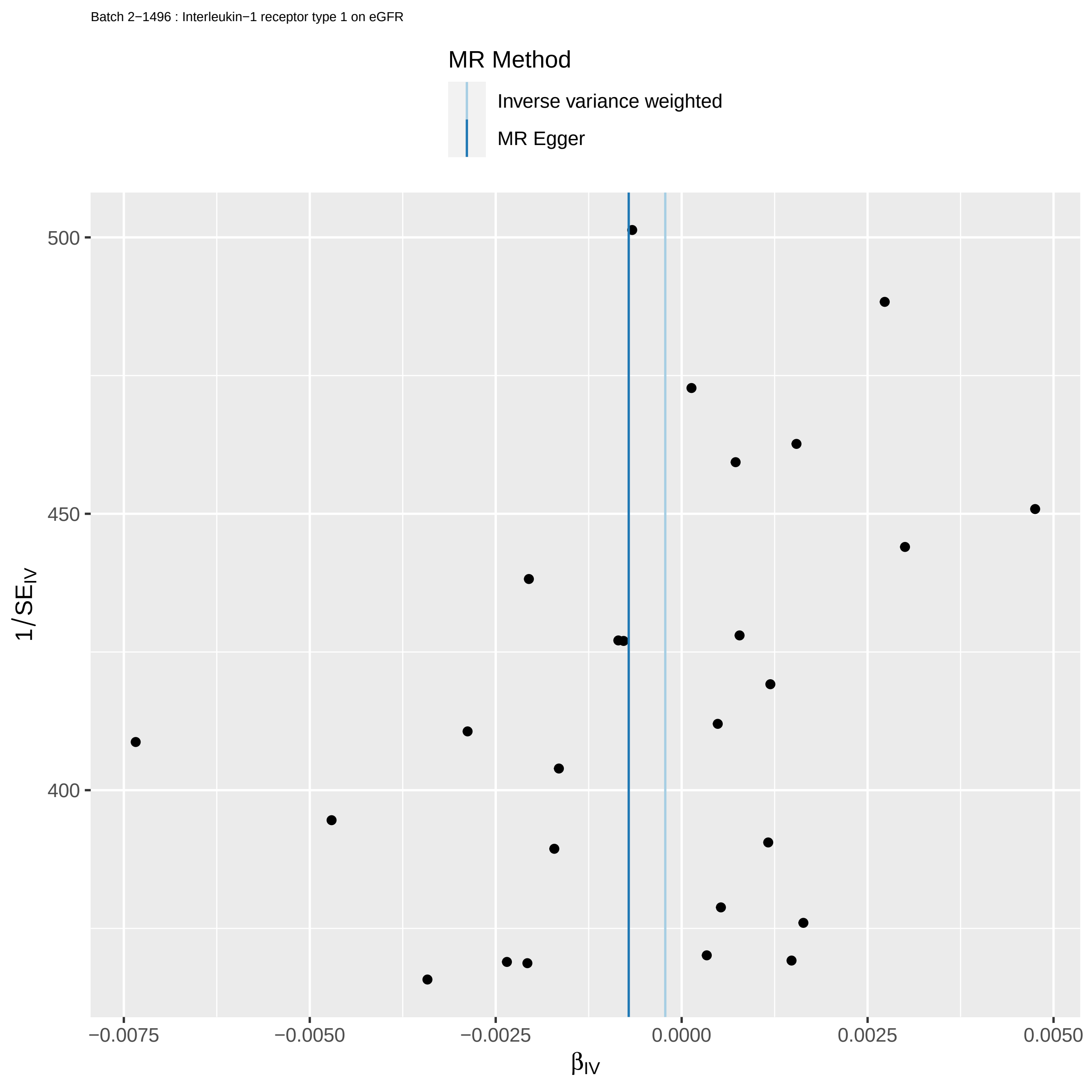
Batch 2−1496 : Interleukin−1 receptor type 1 on eGFR
MR Method
Inverse variance weighted
MR Egger
500
450
400
1/SE_IV
−0.0075
−0.0050
−0.0025
0.0000
0.0025
0.0050
β_IV

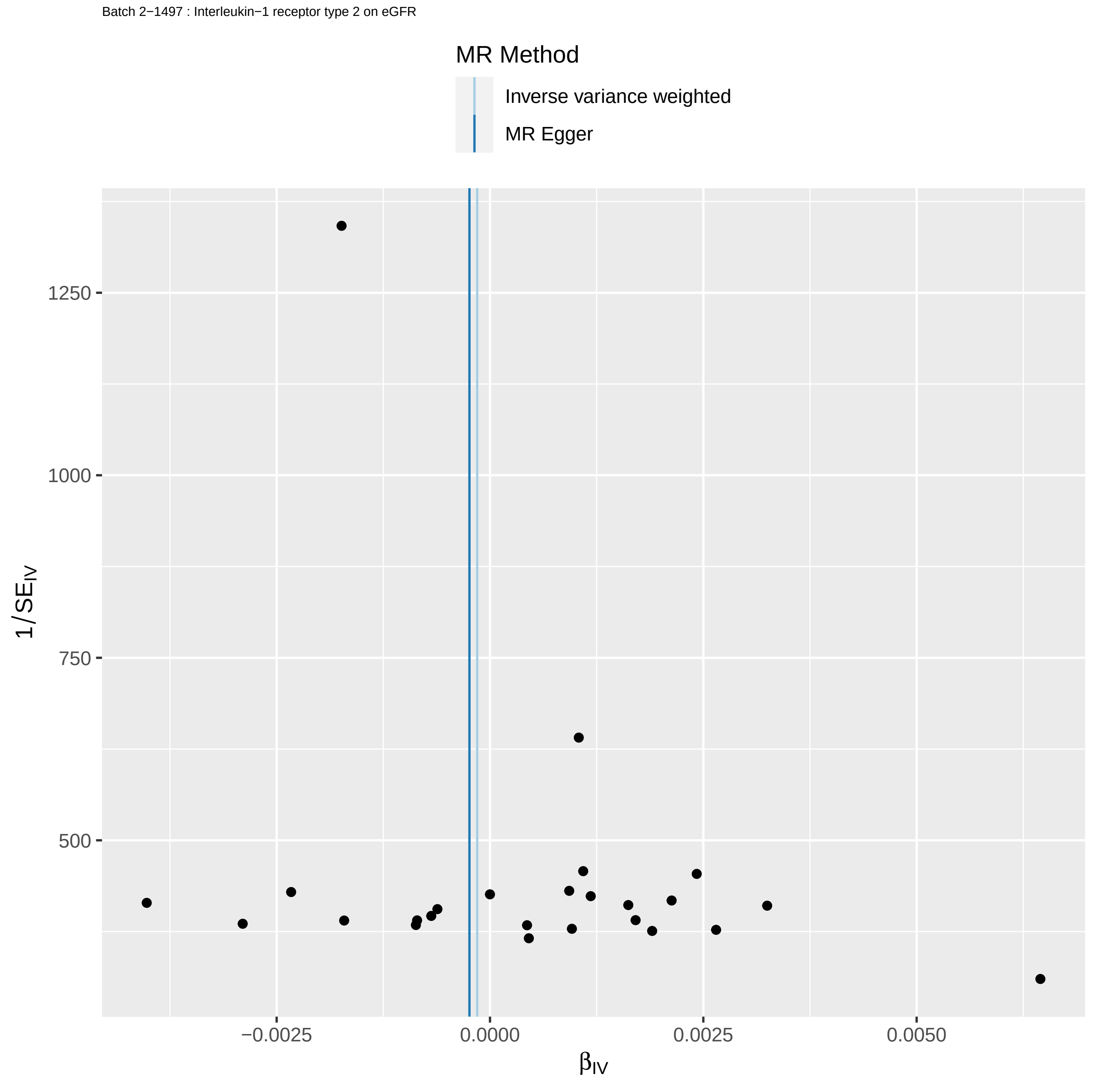

Batch 2−1497 : Interleukin−1 receptor type 2 on eGFR
MR Method
Inverse variance weighted
MR Egger
1250
1000
750
500
1/SE_IV
−0.0025
0.0000
0.0025
0.0050
β_IV

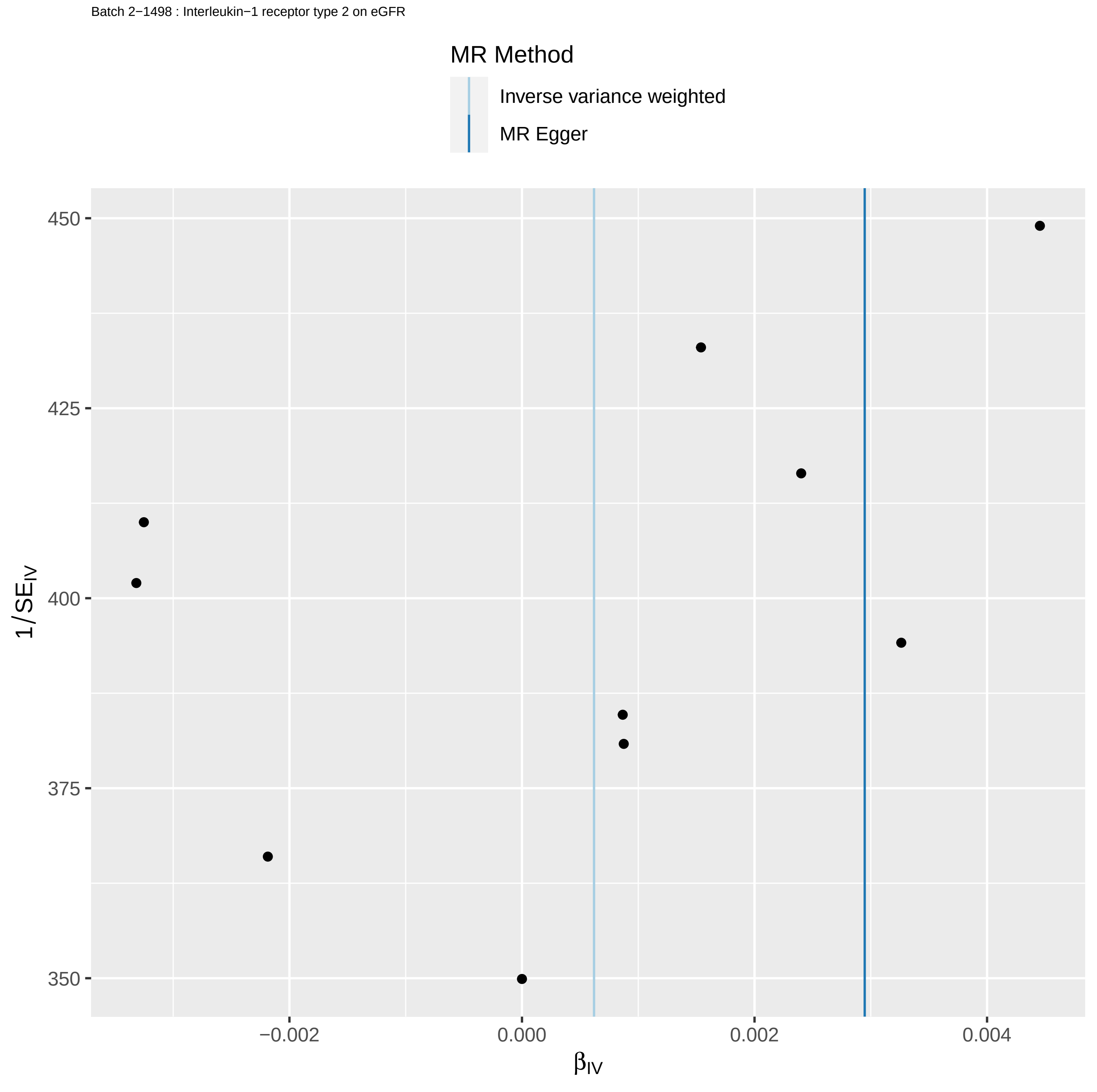

Batch 2−1498 : Interleukin−1 receptor type 2 on eGFR
MR Method
Inverse variance weighted
MR Egger
450
425
400
375
350
1/SE$_{IV}$
−0.002
0.000
0.002
0.004
β$_{IV}$

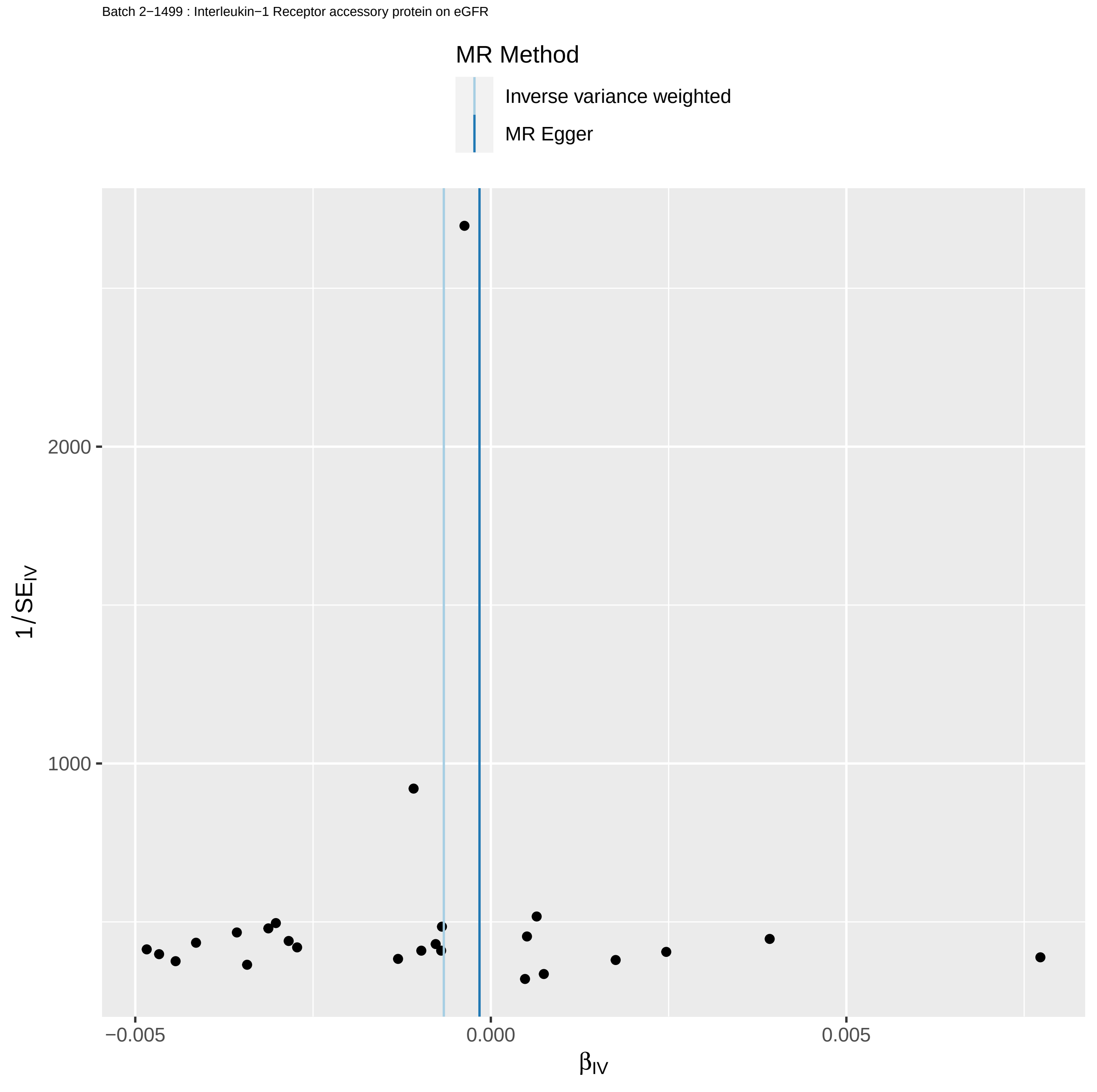

Batch 2−1499 : Interleukin−1 Receptor accessory protein on eGFR
MR Method
Inverse variance weighted
MR Egger
2000
1000
1/SE$_{IV}$
−0.005
0.000
0.005
β$_{IV}$

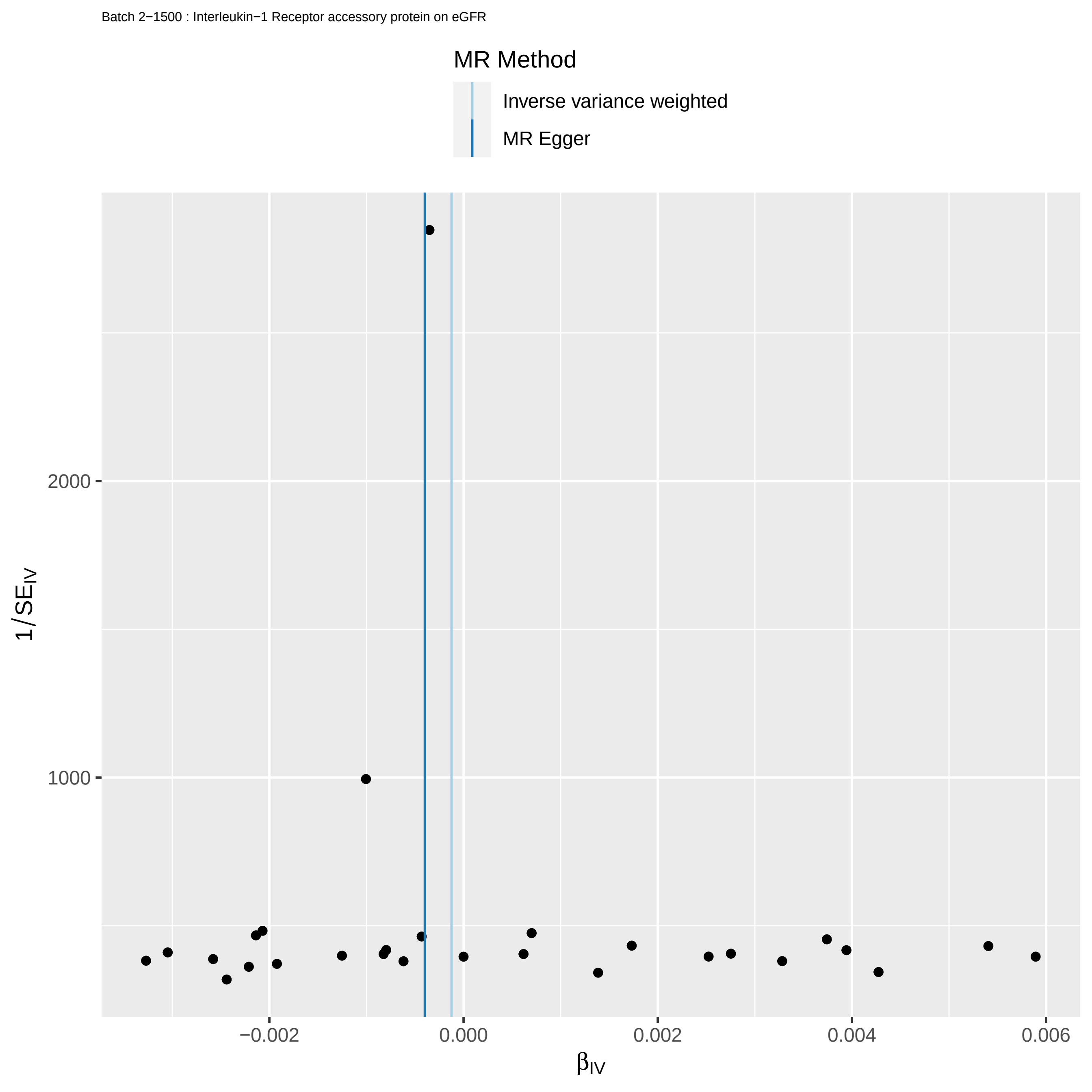

Batch 2−1500 : Interleukin−1 Receptor accessory protein on eGFR
MR Method
Inverse variance weighted
MR Egger
2000
1000
1/SE_IV
−0.002
0.000
0.002
0.004
0.006
β_IV

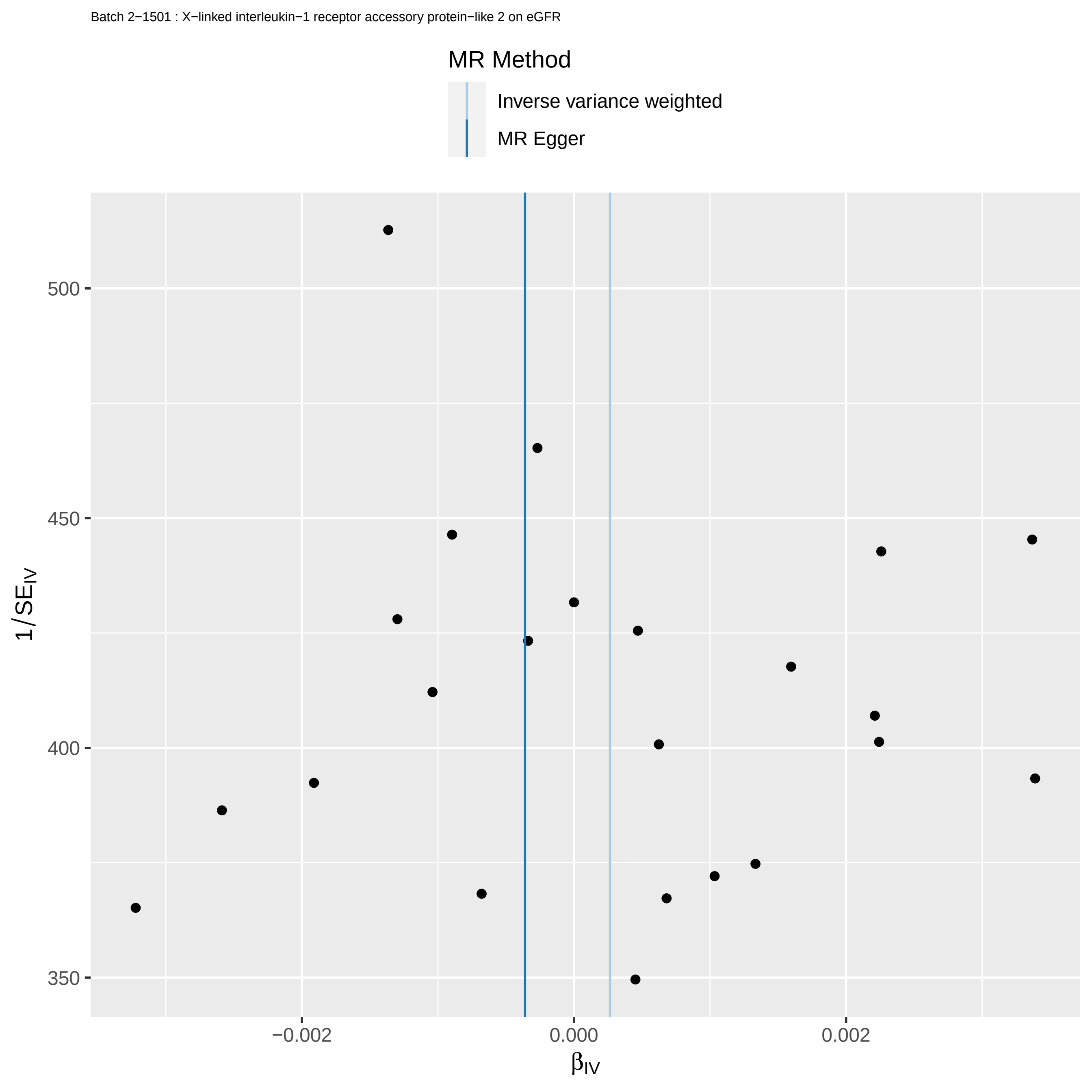
Batch 2−1501 : X−linked interleukin−1 receptor accessory protein−like 2 on eGFR
MR Method
Inverse variance weighted
MR Egger
500
450
400
350
1/SE_IV
−0.002
0.000
0.002
β_IV

Batch 2−1502 : Interleukin−1 receptor−like 1 on eGFR

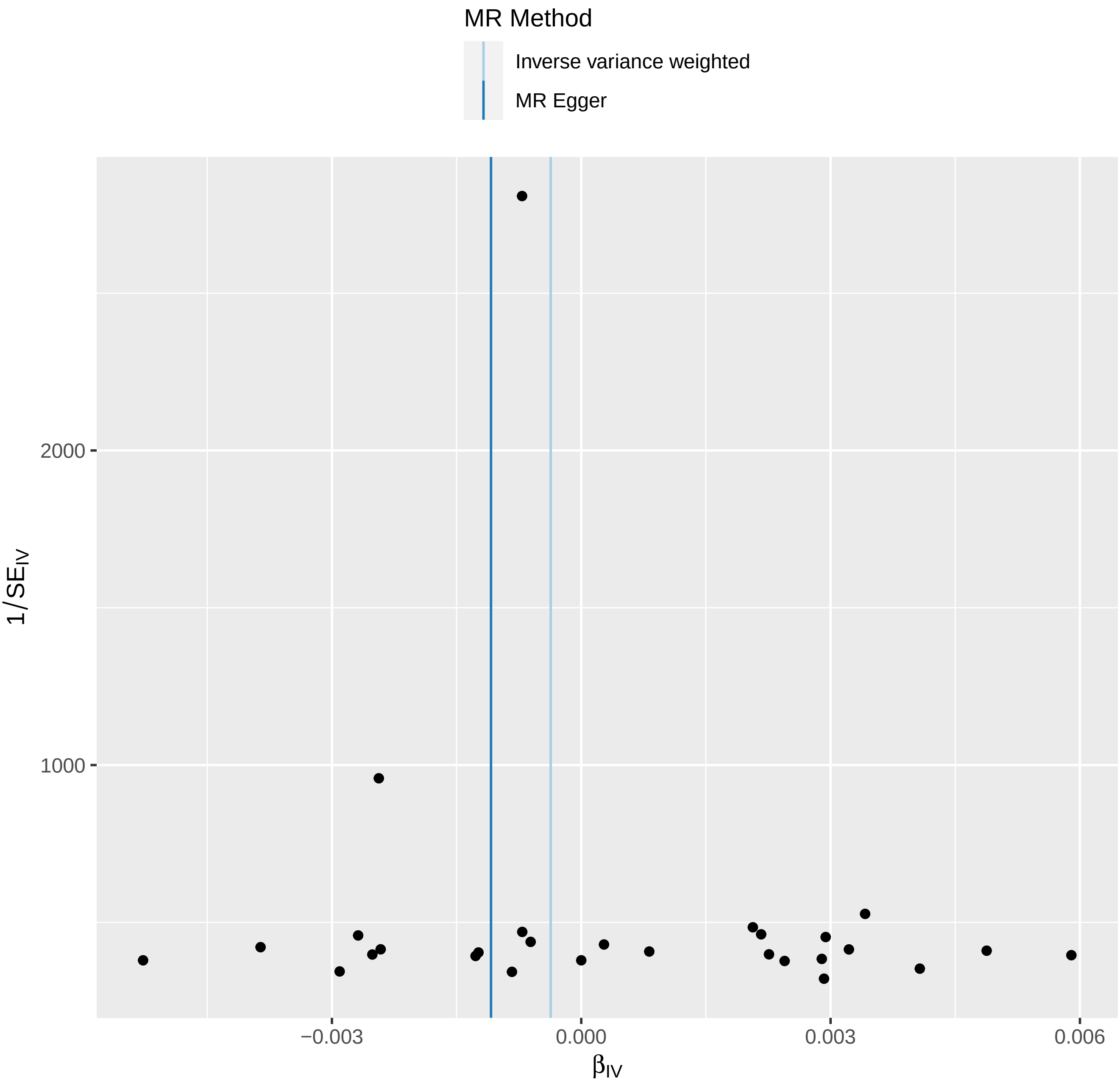

Batch 2−1503 : Interleukin−1 receptor−like 2 on eGFR

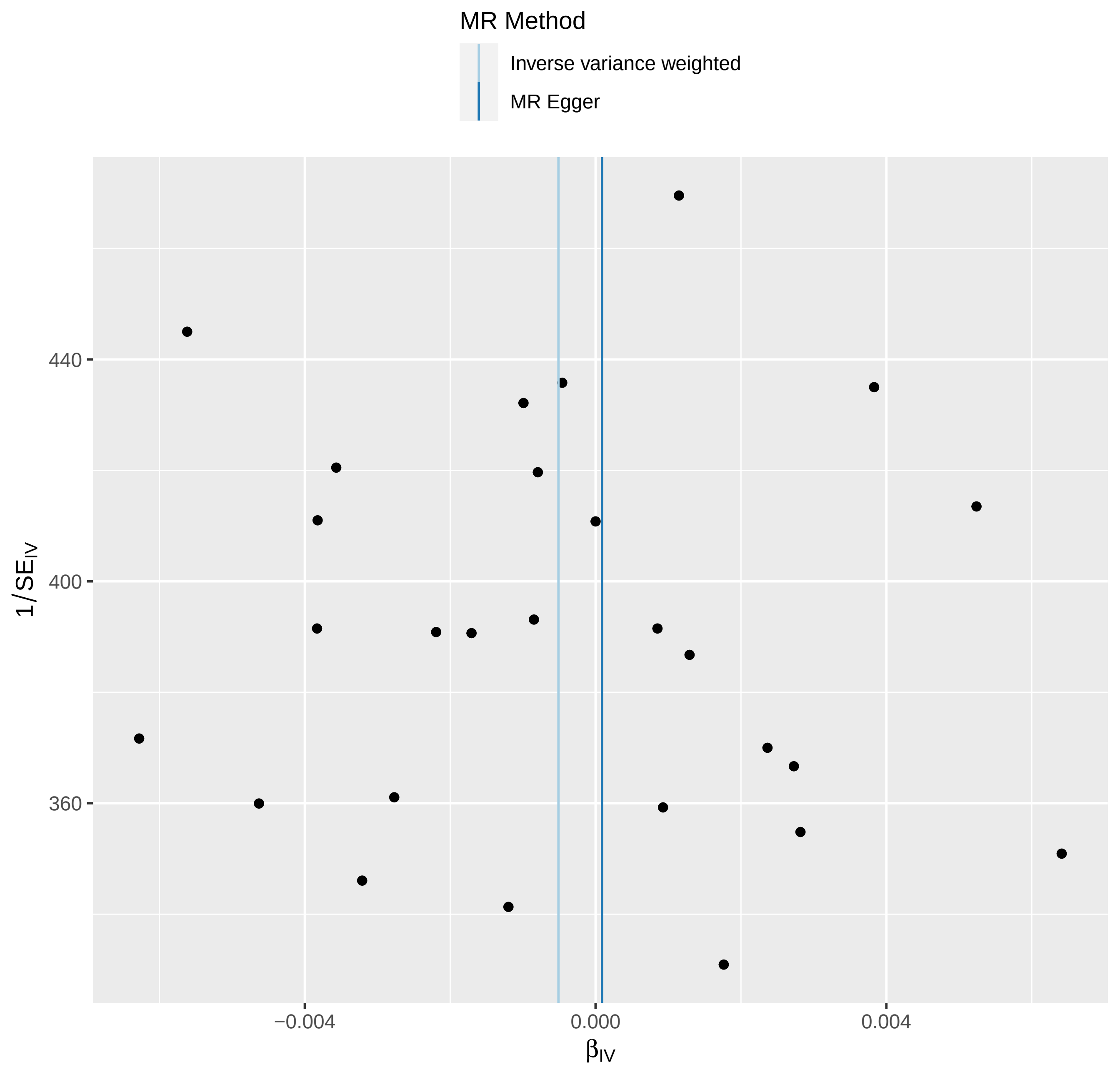

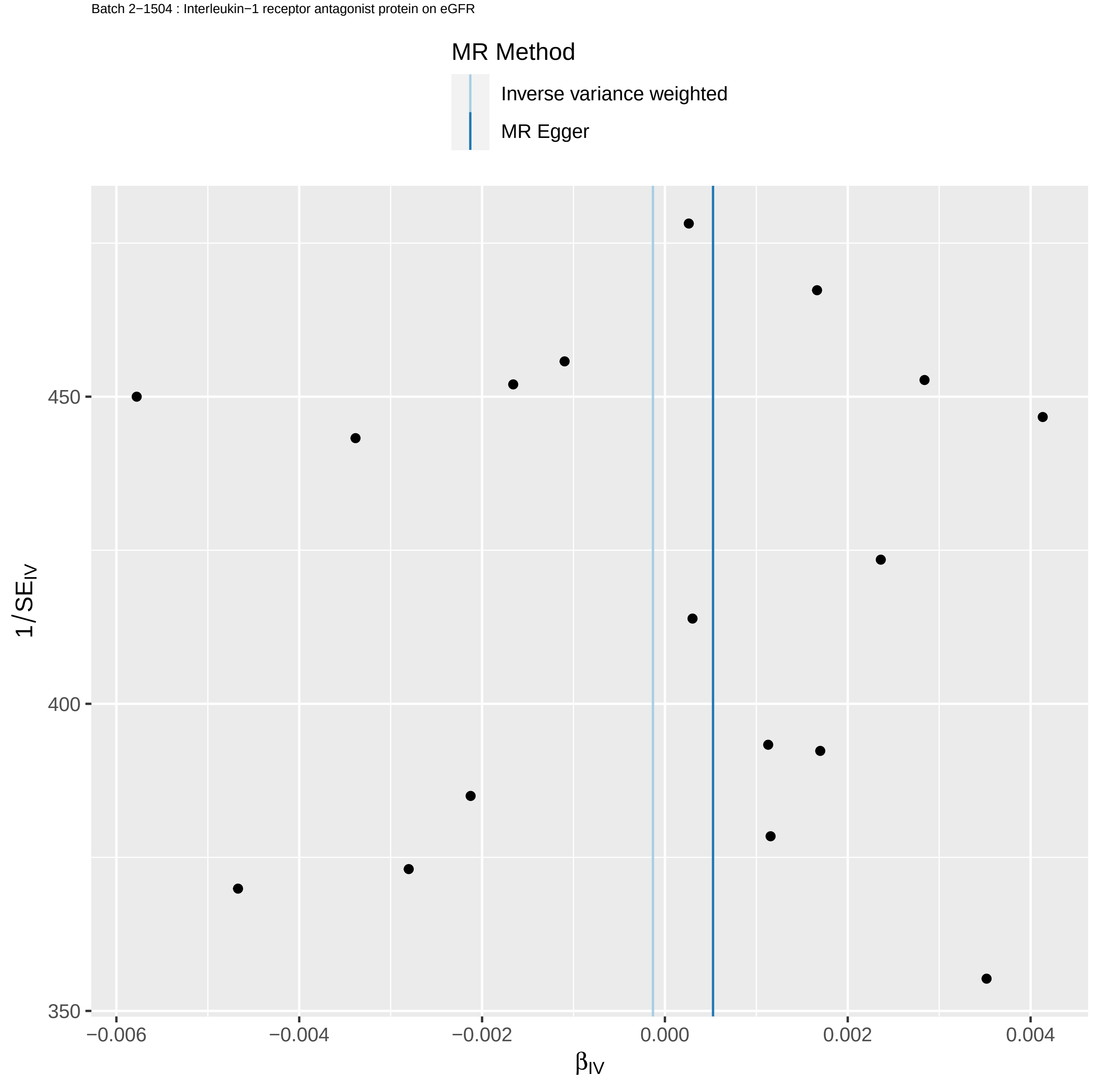
Batch 2−1504 : Interleukin−1 receptor antagonist protein on eGFR
MR Method
Inverse variance weighted
MR Egger
1/SE_IV
450
400
350
−0.006
−0.004
−0.002
0.000
0.002
0.004
β_IV

Batch 2−1505 : Interleukin−20 on eGFR

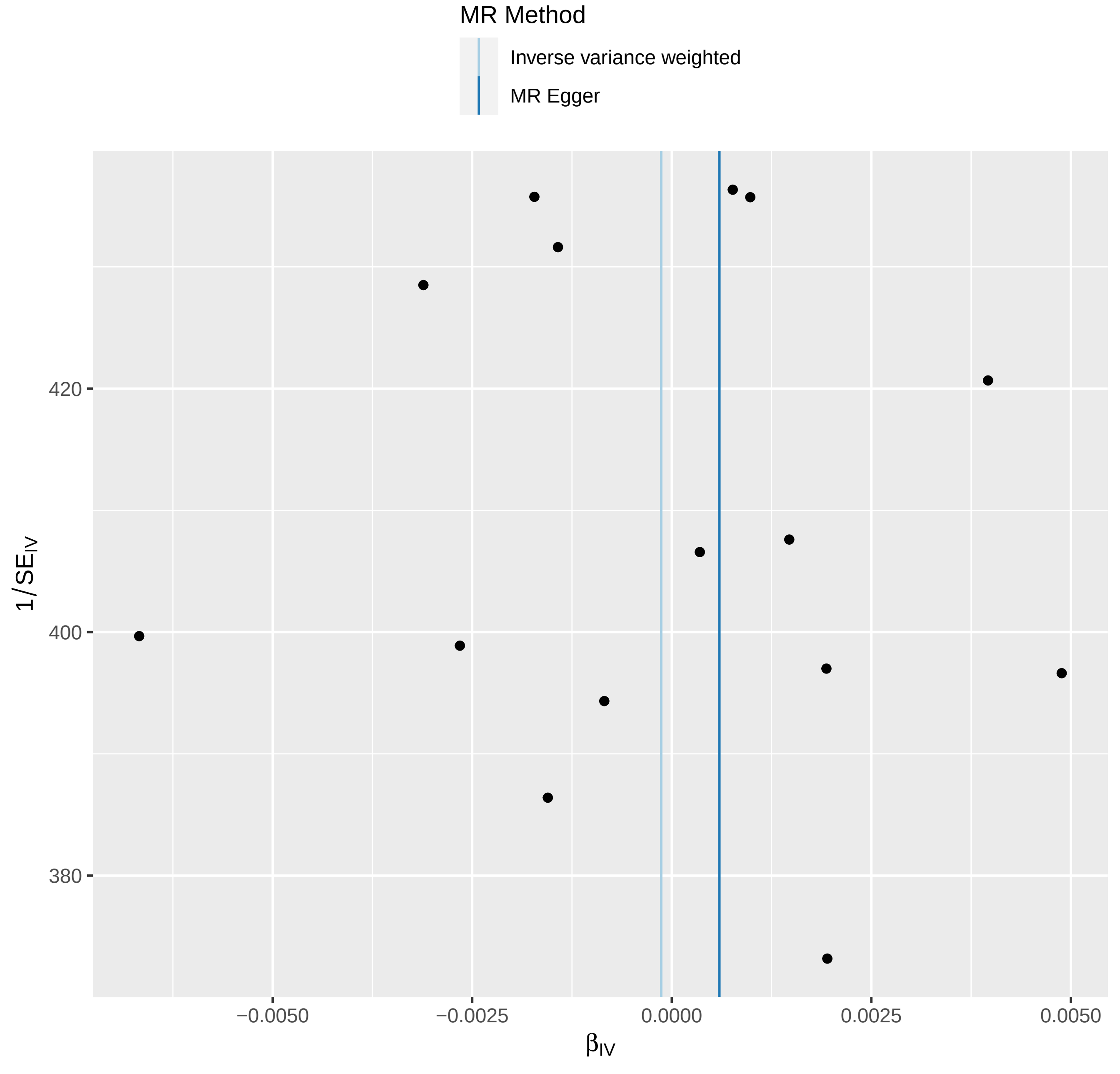

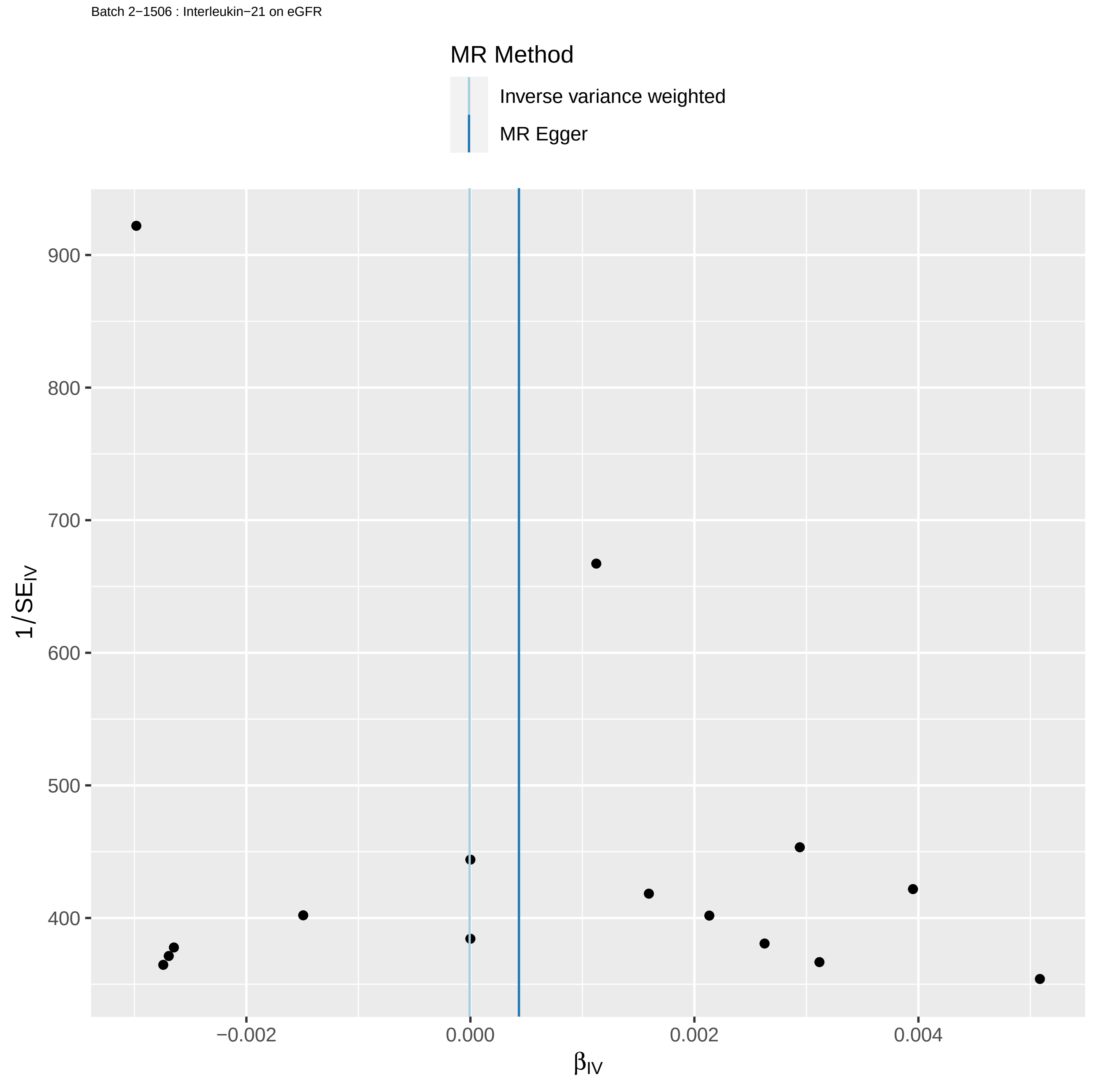

Batch 2−1506 : Interleukin−21 on eGFR
MR Method
Inverse variance weighted
MR Egger
900
800
700
600
500
400
1/SE$_{IV}$
−0.002
0.000
0.002
0.004
β$_{IV}$

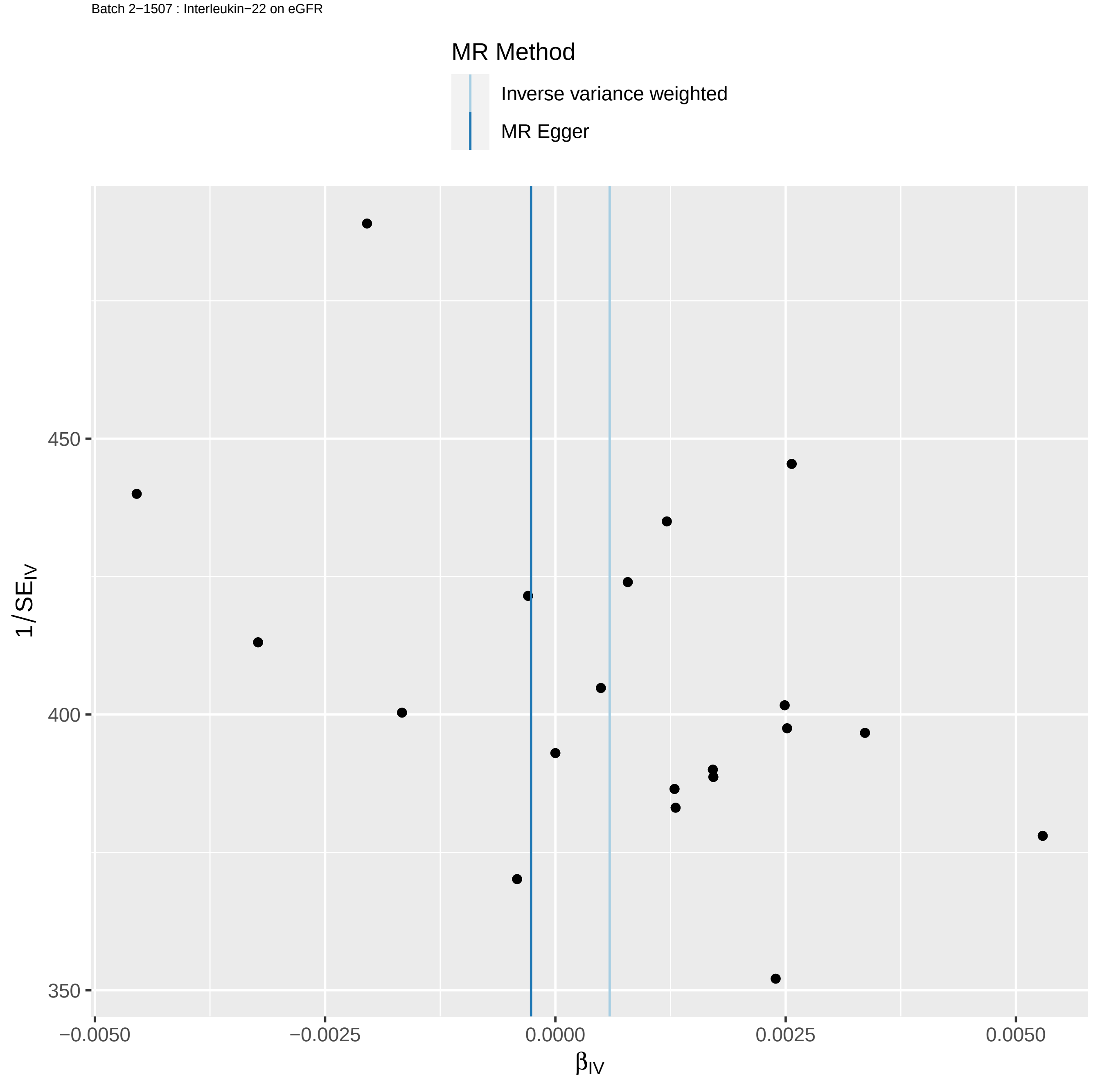

Batch 2−1507 : Interleukin−22 on eGFR
MR Method
Inverse variance weighted
MR Egger
450
400
350
1/SE_IV
−0.0050
−0.0025
0.0000
0.0025
0.0050
β_IV

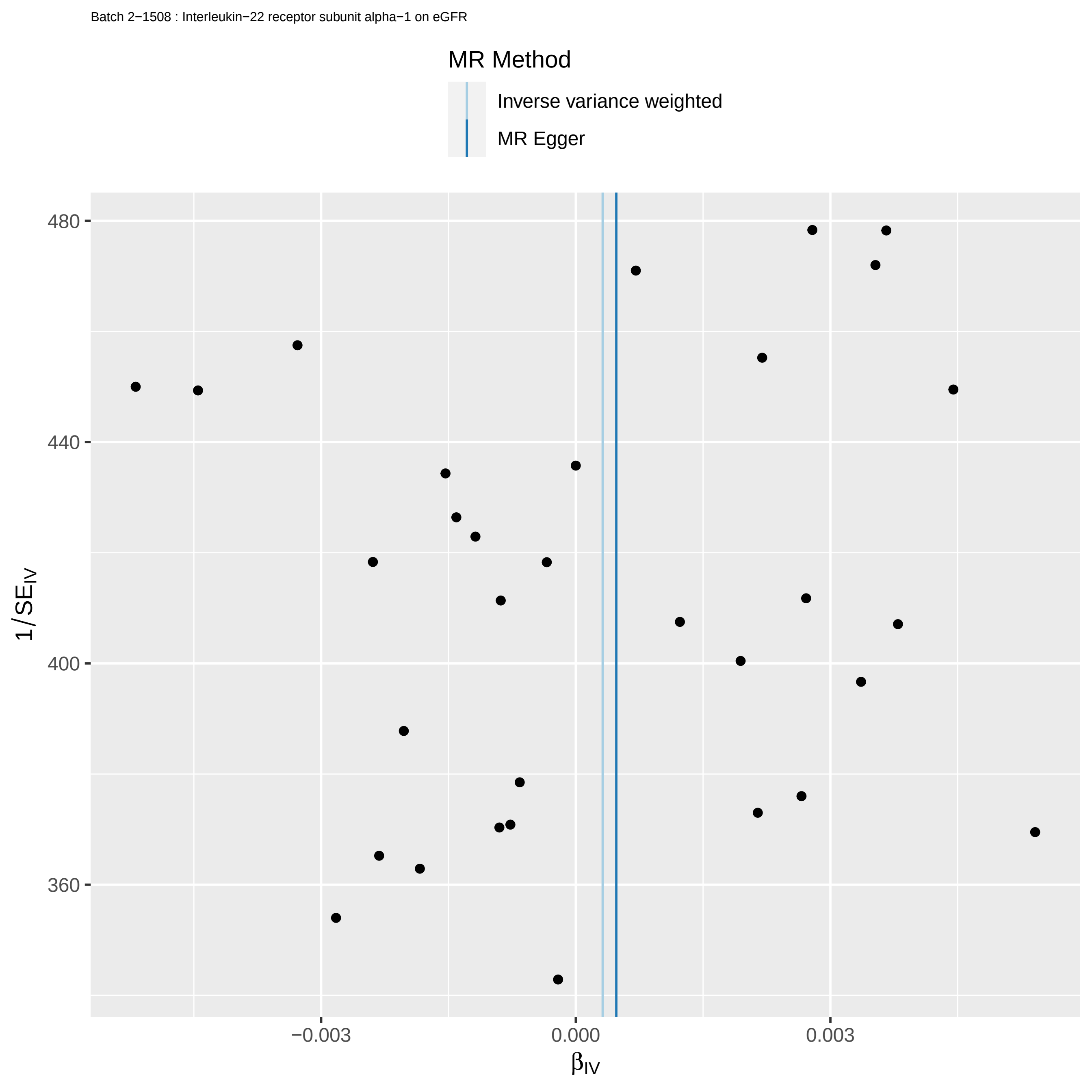

Batch 2−1508 : Interleukin−22 receptor subunit alpha−1 on eGFR
MR Method
Inverse variance weighted
MR Egger
480
440
400
360
$1/SE_{IV}$
−0.003
0.000
0.003
β_{IV}

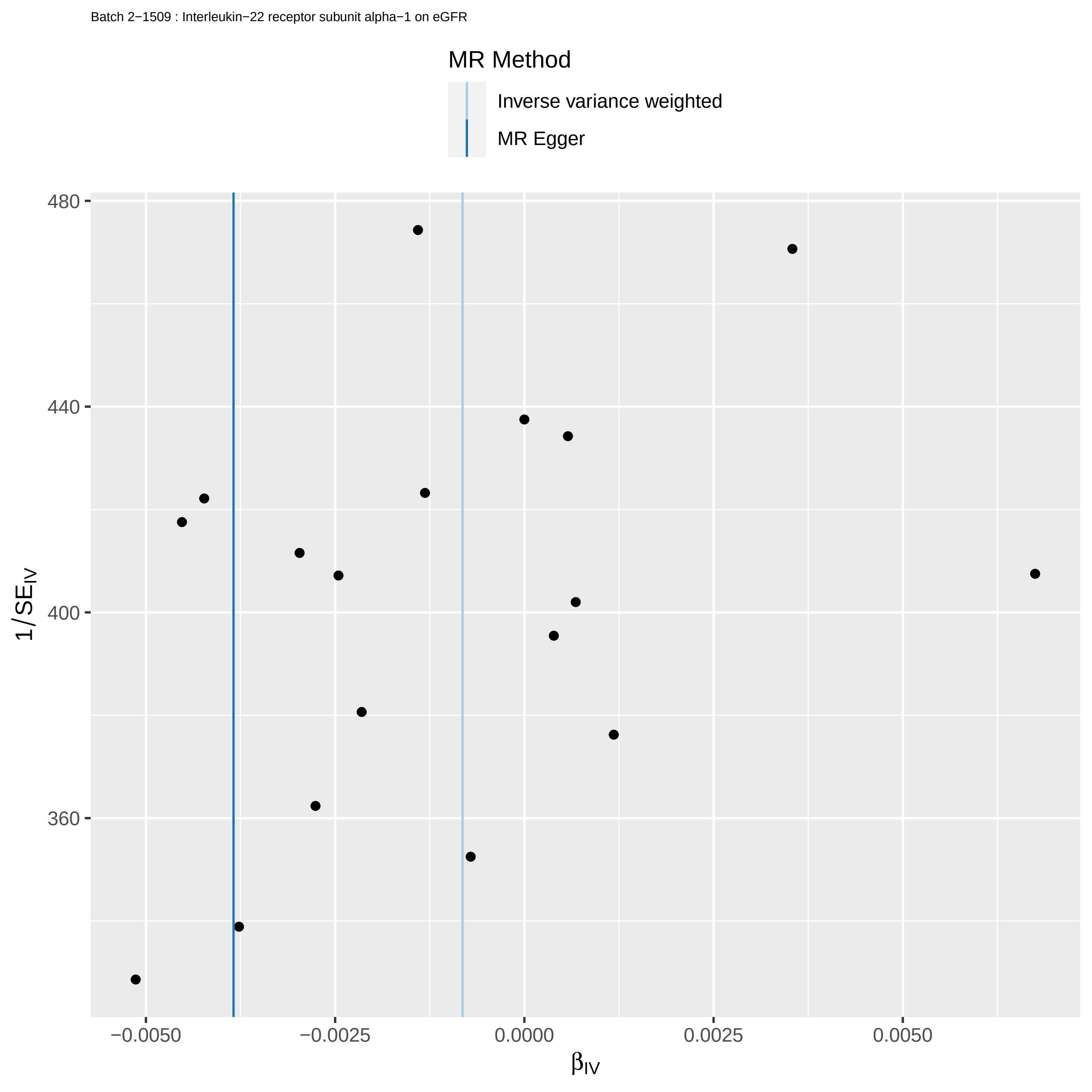
Batch 2−1509 : Interleukin−22 receptor subunit alpha−1 on eGFR
MR Method
Inverse variance weighted
MR Egger
480
440
400
360
1/SE_IV
−0.0050
−0.0025
0.0000
0.0025
0.0050
β_IV

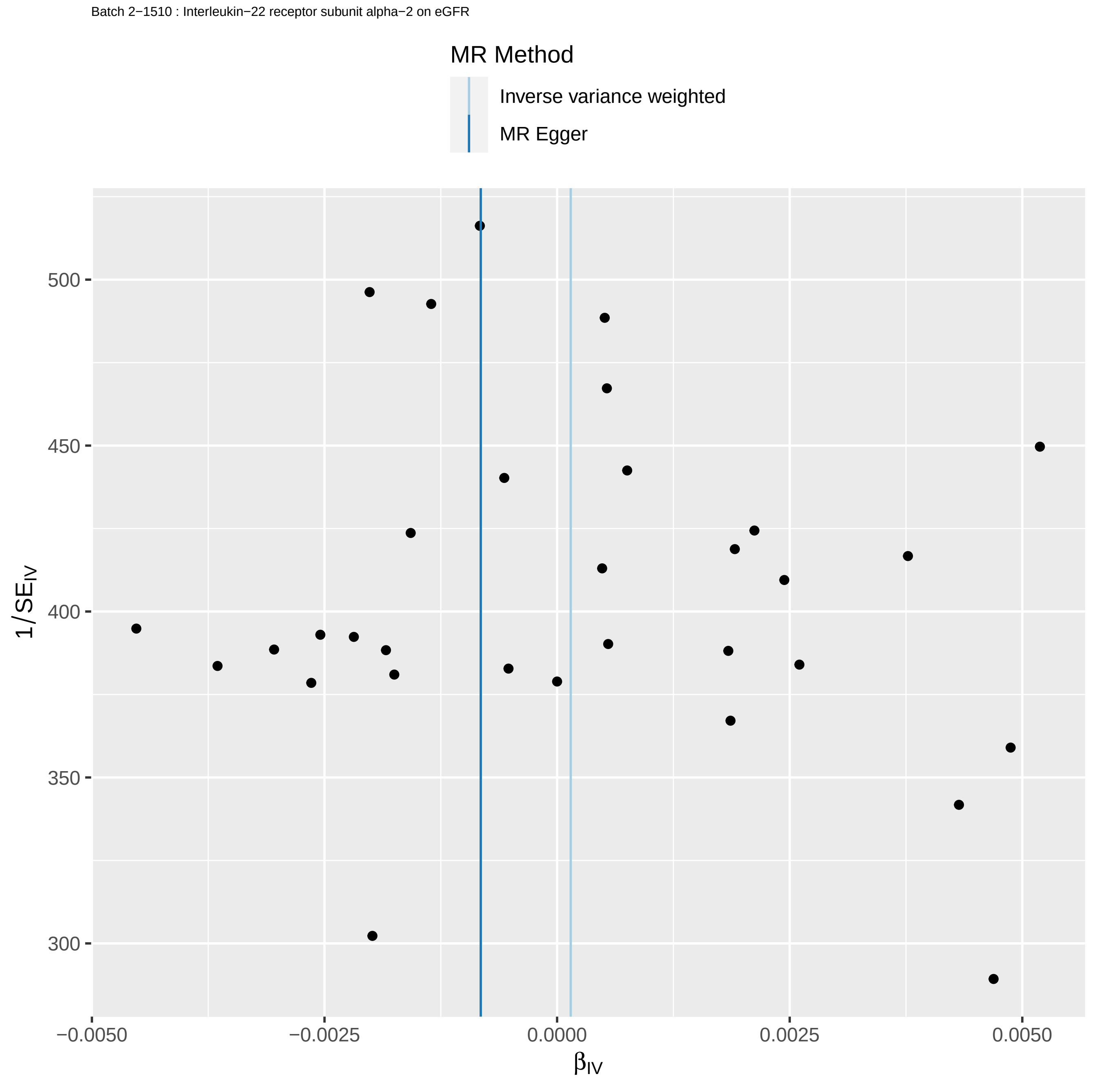

Batch 2−1510 : Interleukin−22 receptor subunit alpha−2 on eGFR
MR Method
Inverse variance weighted
MR Egger
1/SE$_{IV}$
500
450
400
350
300
−0.0050
−0.0025
0.0000
0.0025
0.0050
β$_{IV}$

Batch 2−1511 : Interleukin−22 receptor subunit alpha−2 on eGFR

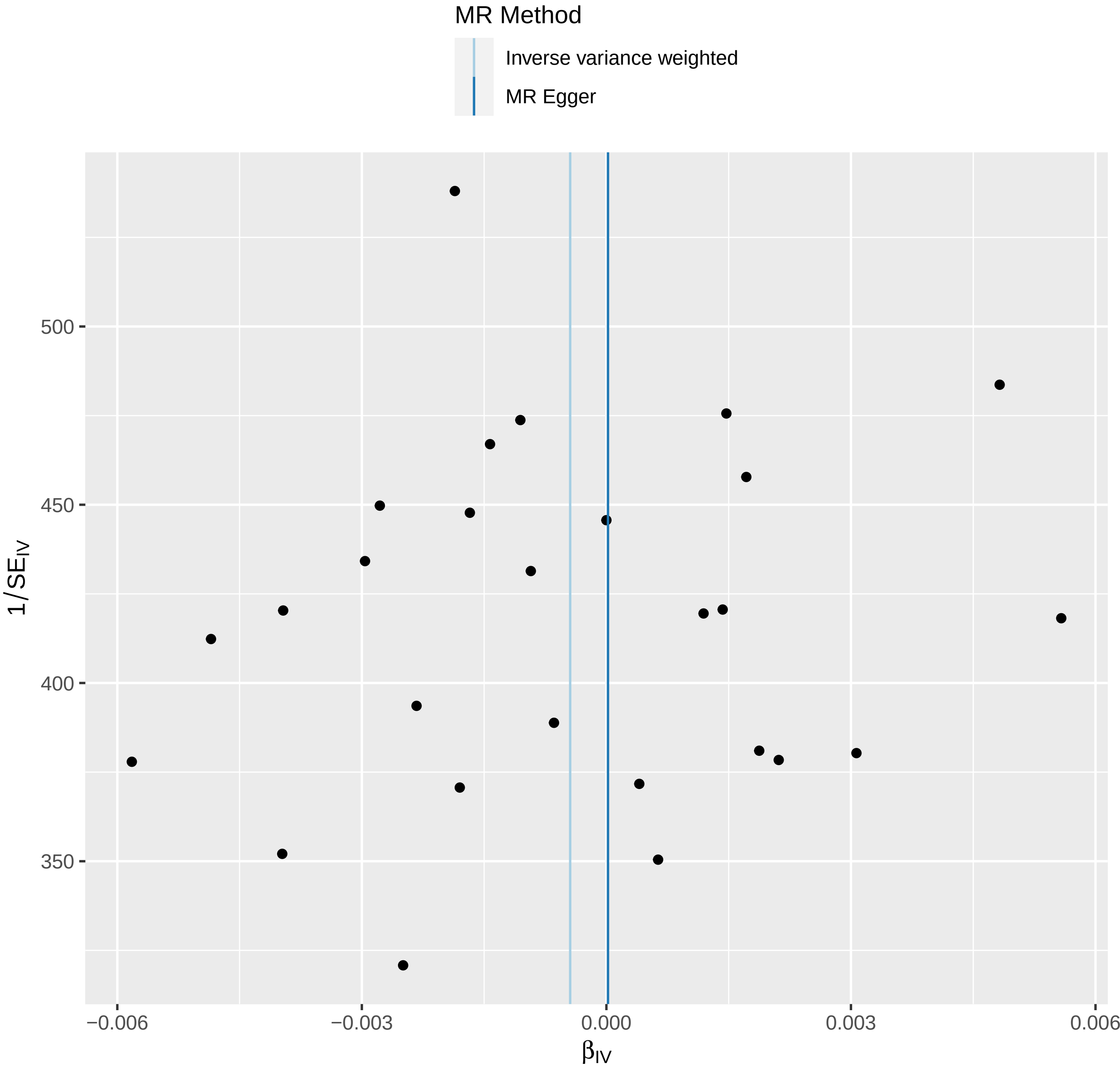

Batch 2−1512 : Interleukin−2 on eGFR

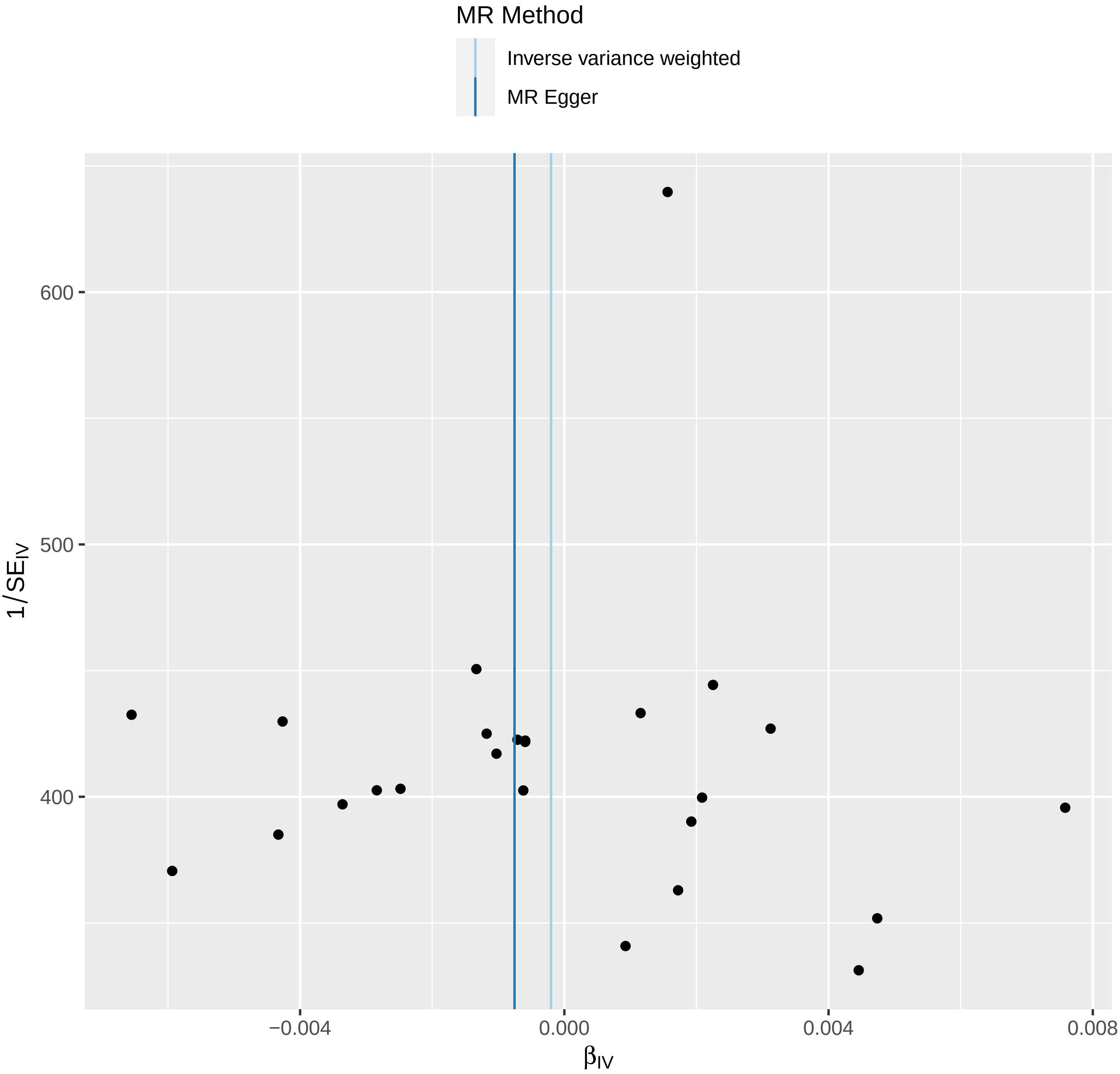

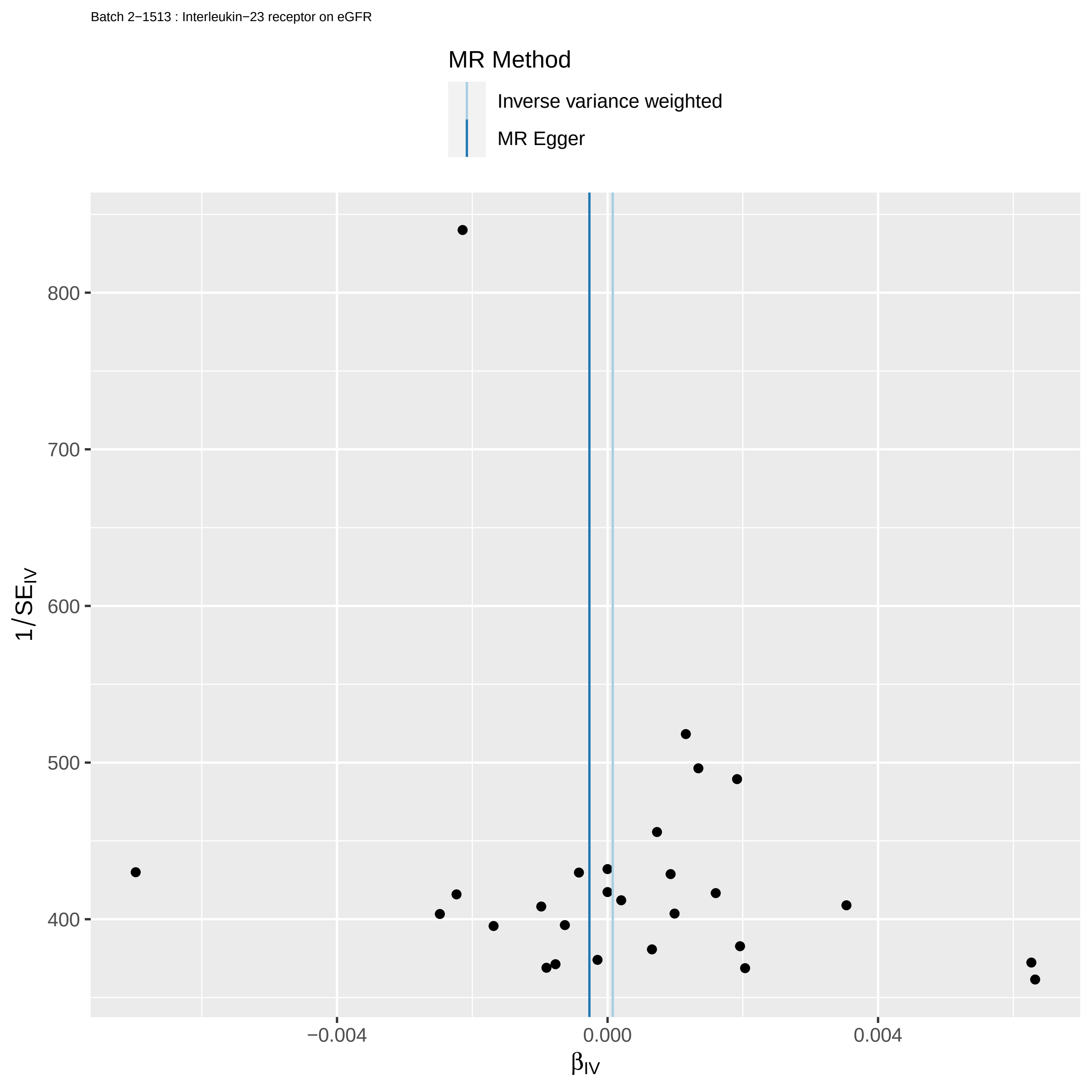

Batch 2−1513 : Interleukin−23 receptor on eGFR
MR Method
Inverse variance weighted
MR Egger
800
700
600
500
400
1/SE_IV
−0.004
0.000
0.004
β_IV

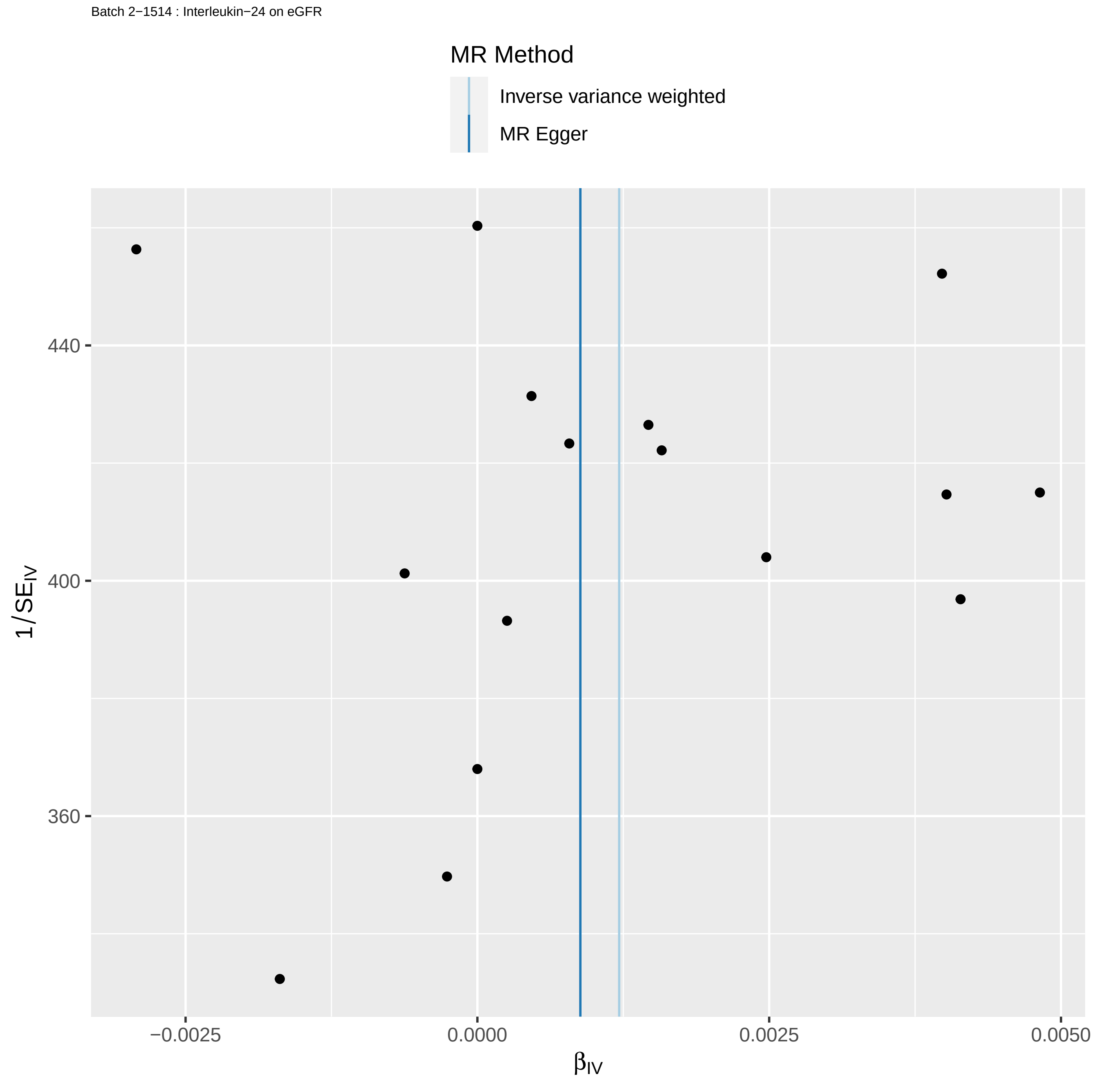
Batch 2−1514 : Interleukin−24 on eGFR
MR Method
Inverse variance weighted
MR Egger
440
400
360
1/SE_IV
−0.0025
0.0000
0.0025
0.0050
β_IV

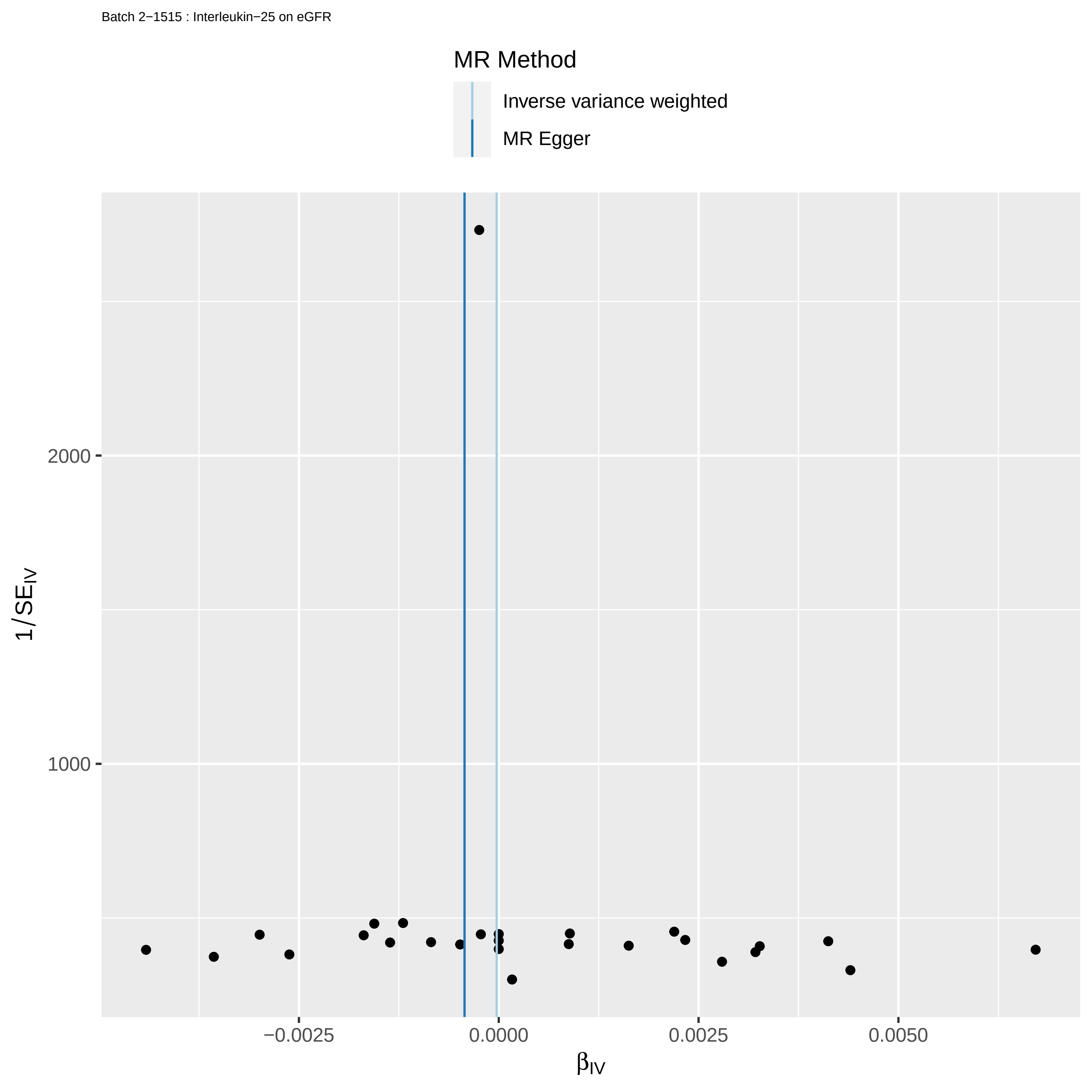

Batch 2−1515 : Interleukin−25 on eGFR
MR Method
Inverse variance weighted
MR Egger
2000
1000
1/SE_IV
−0.0025
0.0000
0.0025
0.0050
β_IV

Batch 2−1516 : Interleukin−27 on eGFR

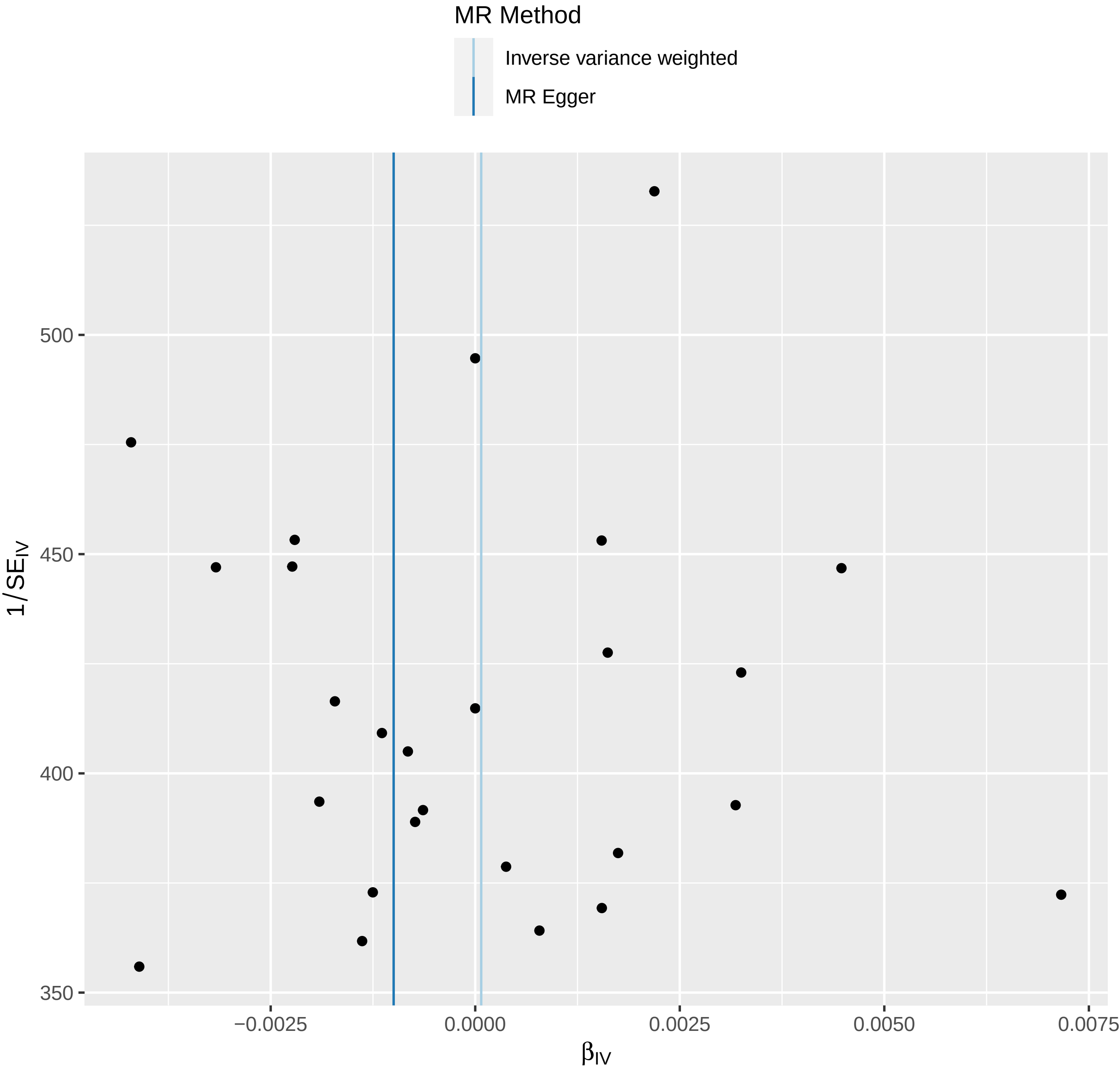

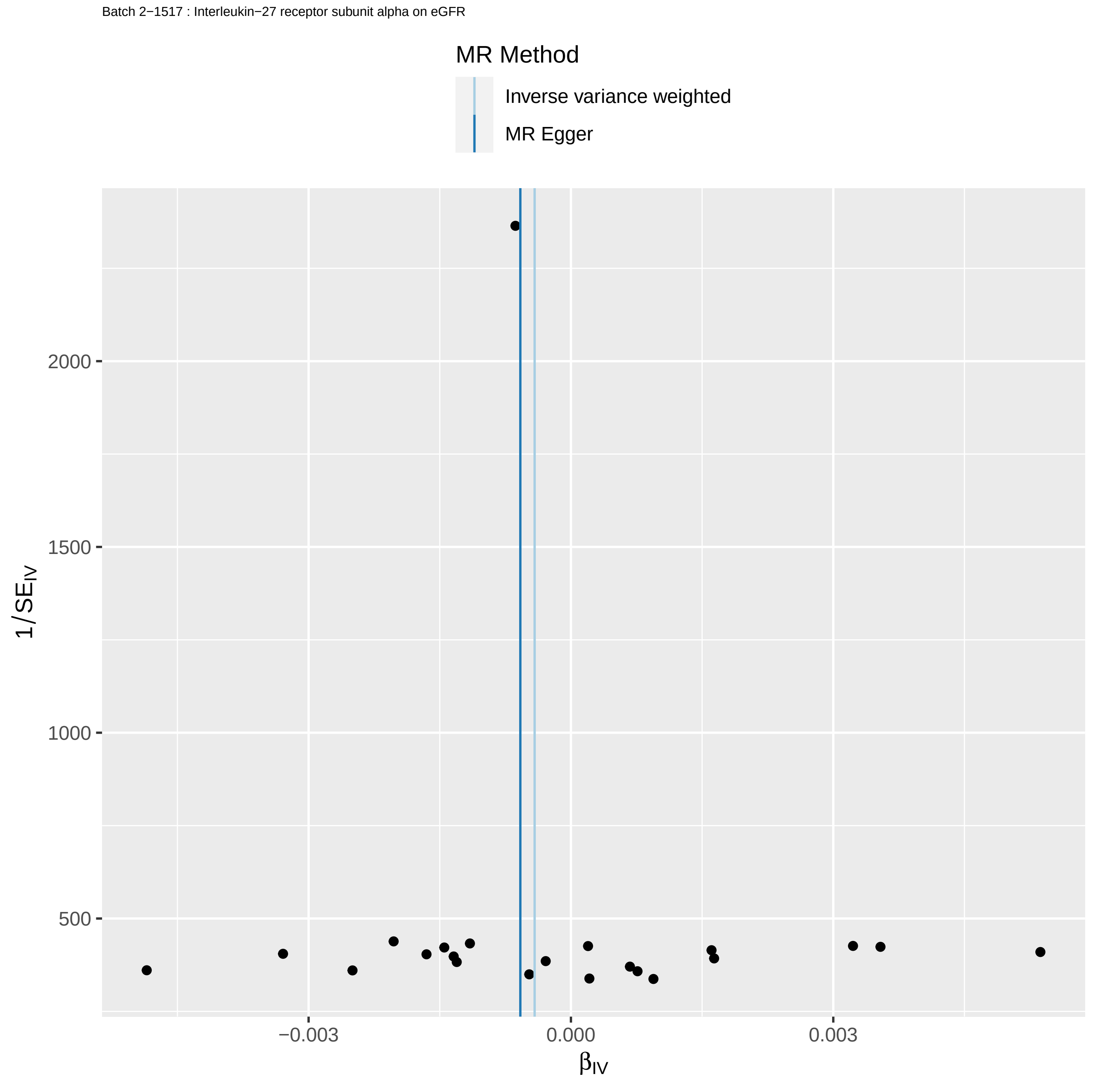
Batch 2−1517 : Interleukin−27 receptor subunit alpha on eGFR
MR Method
Inverse variance weighted
MR Egger
2000
1500
1000
500
1/SE$_{IV}$
−0.003
0.000
0.003
β$_{IV}$

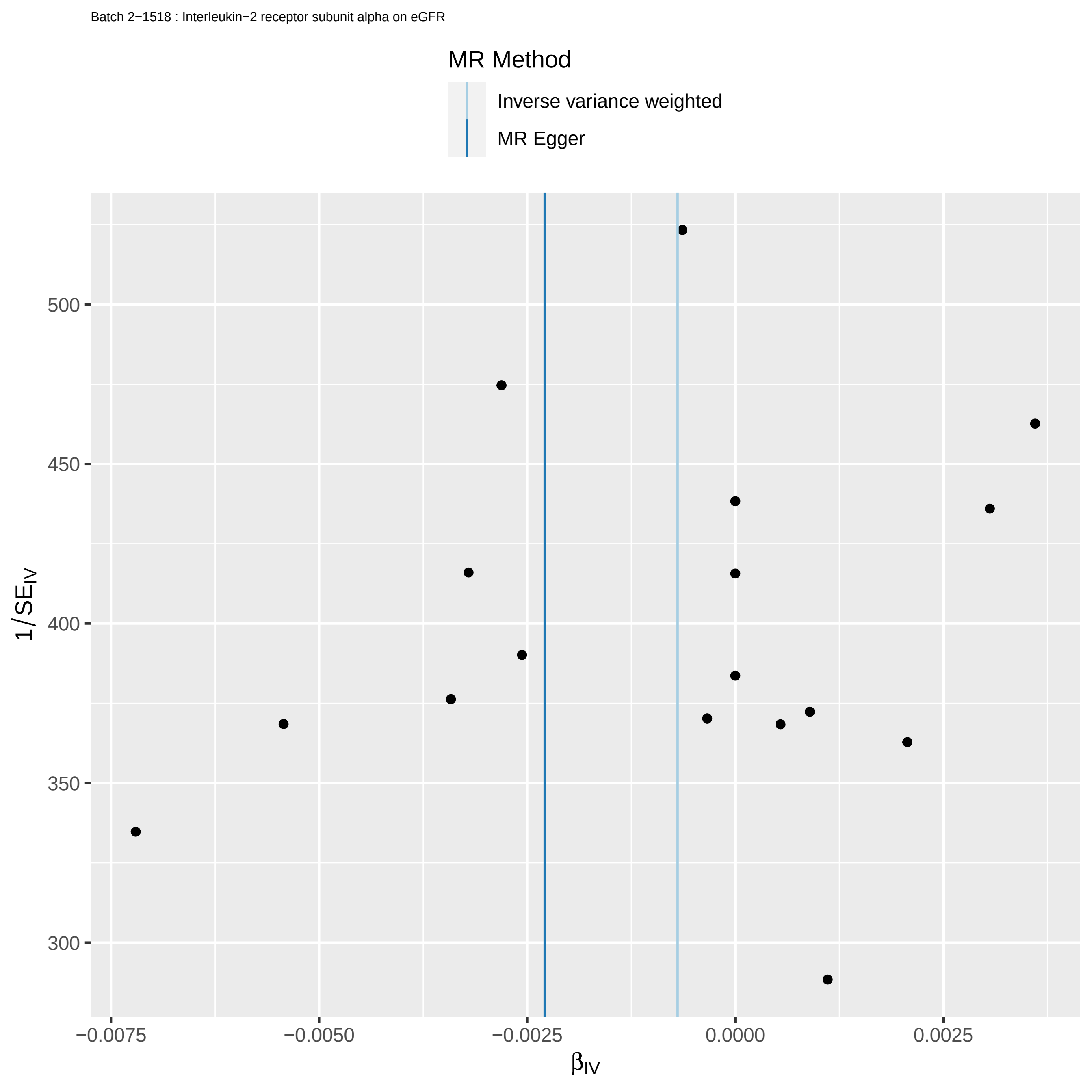

Batch 2−1518 : Interleukin−2 receptor subunit alpha on eGFR
MR Method
Inverse variance weighted
MR Egger
500
450
400
350
300
1/SE_IV
−0.0075
−0.0050
−0.0025
0.0000
0.0025
β_IV

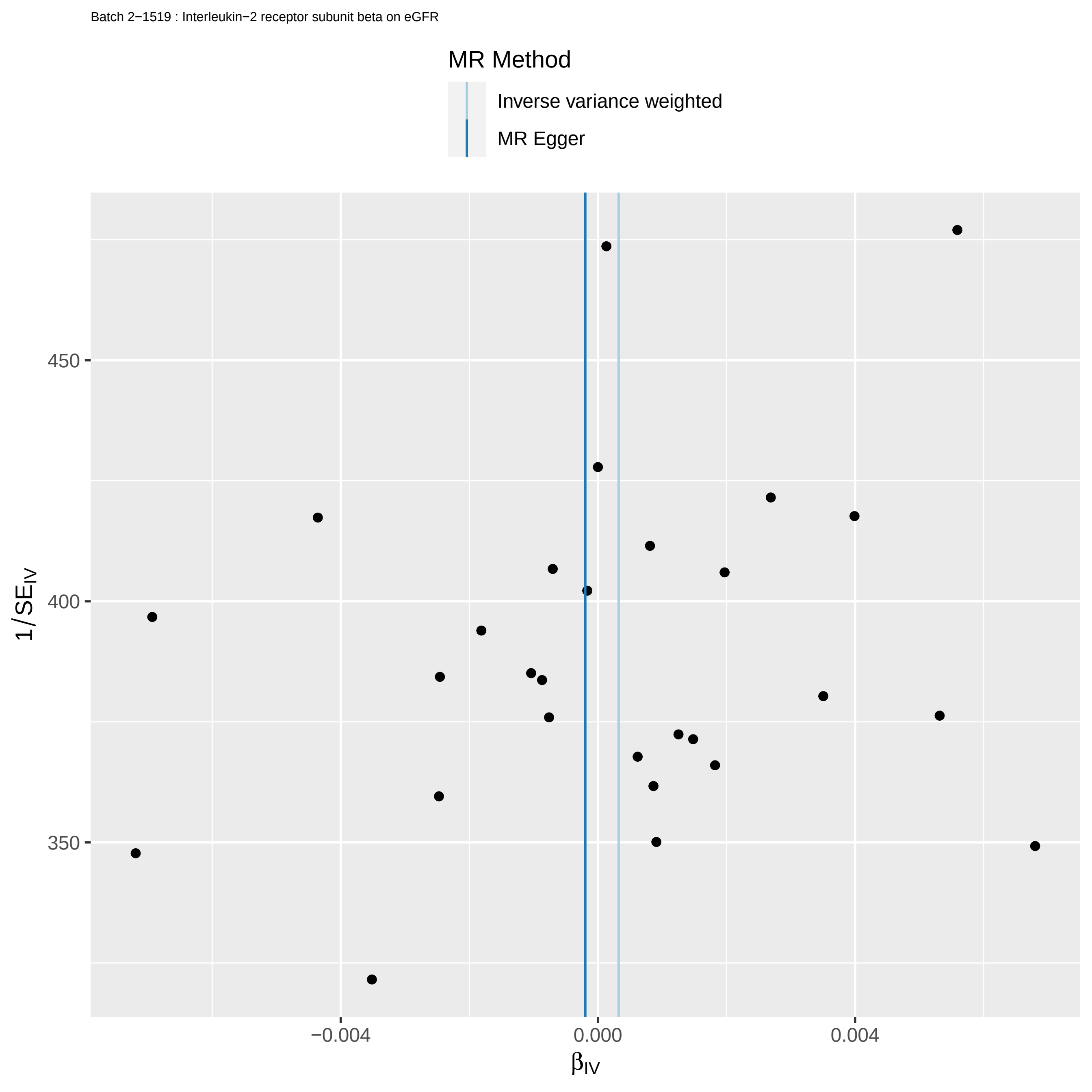

Batch 2−1519 : Interleukin−2 receptor subunit beta on eGFR
MR Method
Inverse variance weighted
MR Egger
450
400
350
1/SE_IV
−0.004
0.000
0.004
β_IV

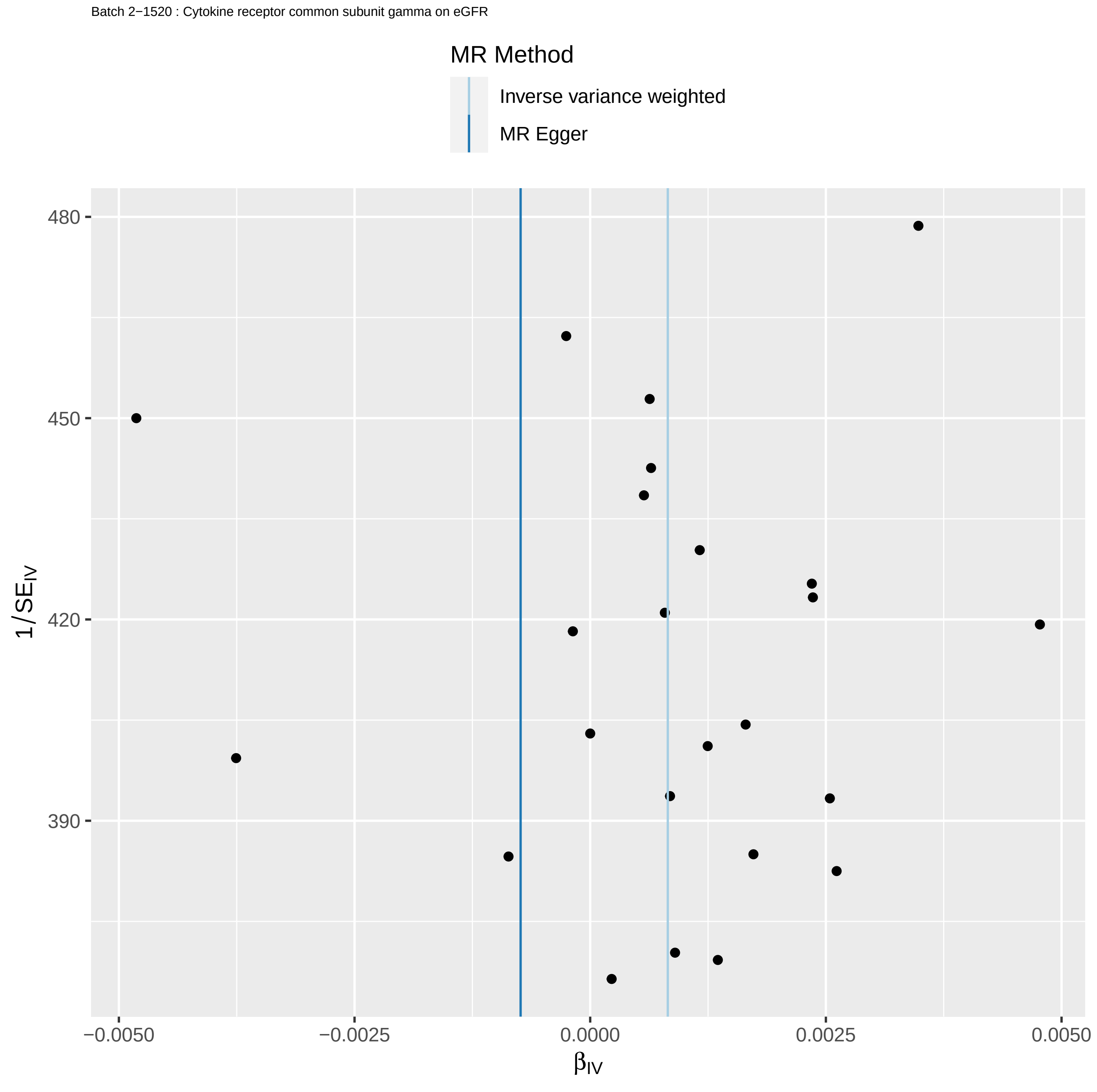

Batch 2−1520 : Cytokine receptor common subunit gamma on eGFR
MR Method
Inverse variance weighted
MR Egger
480
450
420
390
−0.0050
−0.0025
0.0000
0.0025
0.0050
$1/SE_{IV}$
β_{IV}

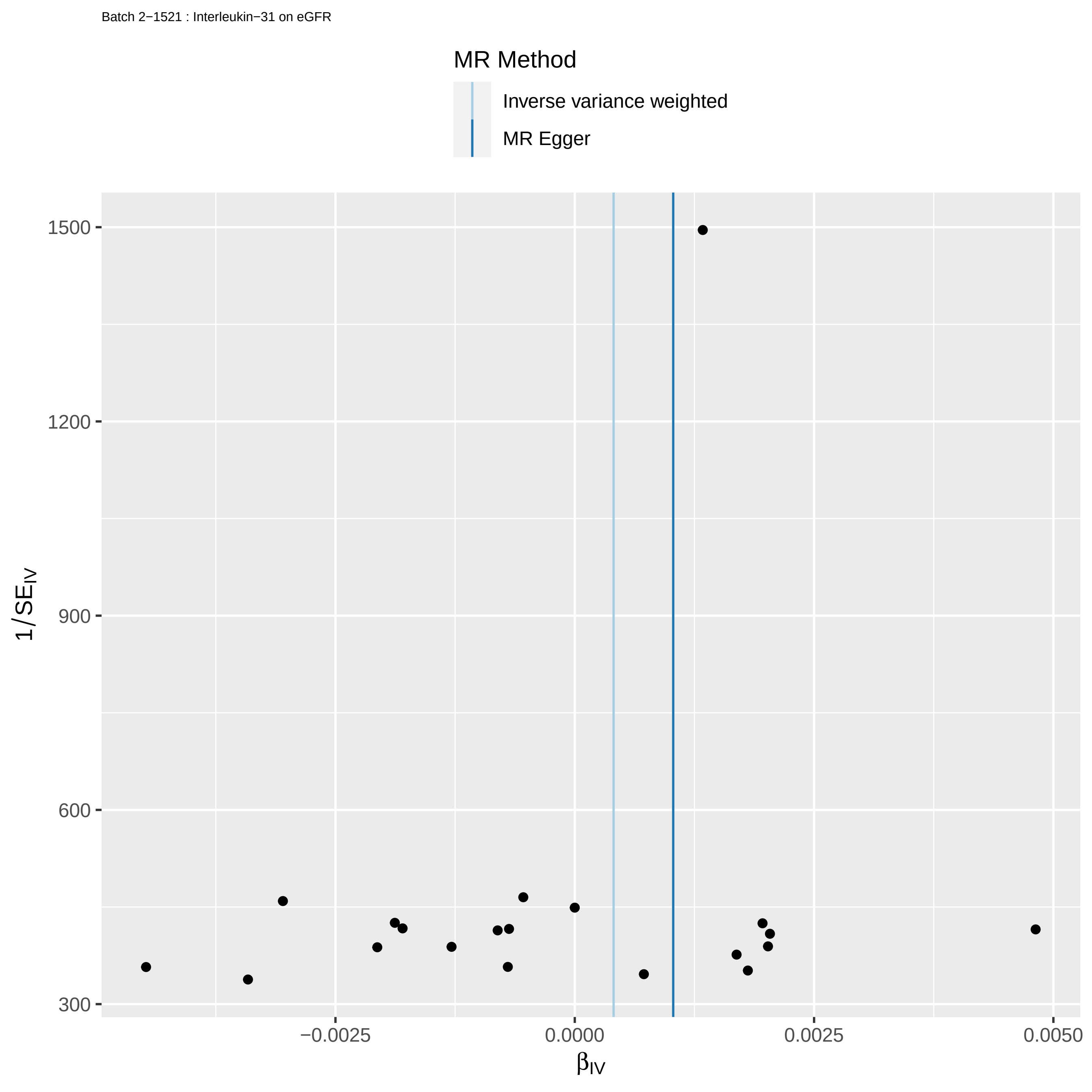
Batch 2−1521 : Interleukin−31 on eGFR
MR Method
Inverse variance weighted
MR Egger
1500
1200
900
600
300
1/SE_IV
−0.0025
0.0000
0.0025
0.0050
β_IV

Batch 2−1522 : Interleukin−31 receptor subunit alpha on eGFR

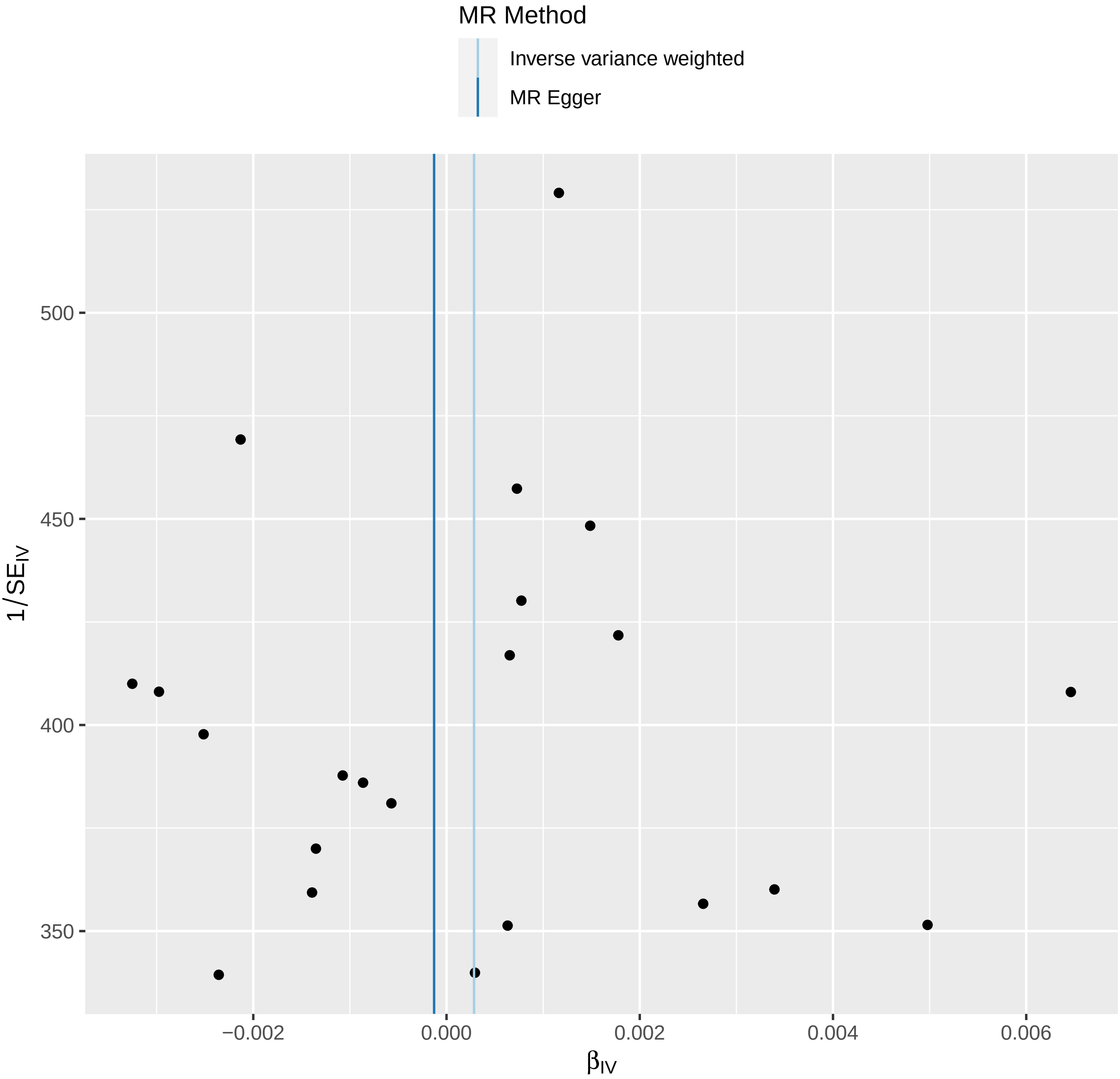

Batch 2−1523 : Interleukin−32 on eGFR

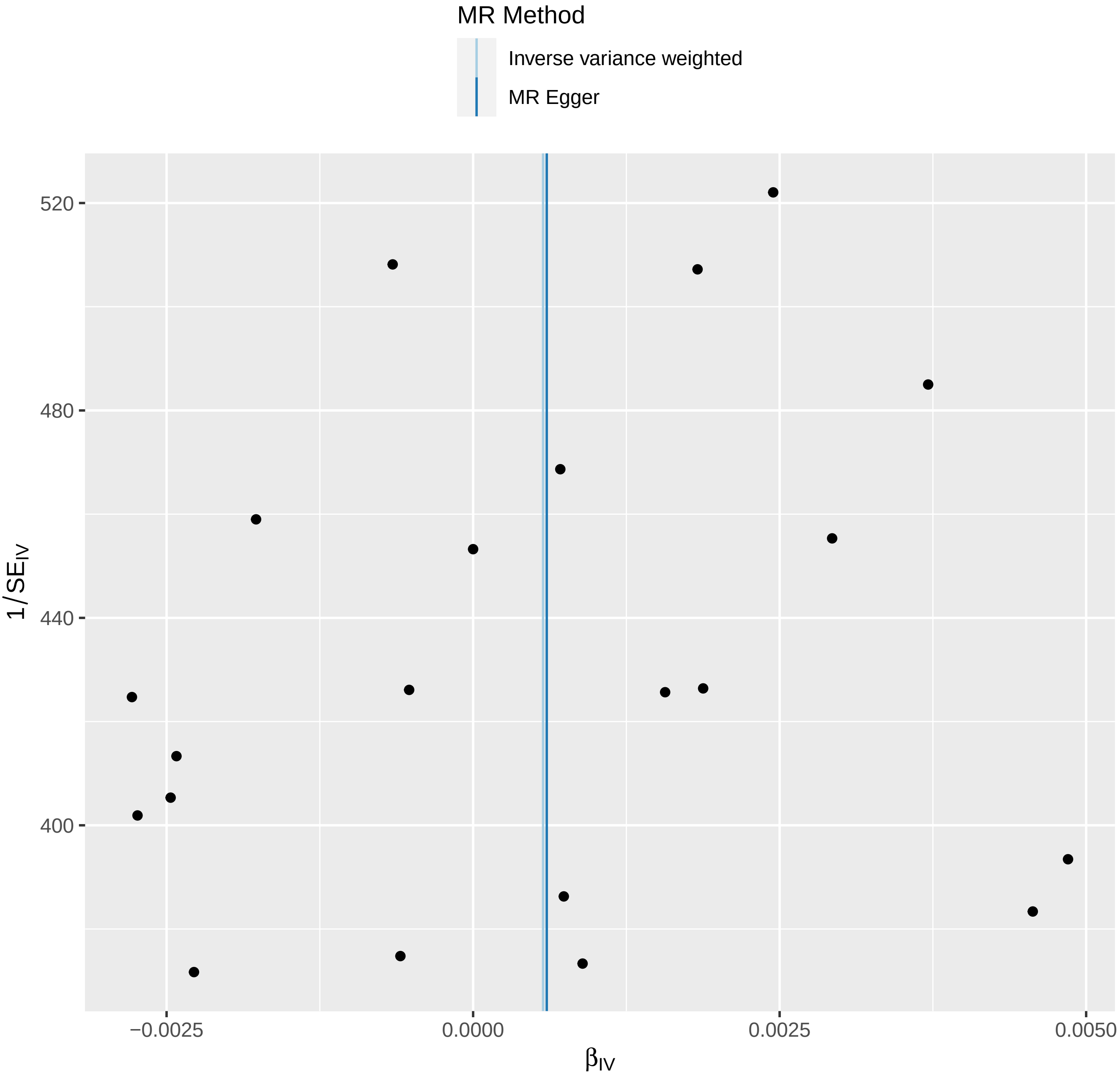

Batch 2−1524 : Interleukin−34 on eGFR

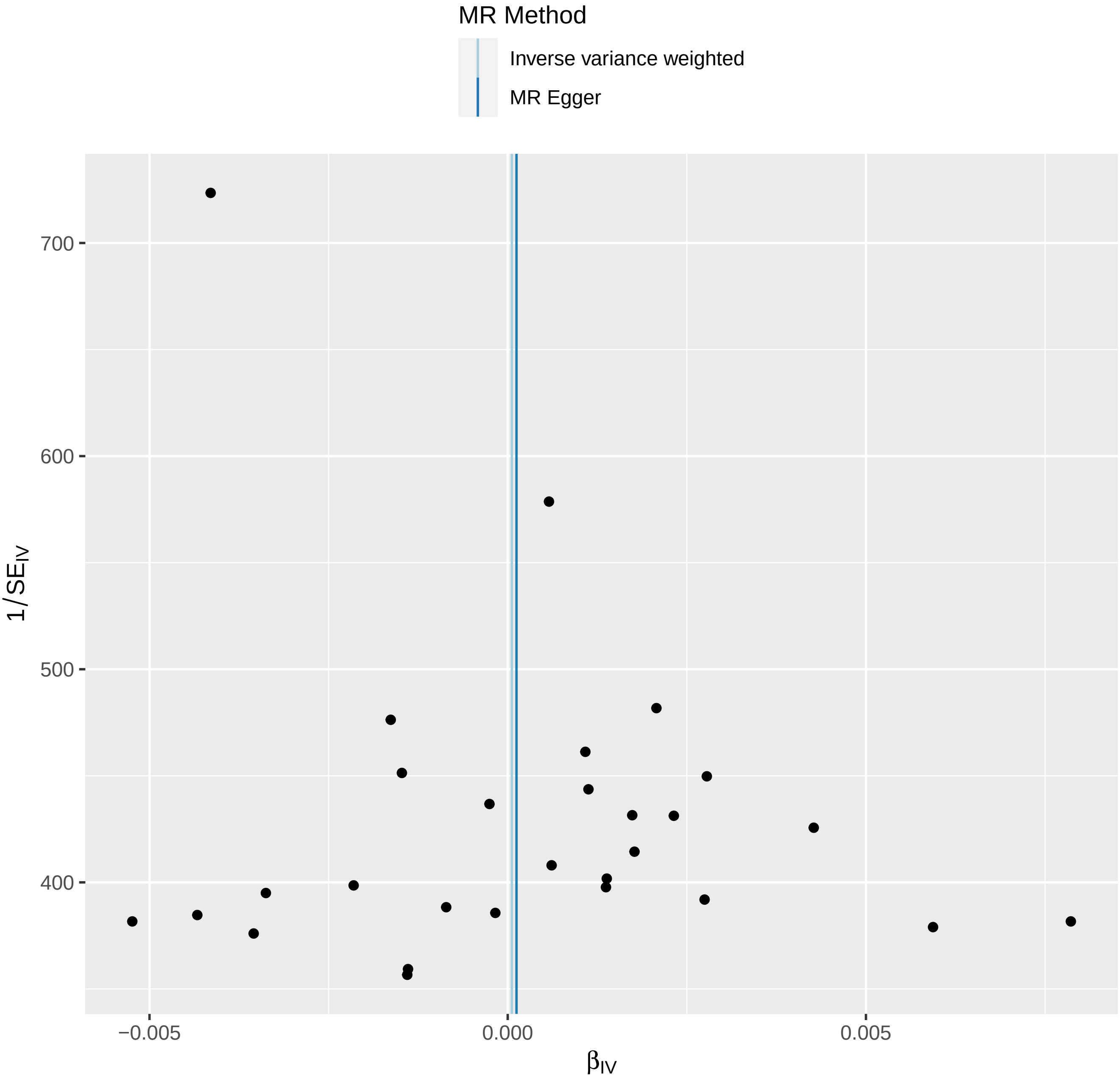

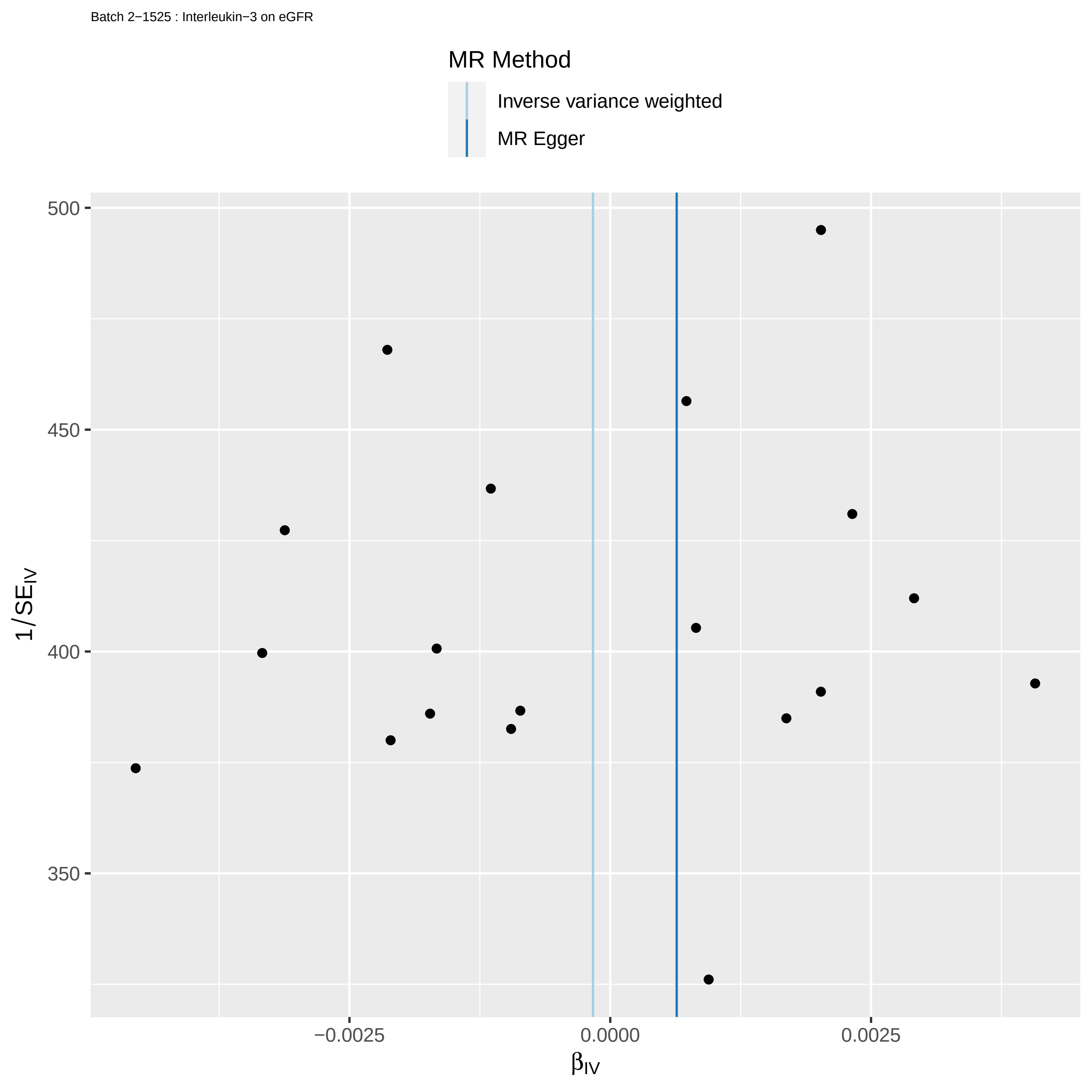

Batch 2−1525 : Interleukin−3 on eGFR
MR Method
Inverse variance weighted
MR Egger
500
450
400
350
1/SE$_{IV}$
−0.0025
0.0000
0.0025
β_{IV}

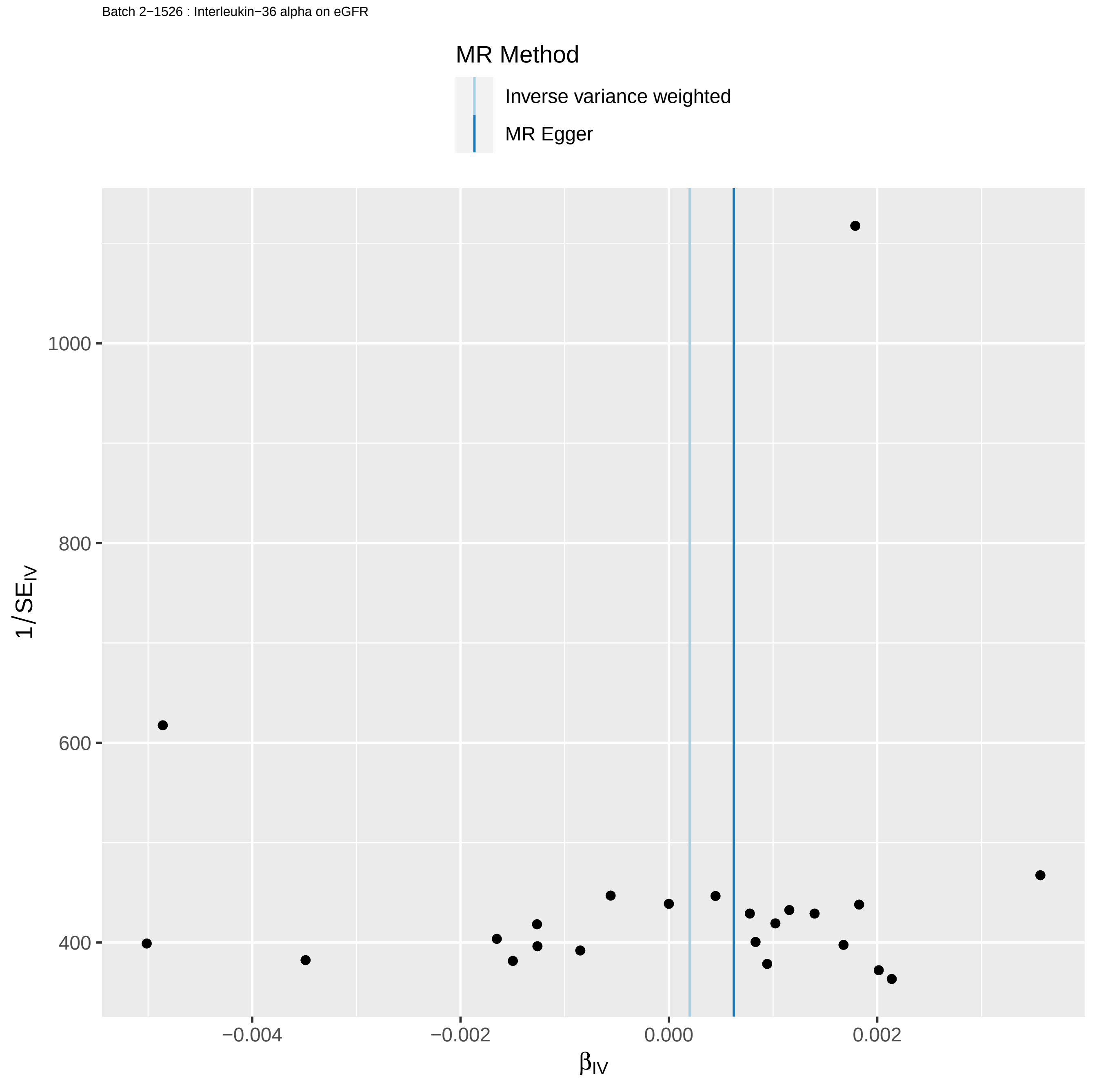

Batch 2−1526 : Interleukin−36 alpha on eGFR
MR Method
Inverse variance weighted
MR Egger
1000
800
600
400
1/SE$_{IV}$
−0.004
−0.002
0.000
0.002
β$_{IV}$

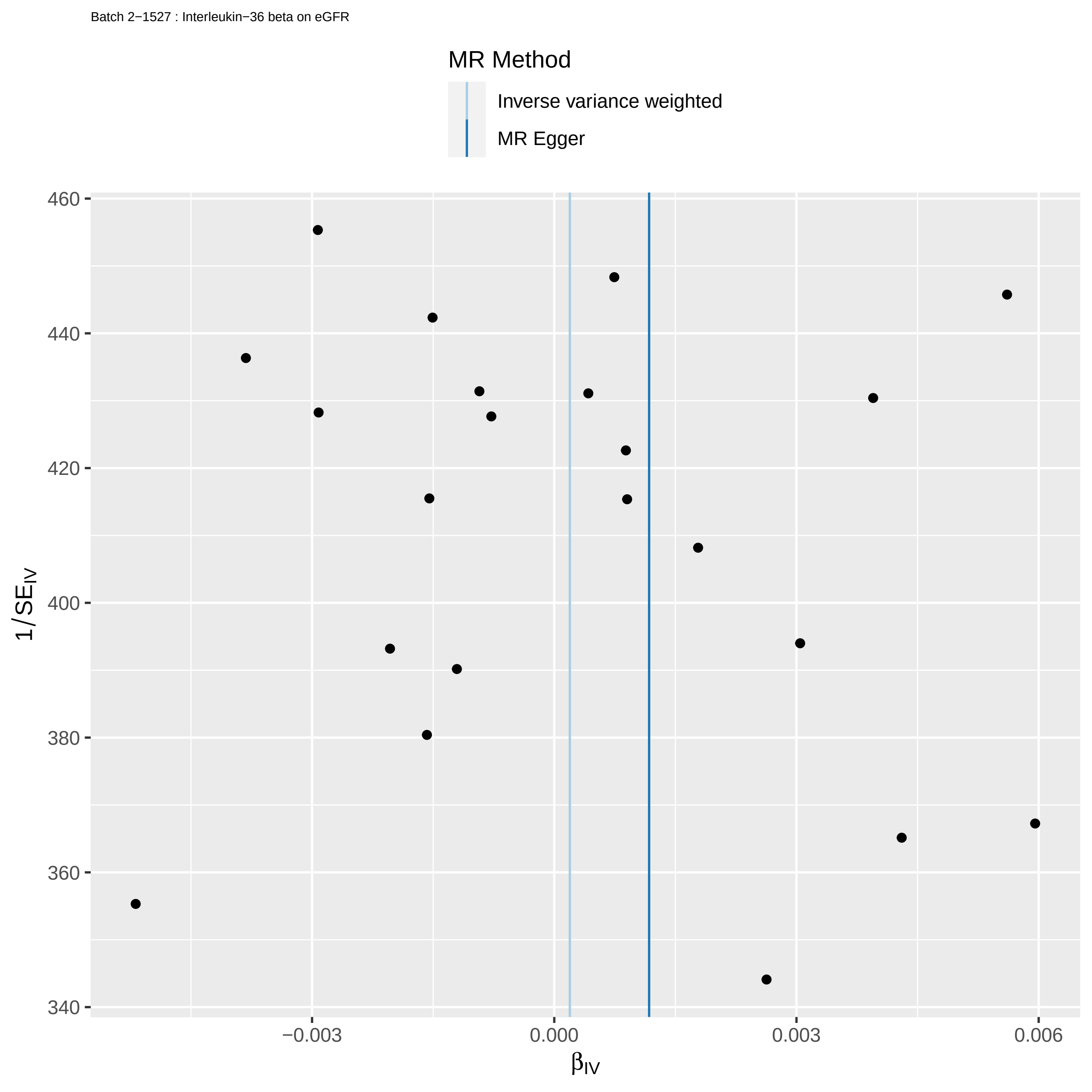

Batch 2−1527 : Interleukin−36 beta on eGFR
MR Method
Inverse variance weighted
MR Egger
460
440
420
400
380
360
340
1/SE_IV
−0.003
0.000
0.003
0.006
β_IV

Batch 2−1528 : Interleukin−36 gamma on eGFR

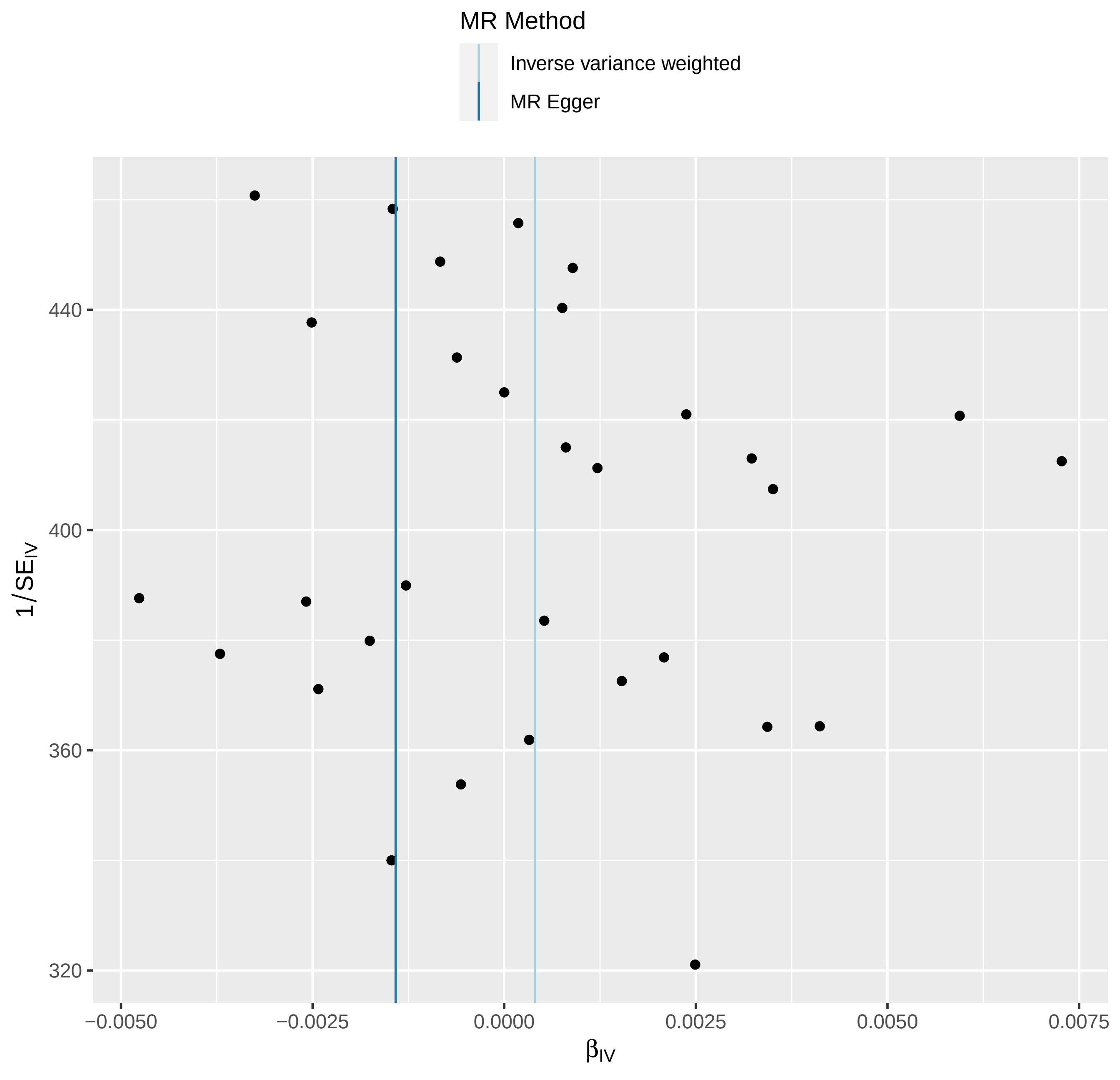

Batch 2−1529 : Interleukin−37 on eGFR

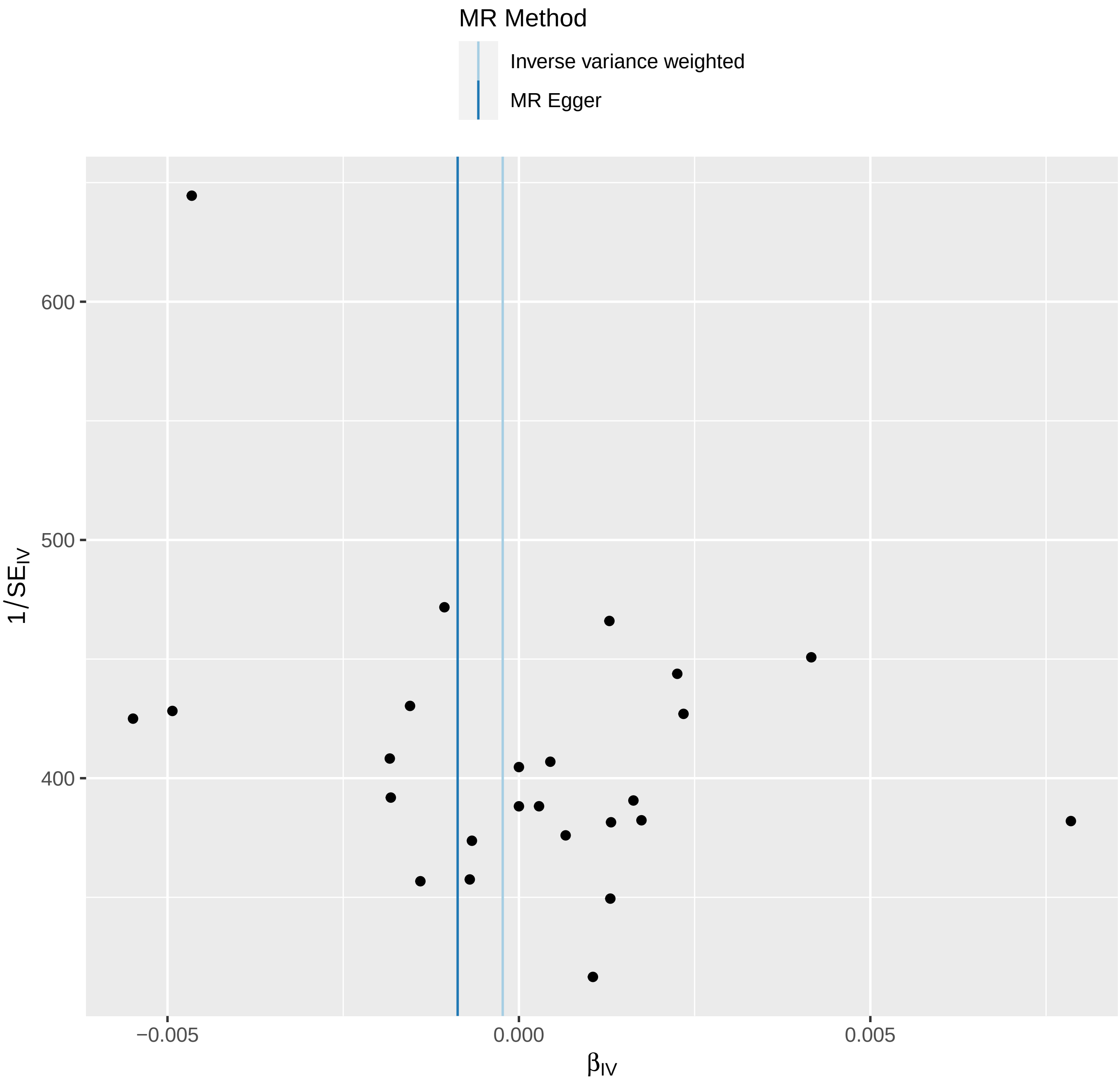

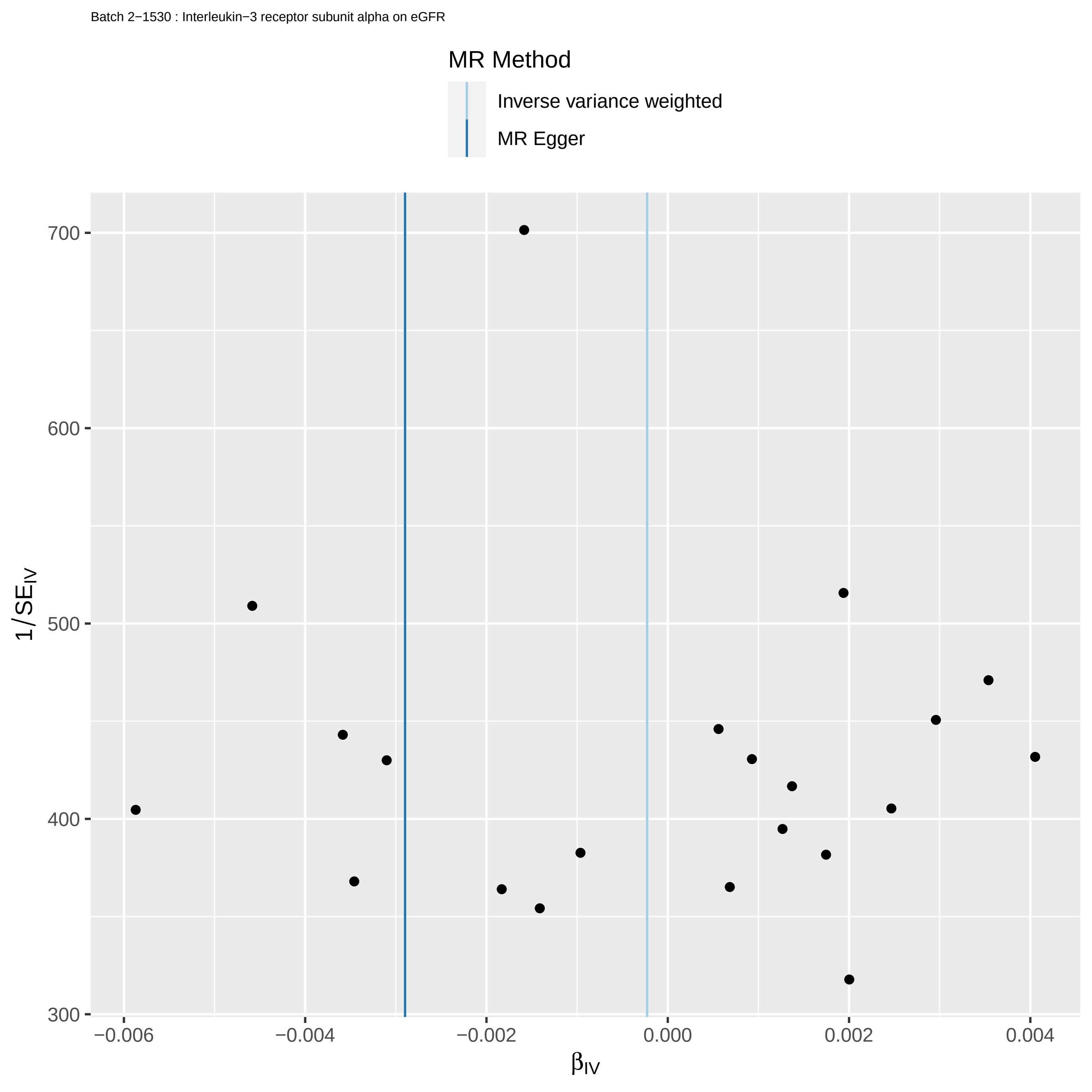

Batch 2−1530 : Interleukin−3 receptor subunit alpha on eGFR
MR Method
Inverse variance weighted
MR Egger
700
600
500
400
300
1/SE_IV
−0.006
−0.004
−0.002
0.000
0.002
0.004
β_IV

Batch 2−1531 : Interleukin−3 receptor subunit alpha on eGFR

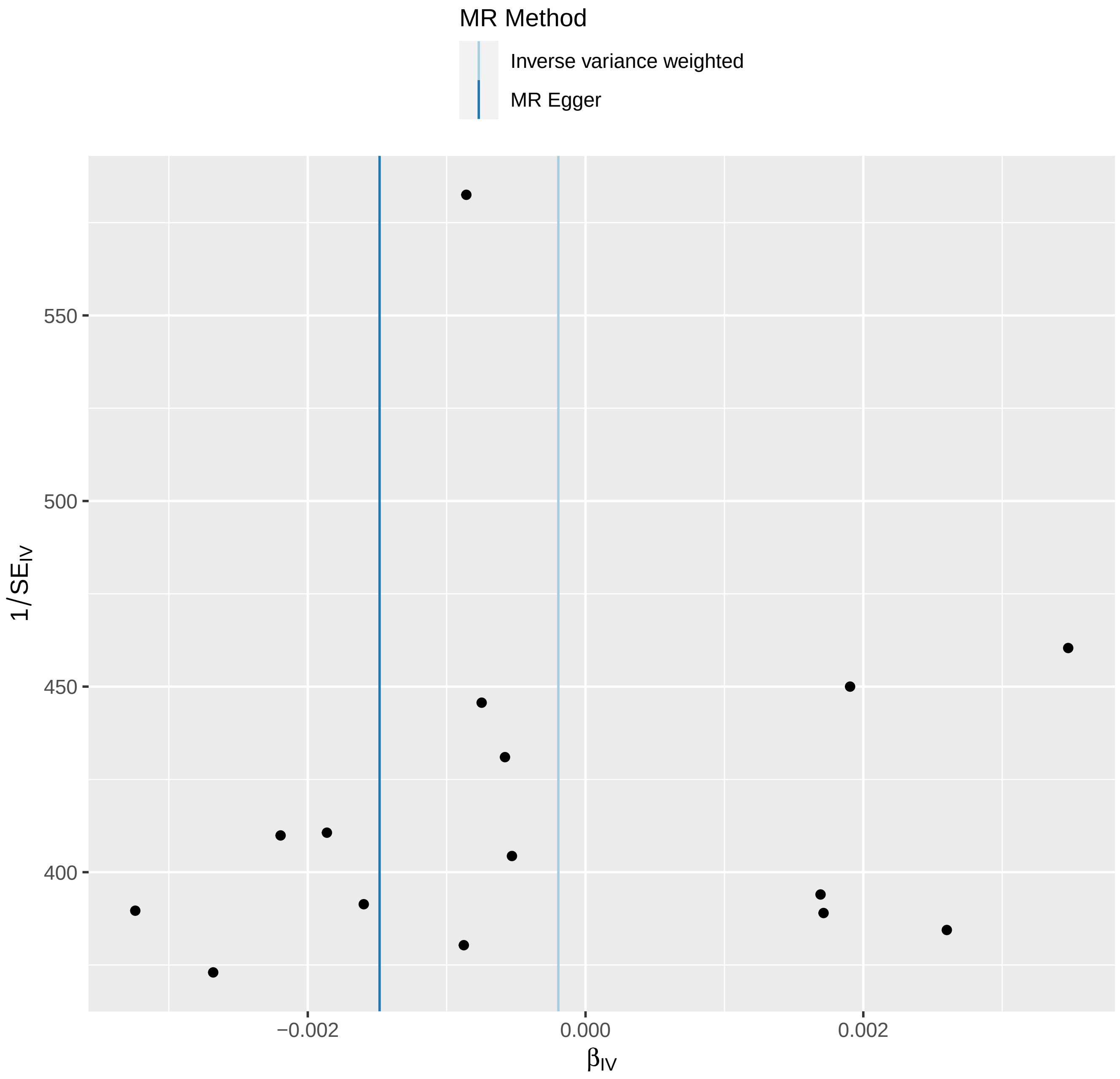

Batch 2−1532 : Interleukin−4 on eGFR

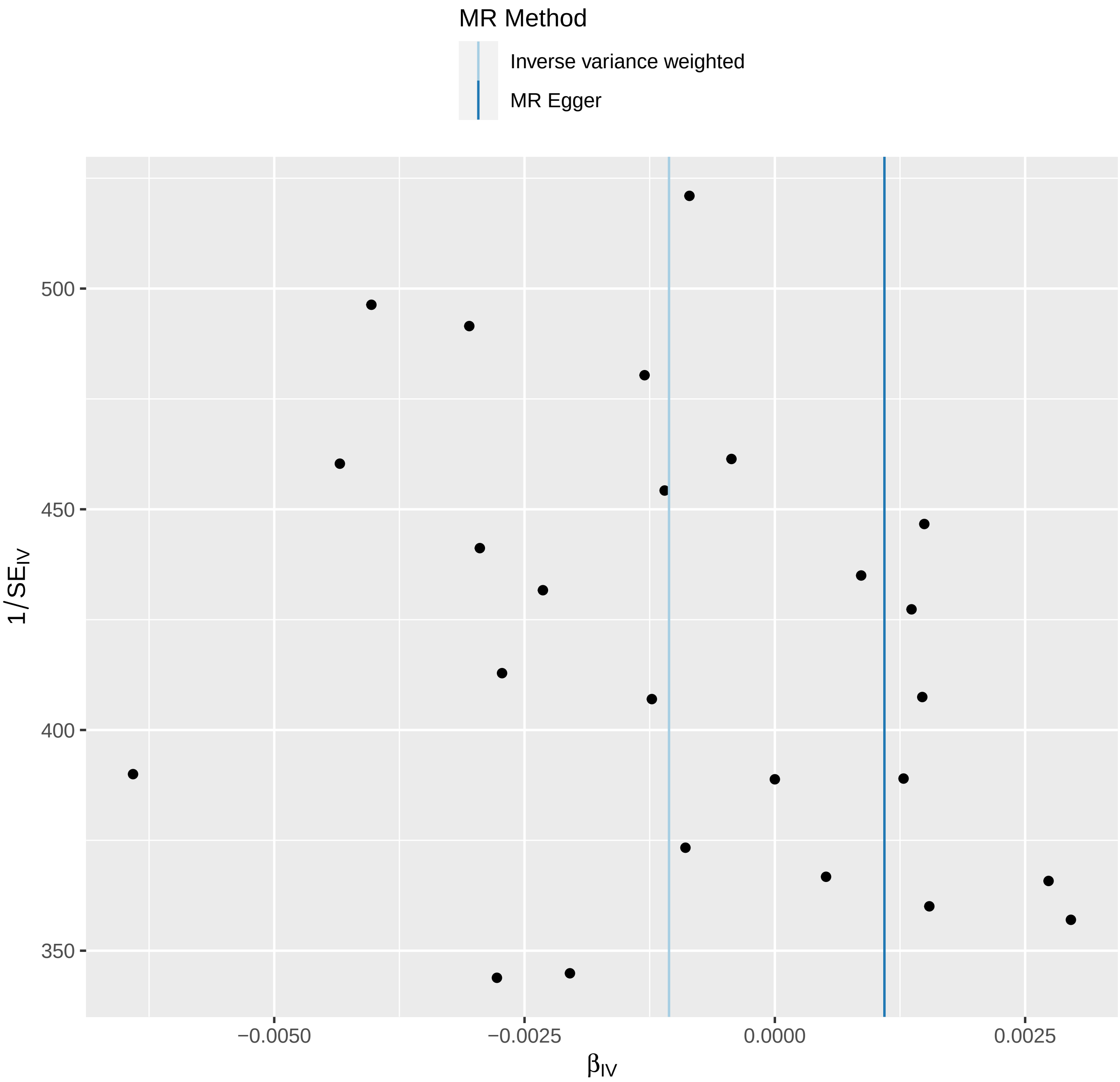

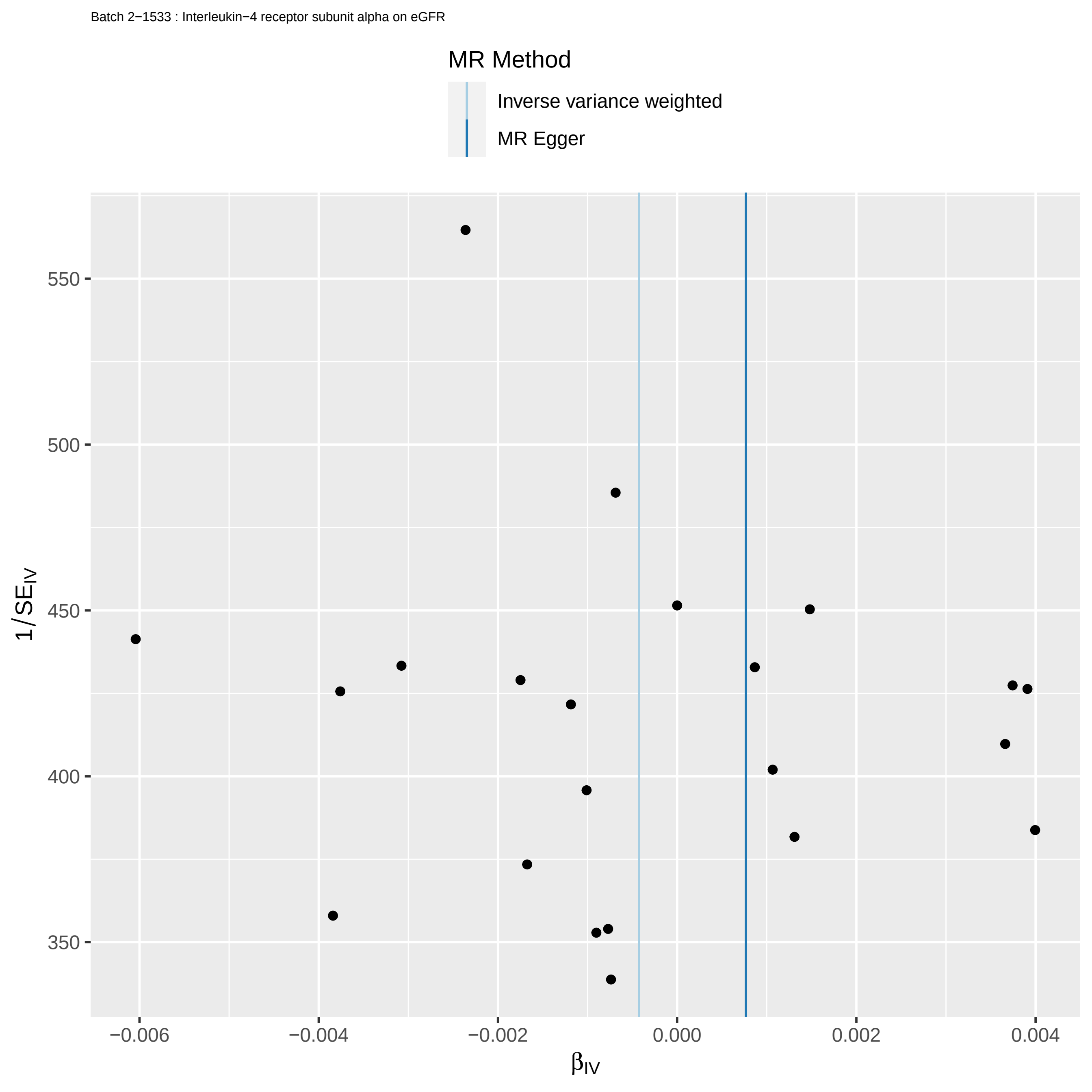
Batch 2−1533 : Interleukin−4 receptor subunit alpha on eGFR
MR Method
Inverse variance weighted
MR Egger
550
500
450
400
350
1/SE$_{IV}$
−0.006
−0.004
−0.002
0.000
0.002
0.004
β_{IV}

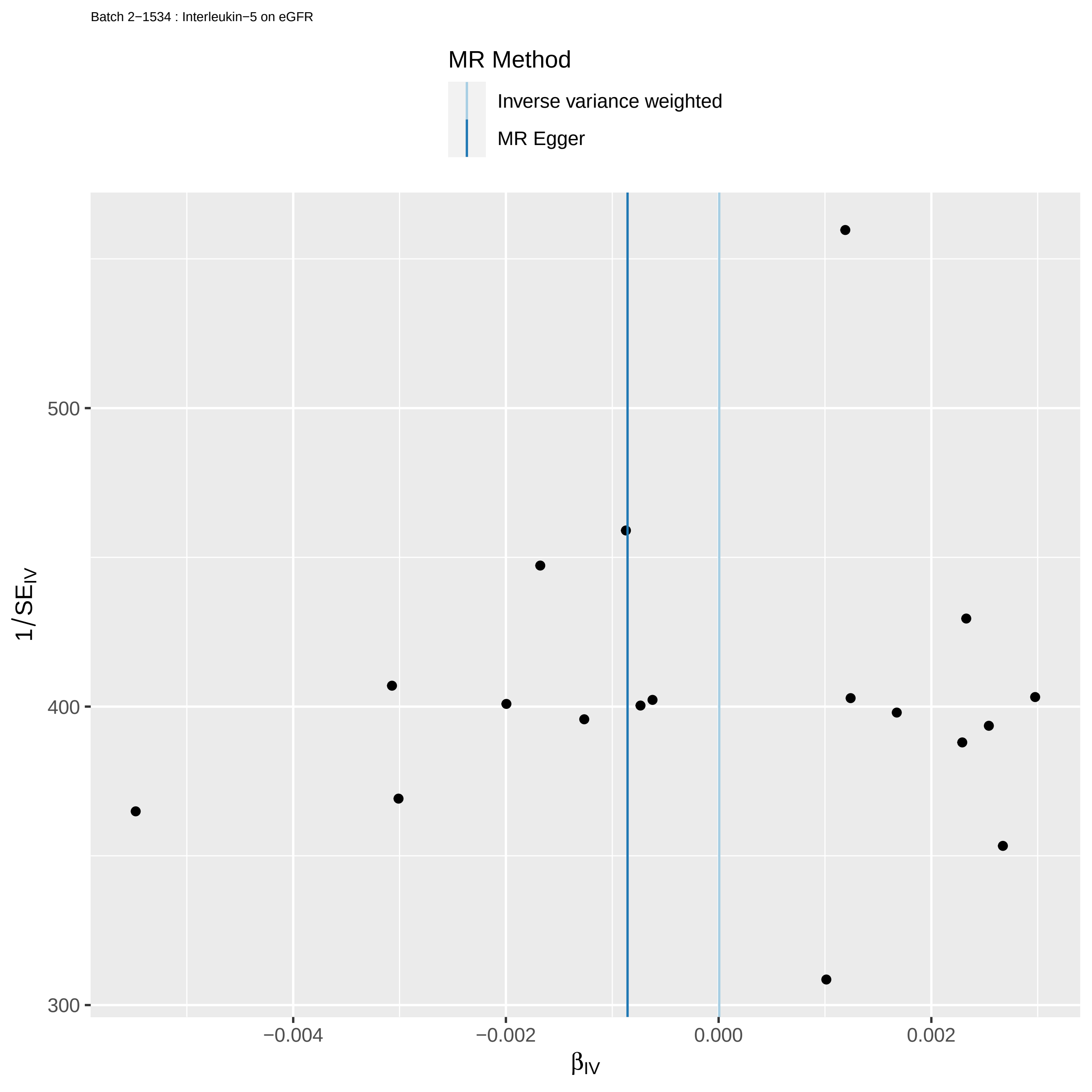

Batch 2−1534 : Interleukin−5 on eGFR
MR Method
Inverse variance weighted
MR Egger
500
400
300
1/SE_IV
−0.004
−0.002
0.000
0.002
β_IV

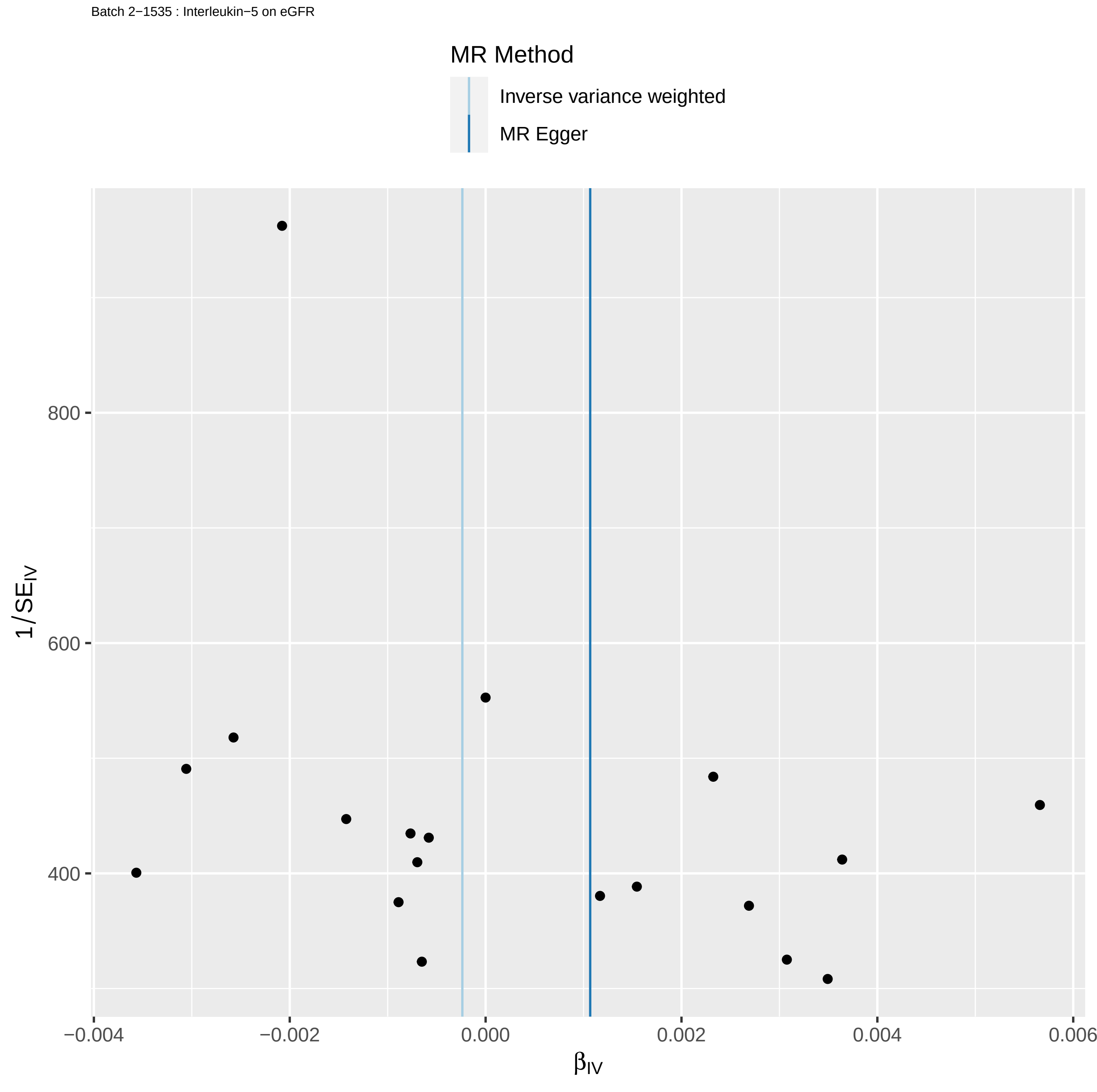

Batch 2−1535 : Interleukin−5 on eGFR
MR Method
Inverse variance weighted
MR Egger
800
600
400
1/SE_IV
−0.004
−0.002
0.000
0.002
0.004
0.006
β_IV

Batch 2−1536 : Interleukin−5 receptor subunit alpha on eGFR

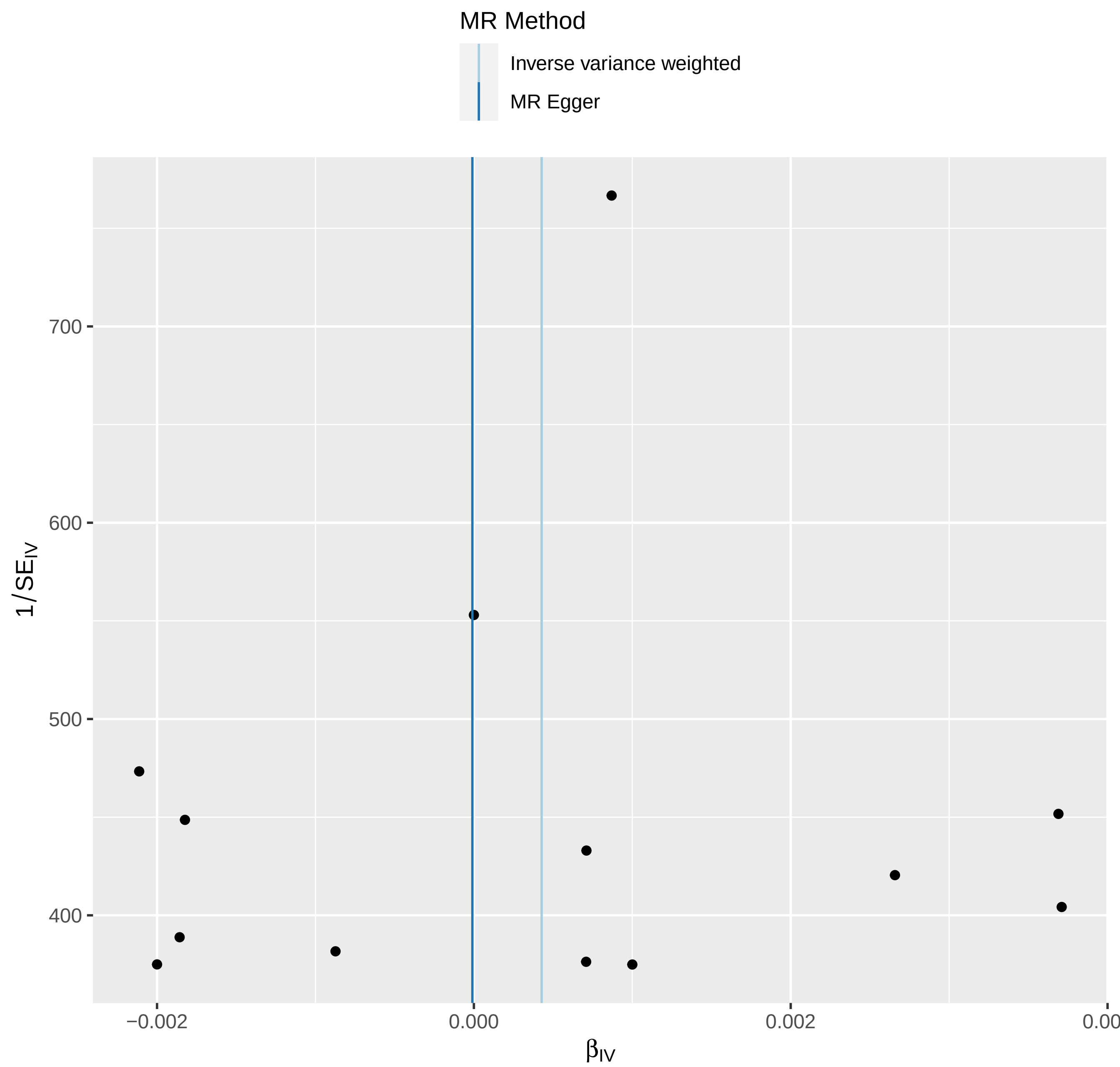

Batch 2−1537 : Interleukin−5 receptor subunit alpha on eGFR

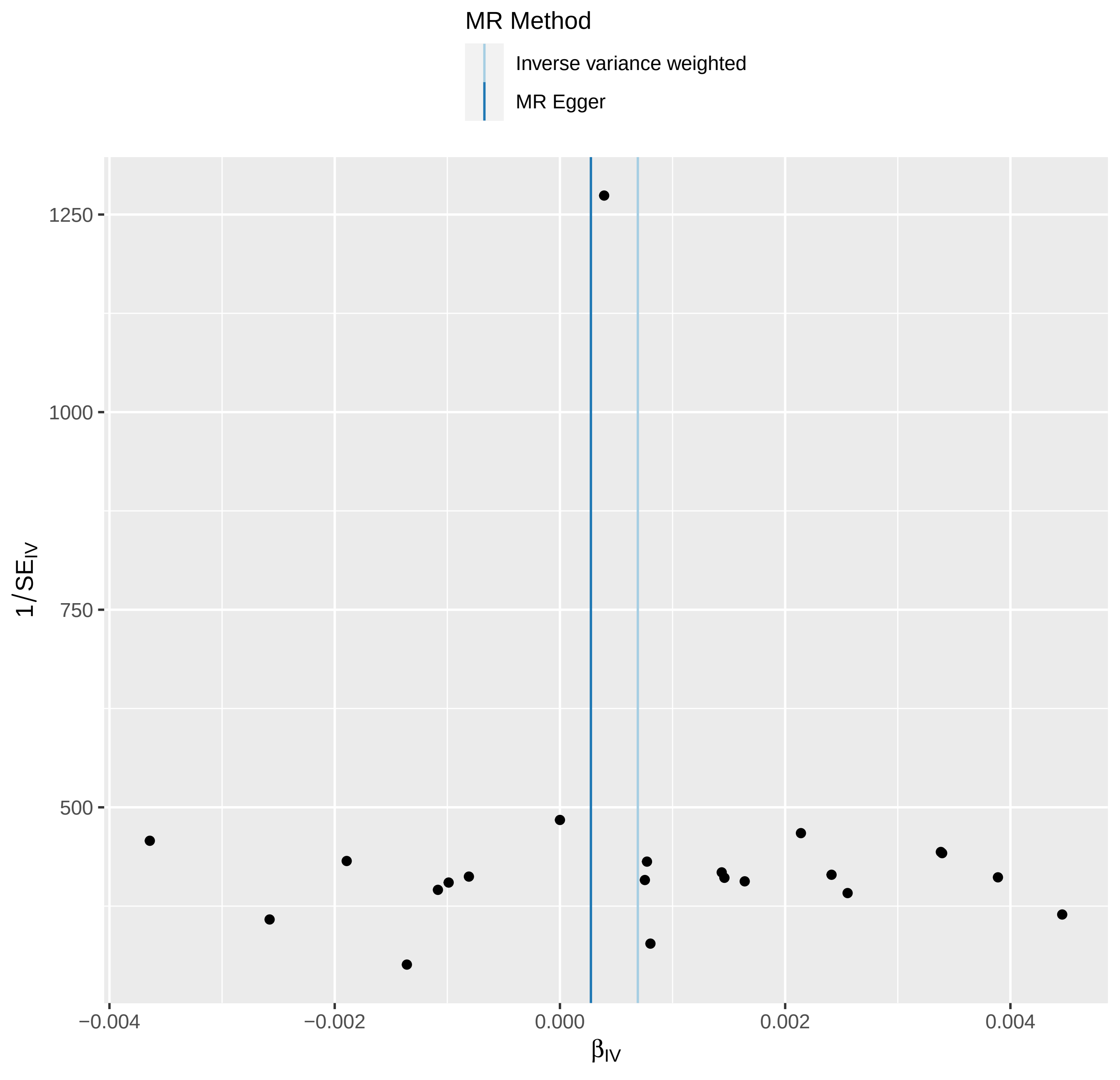

Batch 2−1538 : Interleukin−6 on eGFR

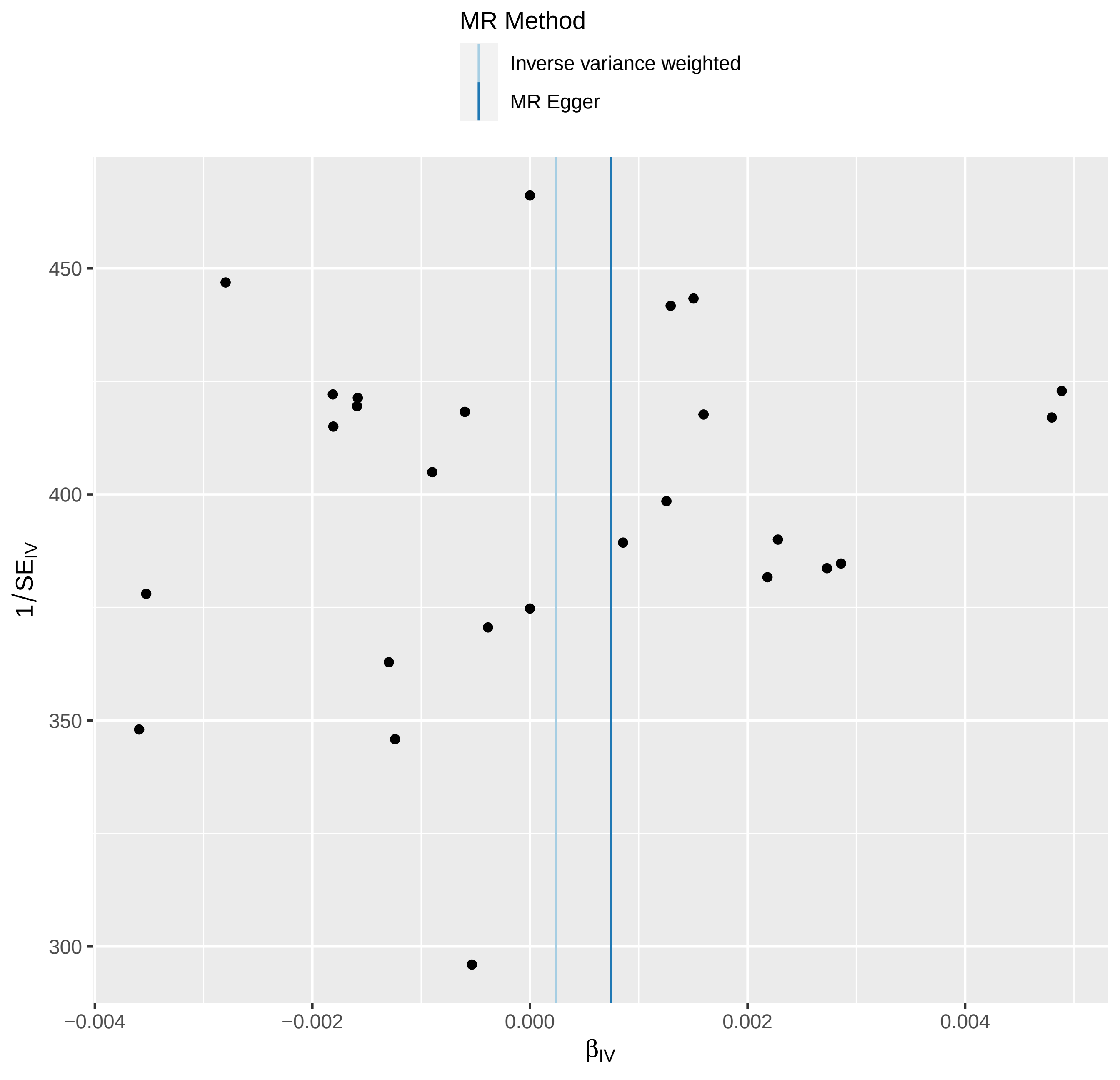

Batch 2−1539 : Interleukin−6 on eGFR

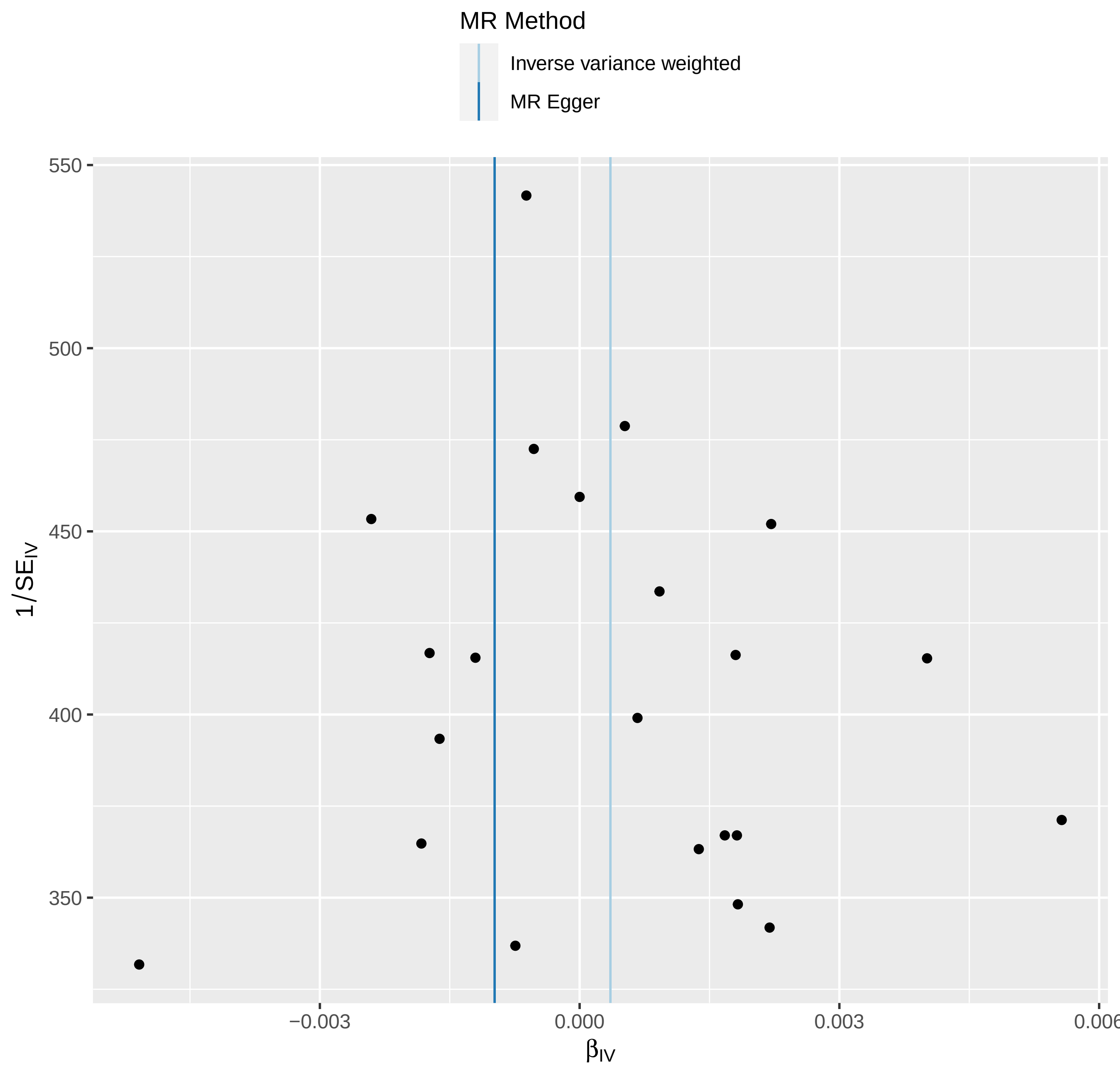

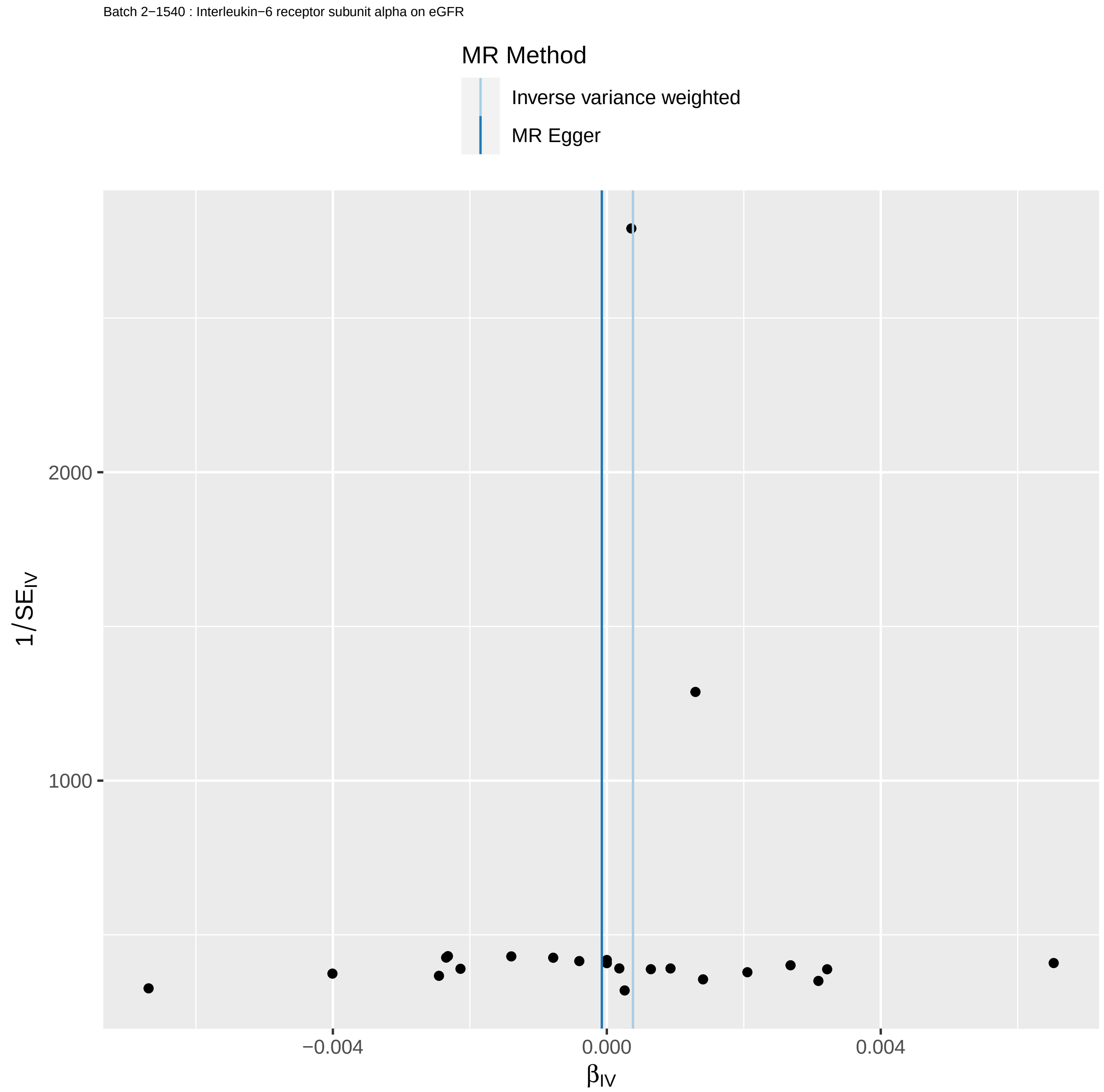
Batch 2−1540 : Interleukin−6 receptor subunit alpha on eGFR
MR Method
Inverse variance weighted
MR Egger
2000
1000
1/SE$_{IV}$
−0.004
0.000
0.004
β$_{IV}$

Batch 2−1541 : Interleukin−6 receptor subunit alpha on eGFR

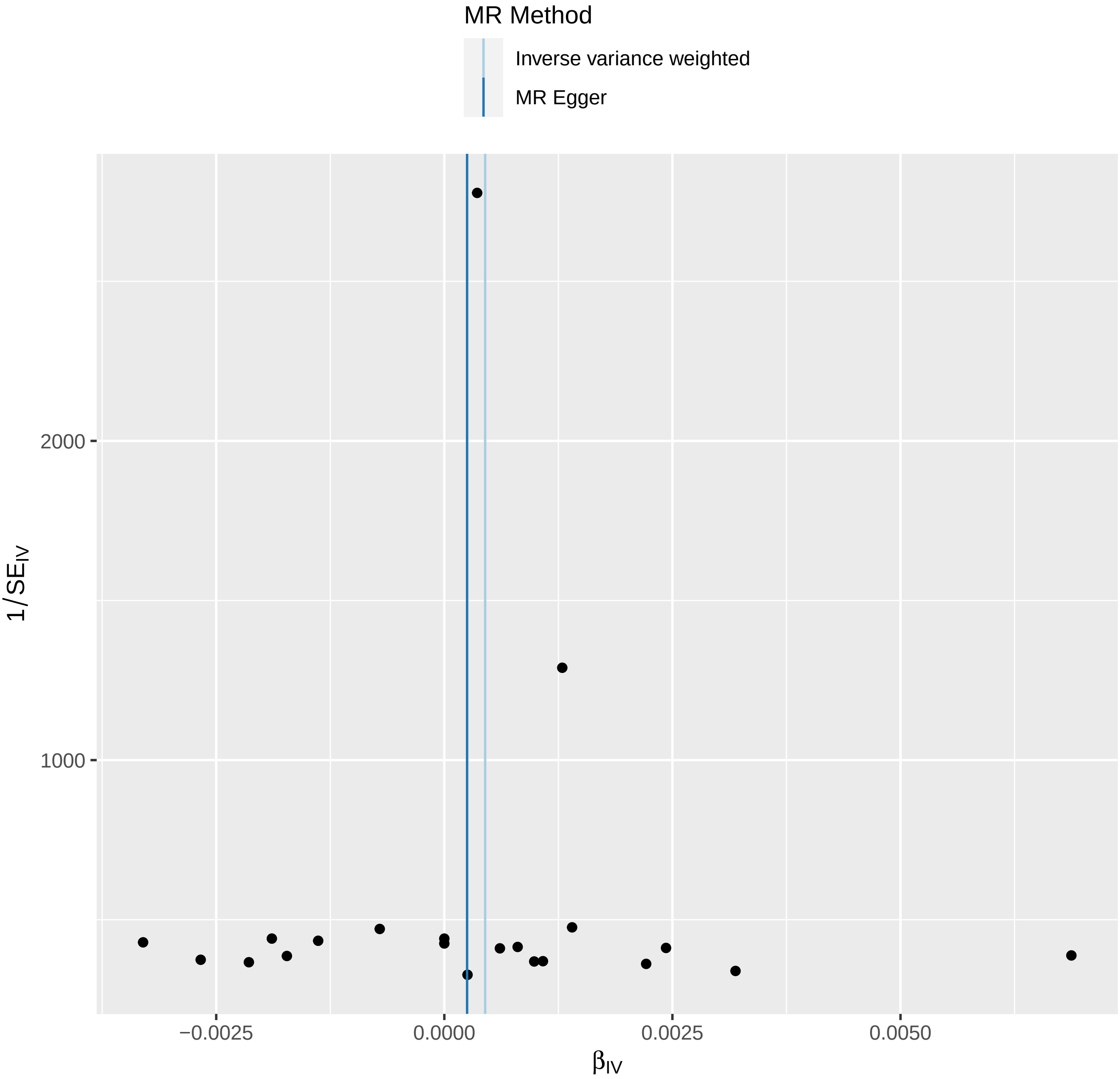

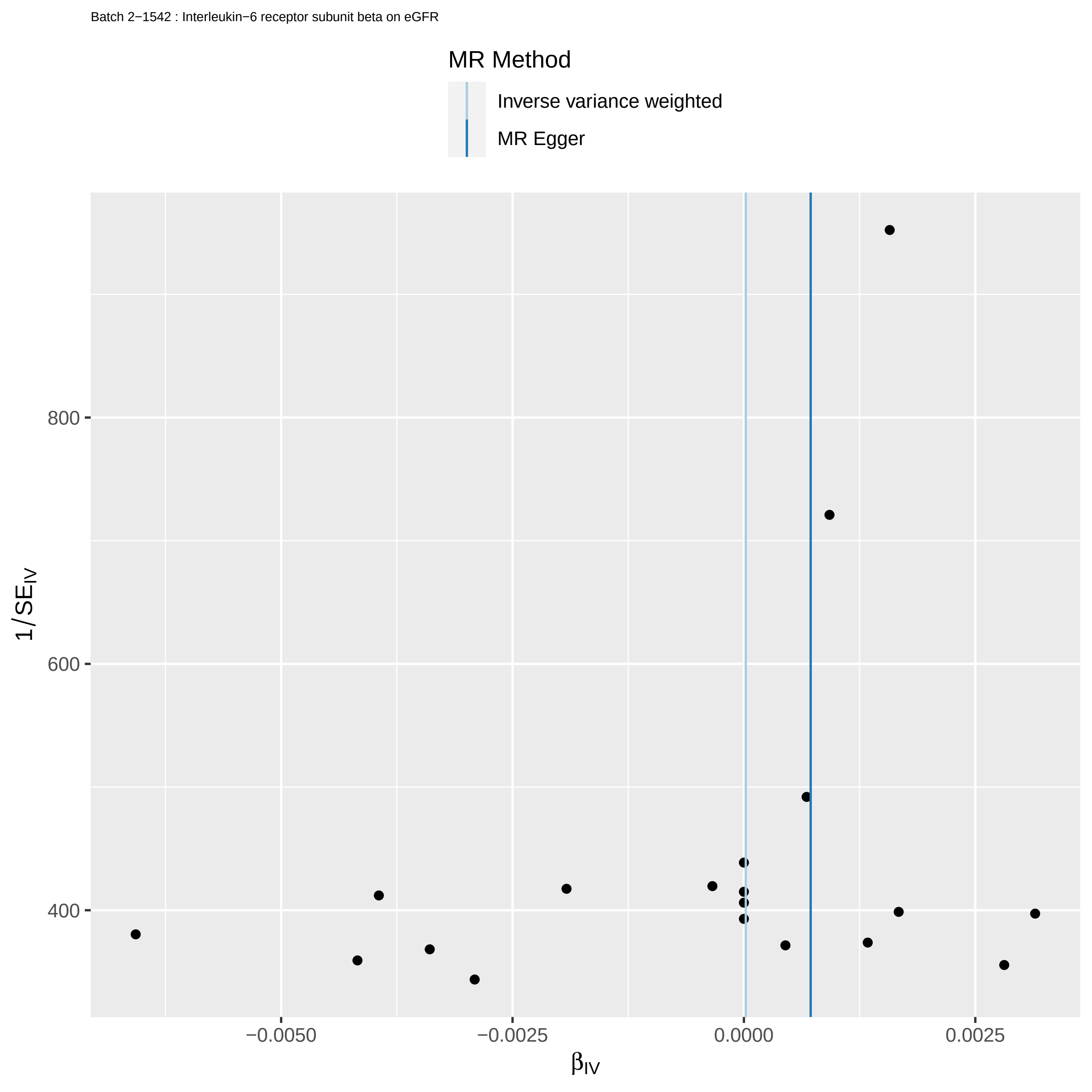

Batch 2−1542 : Interleukin−6 receptor subunit beta on eGFR
MR Method
Inverse variance weighted
MR Egger
800
600
400
1/SE$_{IV}$
−0.0050
−0.0025
0.0000
0.0025
β$_{IV}$

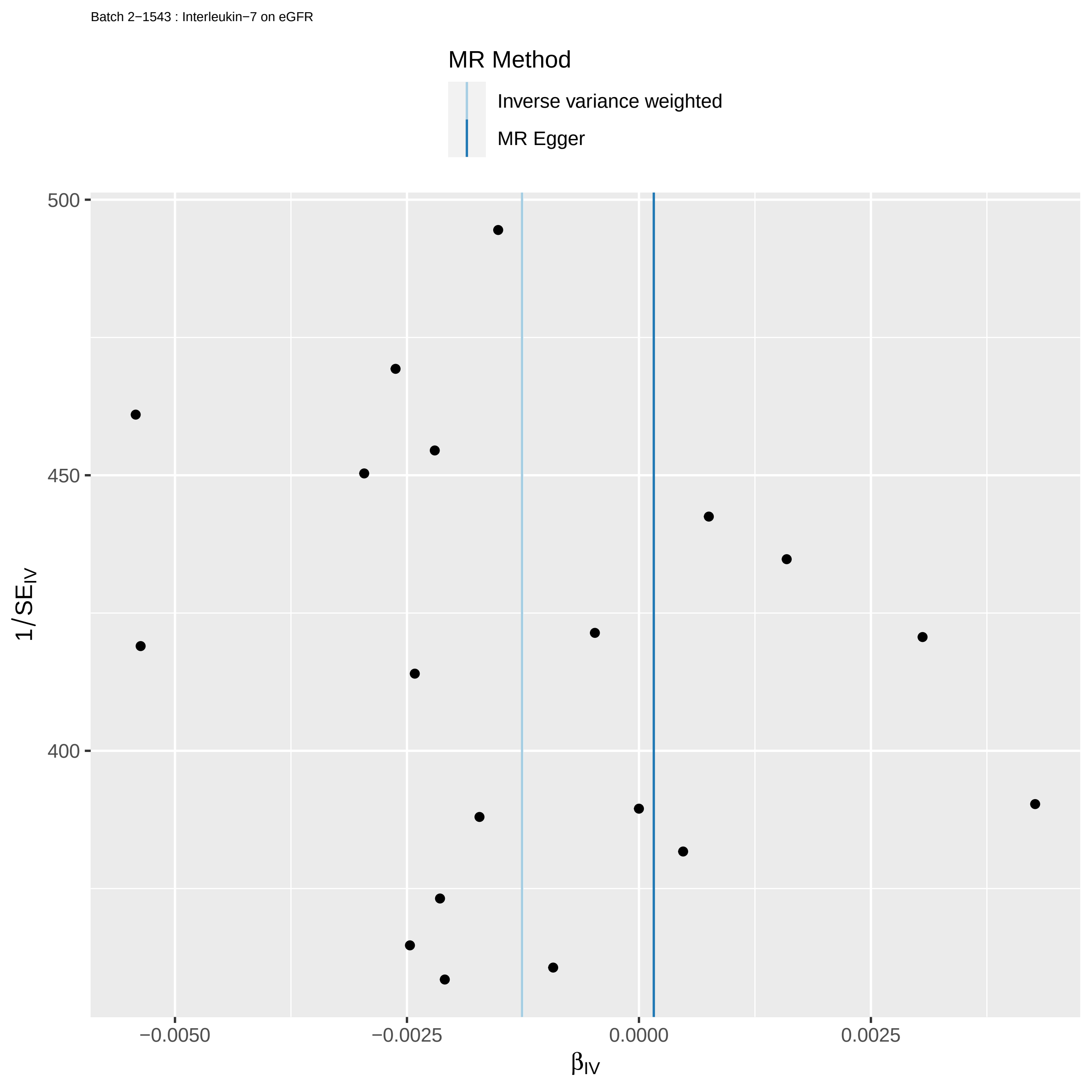

Batch 2−1543 : Interleukin−7 on eGFR
MR Method
Inverse variance weighted
MR Egger
500
450
400
1/SE$_{IV}$
−0.0050
−0.0025
0.0000
0.0025
β$_{IV}$

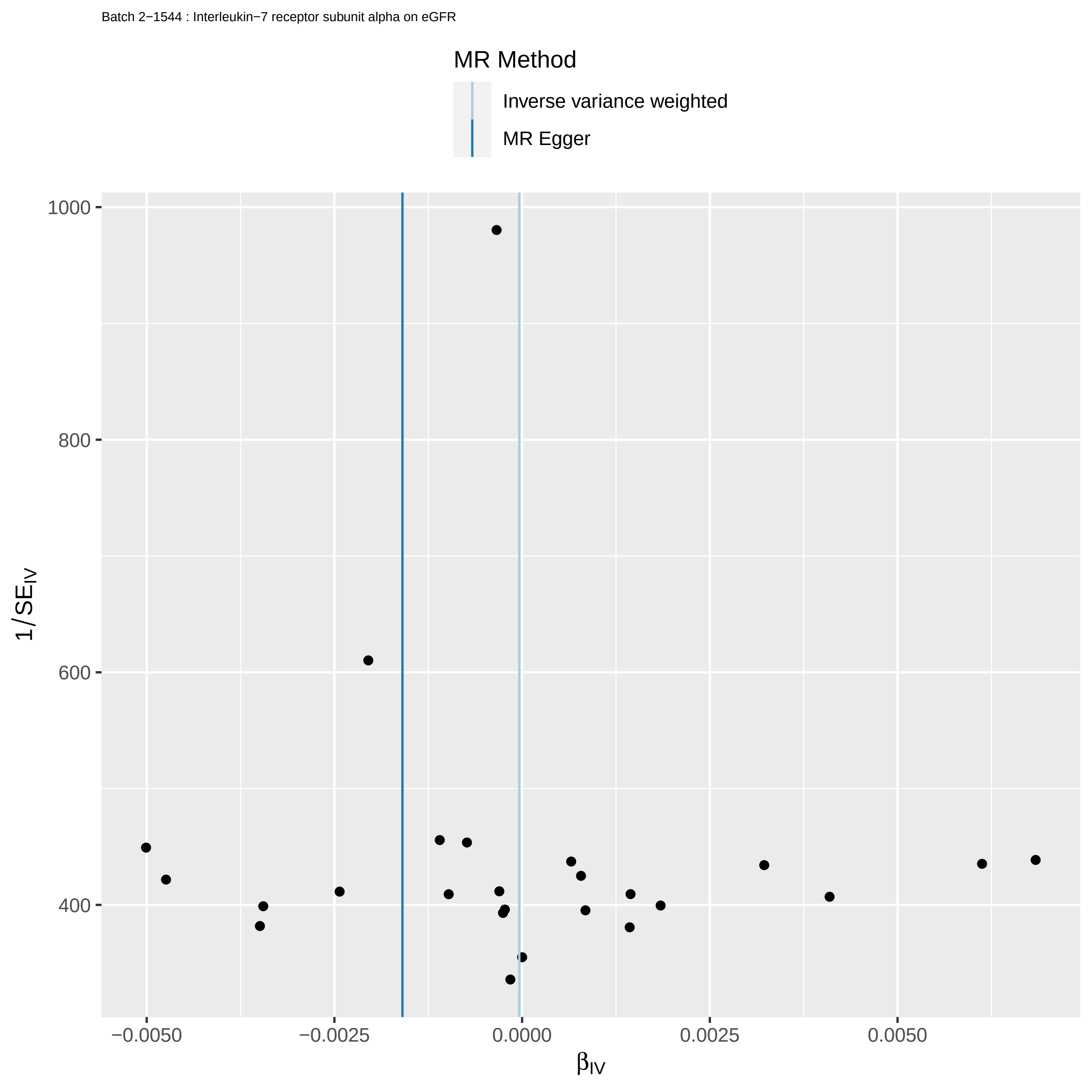

Batch 2−1544 : Interleukin−7 receptor subunit alpha on eGFR
MR Method
Inverse variance weighted
MR Egger
1000
800
600
400
1/SE$_{IV}$
−0.0050
−0.0025
0.0000
0.0025
0.0050
β_{IV}

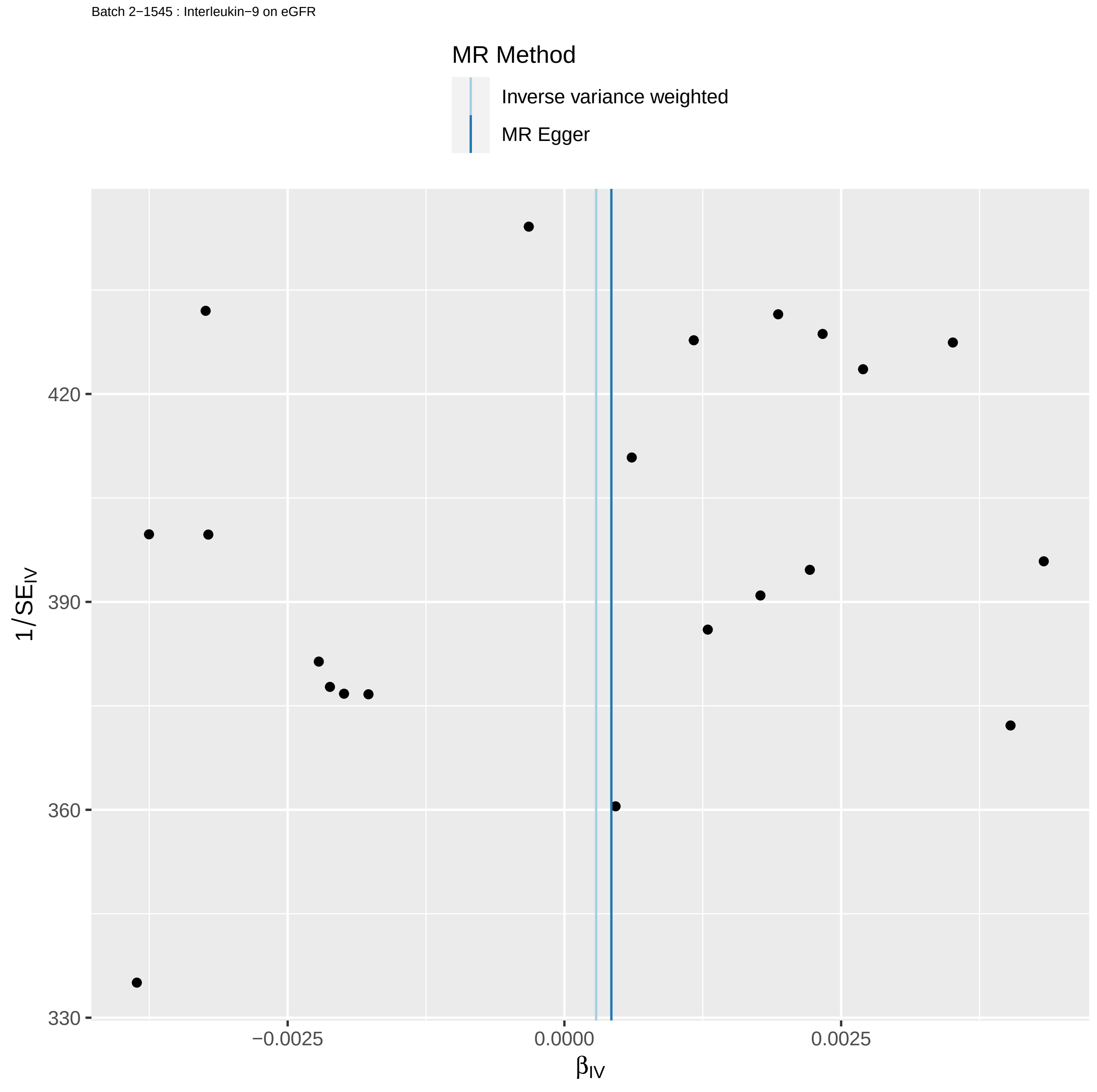

Batch 2−1545 : Interleukin−9 on eGFR
MR Method
Inverse variance weighted
MR Egger
420
390
360
330
1/SE$_{IV}$
−0.0025
0.0000
0.0025
β$_{IV}$

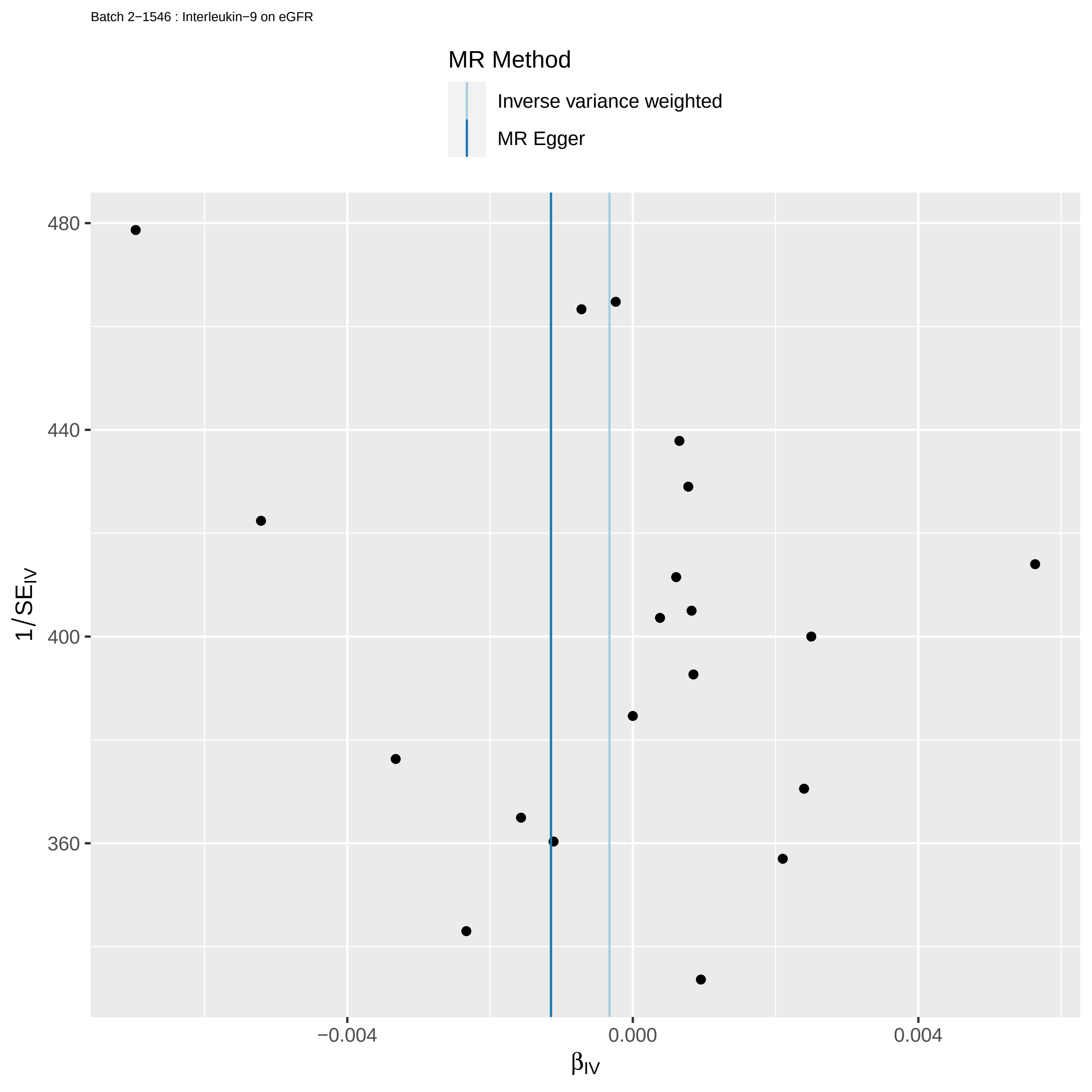

Batch 2−1546 : Interleukin−9 on eGFR
MR Method
Inverse variance weighted
MR Egger
480
440
400
360
1/SE_IV
−0.004
0.000
0.004
β_IV

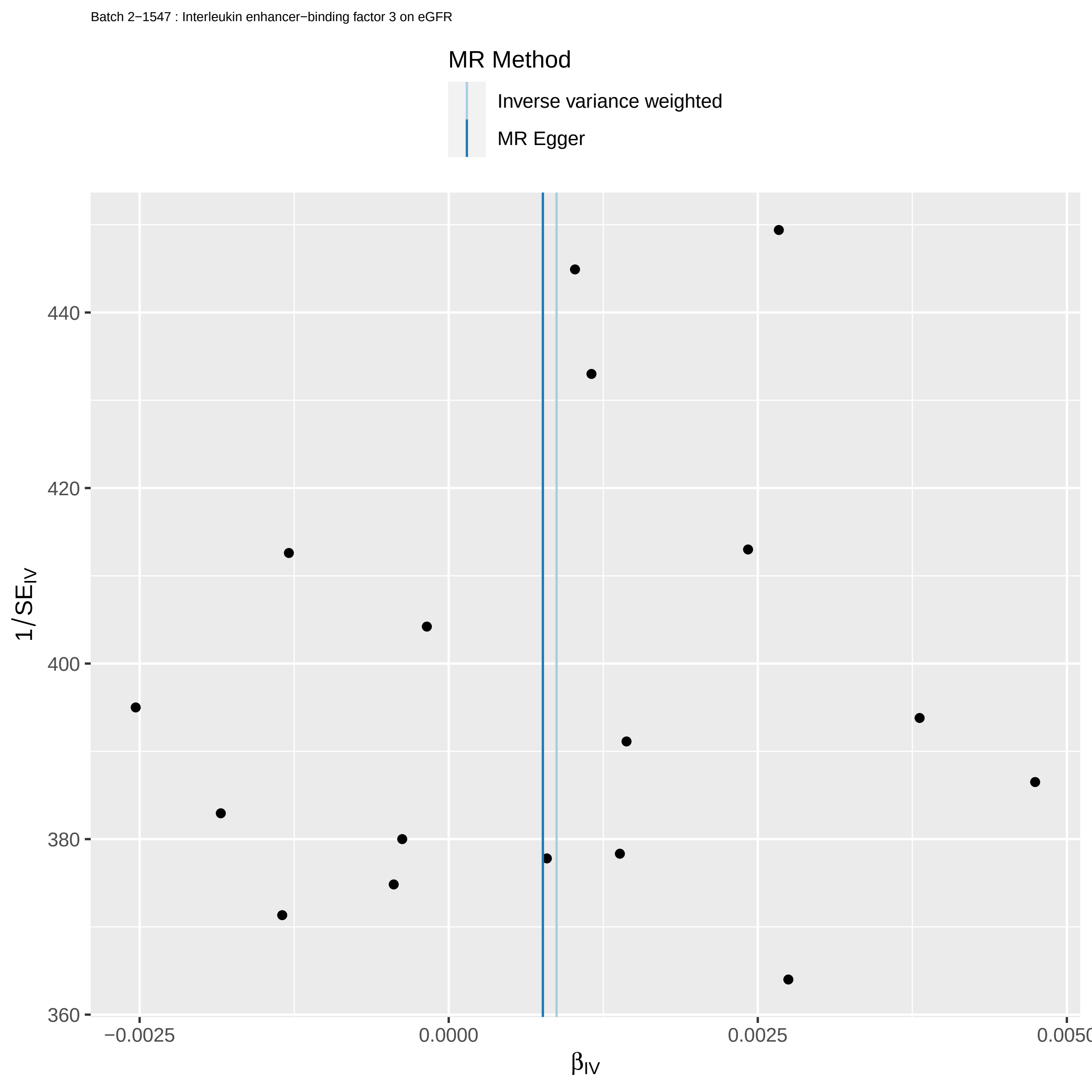
Batch 2−1547 : Interleukin enhancer−binding factor 3 on eGFR
MR Method
Inverse variance weighted
MR Egger
440
420
400
380
360
1/SE_IV
−0.0025
0.0000
0.0025
0.0050
β_IV

Batch 2−1548 : Inositol monophosphatase 3 on eGFR

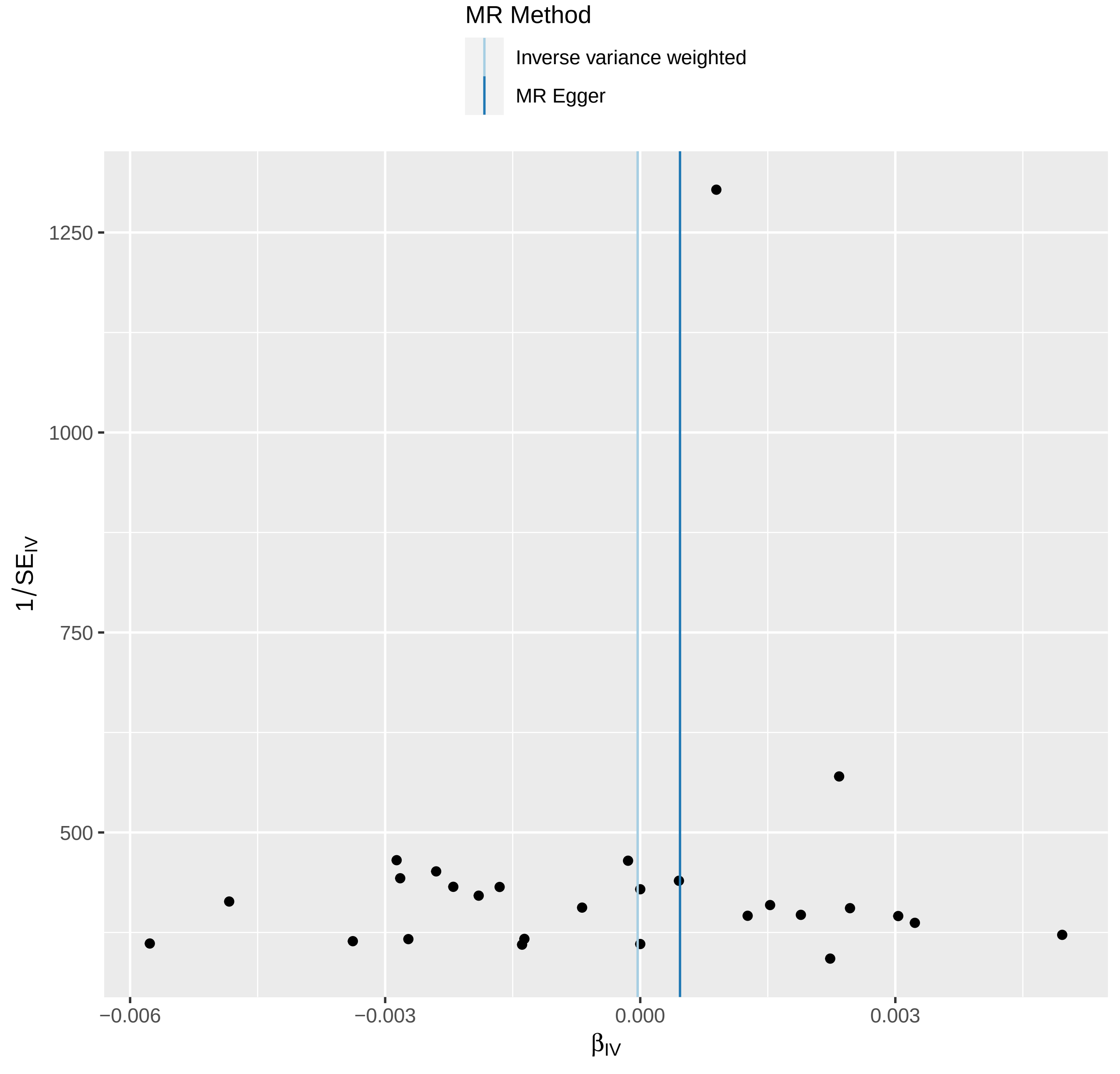

Batch 2−1549 : Inosine−5'−monophosphate dehydrogenase 1 on eGFR

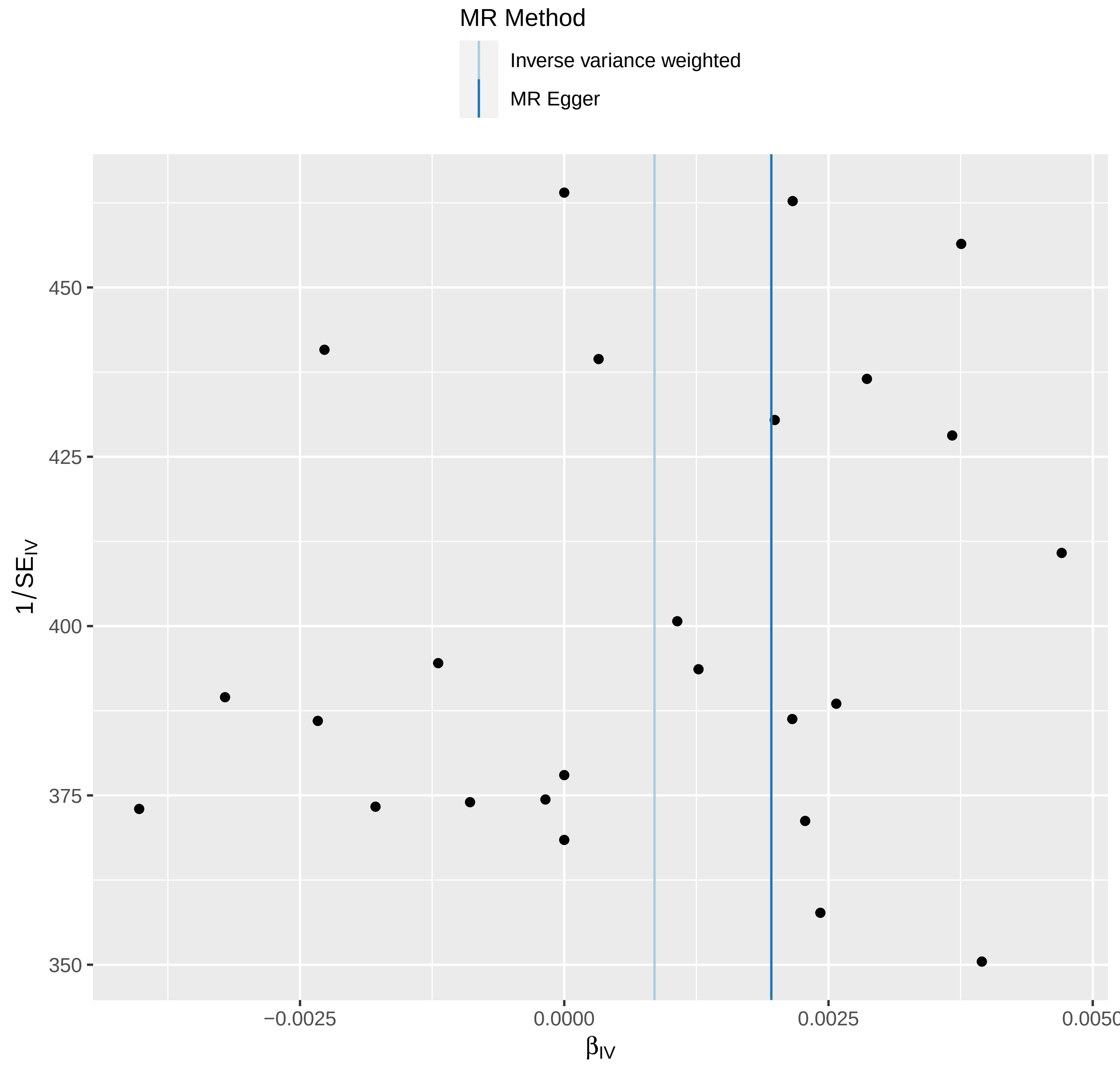

Batch 2−1550 : Inosine−5'−monophosphate dehydrogenase 2 on eGFR

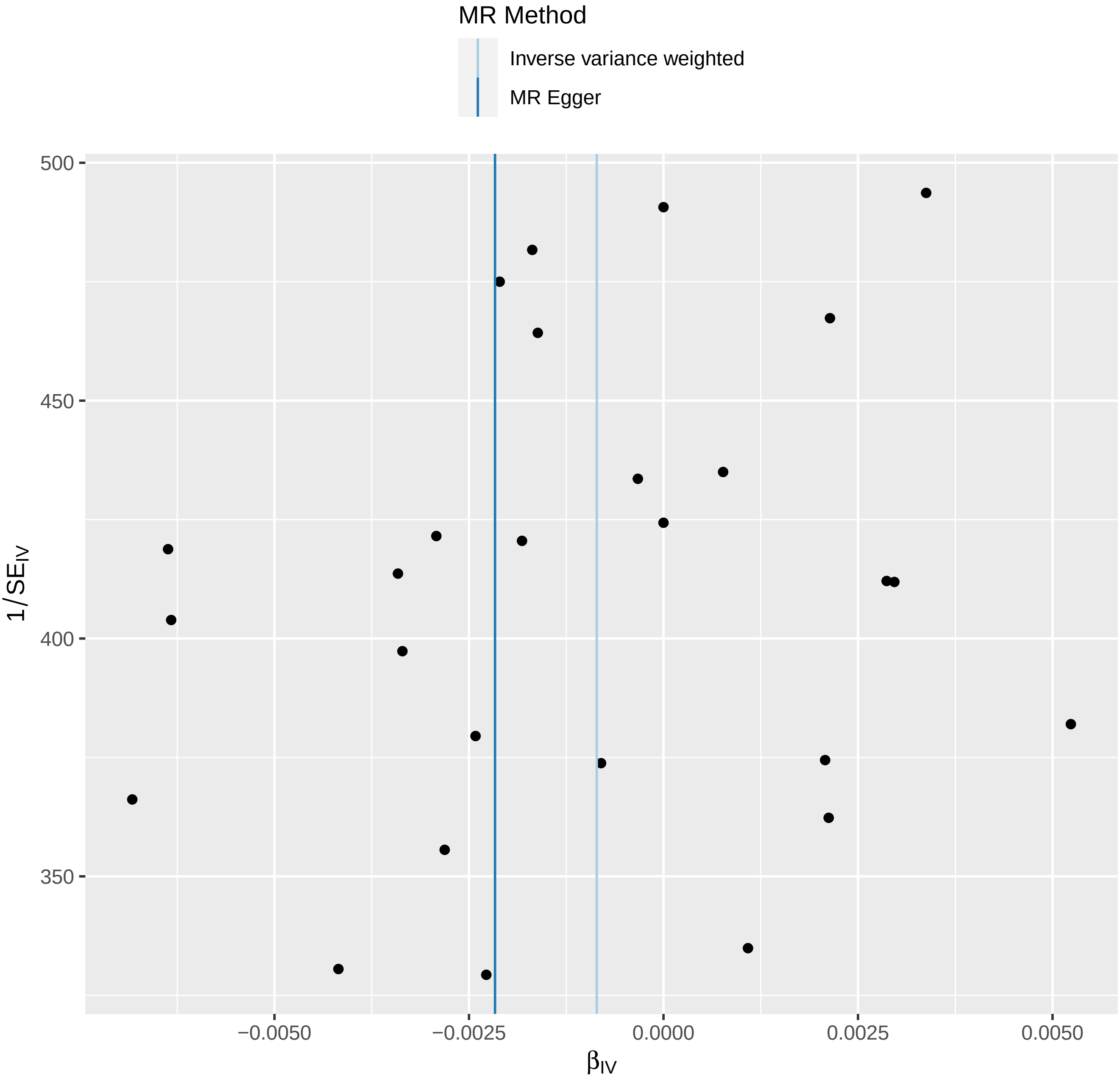

Batch 2−1551 : Alpha−internexin on eGFR

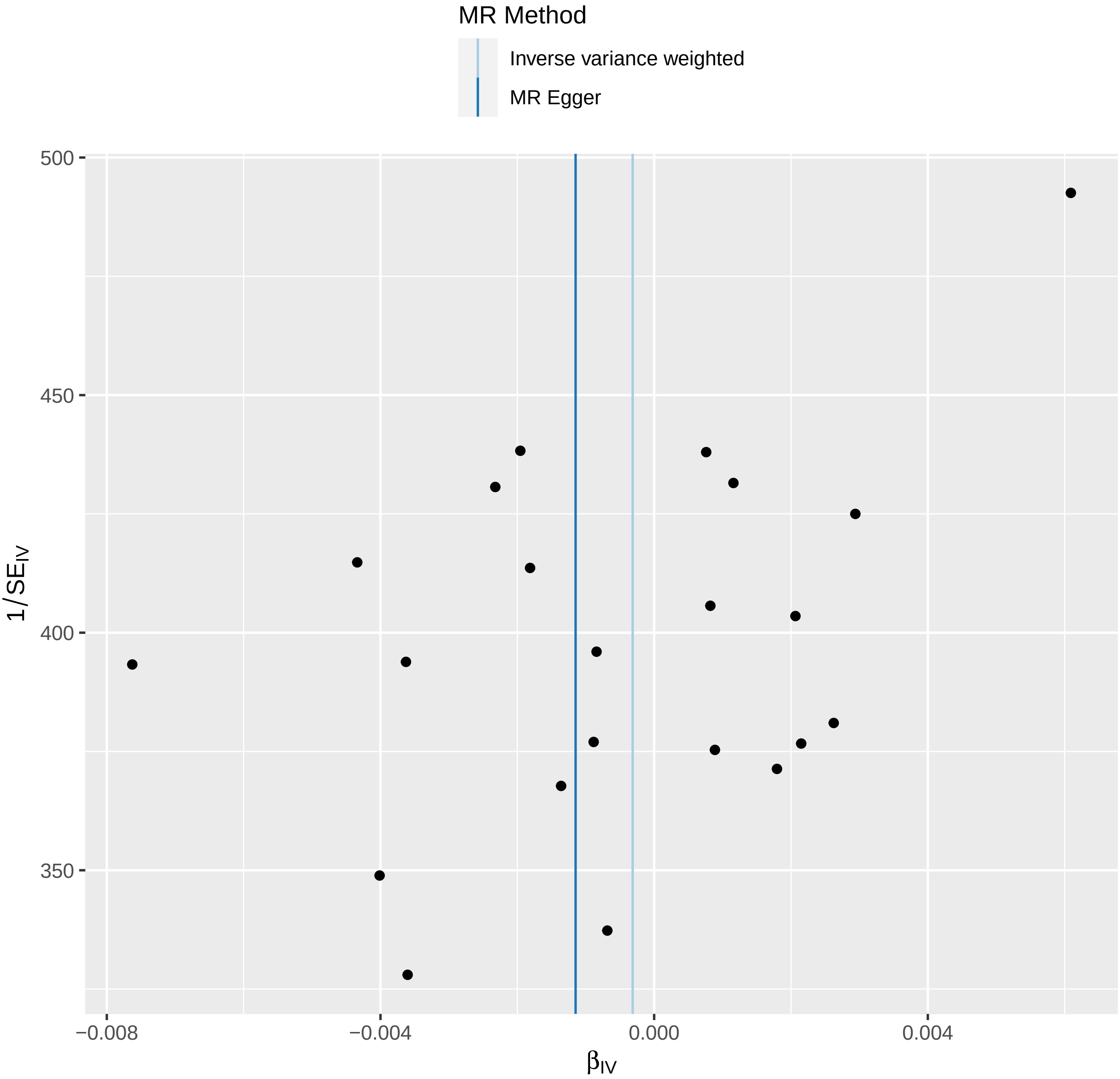

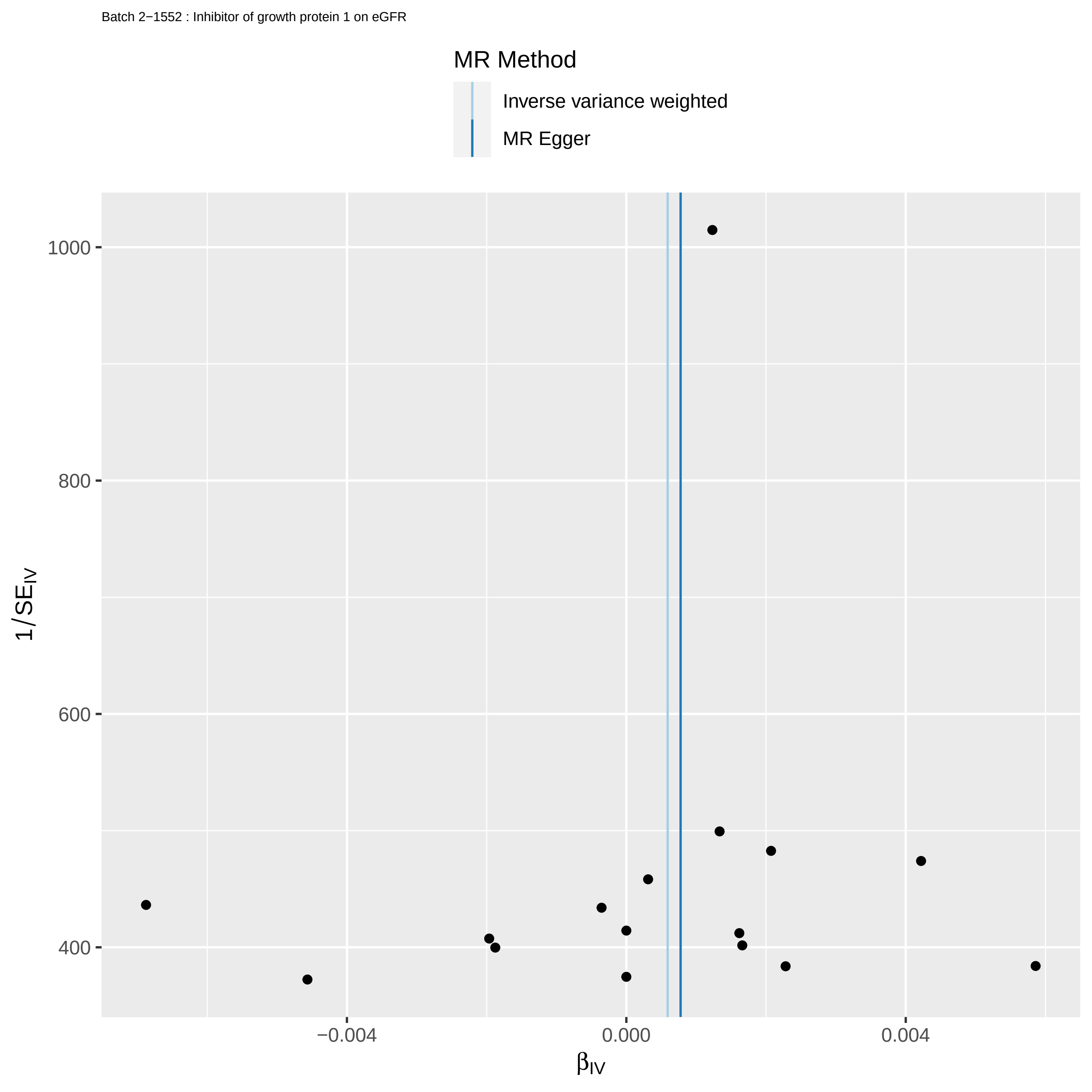
Batch 2−1552 : Inhibitor of growth protein 1 on eGFR
MR Method
Inverse variance weighted
MR Egger
1000
800
600
400
1/SE_IV
−0.004
0.000
0.004
β_IV

Batch 2−1553 : Inhibin beta A chain on eGFR

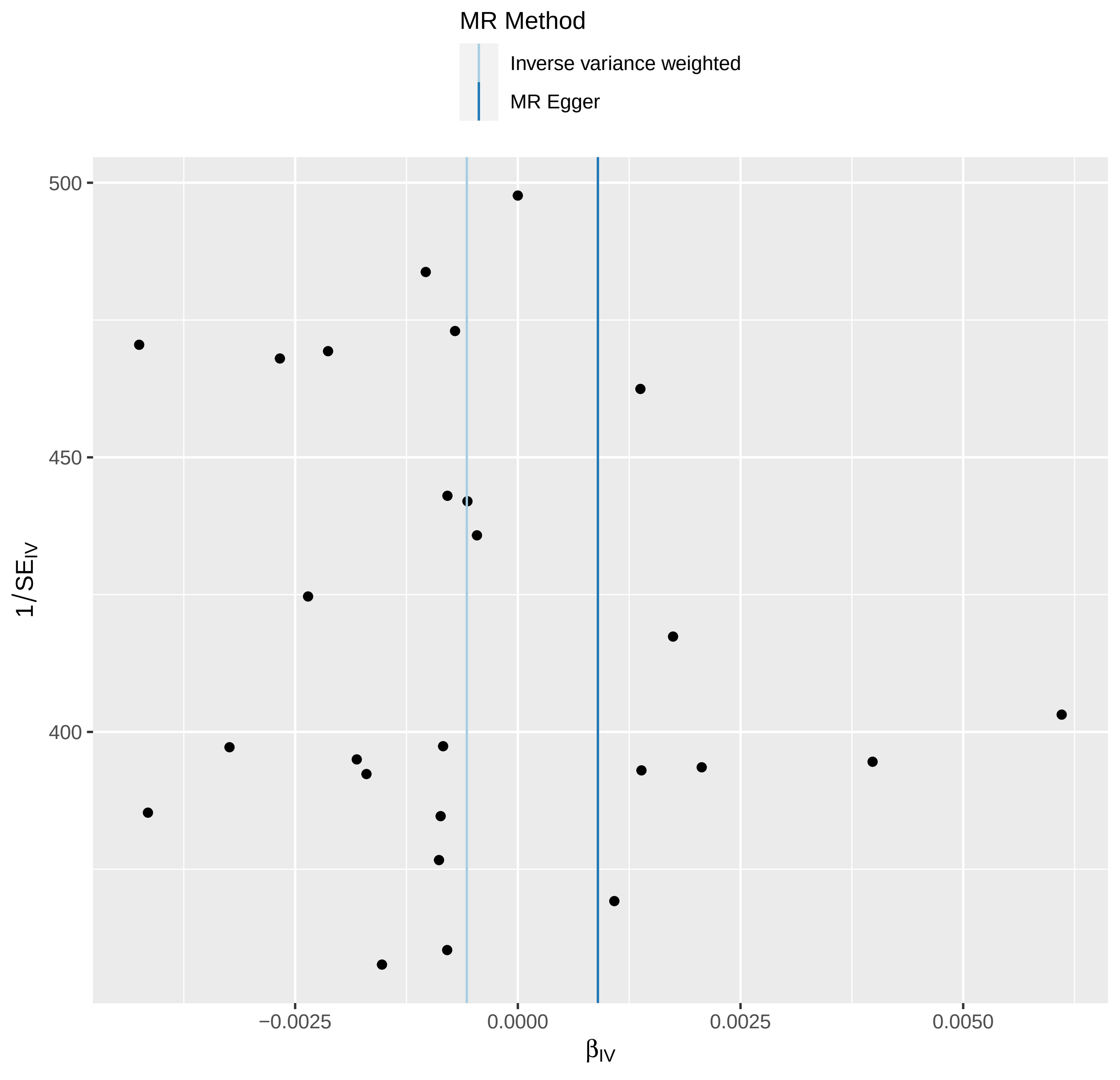

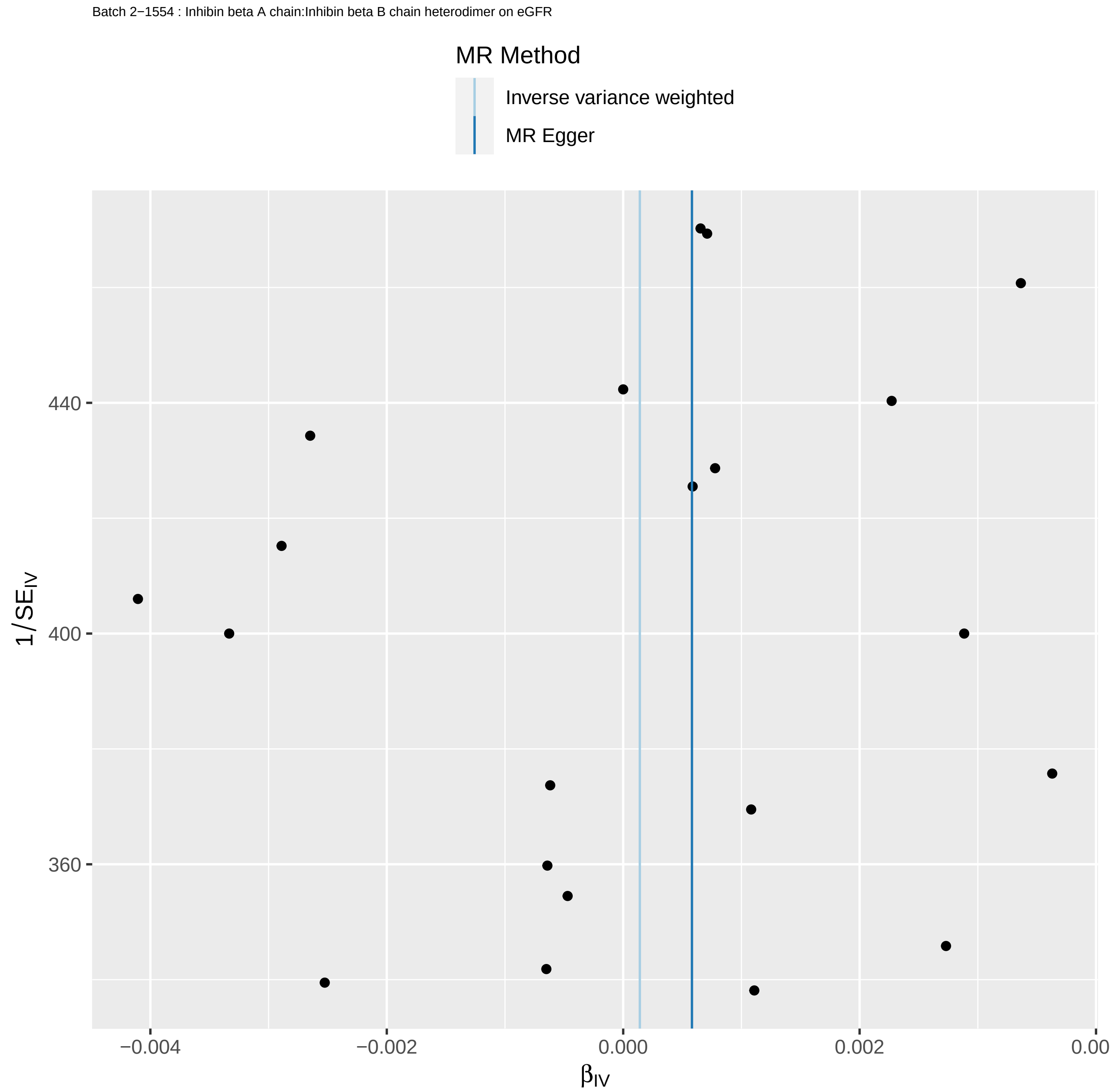

Batch 2−1554 : Inhibin beta A chain:Inhibin beta B chain heterodimer on eGFR
MR Method
Inverse variance weighted
MR Egger
1/SE_IV
440
400
360
−0.004
−0.002
0.000
0.002
0.00
β_IV

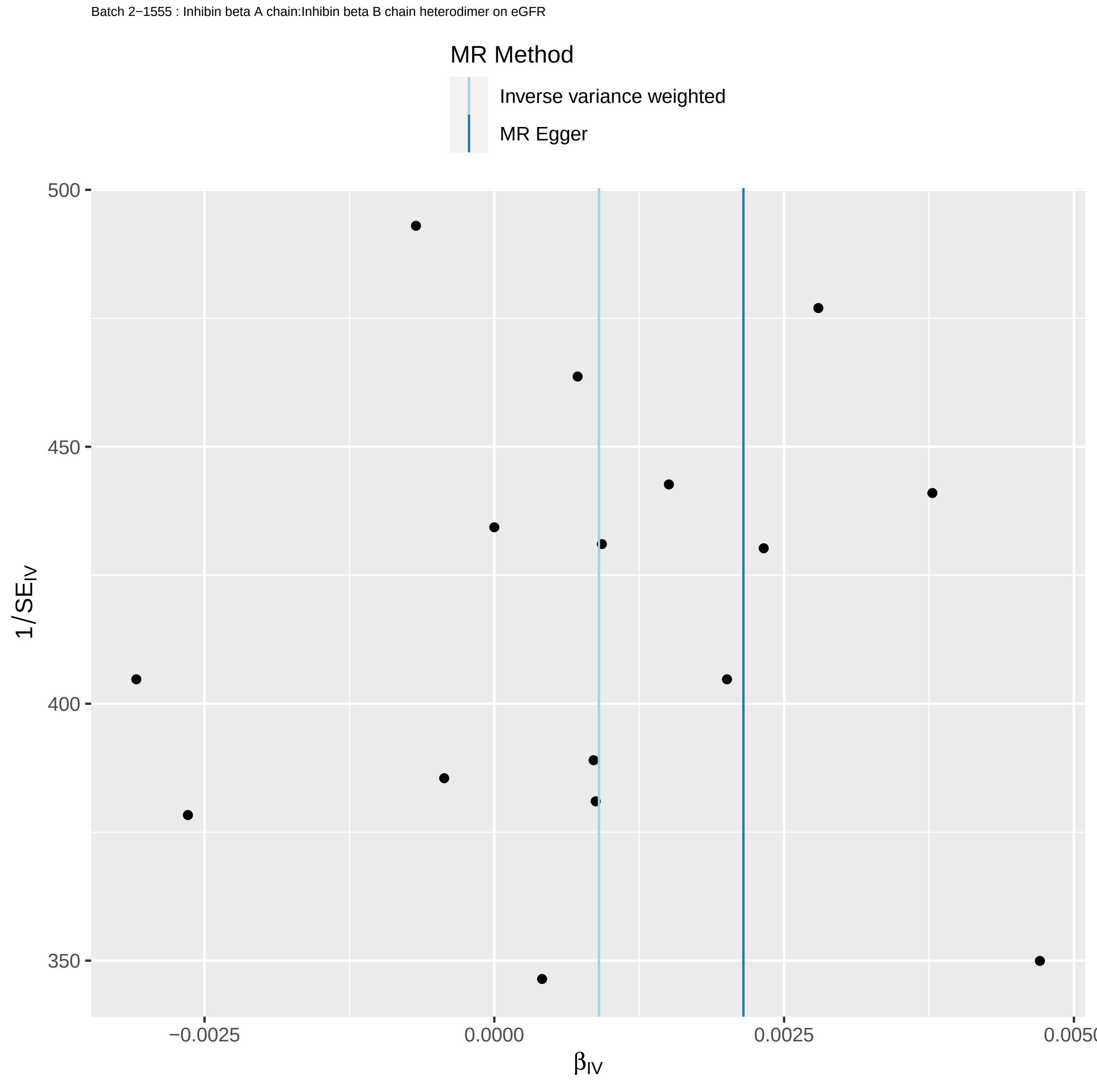

Batch 2−1555 : Inhibin beta A chain:Inhibin beta B chain heterodimer on eGFR
MR Method
Inverse variance weighted
MR Egger
500
450
400
350
1/SE_IV
−0.0025
0.0000
0.0025
0.0050
β_IV

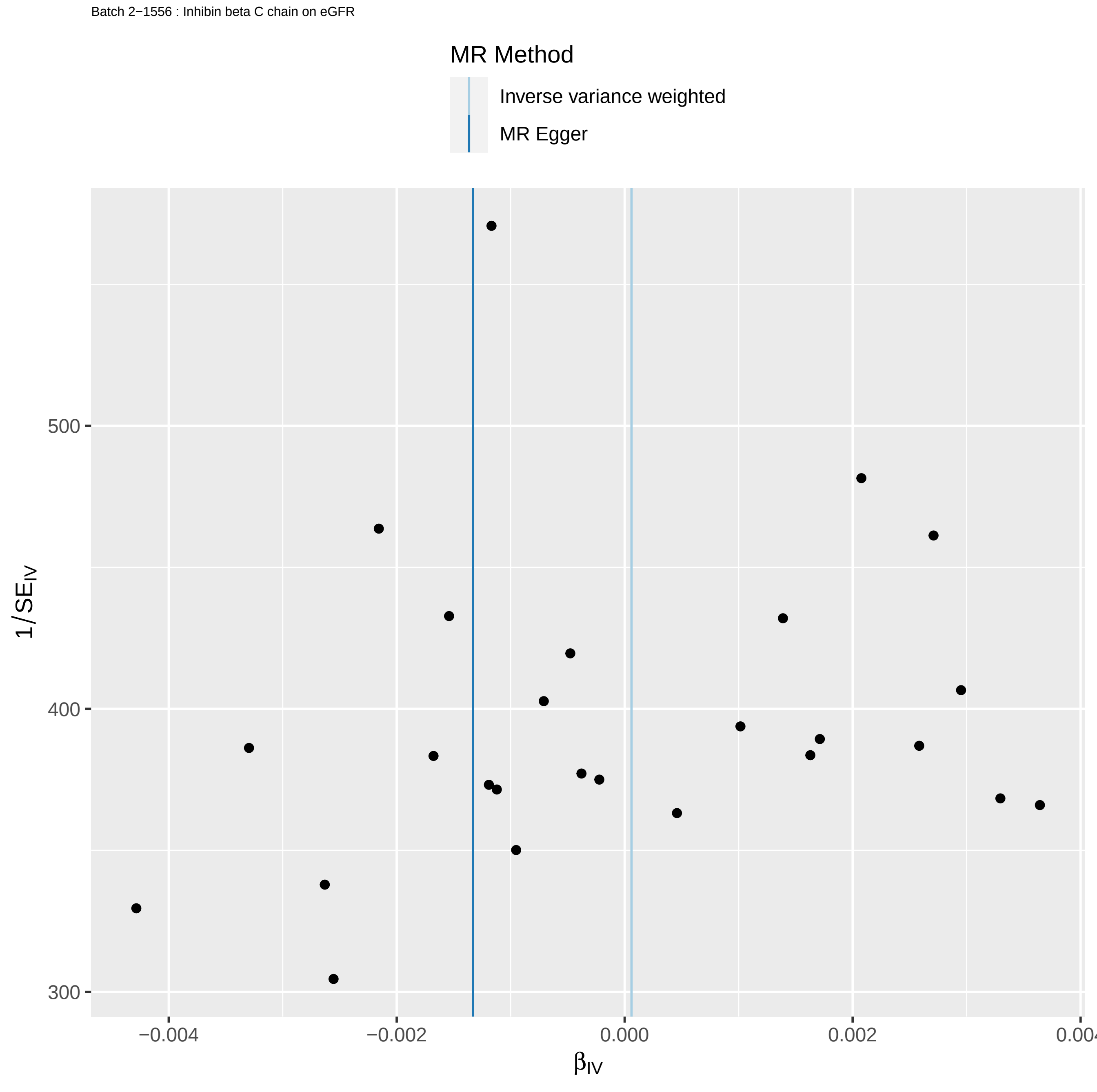

Batch 2−1556 : Inhibin beta C chain on eGFR
MR Method
Inverse variance weighted
MR Egger
500
400
300
1/SE$_{IV}$
−0.004
−0.002
0.000
0.002
β$_{IV}$

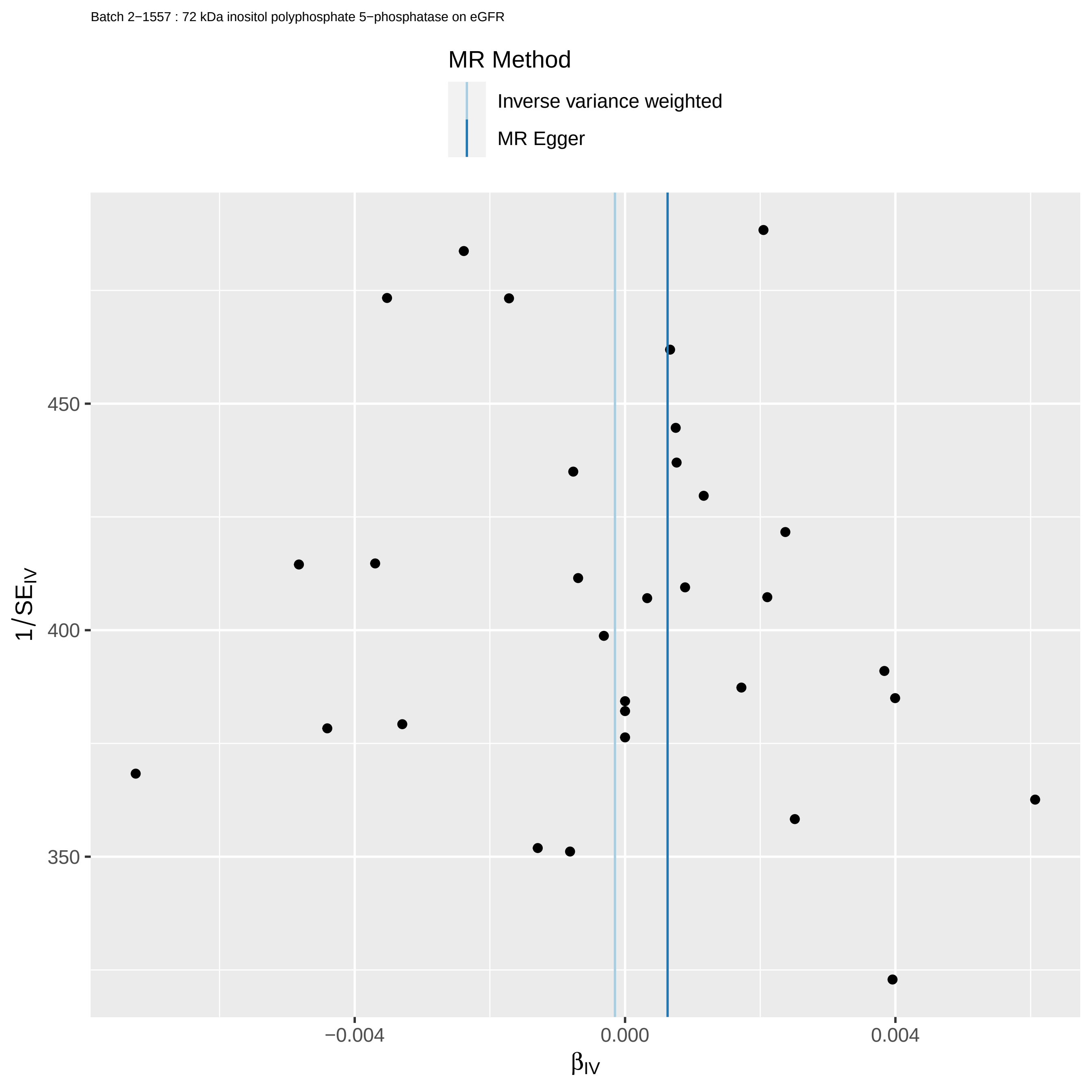

Batch 2−1557 : 72 kDa inositol polyphosphate 5−phosphatase on eGFR
MR Method
Inverse variance weighted
MR Egger
1/SE_IV
450
400
350
−0.004
0.000
0.004
β_IV

Batch 2−1558 : Insulin on eGFR

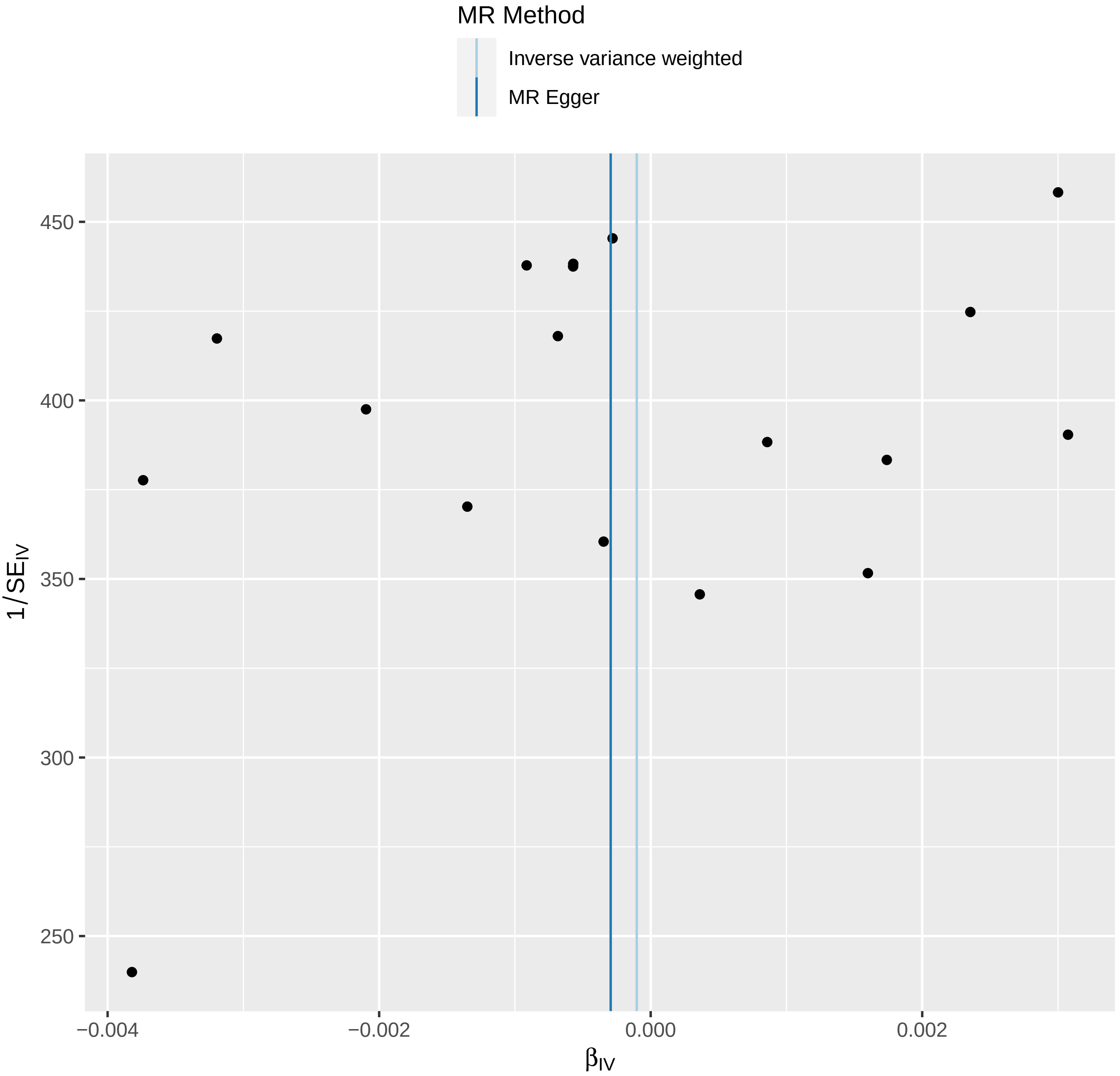

Batch 2−1559 : Insulin−induced gene 1 protein on eGFR

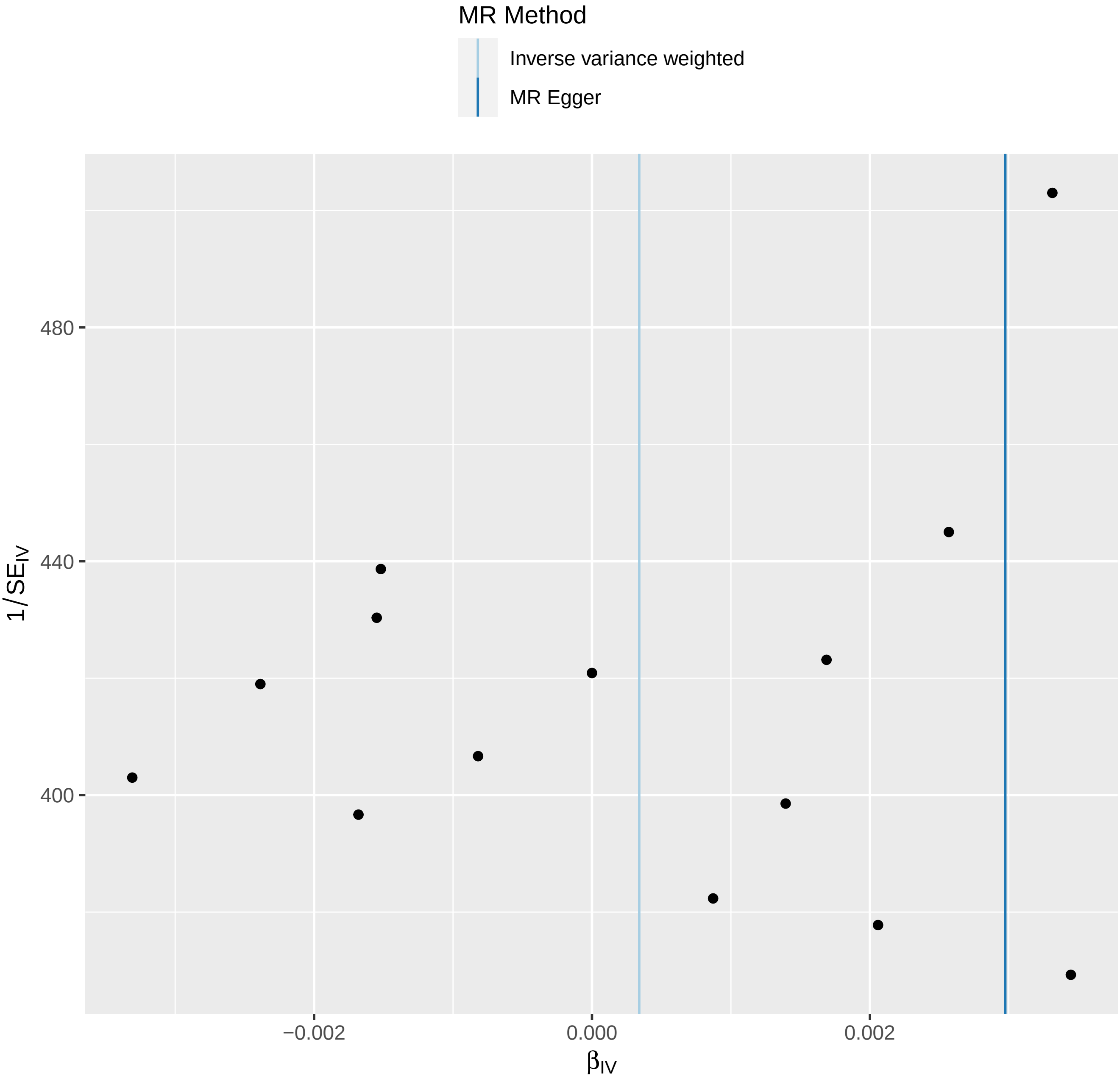

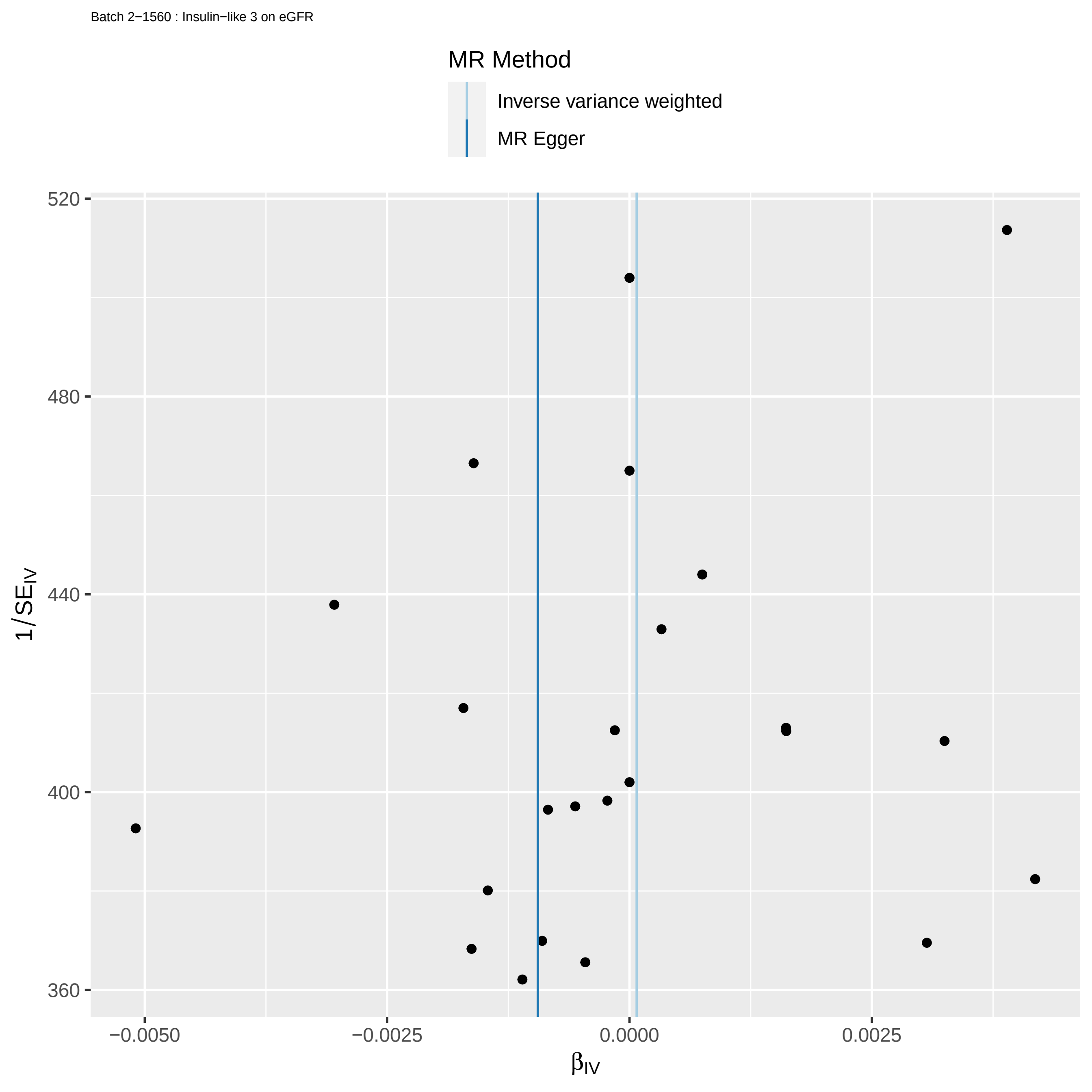

Batch 2−1560 : Insulin−like 3 on eGFR
MR Method
Inverse variance weighted
MR Egger
520
480
440
400
360
1/SE_IV
−0.0050
−0.0025
0.0000
0.0025
β_IV

Batch 2−1561 : Early placenta insulin−like peptide on eGFR

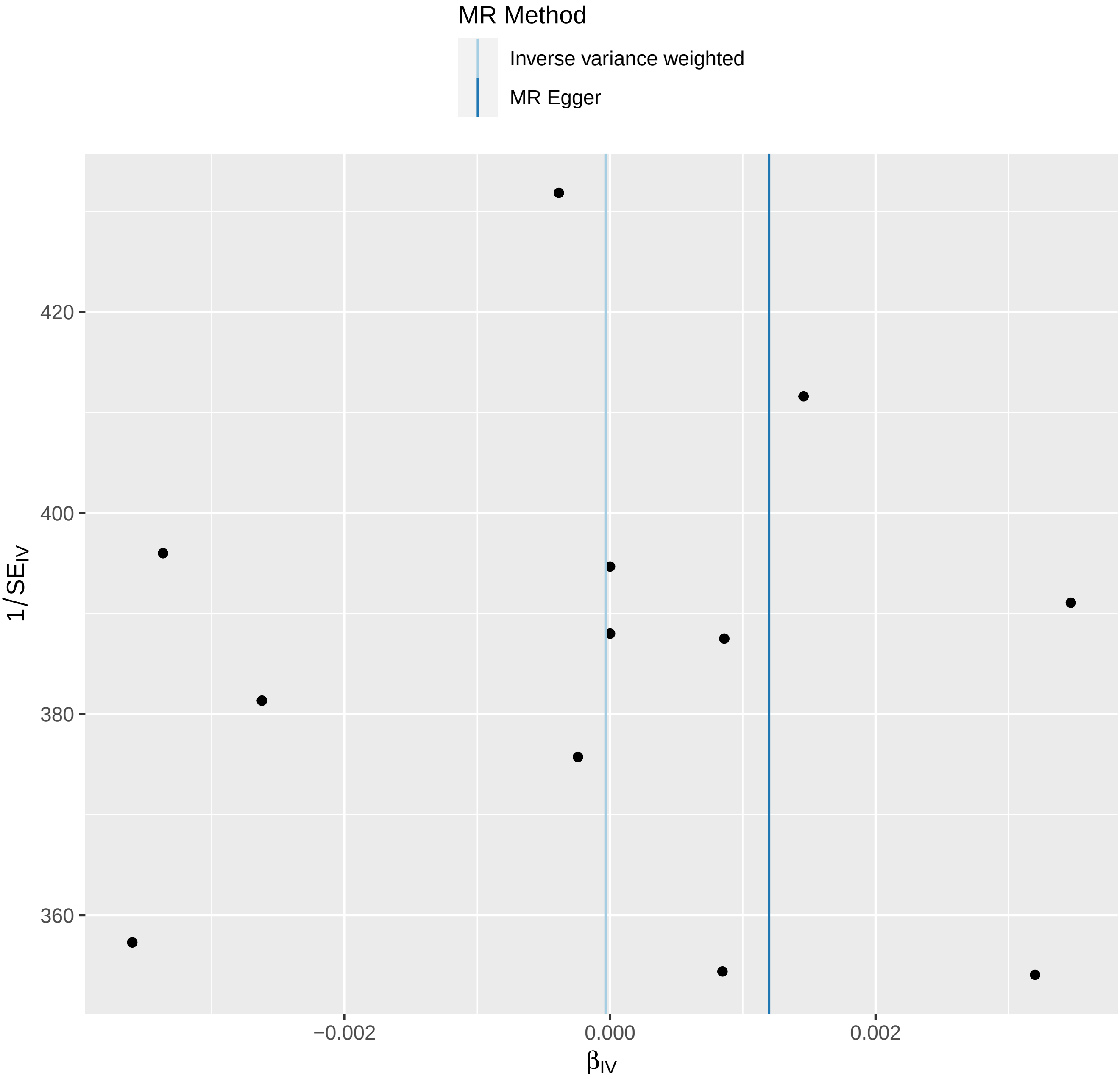

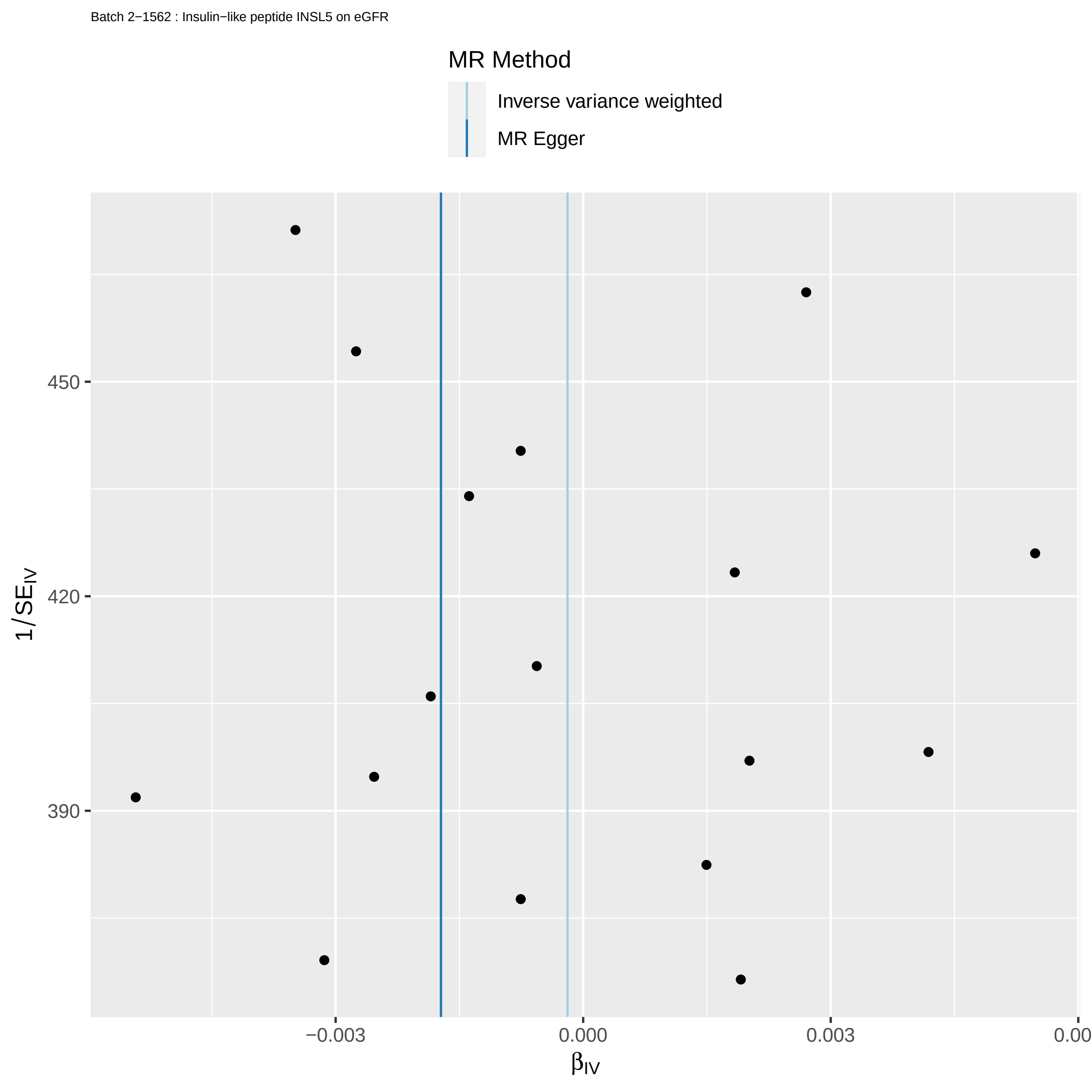
Batch 2−1562 : Insulin−like peptide INSL5 on eGFR
MR Method
Inverse variance weighted
MR Egger
1/SE_IV
450
420
390
−0.003
0.000
0.003
0.00
β_IV

Batch 2−1563 : Insulin−like peptide INSL6 on eGFR

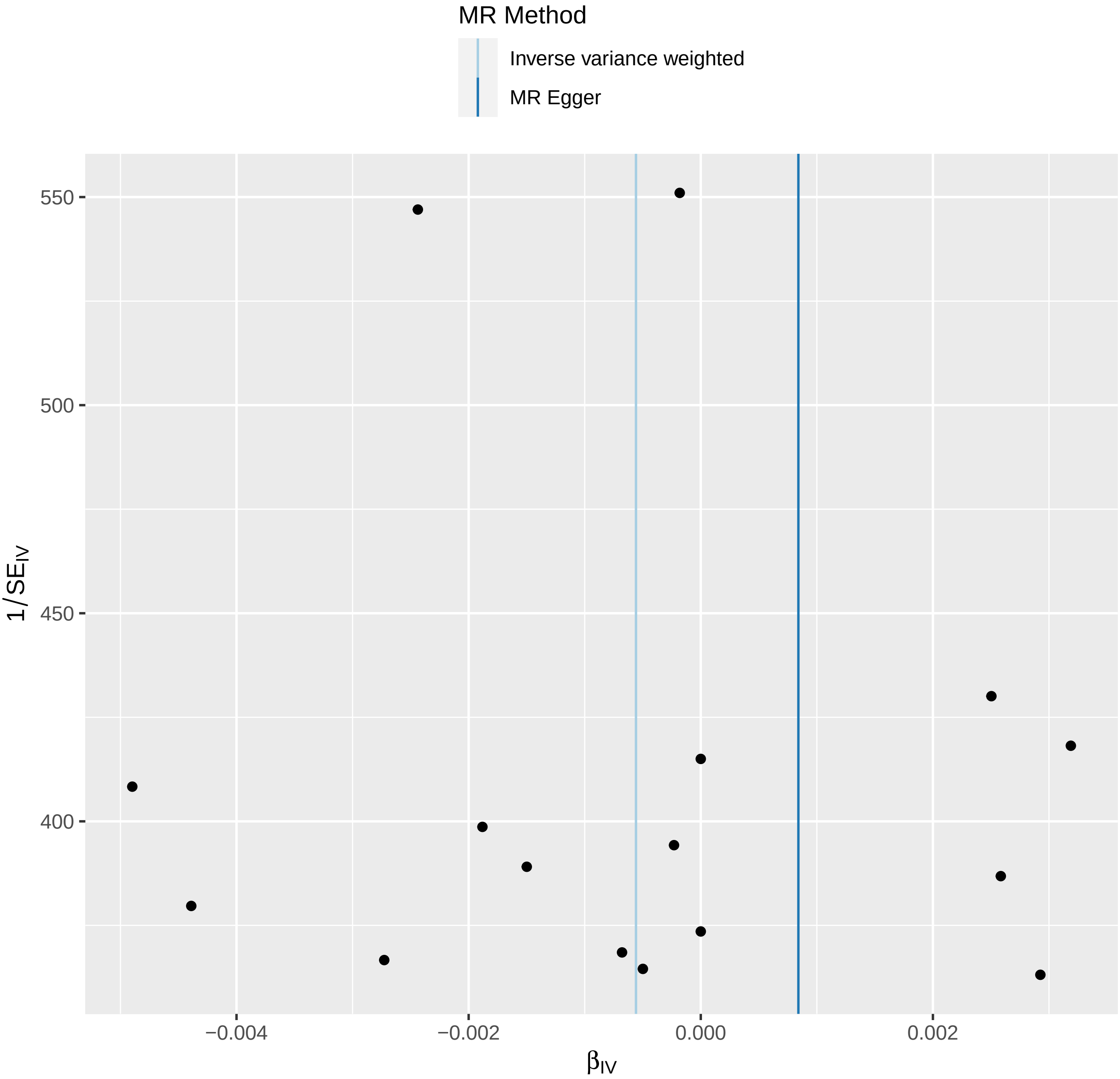

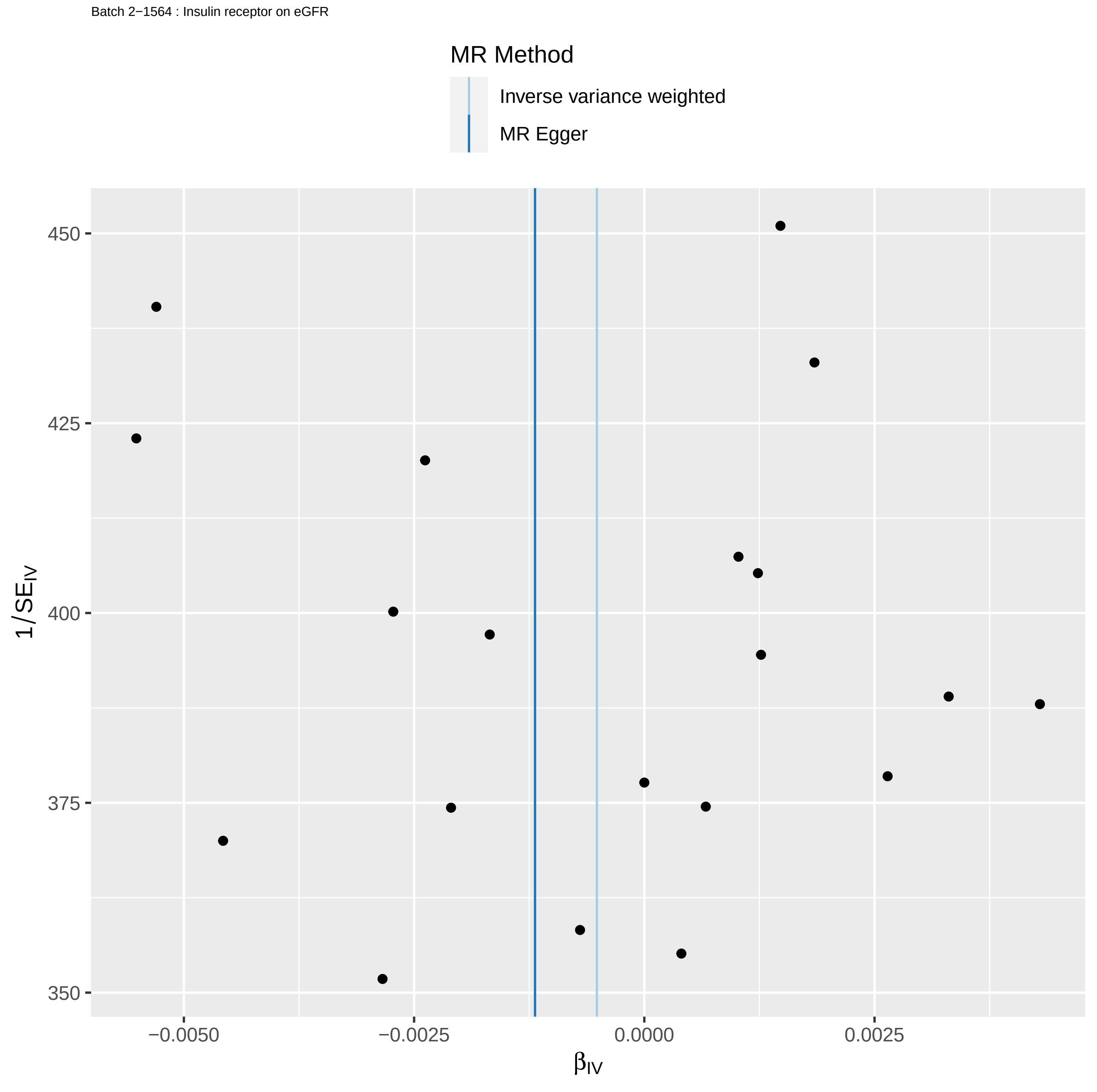

Batch 2−1564 : Insulin receptor on eGFR
MR Method
Inverse variance weighted
MR Egger
1/SE$_{IV}$
450
425
400
375
350
−0.0050
−0.0025
0.0000
0.0025
β_{IV}

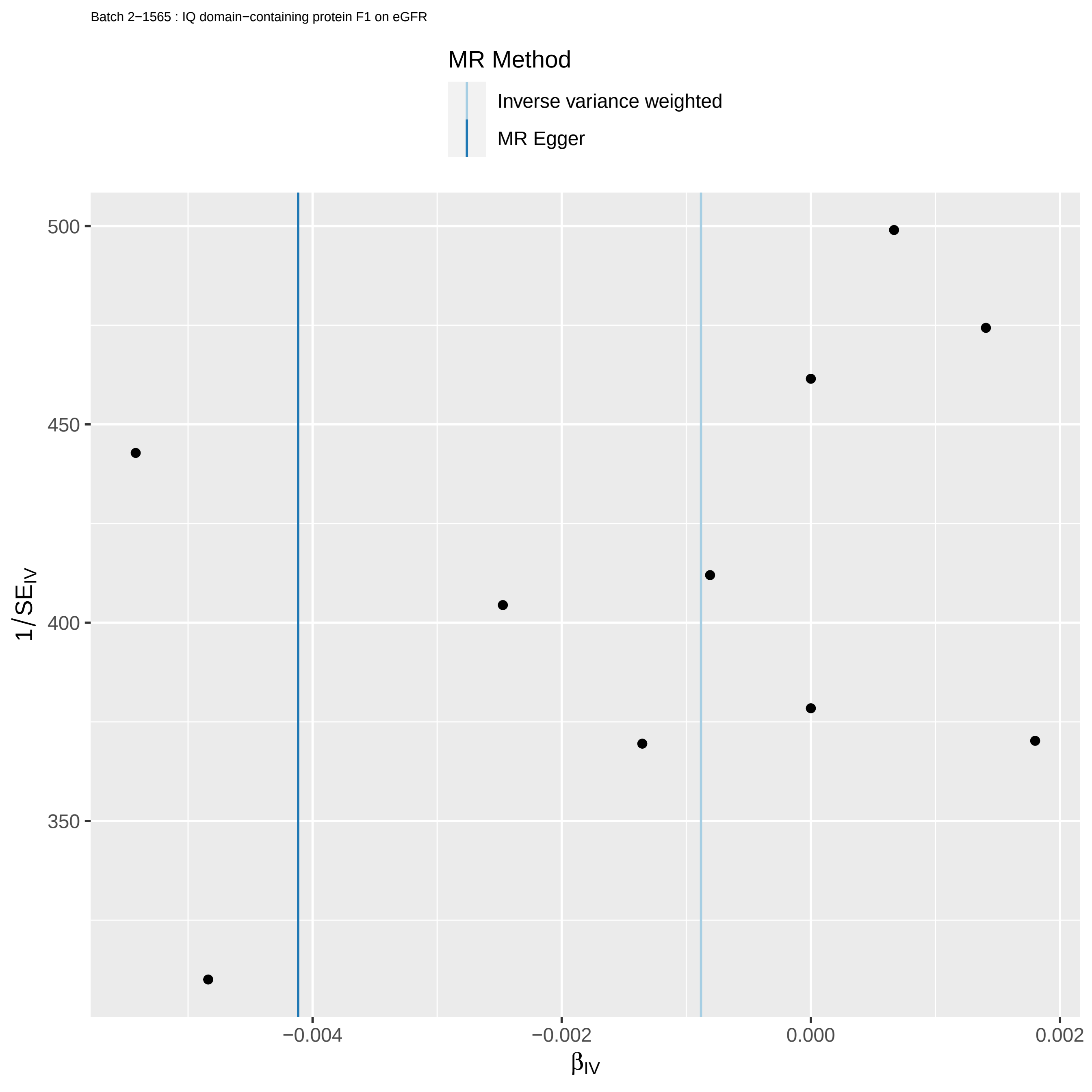

Batch 2−1565 : IQ domain−containing protein F1 on eGFR
MR Method
Inverse variance weighted
MR Egger
500
450
400
350
$1/SE_{IV}$
−0.004
−0.002
0.000
0.002
β_{IV}

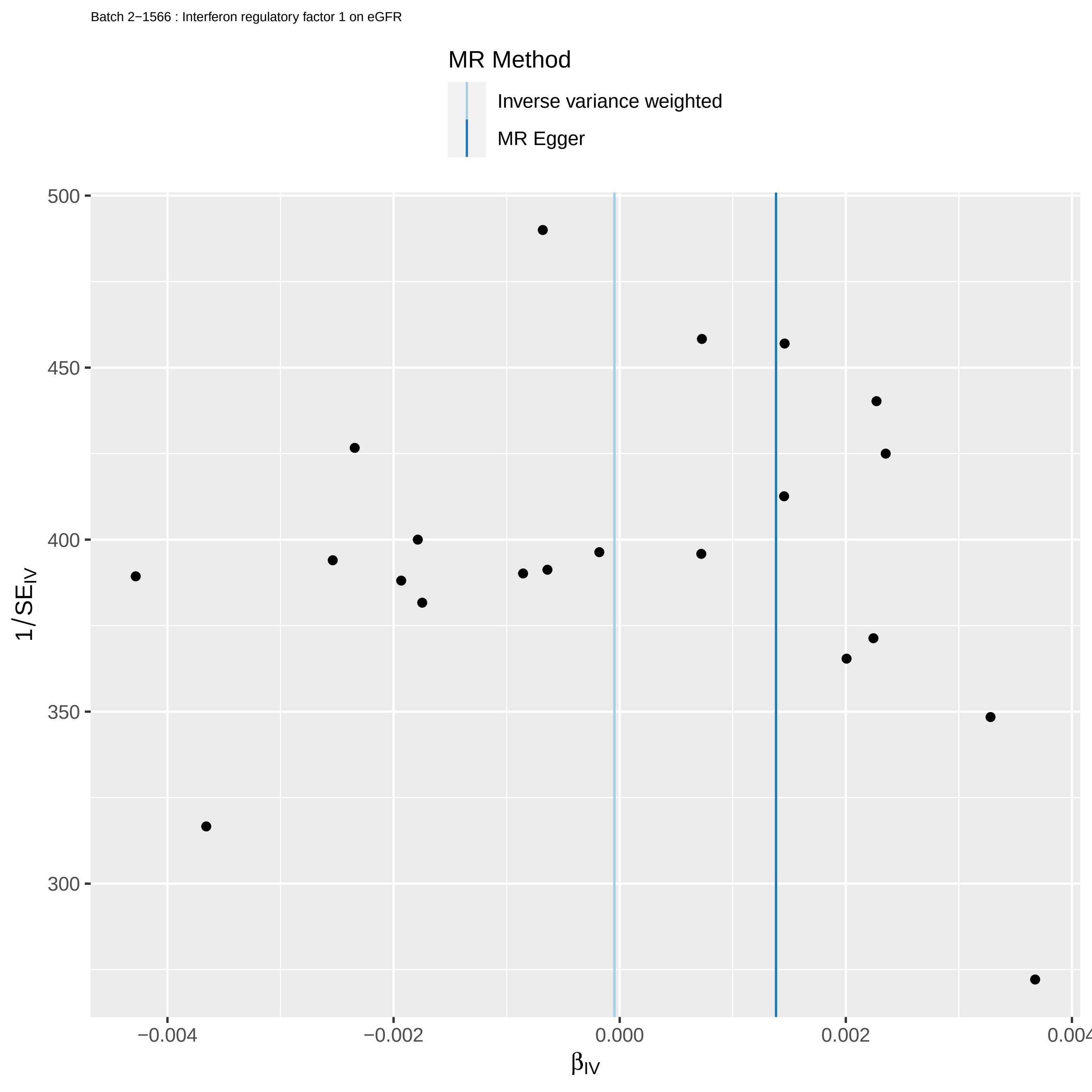
Batch 2−1566 : Interferon regulatory factor 1 on eGFR
MR Method
Inverse variance weighted
MR Egger
500
450
400
350
300
1/SE_IV
−0.004
−0.002
0.000
0.002
0.004
β_IV

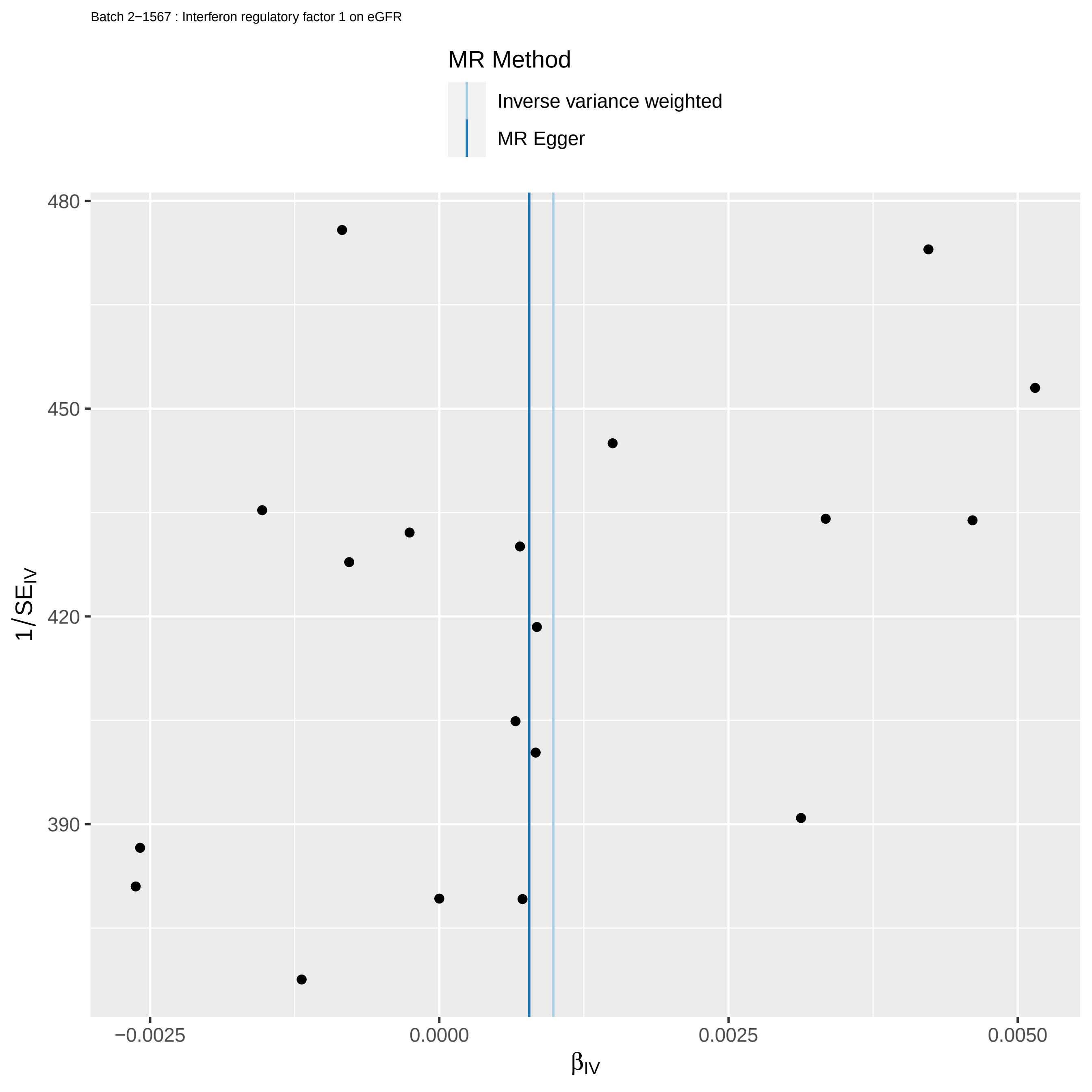

Batch 2−1567 : Interferon regulatory factor 1 on eGFR
MR Method
Inverse variance weighted
MR Egger
480
450
420
390
1/SE_IV
−0.0025
0.0000
0.0025
0.0050
β_IV

Batch 2−1568 : Interferon regulatory factor 2 on eGFR

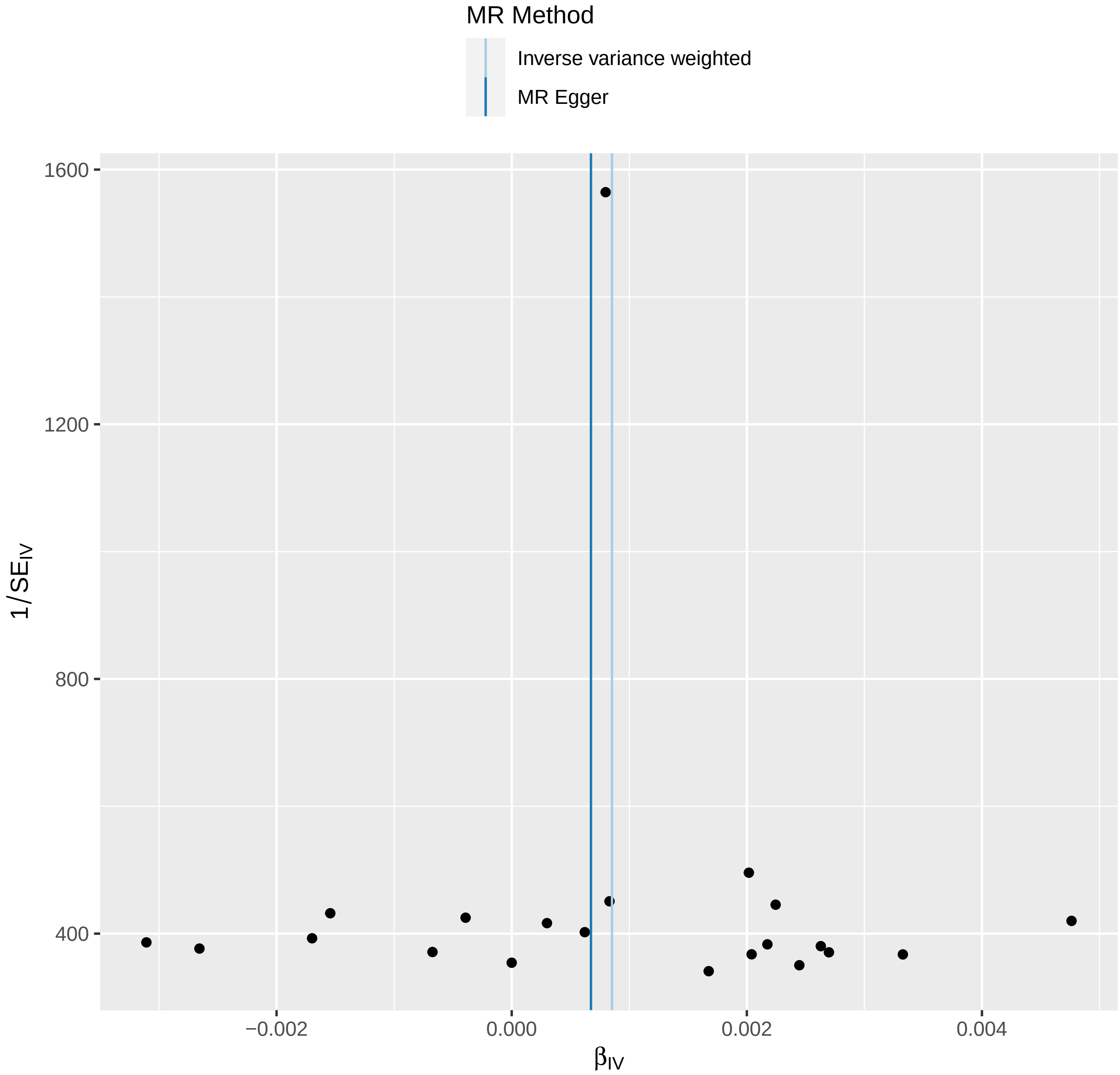

Batch 2−1569 : Interferon regulatory factor 4 on eGFR

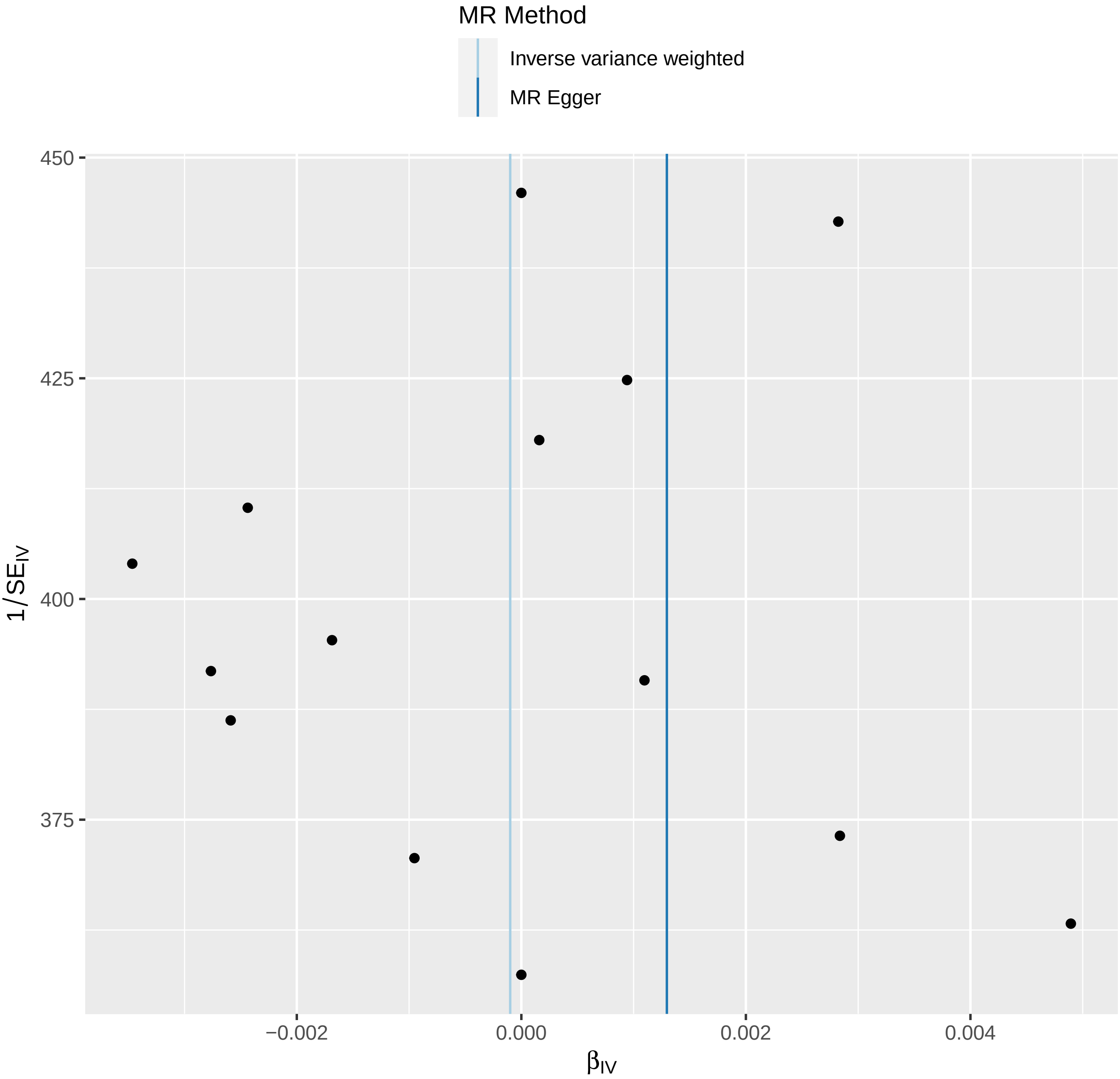

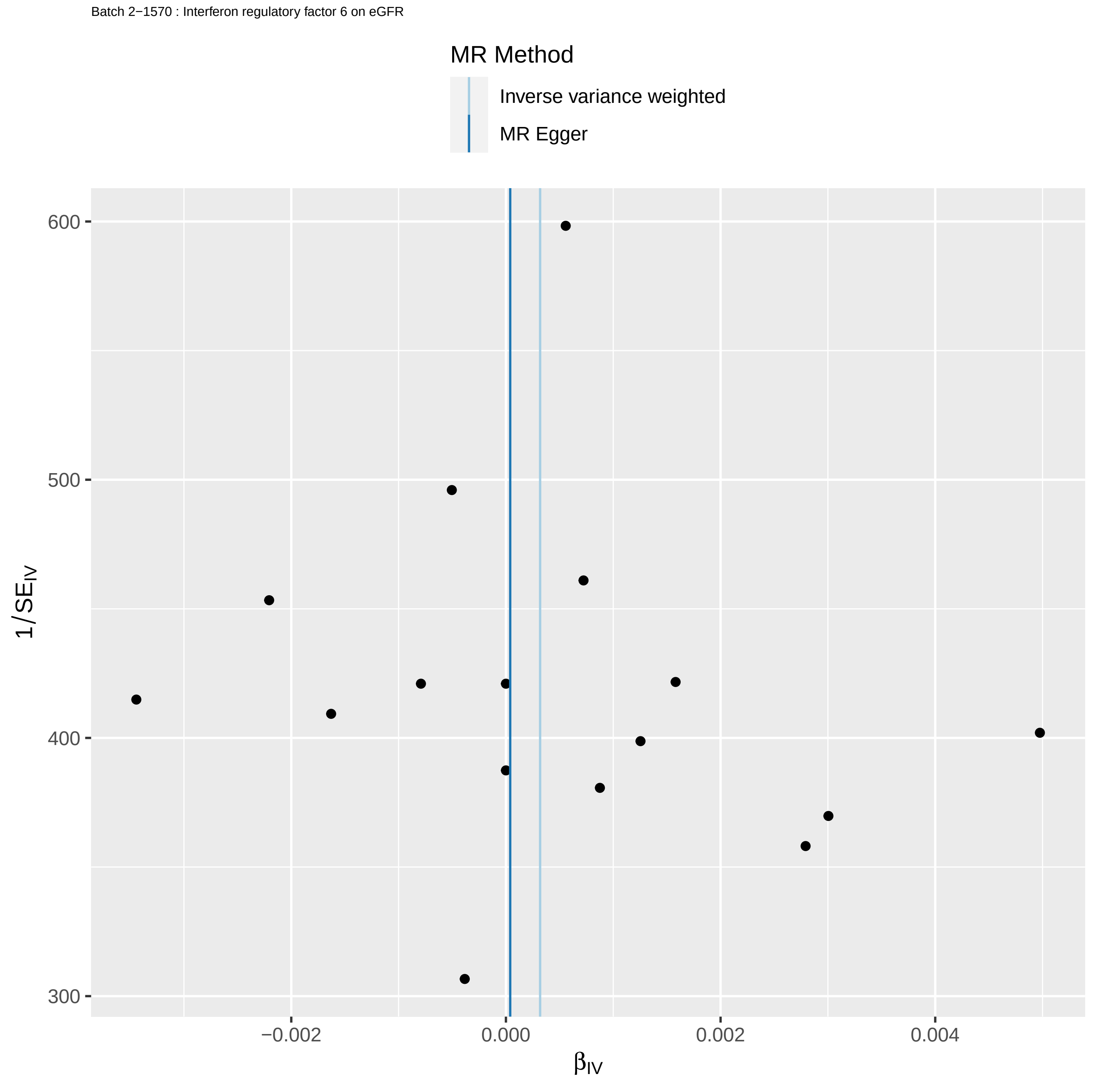
Batch 2−1570 : Interferon regulatory factor 6 on eGFR
MR Method
Inverse variance weighted
MR Egger
600
500
400
300
1/SE$_{IV}$
−0.002
0.000
0.002
0.004
β$_{IV}$

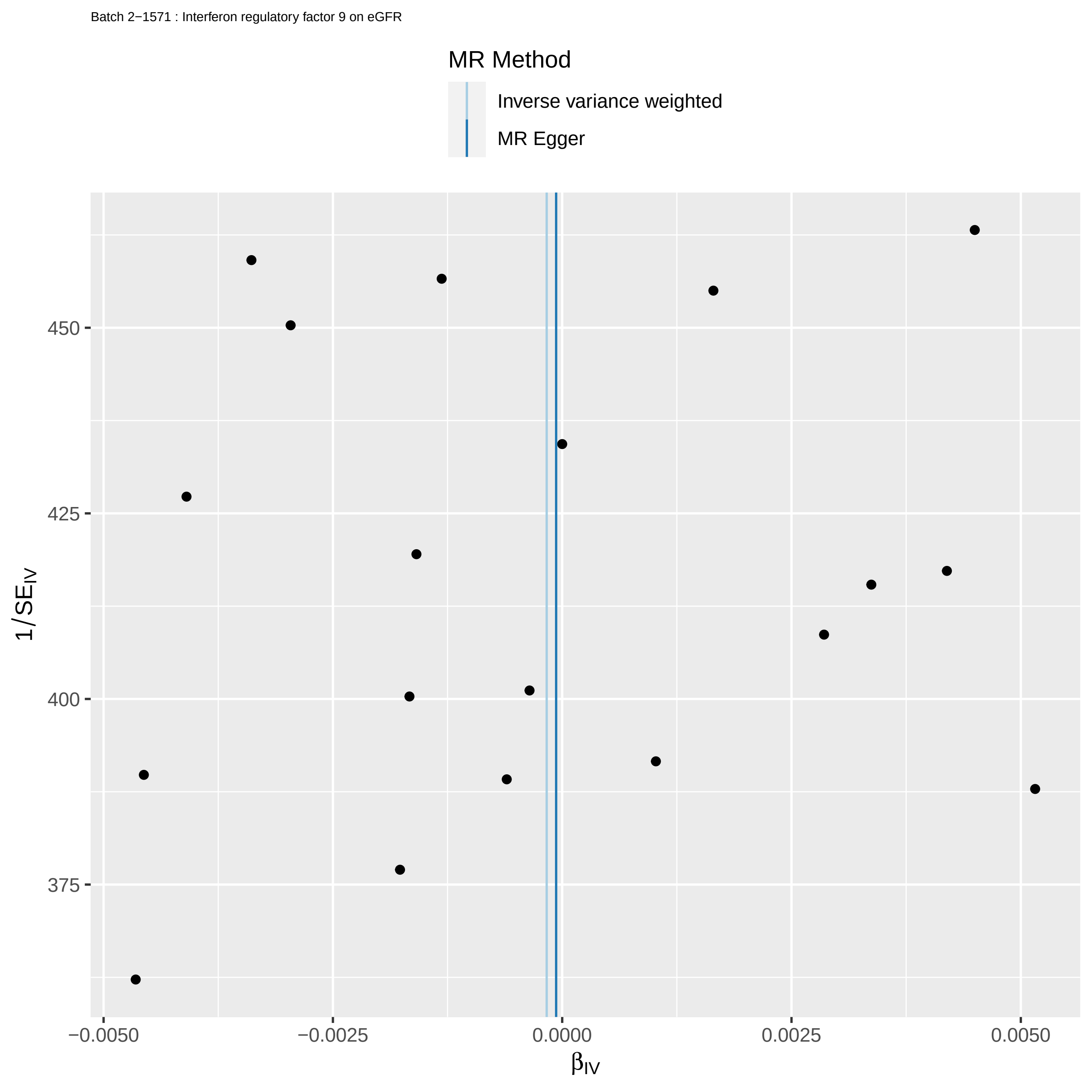
Batch 2−1571 : Interferon regulatory factor 9 on eGFR
MR Method
Inverse variance weighted
MR Egger
1/SE_IV
450
425
400
375
−0.0050
−0.0025
0.0000
0.0025
0.0050
β_IV

Batch 2−1572 : Iron−sulfur cluster assembly enzyme ISCU, mitochondrial on eGFR

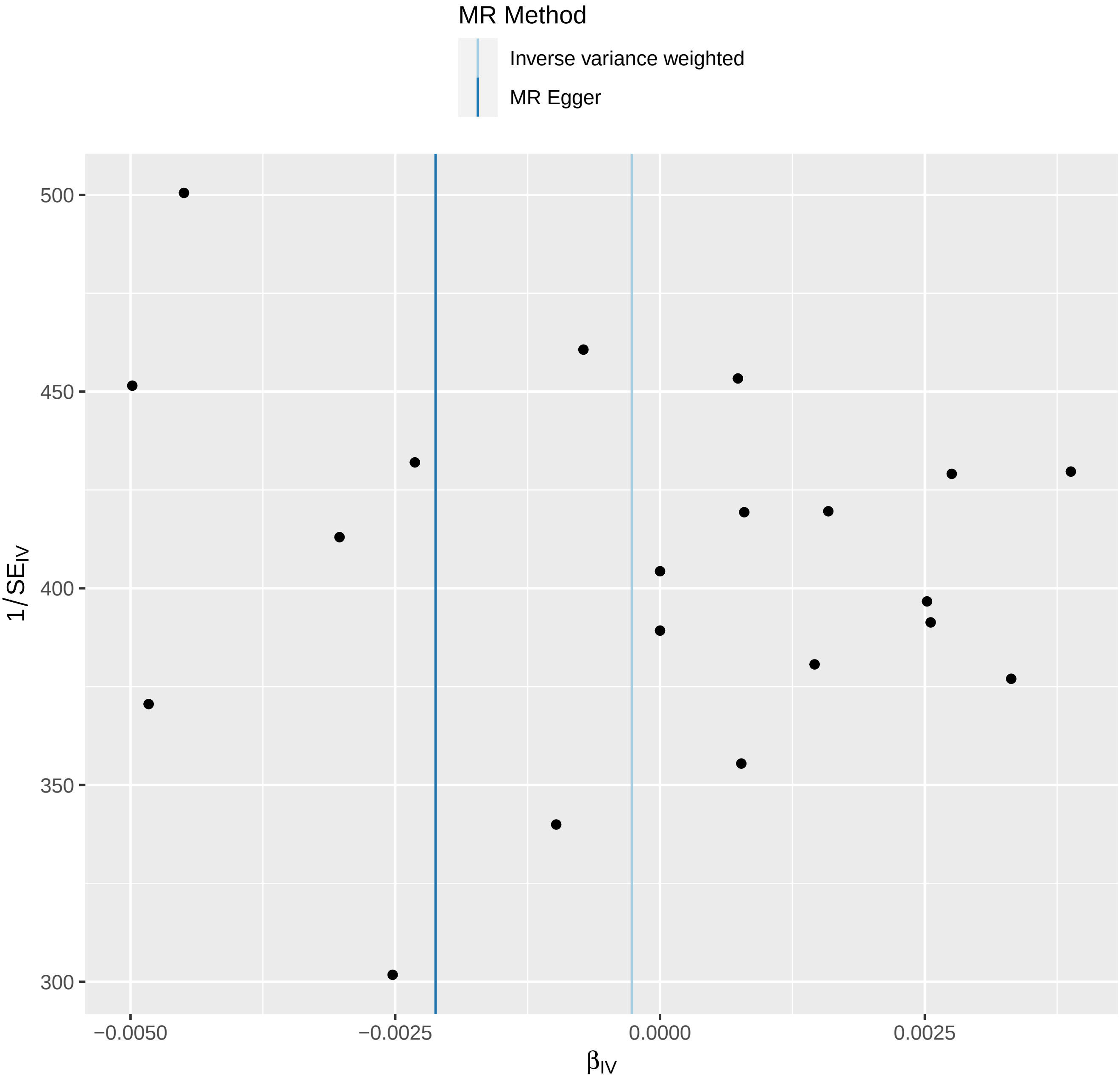

Batch 2−1573 : Ubiquitin−like protein ISG15 on eGFR

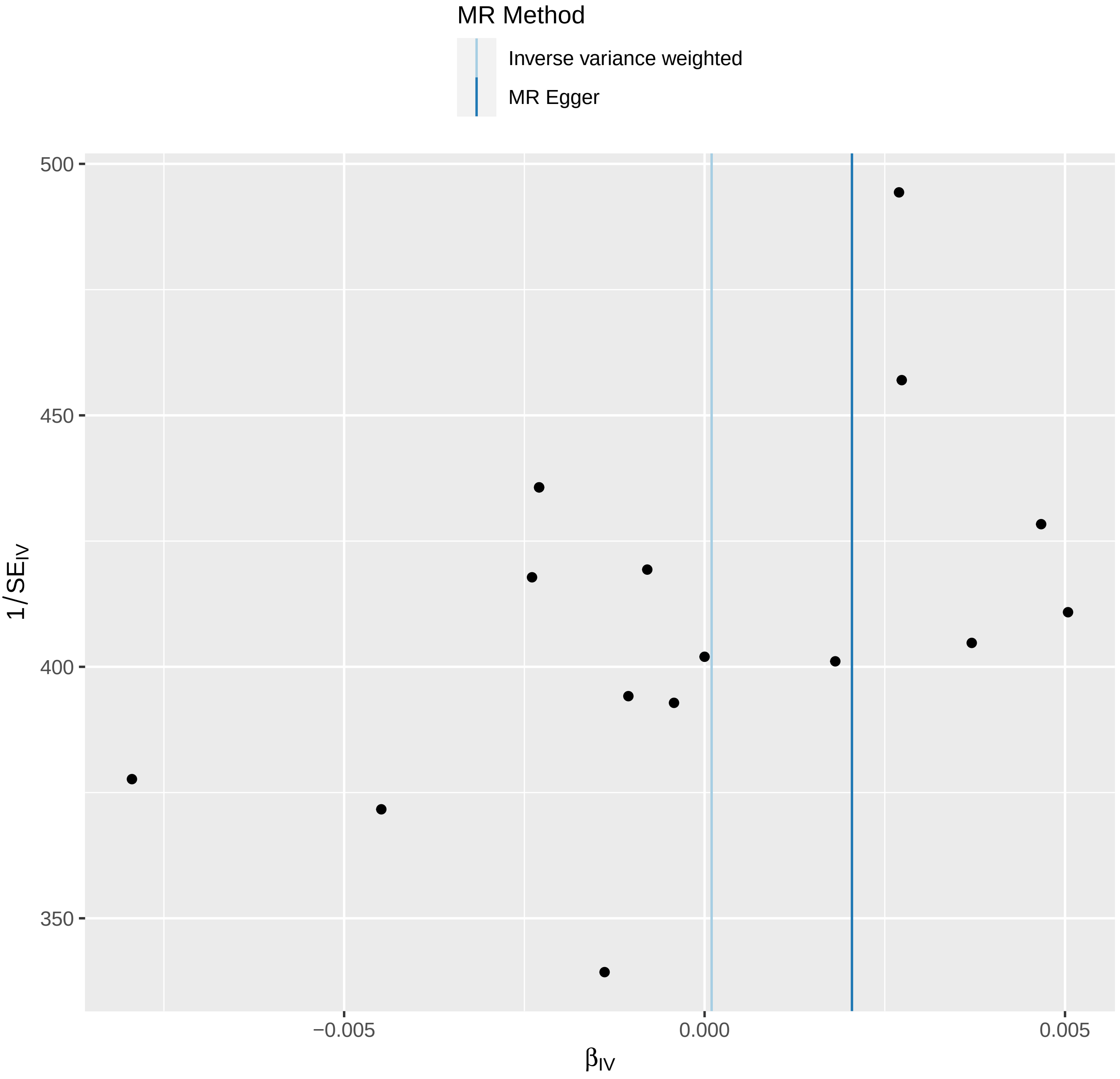

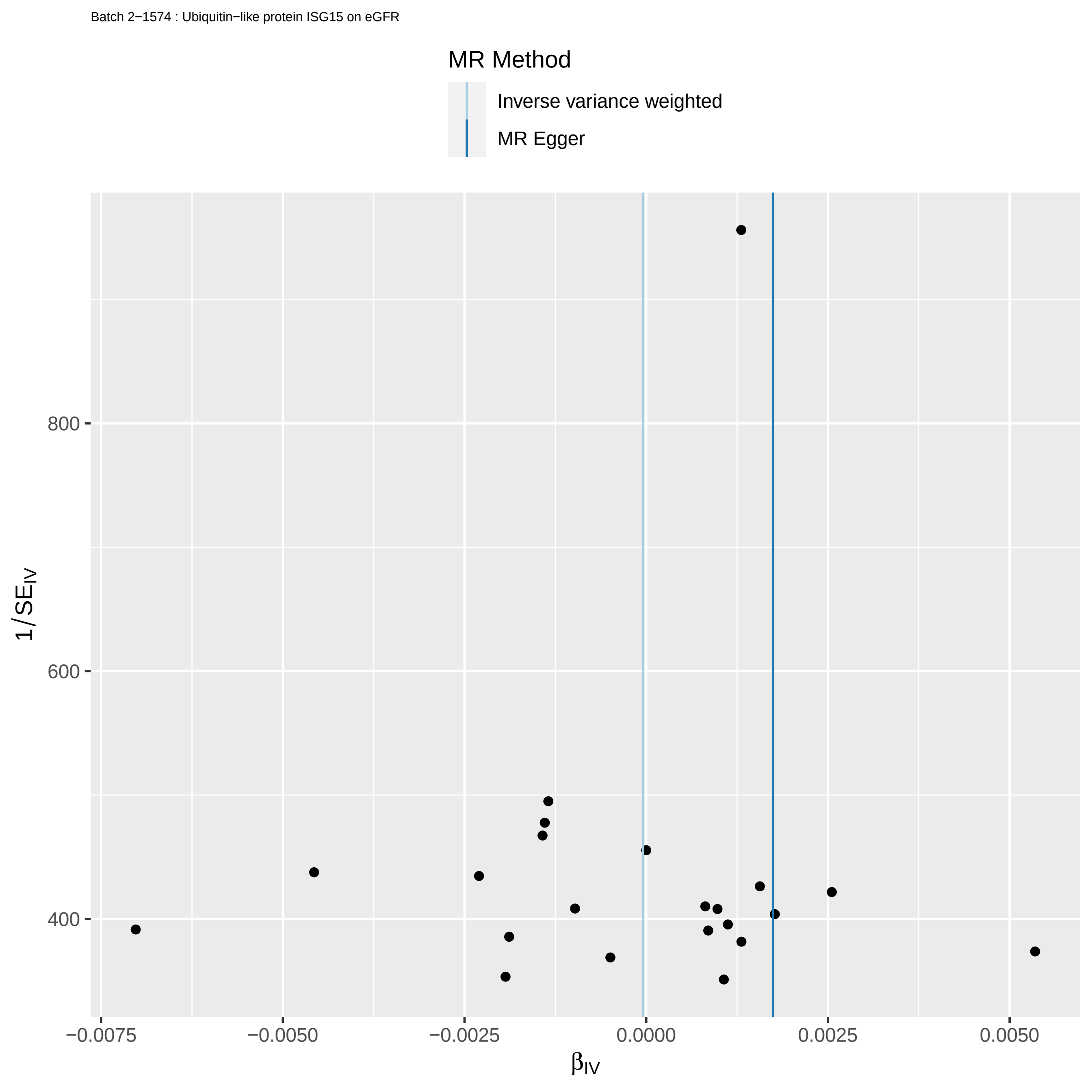

Batch 2−1574 : Ubiquitin−like protein ISG15 on eGFR
MR Method
Inverse variance weighted
MR Egger
800
600
400
1/SE$_{IV}$
−0.0075
−0.0050
−0.0025
0.0000
0.0025
0.0050
β$_{IV}$

Batch 2−1575 : Insulin gene enhancer protein ISL−1 on eGFR

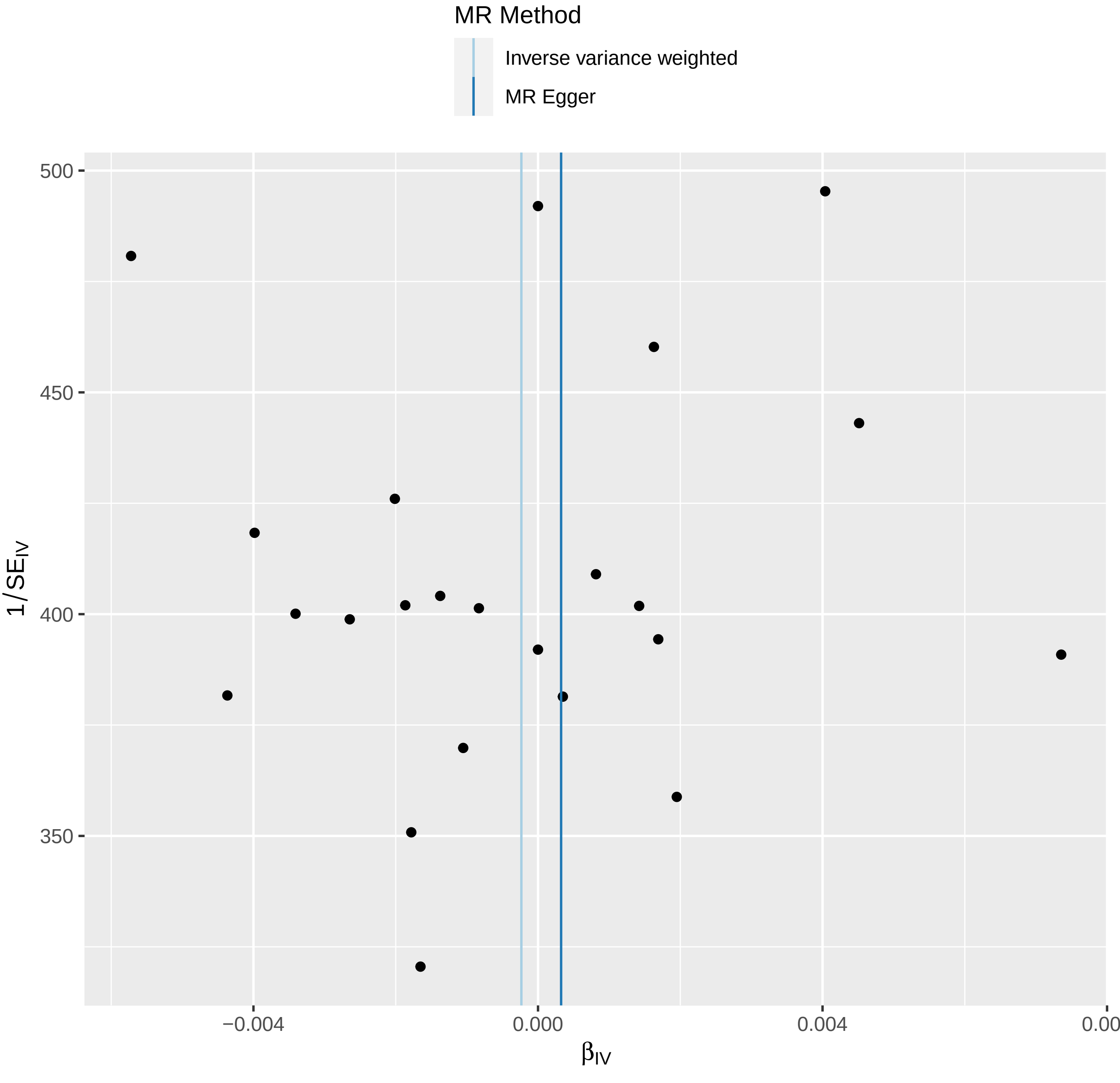

Batch 2−1576 : Immunoglobulin superfamily containing leucine−rich repeat protein 2 on eGFR

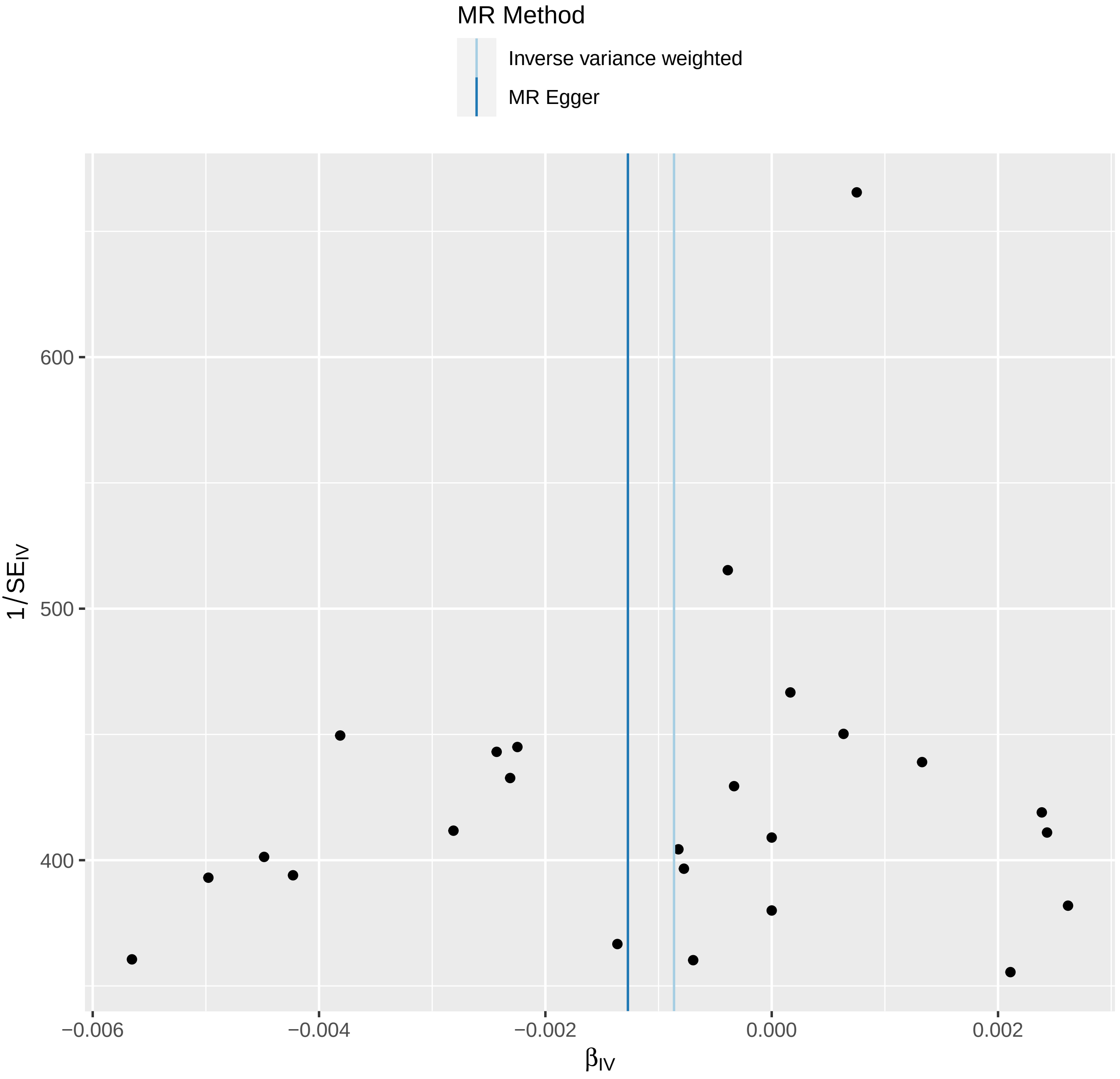

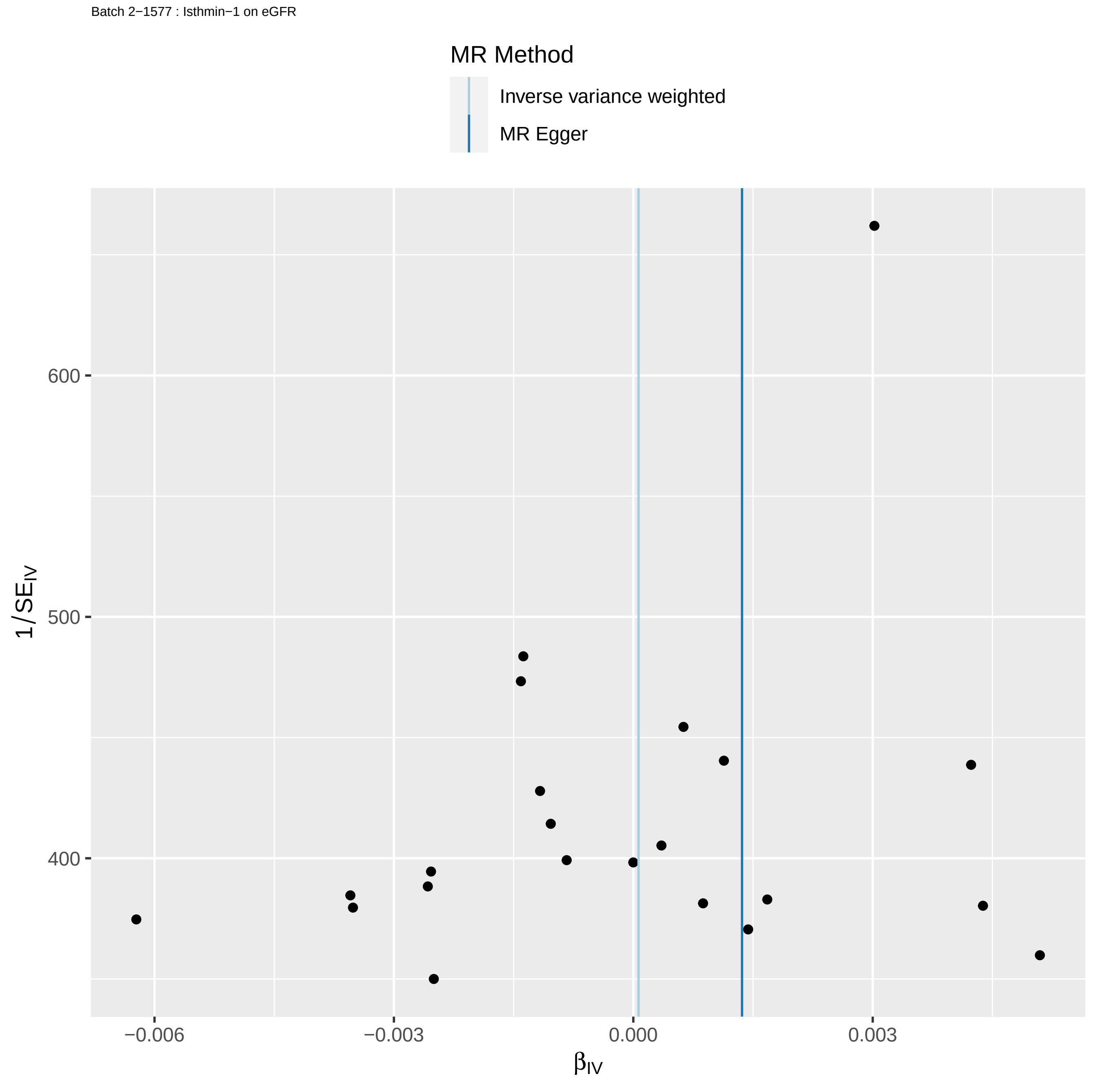
Batch 2−1577 : Isthmin−1 on eGFR
MR Method
Inverse variance weighted
MR Egger
600
500
400
1/SE_IV
−0.006
−0.003
0.000
0.003
β_IV

Batch 2−1578 : Isochorismatase domain−containing protein 1 on eGFR

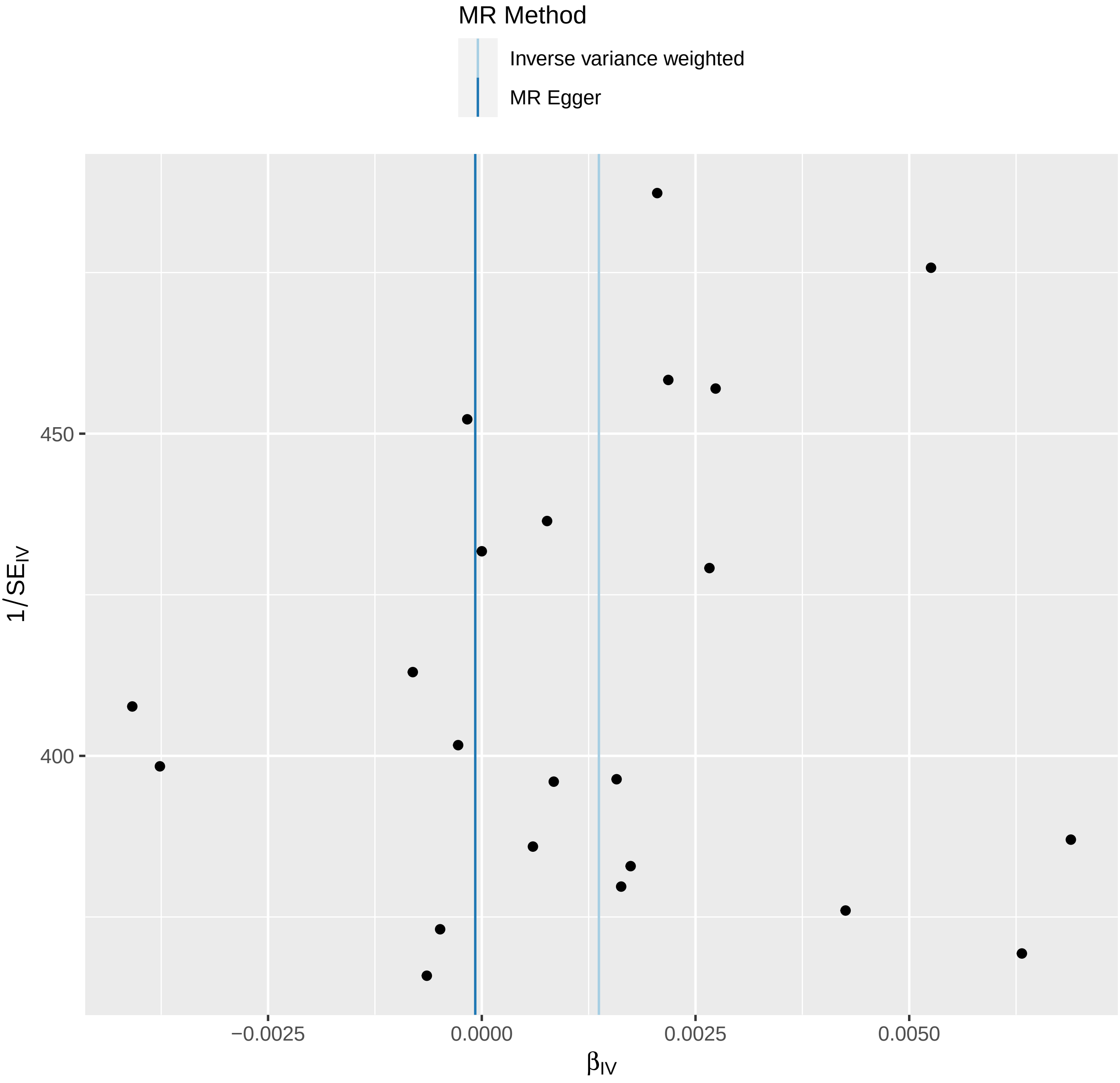

Batch 2−1579 : IST1 homolog on eGFR

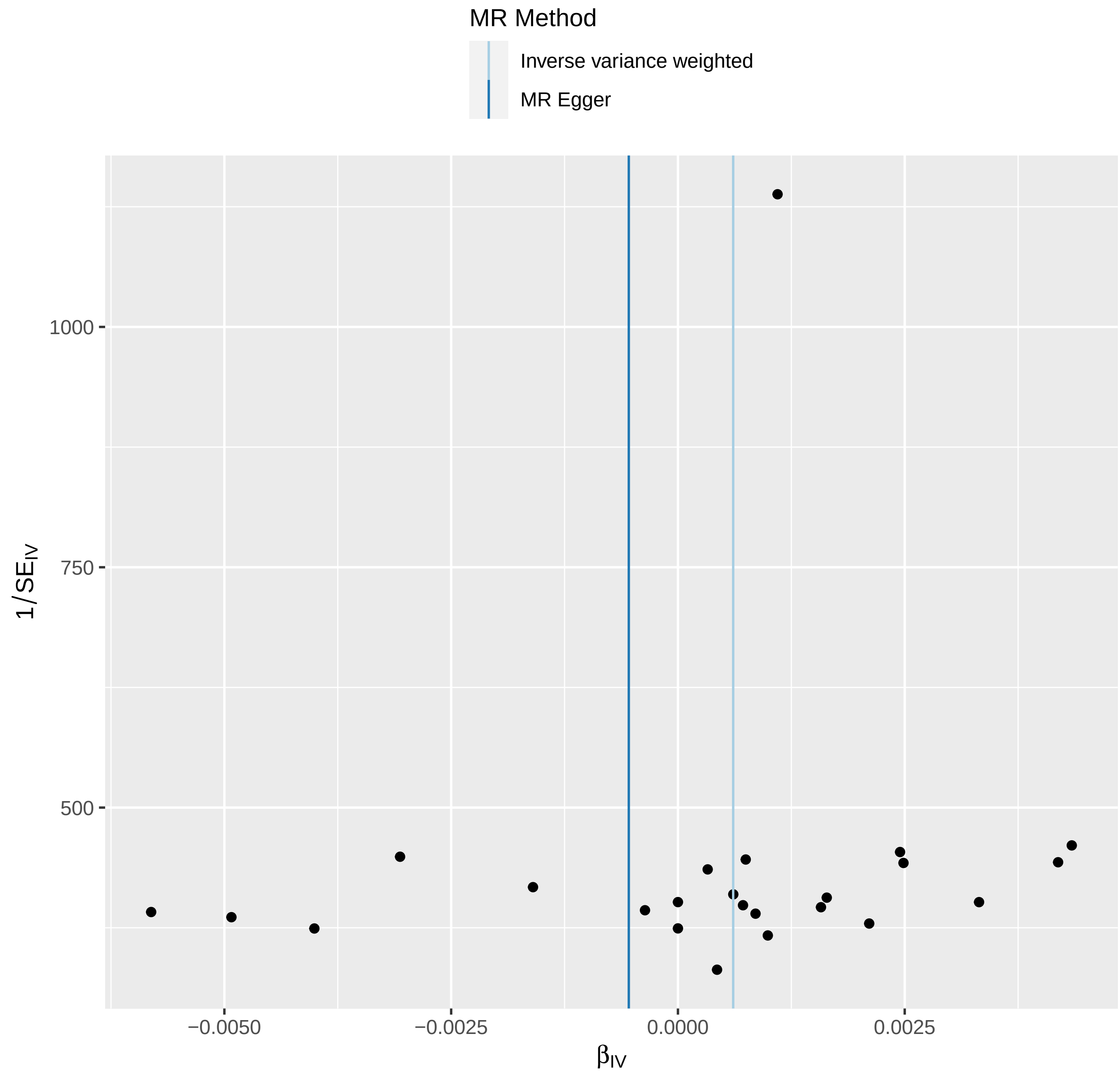

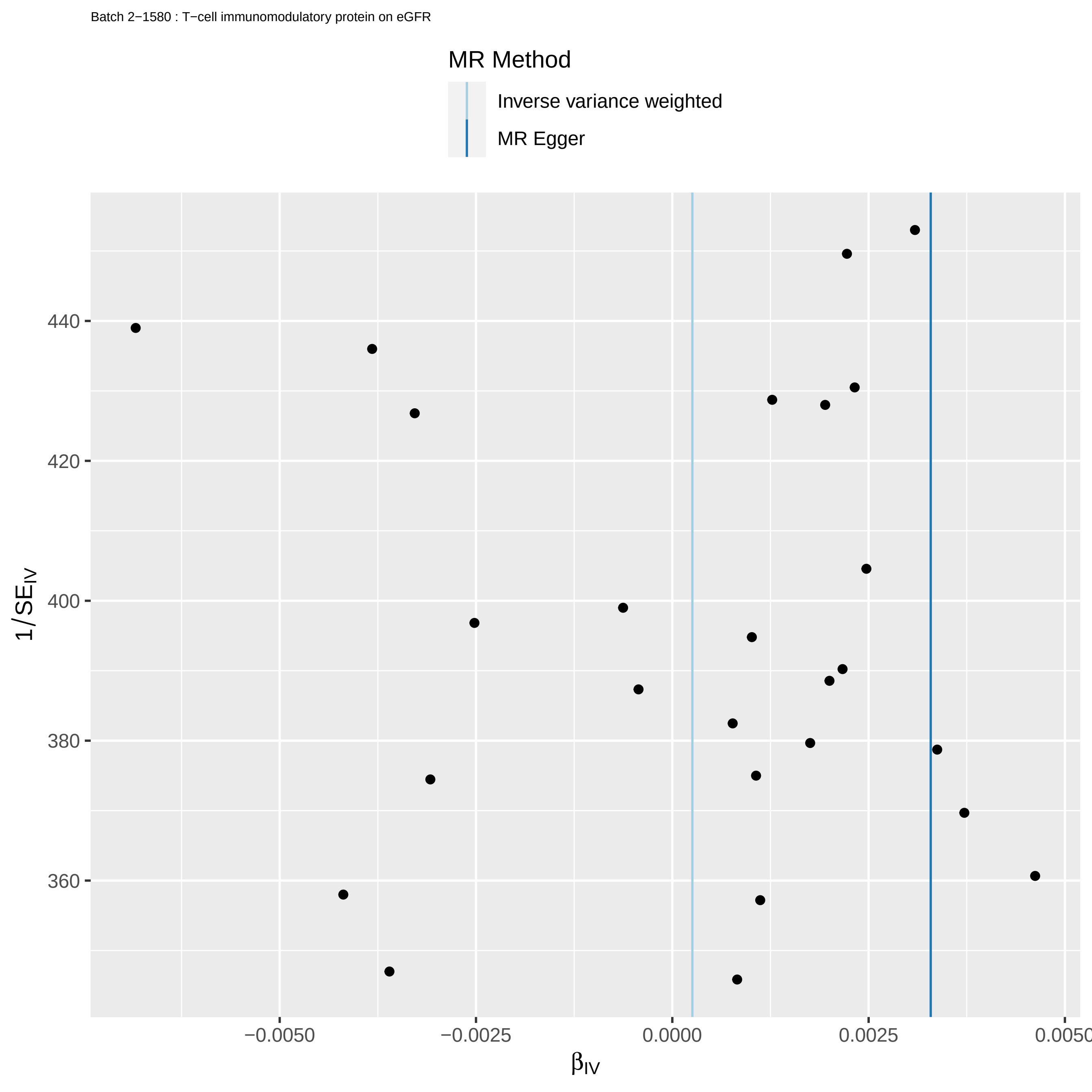

Batch 2−1580 : T−cell immunomodulatory protein on eGFR
MR Method
Inverse variance weighted
MR Egger
440
420
400
380
360
1/SE$_{IV}$
−0.0050
−0.0025
0.0000
0.0025
0.0050
β$_{IV}$

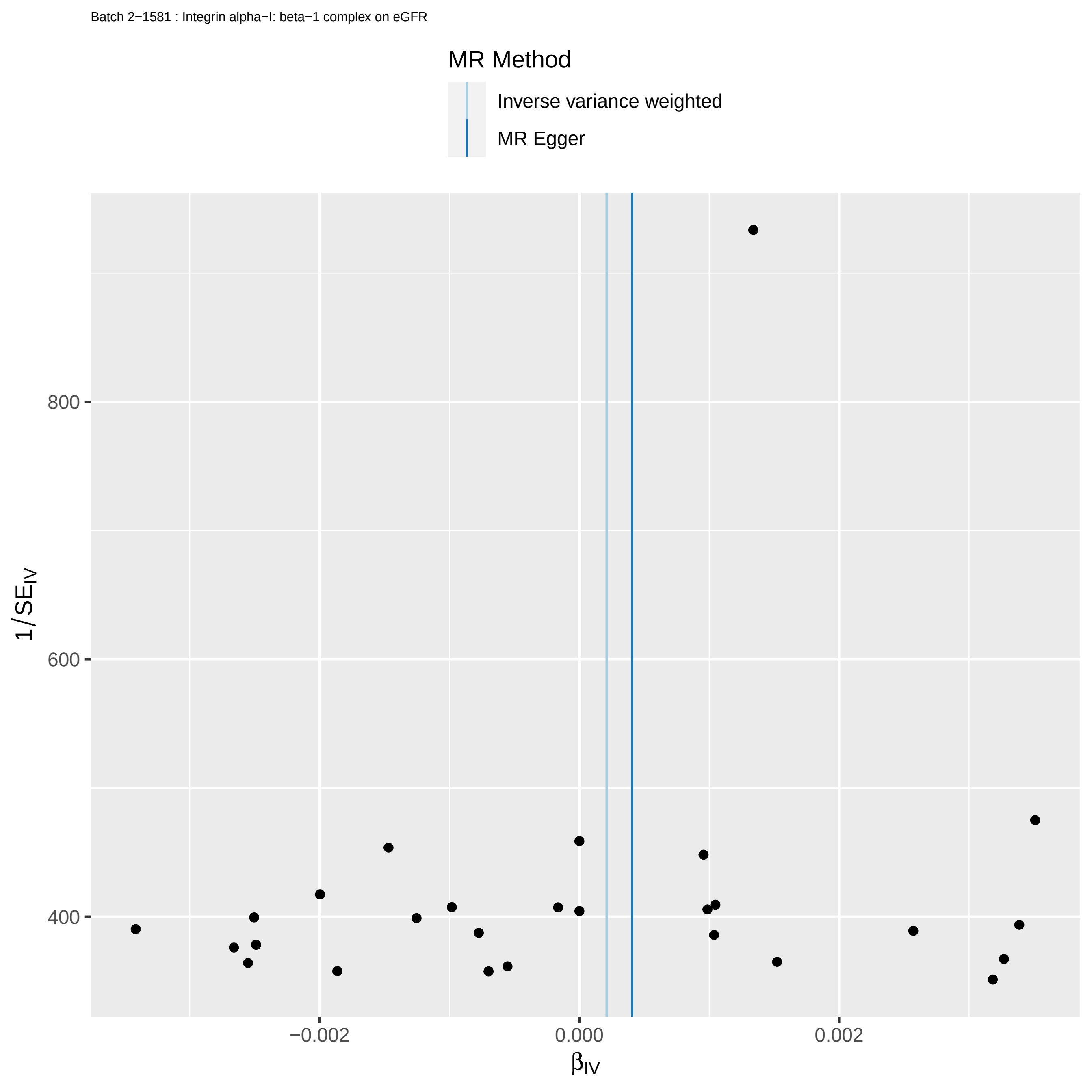

Batch 2−1581 : Integrin alpha−I: beta−1 complex on eGFR
MR Method
Inverse variance weighted
MR Egger
1/SE_IV
800
600
400
−0.002
0.000
0.002
β_IV

Batch 2−1582 : Integrin alpha−5 on eGFR

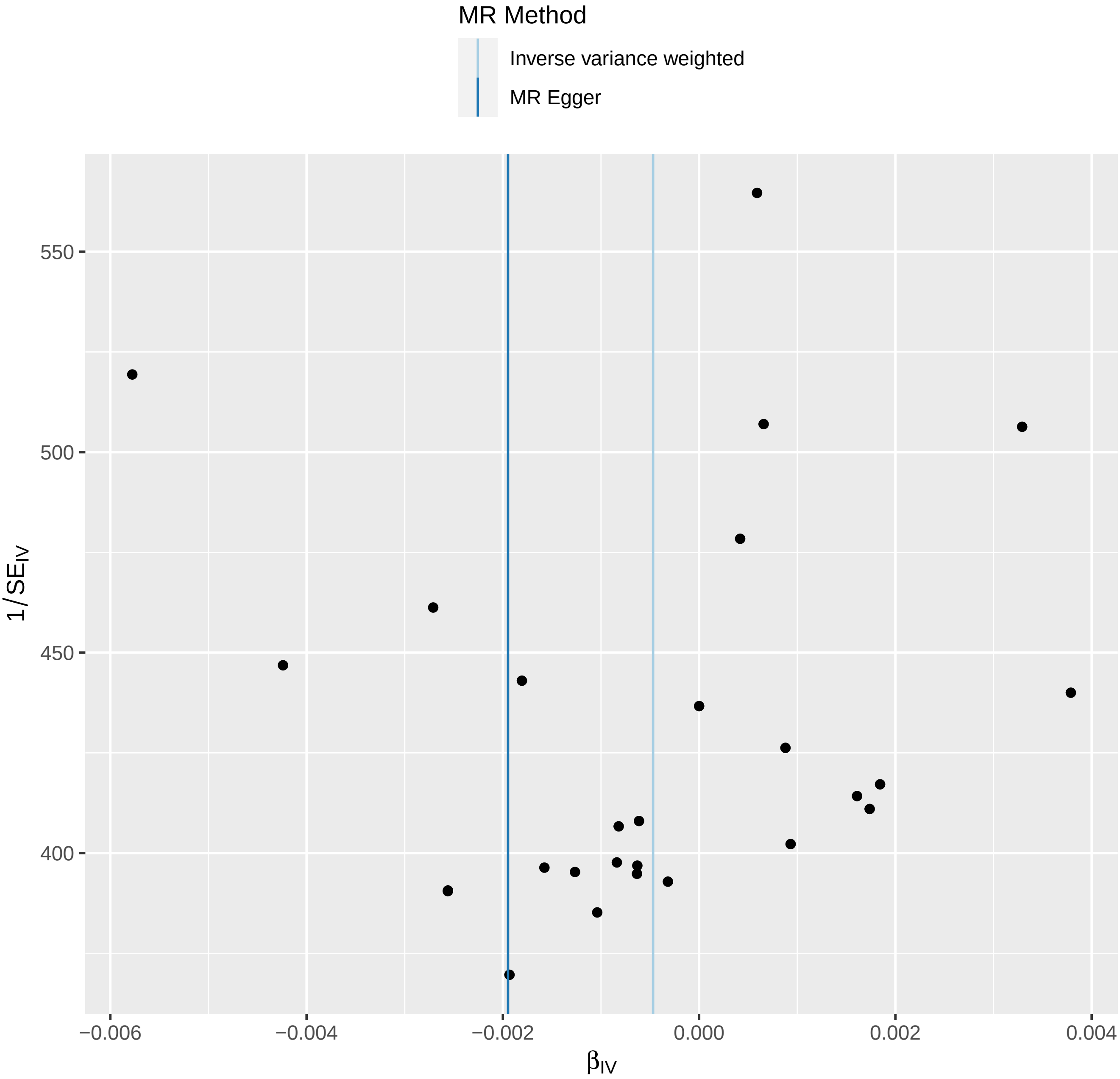

Batch 2−1583 : Integrin alpha−L on eGFR

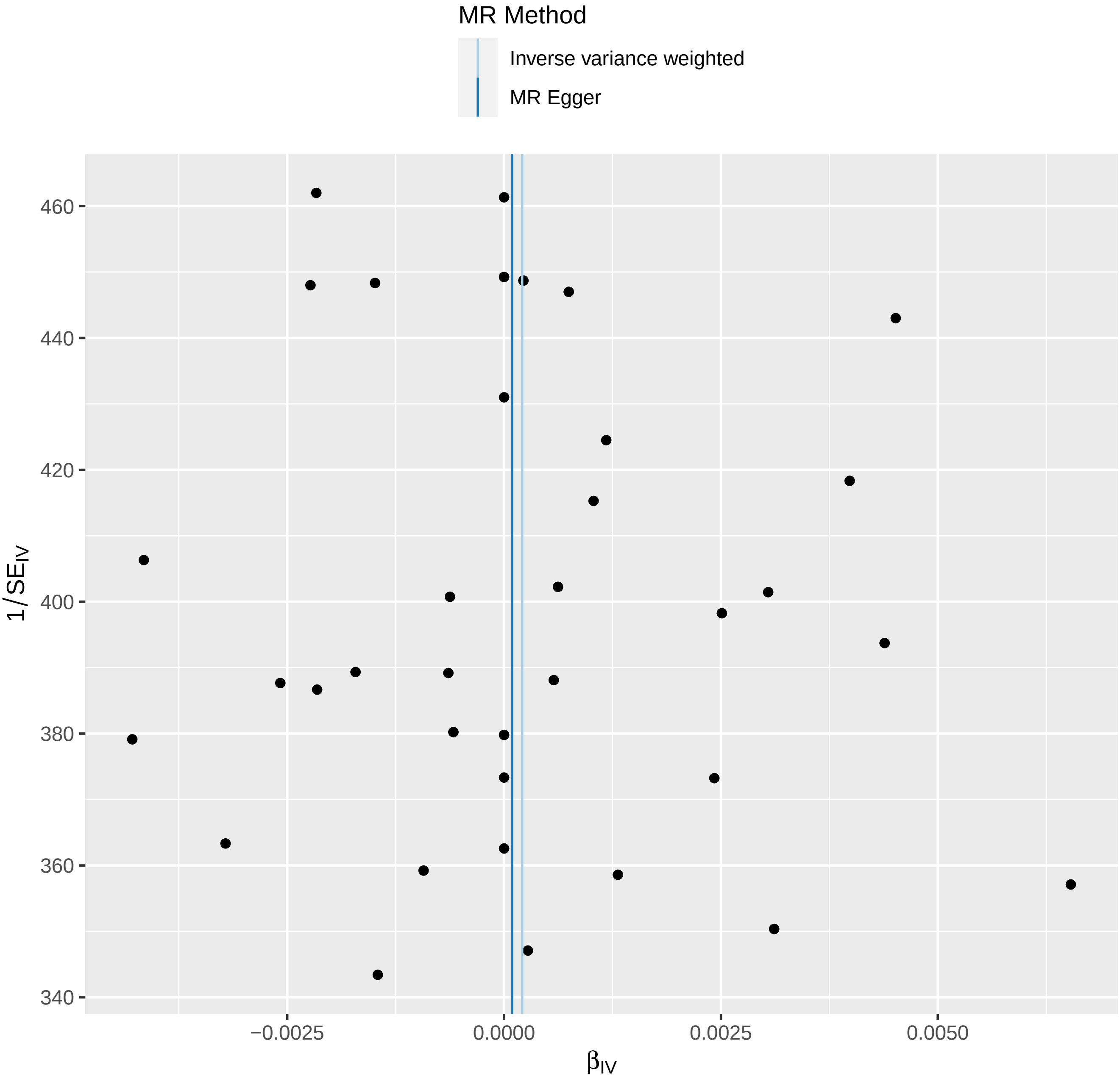

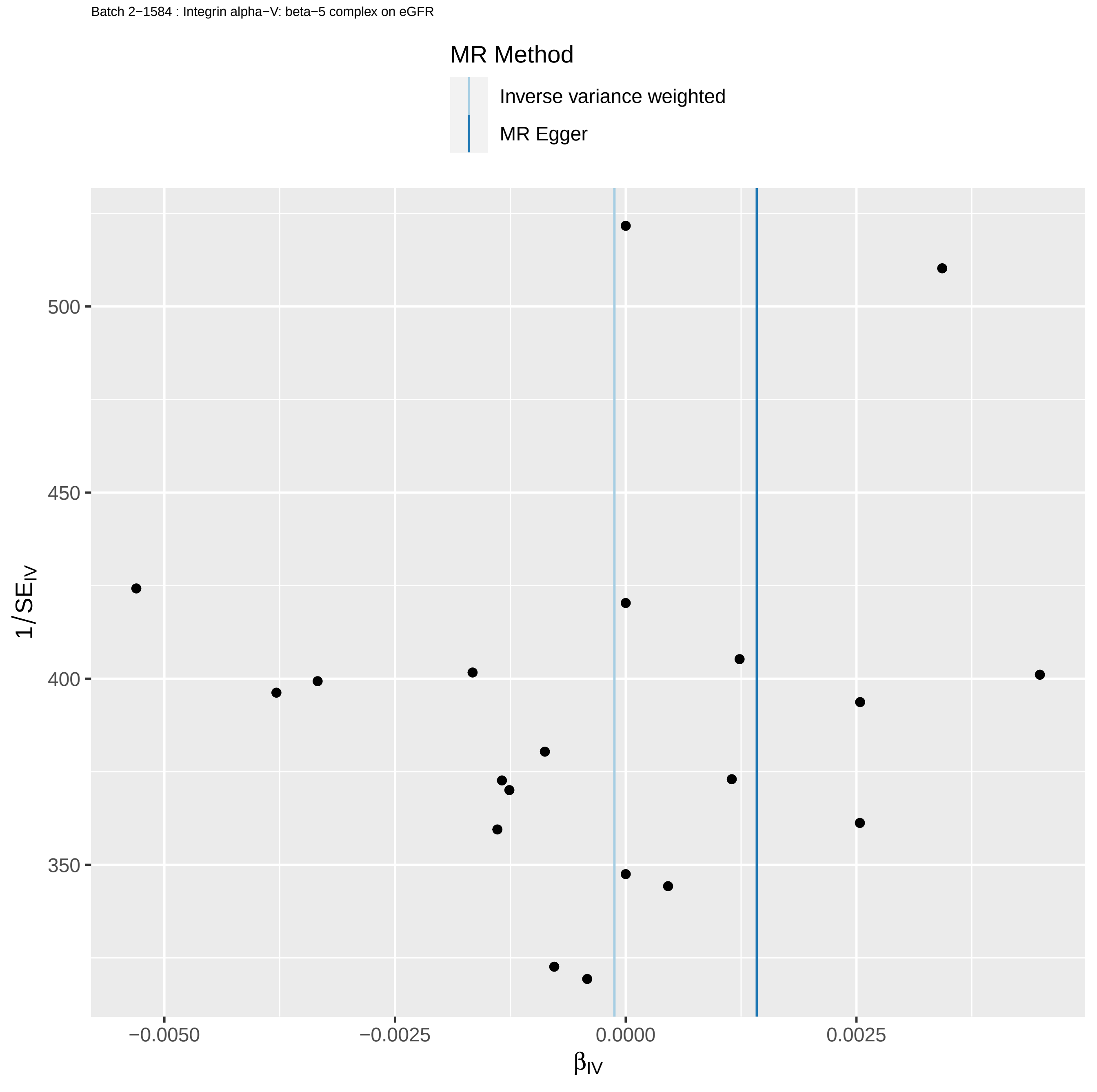

Batch 2−1584 : Integrin alpha−V: beta−5 complex on eGFR
MR Method
Inverse variance weighted
MR Egger
500
450
400
350
1/SE_IV
−0.0050
−0.0025
0.0000
0.0025
β_IV

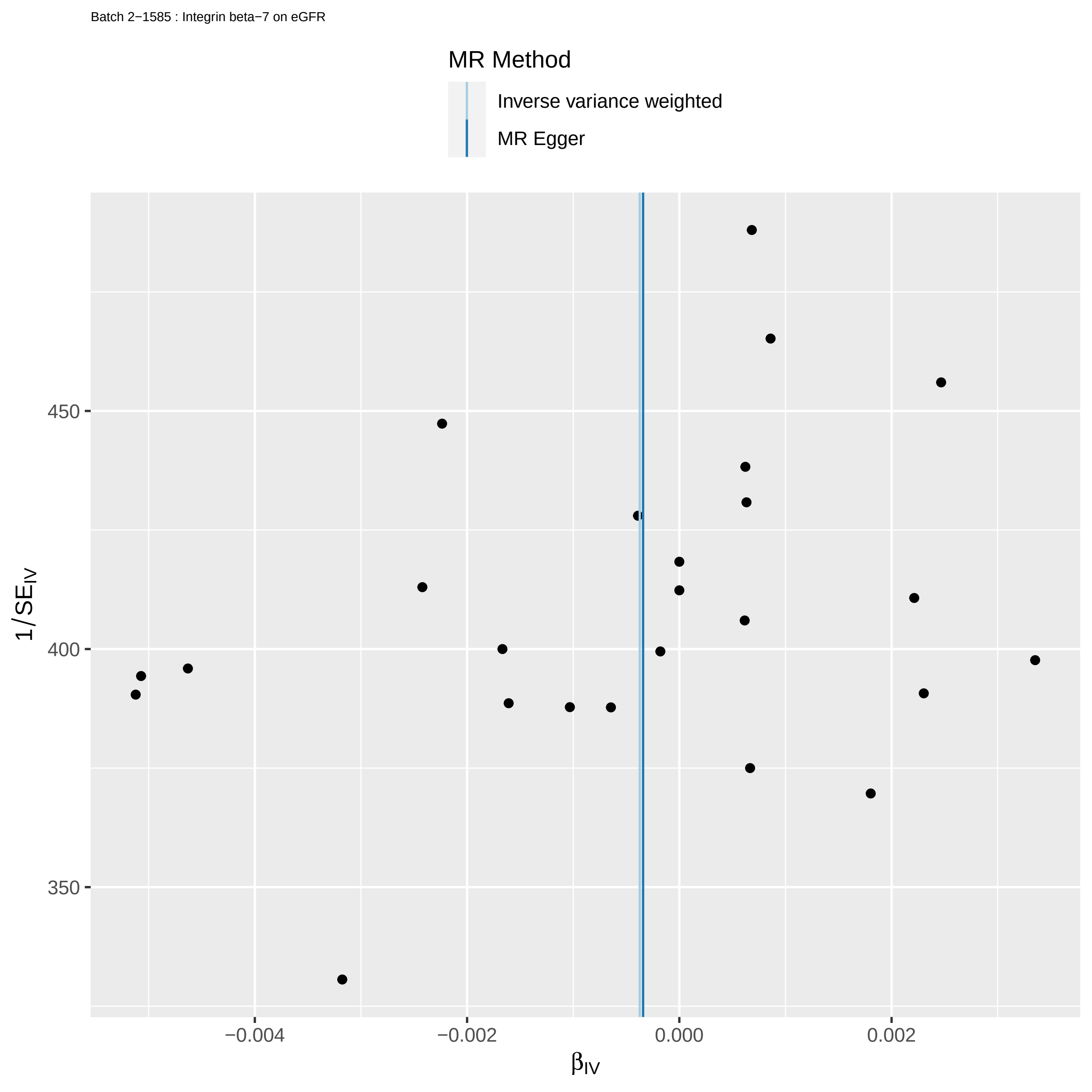

Batch 2−1585 : Integrin beta−7 on eGFR
MR Method
Inverse variance weighted
MR Egger
450
400
350
1/SE$_{IV}$
−0.004
−0.002
0.000
0.002
β$_{IV}$

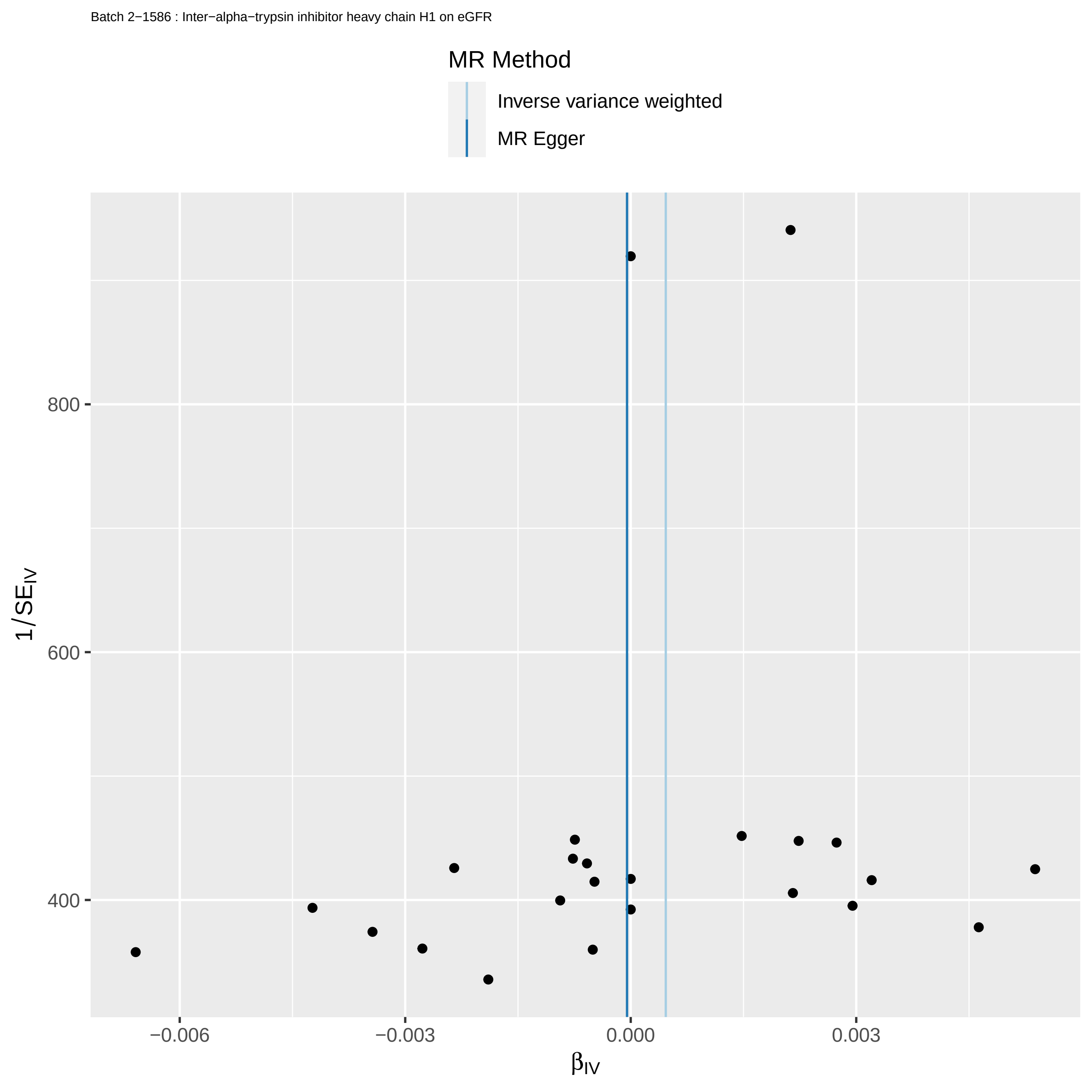
Batch 2−1586 : Inter−alpha−trypsin inhibitor heavy chain H1 on eGFR
MR Method
Inverse variance weighted
MR Egger
800
600
400
1/SE_IV
−0.006
−0.003
0.000
0.003
β_IV

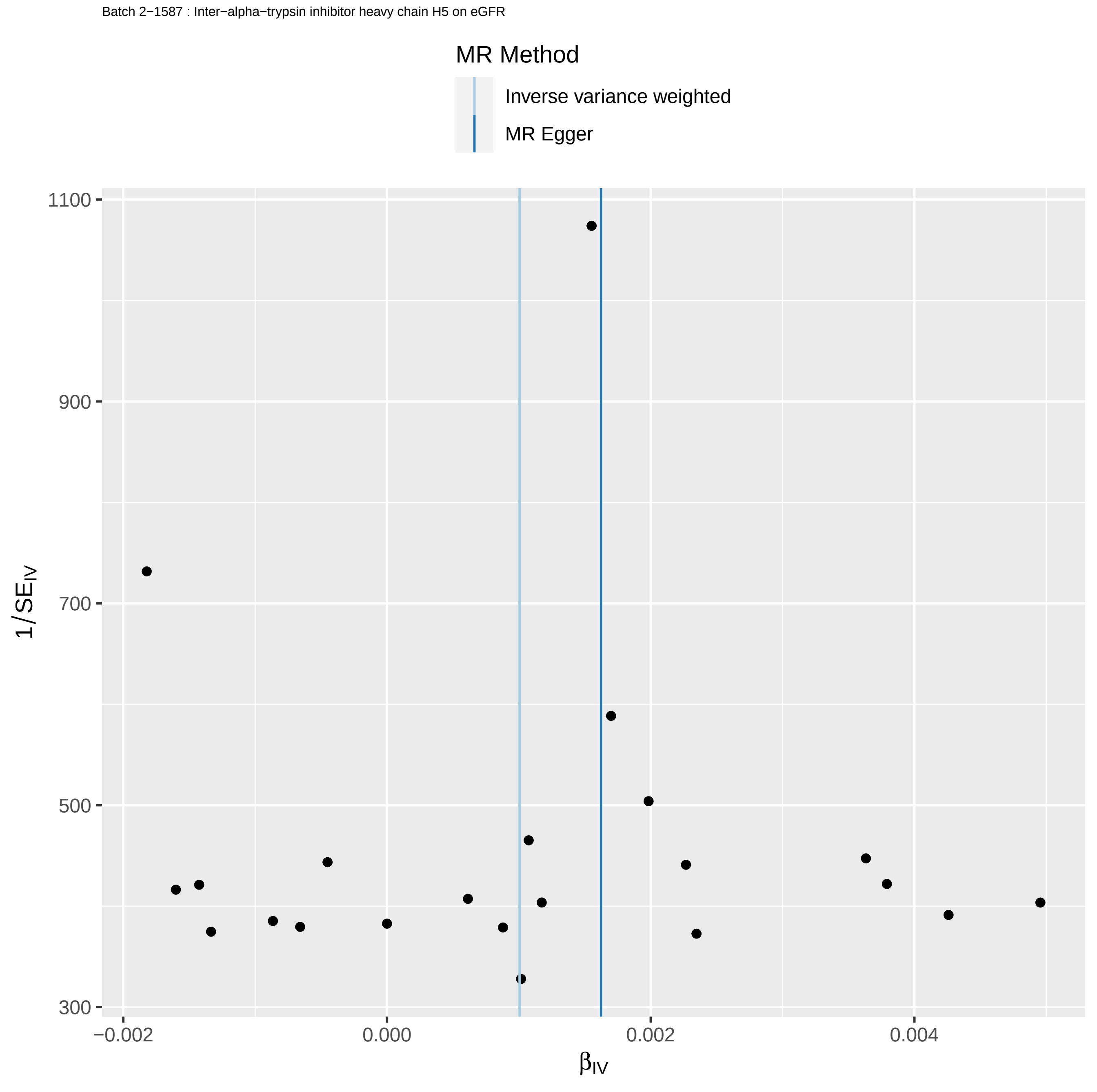

Batch 2−1587 : Inter−alpha−trypsin inhibitor heavy chain H5 on eGFR
MR Method
Inverse variance weighted
MR Egger
1100
900
700
500
300
$1/SE_{IV}$
−0.002
0.000
0.002
0.004
β_{IV}

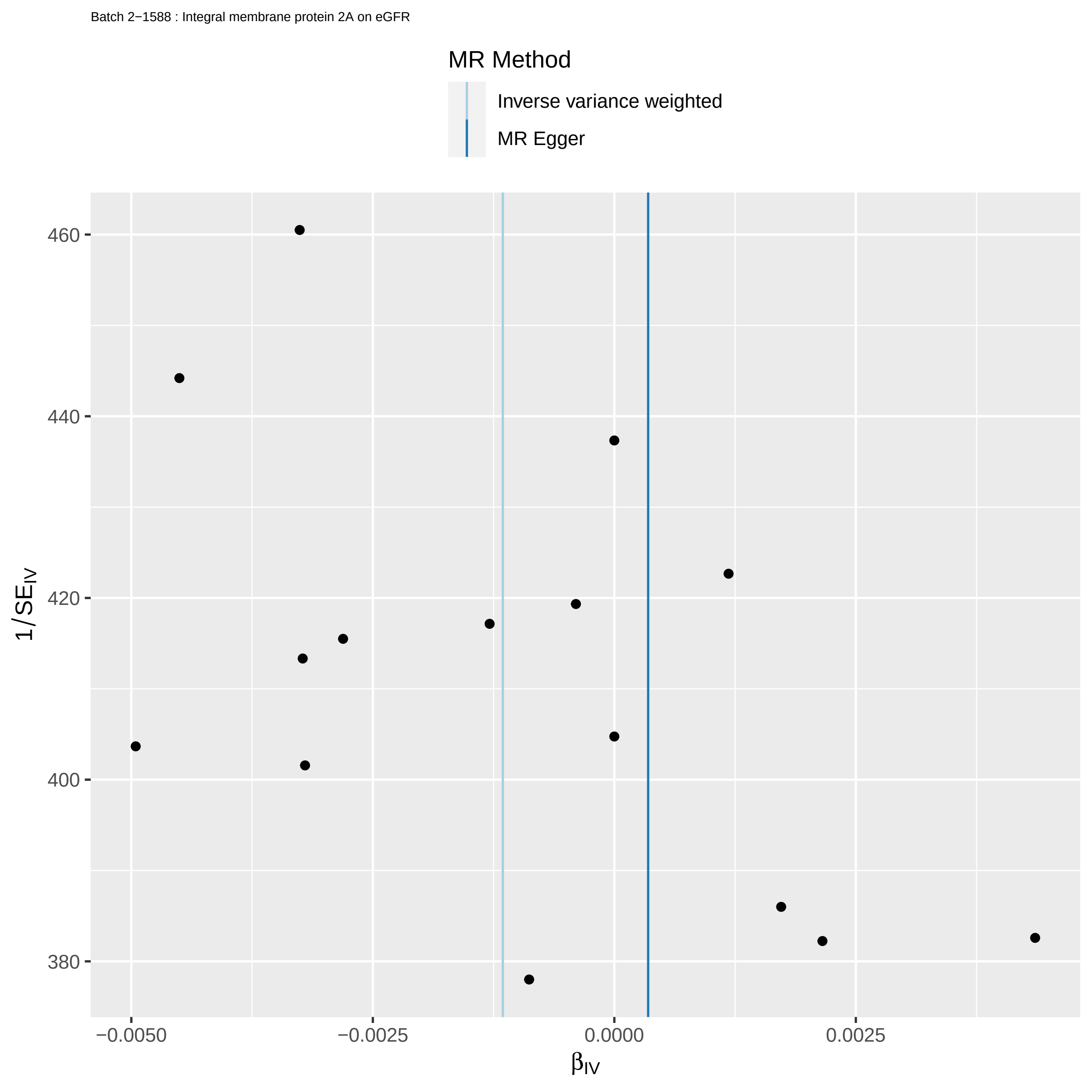

Batch 2−1588 : Integral membrane protein 2A on eGFR
MR Method
Inverse variance weighted
MR Egger
460
440
420
400
380
1/SE_IV
−0.0050
−0.0025
0.0000
0.0025
β_IV

Batch 2−1589 : Integral membrane protein 2A on eGFR

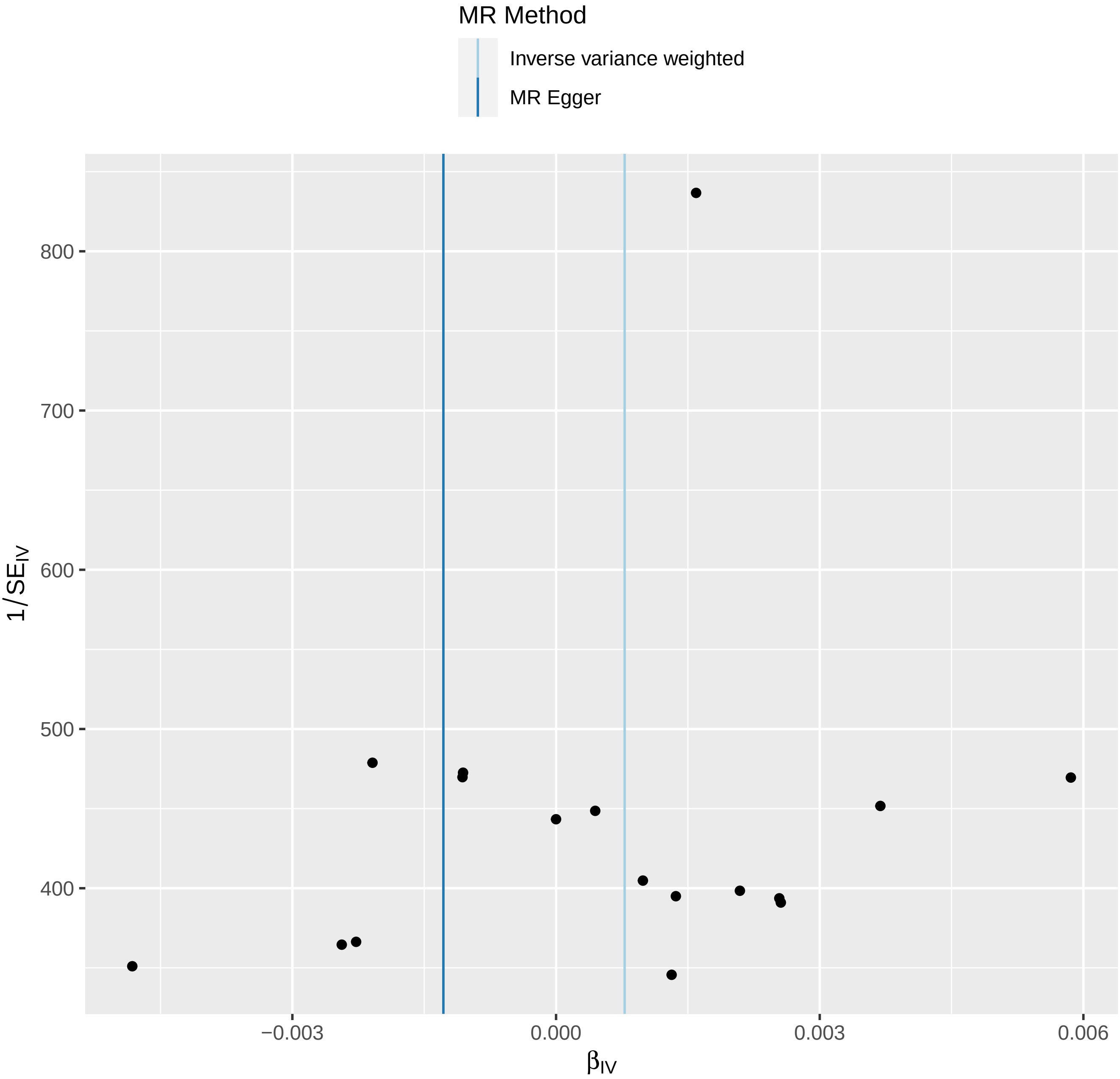

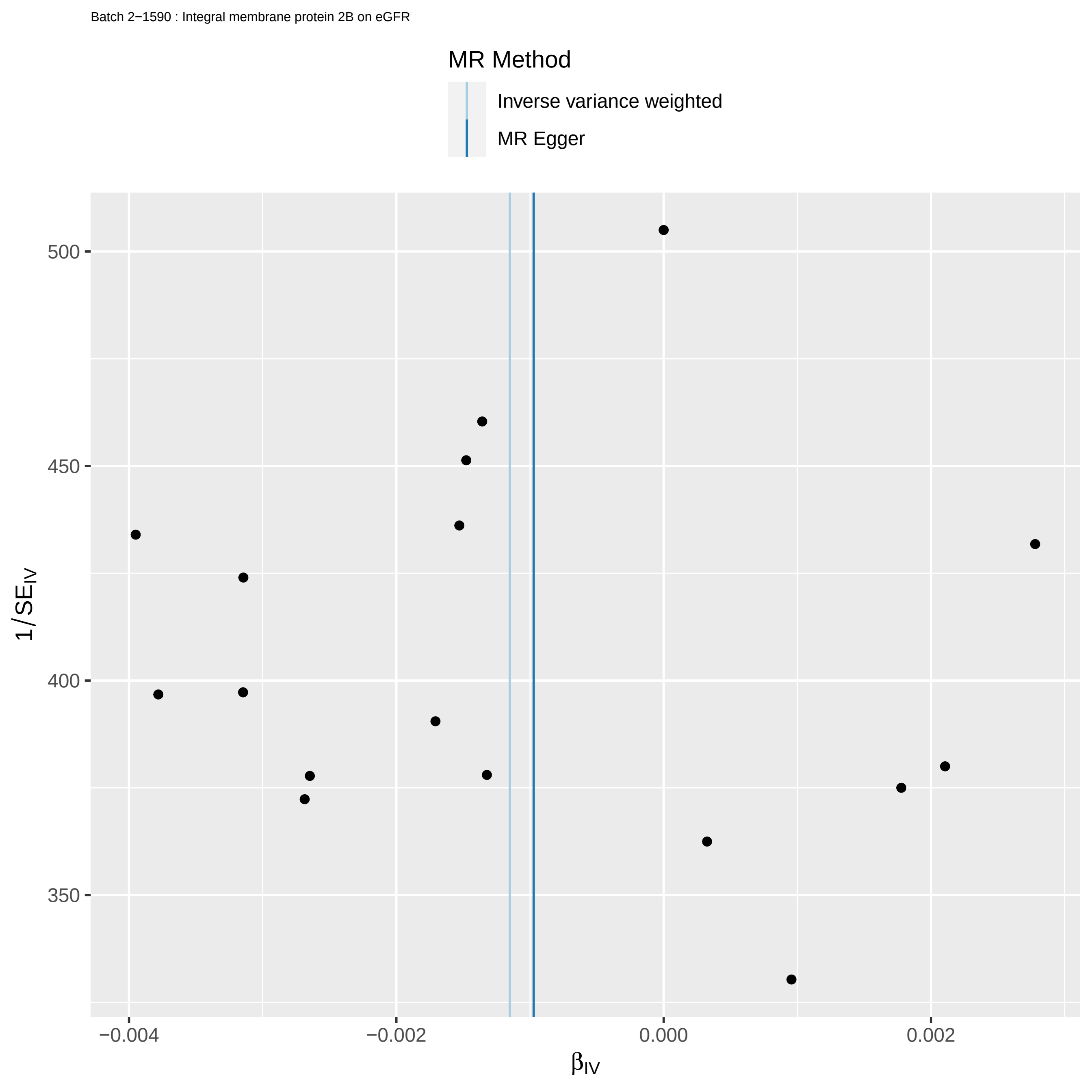

Batch 2−1590 : Integral membrane protein 2B on eGFR
MR Method
Inverse variance weighted
MR Egger
500
450
400
350
1/SE$_{IV}$
−0.004
−0.002
0.000
0.002
β$_{IV}$

Batch 2−1591 : Integral membrane protein 2C on eGFR

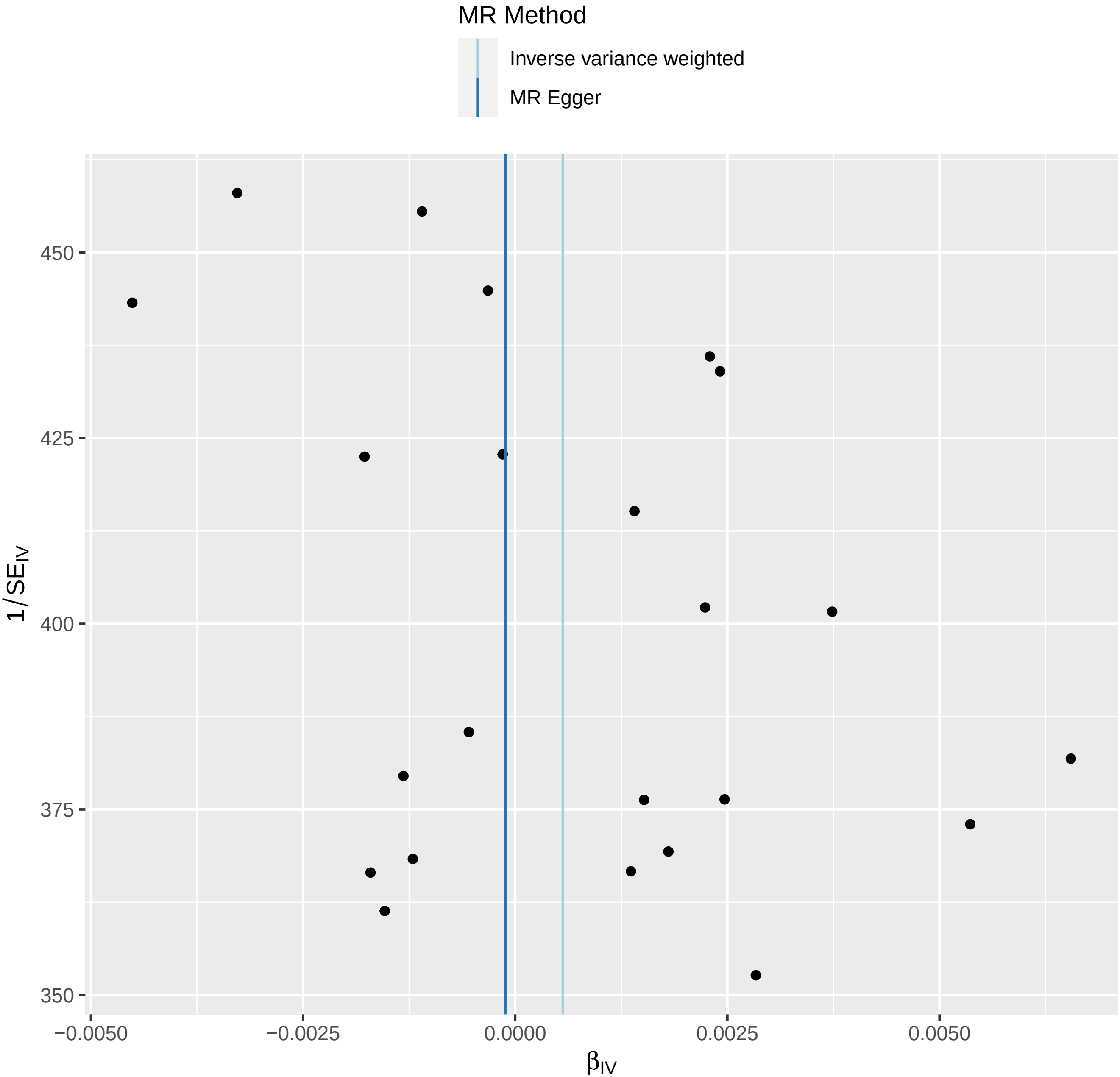

Batch 2−1592 : Integral membrane protein 2C on eGFR

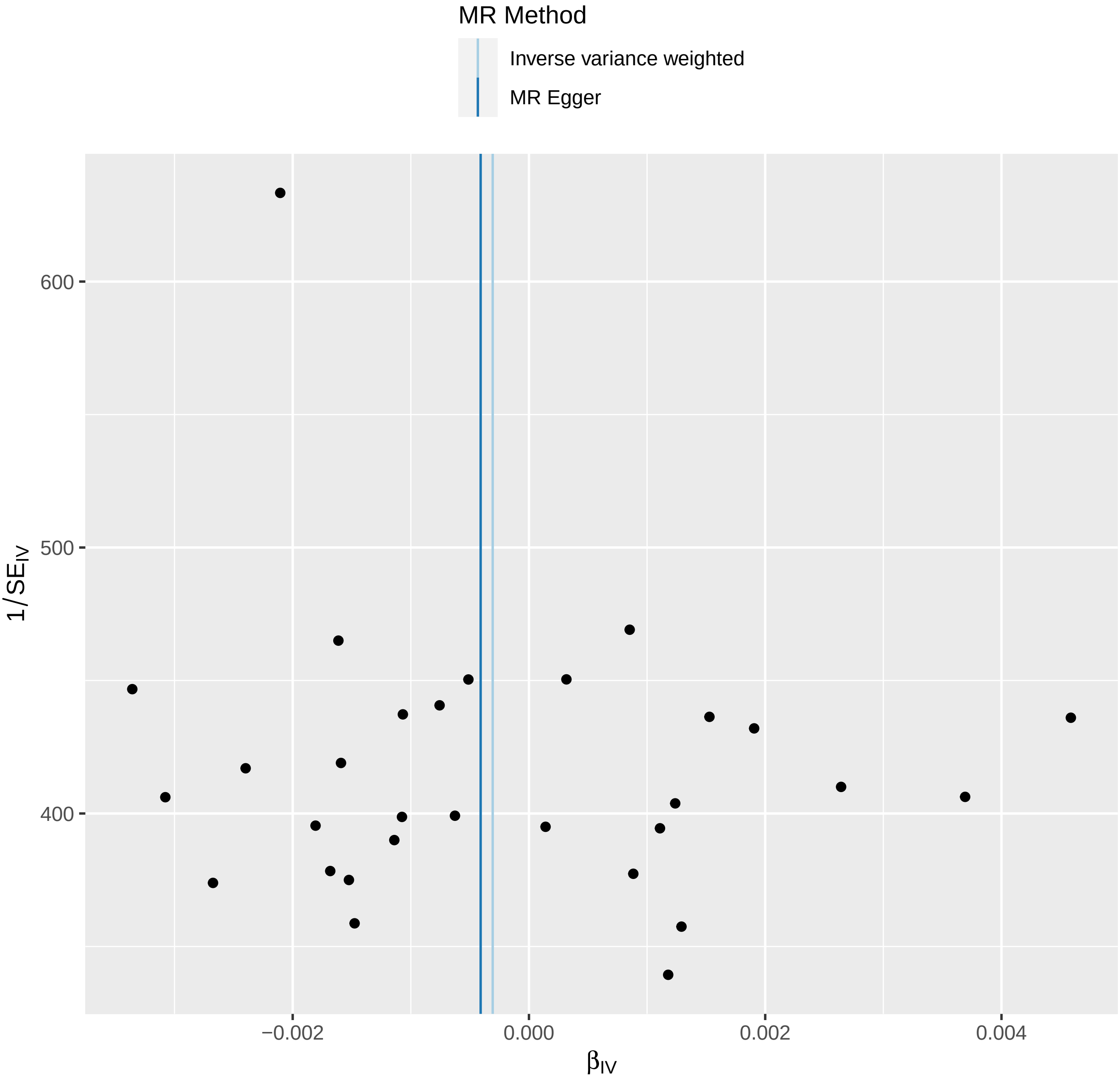

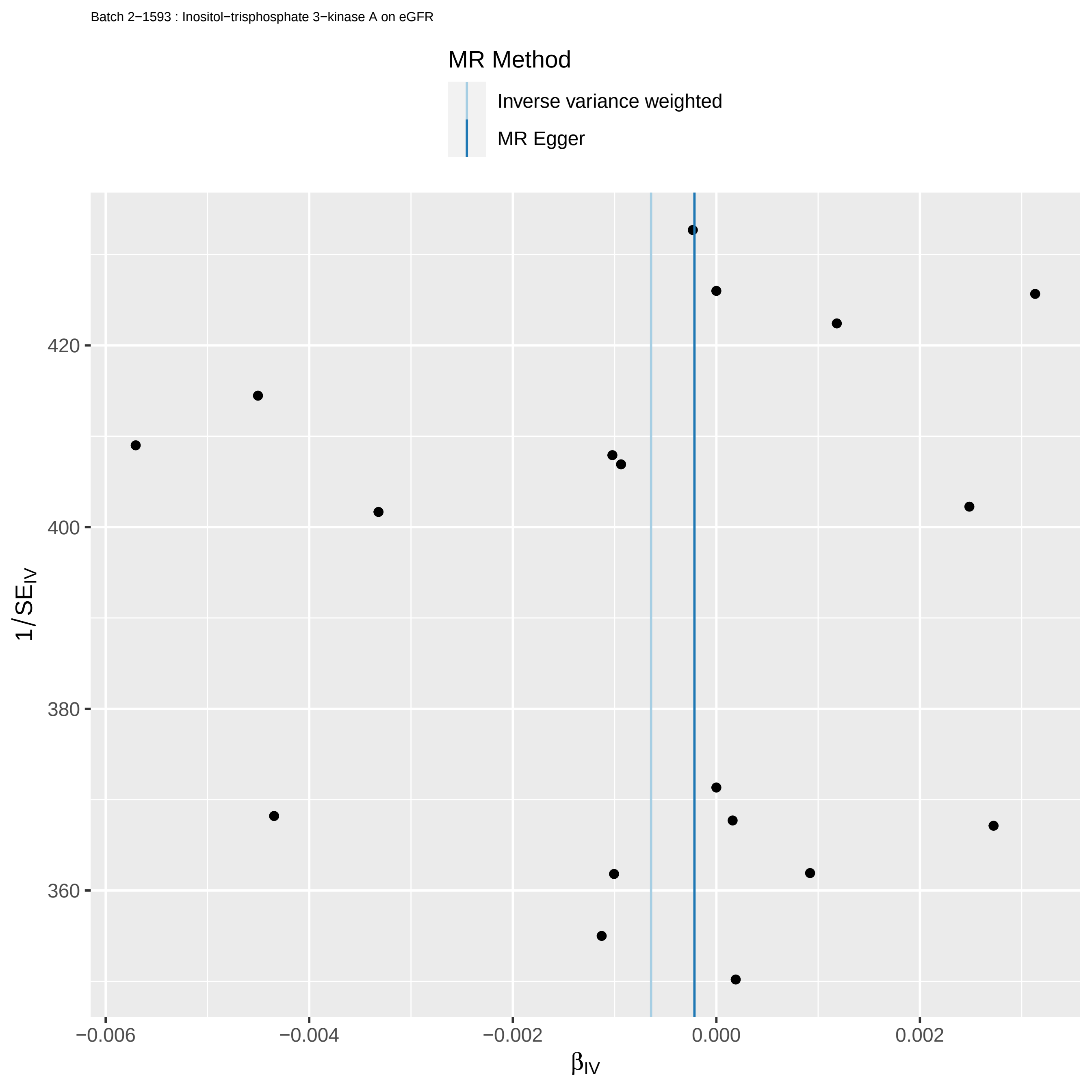

Batch 2−1593 : Inositol−trisphosphate 3−kinase A on eGFR
MR Method
Inverse variance weighted
MR Egger
420
400
380
360
1/SE_IV
−0.006
−0.004
−0.002
0.000
0.002
β_IV

Batch 2−1594 : Inositol 1,4,5−trisphosphate receptor−interacting protein−like 1 on eGFR

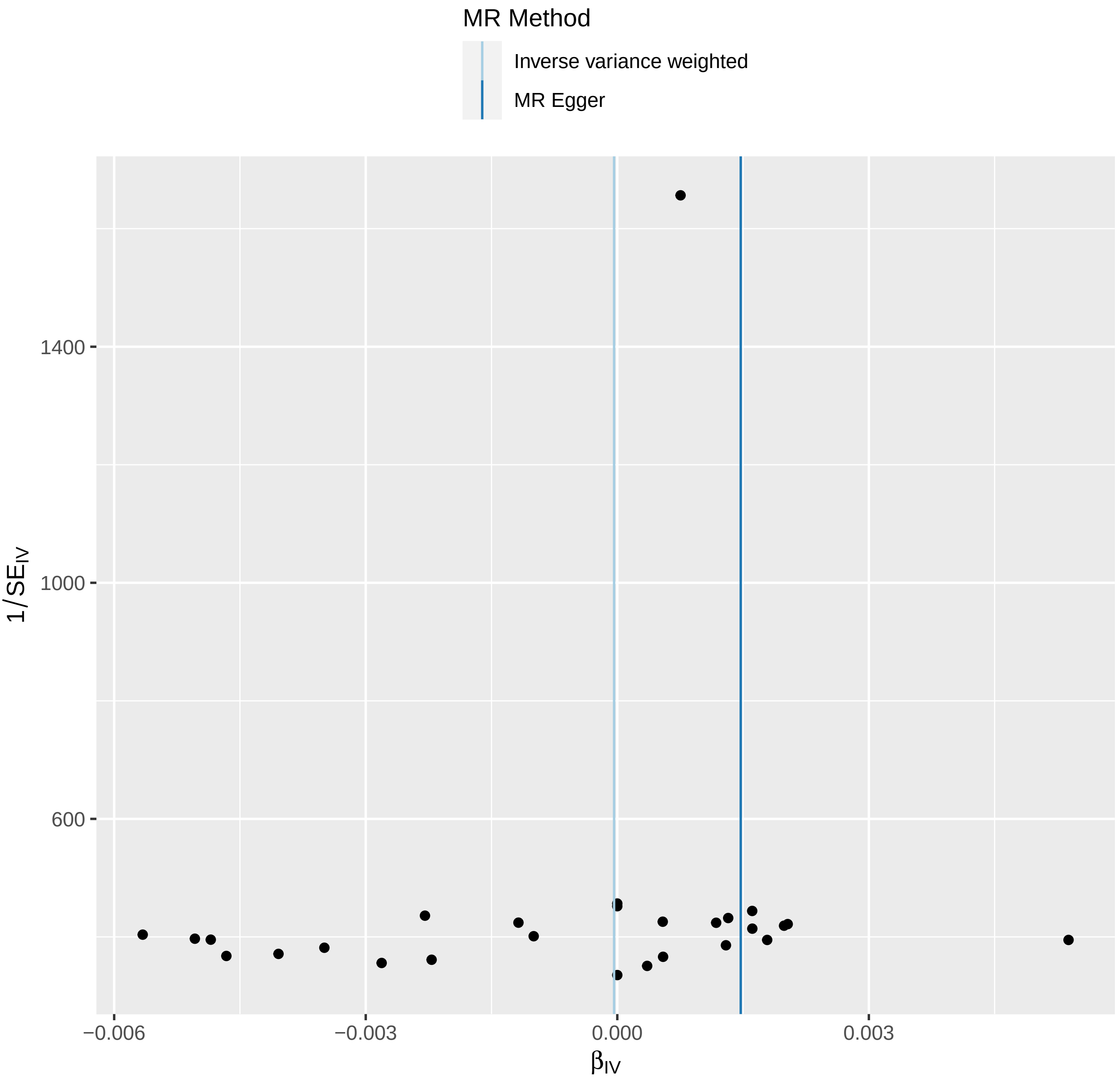

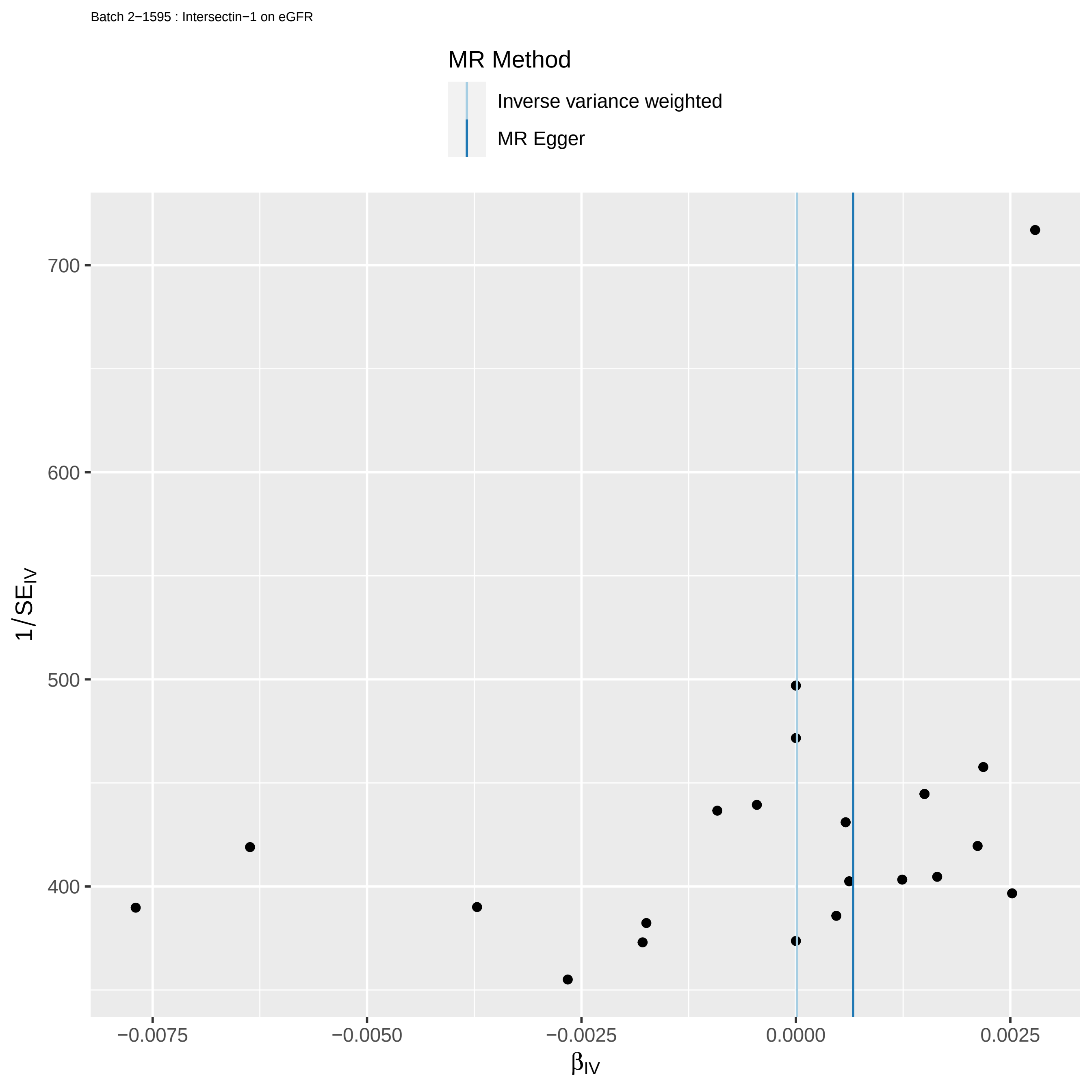
Batch 2−1595 : Intersectin−1 on eGFR
MR Method
Inverse variance weighted
MR Egger
700
600
500
400
1/SE$_{IV}$
−0.0075
−0.0050
−0.0025
0.0000
0.0025
β$_{IV}$

Batch 2−1596 : Protein jagged−1 on eGFR

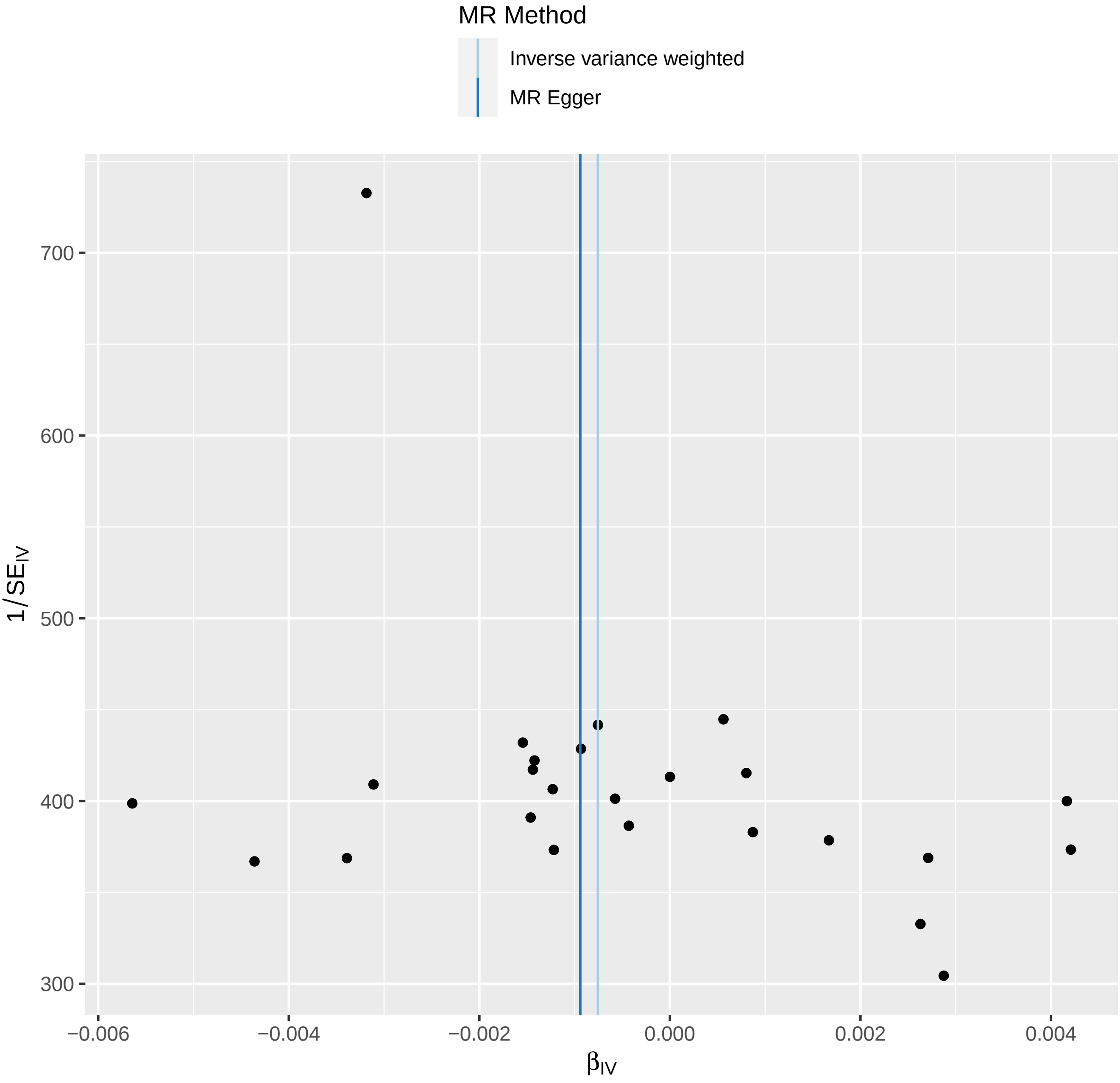

Batch 2−1597 : Protein jagged−2 on eGFR

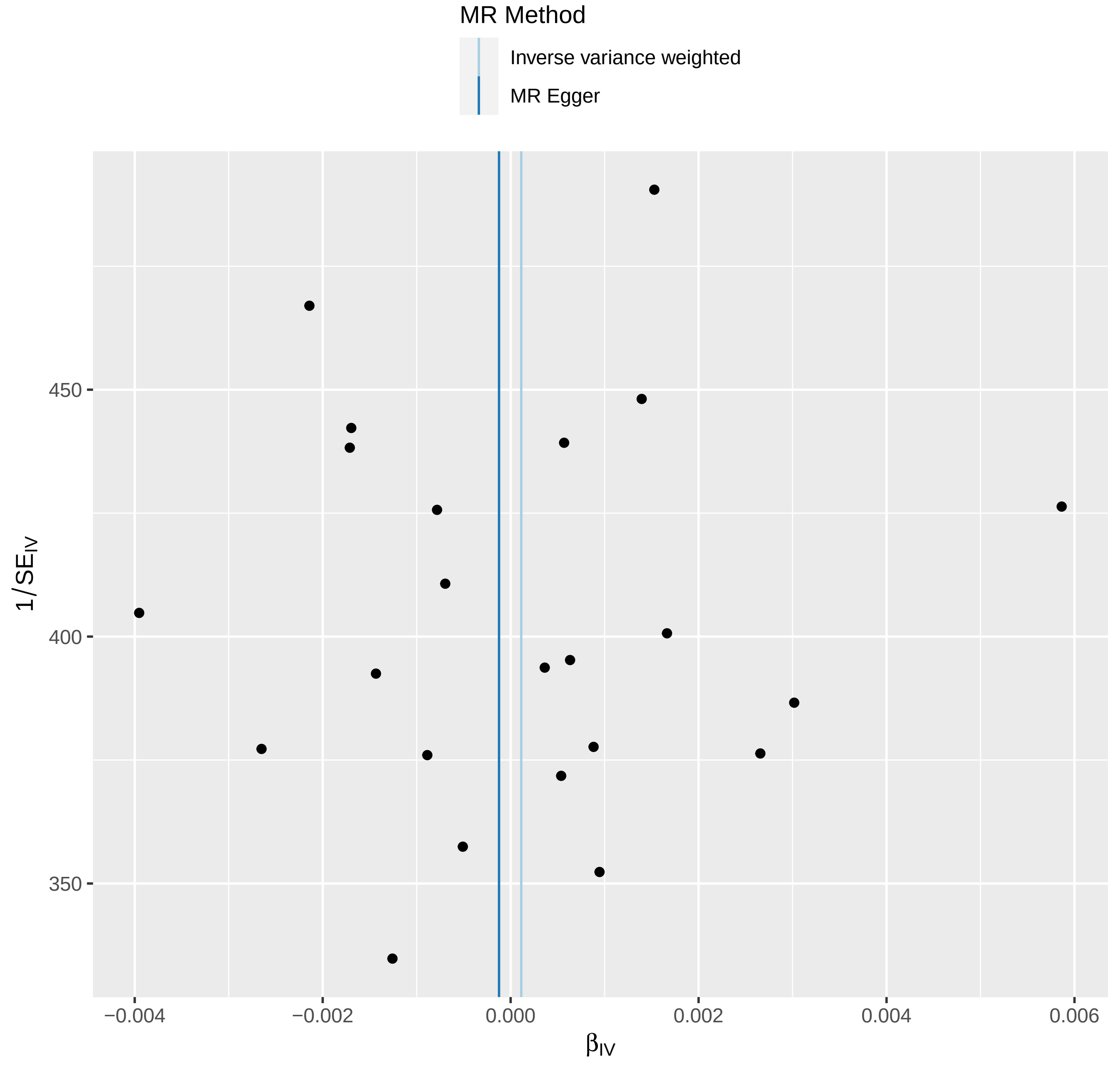

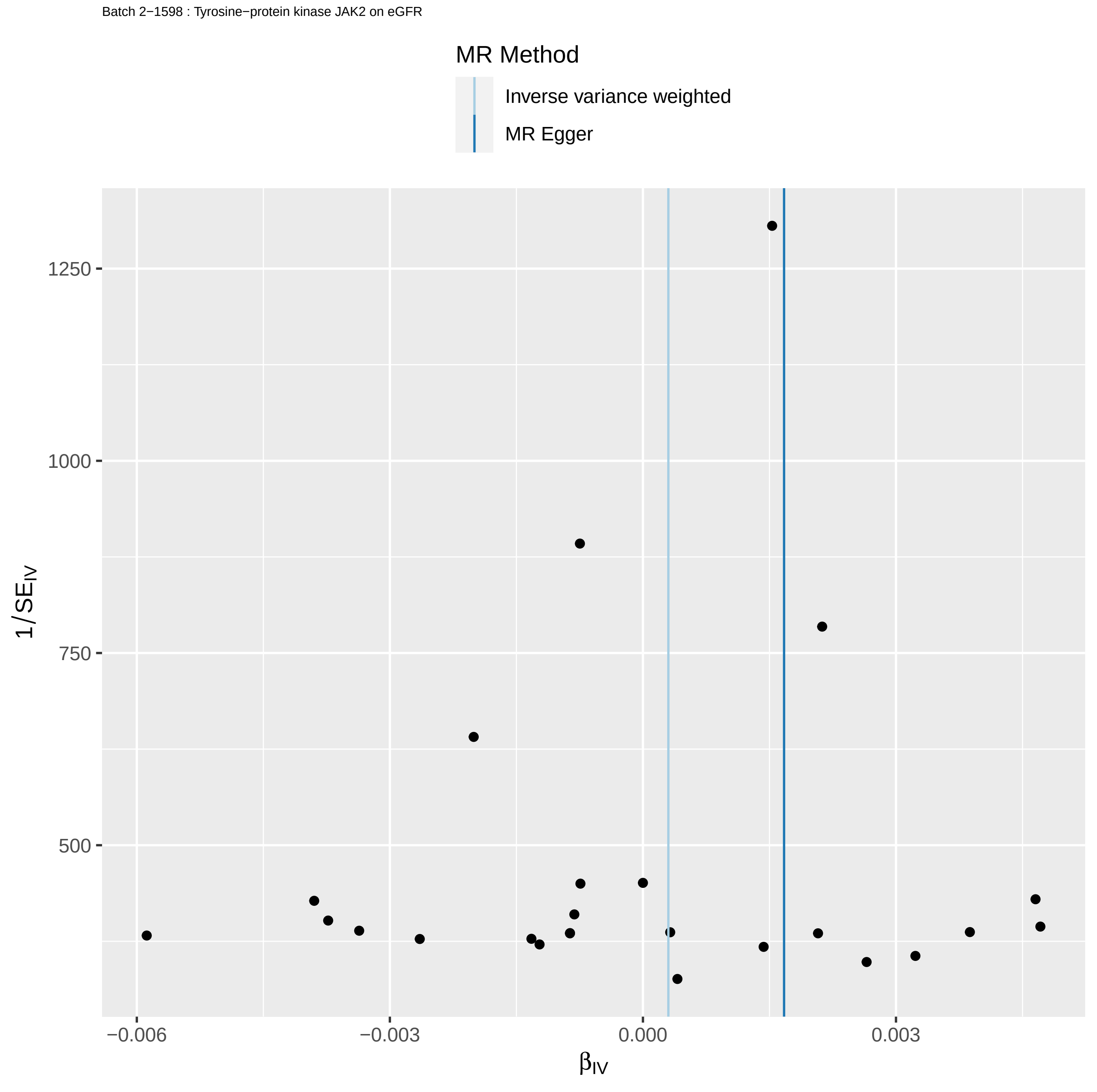

Batch 2−1598 : Tyrosine−protein kinase JAK2 on eGFR
MR Method
Inverse variance weighted
MR Egger
1250
1000
750
500
1/SE_IV
−0.006
−0.003
0.000
0.003
β_IV

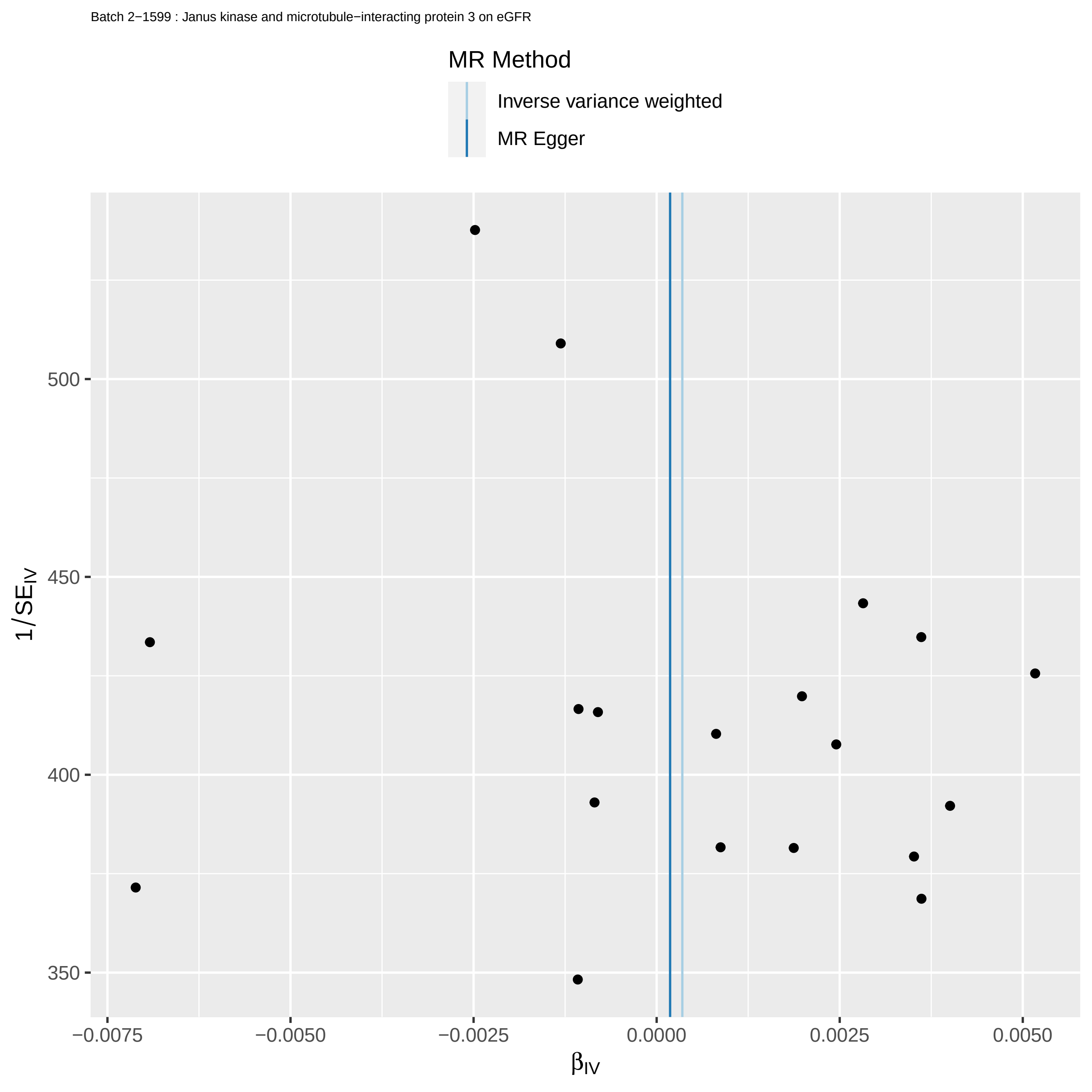
Batch 2−1599 : Janus kinase and microtubule−interacting protein 3 on eGFR
MR Method
Inverse variance weighted
MR Egger
500
450
400
350
1/SE$_{IV}$
−0.0075
−0.0050
−0.0025
0.0000
0.0025
0.0050
β$_{IV}$

Batch 2−1600 : Junctional adhesion molecule B on eGFR

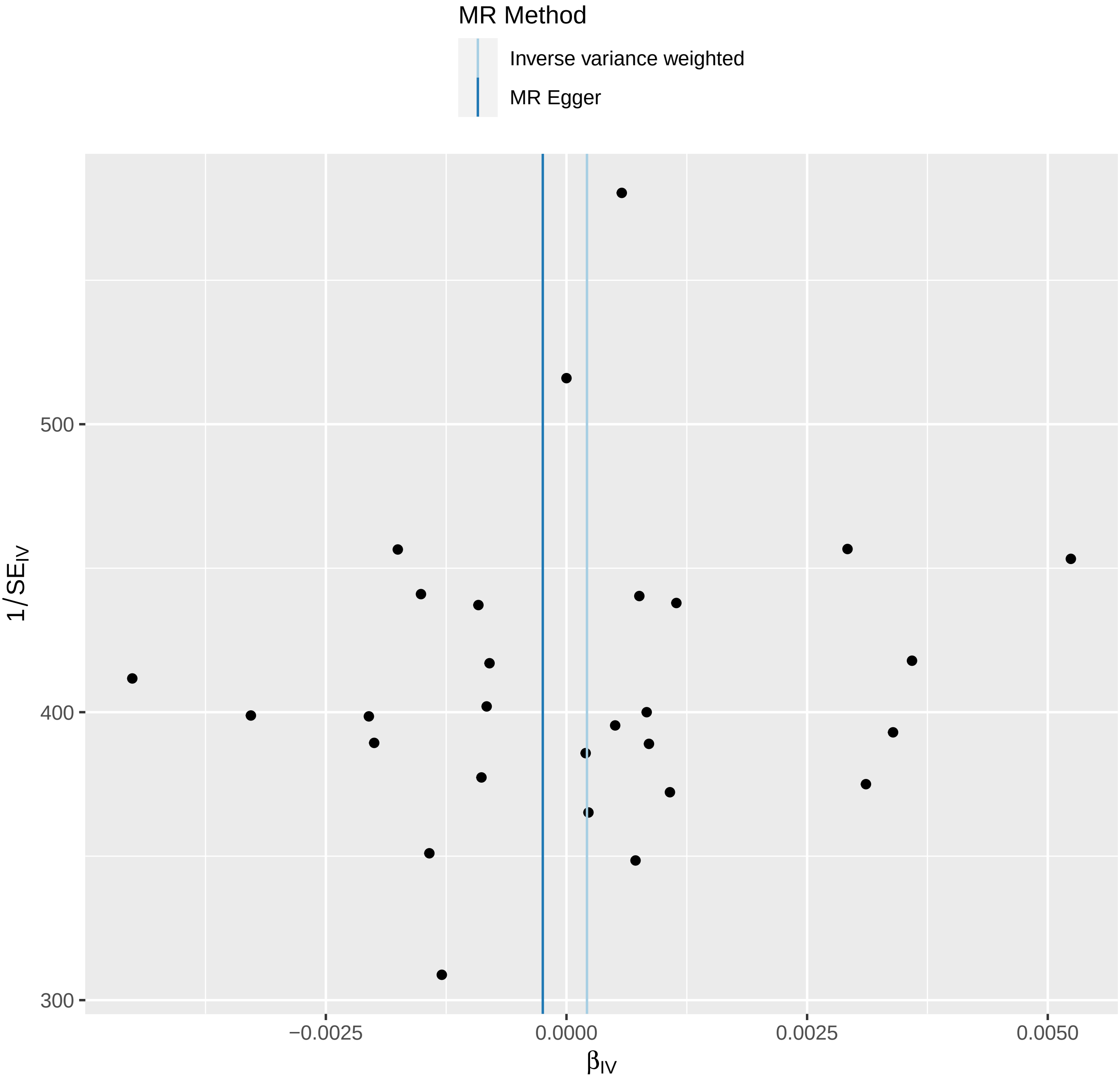

Batch 2−1601 : Junctional adhesion molecule C on eGFR

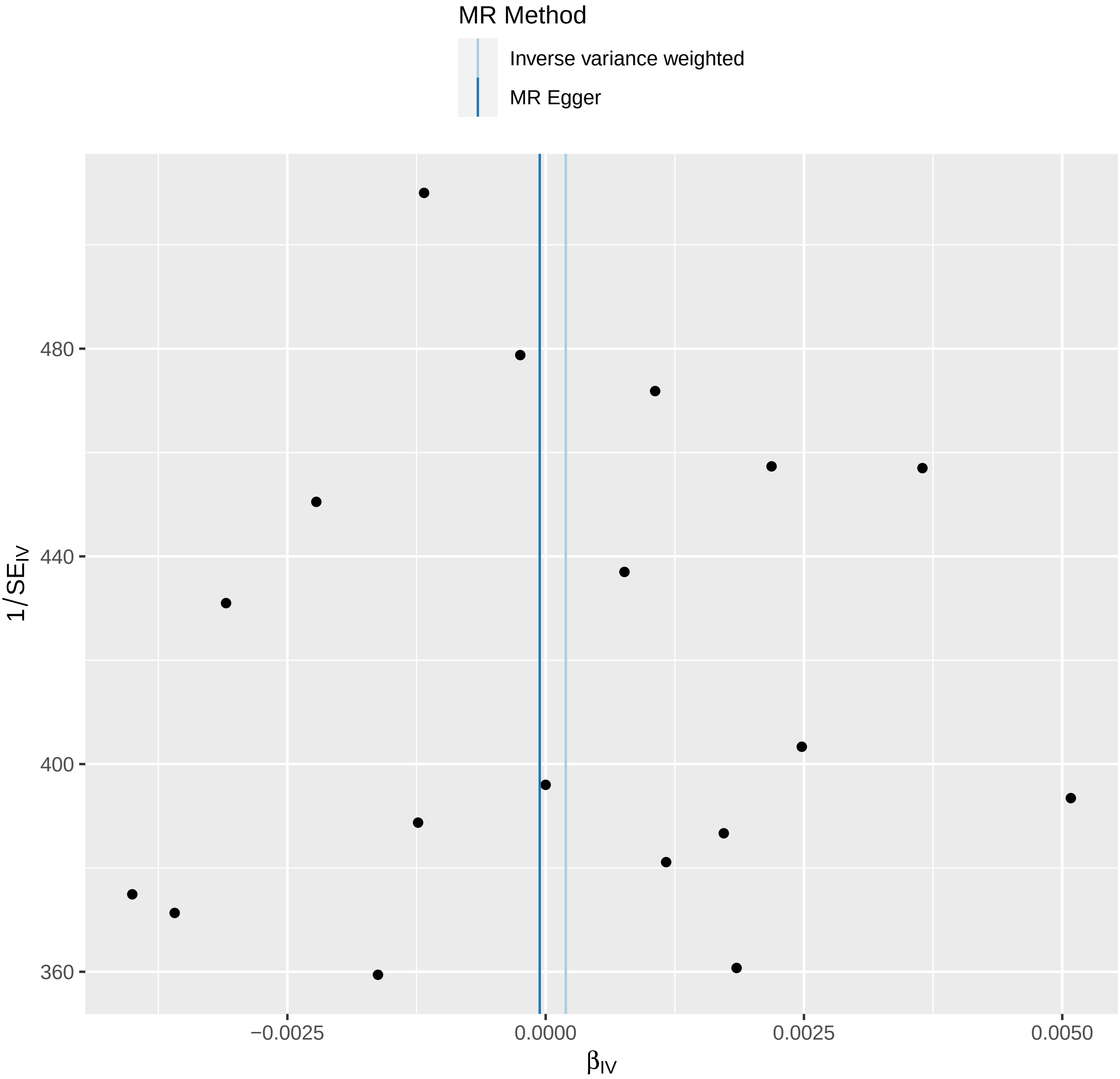

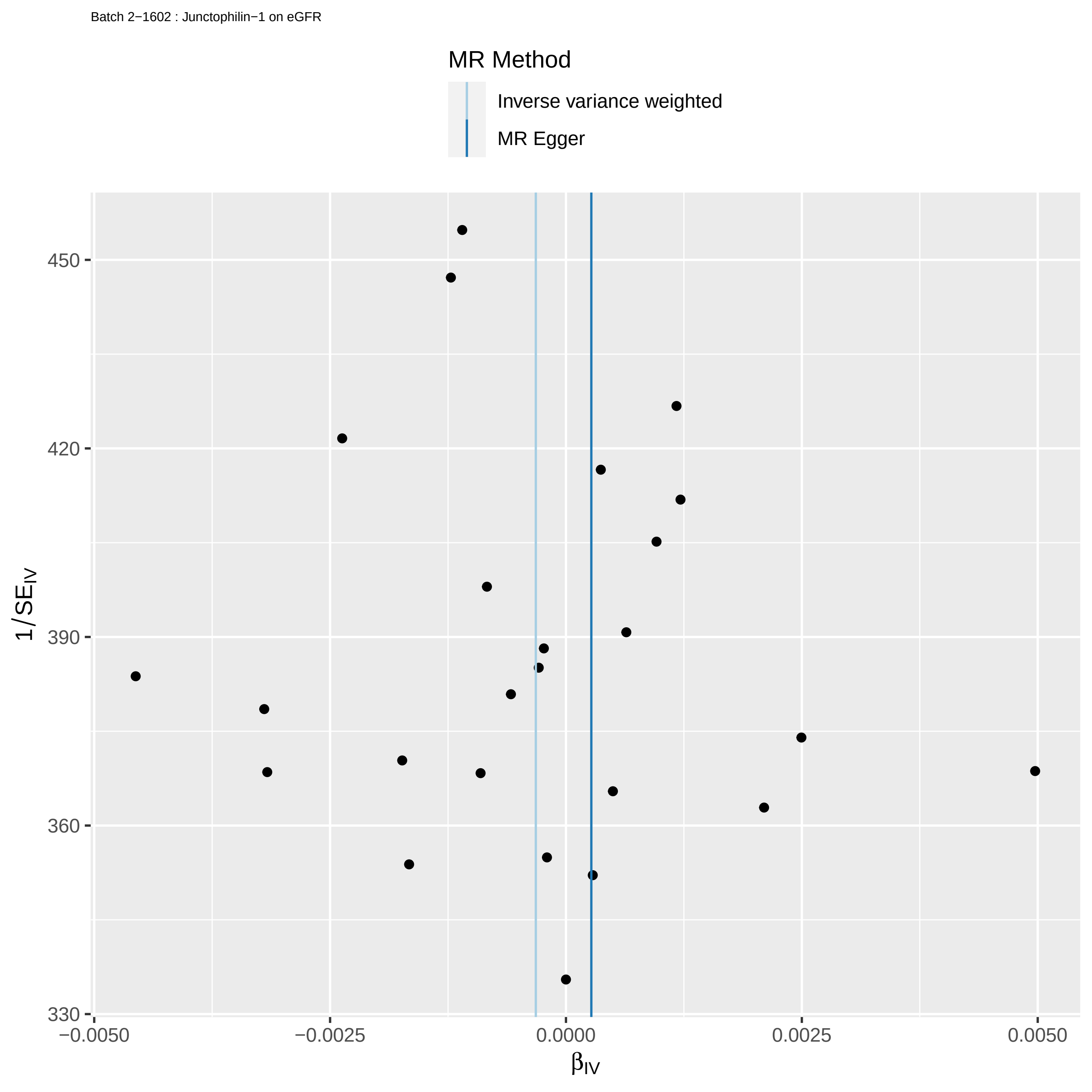
Batch 2−1602 : Junctophilin−1 on eGFR
MR Method
Inverse variance weighted
MR Egger
450
420
390
360
330
1/SE$_{IV}$
−0.0050
−0.0025
0.0000
0.0025
0.0050
β_{IV}

Batch 2−1603 : Junctophilin−3 on eGFR

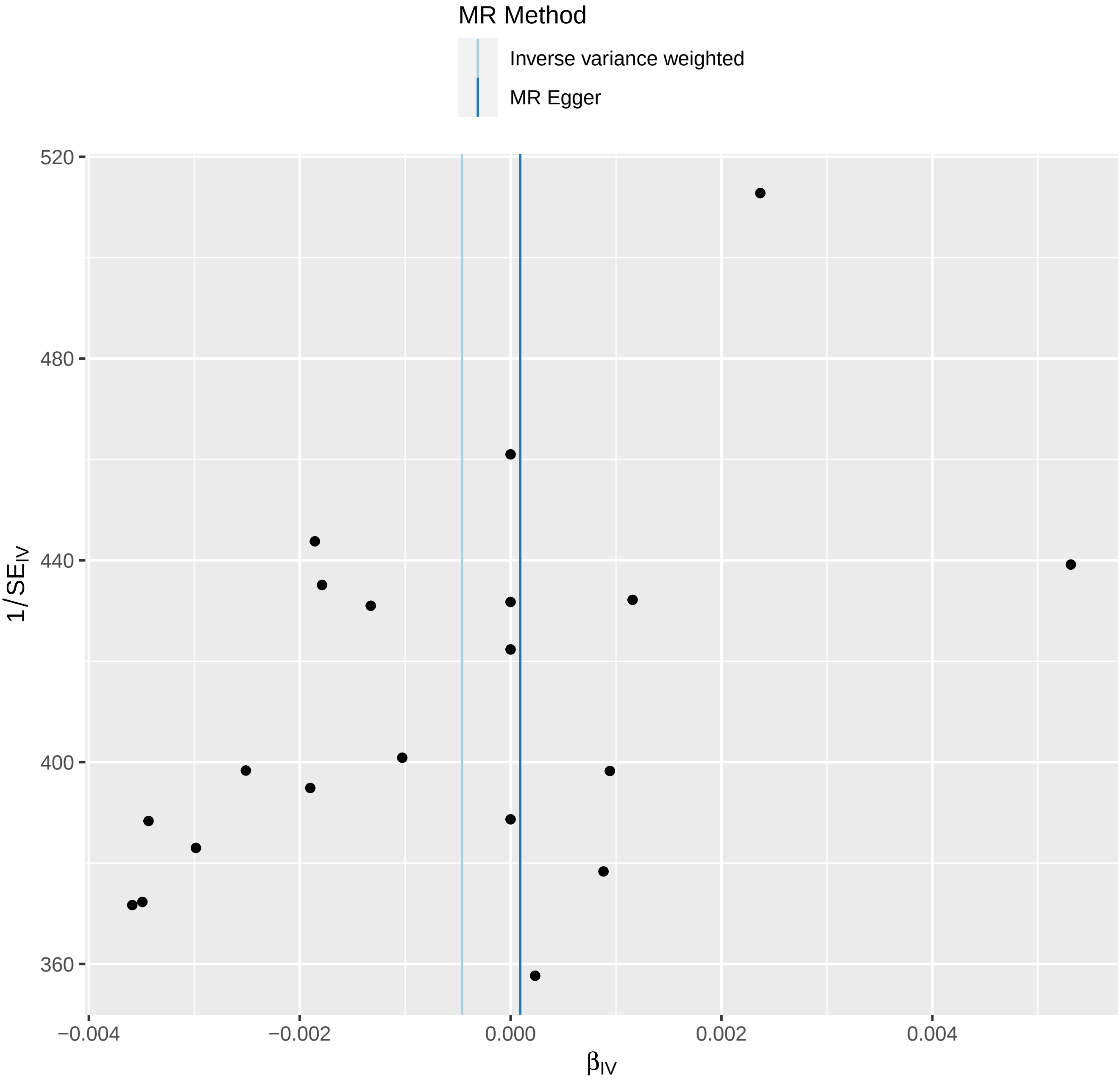

Batch 2−1604 : Protein JTB on eGFR

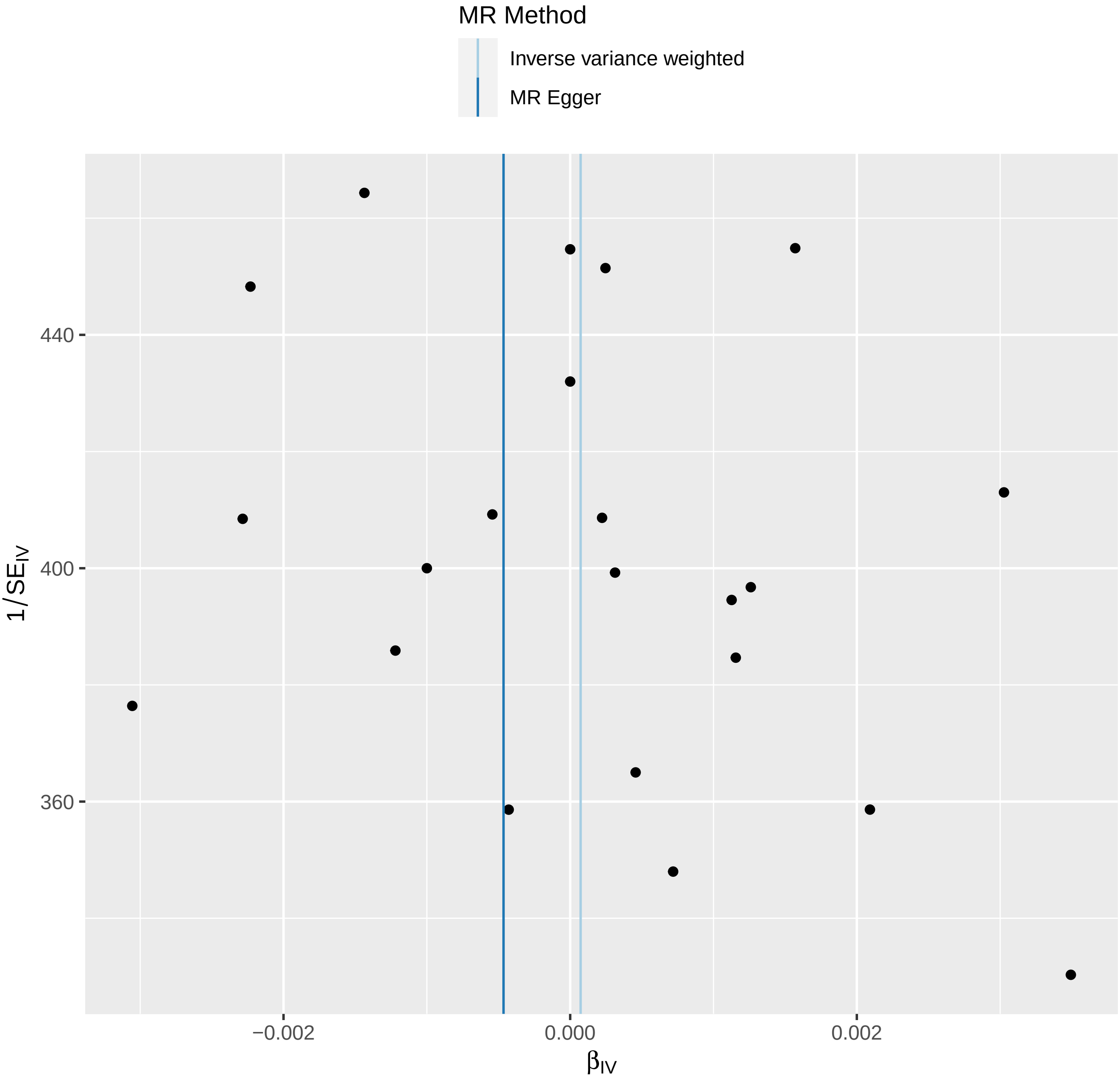

Batch 2−1605 : Transcription factor AP−1 on eGFR

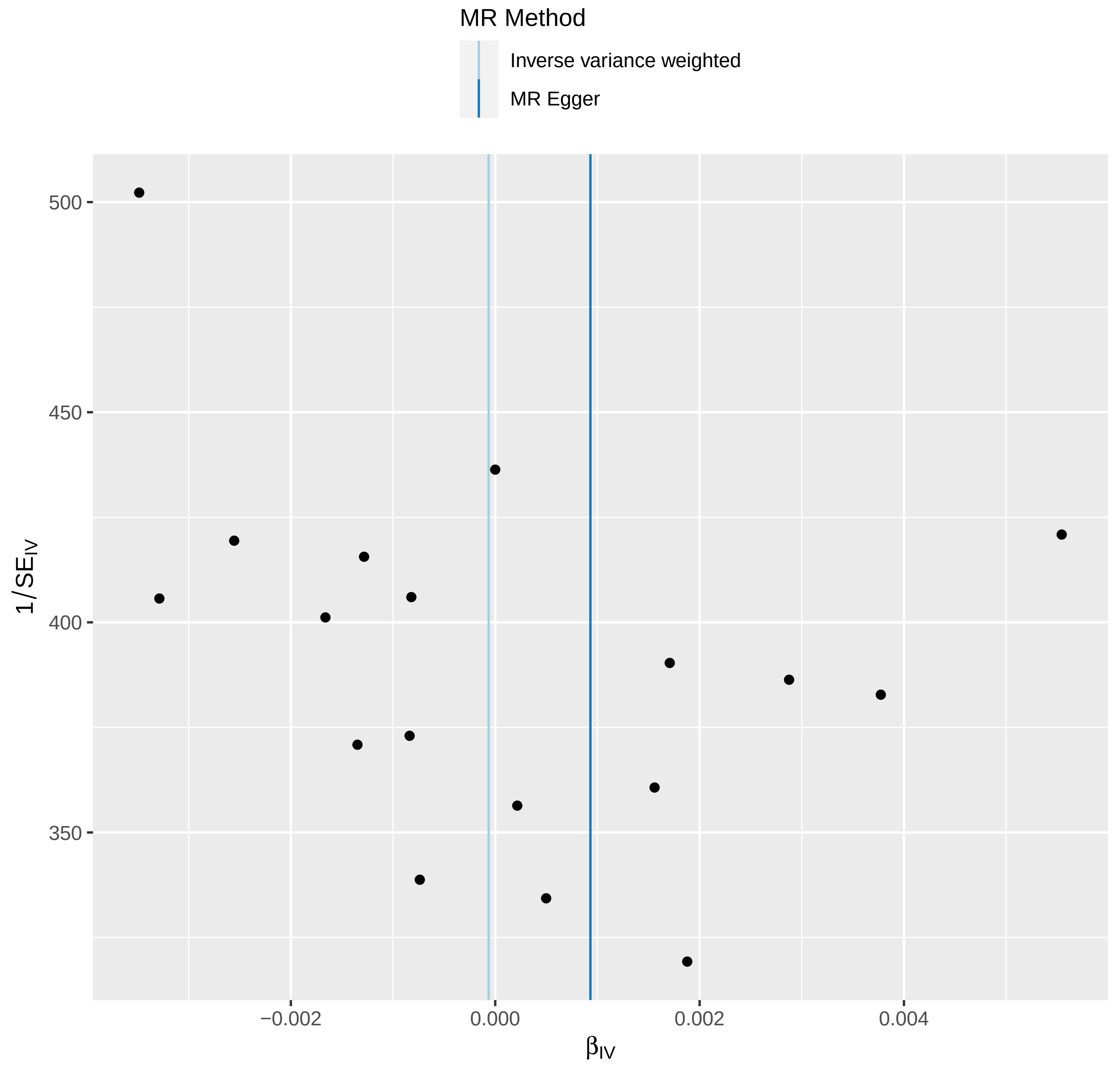

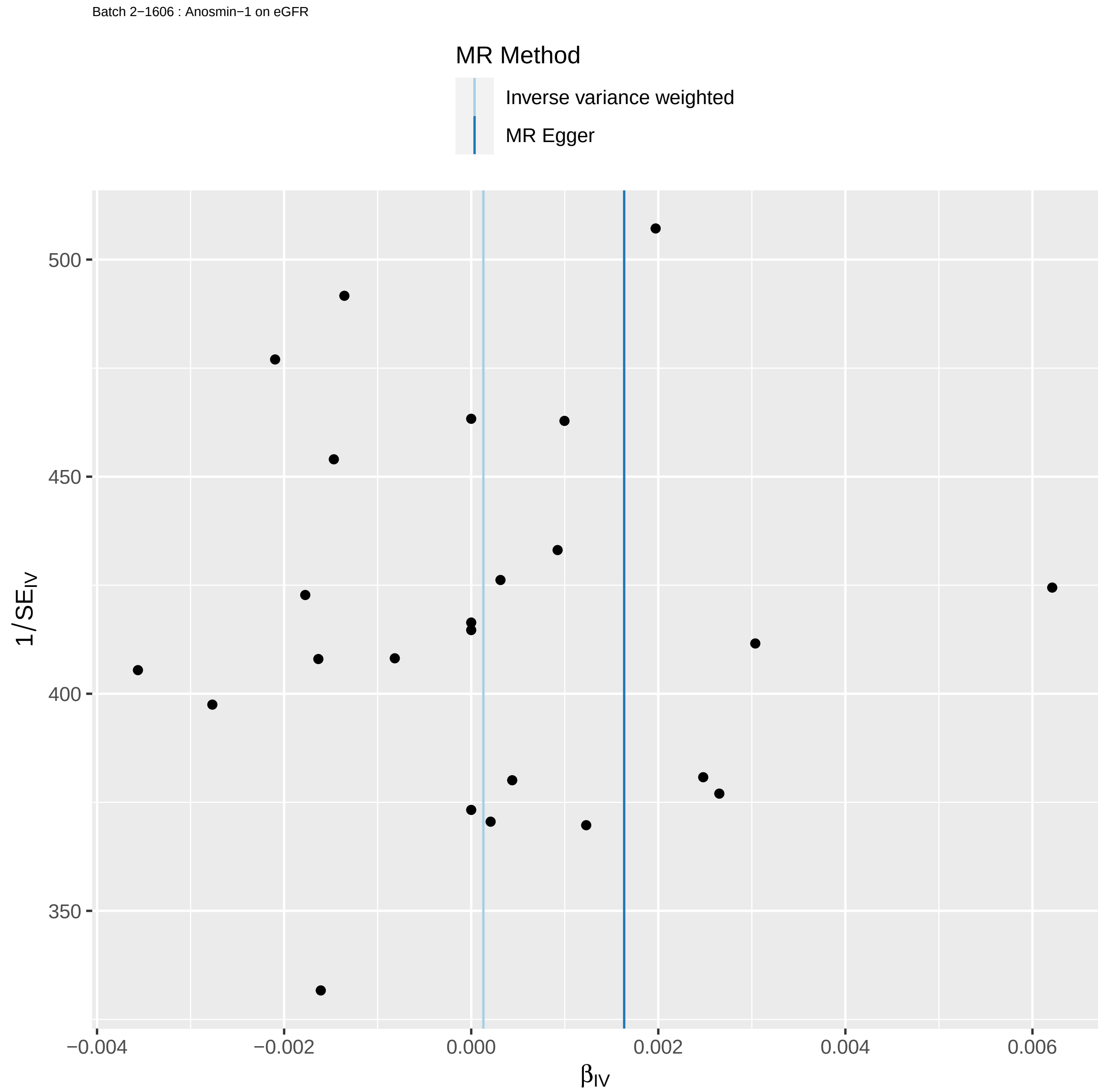
Batch 2−1606 : Anosmin−1 on eGFR
MR Method
Inverse variance weighted
MR Egger
1/SE$_{IV}$
500
450
400
350
−0.004
−0.002
0.000
0.002
0.004
0.006
β$_{IV}$

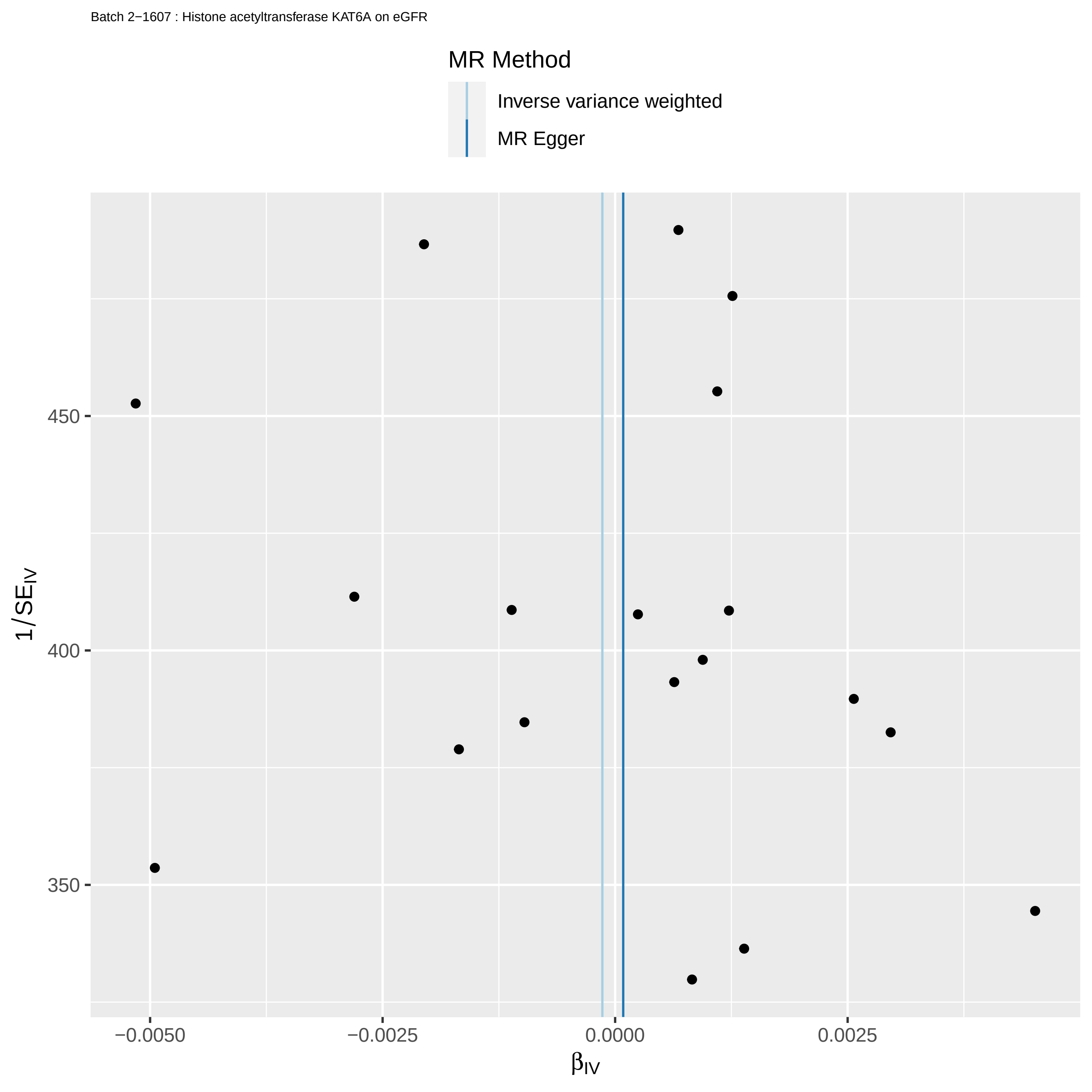
Batch 2−1607 : Histone acetyltransferase KAT6A on eGFR
MR Method
Inverse variance weighted
MR Egger
450
400
350
1/SE_IV
−0.0050
−0.0025
0.0000
0.0025
β_IV

Batch 2−1608 : Potassium voltage−gated channel subfamily A member 10 on eGFR

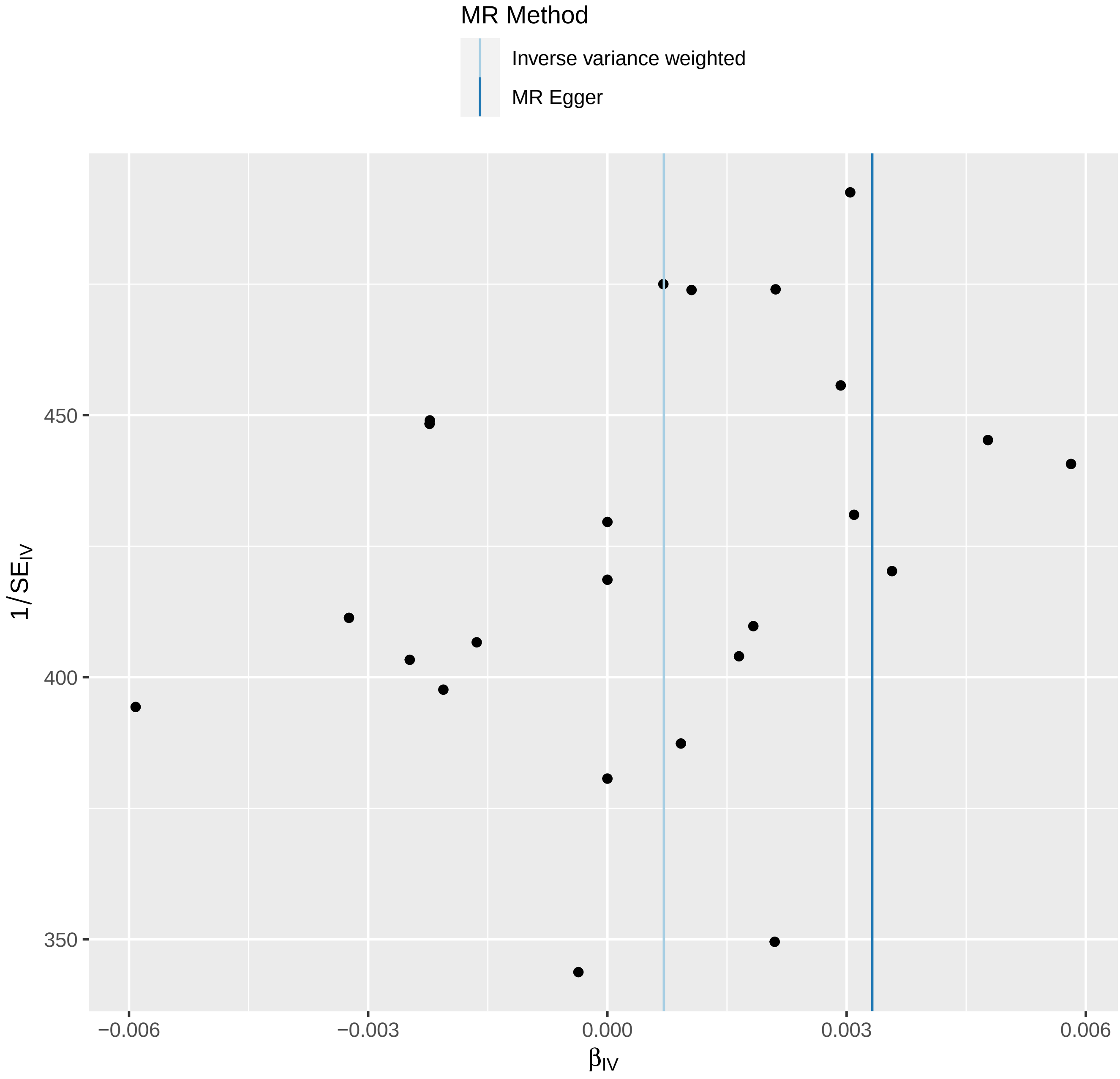

Batch 2−1609 : Voltage−gated potassium channel subunit beta−2 on eGFR

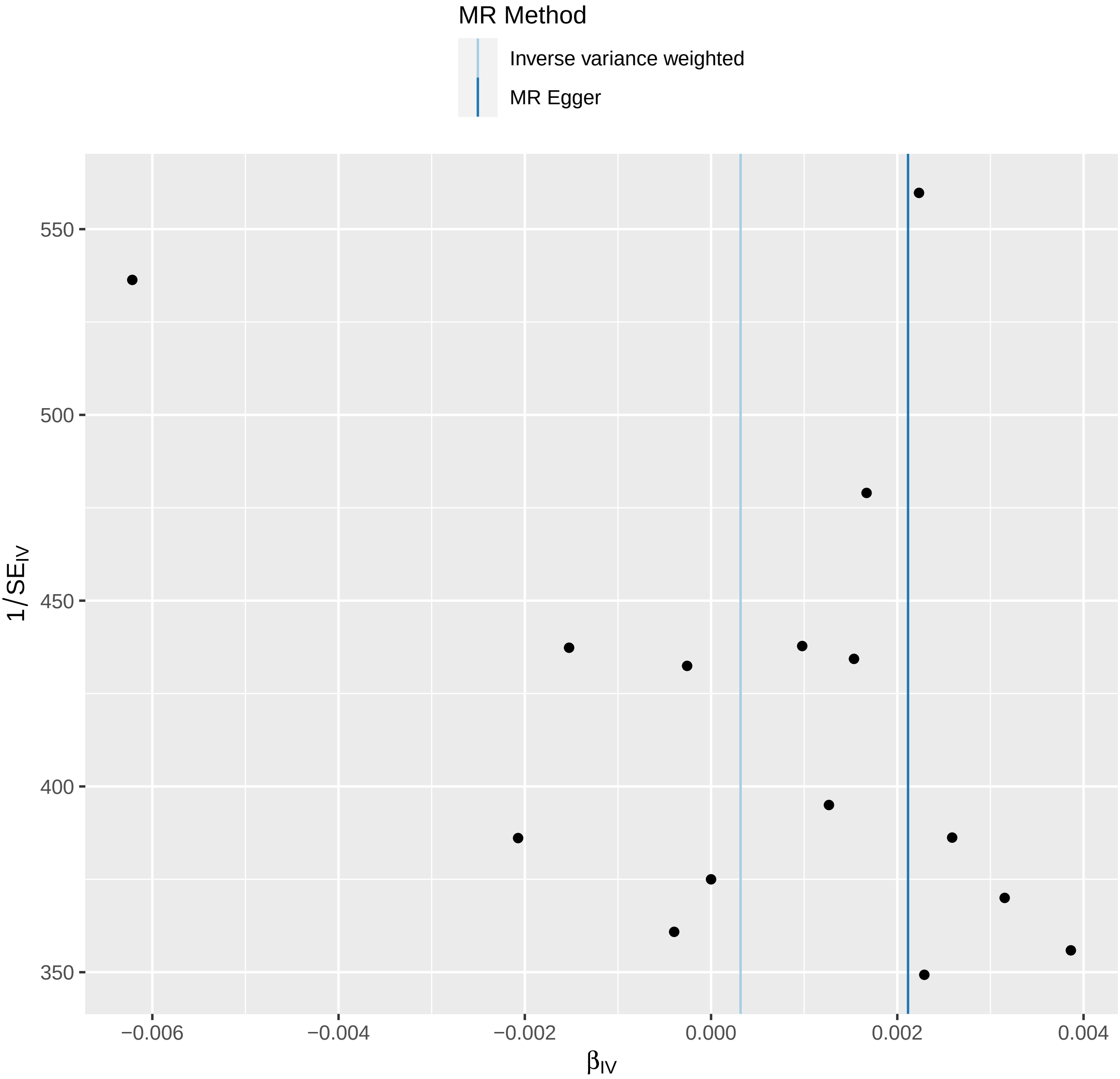

Batch 2−1610 : Potassium voltage−gated channel subfamily E member 2 on eGFR

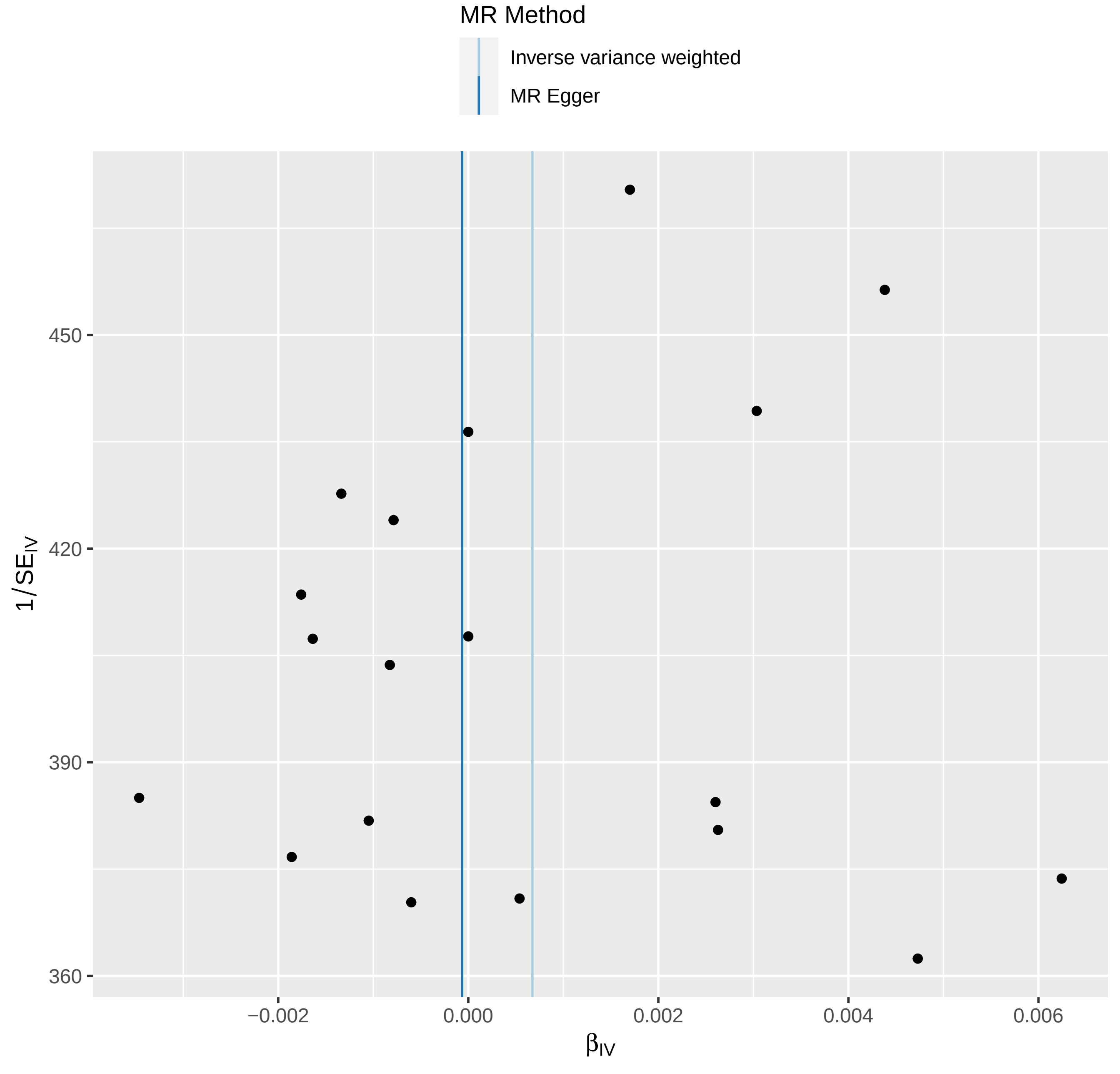

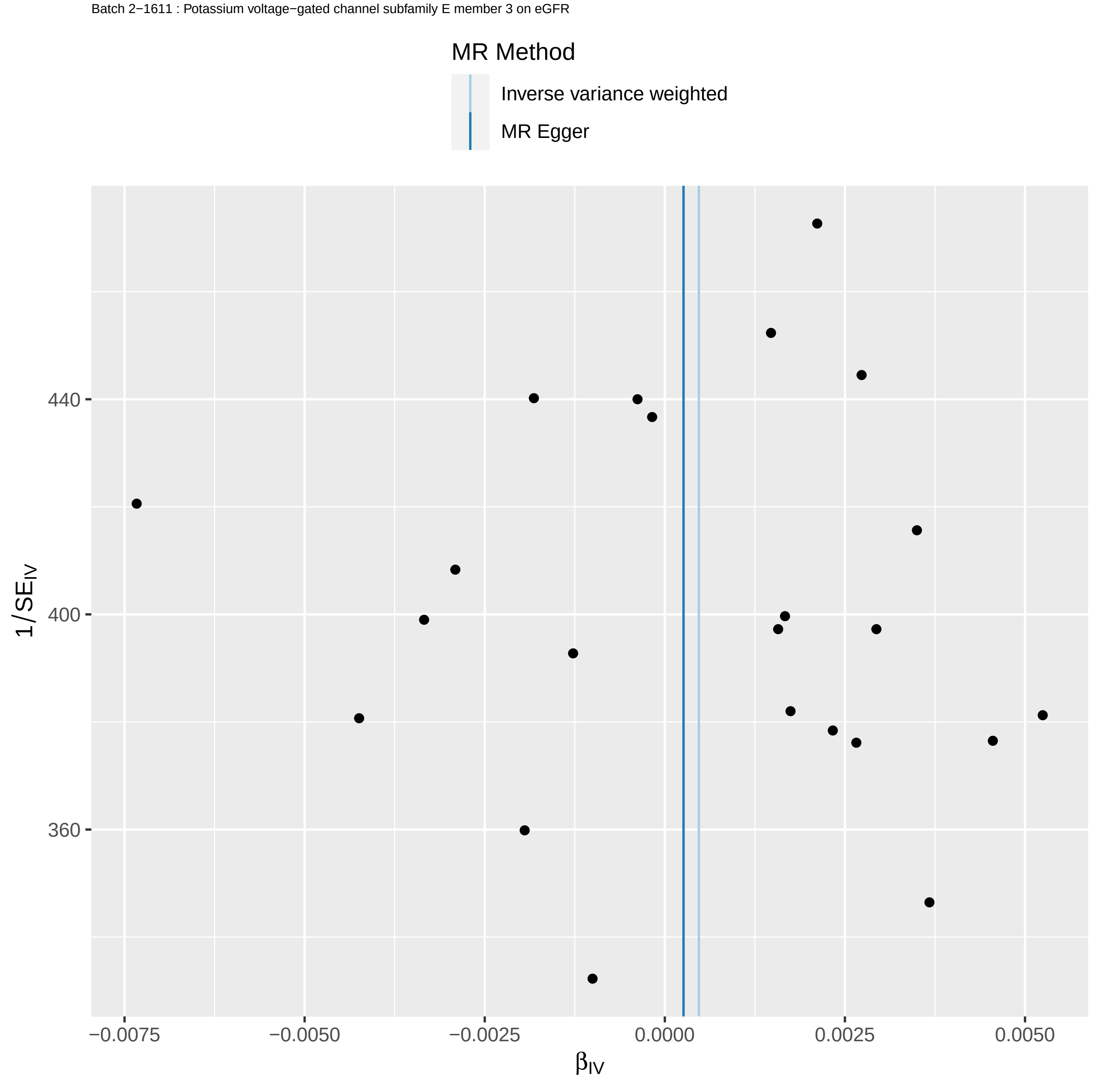
Batch 2−1611 : Potassium voltage−gated channel subfamily E member 3 on eGFR
MR Method
Inverse variance weighted
MR Egger
1/SE_IV
440
400
360
−0.0075
−0.0050
−0.0025
0.0000
0.0025
0.0050
β_IV

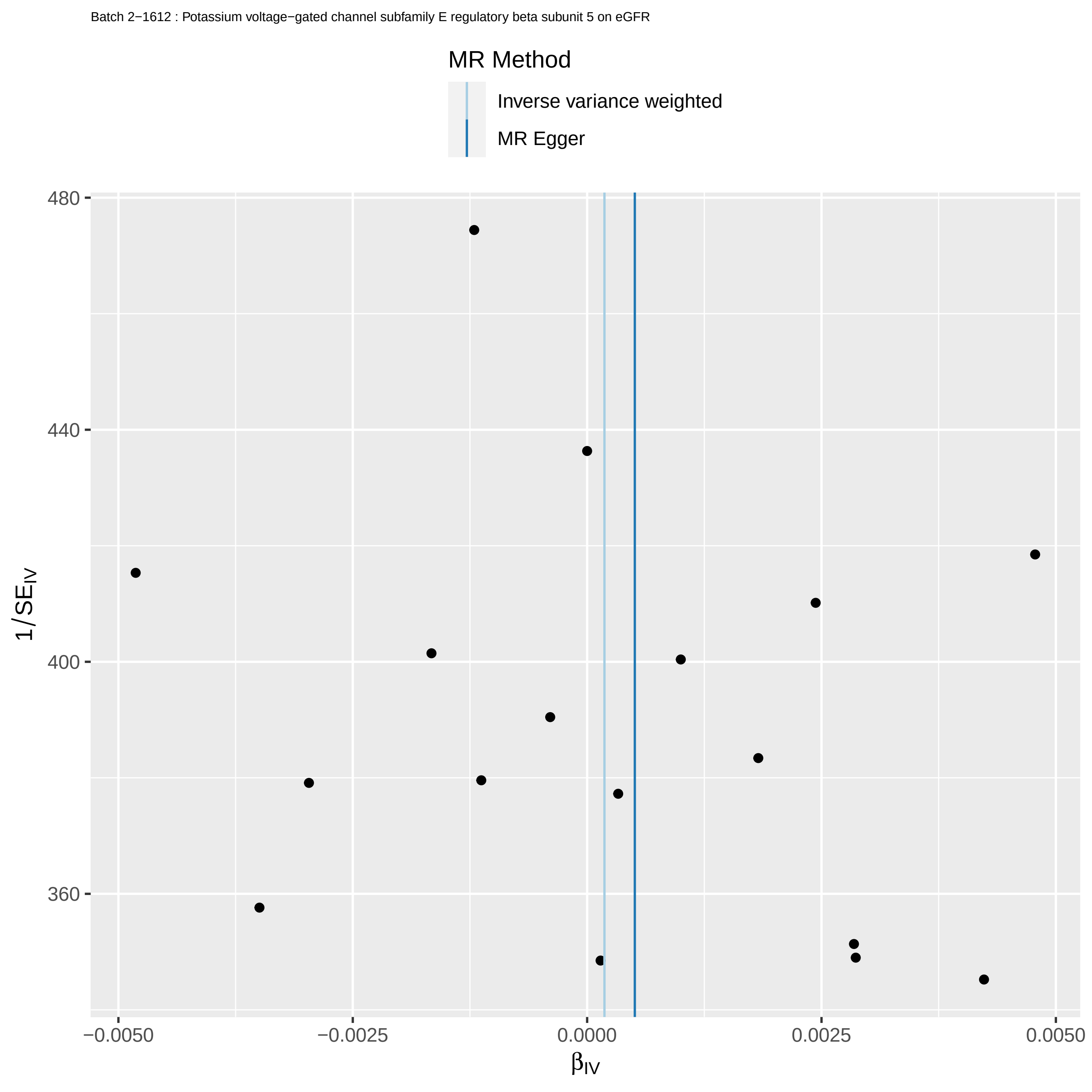
Batch 2−1612 : Potassium voltage−gated channel subfamily E regulatory beta subunit 5 on eGFR
MR Method
Inverse variance weighted
MR Egger
480
440
400
360
1/SE_IV
−0.0050
−0.0025
0.0000
0.0025
0.0050
β_IV

Batch 2−1613 : Potassium voltage−gated channel subfamily E regulatory beta subunit 5 on eGFR

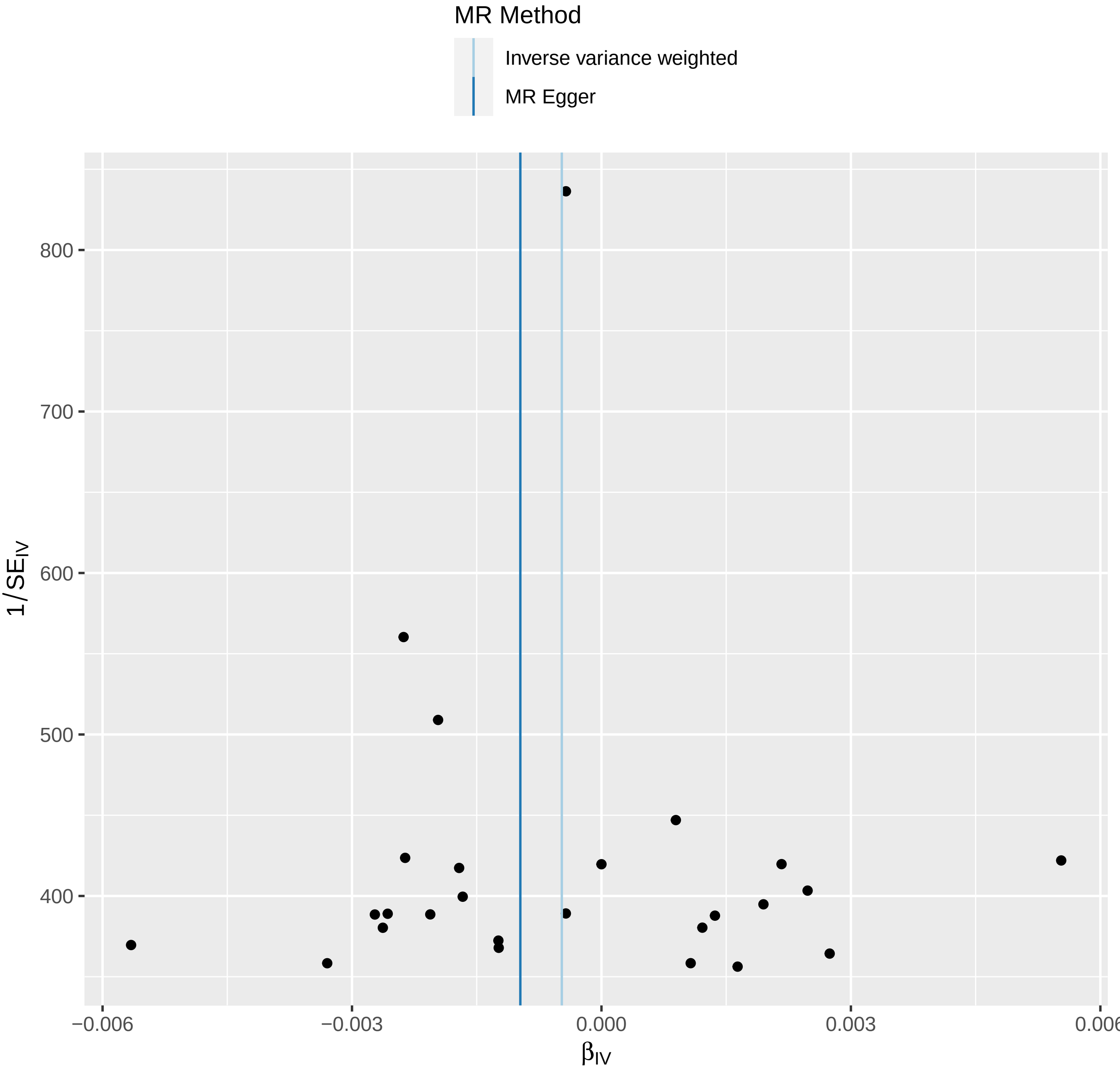

Batch 2−1614 : Potassium voltage−gated channel subfamily F member 1 on eGFR

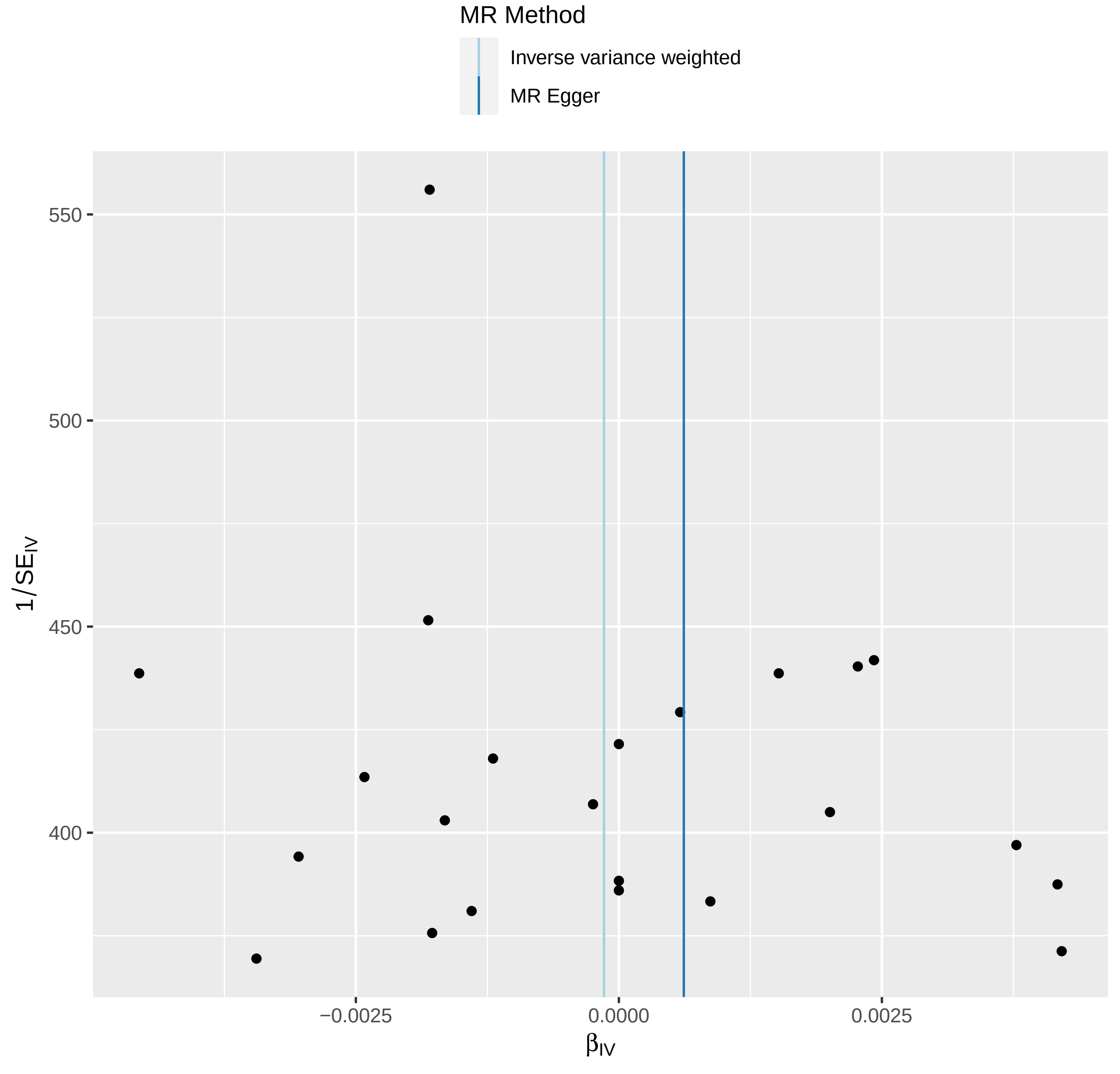

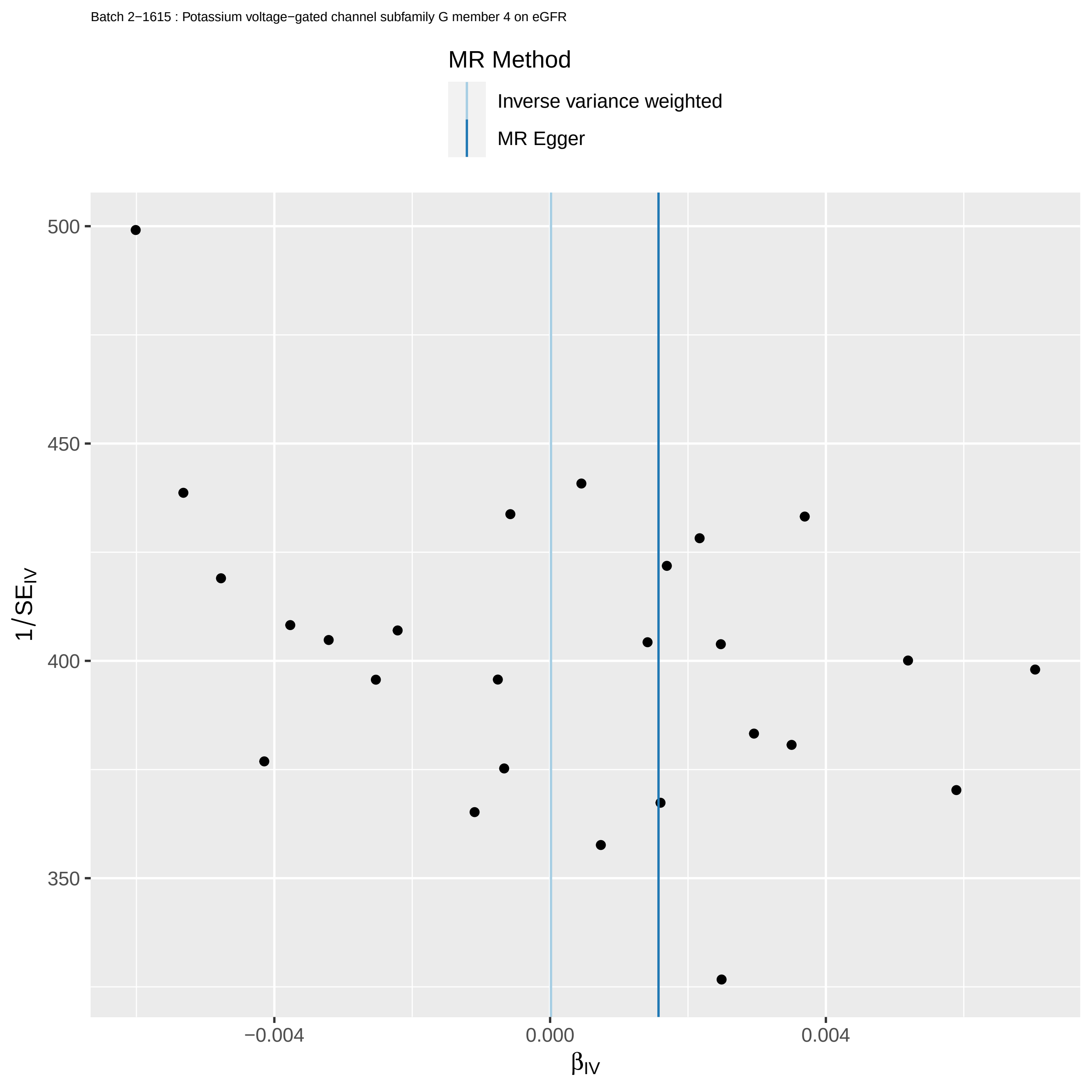
Batch 2−1615 : Potassium voltage−gated channel subfamily G member 4 on eGFR
MR Method
Inverse variance weighted
MR Egger
500
450
400
350
1/SE_IV
−0.004
0.000
0.004
β_IV

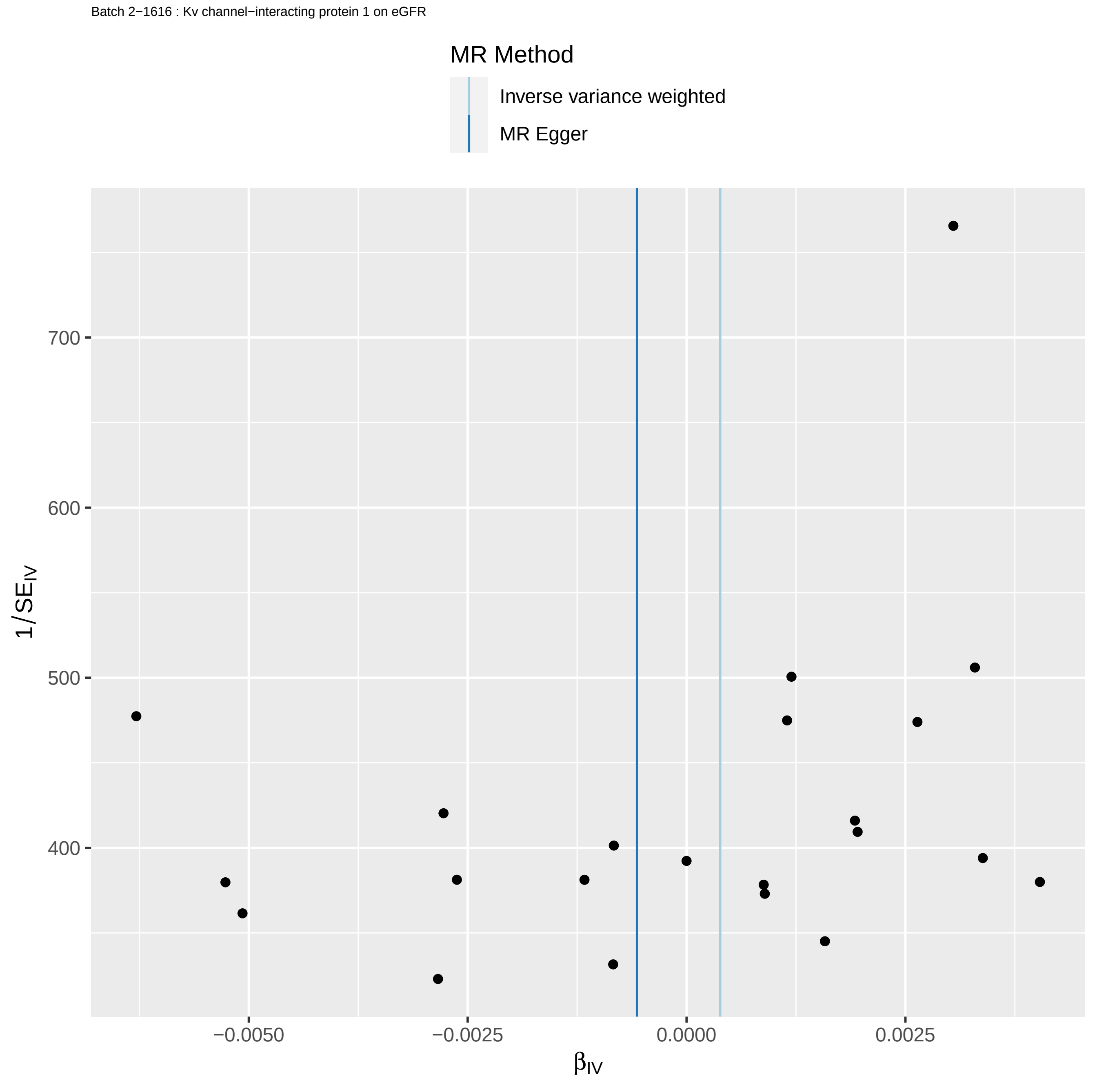

Batch 2−1616 : Kv channel−interacting protein 1 on eGFR
MR Method
Inverse variance weighted
MR Egger
700
600
500
400
1/SE_IV
−0.0050
−0.0025
0.0000
0.0025
β_IV

Batch 2−1617 : Calsenilin on eGFR

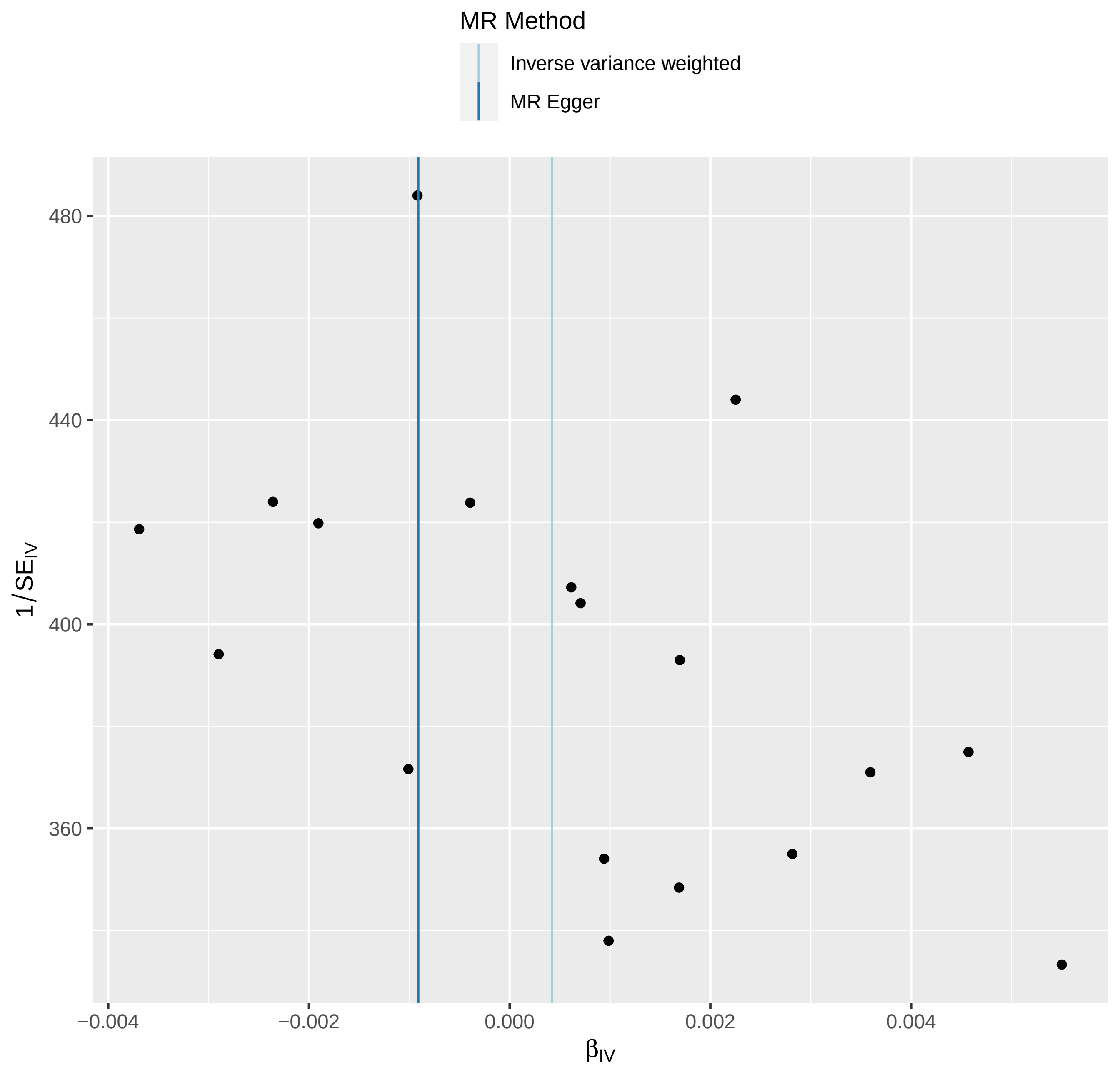

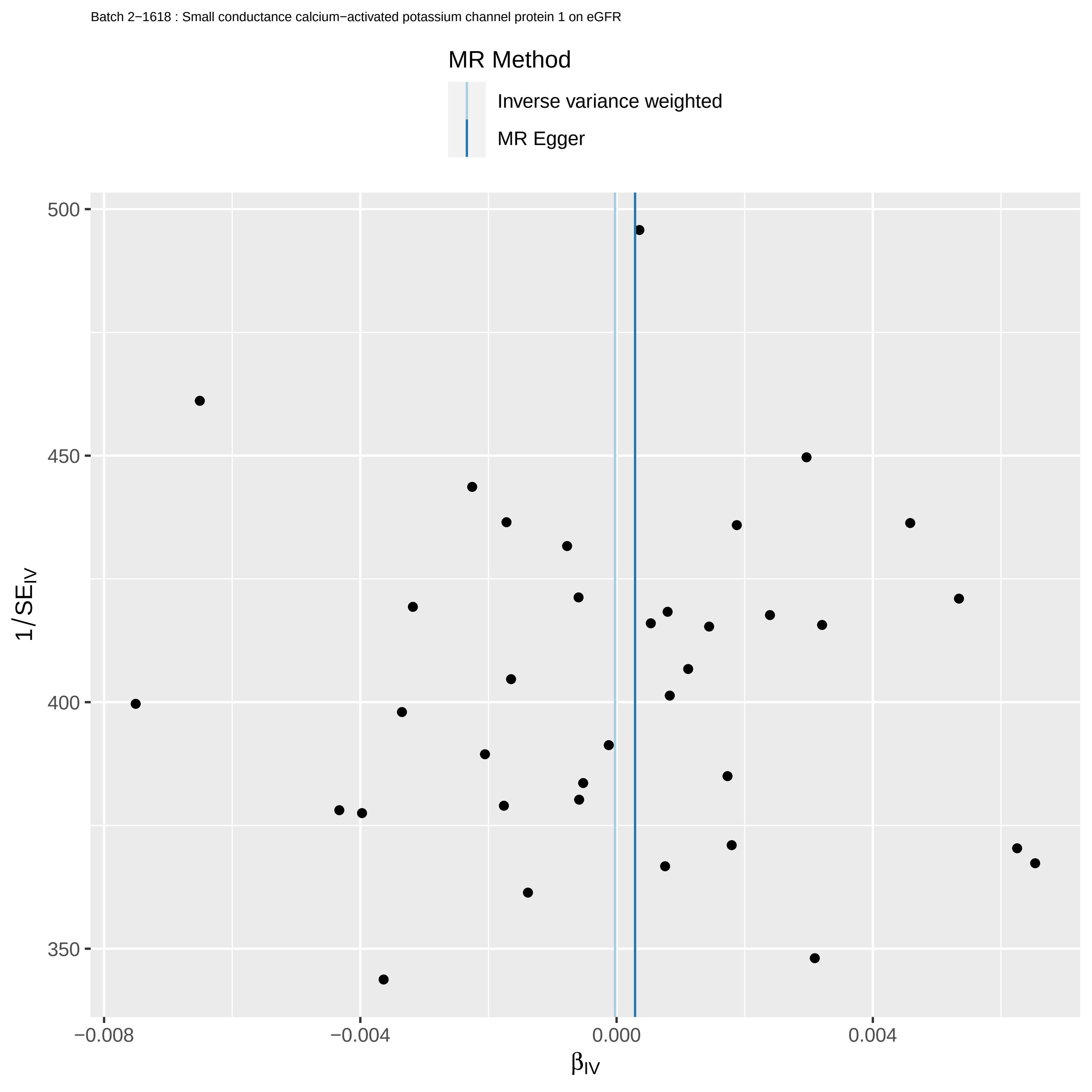
Batch 2−1618 : Small conductance calcium−activated potassium channel protein 1 on eGFR
MR Method
Inverse variance weighted
MR Egger
500
450
400
350
1/SE_IV
−0.008
−0.004
0.000
0.004
β_IV

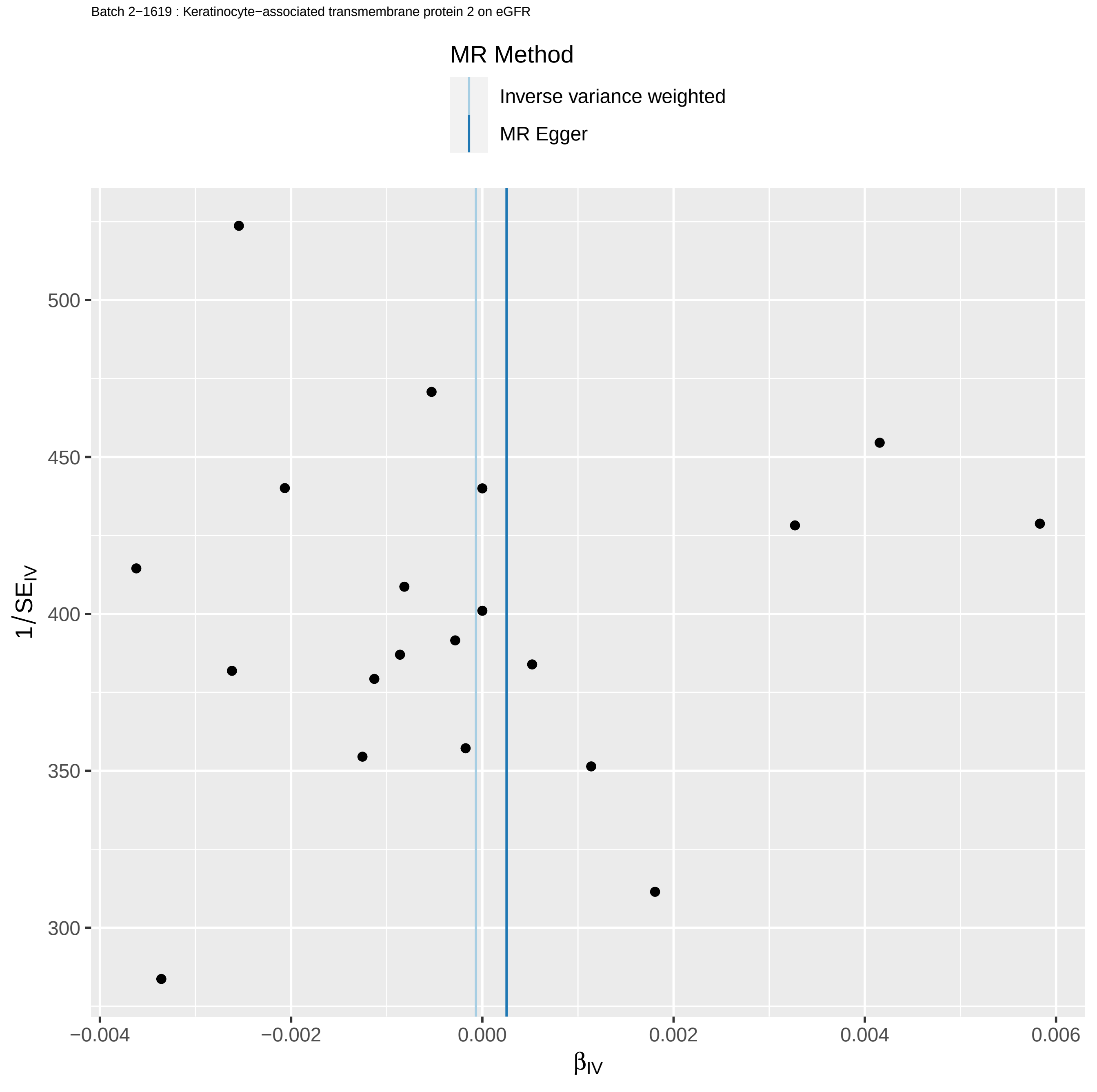

Batch 2−1619 : Keratinocyte−associated transmembrane protein 2 on eGFR
MR Method
Inverse variance weighted
MR Egger
500
450
400
350
300
1/SE_IV
−0.004
−0.002
0.000
0.002
0.004
0.006
β_IV

Batch 2−1620 : BTB/POZ domain−containing protein KCTD5 on eGFR

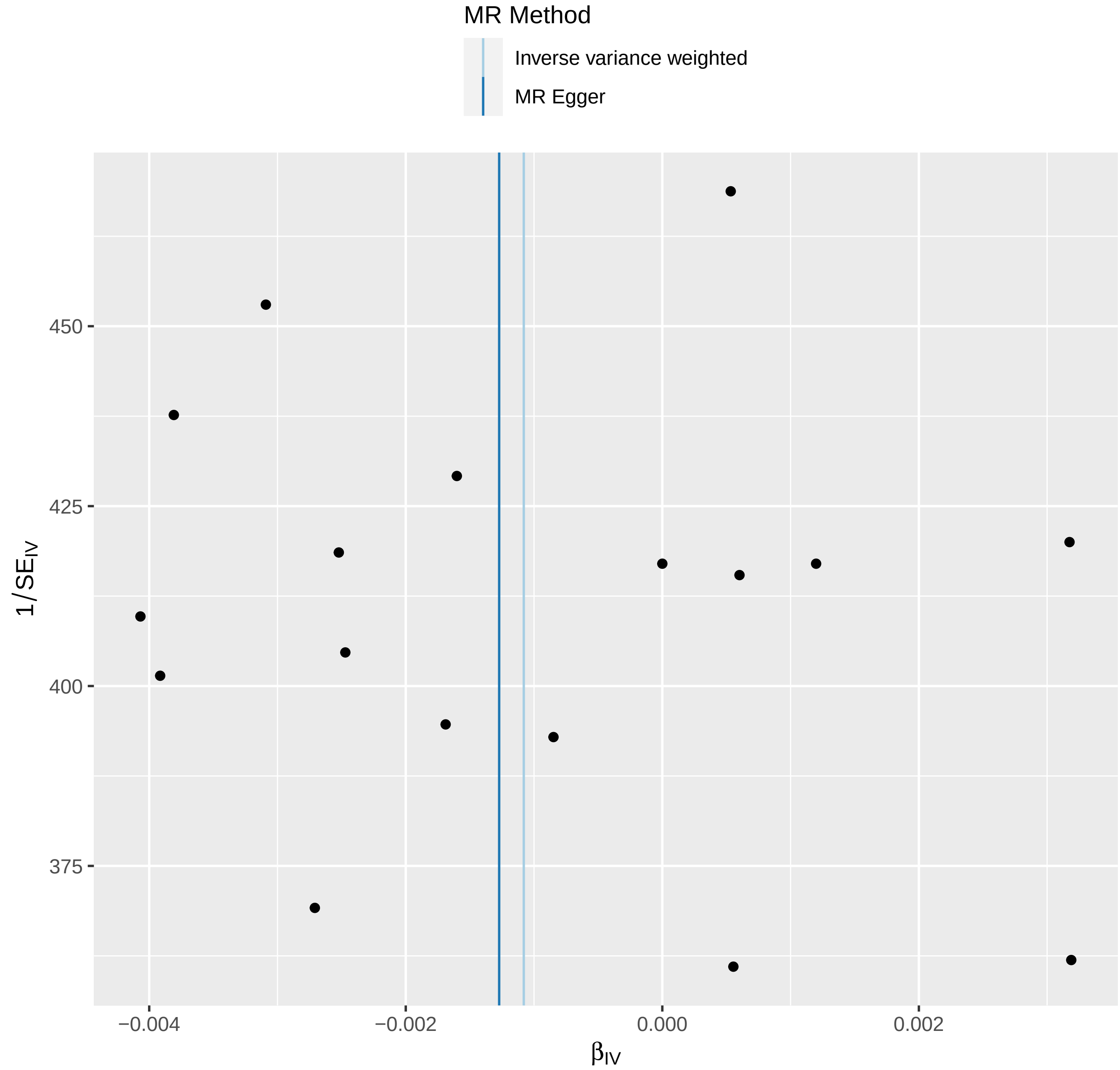

Batch 2−1621 : KDEL motif−containing protein 2 on eGFR

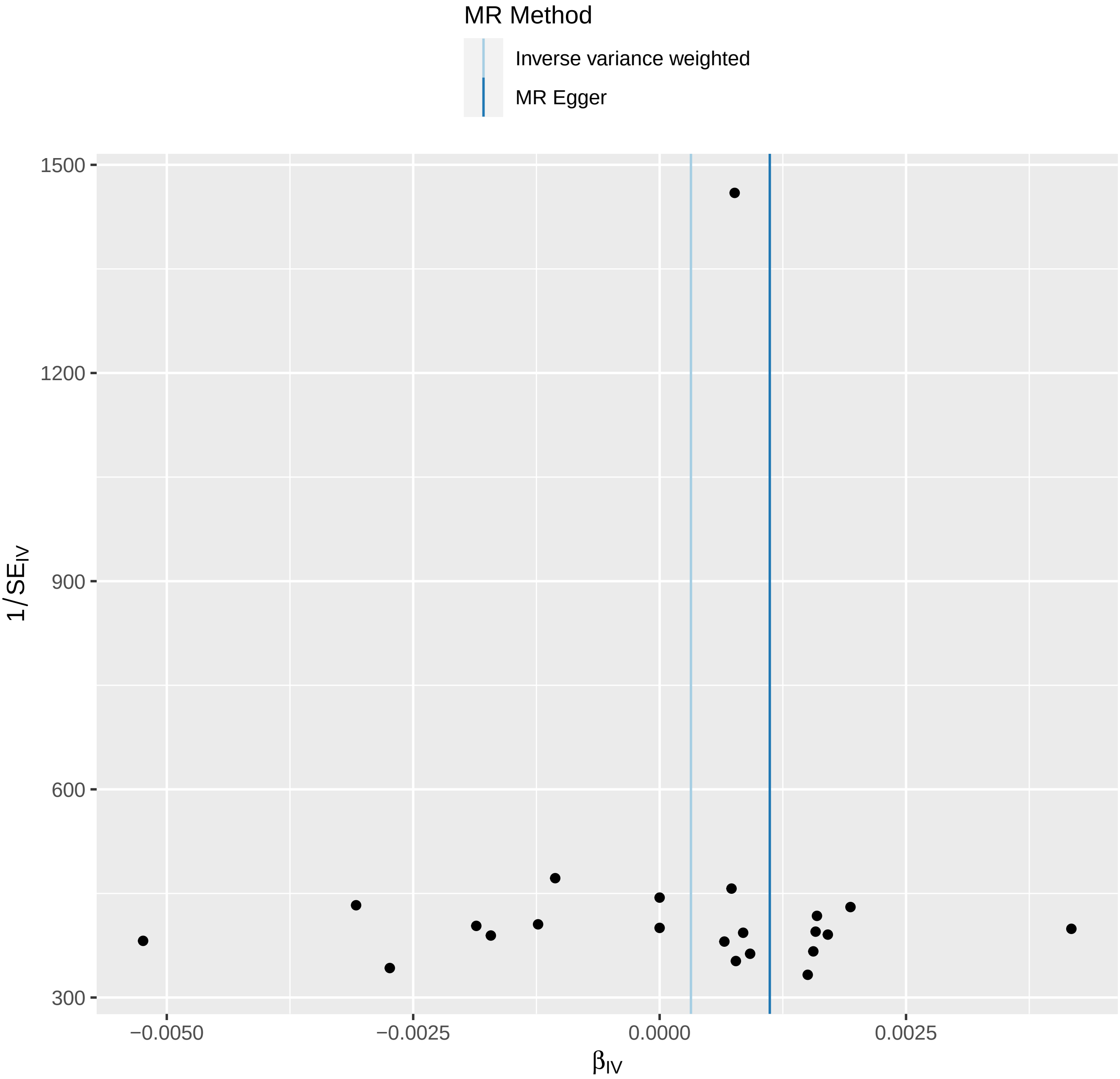

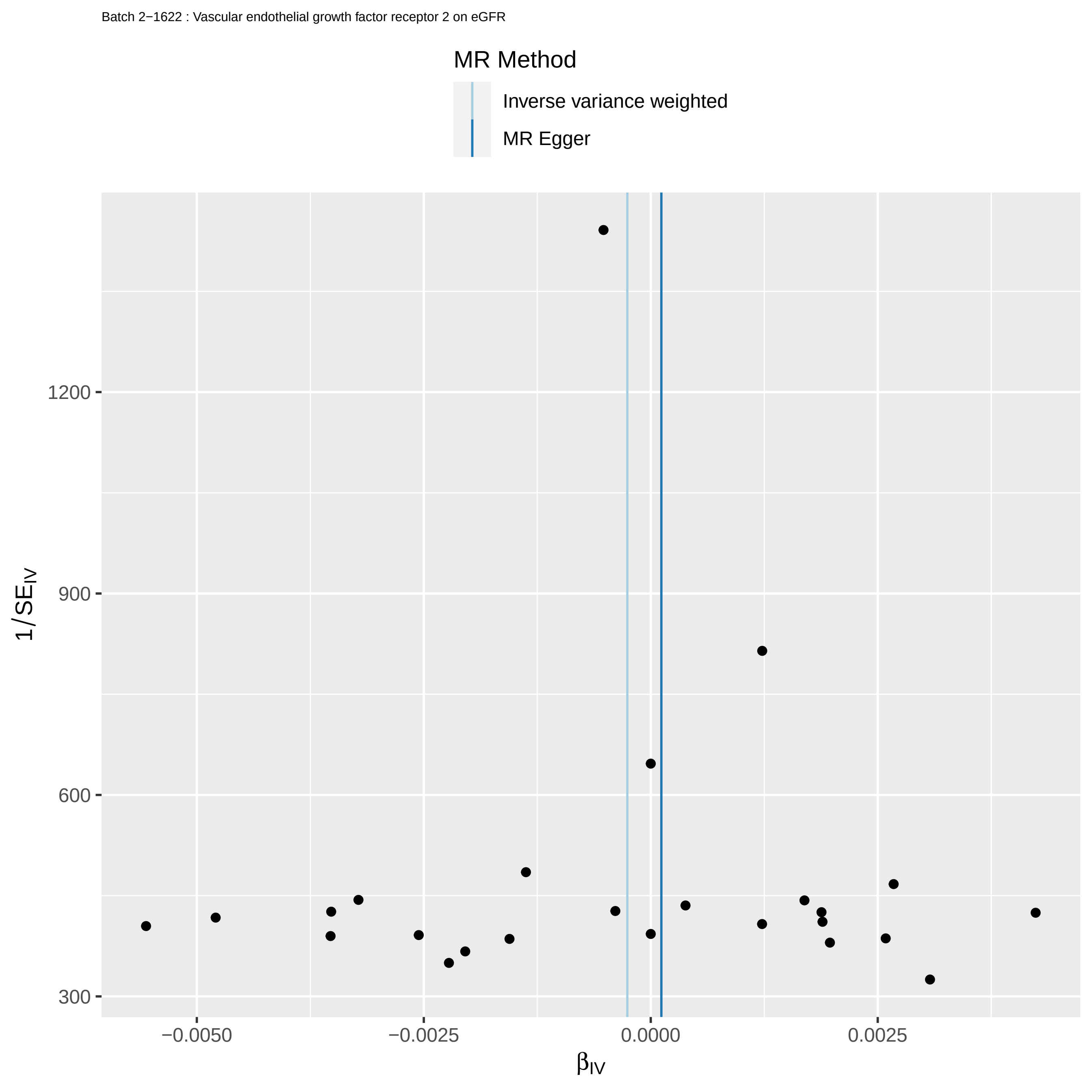

Batch 2−1622 : Vascular endothelial growth factor receptor 2 on eGFR
MR Method
Inverse variance weighted
MR Egger
1200
900
600
300
−0.0050
−0.0025
0.0000
0.0025
$1/SE_{IV}$
β_{IV}

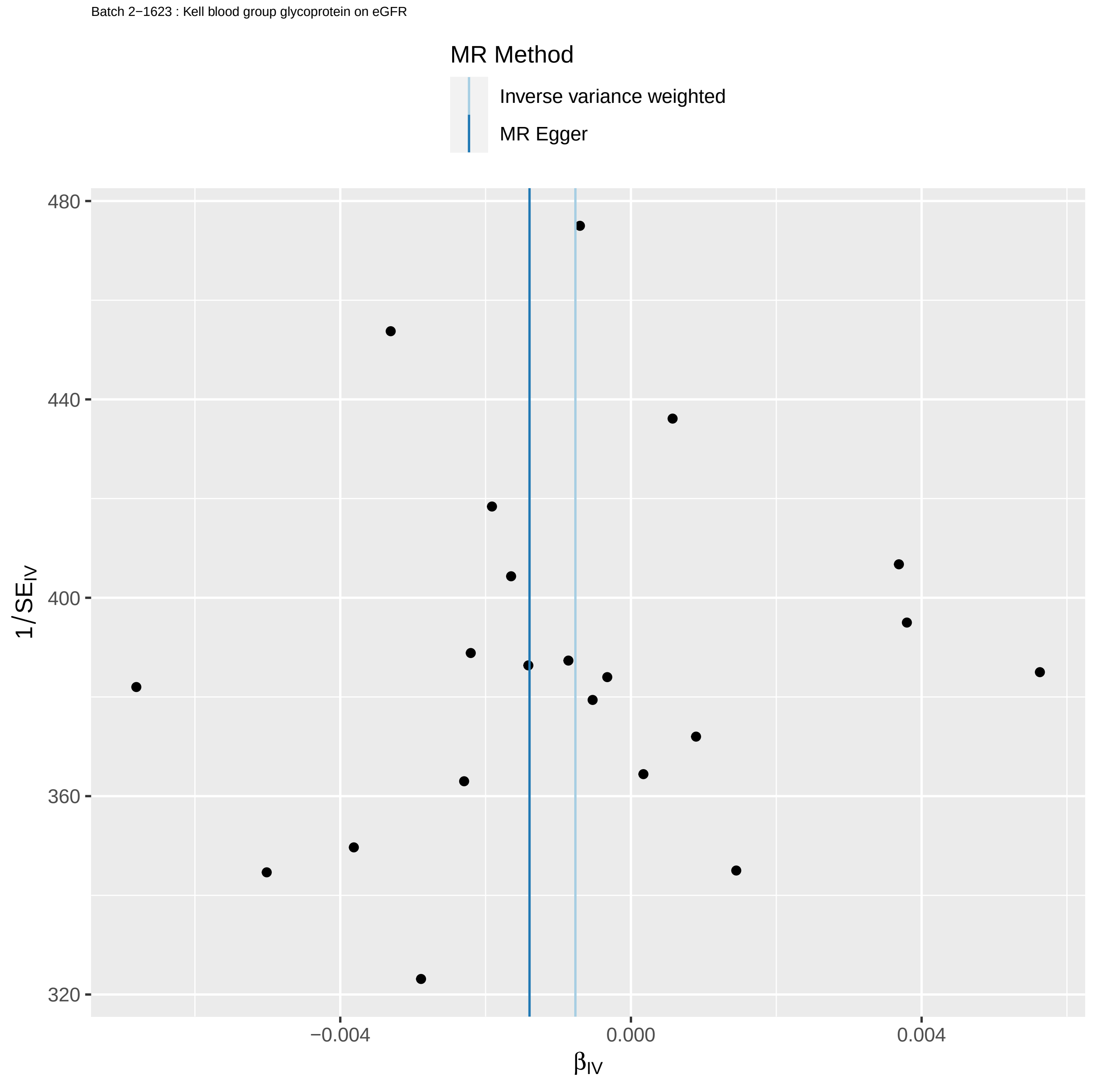

Batch 2−1623 : Kell blood group glycoprotein on eGFR
MR Method
Inverse variance weighted
MR Egger
480
440
400
360
320
1/SE$_{IV}$
−0.004
0.000
0.004
β$_{IV}$

Batch 2−1624 : Uncharacterized protein KIAA0040 on eGFR

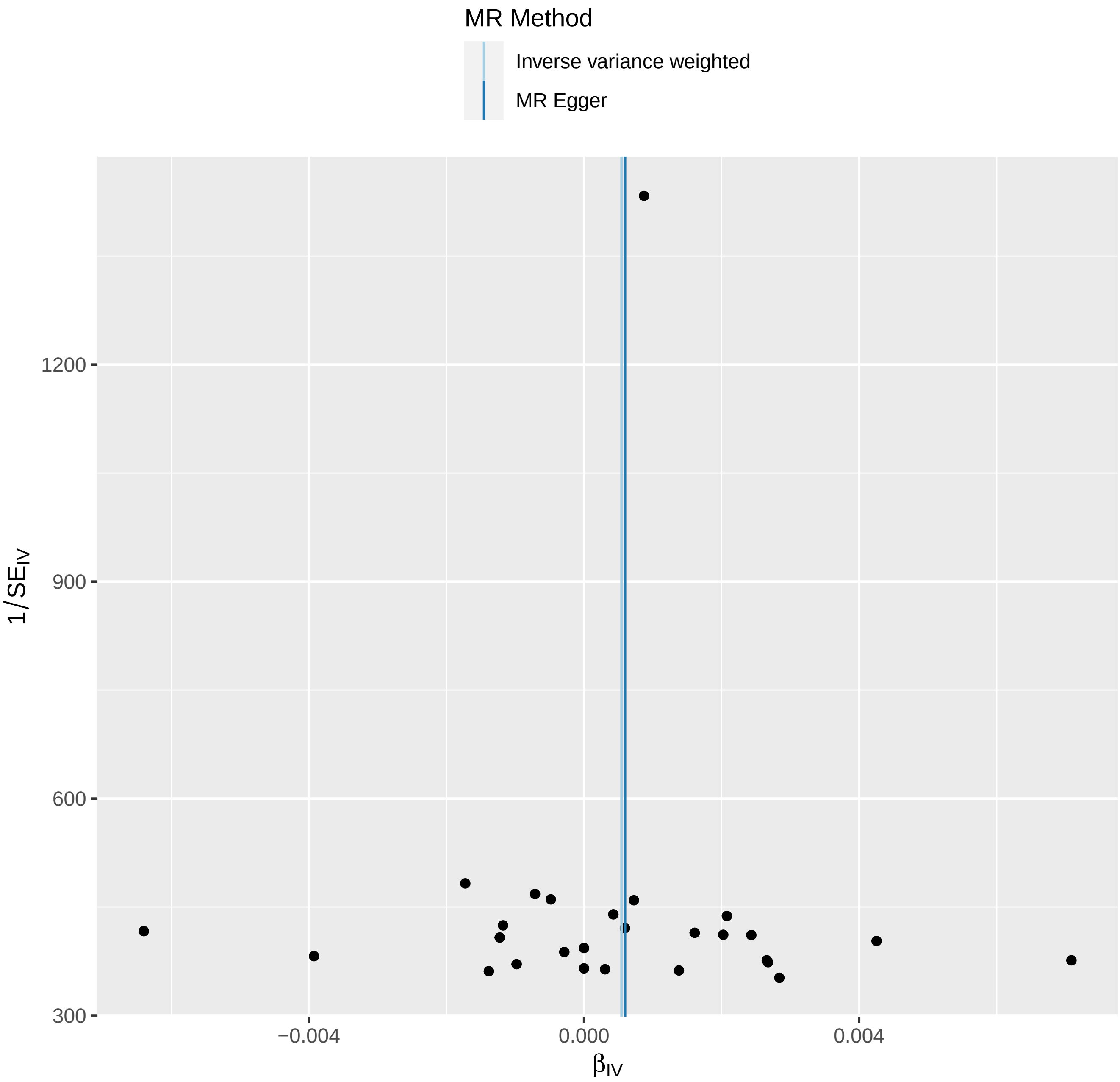

Batch 2−1625 : Dyslexia−associated protein KIAA0319 on eGFR

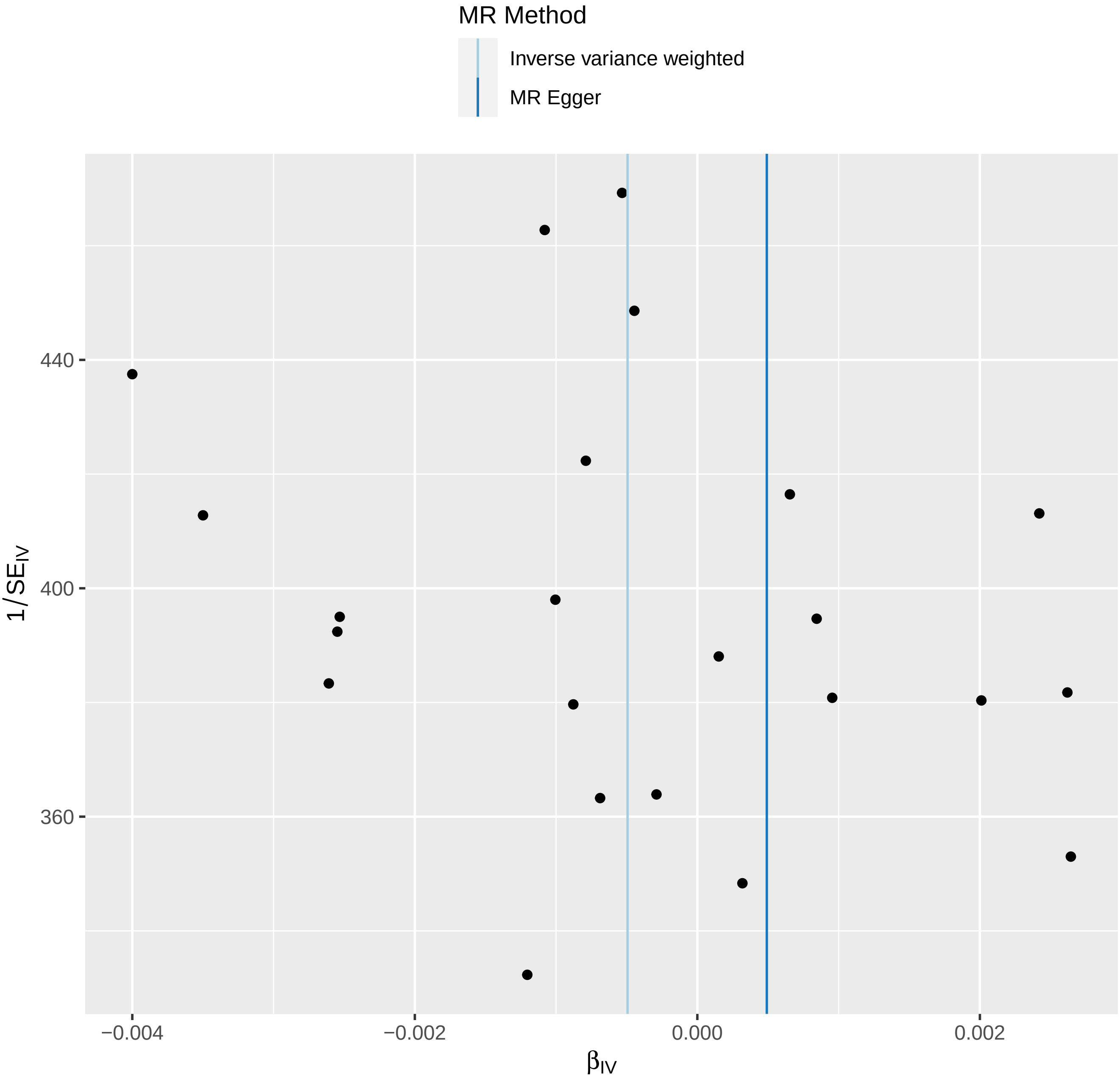

Batch 2−1626 : Dyslexia−associated protein KIAA0319−like protein on eGFR

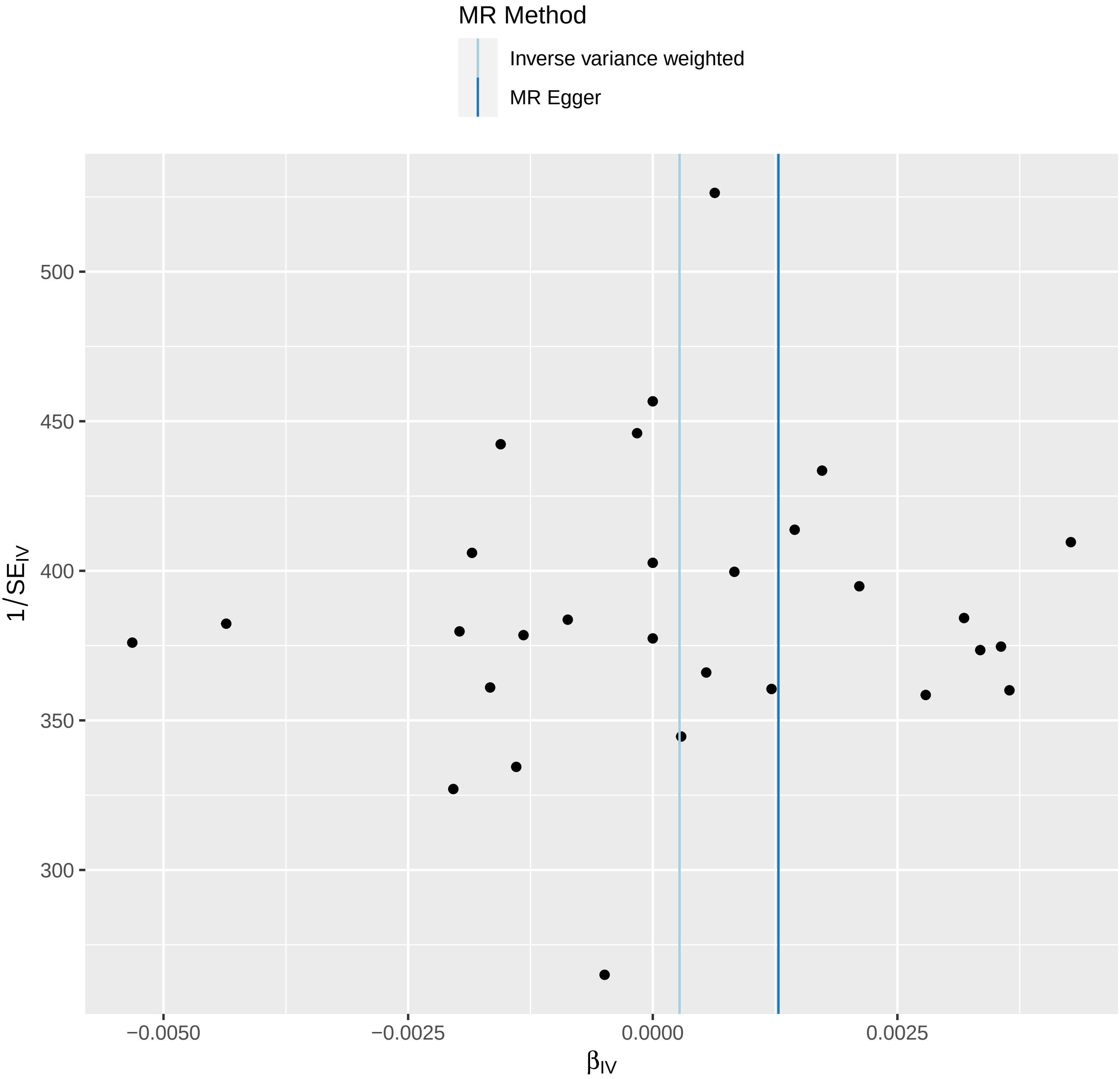

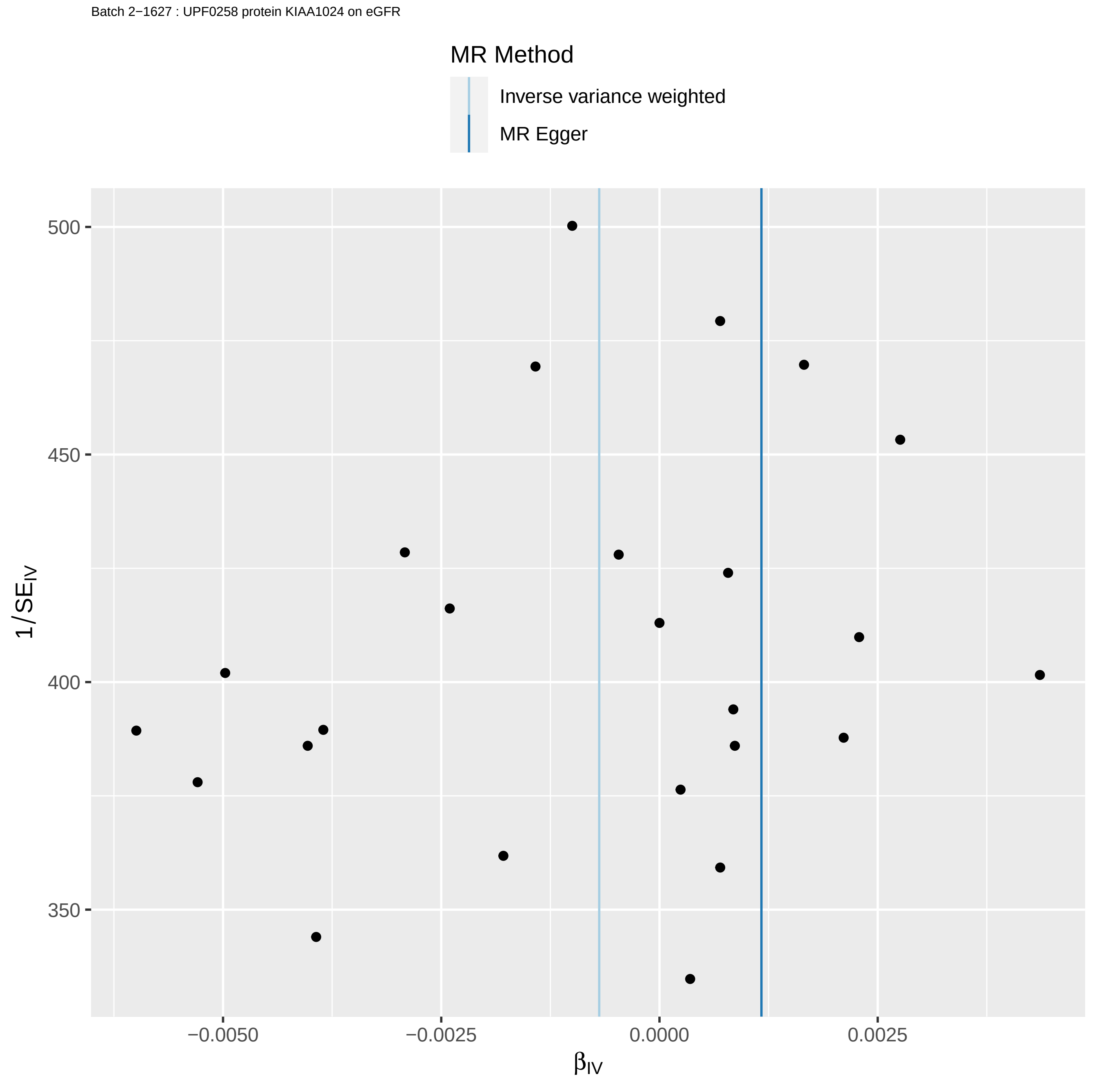

Batch 2−1627 : UPF0258 protein KIAA1024 on eGFR
MR Method
Inverse variance weighted
MR Egger
500
450
400
350
1/SE_IV
−0.0050
−0.0025
0.0000
0.0025
β_IV

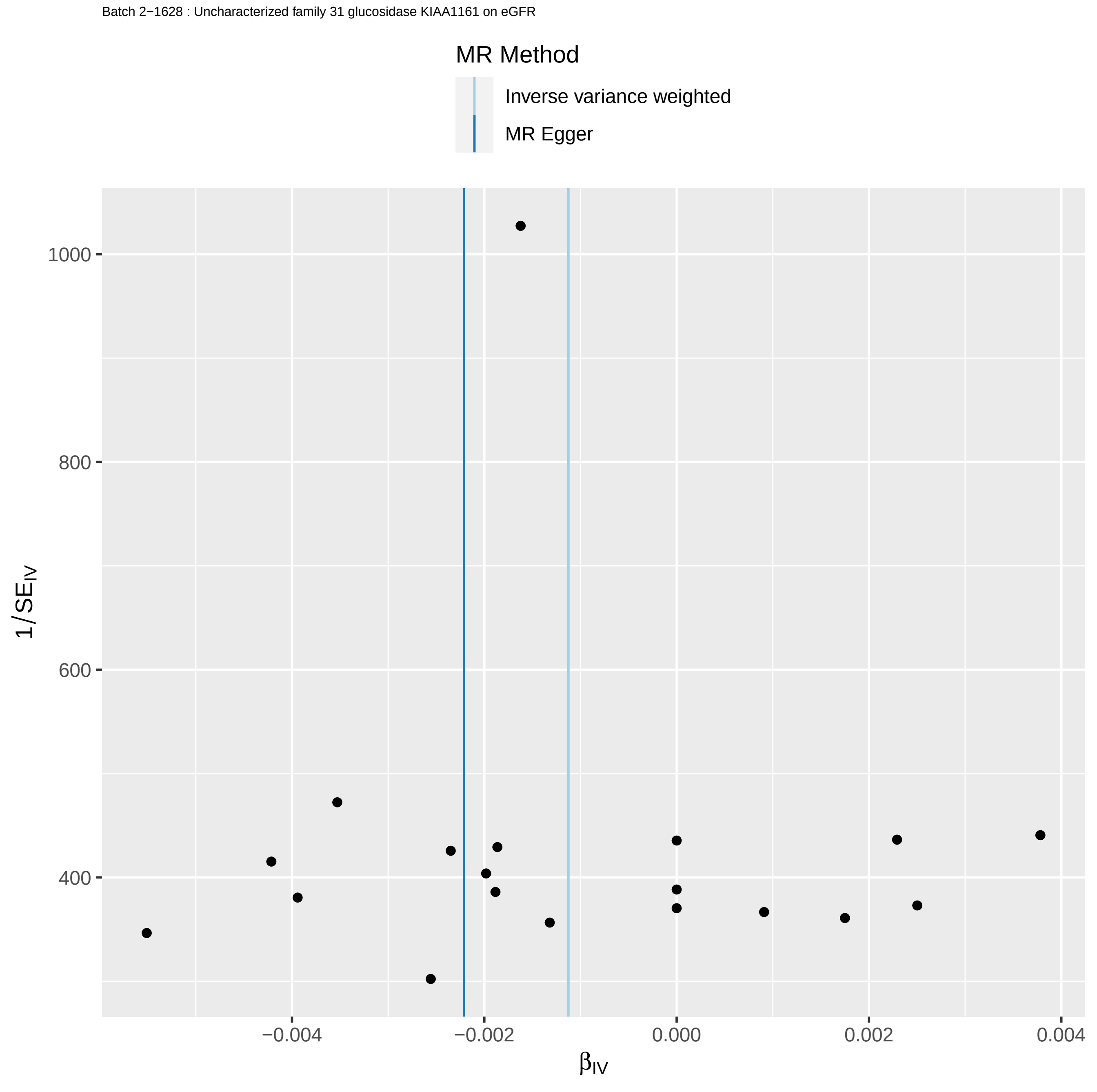
Batch 2−1628 : Uncharacterized family 31 glucosidase KIAA1161 on eGFR
MR Method
Inverse variance weighted
MR Egger
1000
800
600
400
1/SE$_{IV}$
−0.004
−0.002
0.000
0.002
0.004
β$_{IV}$

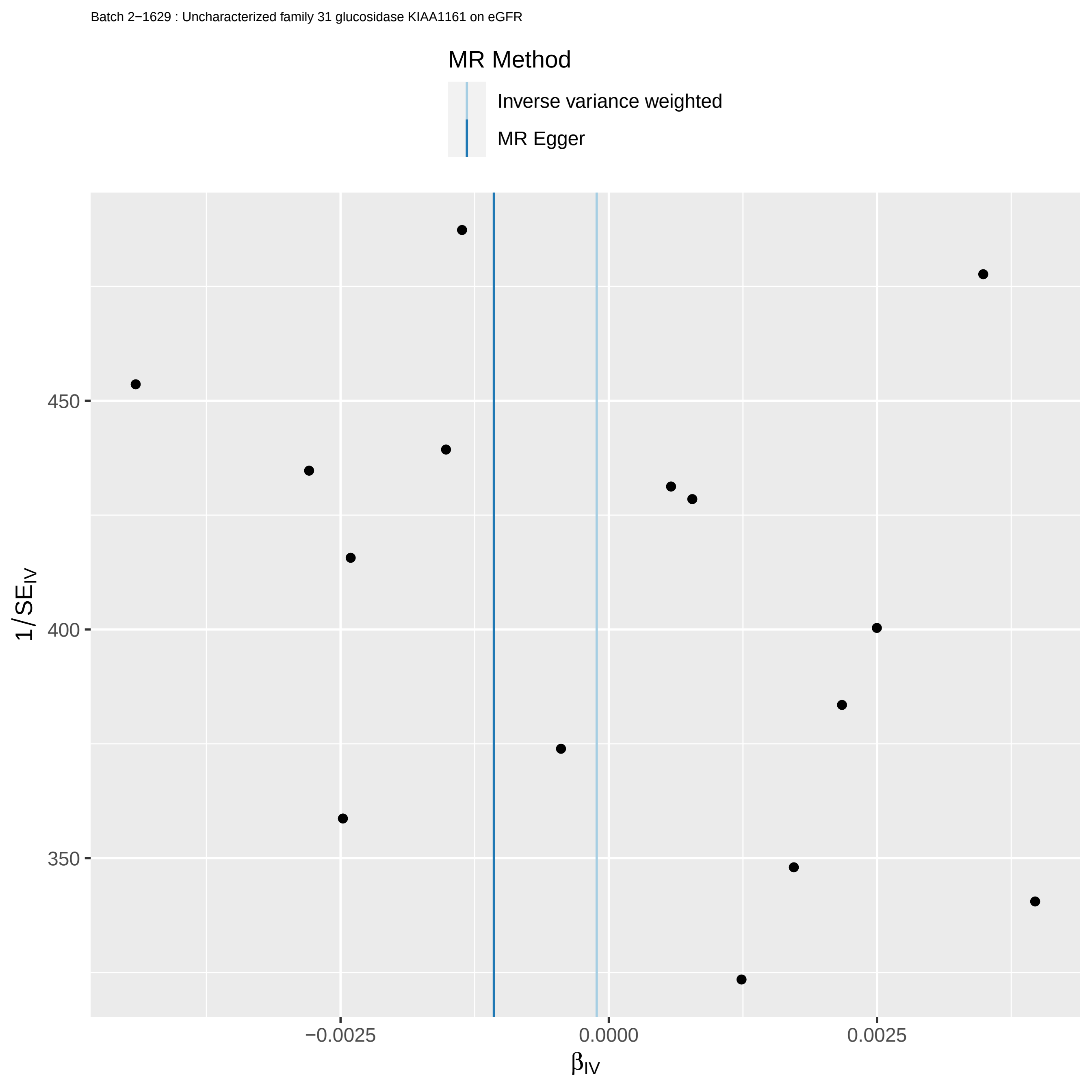
Batch 2−1629 : Uncharacterized family 31 glucosidase KIAA1161 on eGFR
MR Method
Inverse variance weighted
MR Egger
450
400
350
$1/SE_{IV}$
−0.0025
0.0000
0.0025
β_{IV}

Batch 2−1630 : UPF0577 protein KIAA1324 on eGFR

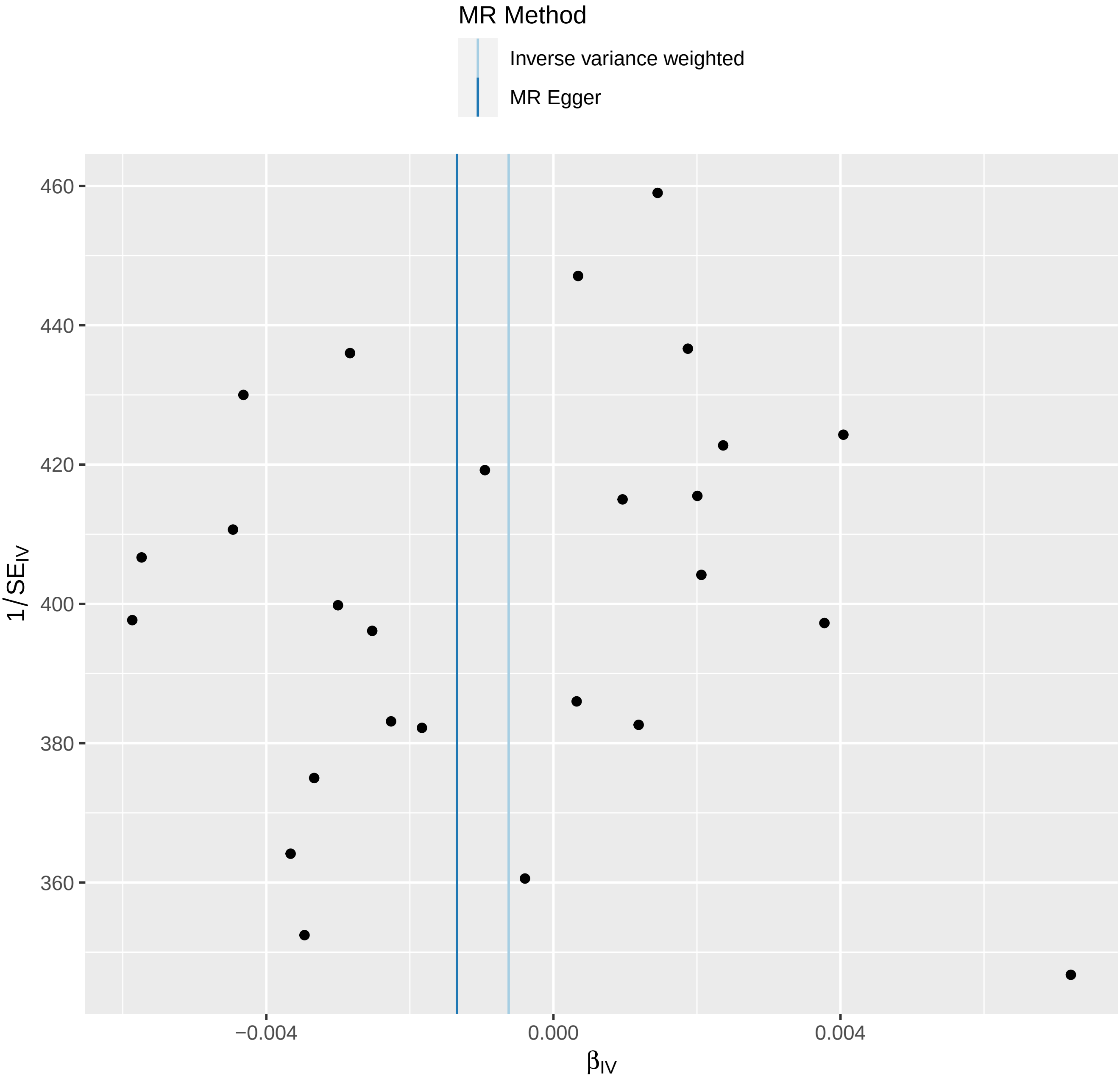

Batch 2−1631 : UPF0577 protein KIAA1324 on eGFR

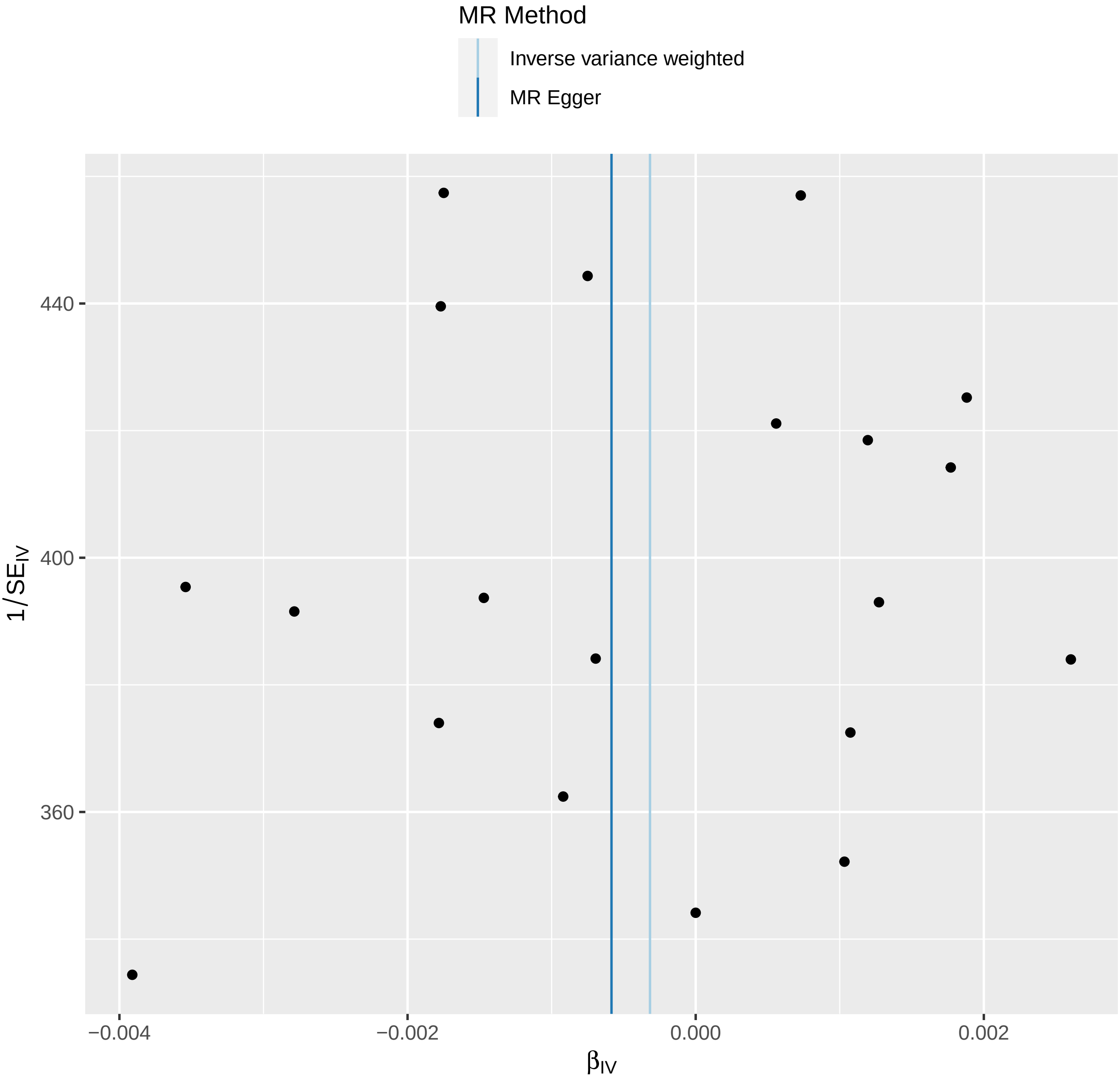

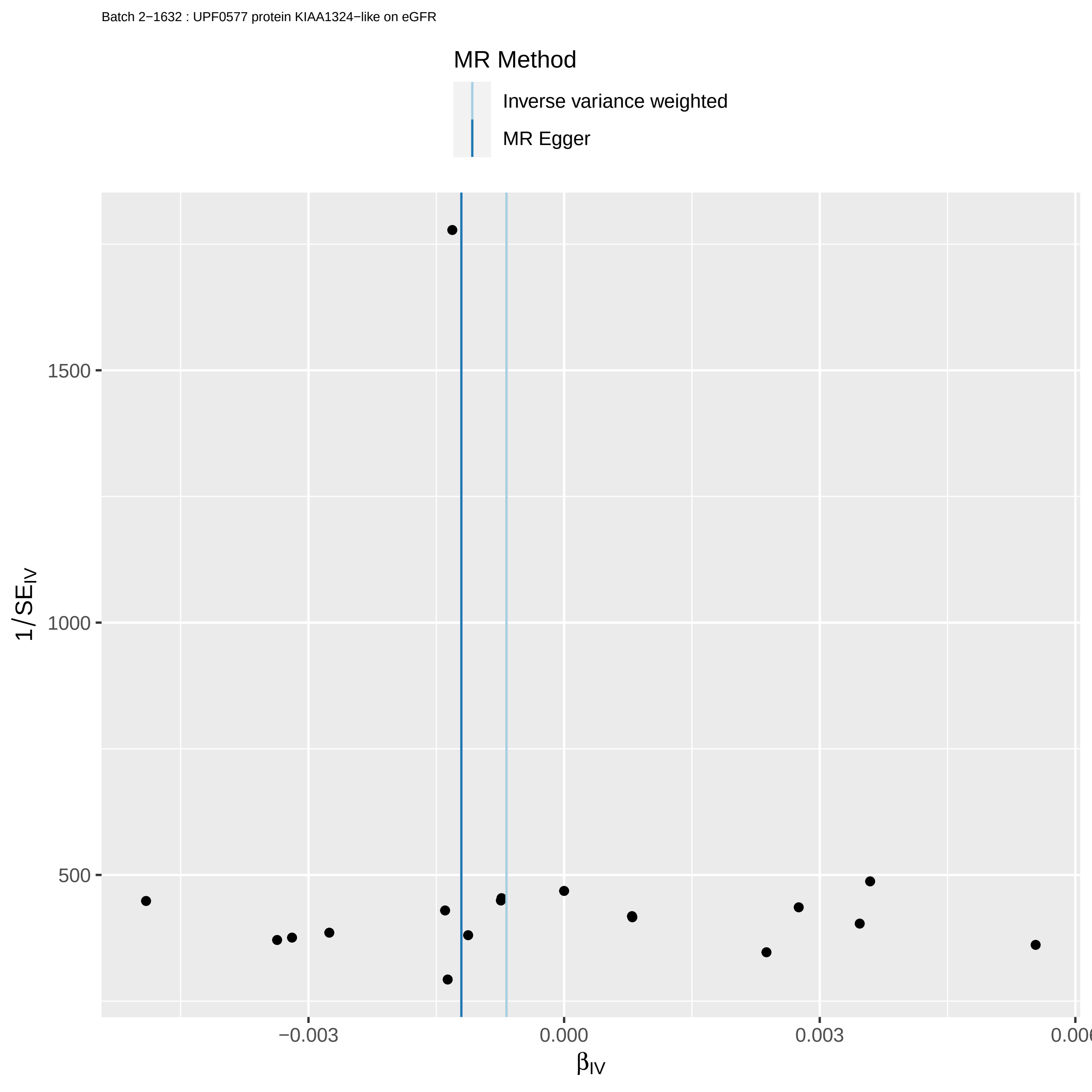

Batch 2−1632 : UPF0577 protein KIAA1324−like on eGFR
MR Method
Inverse variance weighted
MR Egger
1500
1000
500
1/SE$_{IV}$
−0.003
0.000
0.003
0.006
β$_{IV}$

Batch 2−1633 : Uncharacterized protein KIAA1467 on eGFR

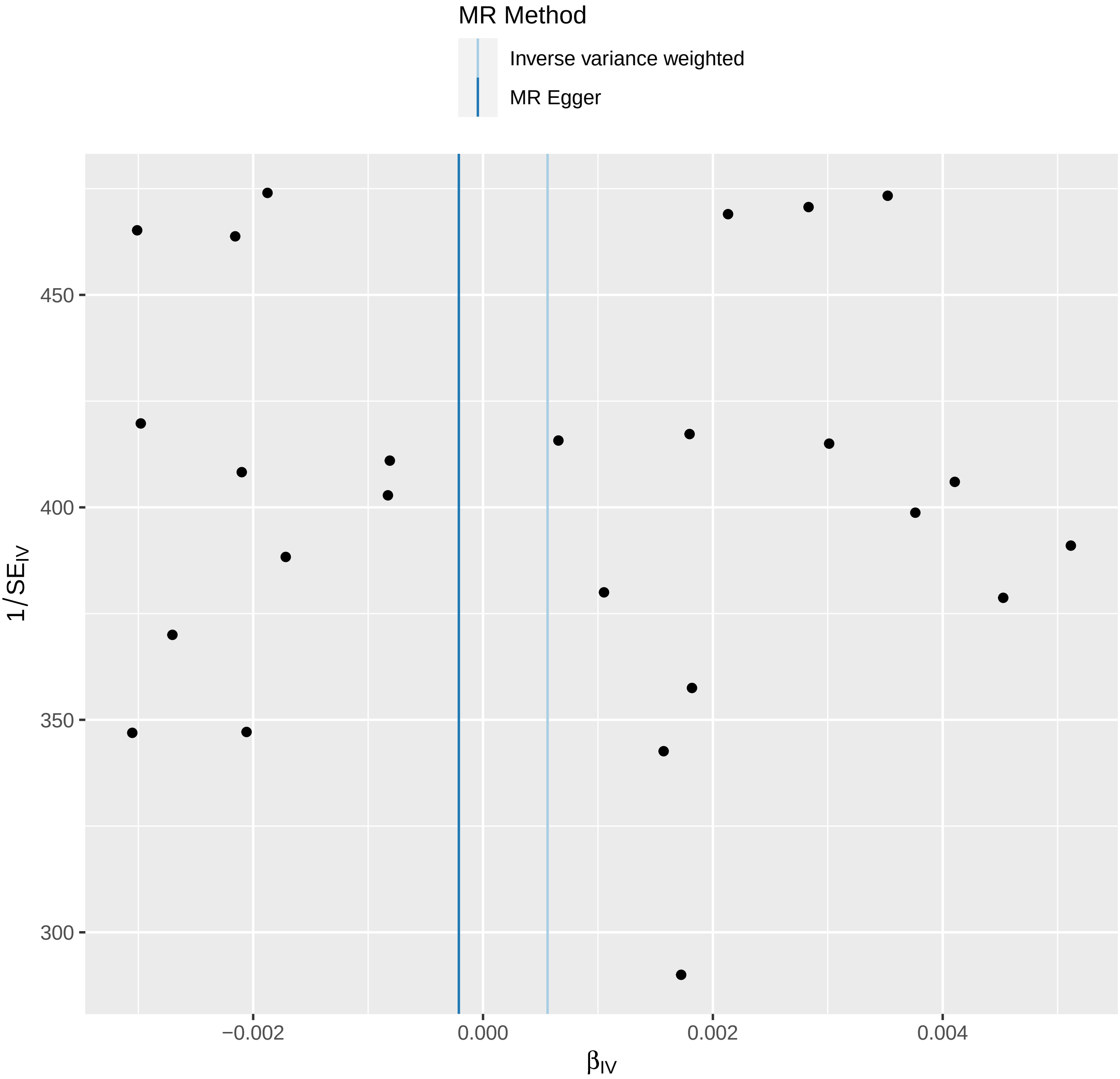

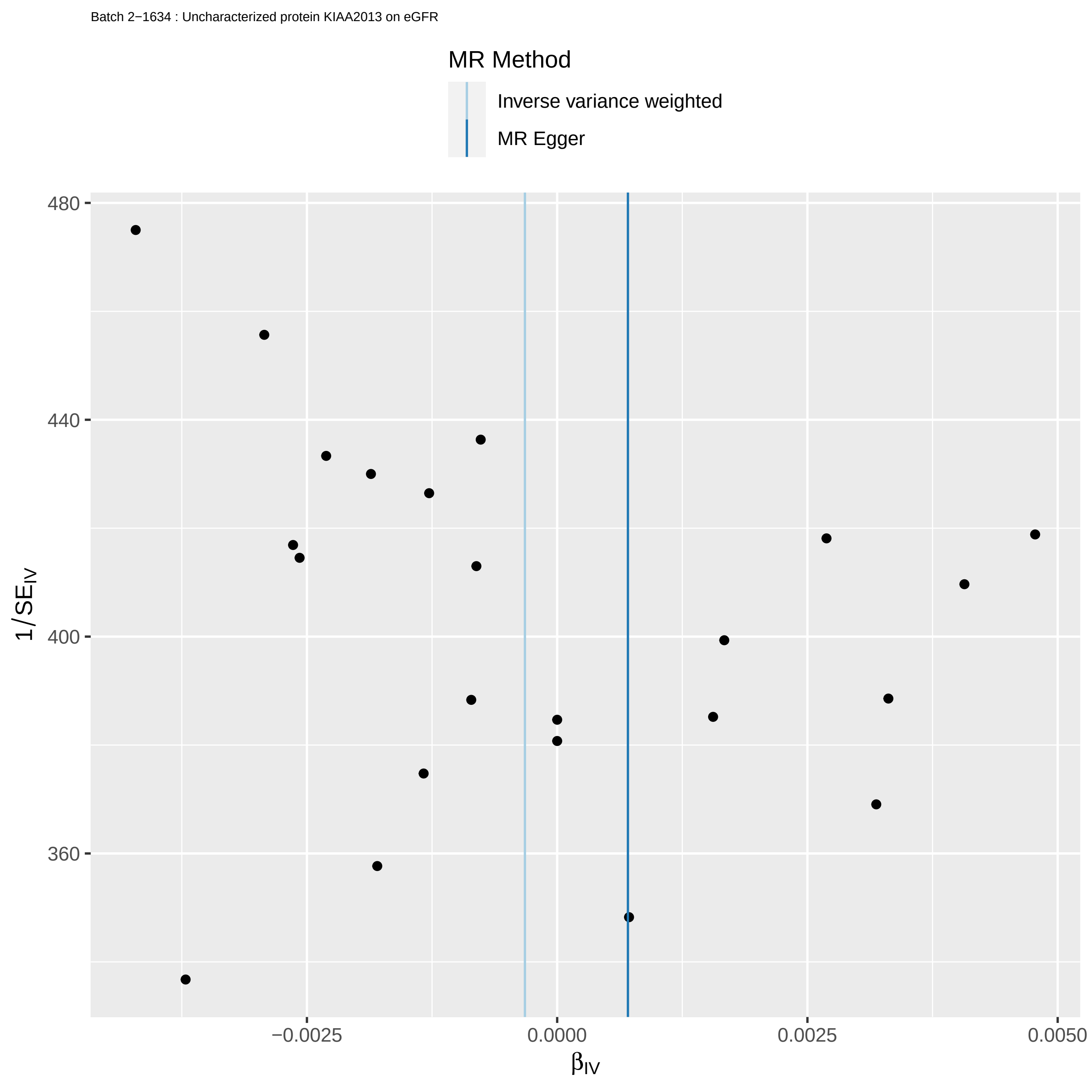

Batch 2−1634 : Uncharacterized protein KIAA2013 on eGFR
MR Method
Inverse variance weighted
MR Egger
480
440
400
360
1/SE$_{IV}$
−0.0025
0.0000
0.0025
0.0050
β$_{IV}$

Batch 2−1635 : Kinesin−like protein KIF16B on eGFR

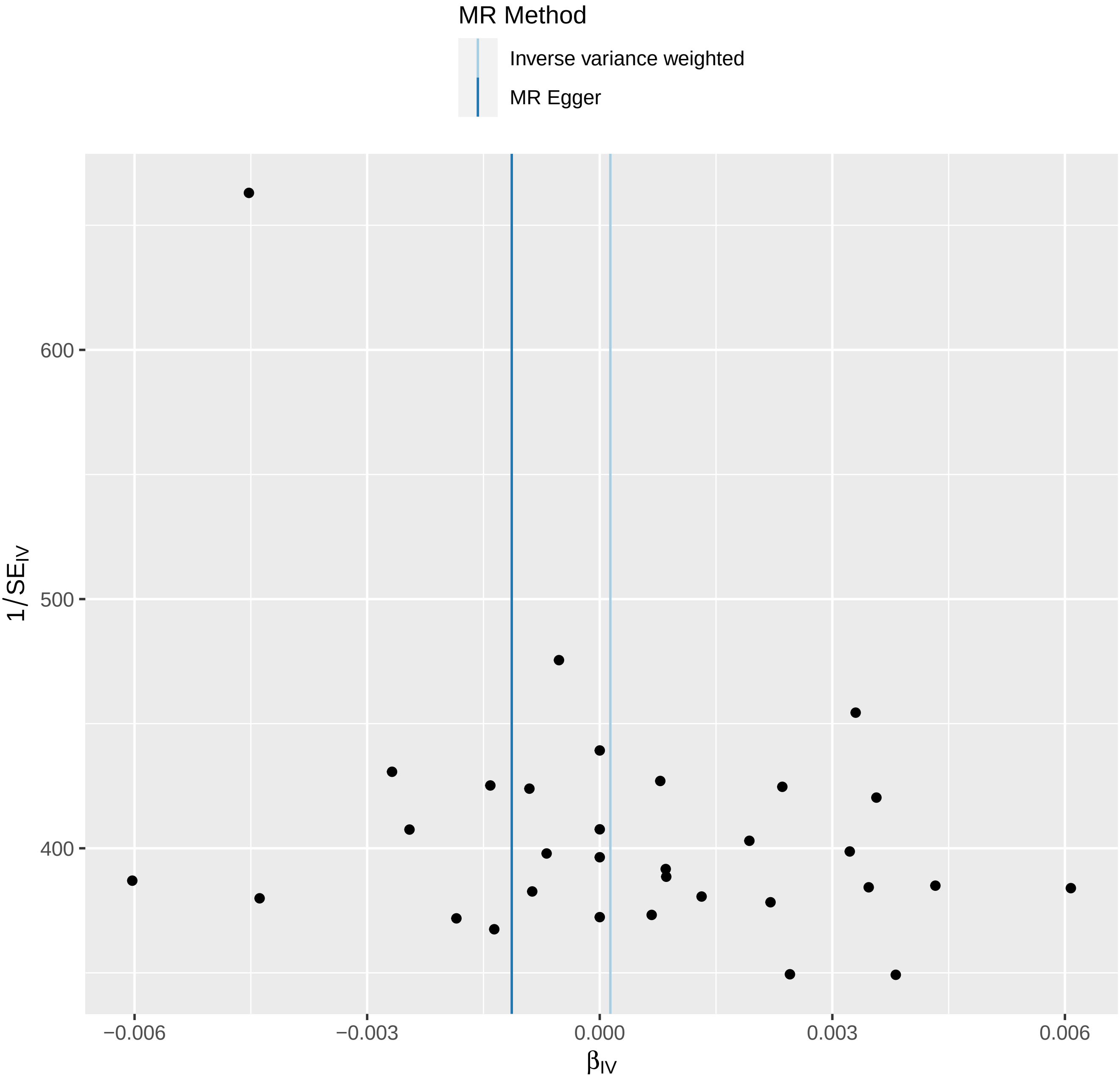

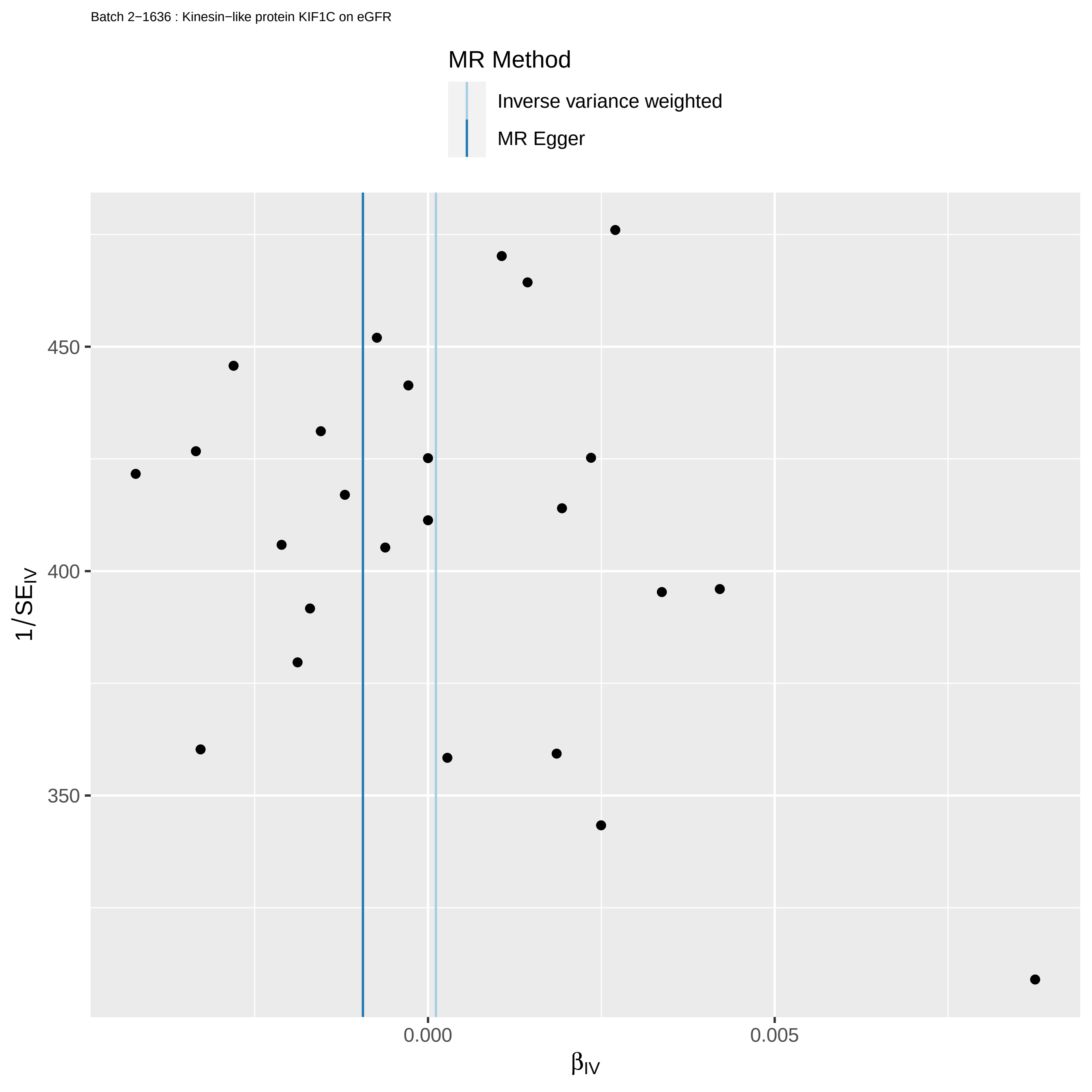

Batch 2−1636 : Kinesin−like protein KIF1C on eGFR
MR Method
Inverse variance weighted
MR Egger
450
400
350
1/SE_IV
0.000
0.005
β_IV

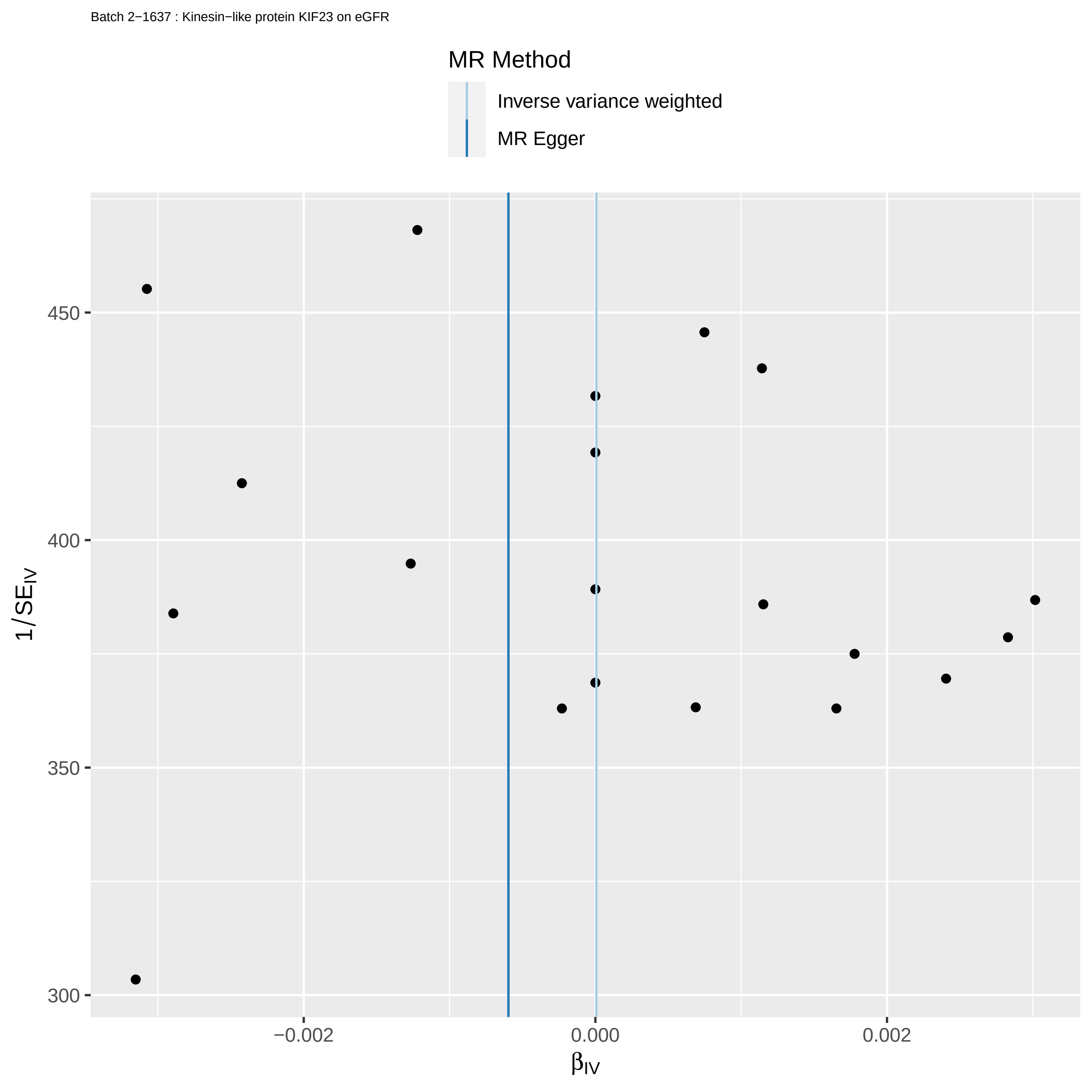
Batch 2−1637 : Kinesin−like protein KIF23 on eGFR
MR Method
Inverse variance weighted
MR Egger
450
400
350
300
1/SE_IV
−0.002
0.000
0.002
β_IV

Batch 2−1638 : DNA/RNA−binding protein KIN17 on eGFR

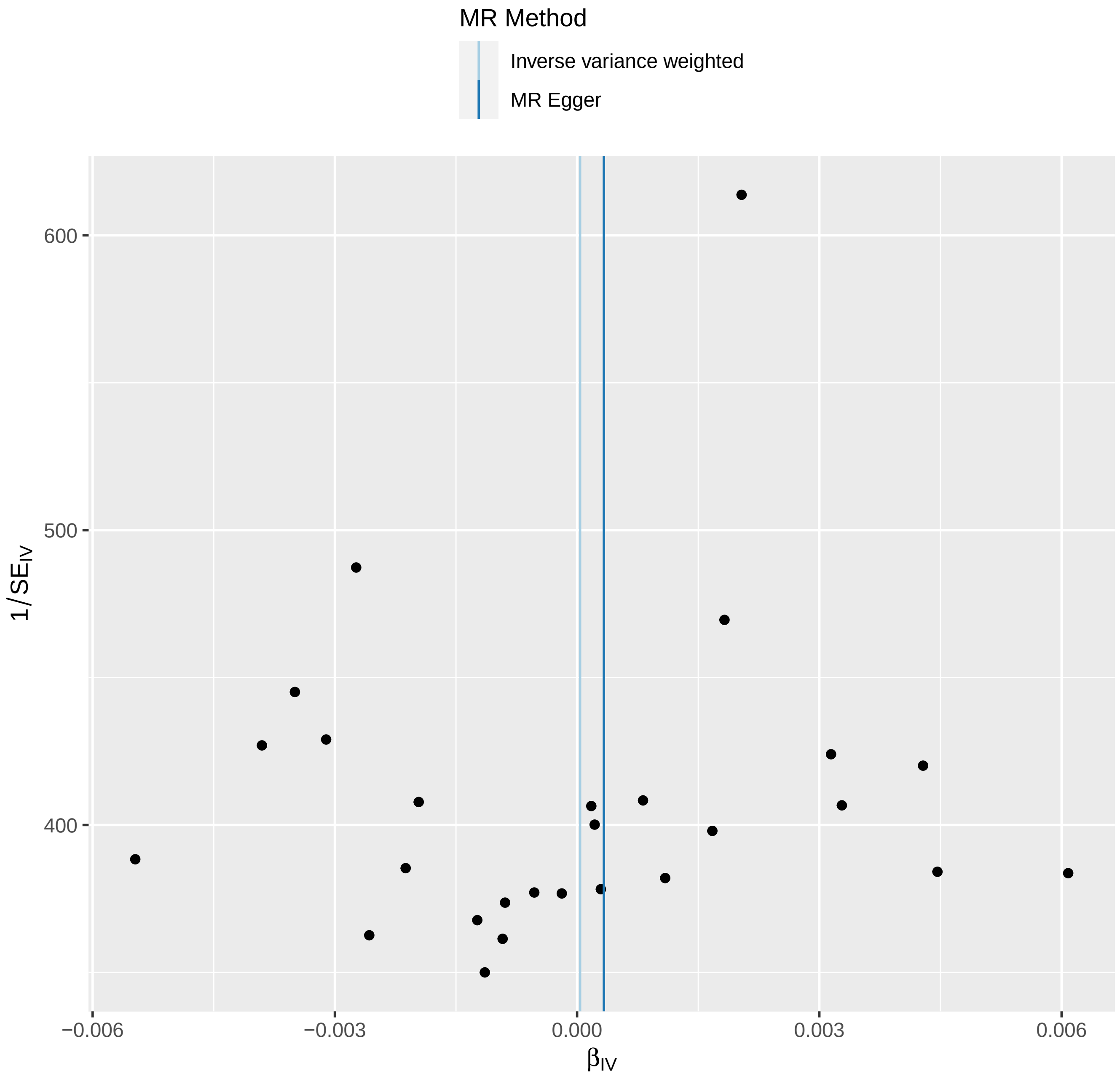

Batch 2−1639 : DNA/RNA−binding protein KIN17 on eGFR

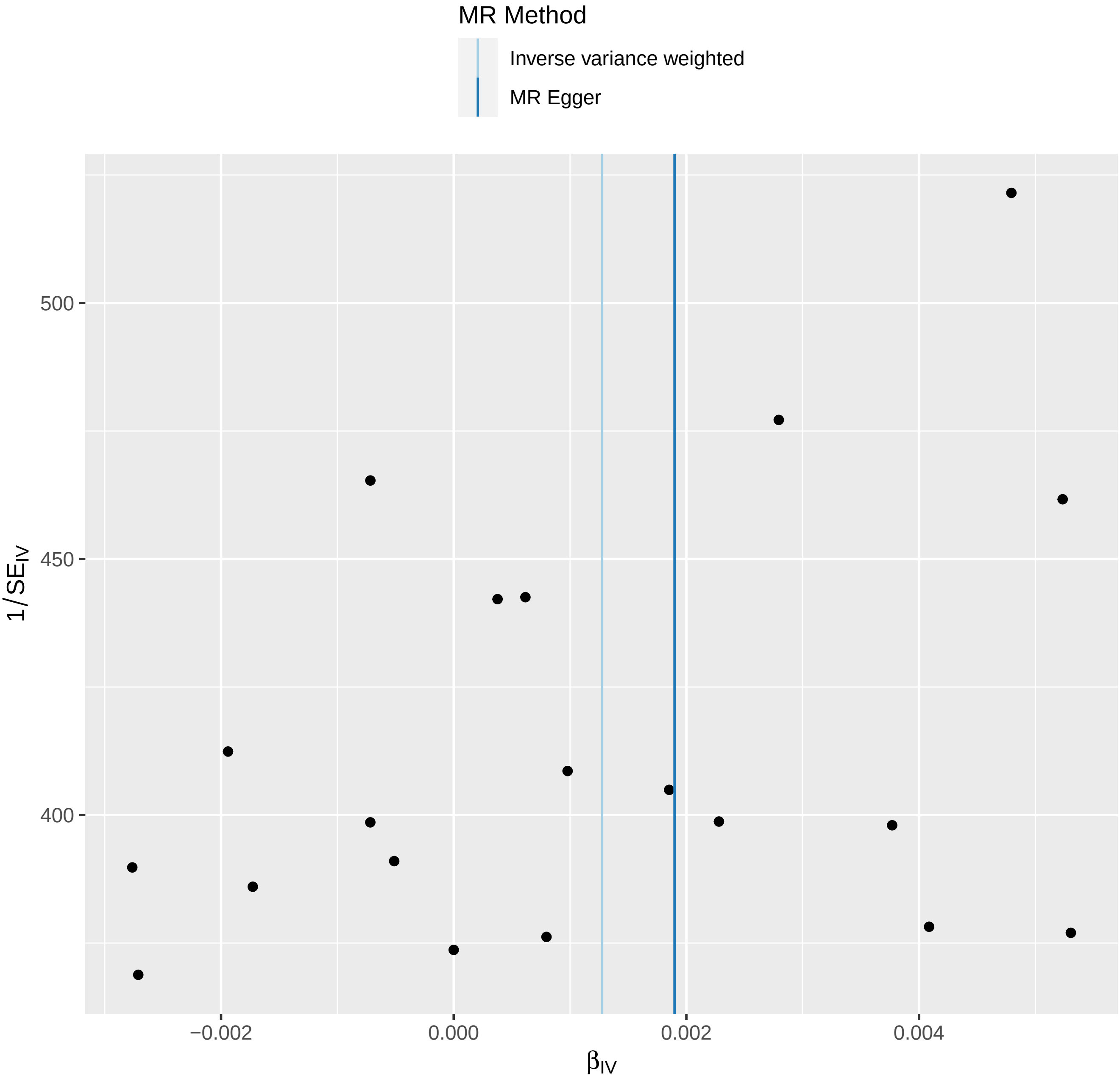

Batch 2−1640 : Killer cell immunoglobulin−like receptor 2DL2 on eGFR

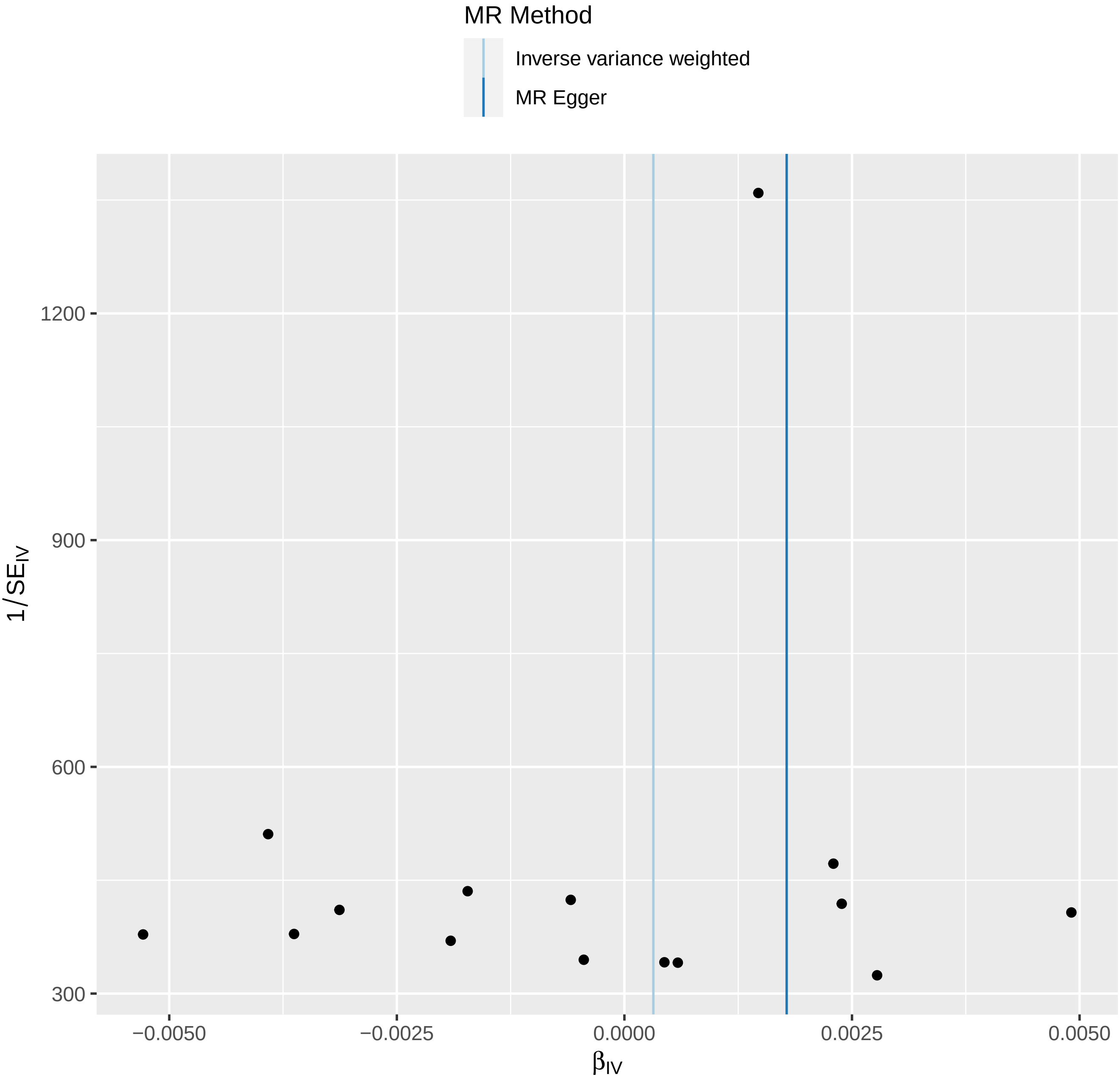

Batch 2−1641 : Killer cell immunoglobulin−like receptor 2DL4 on eGFR

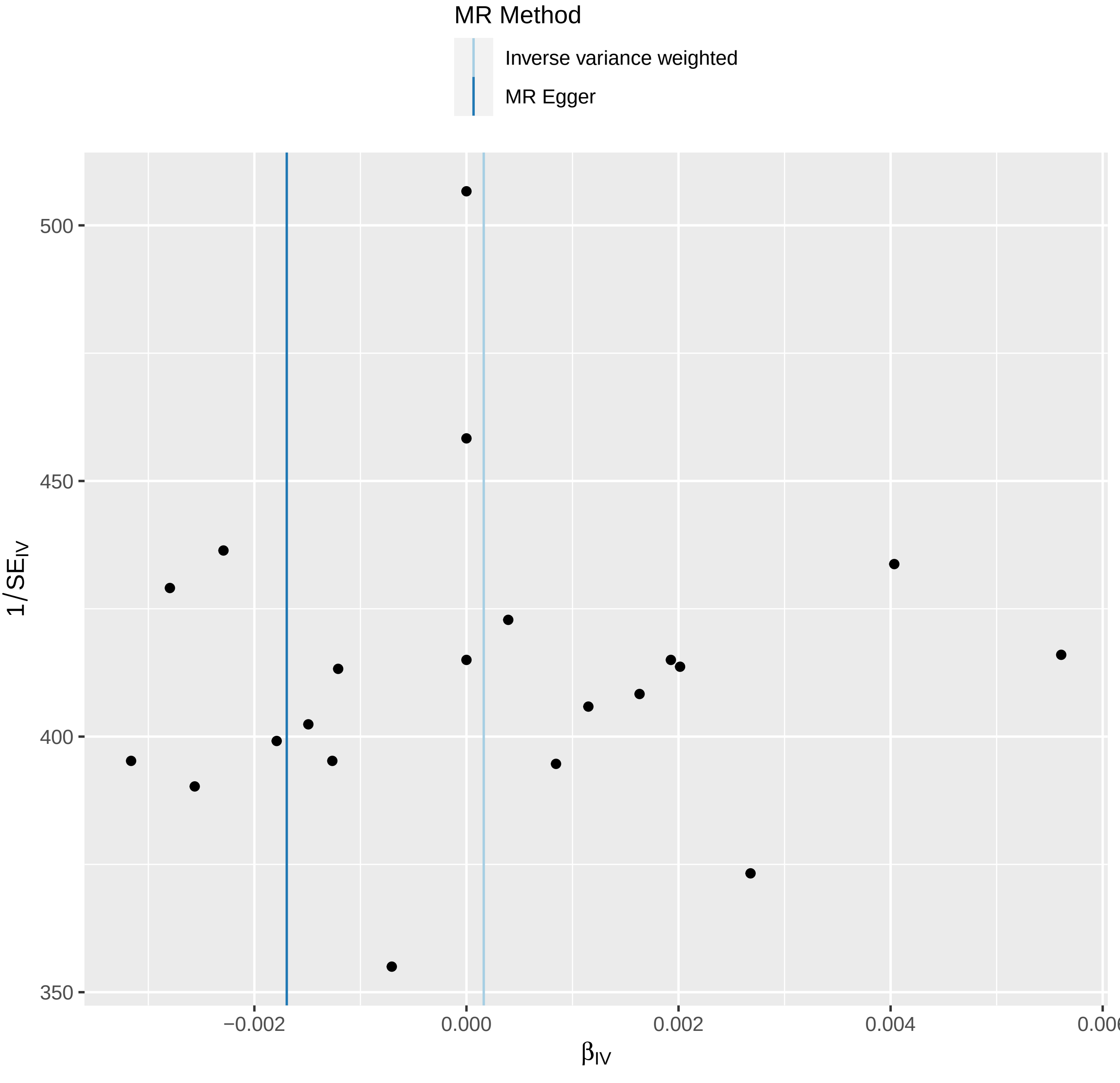

Batch 2−1642 : Killer cell immunoglobulin−like receptor 2DL4 on eGFR

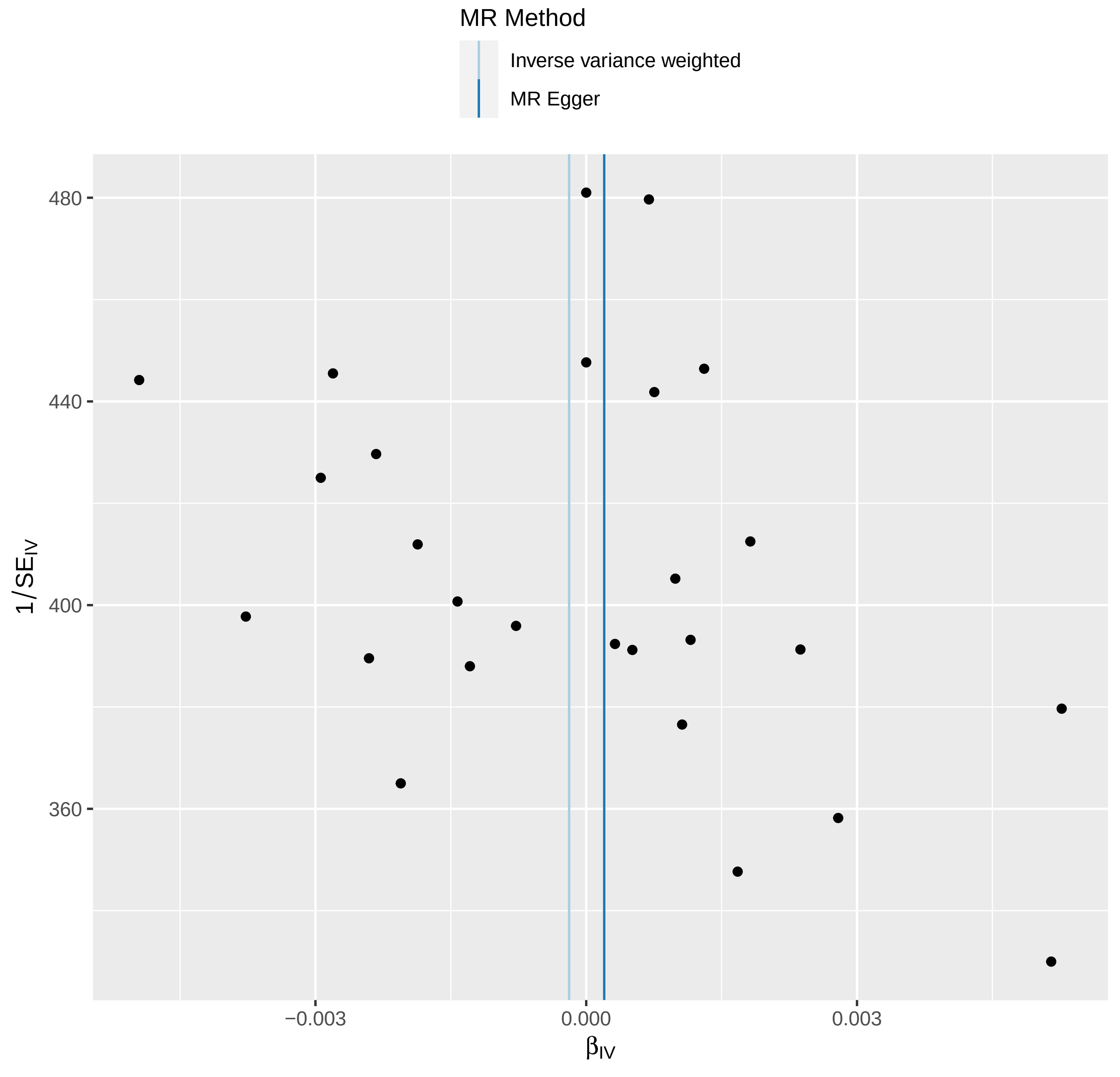

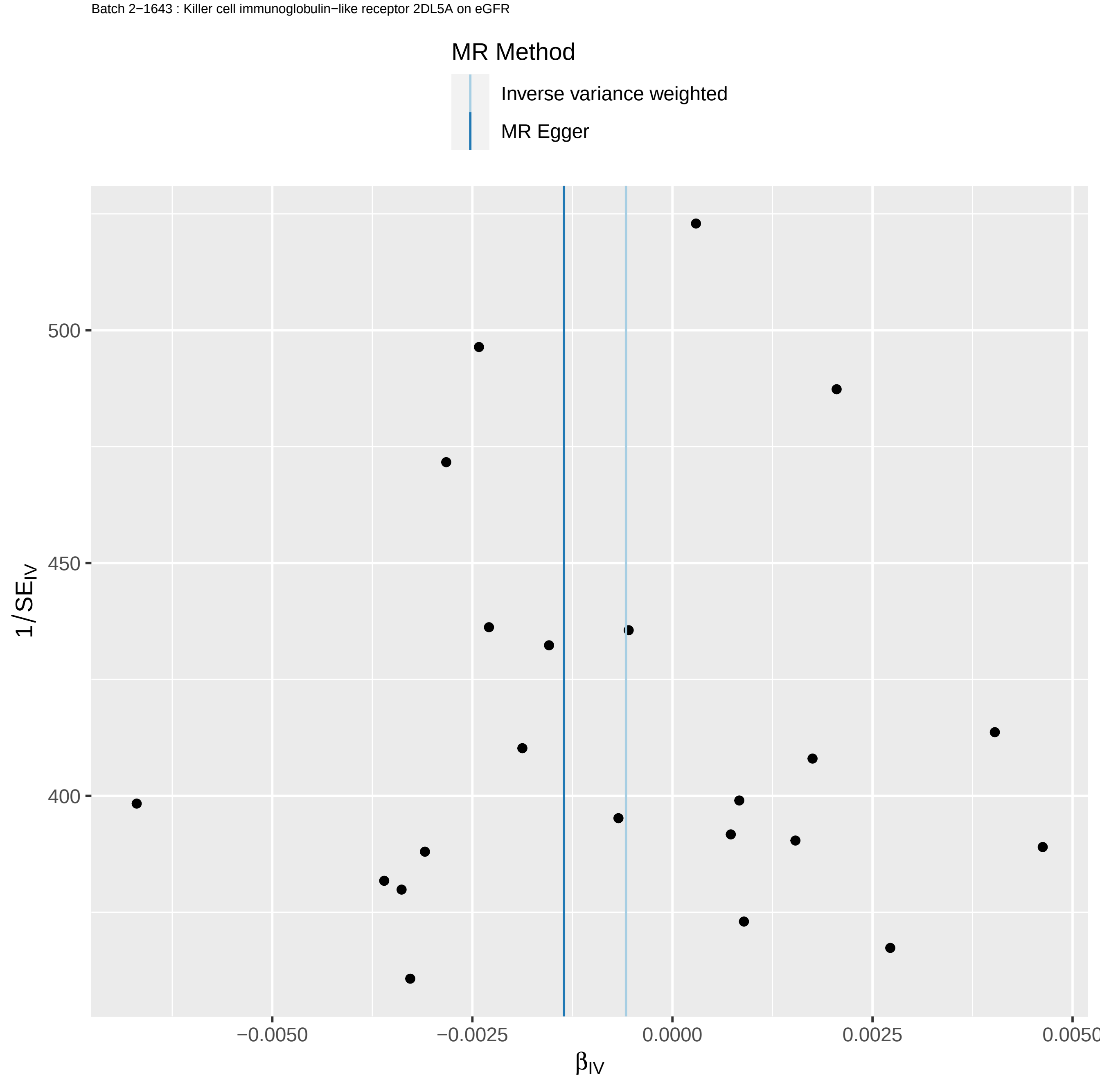

Batch 2−1643 : Killer cell immunoglobulin−like receptor 2DL5A on eGFR
MR Method
Inverse variance weighted
MR Egger
500
450
400
1/SE_IV
−0.0050
−0.0025
0.0000
0.0025
0.0050
β_IV

Batch 2−1644 : Killer cell immunoglobulin−like receptor 2DL5A on eGFR

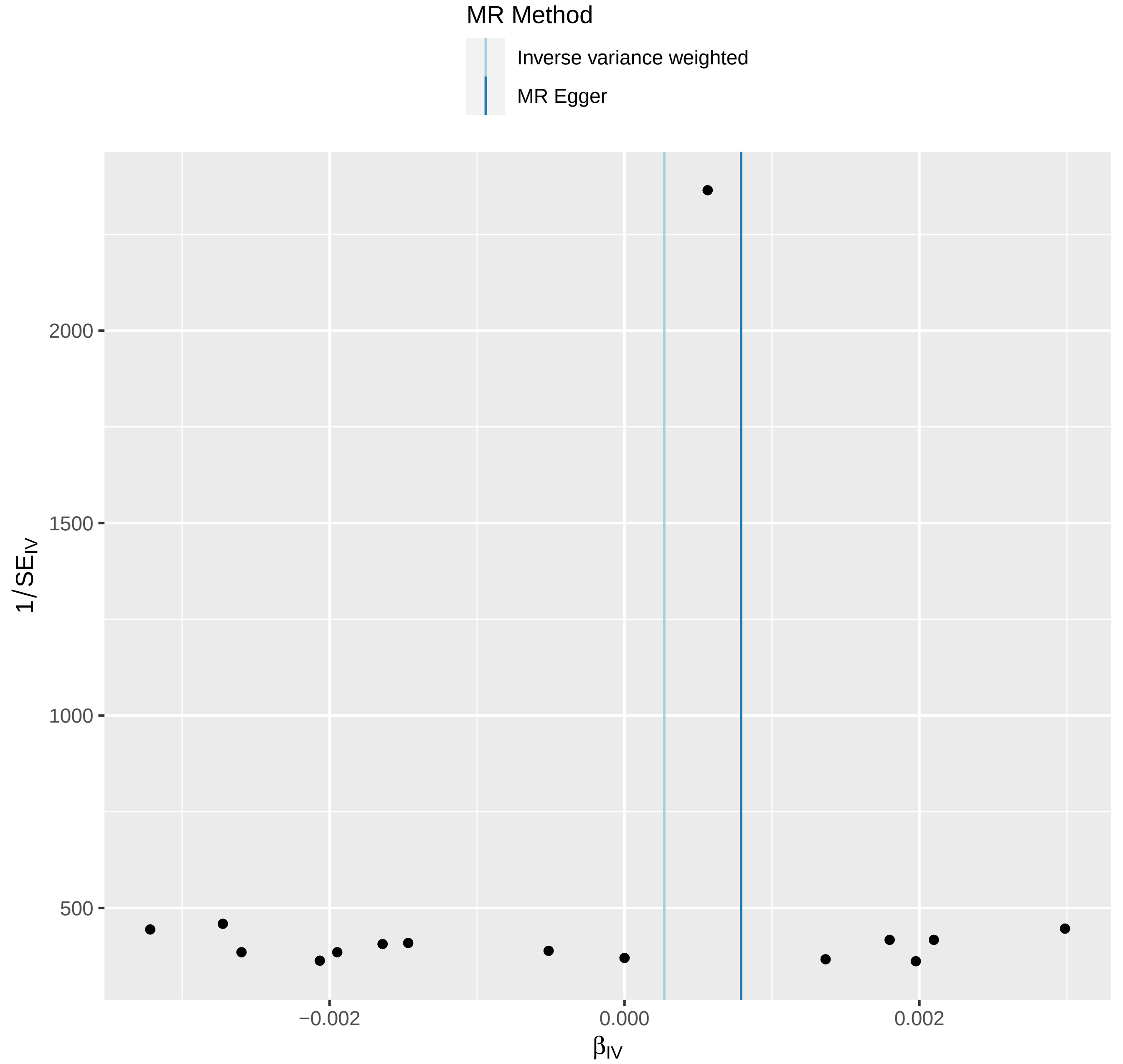

Batch 2−1645 : Killer cell immunoglobulin−like receptor 2DS2 on eGFR

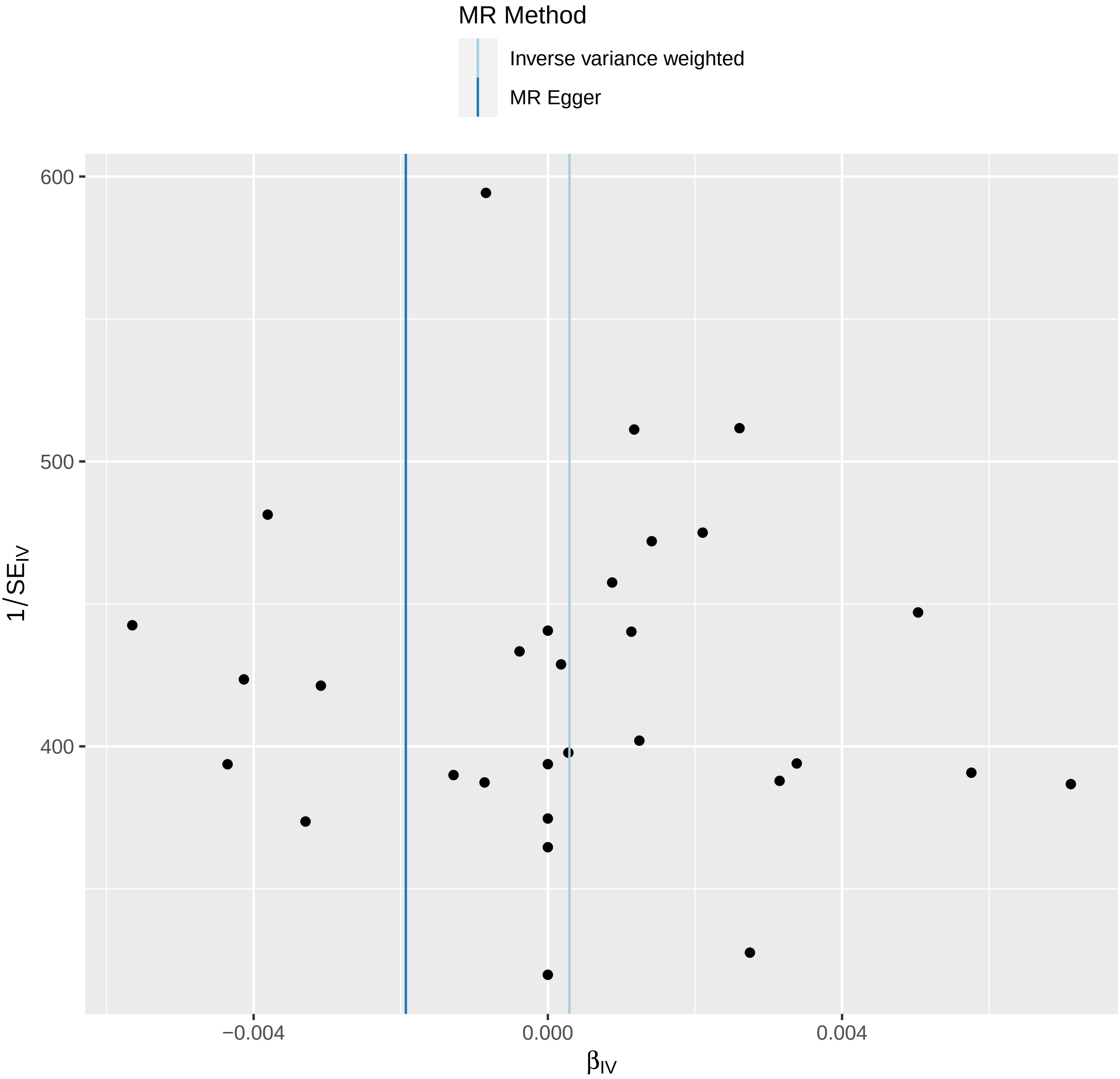

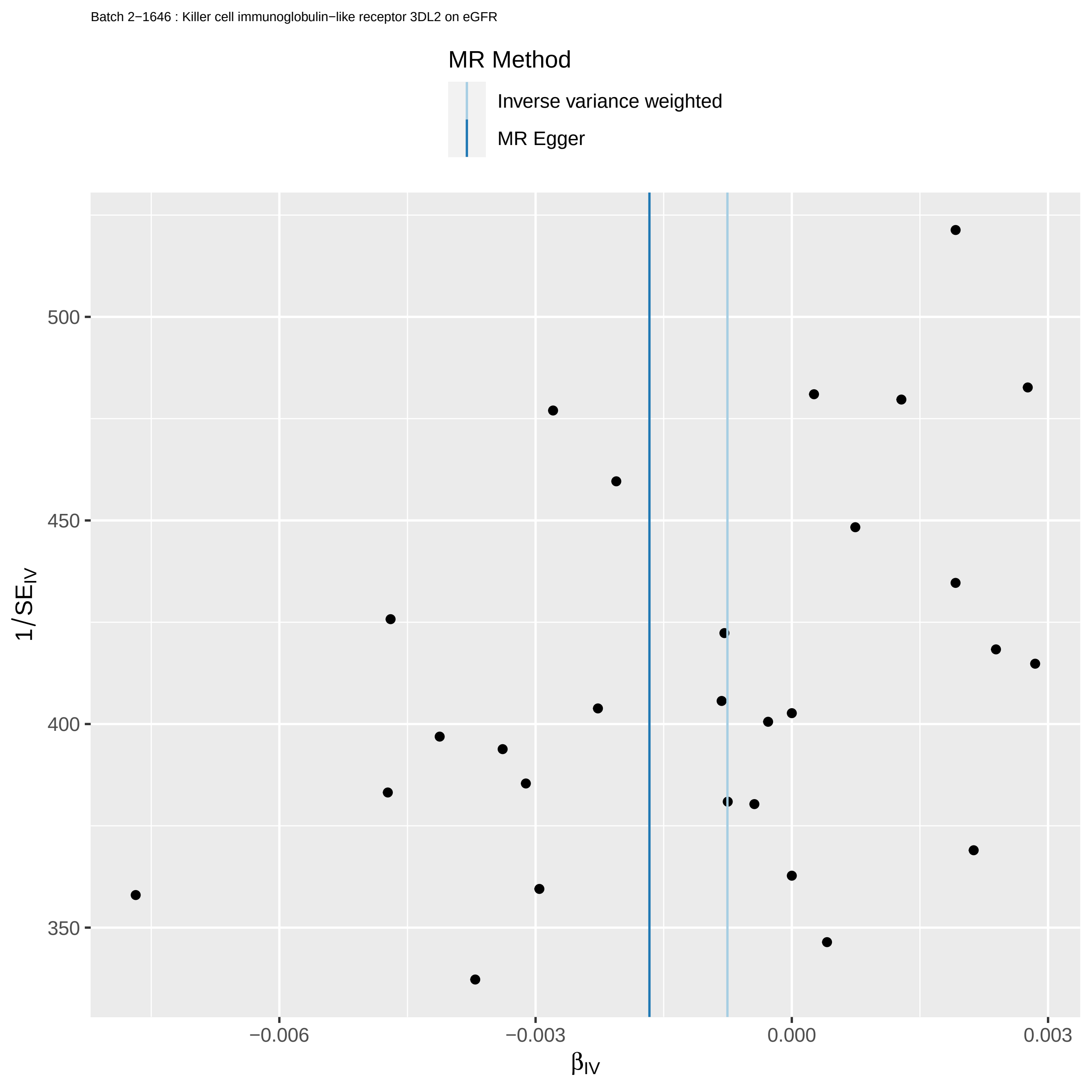

Batch 2−1646 : Killer cell immunoglobulin−like receptor 3DL2 on eGFR
MR Method
Inverse variance weighted
MR Egger
500
450
400
350
1/SE$_{IV}$
−0.006
−0.003
0.000
0.003
β$_{IV}$

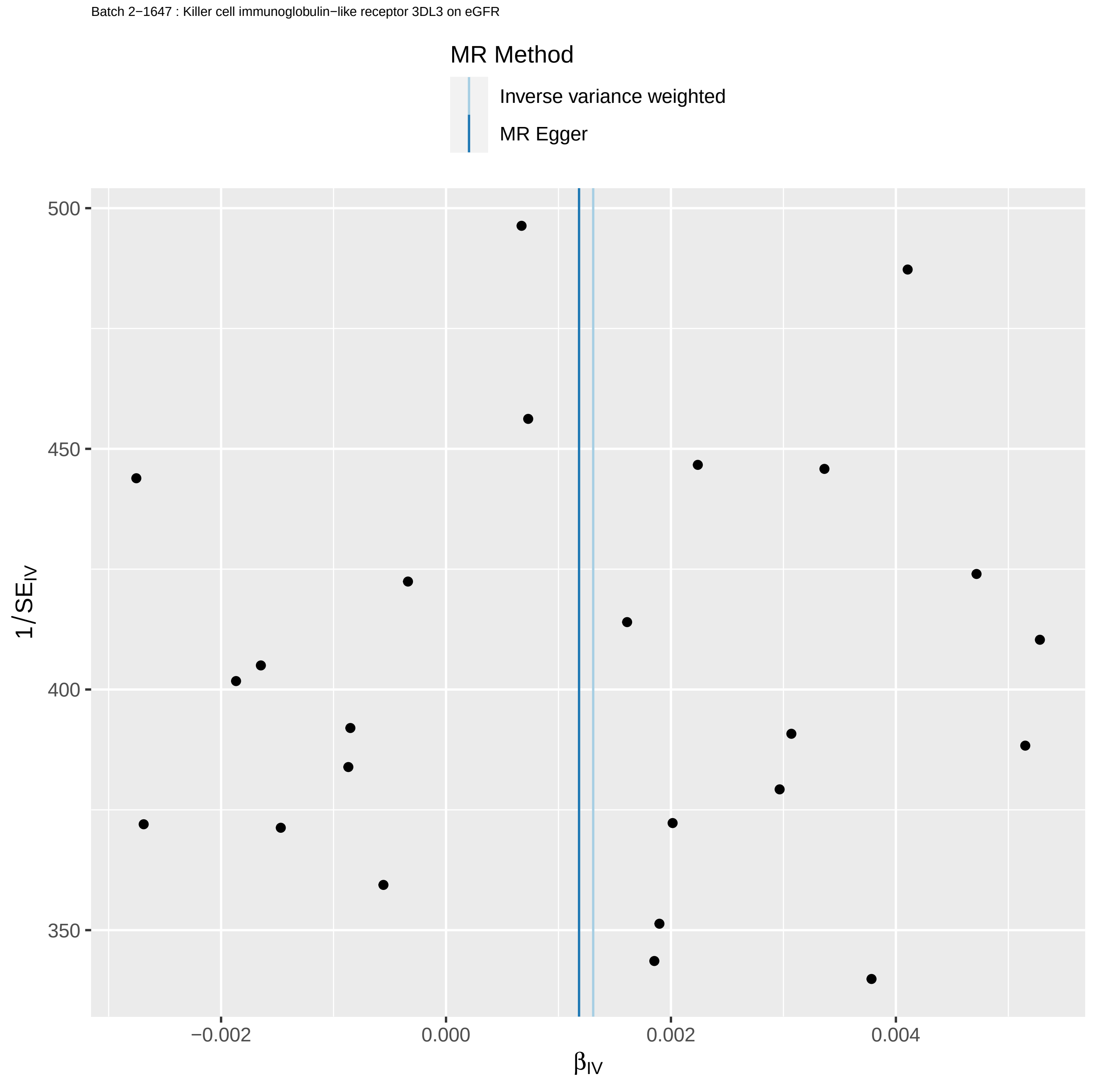
Batch 2−1647 : Killer cell immunoglobulin−like receptor 3DL3 on eGFR
MR Method
Inverse variance weighted
MR Egger
500
450
400
350
1/SE_IV
−0.002
0.000
0.002
0.004
β_IV

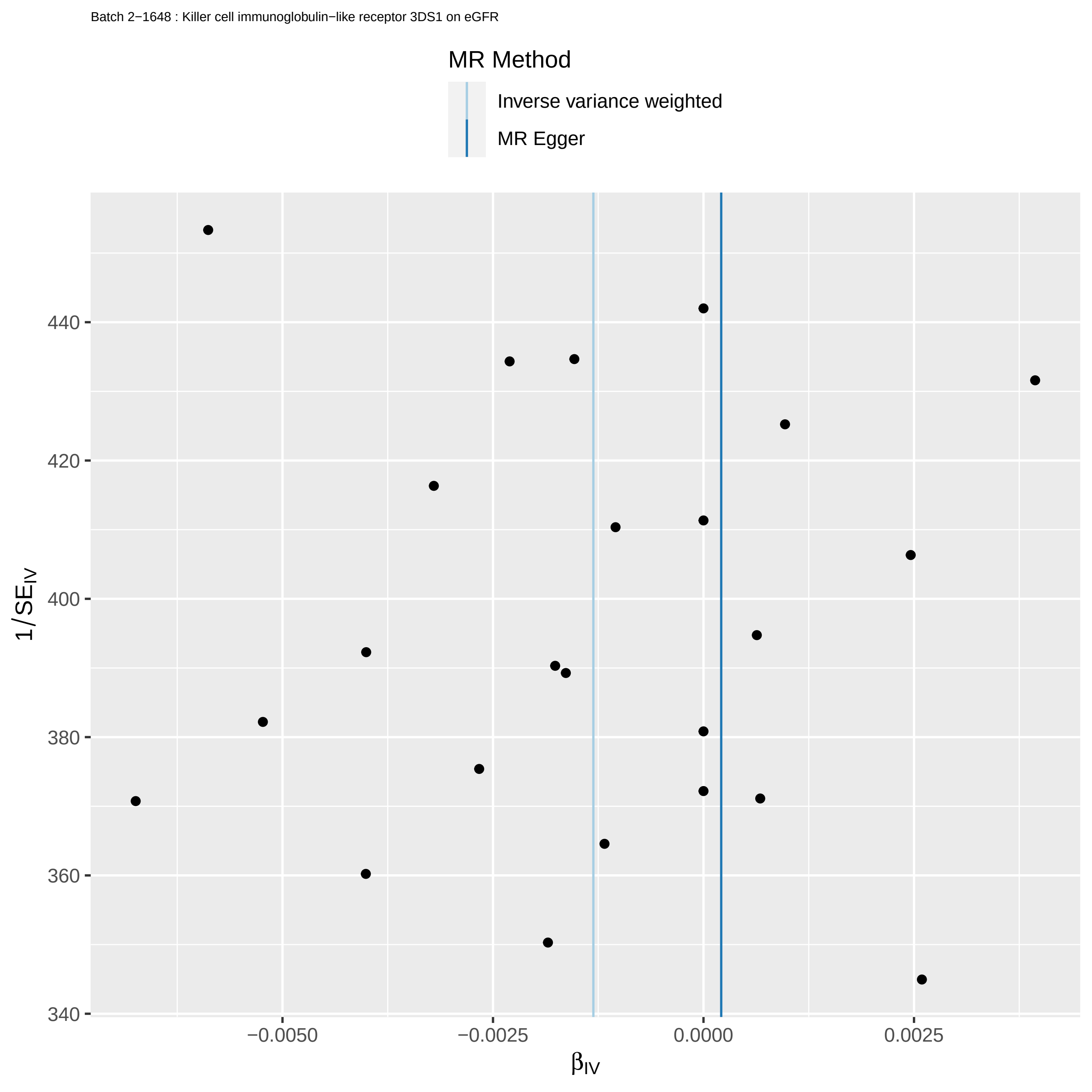

Batch 2−1648 : Killer cell immunoglobulin−like receptor 3DS1 on eGFR
MR Method
Inverse variance weighted
MR Egger
440
420
400
380
360
340
1/SE_IV
−0.0050
−0.0025
0.0000
0.0025
β_IV

Batch 2−1649 : Kin of IRRE−like protein 2 on eGFR

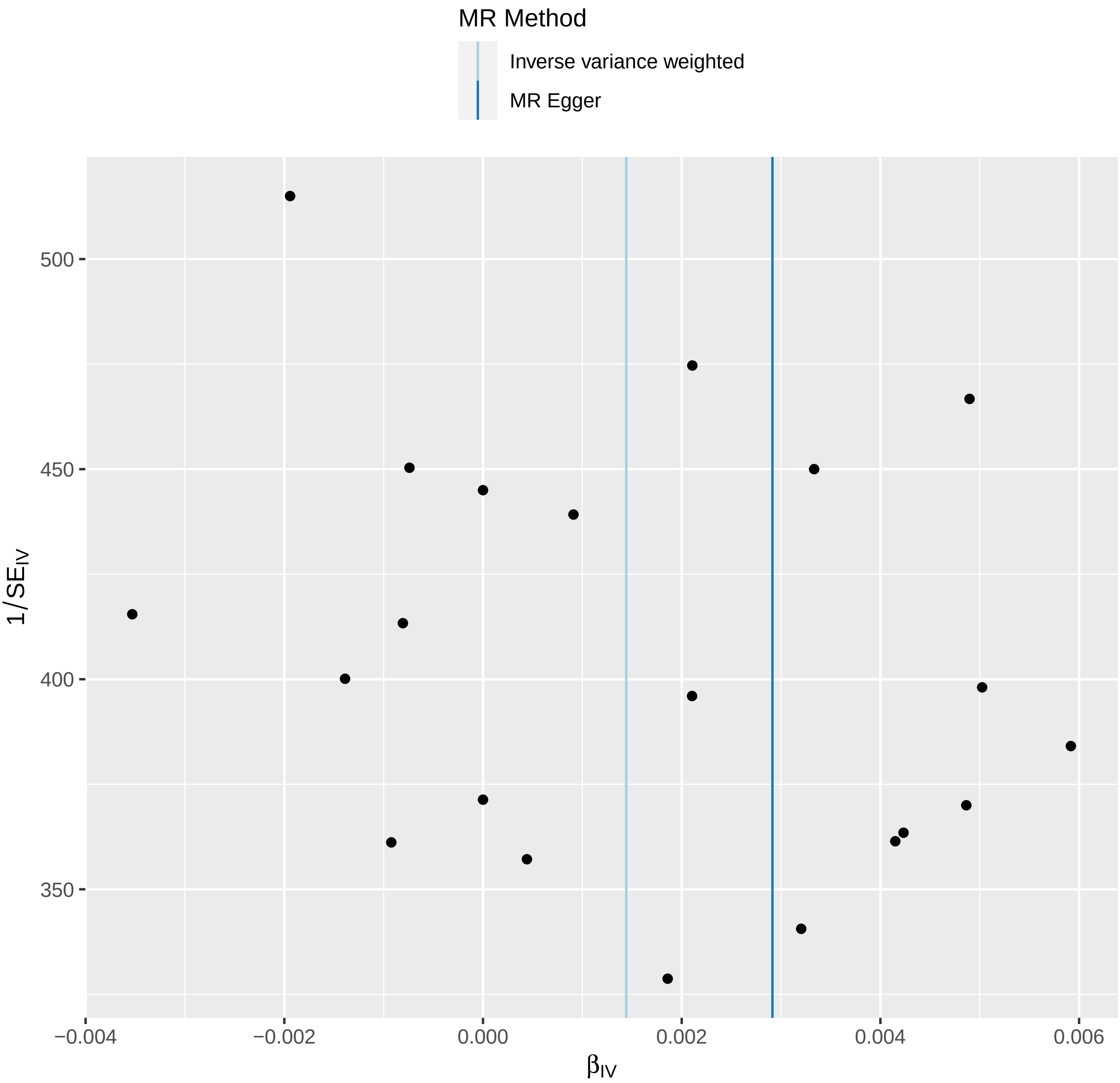

Batch 2−1650 : Kin of IRRE−like protein 2 on eGFR

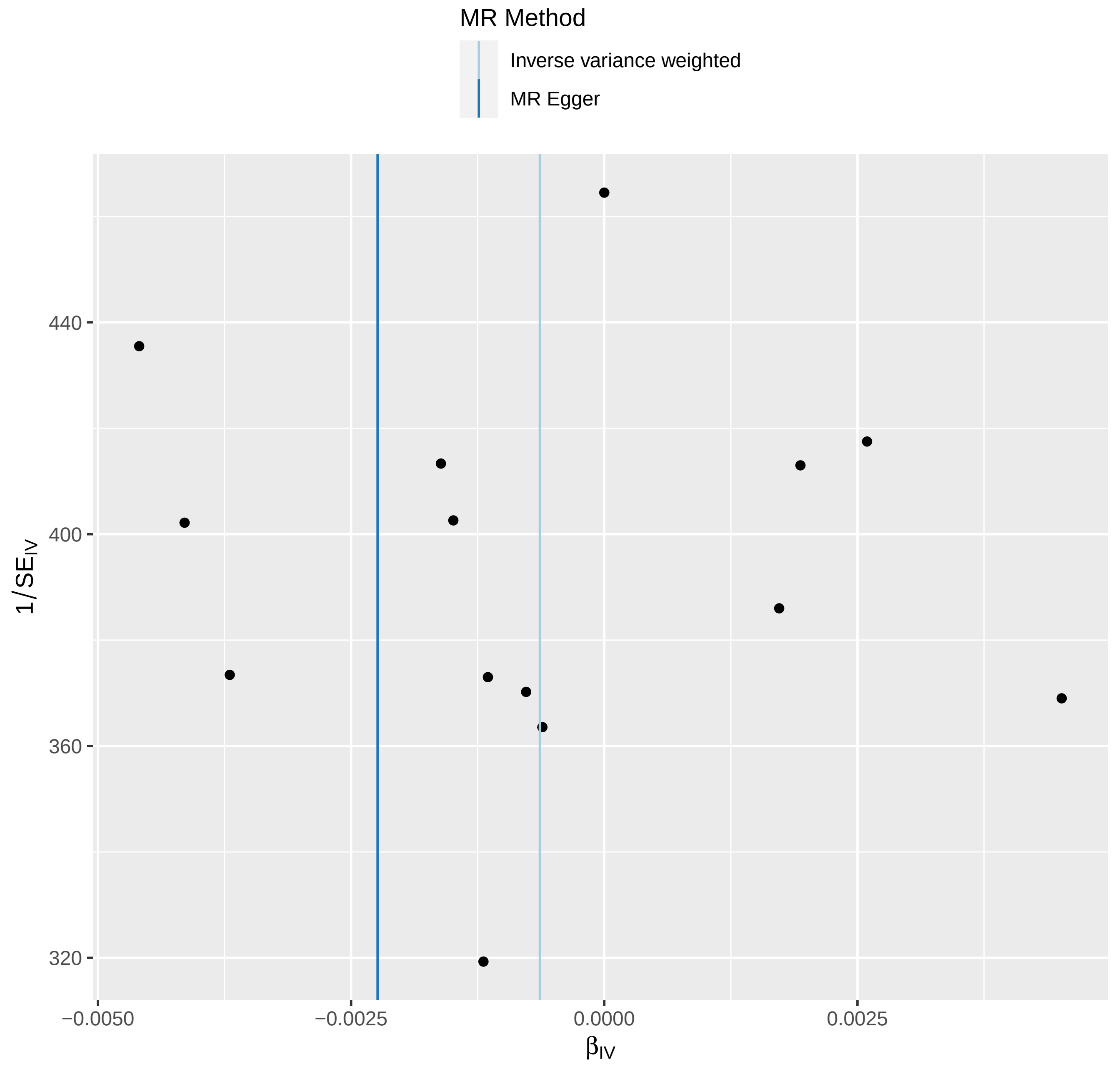

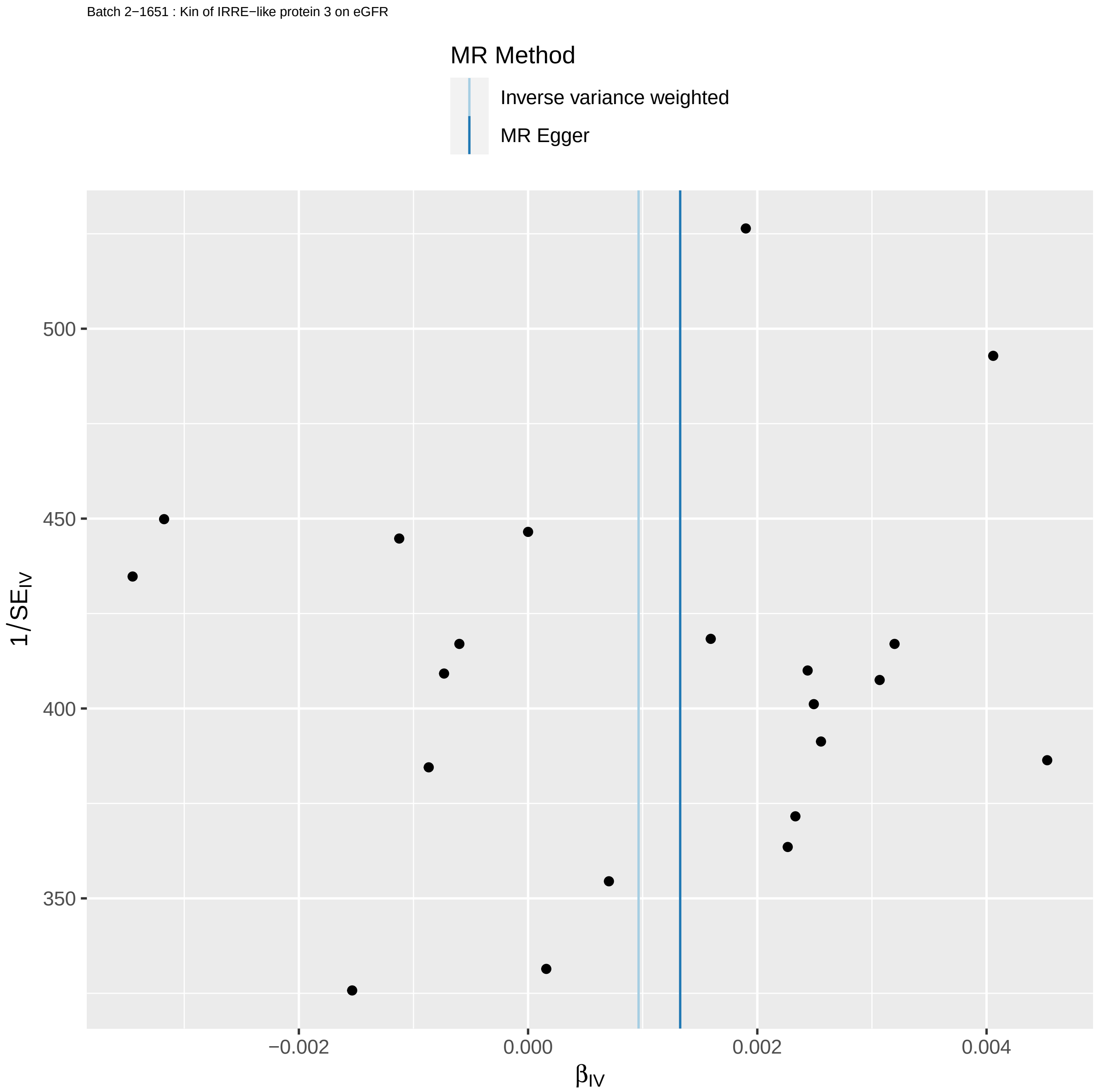

Batch 2−1651 : Kin of IRRE−like protein 3 on eGFR
MR Method
Inverse variance weighted
MR Egger
500
450
400
350
1/SE_IV
−0.002
0.000
0.002
0.004
β_IV

Batch 2−1652 : Mast/stem cell growth factor receptor Kit on eGFR

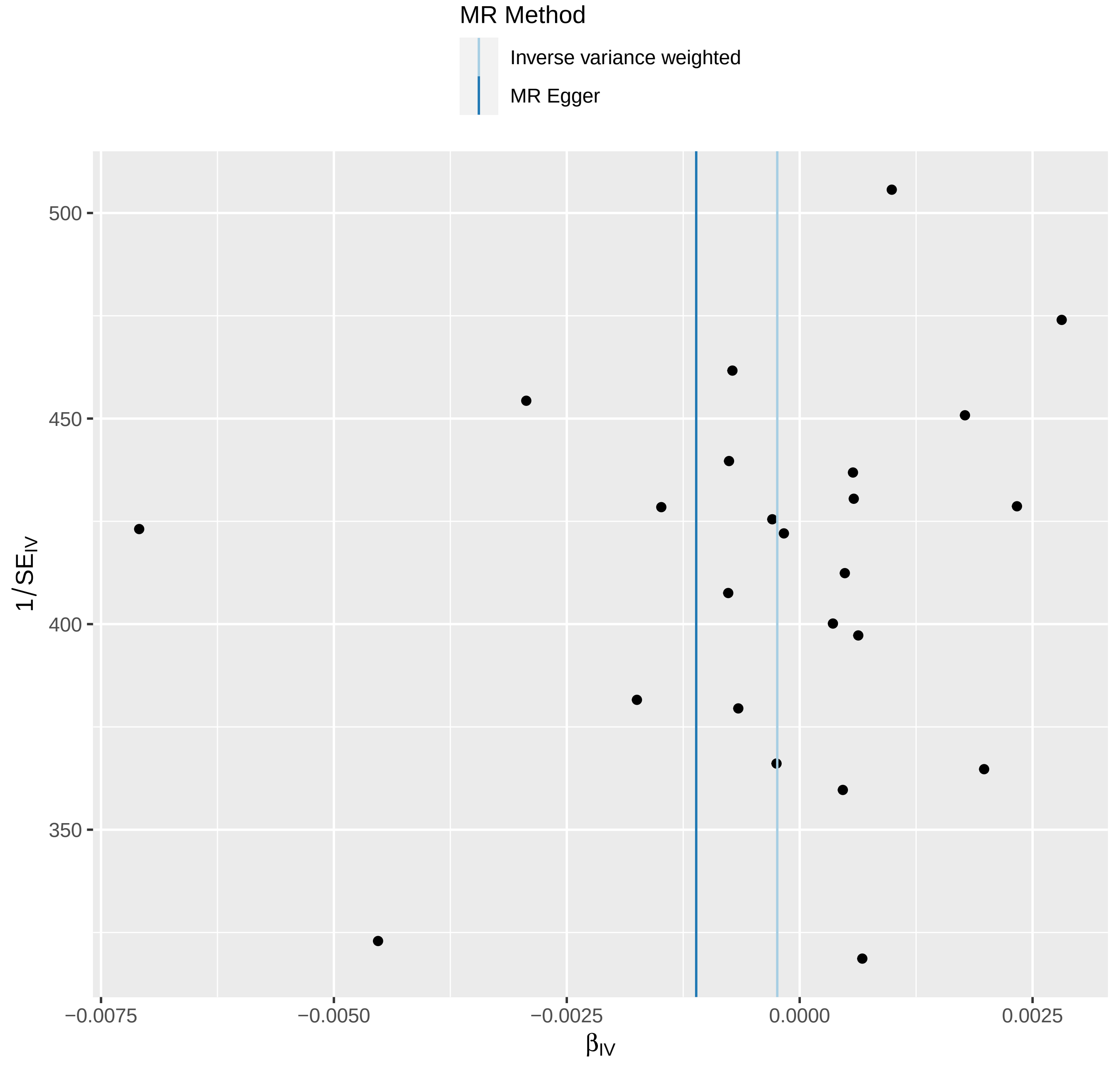

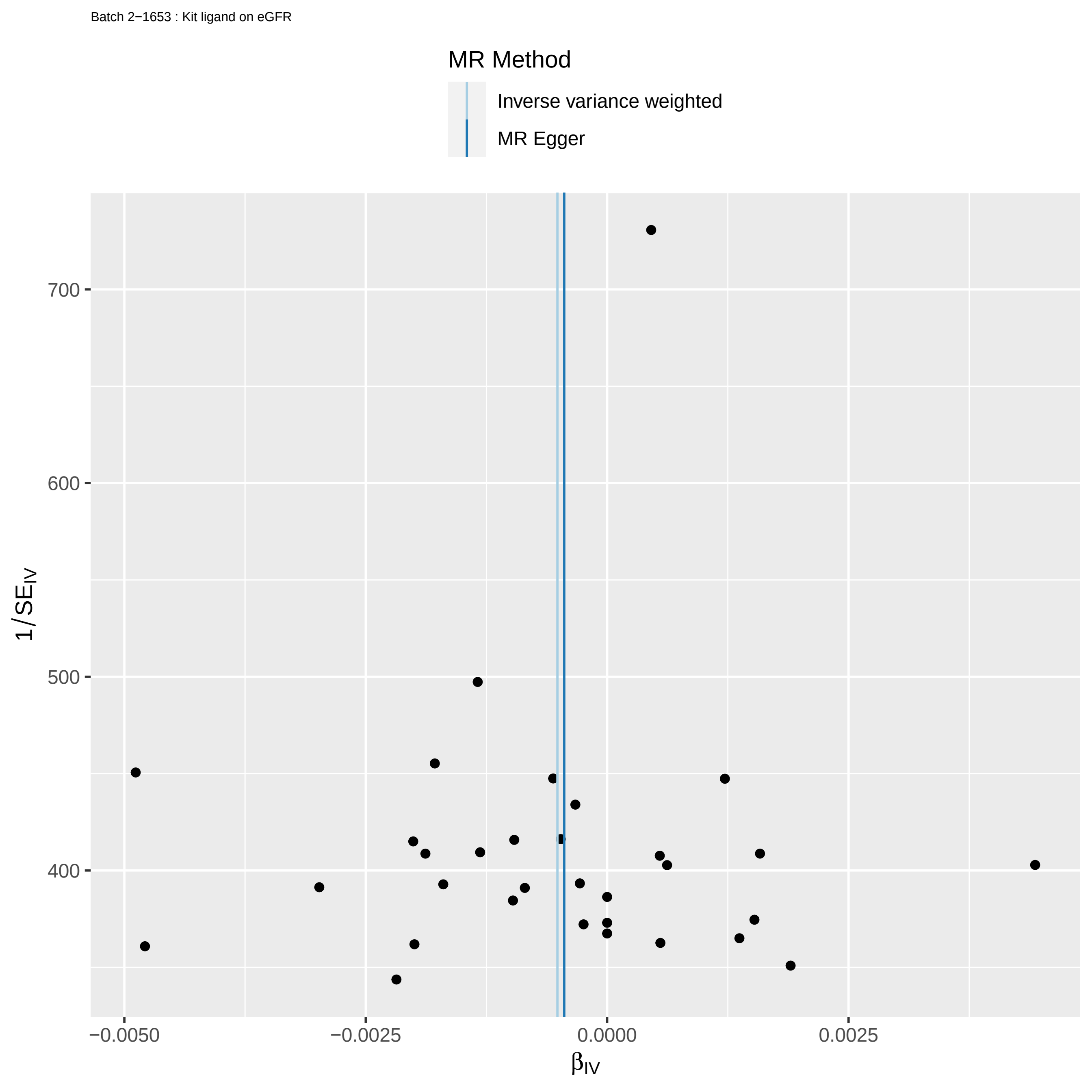

Batch 2−1653 : Kit ligand on eGFR
MR Method
Inverse variance weighted
MR Egger
700
600
500
400
1/SE_IV
−0.0050
−0.0025
0.0000
0.0025
β_IV

Batch 2−1654 : Kelch−like protein 12 on eGFR

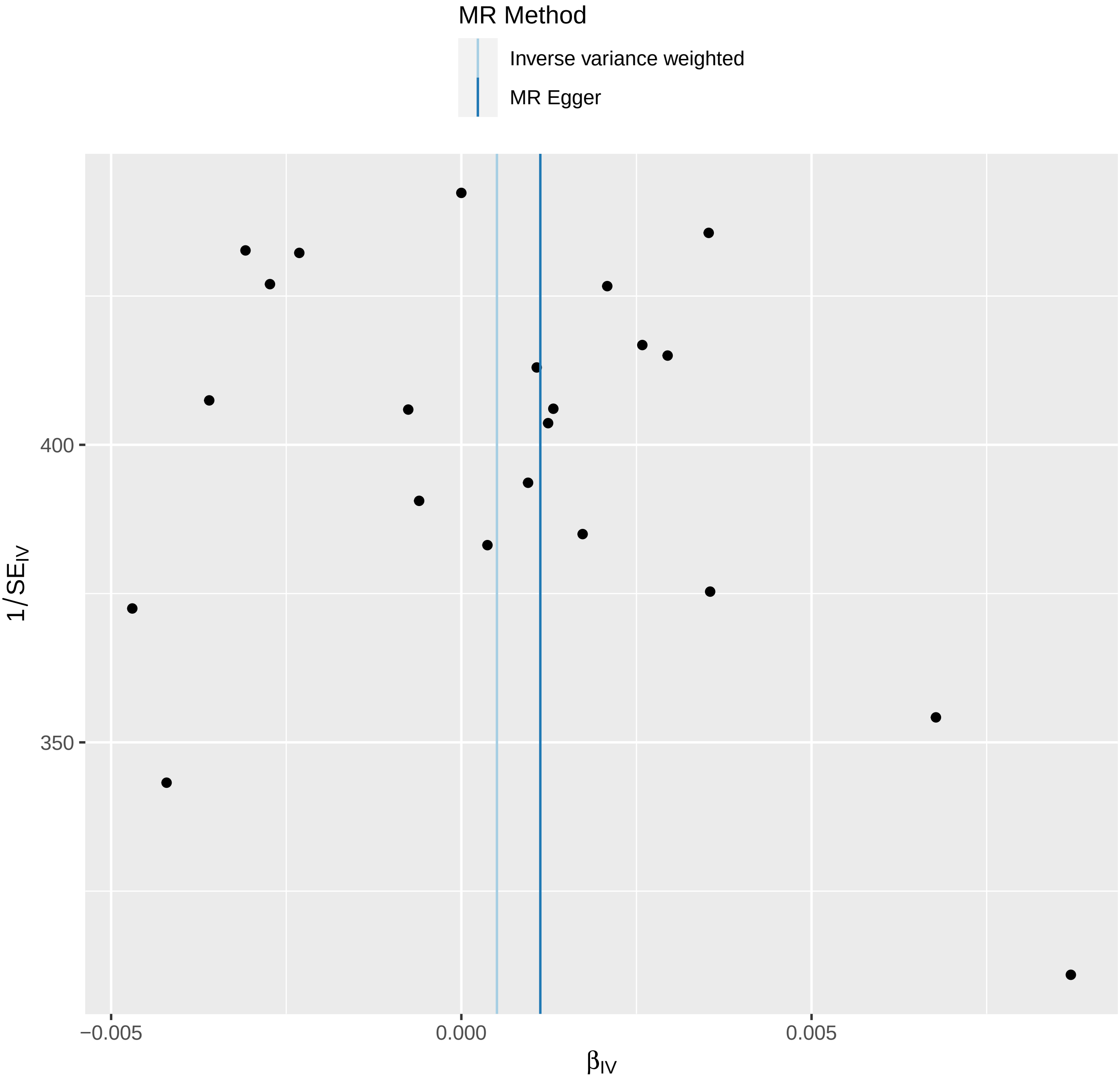

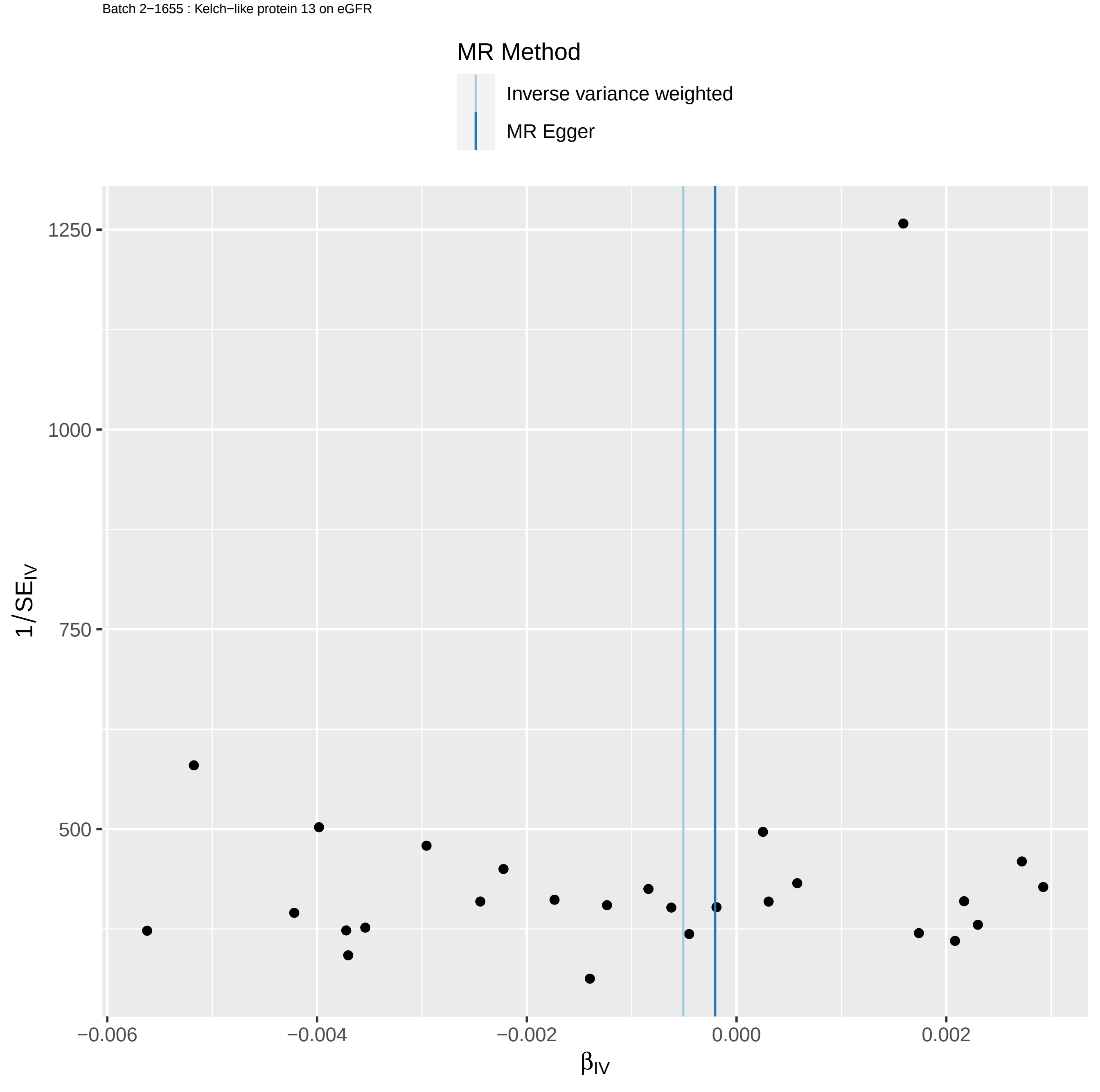

Batch 2−1655 : Kelch−like protein 13 on eGFR
MR Method
Inverse variance weighted
MR Egger
1250
1000
750
500
1/SE$_{IV}$
−0.006
−0.004
−0.002
0.000
0.002
β$_{IV}$

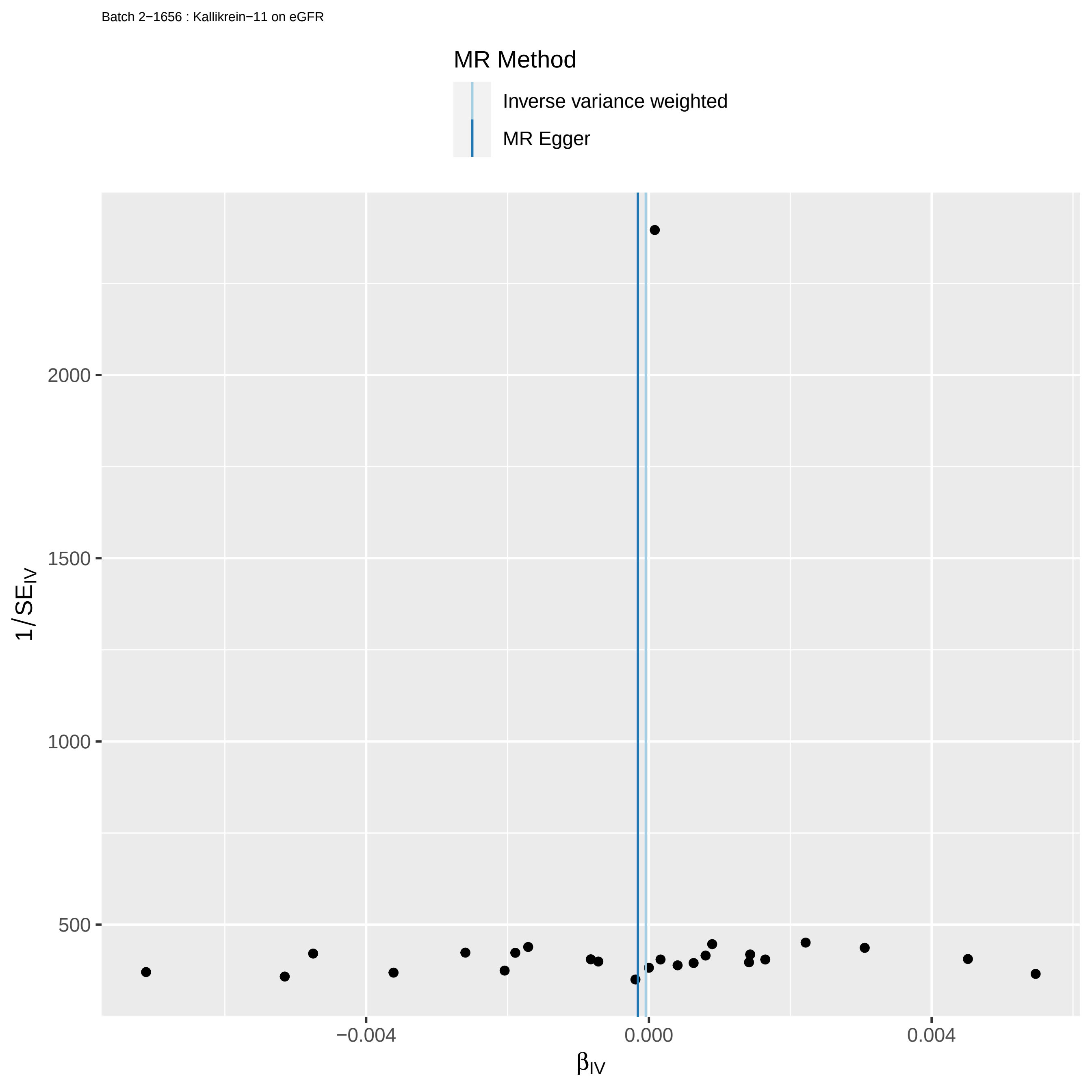
Batch 2−1656 : Kallikrein−11 on eGFR
MR Method
Inverse variance weighted
MR Egger
2000
1500
1000
500
1/SE_IV
−0.004
0.000
0.004
β_IV

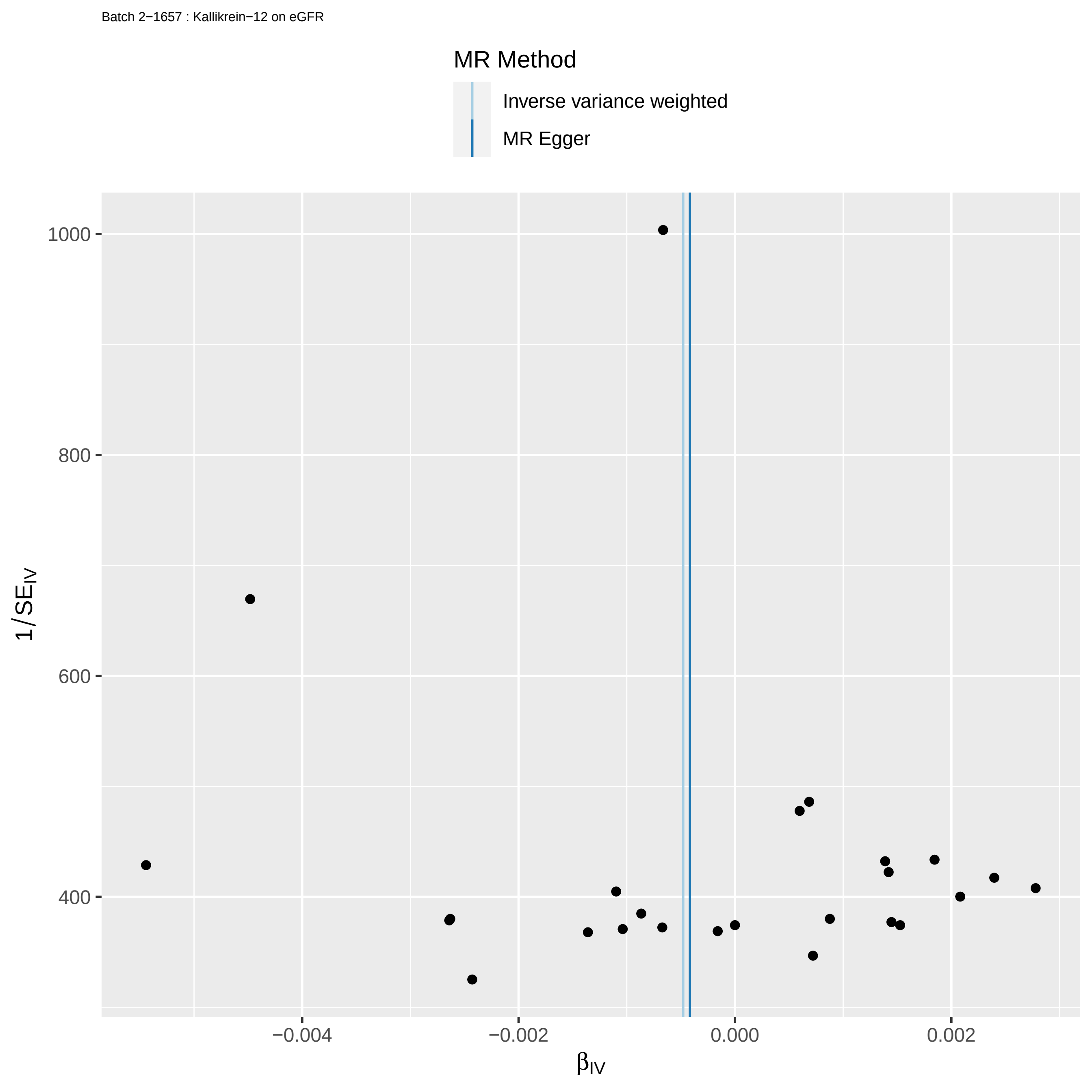
Batch 2−1657 : Kallikrein−12 on eGFR
MR Method
Inverse variance weighted
MR Egger
1000
800
600
400
1/SE$_{IV}$
−0.004
−0.002
0.000
0.002
β$_{IV}$

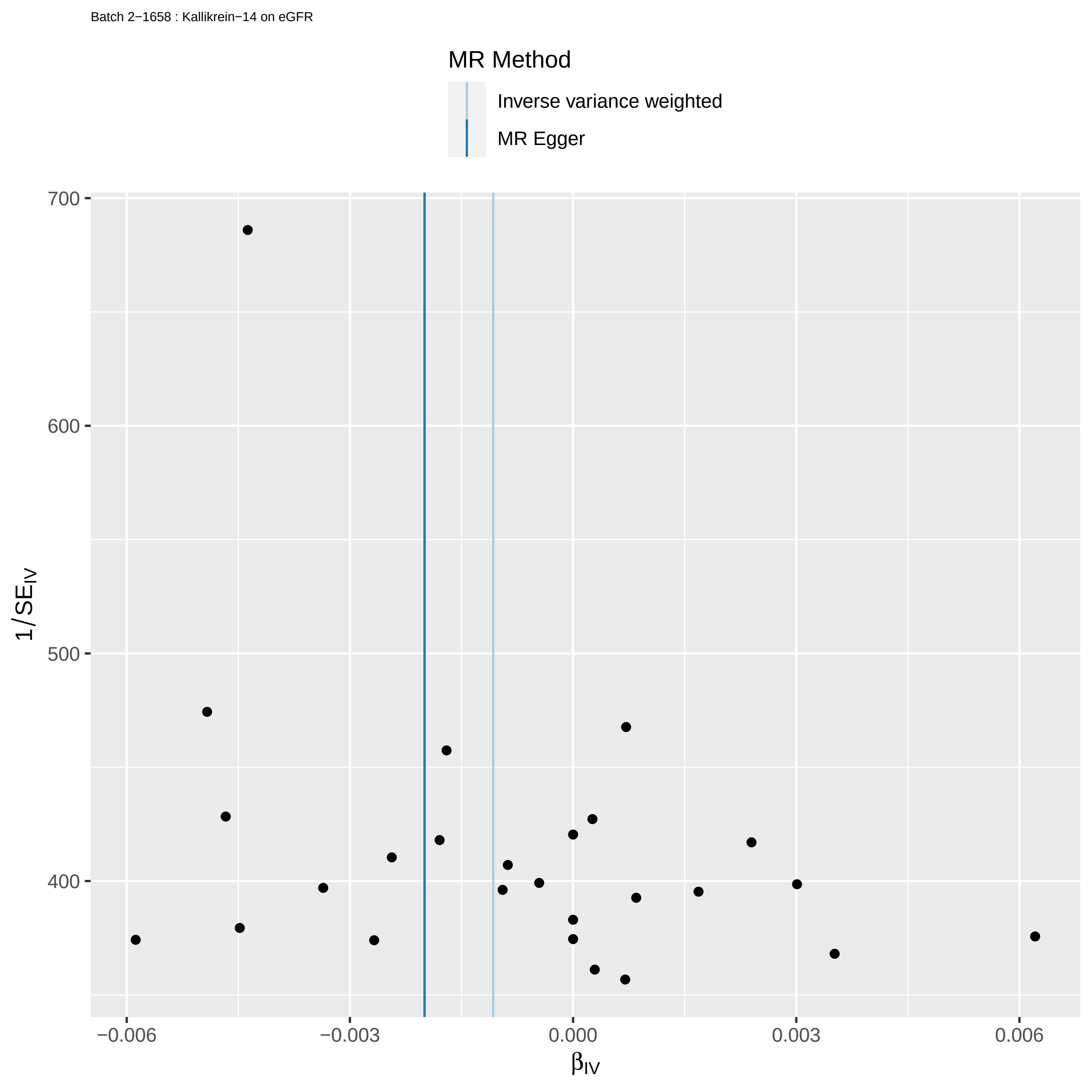

Batch 2−1658 : Kallikrein−14 on eGFR
MR Method
Inverse variance weighted
MR Egger
700
600
500
400
1/SE$_{IV}$
−0.006
−0.003
0.000
0.003
0.006
β$_{IV}$

Batch 2−1659 : Kallikrein−14 on eGFR

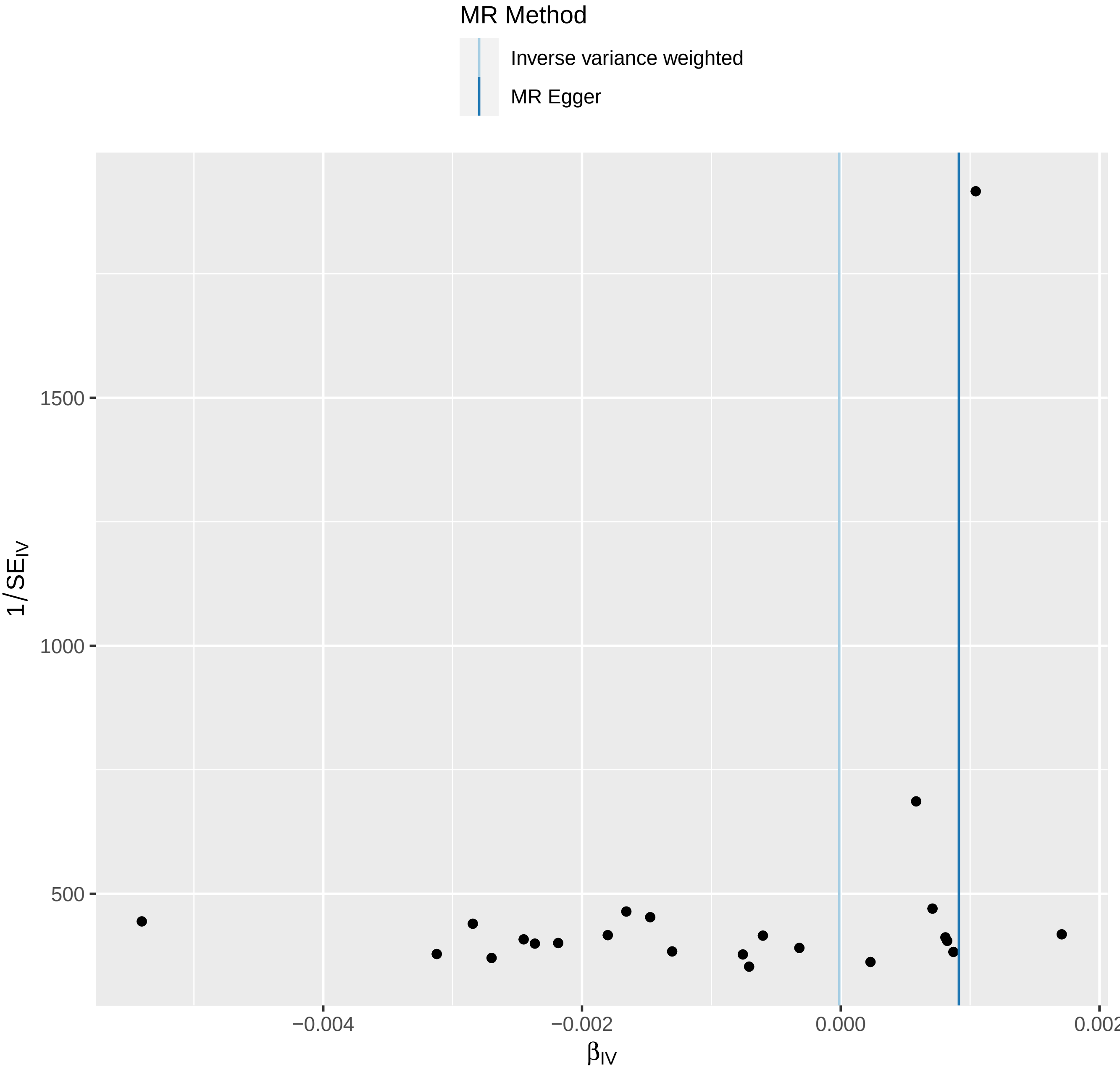

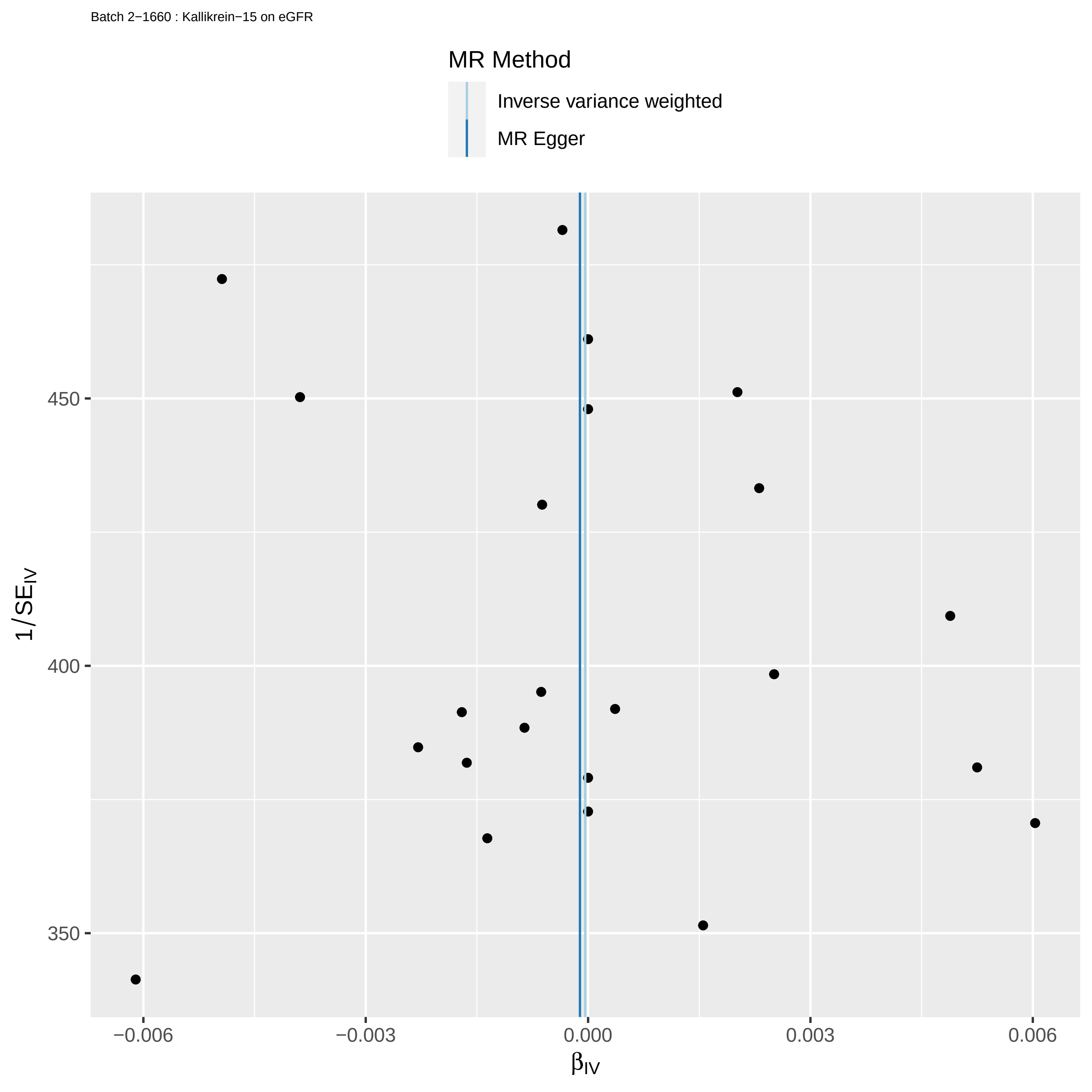
Batch 2−1660 : Kallikrein−15 on eGFR
MR Method
Inverse variance weighted
MR Egger
1/SE_IV
450
400
350
−0.006
−0.003
0.000
0.003
0.006
β_IV

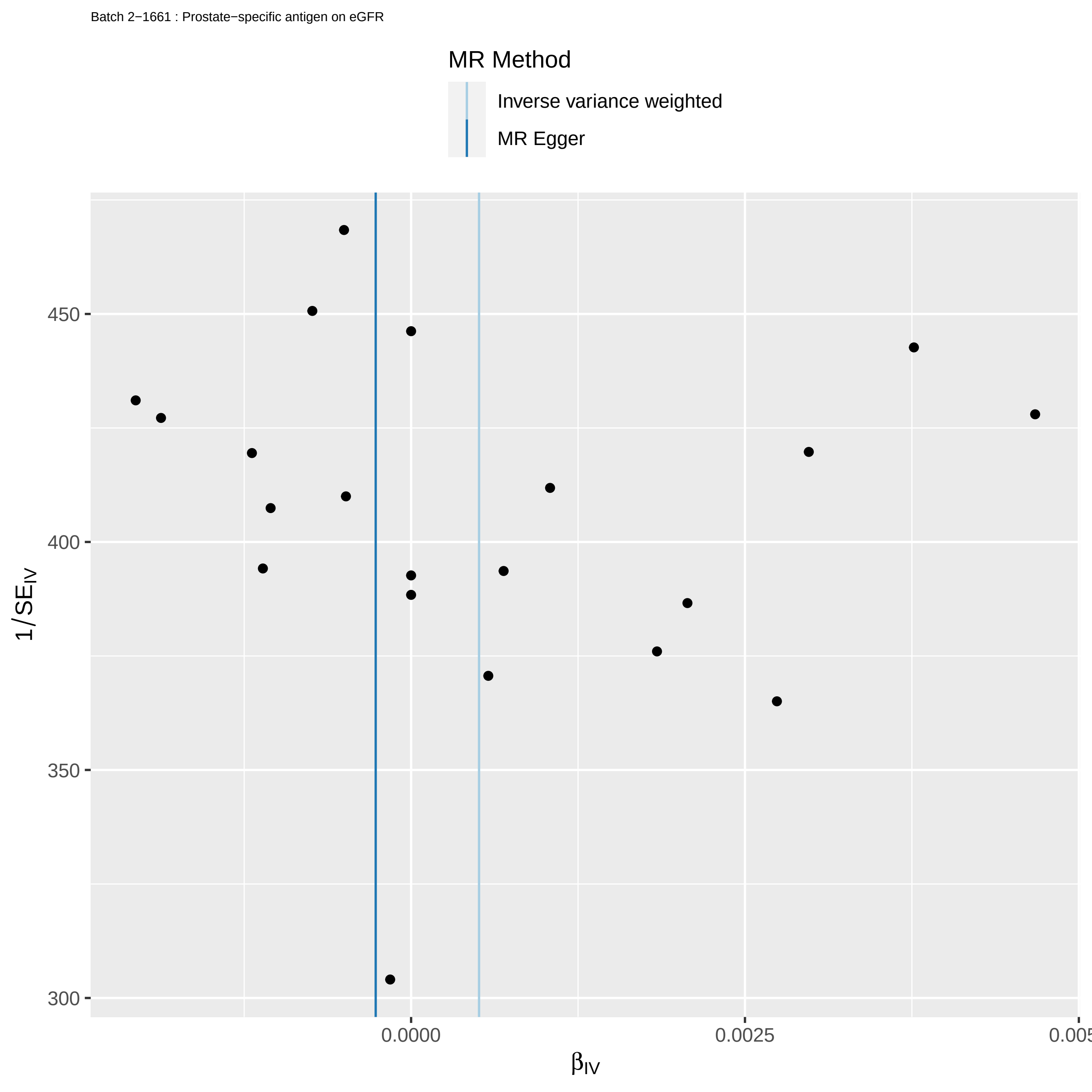

Batch 2−1661 : Prostate−specific antigen on eGFR
MR Method
Inverse variance weighted
MR Egger
450
400
350
300
0.0000
0.0025
0.005
$1/SE_{IV}$
β_{IV}

Batch 2−1662 : Kallikrein−4 on eGFR

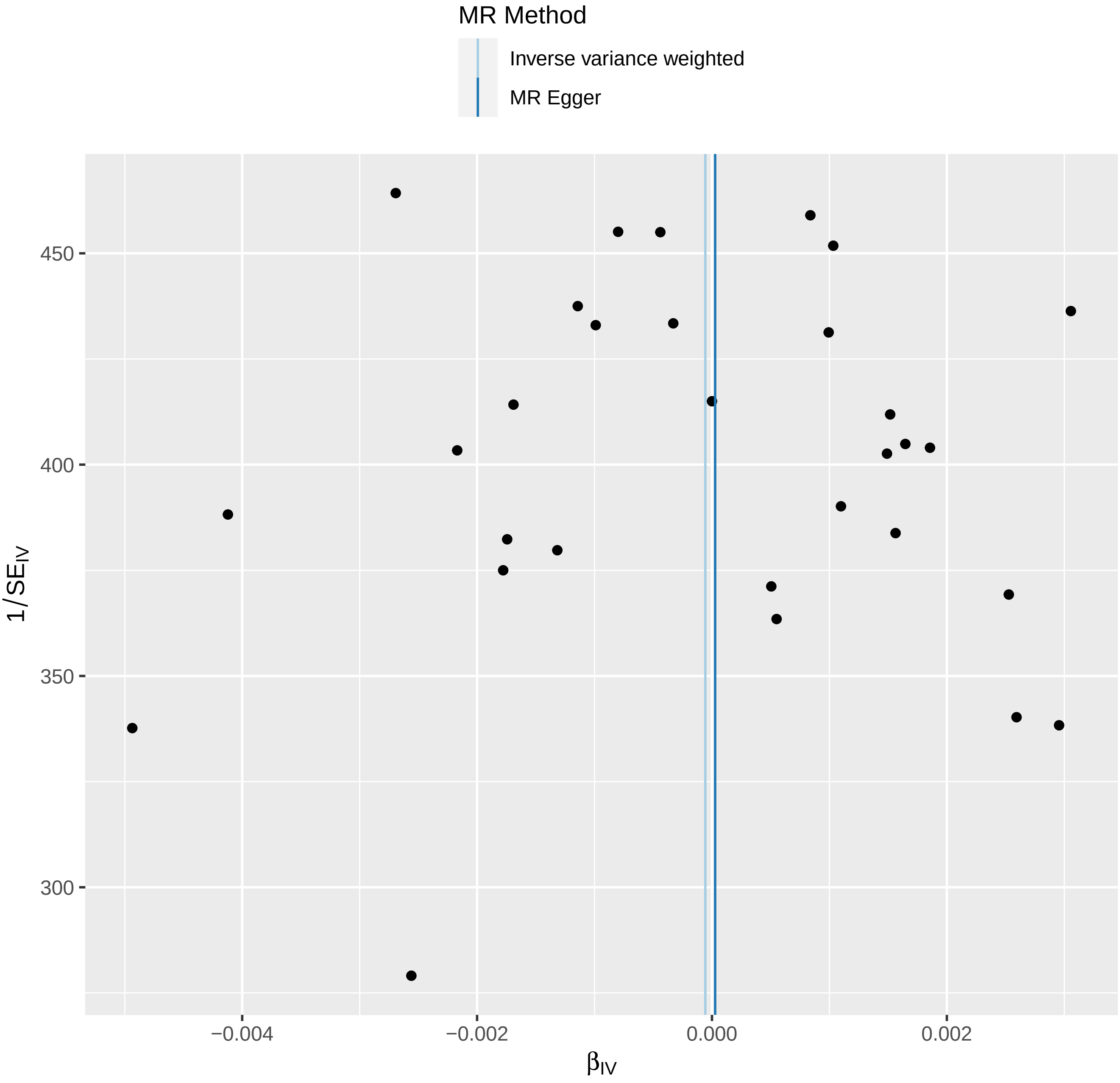

Batch 2−1663 : Kallikrein−5 on eGFR

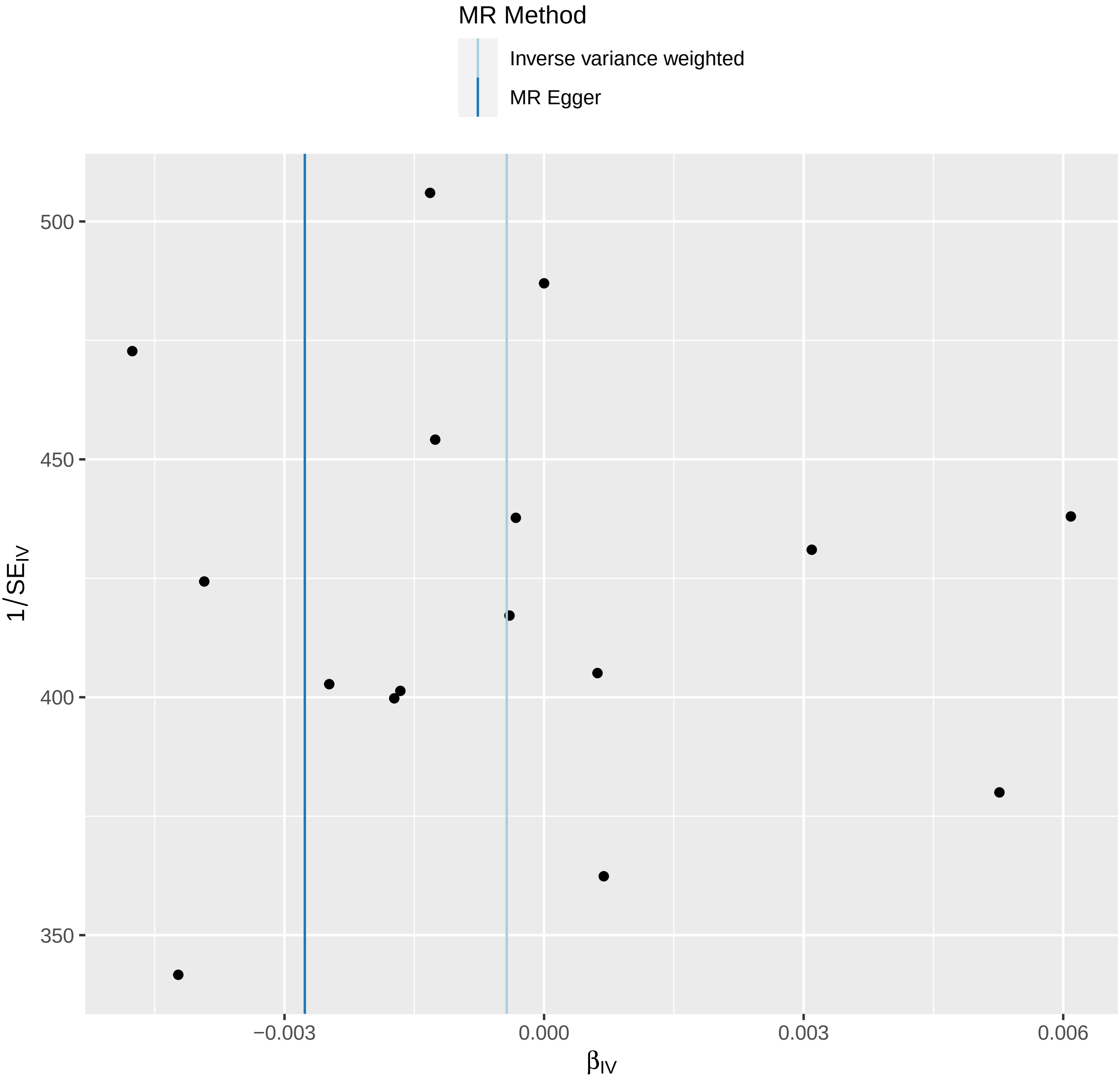

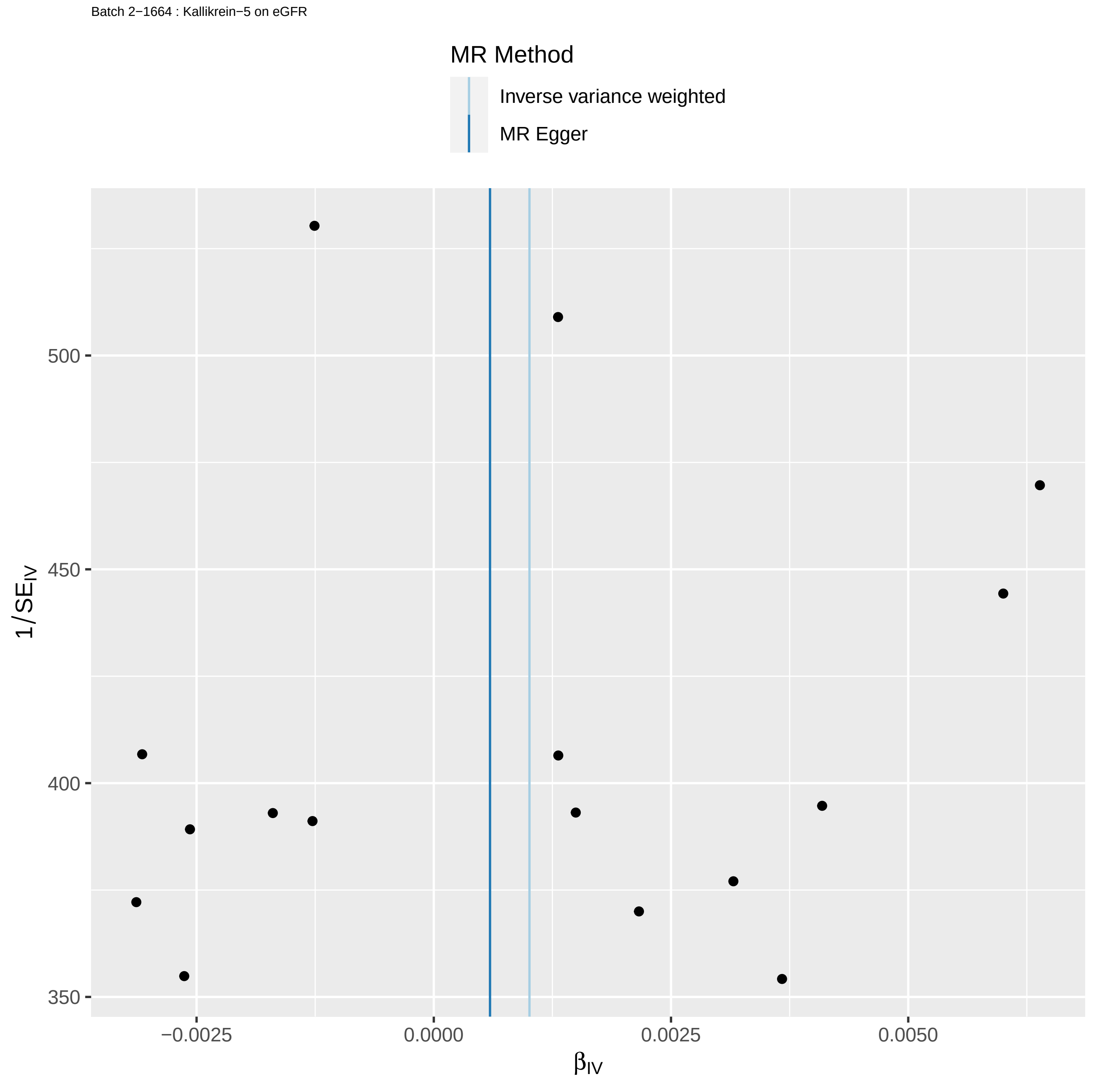
Batch 2−1664 : Kallikrein−5 on eGFR
MR Method
Inverse variance weighted
MR Egger
500
450
400
350
1/SE_IV
−0.0025
0.0000
0.0025
0.0050
β_IV

Batch 2−1665 : Kallikrein−6 on eGFR

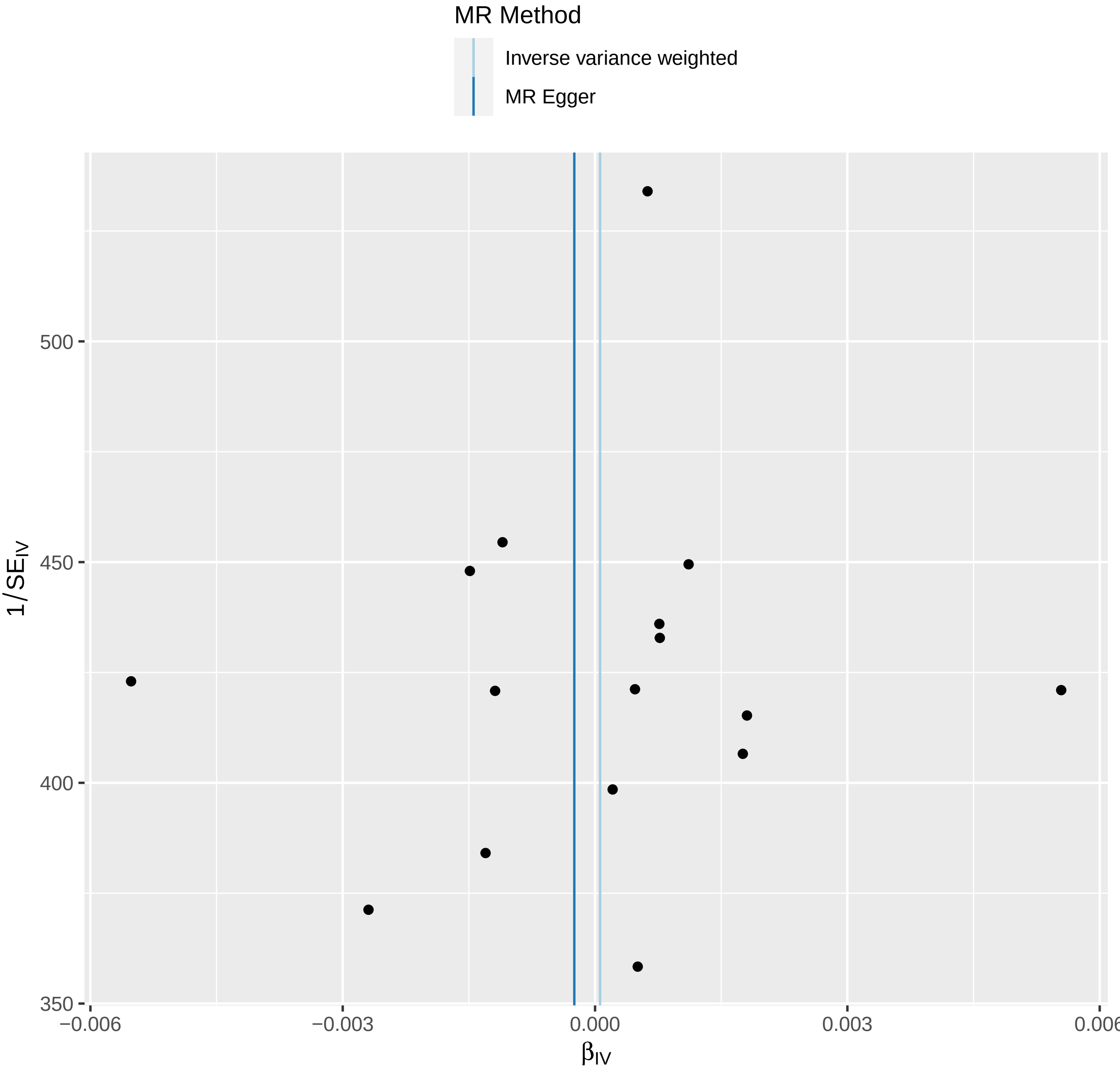

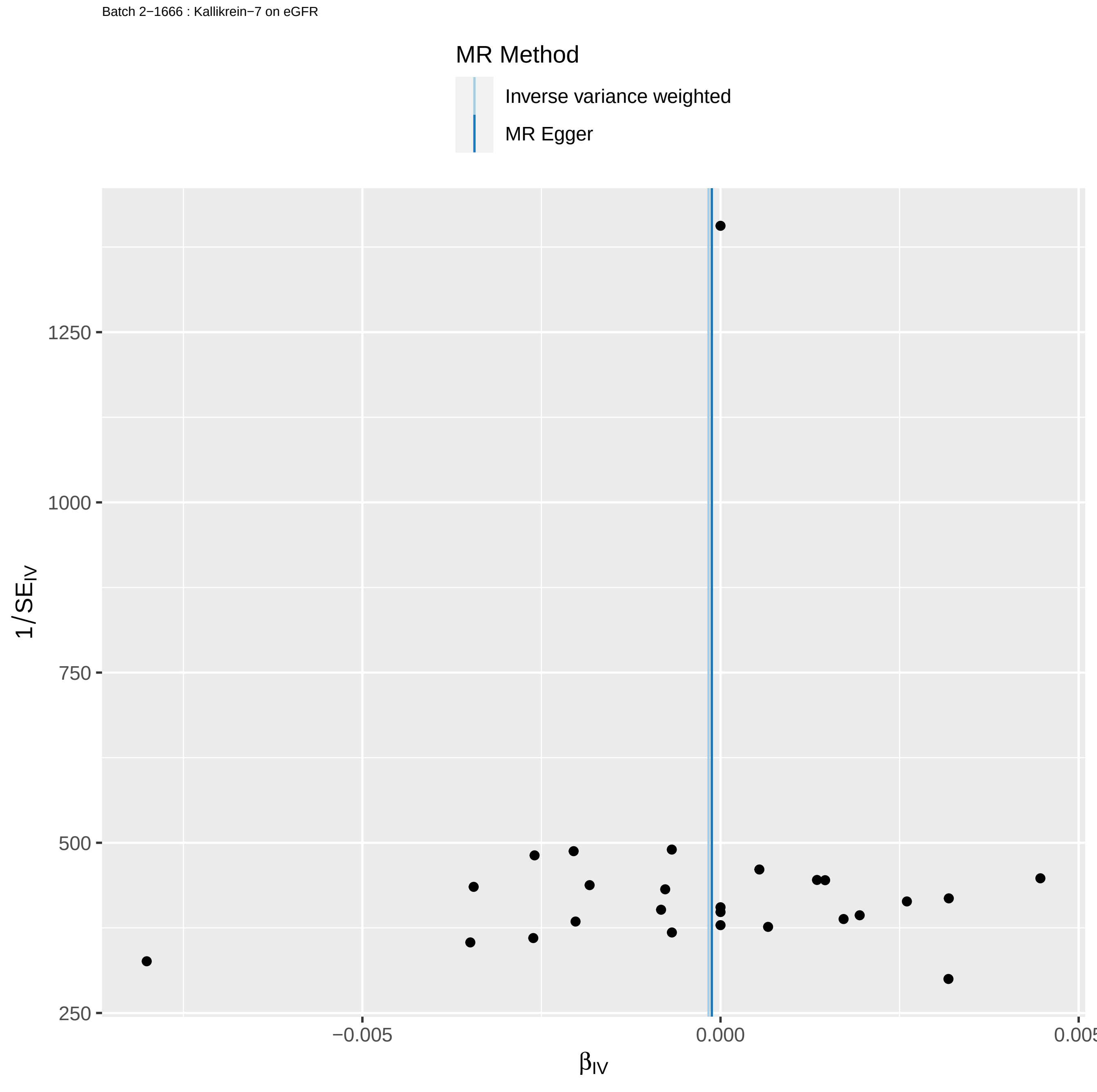

Batch 2−1666 : Kallikrein−7 on eGFR
MR Method
Inverse variance weighted
MR Egger
1250
1000
750
500
250
1/SE$_{IV}$
−0.005
0.000
0.005
β$_{IV}$

Batch 2−1667 : Kallikrein−8 on eGFR

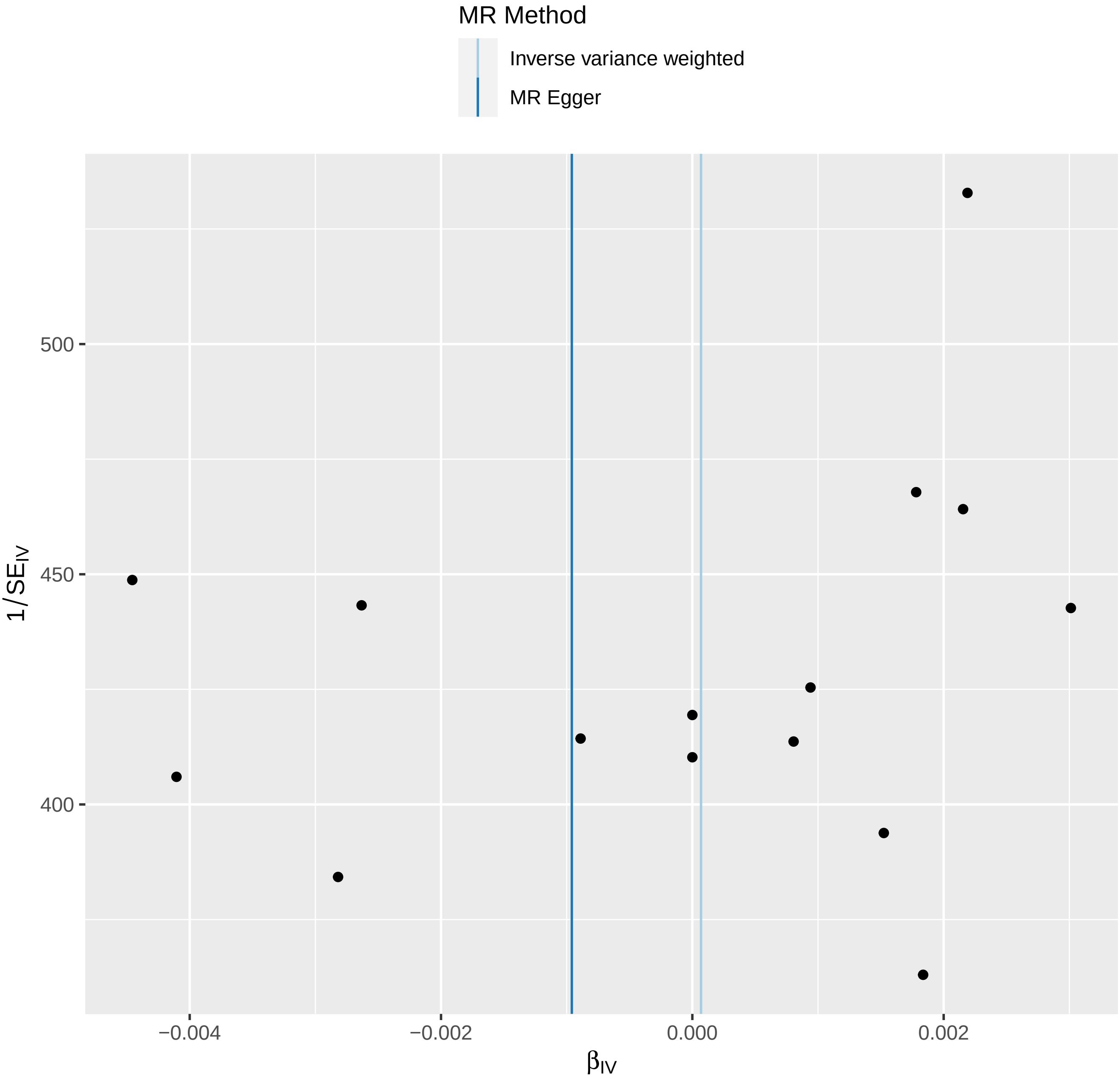

Batch 2−1668 : Kallikrein−8 on eGFR

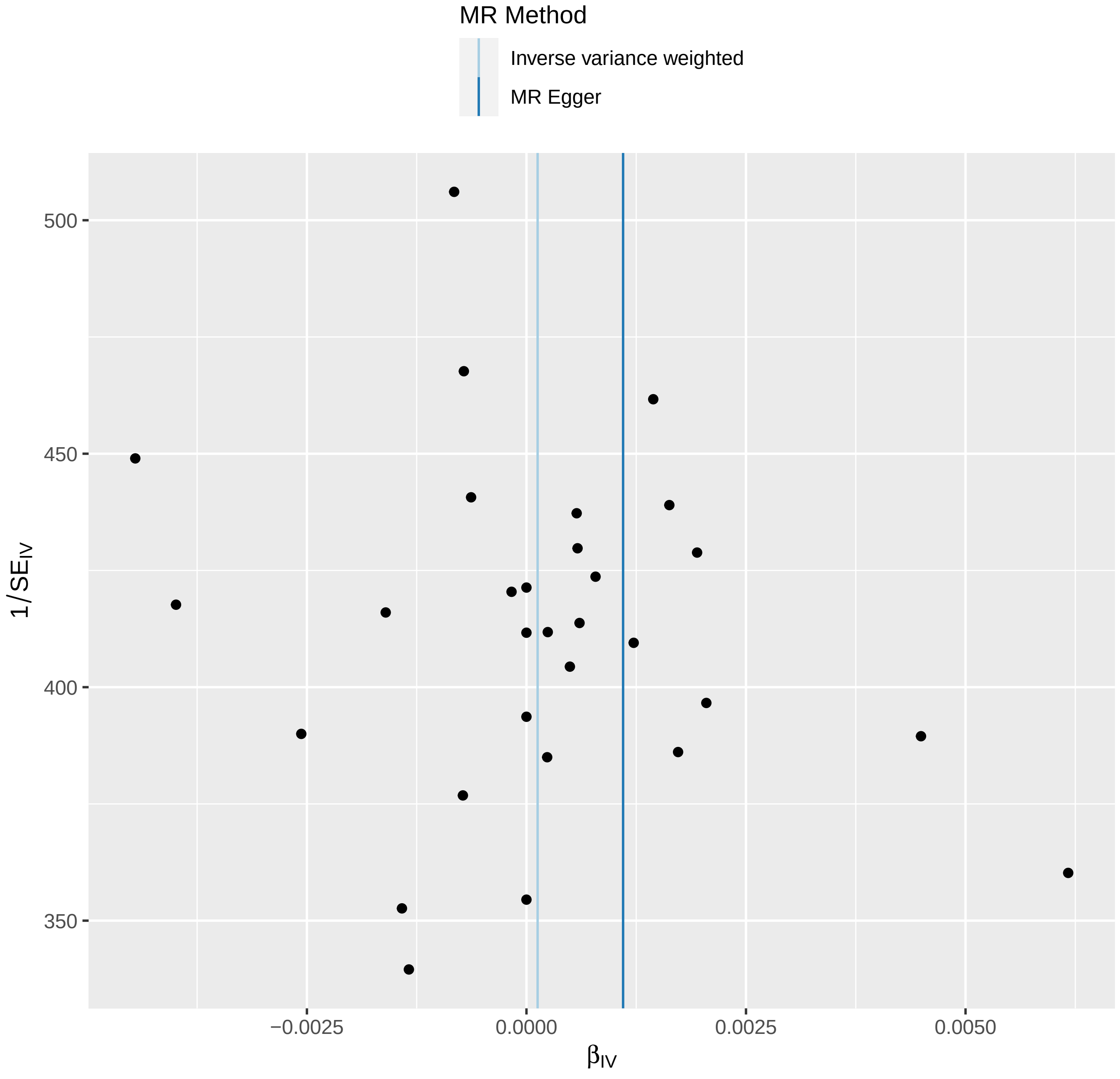

Batch 2−1669 : NKG2−A/NKG2−B type II integral membrane protein on eGFR

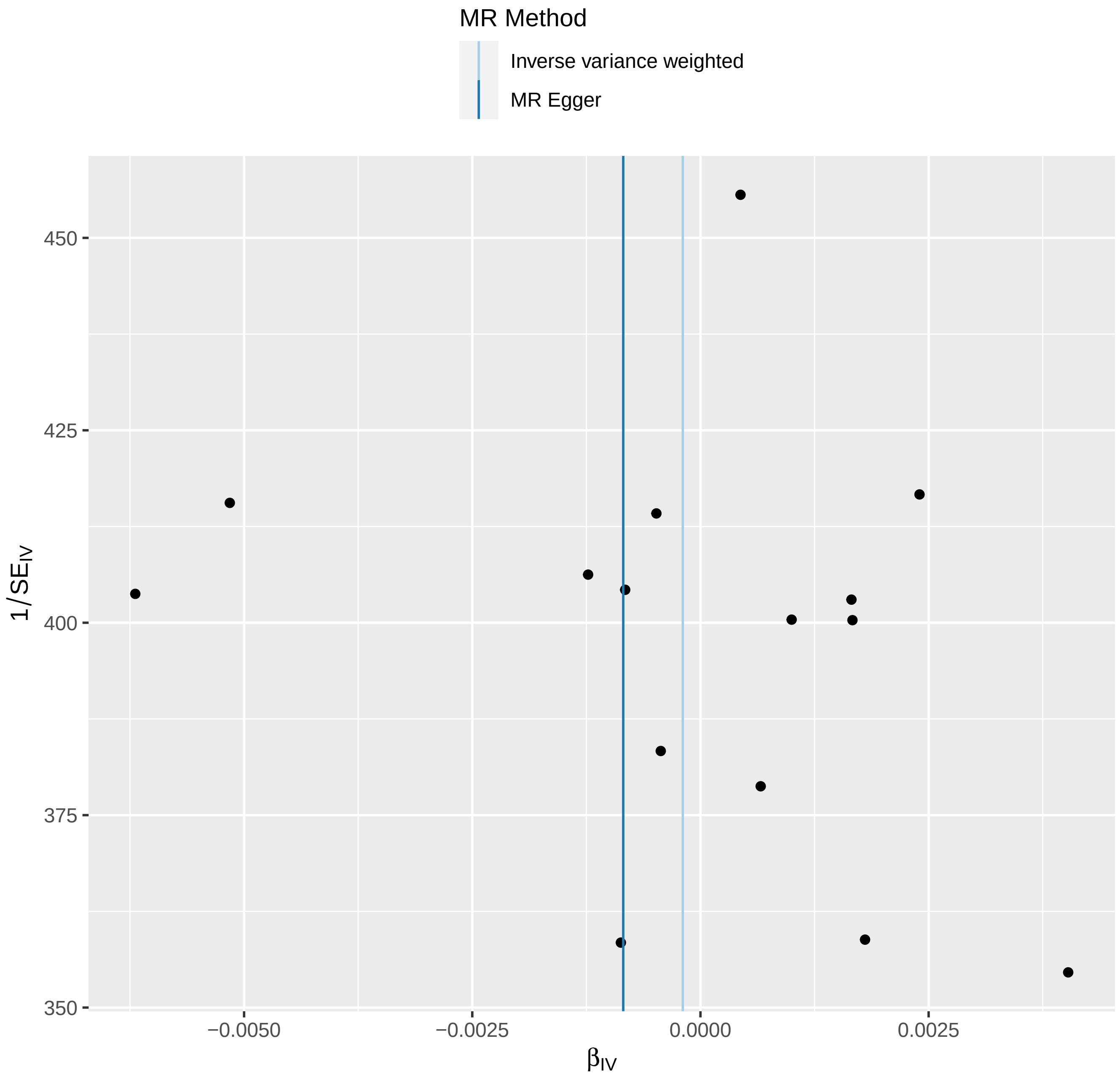

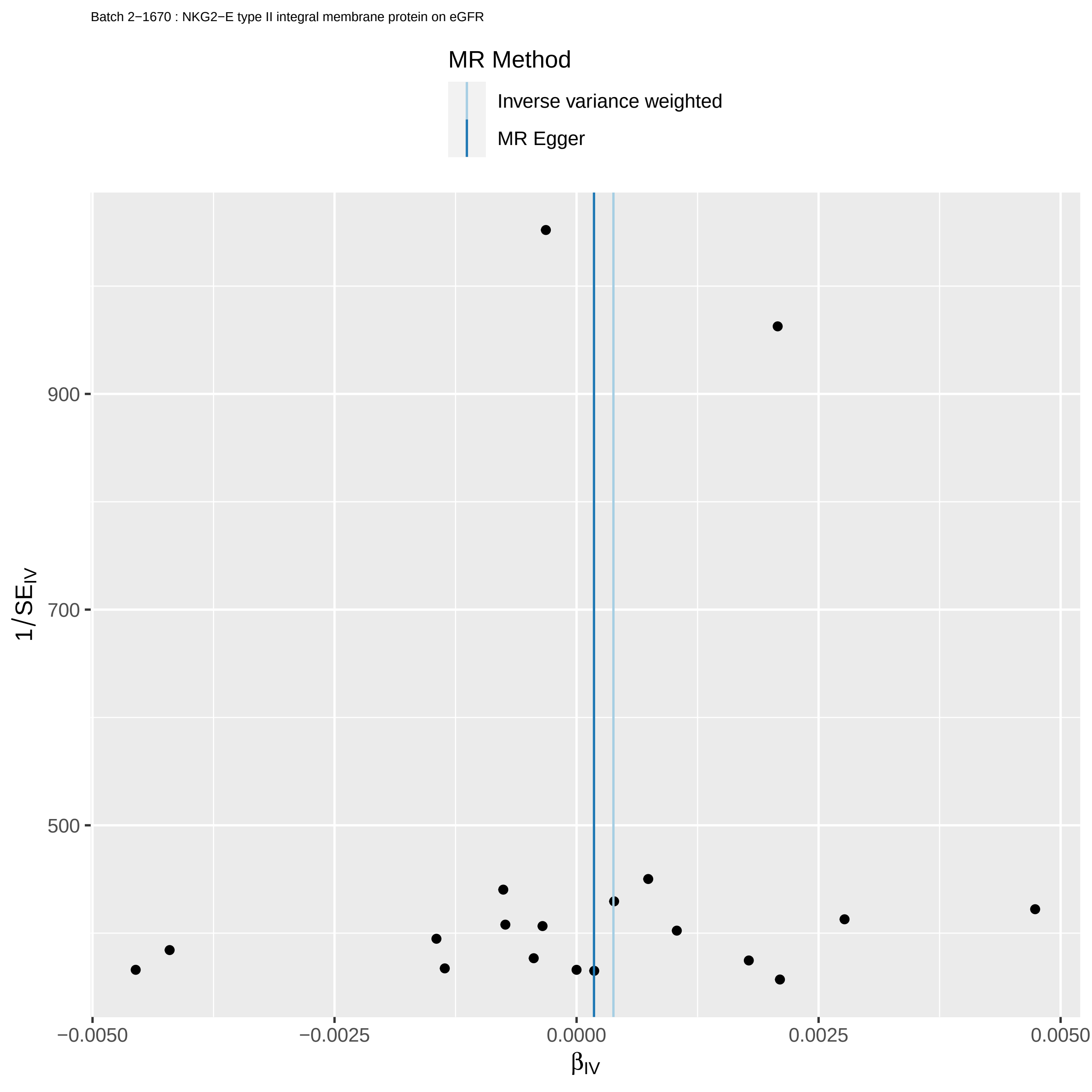

Batch 2−1670 : NKG2−E type II integral membrane protein on eGFR
MR Method
Inverse variance weighted
MR Egger
900
700
500
1/SE$_{IV}$
−0.0050
−0.0025
0.0000
0.0025
0.0050
β$_{IV}$

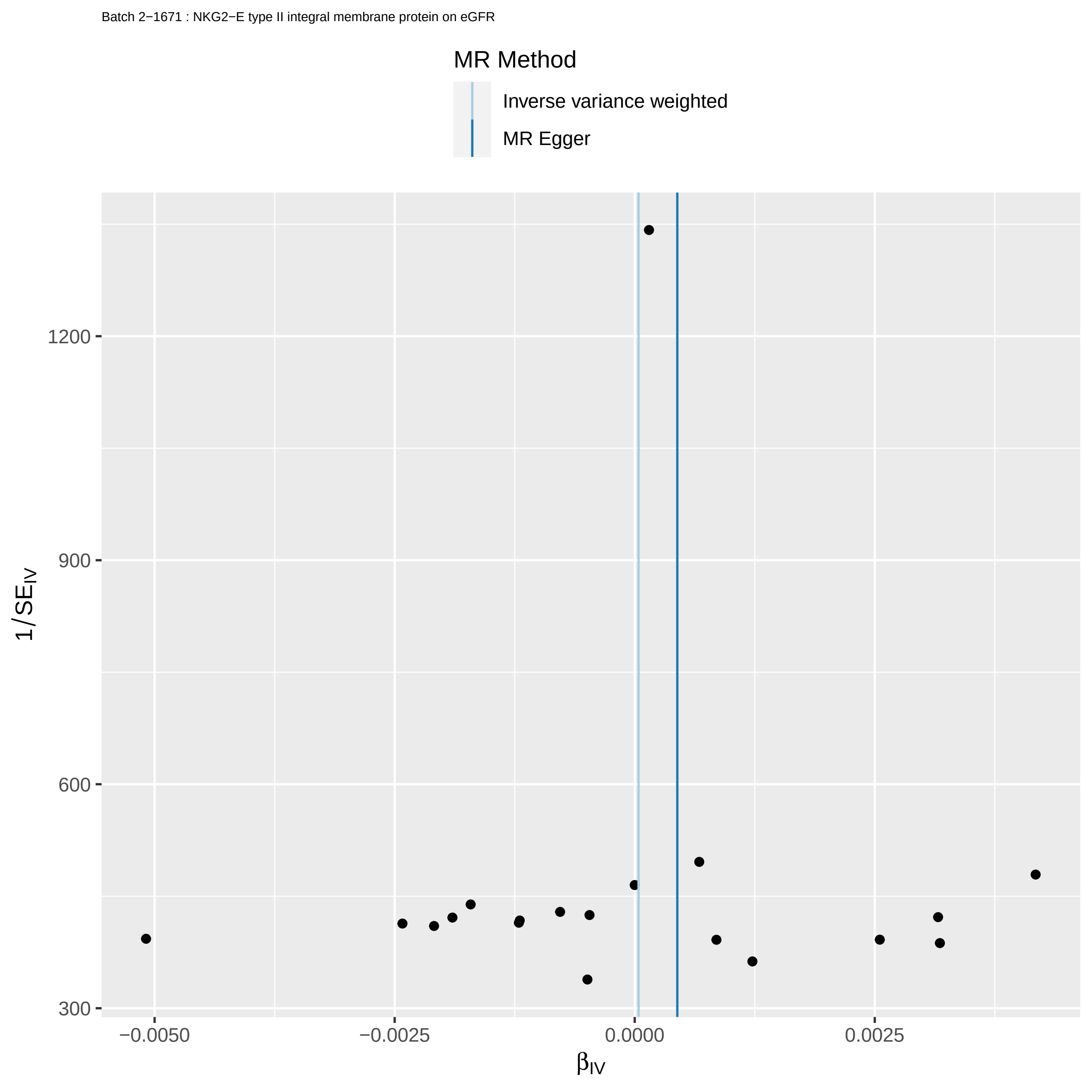
Batch 2−1671 : NKG2−E type II integral membrane protein on eGFR
MR Method
Inverse variance weighted
MR Egger
1200
900
600
300
1/SE$_{IV}$
−0.0050
−0.0025
0.0000
0.0025
β$_{IV}$

Batch 2−1672 : Killer cell lectin−like receptor subfamily F member 1 on eGFR

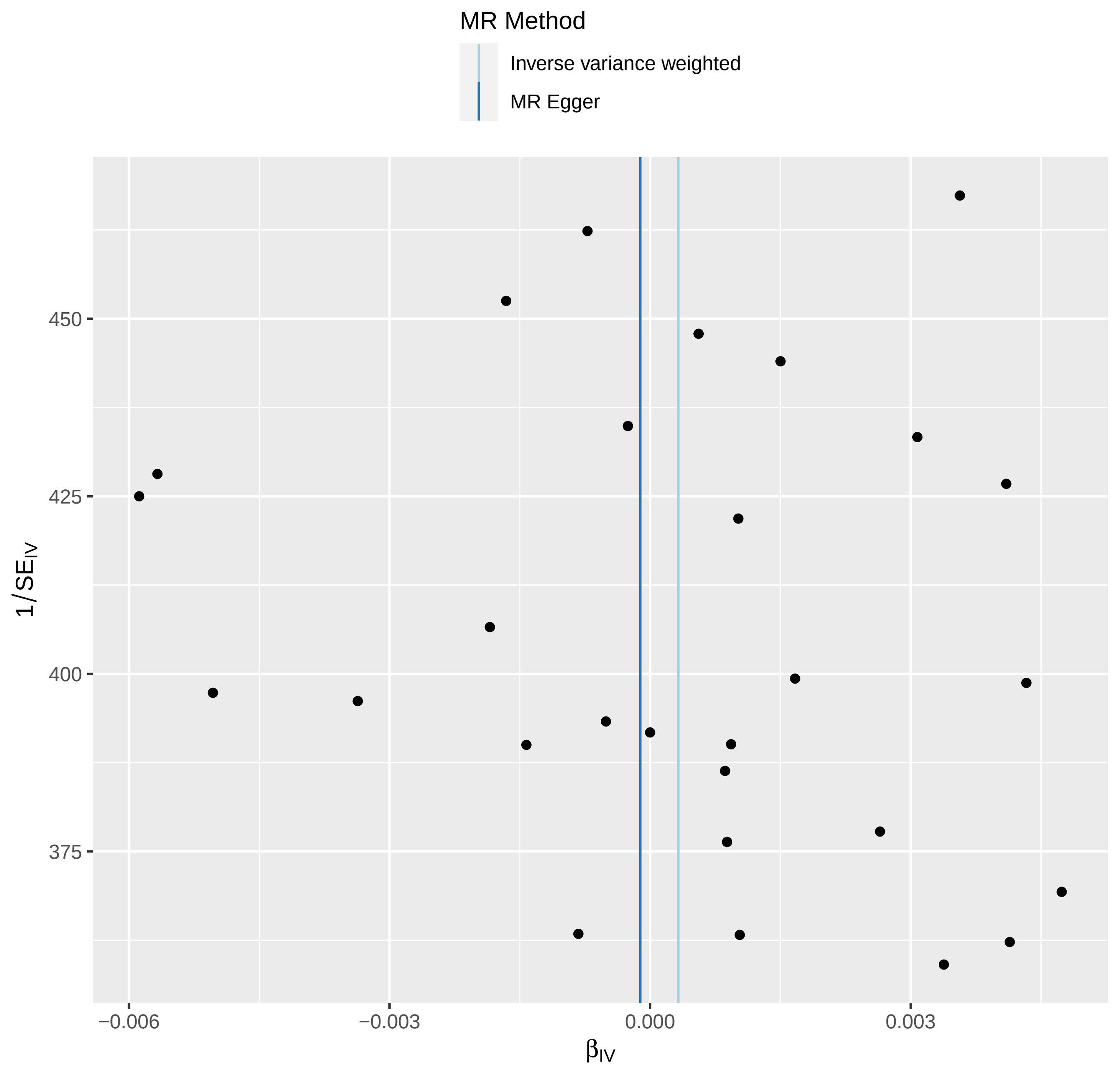

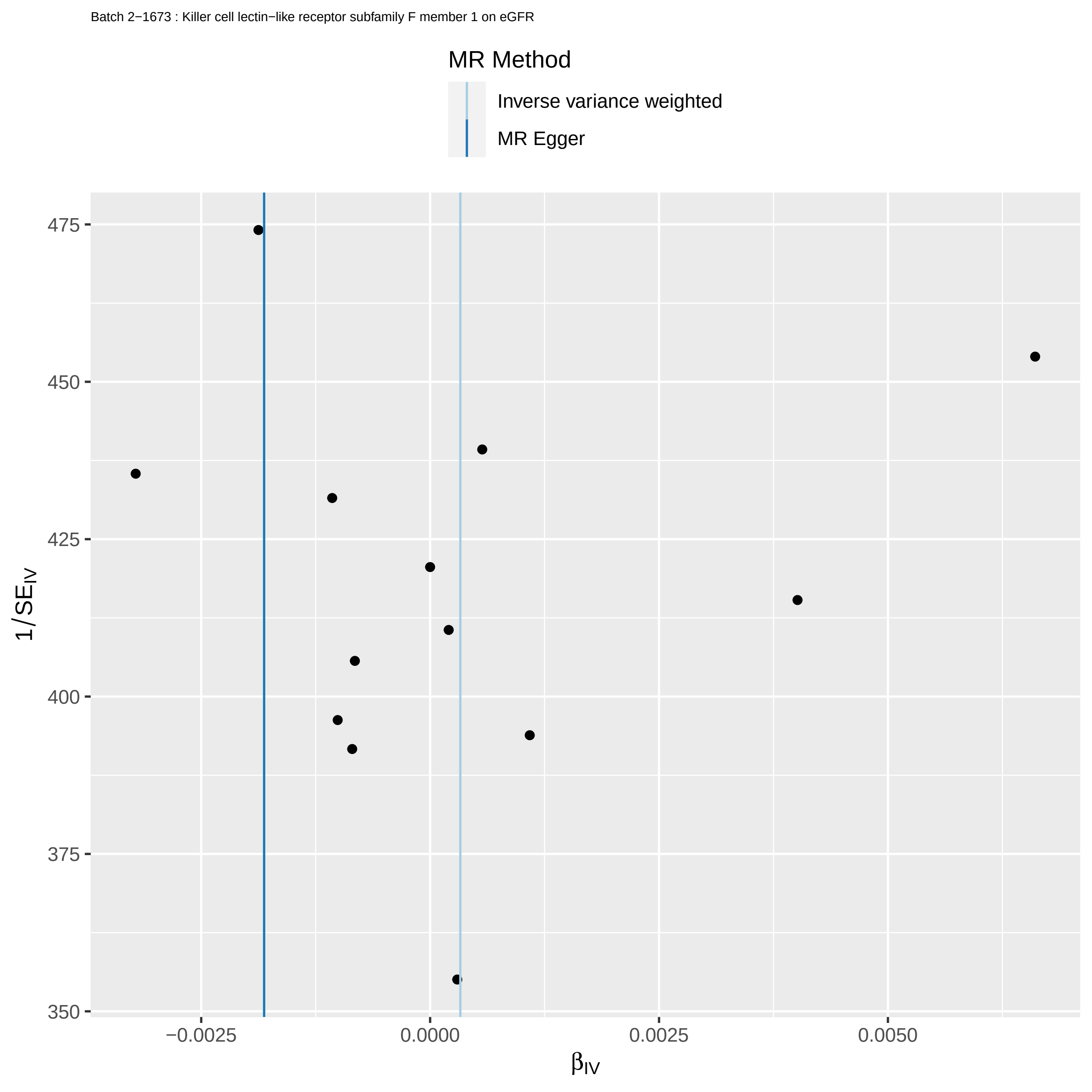

Batch 2−1673 : Killer cell lectin−like receptor subfamily F member 1 on eGFR
MR Method
Inverse variance weighted
MR Egger
475
450
425
400
375
350
1/SE_IV
−0.0025
0.0000
0.0025
0.0050
β_IV

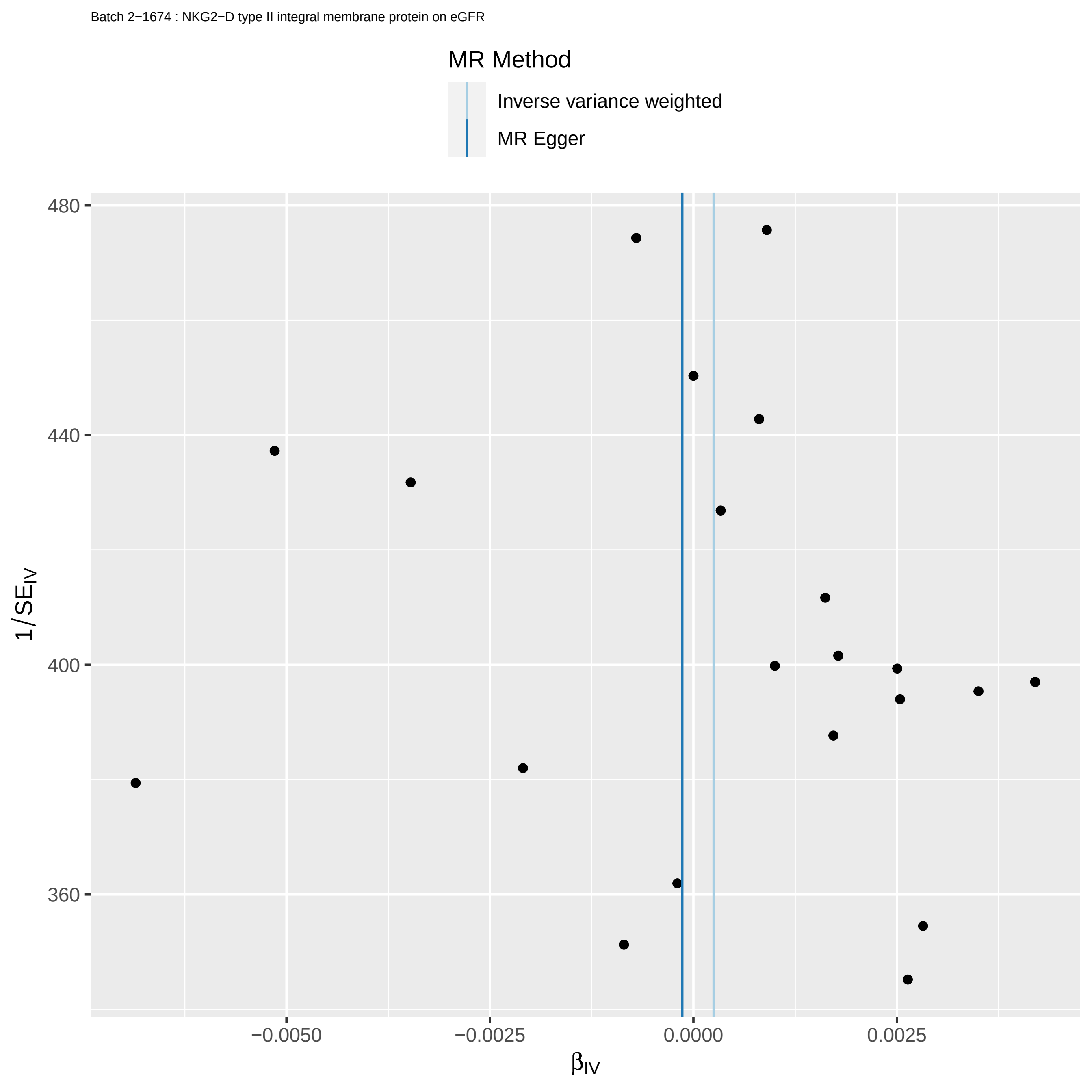
Batch 2−1674 : NKG2−D type II integral membrane protein on eGFR
MR Method
Inverse variance weighted
MR Egger
480
440
400
360
1/SE_IV
−0.0050
−0.0025
0.0000
0.0025
β_IV

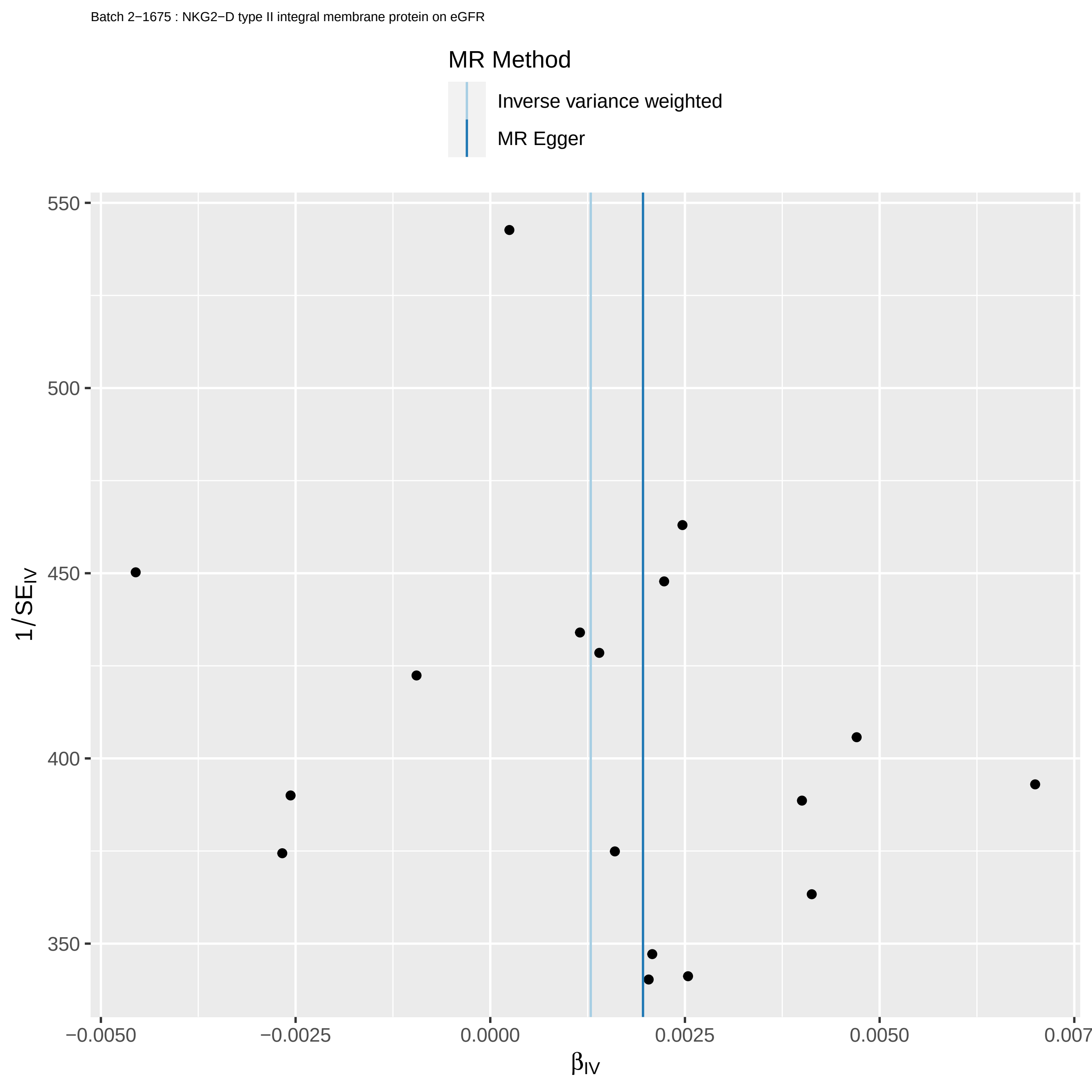

Batch 2−1675 : NKG2−D type II integral membrane protein on eGFR
MR Method
Inverse variance weighted
MR Egger
550
500
450
400
350
1/SE_IV
−0.0050
−0.0025
0.0000
0.0025
0.0050
0.007
β_IV

Batch 2−1676 : Histone−lysine N−methyltransferase 2C on eGFR

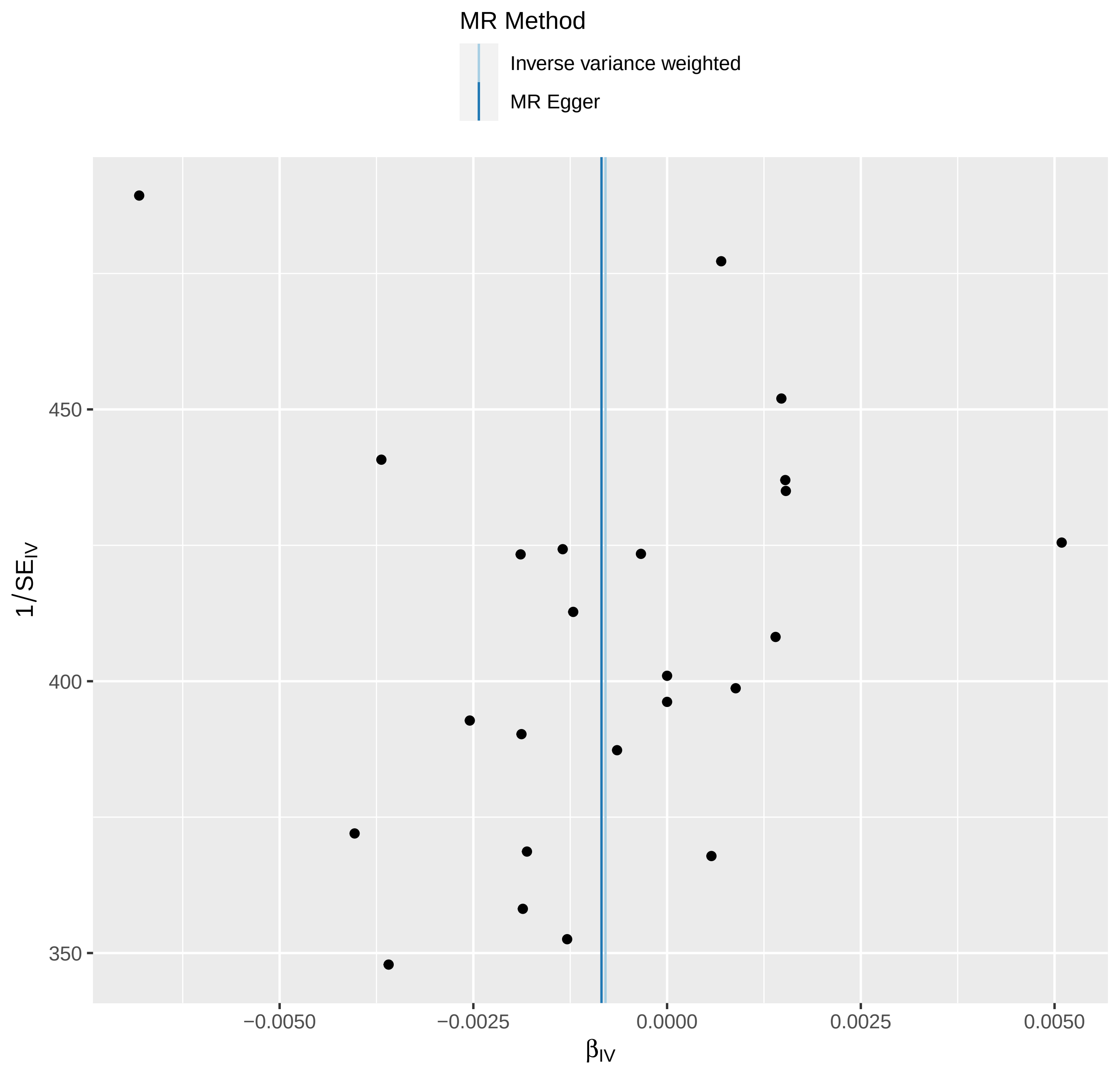

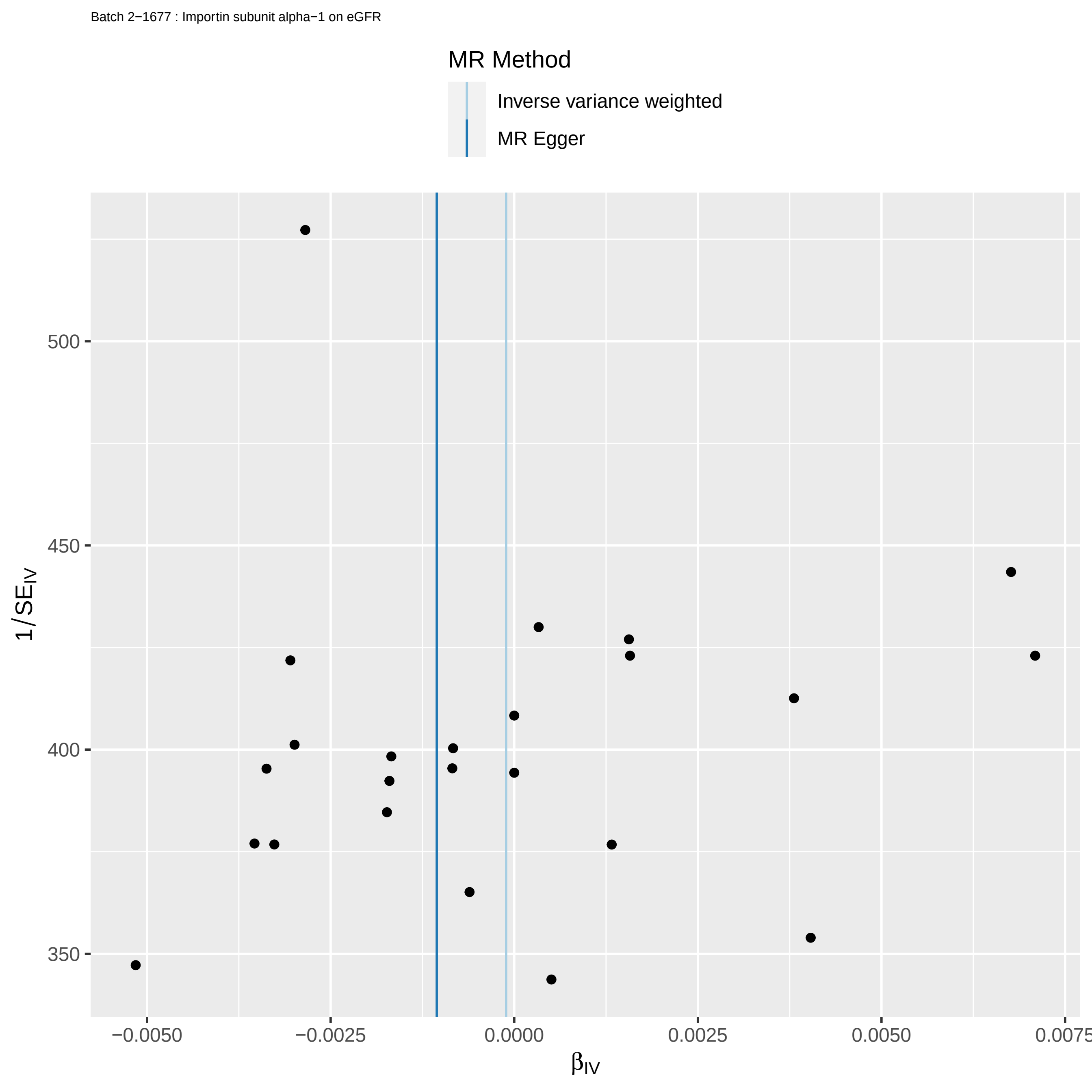

Batch 2−1677 : Importin subunit alpha−1 on eGFR
MR Method
Inverse variance weighted
MR Egger
500
450
400
350
1/SE_IV
−0.0050
−0.0025
0.0000
0.0025
0.0050
0.0075
β_IV

Batch 2−1678 : Importin subunit alpha−3 on eGFR

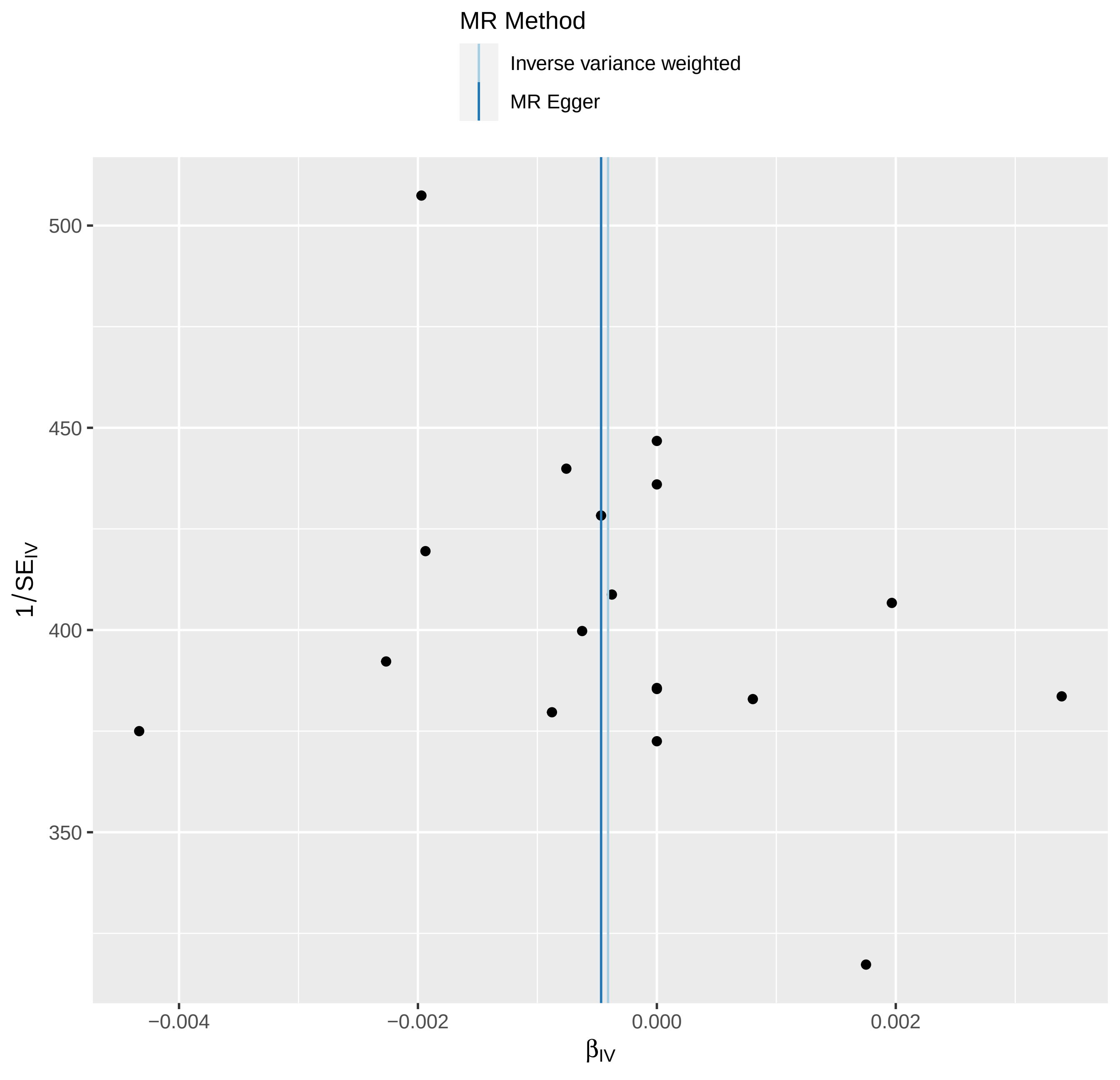

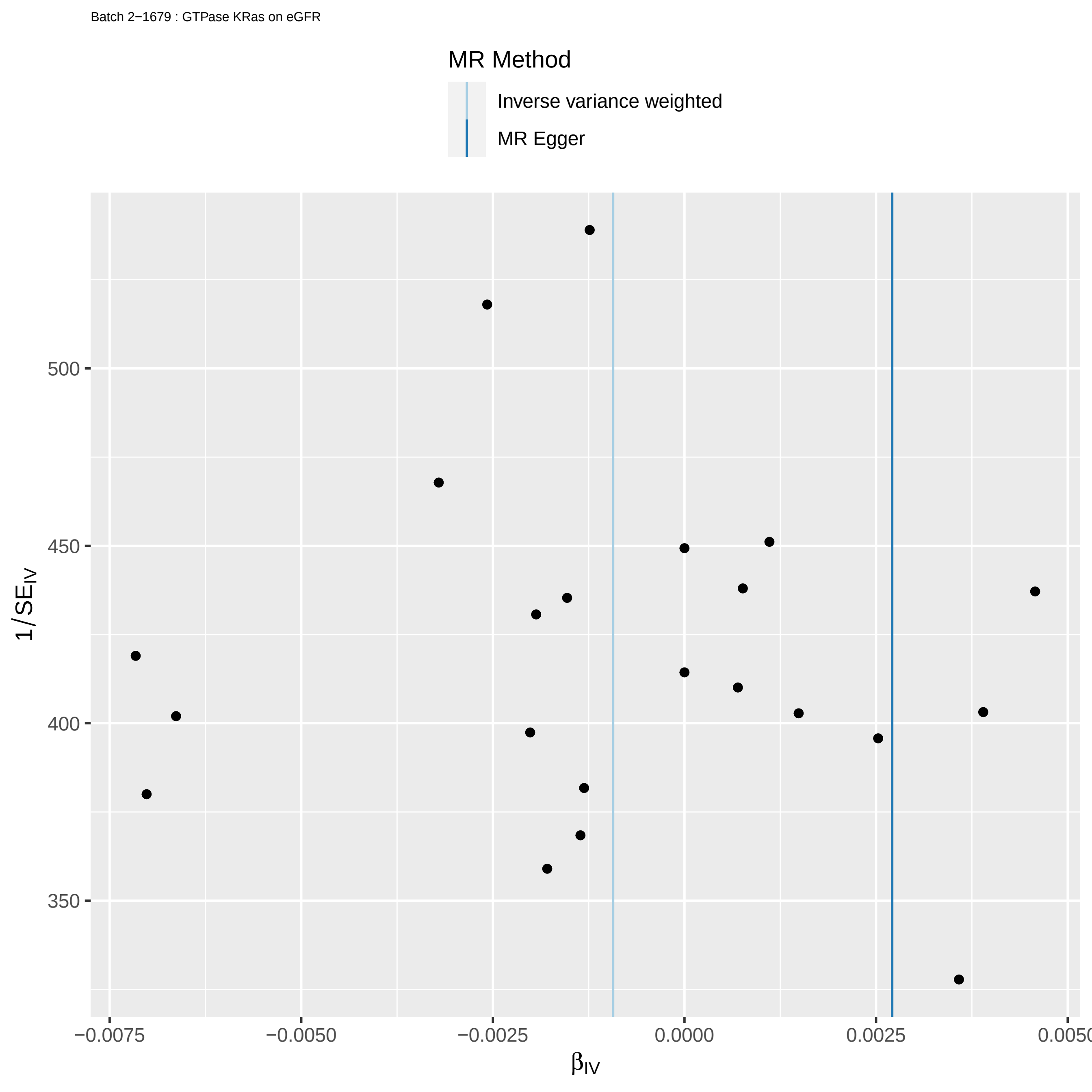

Batch 2−1679 : GTPase KRas on eGFR
MR Method
Inverse variance weighted
MR Egger
500
450
400
350
1/SE$_{IV}$
−0.0075
−0.0050
−0.0025
0.0000
0.0025
0.0050
β_{IV}

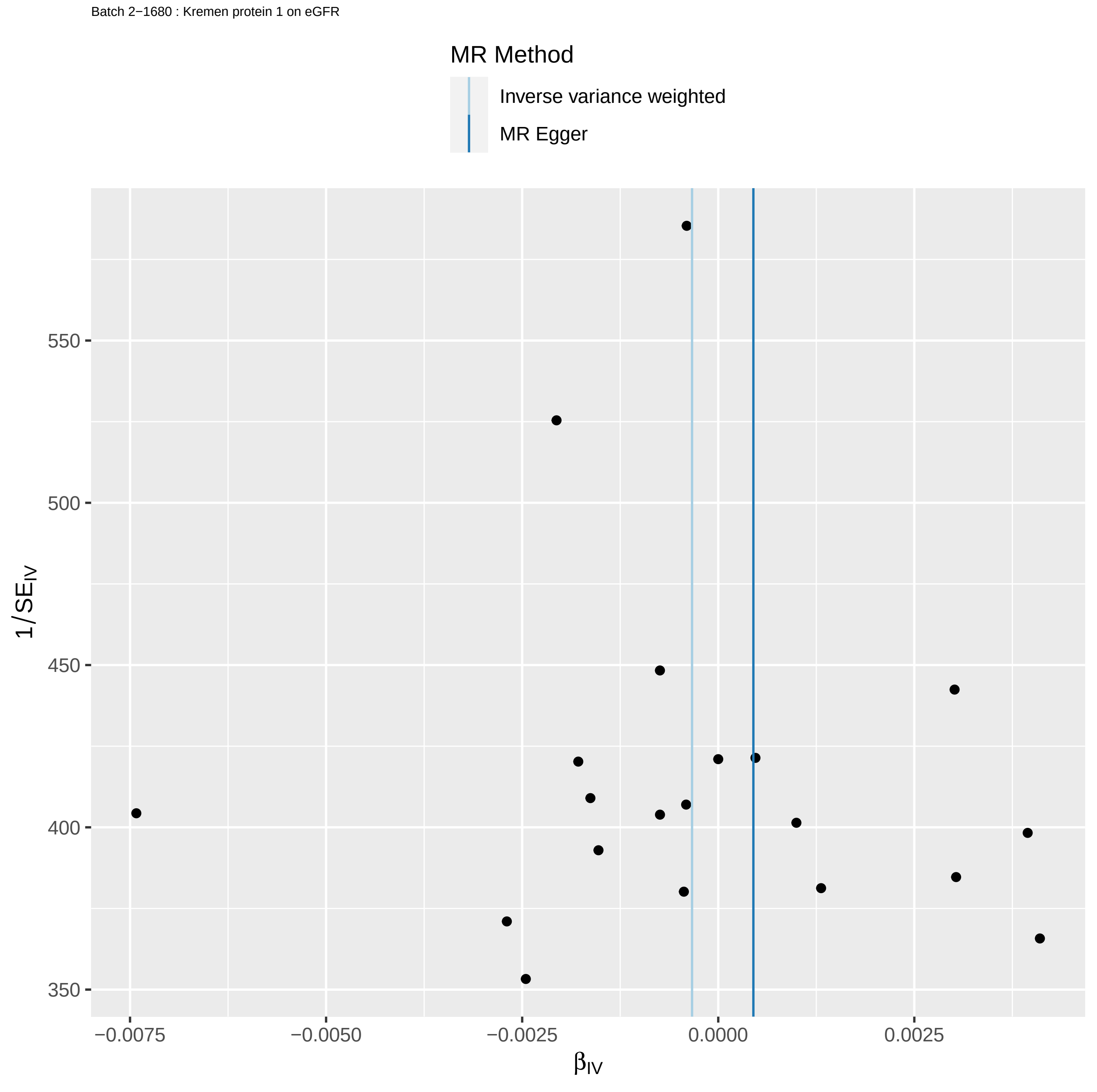

Batch 2−1680 : Kremen protein 1 on eGFR
MR Method
Inverse variance weighted
MR Egger
550
500
450
400
350
1/SE$_{IV}$
−0.0075
−0.0050
−0.0025
0.0000
0.0025
β$_{IV}$

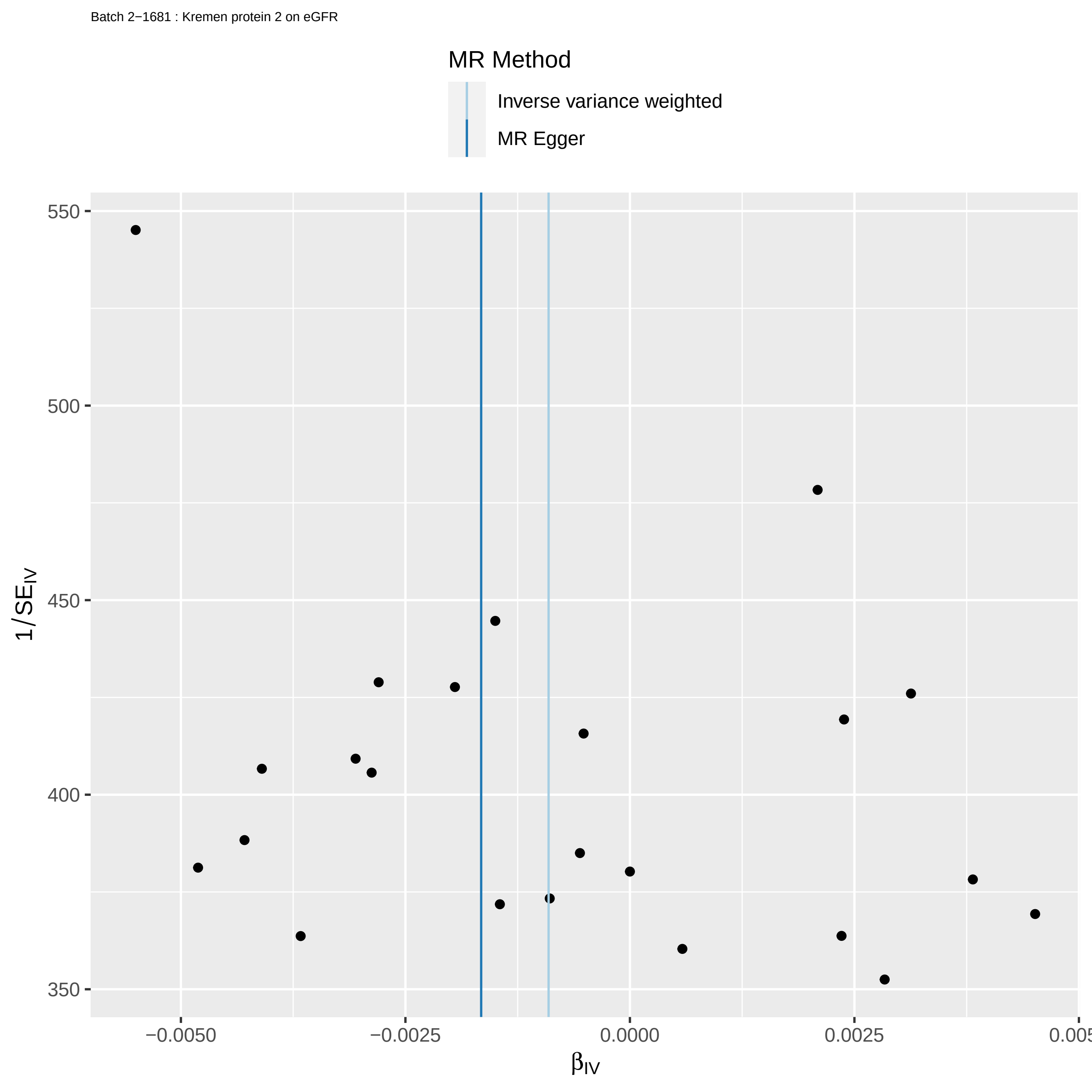
Batch 2−1681 : Kremen protein 2 on eGFR
MR Method
Inverse variance weighted
MR Egger
550
500
450
400
350
1/SE_IV
−0.0050
−0.0025
0.0000
0.0025
0.005
β_IV

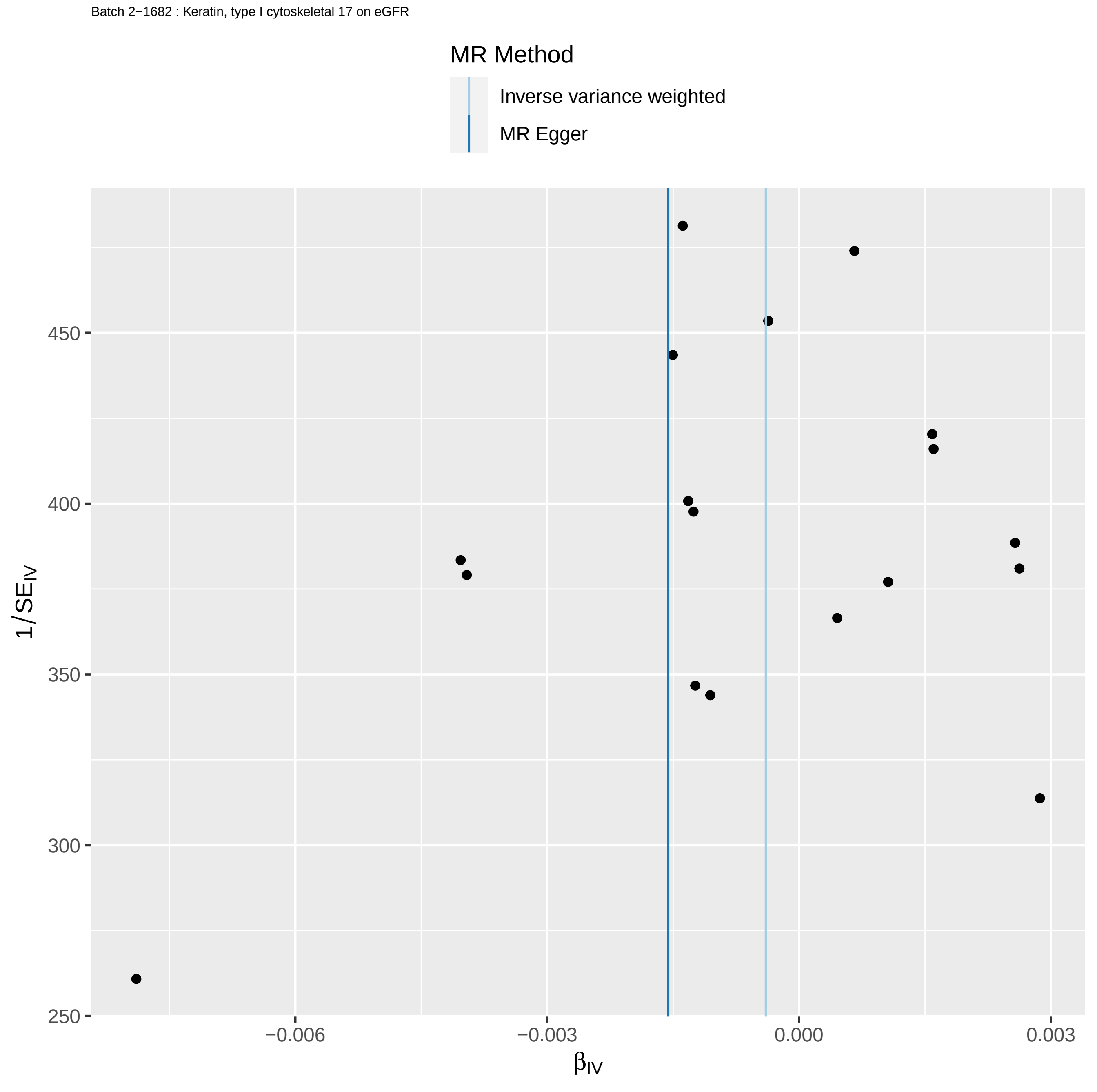
Batch 2−1682 : Keratin, type I cytoskeletal 17 on eGFR
MR Method
Inverse variance weighted
MR Egger
450
400
350
300
250
−0.006
−0.003
0.000
0.003
1/SE_IV
β_IV

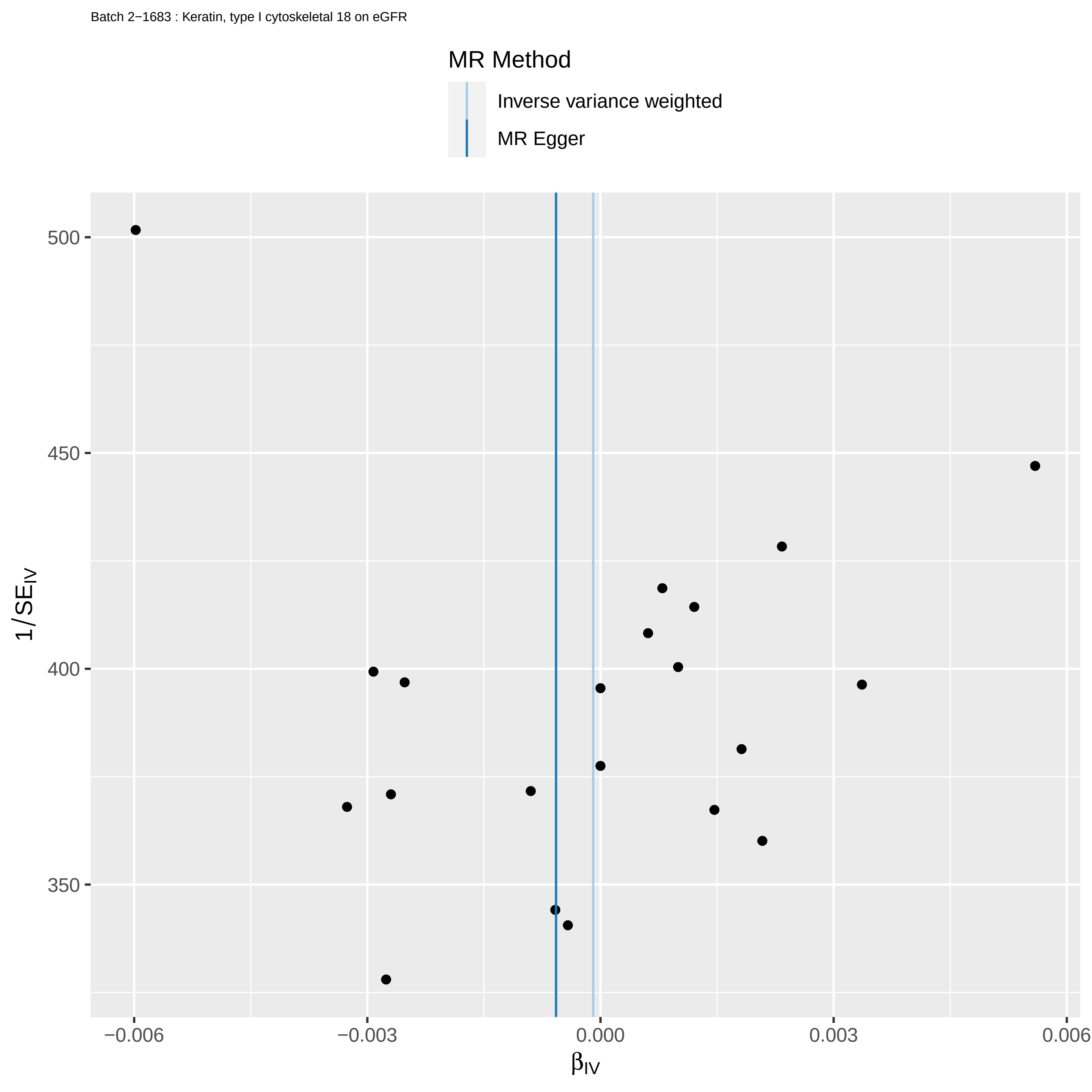
Batch 2−1683 : Keratin, type I cytoskeletal 18 on eGFR
MR Method
Inverse variance weighted
MR Egger
500
450
400
350
1/SE_IV
−0.006
−0.003
0.000
0.003
0.006
β_IV

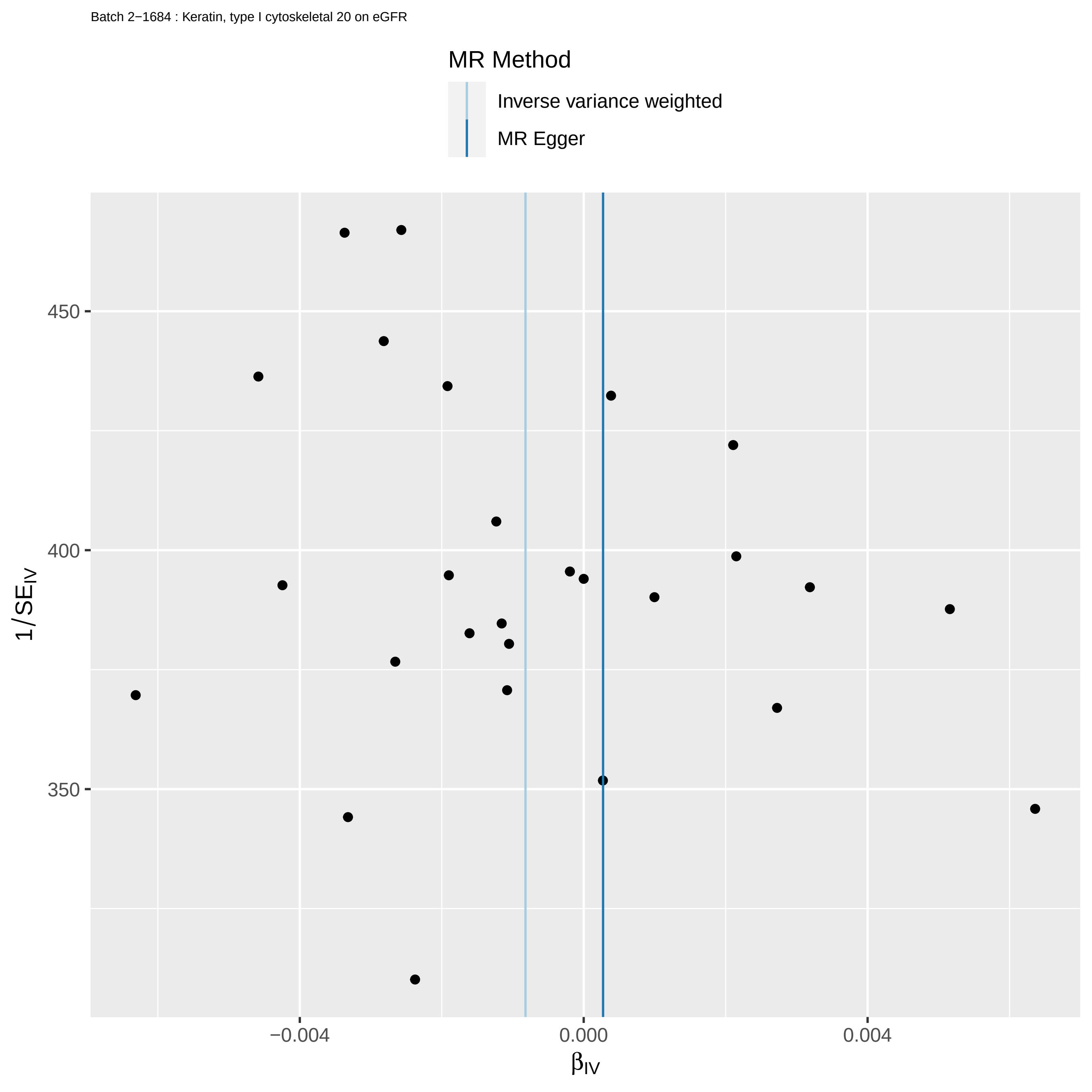
Batch 2−1684 : Keratin, type I cytoskeletal 20 on eGFR
MR Method
Inverse variance weighted
MR Egger
450
400
350
1/SE$_{IV}$
−0.004
0.000
0.004
β_{IV}

Batch 2−1685 : Keratin, type II cytoskeletal 5 on eGFR

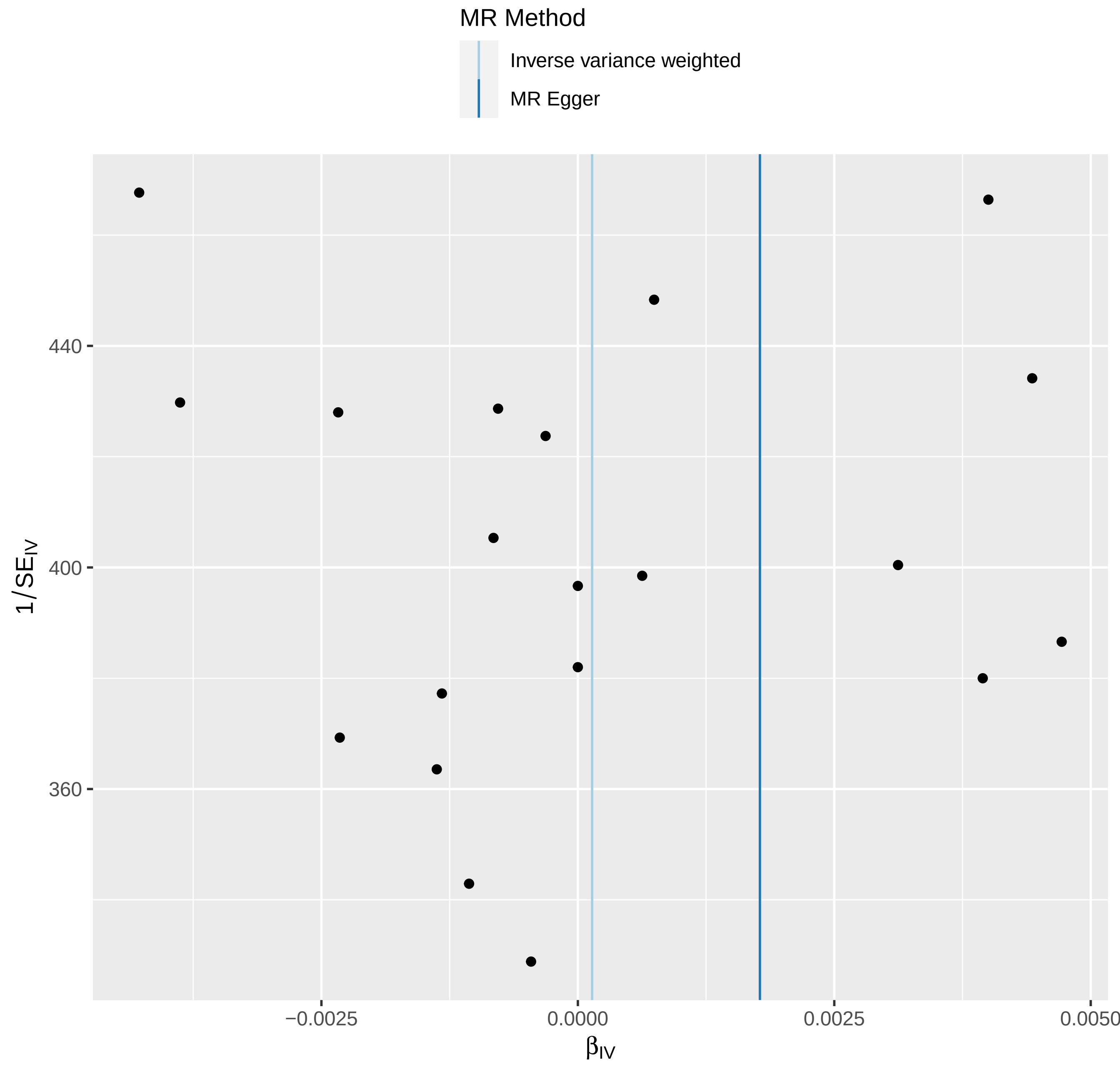

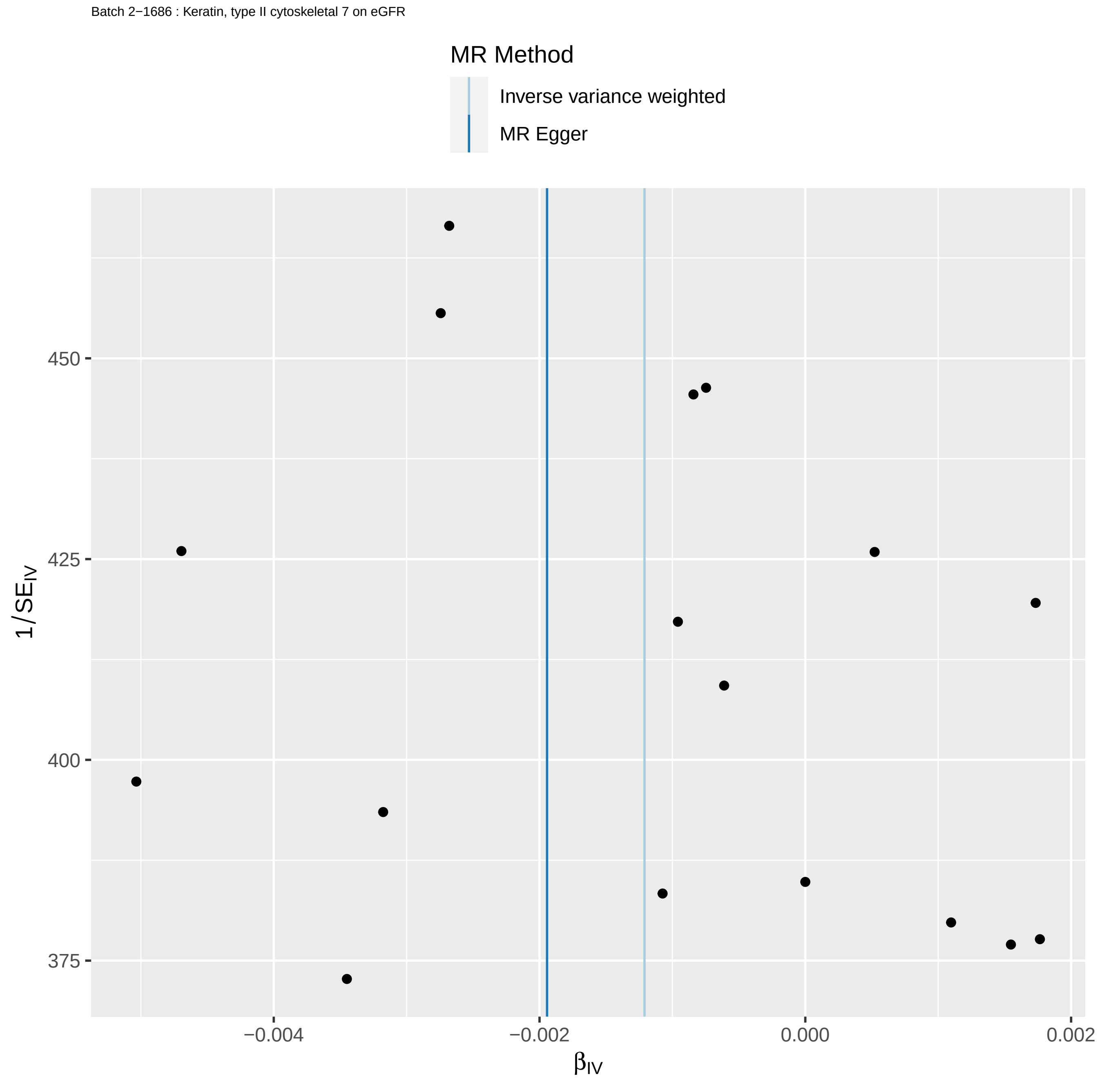

Batch 2−1686 : Keratin, type II cytoskeletal 7 on eGFR
MR Method
Inverse variance weighted
MR Egger
450
425
400
375
1/SE$_{IV}$
−0.004
−0.002
0.000
0.002
β$_{IV}$

Batch 2−1687 : Keratin−associated protein 2−4 on eGFR

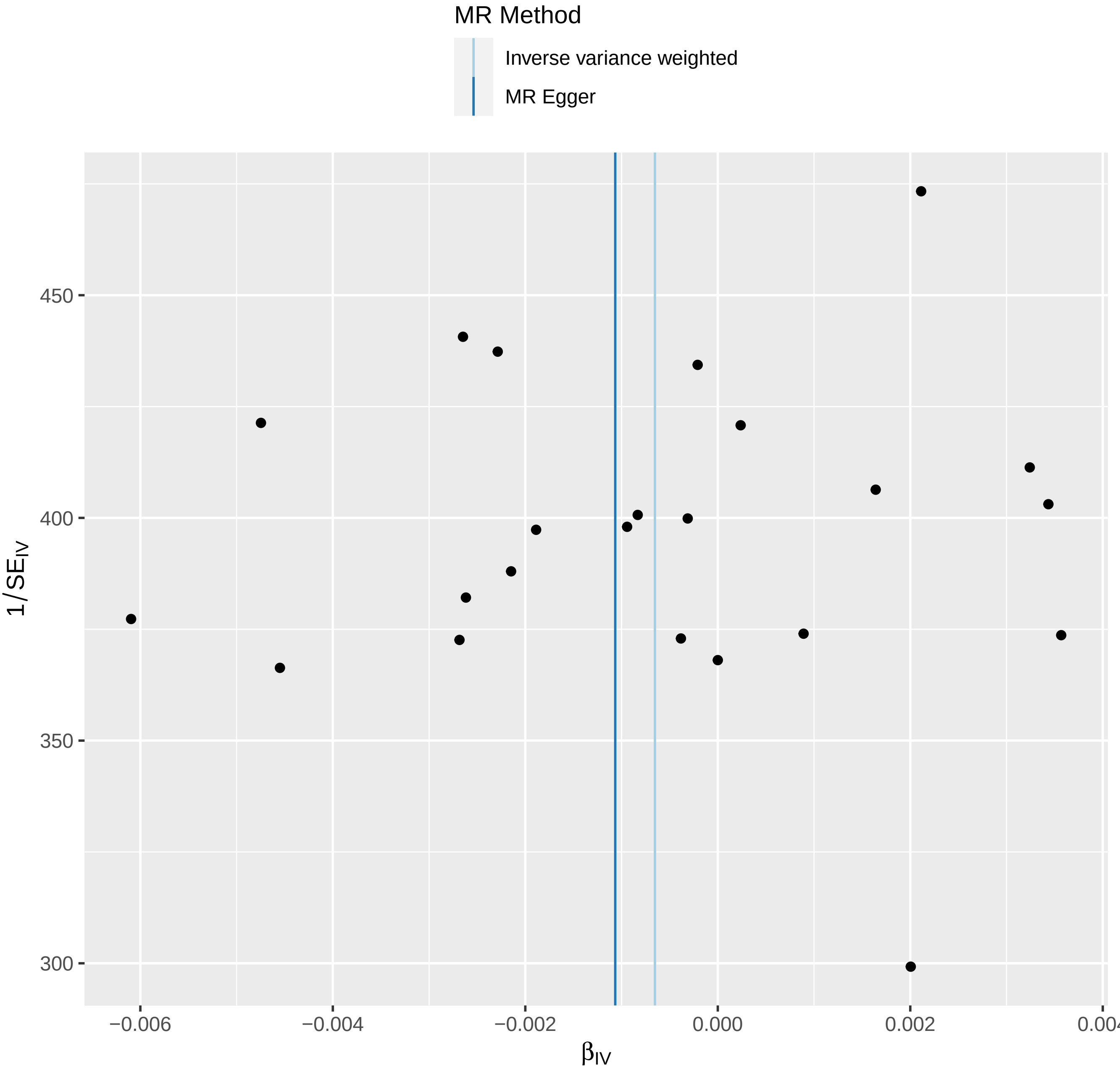

Batch 2−1688 : Keratinocyte differentiation−associated protein on eGFR

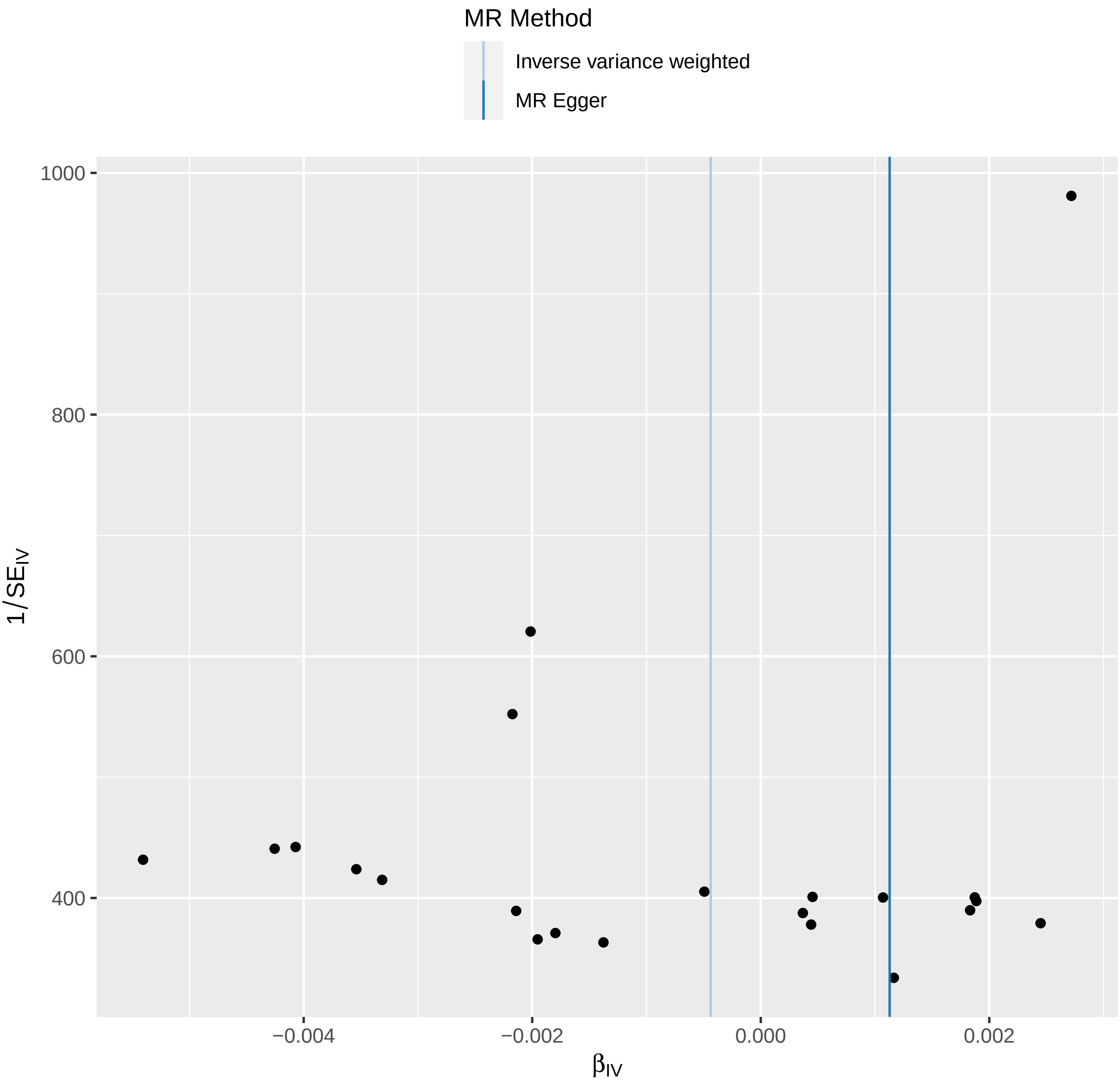

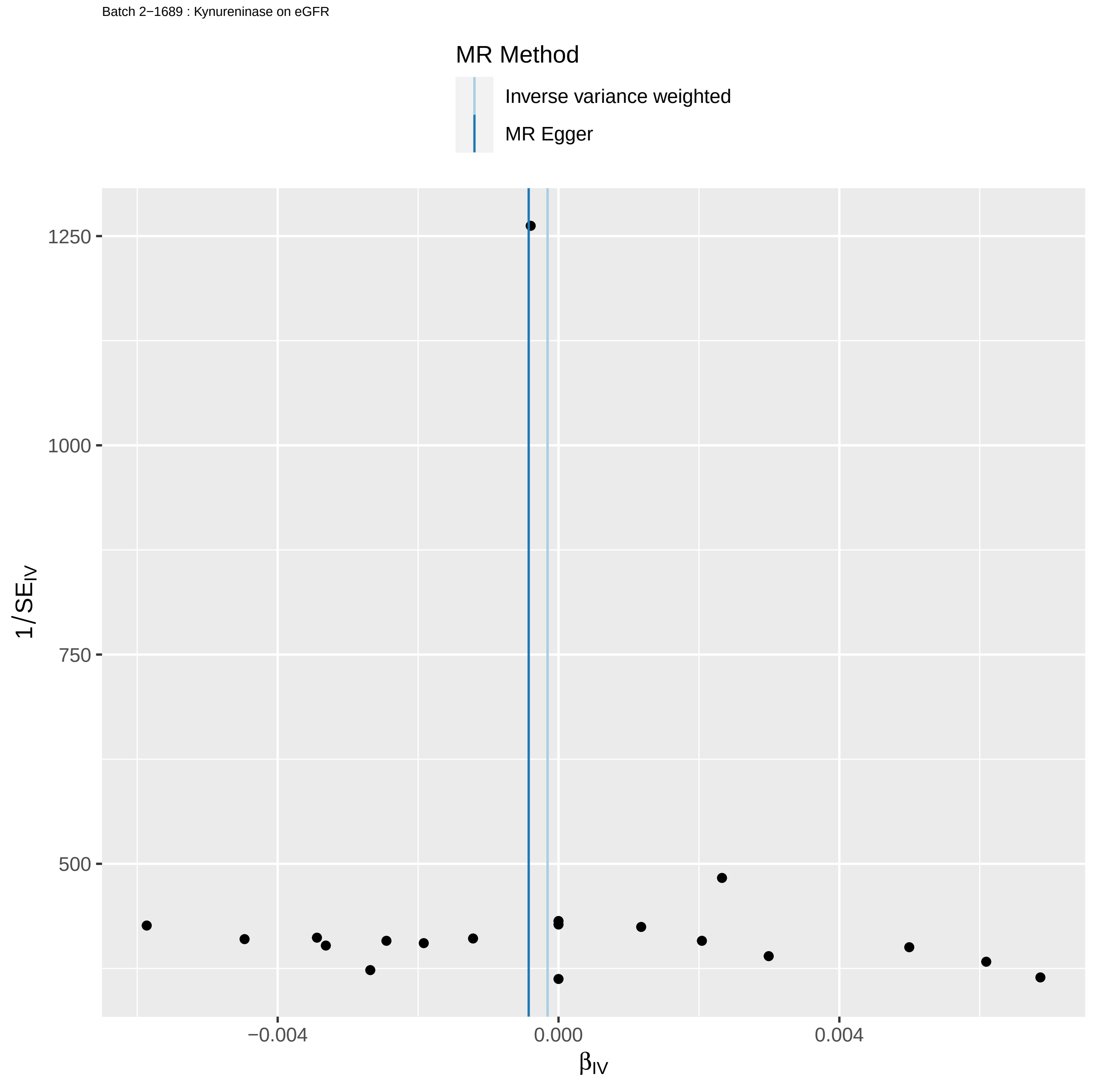
Batch 2−1689 : Kynureninase on eGFR
MR Method
Inverse variance weighted
MR Egger
1250
1000
750
500
1/SE_IV
−0.004
0.000
0.004
β_IV

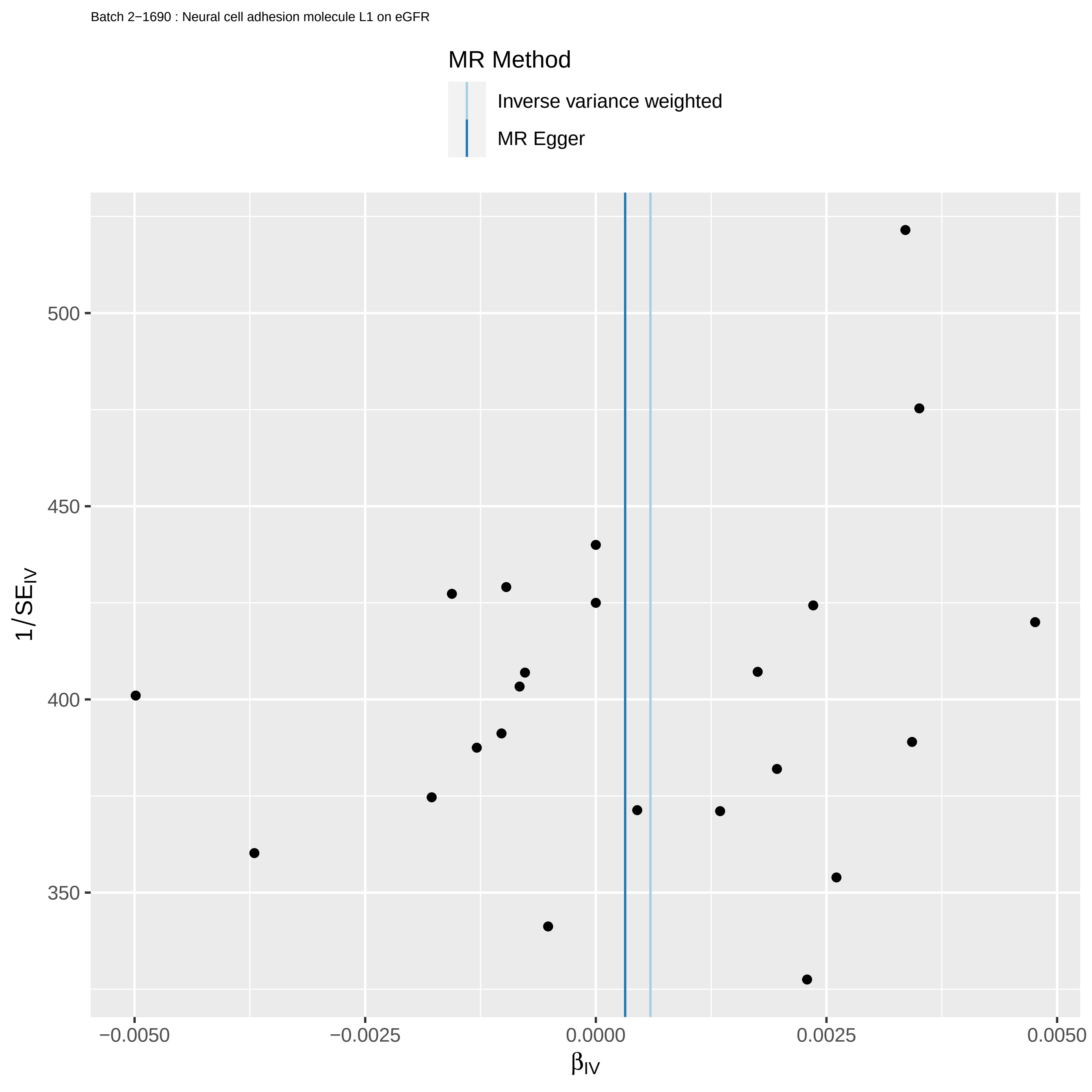
Batch 2−1690 : Neural cell adhesion molecule L1 on eGFR
MR Method
Inverse variance weighted
MR Egger
500
450
400
350
1/SE_IV
−0.0050
−0.0025
0.0000
0.0025
0.0050
β_IV

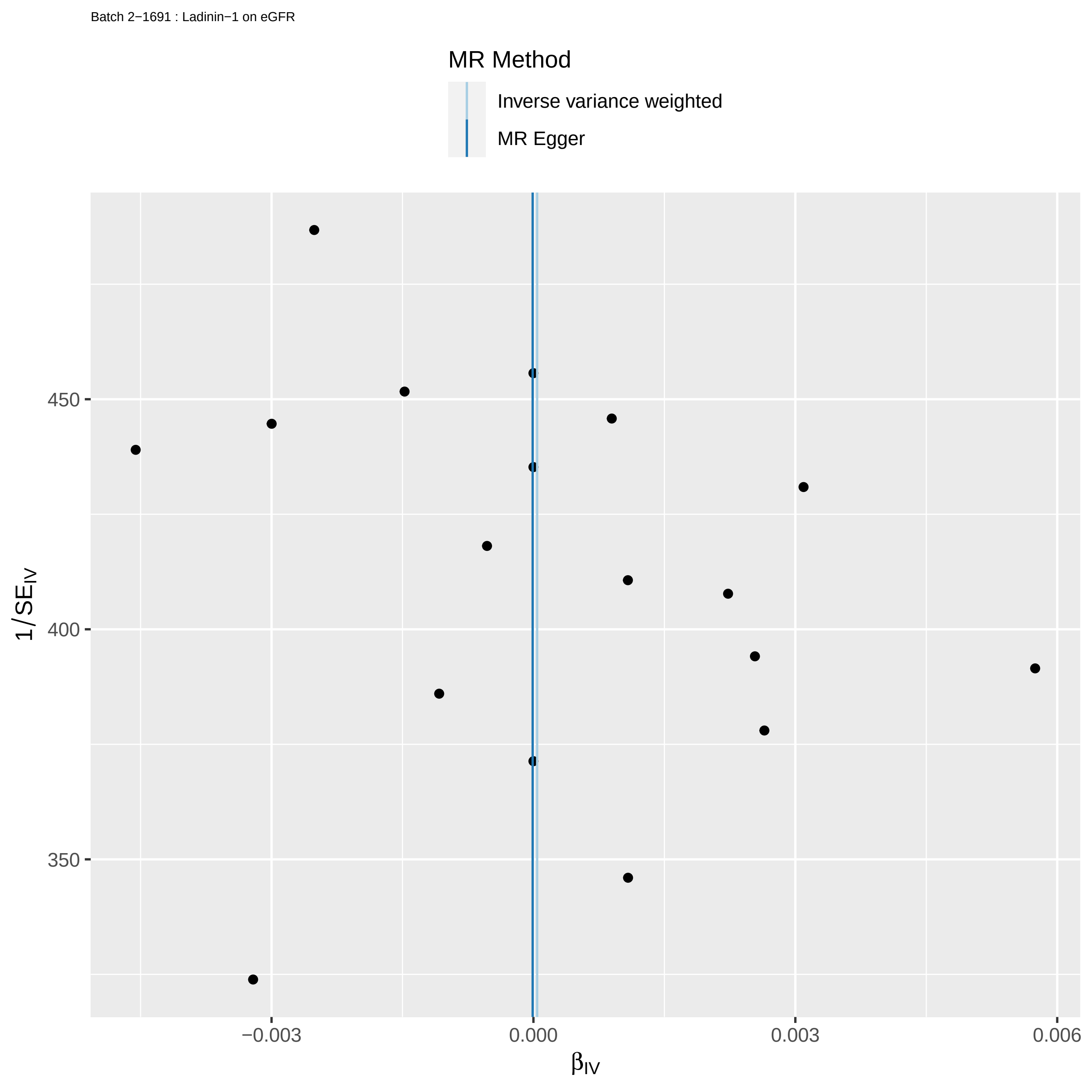

Batch 2−1691 : Ladinin−1 on eGFR
MR Method
Inverse variance weighted
MR Egger
450
400
350
1/SE$_{IV}$
−0.003
0.000
0.003
0.006
β_{IV}

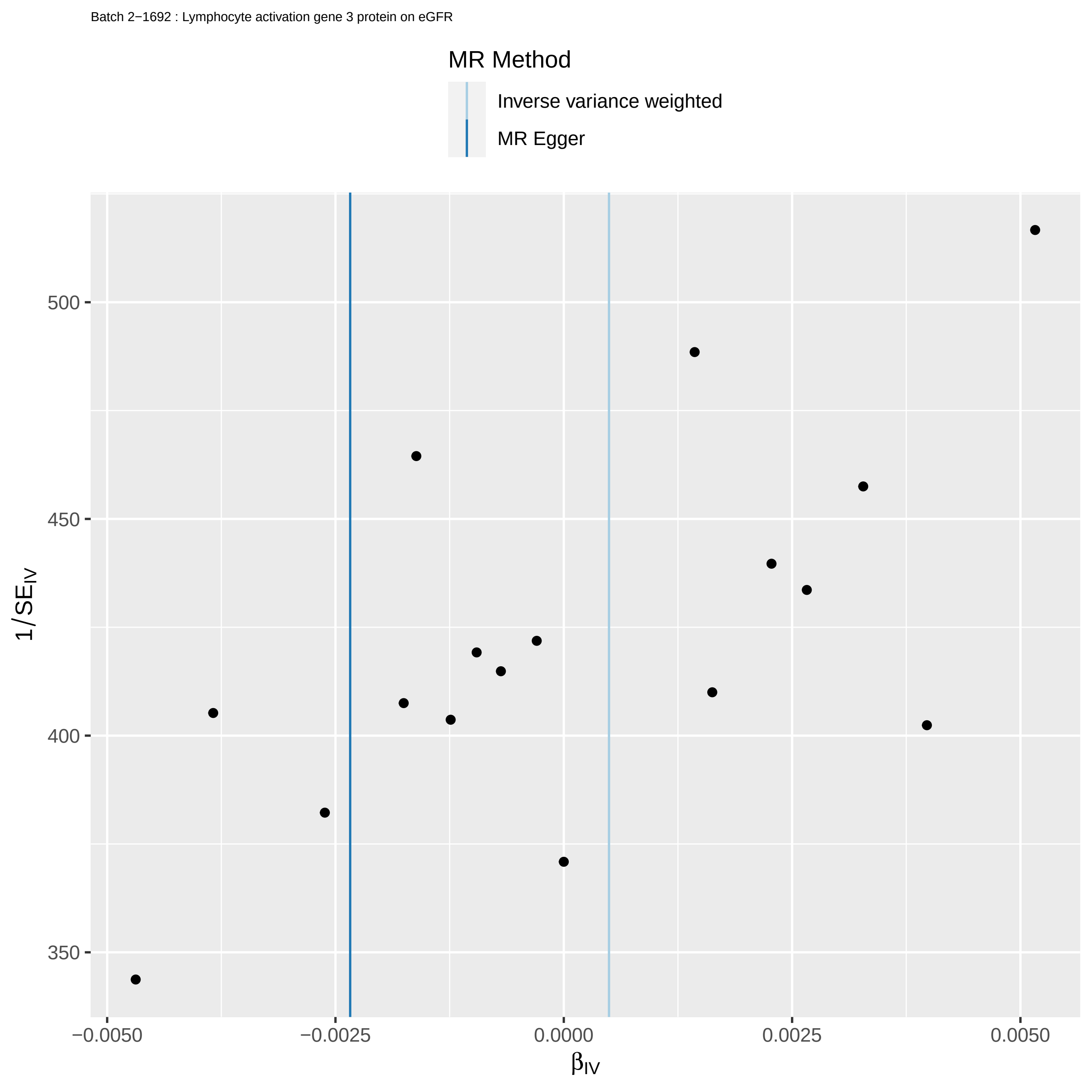
Batch 2−1692 : Lymphocyte activation gene 3 protein on eGFR
MR Method
Inverse variance weighted
MR Egger
500
450
400
350
1/SE$_{IV}$
−0.0050
−0.0025
0.0000
0.0025
0.0050
β$_{IV}$

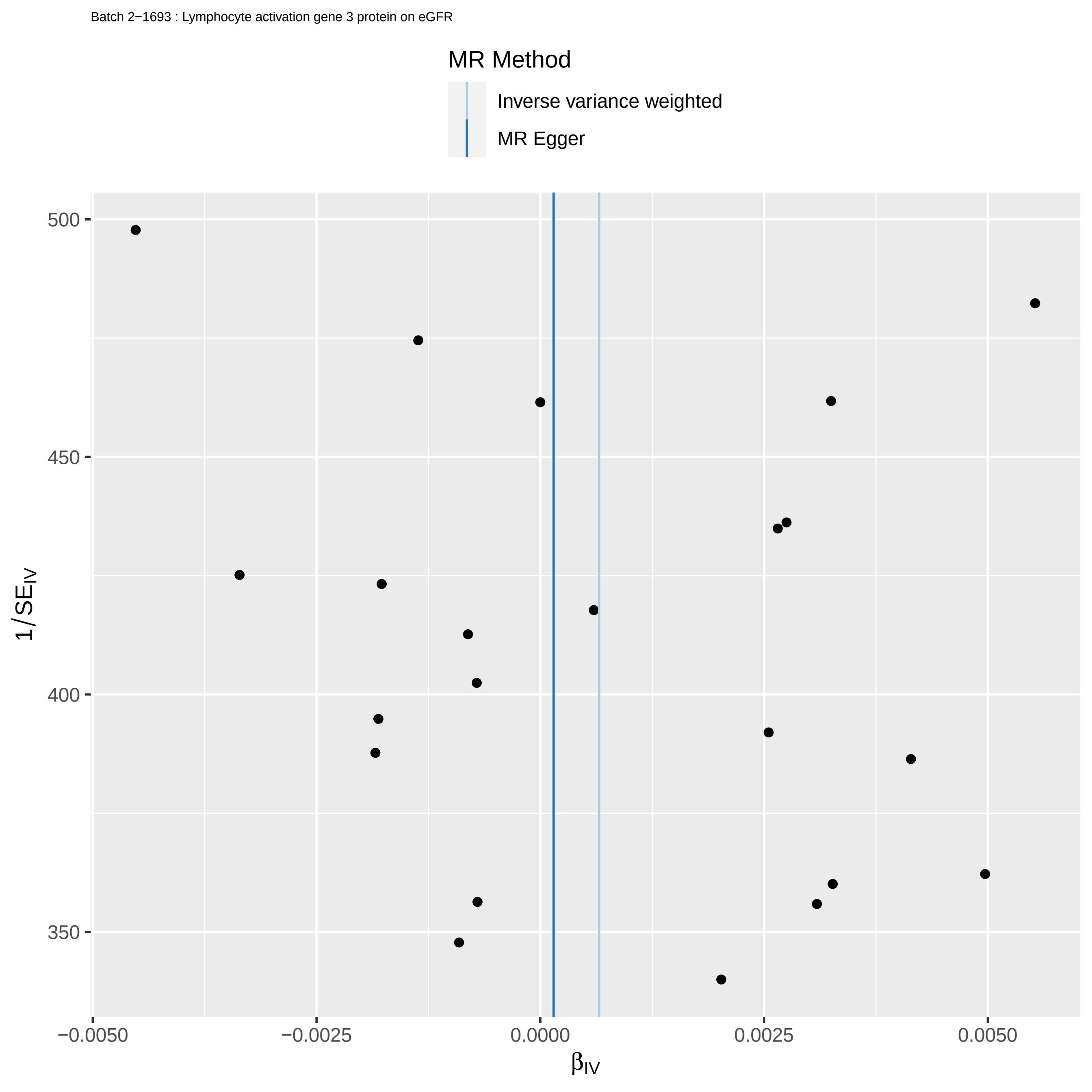

Batch 2−1693 : Lymphocyte activation gene 3 protein on eGFR
MR Method
Inverse variance weighted
MR Egger
500
450
400
350
1/SE$_{IV}$
−0.0050
−0.0025
0.0000
0.0025
0.0050
β$_{IV}$

Batch 2−1694 : Leukocyte−associated immunoglobulin−like receptor 1 on eGFR

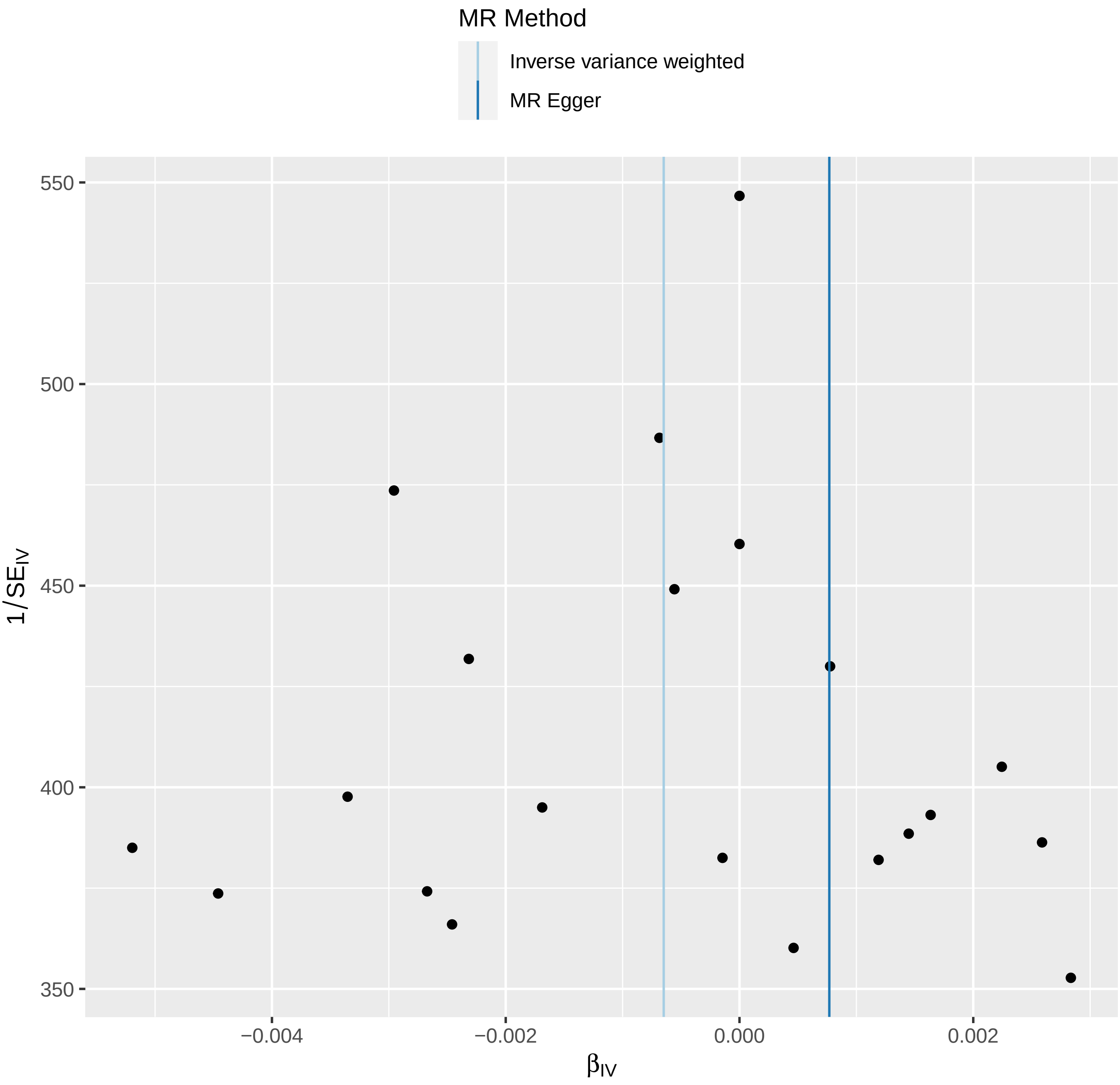

Batch 2−1695 : Laminin on eGFR

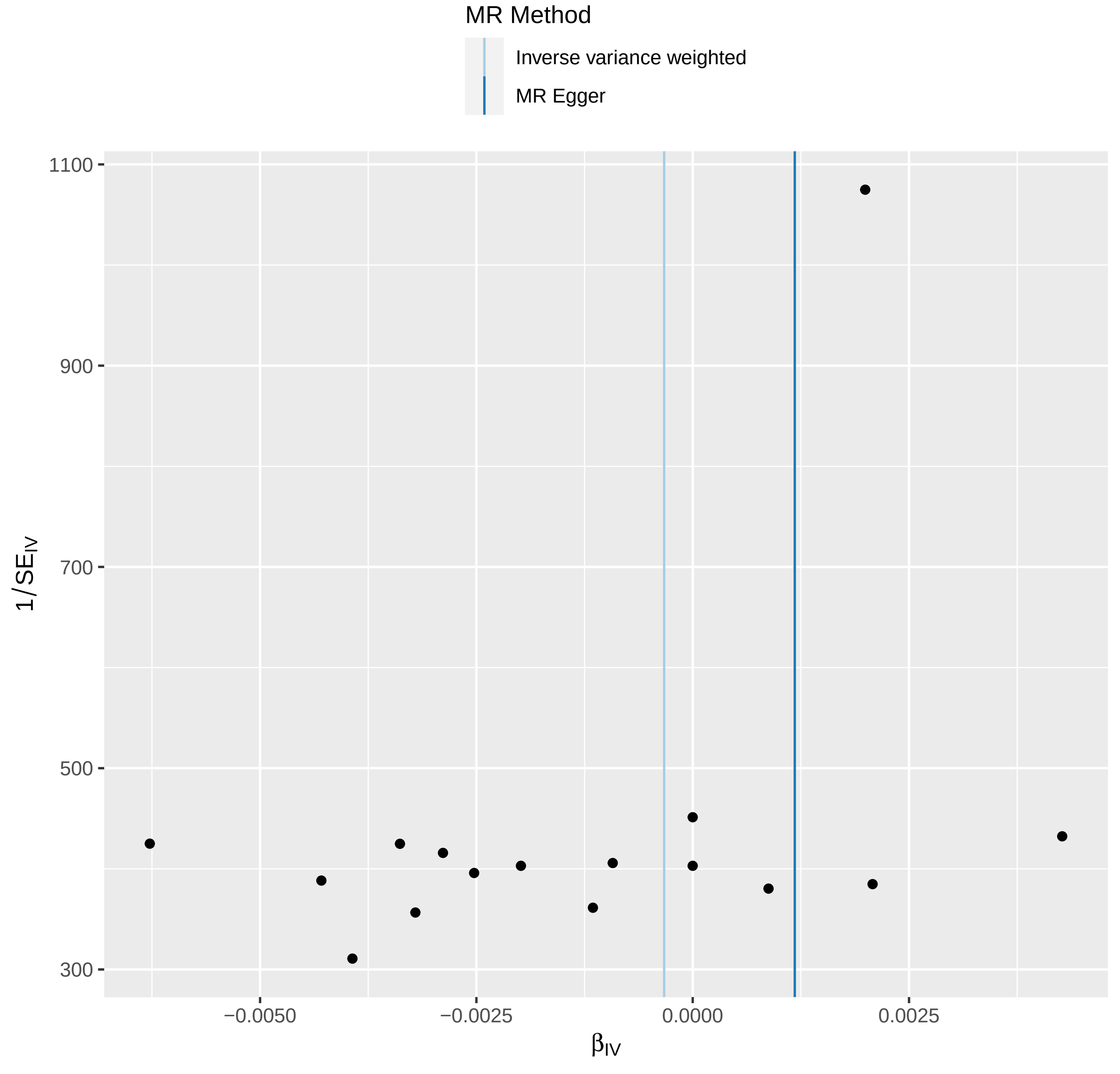

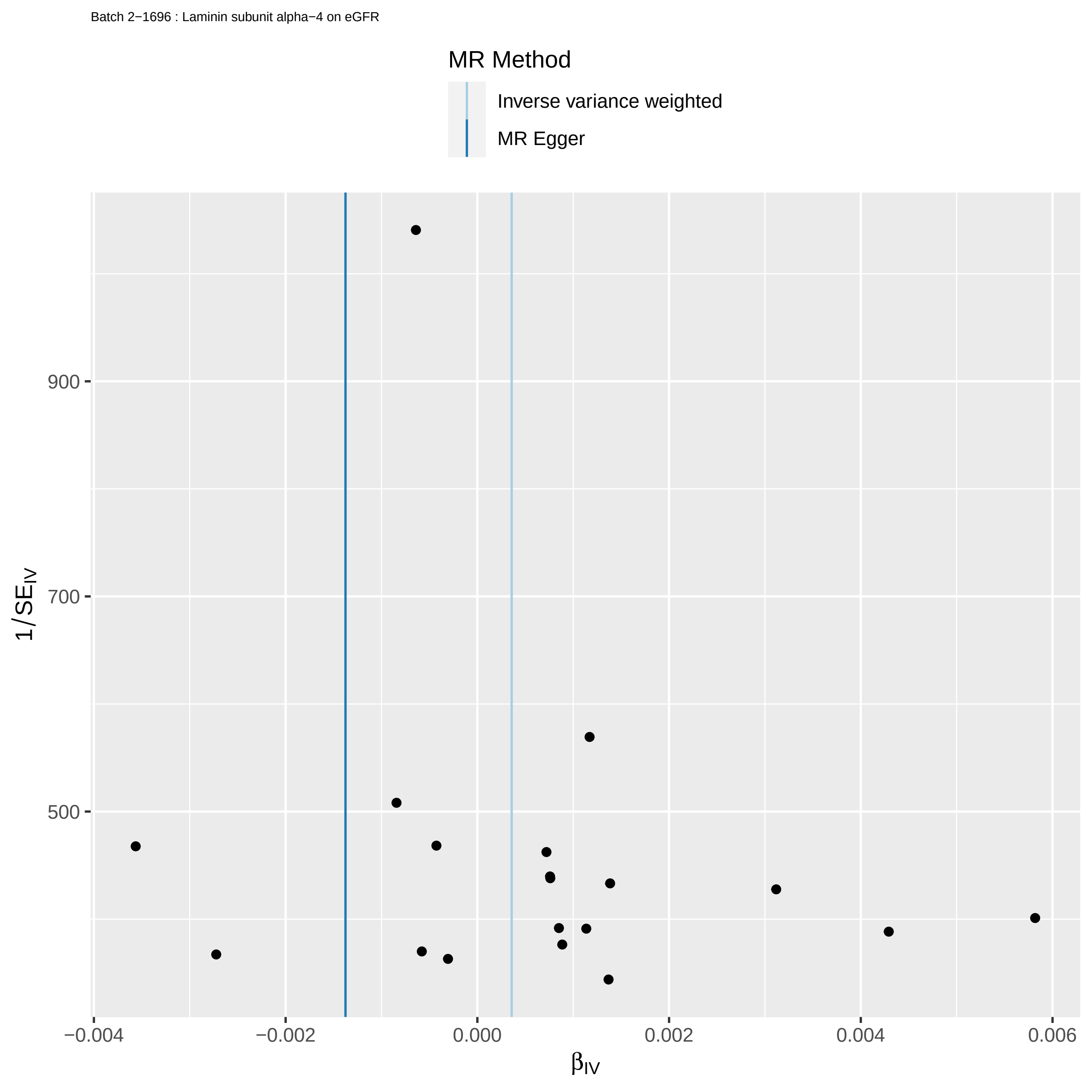

Batch 2−1696 : Laminin subunit alpha−4 on eGFR
MR Method
Inverse variance weighted
MR Egger
900
700
500
1/SE_IV
−0.004
−0.002
0.000
0.002
0.004
0.006
β_IV

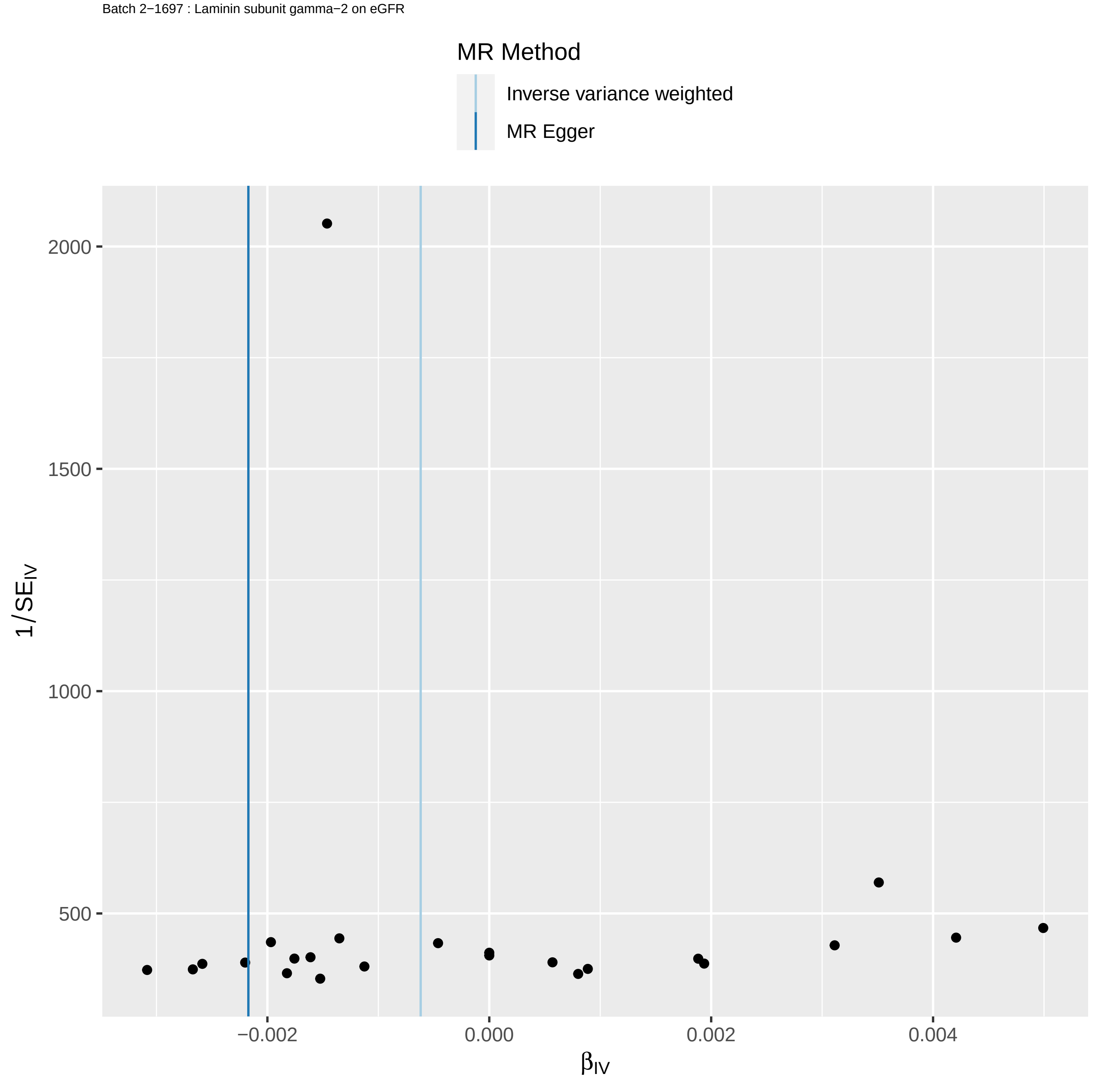

Batch 2−1697 : Laminin subunit gamma−2 on eGFR
MR Method
Inverse variance weighted
MR Egger
2000
1500
1000
500
1/SE$_{IV}$
−0.002
0.000
0.002
0.004
β$_{IV}$

Batch 2−1698 : Lysosome−associated membrane glycoprotein 3 on eGFR

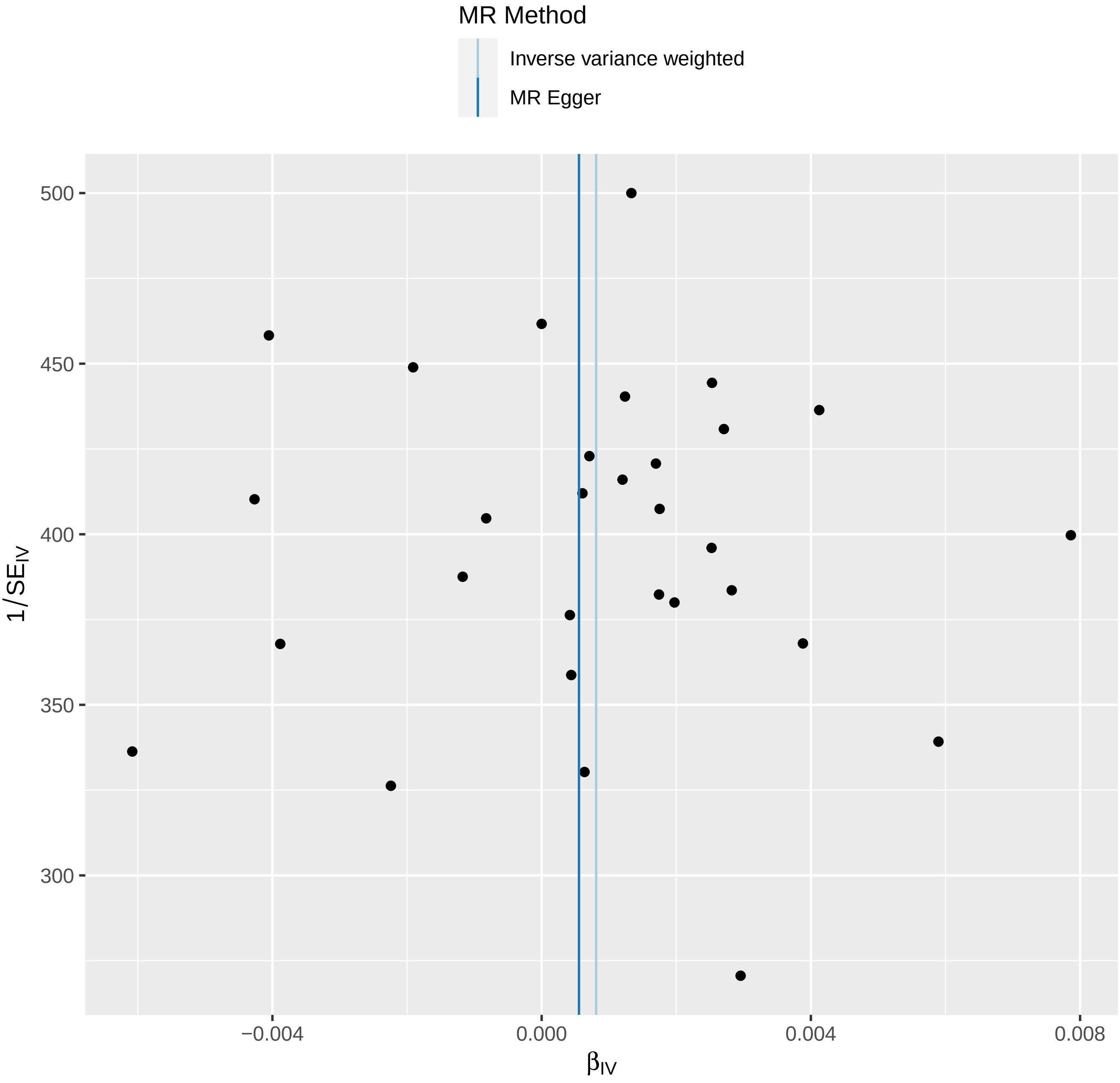

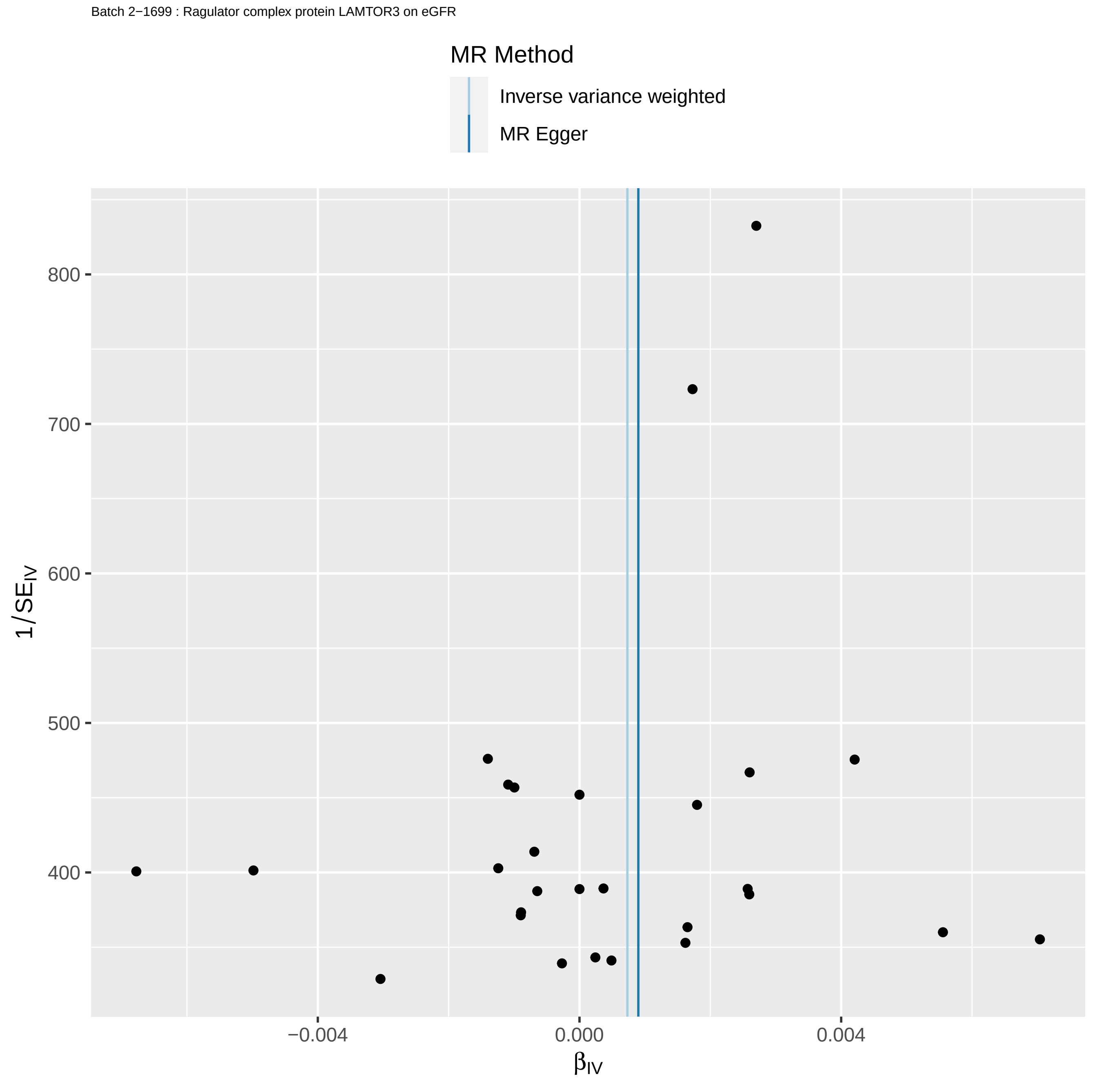
Batch 2−1699 : Ragulator complex protein LAMTOR3 on eGFR
MR Method
Inverse variance weighted
MR Egger
800
700
600
500
400
1/SE$_{IV}$
−0.004
0.000
0.004
β_{IV}

Batch 2−1700 : Glycosyltransferase−like protein LARGE1 on eGFR

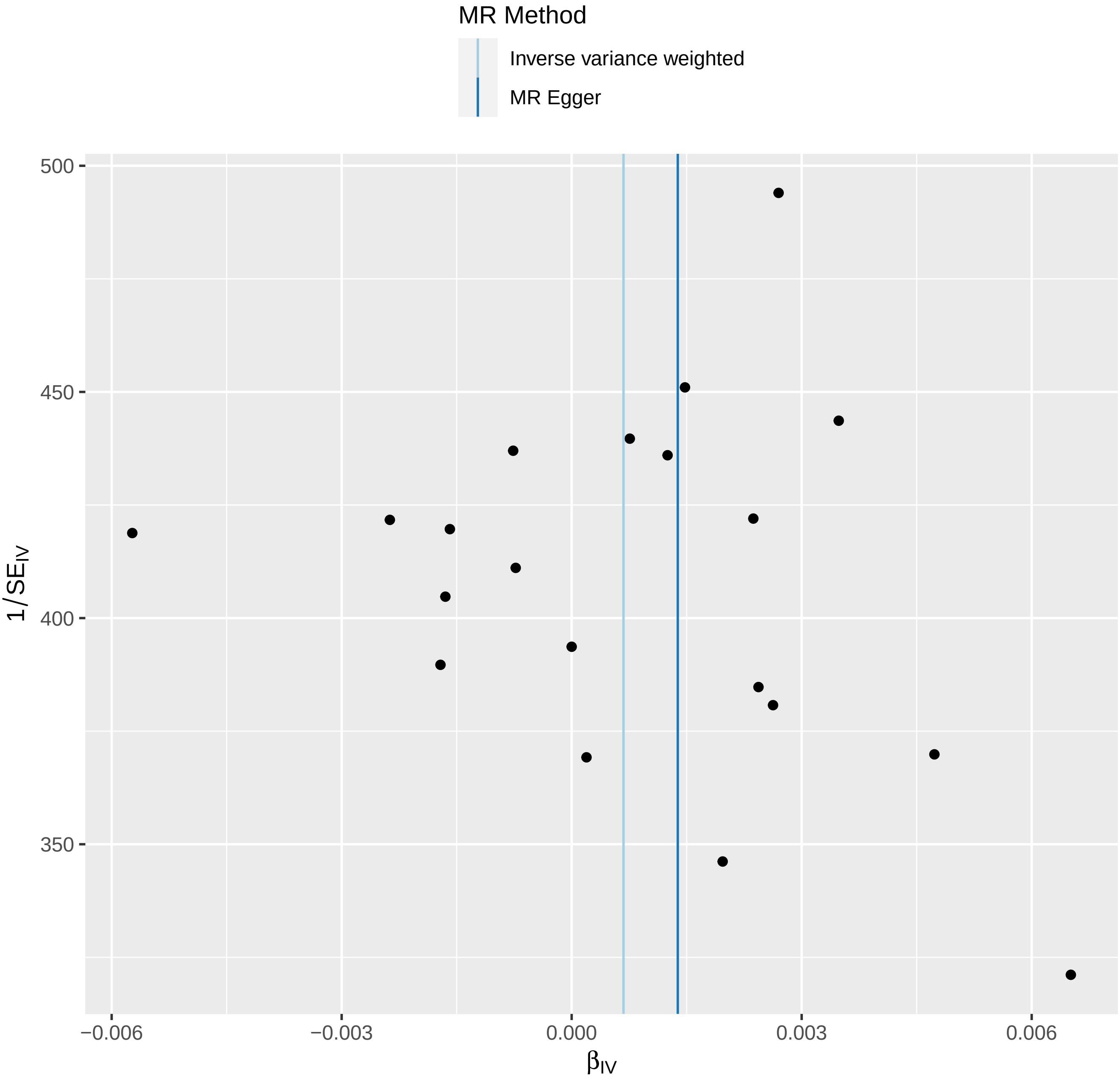

Batch 2−1701 : Linker for activation of T−cells family member 1 on eGFR

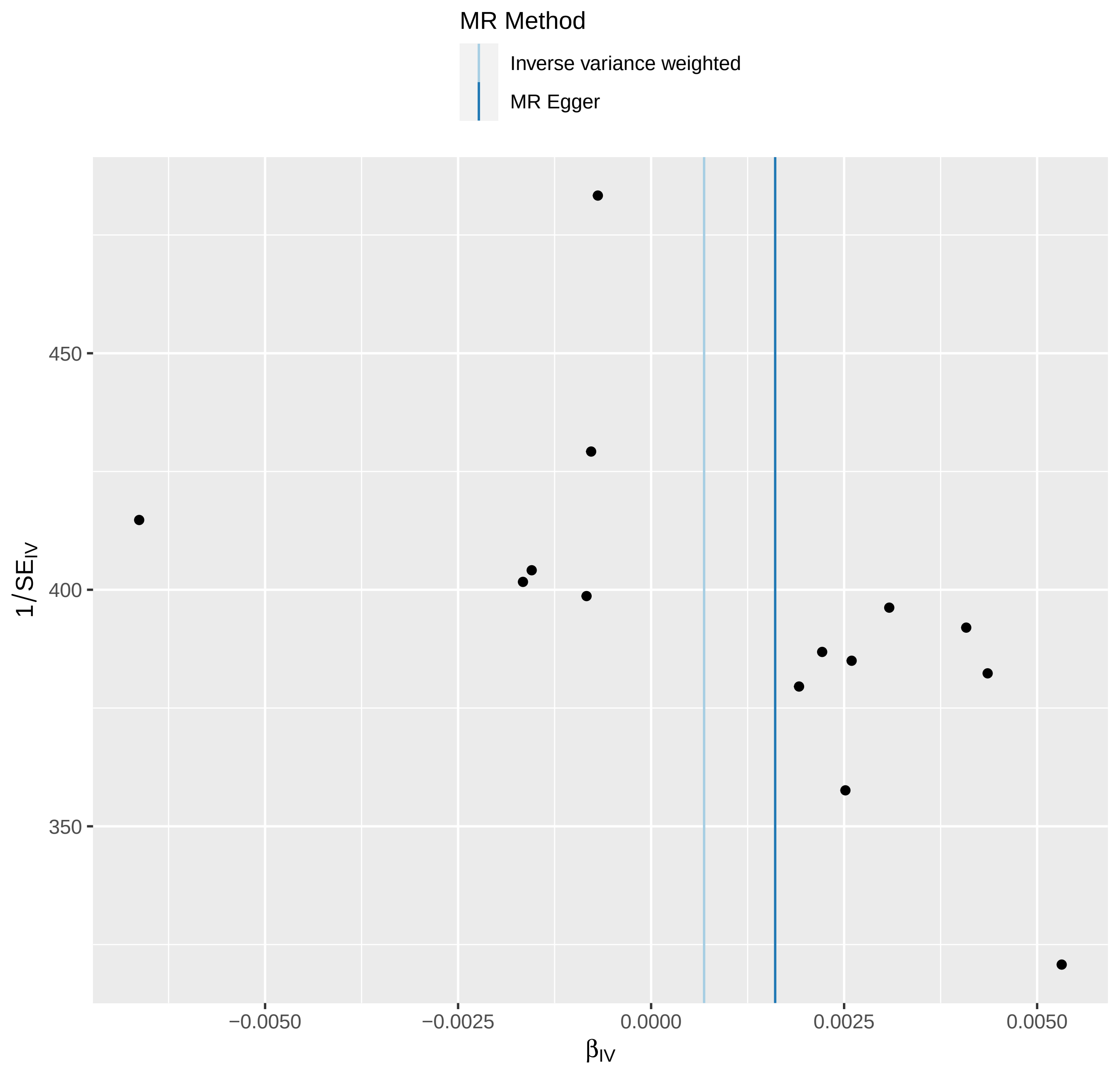

Batch 2−1702 : Linker for activation of T−cells family member 2 on eGFR

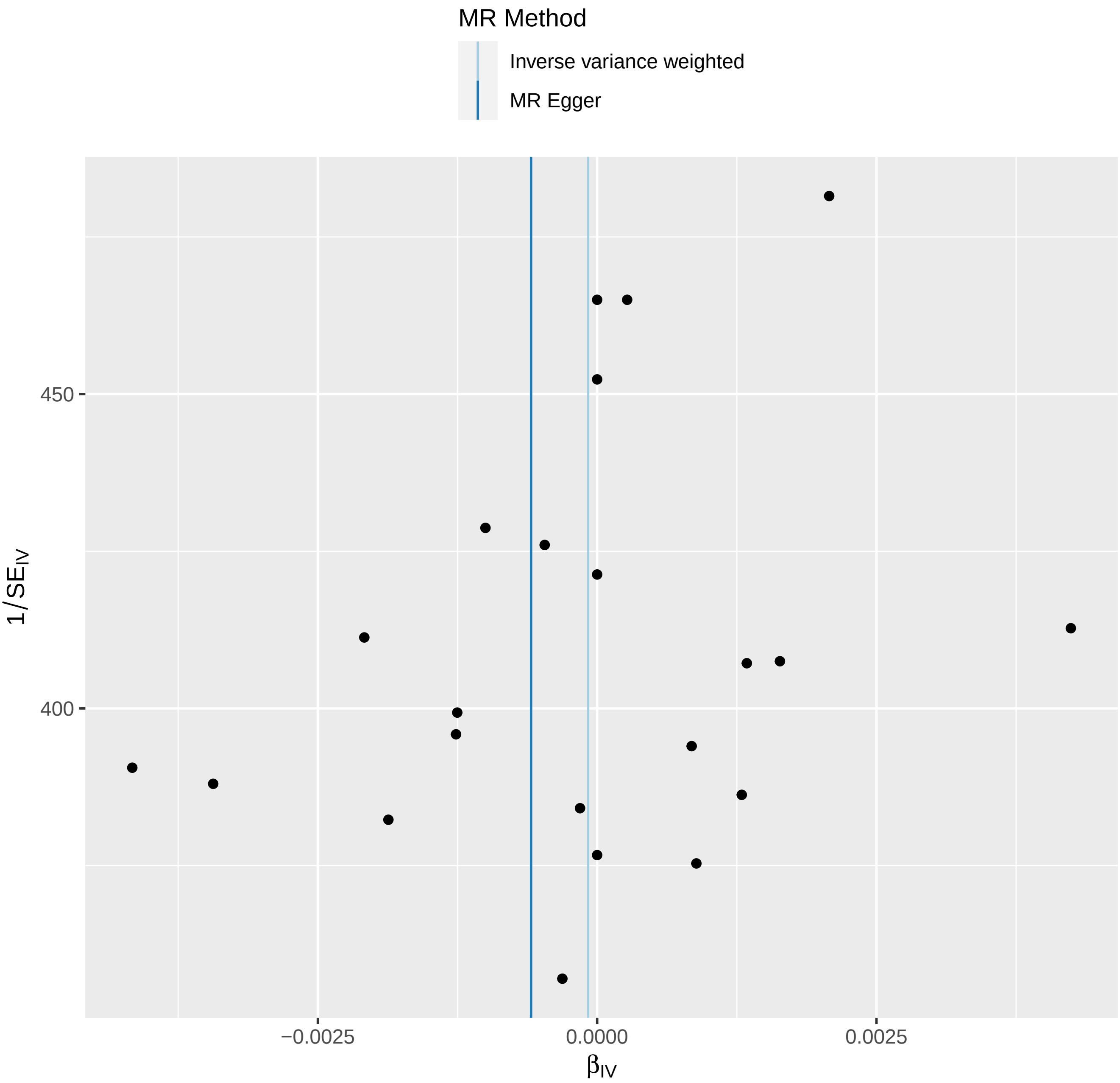

Batch 2−1703 : Layilin on eGFR

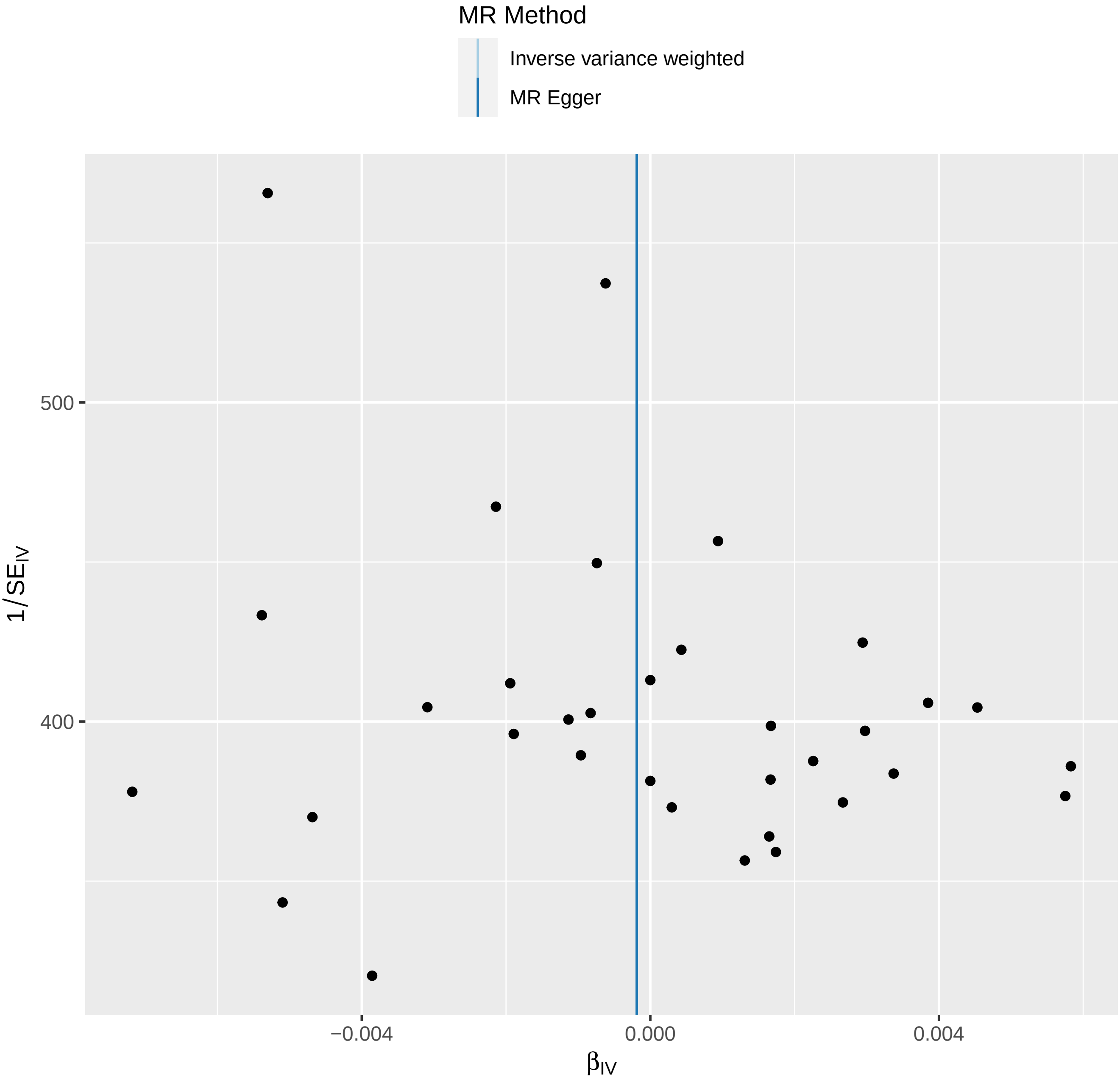

Batch 2−1704 : Tyrosine−protein kinase Lck on eGFR

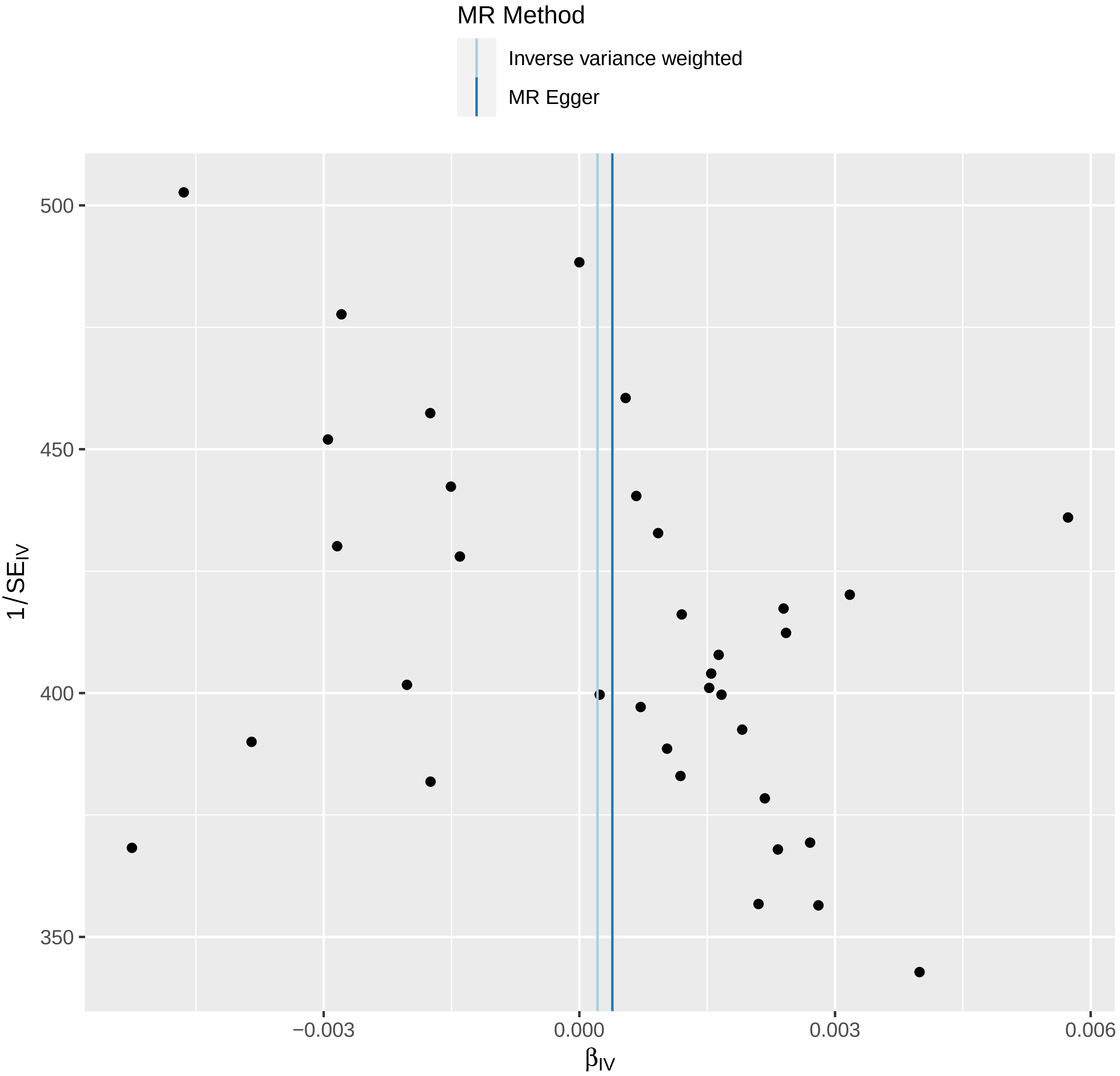

Batch 2−1705 : Tyrosine−protein kinase Lck on eGFR

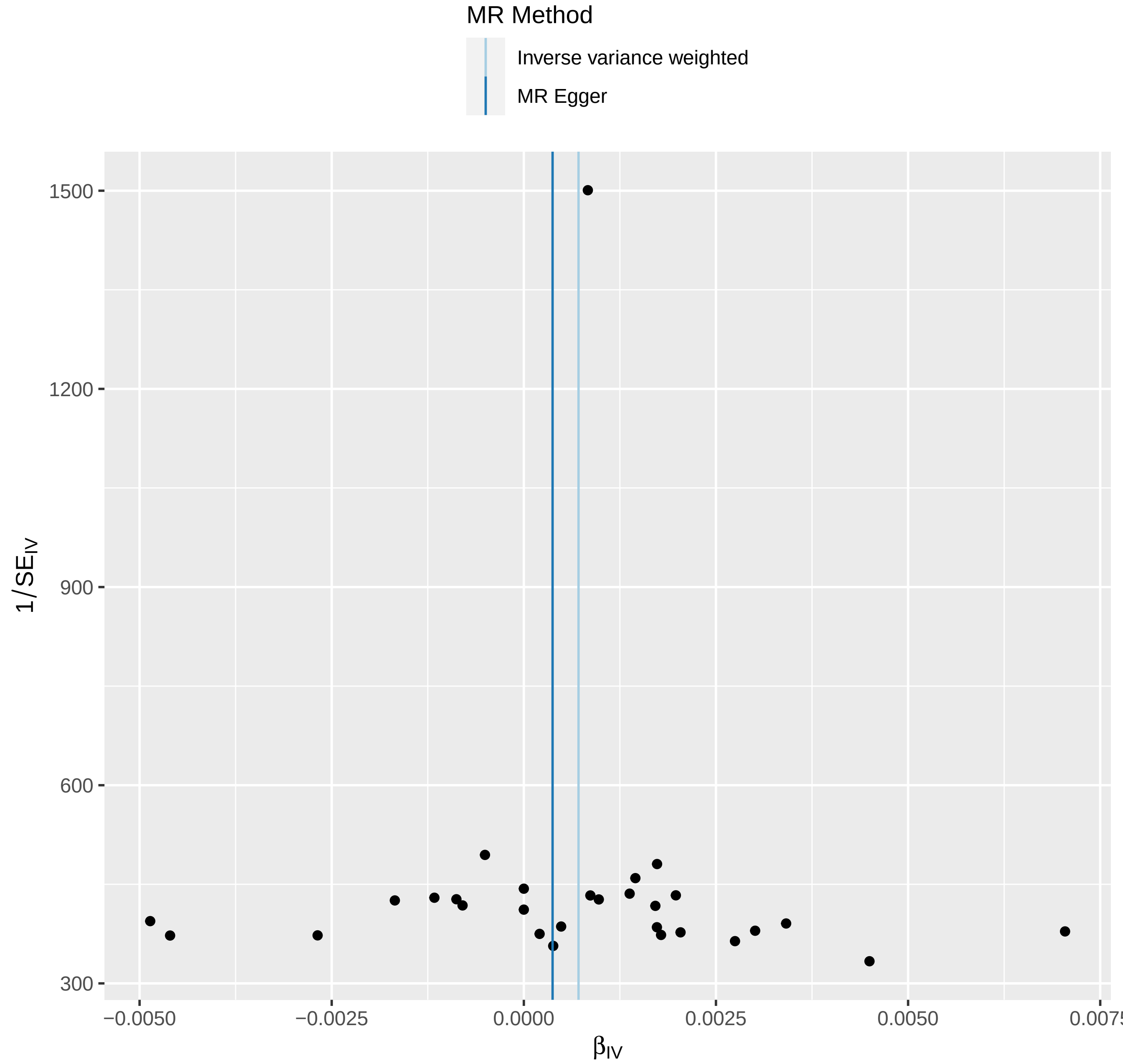

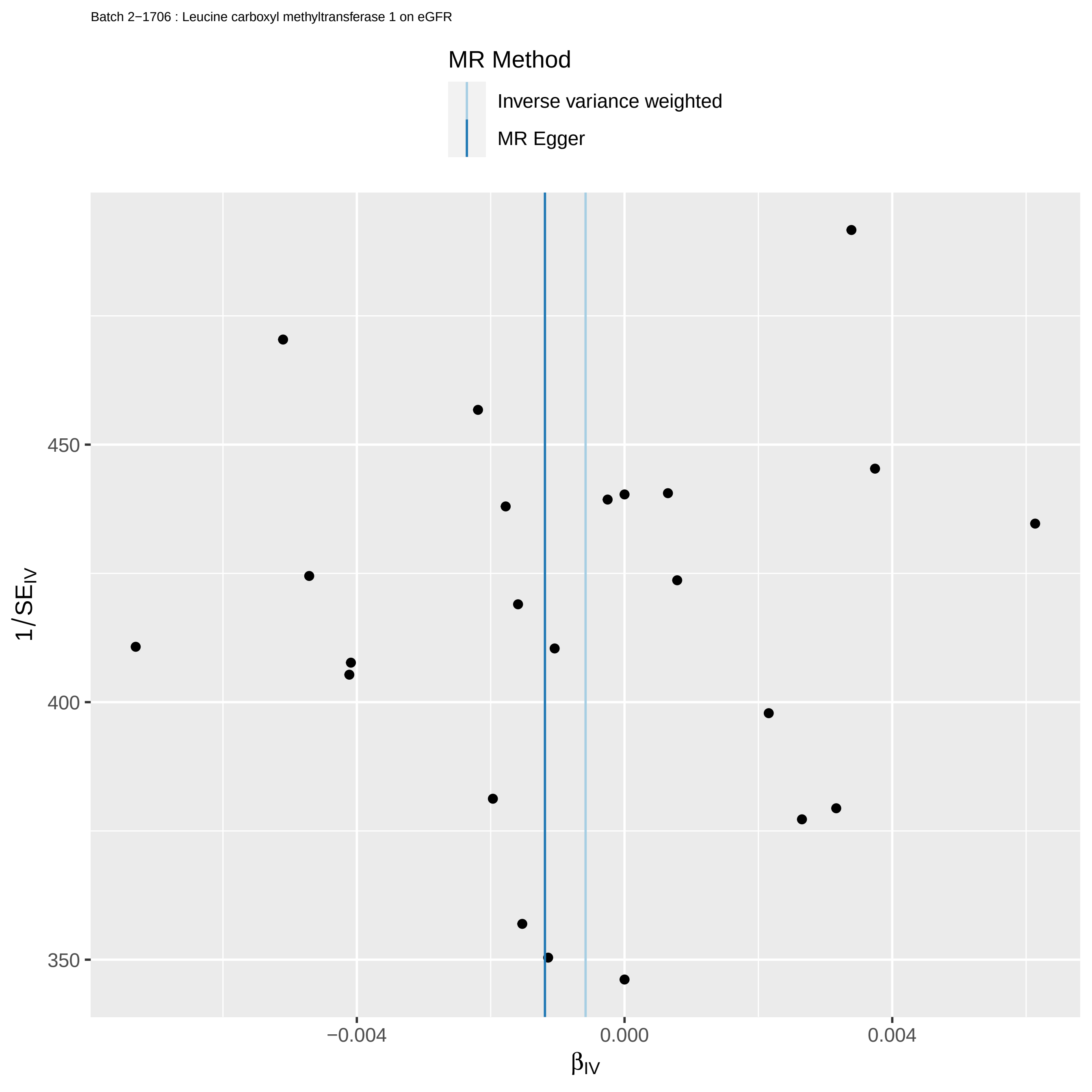

Batch 2−1706 : Leucine carboxyl methyltransferase 1 on eGFR
MR Method
Inverse variance weighted
MR Egger
450
400
350
1/SE_IV
−0.004
0.000
0.004
β_IV

Batch 2−1707 : Epididymal−specific lipocalin−10 on eGFR

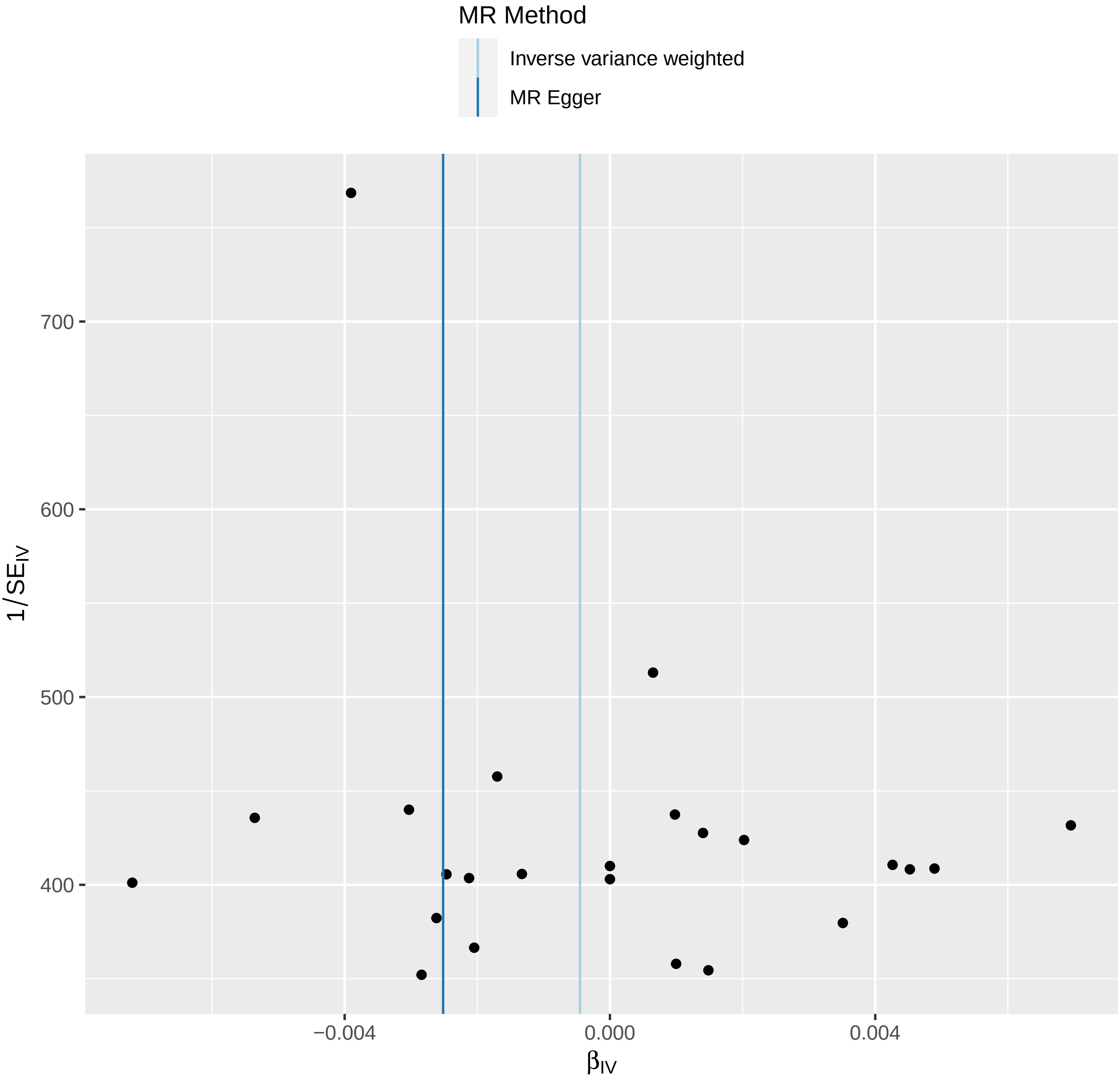

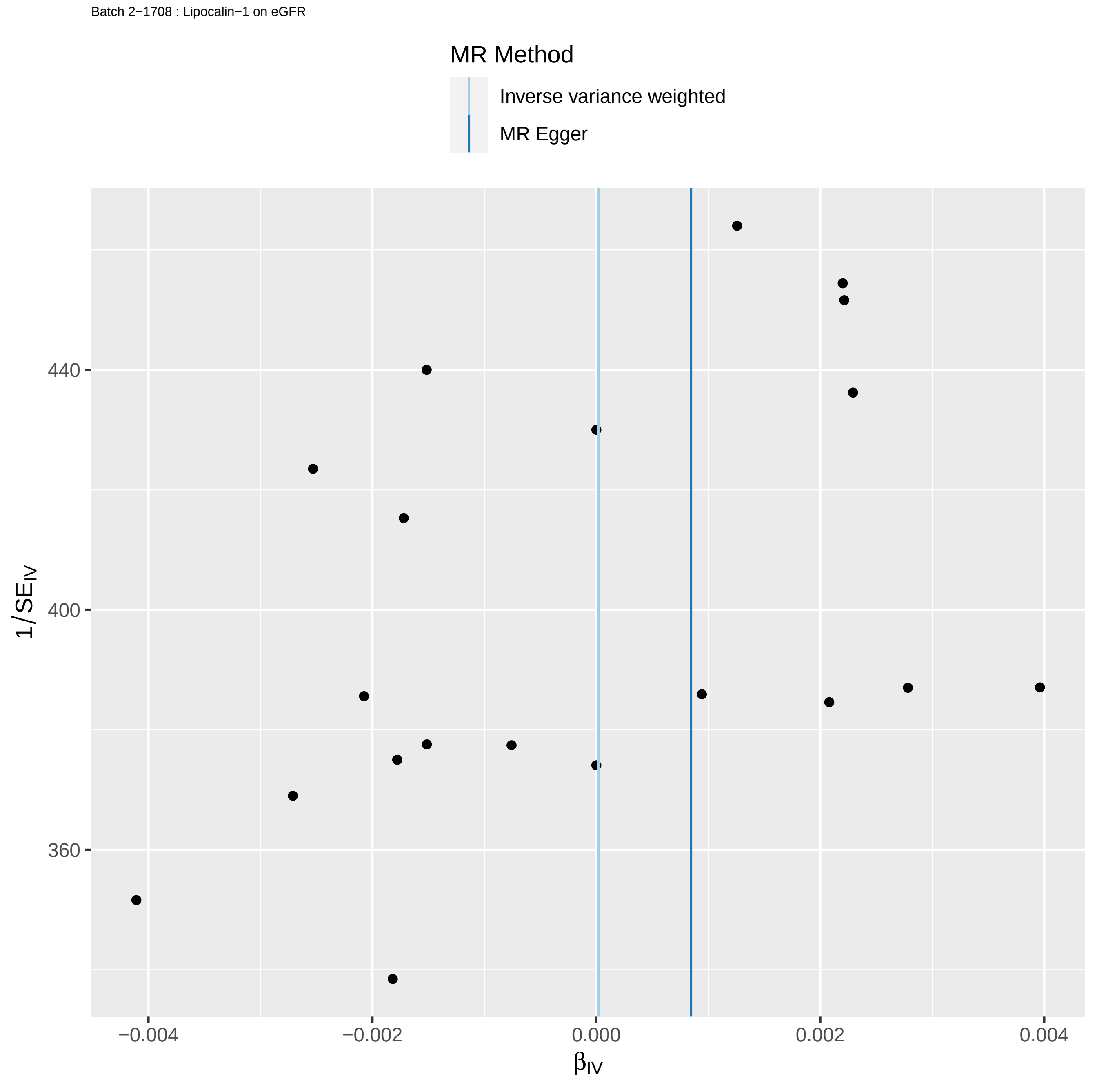
Batch 2−1708 : Lipocalin−1 on eGFR
MR Method
Inverse variance weighted
MR Egger
440
400
360
$1/SE_{IV}$
−0.004
−0.002
0.000
0.002
0.004
β_{IV}

Batch 2−1709 : Neutrophil gelatinase−associated lipocalin on eGFR

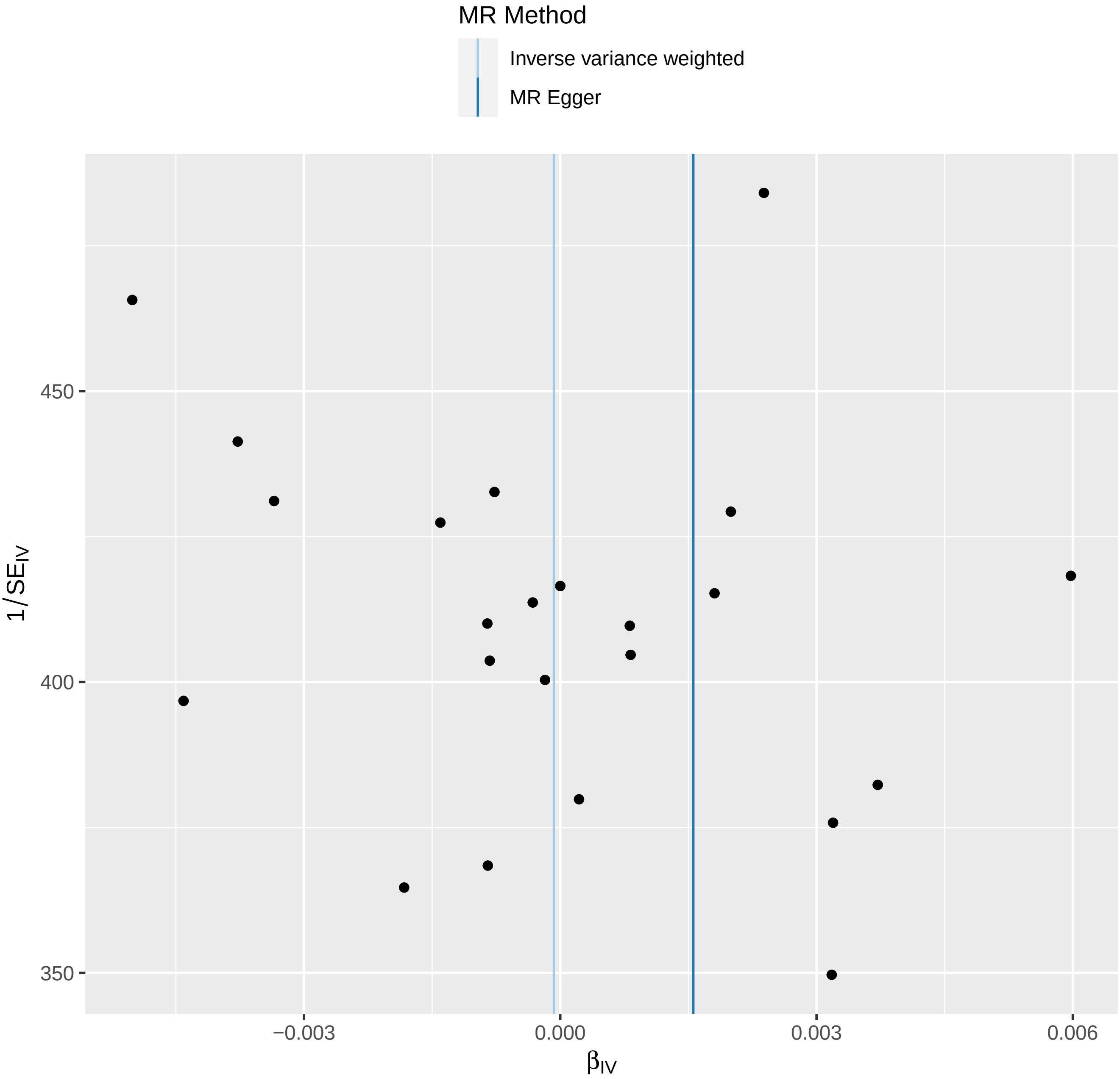

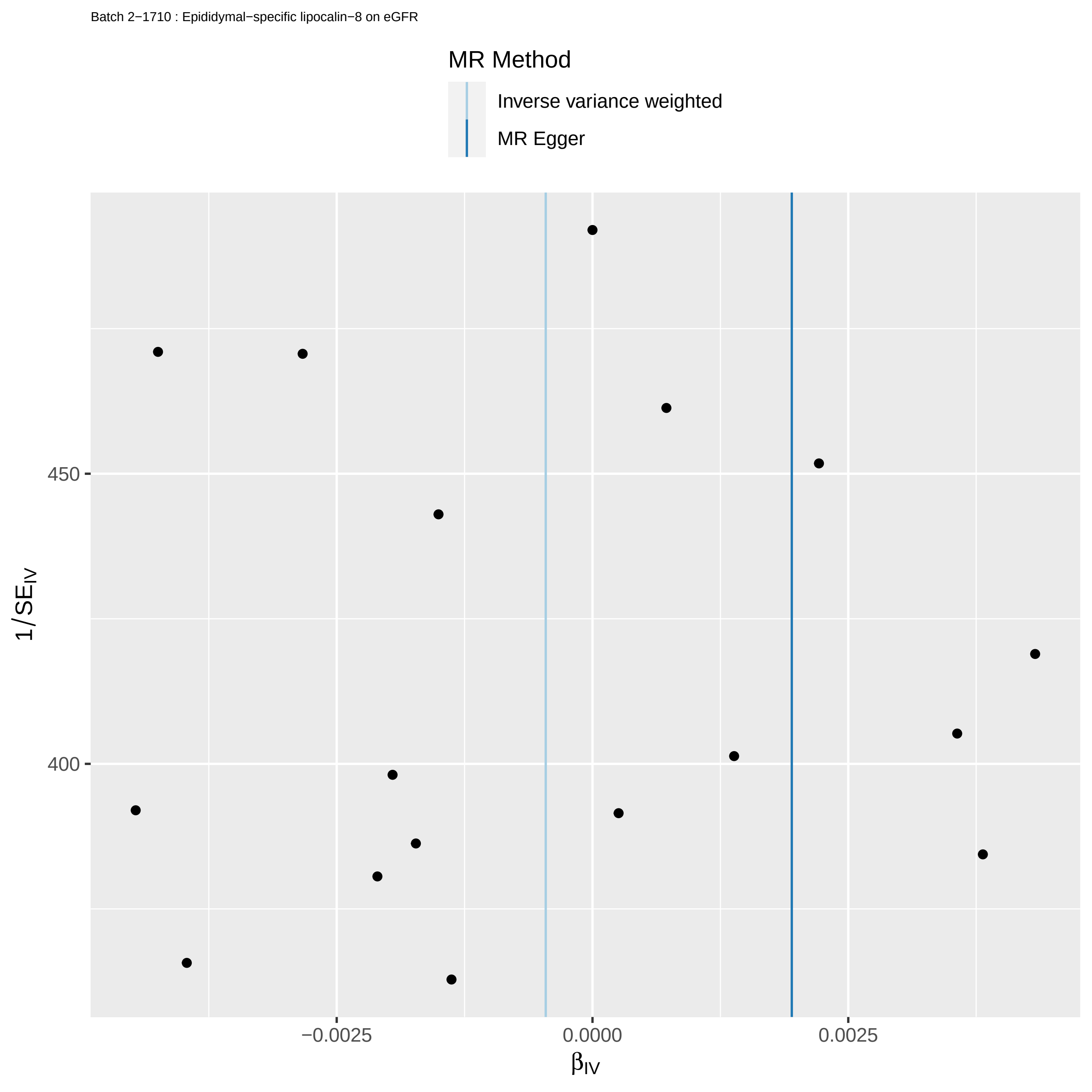

Batch 2−1710 : Epididymal−specific lipocalin−8 on eGFR
MR Method
Inverse variance weighted
MR Egger
450
400
1/SE_IV
−0.0025
0.0000
0.0025
β_IV

Batch 2−1711 : Ligand−dependent nuclear receptor corepressor−like protein on eGFR

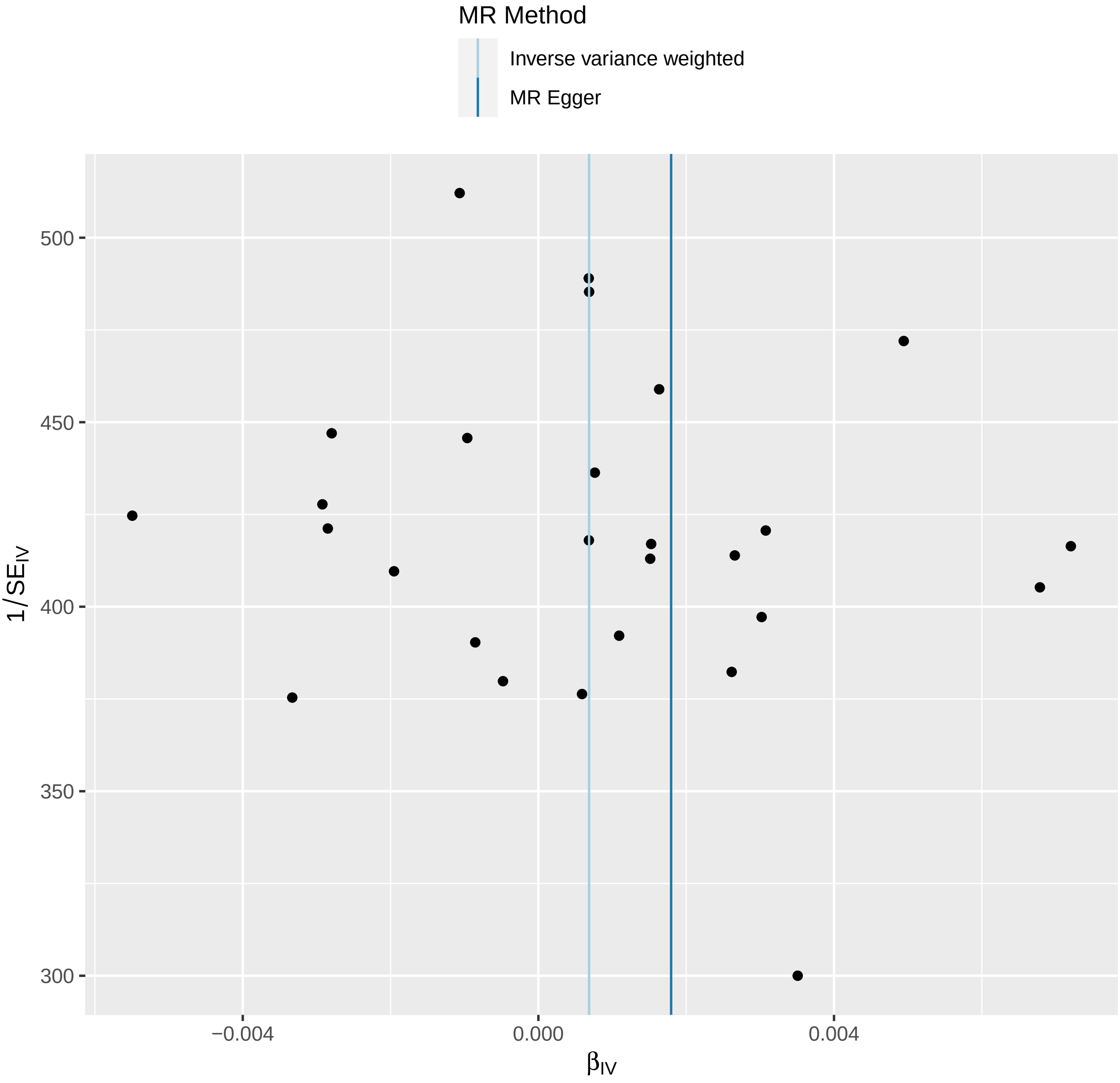

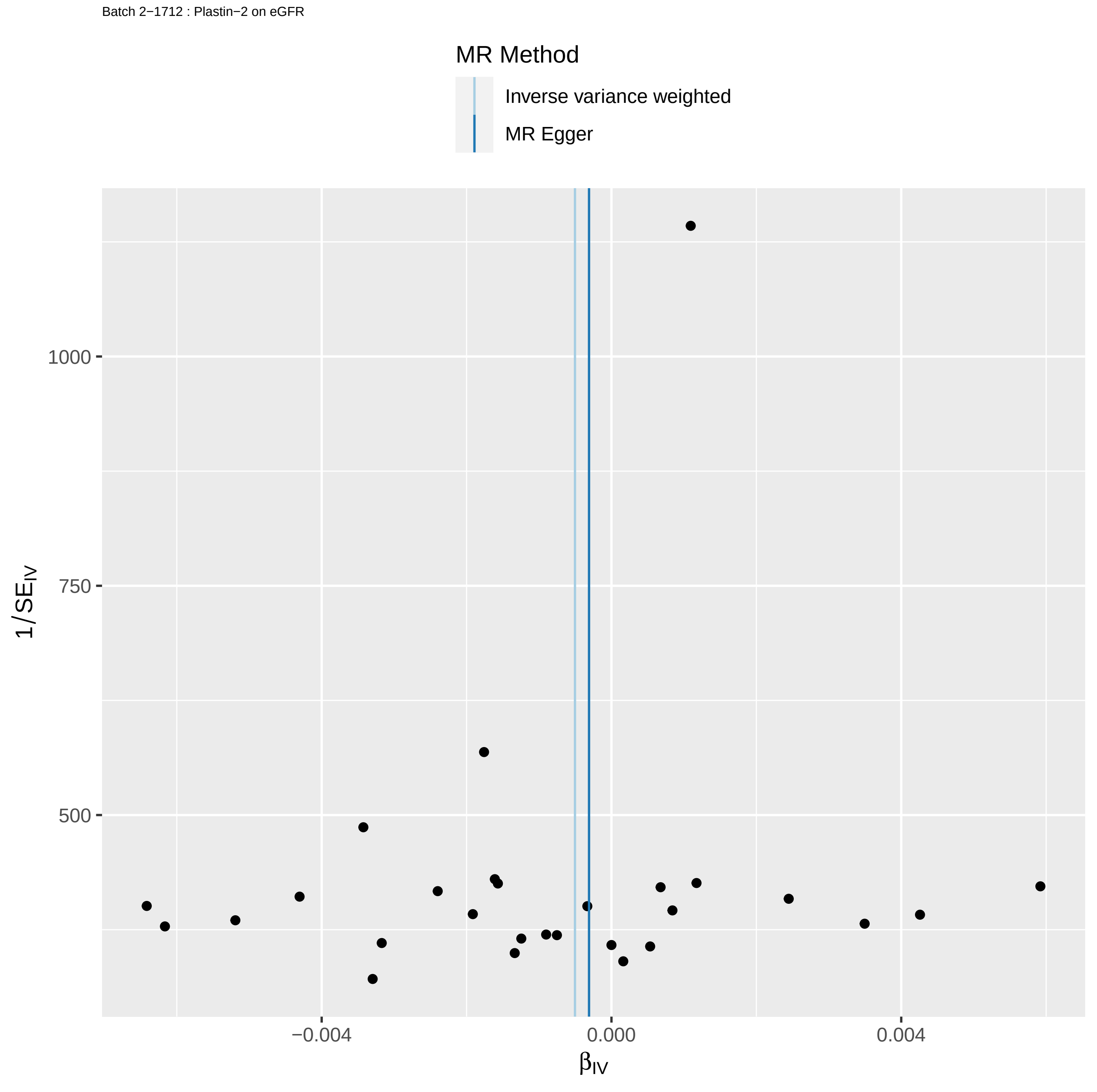
Batch 2−1712 : Plastin−2 on eGFR
MR Method
Inverse variance weighted
MR Egger
1000
750
500
1/SE$_{IV}$
−0.004
0.000
0.004
β_{IV}

Batch 2−1713 : Lactase−phlorizin hydrolase on eGFR

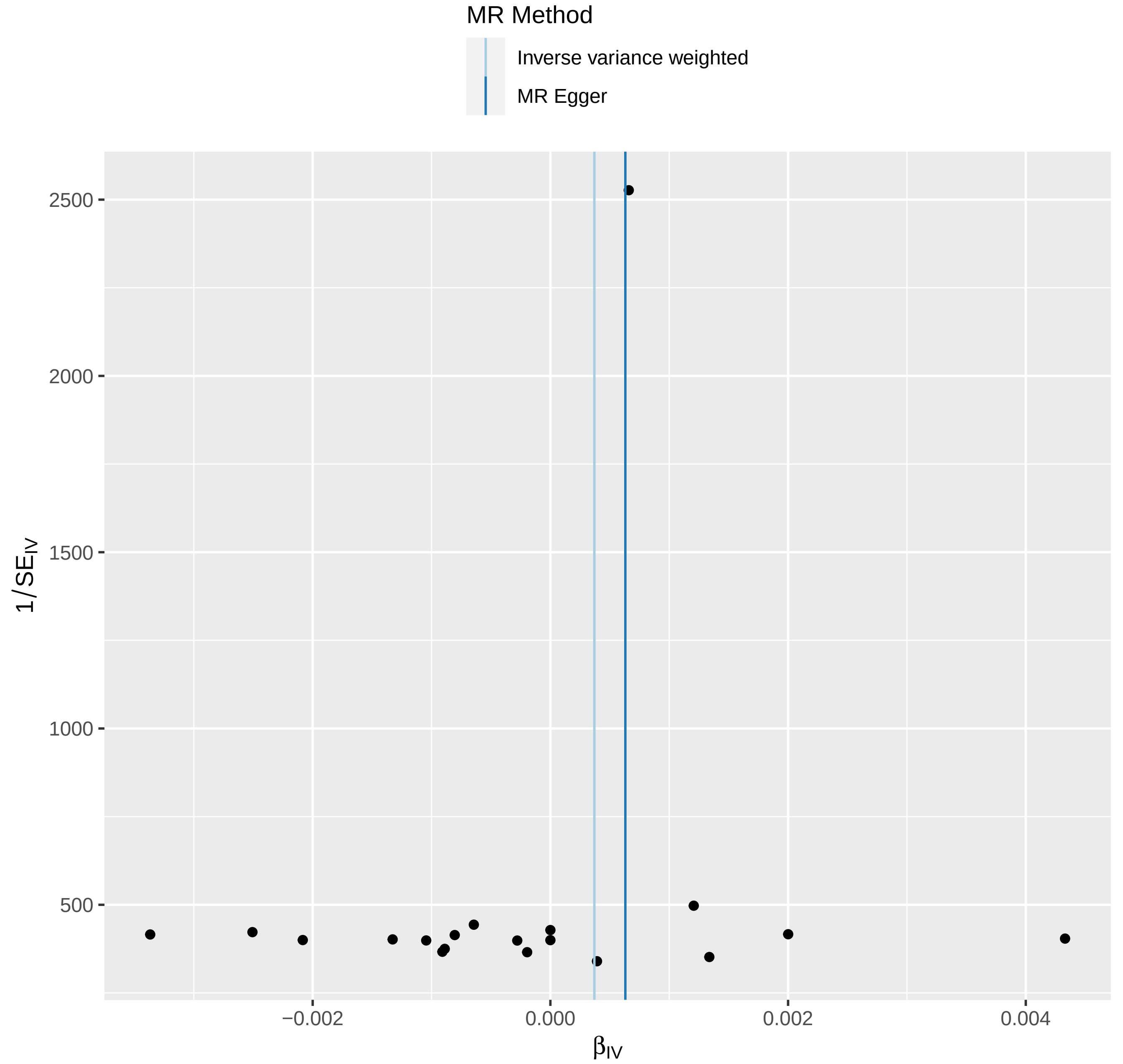

Batch 2−1714 : Lactase−like protein on eGFR

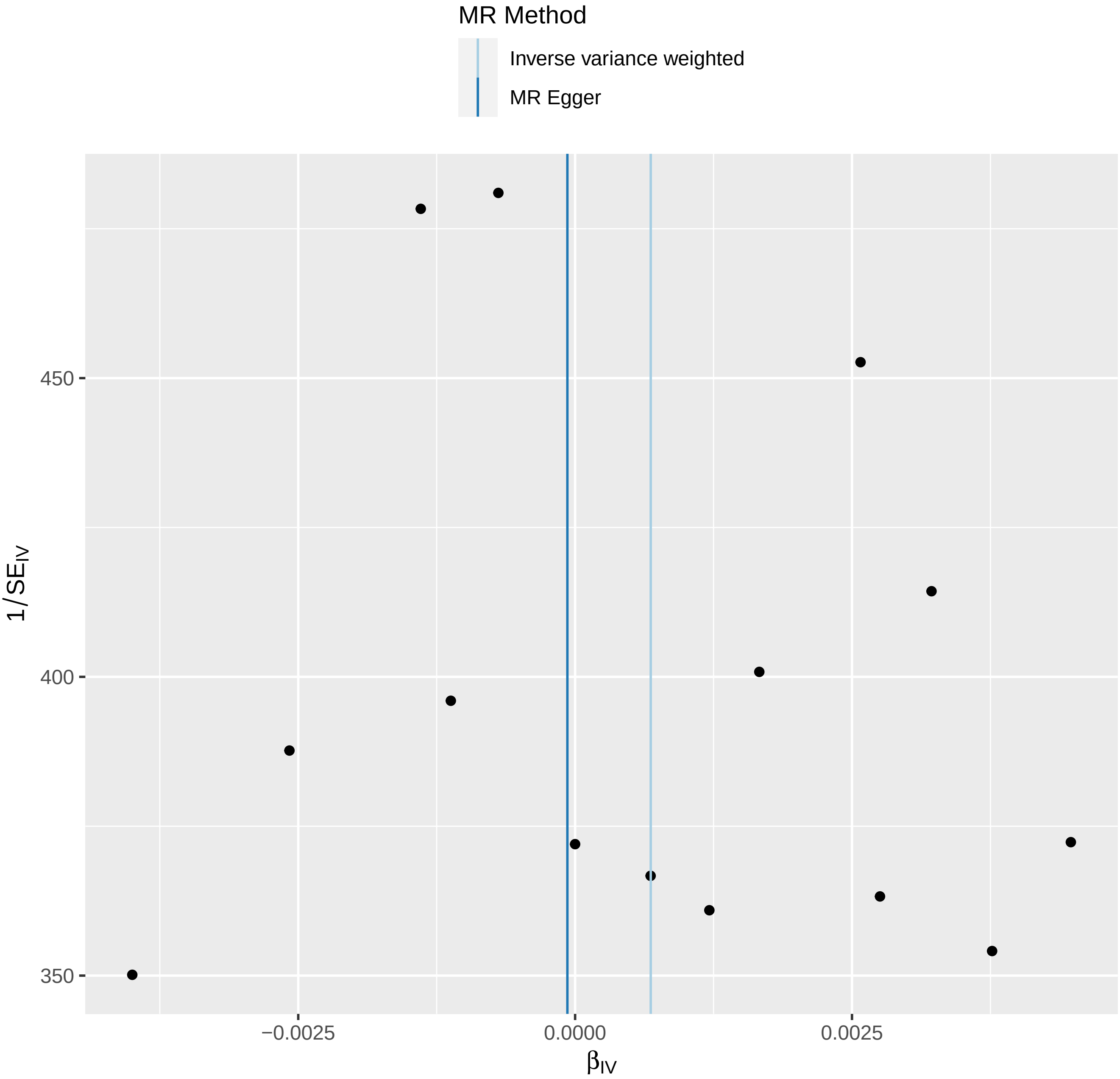

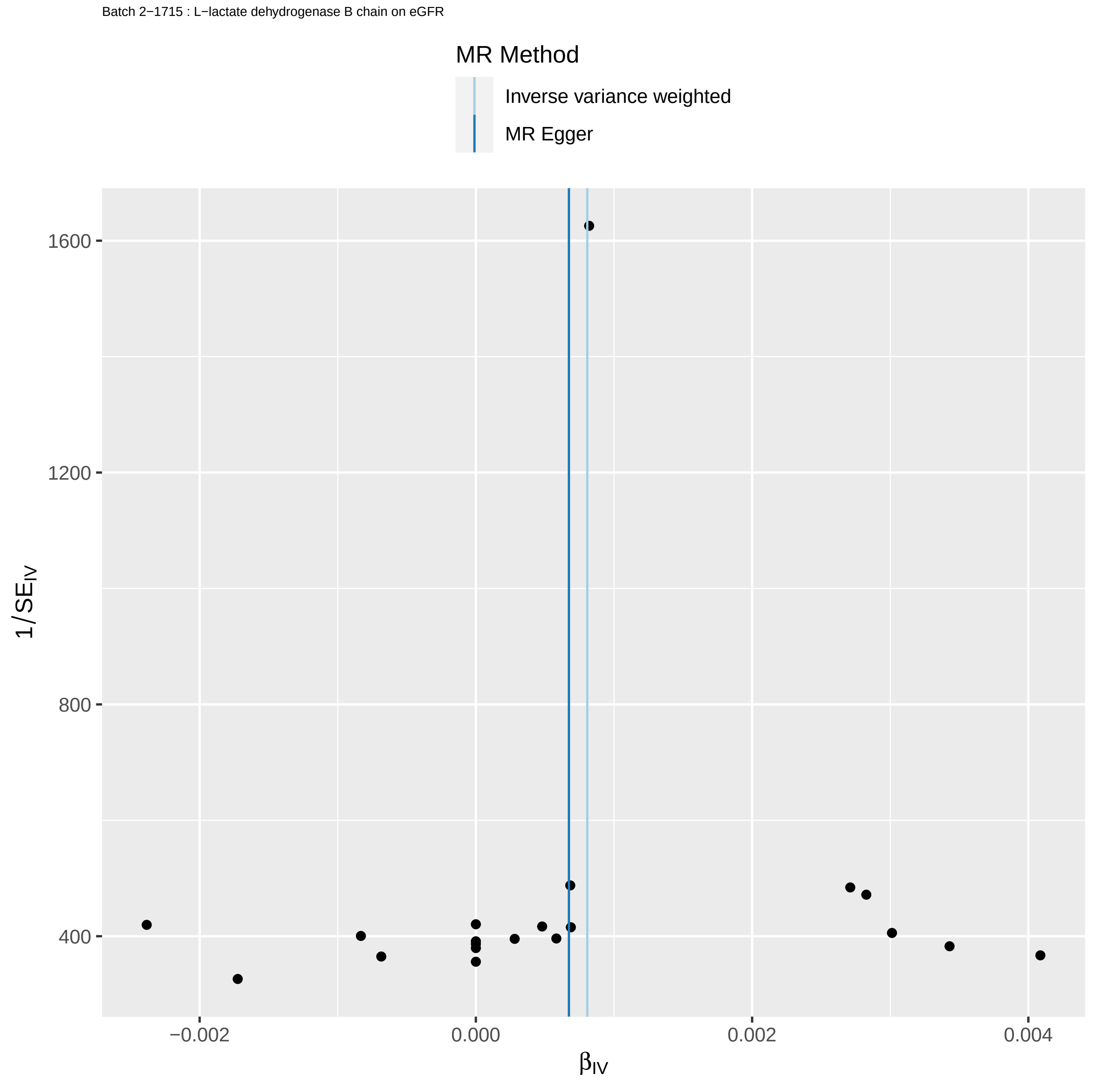

Batch 2−1715 : L−lactate dehydrogenase B chain on eGFR
MR Method
Inverse variance weighted
MR Egger
1600
1200
800
400
1/SE$_{IV}$
−0.002
0.000
0.002
0.004
β$_{IV}$

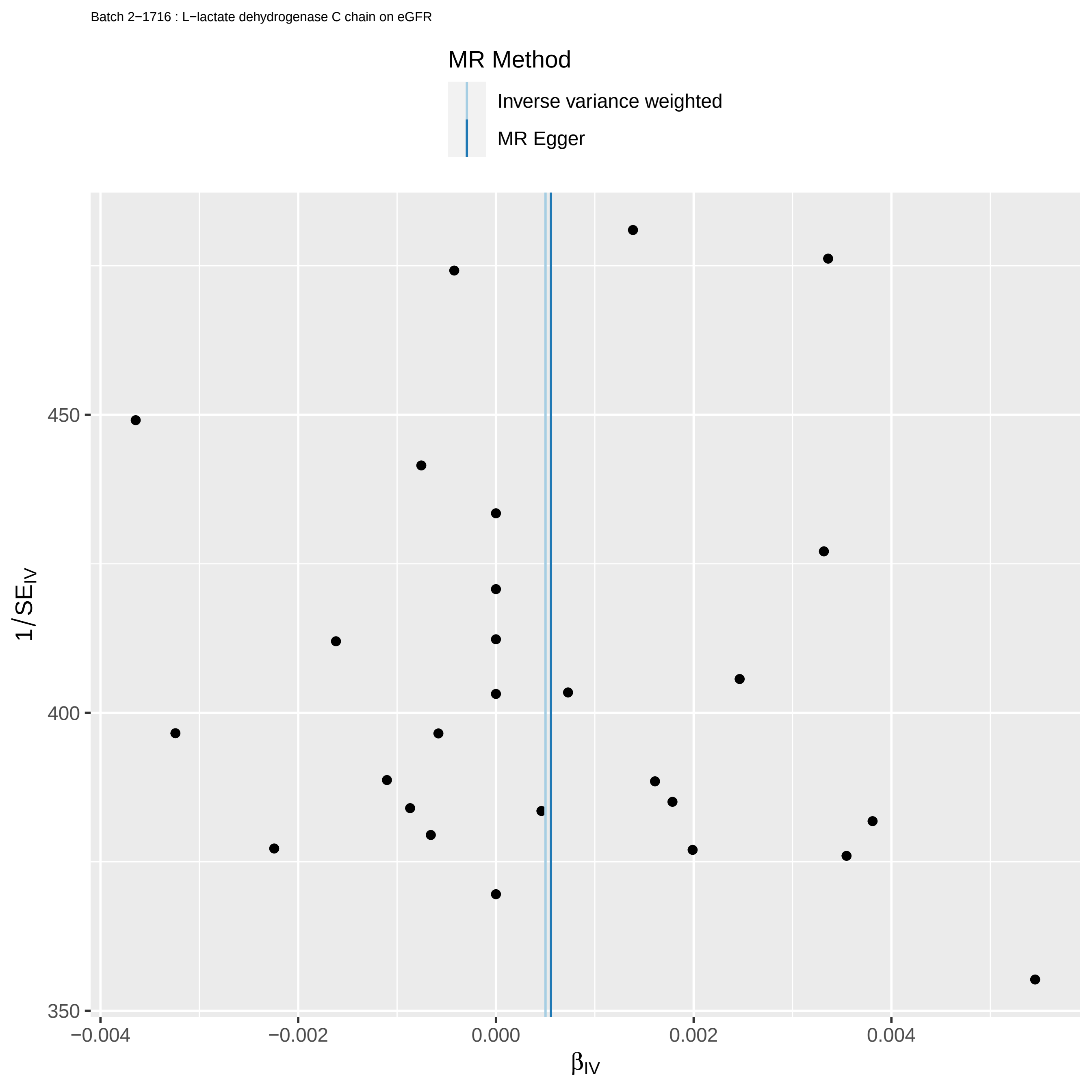
Batch 2−1716 : L−lactate dehydrogenase C chain on eGFR
MR Method
Inverse variance weighted
MR Egger
1/SE_IV
450
400
350
−0.004
−0.002
0.000
0.002
0.004
β_IV

Batch 2−1717 : Low−density lipoprotein receptor on eGFR

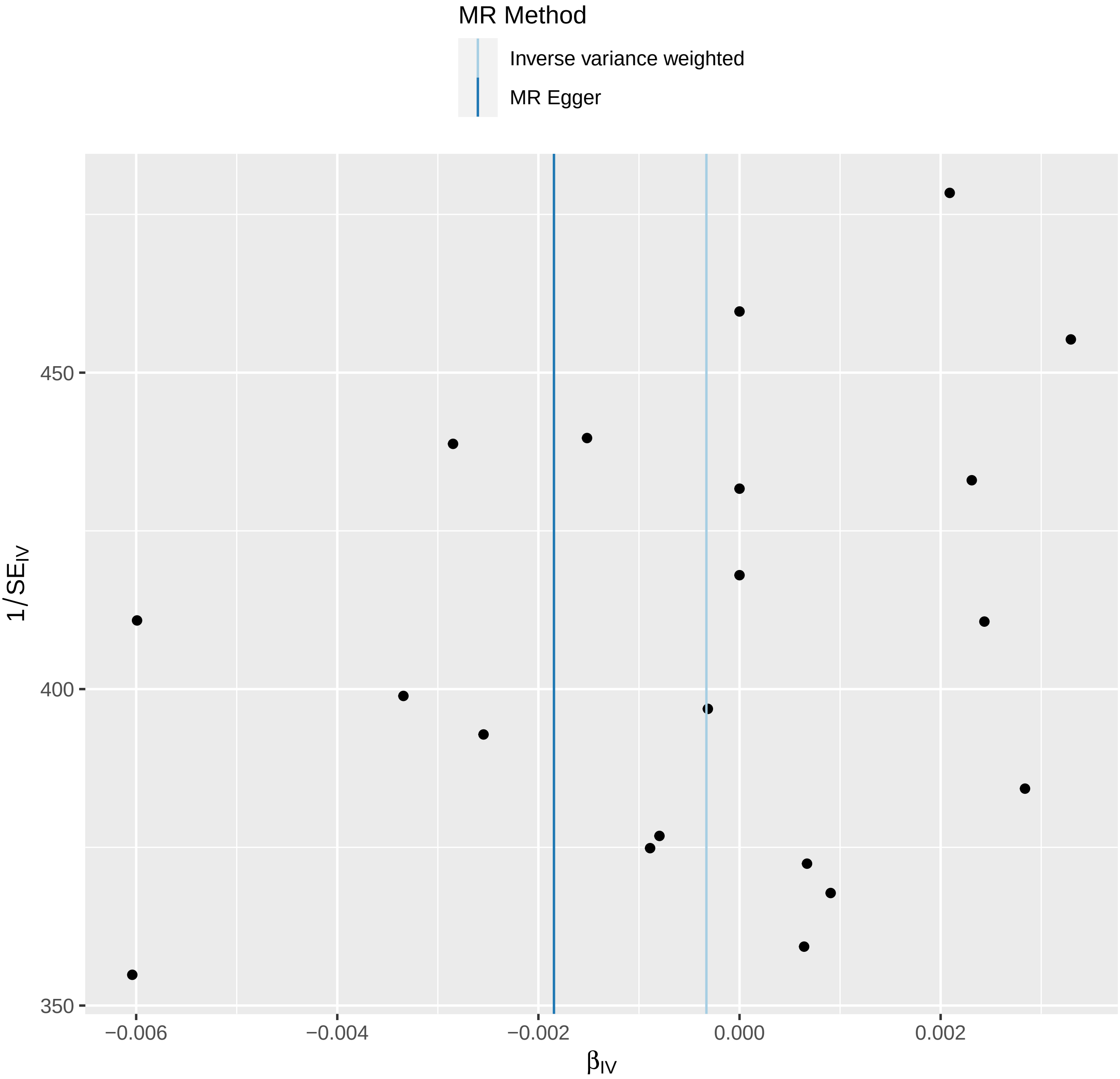

Batch 2−1718 : Protein LDOC1 on eGFR

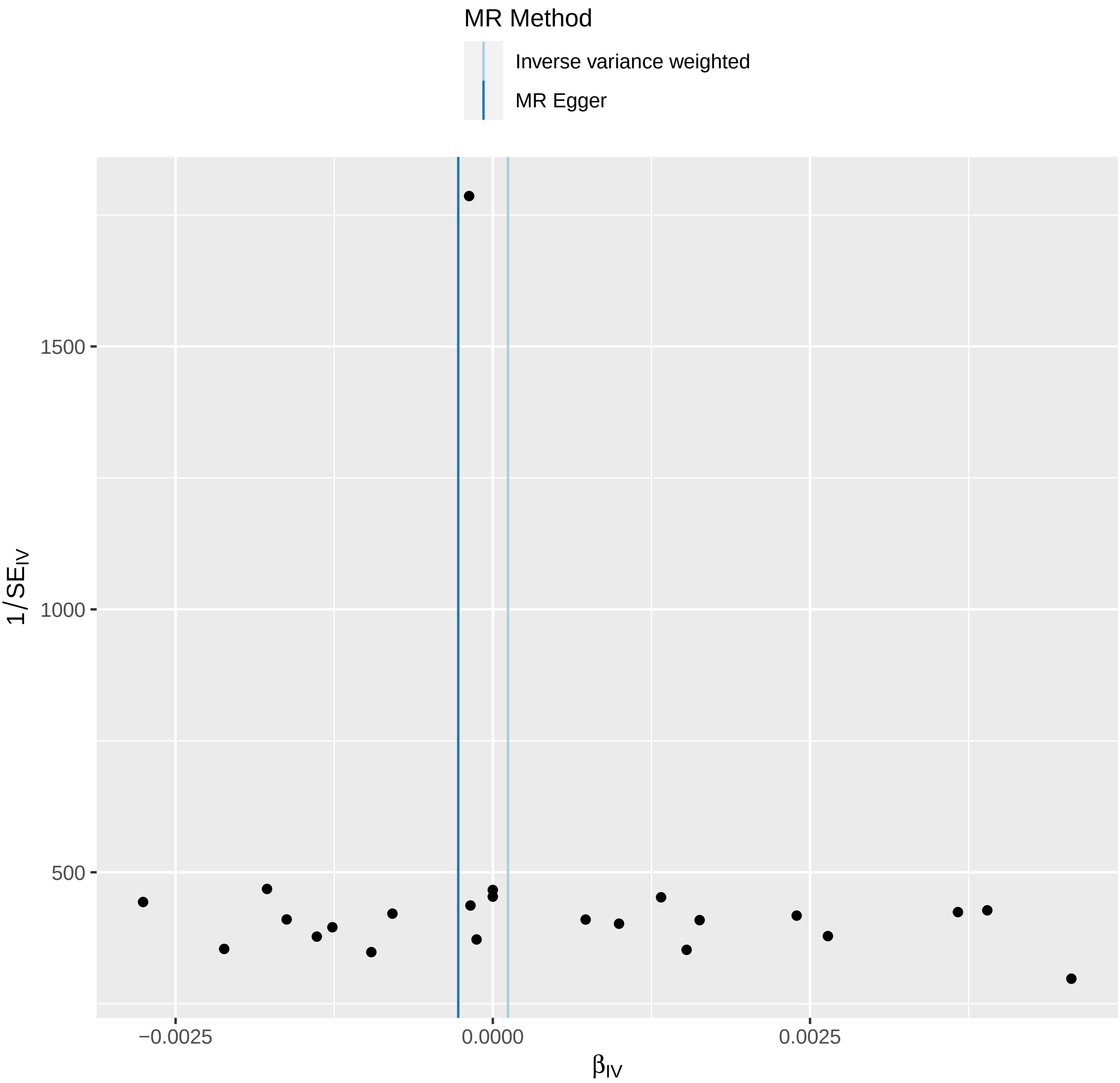

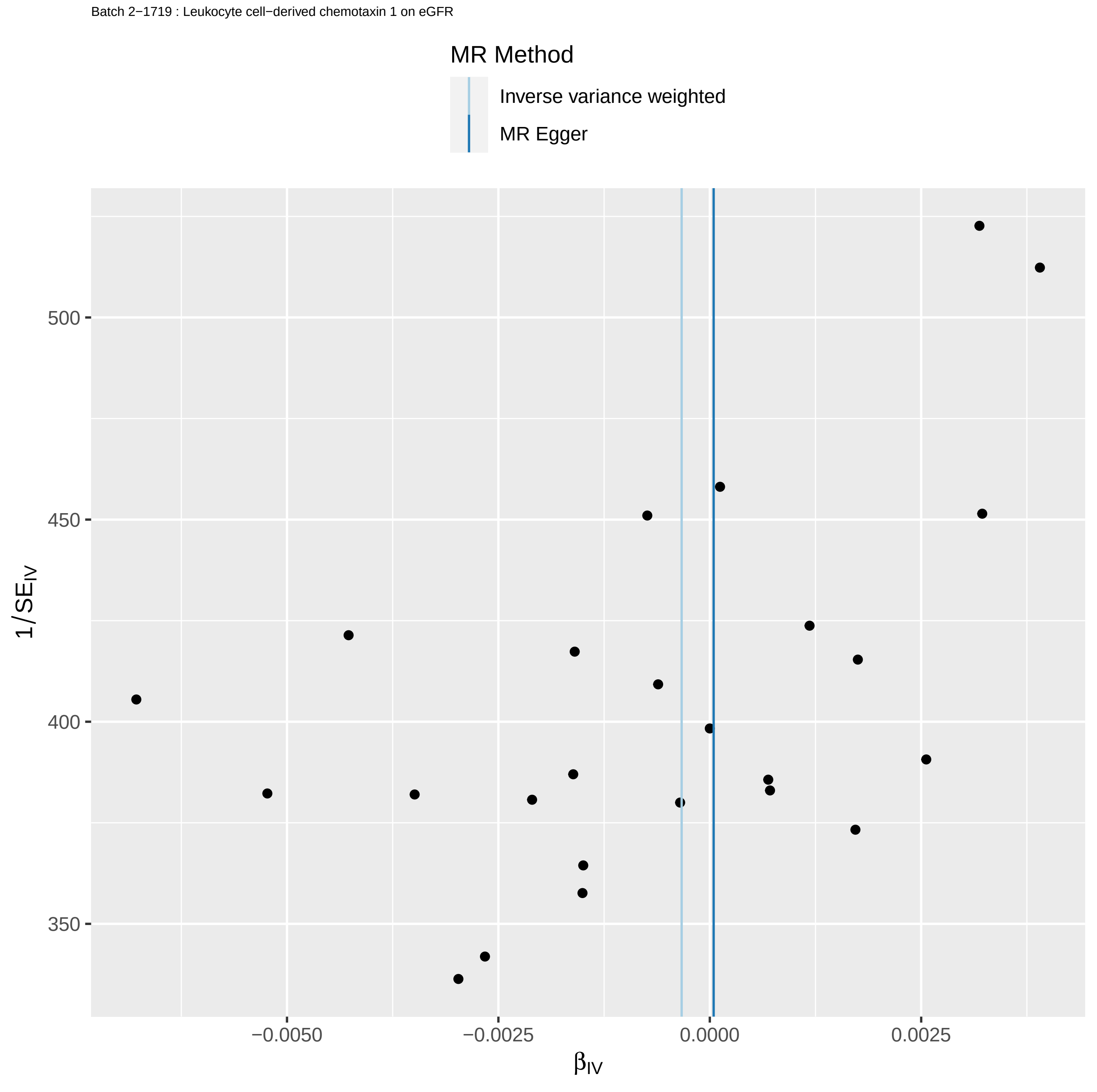

Batch 2−1719 : Leukocyte cell−derived chemotaxin 1 on eGFR
MR Method
Inverse variance weighted
MR Egger
500
450
400
350
1/SE_IV
−0.0050
−0.0025
0.0000
0.0025
β_IV

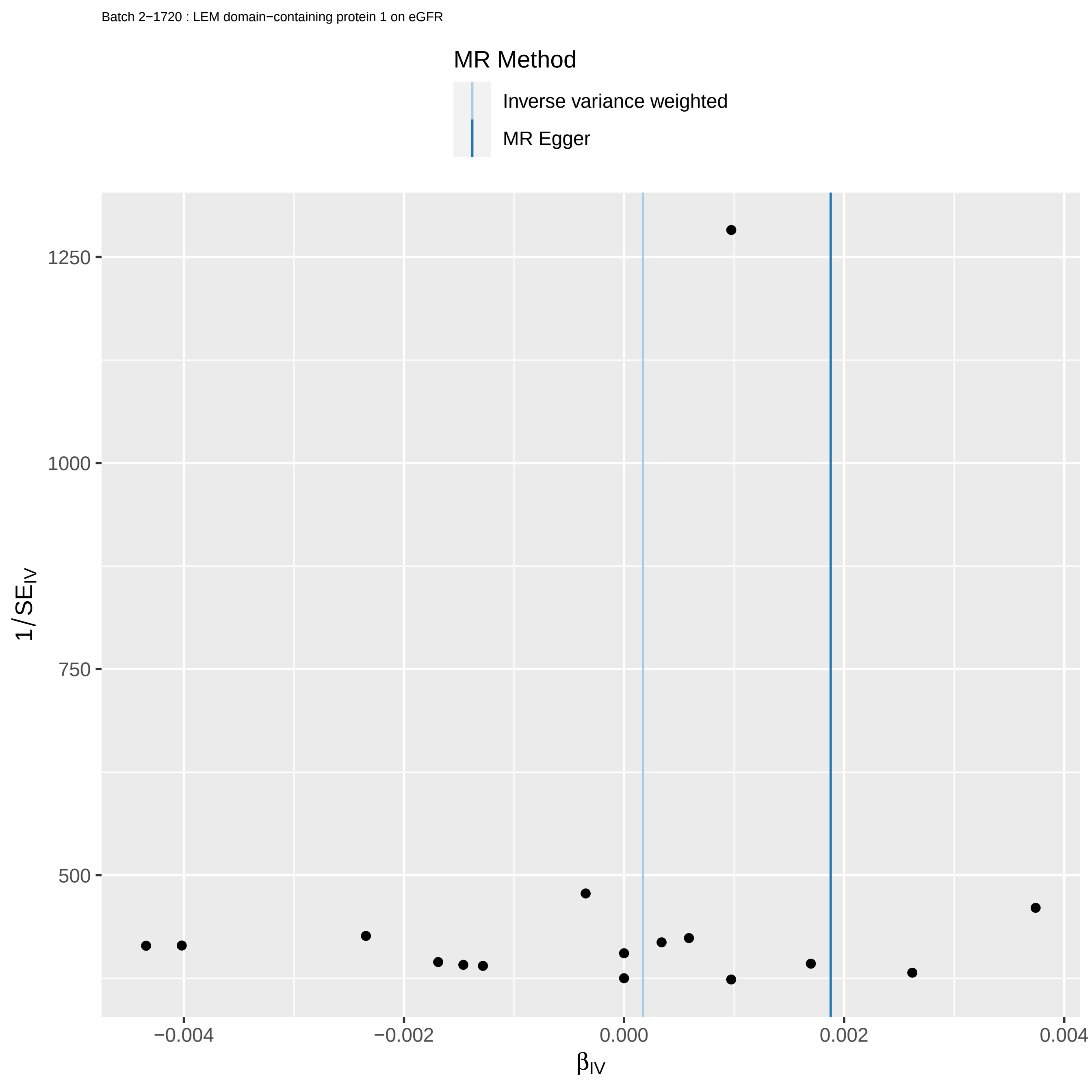

Batch 2−1720 : LEM domain−containing protein 1 on eGFR
MR Method
Inverse variance weighted
MR Egger
1250
1000
750
500
1/SE$_{IV}$
−0.004
−0.002
0.000
0.002
0.004
β_{IV}

Batch 2−1721 : Leptin on eGFR

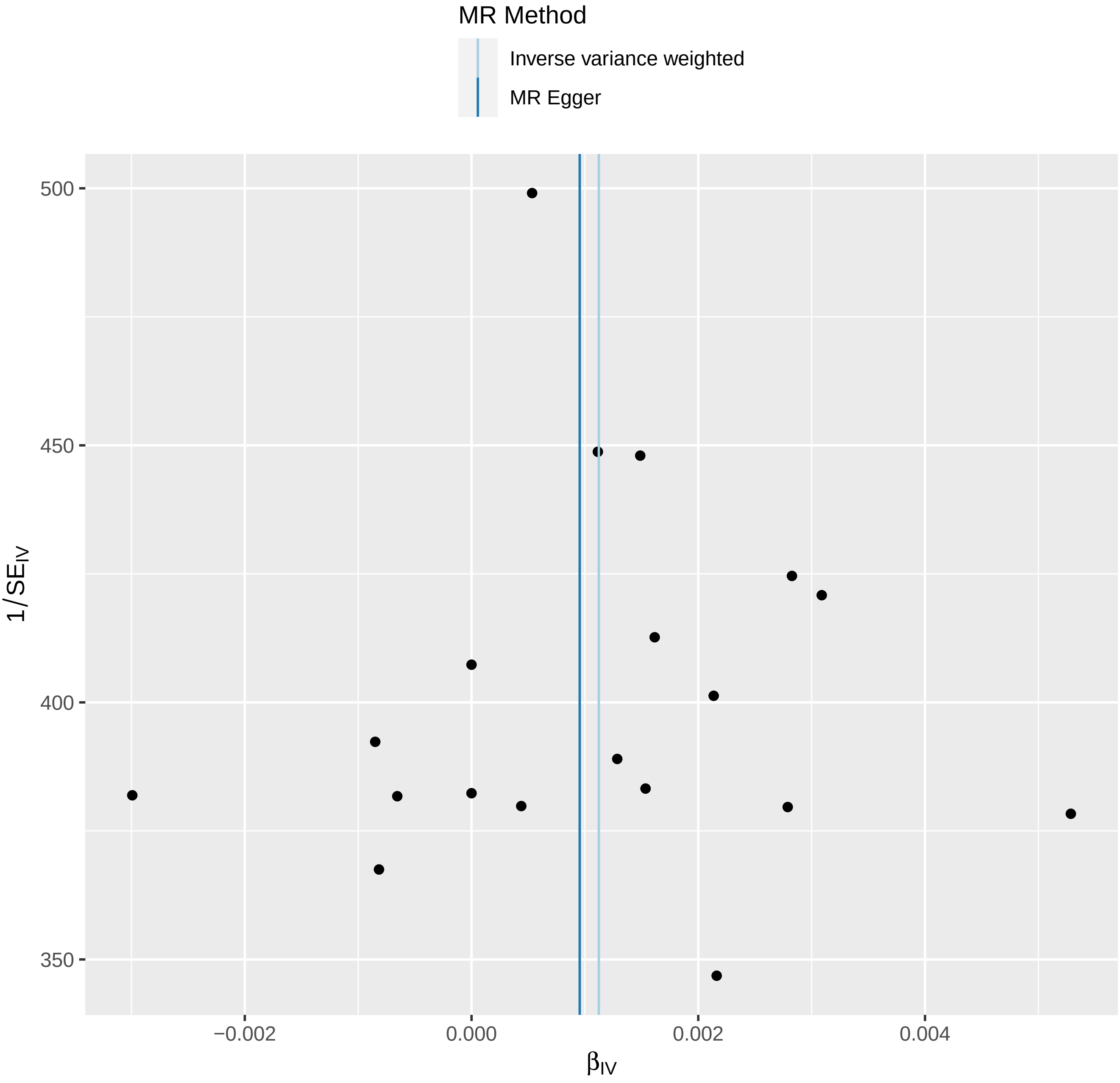

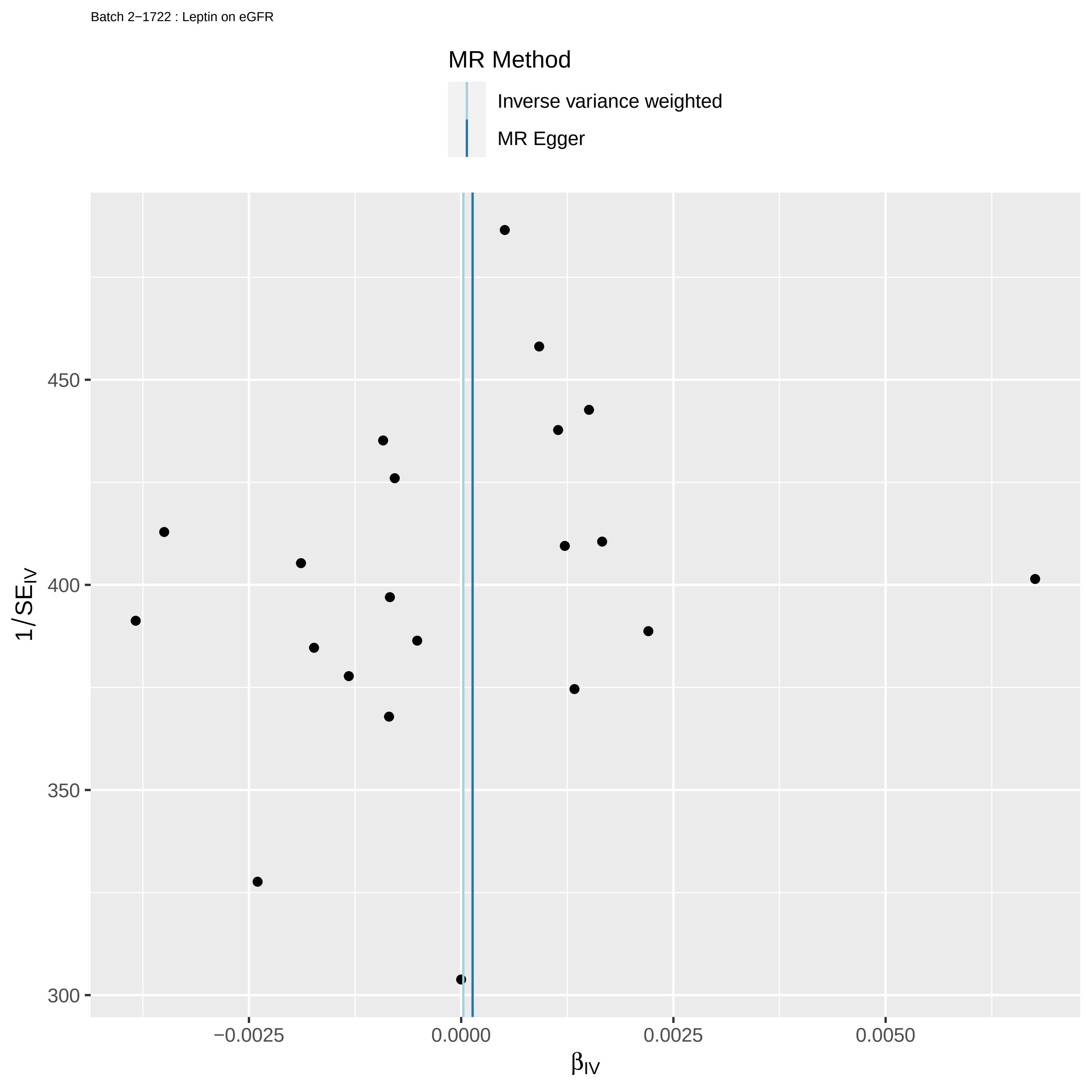
Batch 2−1722 : Leptin on eGFR
MR Method
Inverse variance weighted
MR Egger
450
400
350
300
1/SE_IV
−0.0025
0.0000
0.0025
0.0050
β_IV

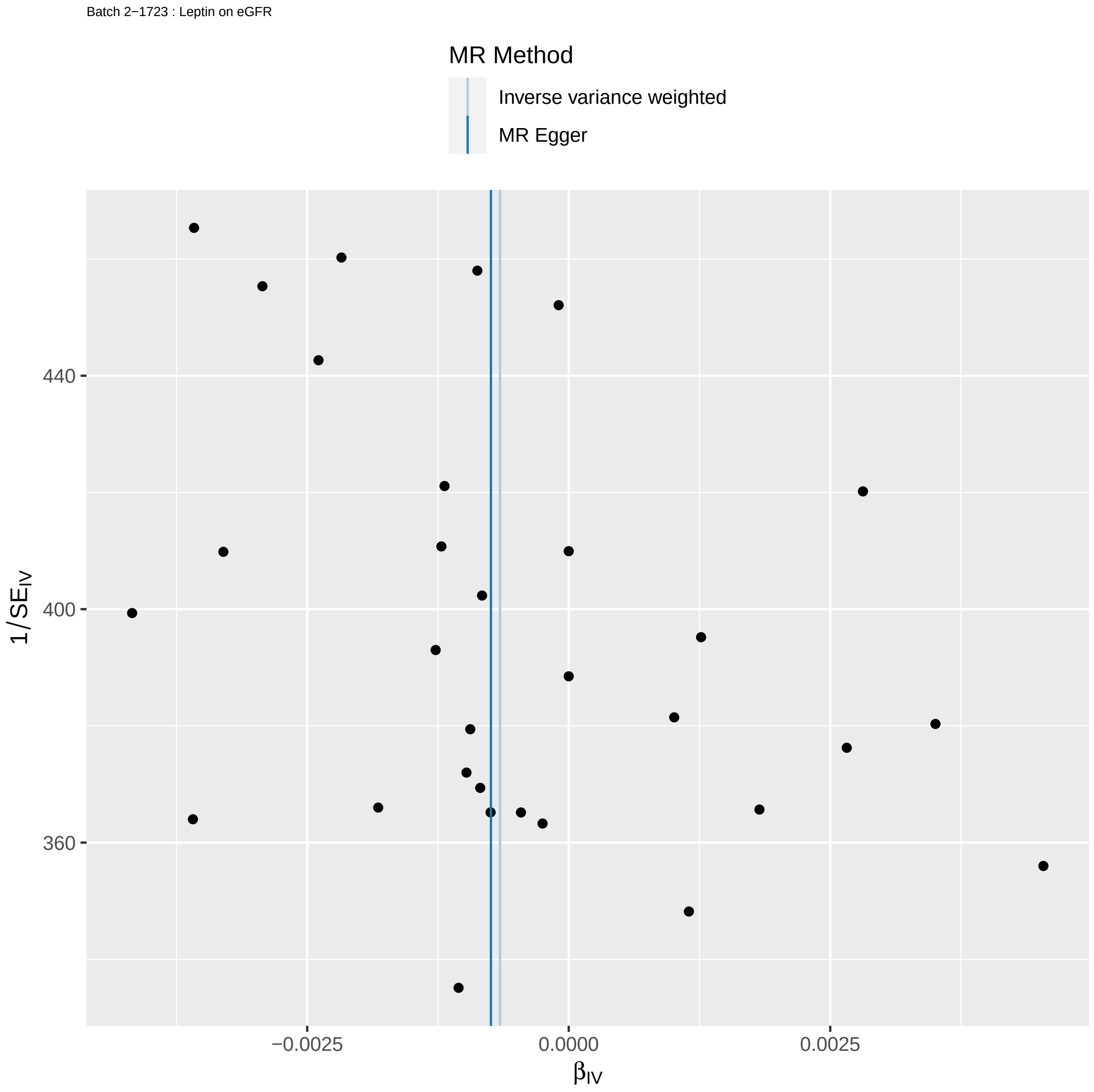
Batch 2−1723 : Leptin on eGFR
MR Method
Inverse variance weighted
MR Egger
440
400
360
−0.0025
0.0000
0.0025
$1/SE_{IV}$
β_{IV}

Batch 2−1724 : Leptin receptor on eGFR

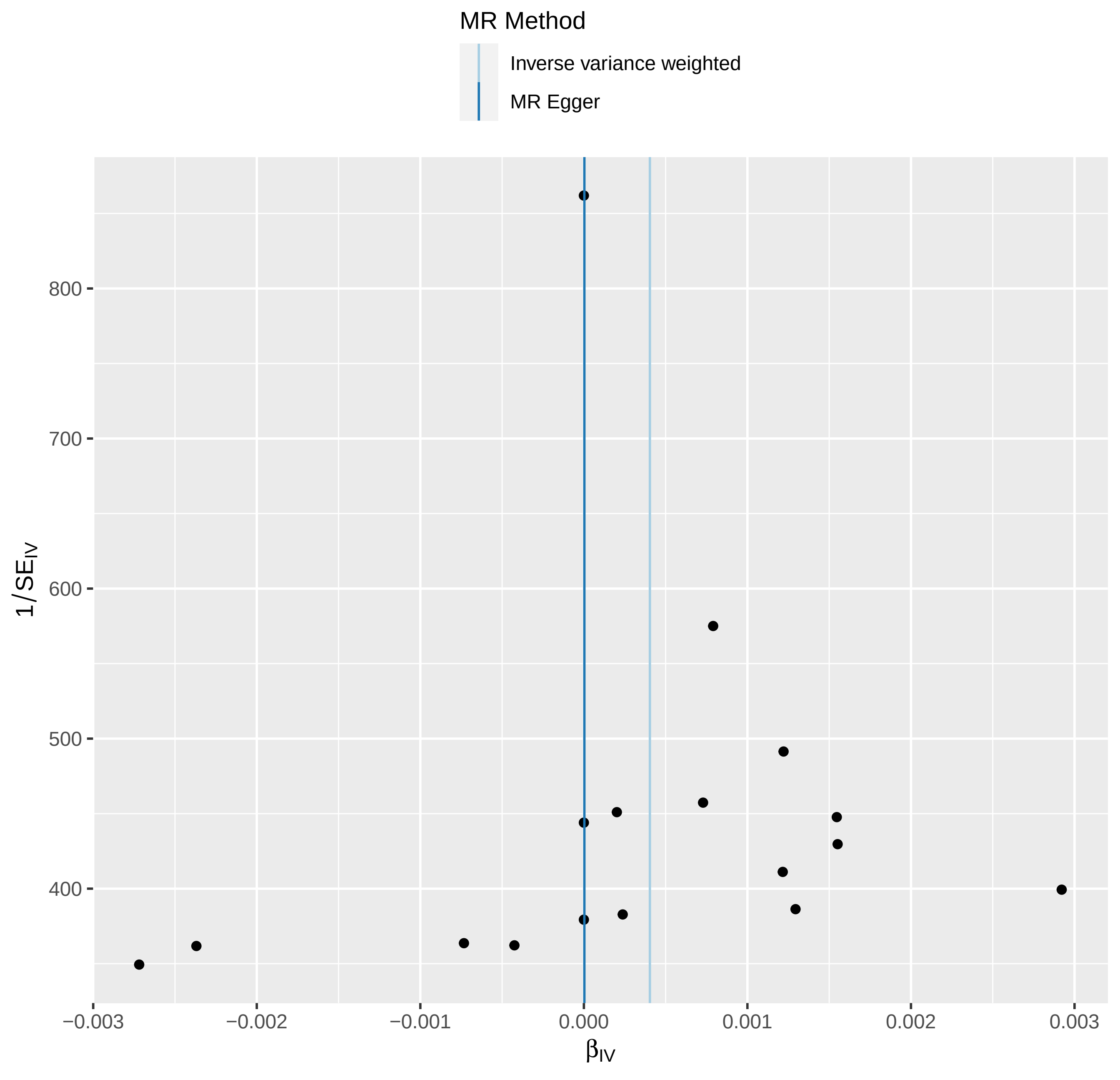

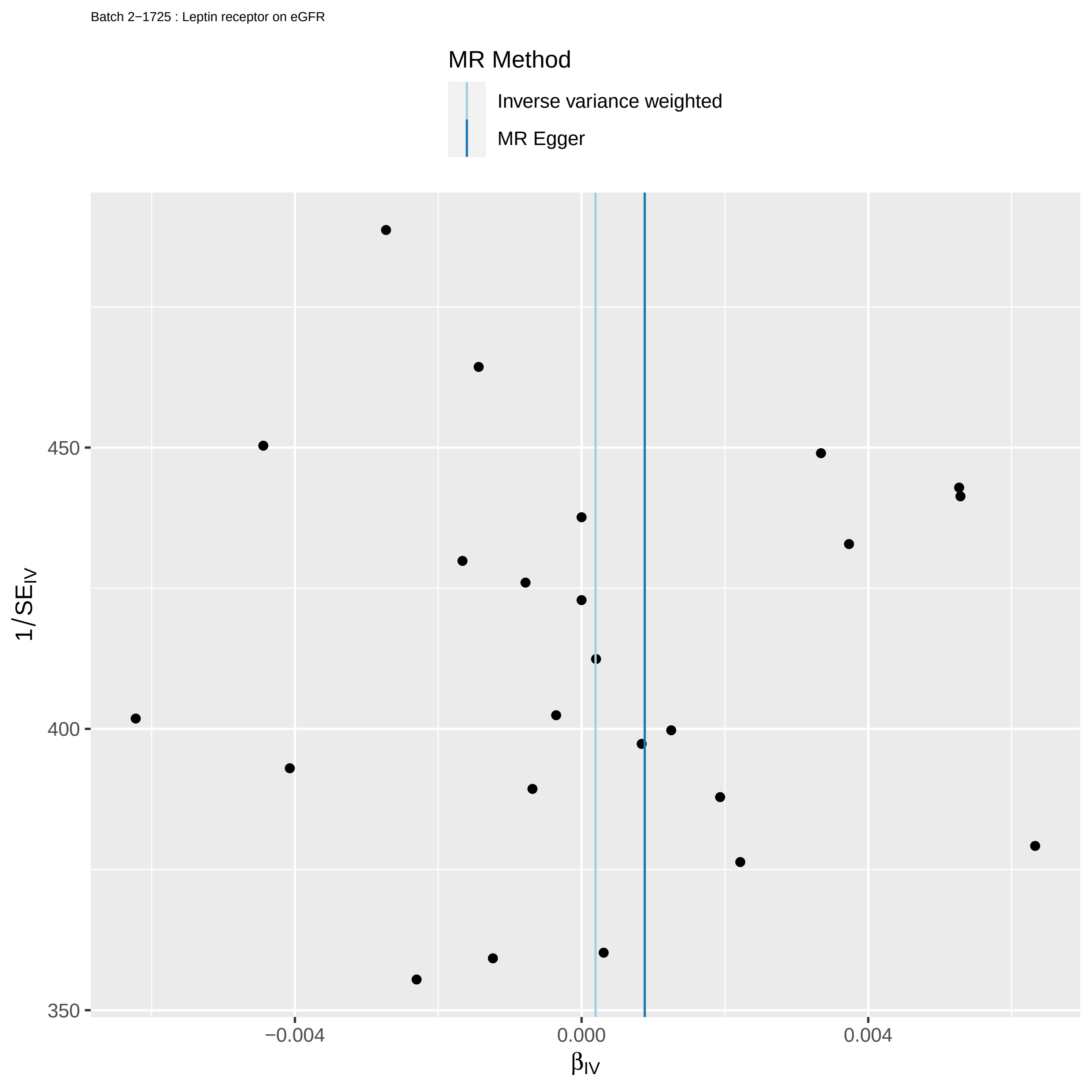
Batch 2−1725 : Leptin receptor on eGFR
MR Method
Inverse variance weighted
MR Egger
450
400
350
1/SE_IV
−0.004
0.000
0.004
β_IV

Batch 2−1726 : Galectin−1 on eGFR

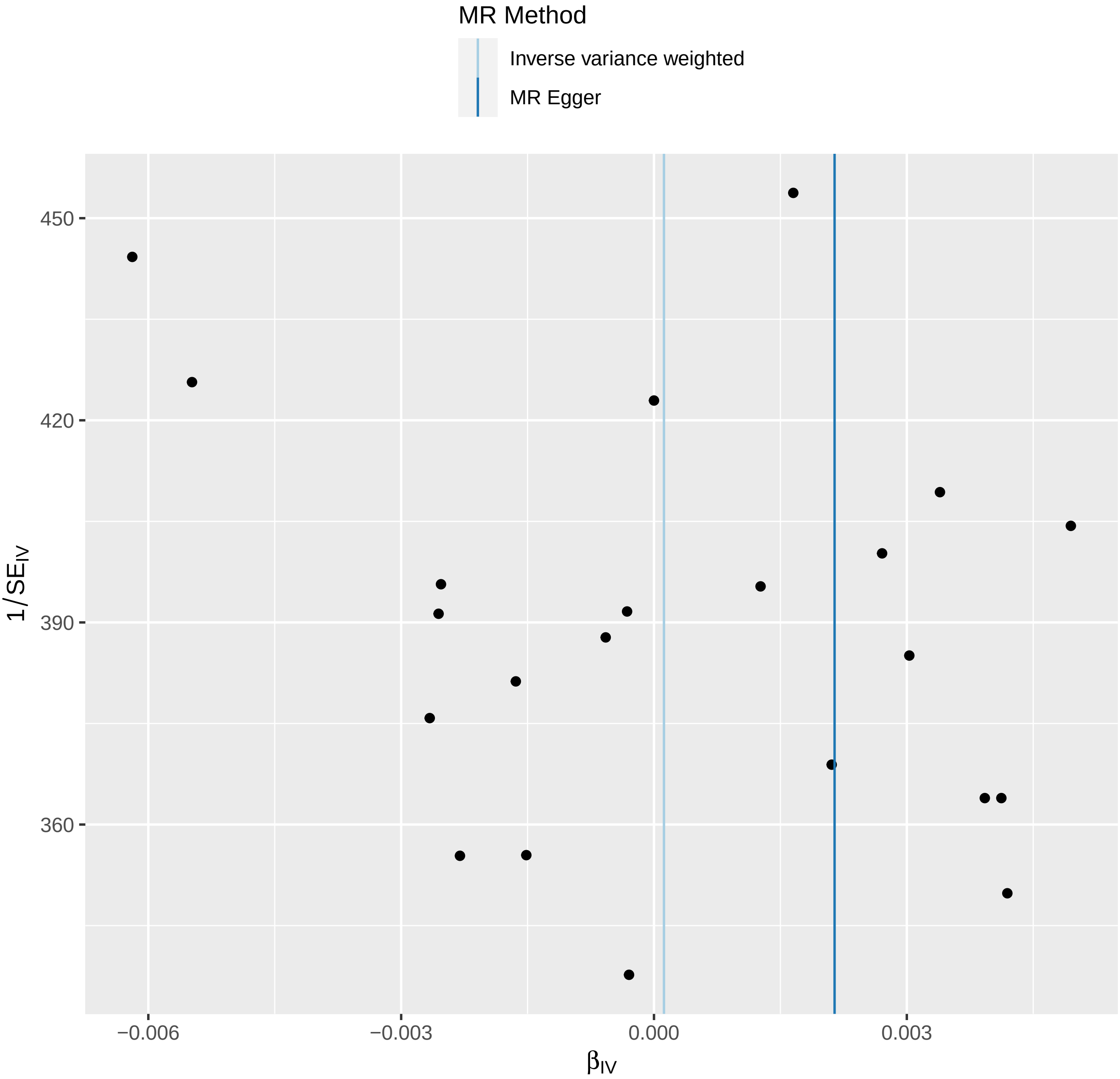

Batch 2−1727 : Galectin−2 on eGFR

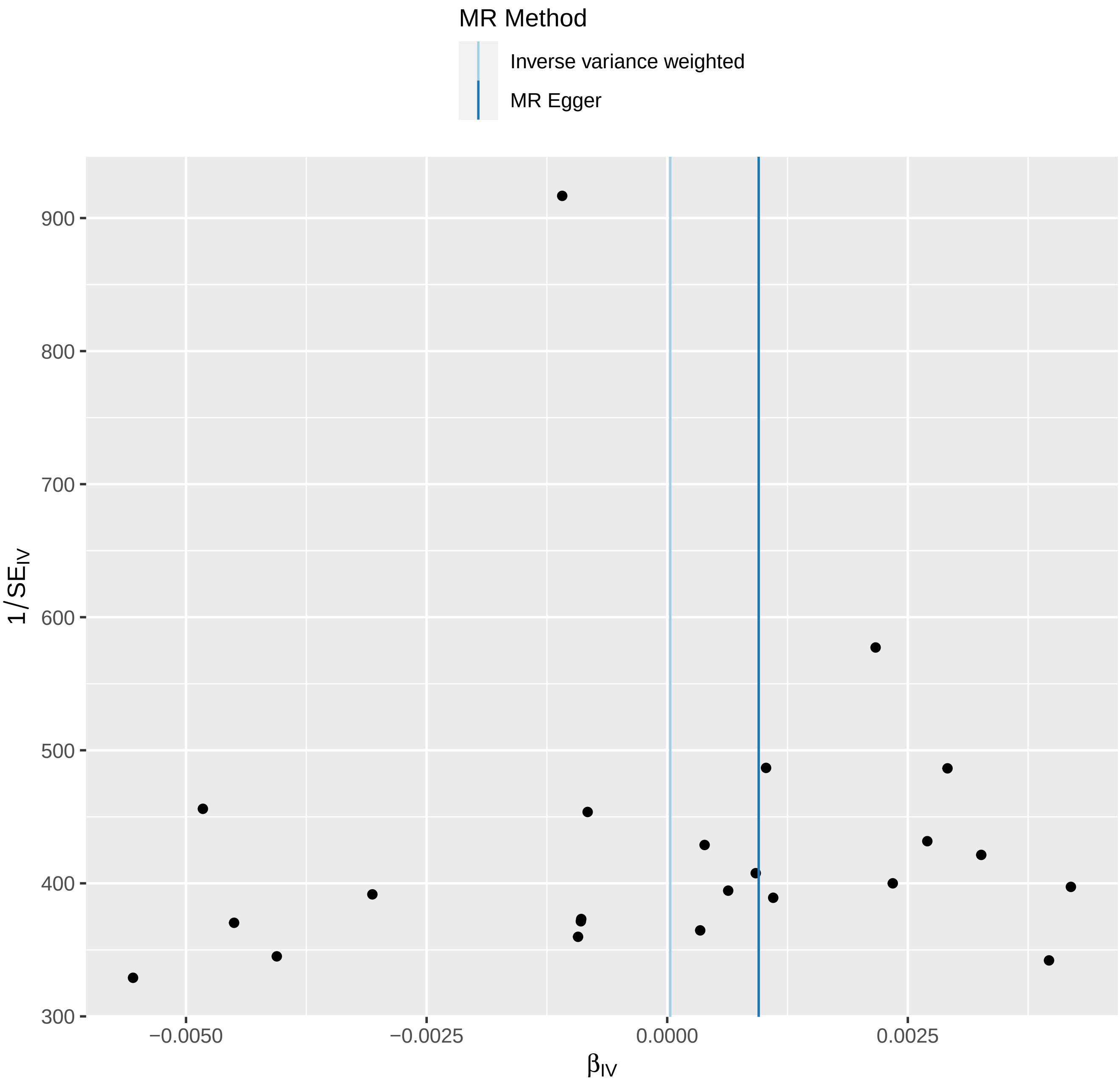

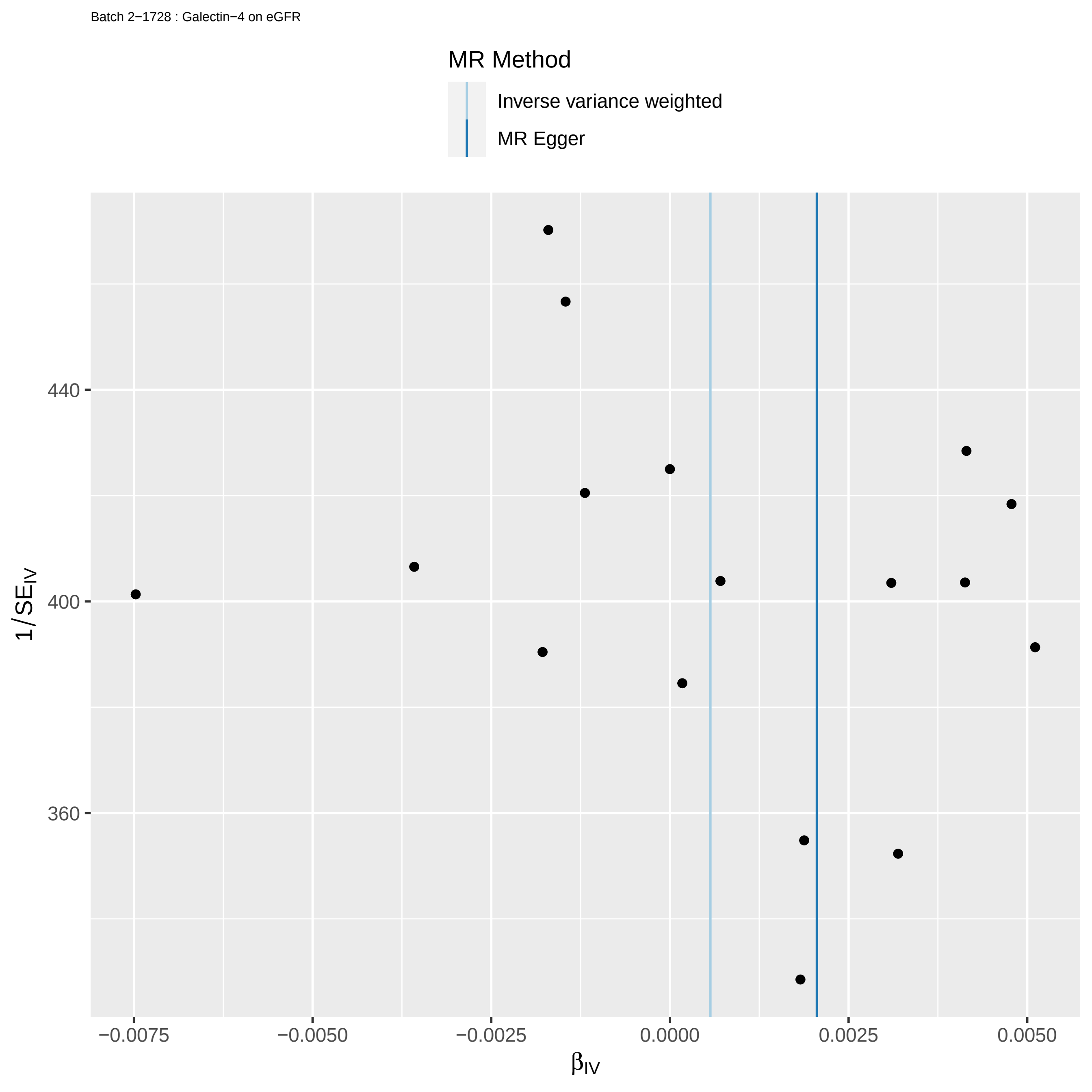
Batch 2−1728 : Galectin−4 on eGFR
MR Method
Inverse variance weighted
MR Egger
440
400
360
1/SE_IV
−0.0075
−0.0050
−0.0025
0.0000
0.0025
0.0050
β_IV

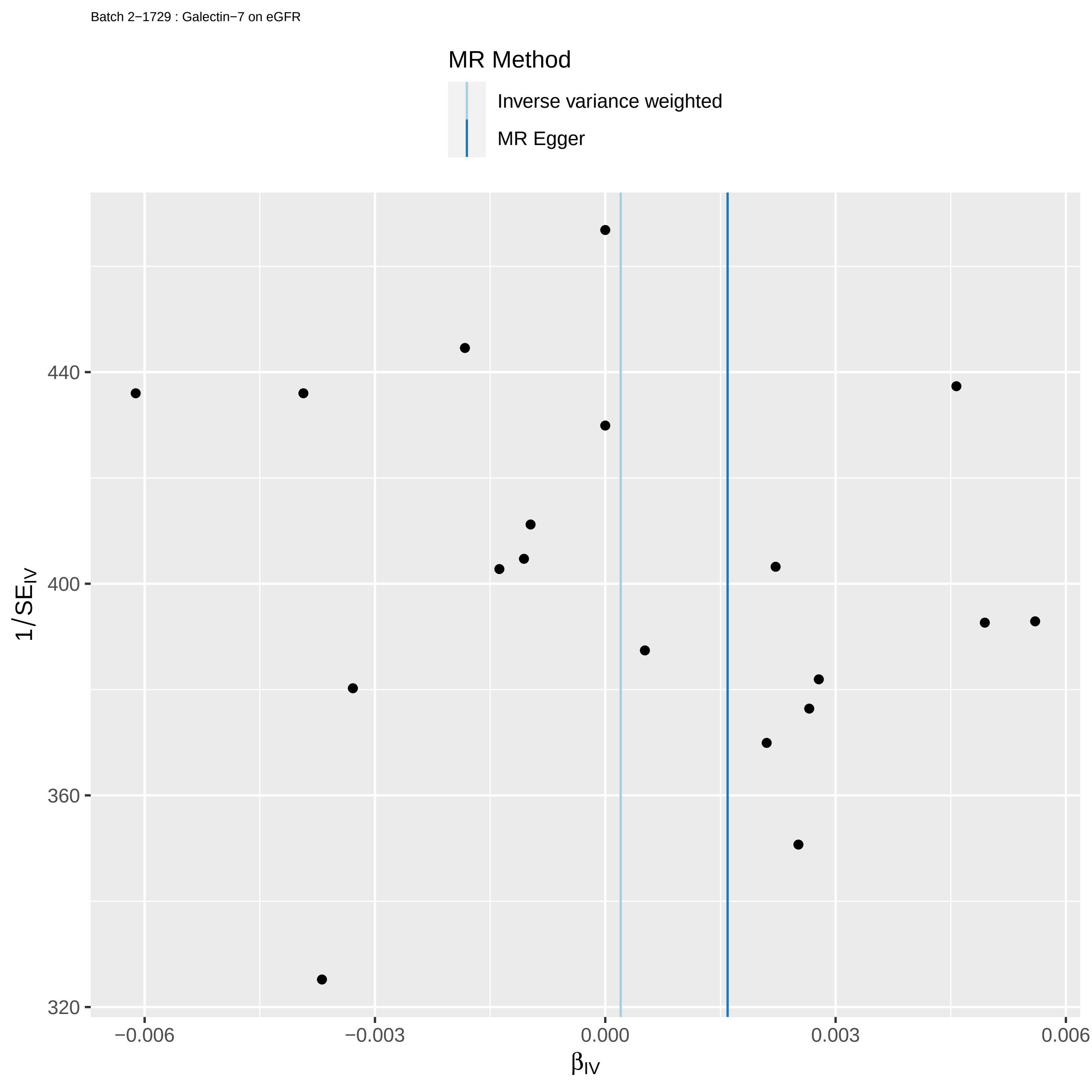

Batch 2−1729 : Galectin−7 on eGFR
MR Method
Inverse variance weighted
MR Egger
440
400
360
320
1/SE$_{IV}$
−0.006
−0.003
0.000
0.003
0.006
β$_{IV}$

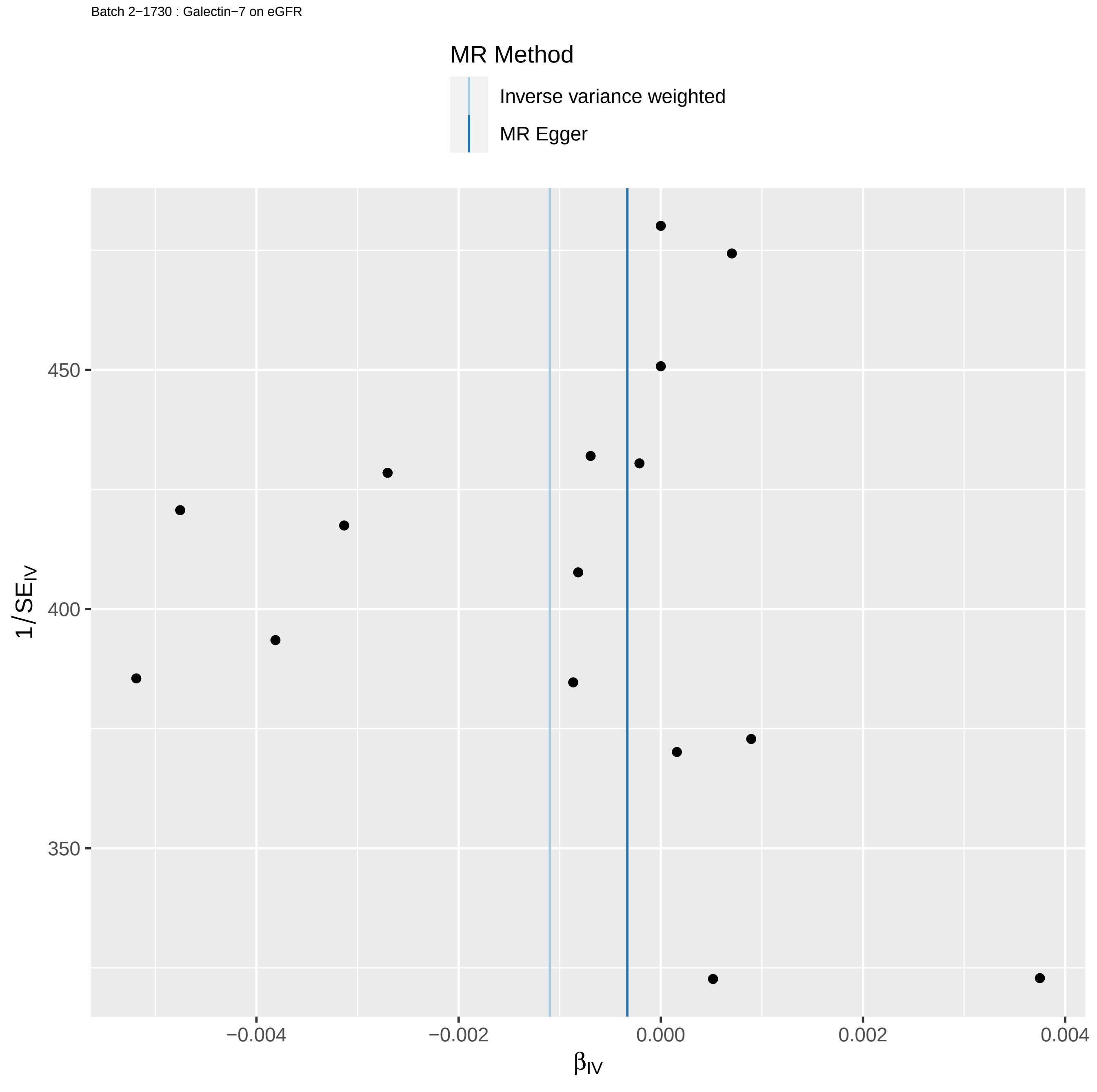
Batch 2−1730 : Galectin−7 on eGFR
MR Method
Inverse variance weighted
MR Egger
450
400
350
1/SE$_{IV}$
−0.004
−0.002
0.000
0.002
0.004
β$_{IV}$

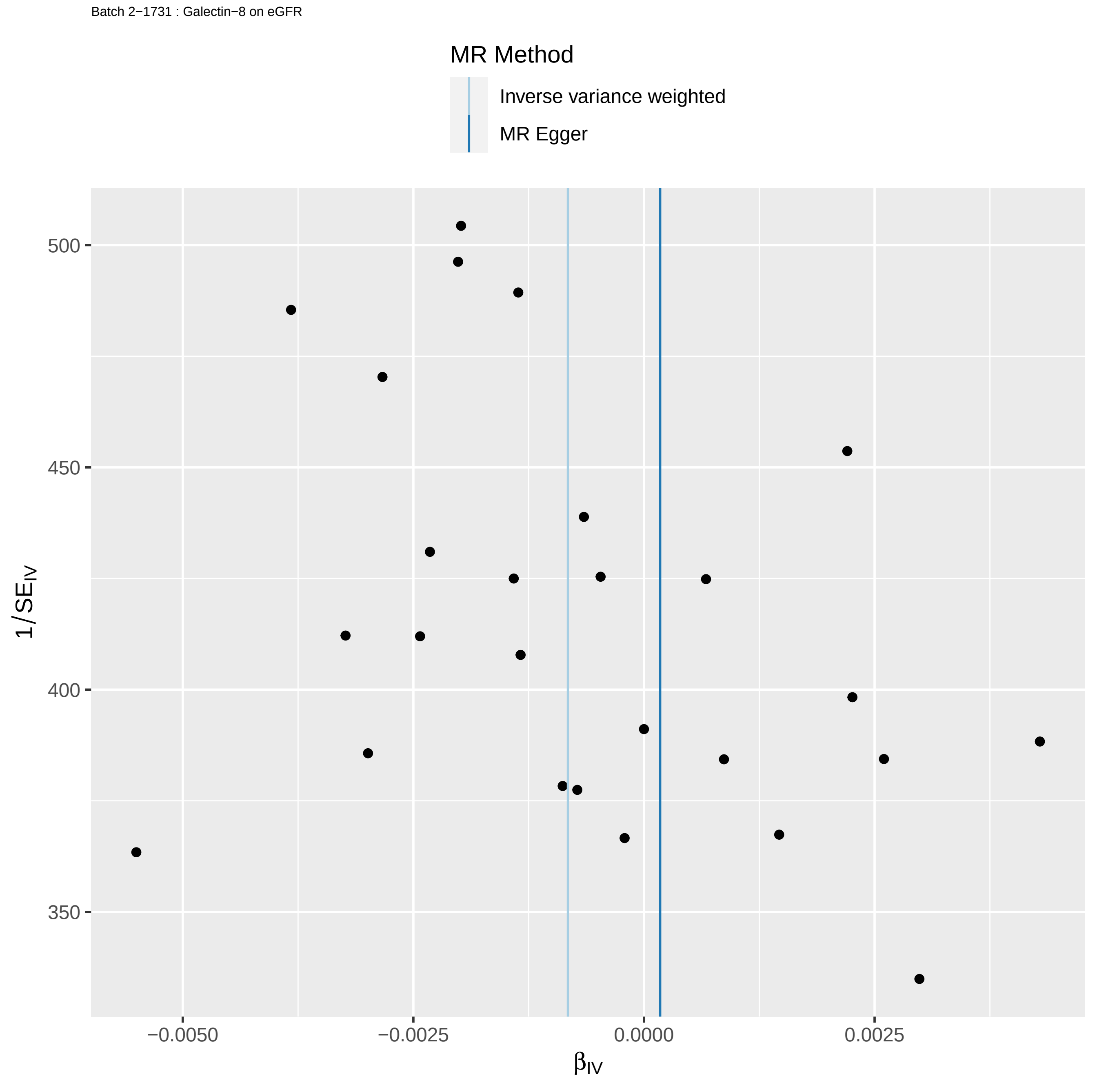

Batch 2−1731 : Galectin−8 on eGFR
MR Method
Inverse variance weighted
MR Egger
500
450
400
350
1/SE_IV
−0.0050
−0.0025
0.0000
0.0025
β_IV

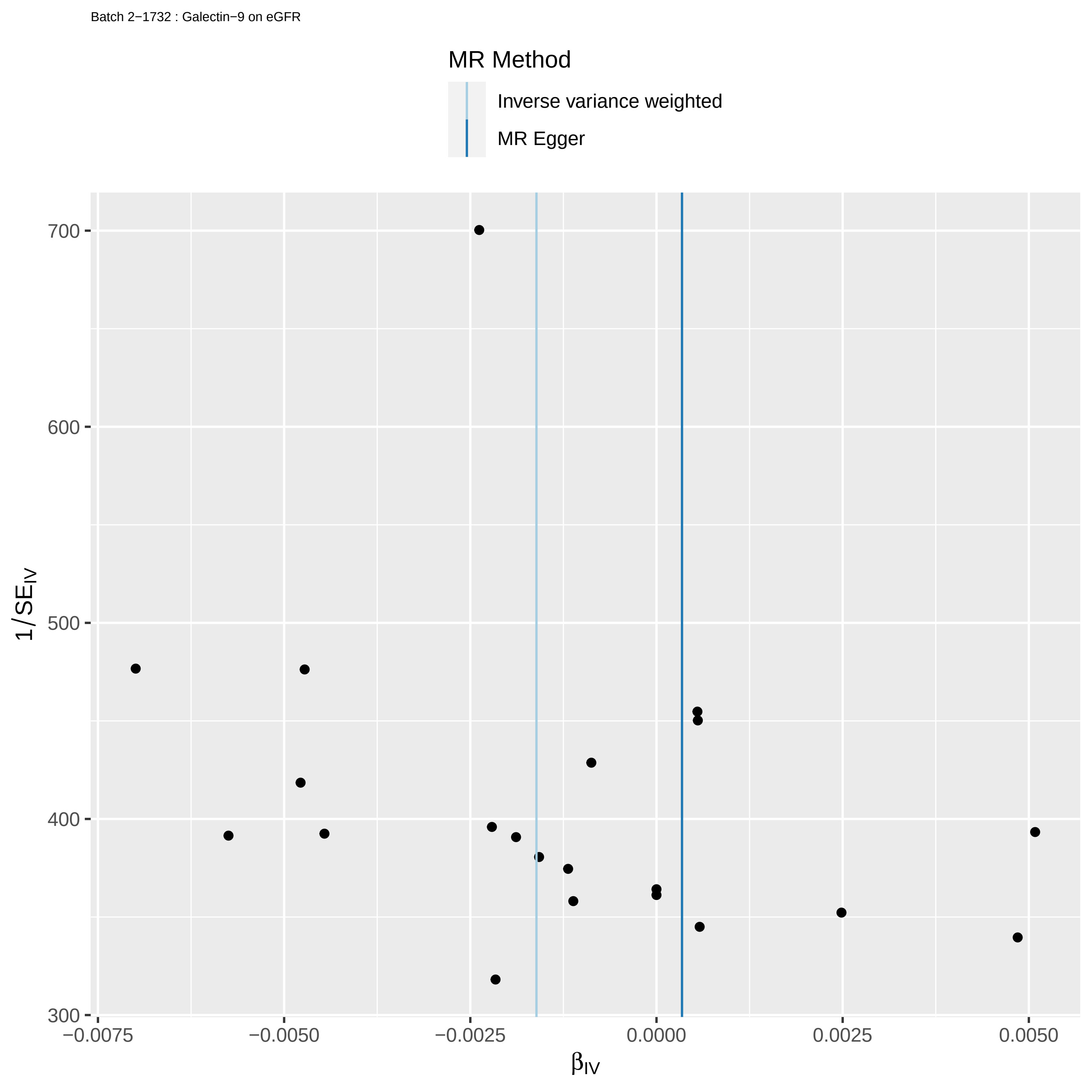

Batch 2−1732 : Galectin−9 on eGFR
MR Method
Inverse variance weighted
MR Egger
700
600
500
400
300
1/SE_IV
−0.0075
−0.0050
−0.0025
0.0000
0.0025
0.0050
β_IV

Batch 2−1733 : Leucine−rich repeat LGI family member 3 on eGFR

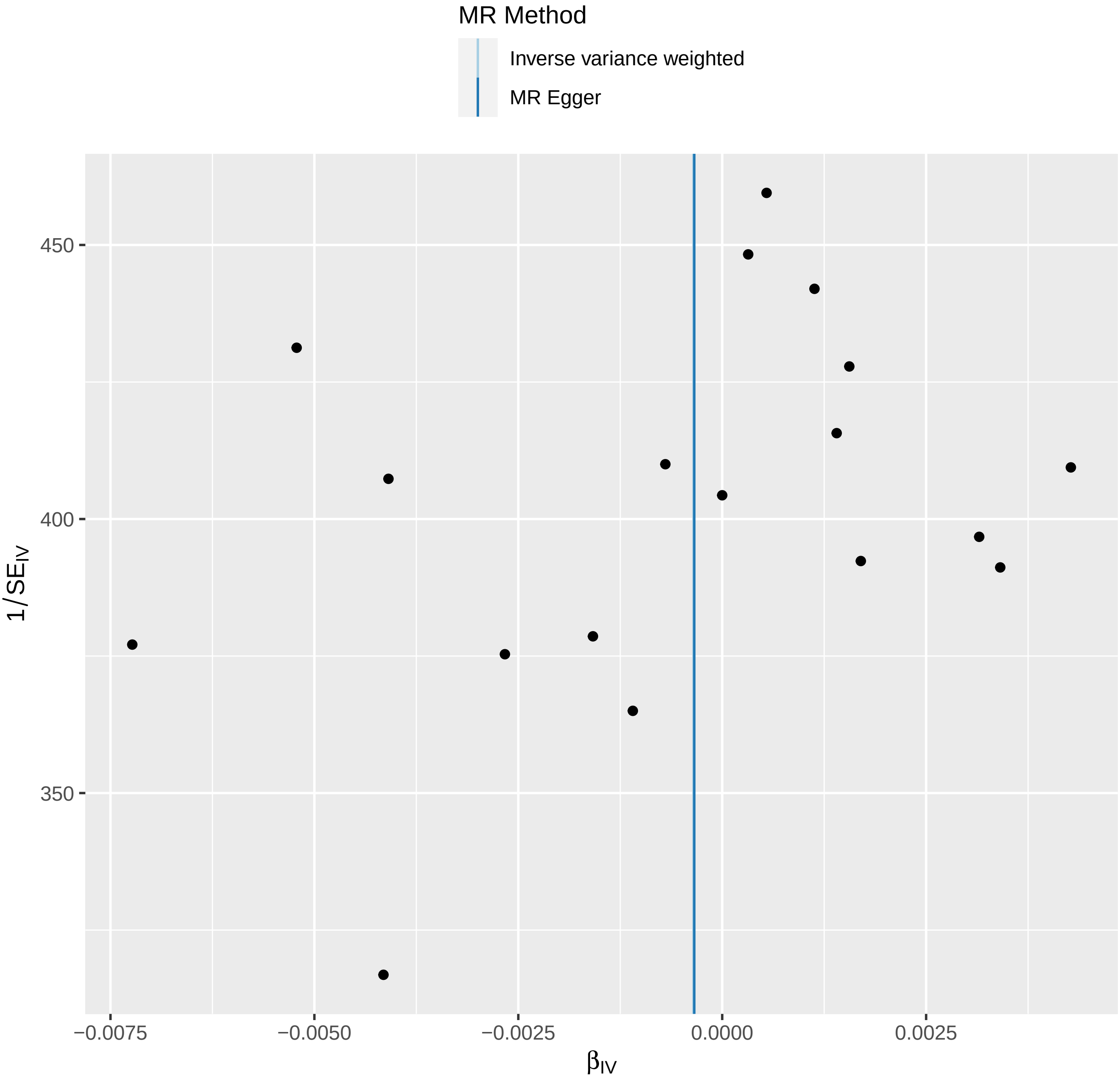

Batch 2−1734 : Legumain on eGFR

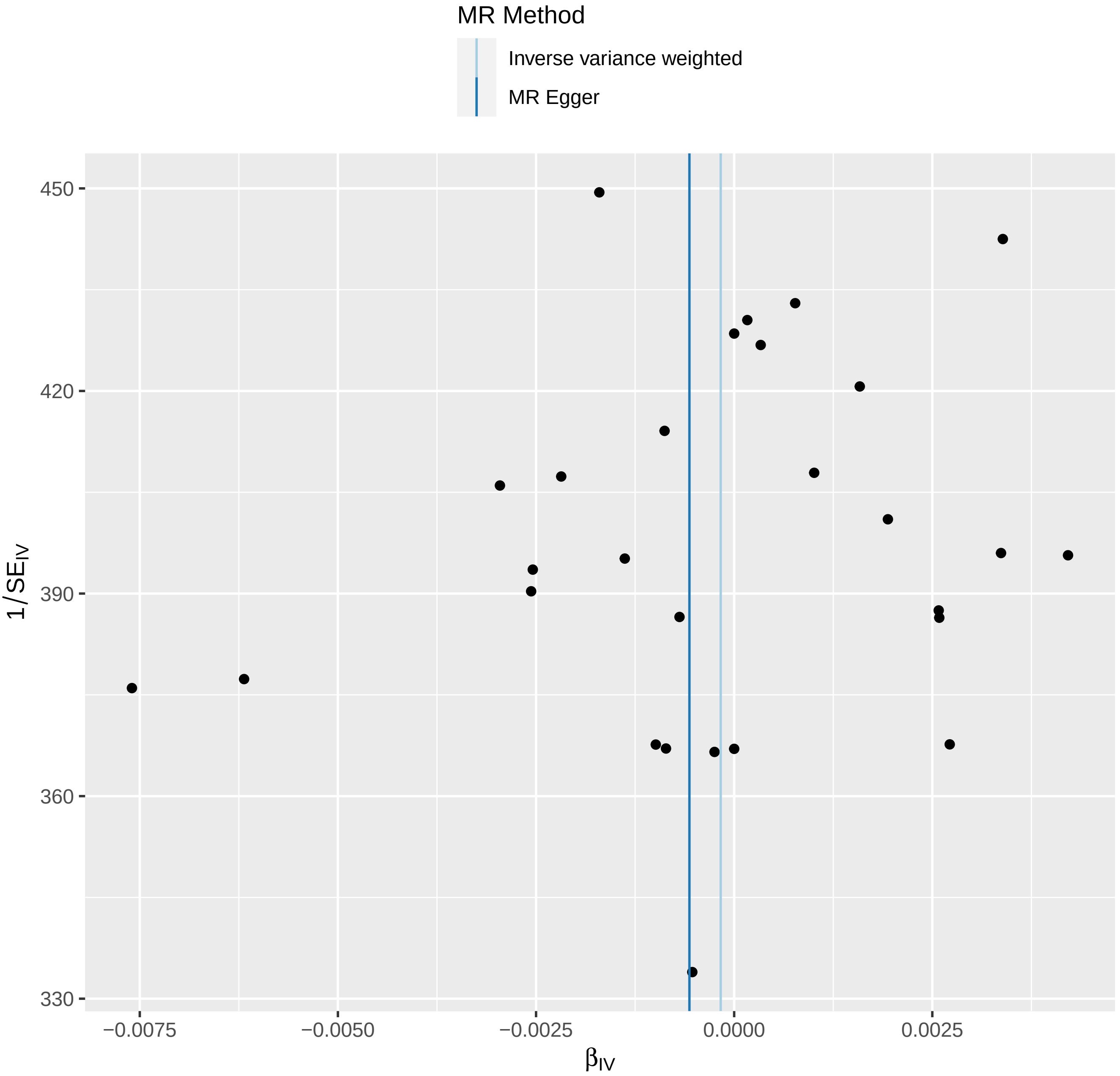

Batch 2−1735 : Lutropin subunit beta on eGFR

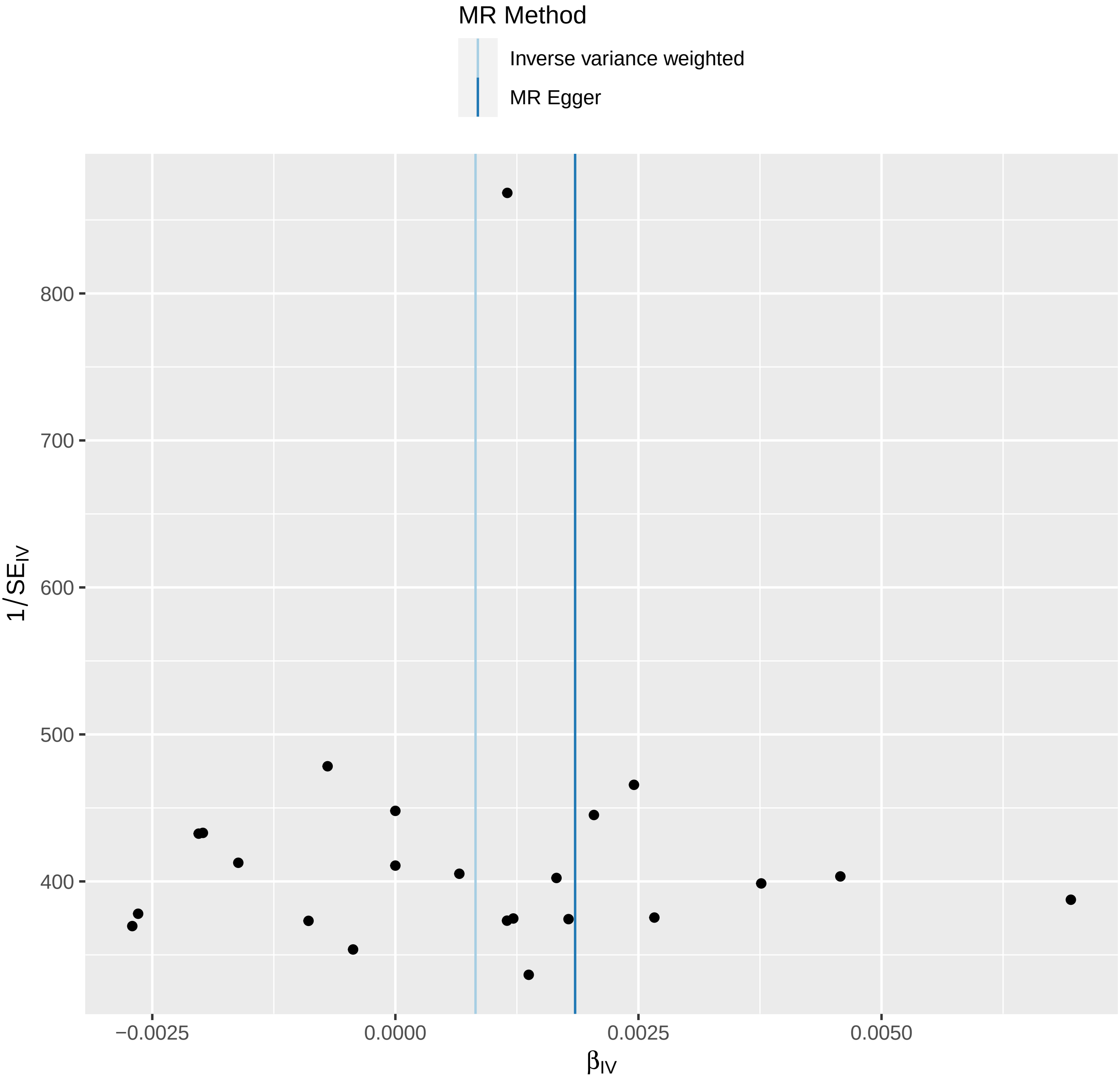

Batch 2−1736 : Leukemia inhibitory factor on eGFR

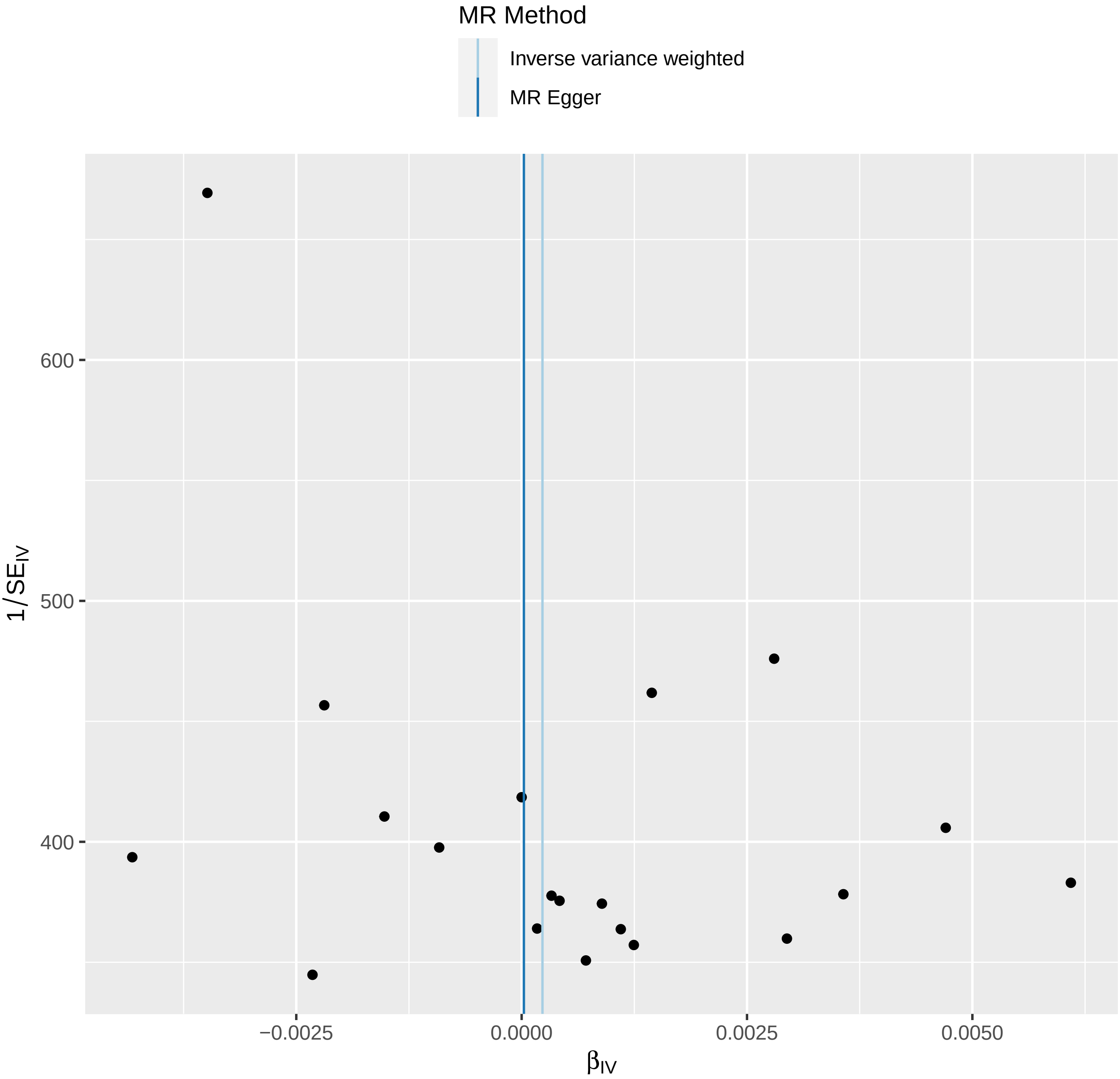

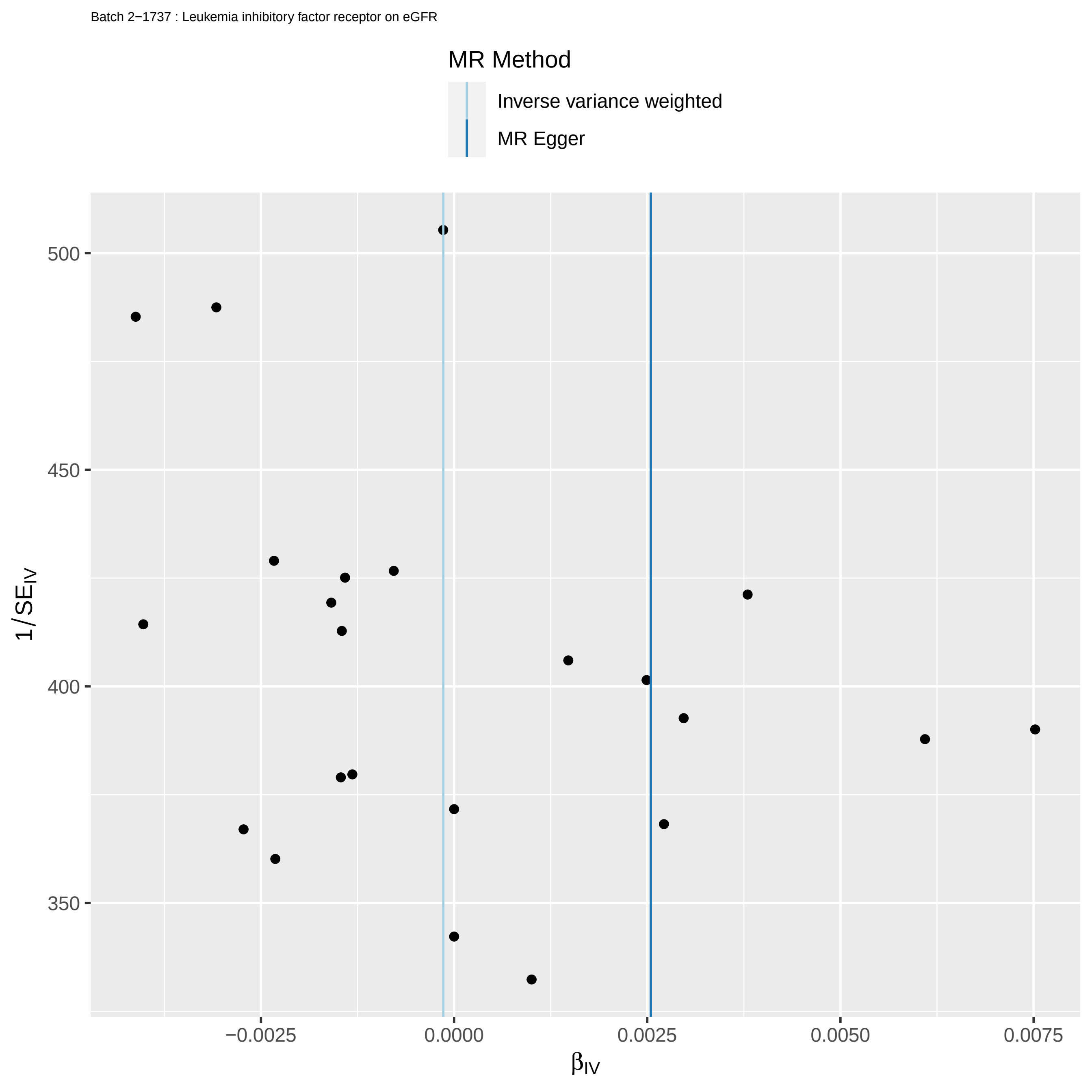
Batch 2−1737 : Leukemia inhibitory factor receptor on eGFR
MR Method
Inverse variance weighted
MR Egger
500
450
400
350
1/SE_IV
−0.0025
0.0000
0.0025
0.0050
0.0075
β_IV

Batch 2−1738 : Leukocyte immunoglobulin−like receptor subfamily A member 4 on eGFR

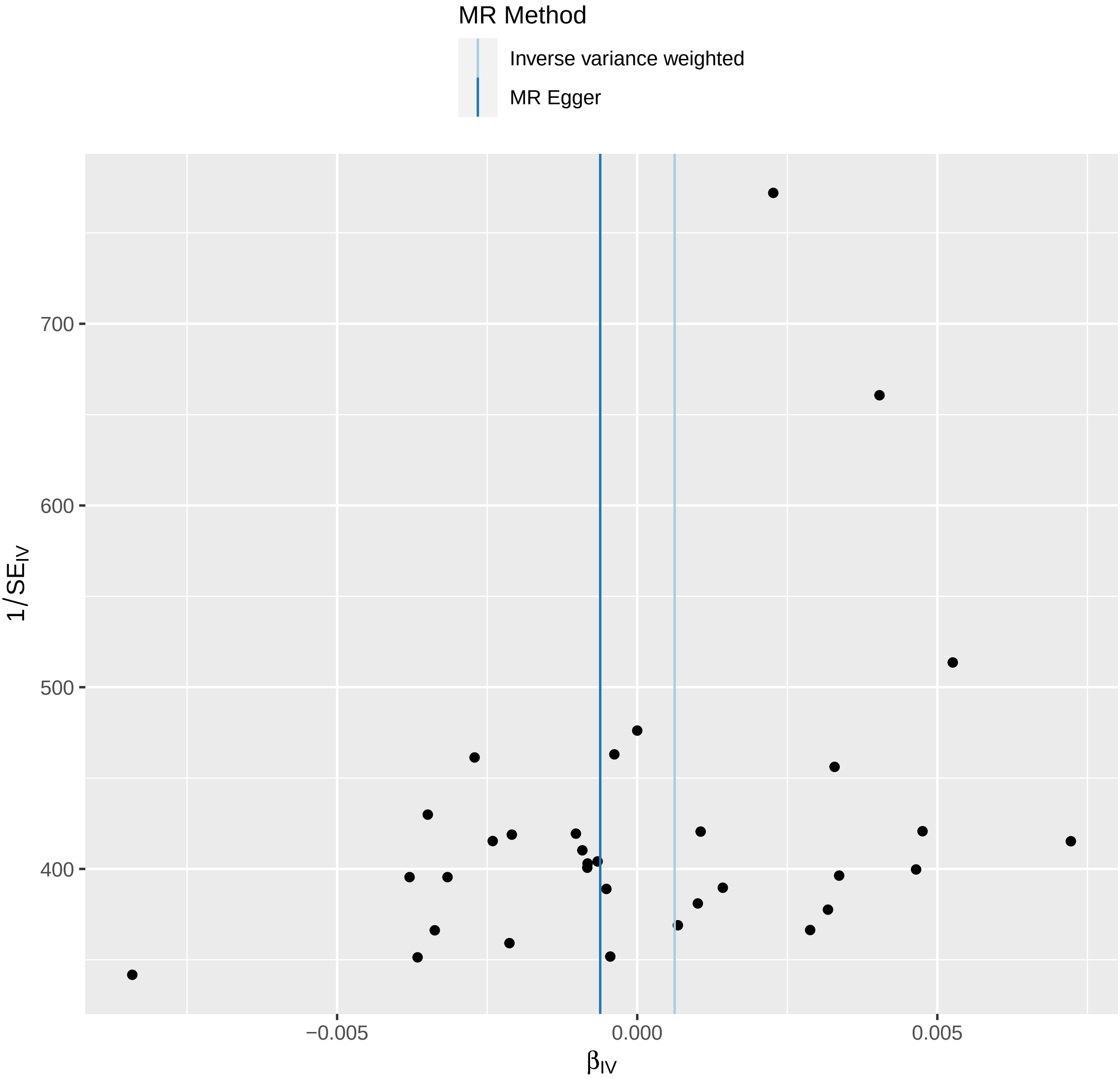

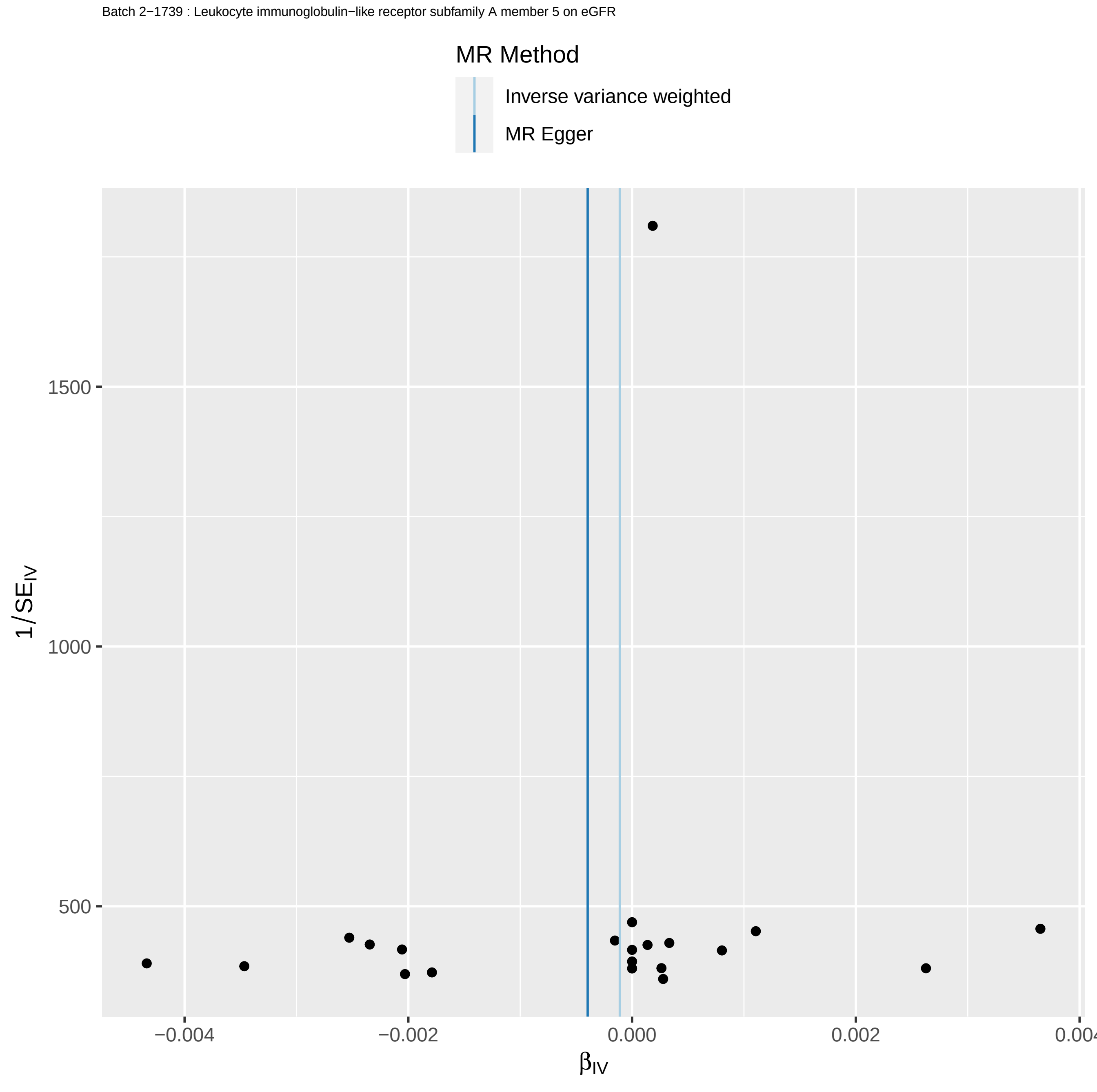

Batch 2−1739 : Leukocyte immunoglobulin−like receptor subfamily A member 5 on eGFR
MR Method
Inverse variance weighted
MR Egger
1500
1000
500
1/SE$_{IV}$
−0.004
−0.002
0.000
0.002
0.004
β$_{IV}$

Batch 2−1740 : Leukocyte immunoglobulin−like receptor subfamily A member 5 on eGFR

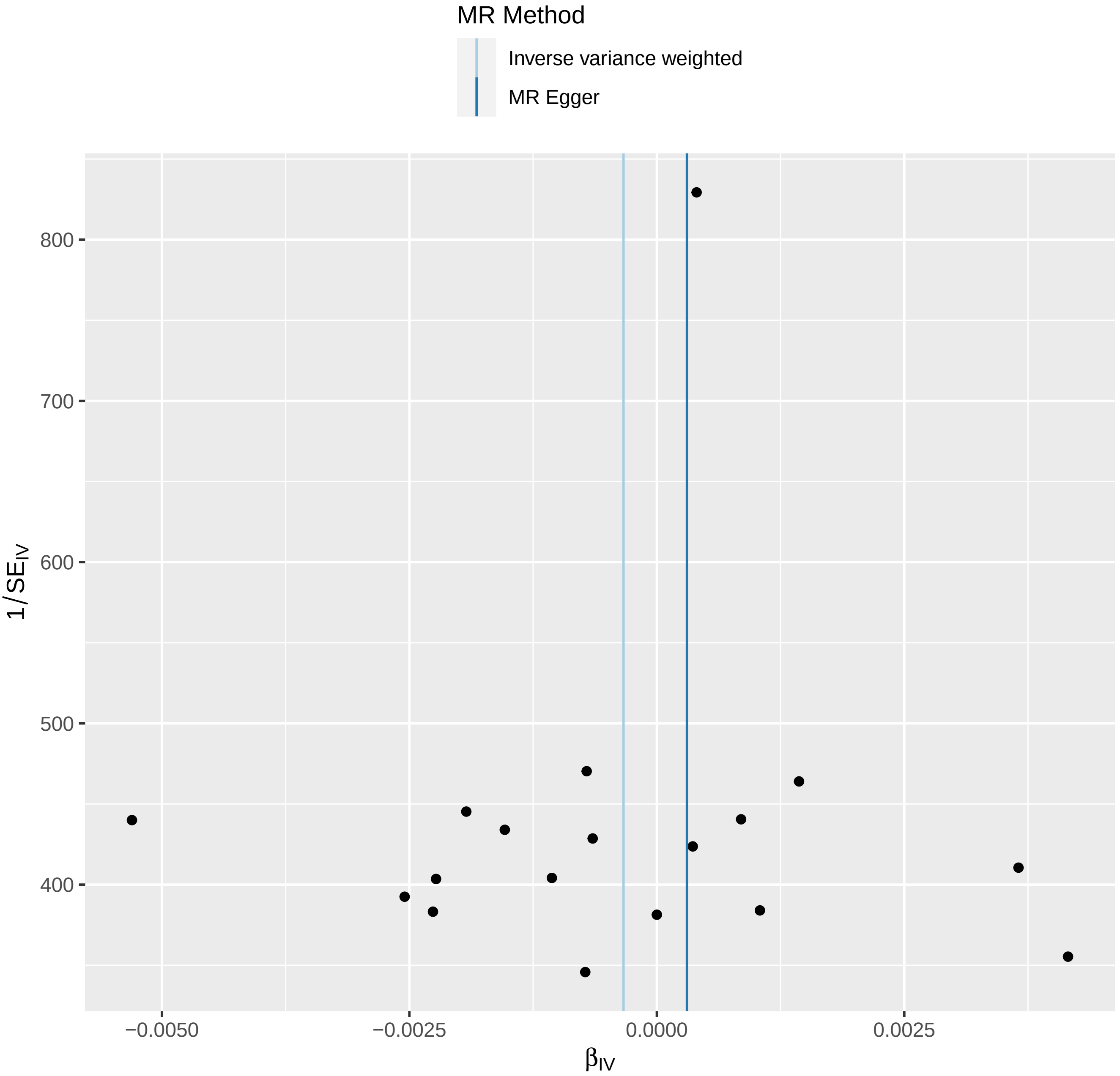

Batch 2−1741 : Leukocyte immunoglobulin−like receptor subfamily A member 6 on eGFR

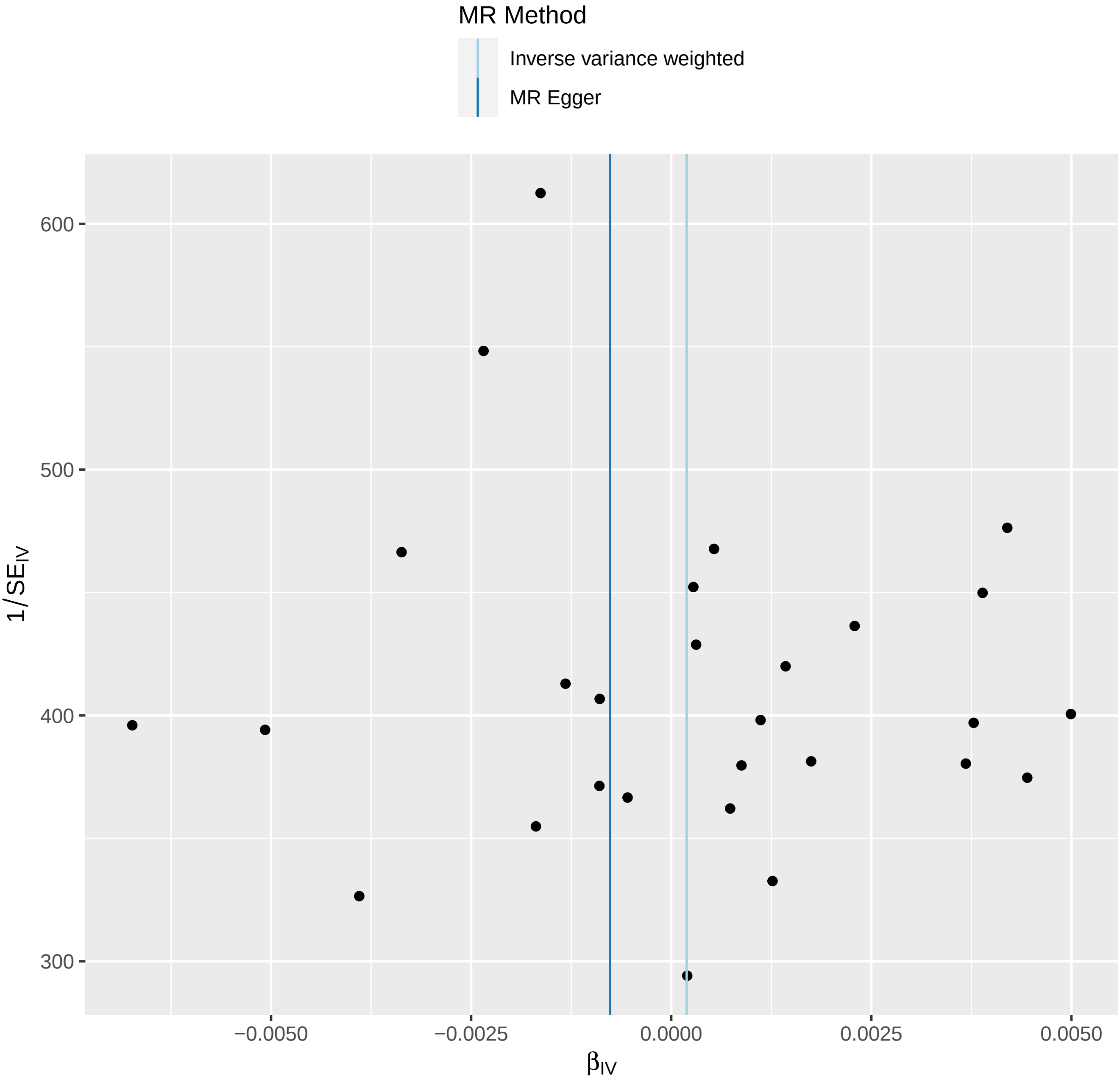

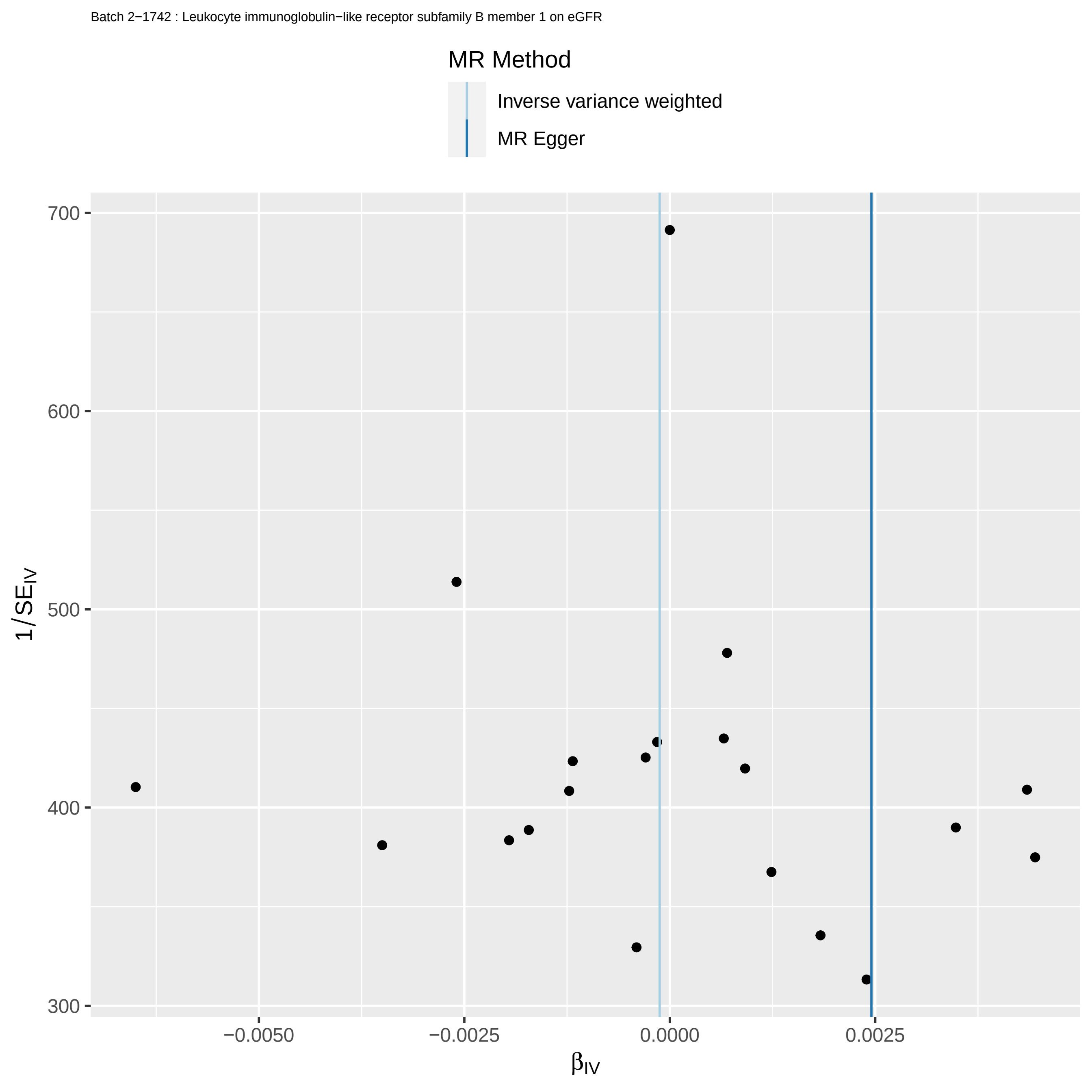

Batch 2−1742 : Leukocyte immunoglobulin−like receptor subfamily B member 1 on eGFR
MR Method
Inverse variance weighted
MR Egger
700
600
500
400
300
1/SE_IV
−0.0050
−0.0025
0.0000
0.0025
β_IV

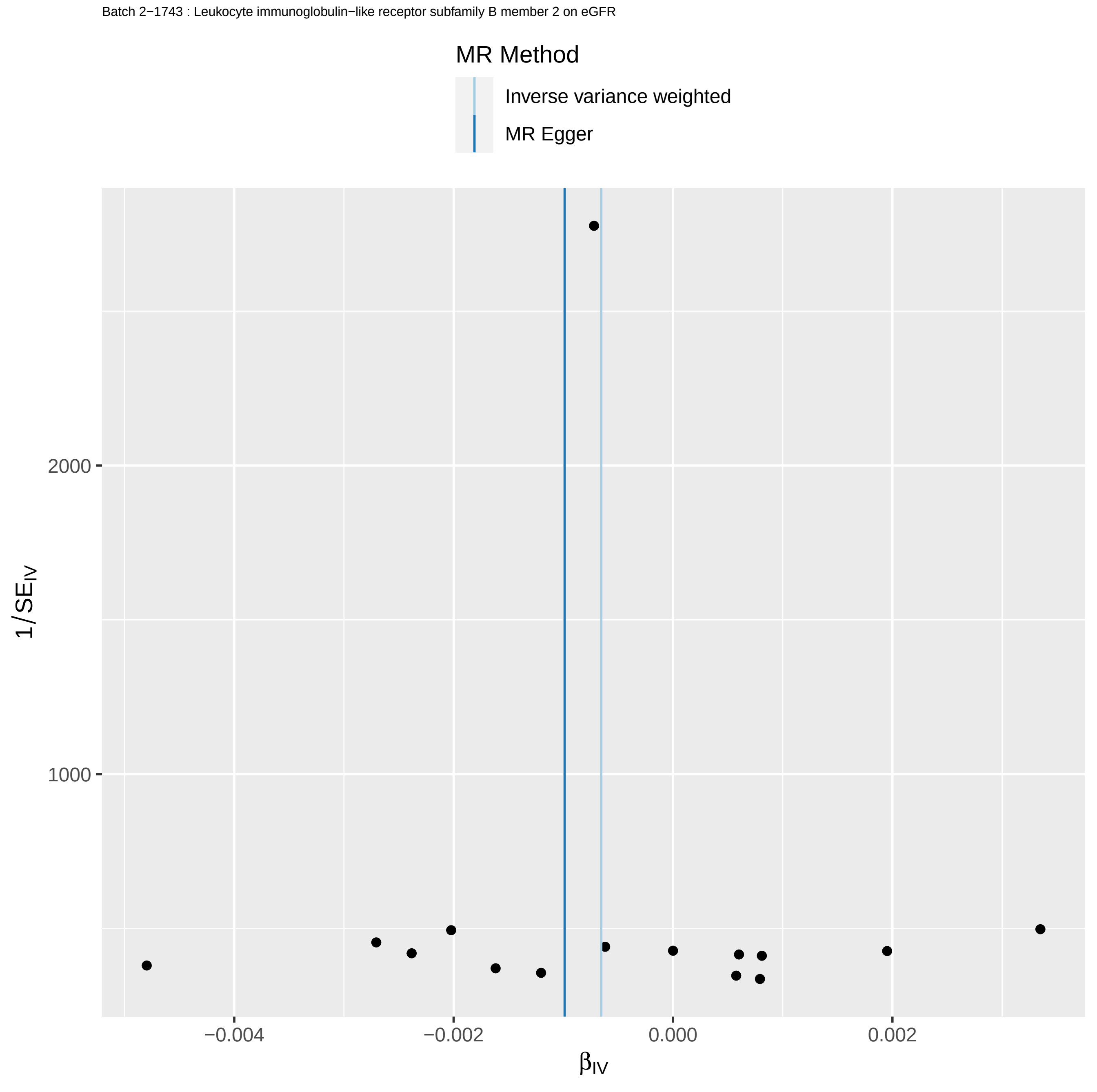
Batch 2−1743 : Leukocyte immunoglobulin−like receptor subfamily B member 2 on eGFR
MR Method
Inverse variance weighted
MR Egger
2000
1000
1/SE_IV
−0.004
−0.002
0.000
0.002
β_IV

Batch 2−1744 : Leukocyte immunoglobulin−like receptor subfamily B member 2 on eGFR

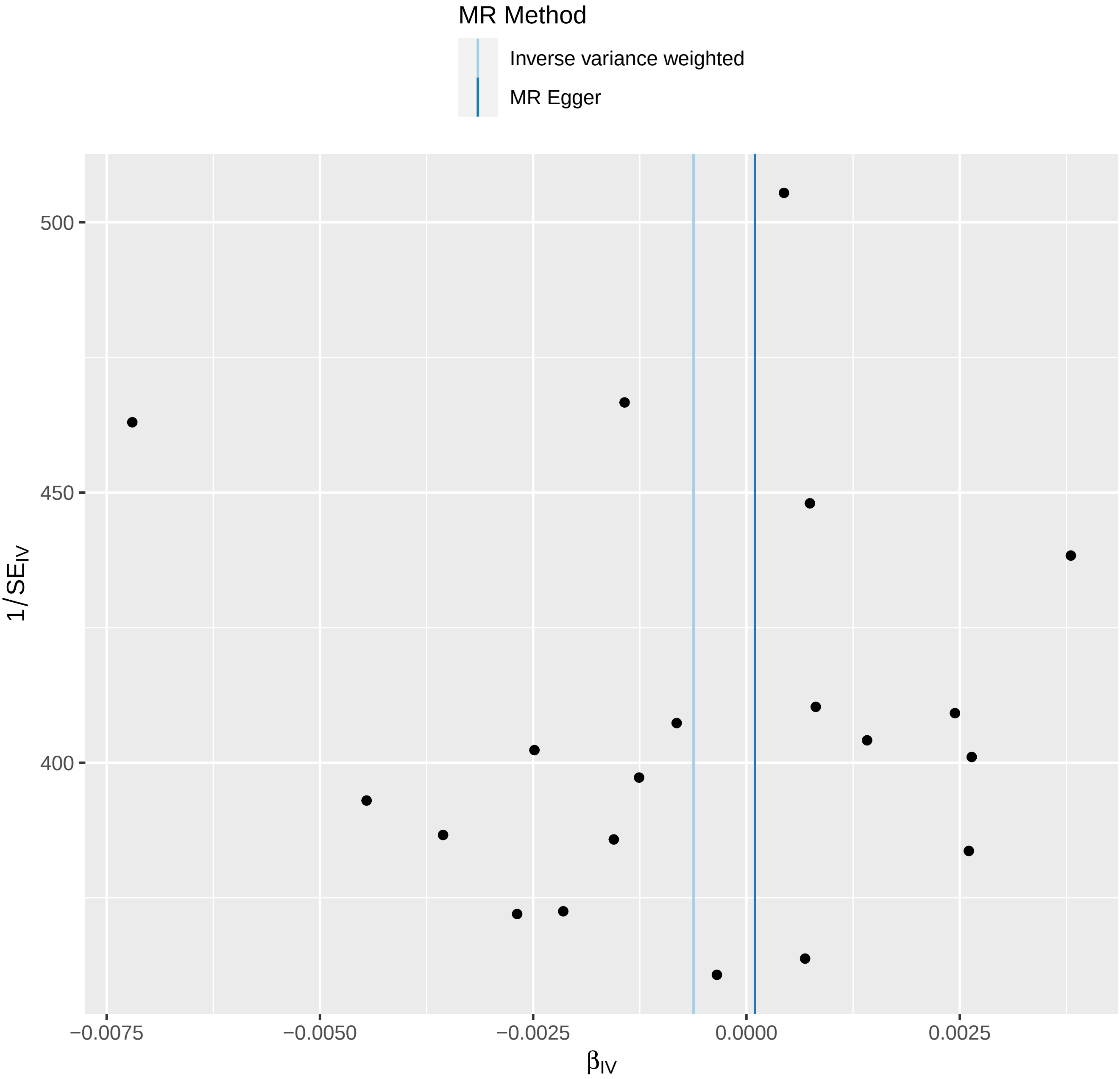

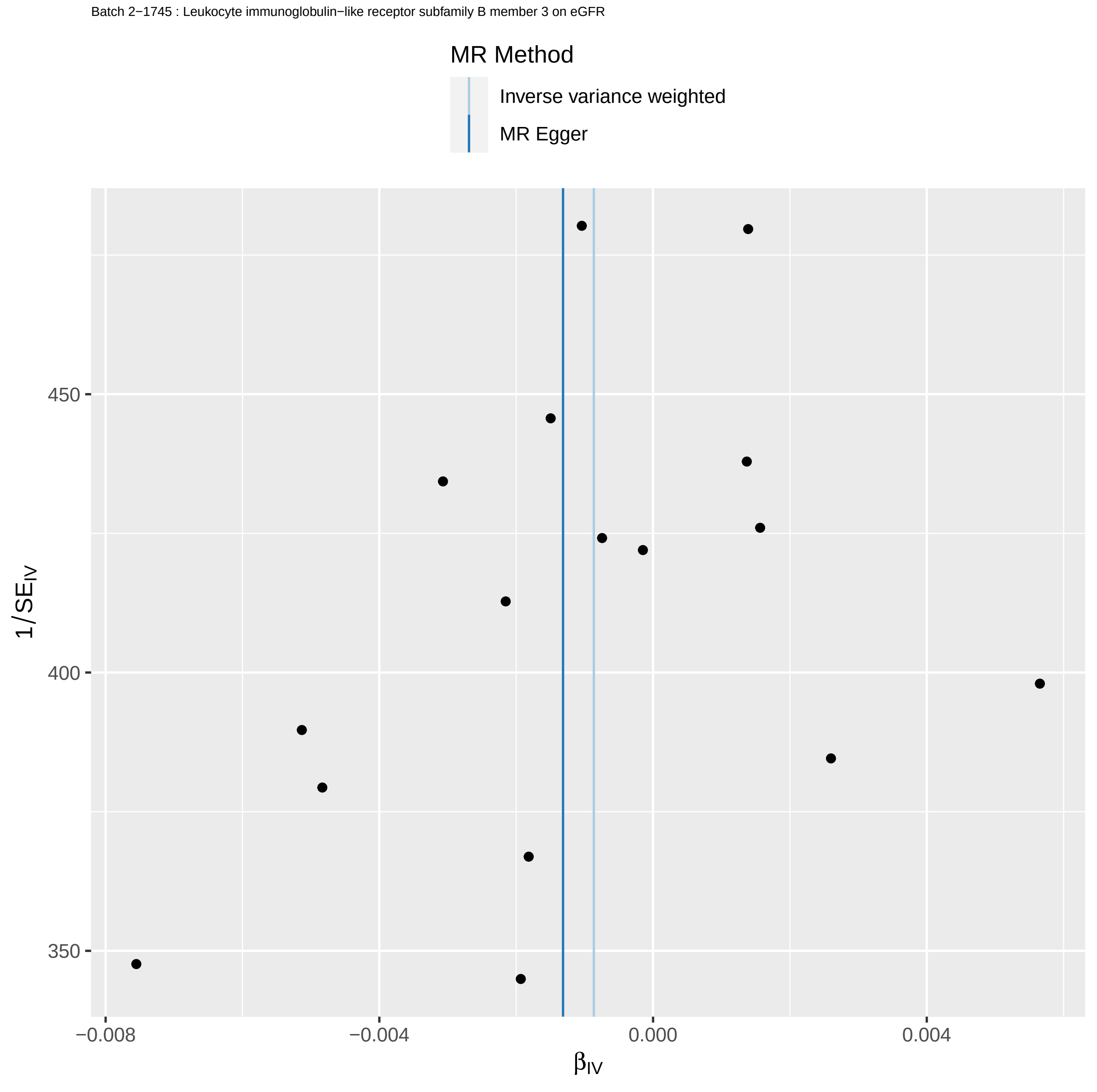
Batch 2−1745 : Leukocyte immunoglobulin−like receptor subfamily B member 3 on eGFR
MR Method
Inverse variance weighted
MR Egger
450
400
350
1/SE_IV
−0.008
−0.004
0.000
0.004
β_IV

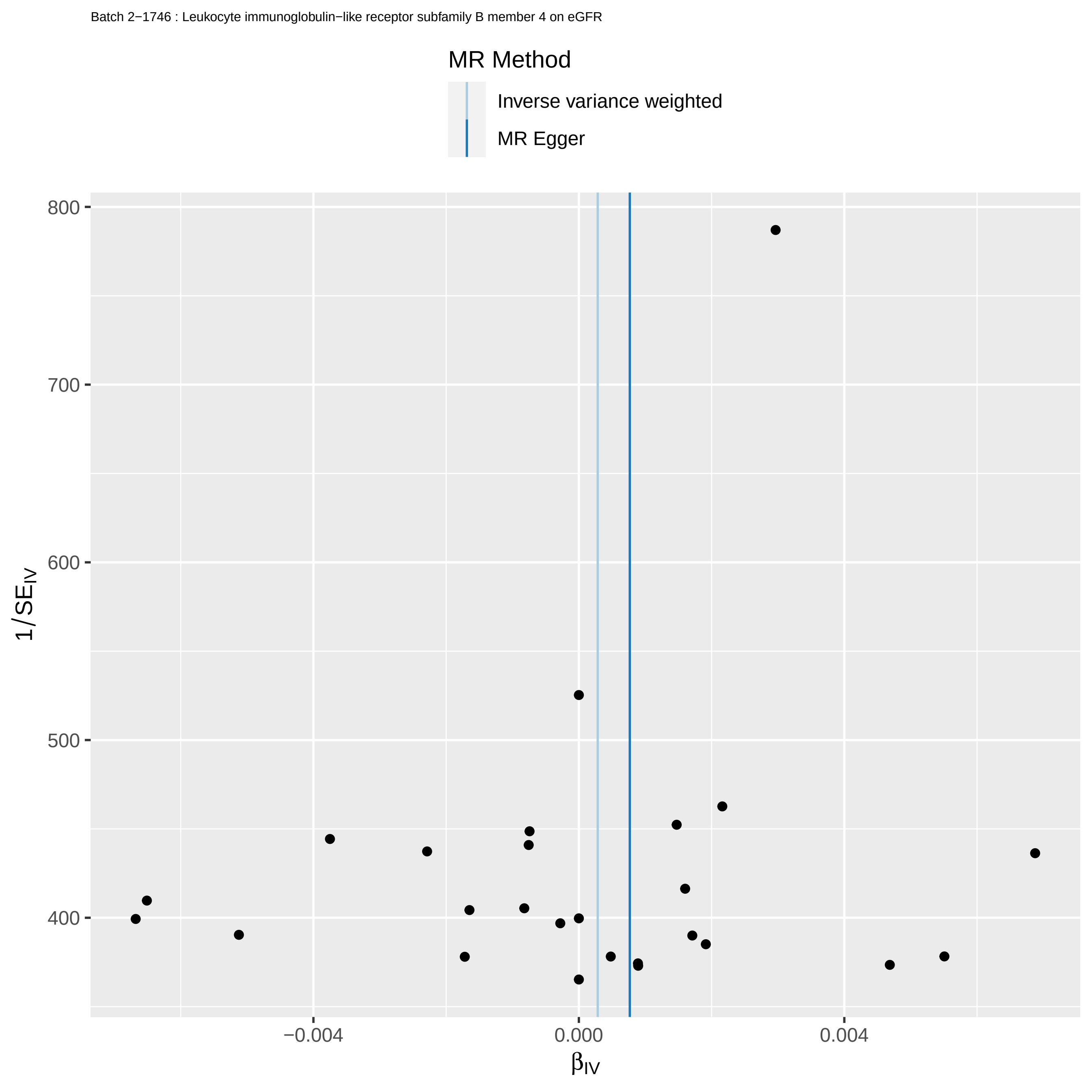

Batch 2−1746 : Leukocyte immunoglobulin−like receptor subfamily B member 4 on eGFR
MR Method
Inverse variance weighted
MR Egger
800
700
600
500
400
1/SE$_{IV}$
−0.004
0.000
0.004
β$_{IV}$

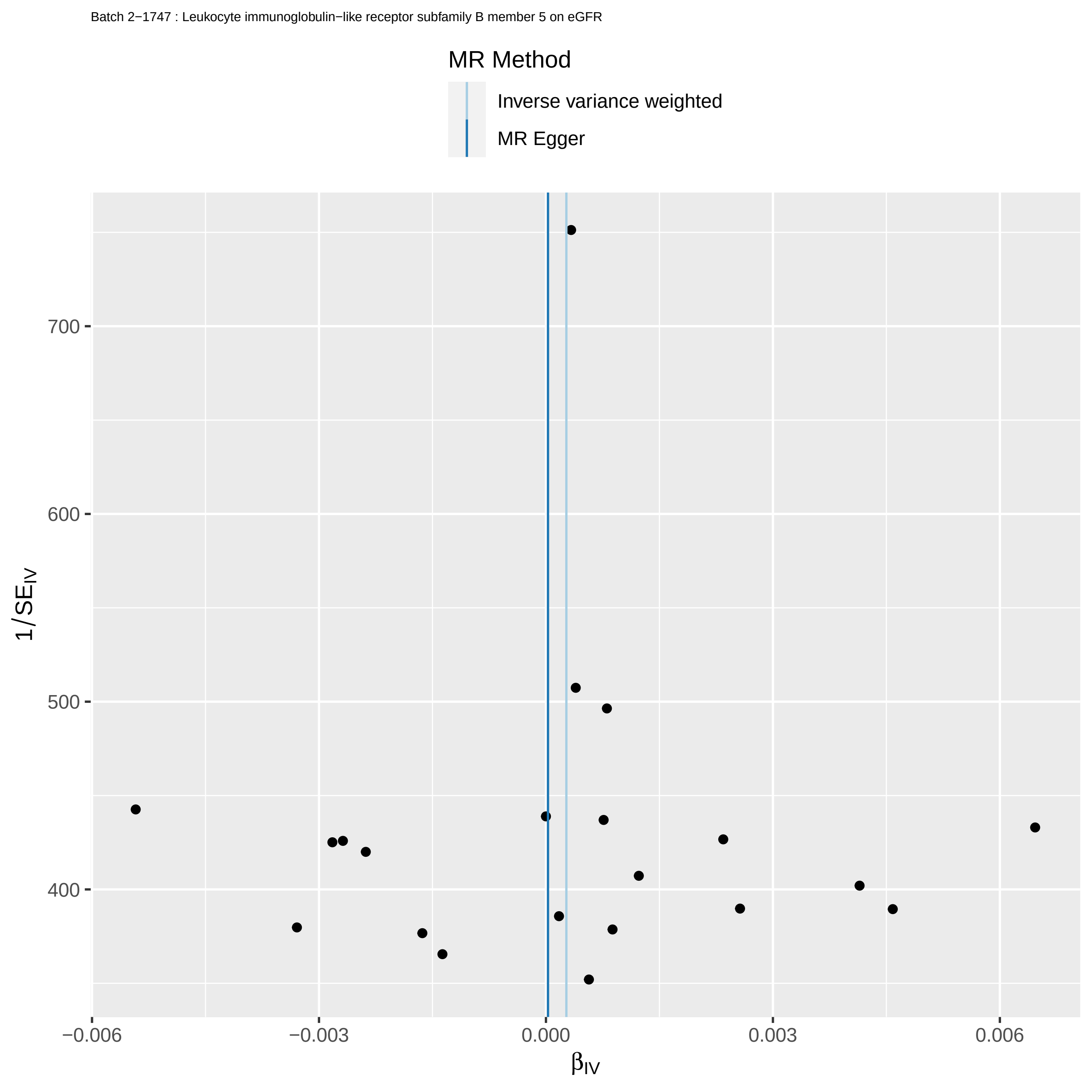

Batch 2−1747 : Leukocyte immunoglobulin−like receptor subfamily B member 5 on eGFR
MR Method
Inverse variance weighted
MR Egger
700
600
500
400
1/SE_IV
−0.006
−0.003
0.000
0.003
0.006
β_IV

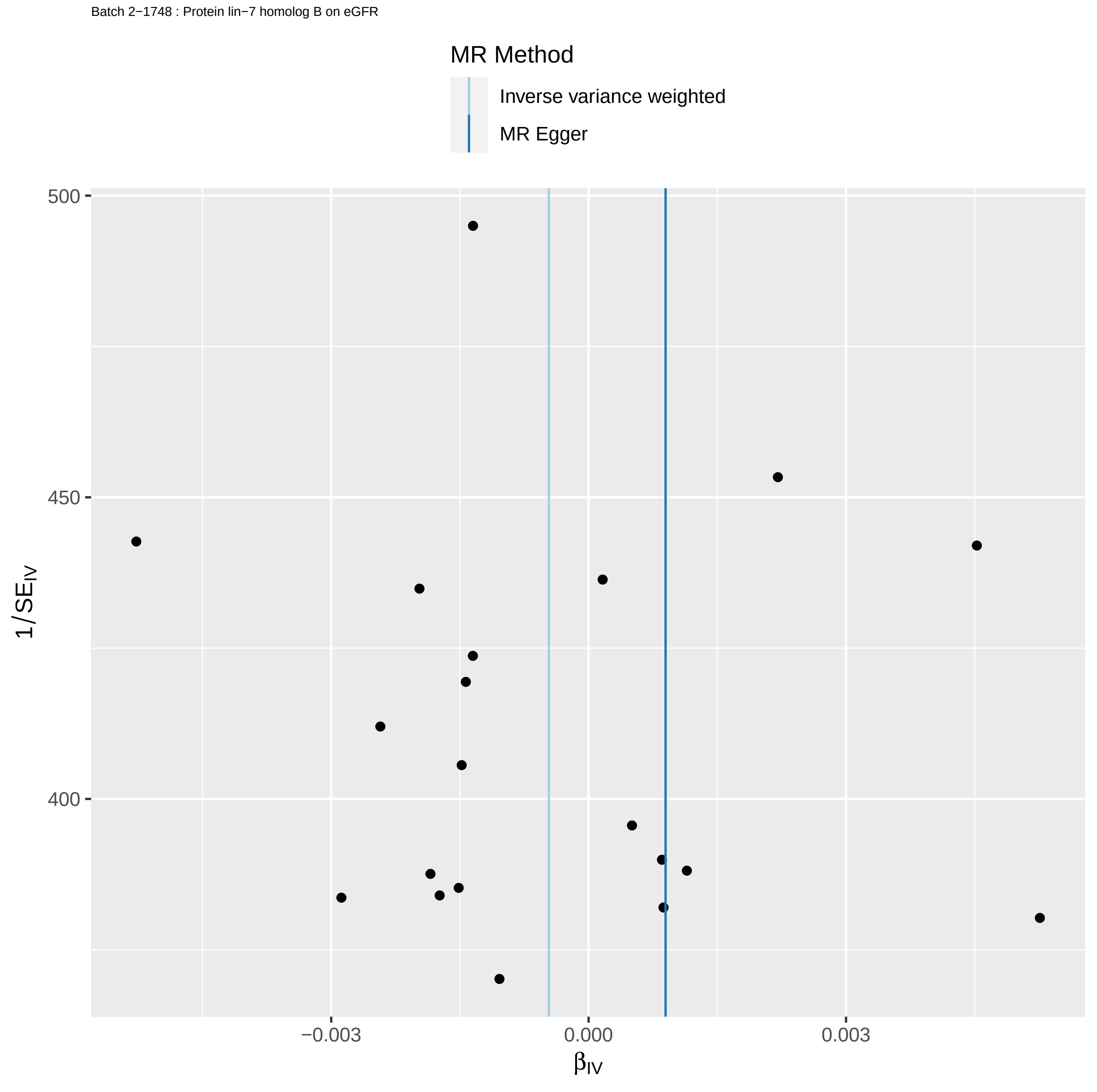

Batch 2−1748 : Protein lin−7 homolog B on eGFR
MR Method
Inverse variance weighted
MR Egger
500
450
400
1/SE_IV
−0.003
0.000
0.003
β_IV

Batch 2−1749 : Leucine−rich repeat and immunoglobulin−like domain−containing nogo receptor−interacting protein 1 on eGFR

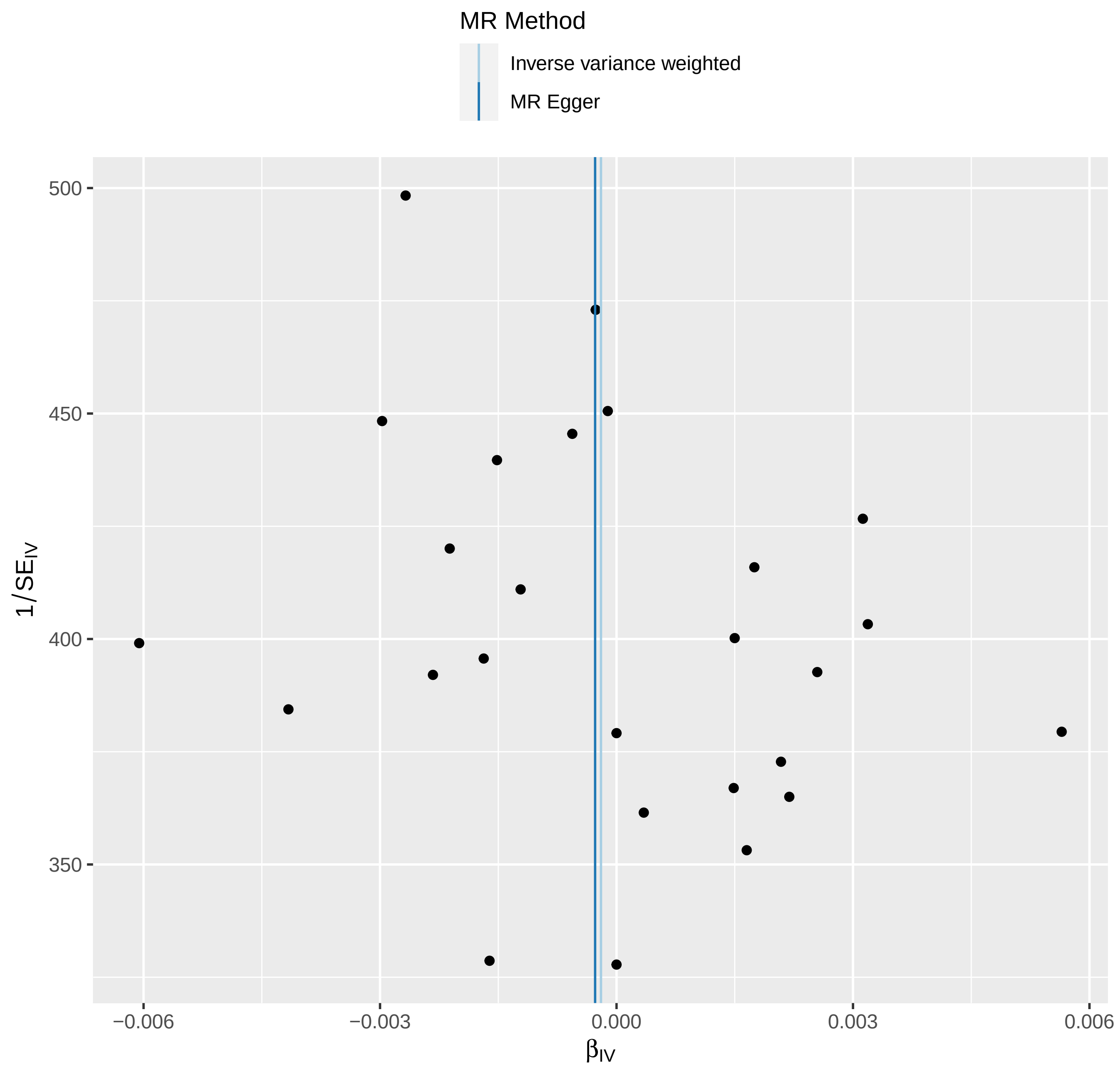

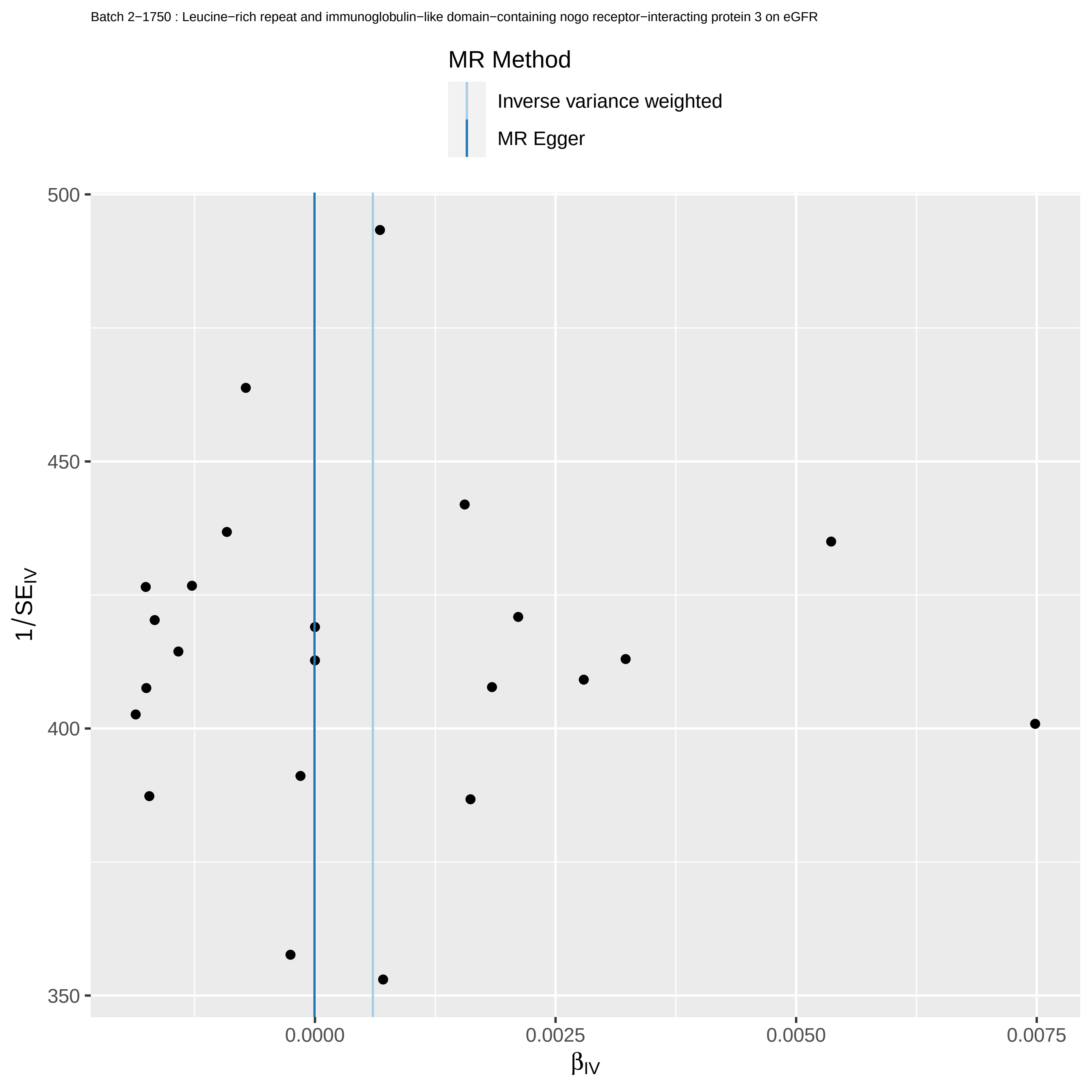
Batch 2−1750 : Leucine−rich repeat and immunoglobulin−like domain−containing nogo receptor−interacting protein 3 on eGFR
MR Method
Inverse variance weighted
MR Egger
500
450
400
350
0.0000
0.0025
0.0050
0.0075
$1/SE_{IV}$
β_{IV}

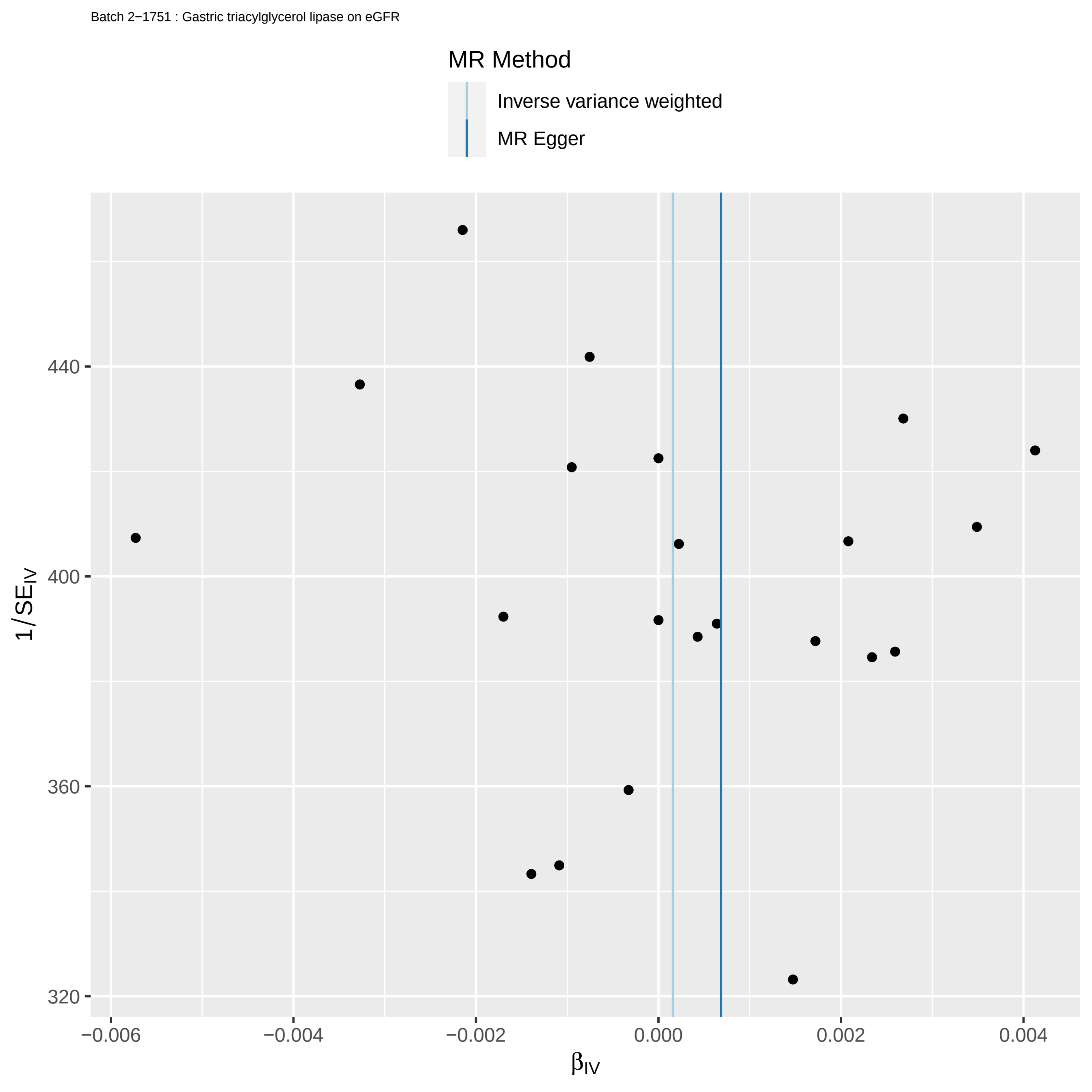

Batch 2−1751 : Gastric triacylglycerol lipase on eGFR
MR Method
Inverse variance weighted
MR Egger
440
400
360
320
1/SE$_{IV}$
−0.006
−0.004
−0.002
0.000
0.002
0.004
β$_{IV}$

Batch 2−1752 : Lipase member K on eGFR

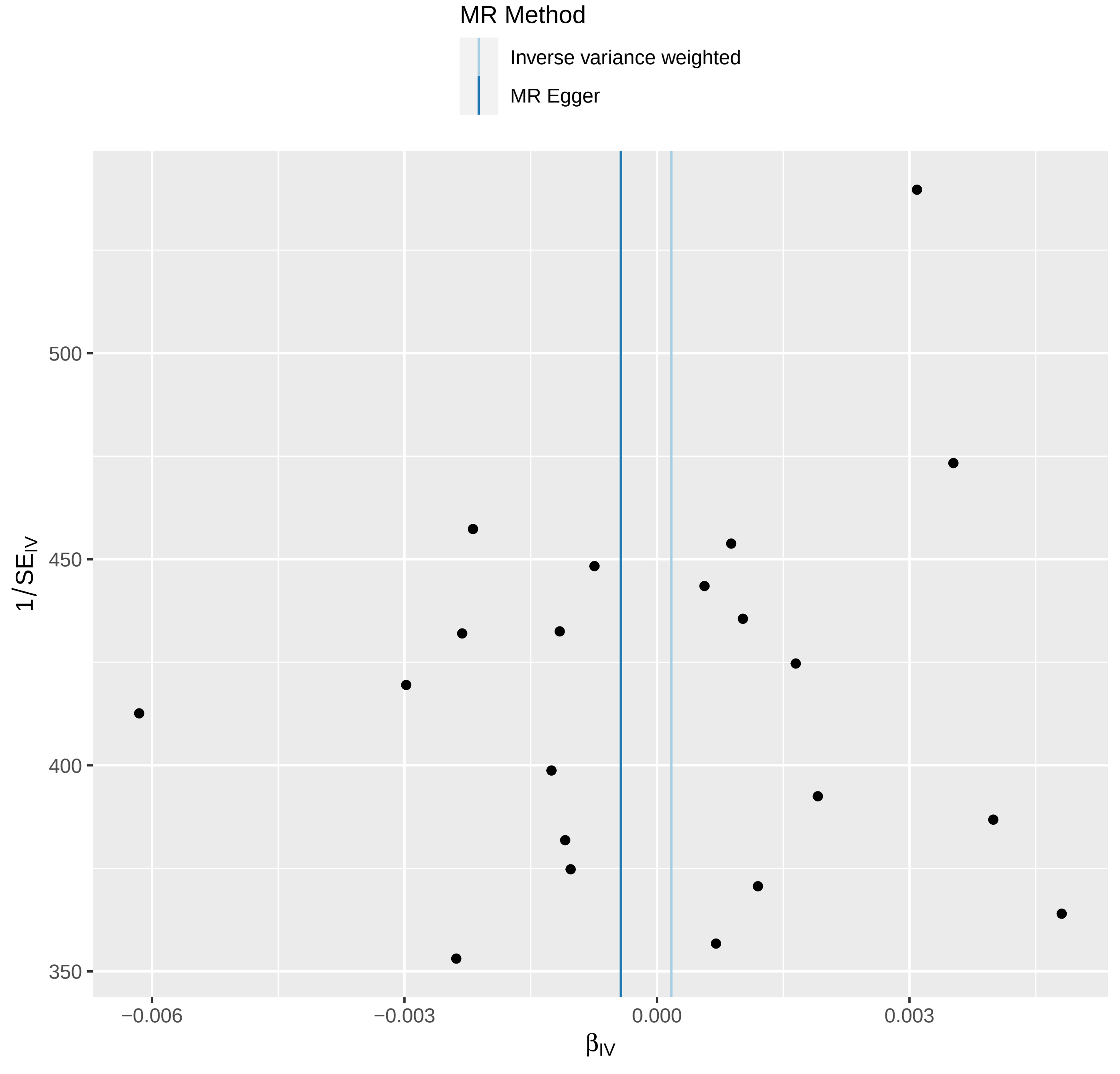

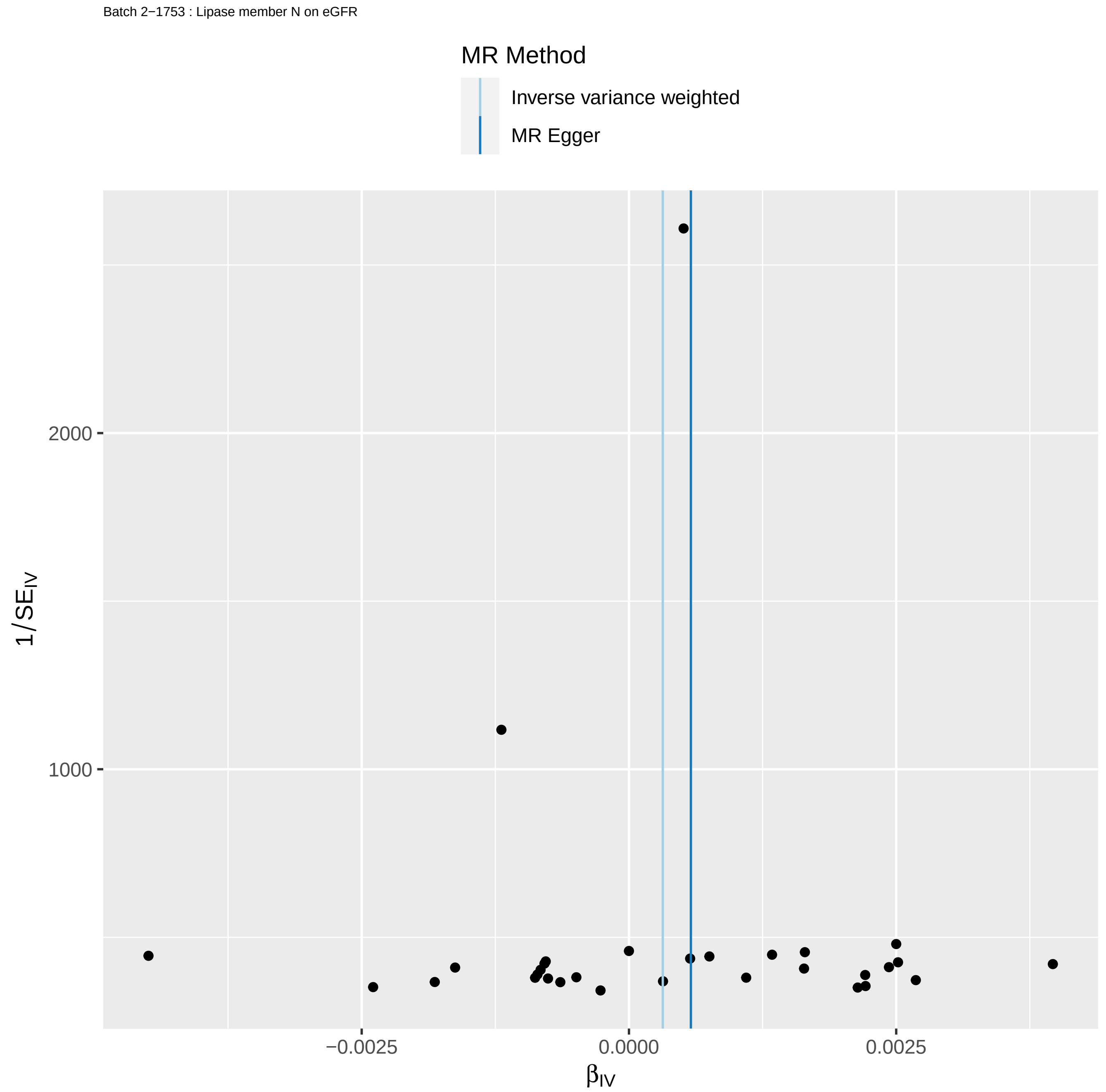

Batch 2−1753 : Lipase member N on eGFR
MR Method
Inverse variance weighted
MR Egger
2000
1000
1/SE_IV
−0.0025
0.0000
0.0025
β_IV

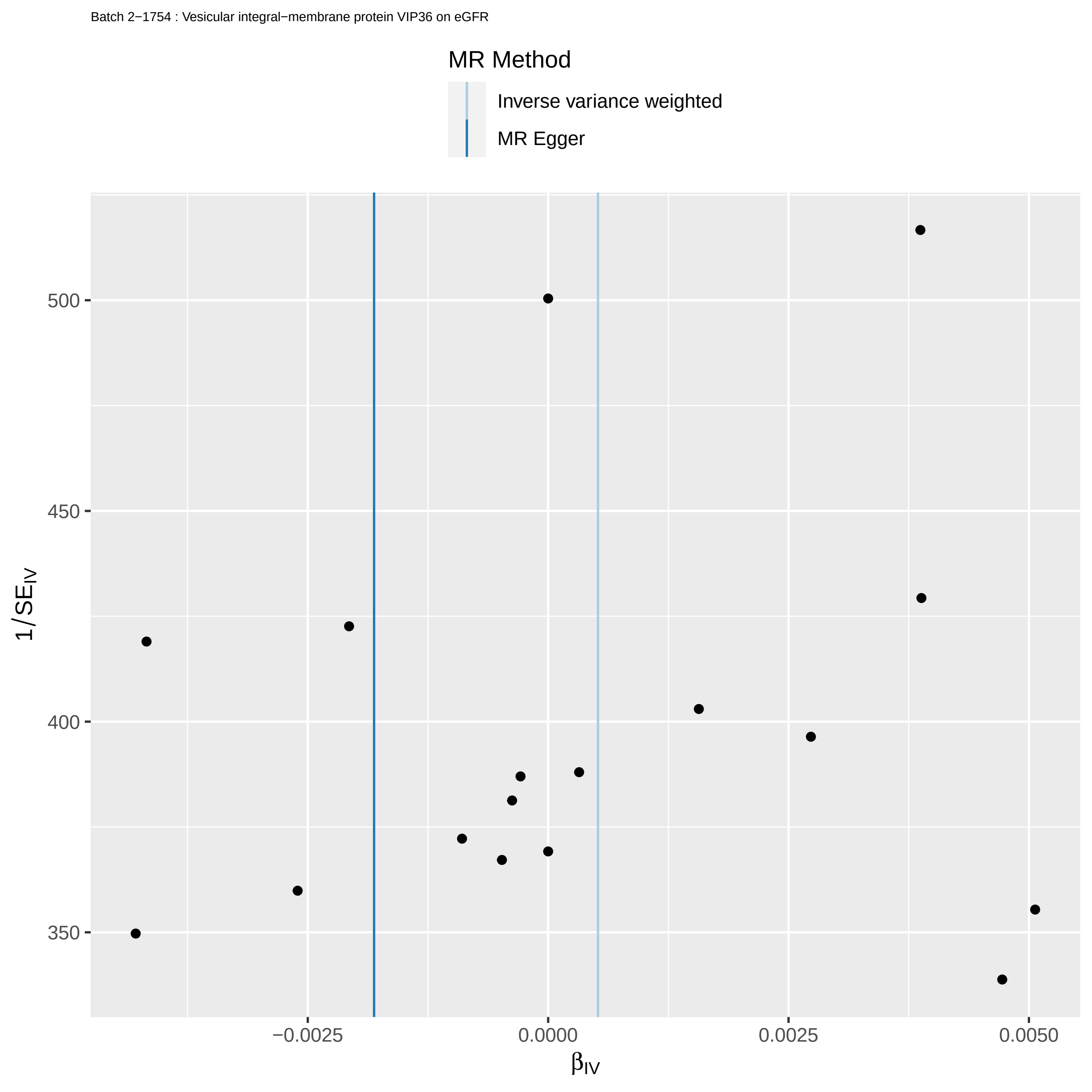

Batch 2−1754 : Vesicular integral−membrane protein VIP36 on eGFR
MR Method
Inverse variance weighted
MR Egger
500
450
400
350
1/SE$_{IV}$
−0.0025
0.0000
0.0025
0.0050
β$_{IV}$

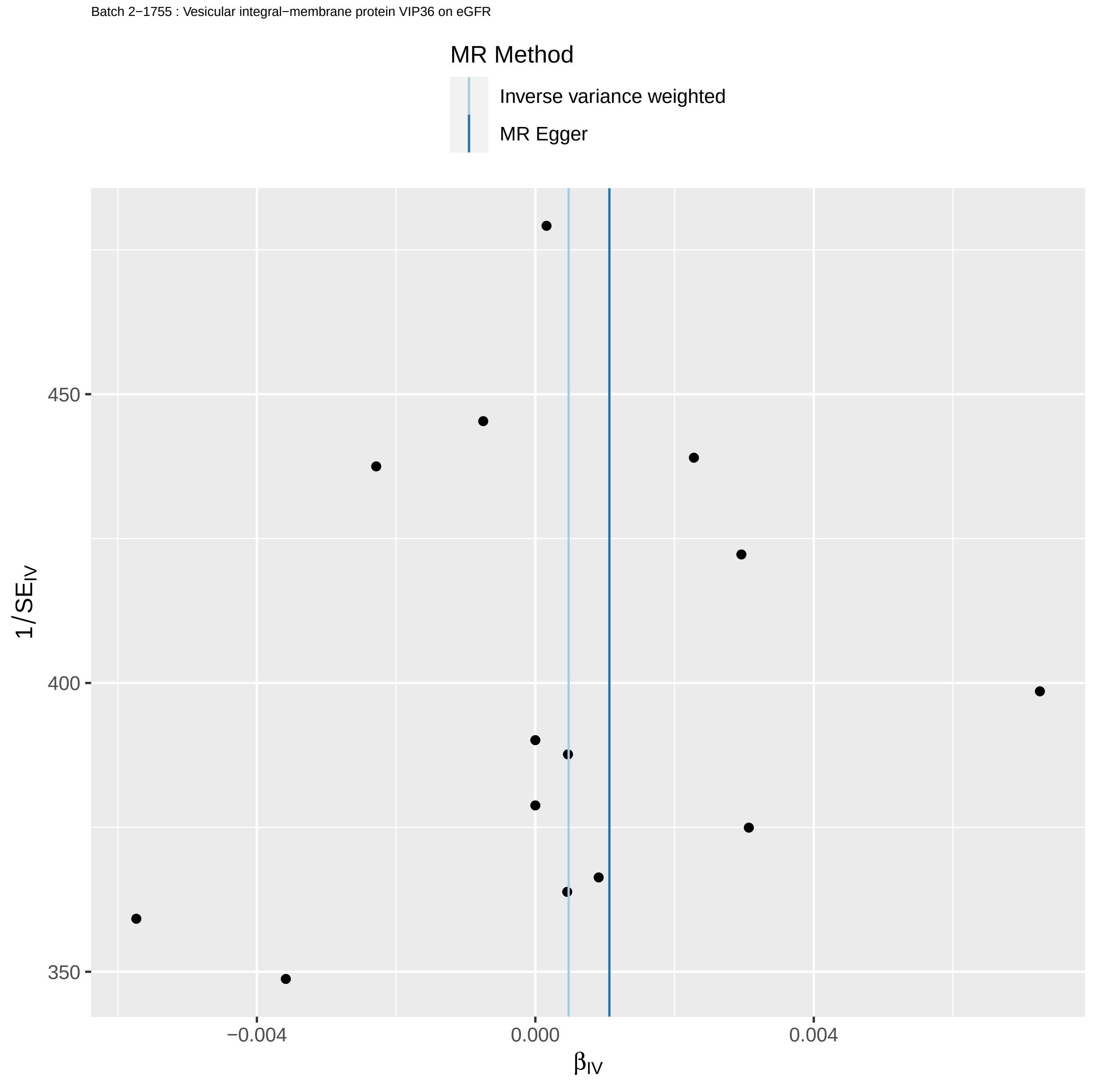

Batch 2−1755 : Vesicular integral−membrane protein VIP36 on eGFR
MR Method
Inverse variance weighted
MR Egger
450
400
350
1/SE_IV
−0.004
0.000
0.004
β_IV

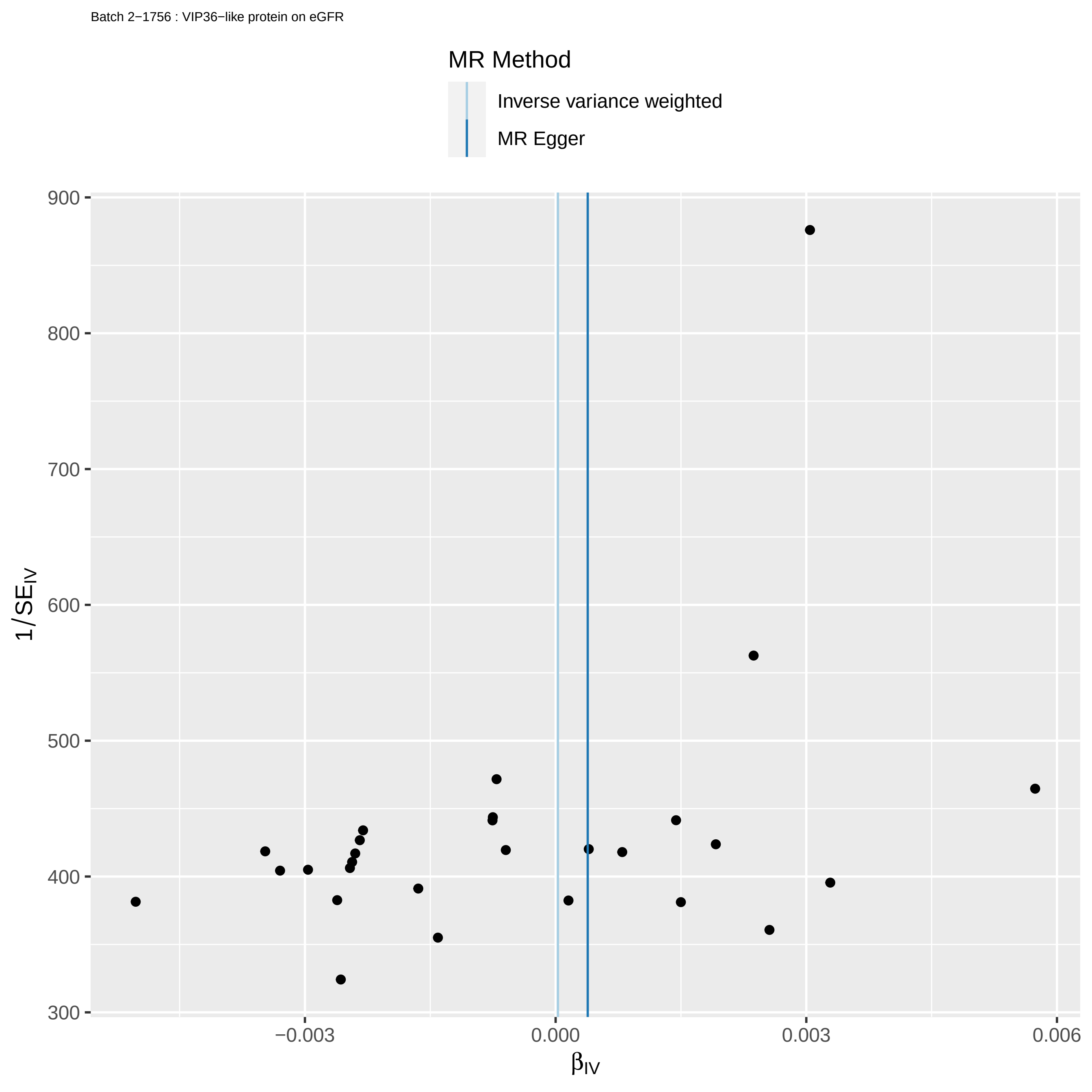
Batch 2−1756 : VIP36−like protein on eGFR
MR Method
Inverse variance weighted
MR Egger
900
800
700
600
500
400
300
1/SE_IV
−0.003
0.000
0.003
0.006
β_IV

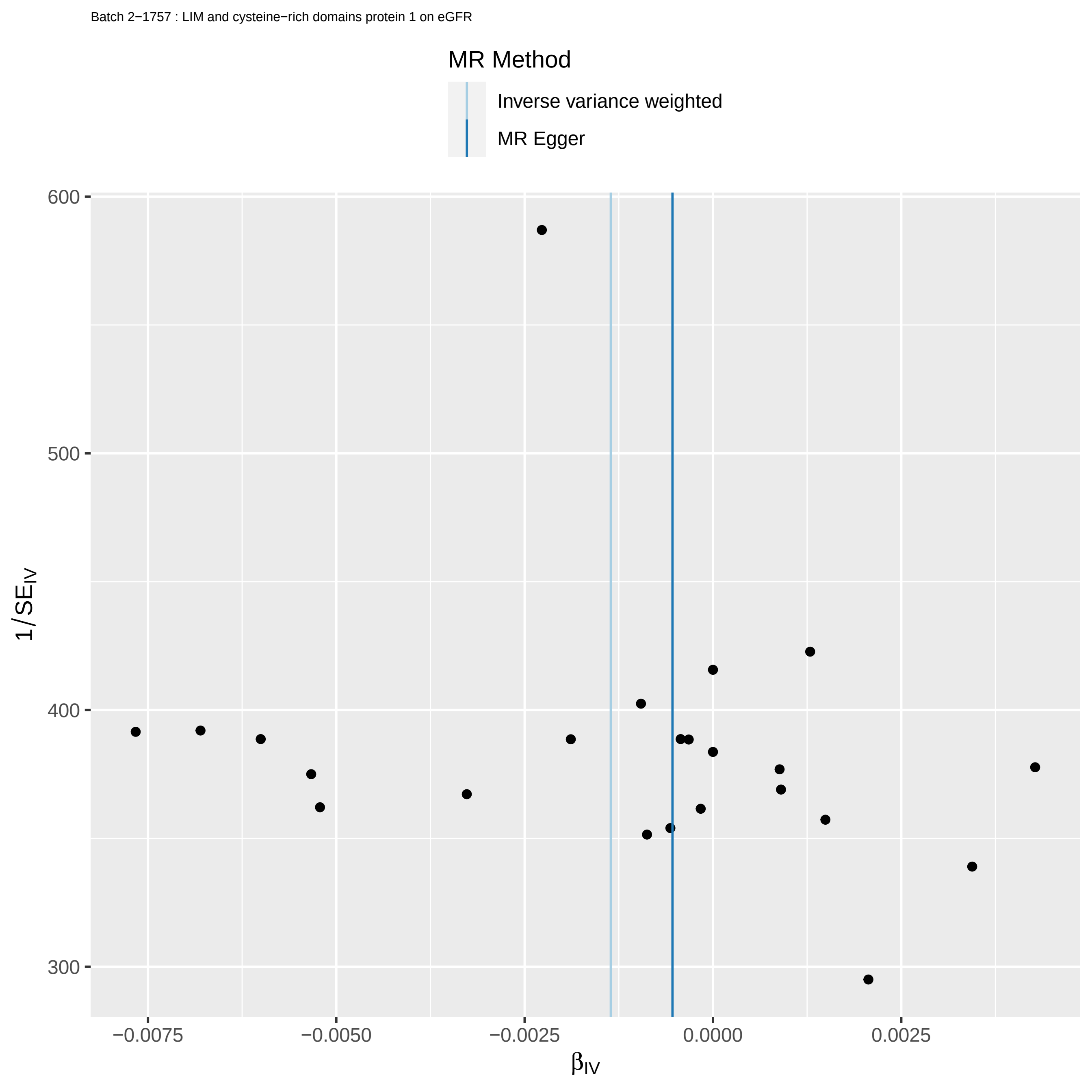

Batch 2−1757 : LIM and cysteine−rich domains protein 1 on eGFR
MR Method
Inverse variance weighted
MR Egger
600
500
400
300
1/SE_IV
−0.0075
−0.0050
−0.0025
0.0000
0.0025
β_IV

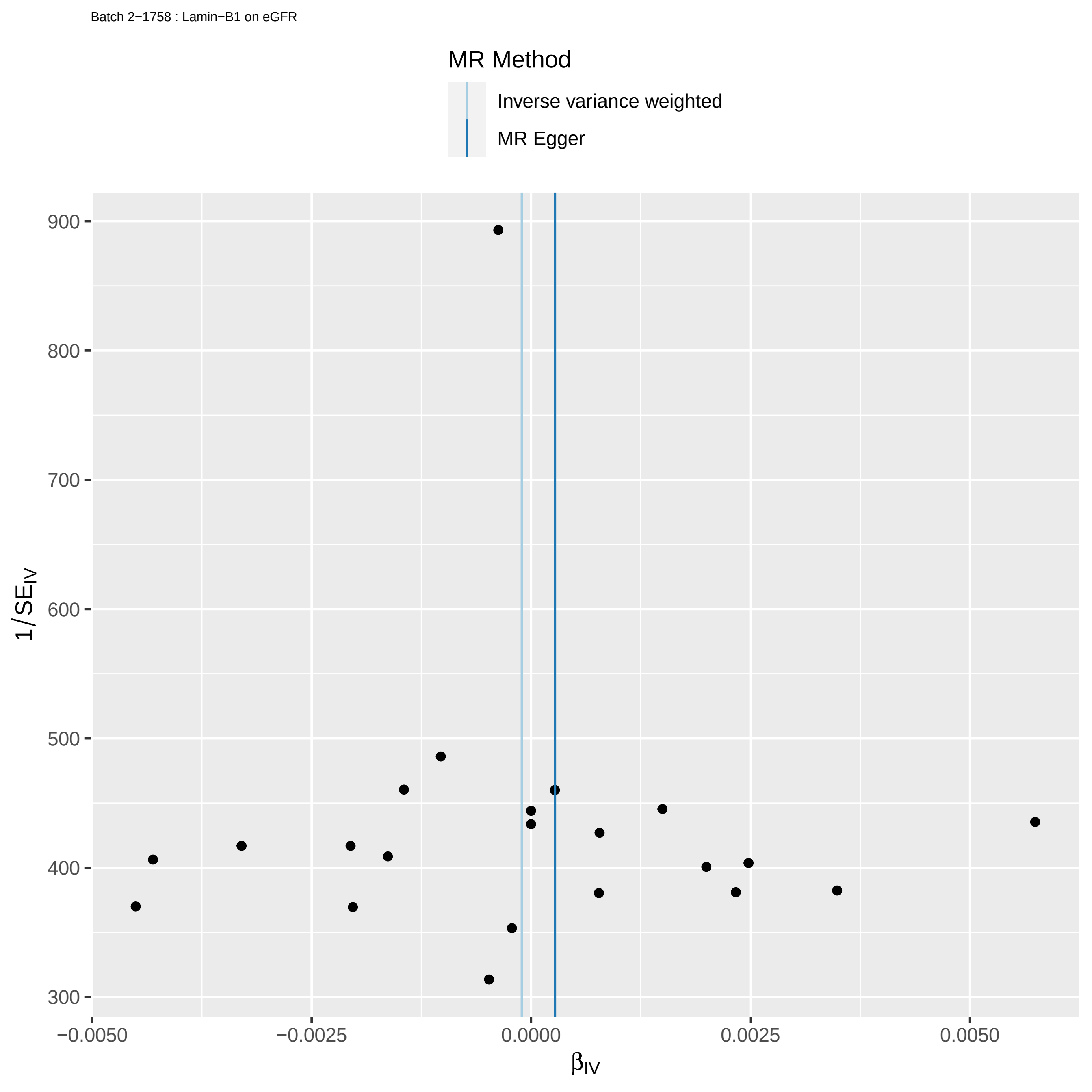

Batch 2−1758 : Lamin−B1 on eGFR
MR Method
Inverse variance weighted
MR Egger
900
800
700
600
500
400
300
1/SE$_{IV}$
−0.0050
−0.0025
0.0000
0.0025
0.0050
β$_{IV}$

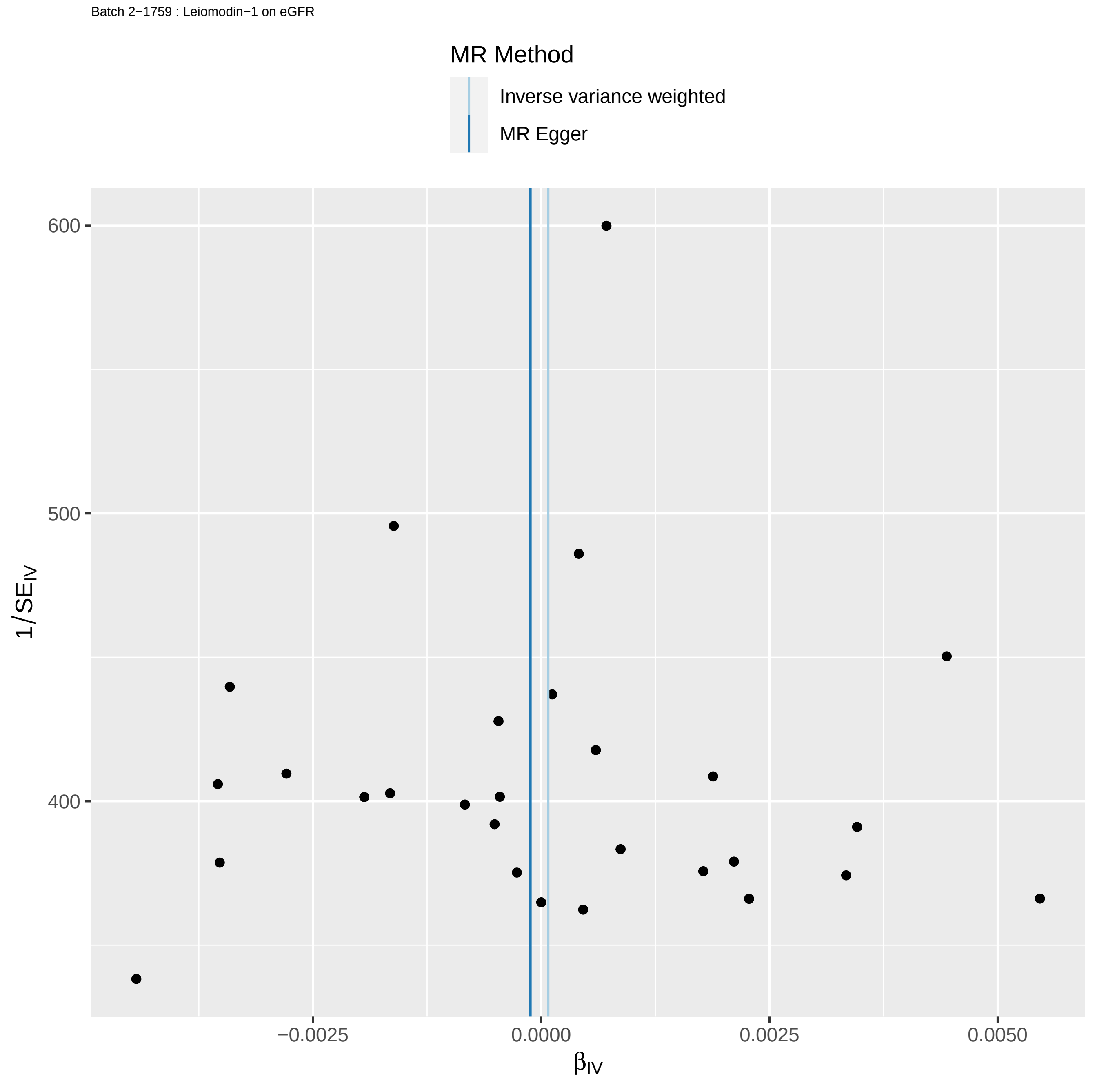
Batch 2−1759 : Leiomodin−1 on eGFR
MR Method
Inverse variance weighted
MR Egger
600
500
400
1/SE$_{IV}$
−0.0025
0.0000
0.0025
0.0050
β$_{IV}$

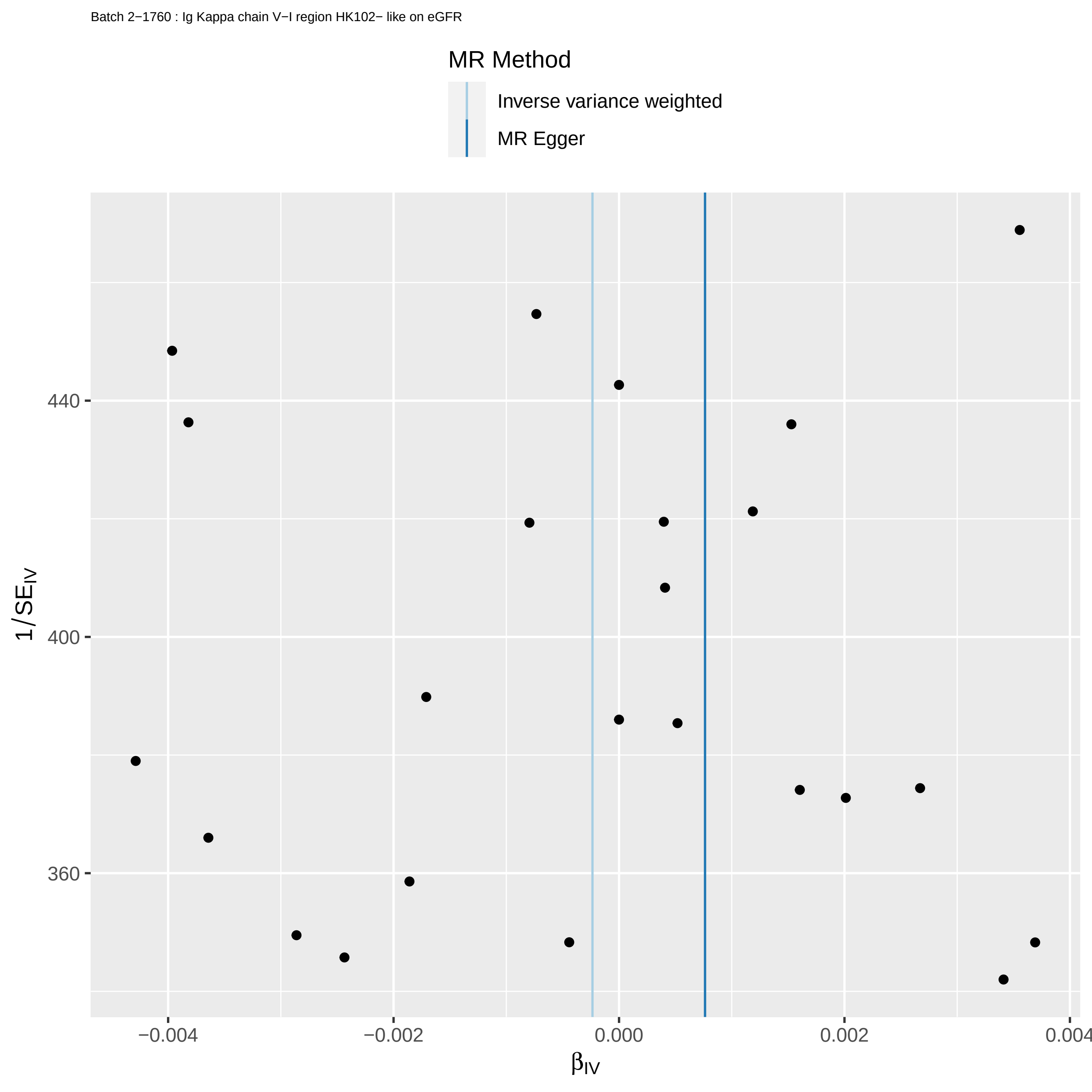
Batch 2−1760 : Ig Kappa chain V−I region HK102− like on eGFR
MR Method
Inverse variance weighted
MR Egger
1/SE_IV
440
400
360
−0.004
−0.002
0.000
0.002
0.004
β_IV

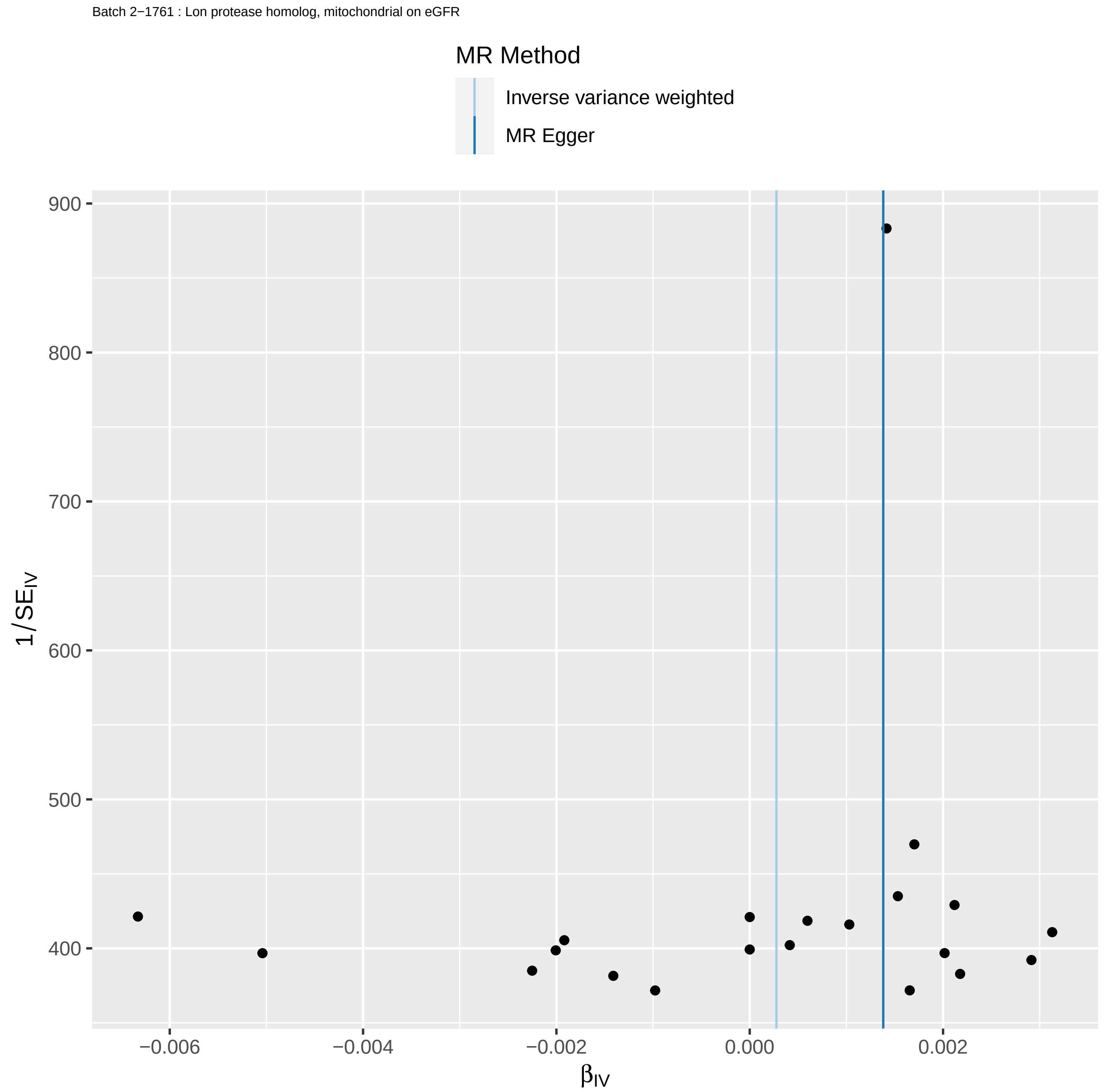
Batch 2−1761 : Lon protease homolog, mitochondrial on eGFR
MR Method
Inverse variance weighted
MR Egger
900
800
700
600
500
400
1/SE_IV
−0.006
−0.004
−0.002
0.000
0.002
β_IV

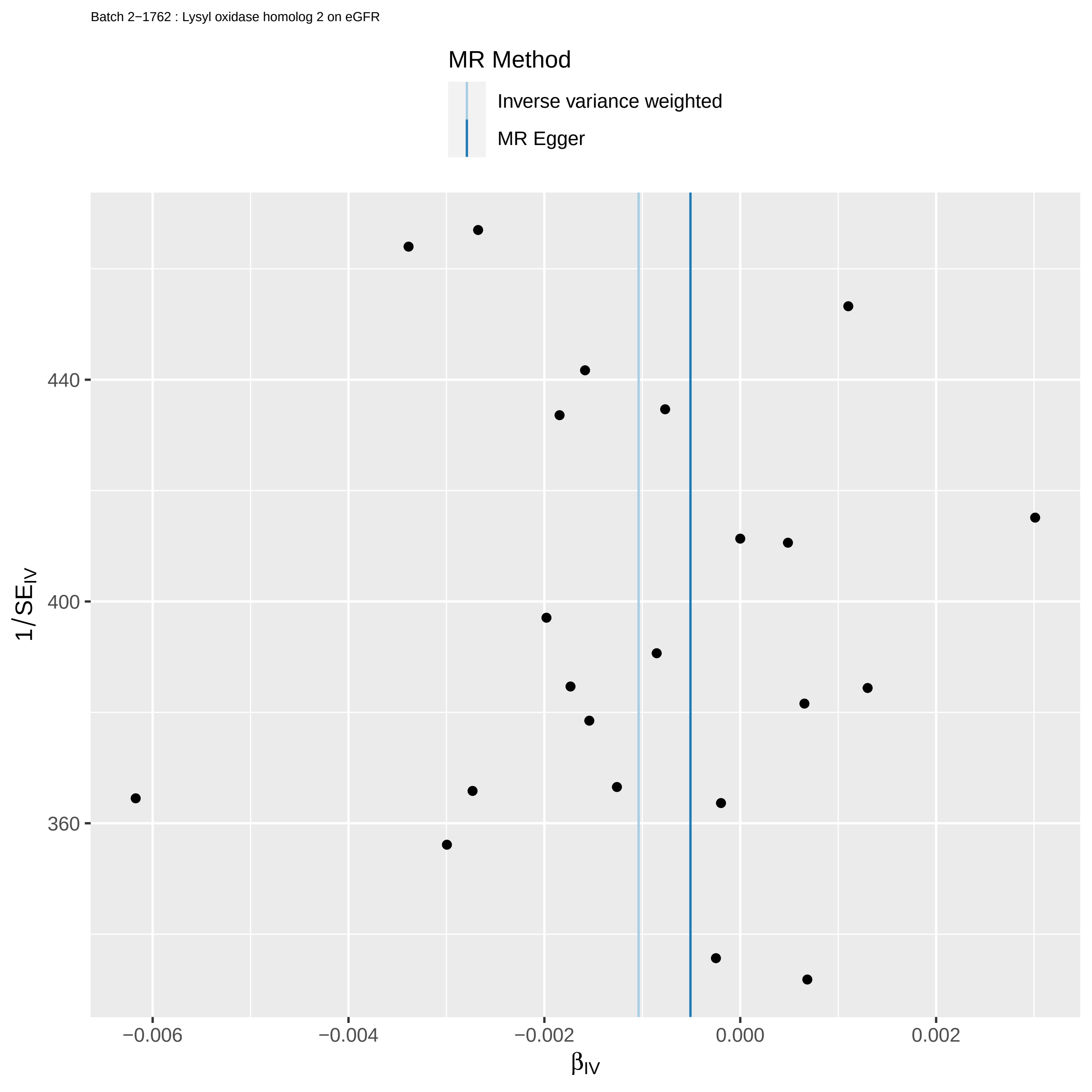
Batch 2−1762 : Lysyl oxidase homolog 2 on eGFR
MR Method
Inverse variance weighted
MR Egger
1/SE_IV
440
400
360
−0.006
−0.004
−0.002
0.000
0.002
β_IV

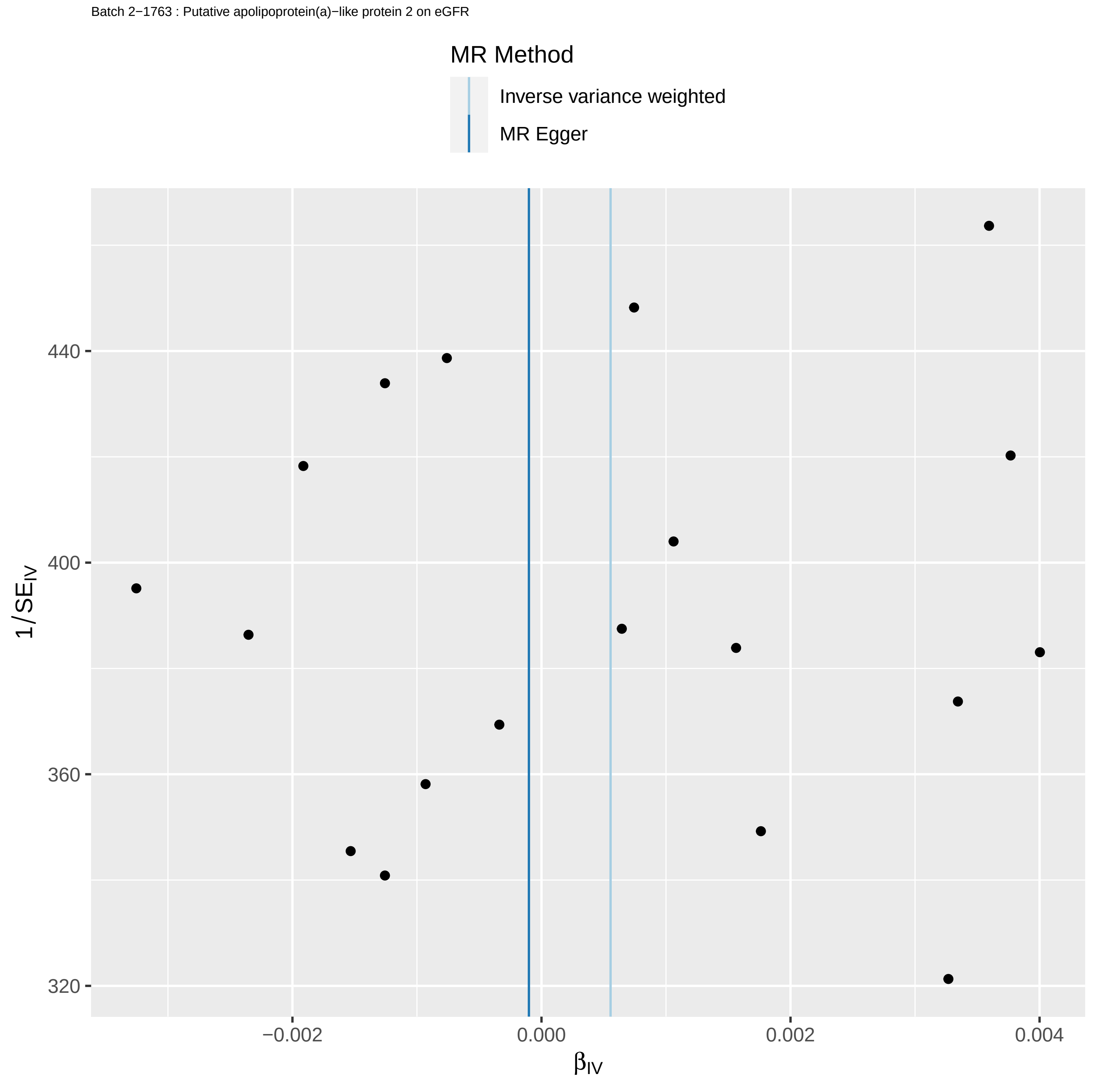

Batch 2−1763 : Putative apolipoprotein(a)−like protein 2 on eGFR
MR Method
Inverse variance weighted
MR Egger
440
400
360
320
1/SE_IV
−0.002
0.000
0.002
0.004
β_IV

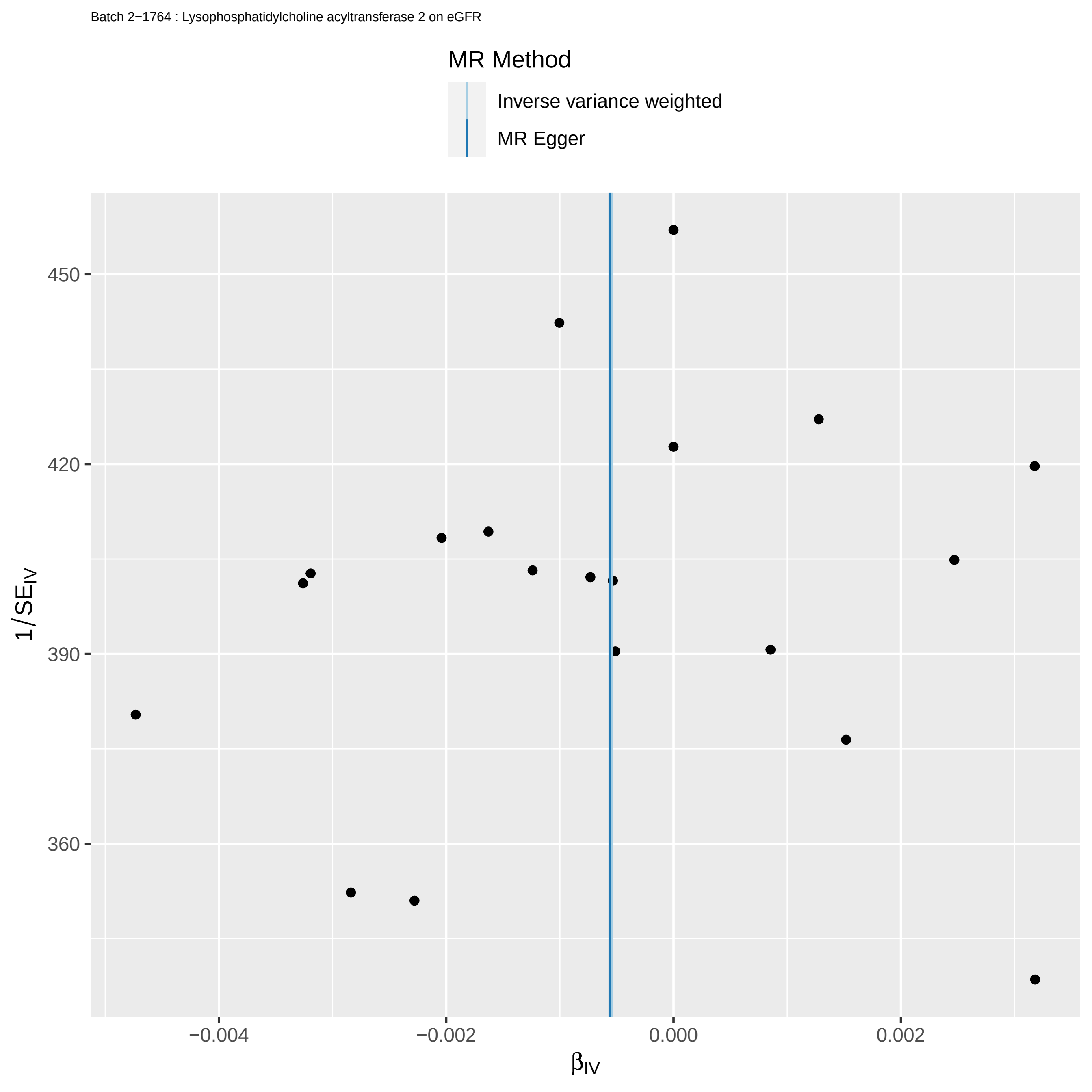

Batch 2−1764 : Lysophosphatidylcholine acyltransferase 2 on eGFR
MR Method
Inverse variance weighted
MR Egger
450
420
390
360
1/SE_IV
−0.004
−0.002
0.000
0.002
β_IV

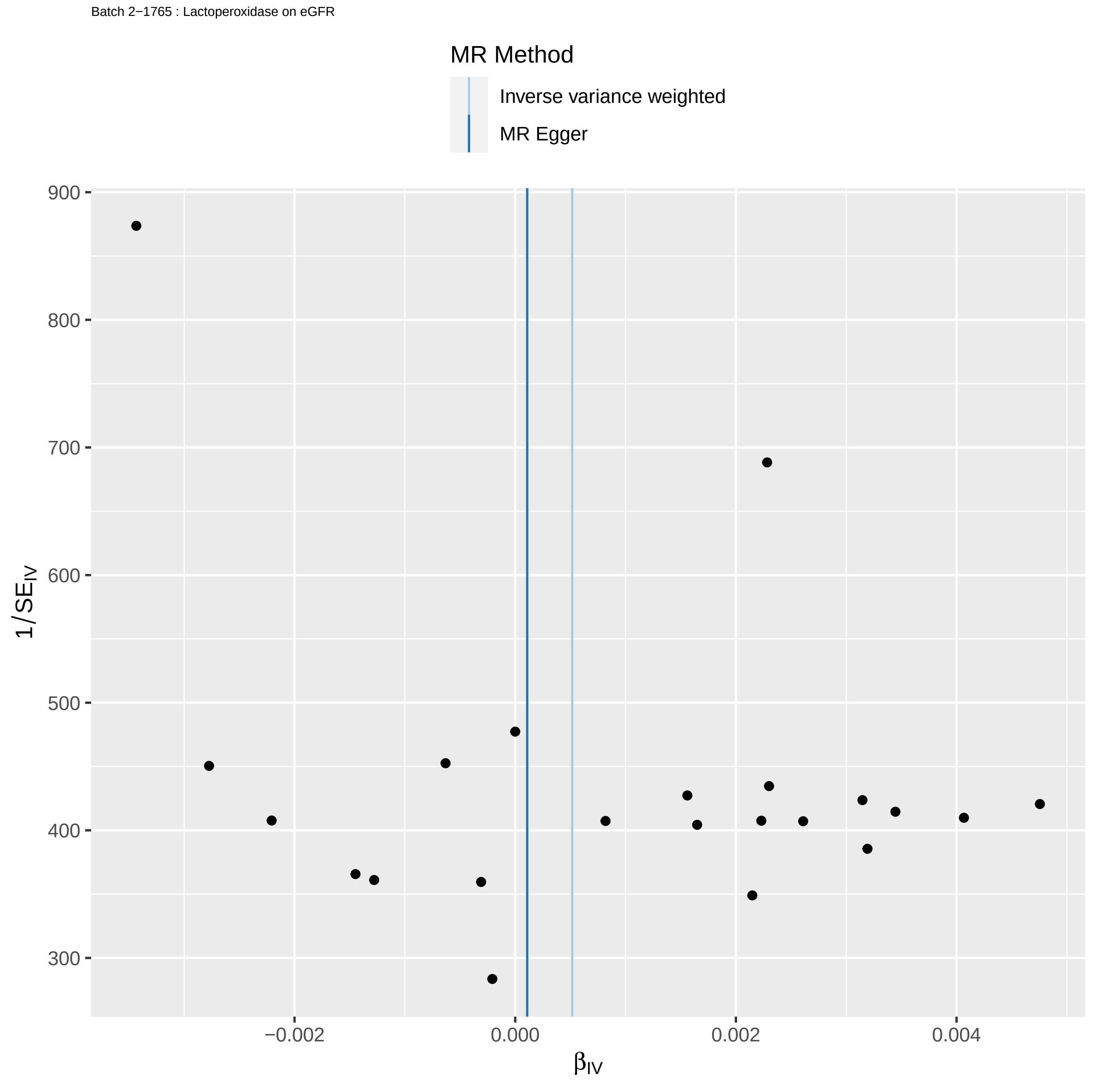

Batch 2−1765 : Lactoperoxidase on eGFR
MR Method
Inverse variance weighted
MR Egger
900
800
700
600
500
400
300
1/SE_IV
−0.002
0.000
0.002
0.004
β_IV

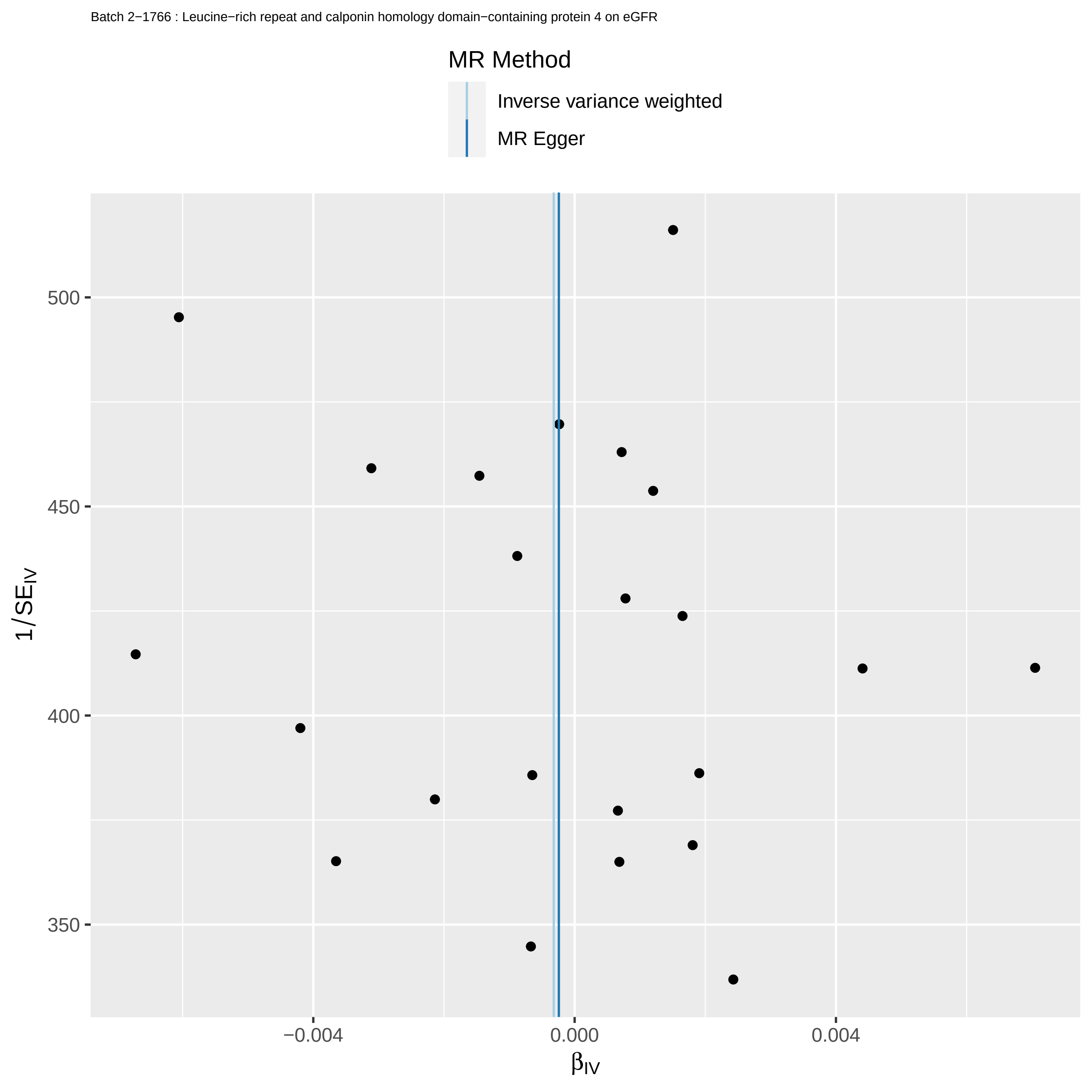

Batch 2−1766 : Leucine−rich repeat and calponin homology domain−containing protein 4 on eGFR
MR Method
Inverse variance weighted
MR Egger
500
450
400
350
1/SE$_{IV}$
−0.004
0.000
0.004
β$_{IV}$

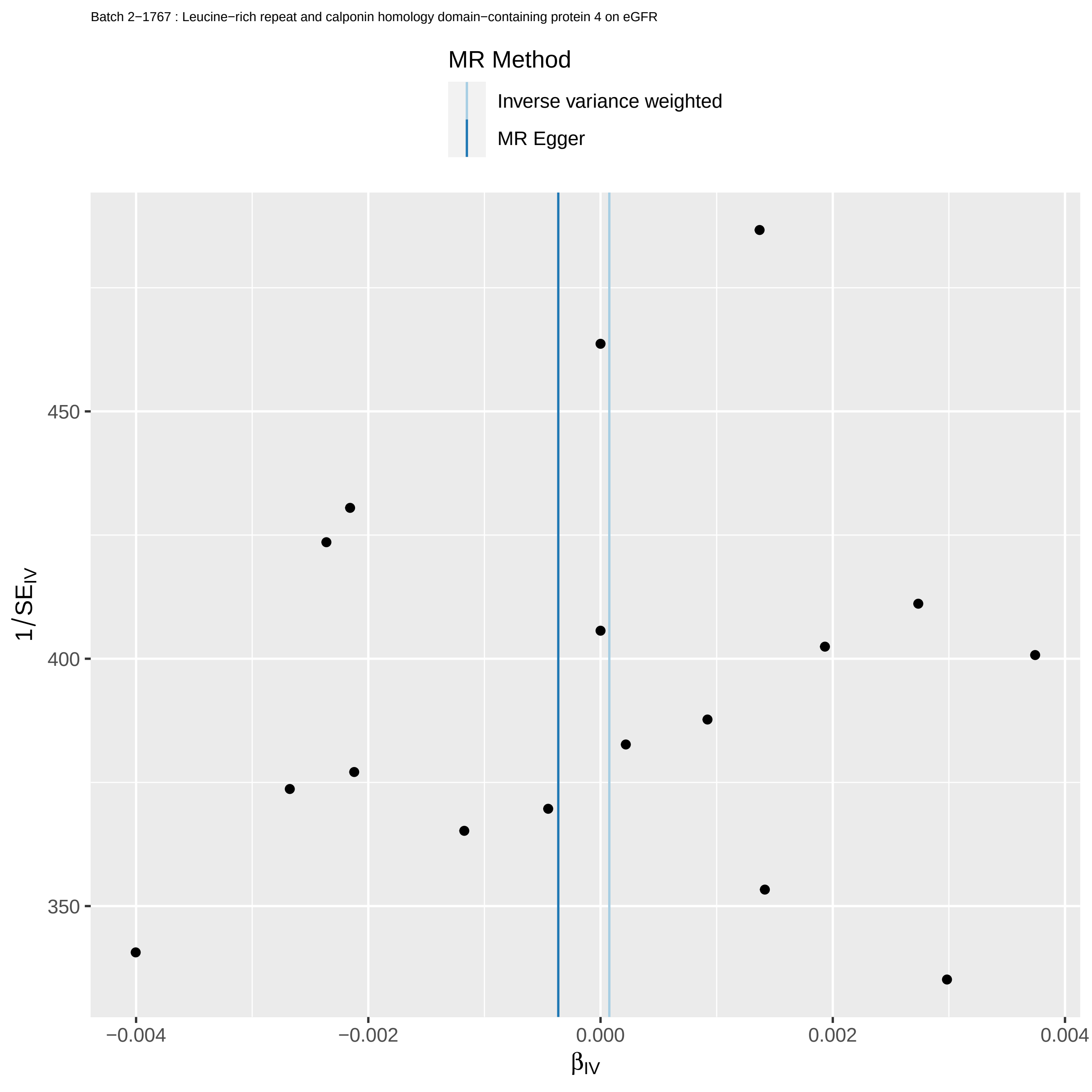
Batch 2−1767 : Leucine−rich repeat and calponin homology domain−containing protein 4 on eGFR
MR Method
Inverse variance weighted
MR Egger
450
400
350
1/SE$_{IV}$
−0.004
−0.002
0.000
0.002
0.004
β$_{IV}$

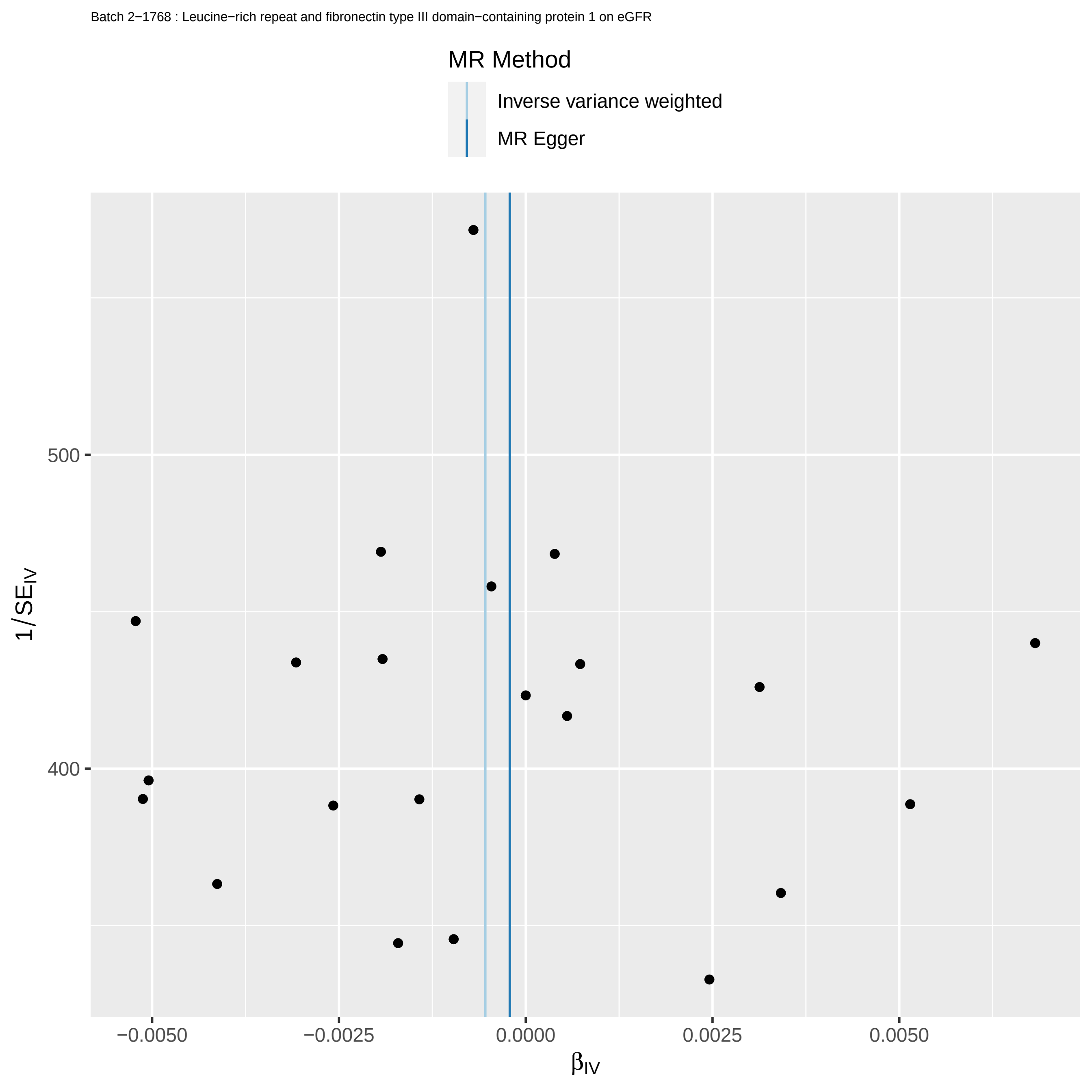
Batch 2−1768 : Leucine−rich repeat and fibronectin type III domain−containing protein 1 on eGFR
MR Method
Inverse variance weighted
MR Egger
500
400
1/SE_IV
−0.0050
−0.0025
0.0000
0.0025
0.0050
β_IV

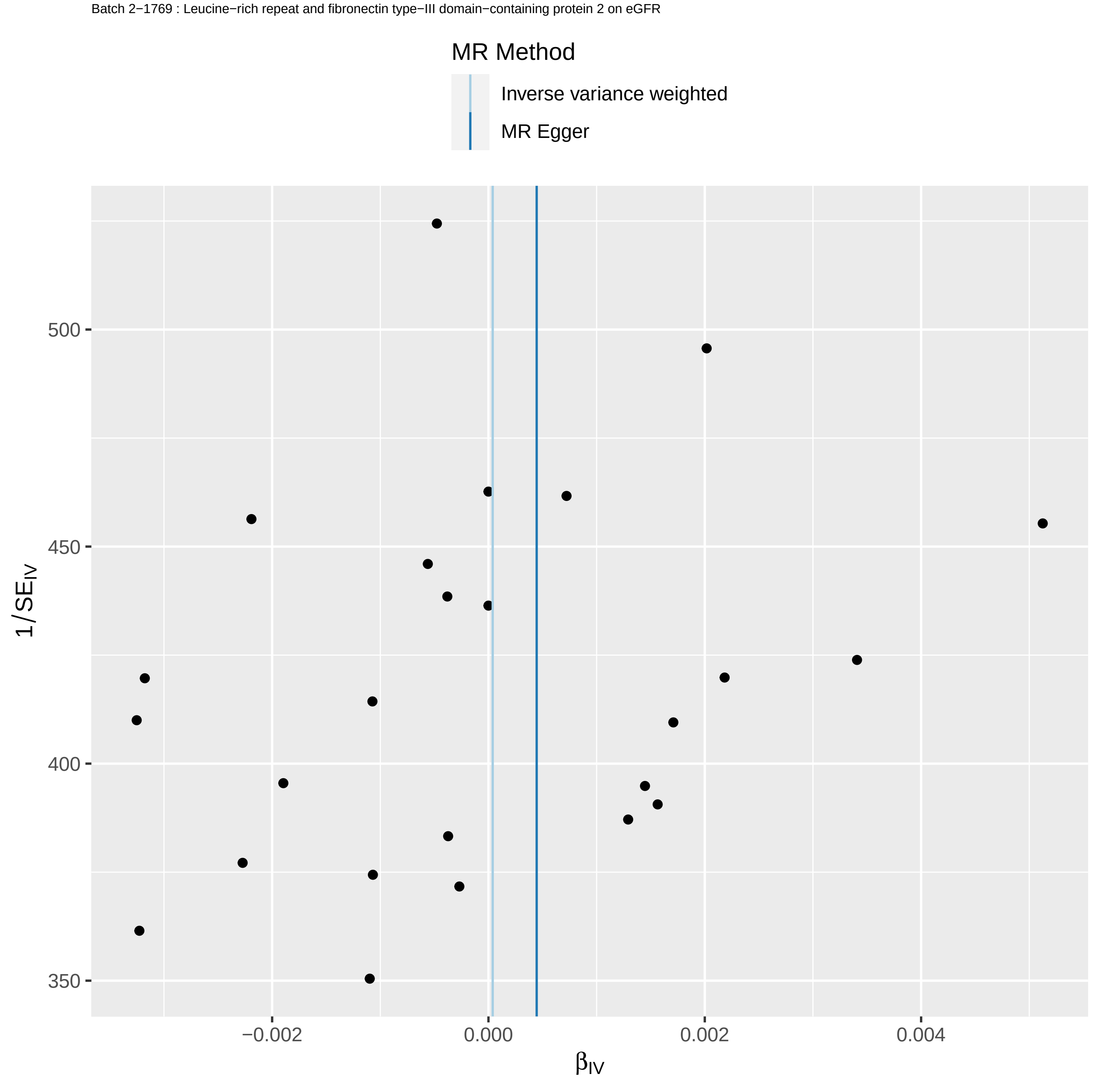

Batch 2−1769 : Leucine−rich repeat and fibronectin type−III domain−containing protein 2 on eGFR
MR Method
Inverse variance weighted
MR Egger
500
450
400
350
1/SE$_{IV}$
−0.002
0.000
0.002
0.004
β$_{IV}$

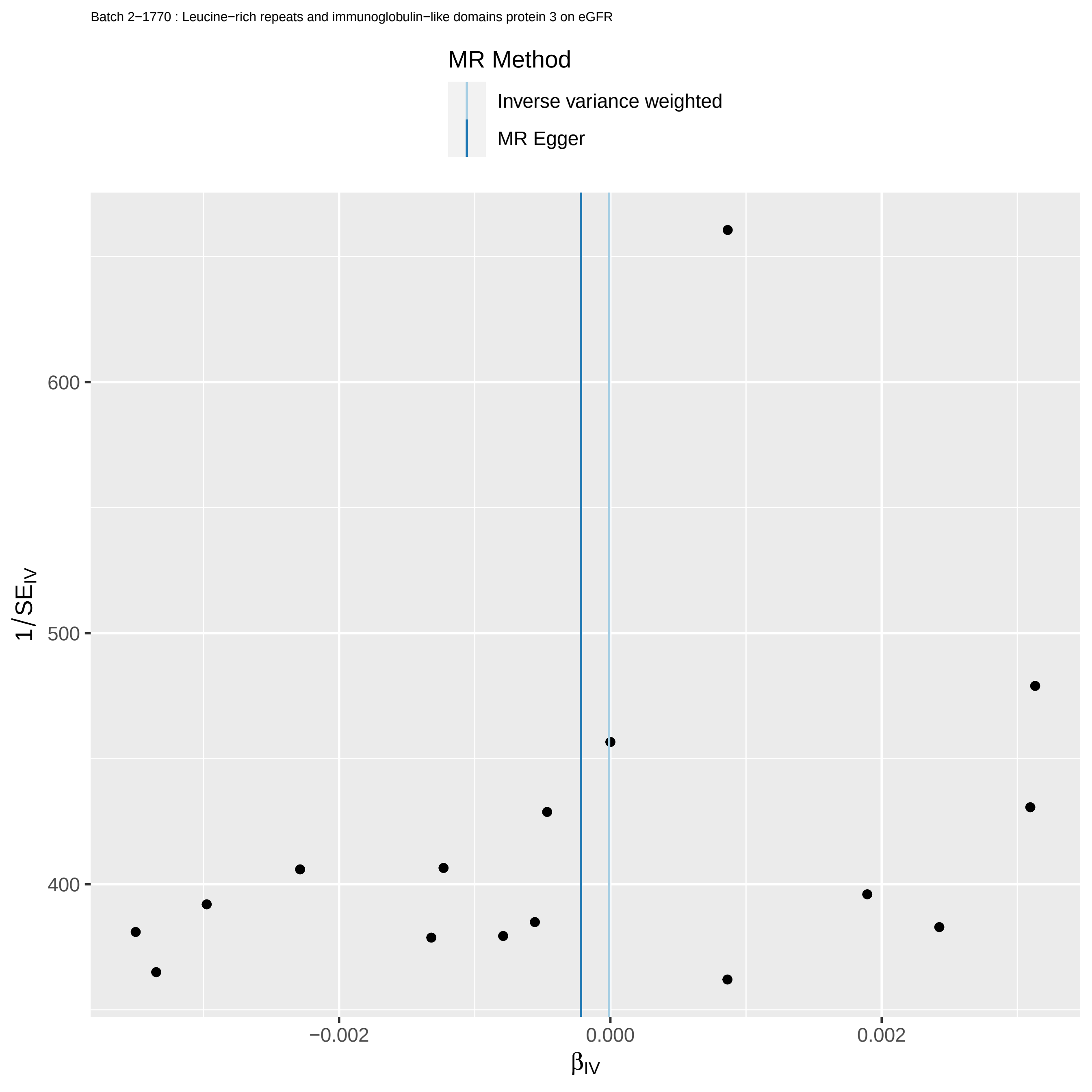

Batch 2−1770 : Leucine−rich repeats and immunoglobulin−like domains protein 3 on eGFR
MR Method
Inverse variance weighted
MR Egger
600
500
400
1/SE_IV
−0.002
0.000
0.002
β_IV

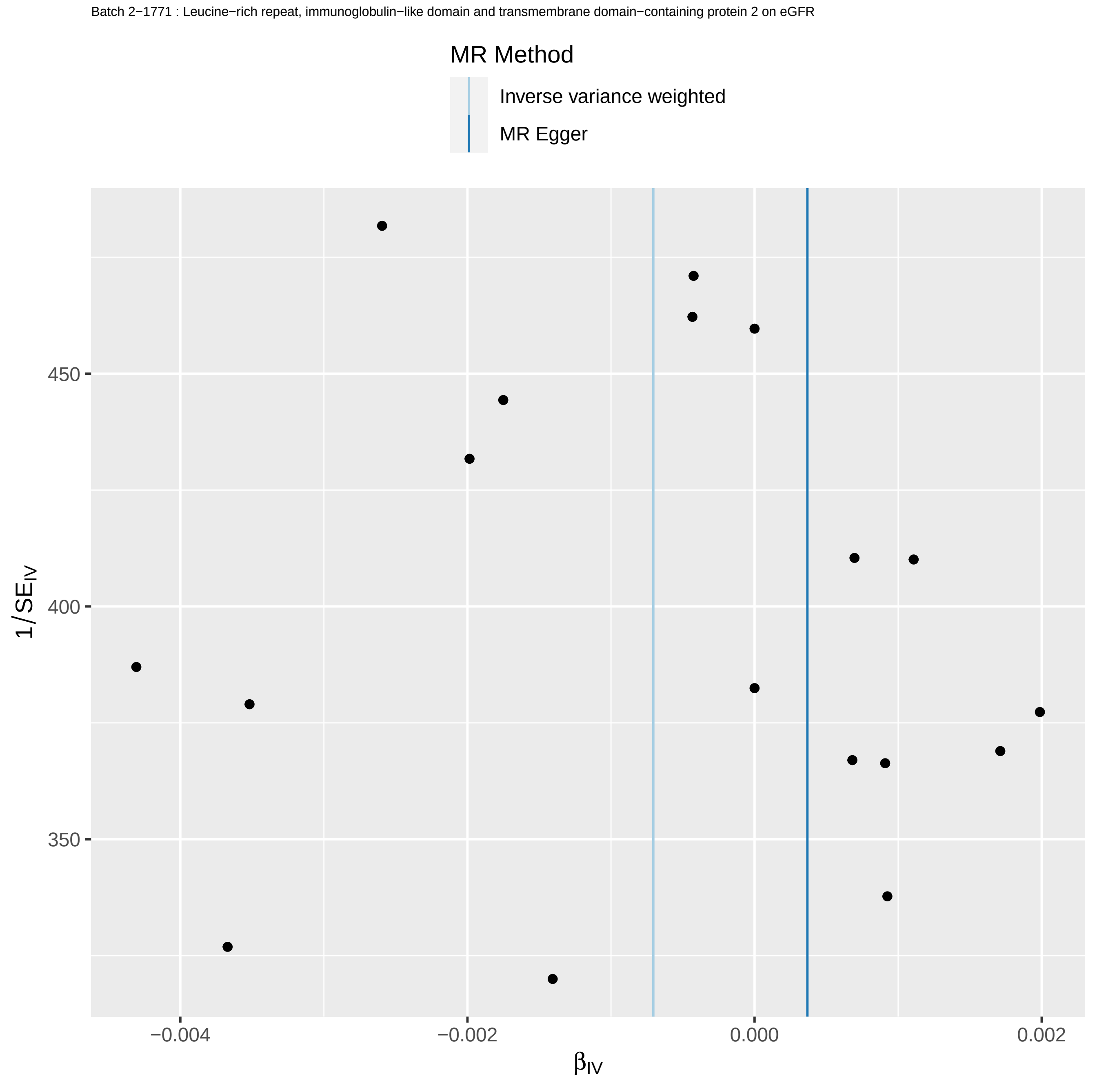
Batch 2−1771 : Leucine−rich repeat, immunoglobulin−like domain and transmembrane domain−containing protein 2 on eGFR
MR Method
Inverse variance weighted
MR Egger
450
400
350
1/SE_IV
−0.004
−0.002
0.000
0.002
β_IV

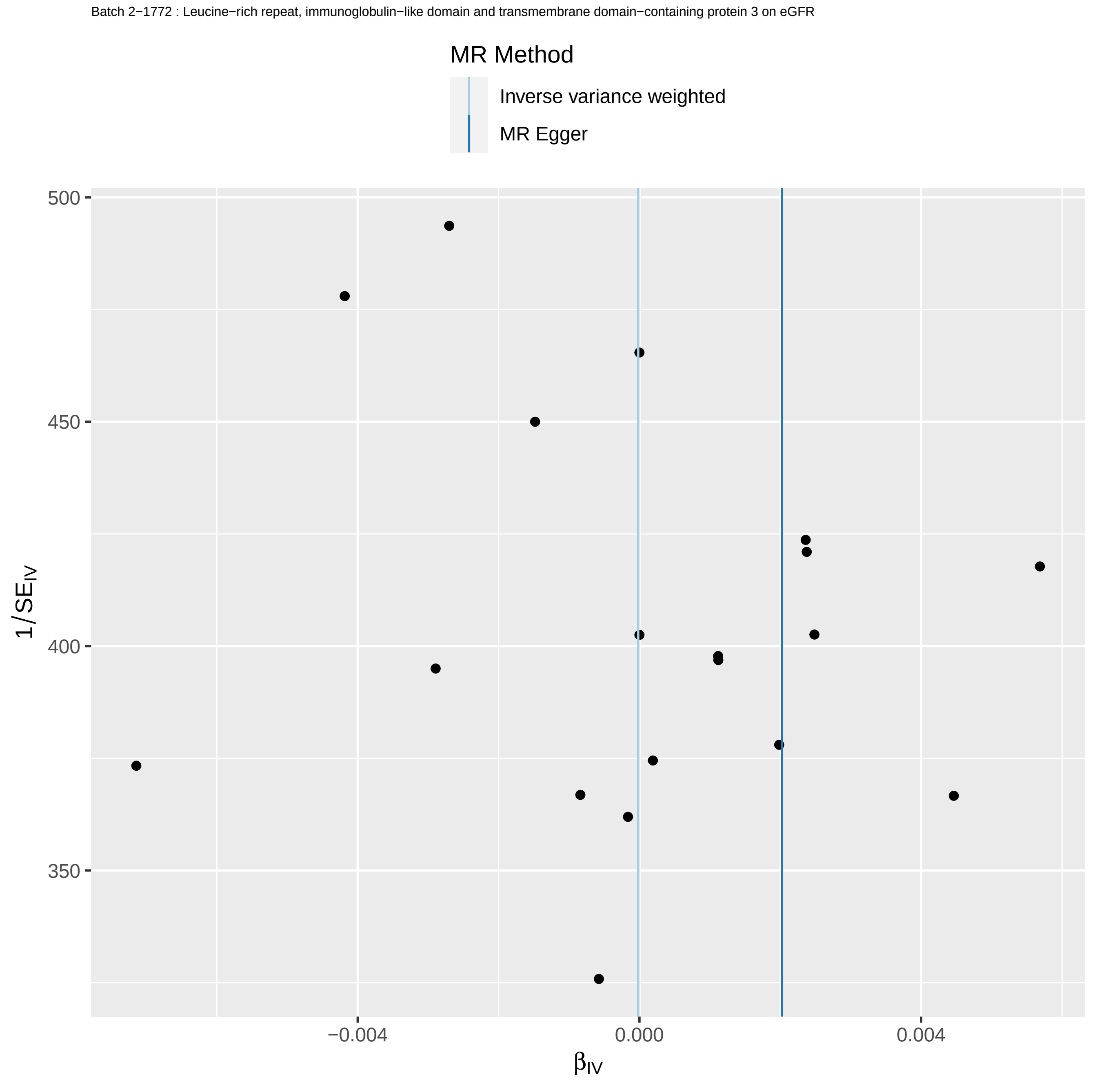
Batch 2−1772 : Leucine−rich repeat, immunoglobulin−like domain and transmembrane domain−containing protein 3 on eGFR
MR Method
Inverse variance weighted
MR Egger
500
450
400
350
$1/SE_{IV}$
−0.004
0.000
0.004
β_{IV}

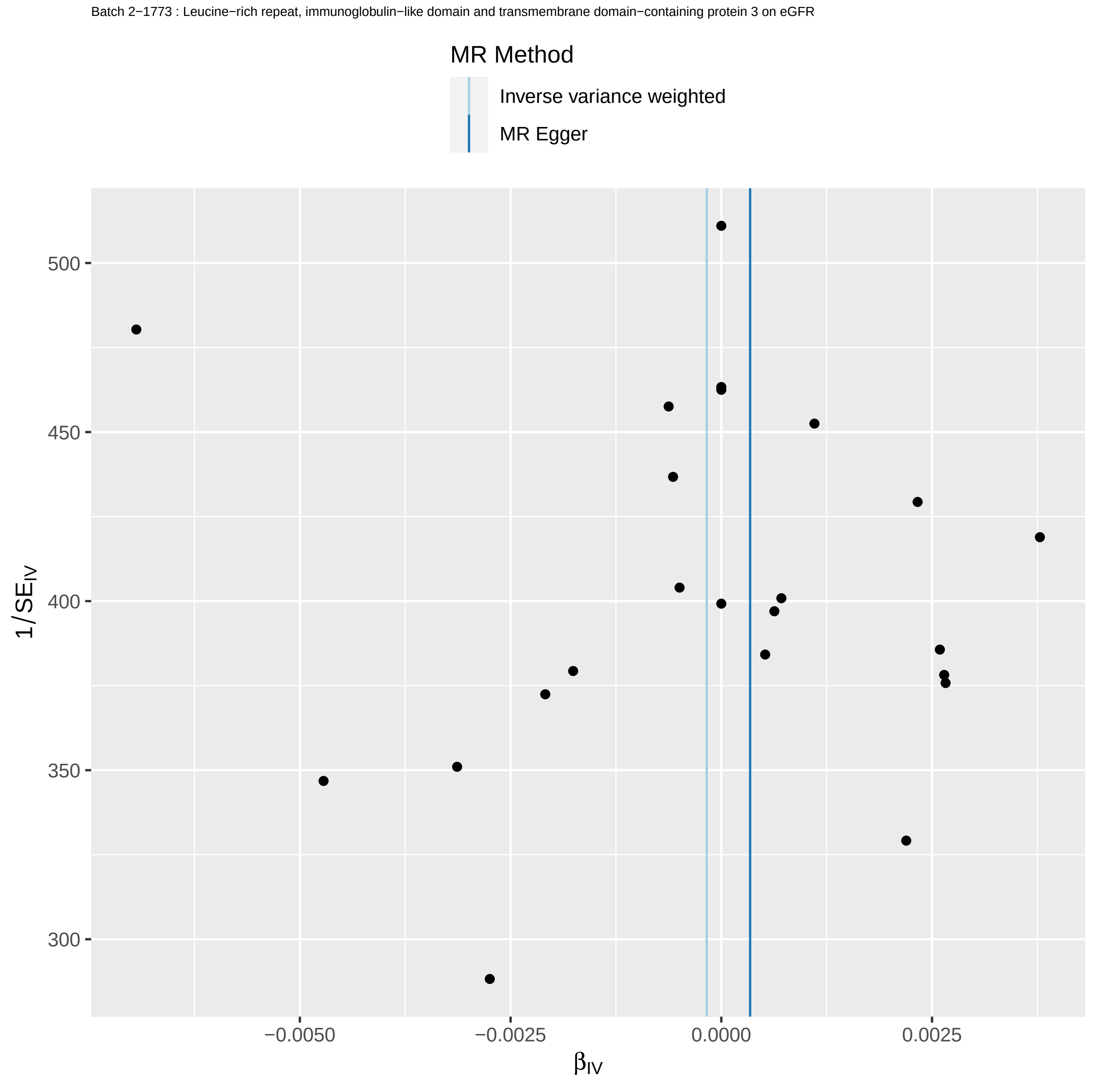

Batch 2−1773 : Leucine−rich repeat, immunoglobulin−like domain and transmembrane domain−containing protein 3 on eGFR
MR Method
Inverse variance weighted
MR Egger
500
450
400
350
300
1/SE$_{IV}$
−0.0050
−0.0025
0.0000
0.0025
β$_{IV}$

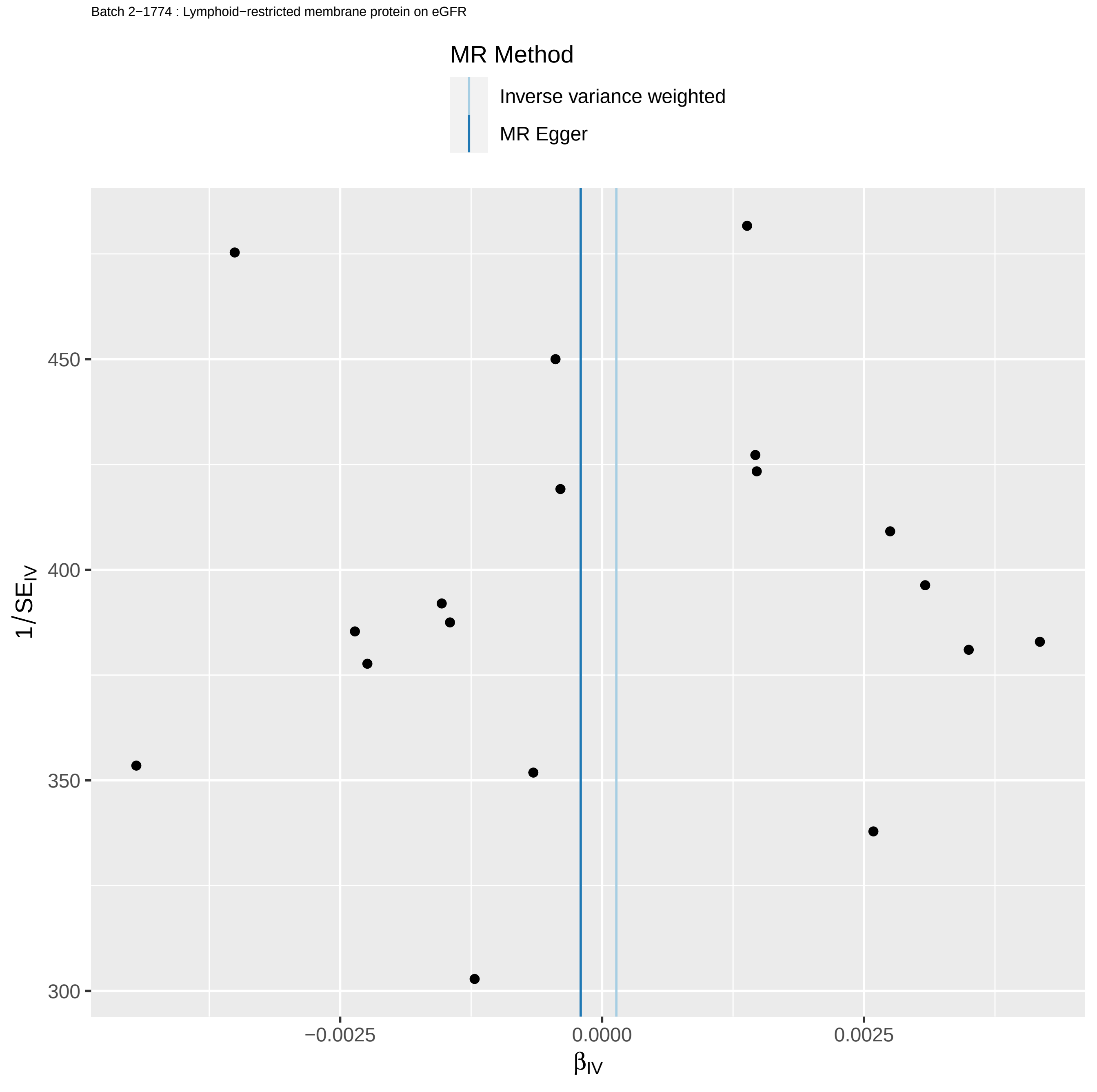

Batch 2−1774 : Lymphoid−restricted membrane protein on eGFR
MR Method
Inverse variance weighted
MR Egger
450
400
350
300
1/SE$_{IV}$
−0.0025
0.0000
0.0025
β$_{IV}$

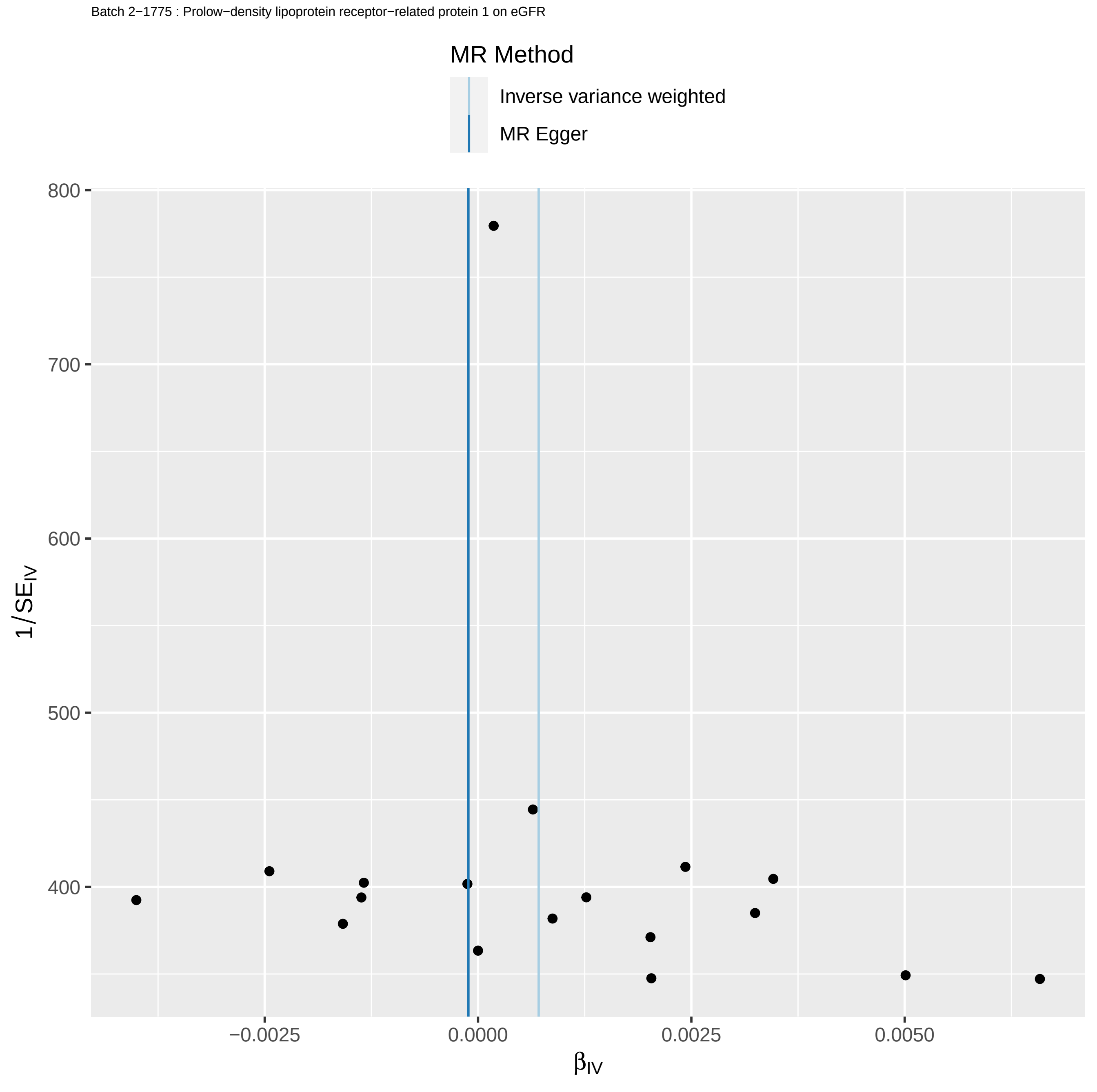

Batch 2−1775 : Prolow−density lipoprotein receptor−related protein 1 on eGFR
MR Method
Inverse variance weighted
MR Egger
800
700
600
500
400
1/SE_IV
−0.0025
0.0000
0.0025
0.0050
β_IV

Batch 2−1776 : Low−density lipoprotein receptor−related protein 1, soluble on eGFR

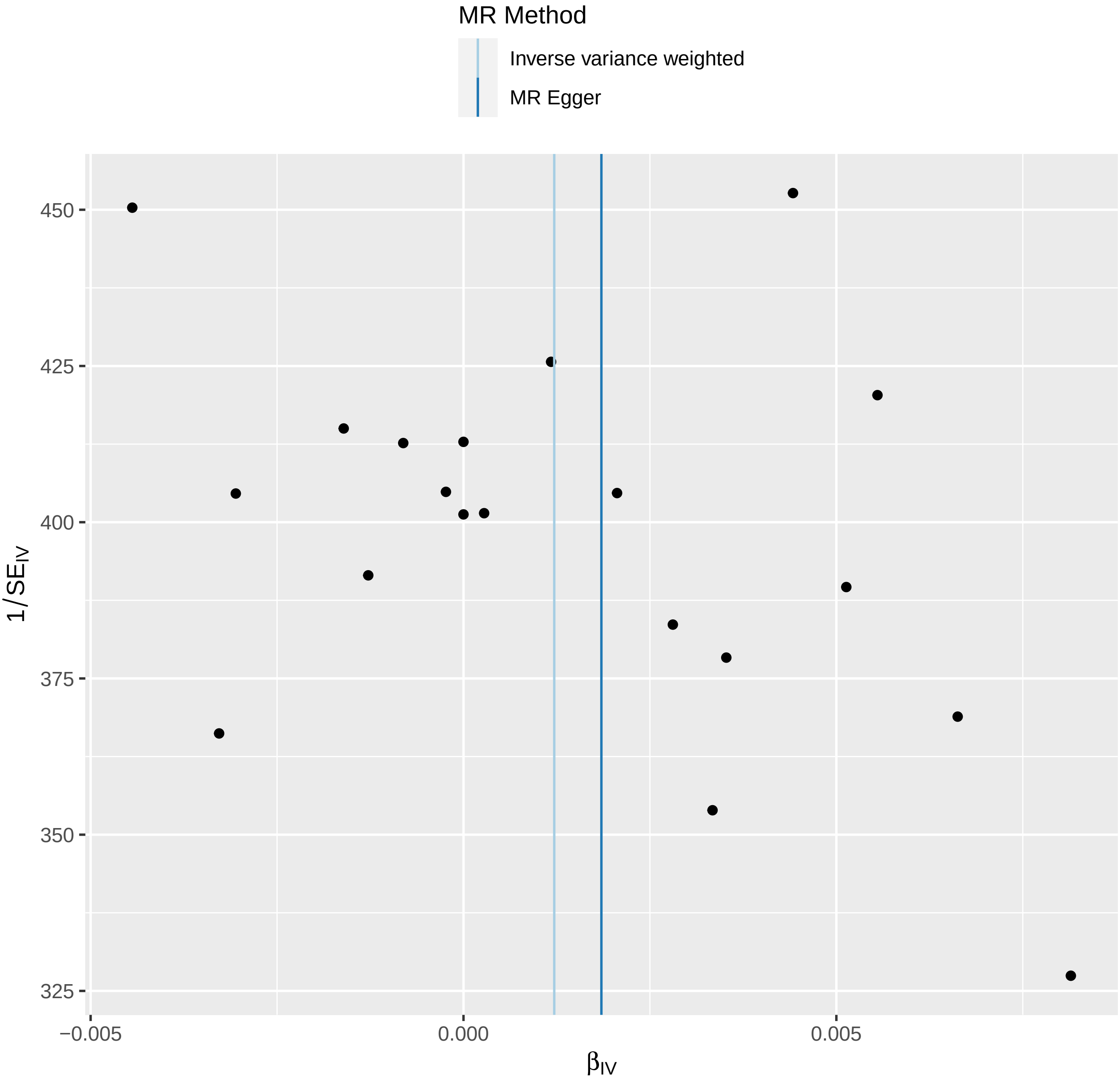

Batch 2−1777 : Low−density lipoprotein receptor−related protein 1, soluble on eGFR

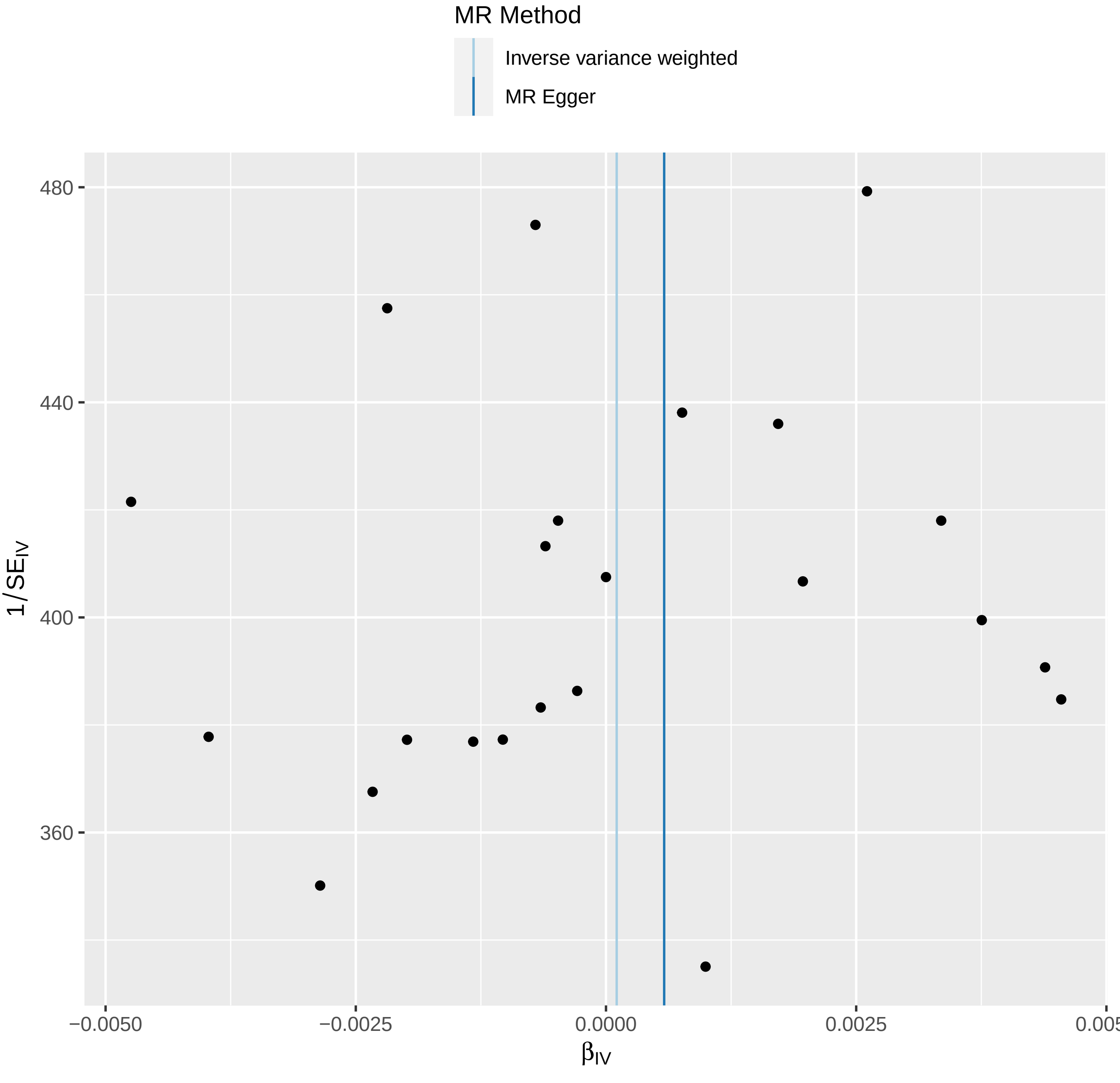

Batch 2−1778 : Low−density lipoprotein receptor−related protein 1B on eGFR

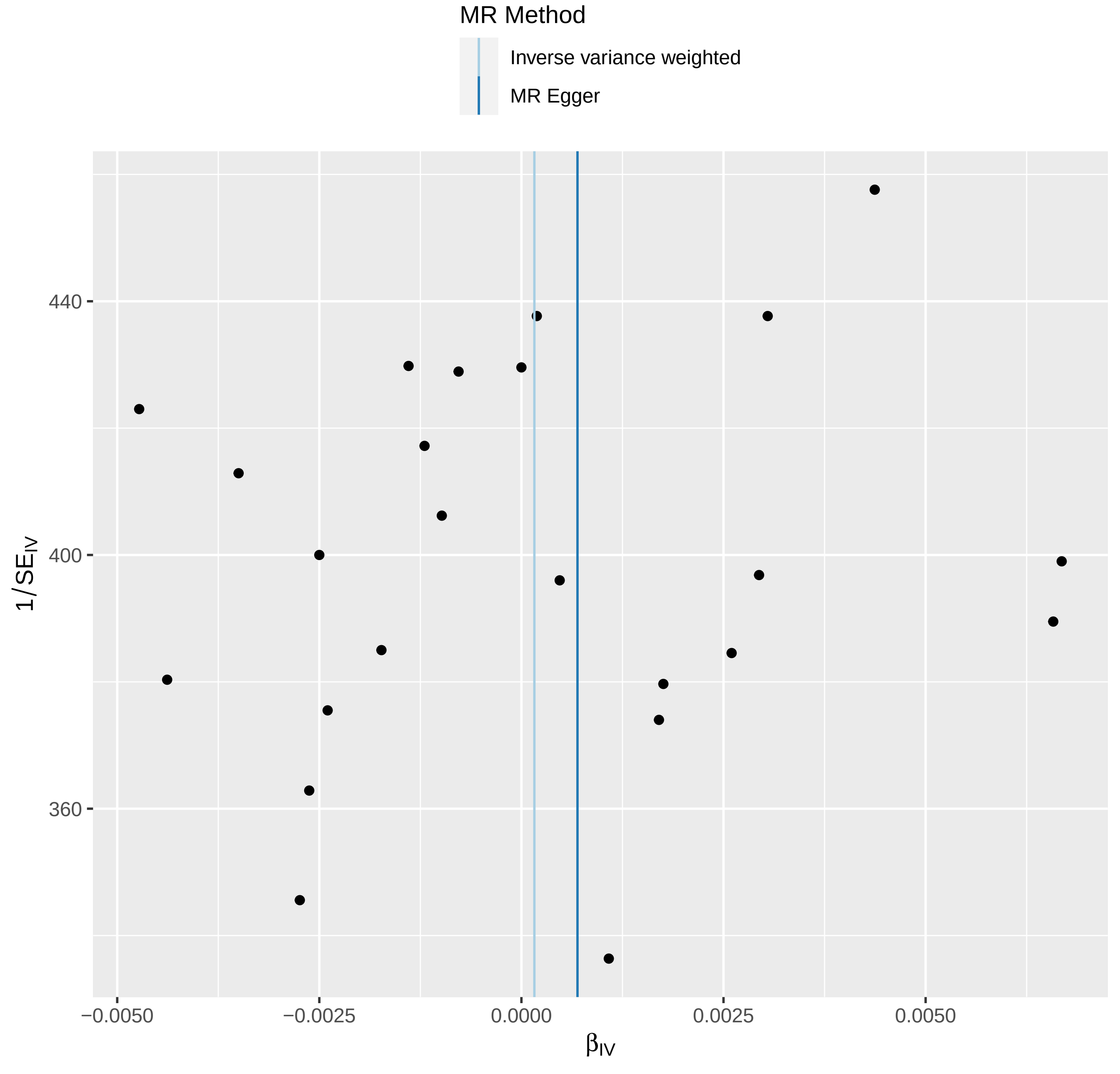

Batch 2−1779 : Low−density lipoprotein receptor−related protein 1B on eGFR

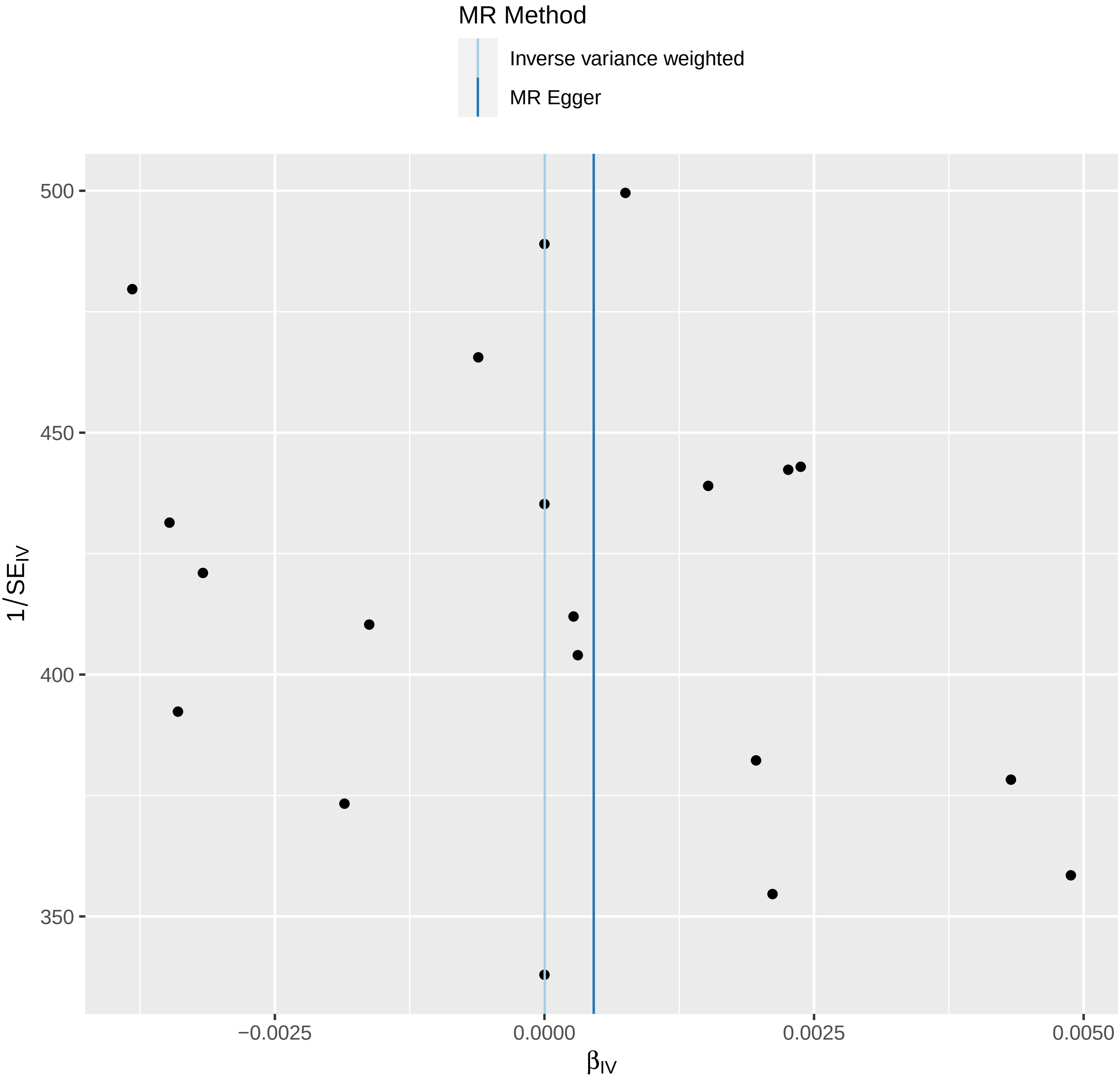

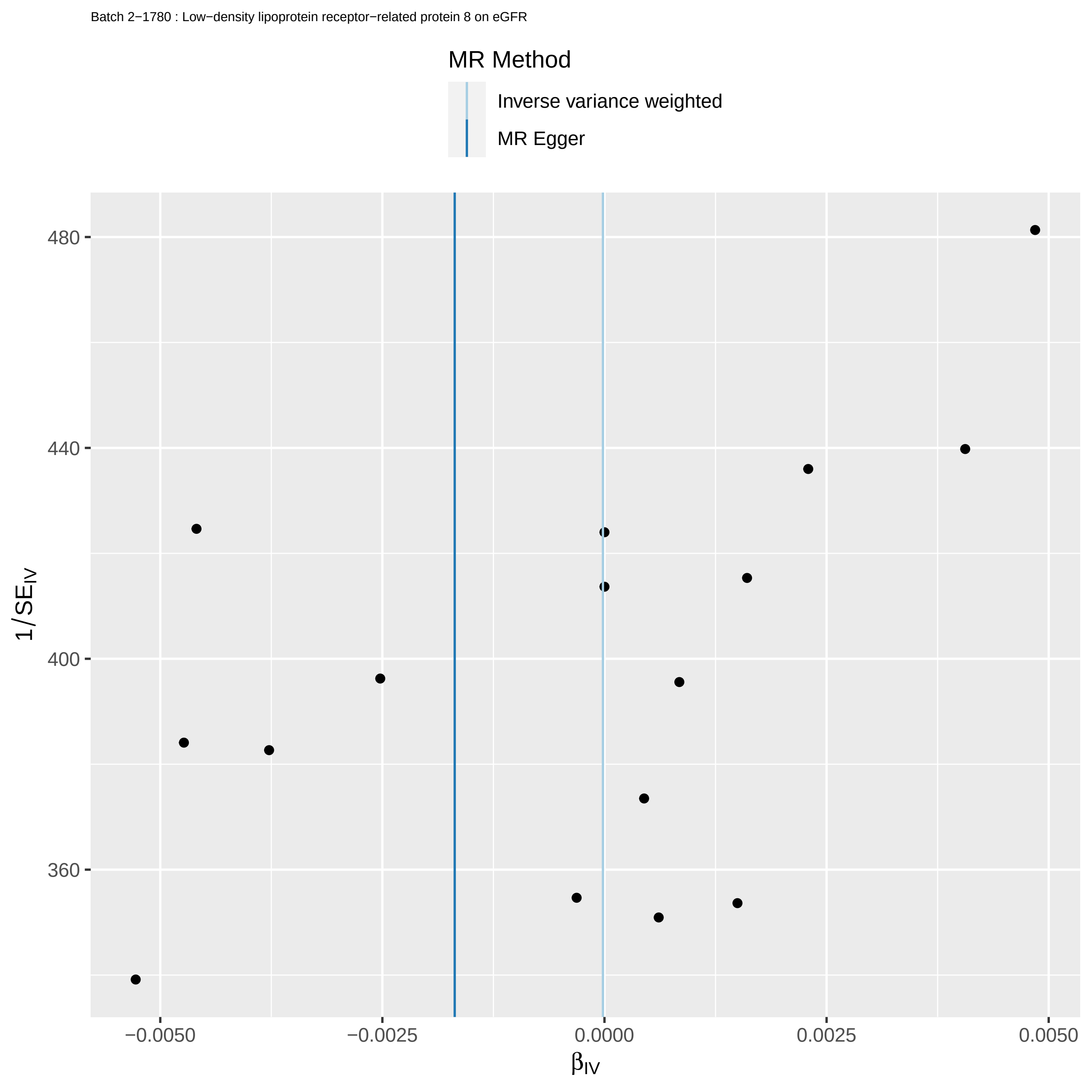

Batch 2−1780 : Low−density lipoprotein receptor−related protein 8 on eGFR
MR Method
Inverse variance weighted
MR Egger
480
440
400
360
1/SE_IV
−0.0050
−0.0025
0.0000
0.0025
0.0050
β_IV

Batch 2−1781 : alpha−2−macroglobulin receptor−associated protein on eGFR

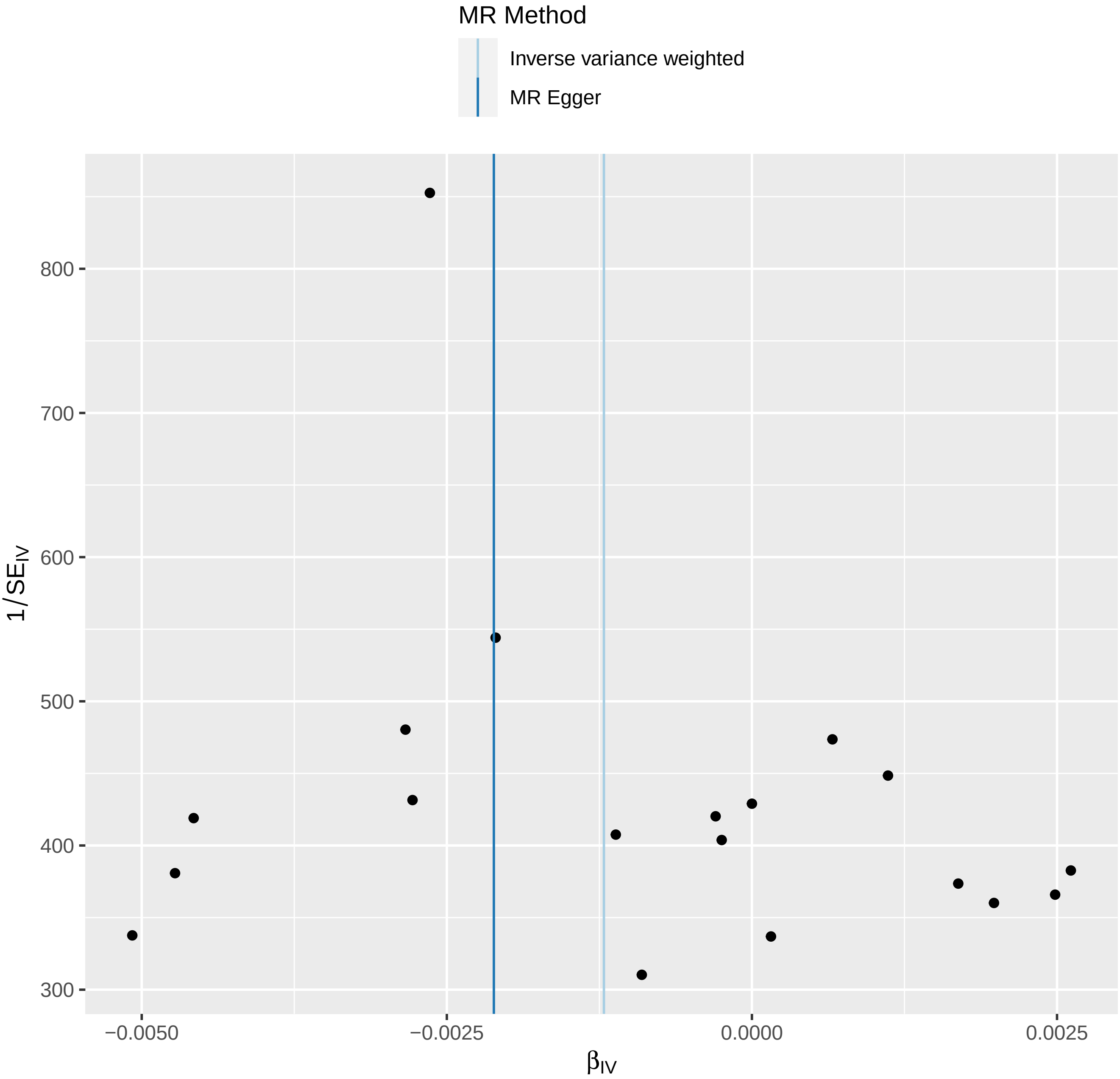

Batch 2−1782 : alpha−2−macroglobulin receptor−associated protein on eGFR

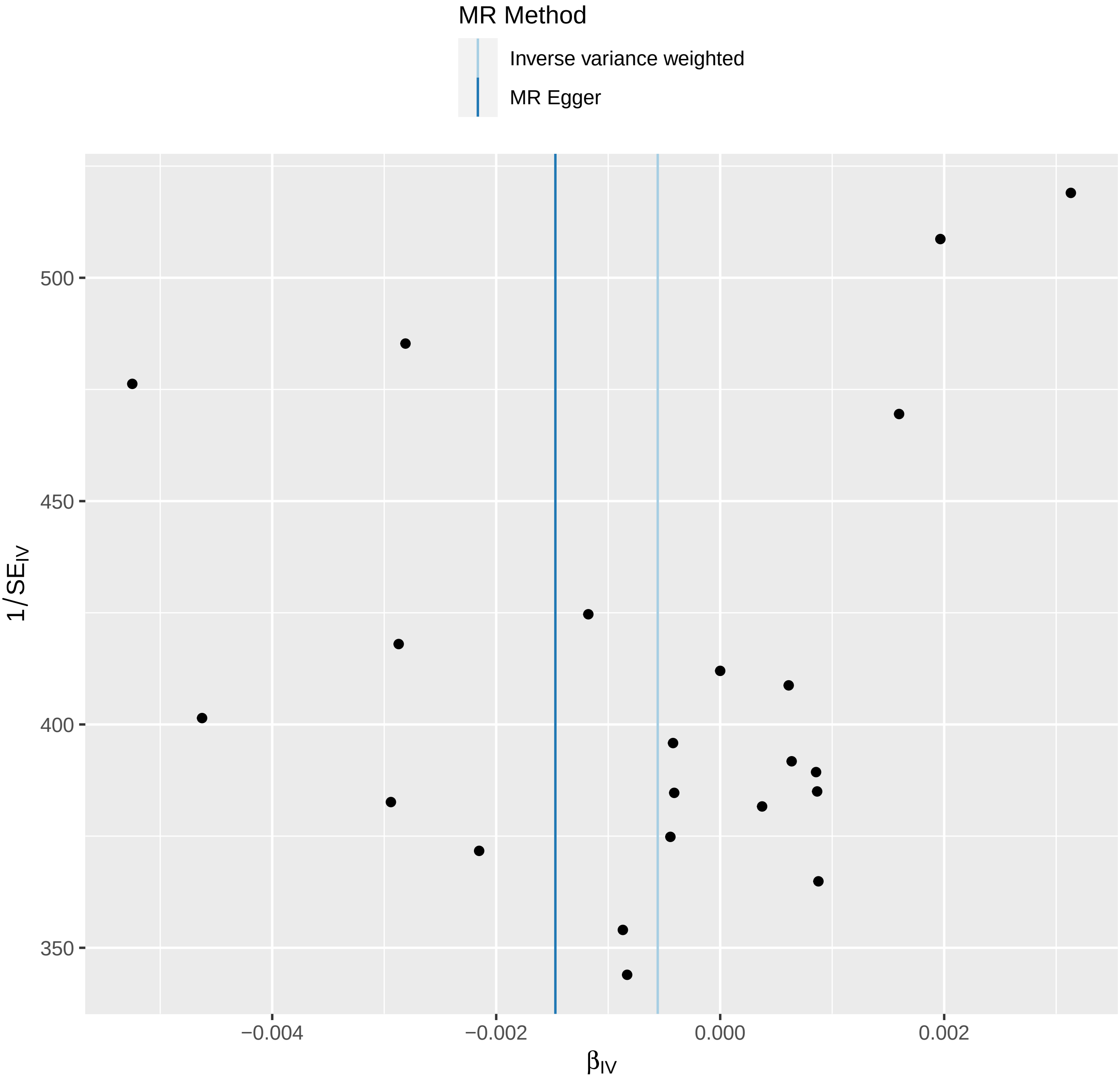

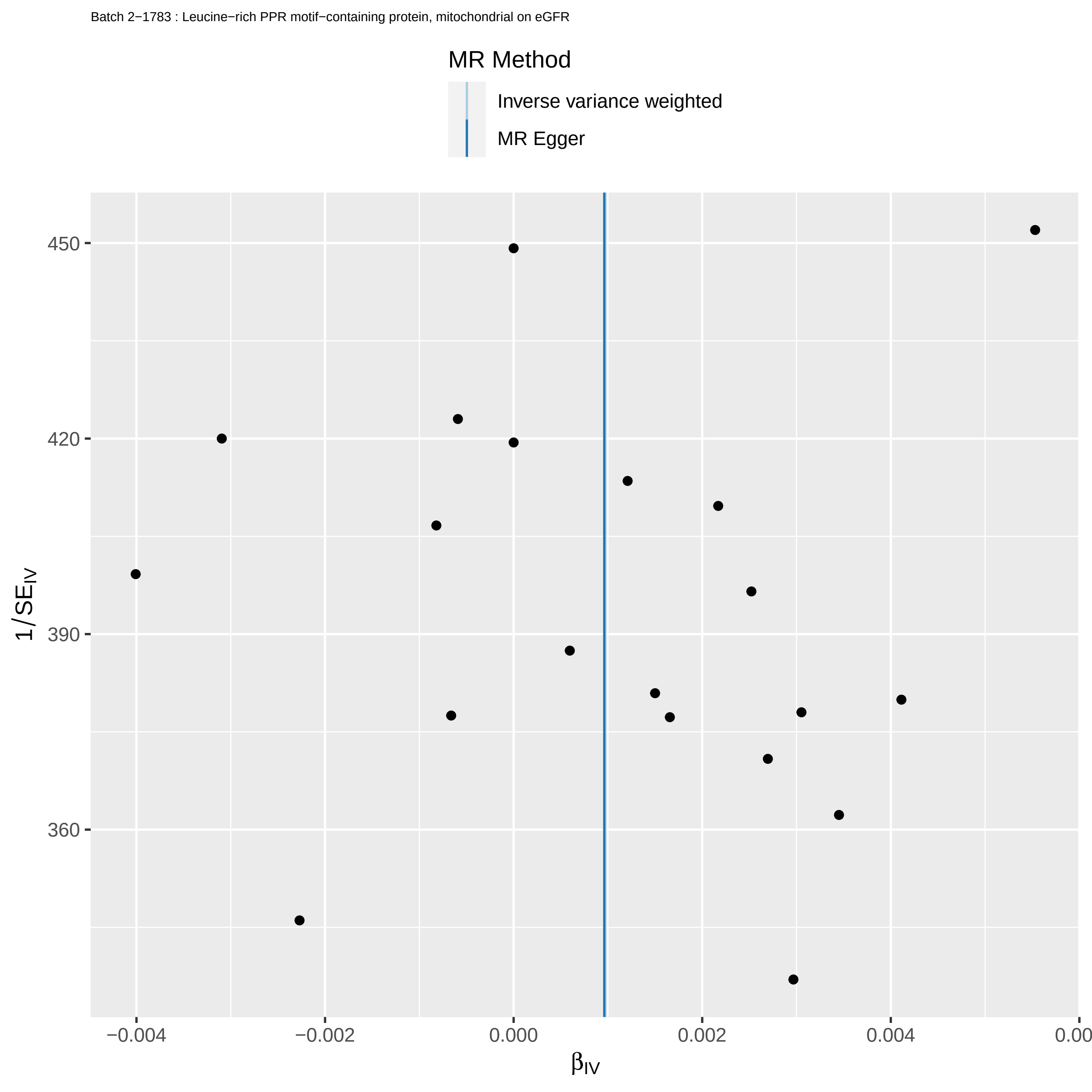
Batch 2−1783 : Leucine−rich PPR motif−containing protein, mitochondrial on eGFR
MR Method
Inverse variance weighted
MR Egger
450
420
390
360
1/SE$_{IV}$
−0.004
−0.002
0.000
0.002
0.004
0.00
β$_{IV}$

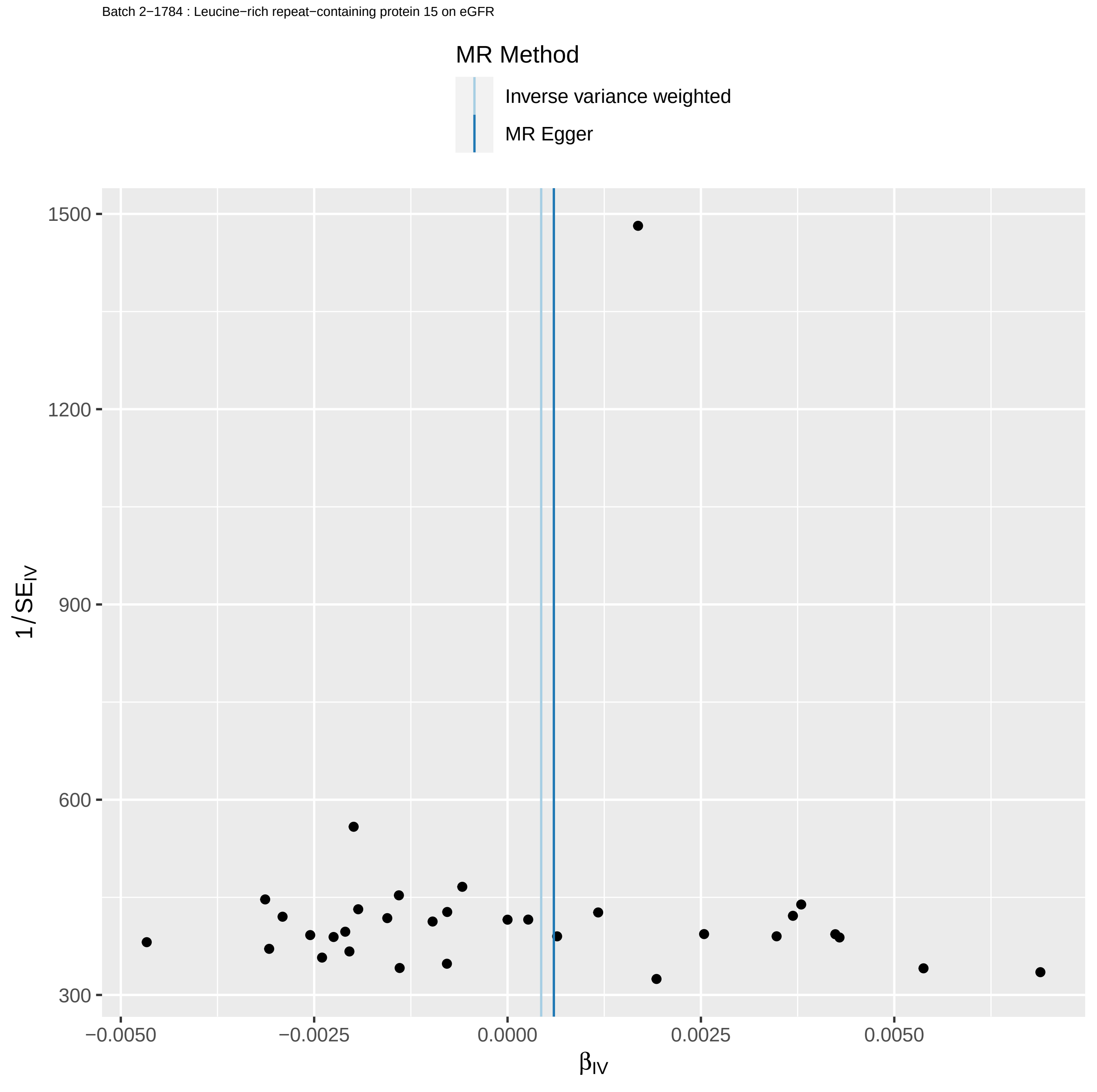

Batch 2−1784 : Leucine−rich repeat−containing protein 15 on eGFR
MR Method
Inverse variance weighted
MR Egger
1500
1200
900
600
300
1/SE$_{IV}$
−0.0050
−0.0025
0.0000
0.0025
0.0050
β$_{IV}$

Batch 2−1785 : Leucine−rich repeat−containing protein 19 on eGFR

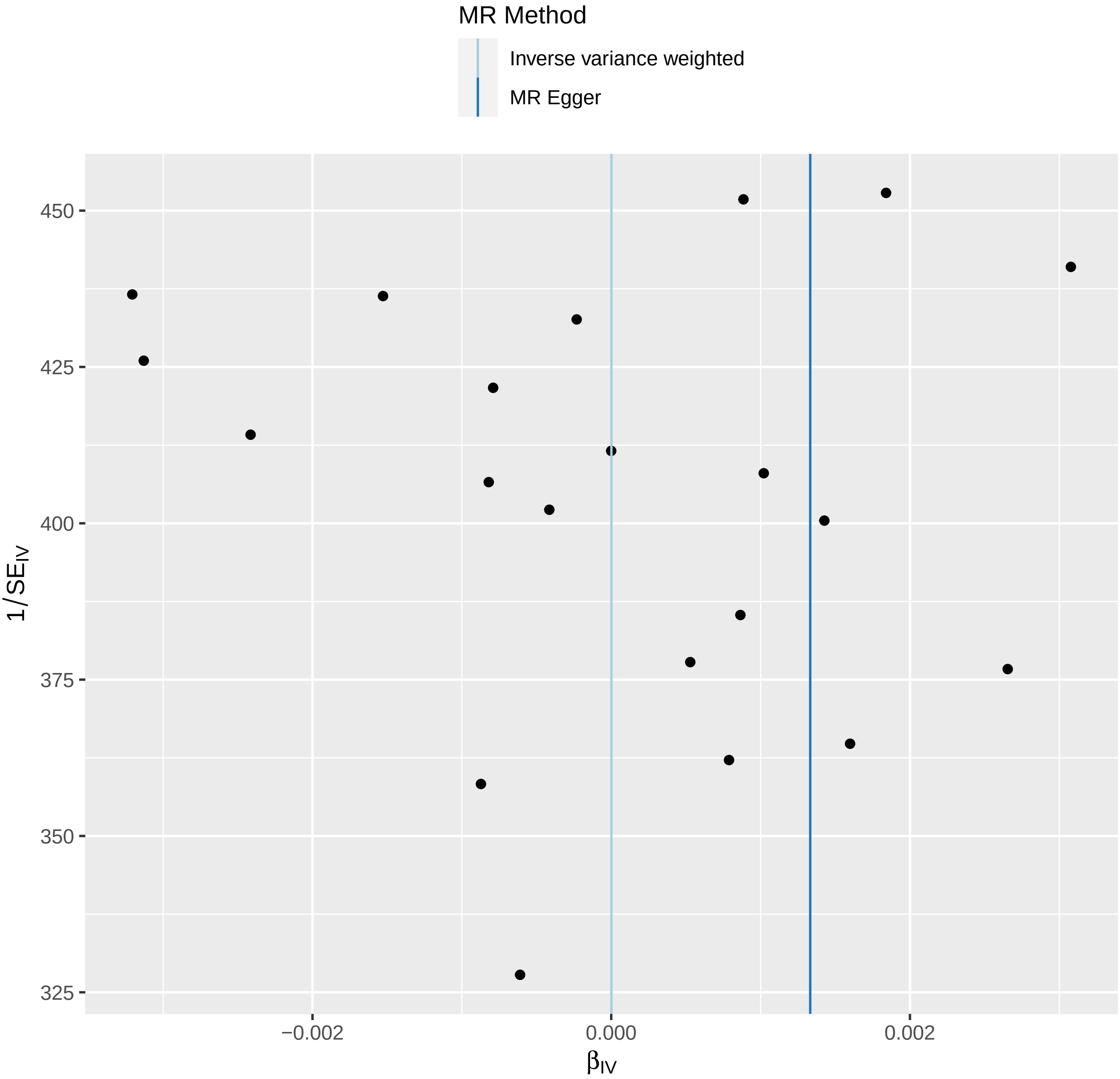

Batch 2−1786 : Leucine−rich repeat−containing protein 3 on eGFR

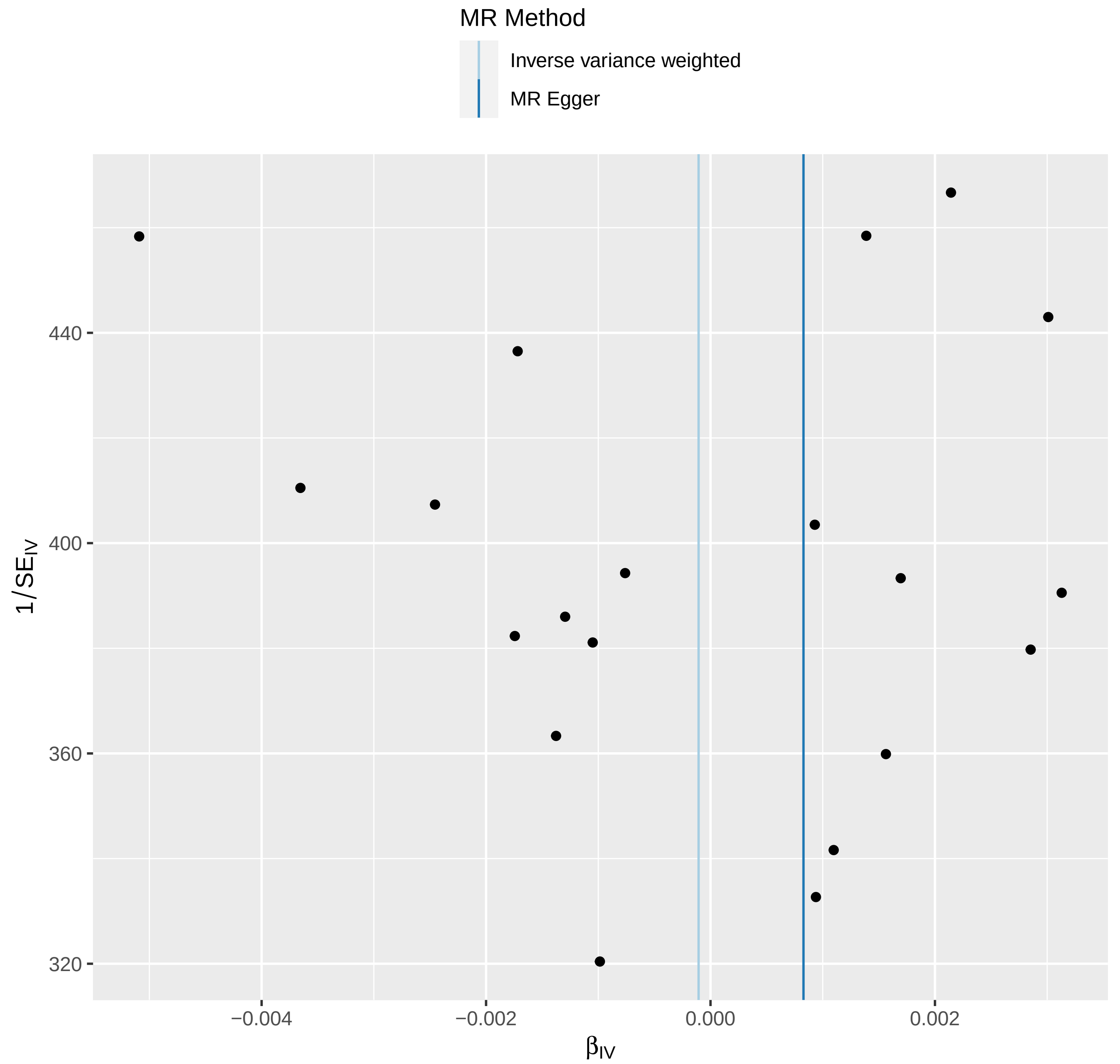

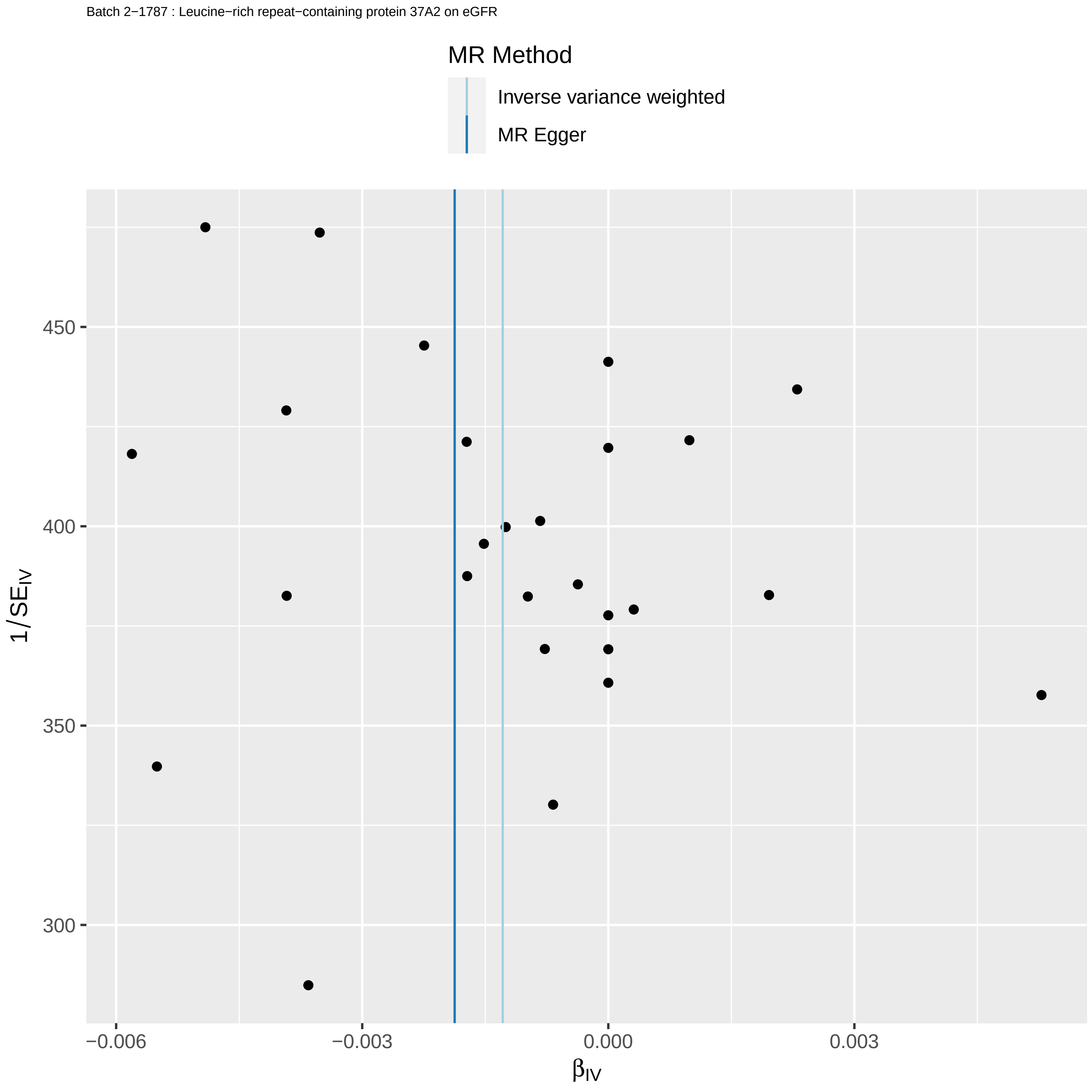

Batch 2−1787 : Leucine−rich repeat−containing protein 37A2 on eGFR
MR Method
Inverse variance weighted
MR Egger
450
400
350
300
1/SE$_{IV}$
−0.006
−0.003
0.000
0.003
β_{IV}

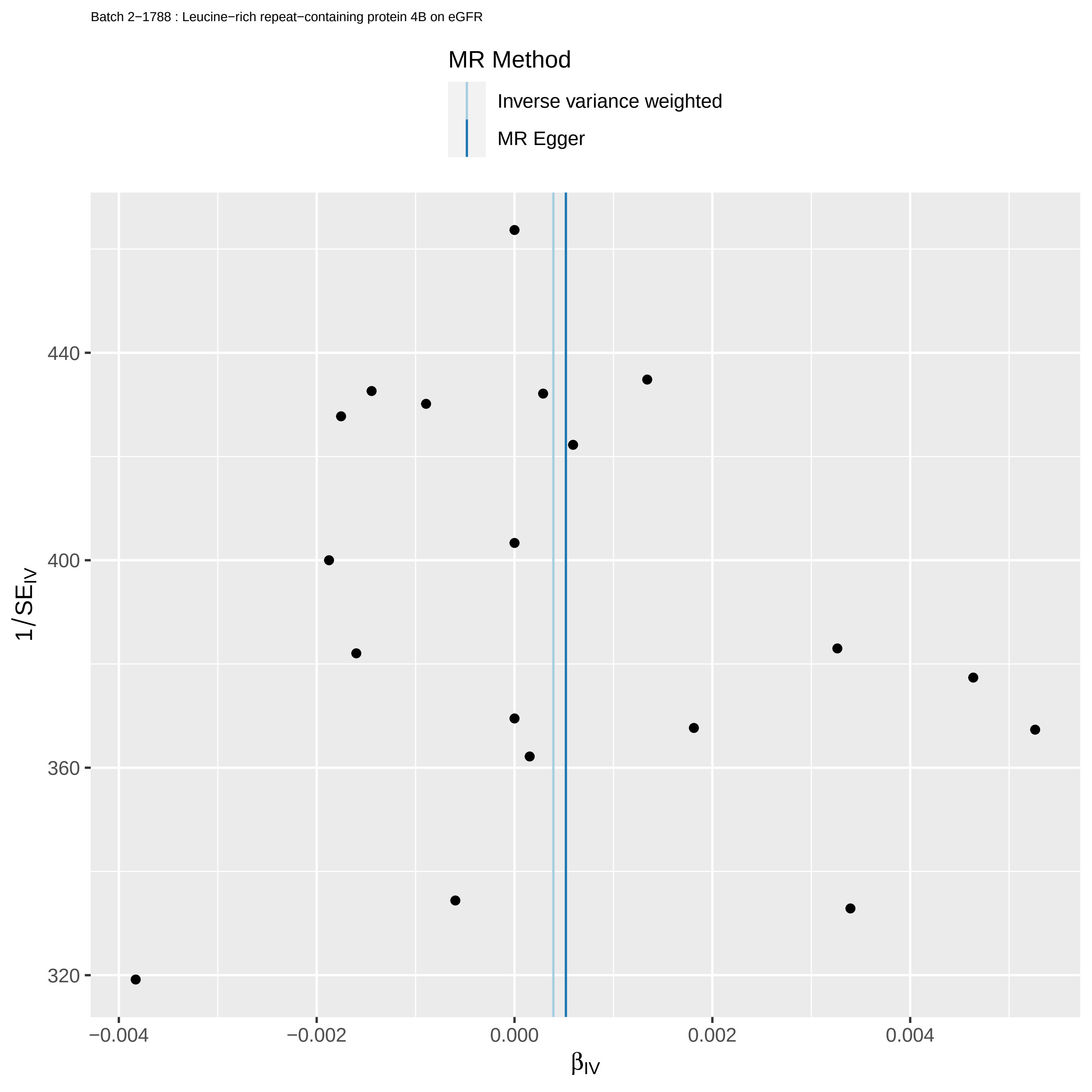
Batch 2−1788 : Leucine−rich repeat−containing protein 4B on eGFR
MR Method
Inverse variance weighted
MR Egger
440
400
360
320
1/SE_IV
−0.004
−0.002
0.000
0.002
0.004
β_IV

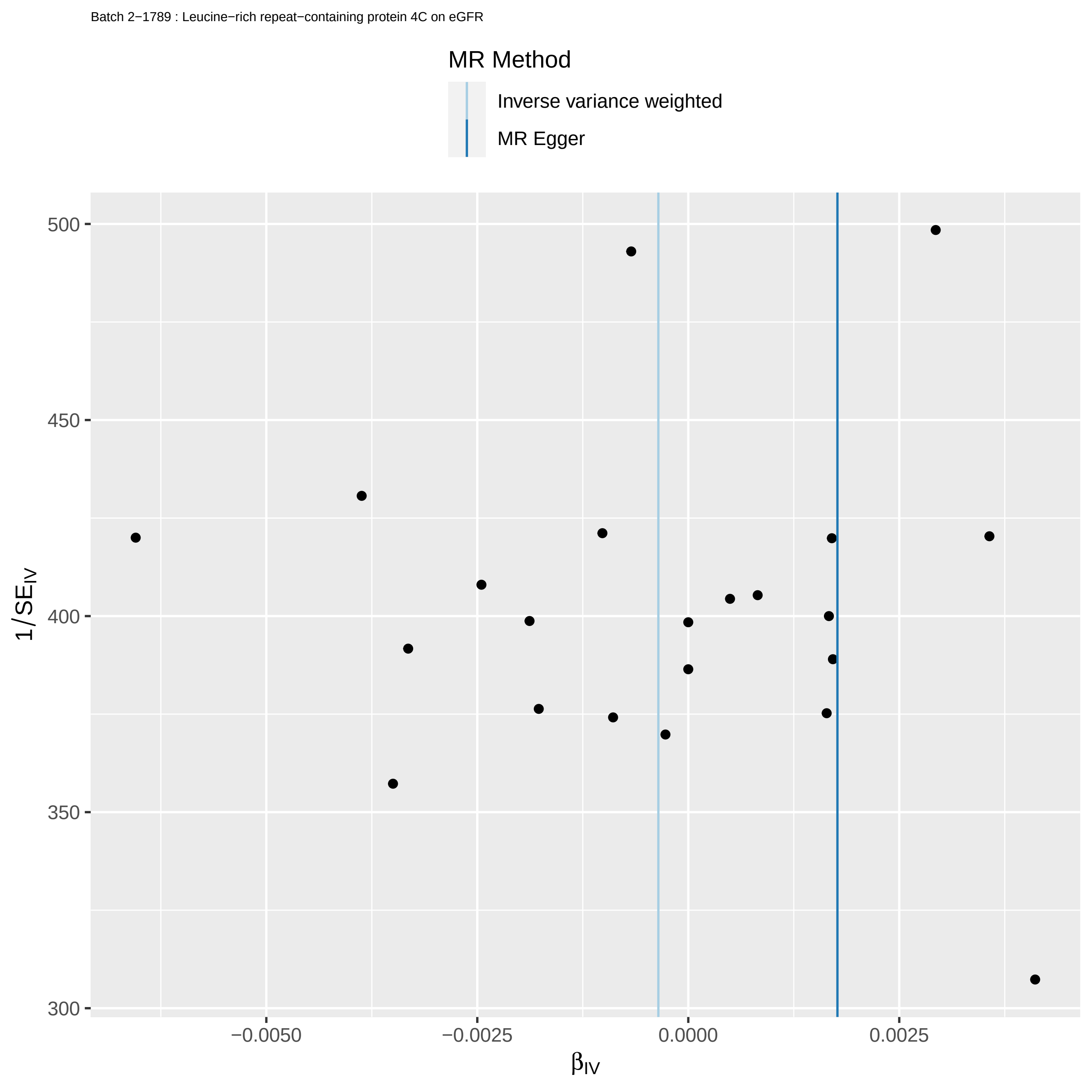

Batch 2−1789 : Leucine−rich repeat−containing protein 4C on eGFR
MR Method
Inverse variance weighted
MR Egger
500
450
400
350
300
1/SE_IV
−0.0050
−0.0025
0.0000
0.0025
β_IV

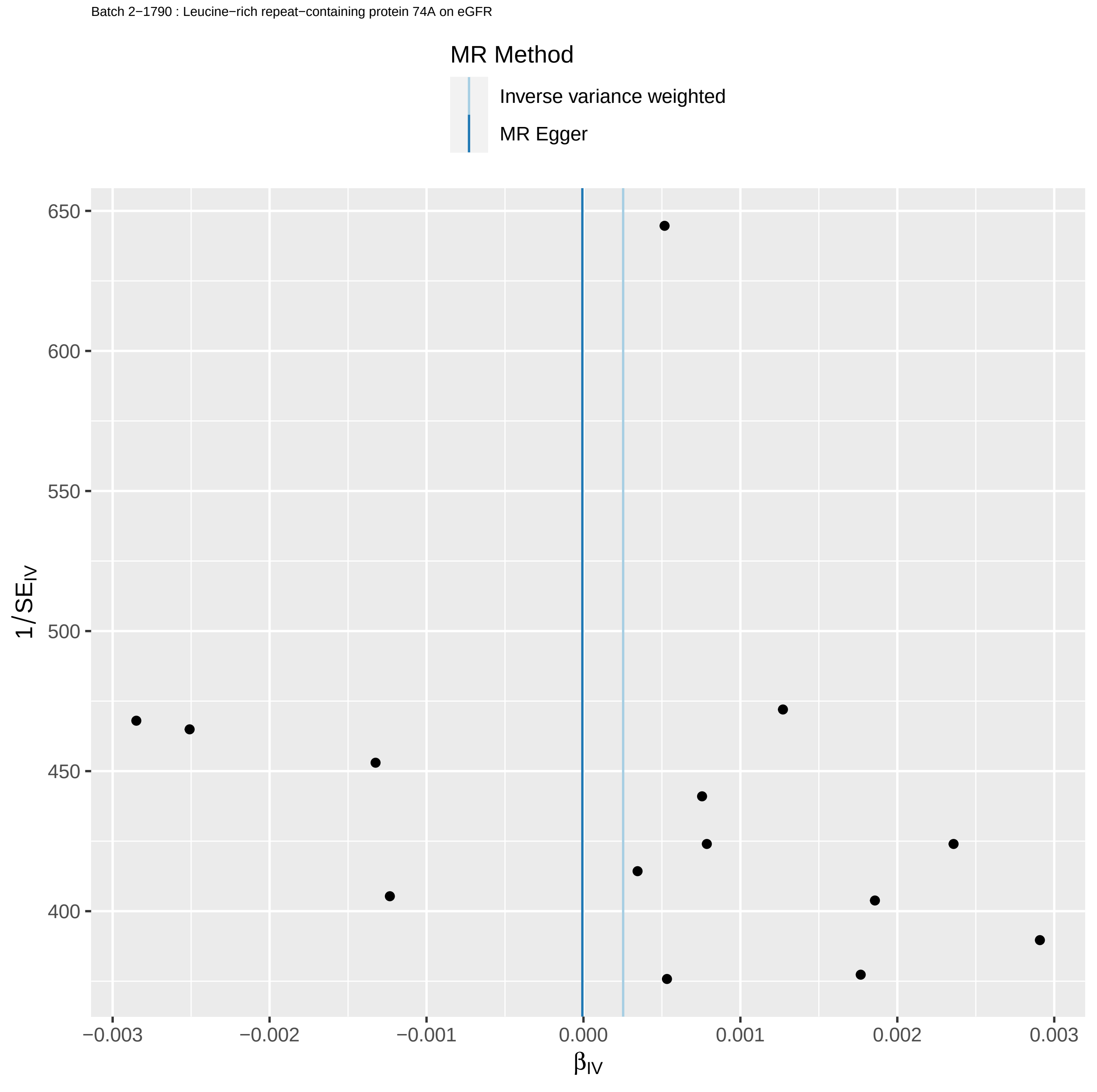

Batch 2−1790 : Leucine−rich repeat−containing protein 74A on eGFR
MR Method
Inverse variance weighted
MR Egger
650
600
550
500
450
400
1/SE_IV
−0.003
−0.002
−0.001
0.000
0.001
0.002
0.003
β_IV

Batch 2−1791 : Leucine−rich repeat serine/threonine−protein kinase 2 on eGFR

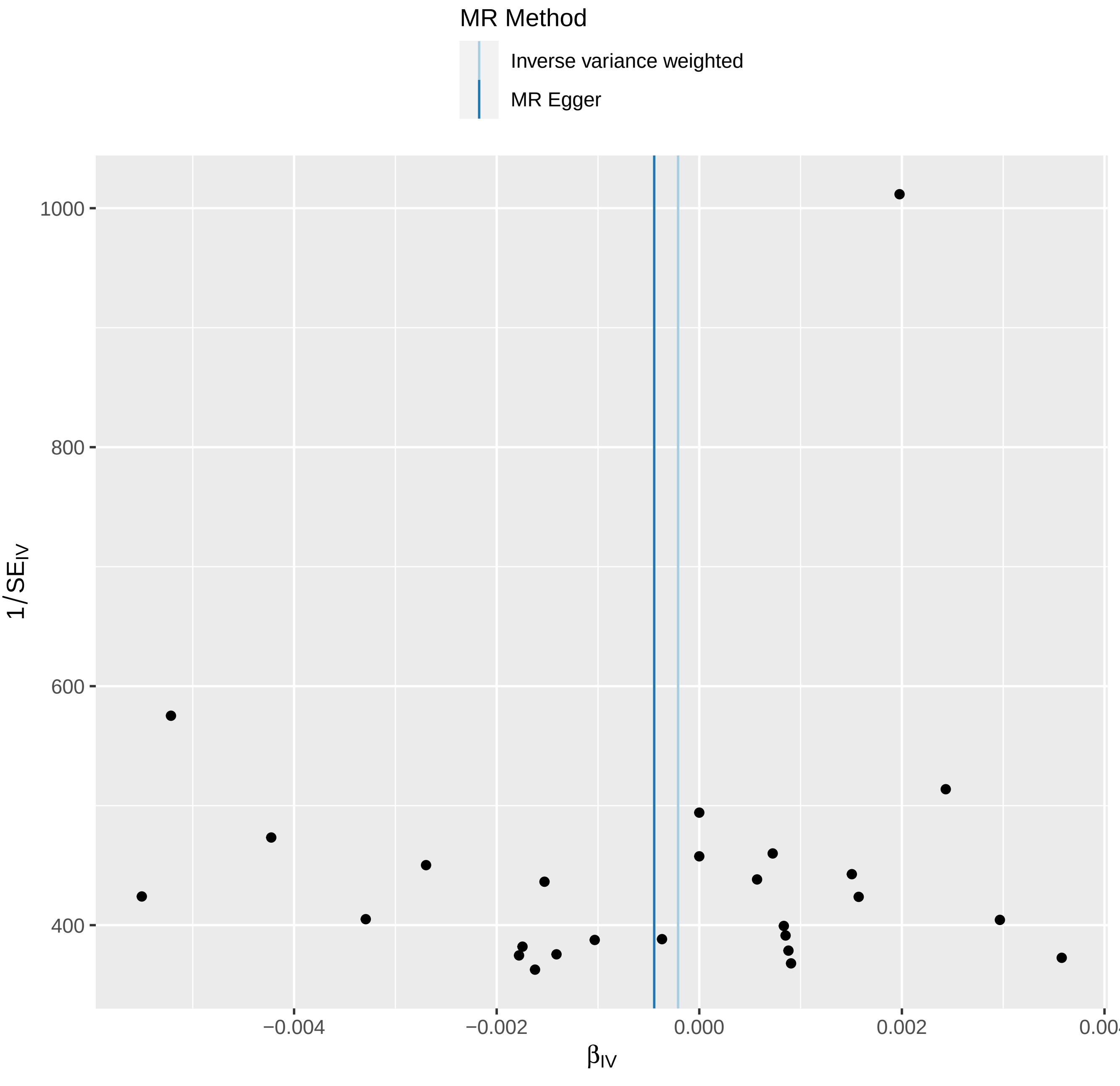

Batch 2−1792 : Leucine−rich repeat neuronal protein 1 on eGFR

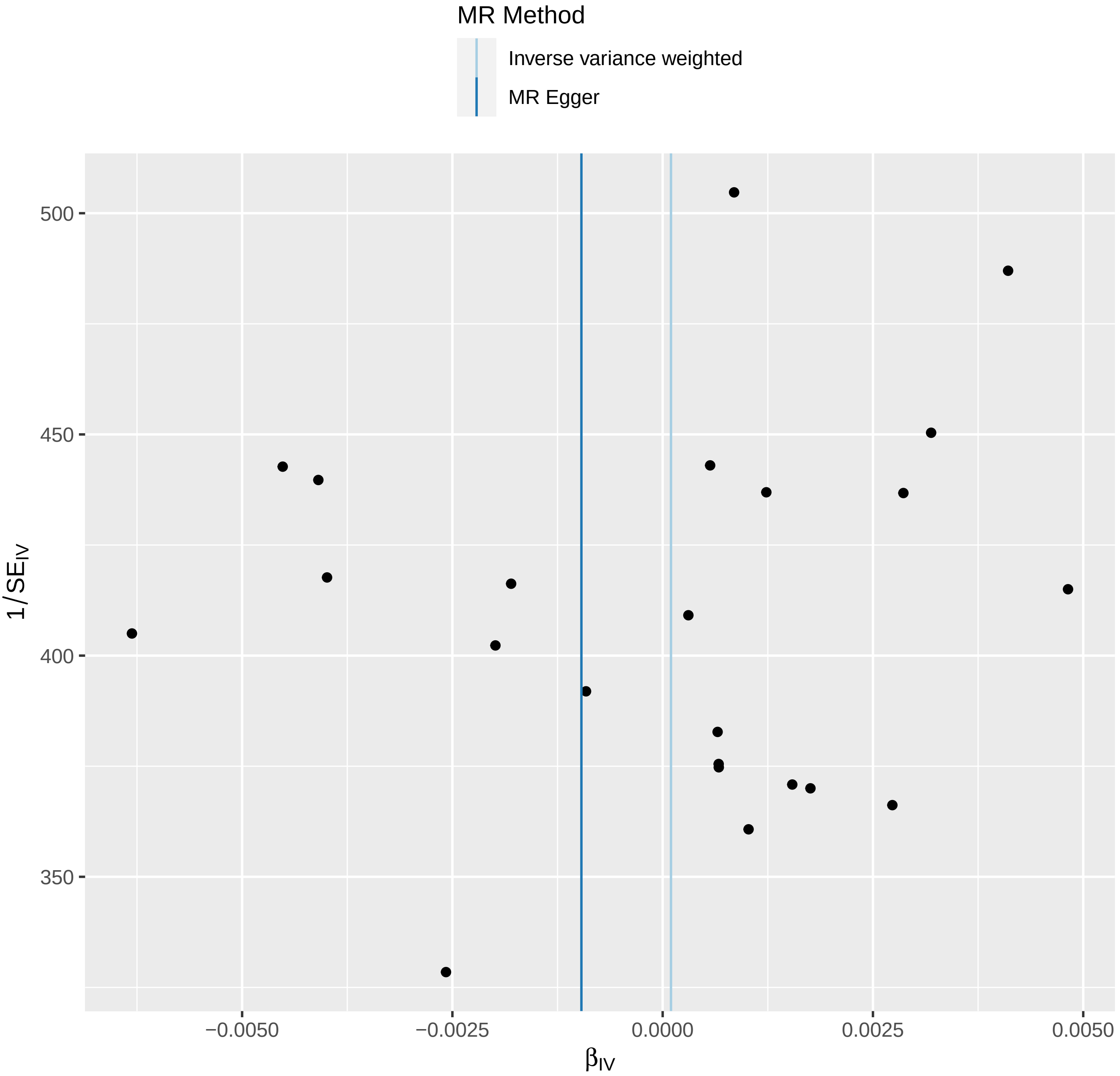

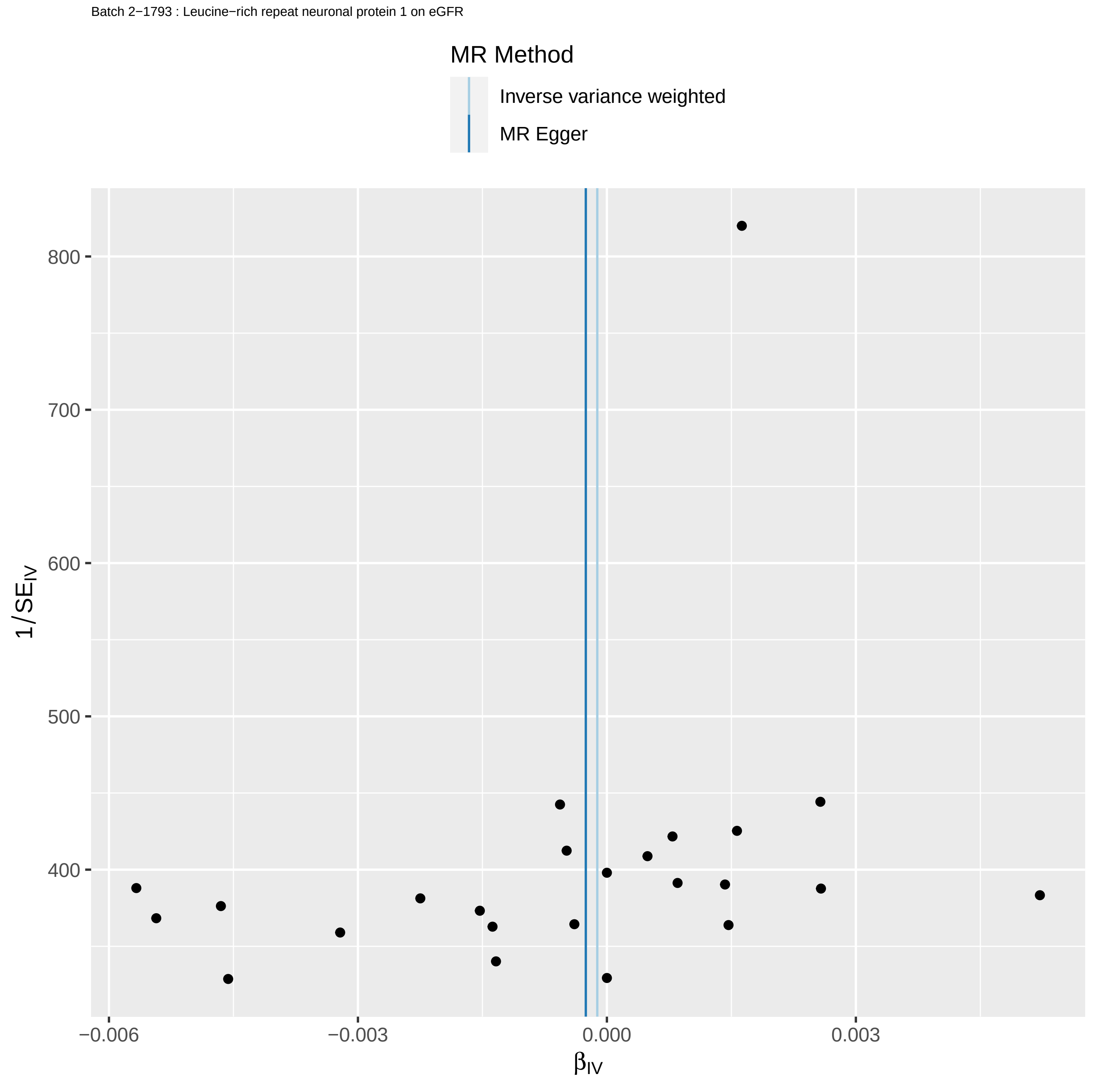
Batch 2−1793 : Leucine−rich repeat neuronal protein 1 on eGFR
MR Method
Inverse variance weighted
MR Egger
800
700
600
500
400
1/SE_IV
−0.006
−0.003
0.000
0.003
β_IV

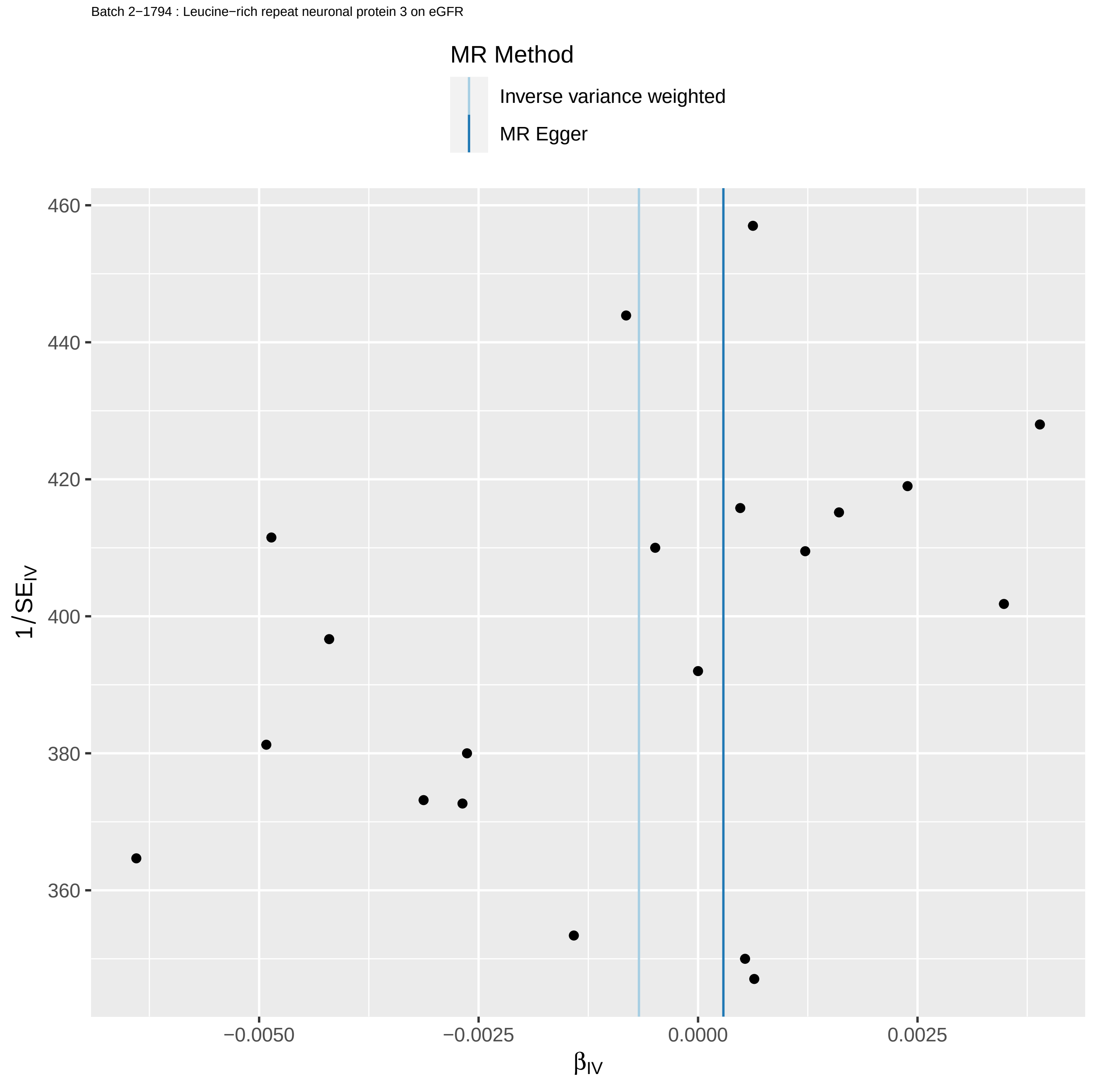

Batch 2−1794 : Leucine−rich repeat neuronal protein 3 on eGFR
MR Method
Inverse variance weighted
MR Egger
460
440
420
400
380
360
1/SE$_{IV}$
−0.0050
−0.0025
0.0000
0.0025
β$_{IV}$

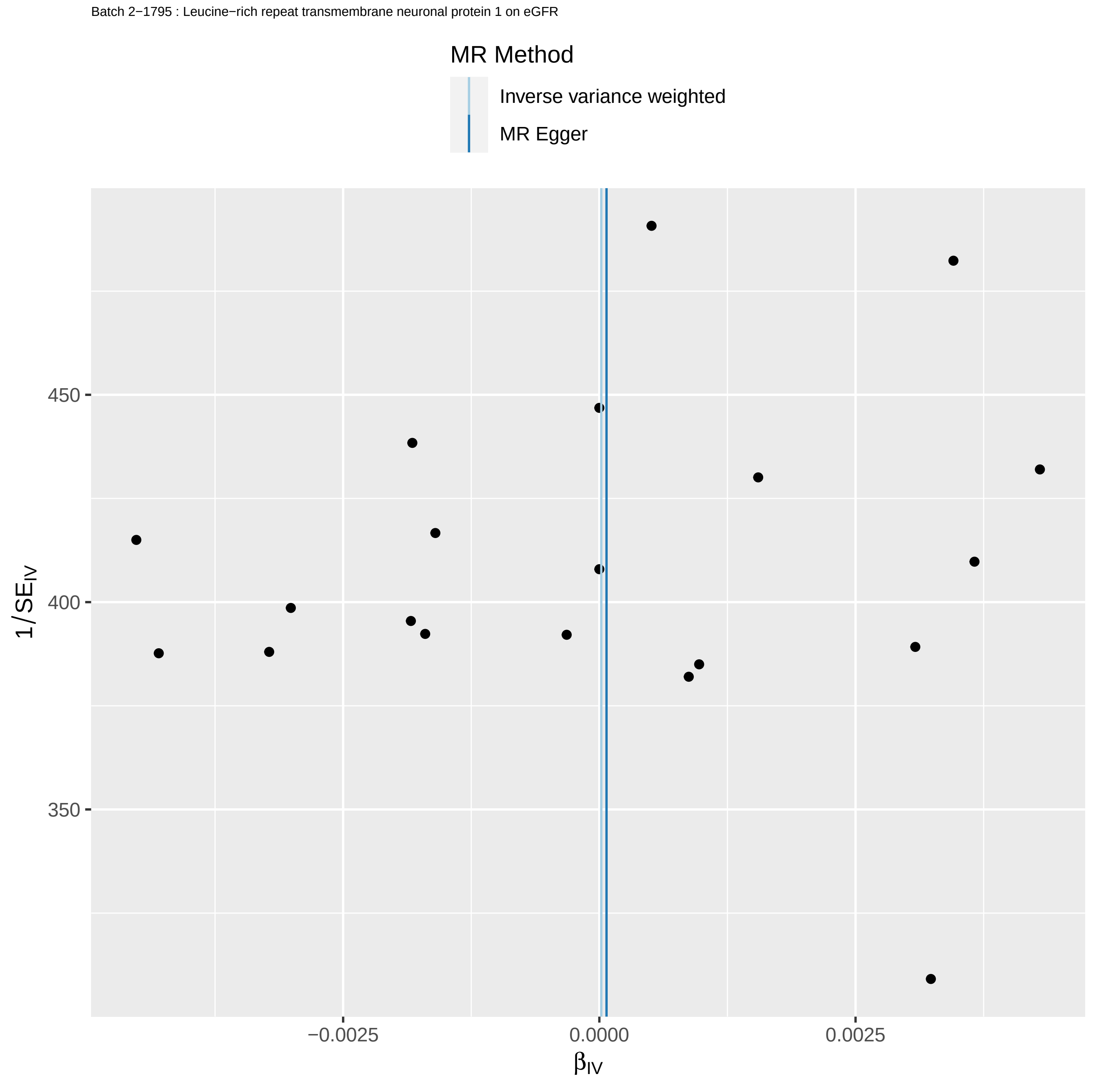
Batch 2−1795 : Leucine−rich repeat transmembrane neuronal protein 1 on eGFR
MR Method
Inverse variance weighted
MR Egger
450
400
350
1/SE_IV
−0.0025
0.0000
0.0025
β_IV

Batch 2−1796 : Leucine−rich repeat transmembrane neuronal protein 2 on eGFR

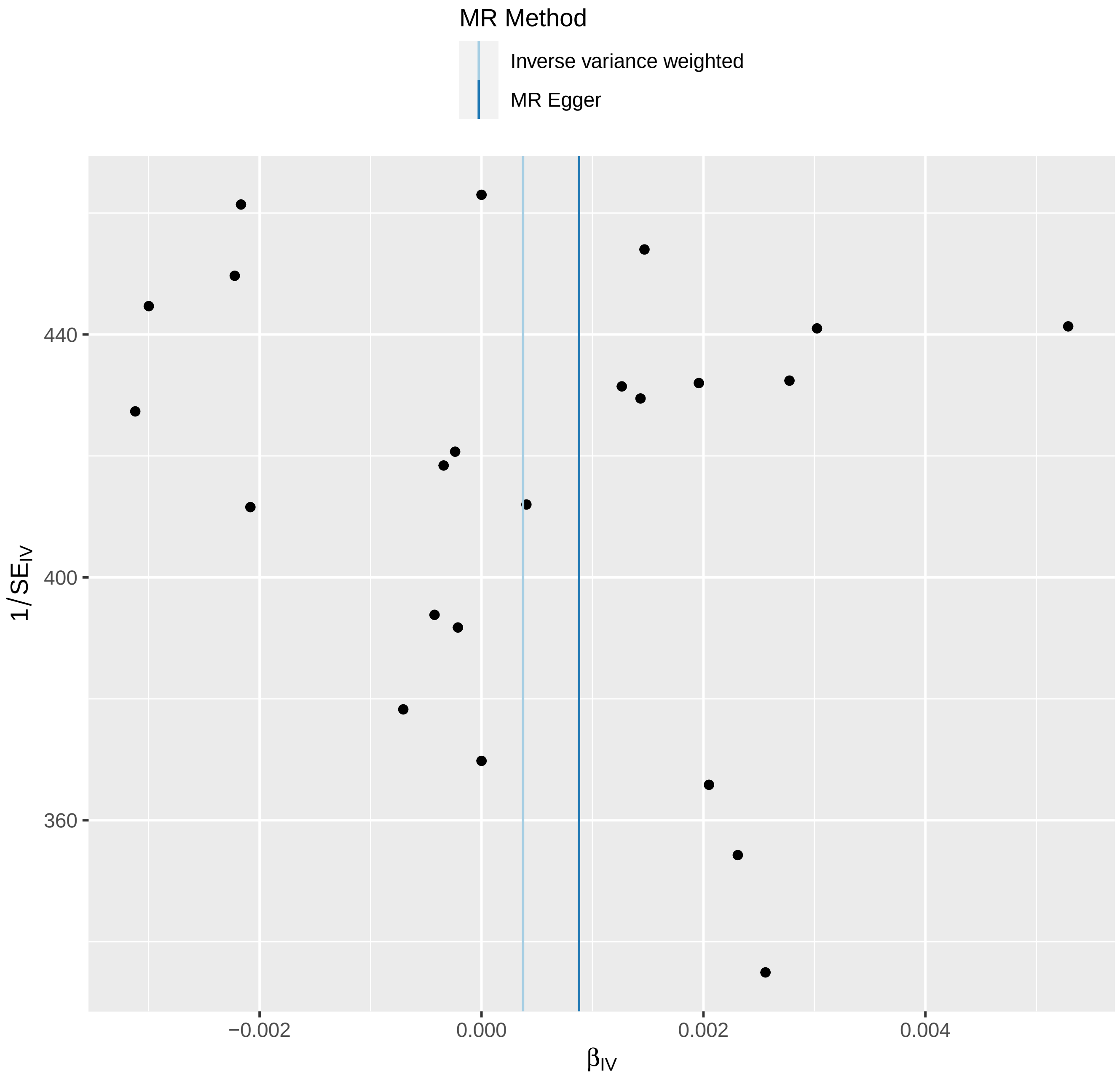

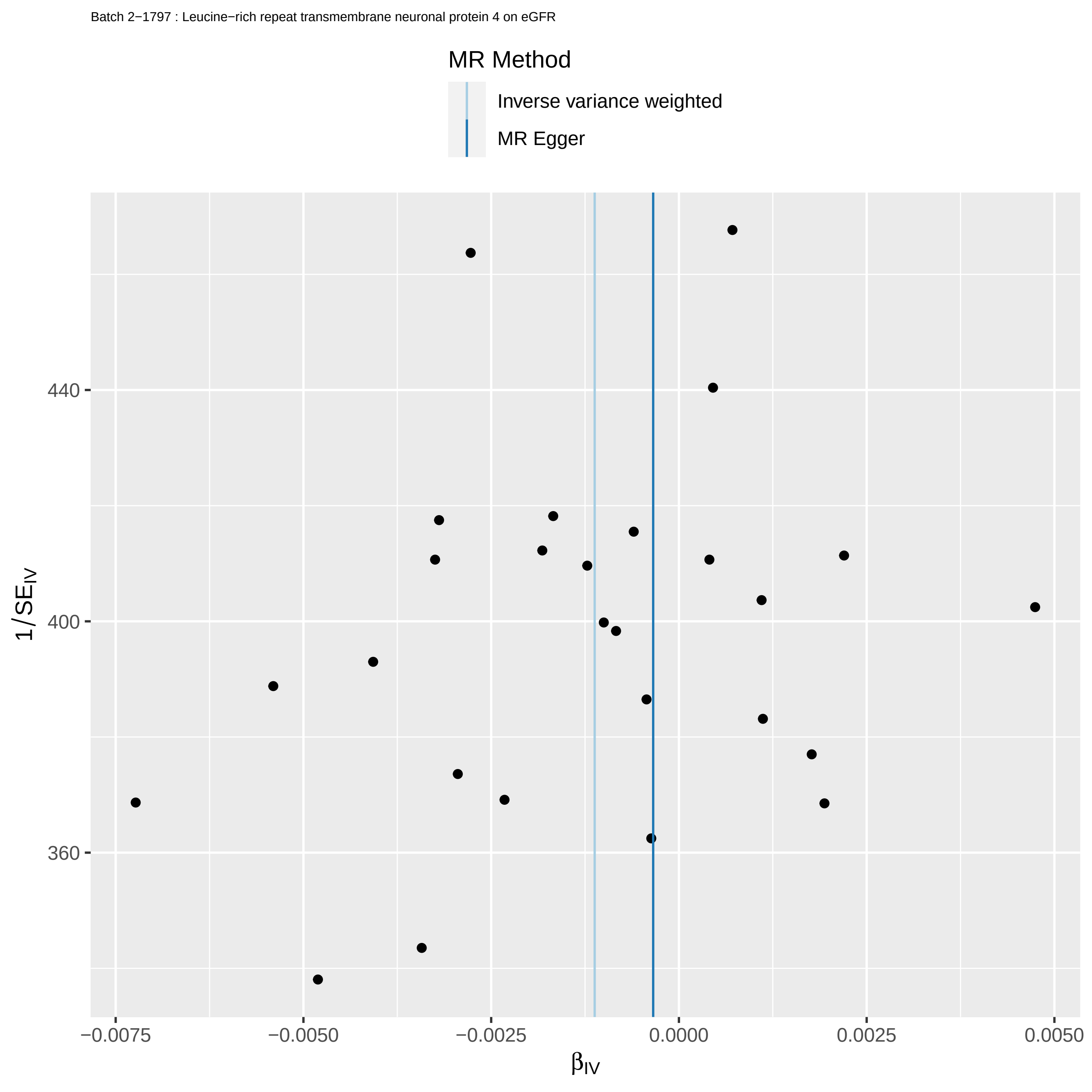

Batch 2−1797 : Leucine−rich repeat transmembrane neuronal protein 4 on eGFR
MR Method
Inverse variance weighted
MR Egger
1/SE$_{IV}$
440
400
360
−0.0075
−0.0050
−0.0025
0.0000
0.0025
0.0050
β$_{IV}$

Batch 2−1798 : Leucine−rich repeat transmembrane neuronal protein 4 on eGFR

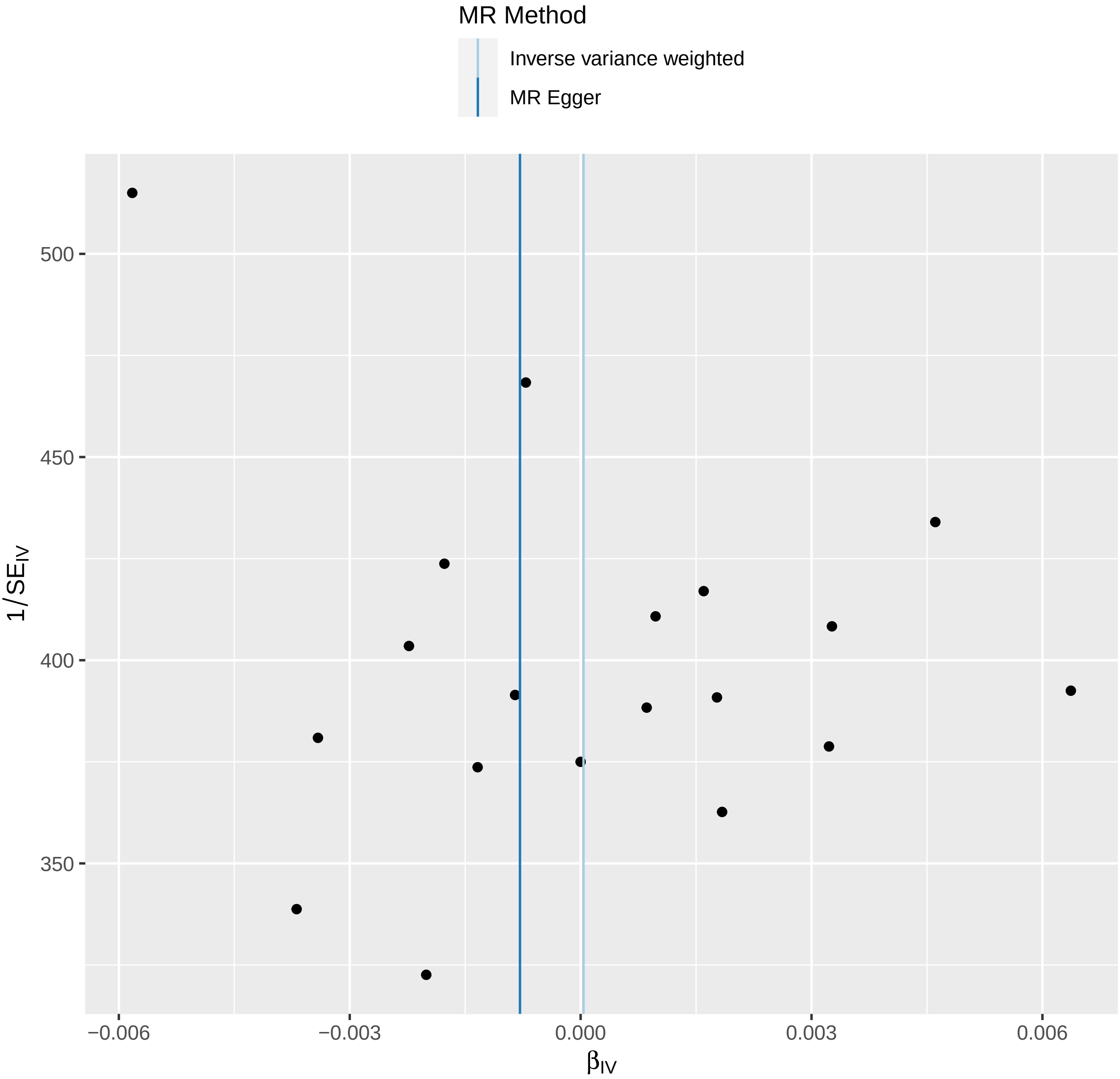

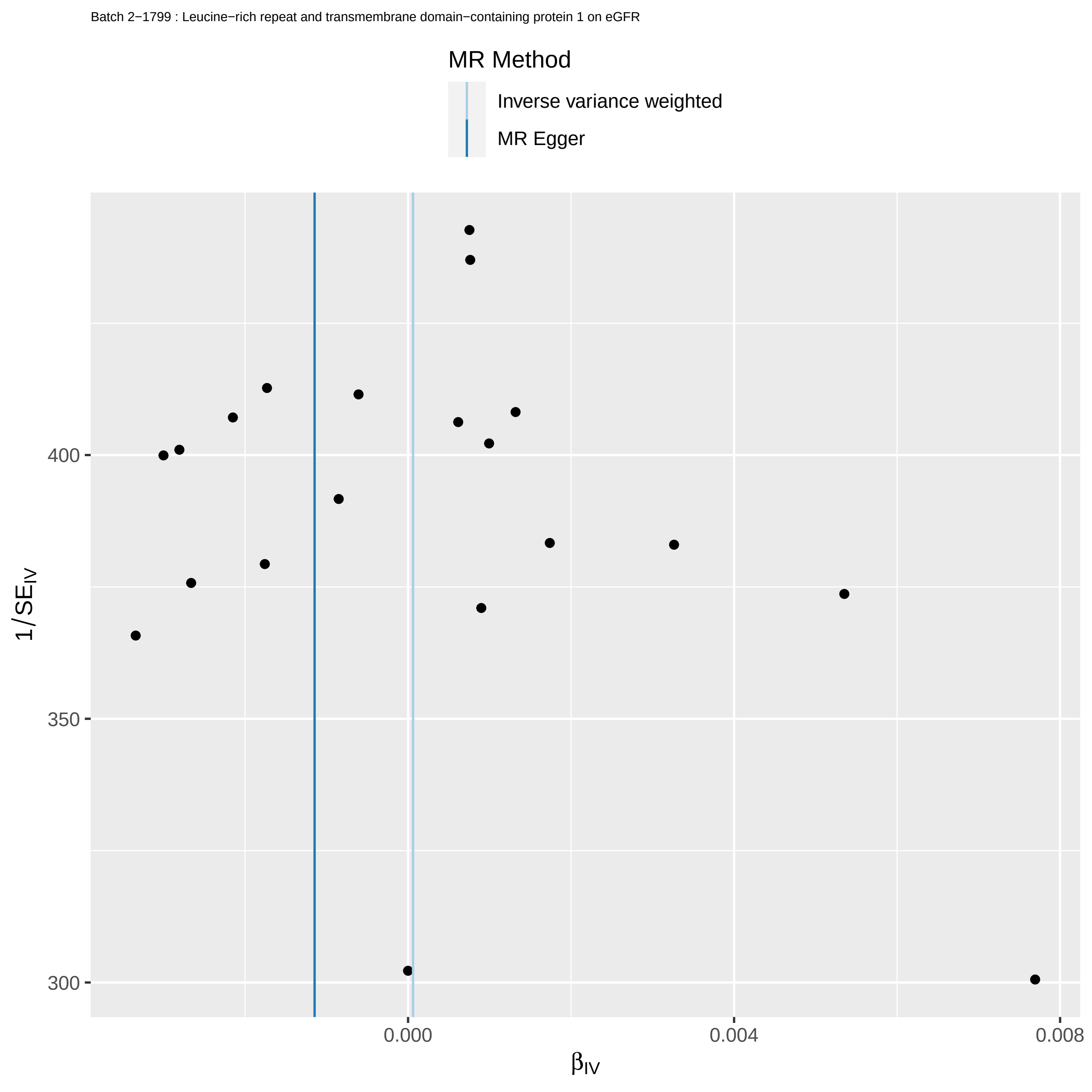

Batch 2−1799 : Leucine−rich repeat and transmembrane domain−containing protein 1 on eGFR
MR Method
Inverse variance weighted
MR Egger
400
350
300
1/SE_IV
0.000
0.004
0.008
β_IV

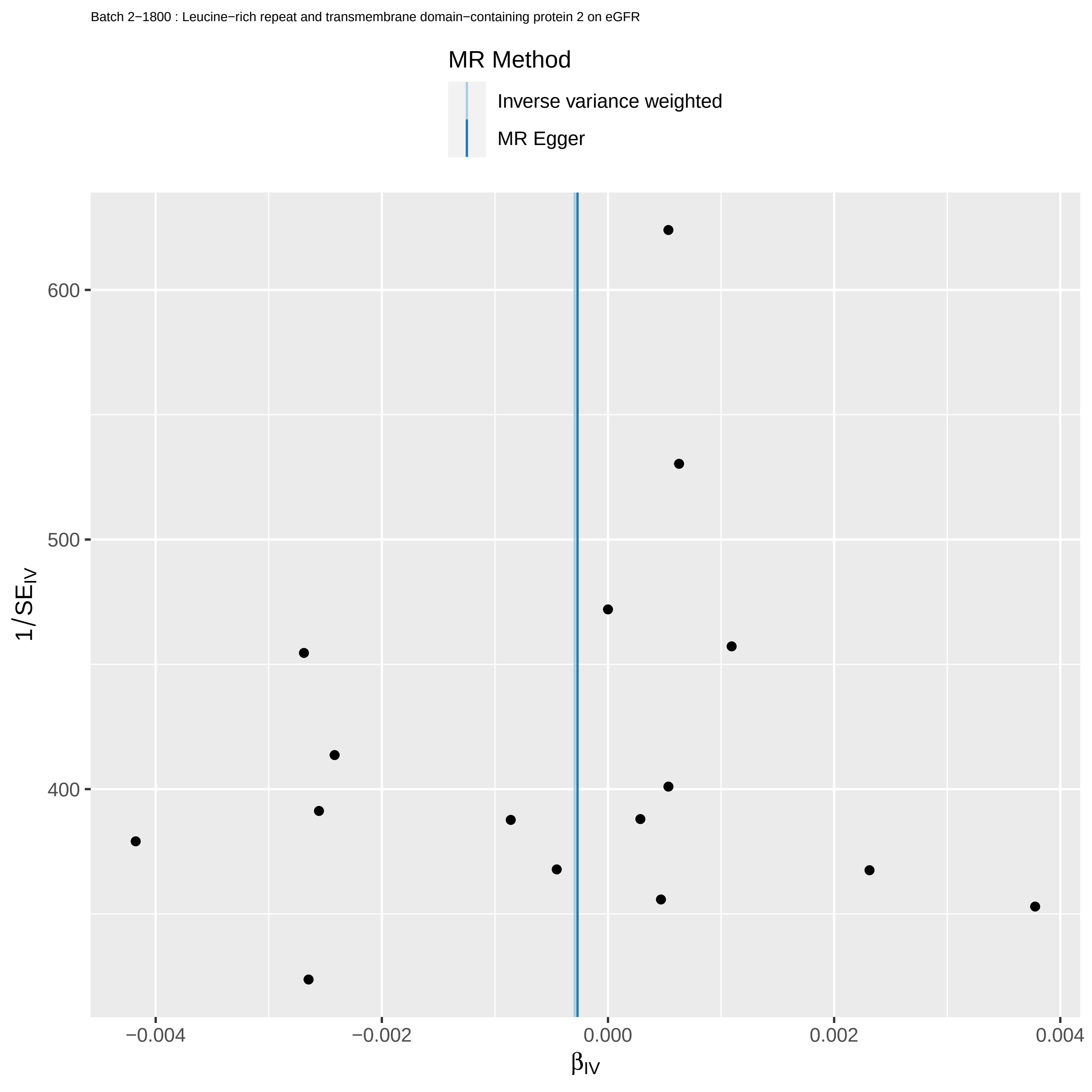
Batch 2−1800 : Leucine−rich repeat and transmembrane domain−containing protein 2 on eGFR
MR Method
Inverse variance weighted
MR Egger
600
500
400
1/SE_IV
−0.004
−0.002
0.000
0.002
0.004
β_IV

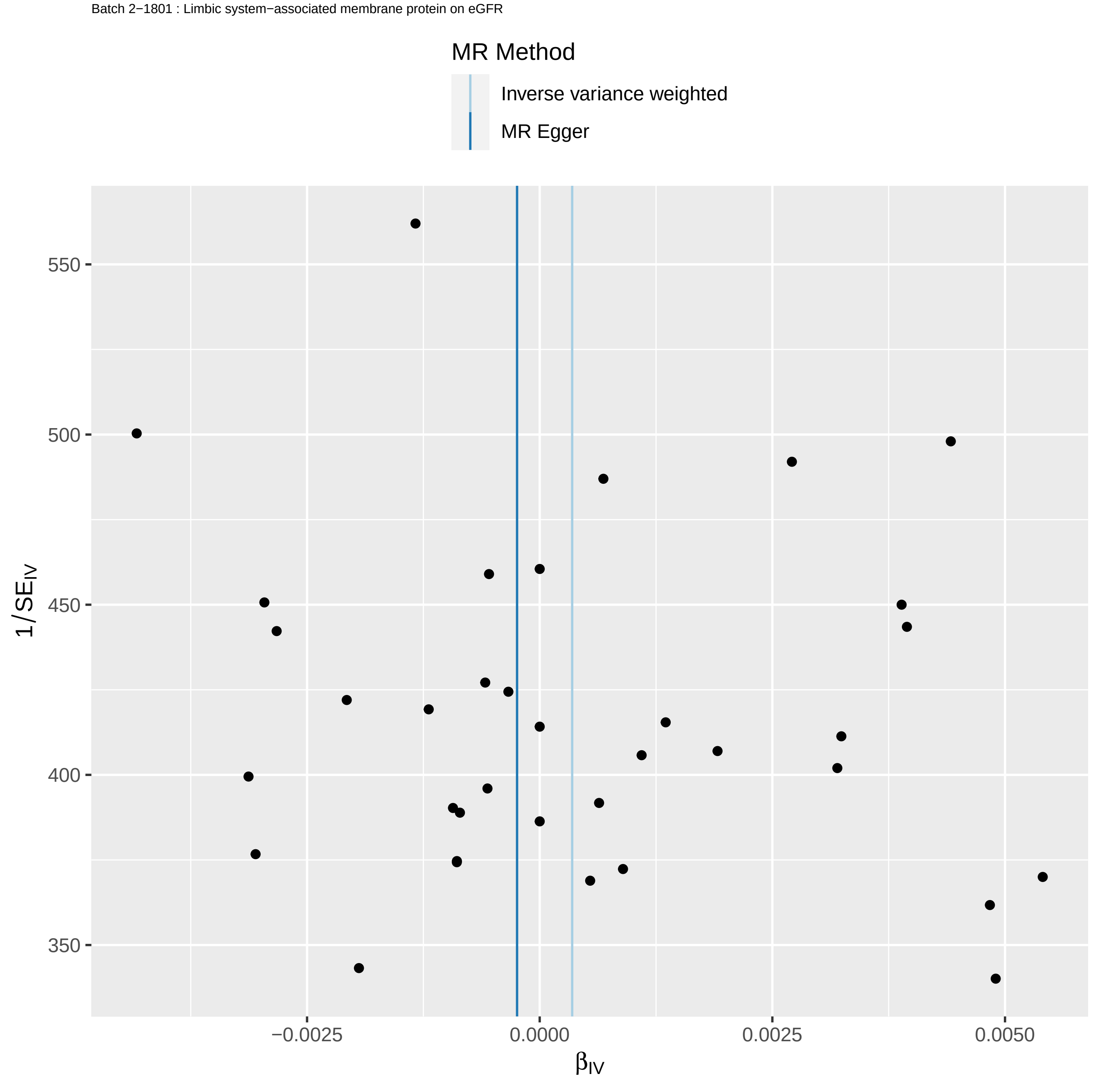

Batch 2−1801 : Limbic system−associated membrane protein on eGFR
MR Method
Inverse variance weighted
MR Egger
550
500
450
400
350
1/SE_IV
−0.0025
0.0000
0.0025
0.0050
β_IV

Batch 2−1802 : Leukocyte−specific transcript 1 protein on eGFR

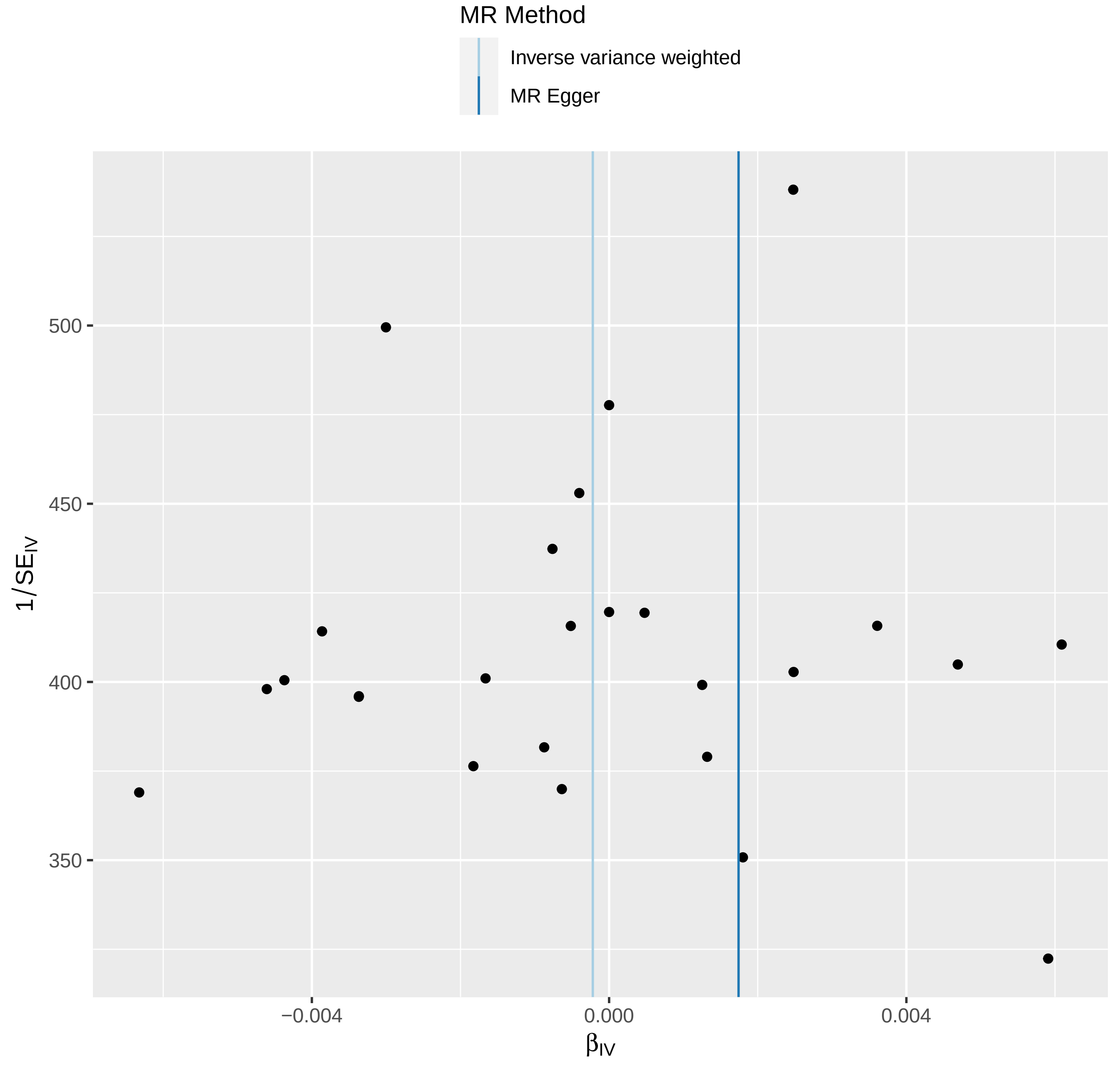

Batch 2−1803 : Lymphotoxin−alpha on eGFR

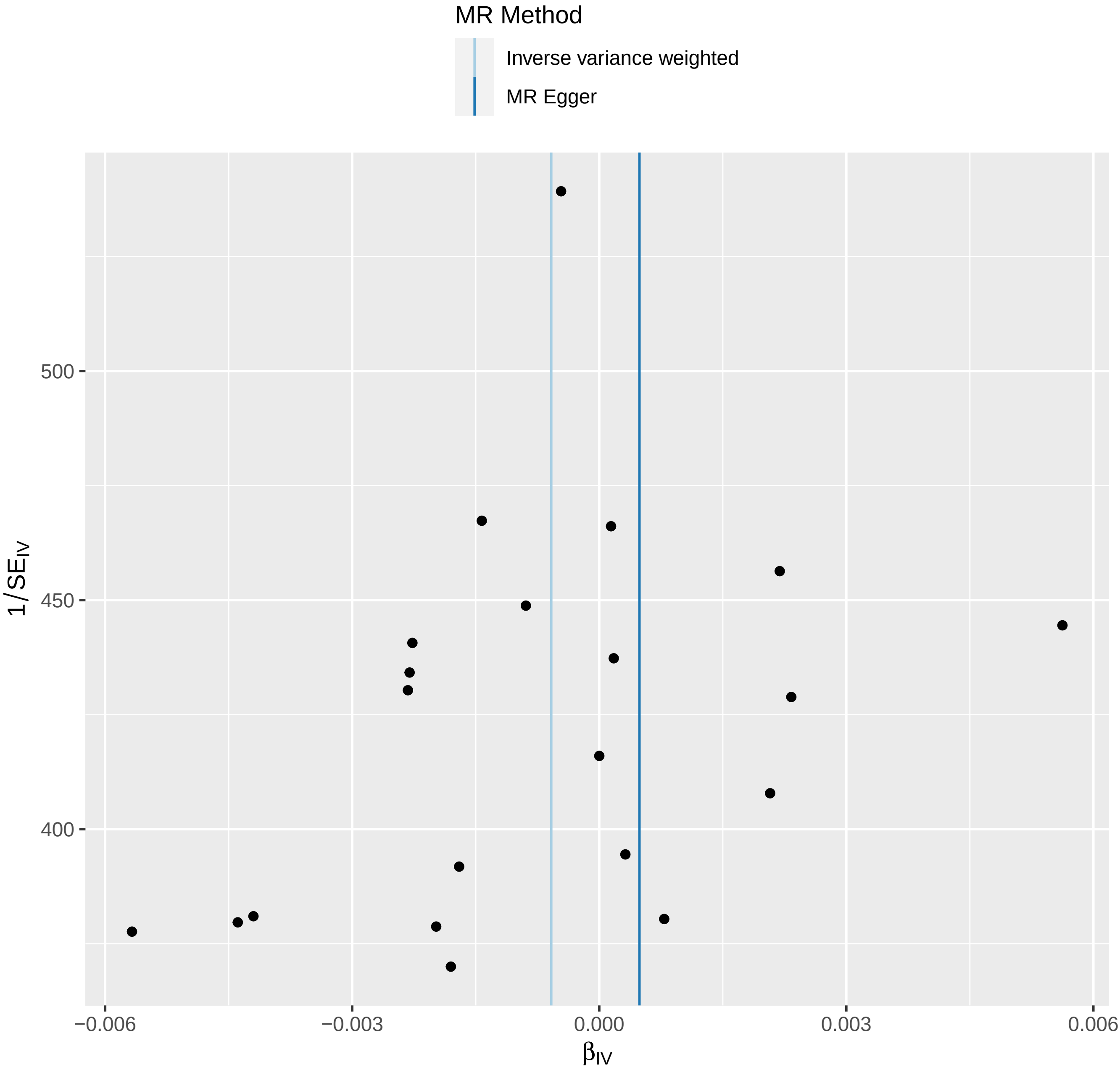

Batch 2−1804 : Leukotriene A−4 hydrolase on eGFR

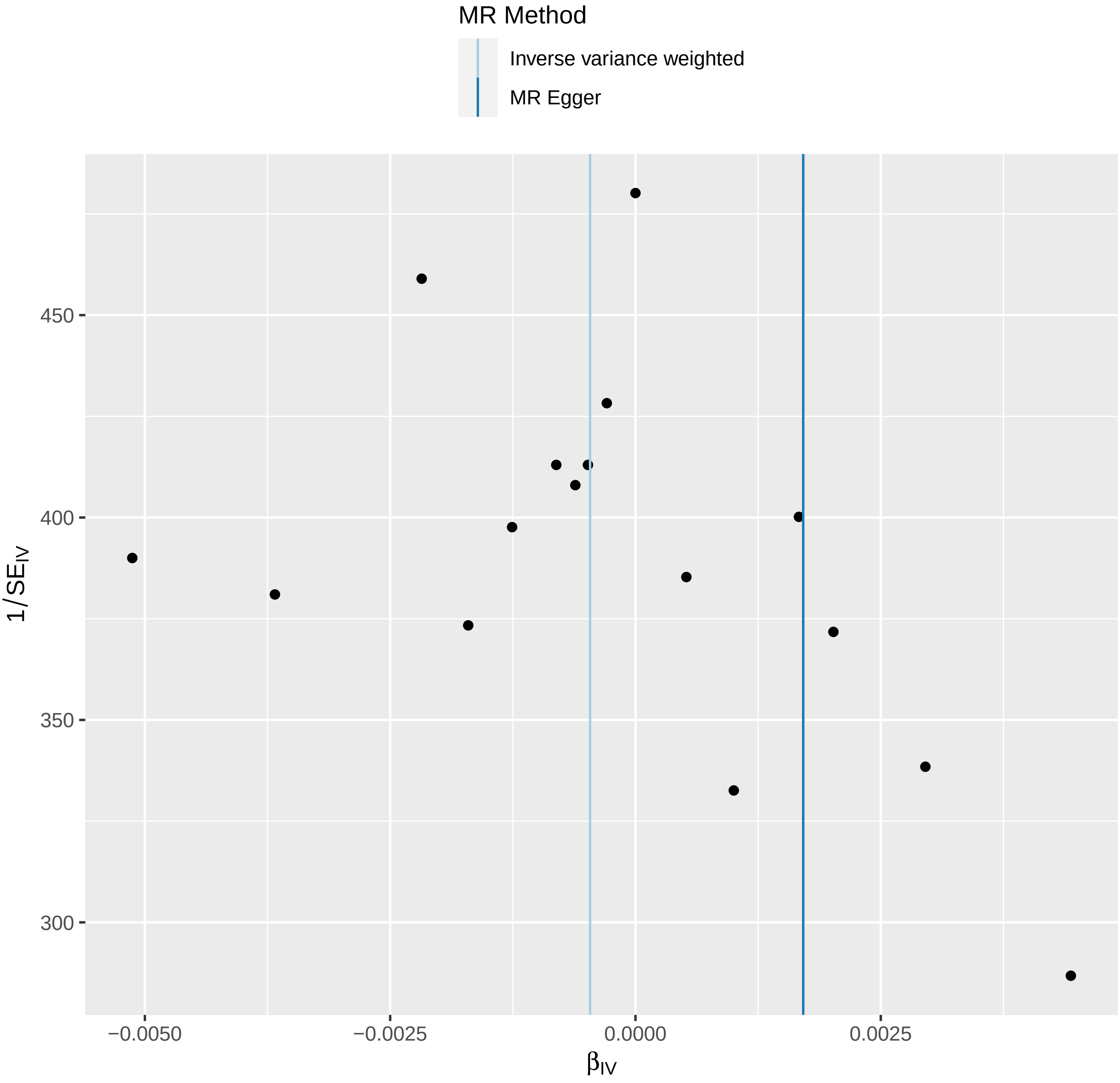

Batch 2−1805 : Lymphotoxin alpha2:beta1 on eGFR

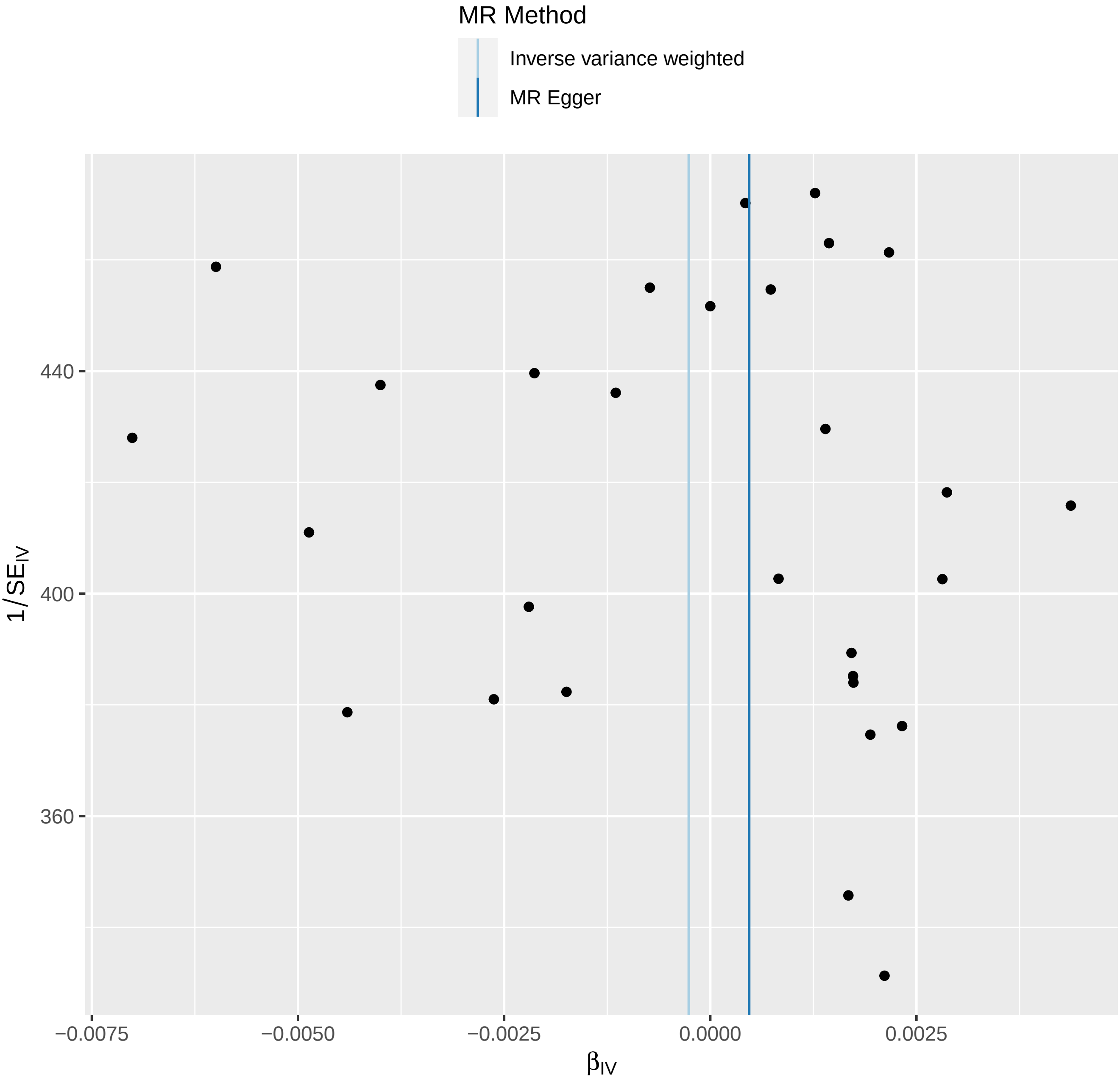

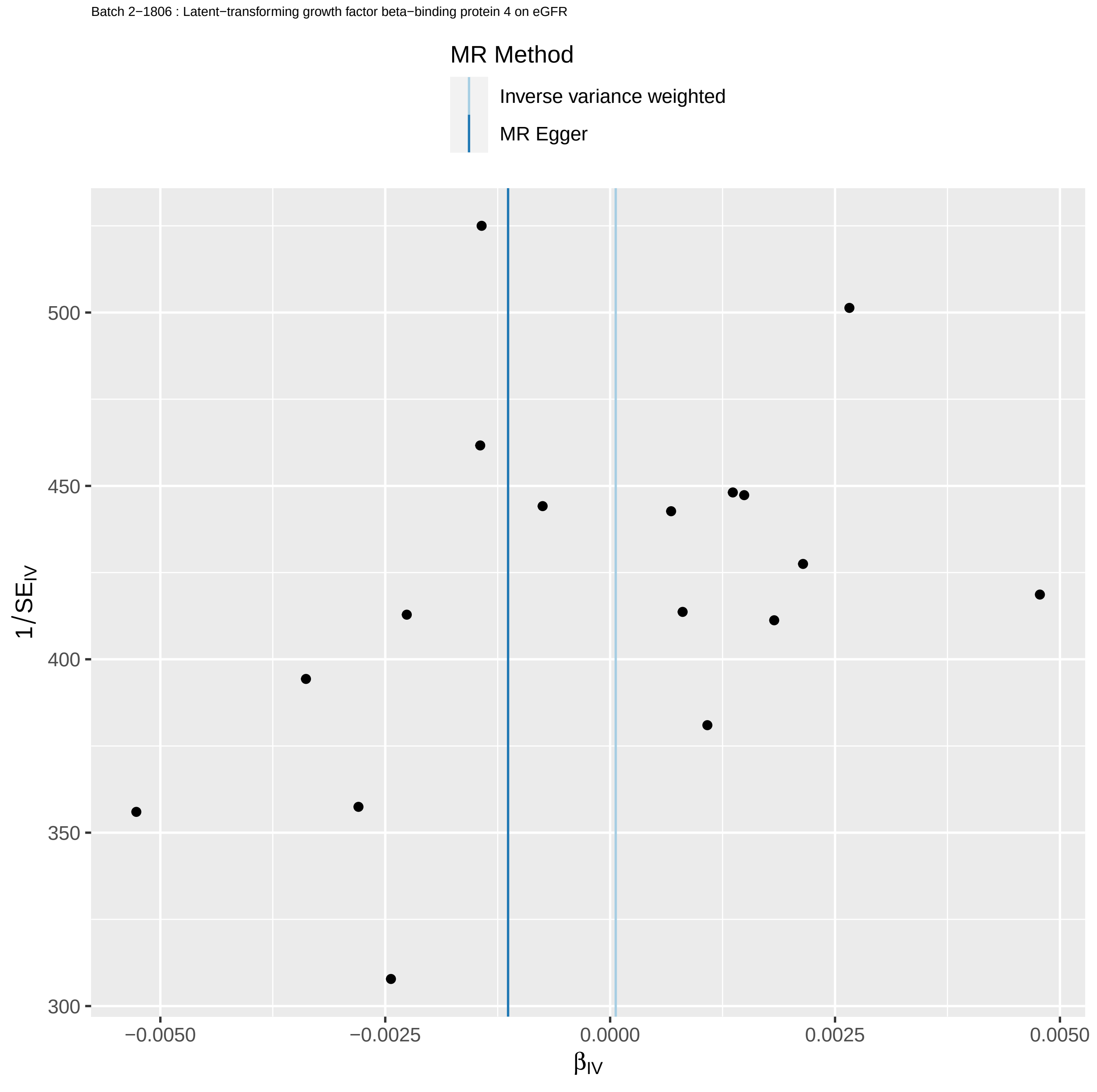
Batch 2−1806 : Latent−transforming growth factor beta−binding protein 4 on eGFR
MR Method
Inverse variance weighted
MR Egger
500
450
400
350
300
1/SE_IV
−0.0050
−0.0025
0.0000
0.0025
0.0050
β_IV

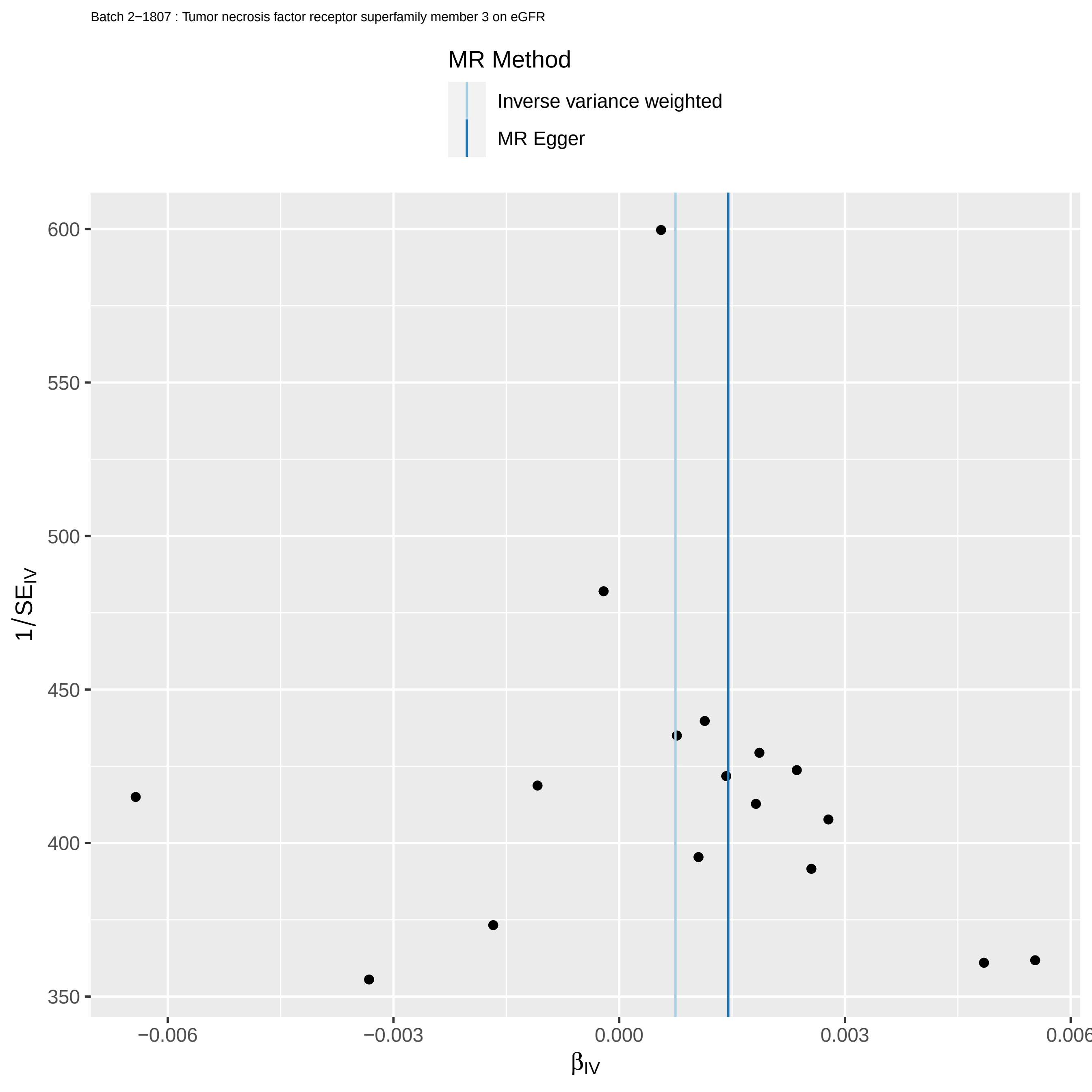

Batch 2−1807 : Tumor necrosis factor receptor superfamily member 3 on eGFR
MR Method
Inverse variance weighted
MR Egger
600
550
500
450
400
350
1/SE_IV
−0.006
−0.003
0.000
0.003
0.006
β_IV

Batch 2−1808 : Lactotransferrin on eGFR

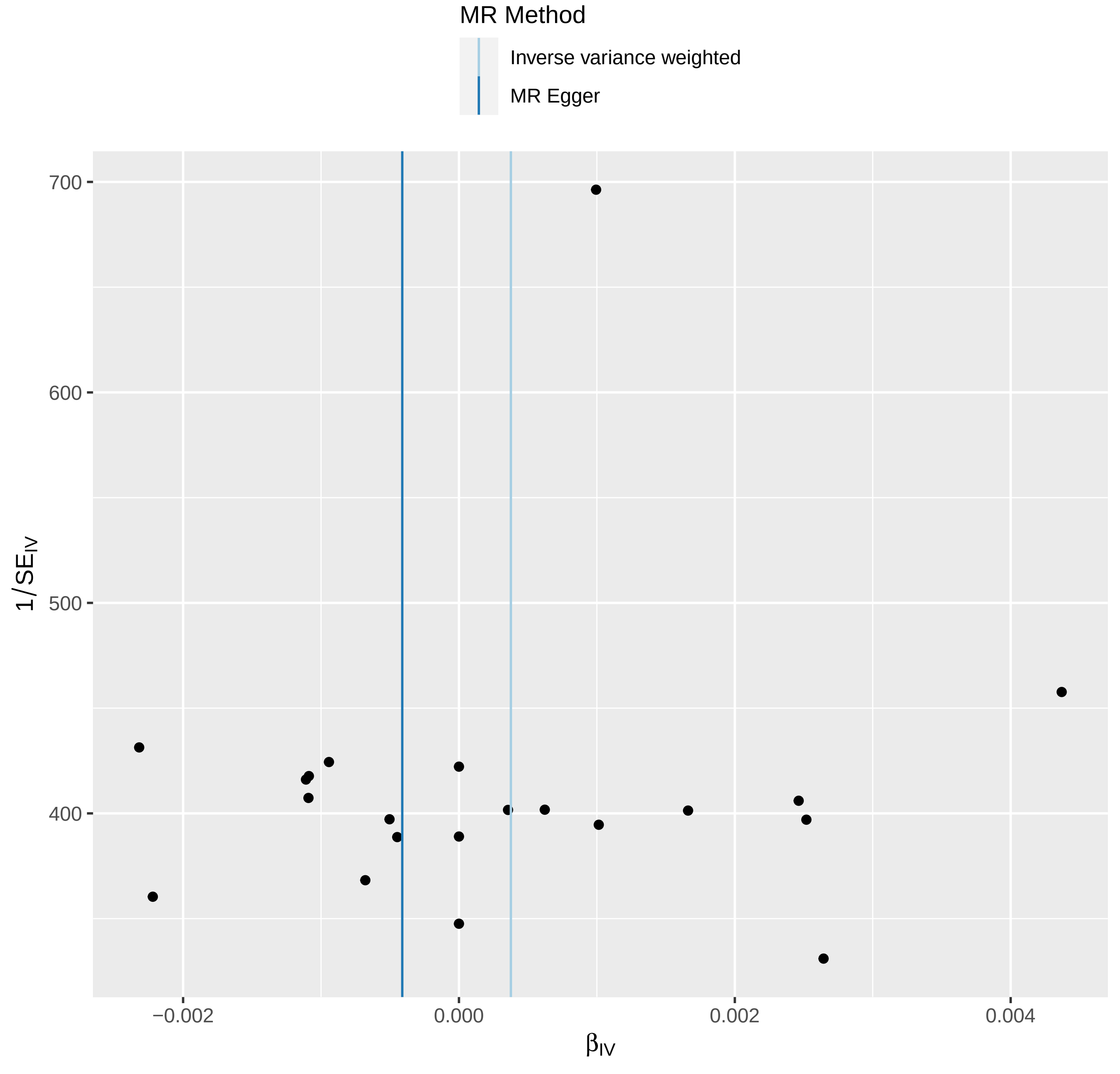

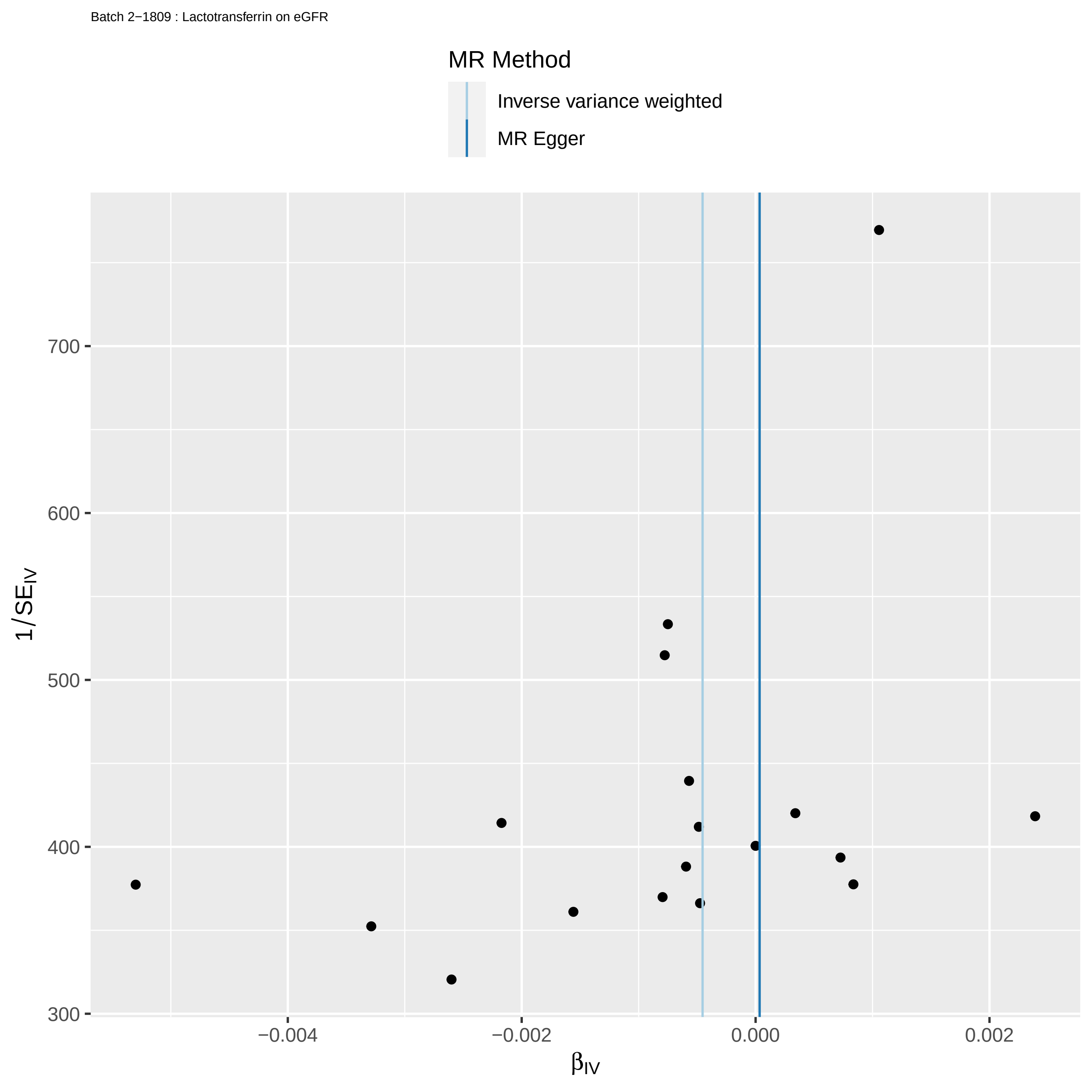

Batch 2−1809 : Lactotransferrin on eGFR
MR Method
Inverse variance weighted
MR Egger
700
600
500
400
300
−0.004
−0.002
0.000
0.002
$1/SE_{IV}$
β_{IV}

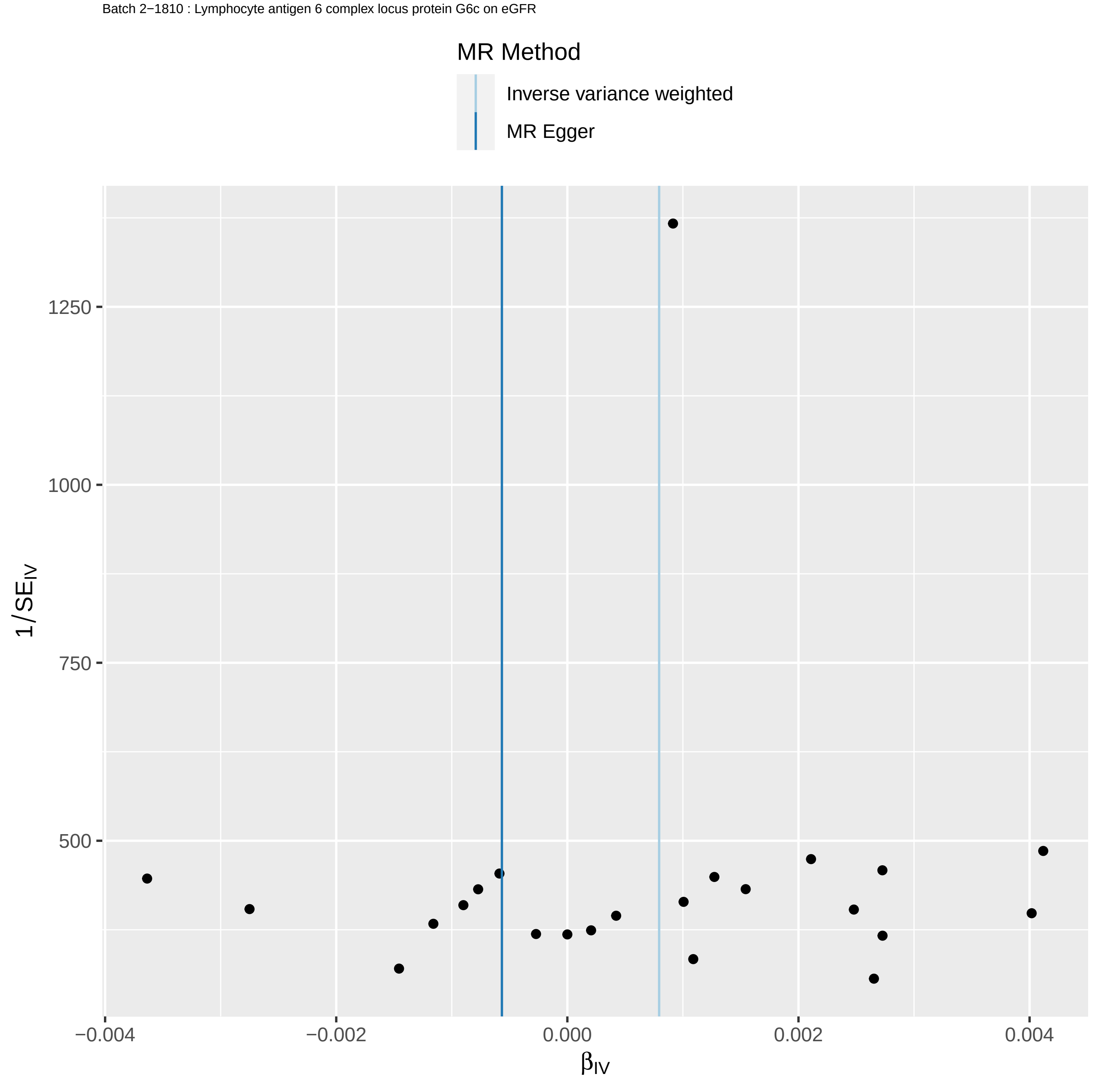

Batch 2−1810 : Lymphocyte antigen 6 complex locus protein G6c on eGFR
MR Method
Inverse variance weighted
MR Egger
1250
1000
750
500
1/SE_IV
−0.004
−0.002
0.000
0.002
0.004
β_IV

Batch 2−1811 : Lymphocyte antigen 6 complex locus protein G6d on eGFR

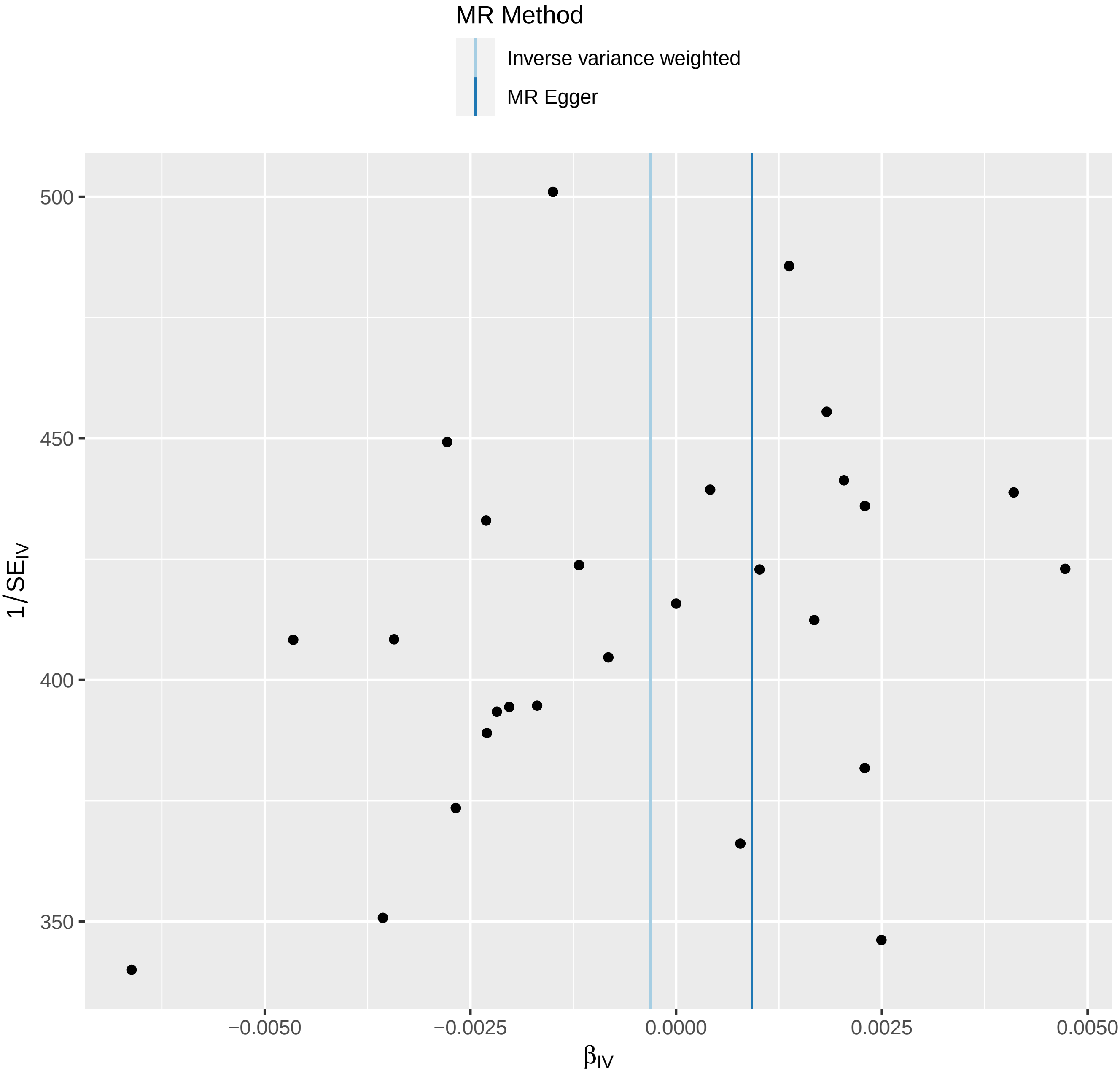

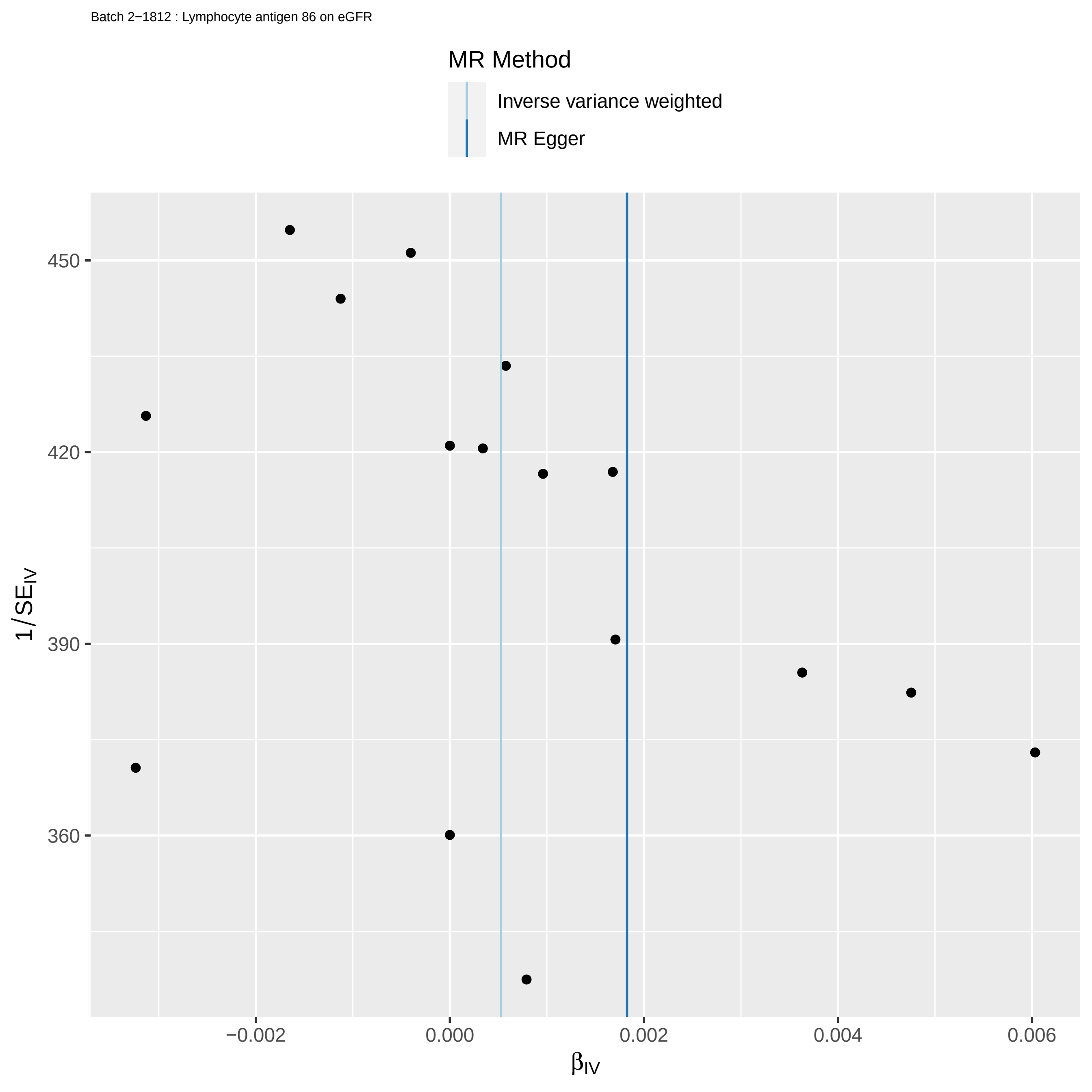

Batch 2−1812 : Lymphocyte antigen 86 on eGFR
MR Method
Inverse variance weighted
MR Egger
450
420
390
360
1/SE_IV
−0.002
0.000
0.002
0.004
0.006
β_IV

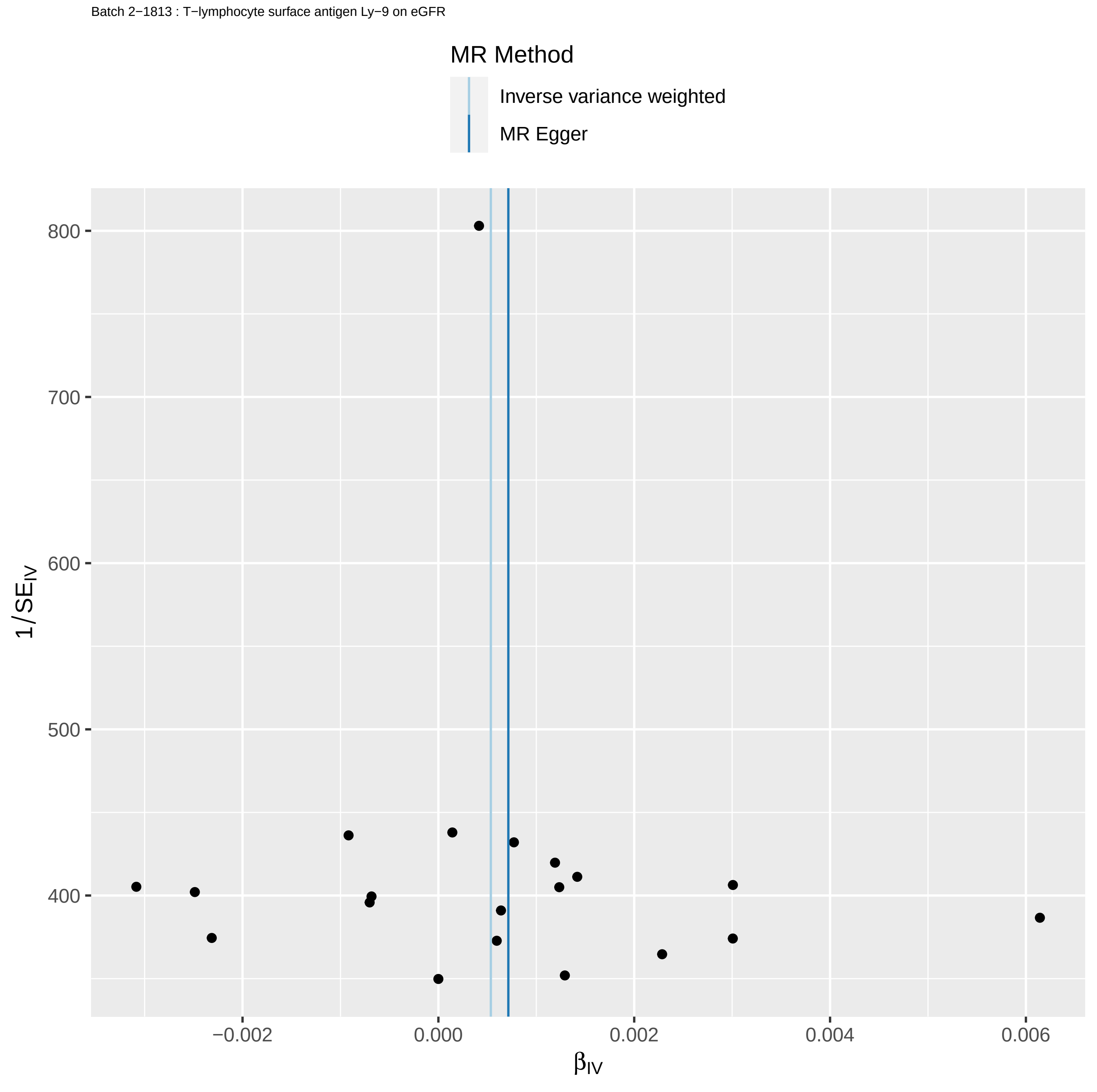
Batch 2−1813 : T−lymphocyte surface antigen Ly−9 on eGFR
MR Method
Inverse variance weighted
MR Egger
800
700
600
500
400
1/SE$_{IV}$
−0.002
0.000
0.002
0.004
0.006
β$_{IV}$

Batch 2−1814 : Lysozyme g−like protein 1 on eGFR

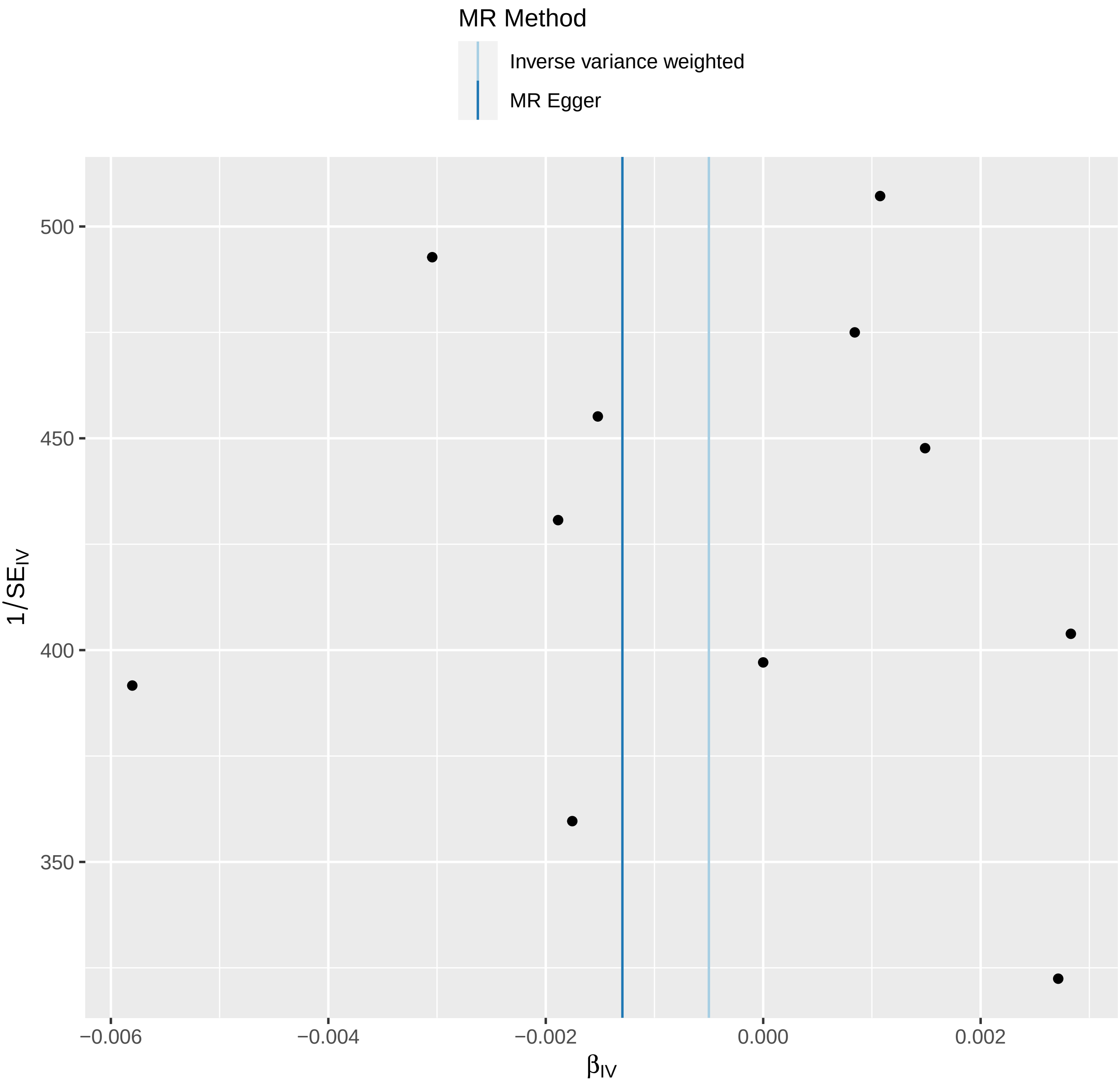

Batch 2−1815 : Tyrosine−protein kinase Lyn, isoform B on eGFR

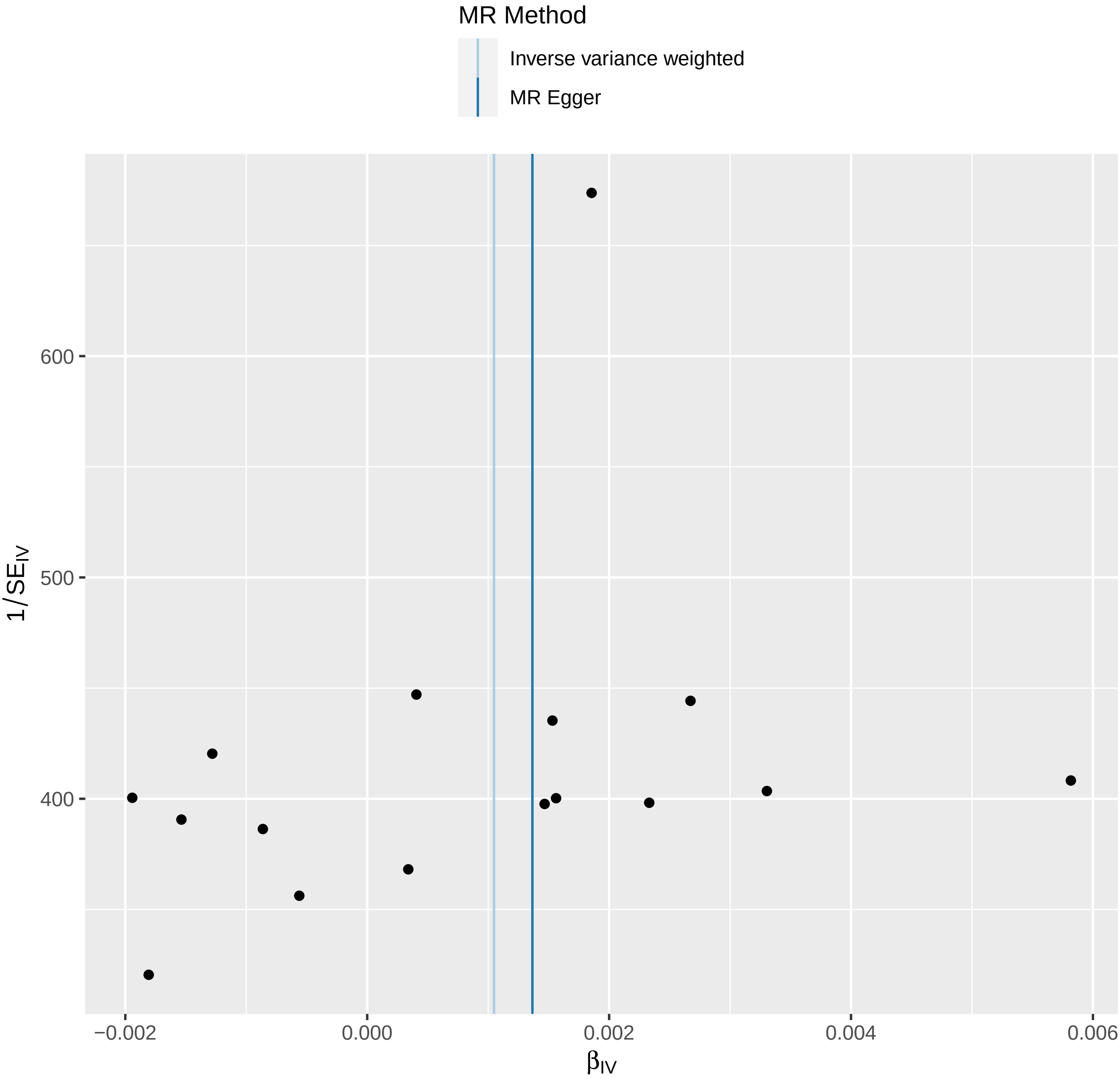

Batch 2−1816 : Tyrosine−protein kinase Lyn on eGFR

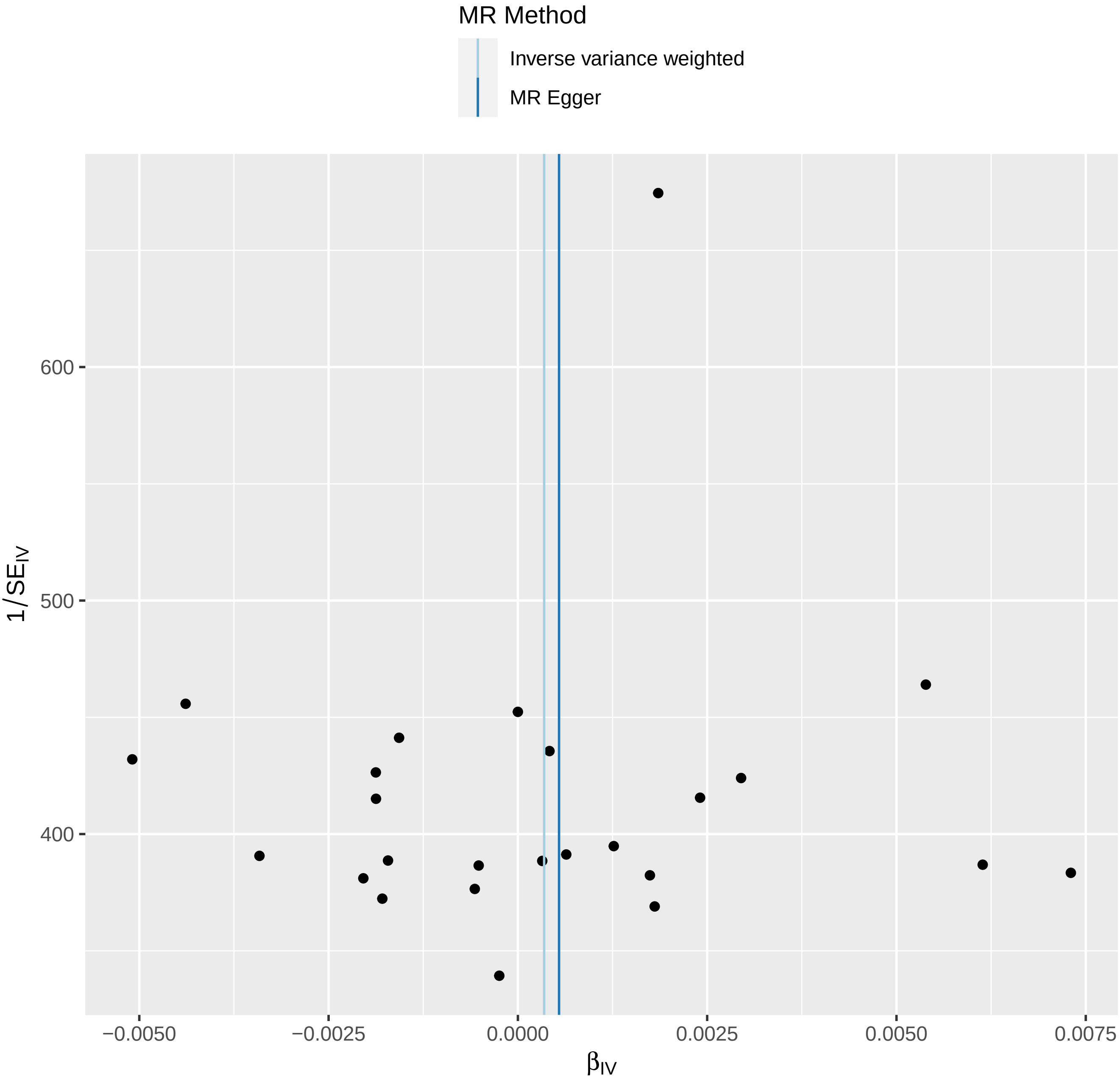

Batch 2−1817 : Ly6/PLAUR domain−containing protein 3 on eGFR

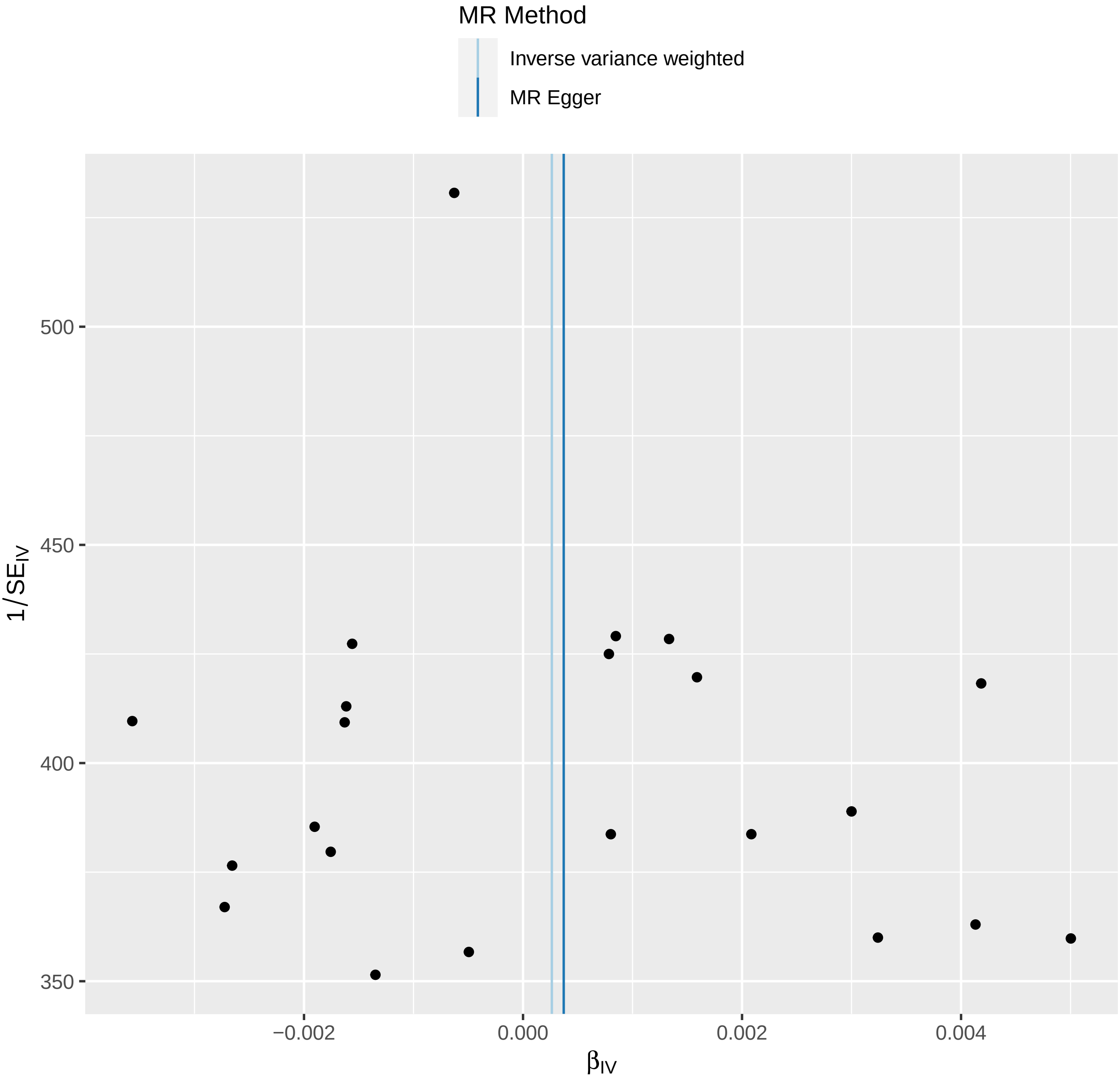

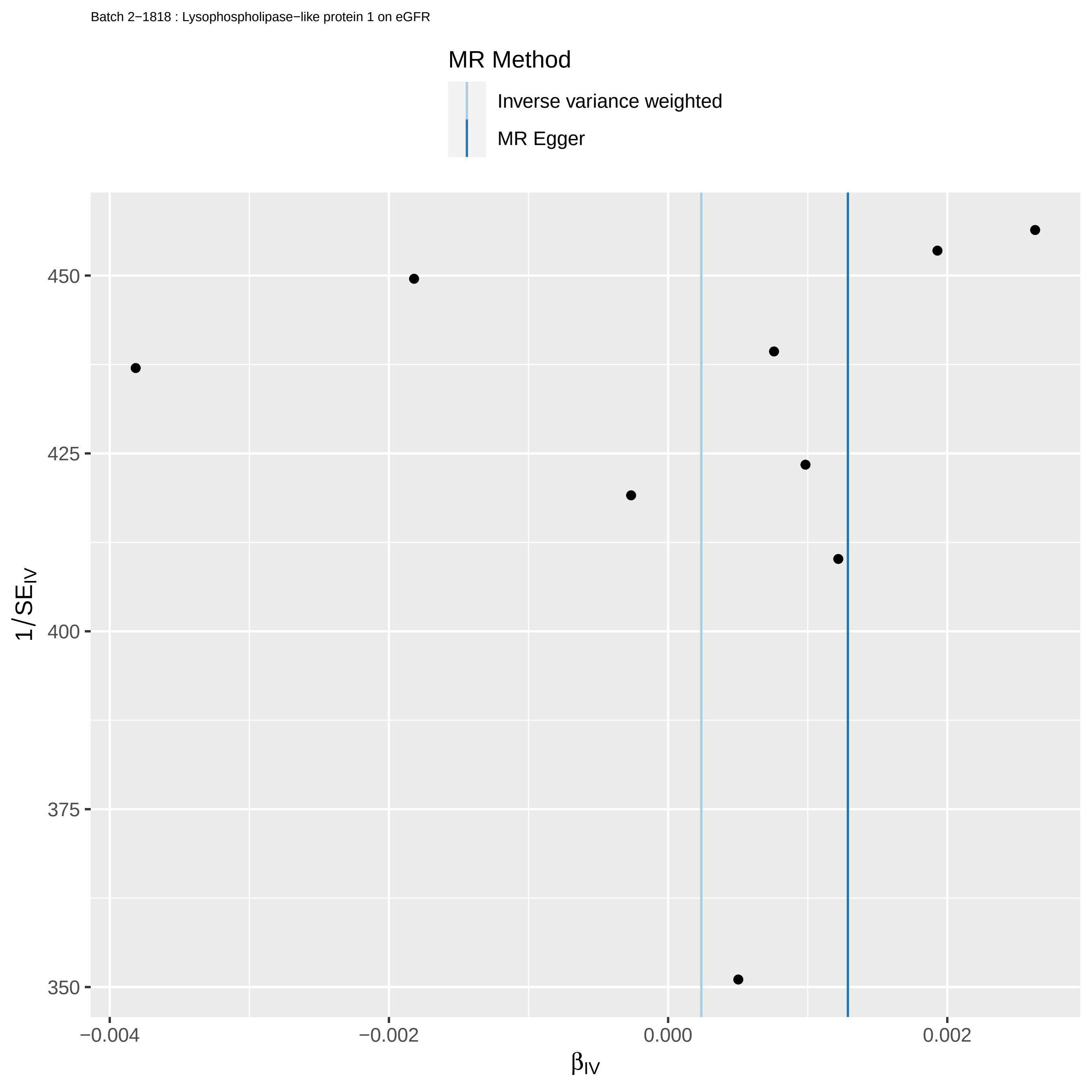

Batch 2−1818 : Lysophospholipase−like protein 1 on eGFR
MR Method
Inverse variance weighted
MR Egger
450
425
400
375
350
1/SE_IV
−0.004
−0.002
0.000
0.002
β_IV

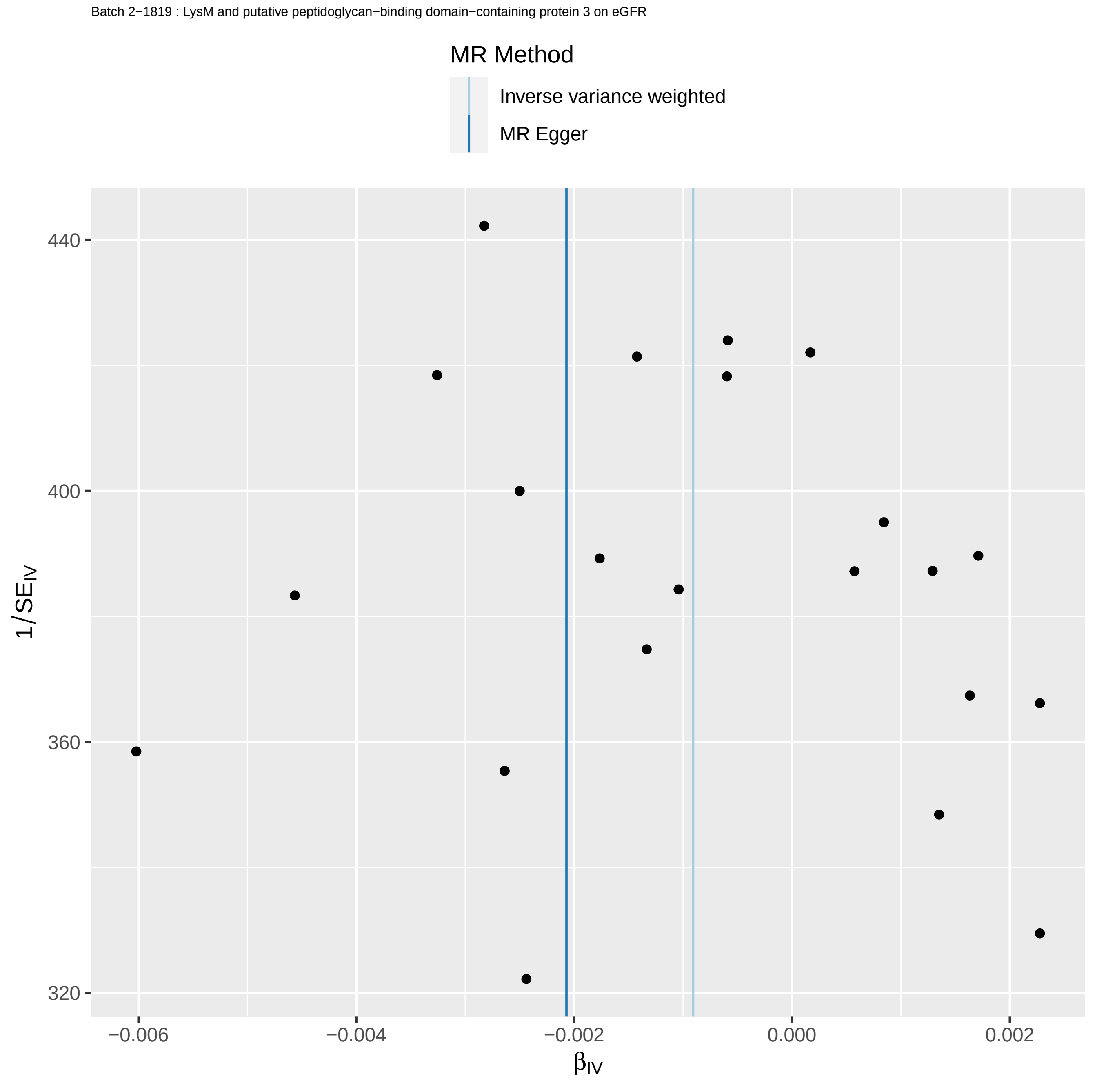
Batch 2−1819 : LysM and putative peptidoglycan−binding domain−containing protein 3 on eGFR
MR Method
Inverse variance weighted
MR Egger
440
400
360
320
1/SE_IV
−0.006
−0.004
−0.002
0.000
0.002
β_IV

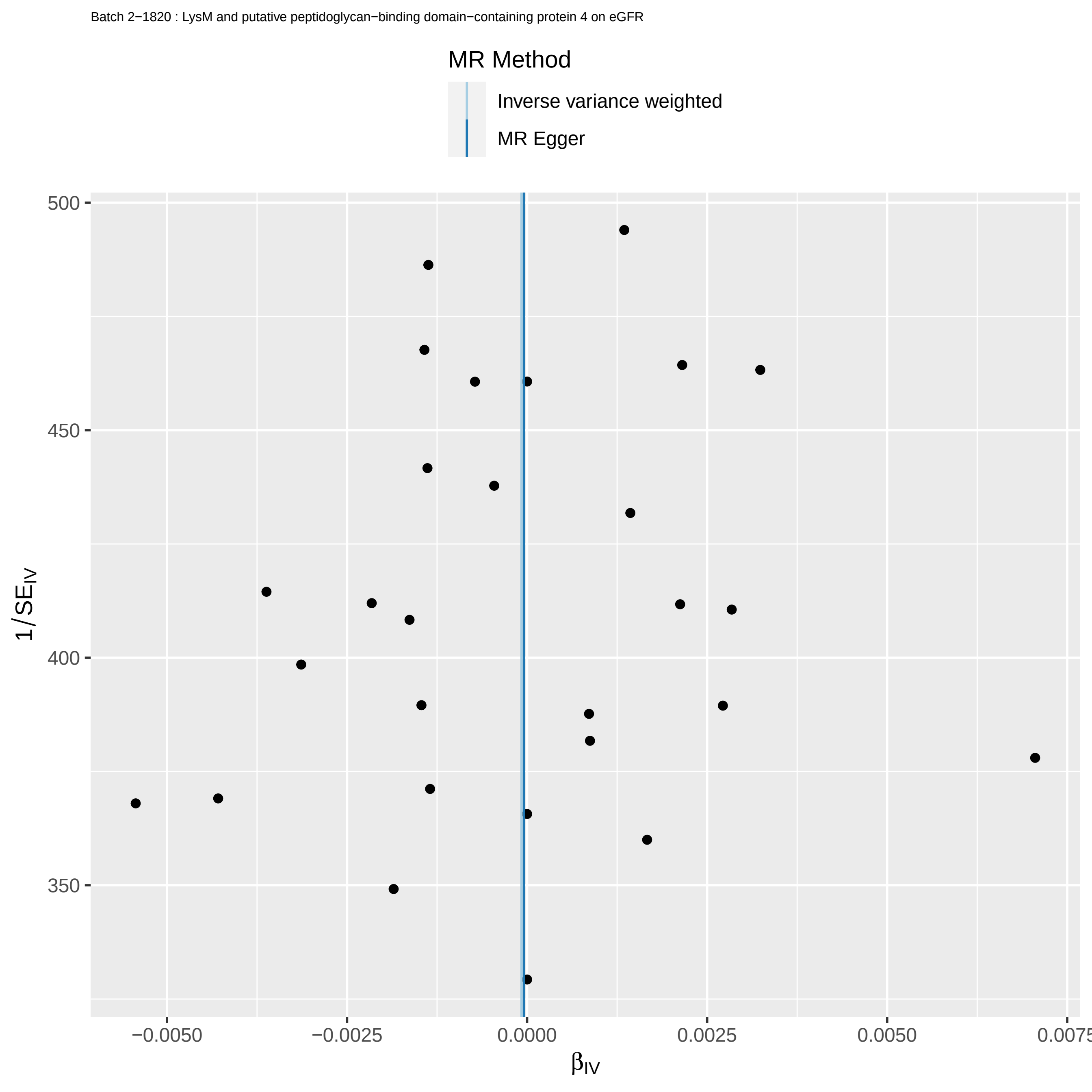

Batch 2−1820 : LysM and putative peptidoglycan−binding domain−containing protein 4 on eGFR
MR Method
Inverse variance weighted
MR Egger
500
450
400
350
1/SE_IV
−0.0050
−0.0025
0.0000
0.0025
0.0050
0.0075
β_IV

Batch 2−1821 : Lysozyme C on eGFR

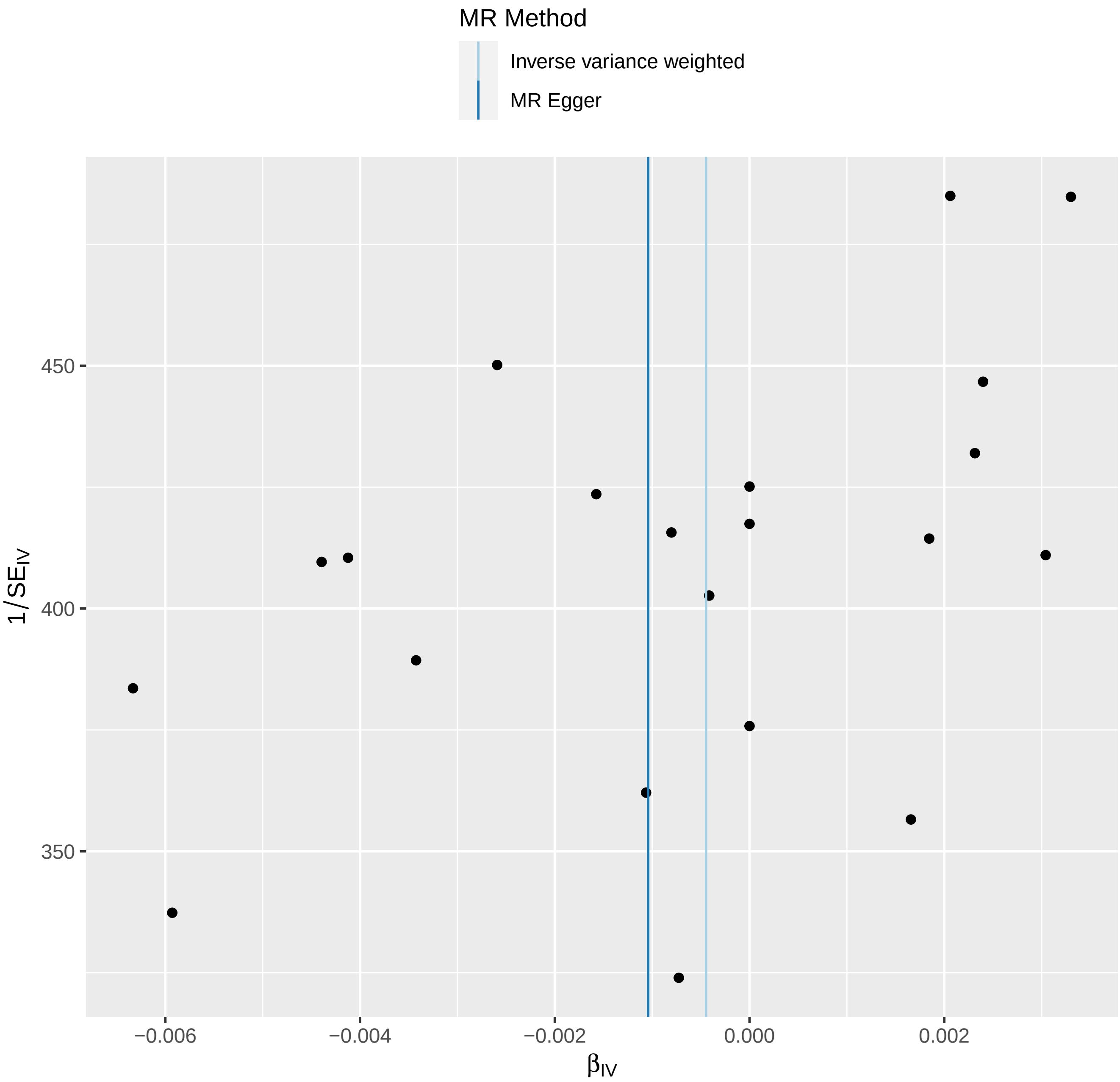

Batch 2−1822 : Lysozyme−like protein 2 on eGFR

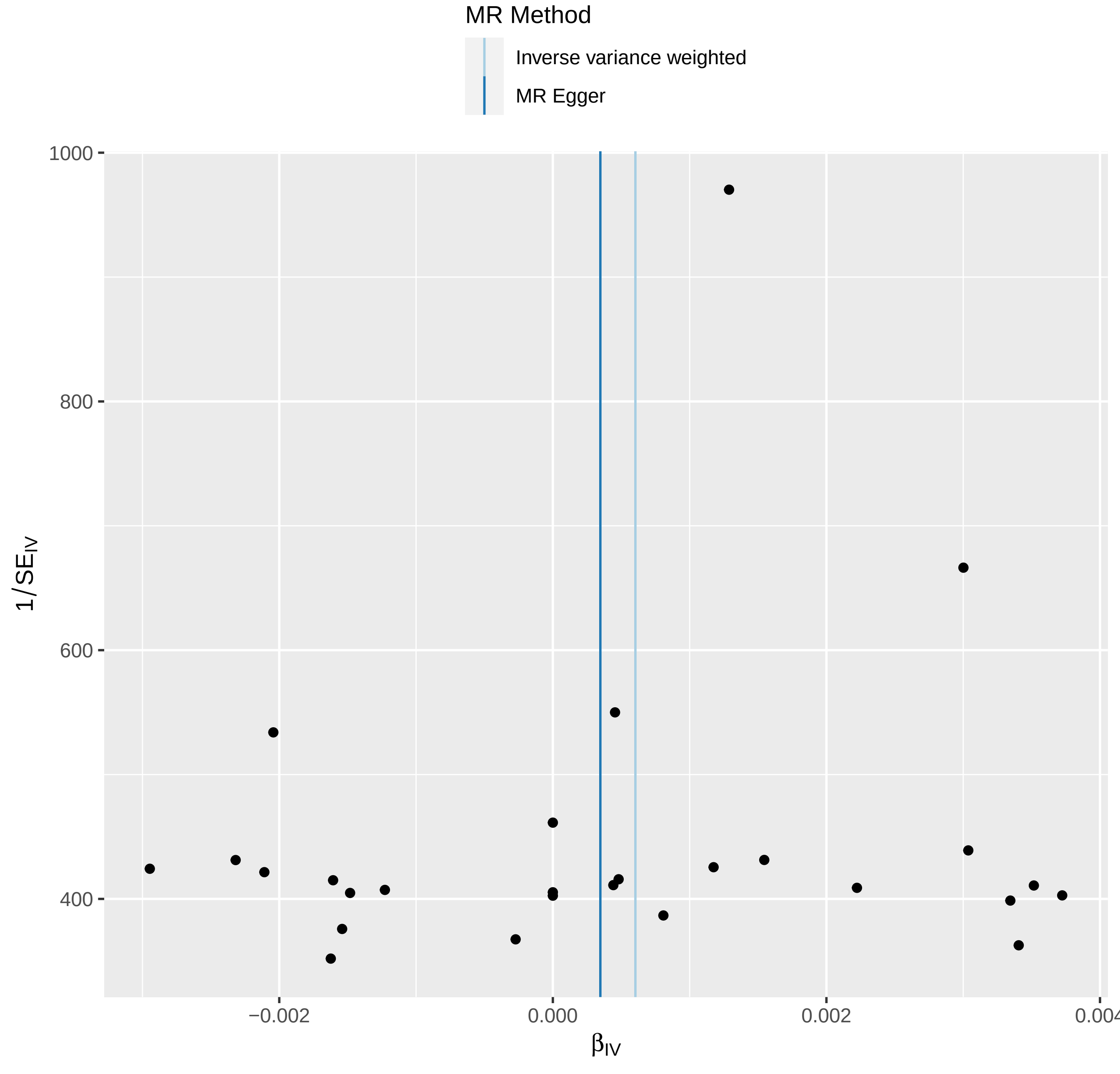

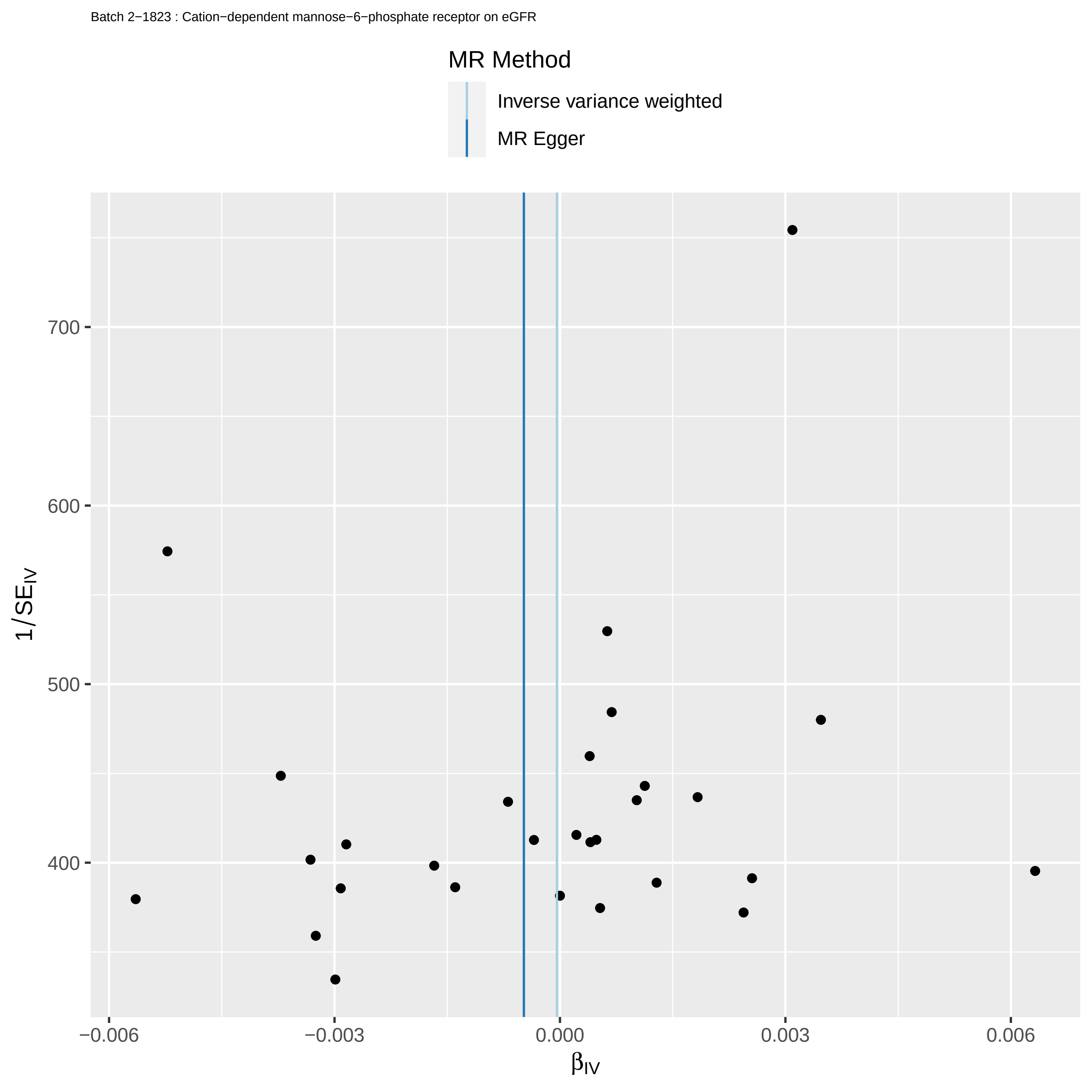

Batch 2−1823 : Cation−dependent mannose−6−phosphate receptor on eGFR
MR Method
Inverse variance weighted
MR Egger
700
600
500
400
1/SE$_{IV}$
−0.006
−0.003
0.000
0.003
0.006
β_{IV}

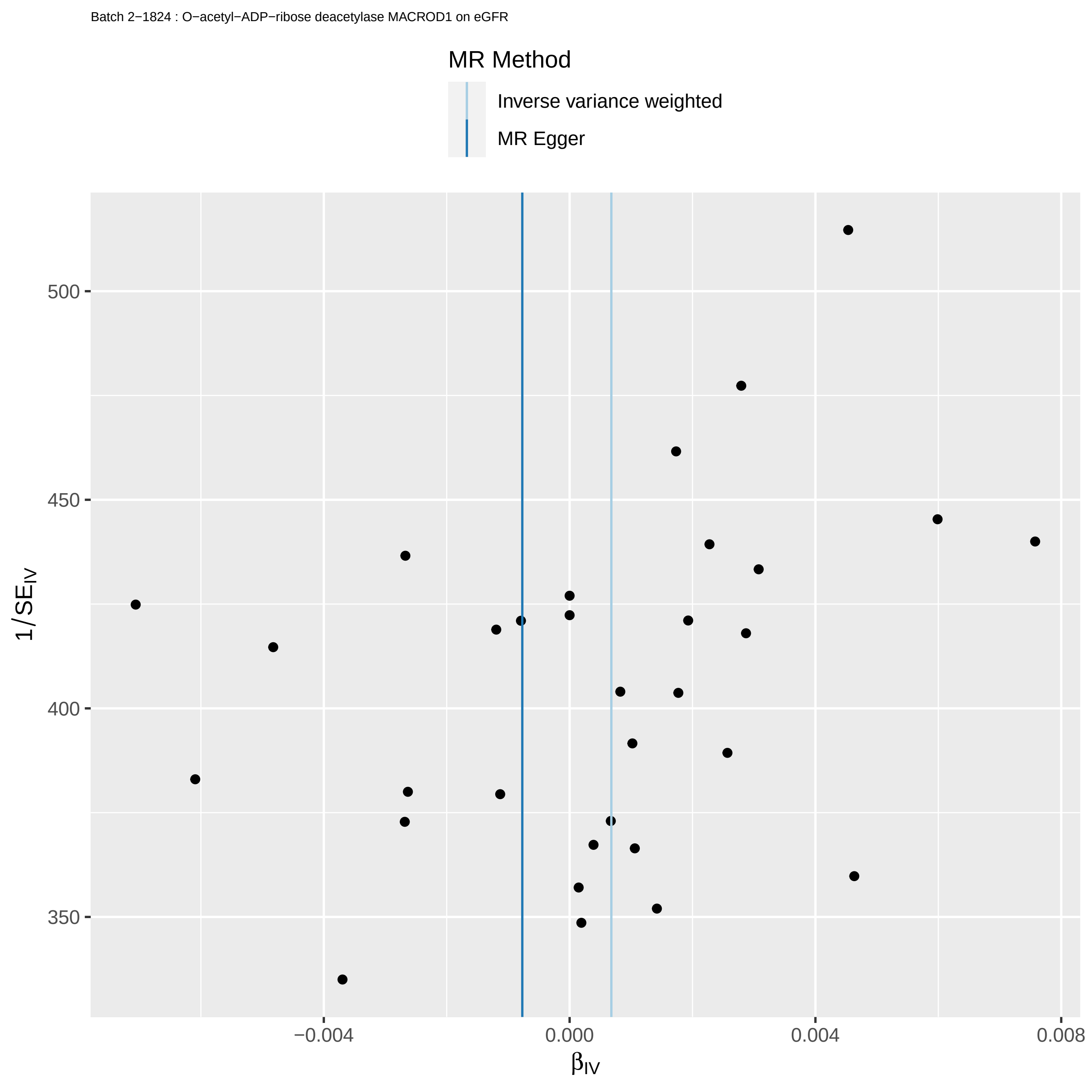

Batch 2−1824 : O−acetyl−ADP−ribose deacetylase MACROD1 on eGFR
MR Method
Inverse variance weighted
MR Egger
500
450
400
350
1/SE_IV
−0.004
0.000
0.004
0.008
β_IV

Batch 2−1825 : Mitotic spindle assembly checkpoint protein MAD1 on eGFR

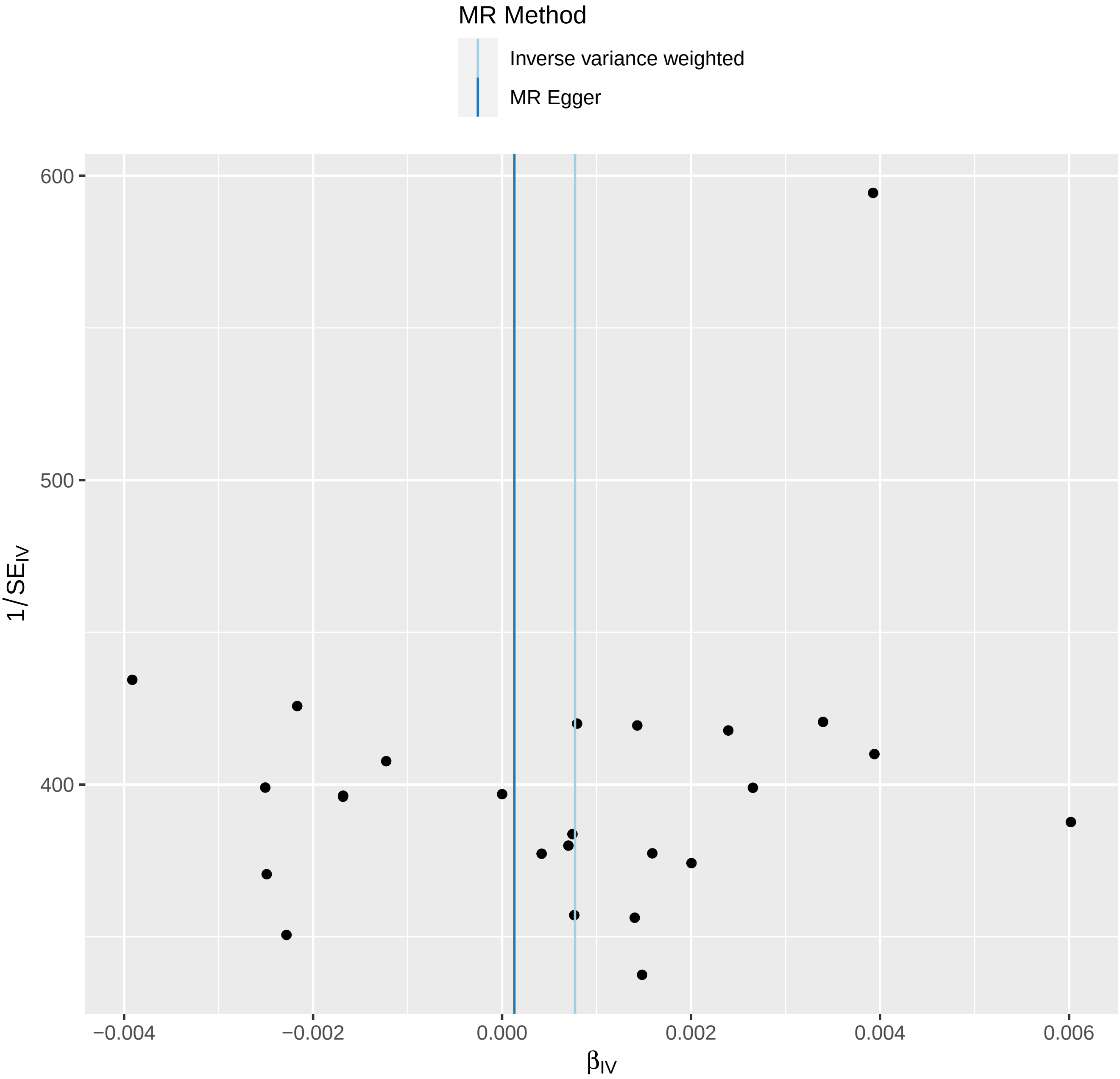

Batch 2−1826 : Mucosal addressin cell adhesion molecule 1 on eGFR

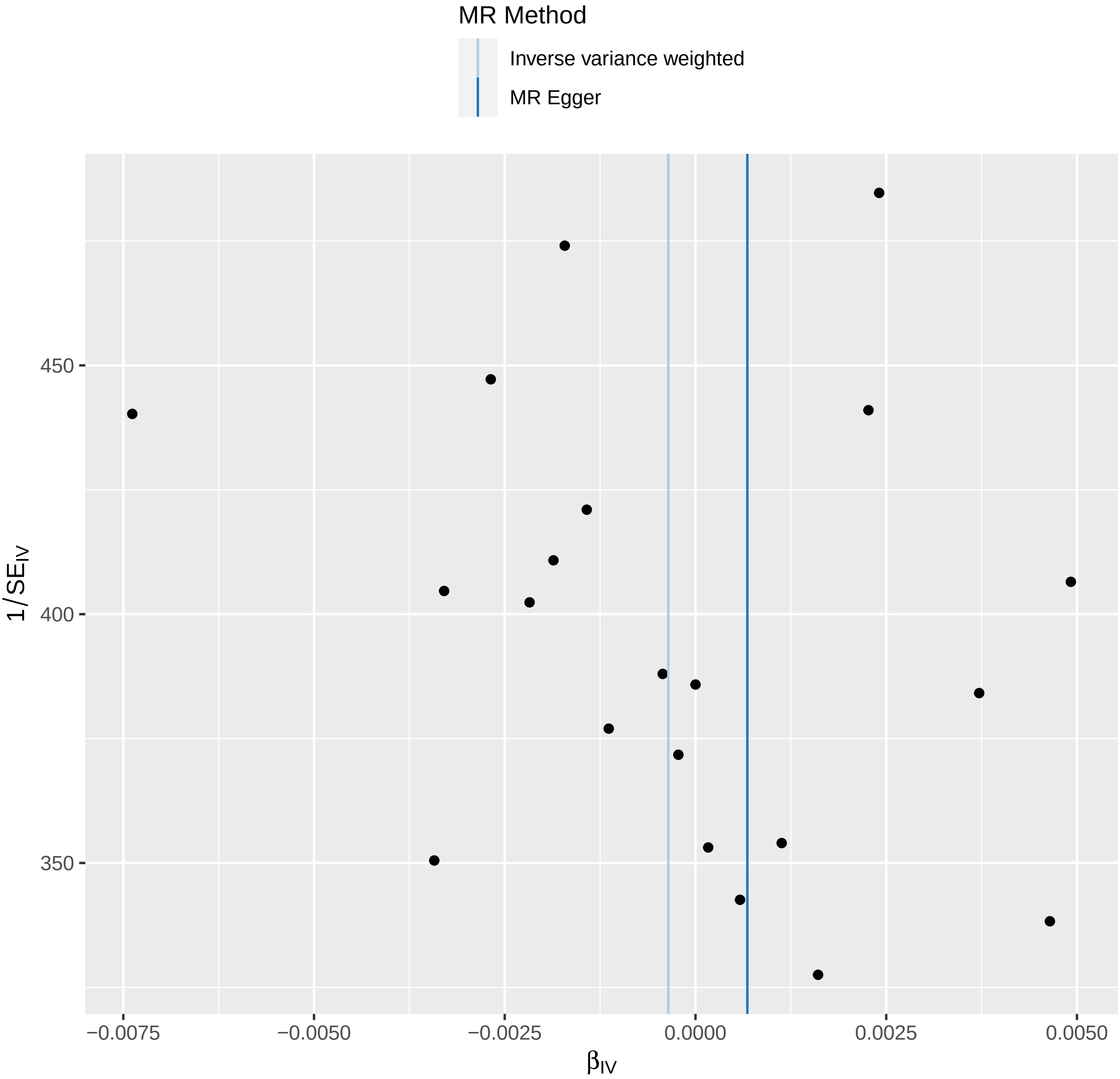

Batch 2−1827 : Melanoma−associated antigen 10 on eGFR

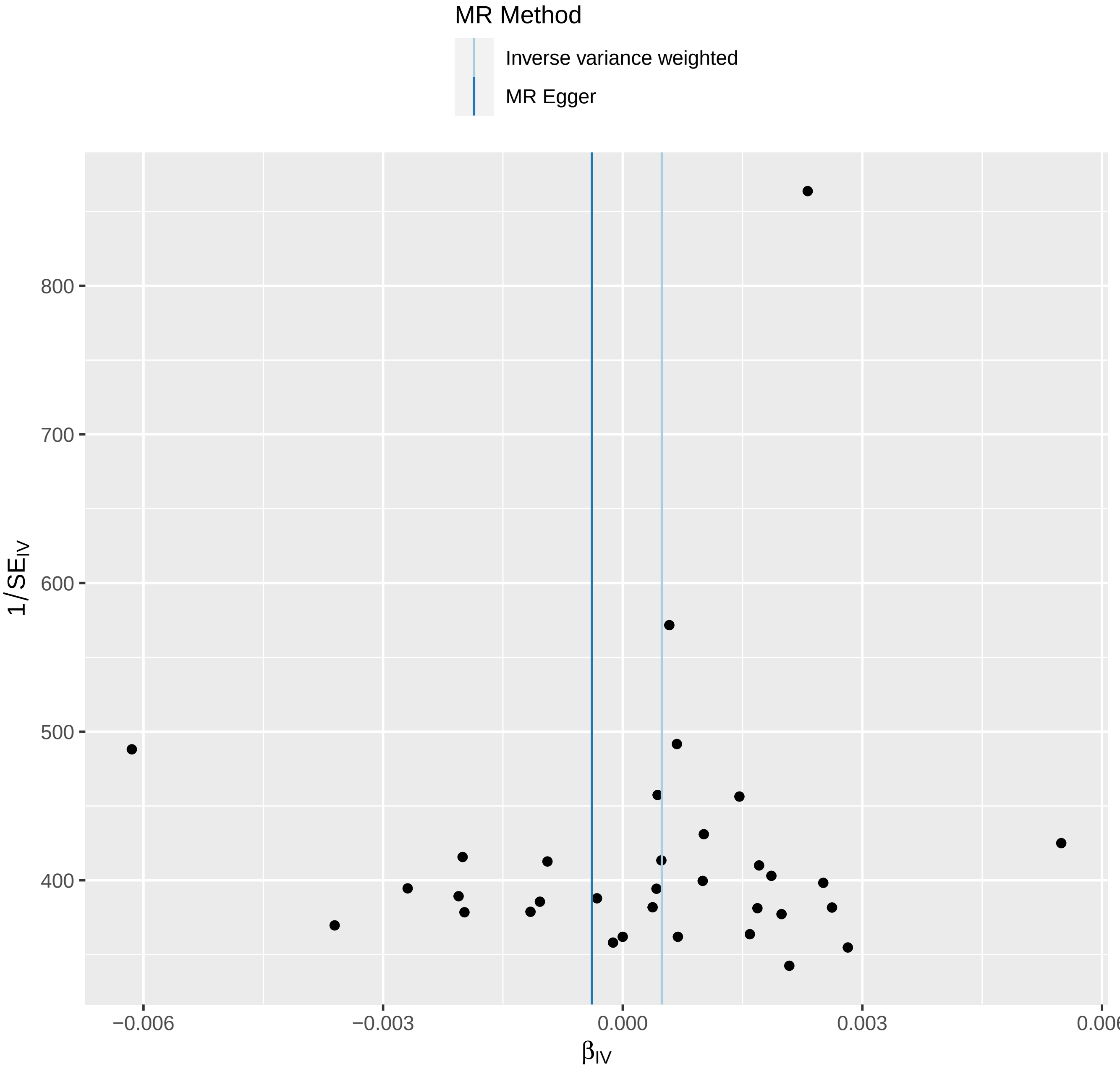

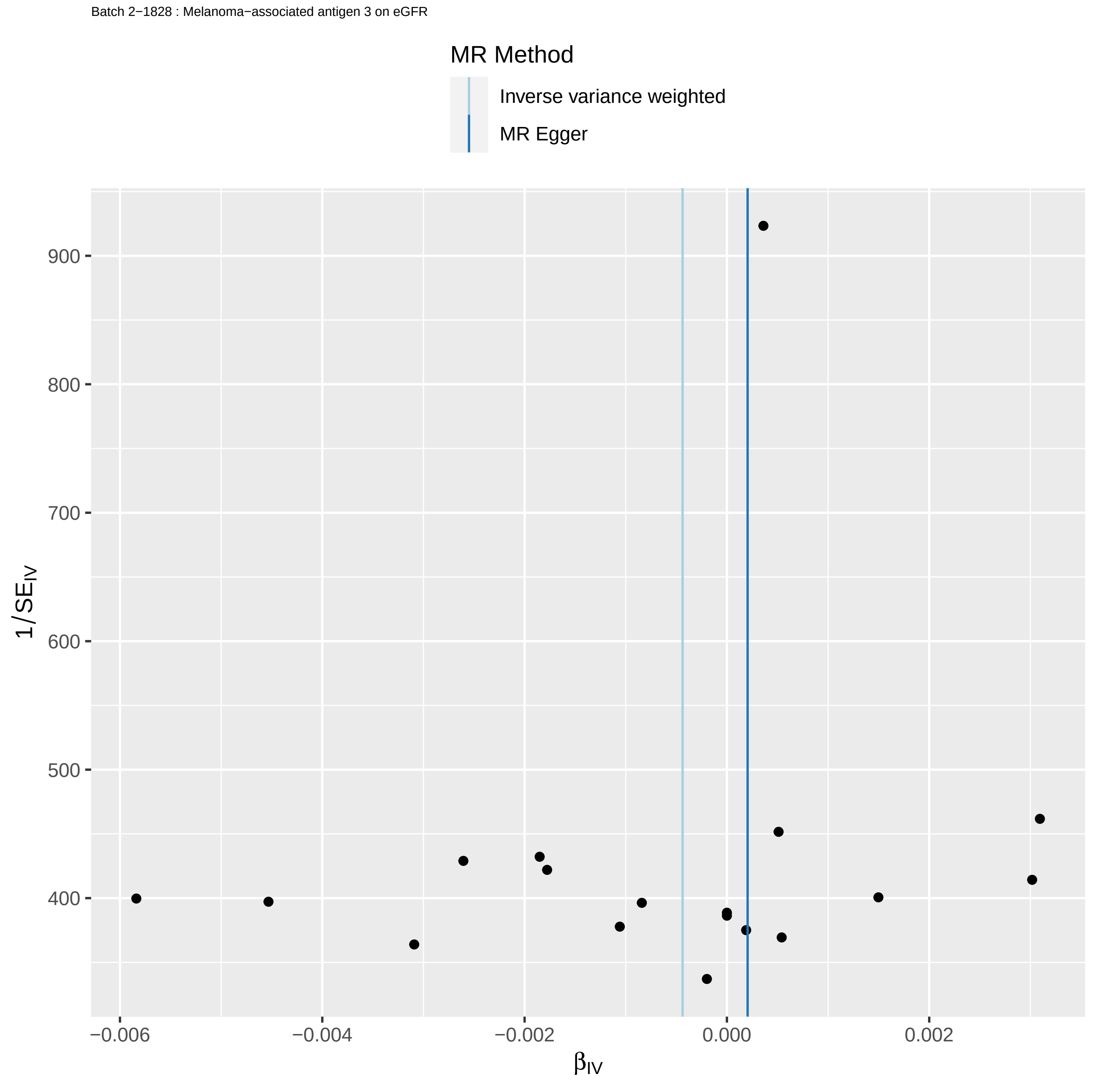
Batch 2−1828 : Melanoma−associated antigen 3 on eGFR
MR Method
Inverse variance weighted
MR Egger
900
800
700
600
500
400
1/SE$_{IV}$
−0.006
−0.004
−0.002
0.000
0.002
β$_{IV}$

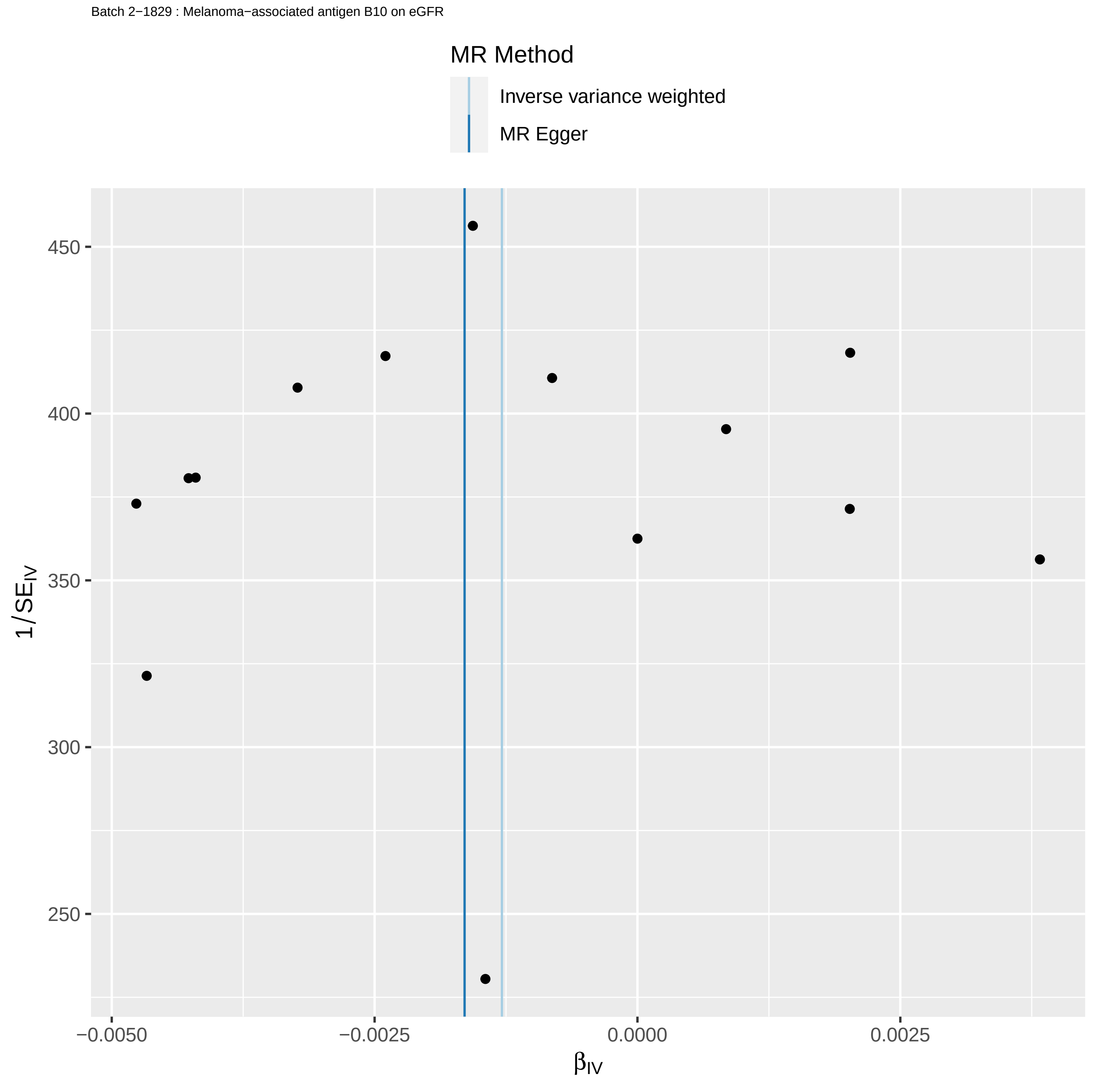

Batch 2−1829 : Melanoma−associated antigen B10 on eGFR
MR Method
Inverse variance weighted
MR Egger
450
400
350
300
250
1/SE$_{IV}$
−0.0050
−0.0025
0.0000
0.0025
β$_{IV}$

Batch 2−1830 : Membrane−associated guanylate kinase, WW and PDZ domain−containing protein 2 on eGFR

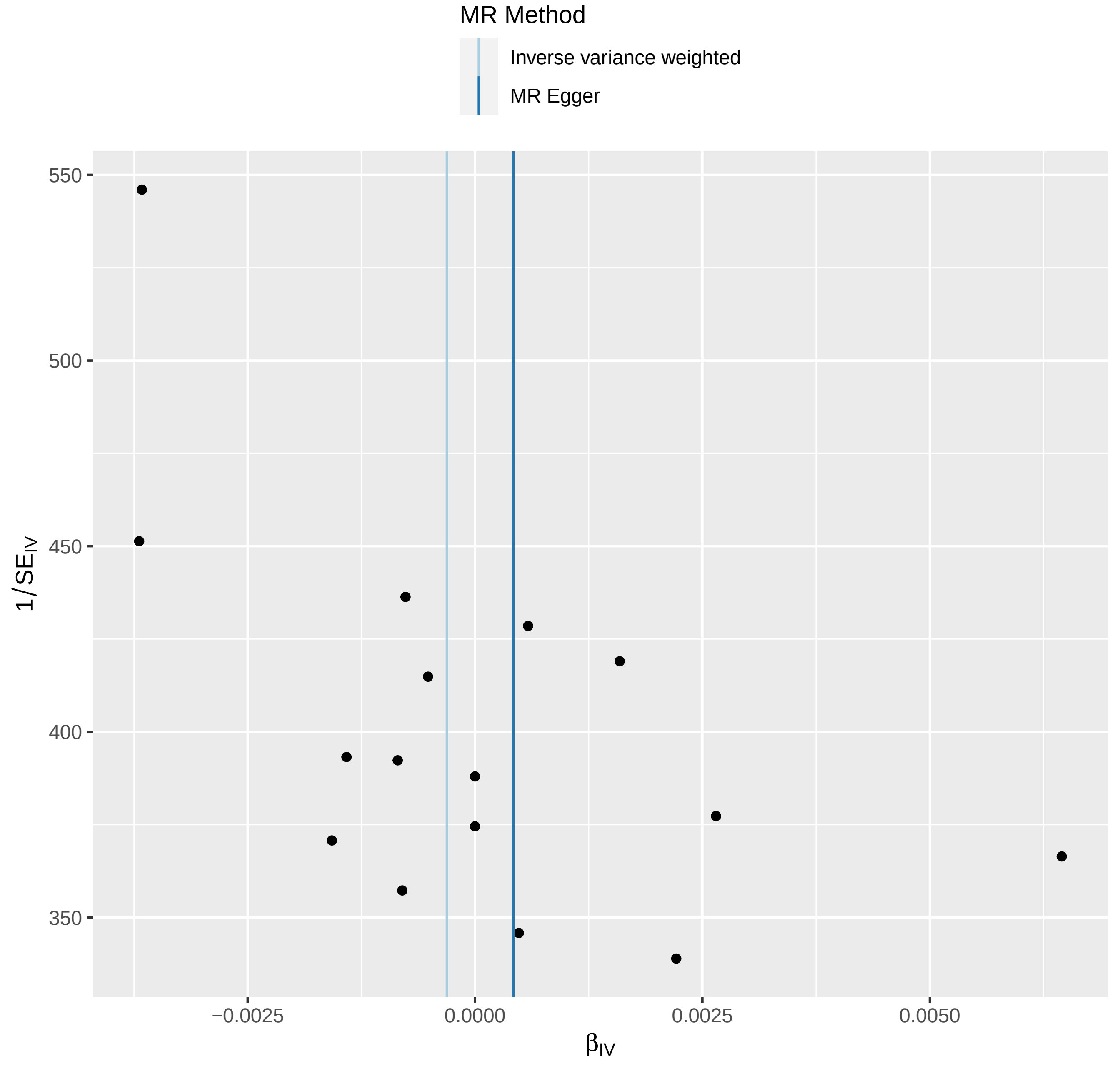

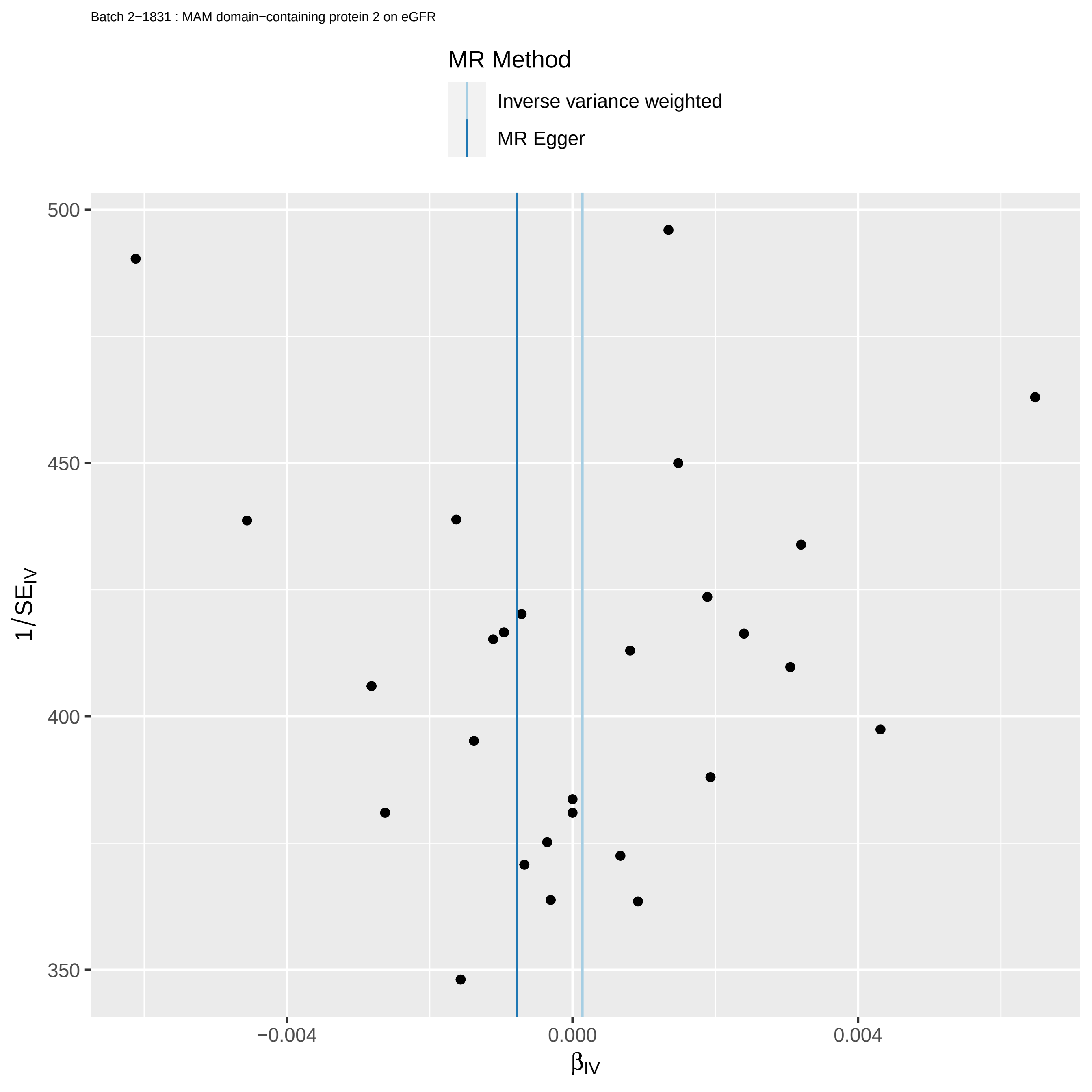

Batch 2−1831 : MAM domain−containing protein 2 on eGFR
MR Method
Inverse variance weighted
MR Egger
500
450
400
350
1/SE$_{IV}$
−0.004
0.000
0.004
β_{IV}

Batch 2−1832 : Mannosyl−oligosaccharide 1,2−alpha−mannosidase IA on eGFR

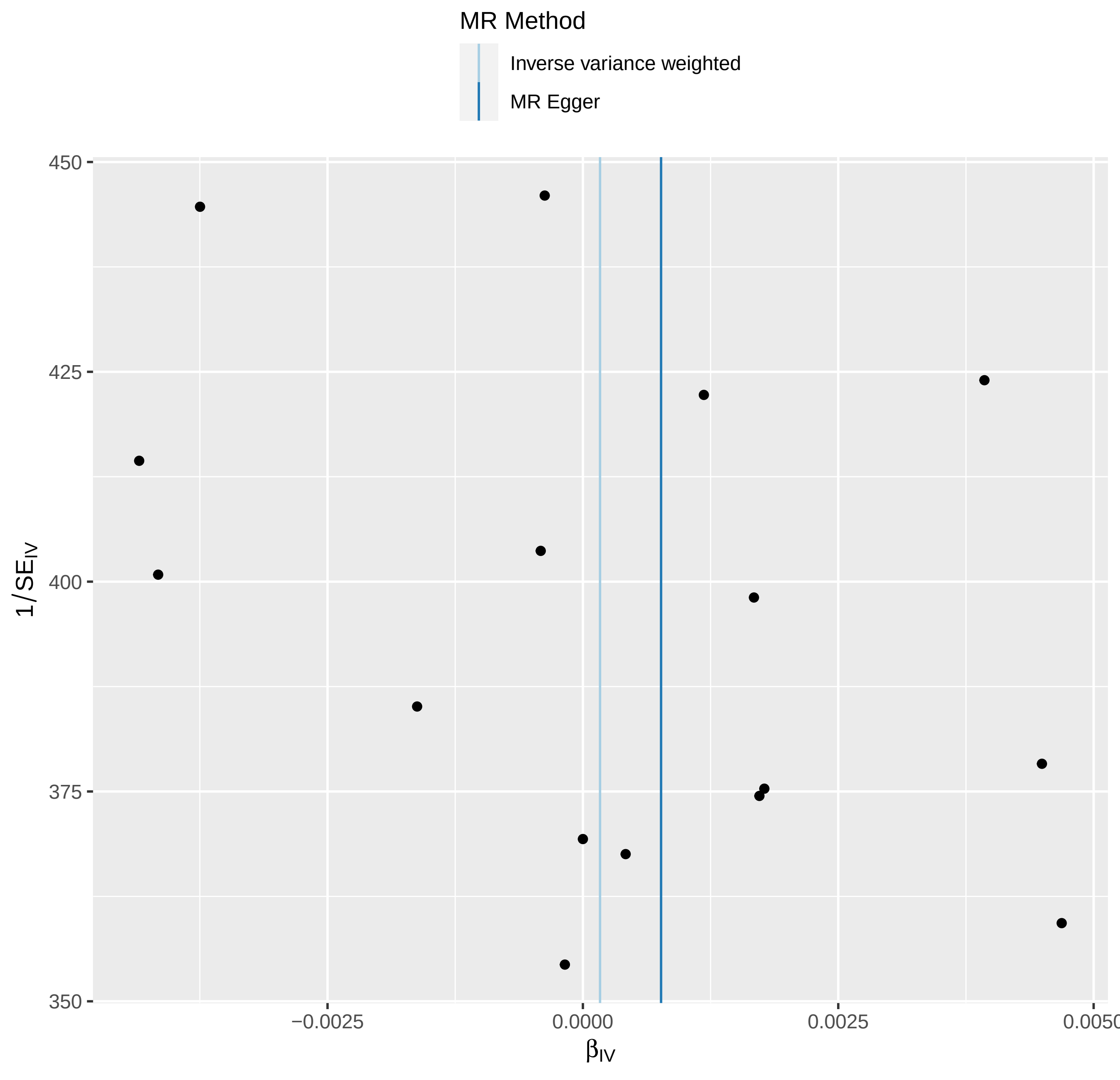

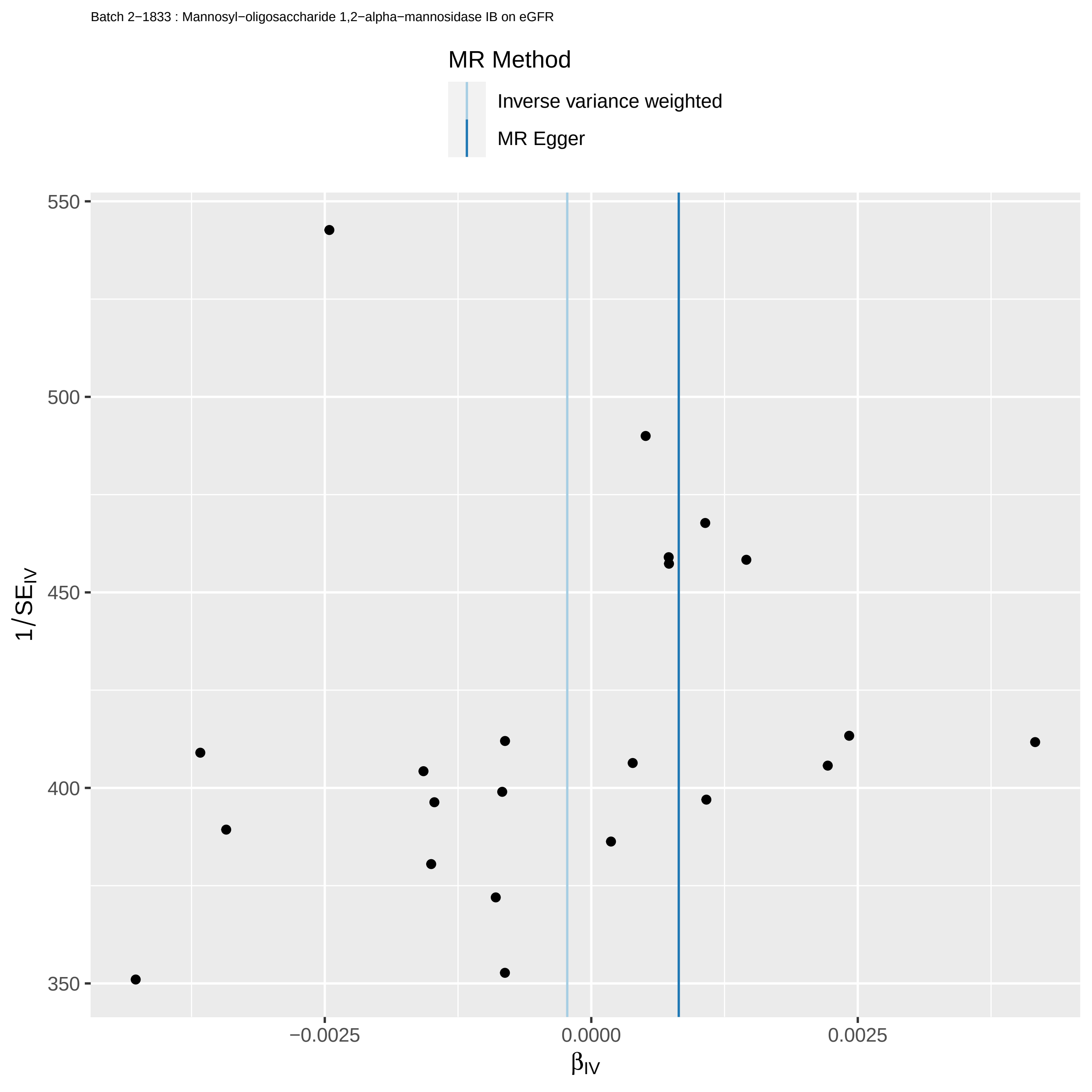

Batch 2−1833 : Mannosyl−oligosaccharide 1,2−alpha−mannosidase IB on eGFR
MR Method
Inverse variance weighted
MR Egger
550
500
450
400
350
1/SE_IV
−0.0025
0.0000
0.0025
β_IV

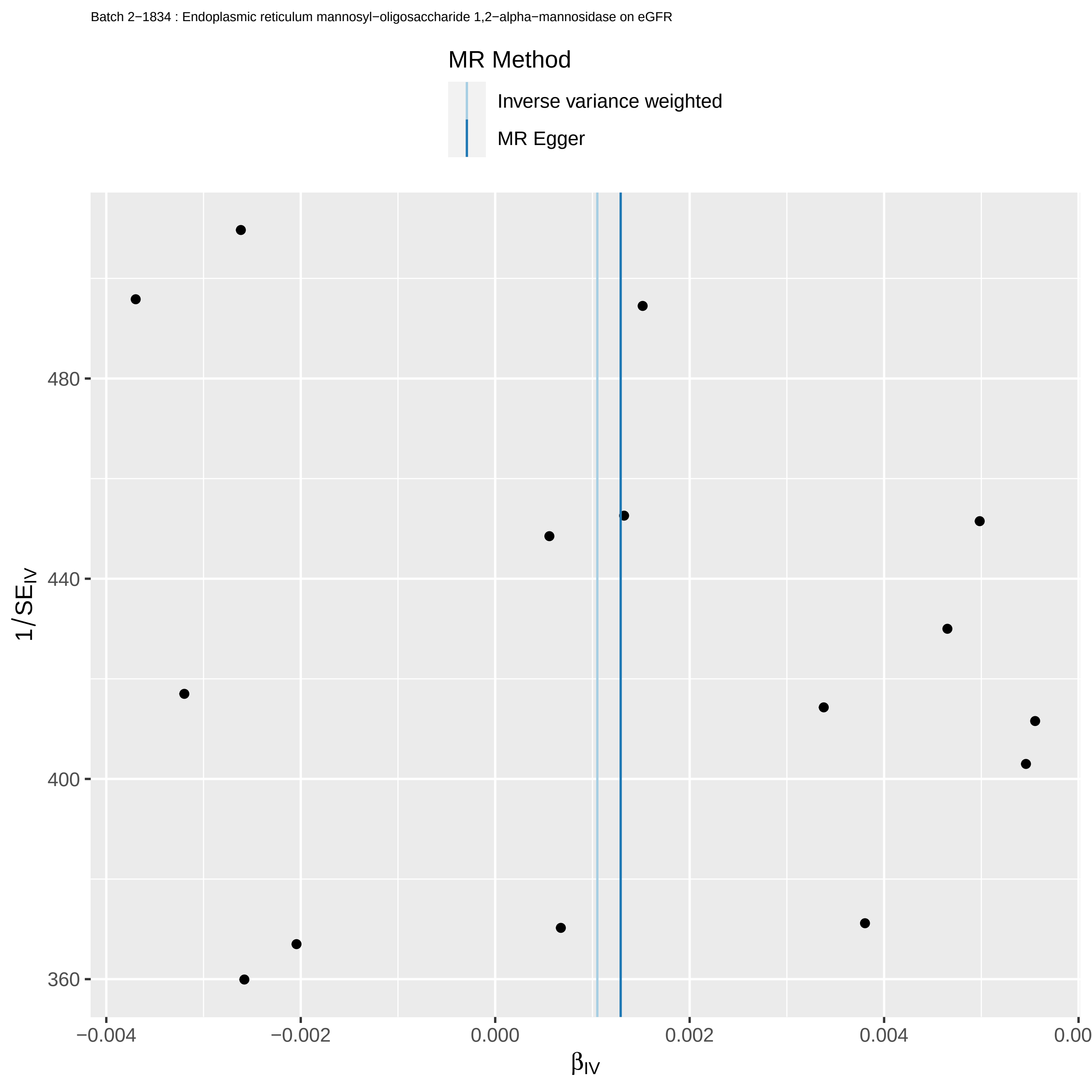

Batch 2−1834 : Endoplasmic reticulum mannosyl−oligosaccharide 1,2−alpha−mannosidase on eGFR
MR Method
Inverse variance weighted
MR Egger
480
440
400
360
1/SE_IV
−0.004
−0.002
0.000
0.002
0.004
0.00
β_IV

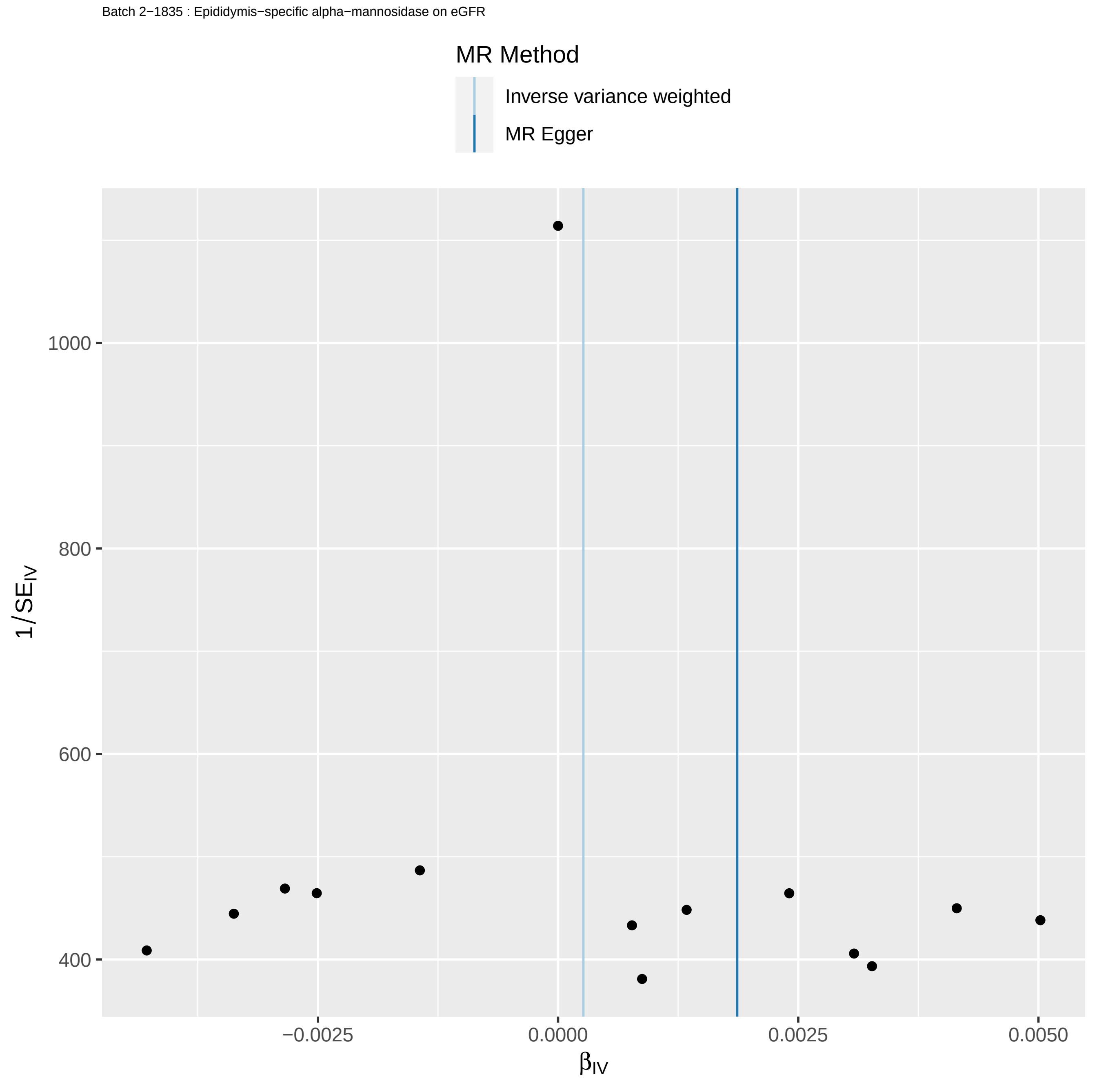

Batch 2−1835 : Epididymis−specific alpha−mannosidase on eGFR
MR Method
Inverse variance weighted
MR Egger
1000
800
600
400
1/SE_IV
−0.0025
0.0000
0.0025
0.0050
β_IV

Batch 2−1836 : Beta−mannosidase on eGFR

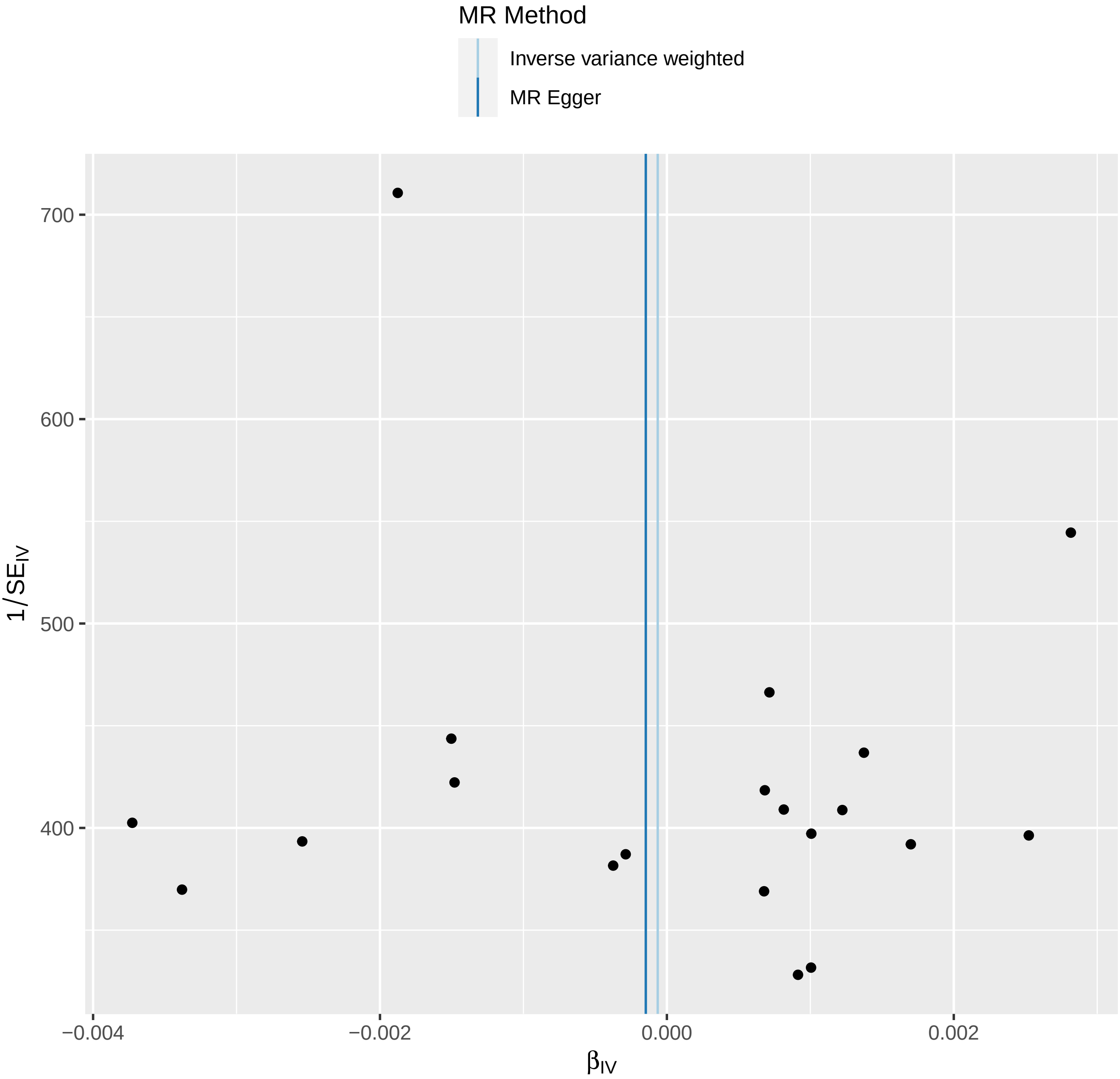

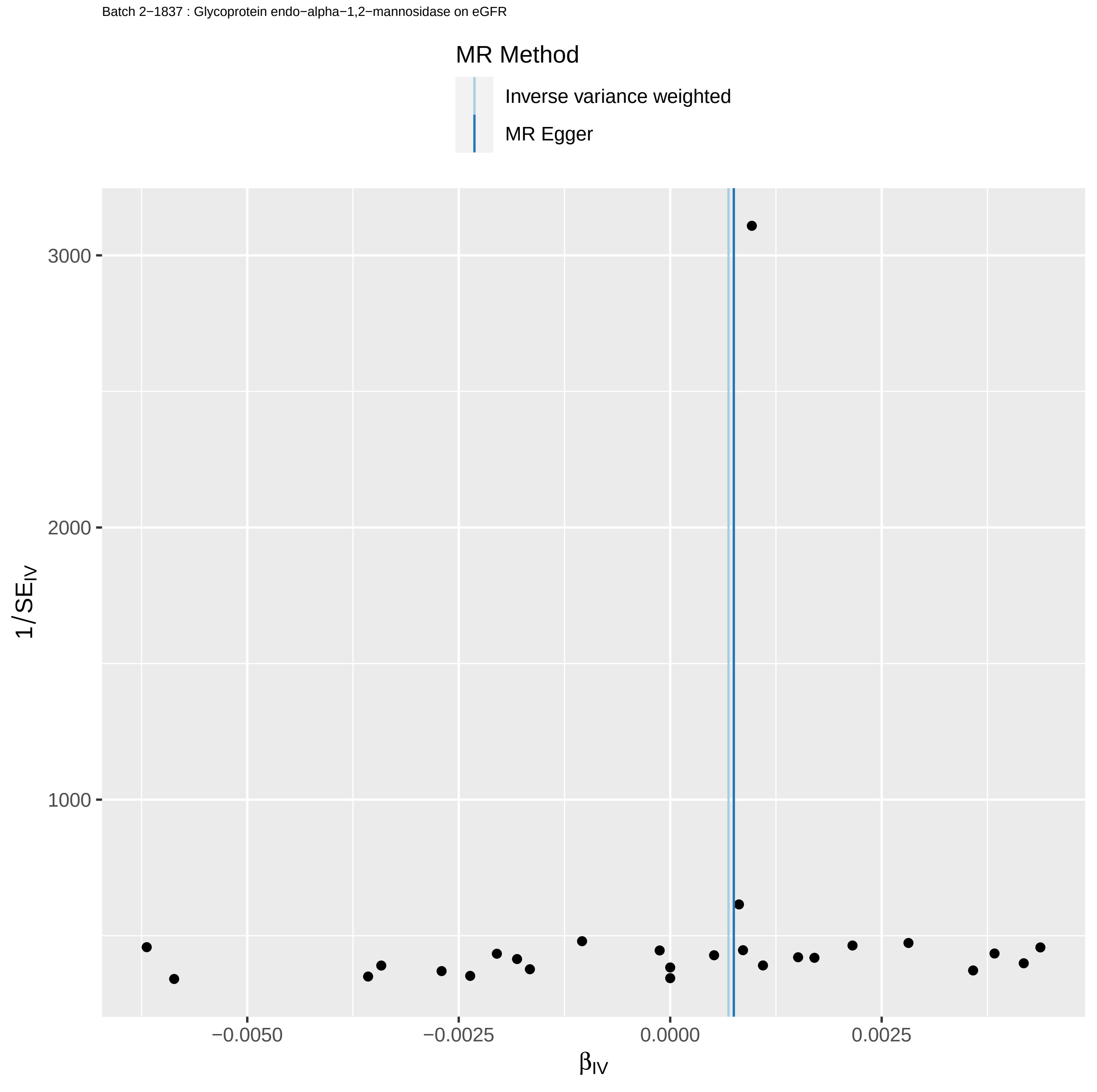
Batch 2−1837 : Glycoprotein endo−alpha−1,2−mannosidase on eGFR
MR Method
Inverse variance weighted
MR Egger
3000
2000
1000
1/SE_IV
−0.0050
−0.0025
0.0000
0.0025
β_IV

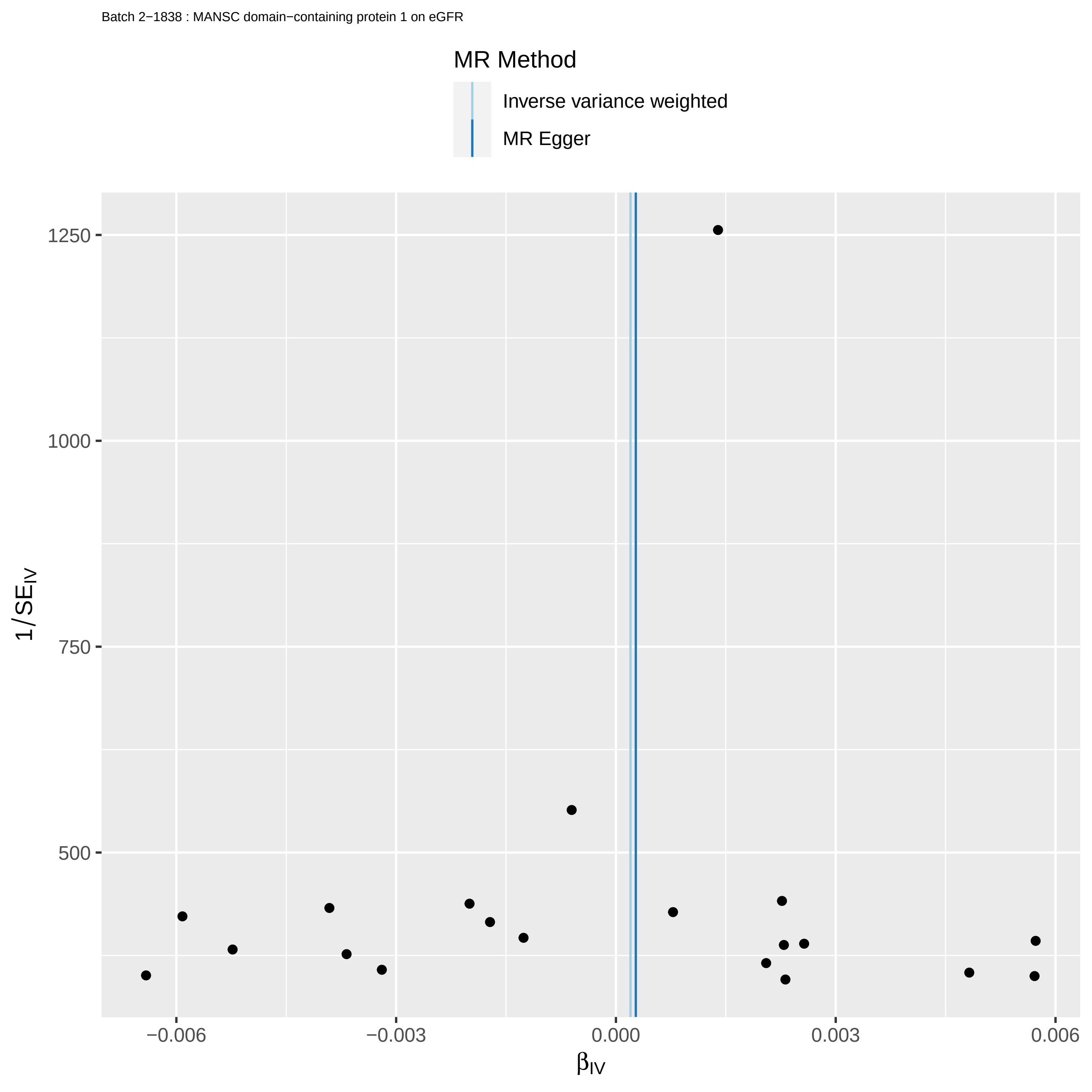

Batch 2−1838 : MANSC domain−containing protein 1 on eGFR
MR Method
Inverse variance weighted
MR Egger
1250
1000
750
500
1/SE$_{IV}$
−0.006
−0.003
0.000
0.003
0.006
β_{IV}

Batch 2−1839 : MANSC domain−containing protein 4 on eGFR

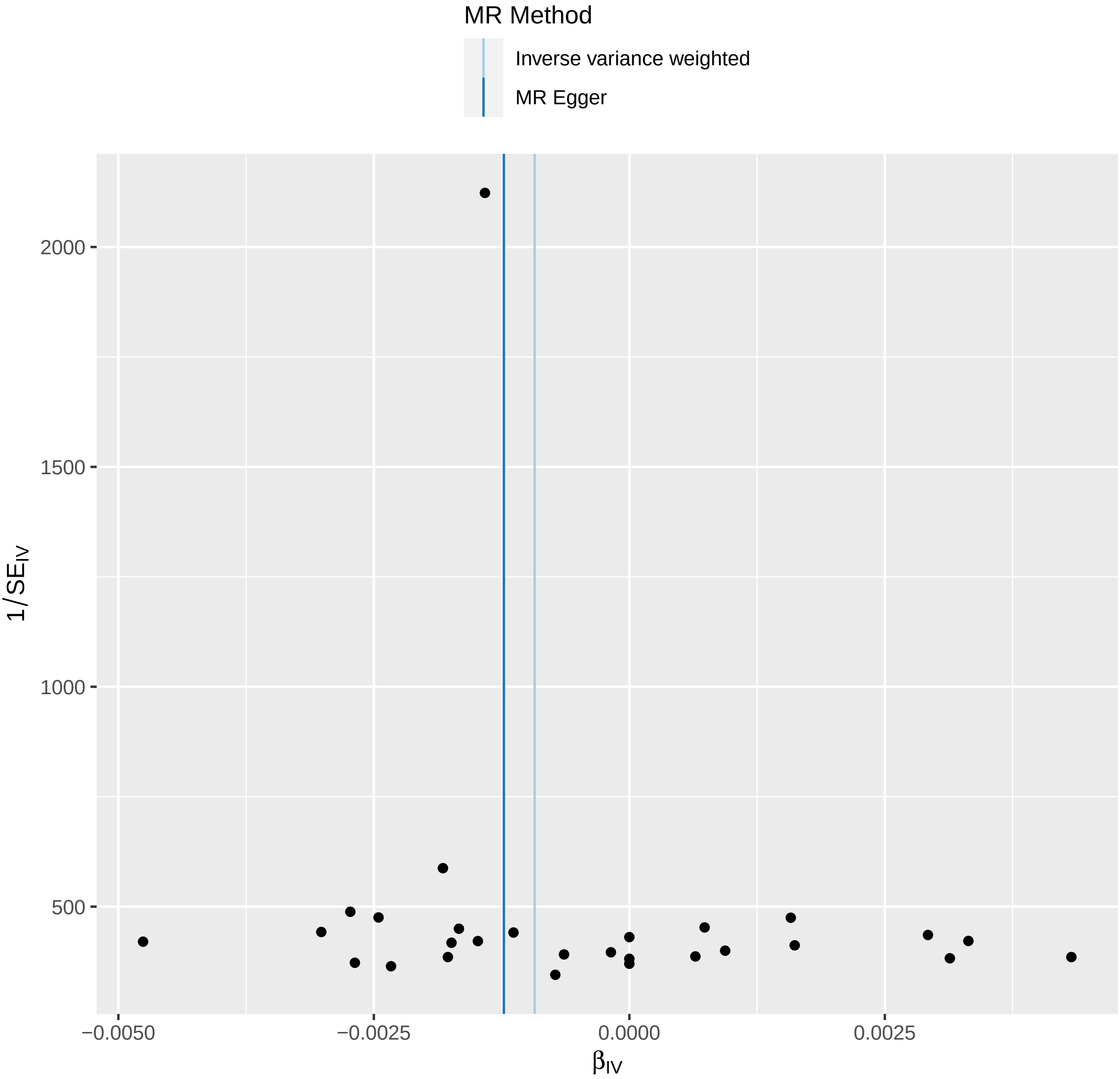

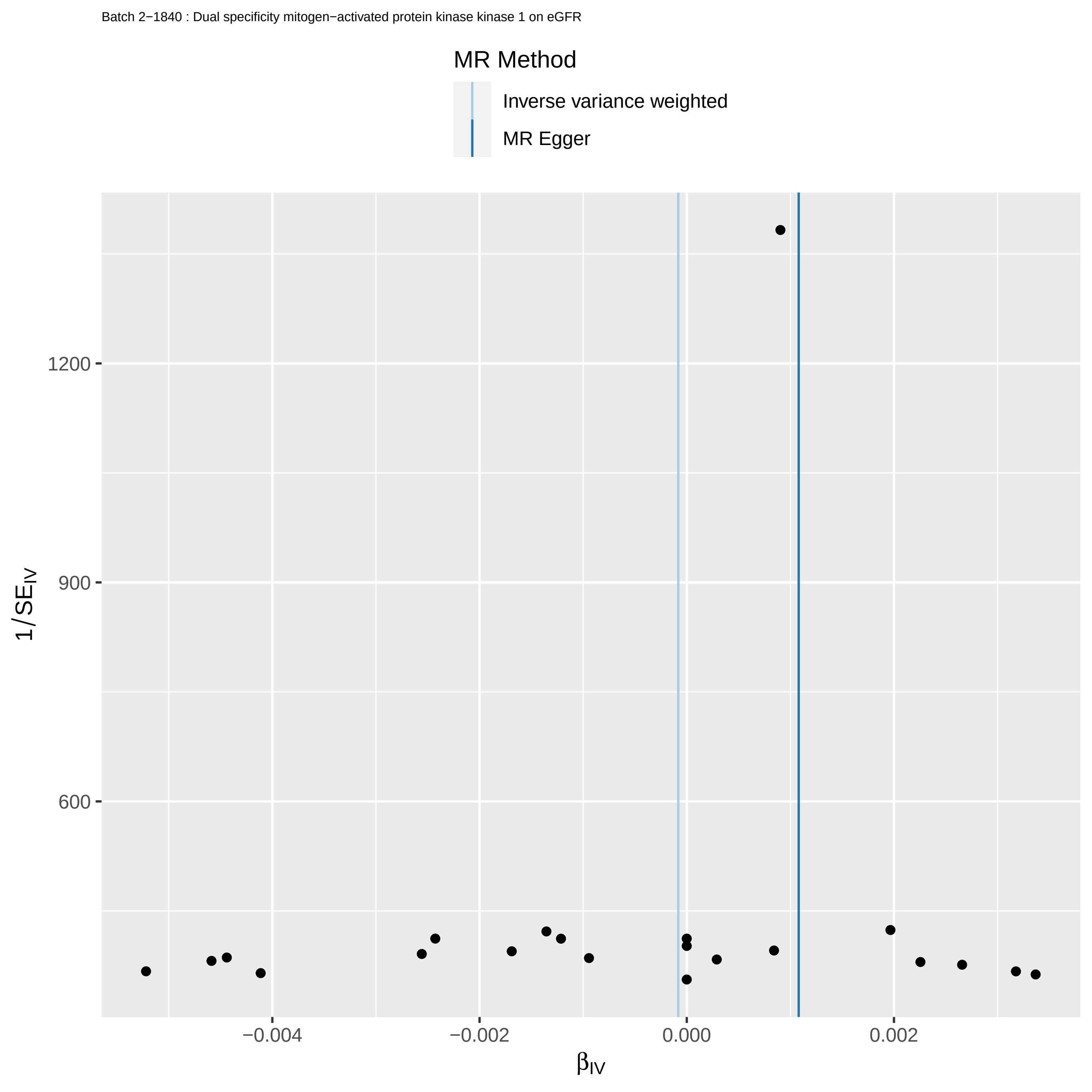

Batch 2−1840 : Dual specificity mitogen−activated protein kinase kinase 1 on eGFR
MR Method
Inverse variance weighted
MR Egger
1200
900
600
1/SE$_{IV}$
−0.004
−0.002
0.000
0.002
β$_{IV}$

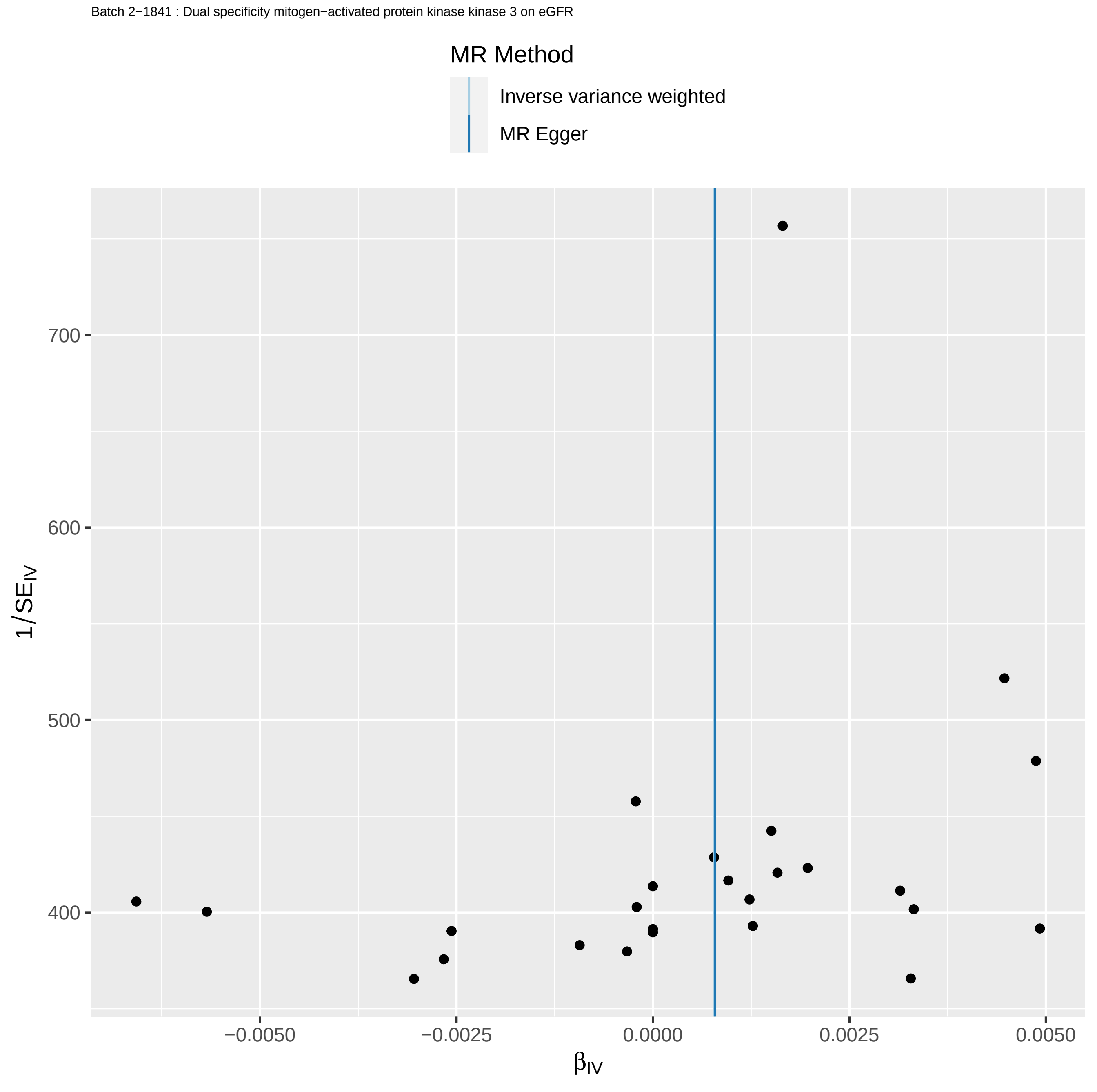

Batch 2−1841 : Dual specificity mitogen−activated protein kinase kinase 3 on eGFR
MR Method
Inverse variance weighted
MR Egger
700
600
500
400
1/SE$_{IV}$
−0.0050
−0.0025
0.0000
0.0025
0.0050
β$_{IV}$

Batch 2−1842 : Dual specificity mitogen−activated protein kinase kinase 4 on eGFR

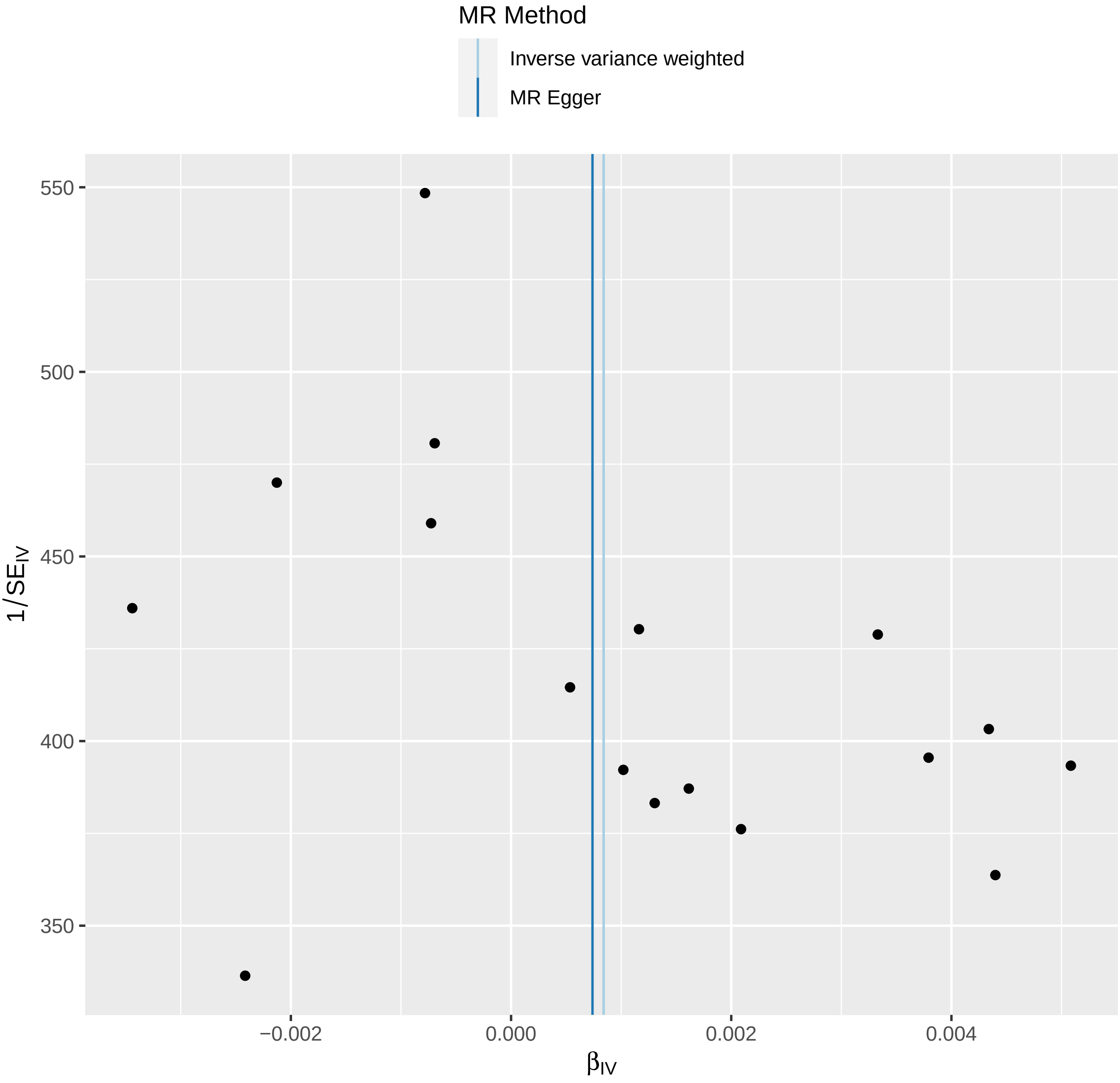

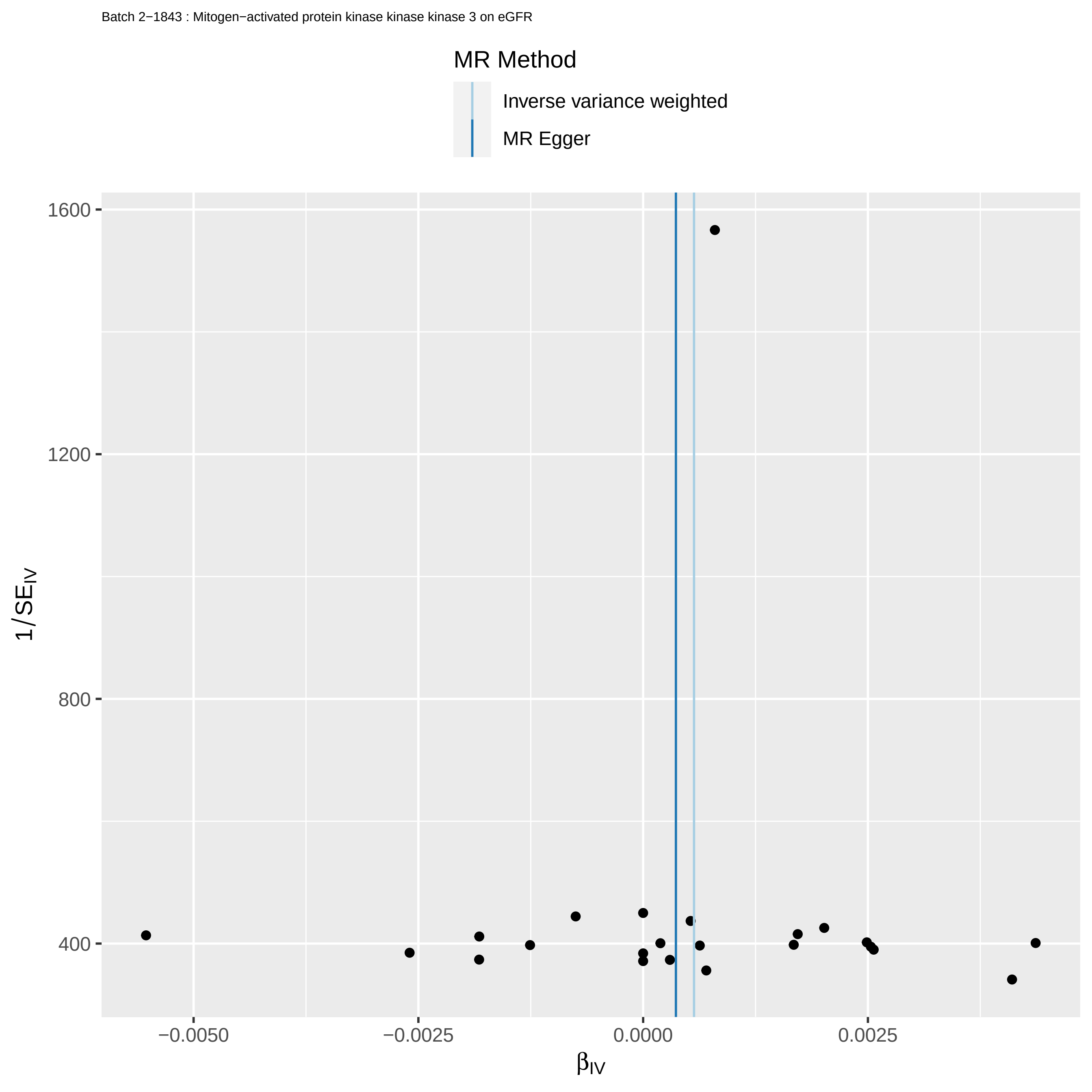
Batch 2−1843 : Mitogen−activated protein kinase kinase kinase 3 on eGFR
MR Method
Inverse variance weighted
MR Egger
1600
1200
800
400
1/SE_IV
−0.0050
−0.0025
0.0000
0.0025
β_IV

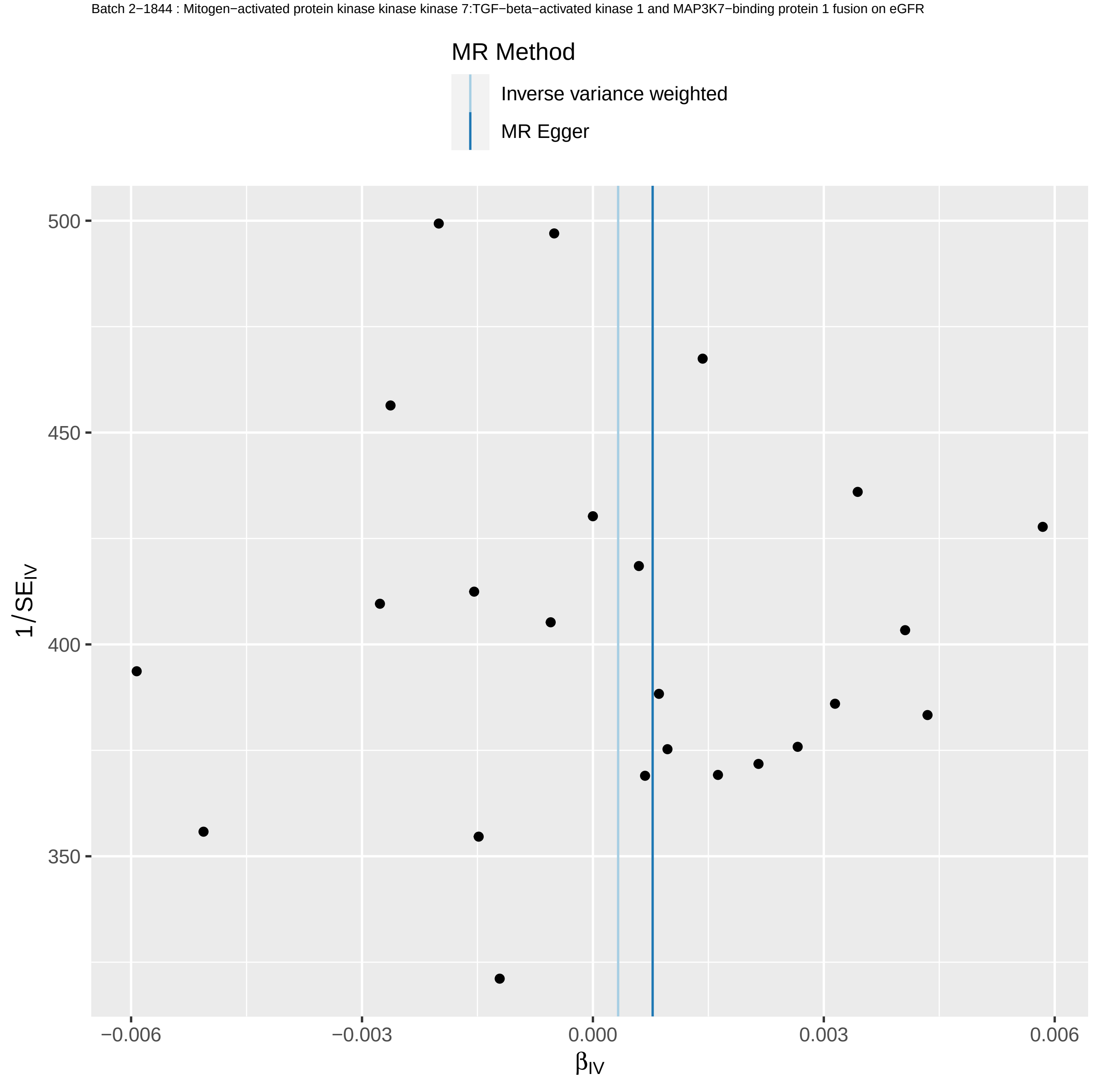
Batch 2−1844 : Mitogen−activated protein kinase kinase kinase 7:TGF−beta−activated kinase 1 and MAP3K7−binding protein 1 fusion on eGFR
MR Method
Inverse variance weighted
MR Egger
500
450
400
350
−0.006
−0.003
0.000
0.003
0.006
1/SE_IV
β_IV

Batch 2−1845 : Mitogen−activated protein kinase 1 on eGFR

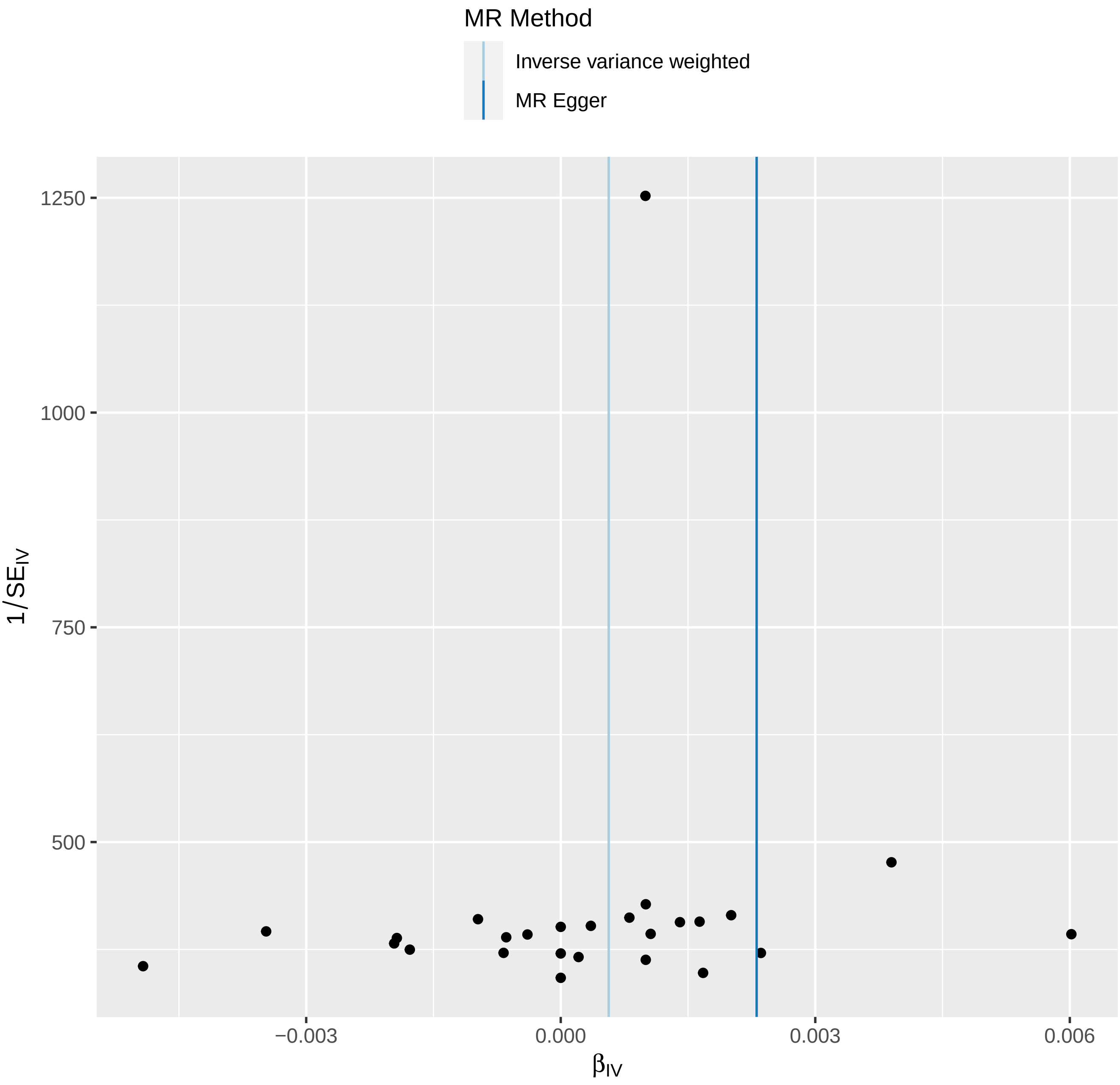

Batch 2−1846 : Mitogen−activated protein kinase 13 on eGFR

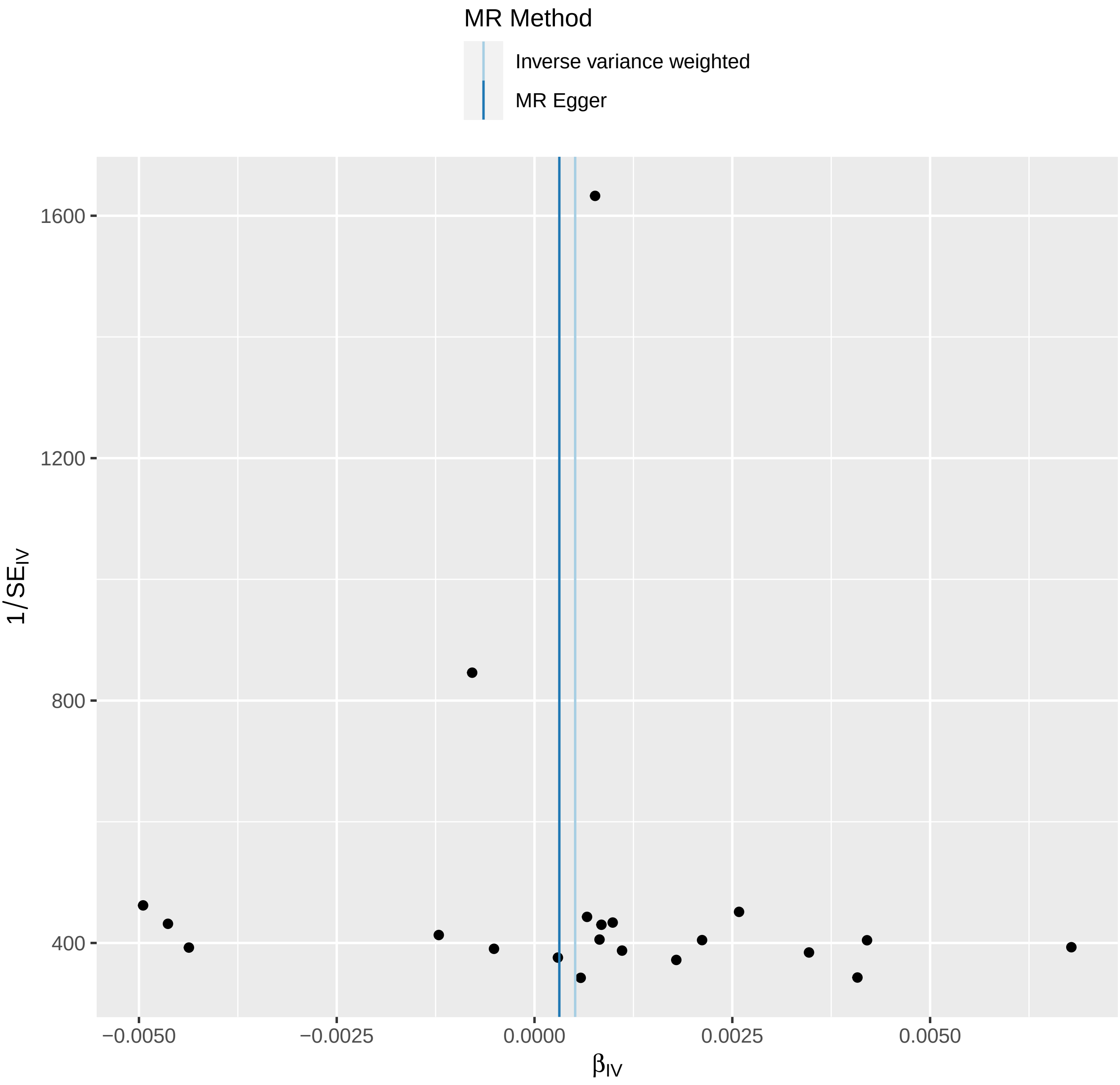

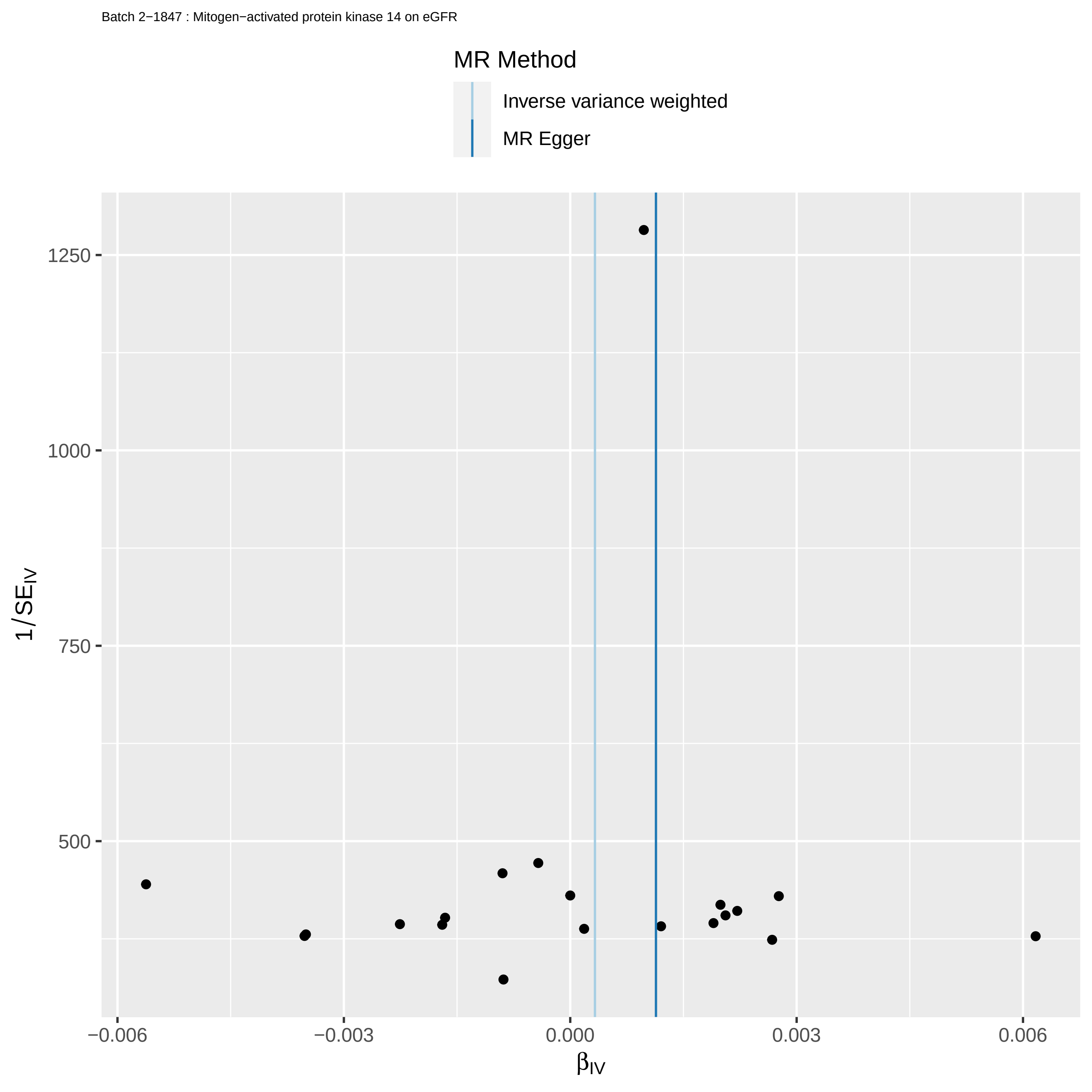

Batch 2−1847 : Mitogen−activated protein kinase 14 on eGFR
MR Method
Inverse variance weighted
MR Egger
1250
1000
750
500
1/SE_IV
−0.006
−0.003
0.000
0.003
0.006
β_IV

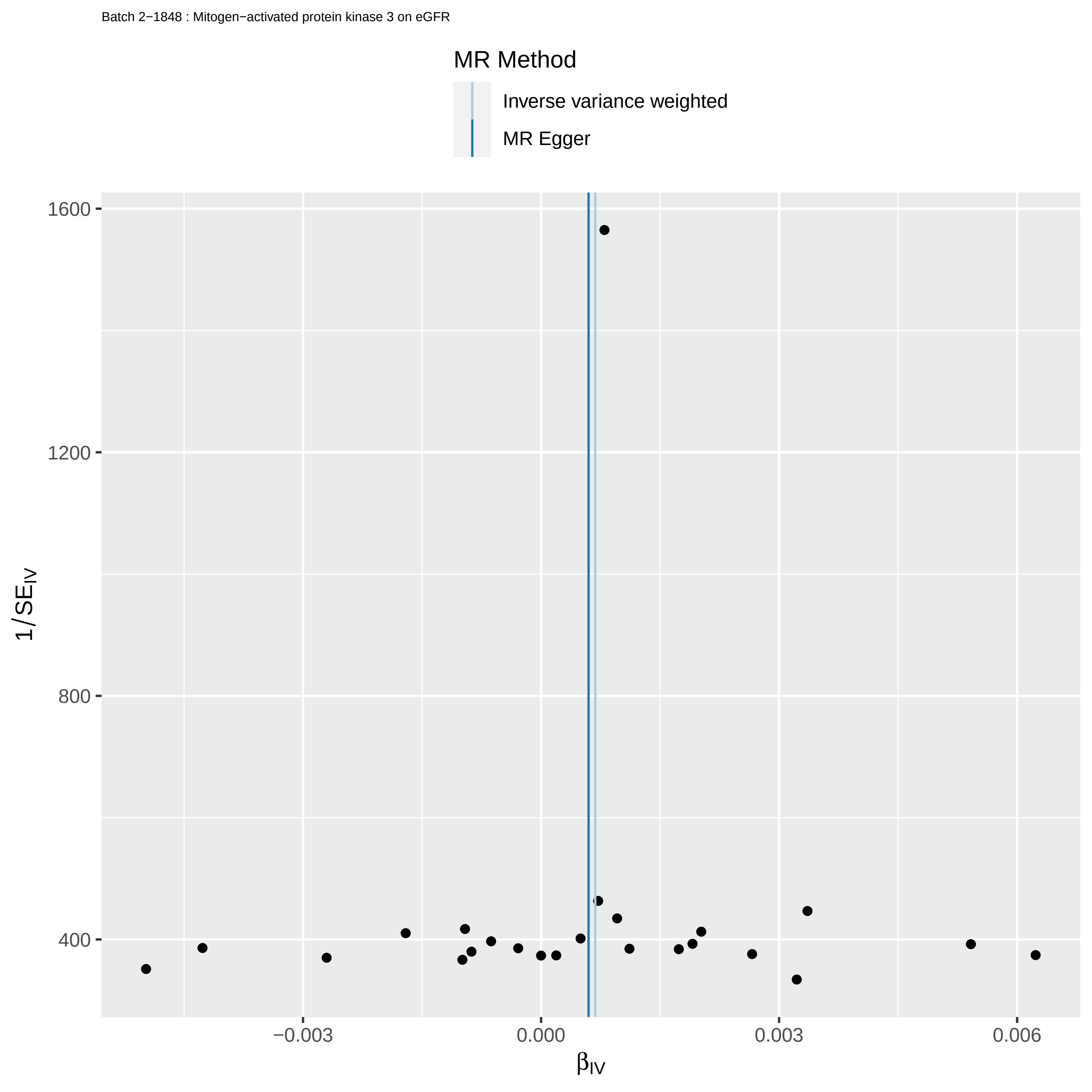

Batch 2−1848 : Mitogen−activated protein kinase 3 on eGFR
MR Method
Inverse variance weighted
MR Egger
1600
1200
800
400
1/SE_IV
−0.003
0.000
0.003
0.006
β_IV

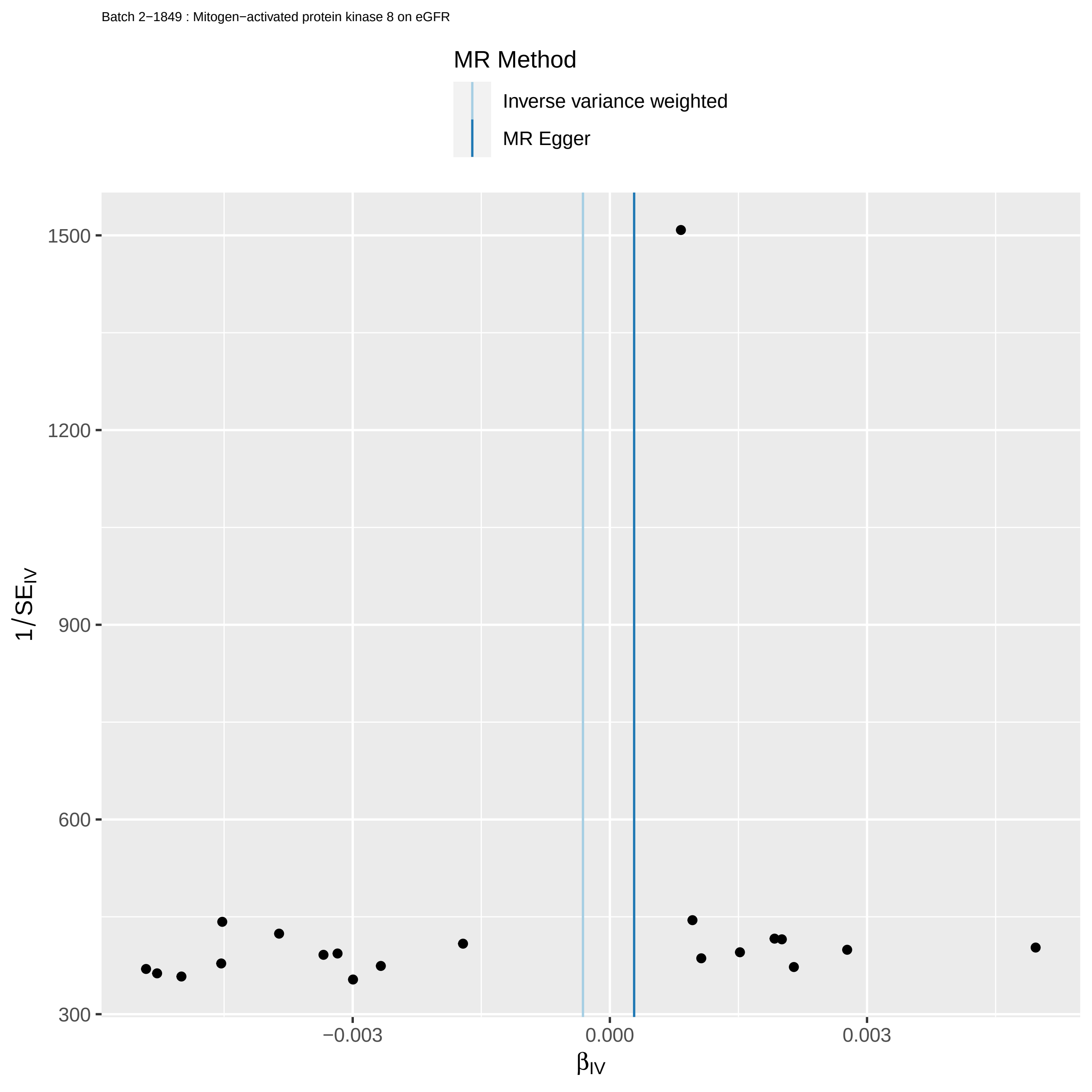
Batch 2−1849 : Mitogen−activated protein kinase 8 on eGFR
MR Method
Inverse variance weighted
MR Egger
1/SE_IV
1500
1200
900
600
300
−0.003
0.000
0.003
β_IV

Batch 2−1850 : Mitogen−activated protein kinase 9 on eGFR

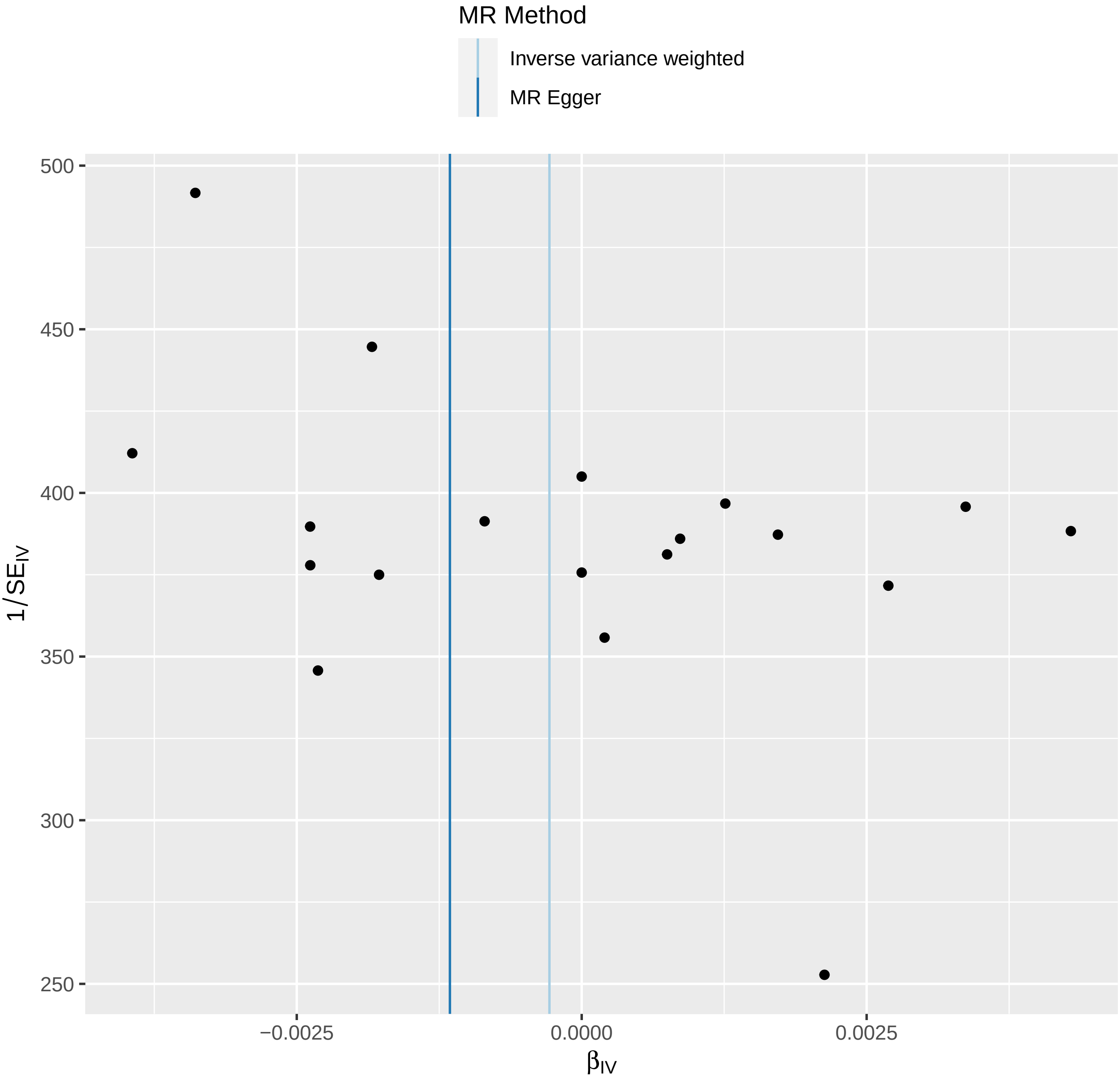

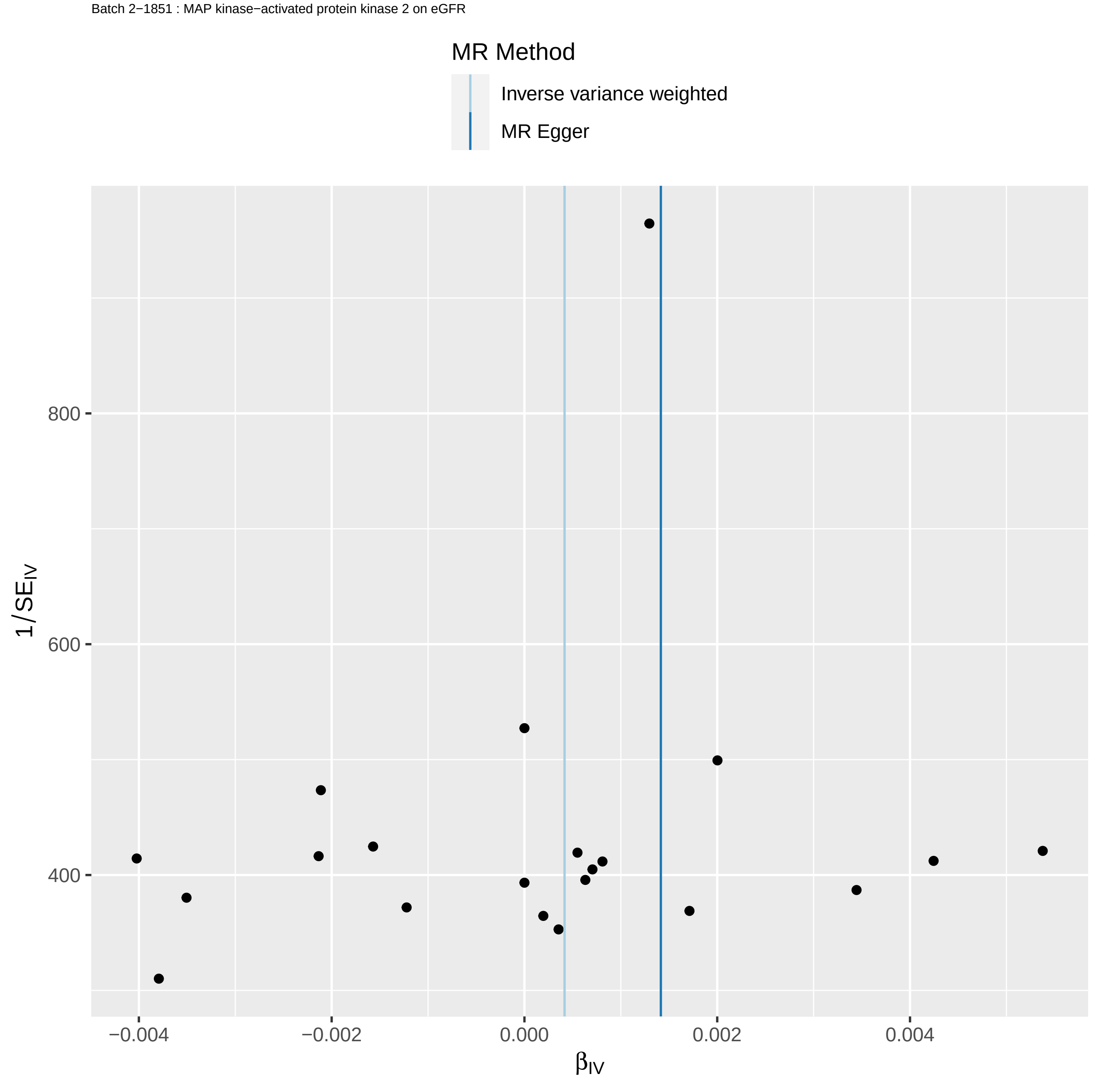

Batch 2−1851 : MAP kinase−activated protein kinase 2 on eGFR
MR Method
Inverse variance weighted
MR Egger
800
600
400
1/SE_IV
−0.004
−0.002
0.000
0.002
0.004
β_IV

Batch 2−1852 : MAP kinase−activated protein kinase 3 on eGFR

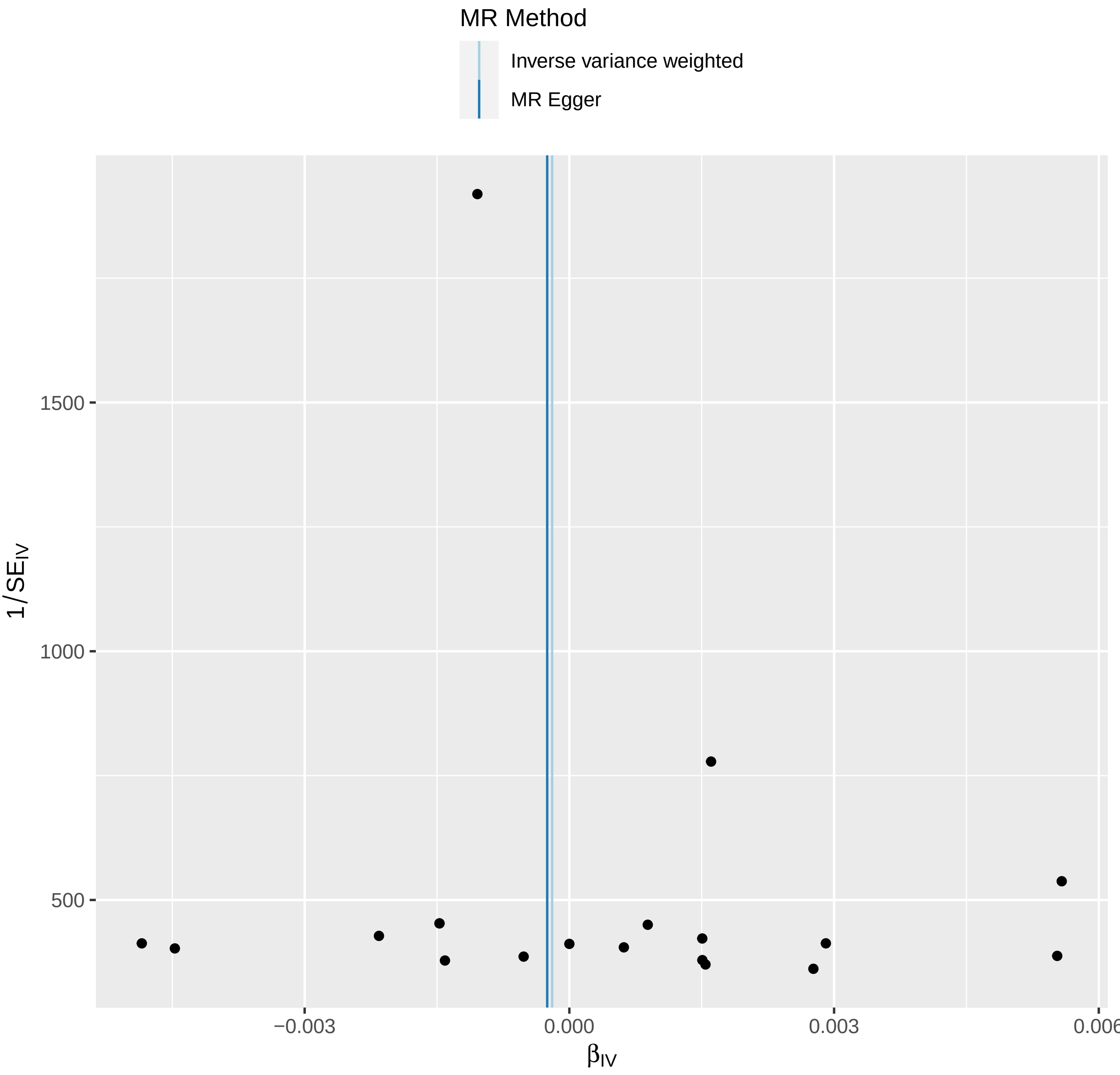

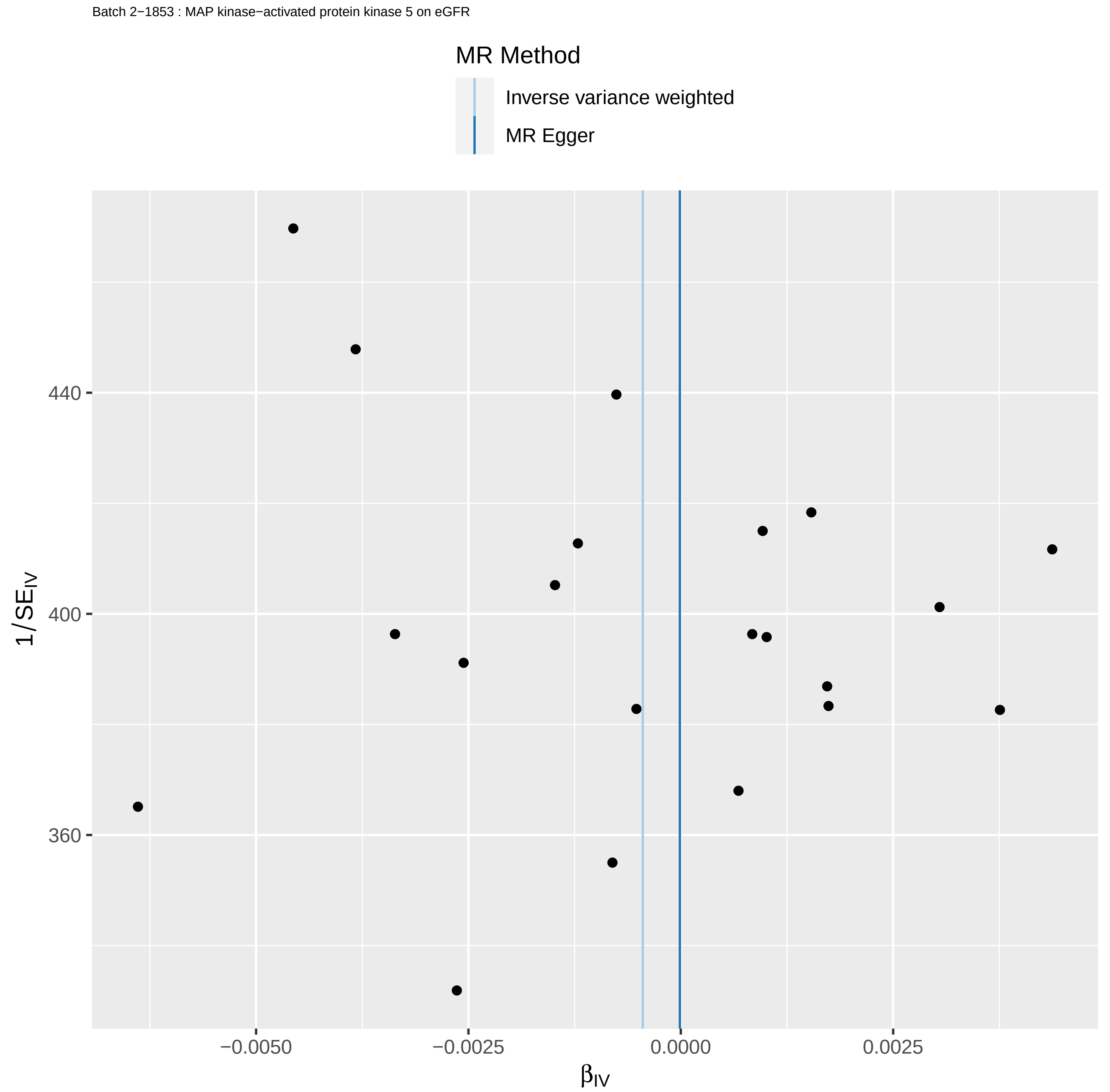
Batch 2−1853 : MAP kinase−activated protein kinase 5 on eGFR
MR Method
Inverse variance weighted
MR Egger
440
400
360
1/SE_IV
−0.0050
−0.0025
0.0000
0.0025
β_IV

Batch 2−1854 : MAP kinase−activated protein kinase 5 on eGFR

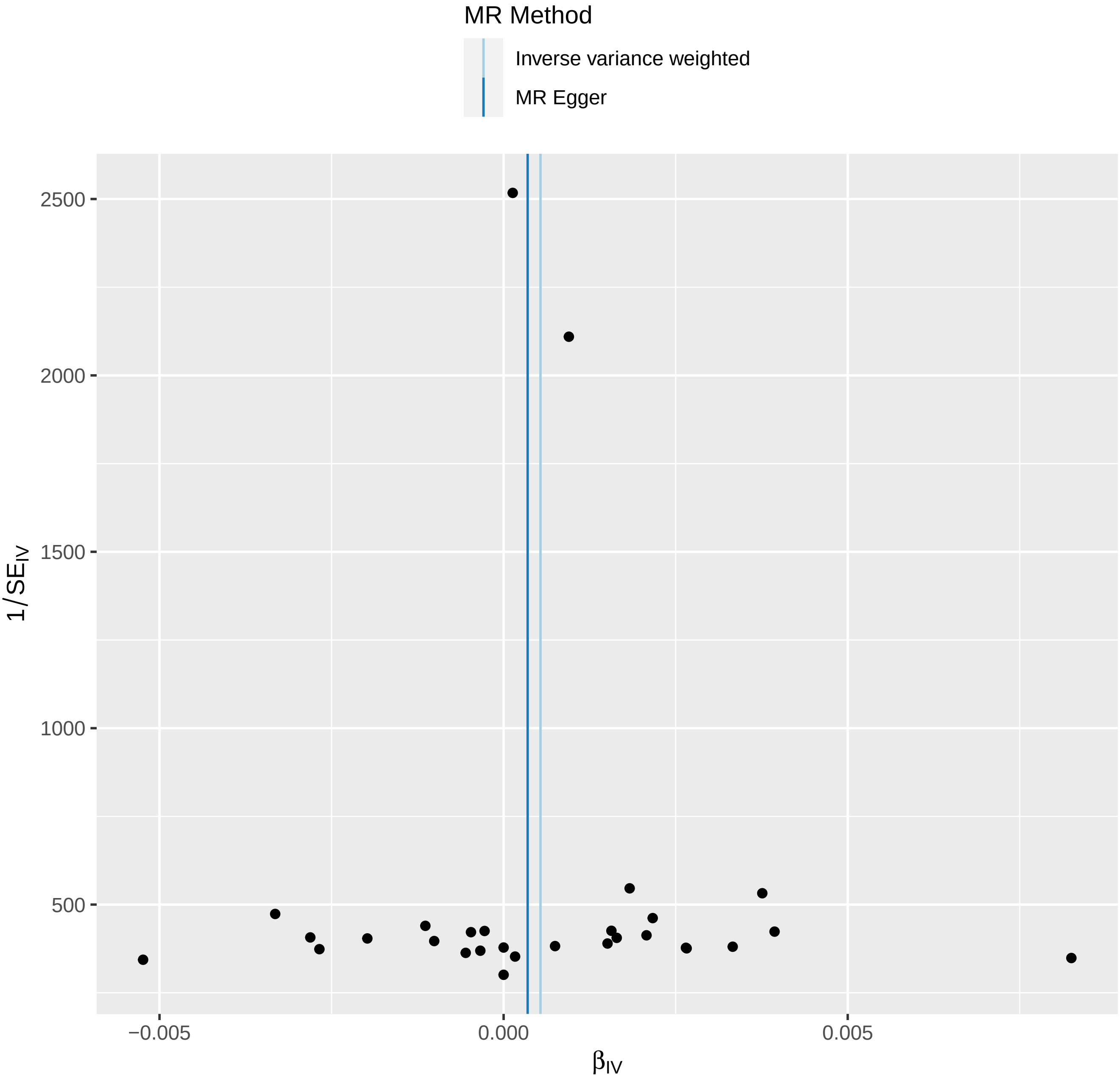

Batch 2−1855 : Mannan−binding lectin serine protease 1 on eGFR

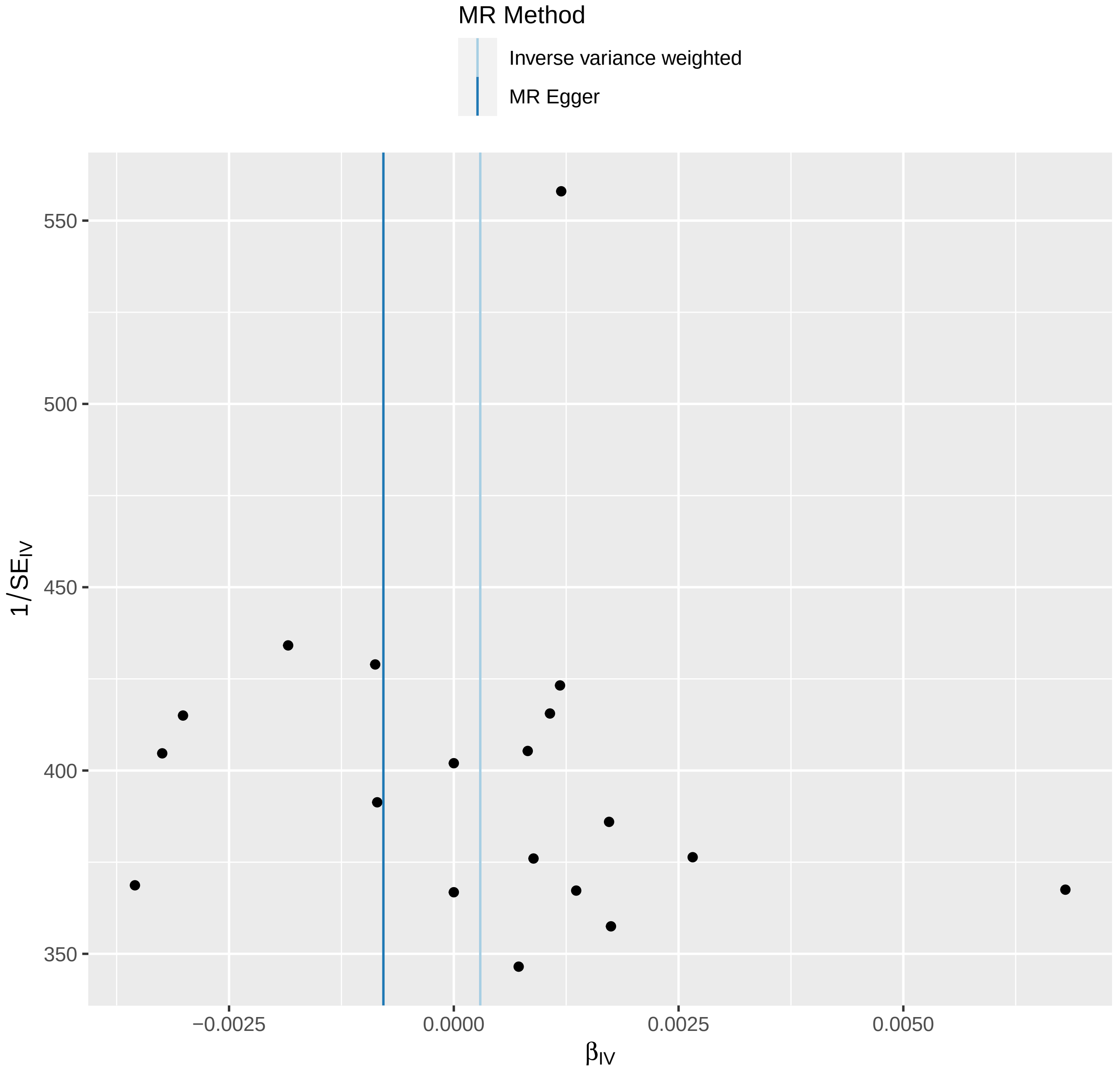

Batch 2−1856 : Megakaryocyte−associated tyrosine−protein kinase on eGFR

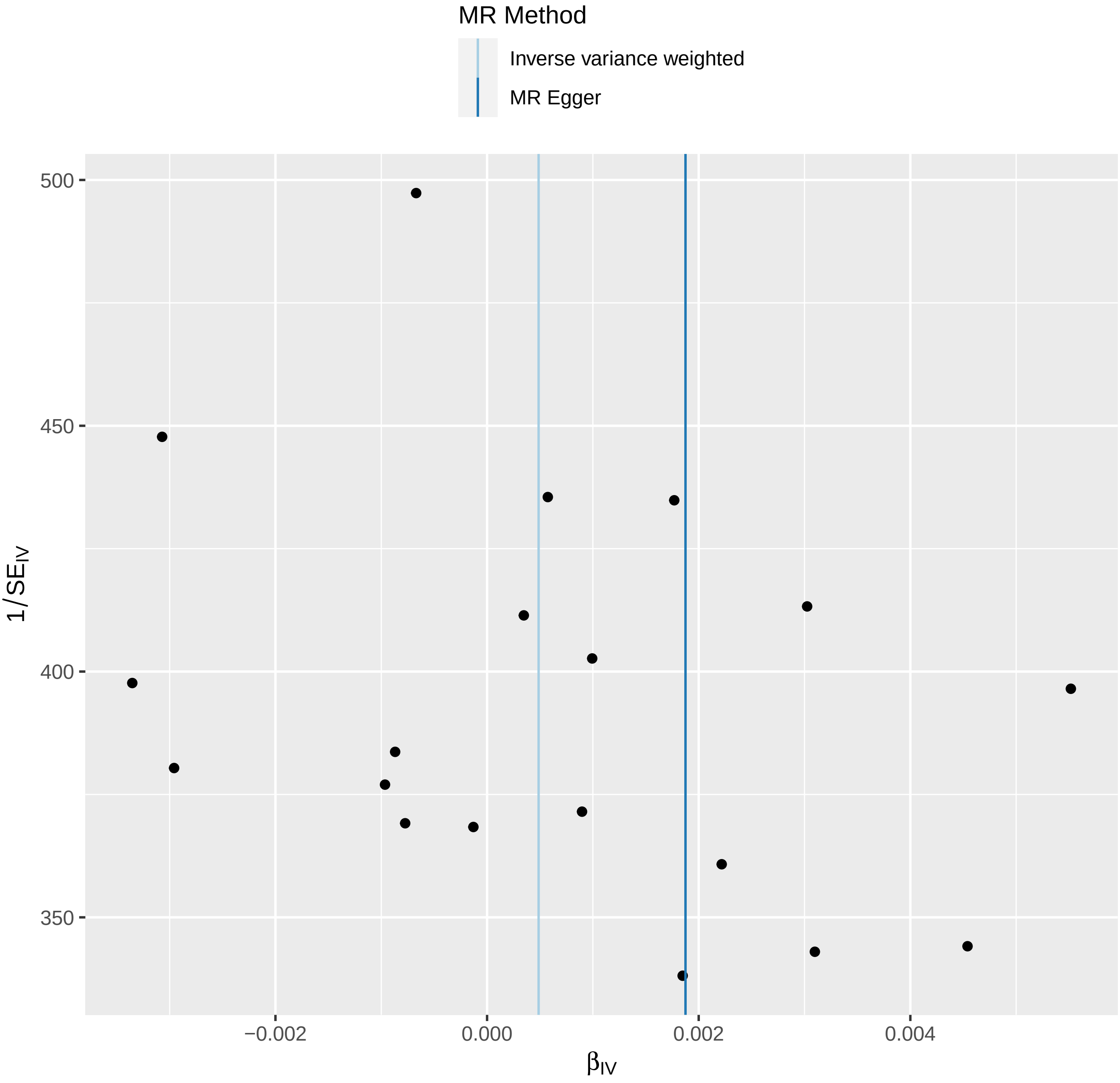

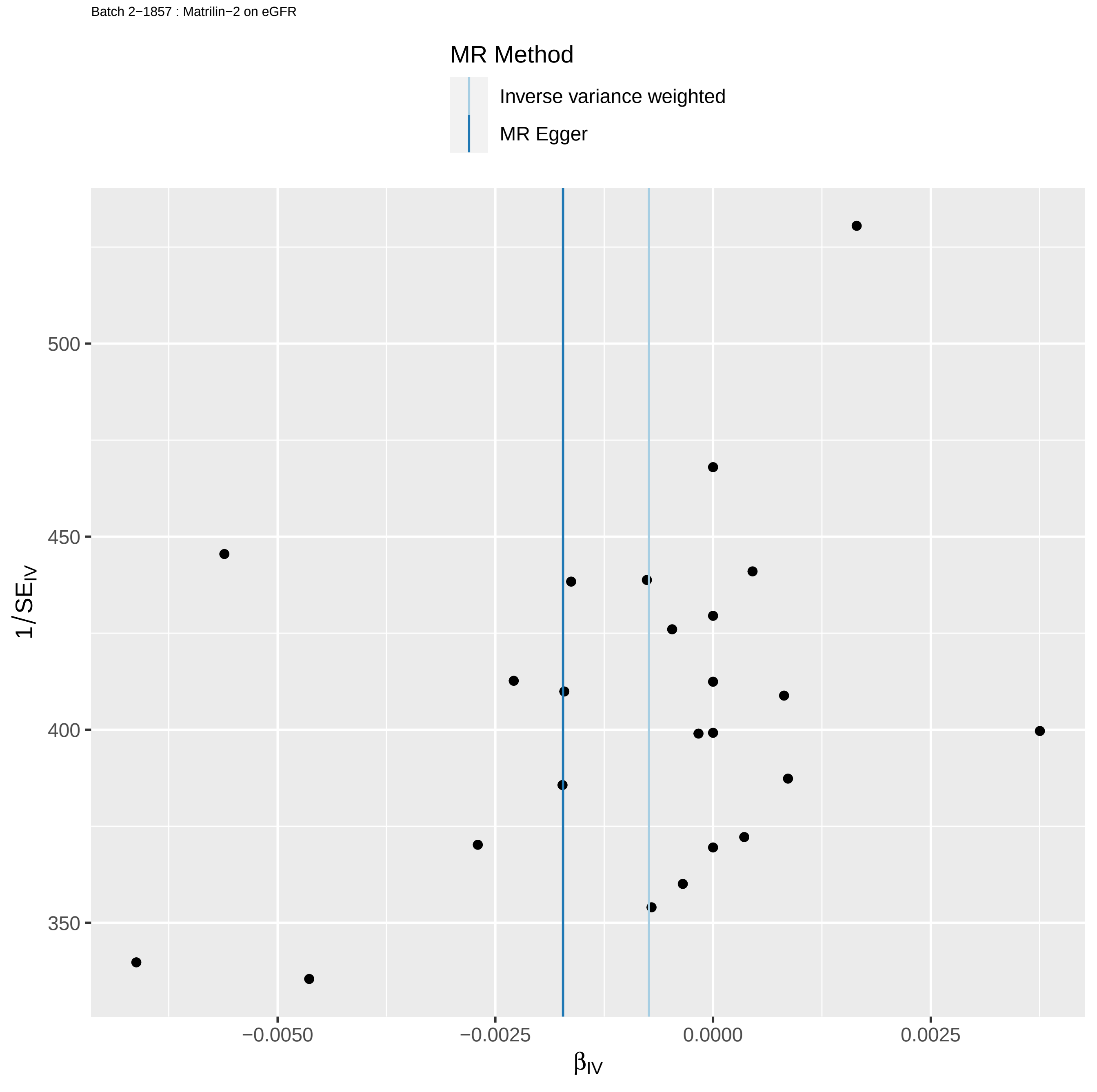

Batch 2−1857 : Matrilin−2 on eGFR
MR Method
Inverse variance weighted
MR Egger
500
450
400
350
1/SE_IV
−0.0050
−0.0025
0.0000
0.0025
β_IV

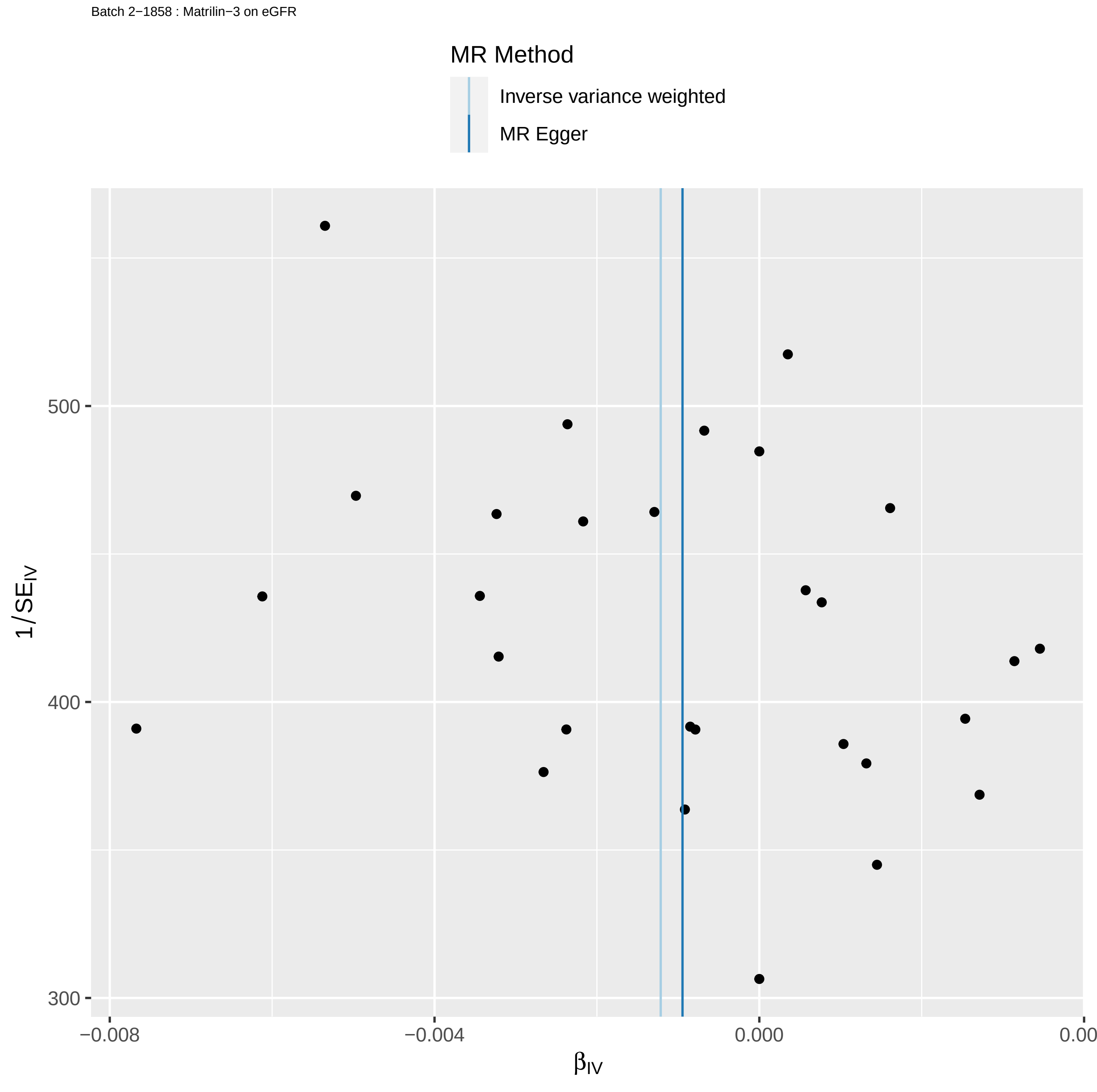
Batch 2−1858 : Matrilin−3 on eGFR
MR Method
Inverse variance weighted
MR Egger
1/SE_IV
500
400
300
−0.008
−0.004
0.000
0.00
β_IV

Batch 2−1859 : Matrilin−4 on eGFR

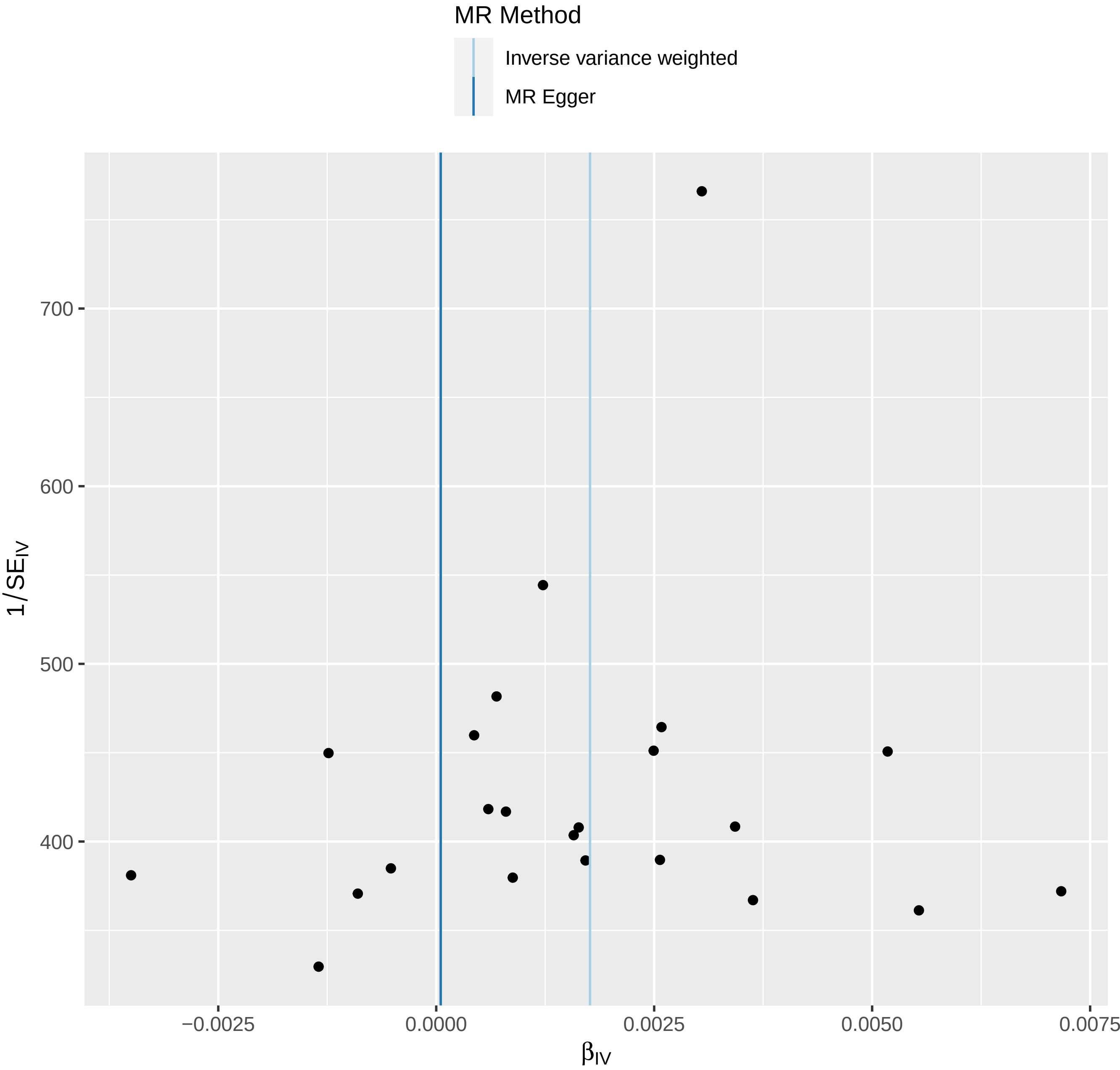

Batch 2−1860 : Myc−associated zinc finger protein on eGFR

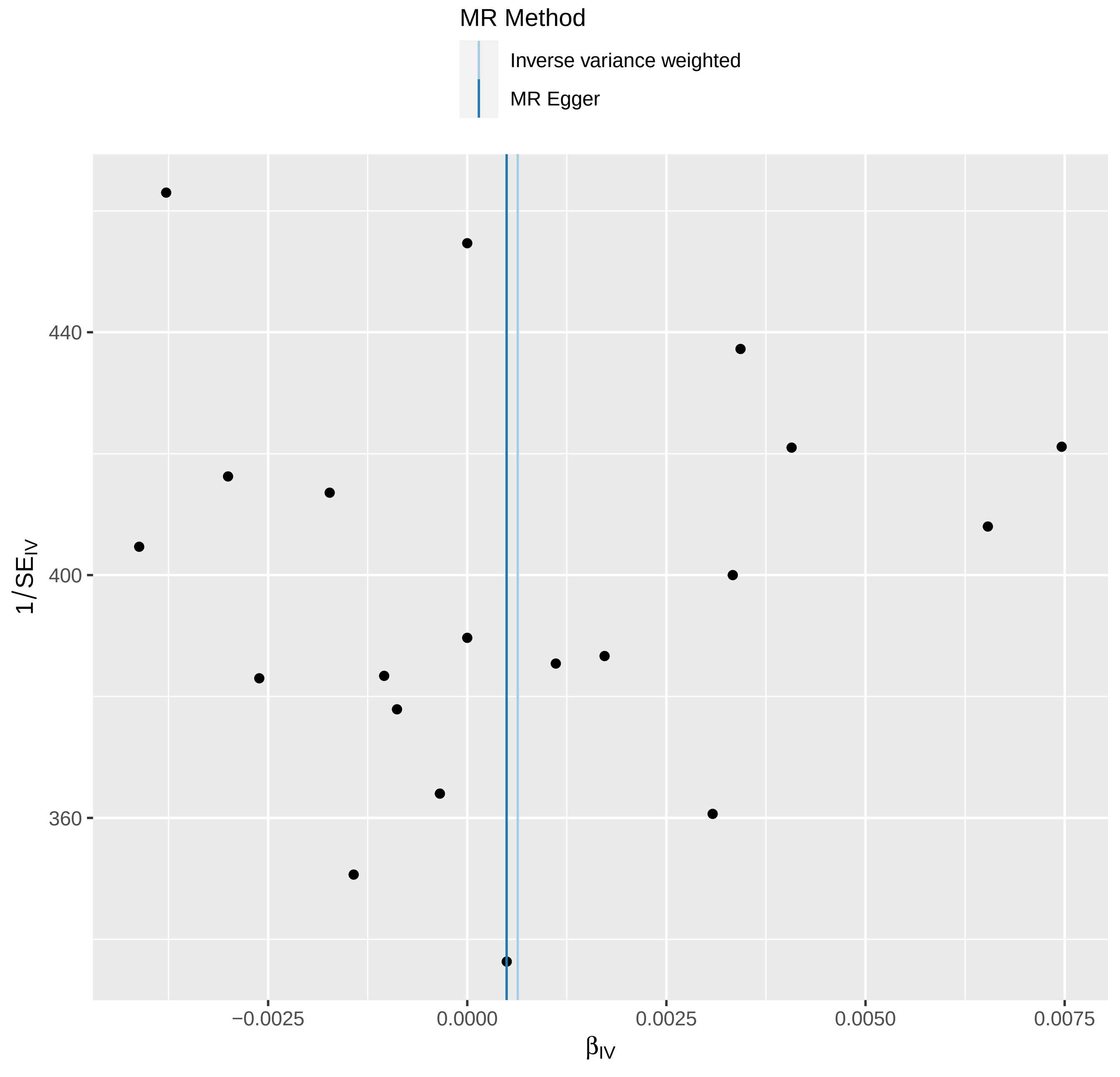

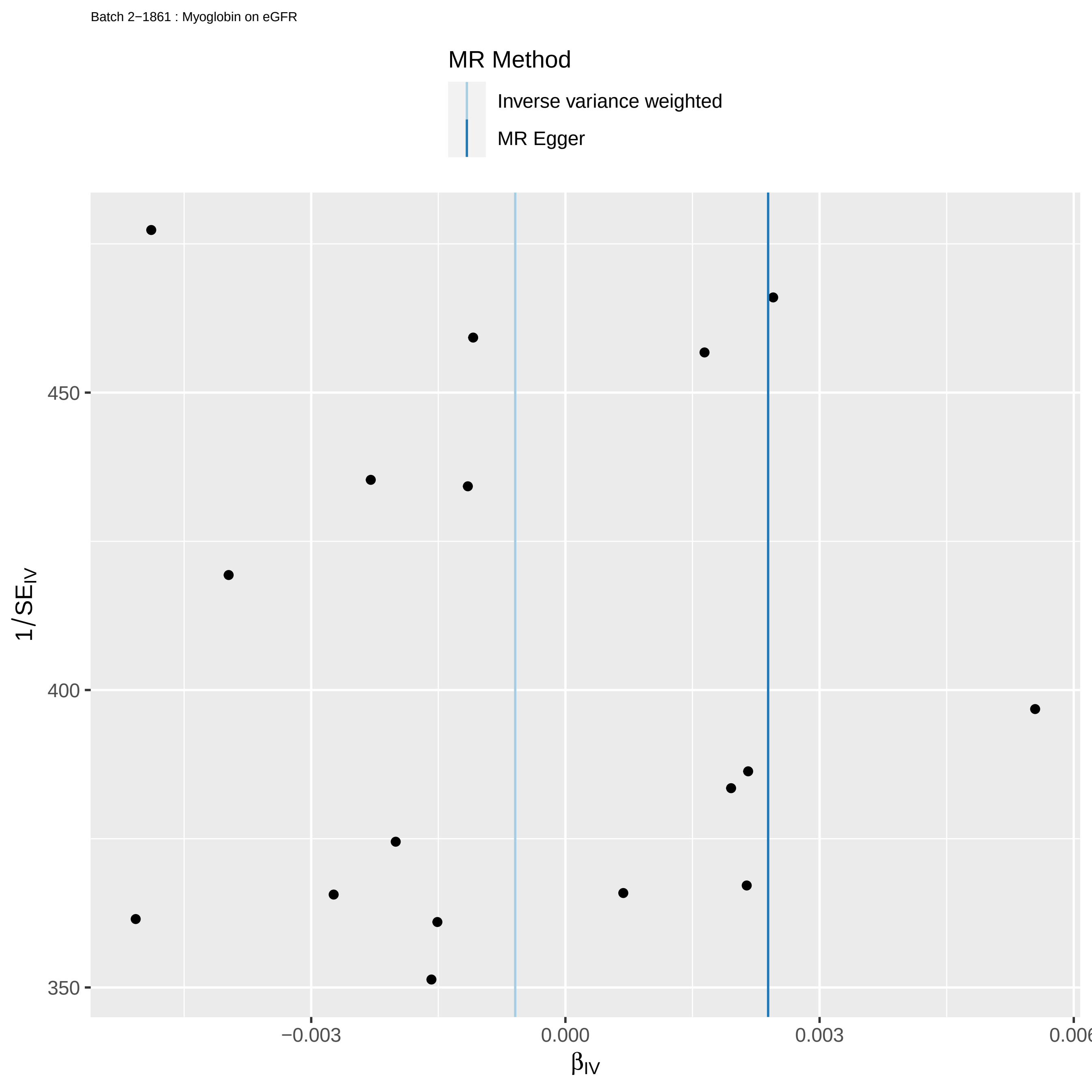

Batch 2−1861 : Myoglobin on eGFR
MR Method
Inverse variance weighted
MR Egger
450
400
350
1/SE$_{IV}$
−0.003
0.000
0.003
0.006
β$_{IV}$

Batch 2−1862 : Methyl−CpG−binding domain protein 4 on eGFR

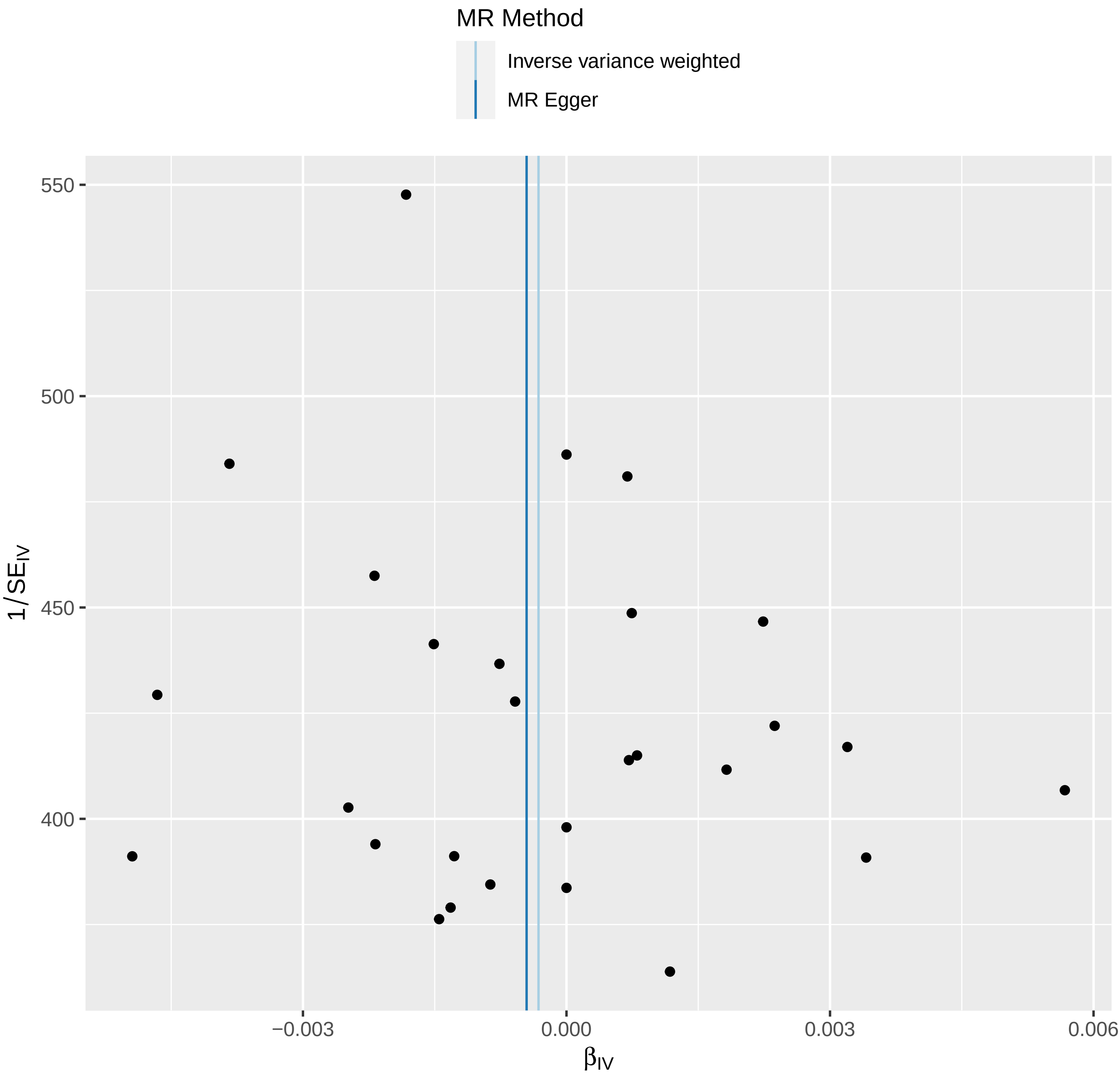

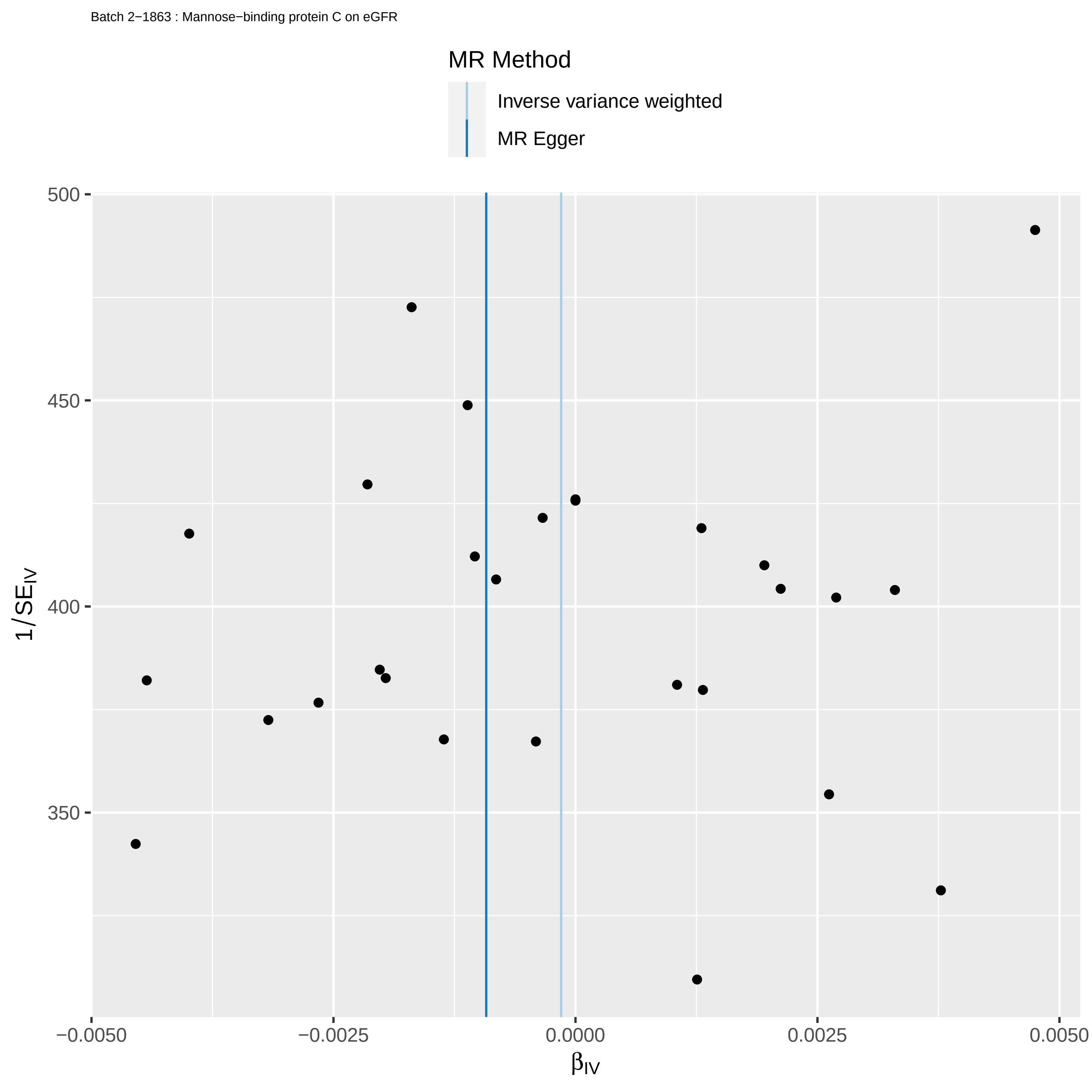
Batch 2−1863 : Mannose−binding protein C on eGFR
MR Method
Inverse variance weighted
MR Egger
500
450
400
350
1/SE$_{IV}$
−0.0050
−0.0025
0.0000
0.0025
0.0050
β$_{IV}$

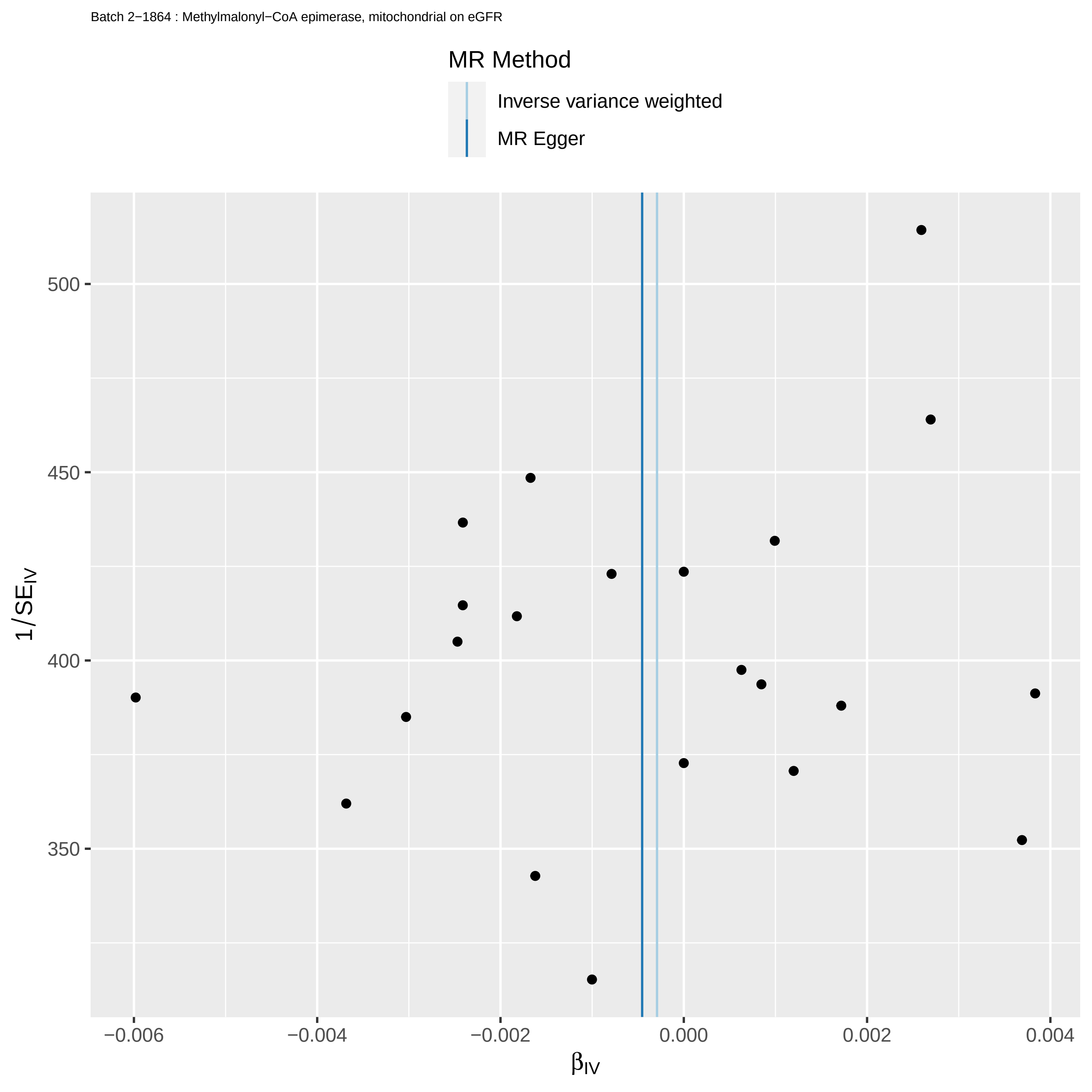

Batch 2−1864 : Methylmalonyl−CoA epimerase, mitochondrial on eGFR
MR Method
Inverse variance weighted
MR Egger
1/SE$_{IV}$
500
450
400
350
−0.006
−0.004
−0.002
0.000
0.002
0.004
β_{IV}

Batch 2−1865 : Mast cell−expressed membrane protein 1 on eGFR

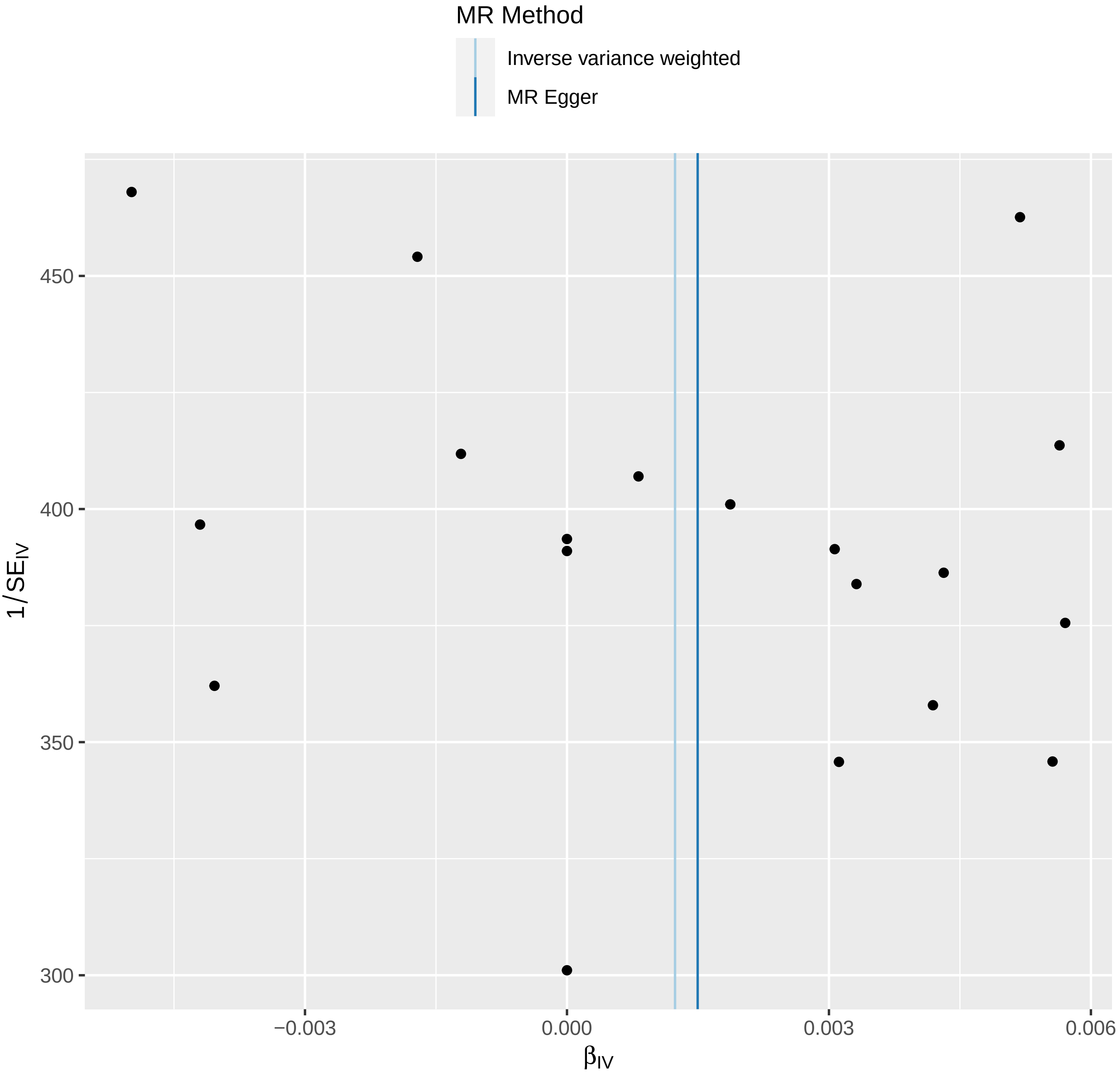

Batch 2−1866 : Guanine nucleotide exchange factor DBS on eGFR

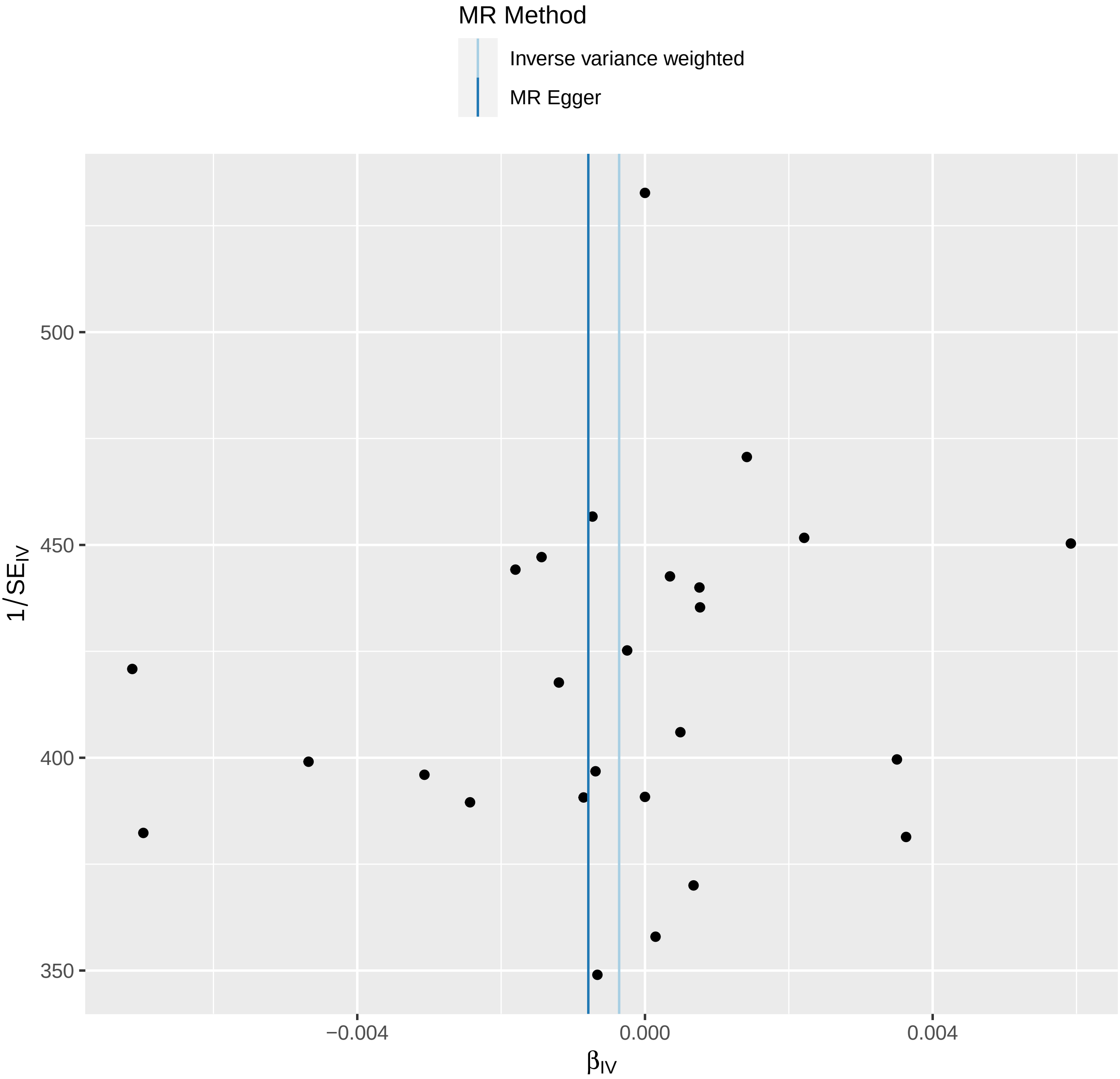

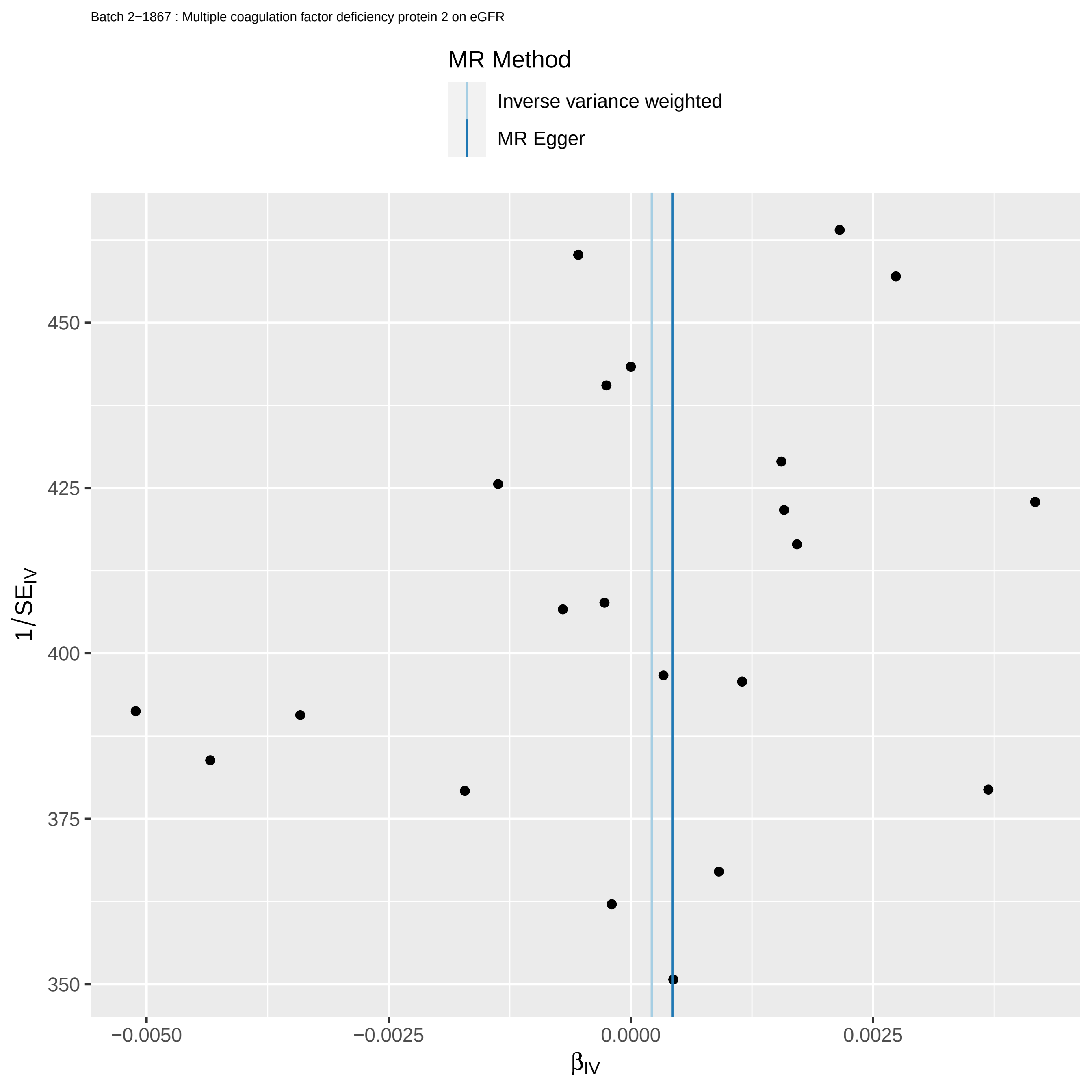
Batch 2−1867 : Multiple coagulation factor deficiency protein 2 on eGFR
MR Method
Inverse variance weighted
MR Egger
450
425
400
375
350
1/SE_IV
−0.0050
−0.0025
0.0000
0.0025
β_IV

Batch 2−1868 : Induced myeloid leukemia cell differentiation protein Mcl−1 on eGFR

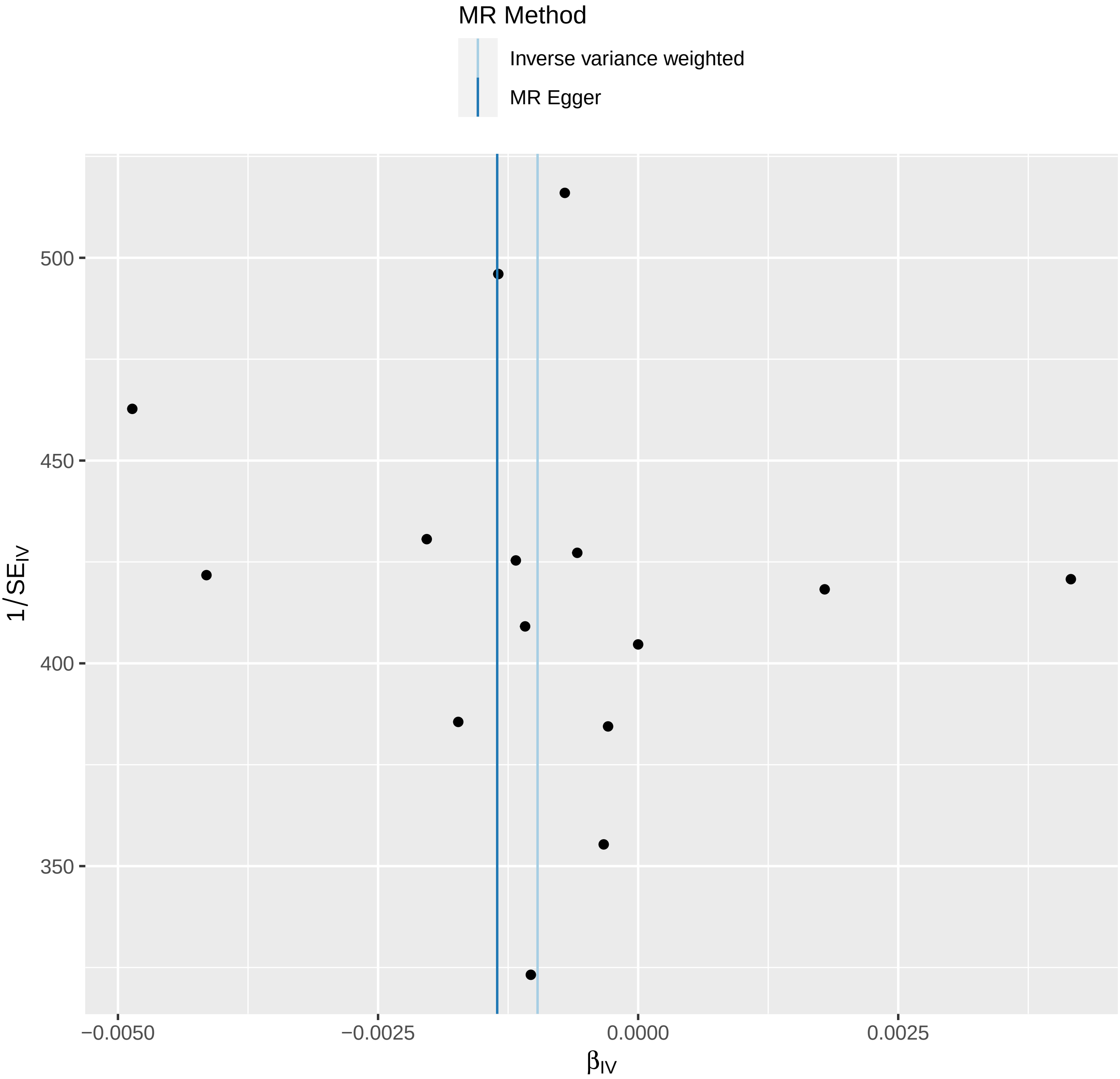

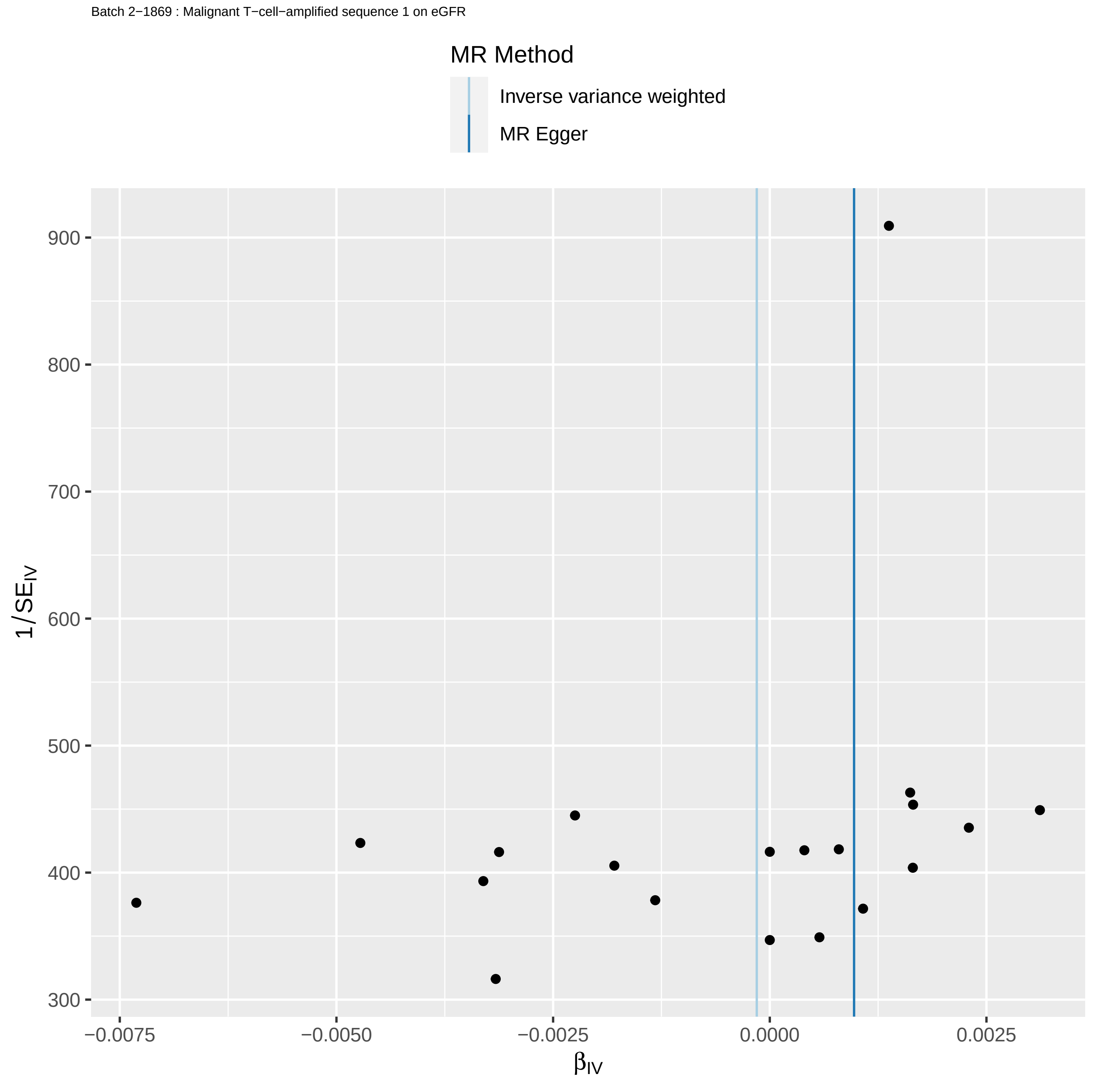

Batch 2−1869 : Malignant T−cell−amplified sequence 1 on eGFR
MR Method
Inverse variance weighted
MR Egger
900
800
700
600
500
400
300
1/SE_IV
−0.0075
−0.0050
−0.0025
0.0000
0.0025
β_IV

Batch 2−1870 : Malate dehydrogenase, cytoplasmic on eGFR

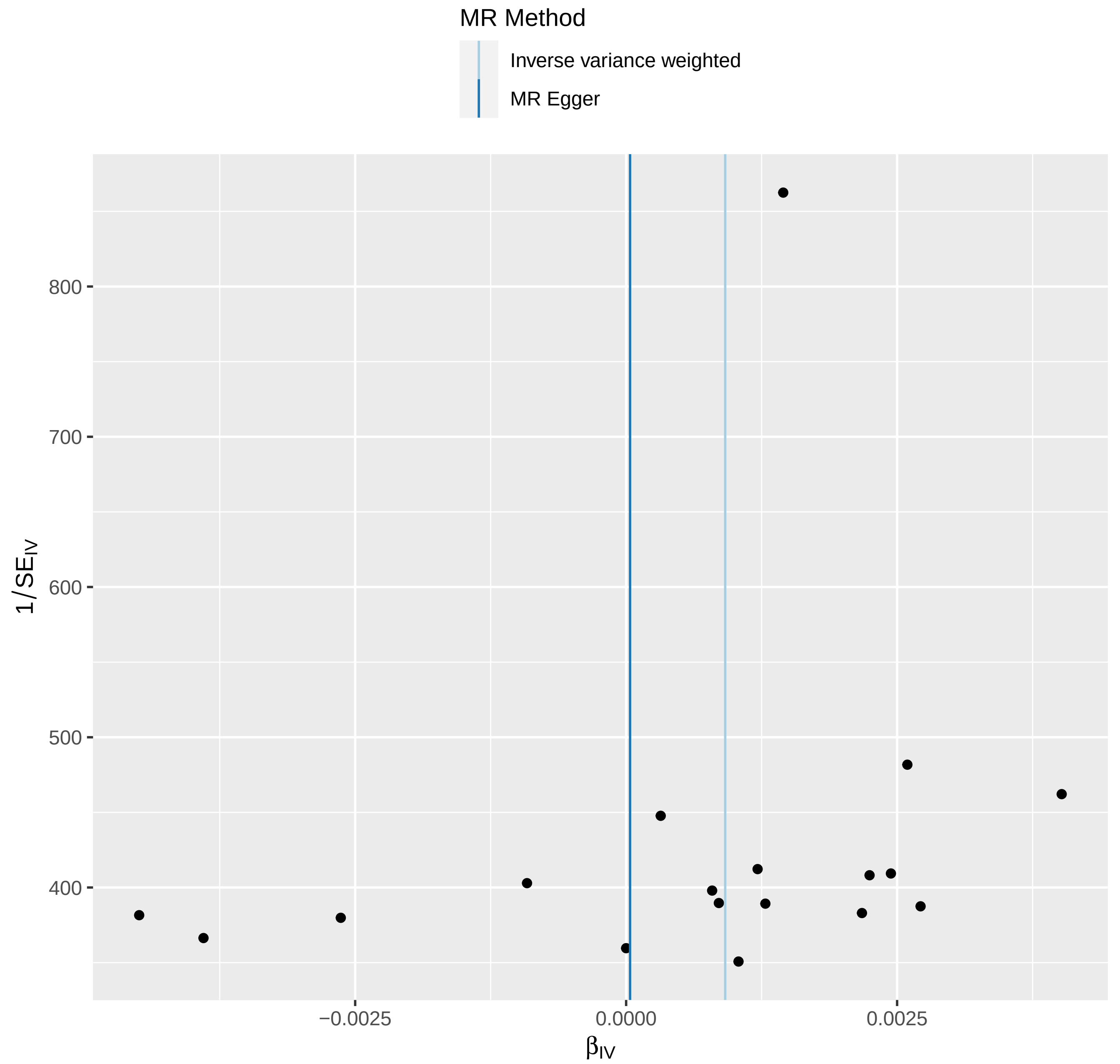

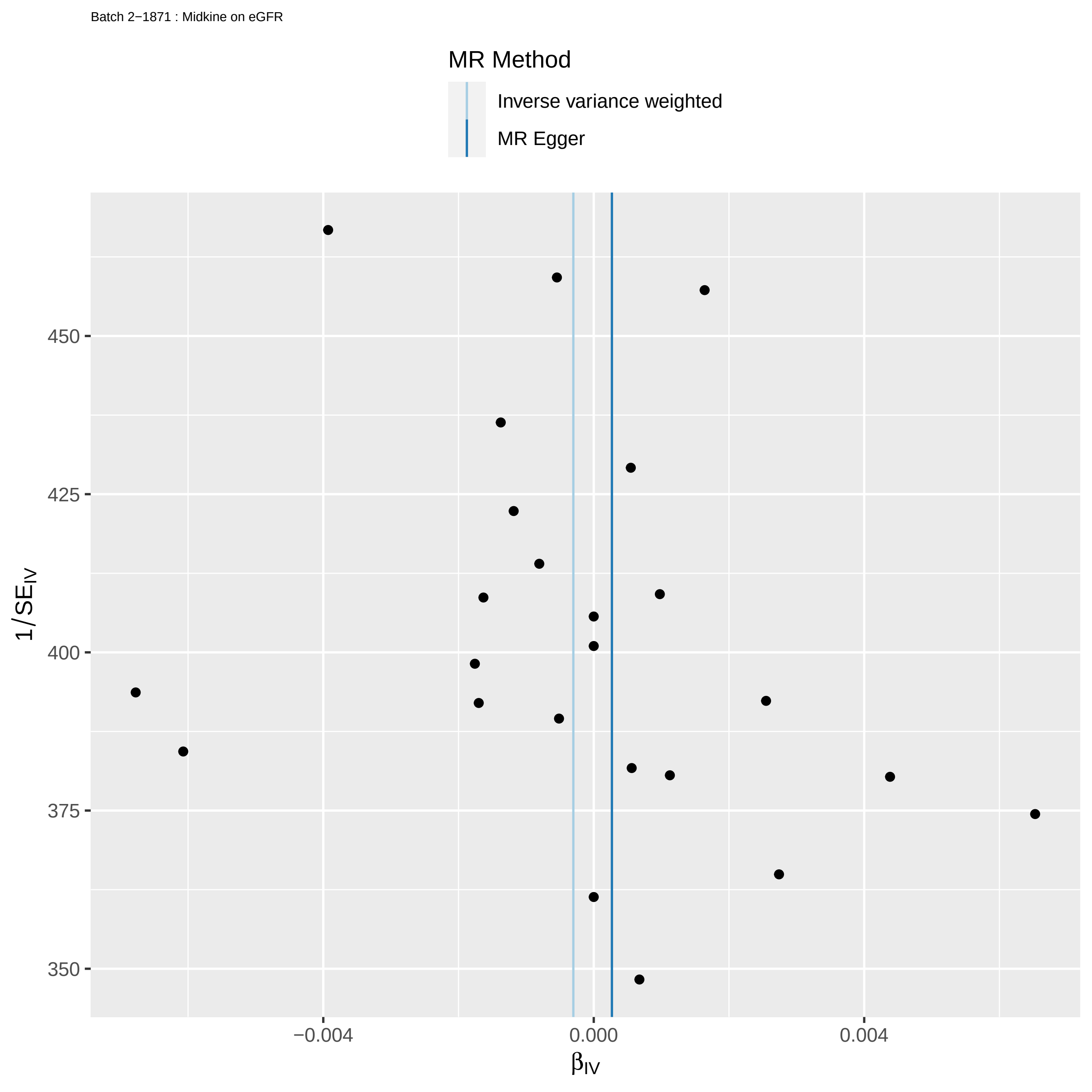

Batch 2−1871 : Midkine on eGFR
MR Method
Inverse variance weighted
MR Egger
450
425
400
375
350
1/SE$_{IV}$
−0.004
0.000
0.004
β$_{IV}$

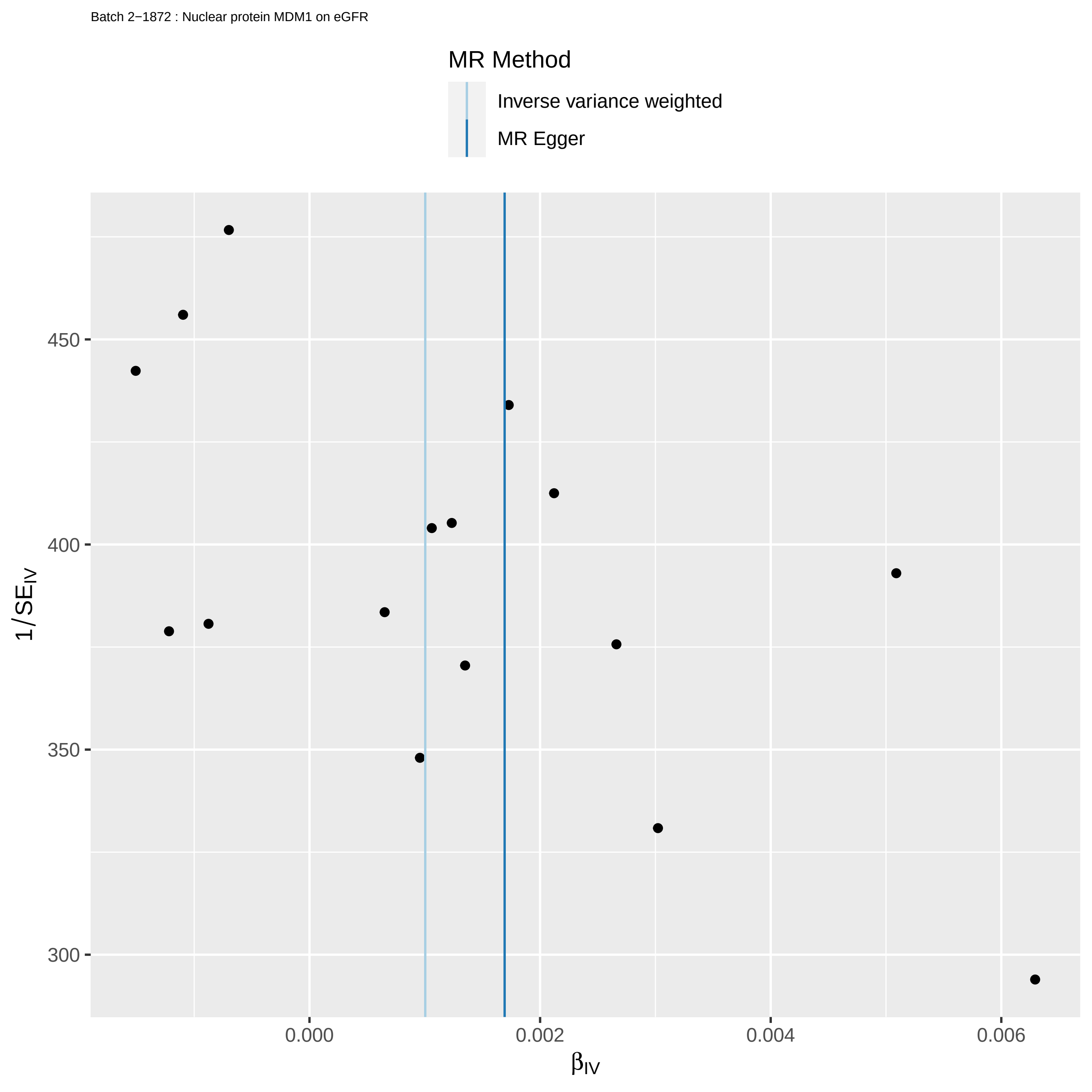

Batch 2−1872 : Nuclear protein MDM1 on eGFR
MR Method
Inverse variance weighted
MR Egger
450
400
350
300
1/SE_IV
0.000
0.002
0.004
0.006
β_IV

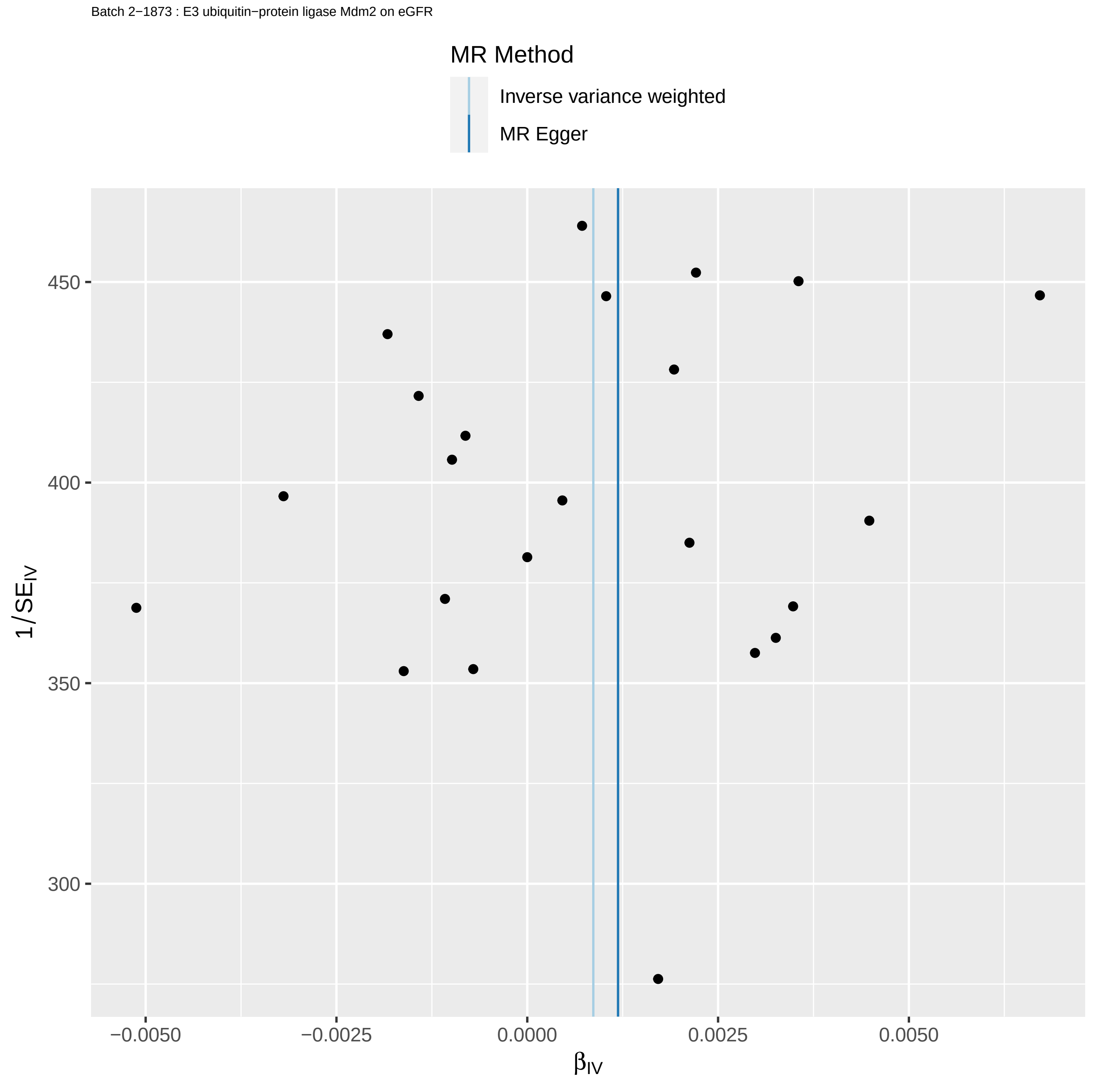

Batch 2−1873 : E3 ubiquitin−protein ligase Mdm2 on eGFR
MR Method
Inverse variance weighted
MR Egger
450
400
350
300
1/SE_IV
−0.0050
−0.0025
0.0000
0.0025
0.0050
β_IV

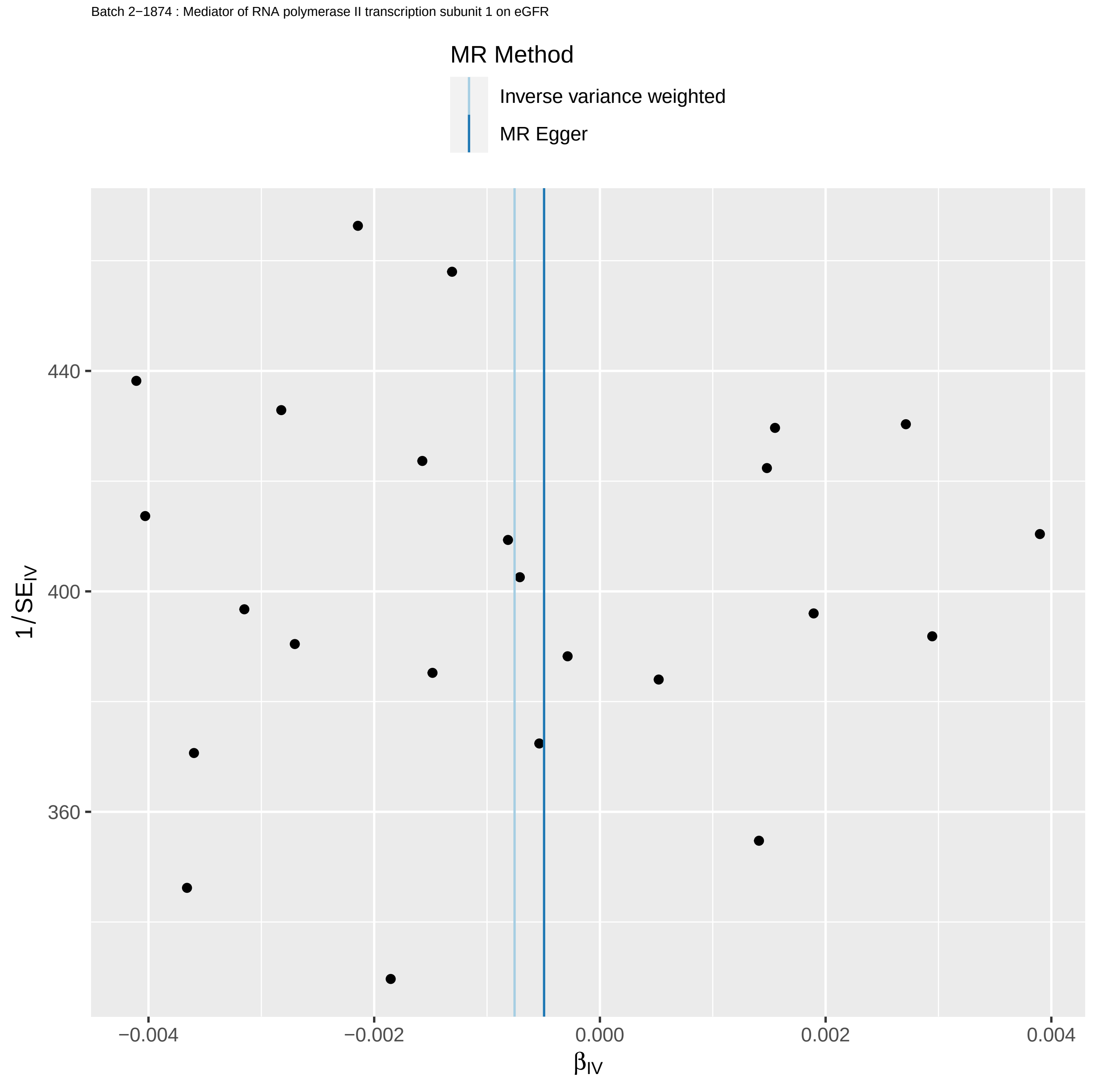

Batch 2−1874 : Mediator of RNA polymerase II transcription subunit 1 on eGFR
MR Method
Inverse variance weighted
MR Egger
440
400
360
1/SE$_{IV}$
−0.004
−0.002
0.000
0.002
0.004
β$_{IV}$

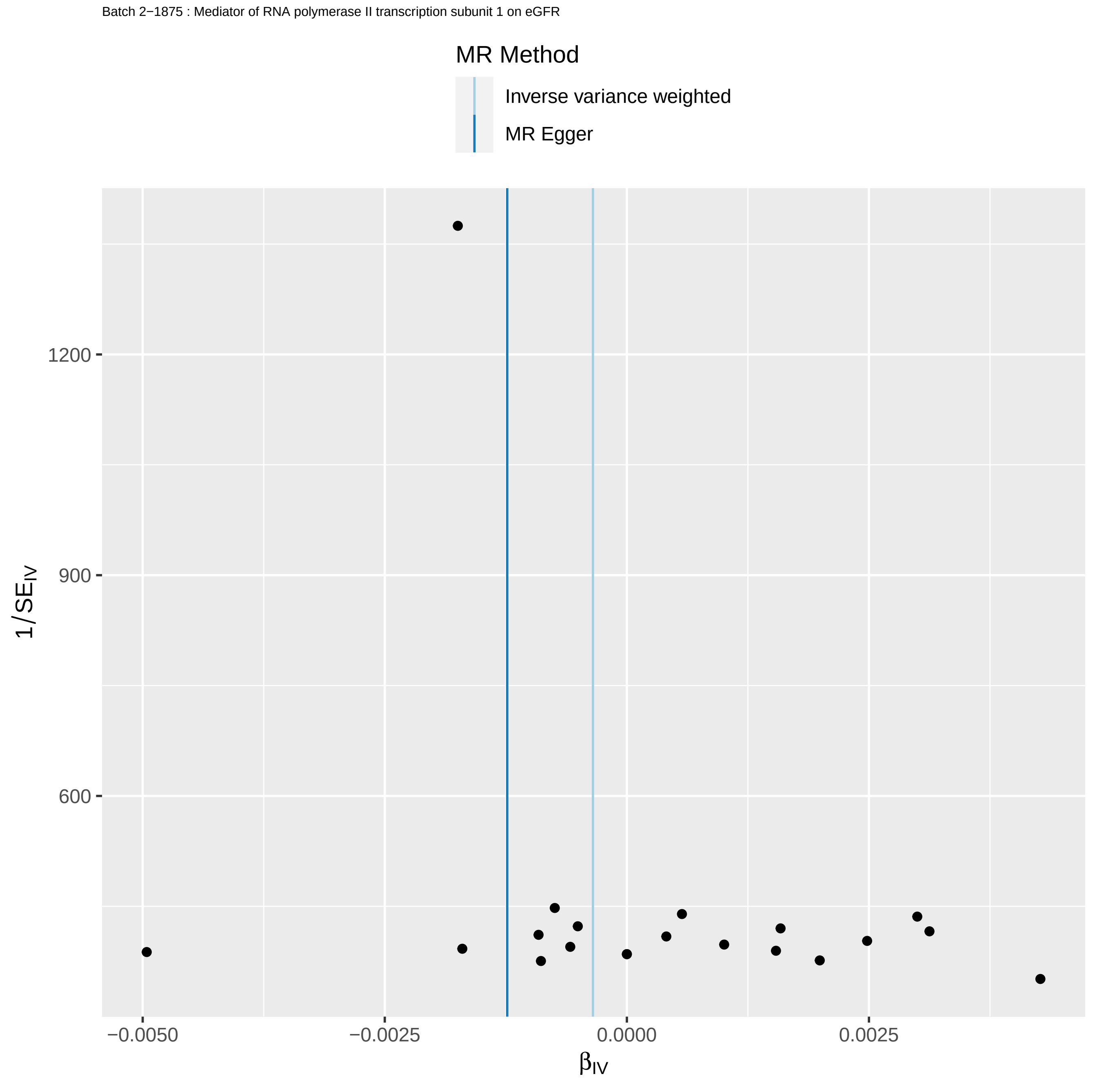

Batch 2−1875 : Mediator of RNA polymerase II transcription subunit 1 on eGFR
MR Method
Inverse variance weighted
MR Egger
1200
900
600
1/SE$_{IV}$
−0.0050
−0.0025
0.0000
0.0025
β$_{IV}$

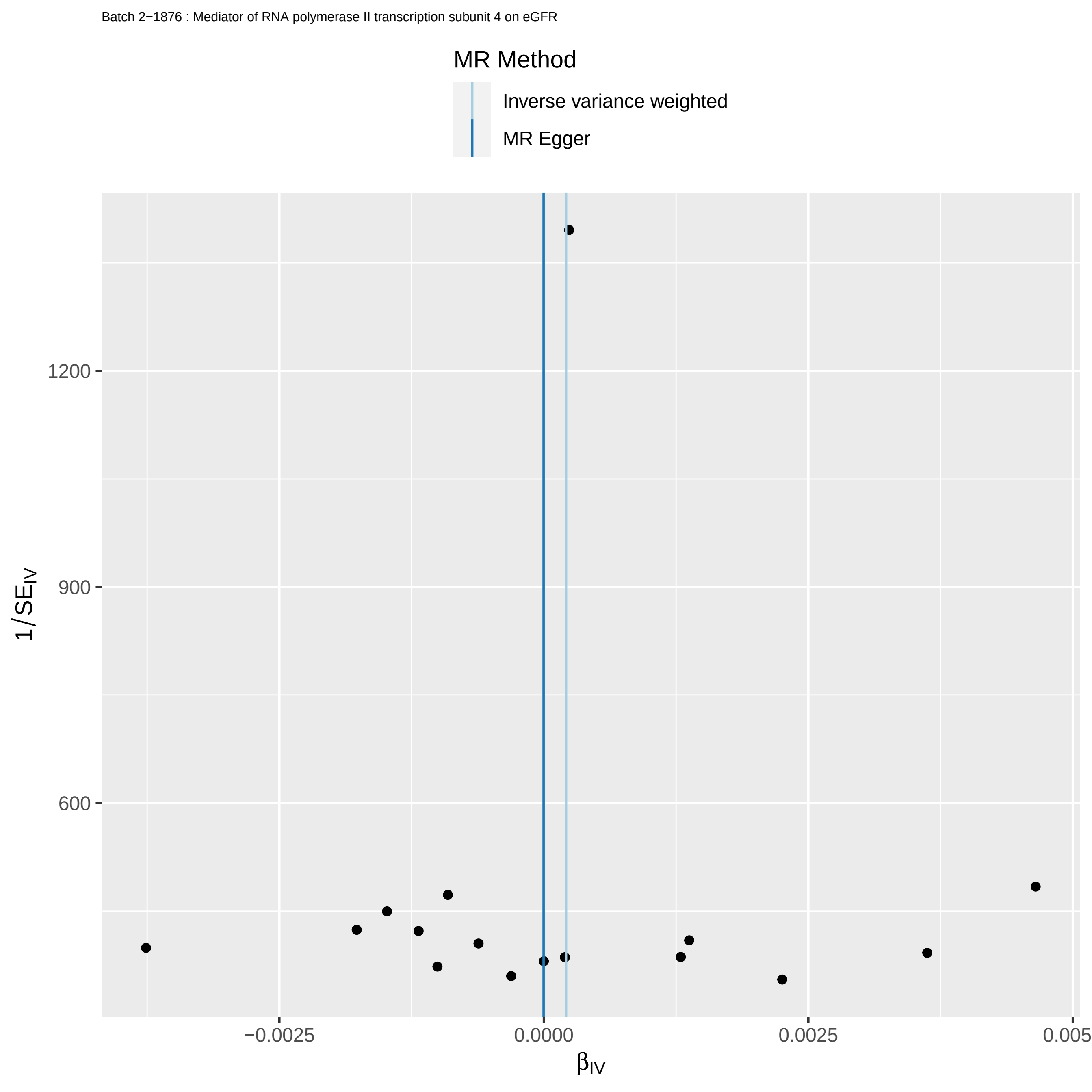

Batch 2−1876 : Mediator of RNA polymerase II transcription subunit 4 on eGFR
MR Method
Inverse variance weighted
MR Egger
1200
900
600
−0.0025
0.0000
0.0025
0.005
1/SE_IV
β_IV

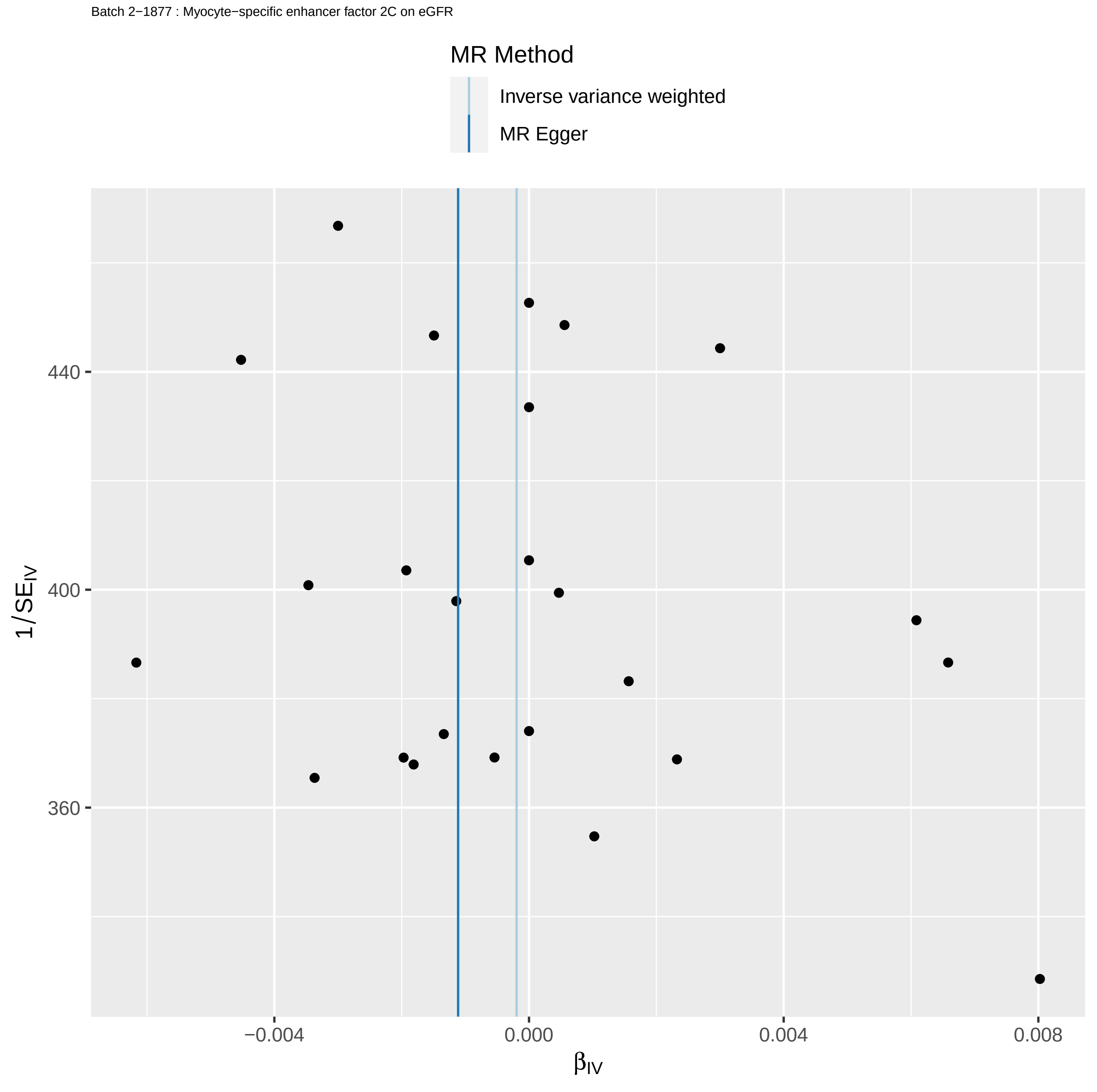
Batch 2−1877 : Myocyte−specific enhancer factor 2C on eGFR
MR Method
Inverse variance weighted
MR Egger
440
400
360
1/SE_IV
−0.004
0.000
0.004
0.008
β_IV

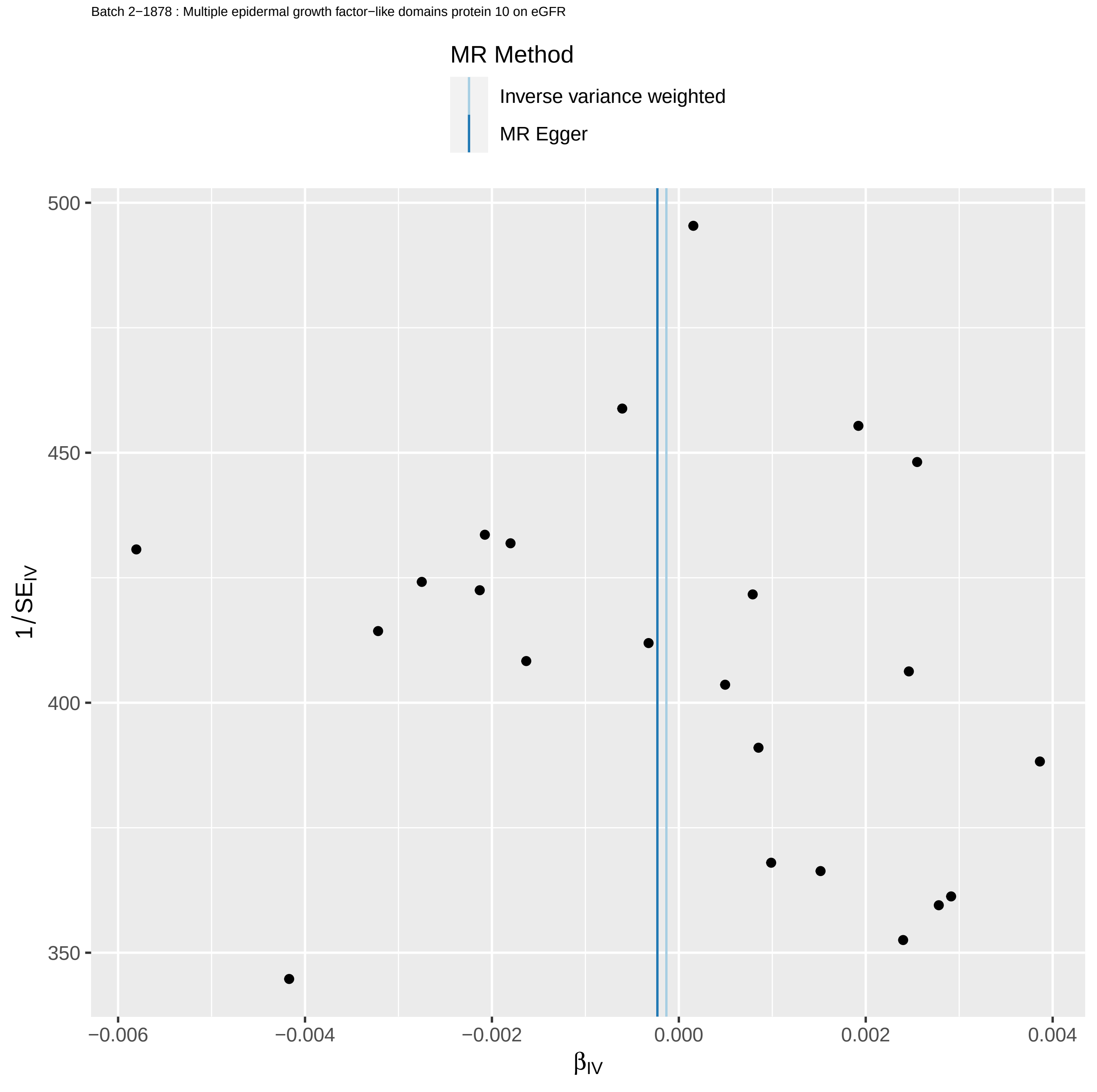

Batch 2−1878 : Multiple epidermal growth factor−like domains protein 10 on eGFR
MR Method
Inverse variance weighted
MR Egger
500
450
400
350
1/SE_IV
−0.006
−0.004
−0.002
0.000
0.002
0.004
β_IV

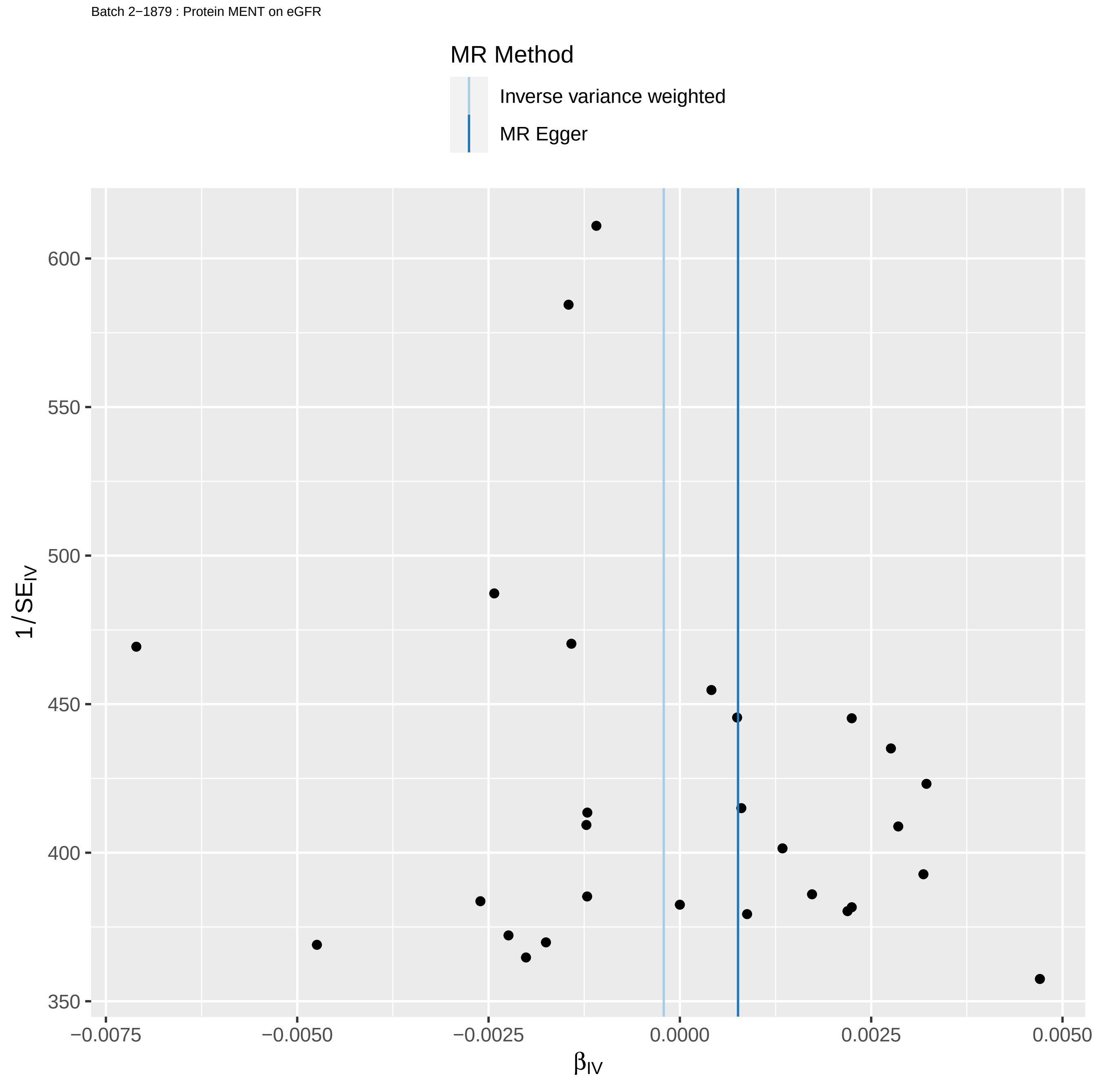

Batch 2−1879 : Protein MENT on eGFR
MR Method
Inverse variance weighted
MR Egger
600
550
500
450
400
350
1/SE_IV
−0.0075
−0.0050
−0.0025
0.0000
0.0025
0.0050
β_IV

Batch 2−1880 : Matrix extracellular phosphoglycoprotein on eGFR

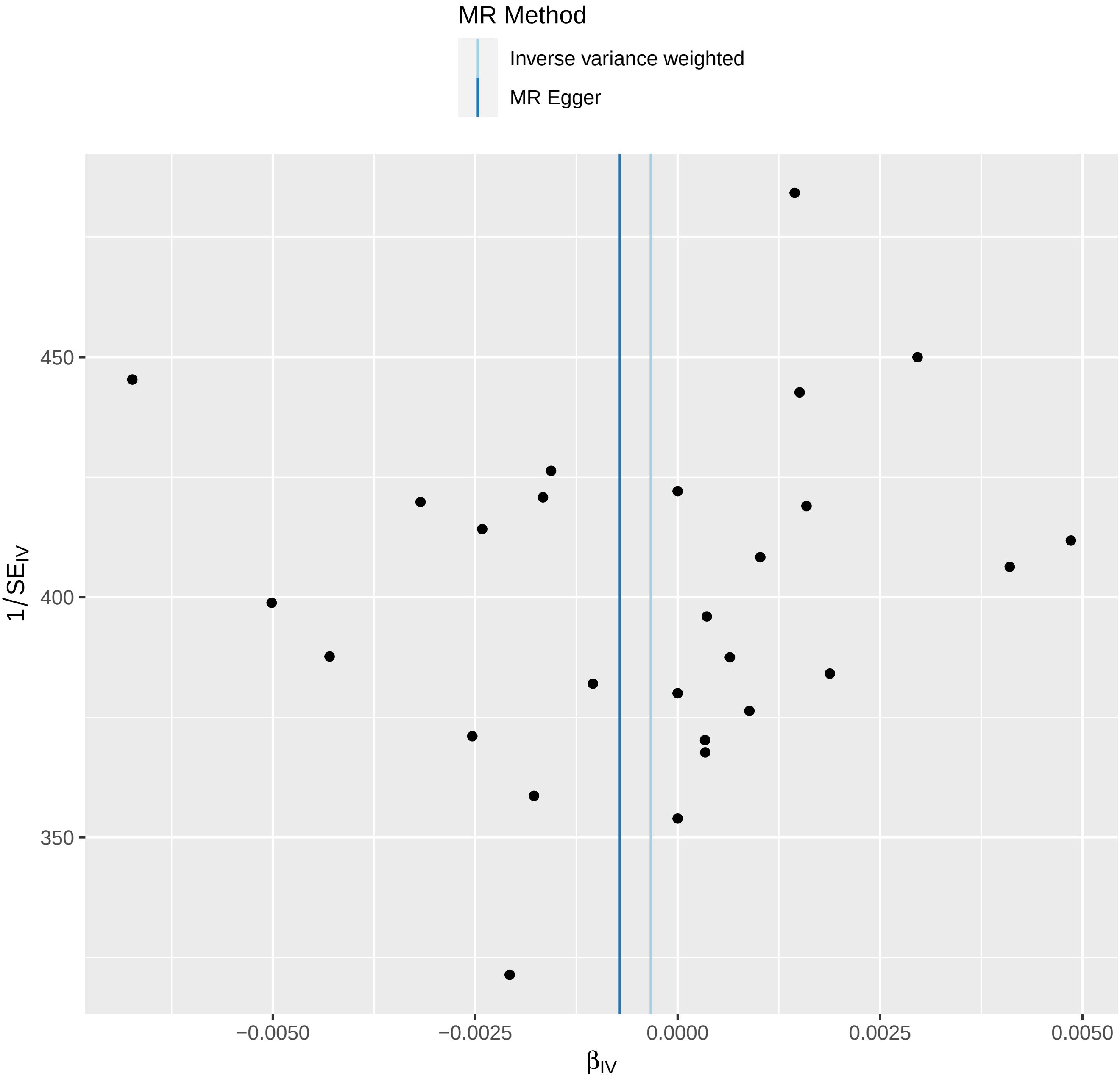

Batch 2−1881 : LDLR chaperone MESD on eGFR

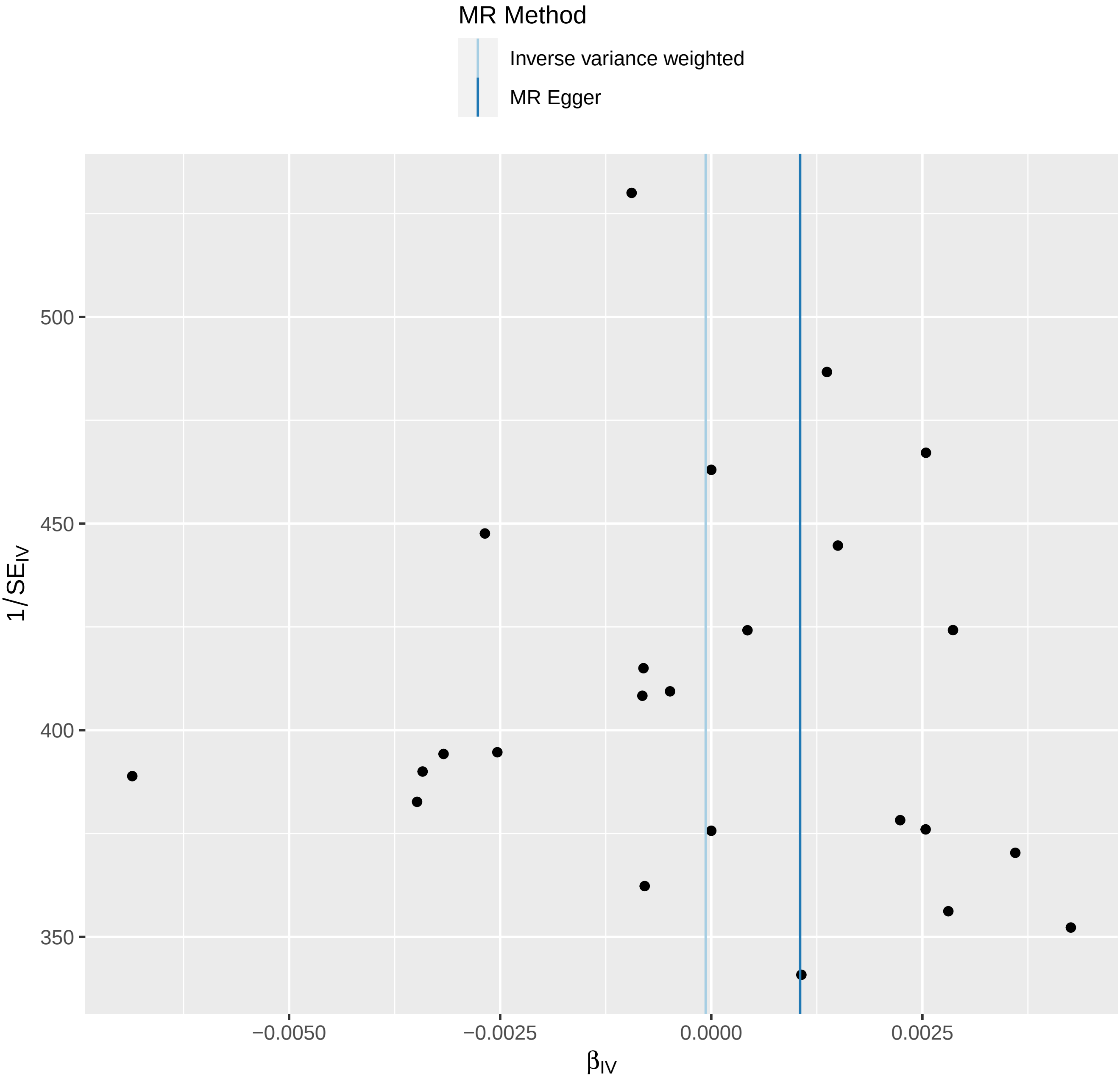

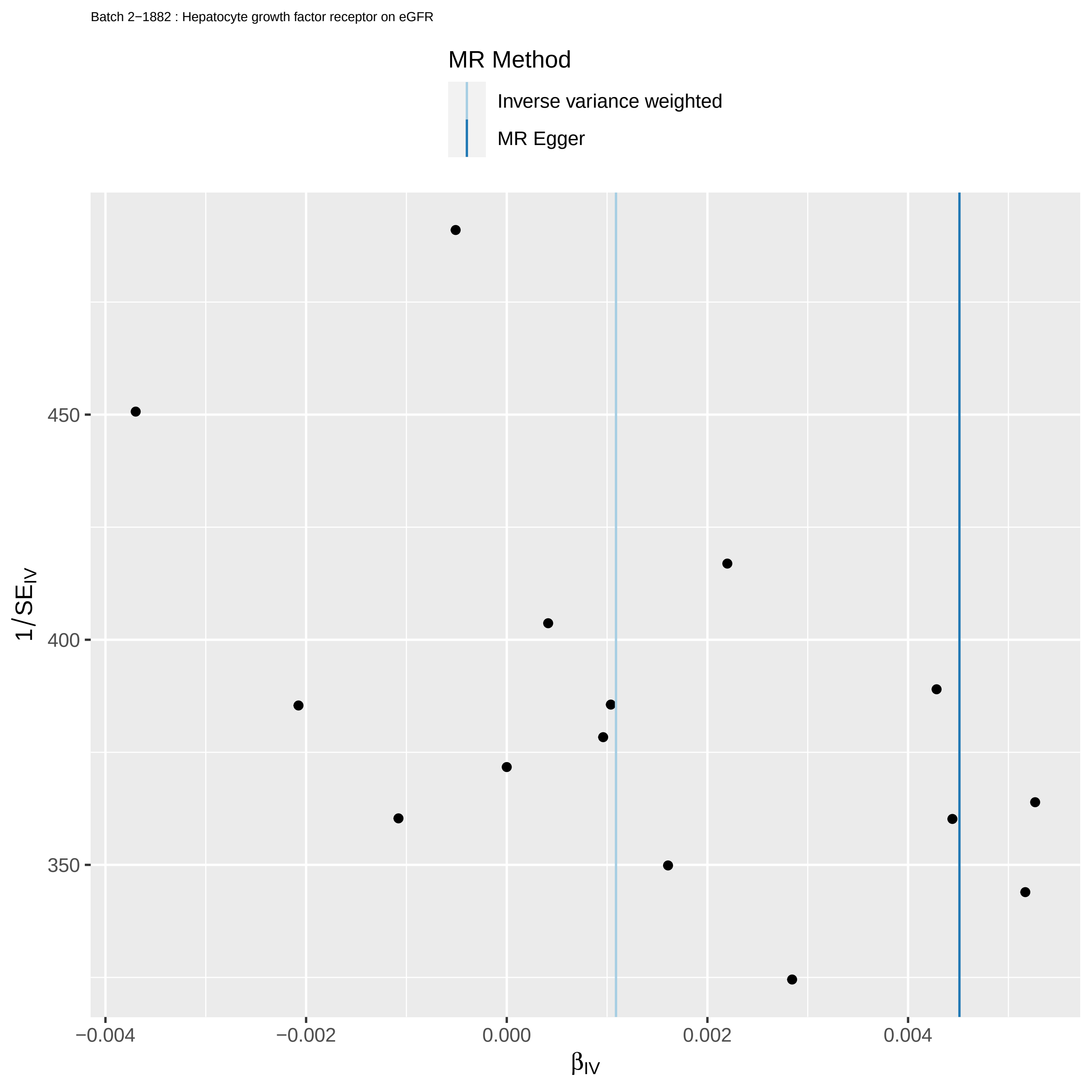
Batch 2−1882 : Hepatocyte growth factor receptor on eGFR
MR Method
Inverse variance weighted
MR Egger
450
400
350
1/SE_IV
−0.004
−0.002
0.000
0.002
0.004
β_IV

Batch 2−1883 : tRNA (guanine−N(7)−)−methyltransferase on eGFR

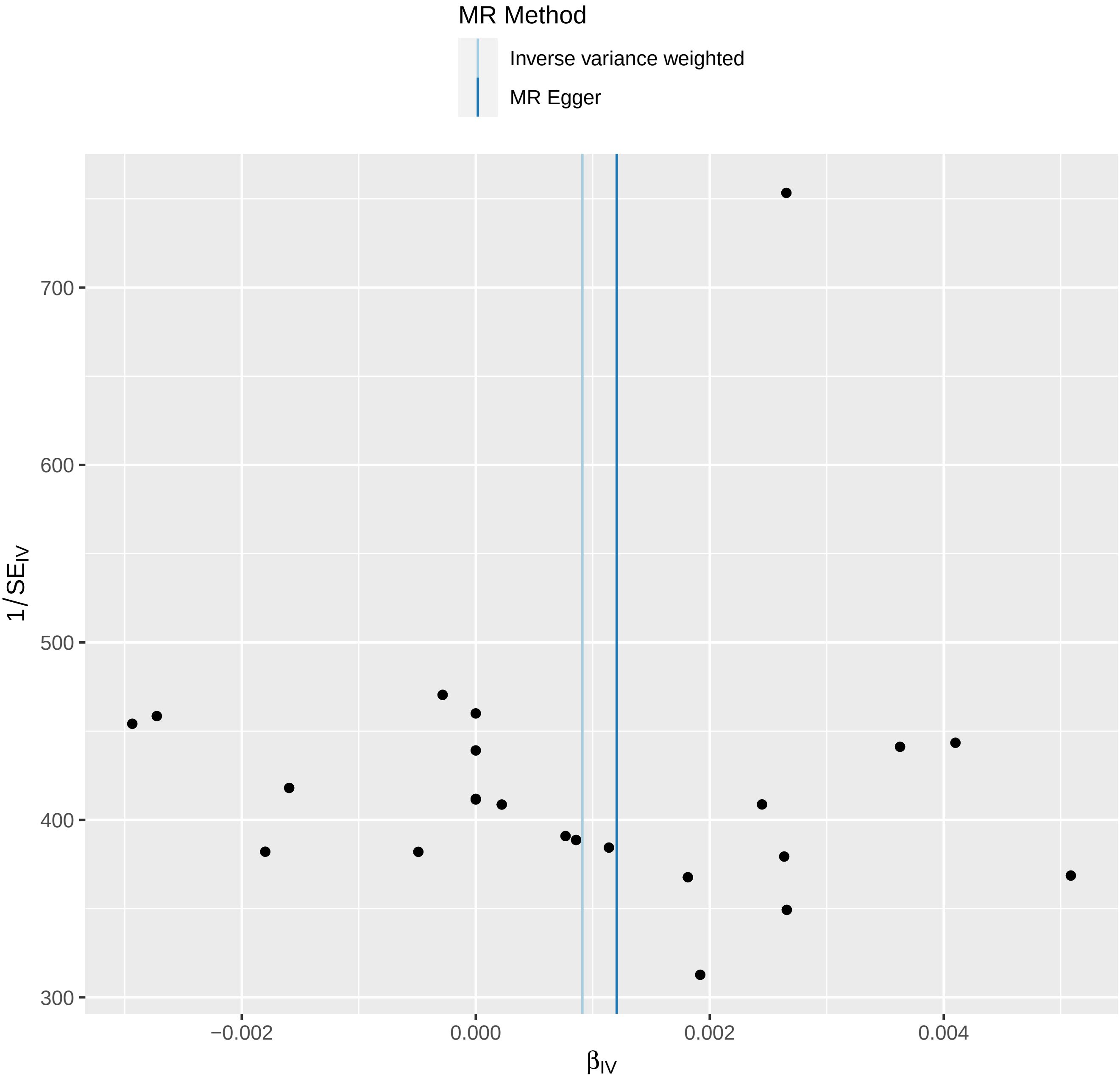

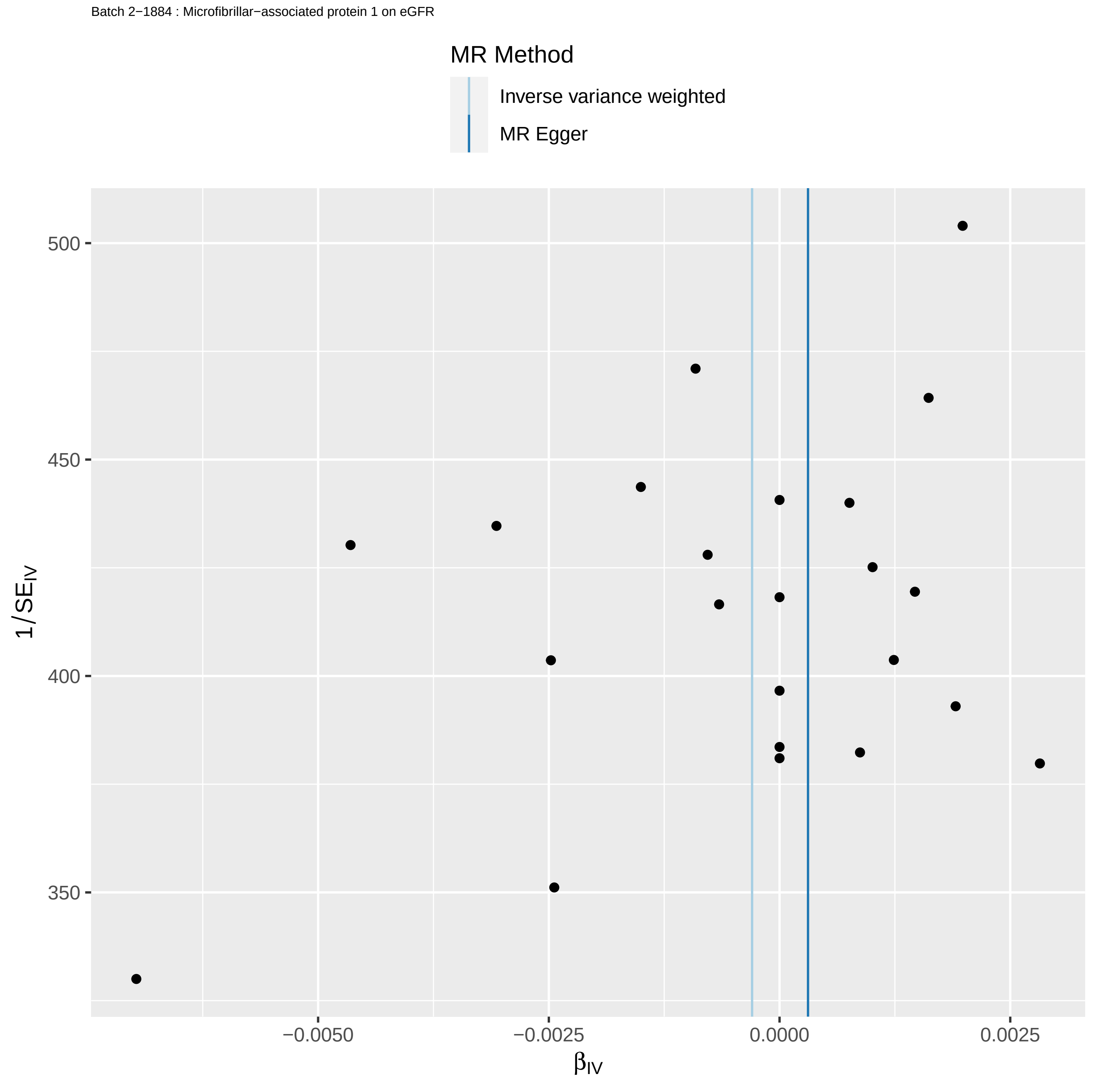

Batch 2−1884 : Microfibrillar−associated protein 1 on eGFR
MR Method
Inverse variance weighted
MR Egger
500
450
400
350
1/SE$_{IV}$
−0.0050
−0.0025
0.0000
0.0025
β_{IV}

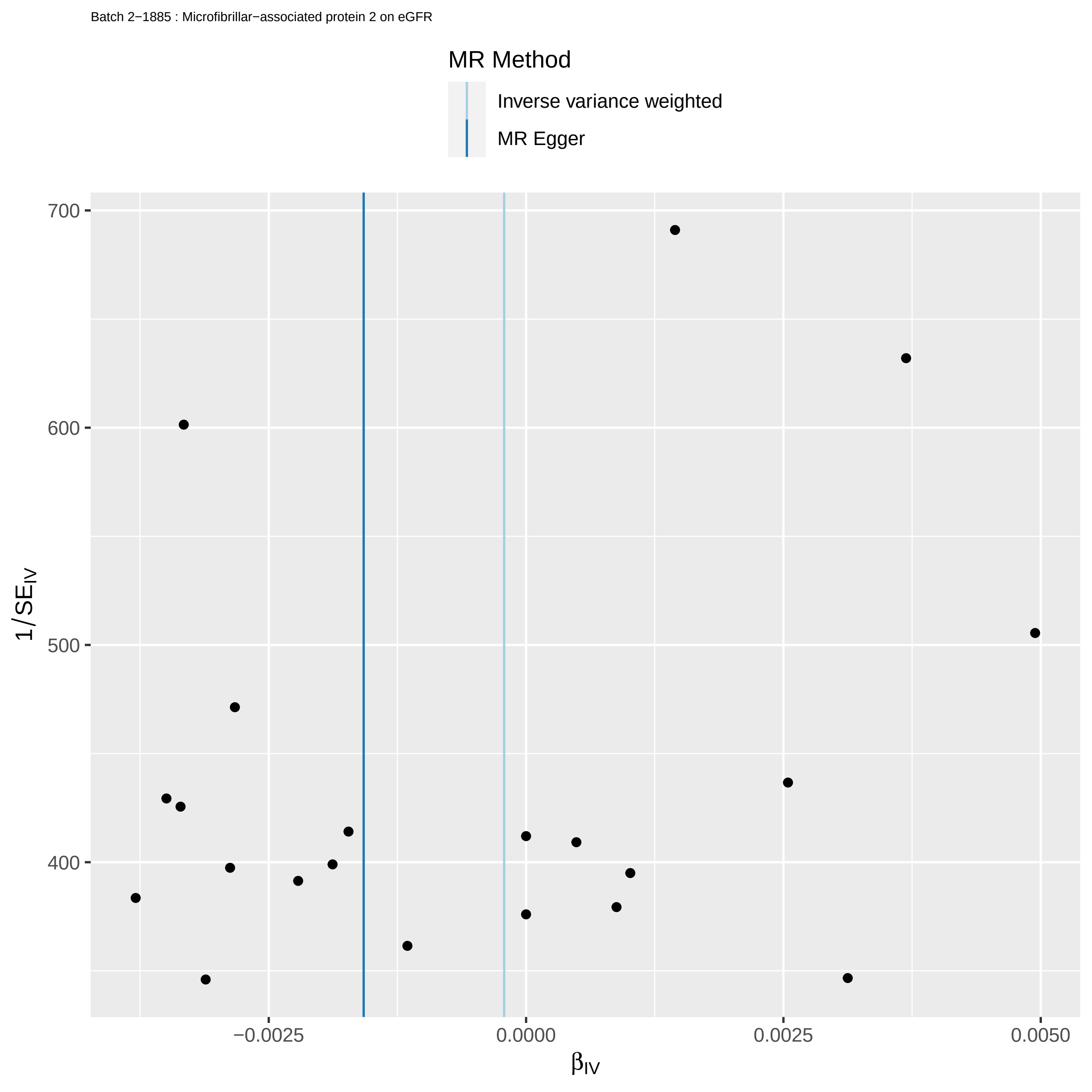

Batch 2−1885 : Microfibrillar−associated protein 2 on eGFR
MR Method
Inverse variance weighted
MR Egger
700
600
500
400
1/SE_IV
−0.0025
0.0000
0.0025
0.0050
β_IV

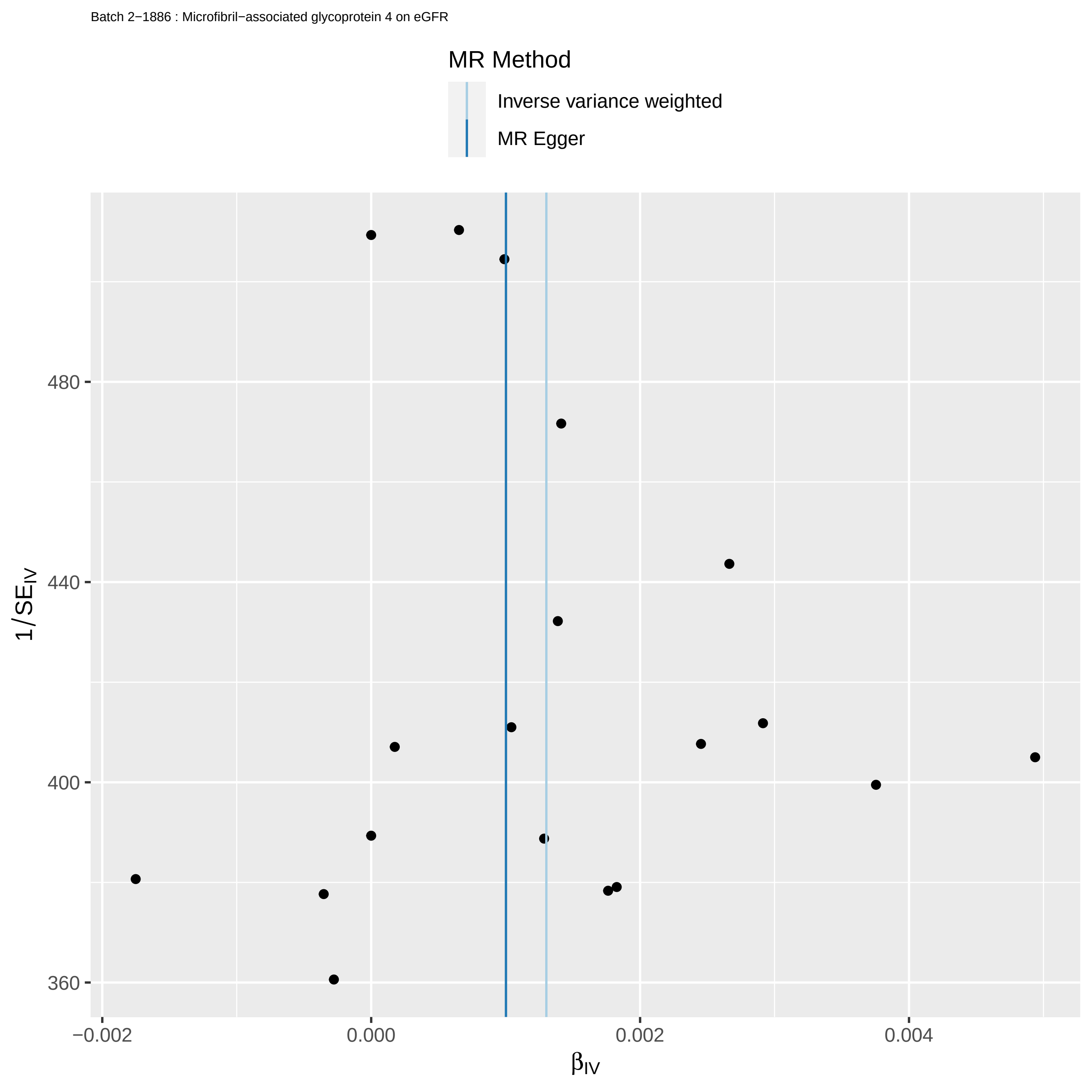
Batch 2−1886 : Microfibril−associated glycoprotein 4 on eGFR
MR Method
Inverse variance weighted
MR Egger
480
440
400
360
1/SE_IV
−0.002
0.000
0.002
0.004
β_IV

Batch 2−1887 : Microfibrillar−associated protein 5 on eGFR

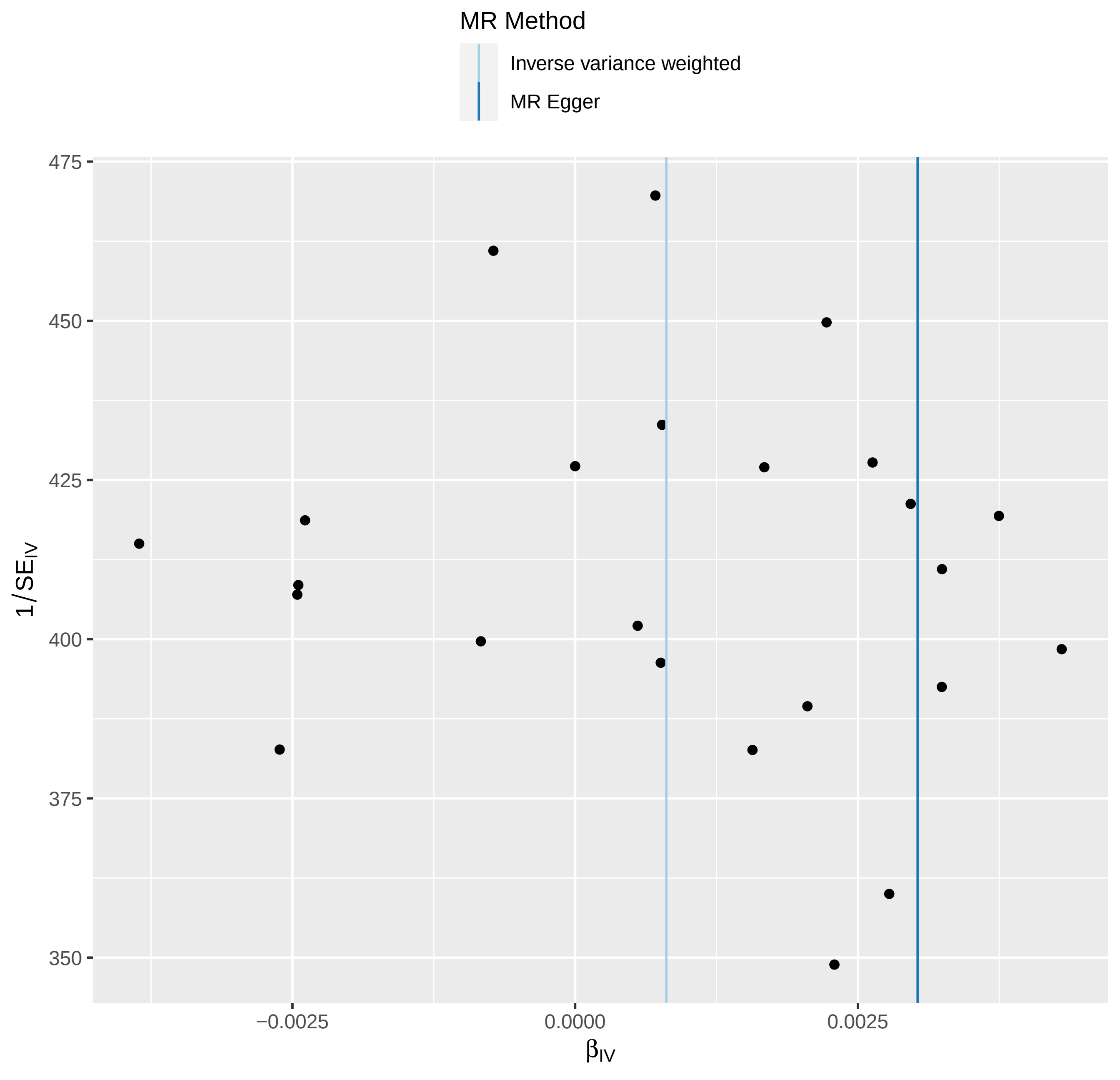

Batch 2−1888 : Lactadherin on eGFR

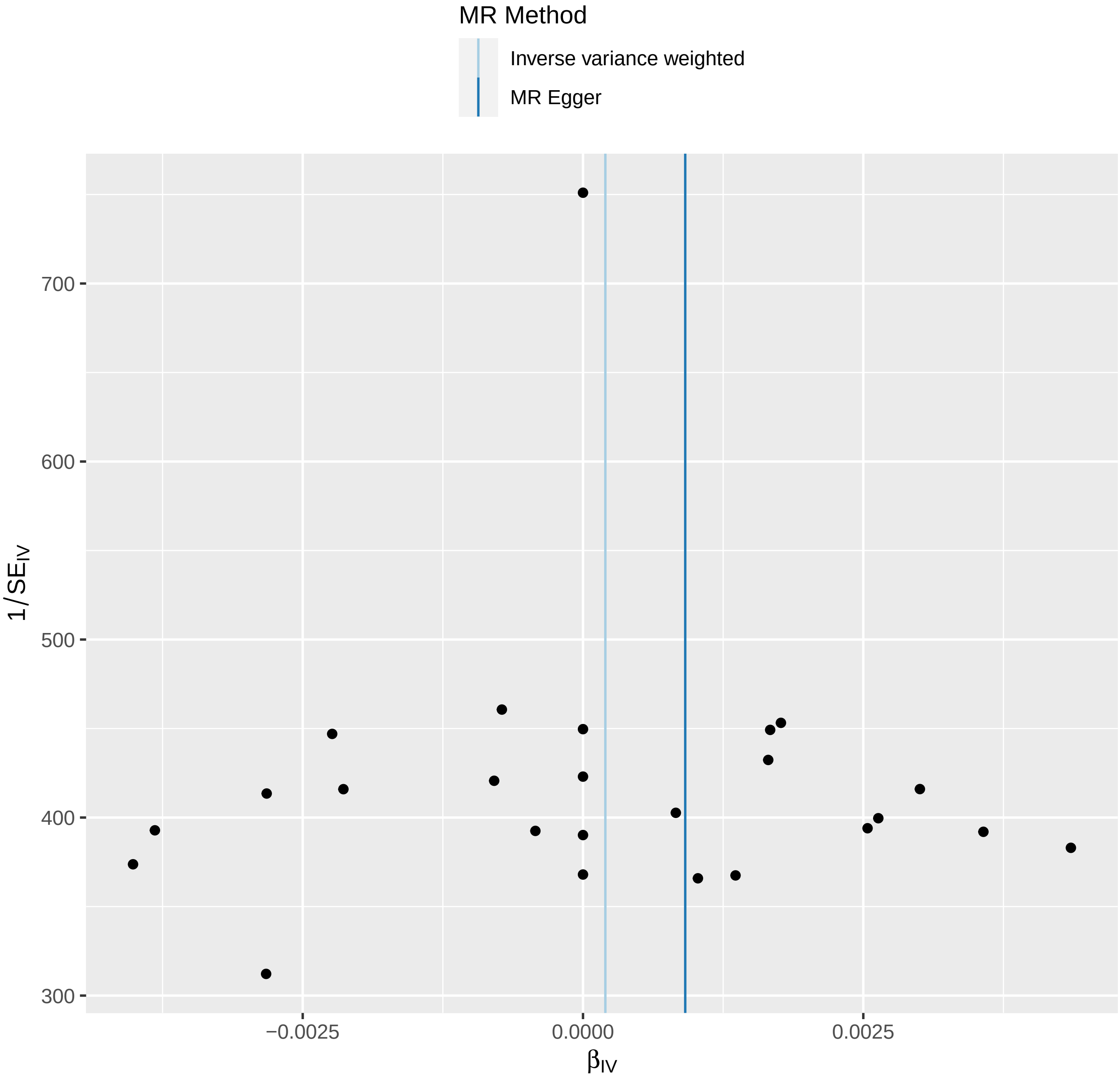

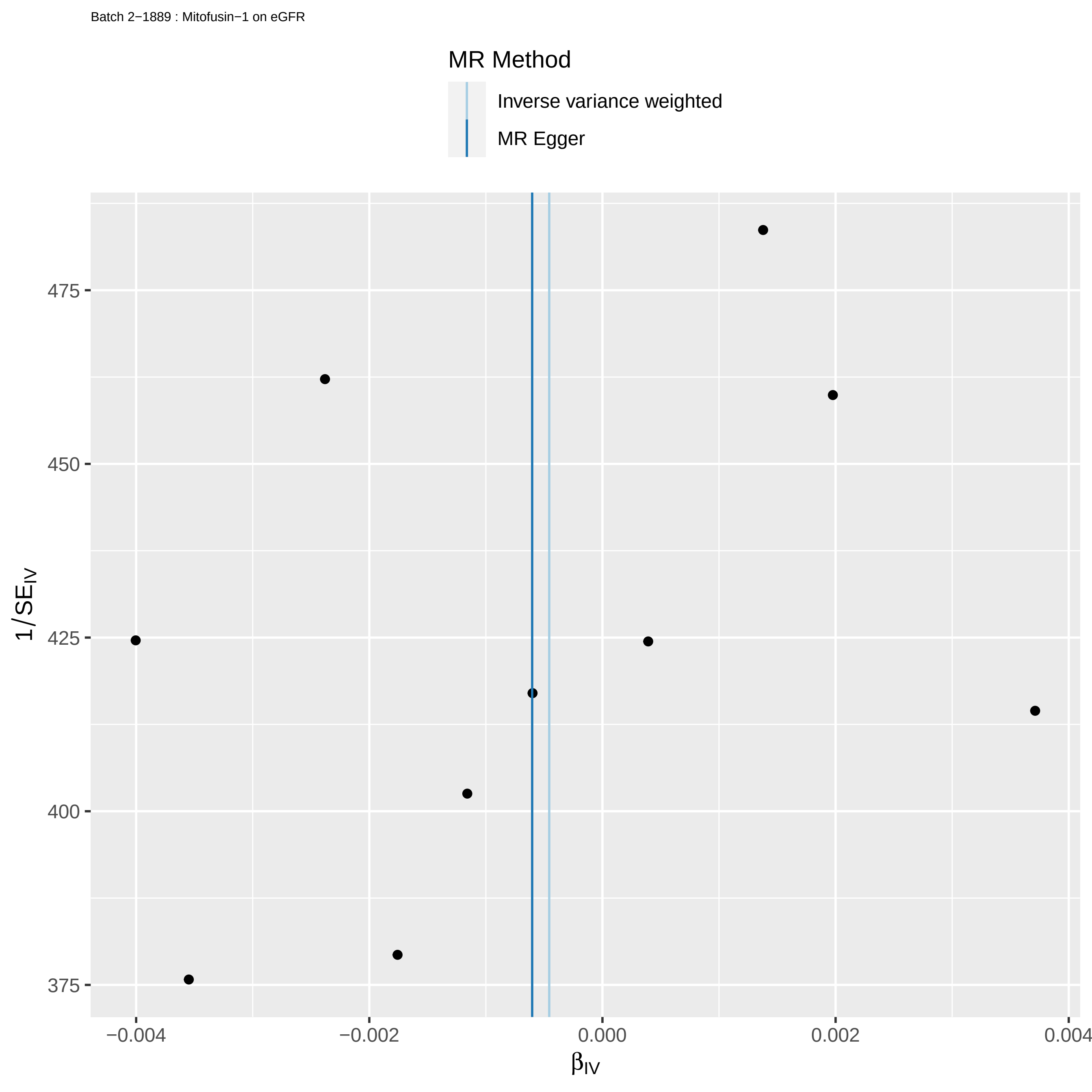
Batch 2−1889 : Mitofusin−1 on eGFR
MR Method
Inverse variance weighted
MR Egger
475
450
425
400
375
1/SE_IV
−0.004
−0.002
0.000
0.002
0.004
β_IV

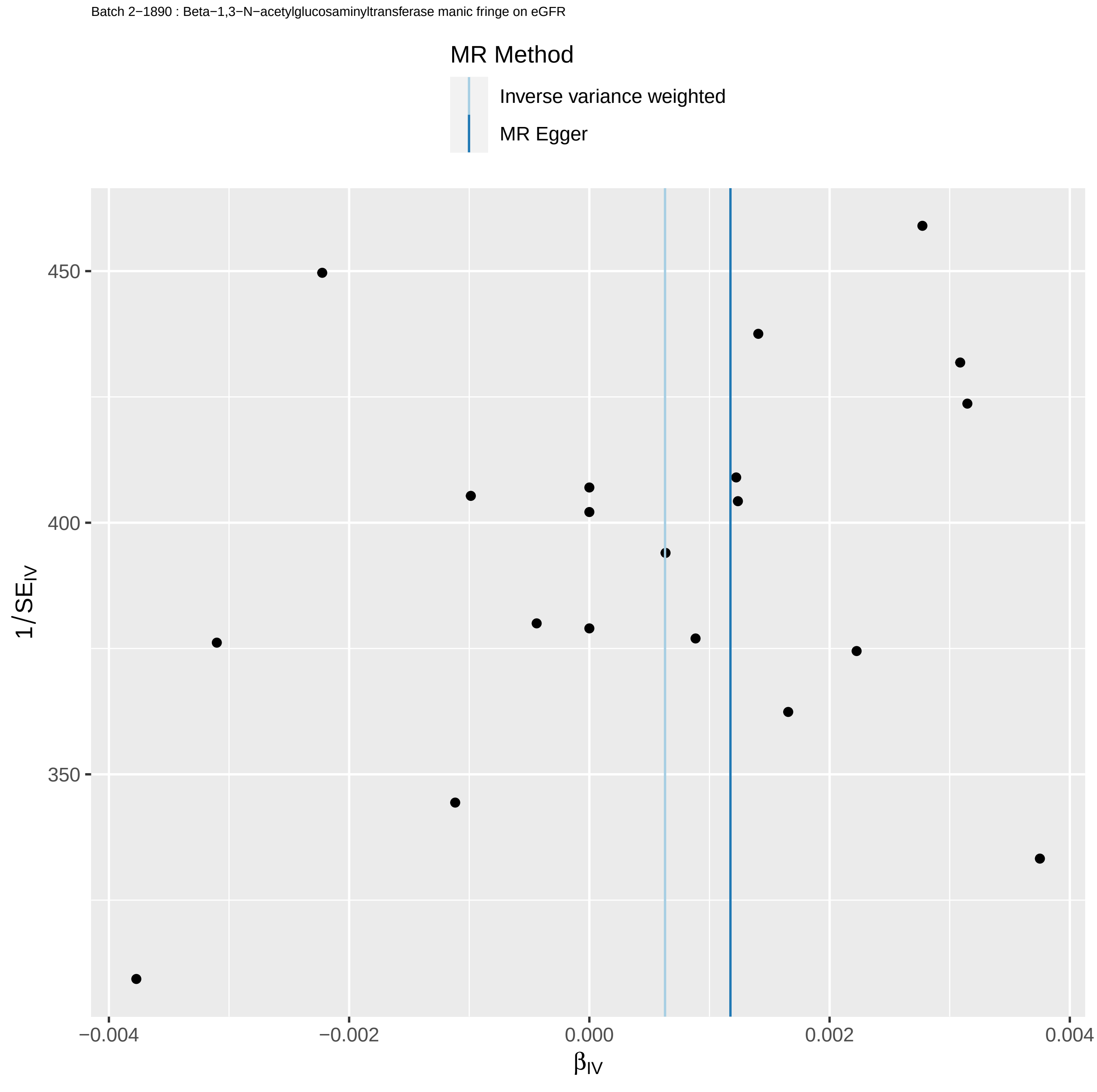
Batch 2−1890 : Beta−1,3−N−acetylglucosaminyltransferase manic fringe on eGFR
MR Method
Inverse variance weighted
MR Egger
450
400
350
1/SE_IV
−0.004
−0.002
0.000
0.002
0.004
β_IV

Batch 2−1891 : MAX gene−associated protein on eGFR

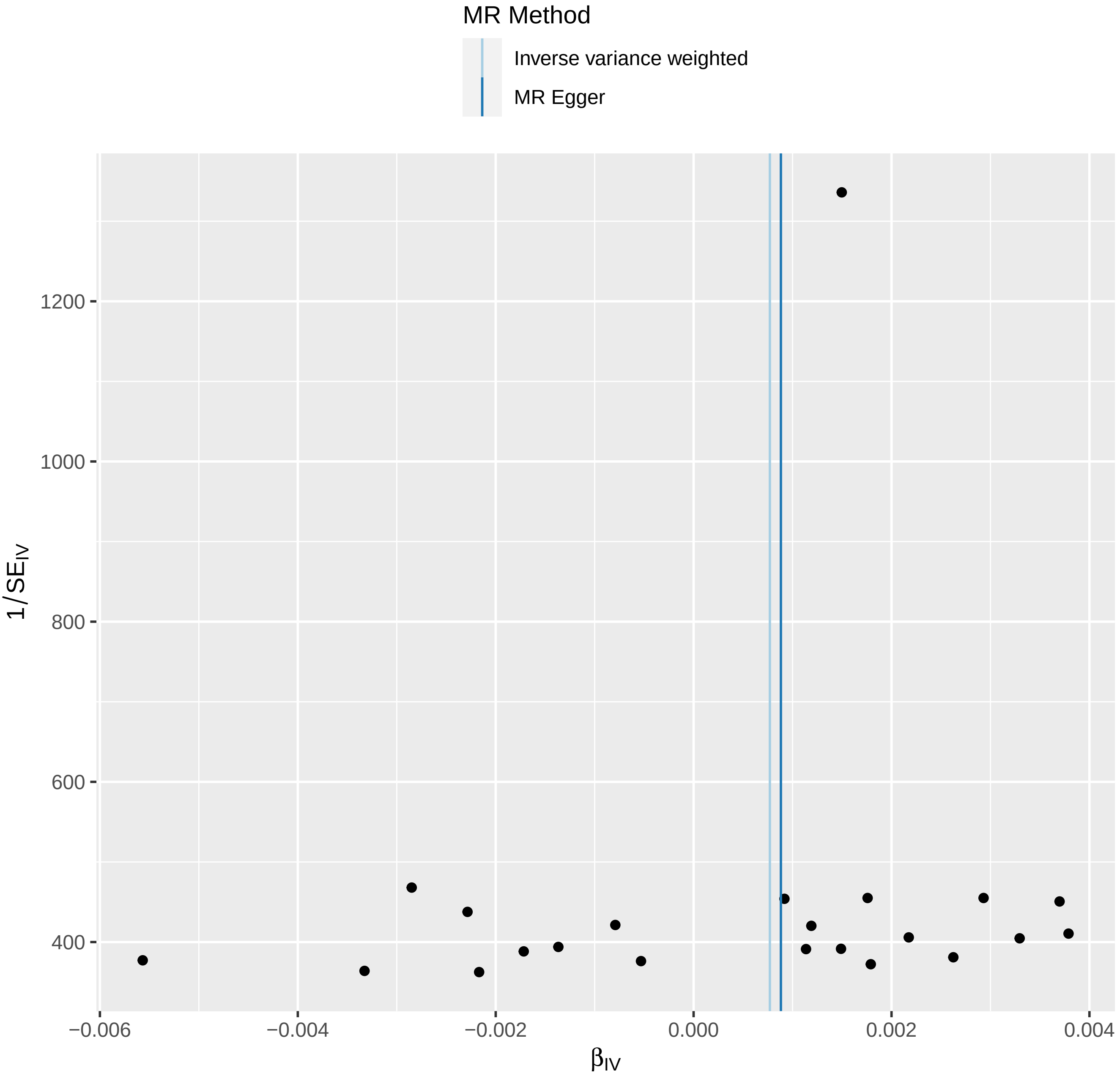

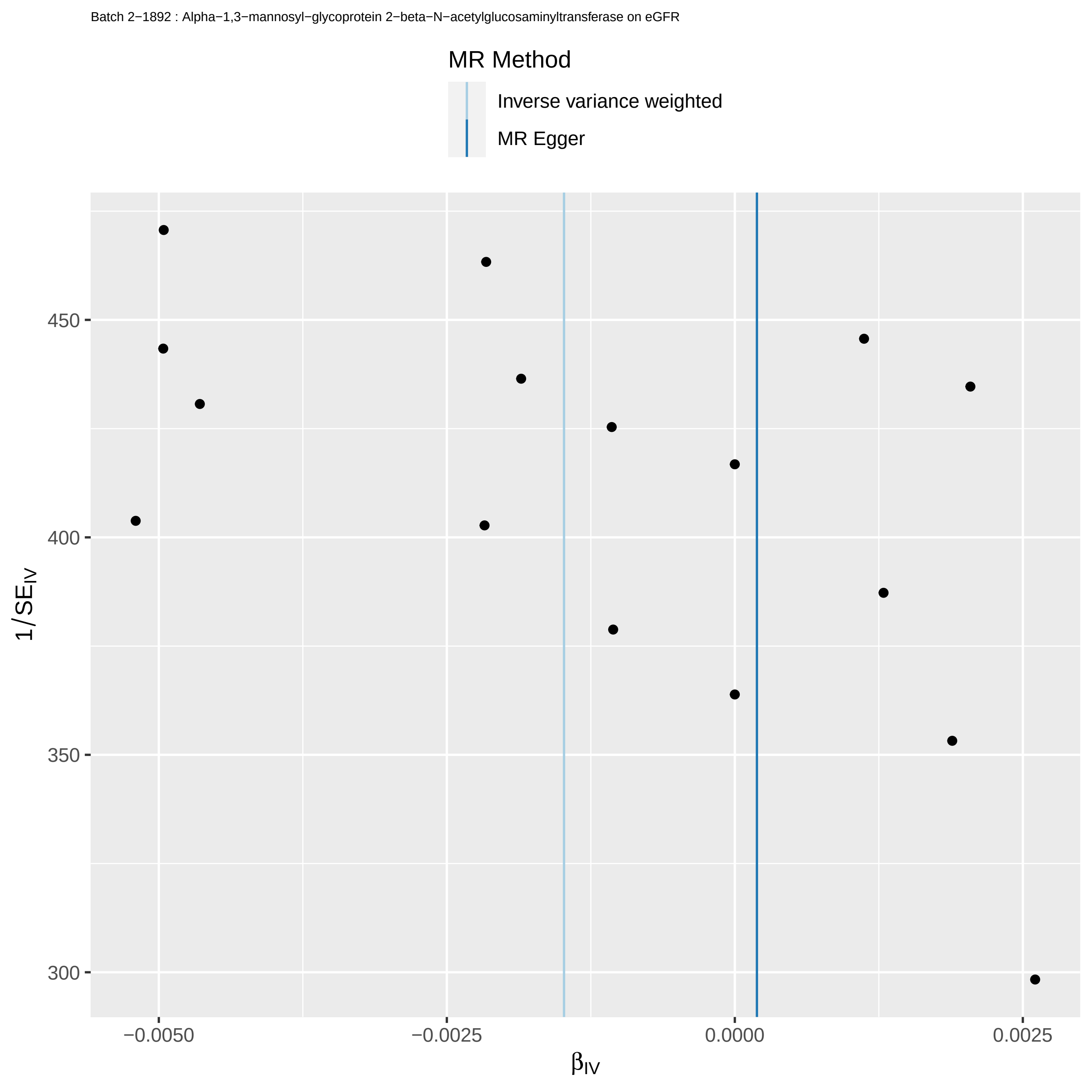
Batch 2−1892 : Alpha−1,3−mannosyl−glycoprotein 2−beta−N−acetylglucosaminyltransferase on eGFR
MR Method
Inverse variance weighted
MR Egger
450
400
350
300
1/SE_IV
−0.0050
−0.0025
0.0000
0.0025
β_IV

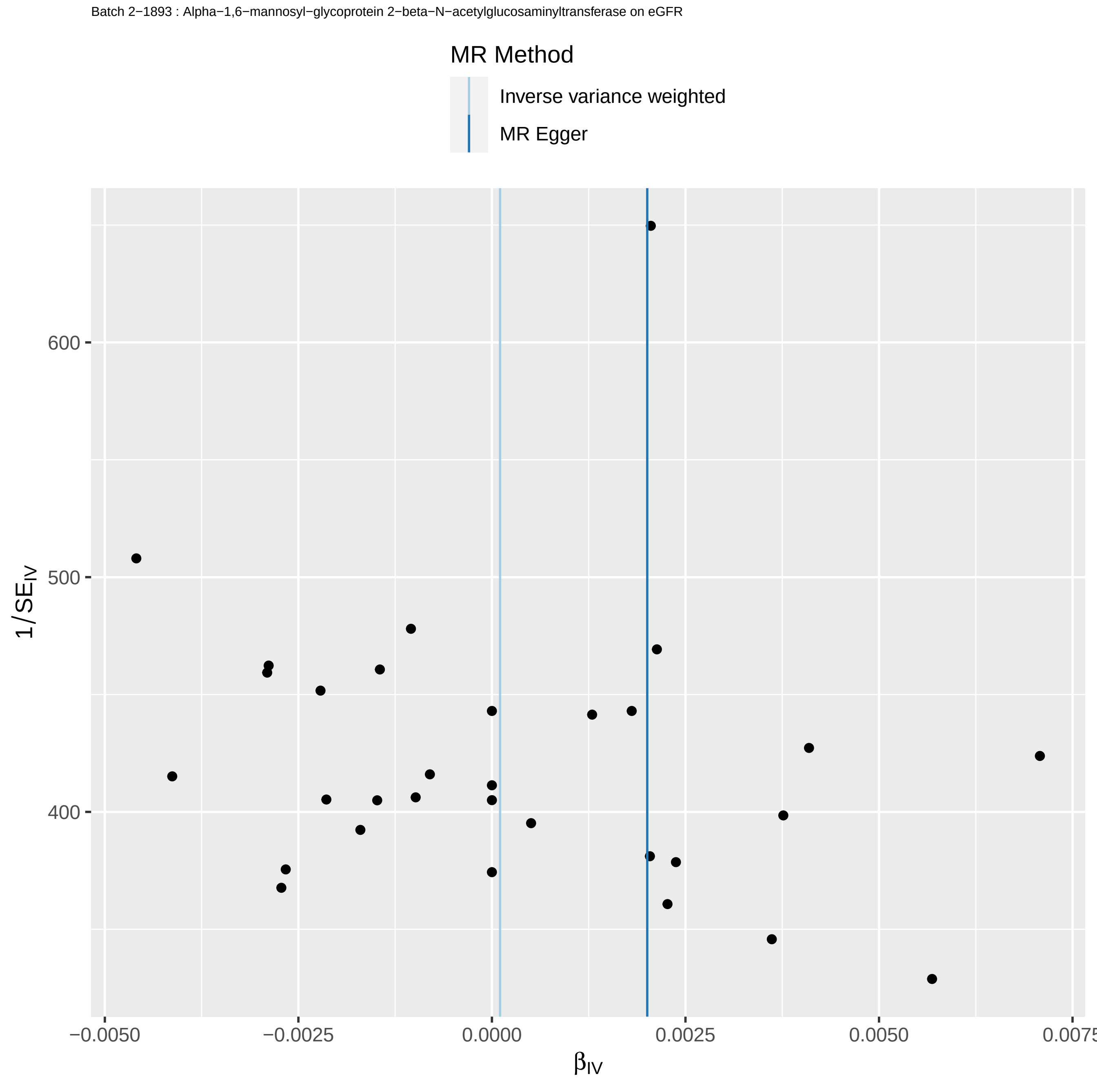

Batch 2−1893 : Alpha−1,6−mannosyl−glycoprotein 2−beta−N−acetylglucosaminyltransferase on eGFR
MR Method
Inverse variance weighted
MR Egger
600
500
400
1/SE_IV
−0.0050
−0.0025
0.0000
0.0025
0.0050
0.0075
β_IV

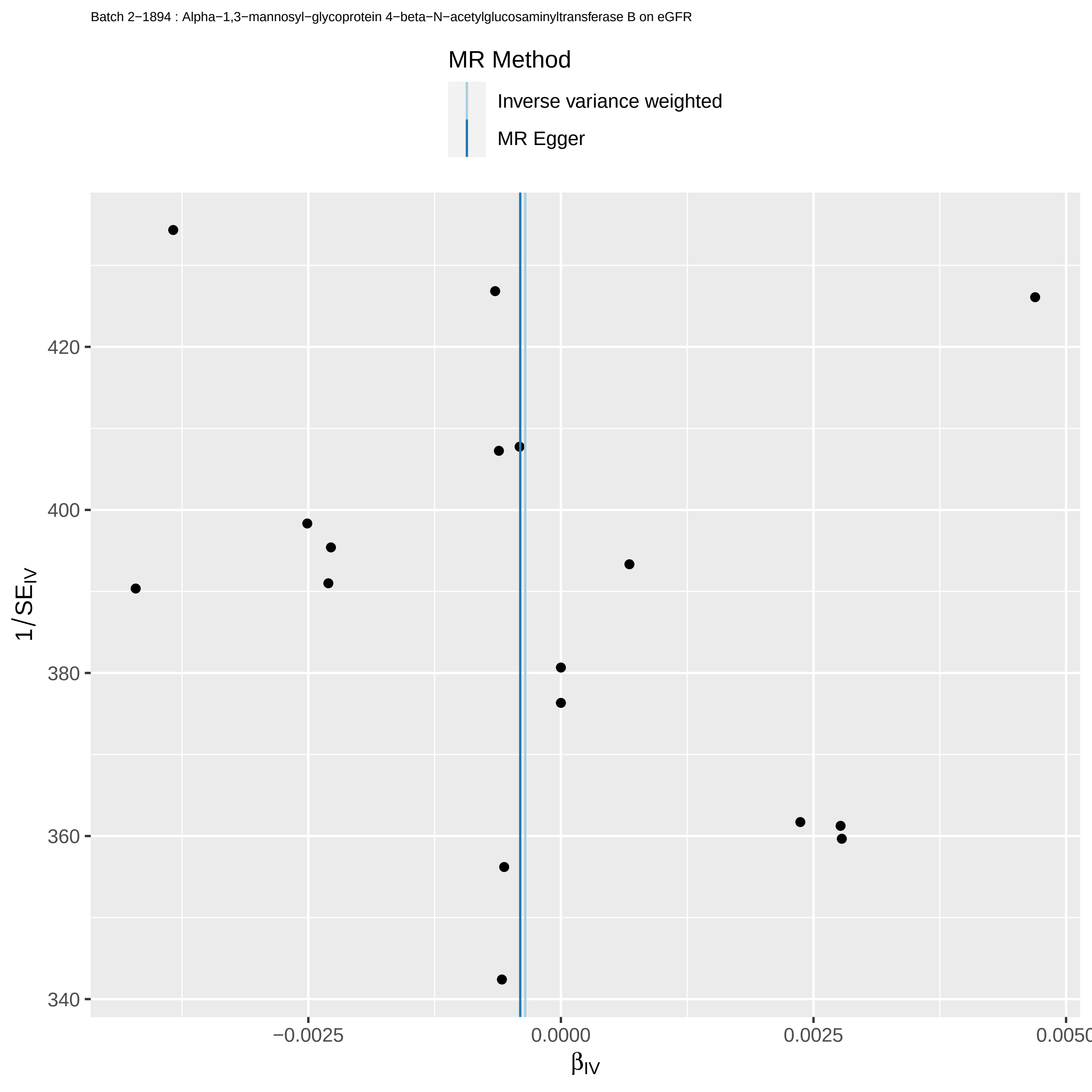
Batch 2−1894 : Alpha−1,3−mannosyl−glycoprotein 4−beta−N−acetylglucosaminyltransferase B on eGFR
MR Method
Inverse variance weighted
MR Egger
1/SE$_{IV}$
420
400
380
360
340
−0.0025
0.0000
0.0025
0.0050
β$_{IV}$

Batch 2−1895 : Alpha−1,3−mannosyl−glycoprotein 4−beta−N−acetylglucosaminyltransferase C on eGFR

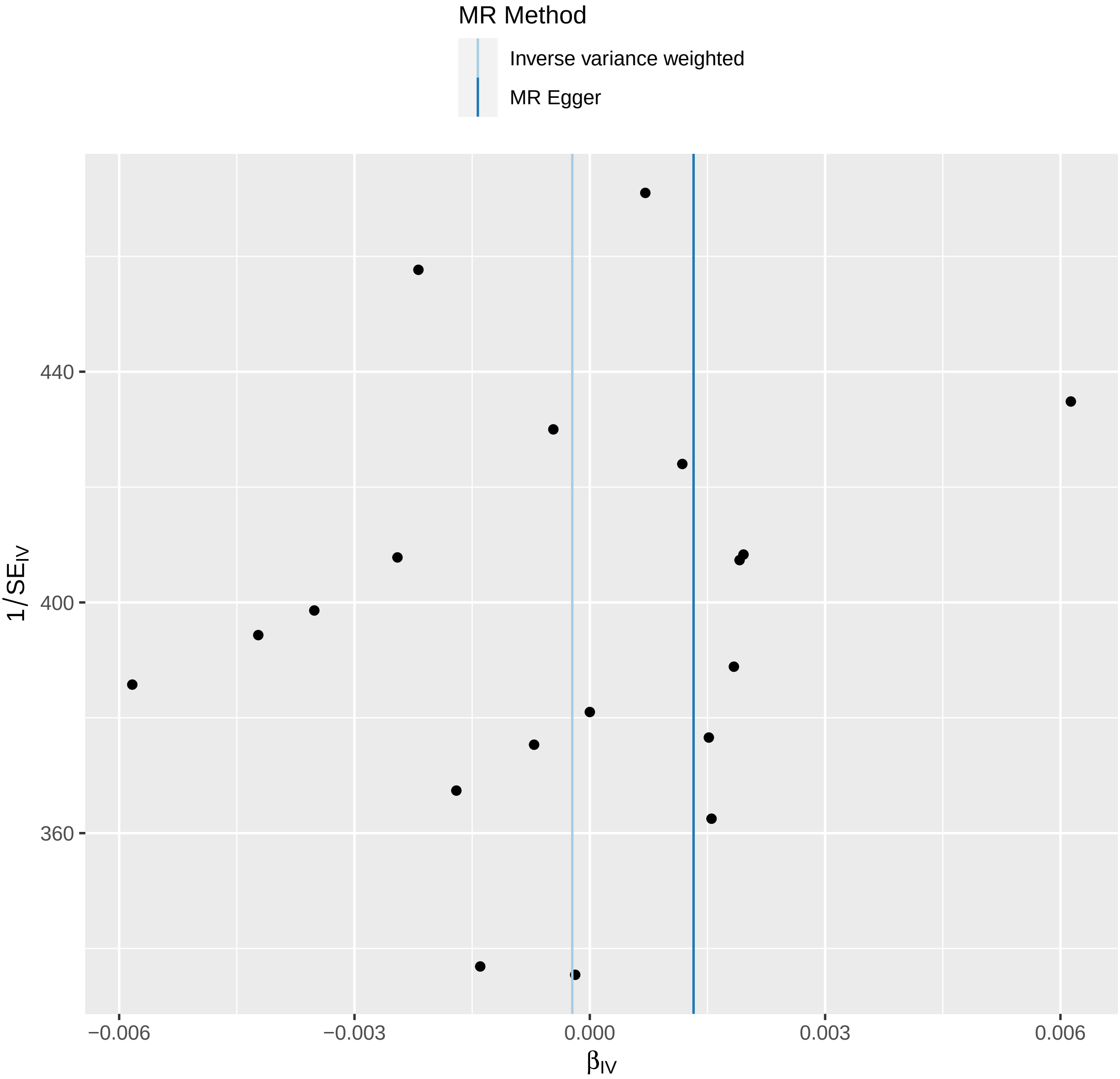

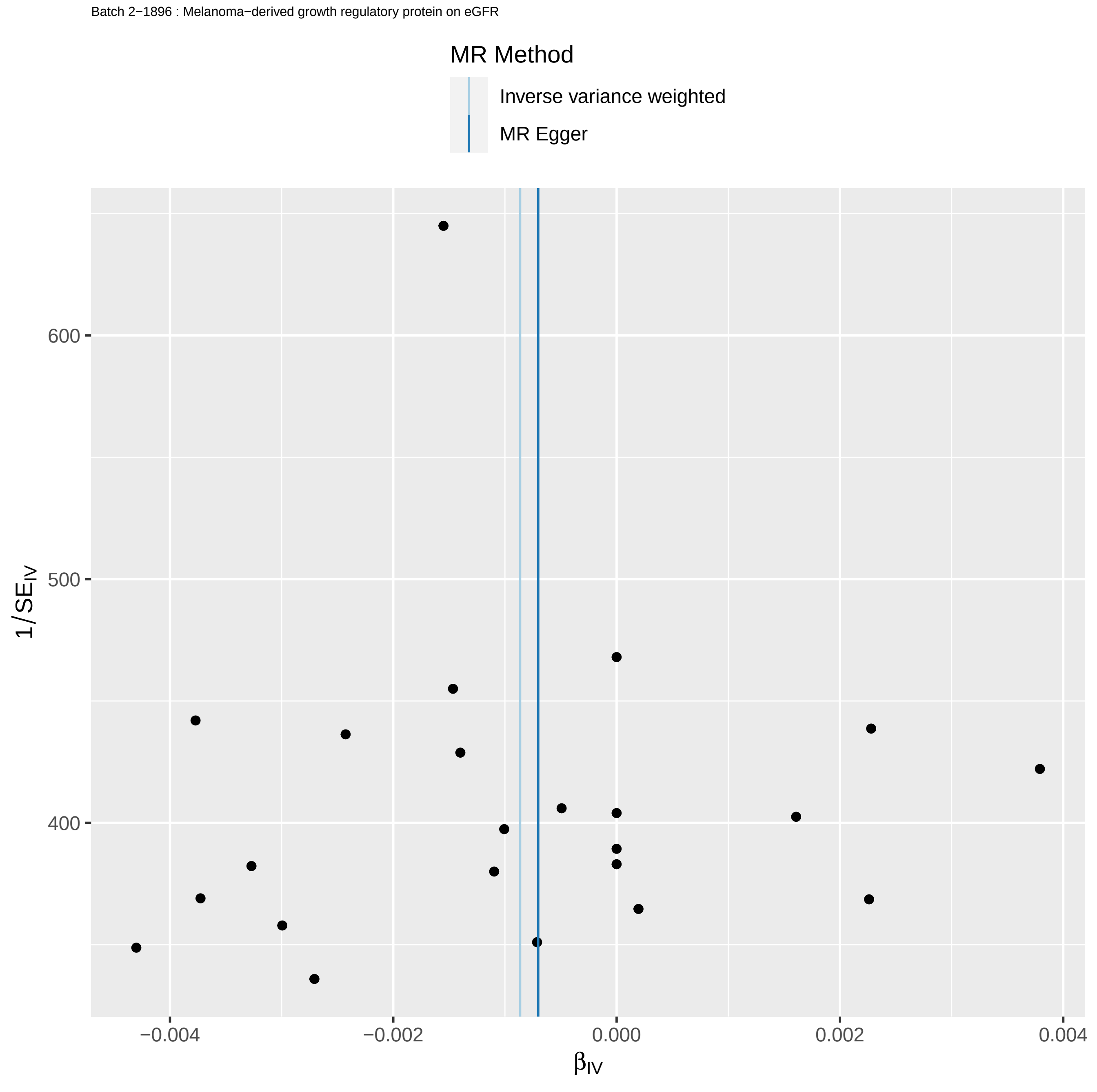
Batch 2−1896 : Melanoma−derived growth regulatory protein on eGFR
MR Method
Inverse variance weighted
MR Egger
600
500
400
1/SE_IV
−0.004
−0.002
0.000
0.002
0.004
β_IV

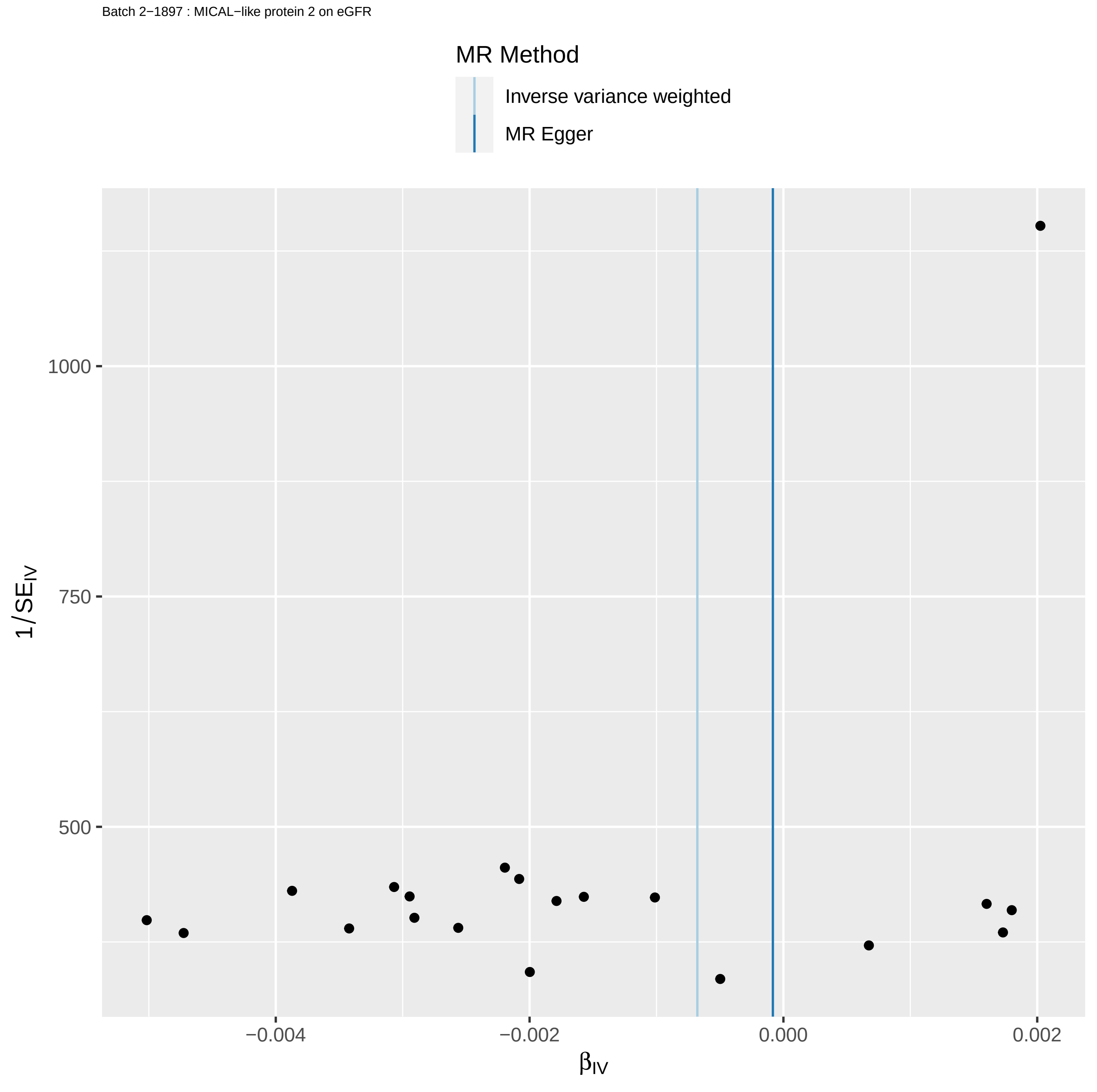
Batch 2−1897 : MICAL−like protein 2 on eGFR
MR Method
Inverse variance weighted
MR Egger
1000
750
500
1/SE_IV
−0.004
−0.002
0.000
0.002
β_IV

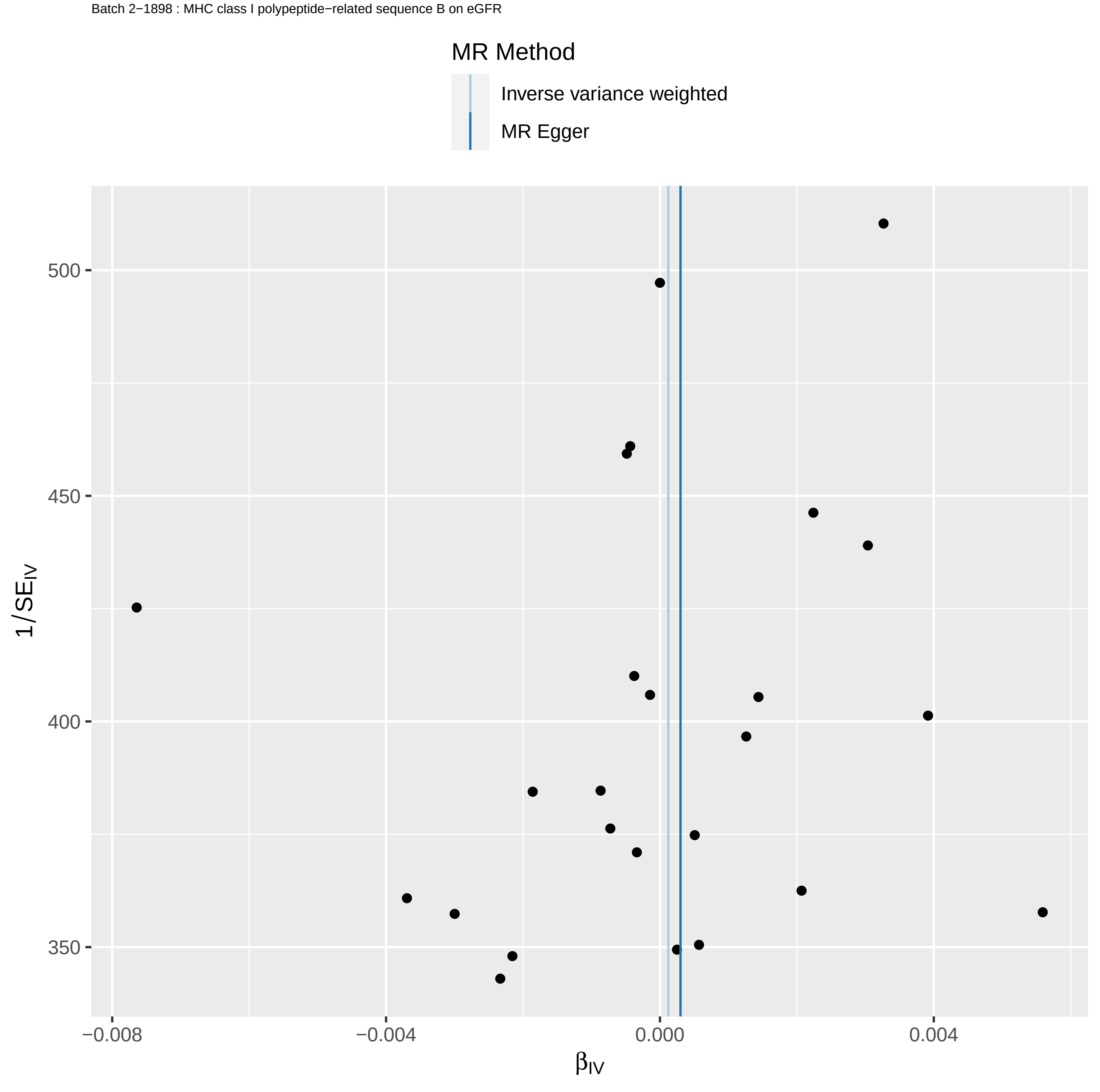
Batch 2−1898 : MHC class I polypeptide−related sequence B on eGFR
MR Method
Inverse variance weighted
MR Egger
500
450
400
350
1/SE_IV
−0.008
−0.004
0.000
0.004
β_IV

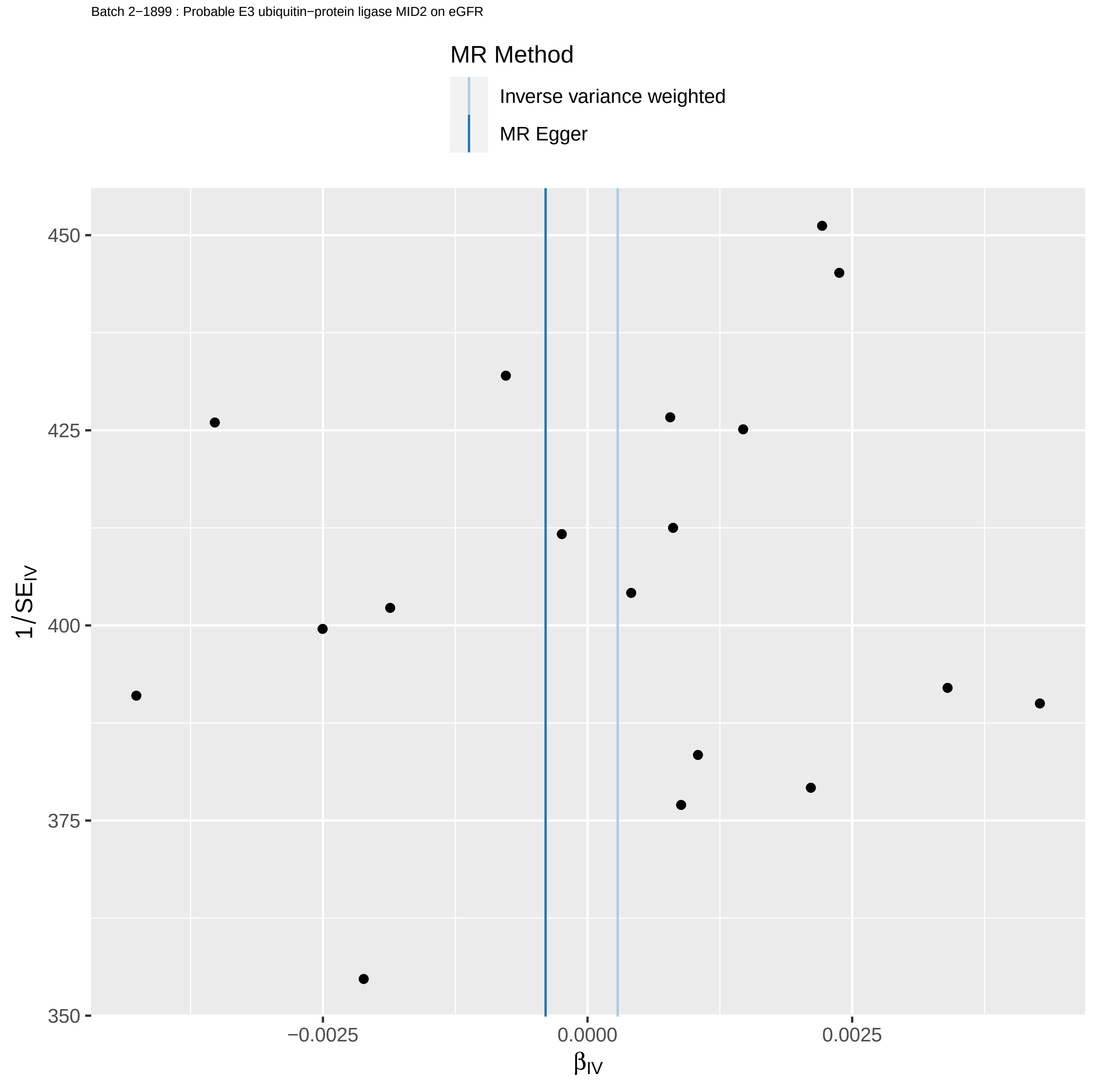
Batch 2−1899 : Probable E3 ubiquitin−protein ligase MID2 on eGFR
MR Method
Inverse variance weighted
MR Egger
450
425
400
375
350
−0.0025
0.0000
0.0025
$1/SE_{IV}$
β_{IV}

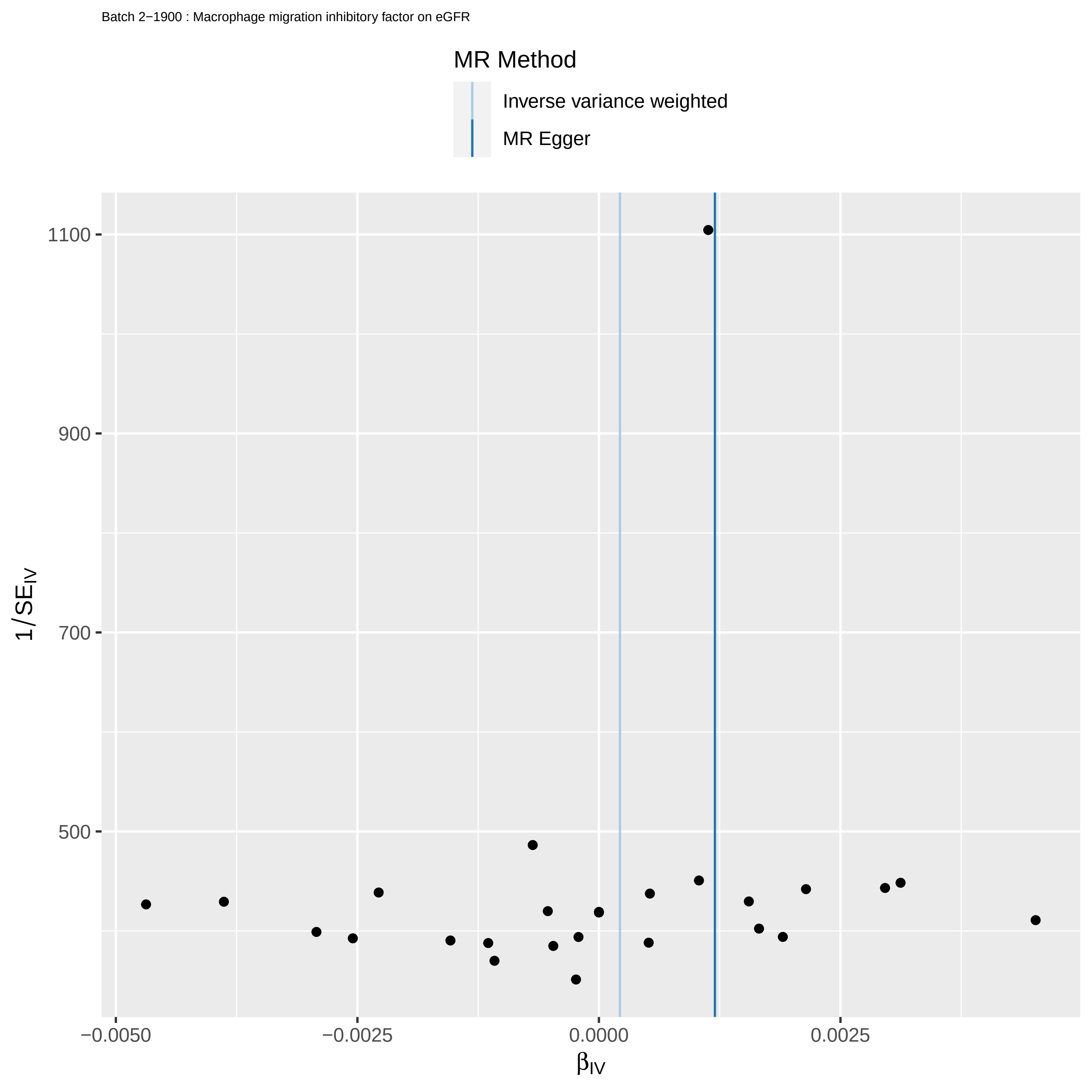

Batch 2−1900 : Macrophage migration inhibitory factor on eGFR
MR Method
Inverse variance weighted
MR Egger
1100
900
700
500
1/SE_IV
−0.0050
−0.0025
0.0000
0.0025
β_IV

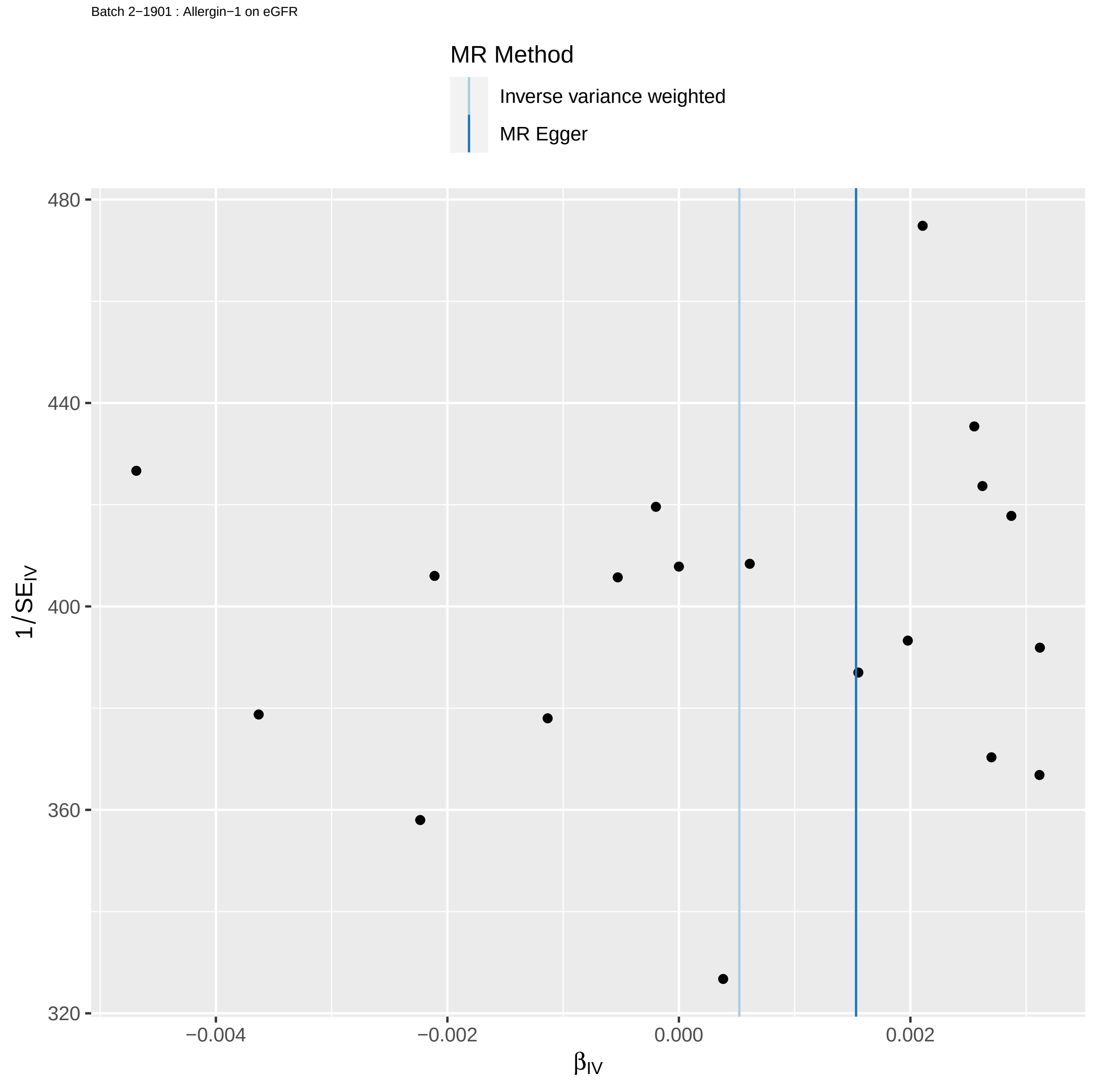
Batch 2−1901 : Allergin−1 on eGFR
MR Method
Inverse variance weighted
MR Egger
480
440
400
360
320
1/SE$_{IV}$
−0.004
−0.002
0.000
0.002
β$_{IV}$

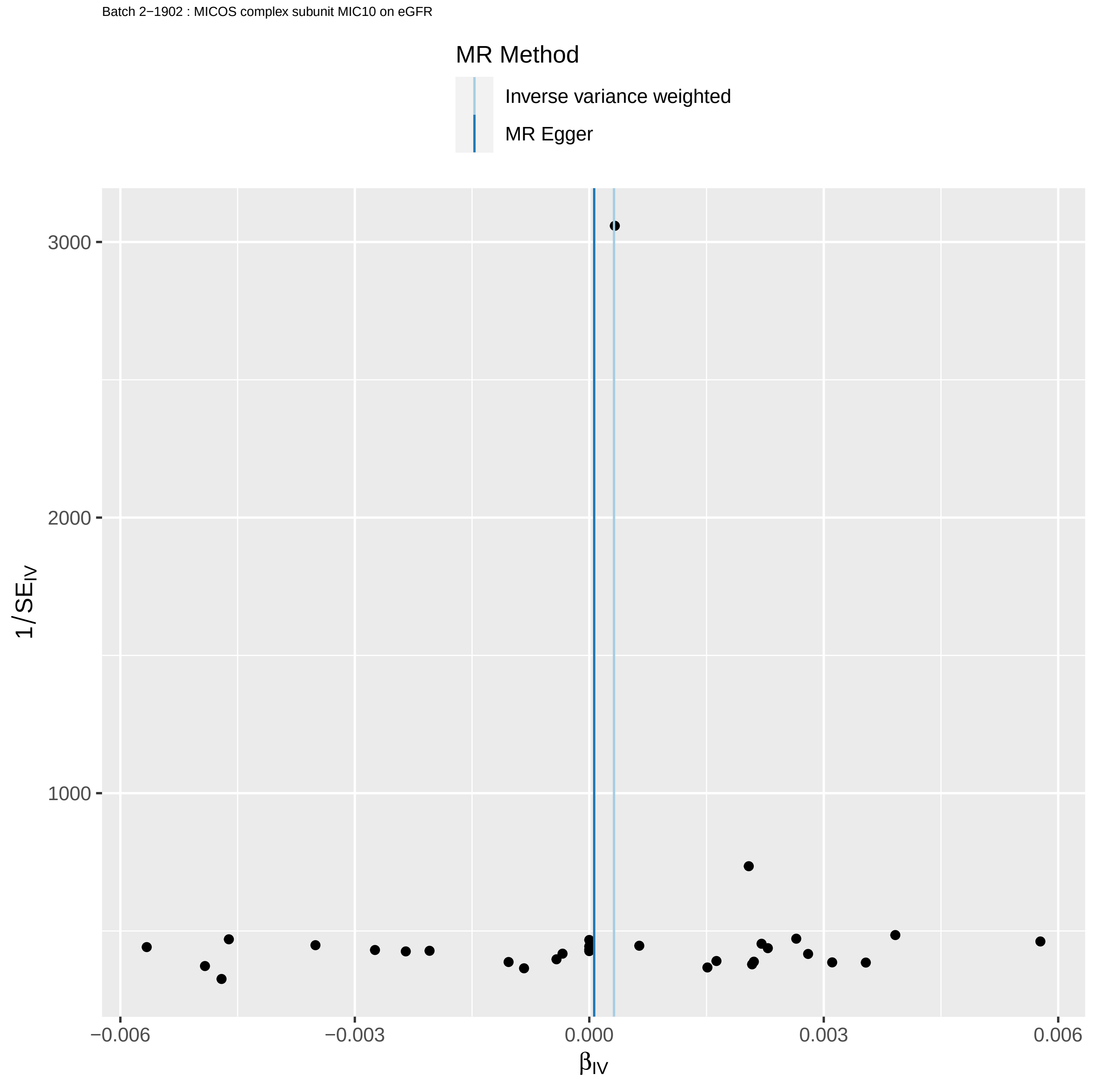

Batch 2−1902 : MICOS complex subunit MIC10 on eGFR
MR Method
Inverse variance weighted
MR Egger
3000
2000
1000
1/SE$_{IV}$
−0.006
−0.003
0.000
0.003
0.006
β_{IV}

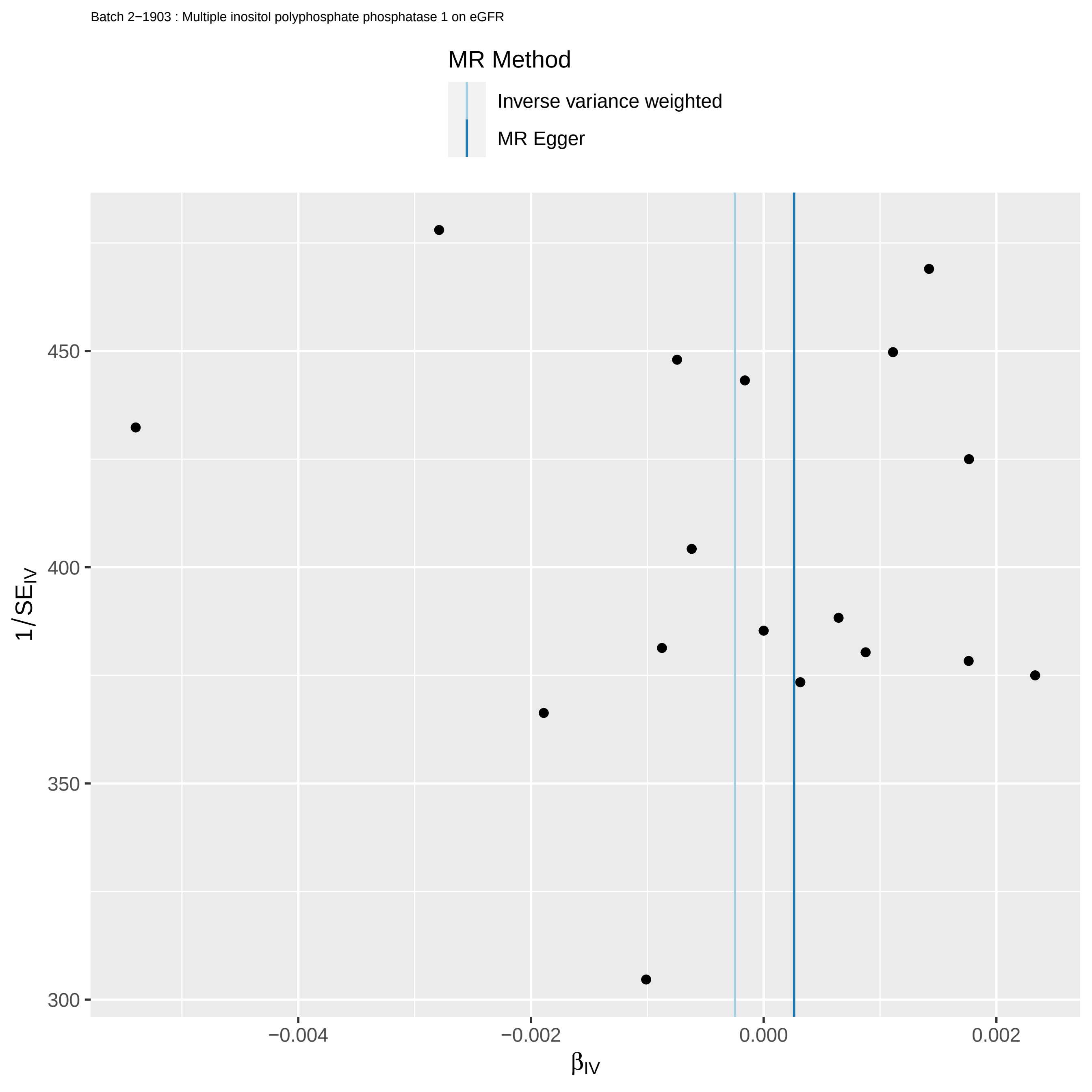

Batch 2−1903 : Multiple inositol polyphosphate phosphatase 1 on eGFR
MR Method
Inverse variance weighted
MR Egger
450
400
350
300
1/SE_IV
−0.004
−0.002
0.000
0.002
β_IV

Batch 2−1904 : Malectin on eGFR

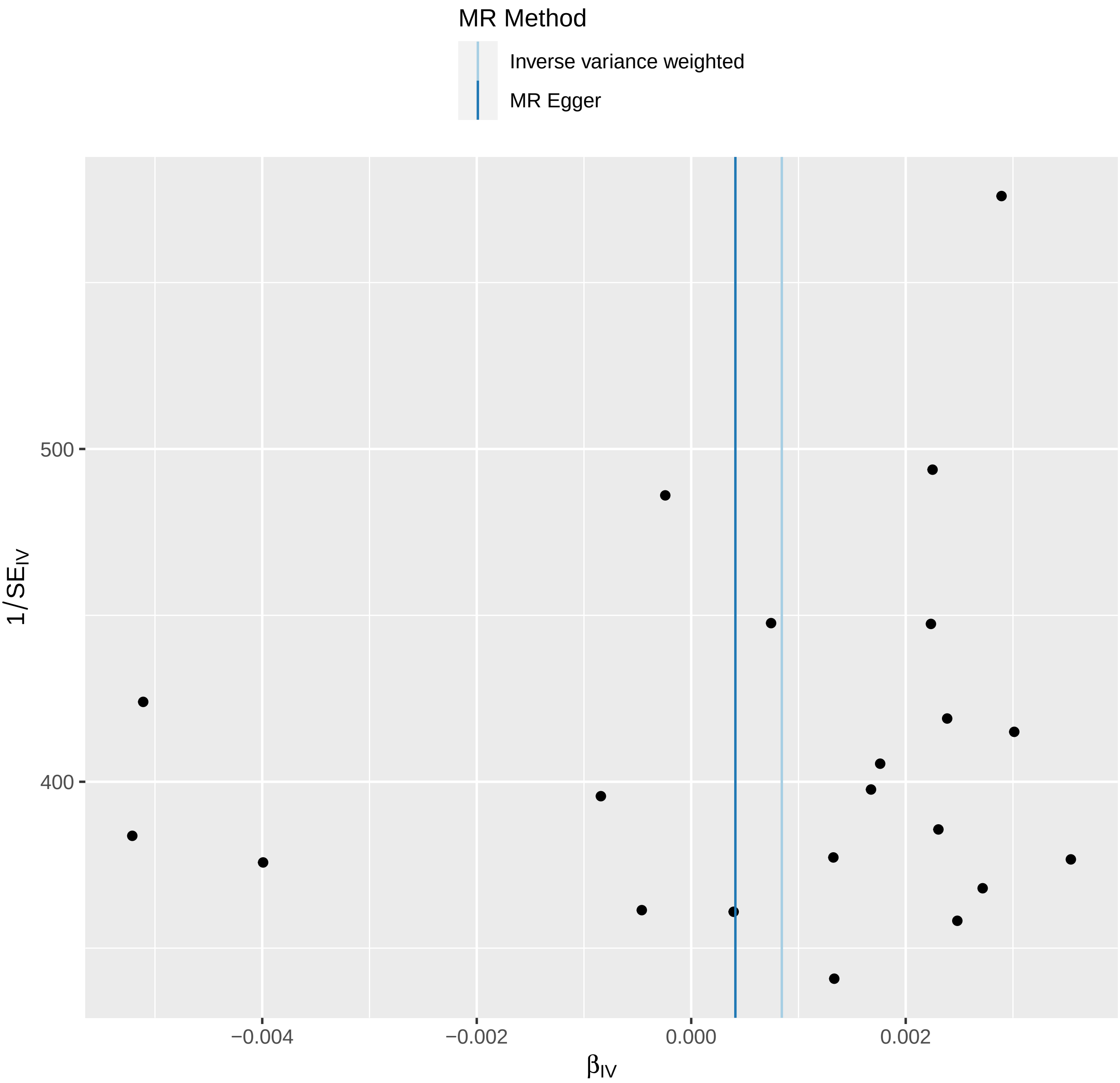

Batch 2−1905 : Histone−lysine N−methyltransferase 2D on eGFR

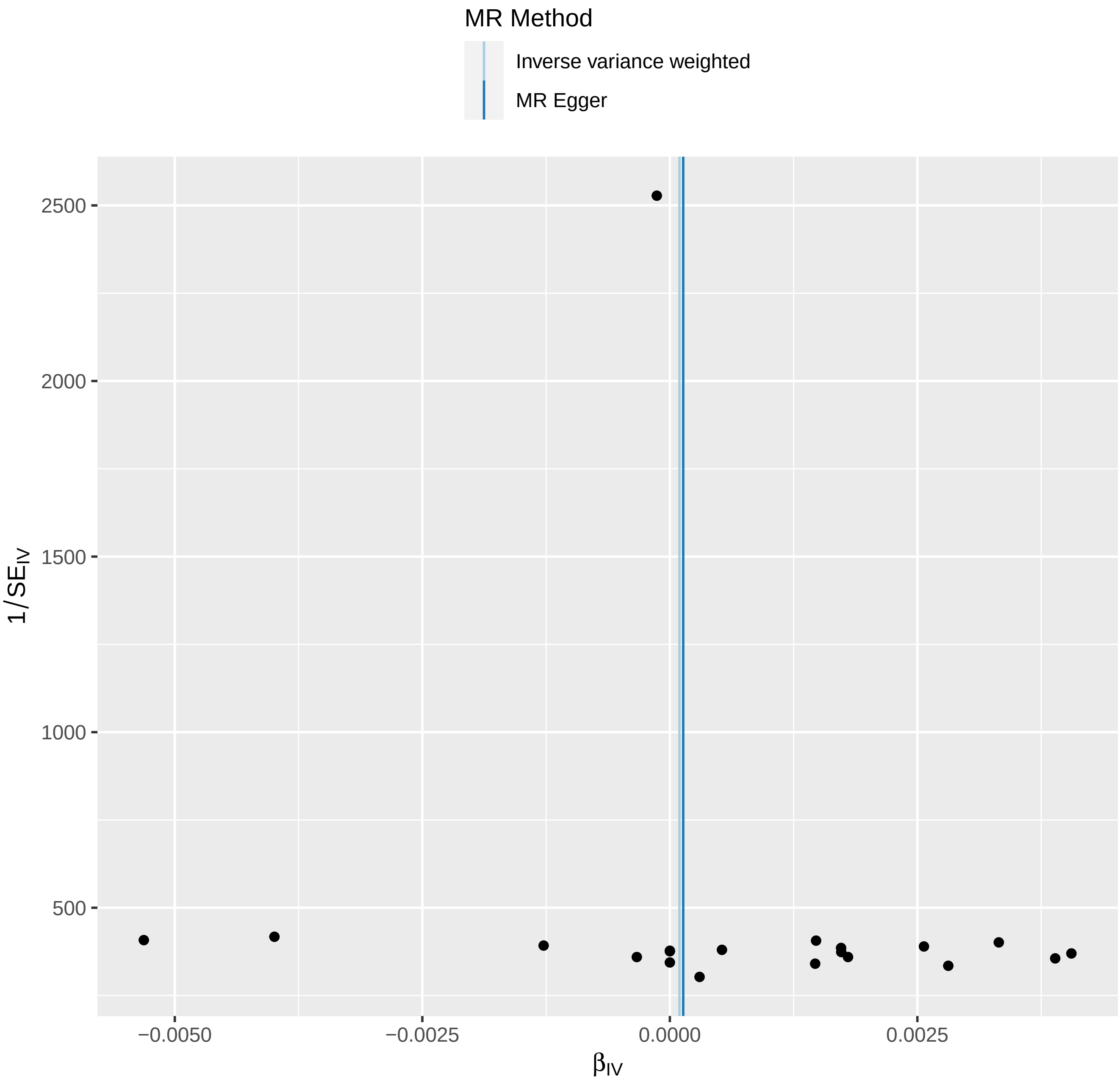

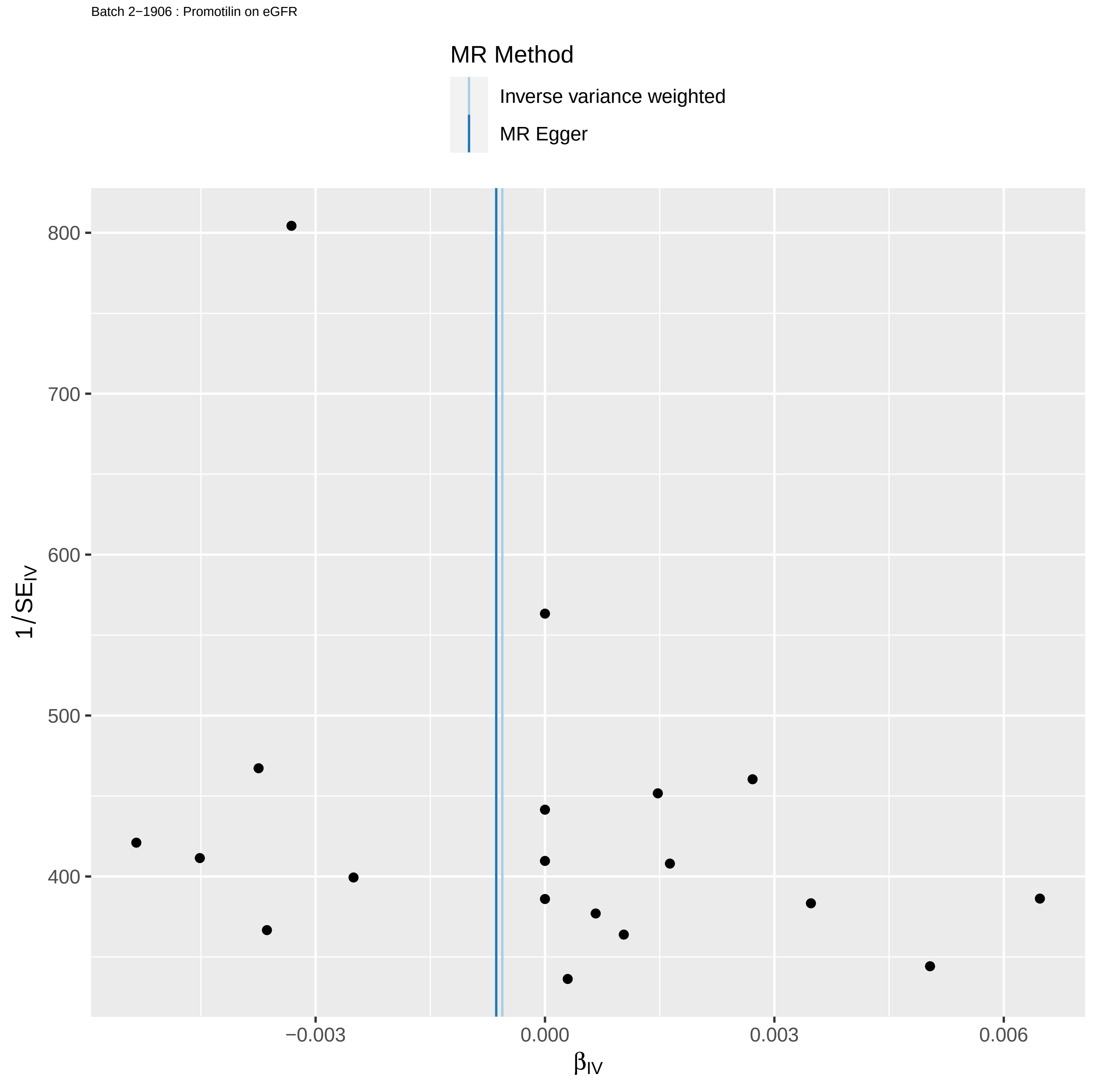
Batch 2−1906 : Promotilin on eGFR
MR Method
Inverse variance weighted
MR Egger
800
700
600
500
400
1/SE_IV
−0.003
0.000
0.003
0.006
β_IV

Batch 2−1907 : Malonyl−CoA decarboxylase, mitochondrial on eGFR

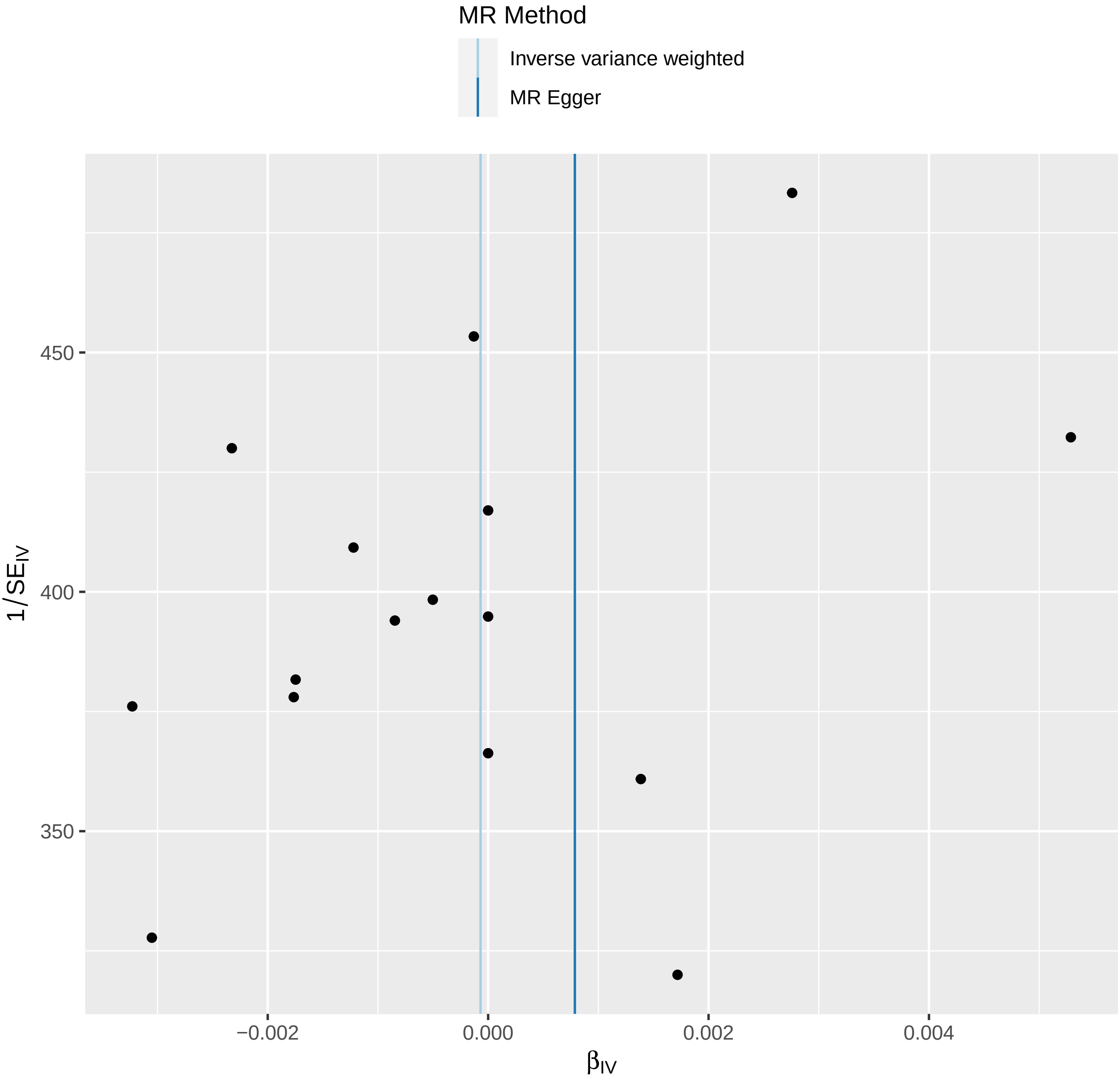

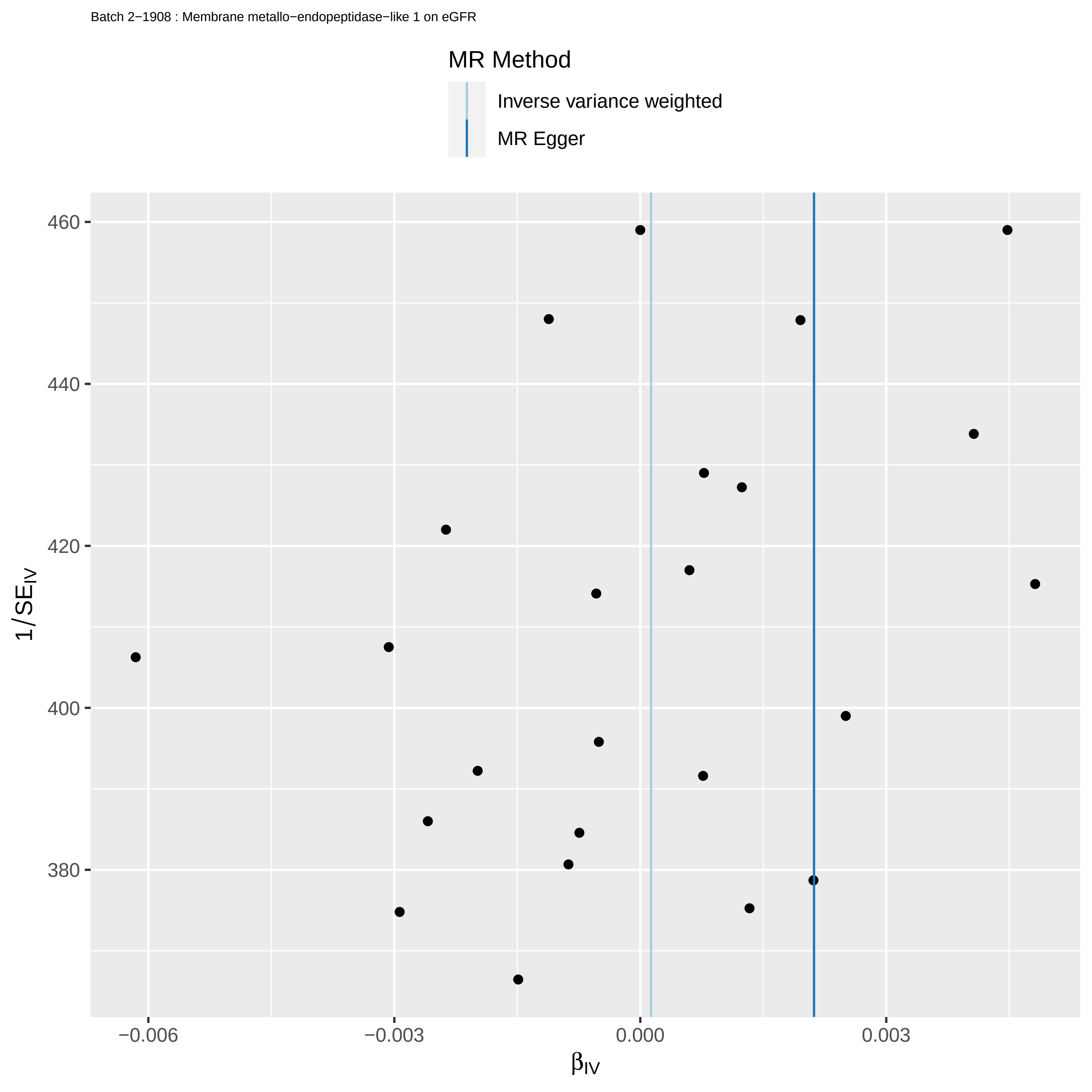

Batch 2−1908 : Membrane metallo−endopeptidase−like 1 on eGFR
MR Method
Inverse variance weighted
MR Egger
460
440
420
400
380
1/SE_IV
−0.006
−0.003
0.000
0.003
β_IV

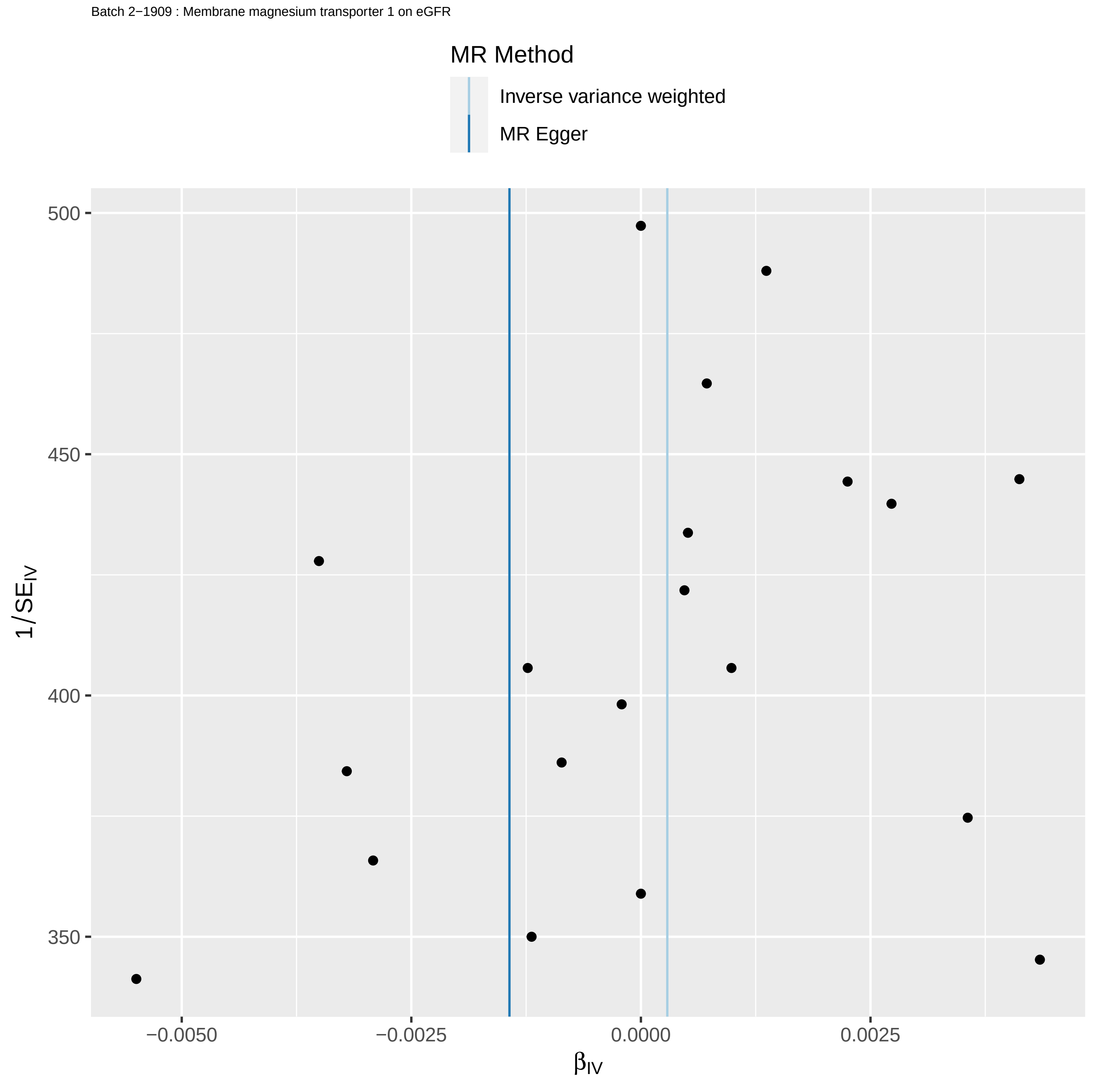

Batch 2−1909 : Membrane magnesium transporter 1 on eGFR
MR Method
Inverse variance weighted
MR Egger
500
450
400
350
1/SE_IV
−0.0050
−0.0025
0.0000
0.0025
β_IV

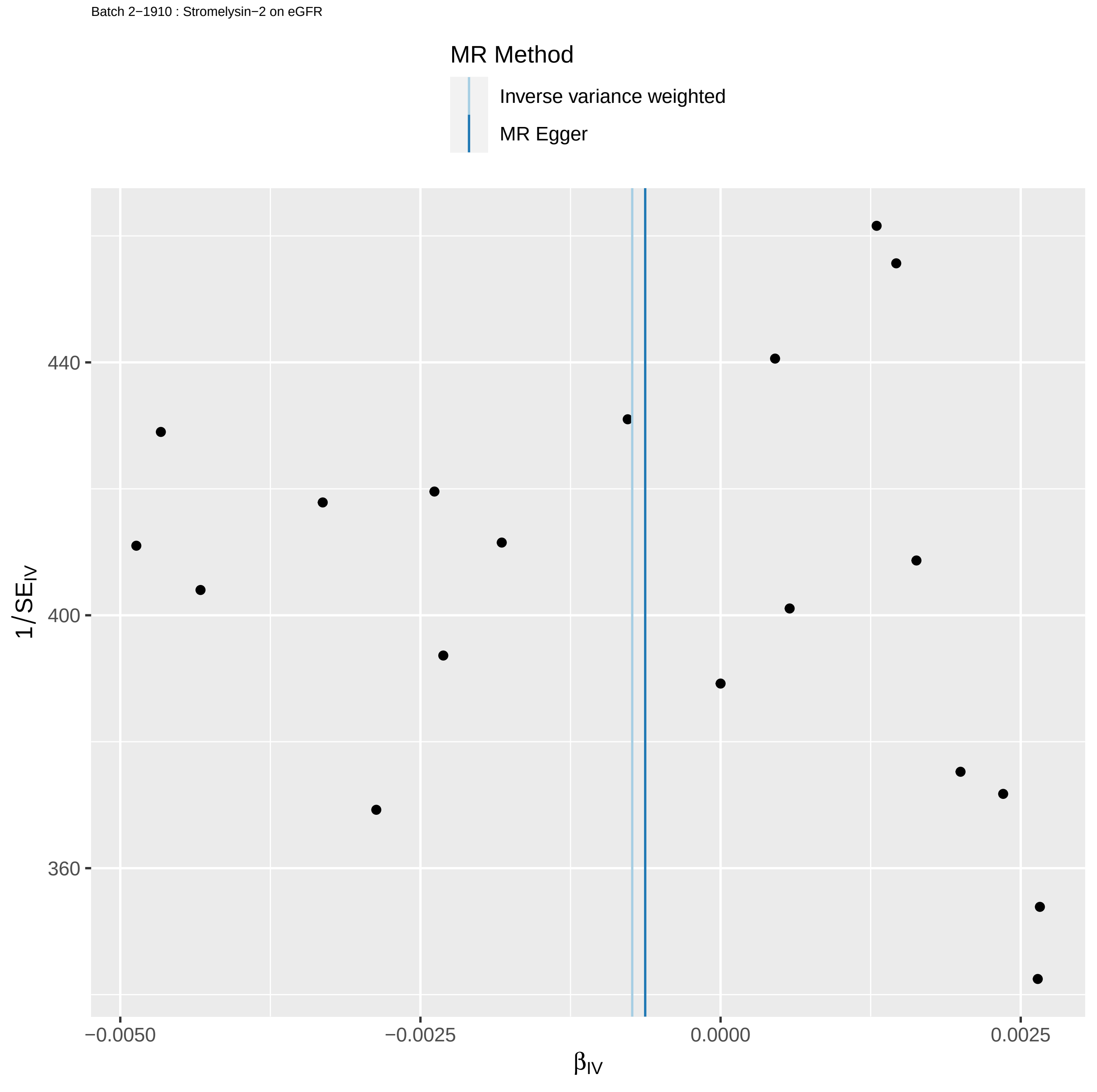

Batch 2−1910 : Stromelysin−2 on eGFR
MR Method
Inverse variance weighted
MR Egger
440
400
360
1/SE_IV
−0.0050
−0.0025
0.0000
0.0025
β_IV

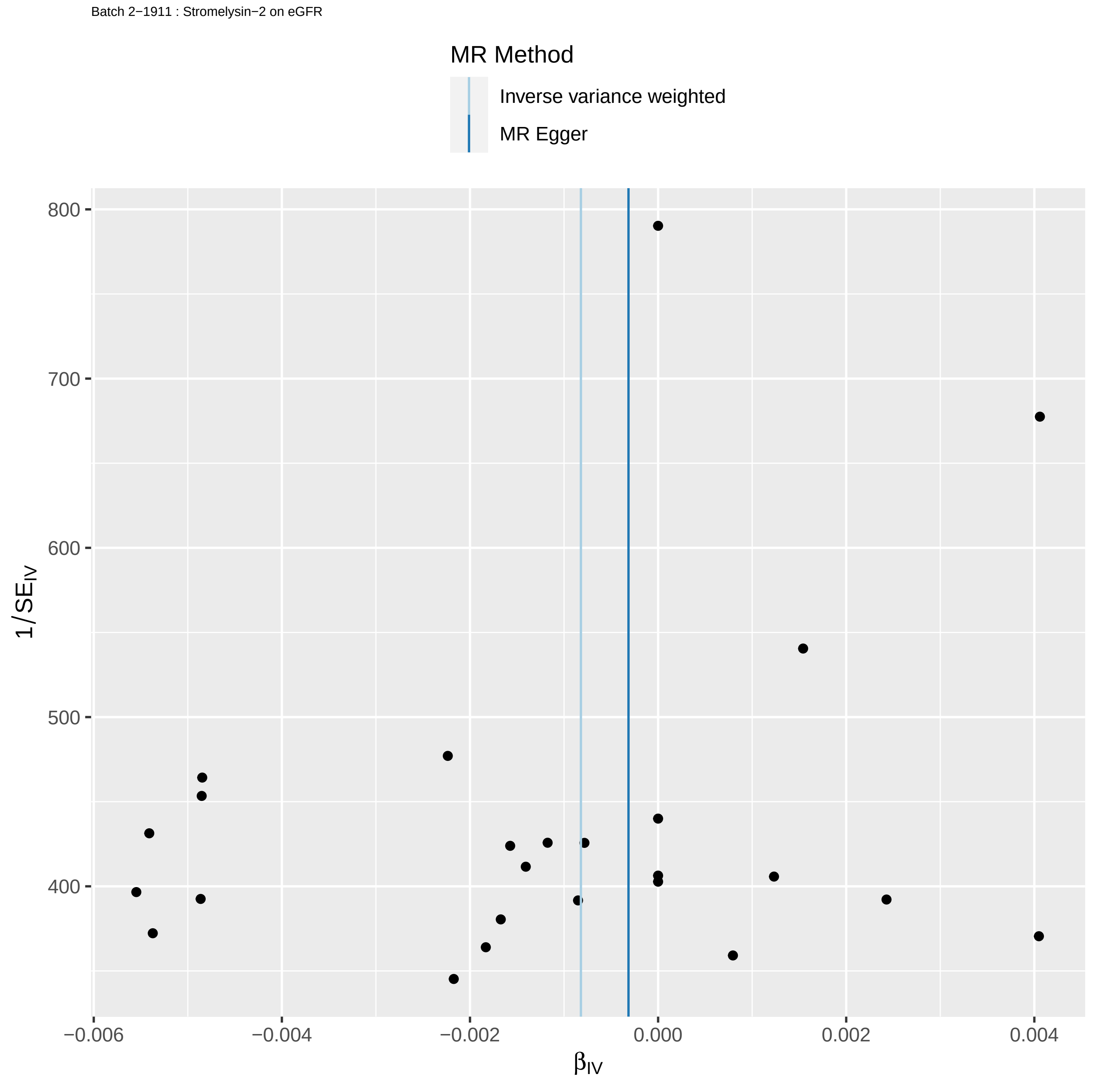
Batch 2−1911 : Stromelysin−2 on eGFR
MR Method
Inverse variance weighted
MR Egger
800
700
600
500
400
1/SE_IV
−0.006
−0.004
−0.002
0.000
0.002
0.004
β_IV

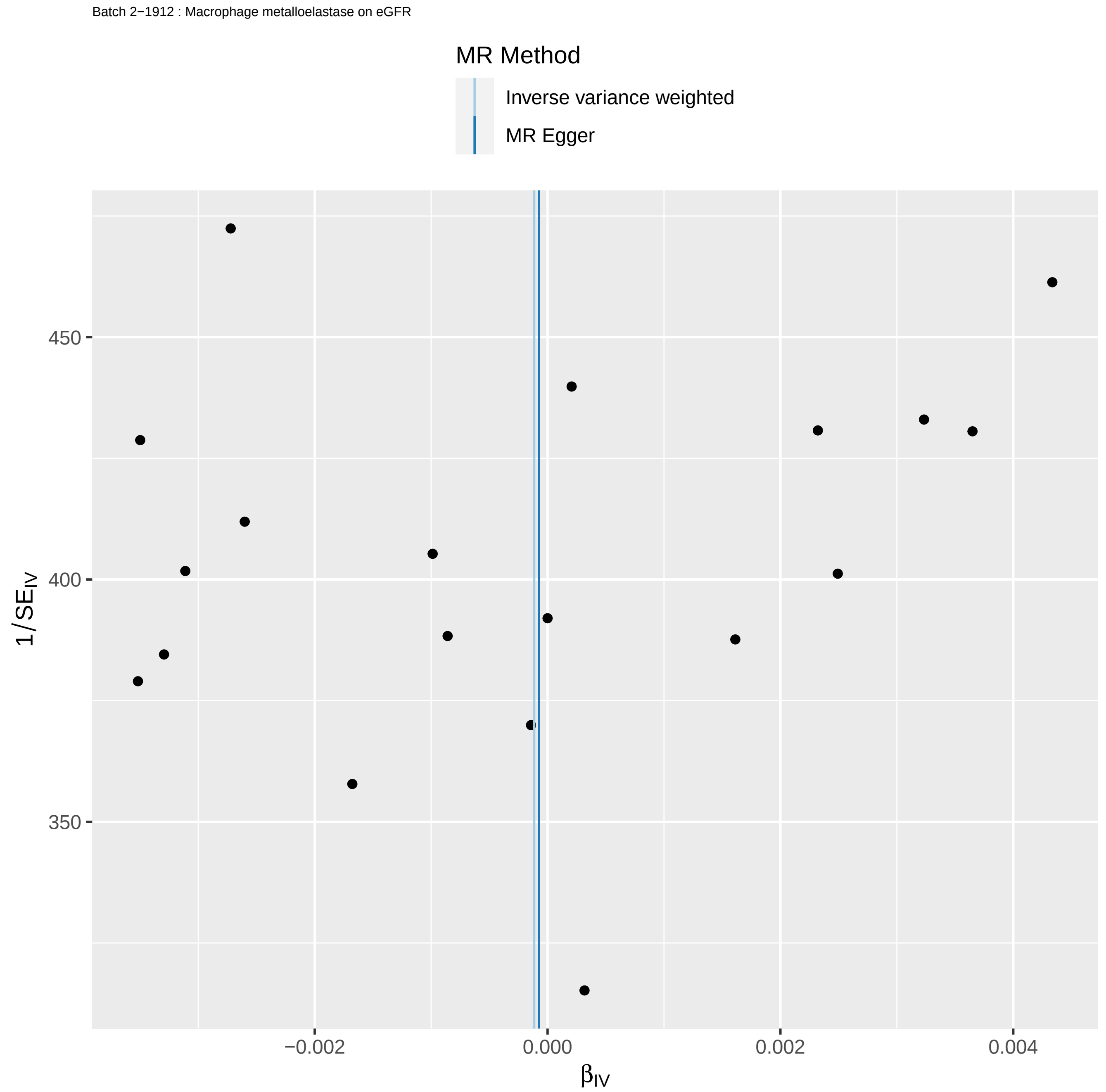
Batch 2−1912 : Macrophage metalloelastase on eGFR
MR Method
Inverse variance weighted
MR Egger
450
400
350
1/SE$_{IV}$
−0.002
0.000
0.002
0.004
β$_{IV}$

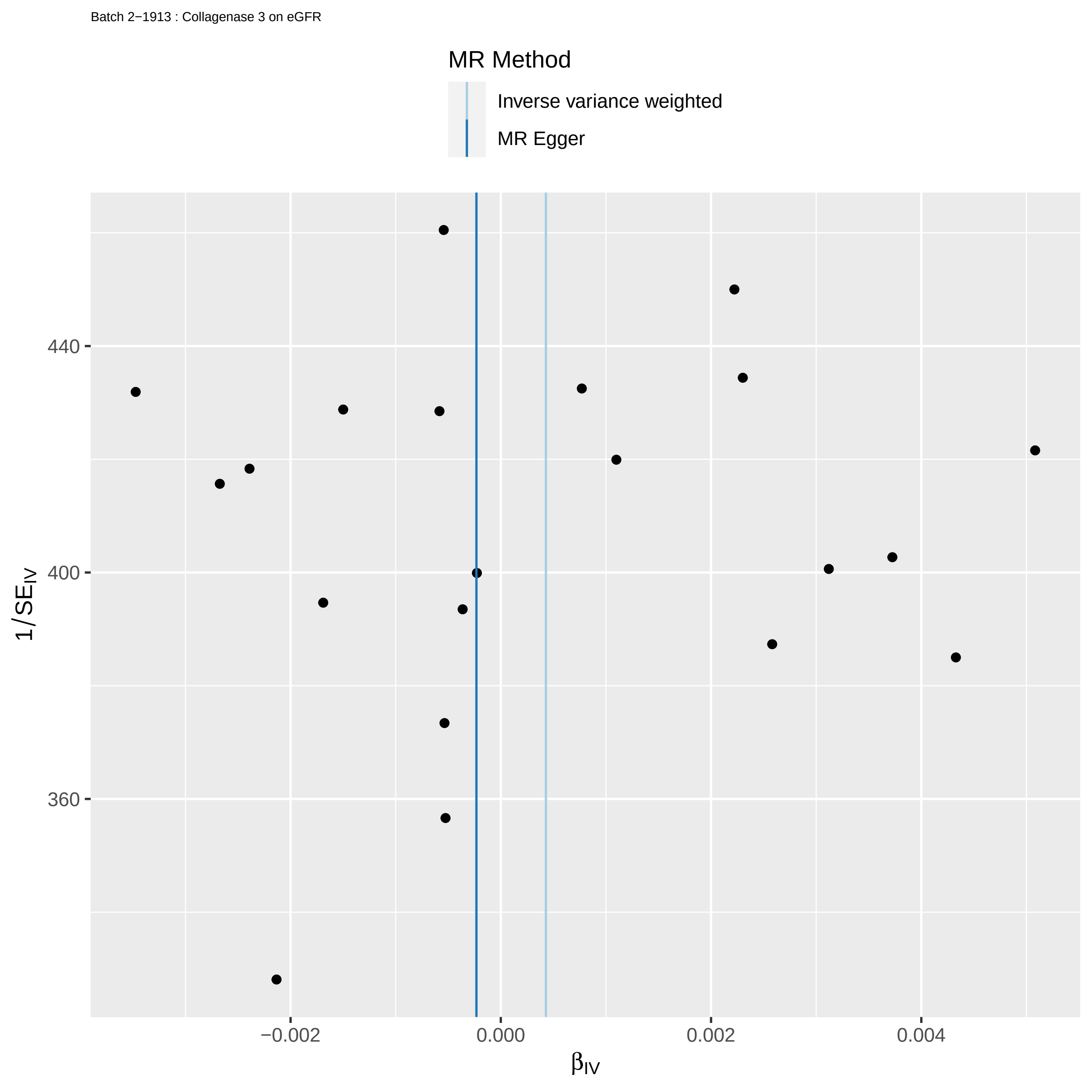
Batch 2−1913 : Collagenase 3 on eGFR
MR Method
Inverse variance weighted
MR Egger
440
400
360
1/SE_IV
−0.002
0.000
0.002
0.004
β_IV

Batch 2−1914 : Matrix metalloproteinase−14 on eGFR

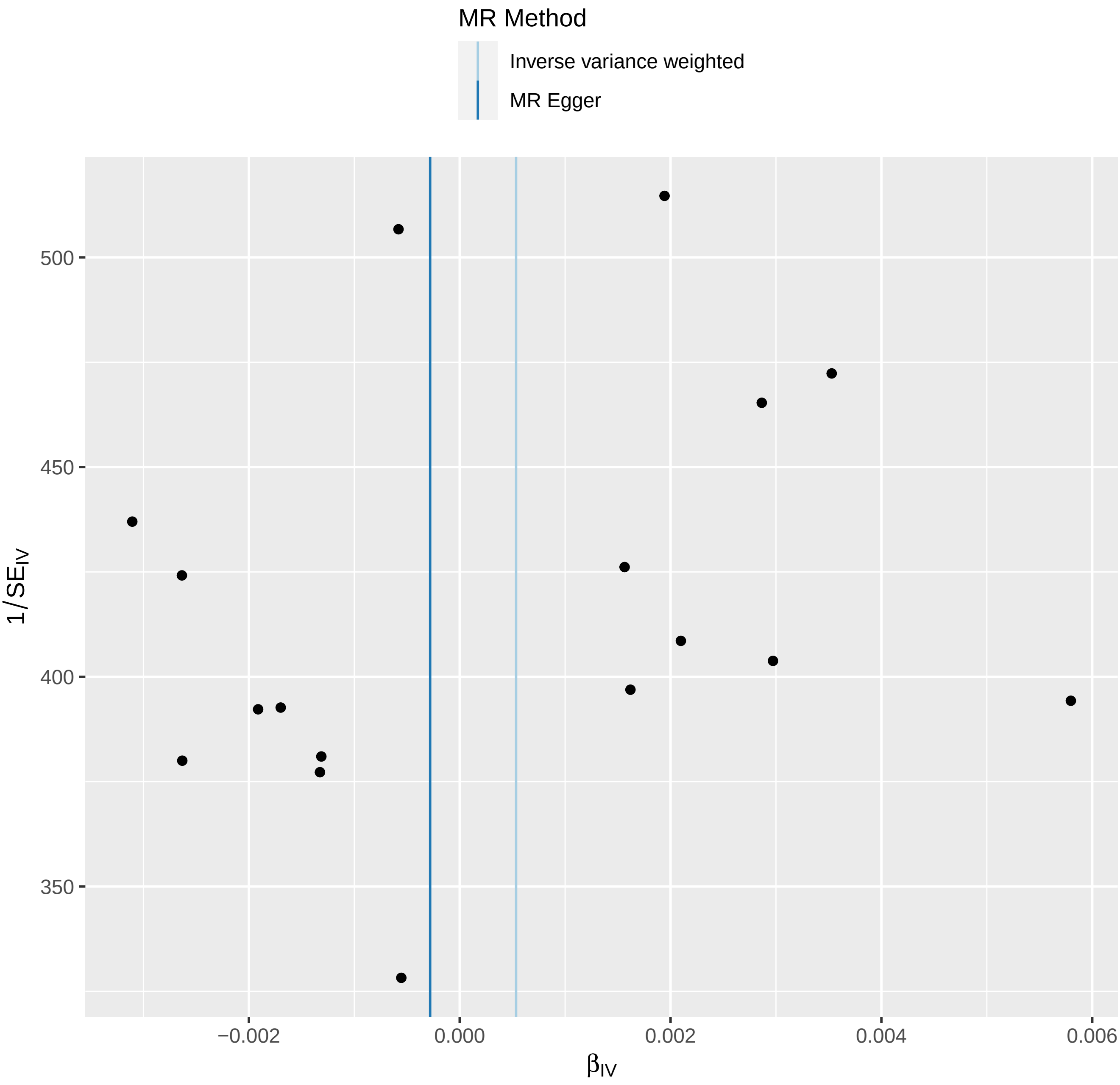

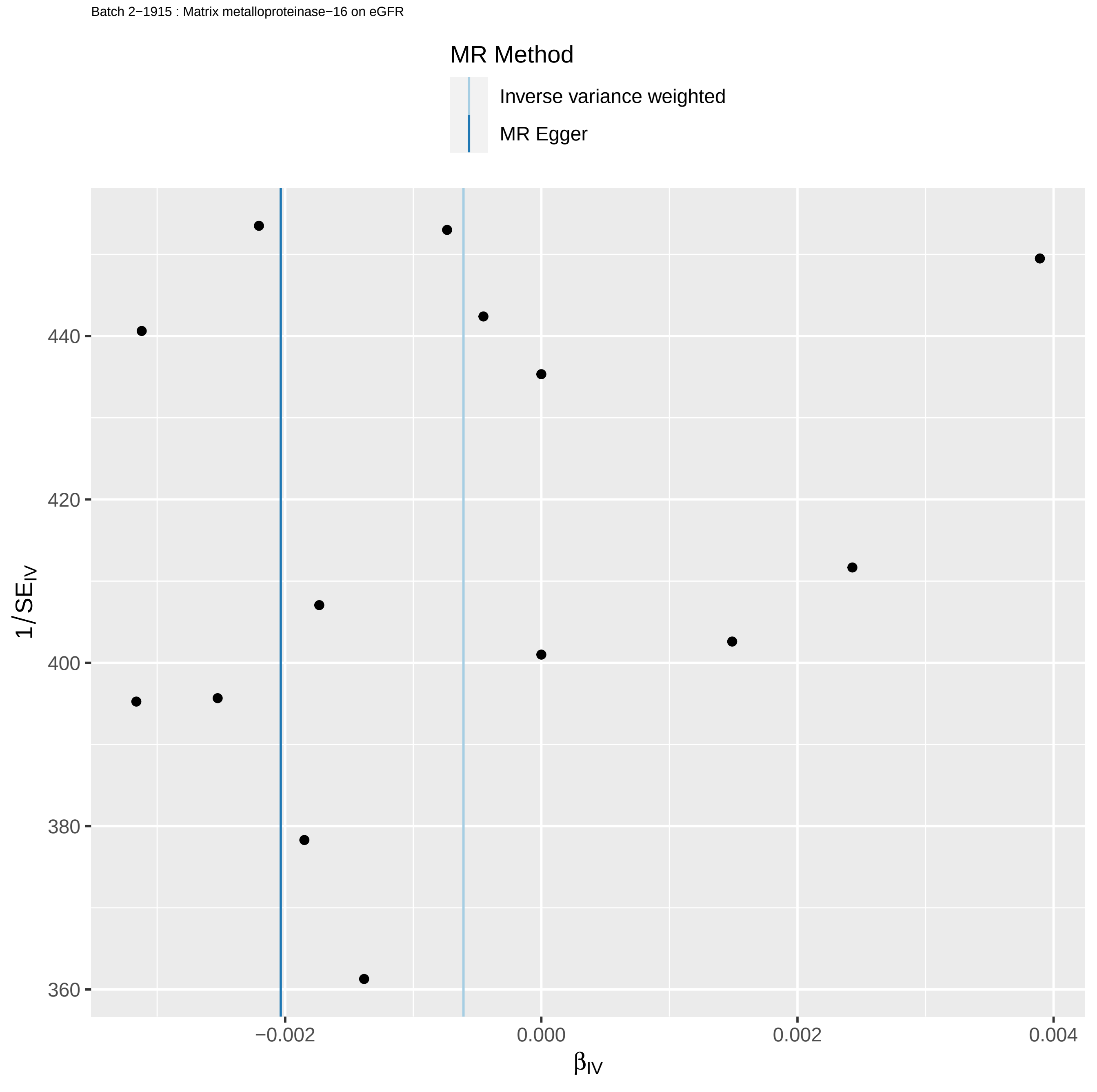
Batch 2−1915 : Matrix metalloproteinase−16 on eGFR
MR Method
Inverse variance weighted
MR Egger
440
420
400
380
360
1/SE_IV
−0.002
0.000
0.002
0.004
β_IV

Batch 2−1916 : Matrix metalloproteinase−16 on eGFR

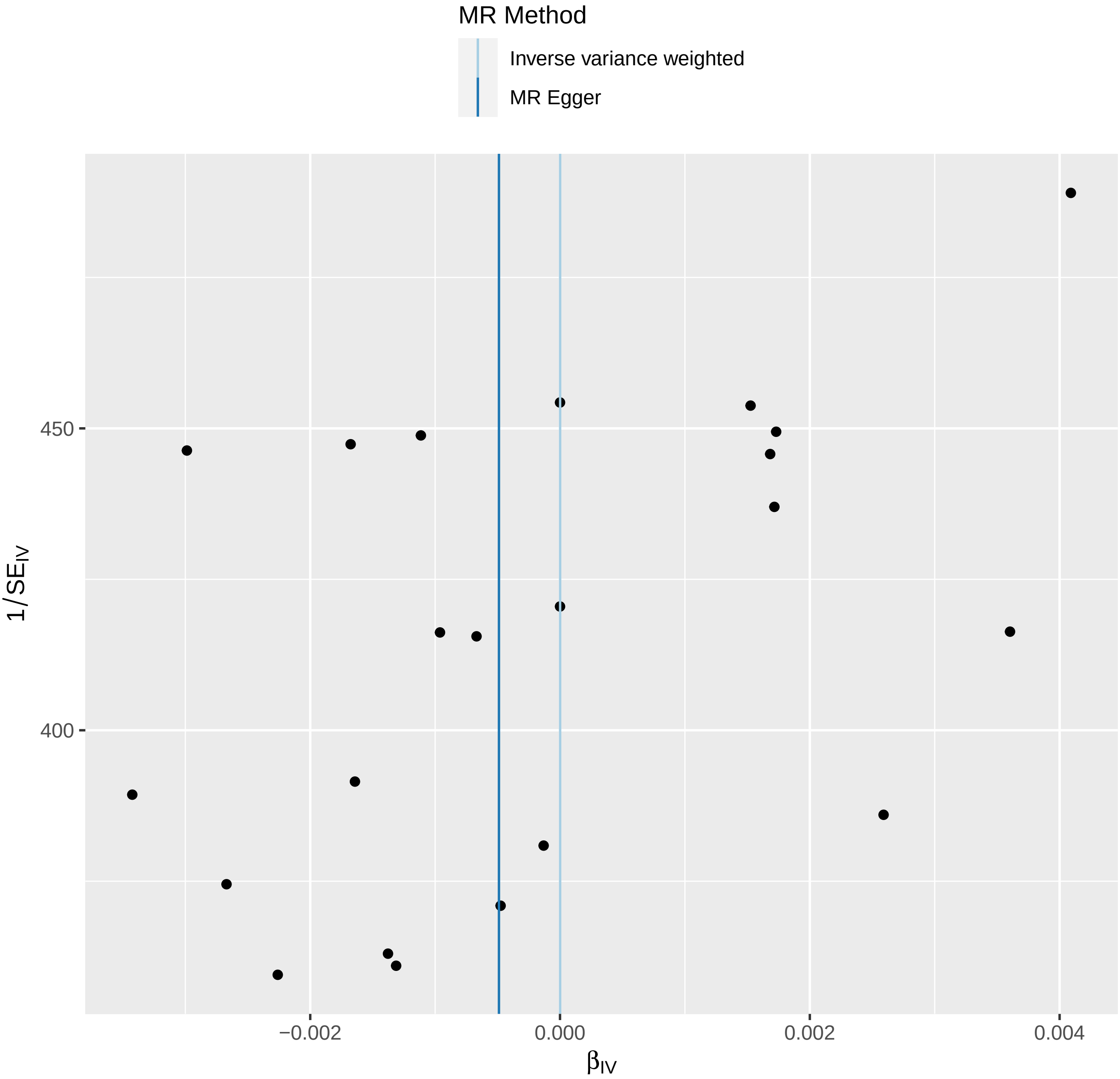

Batch 2−1917 : Matrix metalloproteinase−17 on eGFR

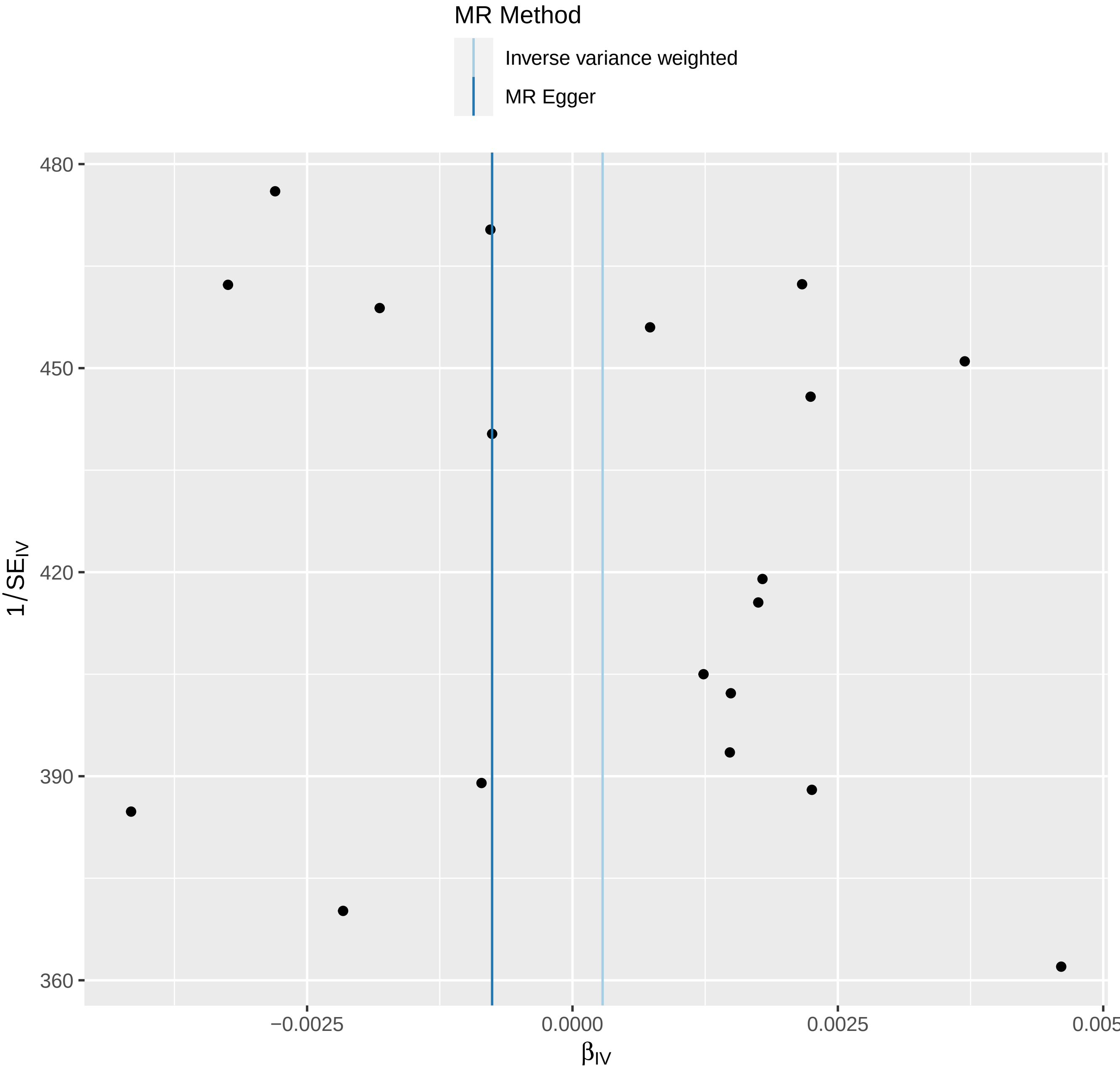

Batch 2−1918 : 72 kDa type IV collagenase on eGFR

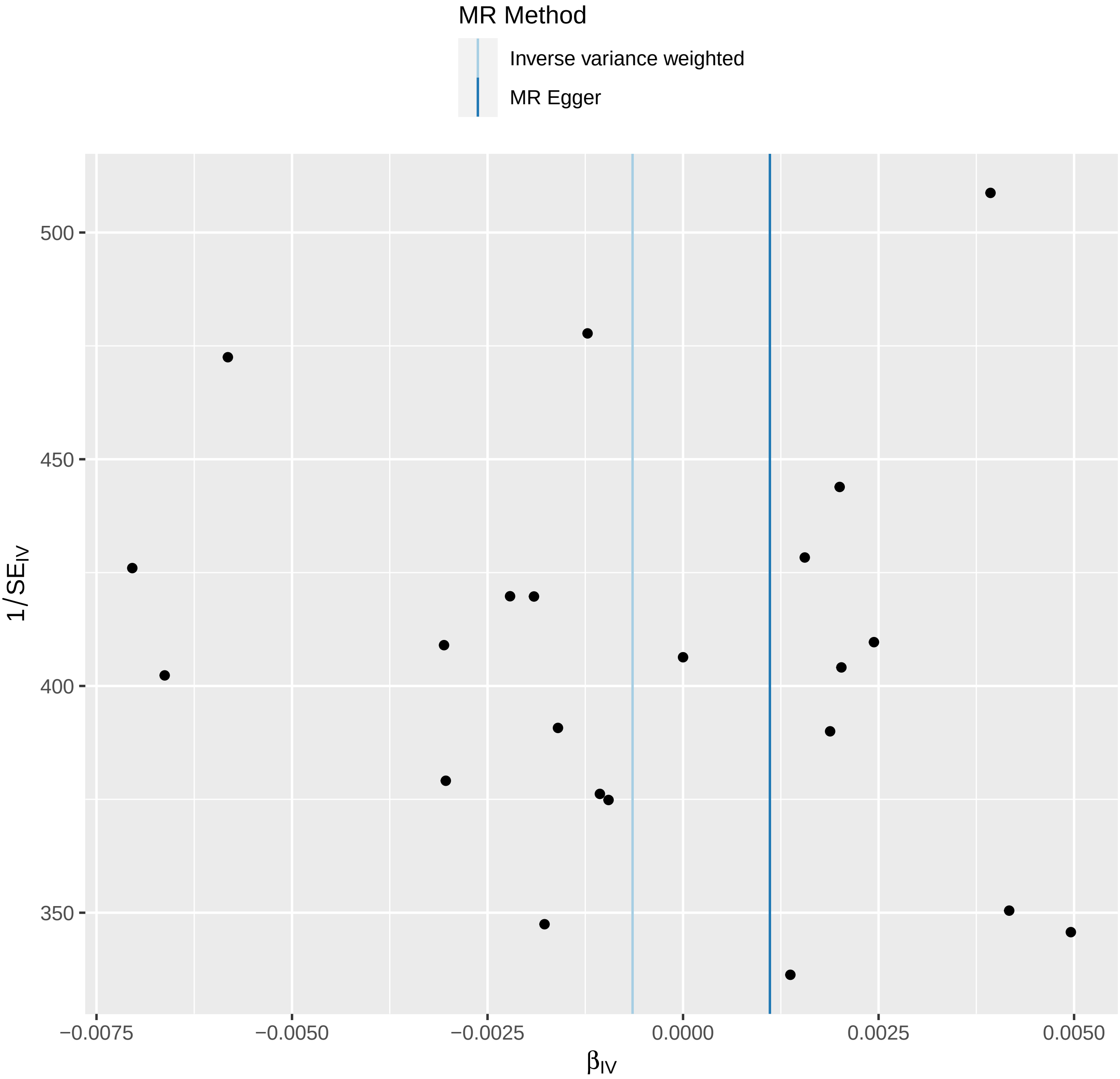

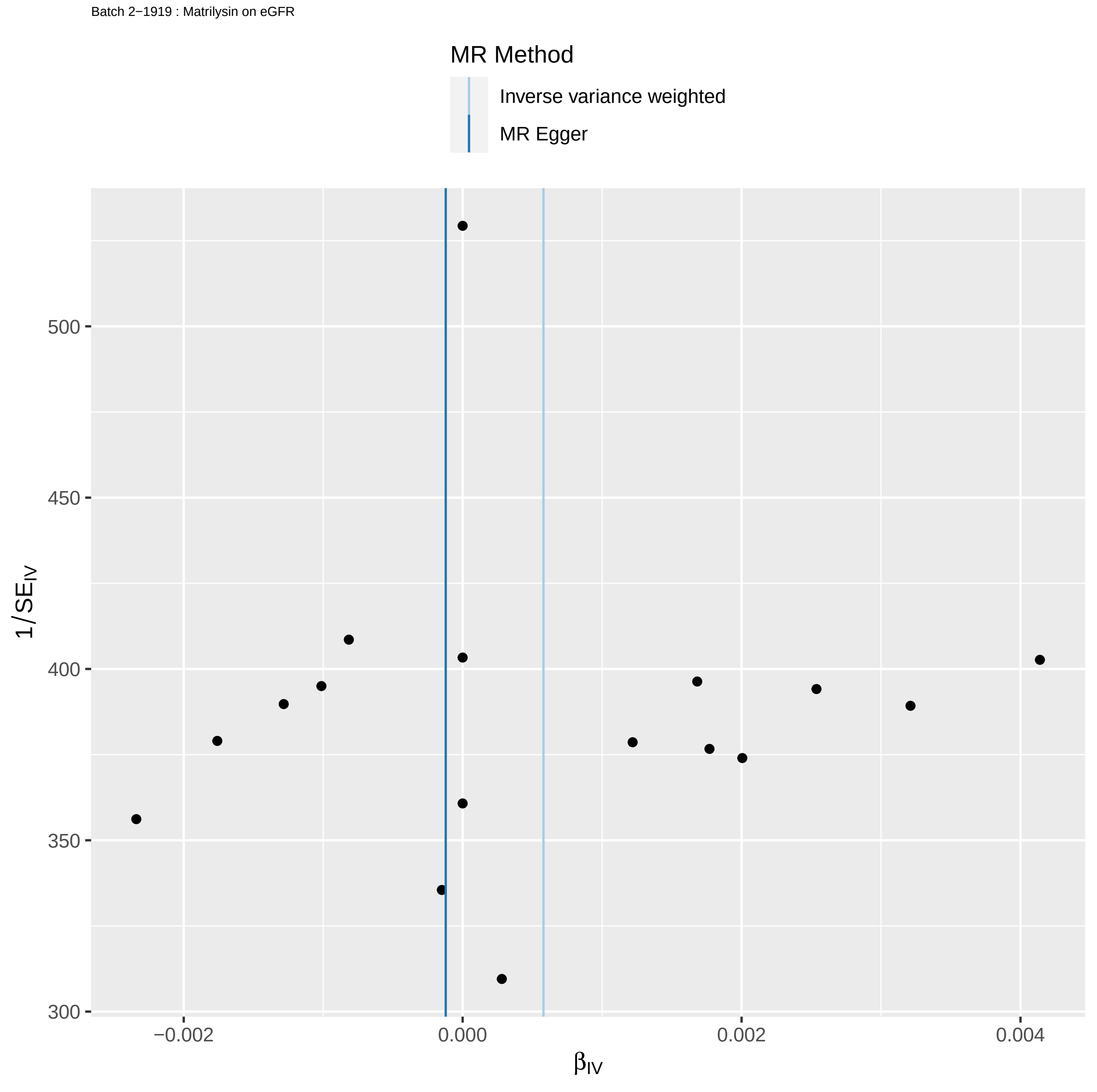

Batch 2−1919 : Matrilysin on eGFR
MR Method
Inverse variance weighted
MR Egger
1/SE_IV
500
450
400
350
300
−0.002
0.000
0.002
0.004
β_IV

Batch 2−1920 : Neutrophil collagenase on eGFR

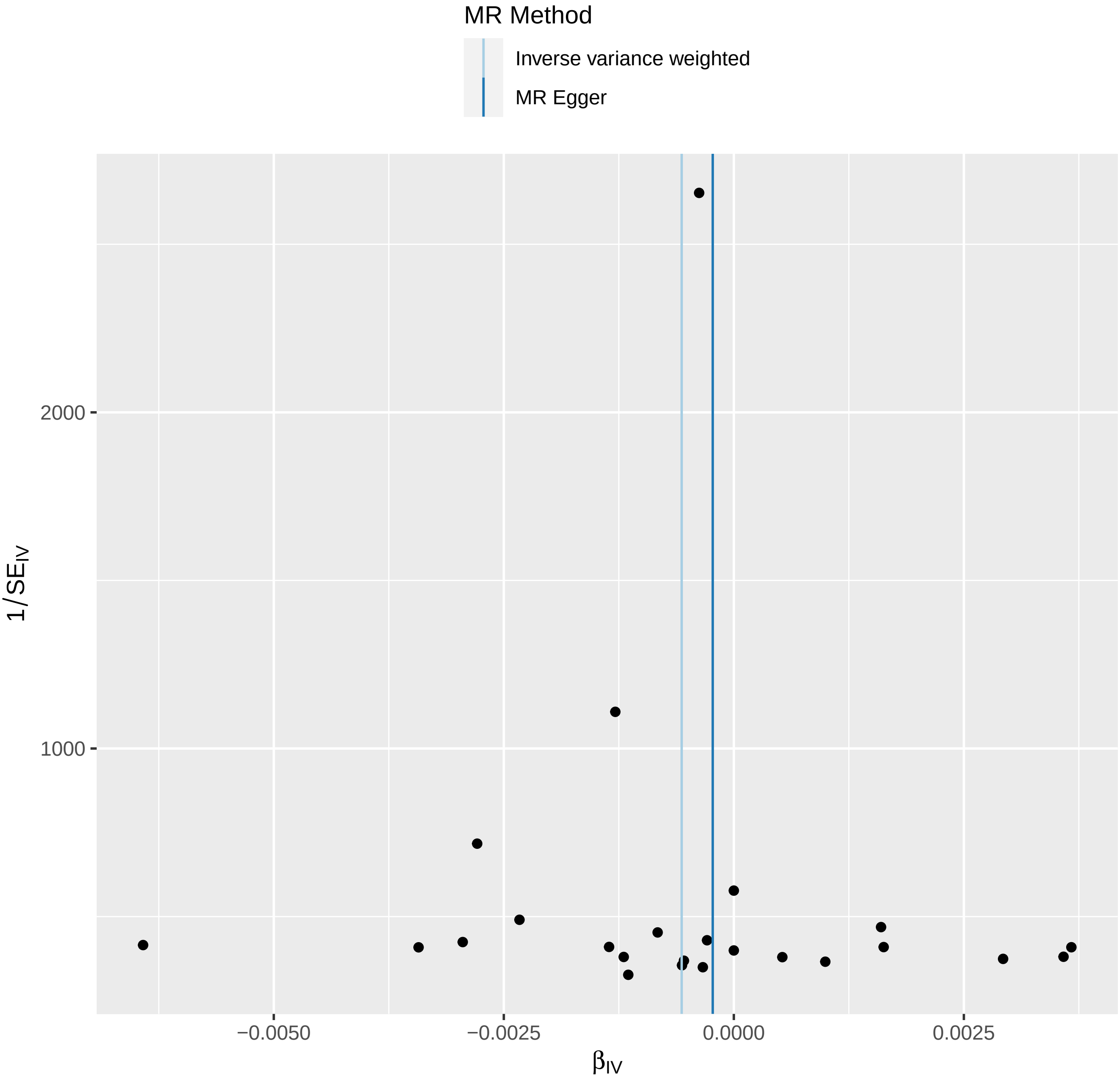

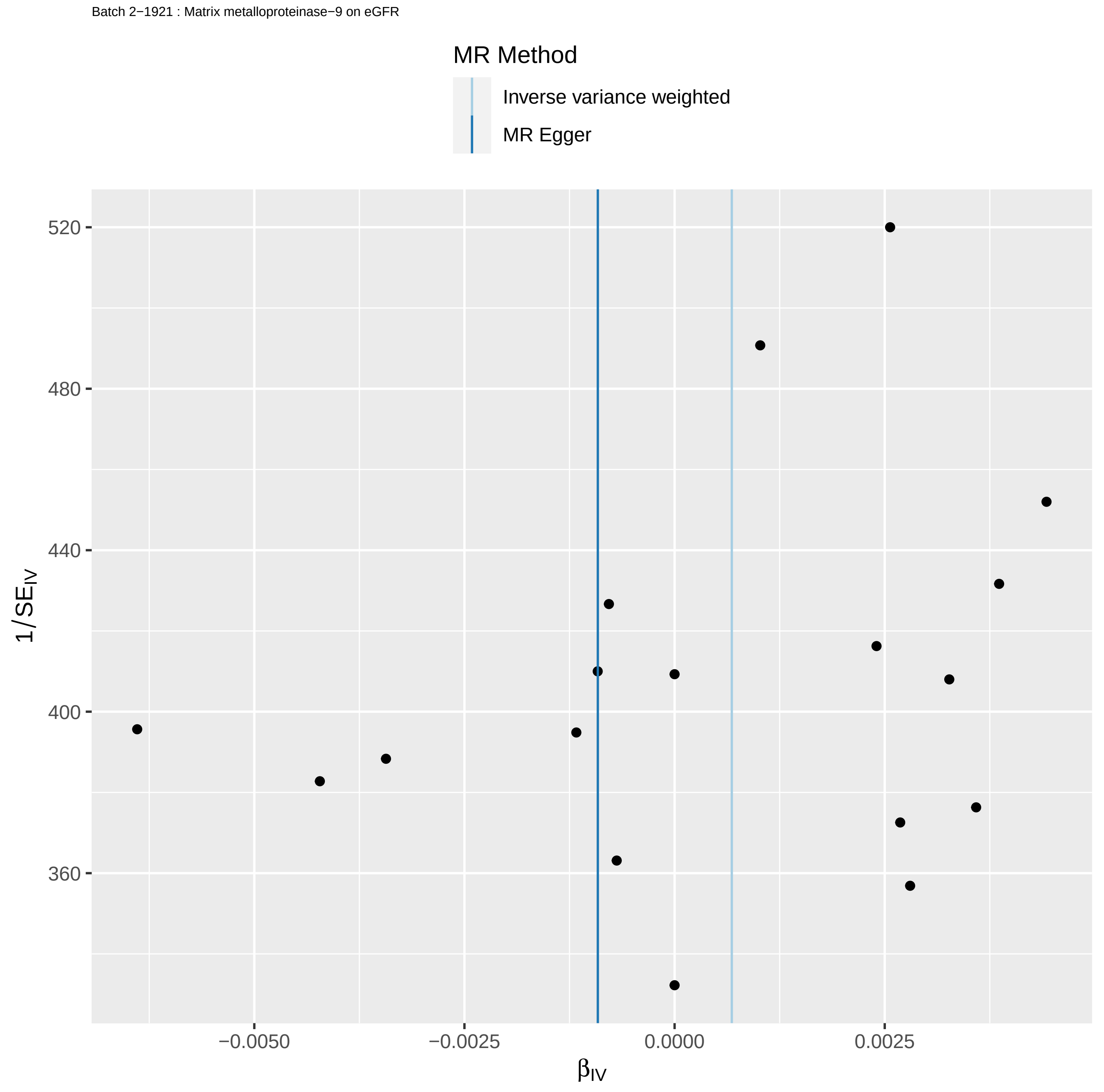
Batch 2−1921 : Matrix metalloproteinase−9 on eGFR
MR Method
Inverse variance weighted
MR Egger
520
480
440
400
360
1/SE_IV
−0.0050
−0.0025
0.0000
0.0025
β_IV

Batch 2−1922 : Multimerin−2 on eGFR

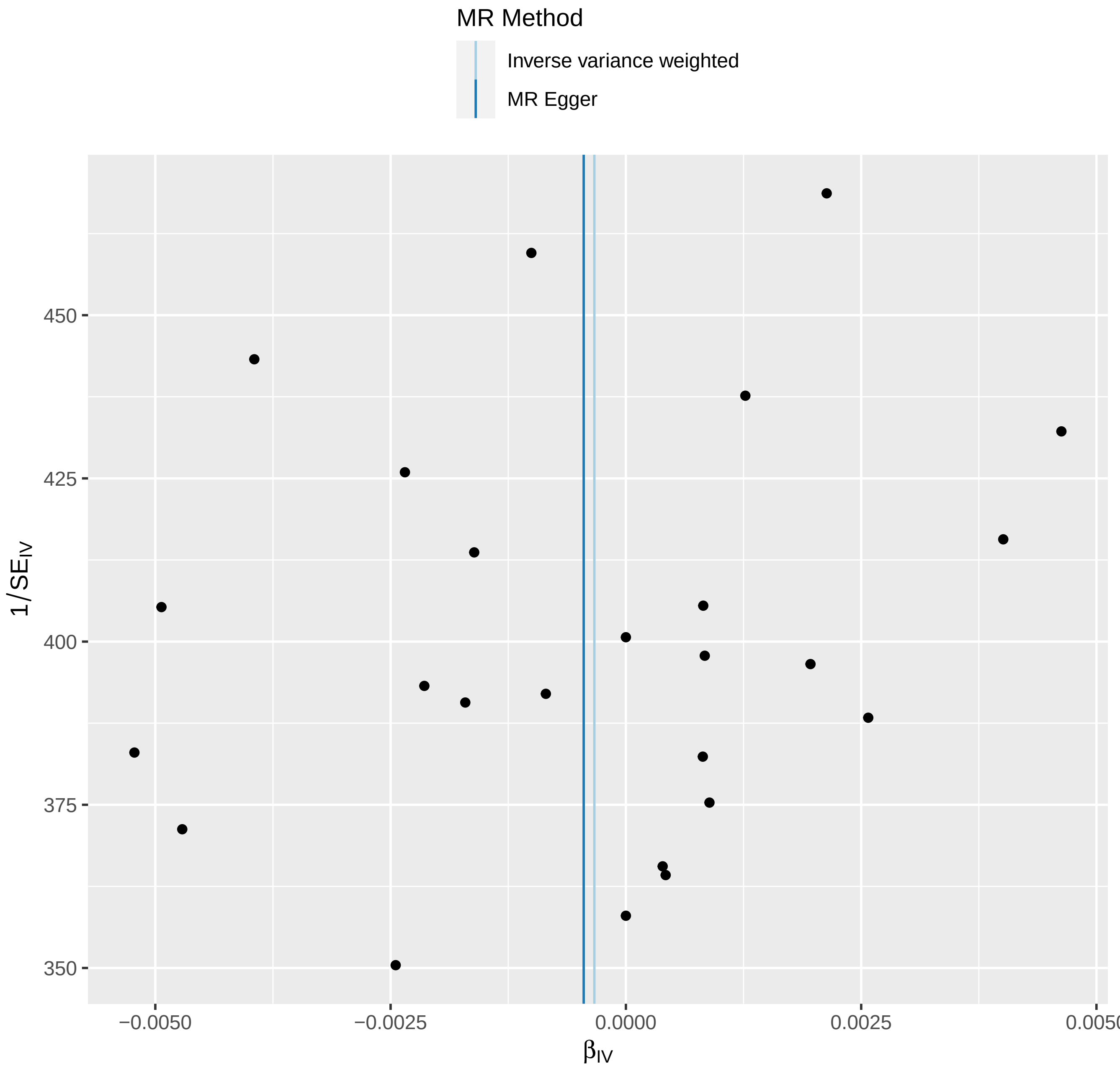

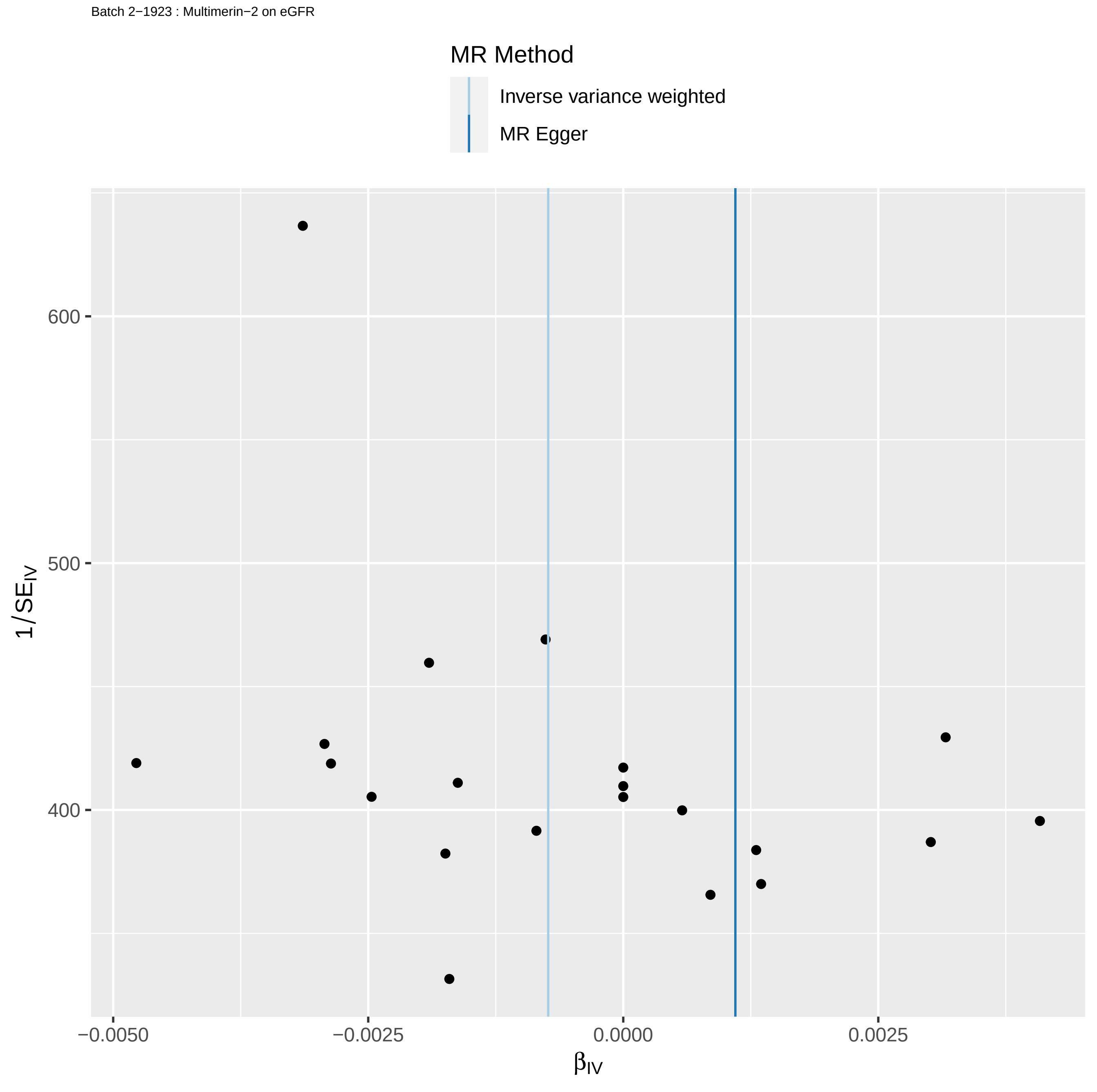
Batch 2−1923 : Multimerin−2 on eGFR
MR Method
Inverse variance weighted
MR Egger
600
500
400
1/SE_IV
−0.0050
−0.0025
0.0000
0.0025
β_IV

Batch 2−1924 : Motor neuron and pancreas homeobox protein 1 on eGFR

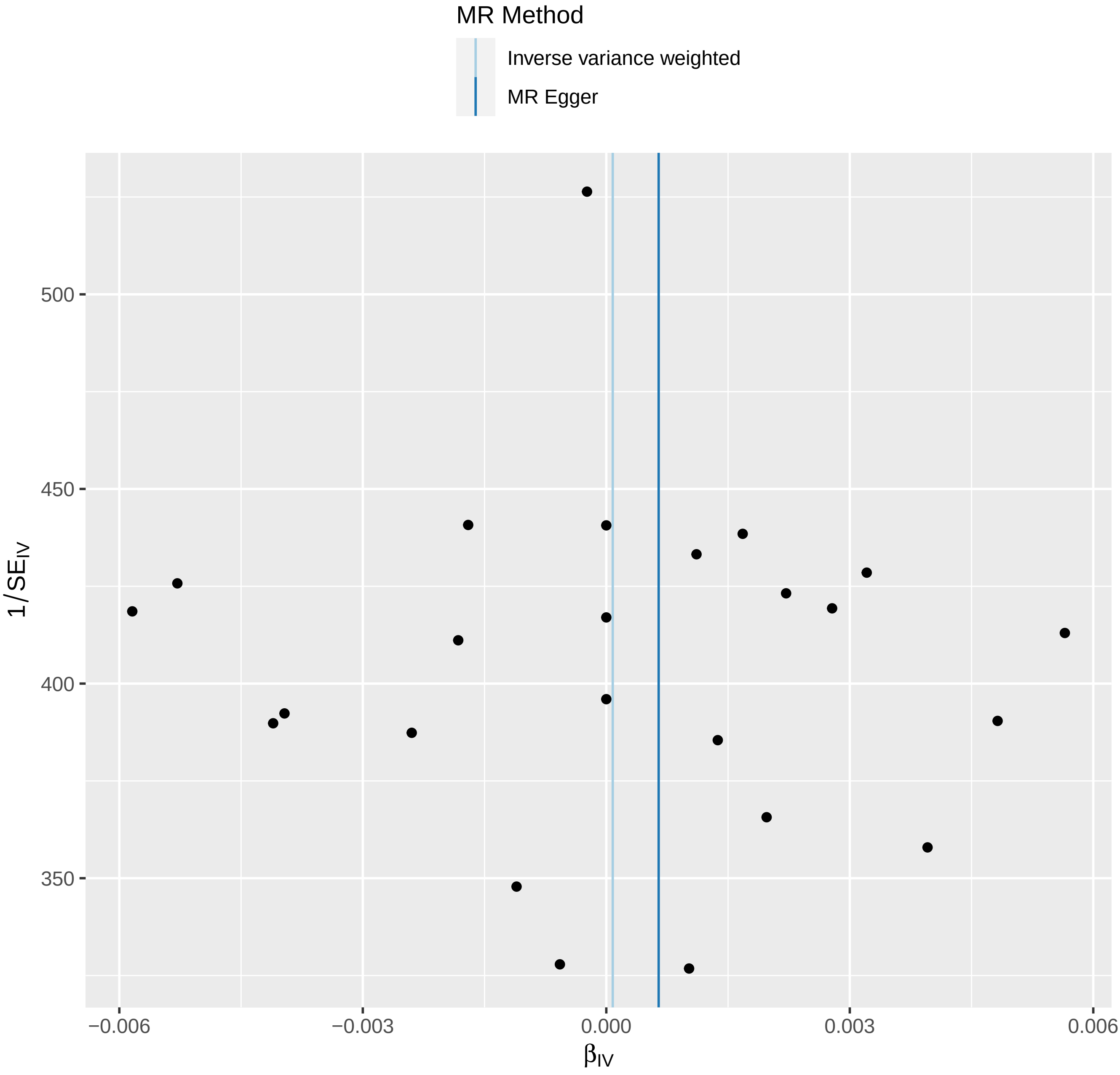

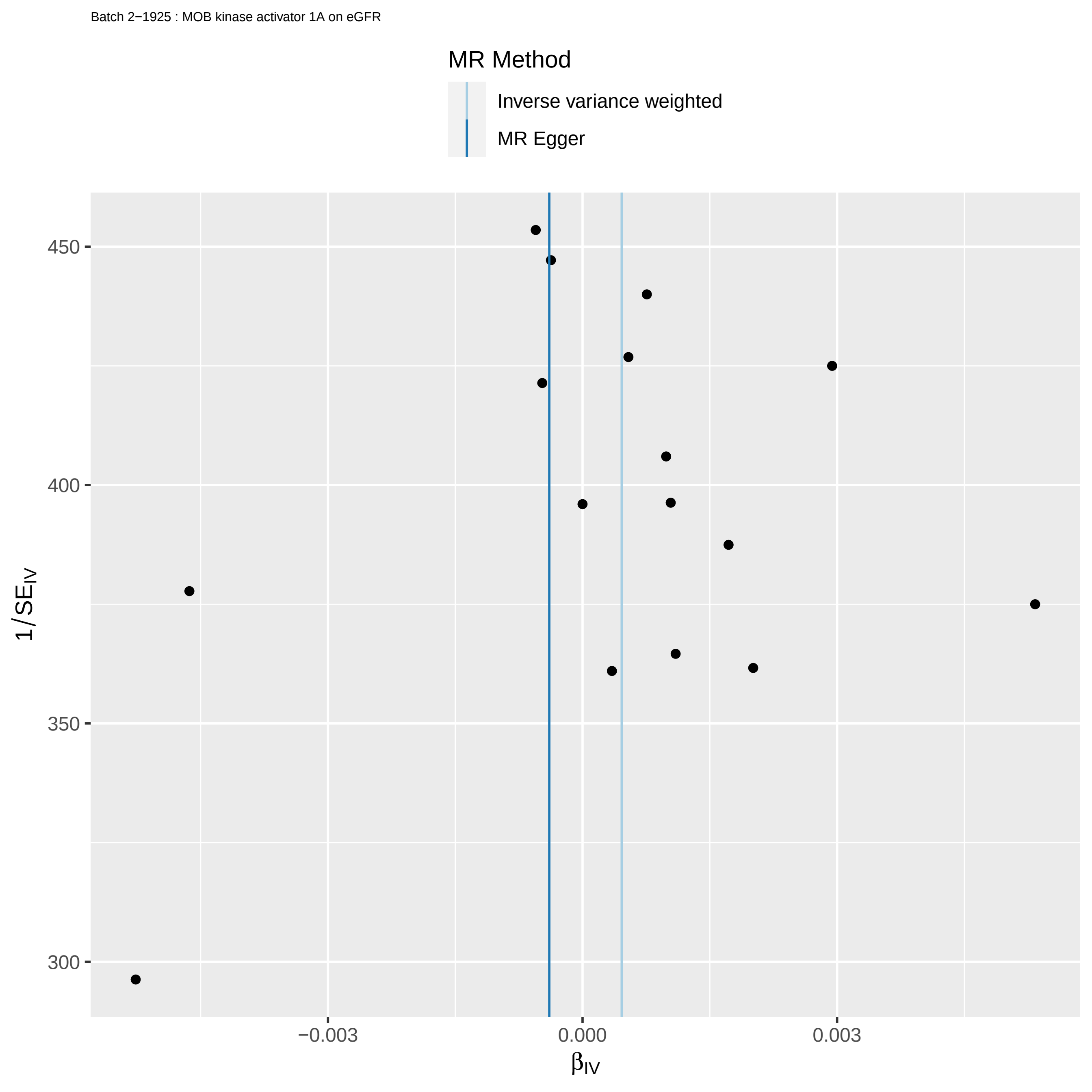

Batch 2−1925 : MOB kinase activator 1A on eGFR
MR Method
Inverse variance weighted
MR Egger
450
400
350
300
1/SE_IV
−0.003
0.000
0.003
β_IV

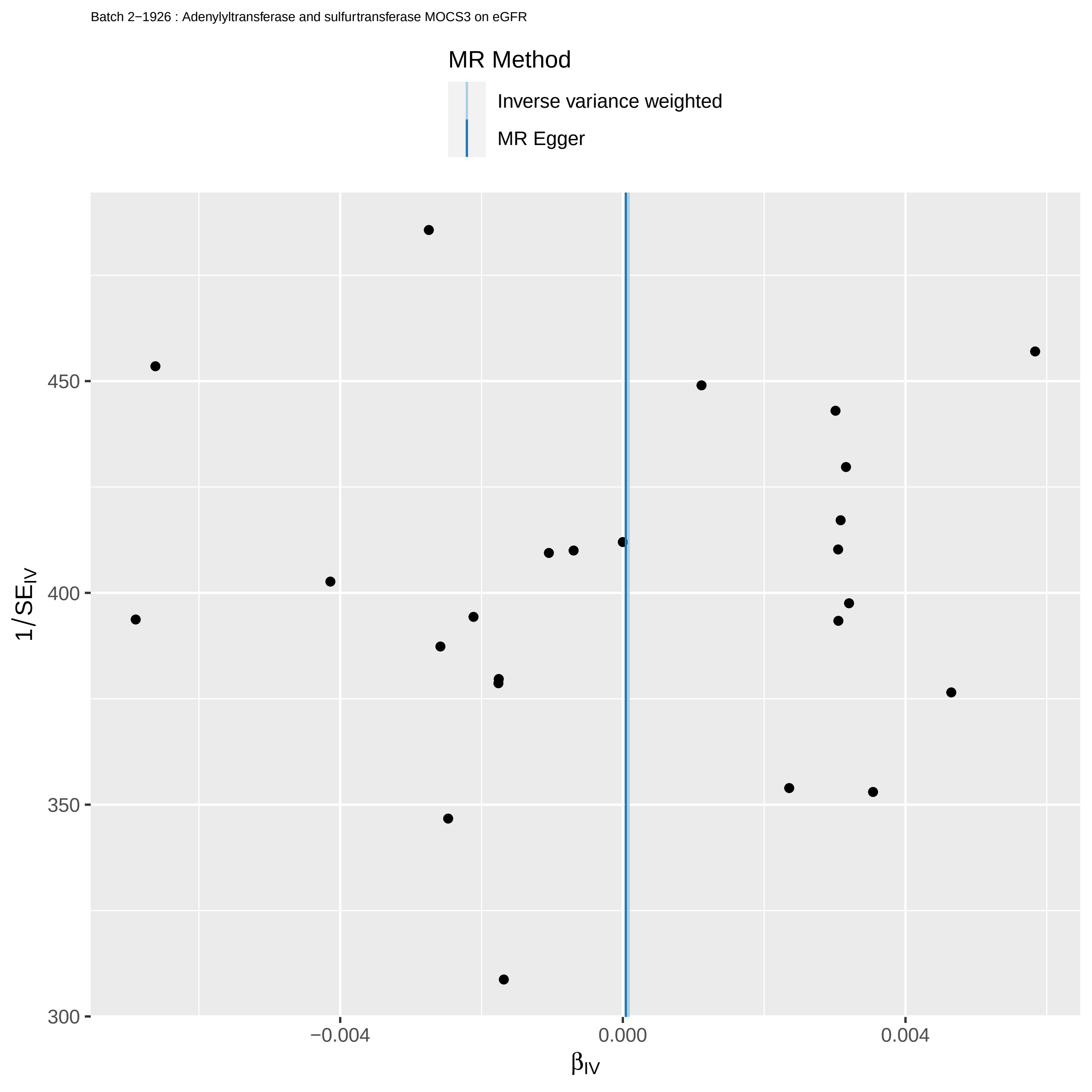

Batch 2−1926 : Adenylyltransferase and sulfurtransferase MOCS3 on eGFR
MR Method
Inverse variance weighted
MR Egger
1/SE_IV
450
400
350
300
−0.004
0.000
0.004
β_IV

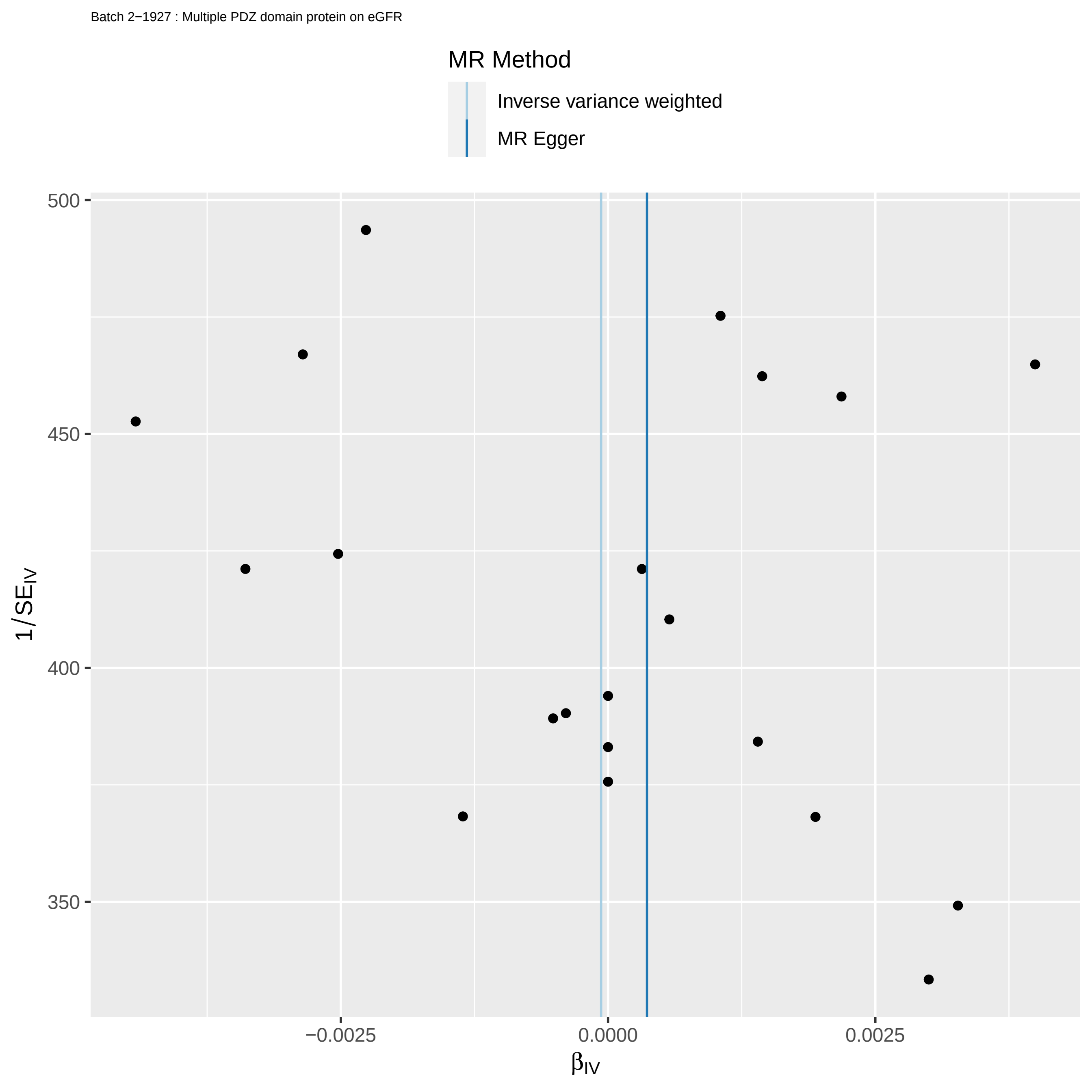
Batch 2−1927 : Multiple PDZ domain protein on eGFR
MR Method
Inverse variance weighted
MR Egger
500
450
400
350
1/SE$_{IV}$
−0.0025
0.0000
0.0025
β_{IV}

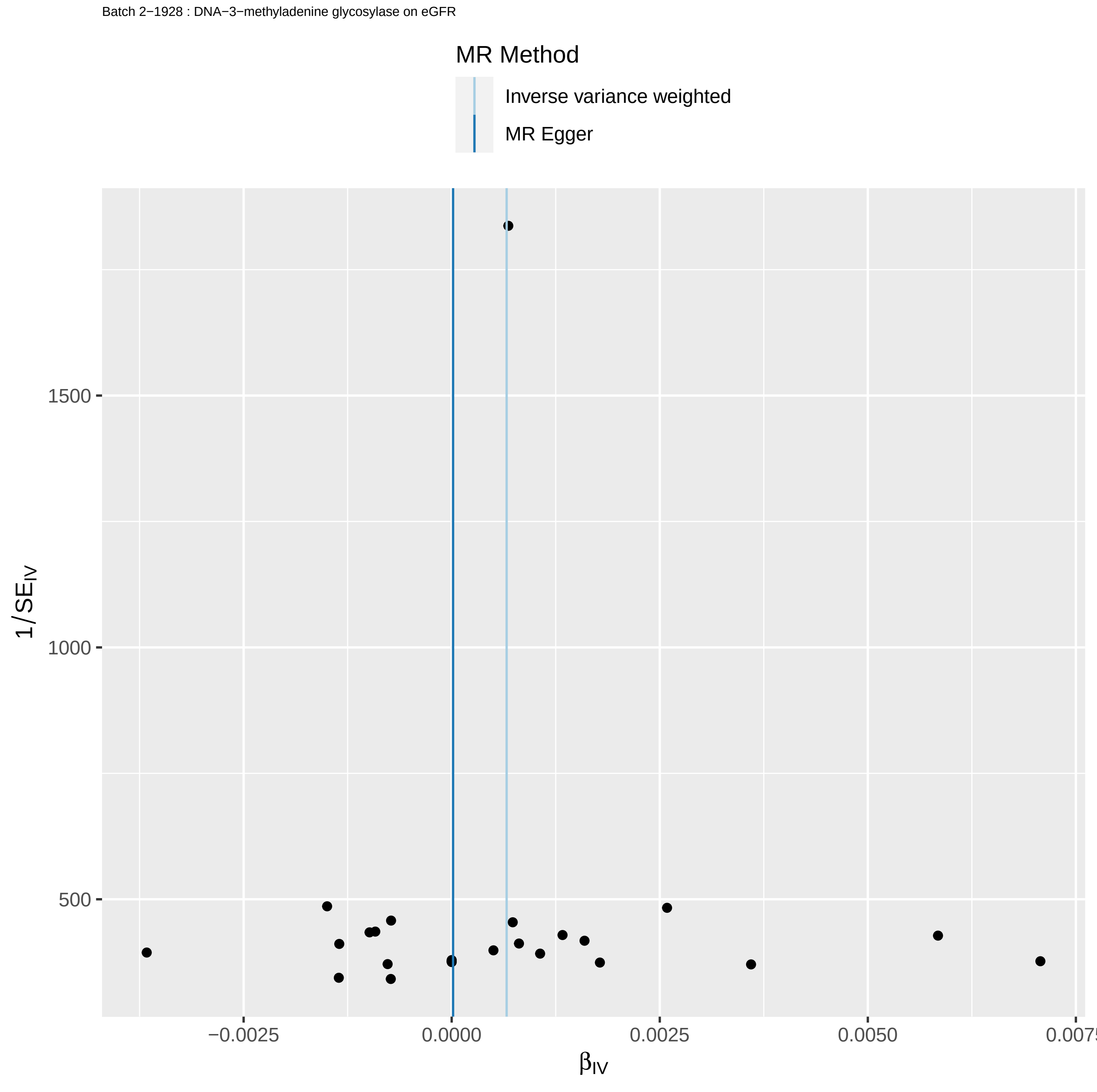
Batch 2−1928 : DNA−3−methyladenine glycosylase on eGFR
MR Method
Inverse variance weighted
MR Egger
1500
1000
500
1/SE_IV
−0.0025
0.0000
0.0025
0.0050
0.0075
β_IV

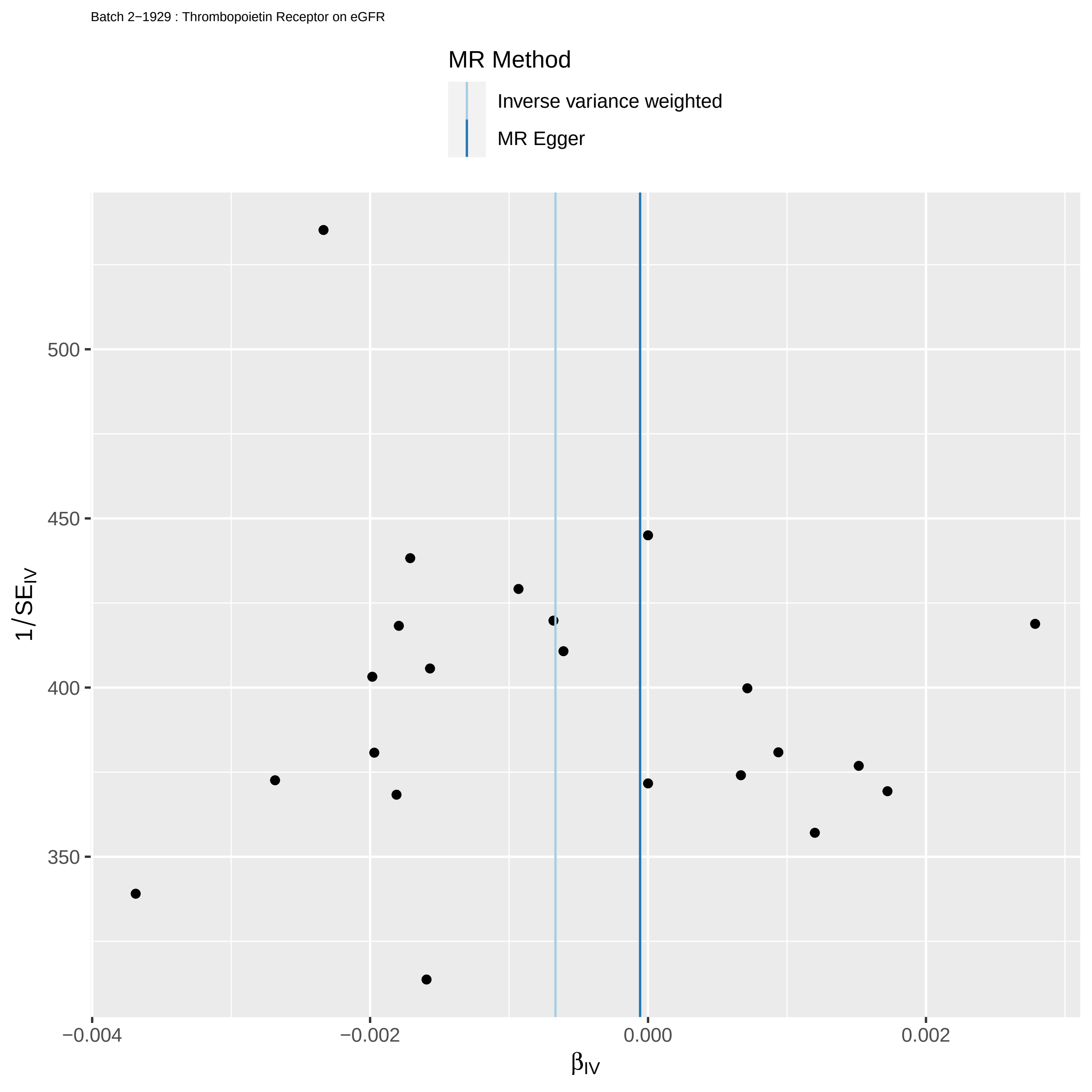
Batch 2−1929 : Thrombopoietin Receptor on eGFR
MR Method
Inverse variance weighted
MR Egger
500
450
400
350
1/SE$_{IV}$
−0.004
−0.002
0.000
0.002
β$_{IV}$

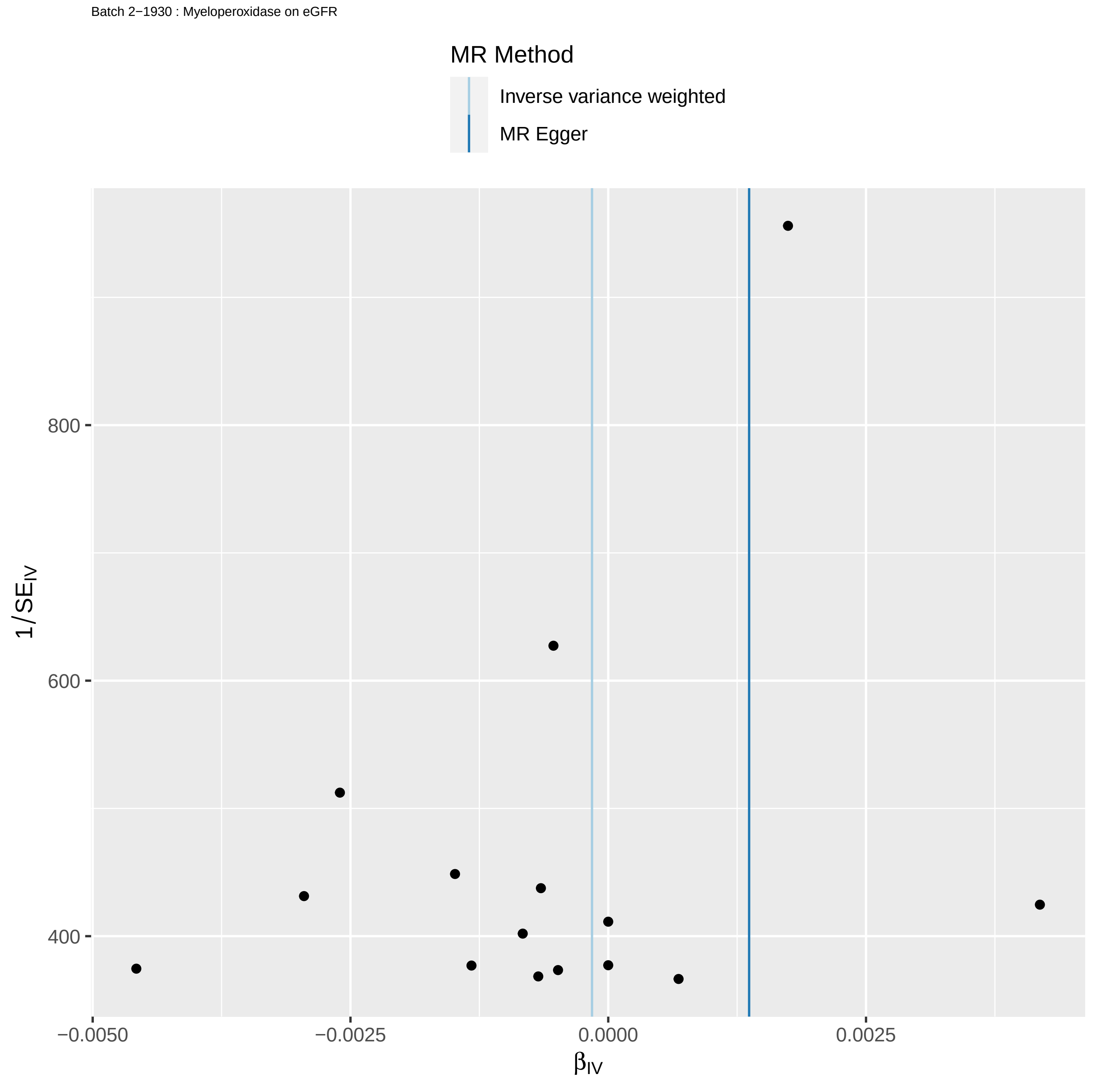
Batch 2−1930 : Myeloperoxidase on eGFR
MR Method
Inverse variance weighted
MR Egger
800
600
400
1/SE_IV
−0.0050
−0.0025
0.0000
0.0025
β_IV

Batch 2−1931 : MAGUK p55 subfamily member 6 on eGFR

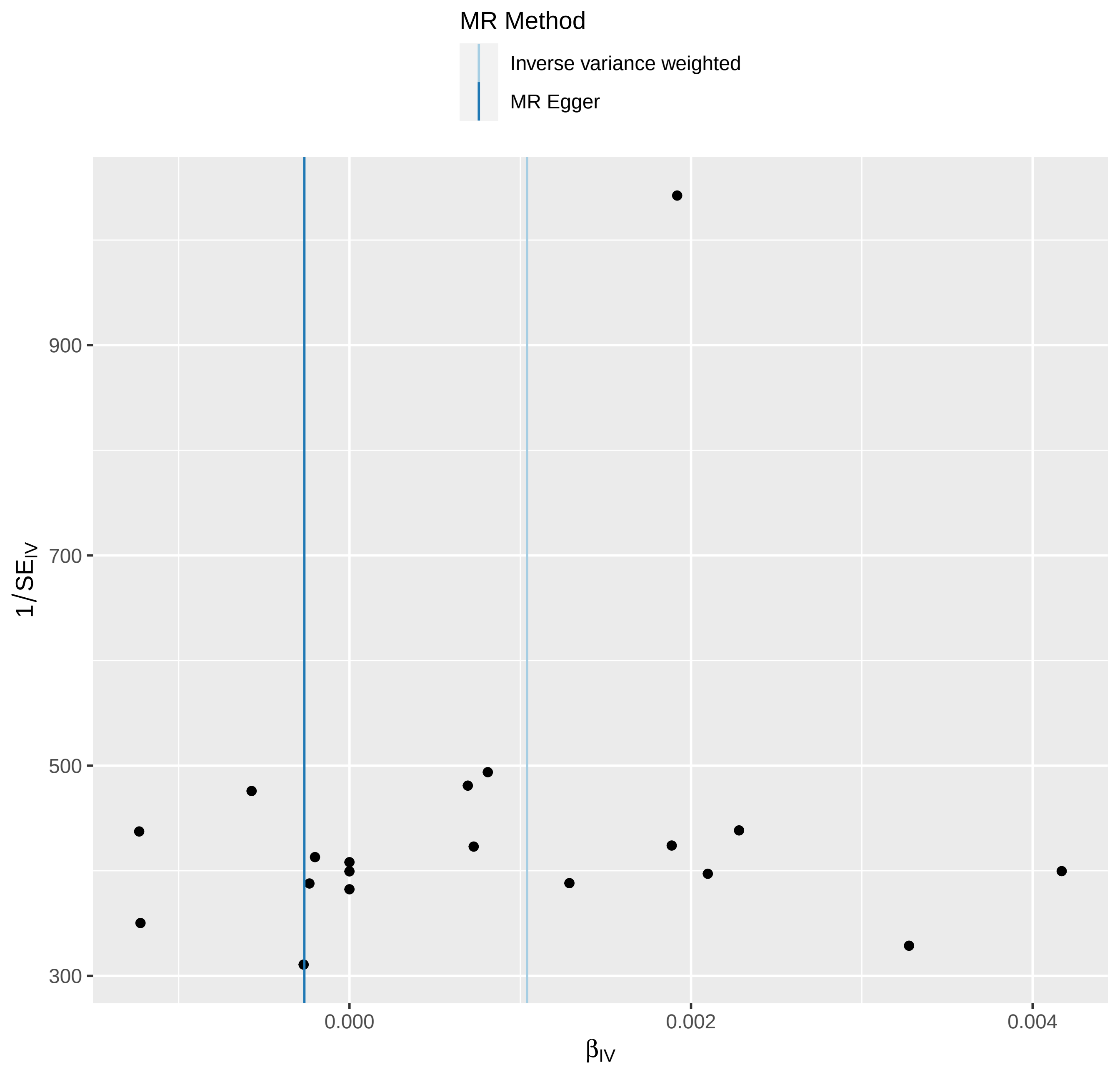

Batch 2−1932 : MAGUK p55 subfamily member 7 on eGFR

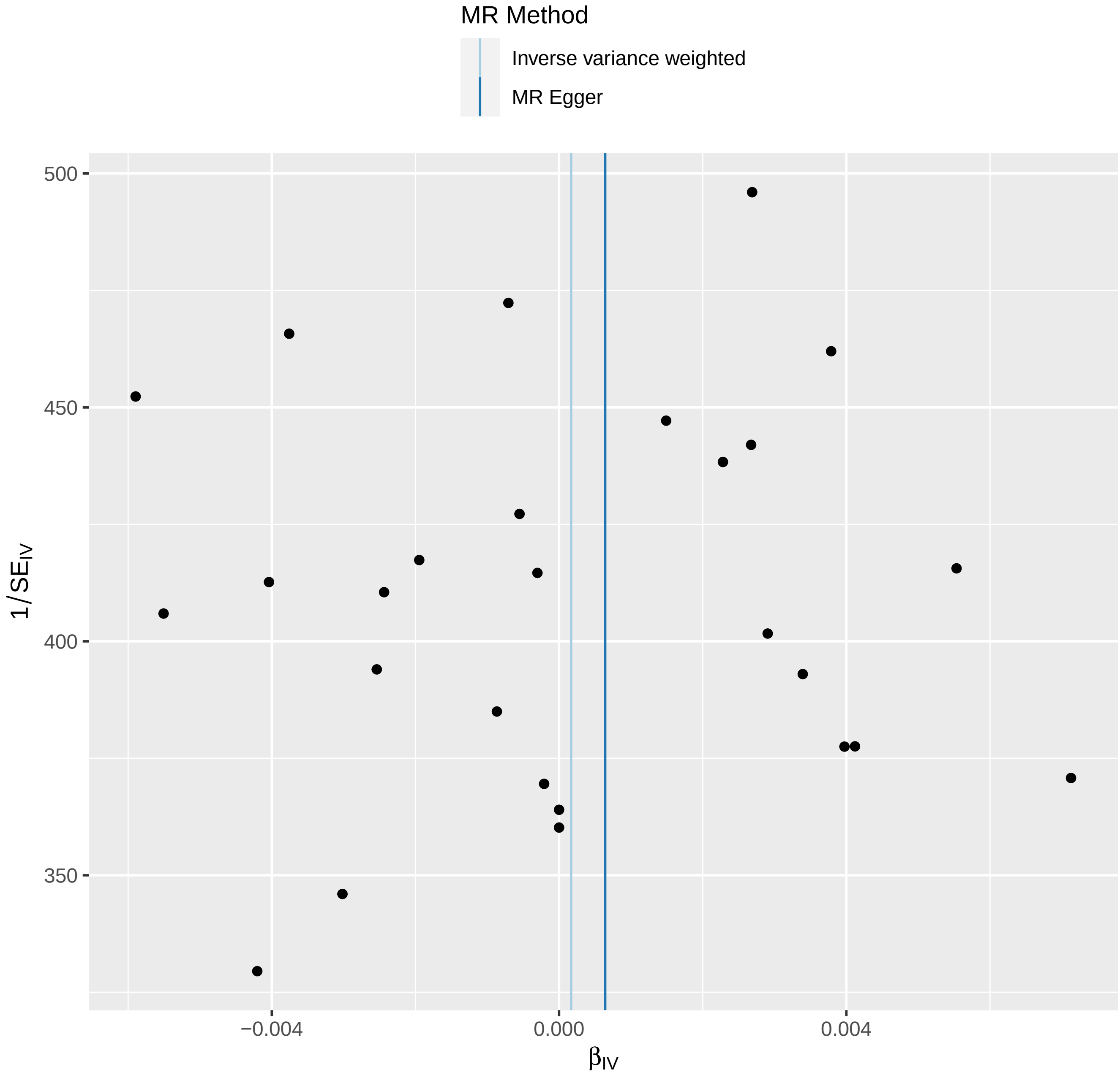

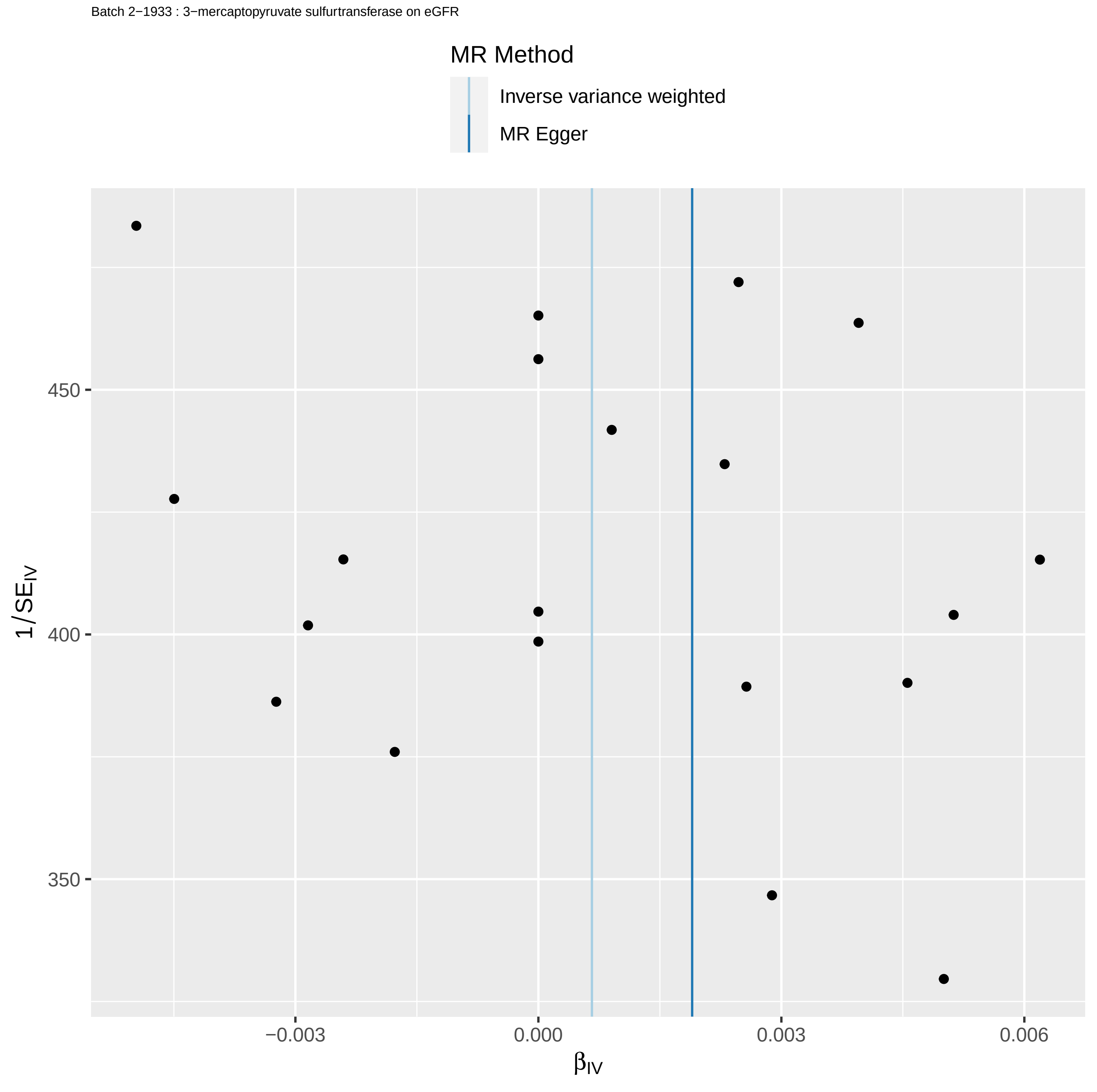
Batch 2−1933 : 3−mercaptopyruvate sulfurtransferase on eGFR
MR Method
Inverse variance weighted
MR Egger
450
400
350
1/SE_IV
−0.003
0.000
0.003
0.006
β_IV

Batch 2−1934 : Myelin protein P0 on eGFR

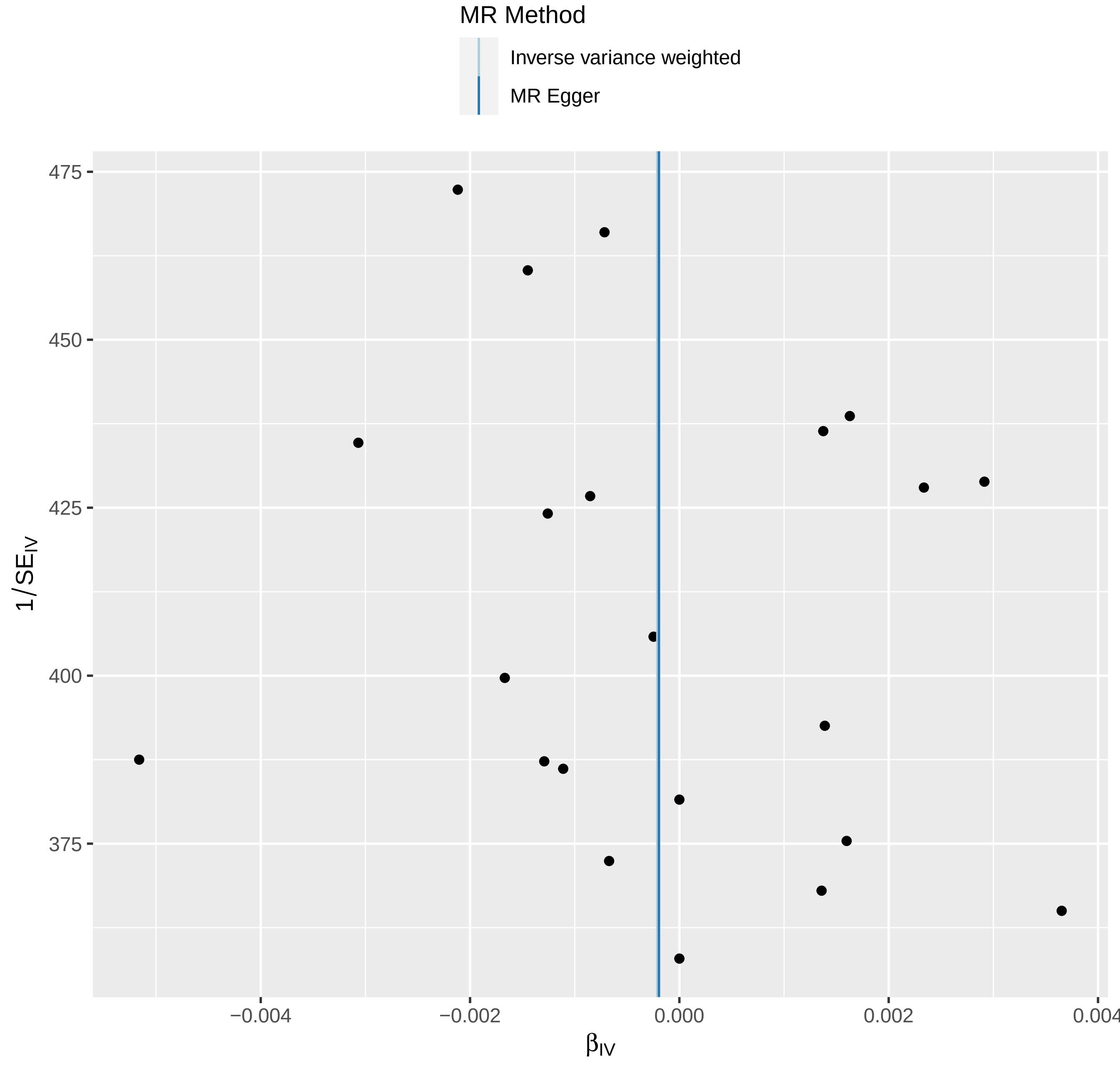

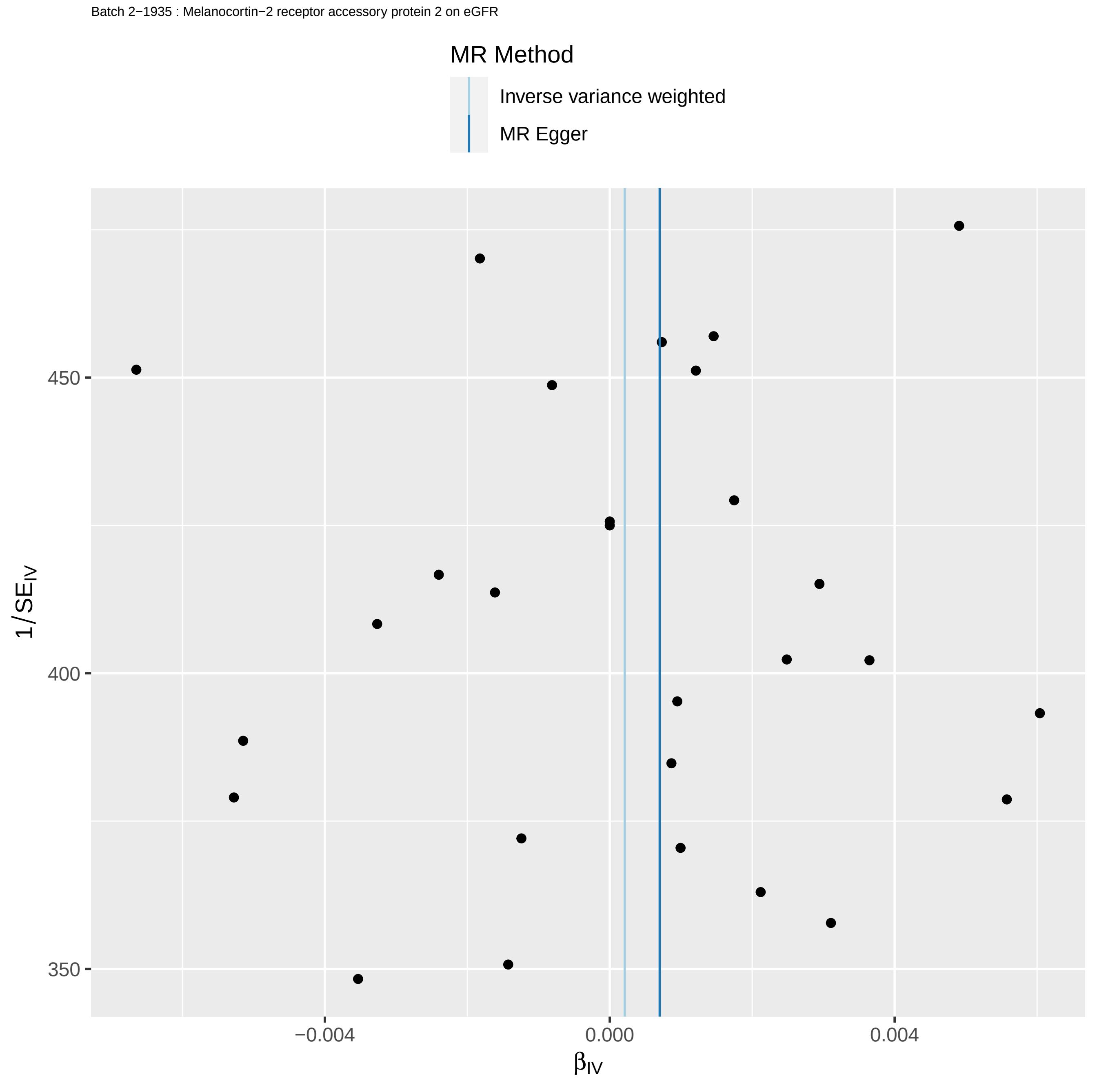

Batch 2−1935 : Melanocortin−2 receptor accessory protein 2 on eGFR
MR Method
Inverse variance weighted
MR Egger
450
400
350
1/SE_IV
−0.004
0.000
0.004
β_IV

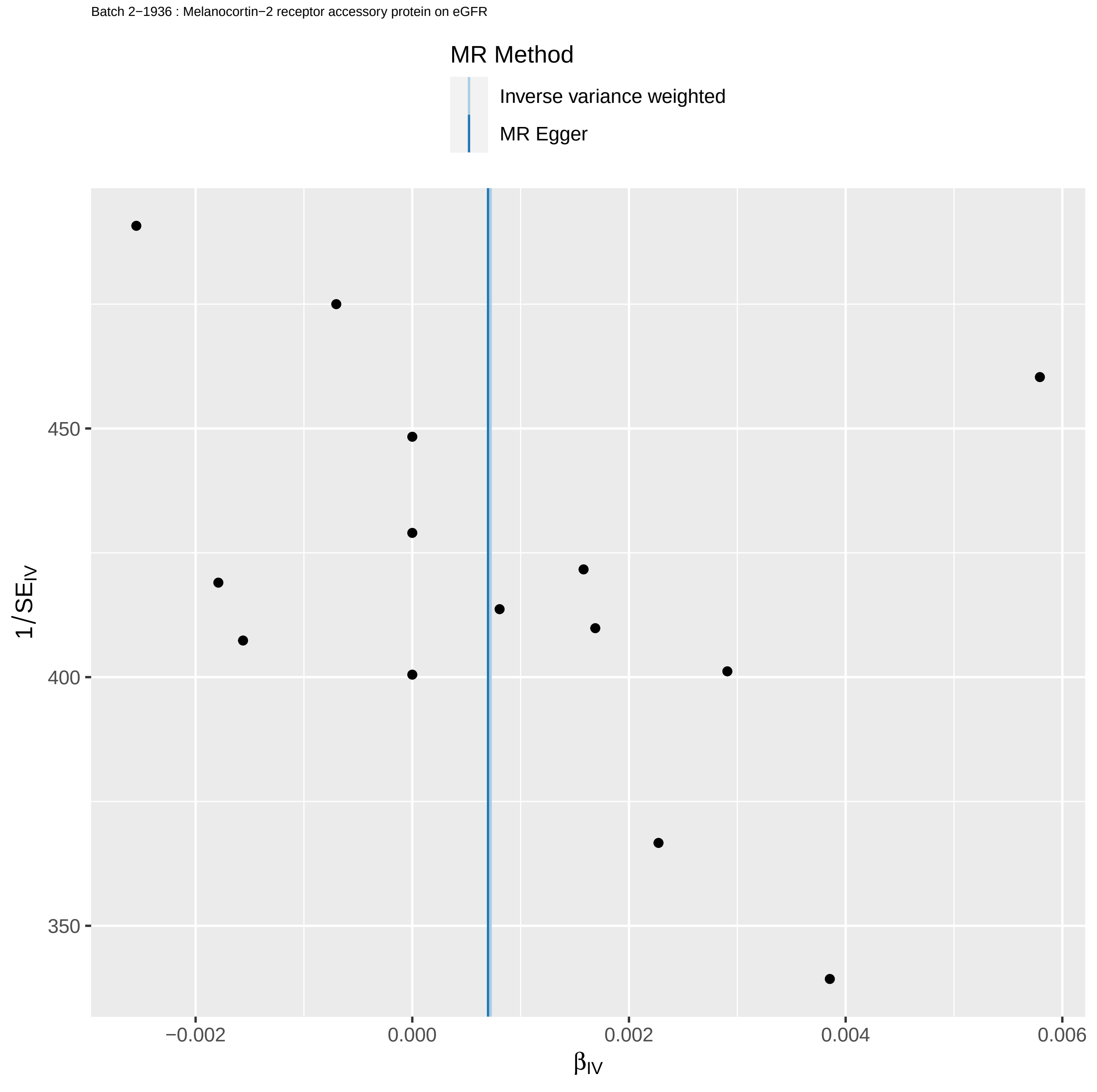
Batch 2−1936 : Melanocortin−2 receptor accessory protein on eGFR
MR Method
Inverse variance weighted
MR Egger
450
400
350
1/SE$_{IV}$
−0.002
0.000
0.002
0.004
0.006
β$_{IV}$

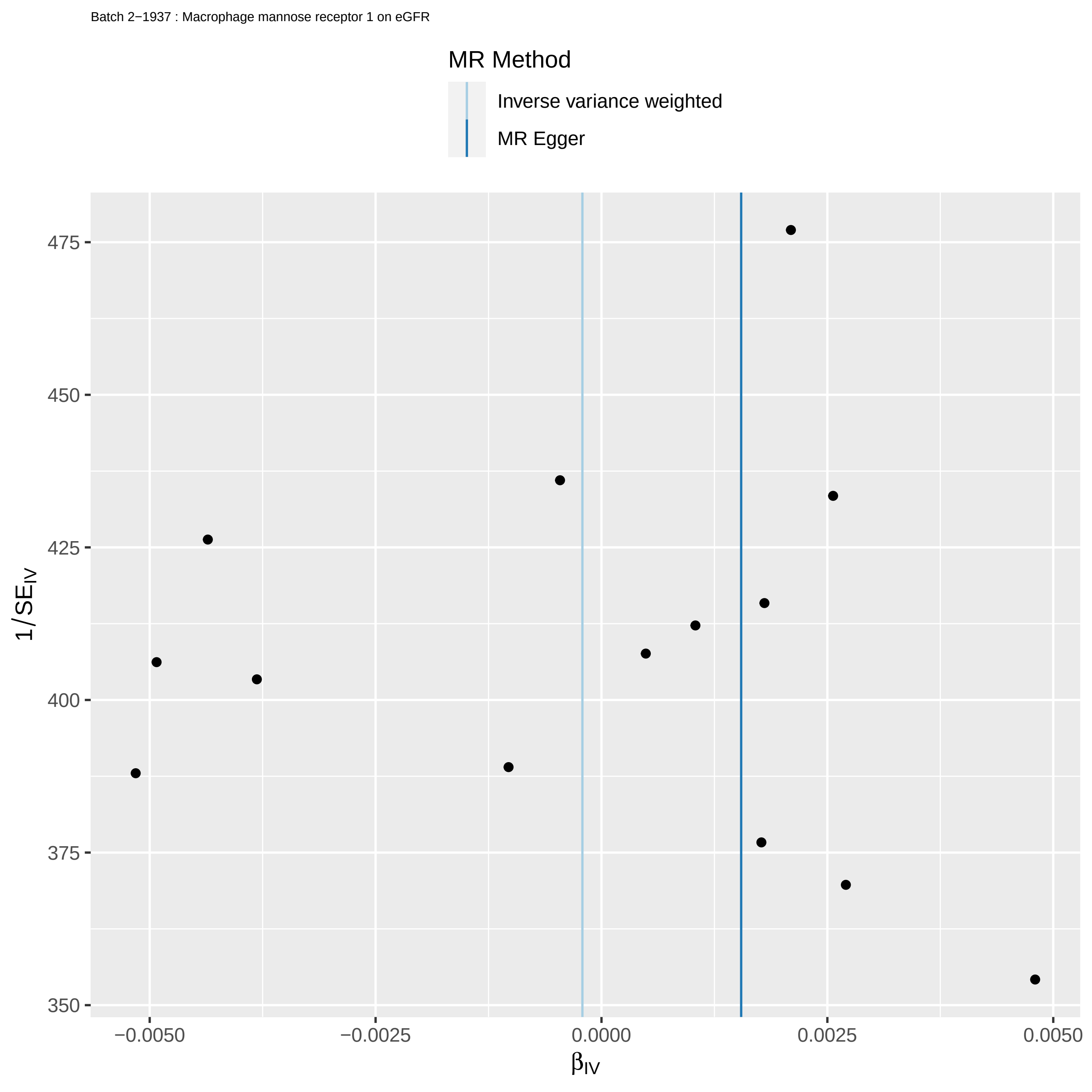
Batch 2−1937 : Macrophage mannose receptor 1 on eGFR
MR Method
Inverse variance weighted
MR Egger
475
450
425
400
375
350
1/SE_IV
−0.0050
−0.0025
0.0000
0.0025
0.0050
β_IV

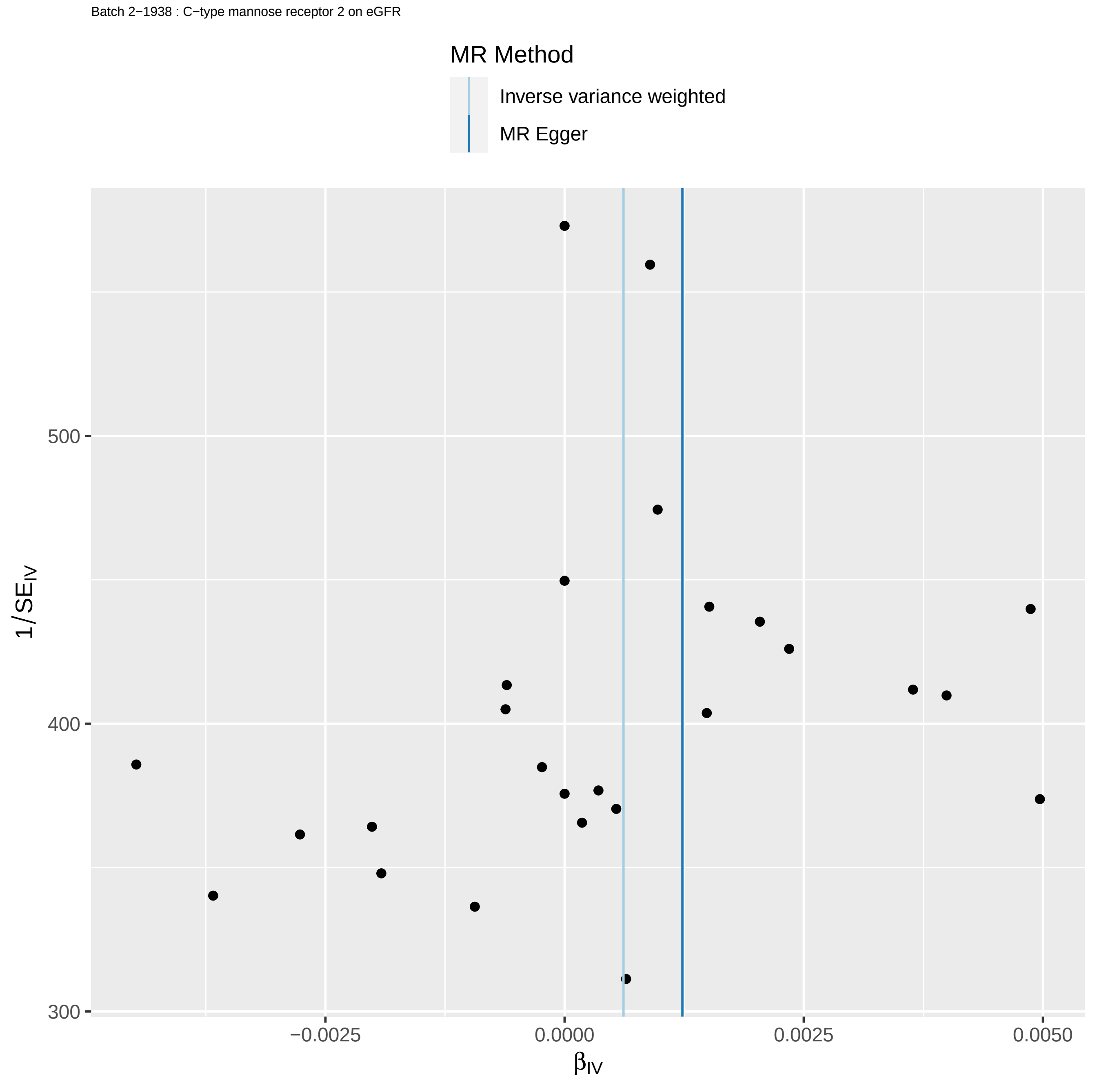

Batch 2−1938 : C−type mannose receptor 2 on eGFR
MR Method
Inverse variance weighted
MR Egger
1/SE_IV
500
400
300
−0.0025
0.0000
0.0025
0.0050
β_IV

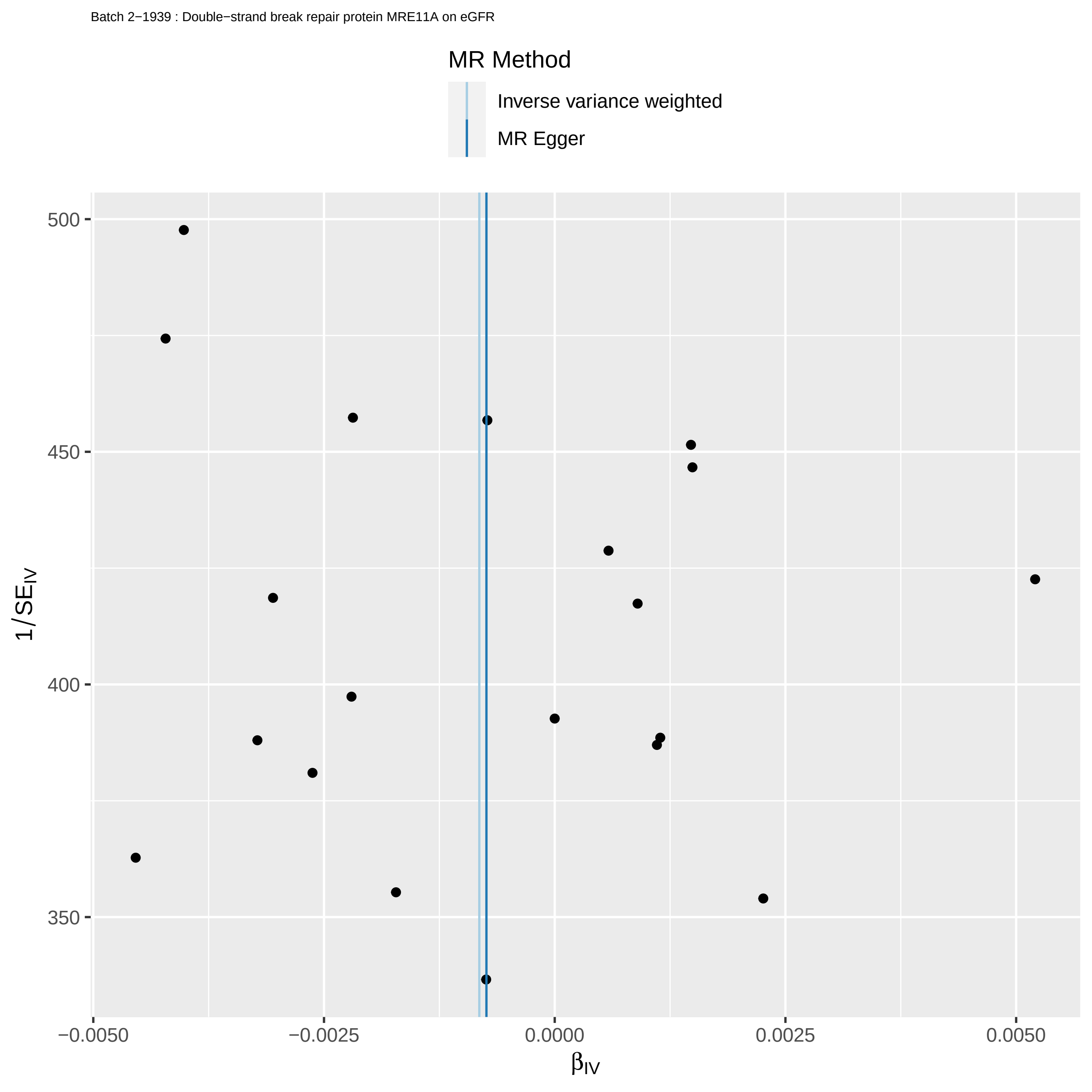
Batch 2−1939 : Double−strand break repair protein MRE11A on eGFR
MR Method
Inverse variance weighted
MR Egger
500
450
400
350
1/SE_IV
−0.0050
−0.0025
0.0000
0.0025
0.0050
β_IV

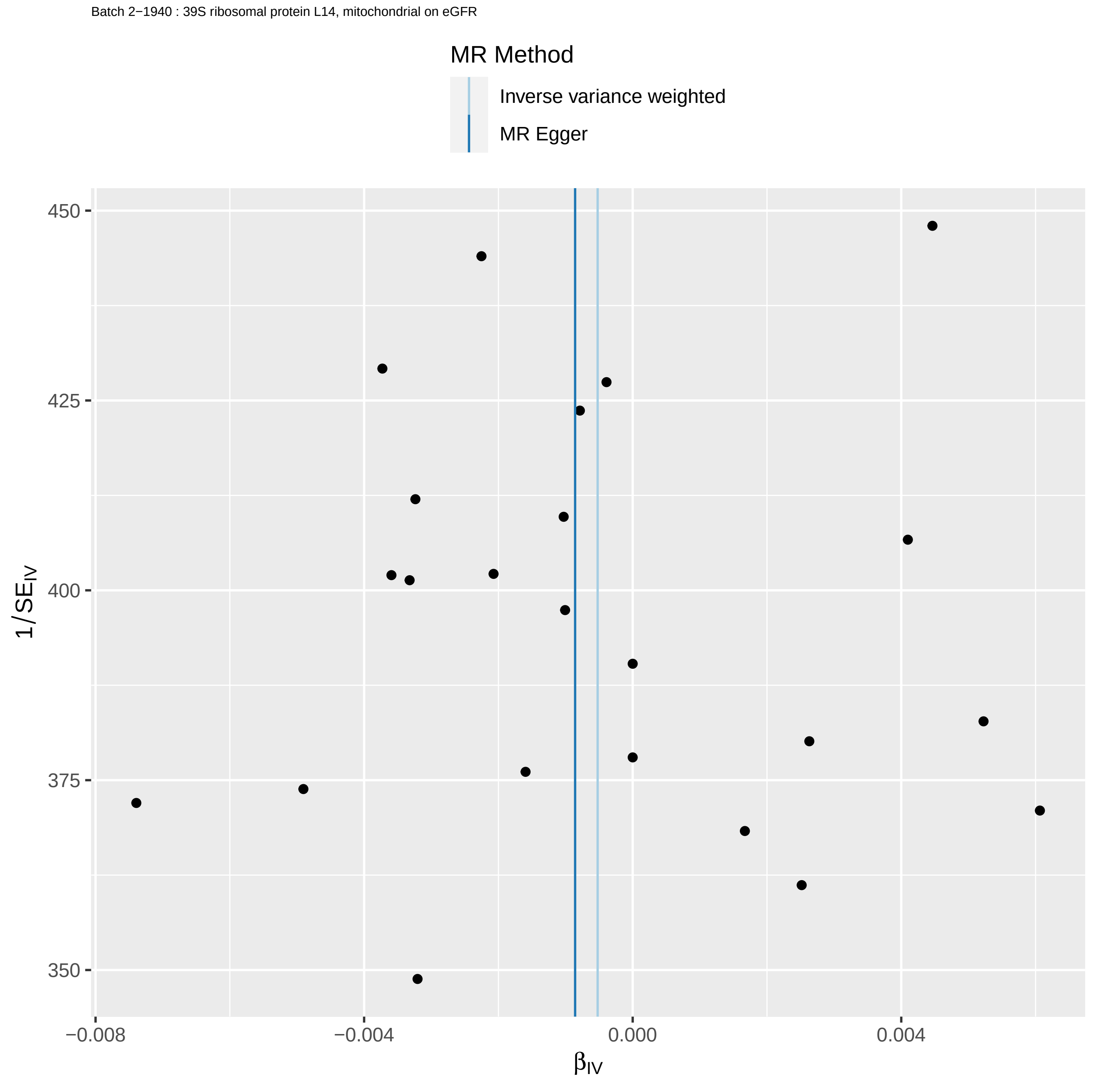
Batch 2−1940 : 39S ribosomal protein L14, mitochondrial on eGFR
MR Method
Inverse variance weighted
MR Egger
450
425
400
375
350
1/SE_IV
−0.008
−0.004
0.000
0.004
β_IV

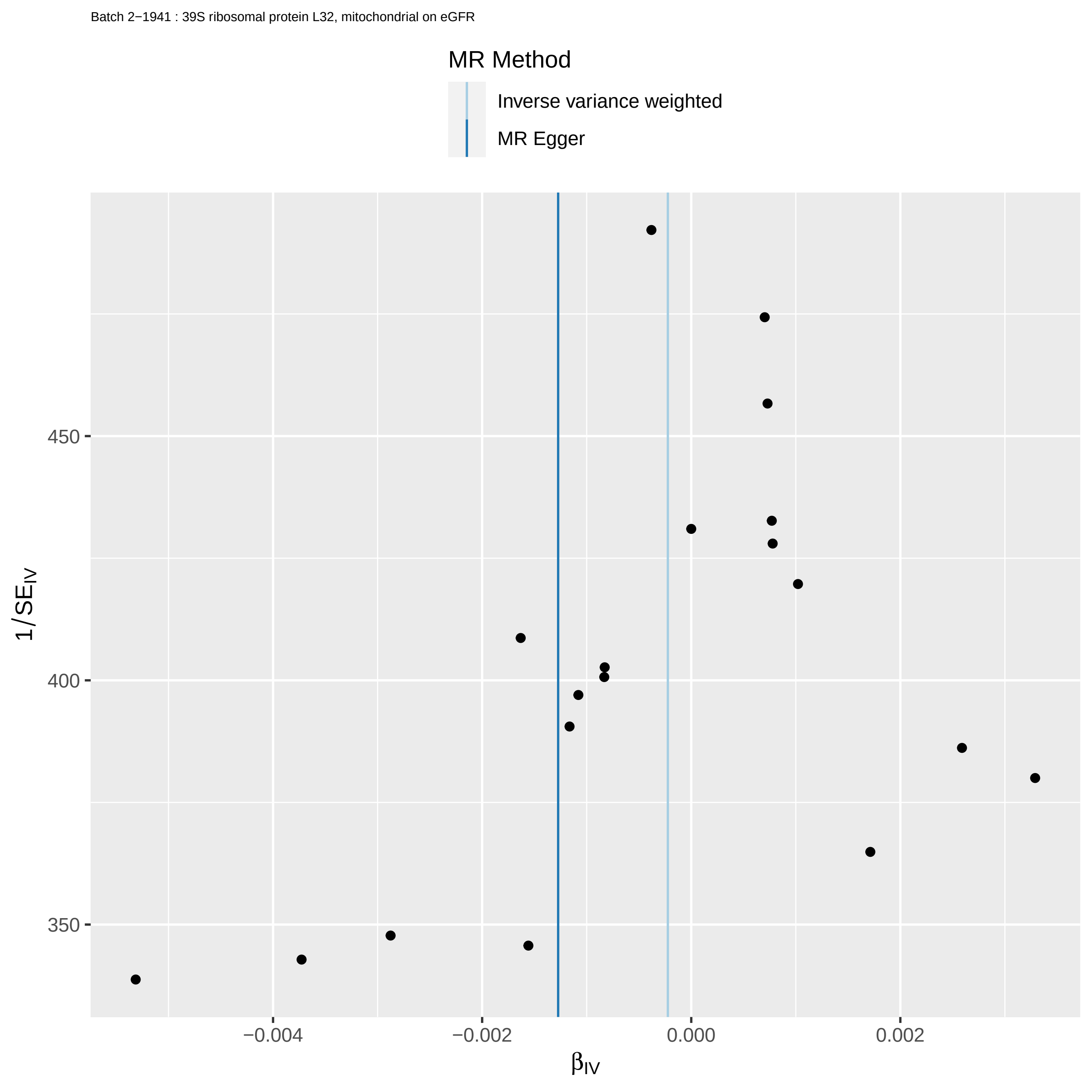

Batch 2−1941 : 39S ribosomal protein L32, mitochondrial on eGFR
MR Method
Inverse variance weighted
MR Egger
450
400
350
1/SE$_{IV}$
−0.004
−0.002
0.000
0.002
β$_{IV}$

Batch 2−1942 : 39S ribosomal protein L33, mitochondrial on eGFR

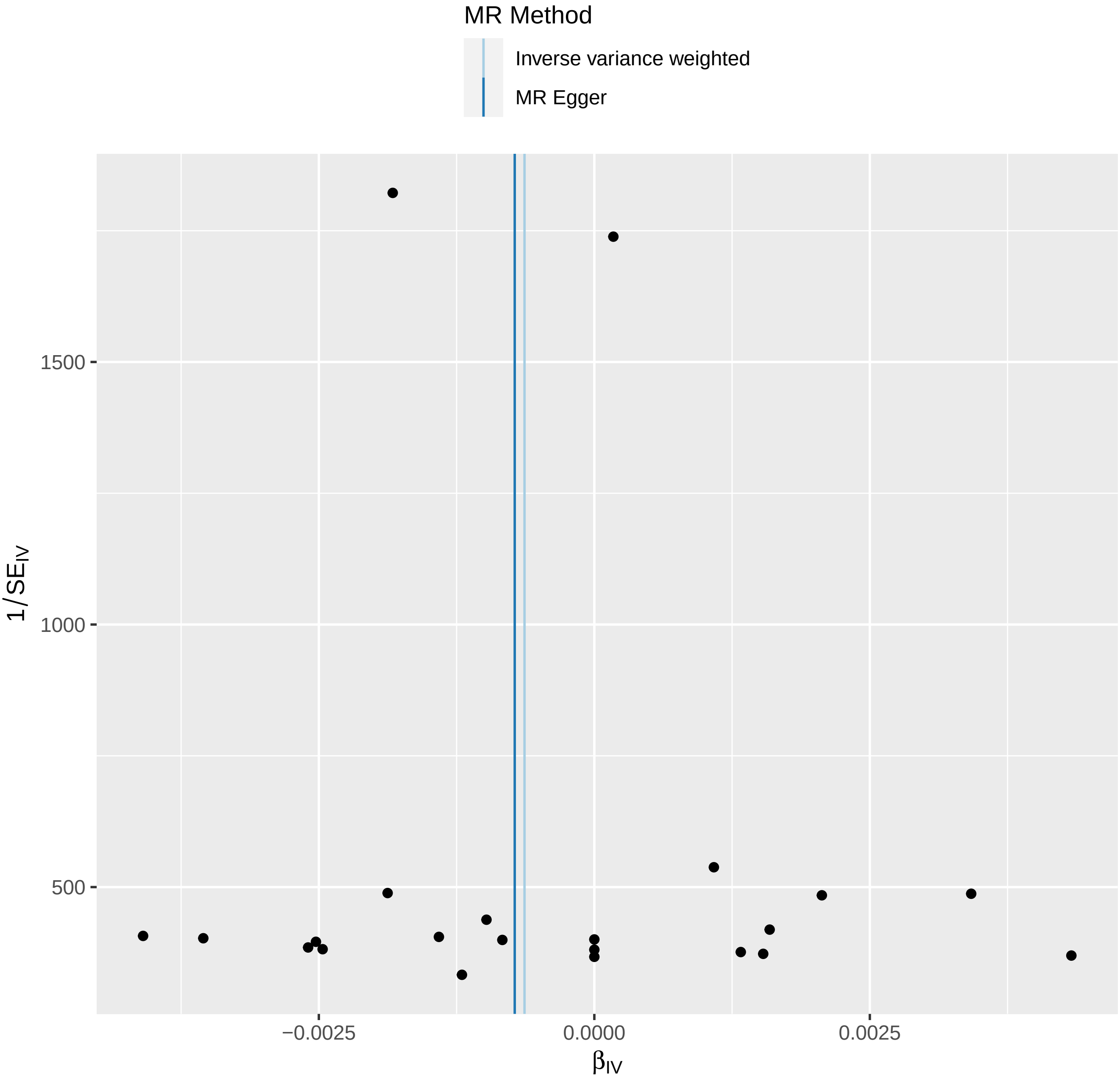

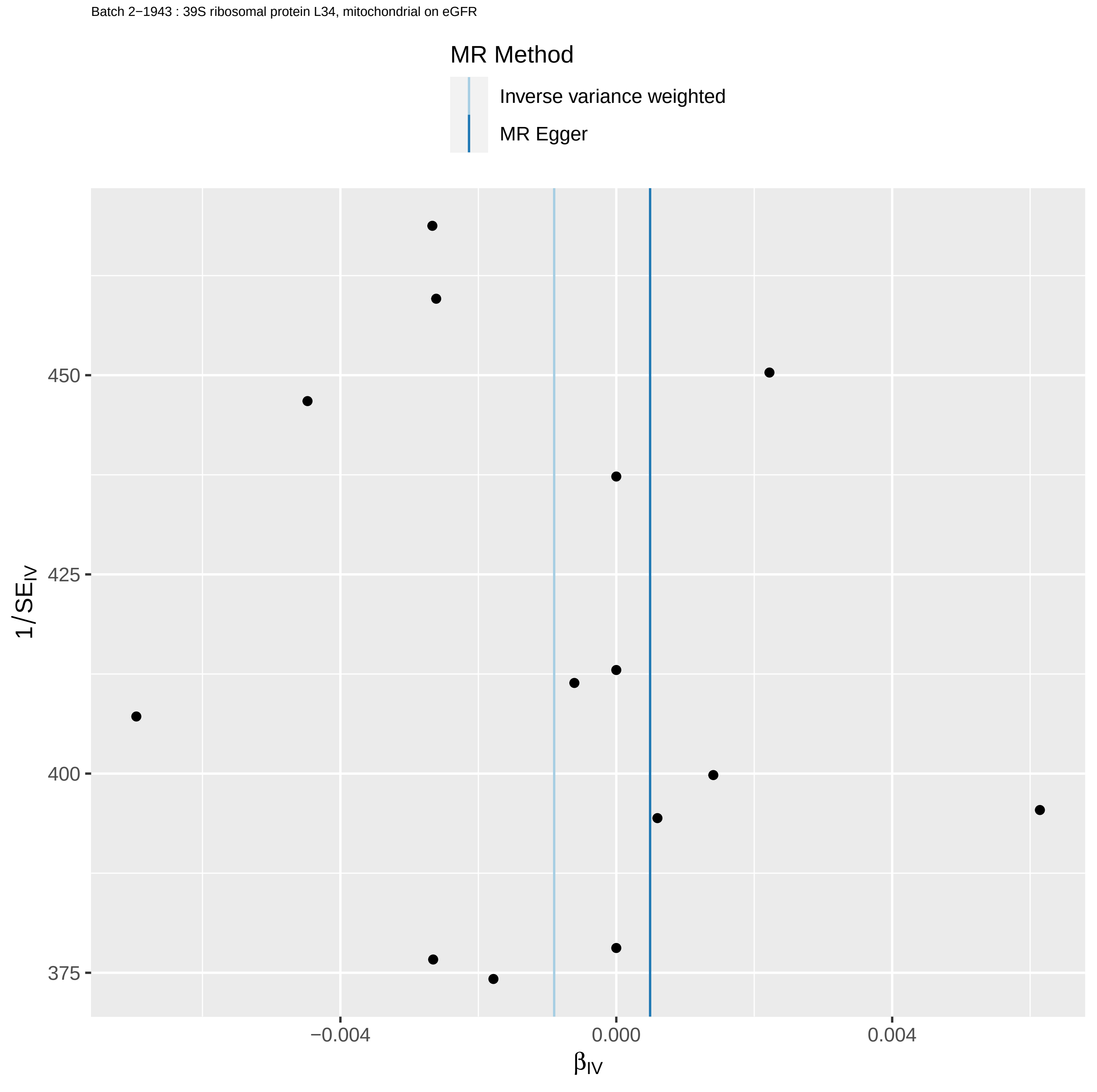
Batch 2−1943 : 39S ribosomal protein L34, mitochondrial on eGFR
MR Method
Inverse variance weighted
MR Egger
1/SE_IV
450
425
400
375
−0.004
0.000
0.004
β_IV

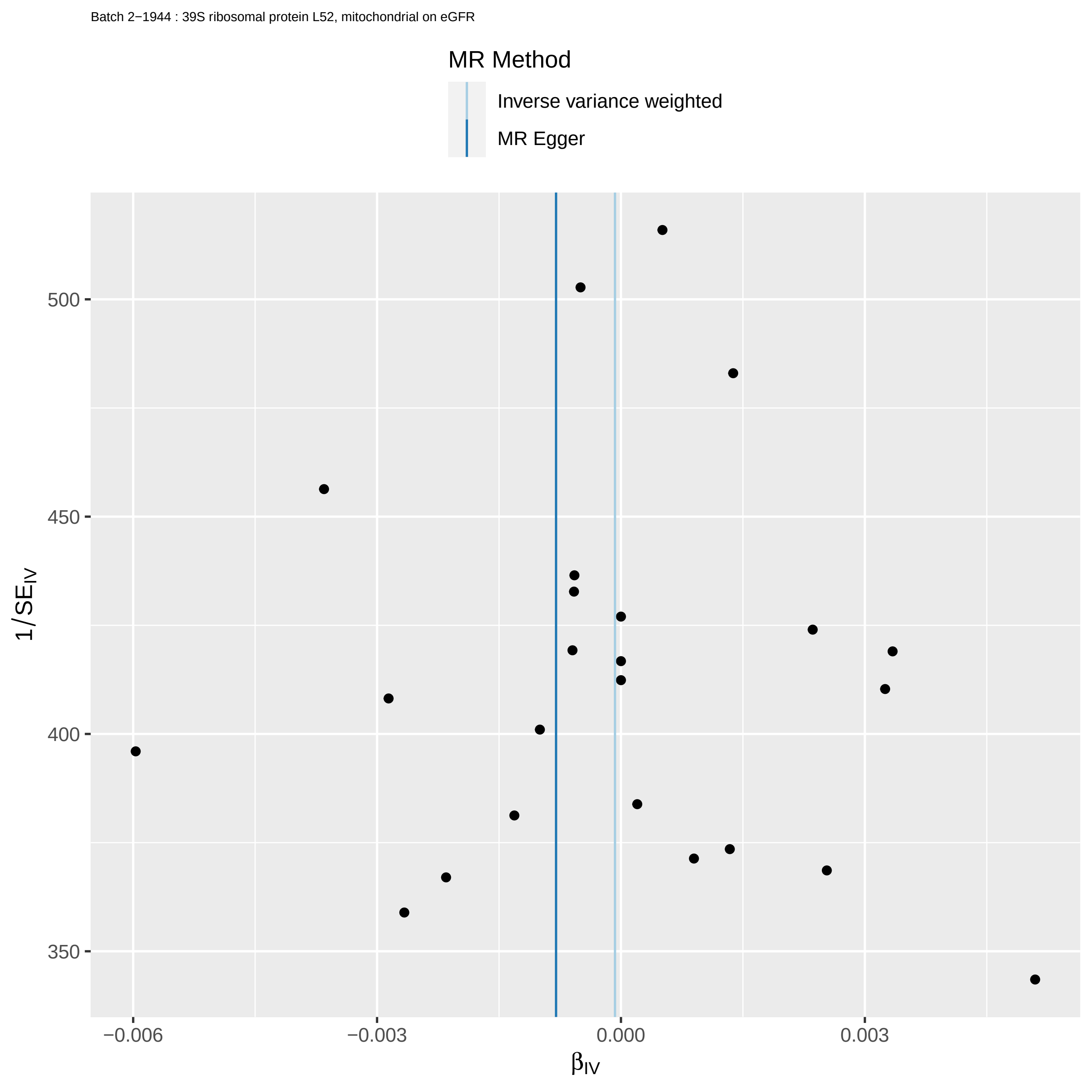

Batch 2−1944 : 39S ribosomal protein L52, mitochondrial on eGFR
MR Method
Inverse variance weighted
MR Egger
500
450
400
350
1/SE_IV
−0.006
−0.003
0.000
0.003
β_IV

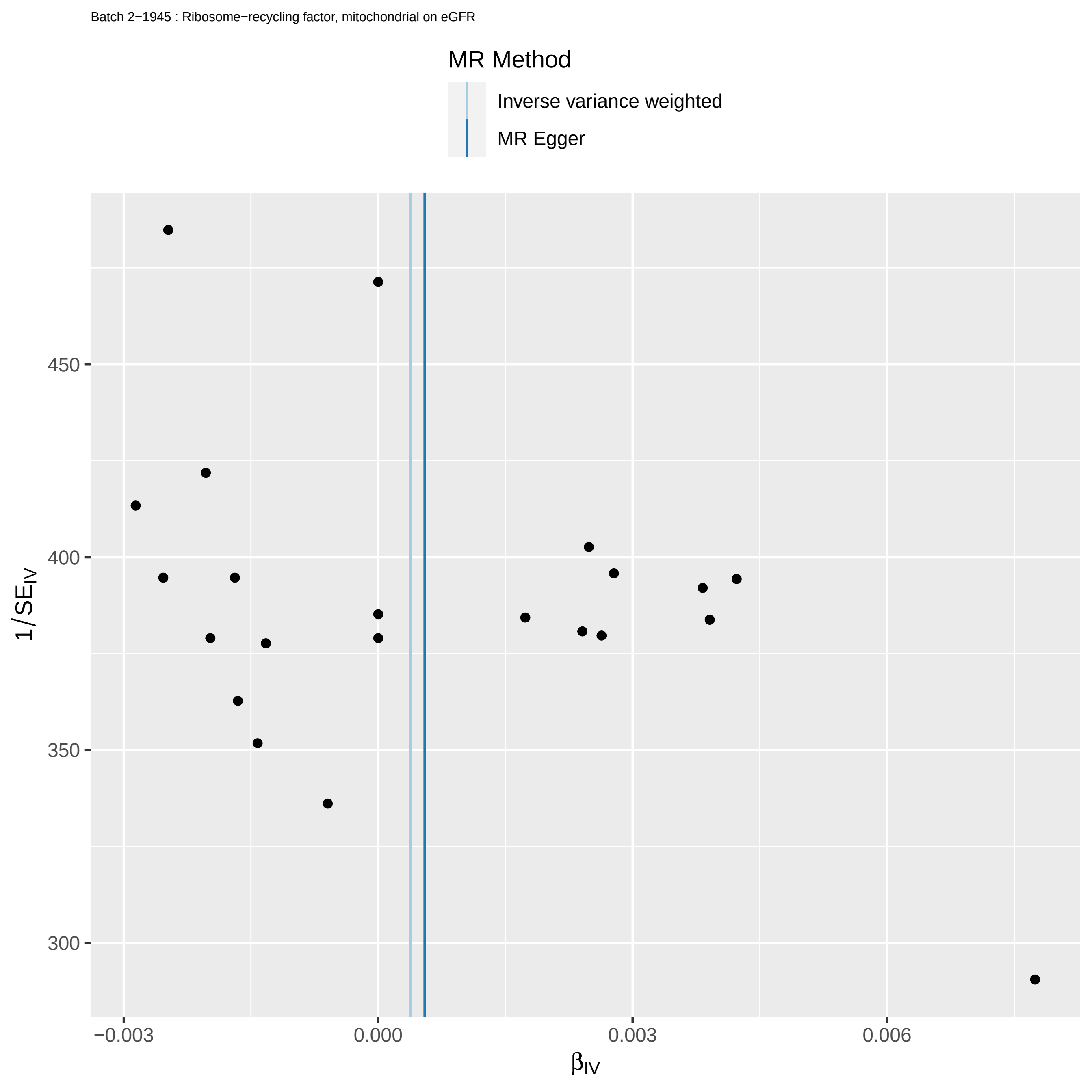

Batch 2−1945 : Ribosome−recycling factor, mitochondrial on eGFR
MR Method
Inverse variance weighted
MR Egger
450
400
350
300
1/SE_IV
−0.003
0.000
0.003
0.006
β_IV

Batch 2−1946 : Protein MRVI1 on eGFR

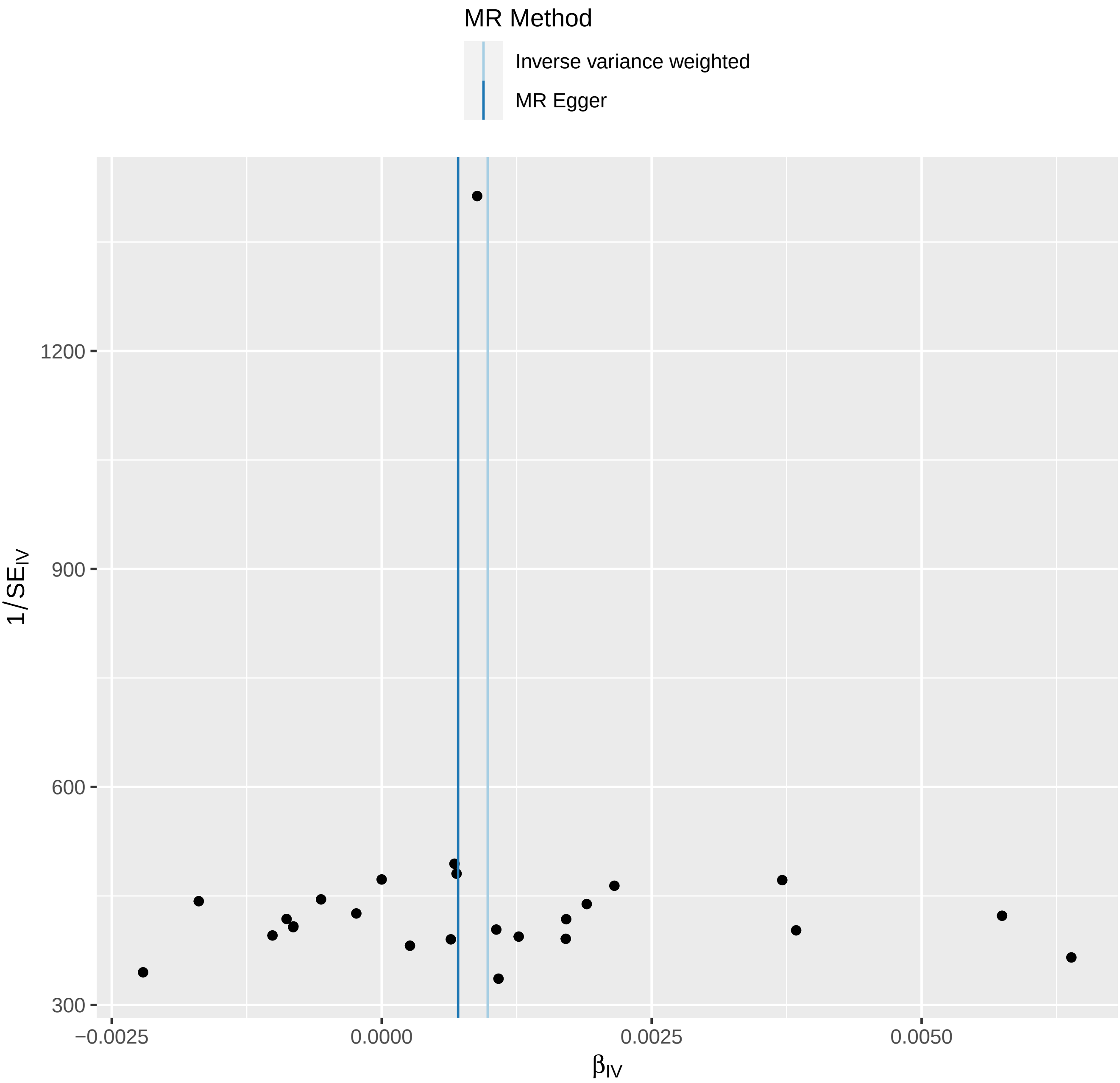

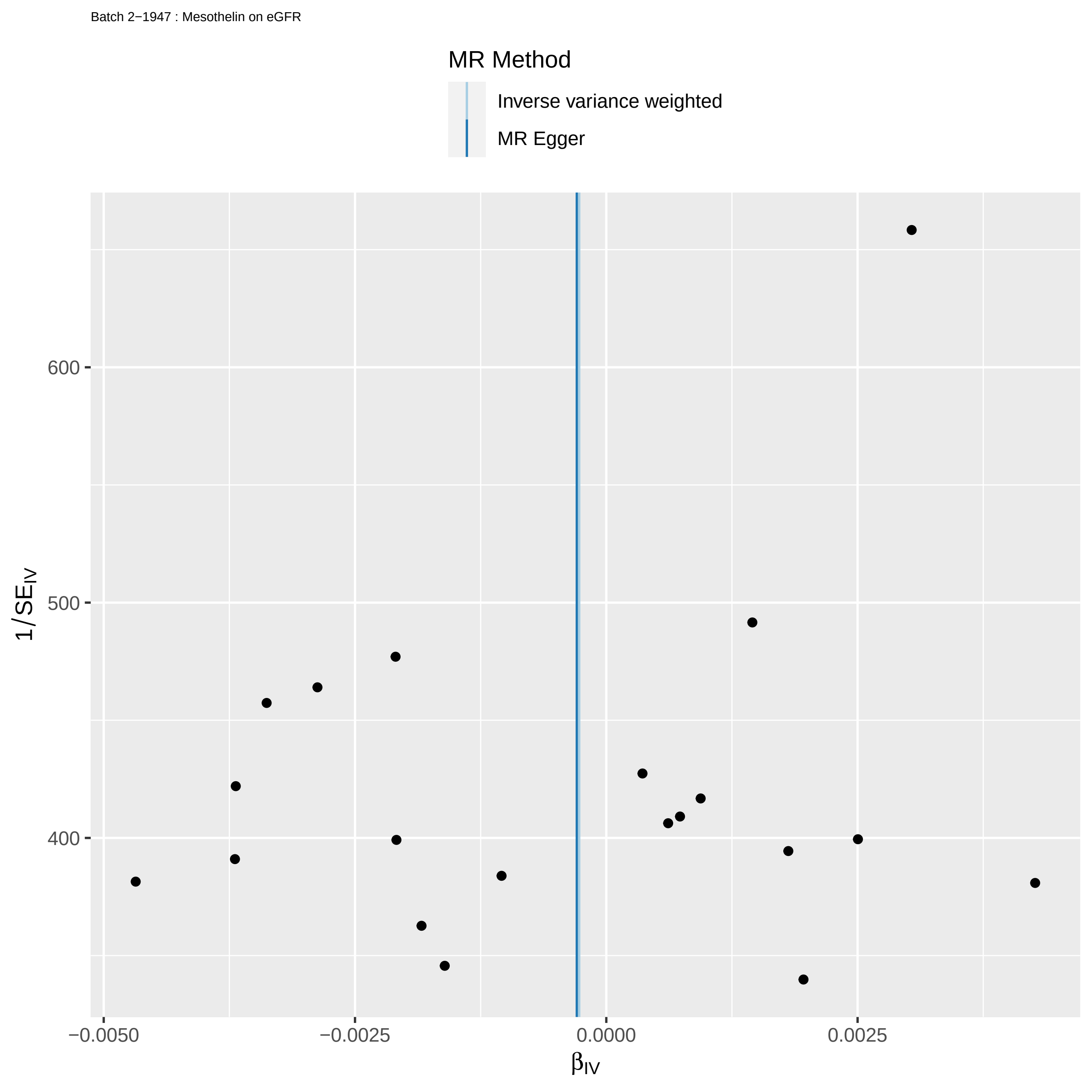
Batch 2−1947 : Mesothelin on eGFR
MR Method
Inverse variance weighted
MR Egger
600
500
400
1/SE$_{IV}$
−0.0050
−0.0025
0.0000
0.0025
β$_{IV}$

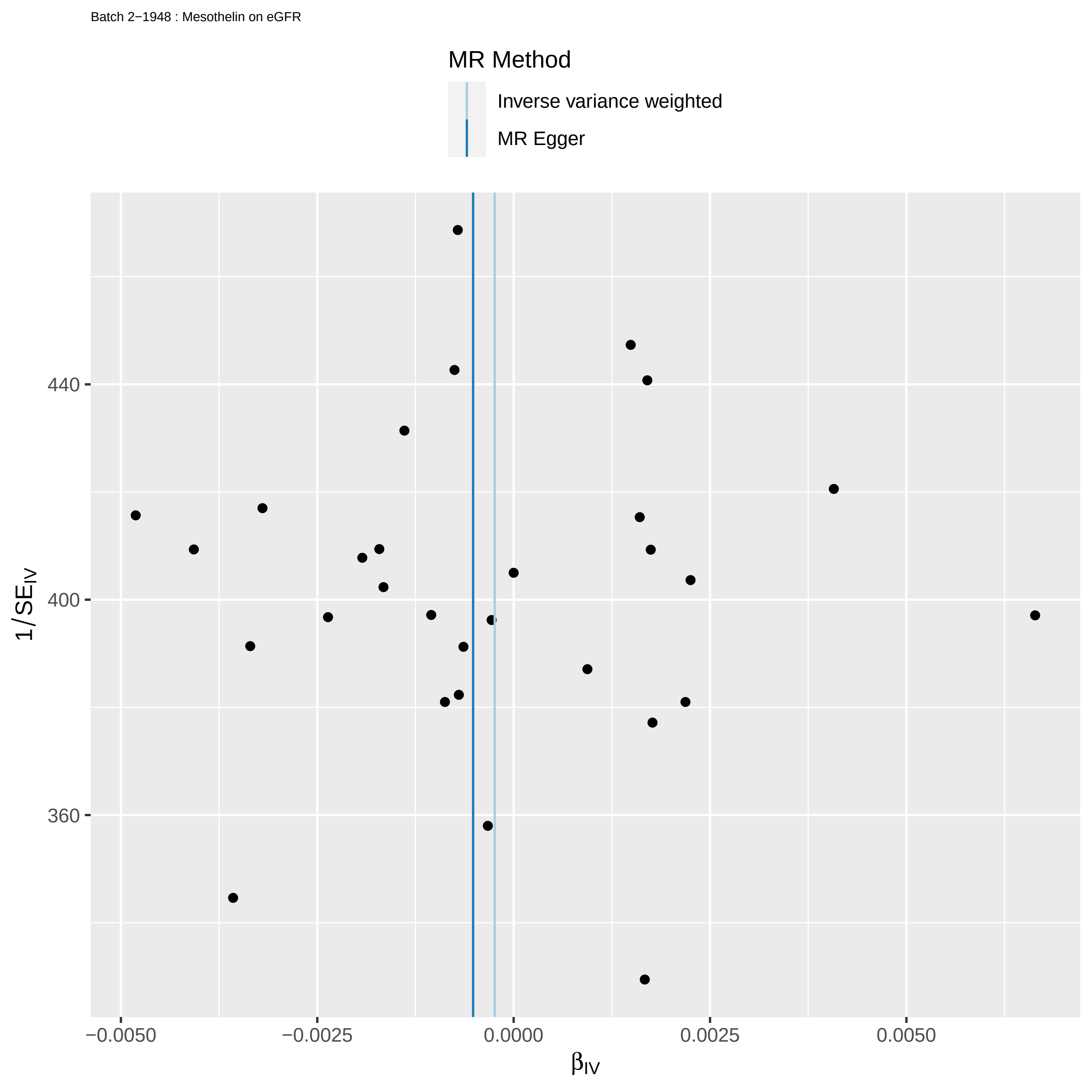
Batch 2−1948 : Mesothelin on eGFR
MR Method
Inverse variance weighted
MR Egger
440
400
360
1/SE$_{IV}$
−0.0050
−0.0025
0.0000
0.0025
0.0050
β$_{IV}$

Batch 2−1949 : Beta−microseminoprotein on eGFR

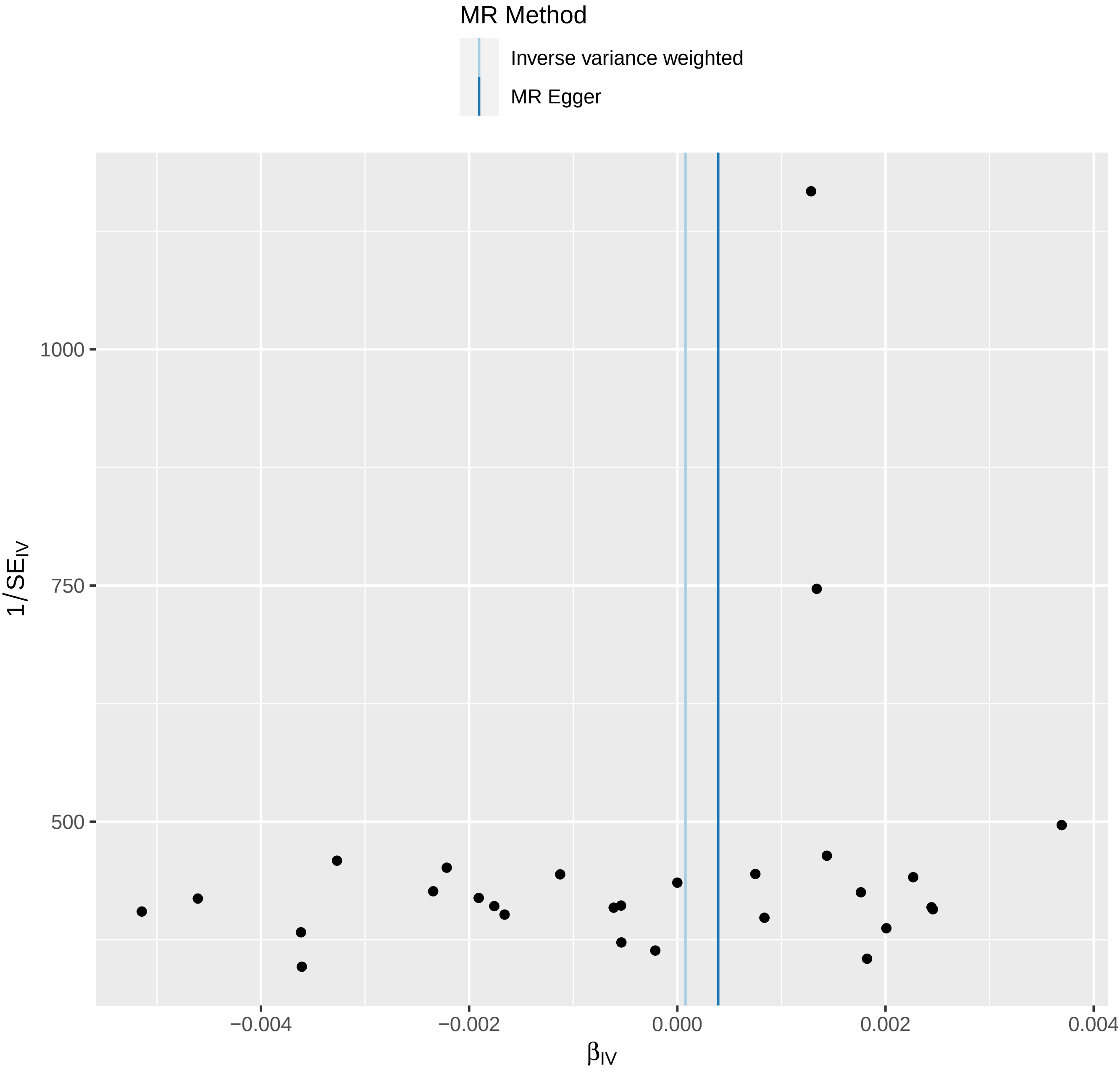

Batch 2−1950 : Prostate−associated microseminoprotein on eGFR

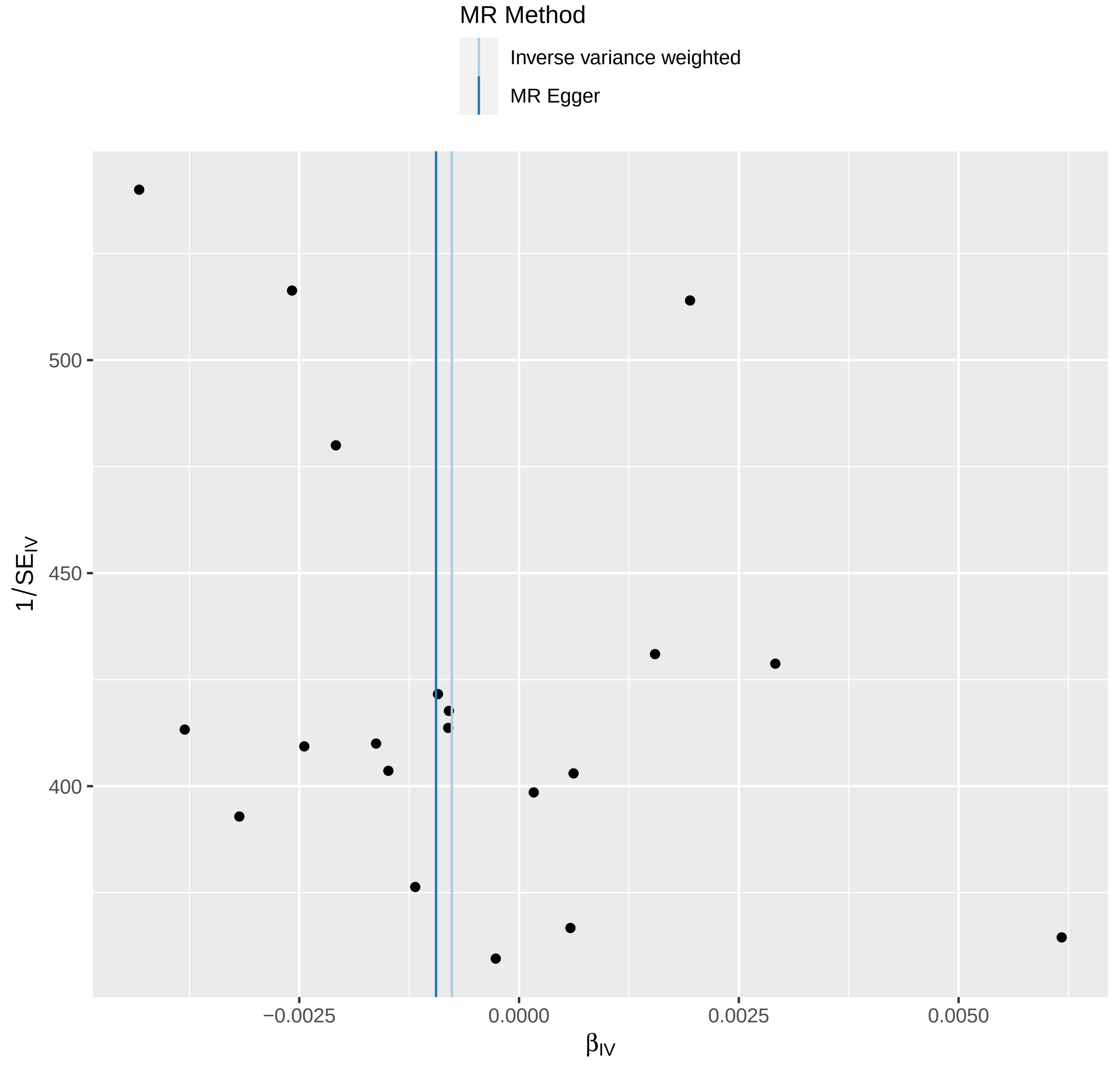

Batch 2−1951 : Macrophage scavenger receptor types I and II on eGFR

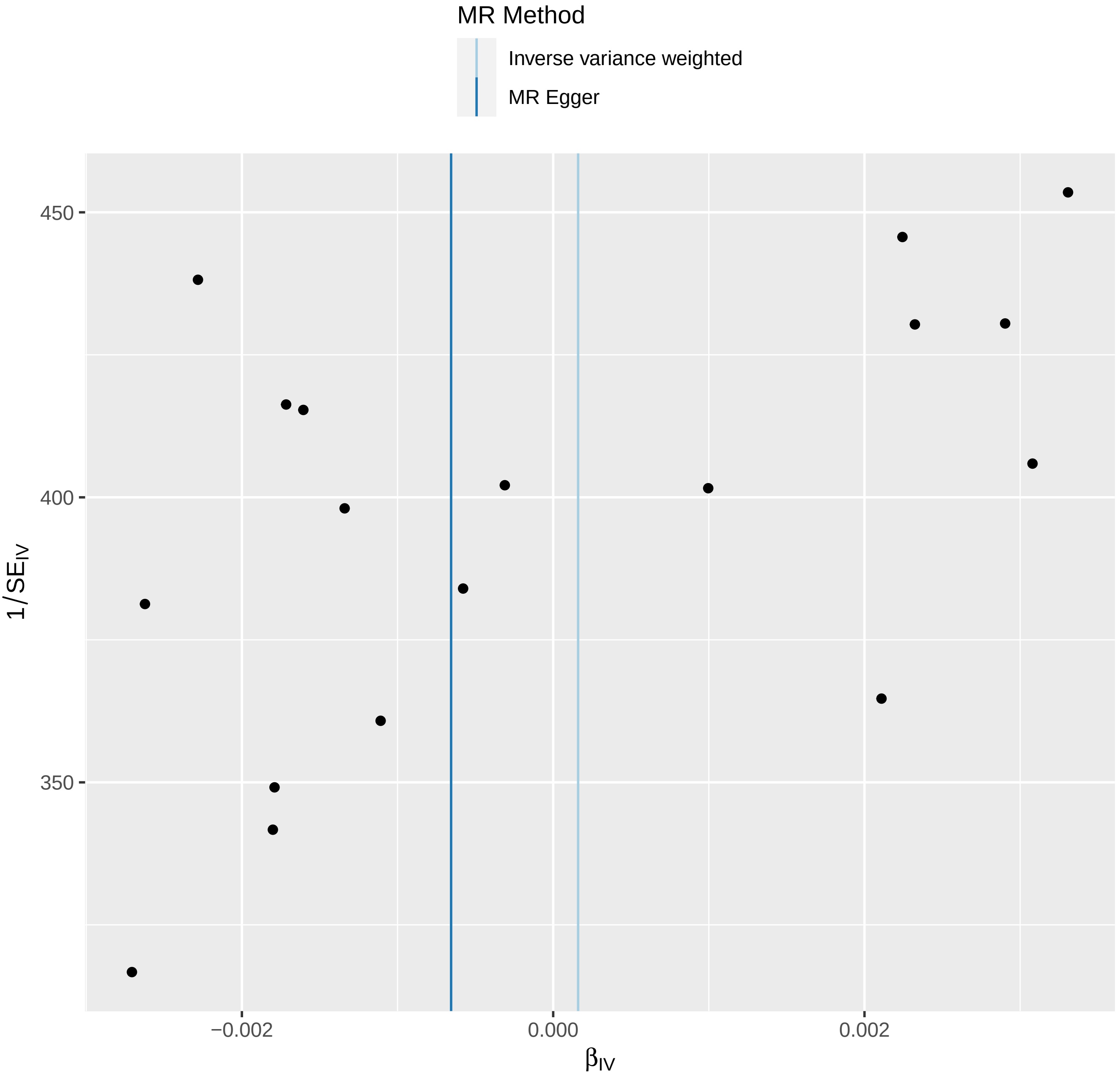

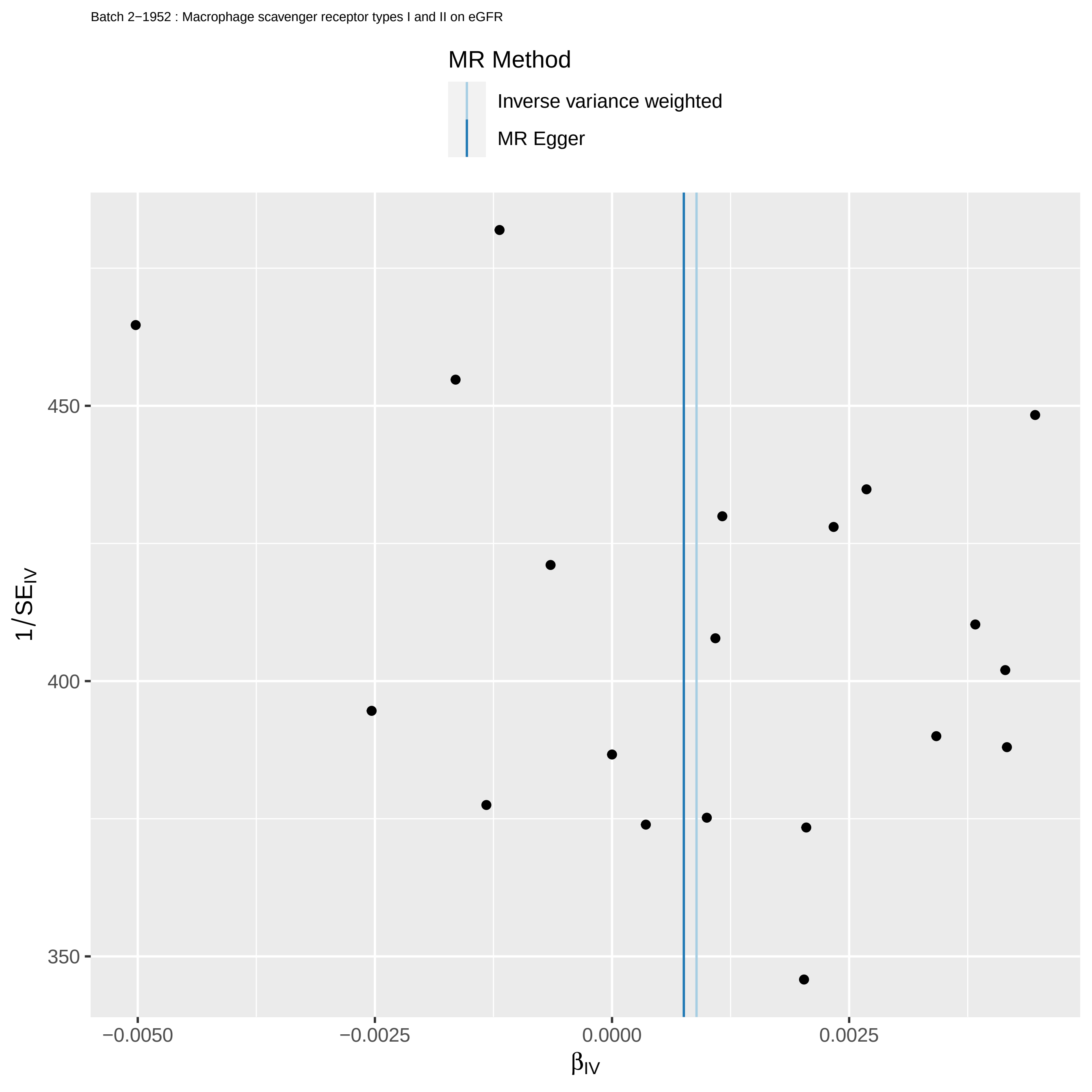

Batch 2−1952 : Macrophage scavenger receptor types I and II on eGFR
MR Method
Inverse variance weighted
MR Egger
450
400
350
−0.0050
−0.0025
0.0000
0.0025
$1/SE_{IV}$
β_{IV}

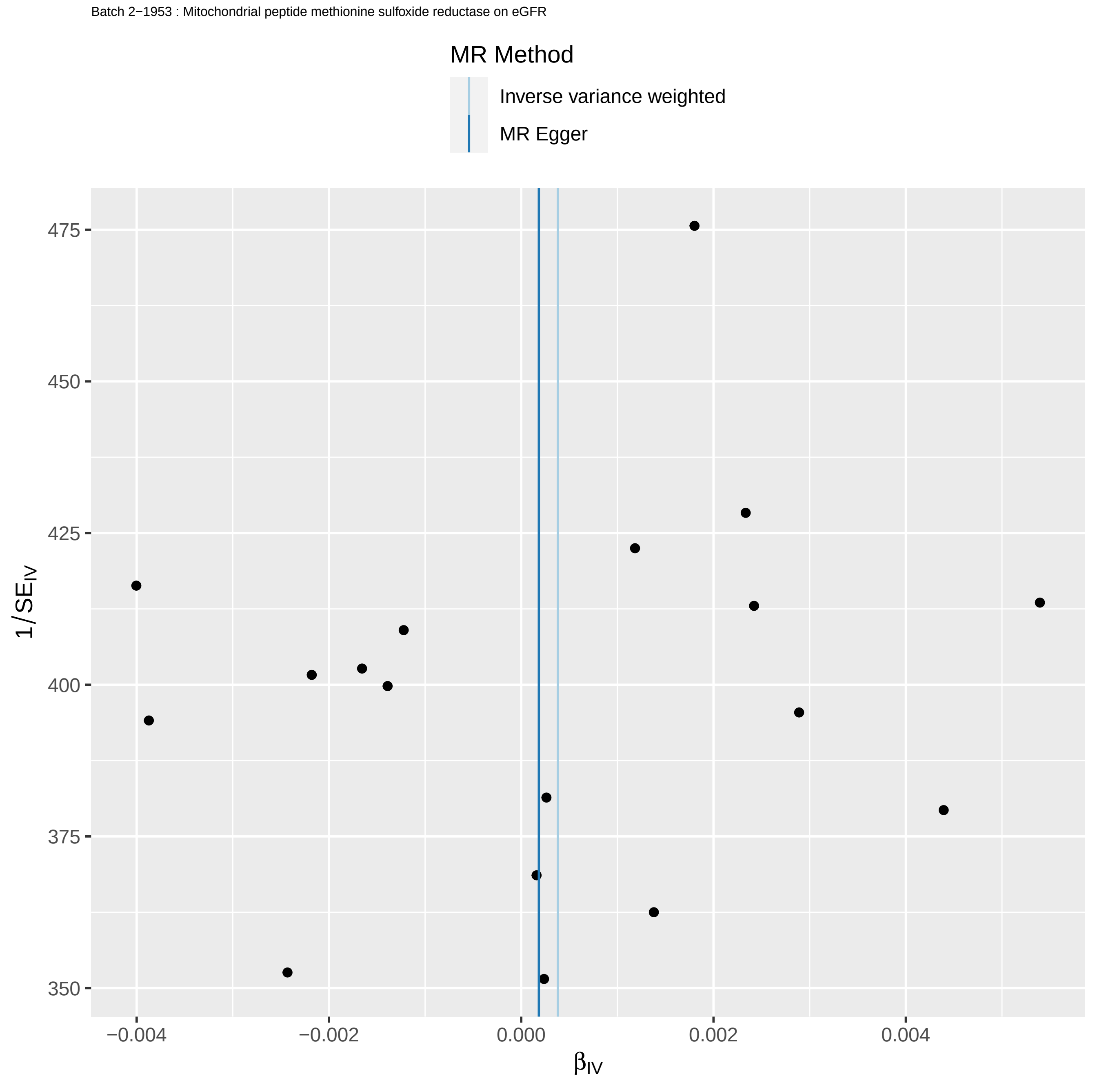
Batch 2−1953 : Mitochondrial peptide methionine sulfoxide reductase on eGFR
MR Method
Inverse variance weighted
MR Egger
475
450
425
400
375
350
1/SE$_{IV}$
−0.004
−0.002
0.000
0.002
0.004
β$_{IV}$

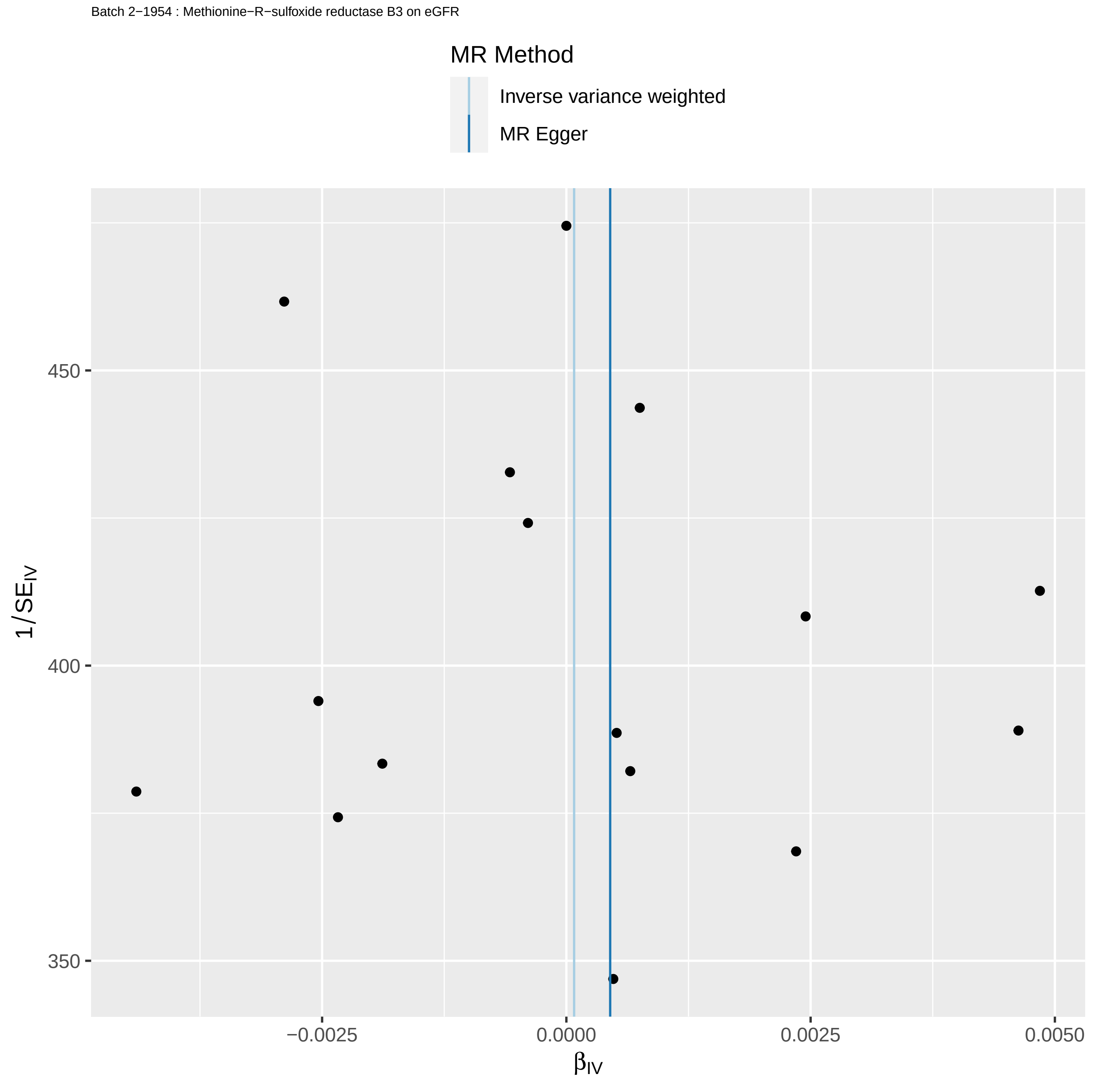
Batch 2−1954 : Methionine−R−sulfoxide reductase B3 on eGFR
MR Method
Inverse variance weighted
MR Egger
450
400
350
1/SE_IV
−0.0025
0.0000
0.0025
0.0050
β_IV

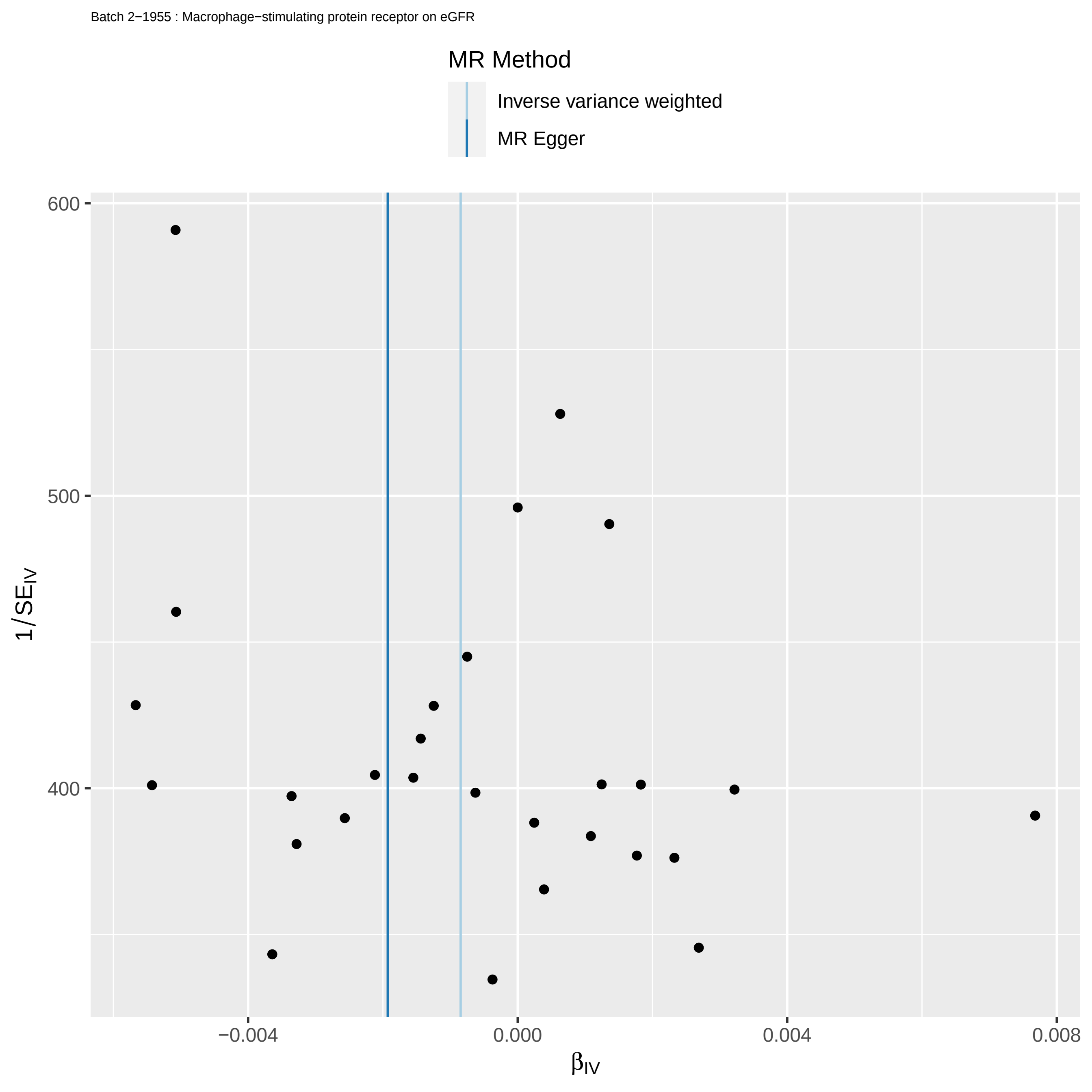
Batch 2−1955 : Macrophage−stimulating protein receptor on eGFR
MR Method
Inverse variance weighted
MR Egger
600
500
400
1/SE_IV
−0.004
0.000
0.004
0.008
β_IV

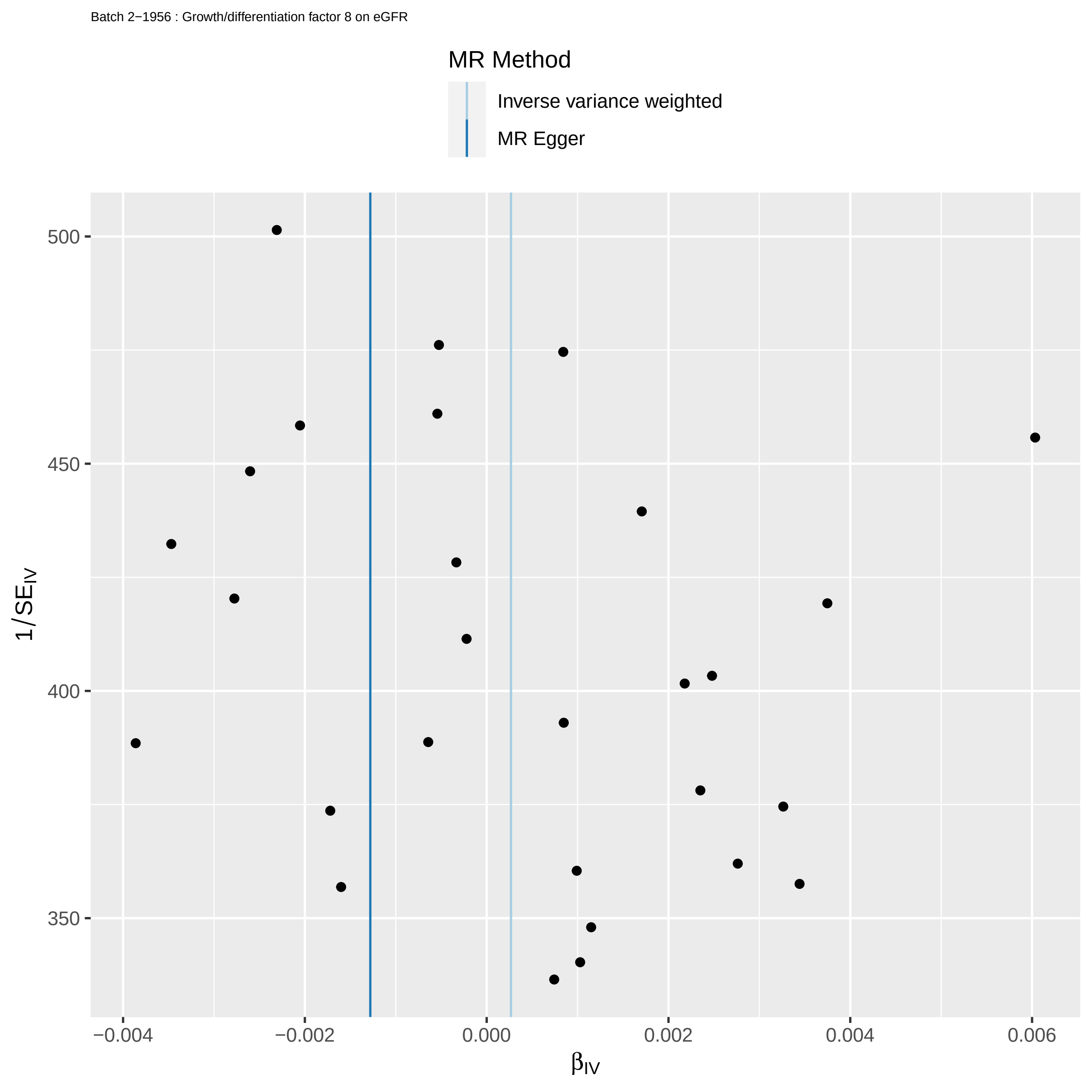

Batch 2−1956 : Growth/differentiation factor 8 on eGFR
MR Method
Inverse variance weighted
MR Egger
500
450
400
350
1/SE_IV
−0.004
−0.002
0.000
0.002
0.004
0.006
β_IV

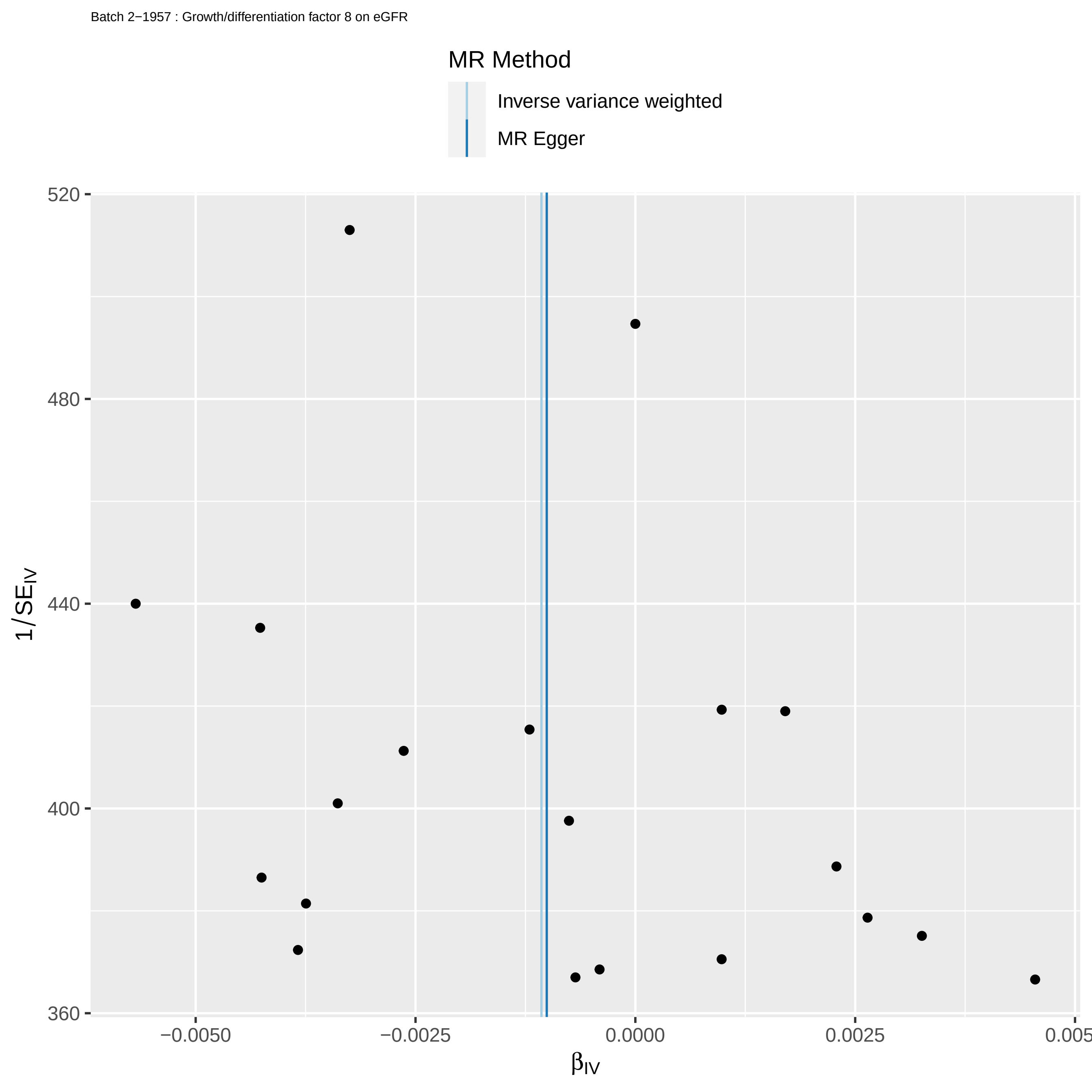

Batch 2−1957 : Growth/differentiation factor 8 on eGFR
MR Method
Inverse variance weighted
MR Egger
520
480
440
400
360
1/SE_IV
−0.0050
−0.0025
0.0000
0.0025
0.005
β_IV

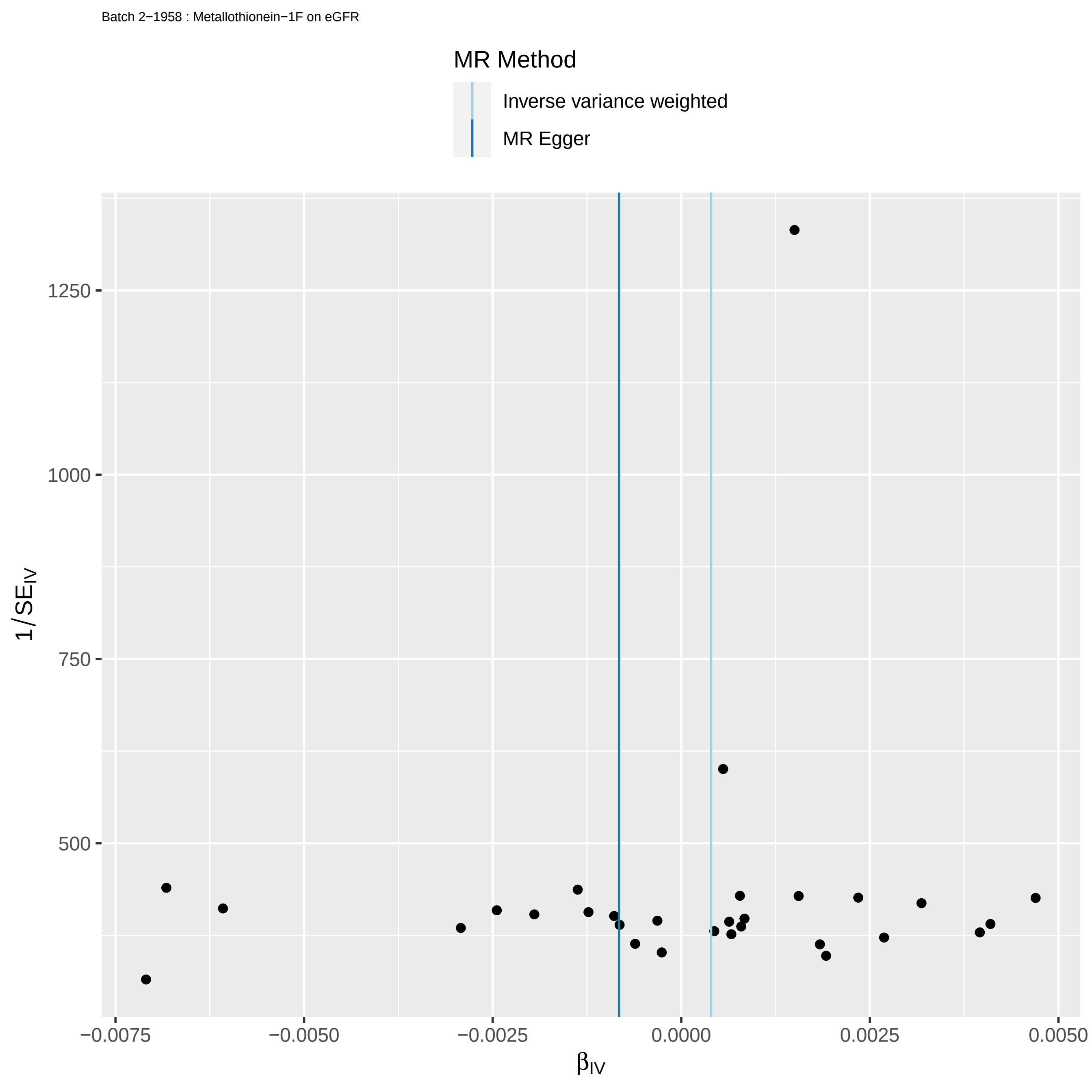

Batch 2−1958 : Metallothionein−1F on eGFR
MR Method
Inverse variance weighted
MR Egger
1250
1000
750
500
1/SE$_{IV}$
−0.0075
−0.0050
−0.0025
0.0000
0.0025
0.0050
β$_{IV}$

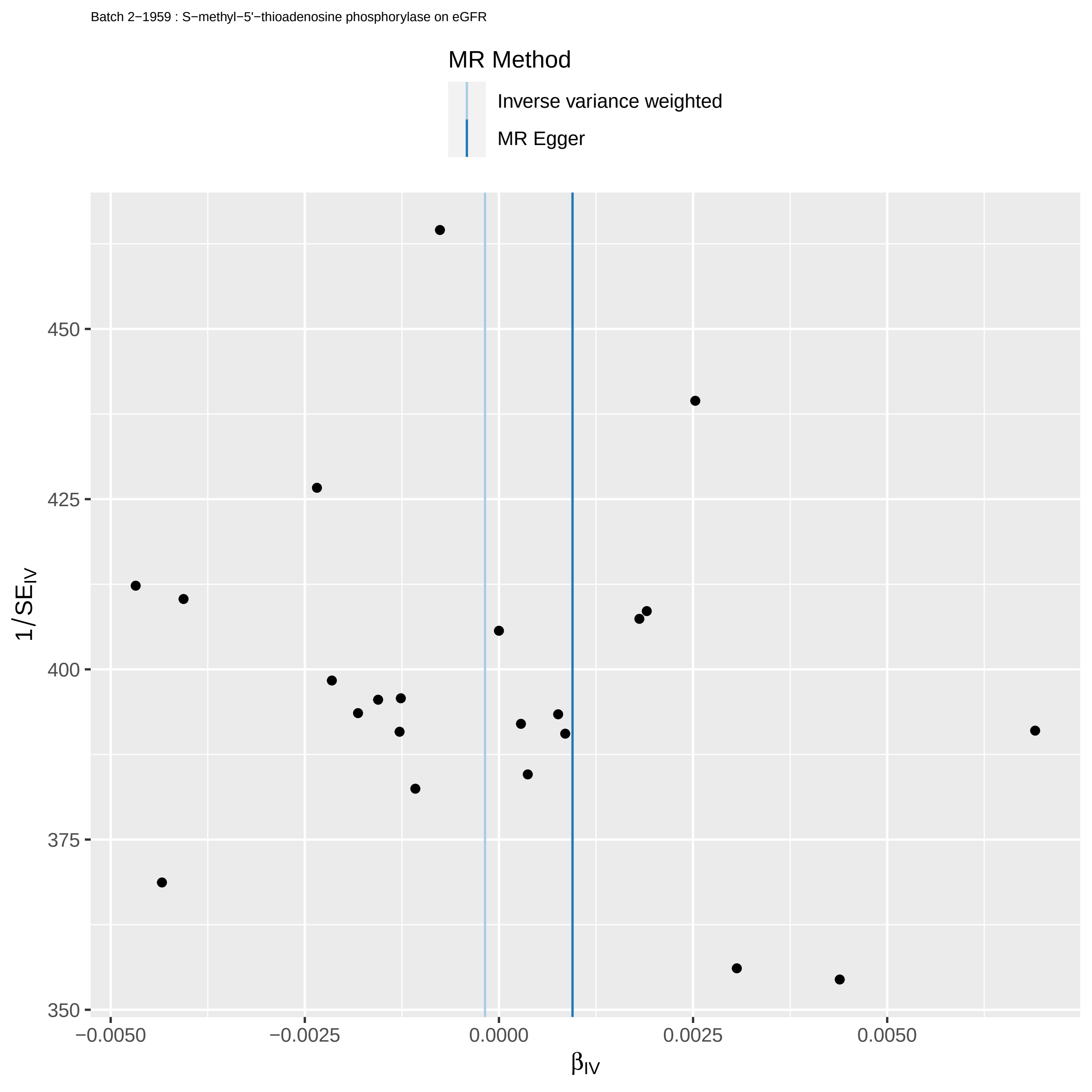

Batch 2−1959 : S−methyl−5'−thioadenosine phosphorylase on eGFR
MR Method
Inverse variance weighted
MR Egger
450
425
400
375
350
1/SE_IV
−0.0050
−0.0025
0.0000
0.0025
0.0050
β_IV

Batch 2−1960 : Protein p13 MTCP−1 on eGFR

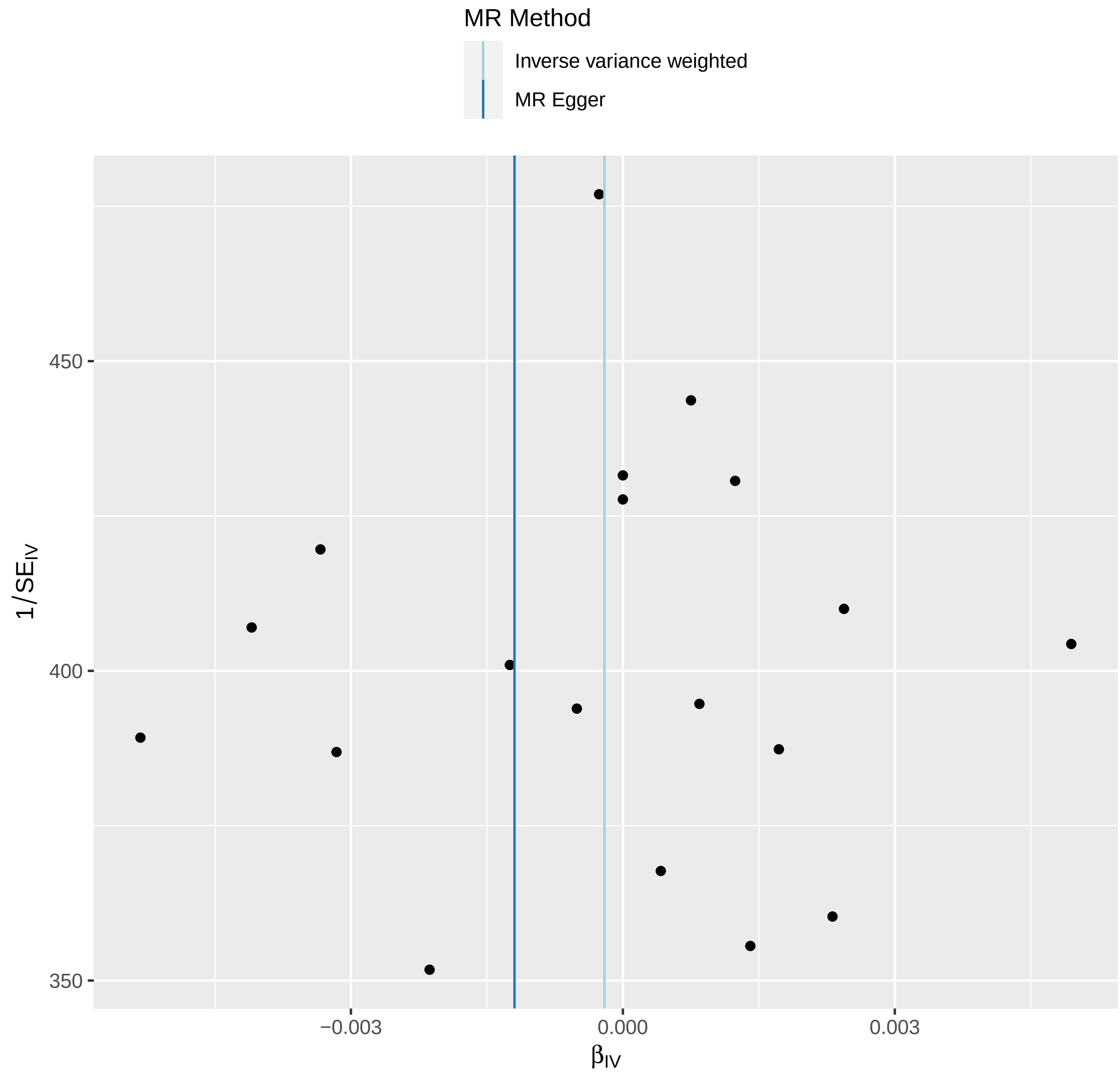

Batch 2−1961 : Mitochondrial fission regulator 1 on eGFR

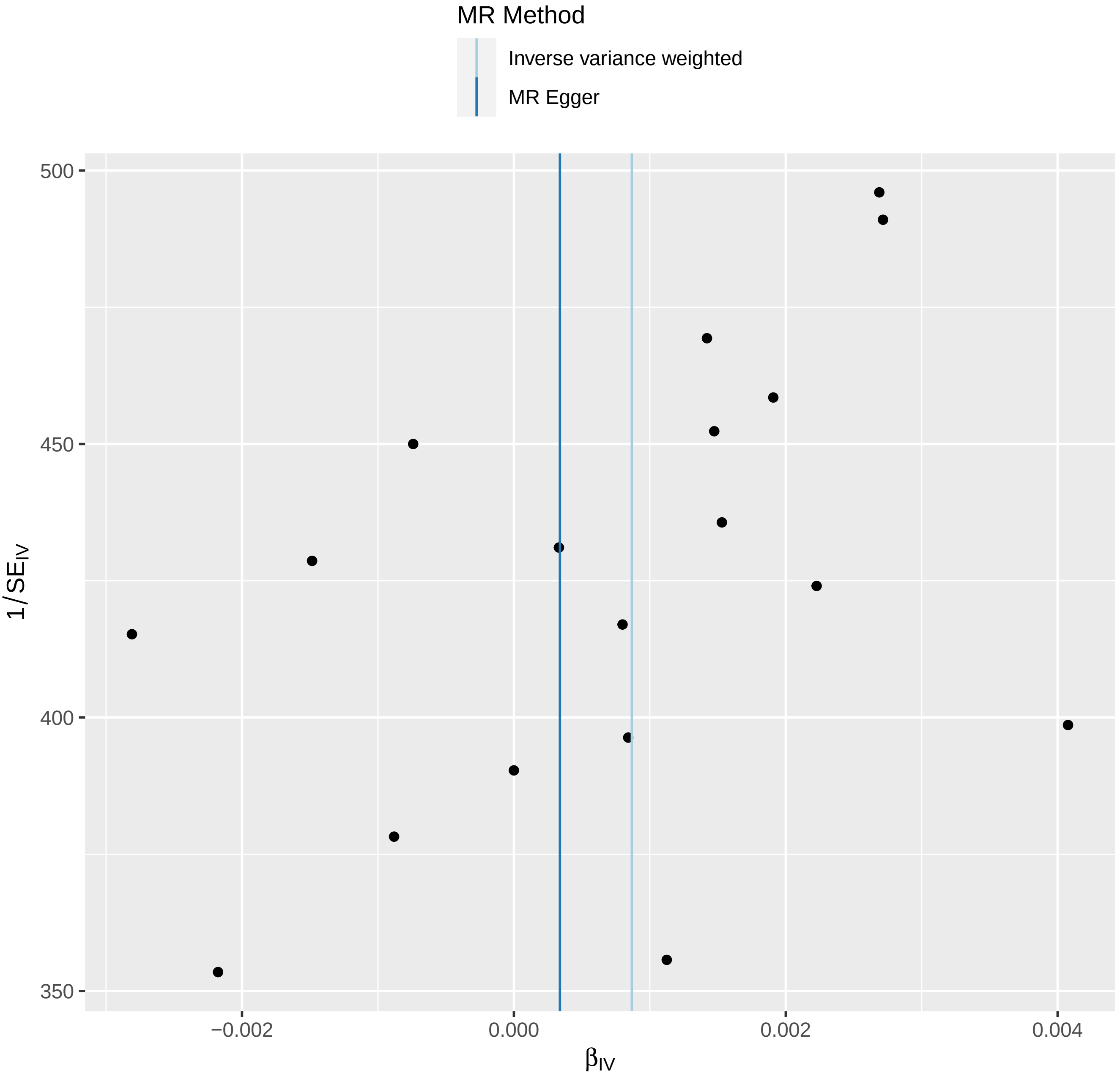

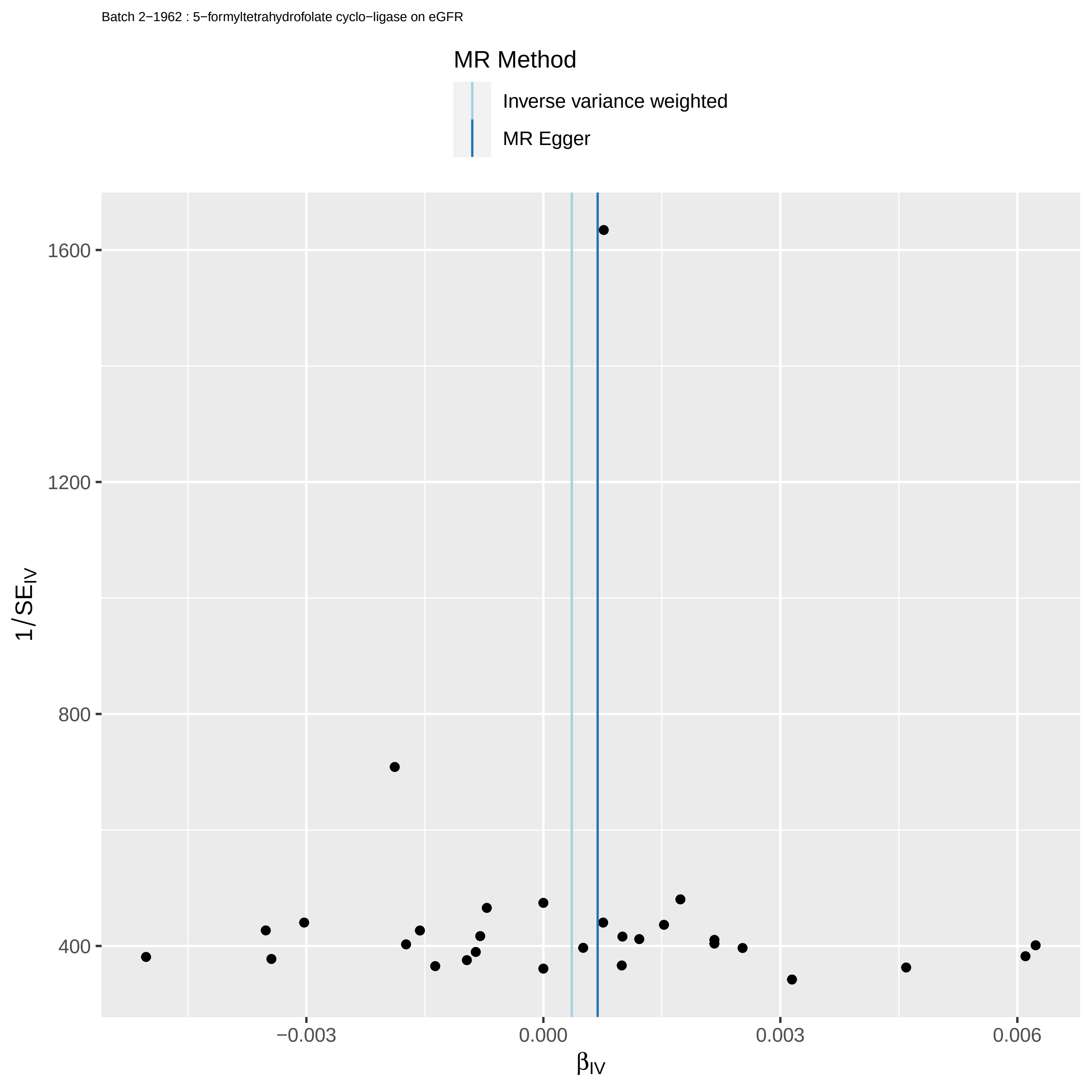
Batch 2−1962 : 5−formyltetrahydrofolate cyclo−ligase on eGFR
MR Method
Inverse variance weighted
MR Egger
1600
1200
800
400
1/SE$_{IV}$
−0.003
0.000
0.003
0.006
β$_{IV}$

Batch 2−1963 : Myotubularin−related protein 1 on eGFR

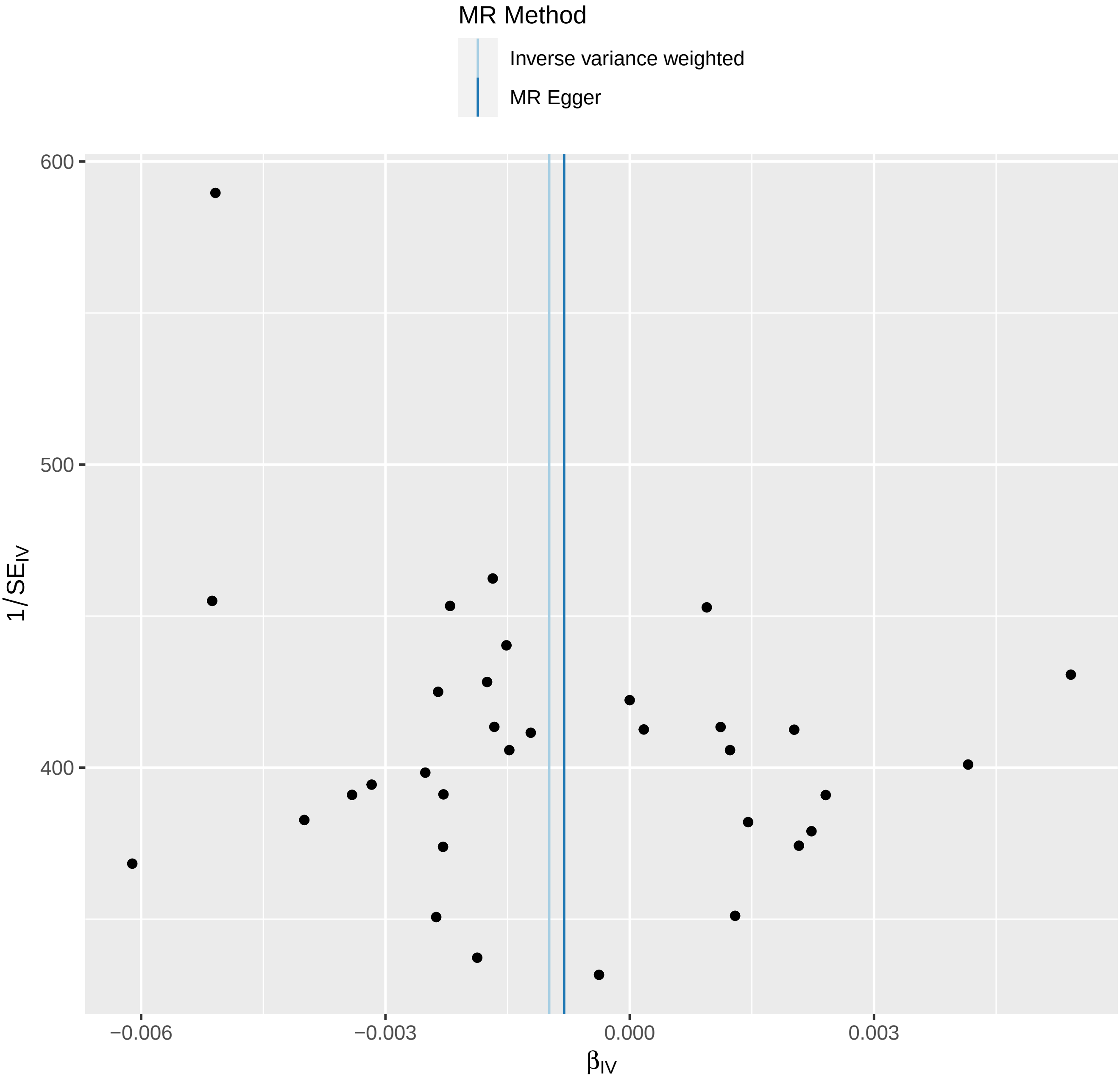

Batch 2−1964 : Poly(A) RNA polymerase, mitochondrial on eGFR

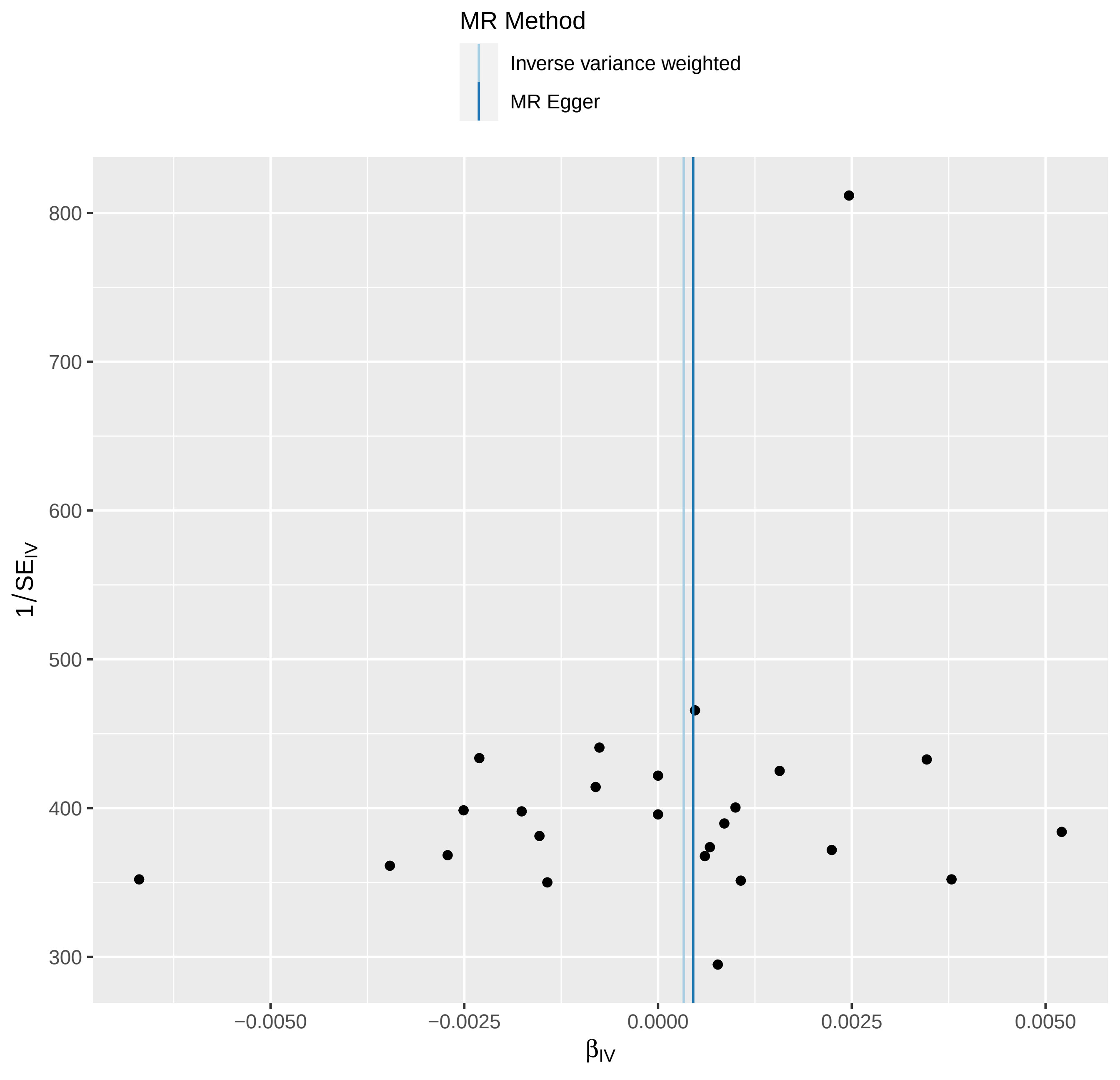

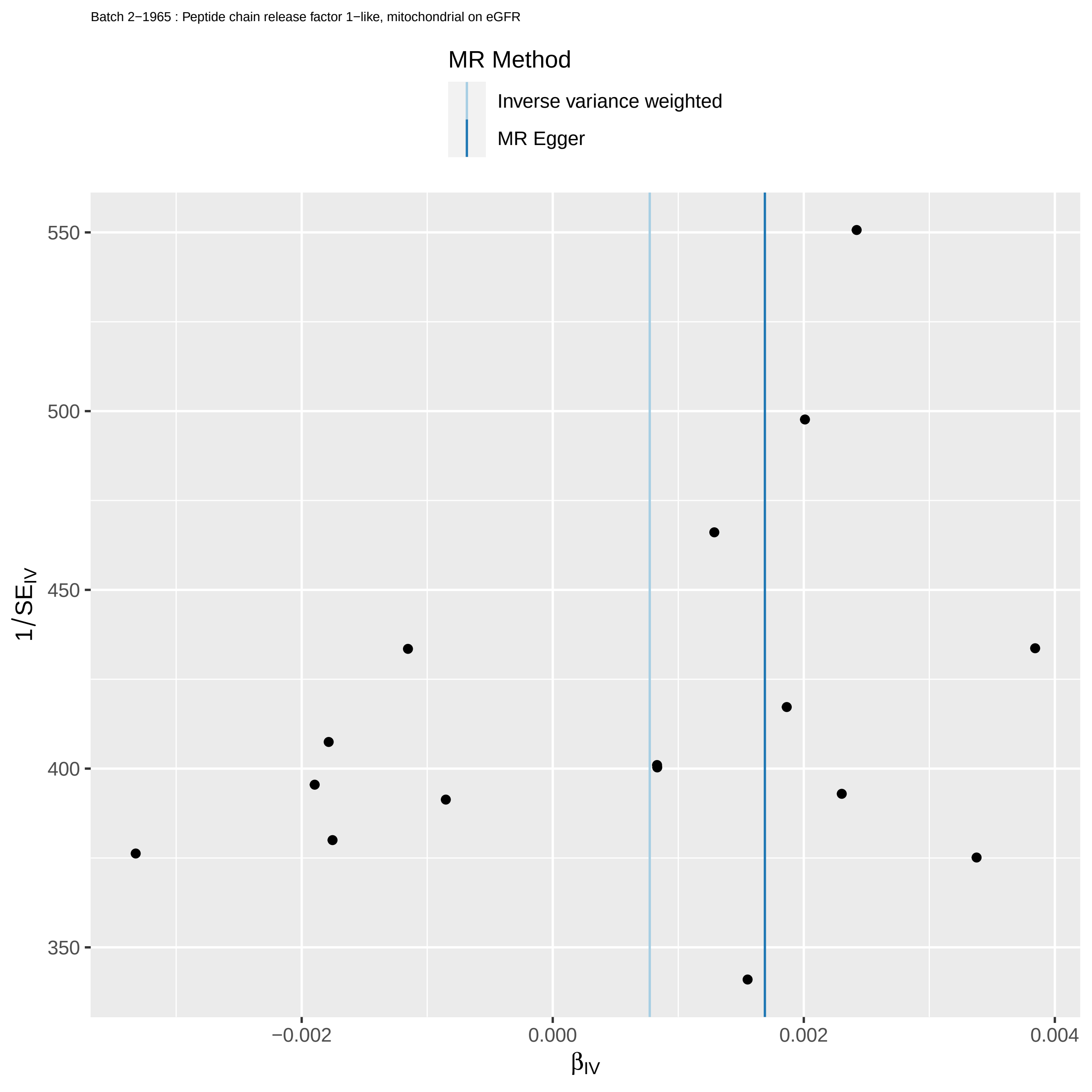
Batch 2−1965 : Peptide chain release factor 1−like, mitochondrial on eGFR
MR Method
Inverse variance weighted
MR Egger
550
500
450
400
350
1/SE$_{IV}$
−0.002
0.000
0.002
0.004
β$_{IV}$

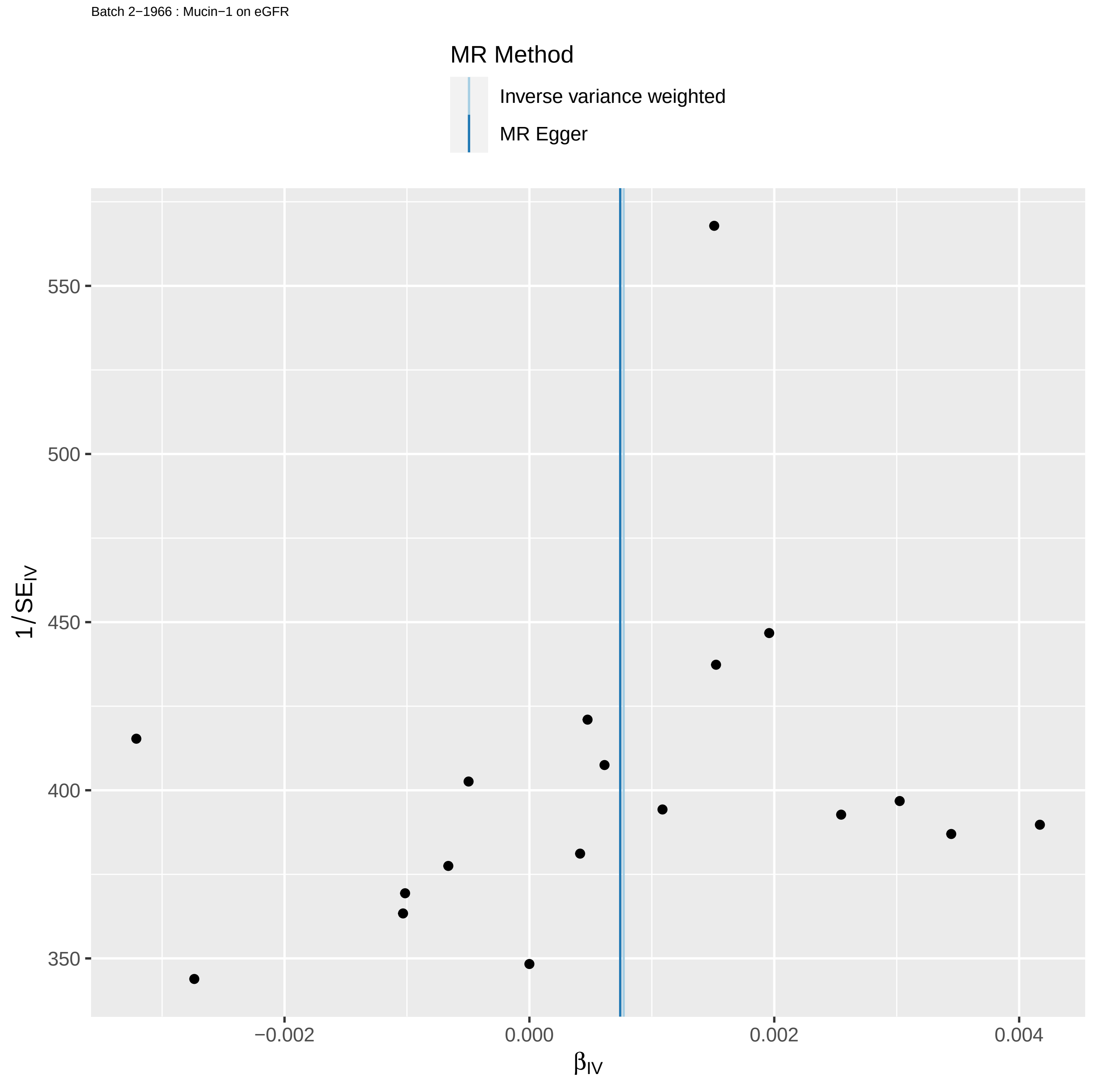
Batch 2−1966 : Mucin−1 on eGFR
MR Method
Inverse variance weighted
MR Egger
550
500
450
400
350
1/SE_IV
−0.002
0.000
0.002
0.004
β_IV

Batch 2−1967 : Mucin−1 on eGFR

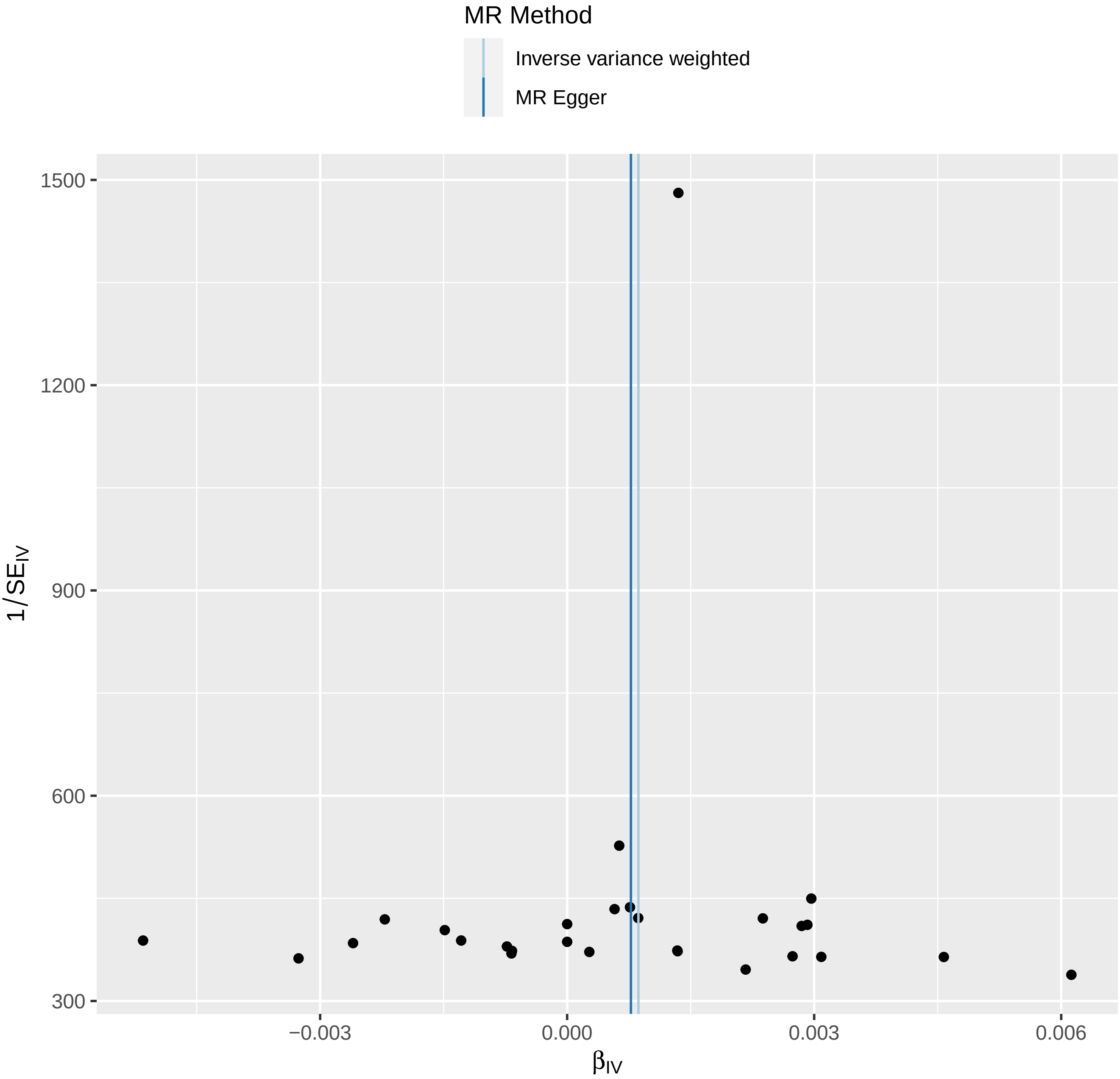

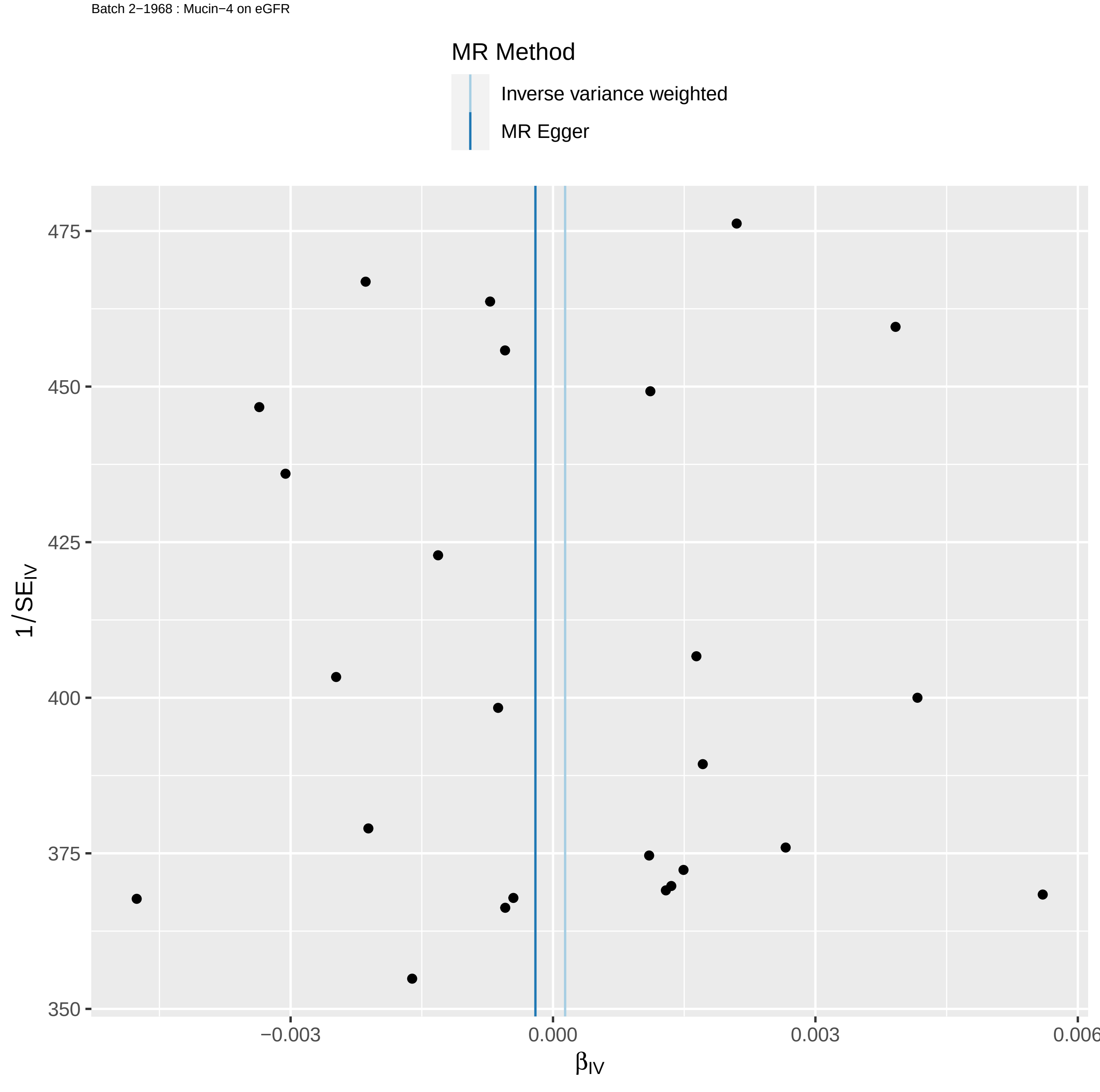
Batch 2−1968 : Mucin−4 on eGFR
MR Method
Inverse variance weighted
MR Egger
475
450
425
400
375
350
1/SE$_{IV}$
−0.003
0.000
0.003
0.006
β$_{IV}$

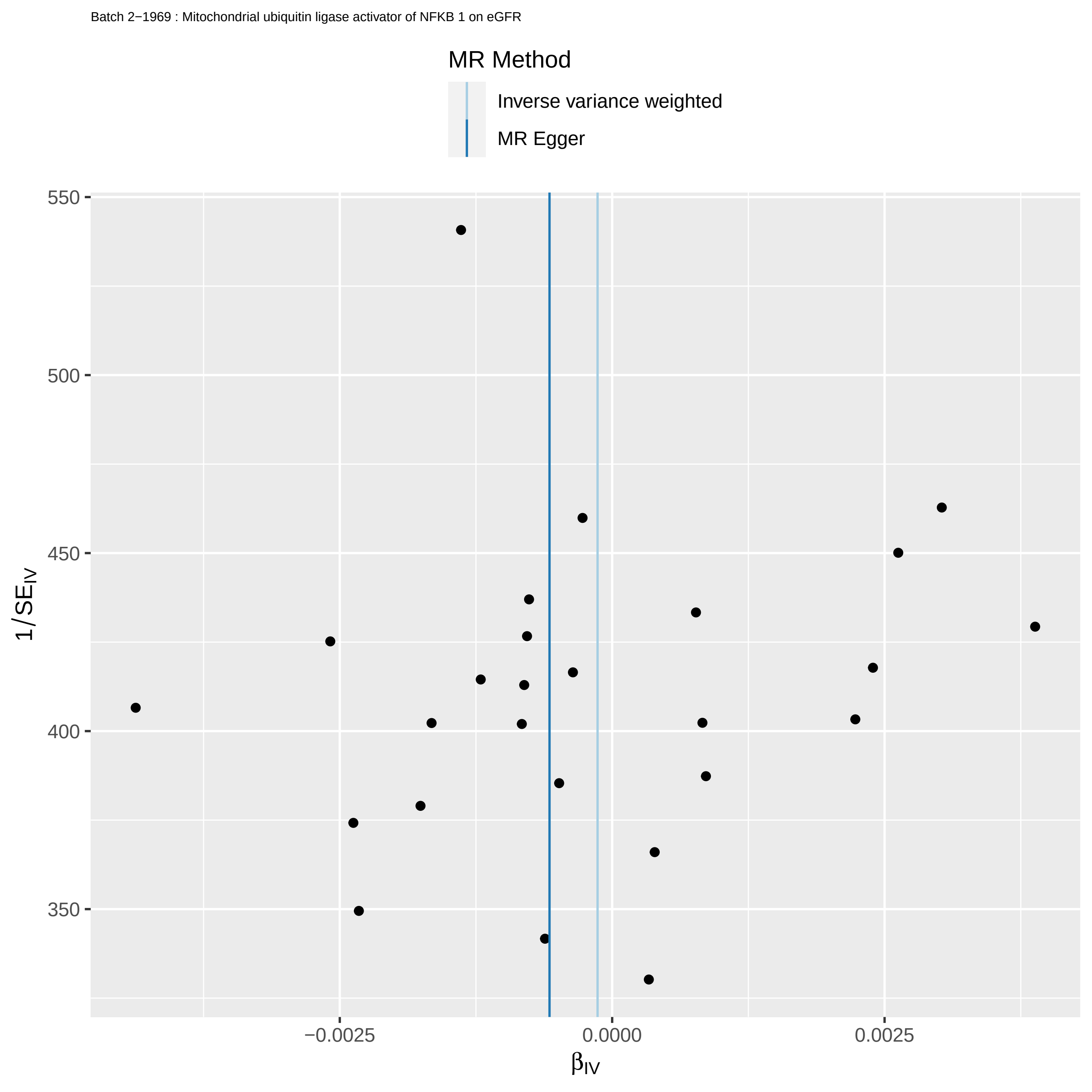

Batch 2−1969 : Mitochondrial ubiquitin ligase activator of NFKB 1 on eGFR
MR Method
Inverse variance weighted
MR Egger
550
500
450
400
350
1/SE_IV
−0.0025
0.0000
0.0025
β_IV

Batch 2−1970 : Mitochondrial ubiquitin ligase activator of NFKB 1 on eGFR

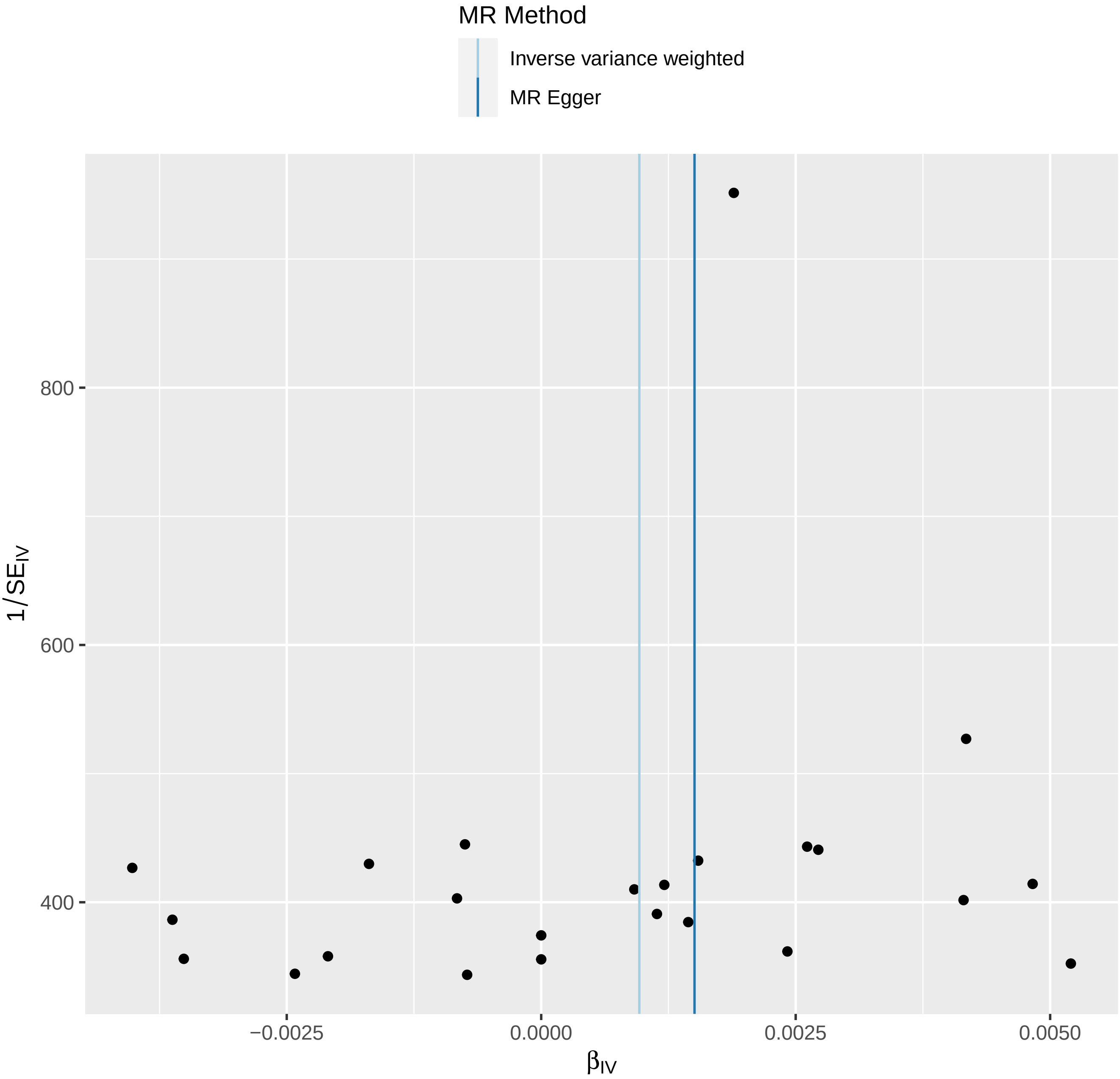

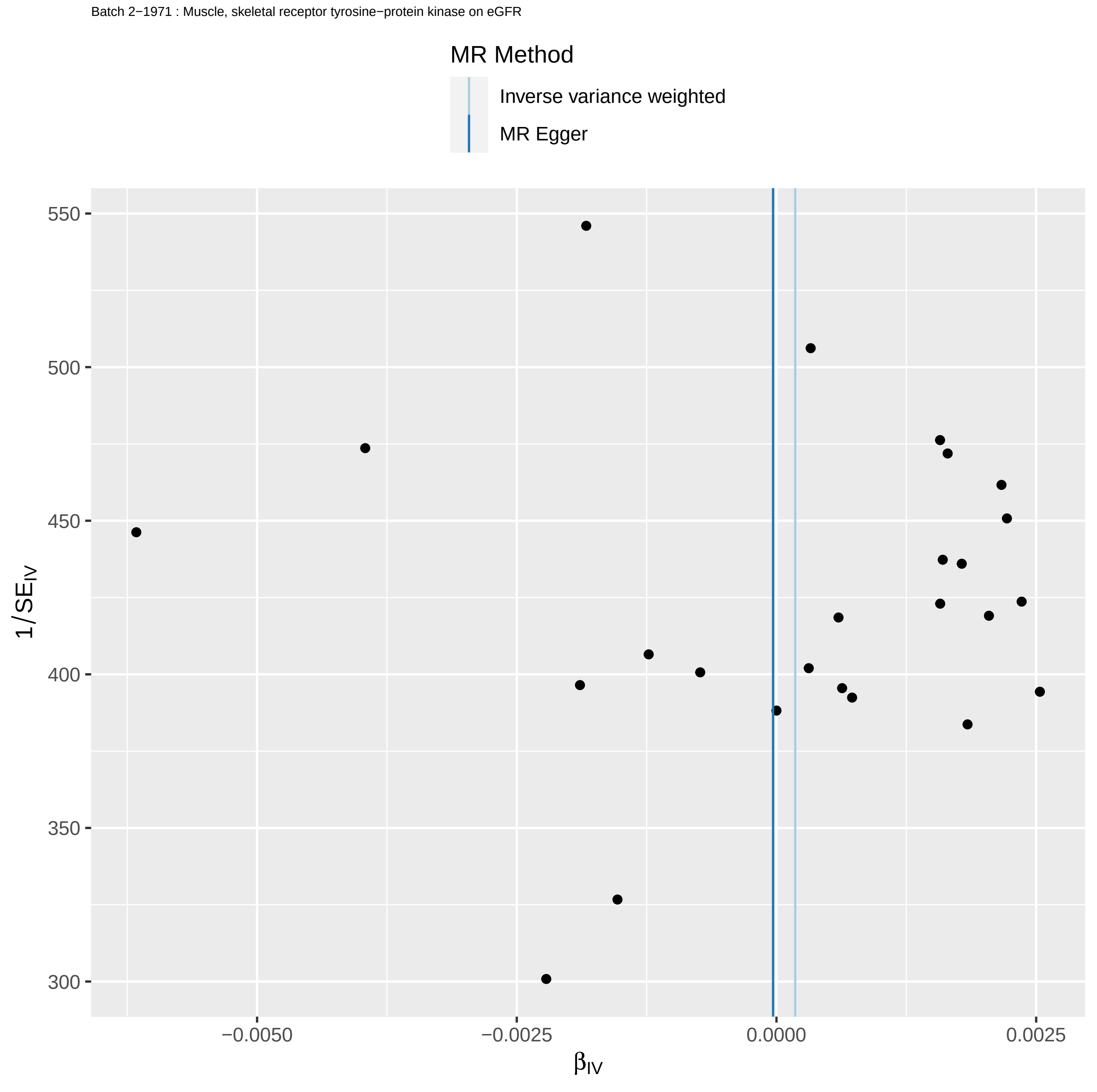

Batch 2−1971 : Muscle, skeletal receptor tyrosine−protein kinase on eGFR
MR Method
Inverse variance weighted
MR Egger
550
500
450
400
350
300
1/SE_IV
−0.0050
−0.0025
0.0000
0.0025
β_IV

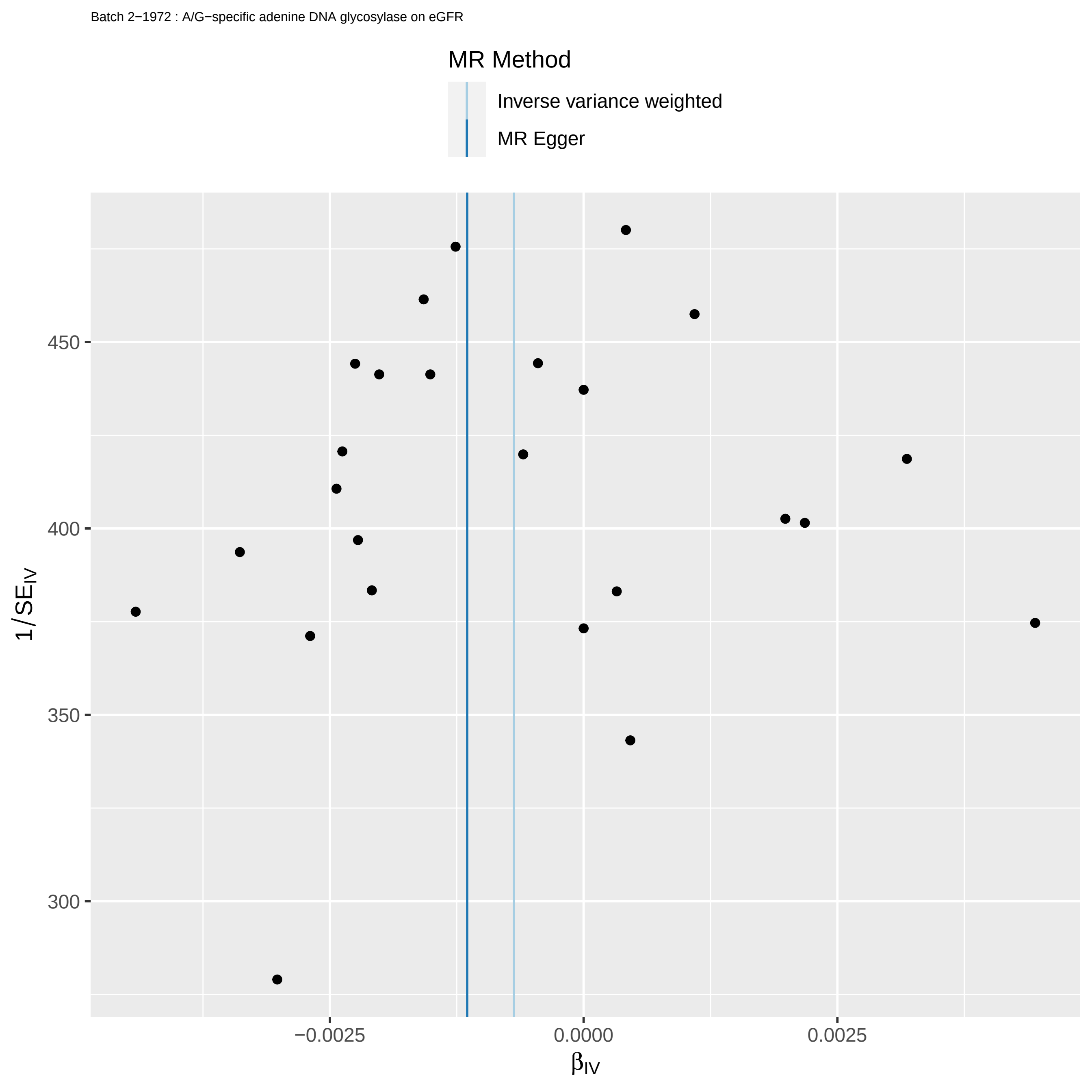

Batch 2−1972 : A/G−specific adenine DNA glycosylase on eGFR
MR Method
Inverse variance weighted
MR Egger
450
400
350
300
1/SE_IV
−0.0025
0.0000
0.0025
β_IV

Batch 2−1973 : MAX−interacting protein 1 on eGFR

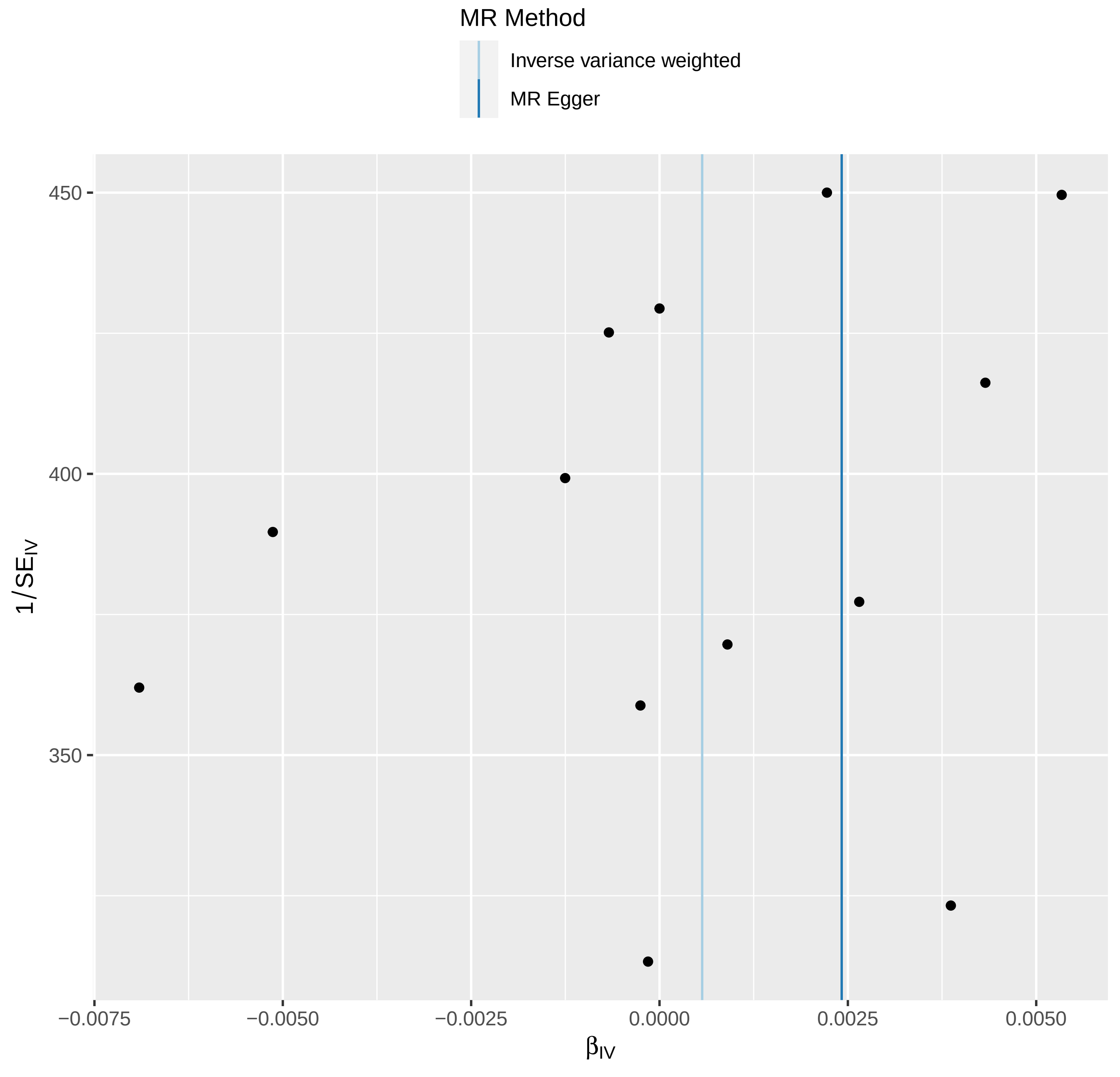

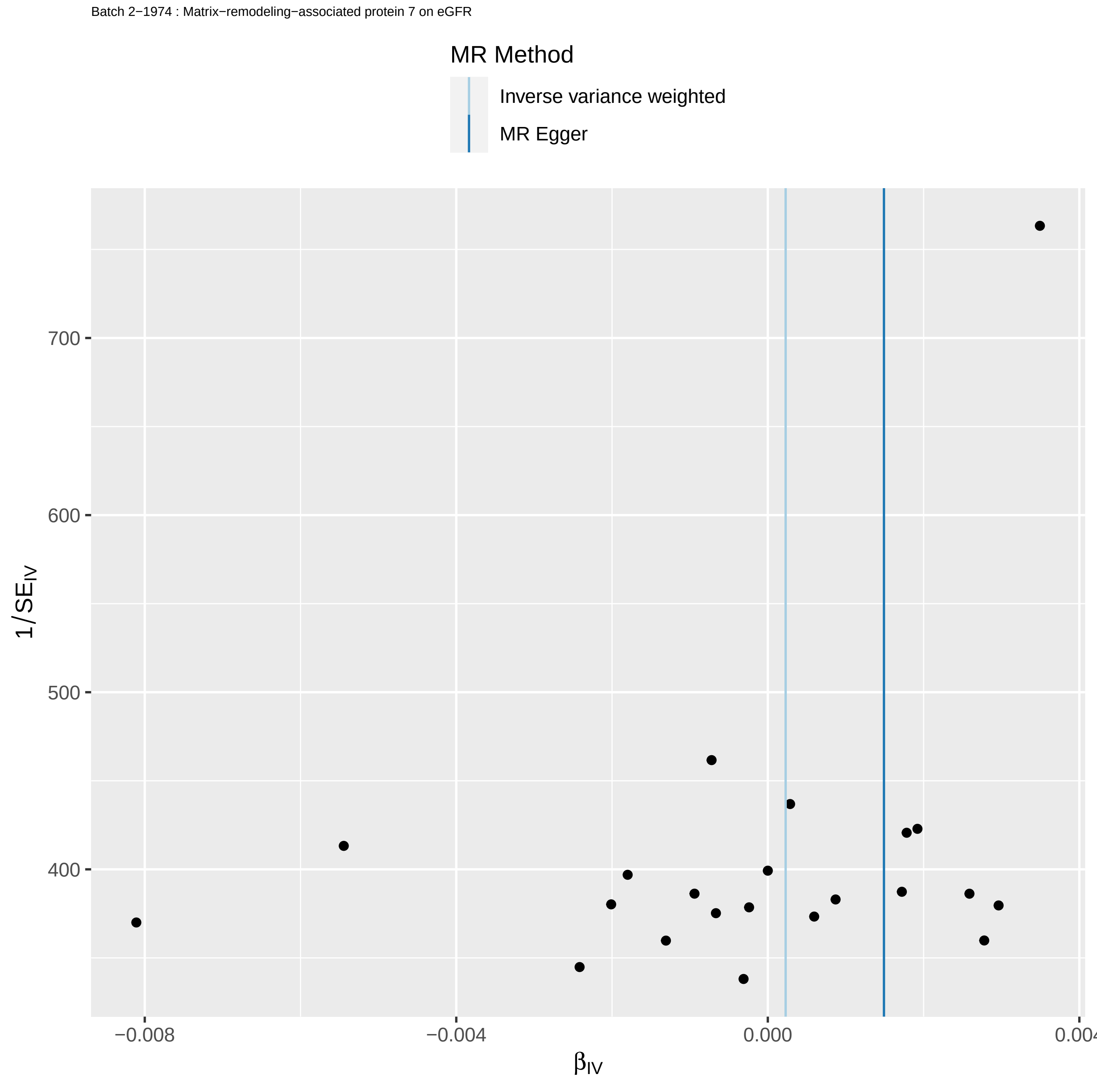

Batch 2−1974 : Matrix−remodeling−associated protein 7 on eGFR
MR Method
Inverse variance weighted
MR Egger
700
600
500
400
1/SE$_{IV}$
−0.008
−0.004
0.000
0.004
β$_{IV}$

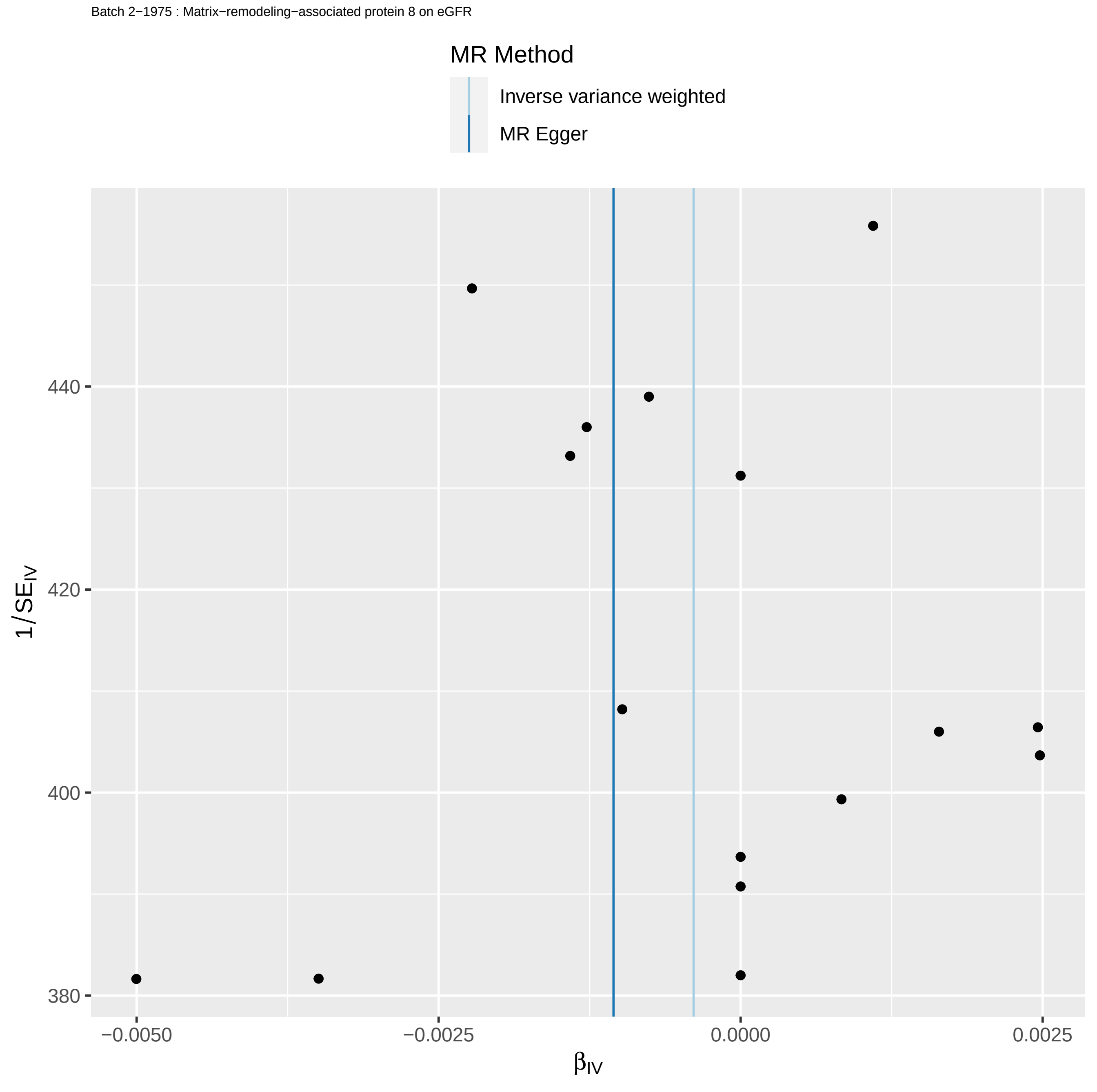

Batch 2−1975 : Matrix−remodeling−associated protein 8 on eGFR
MR Method
Inverse variance weighted
MR Egger
440
420
400
380
1/SE_IV
−0.0050
−0.0025
0.0000
0.0025
β_IV

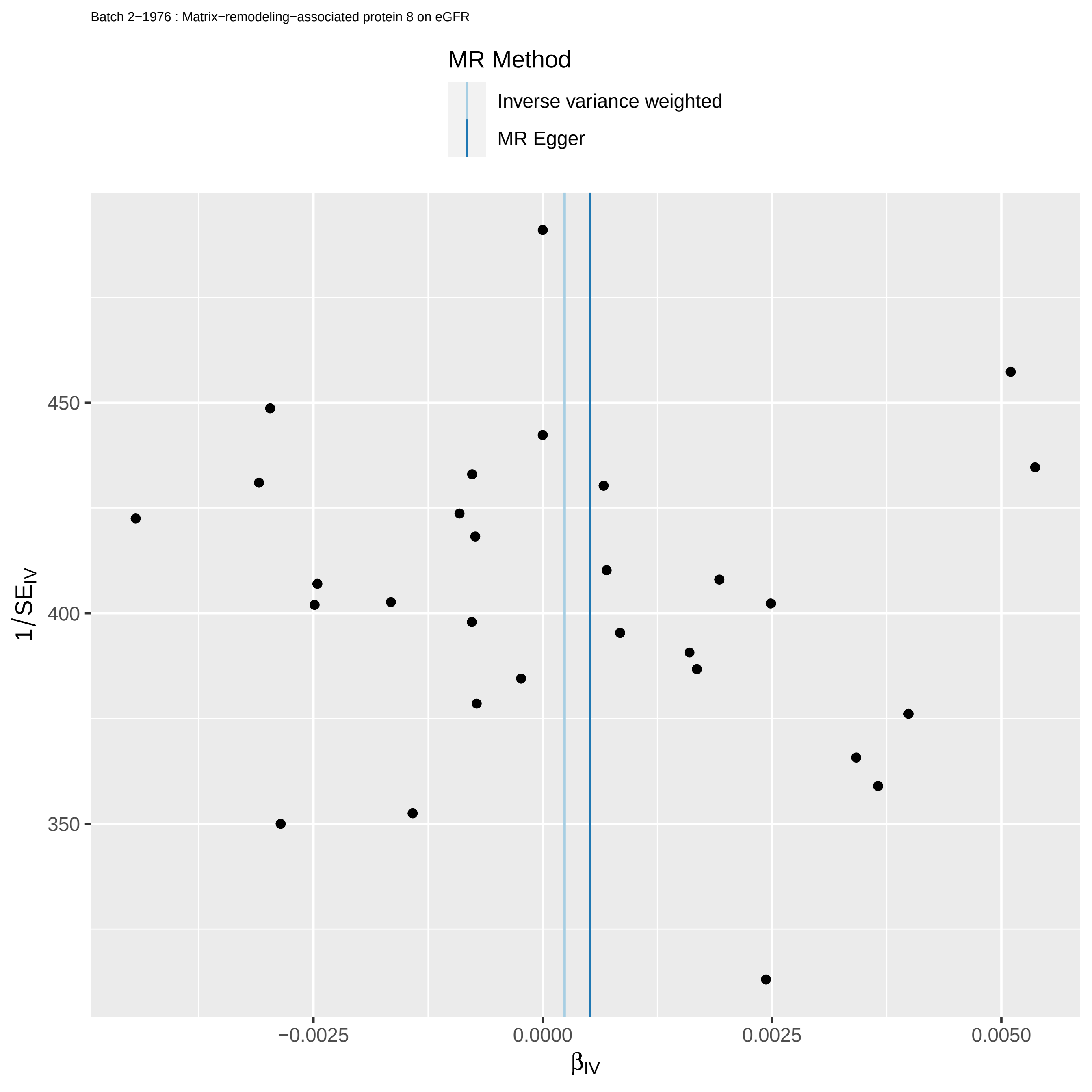

Batch 2−1976 : Matrix−remodeling−associated protein 8 on eGFR
MR Method
Inverse variance weighted
MR Egger
450
400
350
1/SE_IV
−0.0025
0.0000
0.0025
0.0050
β_IV

Batch 2−1977 : Myosin−binding protein C, slow−type on eGFR

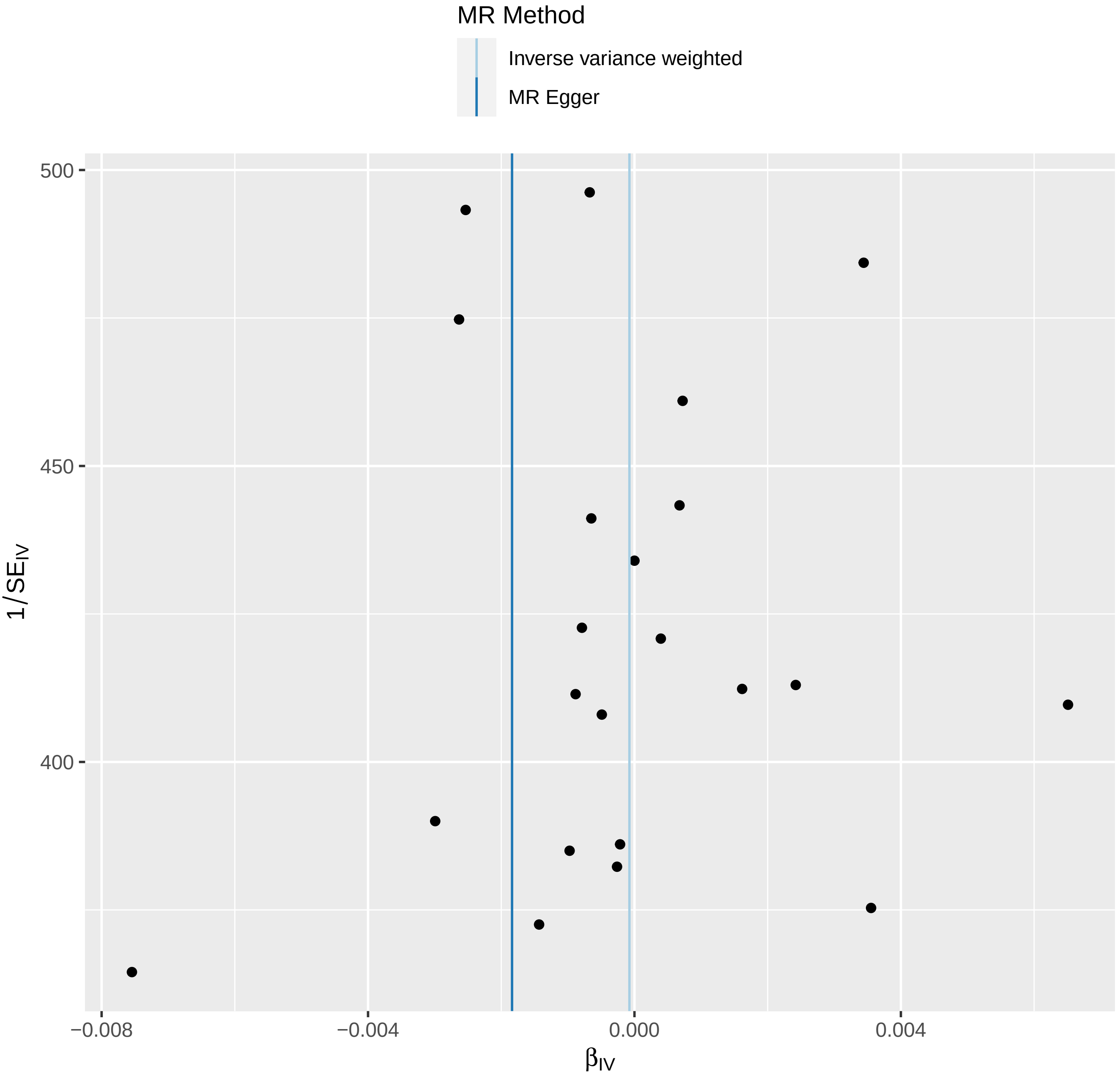

Batch 2−1978 : Myc proto−oncogene protein on eGFR

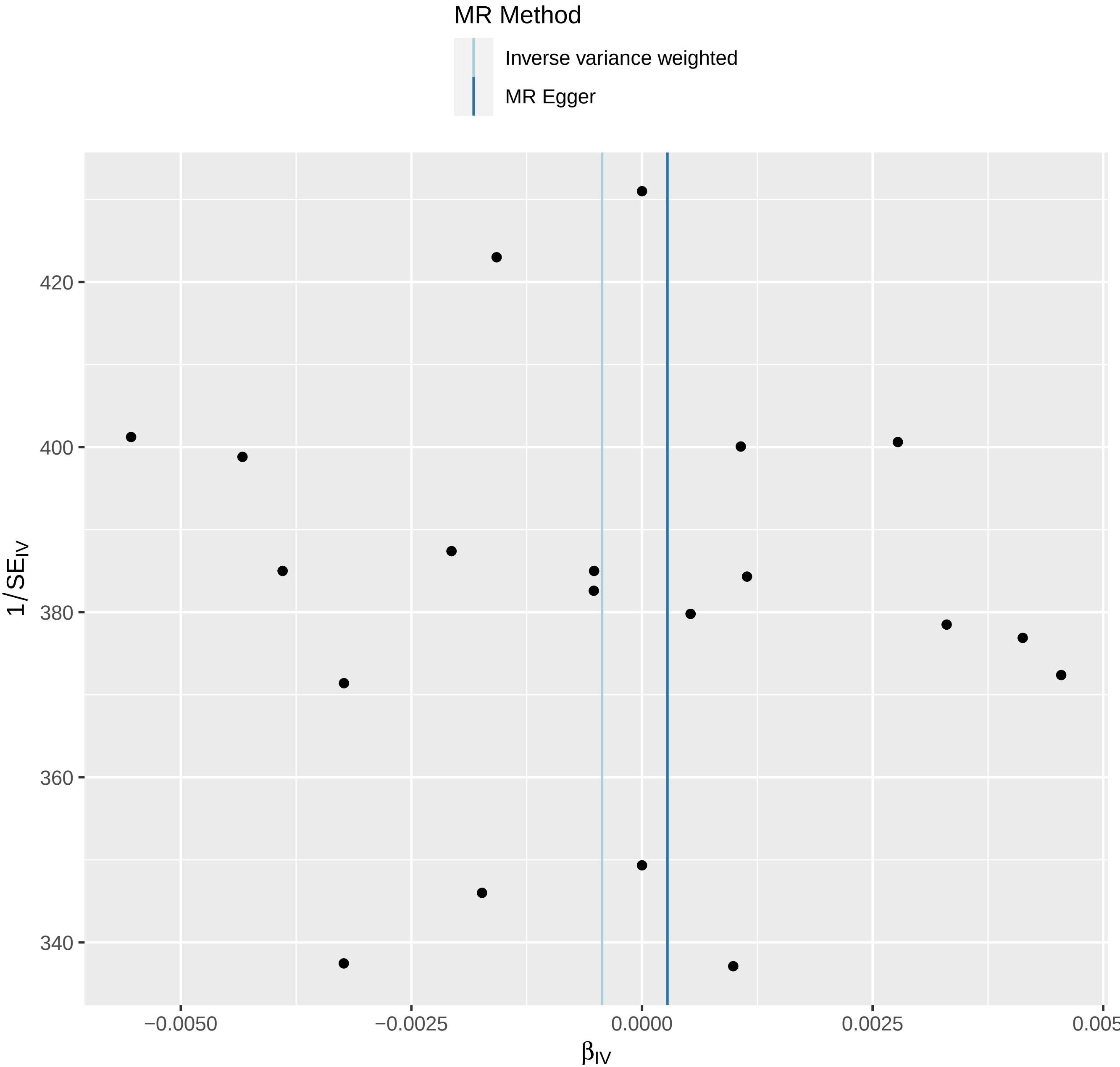

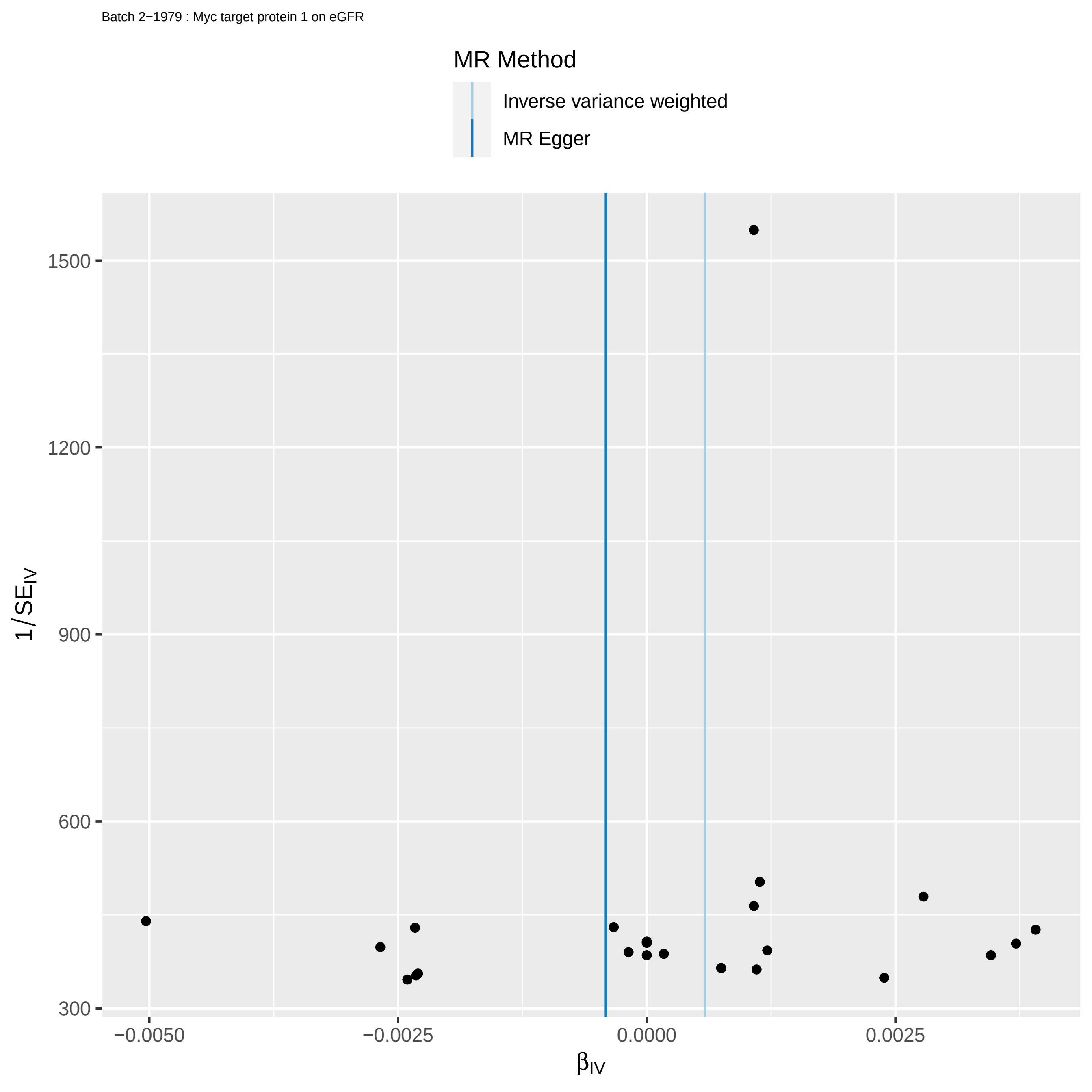

Batch 2−1979 : Myc target protein 1 on eGFR
MR Method
Inverse variance weighted
MR Egger
1500
1200
900
600
300
1/SE_IV
−0.0050
−0.0025
0.0000
0.0025
β_IV

Batch 2−1980 : Unconventional myosin−VI on eGFR

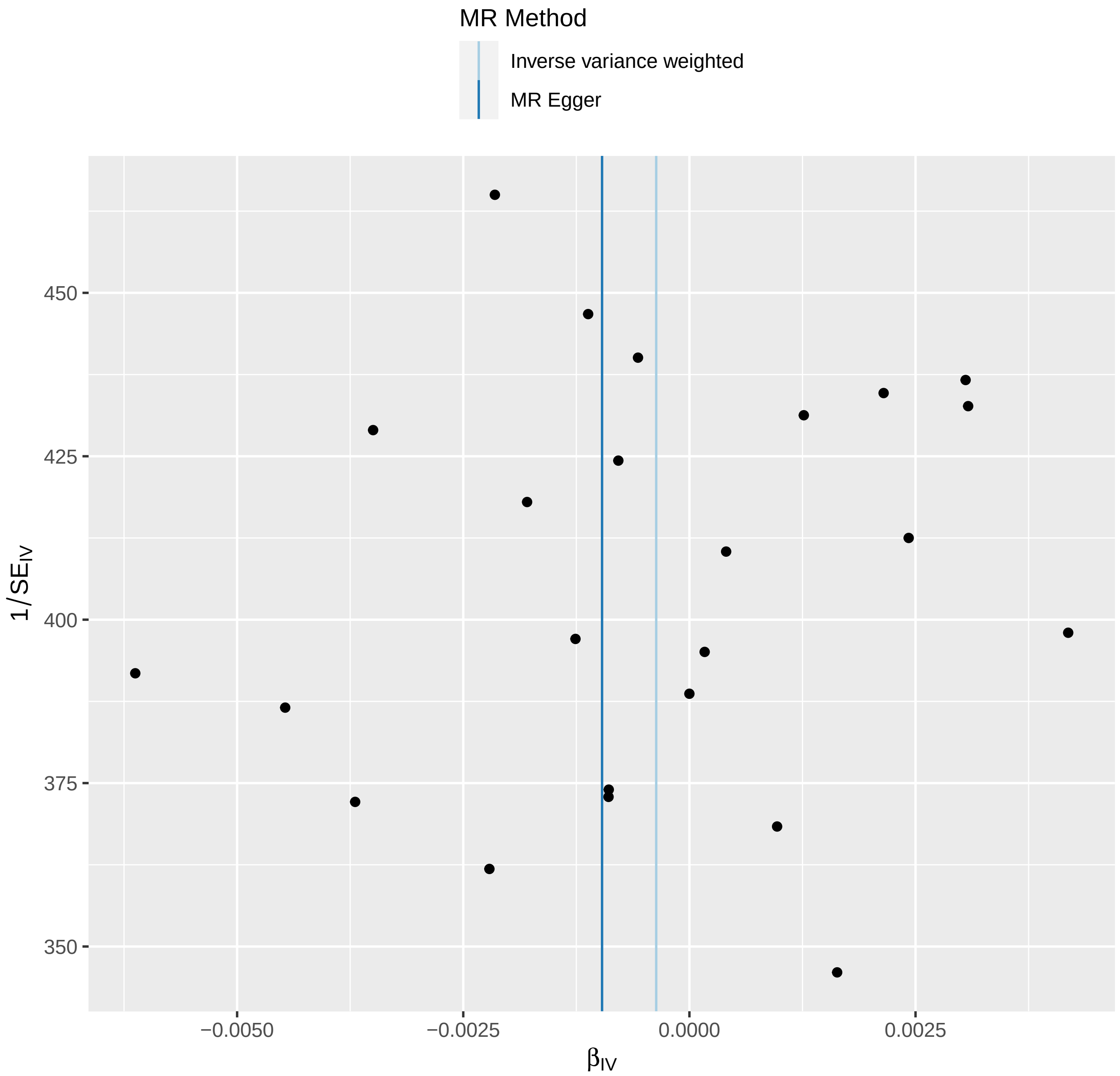

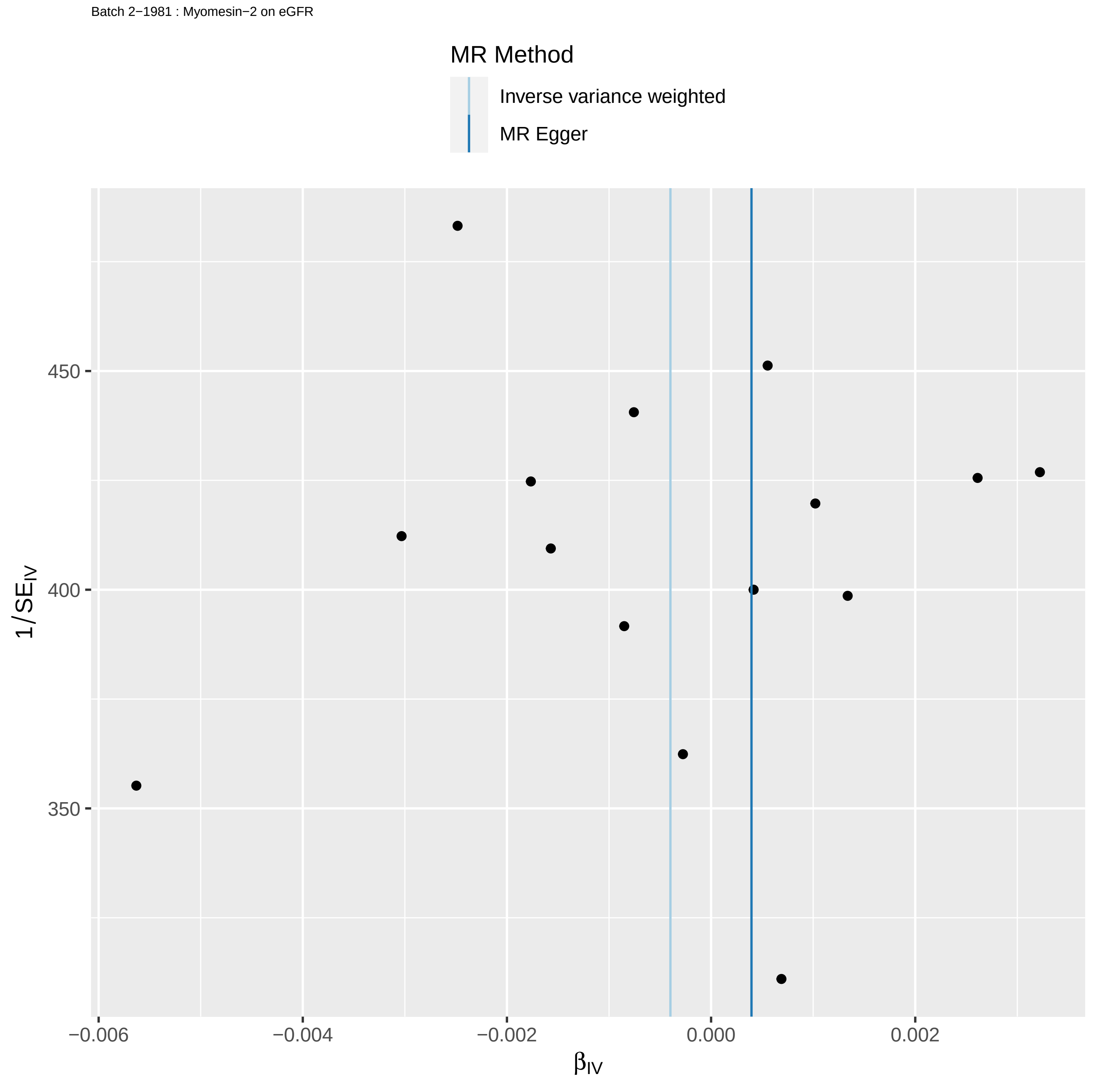
Batch 2−1981 : Myomesin−2 on eGFR
MR Method
Inverse variance weighted
MR Egger
450
400
350
1/SE_IV
−0.006
−0.004
−0.002
0.000
0.002
β_IV

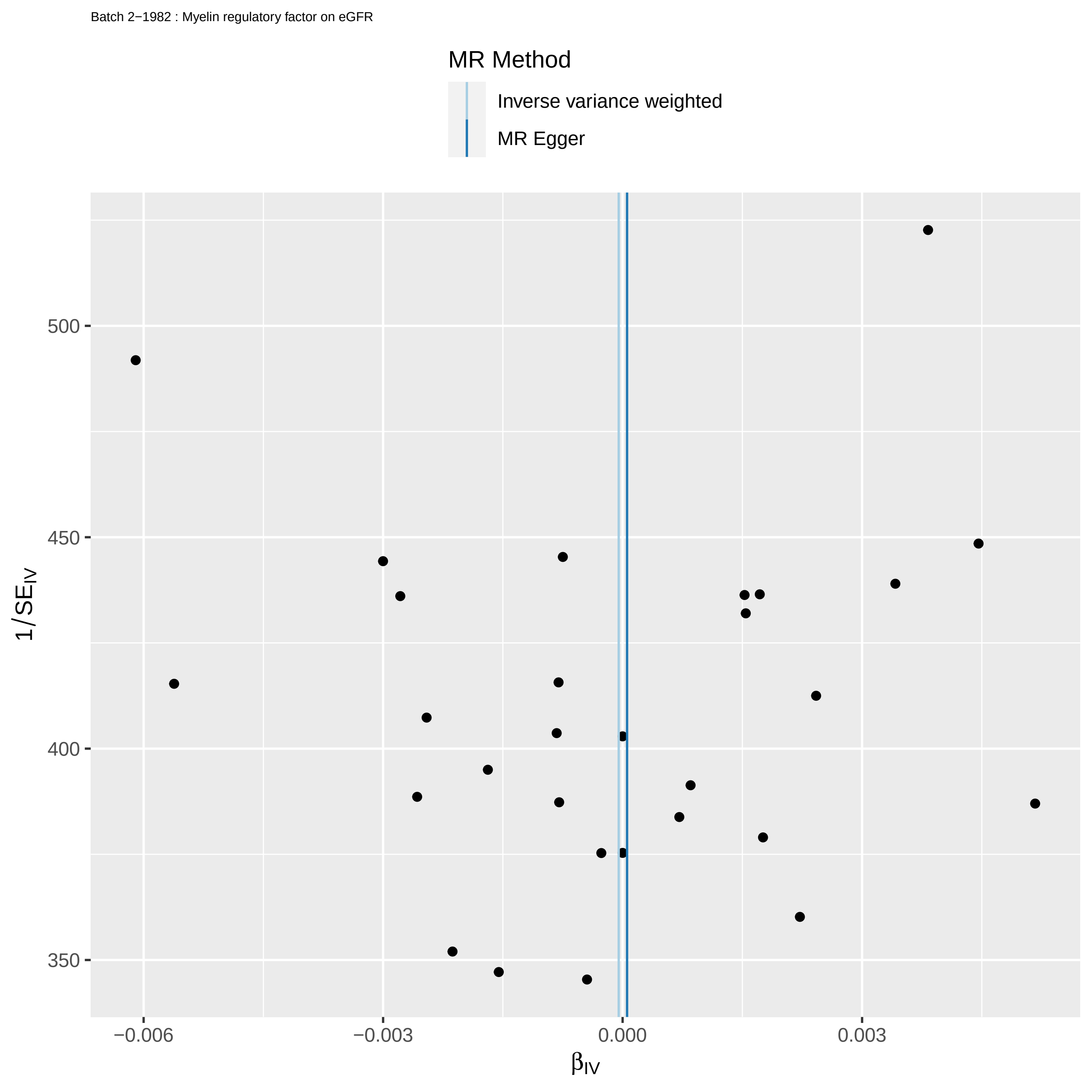
Batch 2−1982 : Myelin regulatory factor on eGFR
MR Method
Inverse variance weighted
MR Egger
500
450
400
350
1/SE_IV
−0.006
−0.003
0.000
0.003
β_IV

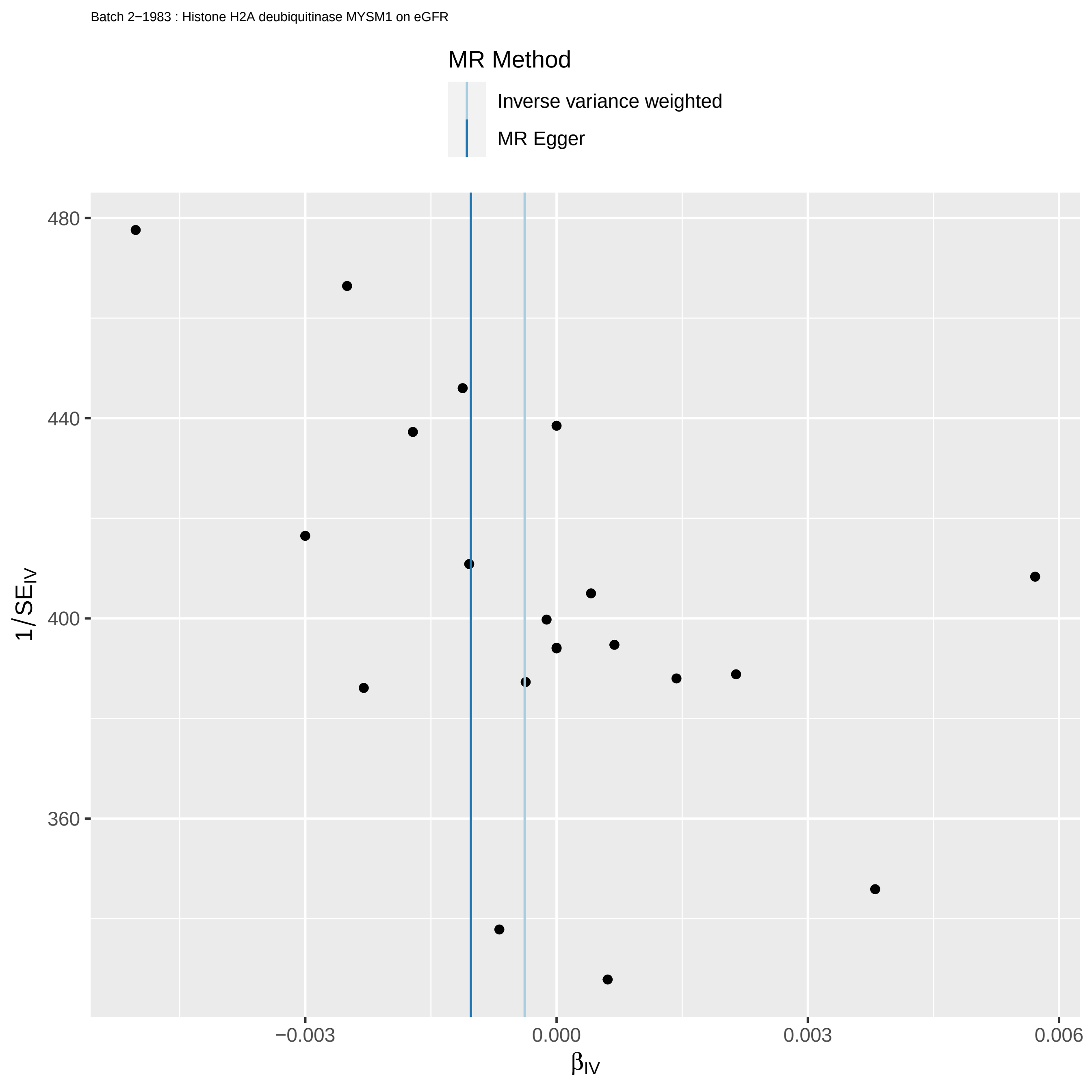

Batch 2−1983 : Histone H2A deubiquitinase MYSM1 on eGFR
MR Method
Inverse variance weighted
MR Egger
480
440
400
360
1/SE_IV
−0.003
0.000
0.003
0.006
β_IV

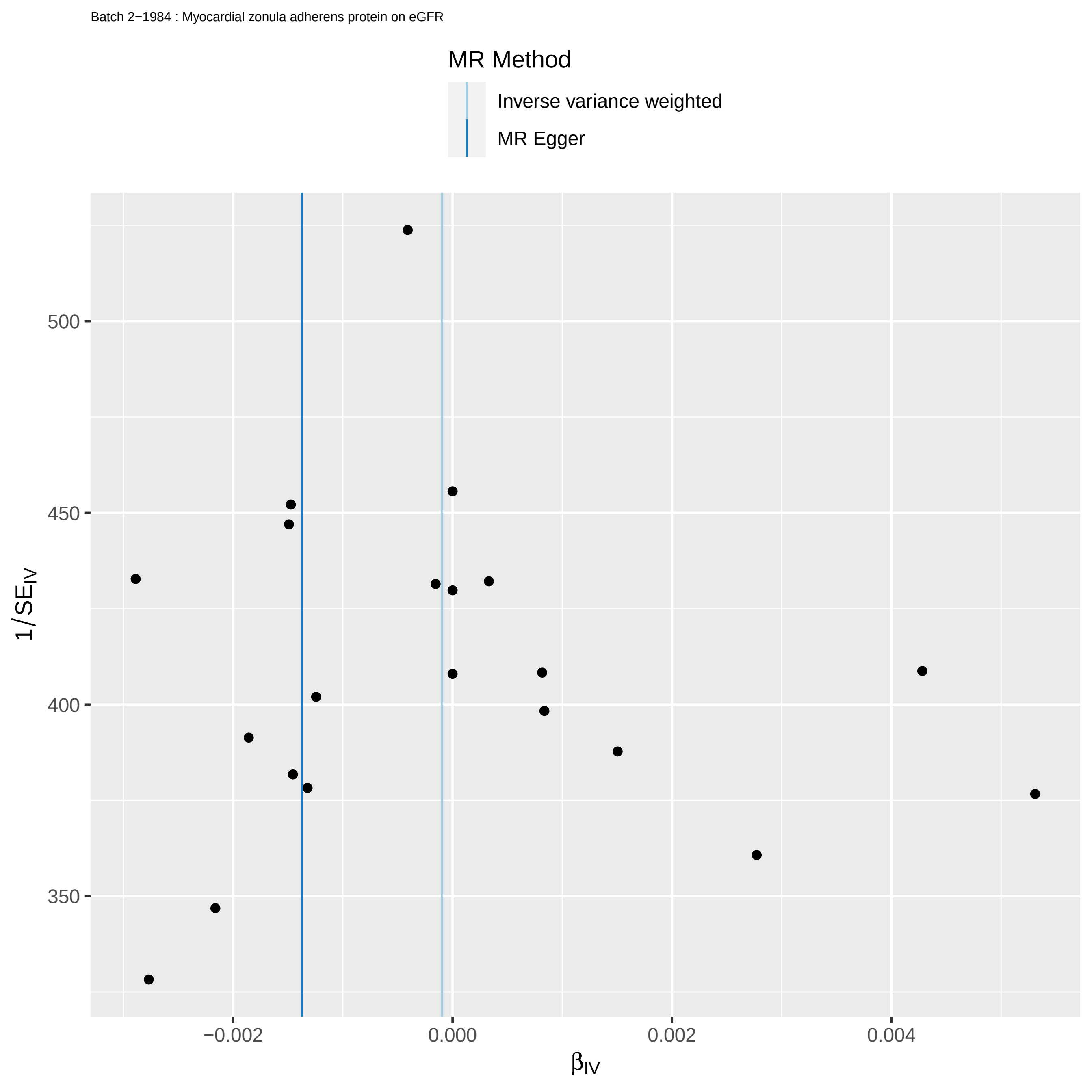
Batch 2−1984 : Myocardial zonula adherens protein on eGFR
MR Method
Inverse variance weighted
MR Egger
500
450
400
350
1/SE$_{IV}$
−0.002
0.000
0.002
0.004
β$_{IV}$

Batch 2−1985 : Myocardial zonula adherens protein on eGFR

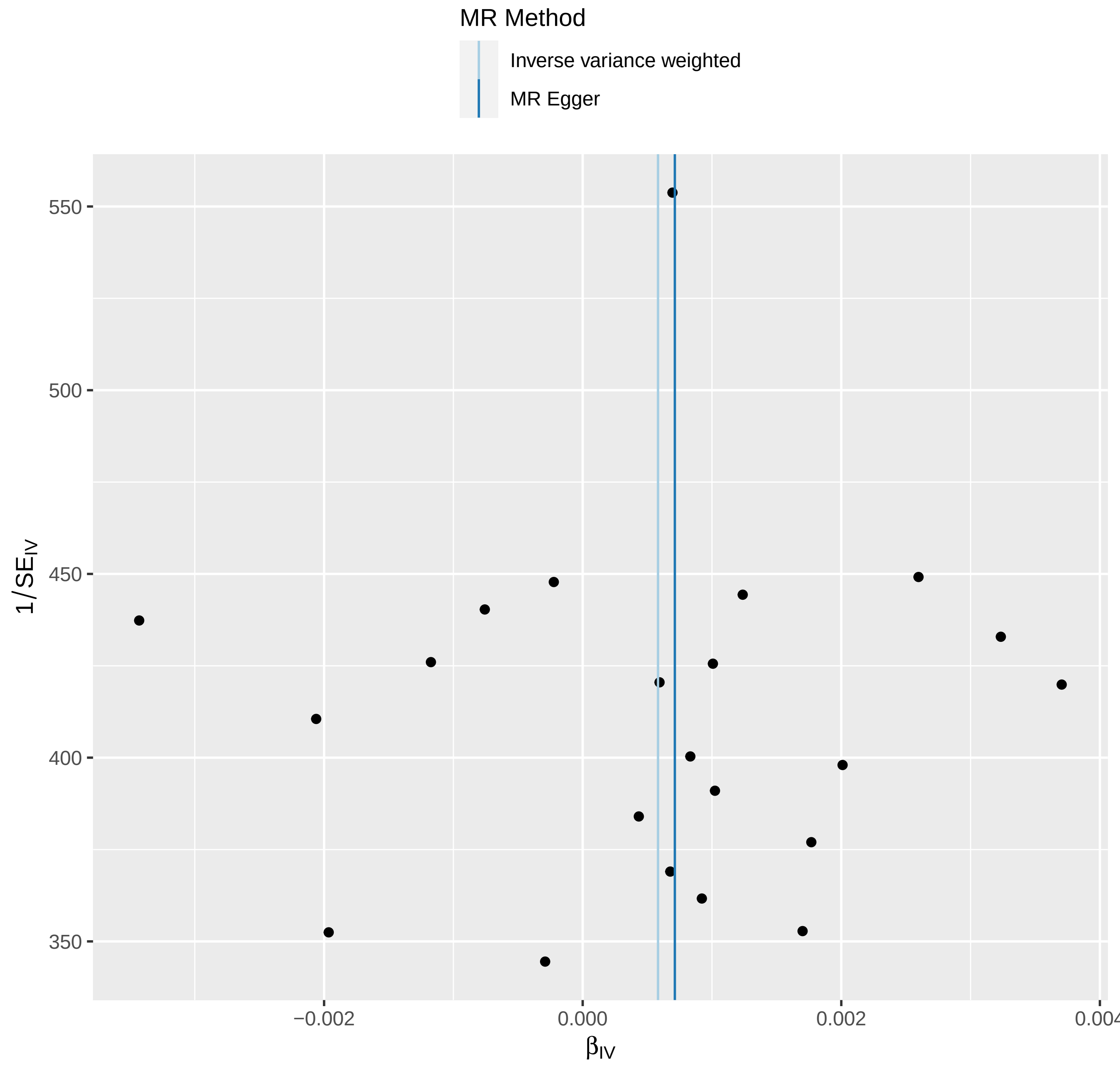

Batch 2−1986 : Myocardial zonula adherens protein on eGFR

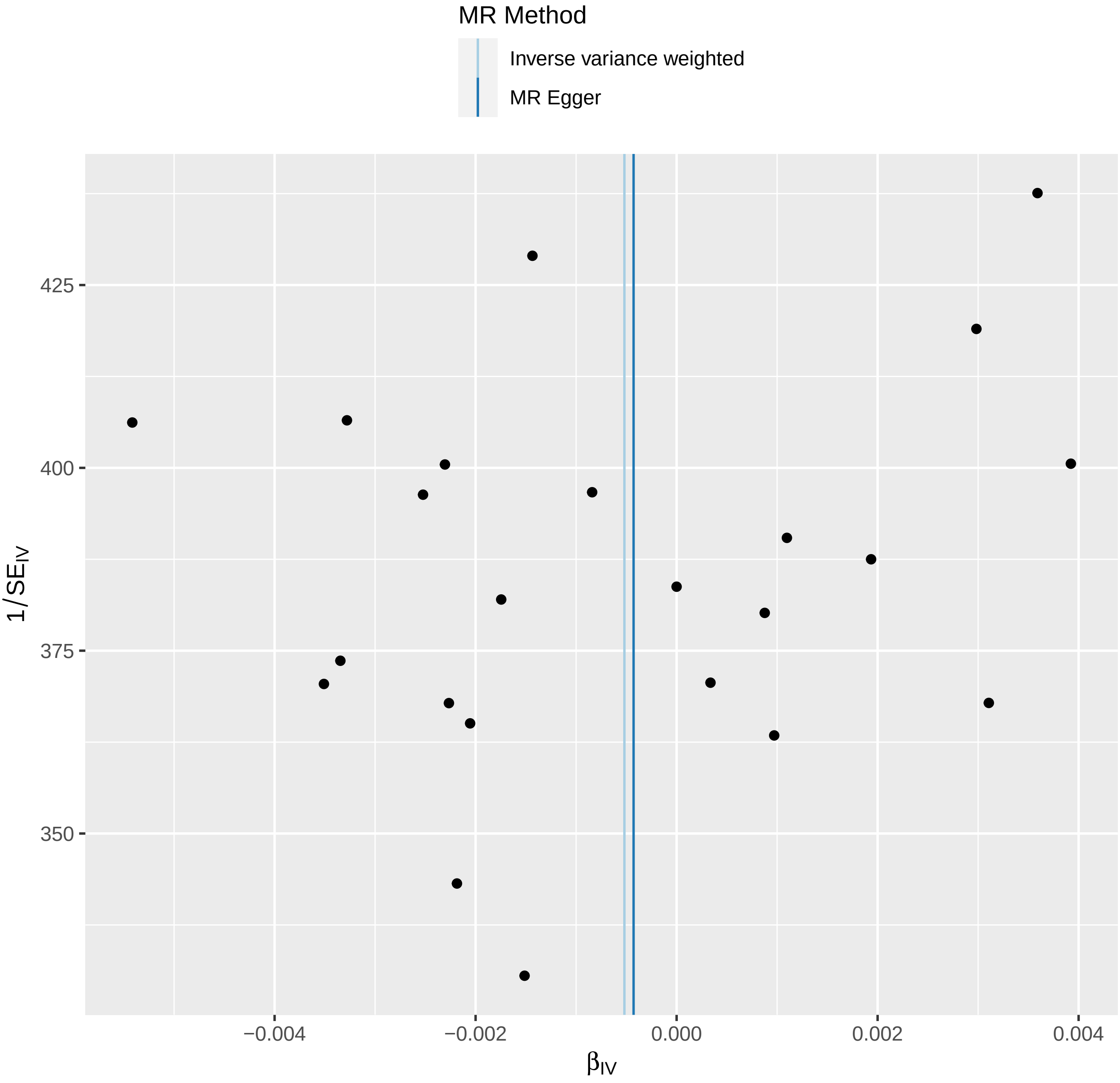

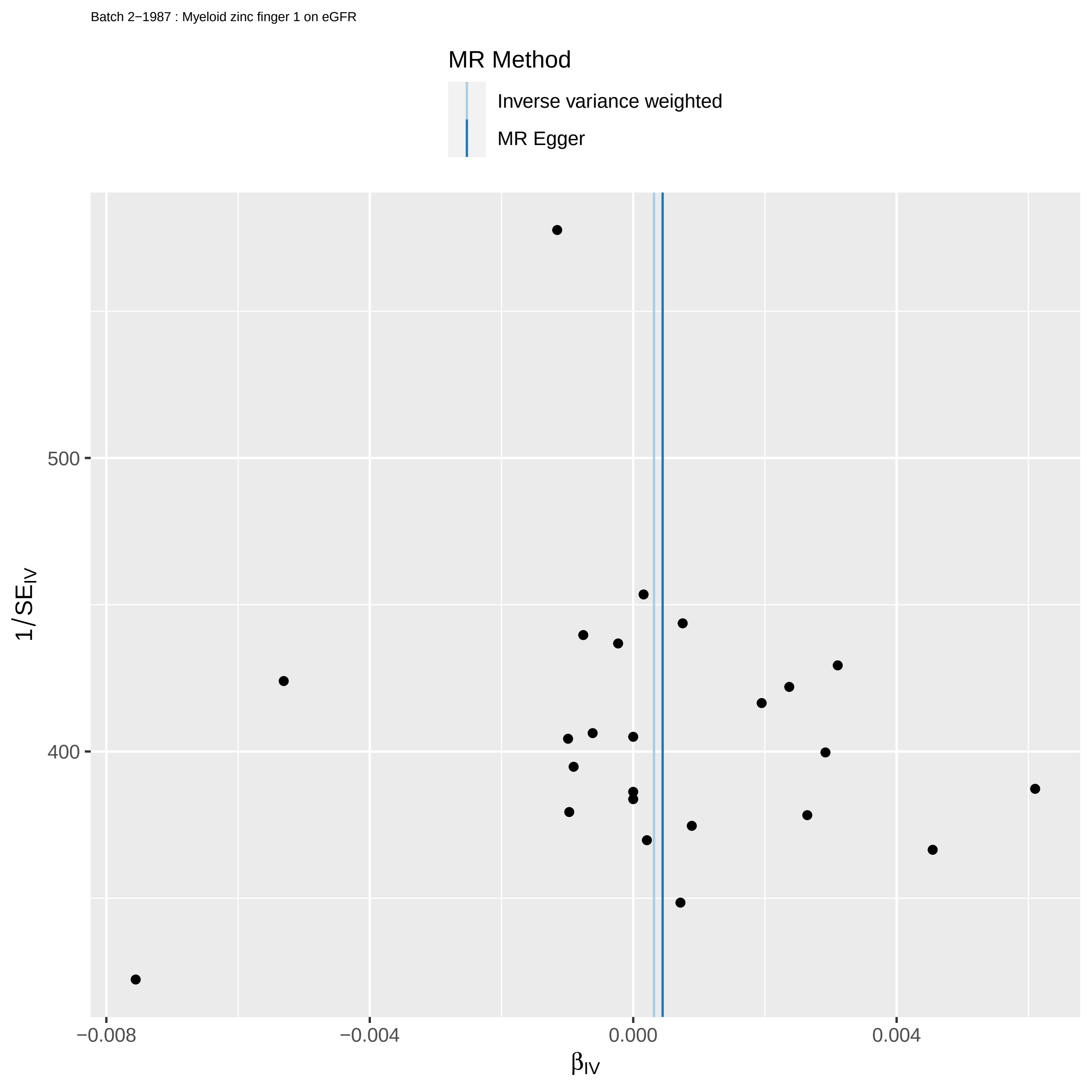

Batch 2−1987 : Myeloid zinc finger 1 on eGFR
MR Method
Inverse variance weighted
MR Egger
500
400
1/SE_IV
−0.008
−0.004
0.000
0.004
β_IV

Batch 2−1988 : Mitotic−spindle organizing protein 1 on eGFR

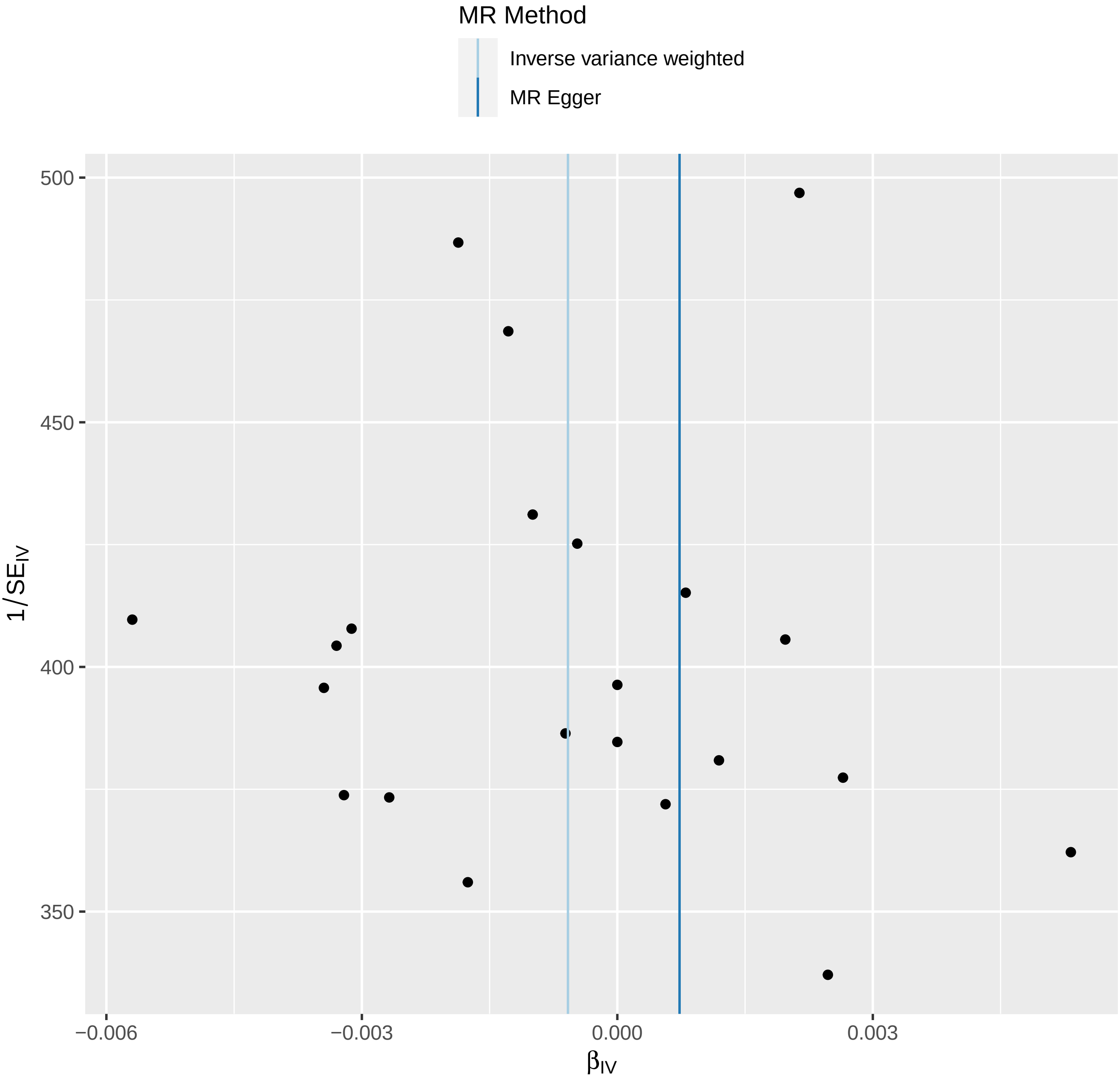

Batch 2−1989 : HemK methyltransferase family member 2 on eGFR

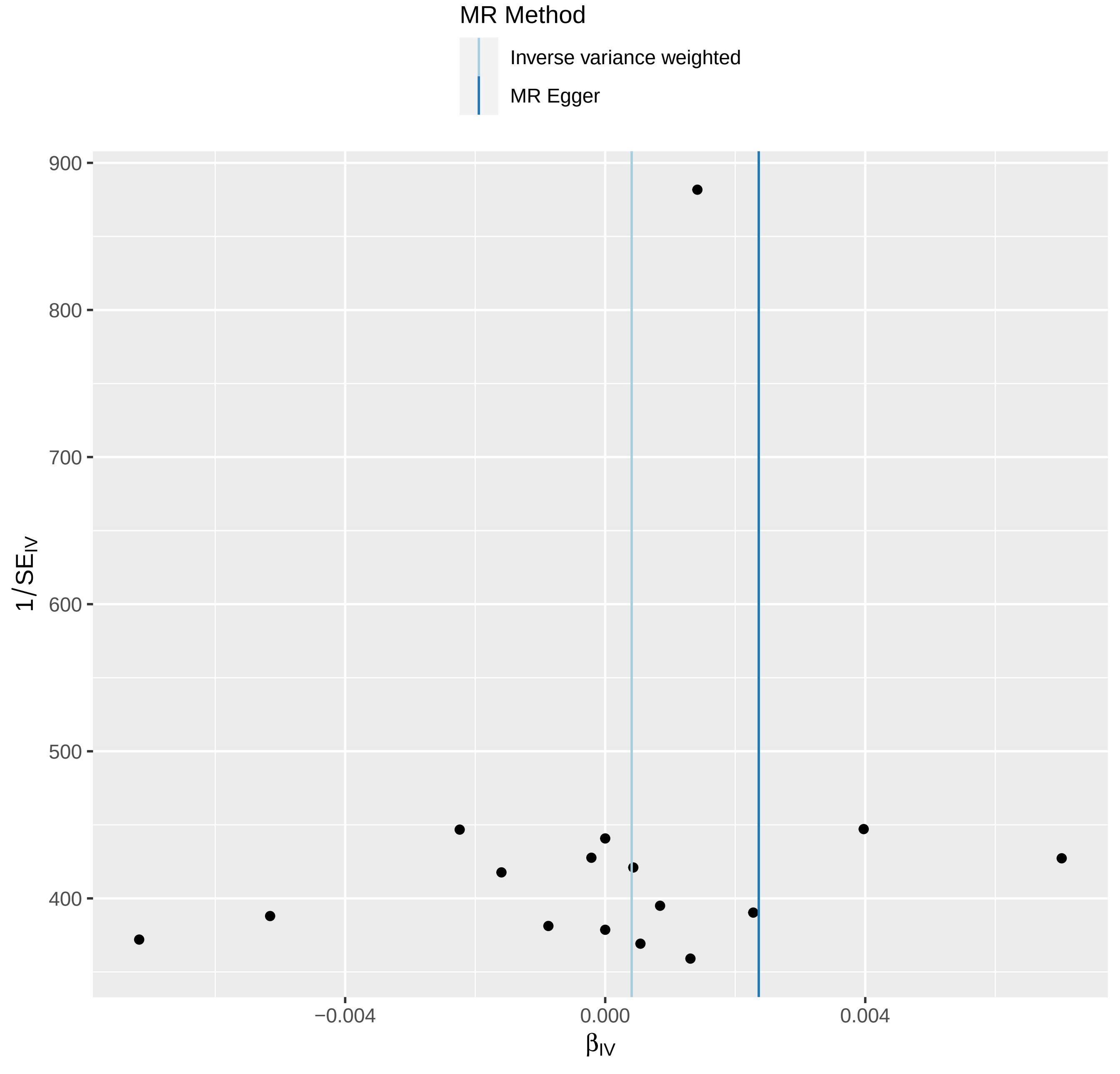

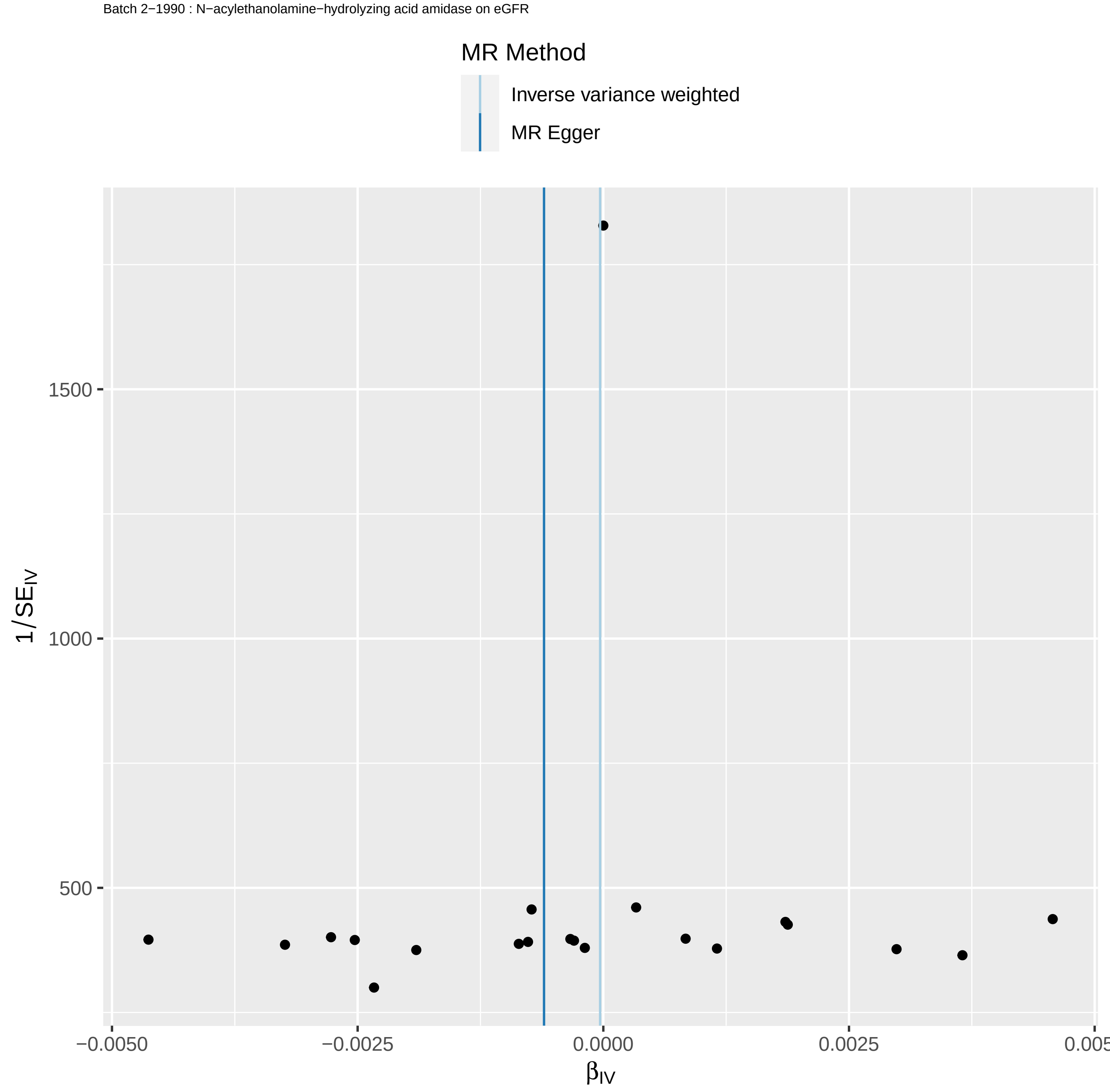

Batch 2−1990 : N−acylethanolamine−hydrolyzing acid amidase on eGFR
MR Method
Inverse variance weighted
MR Egger
1500
1000
500
1/SE_IV
−0.0050
−0.0025
0.0000
0.0025
0.005
β_IV

Batch 2−1991 : N−acetylated−alpha−linked acidic dipeptidase 2 on eGFR

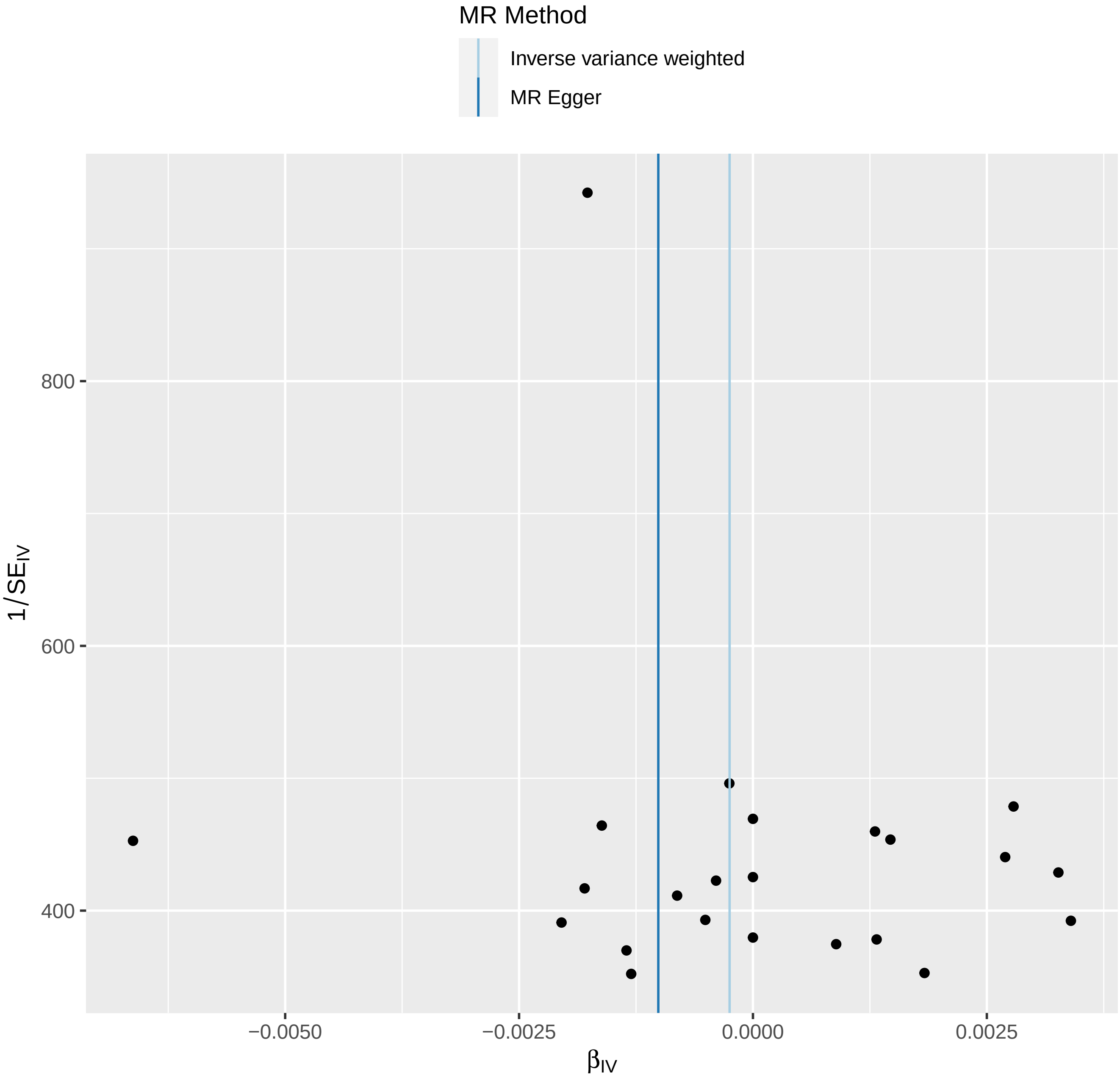

Batch 2−1992 : NGFI−A−binding protein 1 on eGFR

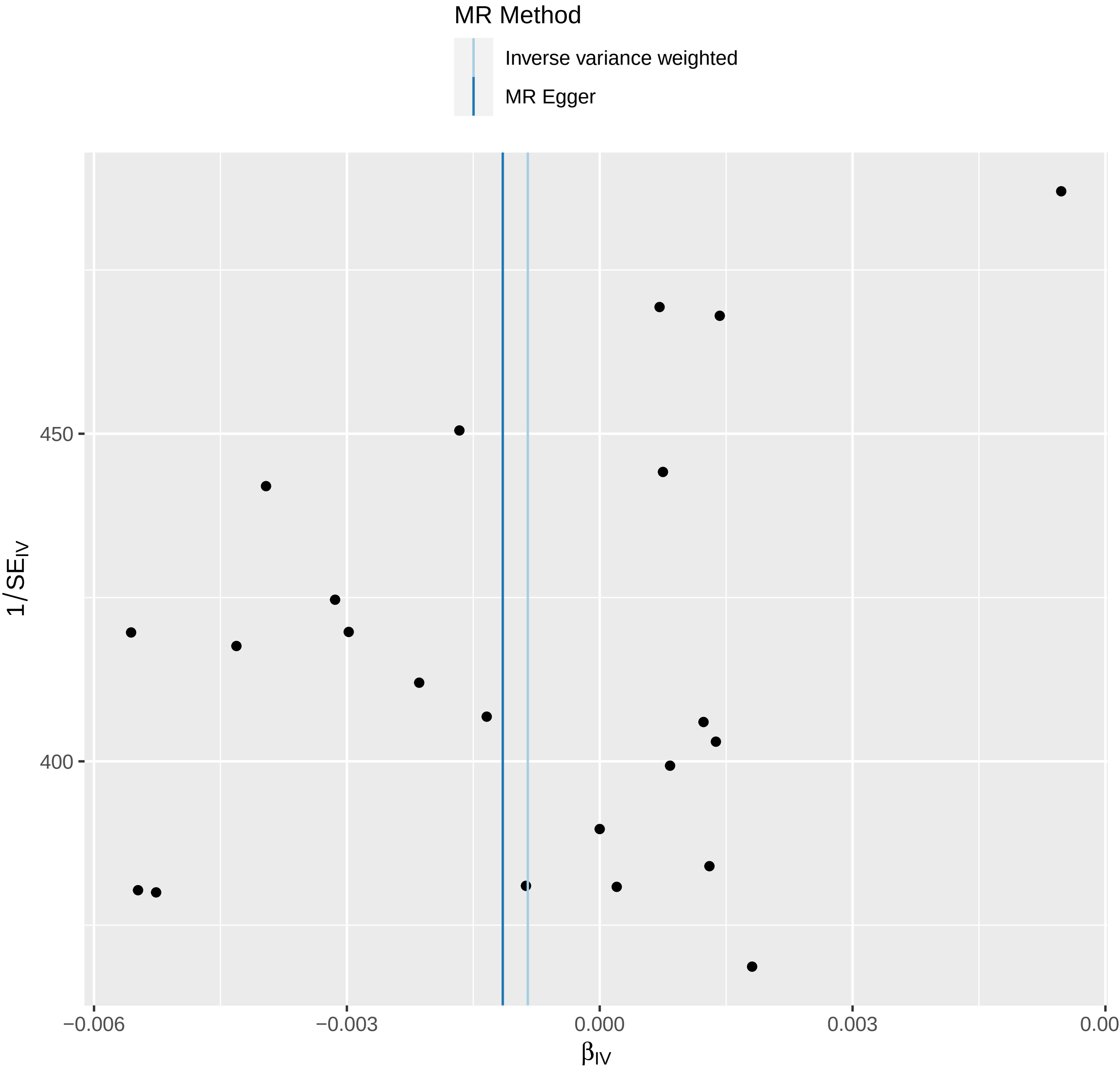

Batch 2−1993 : NGFI−A−binding protein 2 on eGFR

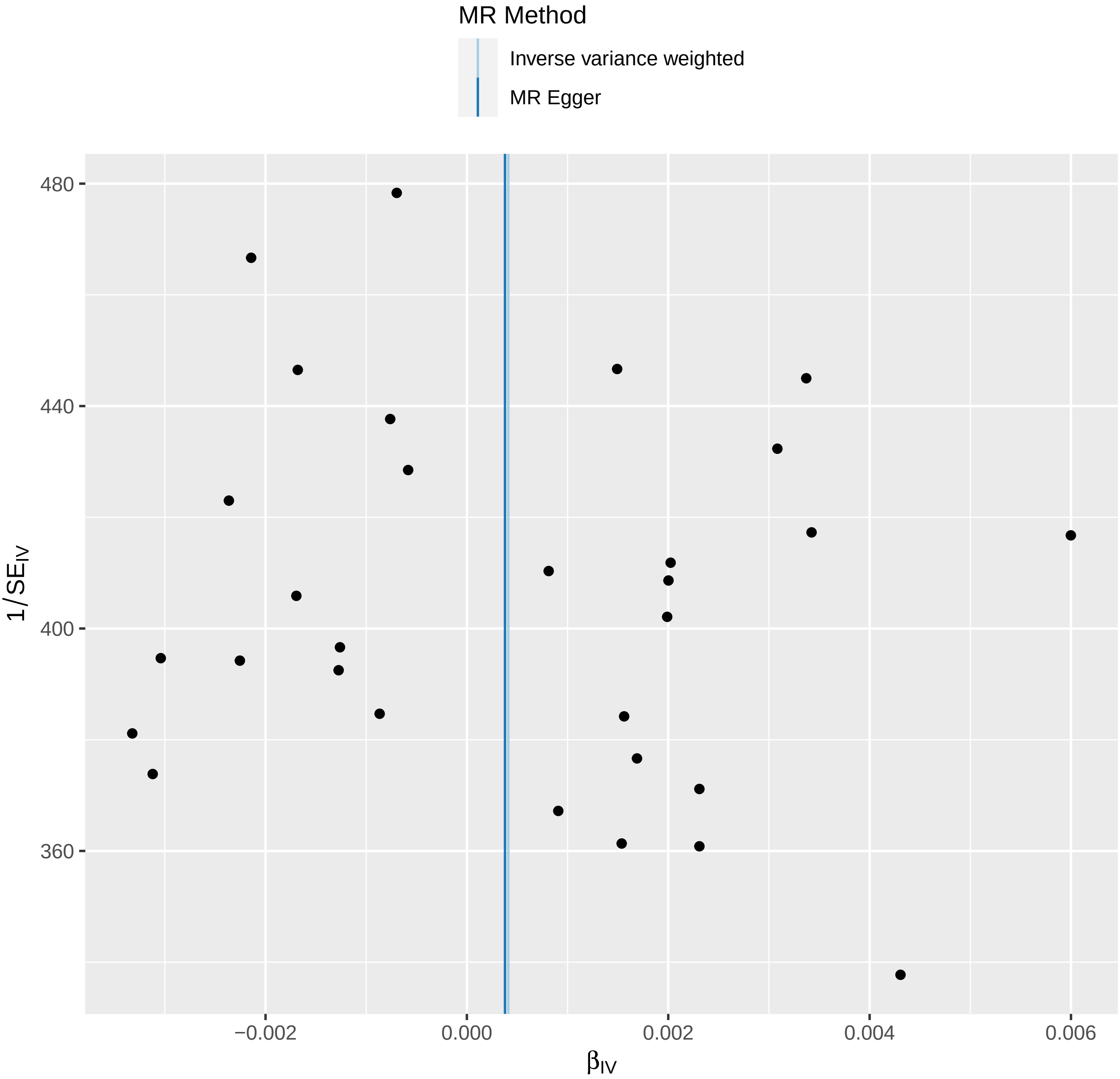

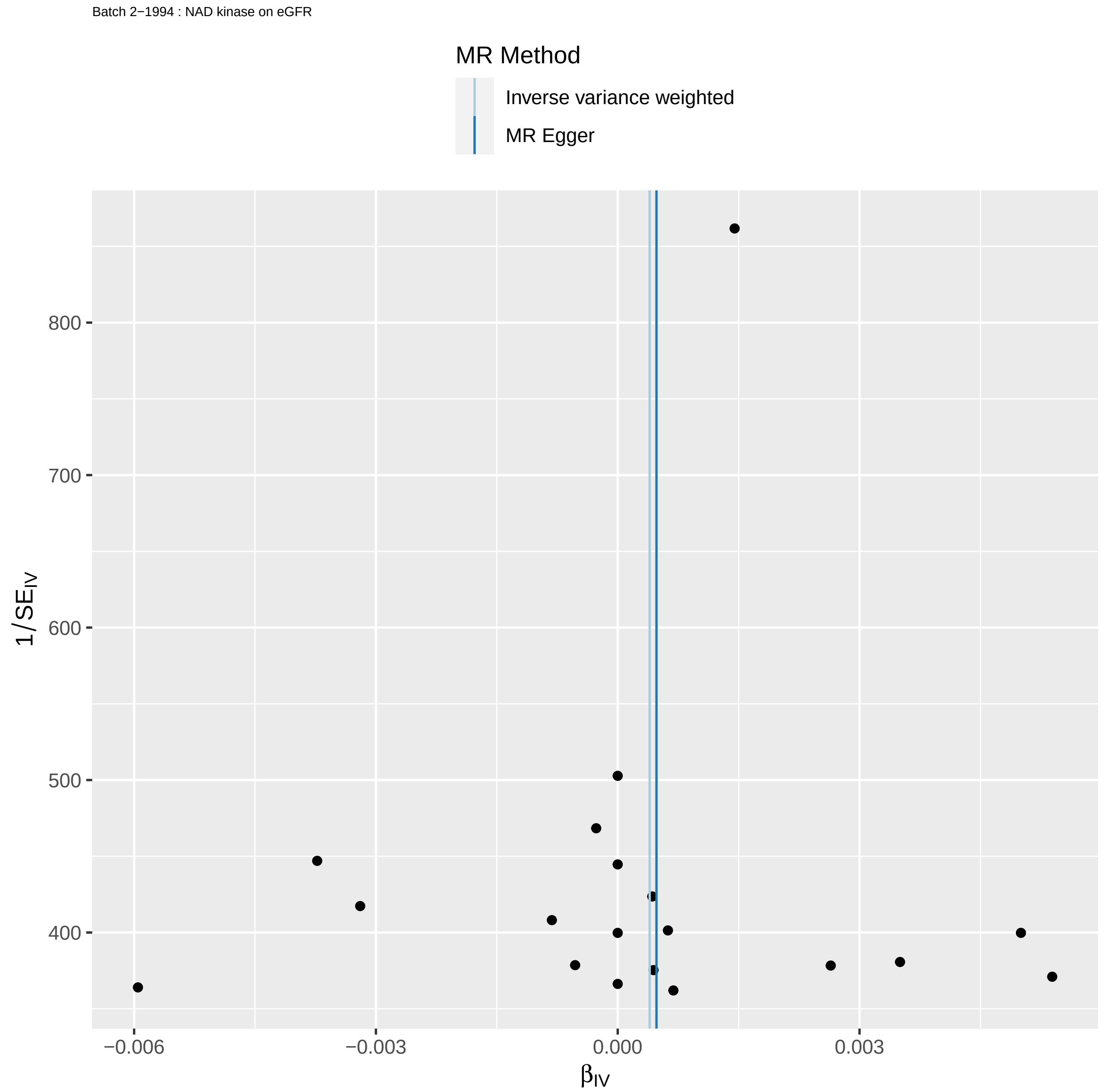
Batch 2−1994 : NAD kinase on eGFR
MR Method
Inverse variance weighted
MR Egger
800
700
600
500
400
$1/SE_{IV}$
−0.006
−0.003
0.000
0.003
β_{IV}

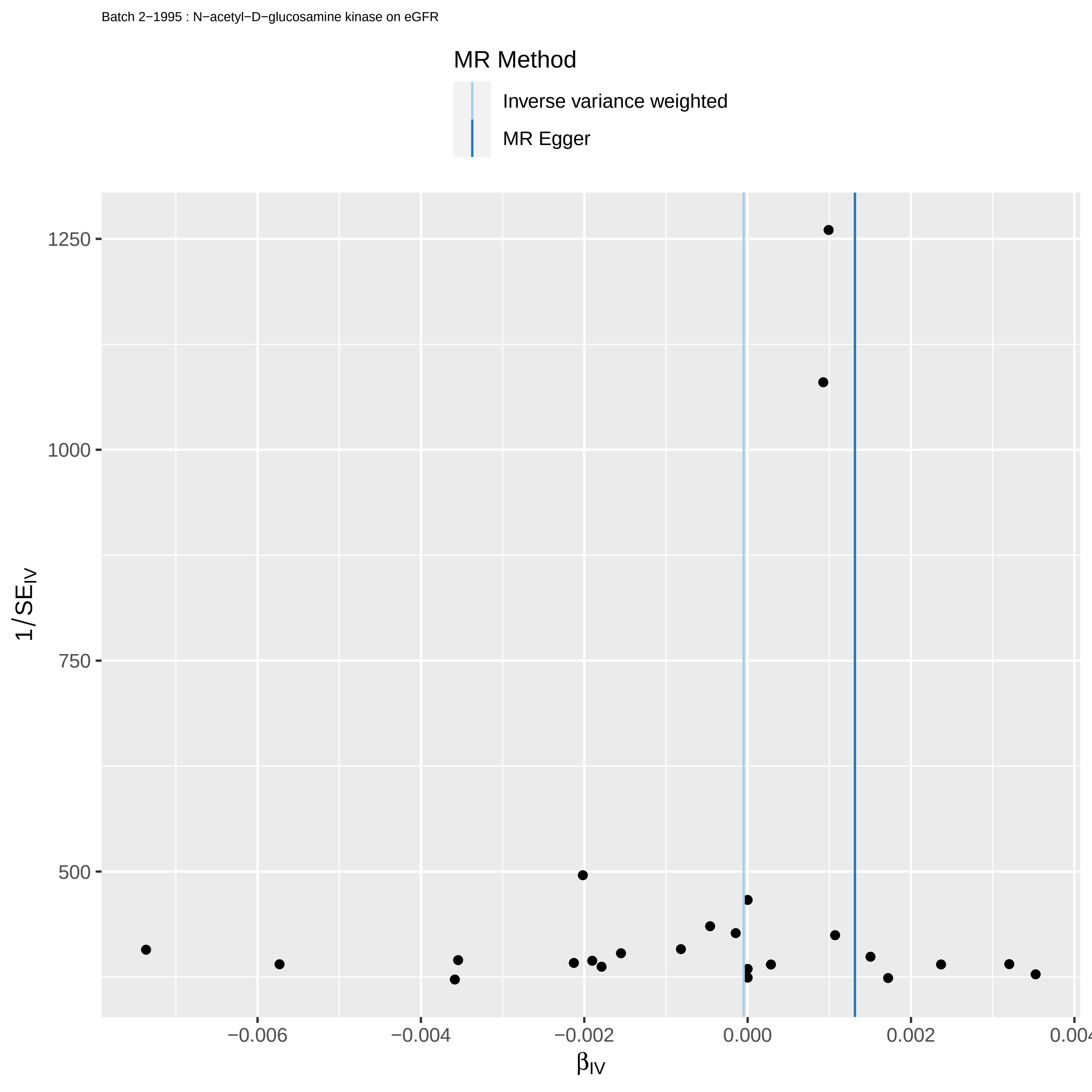

Batch 2−1995 : N−acetyl−D−glucosamine kinase on eGFR
MR Method
Inverse variance weighted
MR Egger
1250
1000
750
500
1/SE_IV
−0.006
−0.004
−0.002
0.000
0.002
0.004
β_IV

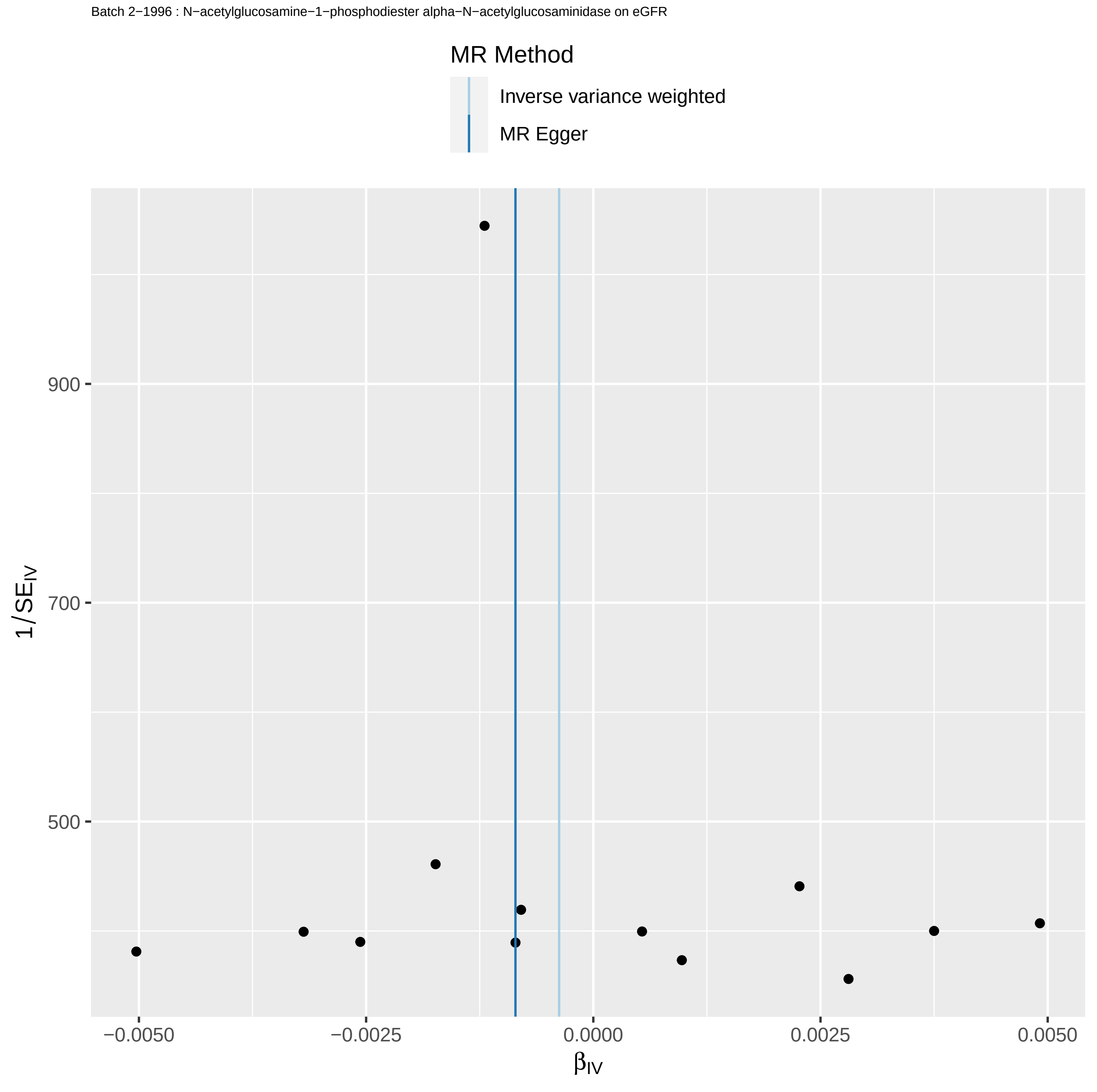
Batch 2−1996 : N−acetylglucosamine−1−phosphodiester alpha−N−acetylglucosaminidase on eGFR
MR Method
Inverse variance weighted
MR Egger
900
700
500
1/SE_IV
−0.0050
−0.0025
0.0000
0.0025
0.0050
β_IV

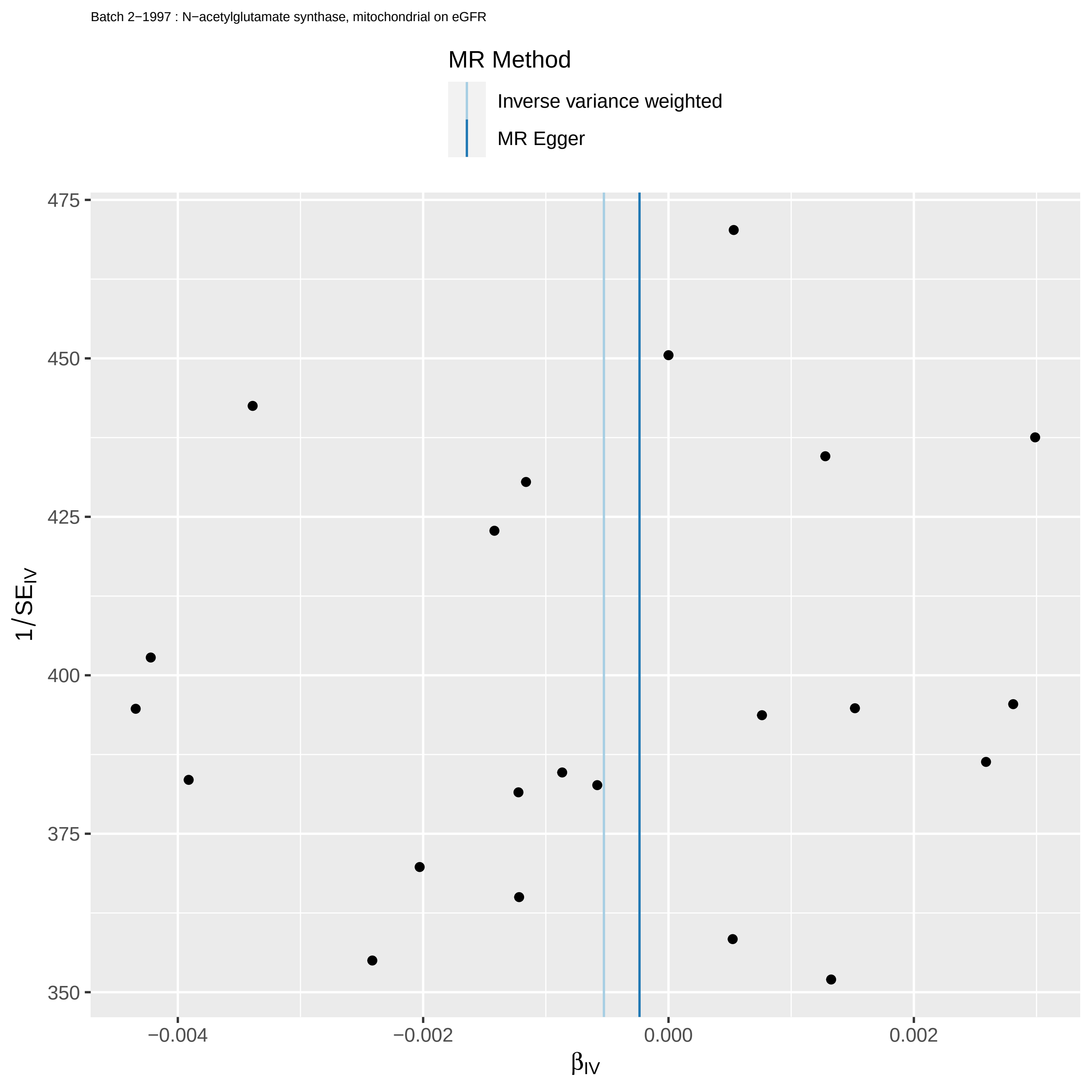

Batch 2−1997 : N−acetylglutamate synthase, mitochondrial on eGFR
MR Method
Inverse variance weighted
MR Egger
475
450
425
400
375
350
1/SE_IV
−0.004
−0.002
0.000
0.002
β_IV

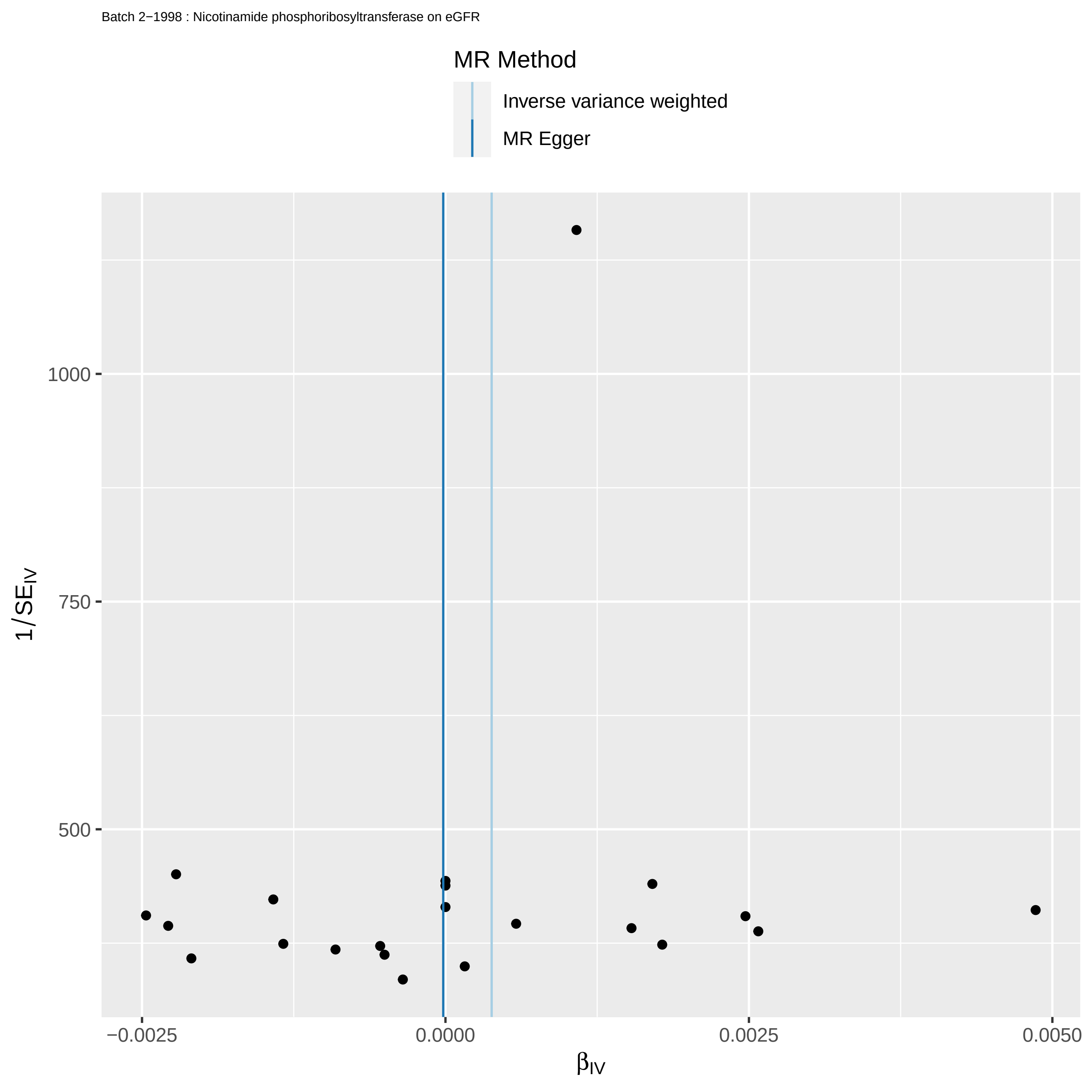
Batch 2−1998 : Nicotinamide phosphoribosyltransferase on eGFR
MR Method
Inverse variance weighted
MR Egger
1000
750
500
1/SE$_{IV}$
−0.0025
0.0000
0.0025
0.0050
β_{IV}

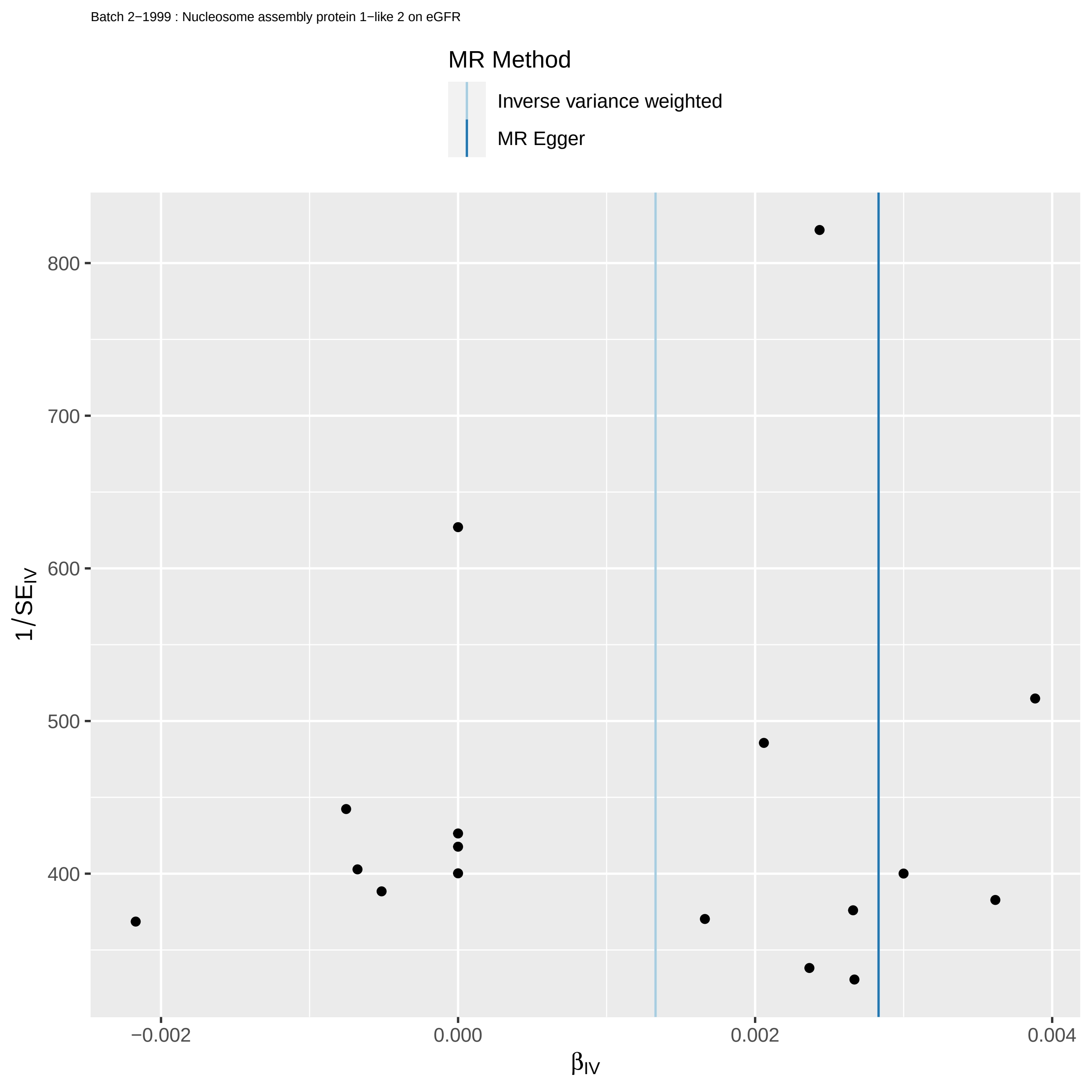
Batch 2−1999 : Nucleosome assembly protein 1−like 2 on eGFR
MR Method
Inverse variance weighted
MR Egger
800
700
600
500
400
1/SE_IV
−0.002
0.000
0.002
0.004
β_IV

Batch 2−2000 : Beta−soluble NSF attachment protein on eGFR

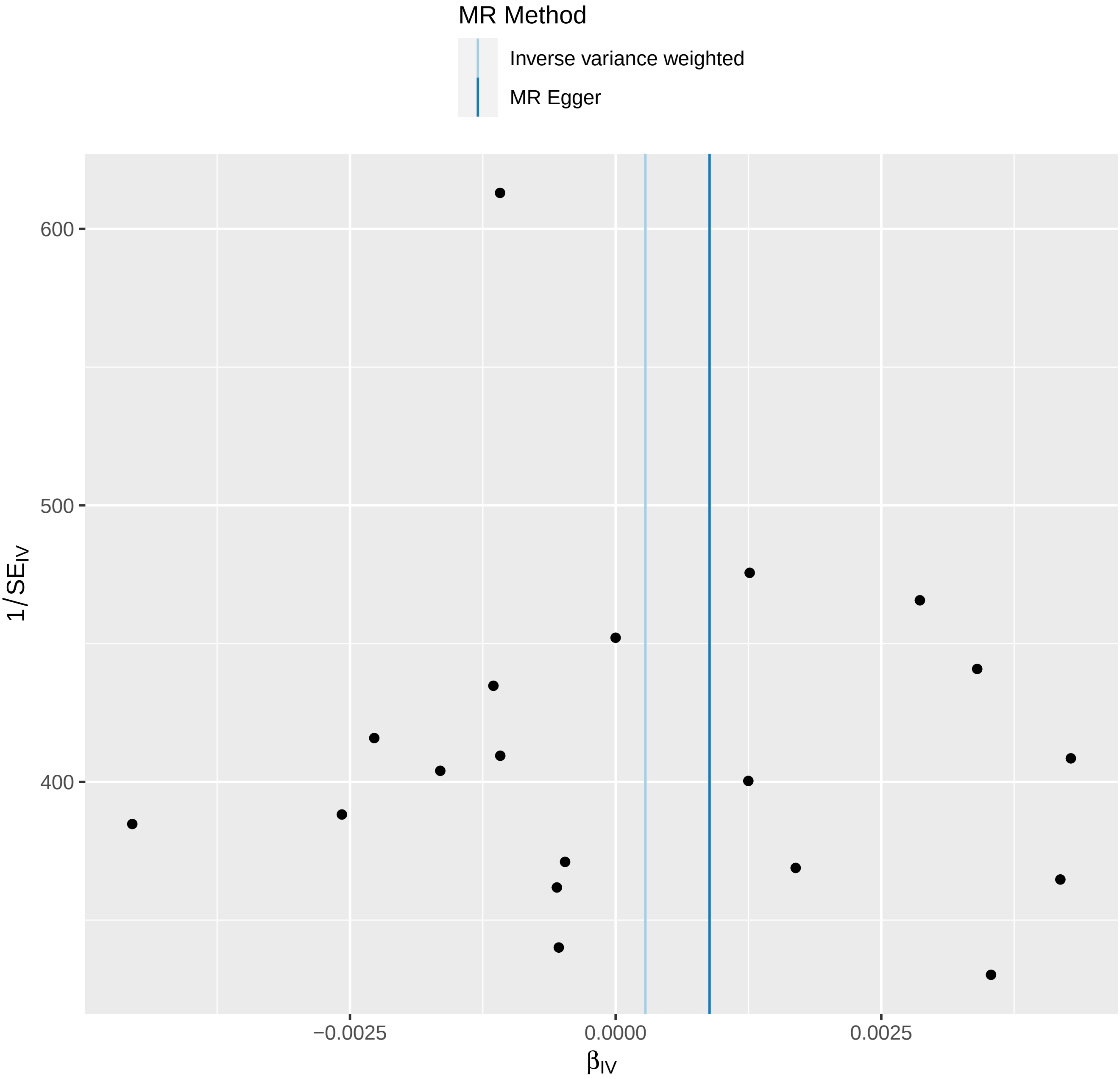

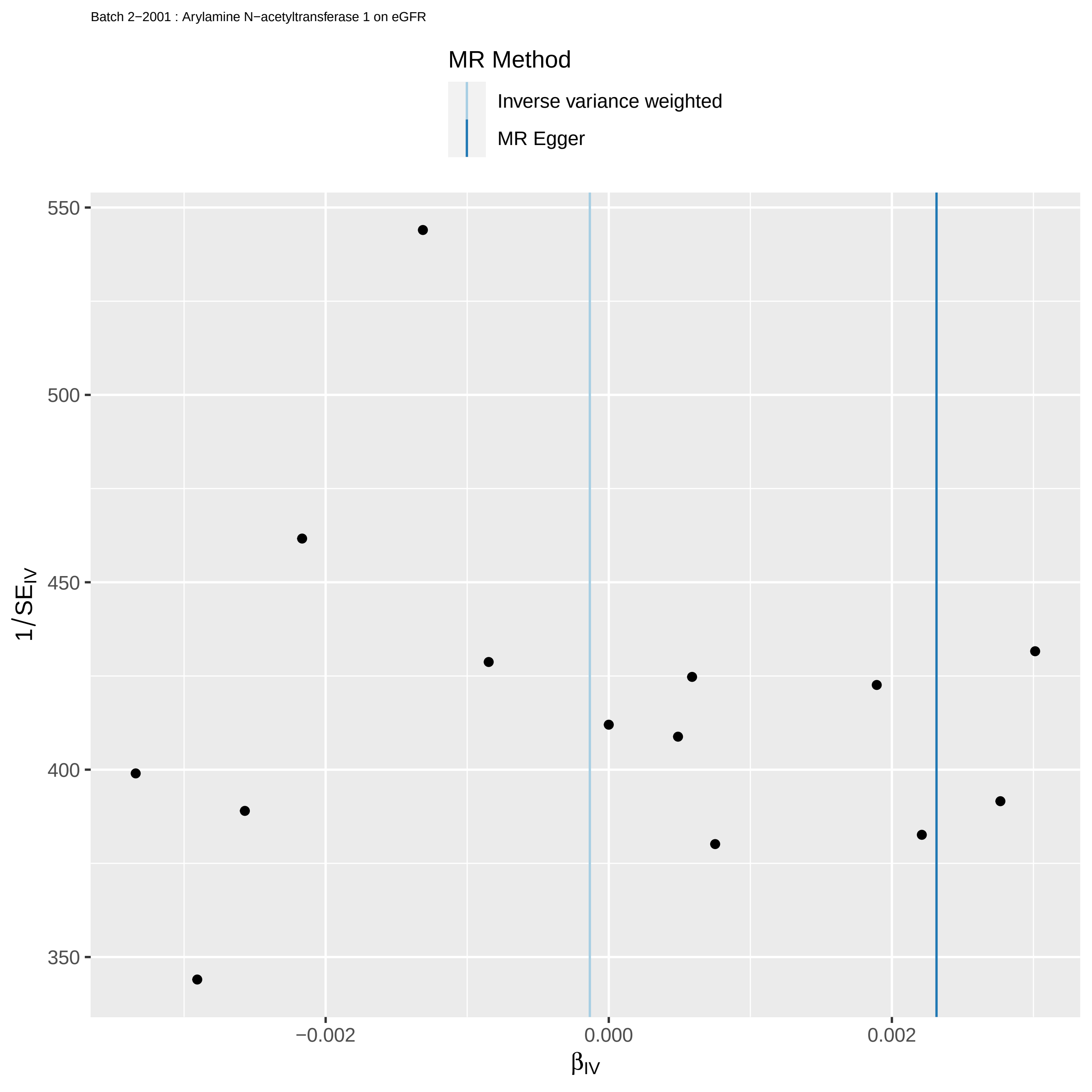
Batch 2−2001 : Arylamine N−acetyltransferase 1 on eGFR
MR Method
Inverse variance weighted
MR Egger
550
500
450
400
350
1/SE_IV
−0.002
0.000
0.002
β_IV

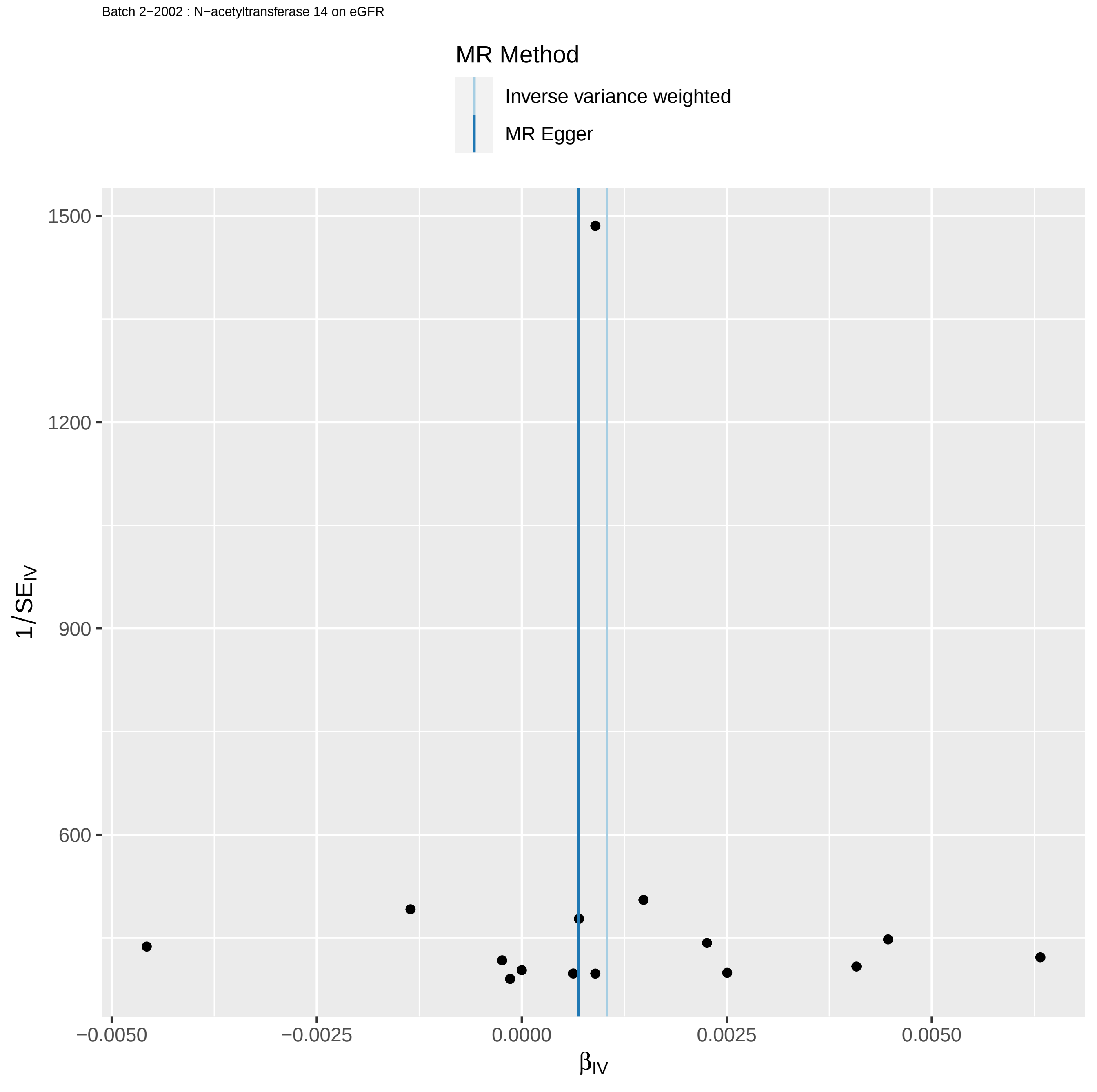
Batch 2−2002 : N−acetyltransferase 14 on eGFR
MR Method
Inverse variance weighted
MR Egger
1500
1200
900
600
1/SE$_{IV}$
−0.0050
−0.0025
0.0000
0.0025
0.0050
β_{IV}

Batch 2−2003 : Neuroblastoma suppressor of tumorigenicity 1 on eGFR

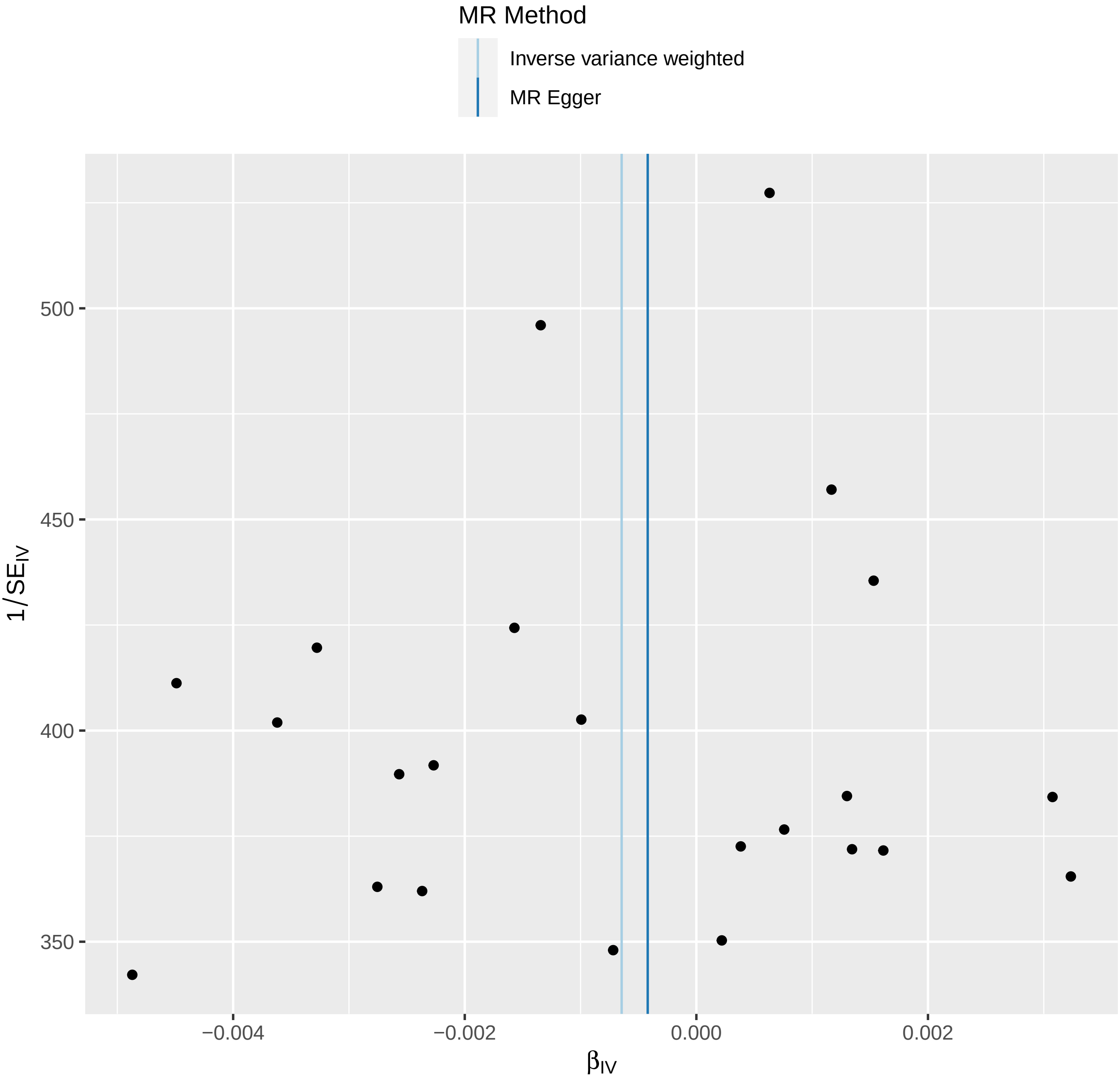

Batch 2−2004 : Next to BRCA1 gene 1 protein on eGFR

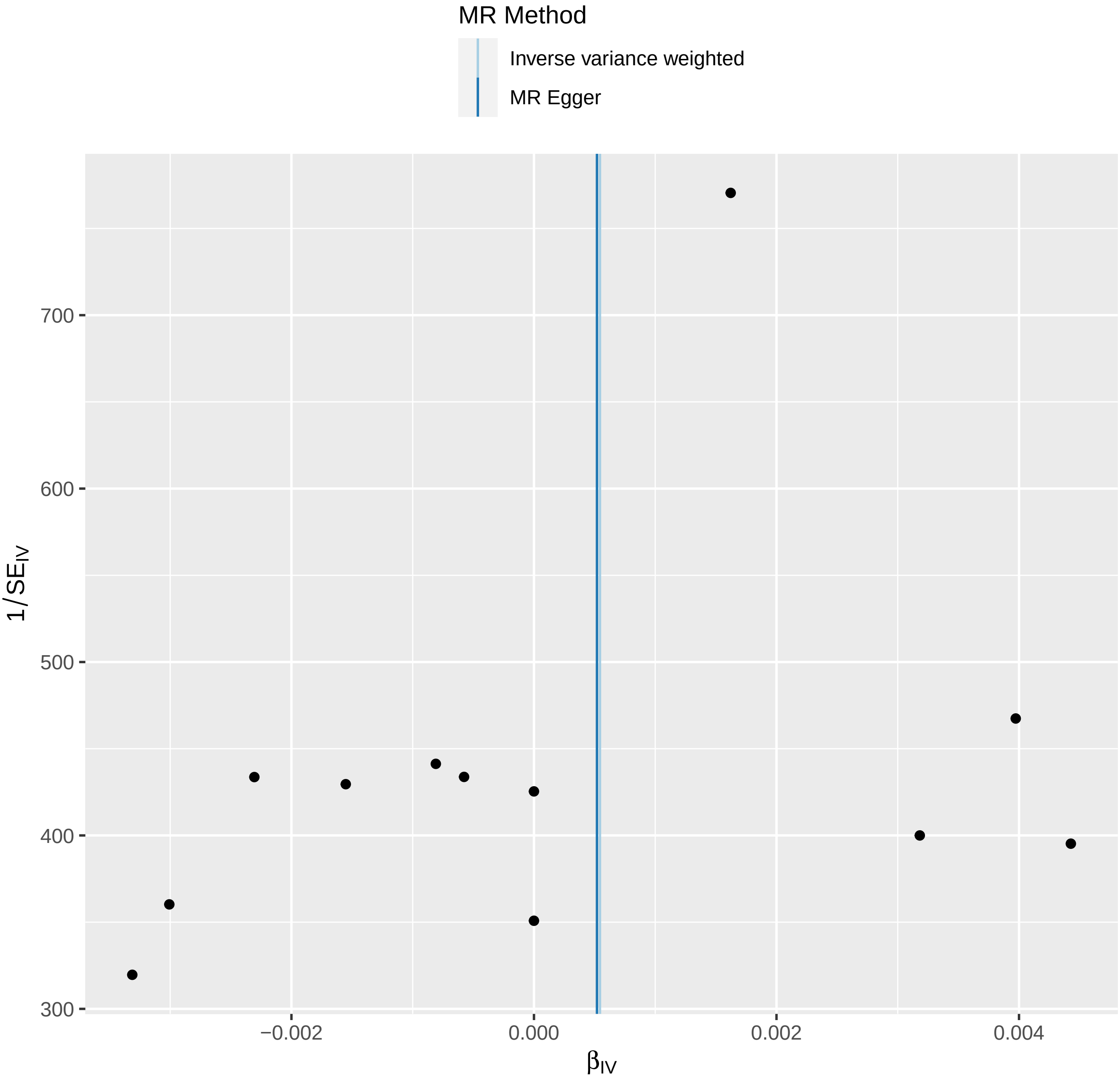

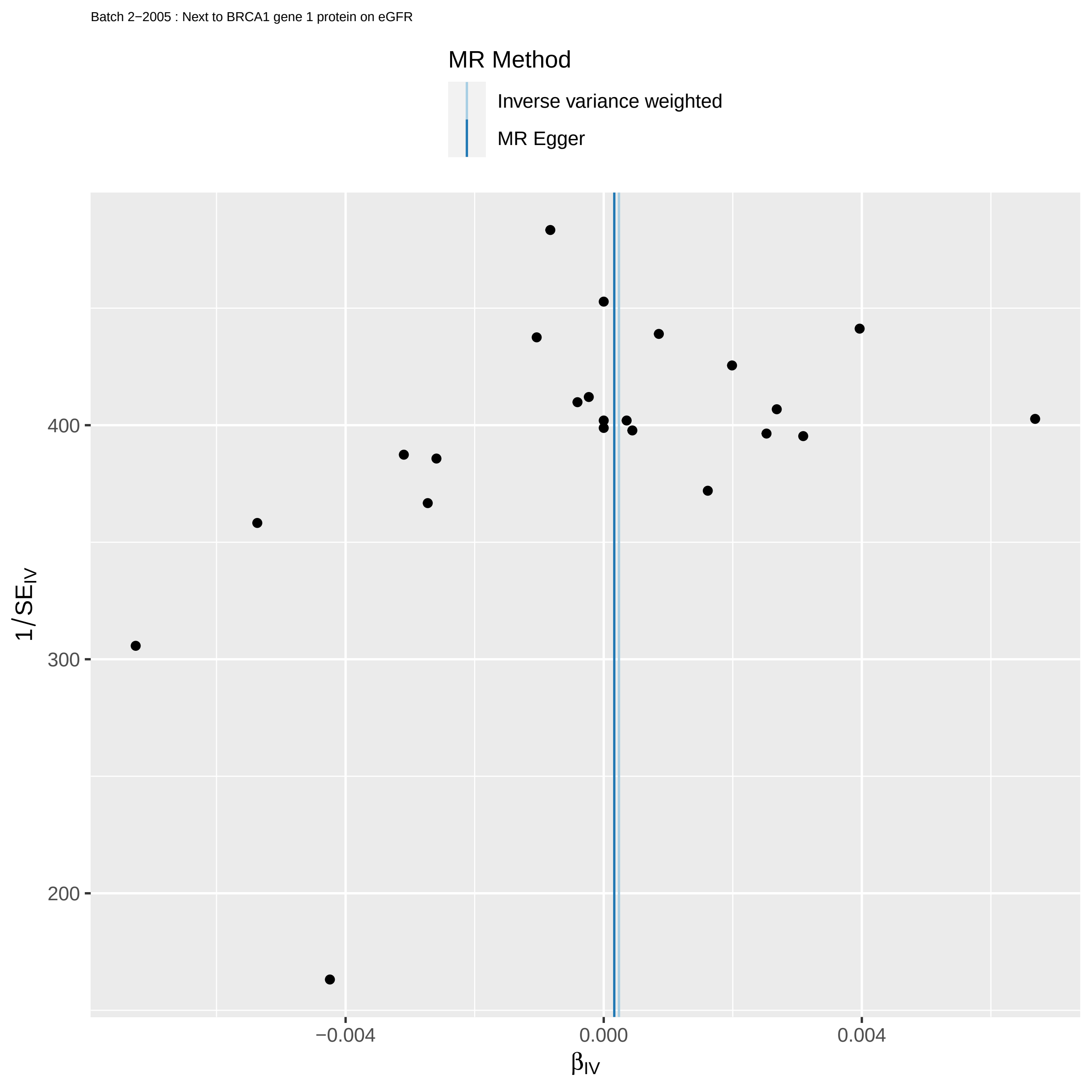
Batch 2−2005 : Next to BRCA1 gene 1 protein on eGFR
MR Method
Inverse variance weighted
MR Egger
1/SE_IV
400
300
200
−0.004
0.000
0.004
β_IV

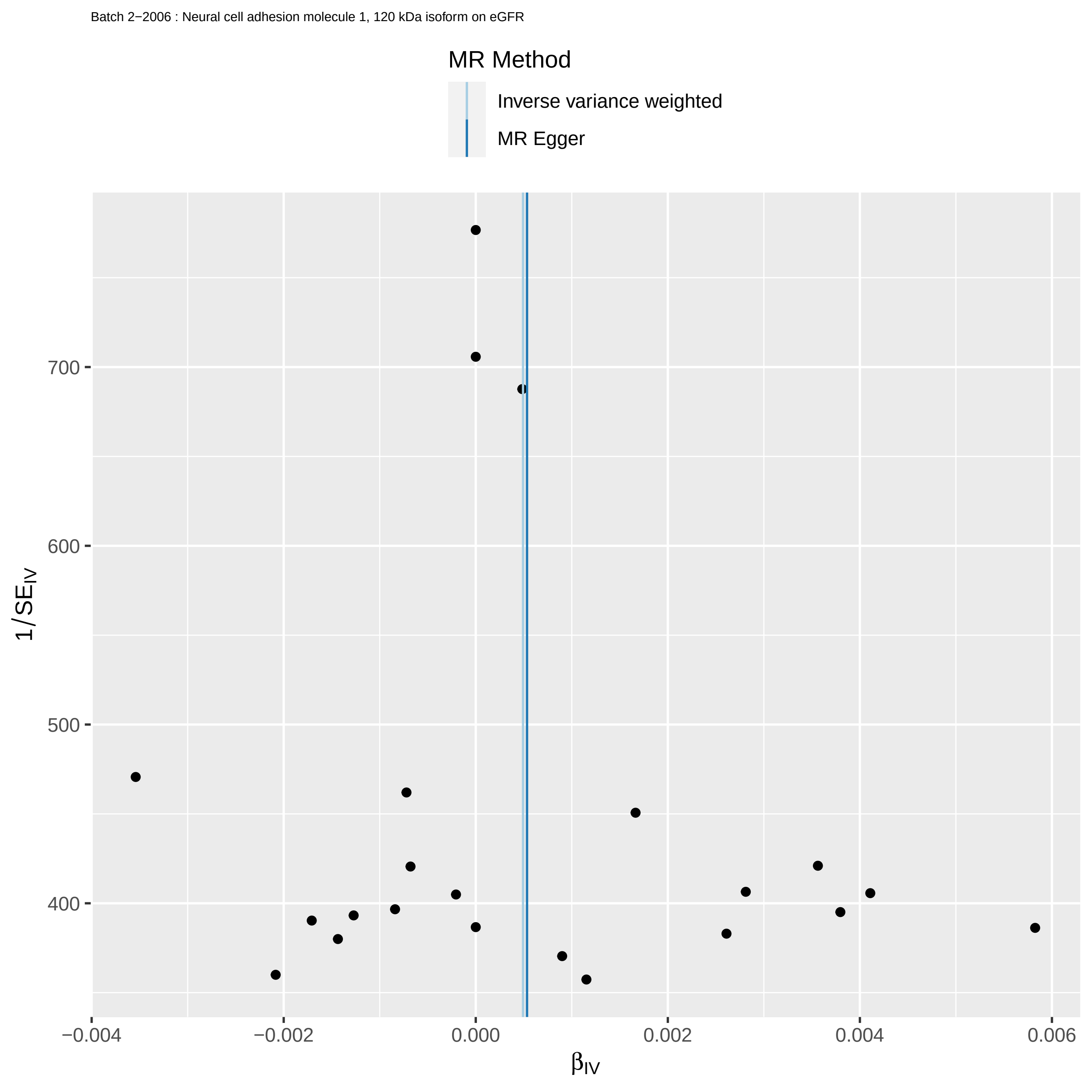

Batch 2−2006 : Neural cell adhesion molecule 1, 120 kDa isoform on eGFR
MR Method
Inverse variance weighted
MR Egger
700
600
500
400
1/SE_IV
−0.004
−0.002
0.000
0.002
0.004
0.006
β_IV

Batch 2−2007 : Neural cell adhesion molecule 1 on eGFR

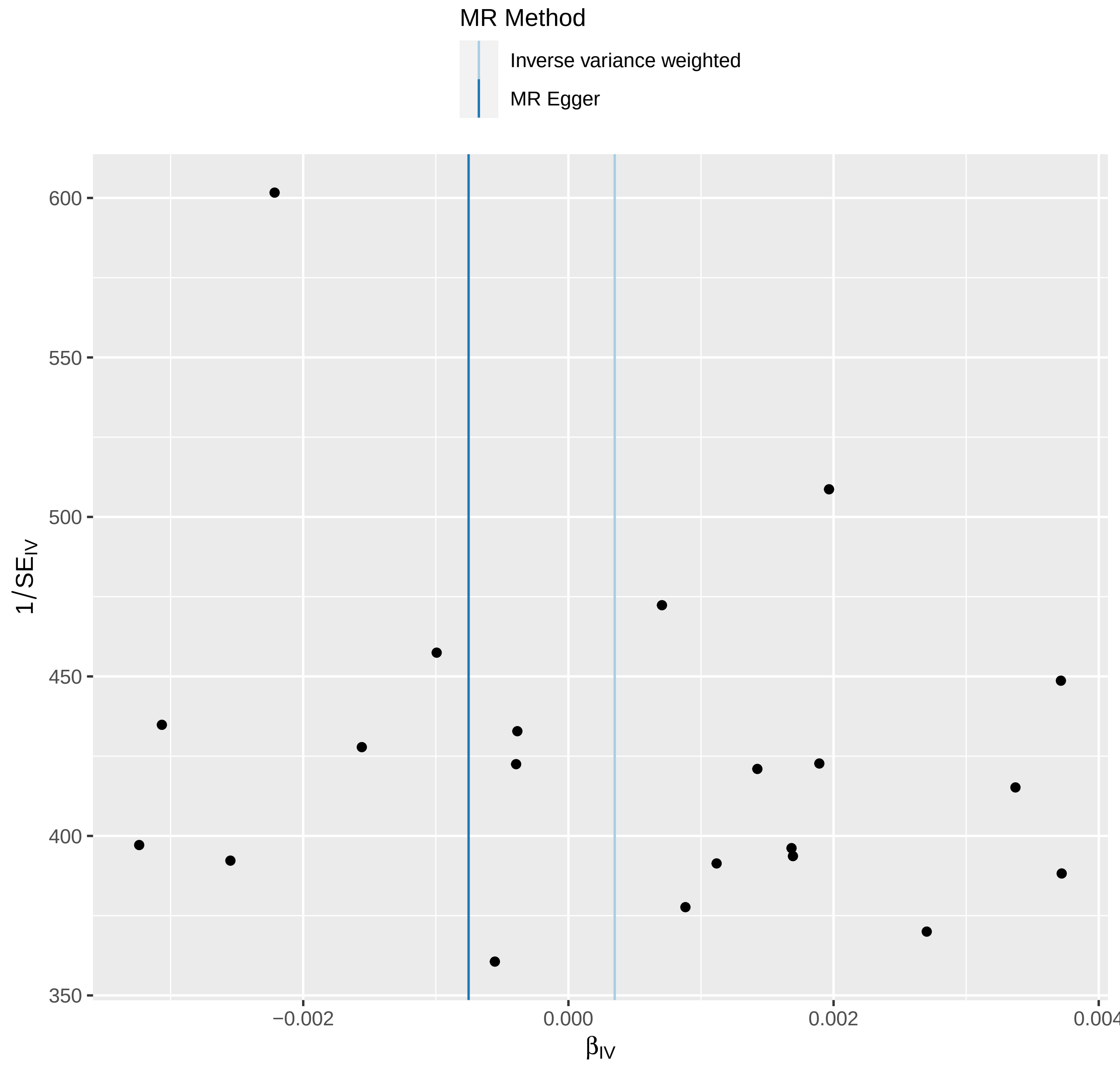

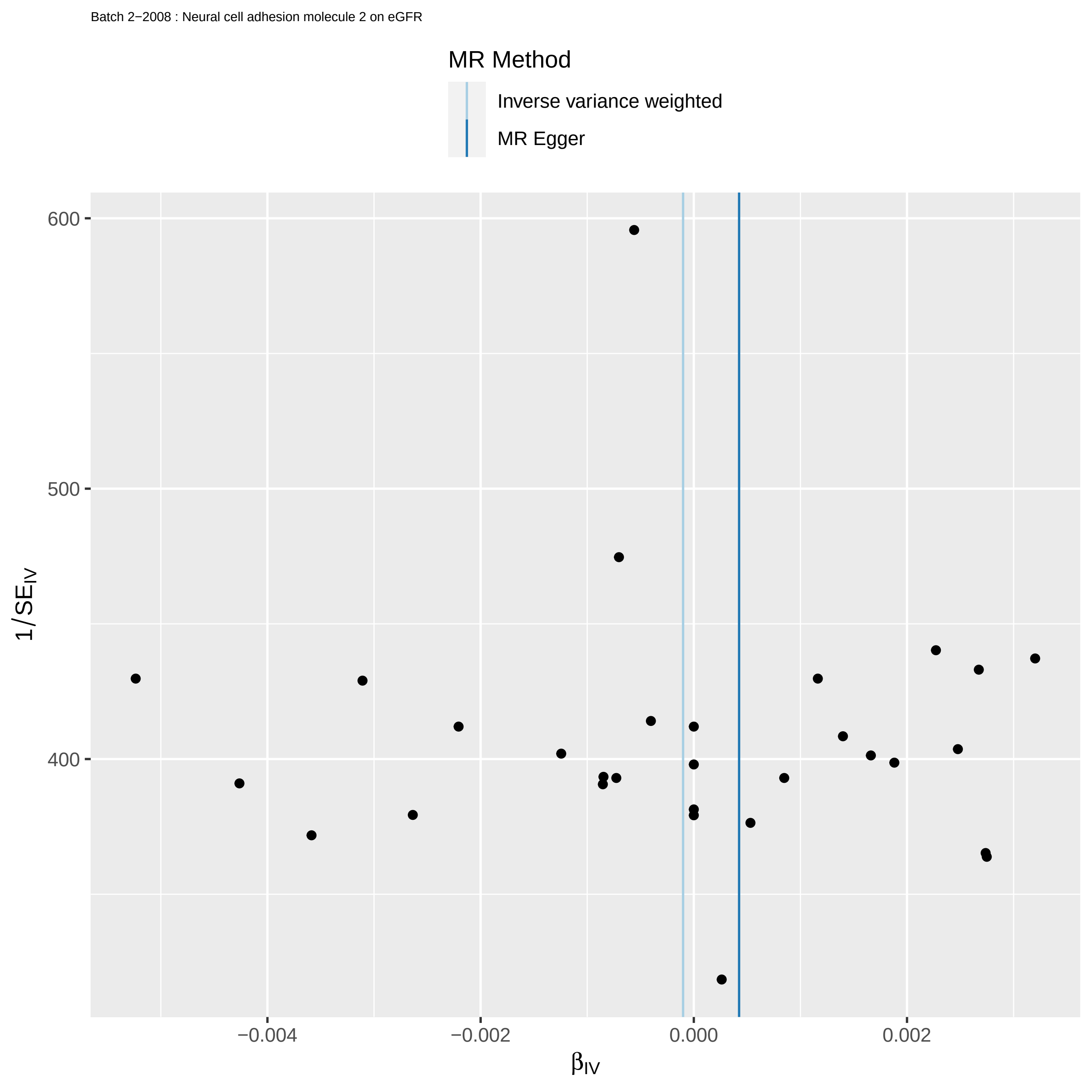

Batch 2−2008 : Neural cell adhesion molecule 2 on eGFR
MR Method
Inverse variance weighted
MR Egger
600
500
400
−0.004
−0.002
0.000
0.002
1/SE_IV
β_IV

Batch 2−2009 : Neutrophil cytosol factor 2 on eGFR

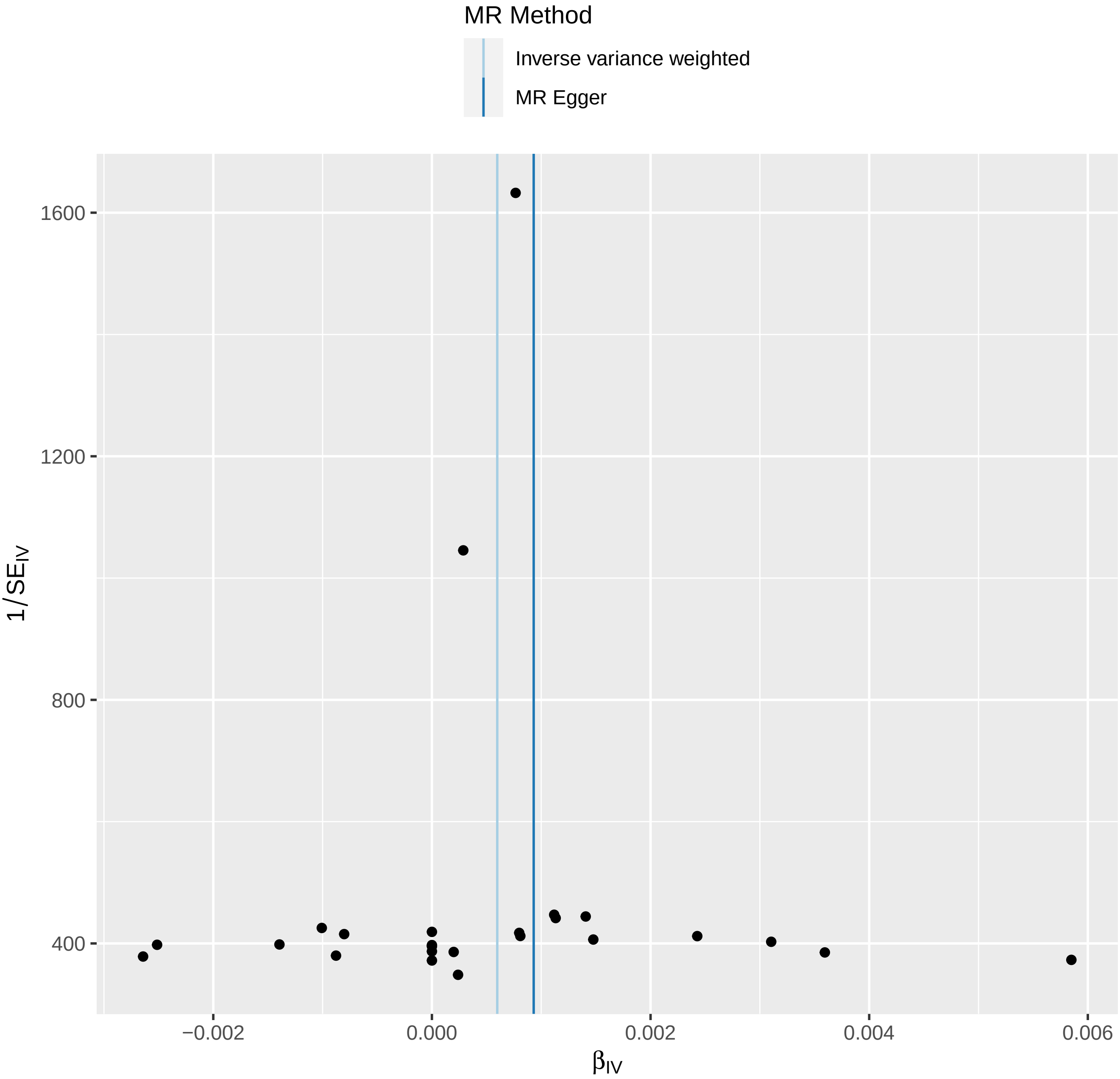

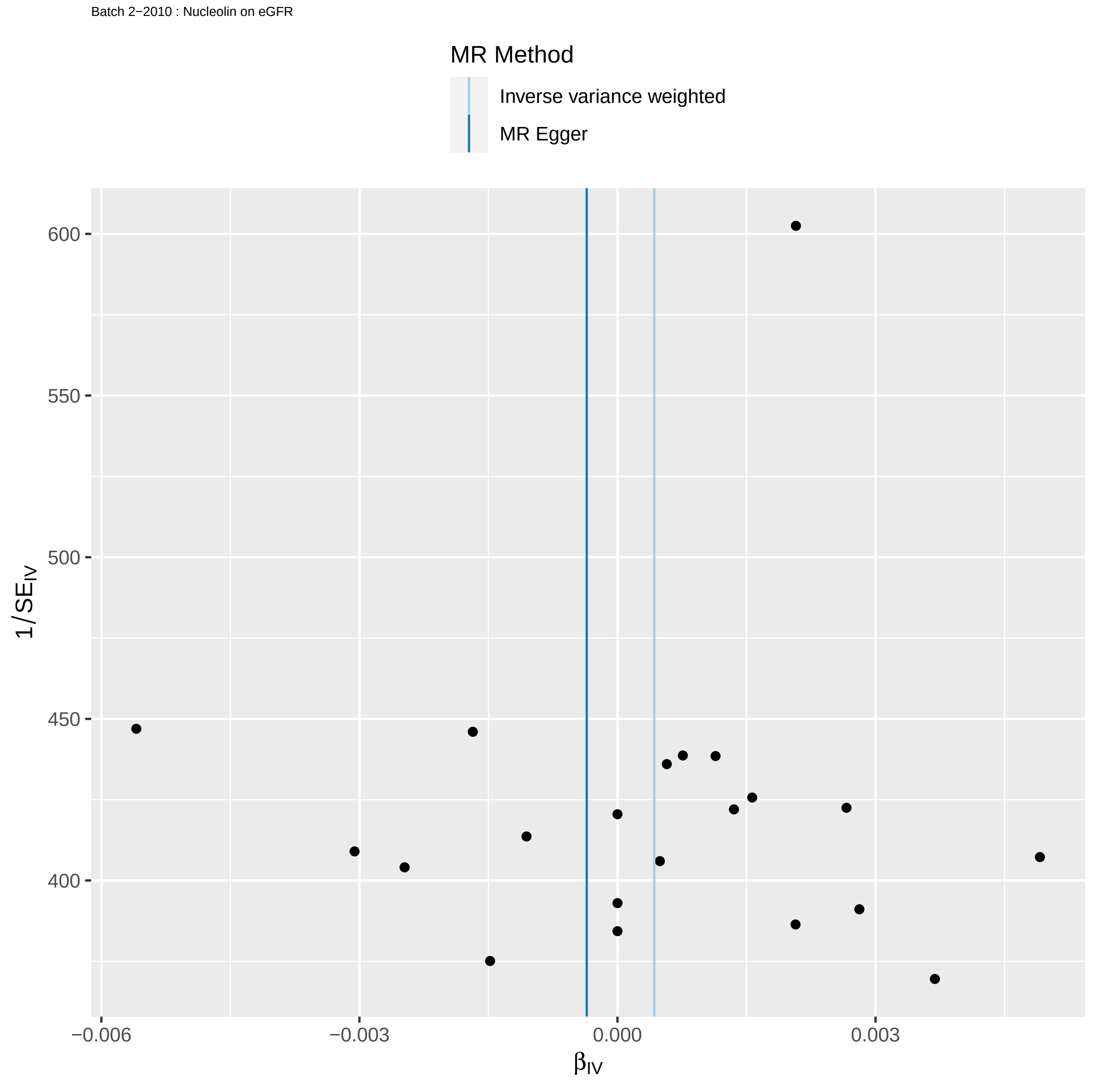

Batch 2−2010 : Nucleolin on eGFR
MR Method
Inverse variance weighted
MR Egger
600
550
500
450
400
1/SE_IV
−0.006
−0.003
0.000
0.003
β_IV

Batch 2−2011 : Noncompact myelin−associated protein on eGFR

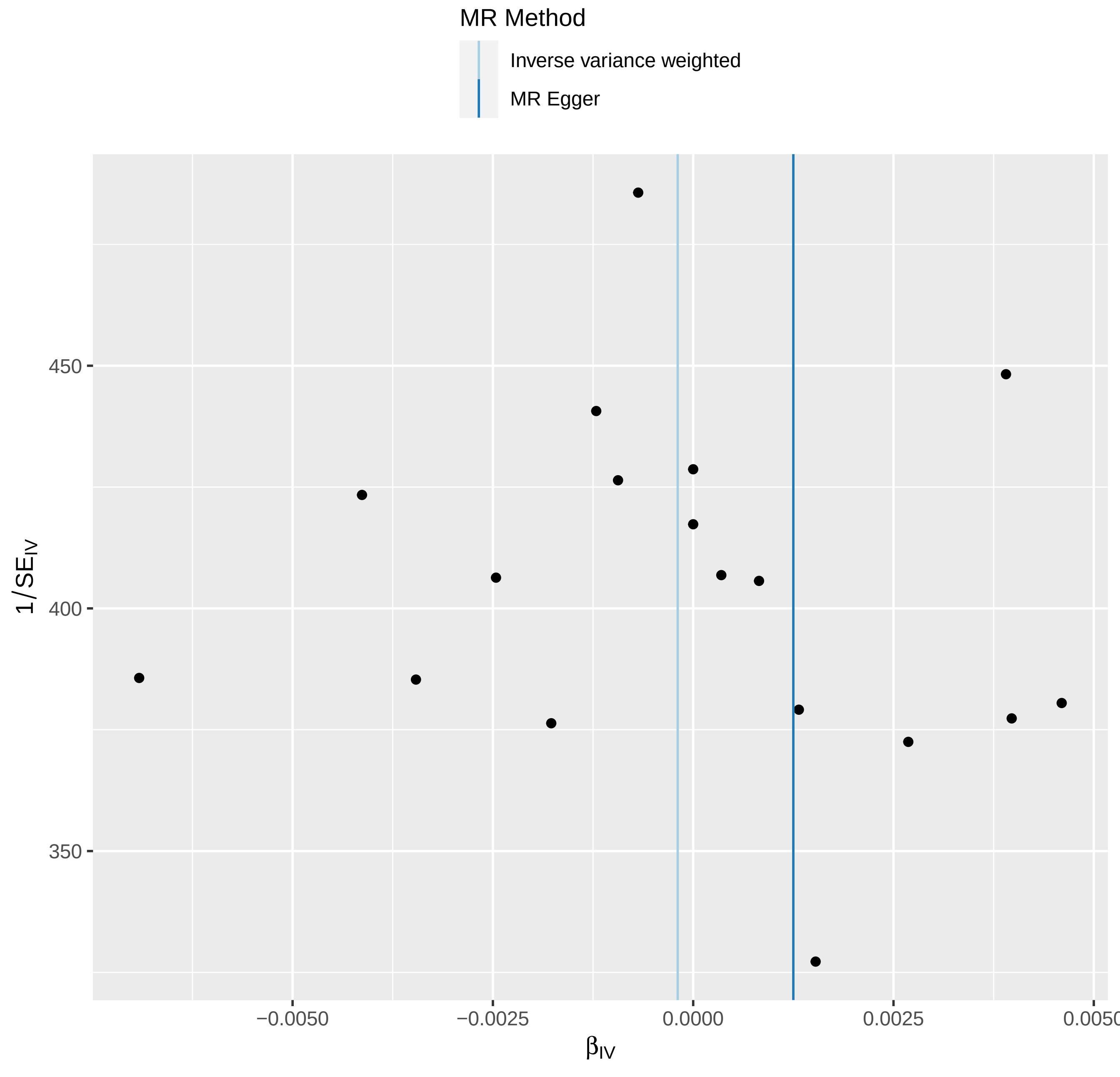

Batch 2−2012 : Nuclear receptor coactivator 2 on eGFR

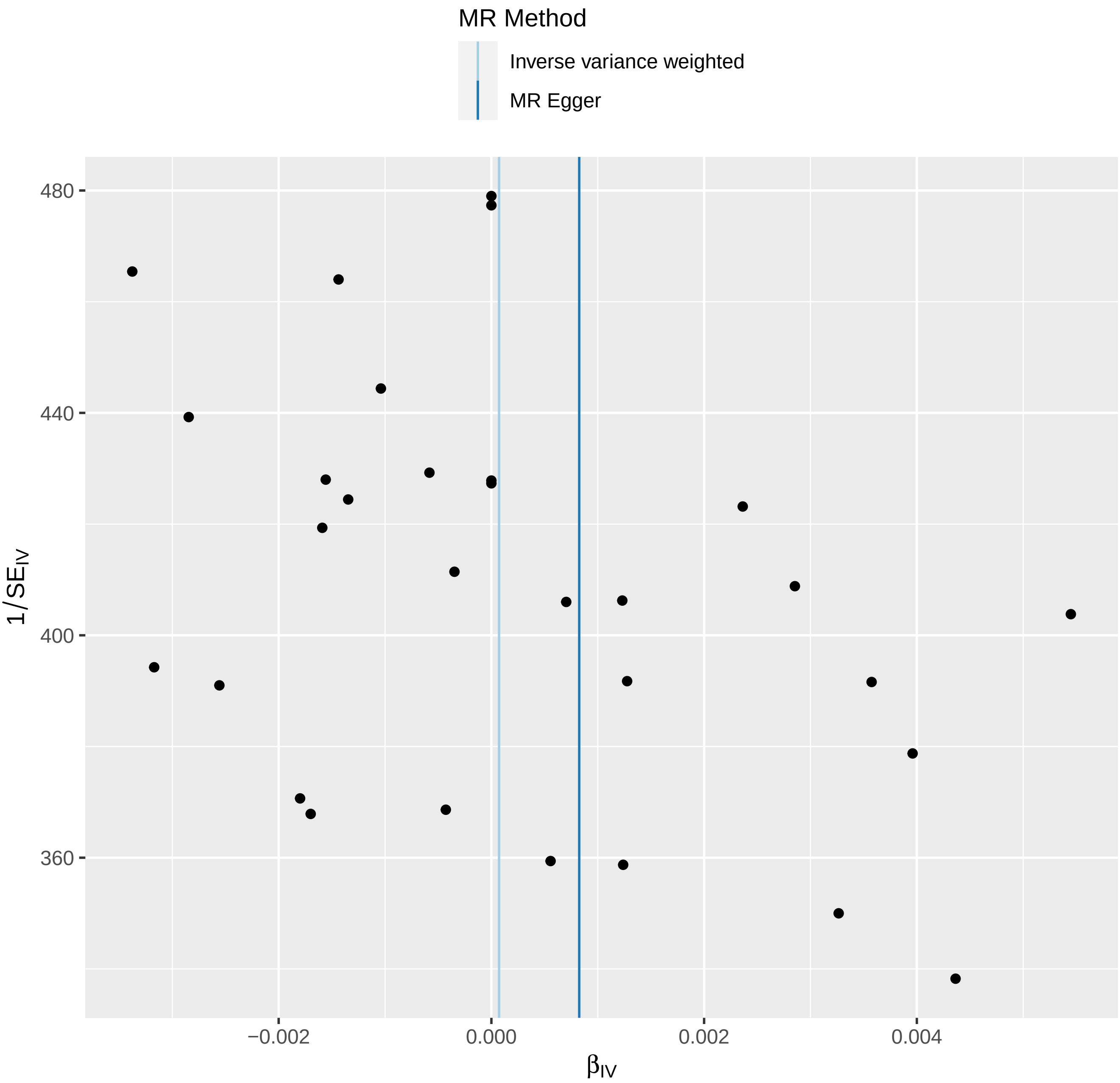

Batch 2−2013 : Natural cytotoxicity triggering receptor 1 on eGFR

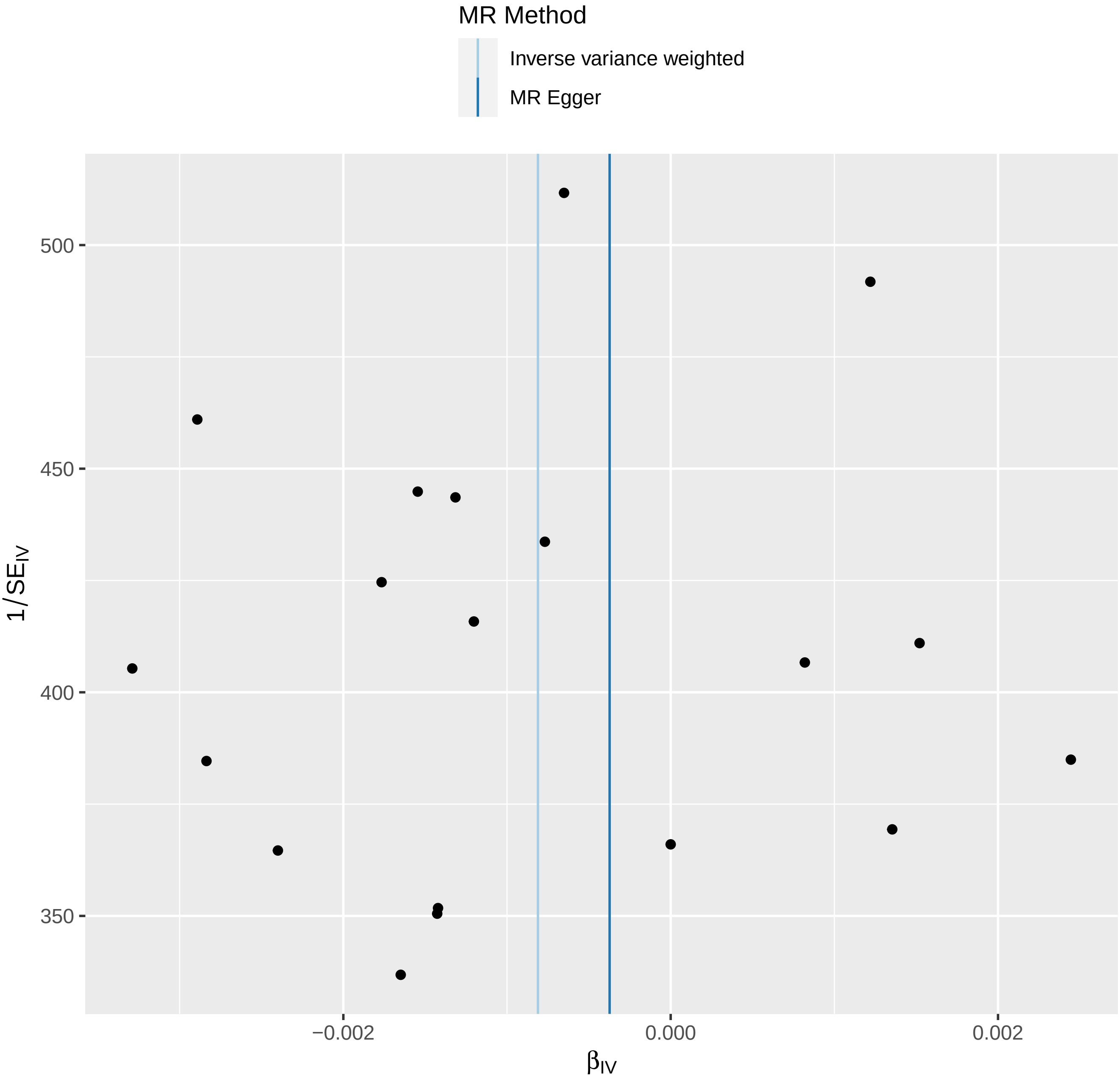

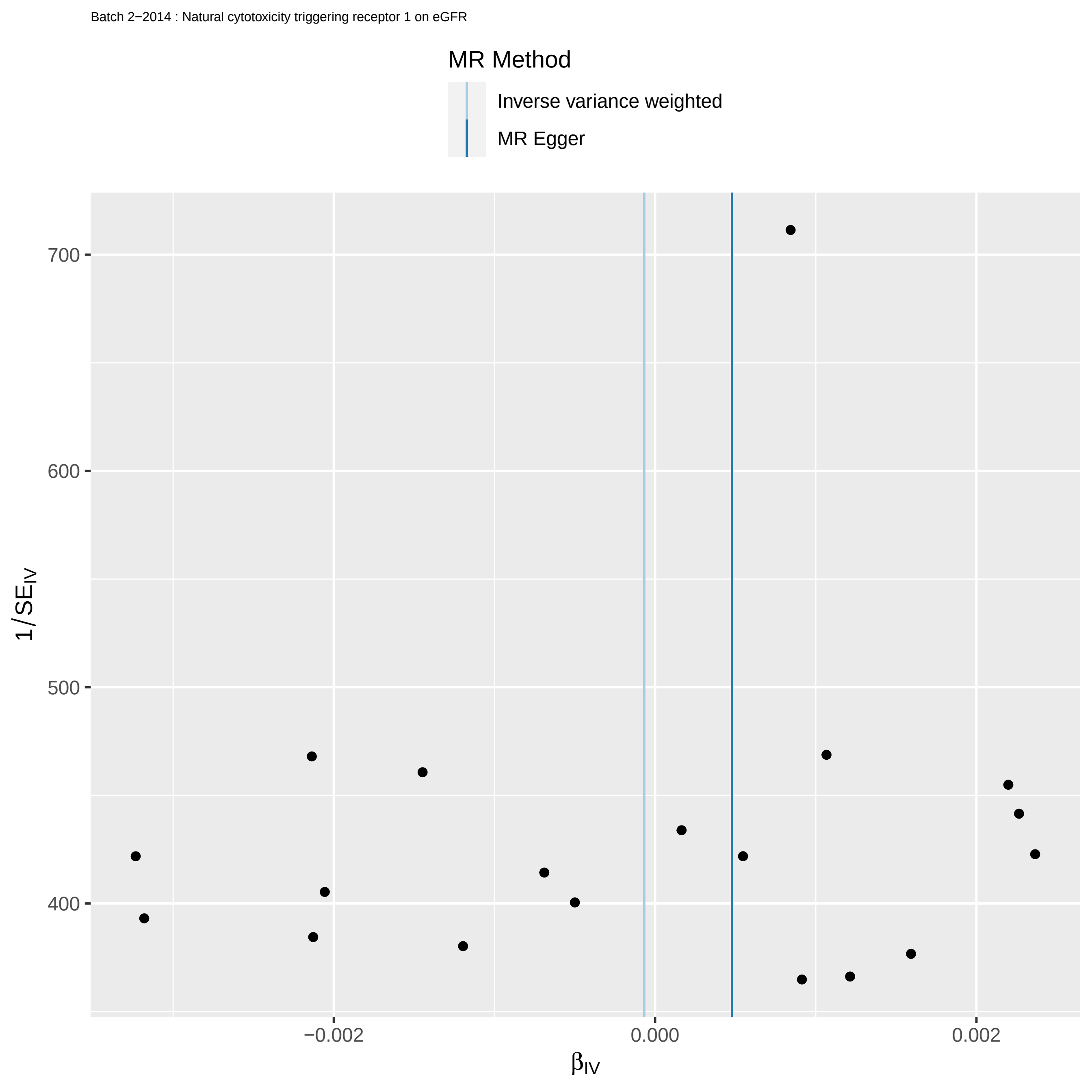

Batch 2−2014 : Natural cytotoxicity triggering receptor 1 on eGFR
MR Method
Inverse variance weighted
MR Egger
700
600
500
400
1/SE$_{IV}$
−0.002
0.000
0.002
β$_{IV}$

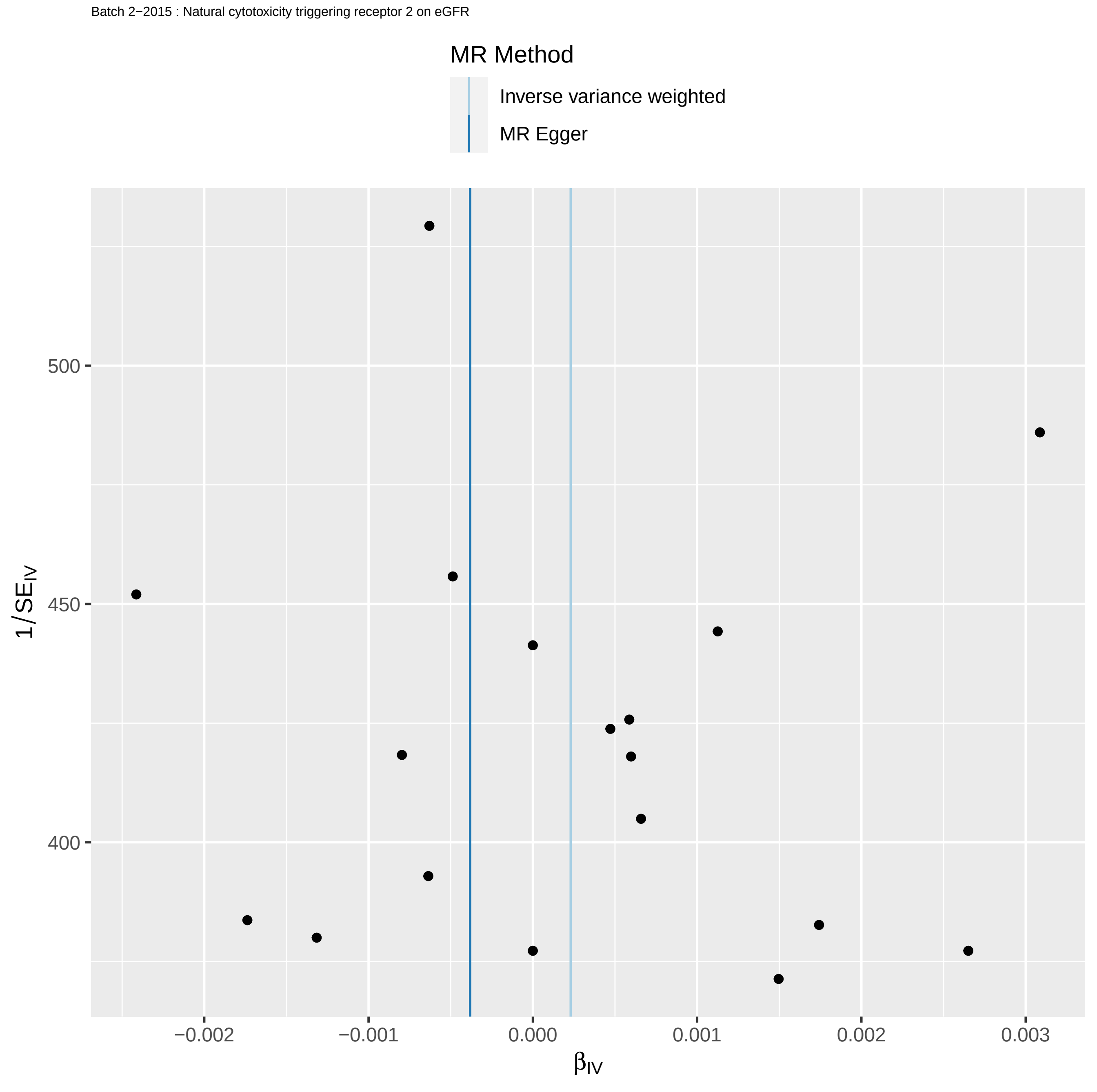

Batch 2−2015 : Natural cytotoxicity triggering receptor 2 on eGFR
MR Method
Inverse variance weighted
MR Egger
500
450
400
1/SE$_{IV}$
−0.002
−0.001
0.000
0.001
0.002
0.003
β$_{IV}$

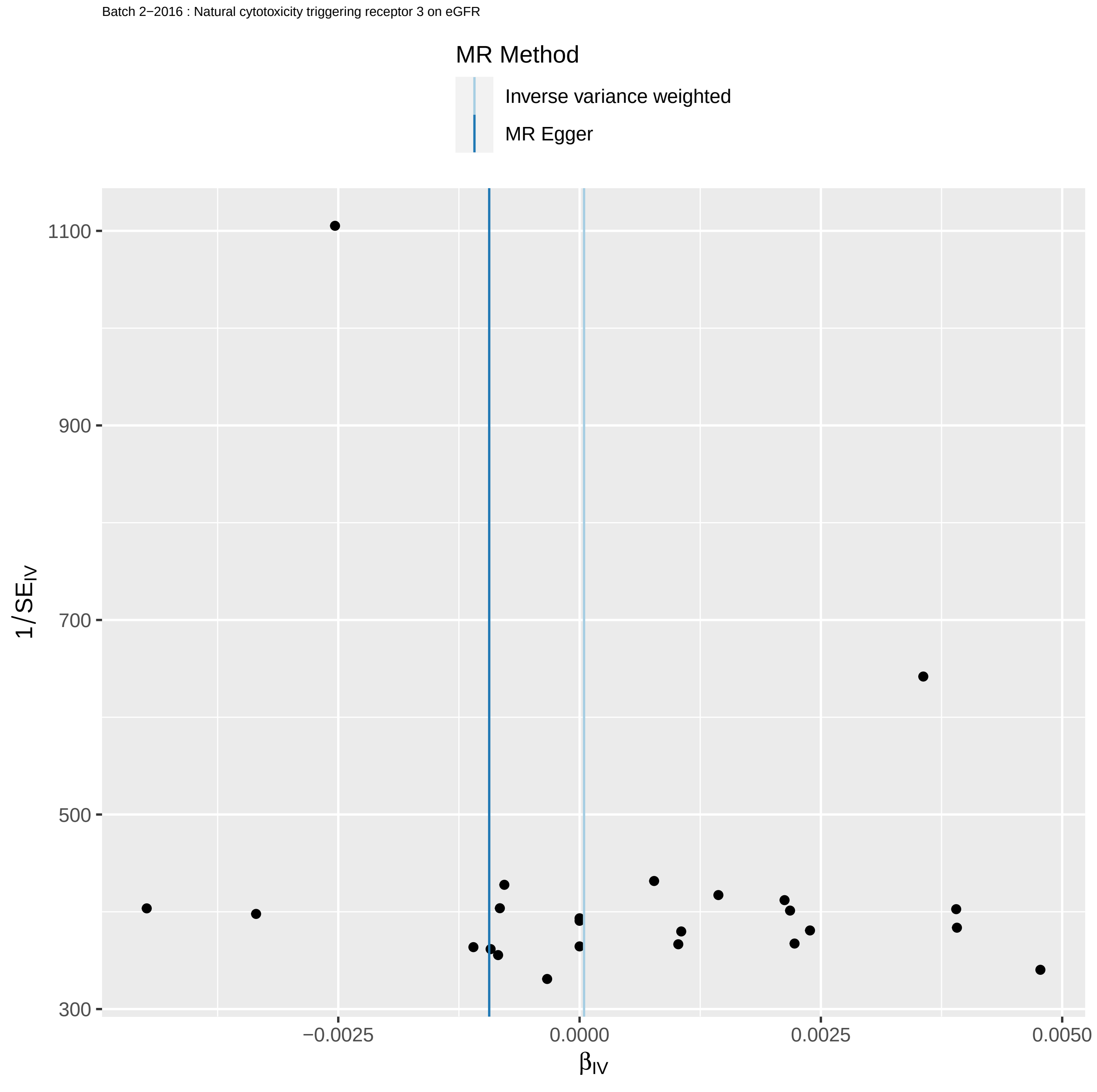

Batch 2−2016 : Natural cytotoxicity triggering receptor 3 on eGFR
MR Method
Inverse variance weighted
MR Egger
1100
900
700
500
300
1/SE$_{IV}$
−0.0025
0.0000
0.0025
0.0050
β_{IV}

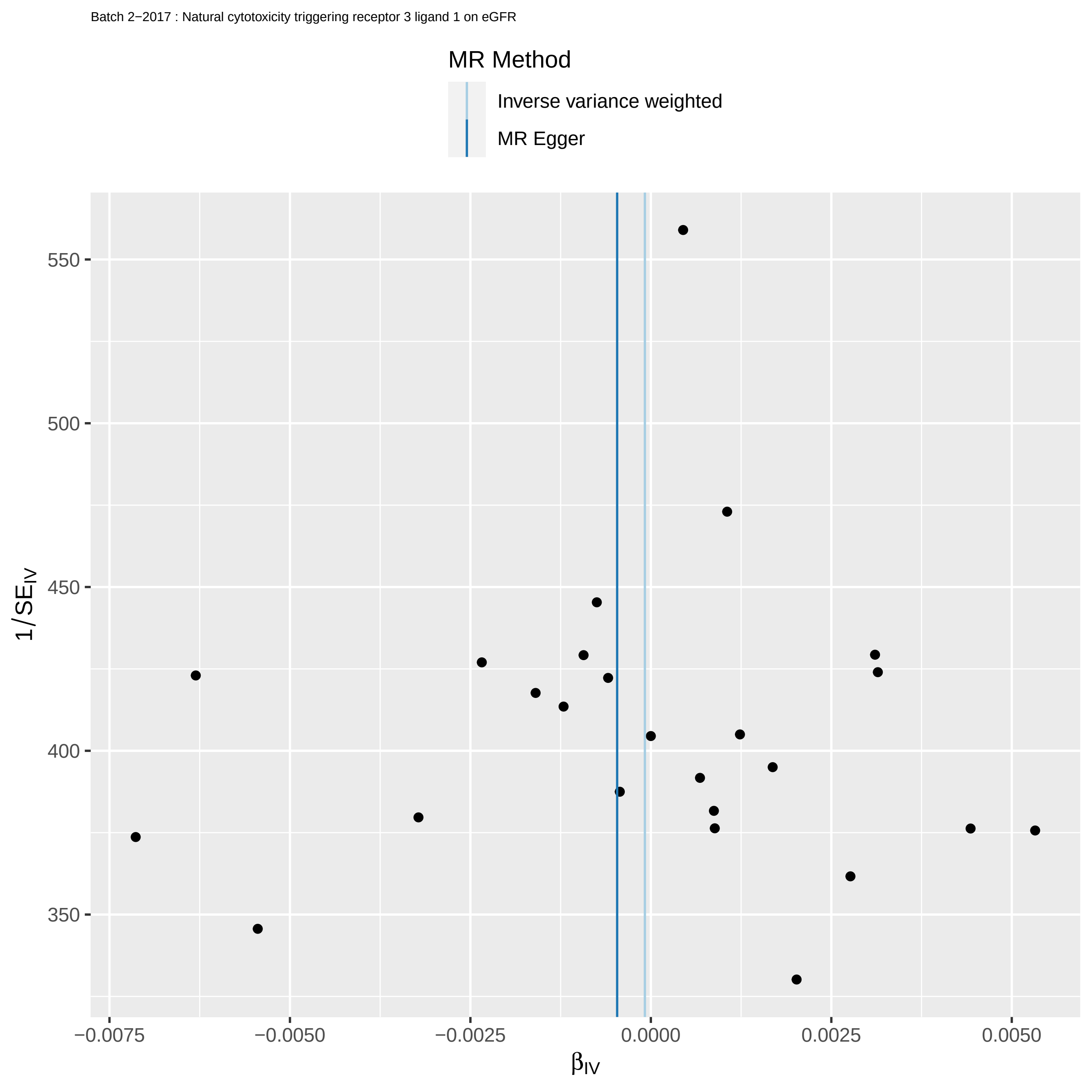

Batch 2−2017 : Natural cytotoxicity triggering receptor 3 ligand 1 on eGFR
MR Method
Inverse variance weighted
MR Egger
550
500
450
400
350
1/SE_IV
−0.0075
−0.0050
−0.0025
0.0000
0.0025
0.0050
β_IV

Batch 2−2018 : Kinetochore protein NDC80 homolog on eGFR

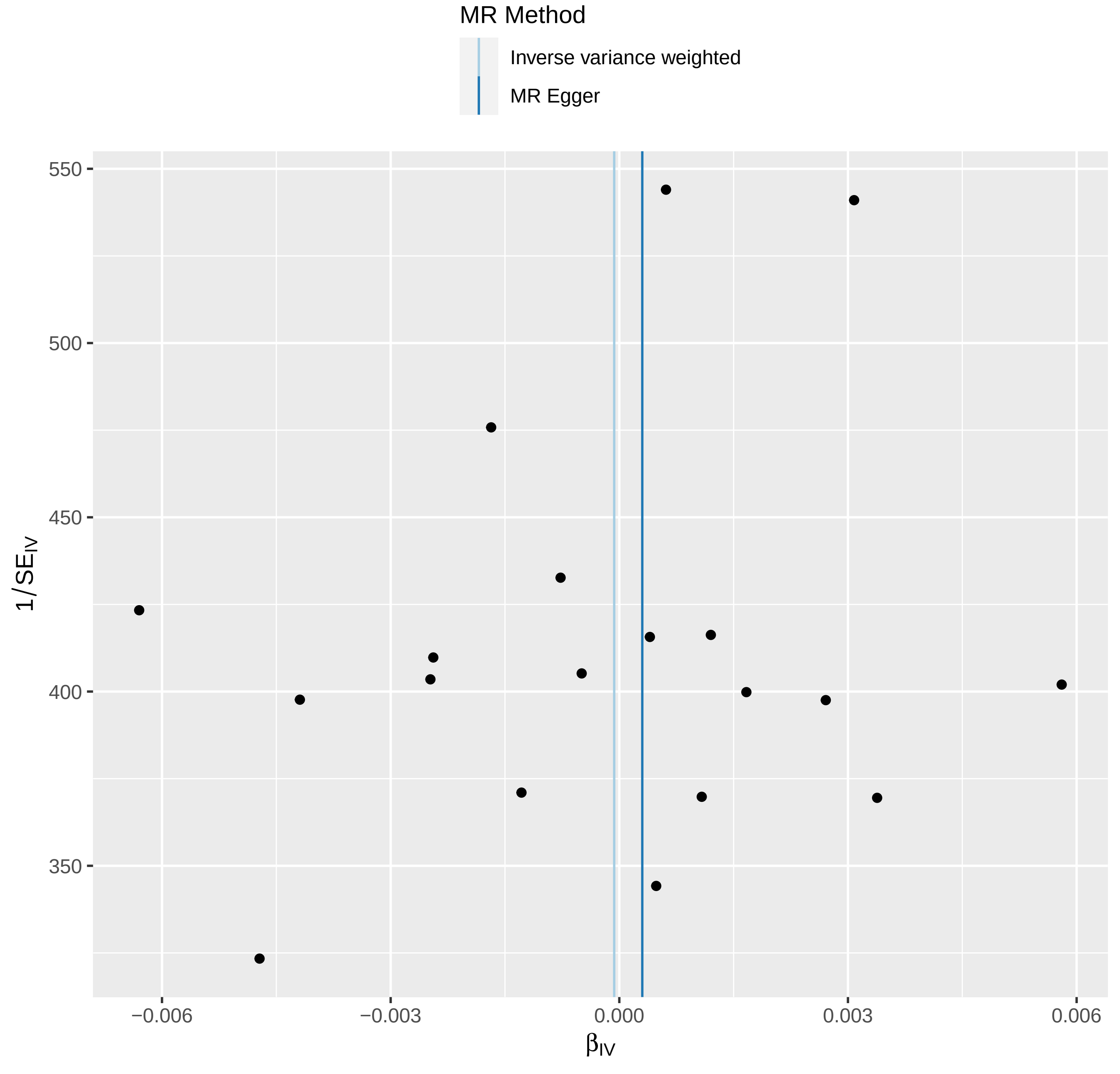

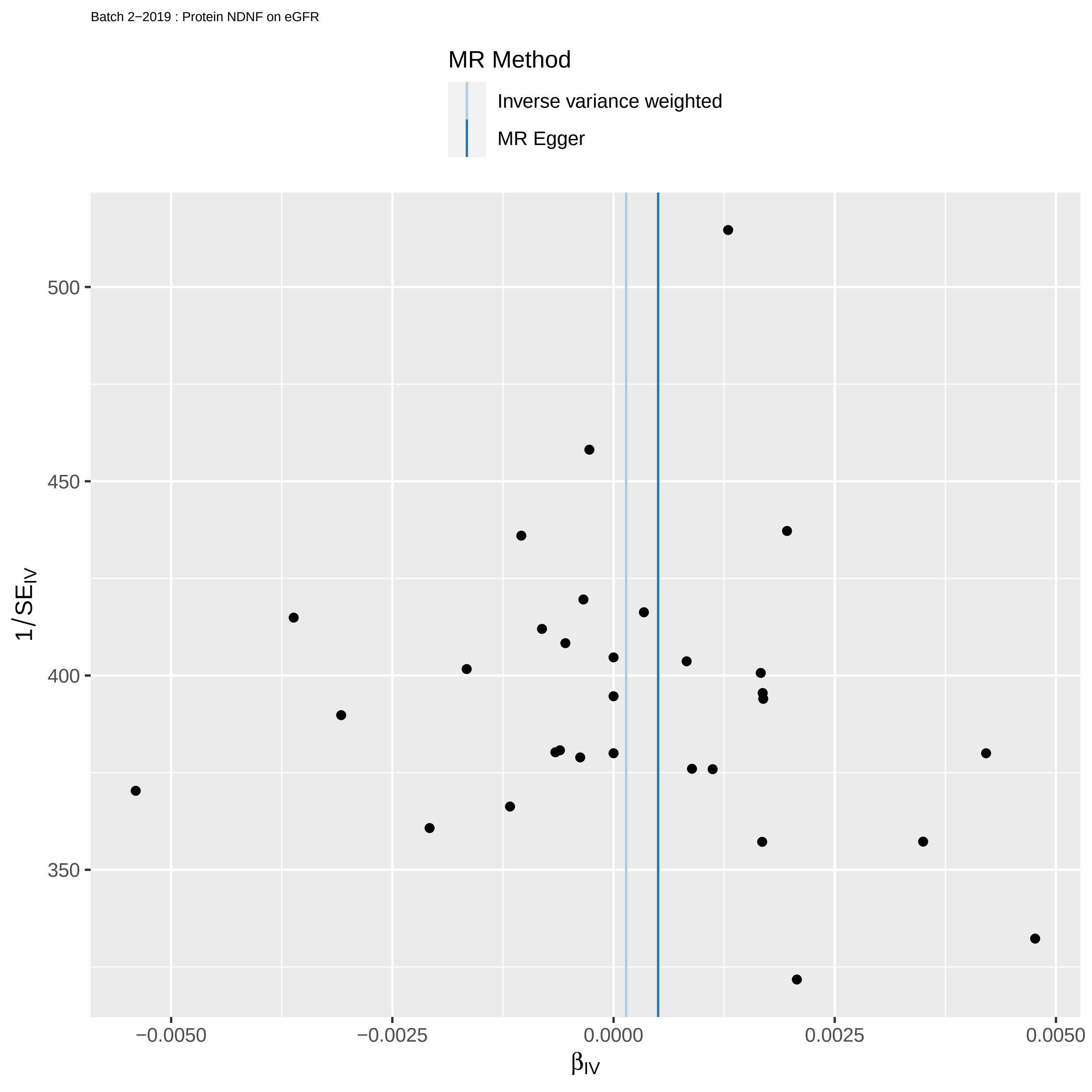
Batch 2−2019 : Protein NDNF on eGFR
MR Method
Inverse variance weighted
MR Egger
500
450
400
350
1/SE$_{IV}$
−0.0050
−0.0025
0.0000
0.0025
0.0050
β$_{IV}$

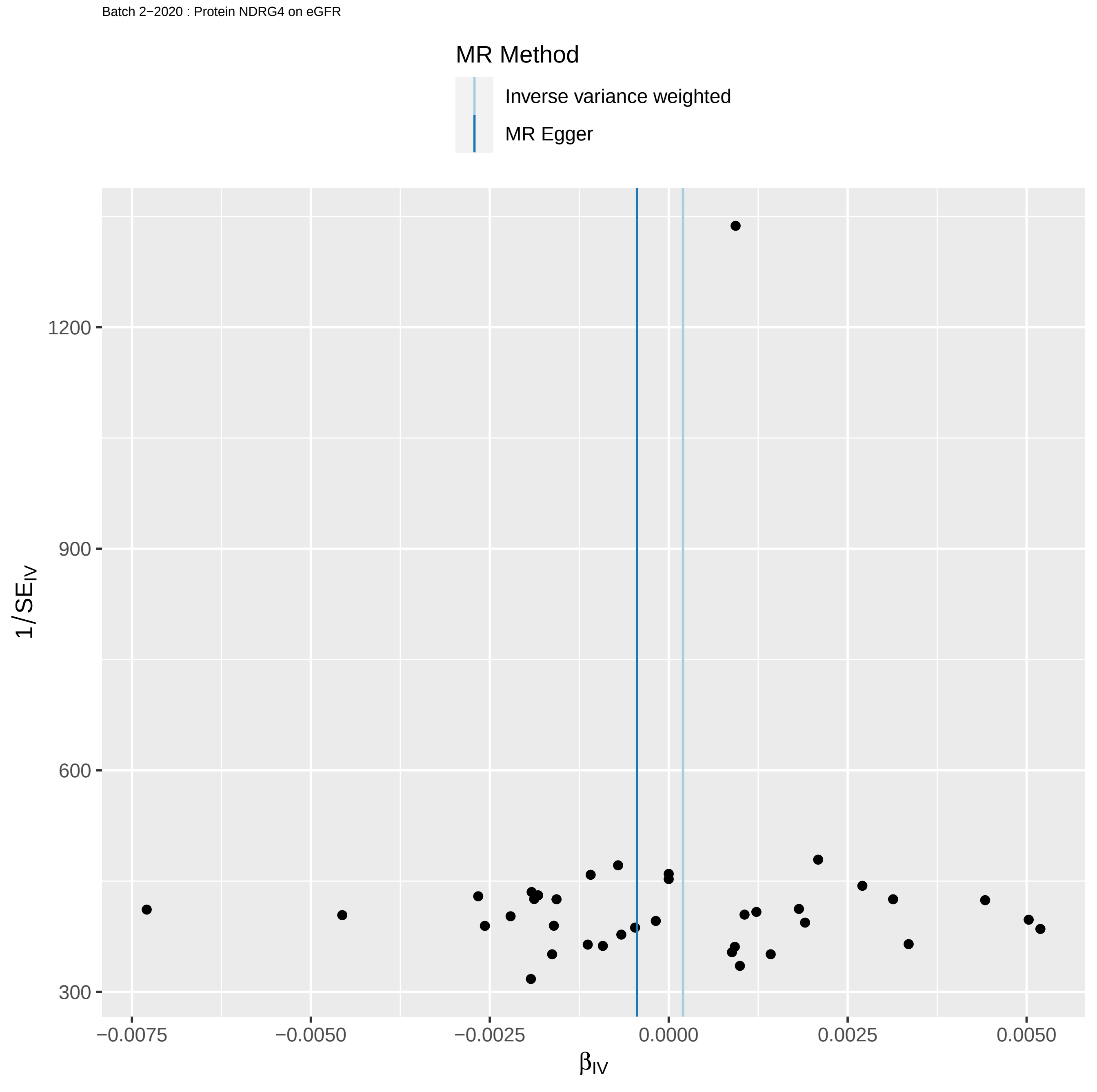
Batch 2−2020 : Protein NDRG4 on eGFR
MR Method
Inverse variance weighted
MR Egger
1200
900
600
300
1/SE_IV
−0.0075
−0.0050
−0.0025
0.0000
0.0025
0.0050
β_IV

Batch 2−2021 : Bifunctional heparan sulfate N−deacetylase/N−sulfotransferase 1 on eGFR

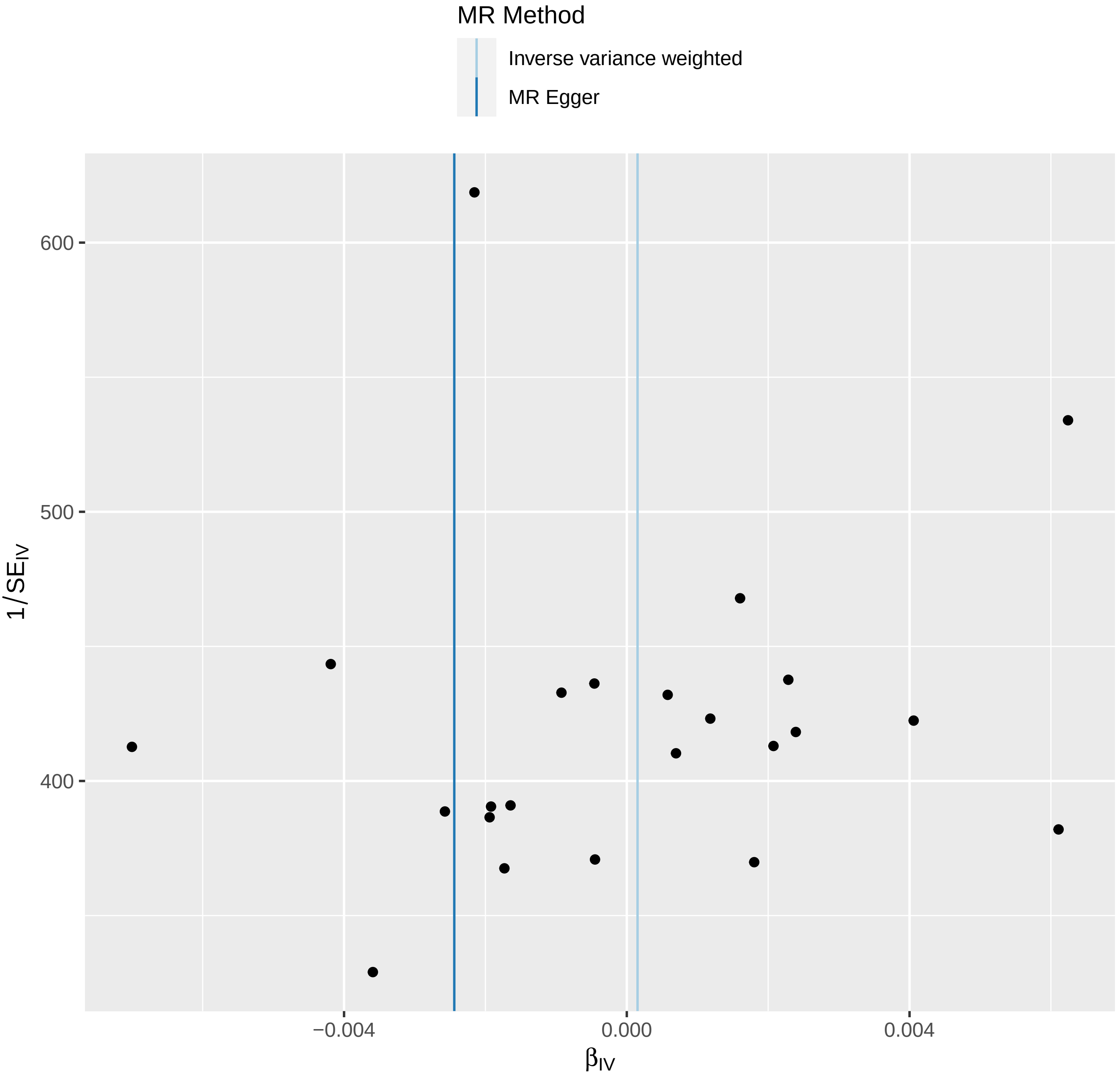

Batch 2−2022 : NADH dehydrogenase [ubiquinone] 1 beta subcomplex subunit 11, mitochondrial on eGFR

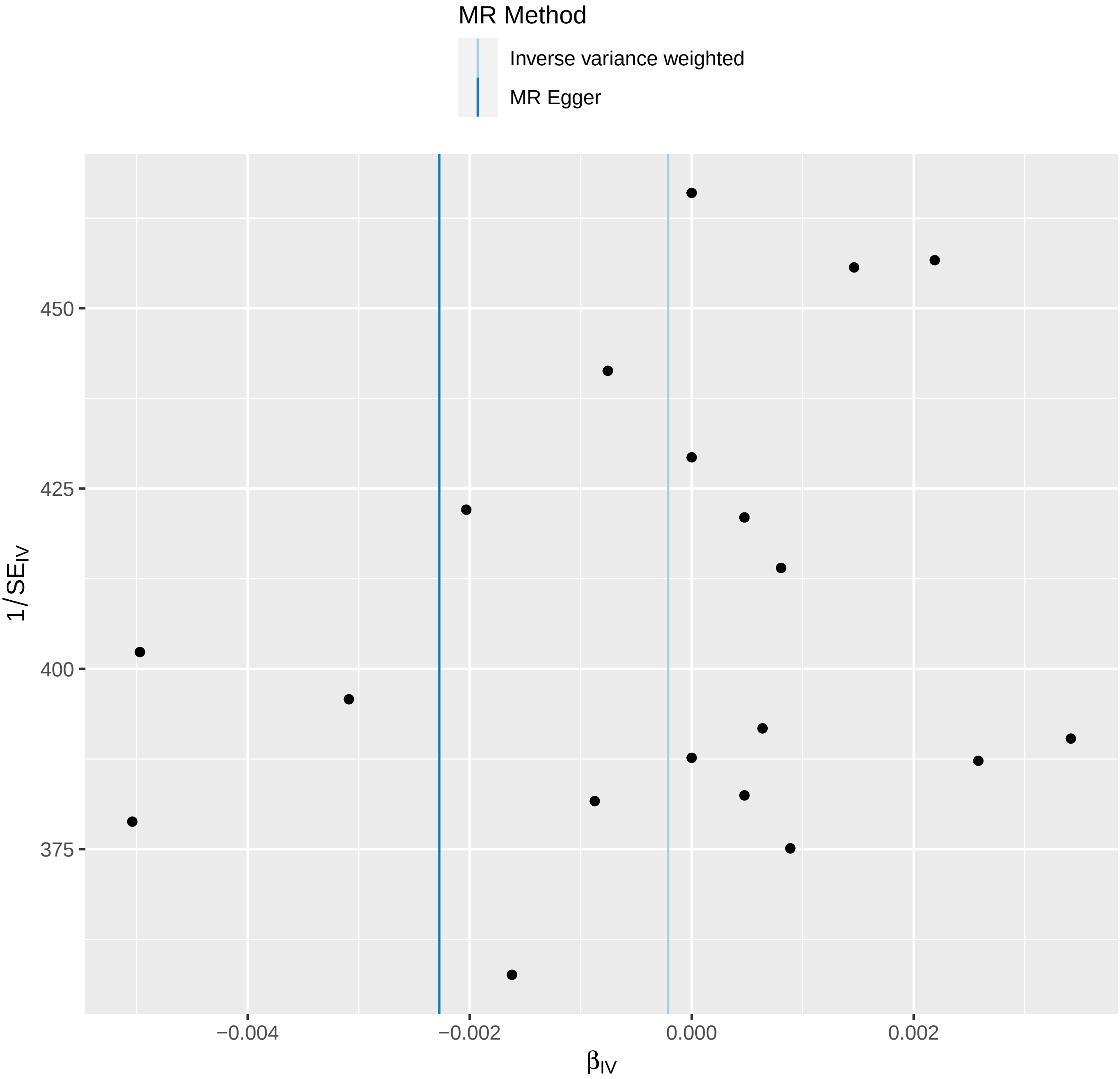

Batch 2−2023 : NADH dehydrogenase [ubiquinone] 1 beta subcomplex subunit 4 on eGFR

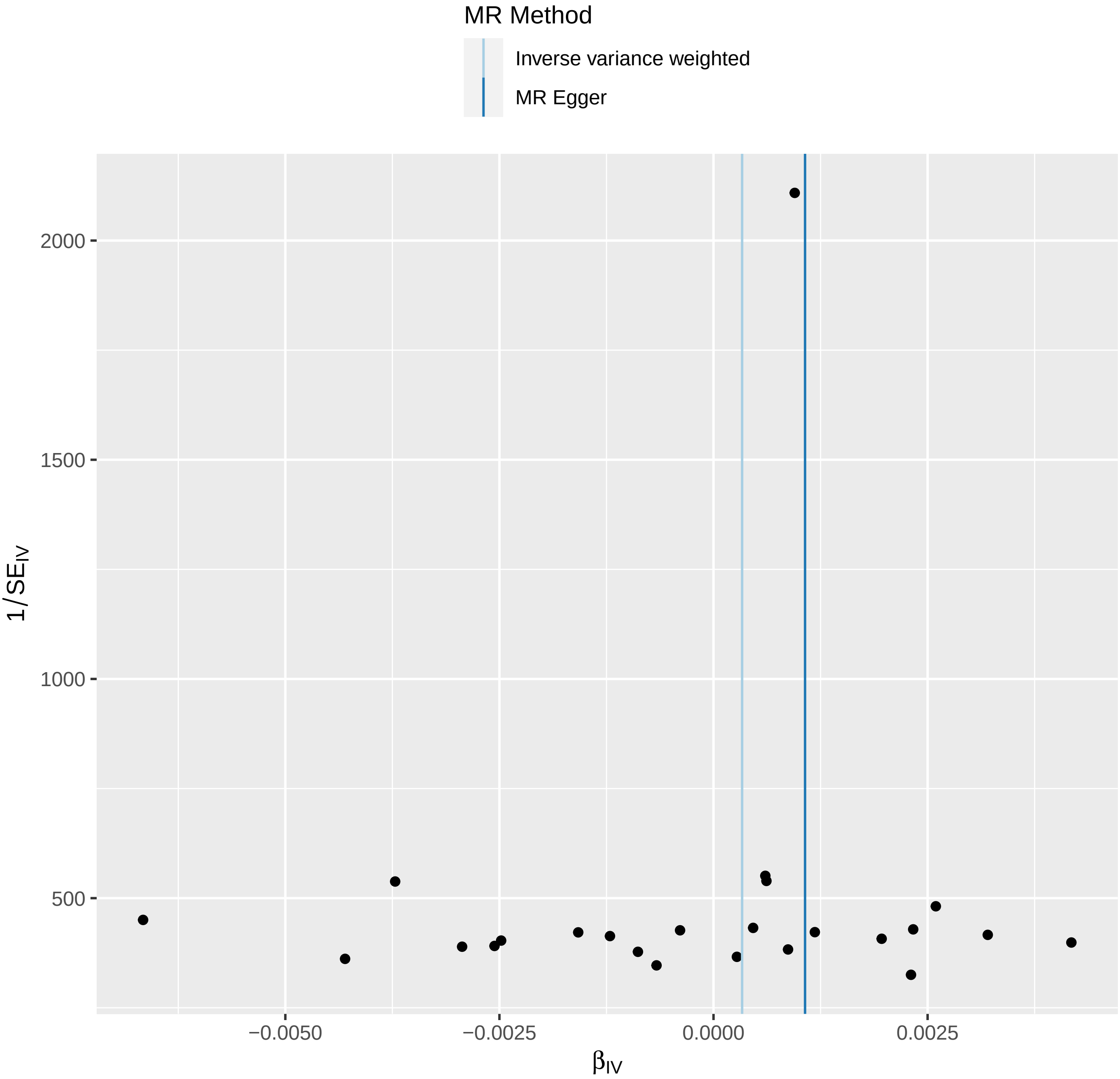

Batch 2−2024 : NADH dehydrogenase [ubiquinone] 1 beta subcomplex subunit 8, mitochondrial on eGFR

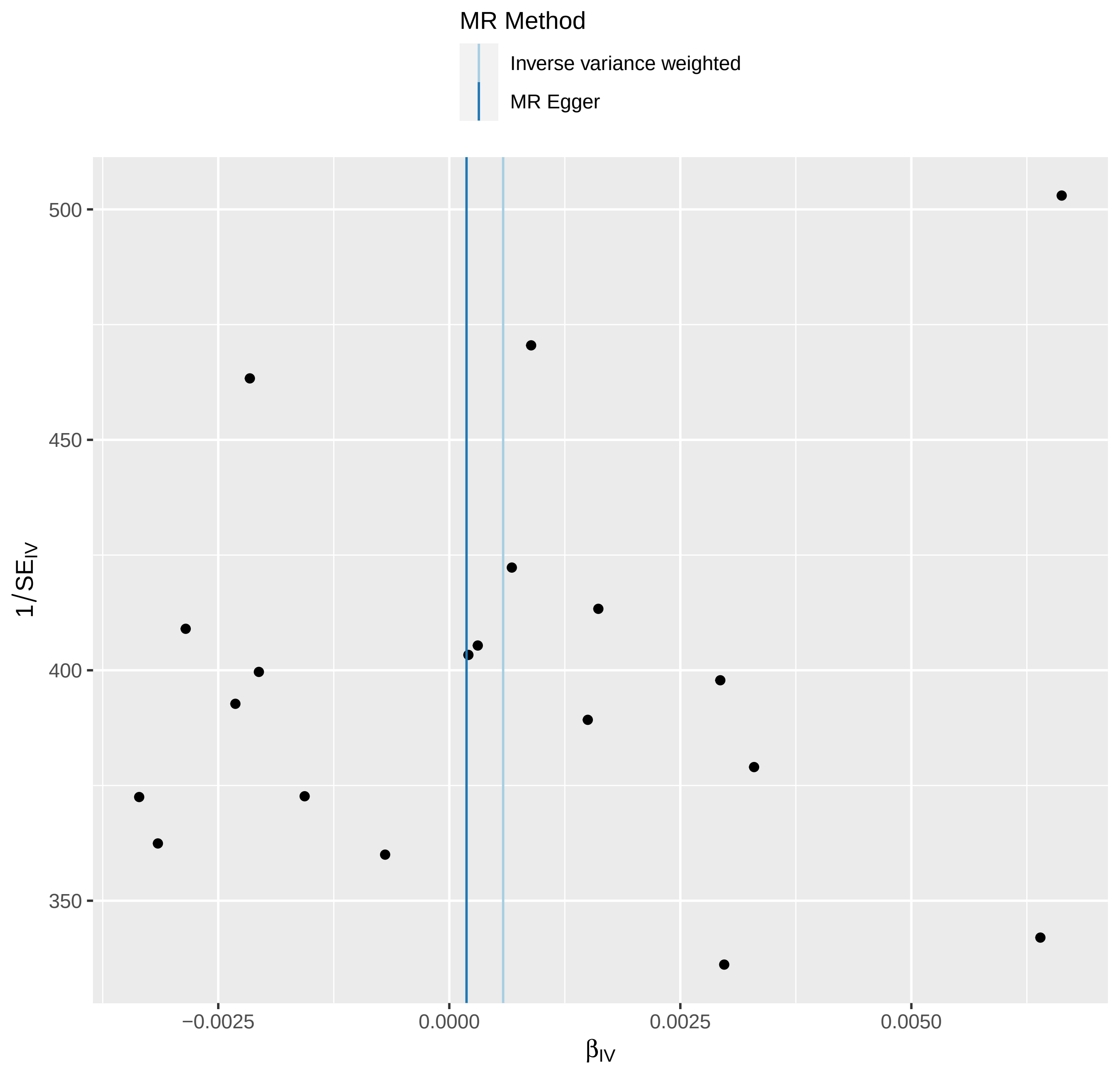

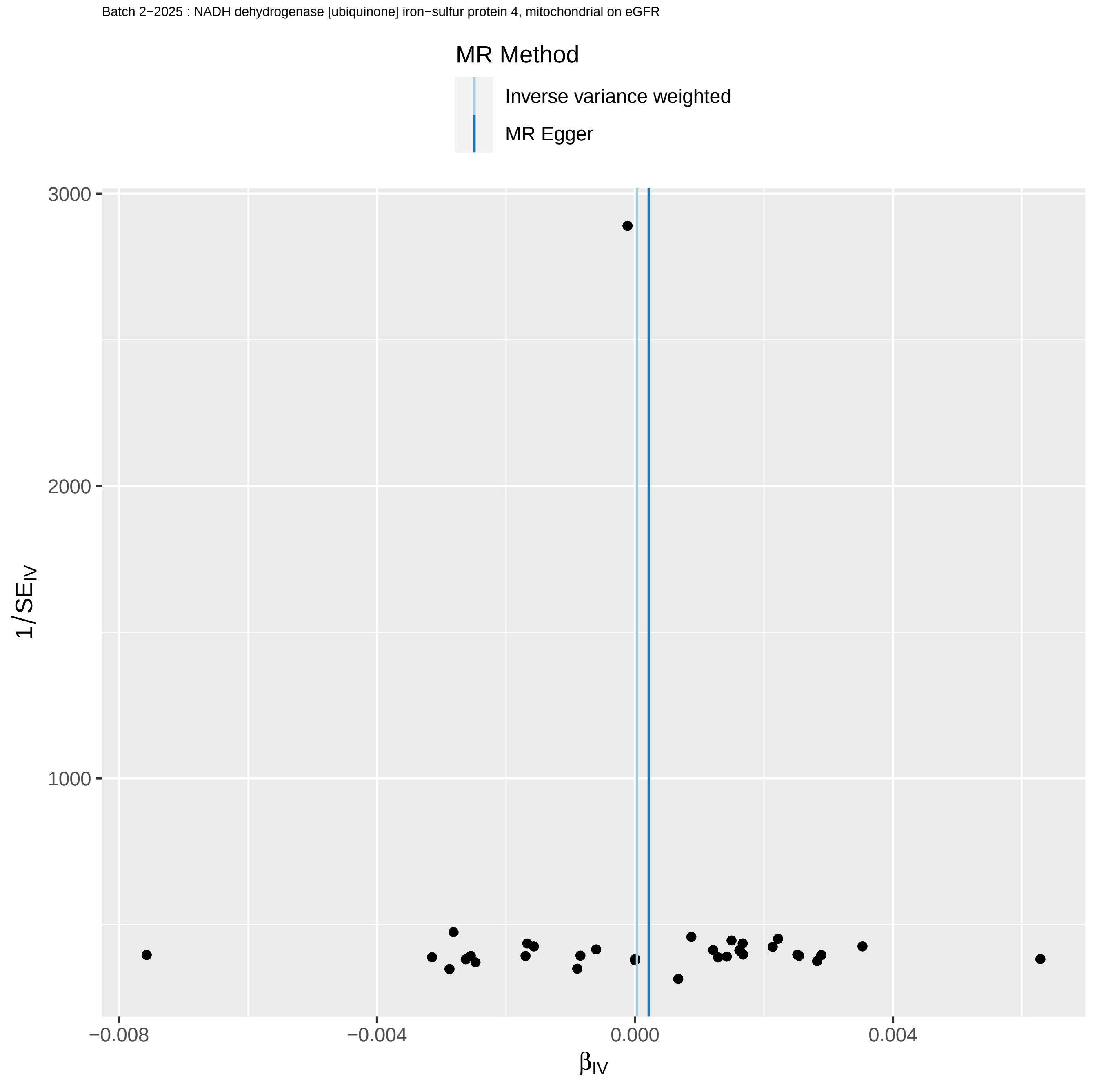
Batch 2−2025 : NADH dehydrogenase [ubiquinone] iron−sulfur protein 4, mitochondrial on eGFR
MR Method
Inverse variance weighted
MR Egger
3000
2000
1000
1/SE_IV
−0.008
−0.004
0.000
0.004
β_IV

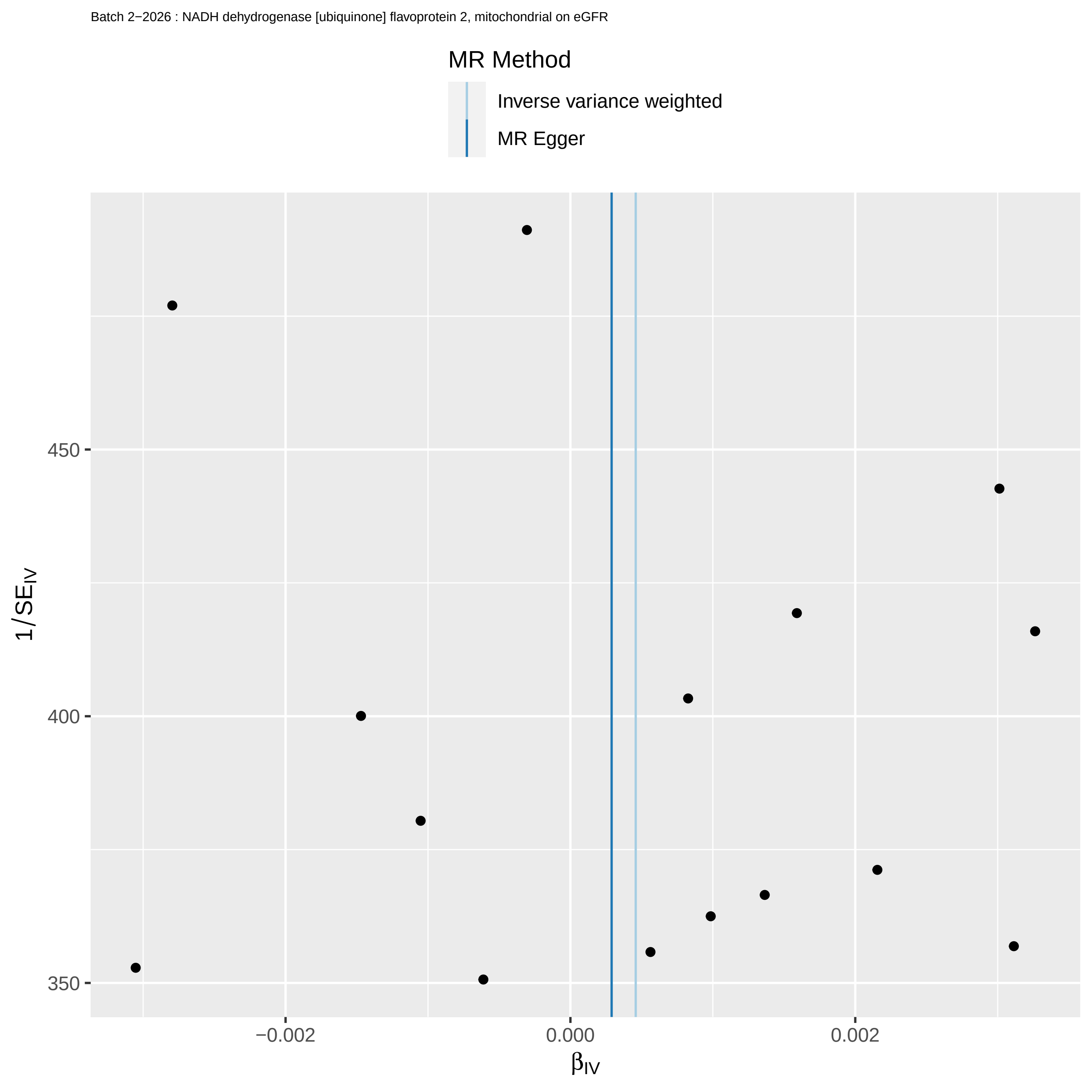
Batch 2−2026 : NADH dehydrogenase [ubiquinone] flavoprotein 2, mitochondrial on eGFR
MR Method
Inverse variance weighted
MR Egger
450
400
350
1/SE_IV
−0.002
0.000
0.002
β_IV

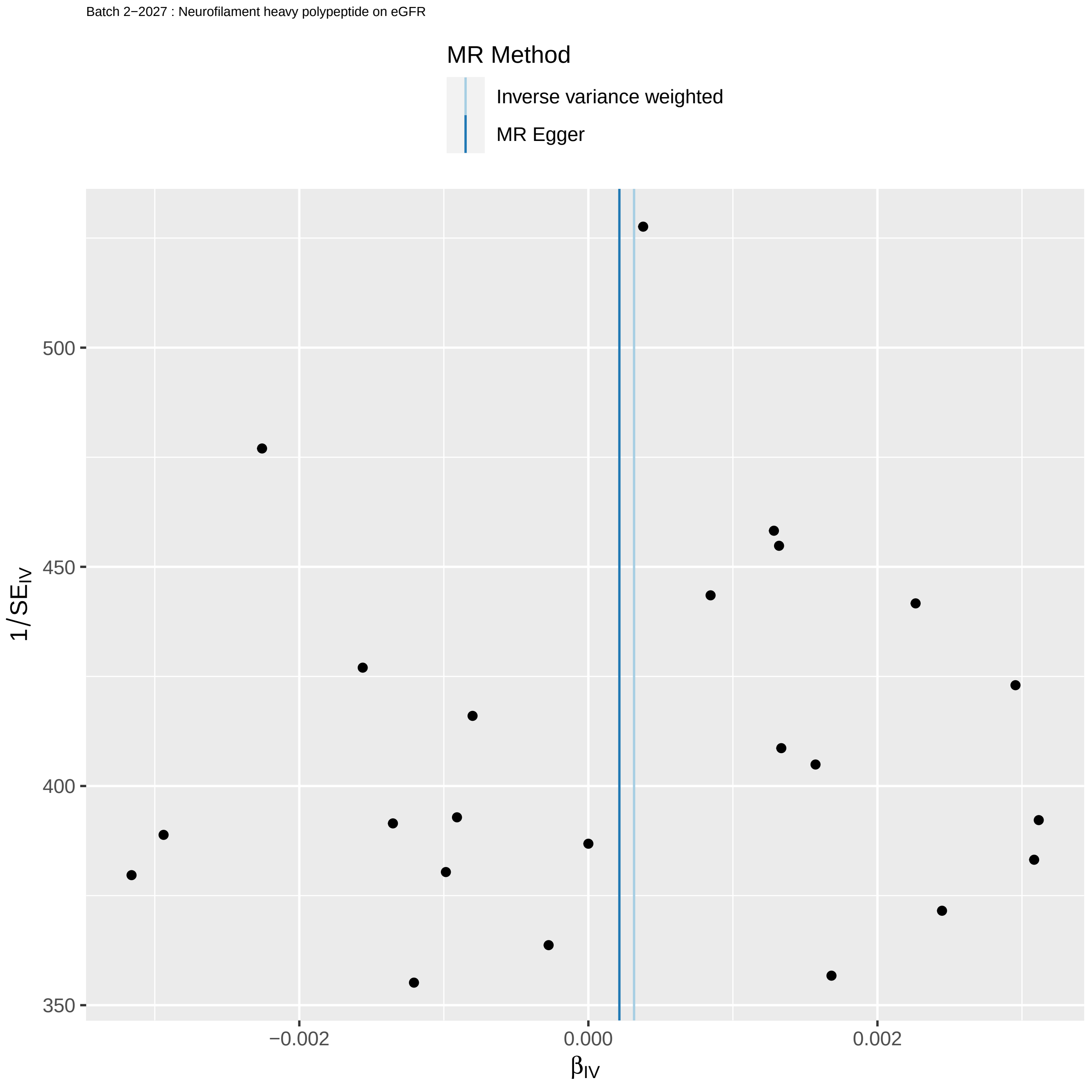

Batch 2−2027 : Neurofilament heavy polypeptide on eGFR
MR Method
Inverse variance weighted
MR Egger
500
450
400
350
1/SE_IV
−0.002
0.000
0.002
β_IV

Batch 2−2028 : Neuronal growth regulator 1 on eGFR

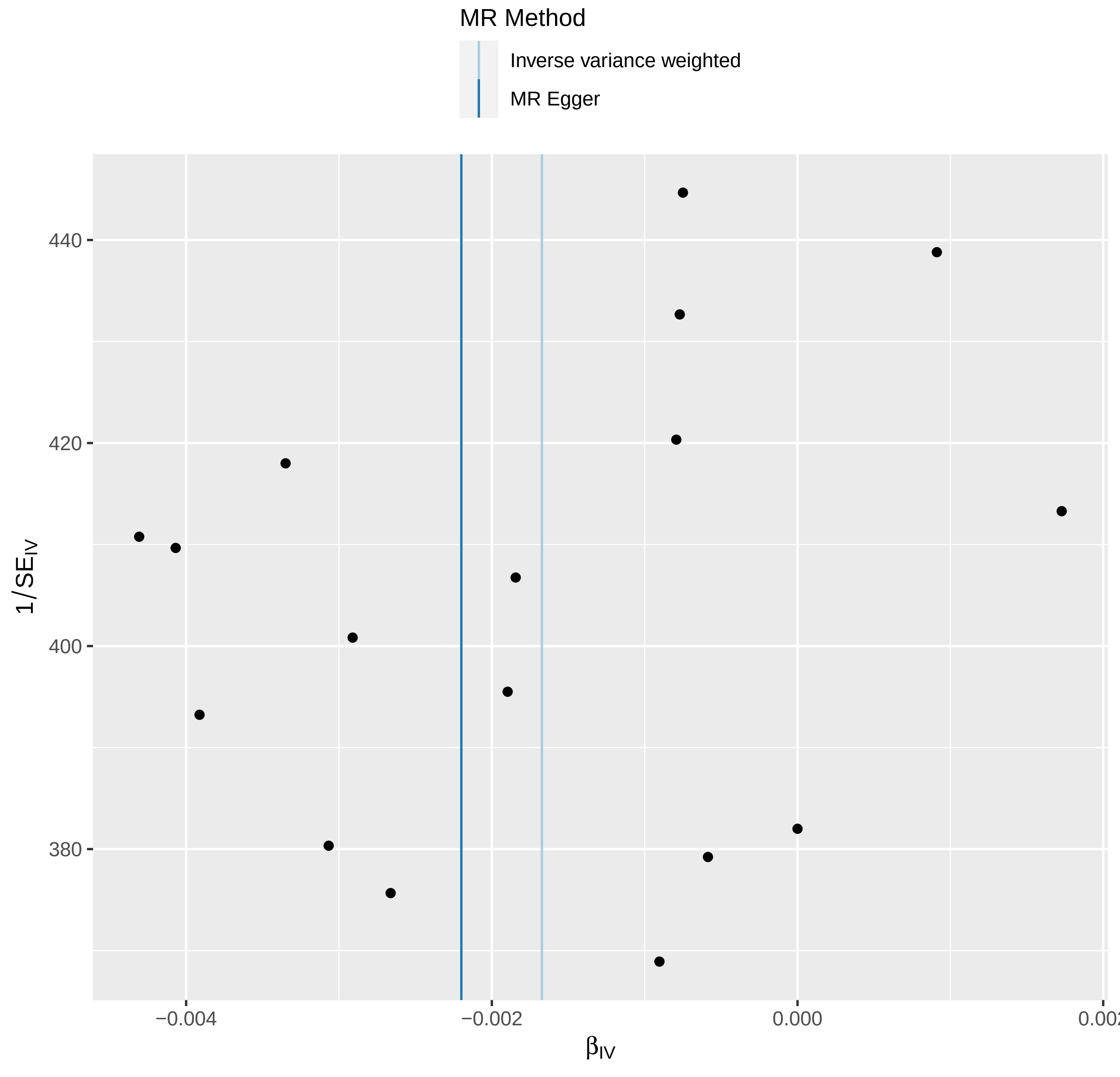

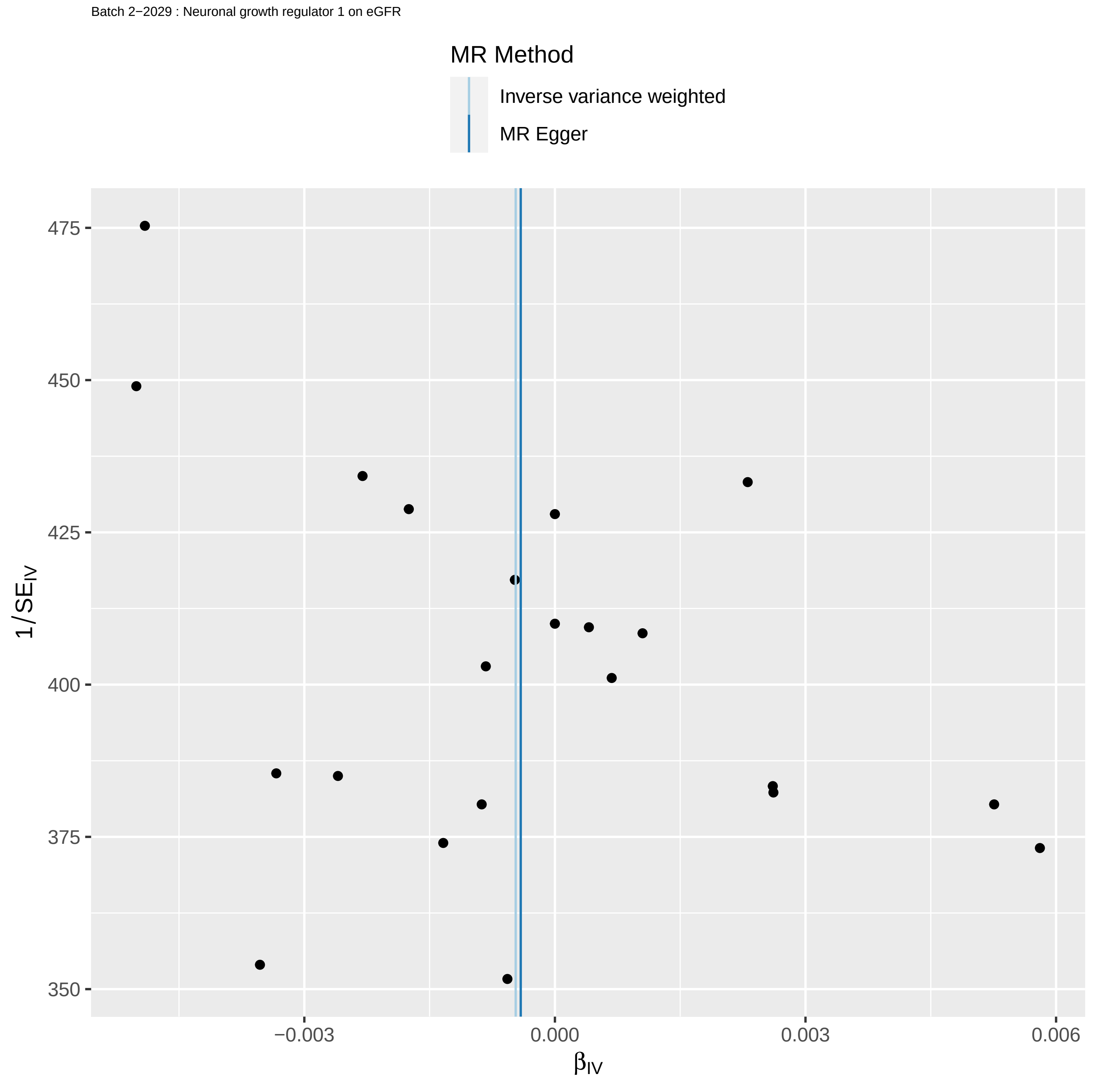

Batch 2−2029 : Neuronal growth regulator 1 on eGFR
MR Method
Inverse variance weighted
MR Egger
475
450
425
400
375
350
1/SE_IV
−0.003
0.000
0.003
0.006
β_IV

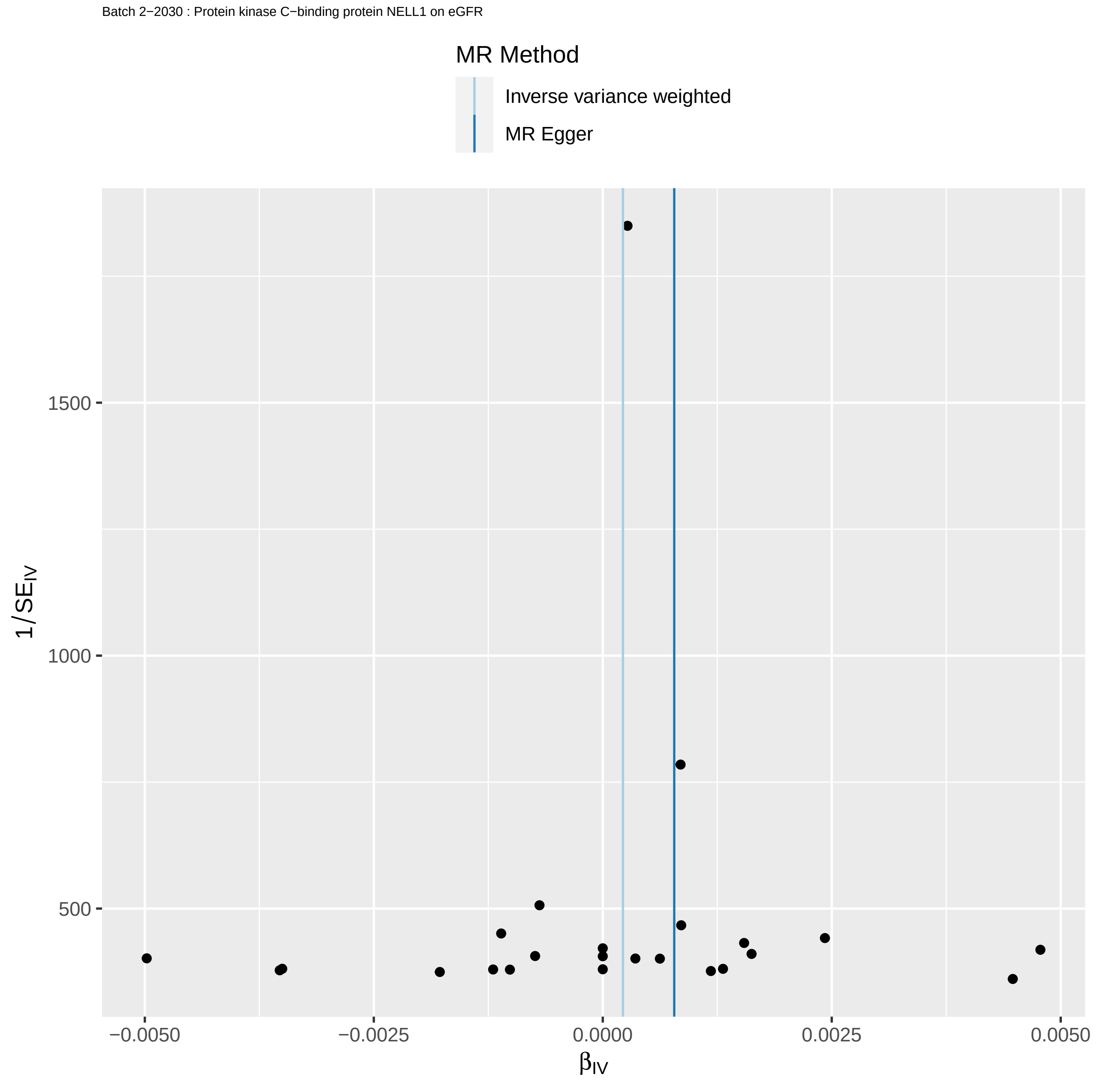
Batch 2−2030 : Protein kinase C−binding protein NELL1 on eGFR
MR Method
Inverse variance weighted
MR Egger
1500
1000
500
1/SE$_{IV}$
−0.0050
−0.0025
0.0000
0.0025
0.0050
β$_{IV}$

Batch 2−2031 : Protein kinase C−binding protein NELL2 on eGFR

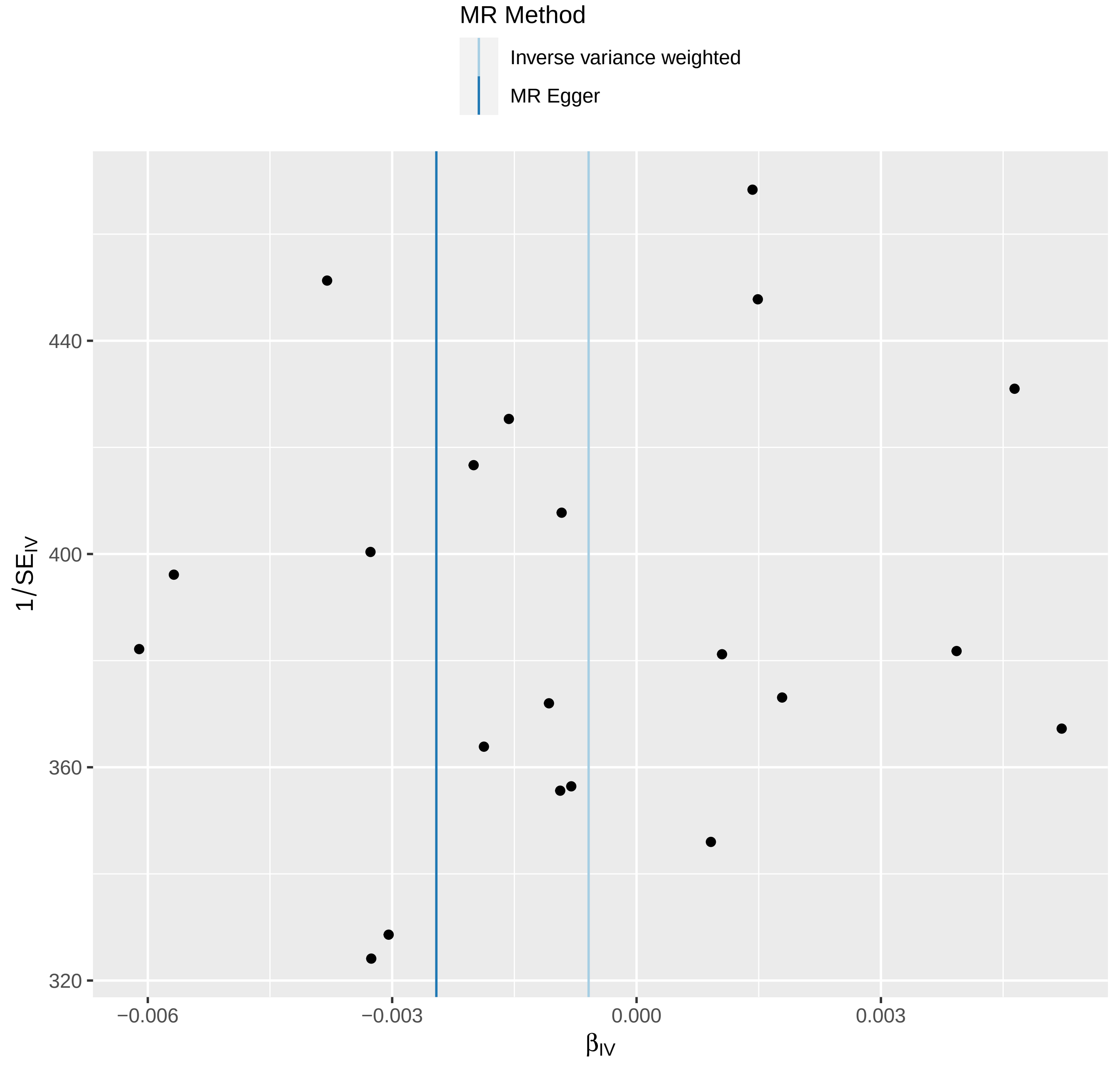

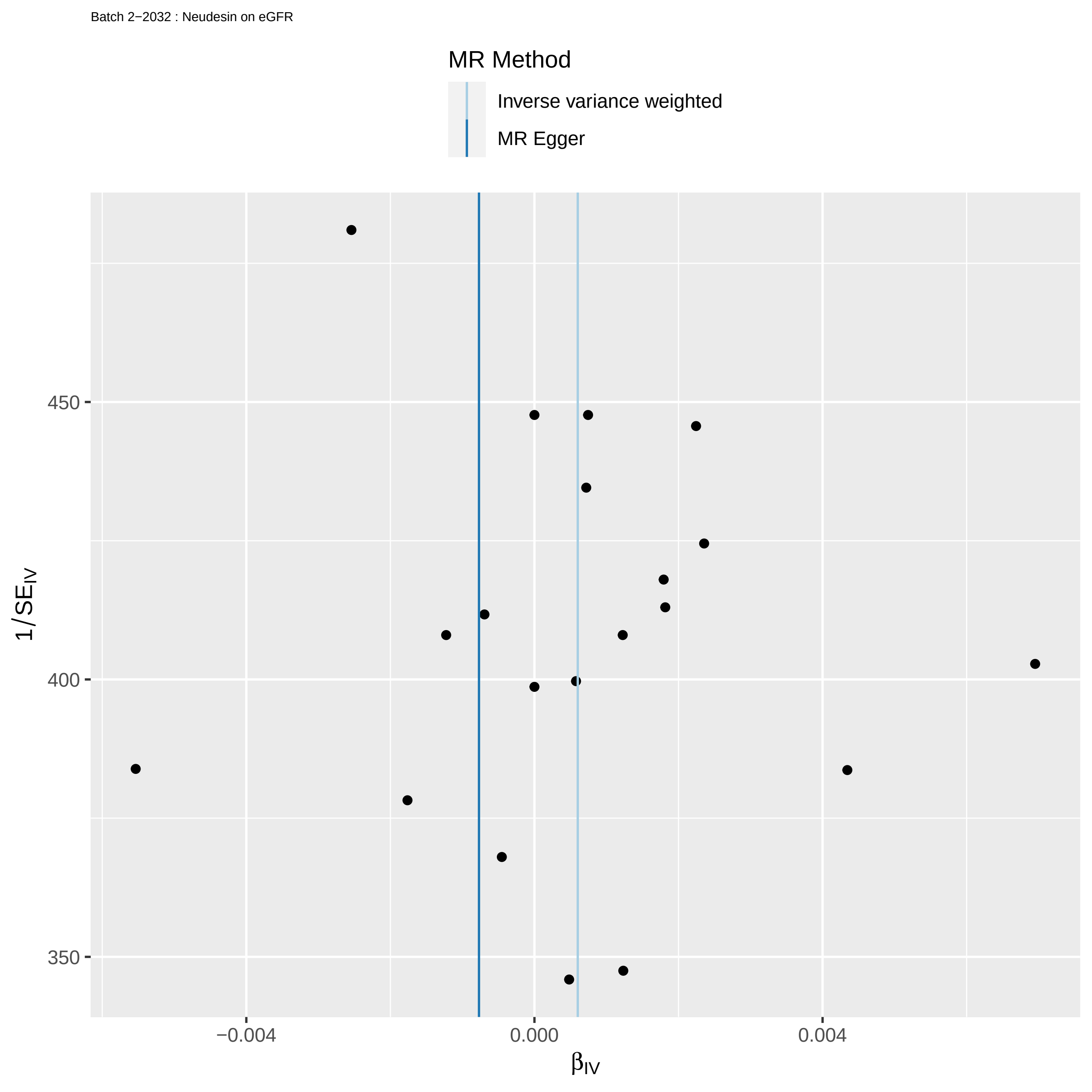

Batch 2−2032 : Neudesin on eGFR
MR Method
Inverse variance weighted
MR Egger
450
400
350
1/SE$_{IV}$
−0.004
0.000
0.004
β$_{IV}$

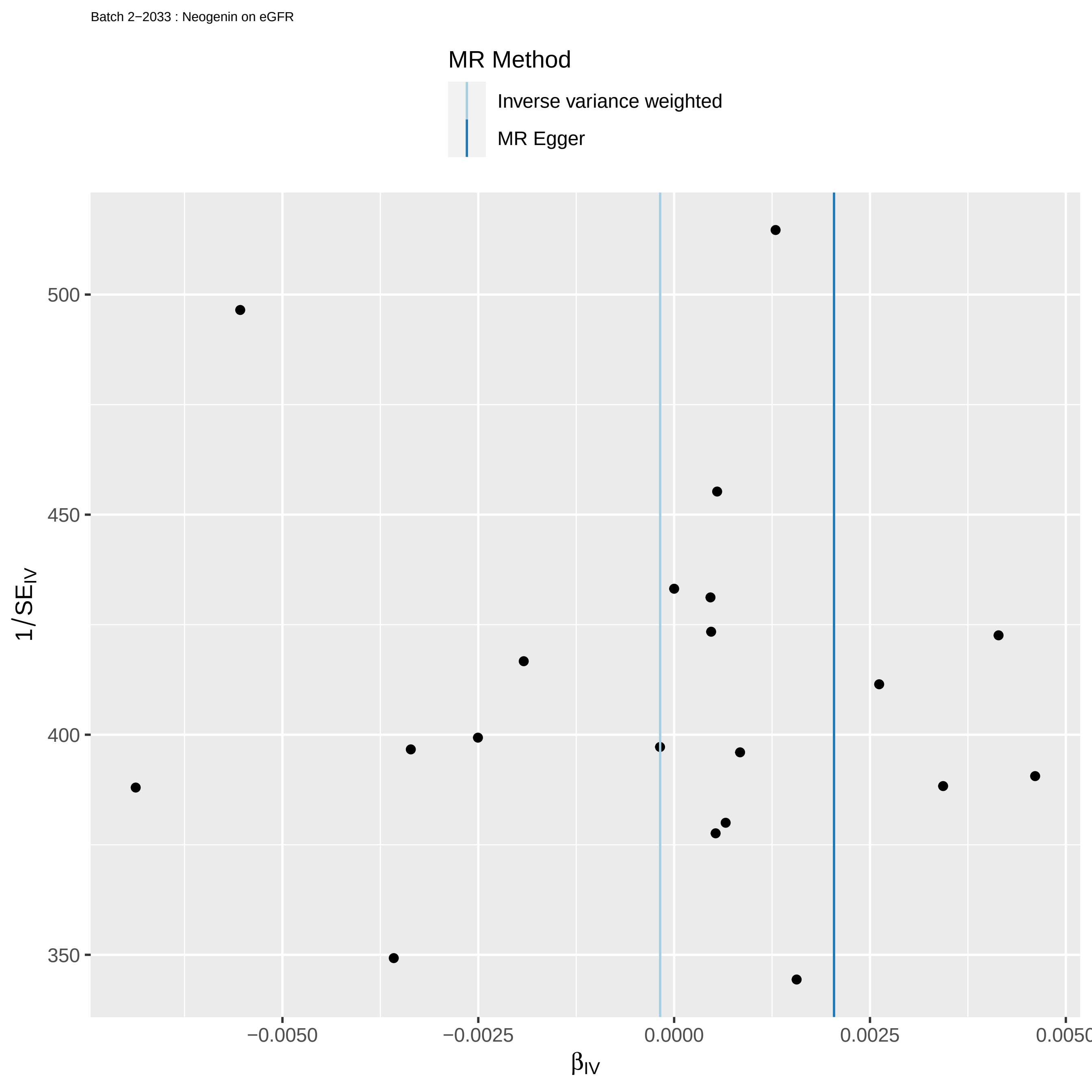

Batch 2−2033 : Neogenin on eGFR
MR Method
Inverse variance weighted
MR Egger
500
450
400
350
1/SE_IV
−0.0050
−0.0025
0.0000
0.0025
0.0050
β_IV

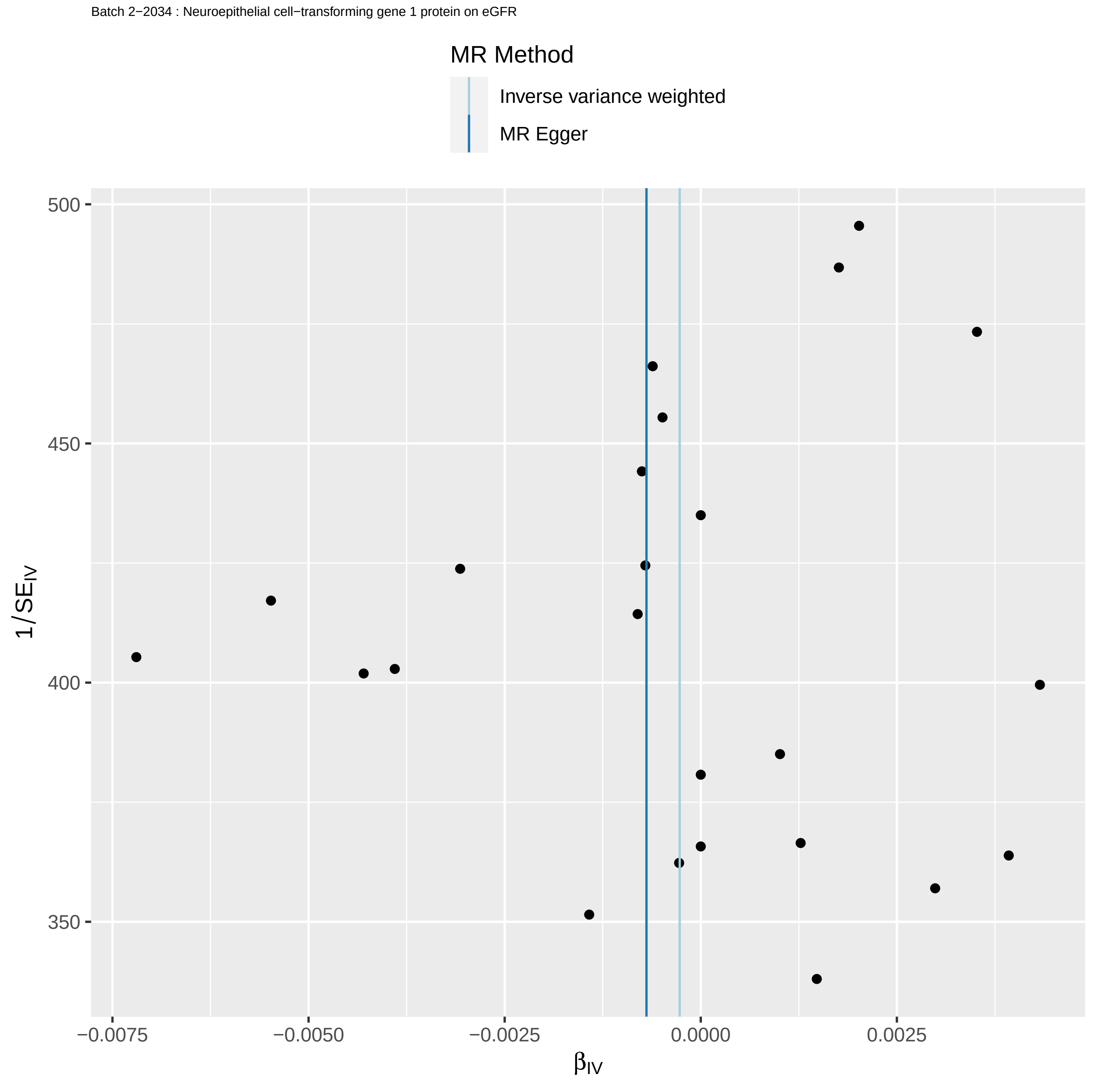

Batch 2−2034 : Neuroepithelial cell−transforming gene 1 protein on eGFR
MR Method
Inverse variance weighted
MR Egger
500
450
400
350
1/SE_IV
−0.0075
−0.0050
−0.0025
0.0000
0.0025
β_IV

Batch 2−2035 : Neuropilin and tolloid−like protein 1 on eGFR

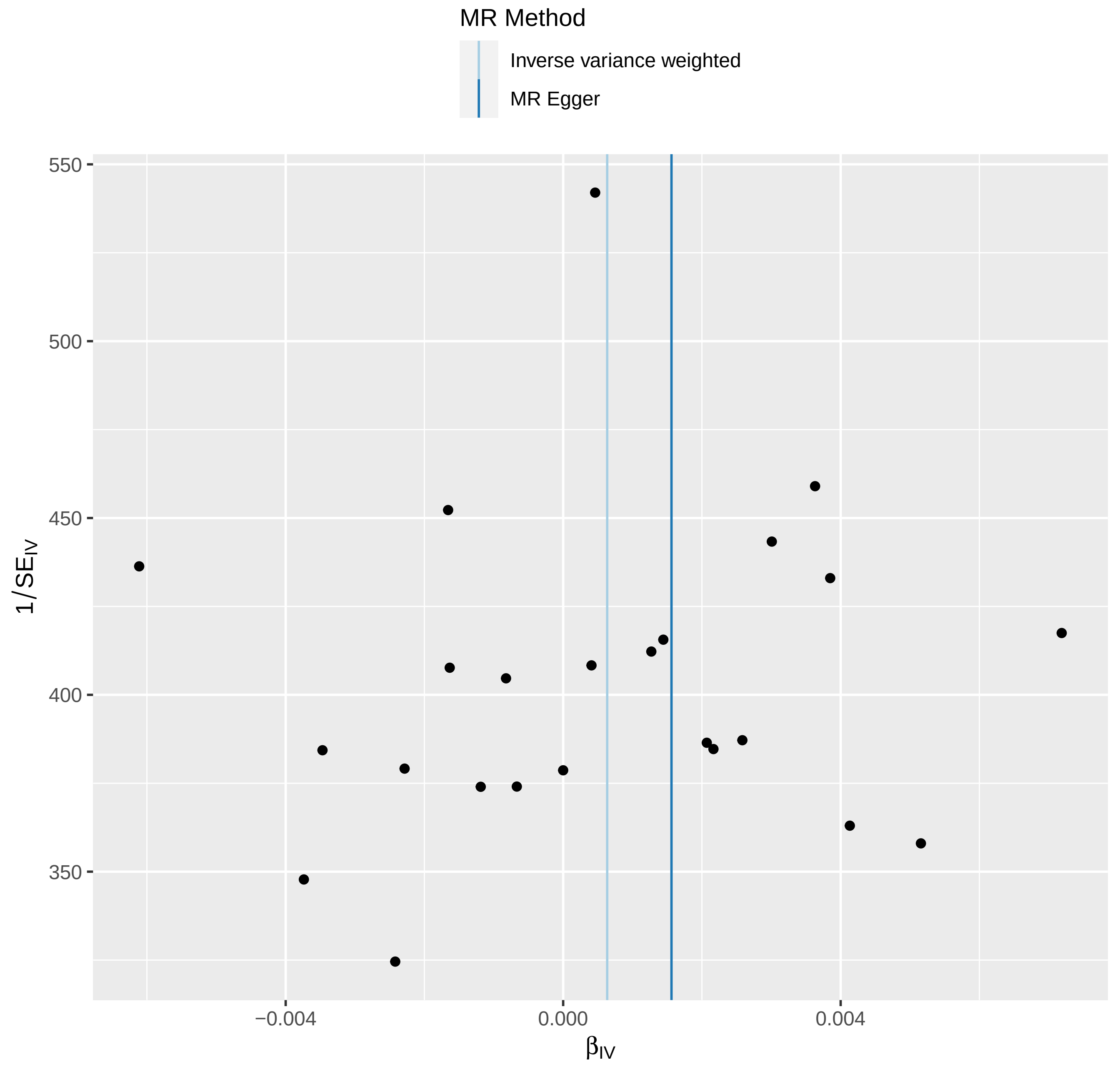

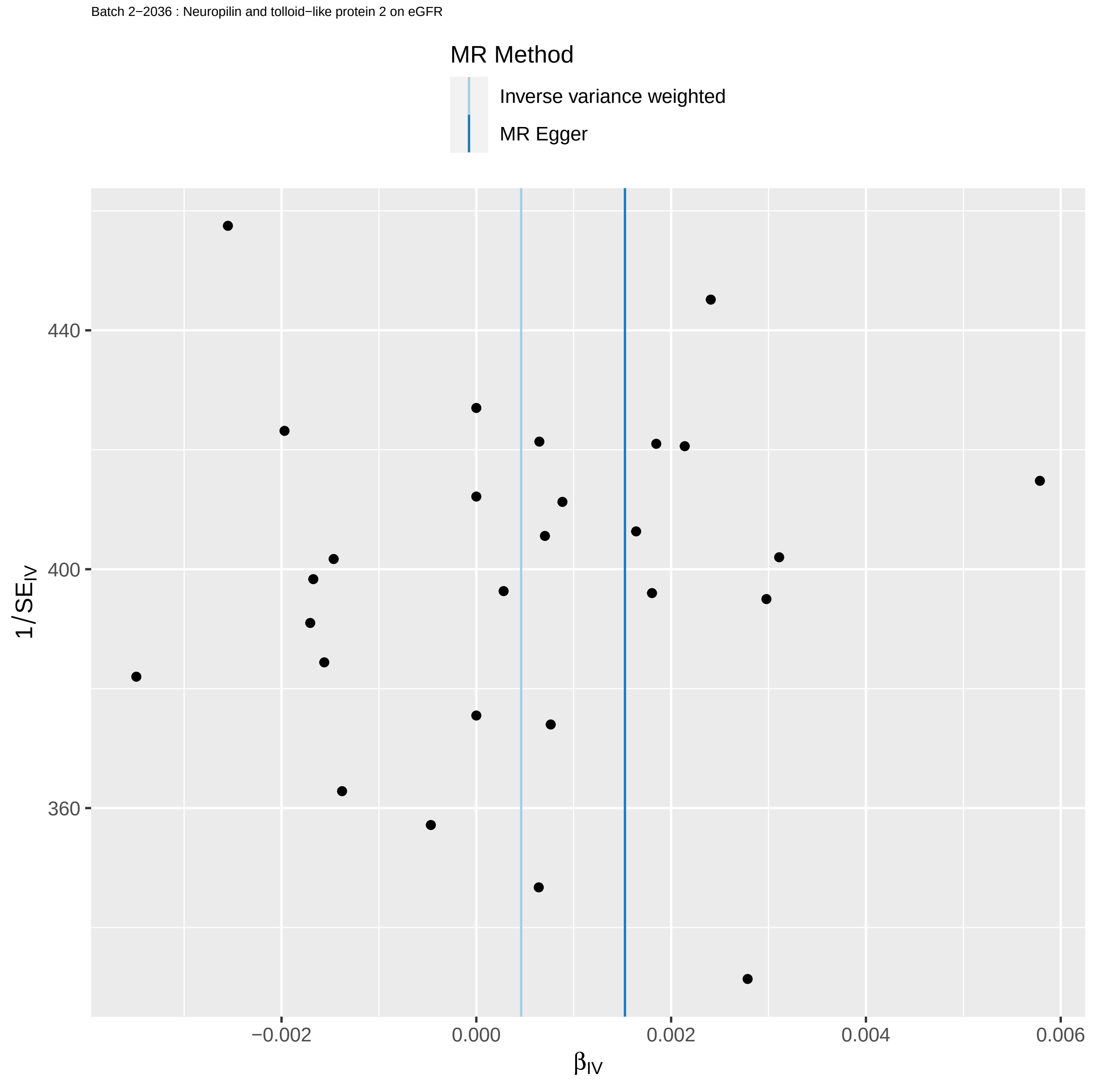
Batch 2−2036 : Neuropilin and tolloid−like protein 2 on eGFR
MR Method
Inverse variance weighted
MR Egger
440
400
360
1/SE_IV
−0.002
0.000
0.002
0.004
0.006
β_IV

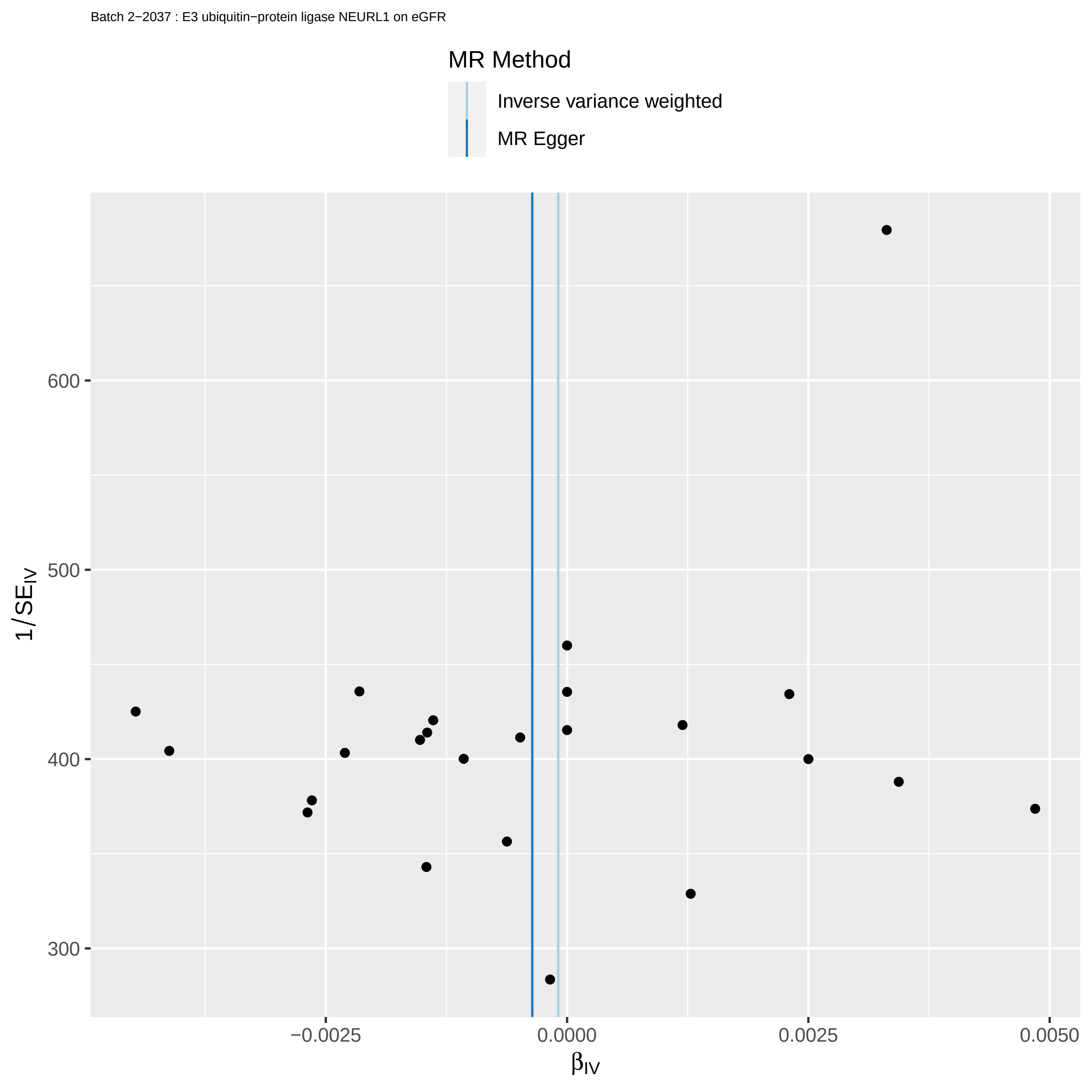

Batch 2−2037 : E3 ubiquitin−protein ligase NEURL1 on eGFR
MR Method
Inverse variance weighted
MR Egger
600
500
400
300
1/SE$_{IV}$
−0.0025
0.0000
0.0025
0.0050
β_{IV}

Batch 2−2038 : Neuralized−like protein 4 on eGFR

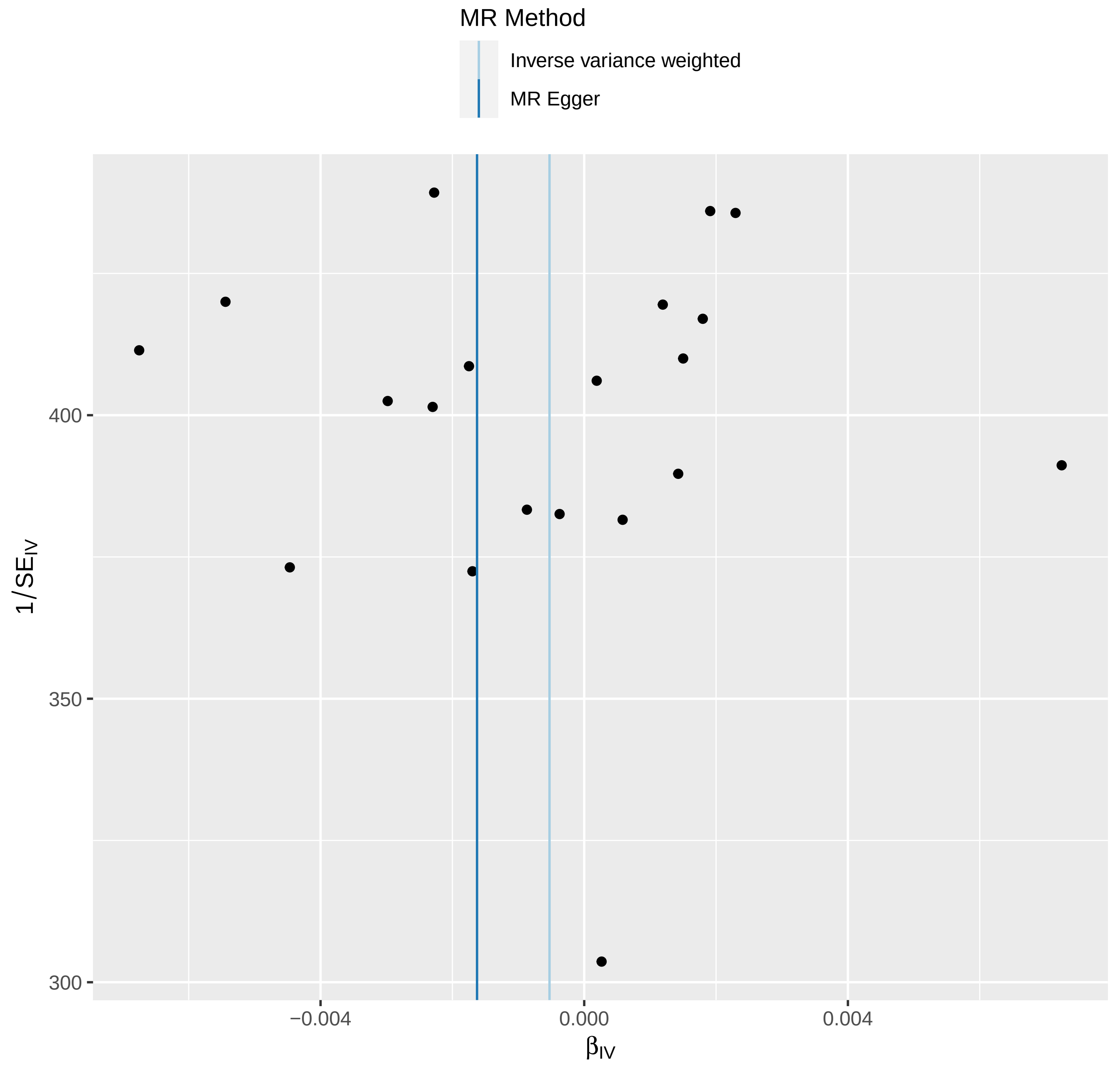

Batch 2−2039 : Neurofascin on eGFR

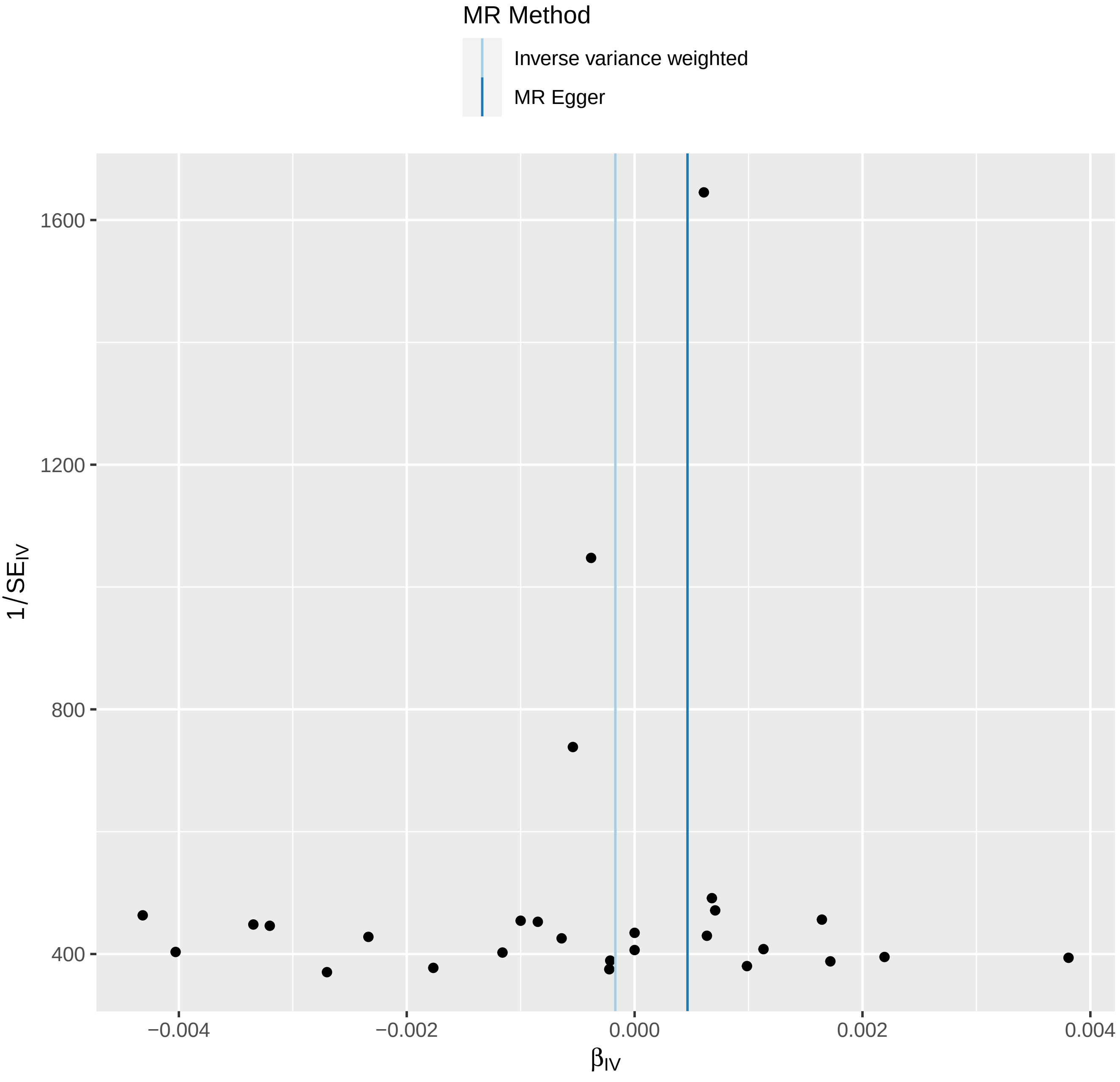

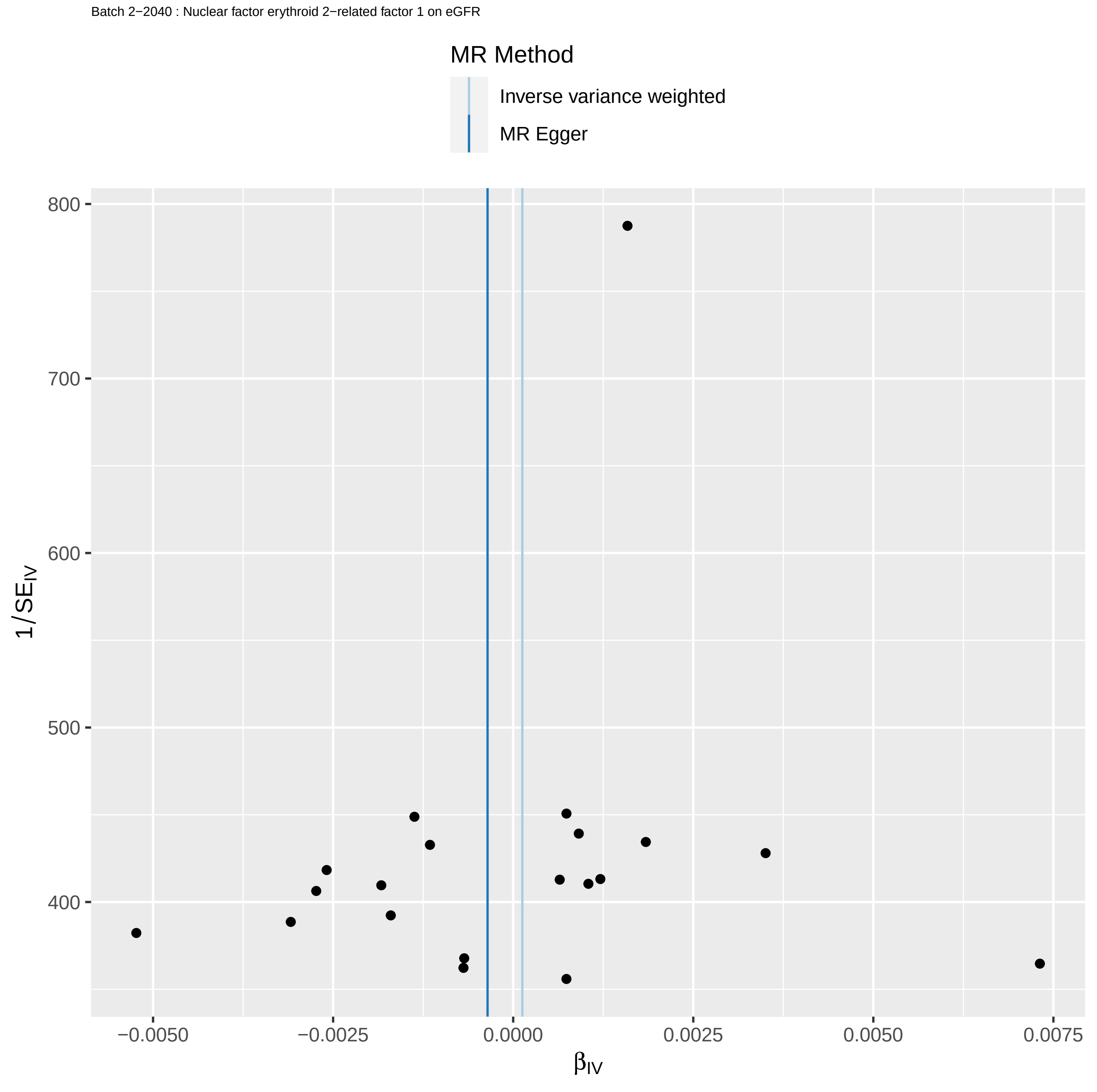

Batch 2−2040 : Nuclear factor erythroid 2−related factor 1 on eGFR
MR Method
Inverse variance weighted
MR Egger
800
700
600
500
400
1/SE$_{IV}$
−0.0050
−0.0025
0.0000
0.0025
0.0050
0.0075
β$_{IV}$

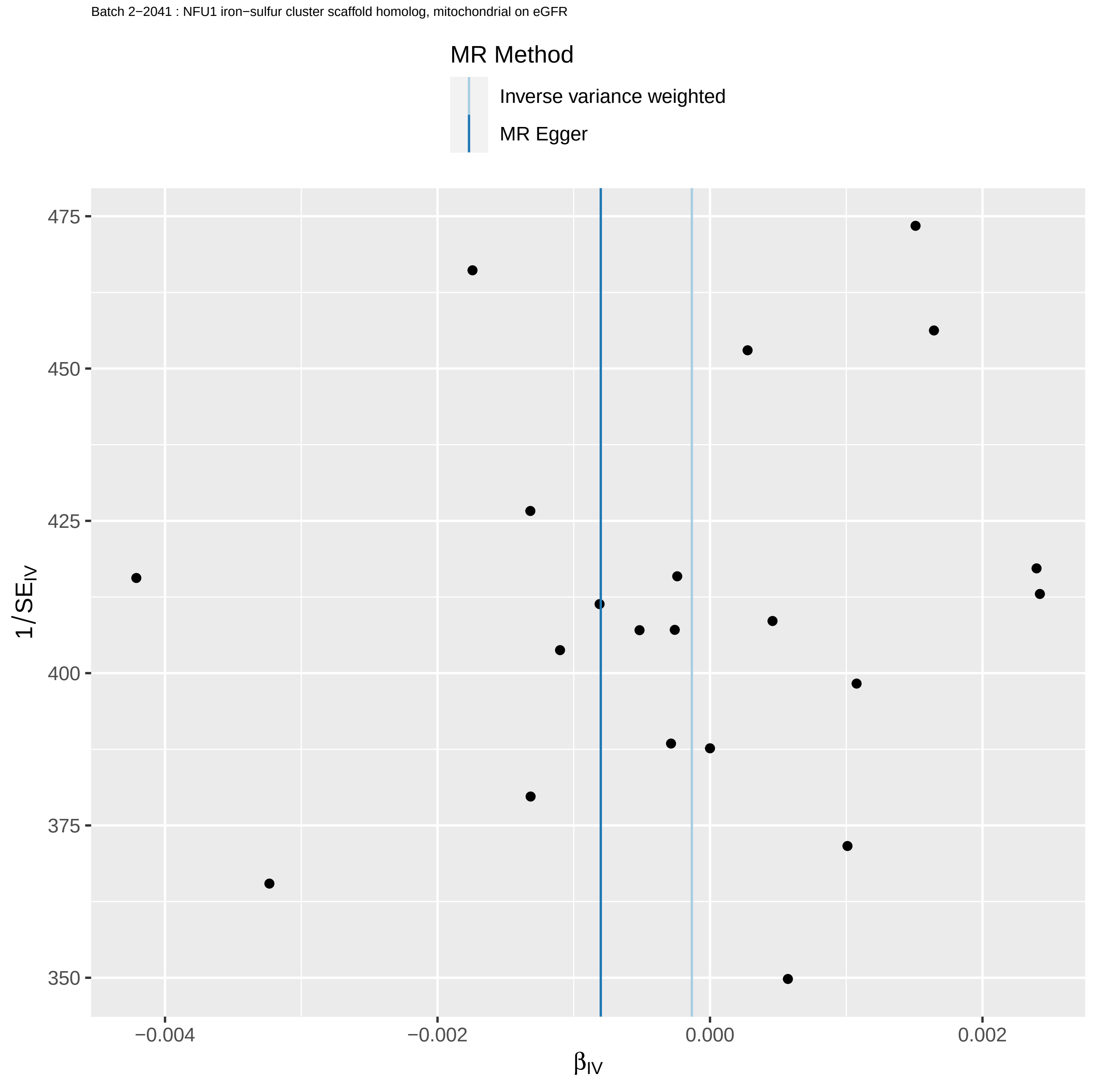

Batch 2−2041 : NFU1 iron−sulfur cluster scaffold homolog, mitochondrial on eGFR
MR Method
Inverse variance weighted
MR Egger
475
450
425
400
375
350
1/SE_IV
−0.004
−0.002
0.000
0.002
β_IV

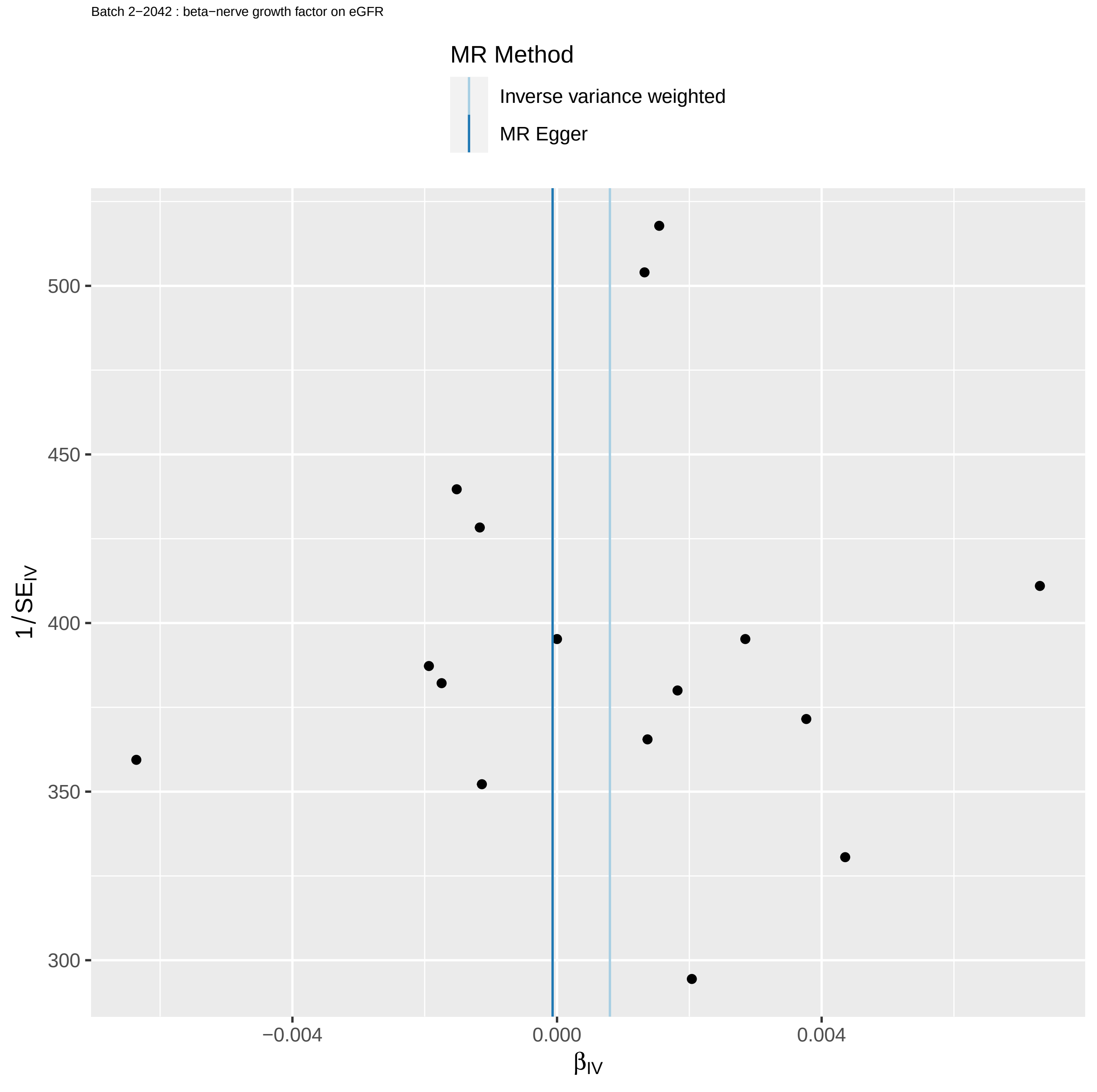

Batch 2−2042 : beta−nerve growth factor on eGFR
MR Method
Inverse variance weighted
MR Egger
500
450
400
350
300
−0.004
0.000
0.004
β_{IV}
$1/SE_{IV}$

Batch 2−2043 : Tumor necrosis factor receptor superfamily member 16 on eGFR

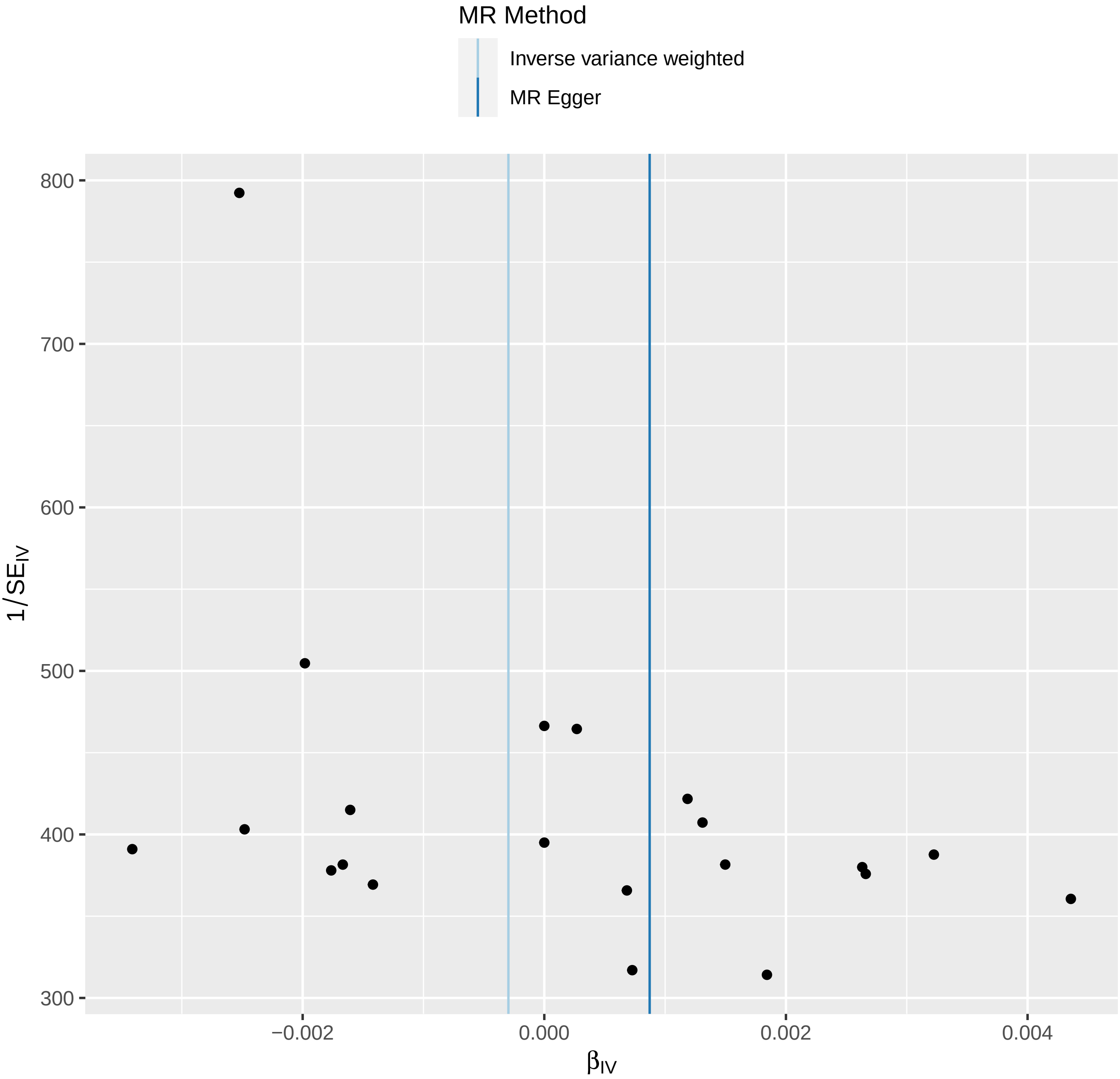

Batch 2−2044 : Tumor necrosis factor receptor superfamily member 16 on eGFR

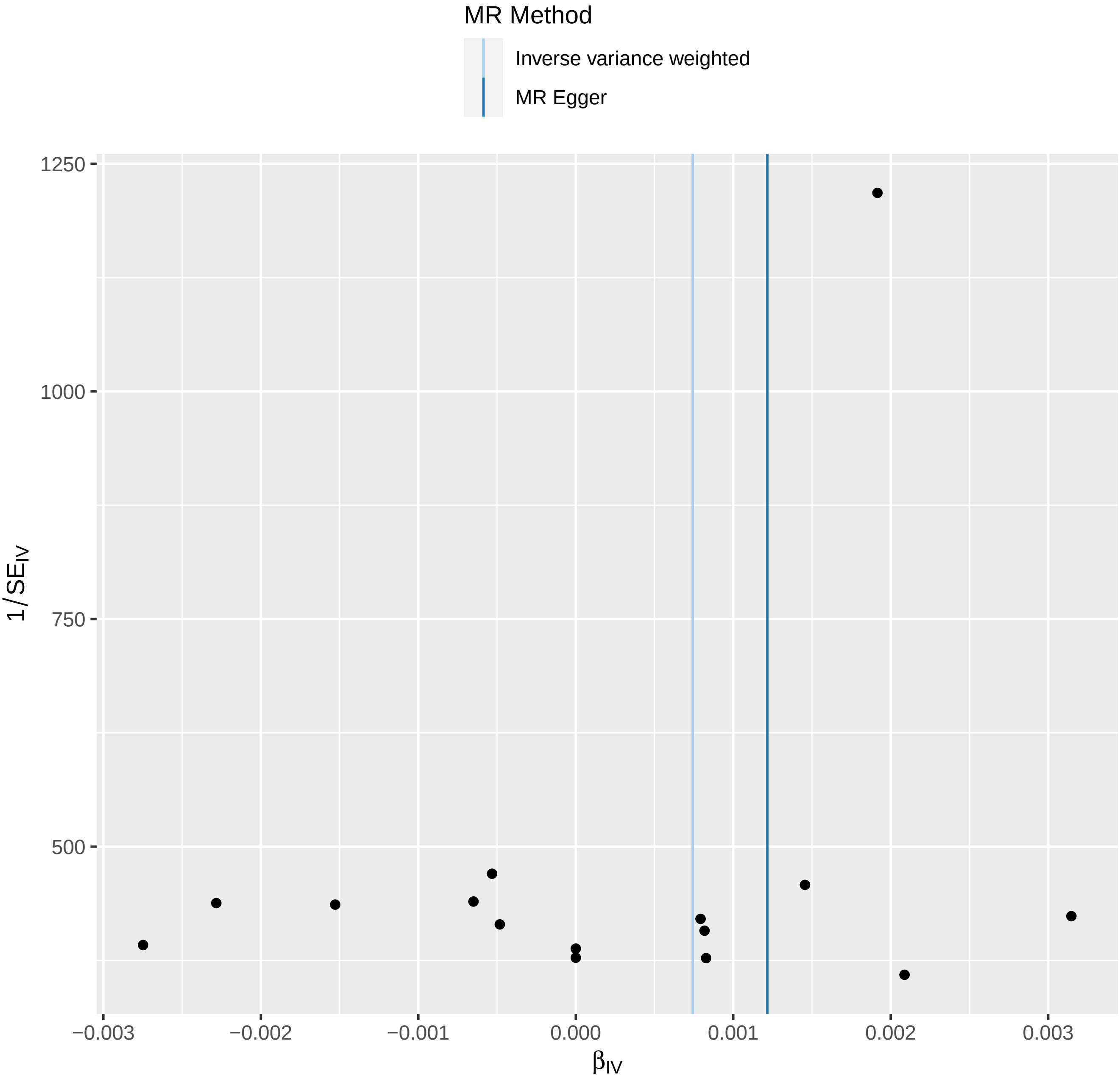

Batch 2−2045 : Neugrin on eGFR

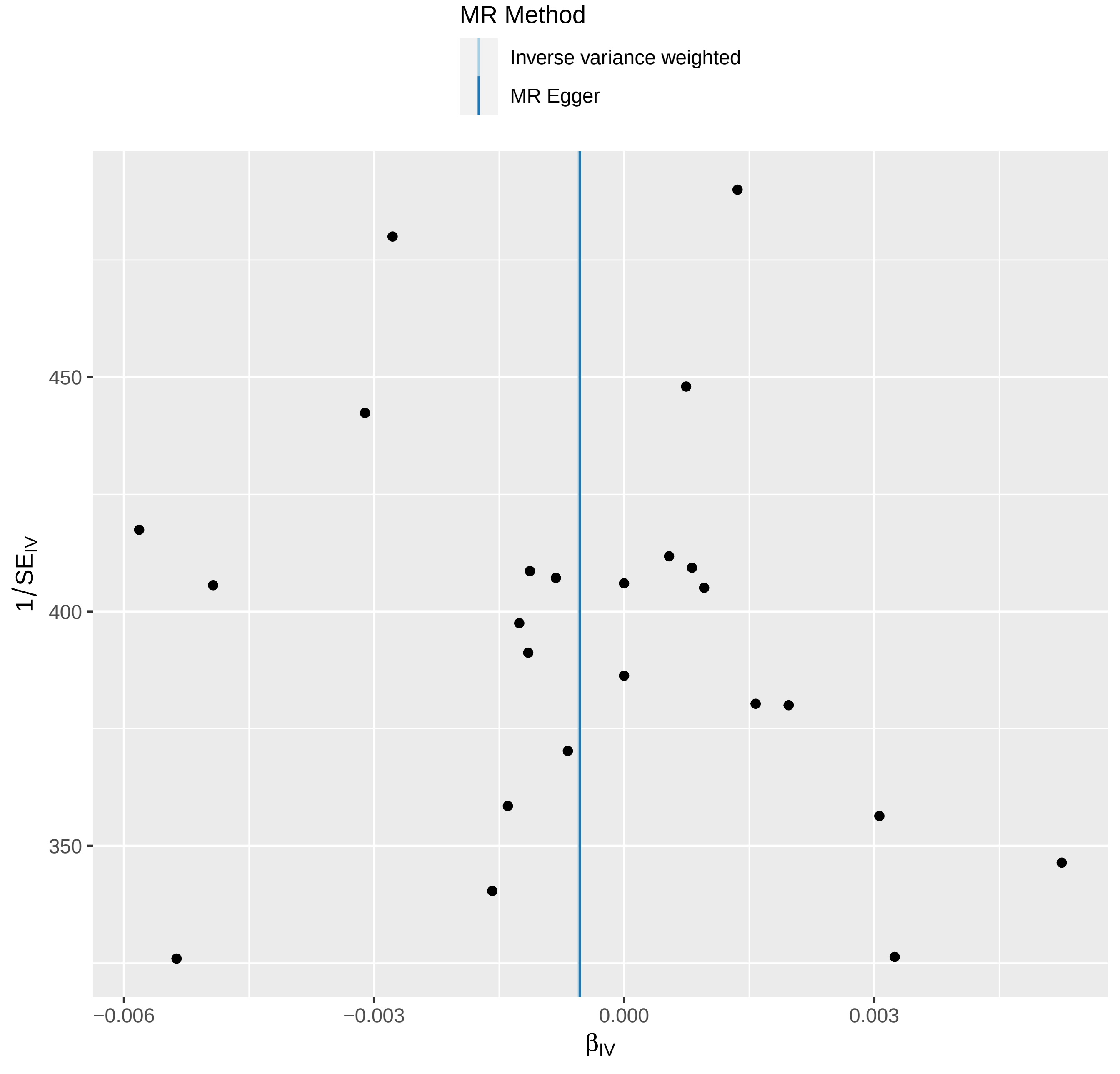

Batch 2−2046 : Non−homologous end−joining factor 1 on eGFR

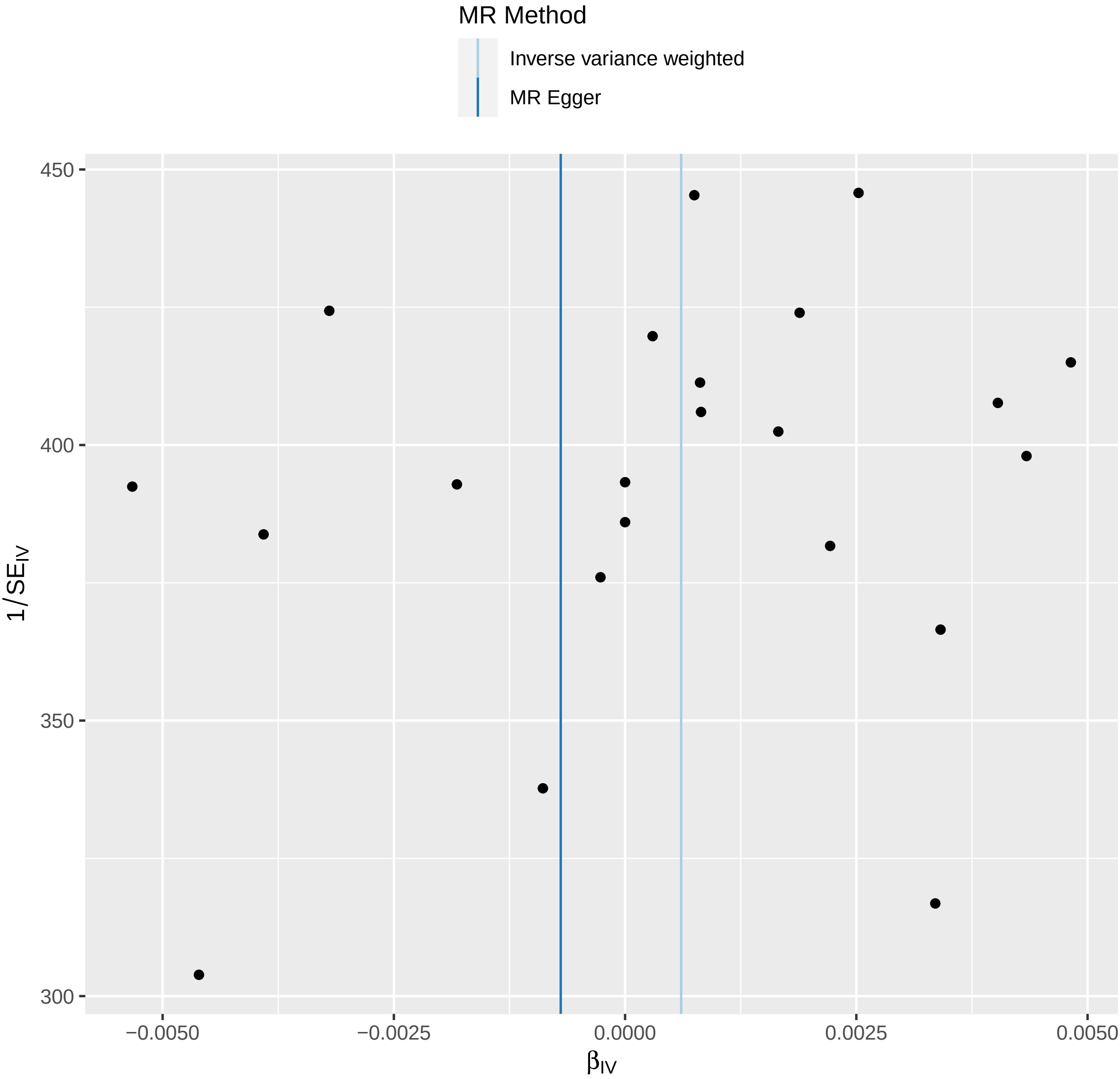

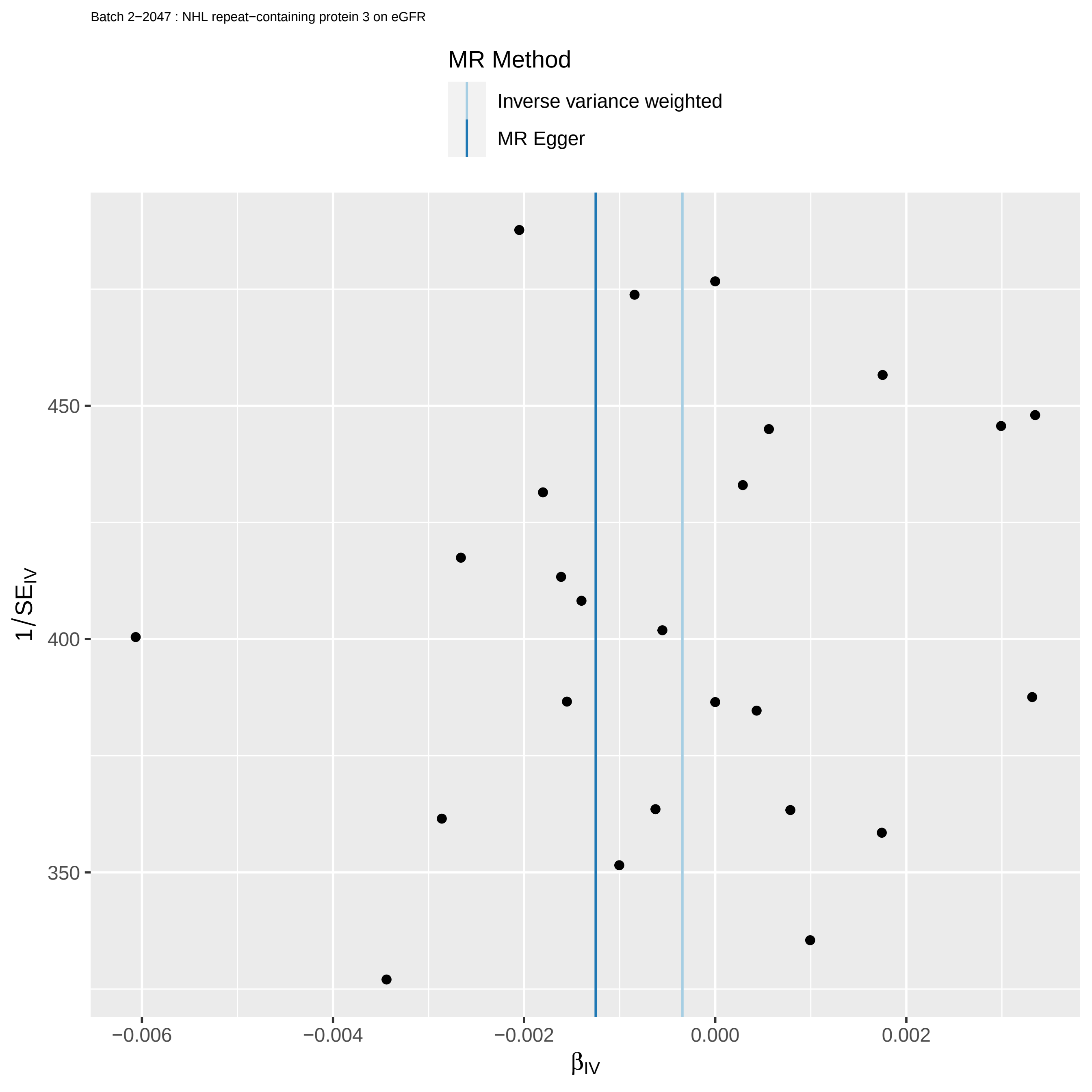

Batch 2−2047 : NHL repeat−containing protein 3 on eGFR
MR Method
Inverse variance weighted
MR Egger
450
400
350
1/SE$_{IV}$
−0.006
−0.004
−0.002
0.000
0.002
β$_{IV}$

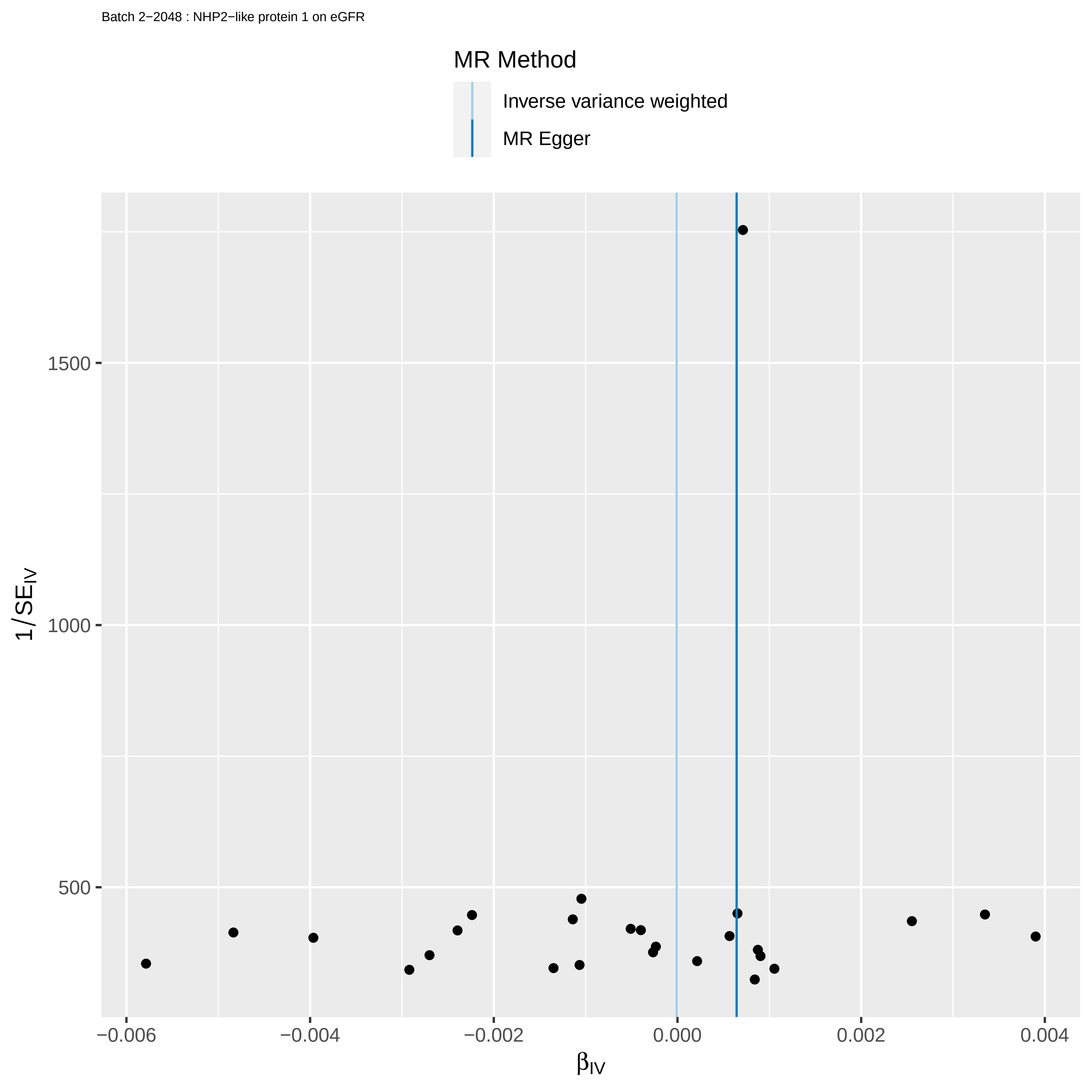

Batch 2−2048 : NHP2−like protein 1 on eGFR
MR Method
Inverse variance weighted
MR Egger
1500
1000
500
1/SE_IV
−0.006
−0.004
−0.002
0.000
0.002
0.004
β_IV

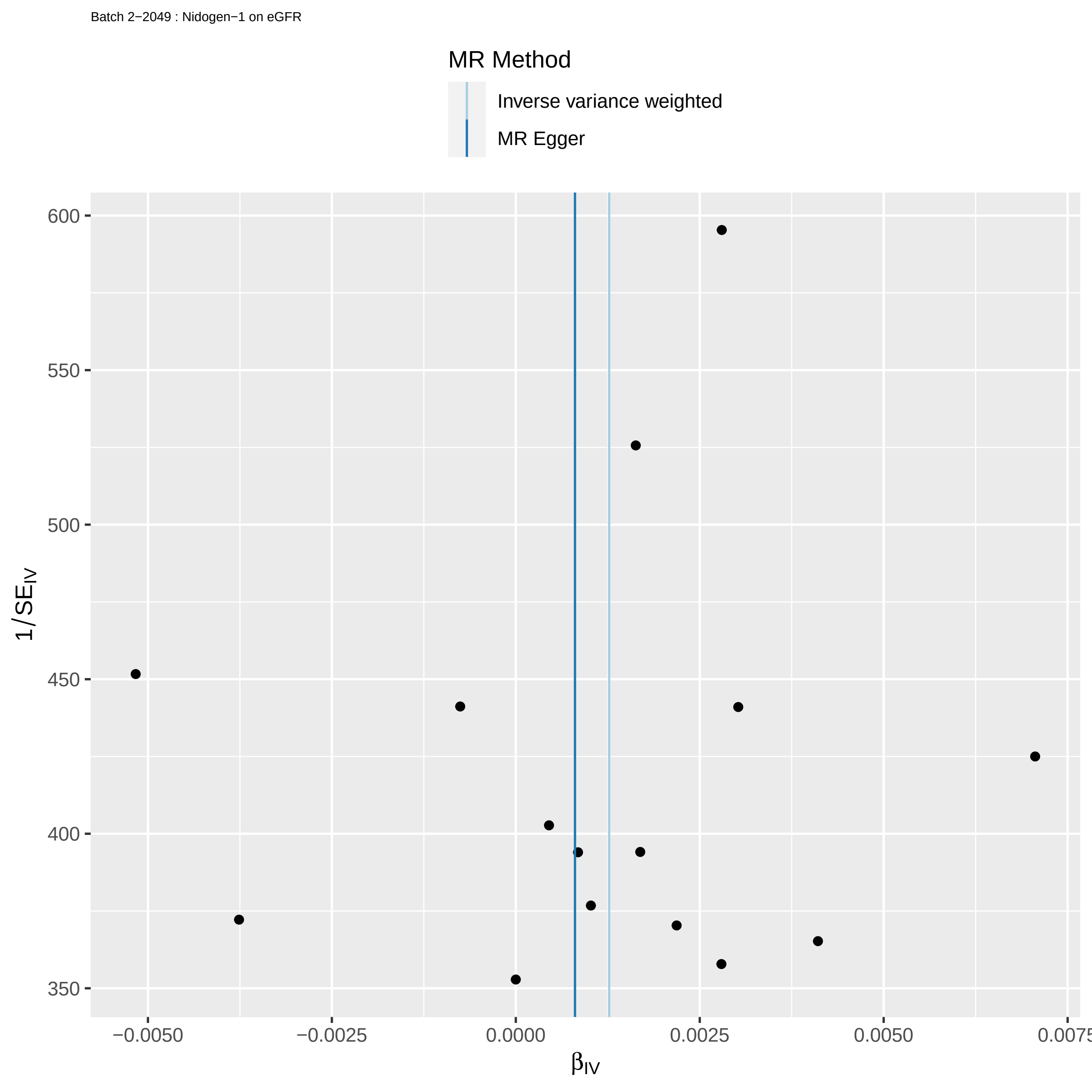

Batch 2−2049 : Nidogen−1 on eGFR
MR Method
Inverse variance weighted
MR Egger
600
550
500
450
400
350
1/SE$_{IV}$
−0.0050
−0.0025
0.0000
0.0025
0.0050
0.0075
β$_{IV}$

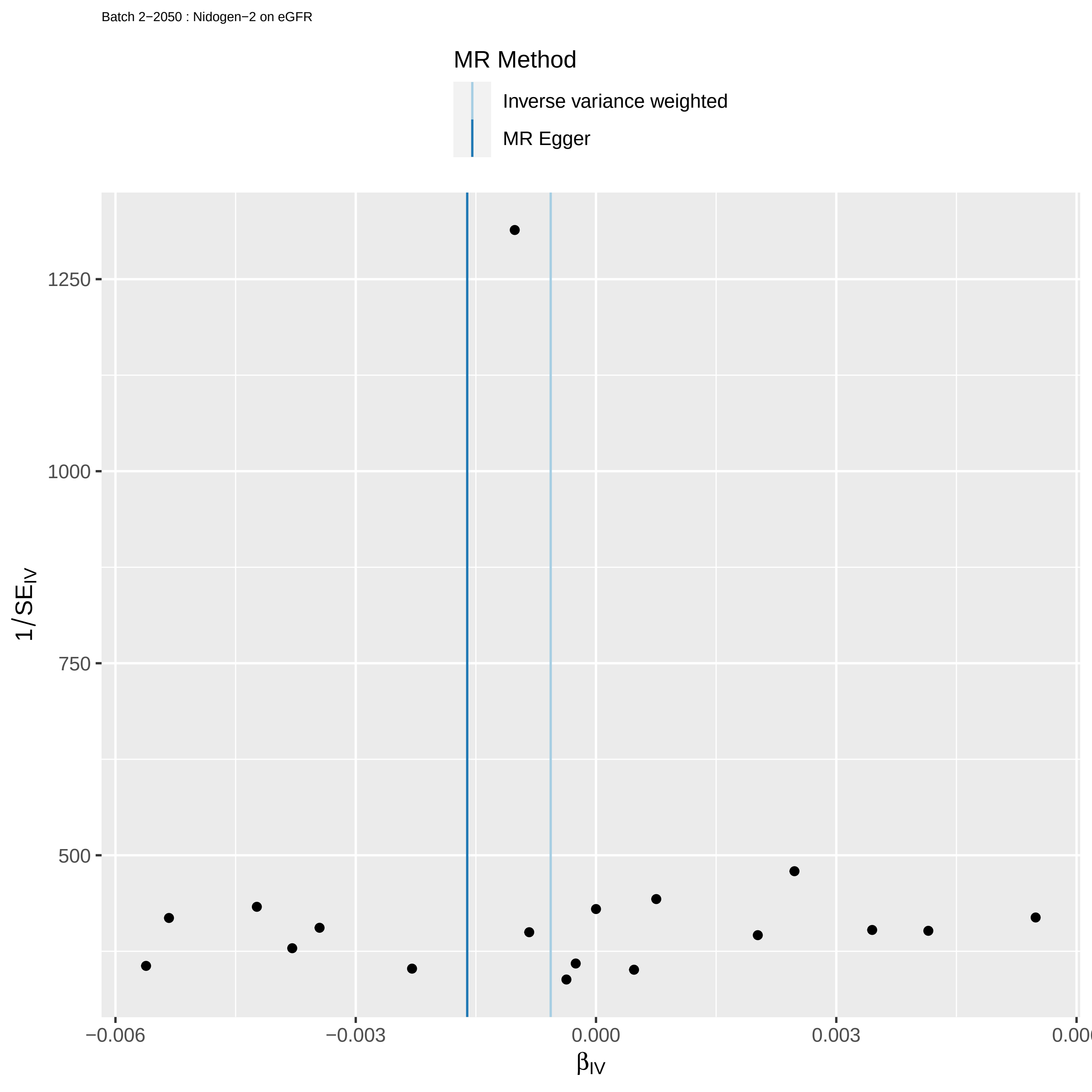

Batch 2−2050 : Nidogen−2 on eGFR
MR Method
Inverse variance weighted
MR Egger
1250
1000
750
500
1/SE_IV
−0.006
−0.003
0.000
0.003
β_IV

Batch 2−2051 : Magnesium transporter NIPA4 on eGFR

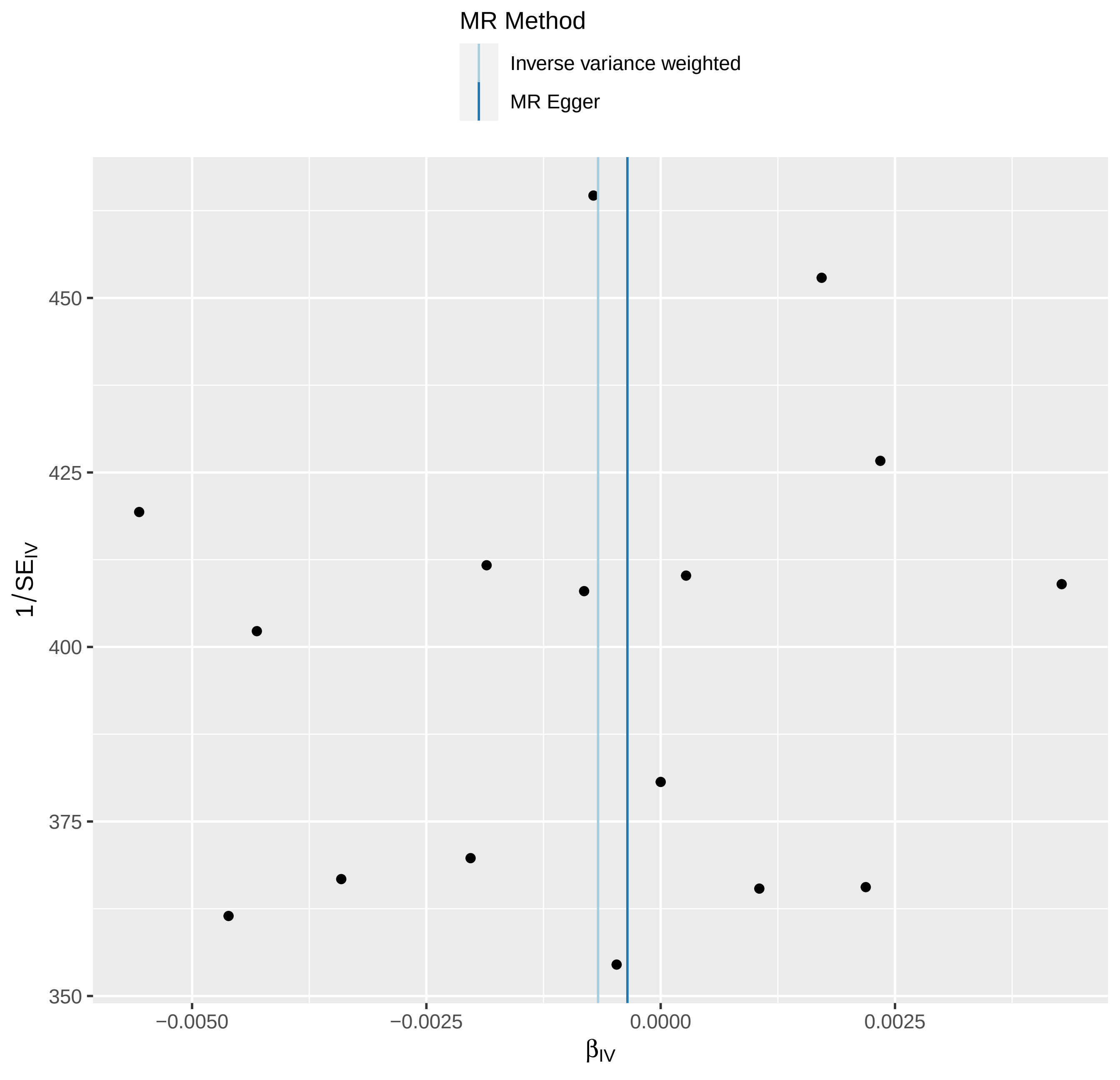

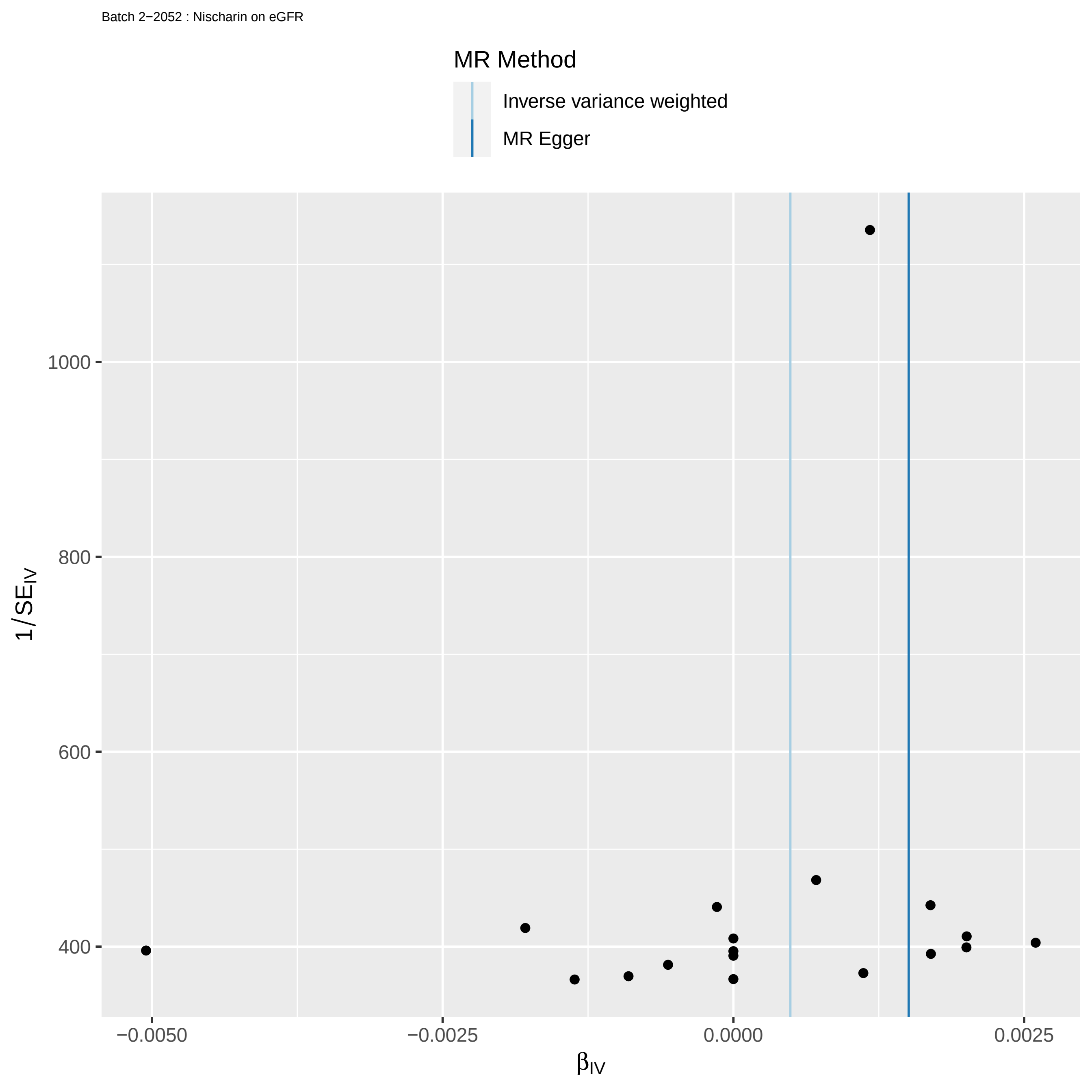
Batch 2−2052 : Nischarin on eGFR
MR Method
Inverse variance weighted
MR Egger
1000
800
600
400
1/SE_IV
−0.0050
−0.0025
0.0000
0.0025
β_IV

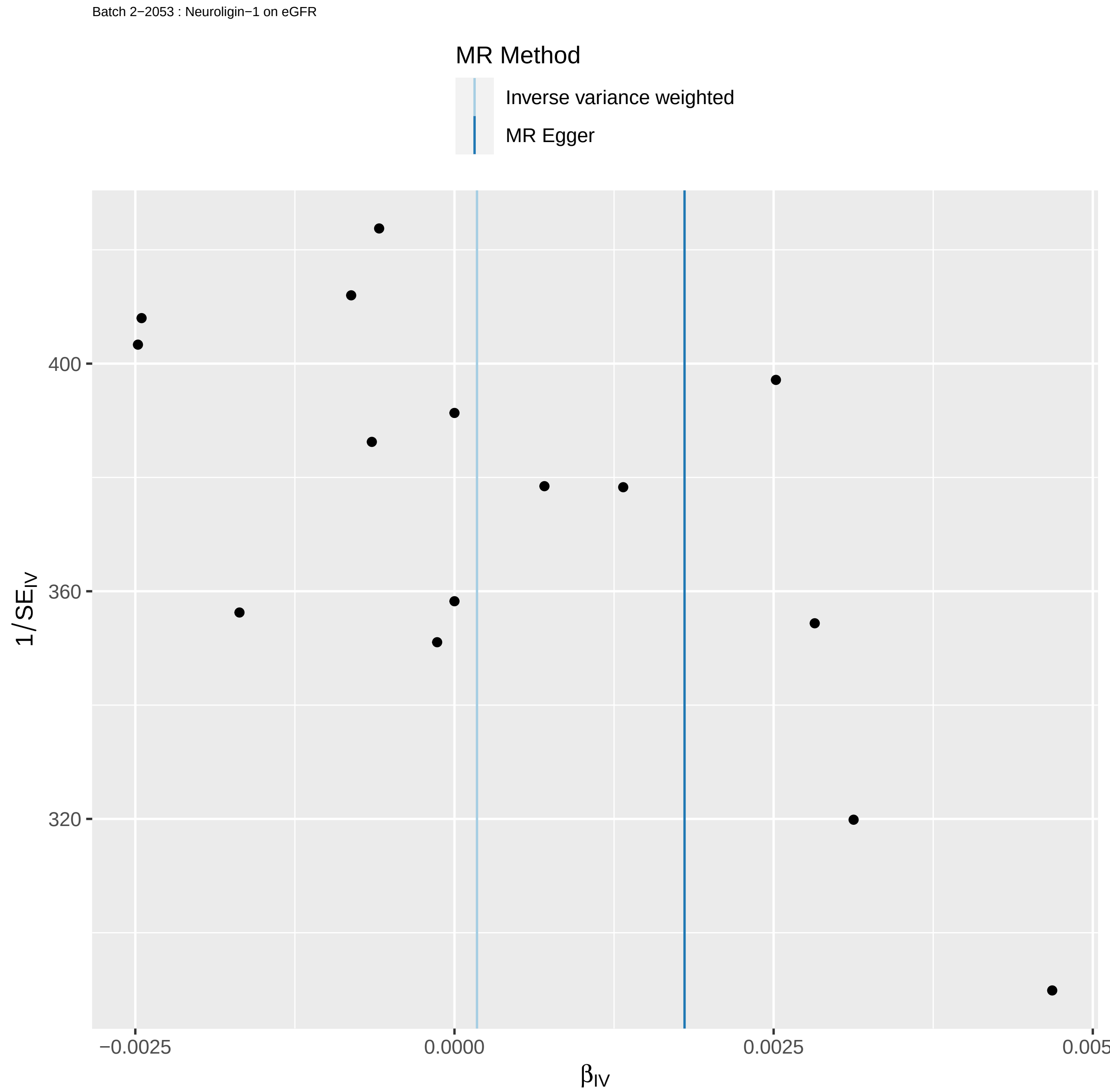

Batch 2−2053 : Neuroligin−1 on eGFR
MR Method
Inverse variance weighted
MR Egger
400
360
320
1/SE$_{IV}$
−0.0025
0.0000
0.0025
0.005
β$_{IV}$

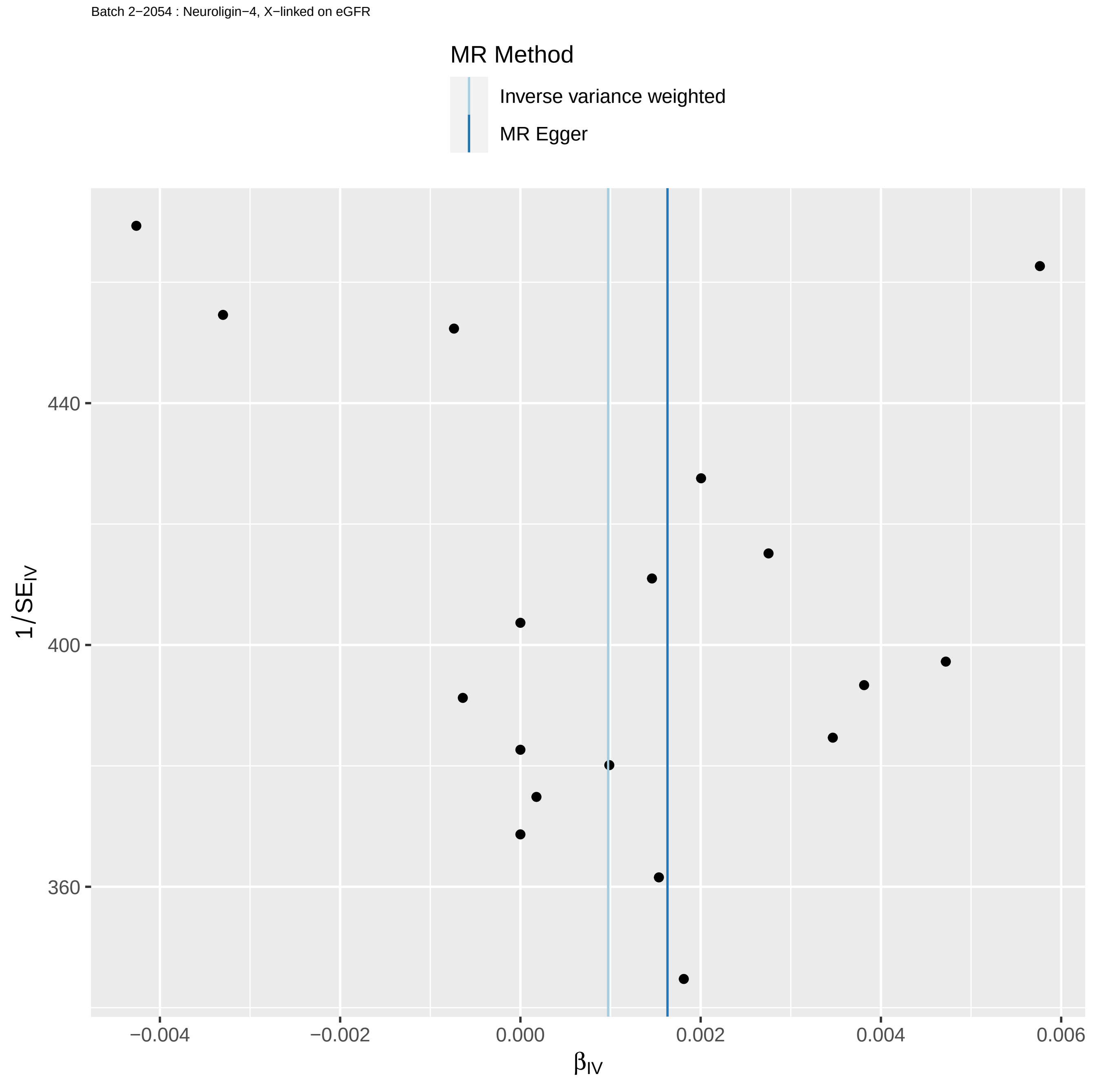

Batch 2−2054 : Neuroligin−4, X−linked on eGFR
MR Method
Inverse variance weighted
MR Egger
440
400
360
1/SE_IV
−0.004
−0.002
0.000
0.002
0.004
0.006
β_IV

Batch 2−2055 : NACHT, LRR and PYD domains−containing protein 10 on eGFR

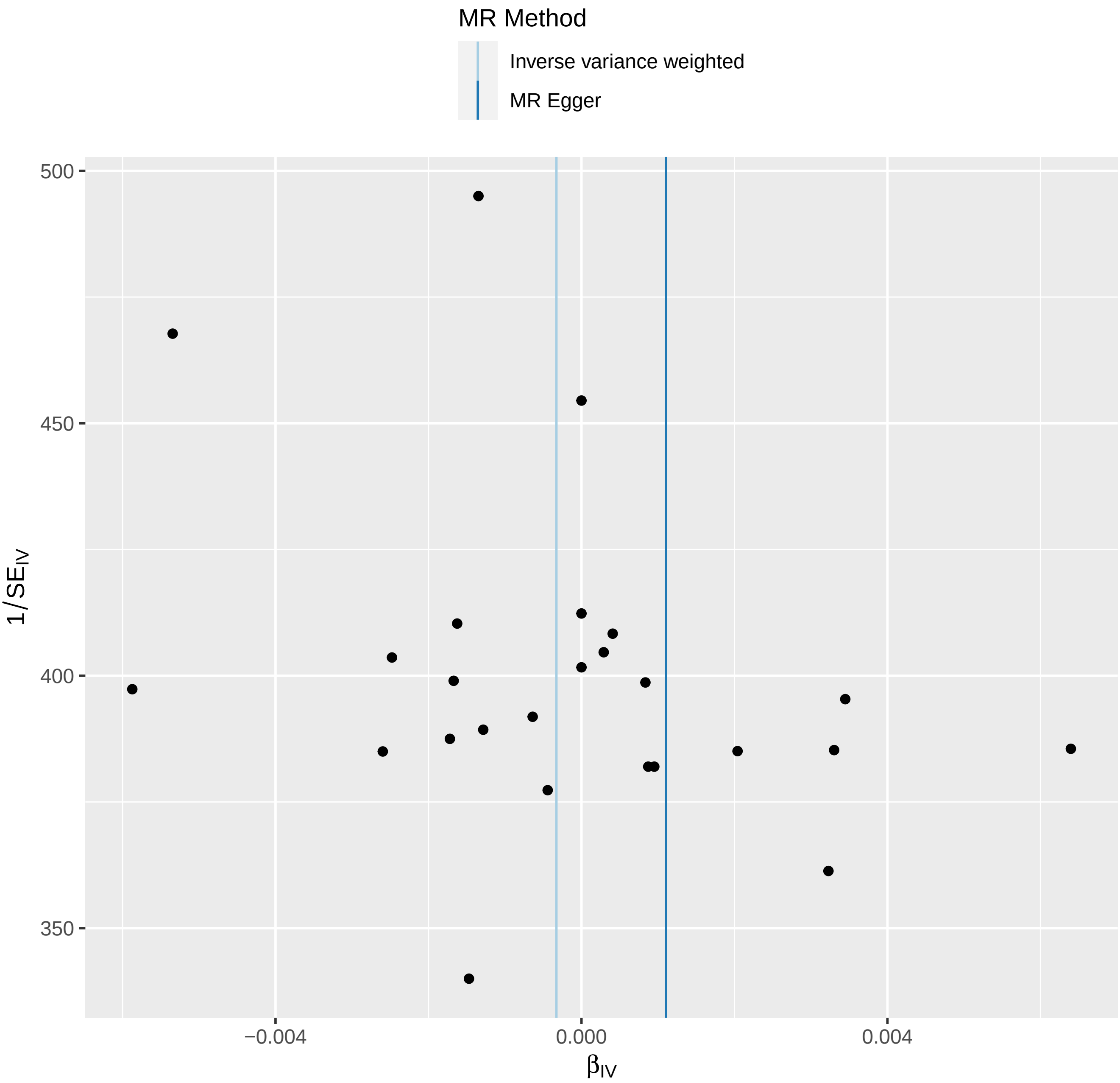

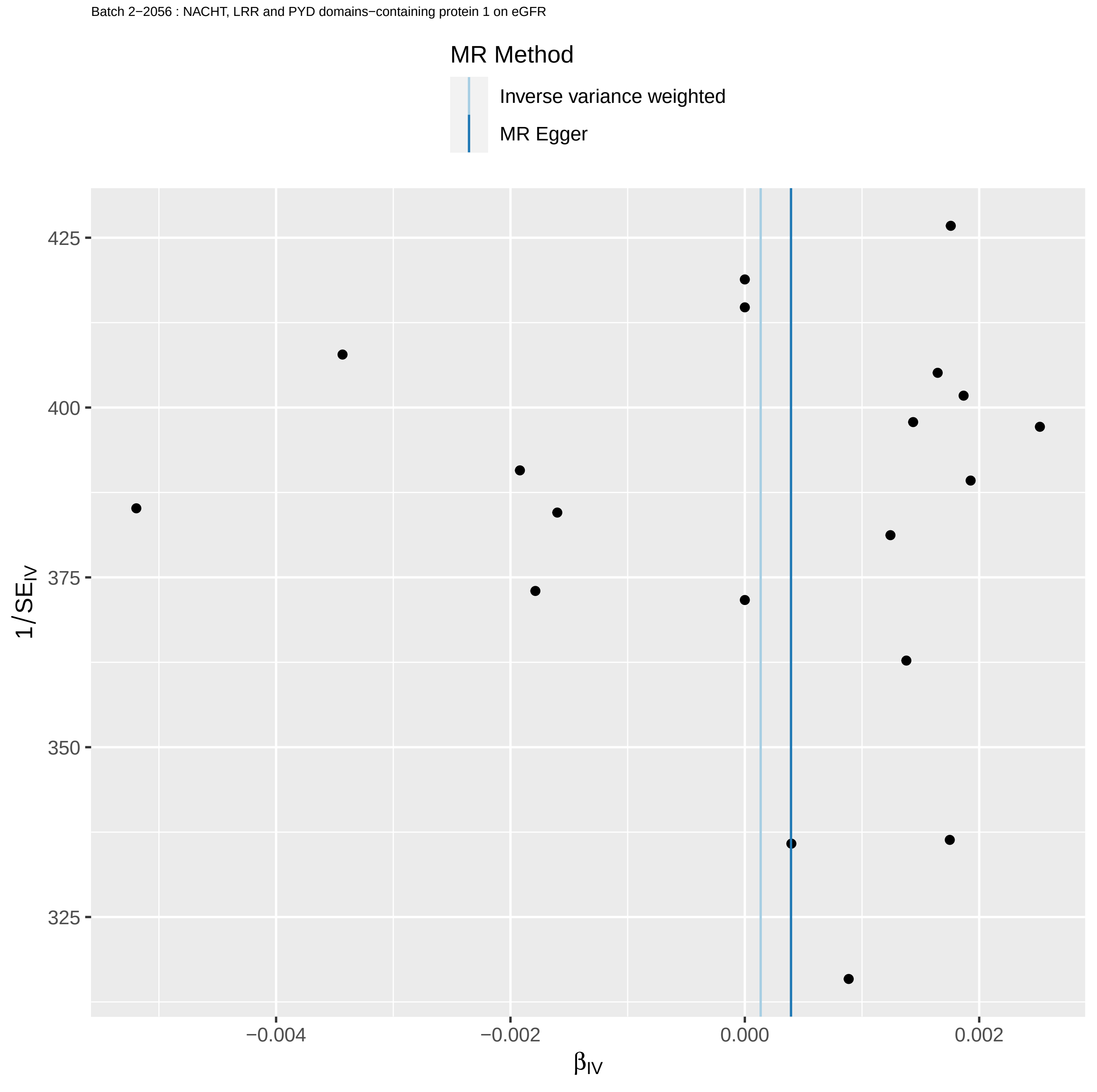

Batch 2−2056 : NACHT, LRR and PYD domains−containing protein 1 on eGFR
MR Method
Inverse variance weighted
MR Egger
425
400
375
350
325
1/SE$_{IV}$
−0.004
−0.002
0.000
0.002
β_{IV}

Batch 2−2057 : NACHT, LRR and PYD domains−containing protein 4 on eGFR

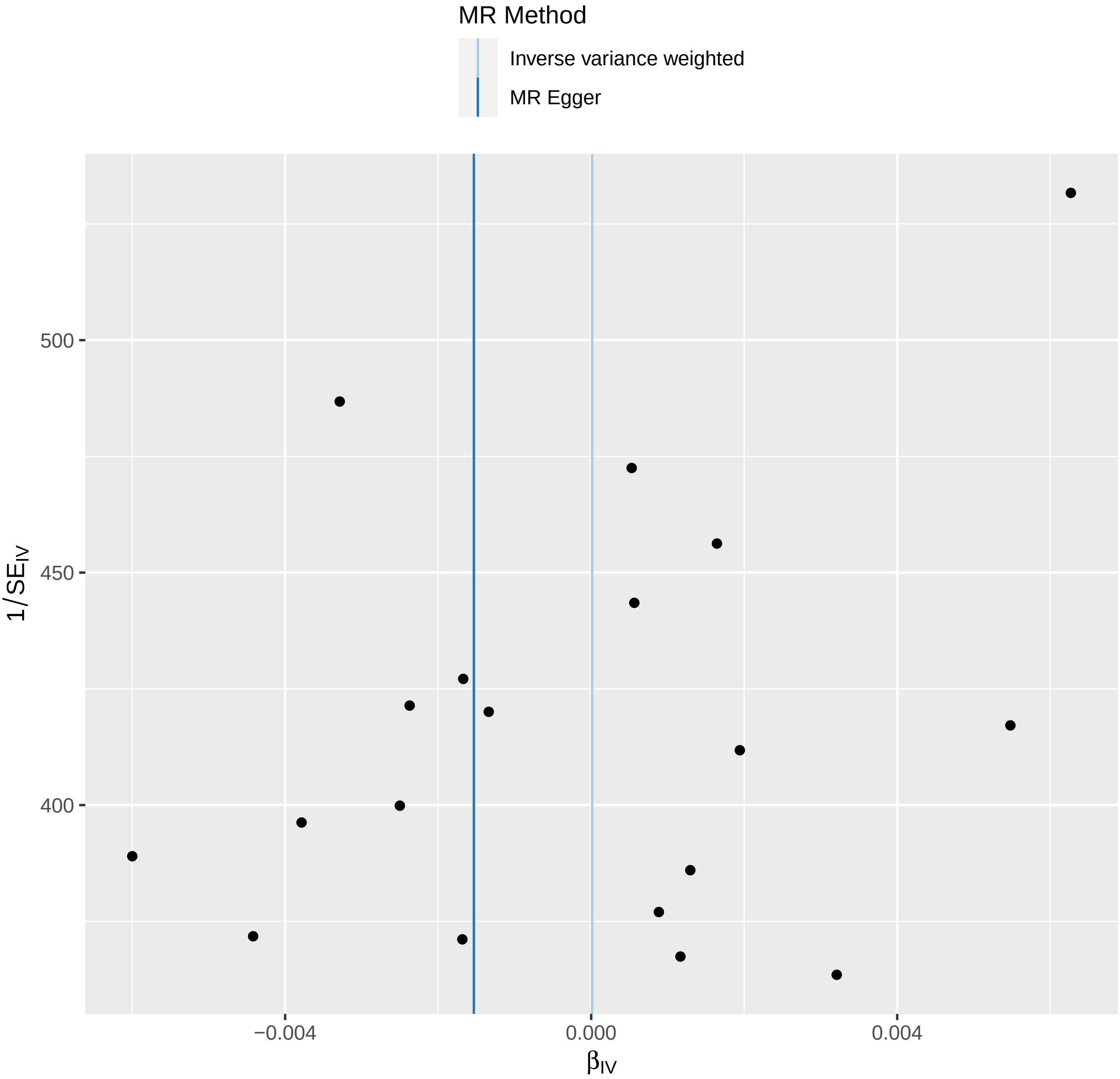

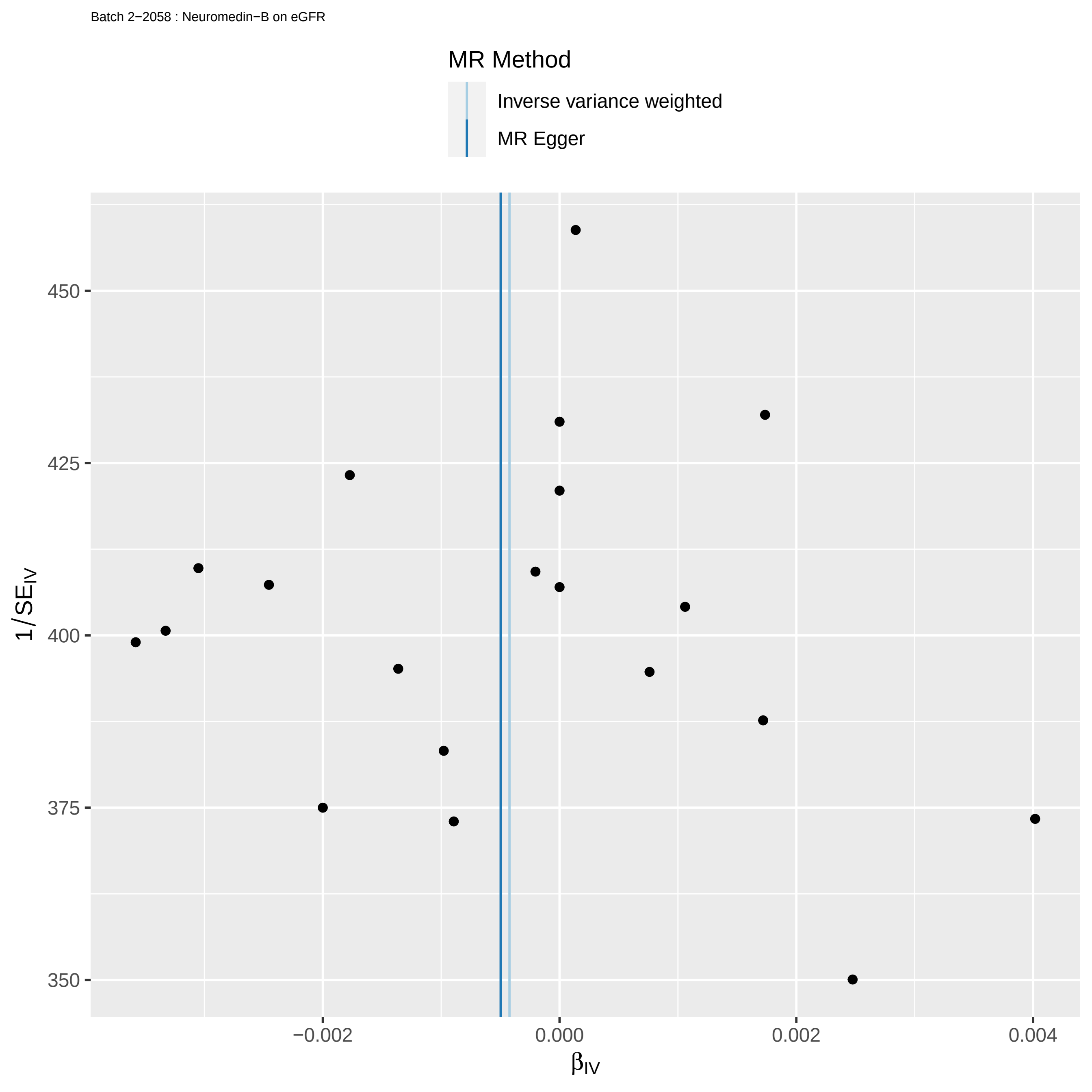
Batch 2−2058 : Neuromedin−B on eGFR
MR Method
Inverse variance weighted
MR Egger
450
425
400
375
350
1/SE$_{IV}$
−0.002
0.000
0.002
0.004
β$_{IV}$

Batch 2−2059 : Nucleoside diphosphate kinase A on eGFR

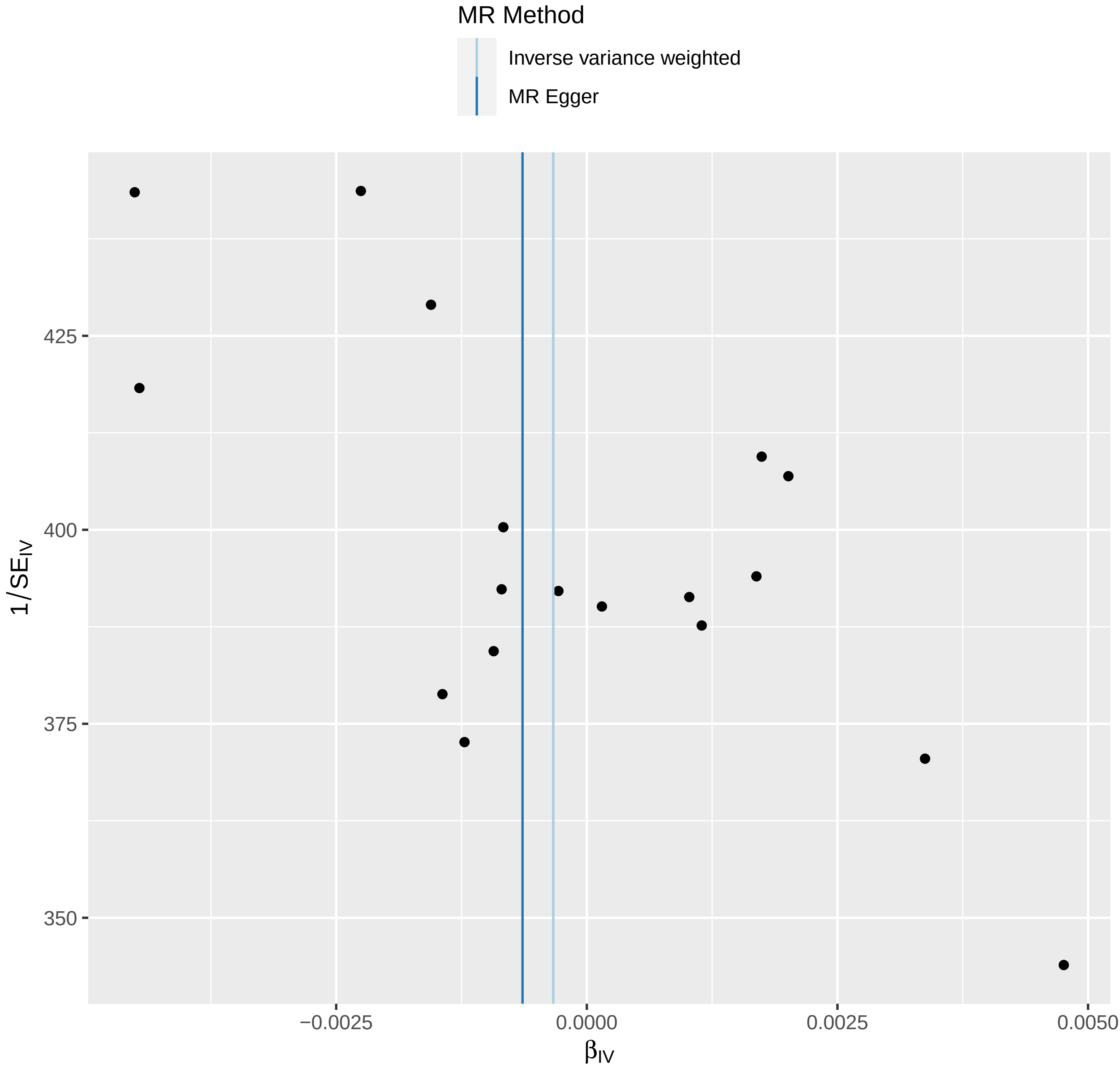

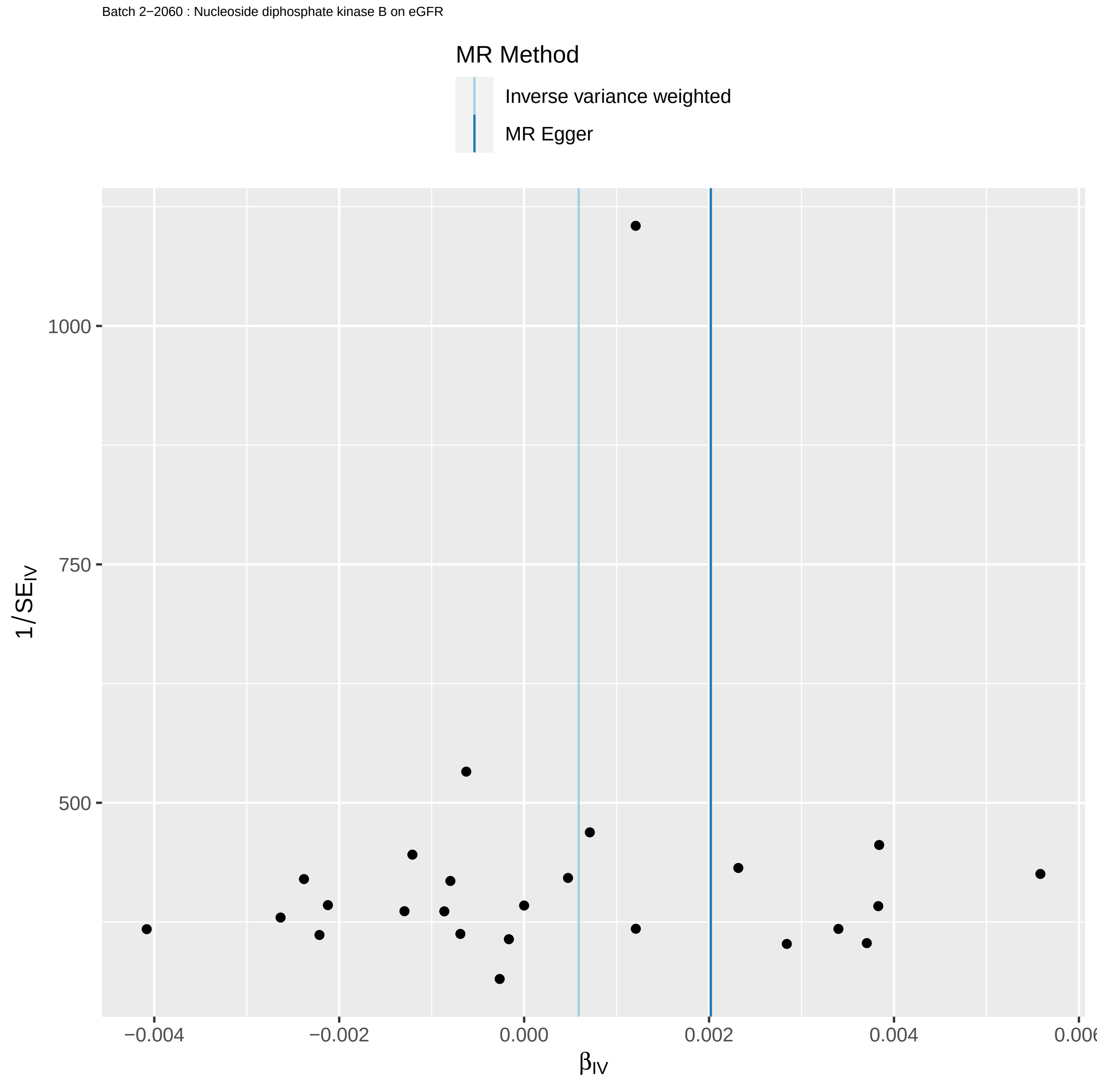

Batch 2−2060 : Nucleoside diphosphate kinase B on eGFR
MR Method
Inverse variance weighted
MR Egger
1000
750
500
1/SE_IV
−0.004
−0.002
0.000
0.002
0.004
β_IV

Batch 2−2061 : Normal mucosa of esophagus−specific gene 1 protein on eGFR

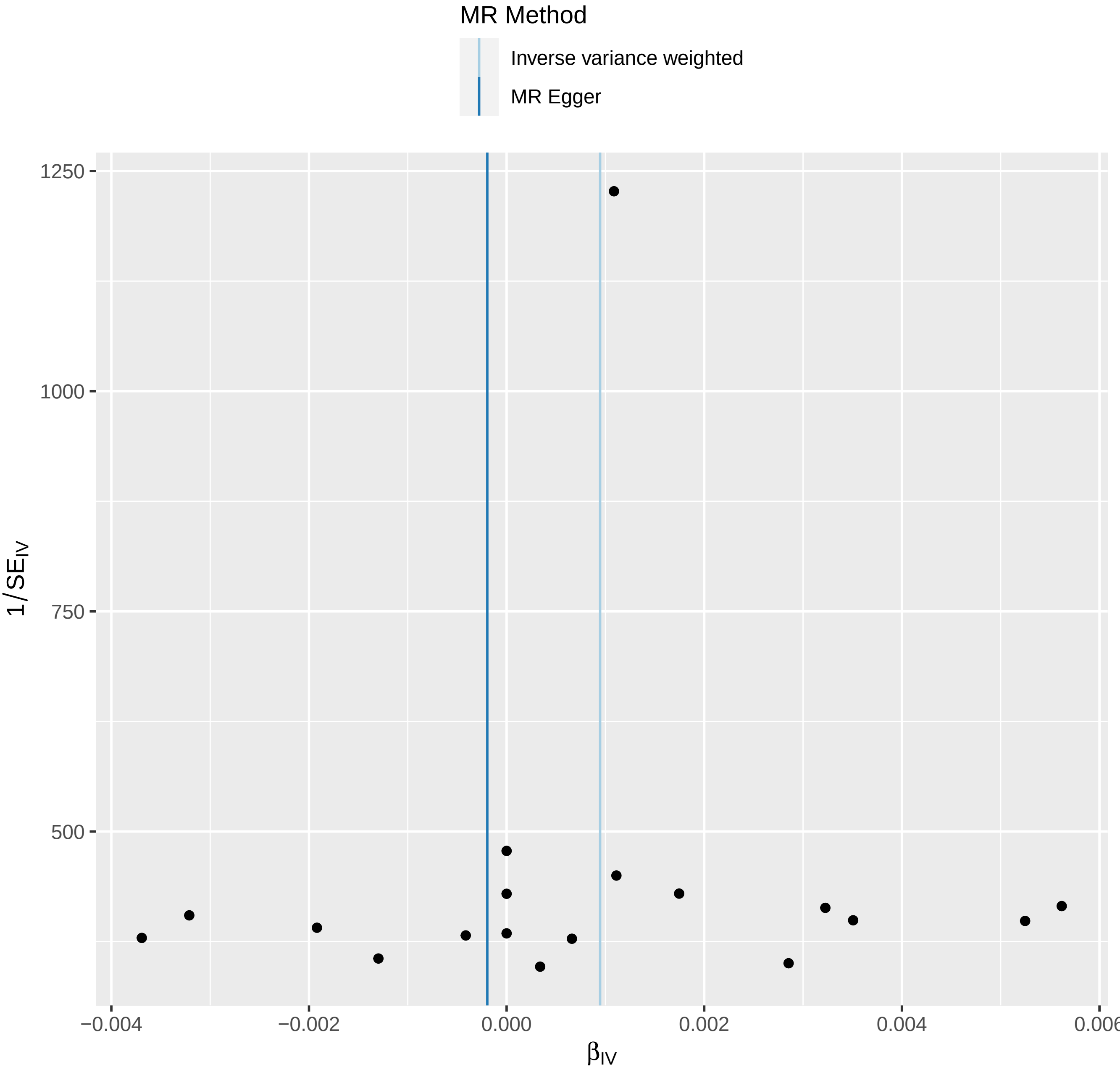

Batch 2−2062 : NmrA−like family domain−containing protein 1 on eGFR

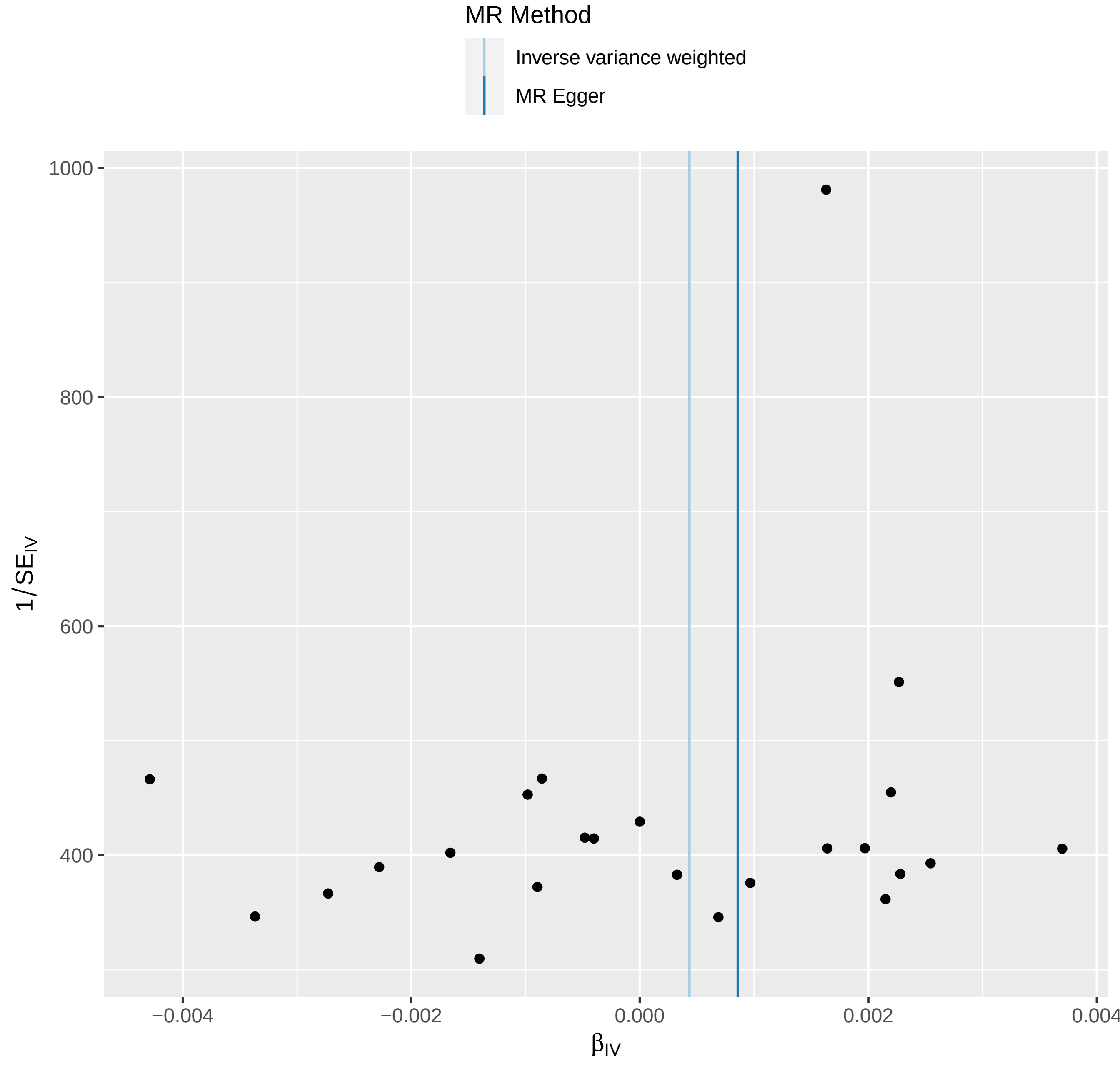

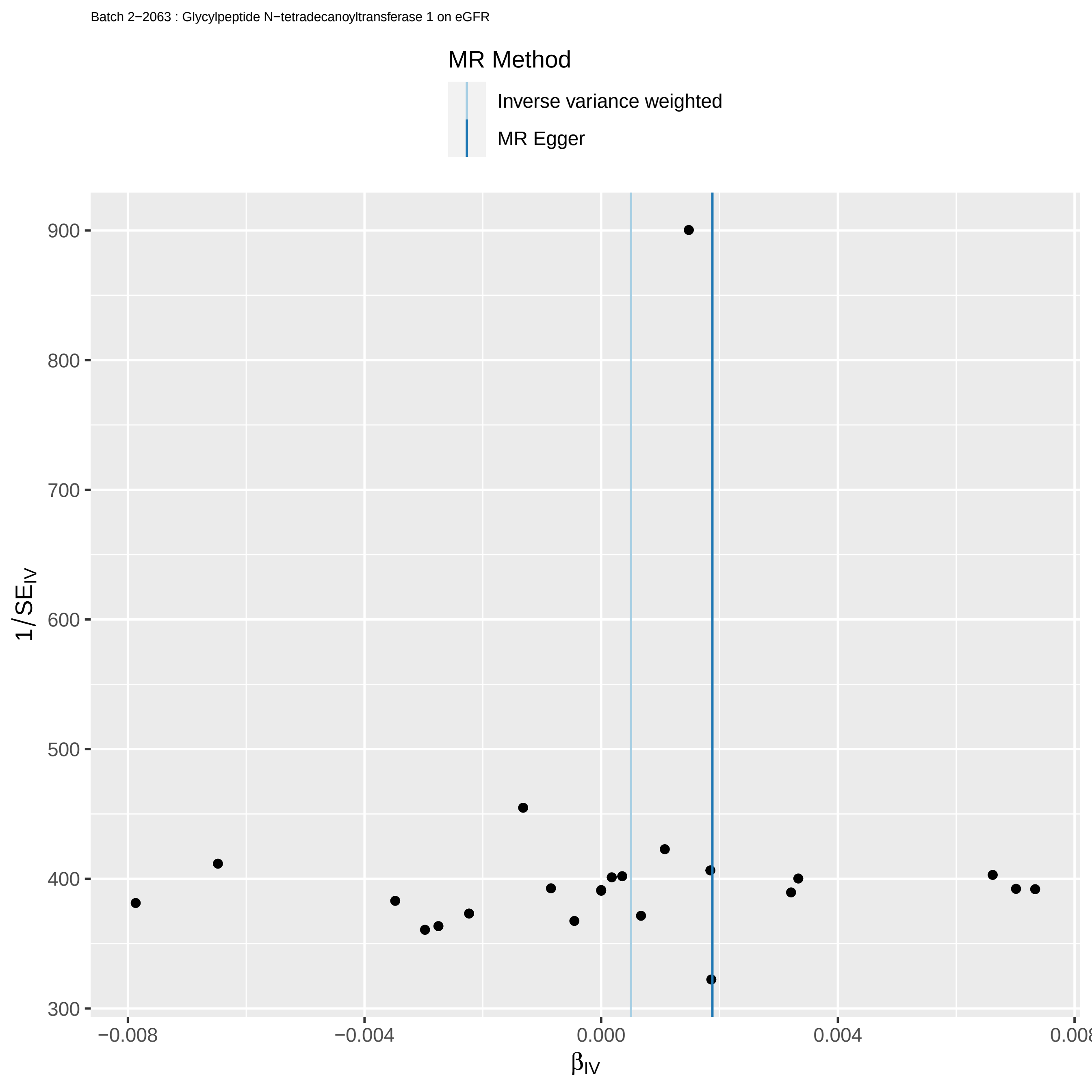
Batch 2−2063 : Glycylpeptide N−tetradecanoyltransferase 1 on eGFR
MR Method
Inverse variance weighted
MR Egger
900
800
700
600
500
400
300
1/SE_IV
−0.008
−0.004
0.000
0.004
0.008
β_IV

Batch 2−2064 : Noggin on eGFR

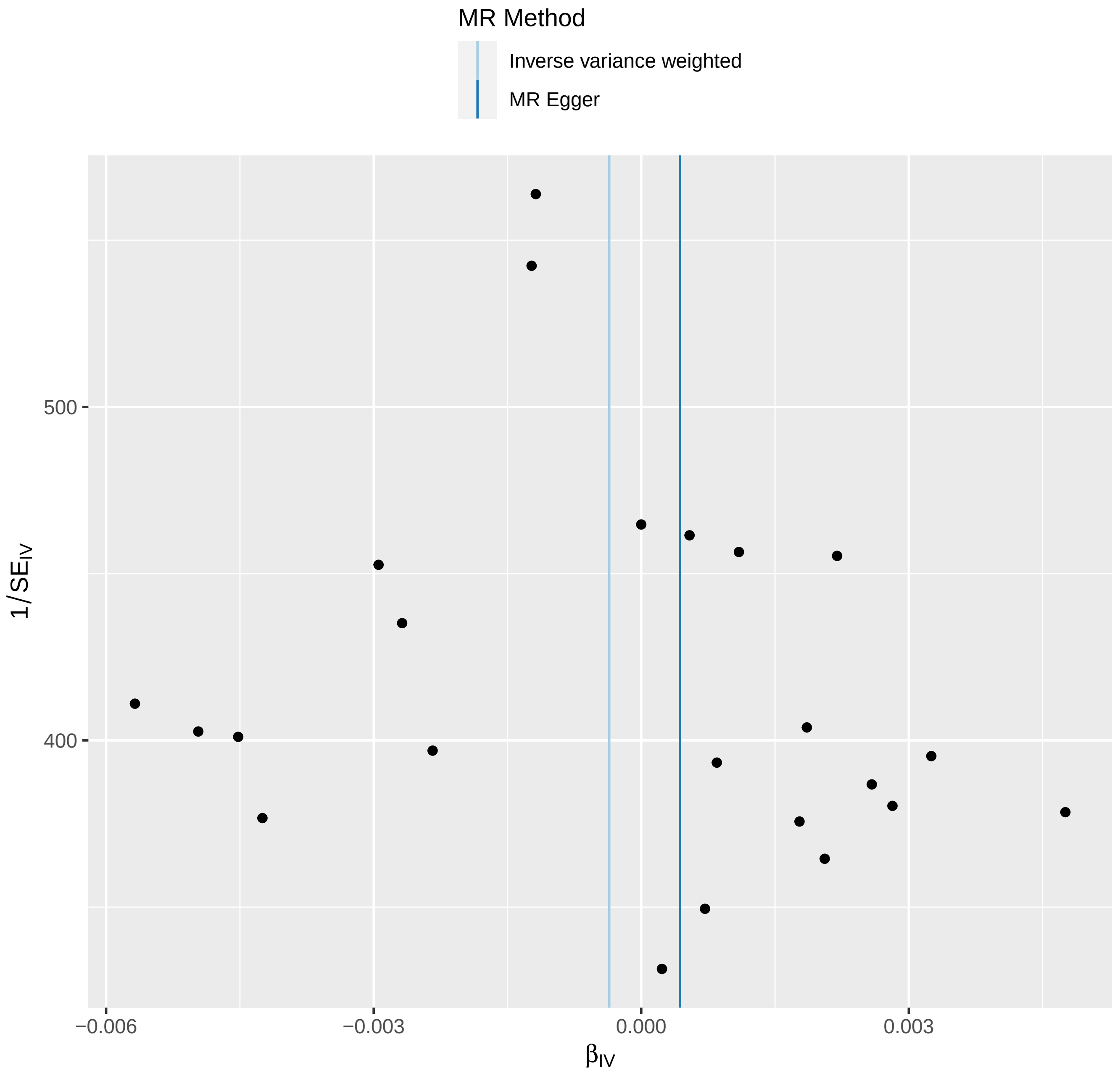

Batch 2−2065 : NoneX on eGFR

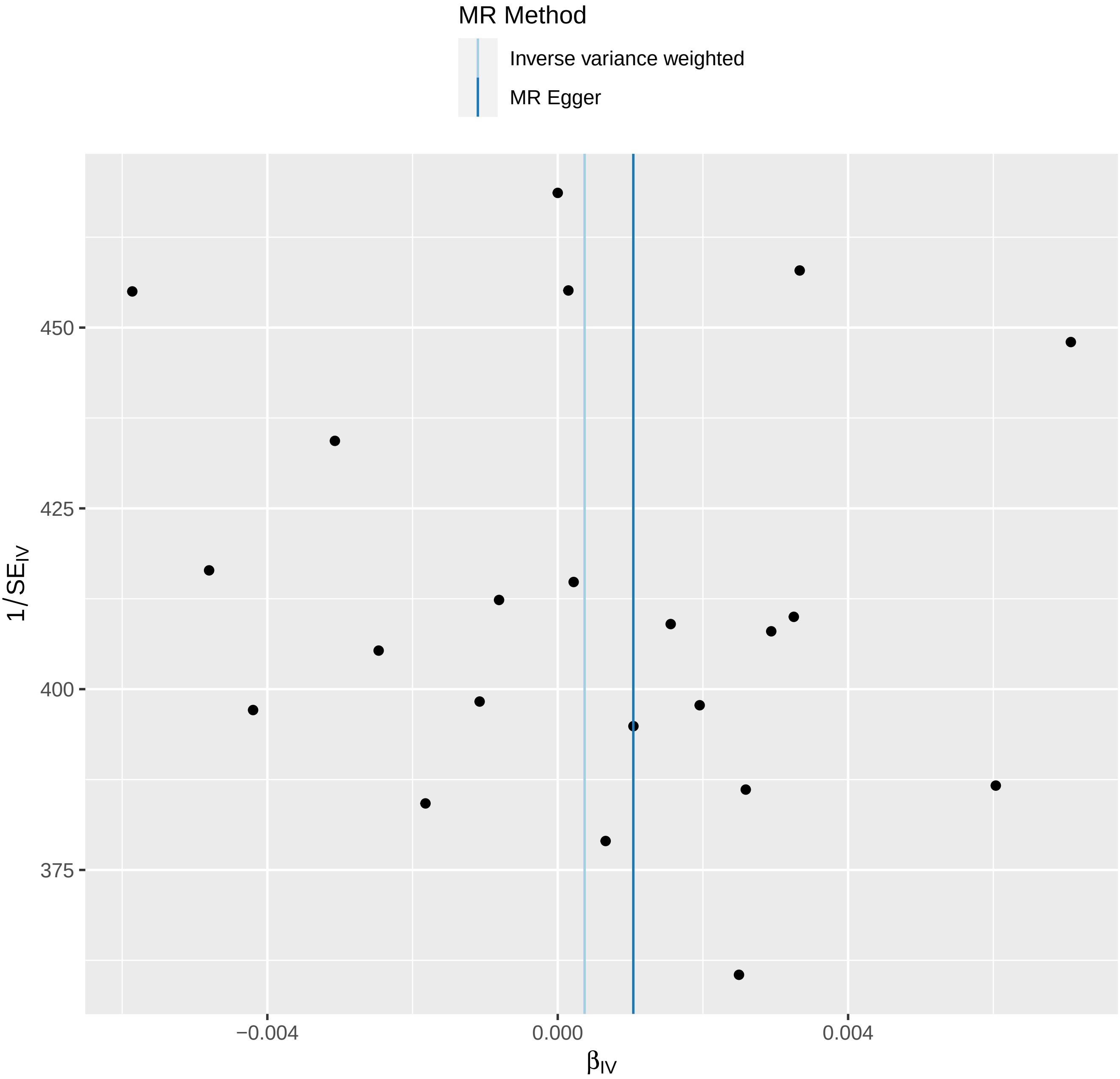

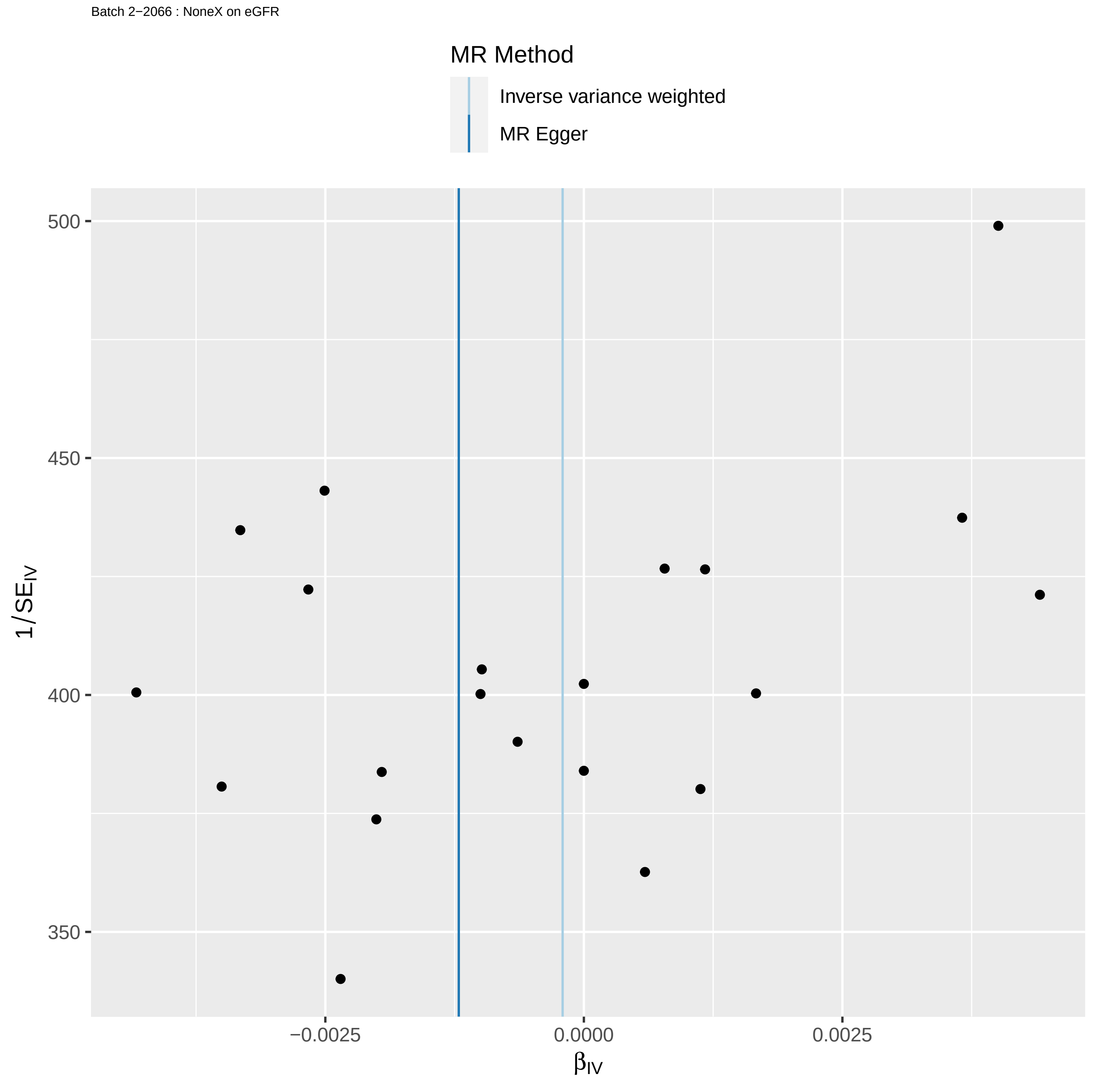

Batch 2−2066 : NoneX on eGFR
MR Method
Inverse variance weighted
MR Egger
500
450
400
350
1/SE_IV
−0.0025
0.0000
0.0025
β_IV

Batch 2−2067 : Neurogenic locus notch homolog protein 1 on eGFR

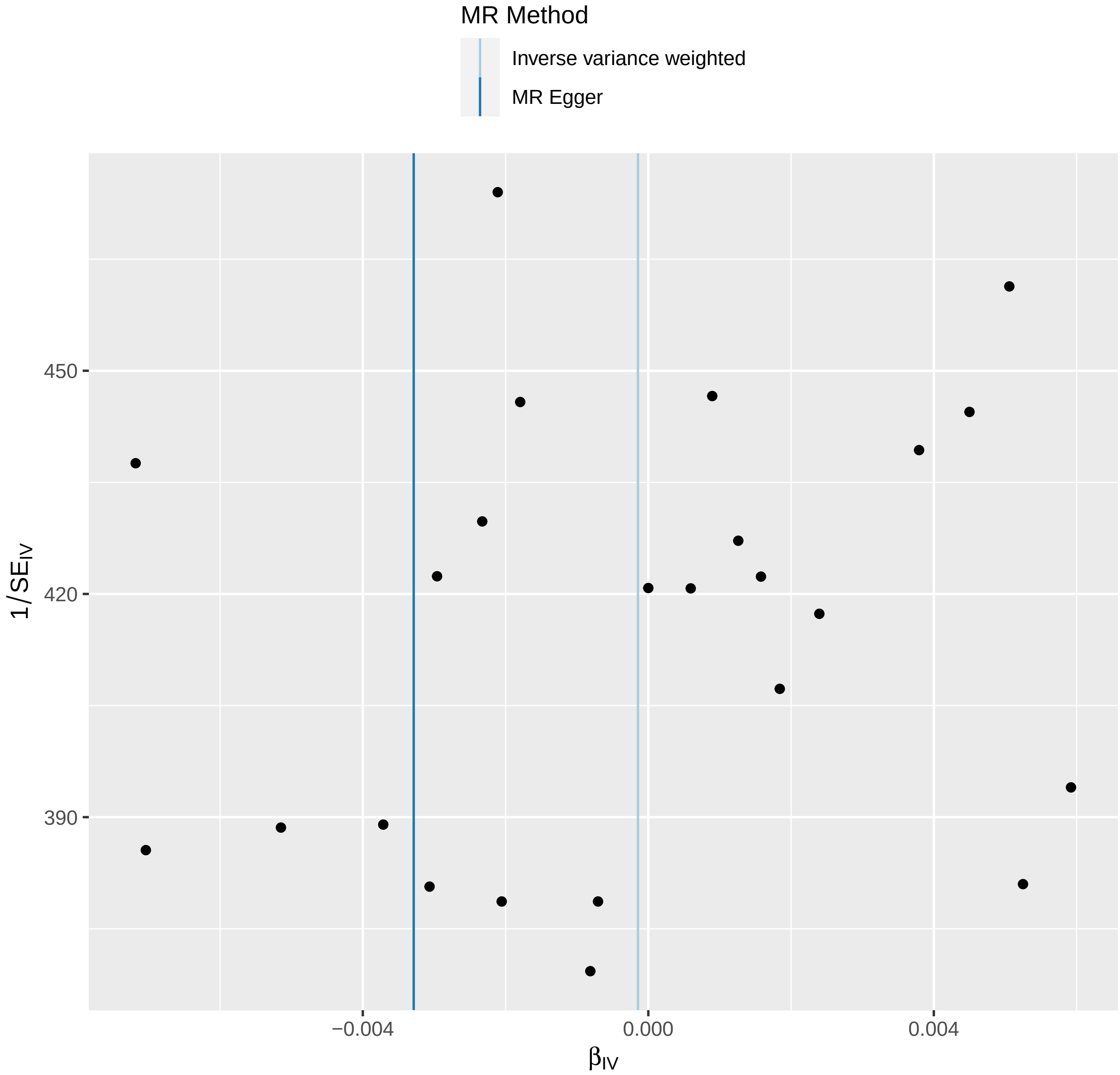

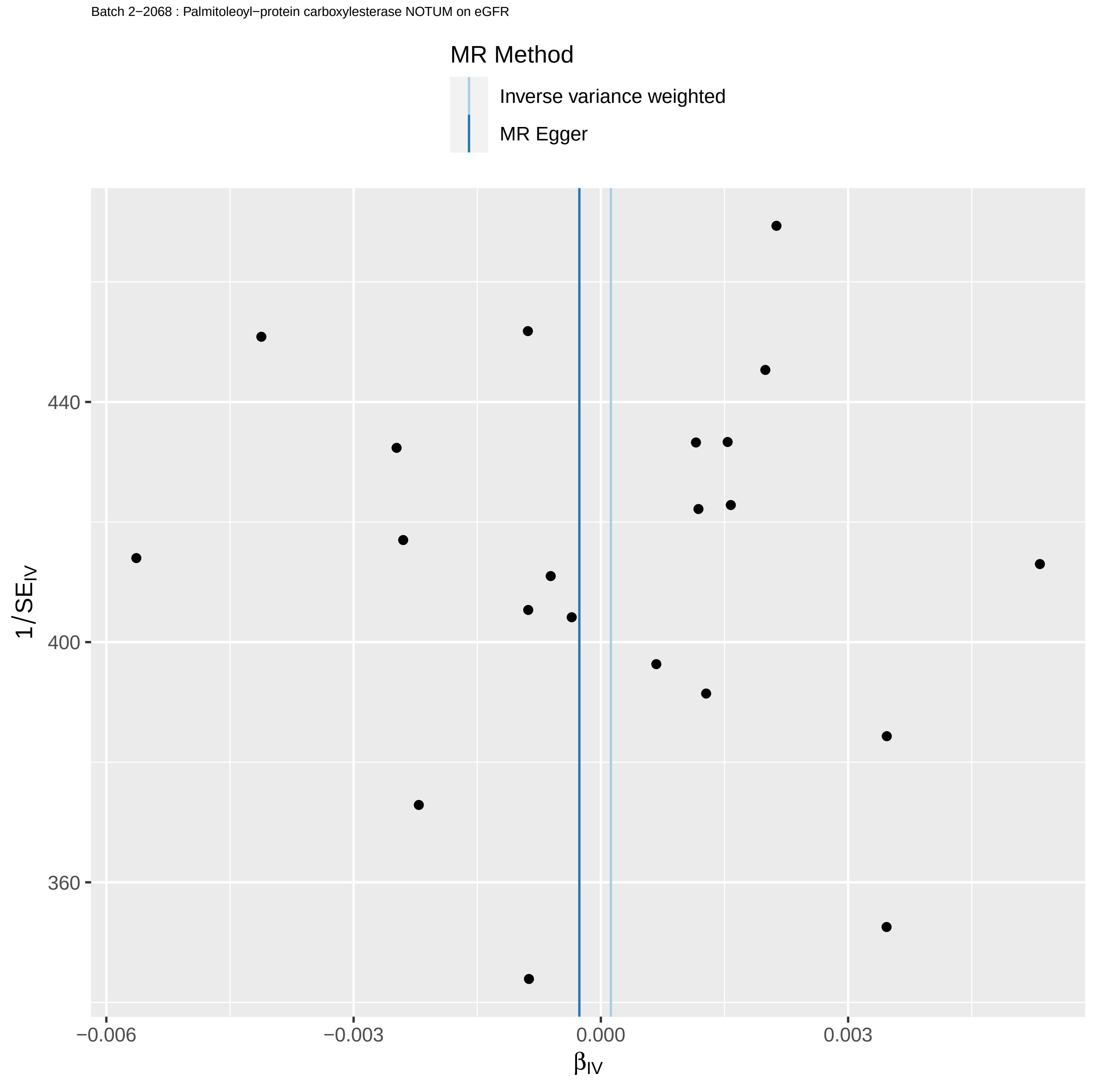

Batch 2−2068 : Palmitoleoyl−protein carboxylesterase NOTUM on eGFR
MR Method
Inverse variance weighted
MR Egger
1/SE_IV
440
400
360
−0.006
−0.003
0.000
0.003
β_IV

Batch 2−2069 : Protein NOV homolog on eGFR

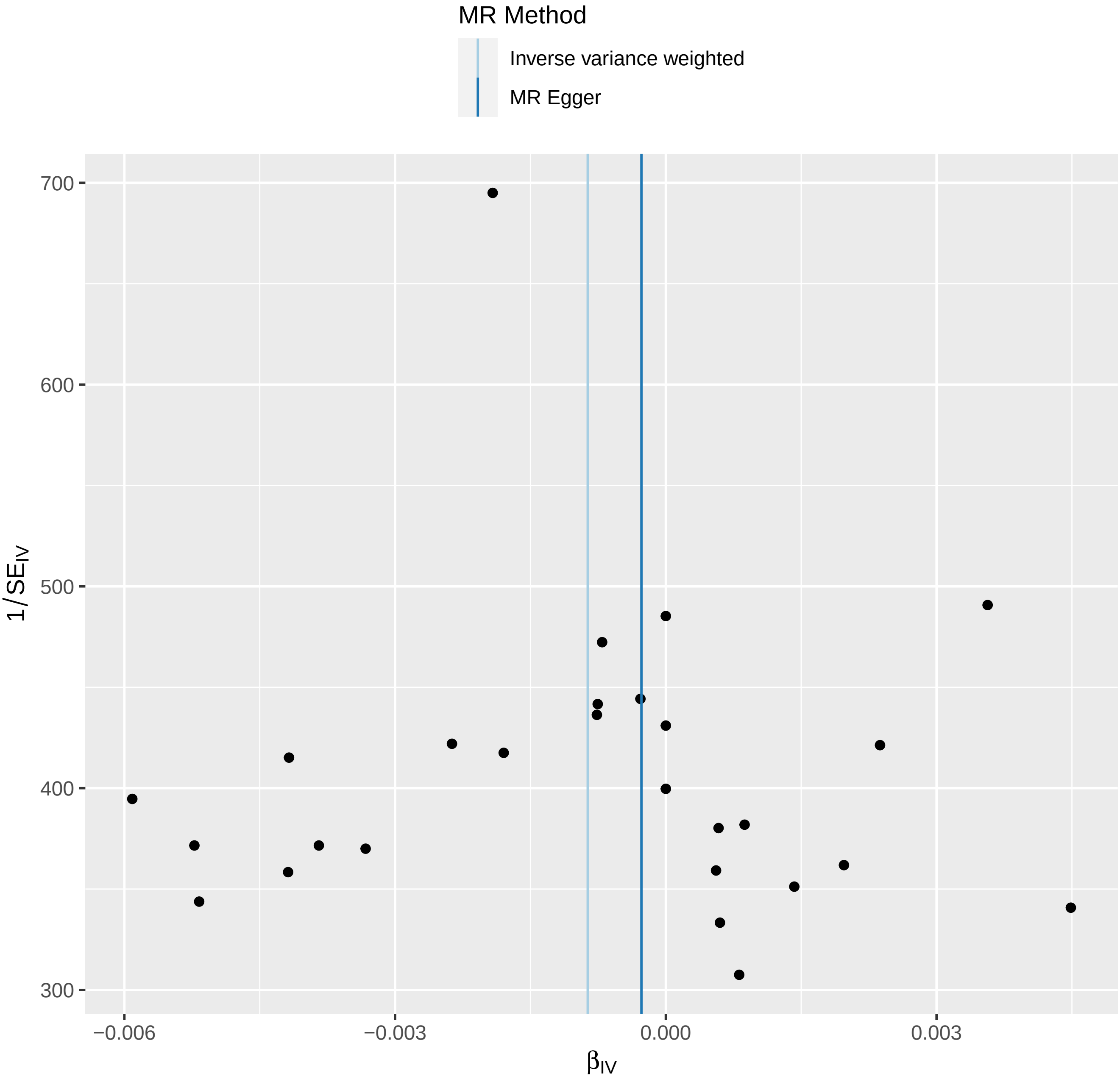

Batch 2−2070 : RNA−binding protein Nova−1 on eGFR

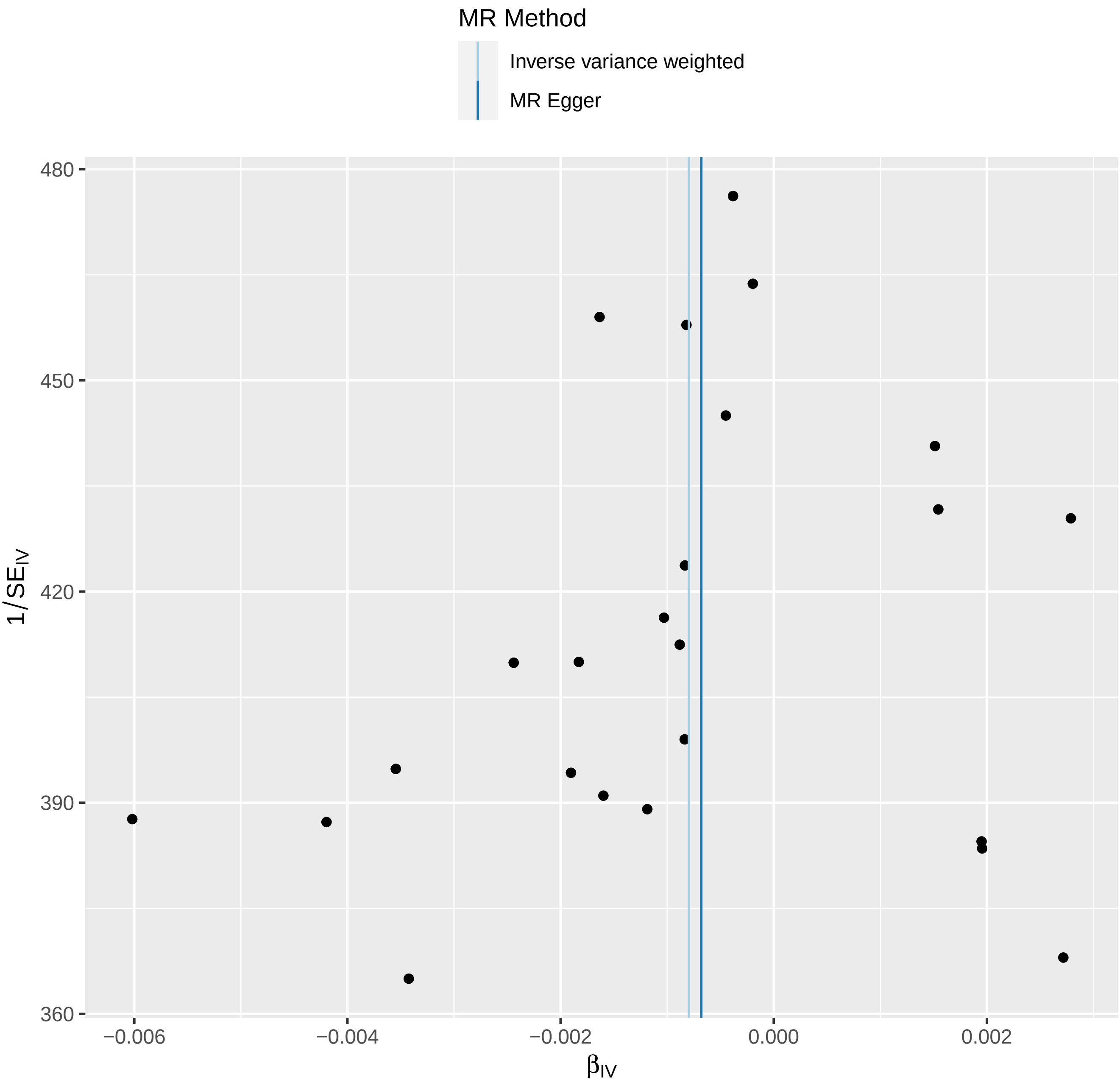

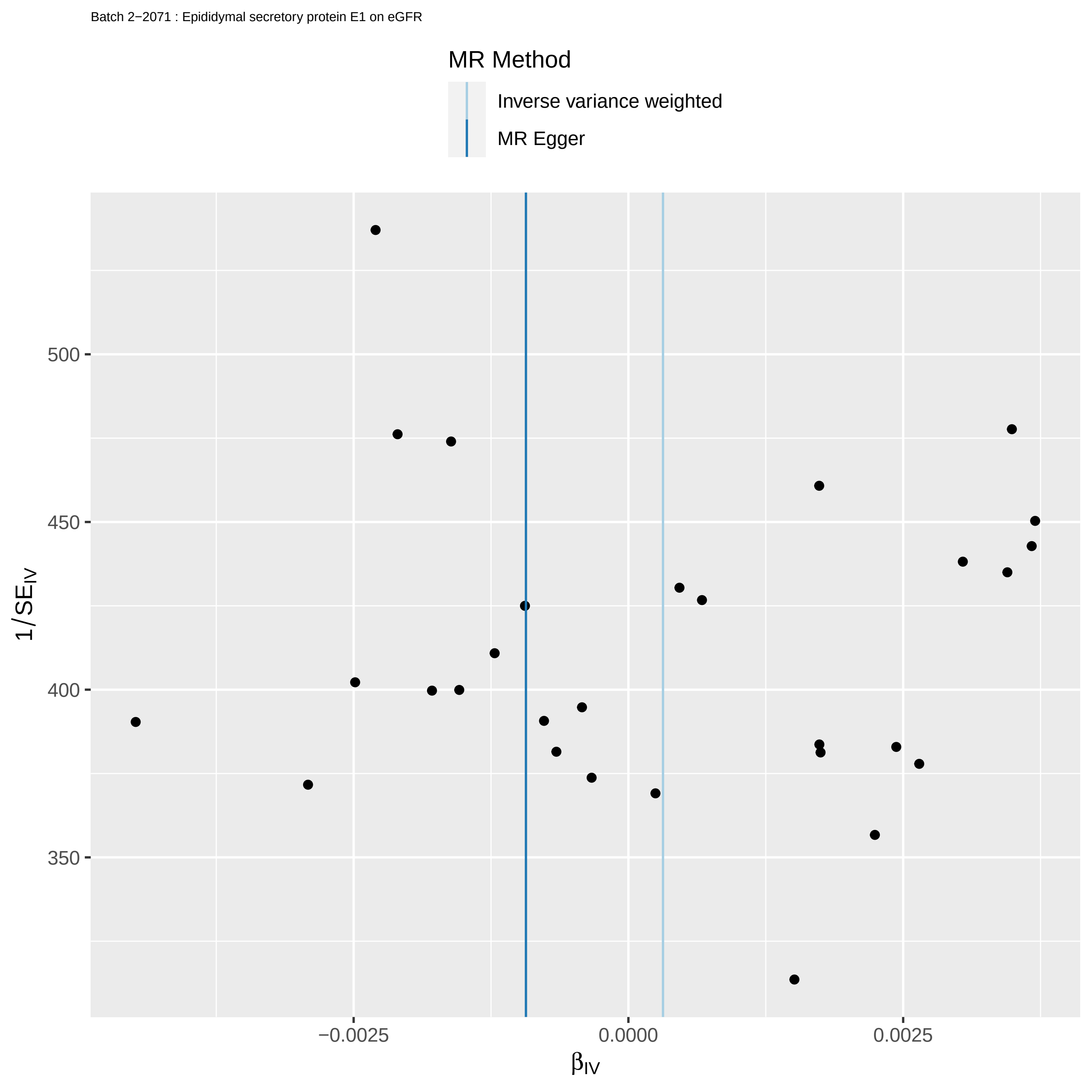
Batch 2−2071 : Epididymal secretory protein E1 on eGFR
MR Method
Inverse variance weighted
MR Egger
500
450
400
350
1/SE_IV
−0.0025
0.0000
0.0025
β_IV

Batch 2−2072 : Neural proliferation differentiation and control protein 1 on eGFR

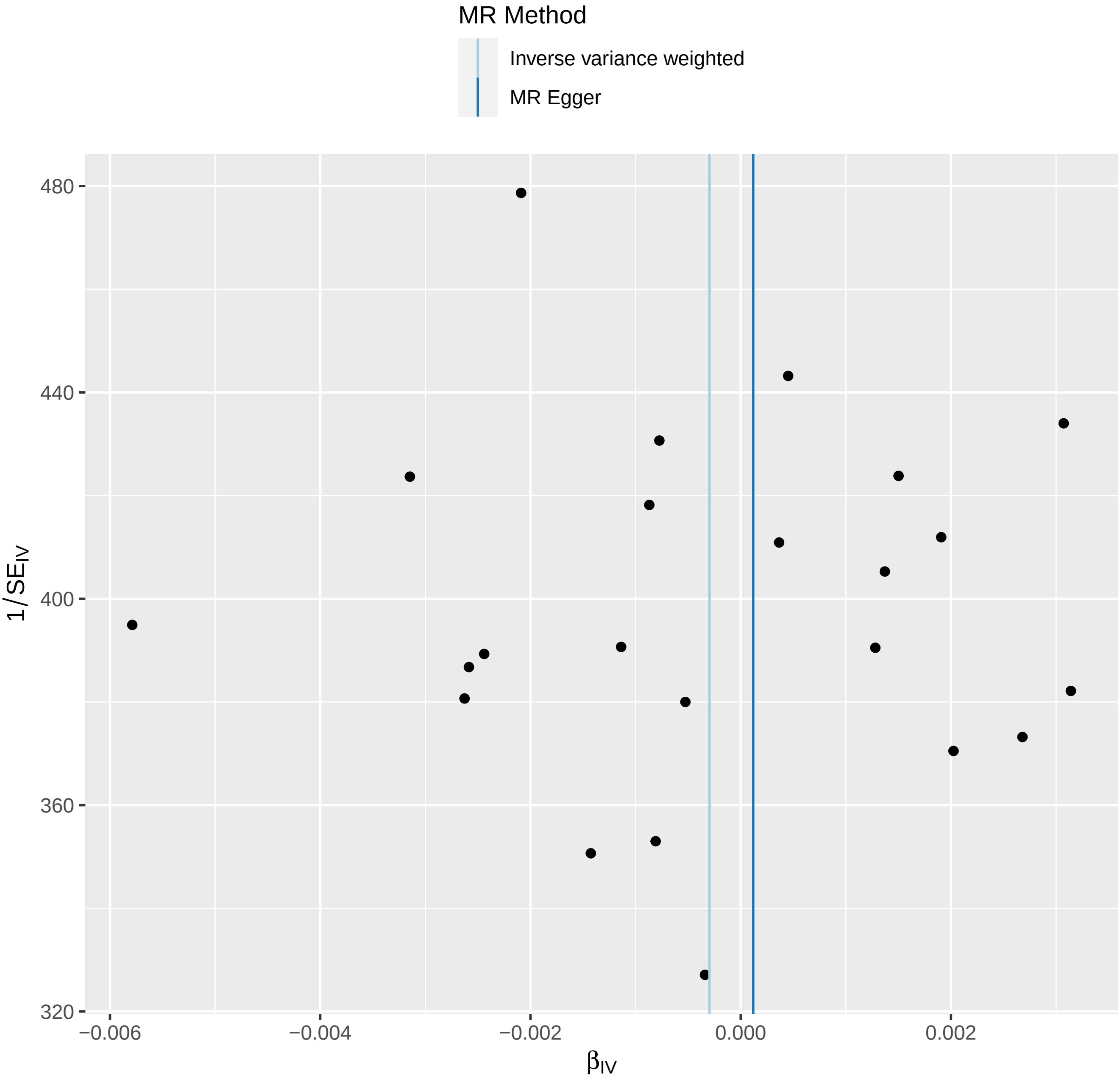

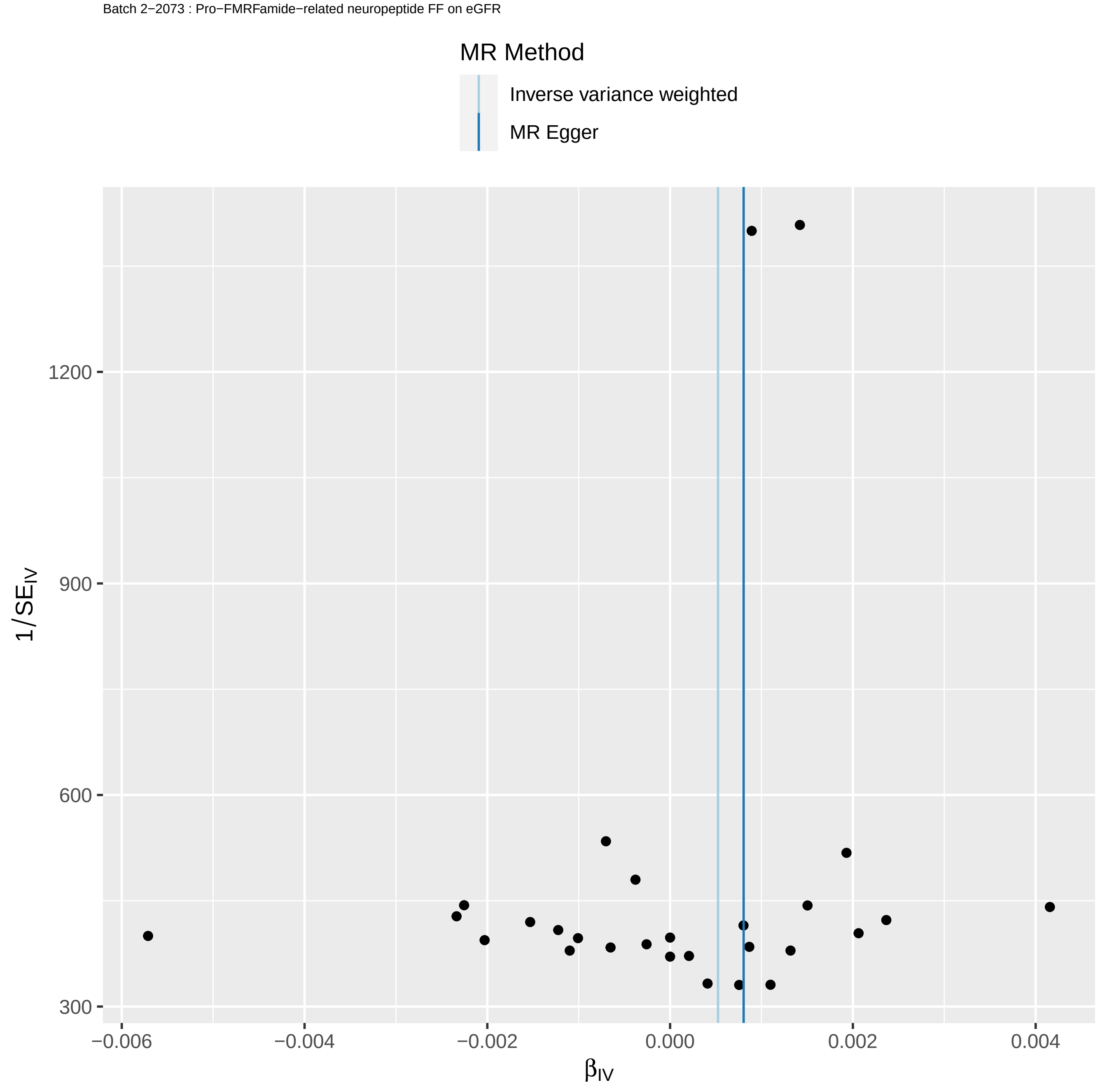
Batch 2−2073 : Pro−FMRFamide−related neuropeptide FF on eGFR
MR Method
Inverse variance weighted
MR Egger
1200
900
600
300
1/SE_IV
−0.006
−0.004
−0.002
0.000
0.002
0.004
β_IV

Batch 2−2074 : Nuclear pore complex−interacting protein family member B3 on eGFR

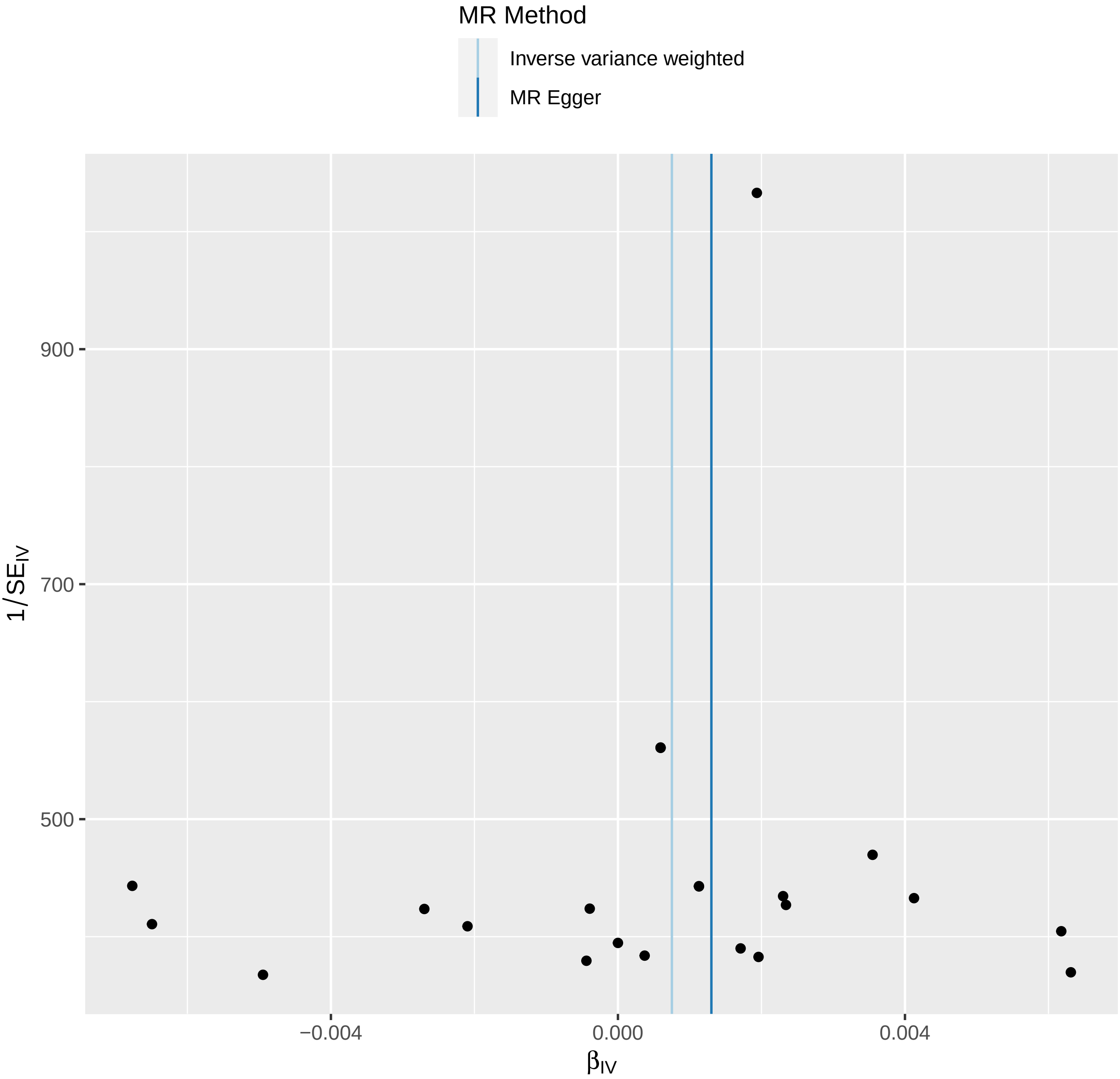

Batch 2−2075 : Nuclear protein localization protein 4 homolog on eGFR

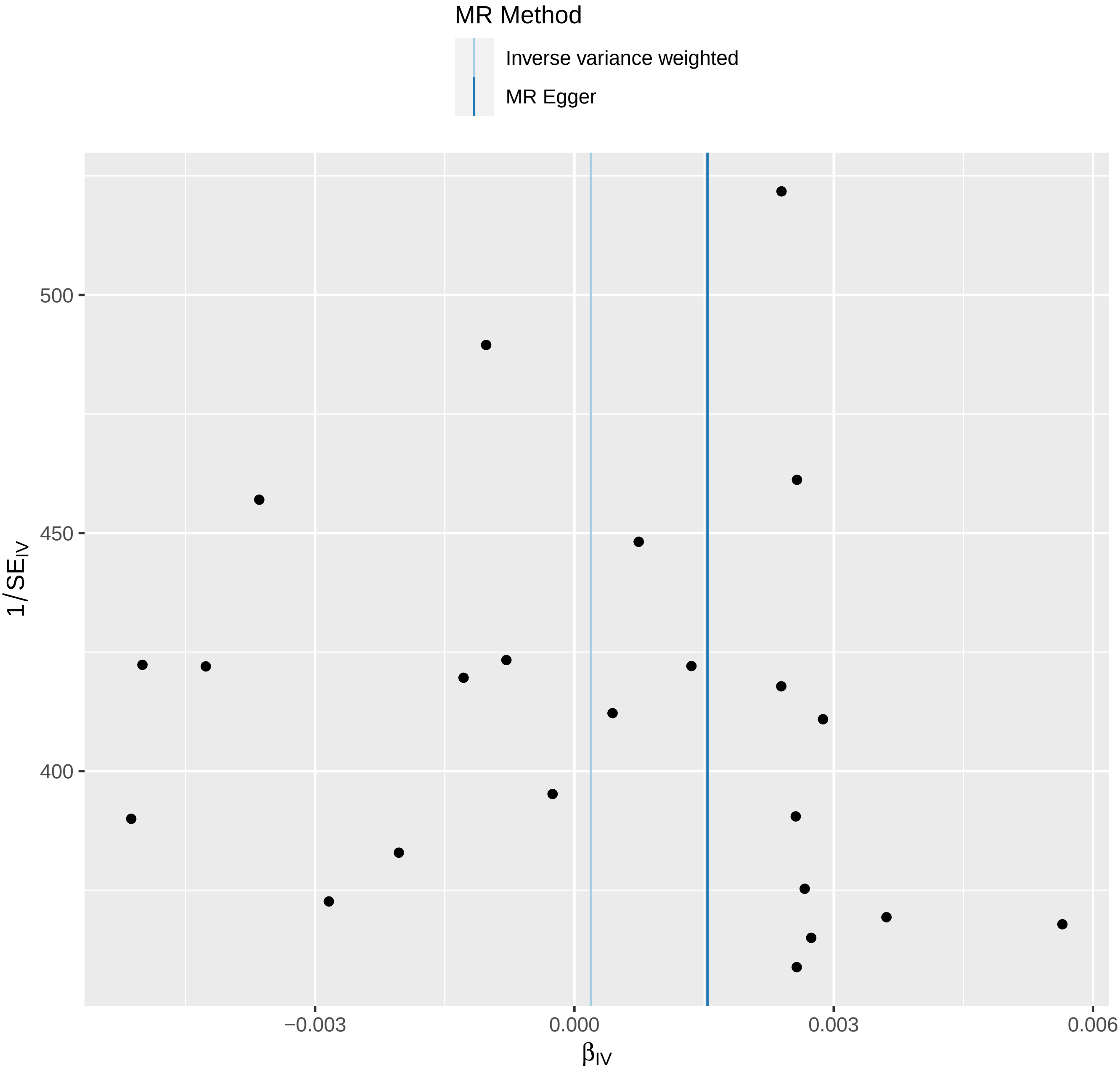

Batch 2−2076 : Atrial natriuretic factor on eGFR

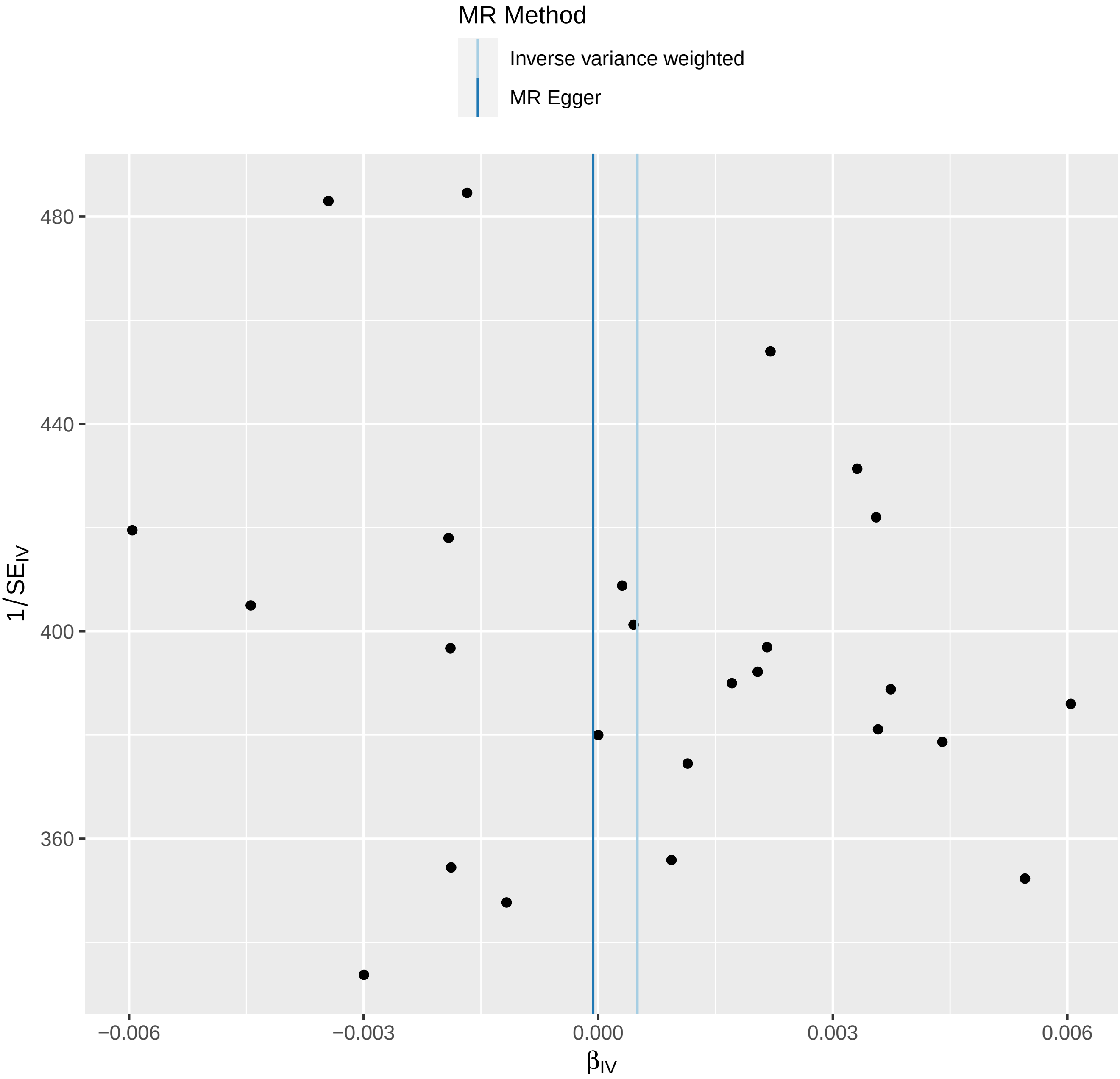

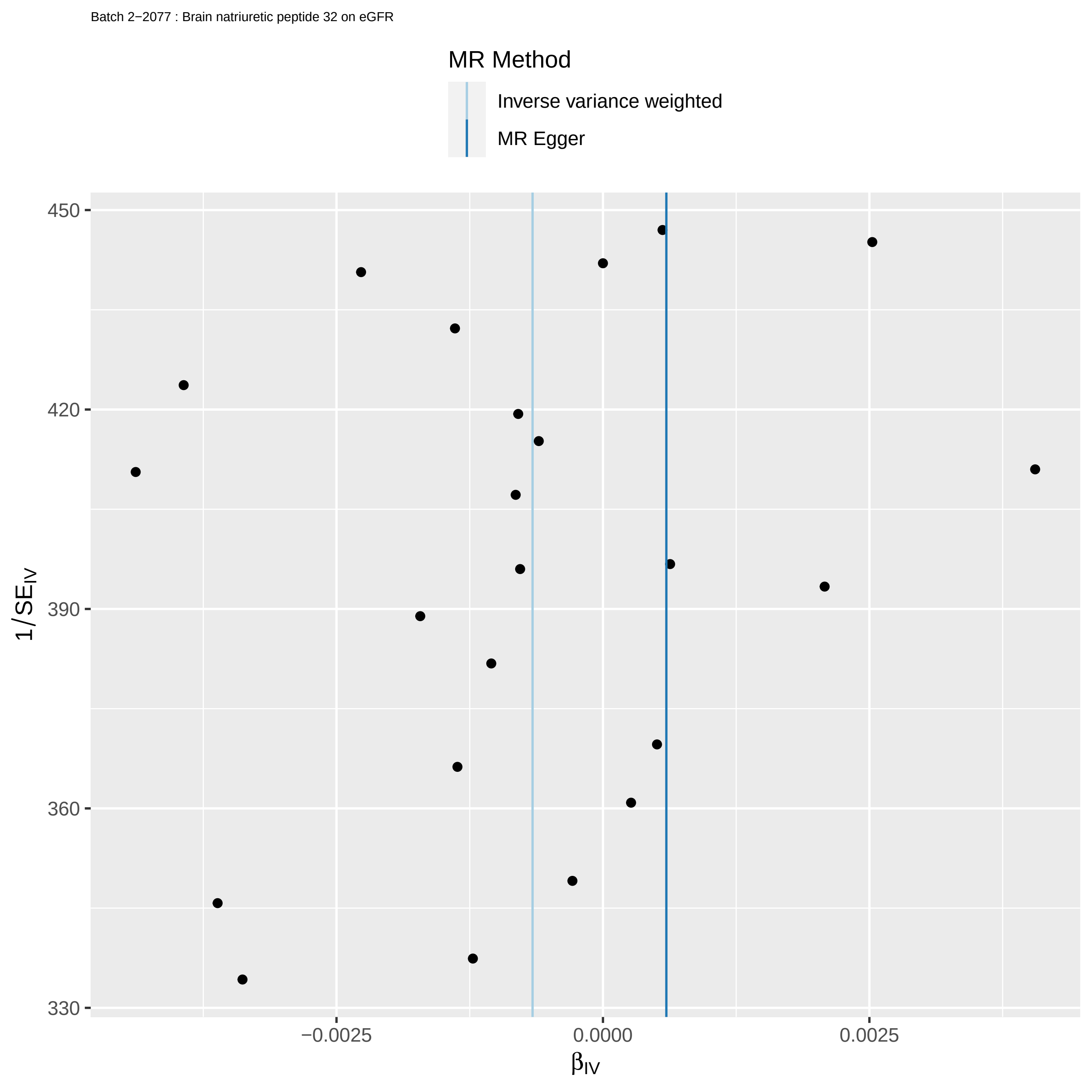
Batch 2−2077 : Brain natriuretic peptide 32 on eGFR
MR Method
Inverse variance weighted
MR Egger
450
420
390
360
330
1/SE_IV
−0.0025
0.0000
0.0025
β_IV

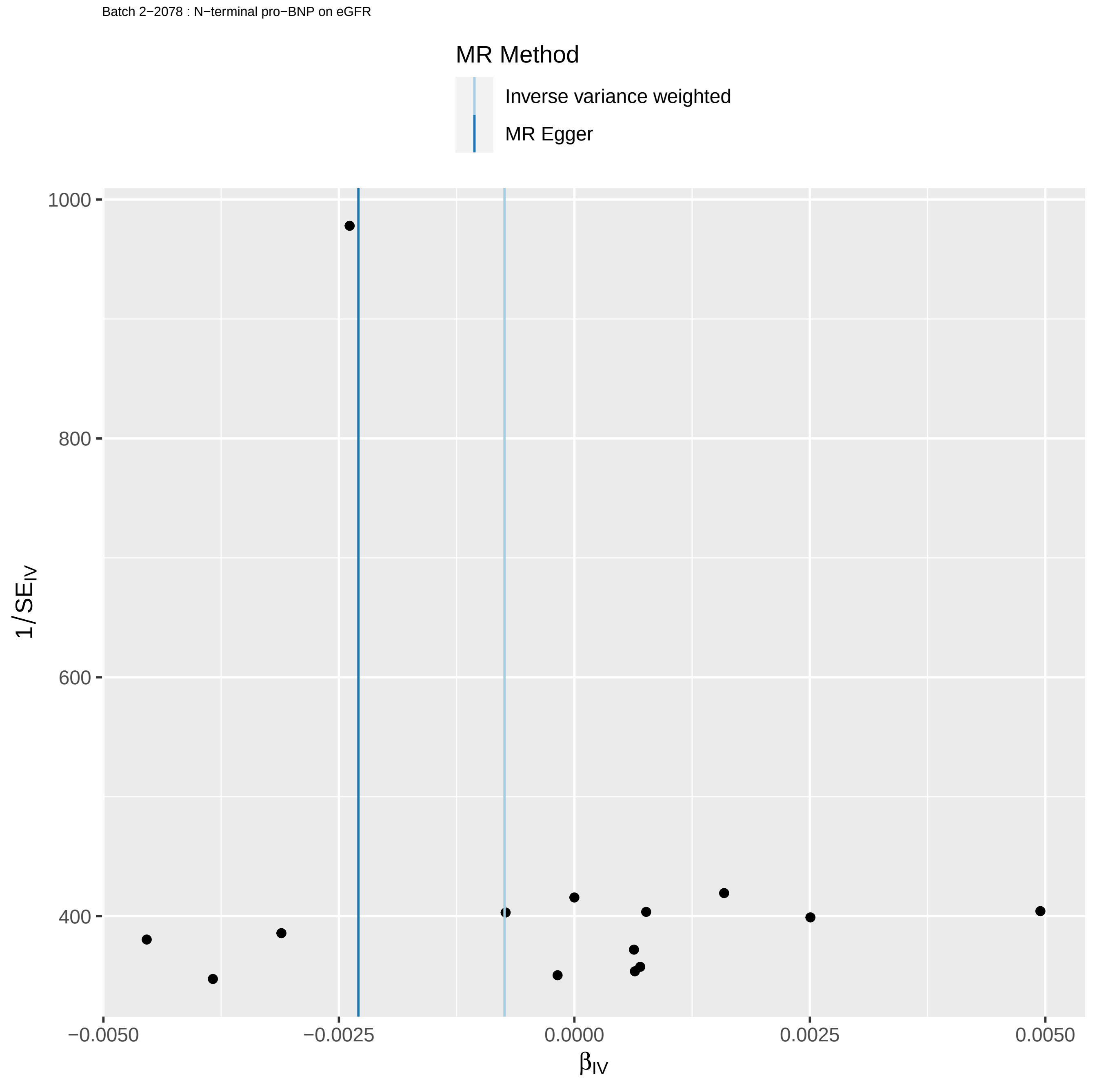

Batch 2−2078 : N−terminal pro−BNP on eGFR
MR Method
Inverse variance weighted
MR Egger
1000
800
600
400
−0.0050
−0.0025
0.0000
0.0025
0.0050
$1/SE_{IV}$
β_{IV}

Batch 2−2079 : Neuropeptide S on eGFR

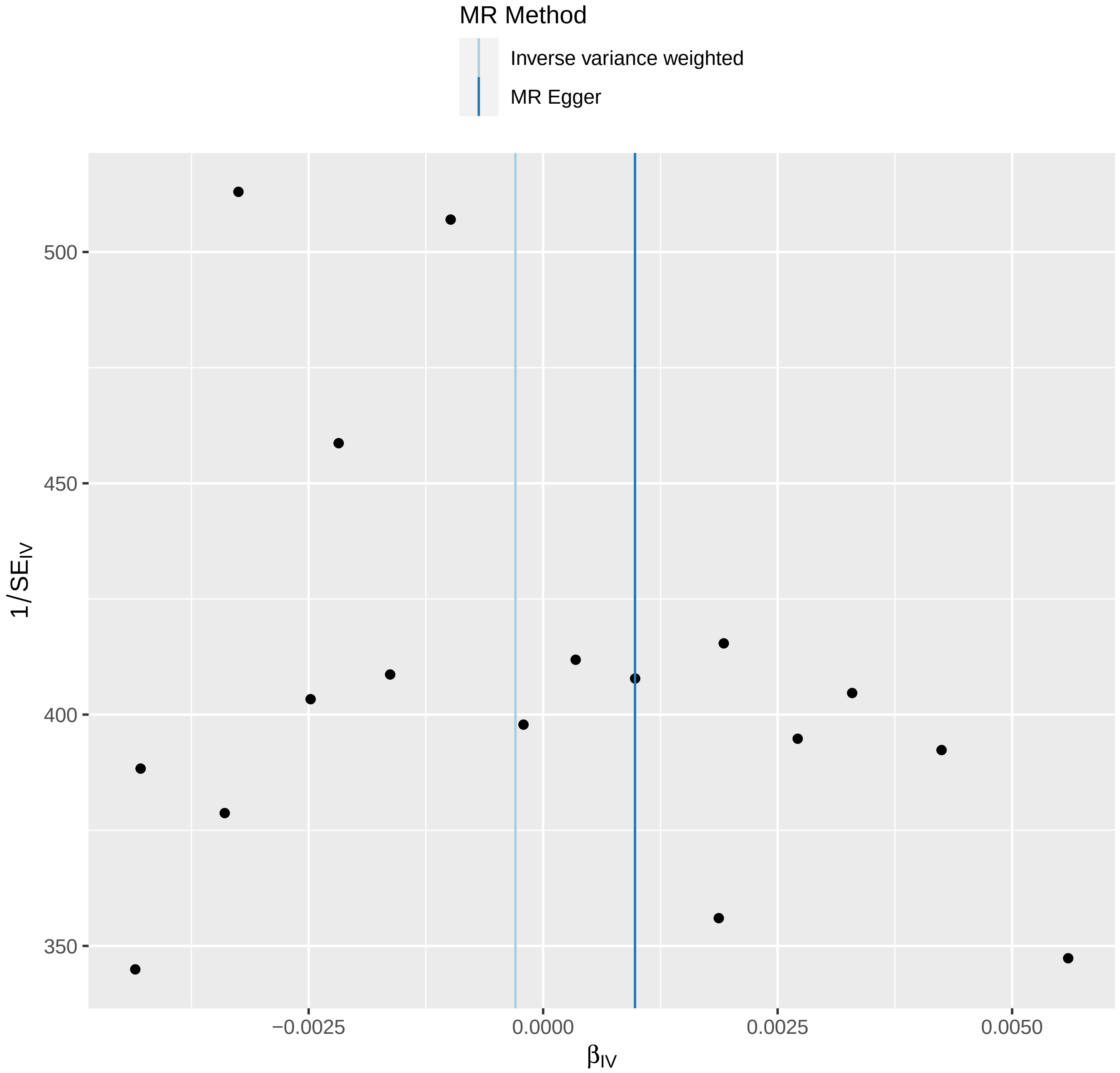

Batch 2−2080 : Neuronal pentraxin−1 on eGFR

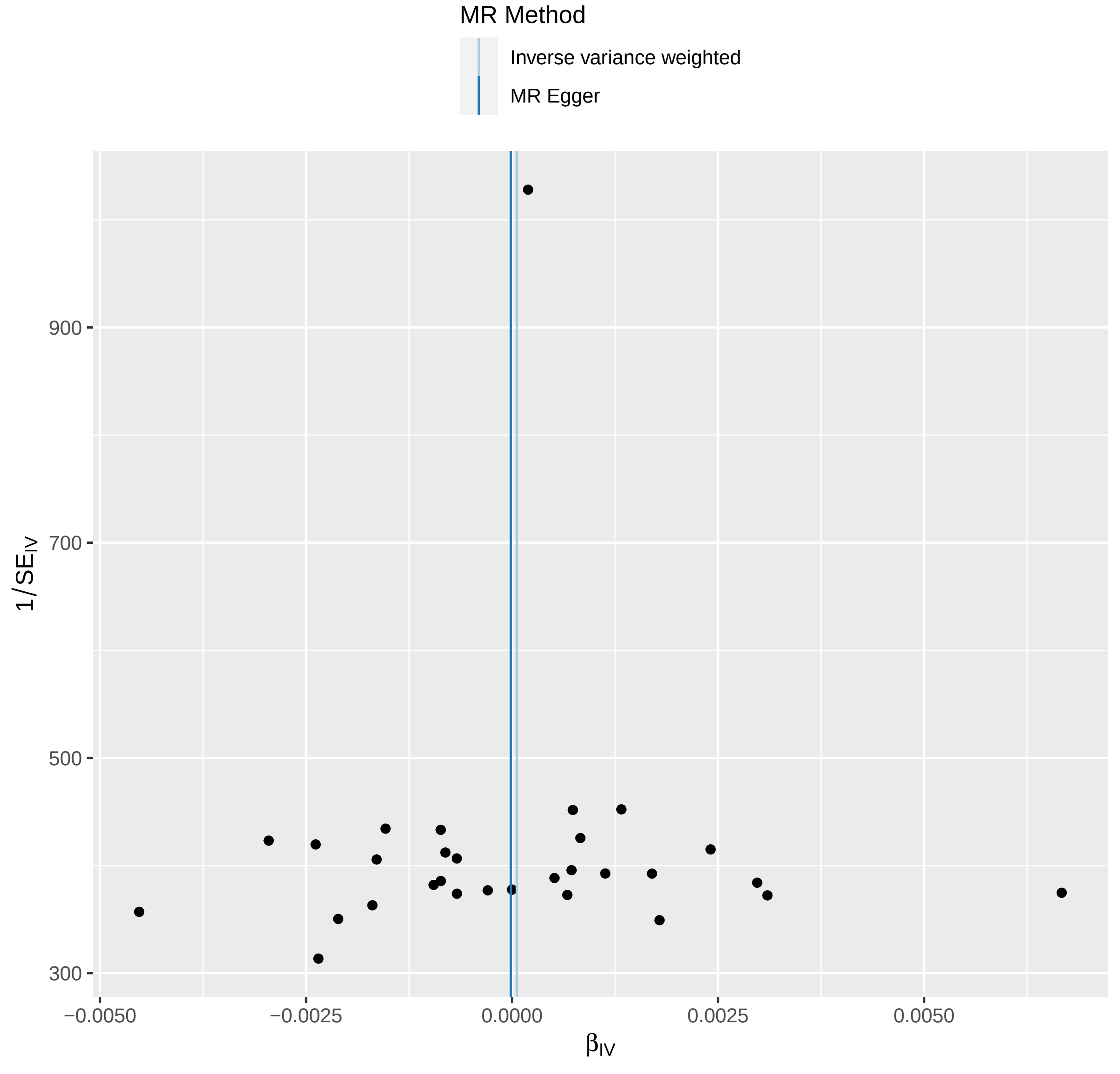

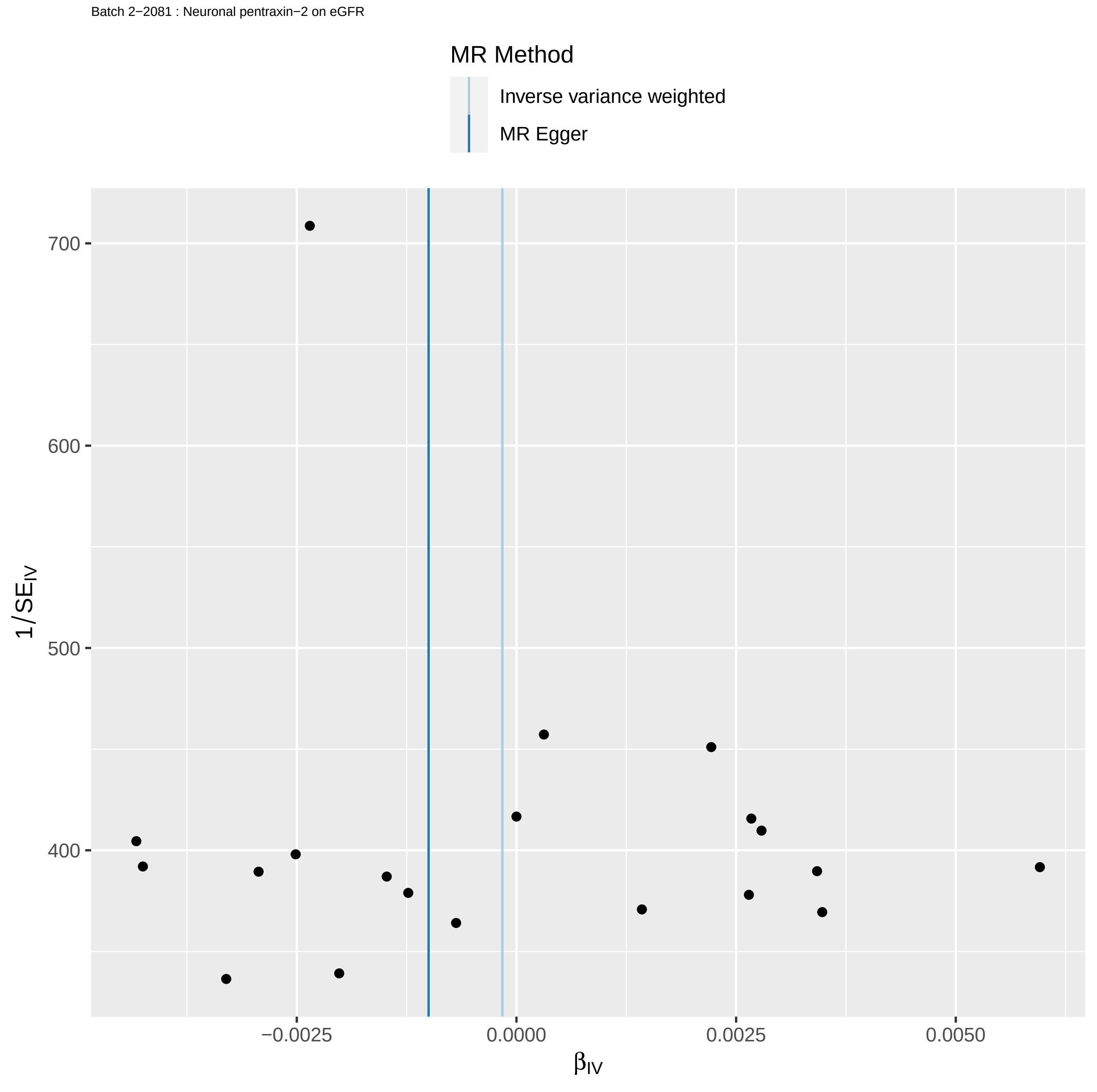

Batch 2−2081 : Neuronal pentraxin−2 on eGFR
MR Method
Inverse variance weighted
MR Egger
700
600
500
400
1/SE_IV
−0.0025
0.0000
0.0025
0.0050
β_IV

Batch 2−2082 : Neuropeptide W on eGFR

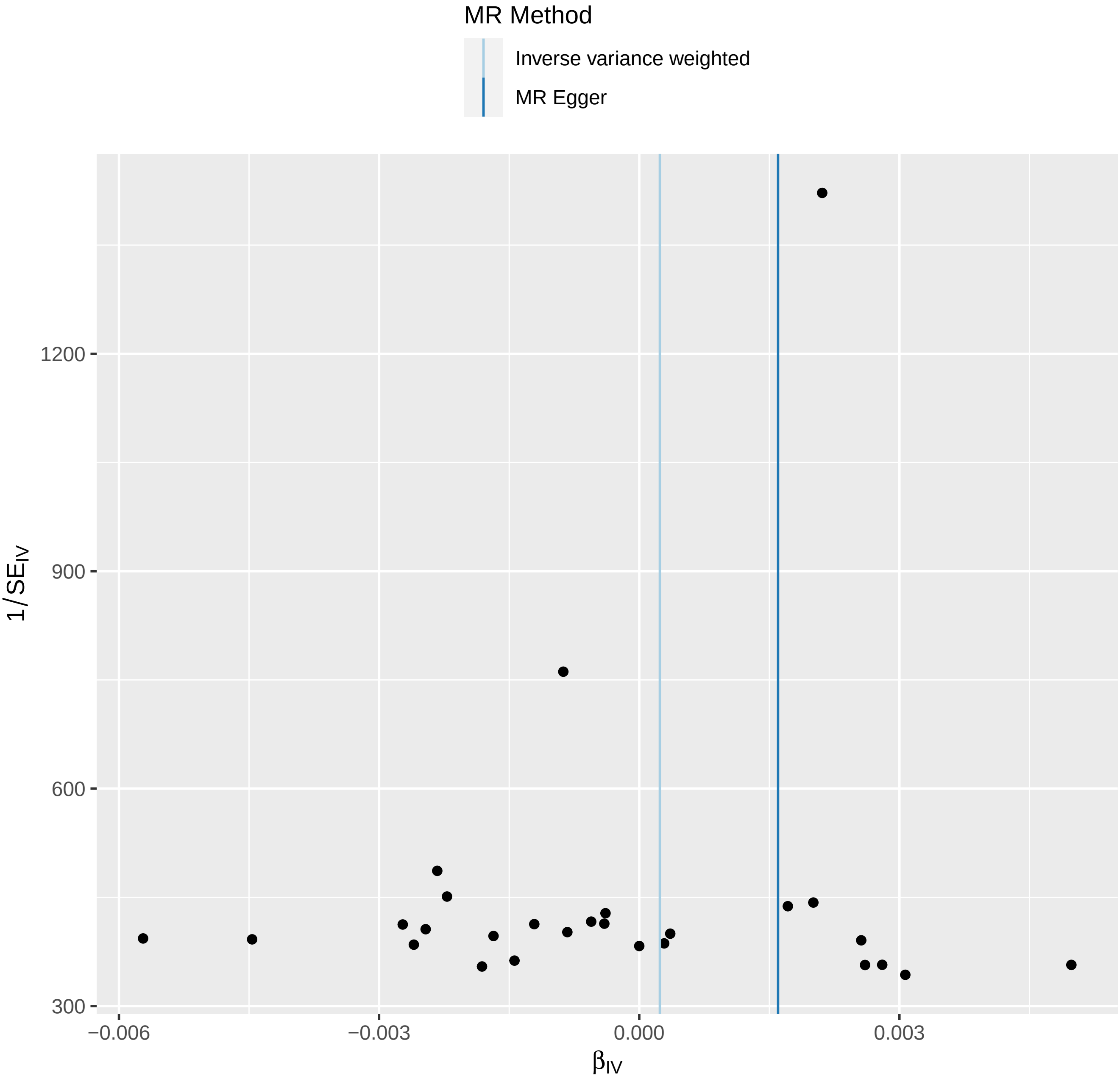

Batch 2−2083 : NAD(P)H dehydrogenase [quinone] 1 on eGFR

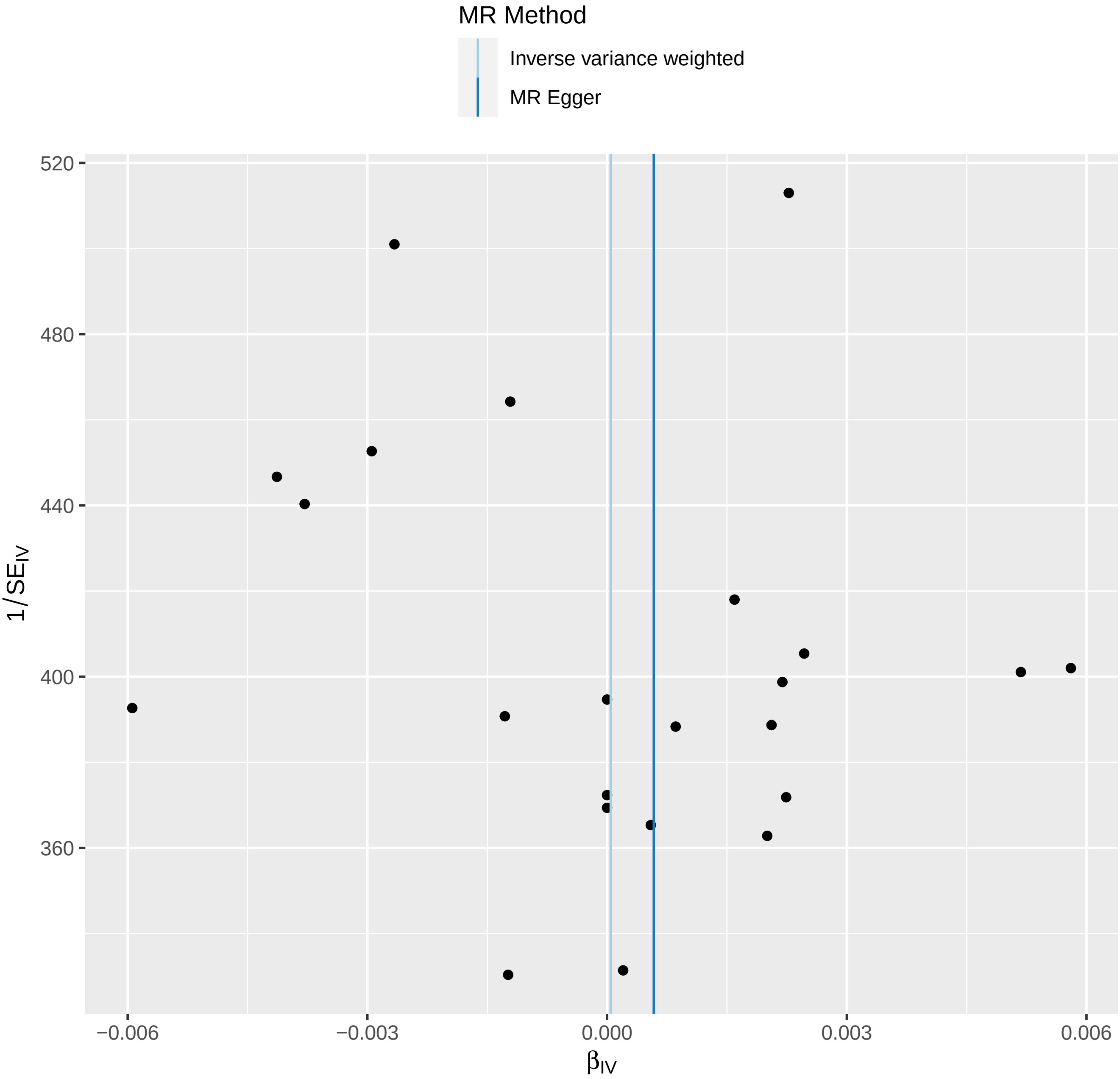

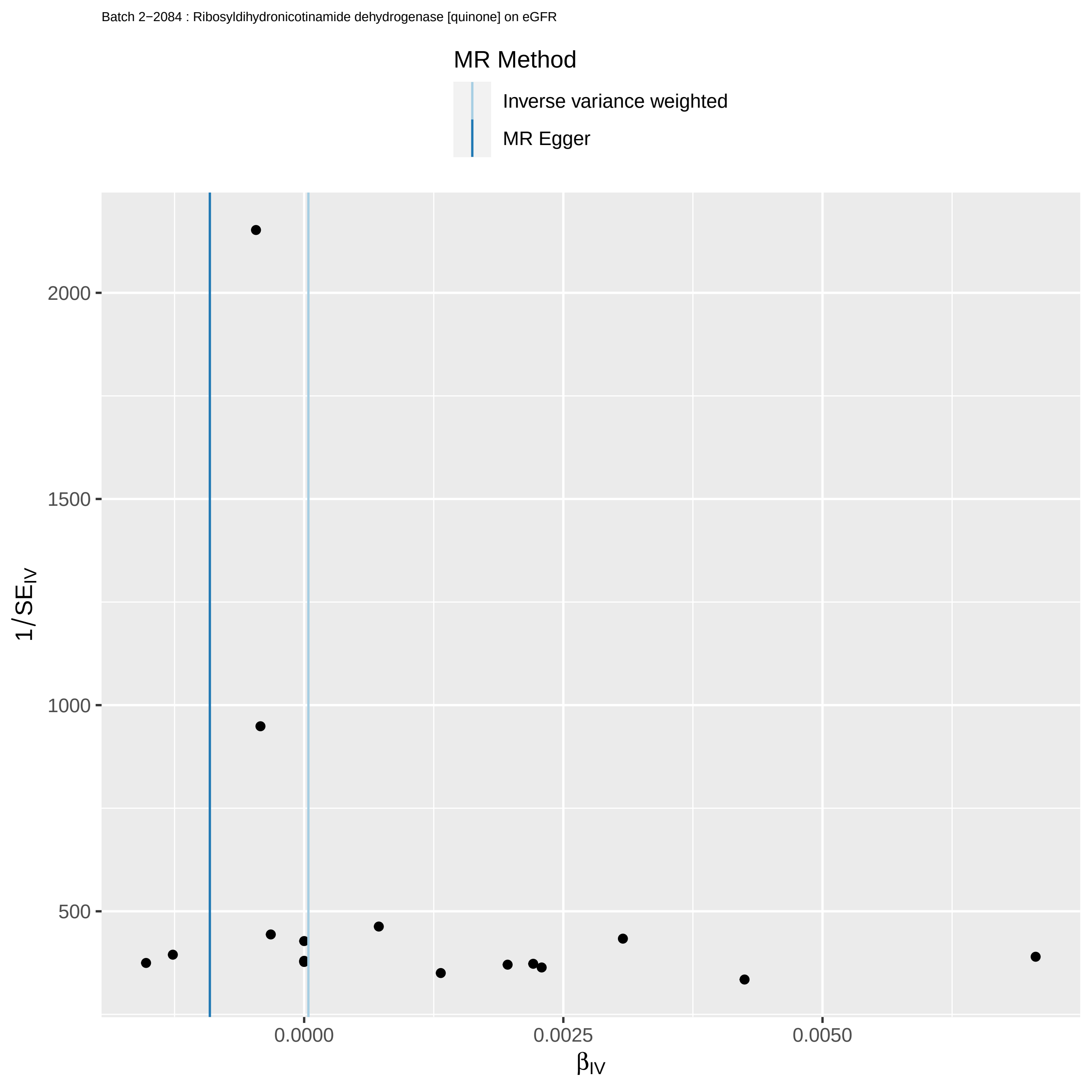
Batch 2−2084 : Ribosyldihydronicotinamide dehydrogenase [quinone] on eGFR
MR Method
Inverse variance weighted
MR Egger
2000
1500
1000
500
1/SE_IV
0.0000
0.0025
0.0050
β_IV

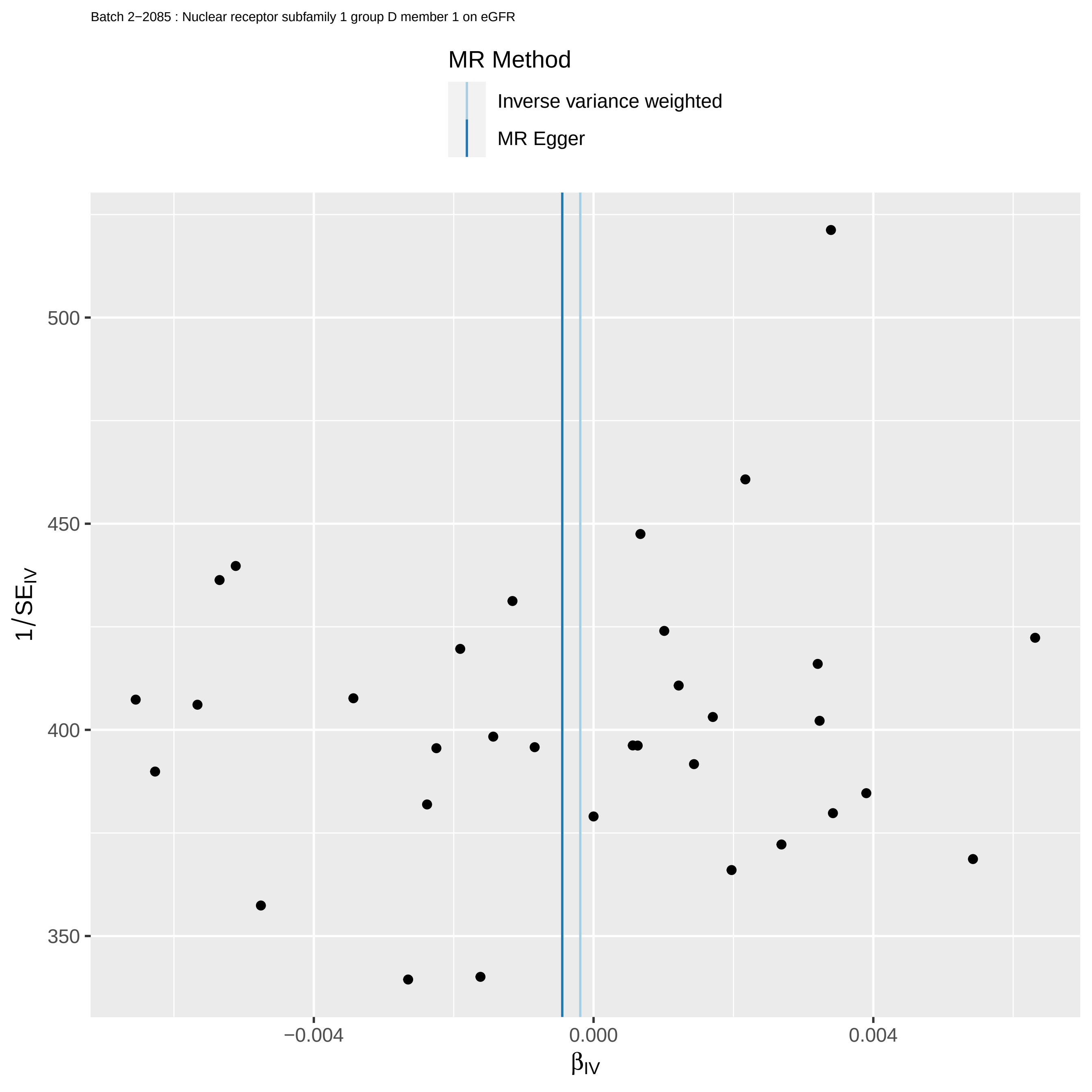
Batch 2−2085 : Nuclear receptor subfamily 1 group D member 1 on eGFR
MR Method
Inverse variance weighted
MR Egger
500
450
400
350
1/SE_IV
−0.004
0.000
0.004
β_IV

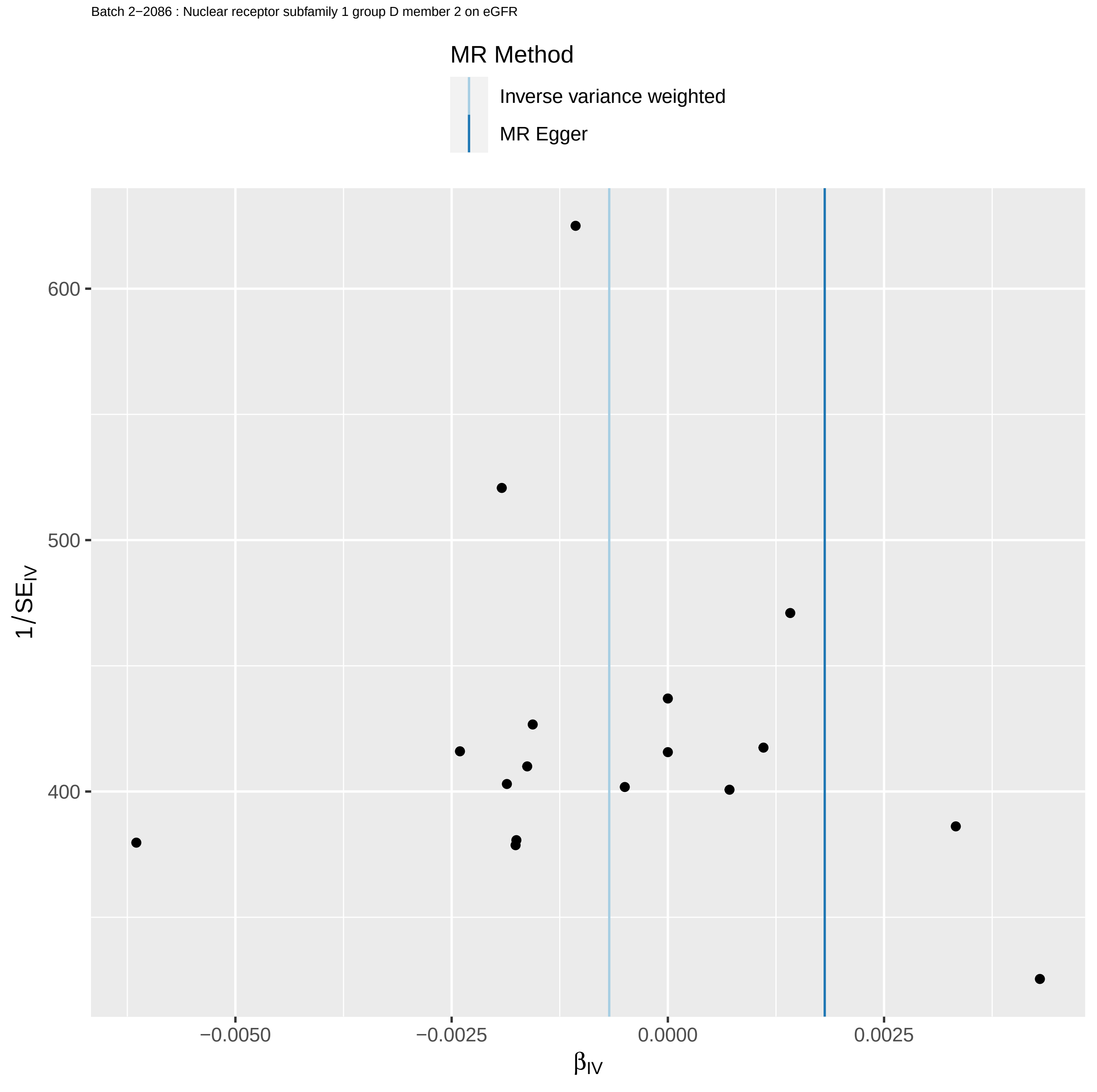
Batch 2−2086 : Nuclear receptor subfamily 1 group D member 2 on eGFR
MR Method
Inverse variance weighted
MR Egger
600
500
400
1/SE_IV
−0.0050
−0.0025
0.0000
0.0025
β_IV

Batch 2−2087 : Oxysterols receptor LXR−beta on eGFR

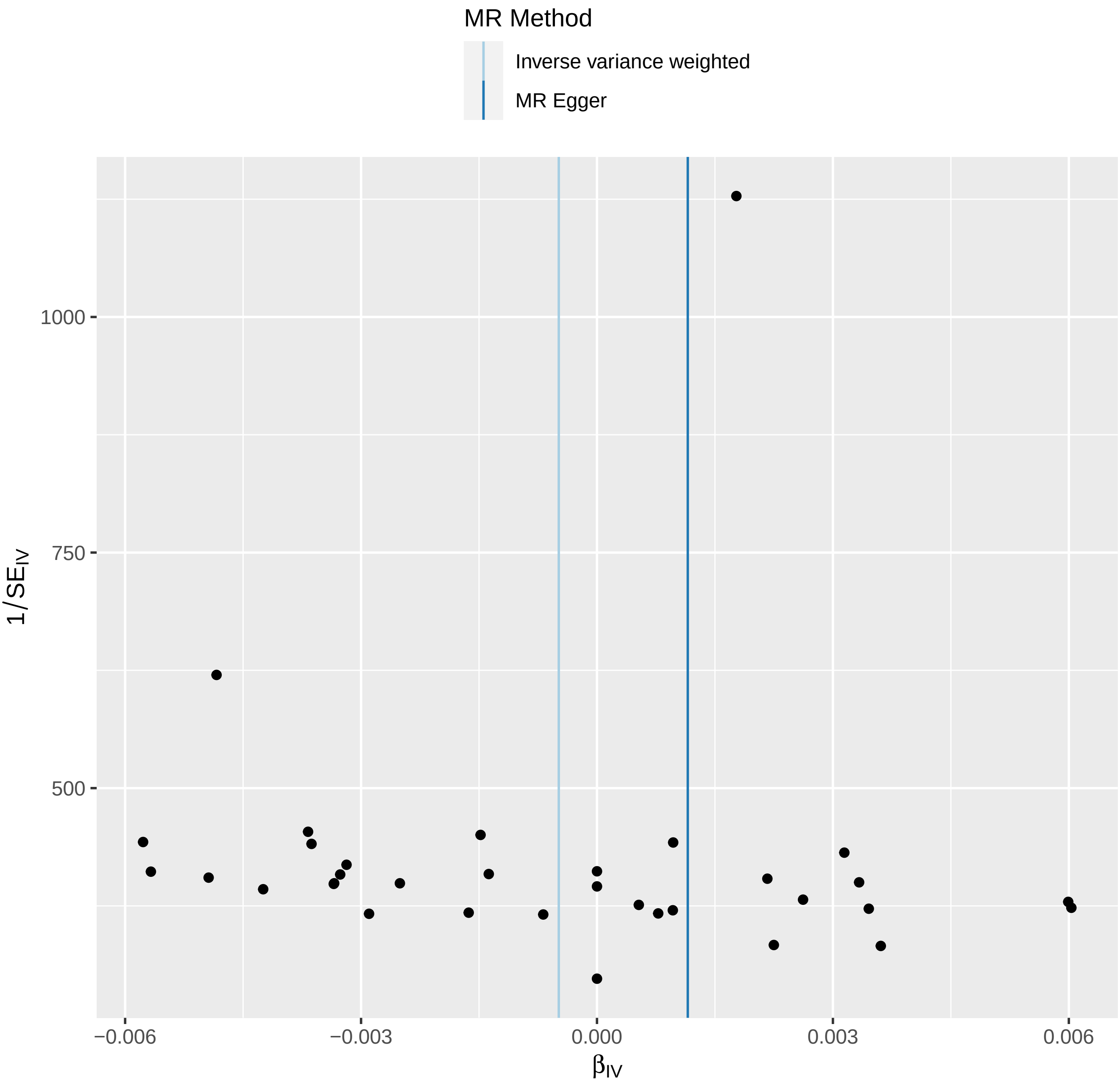

Batch 2−2088 : Glucocorticoid receptor on eGFR

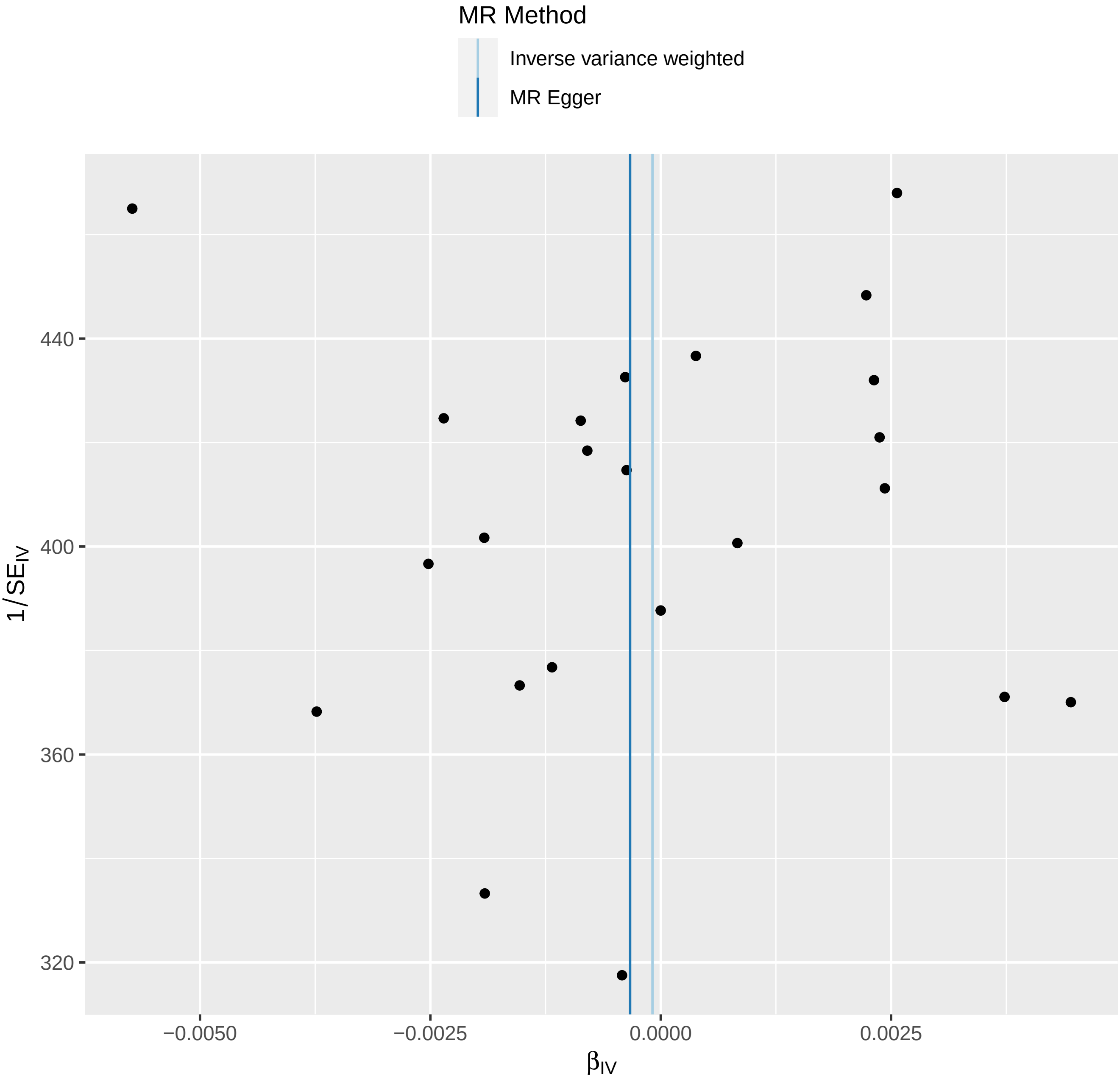

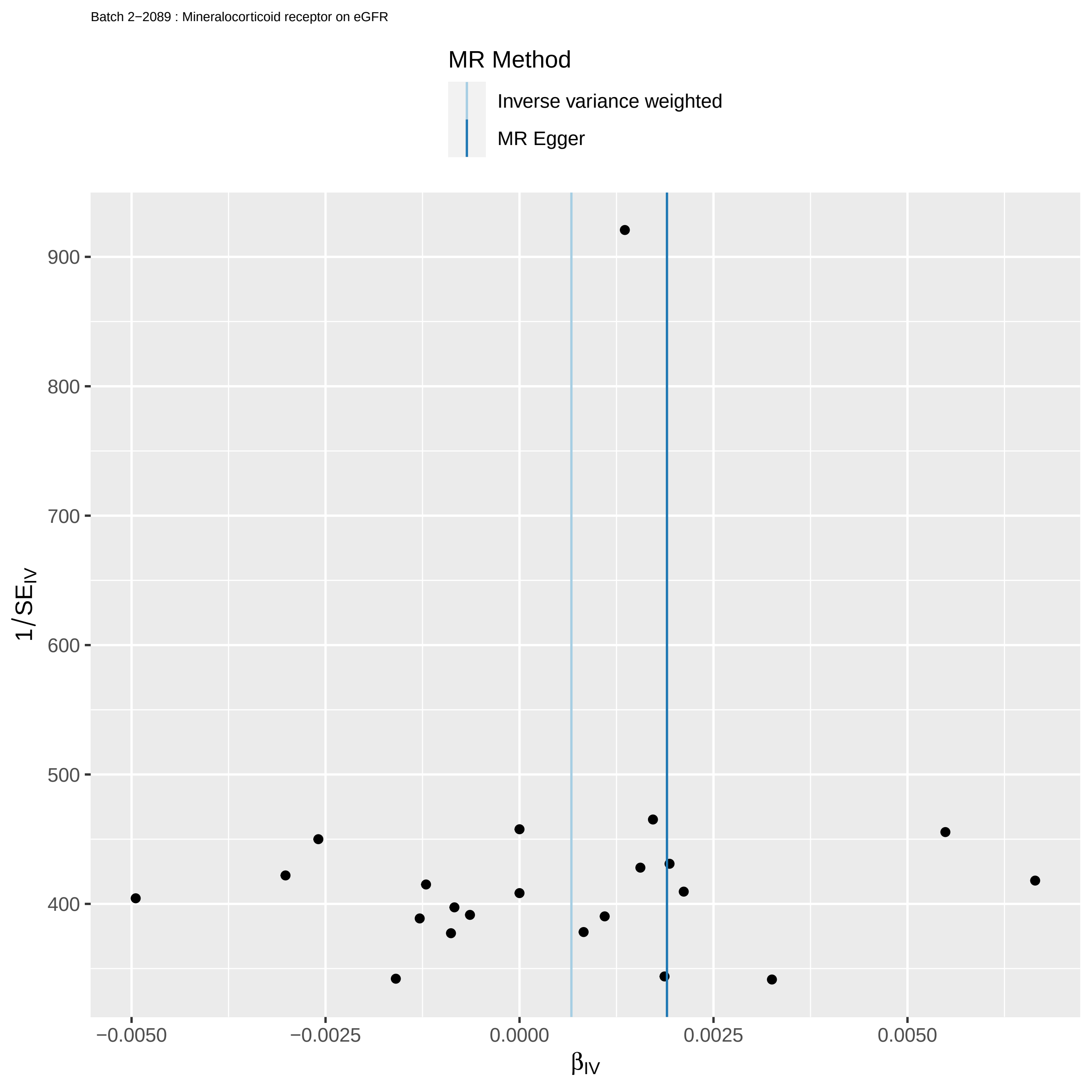
Batch 2−2089 : Mineralocorticoid receptor on eGFR
MR Method
Inverse variance weighted
MR Egger
900
800
700
600
500
400
1/SE$_{IV}$
−0.0050
−0.0025
0.0000
0.0025
0.0050
β$_{IV}$

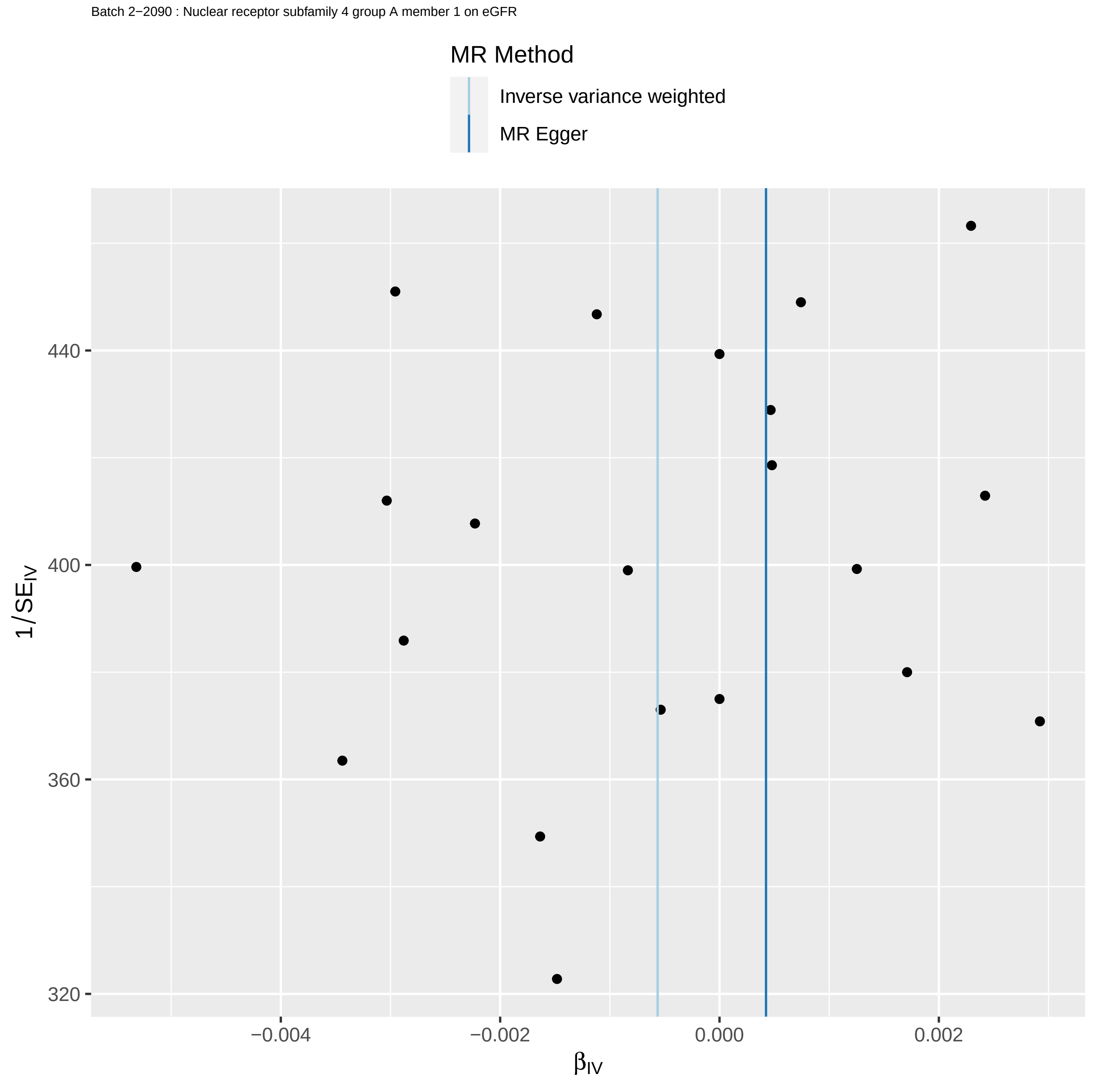

Batch 2−2090 : Nuclear receptor subfamily 4 group A member 1 on eGFR
MR Method
Inverse variance weighted
MR Egger
440
400
360
320
1/SE$_{IV}$
−0.004
−0.002
0.000
0.002
β$_{IV}$

Batch 2−2091 : Nuclear receptor subfamily 5 group A member 2 on eGFR

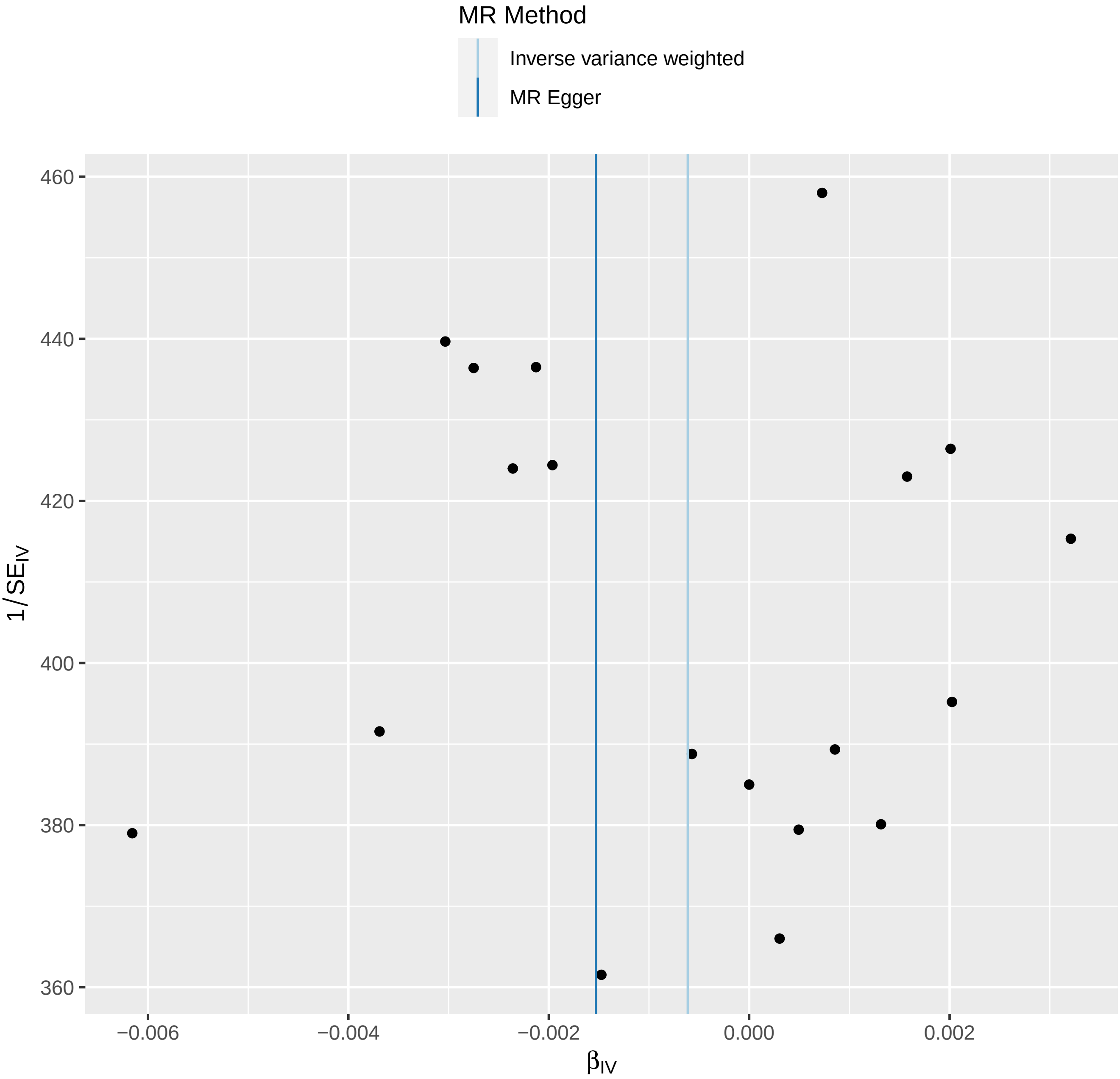

Batch 2−2092 : Nutritionally−regulated adipose and cardiac enriched protein homolog on eGFR

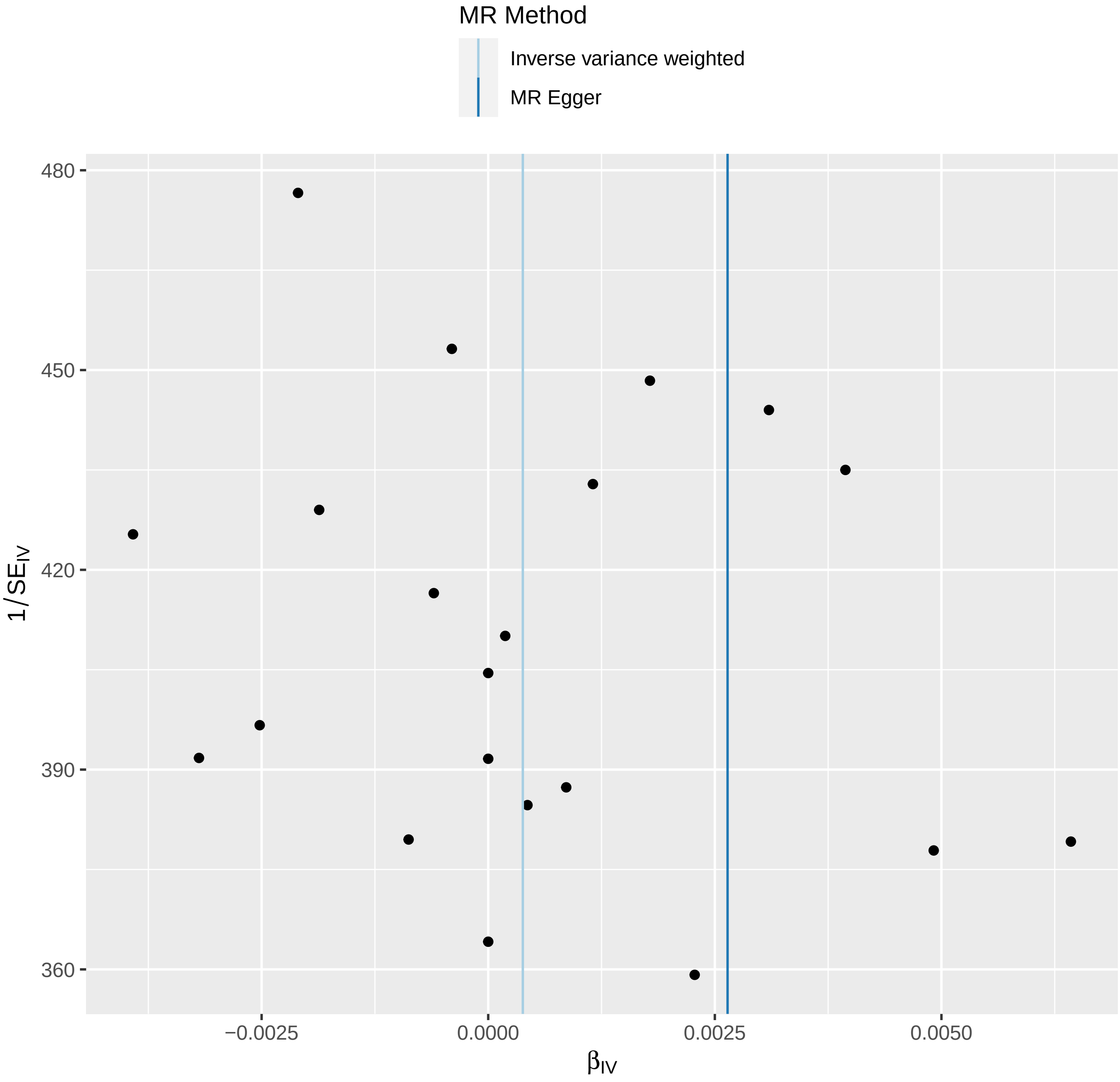

Batch 2−2093 : Nuclear receptor−binding protein on eGFR

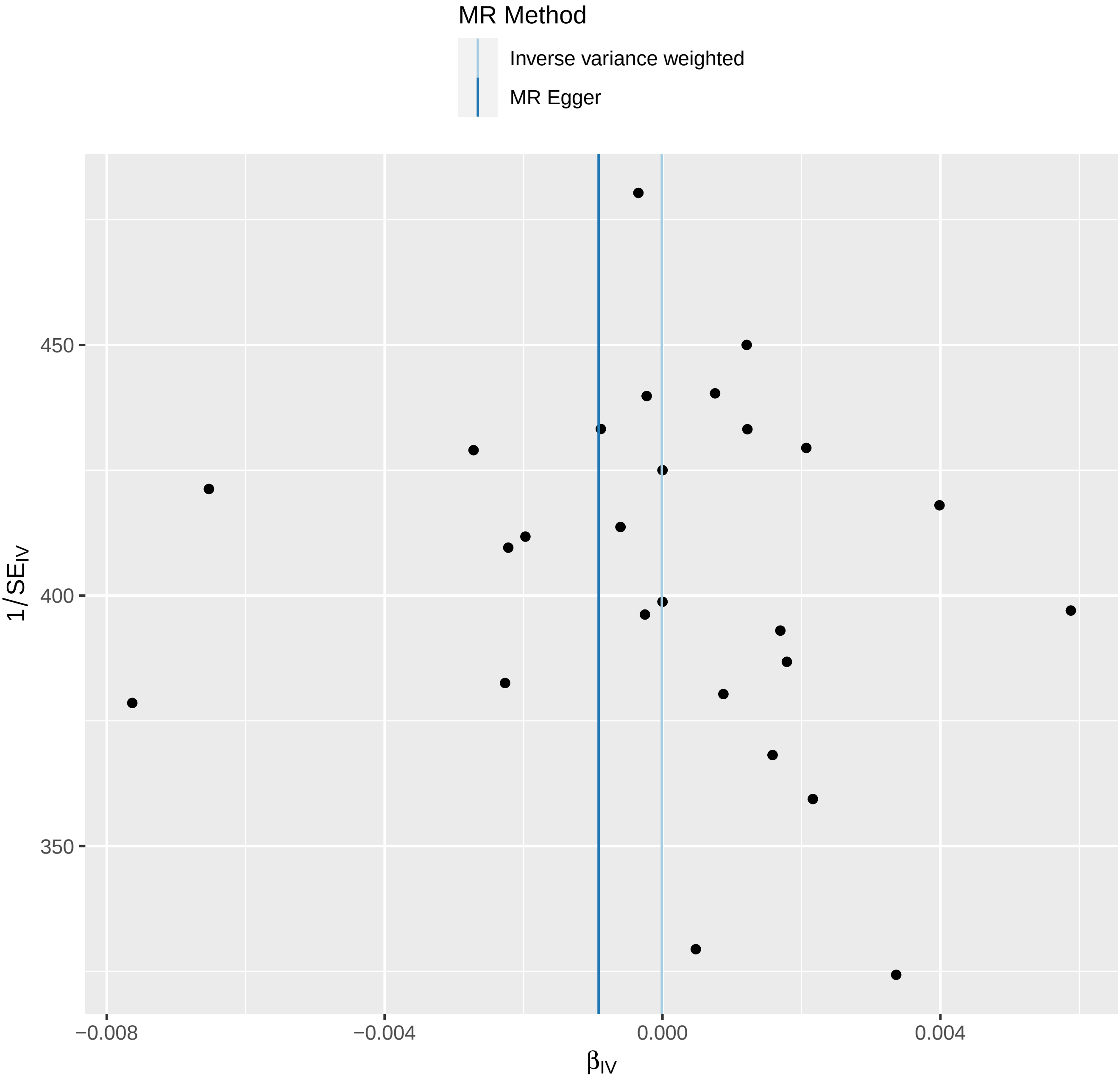

Batch 2−2094 : Neuronal cell adhesion molecule on eGFR

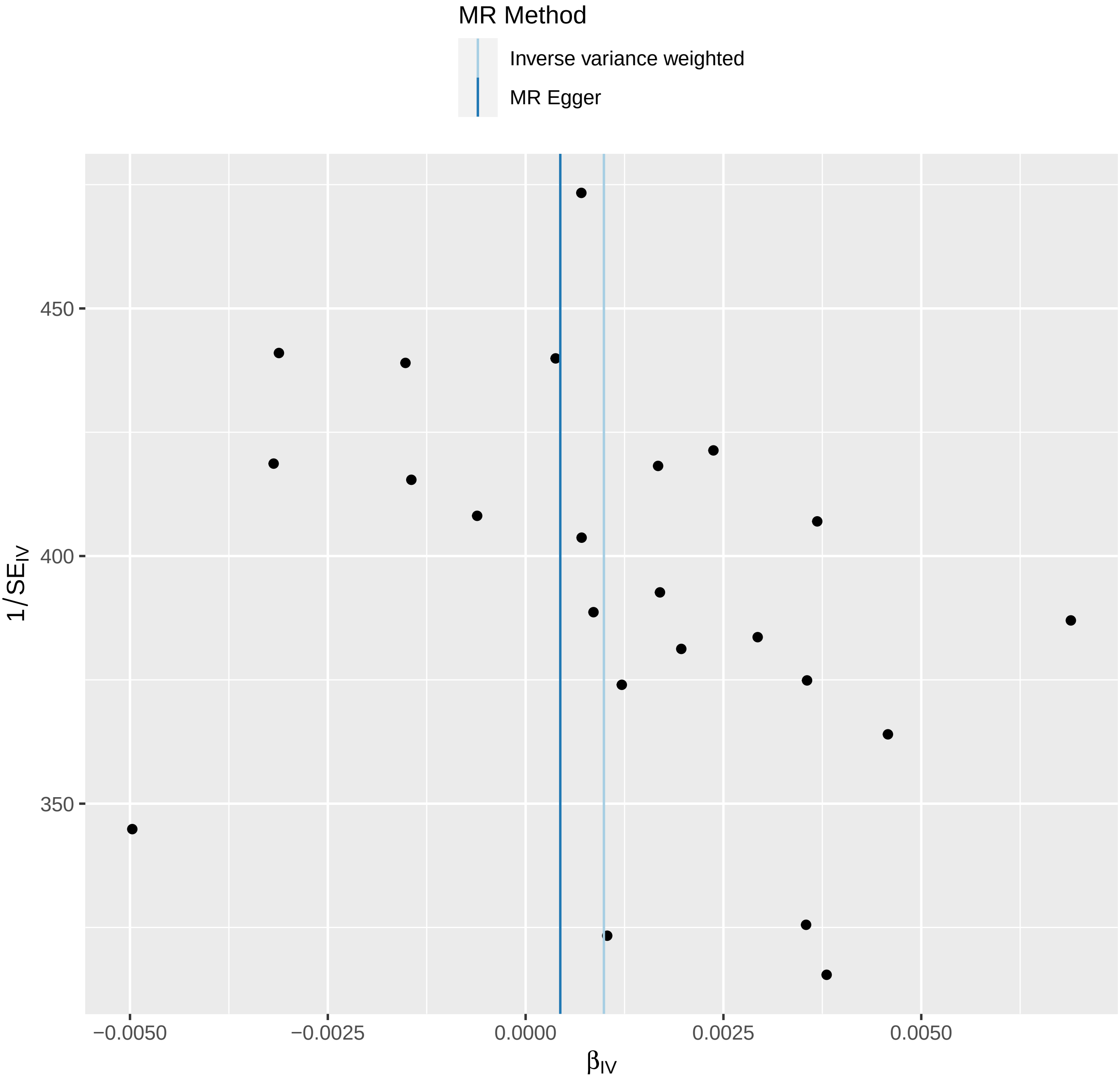

Batch 2−2095 : Neuregulin−1 on eGFR

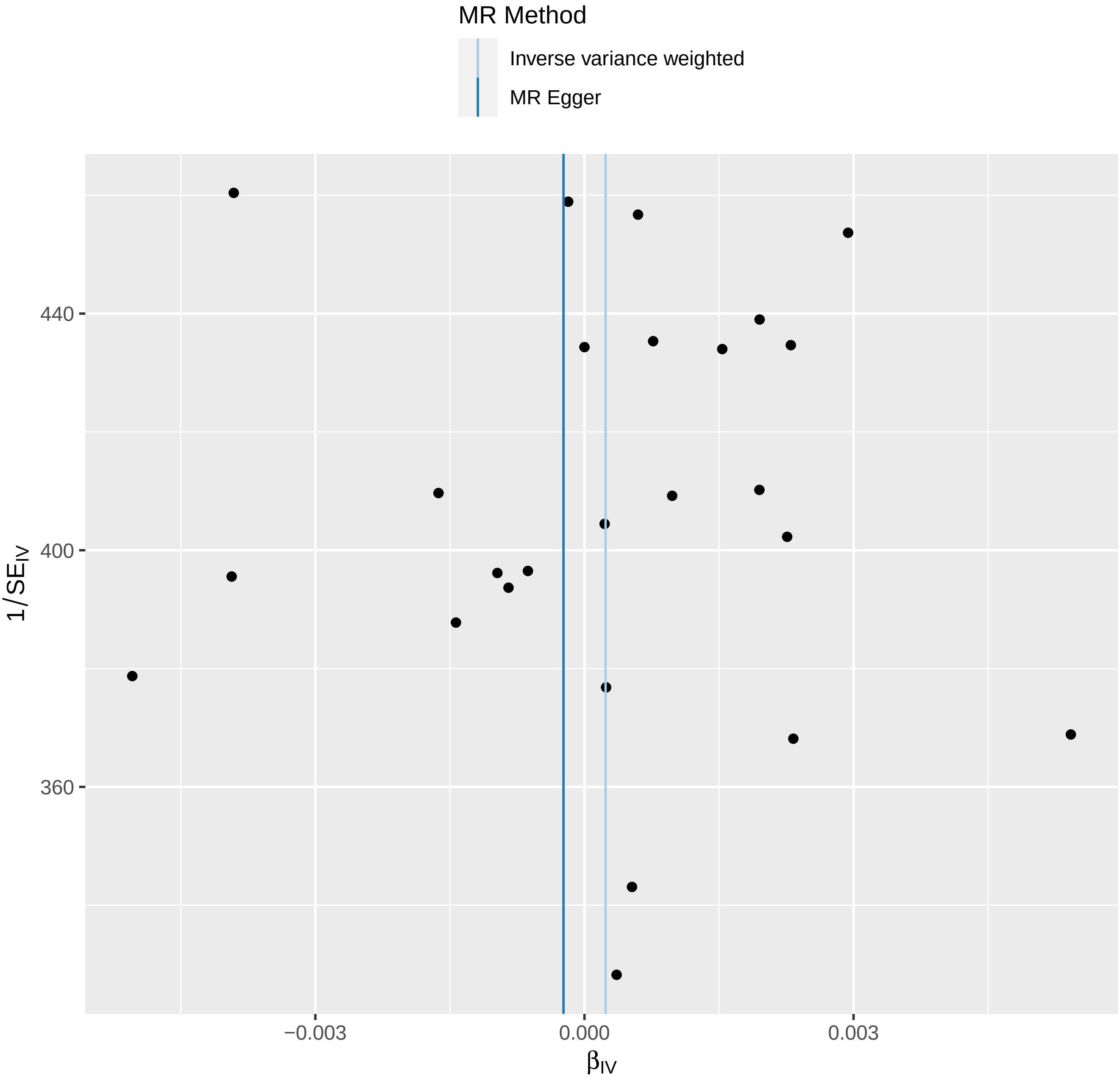

Batch 2−2096 : Pro−neuregulin−2, membrane−bound isoform on eGFR

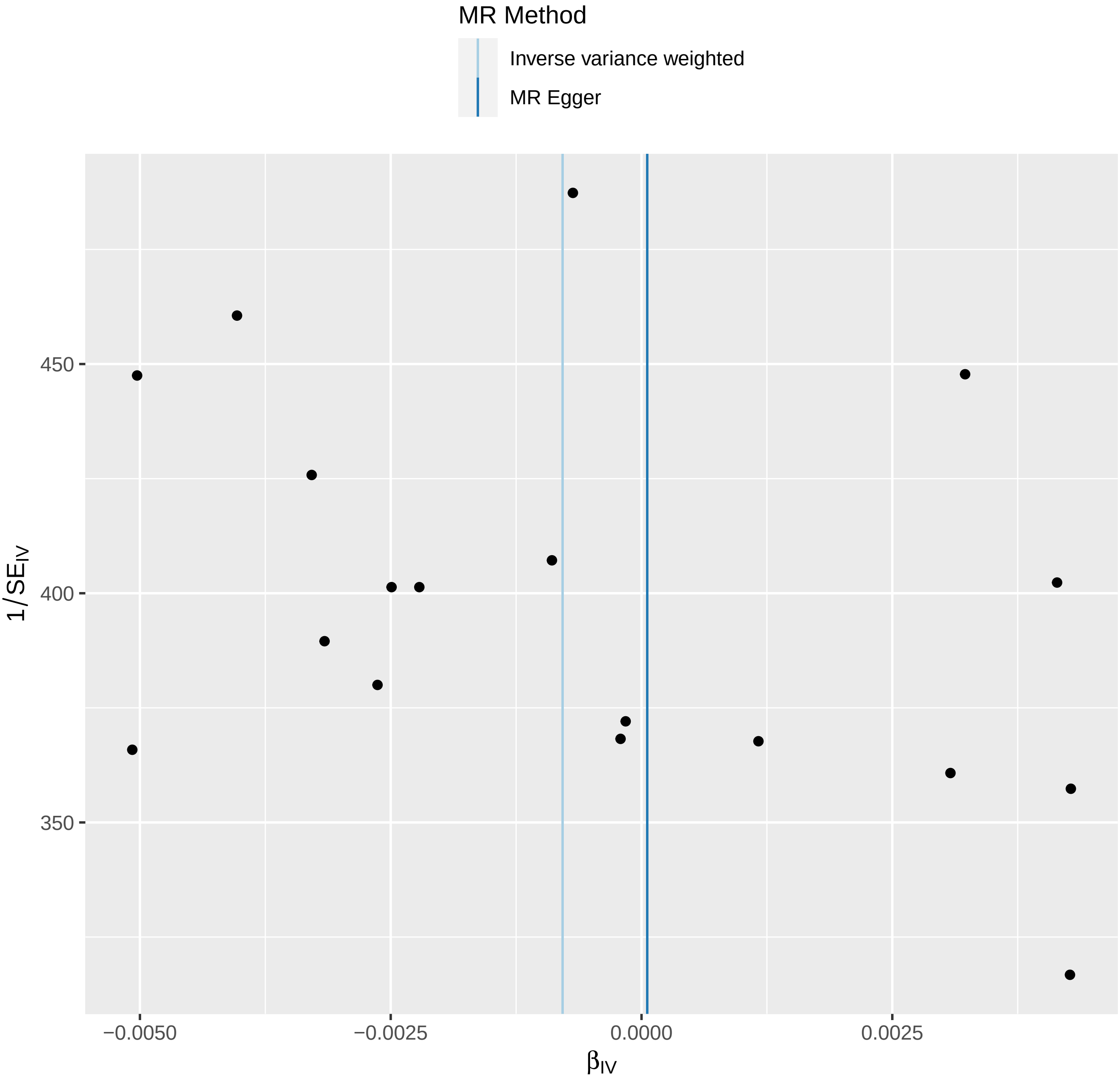

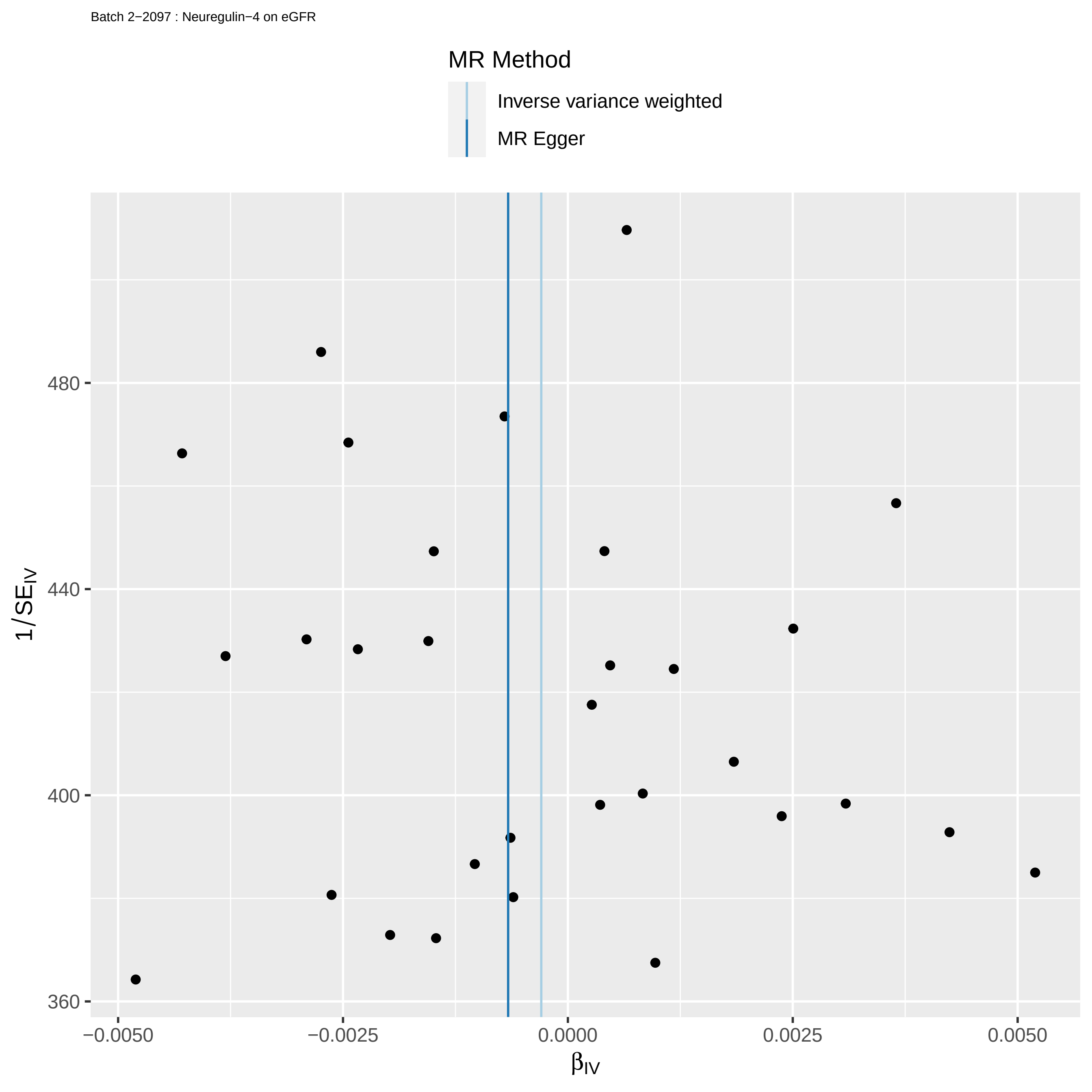

Batch 2−2097 : Neuregulin−4 on eGFR
MR Method
Inverse variance weighted
MR Egger
1/SE_IV
480
440
400
360
−0.0050
−0.0025
0.0000
0.0025
0.0050
β_IV

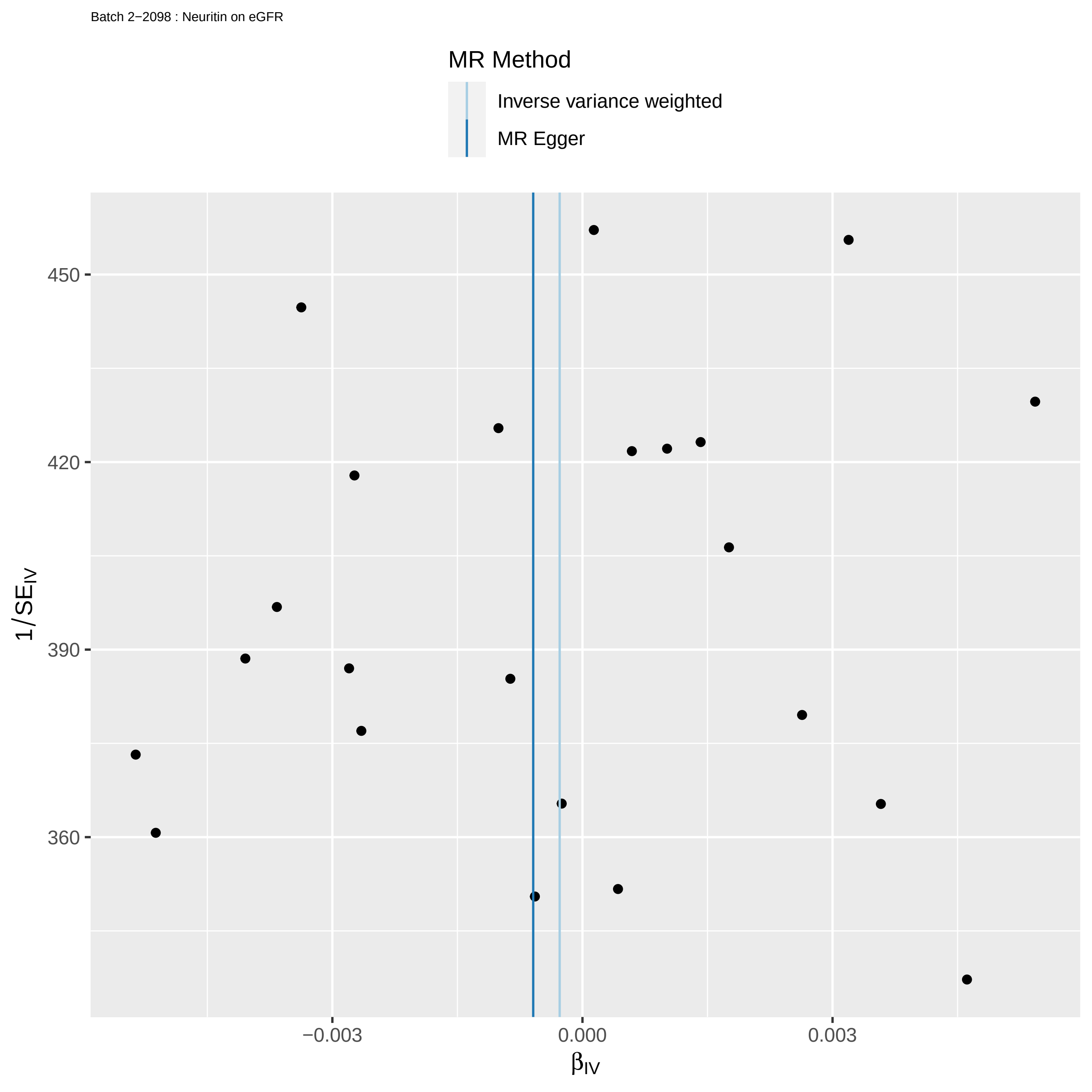
Batch 2−2098 : Neuritin on eGFR
MR Method
Inverse variance weighted
MR Egger
450
420
390
360
$1/SE_{IV}$
−0.003
0.000
0.003
β_{IV}

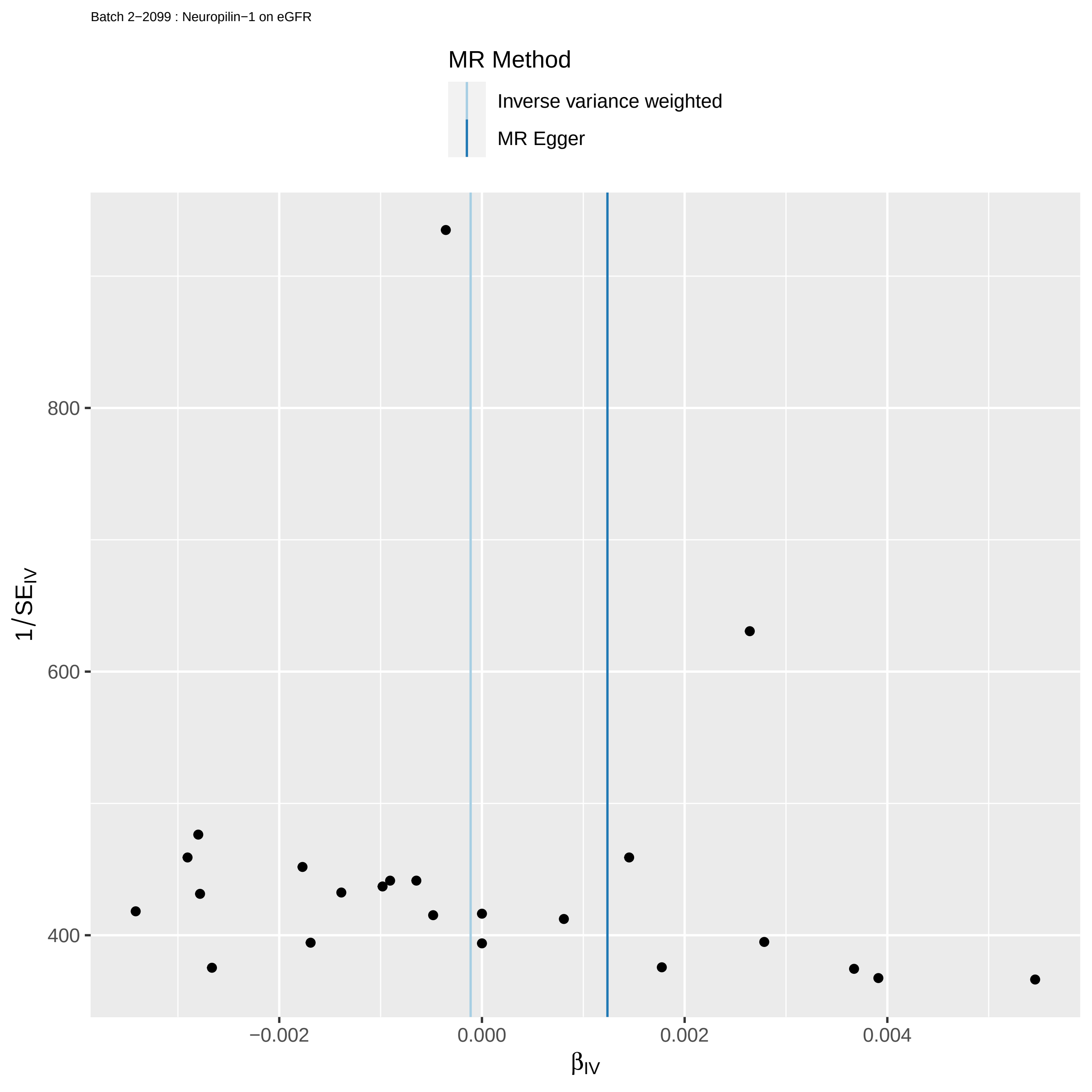
Batch 2−2099 : Neuropilin−1 on eGFR
MR Method
Inverse variance weighted
MR Egger
800
600
400
1/SE$_{IV}$
−0.002
0.000
0.002
0.004
β$_{IV}$

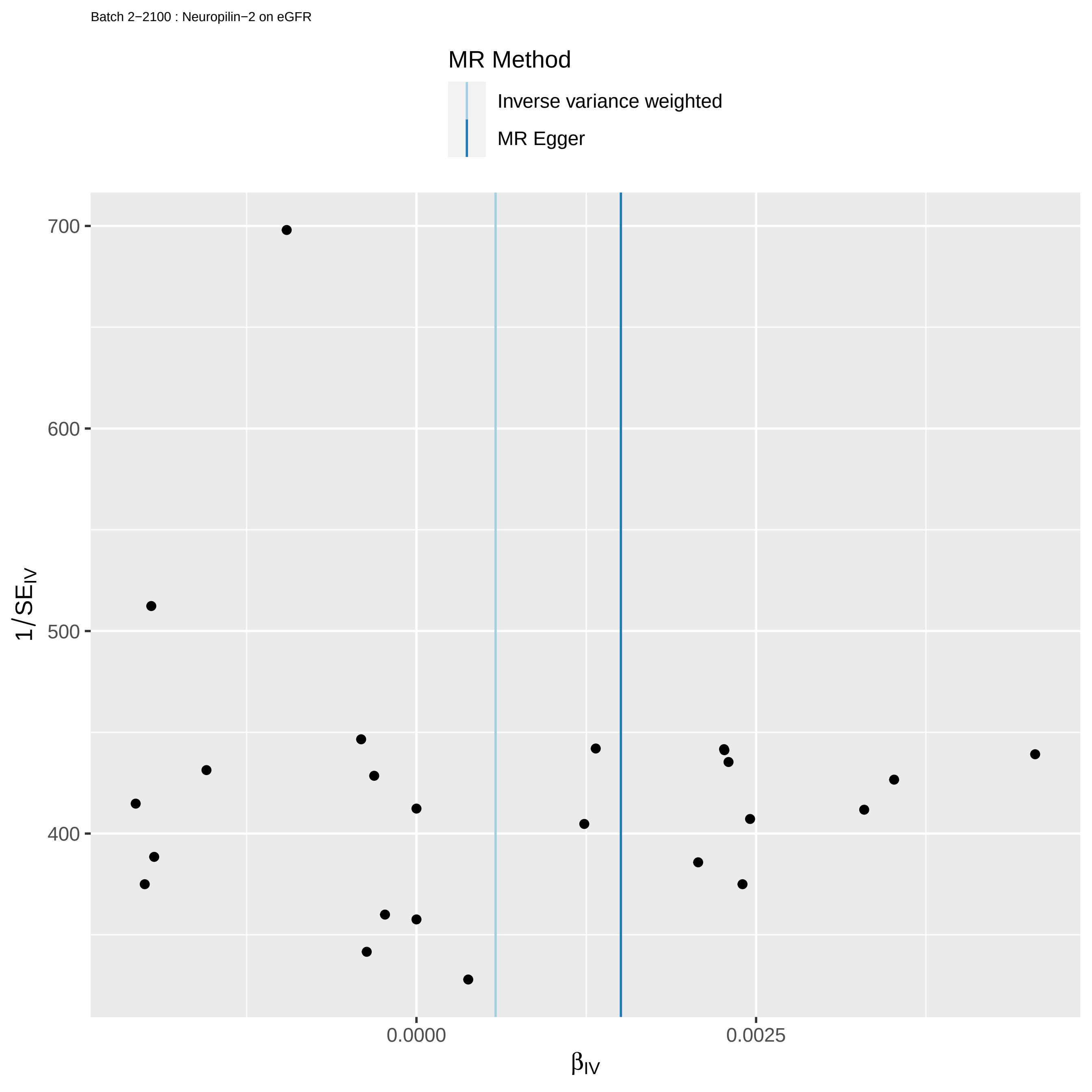

Batch 2−2100 : Neuropilin−2 on eGFR
MR Method
Inverse variance weighted
MR Egger
700
600
500
400
1/SE_IV
0.0000
0.0025
β_IV

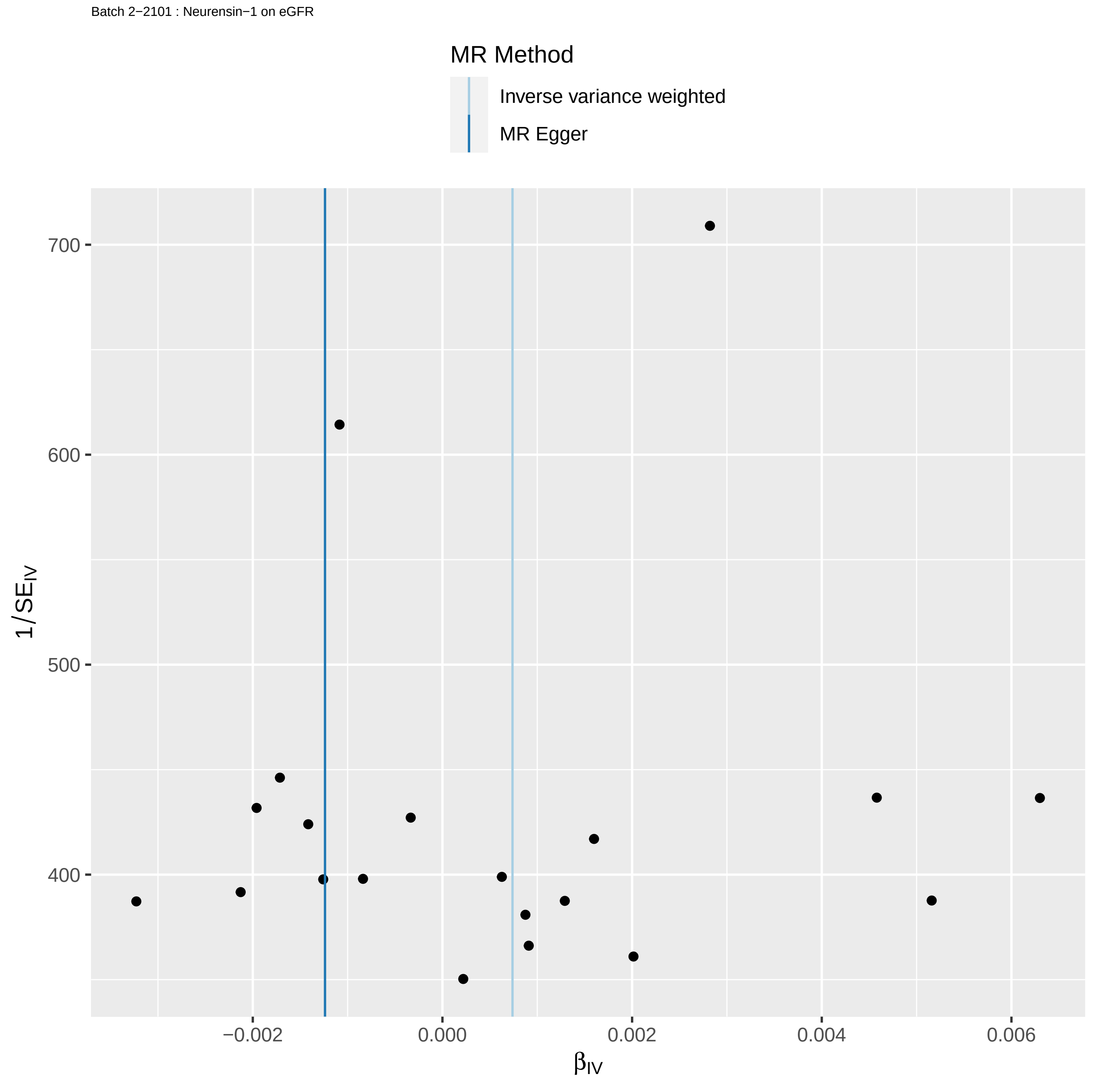

Batch 2−2101 : Neurensin−1 on eGFR
MR Method
Inverse variance weighted
MR Egger
700
600
500
400
1/SE_IV
−0.002
0.000
0.002
0.004
0.006
β_IV

Batch 2−2102 : Neurexin−1−beta on eGFR

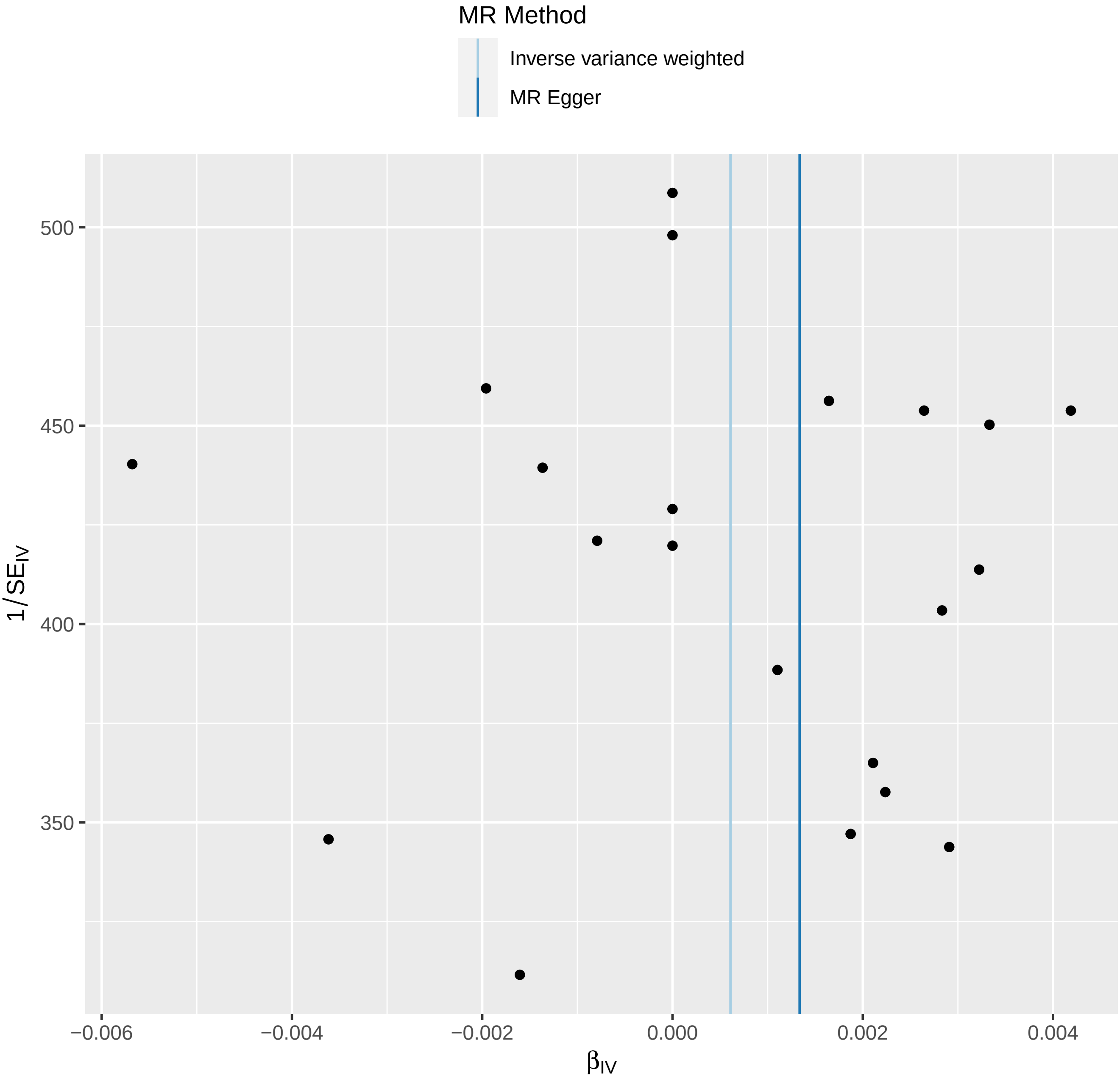

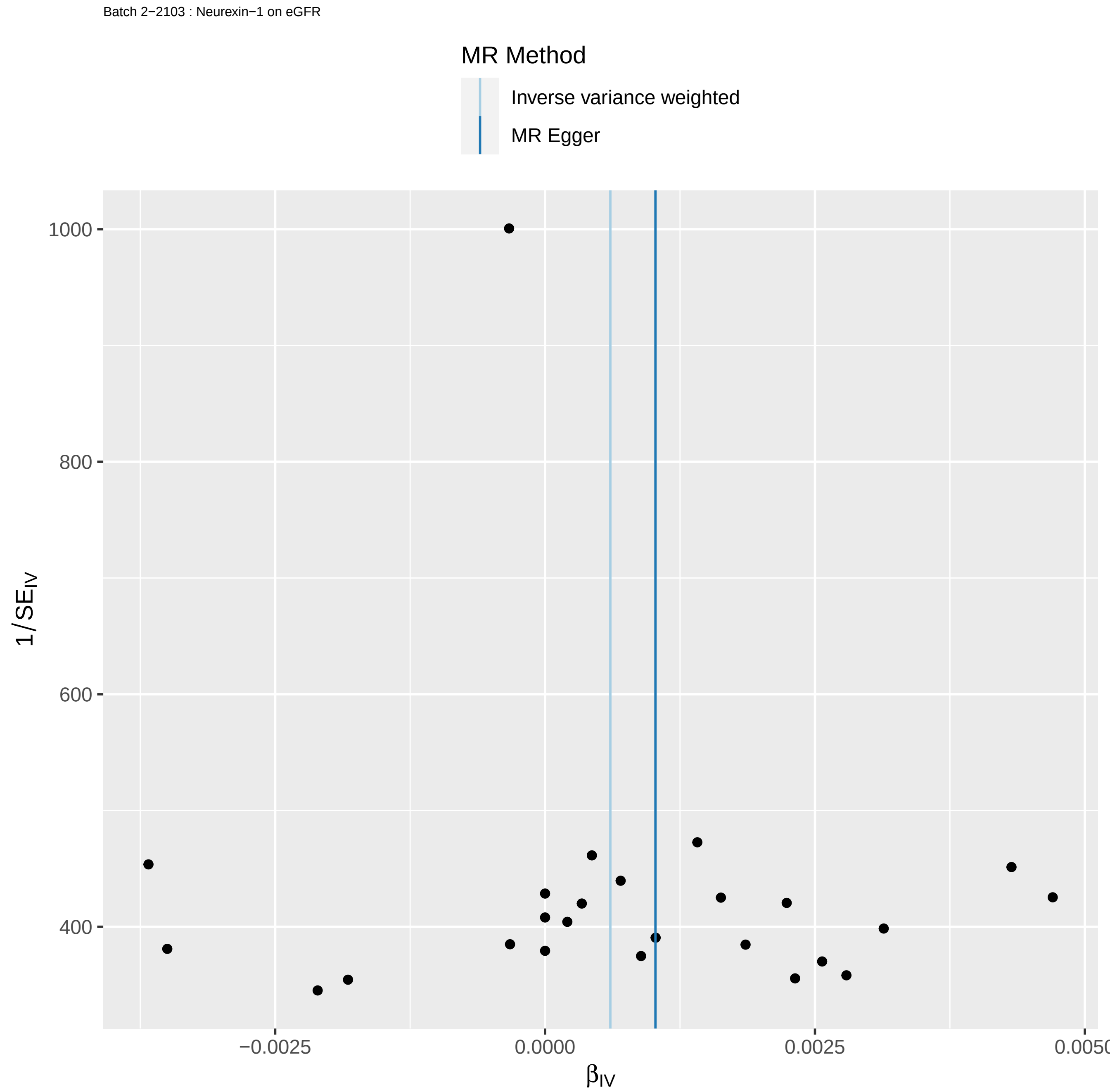

Batch 2−2103 : Neurexin−1 on eGFR
MR Method
Inverse variance weighted
MR Egger
1000
800
600
400
1/SE_IV
−0.0025
0.0000
0.0025
0.0050
β_IV

Batch 2−2104 : Neurexin−2−beta on eGFR

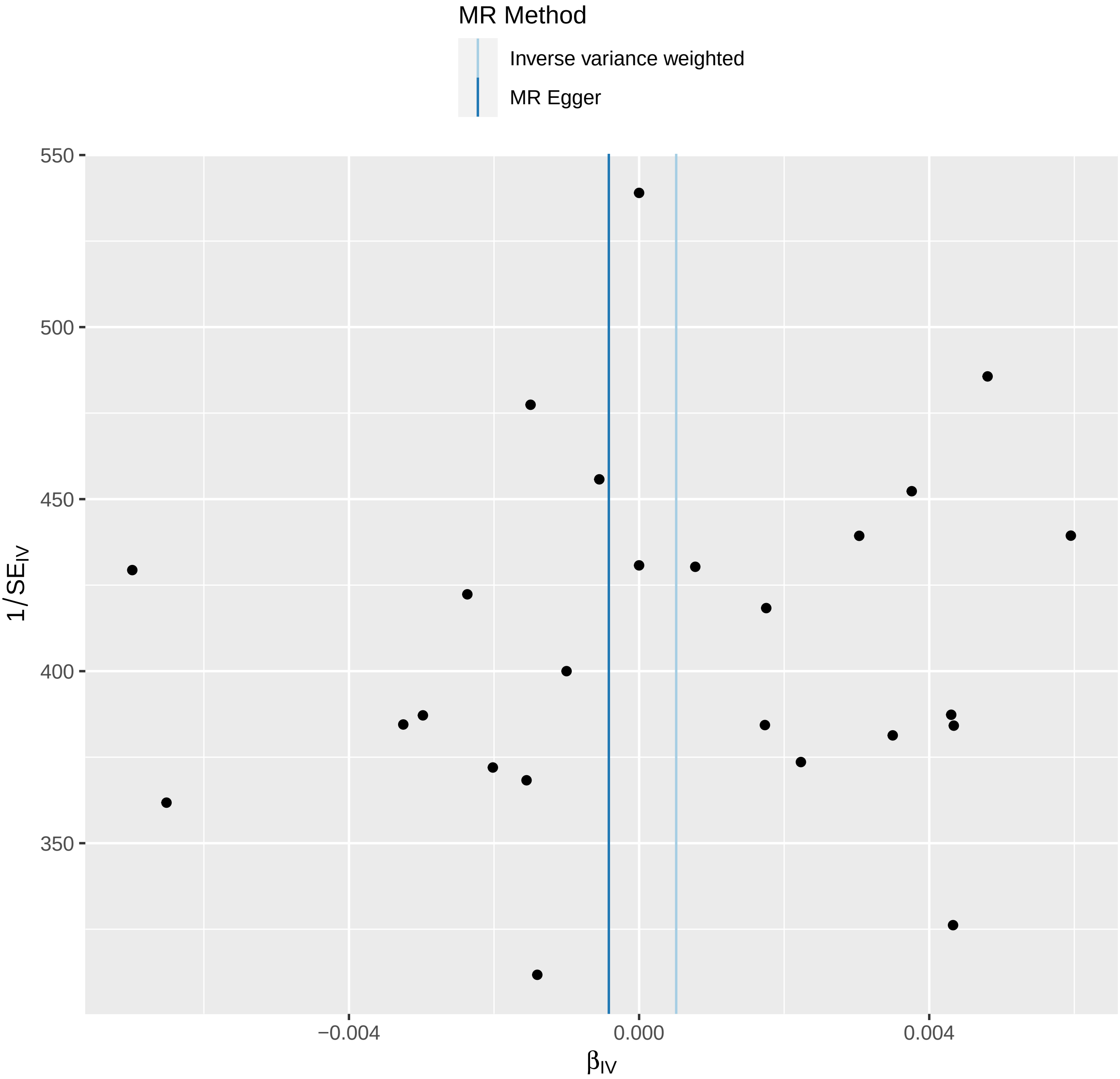

Batch 2−2105 : Neurexin−3−beta on eGFR

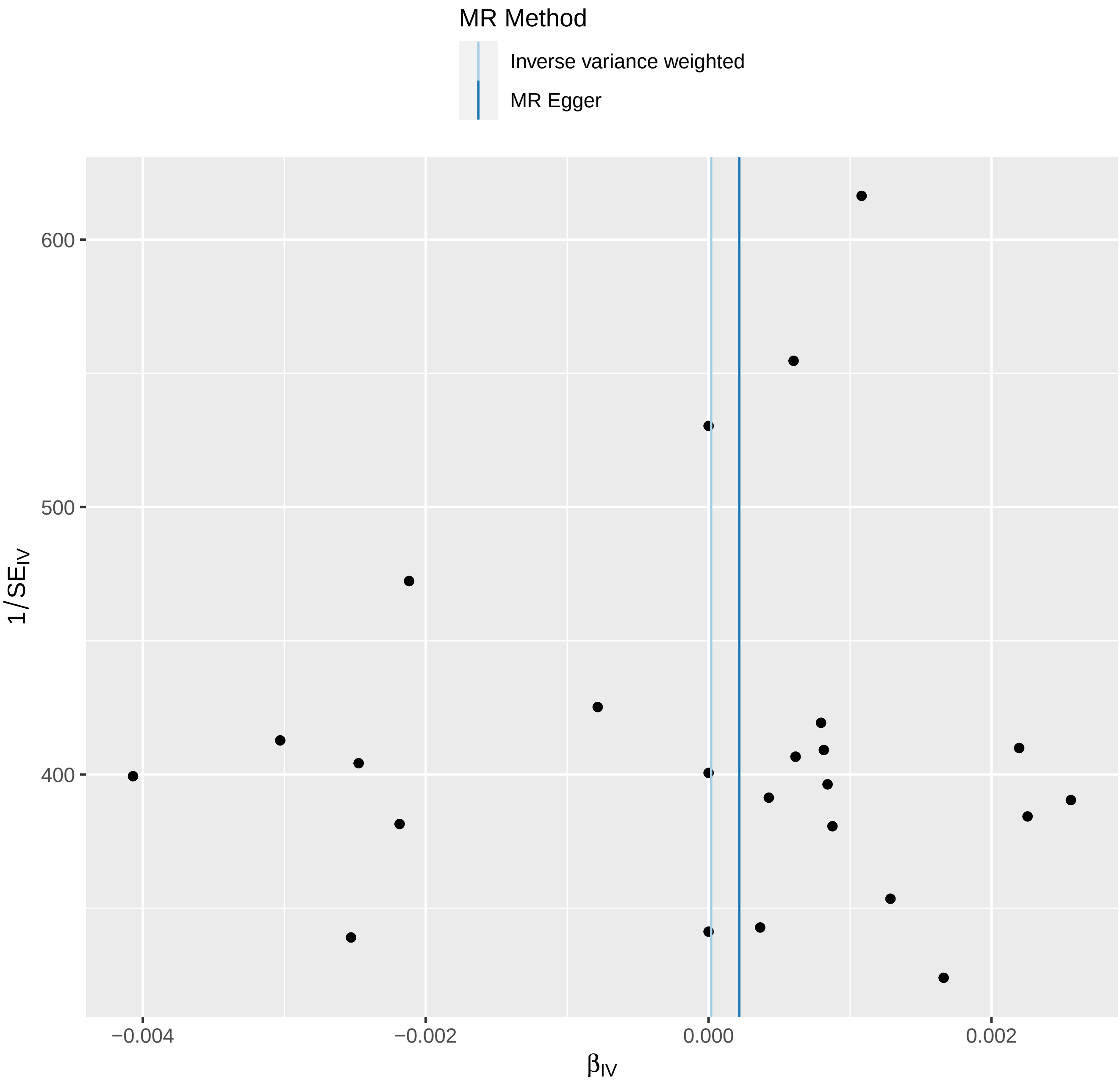

Batch 2−2106 : Neurexin−3−beta on eGFR

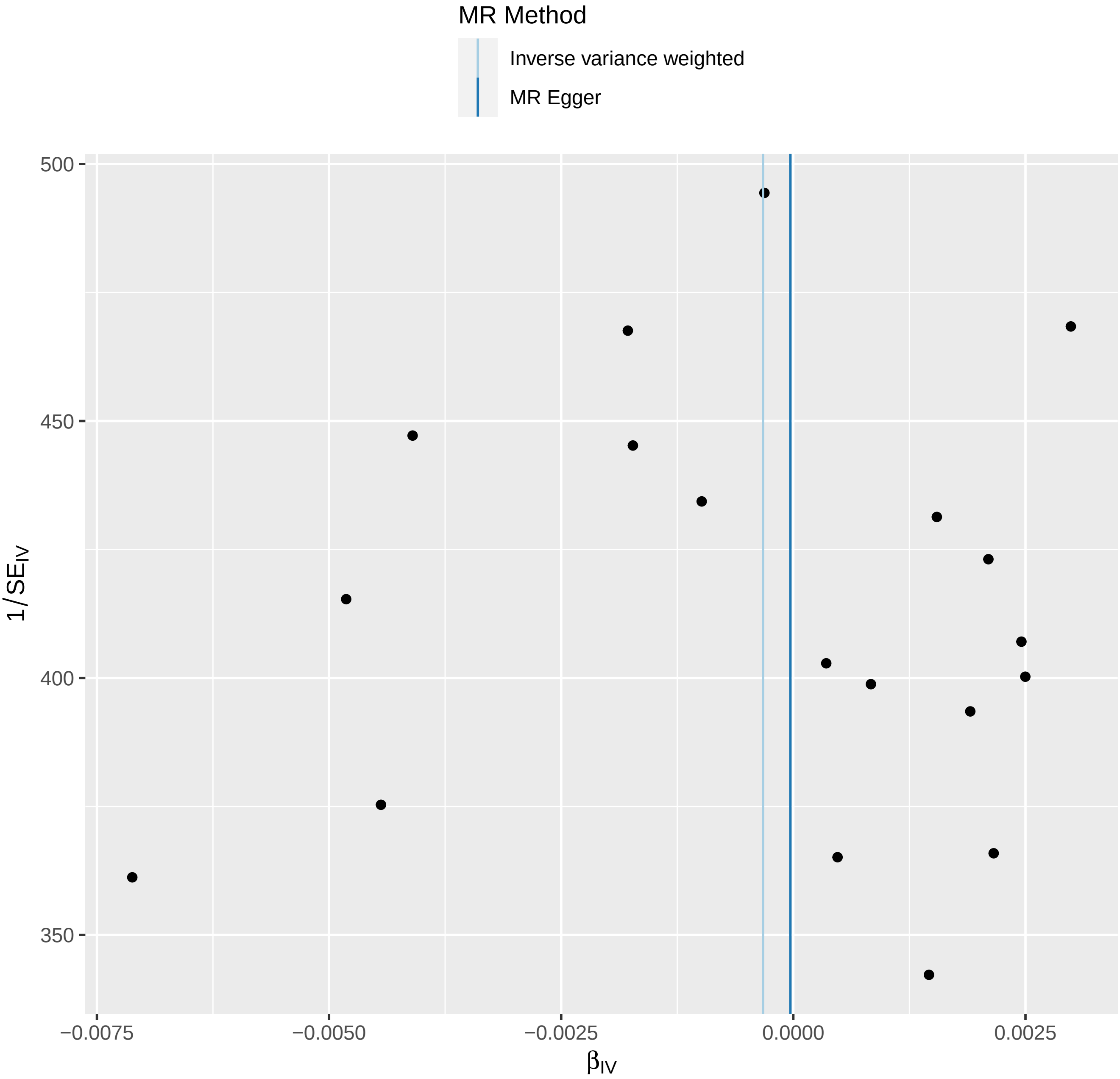

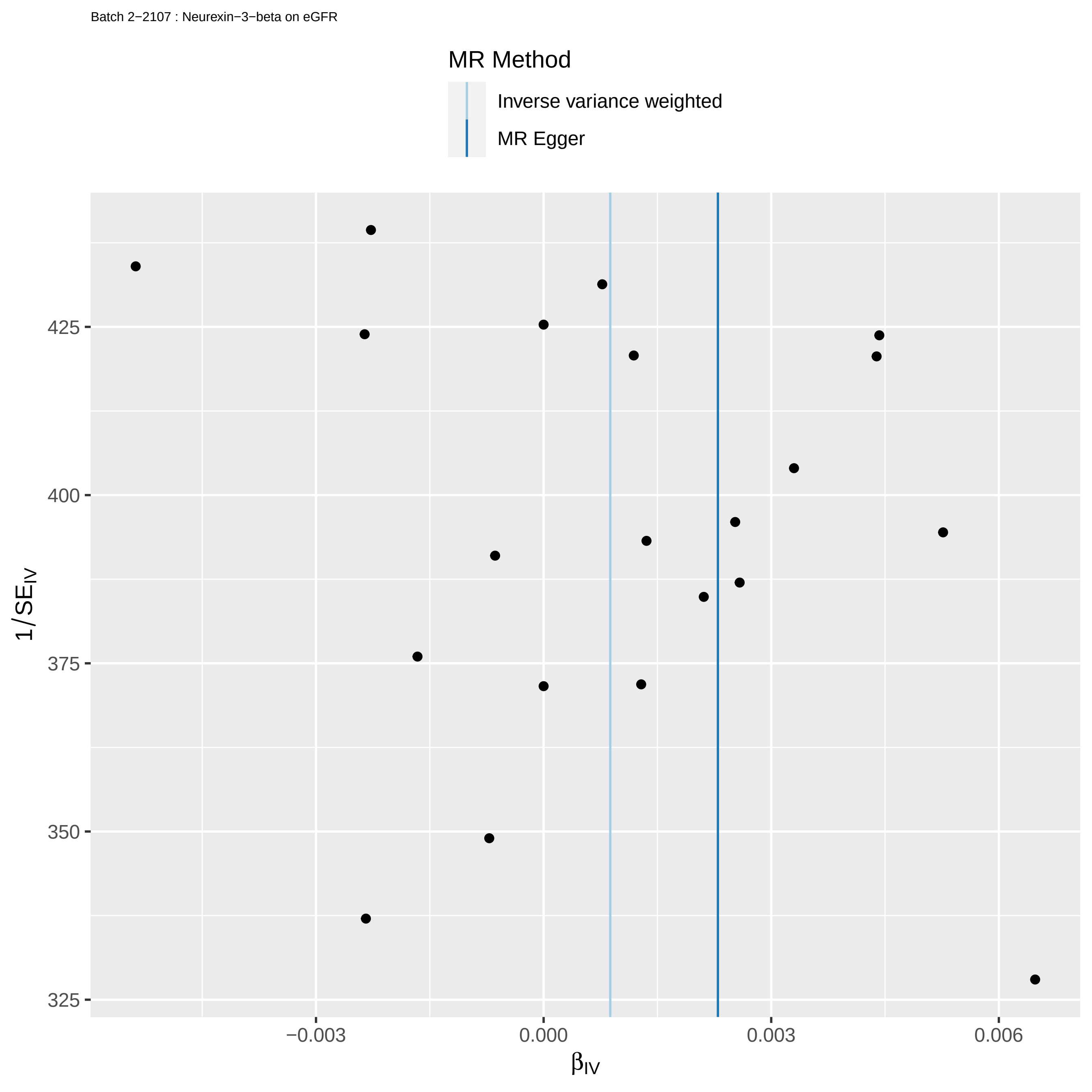

Batch 2−2107 : Neurexin−3−beta on eGFR
MR Method
Inverse variance weighted
MR Egger
425
400
375
350
325
1/SE$_{IV}$
−0.003
0.000
0.003
0.006
β$_{IV}$

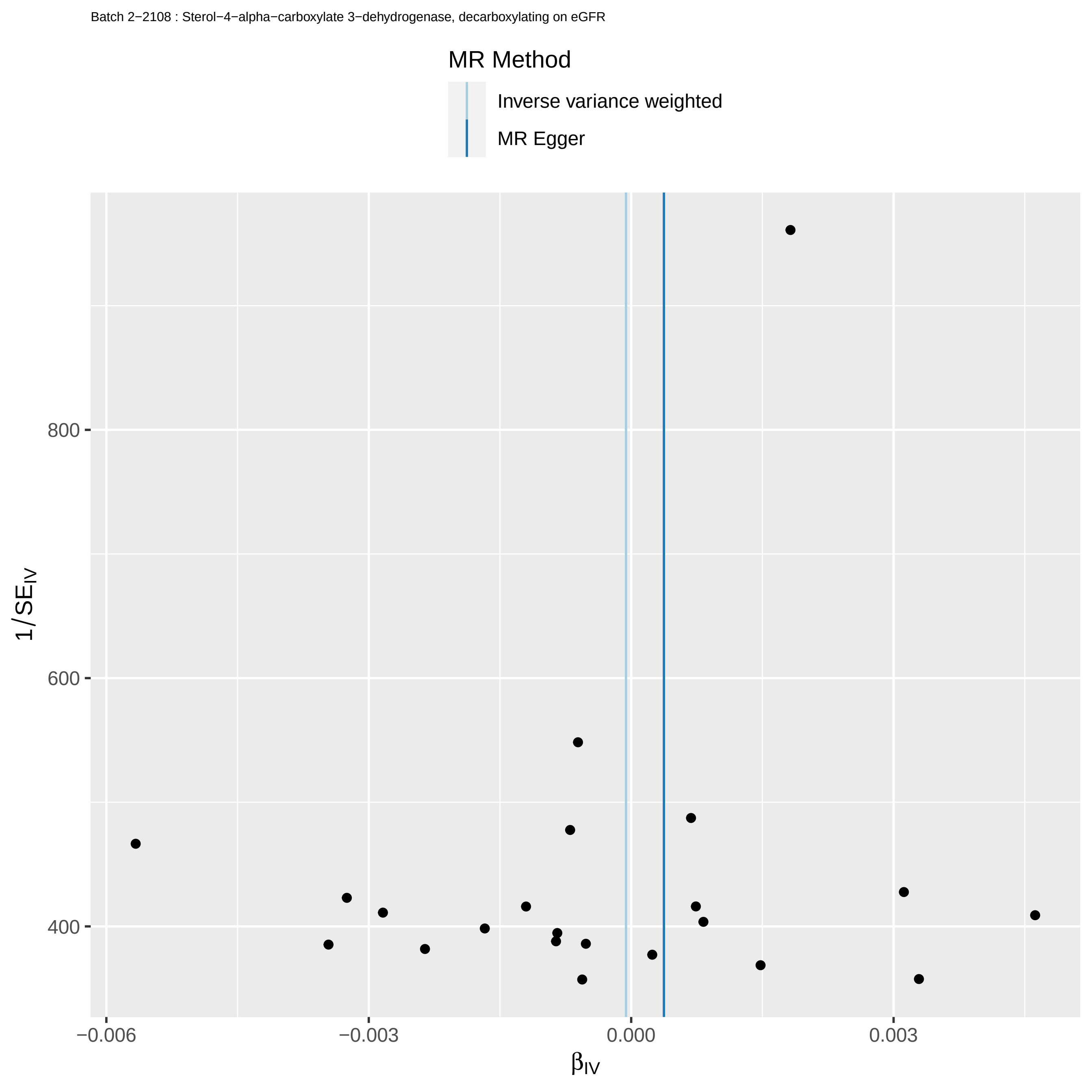
Batch 2−2108 : Sterol−4−alpha−carboxylate 3−dehydrogenase, decarboxylating on eGFR
MR Method
Inverse variance weighted
MR Egger
800
600
400
1/SE$_{IV}$
−0.006
−0.003
0.000
0.003
β_{IV}

Batch 2−2109 : Vesicle−fusing ATPase on eGFR

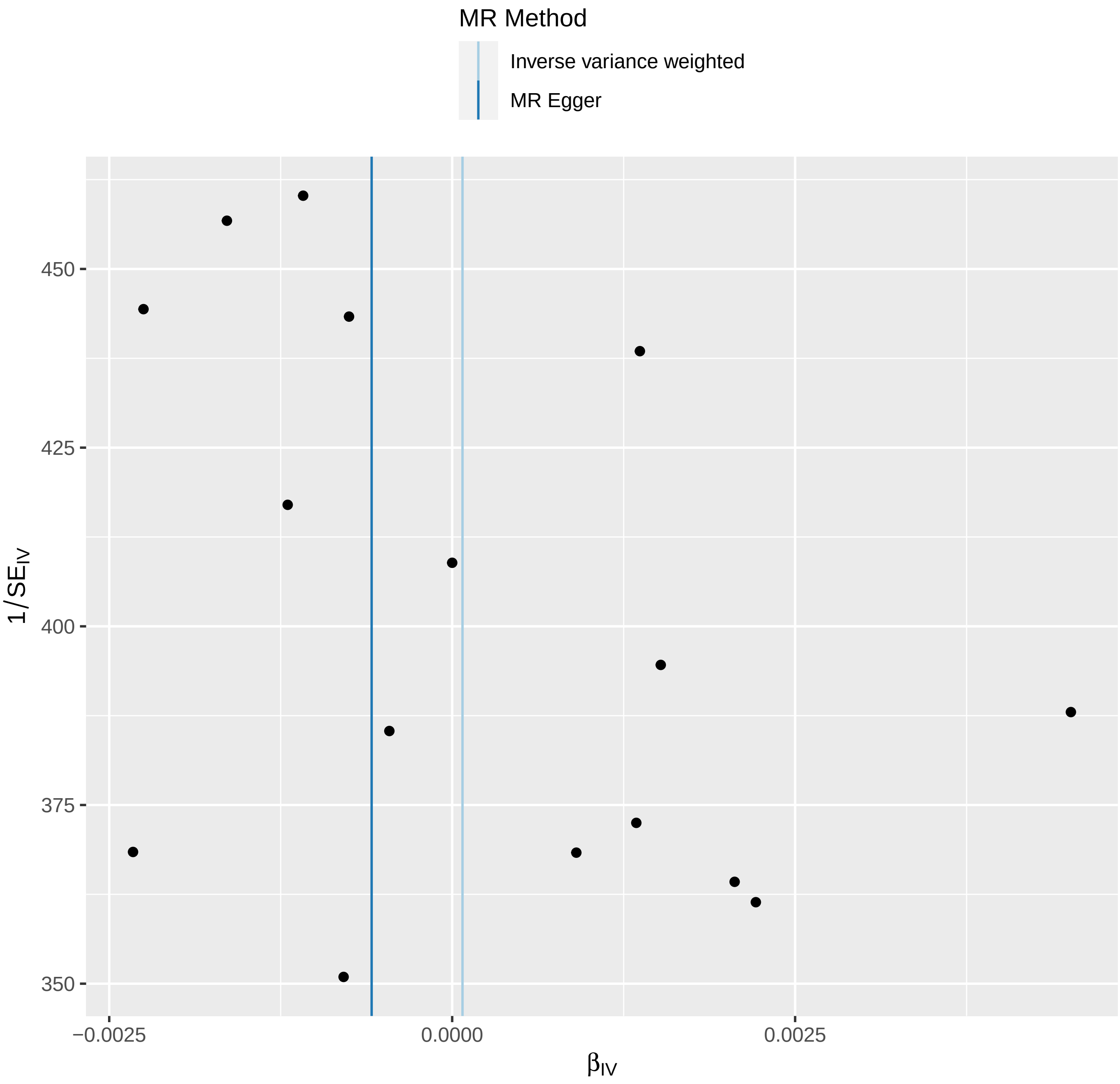

Batch 2−2110 : Neuron−specific protein family member 1 on eGFR

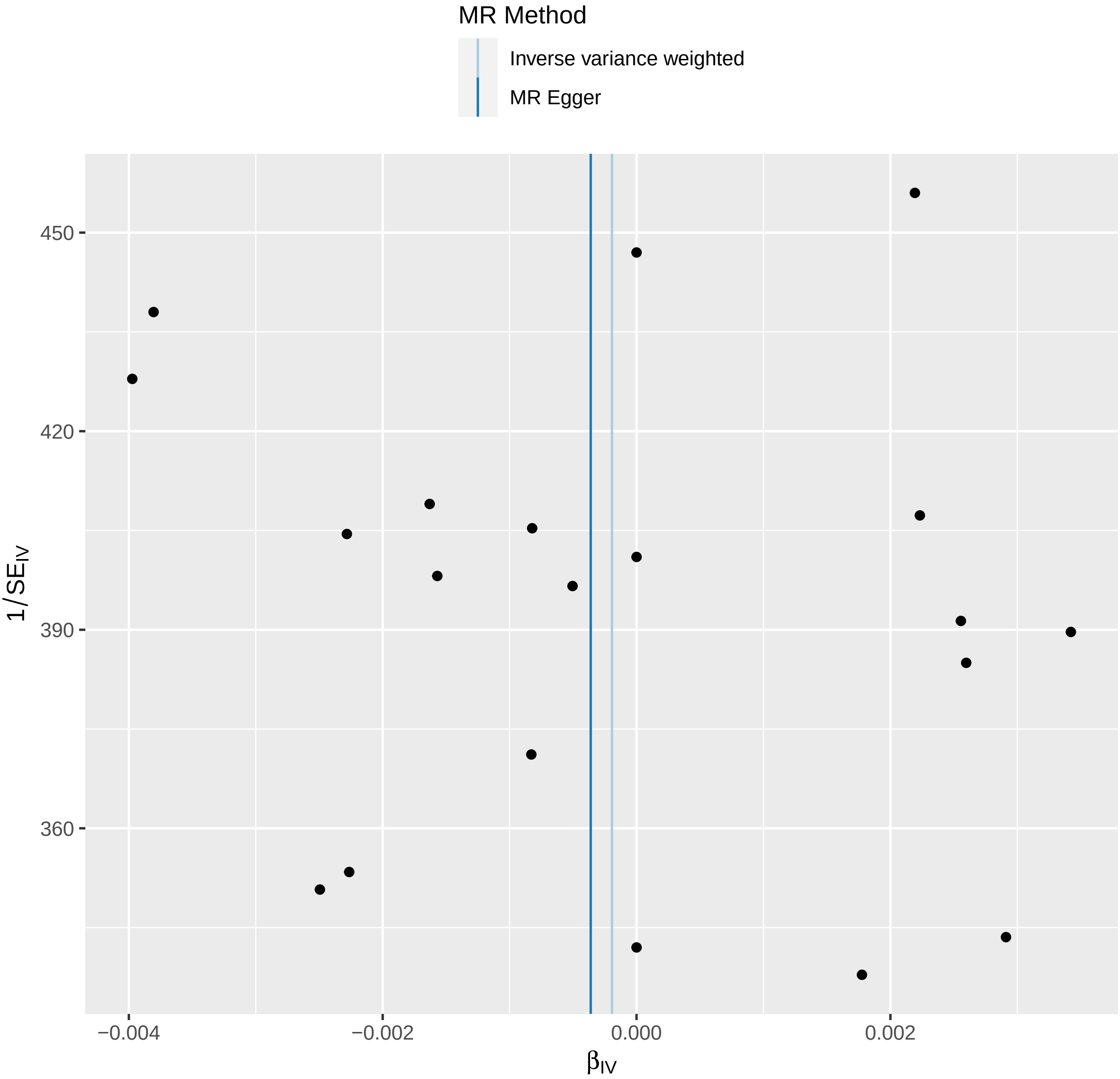

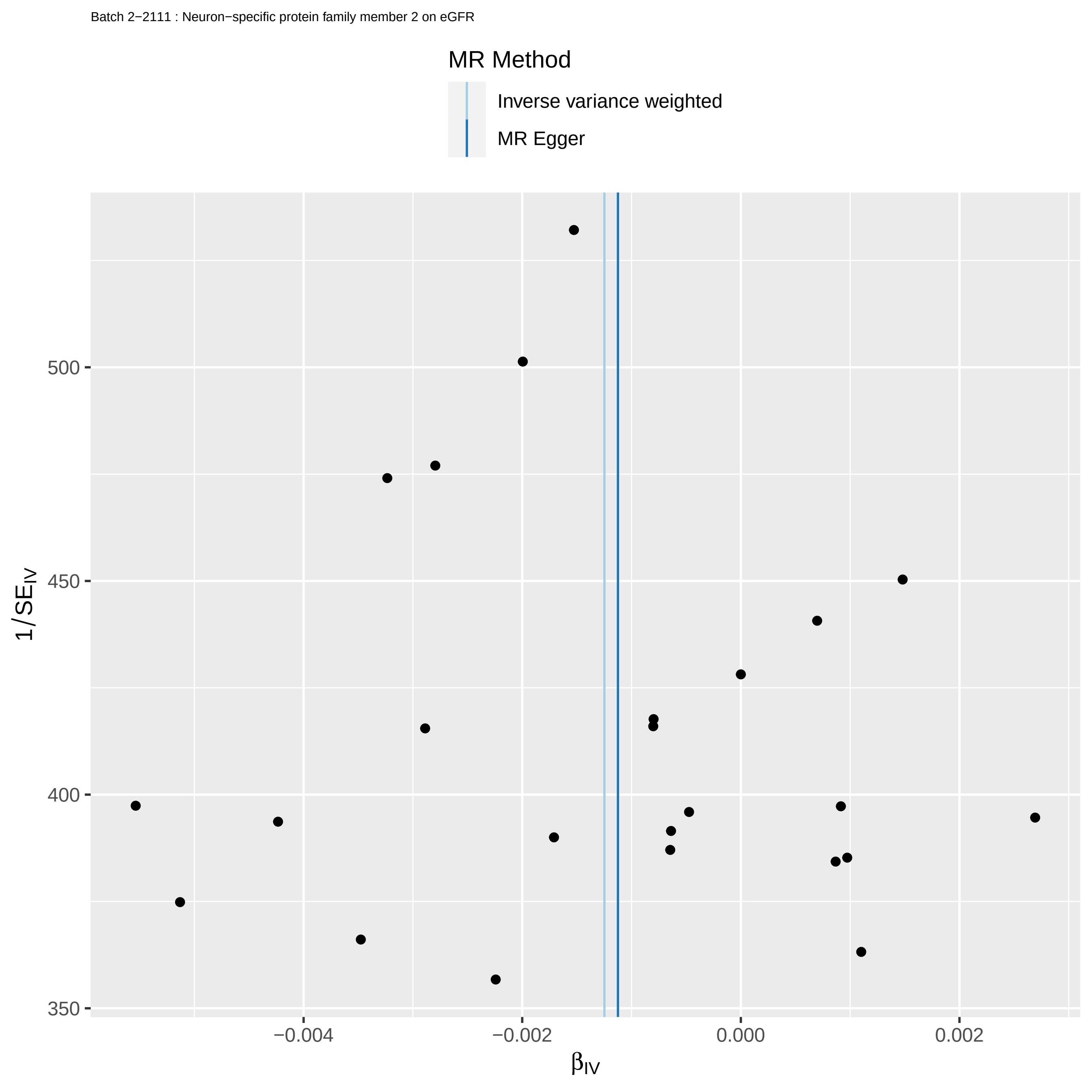
Batch 2−2111 : Neuron−specific protein family member 2 on eGFR
MR Method
Inverse variance weighted
MR Egger
500
450
400
350
1/SE_IV
−0.004
−0.002
0.000
0.002
β_IV

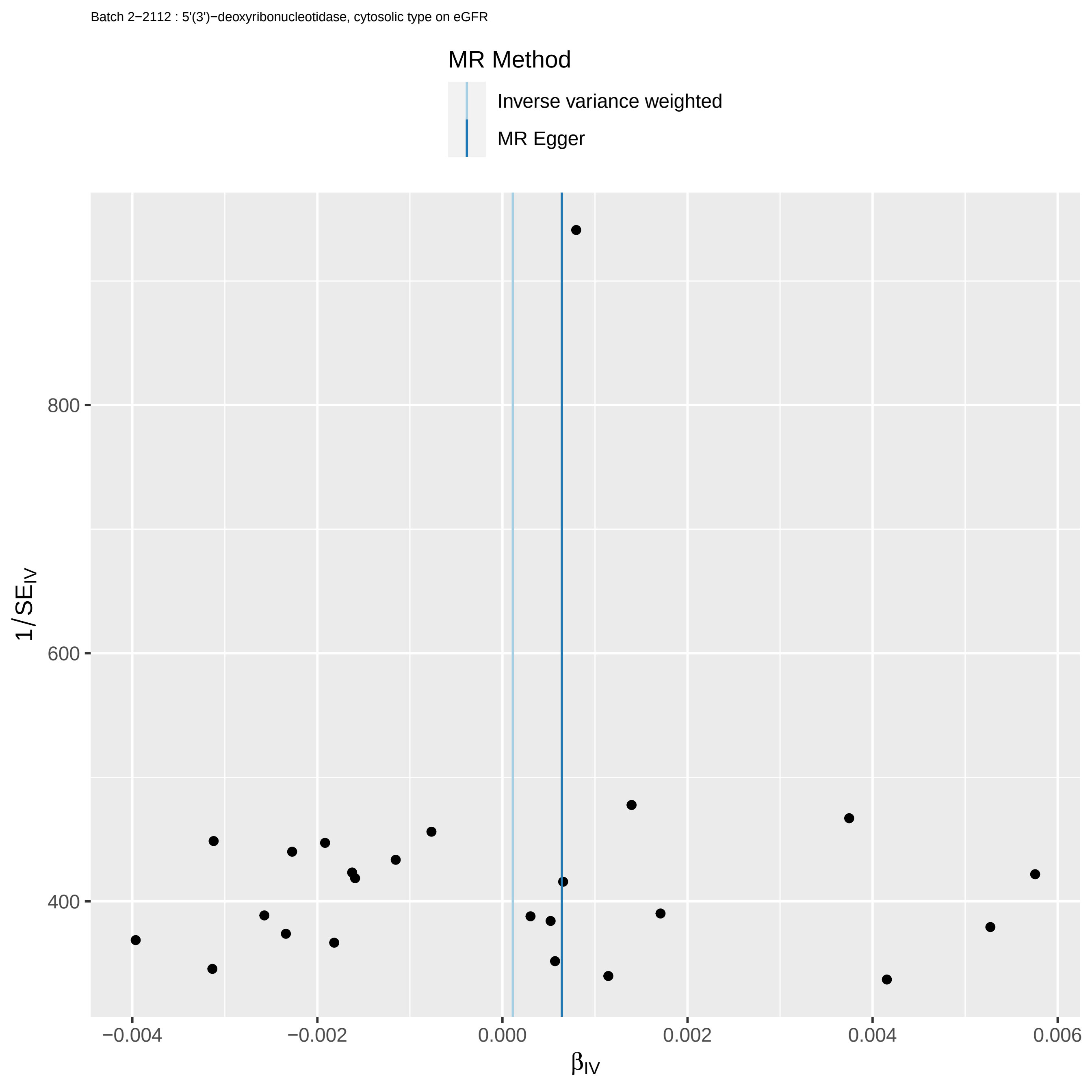

Batch 2−2112 : 5'(3')−deoxyribonucleotidase, cytosolic type on eGFR
MR Method
Inverse variance weighted
MR Egger
800
600
400
1/SE_IV
−0.004
−0.002
0.000
0.002
0.004
0.006
β_IV

Batch 2−2113 : 5'−Nucleotidase on eGFR

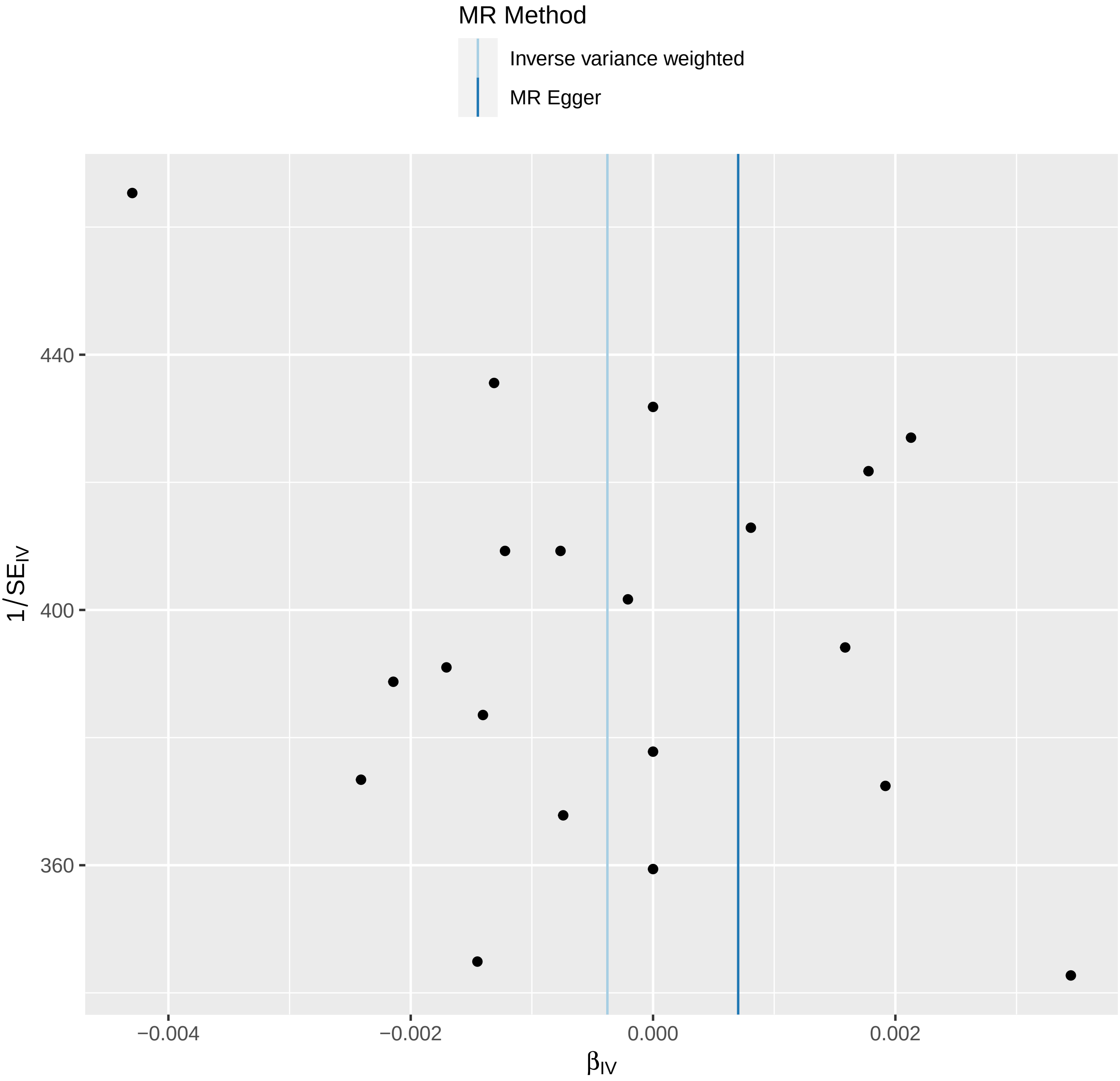

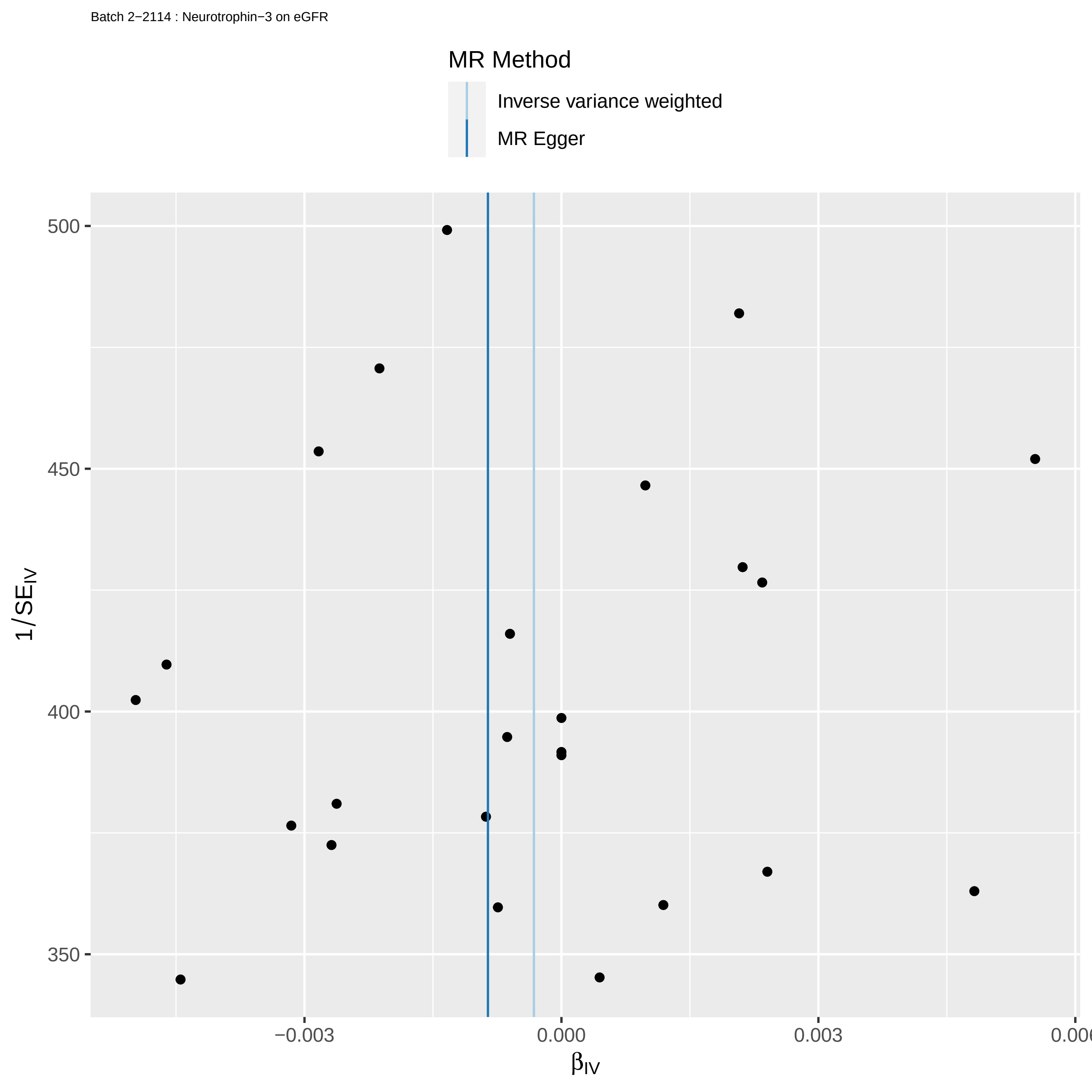
Batch 2−2114 : Neurotrophin−3 on eGFR
MR Method
Inverse variance weighted
MR Egger
500
450
400
350
1/SE$_{IV}$
−0.003
0.000
0.003
0.006
β$_{IV}$

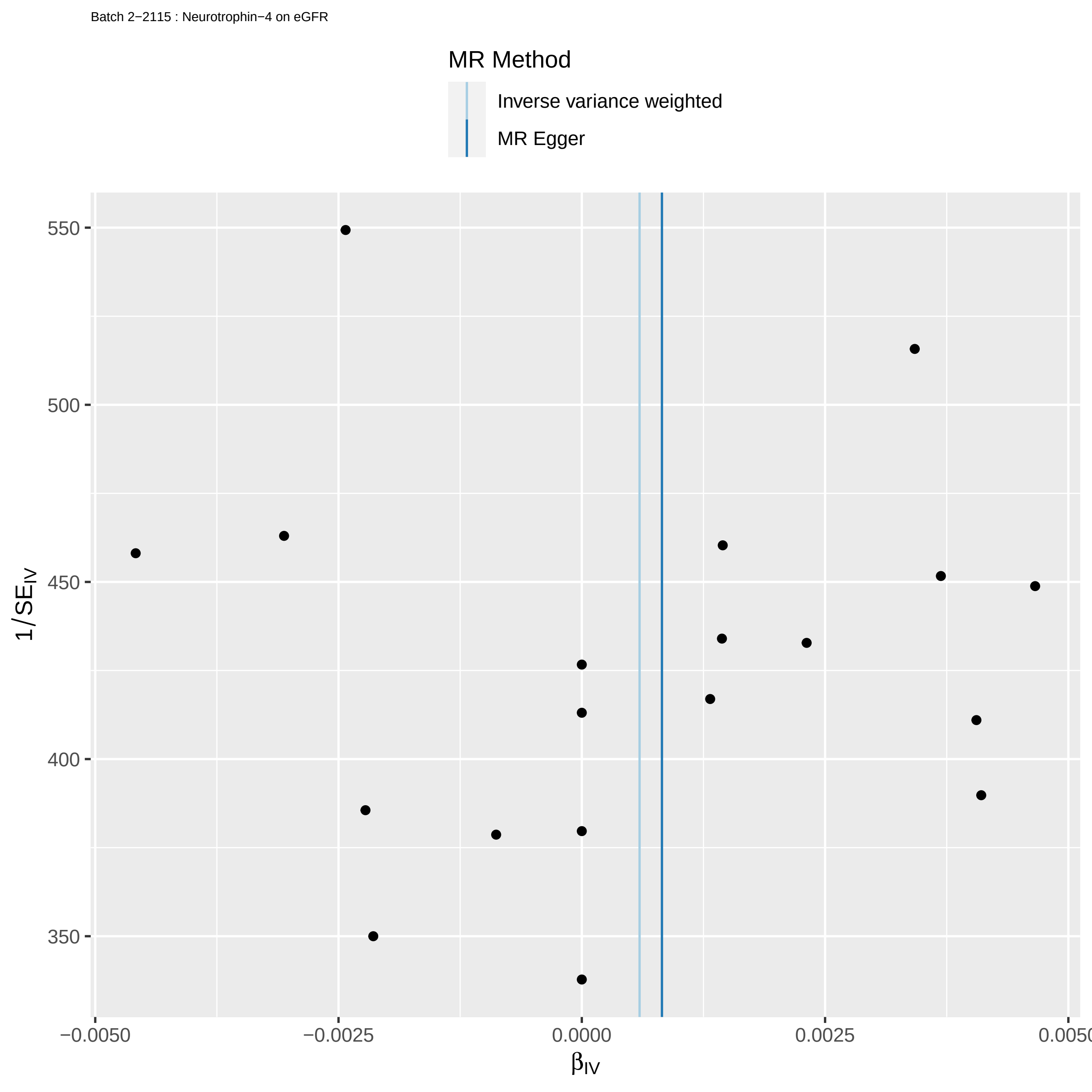

Batch 2−2115 : Neurotrophin−4 on eGFR
MR Method
Inverse variance weighted
MR Egger
550
500
450
400
350
1/SE_IV
−0.0050
−0.0025
0.0000
0.0025
0.0050
β_IV

Batch 2−2116 : N−terminal Xaa−Pro−Lys N−methyltransferase 1 on eGFR

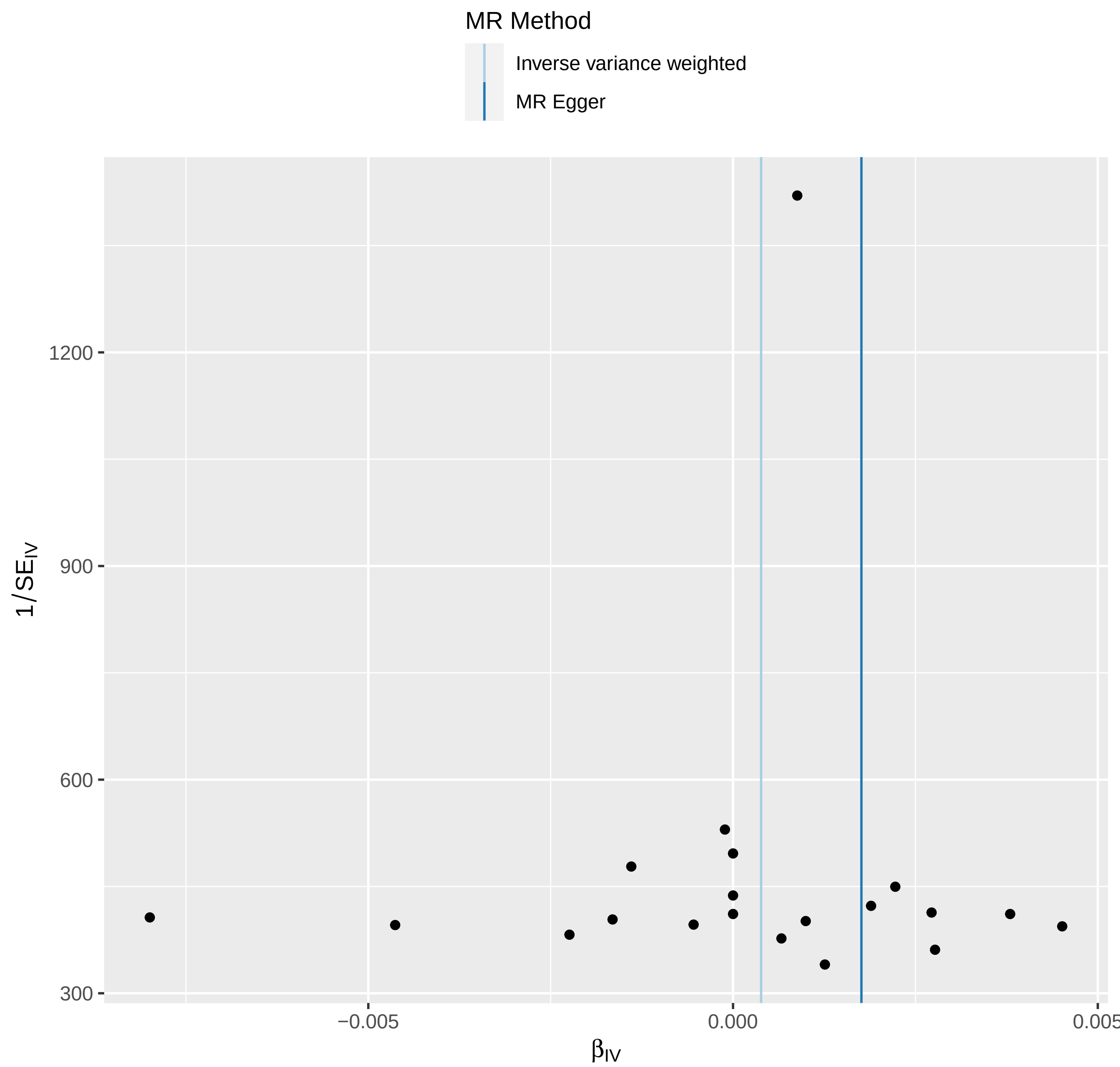

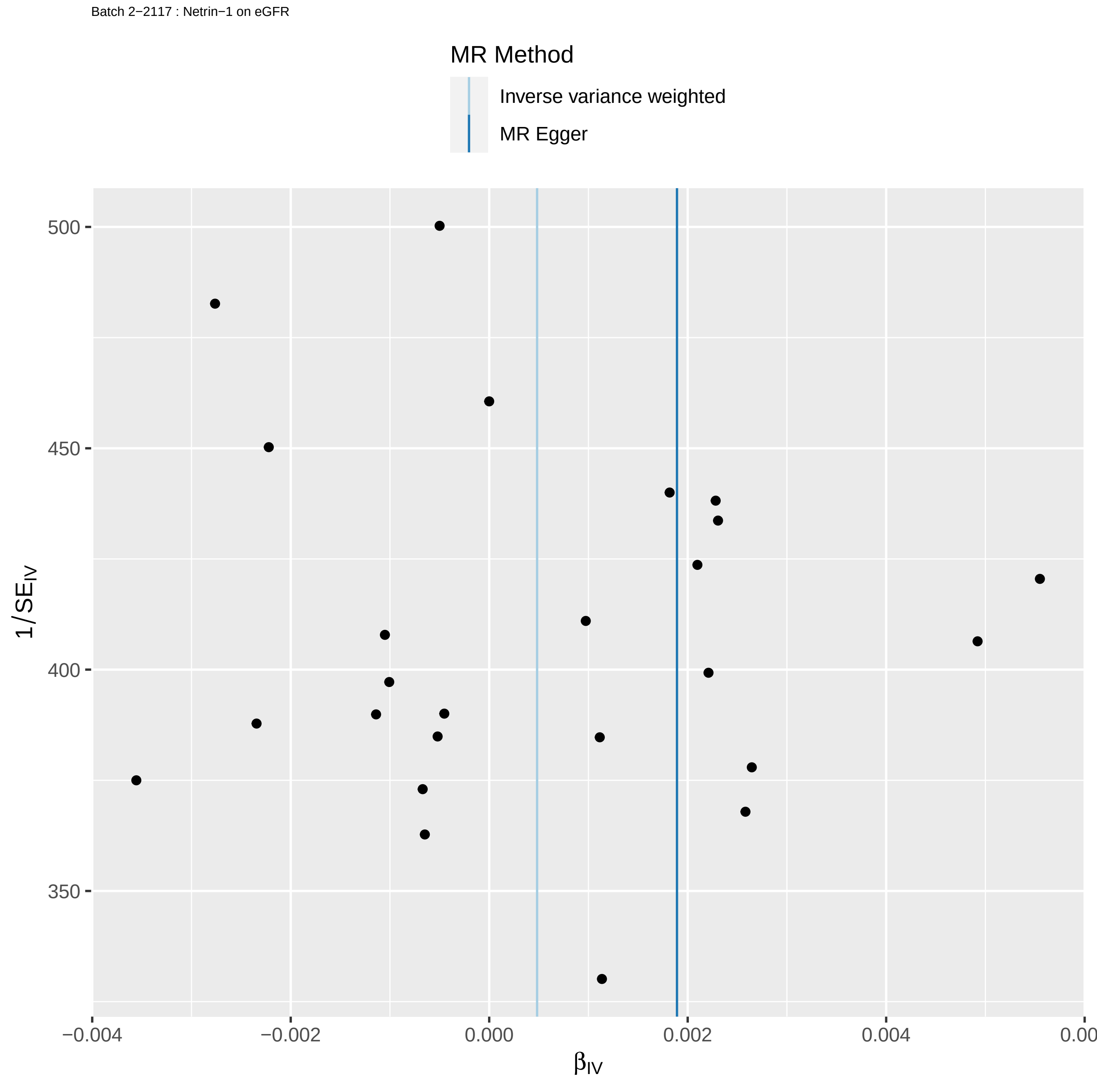
Batch 2−2117 : Netrin−1 on eGFR
MR Method
Inverse variance weighted
MR Egger
500
450
400
350
1/SE$_{IV}$
−0.004
−0.002
0.000
0.002
0.004
β$_{IV}$

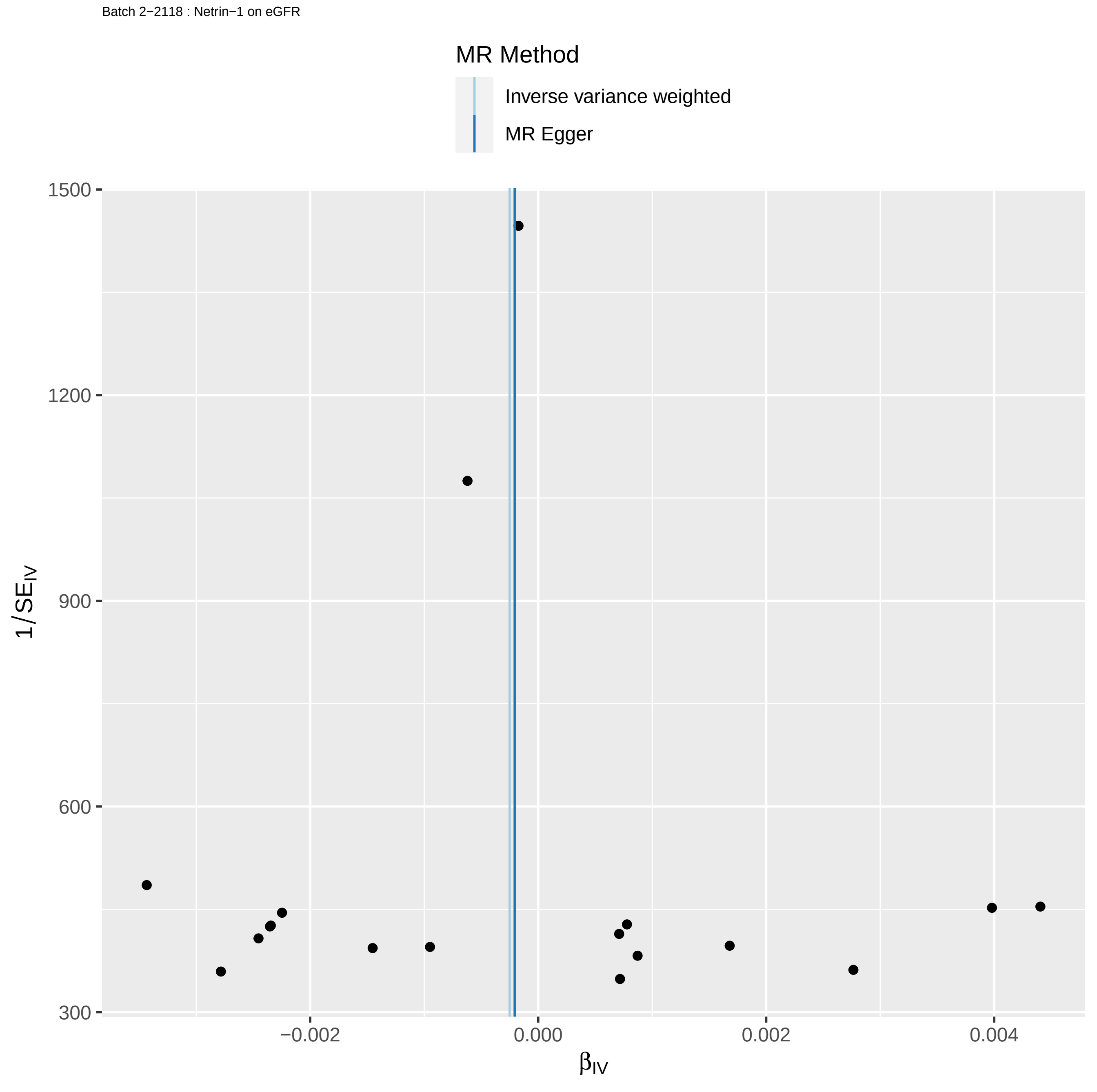

Batch 2−2118 : Netrin−1 on eGFR
MR Method
Inverse variance weighted
MR Egger
1500
1200
900
600
300
1/SE_IV
−0.002
0.000
0.002
0.004
β_IV

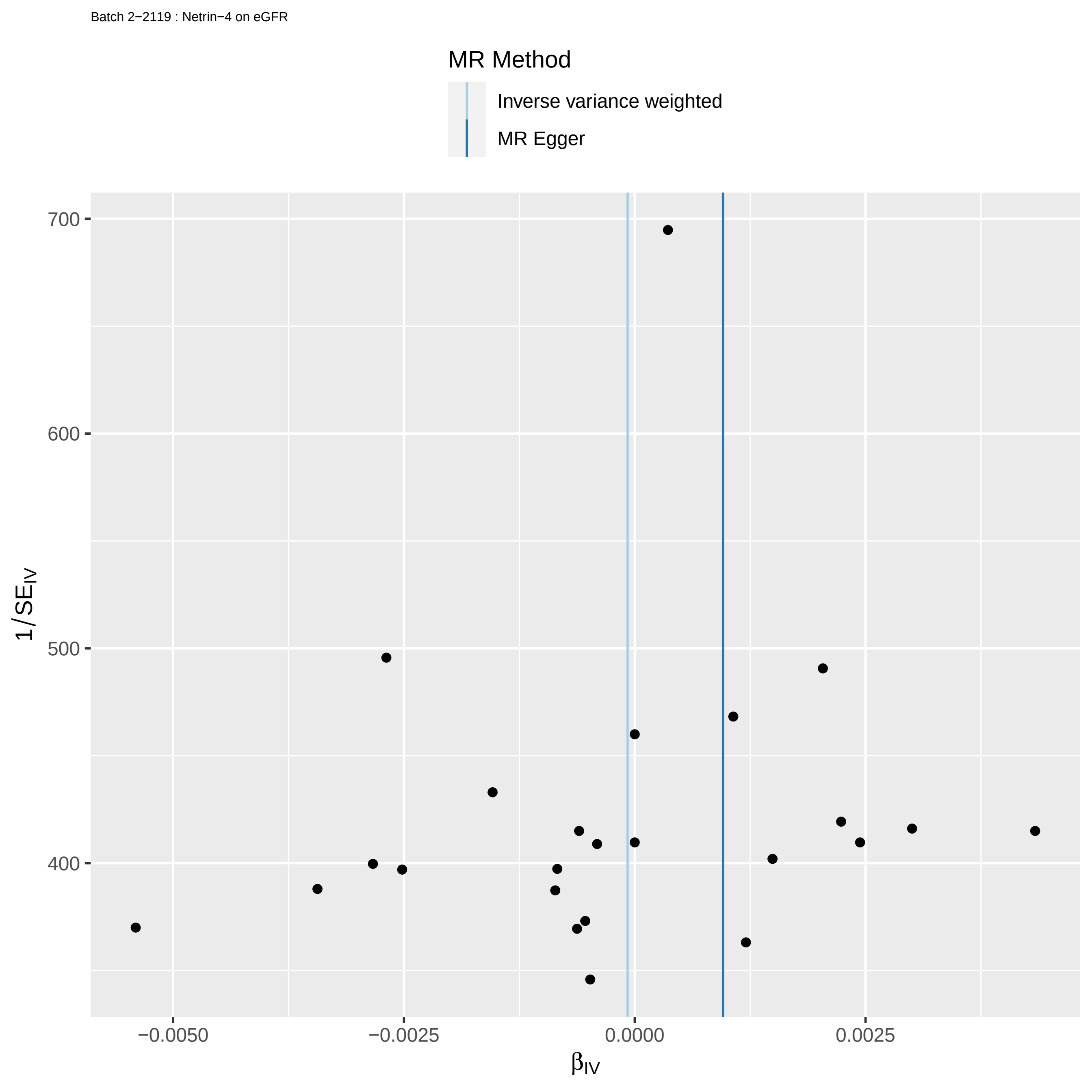
Batch 2−2119 : Netrin−4 on eGFR
MR Method
Inverse variance weighted
MR Egger
700
600
500
400
1/SE$_{IV}$
−0.0050
−0.0025
0.0000
0.0025
β$_{IV}$

Batch 2−2120 : Netrin−G1 on eGFR

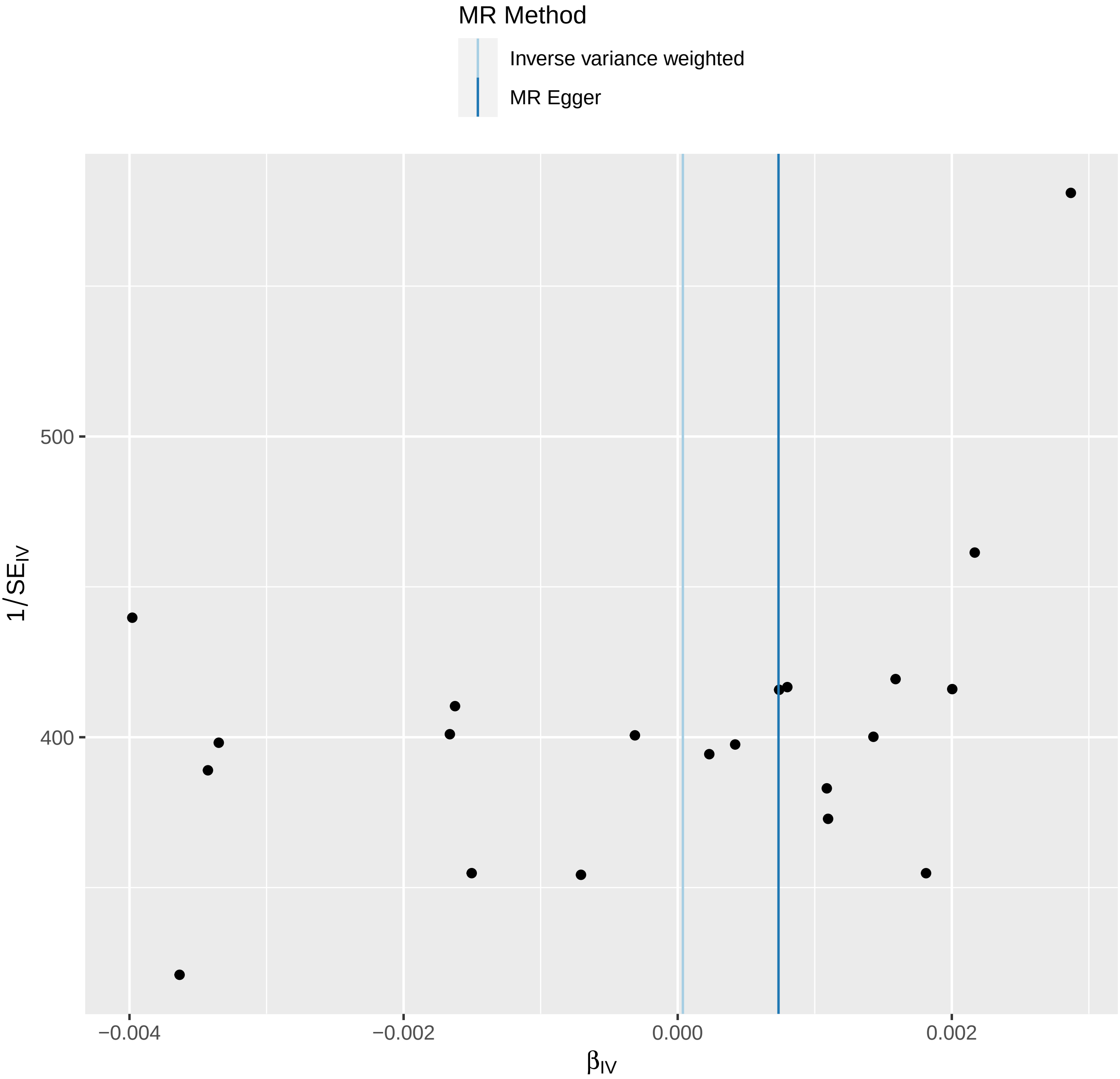

Batch 2−2121 : High affinity nerve growth factor receptor on eGFR

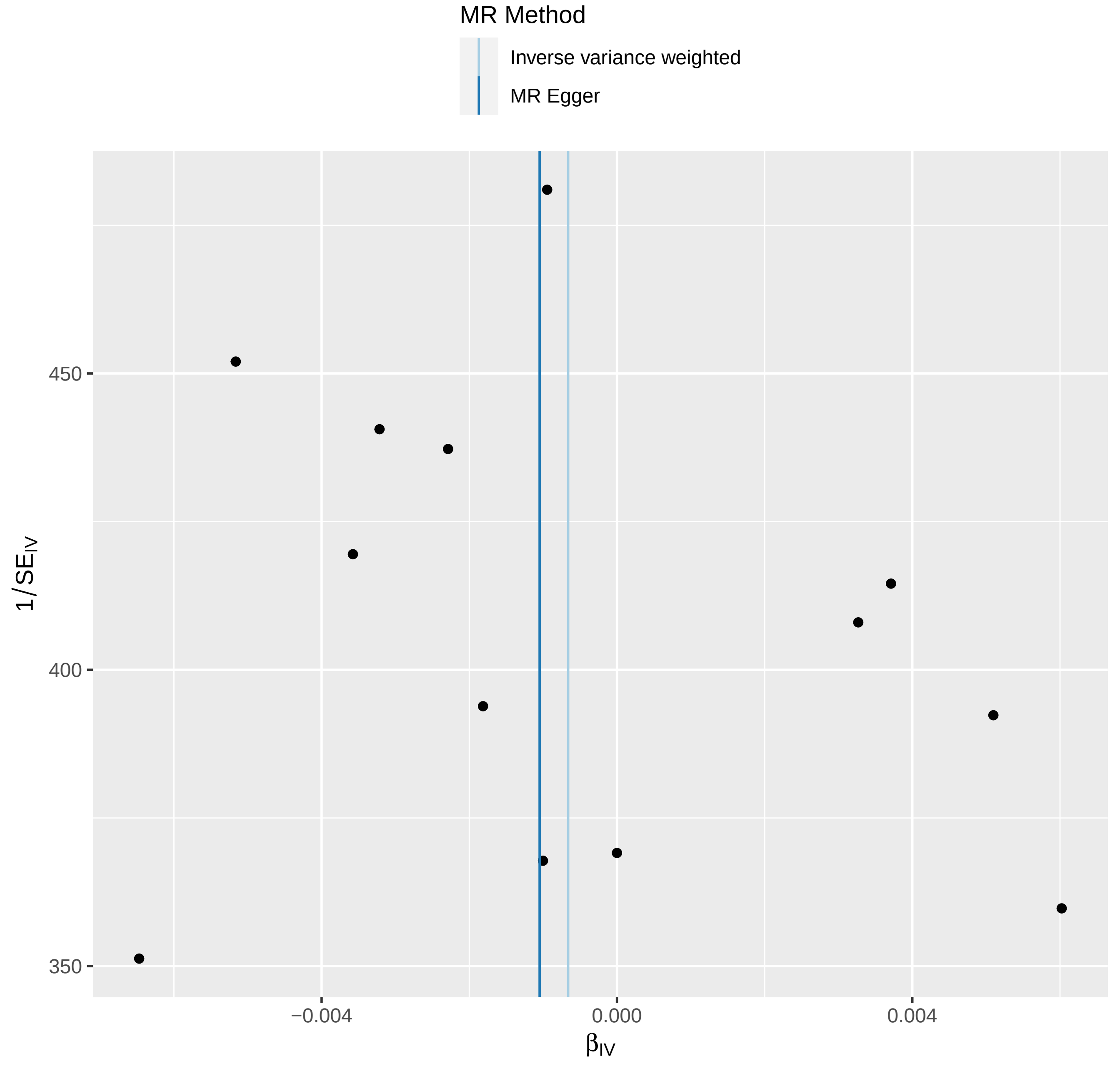

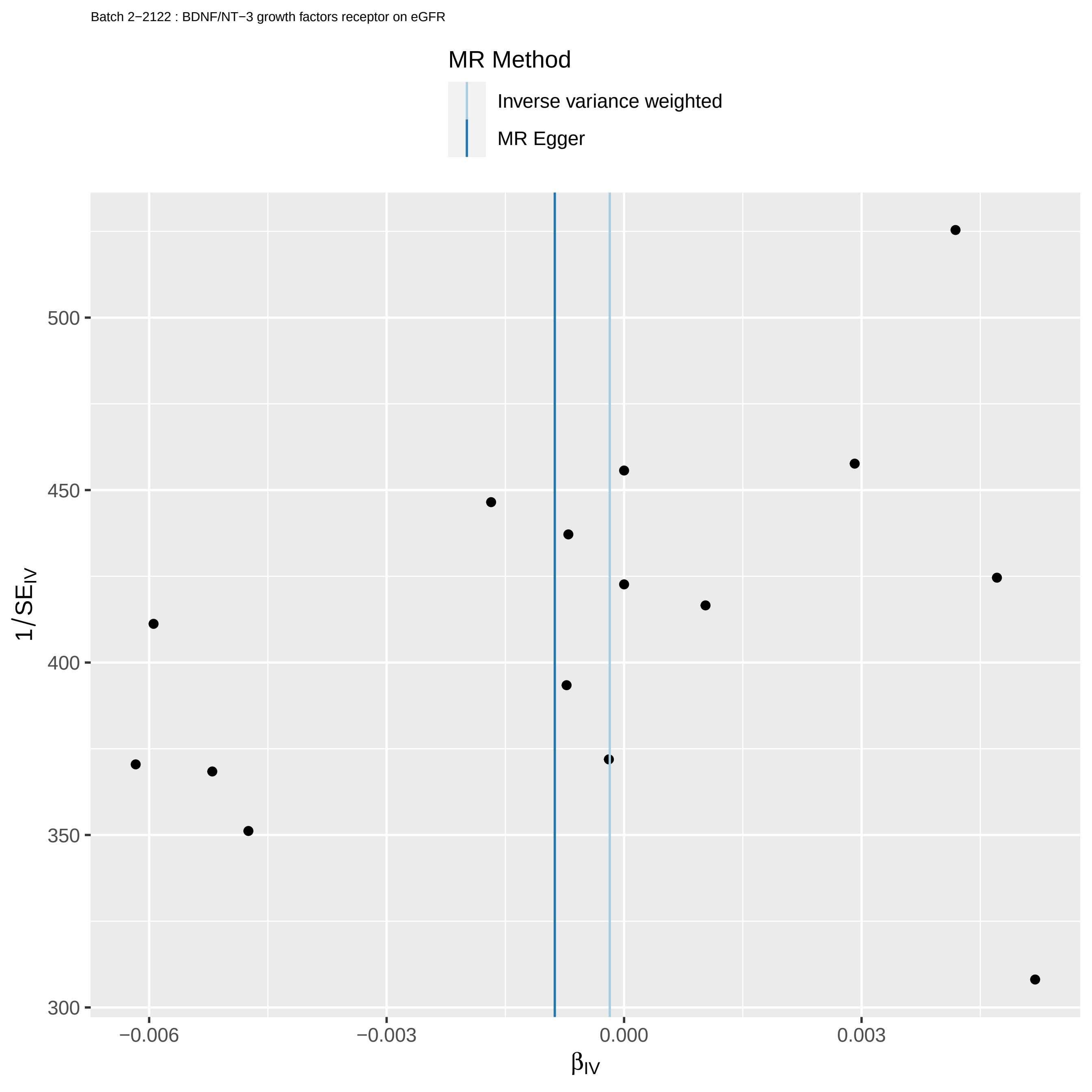

Batch 2−2122 : BDNF/NT−3 growth factors receptor on eGFR
MR Method
Inverse variance weighted
MR Egger
1/SE_IV
500
450
400
350
300
−0.006
−0.003
0.000
0.003
β_IV

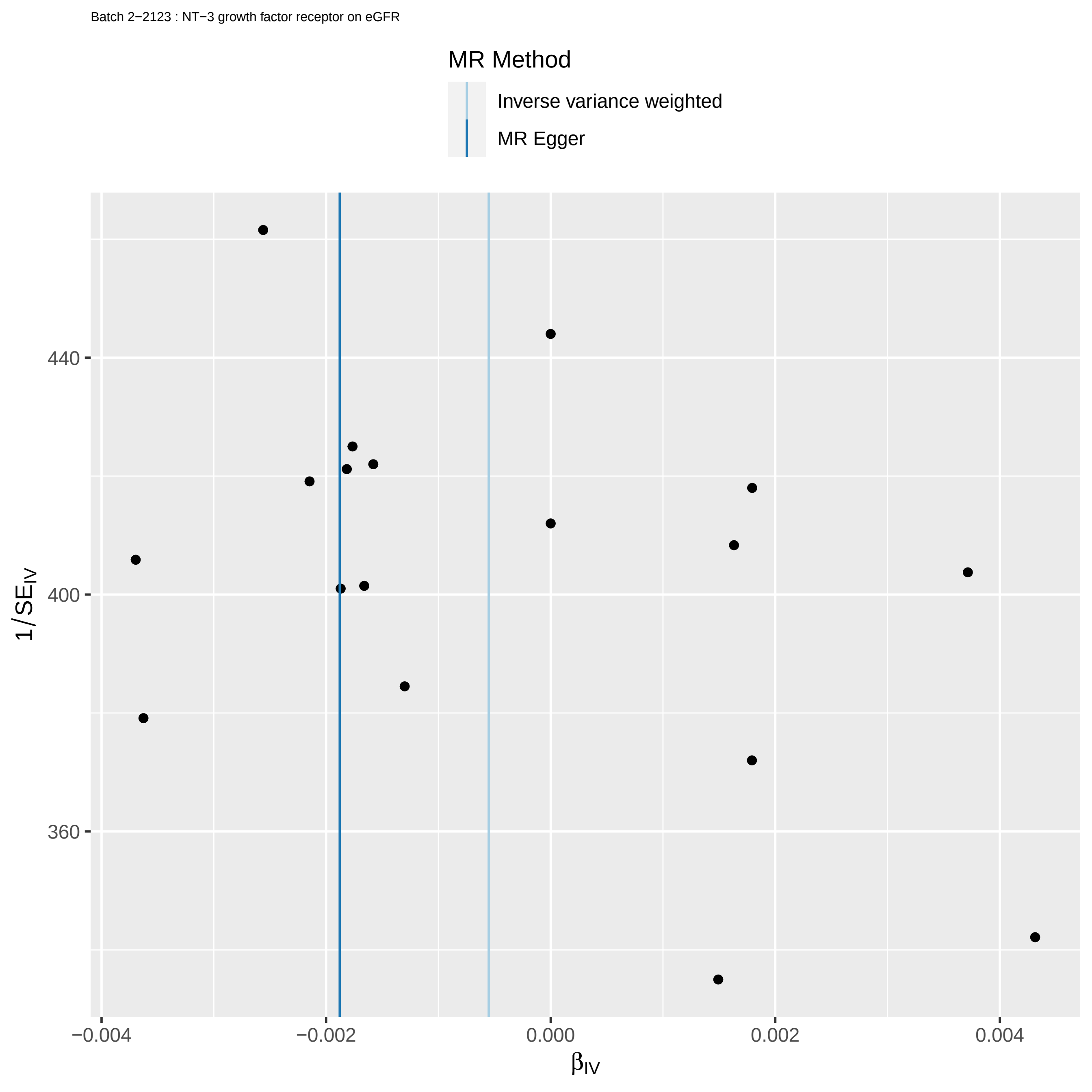
Batch 2−2123 : NT−3 growth factor receptor on eGFR
MR Method
Inverse variance weighted
MR Egger
440
400
360
$1/SE_{IV}$
−0.004
−0.002
0.000
0.002
0.004
β_{IV}

Batch 2−2124 : Neurotensin/neuromedin N on eGFR

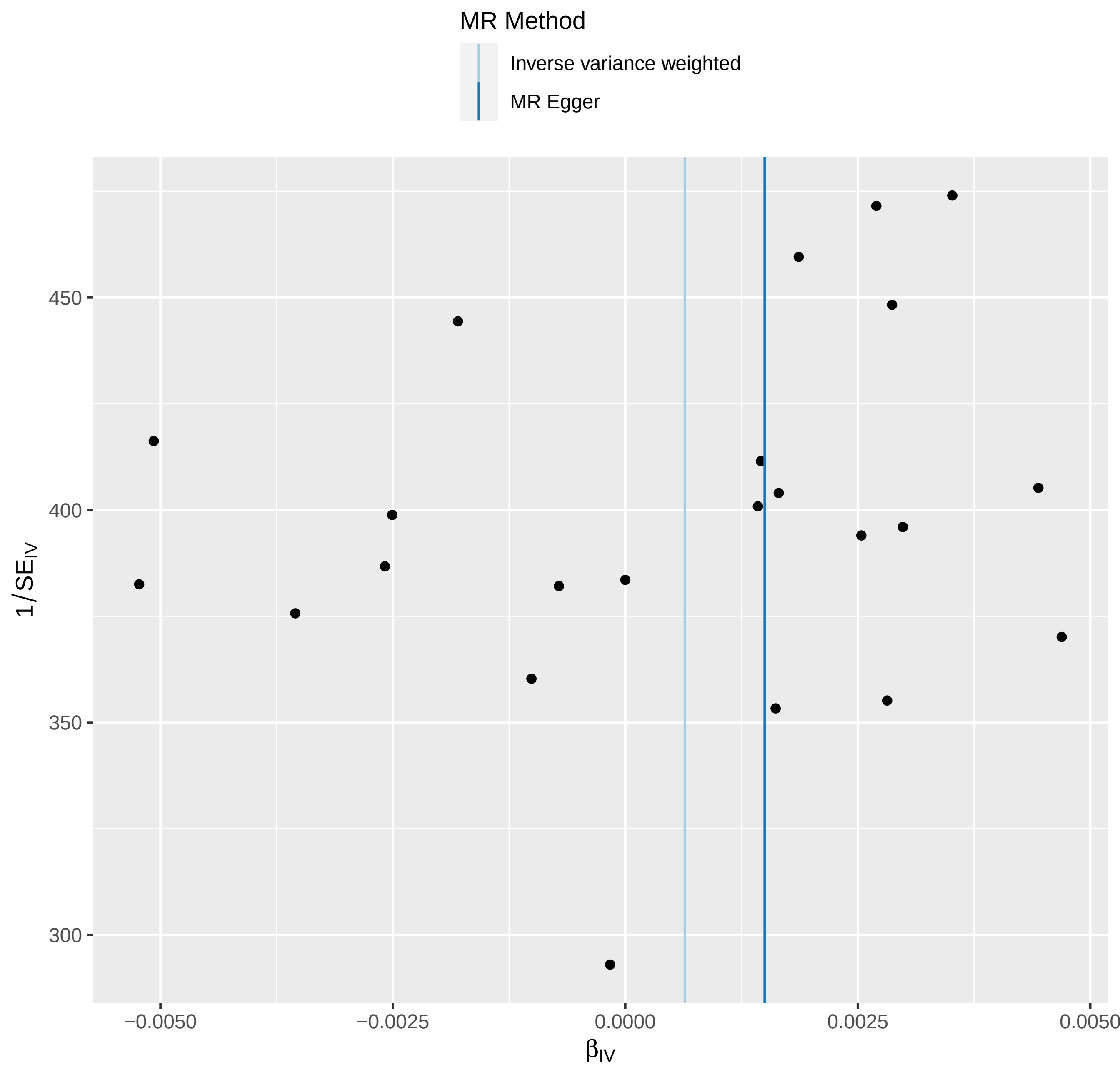

Batch 2−2125 : Nuclear migration protein nudC on eGFR

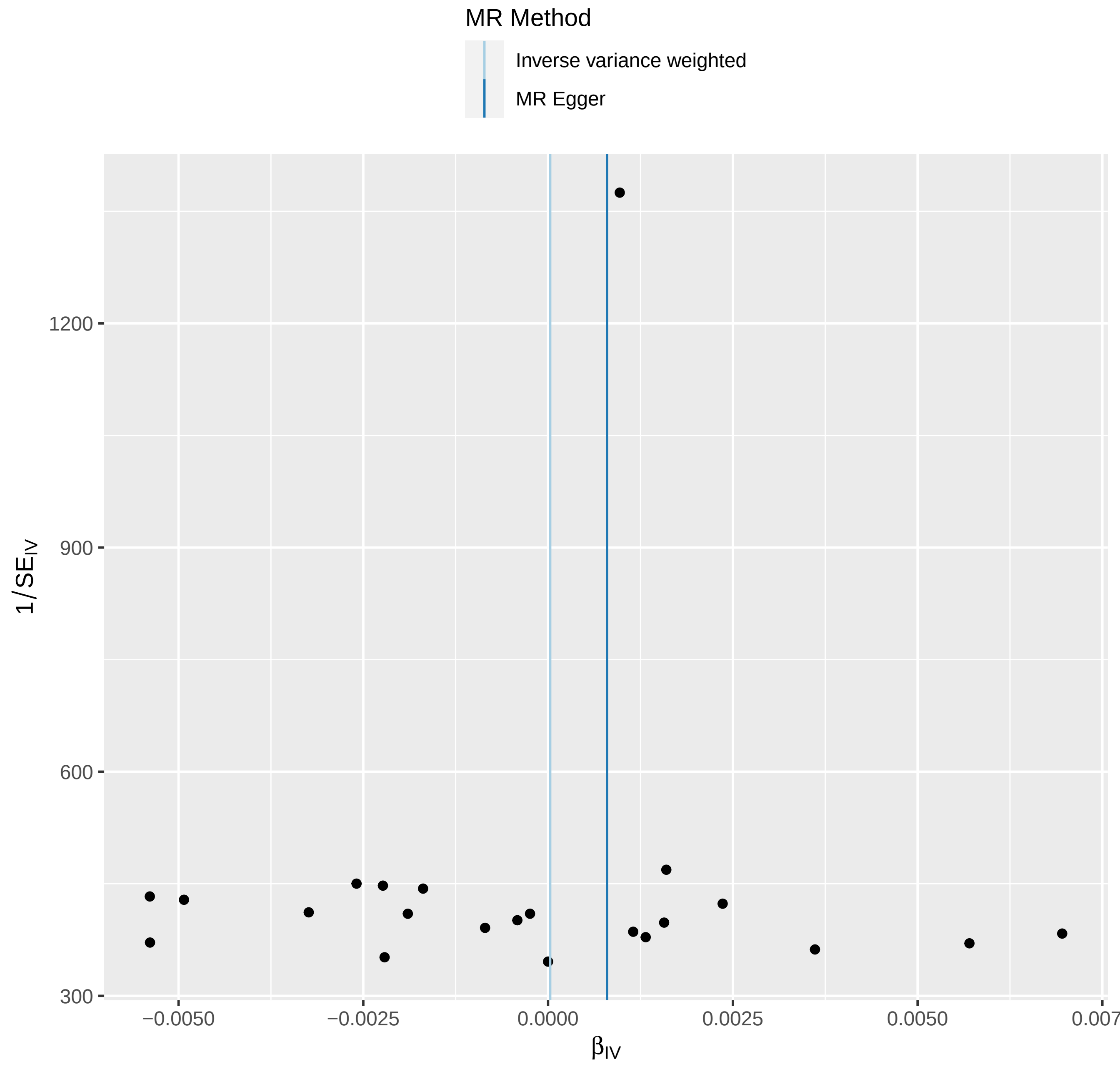

Batch 2−2126 : Peroxisomal NADH pyrophosphatase NUDT12 on eGFR

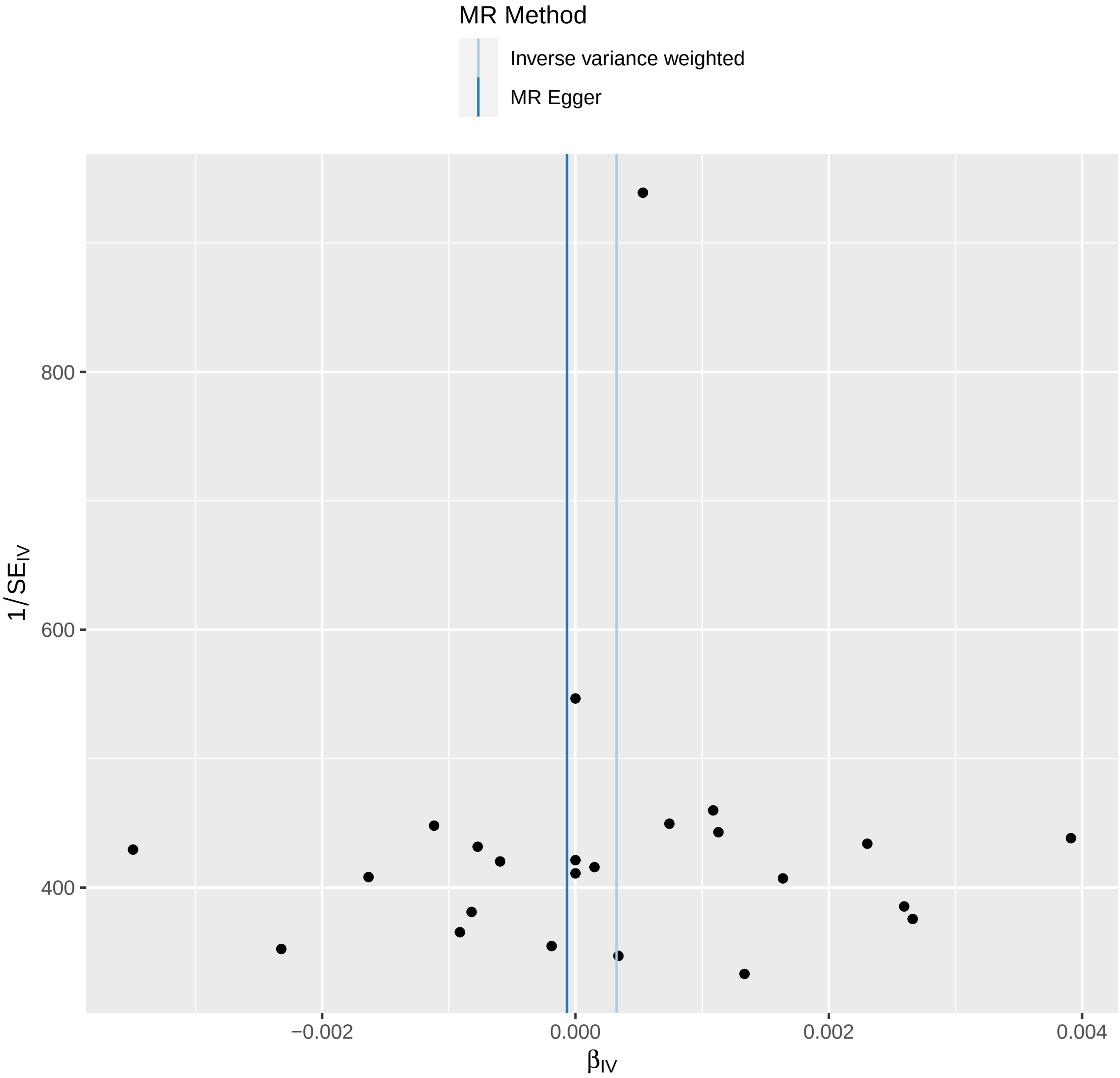

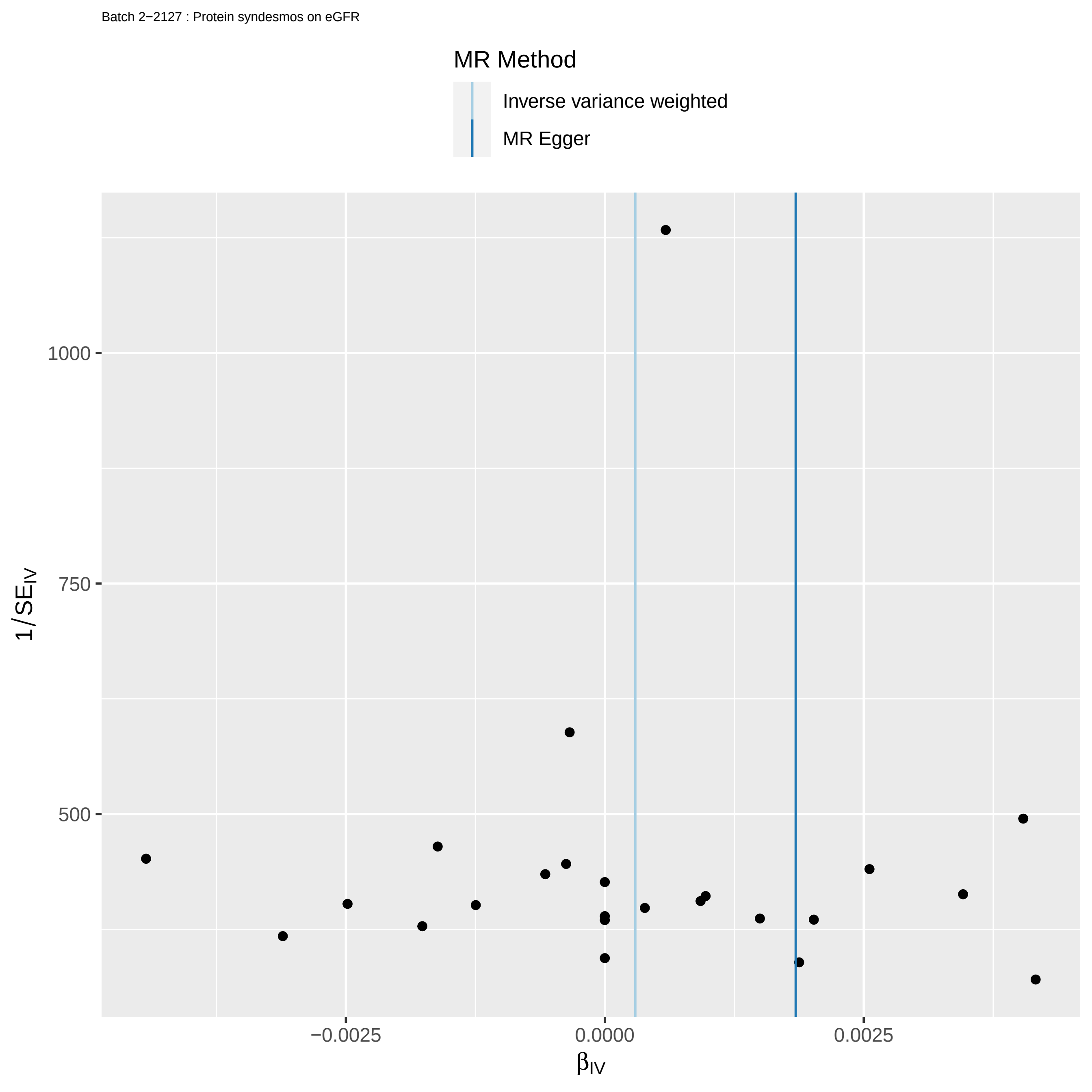

Batch 2−2127 : Protein syndesmos on eGFR
MR Method
Inverse variance weighted
MR Egger
1000
750
500
1/SE$_{IV}$
−0.0025
0.0000
0.0025
β$_{IV}$

Batch 2−2128 : Nucleoside diphosphate−linked moiety X motif 8, mitochondrial on eGFR

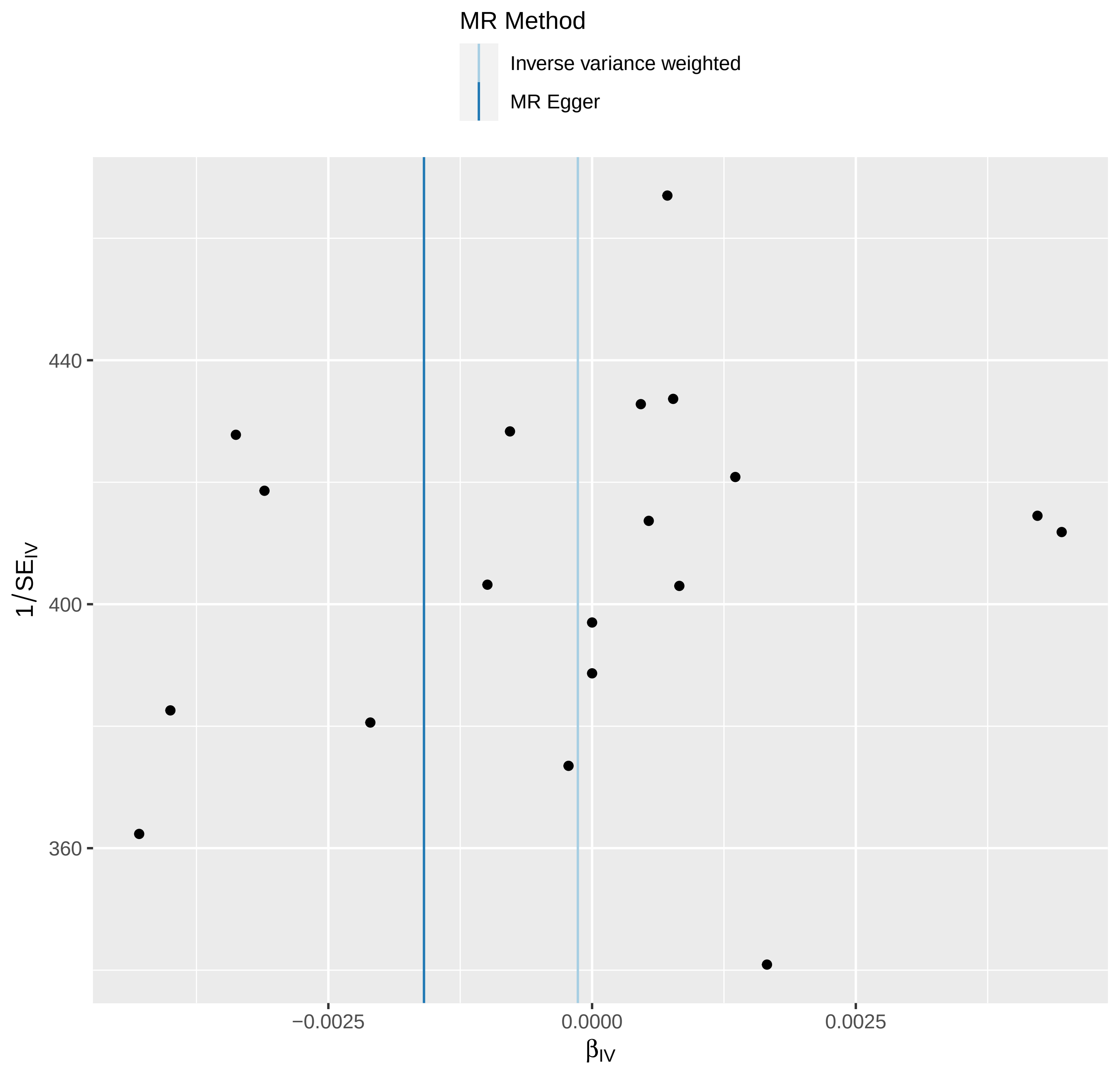

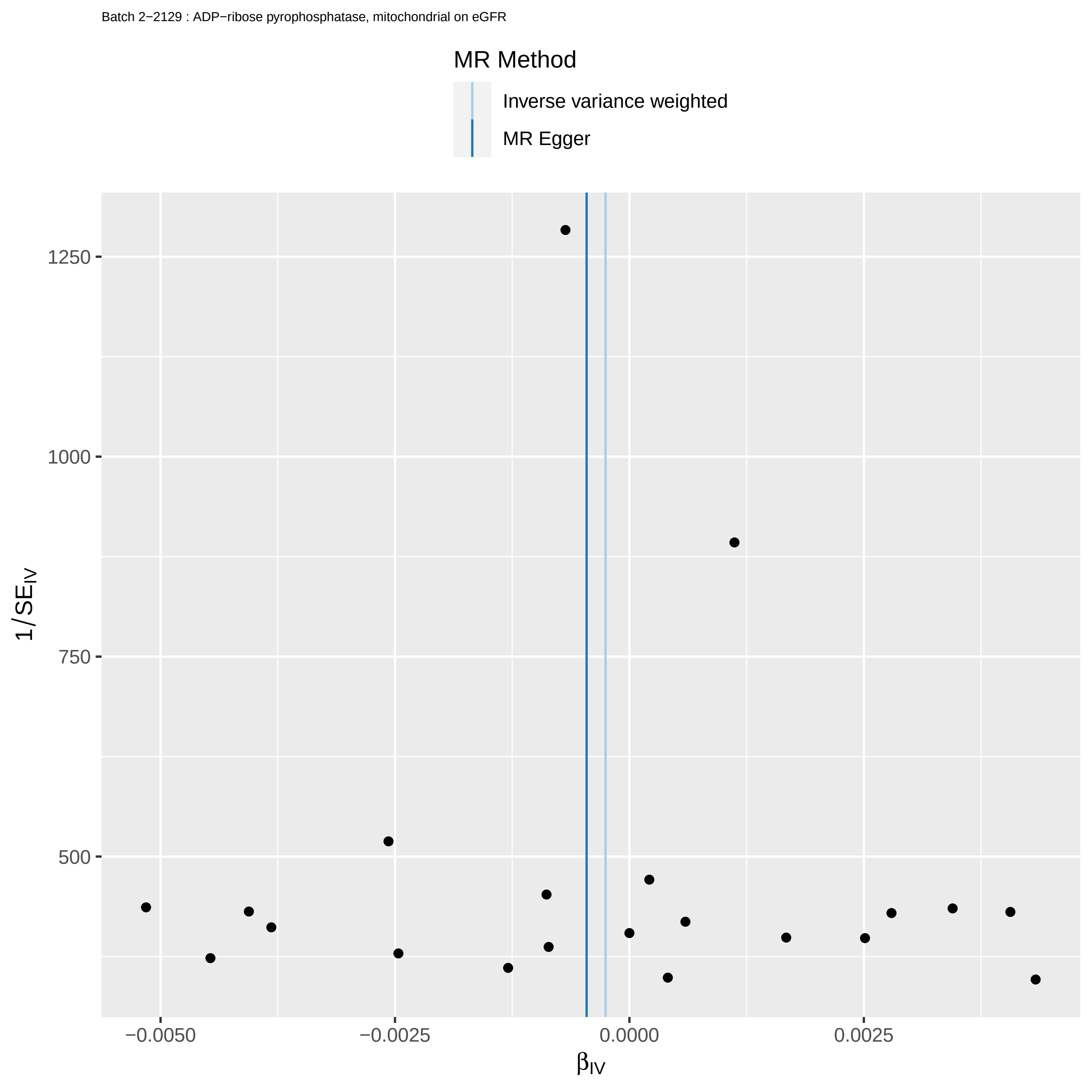

Batch 2−2129 : ADP−ribose pyrophosphatase, mitochondrial on eGFR
MR Method
Inverse variance weighted
MR Egger
1250
1000
750
500
1/SE$_{IV}$
−0.0050
−0.0025
0.0000
0.0025
β$_{IV}$

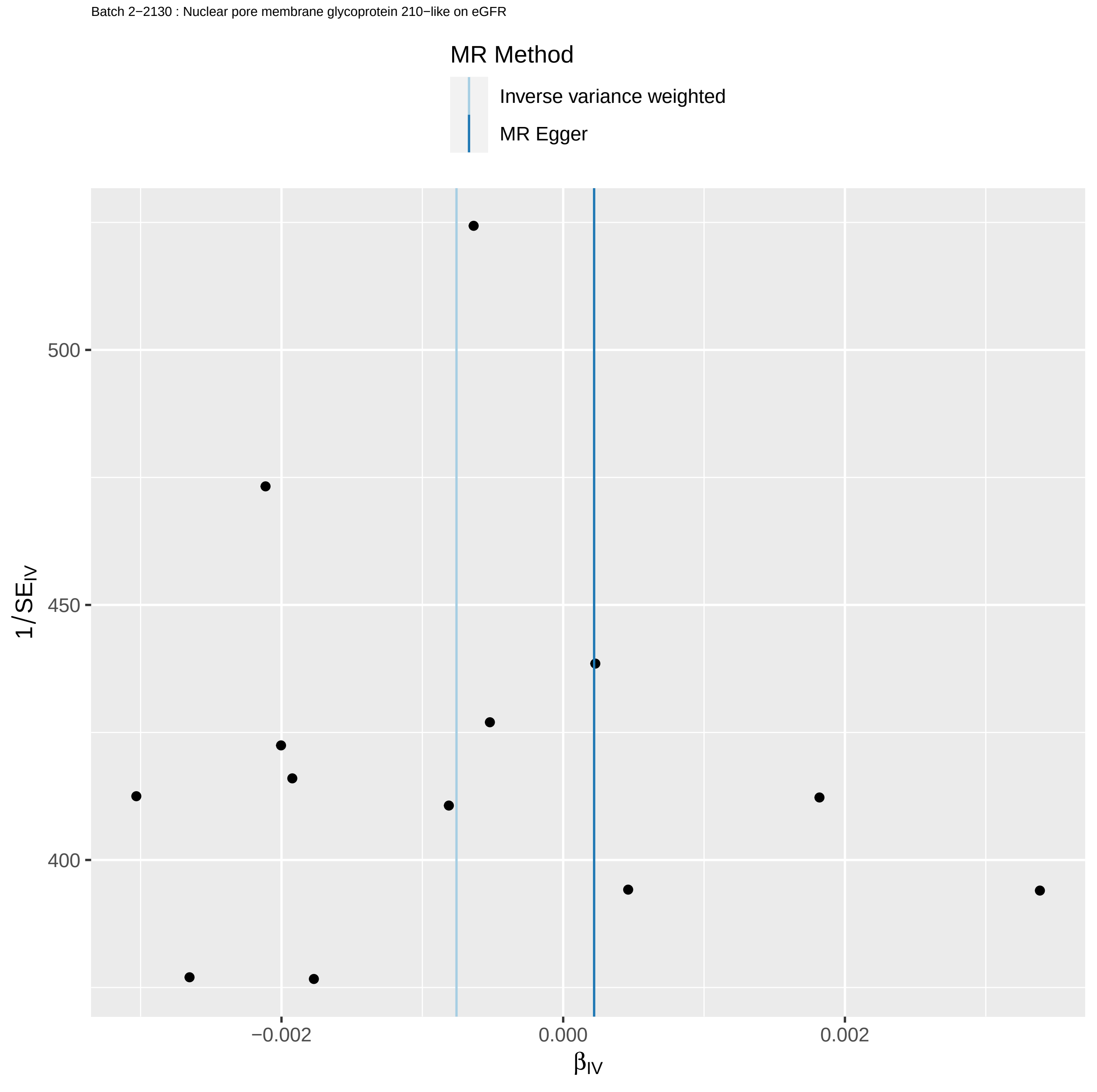

Batch 2−2130 : Nuclear pore membrane glycoprotein 210−like on eGFR
MR Method
Inverse variance weighted
MR Egger
500
450
400
1/SE$_{IV}$
−0.002
0.000
0.002
β$_{IV}$

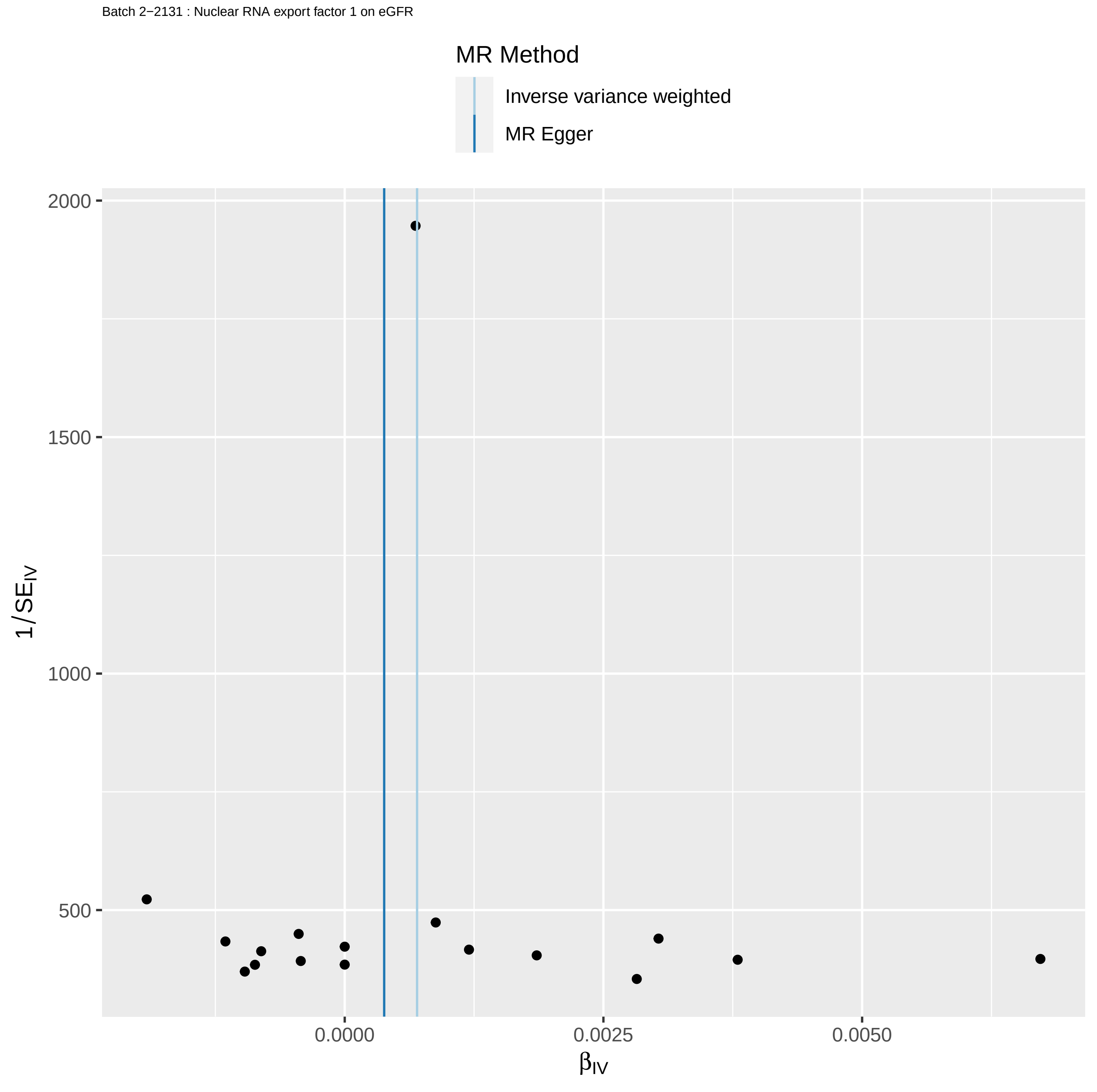

Batch 2−2131 : Nuclear RNA export factor 1 on eGFR
MR Method
Inverse variance weighted
MR Egger
2000
1500
1000
500
1/SE$_{IV}$
0.0000
0.0025
0.0050
β$_{IV}$

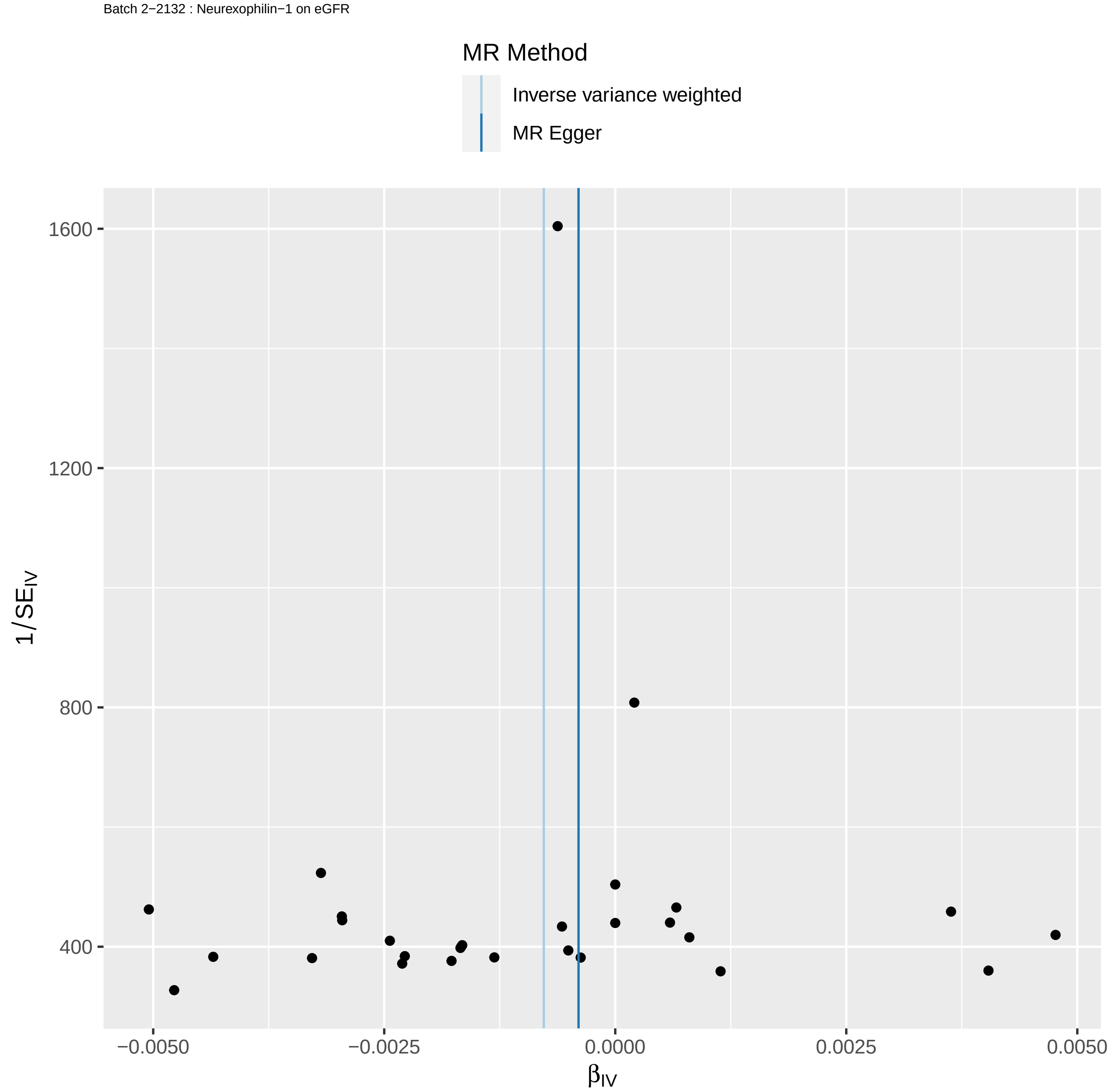

Batch 2−2132 : Neurexophilin−1 on eGFR
MR Method
Inverse variance weighted
MR Egger
1600
1200
800
400
1/SE$_{IV}$
−0.0050
−0.0025
0.0000
0.0025
0.0050
β$_{IV}$

Batch 2−2133 : Neurexophilin−3 on eGFR

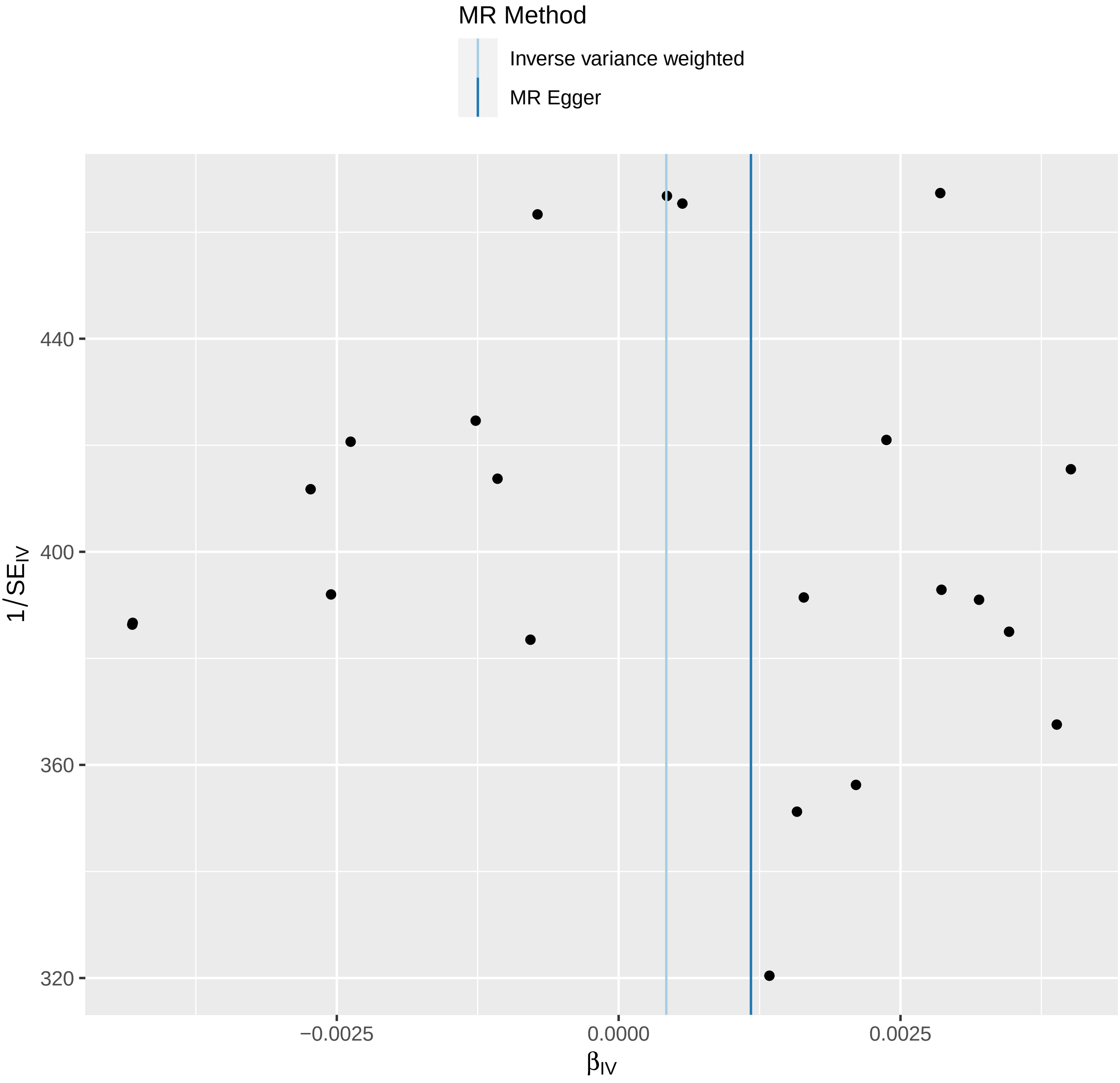

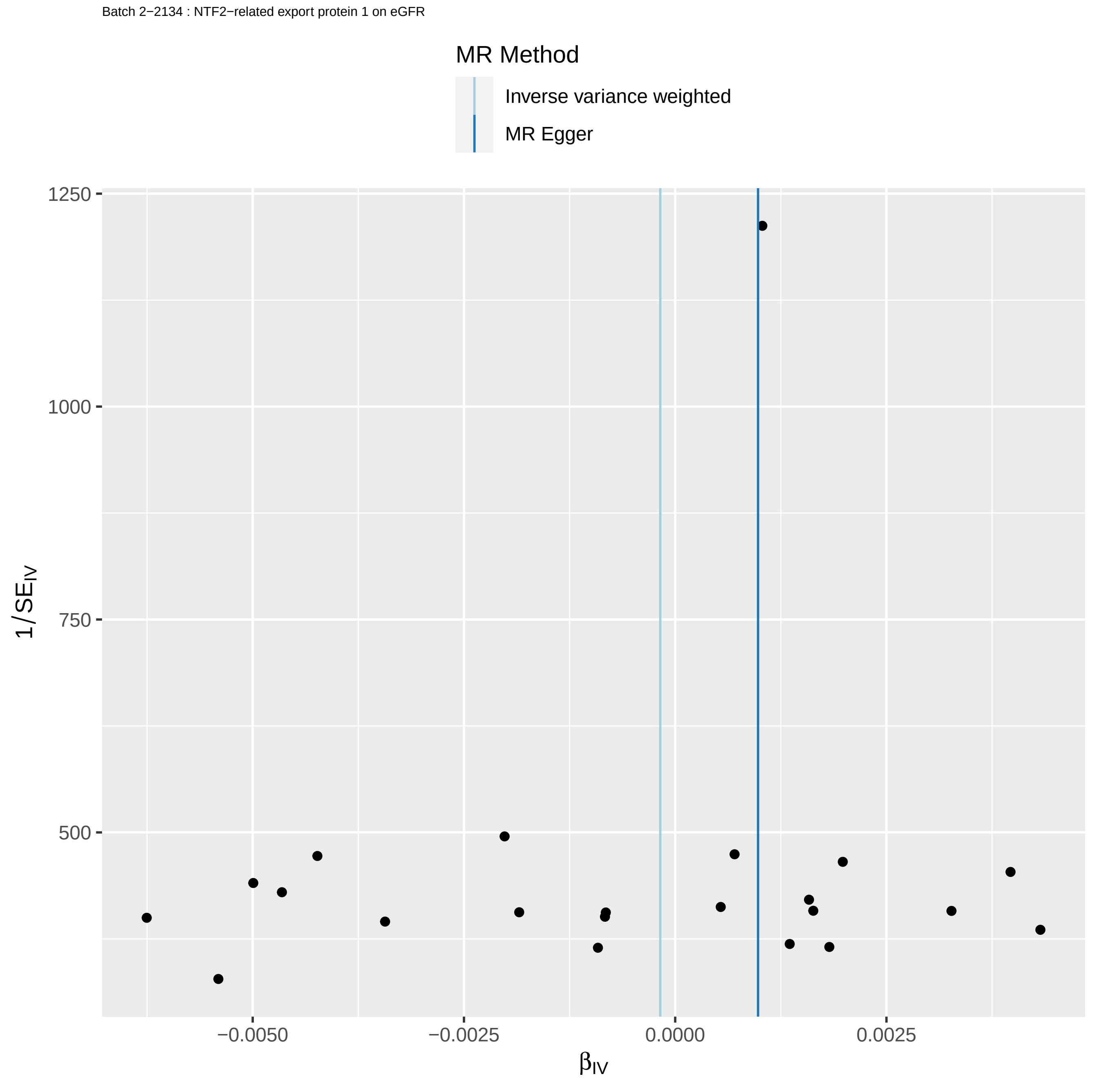
Batch 2−2134 : NTF2−related export protein 1 on eGFR
MR Method
Inverse variance weighted
MR Egger
1250
1000
750
500
1/SE$_{IV}$
−0.0050
−0.0025
0.0000
0.0025
β$_{IV}$

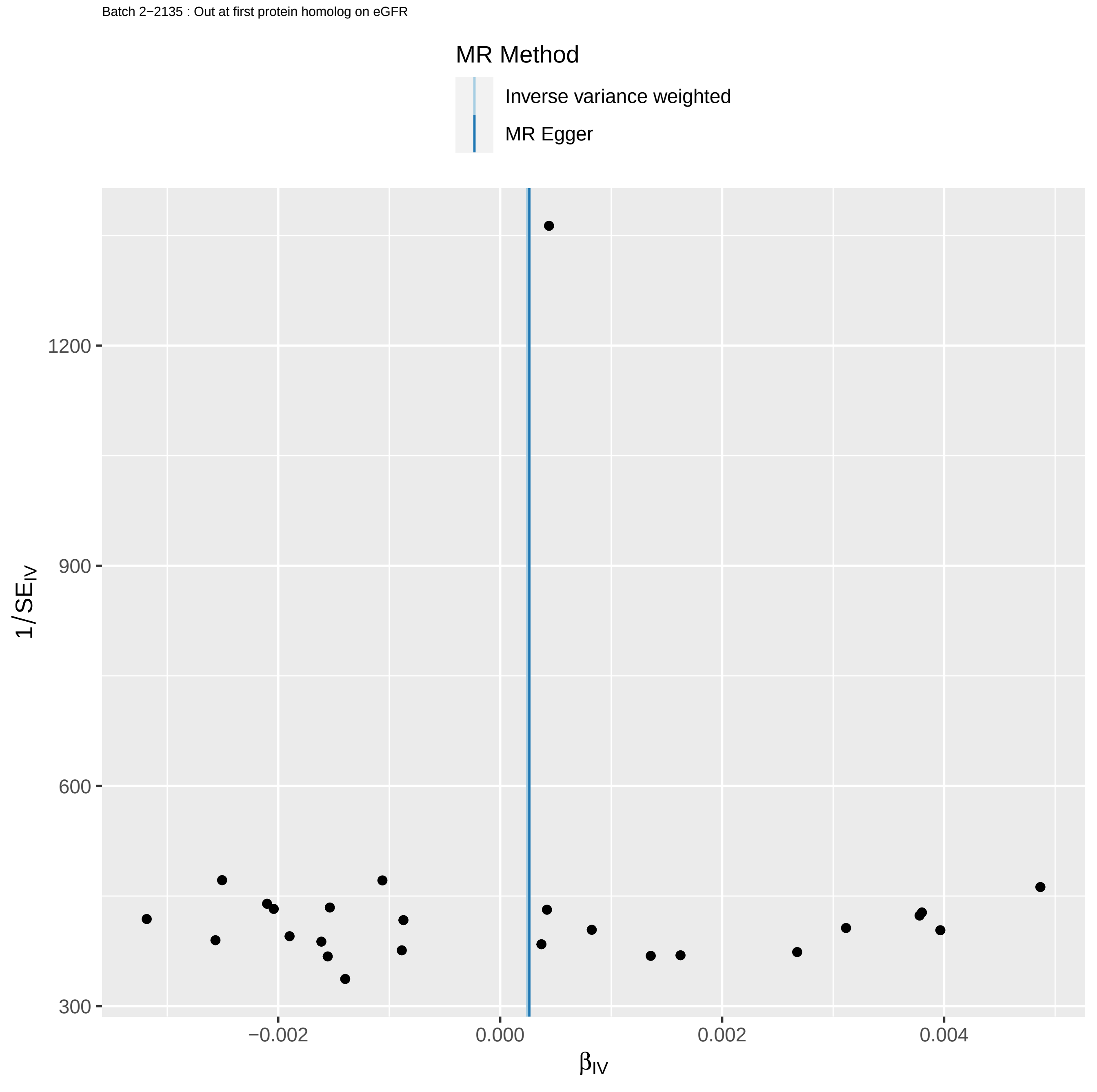
Batch 2−2135 : Out at first protein homolog on eGFR
MR Method
Inverse variance weighted
MR Egger
1200
900
600
300
1/SE_IV
−0.002
0.000
0.002
0.004
β_IV

Batch 2−2136 : 2'−5'−oligoadenylate synthase 1 on eGFR

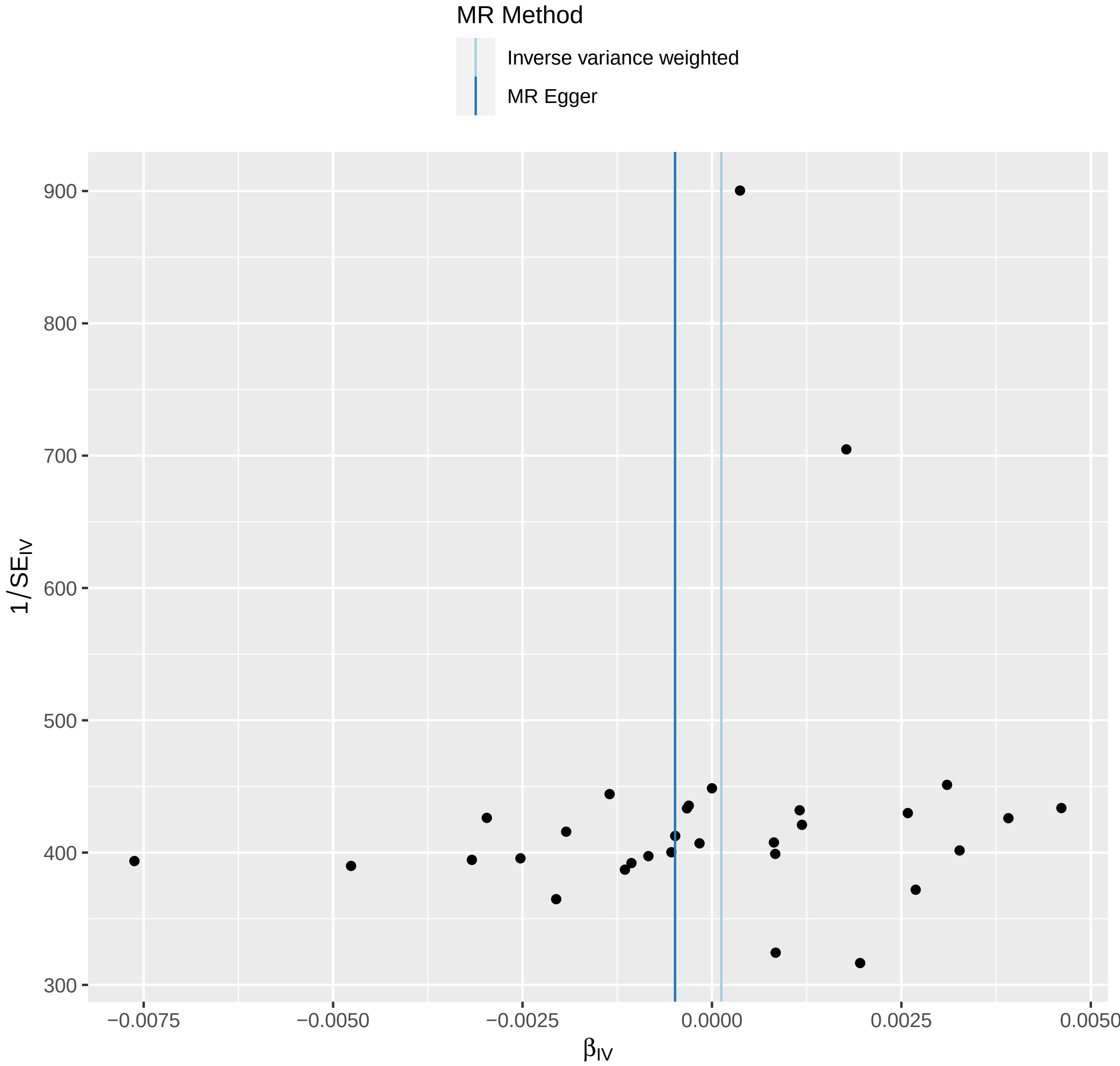

Batch 2−2137 : Ornithine decarboxylase antizyme 1 on eGFR

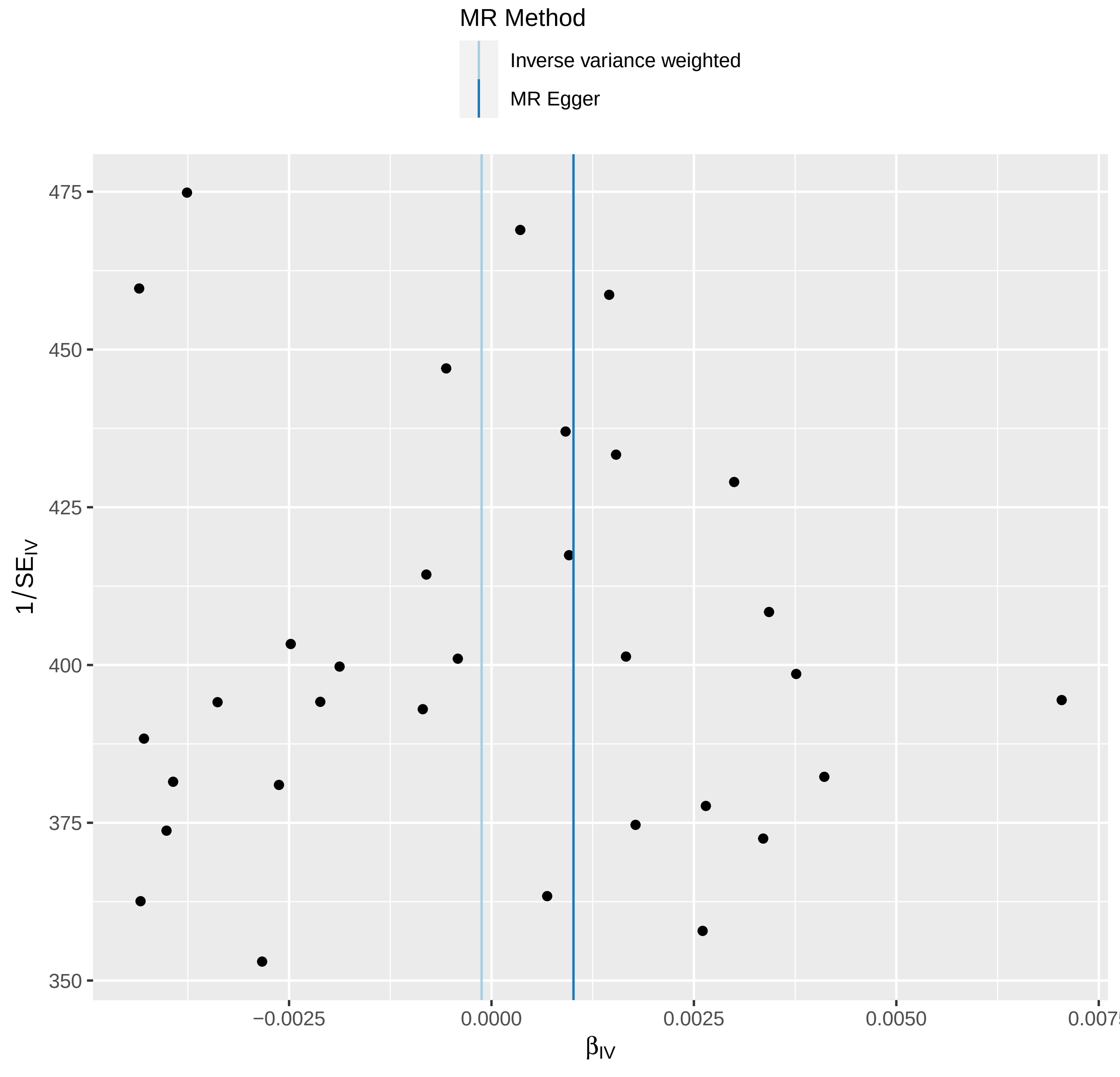

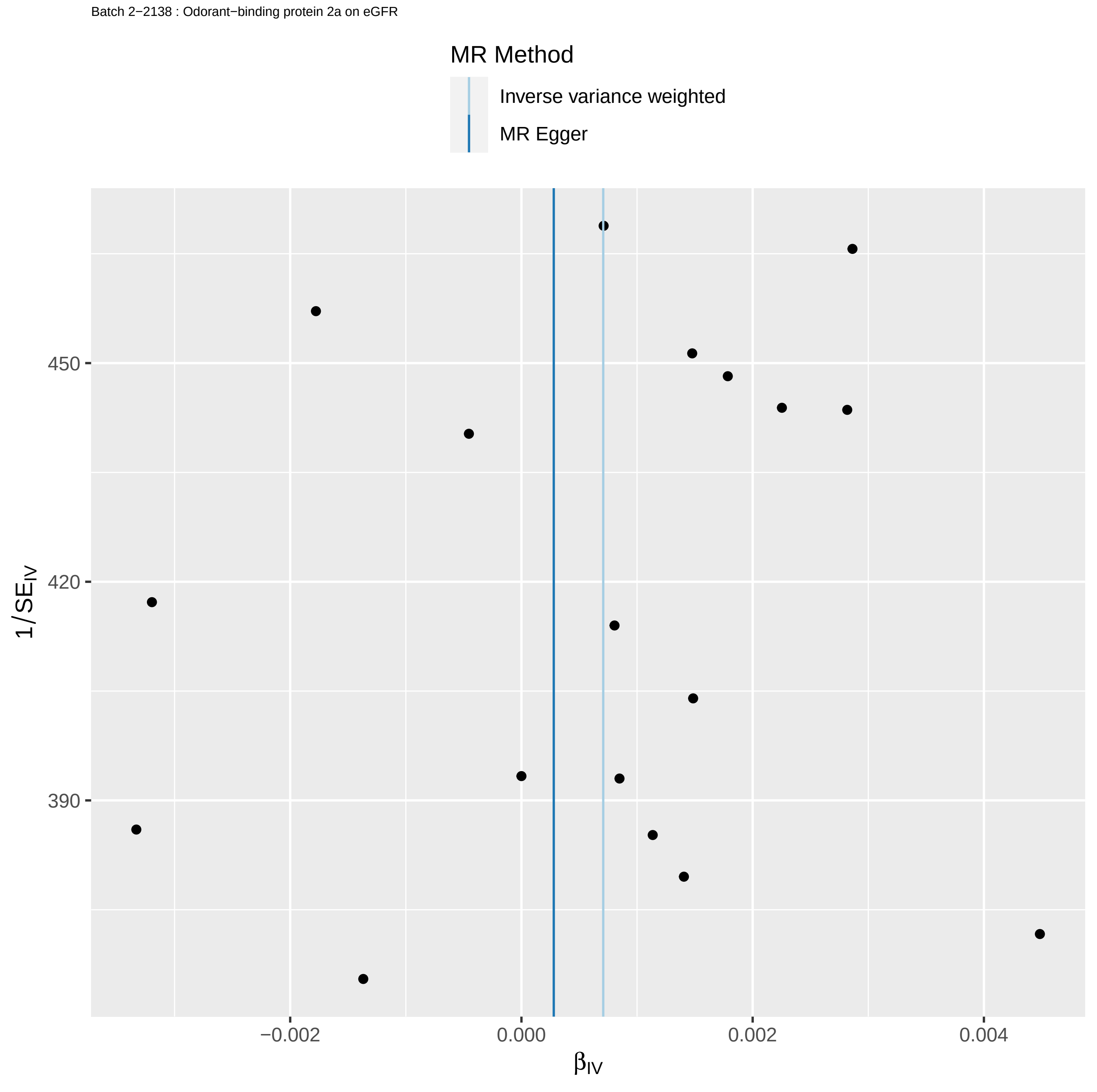
Batch 2−2138 : Odorant−binding protein 2a on eGFR
MR Method
Inverse variance weighted
MR Egger
450
420
390
$1/SE_{IV}$
−0.002
0.000
0.002
0.004
β_{IV}

Batch 2−2139 : Odorant−binding protein 2b on eGFR

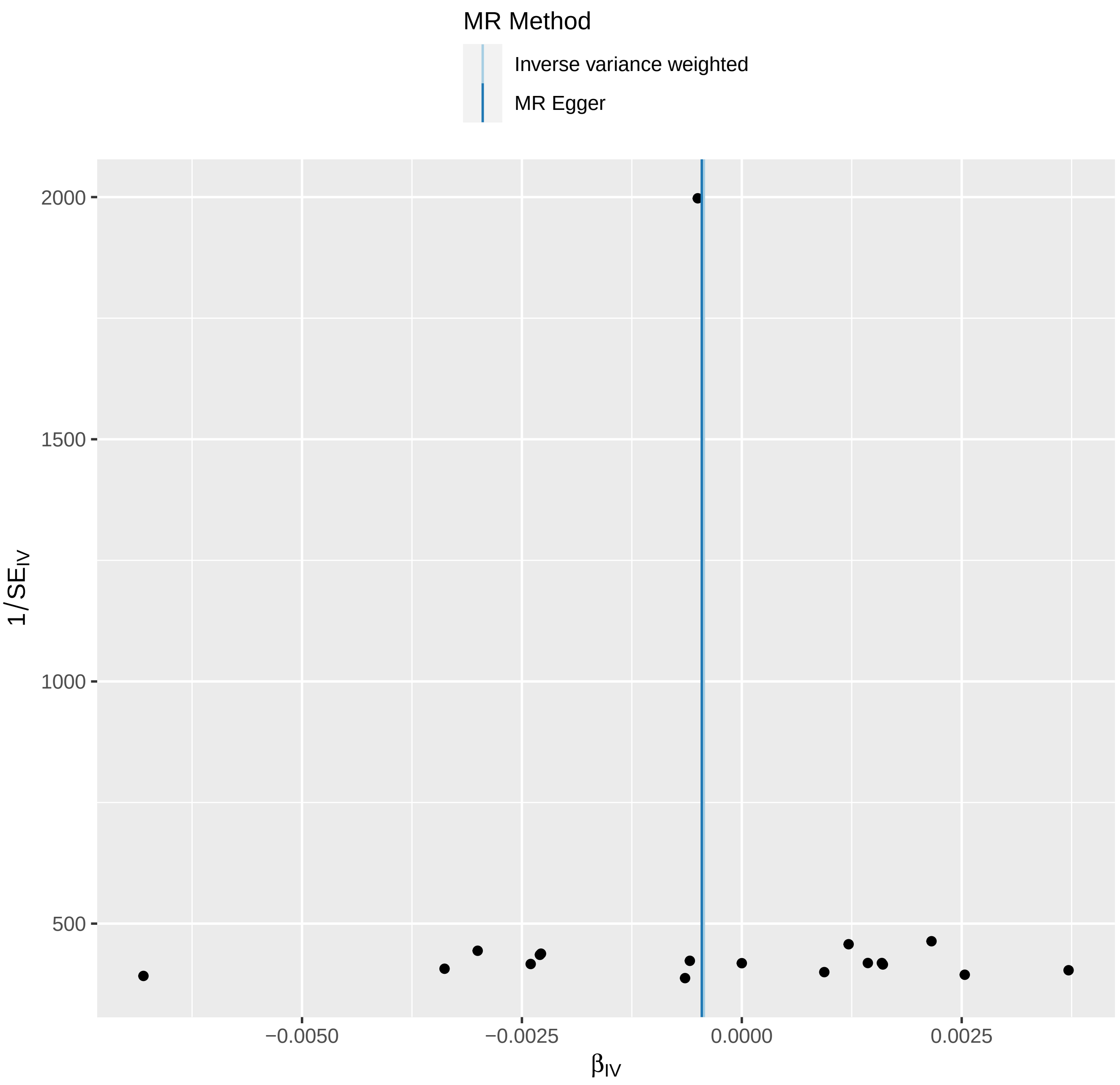

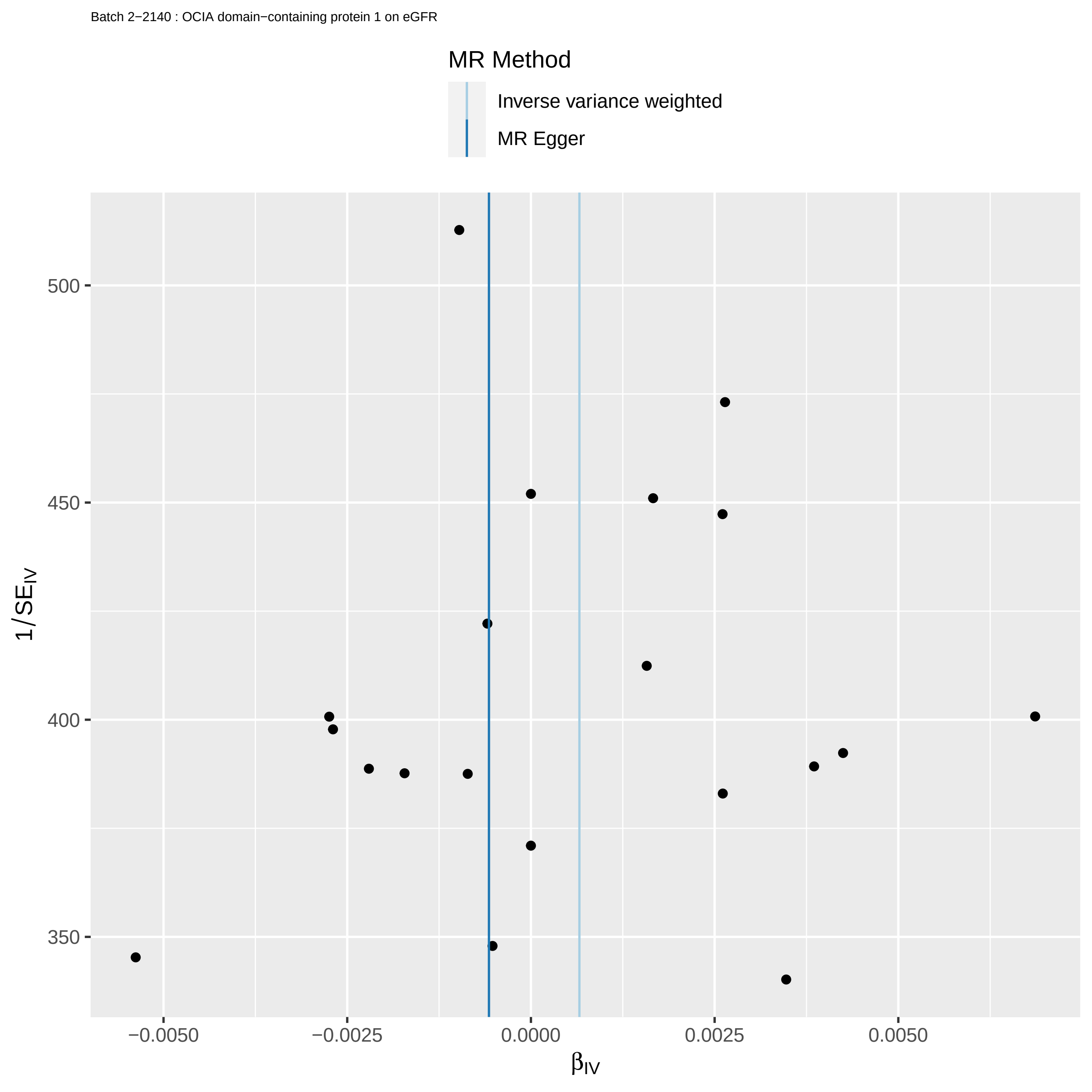

Batch 2−2140 : OCIA domain−containing protein 1 on eGFR
MR Method
Inverse variance weighted
MR Egger
500
450
400
350
1/SE_IV
−0.0050
−0.0025
0.0000
0.0025
0.0050
β_IV

Batch 2−2141 : Inositol polyphosphate 5−phosphatase OCRL−1 on eGFR

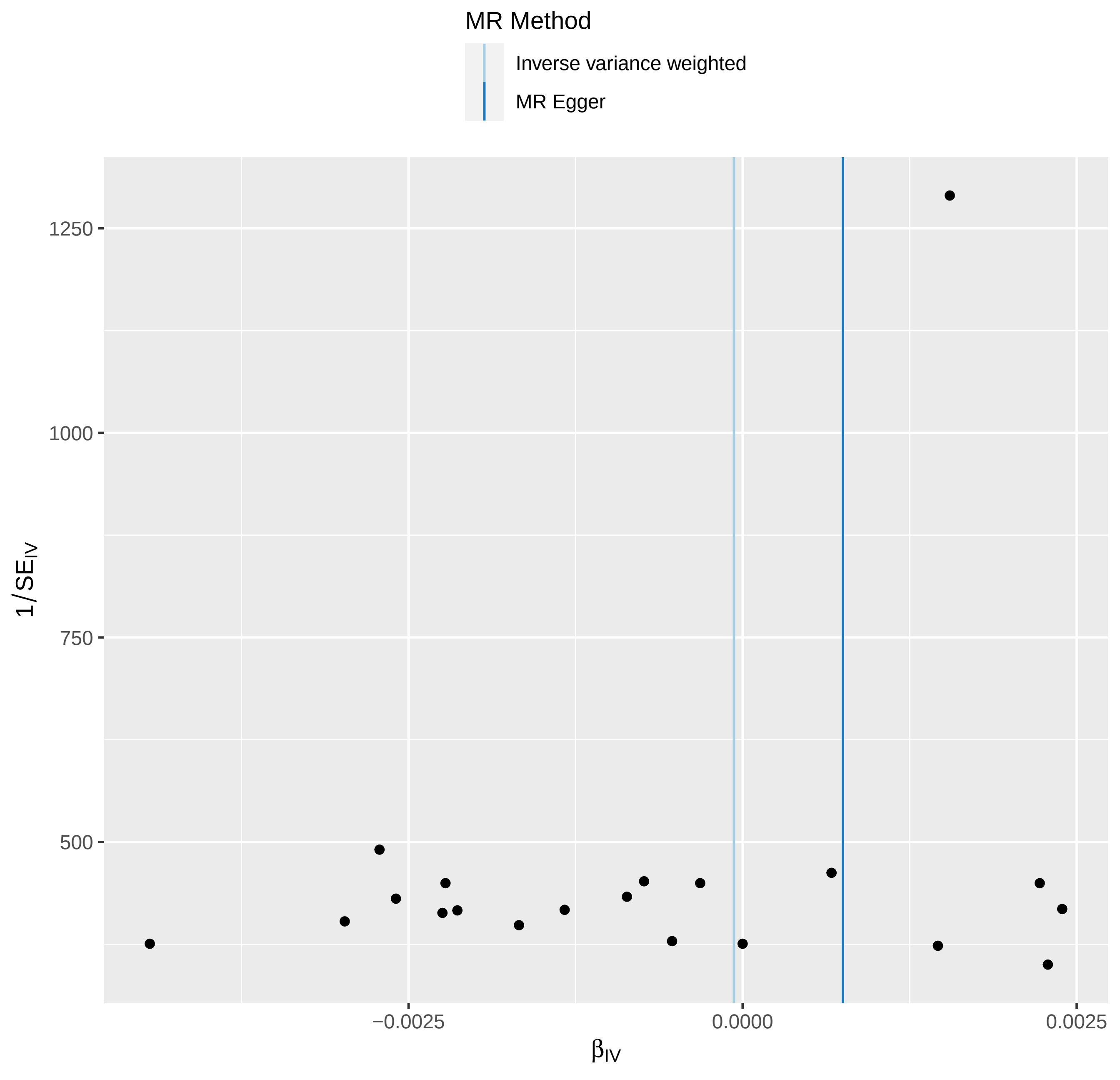

Batch 2−2142 : Ornithine decarboxylase on eGFR

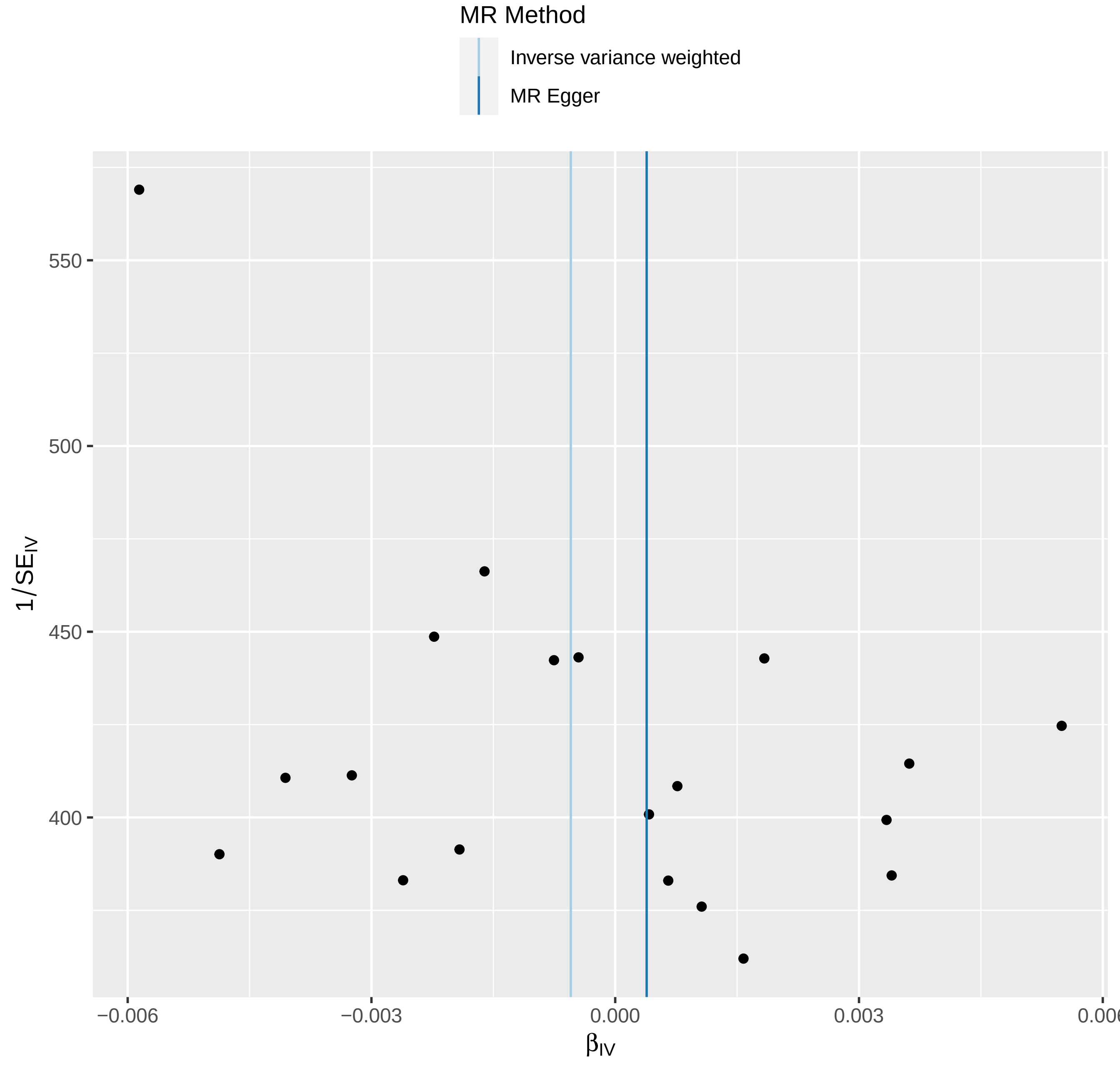

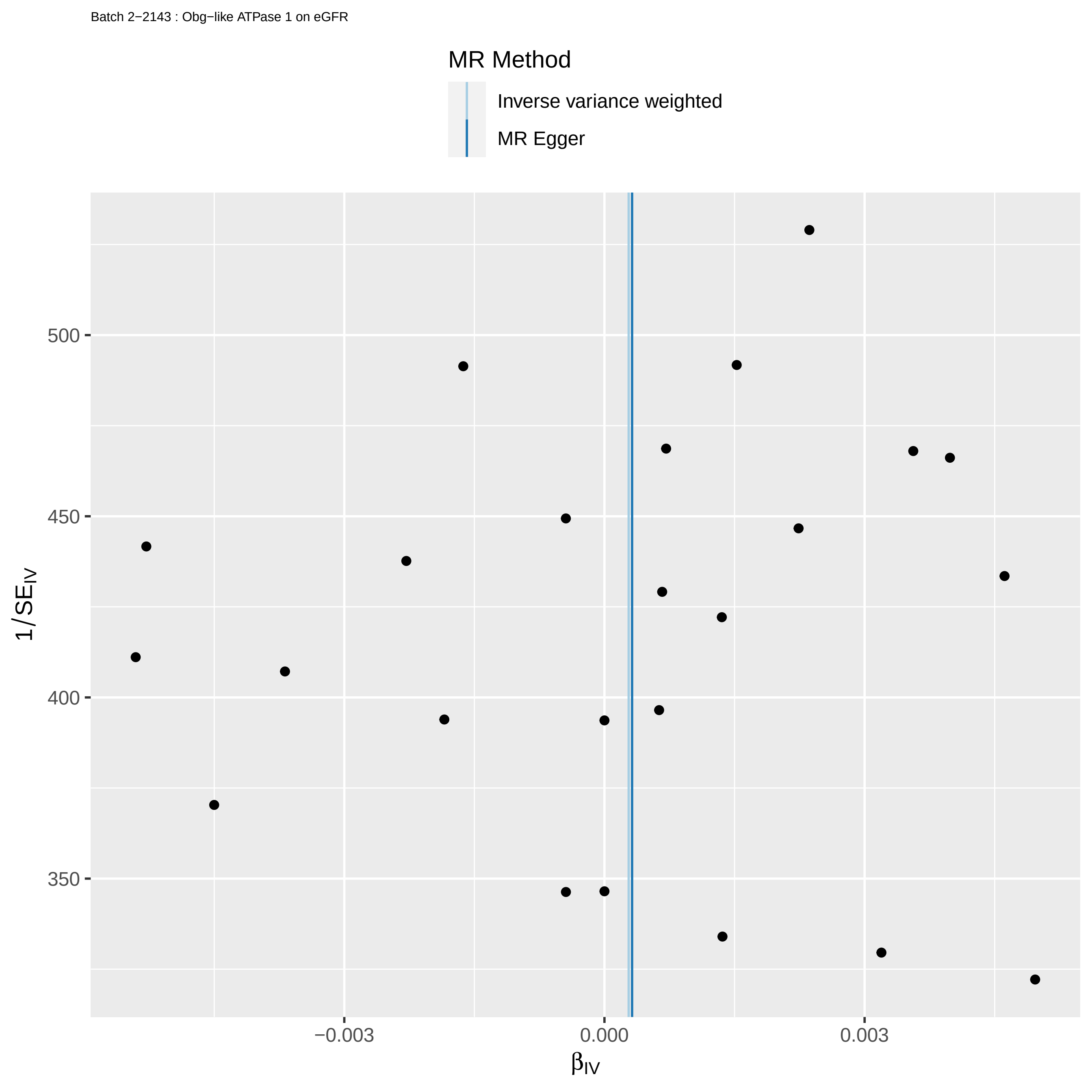
Batch 2−2143 : Obg−like ATPase 1 on eGFR
MR Method
Inverse variance weighted
MR Egger
500
450
400
350
1/SE_IV
−0.003
0.000
0.003
β_IV

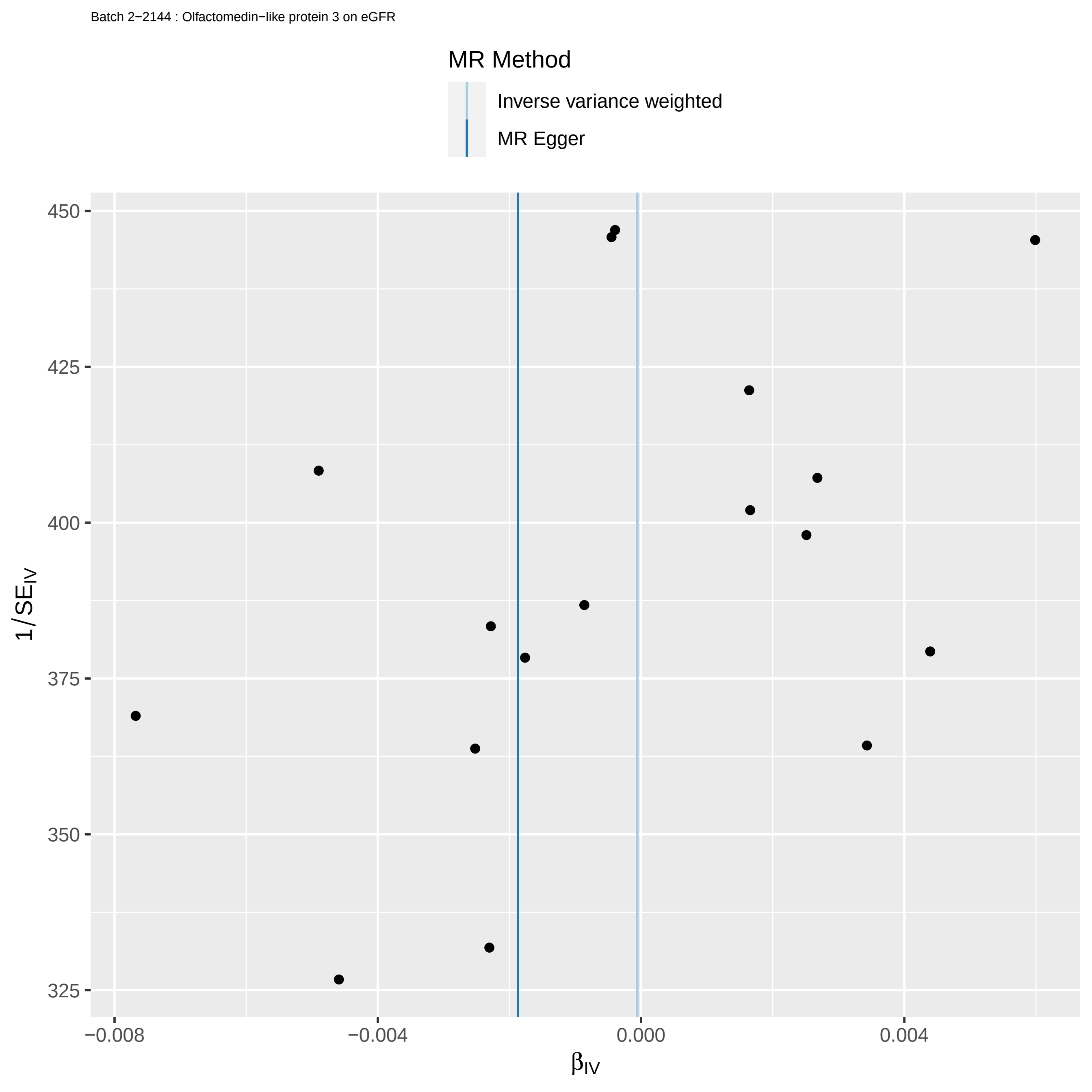

Batch 2−2144 : Olfactomedin−like protein 3 on eGFR
MR Method
Inverse variance weighted
MR Egger
450
425
400
375
350
325
1/SE_IV
−0.008
−0.004
0.000
0.004
β_IV

Batch 2−2145 : Olfactomedin−like protein 3 on eGFR

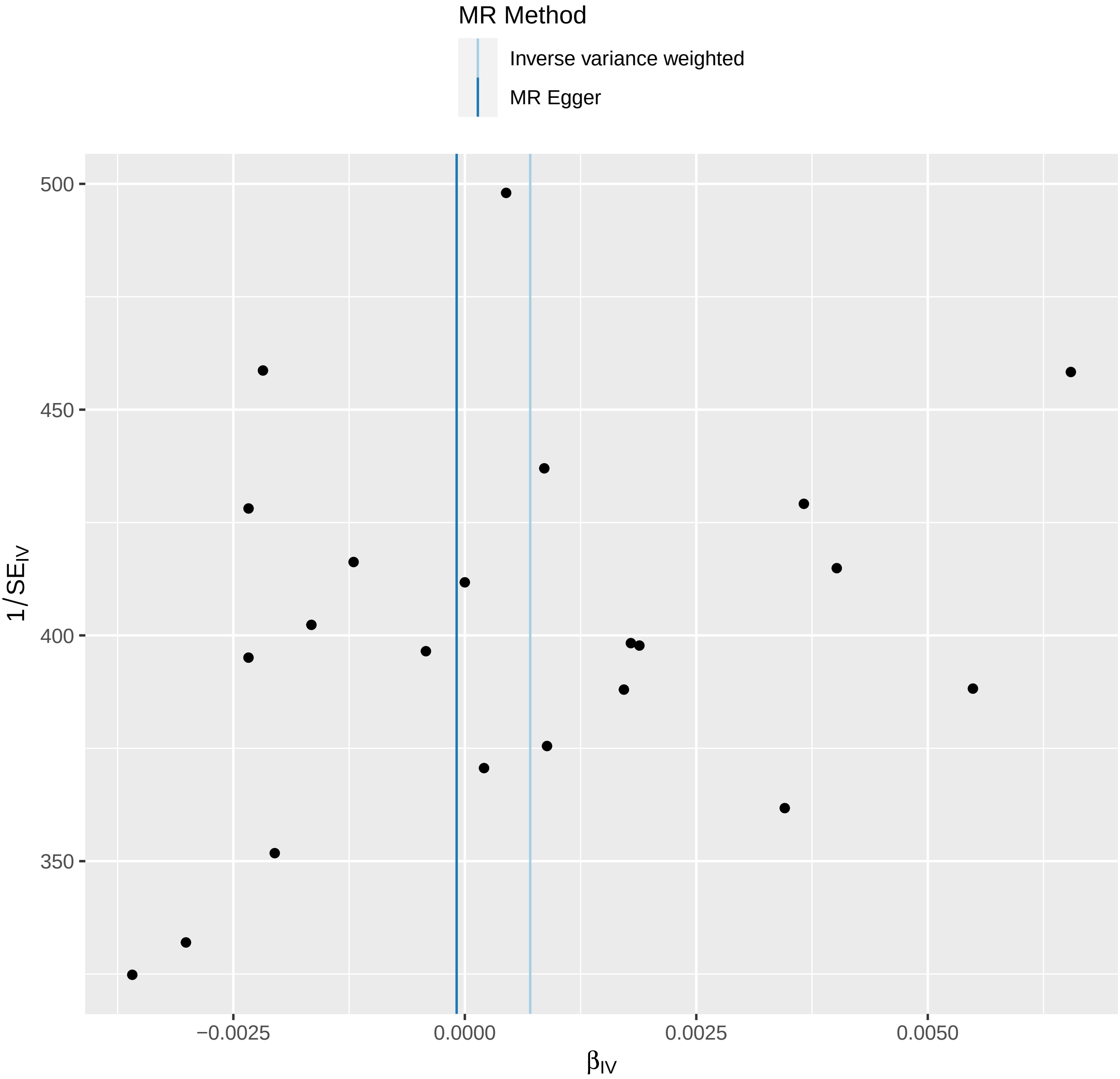

Batch 2−2146 : Oxidized low−density lipoprotein receptor 1 on eGFR

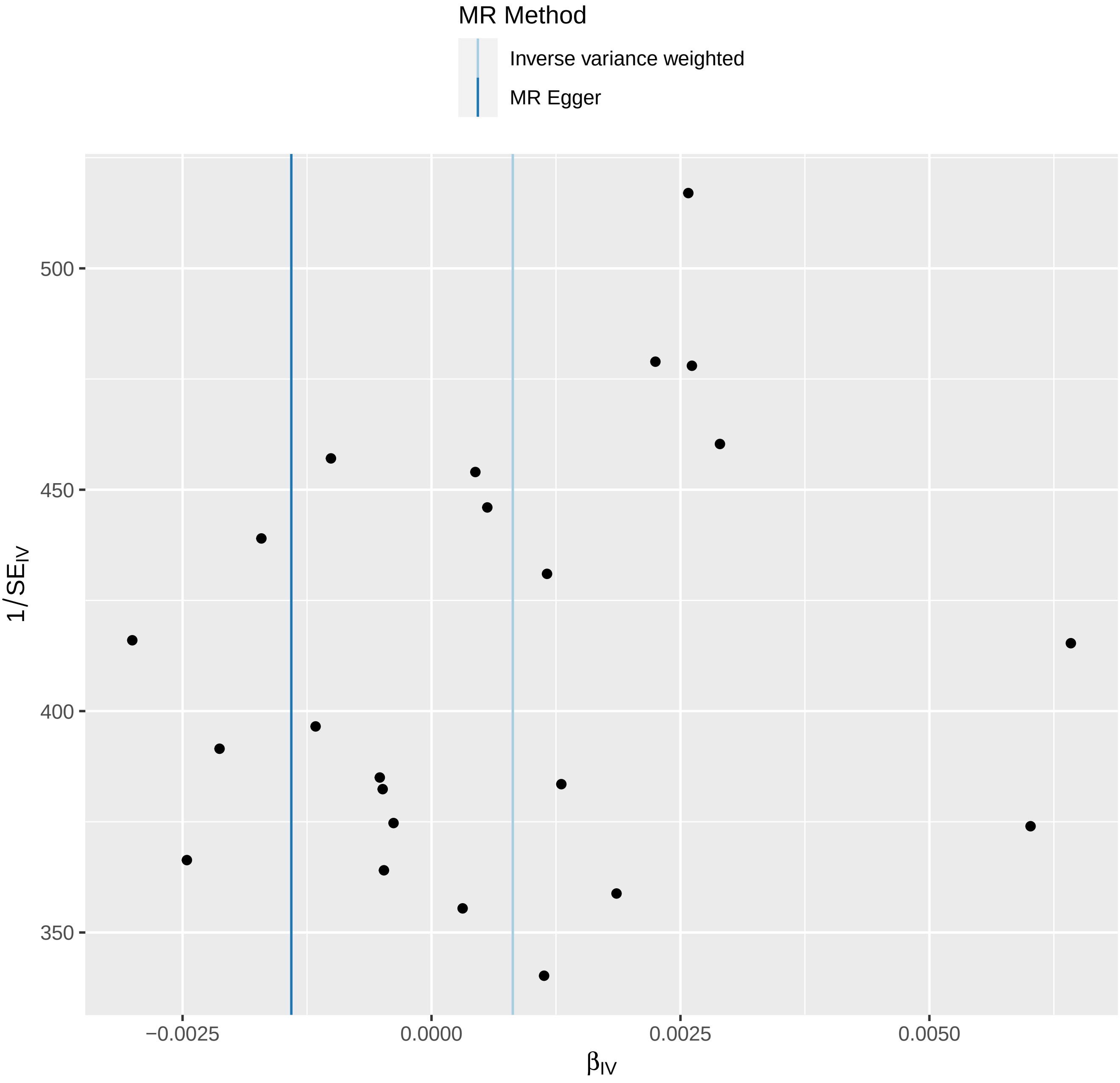

Batch 2−2147 : Osteomodulin on eGFR

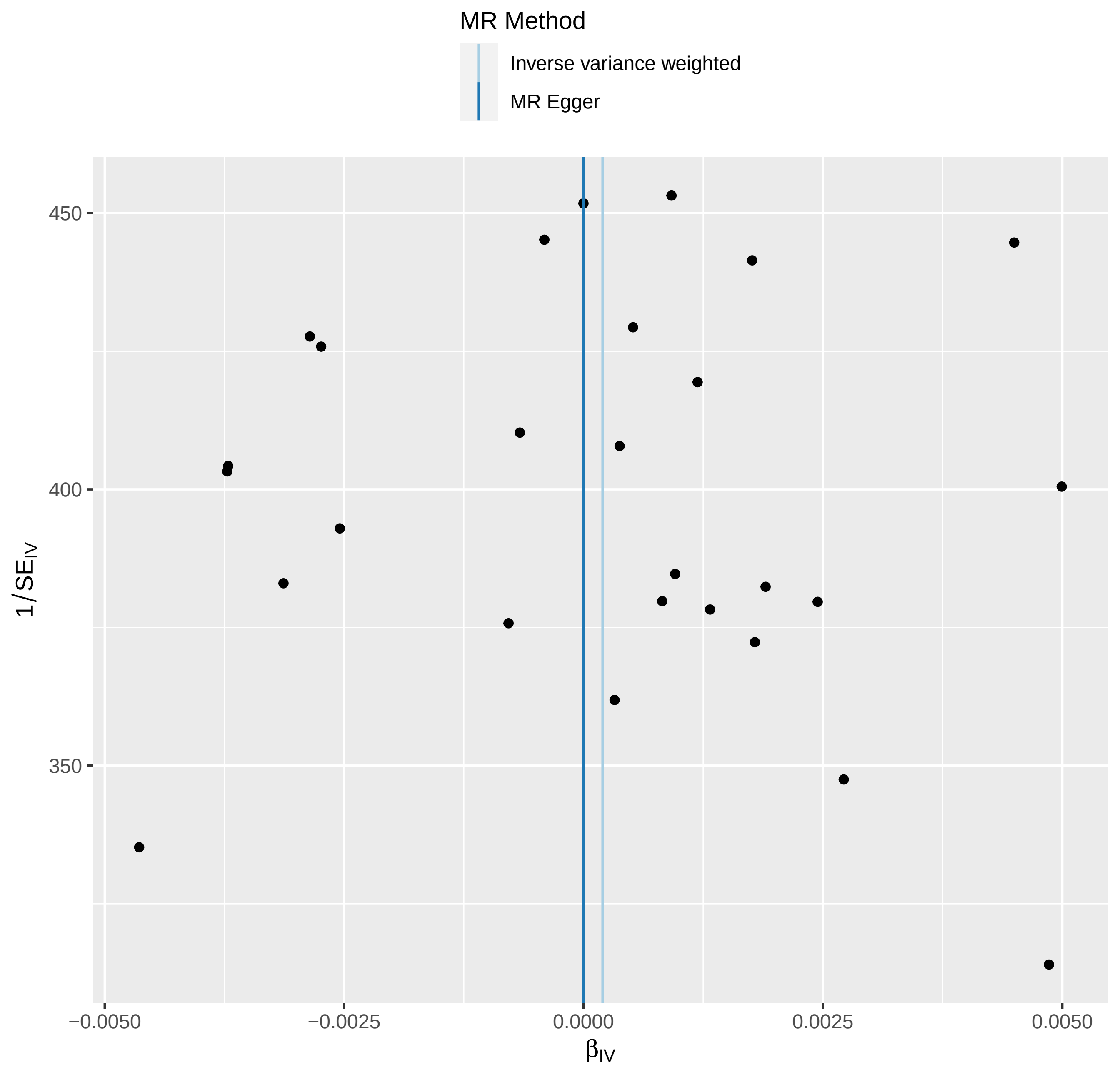

Batch 2−2148 : Opalin on eGFR

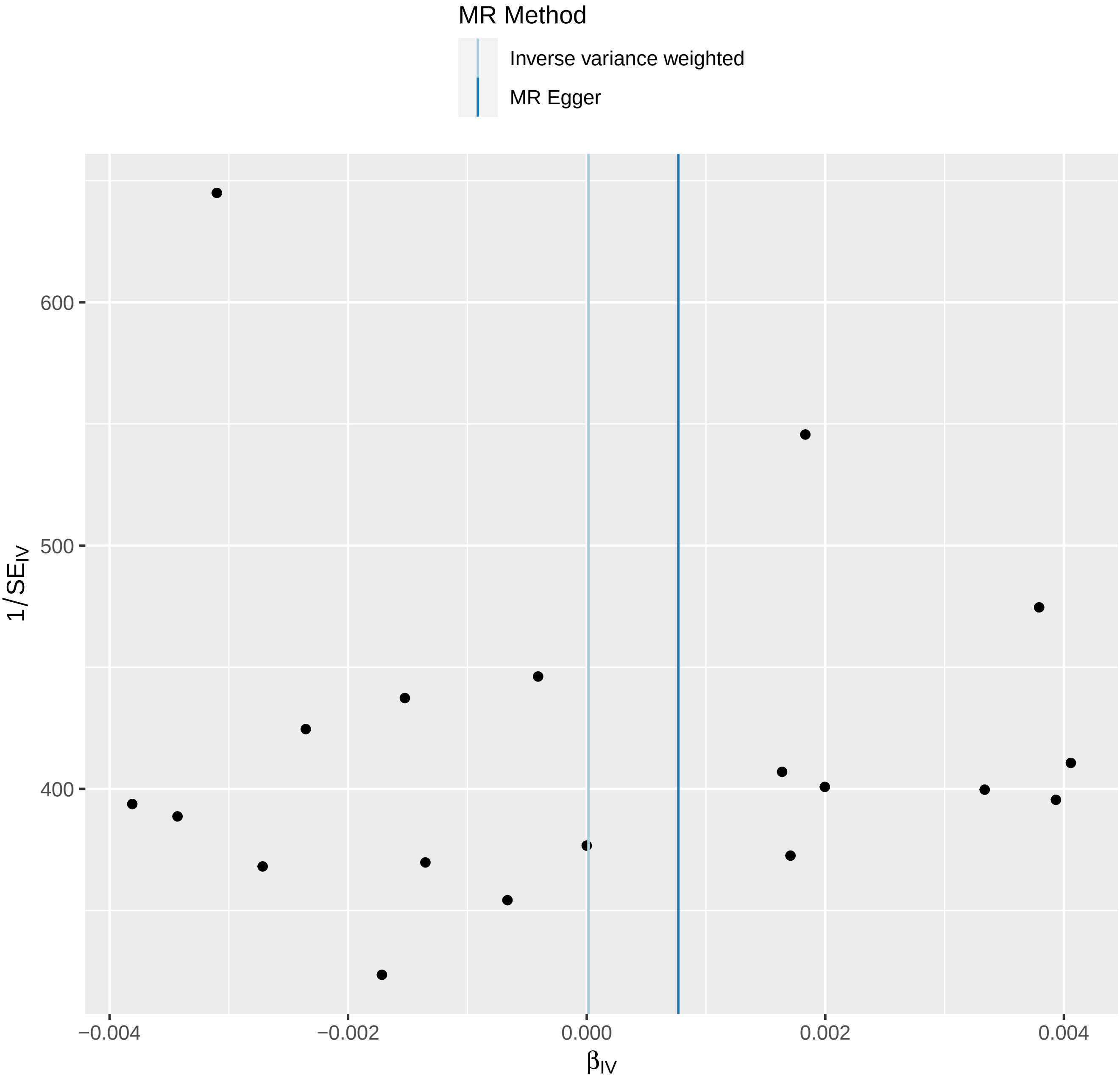

Batch 2−2149 : Opioid−binding protein/cell adhesion molecule on eGFR

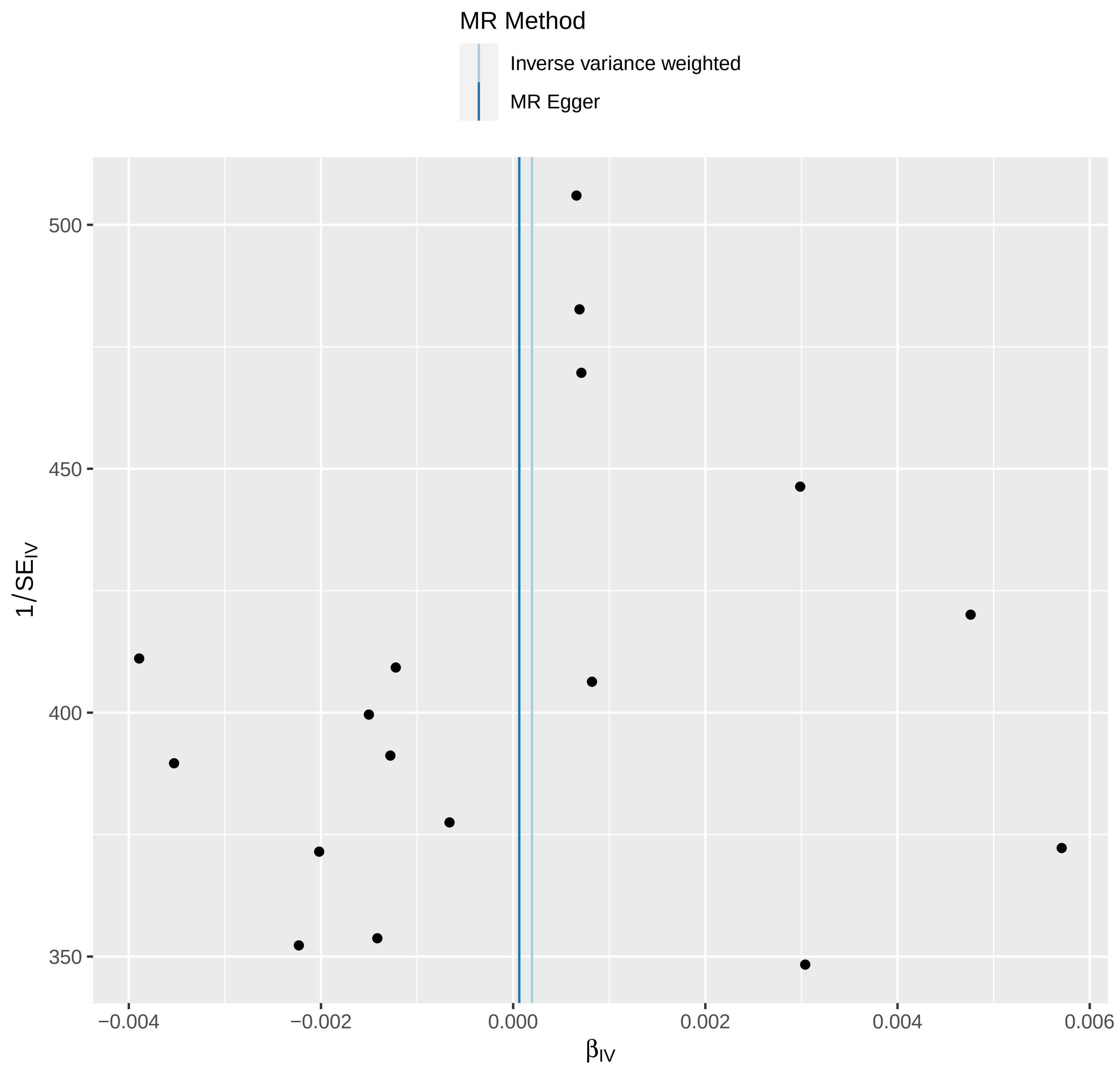

Batch 2−2150 : Oligophrenin−1 on eGFR

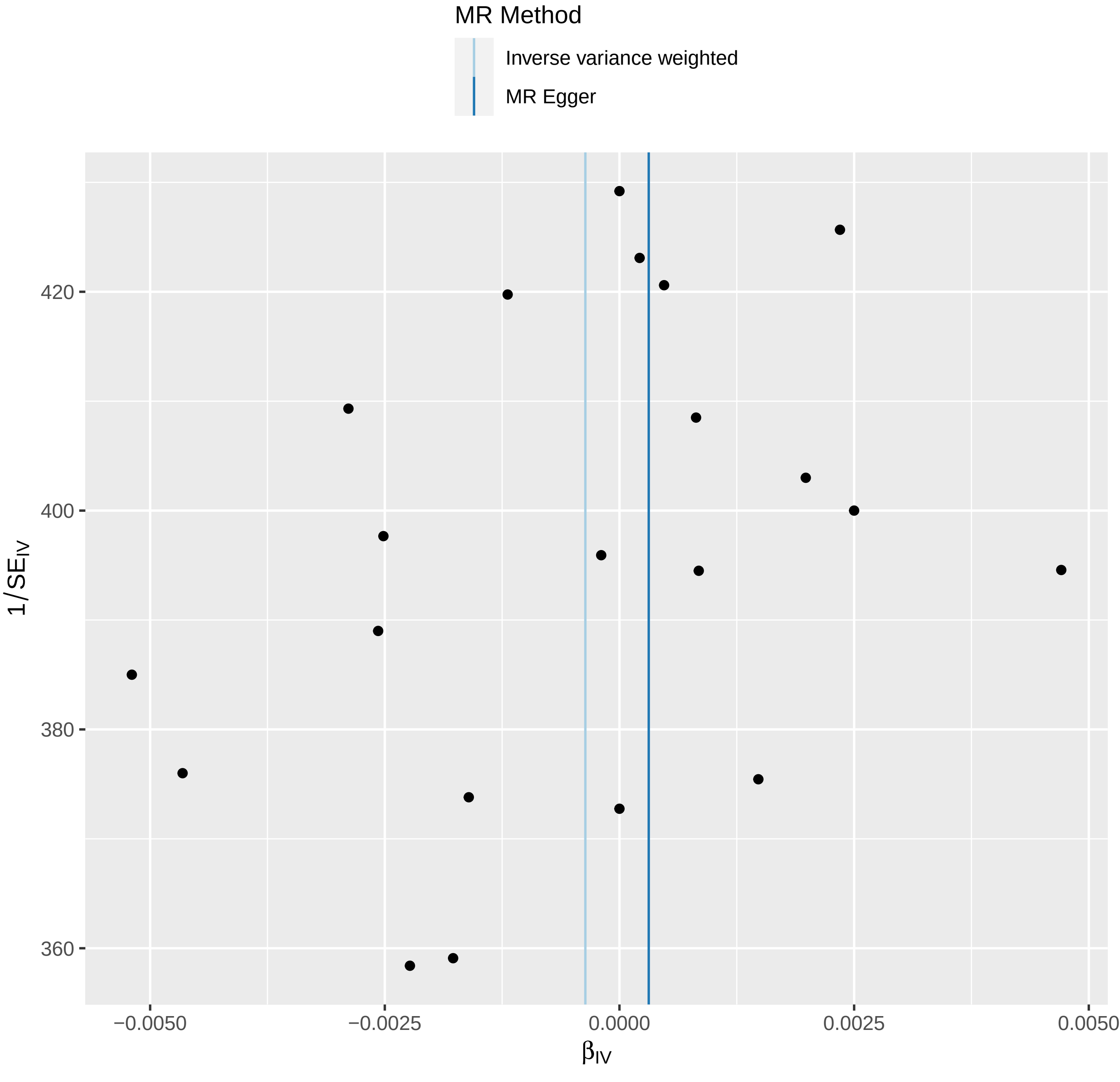

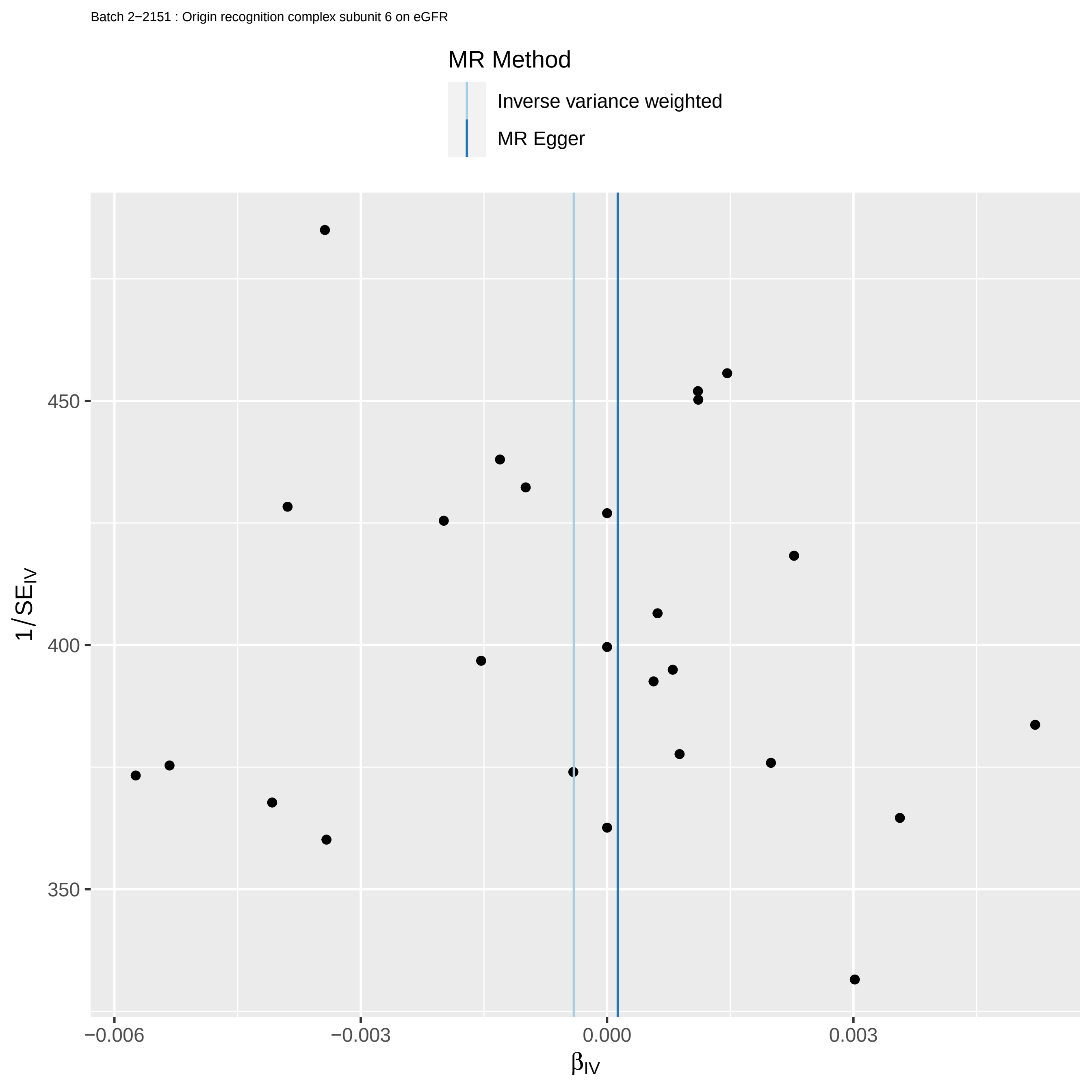
Batch 2−2151 : Origin recognition complex subunit 6 on eGFR
MR Method
Inverse variance weighted
MR Egger
450
400
350
1/SE$_{IV}$
−0.006
−0.003
0.000
0.003
β$_{IV}$

Batch 2−2152 : Oxysterol−binding protein−related protein 11 on eGFR

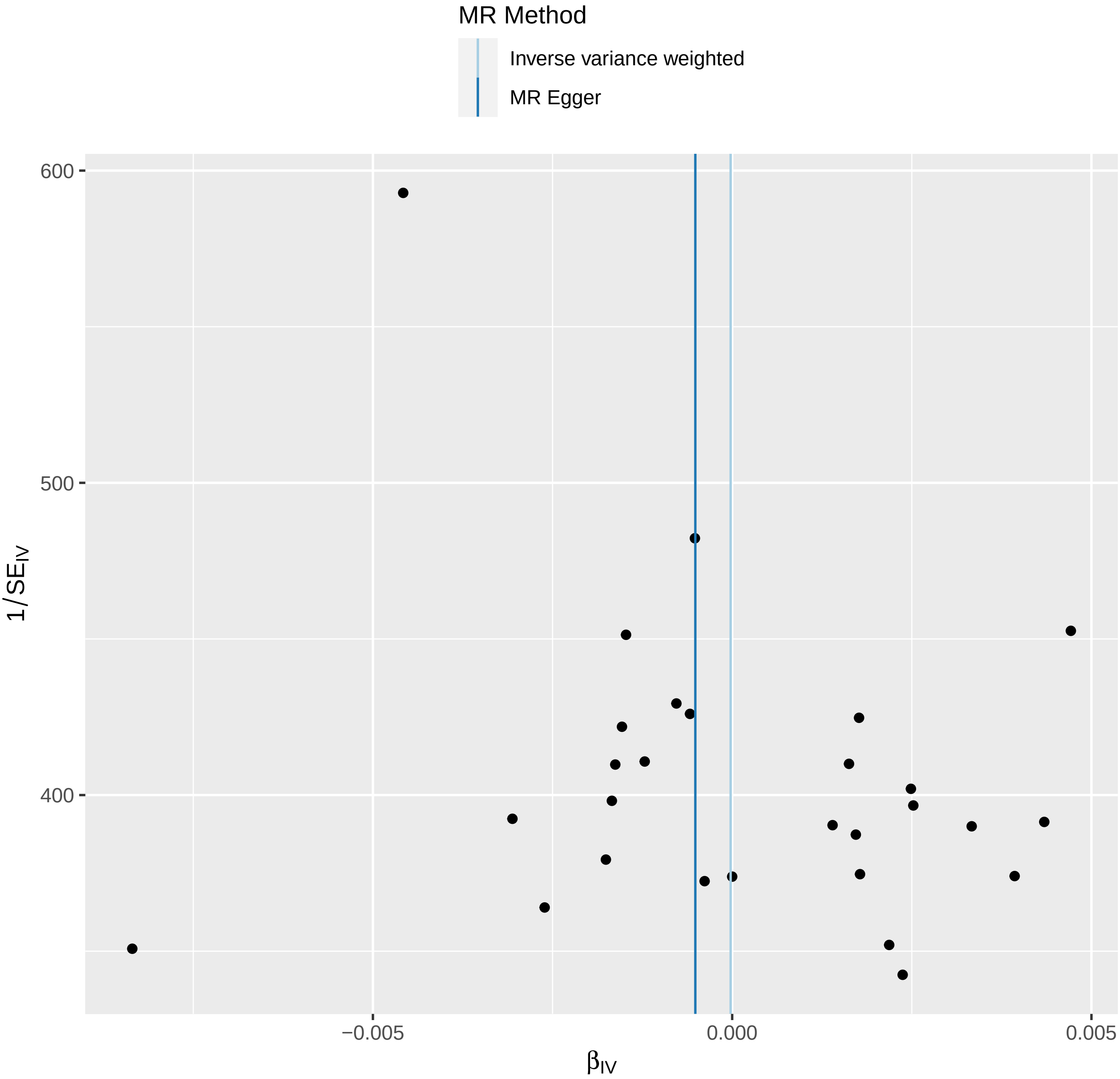

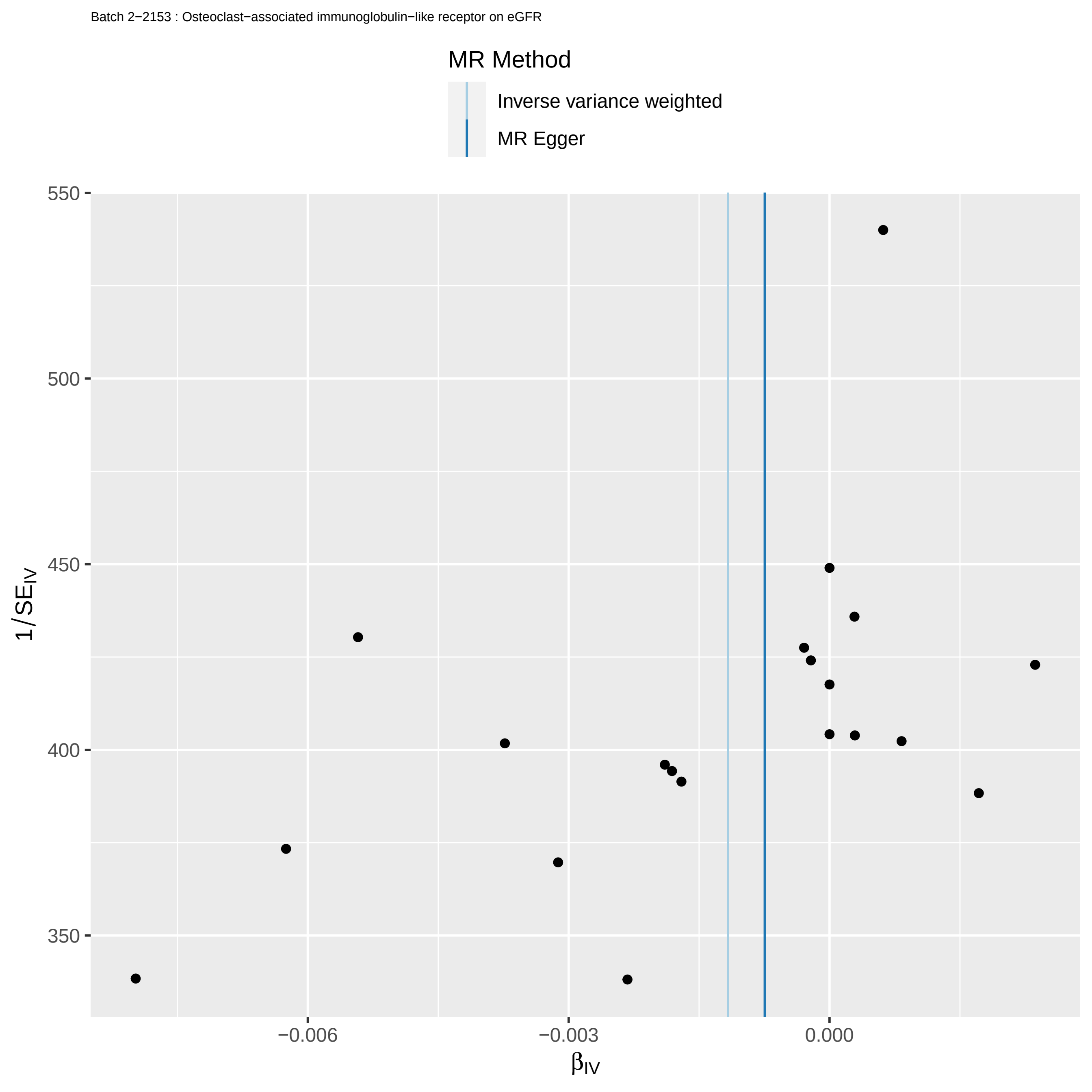
Batch 2−2153 : Osteoclast−associated immunoglobulin−like receptor on eGFR
MR Method
Inverse variance weighted
MR Egger
550
500
450
400
350
1/SE_IV
−0.006
−0.003
0.000
β_IV

Batch 2−2154 : Oncostatin−M on eGFR

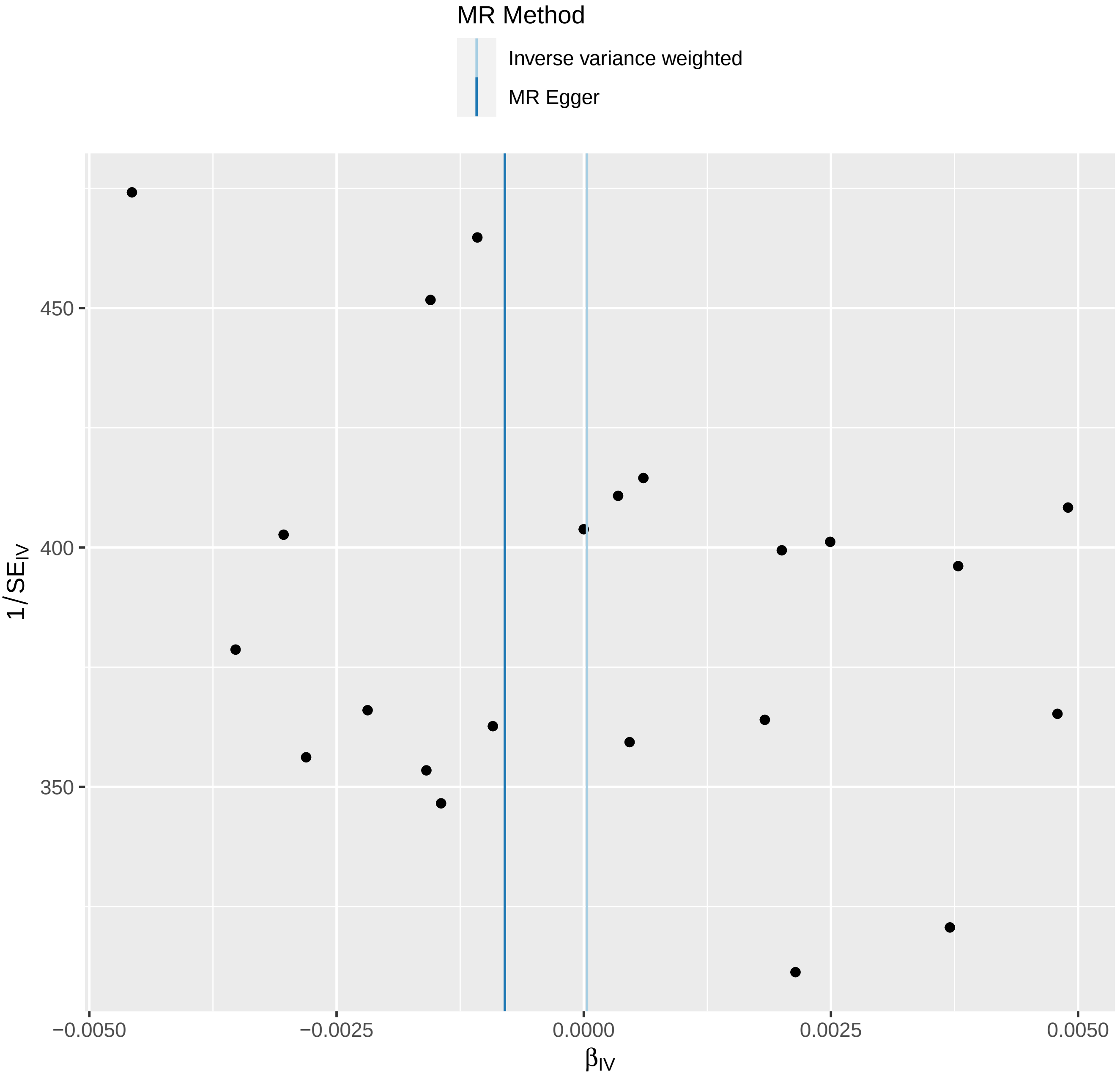

Batch 2−2155 : Oncostatin−M on eGFR

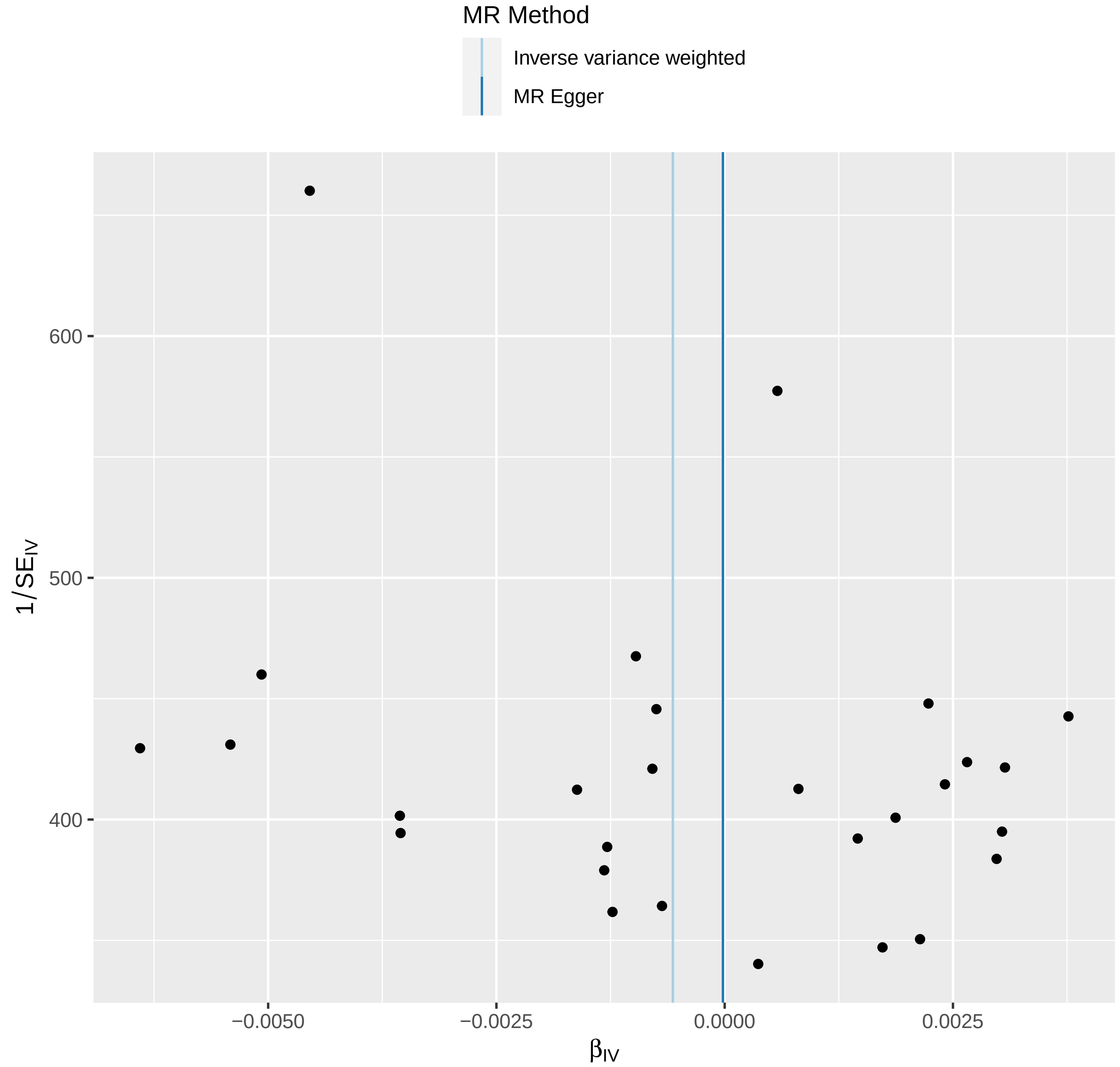

Batch 2−2156 : Oncostatin−M−specific receptor subunit beta on eGFR

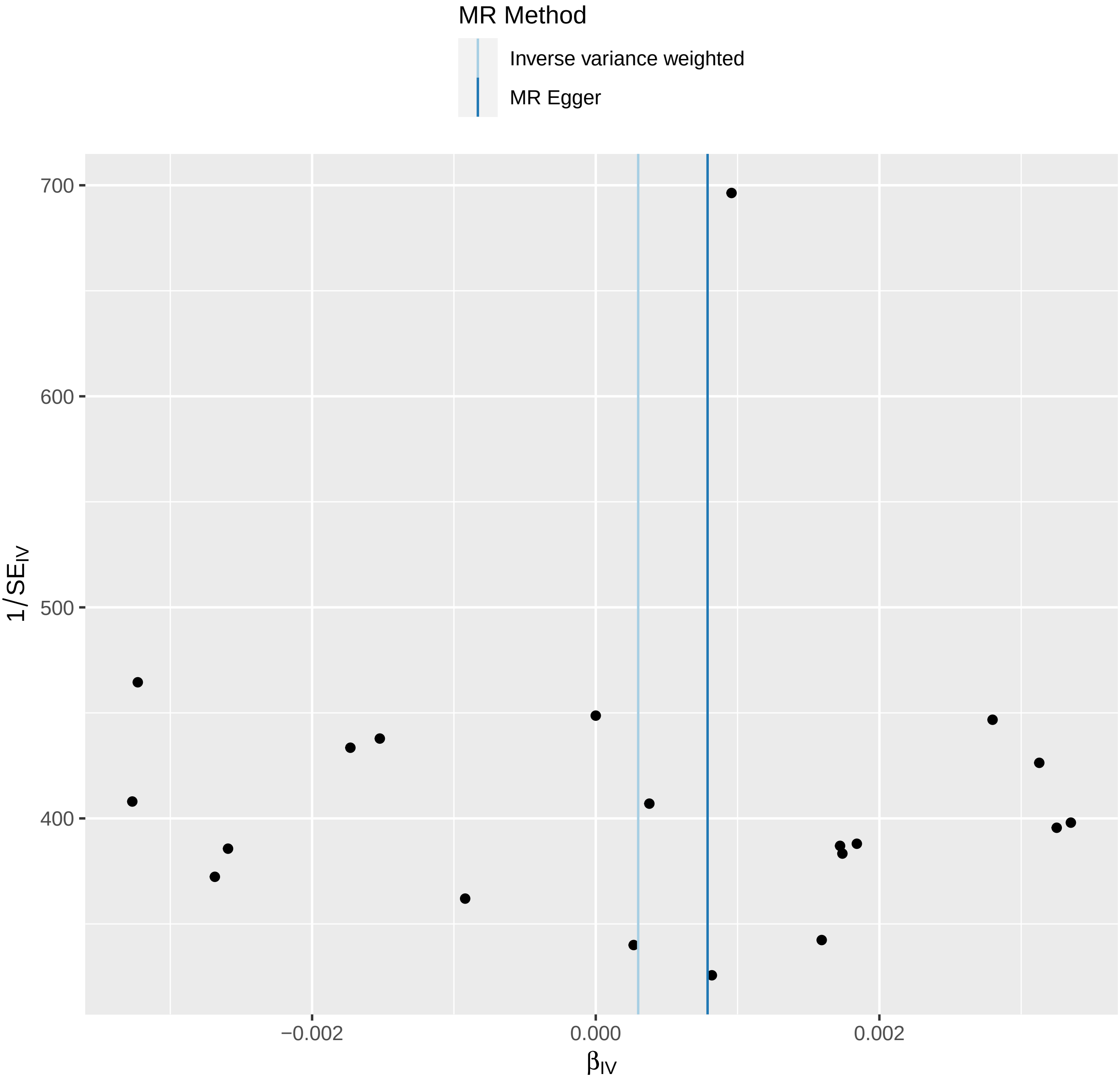

Batch 2−2157 : Osteocrin on eGFR

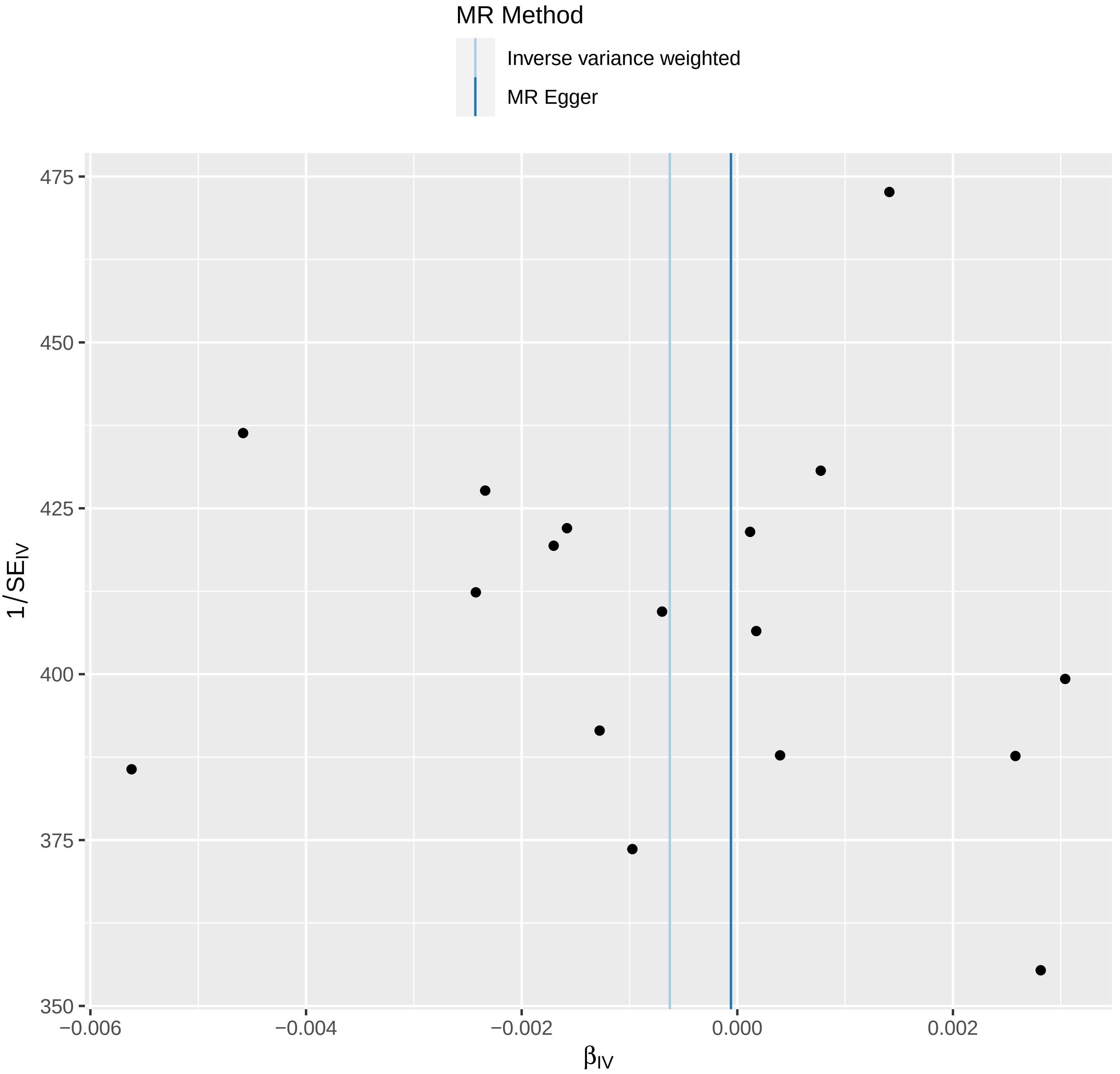

Batch 2−2158 : Ubiquitin thioesterase OTUB2 on eGFR

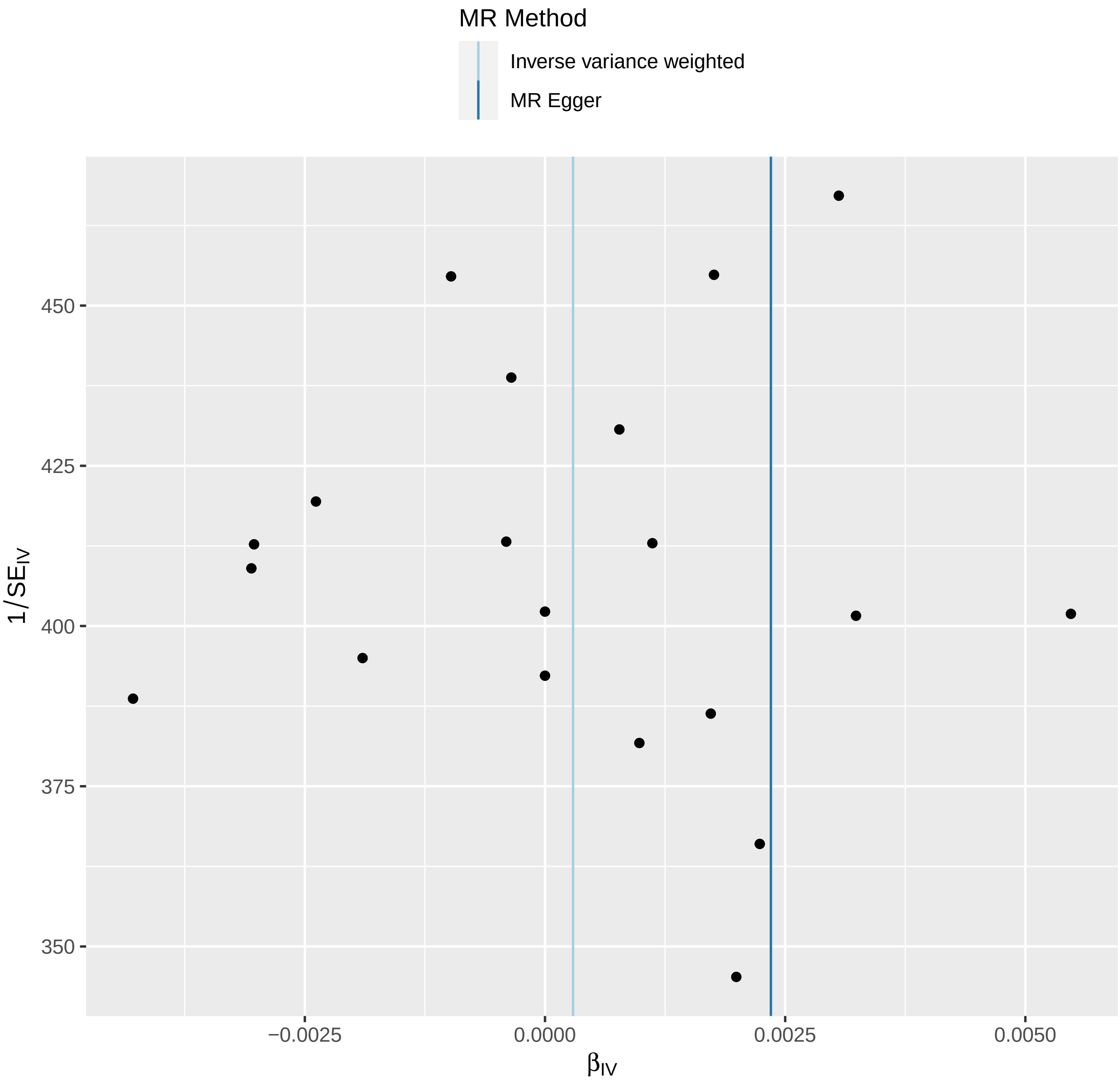

Batch 2−2159 : Oxytocin−neurophysin 1 on eGFR

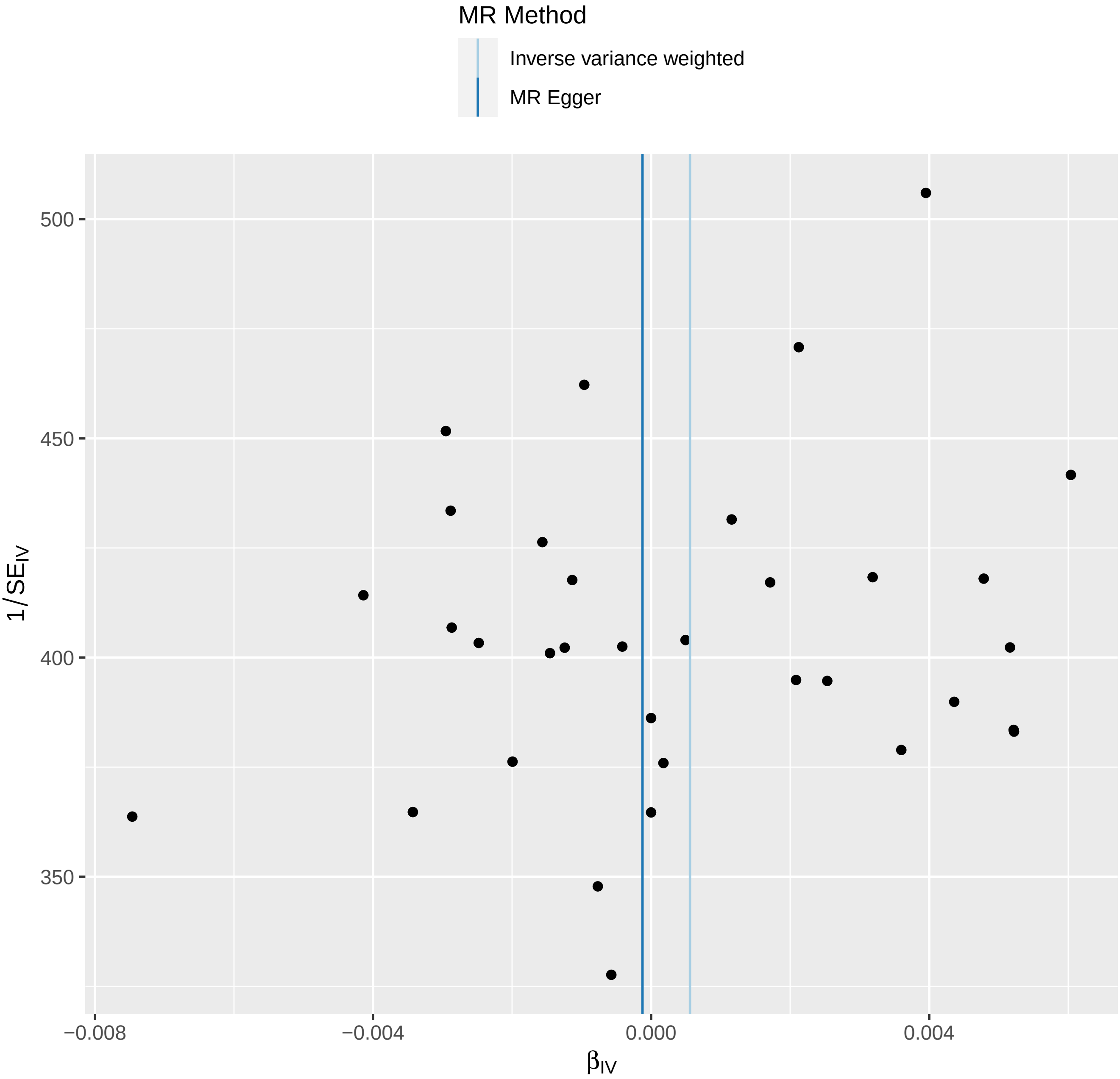

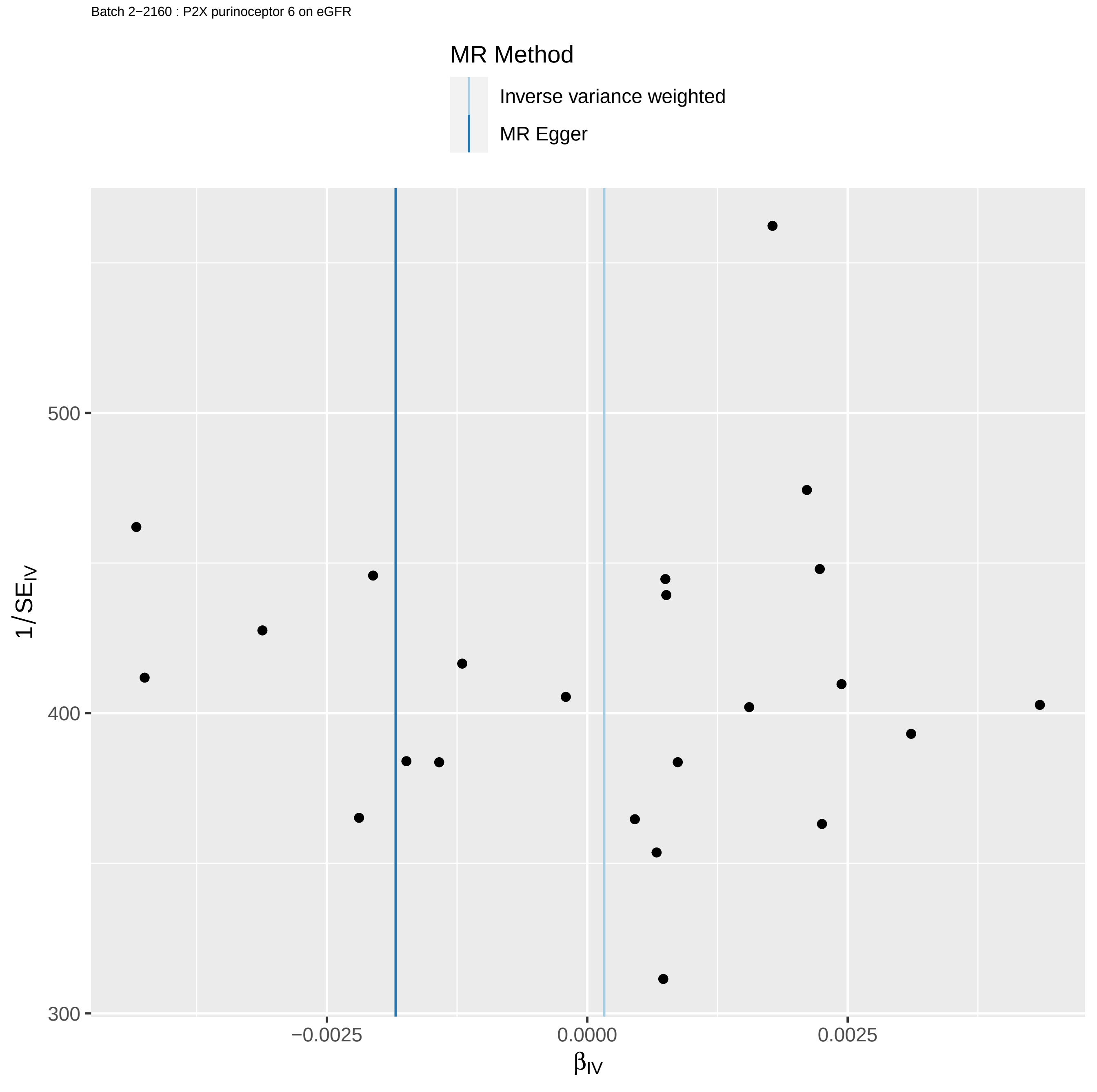

Batch 2−2160 : P2X purinoceptor 6 on eGFR
MR Method
Inverse variance weighted
MR Egger
500
400
300
1/SE$_{IV}$
−0.0025
0.0000
0.0025
β$_{IV}$

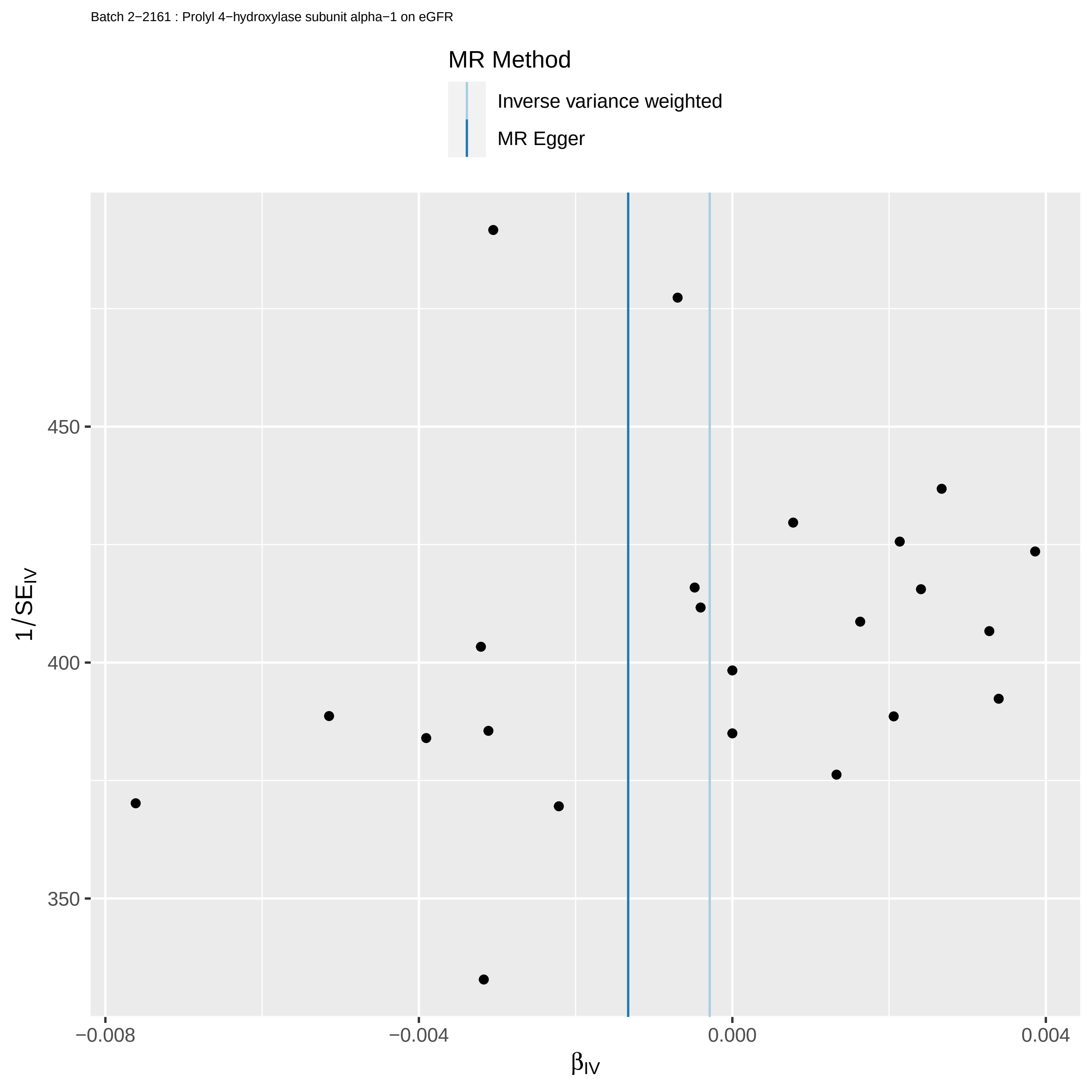

Batch 2−2161 : Prolyl 4−hydroxylase subunit alpha−1 on eGFR
MR Method
Inverse variance weighted
MR Egger
450
400
350
1/SE_IV
−0.008
−0.004
0.000
0.004
β_IV

Batch 2−2162 : Prolyl 4−hydroxylase subunit alpha−2 on eGFR

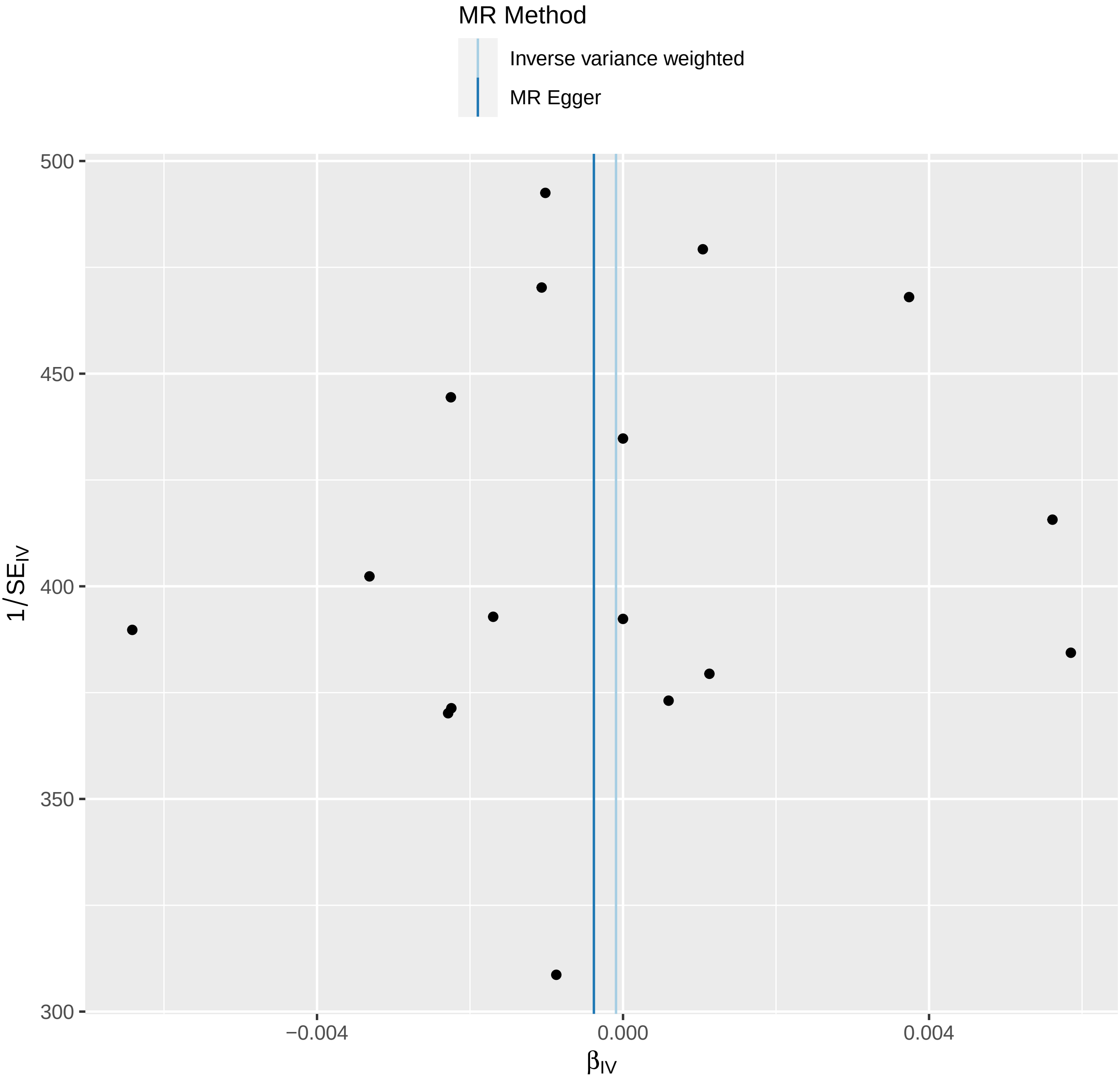

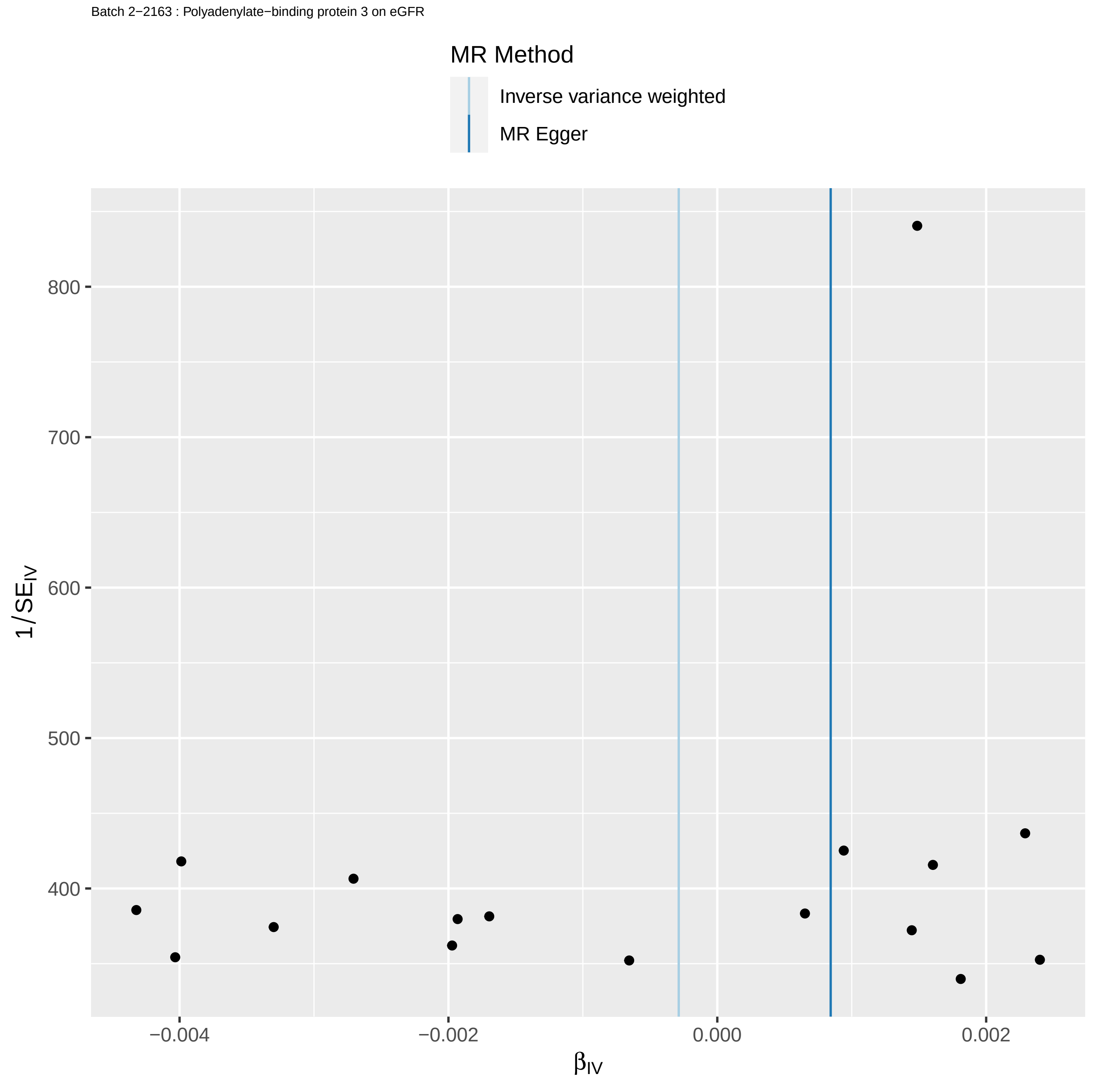
Batch 2−2163 : Polyadenylate−binding protein 3 on eGFR
MR Method
Inverse variance weighted
MR Egger
800
700
600
500
400
1/SE$_{IV}$
−0.004
−0.002
0.000
0.002
β$_{IV}$

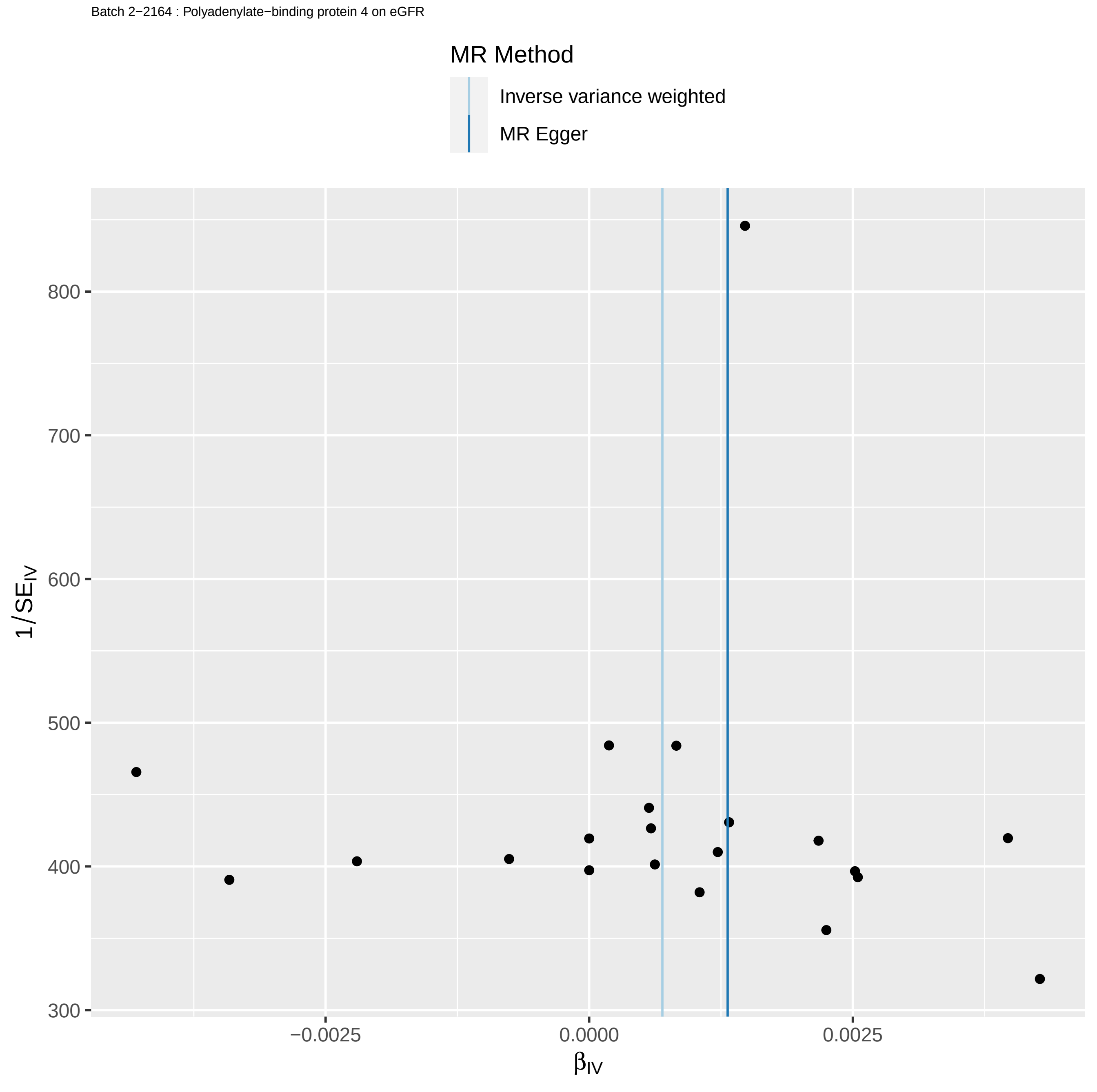
Batch 2−2164 : Polyadenylate−binding protein 4 on eGFR
MR Method
Inverse variance weighted
MR Egger
800
700
600
500
400
300
1/SE_IV
−0.0025
0.0000
0.0025
β_IV

Batch 2−2165 : Marginal zone B− and B1−cell−specific protein on eGFR

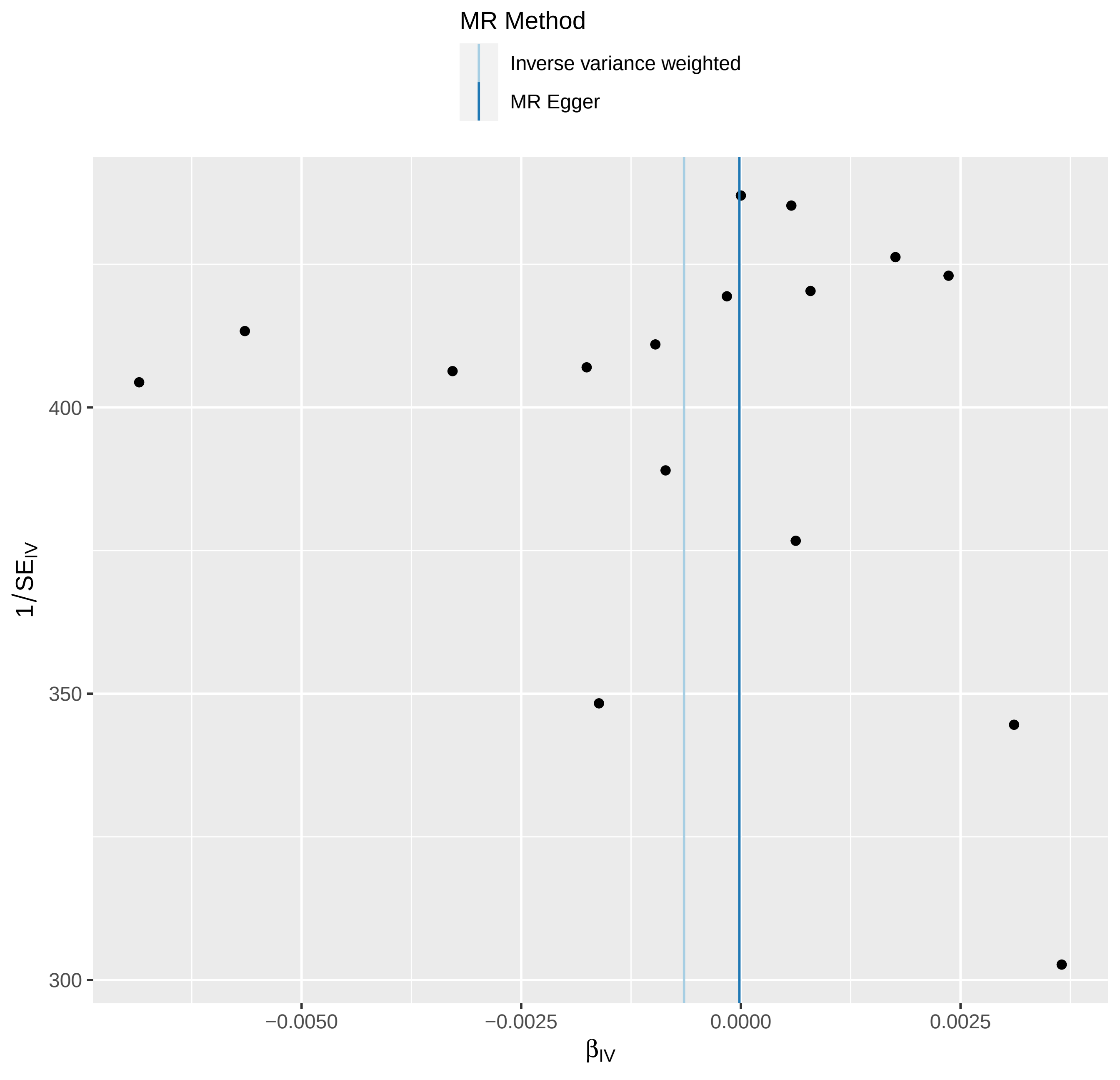

Batch 2−2166 : Platelet−activating factor acetylhydrolase IB subunit beta on eGFR

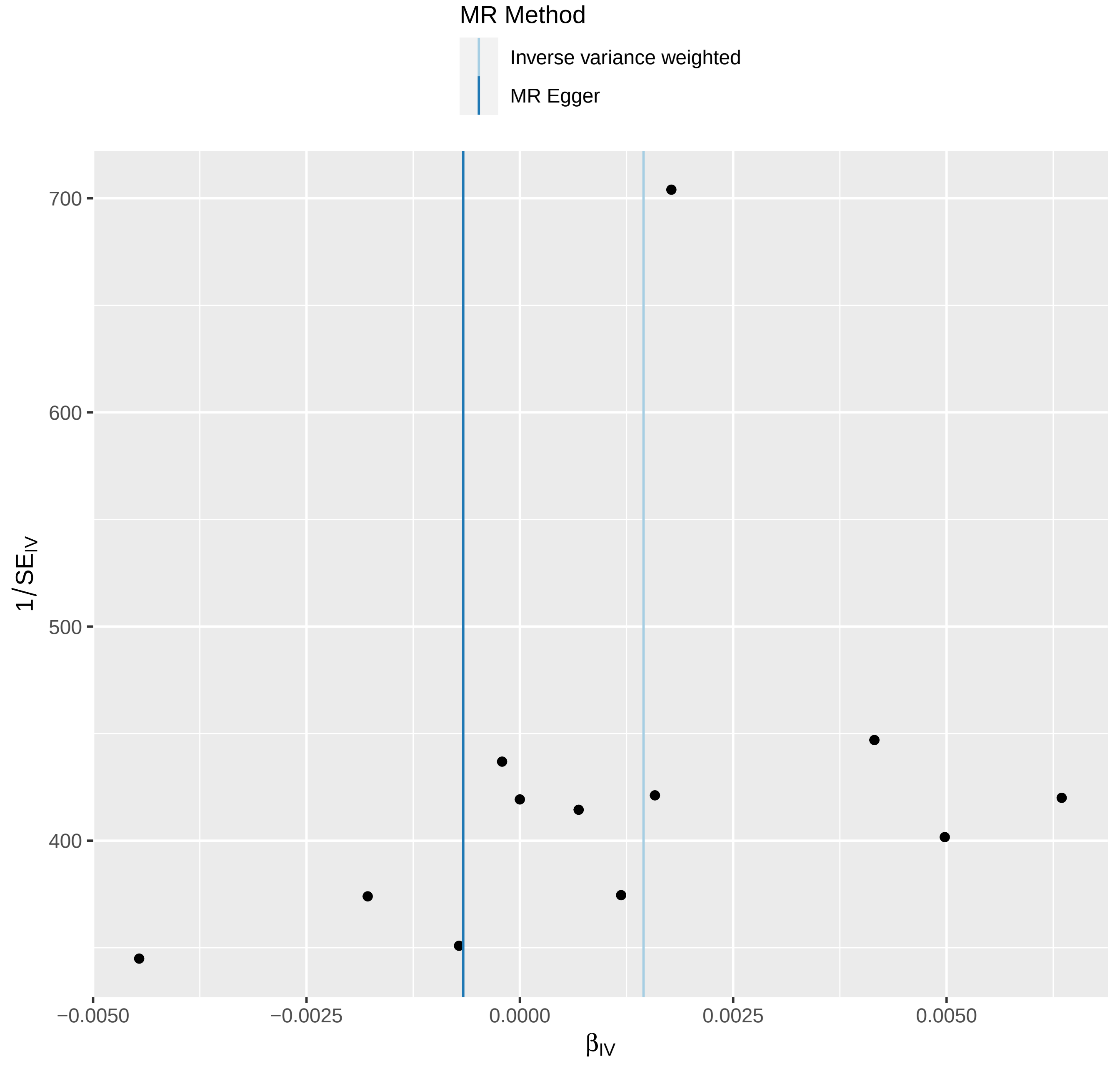

Batch 2−2167 : Multifunctional protein ADE2 on eGFR

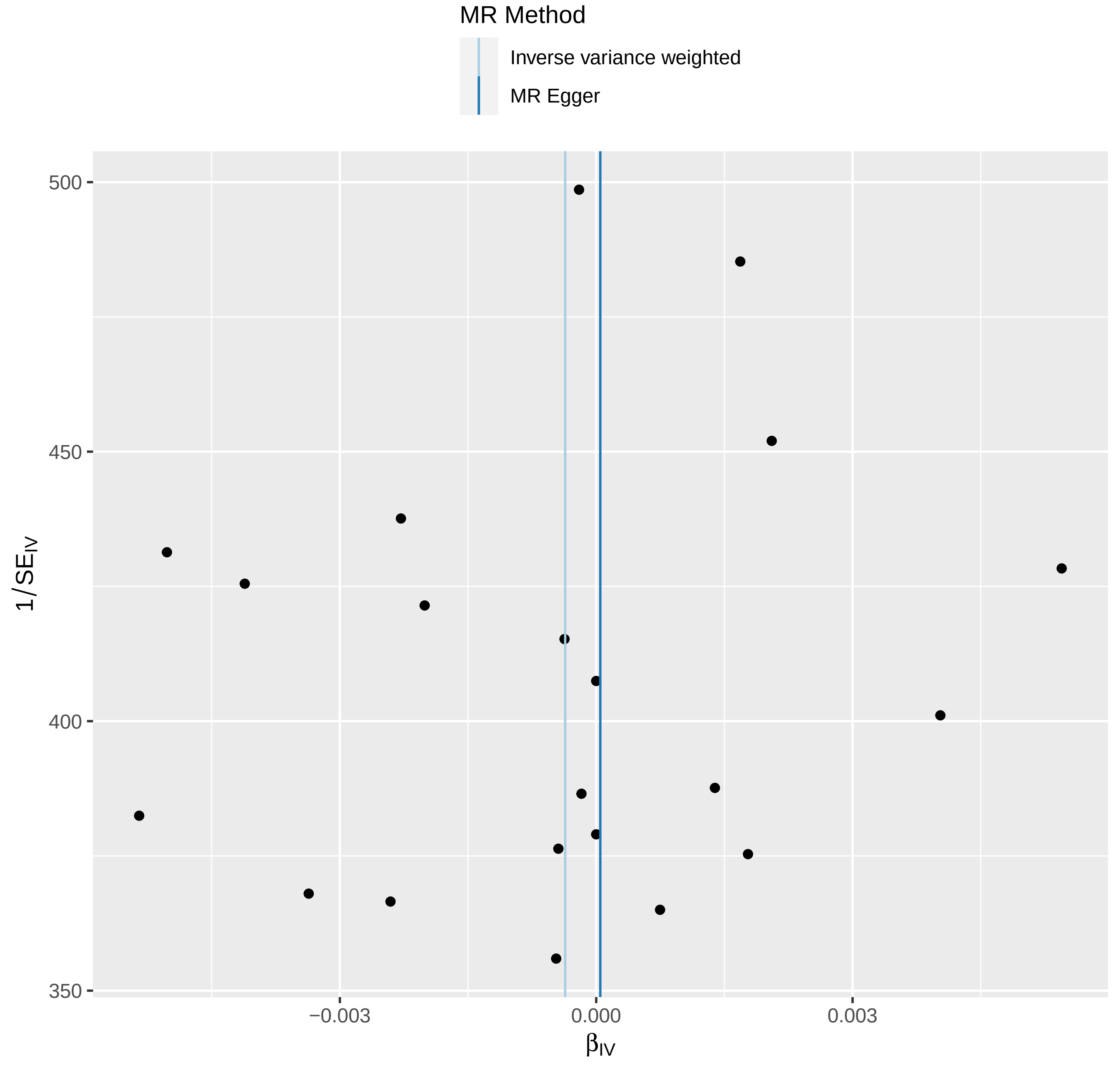

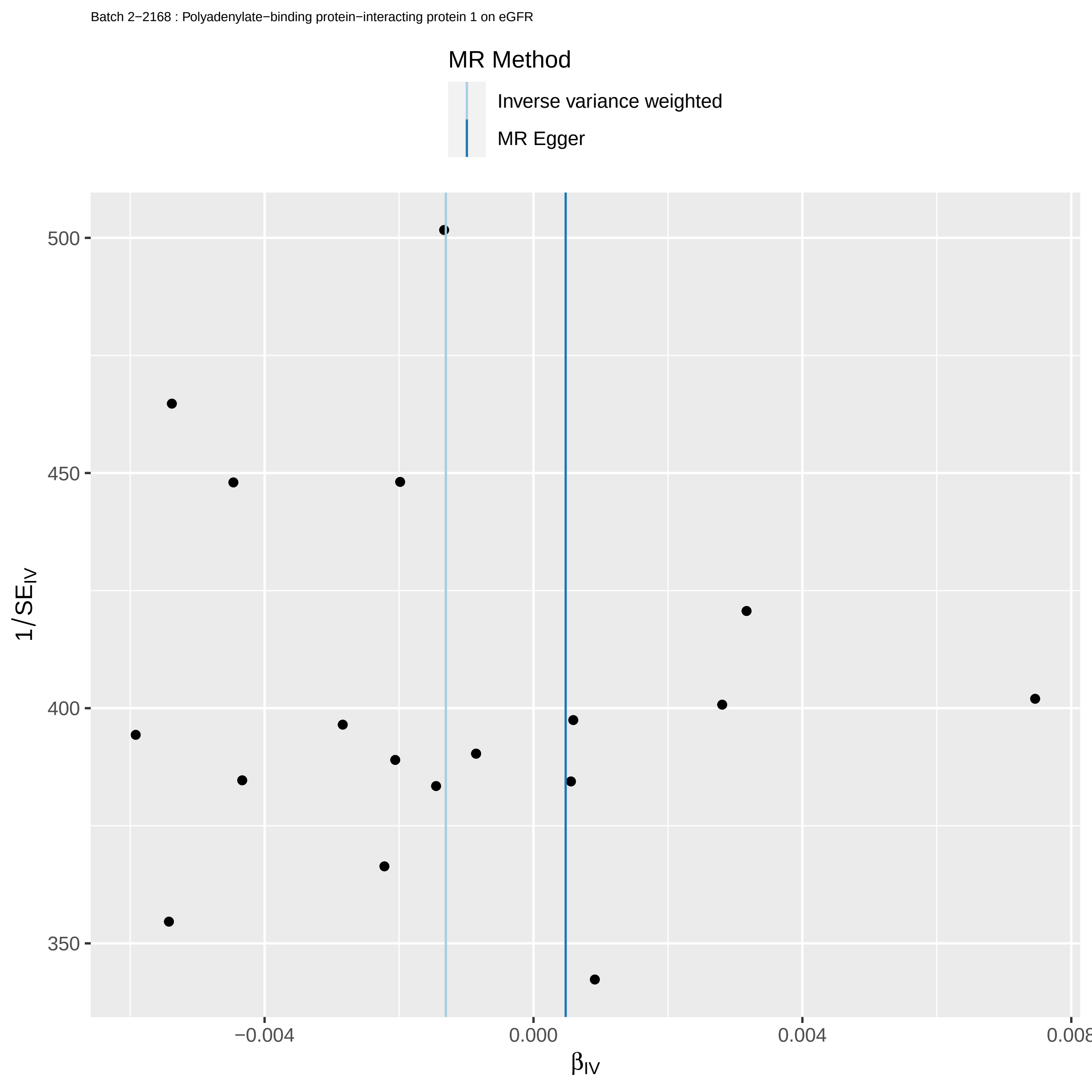

Batch 2−2168 : Polyadenylate−binding protein−interacting protein 1 on eGFR
MR Method
Inverse variance weighted
MR Egger
500
450
400
350
1/SE_IV
−0.004
0.000
0.004
0.008
β_IV

Batch 2−2169 : Serine/threonine−protein kinase PAK 3 on eGFR

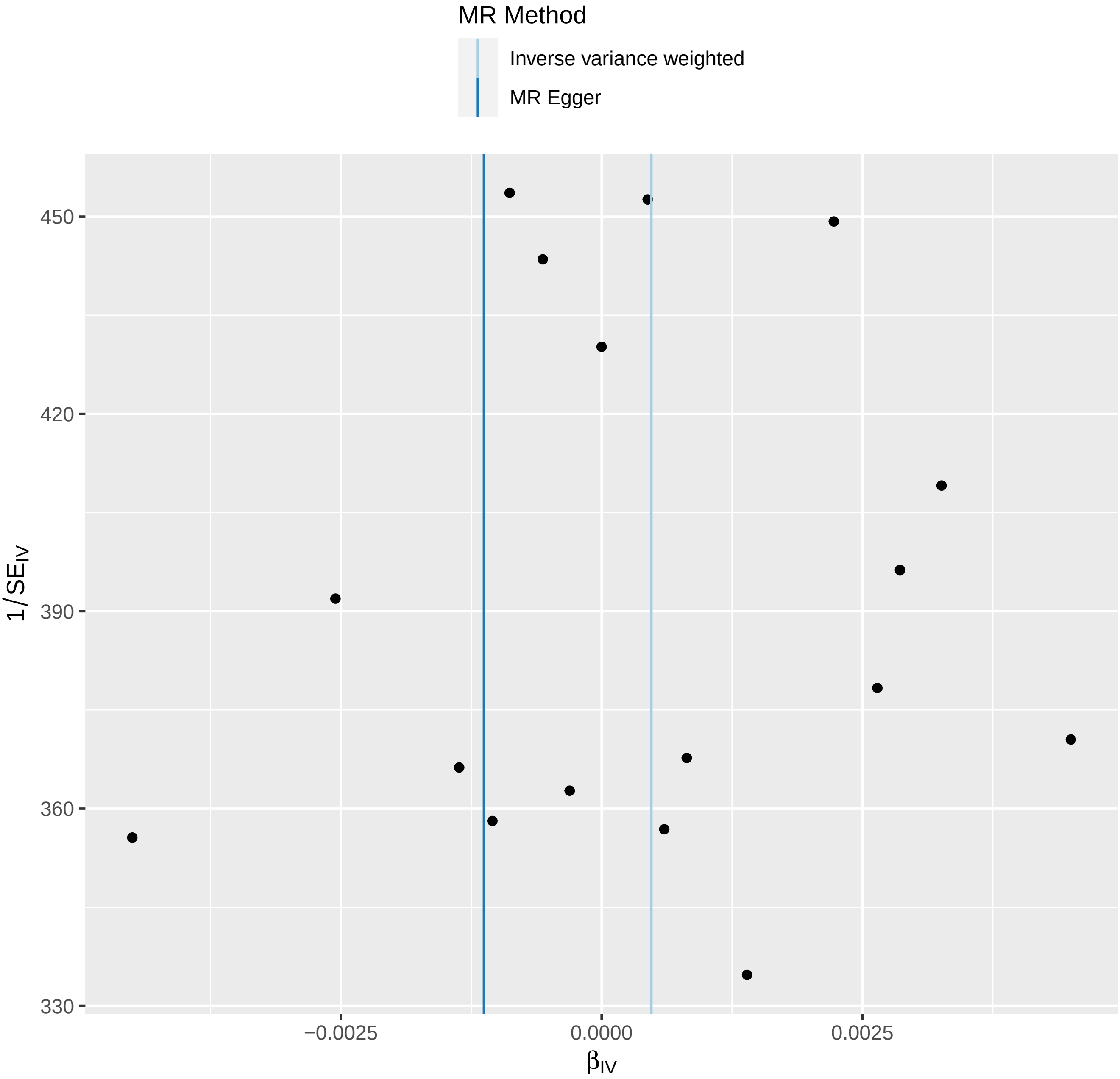

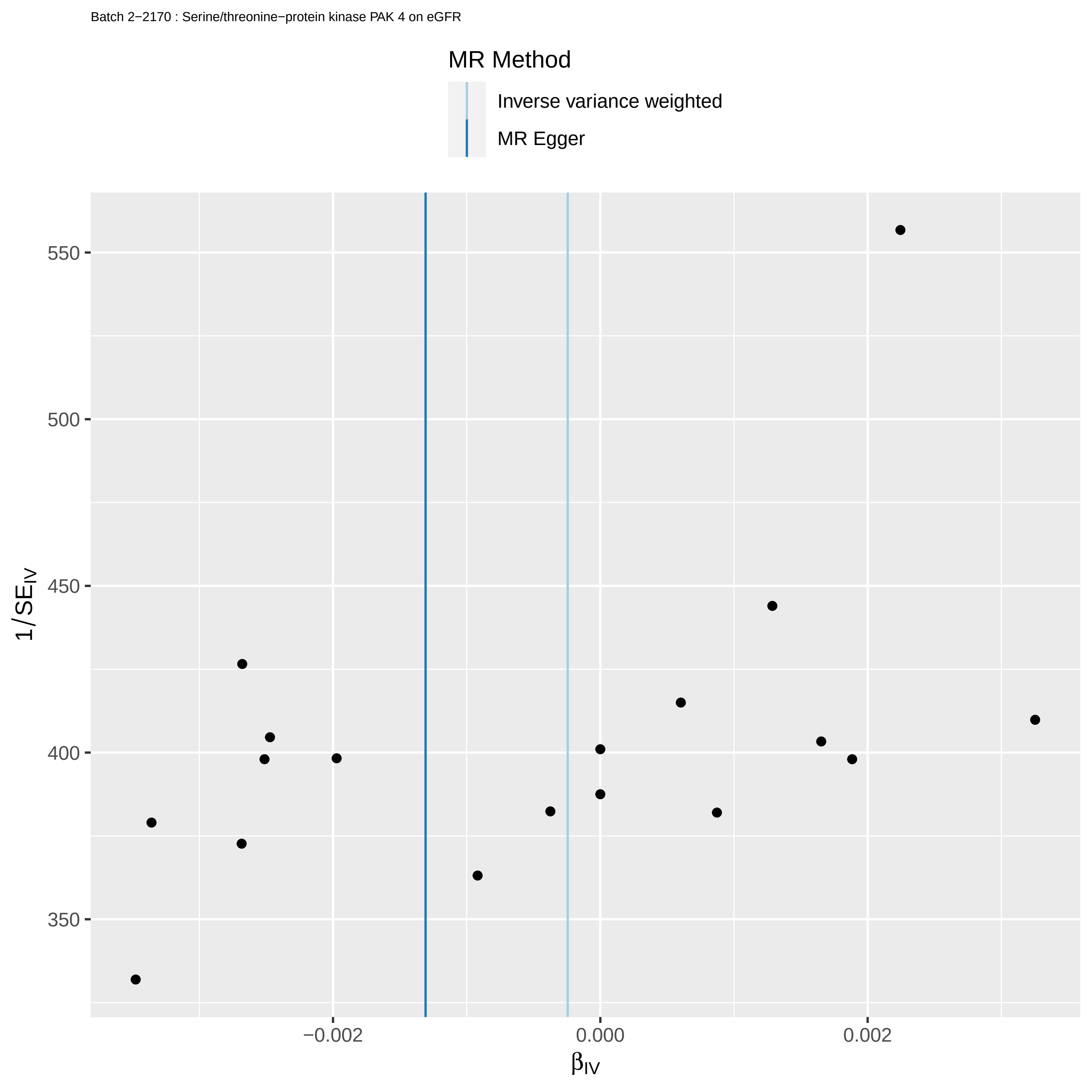

Batch 2−2170 : Serine/threonine−protein kinase PAK 4 on eGFR
MR Method
Inverse variance weighted
MR Egger
550
500
450
400
350
1/SE$_{IV}$
−0.002
0.000
0.002
β_{IV}

Batch 2−2171 : Serine/threonine−protein kinase PAK 7 on eGFR

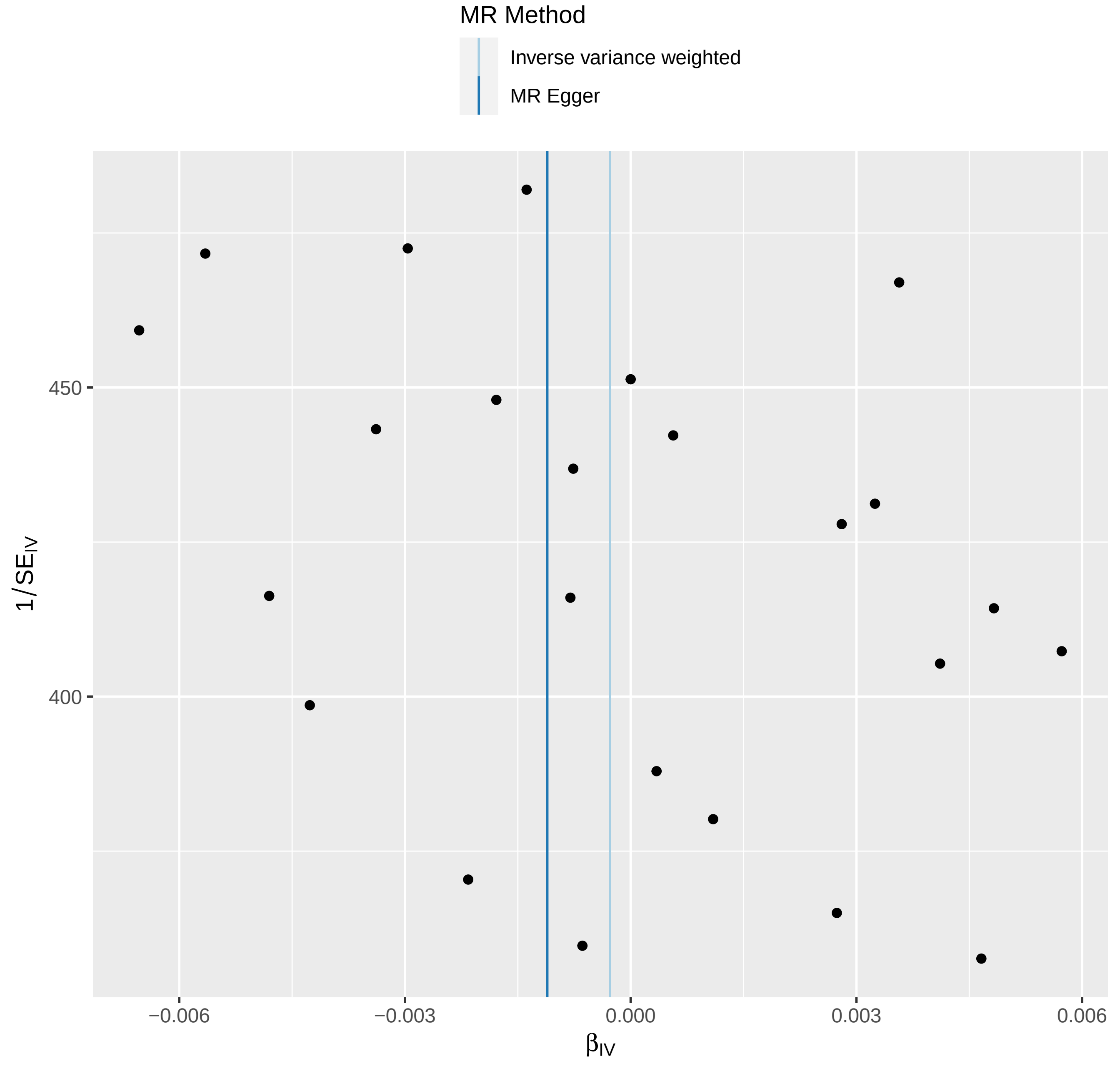

Batch 2−2172 : Peptidyl−glycine alpha−amidating monooxygenase on eGFR

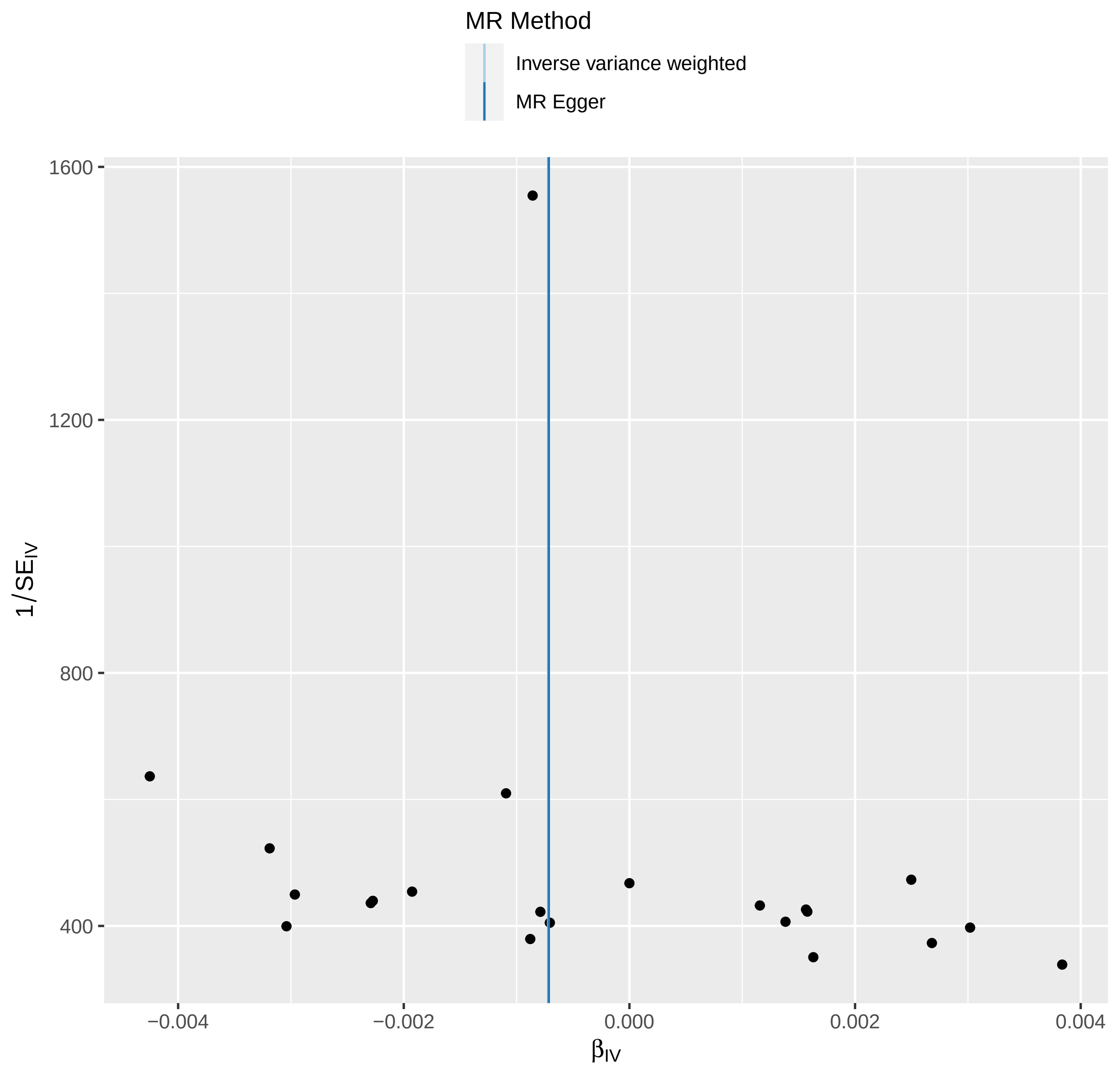

Batch 2−2173 : Pantothenate kinase 3 on eGFR

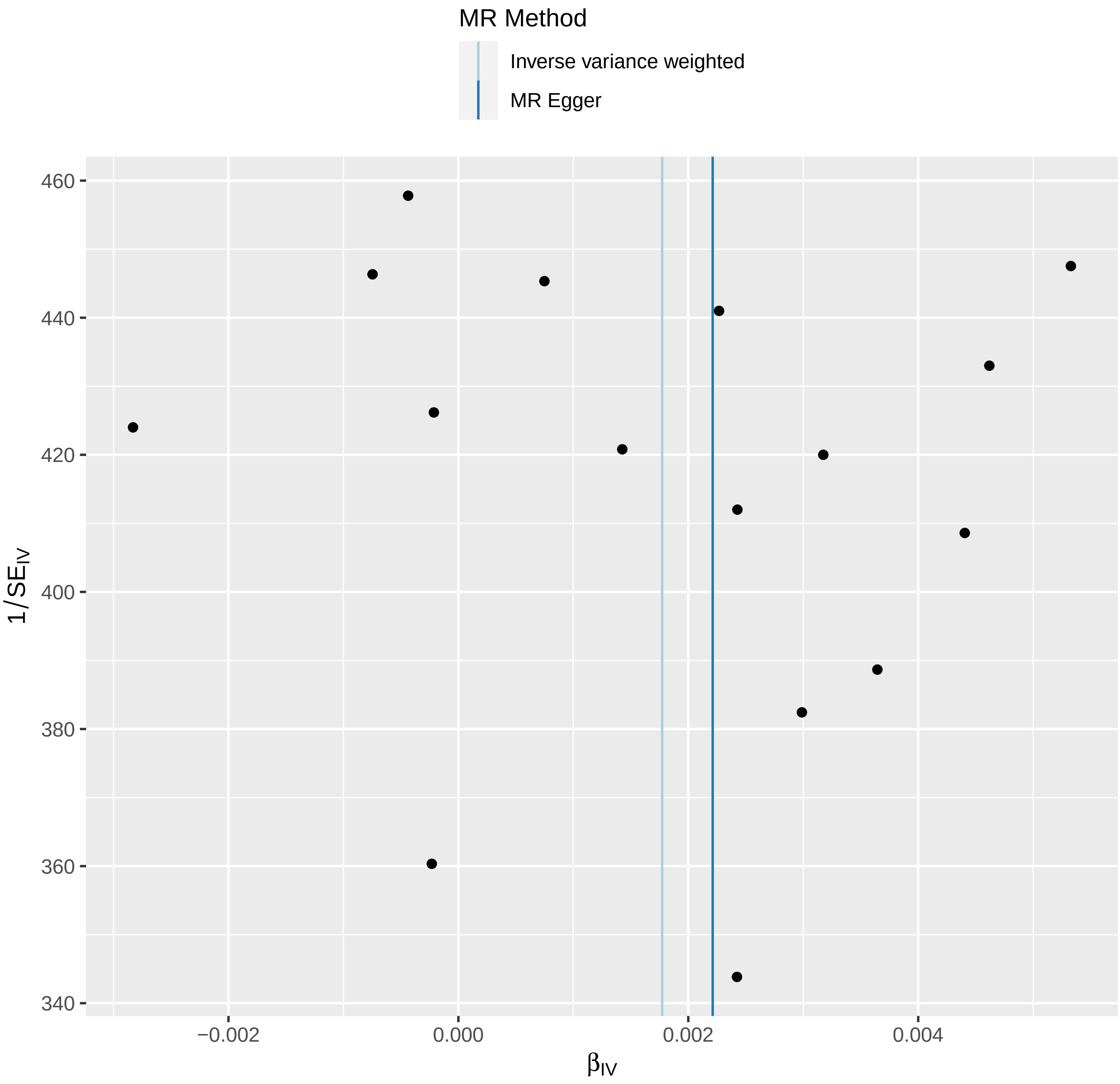

Batch 2−2174 : Papilin on eGFR

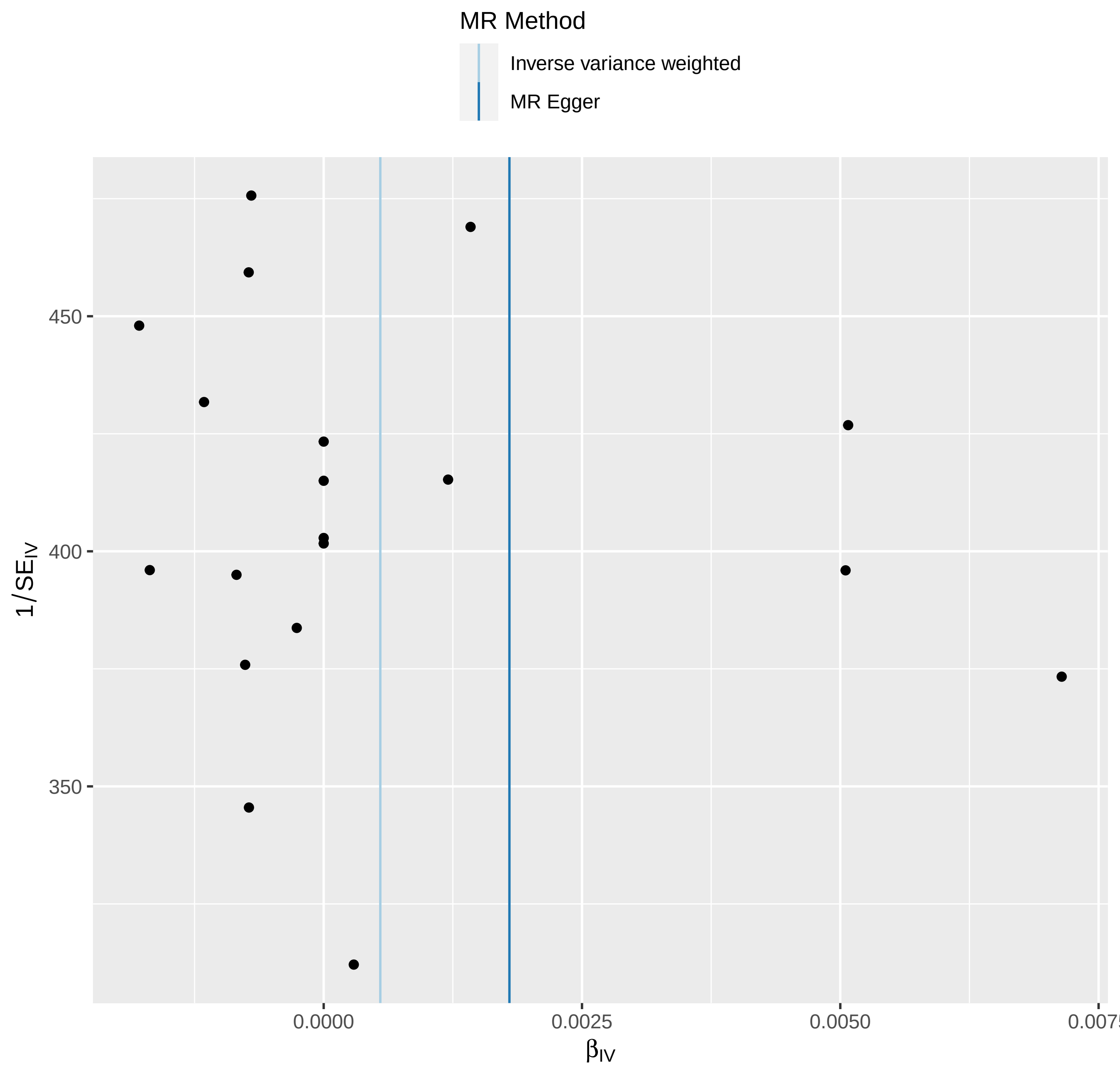

Batch 2−2175 : Poly(A) polymerase gamma on eGFR

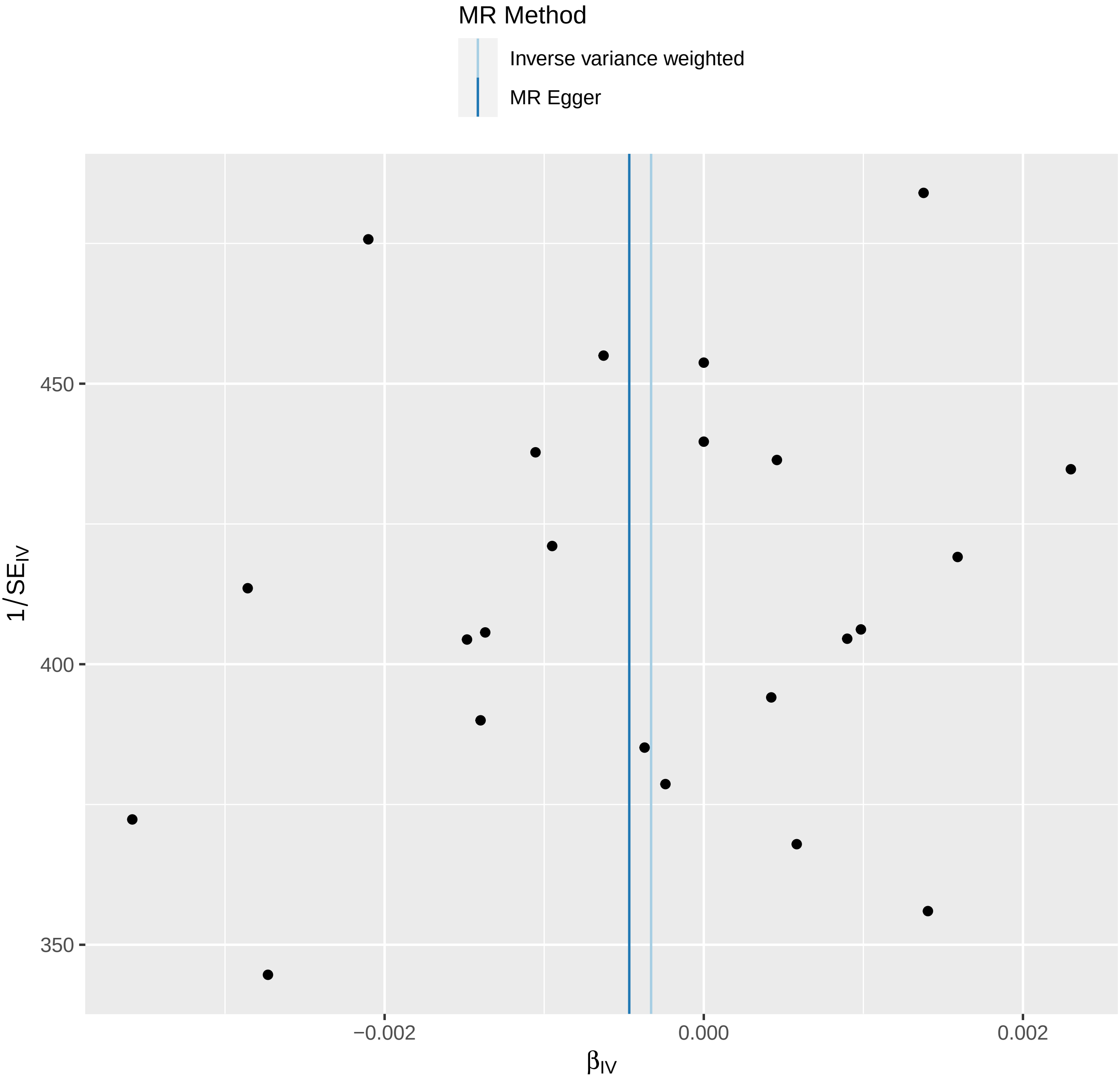

Batch 2−2176 : Pappalysin−2 on eGFR

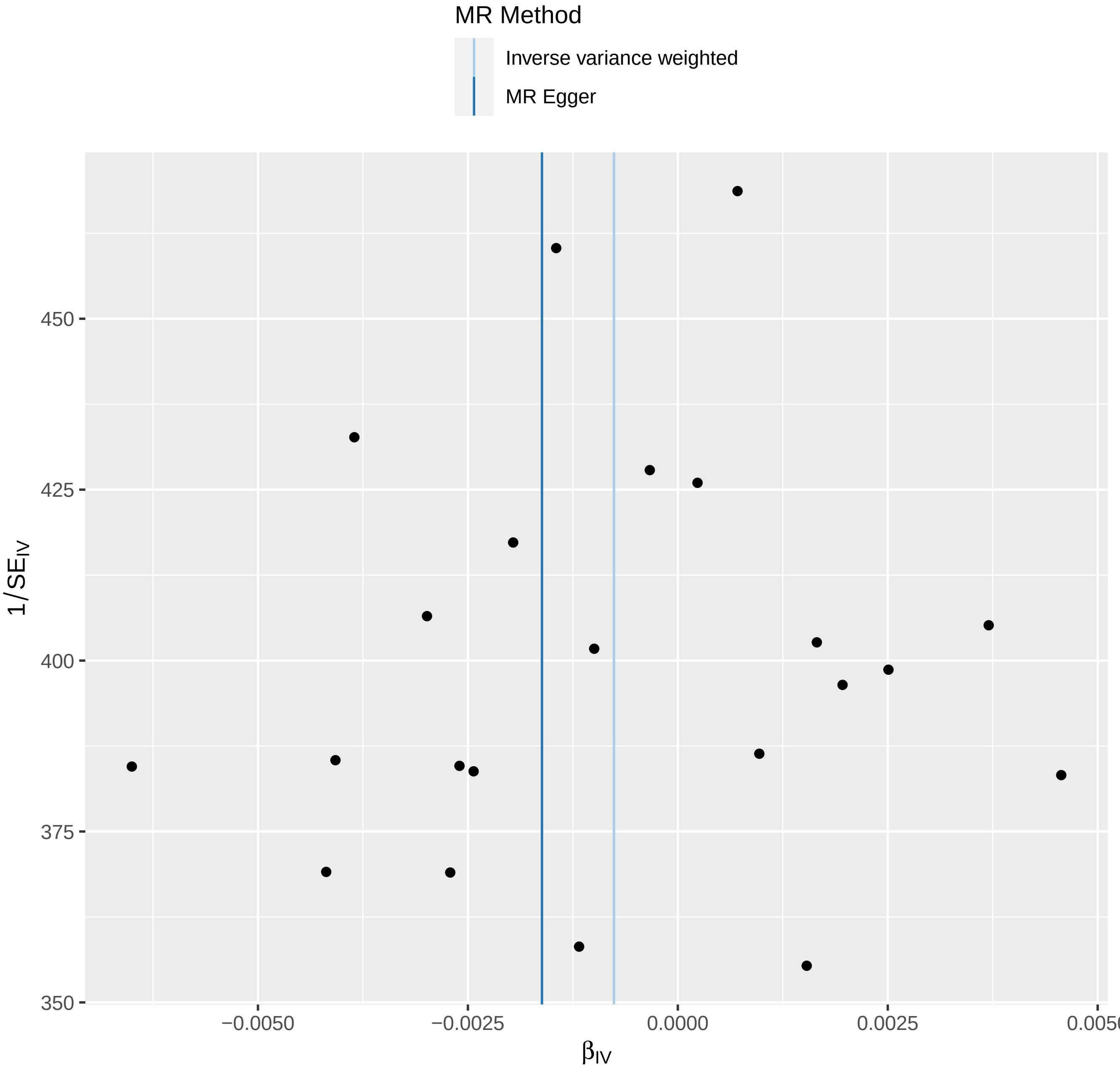

Batch 2−2177 : Pappalysin−1 on eGFR

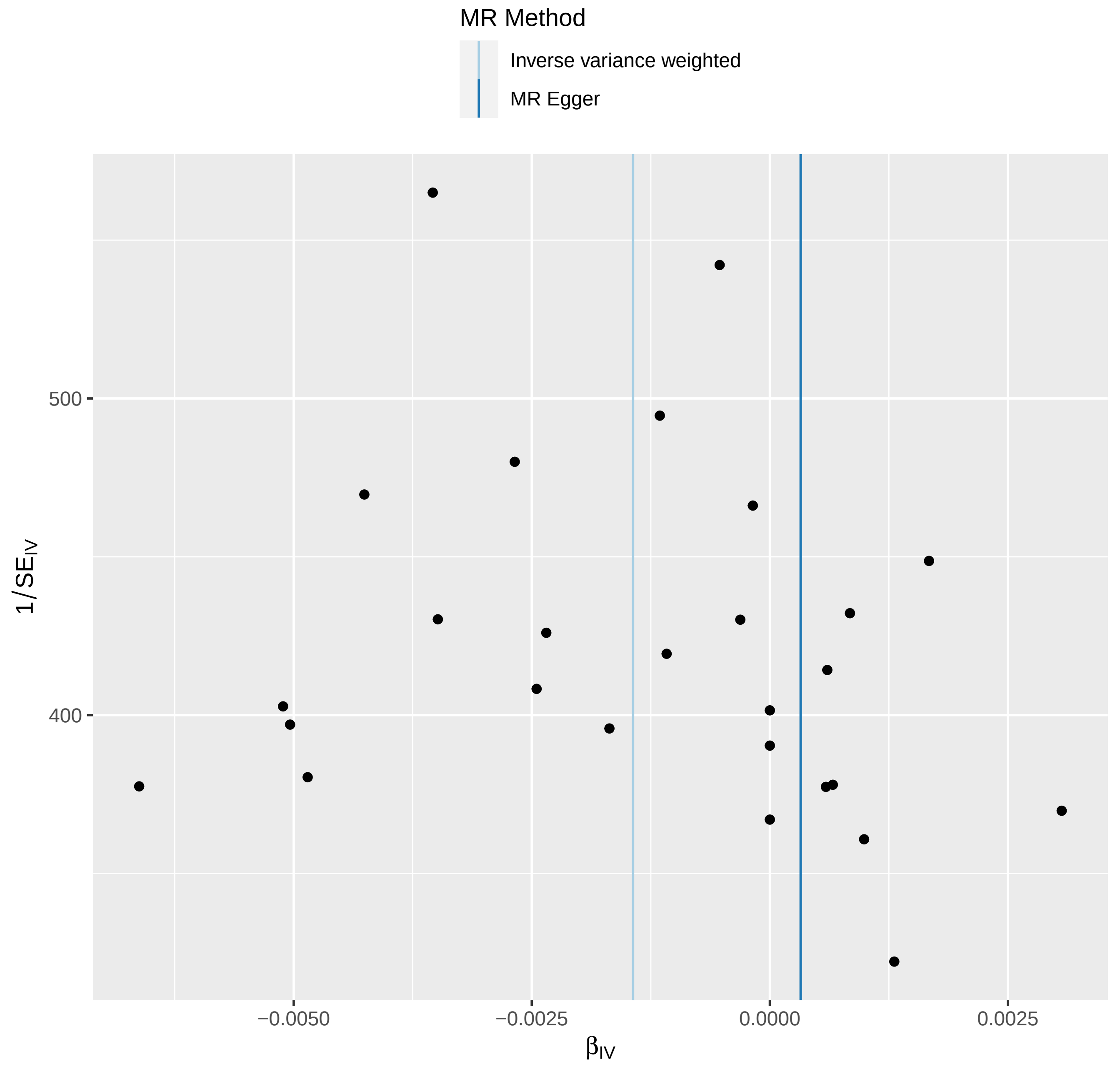

Batch 2−2178 : Bifunctional 3'−phosphoadenosine 5'−phosphosulfate synthase 1 on eGFR

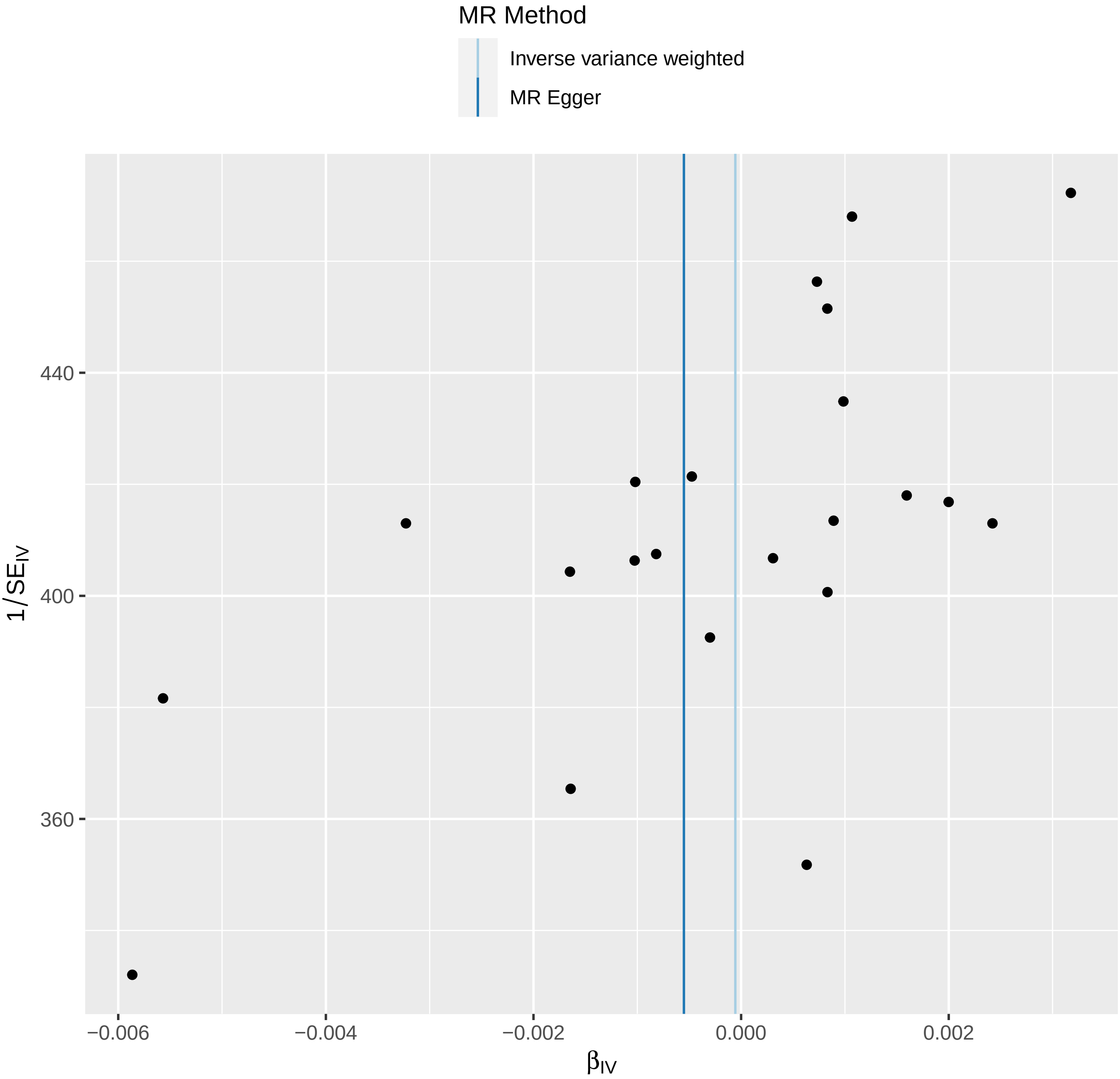

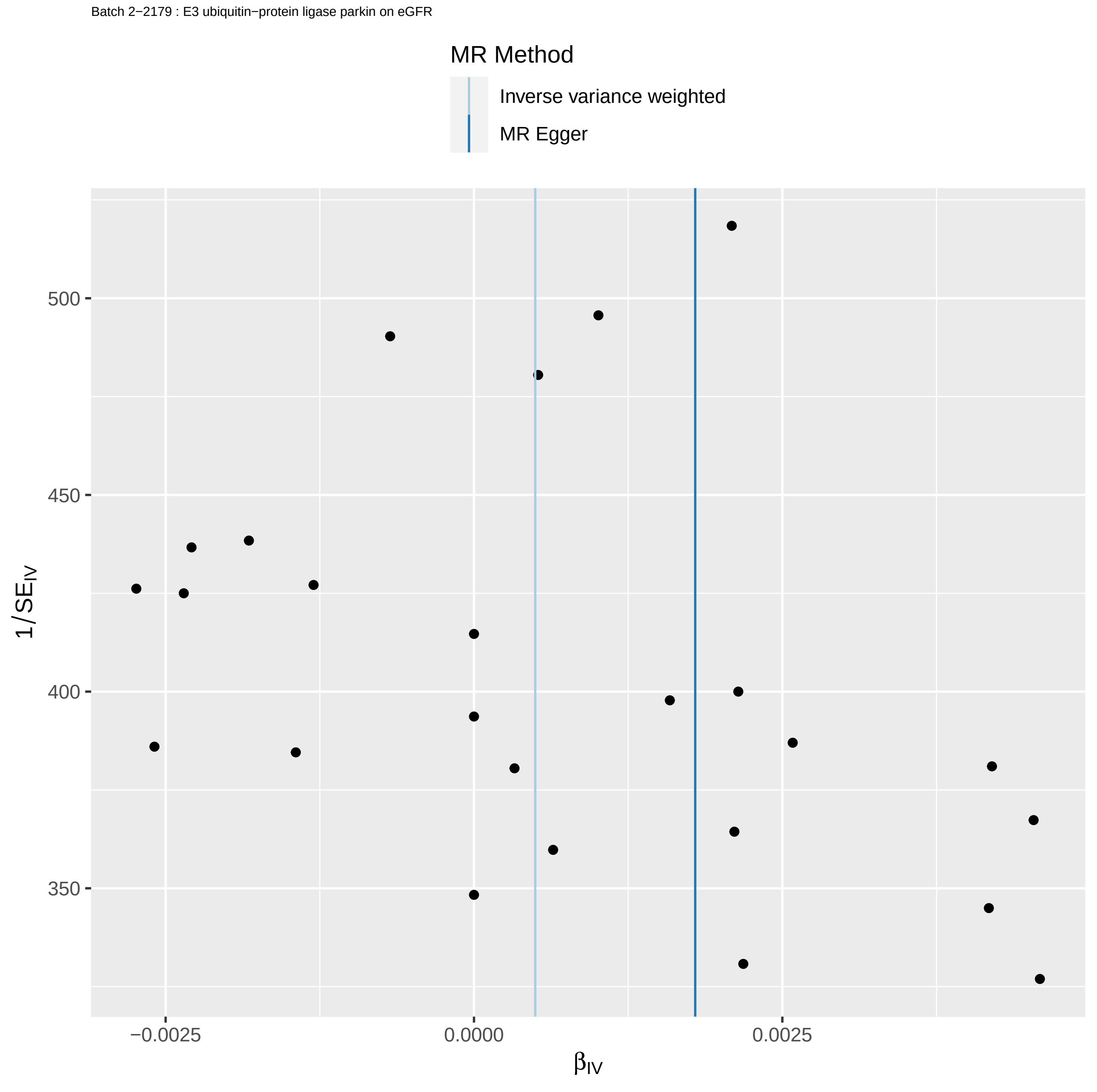
Batch 2−2179 : E3 ubiquitin−protein ligase parkin on eGFR
MR Method
Inverse variance weighted
MR Egger
500
450
400
350
1/SE_IV
−0.0025
0.0000
0.0025
β_IV

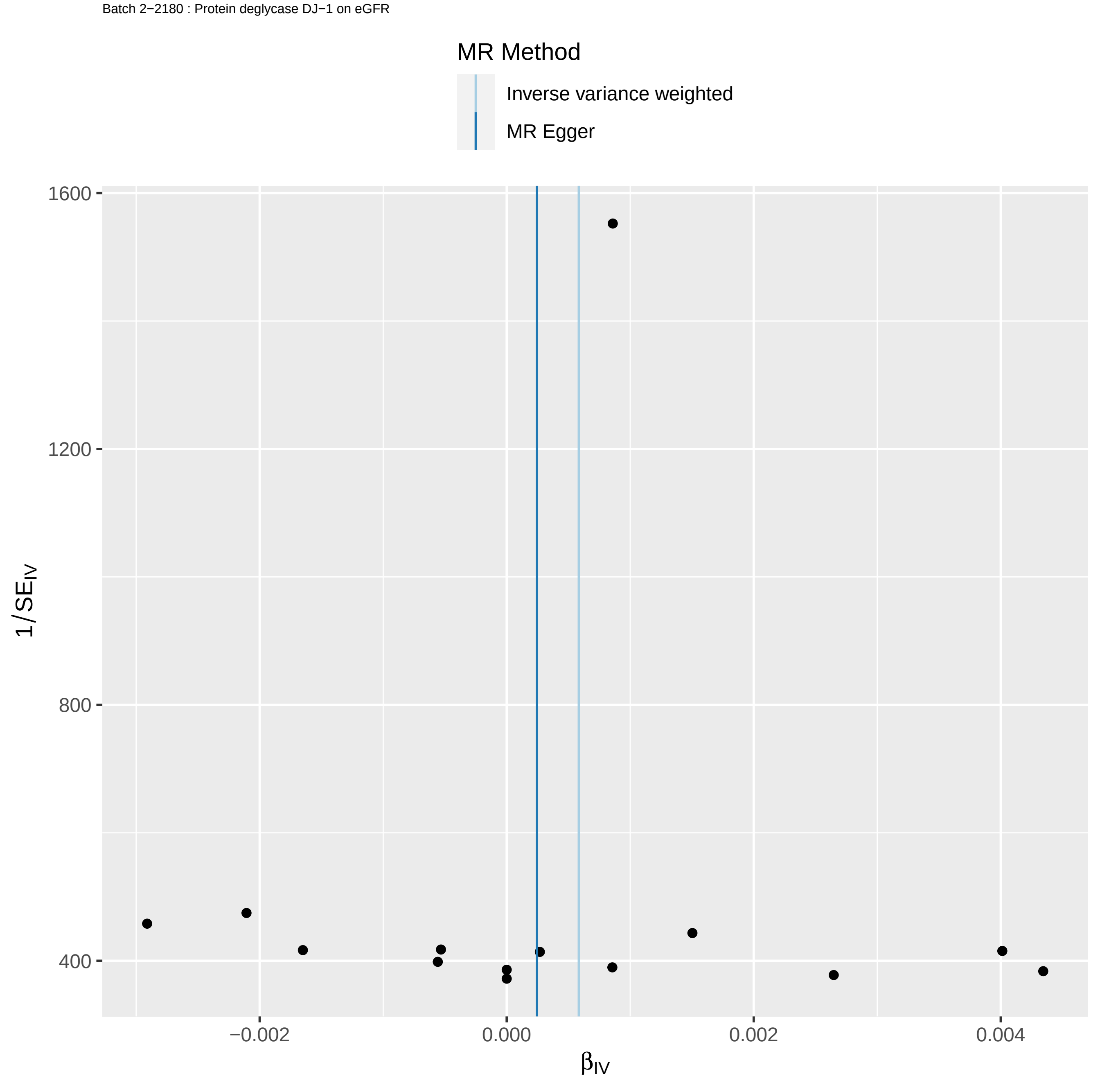

Batch 2−2180 : Protein deglycase DJ−1 on eGFR
MR Method
Inverse variance weighted
MR Egger
1600
1200
800
400
1/SE$_{IV}$
−0.002
0.000
0.002
0.004
β_{IV}

Batch 2−2181 : Protein deglycase DJ−1 on eGFR

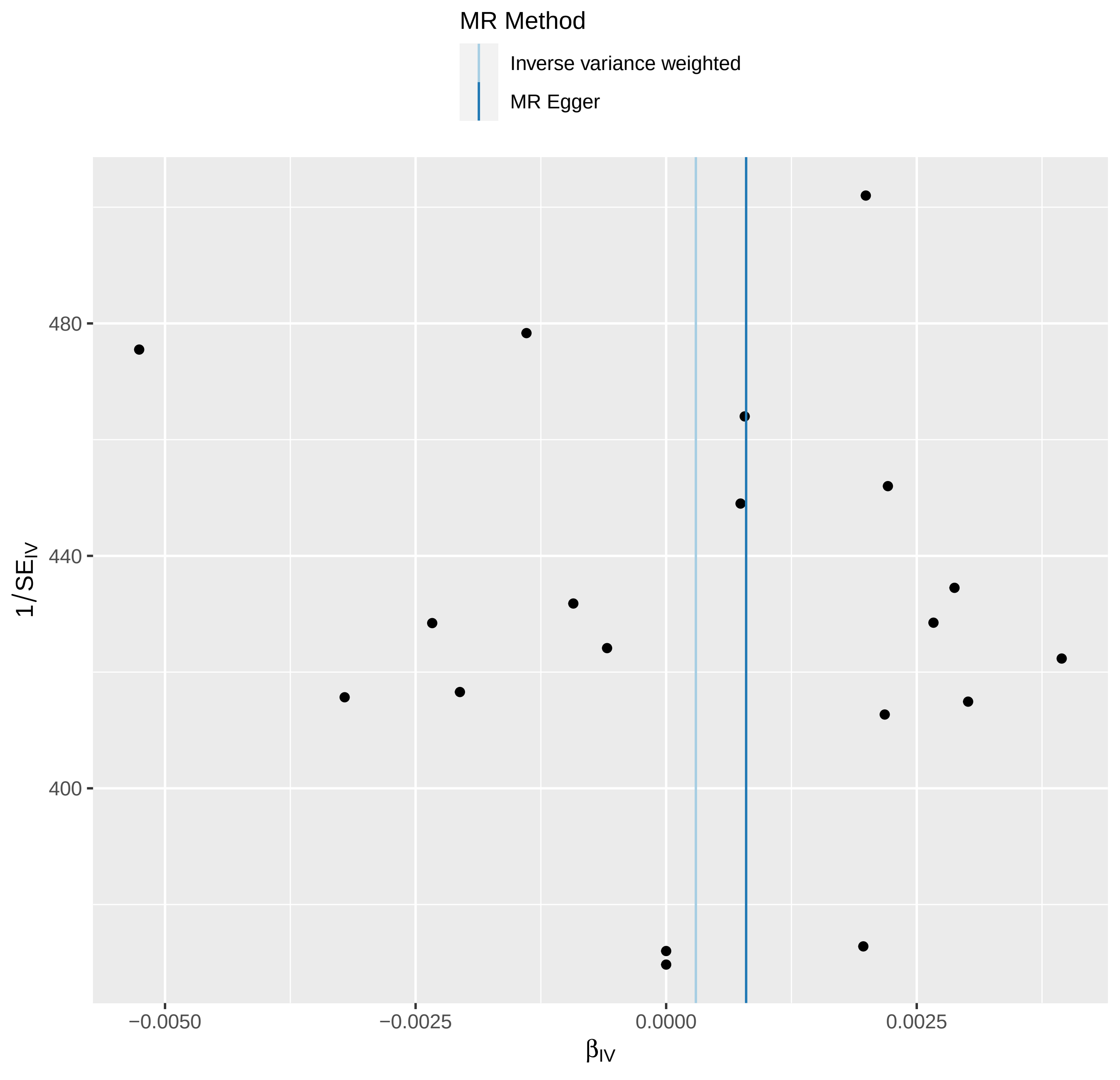

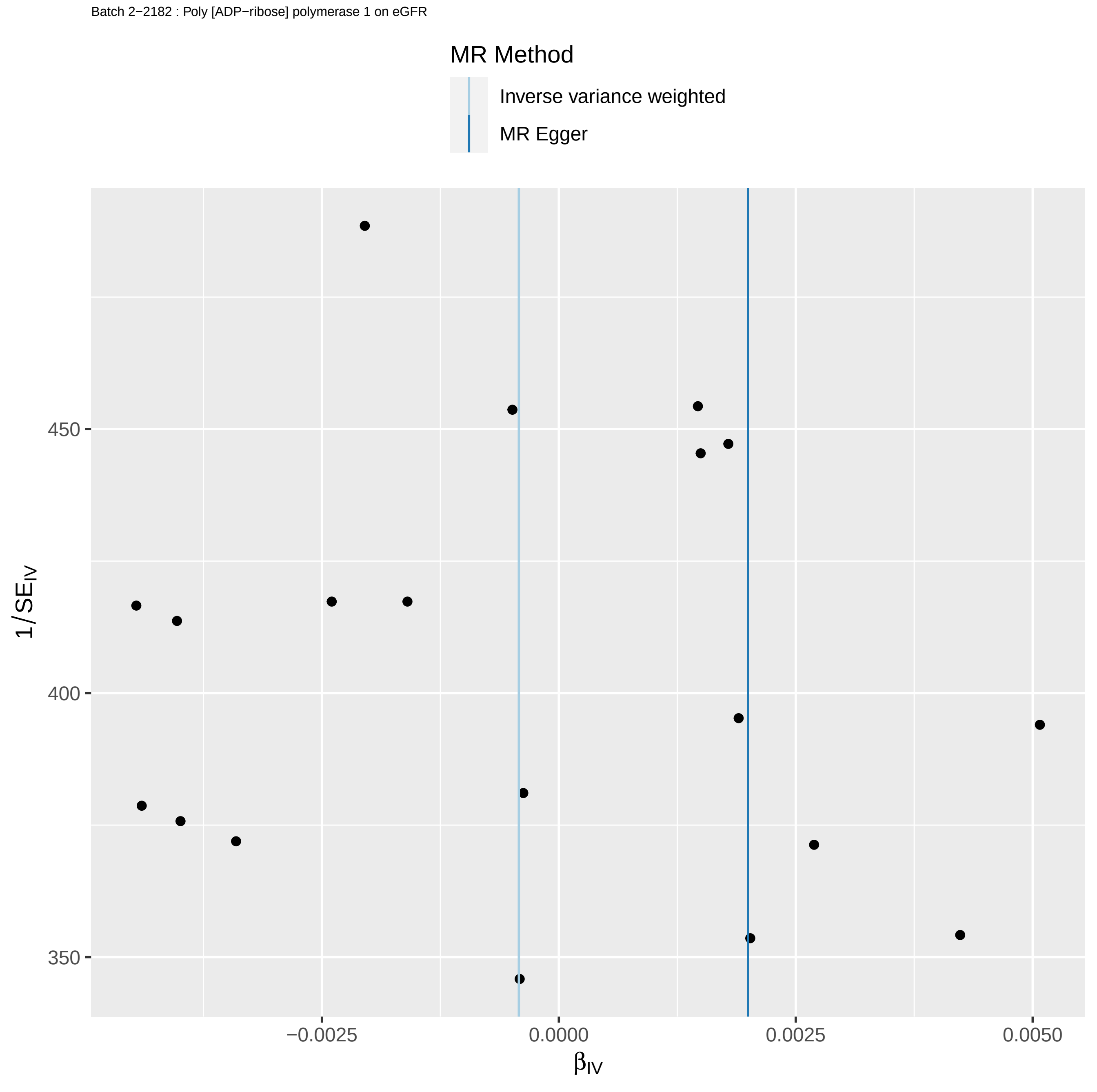

Batch 2−2182 : Poly [ADP−ribose] polymerase 1 on eGFR
MR Method
Inverse variance weighted
MR Egger
450
400
350
1/SE$_{IV}$
−0.0025
0.0000
0.0025
0.0050
β$_{IV}$

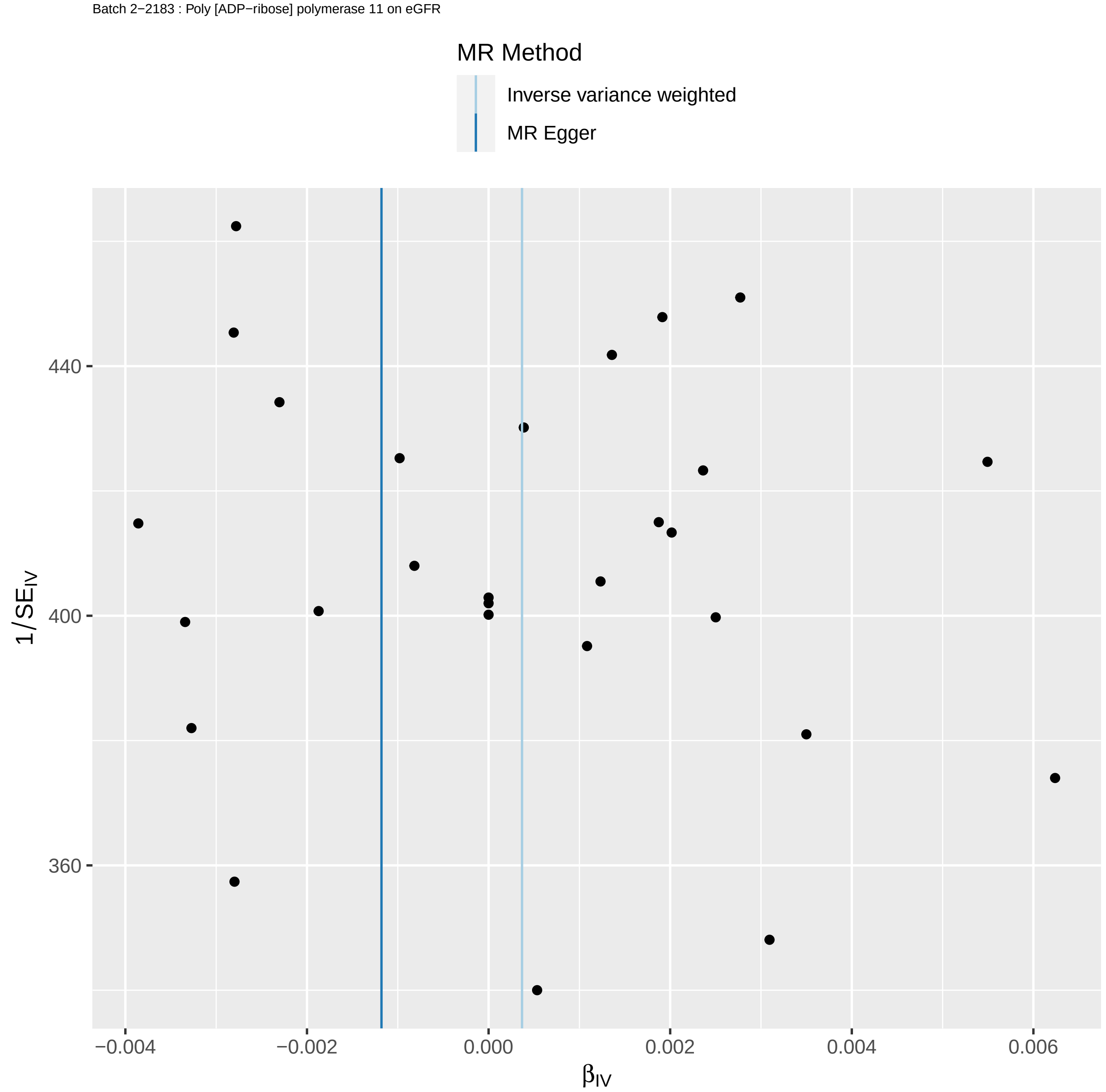

Batch 2−2183 : Poly [ADP−ribose] polymerase 11 on eGFR
MR Method
Inverse variance weighted
MR Egger
440
400
360
1/SE_IV
−0.004
−0.002
0.000
0.002
0.004
0.006
β_IV

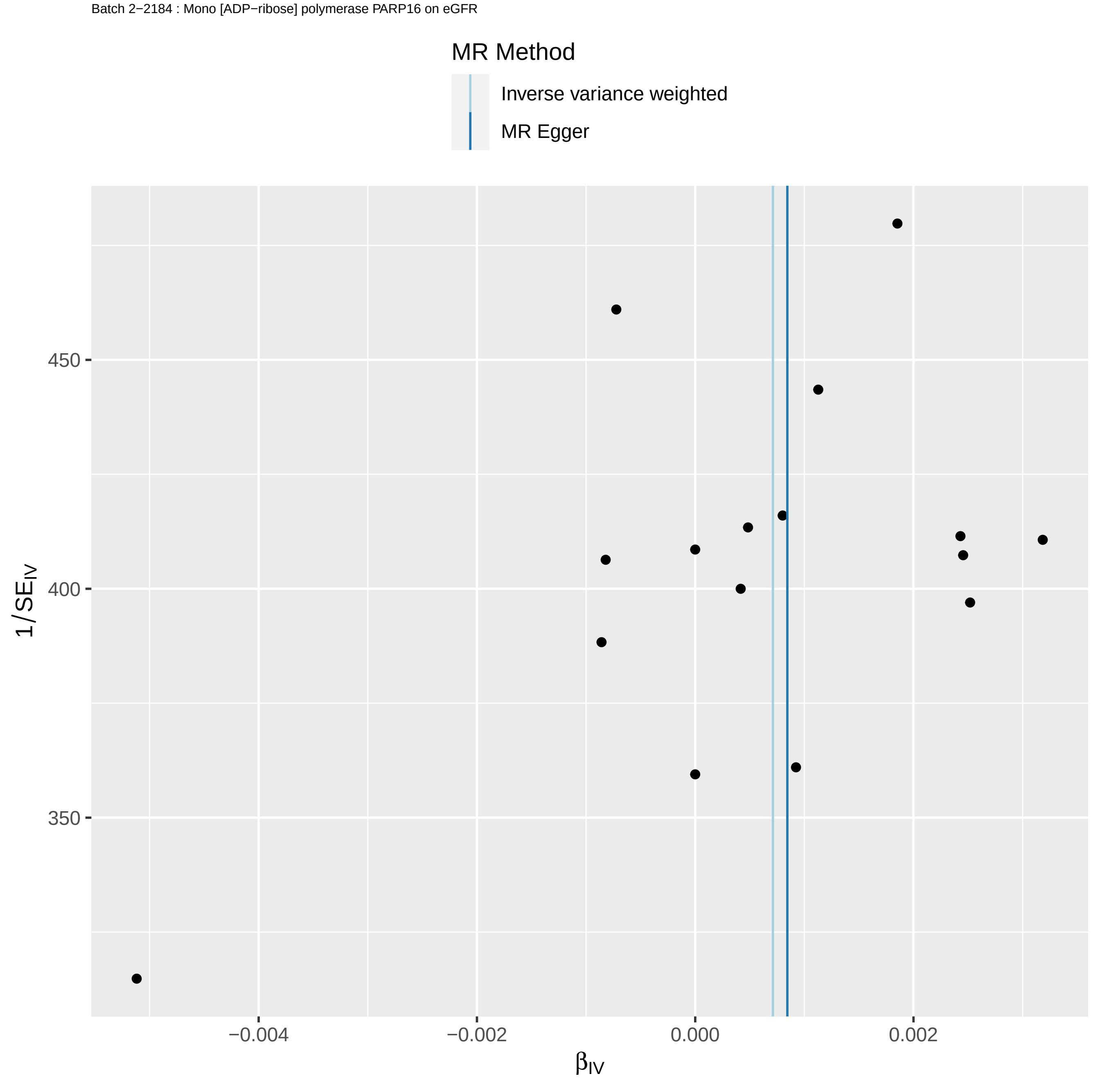

Batch 2−2184 : Mono [ADP−ribose] polymerase PARP16 on eGFR
MR Method
Inverse variance weighted
MR Egger
450
400
350
$1/SE_{IV}$
−0.004
−0.002
0.000
0.002
β_{IV}

Batch 2−2185 : Alpha−parvin on eGFR

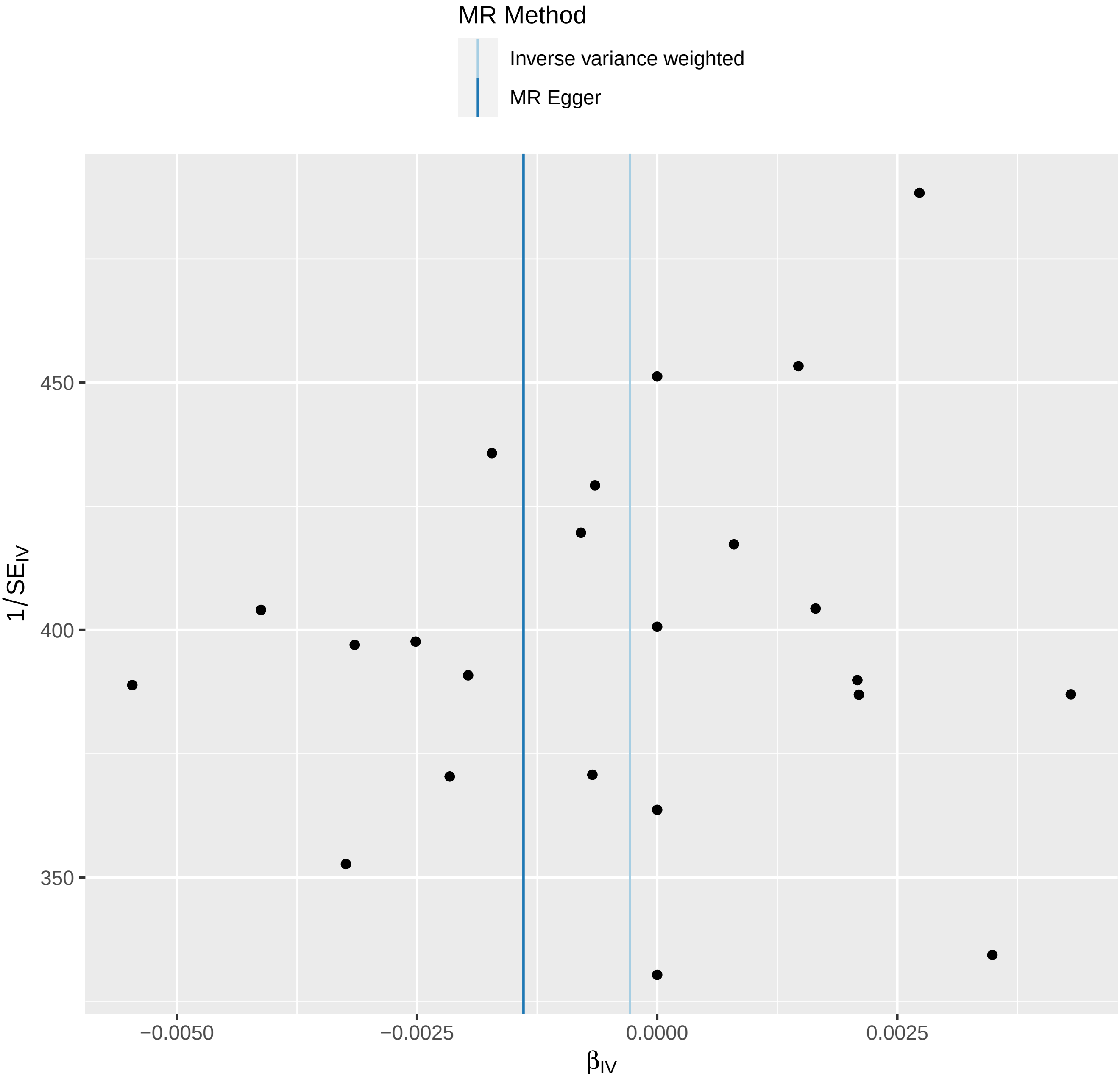

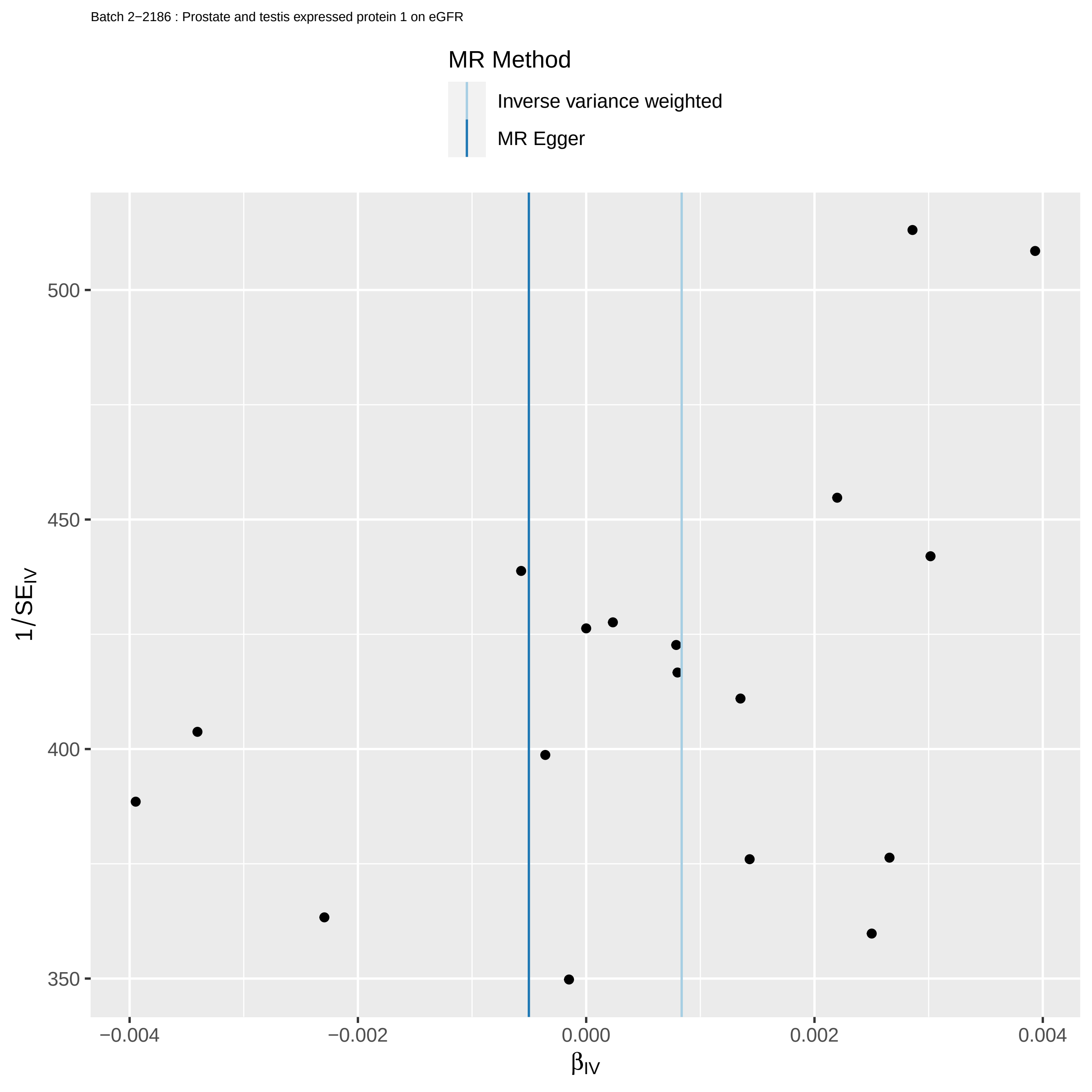

Batch 2−2186 : Prostate and testis expressed protein 1 on eGFR
MR Method
Inverse variance weighted
MR Egger
500
450
400
350
1/SE$_{IV}$
−0.004
−0.002
0.000
0.002
0.004
β$_{IV}$

Batch 2−2187 : Prostate and testis expressed protein 4 on eGFR

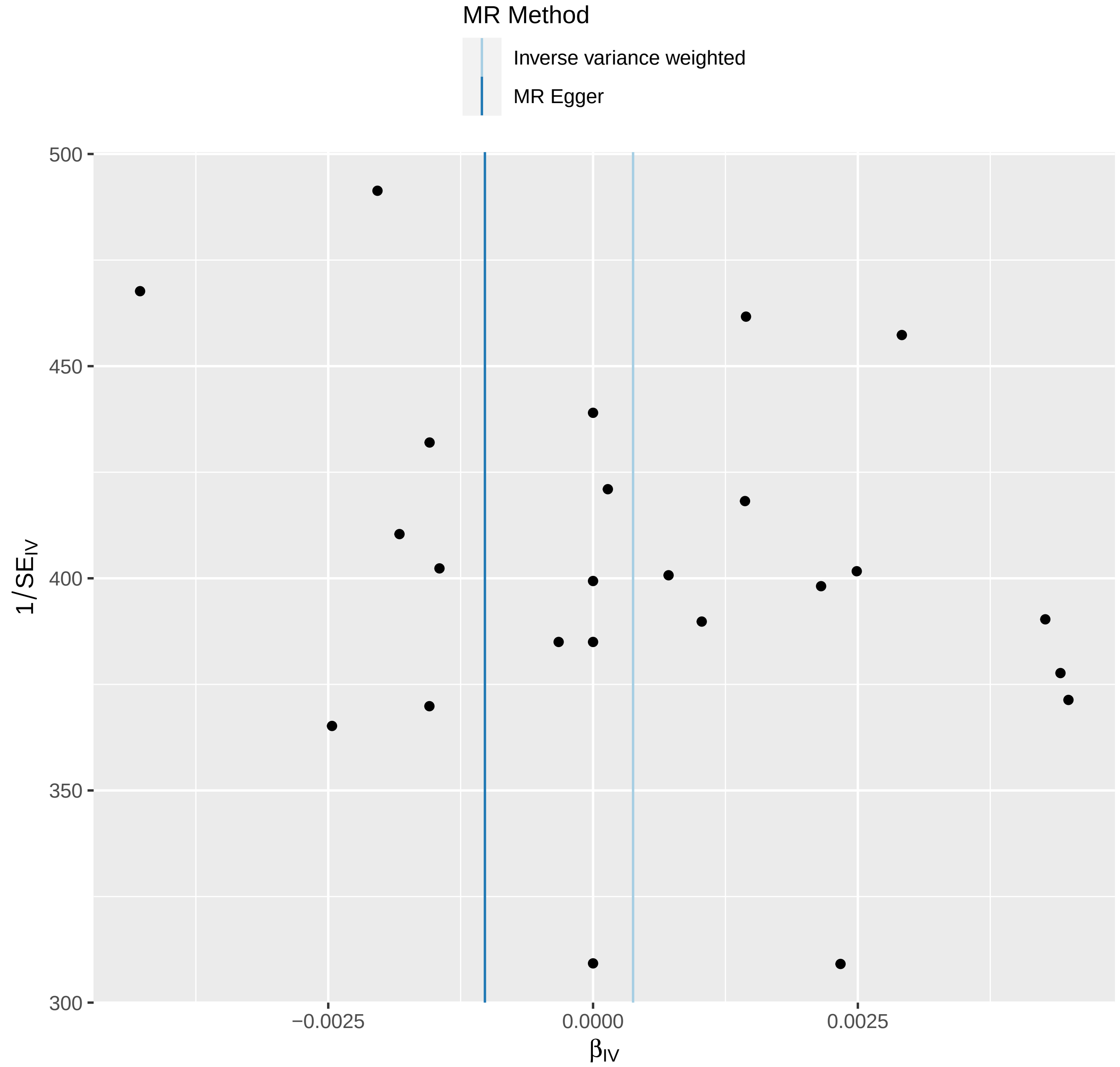

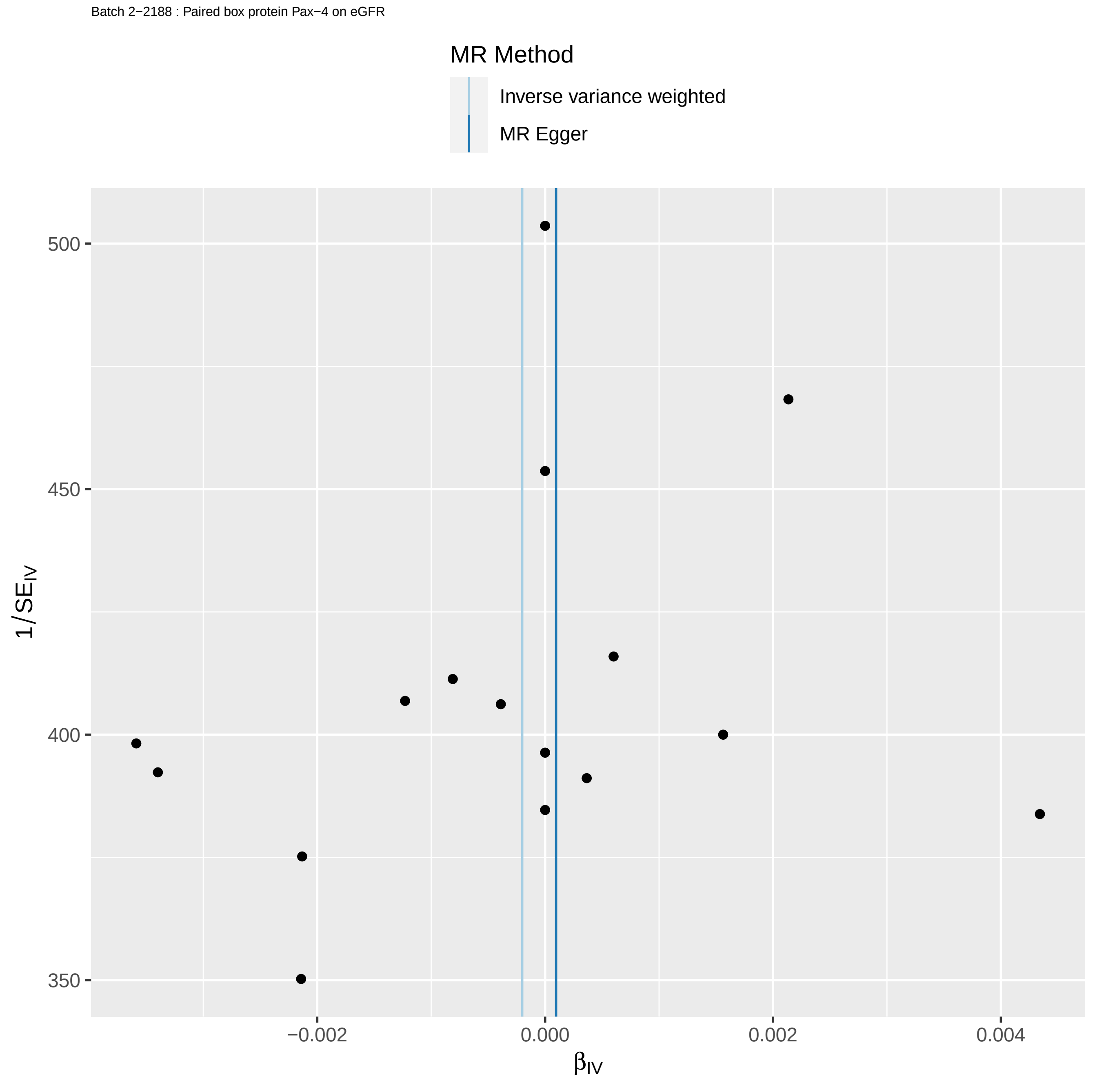
Batch 2−2188 : Paired box protein Pax−4 on eGFR
MR Method
Inverse variance weighted
MR Egger
500
450
400
350
1/SE_IV
−0.002
0.000
0.002
0.004
β_IV

Batch 2−2189 : PAX−interacting protein 1 on eGFR

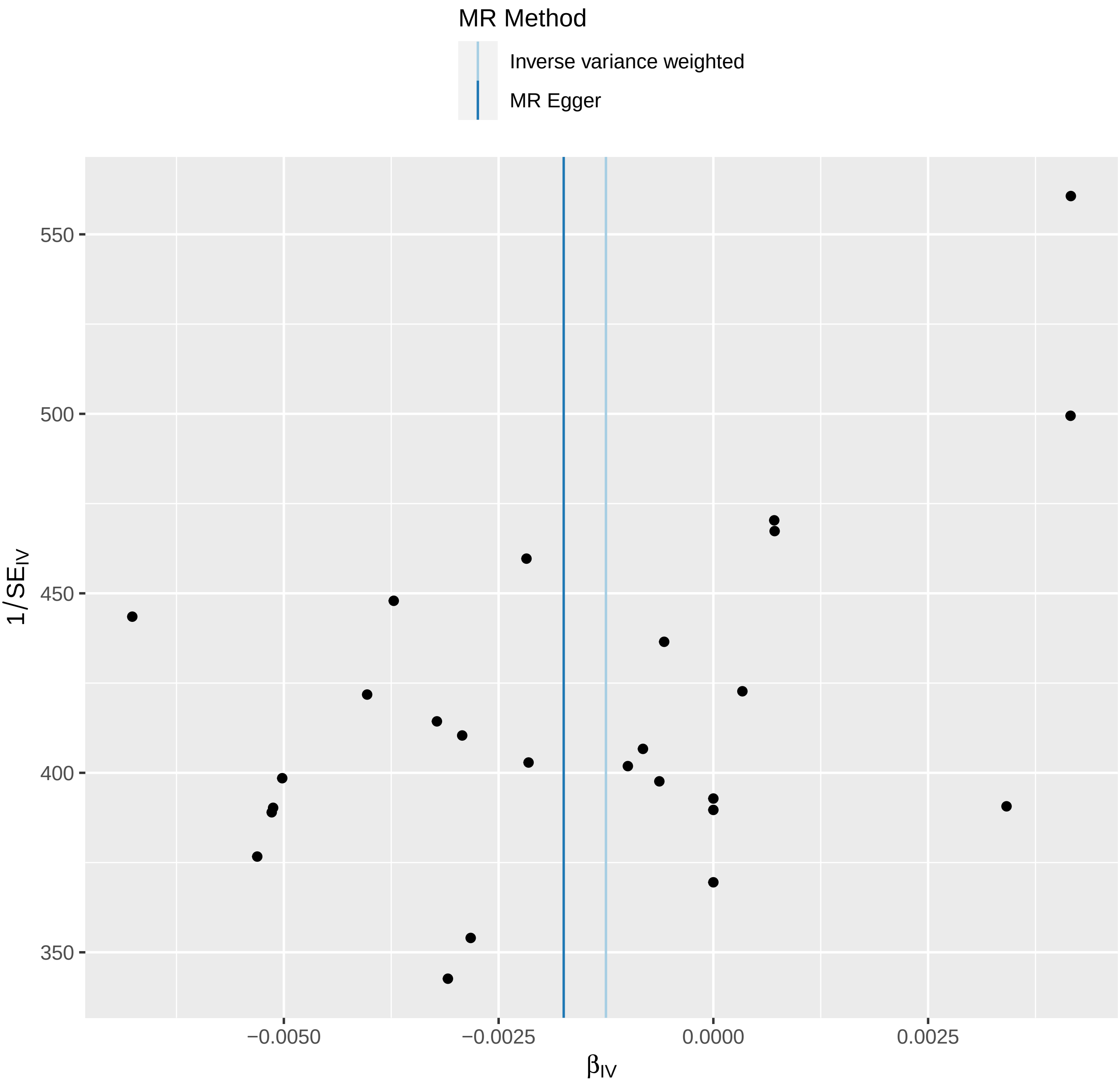

Batch 2−2190 : Pyruvate carboxylase, mitochondrial on eGFR

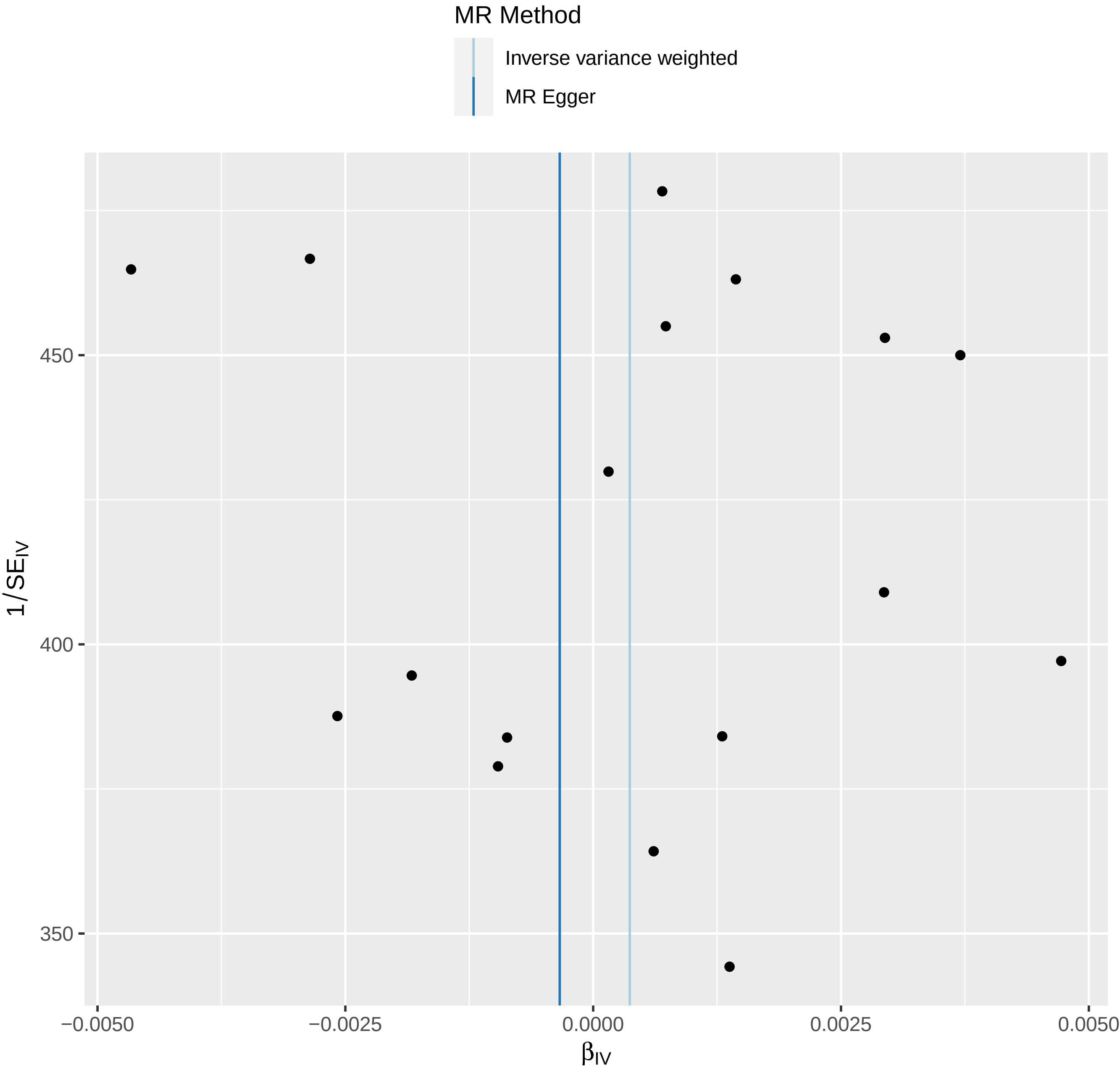

Batch 2−2191 : Pterin−4−alpha−carbinolamine dehydratase on eGFR

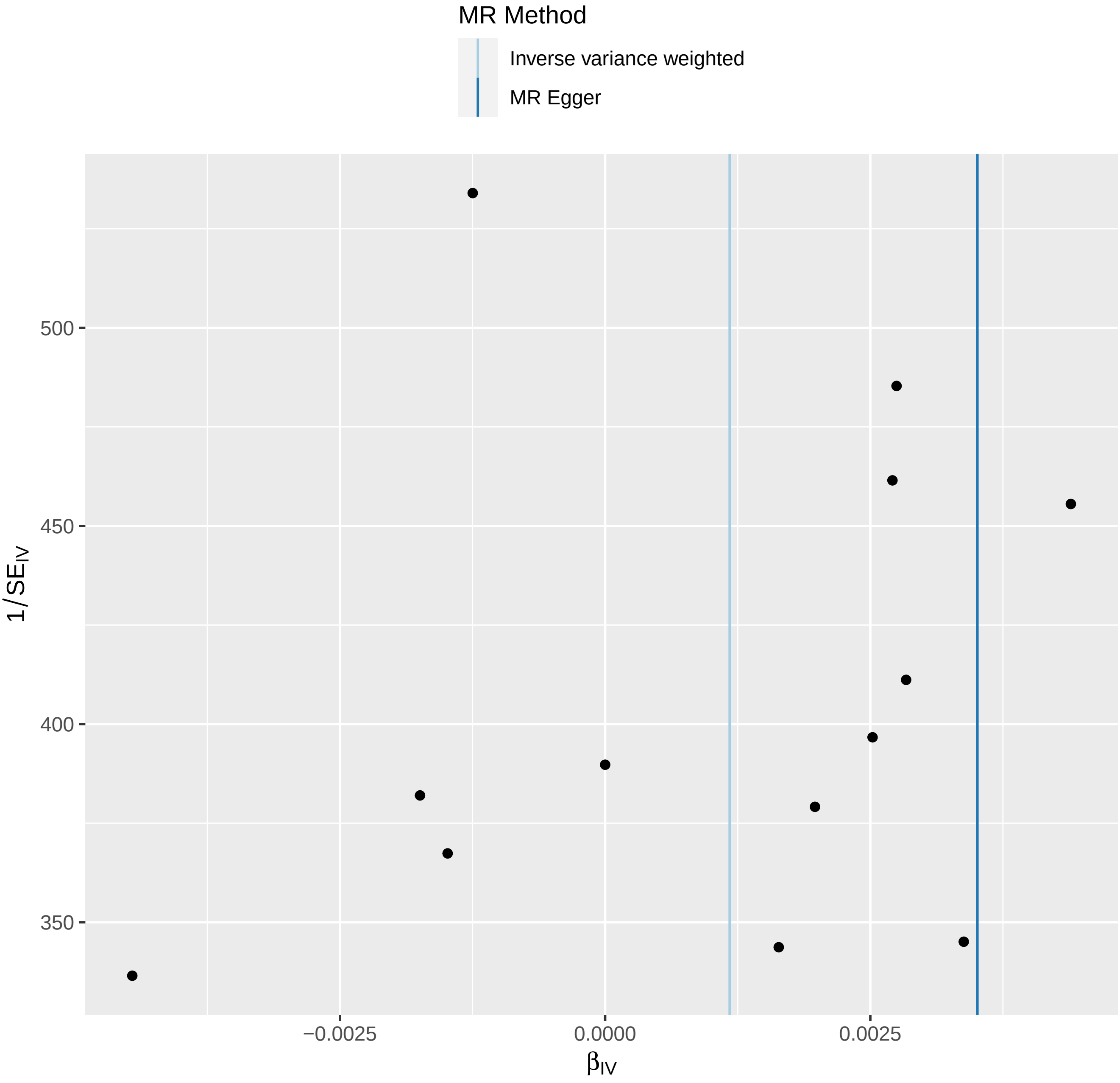

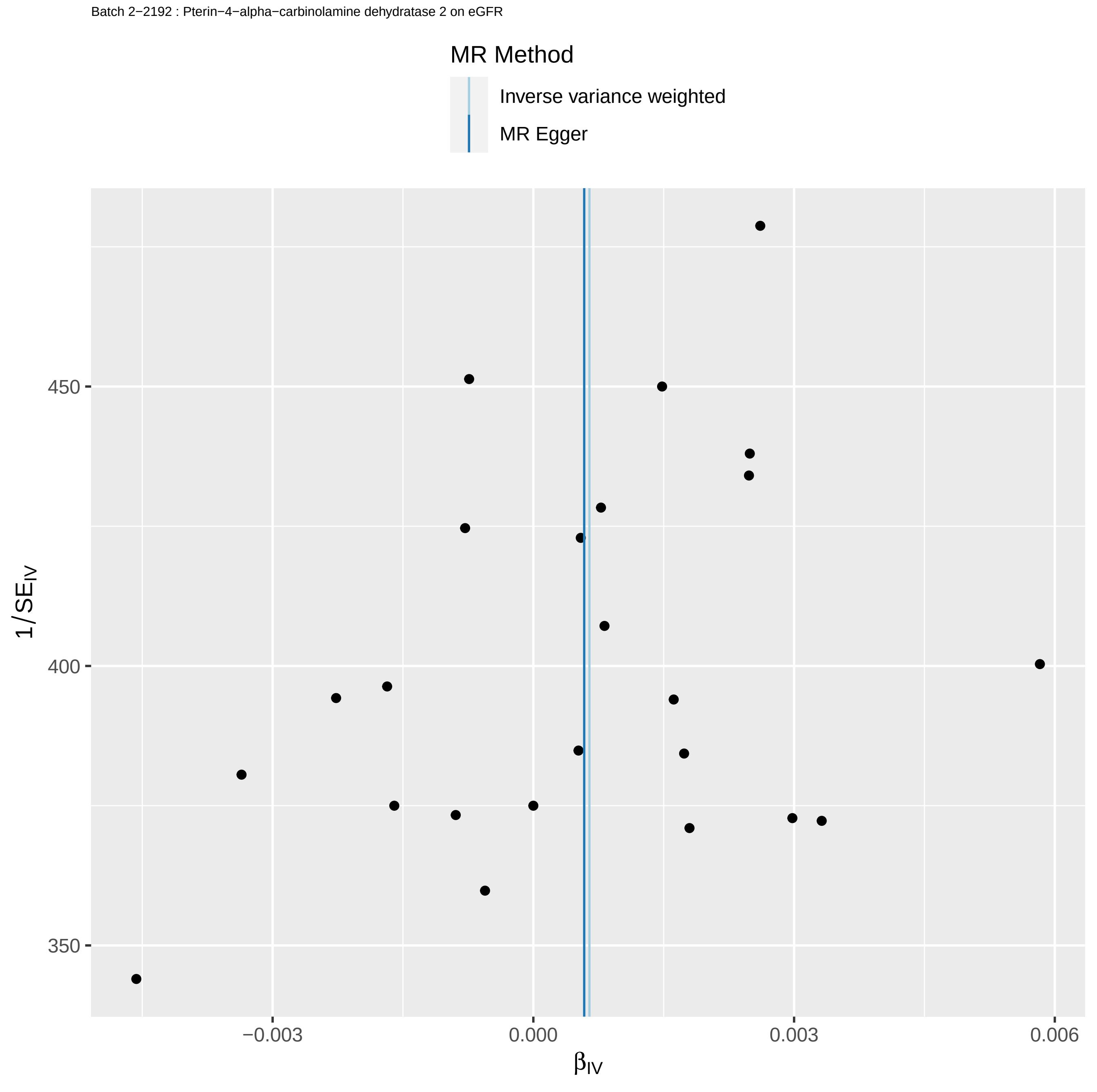

Batch 2−2192 : Pterin−4−alpha−carbinolamine dehydratase 2 on eGFR
MR Method
Inverse variance weighted
MR Egger
450
400
350
1/SE_IV
−0.003
0.000
0.003
0.006
β_IV

Batch 2−2193 : Poly(rC)−binding protein 1 on eGFR

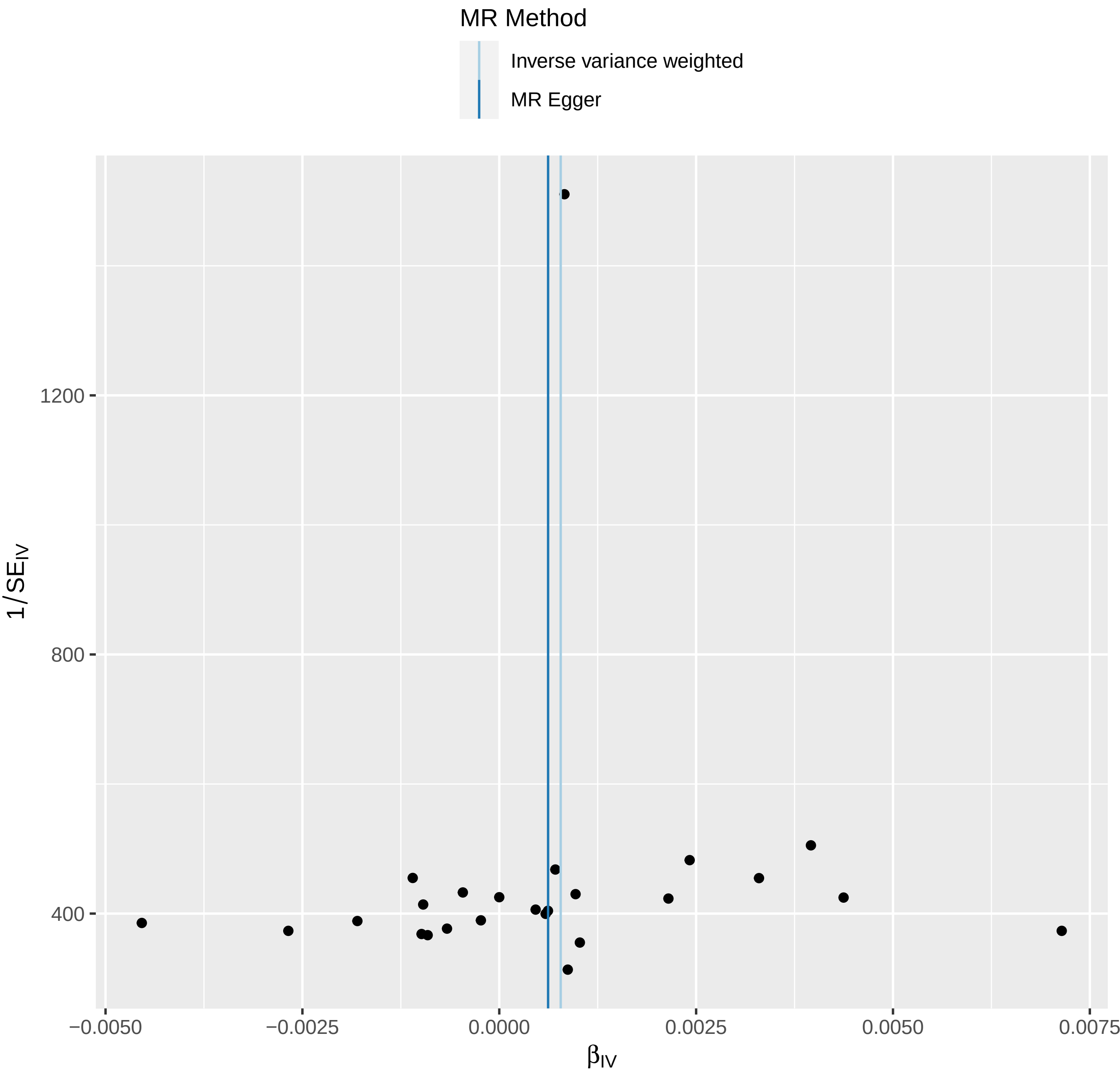

Batch 2−2194 : Protocadherin−10 on eGFR

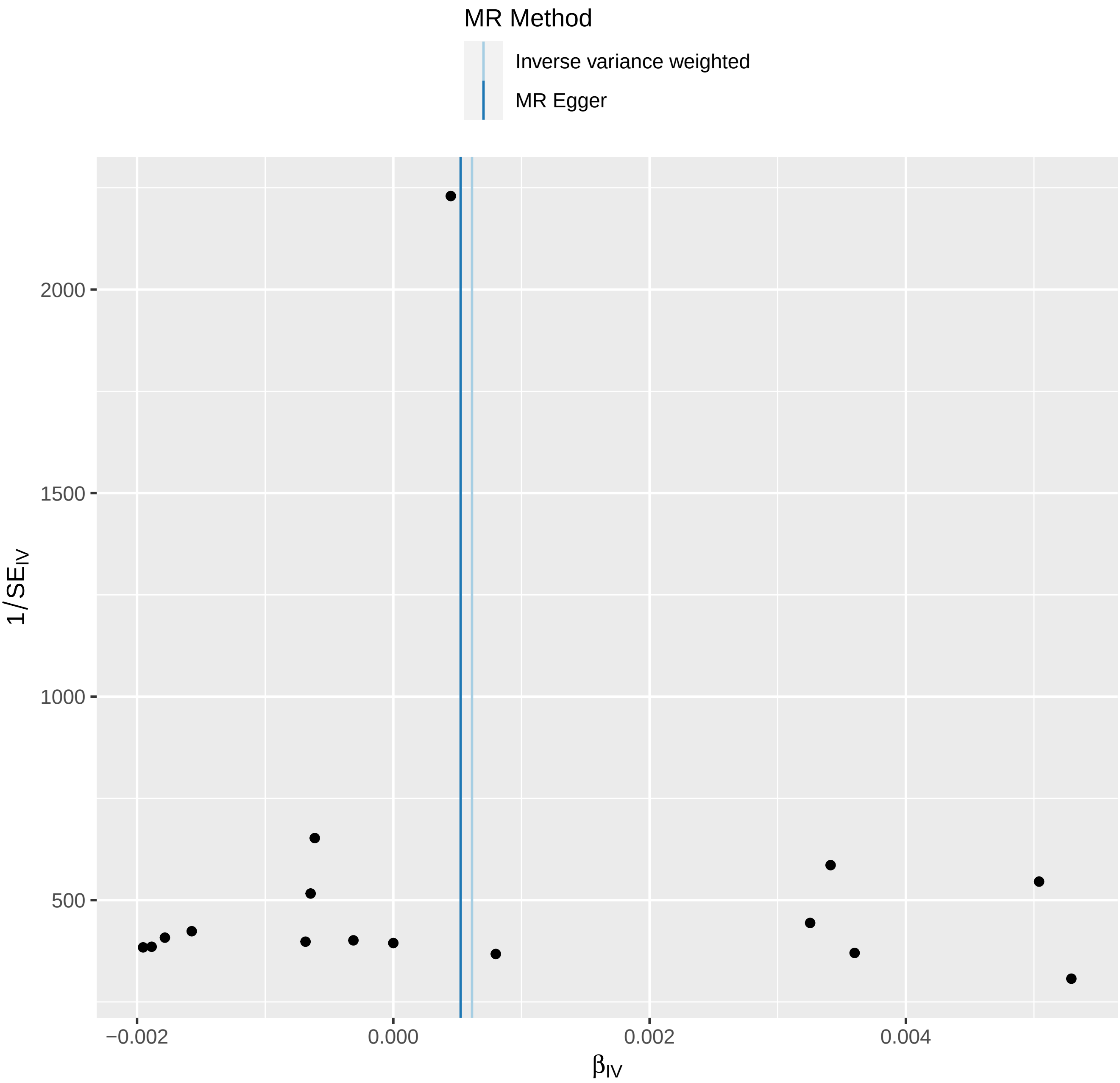

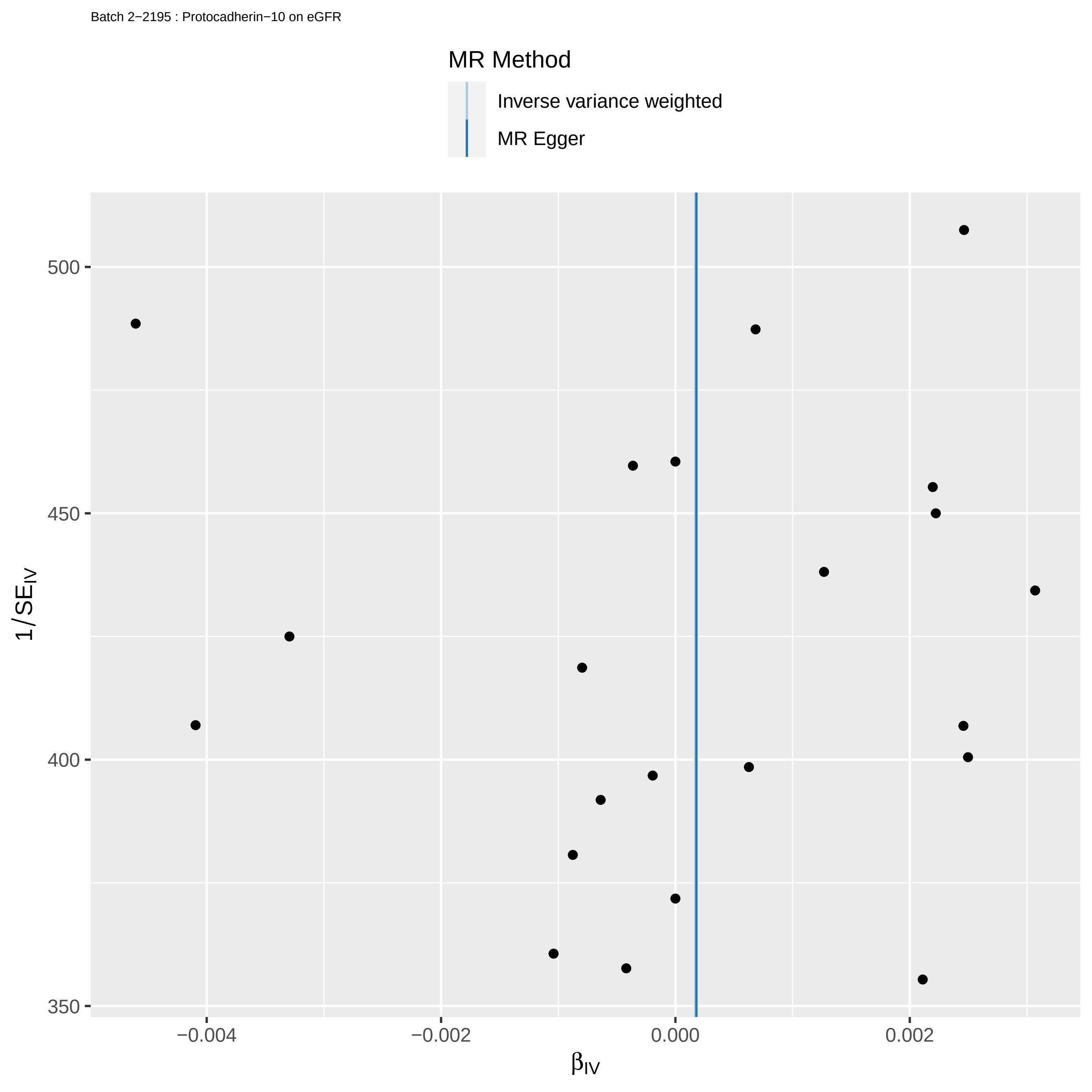

Batch 2−2195 : Protocadherin−10 on eGFR
MR Method
Inverse variance weighted
MR Egger
500
450
400
350
1/SE$_{IV}$
−0.004
−0.002
0.000
0.002
β$_{IV}$

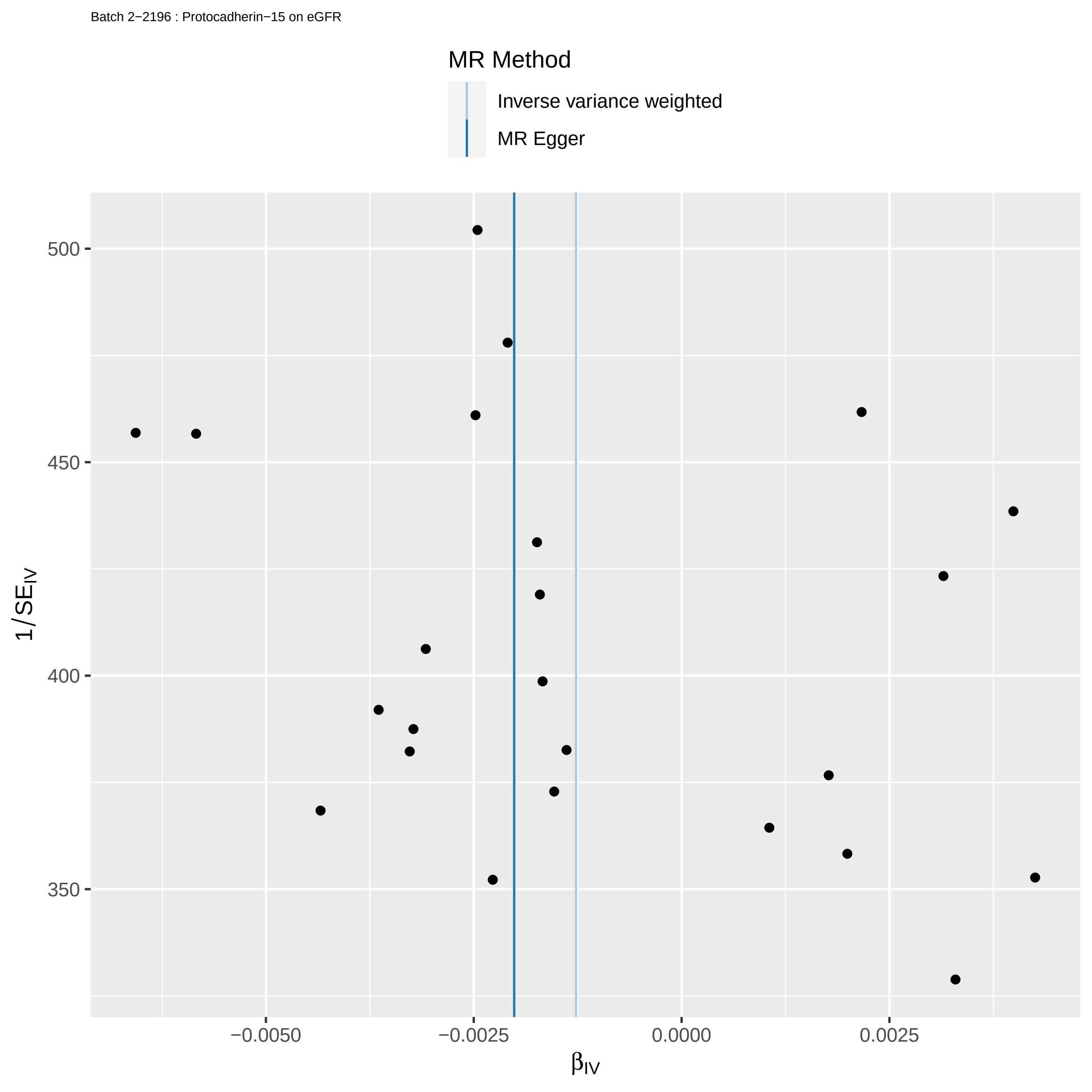
Batch 2−2196 : Protocadherin−15 on eGFR
MR Method
Inverse variance weighted
MR Egger
500
450
400
350
1/SE$_{IV}$
−0.0050
−0.0025
0.0000
0.0025
β$_{IV}$

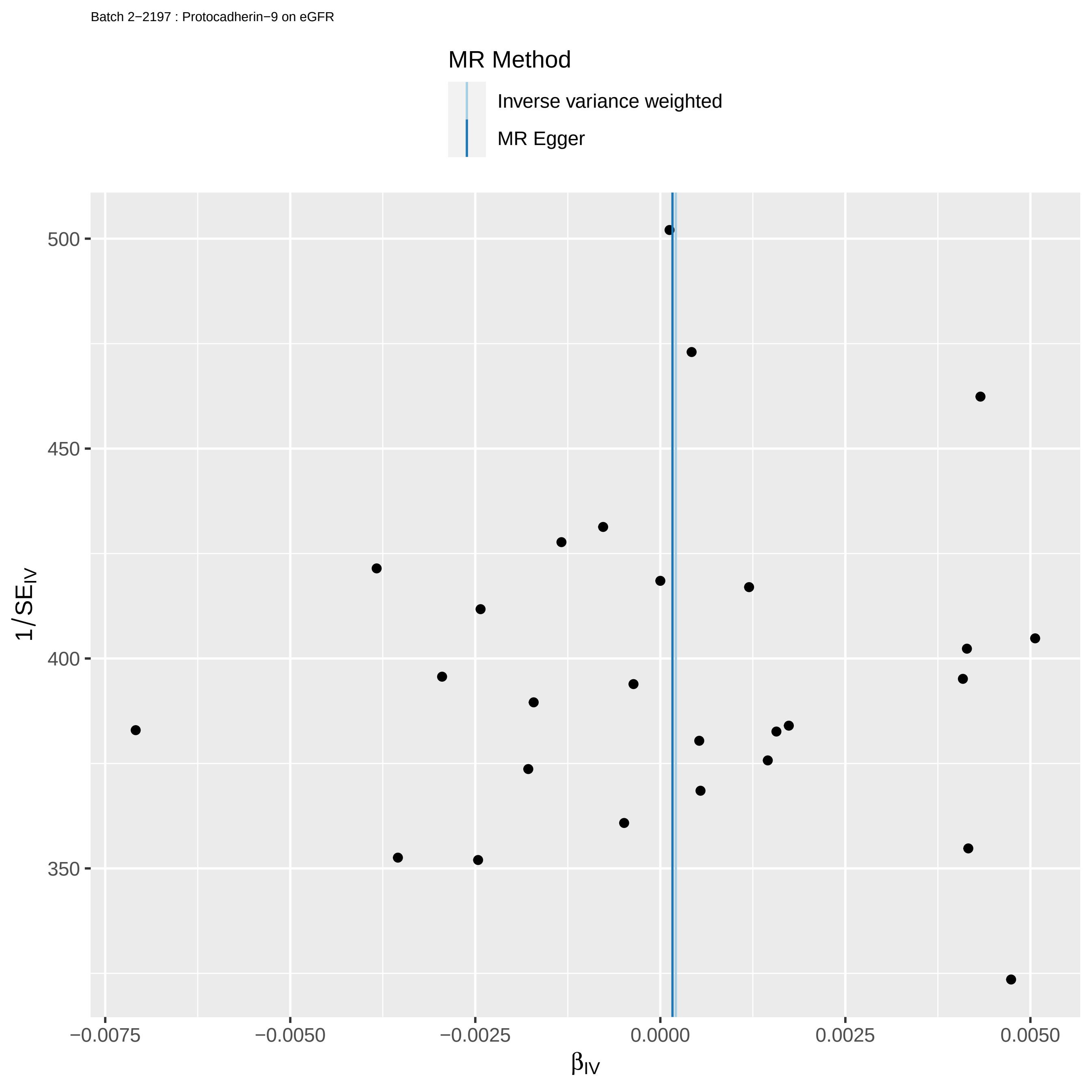

Batch 2−2197 : Protocadherin−9 on eGFR
MR Method
Inverse variance weighted
MR Egger
500
450
400
350
−0.0075
−0.0050
−0.0025
0.0000
0.0025
0.0050
1/SE_IV
β_IV

Batch 2−2198 : Protocadherin alpha−4 on eGFR

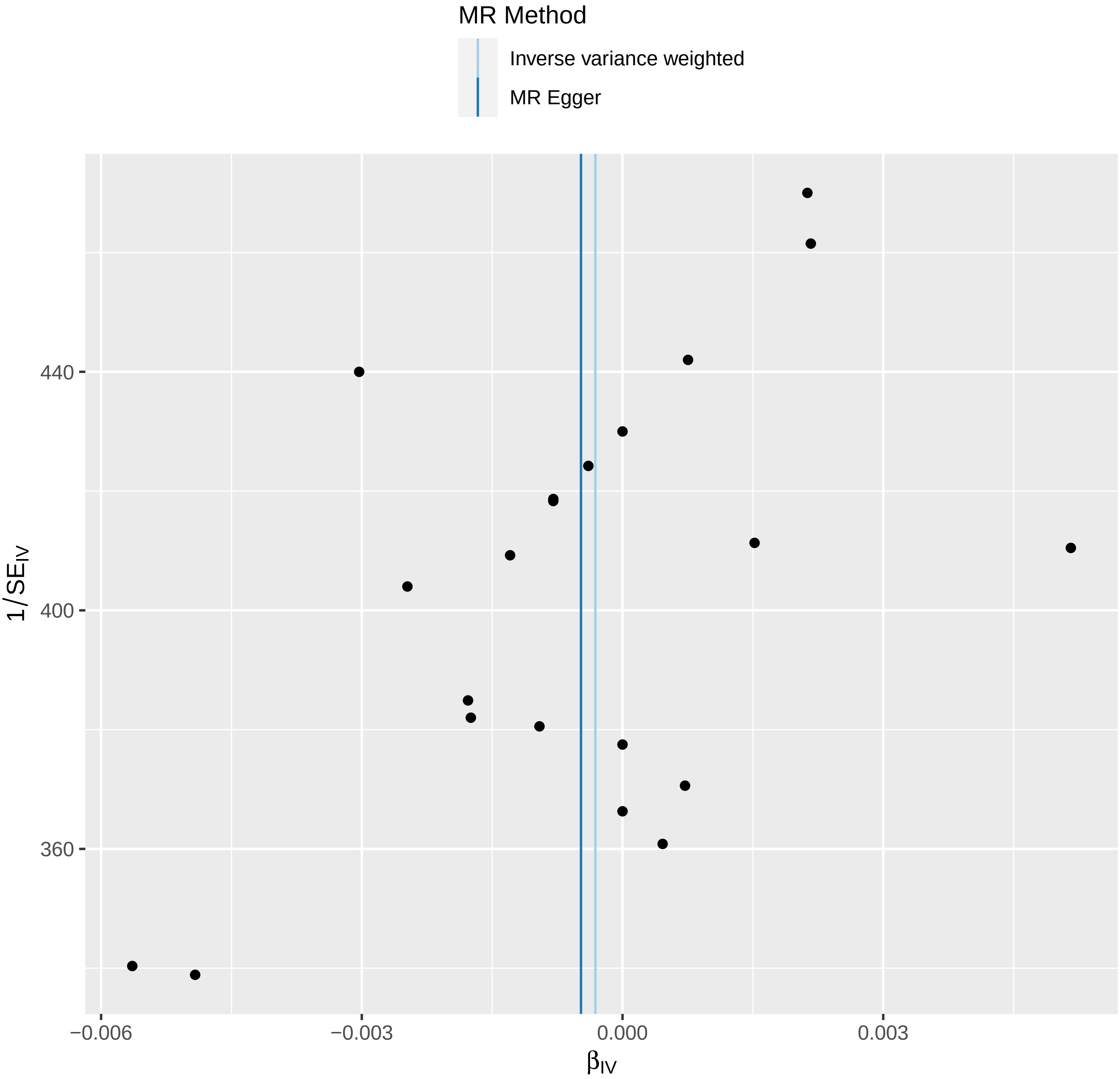

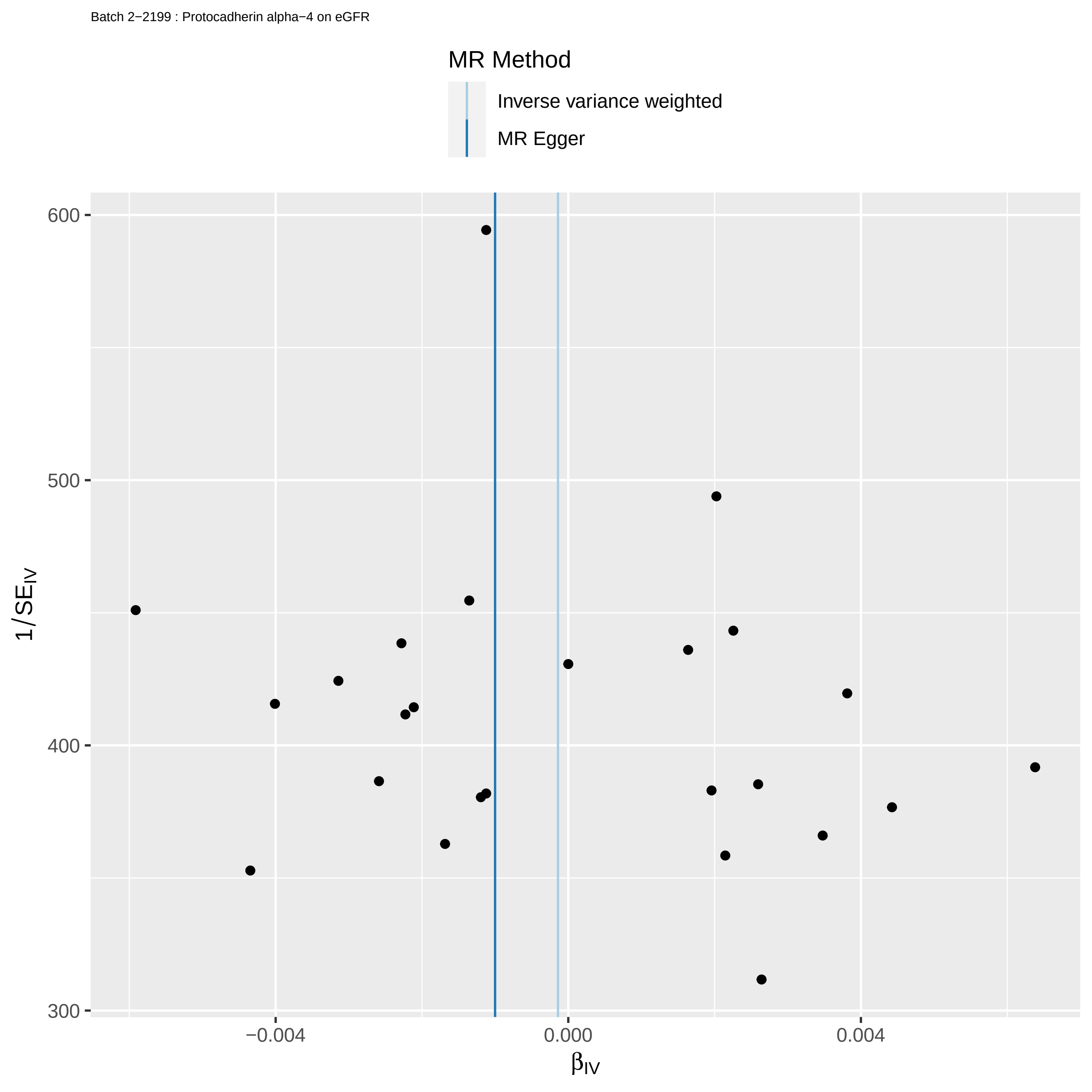

Batch 2−2199 : Protocadherin alpha−4 on eGFR
MR Method
Inverse variance weighted
MR Egger
600
500
400
300
1/SE_IV
−0.004
0.000
0.004
β_IV

Batch 2−2200 : Protocadherin alpha−7 on eGFR

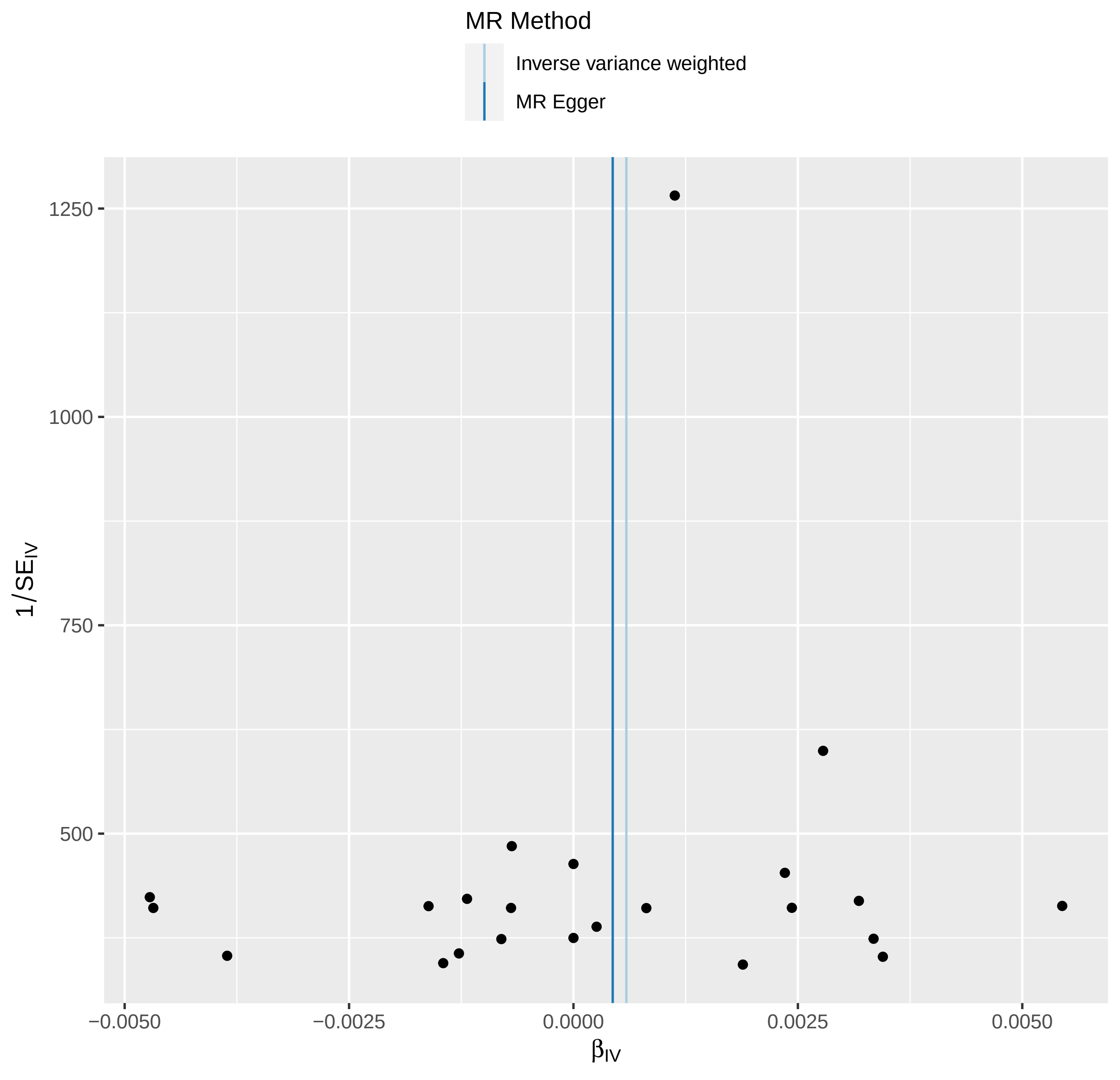

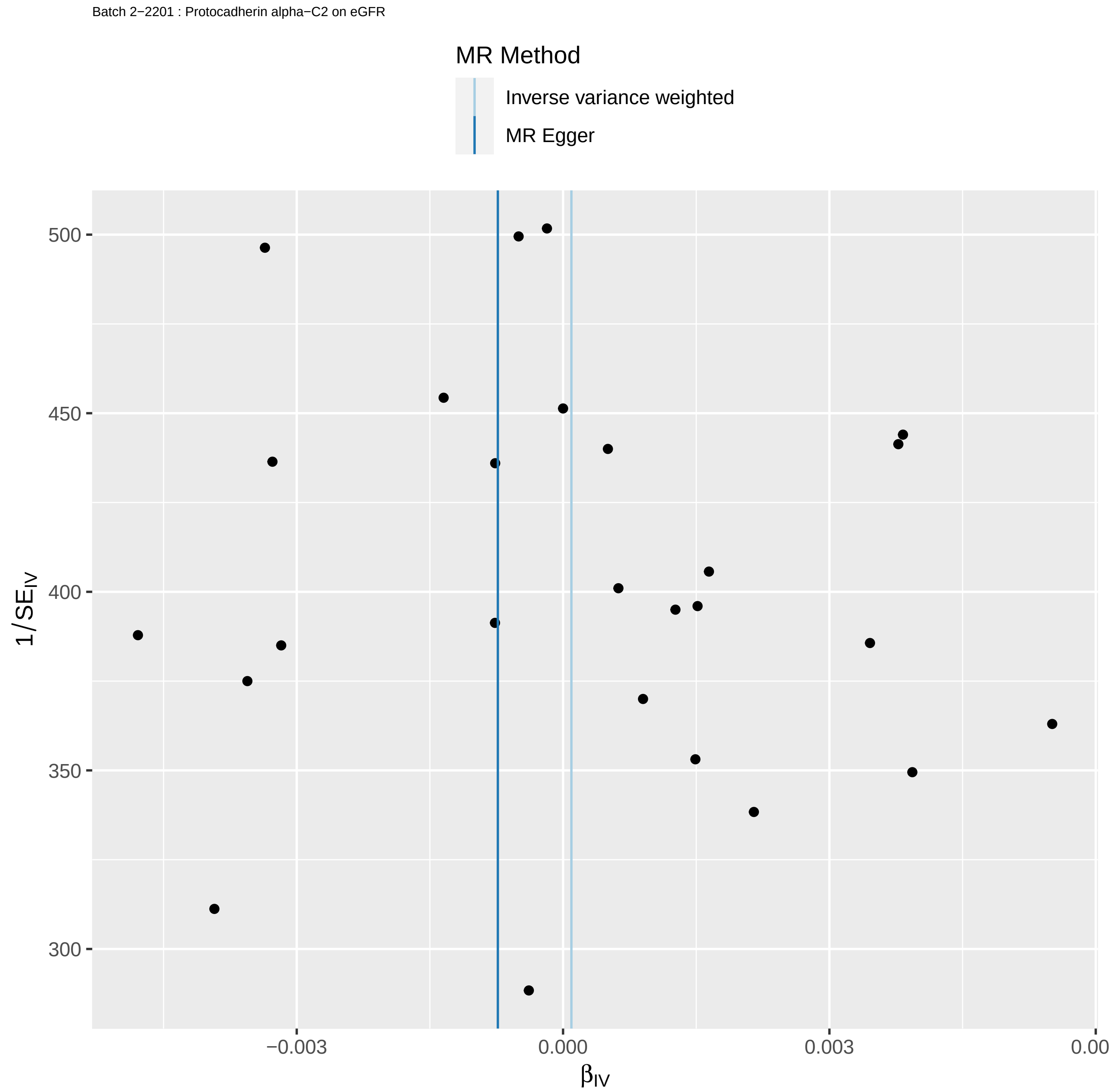

Batch 2−2201 : Protocadherin alpha−C2 on eGFR
MR Method
Inverse variance weighted
MR Egger
500
450
400
350
300
1/SE_IV
−0.003
0.000
0.003
0.00
β_IV

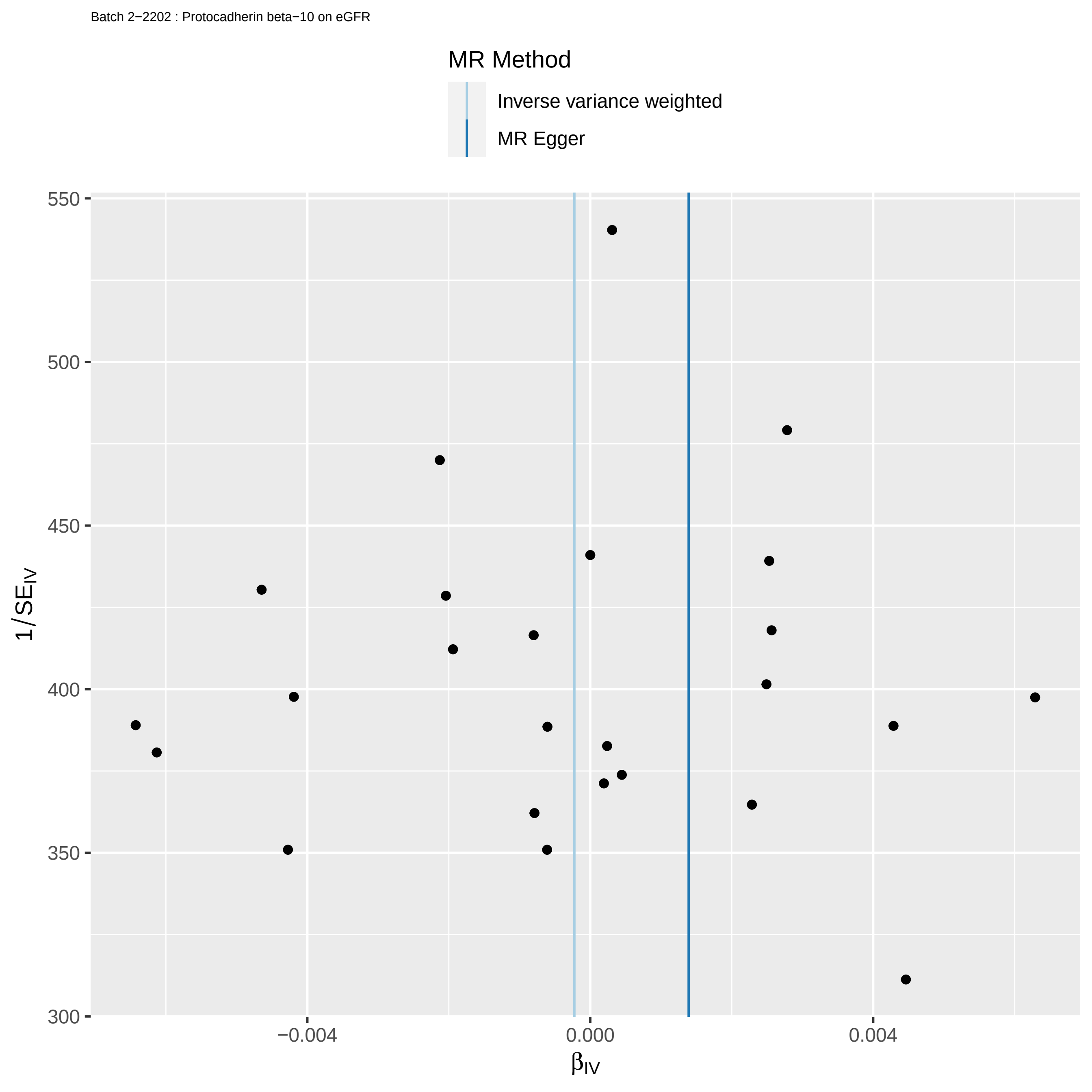

Batch 2−2202 : Protocadherin beta−10 on eGFR
MR Method
Inverse variance weighted
MR Egger
550
500
450
400
350
300
1/SE_IV
−0.004
0.000
0.004
β_IV

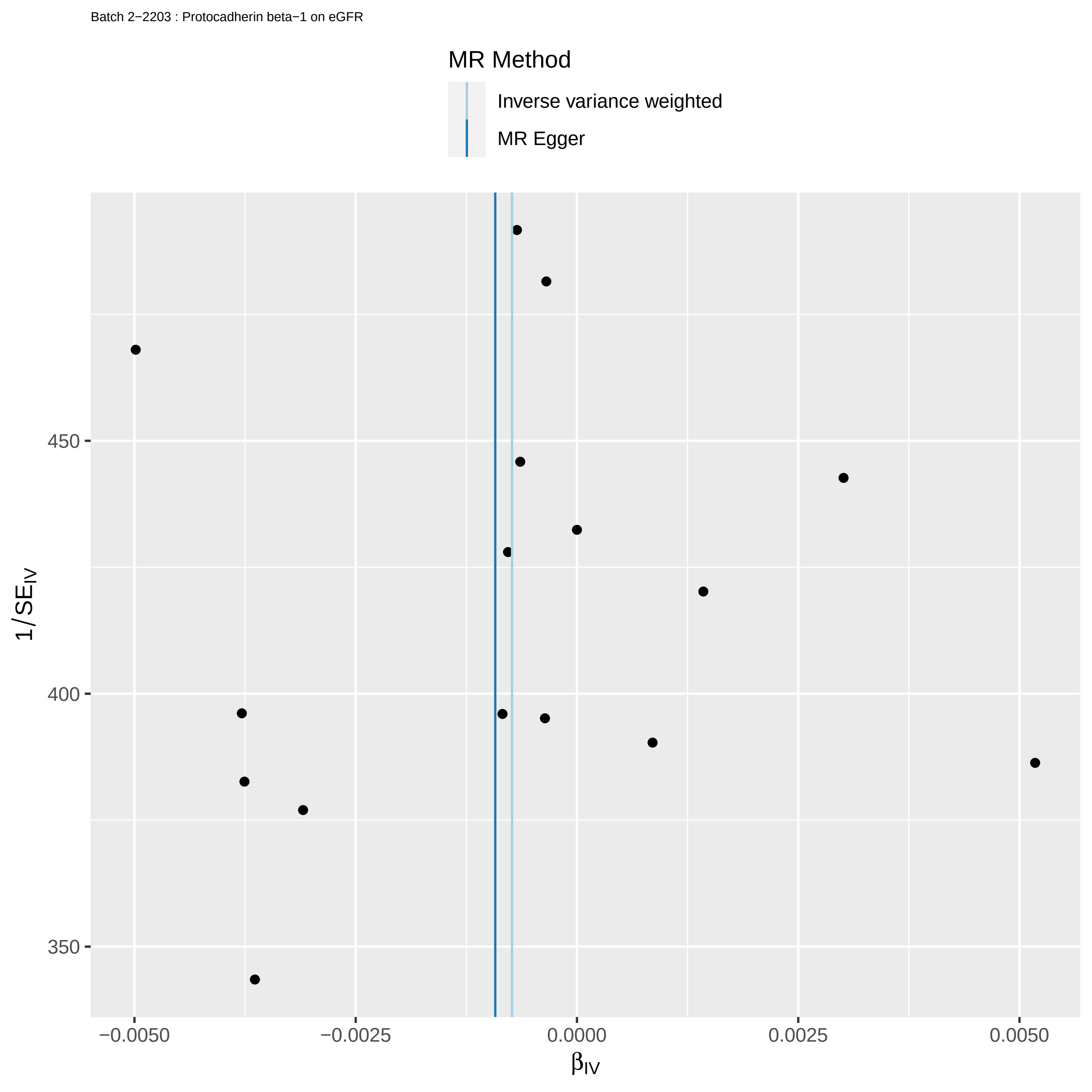

Batch 2−2203 : Protocadherin beta−1 on eGFR
MR Method
Inverse variance weighted
MR Egger
450
400
350
1/SE_IV
−0.0050
−0.0025
0.0000
0.0025
0.0050
β_IV

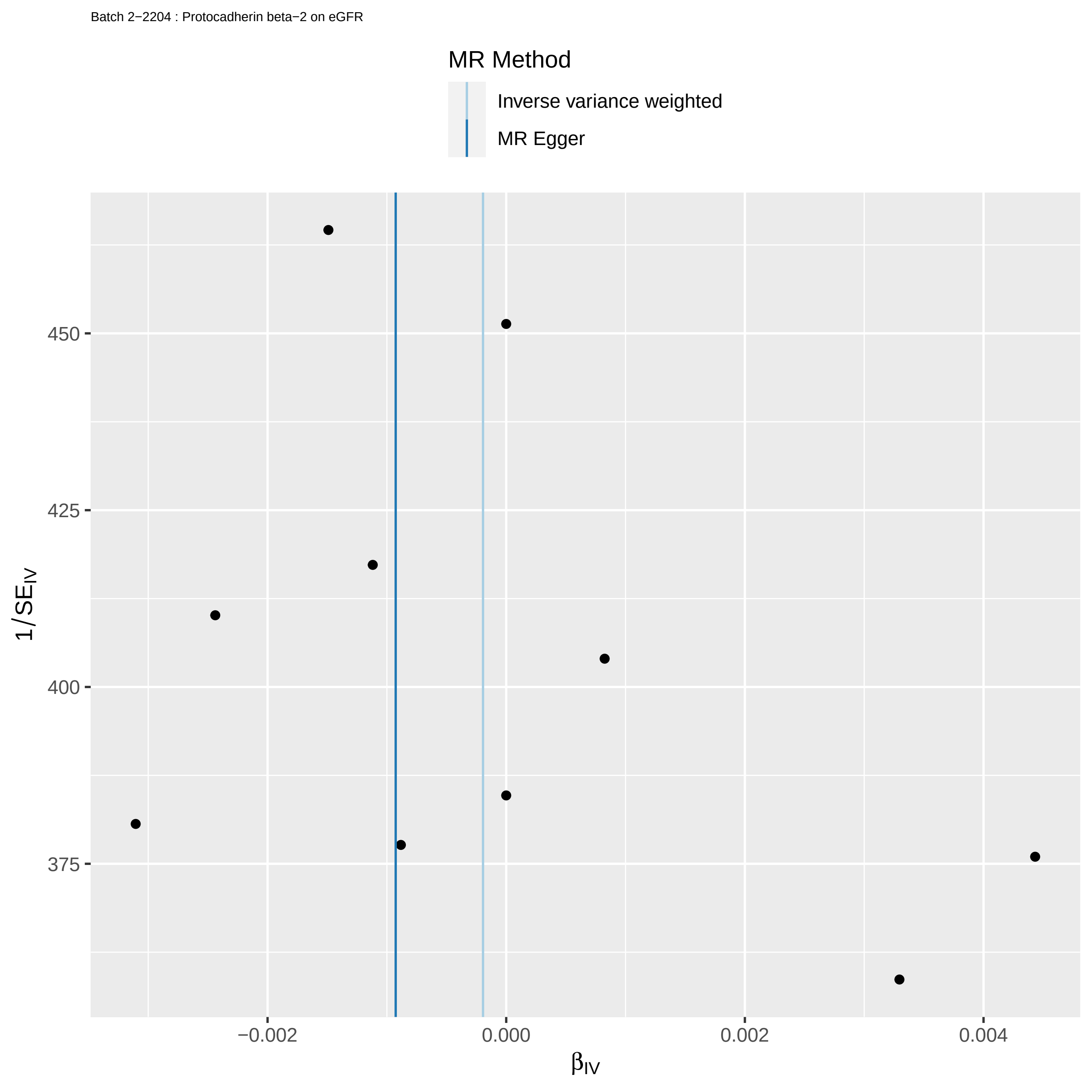
Batch 2−2204 : Protocadherin beta−2 on eGFR
MR Method
Inverse variance weighted
MR Egger
450
425
400
375
1/SE_IV
−0.002
0.000
0.002
0.004
β_IV

Batch 2−2205 : Protocadherin beta−4 on eGFR

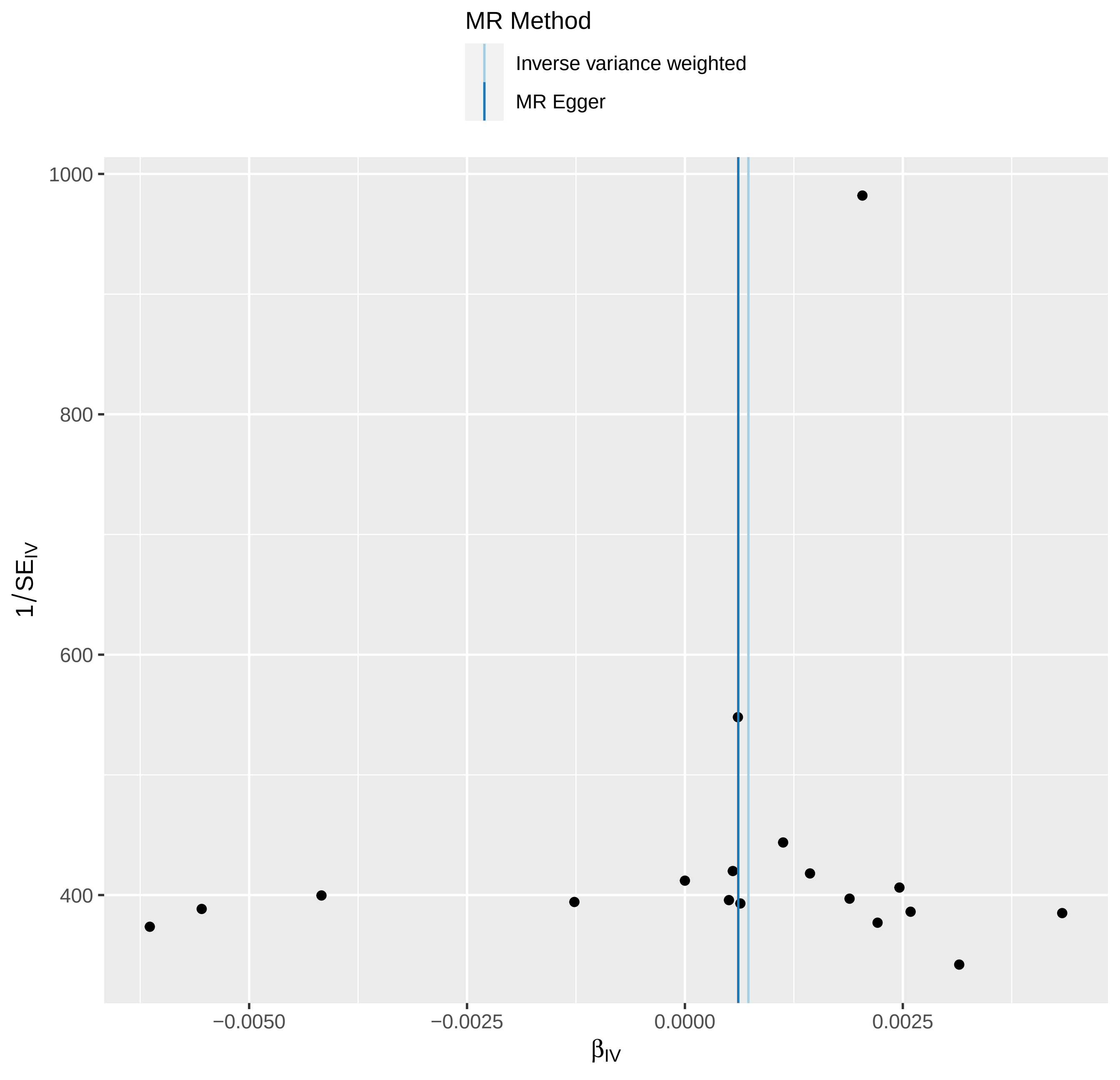

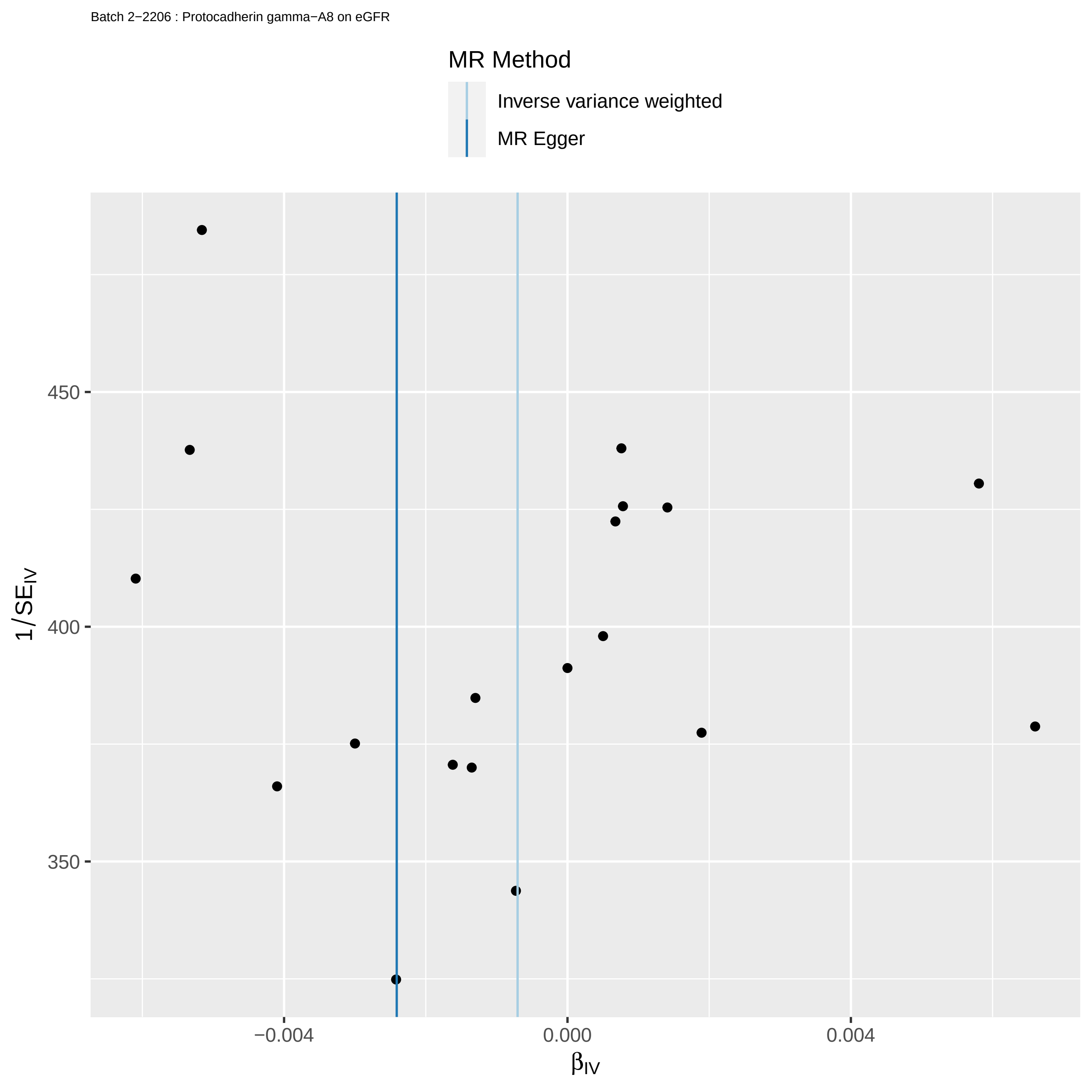

Batch 2−2206 : Protocadherin gamma−A8 on eGFR
MR Method
Inverse variance weighted
MR Egger
450
400
350
−0.004
0.000
0.004
1/SE_IV
β_IV

Batch 2−2207 : Protocadherin gamma−C5 on eGFR

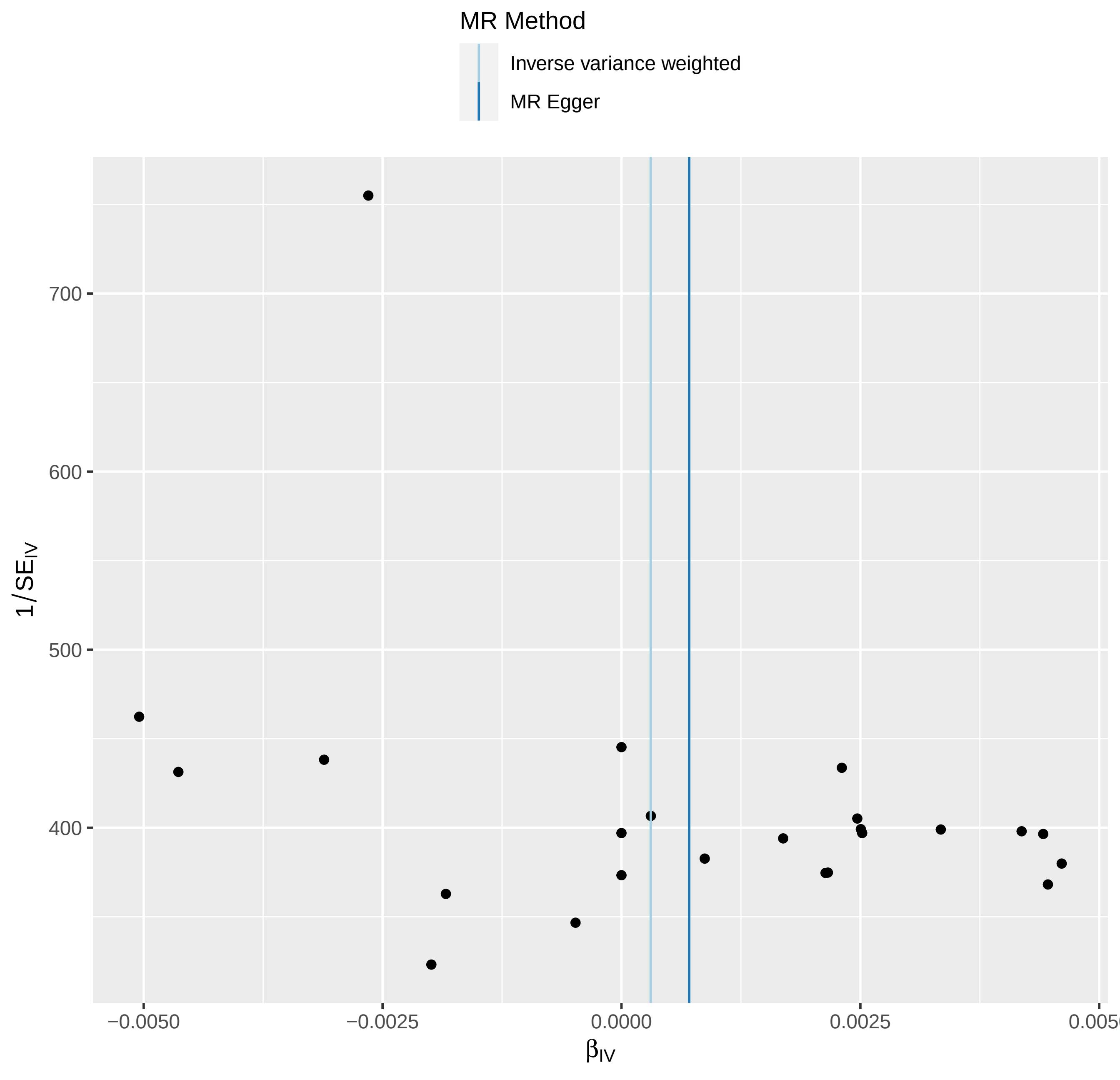

Batch 2−2208 : Proliferating cell nuclear antigen on eGFR

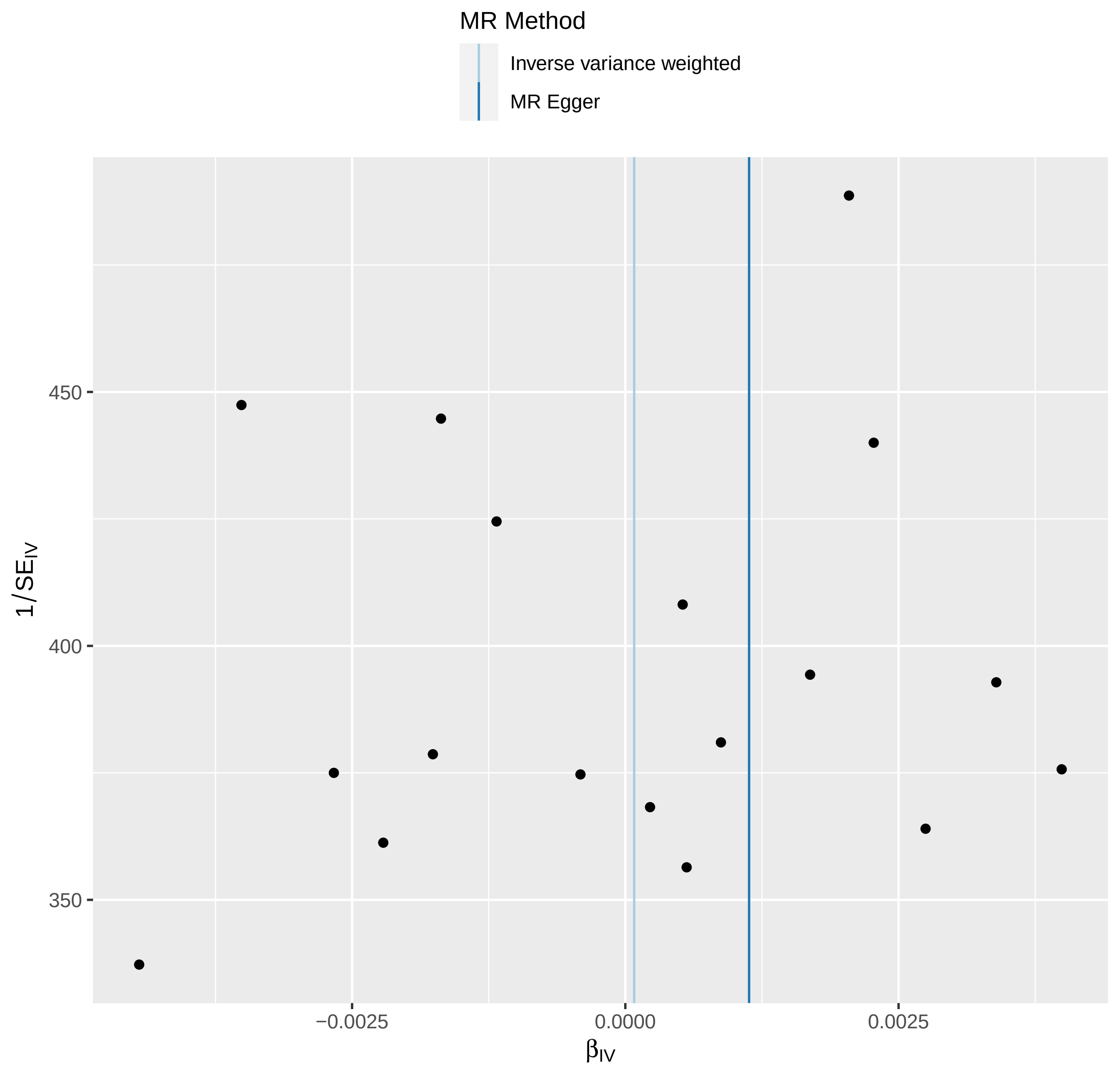

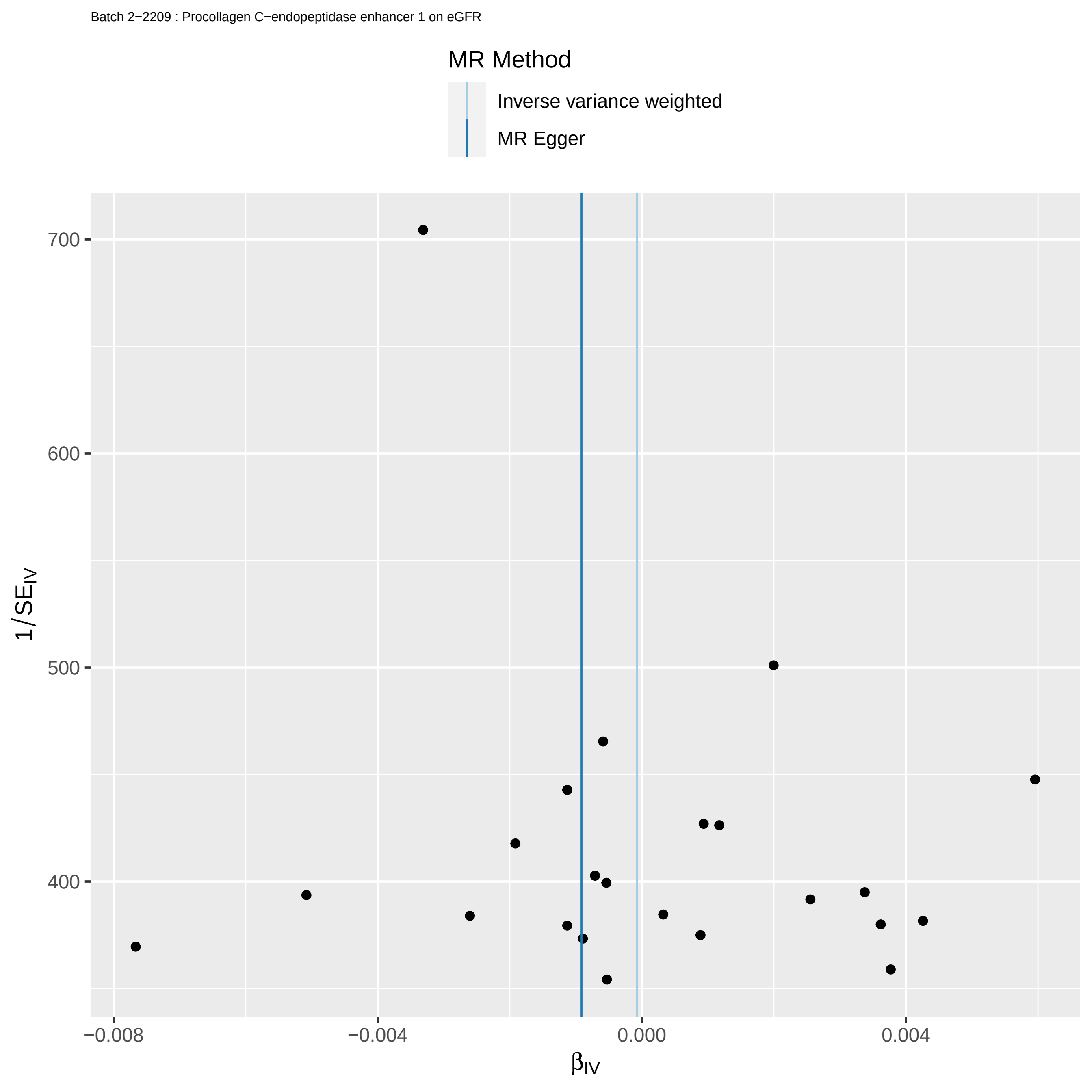
Batch 2−2209 : Procollagen C−endopeptidase enhancer 1 on eGFR
MR Method
Inverse variance weighted
MR Egger
700
600
500
400
$1/SE_{IV}$
−0.008
−0.004
0.000
0.004
β_{IV}

Batch 2−2210 : Procollagen C−endopeptidase enhancer 2 on eGFR

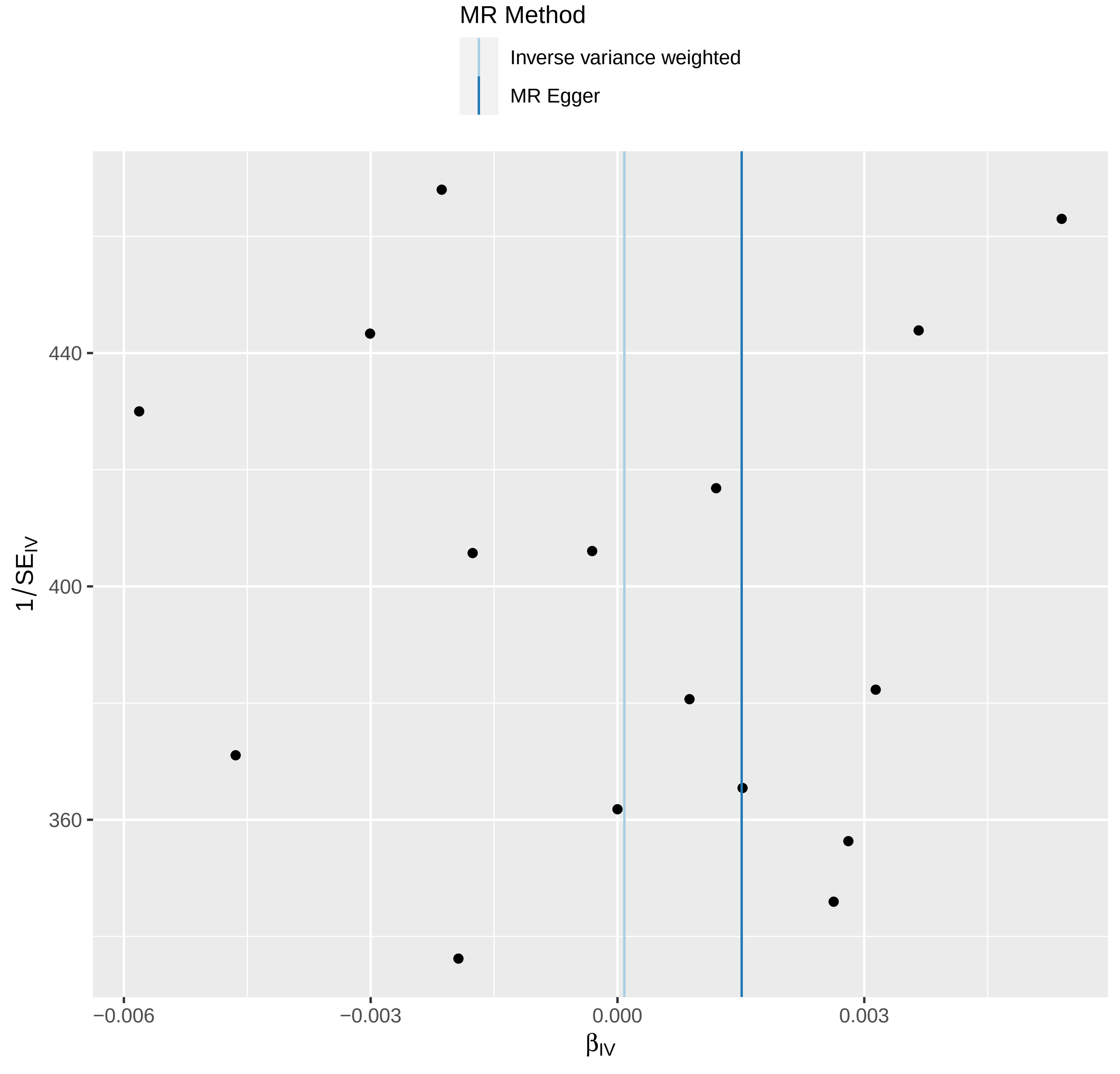

Batch 2−2211 : Neuroendocrine convertase 1 on eGFR

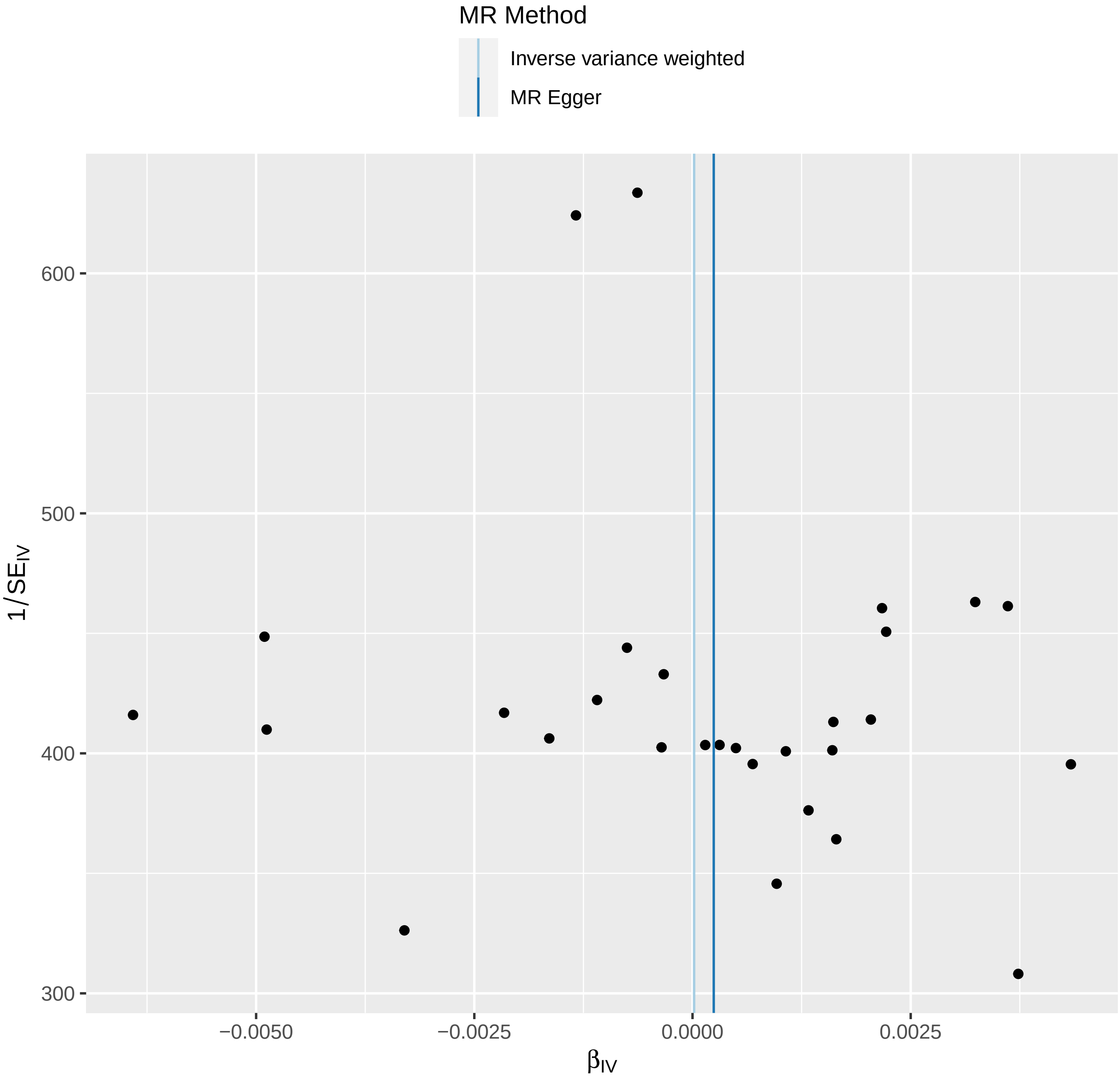

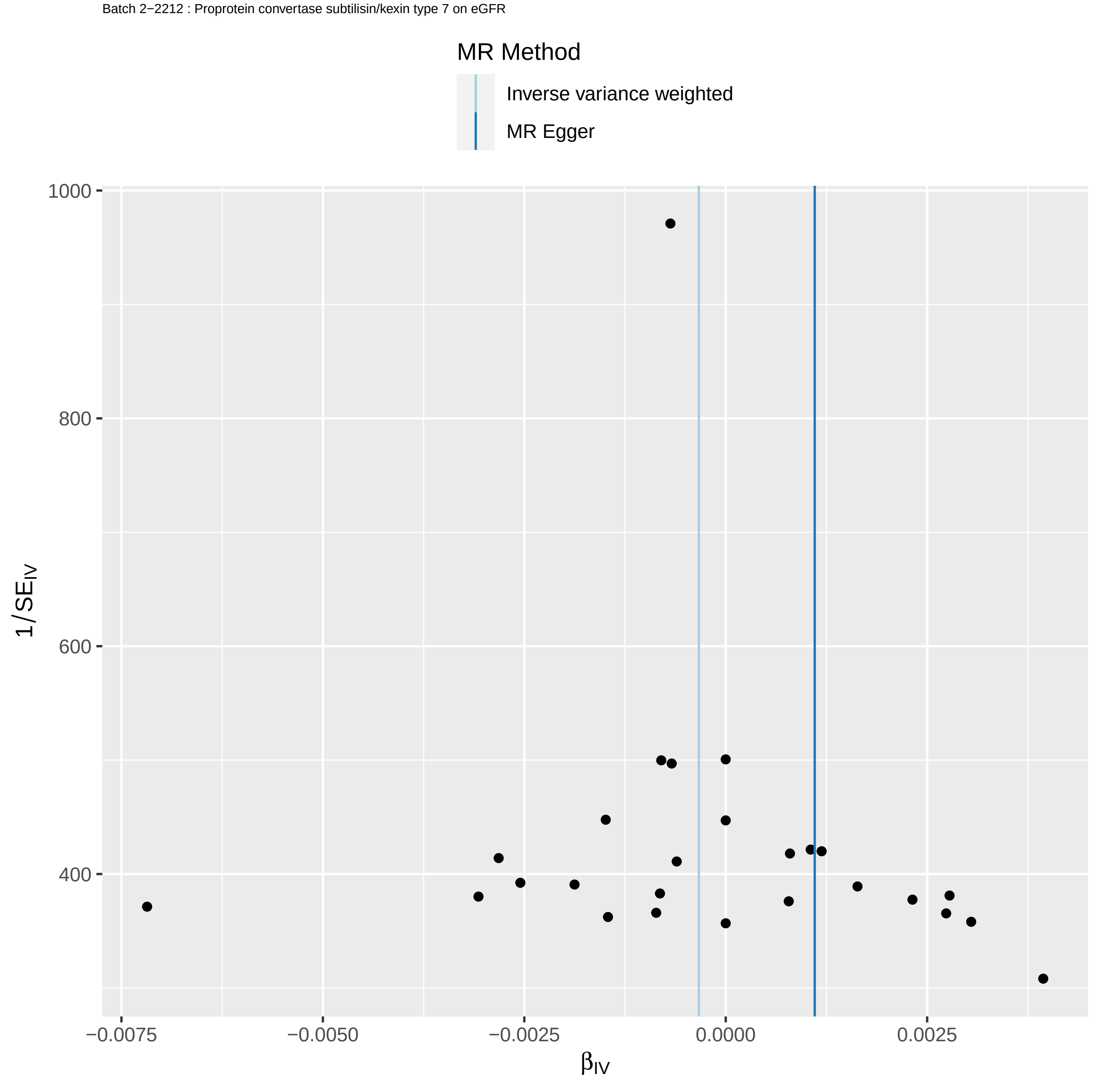
Batch 2−2212 : Proprotein convertase subtilisin/kexin type 7 on eGFR
MR Method
Inverse variance weighted
MR Egger
1000
800
600
400
1/SE_IV
−0.0075
−0.0050
−0.0025
0.0000
0.0025
β_IV

Batch 2−2213 : Prenylcysteine oxidase−like on eGFR

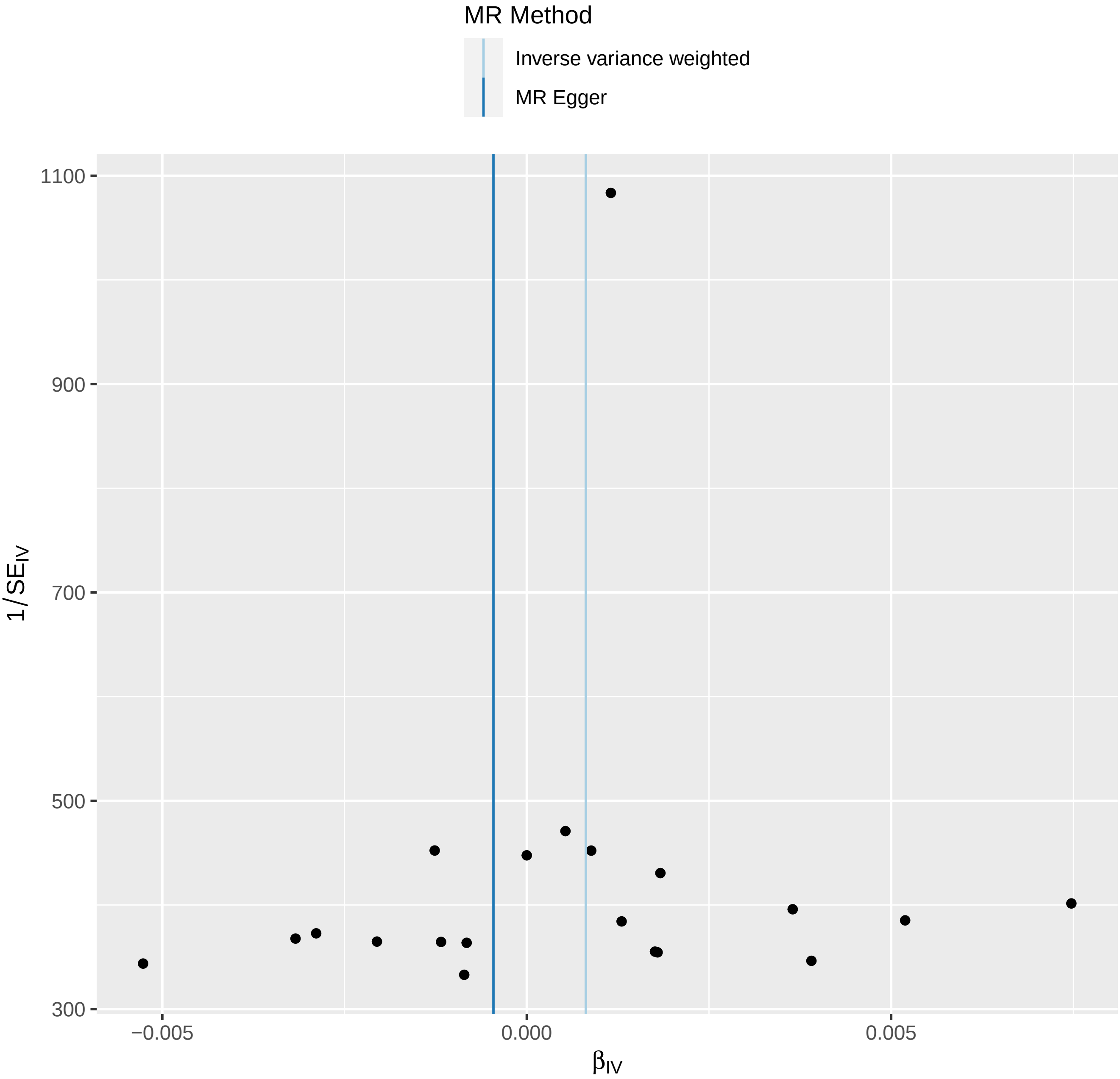

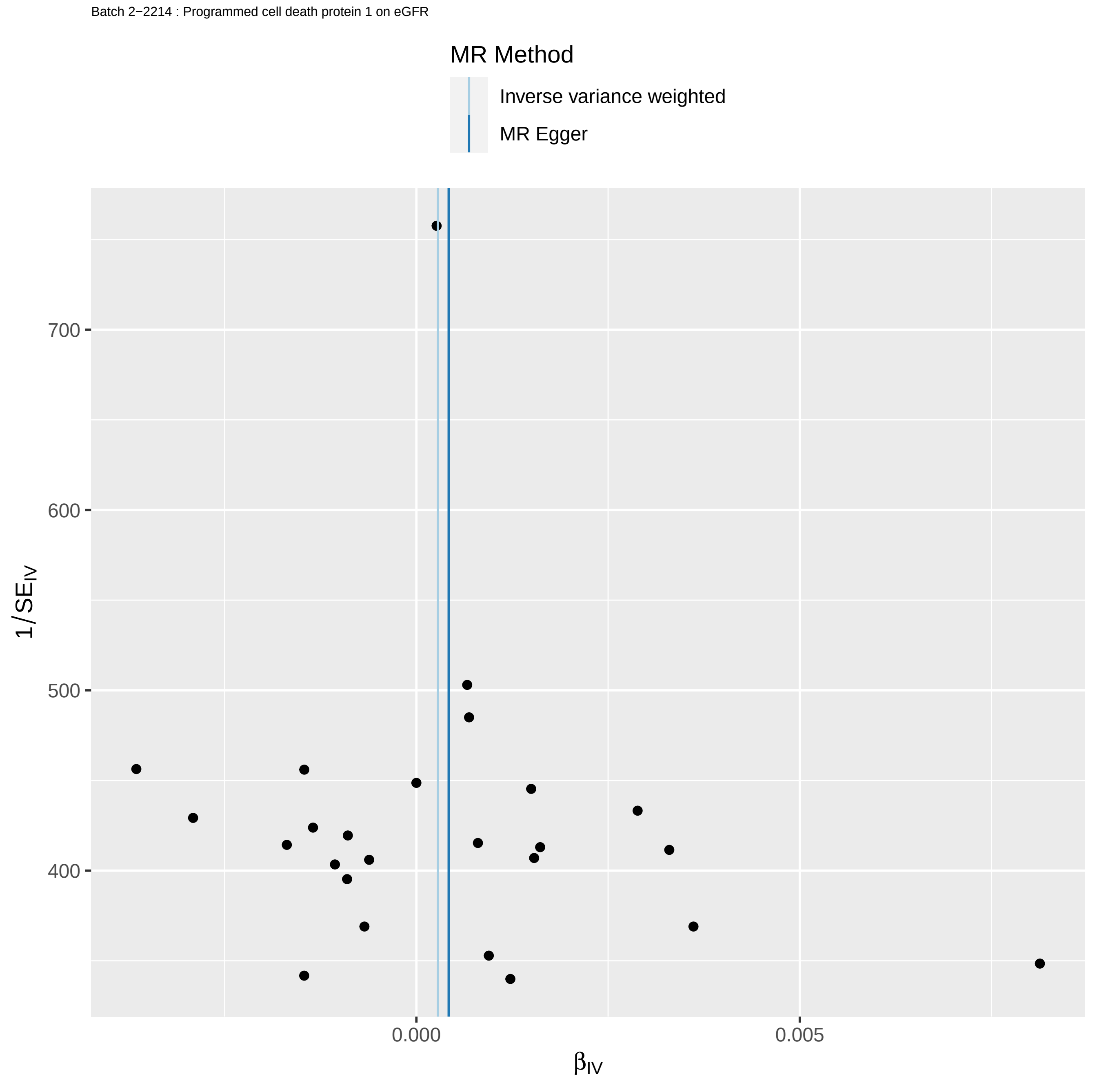

Batch 2−2214 : Programmed cell death protein 1 on eGFR
MR Method
Inverse variance weighted
MR Egger
700
600
500
400
0.000
0.005
$1/SE_{IV}$
β_{IV}

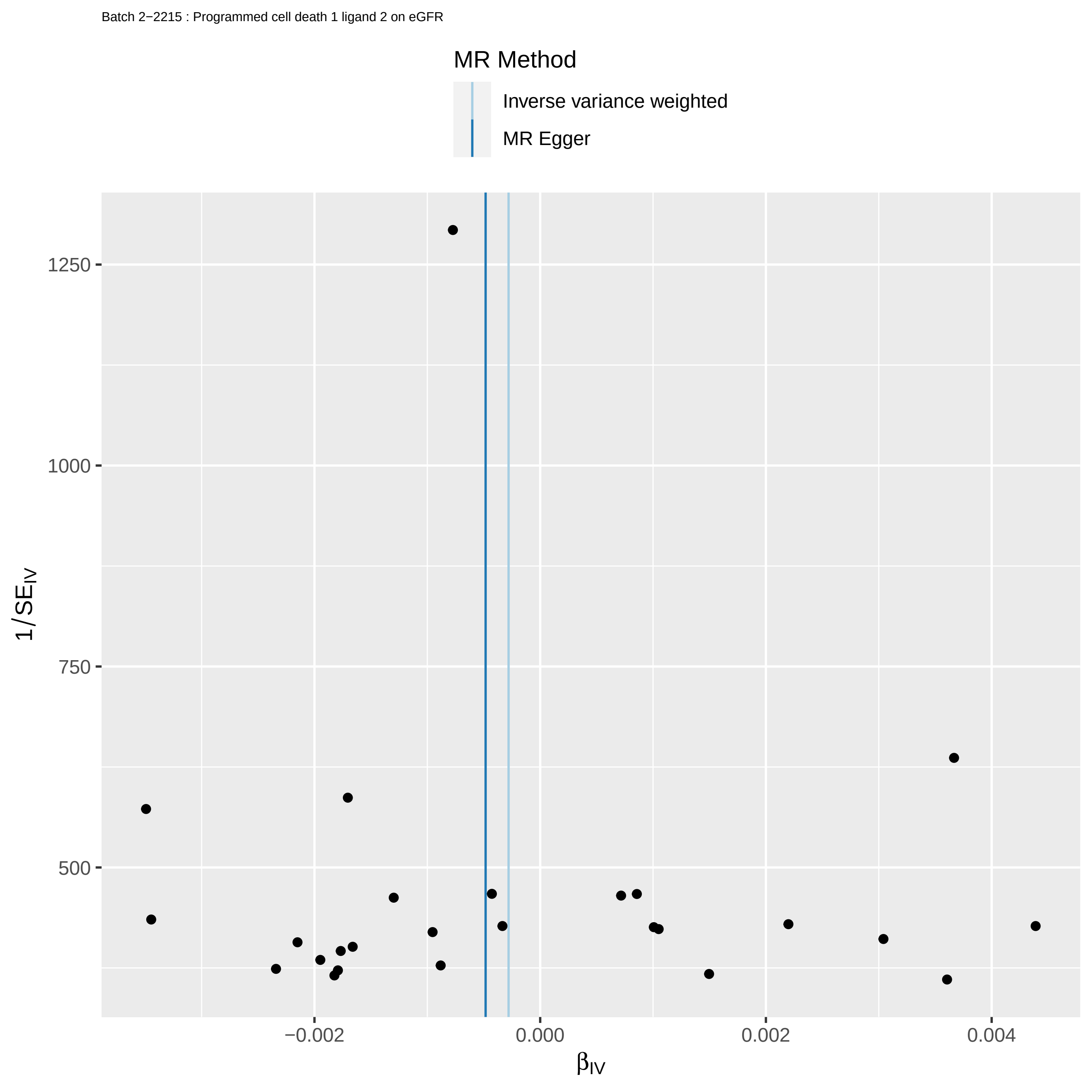
Batch 2−2215 : Programmed cell death 1 ligand 2 on eGFR
MR Method
Inverse variance weighted
MR Egger
1250
1000
750
500
1/SE_IV
−0.002
0.000
0.002
0.004
β_IV

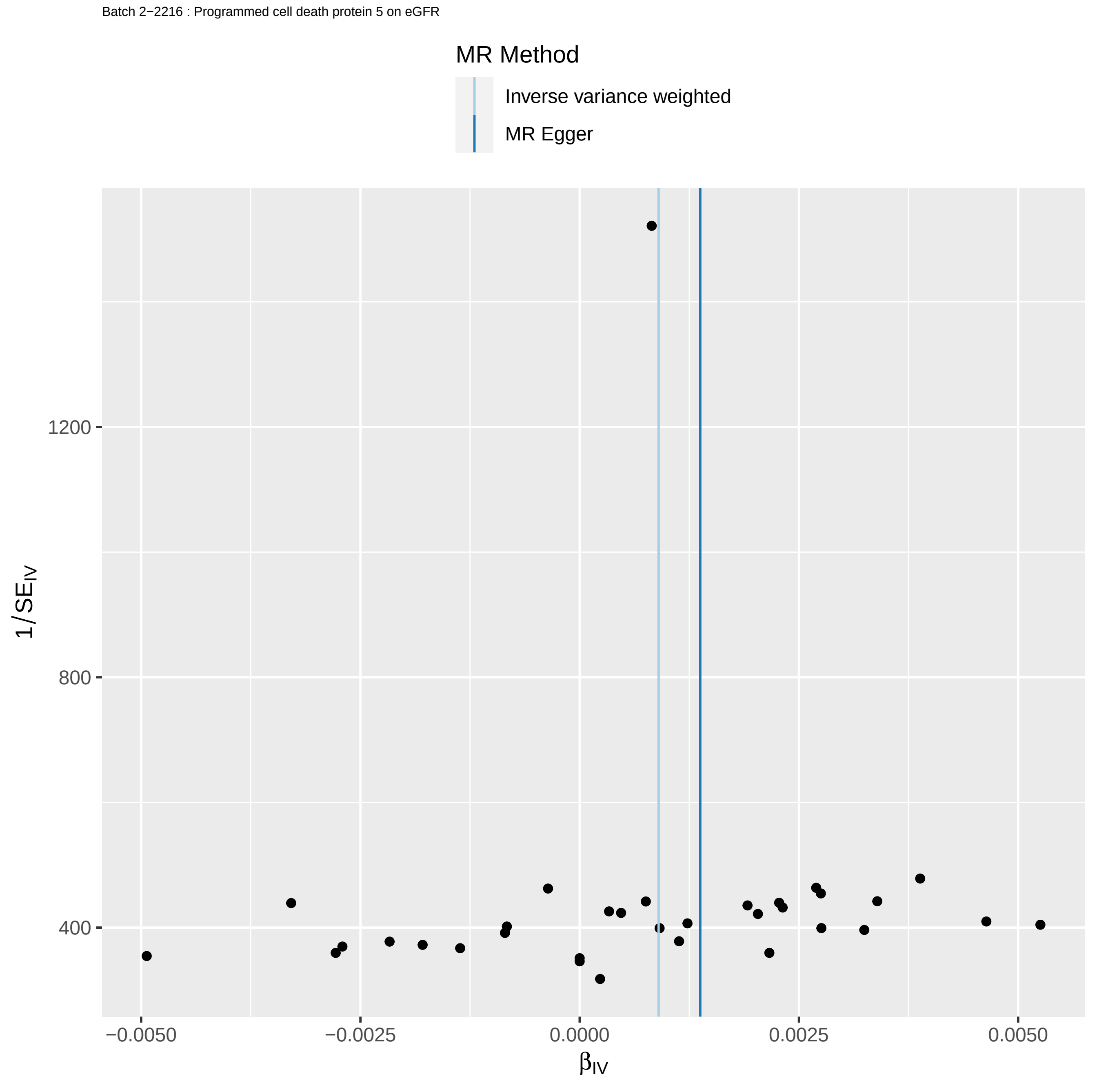

Batch 2−2216 : Programmed cell death protein 5 on eGFR
MR Method
Inverse variance weighted
MR Egger
1200
800
400
1/SE_IV
−0.0050
−0.0025
0.0000
0.0025
0.0050
β_IV

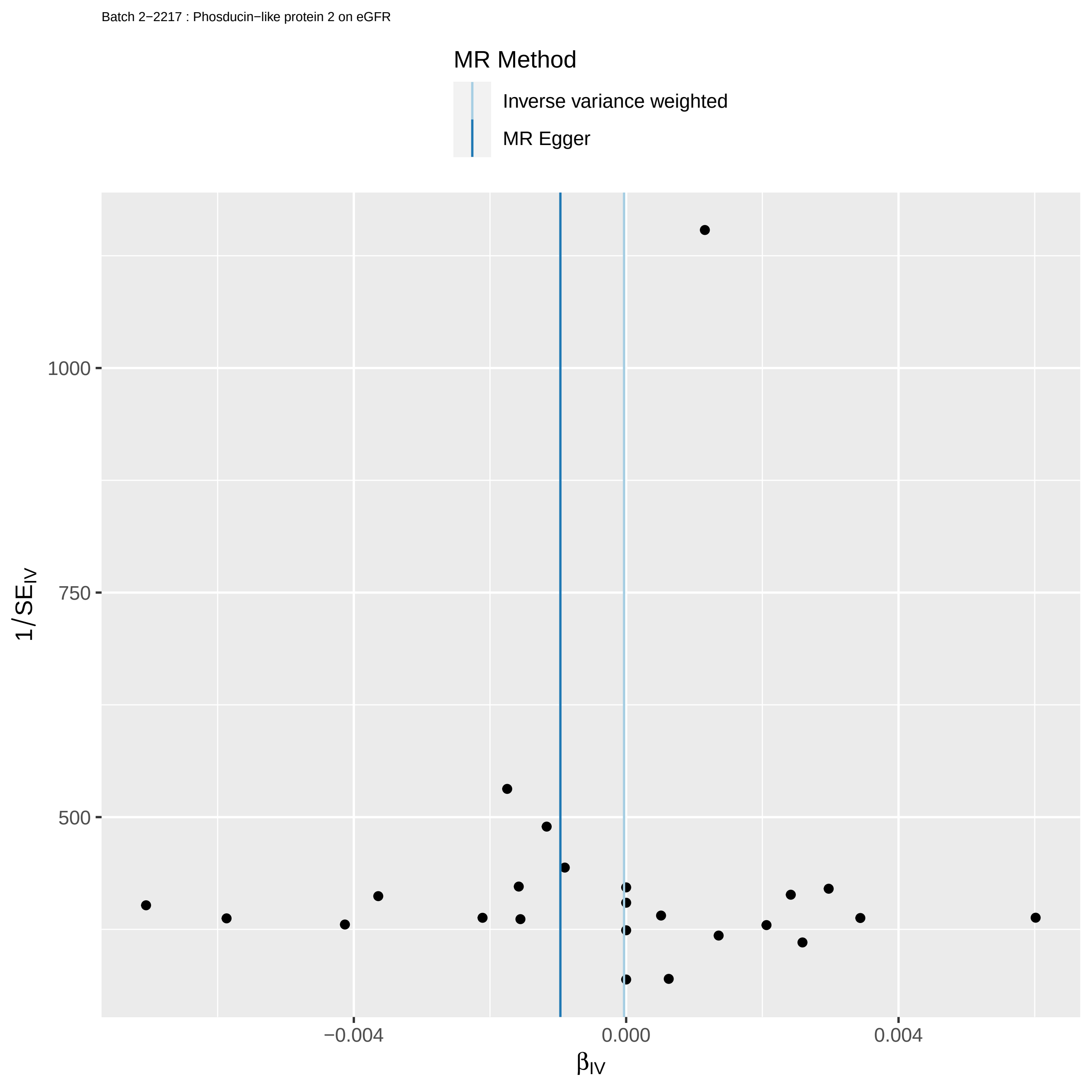

Batch 2−2217 : Phosducin−like protein 2 on eGFR
MR Method
Inverse variance weighted
MR Egger
1000
750
500
1/SE_IV
−0.004
0.000
0.004
β_IV

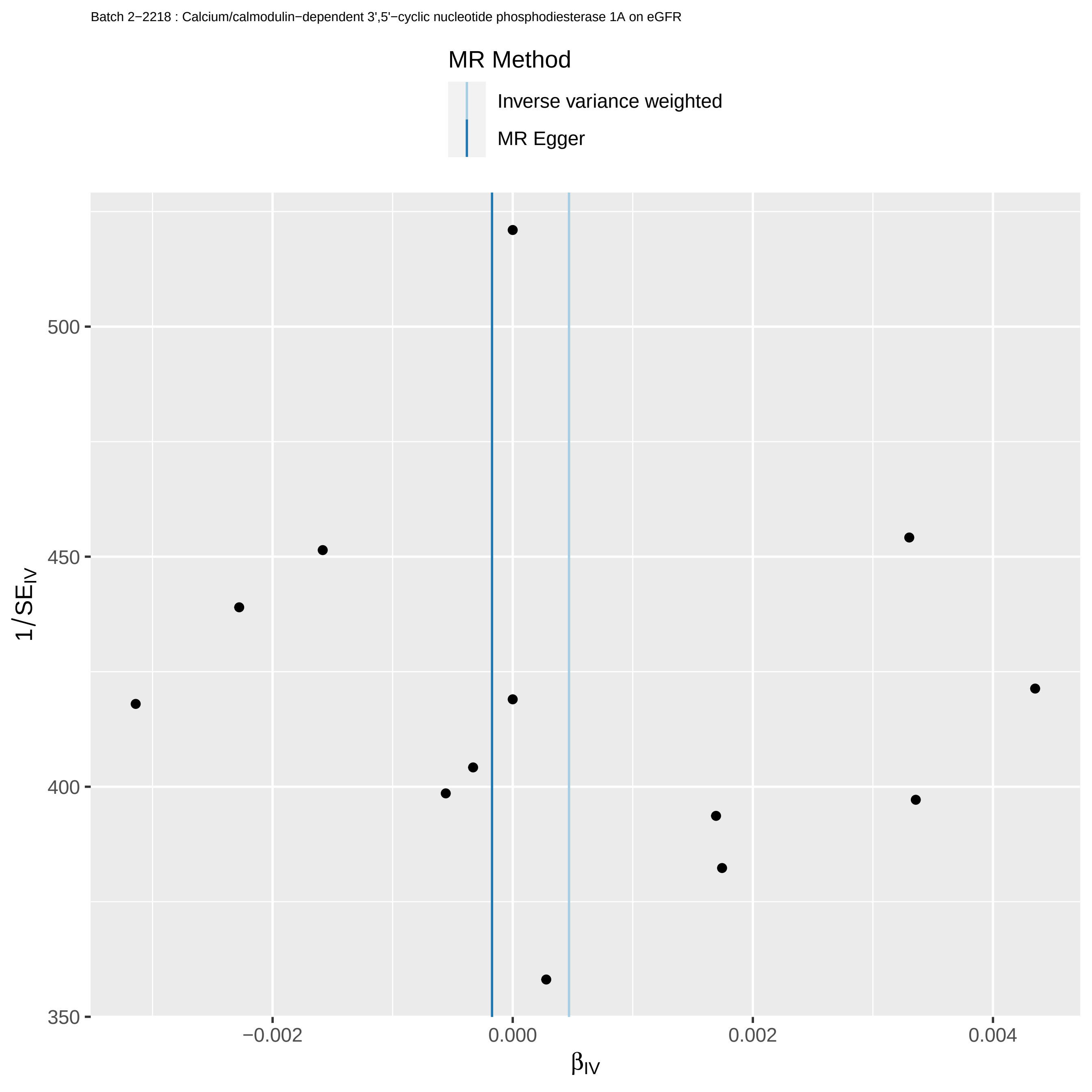
Batch 2−2218 : Calcium/calmodulin−dependent 3',5'−cyclic nucleotide phosphodiesterase 1A on eGFR
MR Method
Inverse variance weighted
MR Egger
500
450
400
350
1/SE_IV
−0.002
0.000
0.002
0.004
β_IV

Batch 2−2219 : cGMP−dependent 3',5'−cyclic phosphodiesterase on eGFR

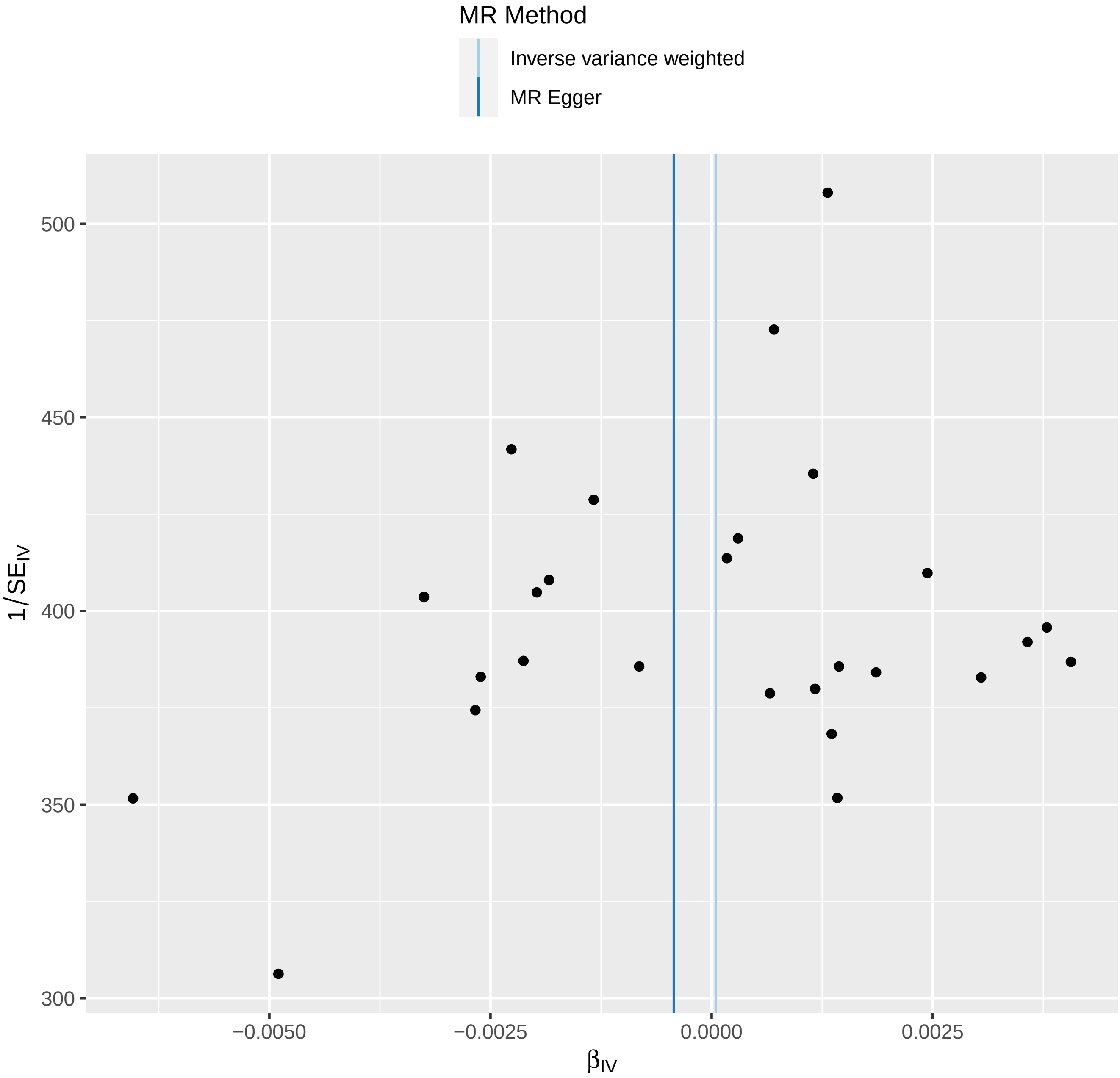

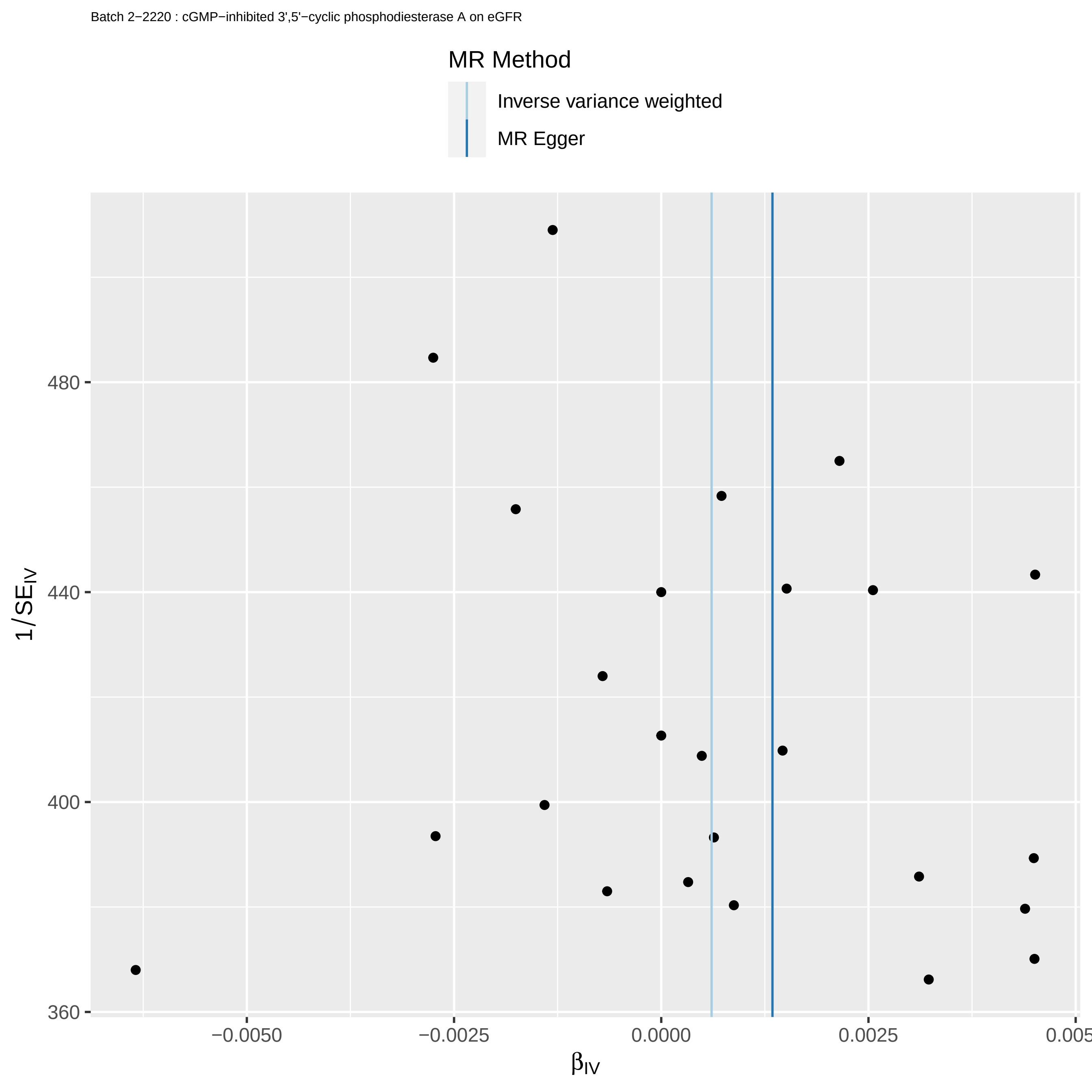

Batch 2−2220 : cGMP−inhibited 3',5'−cyclic phosphodiesterase A on eGFR
MR Method
Inverse variance weighted
MR Egger
480
440
400
360
1/SE_IV
−0.0050
−0.0025
0.0000
0.0025
0.005
β_IV

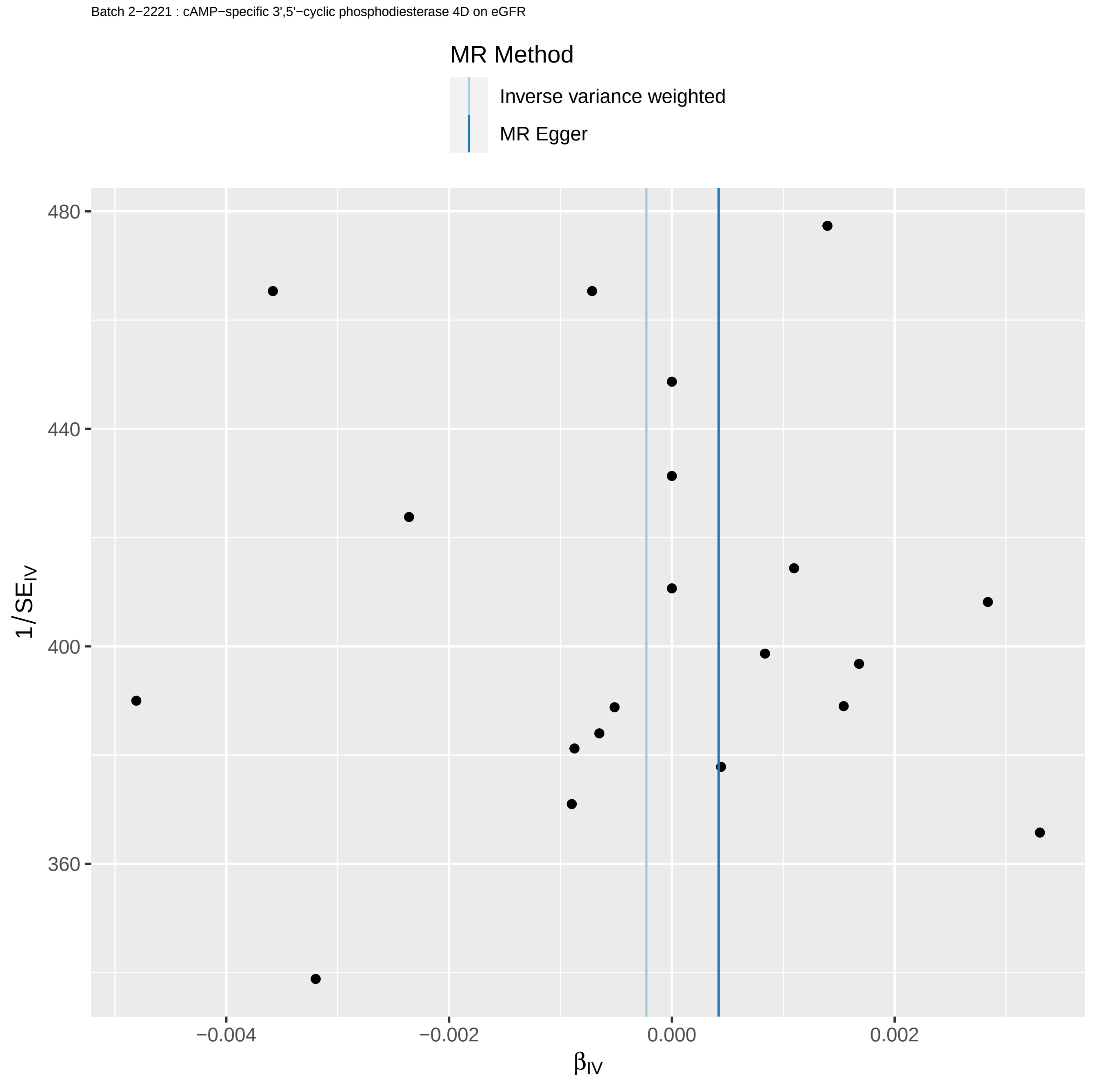
Batch 2−2221 : cAMP−specific 3',5'−cyclic phosphodiesterase 4D on eGFR
MR Method
Inverse variance weighted
MR Egger
480
440
400
360
1/SE_IV
−0.004
−0.002
0.000
0.002
β_IV

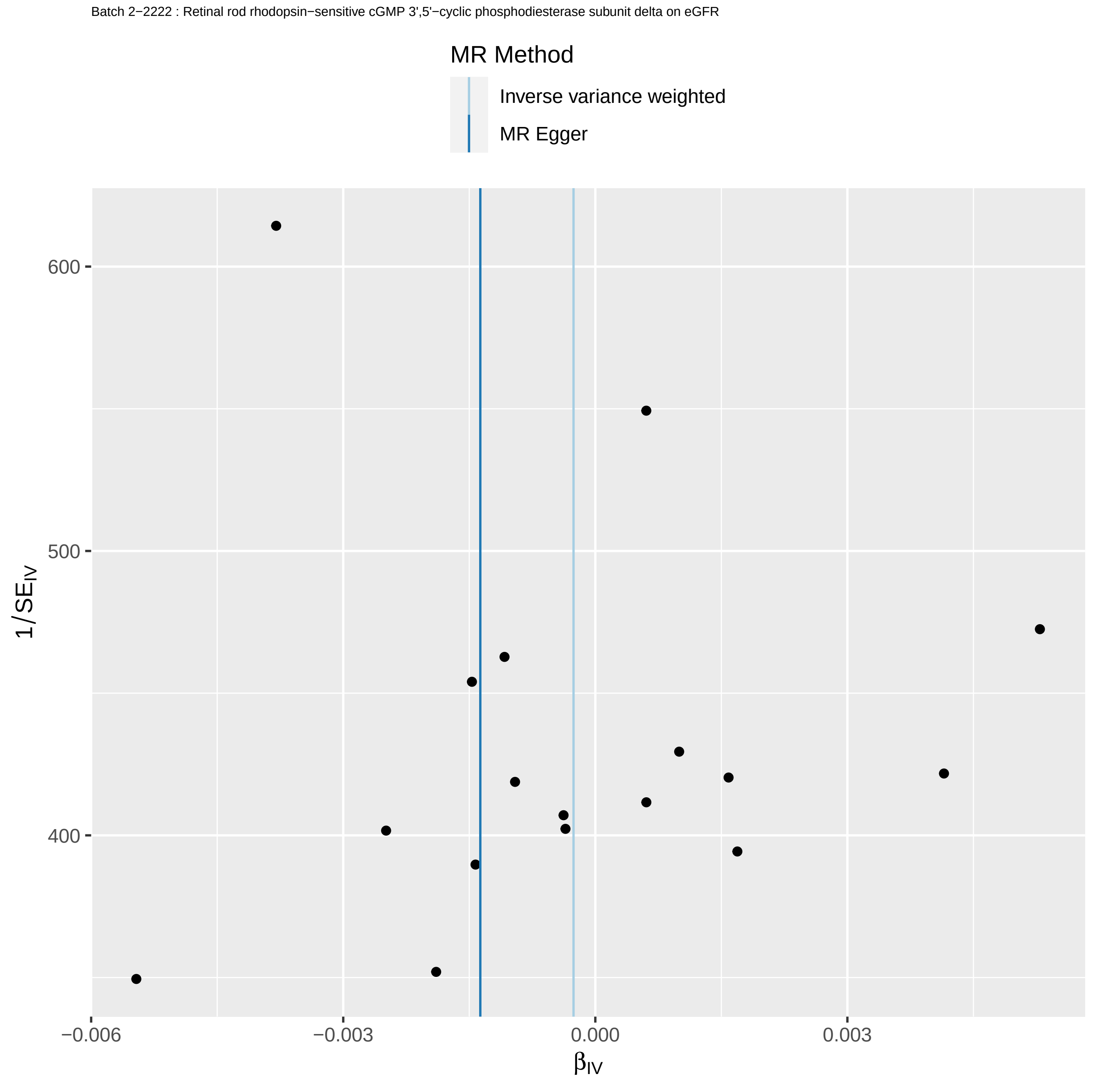
Batch 2−2222 : Retinal rod rhodopsin−sensitive cGMP 3',5'−cyclic phosphodiesterase subunit delta on eGFR
MR Method
Inverse variance weighted
MR Egger
600
500
400
1/SE_IV
−0.006
−0.003
0.000
0.003
β_IV

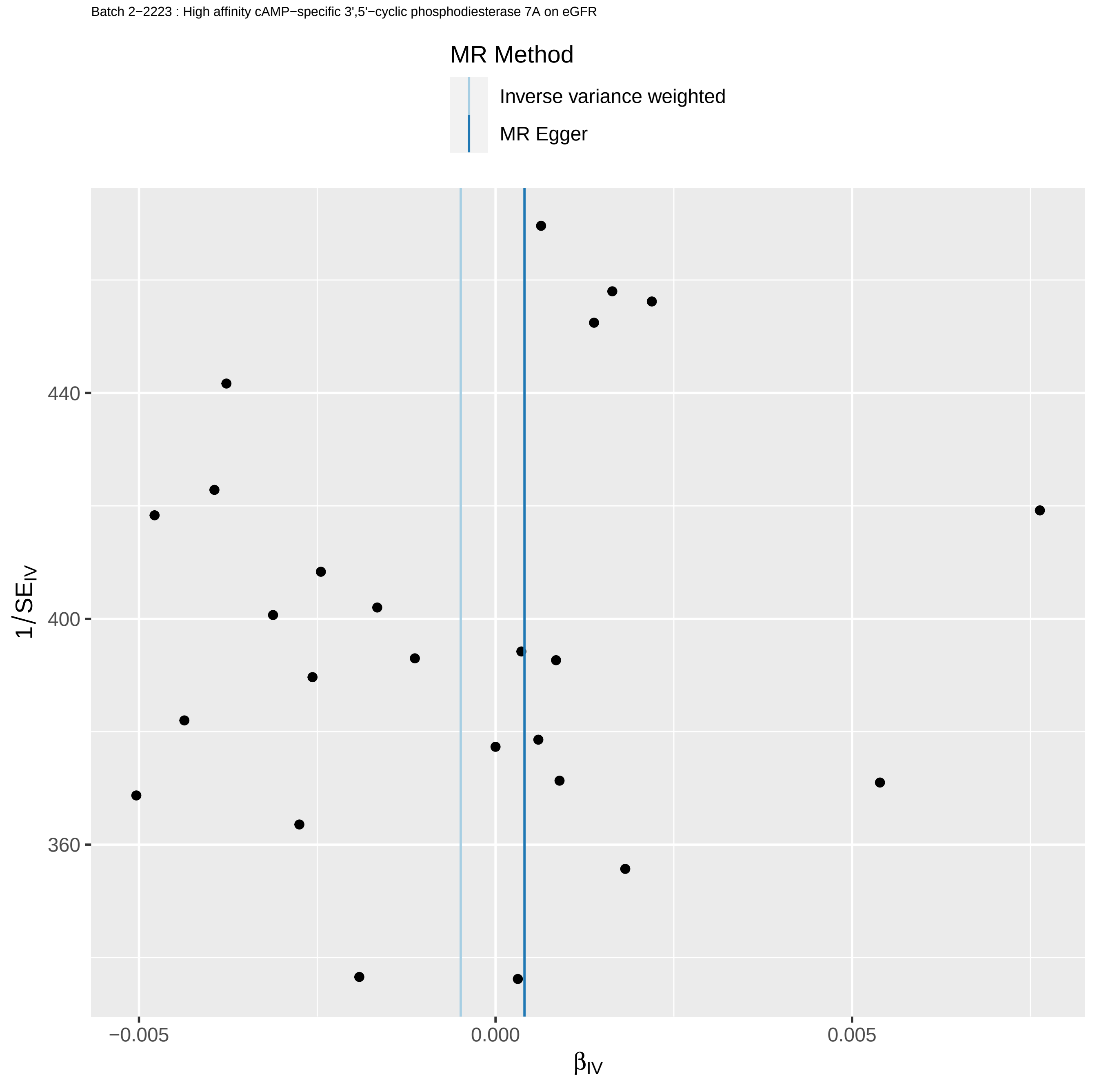
Batch 2−2223 : High affinity cAMP−specific 3',5'−cyclic phosphodiesterase 7A on eGFR
MR Method
Inverse variance weighted
MR Egger
440
400
360
1/SE_IV
−0.005
0.000
0.005
β_IV

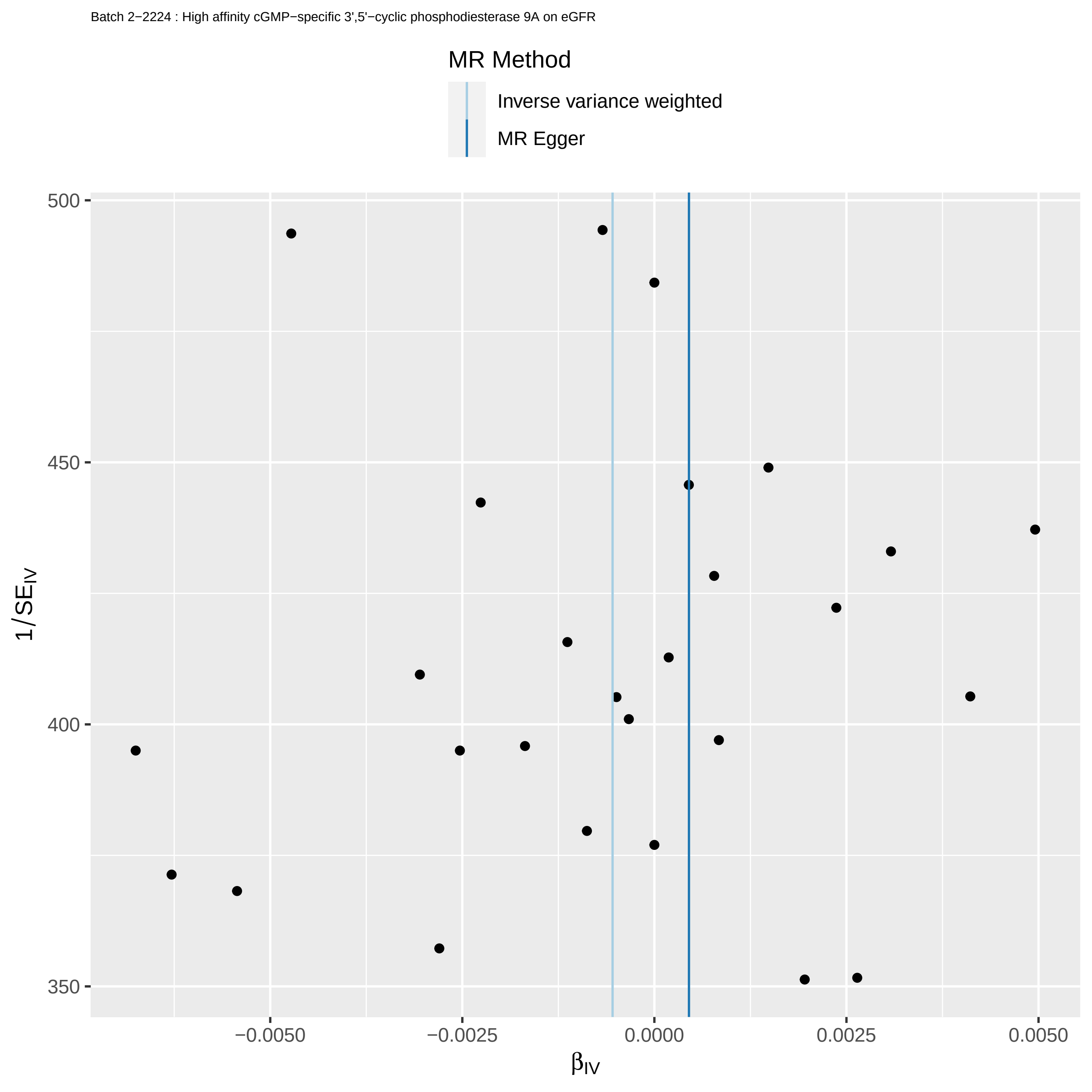

Batch 2−2224 : High affinity cGMP−specific 3',5'−cyclic phosphodiesterase 9A on eGFR
MR Method
Inverse variance weighted
MR Egger
500
450
400
350
1/SE_IV
−0.0050
−0.0025
0.0000
0.0025
0.0050
β_IV

Batch 2−2225 : Platelet−derived growth factor subunit A on eGFR

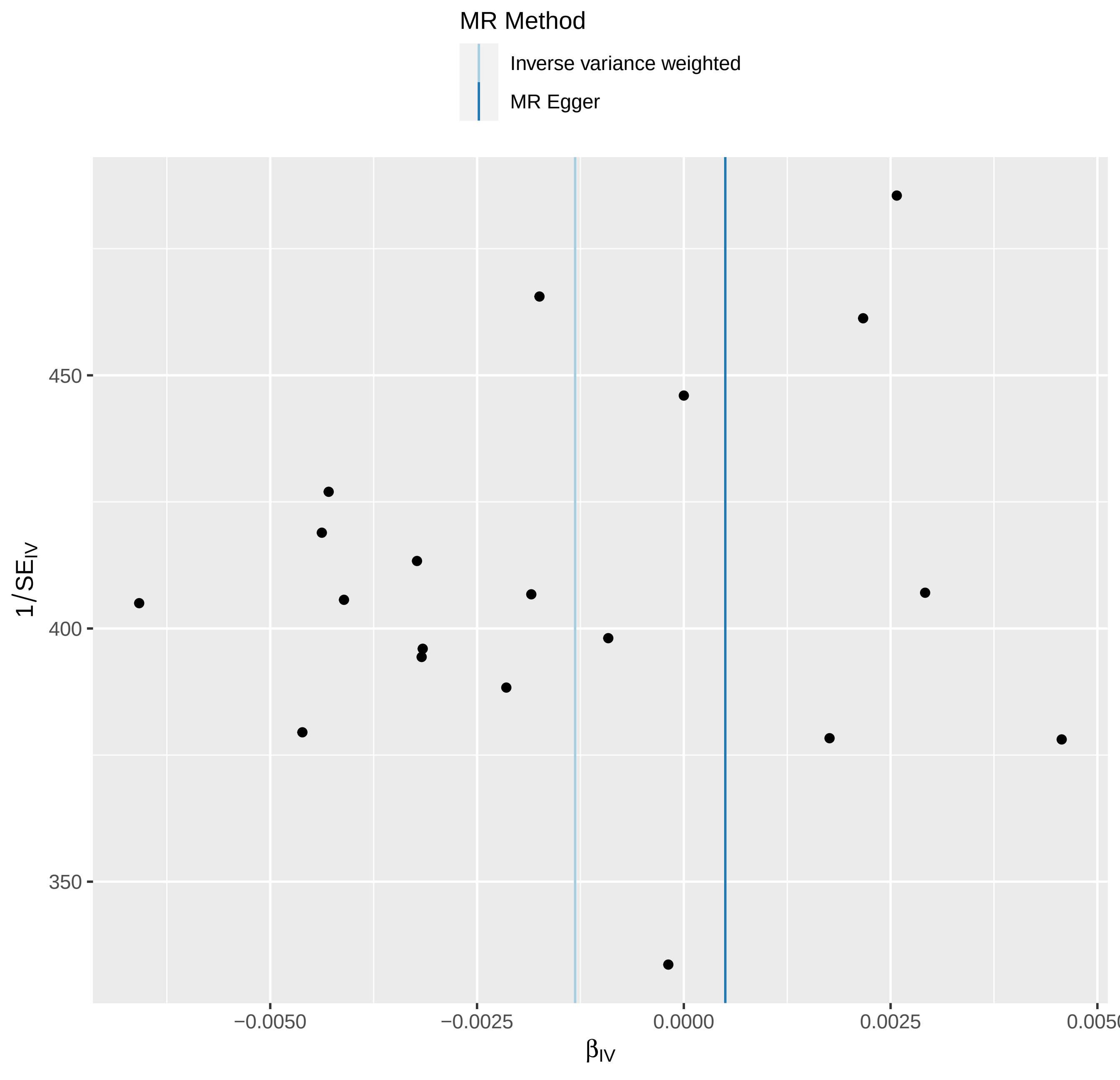

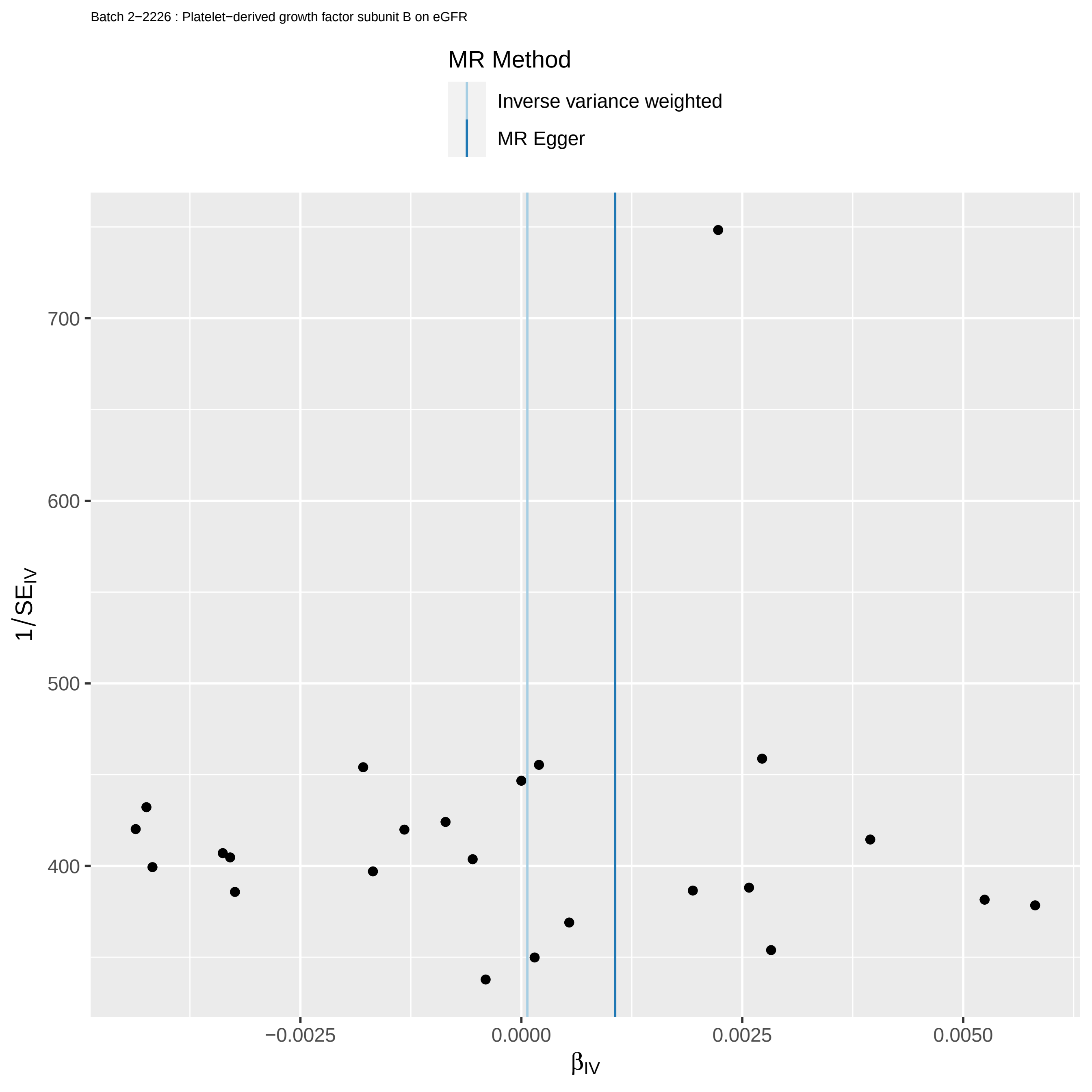

Batch 2−2226 : Platelet−derived growth factor subunit B on eGFR
MR Method
Inverse variance weighted
MR Egger
700
600
500
400
1/SE_IV
−0.0025
0.0000
0.0025
0.0050
β_IV

Batch 2−2227 : Platelet−derived growth factor C on eGFR

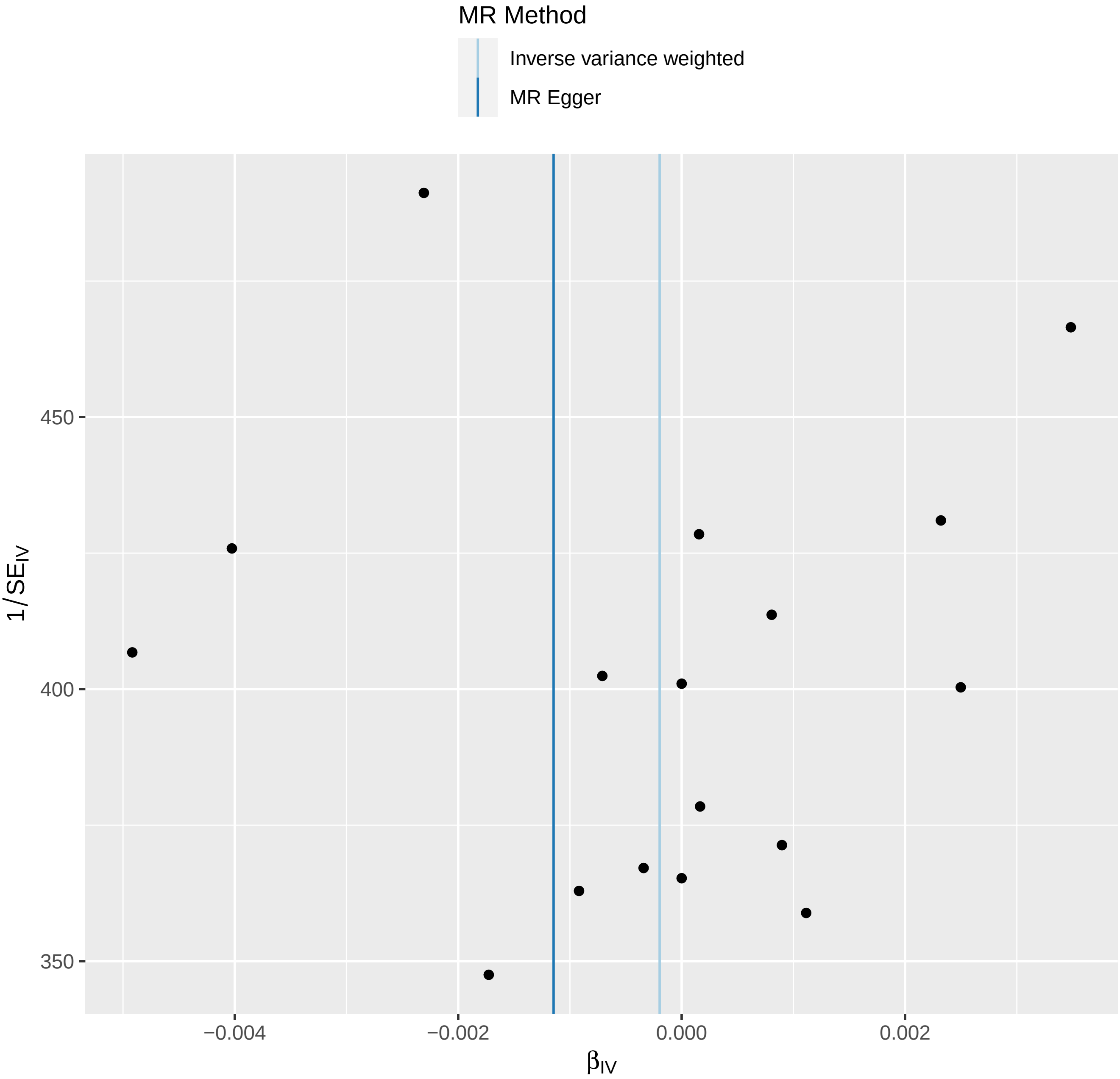

Batch 2−2228 : Platelet−derived growth factor D on eGFR

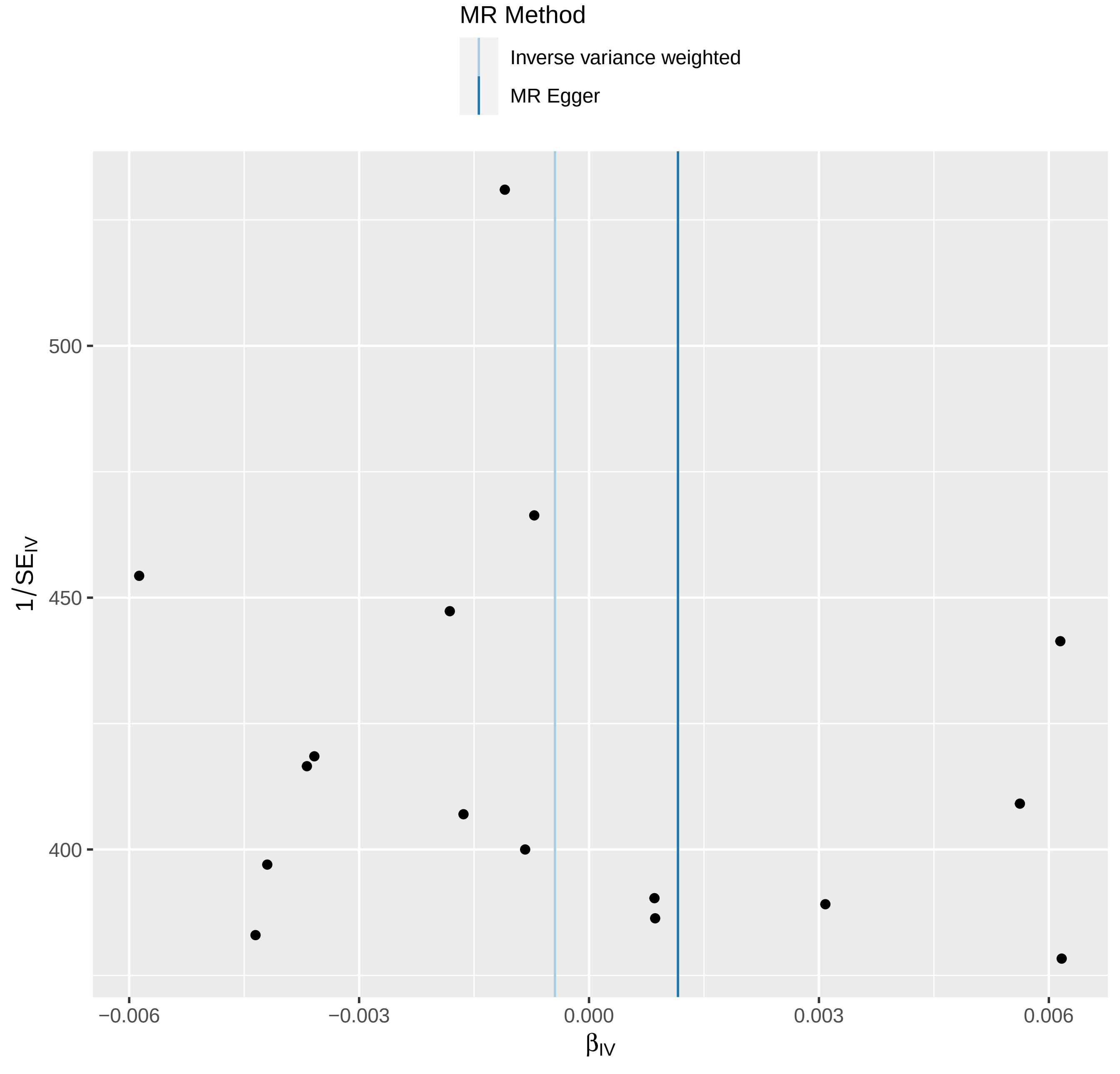

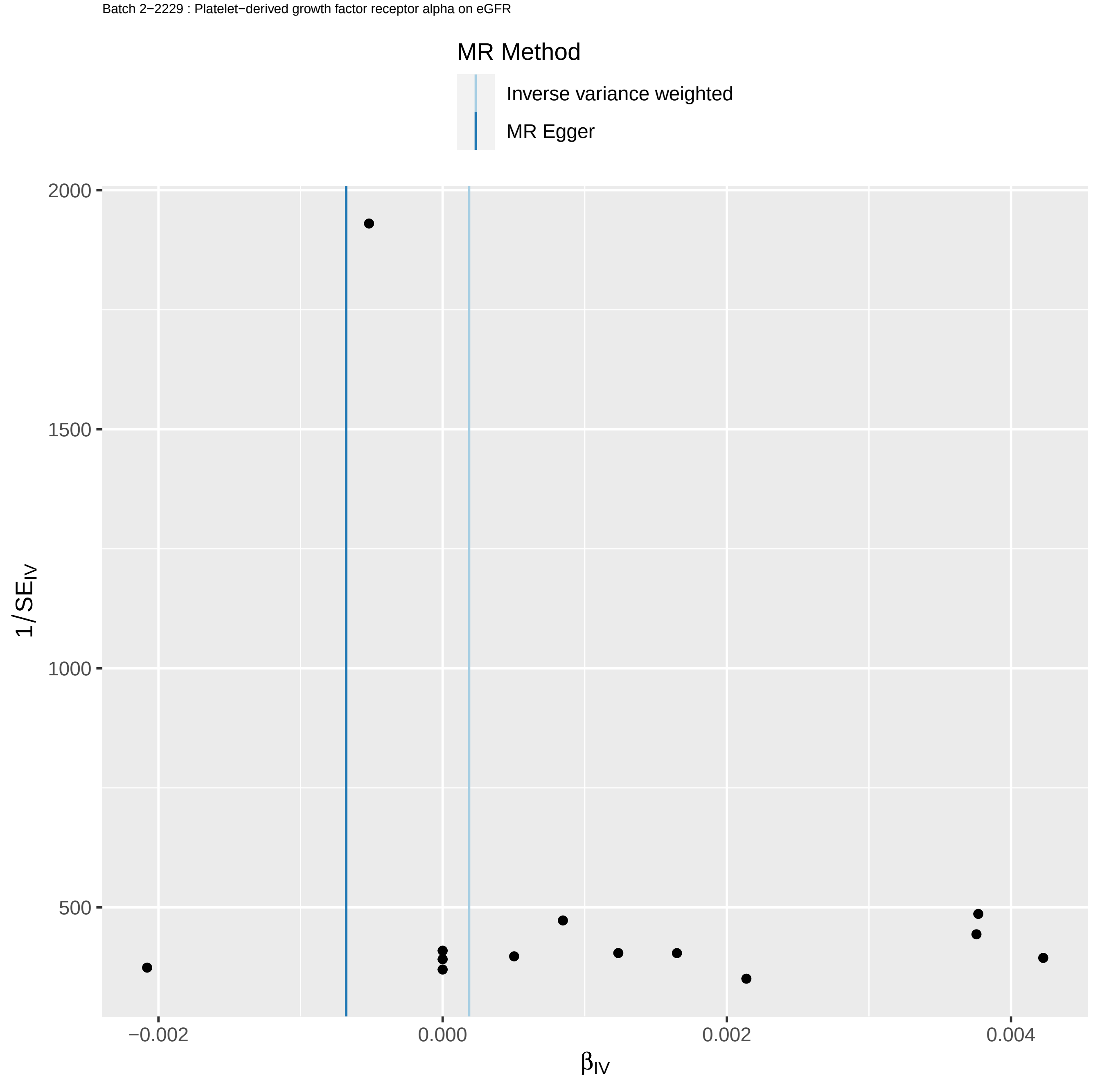
Batch 2−2229 : Platelet−derived growth factor receptor alpha on eGFR
MR Method
Inverse variance weighted
MR Egger
2000
1500
1000
500
1/SE_IV
−0.002
0.000
0.002
0.004
β_IV

Batch 2−2230 : Platelet−derived growth factor receptor beta on eGFR

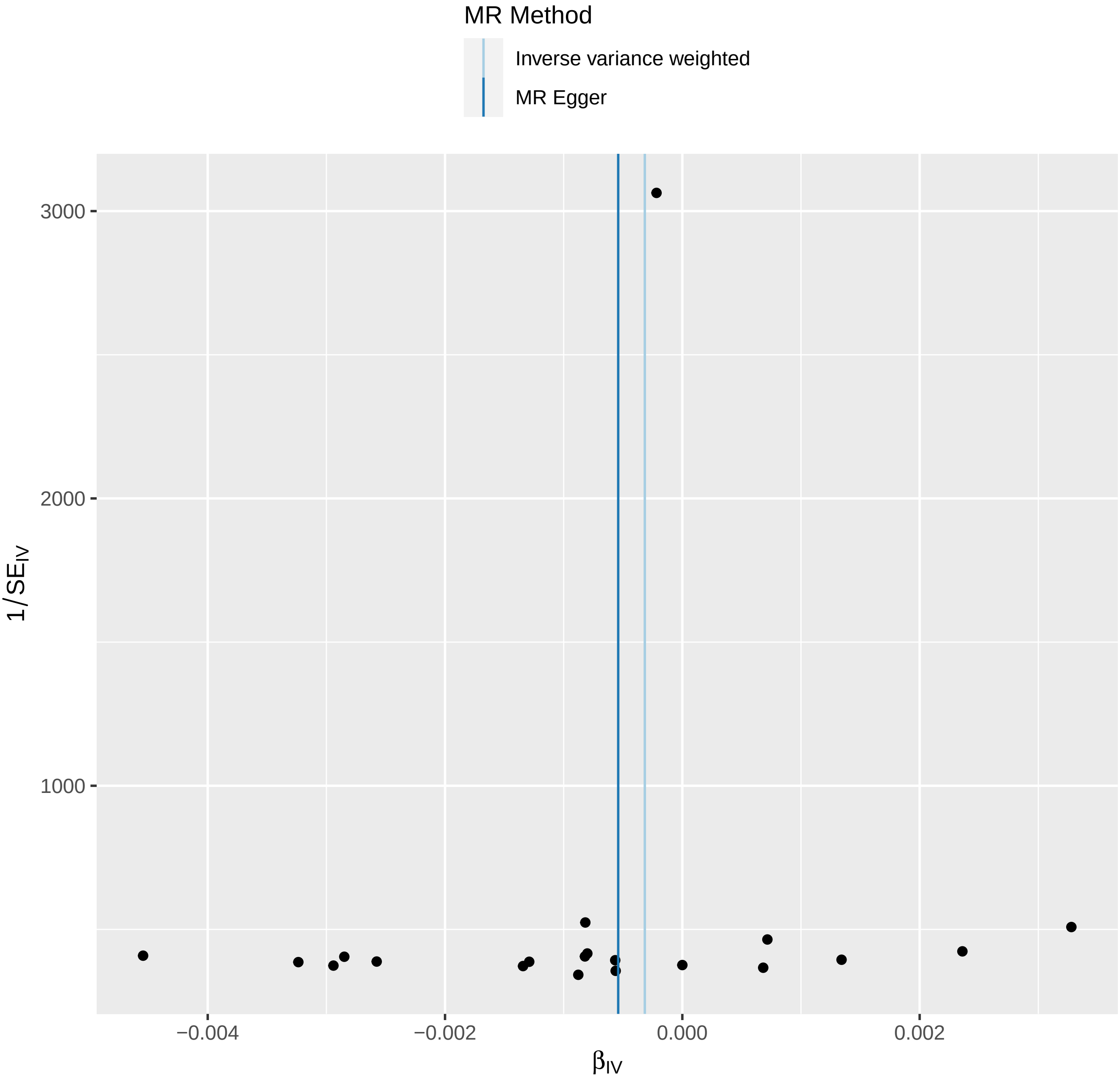

Batch 2−2231 : Platelet−derived growth factor receptor−like protein on eGFR

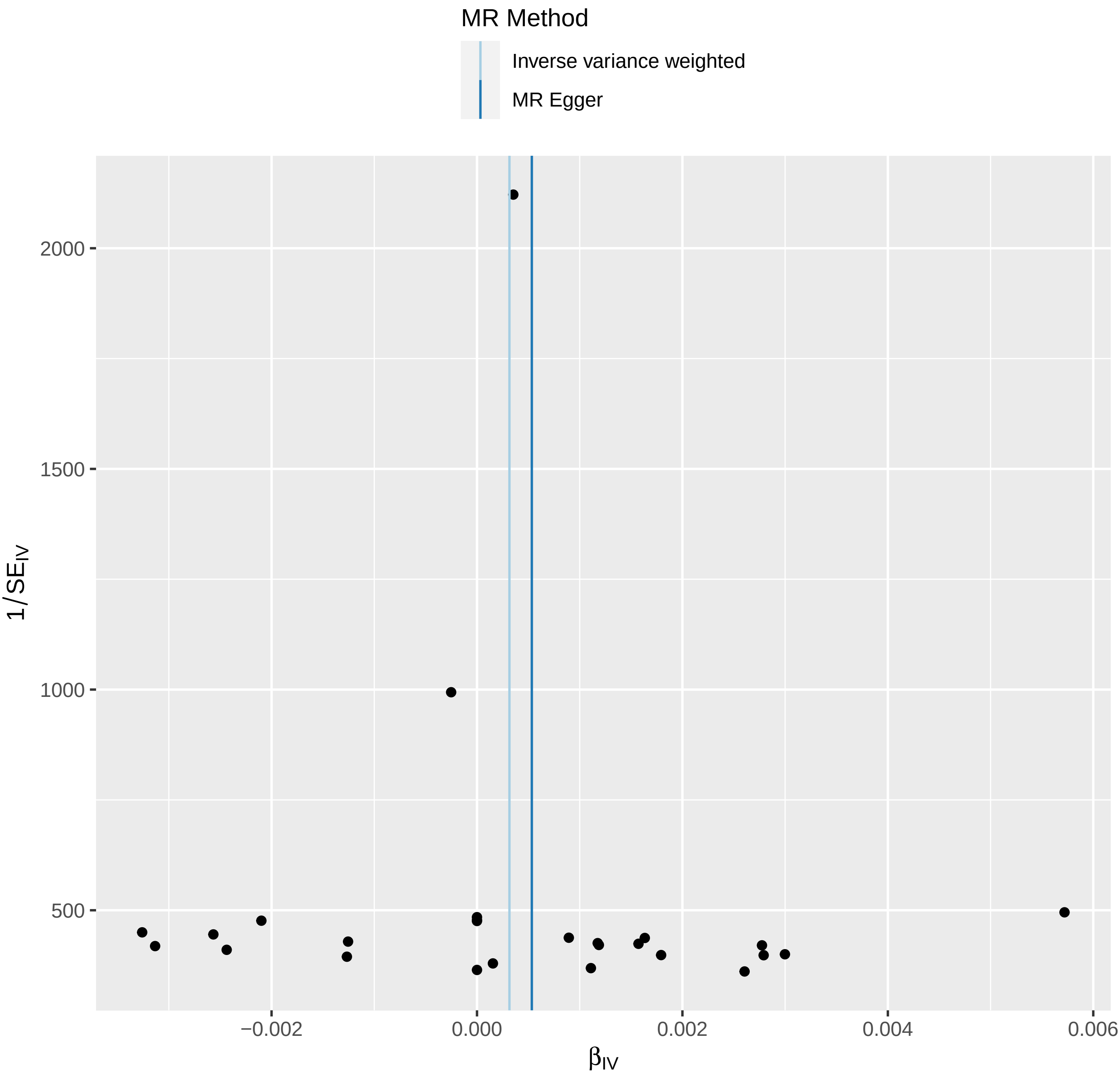

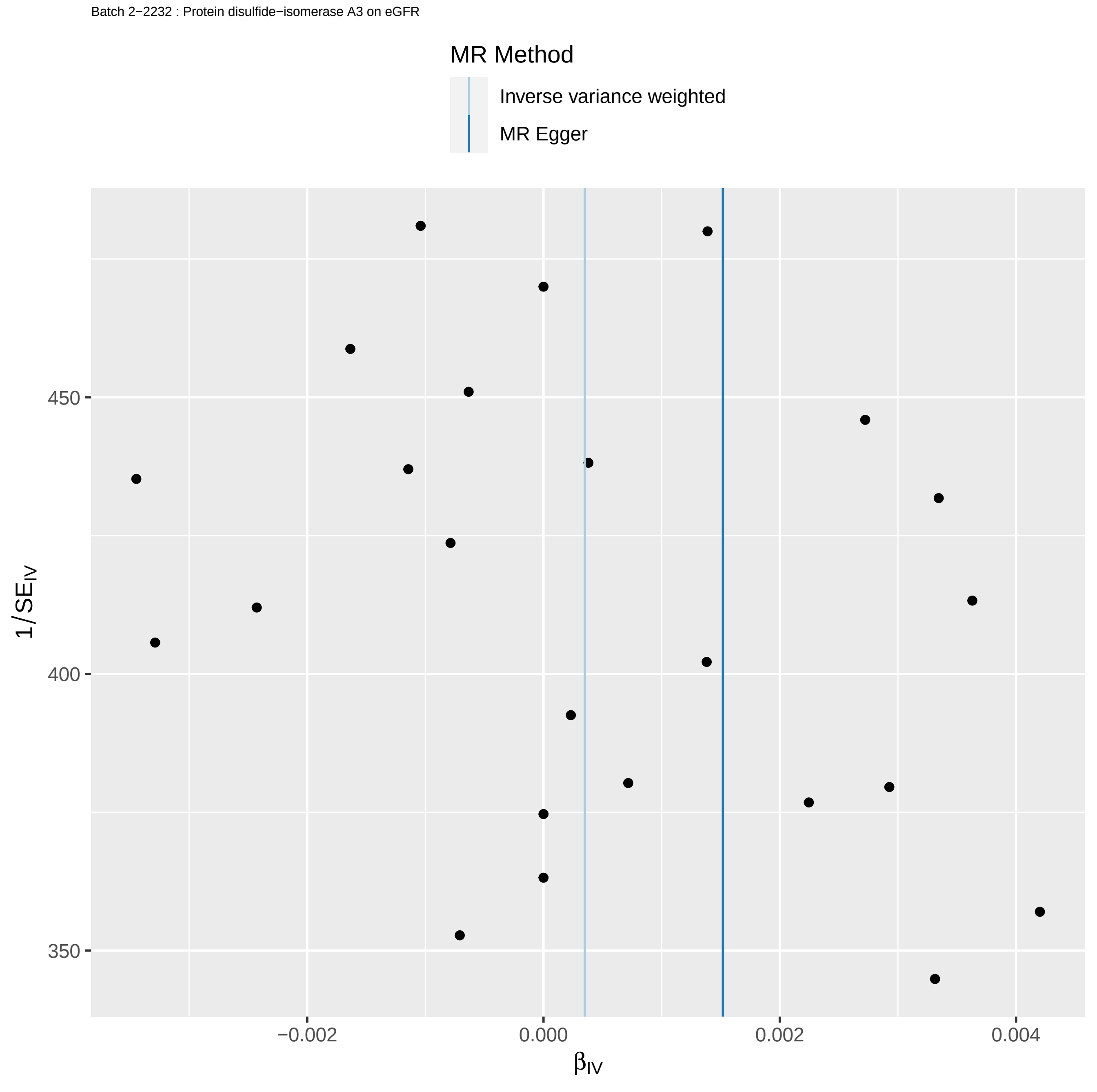

Batch 2−2232 : Protein disulfide−isomerase A3 on eGFR
MR Method
Inverse variance weighted
MR Egger
450
400
350
−0.002
0.000
0.002
0.004
$1/SE_{IV}$
β_{IV}

Batch 2−2233 : Protein disulfide−isomerase A5 on eGFR

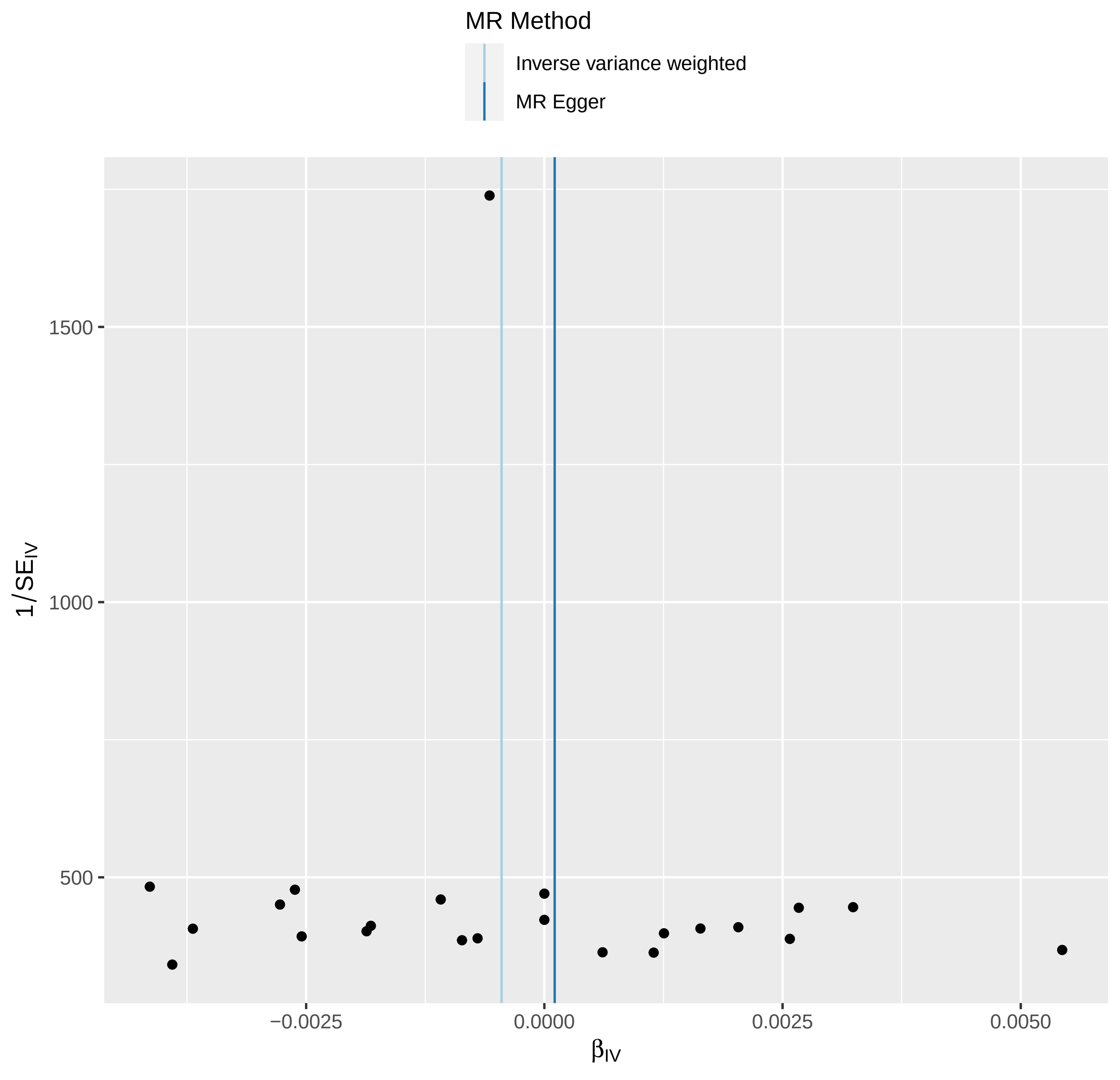

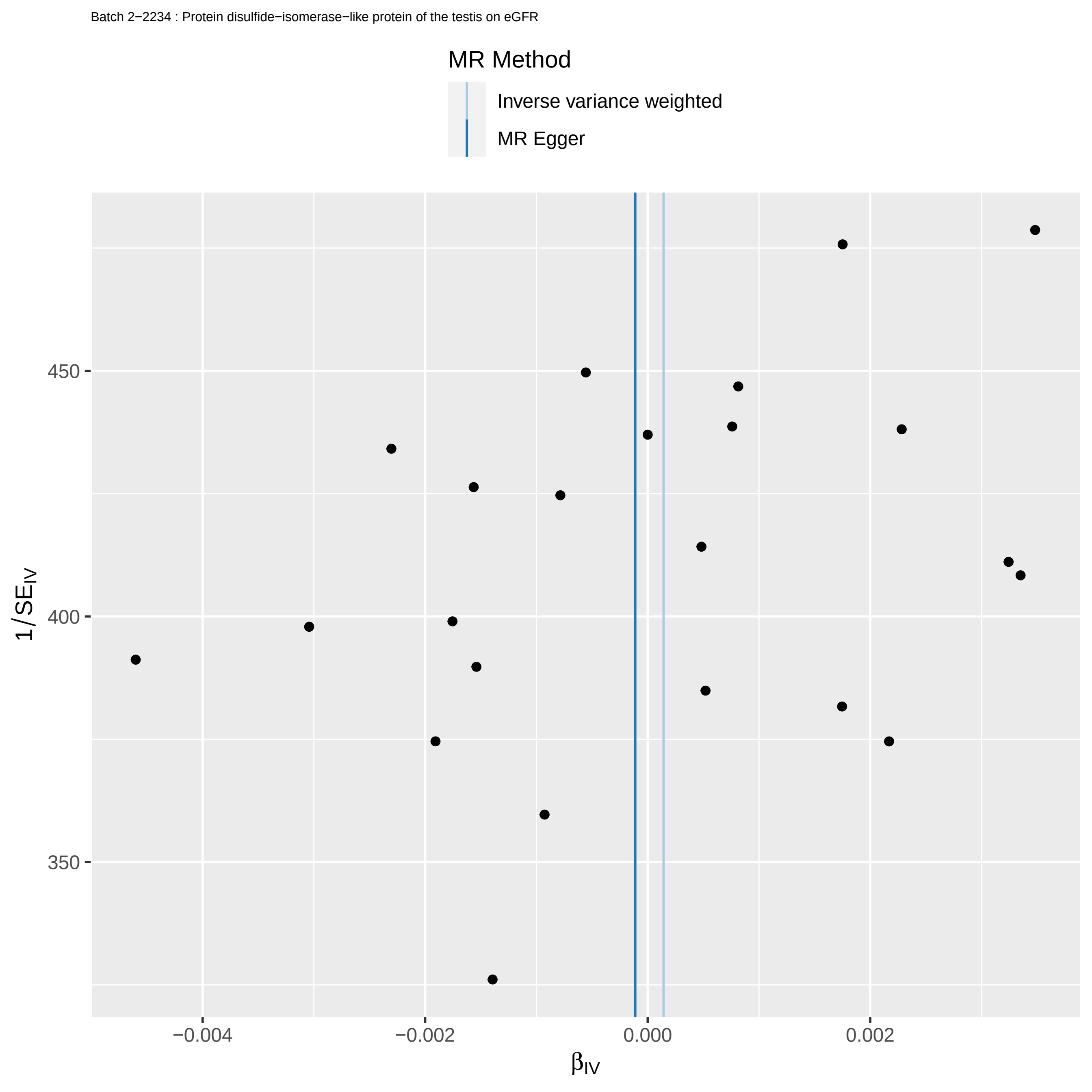

Batch 2−2234 : Protein disulfide−isomerase−like protein of the testis on eGFR
MR Method
Inverse variance weighted
MR Egger
450
400
350
1/SE_IV
−0.004
−0.002
0.000
0.002
β_IV

Batch 2−2235 : [Pyruvate dehydrogenase (acetyl−transferring)] kinase isozyme 1, mitochondrial on eGFR

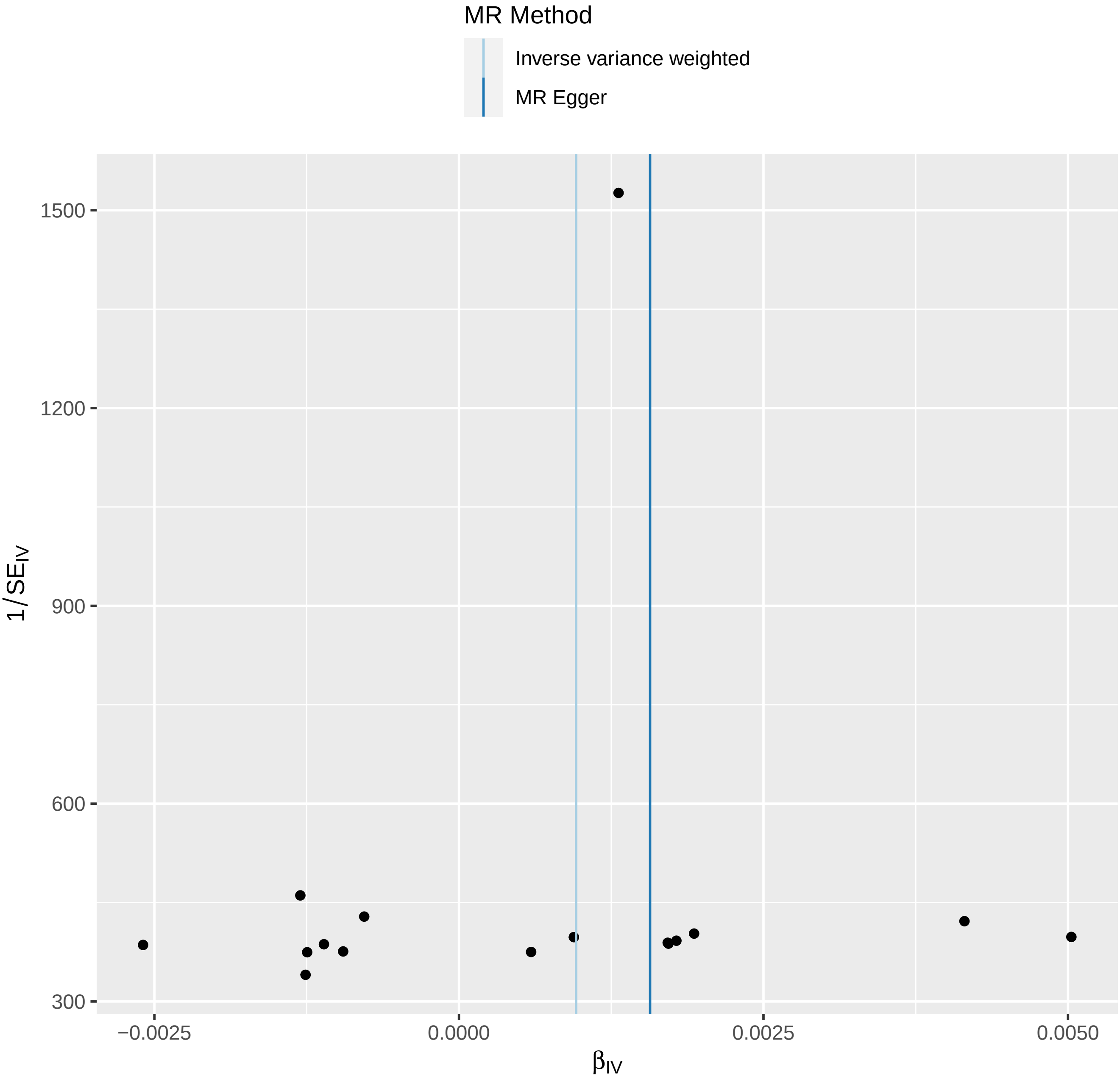

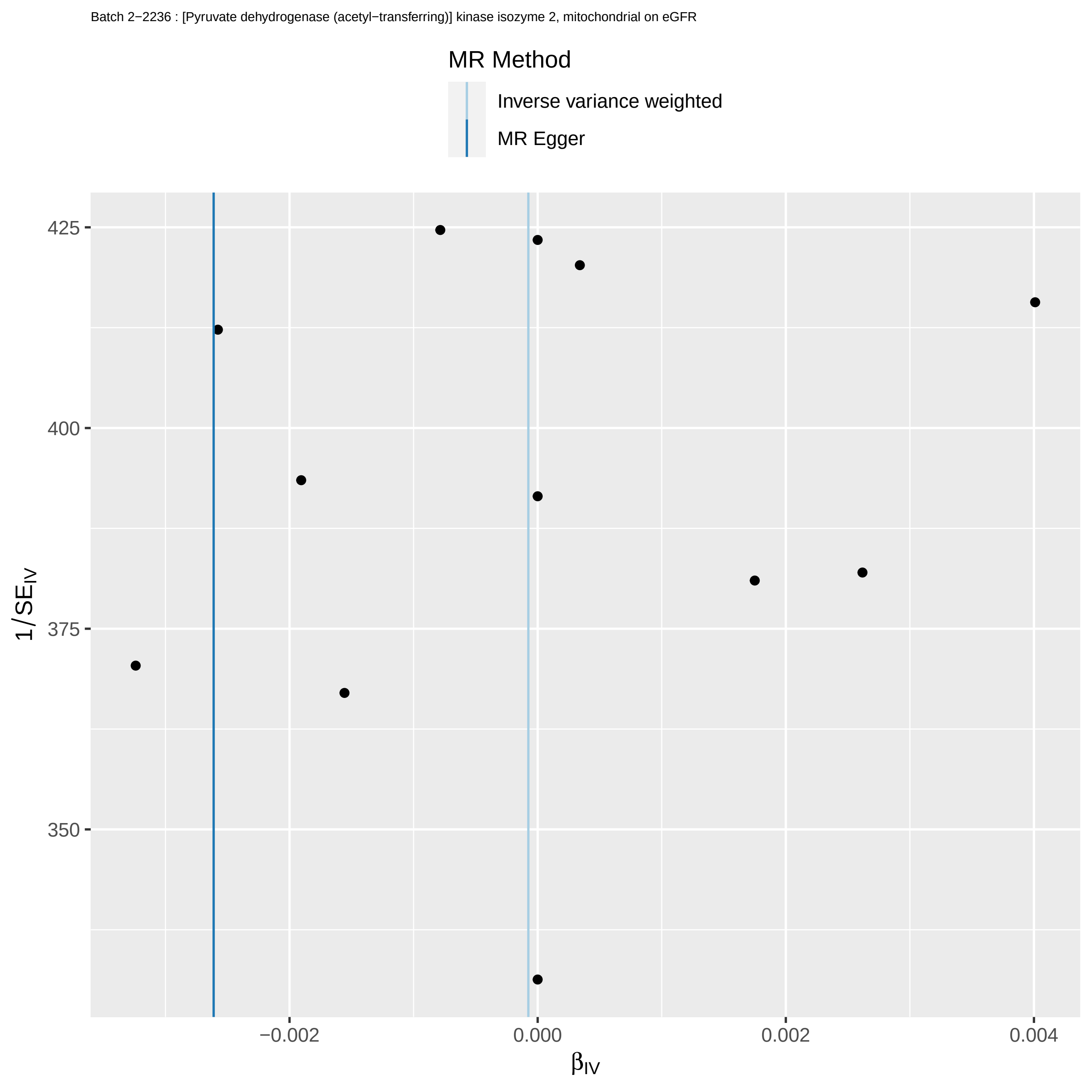

Batch 2−2236 : [Pyruvate dehydrogenase (acetyl−transferring)] kinase isozyme 2, mitochondrial on eGFR
MR Method
Inverse variance weighted
MR Egger
425
400
375
350
1/SE$_{IV}$
−0.002
0.000
0.002
0.004
β$_{IV}$

Batch 2−2237 : PDZ and LIM domain protein 4 on eGFR

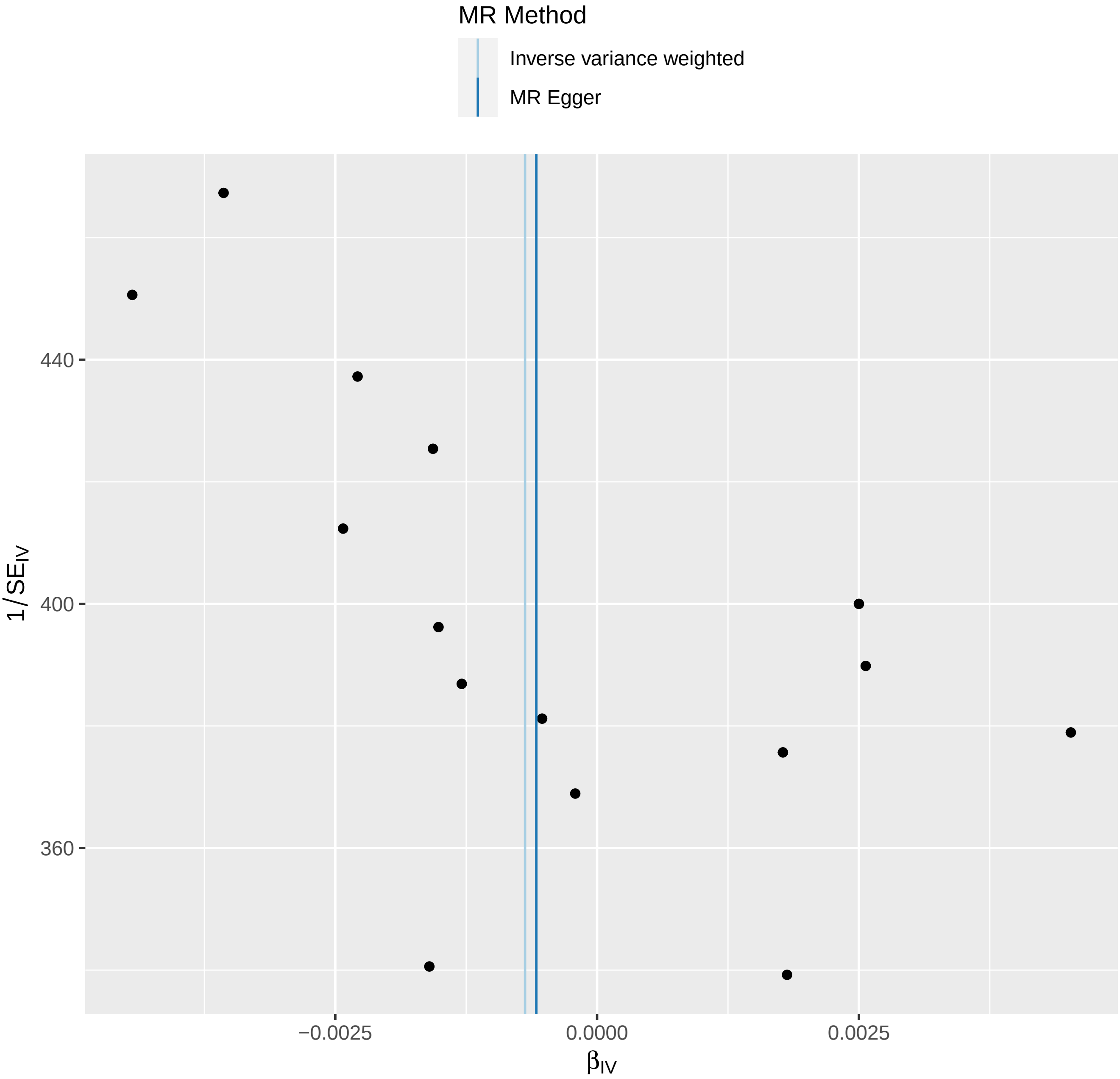

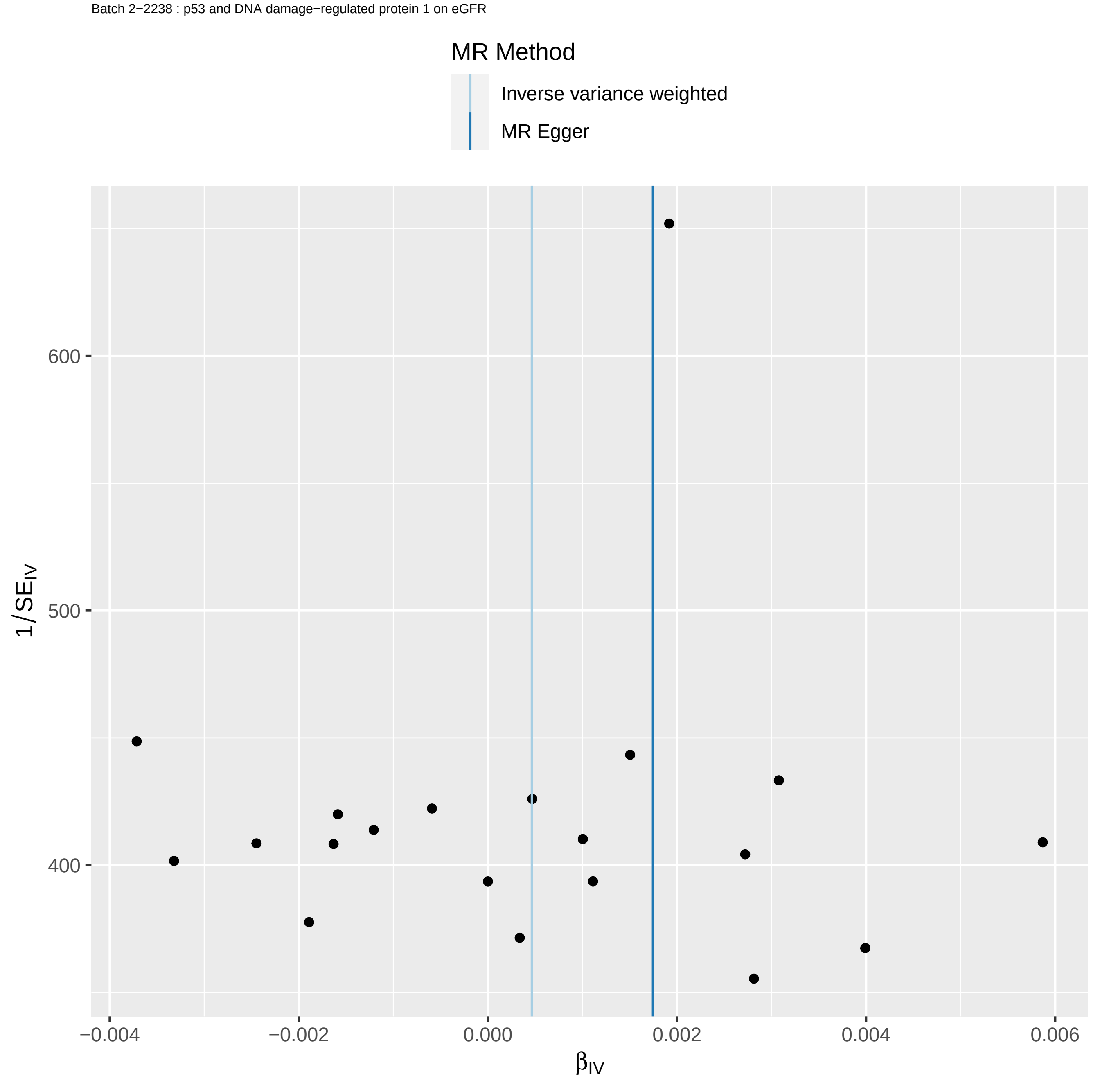

Batch 2−2238 : p53 and DNA damage−regulated protein 1 on eGFR
MR Method
Inverse variance weighted
MR Egger
600
500
400
1/SE$_{IV}$
−0.004
−0.002
0.000
0.002
0.004
0.006
β$_{IV}$

Batch 2−2239 : Pyridoxal kinase on eGFR

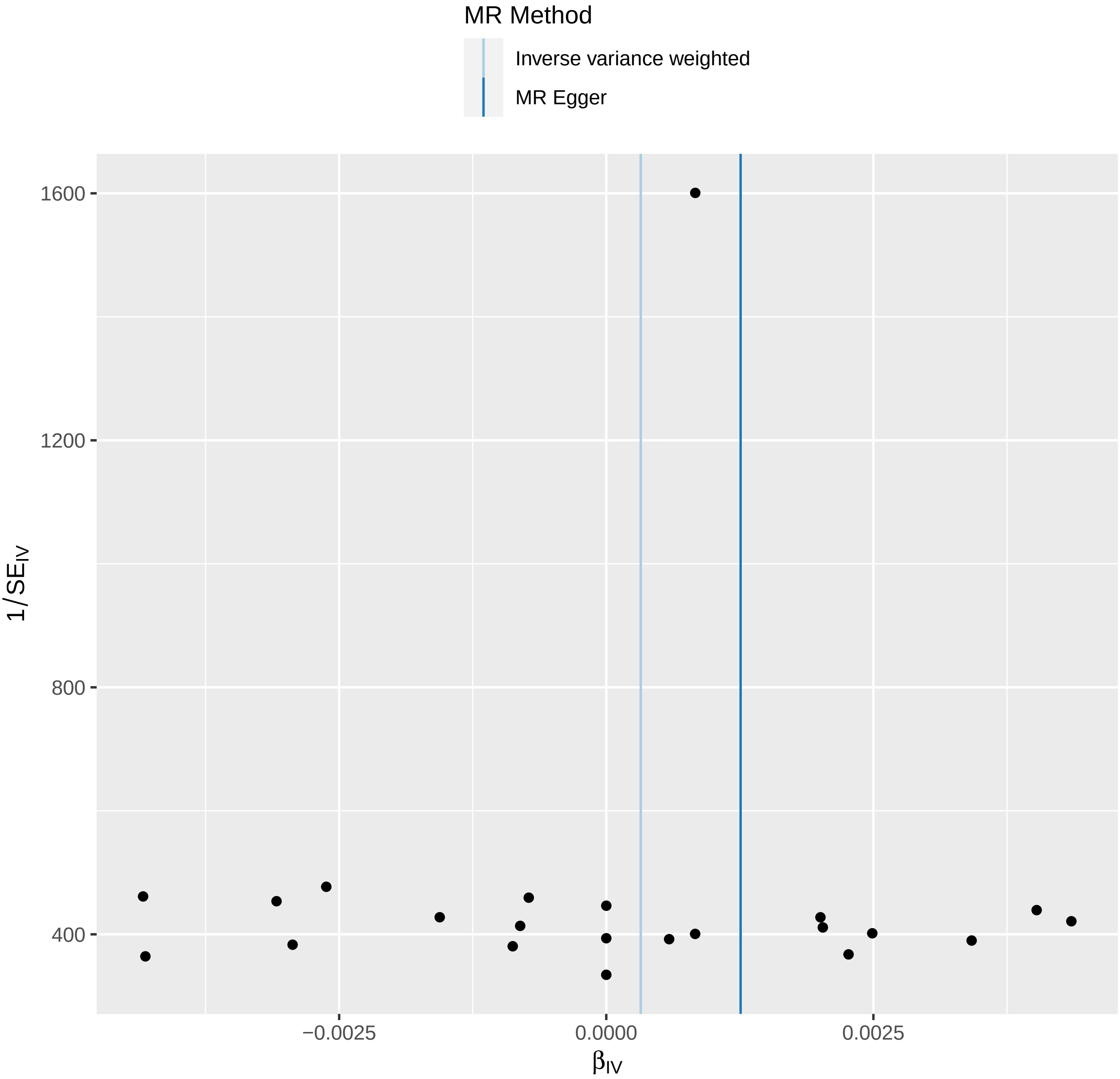

Batch 2−2240 : Pyridoxal phosphate phosphatase on eGFR

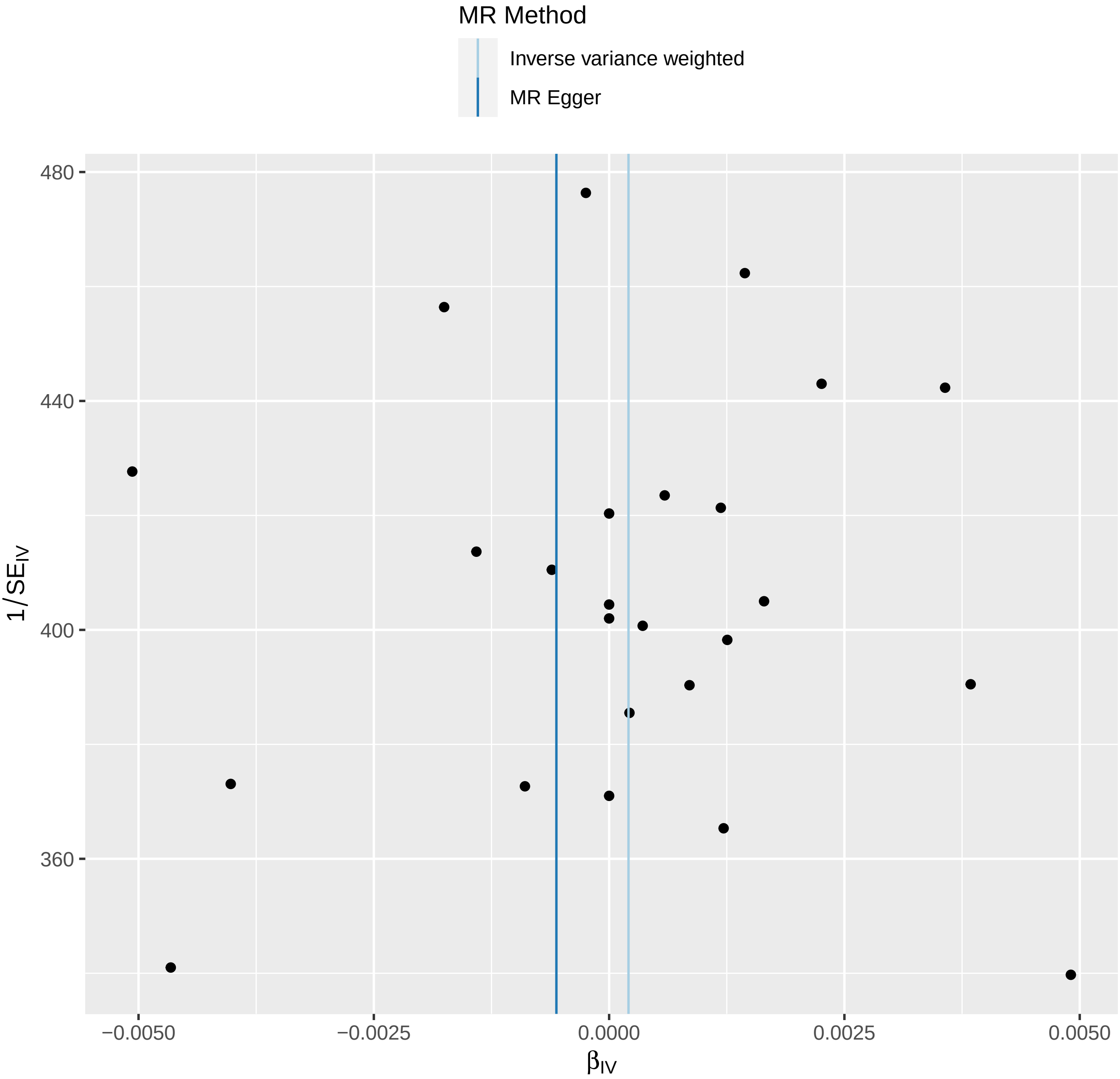

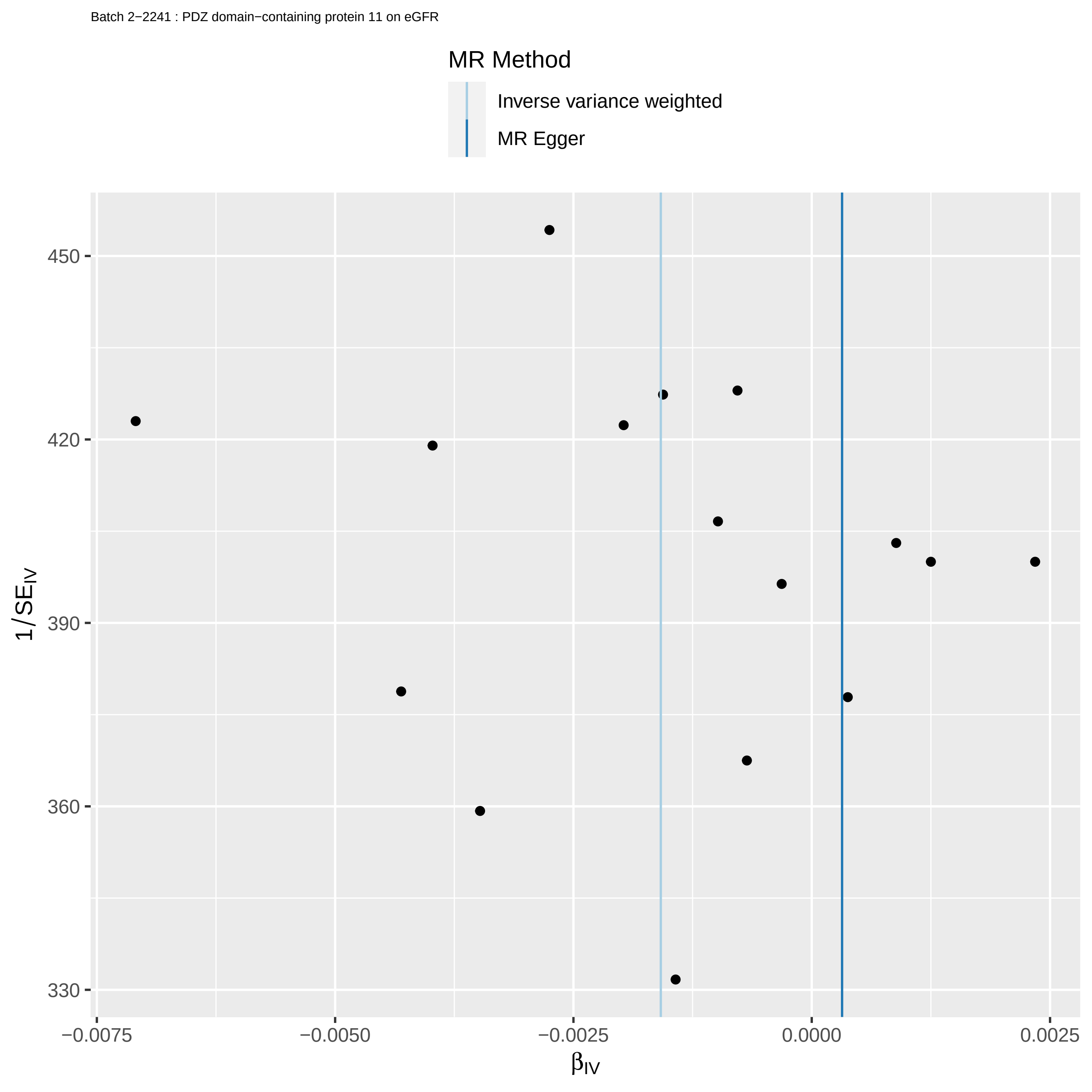
Batch 2−2241 : PDZ domain−containing protein 11 on eGFR
MR Method
Inverse variance weighted
MR Egger
450
420
390
360
330
1/SE$_{IV}$
−0.0075
−0.0050
−0.0025
0.0000
0.0025
β$_{IV}$

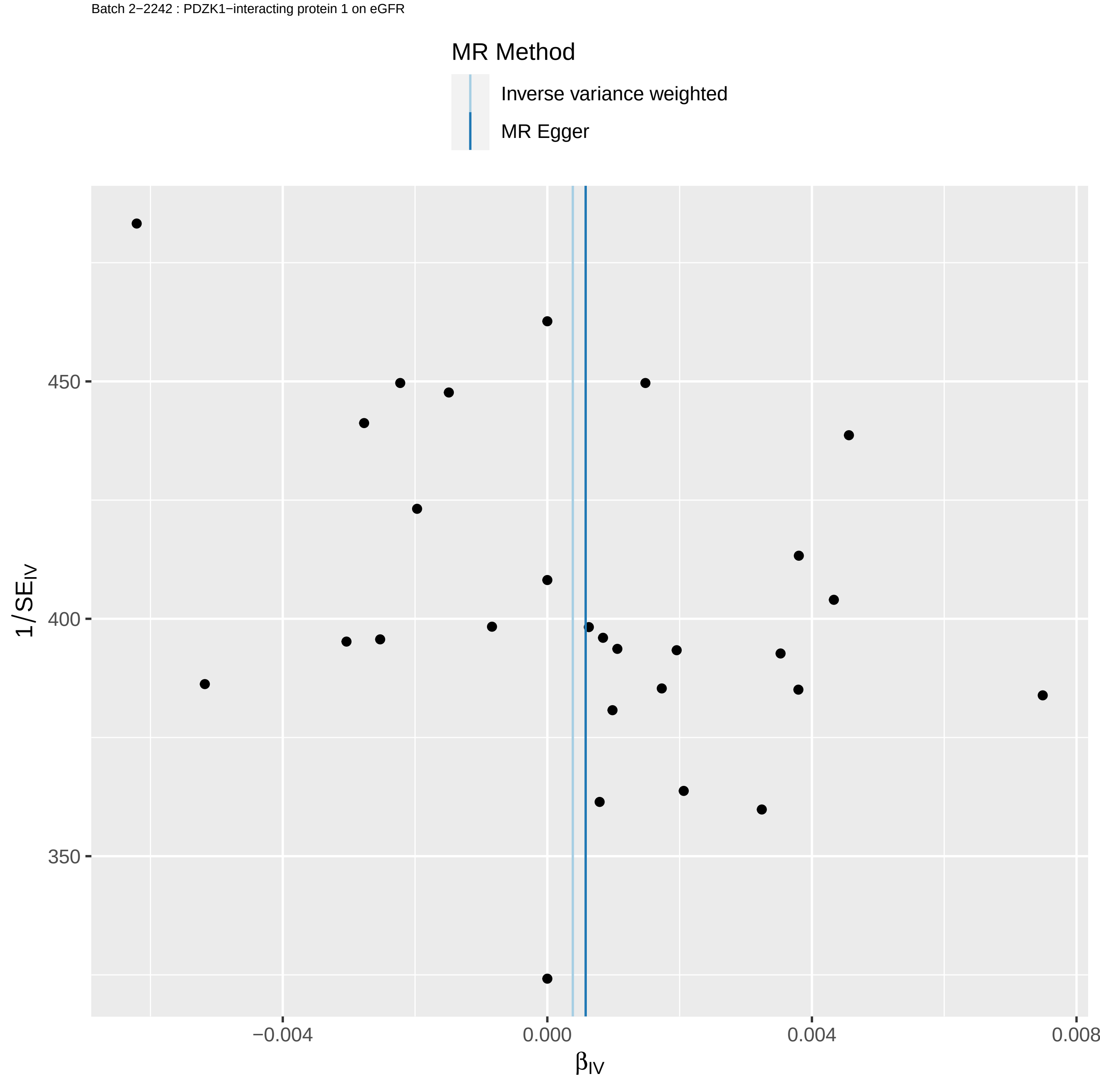

Batch 2−2242 : PDZK1−interacting protein 1 on eGFR
MR Method
Inverse variance weighted
MR Egger
450
400
350
1/SE$_{IV}$
−0.004
0.000
0.004
0.008
β$_{IV}$

Batch 2−2243 : Platelet endothelial aggregation receptor 1 on eGFR

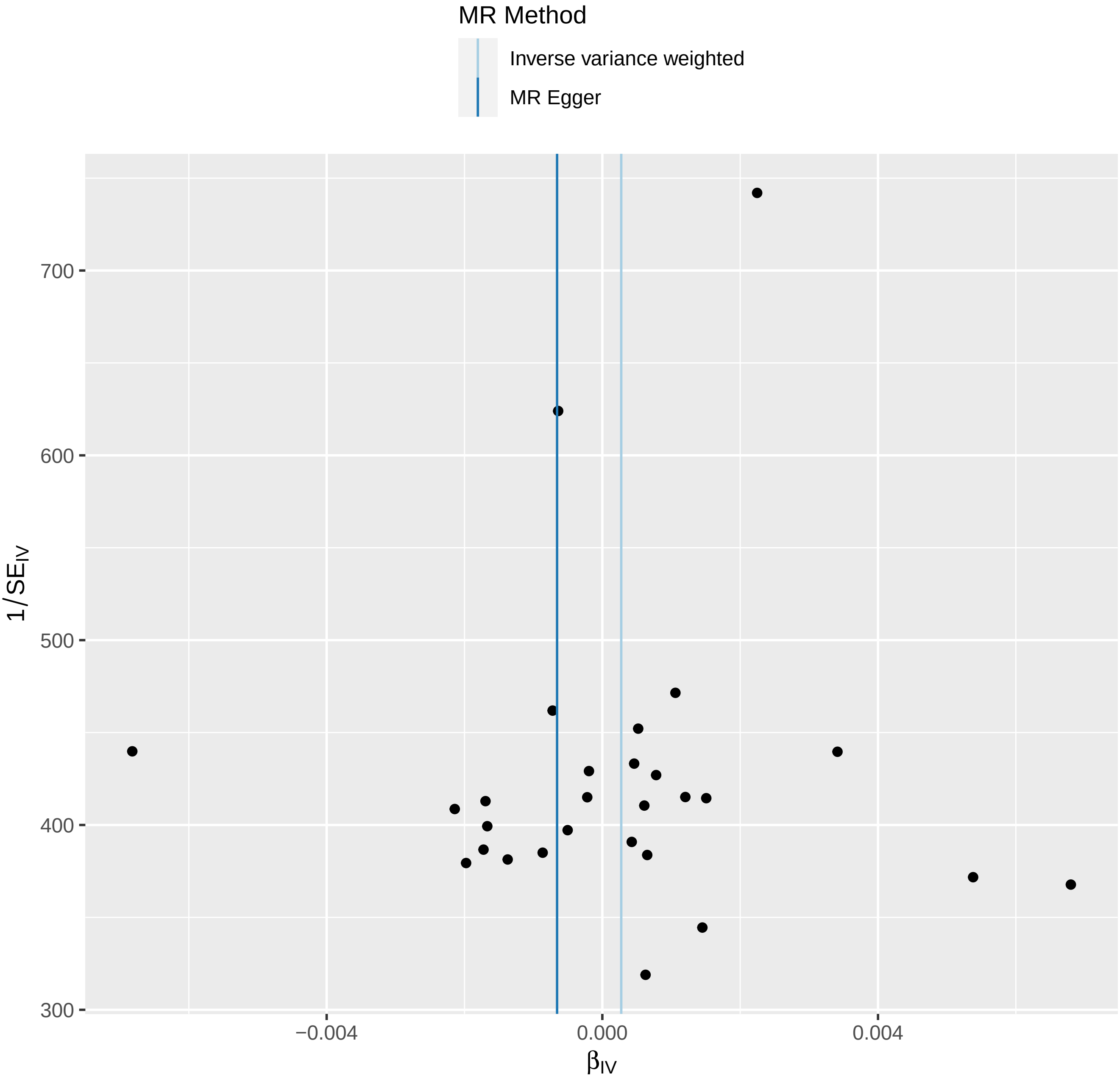

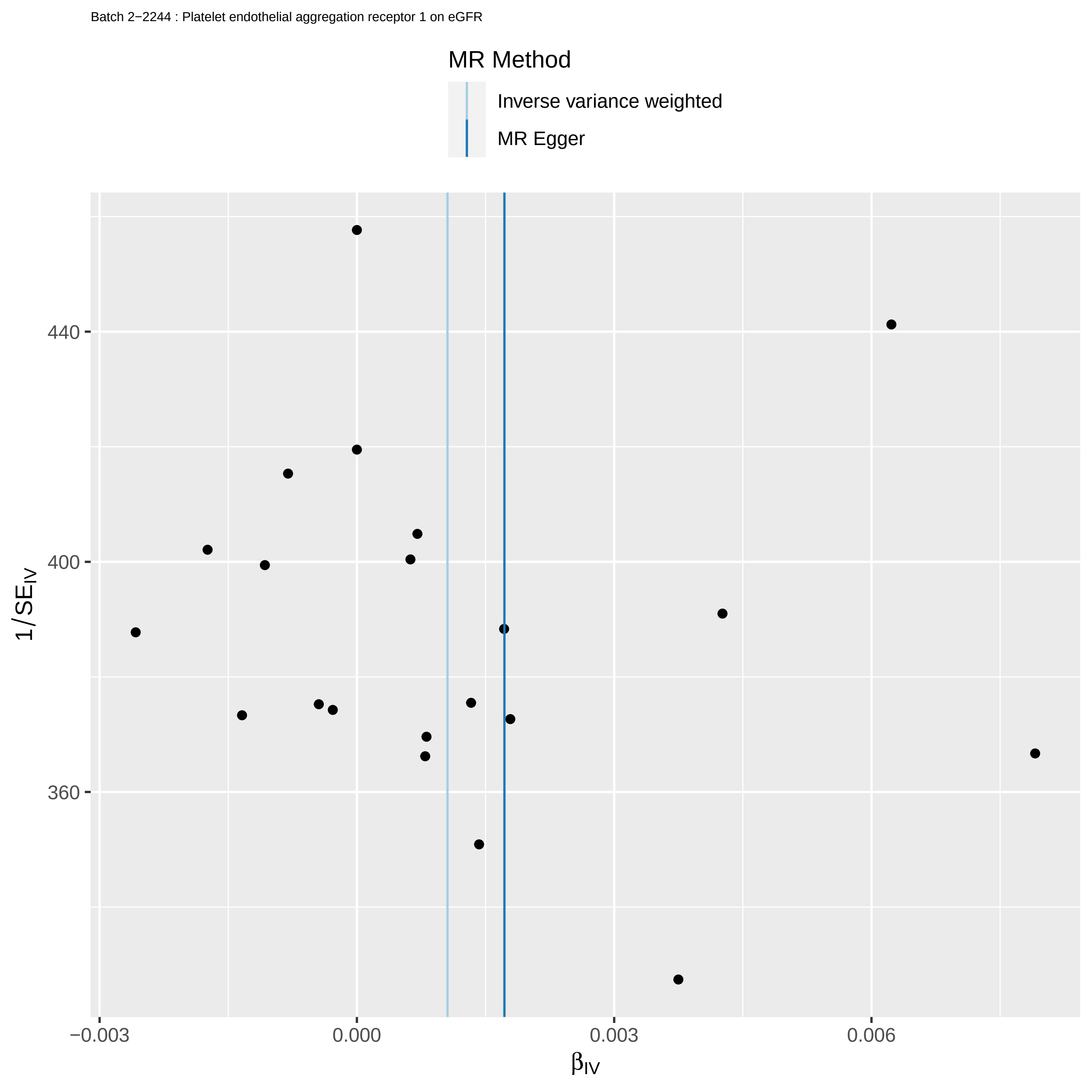
Batch 2−2244 : Platelet endothelial aggregation receptor 1 on eGFR
MR Method
Inverse variance weighted
MR Egger
440
400
360
1/SE_IV
−0.003
0.000
0.003
0.006
β_IV

Batch 2−2245 : Platelet endothelial cell adhesion molecule on eGFR

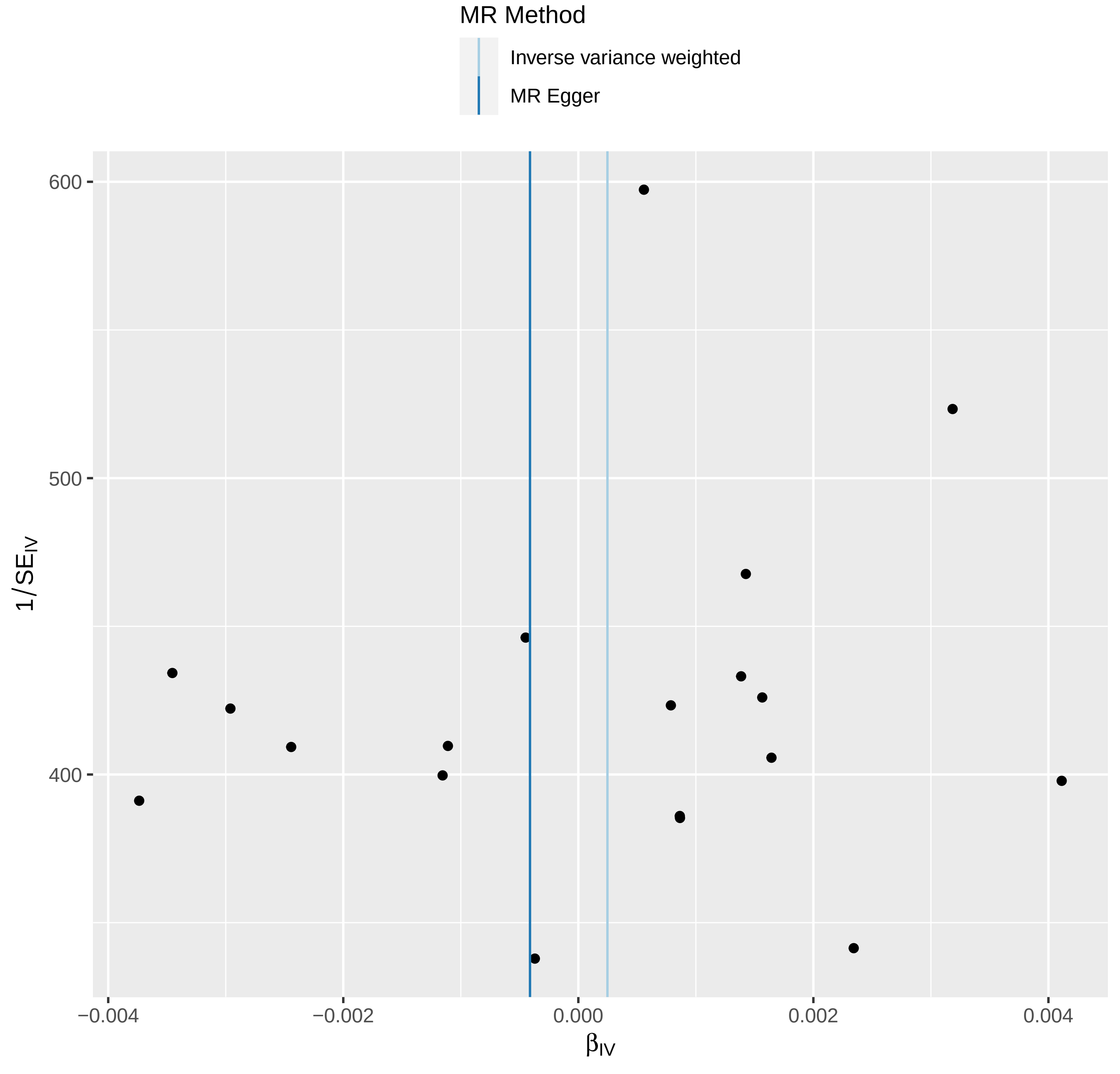

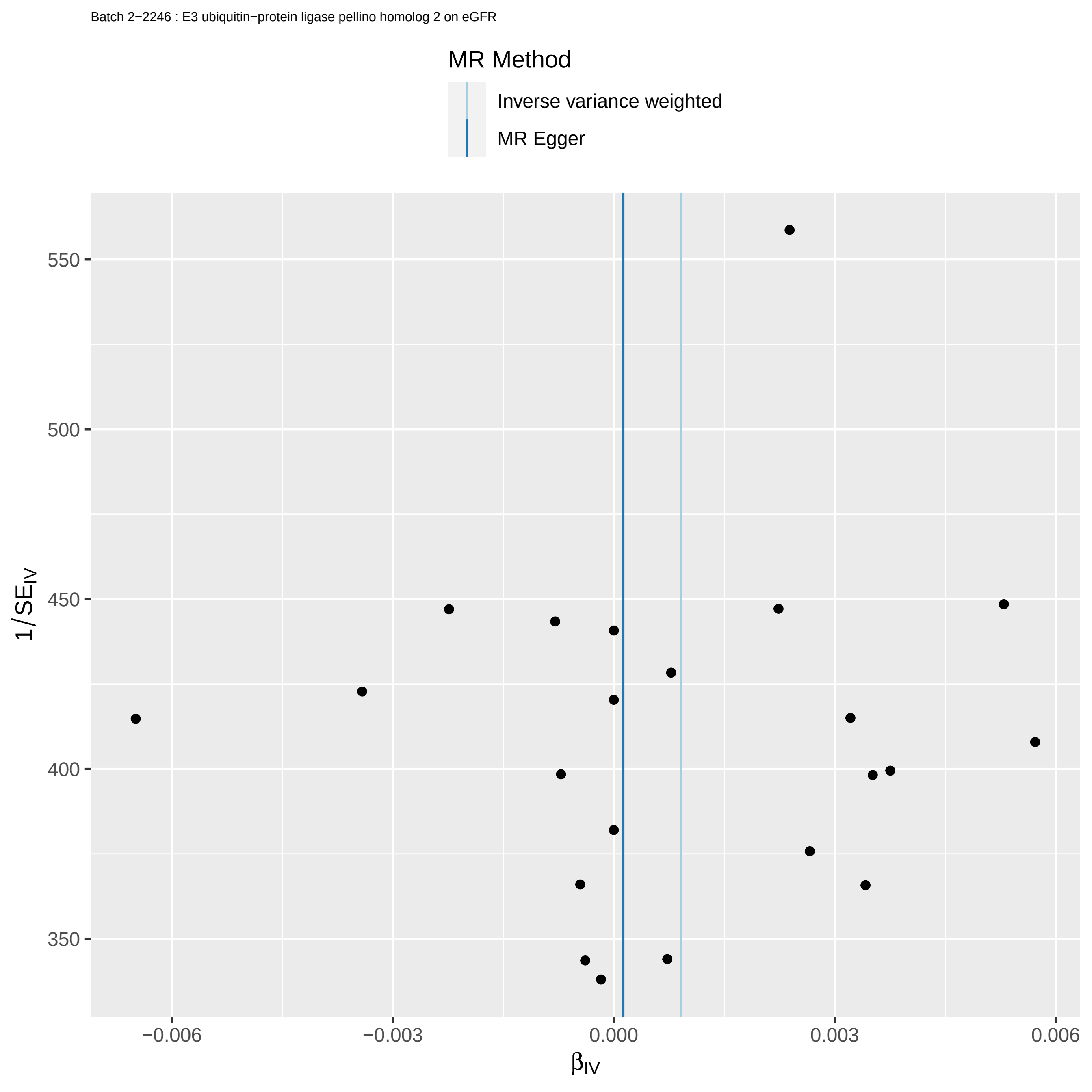

Batch 2−2246 : E3 ubiquitin−protein ligase pellino homolog 2 on eGFR
MR Method
Inverse variance weighted
MR Egger
550
500
450
400
350
$1/SE_{IV}$
−0.006
−0.003
0.000
0.003
0.006
β_{IV}

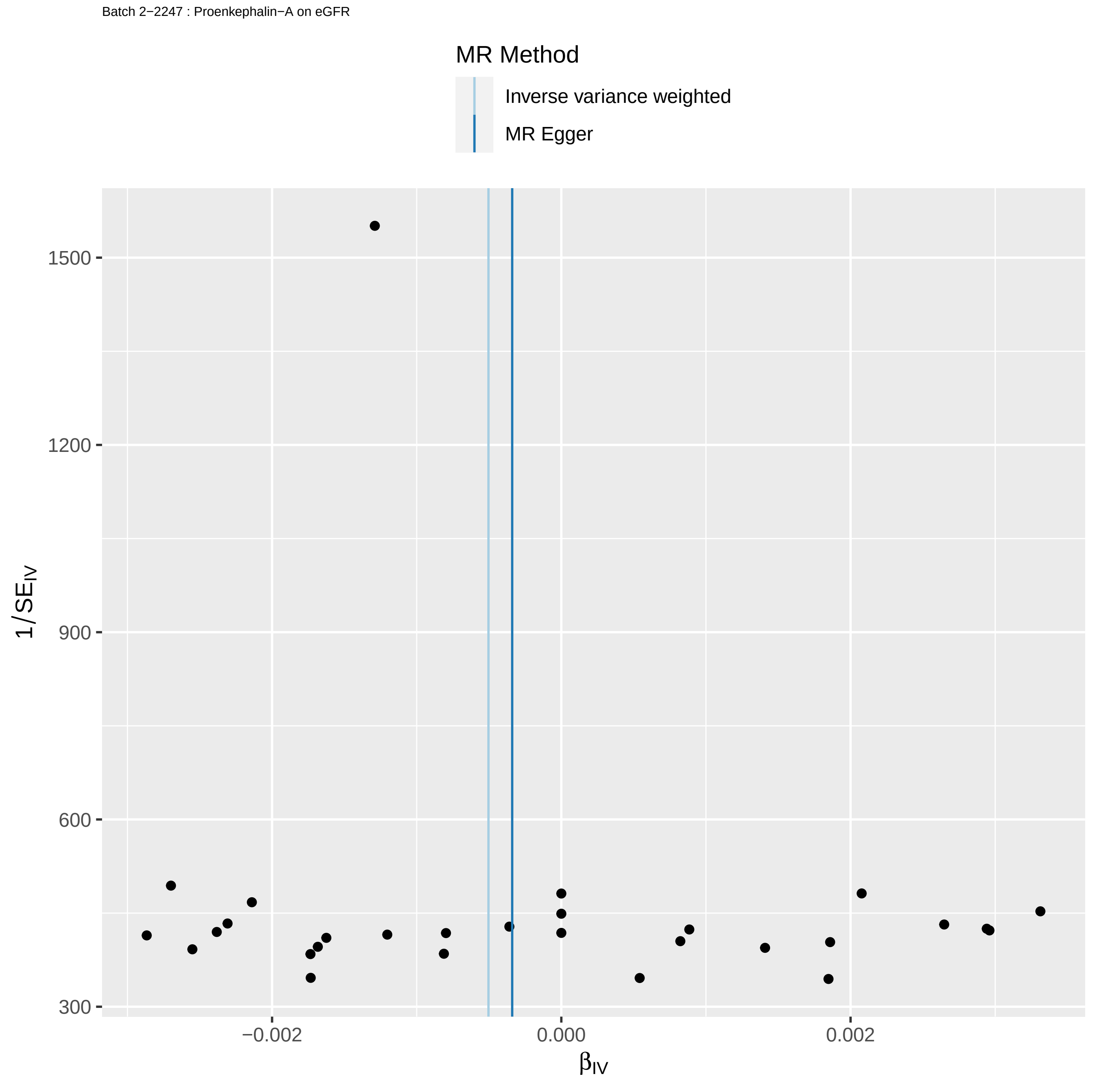

Batch 2−2247 : Proenkephalin−A on eGFR
MR Method
Inverse variance weighted
MR Egger
1500
1200
900
600
300
−0.002
0.000
0.002
$1/SE_{IV}$
β_{IV}

Batch 2−2248 : Pescadillo homolog on eGFR

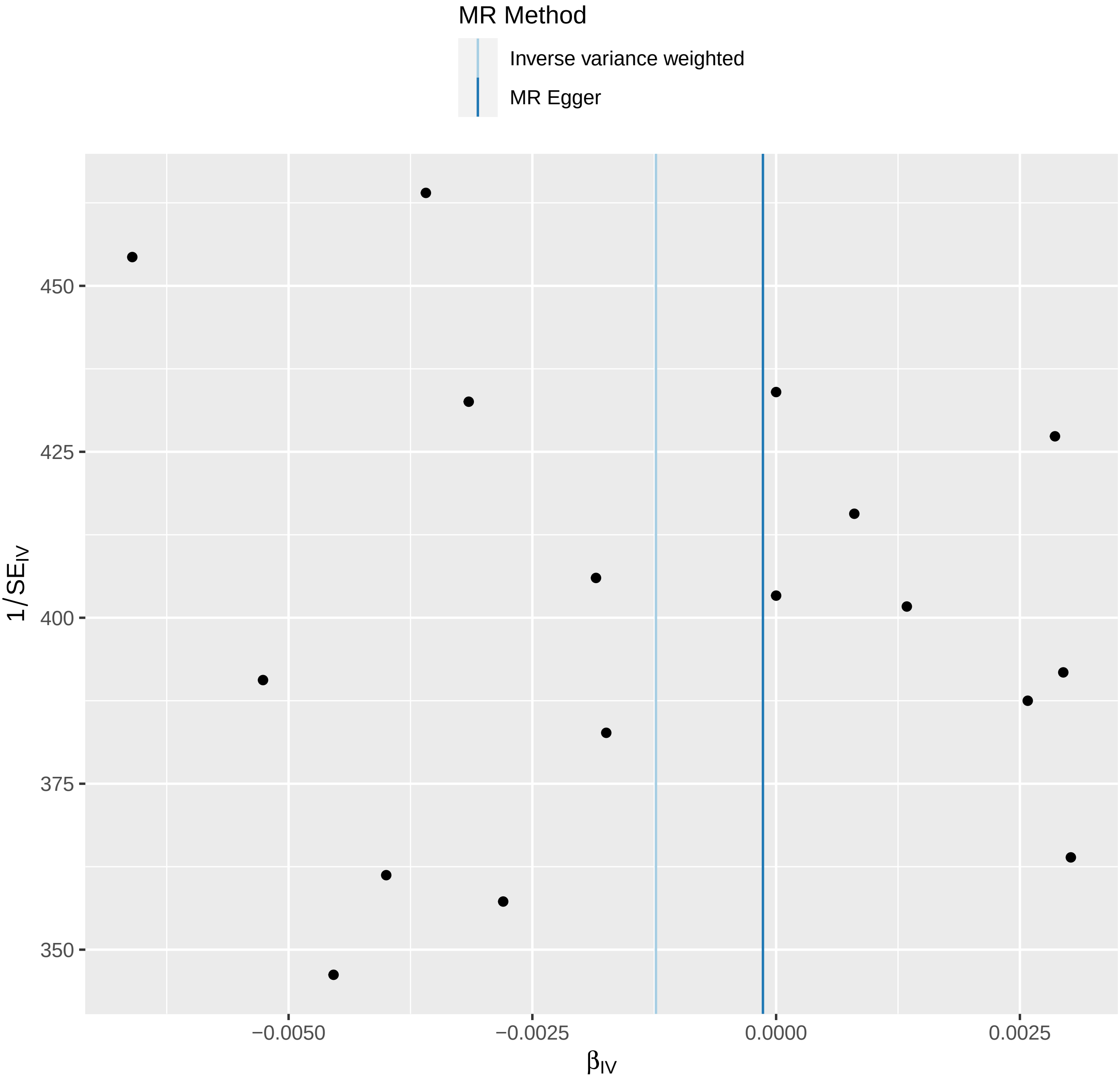

Batch 2−2249 : Peroxisomal membrane protein PEX14 on eGFR

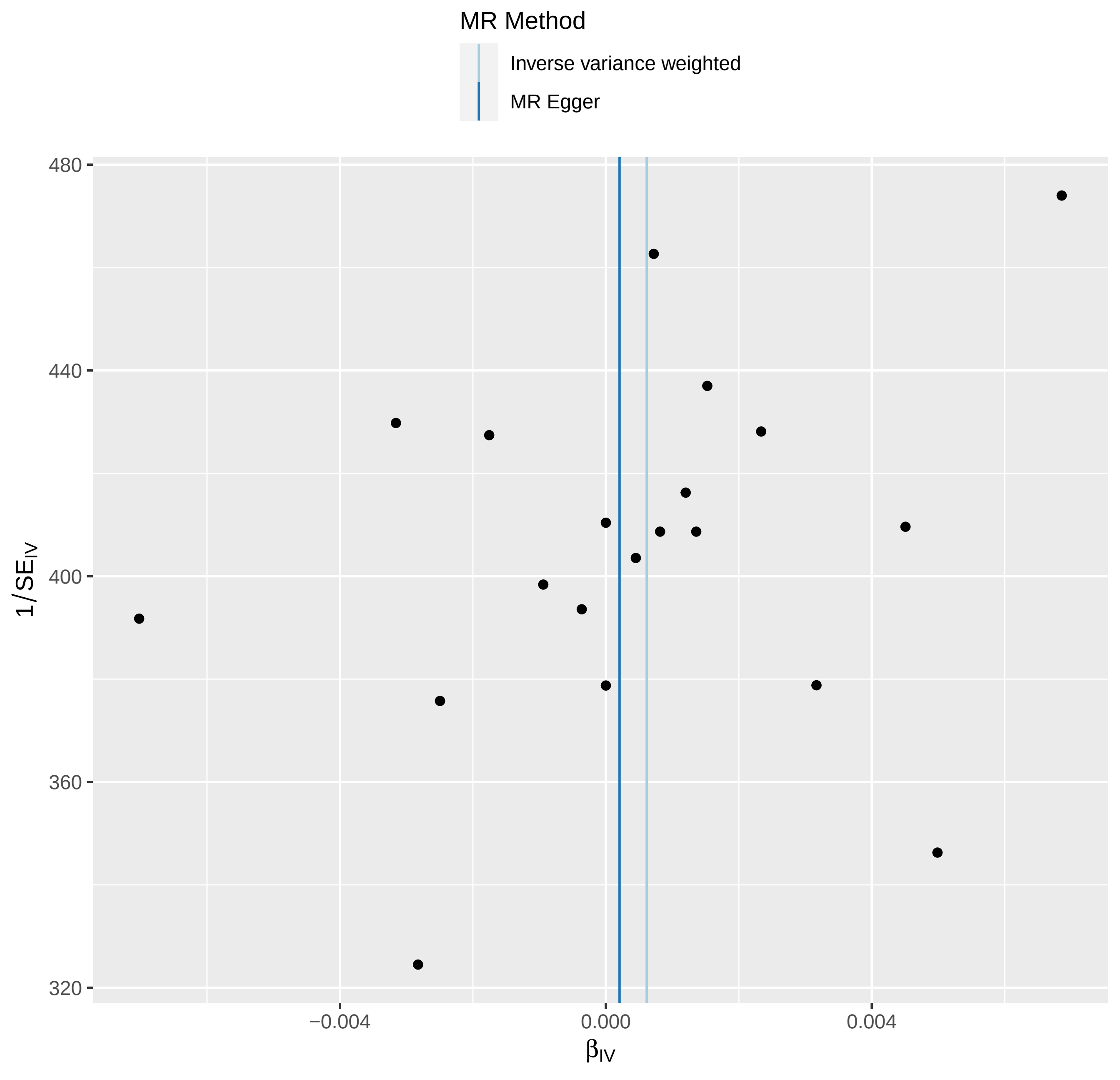

Batch 2−2250 : Peroxisomal targeting signal 1 receptor on eGFR

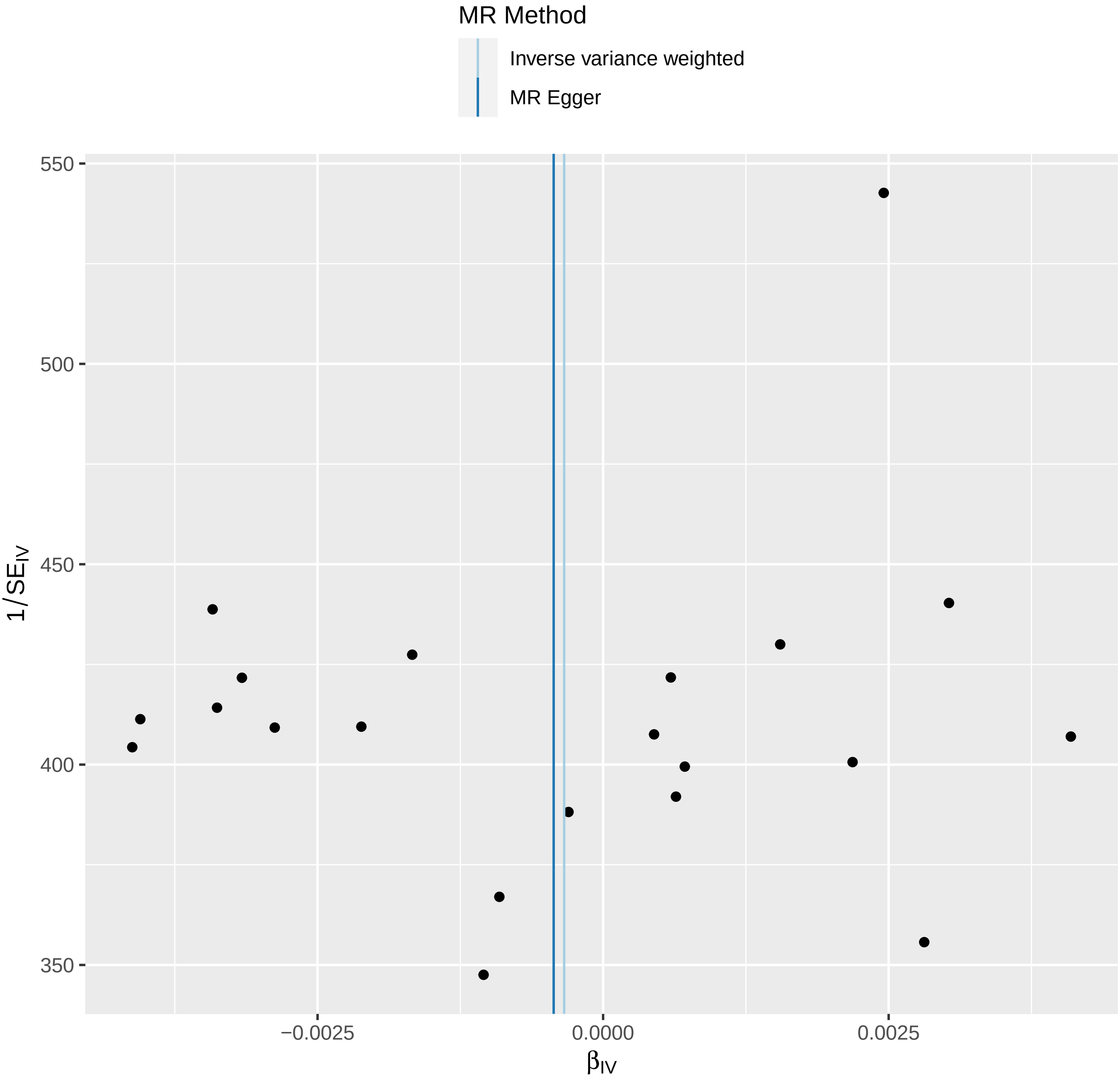

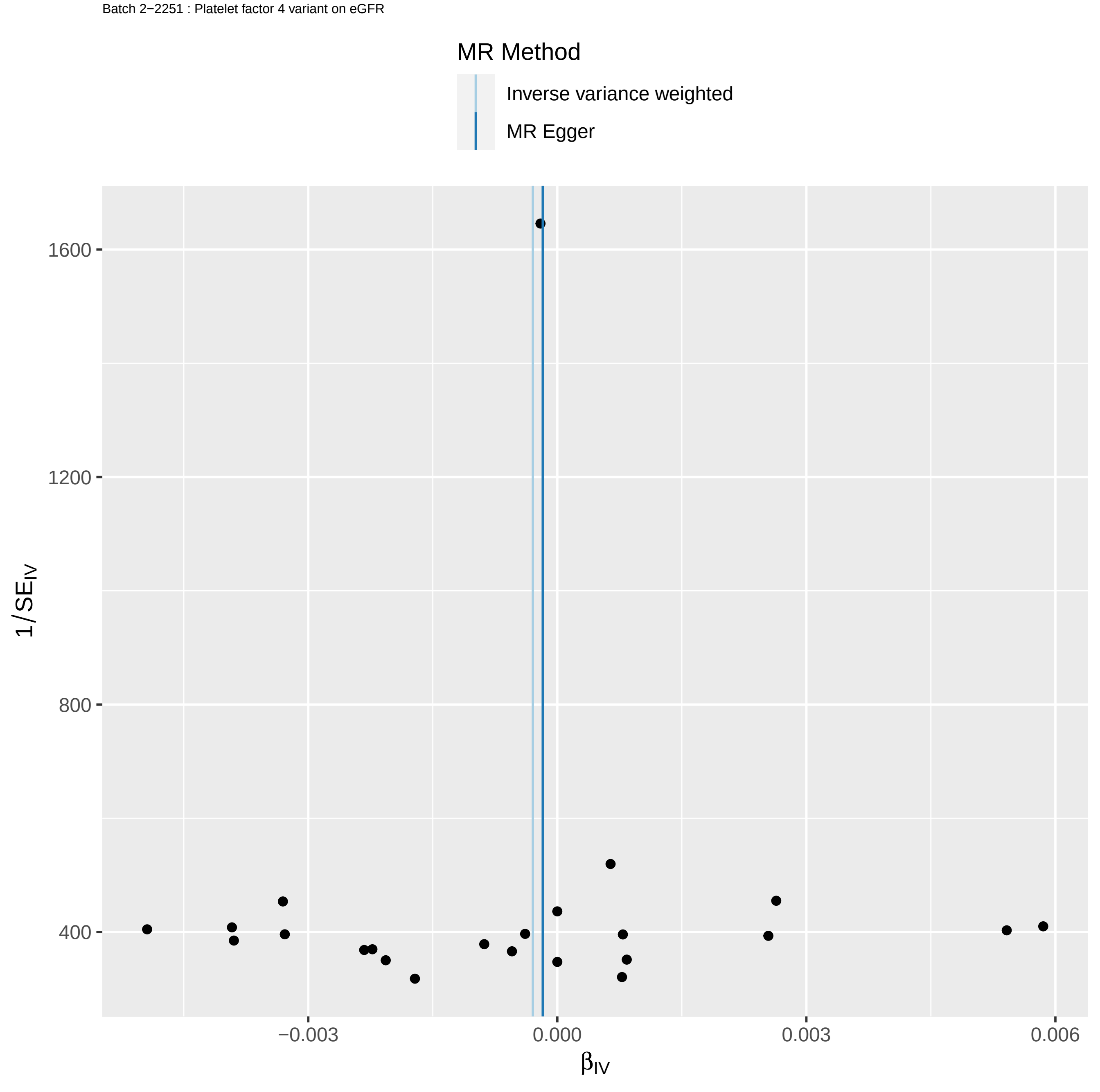

Batch 2−2251 : Platelet factor 4 variant on eGFR
MR Method
Inverse variance weighted
MR Egger
1600
1200
800
400
1/SE$_{IV}$
−0.003
0.000
0.003
0.006
β$_{IV}$

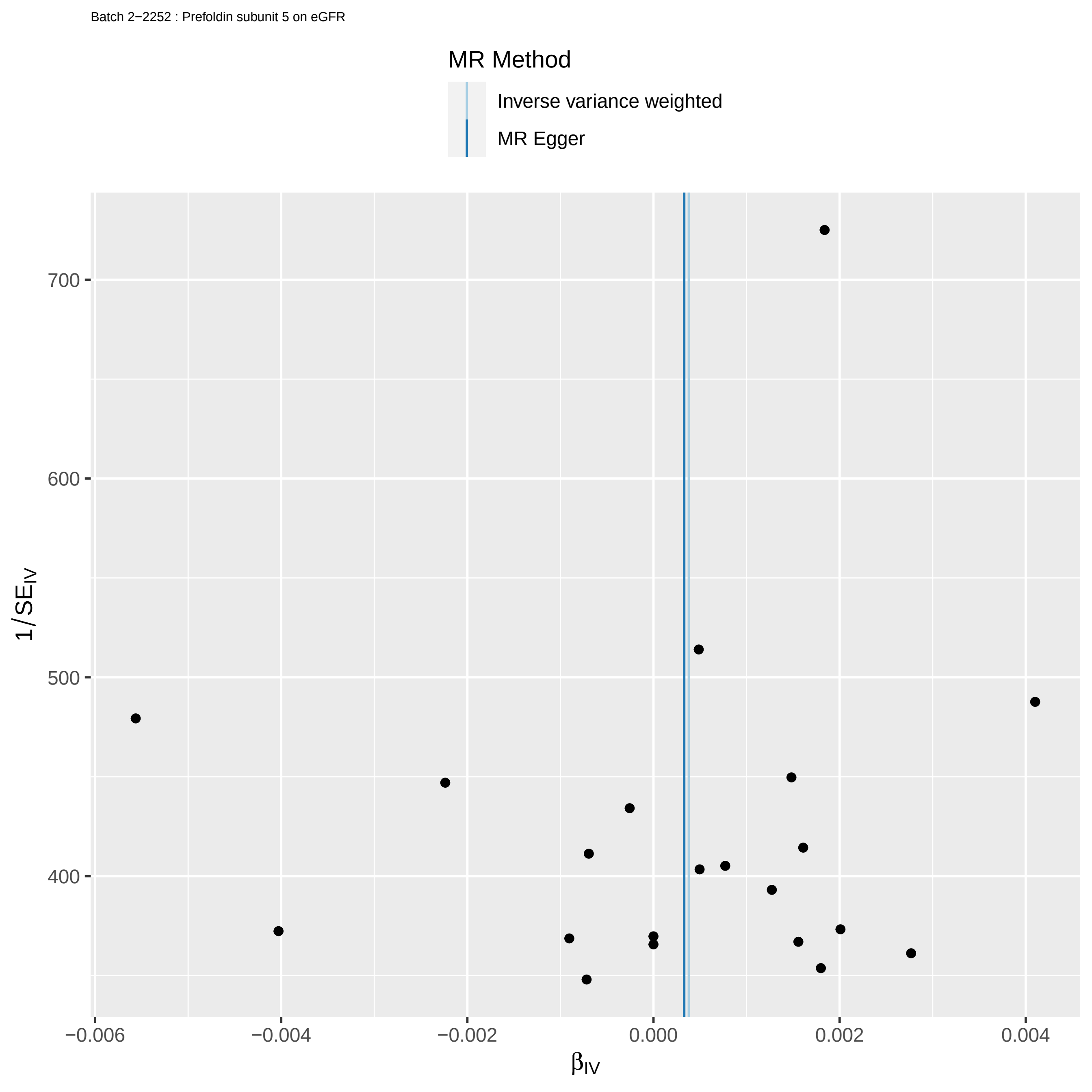
Batch 2−2252 : Prefoldin subunit 5 on eGFR
MR Method
Inverse variance weighted
MR Egger
700
600
500
400
1/SE$_{IV}$
−0.006
−0.004
−0.002
0.000
0.002
0.004
β$_{IV}$

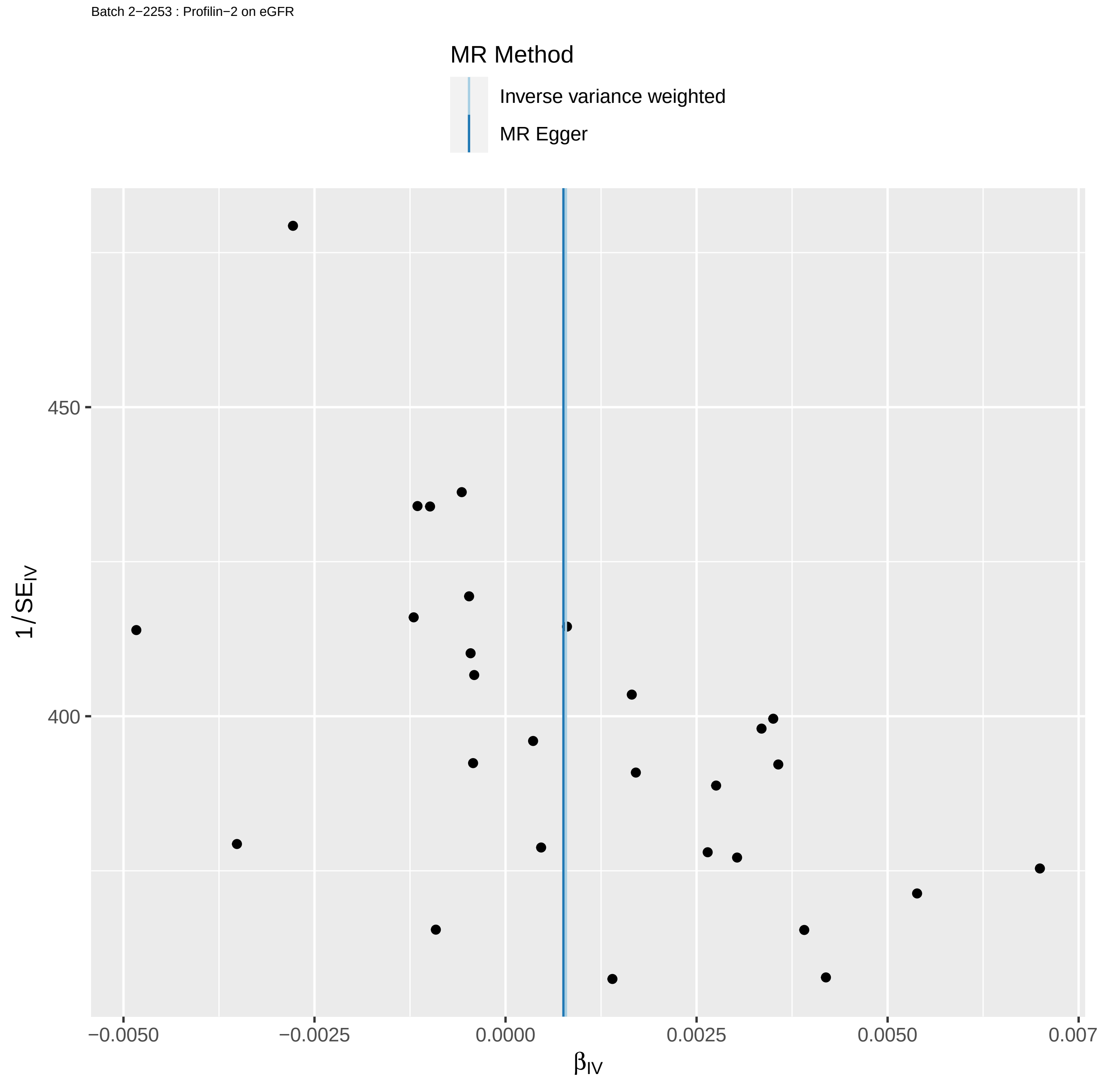
Batch 2−2253 : Profilin−2 on eGFR
MR Method
Inverse variance weighted
MR Egger
1/SE_IV
450
400
−0.0050
−0.0025
0.0000
0.0025
0.0050
0.007
β_IV

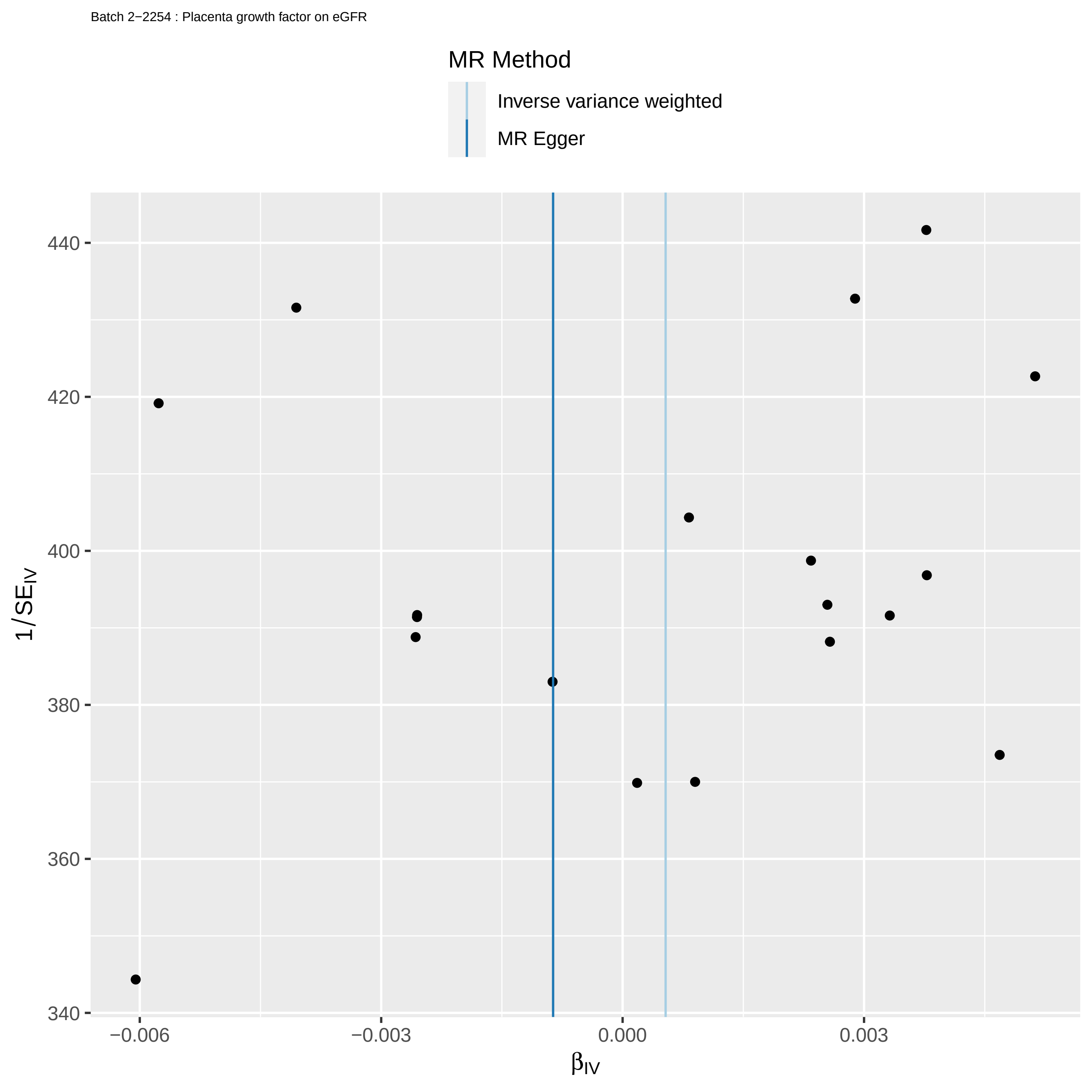
Batch 2−2254 : Placenta growth factor on eGFR
MR Method
Inverse variance weighted
MR Egger
440
420
400
380
360
340
1/SE$_{IV}$
−0.006
−0.003
0.000
0.003
β$_{IV}$

Batch 2−2255 : Phosphoglycerate kinase 1 on eGFR

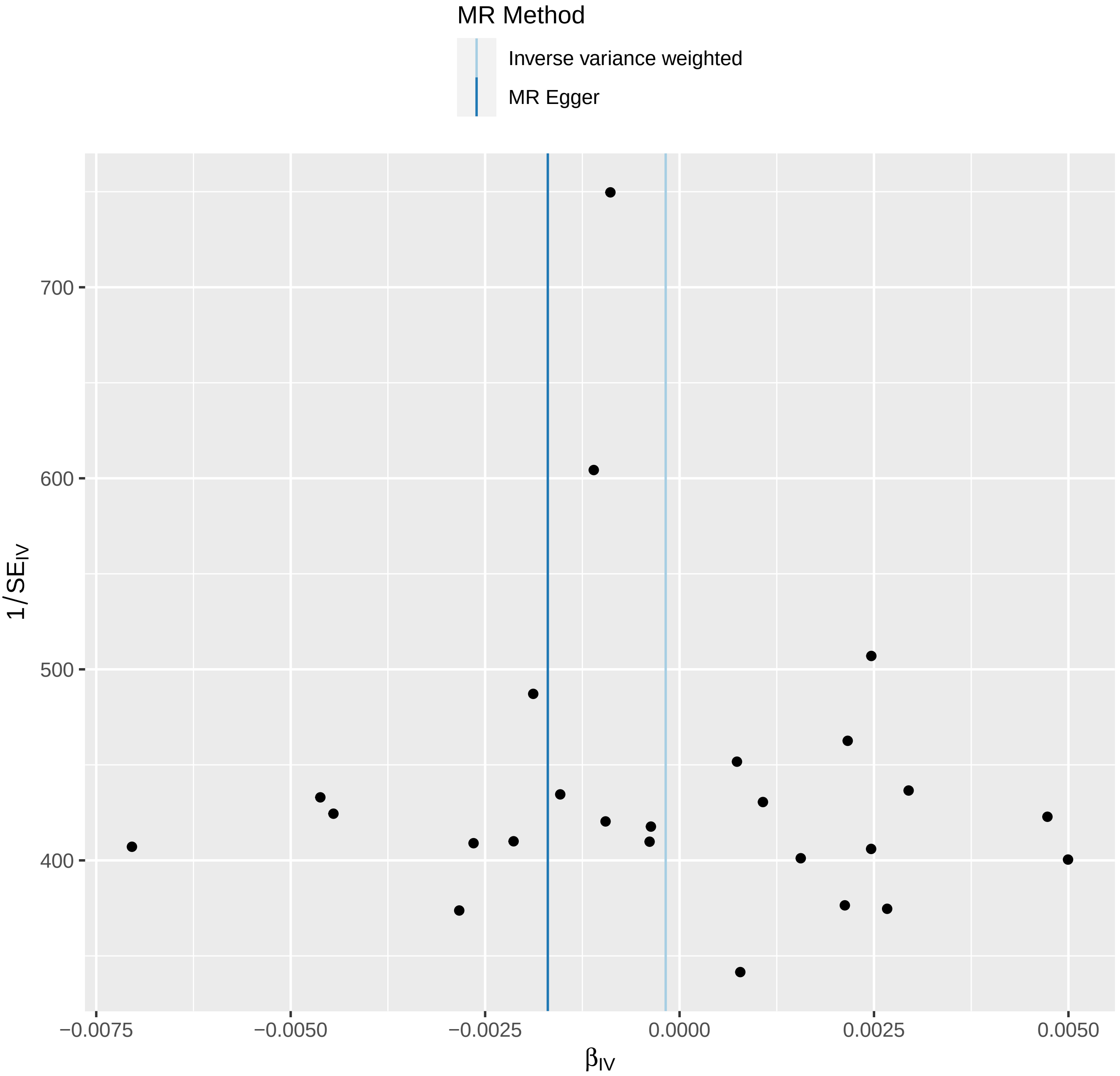

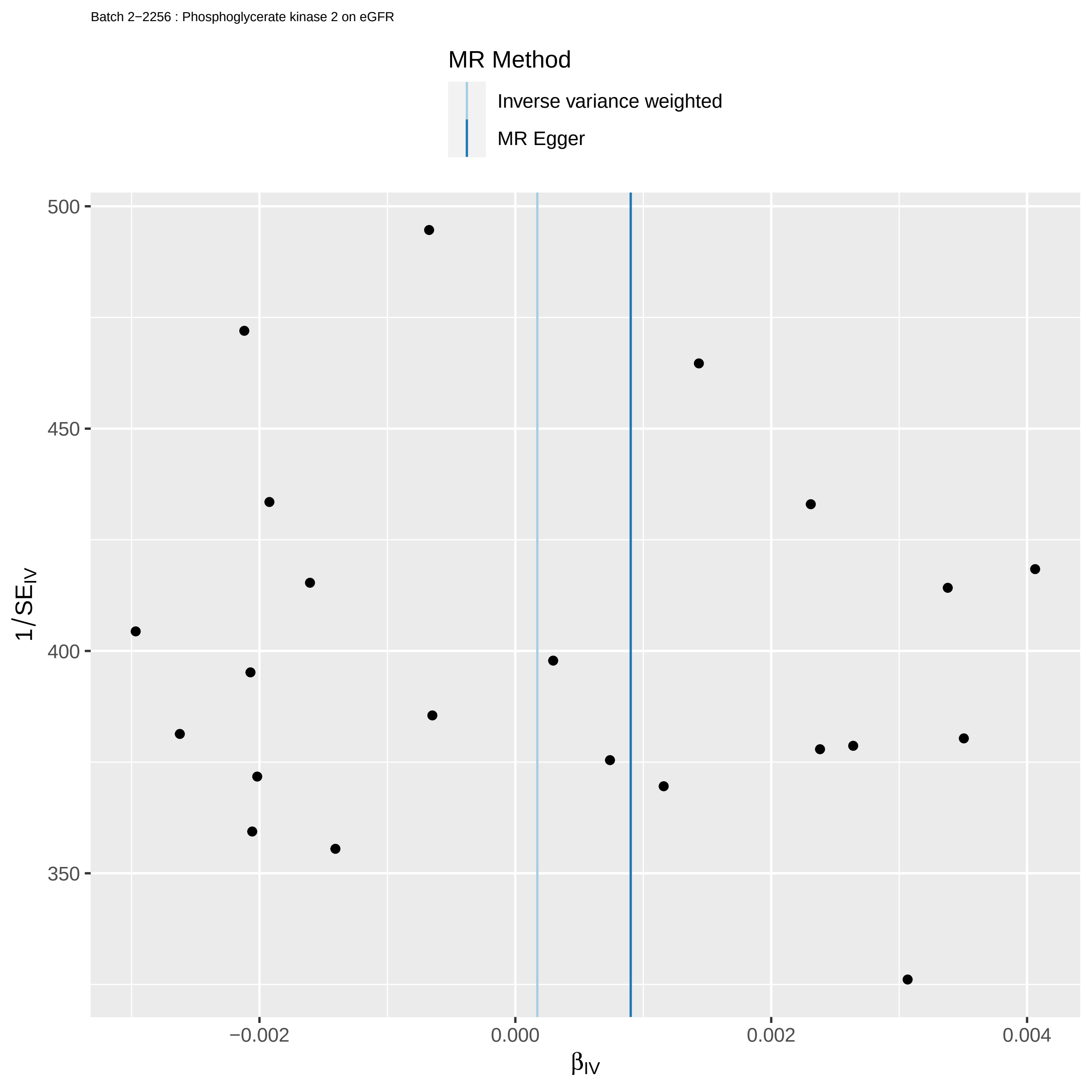
Batch 2−2256 : Phosphoglycerate kinase 2 on eGFR
MR Method
Inverse variance weighted
MR Egger
500
450
400
350
$1/SE_{IV}$
−0.002
0.000
0.002
0.004
β_{IV}

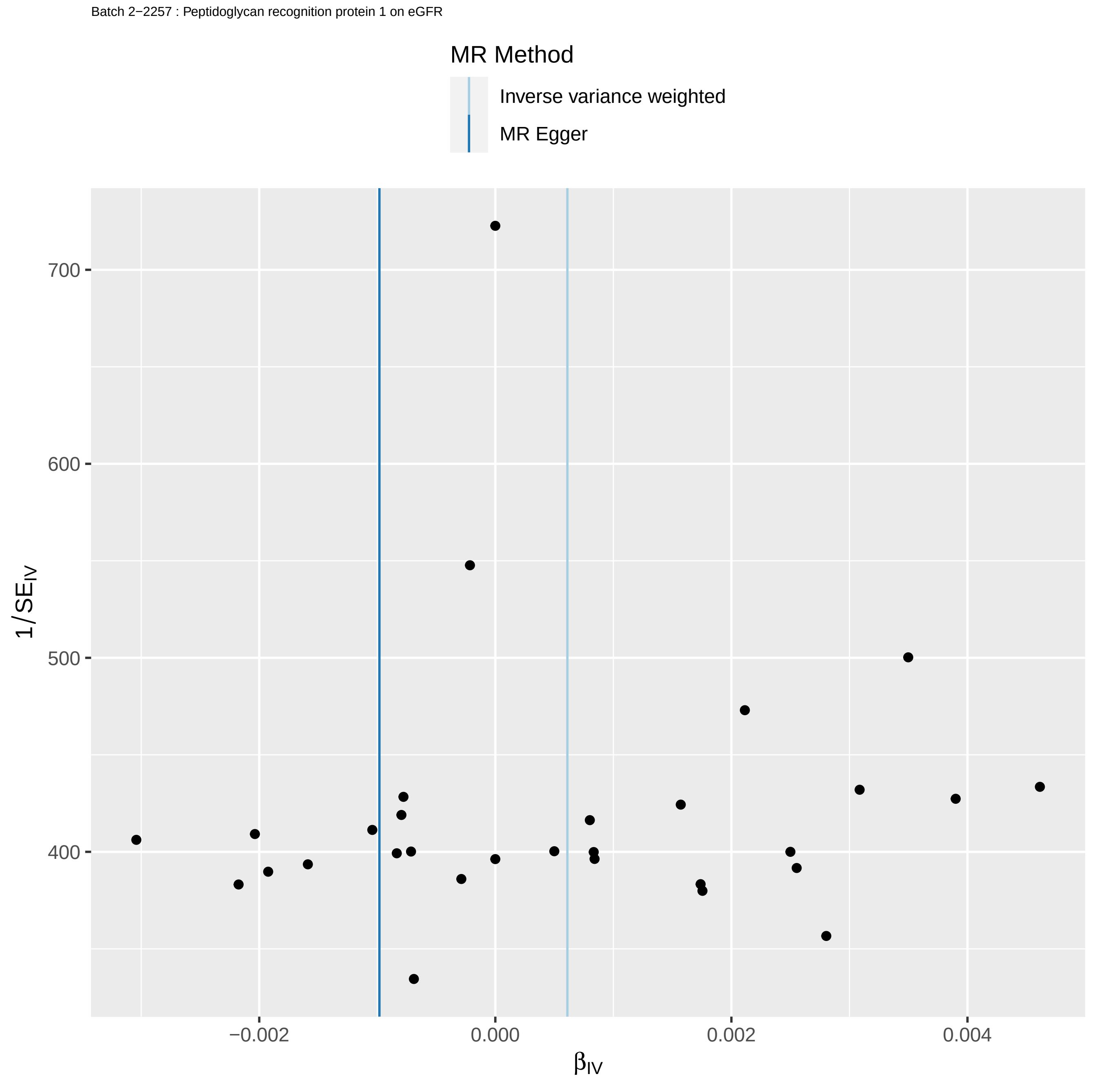

Batch 2−2257 : Peptidoglycan recognition protein 1 on eGFR
MR Method
Inverse variance weighted
MR Egger
700
600
500
400
1/SE$_{IV}$
−0.002
0.000
0.002
0.004
β_{IV}

Batch 2−2258 : Phosphoglucomutase−1 on eGFR

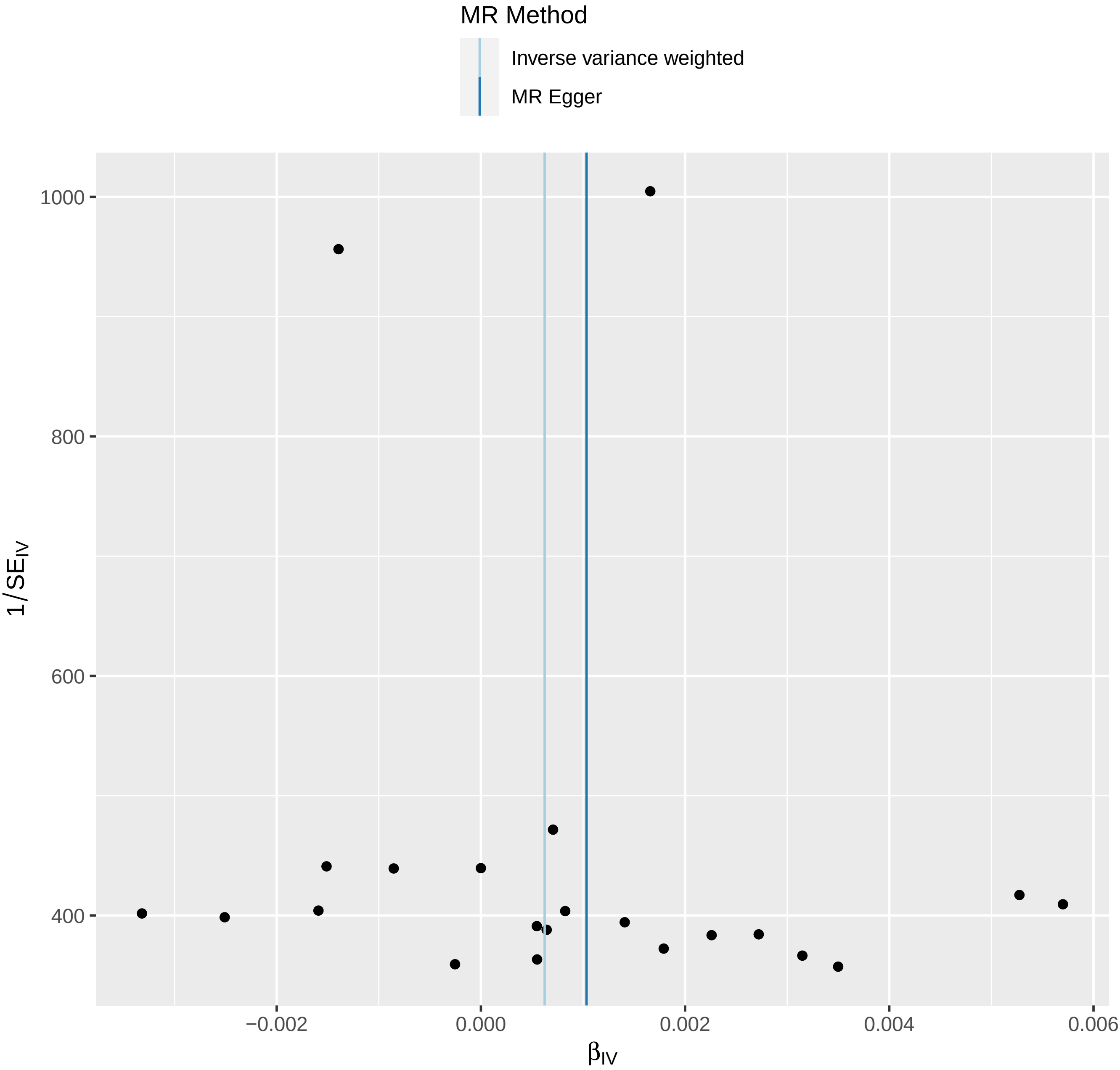

Batch 2−2259 : Membrane−associated progesterone receptor component 1 on eGFR

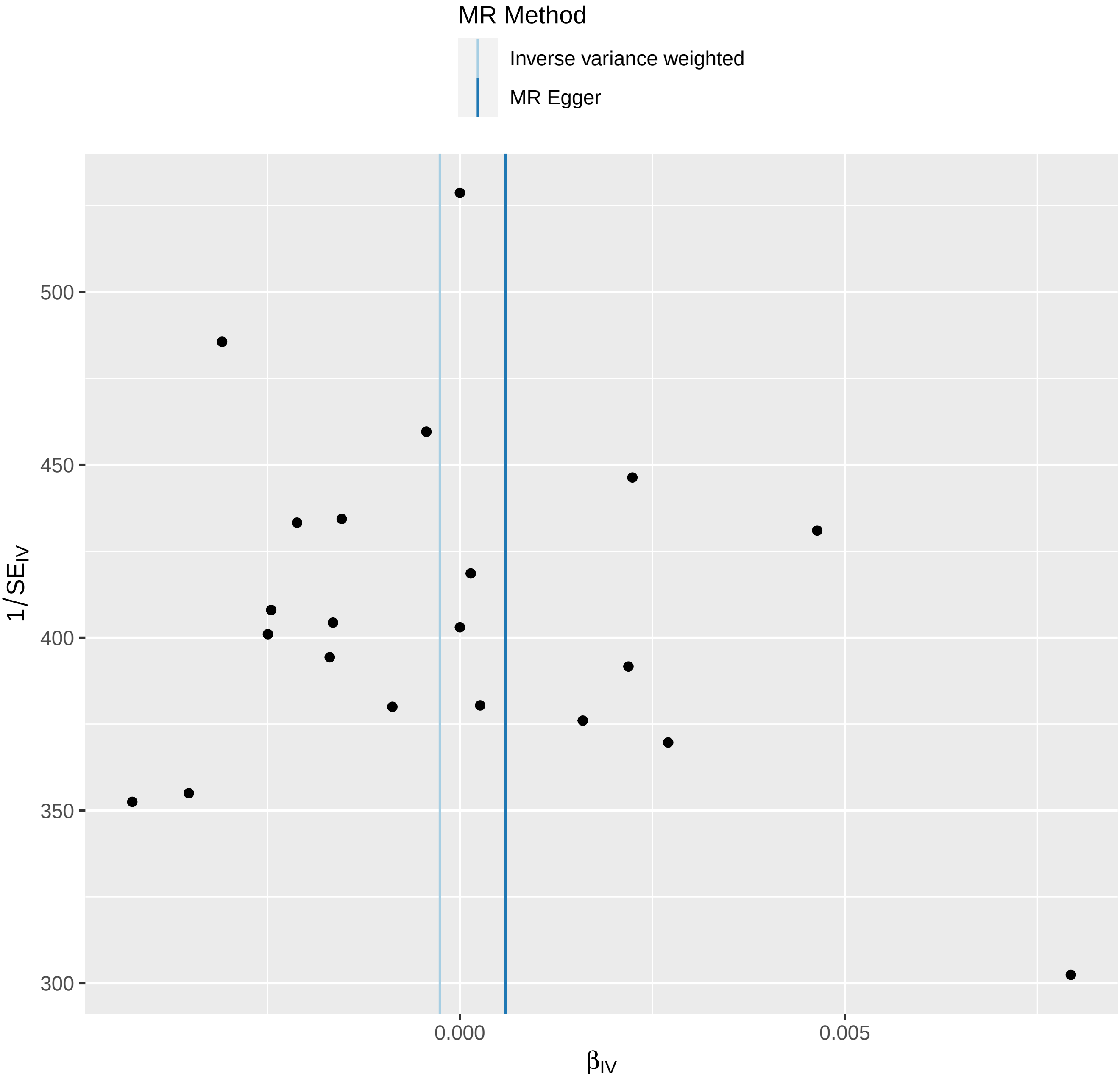

Batch 2−2260 : Membrane−associated progesterone receptor component 2 on eGFR

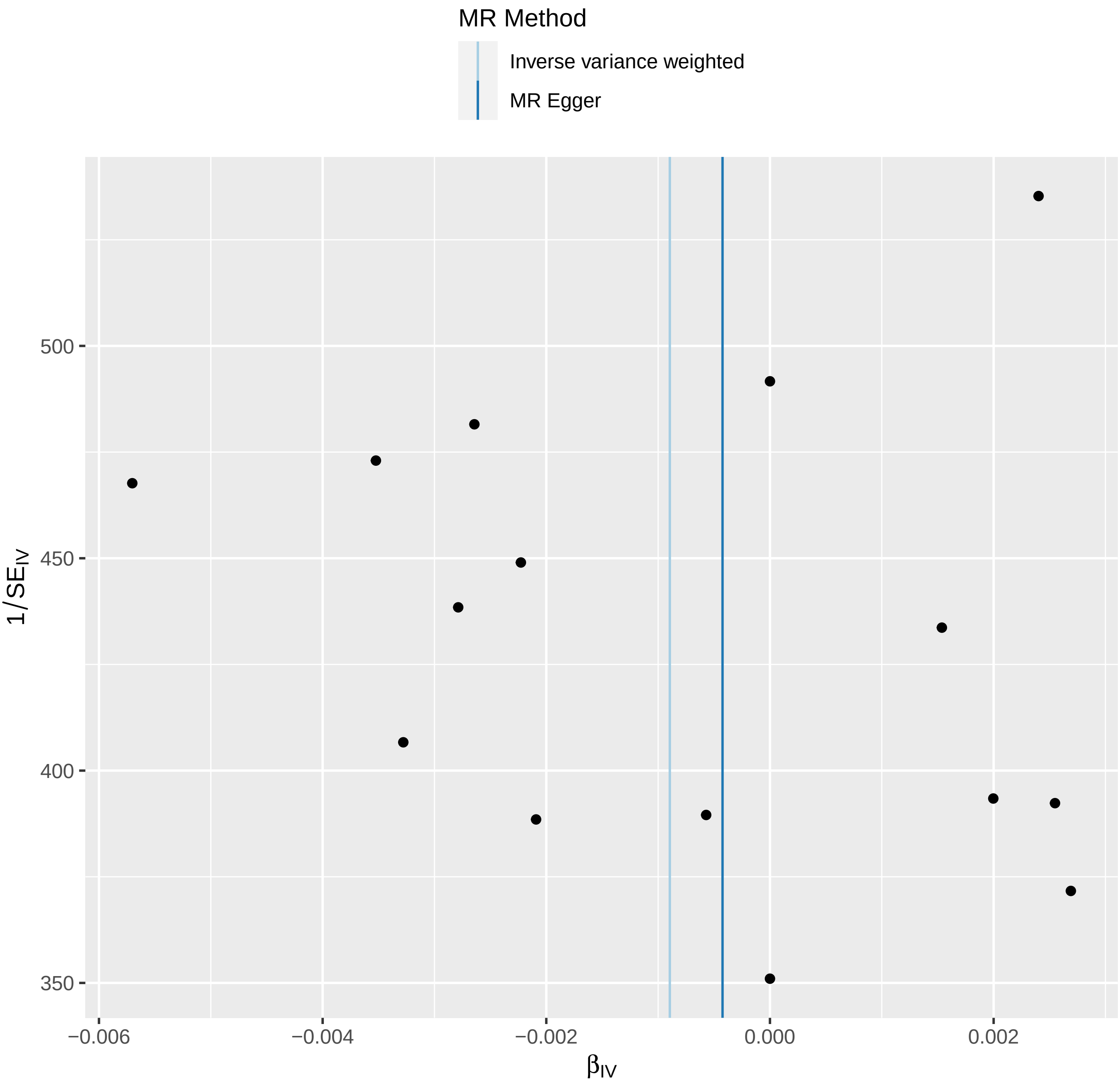

Batch 2−2261 : Membrane−associated progesterone receptor component 2 on eGFR

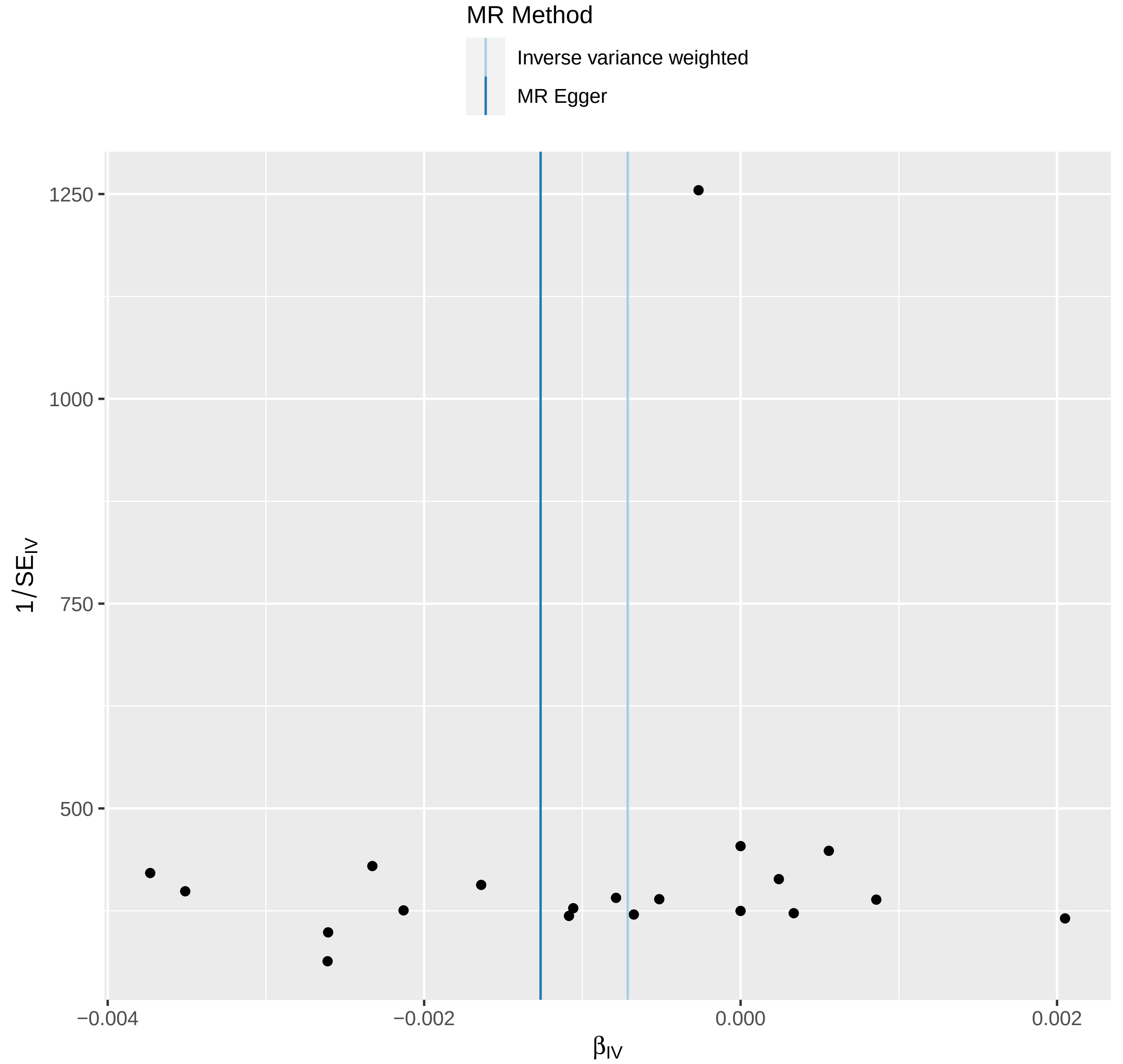

Batch 2−2262 : Membrane−associated progesterone receptor component 2 on eGFR

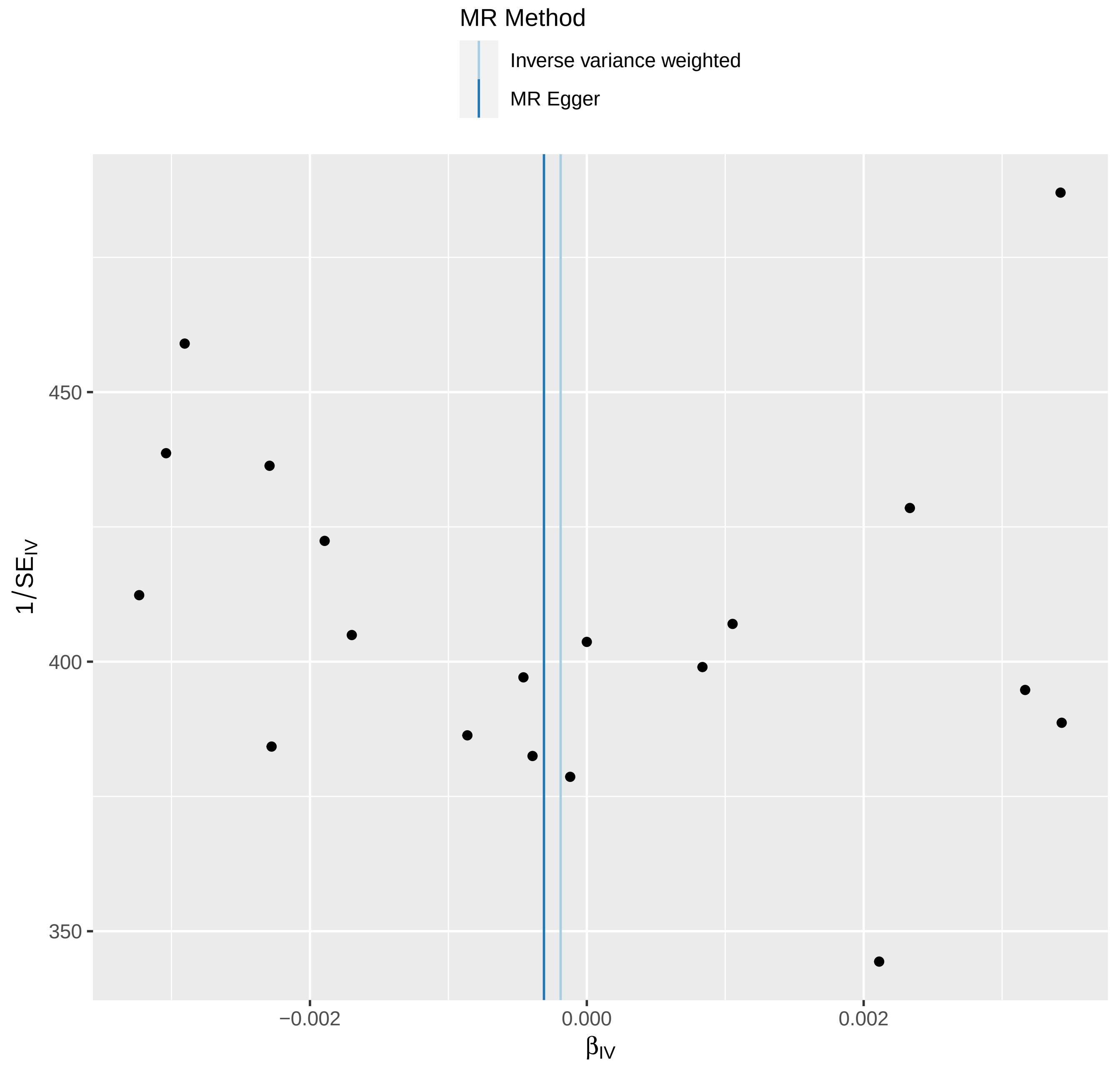

Batch 2−2263 : PHD finger protein 3 on eGFR

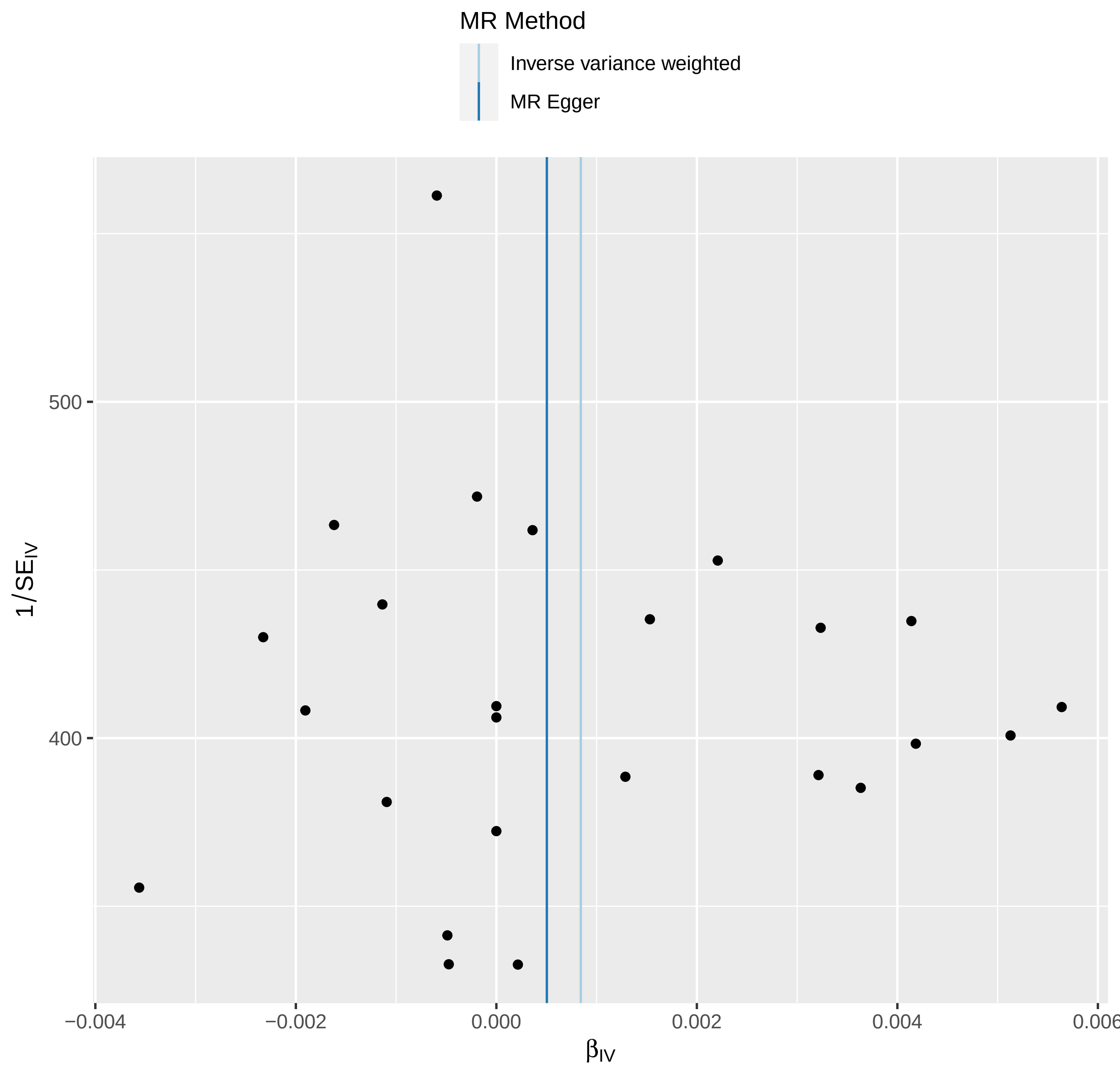

Batch 2−2264 : Peptidase inhibitor 15 on eGFR

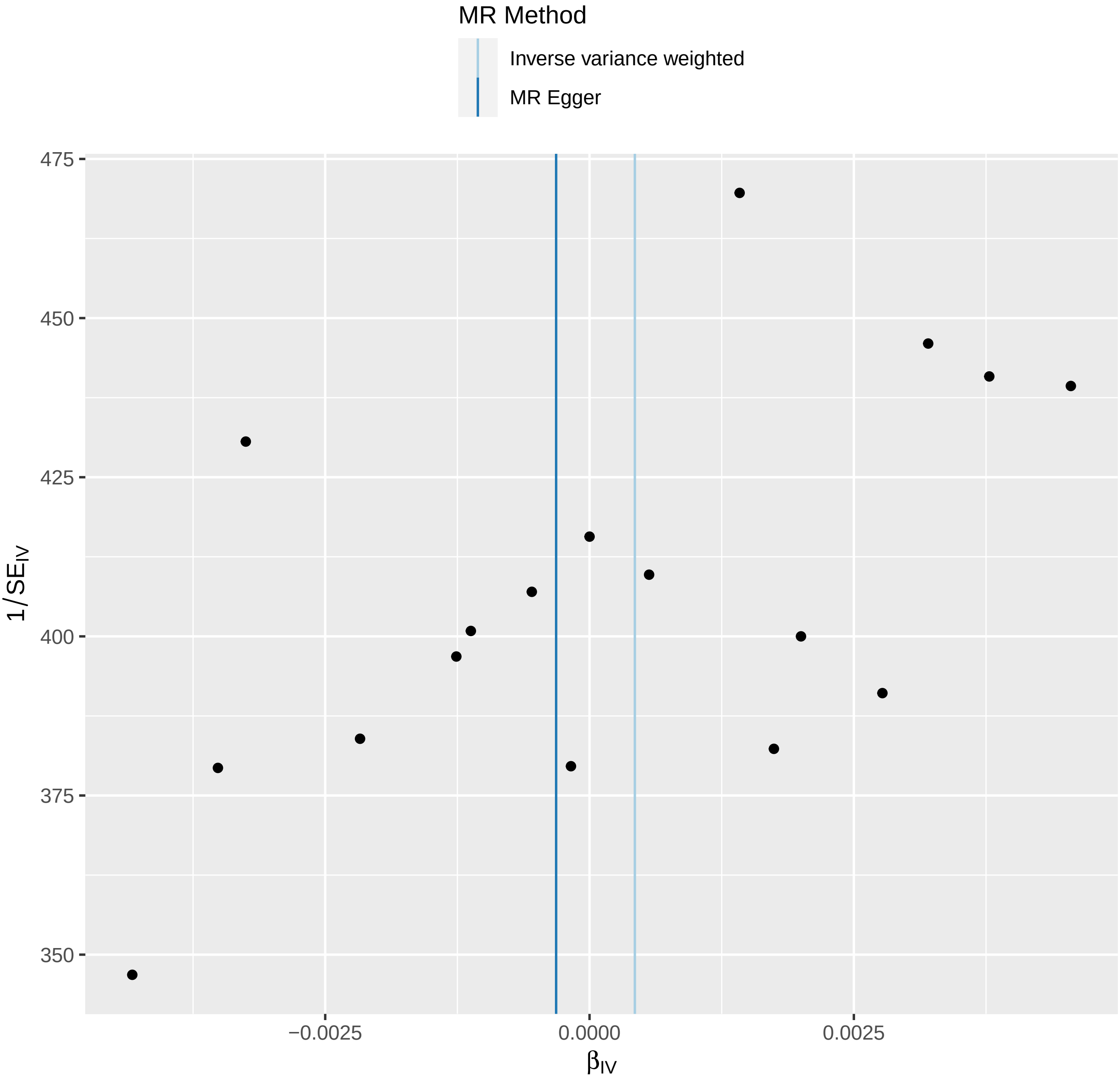

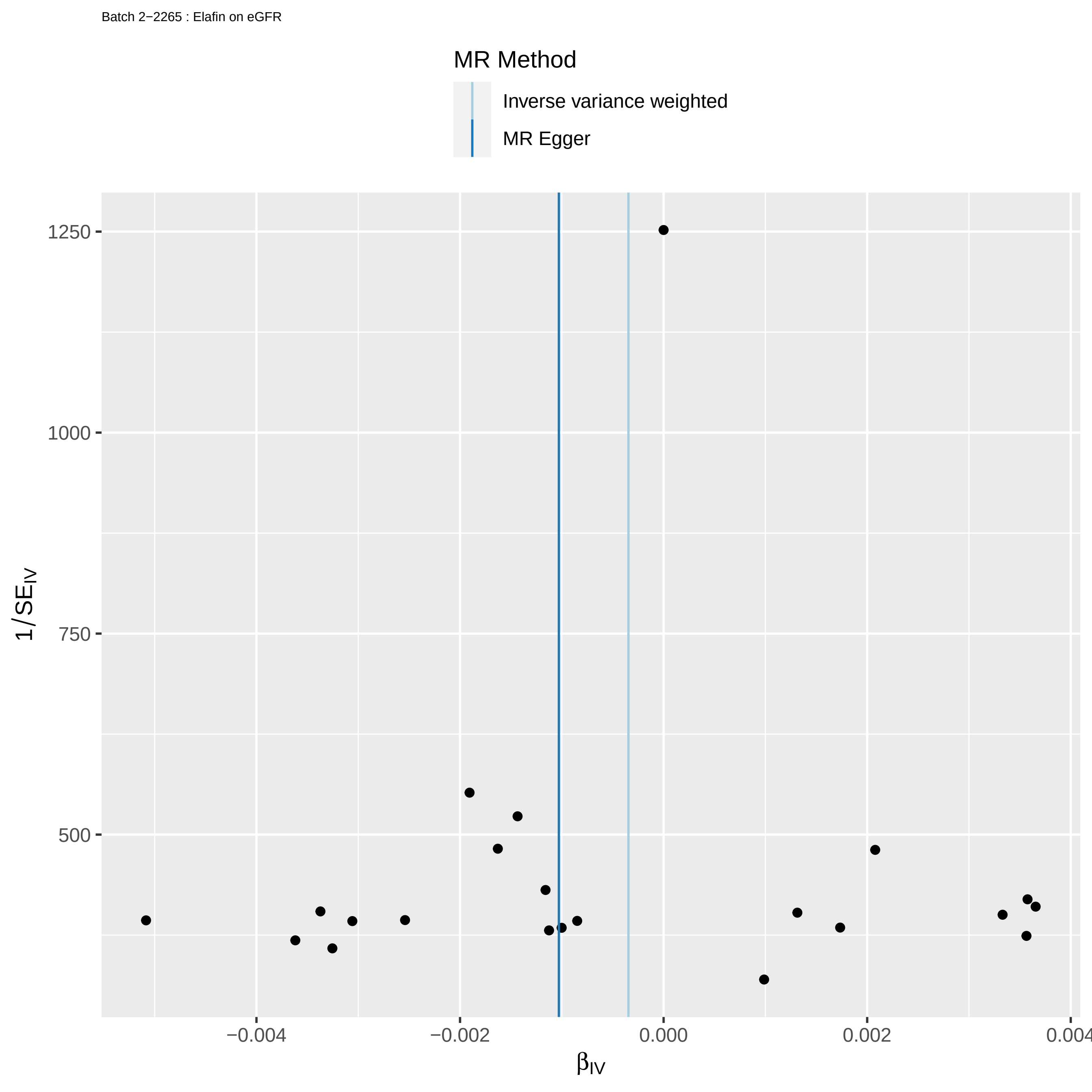
Batch 2−2265 : Elafin on eGFR
MR Method
Inverse variance weighted
MR Egger
1250
1000
750
500
1/SE_IV
−0.004
−0.002
0.000
0.002
0.004
β_IV

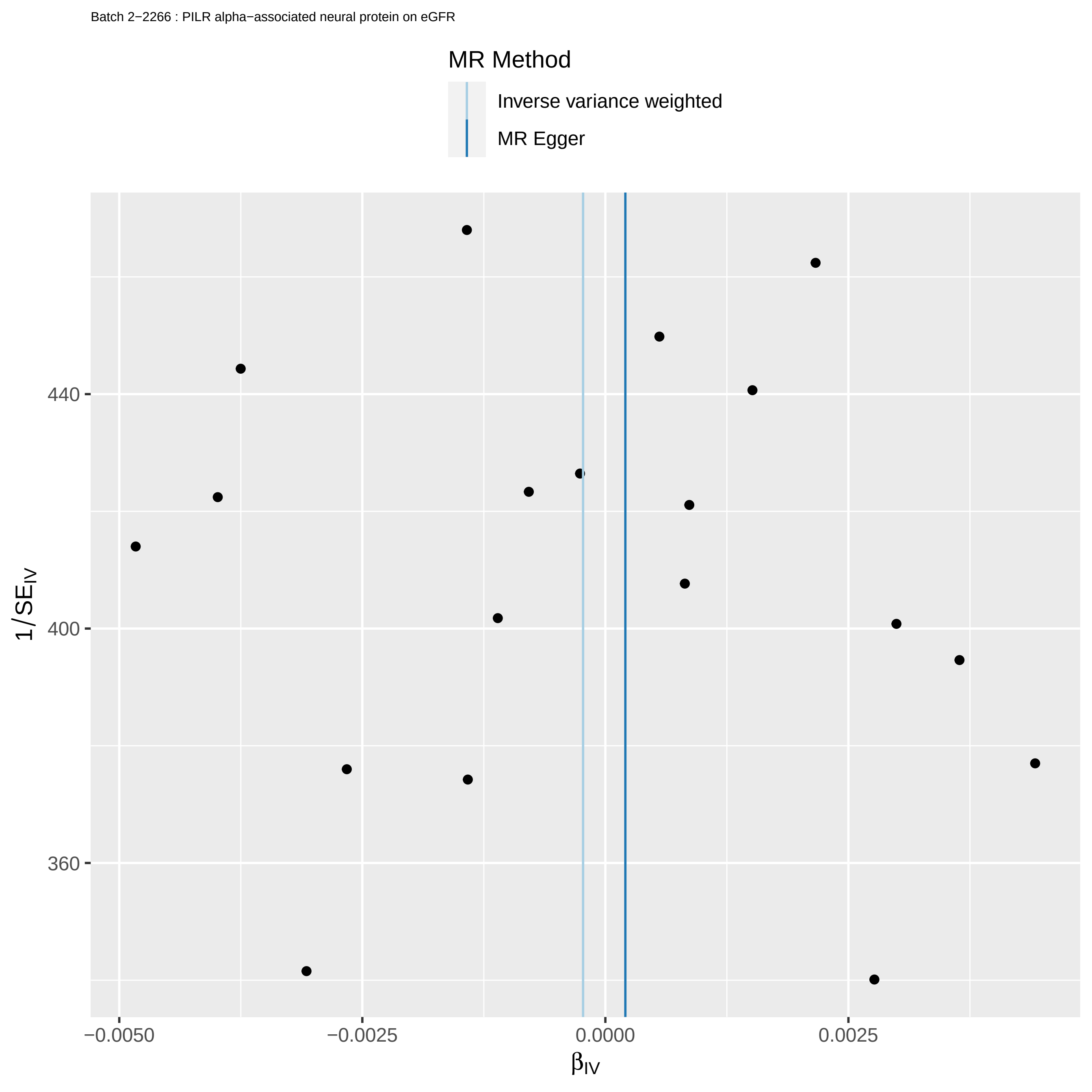

Batch 2−2266 : PILR alpha−associated neural protein on eGFR
MR Method
Inverse variance weighted
MR Egger
440
400
360
1/SE$_{IV}$
−0.0050
−0.0025
0.0000
0.0025
β_{IV}

Batch 2−2267 : PILR alpha−associated neural protein on eGFR

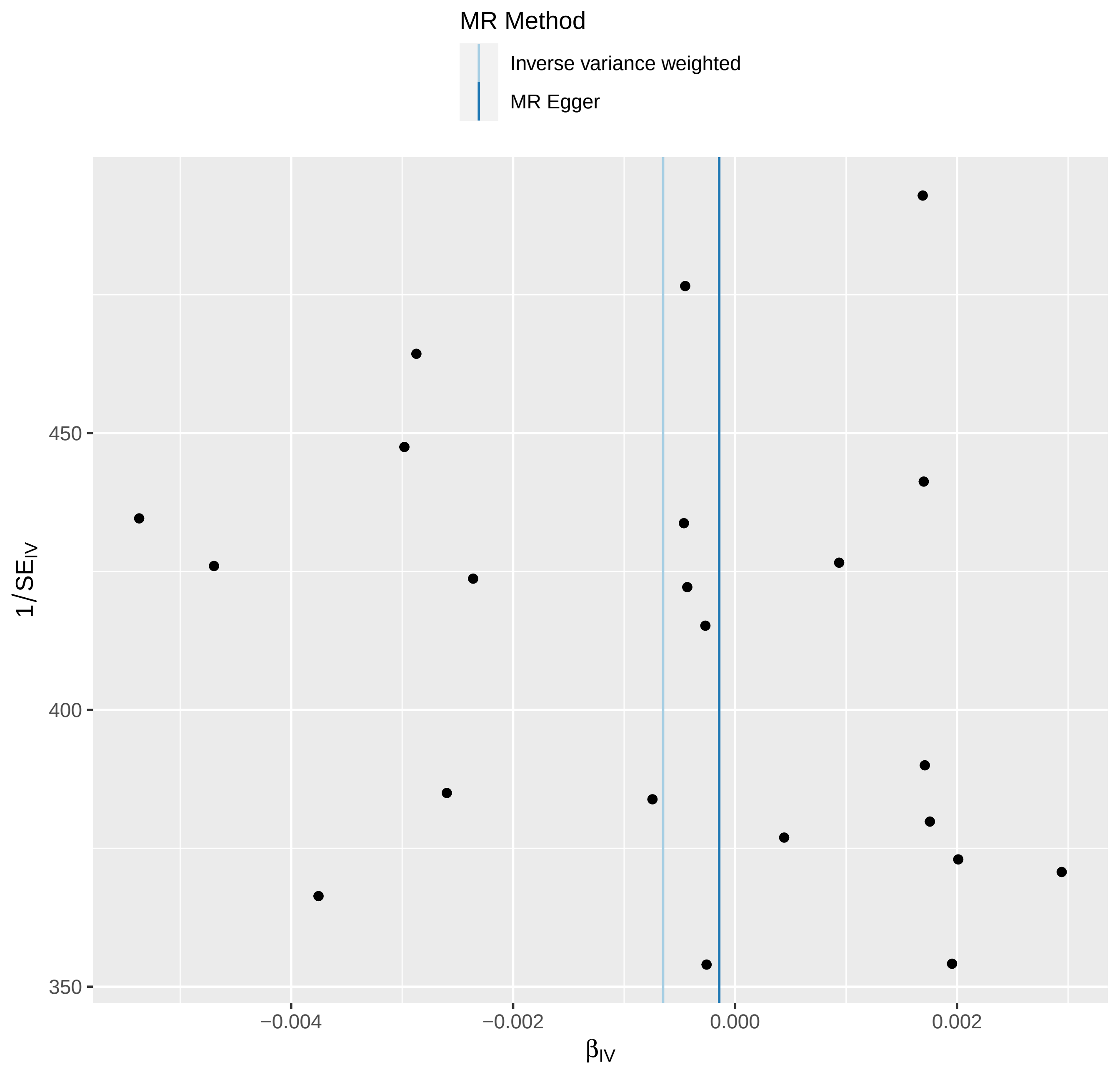

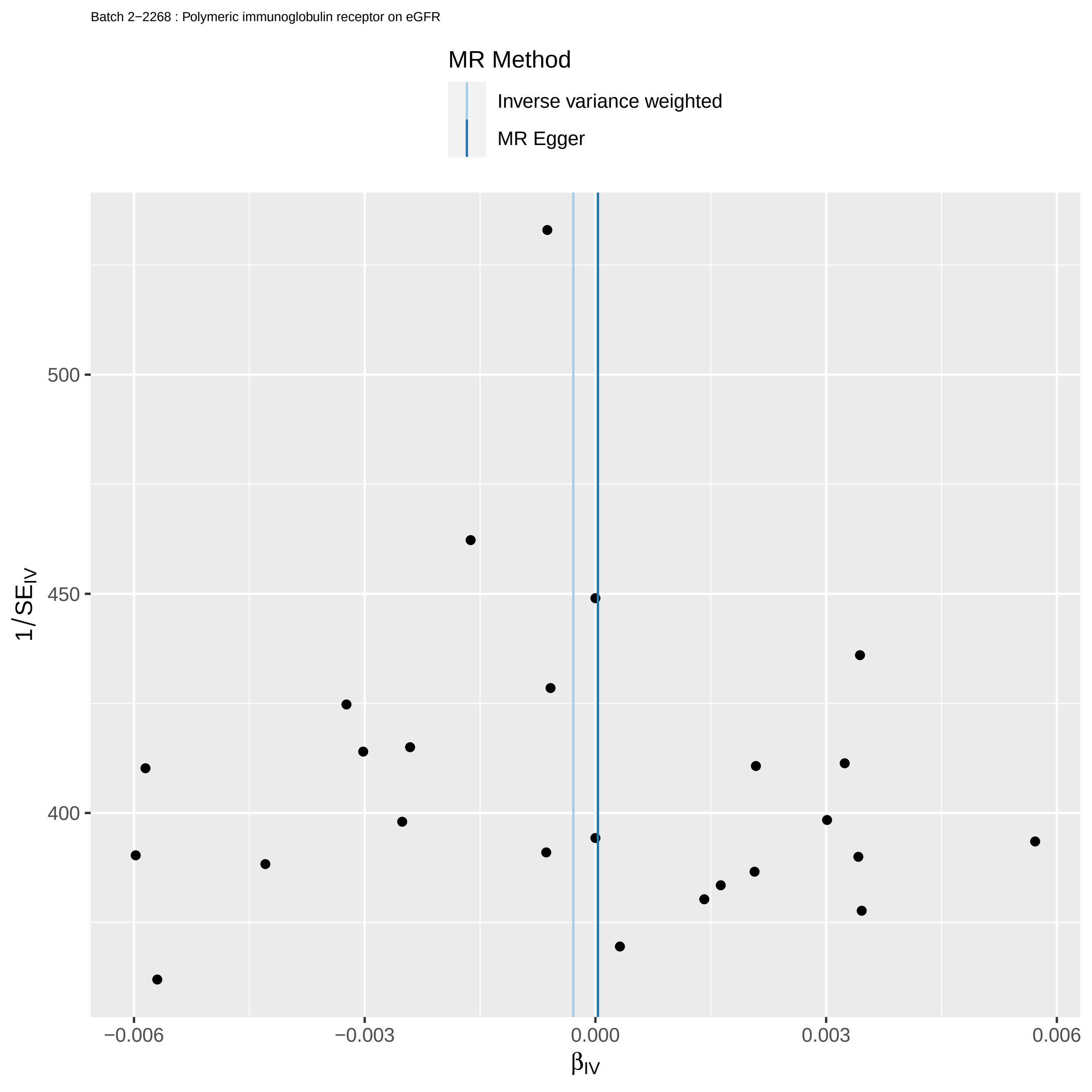

Batch 2−2268 : Polymeric immunoglobulin receptor on eGFR
MR Method
Inverse variance weighted
MR Egger
500
450
400
1/SE_IV
−0.006
−0.003
0.000
0.003
0.006
β_IV

Batch 2−2269 : Phosphatidylinositol 4−phosphate 3−kinase C2 domain−containing subunit alpha on eGFR

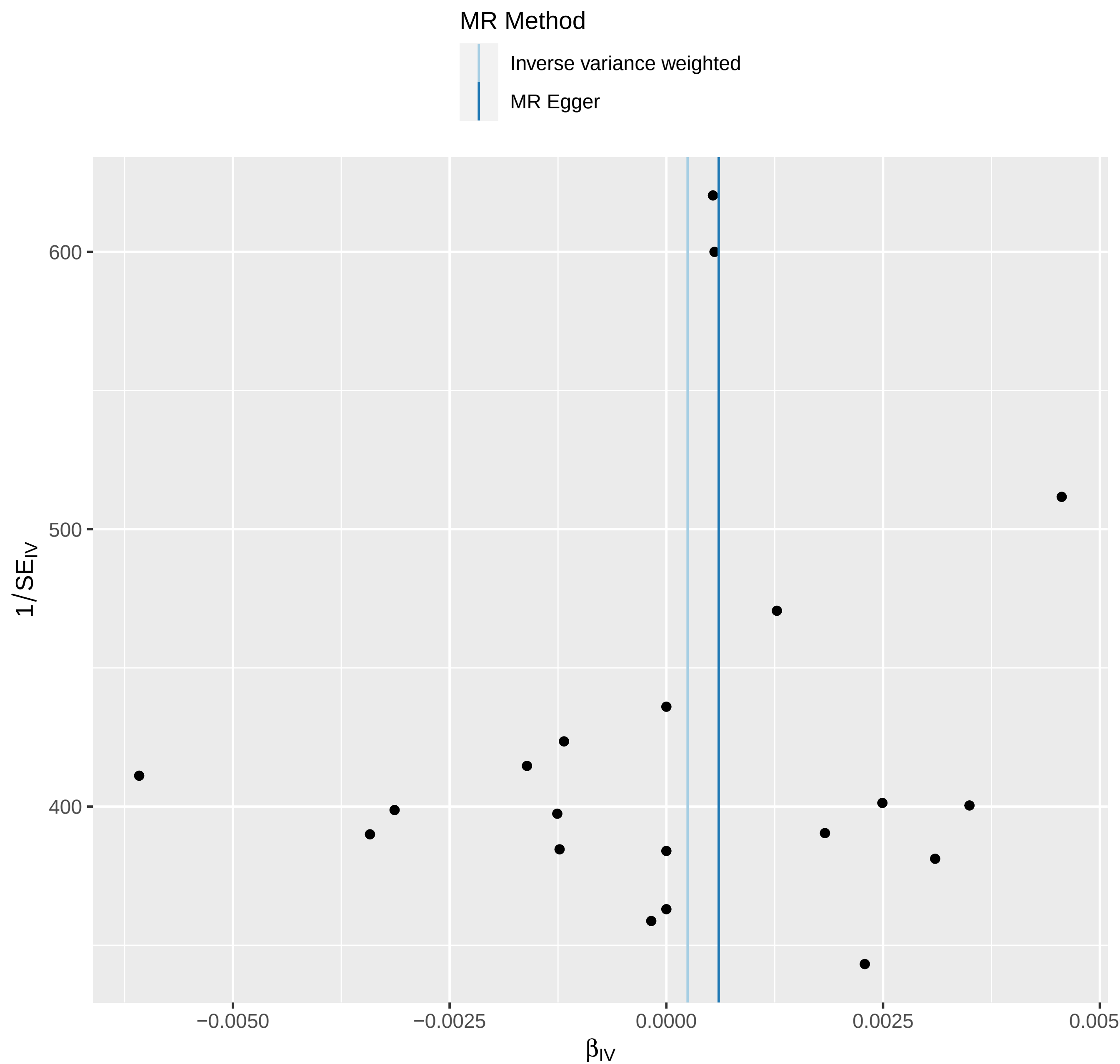

Batch 2−2270 : PIK3CA/PIK3R1 on eGFR

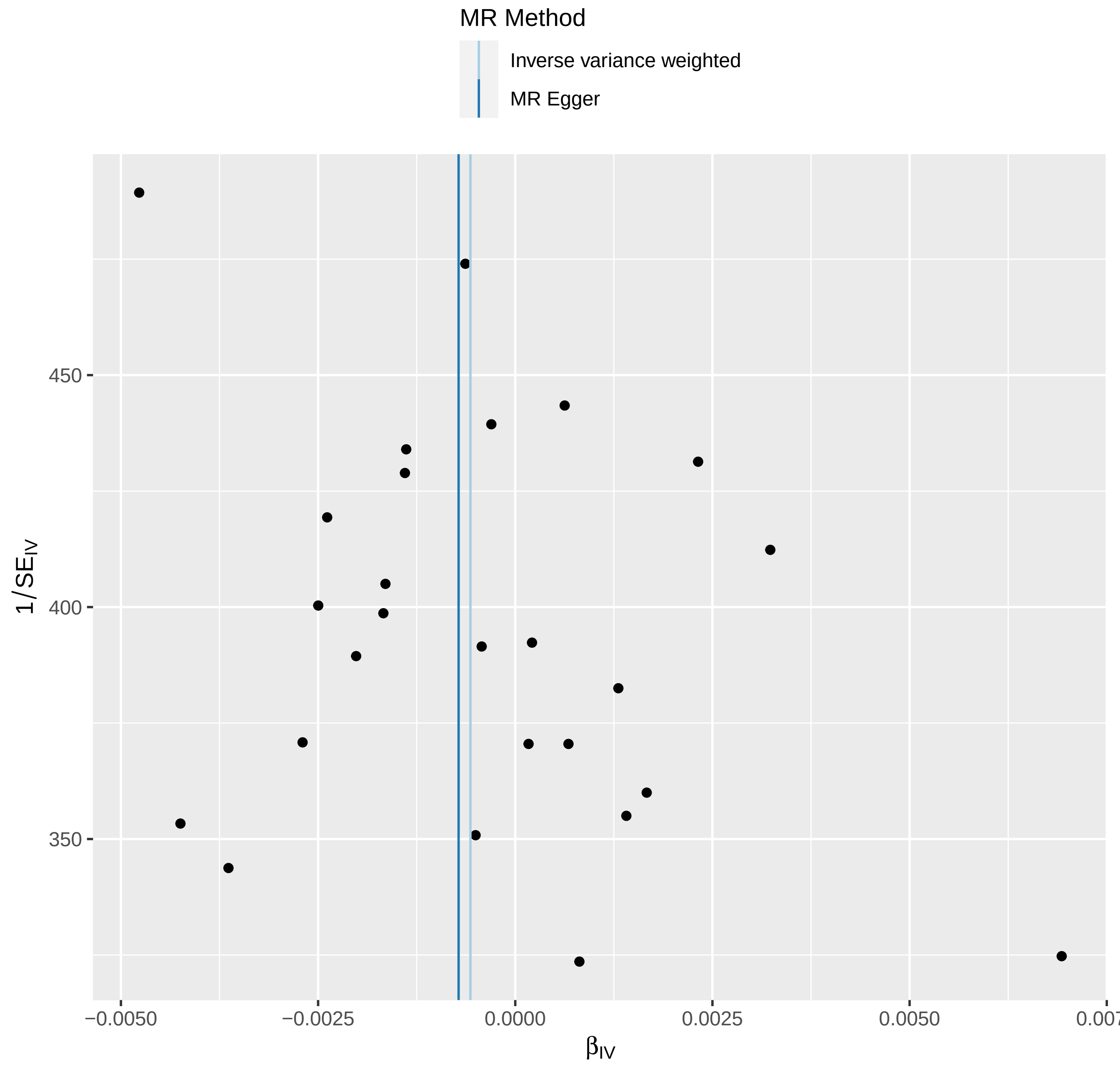

Batch 2−2271 : Phosphatidylinositol 4,5−bisphosphate 3−kinase catalytic subunit gamma isoform on eGFR

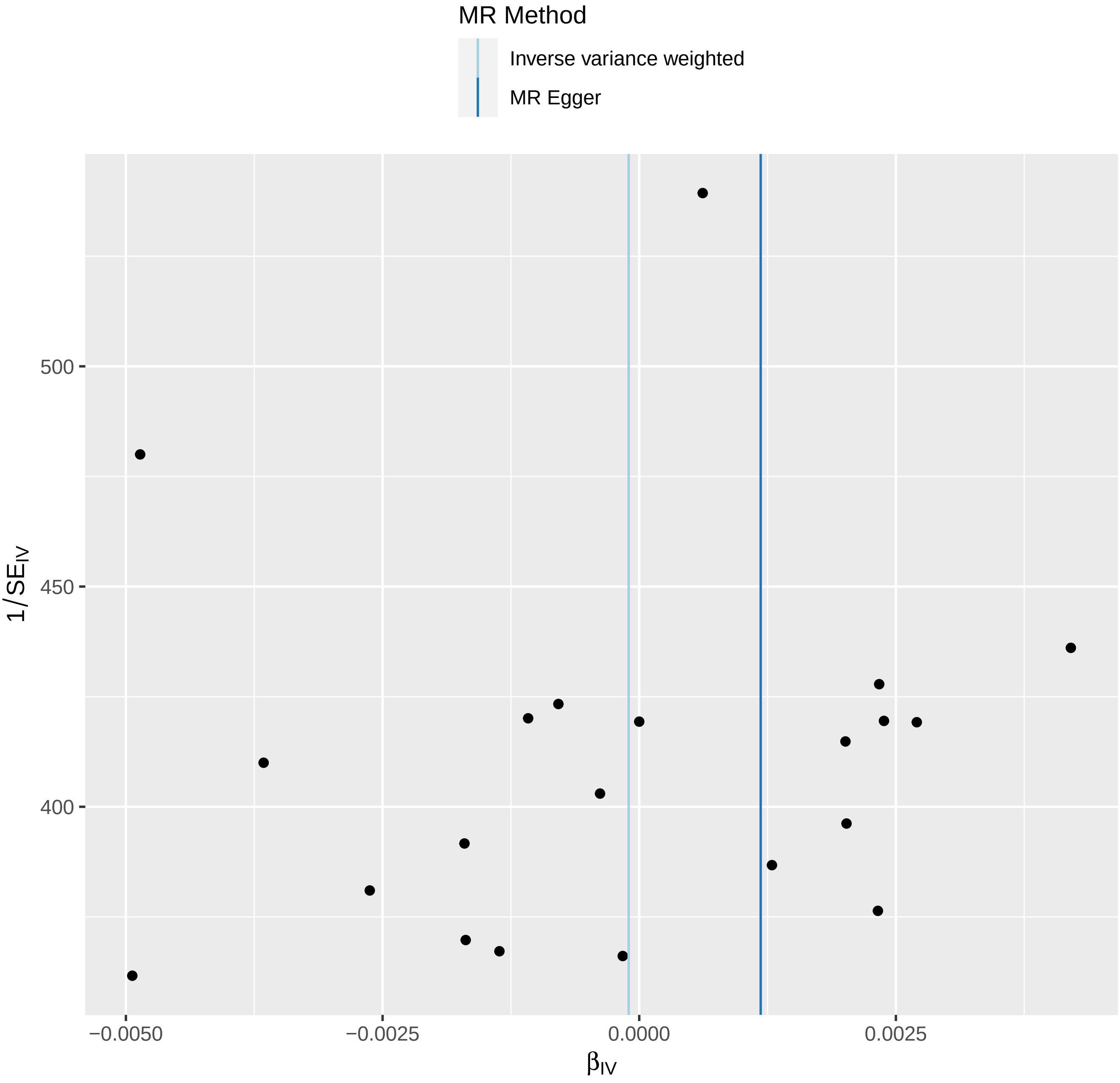

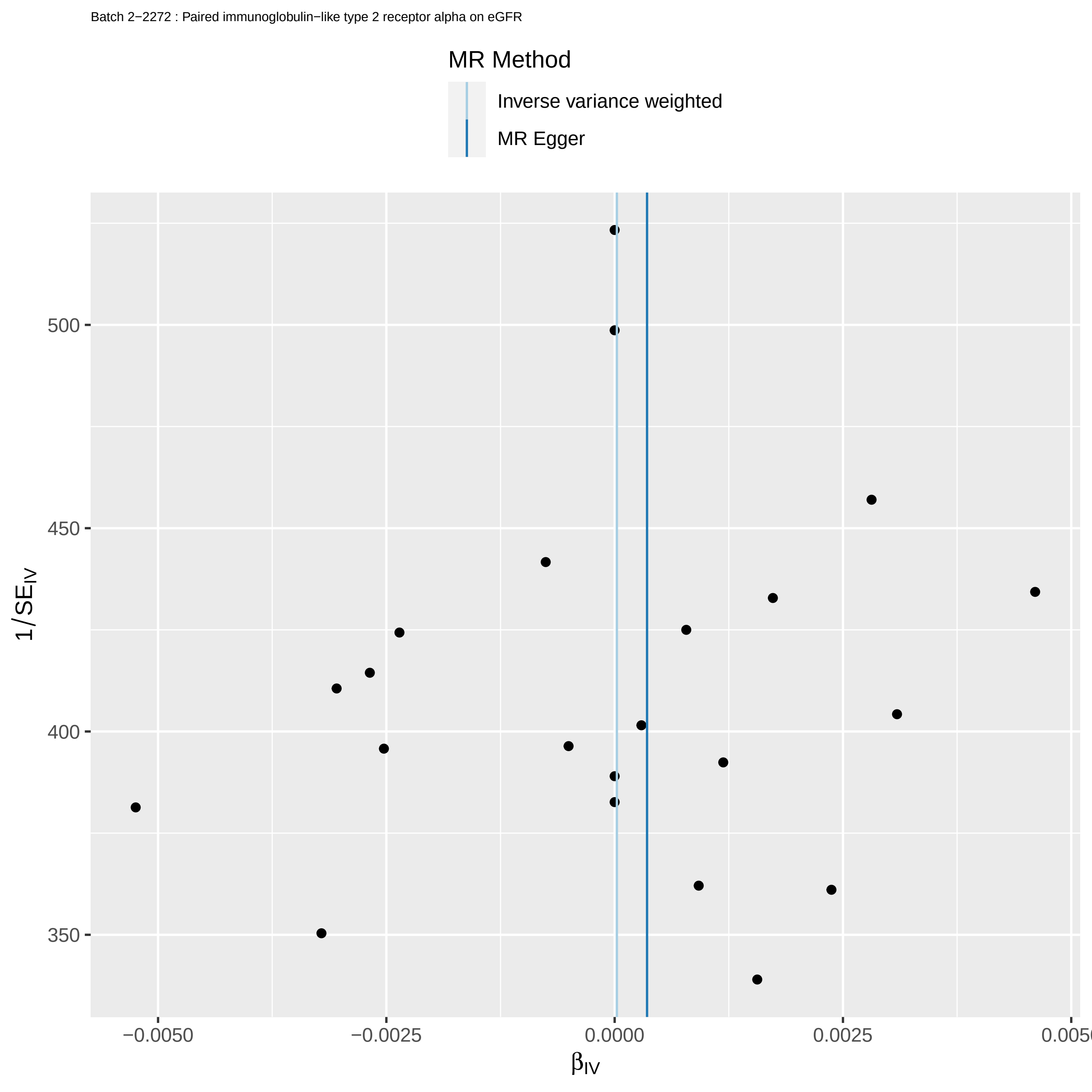

Batch 2−2272 : Paired immunoglobulin−like type 2 receptor alpha on eGFR
MR Method
Inverse variance weighted
MR Egger
500
450
400
350
1/SE$_{IV}$
−0.0050
−0.0025
0.0000
0.0025
0.0050
β$_{IV}$

Batch 2−2273 : Paired immunoglobulin−like type 2 receptor alpha on eGFR

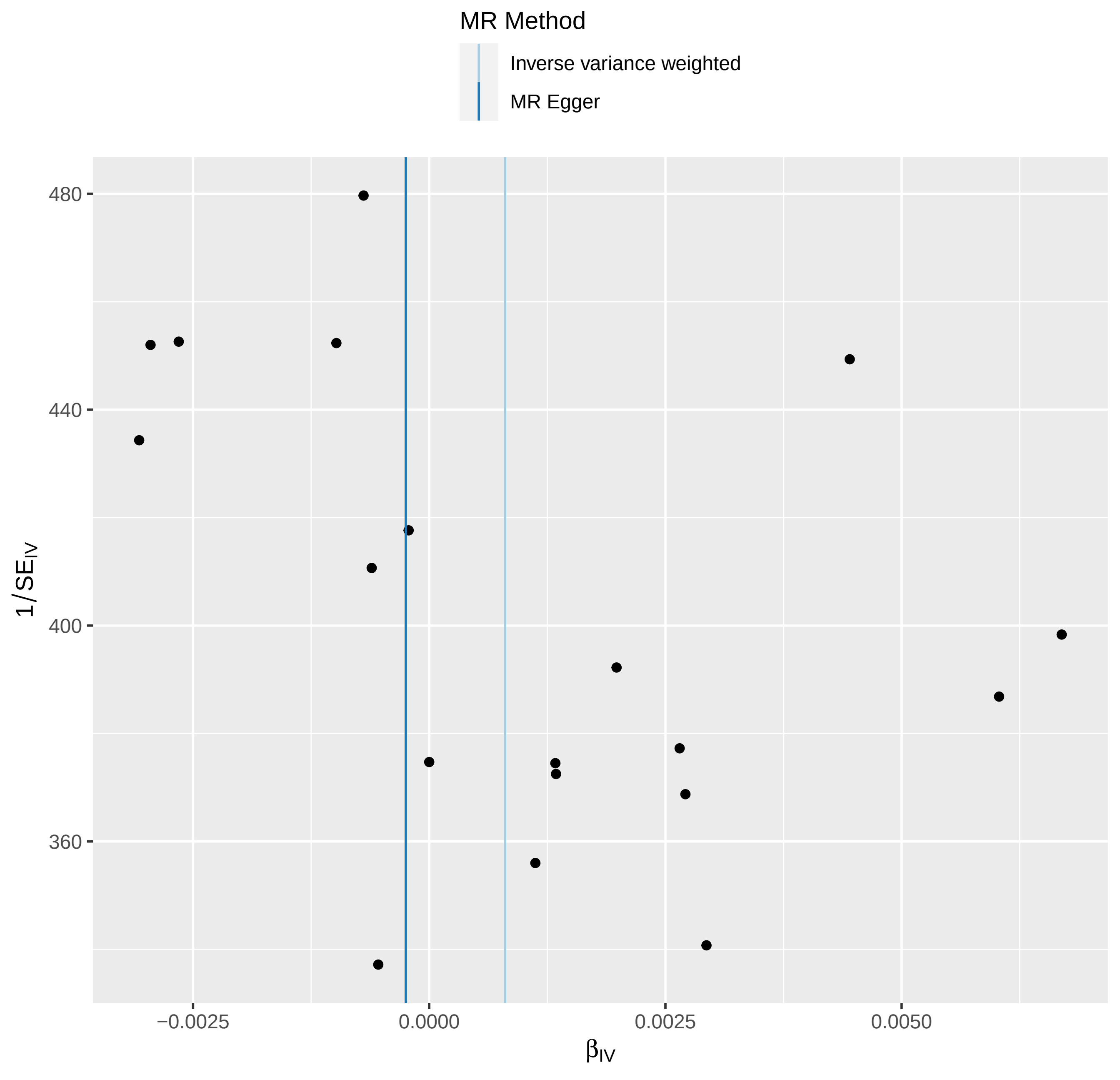

Batch 2−2274 : Serine/threonine−protein kinase pim−1 on eGFR

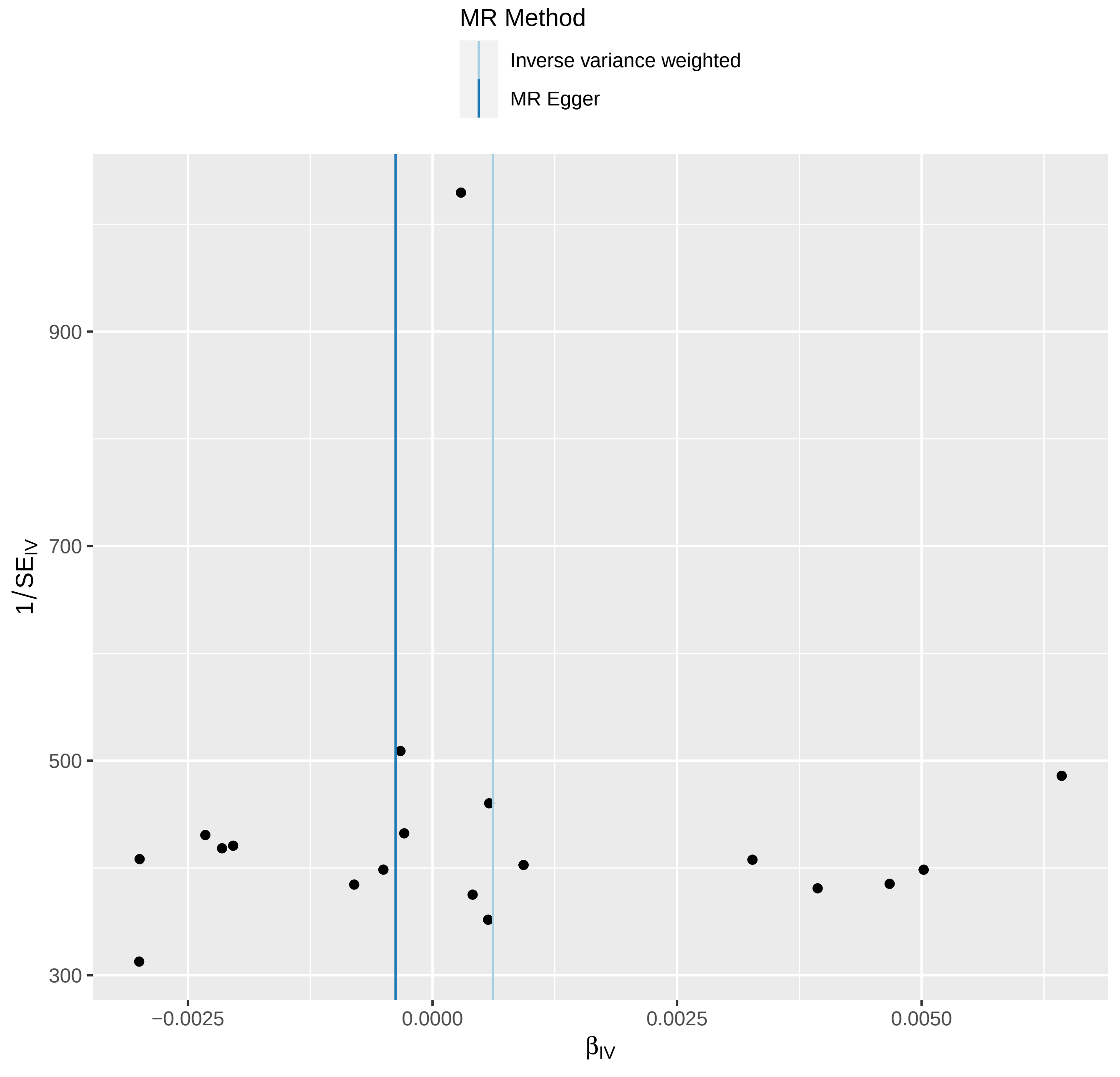

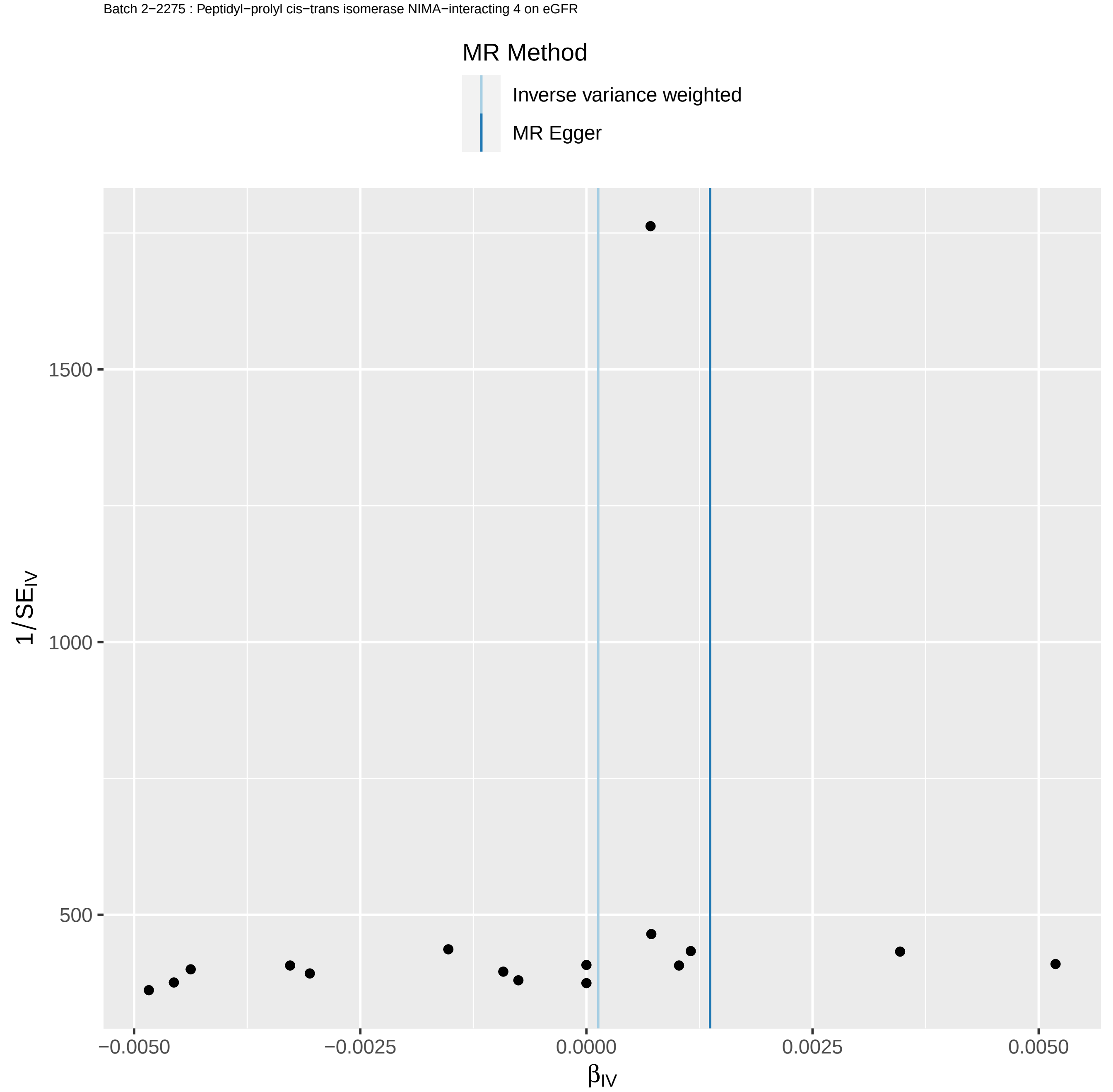
Batch 2−2275 : Peptidyl−prolyl cis−trans isomerase NIMA−interacting 4 on eGFR
MR Method
Inverse variance weighted
MR Egger
1500
1000
500
−0.0050
−0.0025
0.0000
0.0025
0.0050
β_{IV}
$1/SE_{IV}$

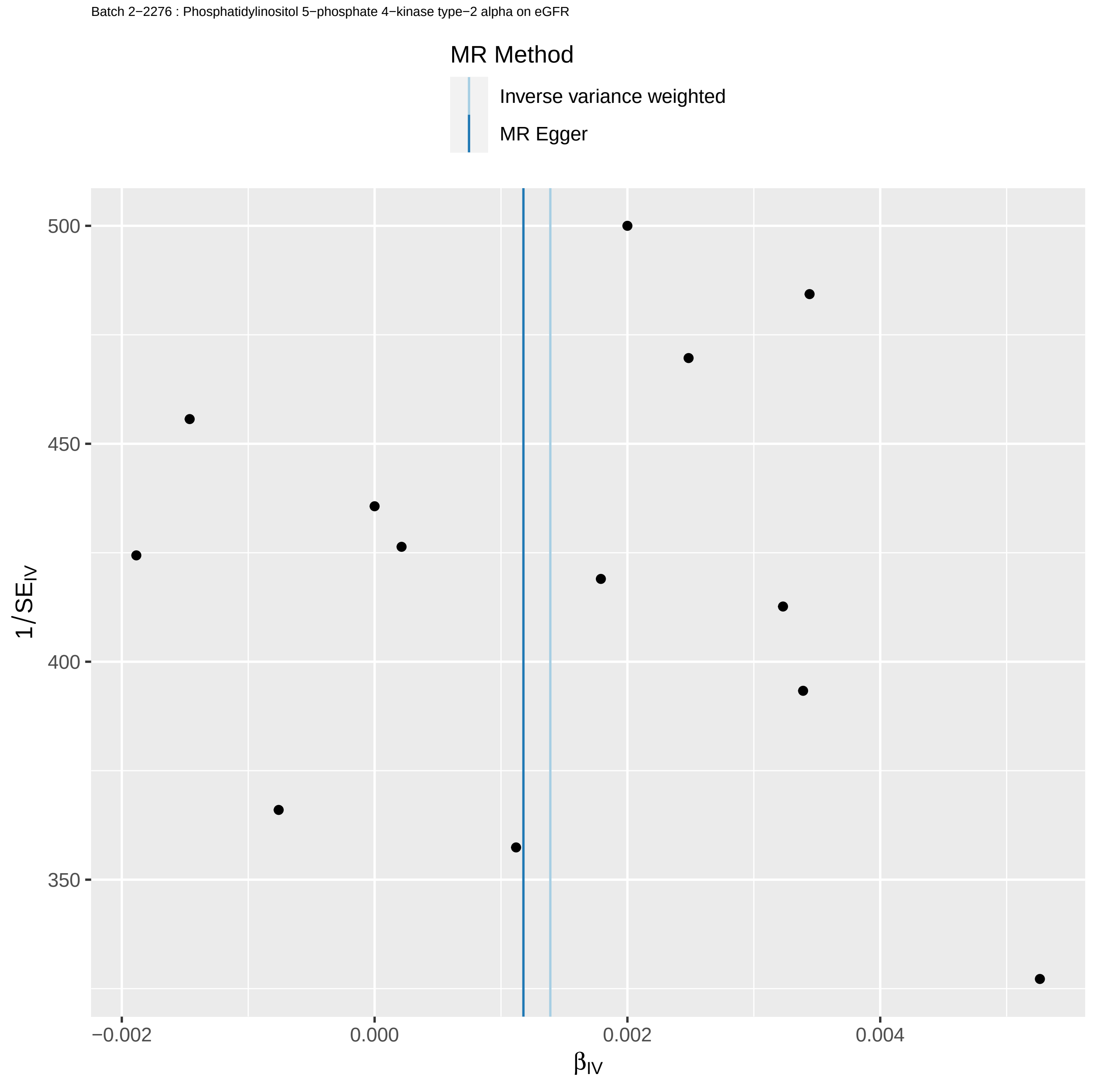

Batch 2−2276 : Phosphatidylinositol 5−phosphate 4−kinase type−2 alpha on eGFR
MR Method
Inverse variance weighted
MR Egger
500
450
400
350
1/SE_IV
−0.002
0.000
0.002
0.004
β_IV

Batch 2−2277 : Pirin on eGFR

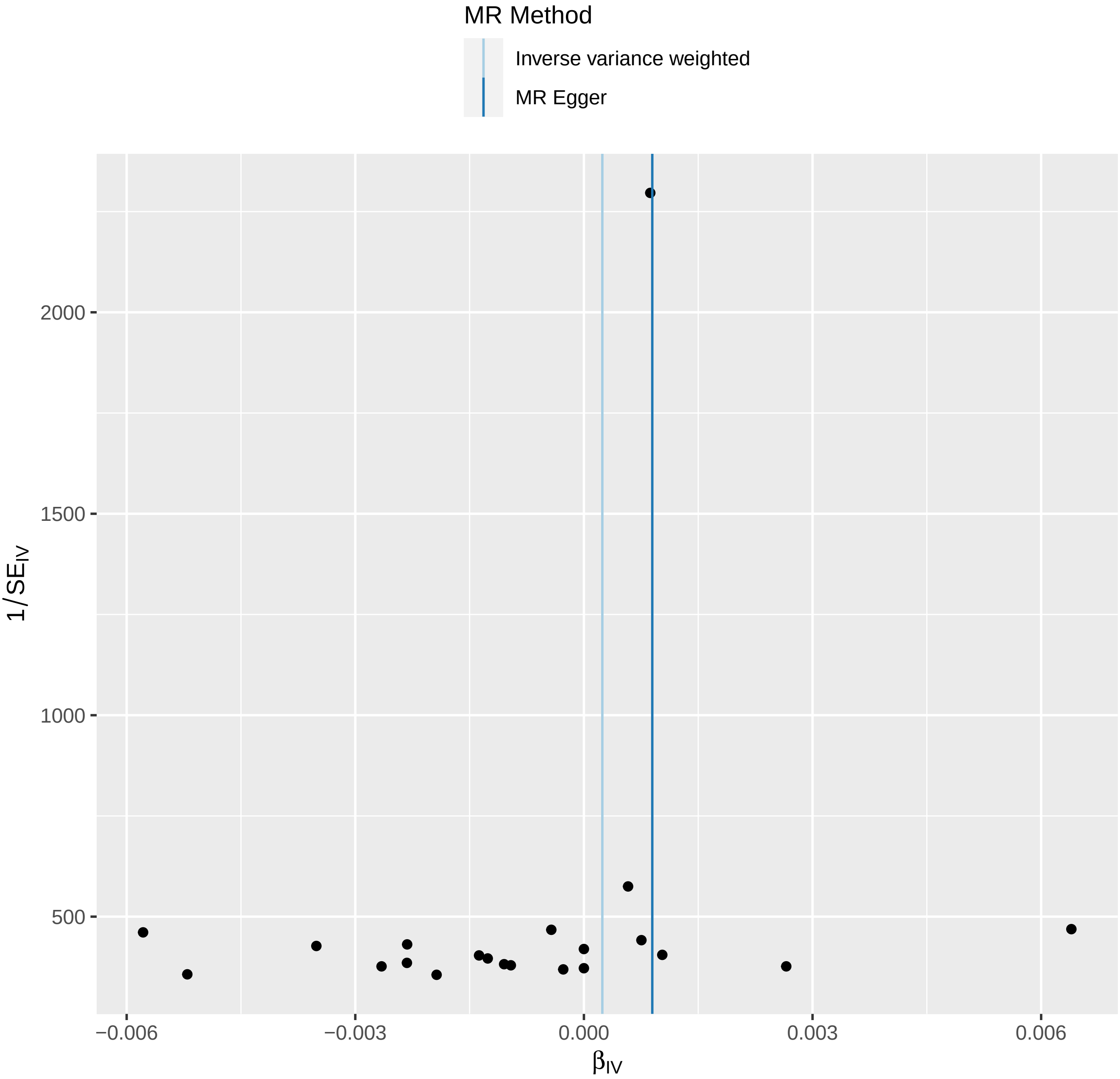

Batch 2−2278 : Phosphatidylinositol transfer protein alpha isoform on eGFR

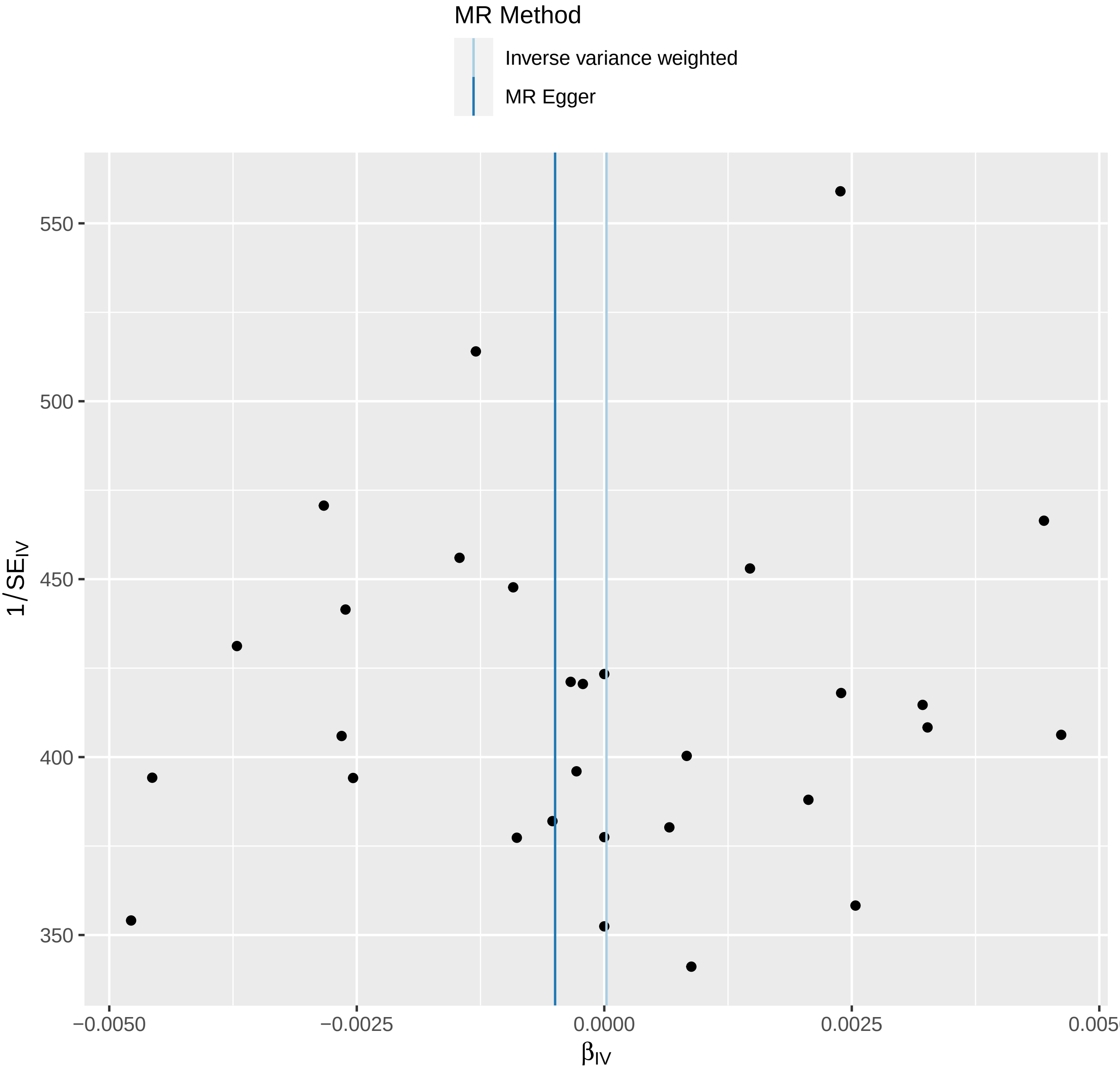

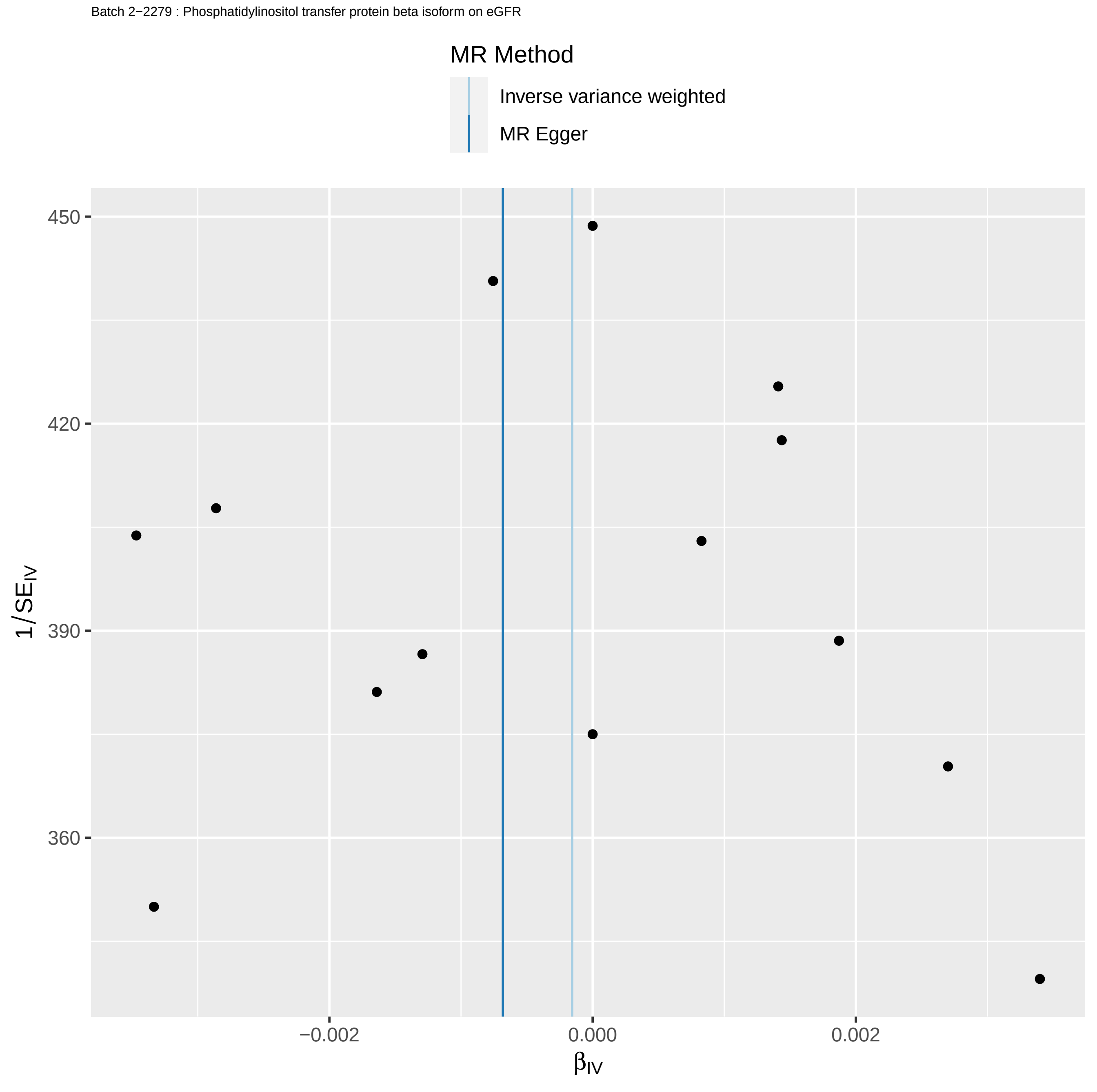

Batch 2−2279 : Phosphatidylinositol transfer protein beta isoform on eGFR
MR Method
Inverse variance weighted
MR Egger
450
420
390
360
1/SE_IV
−0.002
0.000
0.002
β_IV

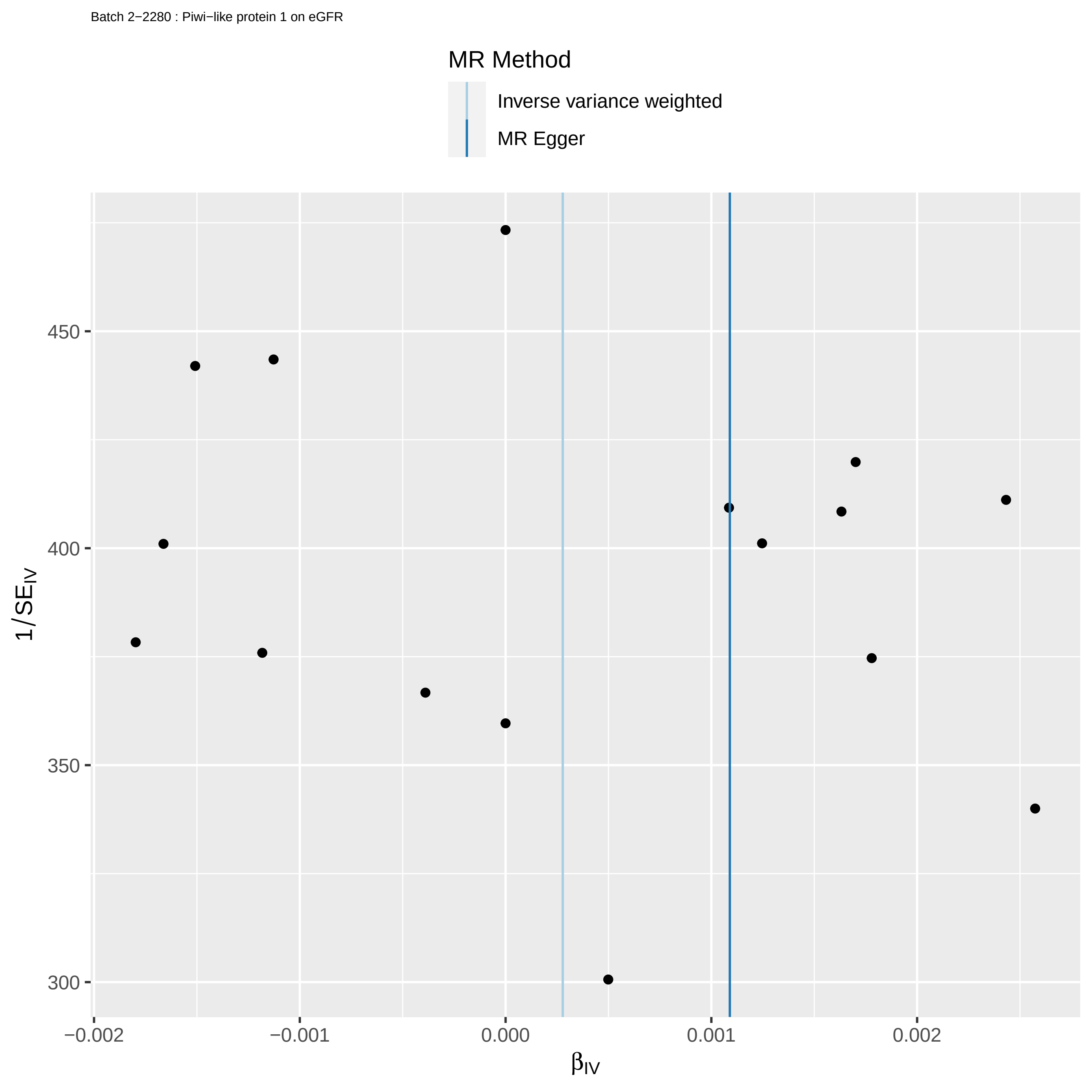

Batch 2−2280 : Piwi−like protein 1 on eGFR
MR Method
Inverse variance weighted
MR Egger
450
400
350
300
1/SE_IV
−0.002
−0.001
0.000
0.001
0.002
β_IV

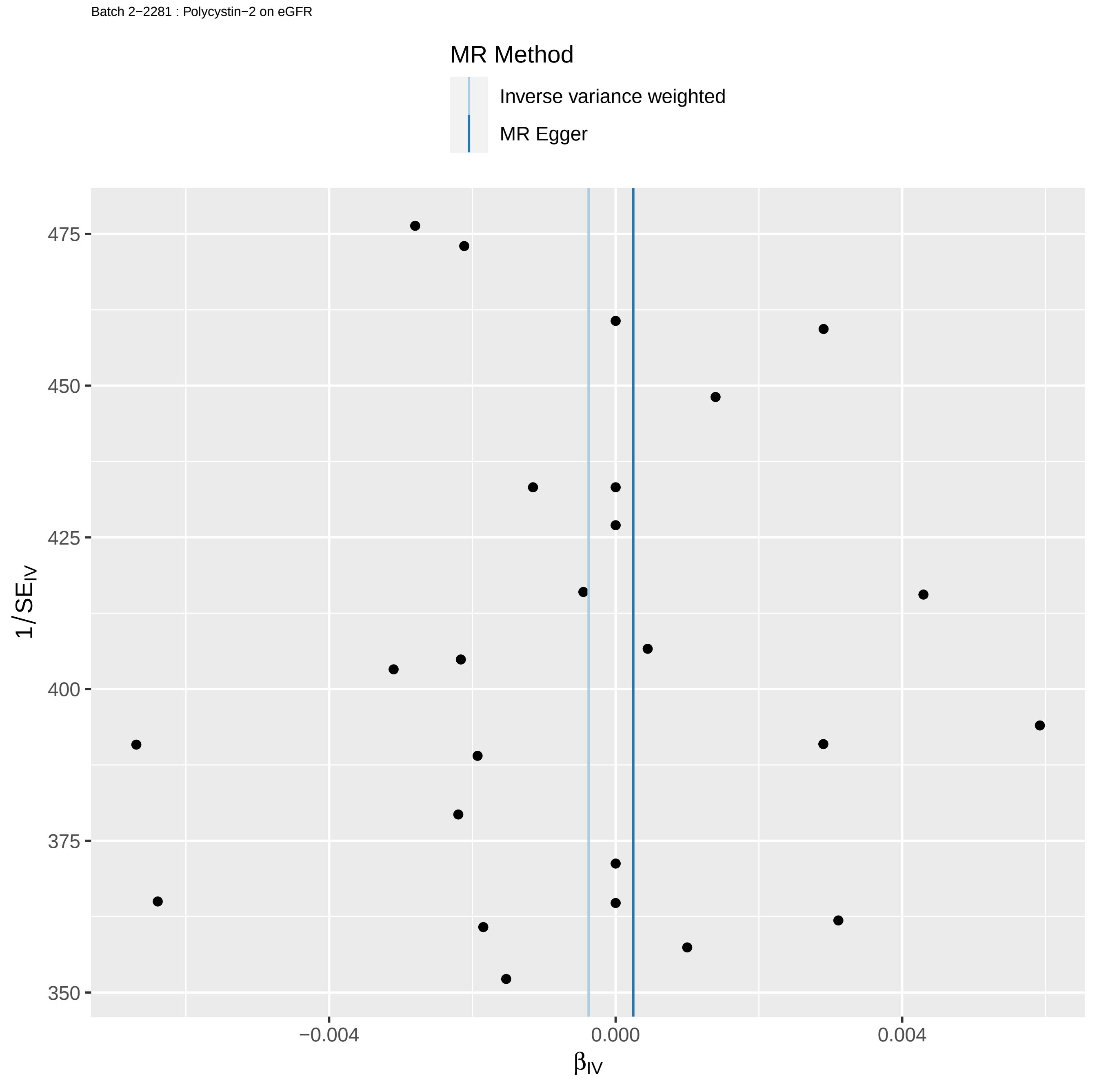

Batch 2−2281 : Polycystin−2 on eGFR
MR Method
Inverse variance weighted
MR Egger
1/SE_IV
475
450
425
400
375
350
−0.004
0.000
0.004
β_IV

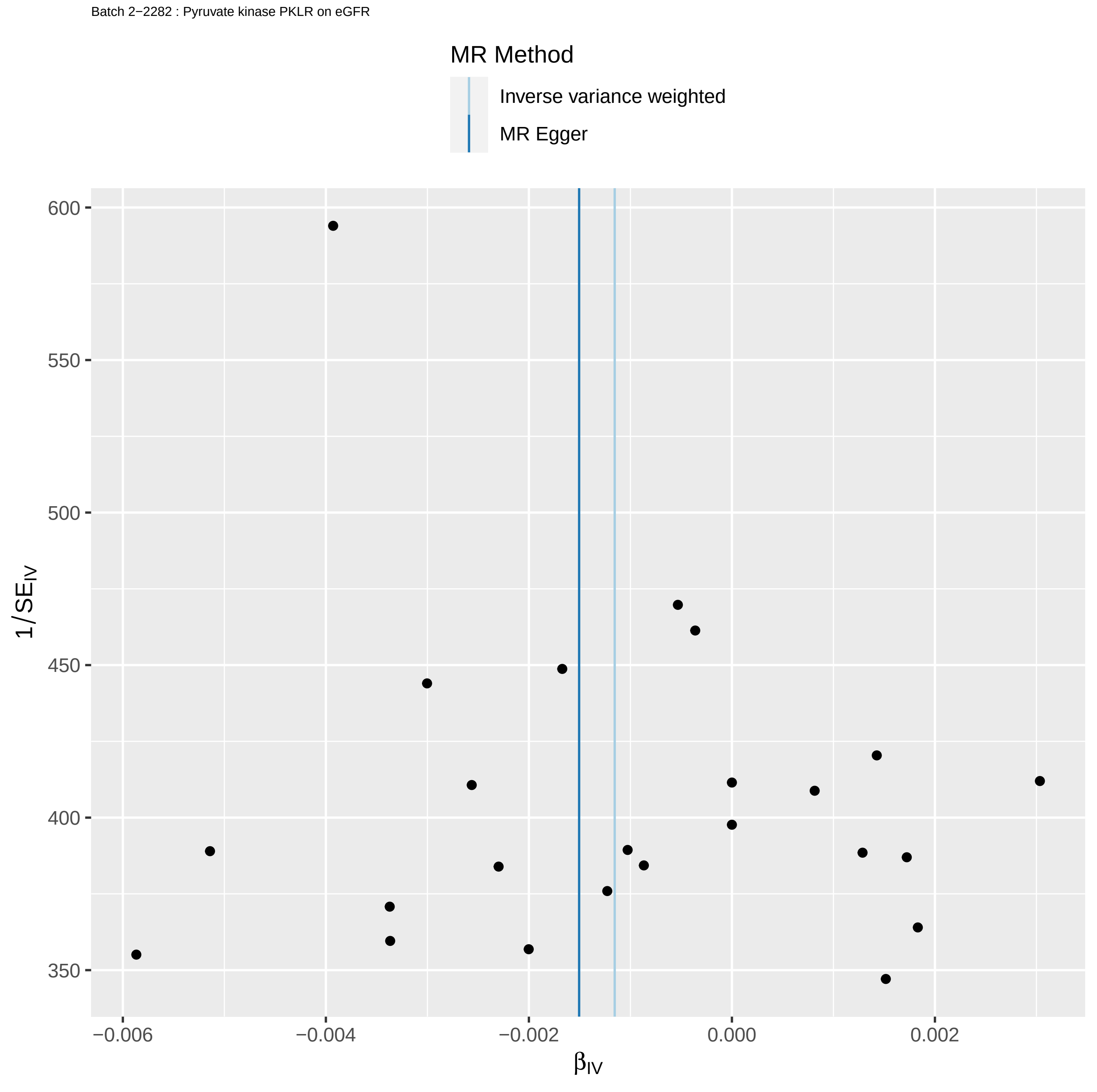

Batch 2−2282 : Pyruvate kinase PKLR on eGFR
MR Method
Inverse variance weighted
MR Egger
600
550
500
450
400
350
1/SE$_{IV}$
−0.006
−0.004
−0.002
0.000
0.002
β$_{IV}$

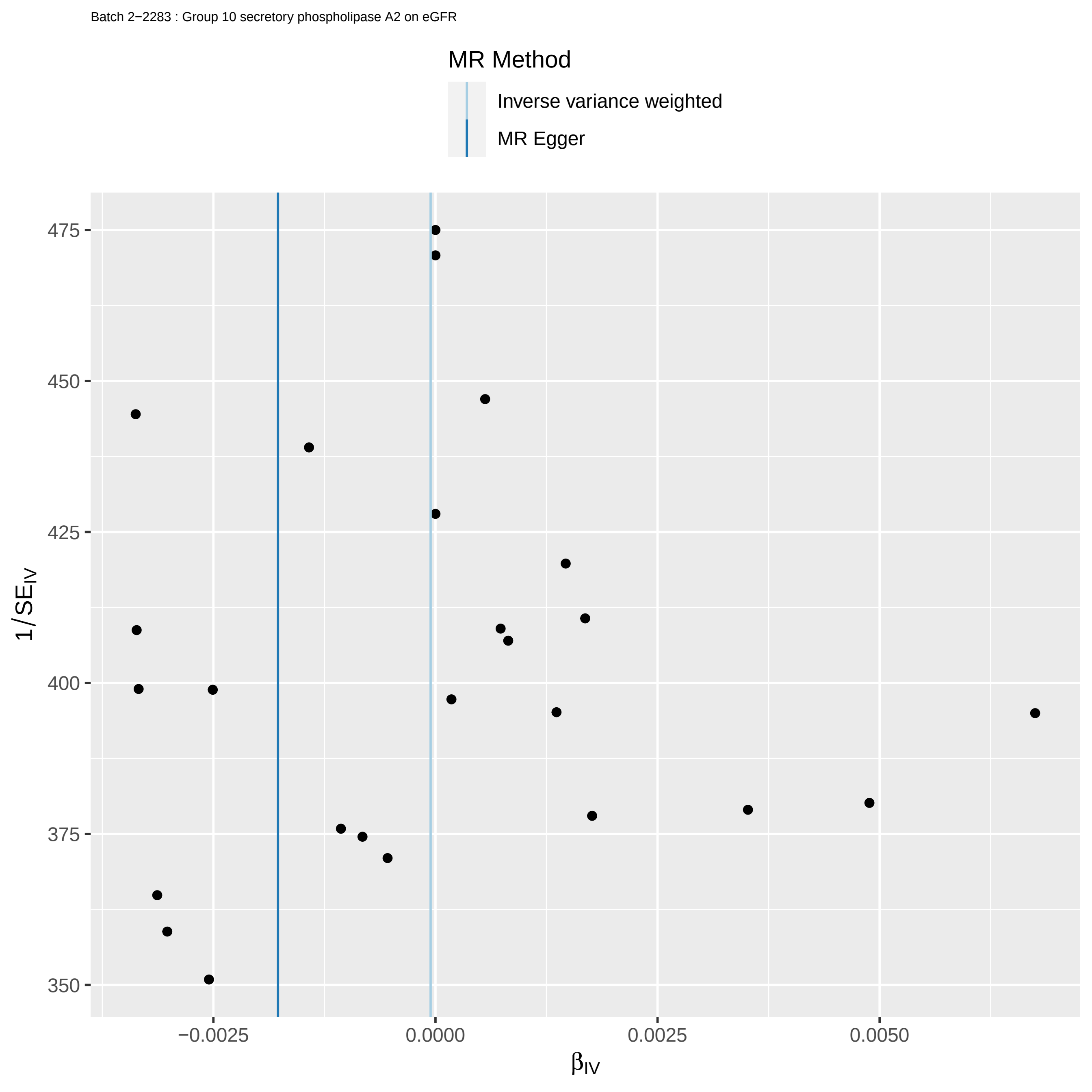

Batch 2−2283 : Group 10 secretory phospholipase A2 on eGFR
MR Method
Inverse variance weighted
MR Egger
475
450
425
400
375
350
1/SE_IV
−0.0025
0.0000
0.0025
0.0050
β_IV

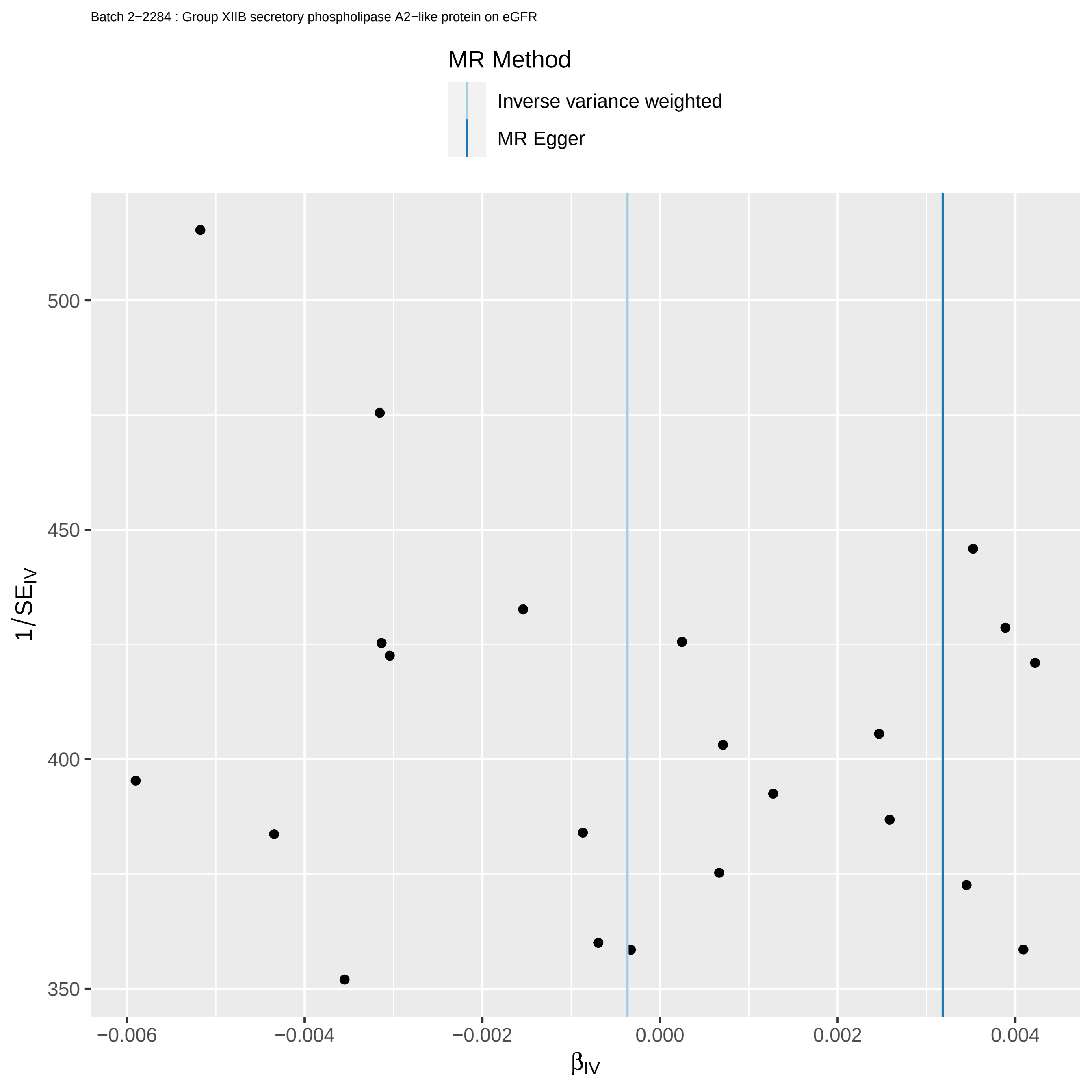

Batch 2−2284 : Group XIIB secretory phospholipase A2−like protein on eGFR
MR Method
Inverse variance weighted
MR Egger
500
450
400
350
1/SE_IV
−0.006
−0.004
−0.002
0.000
0.002
0.004
β_IV

Batch 2−2285 : HRAS−like suppressor 3 on eGFR

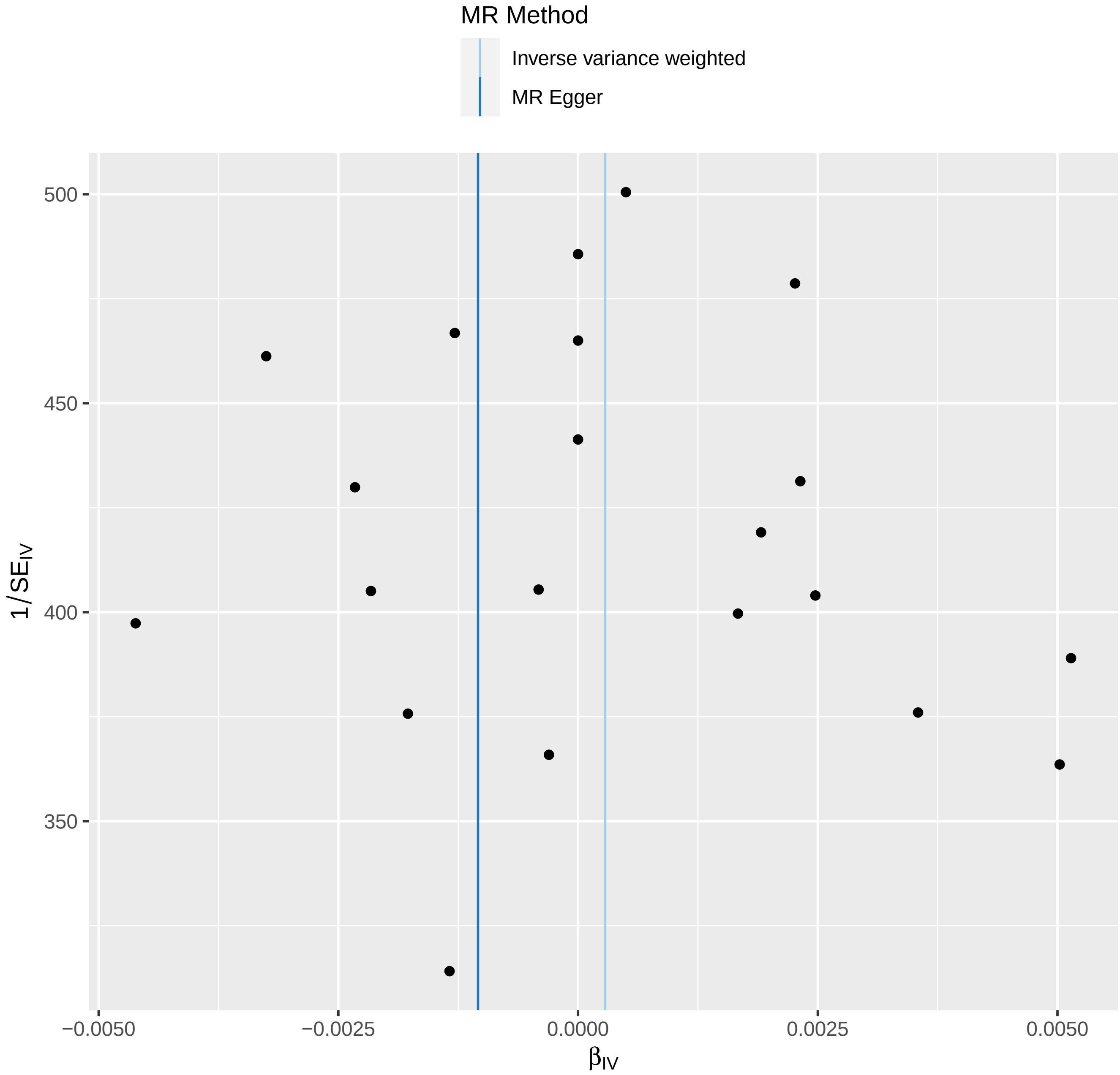

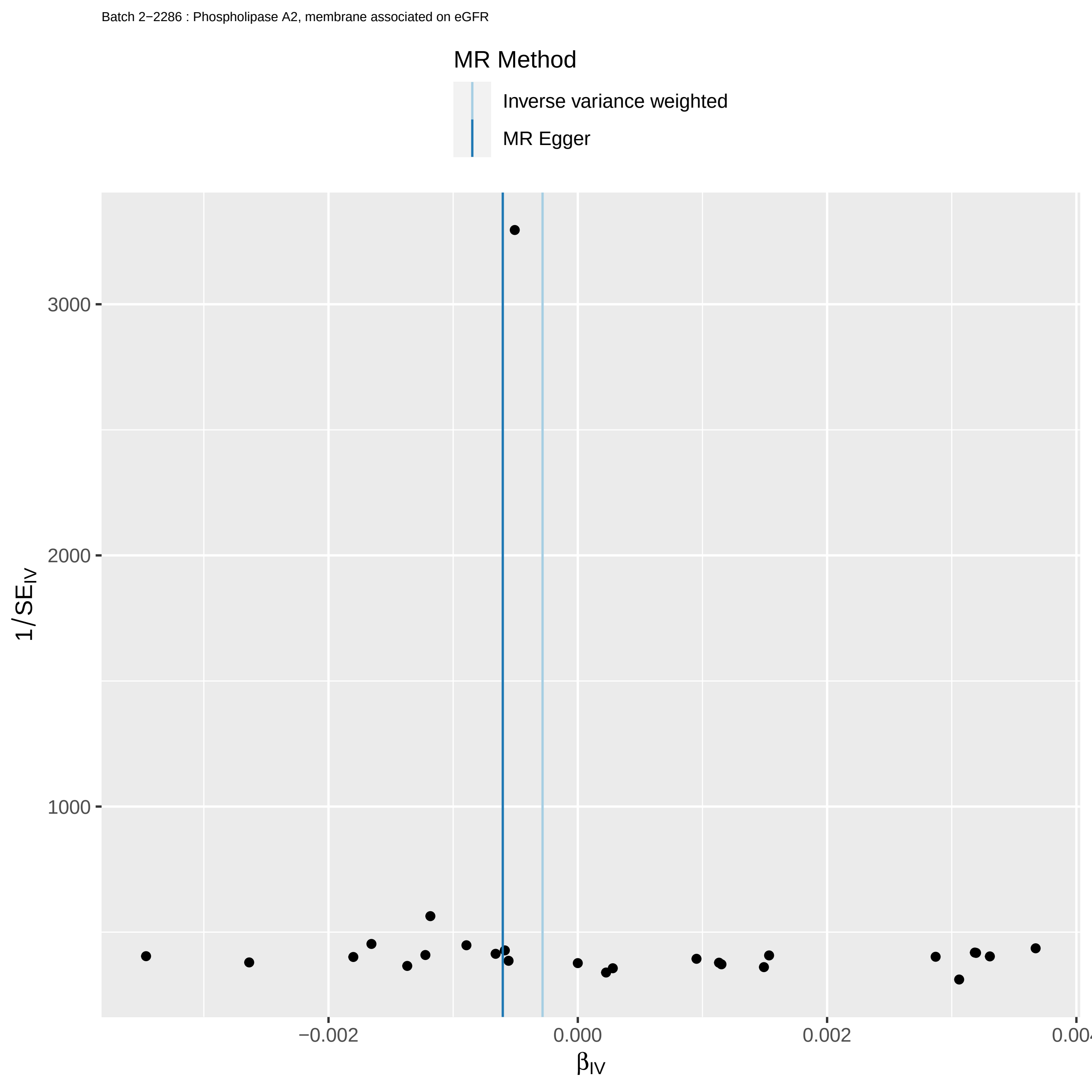

Batch 2−2286 : Phospholipase A2, membrane associated on eGFR
MR Method
Inverse variance weighted
MR Egger
3000
2000
1000
1/SE$_{IV}$
−0.002
0.000
0.002
β$_{IV}$

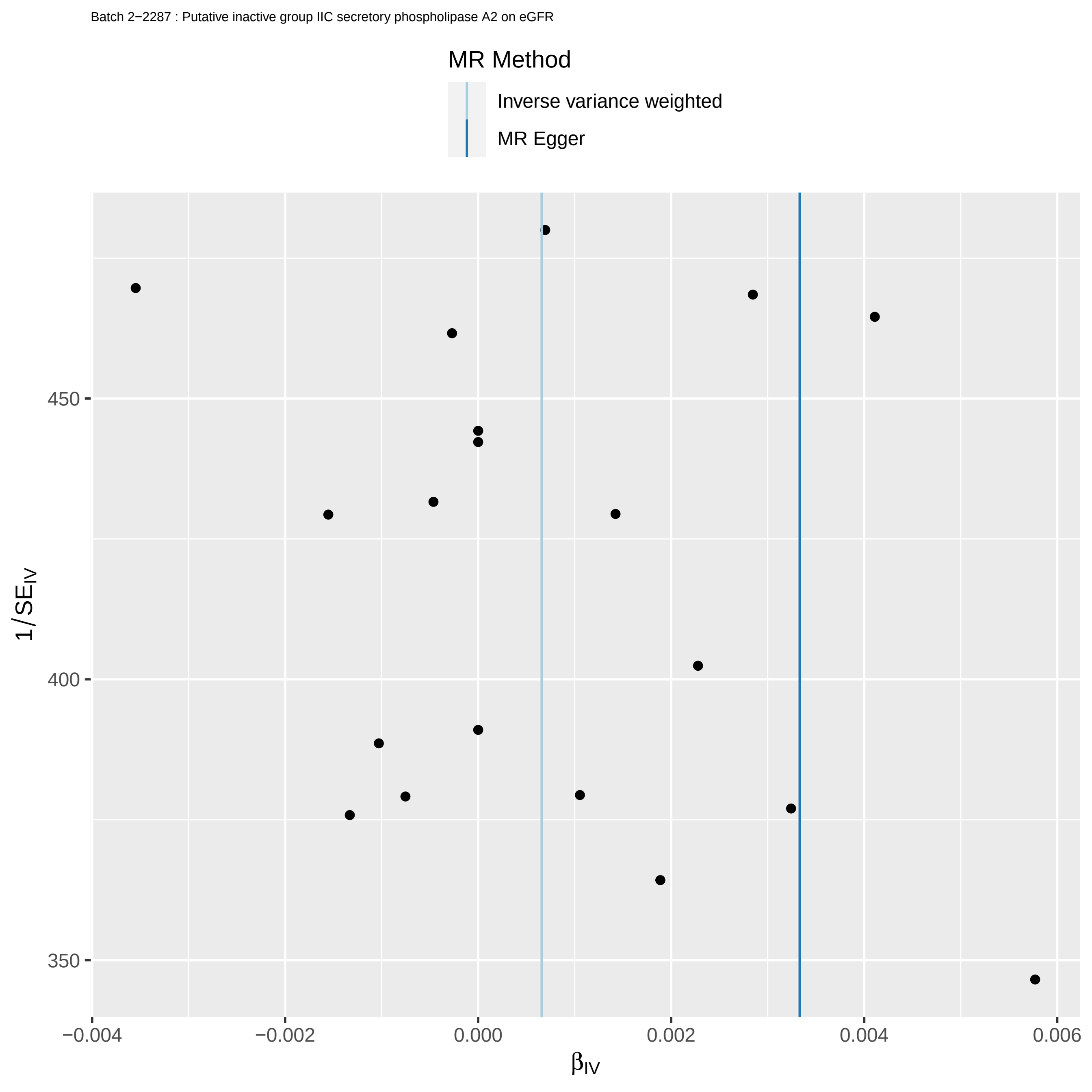

Batch 2−2287 : Putative inactive group IIC secretory phospholipase A2 on eGFR
MR Method
Inverse variance weighted
MR Egger
1/SE_IV
450
400
350
−0.004
−0.002
0.000
0.002
0.004
0.006
β_IV

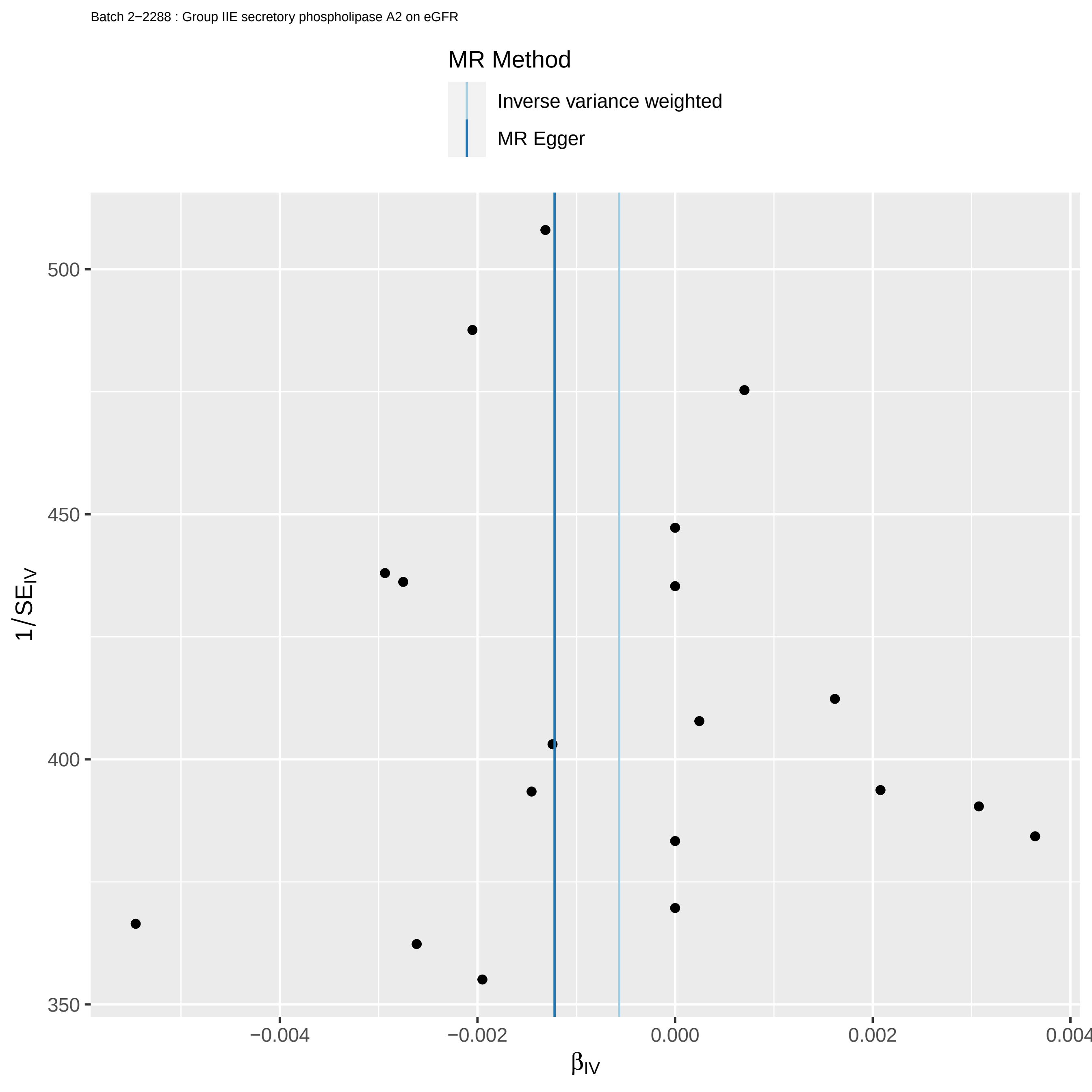
Batch 2−2288 : Group IIE secretory phospholipase A2 on eGFR
MR Method
Inverse variance weighted
MR Egger
500
450
400
350
1/SE_IV
−0.004
−0.002
0.000
0.002
0.004
β_IV

Batch 2−2289 : Group IIE secretory phospholipase A2 on eGFR

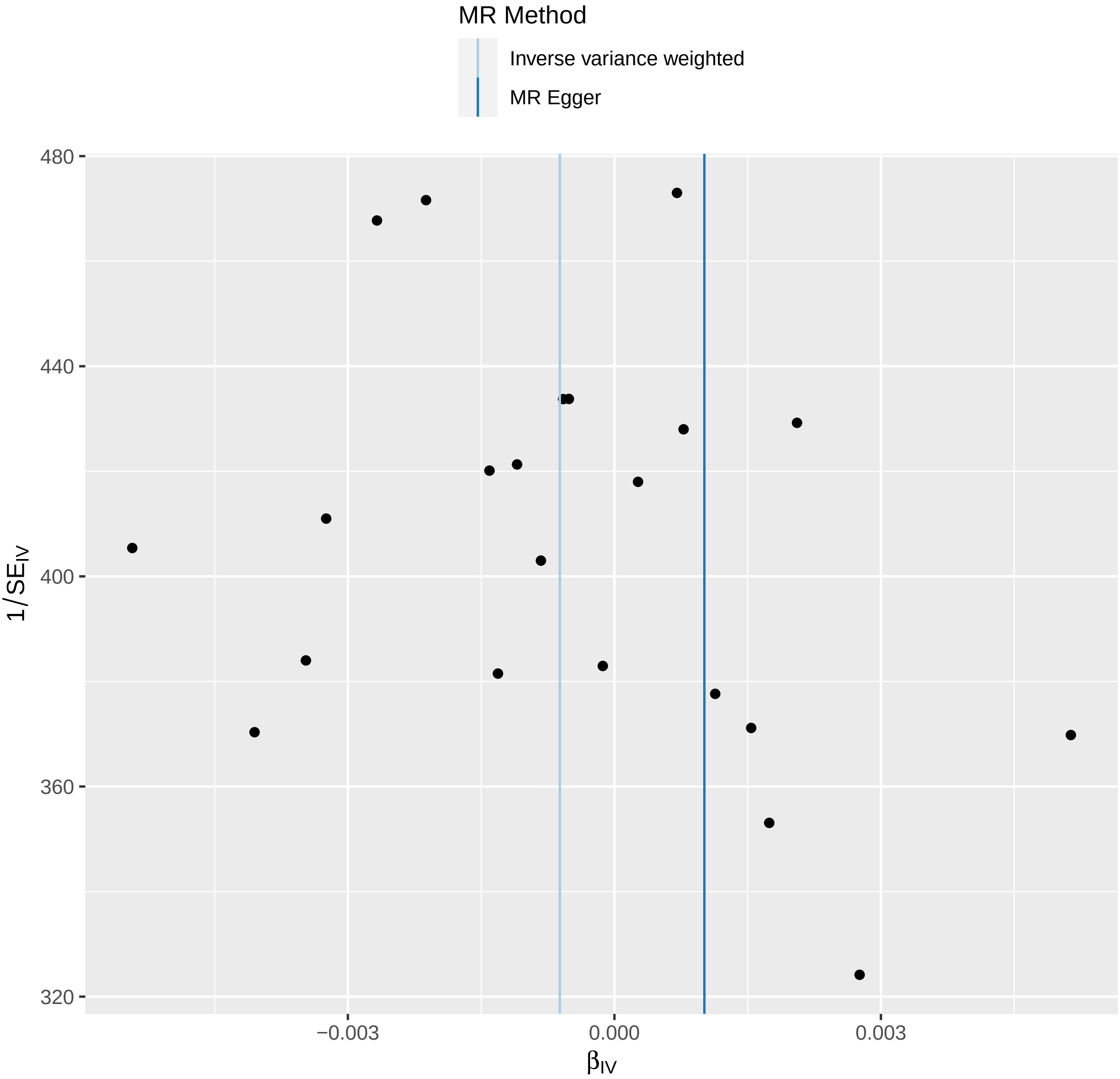

Batch 2−2290 : Calcium−dependent phospholipase A2 on eGFR

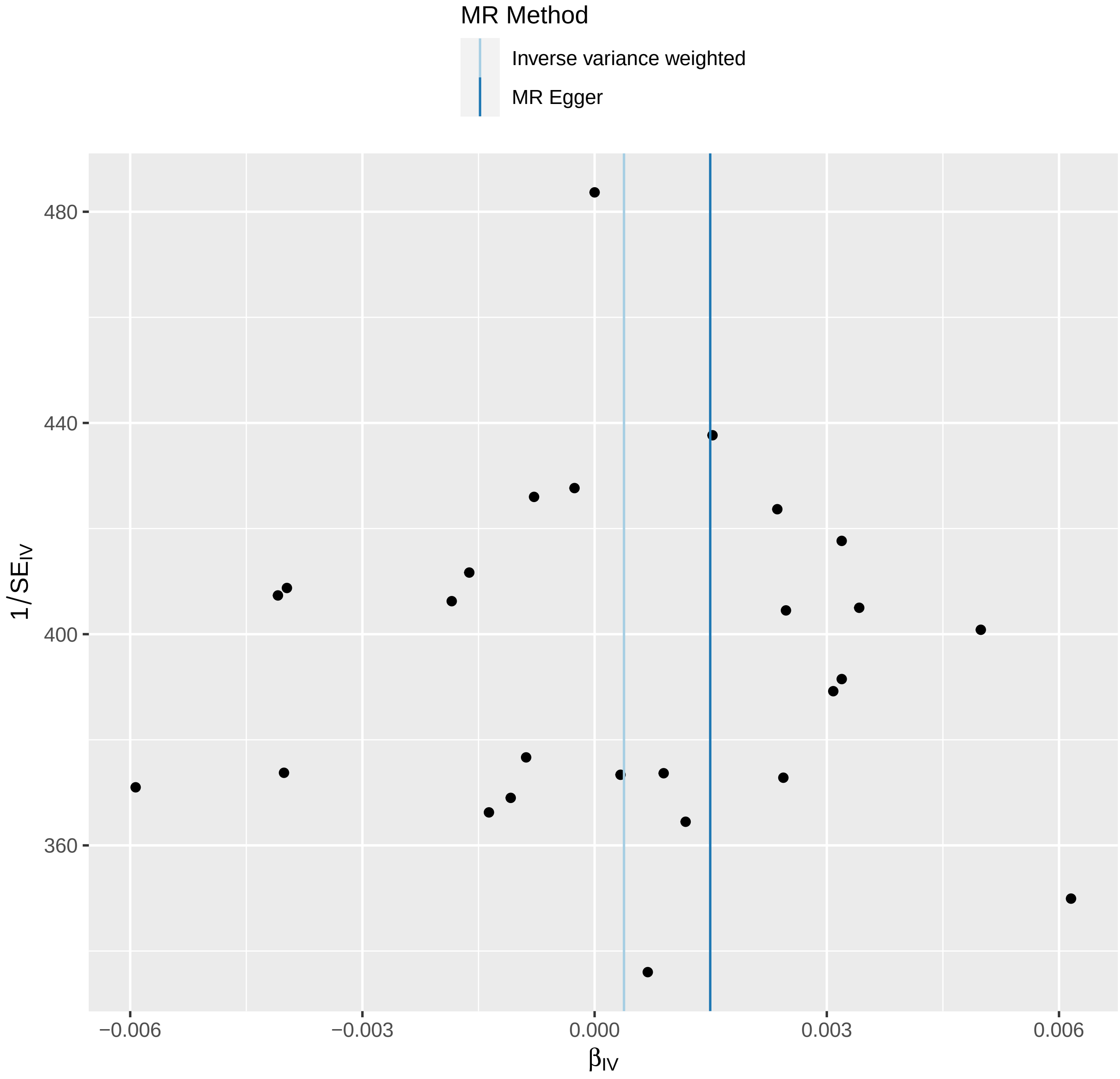

Batch 2−2291 : Secretory phospholipase A2 receptor on eGFR

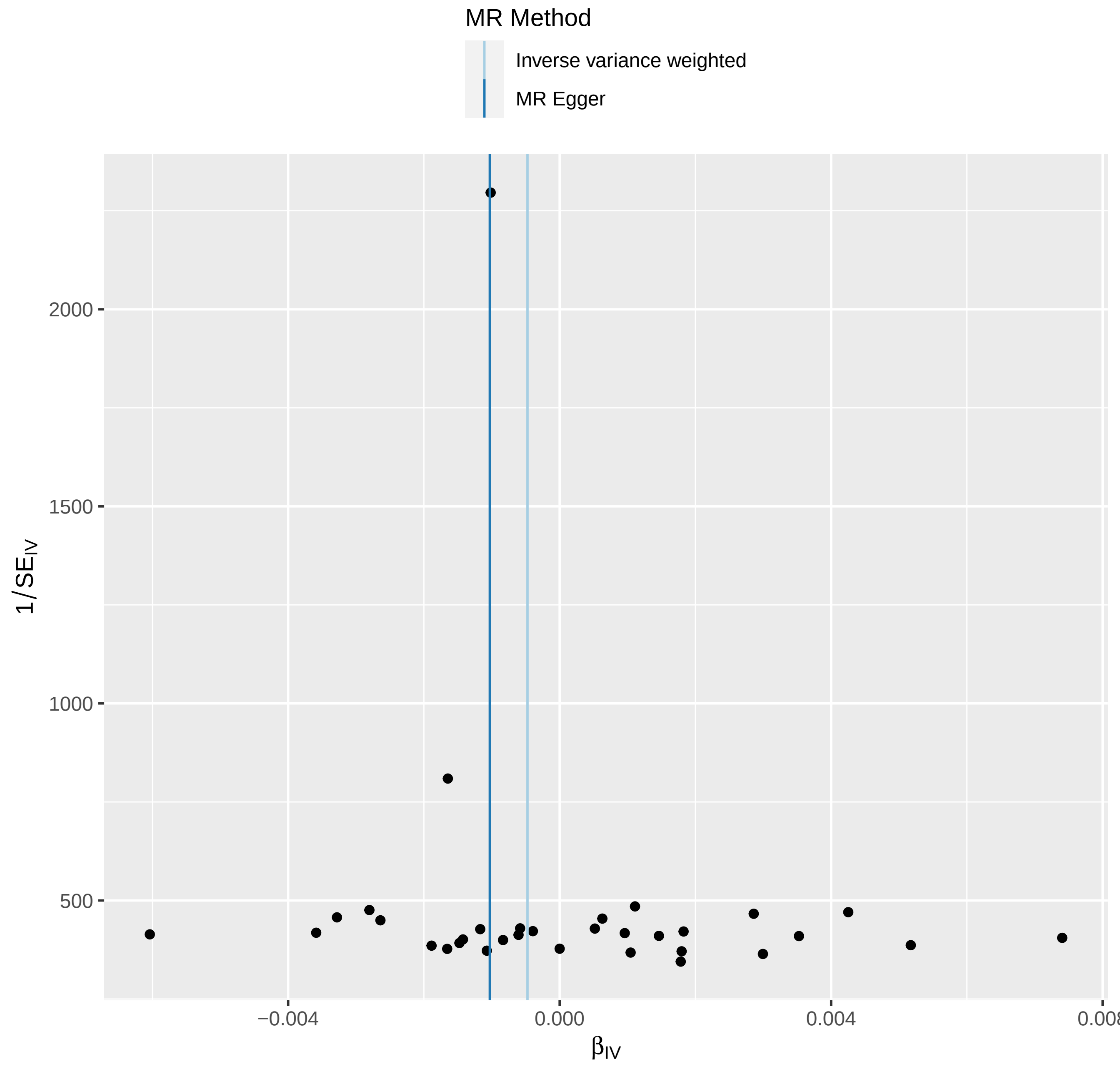

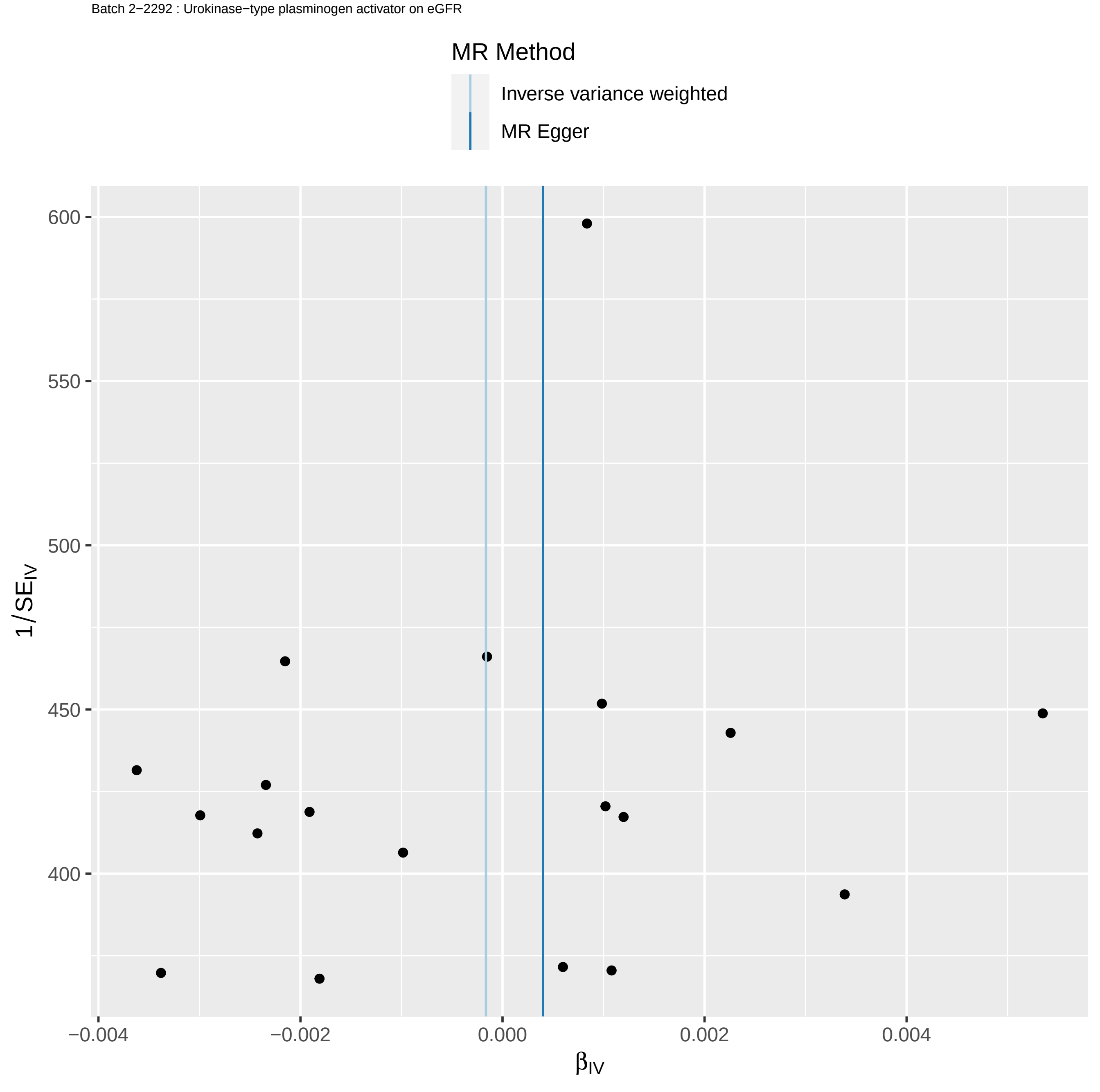

Batch 2−2292 : Urokinase−type plasminogen activator on eGFR
MR Method
Inverse variance weighted
MR Egger
600
550
500
450
400
1/SE_IV
−0.004
−0.002
0.000
0.002
0.004
β_IV

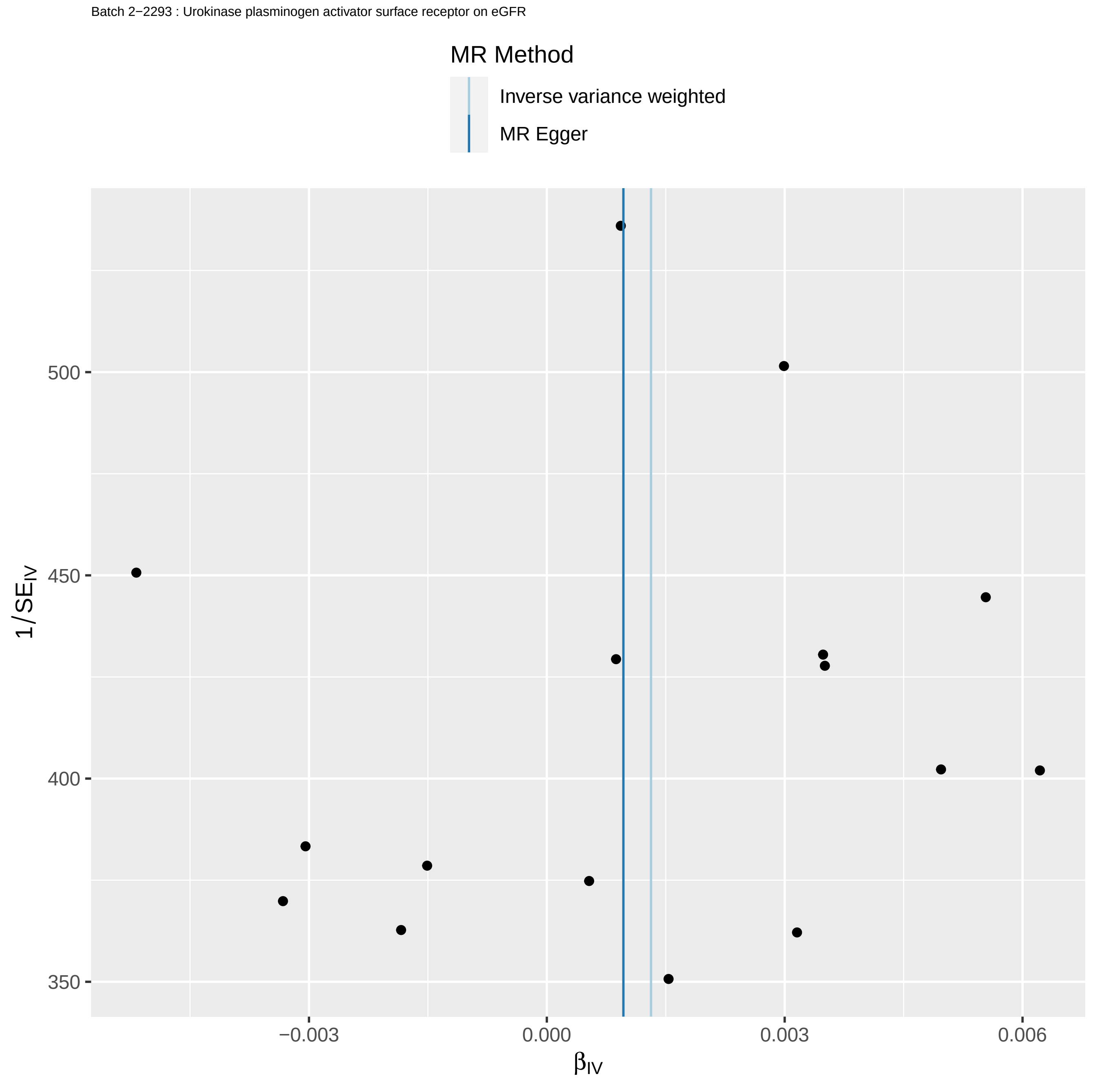
Batch 2−2293 : Urokinase plasminogen activator surface receptor on eGFR
MR Method
Inverse variance weighted
MR Egger
500
450
400
350
1/SE$_{IV}$
−0.003
0.000
0.003
0.006
β_{IV}

Batch 2−2294 : Phospholipase B−like 1 on eGFR

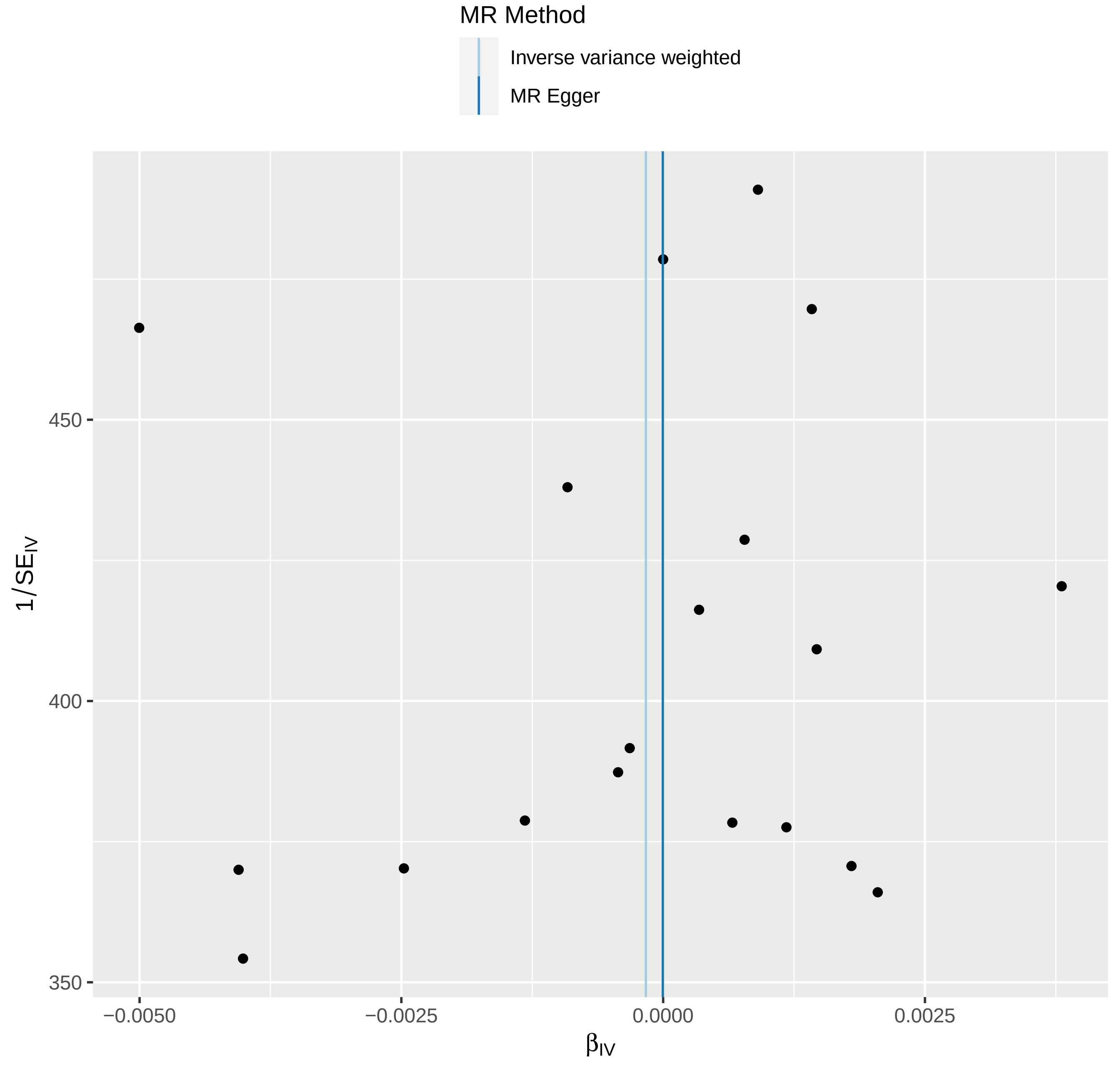

Batch 2−2295 : Putative phospholipase B−like 2 on eGFR

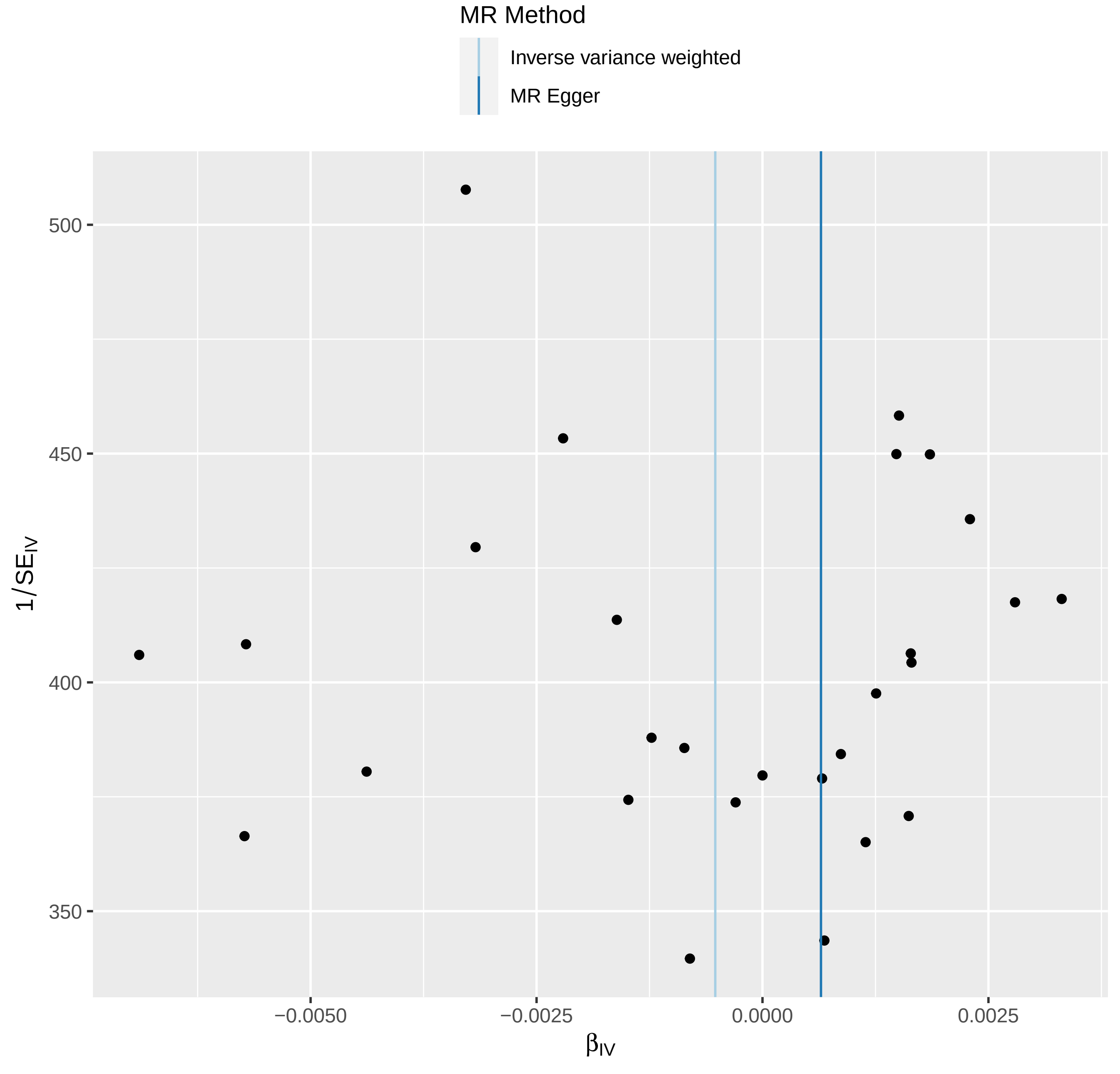

Batch 2−2296 : 1−phosphatidylinositol 4,5−bisphosphate phosphodiesterase gamma−1 on eGFR

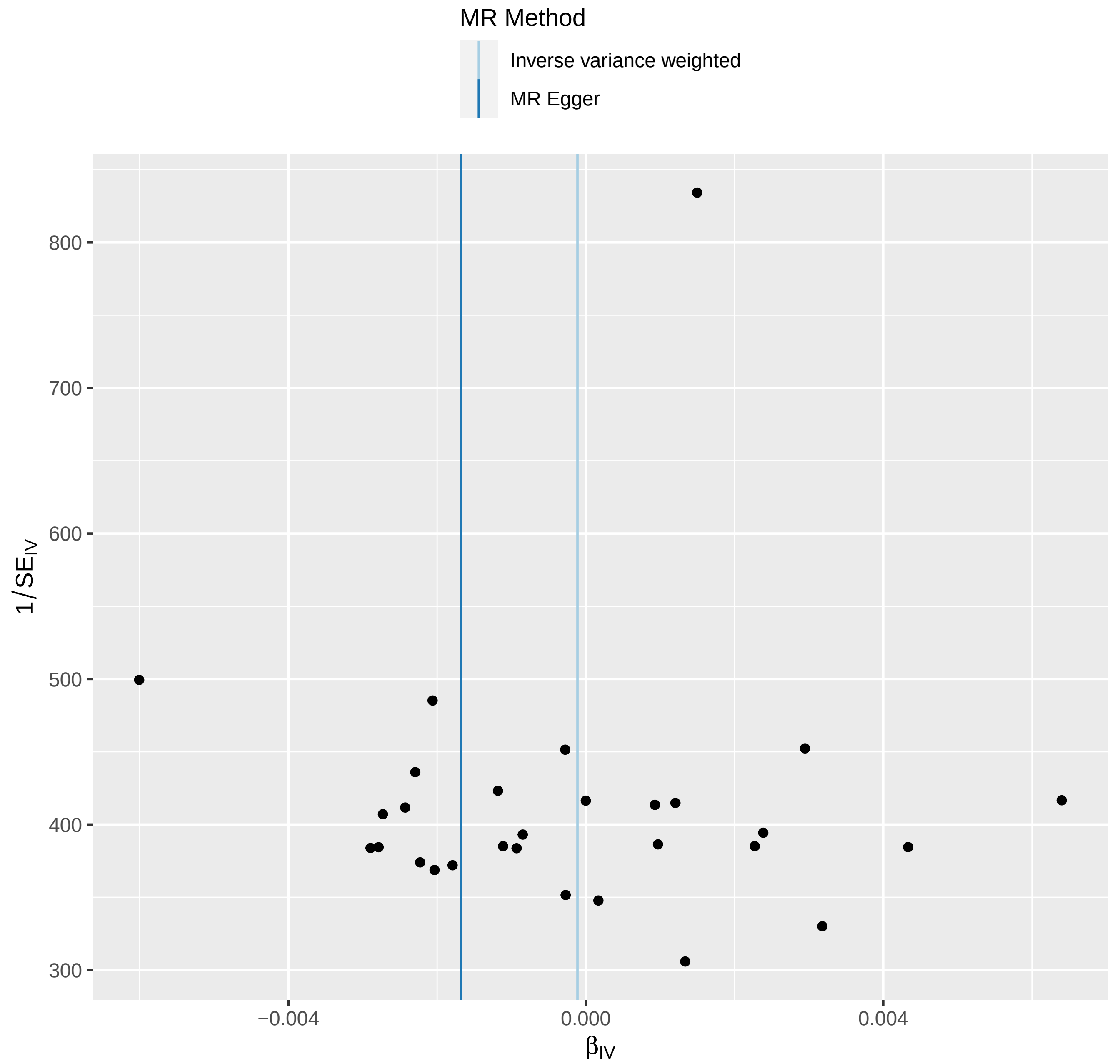

Batch 2−2297 : Phospholipase D3 on eGFR

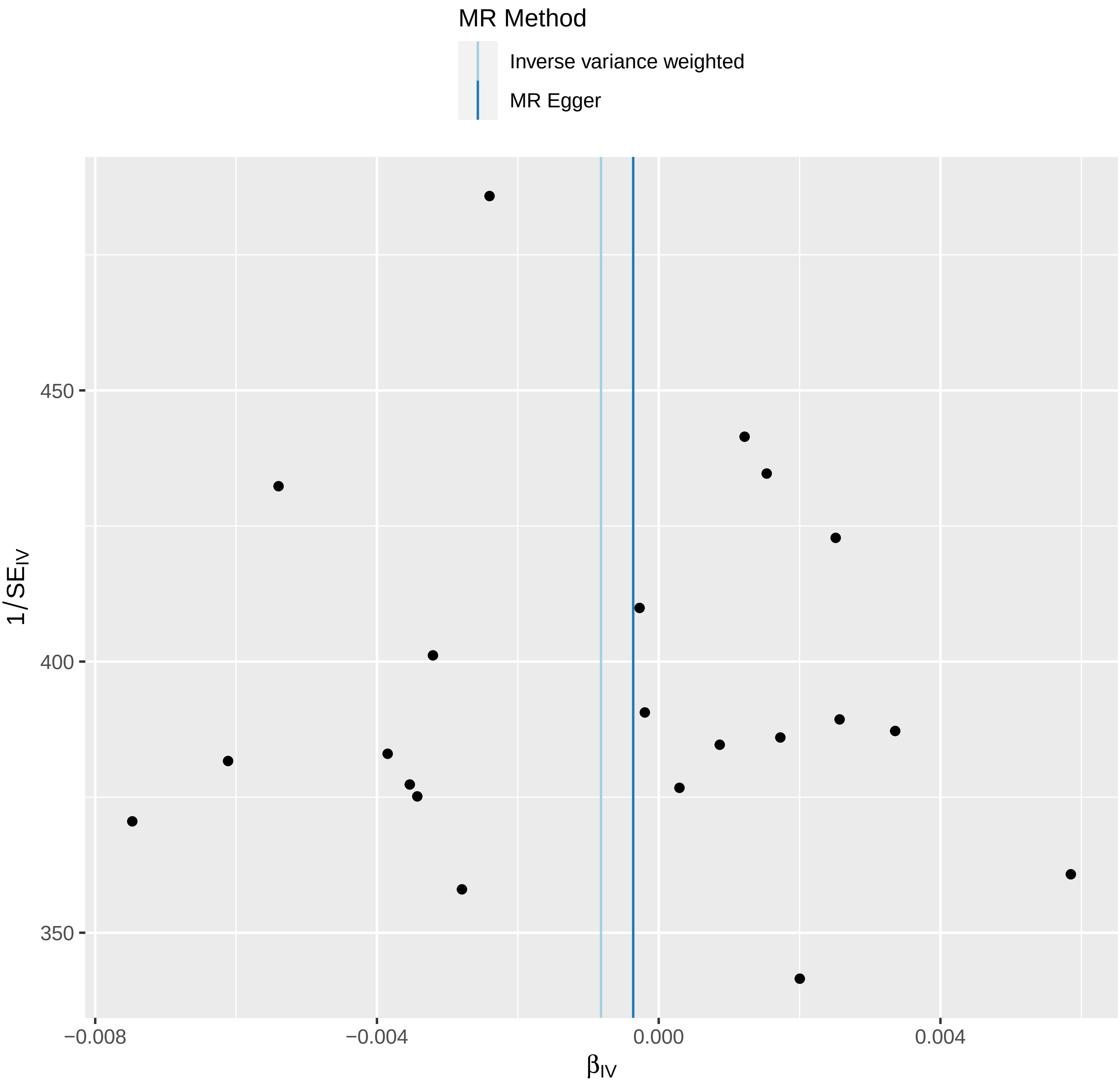

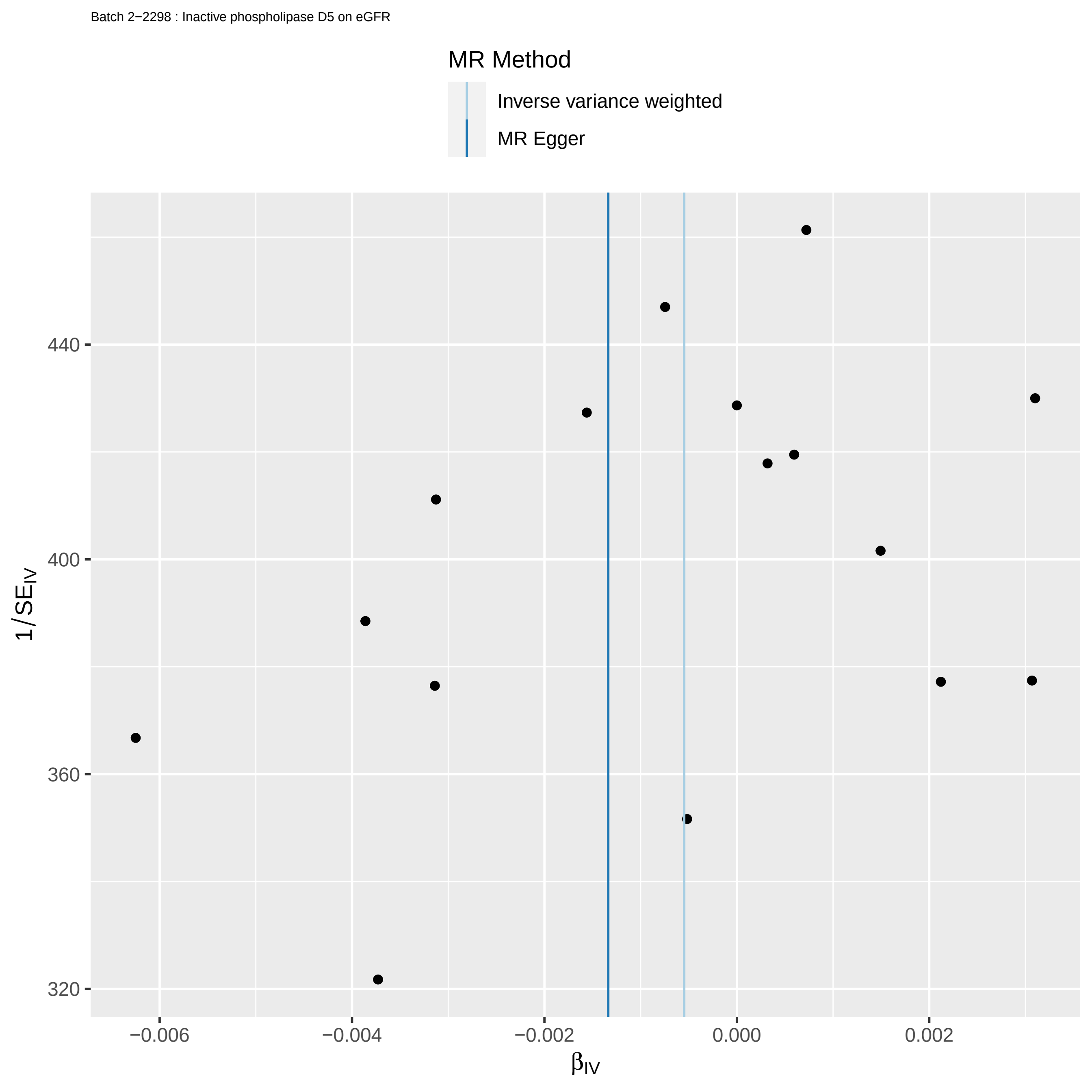

Batch 2−2298 : Inactive phospholipase D5 on eGFR
MR Method
Inverse variance weighted
MR Egger
440
400
360
320
1/SE_IV
−0.006
−0.004
−0.002
0.000
0.002
β_IV

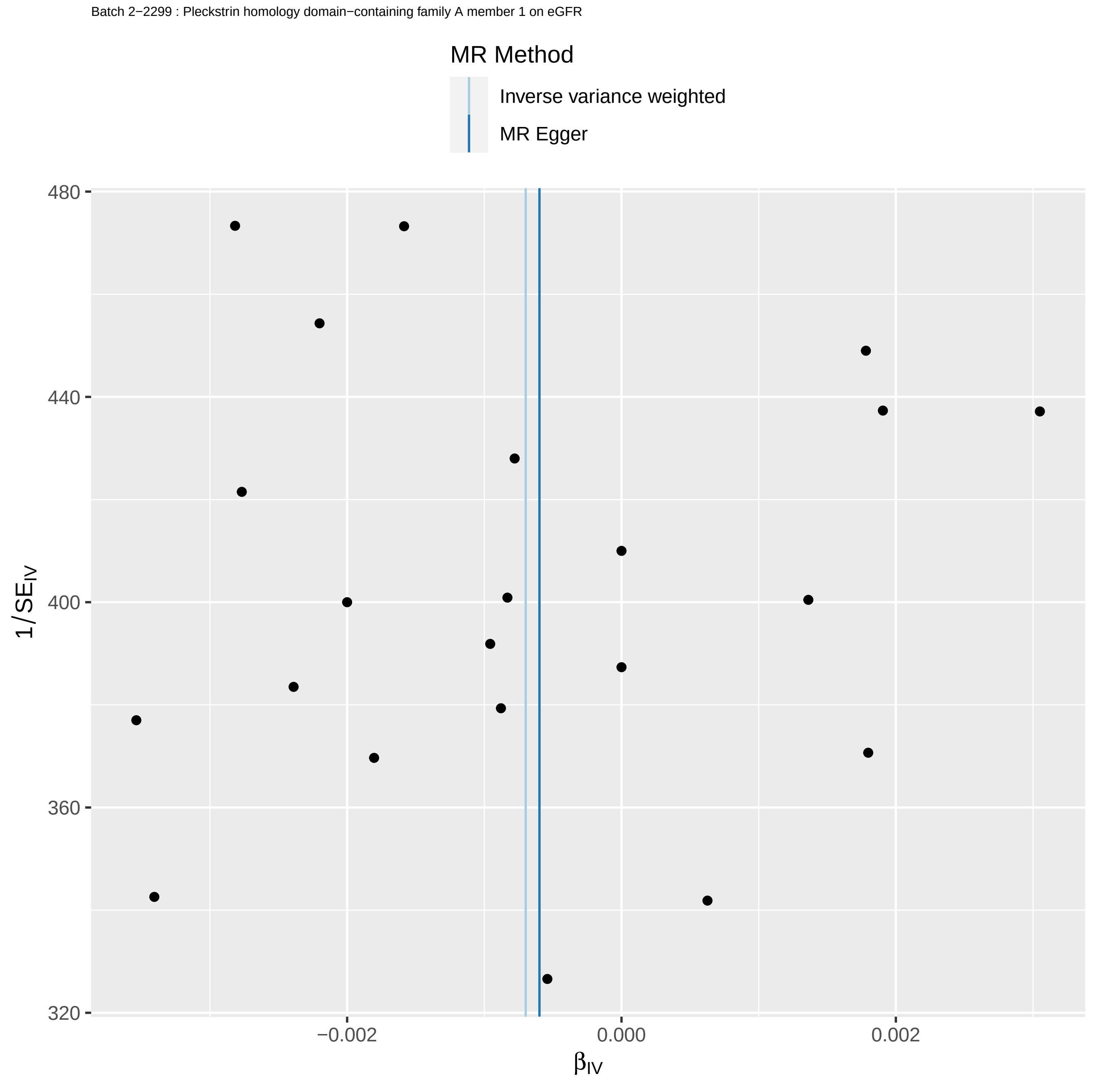

Batch 2−2299 : Pleckstrin homology domain−containing family A member 1 on eGFR
MR Method
Inverse variance weighted
MR Egger
480
440
400
360
320
1/SE$_{IV}$
−0.002
0.000
0.002
β$_{IV}$

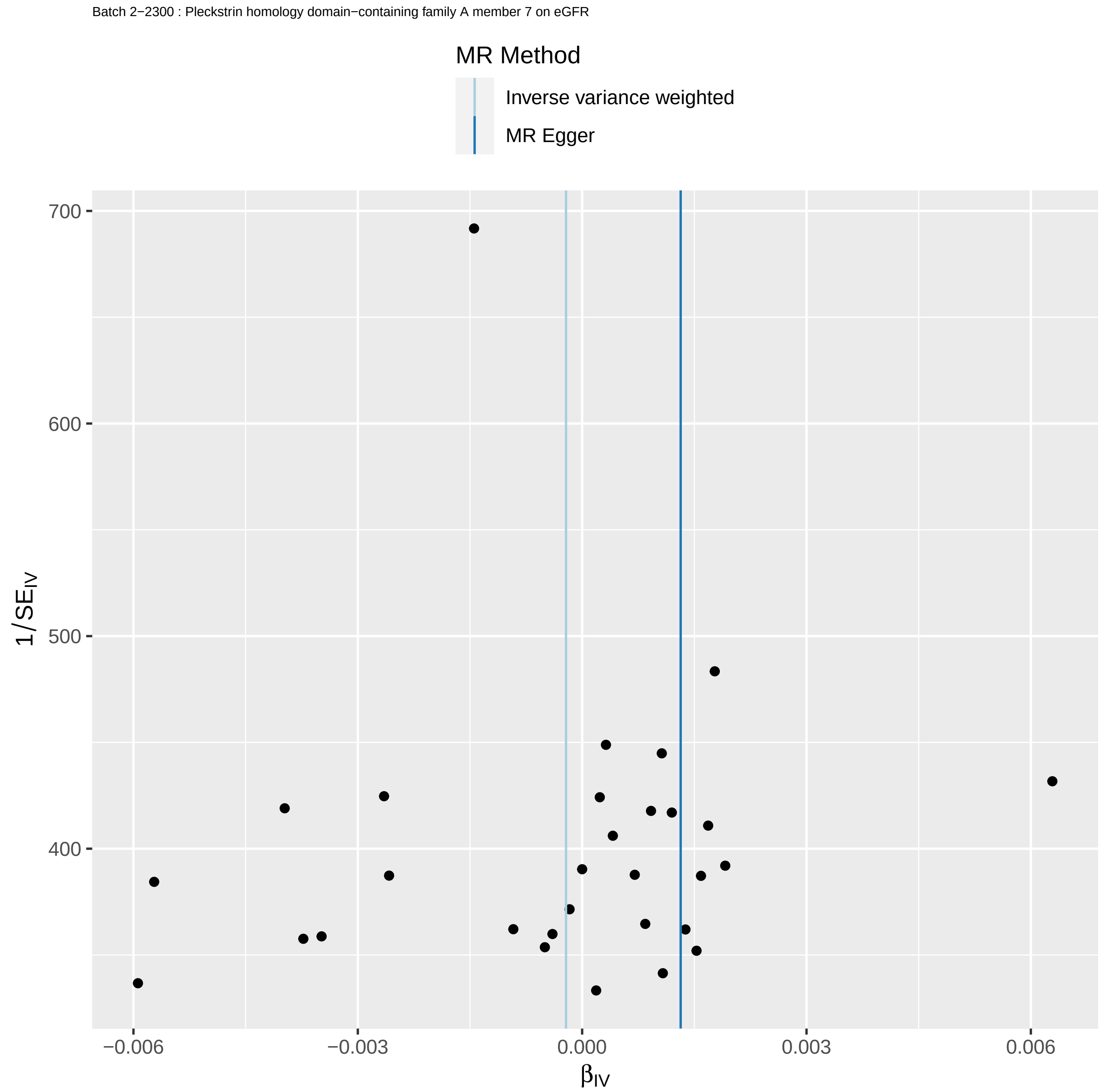
Batch 2−2300 : Pleckstrin homology domain−containing family A member 7 on eGFR
MR Method
Inverse variance weighted
MR Egger
700
600
500
400
1/SE_IV
−0.006
−0.003
0.000
0.003
0.006
β_IV

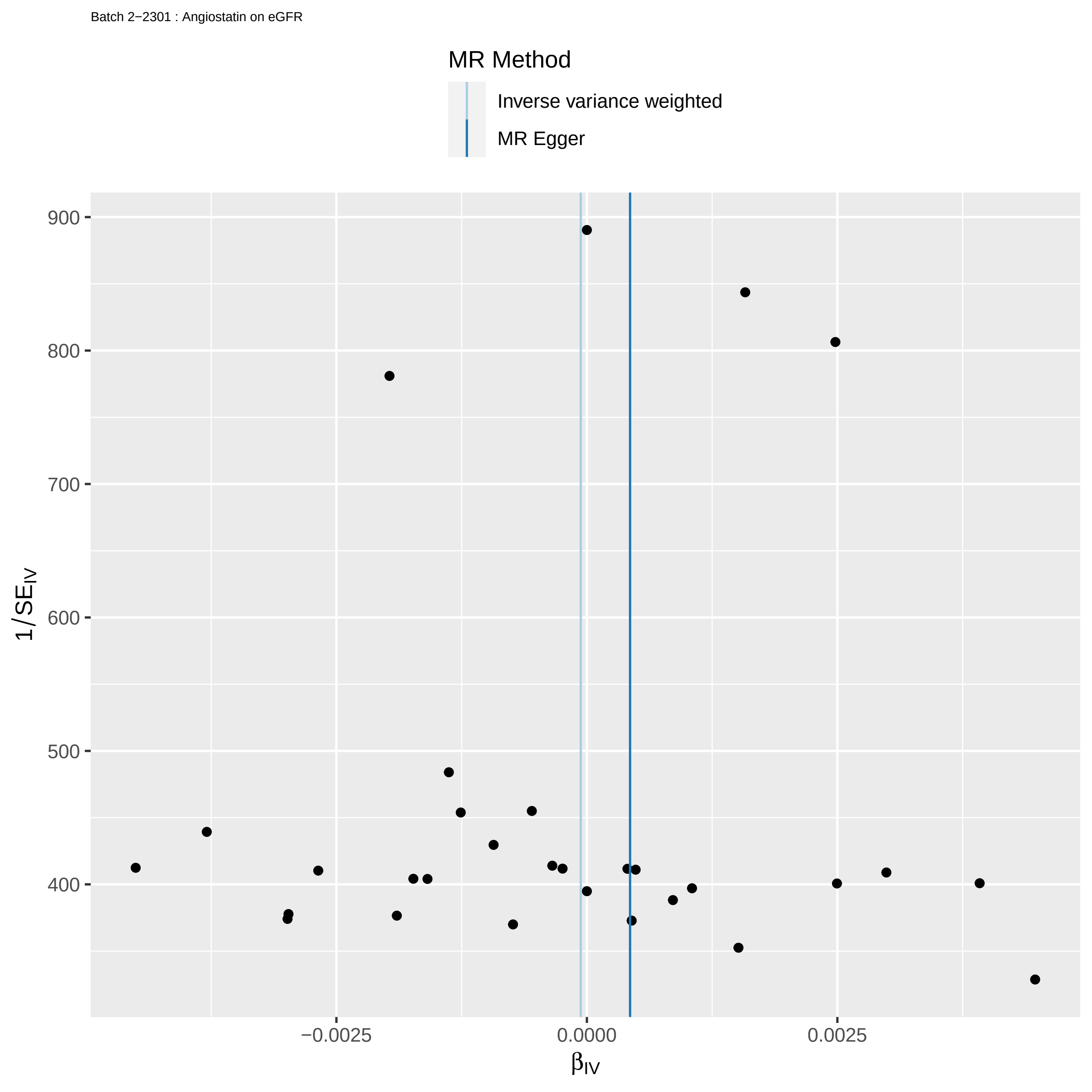

Batch 2−2301 : Angiostatin on eGFR
MR Method
Inverse variance weighted
MR Egger
900
800
700
600
500
400
1/SE_IV
−0.0025
0.0000
0.0025
β_IV

Batch 2−2302 : Plasmin on eGFR

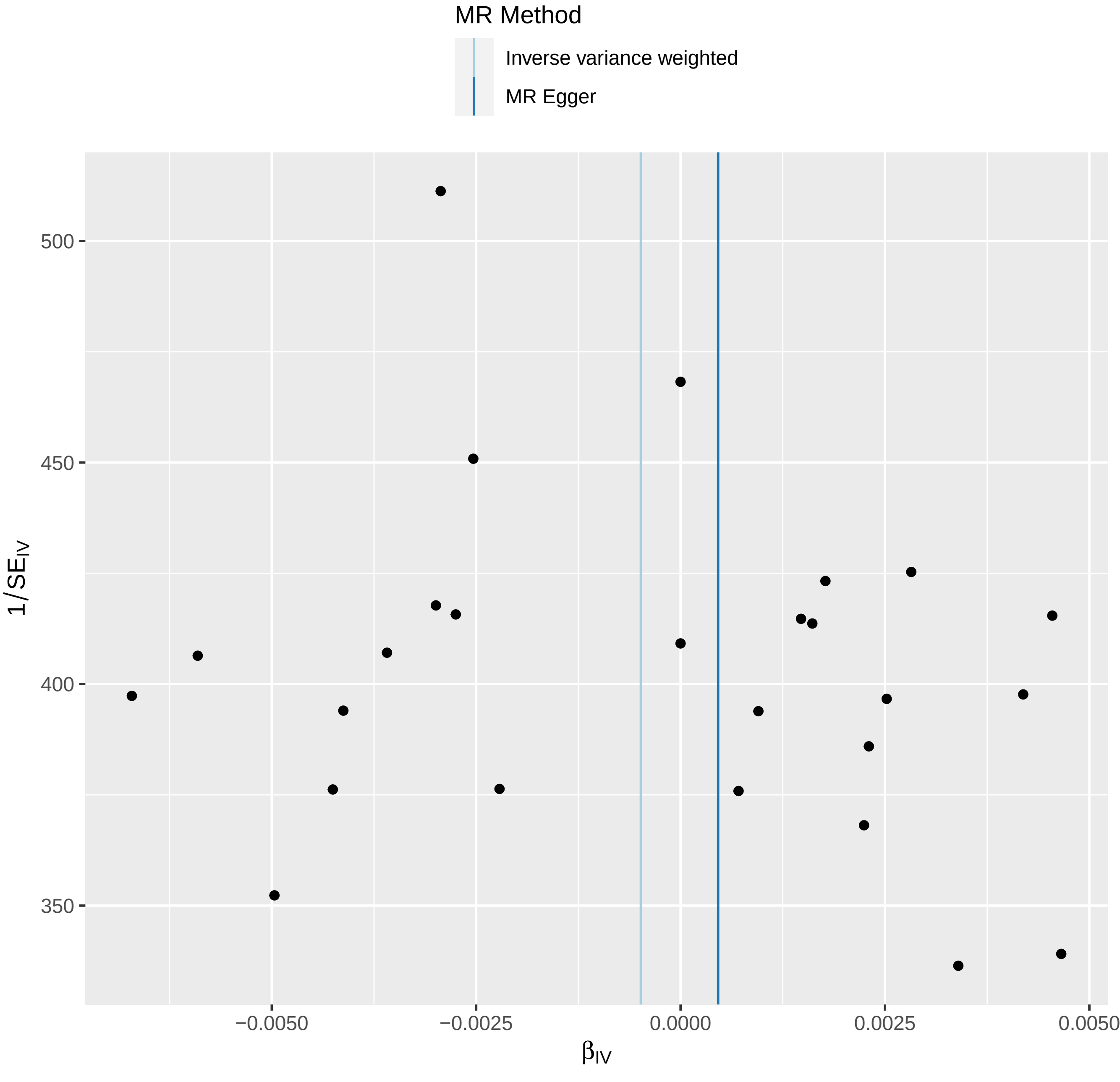

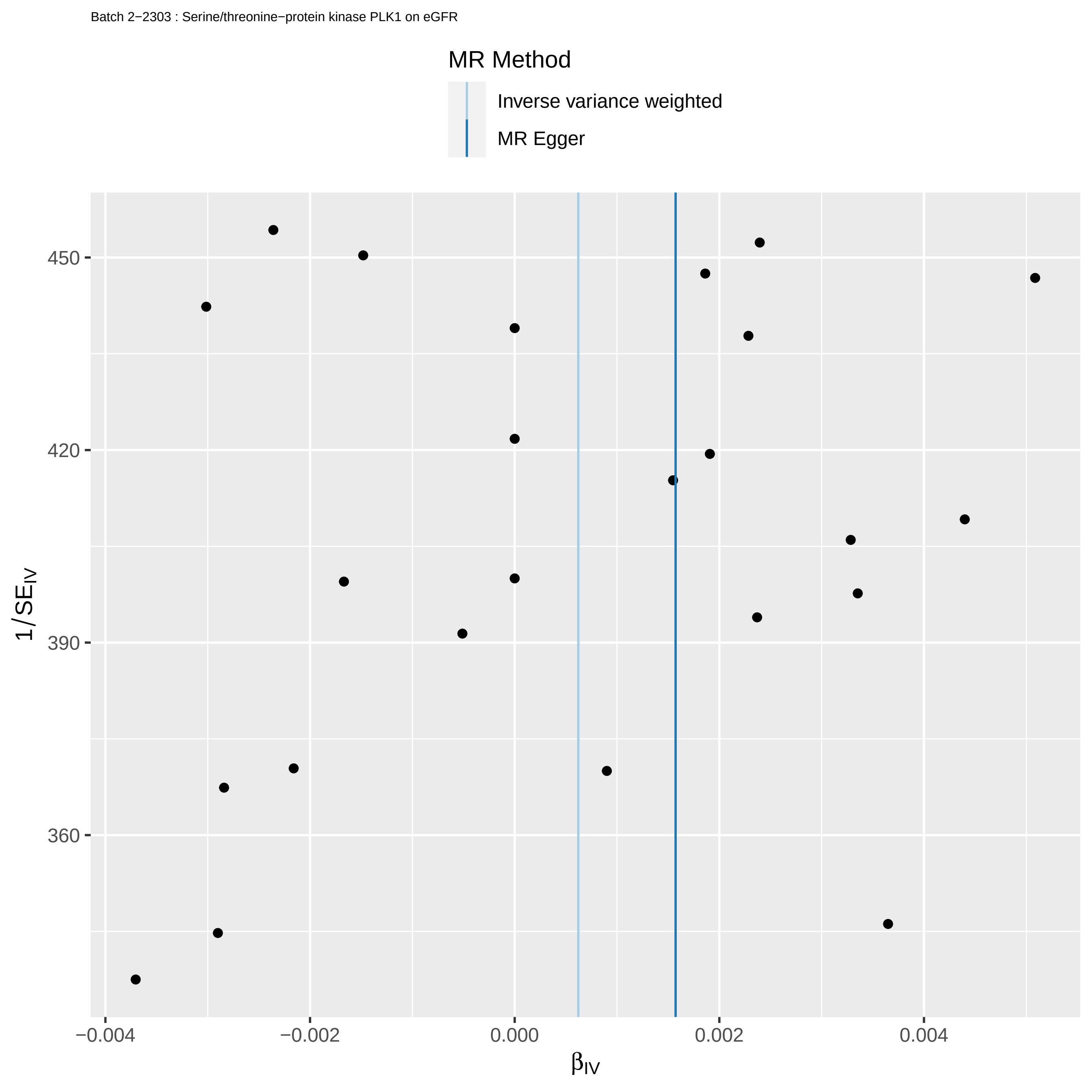

Batch 2−2303 : Serine/threonine−protein kinase PLK1 on eGFR
MR Method
Inverse variance weighted
MR Egger
450
420
390
360
1/SE_IV
−0.004
−0.002
0.000
0.002
0.004
β_IV

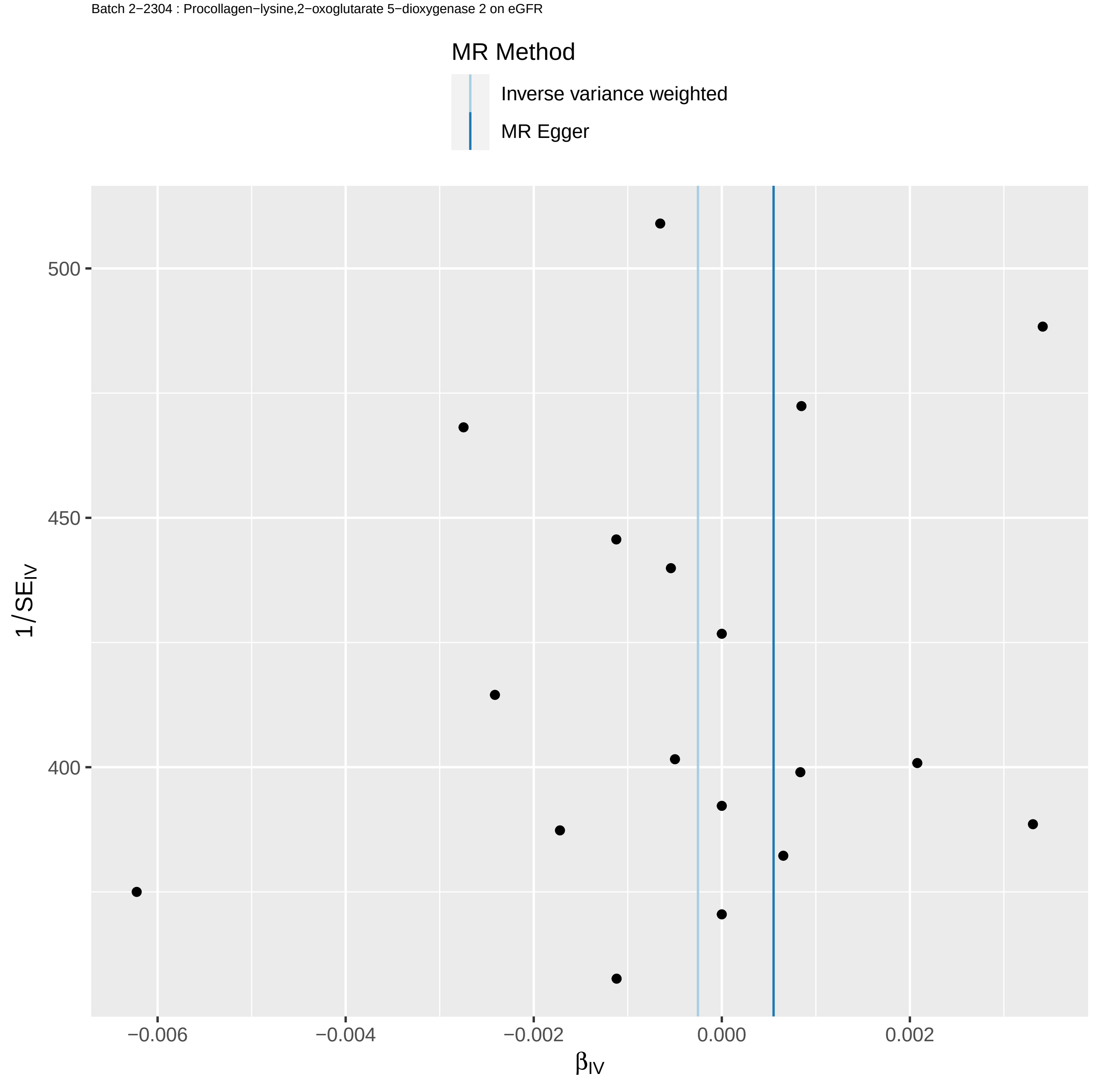

Batch 2−2304 : Procollagen−lysine,2−oxoglutarate 5−dioxygenase 2 on eGFR
MR Method
Inverse variance weighted
MR Egger
500
450
400
$1/SE_{IV}$
−0.006
−0.004
−0.002
0.000
0.002
β_{IV}

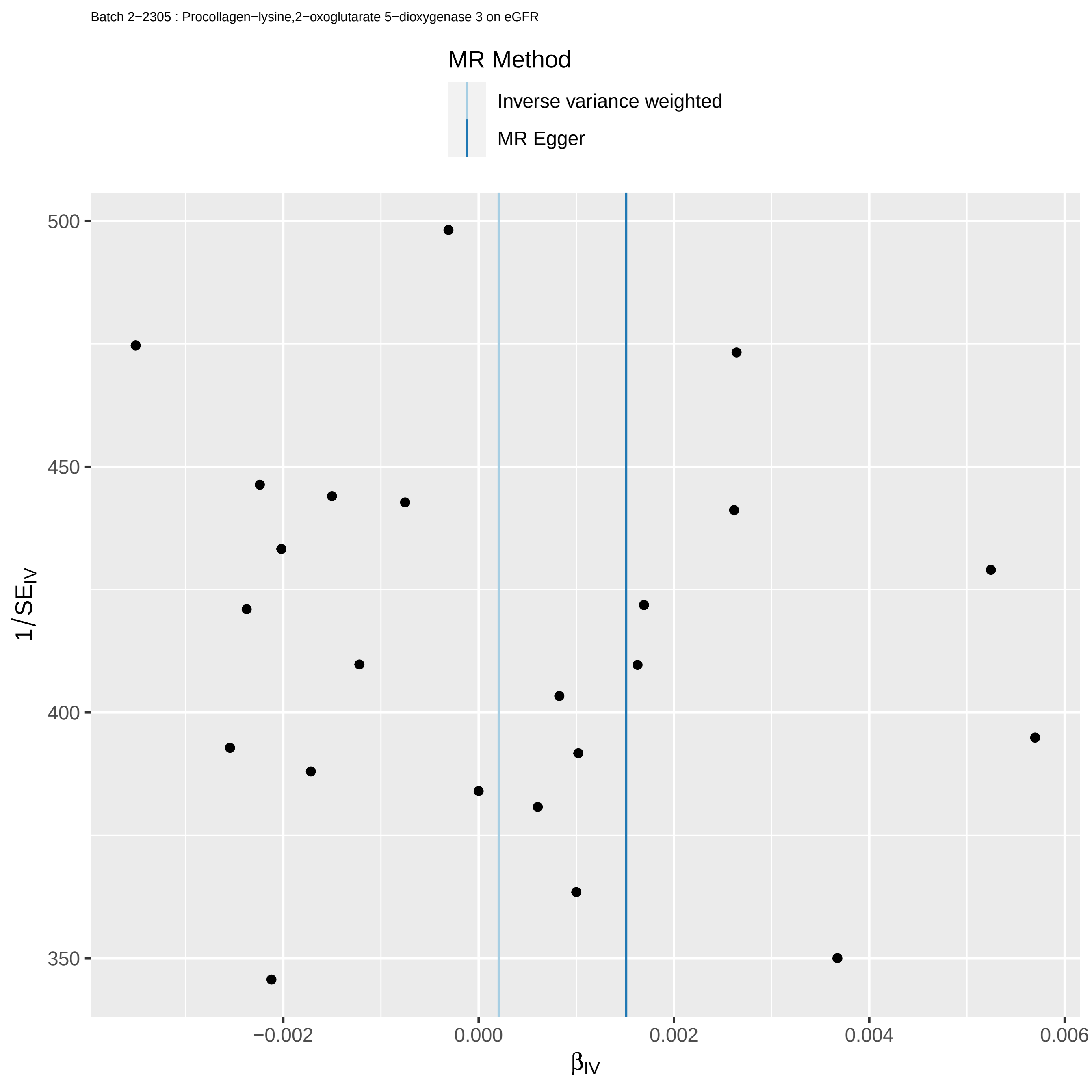

Batch 2−2305 : Procollagen−lysine,2−oxoglutarate 5−dioxygenase 3 on eGFR
MR Method
Inverse variance weighted
MR Egger
500
450
400
350
1/SE_IV
−0.002
0.000
0.002
0.004
0.006
β_IV

Batch 2−2306 : Plastin−1 on eGFR

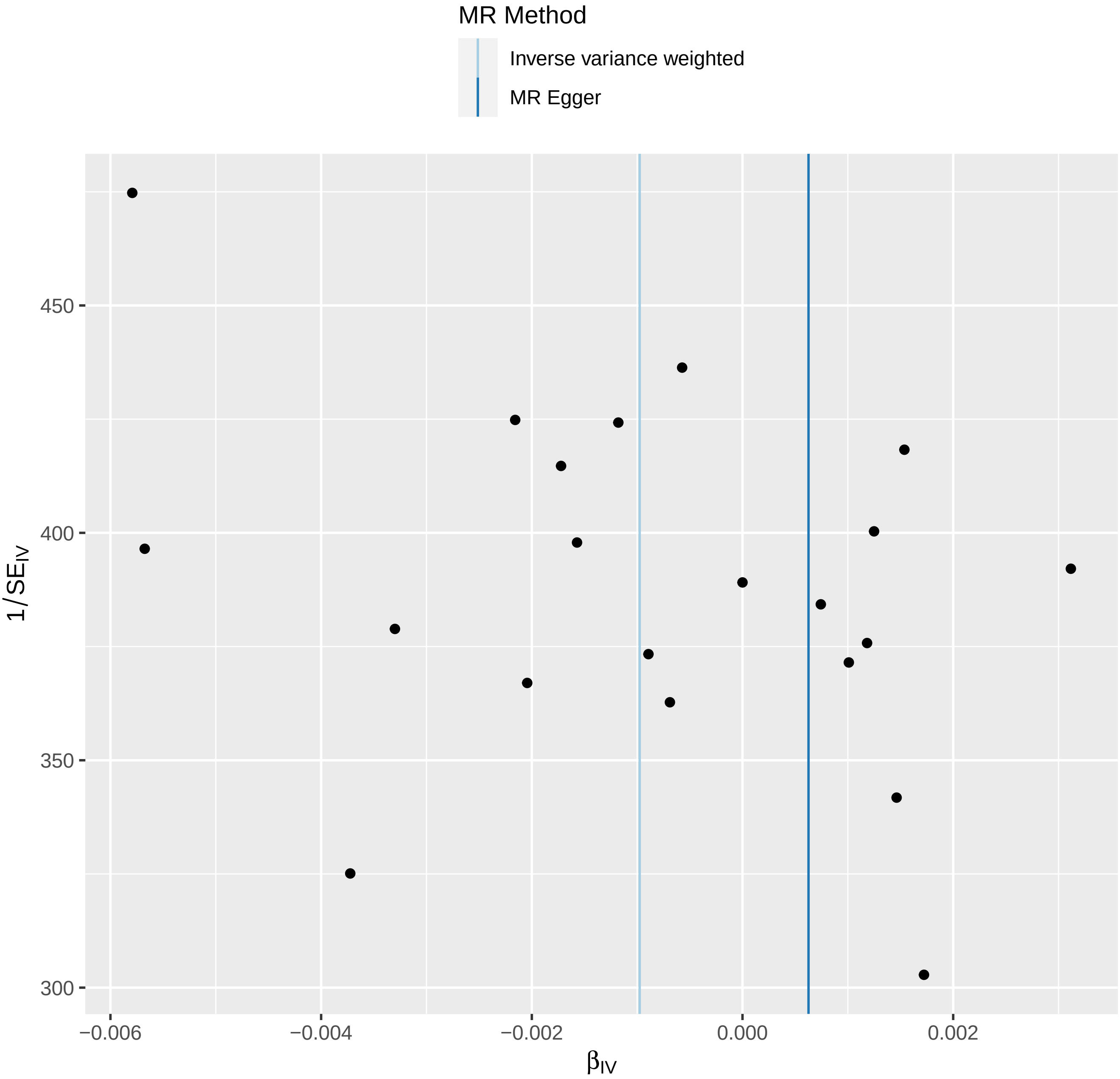

Batch 2−2307 : Plexin−A1 on eGFR

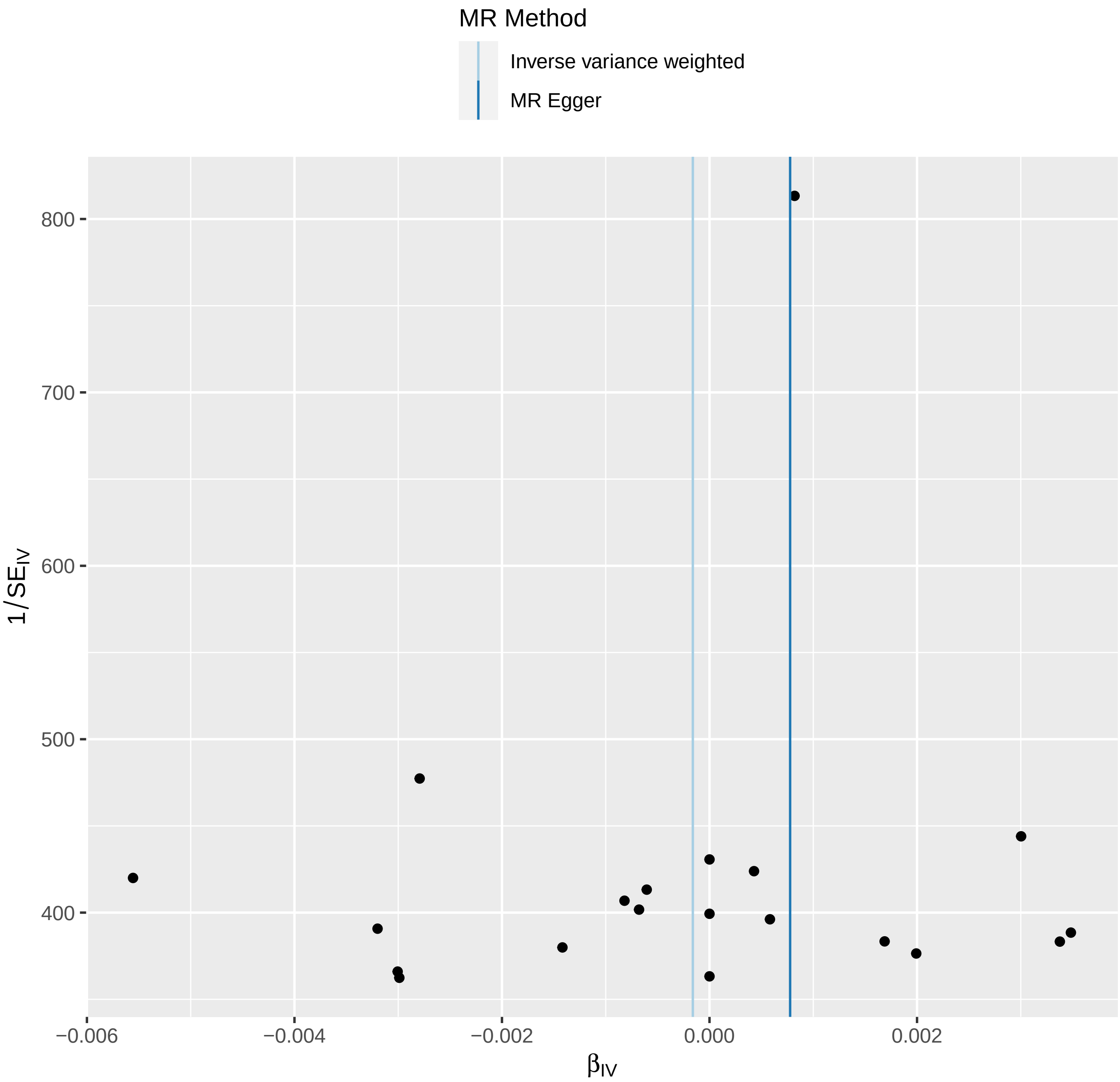

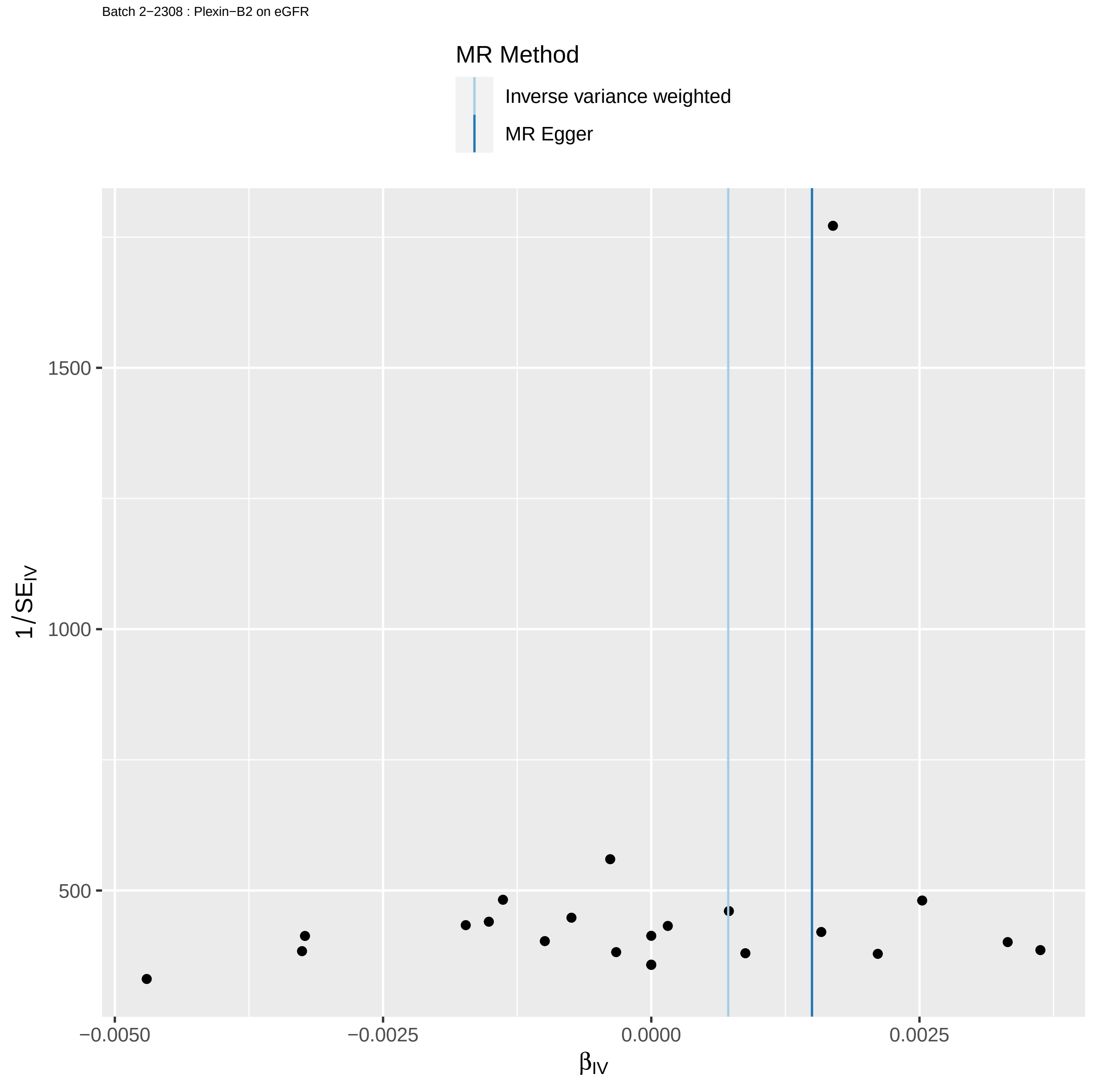

Batch 2−2308 : Plexin−B2 on eGFR
MR Method
Inverse variance weighted
MR Egger
1500
1000
500
−0.0050
−0.0025
0.0000
0.0025
1/SE_IV
β_IV

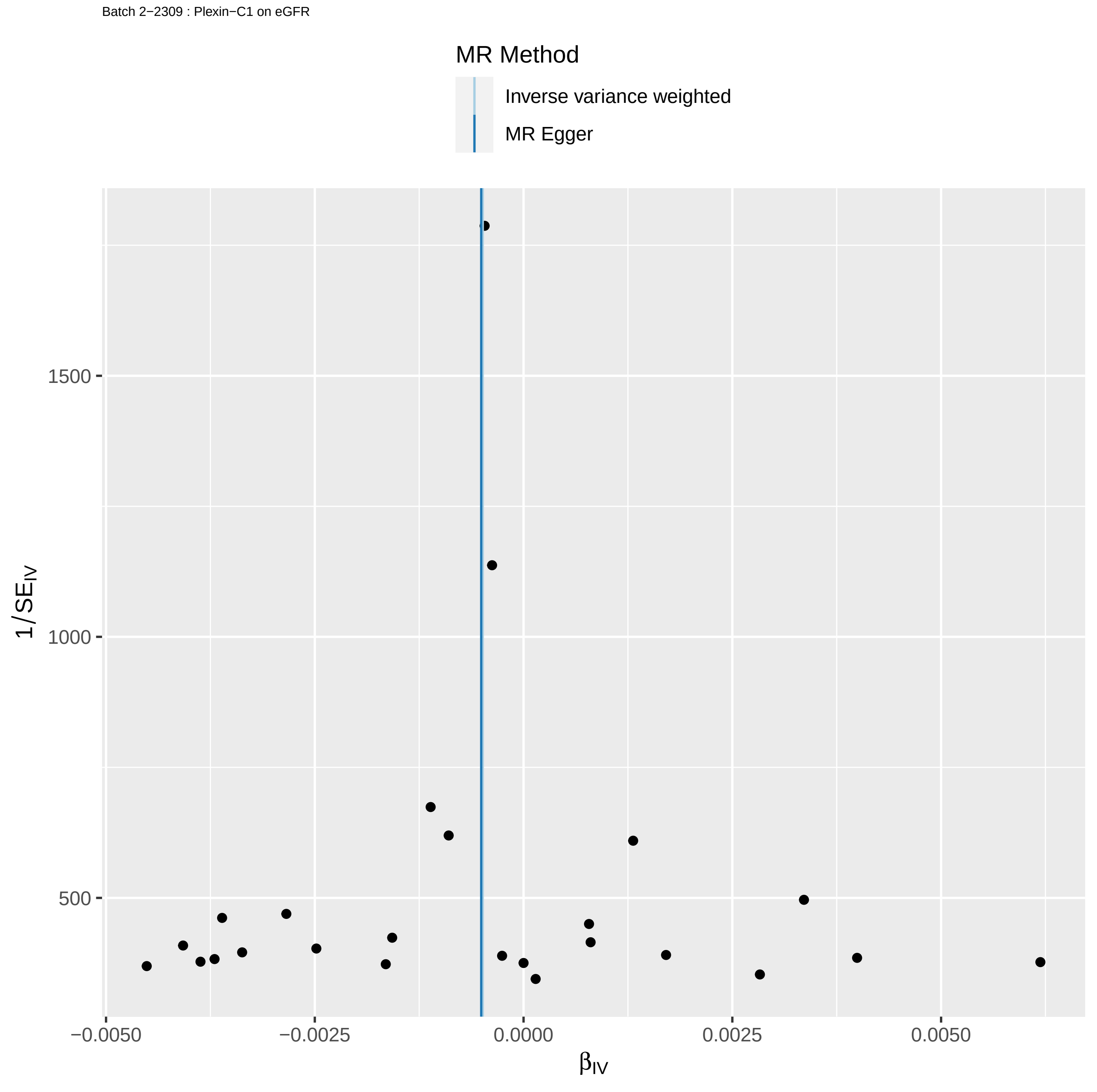
Batch 2−2309 : Plexin−C1 on eGFR
MR Method
Inverse variance weighted
MR Egger
1500
1000
500
$1/SE_{IV}$
−0.0050
−0.0025
0.0000
0.0025
0.0050
β_{IV}

Batch 2−2310 : Melanocyte protein PMEL on eGFR

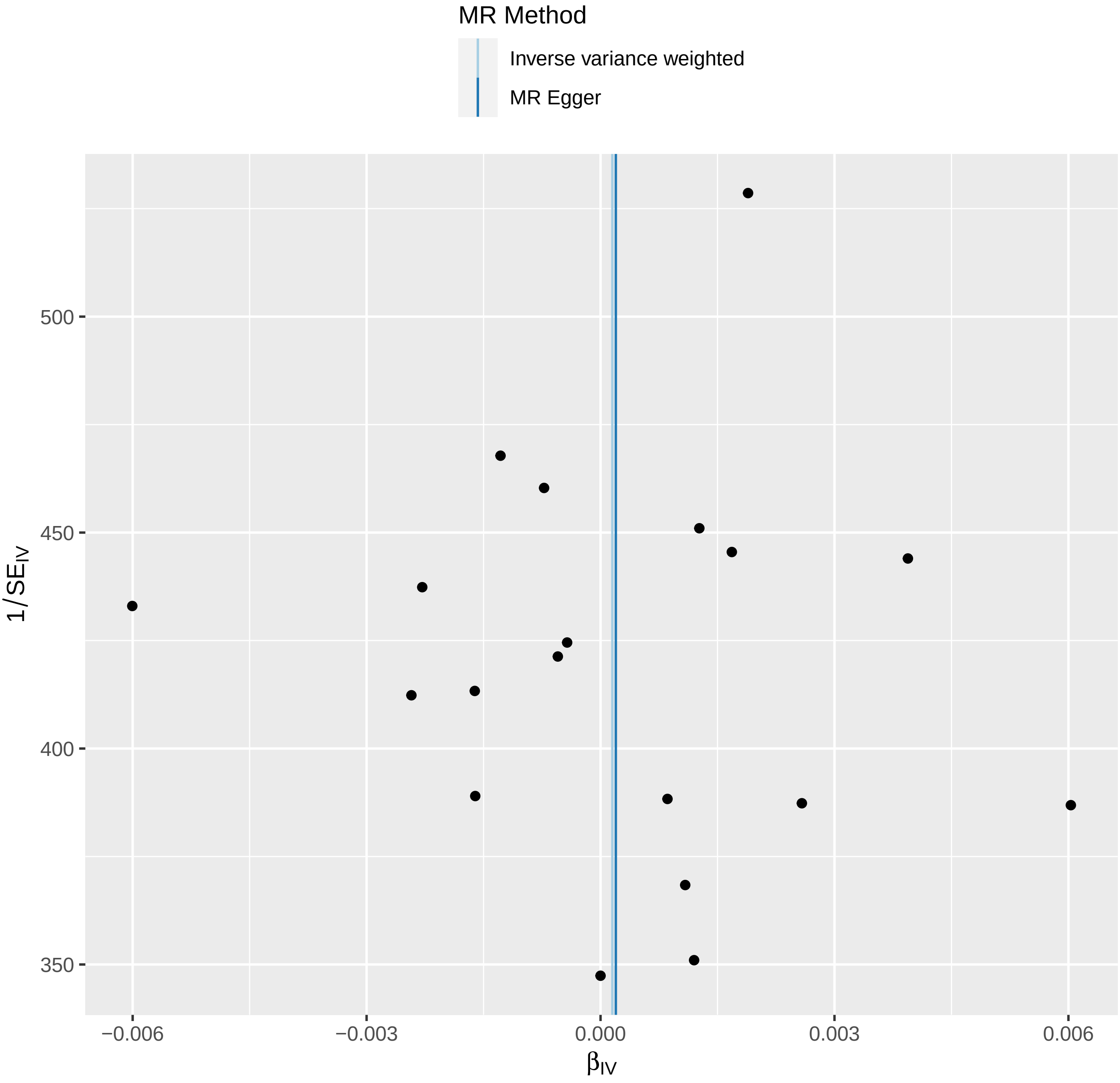

Batch 2−2311 : Protein TMEPAI on eGFR

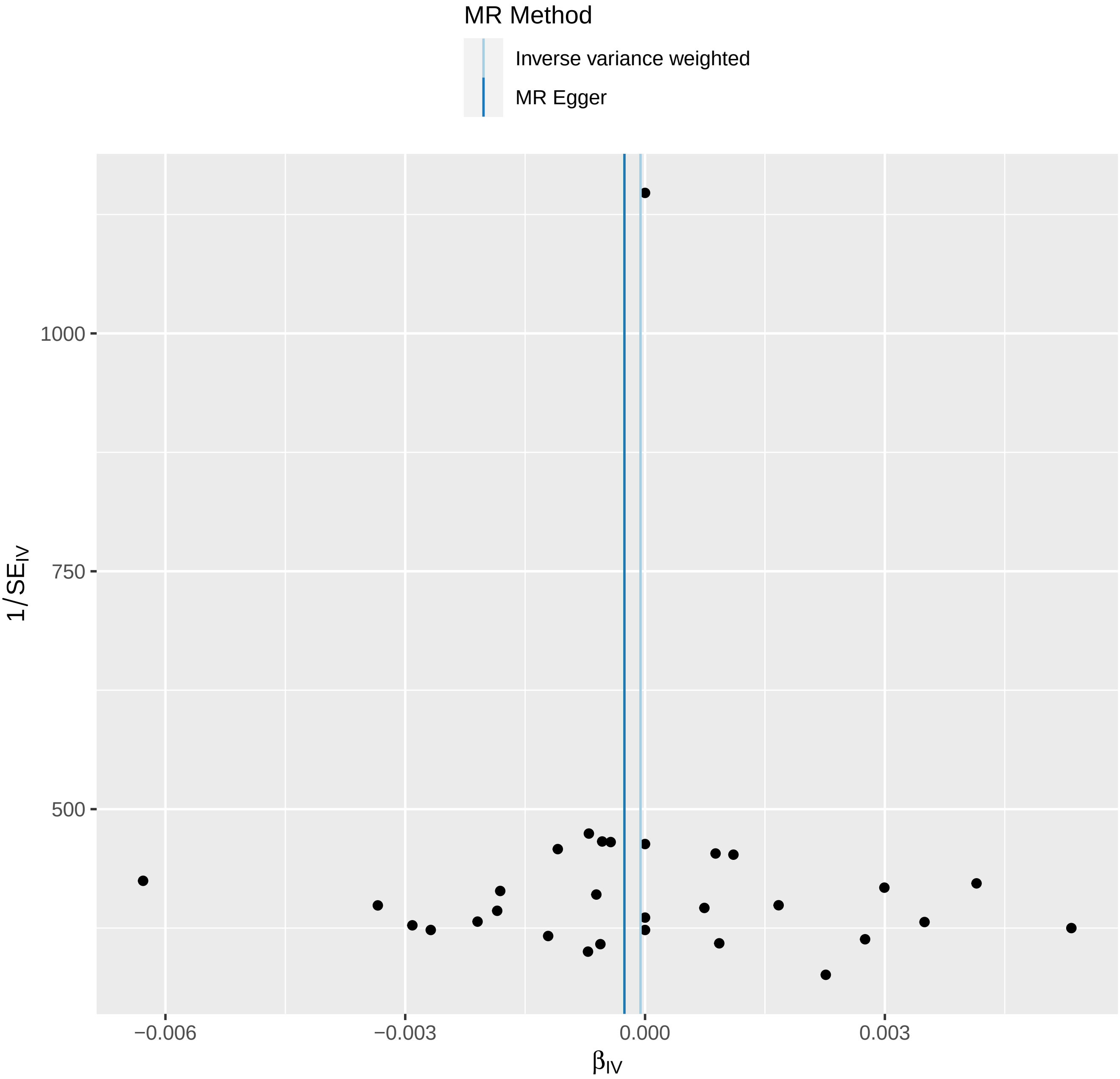

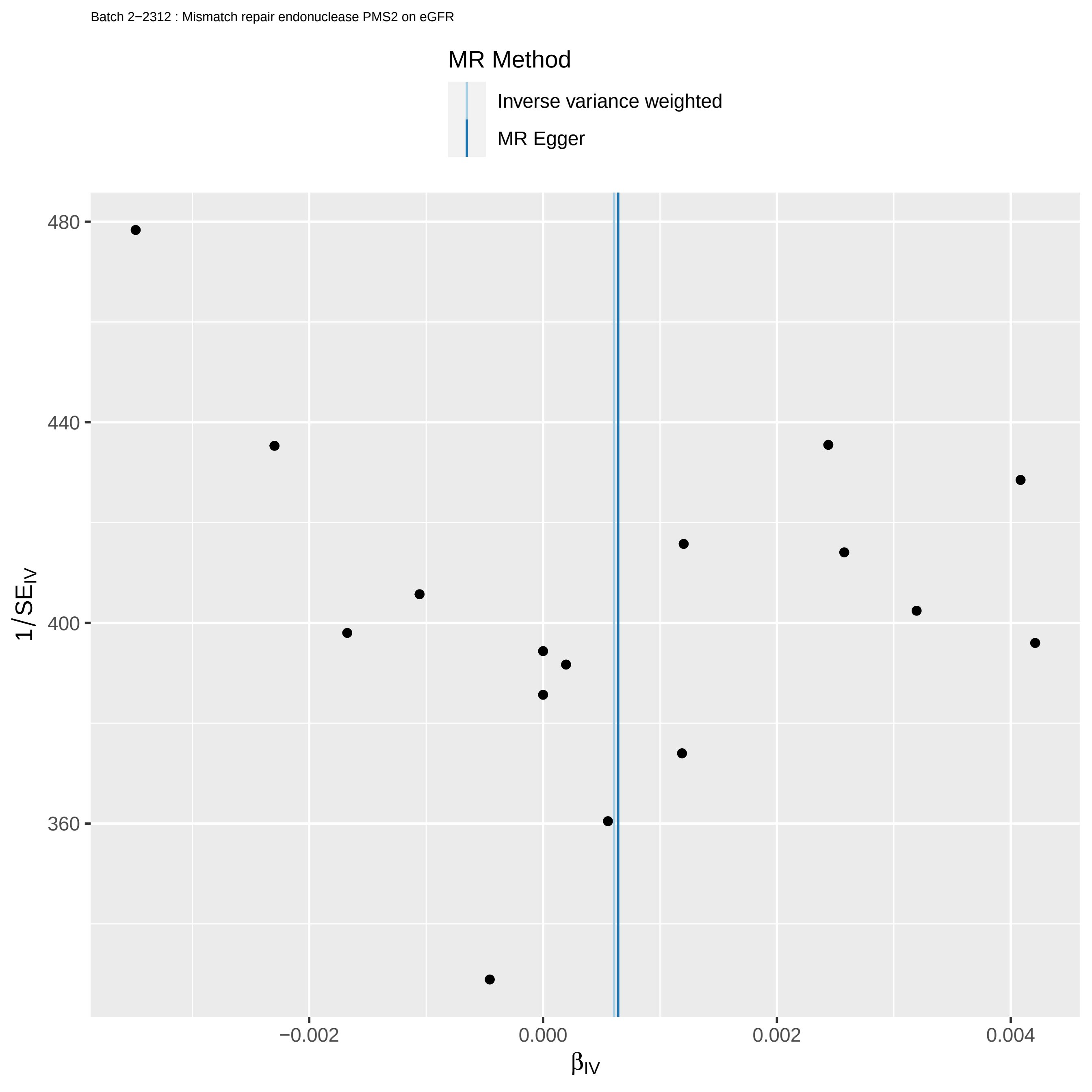
Batch 2−2312 : Mismatch repair endonuclease PMS2 on eGFR
MR Method
Inverse variance weighted
MR Egger
480
440
400
360
1/SE_IV
−0.002
0.000
0.002
0.004
β_IV

Batch 2−2313 : Phosphomevalonate kinase on eGFR

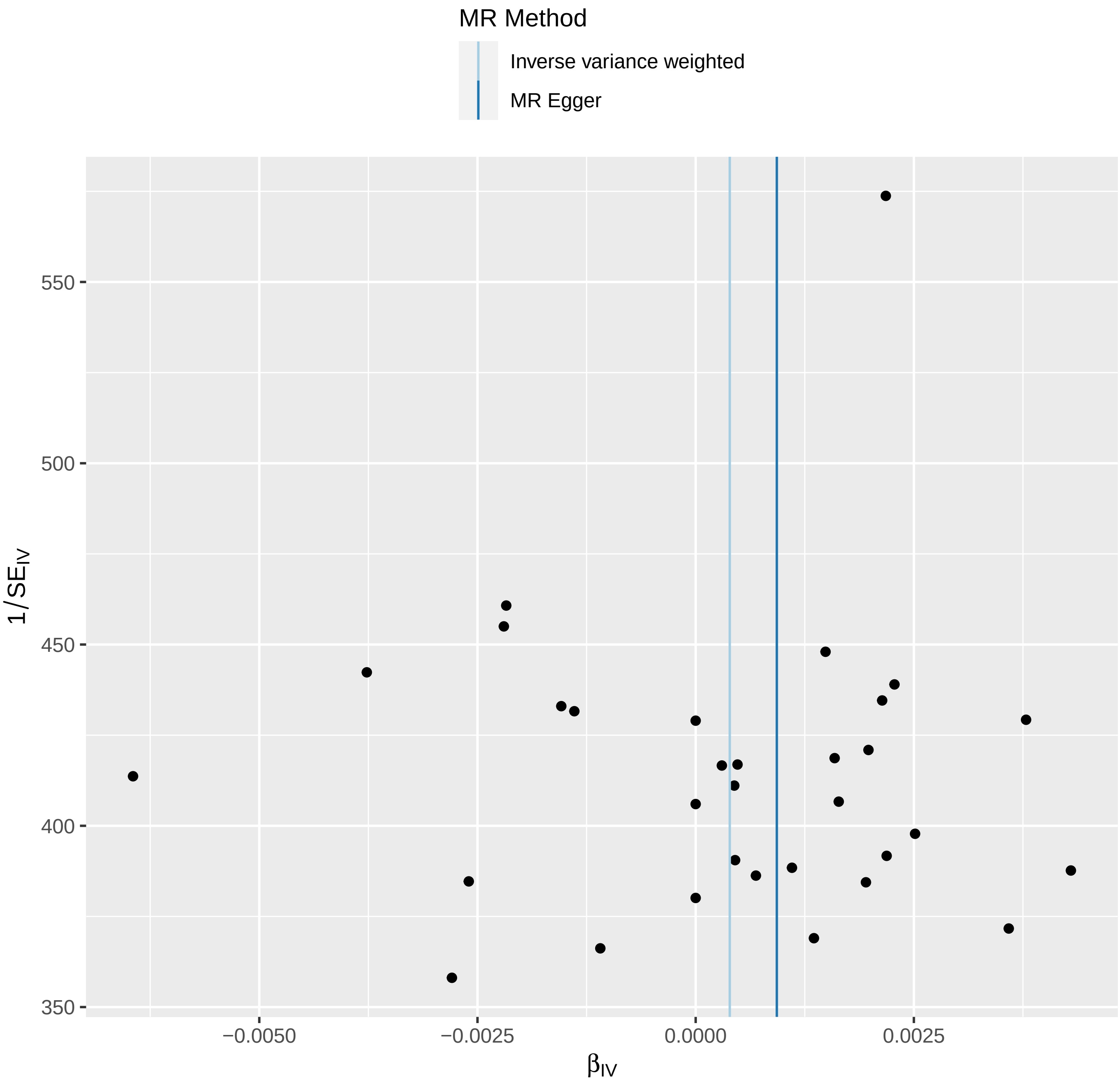

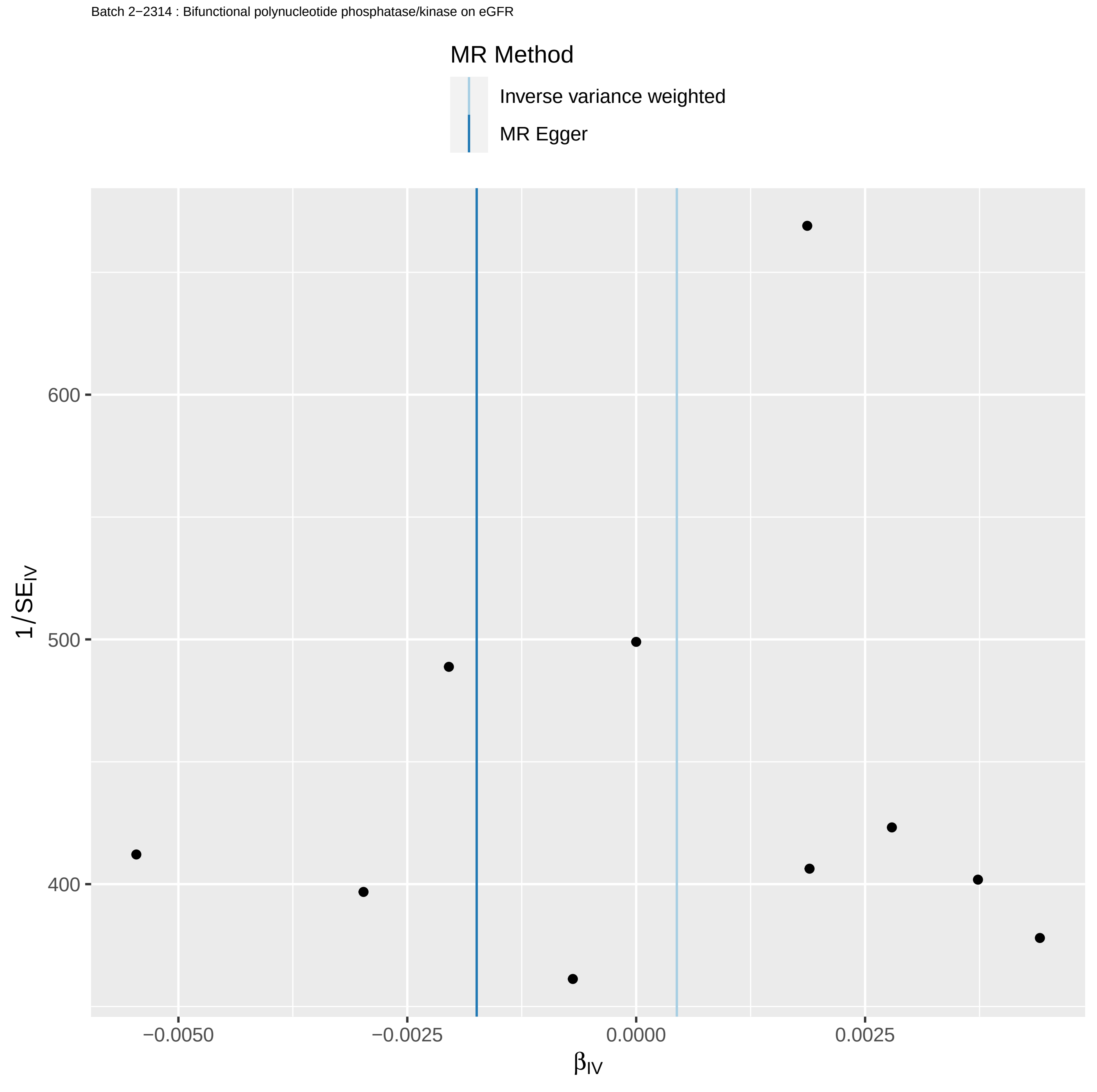
Batch 2−2314 : Bifunctional polynucleotide phosphatase/kinase on eGFR
MR Method
Inverse variance weighted
MR Egger
600
500
400
1/SE_IV
−0.0050
−0.0025
0.0000
0.0025
β_IV

Batch 2−2315 : Inactive pancreatic lipase−related protein 1 on eGFR

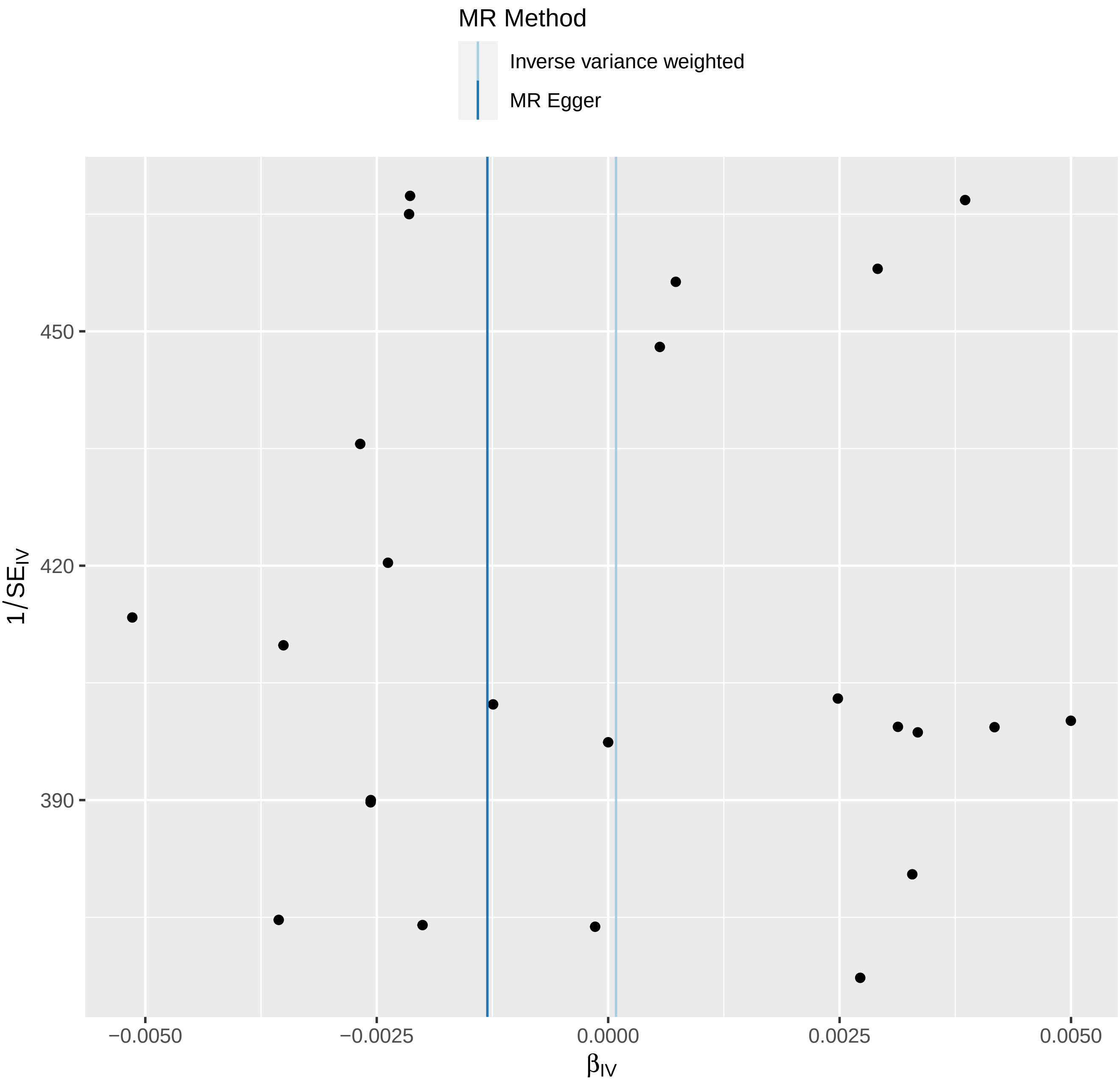

Batch 2−2316 : Purine nucleoside phosphorylase on eGFR

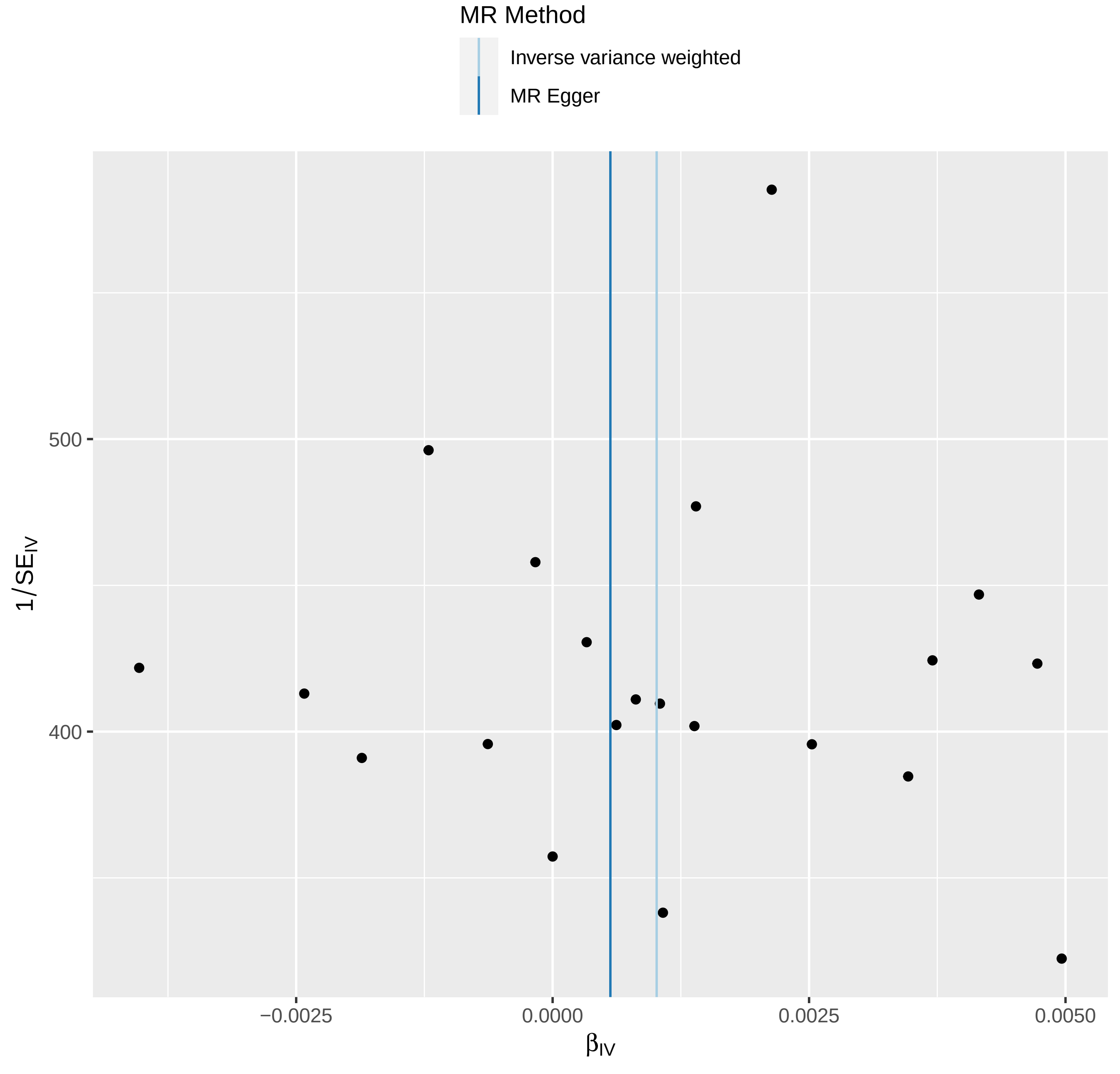

Batch 2−2317 : Podocalyxin on eGFR

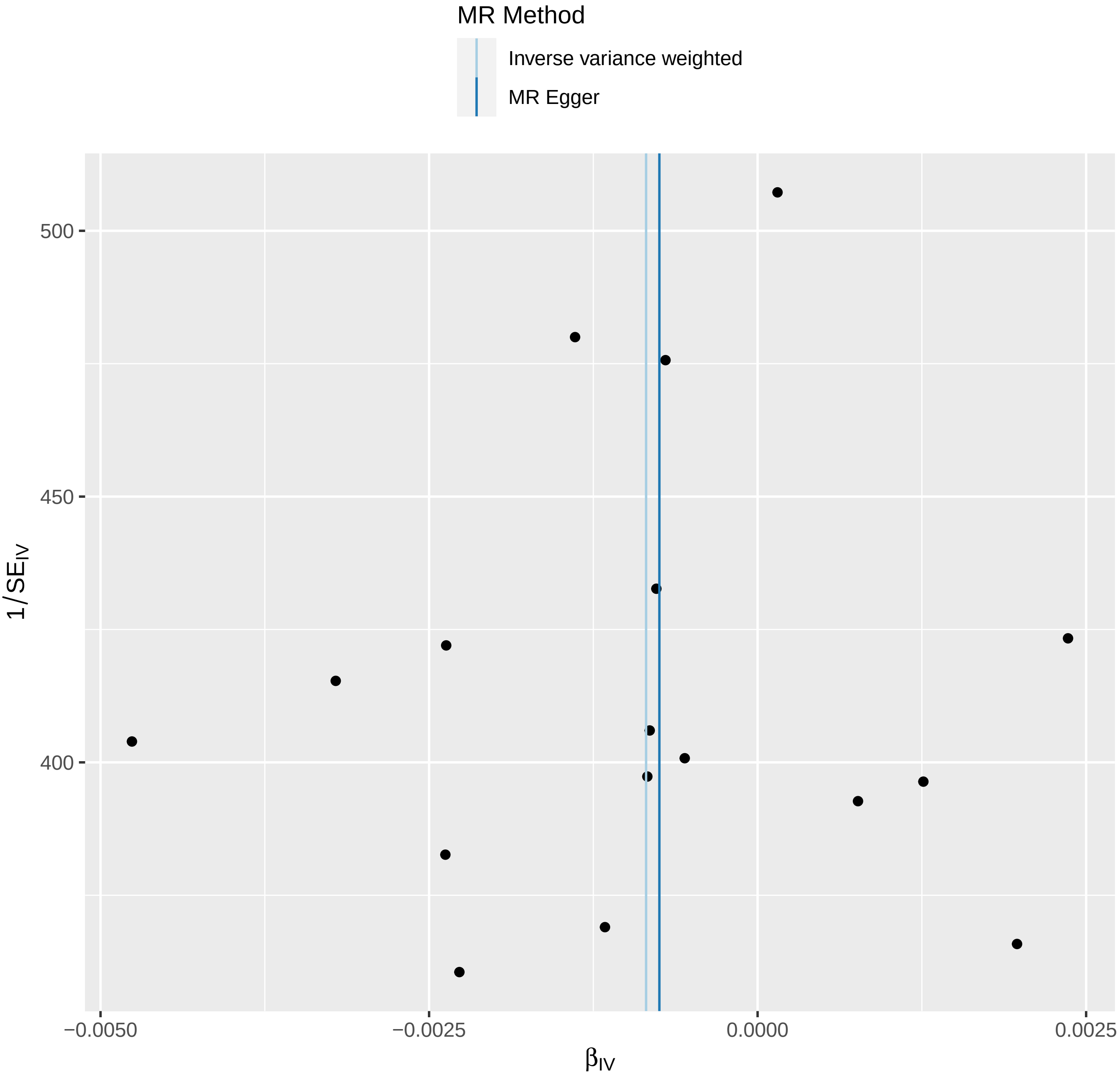

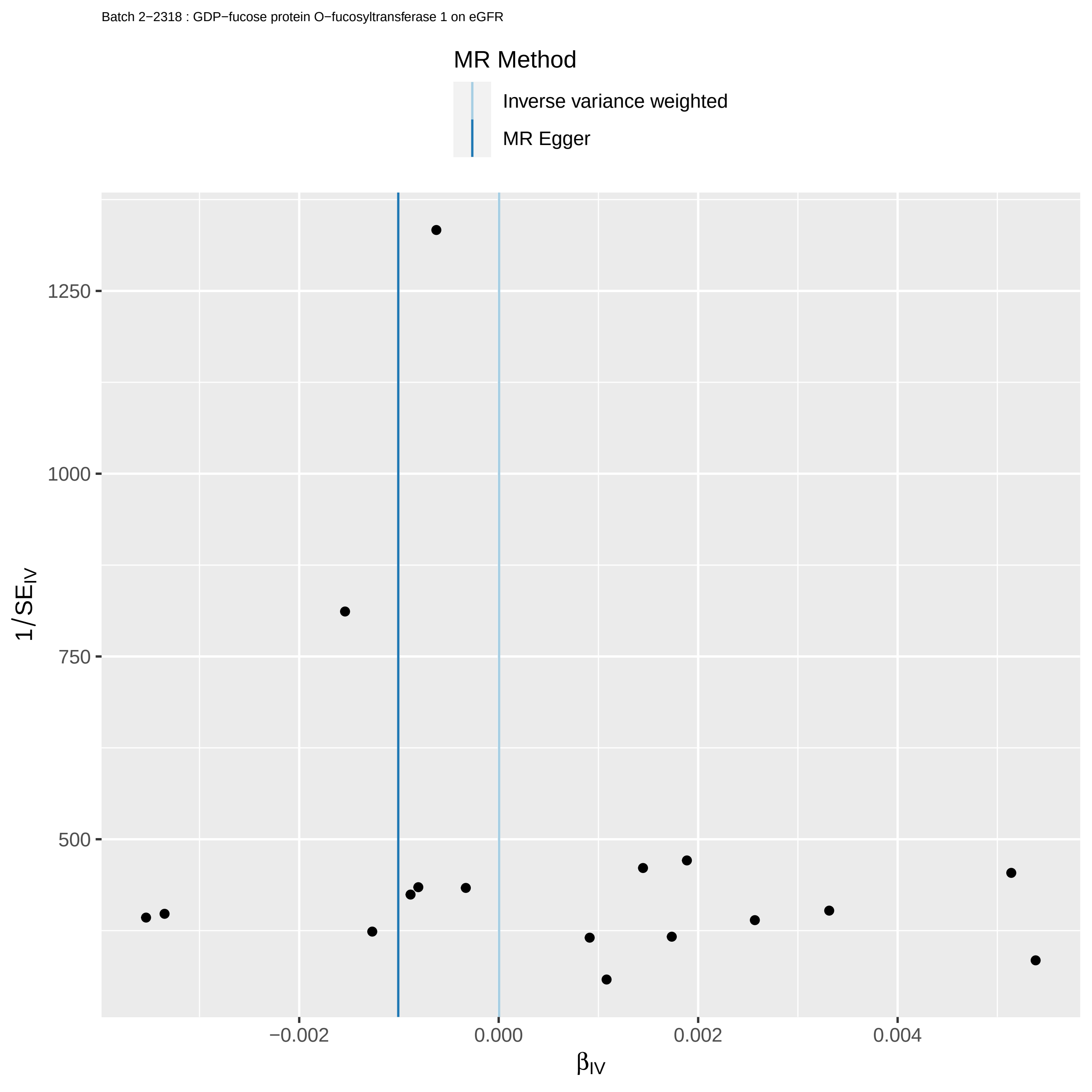

Batch 2−2318 : GDP−fucose protein O−fucosyltransferase 1 on eGFR
MR Method
Inverse variance weighted
MR Egger
1250
1000
750
500
1/SE$_{IV}$
−0.002
0.000
0.002
0.004
β$_{IV}$

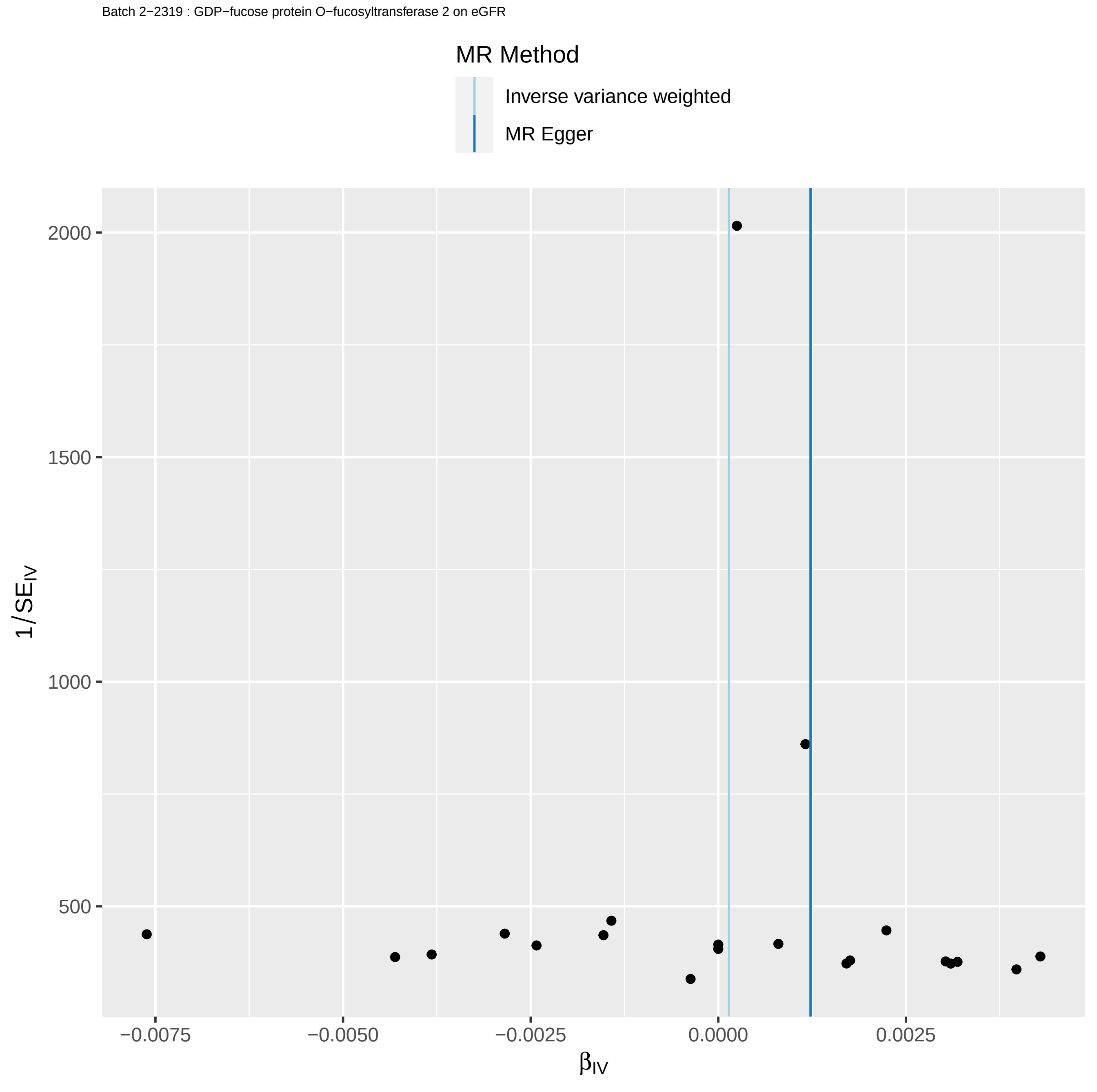
Batch 2−2319 : GDP−fucose protein O−fucosyltransferase 2 on eGFR
MR Method
Inverse variance weighted
MR Egger
2000
1500
1000
500
1/SE_IV
−0.0075
−0.0050
−0.0025
0.0000
0.0025
β_IV

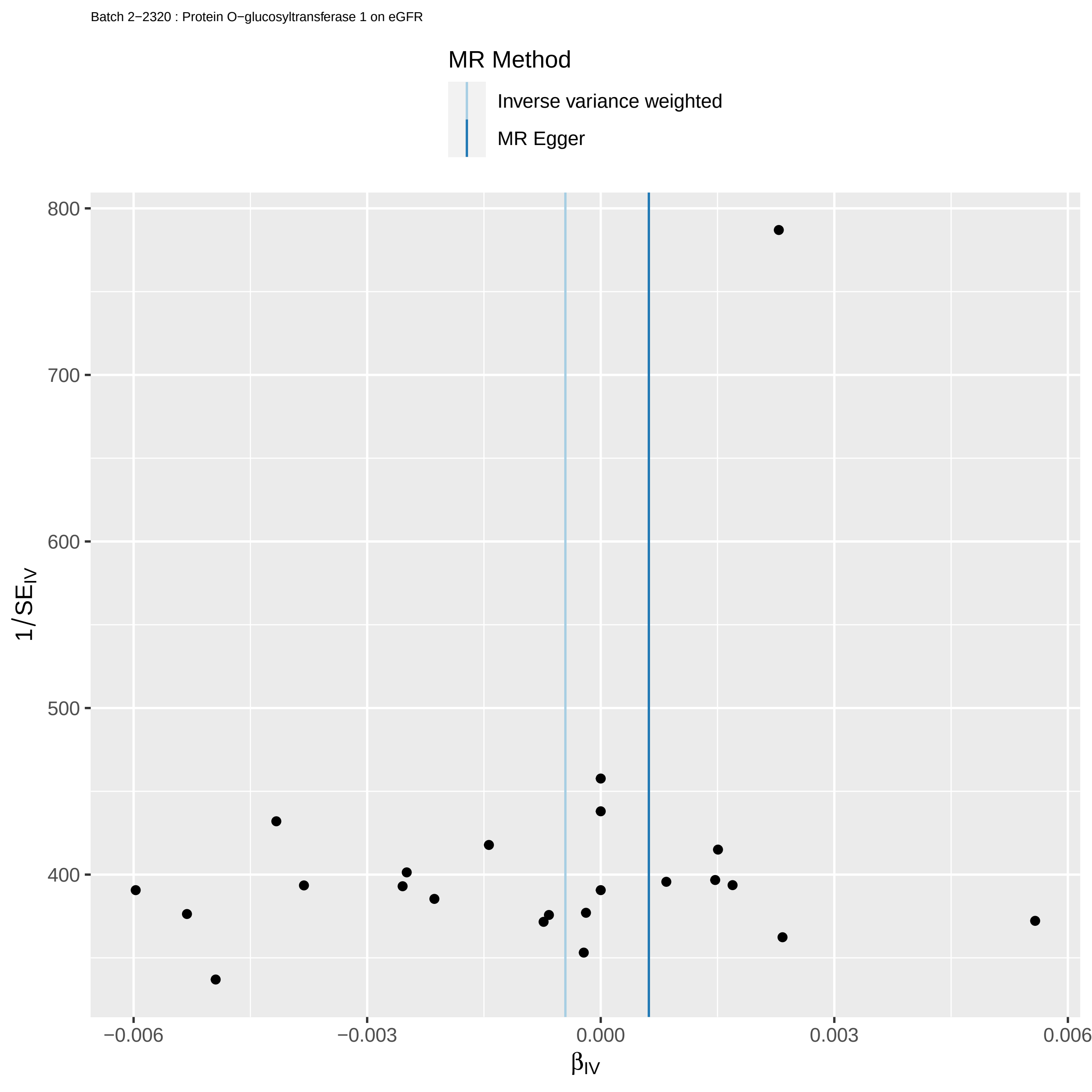
Batch 2−2320 : Protein O−glucosyltransferase 1 on eGFR
MR Method
Inverse variance weighted
MR Egger
800
700
600
500
400
1/SE$_{IV}$
−0.006
−0.003
0.000
0.003
0.006
β$_{IV}$

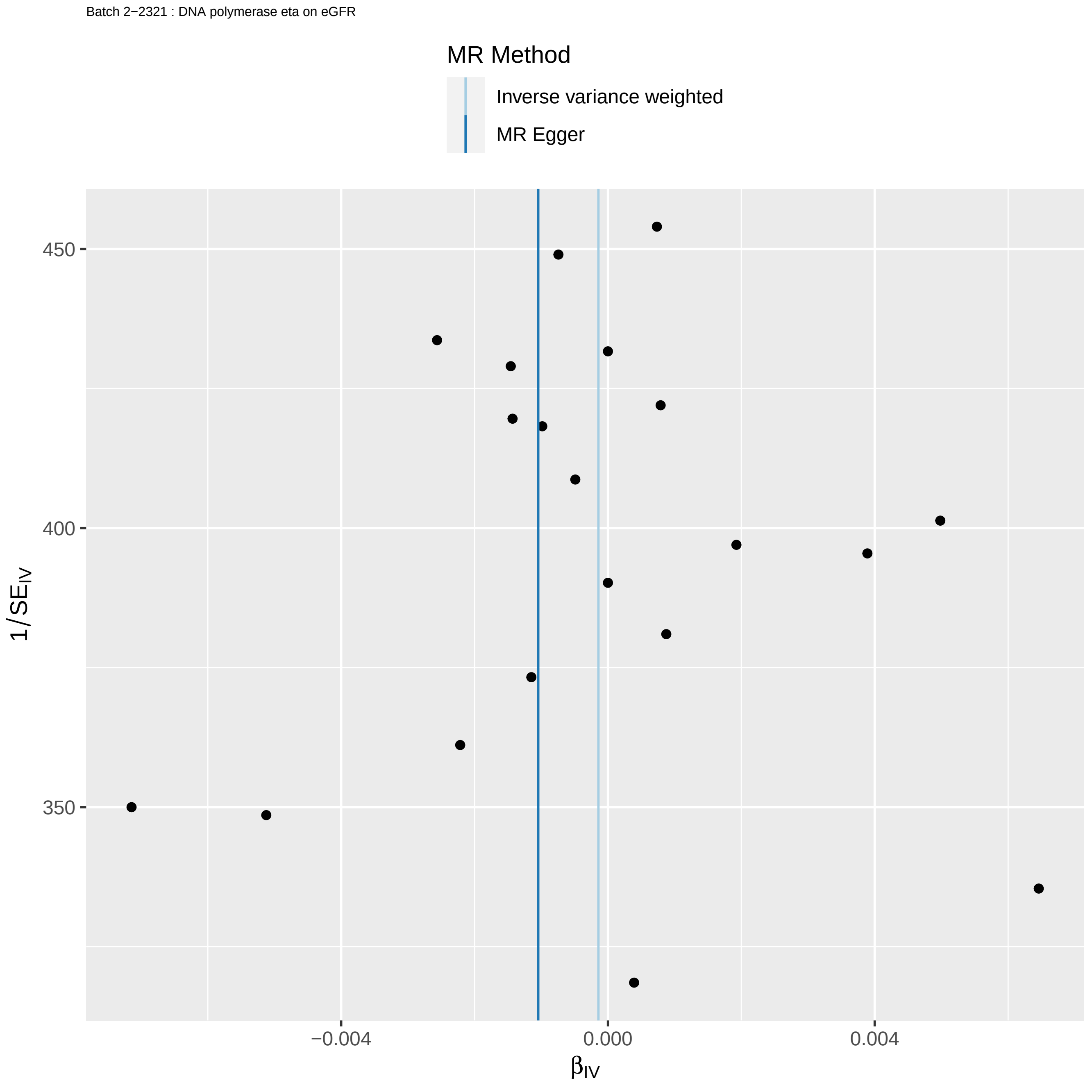

Batch 2−2321 : DNA polymerase eta on eGFR
MR Method
Inverse variance weighted
MR Egger
450
400
350
1/SE_IV
−0.004
0.000
0.004
β_IV

Batch 2−2322 : DNA polymerase iota on eGFR

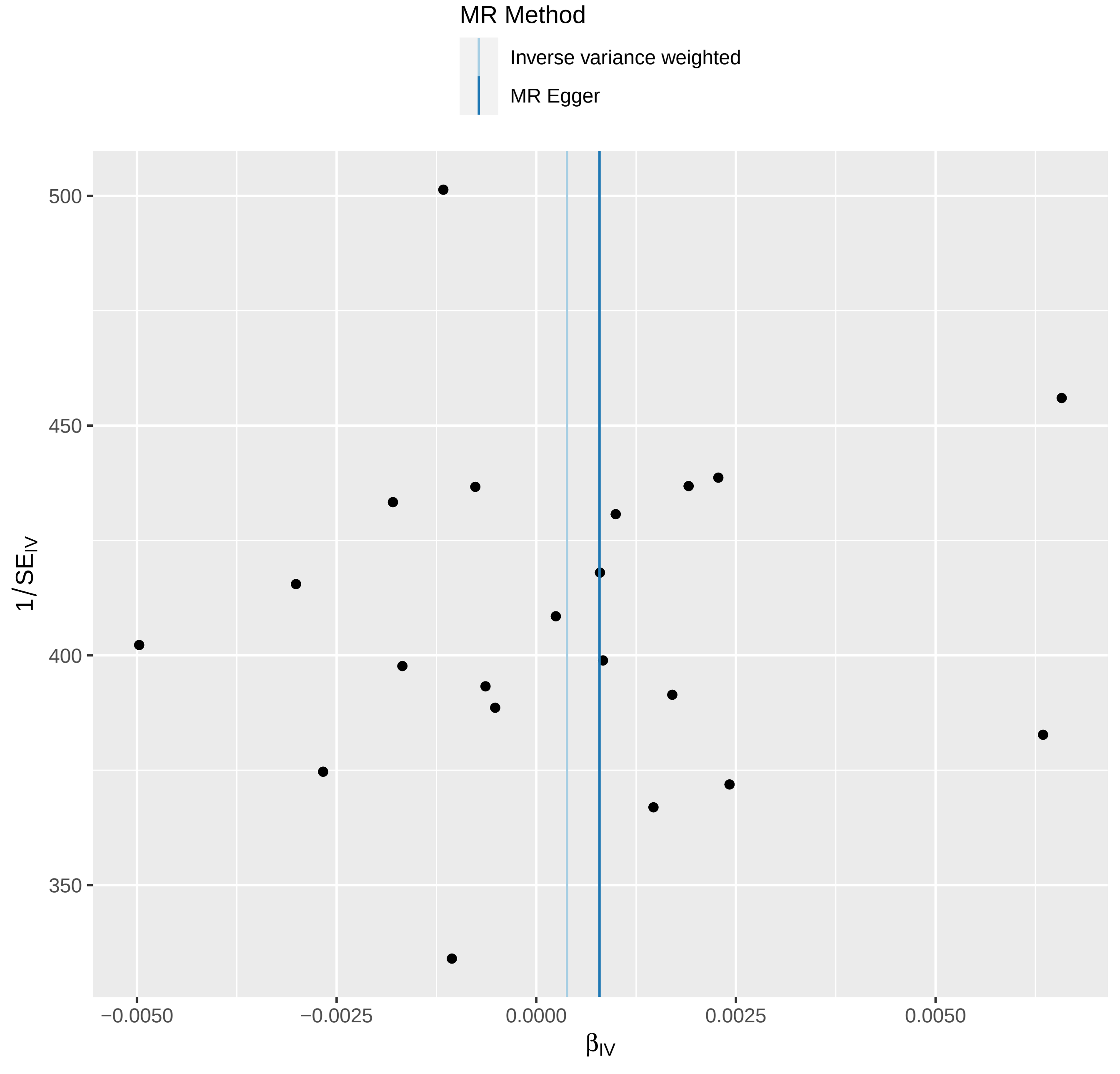

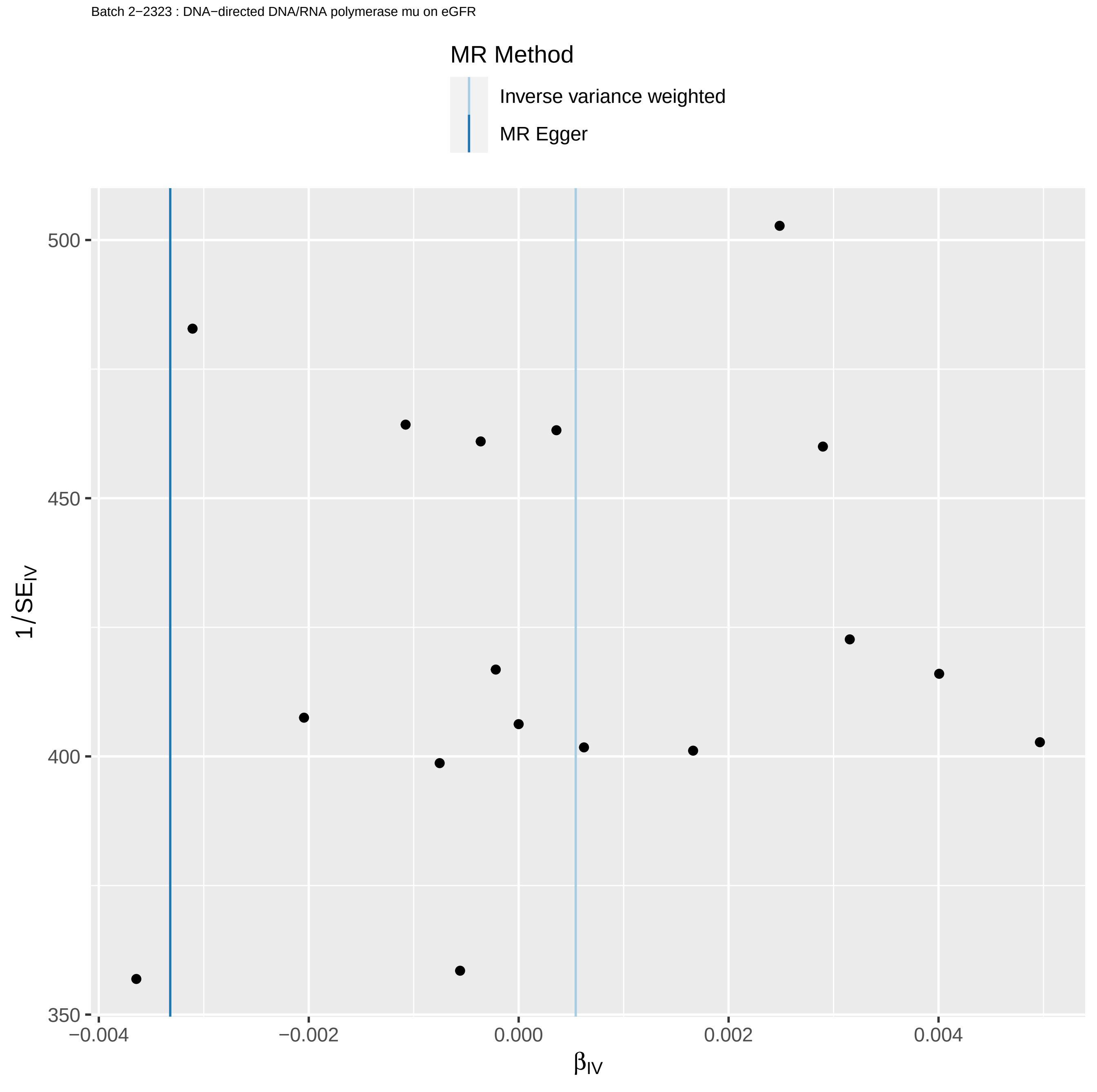

Batch 2−2323 : DNA−directed DNA/RNA polymerase mu on eGFR
MR Method
Inverse variance weighted
MR Egger
1/SE_IV
500
450
400
350
−0.004
−0.002
0.000
0.002
0.004
β_IV

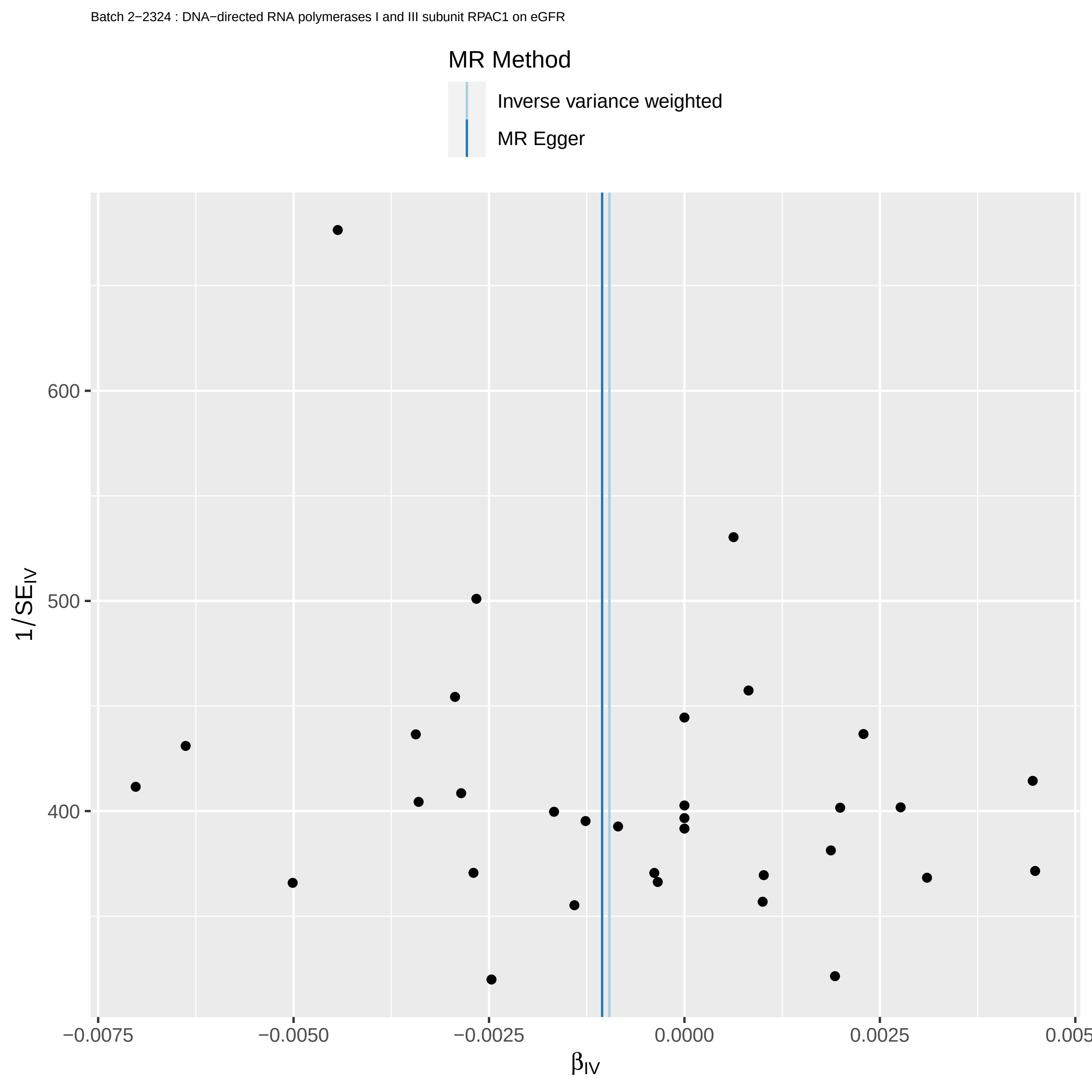

Batch 2−2324 : DNA−directed RNA polymerases I and III subunit RPAC1 on eGFR
MR Method
Inverse variance weighted
MR Egger
1/SE$_{IV}$
600
500
400
−0.0075
−0.0050
−0.0025
0.0000
0.0025
0.005
β_{IV}

Batch 2−2325 : Beta−endorphin on eGFR

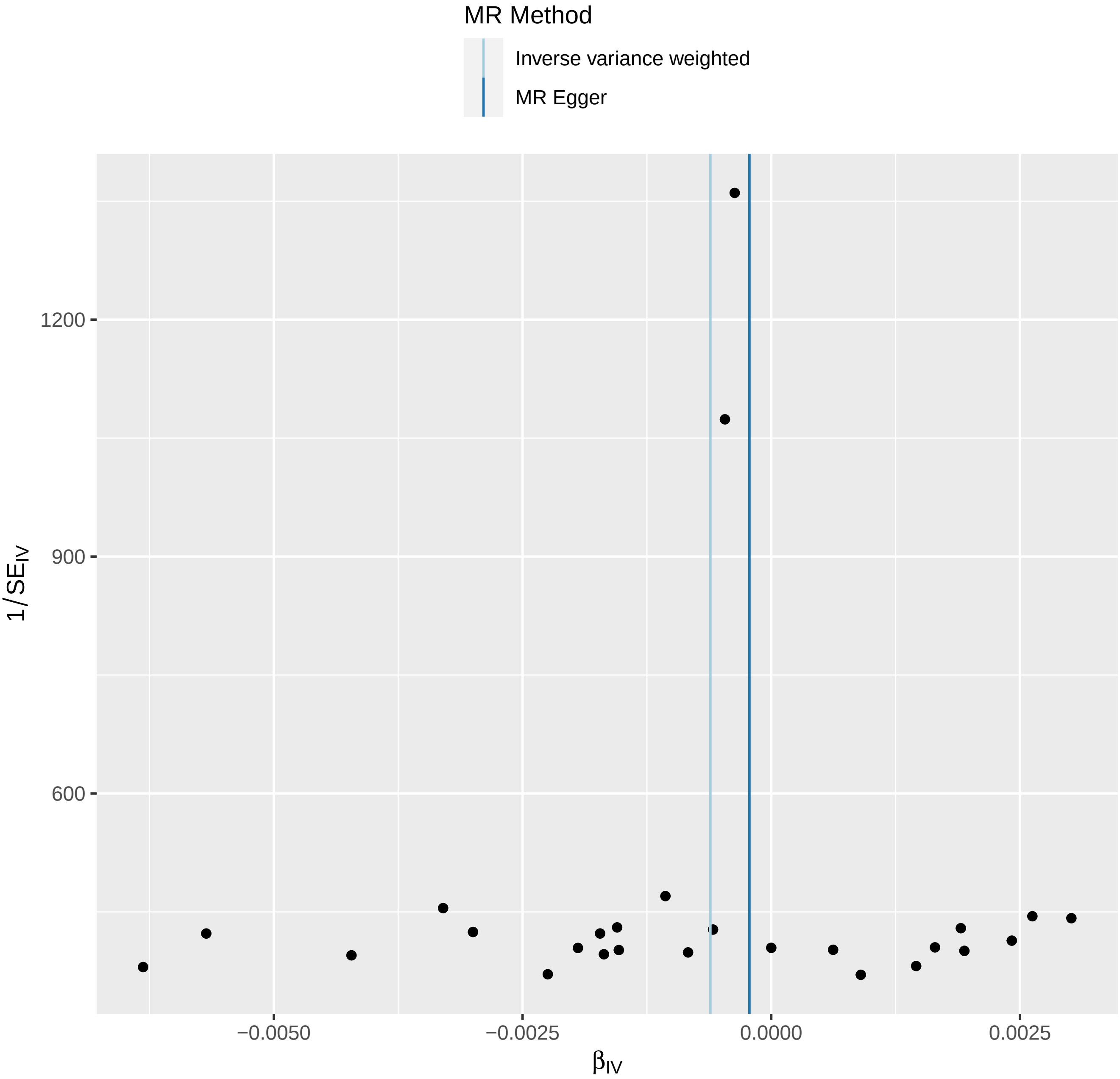

Batch 2−2326 : Corticotropin on eGFR

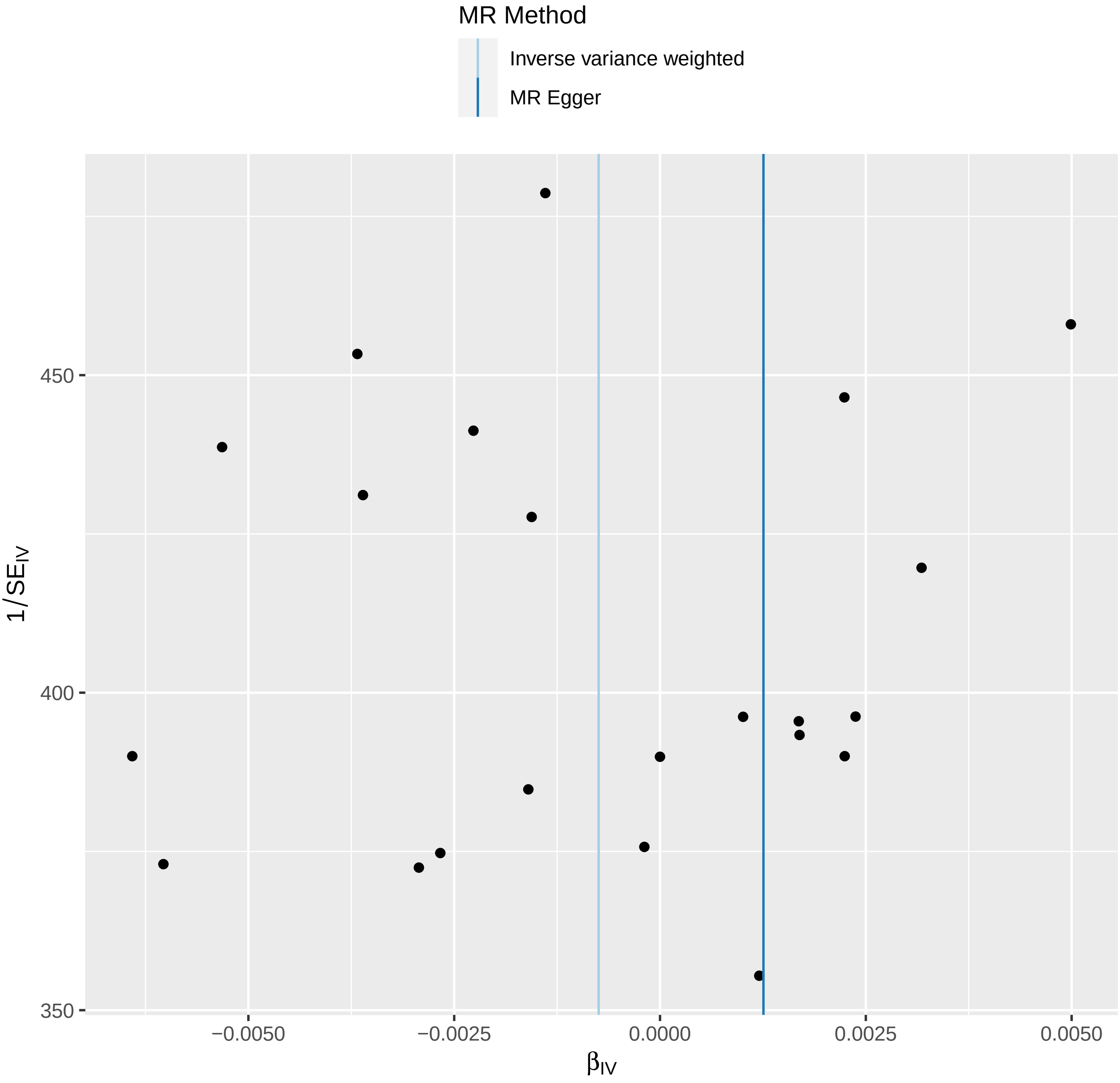

Batch 2−2327 : Pro−opiomelanocortin on eGFR

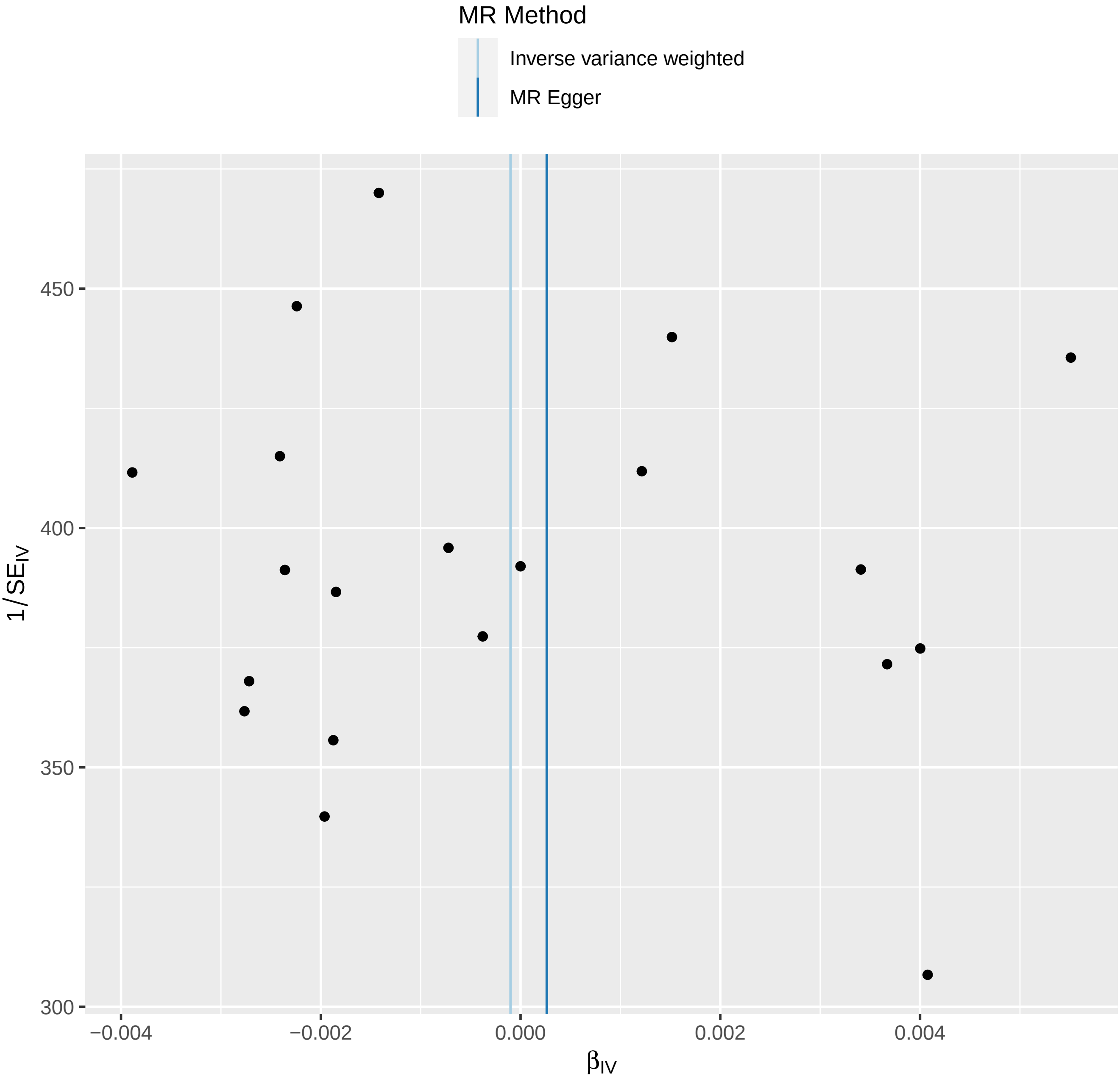

Batch 2−2328 : Protein O−linked−mannose beta−1,2−N−acetylglucosaminyltransferase 1 on eGFR

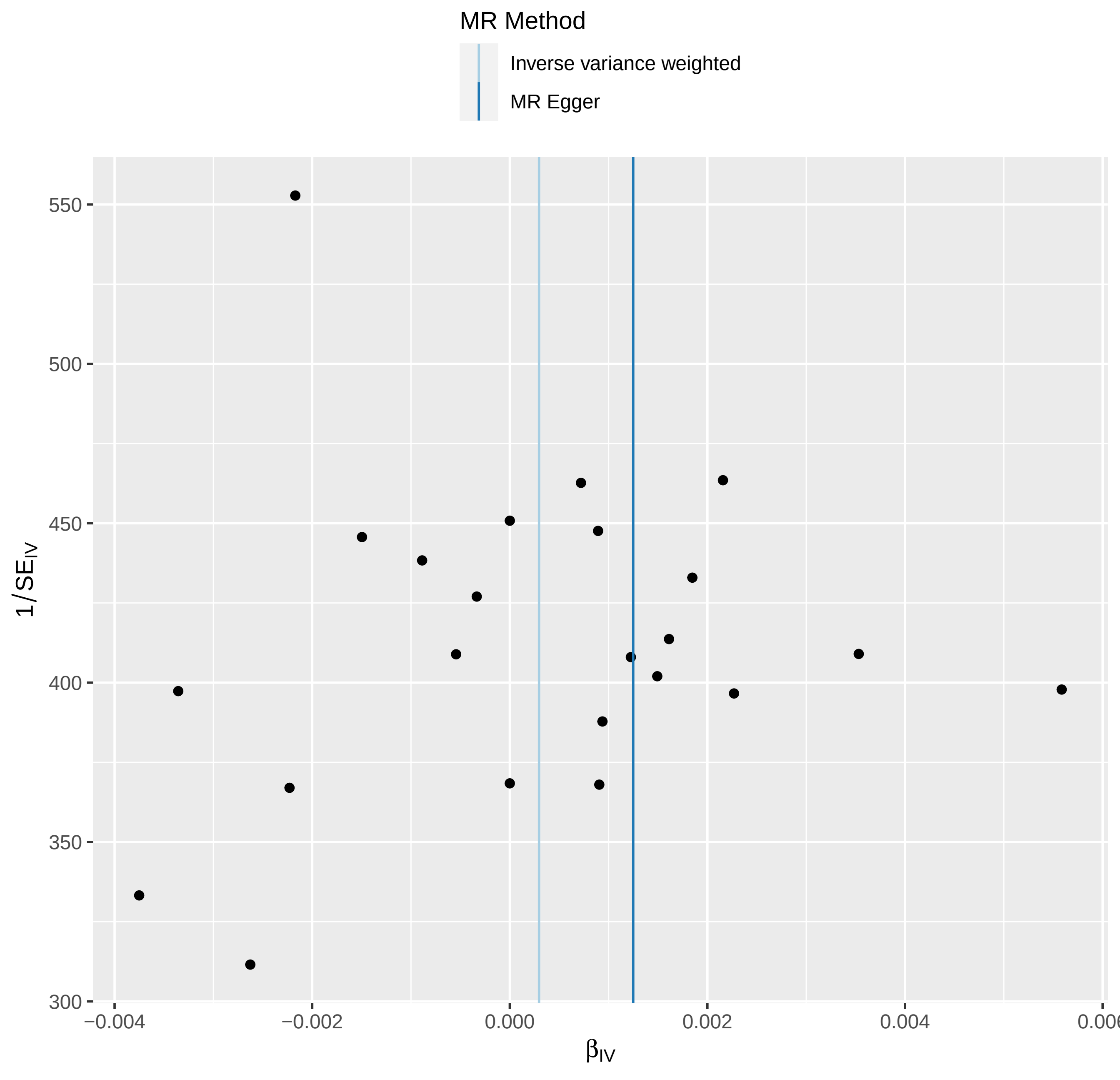

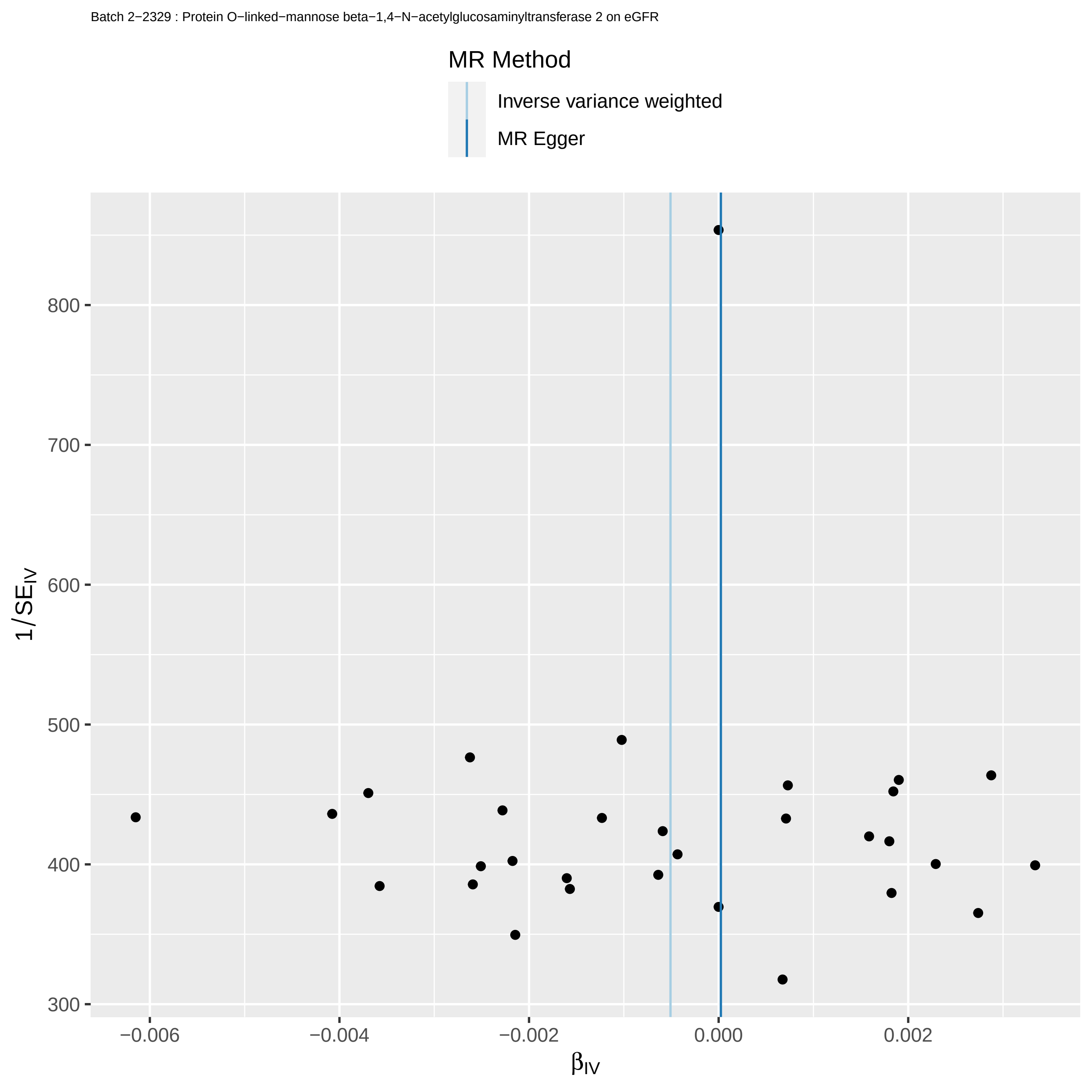

Batch 2−2329 : Protein O−linked−mannose beta−1,4−N−acetylglucosaminyltransferase 2 on eGFR
MR Method
Inverse variance weighted
MR Egger
800
700
600
500
400
300
1/SE_IV
−0.006
−0.004
−0.002
0.000
0.002
β_IV

Batch 2−2330 : NADPH−−cytochrome P450 reductase on eGFR

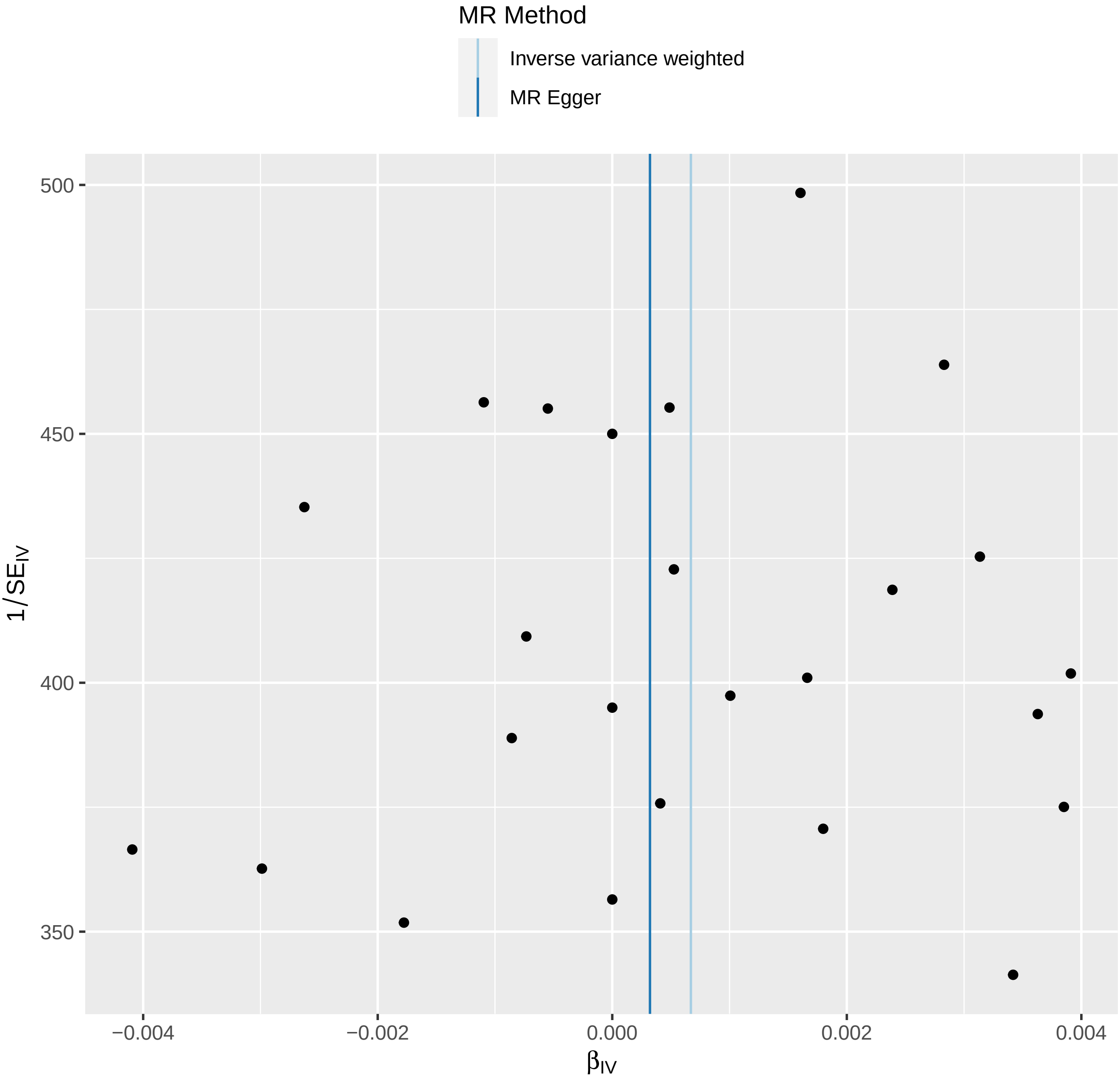

Batch 2−2331 : Periostin on eGFR

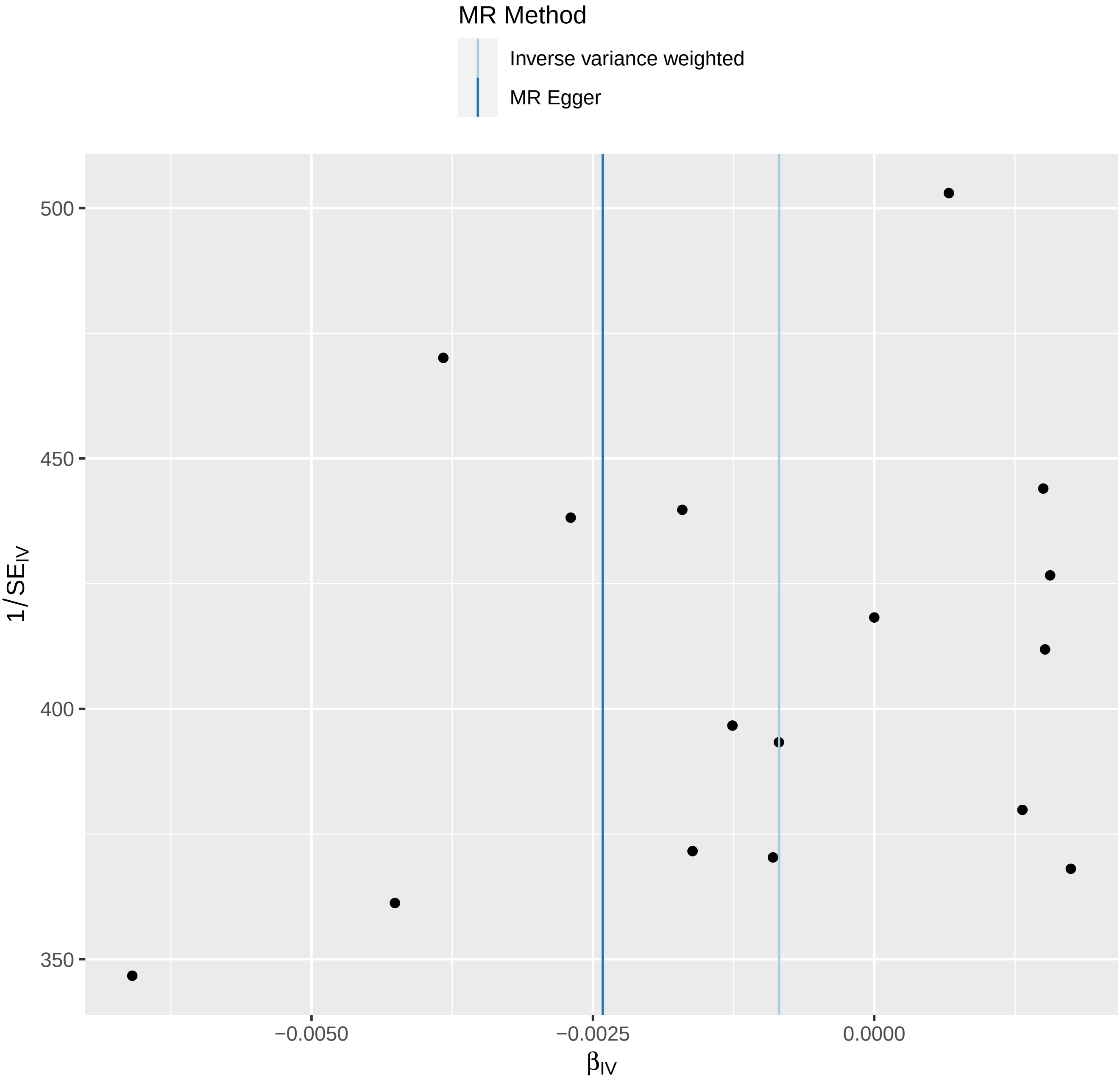

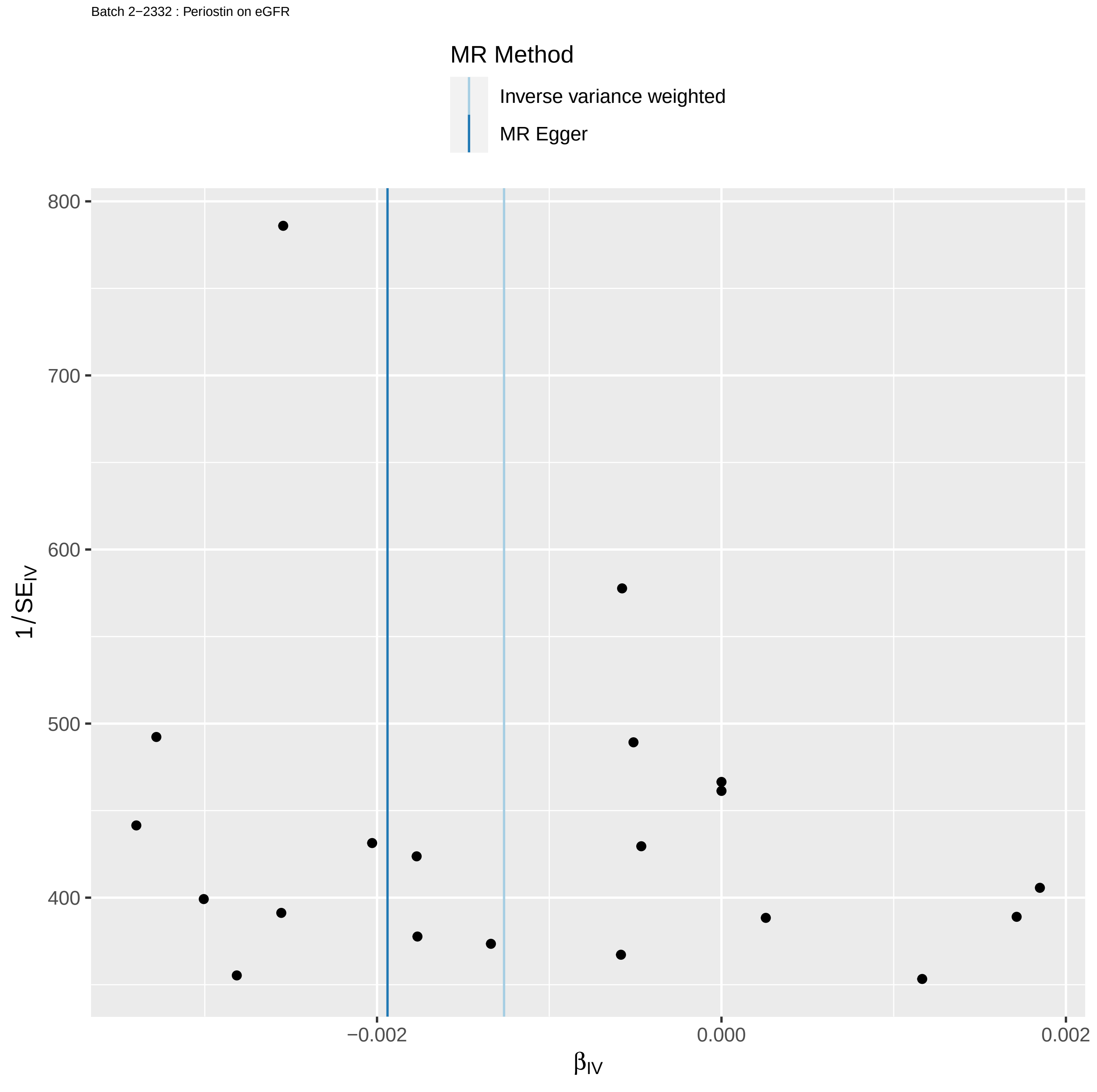

Batch 2−2332 : Periosti on eGFR
MR Method
Inverse variance weighted
MR Egger
800
700
600
500
400
1/SE$_{IV}$
−0.002
0.000
0.002
β$_{IV}$

Batch 2−2333 : Periostin on eGFR

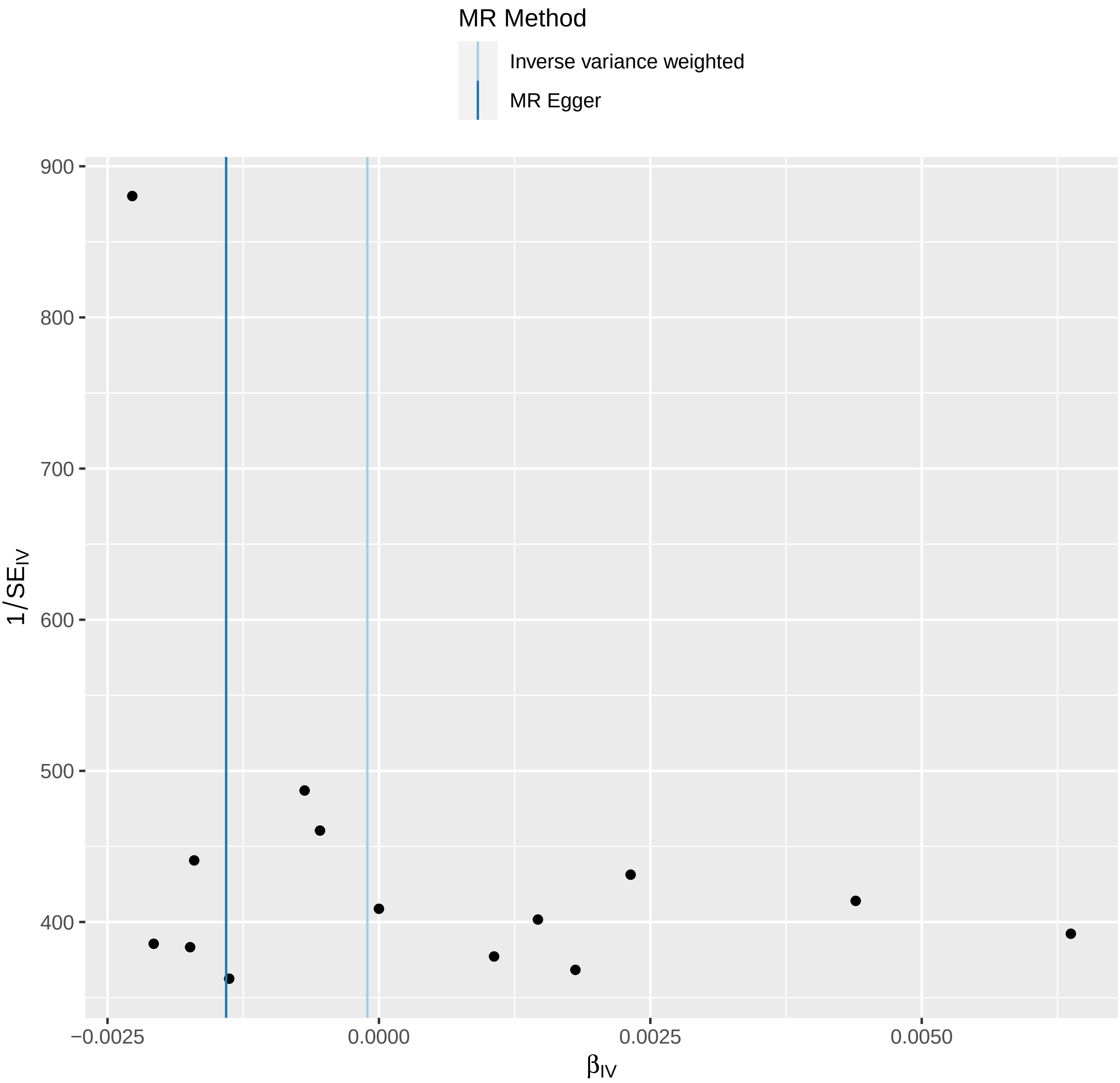

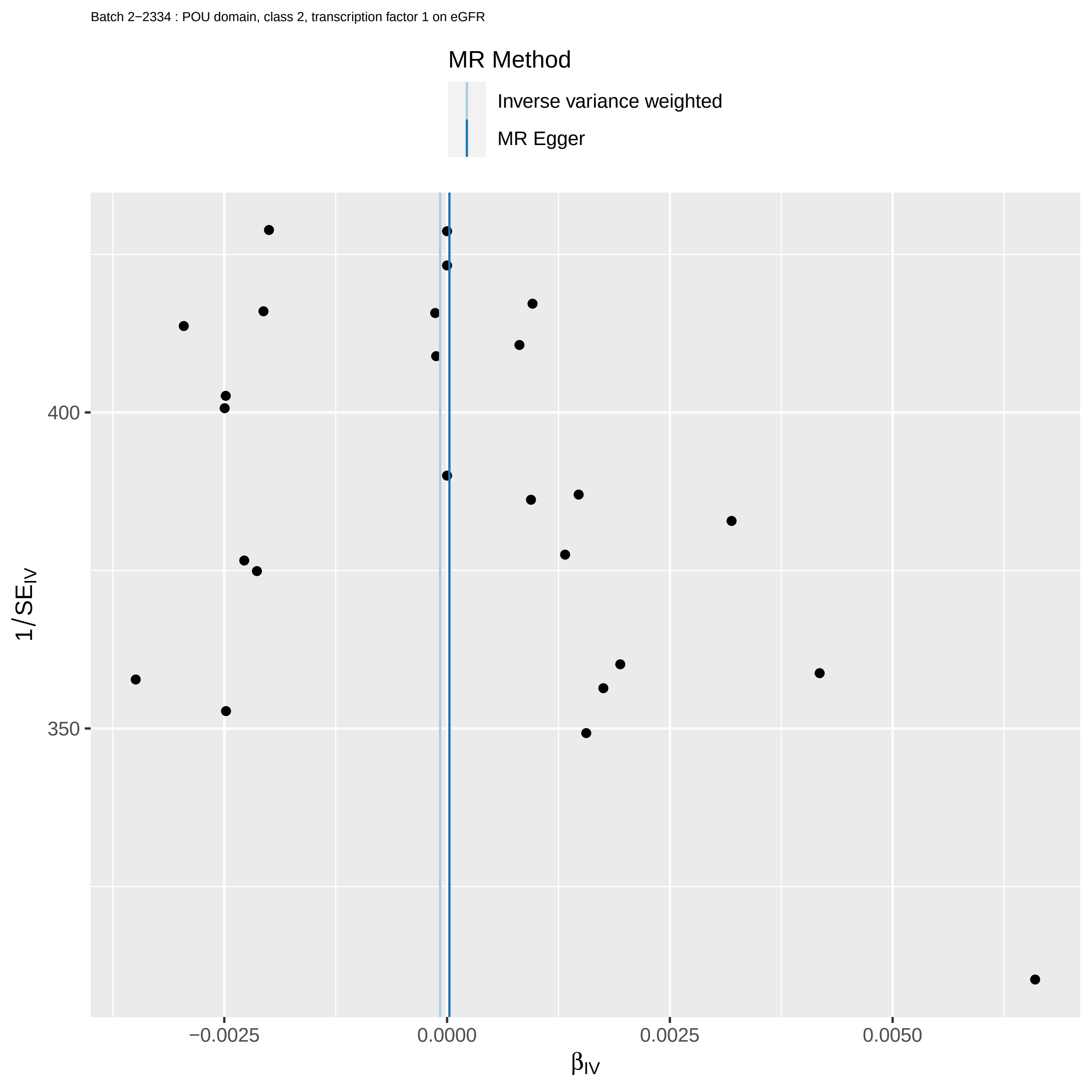

Batch 2−2334 : POU domain, class 2, transcription factor 1 on eGFR
MR Method
Inverse variance weighted
MR Egger
400
350
1/SE$_{IV}$
−0.0025
0.0000
0.0025
0.0050
β$_{IV}$

Batch 2−2335 : Phosphatidate phosphatase PPAPDC1A on eGFR

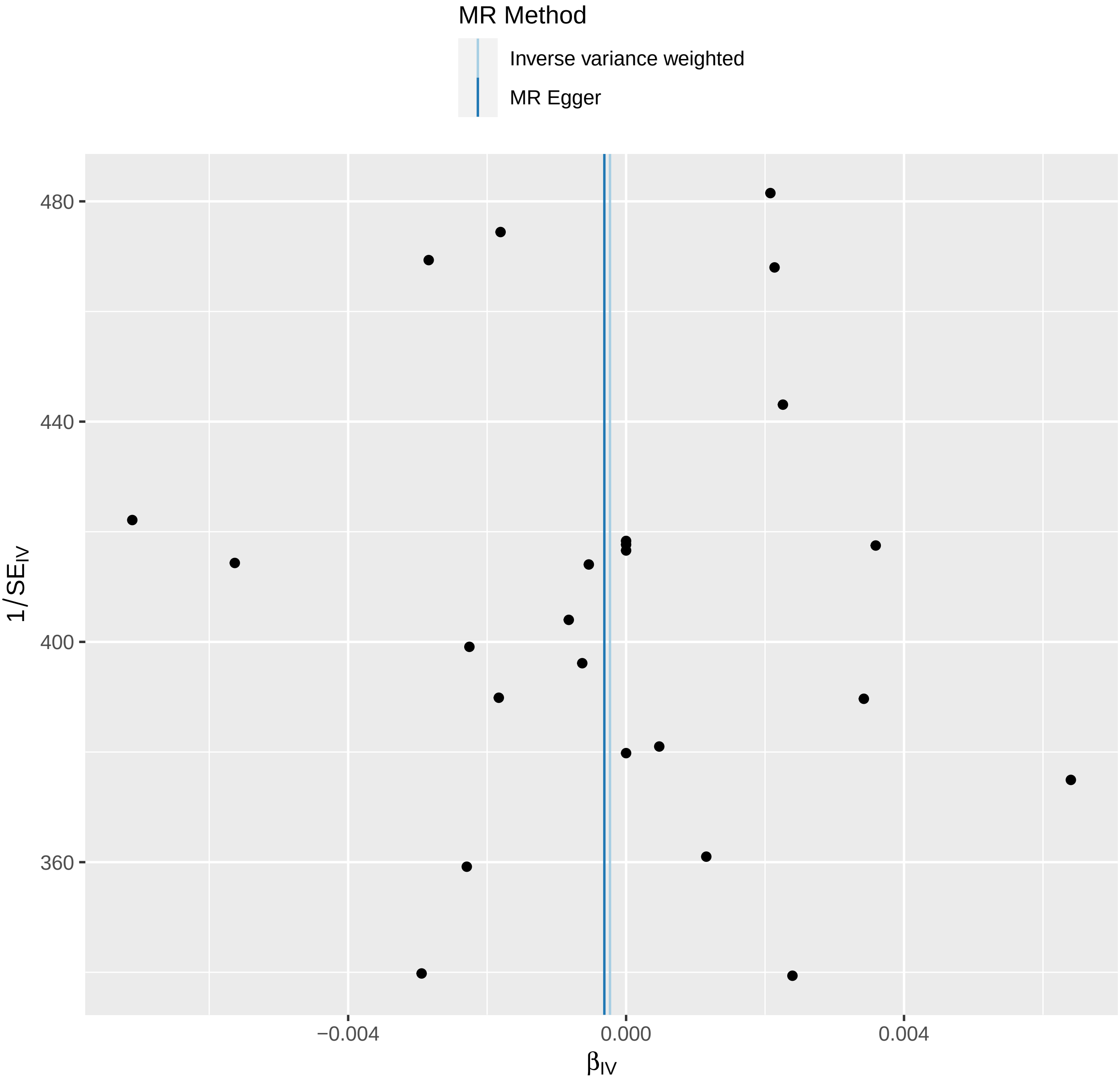

Batch 2−2336 : Peroxisome proliferator−activated receptor alpha on eGFR

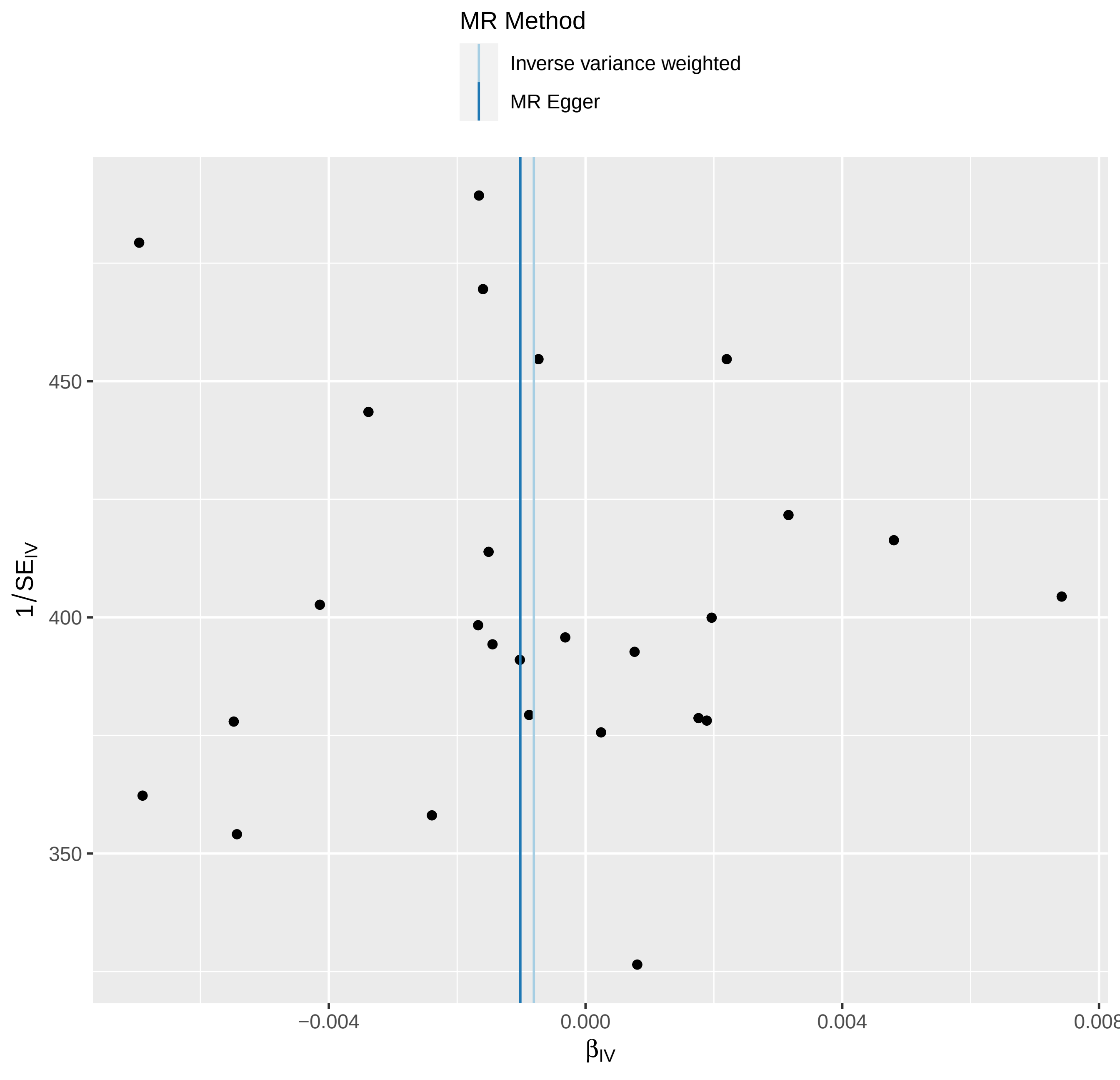

Batch 2−2337 : Neutrophil−activating peptide 2 on eGFR

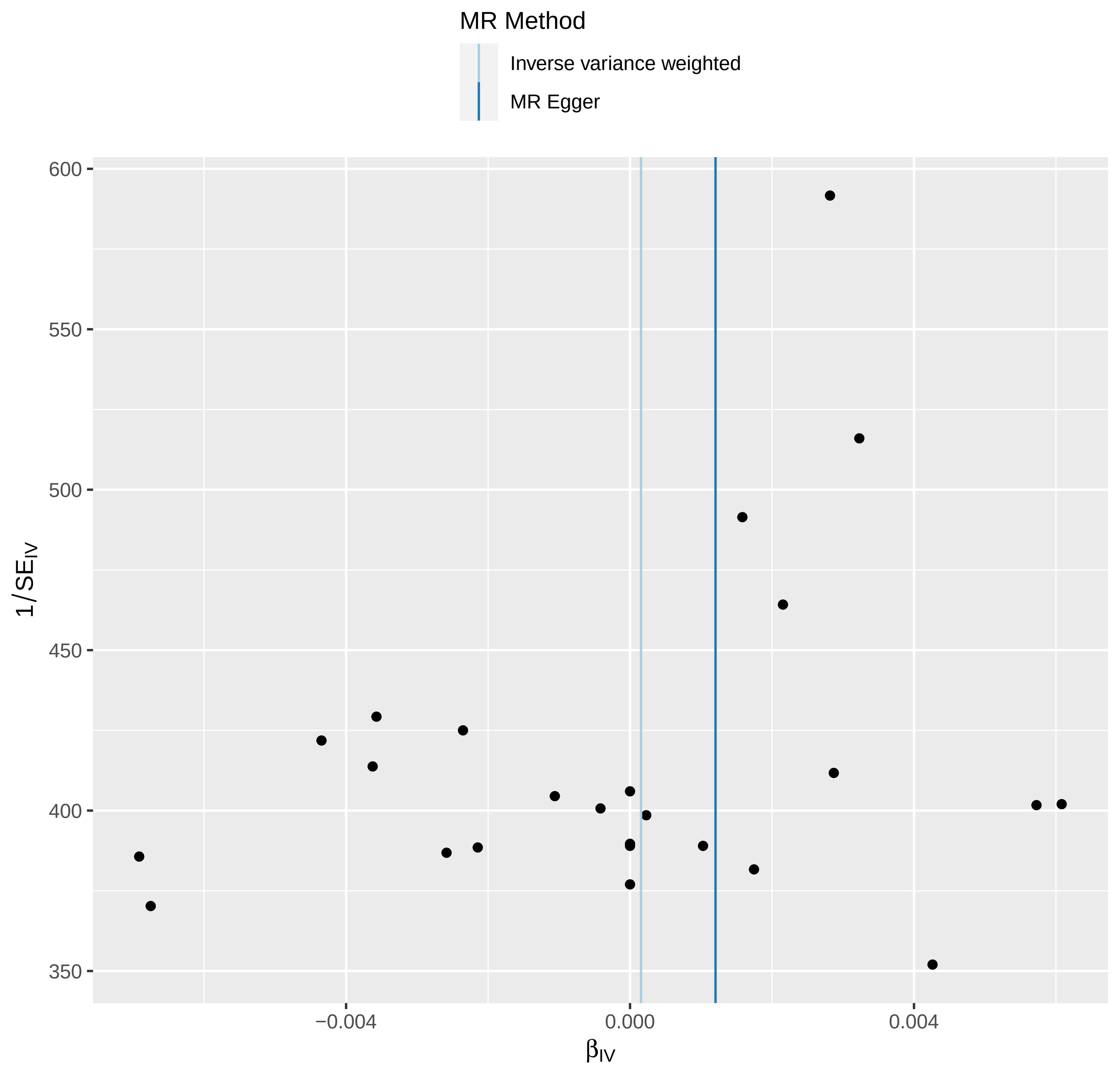

Batch 2−2338 : Phosphopantothenoylcysteine decarboxylase on eGFR

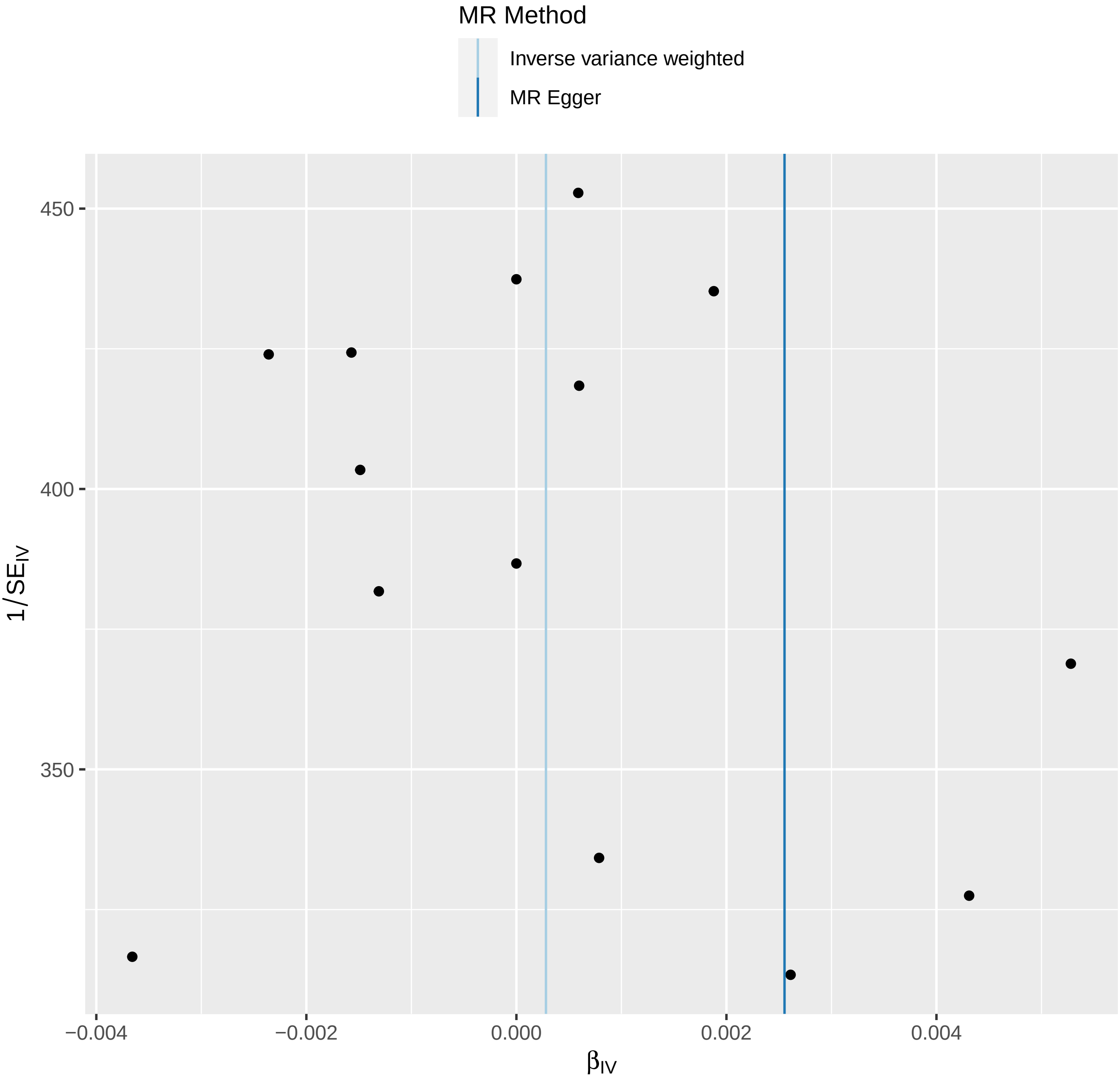

Batch 2−2339 : Peptidyl−prolyl cis−trans isomerase A on eGFR

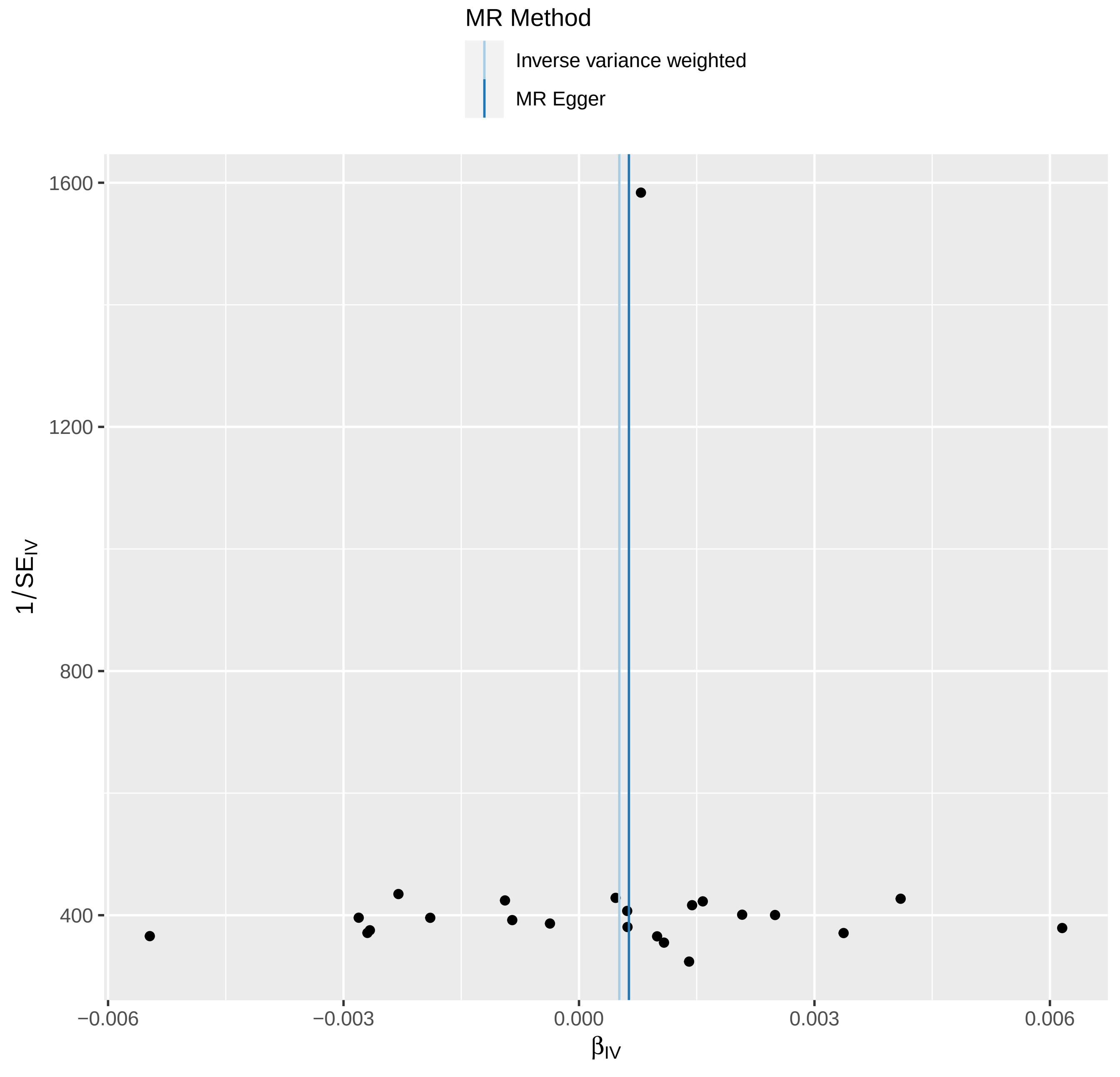

Batch 2−2340 : Peptidyl−prolyl cis−trans isomerase B on eGFR

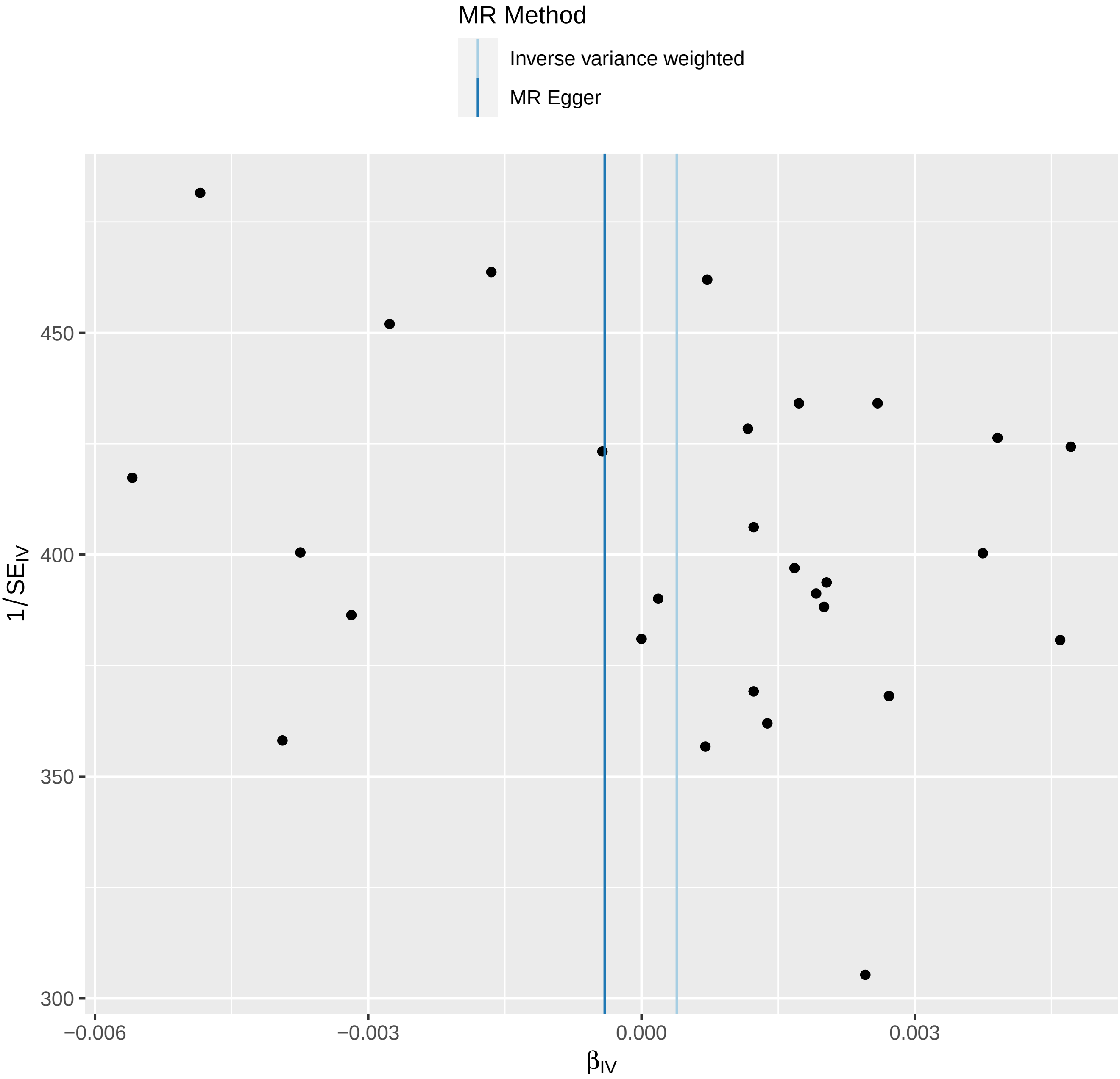

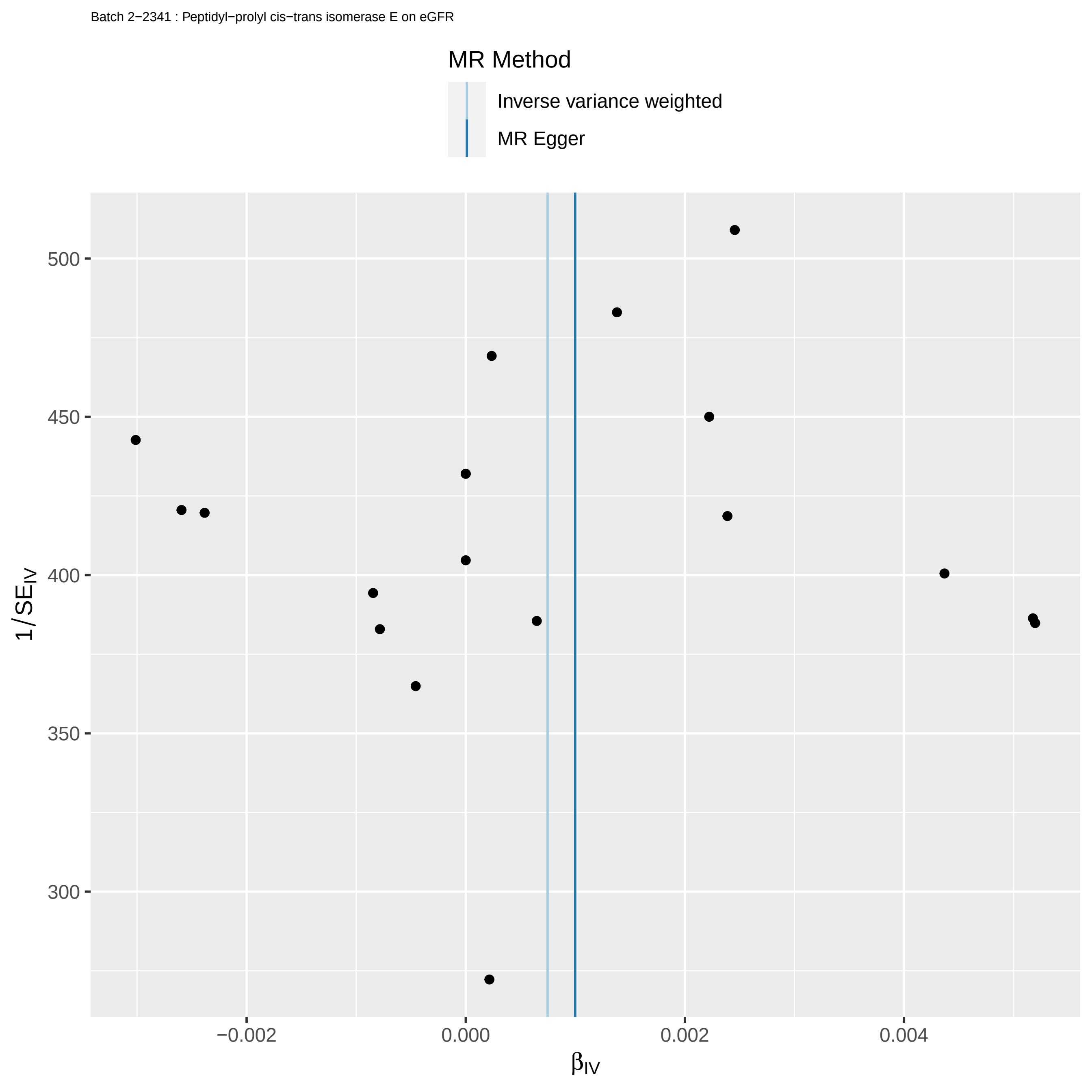

Batch 2−2341 : Peptidyl−prolyl cis−trans isomerase E on eGFR
MR Method
Inverse variance weighted
MR Egger
500
450
400
350
300
1/SE_IV
−0.002
0.000
0.002
0.004
β_IV

Batch 2−2342 : Peptidyl−prolyl cis−trans isomerase H on eGFR

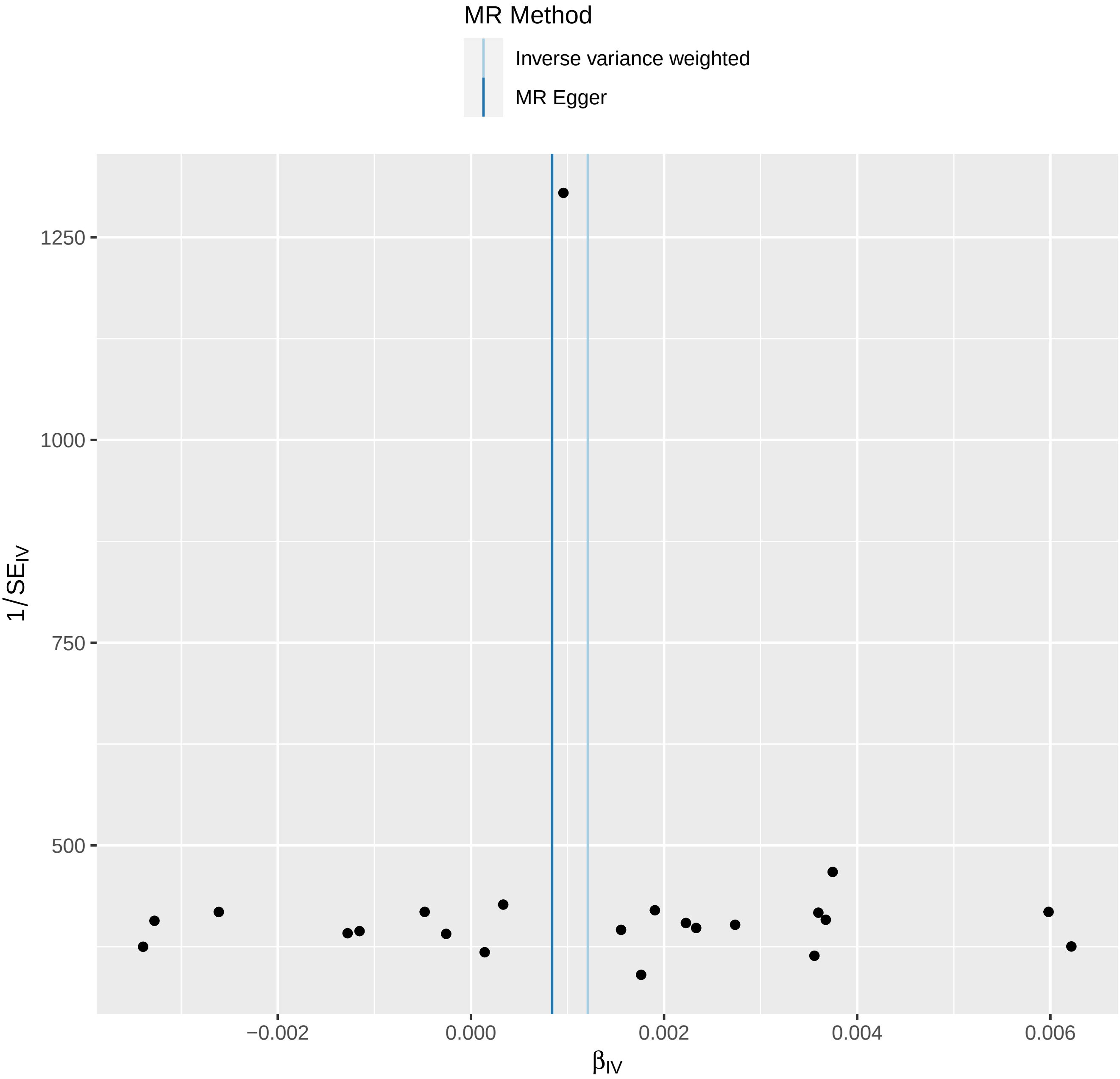

Batch 2−2343 : Peptidyl−prolyl cis−trans isomerase−like 1 on eGFR

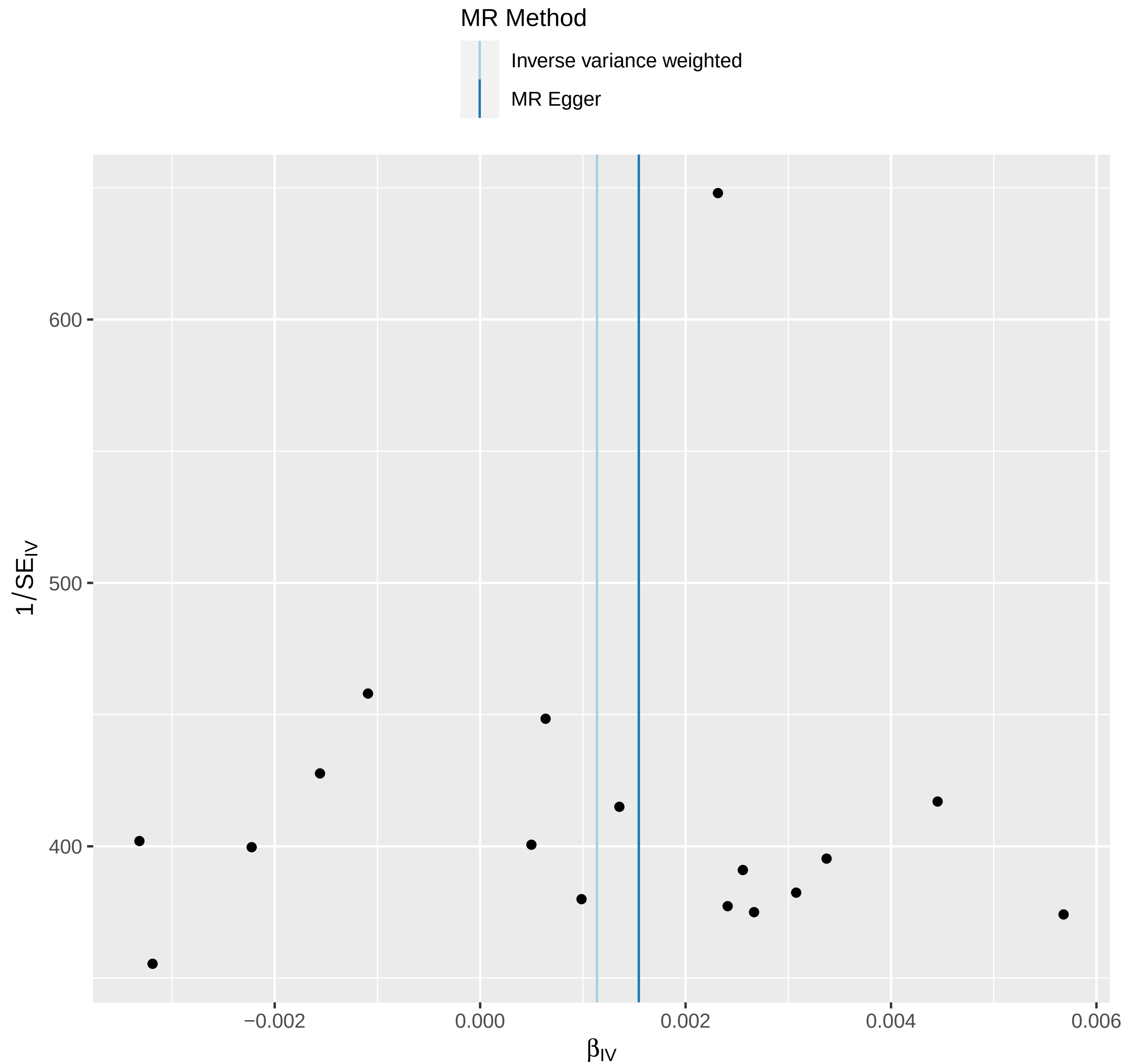

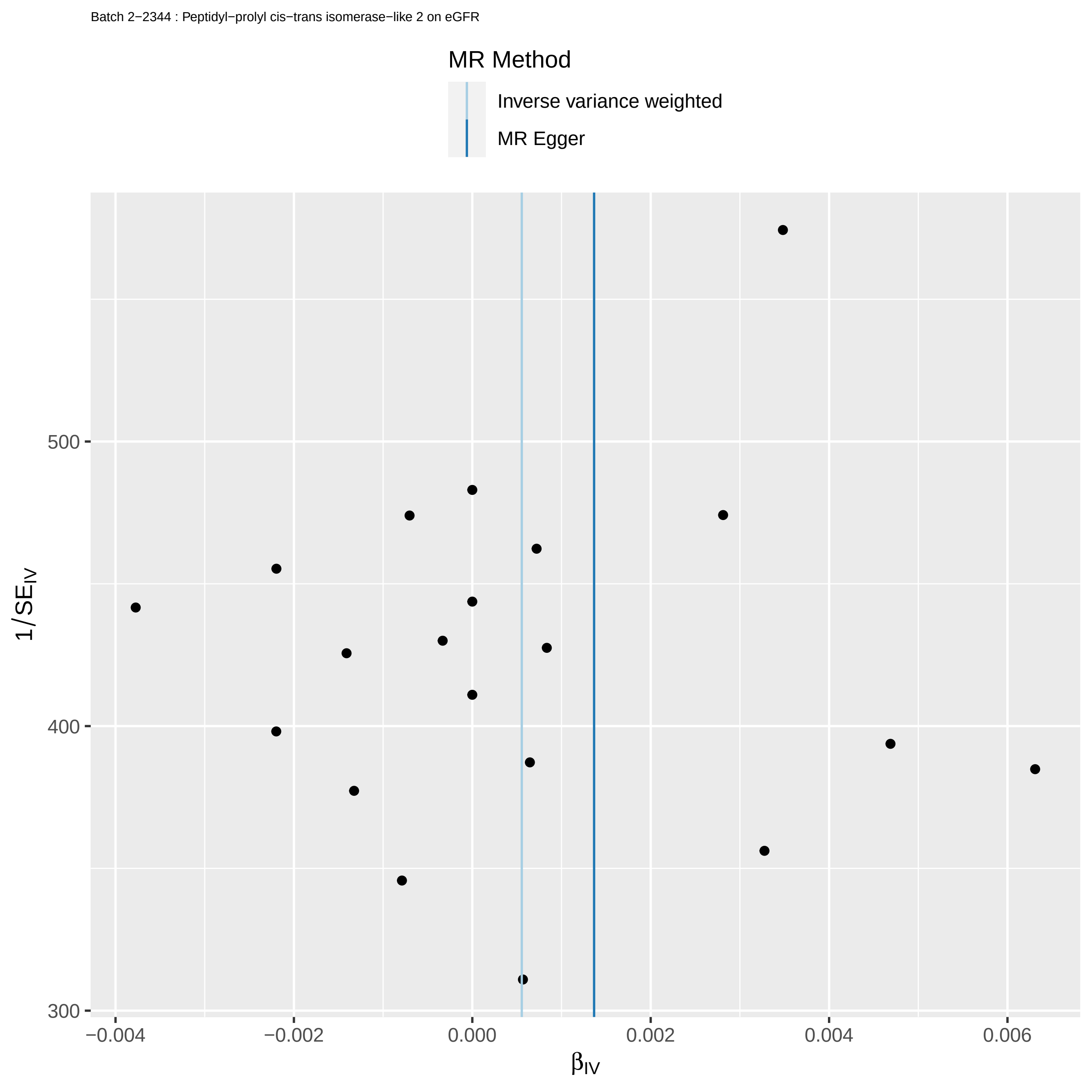
Batch 2−2344 : Peptidyl−prolyl cis−trans isomerase−like 2 on eGFR
MR Method
Inverse variance weighted
MR Egger
500
400
300
1/SE_IV
−0.004
−0.002
0.000
0.002
0.004
0.006
β_IV

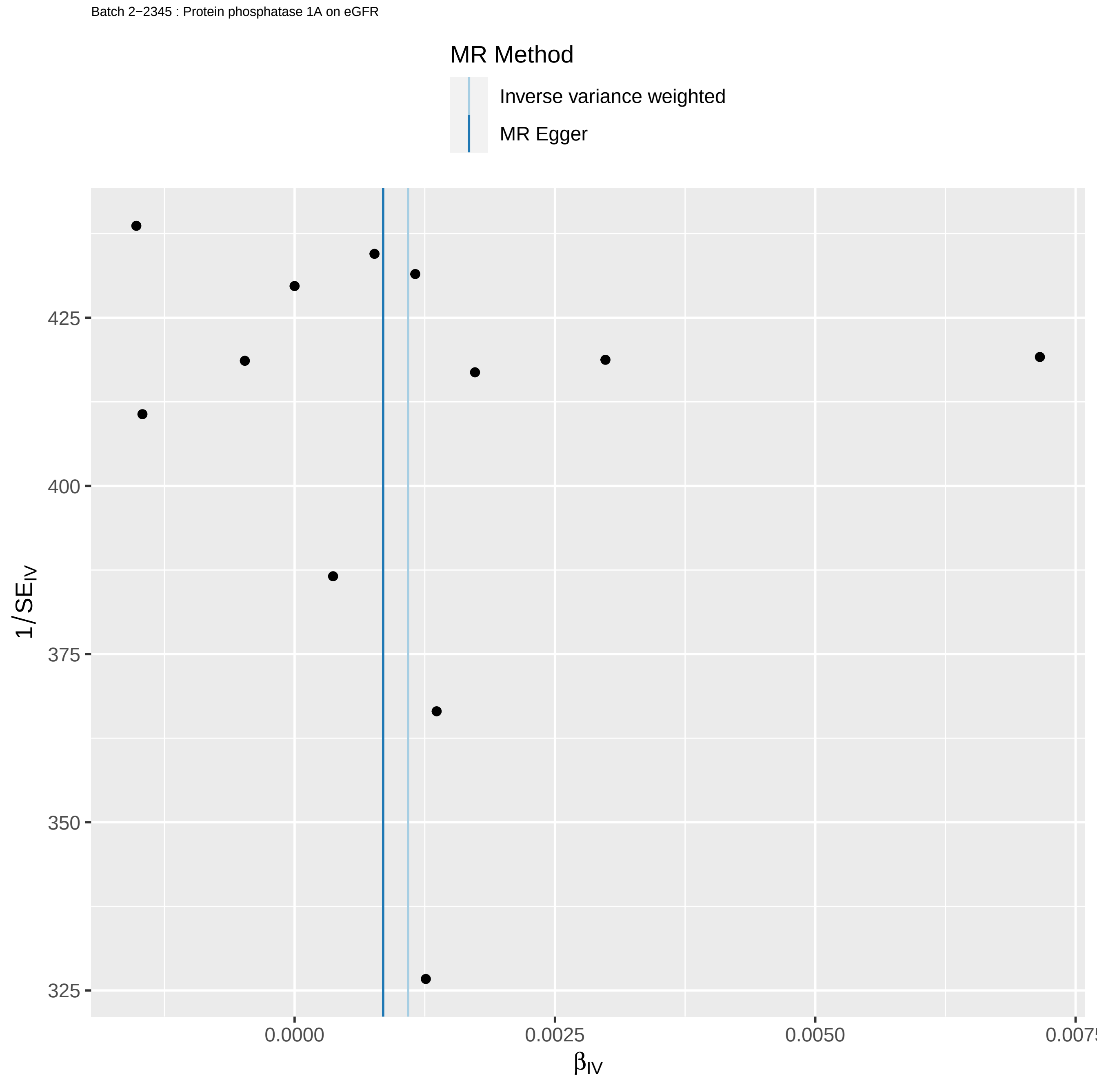

Batch 2−2345 : Protein phosphatase 1A on eGFR
MR Method
Inverse variance weighted
MR Egger
1/SE_IV
425
400
375
350
325
0.0000
0.0025
0.0050
0.0075
β_IV

Batch 2−2346 : Protein phosphatase 1L on eGFR

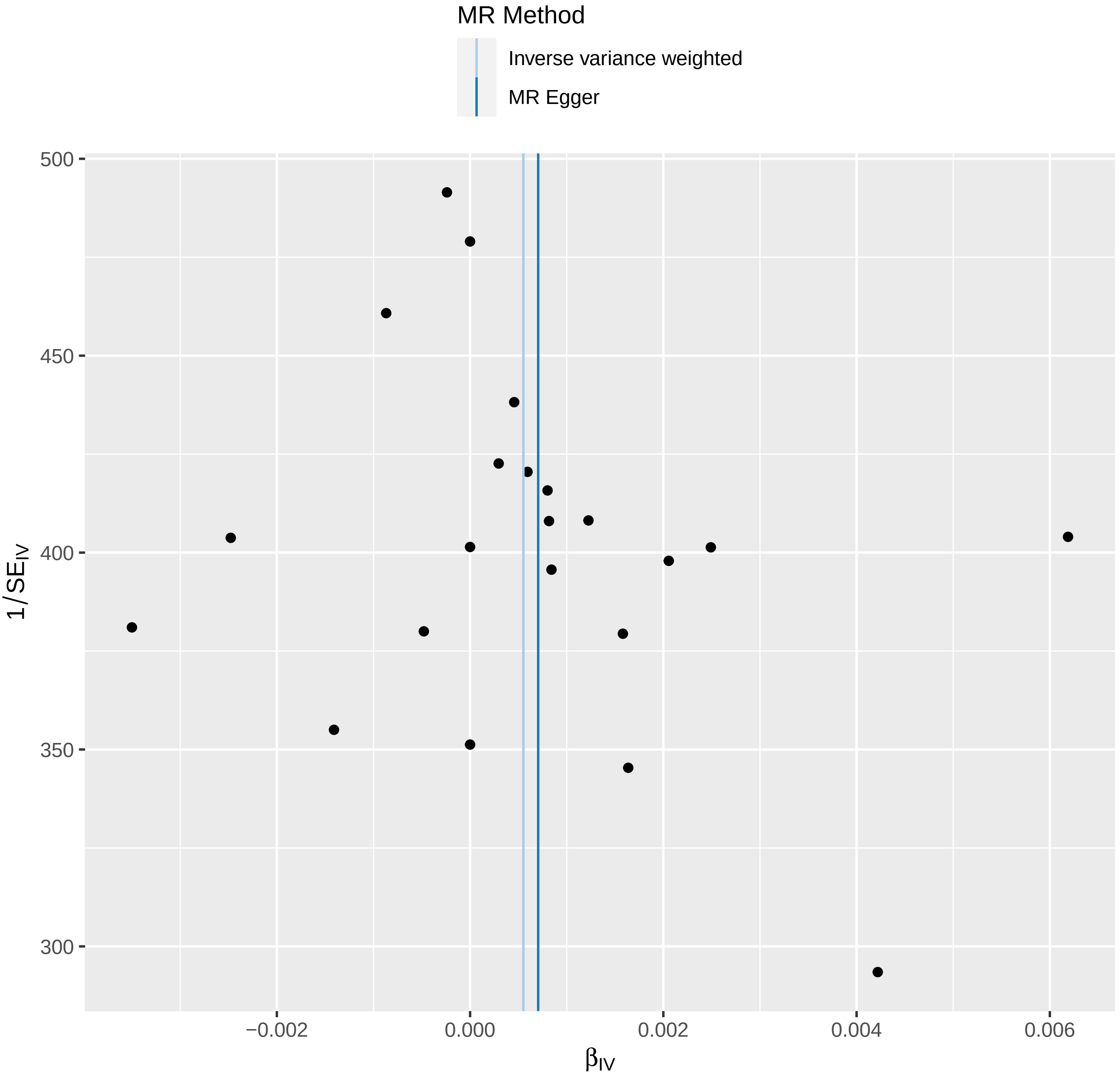

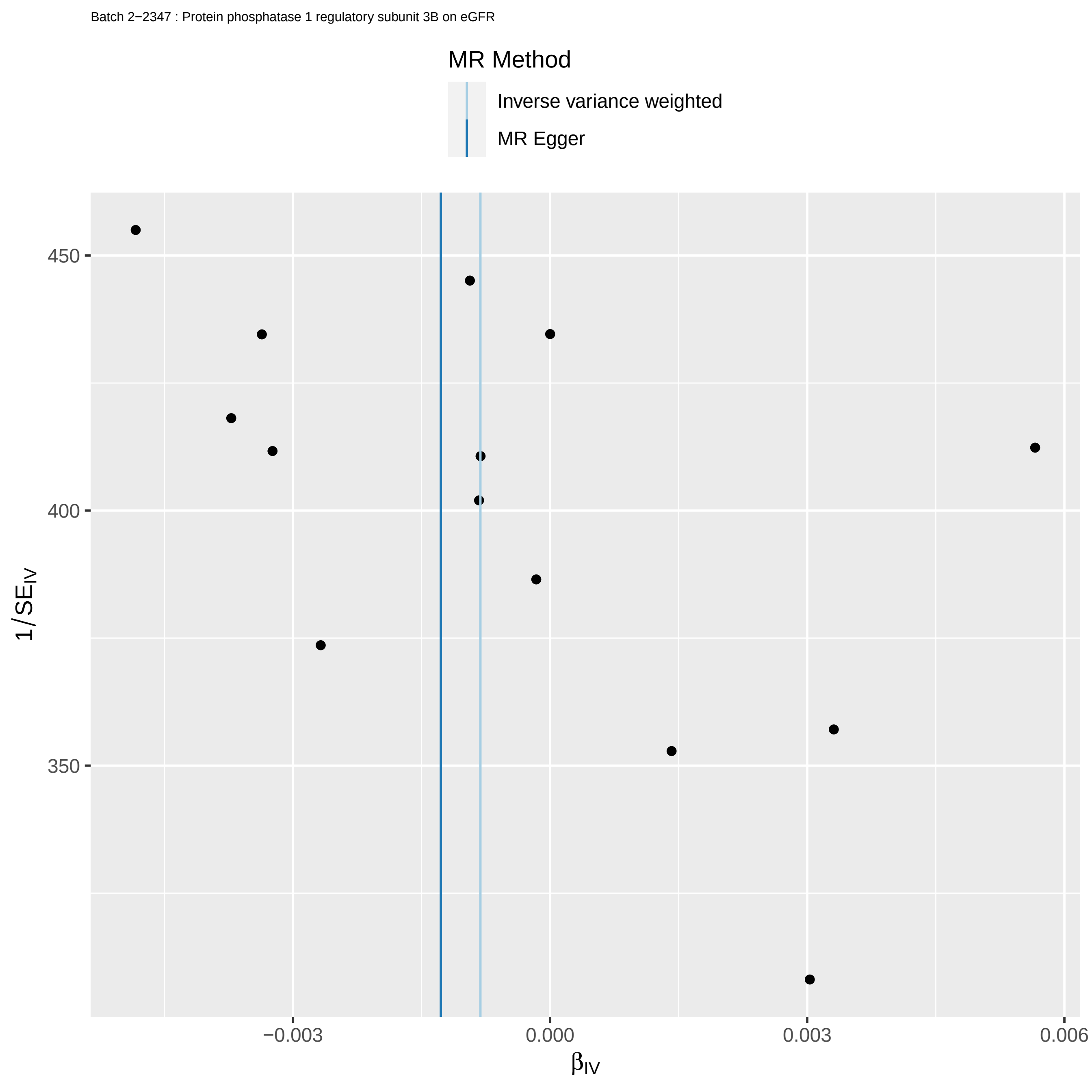
Batch 2−2347 : Protein phosphatase 1 regulatory subunit 3B on eGFR
MR Method
Inverse variance weighted
MR Egger
450
400
350
1/SE_IV
−0.003
0.000
0.003
0.006
β_IV

Batch 2−2348 : Serine/threonine−protein phosphatase 2A 65 kDa regulatory subunit A alpha isoform on eGFR

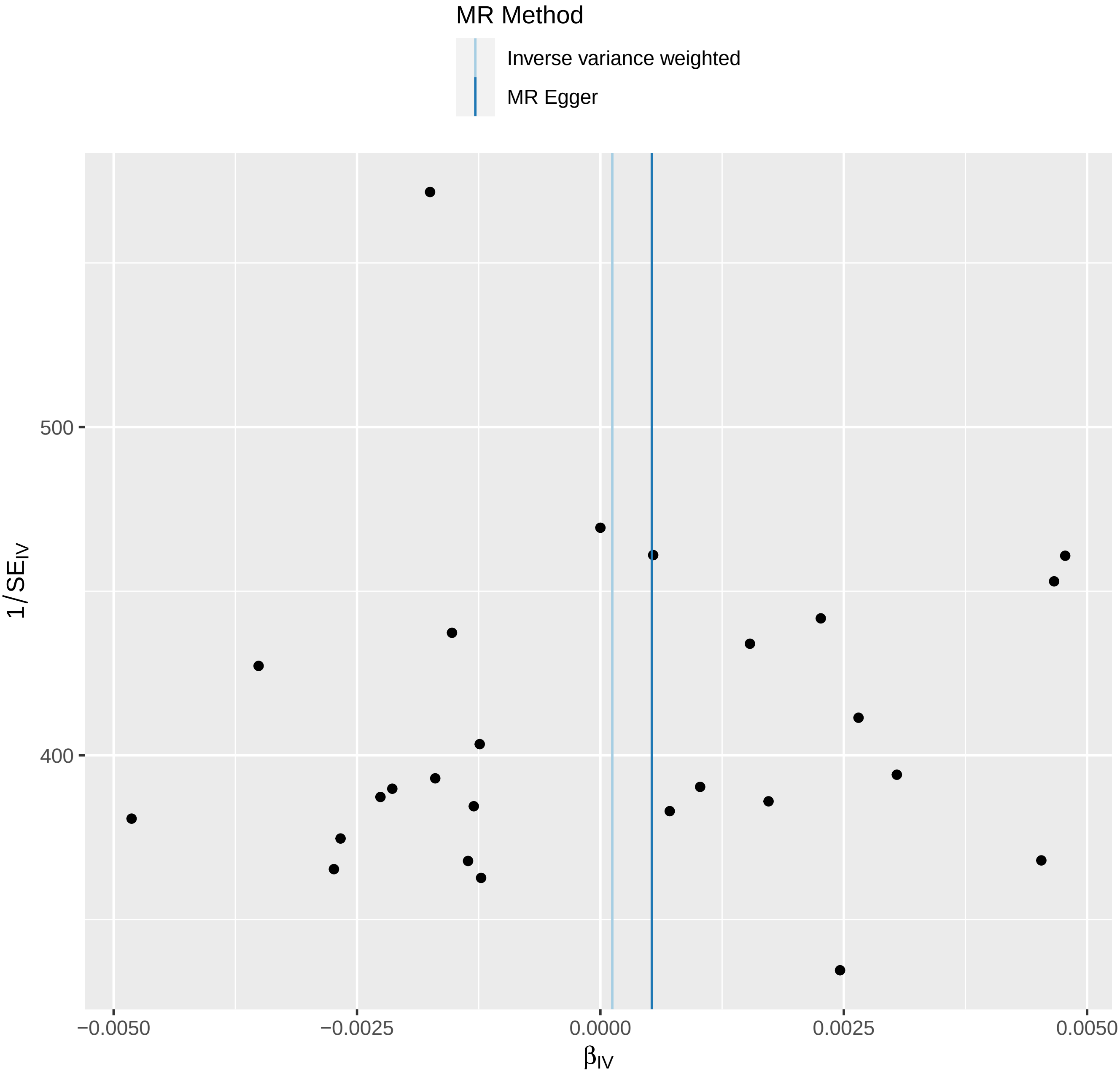

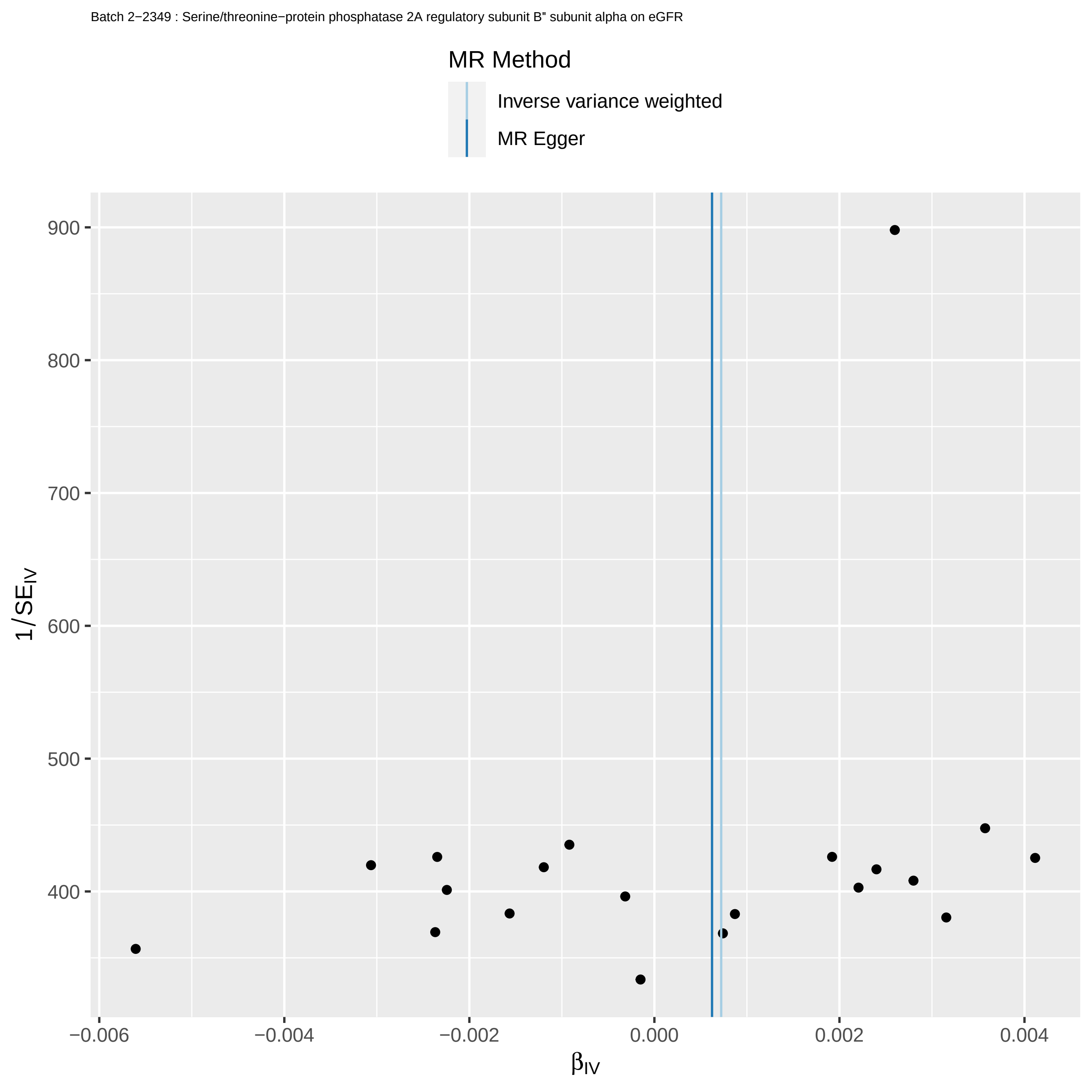

Batch 2−2349 : Serine/threonine−protein phosphatase 2A regulatory subunit B" subunit alpha on eGFR
MR Method
Inverse variance weighted
MR Egger
900
800
700
600
500
400
$1/SE_{IV}$
−0.006
−0.004
−0.002
0.000
0.002
0.004
β_{IV}

Batch 2−2350 : Calcineurin subunit B type 1 on eGFR

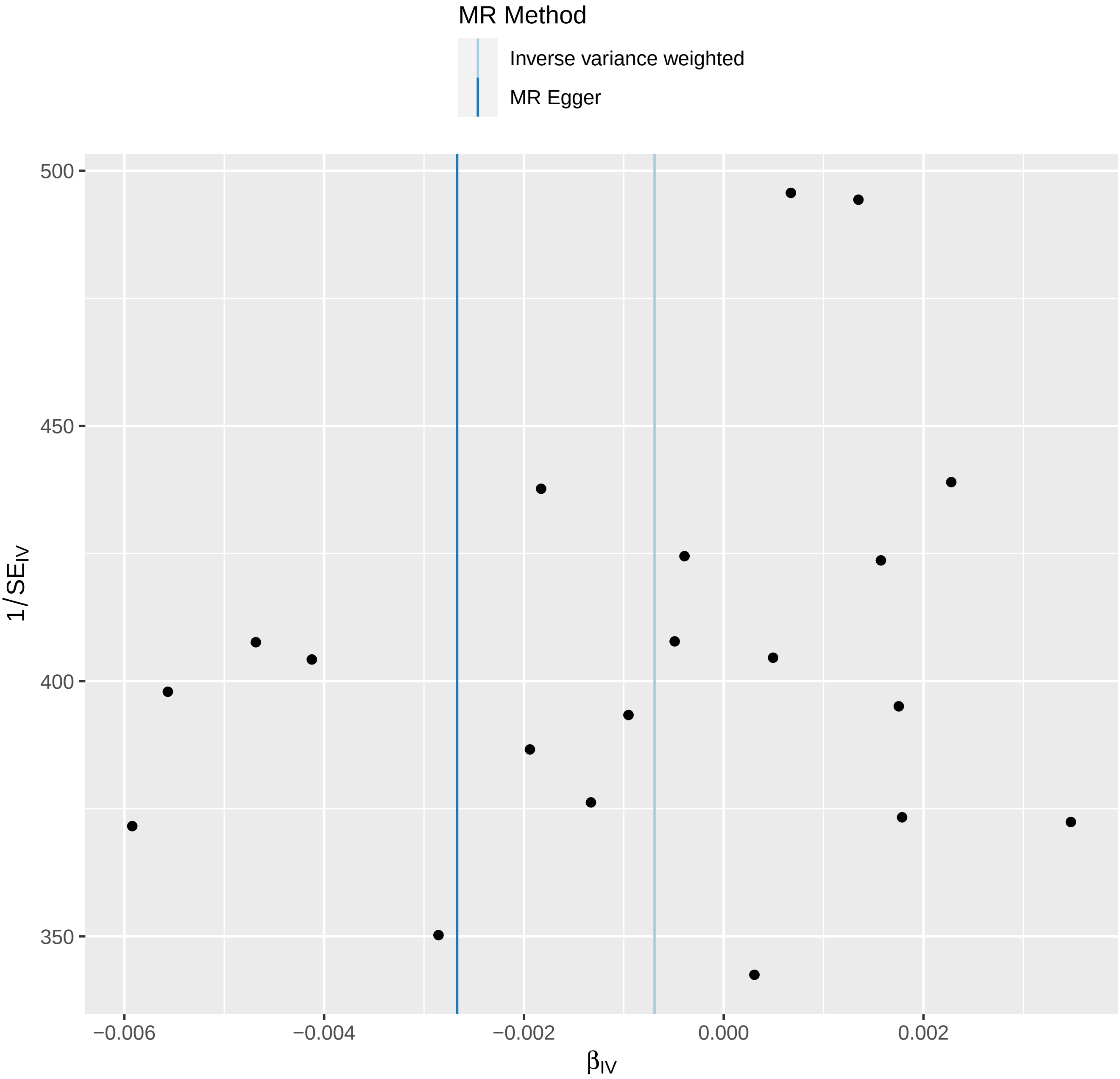

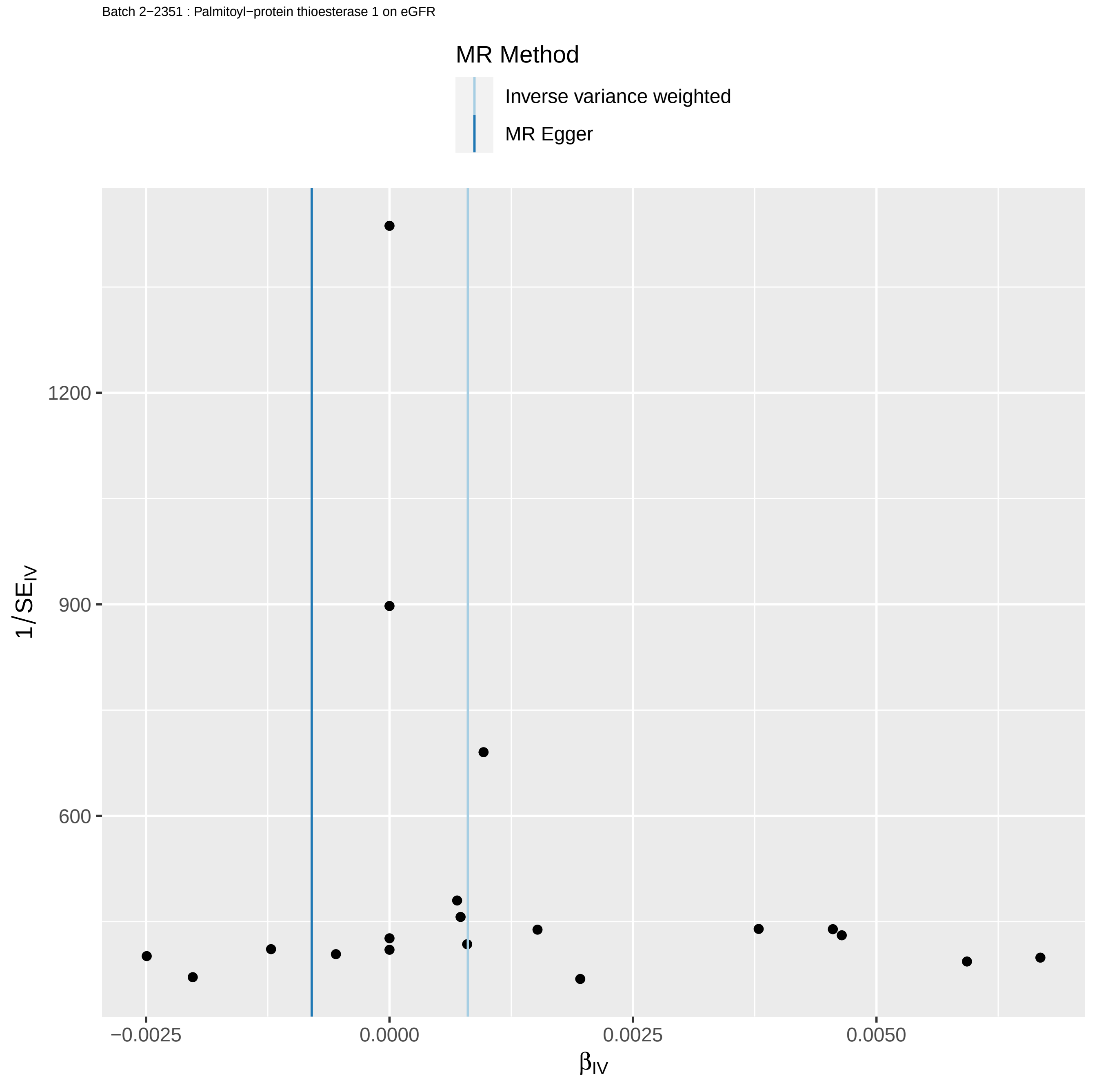
Batch 2−2351 : Palmitoyl−protein thioesterase 1 on eGFR
MR Method
Inverse variance weighted
MR Egger
1200
900
600
1/SE$_{IV}$
−0.0025
0.0000
0.0025
0.0050
β_{IV}

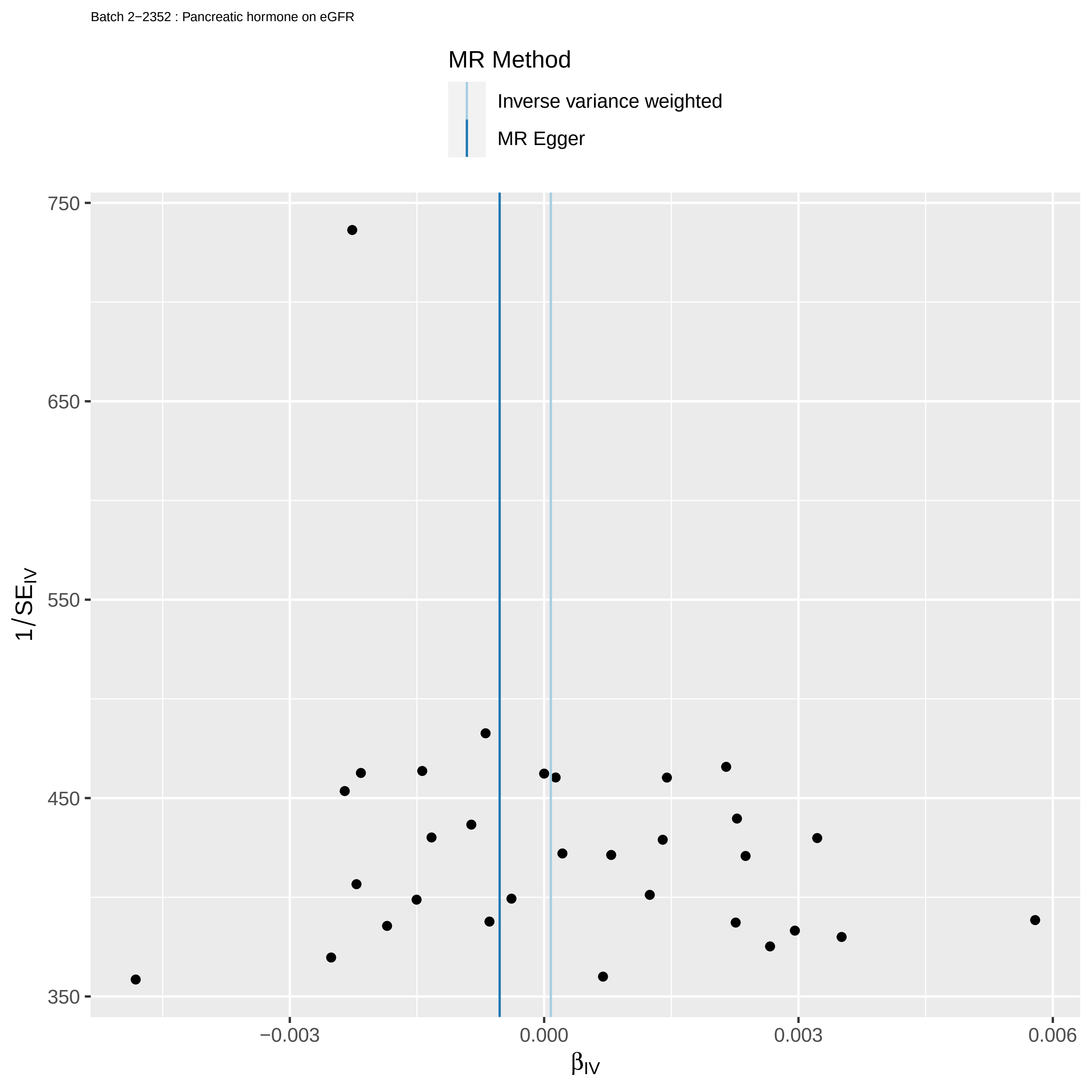
Batch 2−2352 : Pancreatic hormone on eGFR
MR Method
Inverse variance weighted
MR Egger
750
650
550
450
350
1/SE$_{IV}$
−0.003
0.000
0.003
0.006
β$_{IV}$

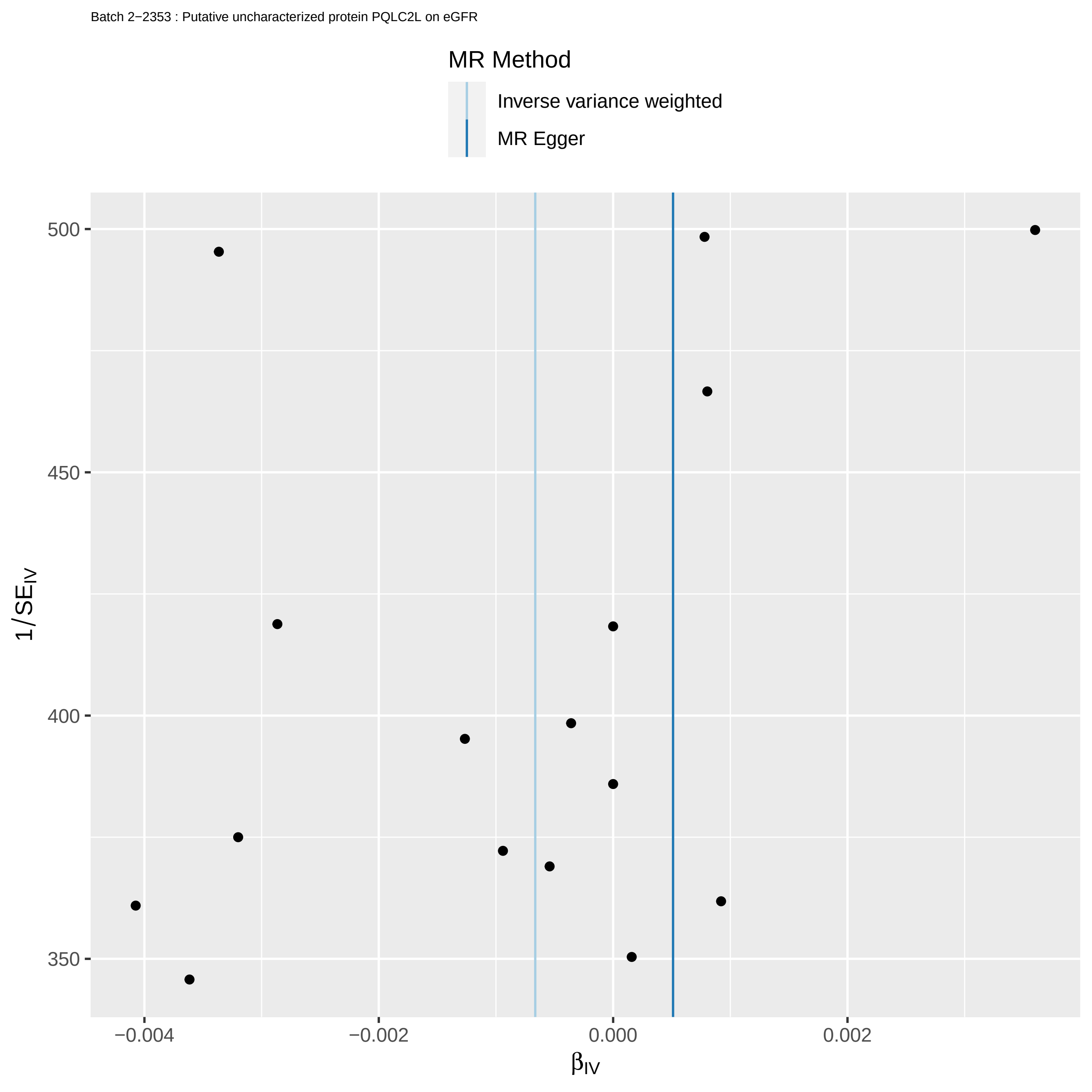

Batch 2−2353 : Putative uncharacterized protein PQLC2L on eGFR
MR Method
Inverse variance weighted
MR Egger
500
450
400
350
1/SE_IV
−0.004
−0.002
0.000
0.002
β_IV

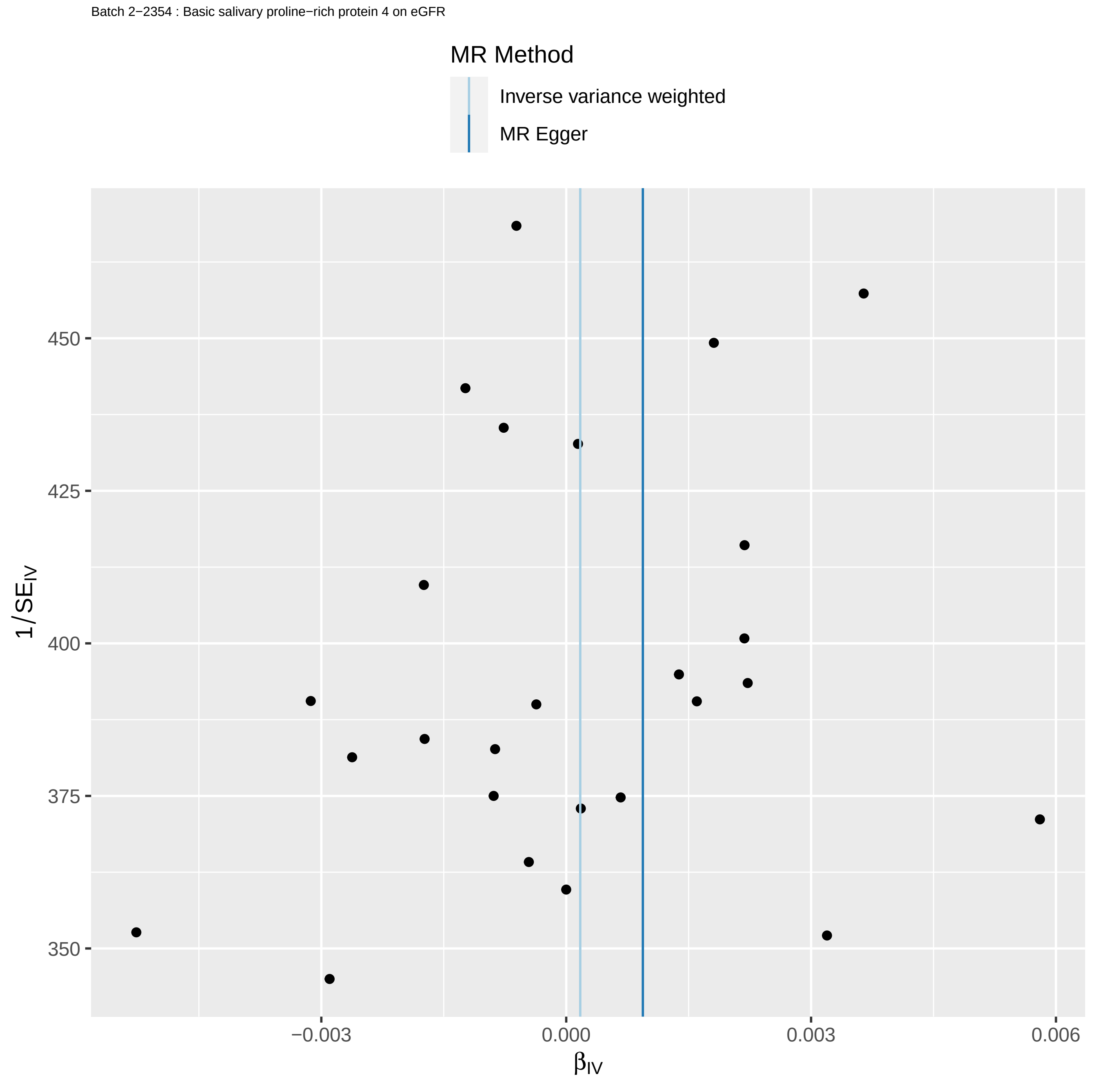

Batch 2−2354 : Basic salivary proline−rich protein 4 on eGFR
MR Method
Inverse variance weighted
MR Egger
450
425
400
375
350
1/SE_IV
−0.003
0.000
0.003
0.006
β_IV

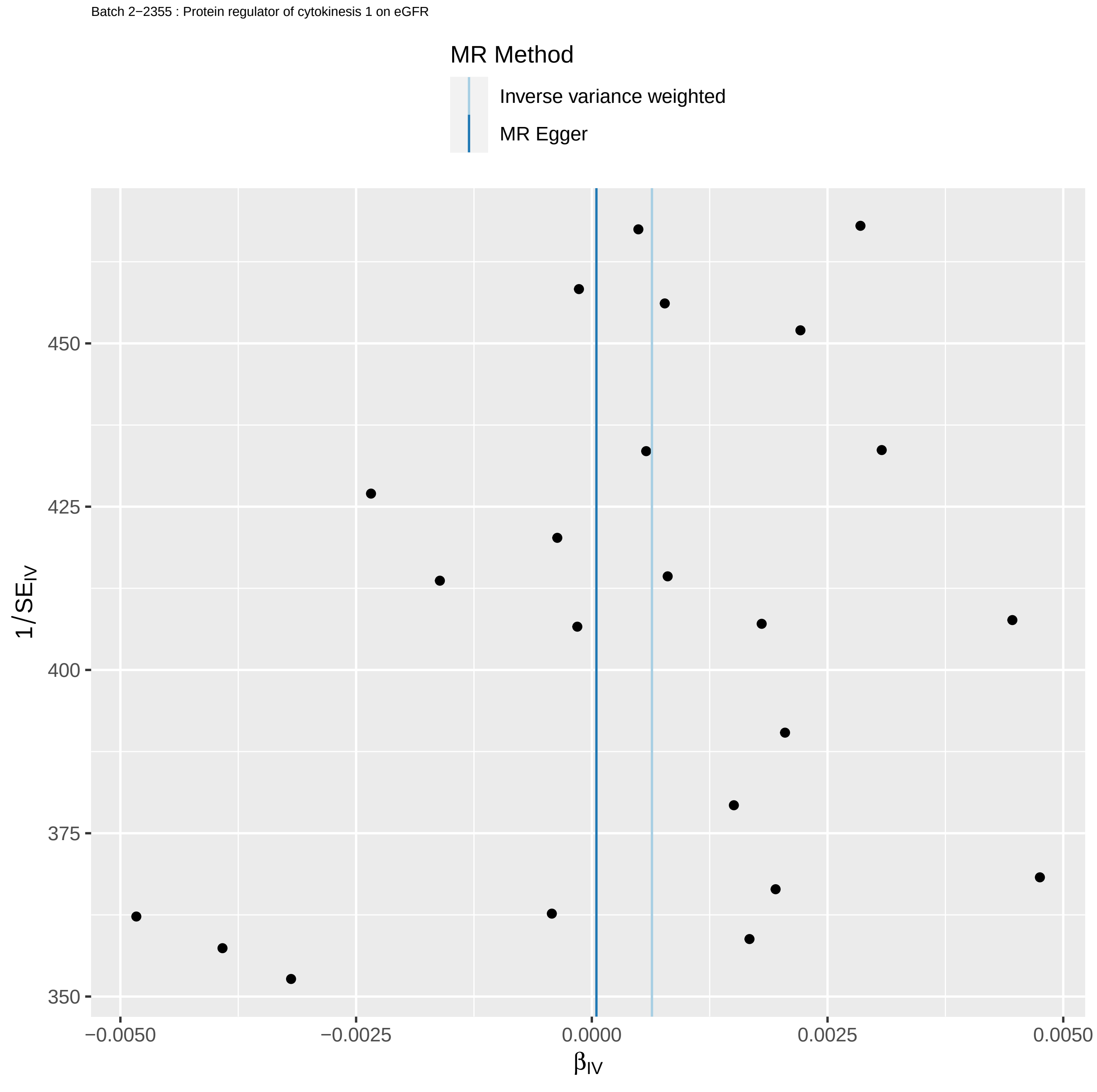

Batch 2−2355 : Protein regulator of cytokinesis 1 on eGFR
MR Method
Inverse variance weighted
MR Egger
450
425
400
375
350
1/SE_IV
−0.0050
−0.0025
0.0000
0.0025
0.0050
β_IV

Batch 2−2356 : Lysosomal Pro−X carboxypeptidase on eGFR

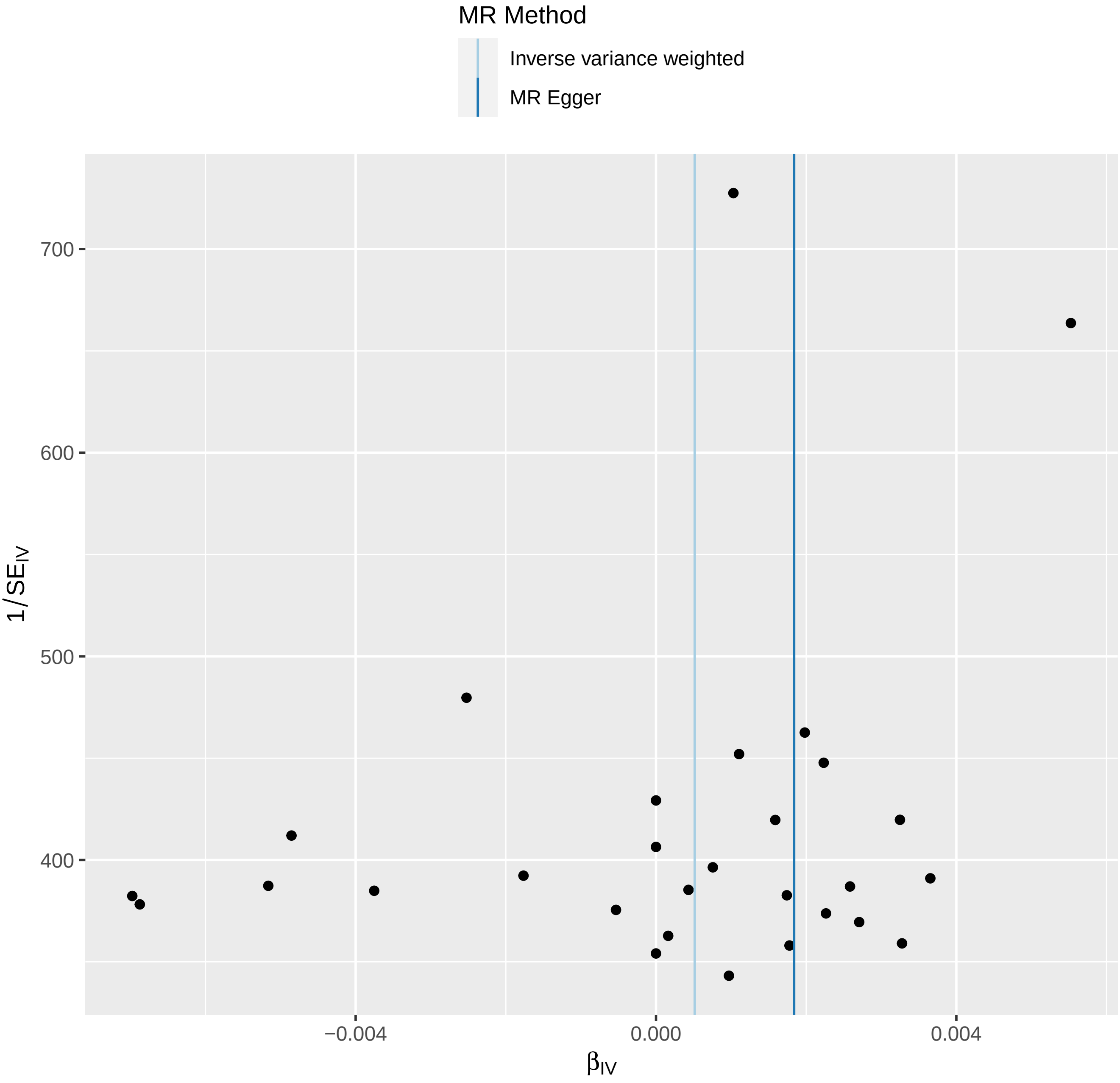

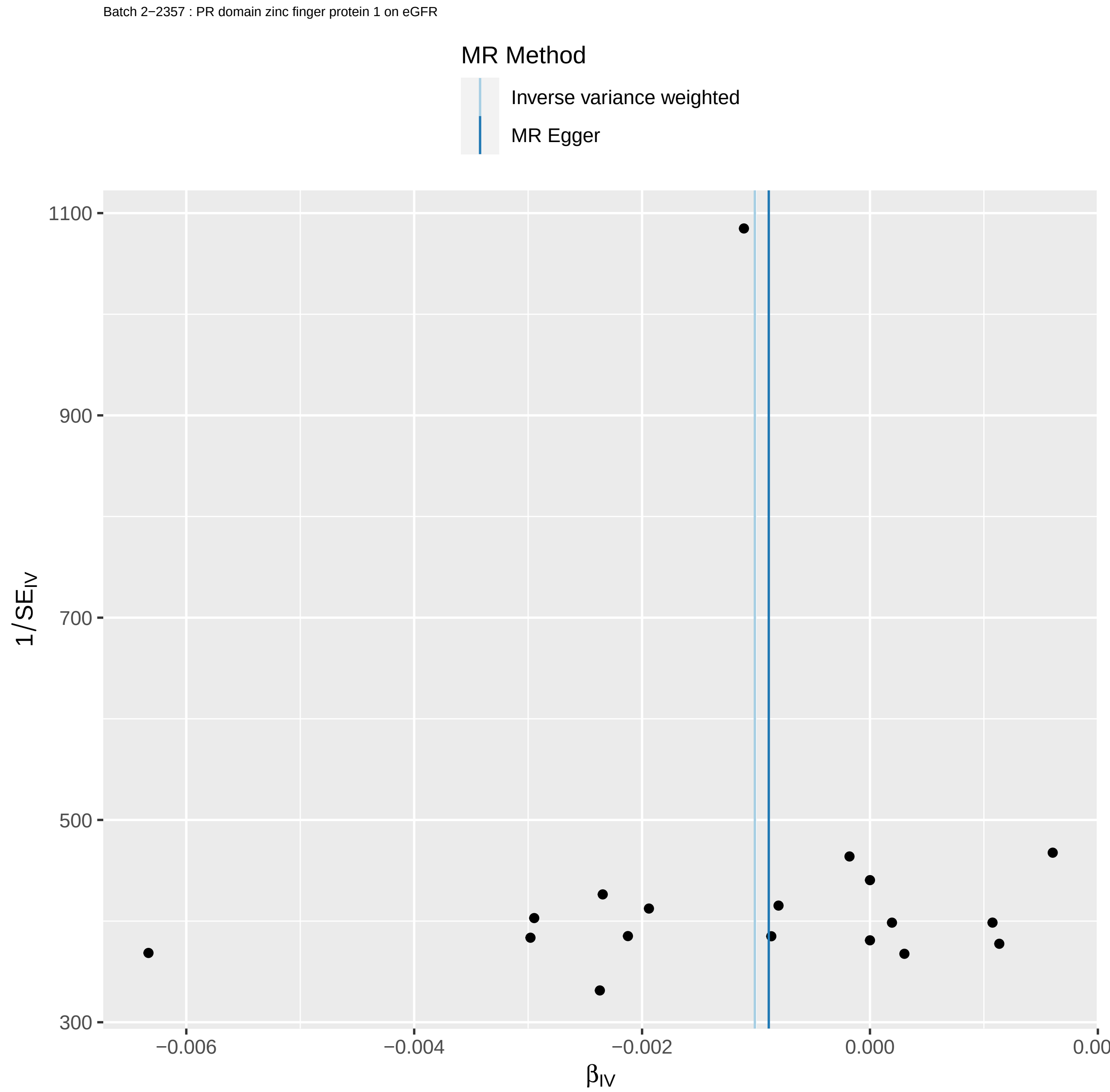

Batch 2−2357 : PR domain zinc finger protein 1 on eGFR
MR Method
Inverse variance weighted
MR Egger
1100
900
700
500
300
1/SE_IV
−0.006
−0.004
−0.002
0.000
0.00
β_IV

Batch 2−2358 : PR domain zinc finger protein 4 on eGFR

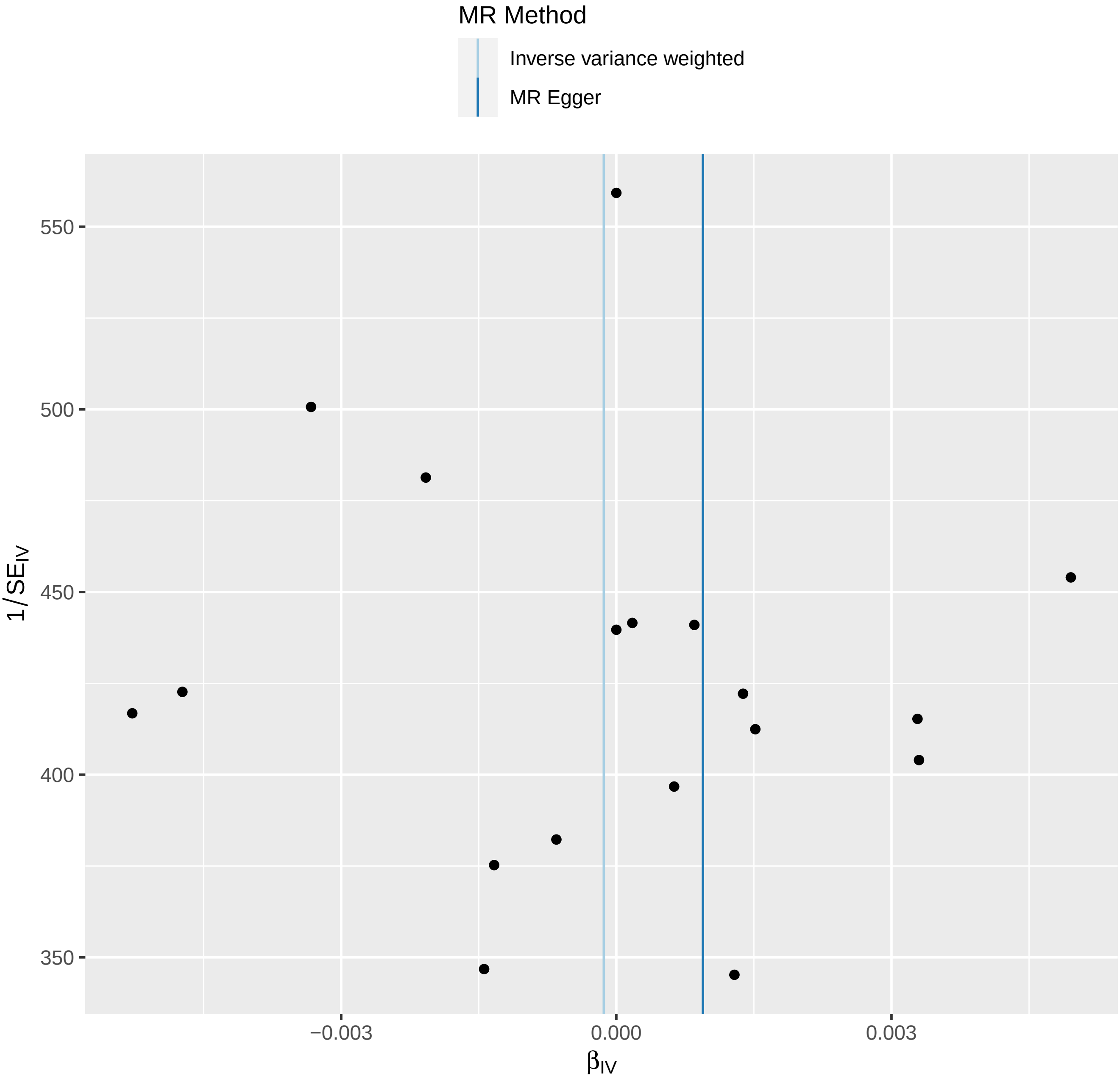

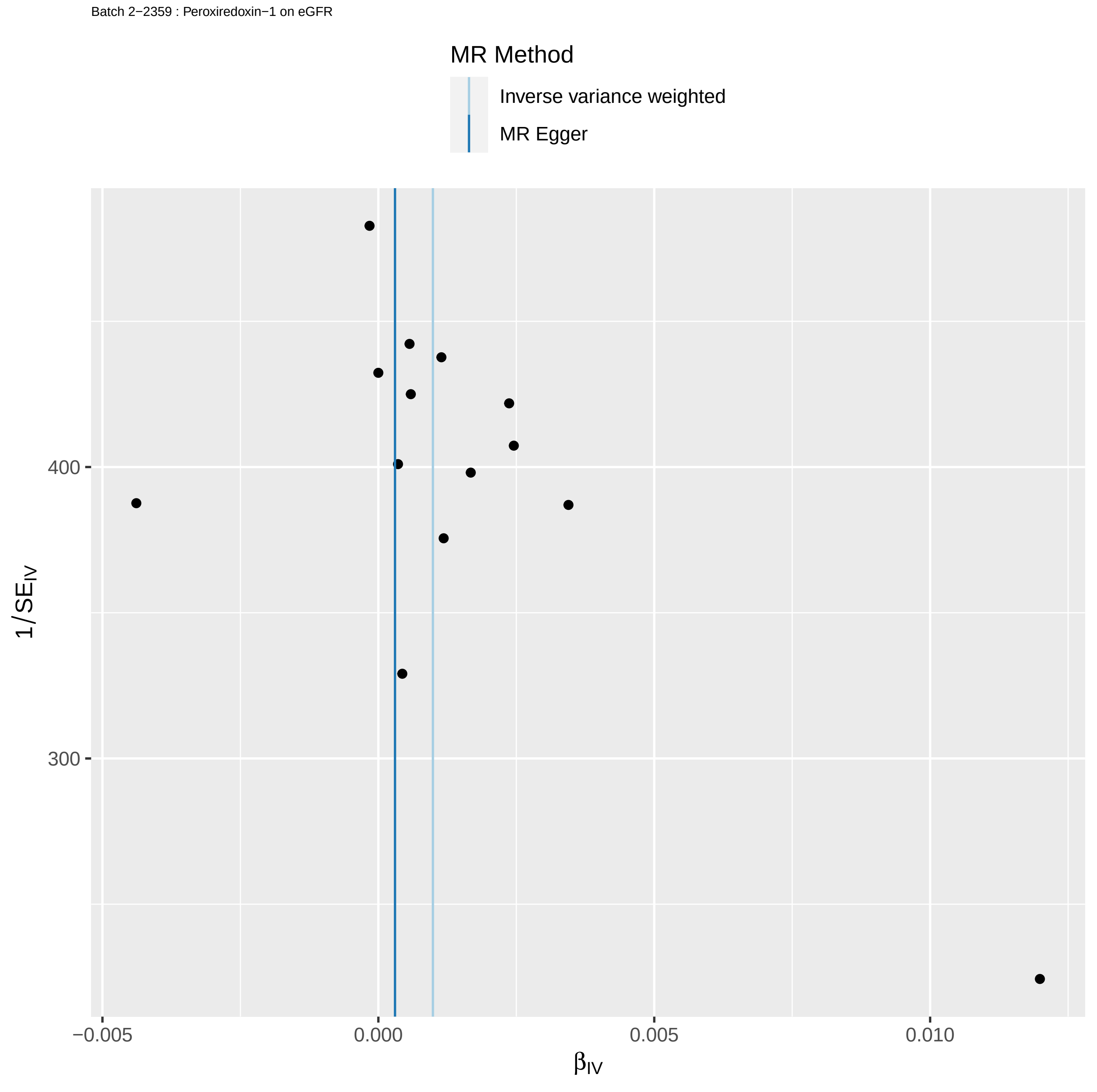

Batch 2−2359 : Peroxiredoxin−1 on eGFR
MR Method
Inverse variance weighted
MR Egger
1/SE_IV
400
300
−0.005
0.000
0.005
0.010
β_IV

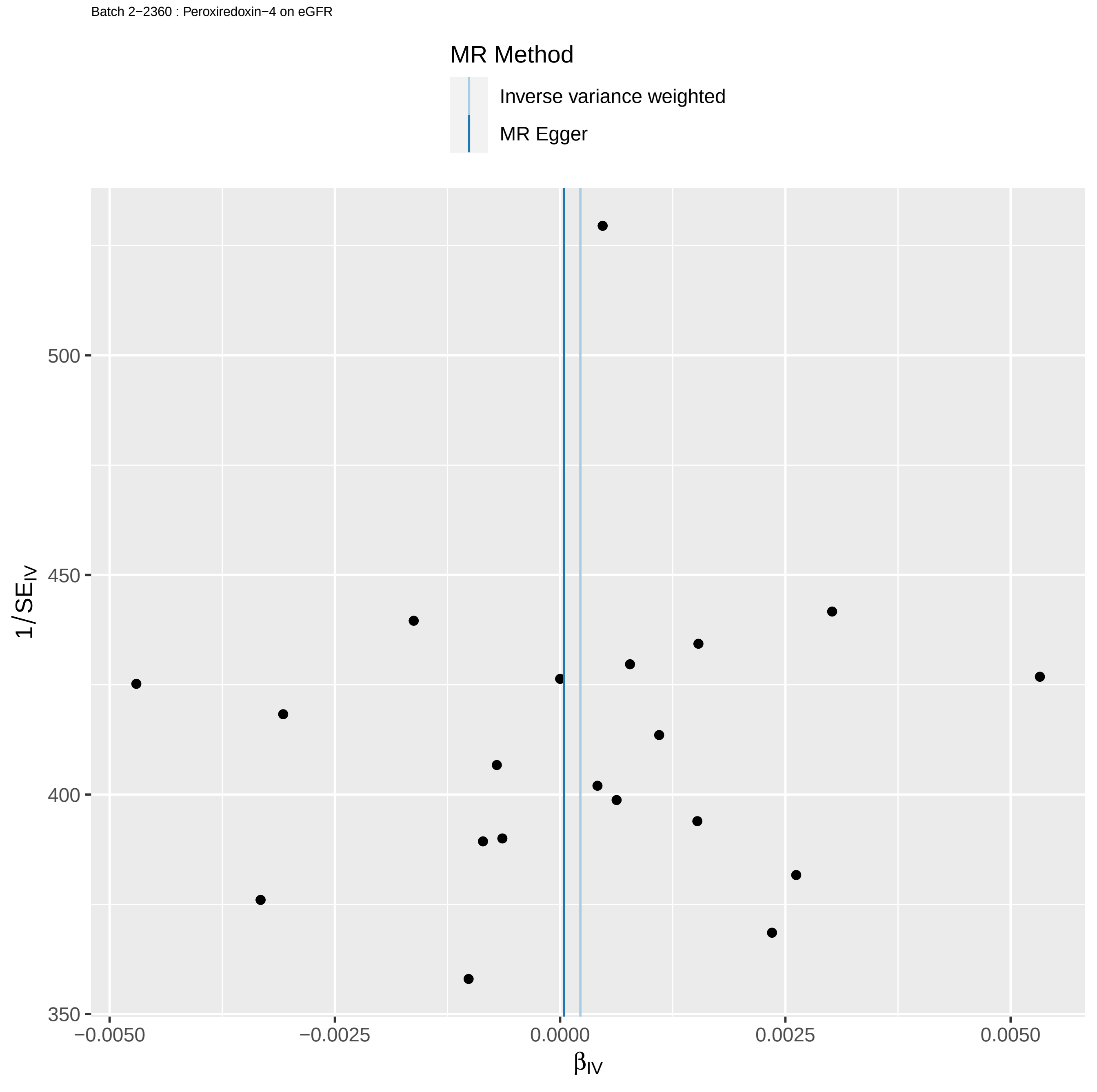

Batch 2−2360 : Peroxiredoxin−4 on eGFR
MR Method
Inverse variance weighted
MR Egger
500
450
400
350
1/SE$_{IV}$
−0.0050
−0.0025
0.0000
0.0025
0.0050
β$_{IV}$

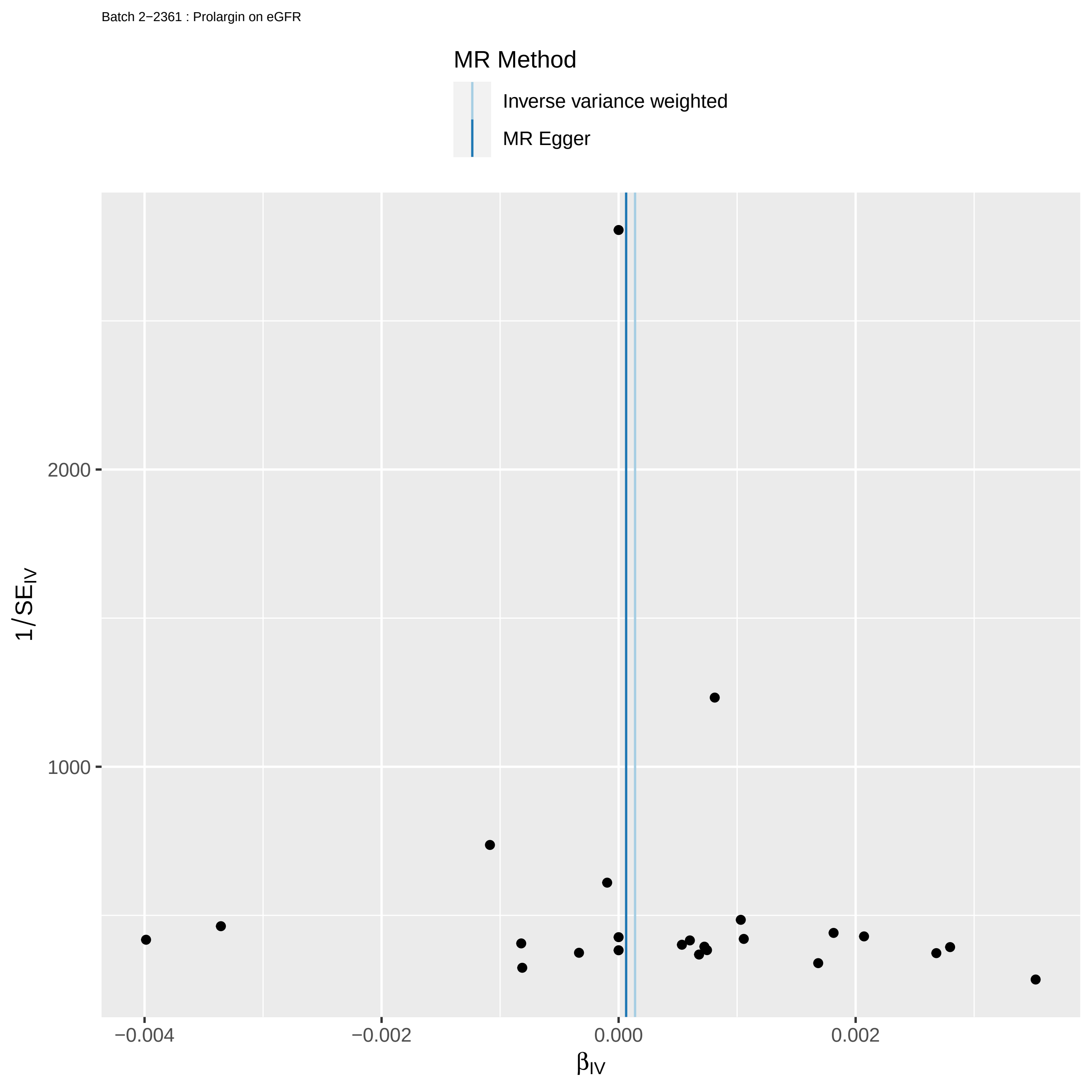

Batch 2−2361 : Prolargin on eGFR
MR Method
Inverse variance weighted
MR Egger
2000
1000
1/SE_IV
−0.004
−0.002
0.000
0.002
β_IV

Batch 2−2362 : Proteoglycan 3 on eGFR

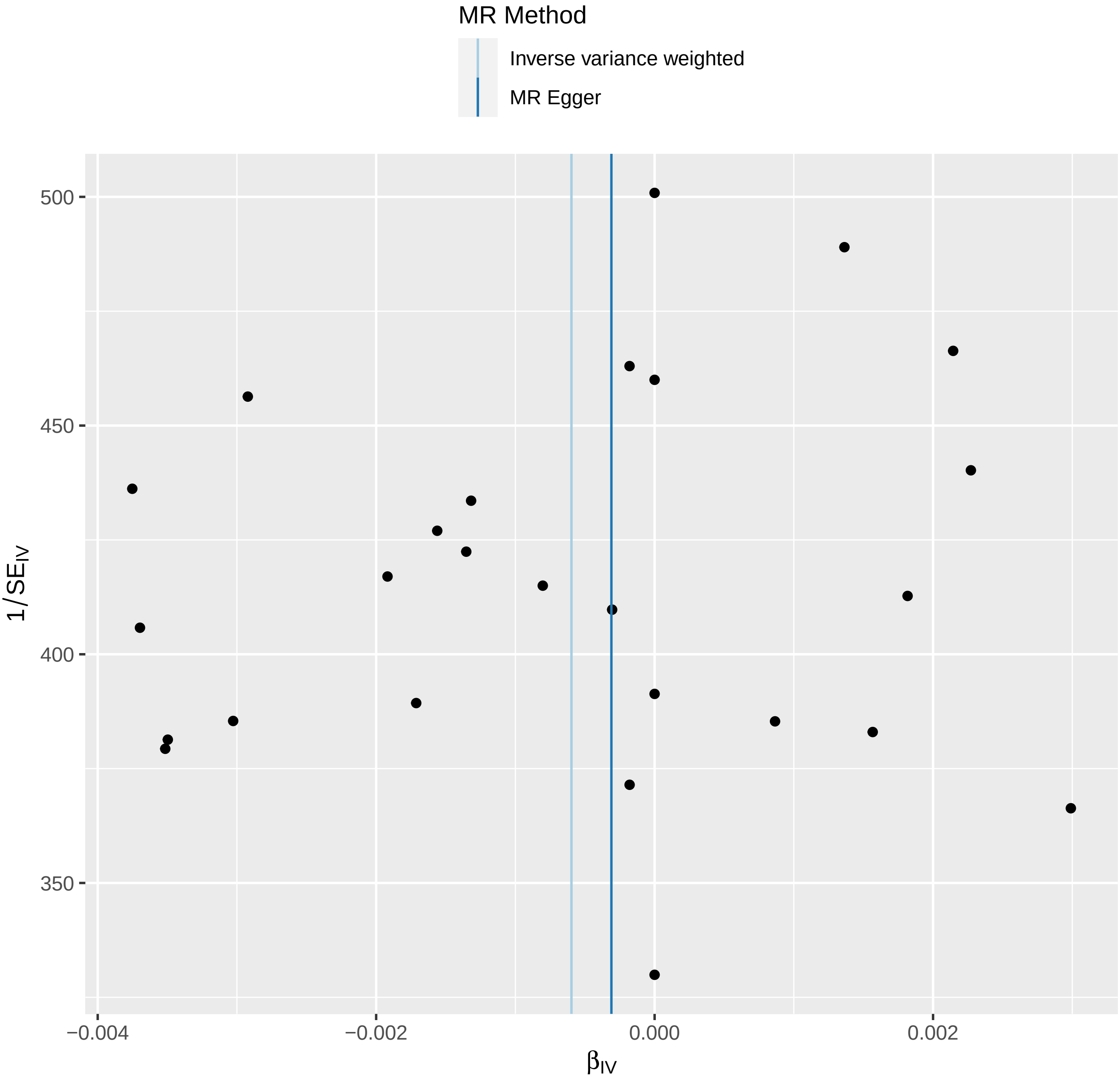

Batch 2−2363 : Salivary acidic proline−rich phosphoprotein 1/2 on eGFR

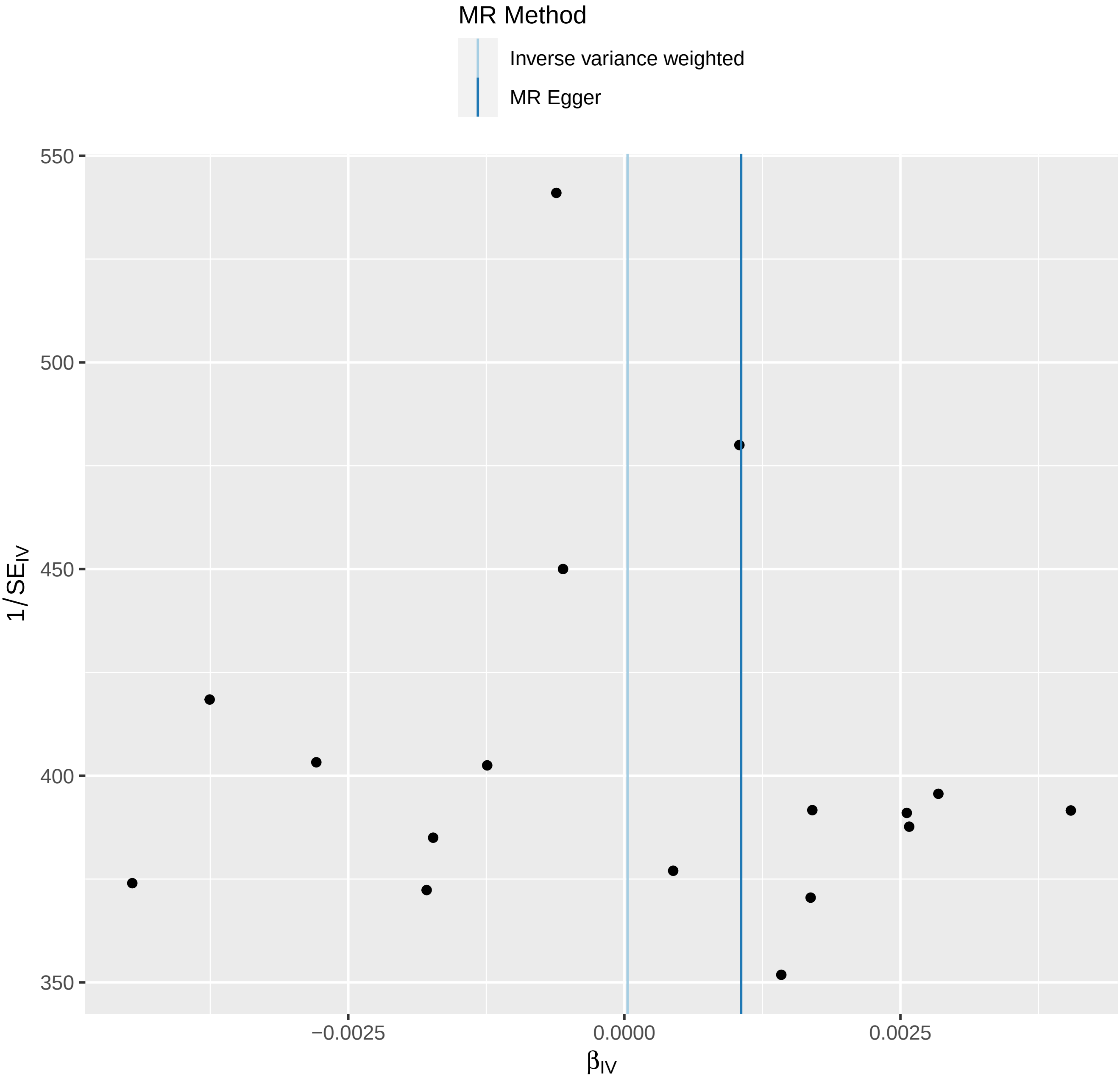

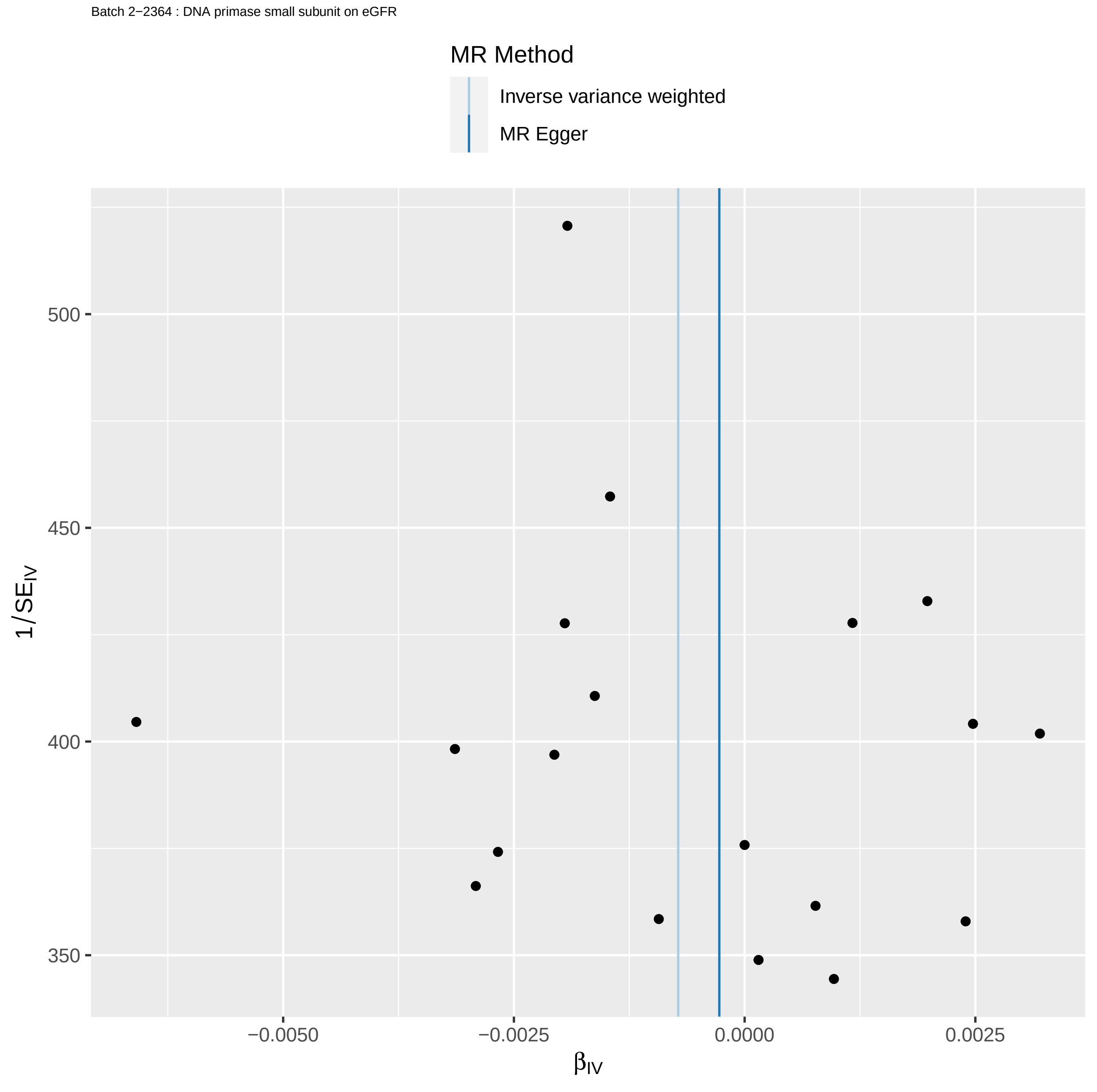

Batch 2−2364 : DNA primase small subunit on eGFR
MR Method
Inverse variance weighted
MR Egger
500
450
400
350
−0.0050
−0.0025
0.0000
0.0025
1/SE_IV
β_IV

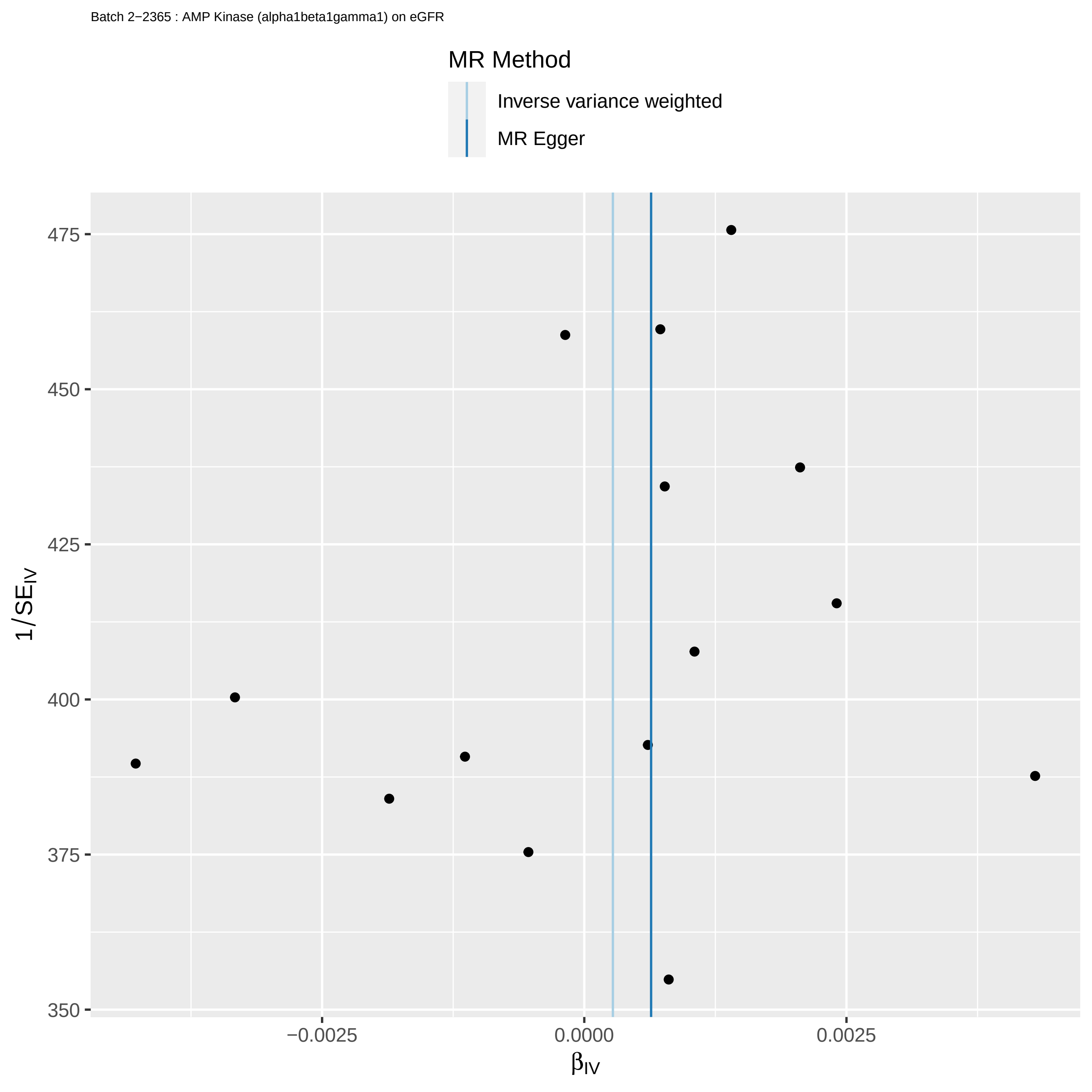

Batch 2−2365 : AMP Kinase (alpha1beta1gamma1) on eGFR
MR Method
Inverse variance weighted
MR Egger
475
450
425
400
375
350
1/SE$_{IV}$
−0.0025
0.0000
0.0025
β$_{IV}$

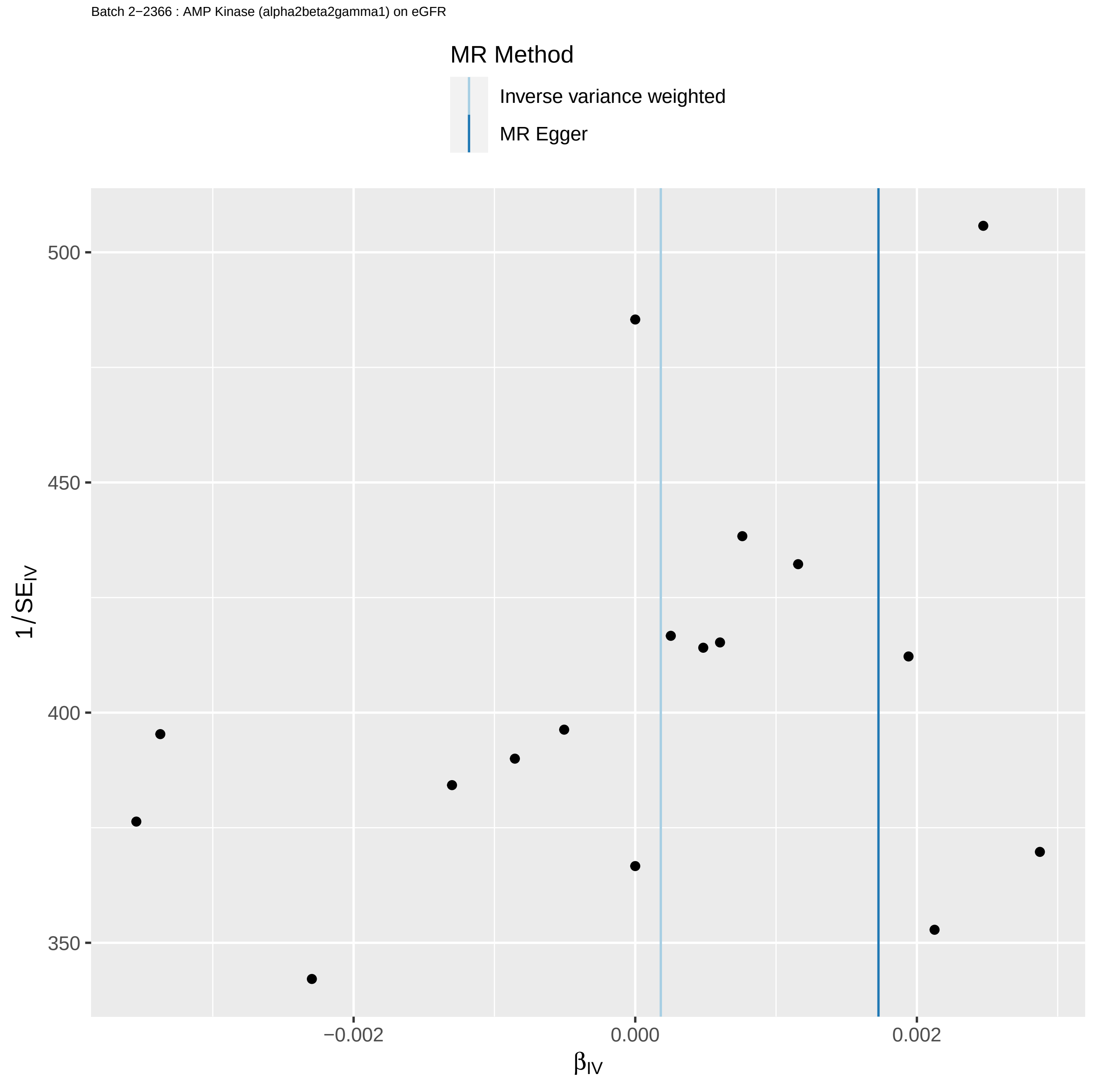

Batch 2−2366 : AMP Kinase (alpha2beta2gamma1) on eGFR
MR Method
Inverse variance weighted
MR Egger
500
450
400
350
1/SE$_{IV}$
−0.002
0.000
0.002
β$_{IV}$

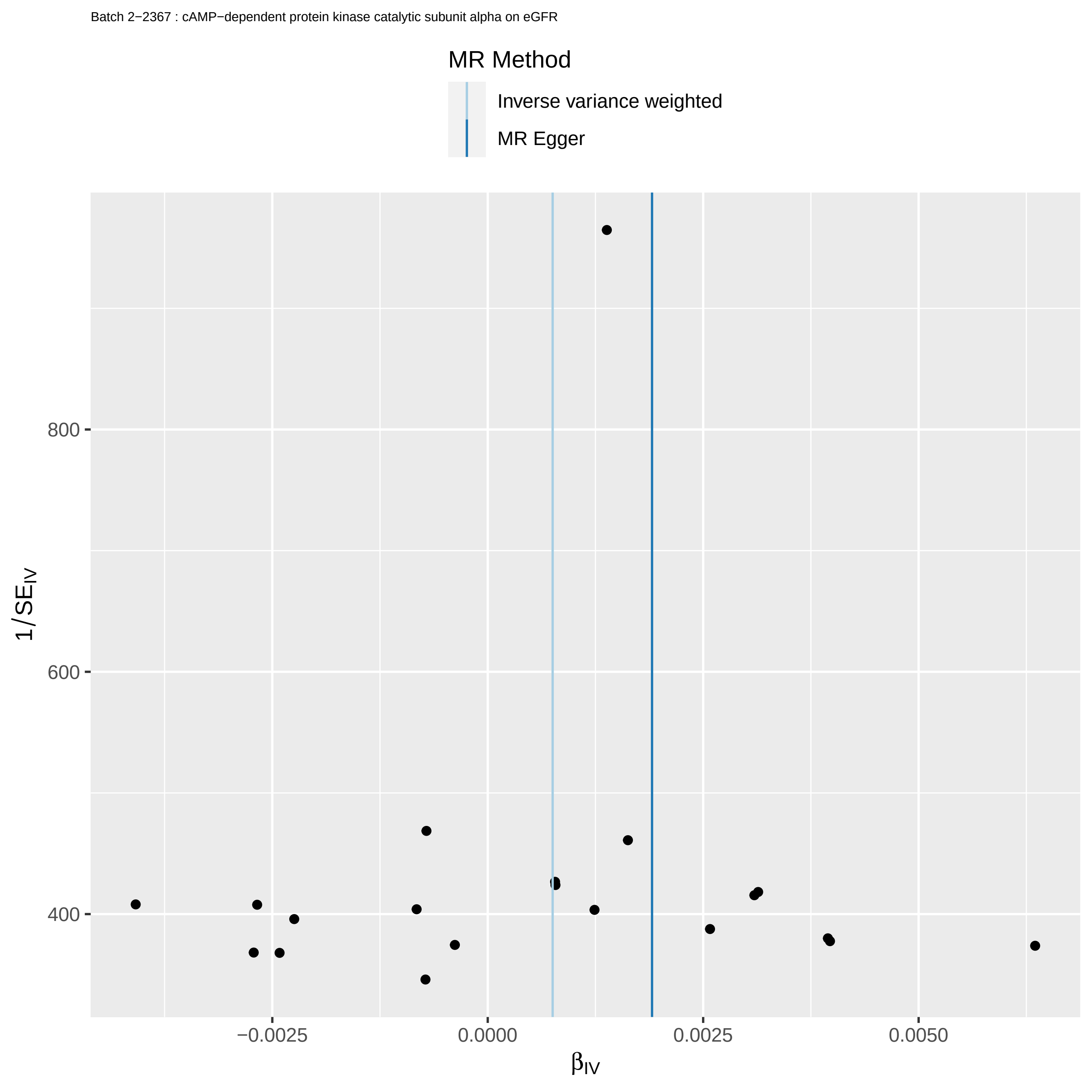

Batch 2−2367 : cAMP−dependent protein kinase catalytic subunit alpha on eGFR
MR Method
Inverse variance weighted
MR Egger
800
600
400
1/SE_IV
−0.0025
0.0000
0.0025
0.0050
β_IV

Batch 2−2368 : cAMP−dependent protein kinase type I−beta regulatory subunit on eGFR

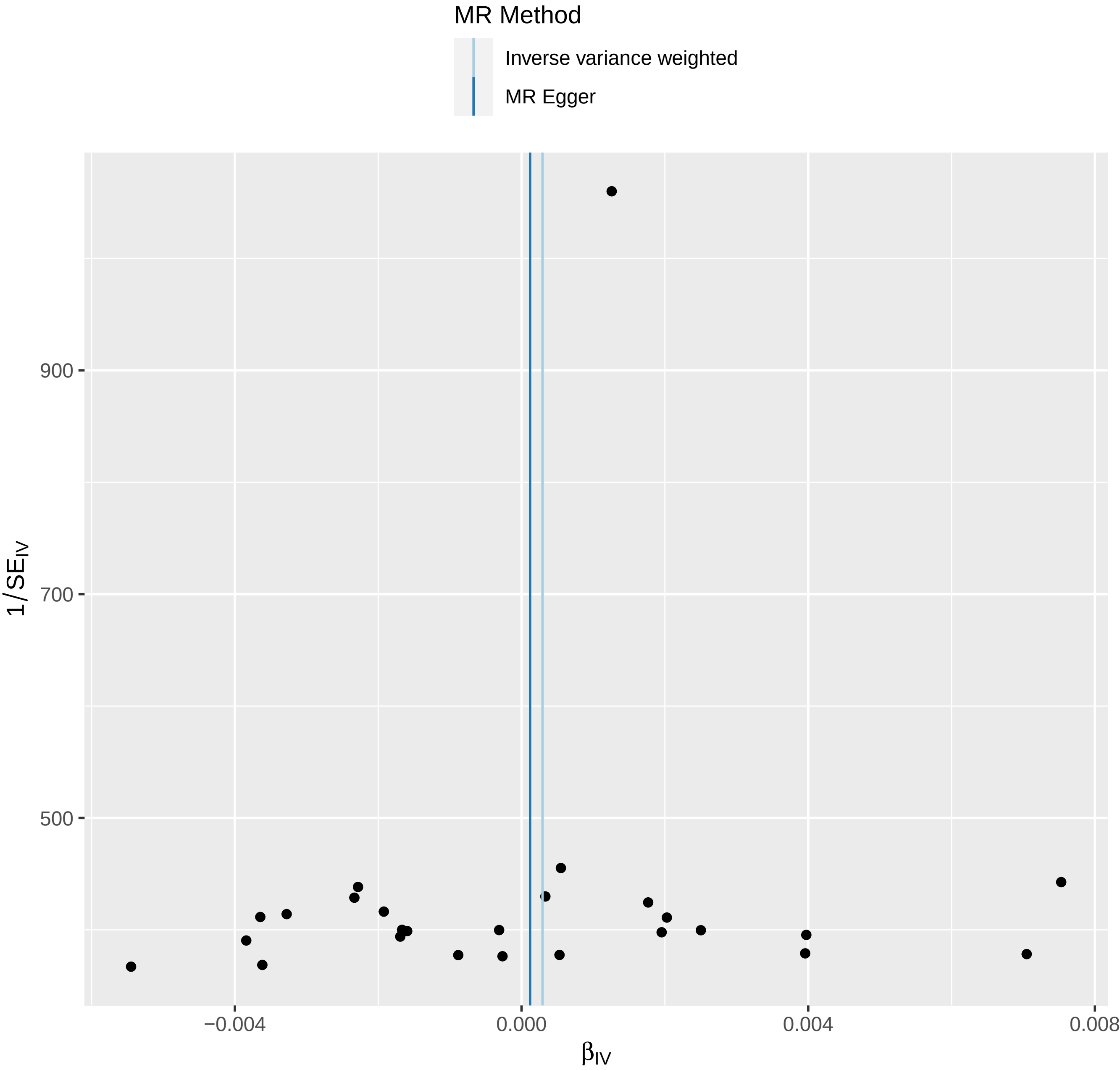

Batch 2−2369 : Protein kinase C alpha type on eGFR

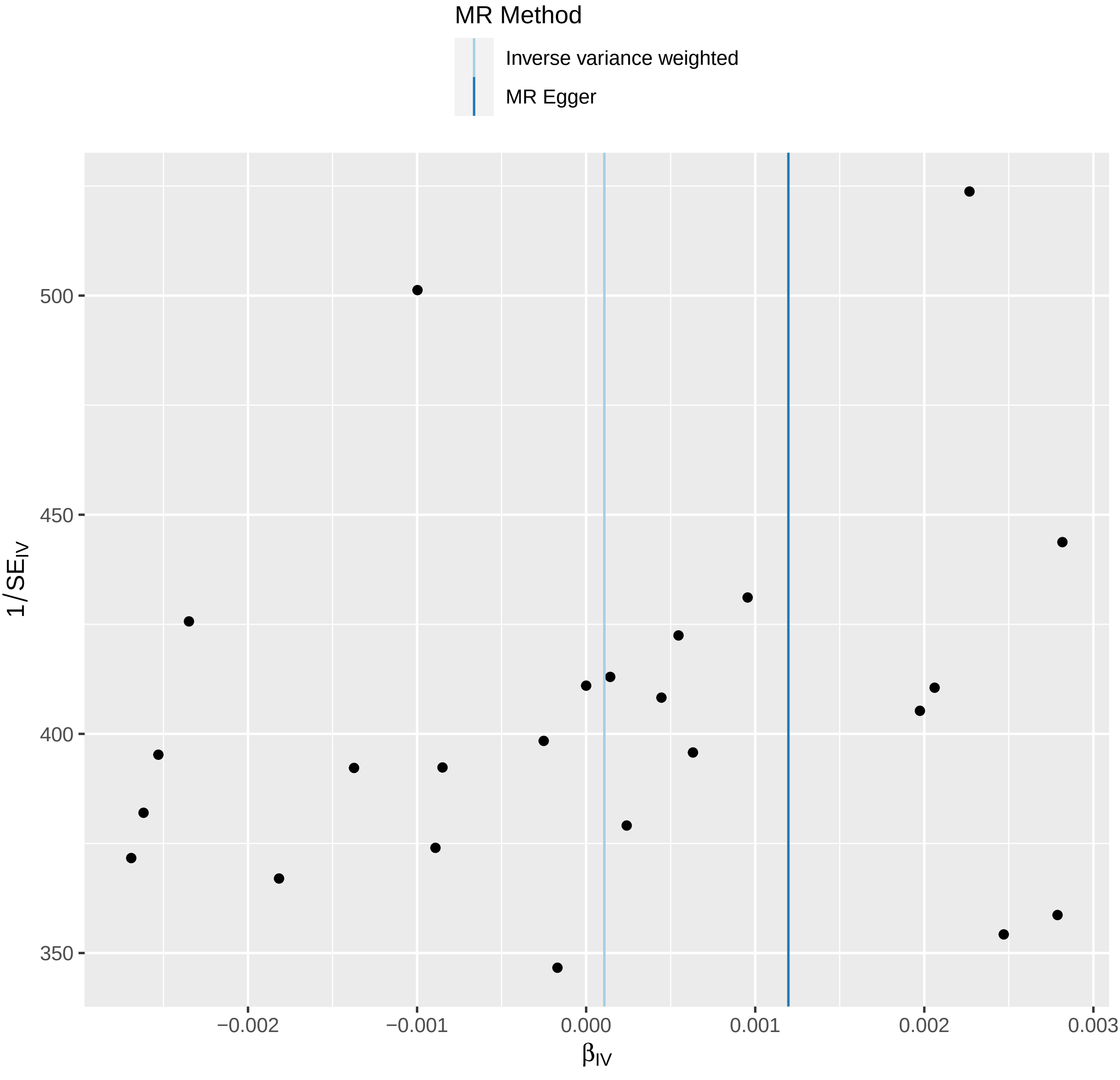

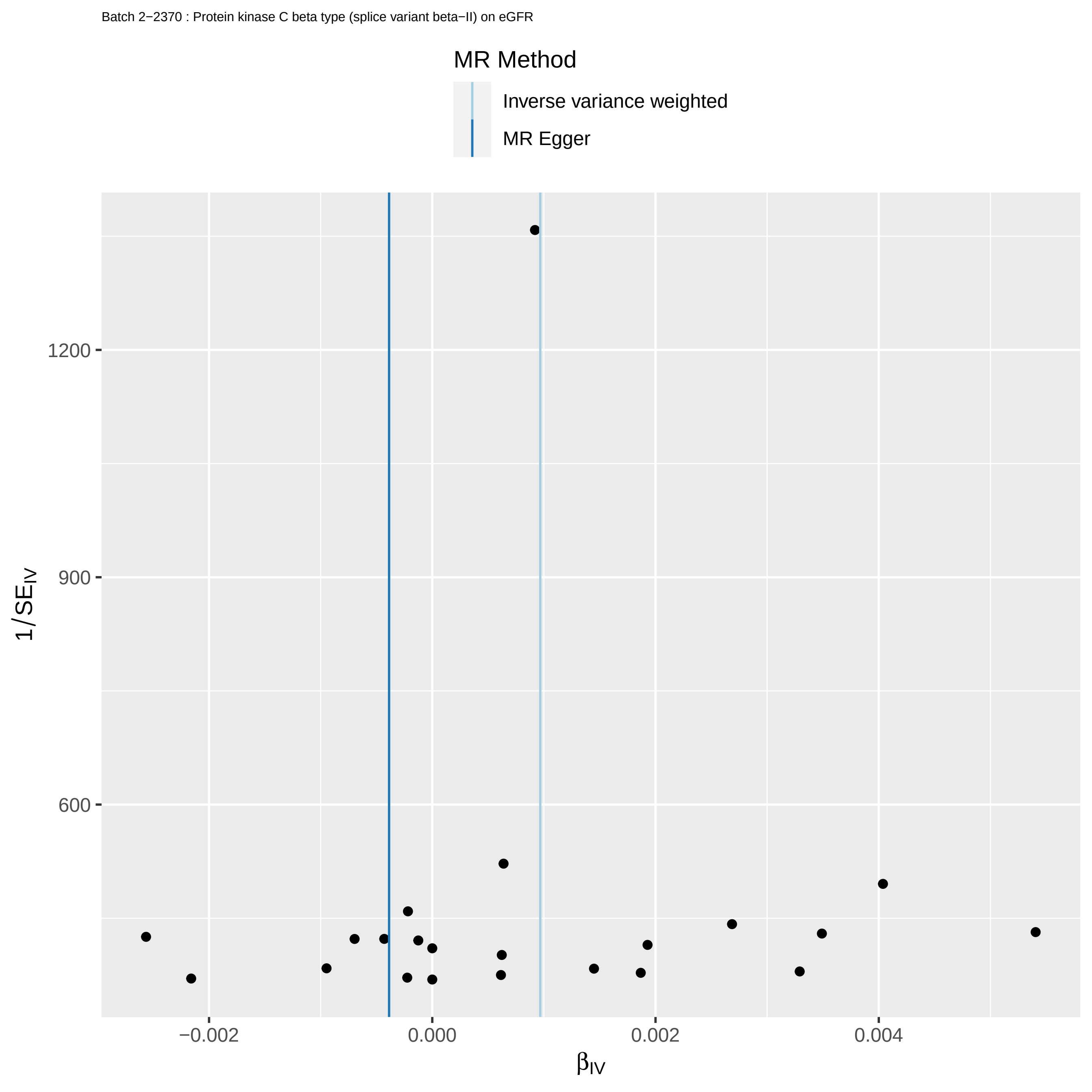
Batch 2−2370 : Protein kinase C beta type (splice variant beta−II) on eGFR
MR Method
Inverse variance weighted
MR Egger
1200
900
600
1/SE_IV
−0.002
0.000
0.002
0.004
β_IV

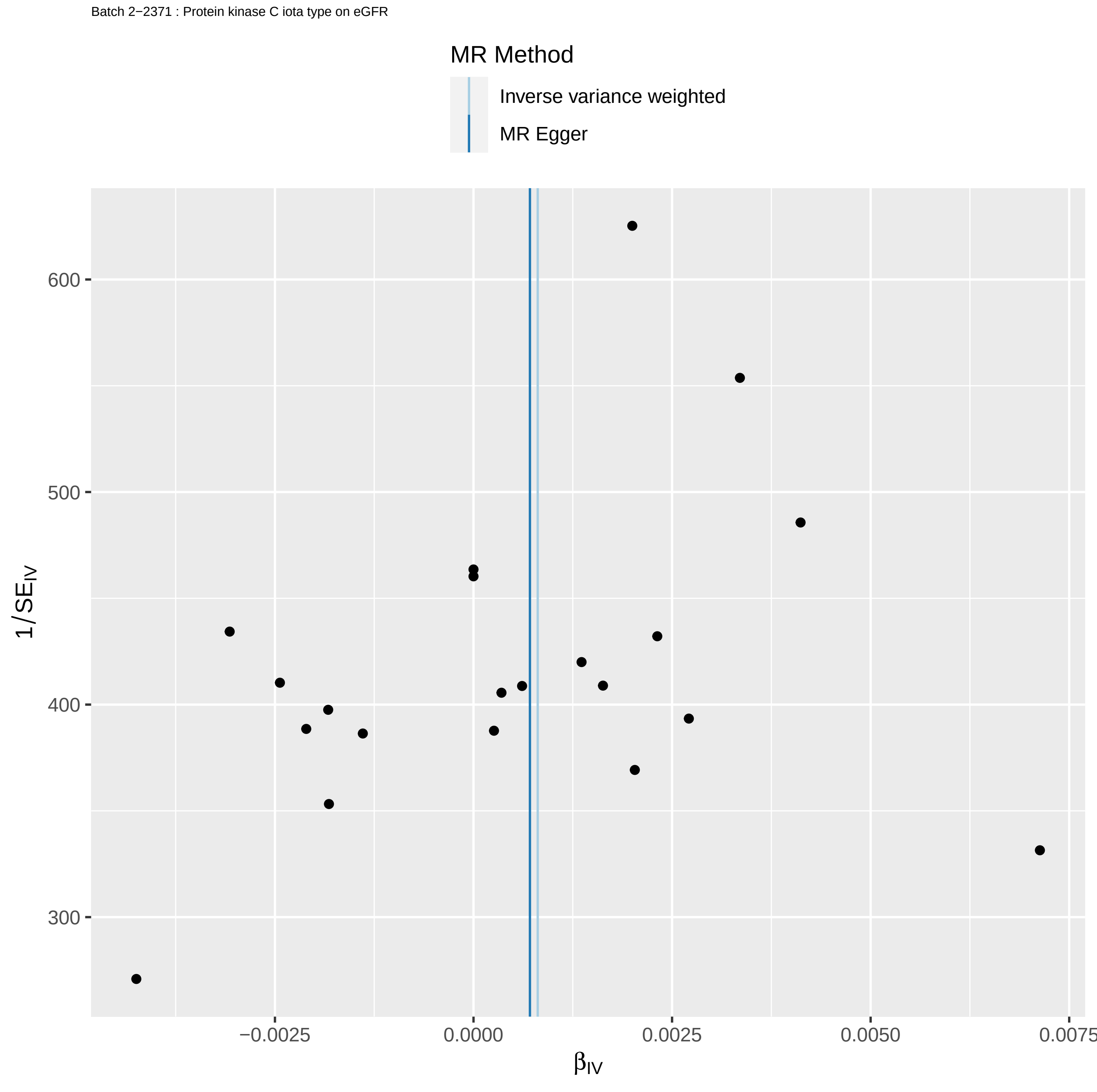

Batch 2−2371 : Protein kinase C iota type on eGFR
MR Method
Inverse variance weighted
MR Egger
600
500
400
300
1/SE_IV
−0.0025
0.0000
0.0025
0.0050
0.0075
β_IV

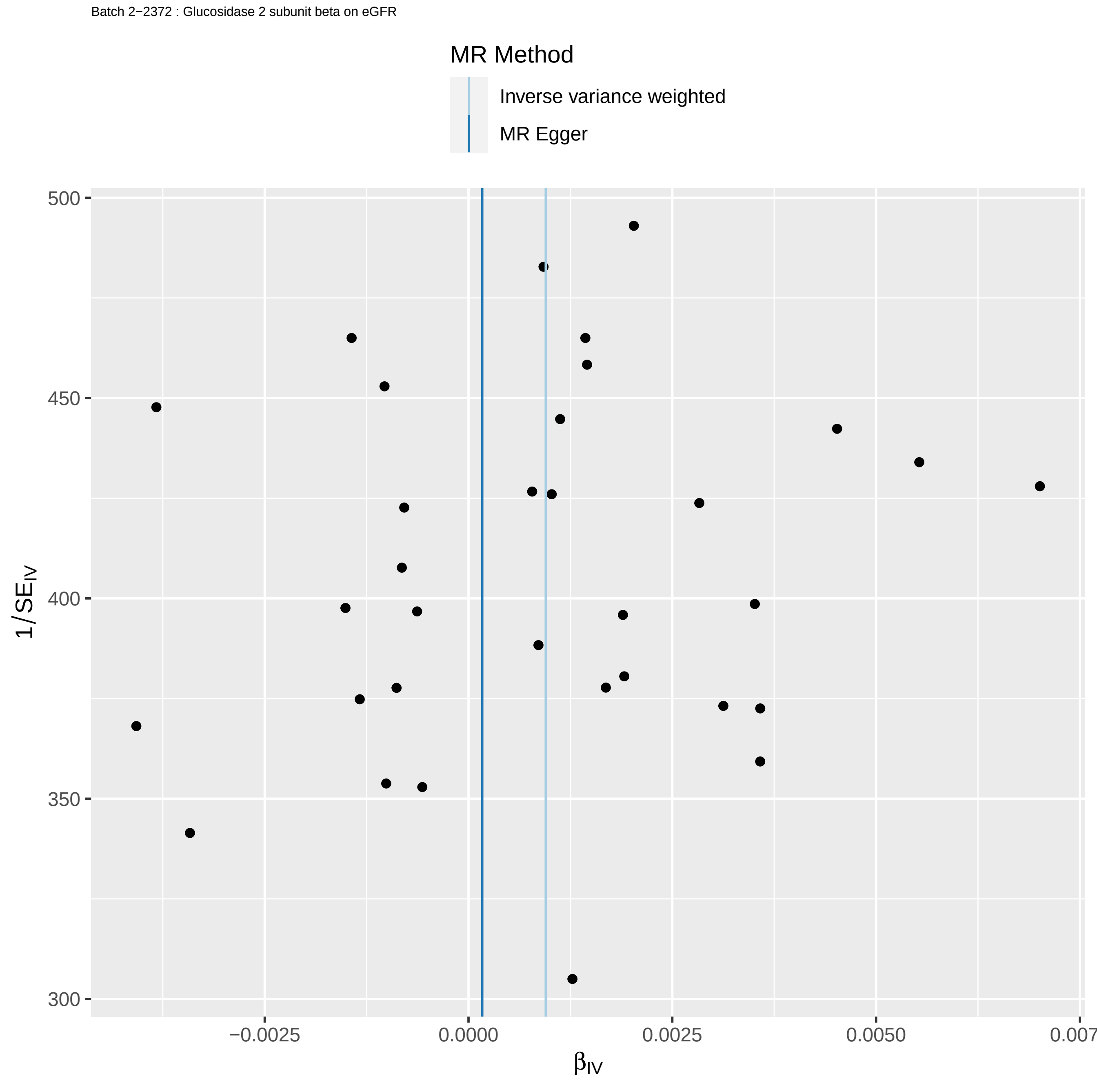

Batch 2−2372 : Glucosidase 2 subunit beta on eGFR
MR Method
Inverse variance weighted
MR Egger
500
450
400
350
300
1/SE_IV
−0.0025
0.0000
0.0025
0.0050
0.007
β_IV

Batch 2−2373 : Protein kinase C zeta type on eGFR

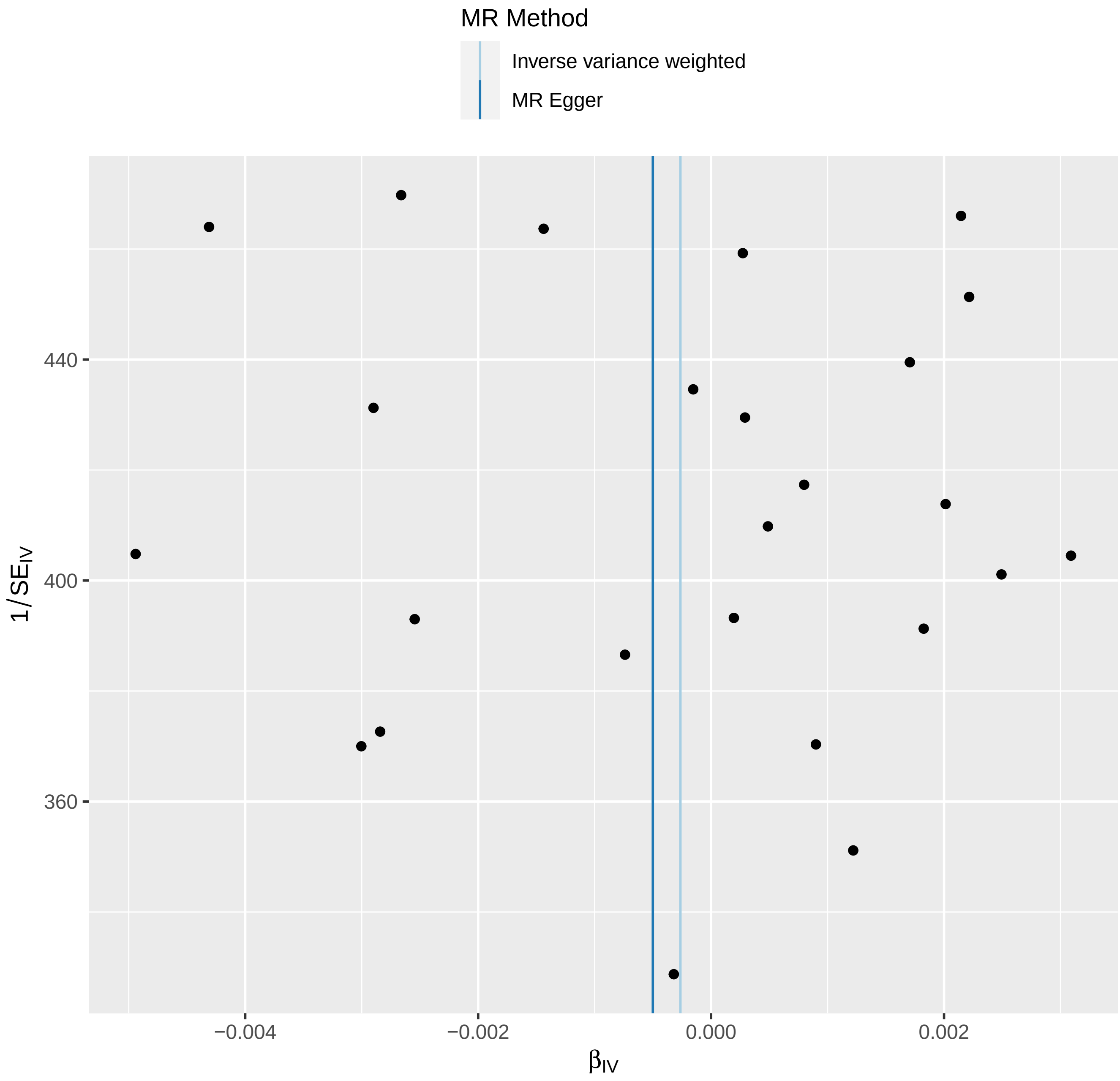

Batch 2−2374 : cGMP−dependent protein kinase 1, beta isozyme on eGFR

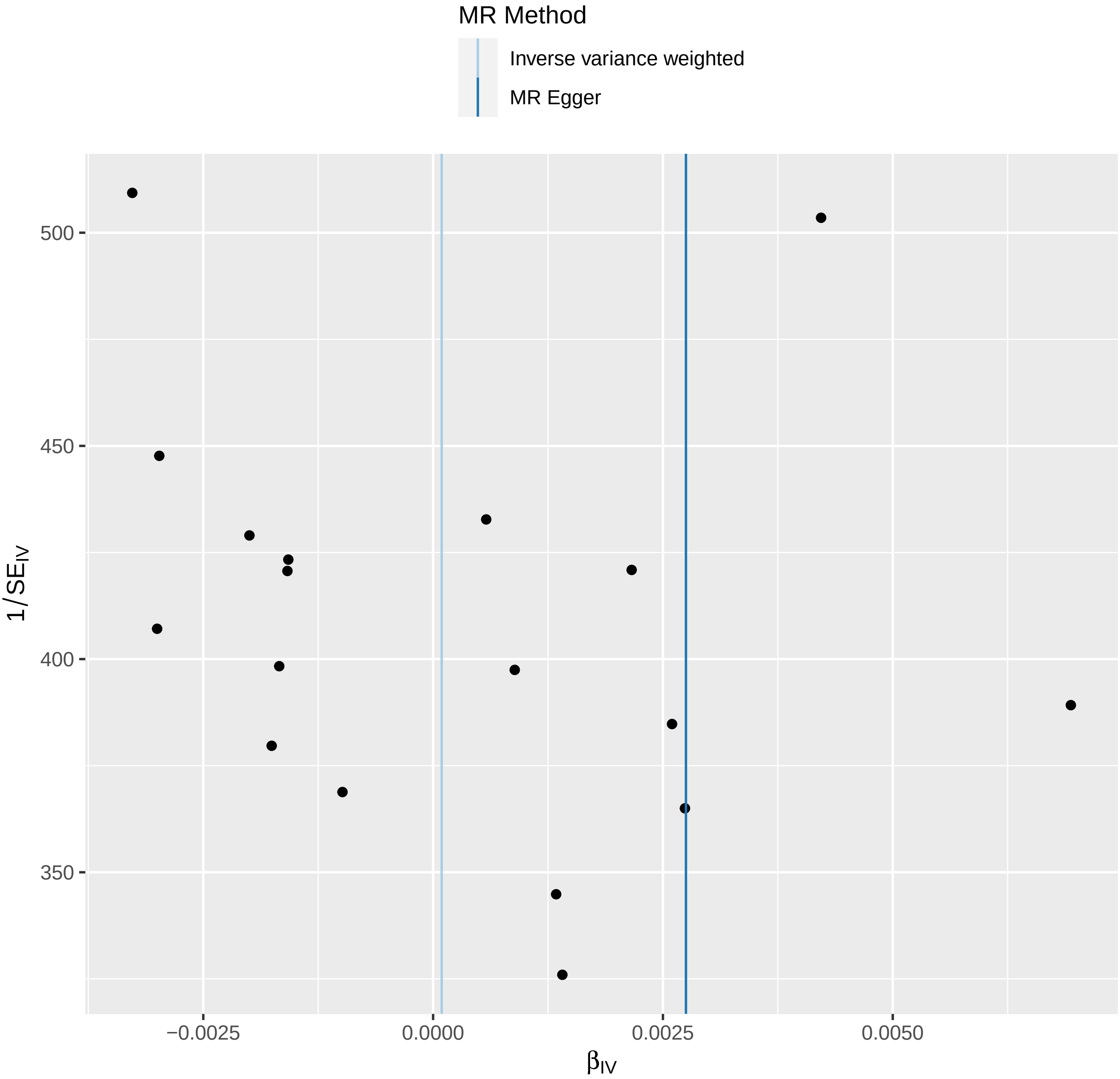

Batch 2−2375 : Prolactin on eGFR

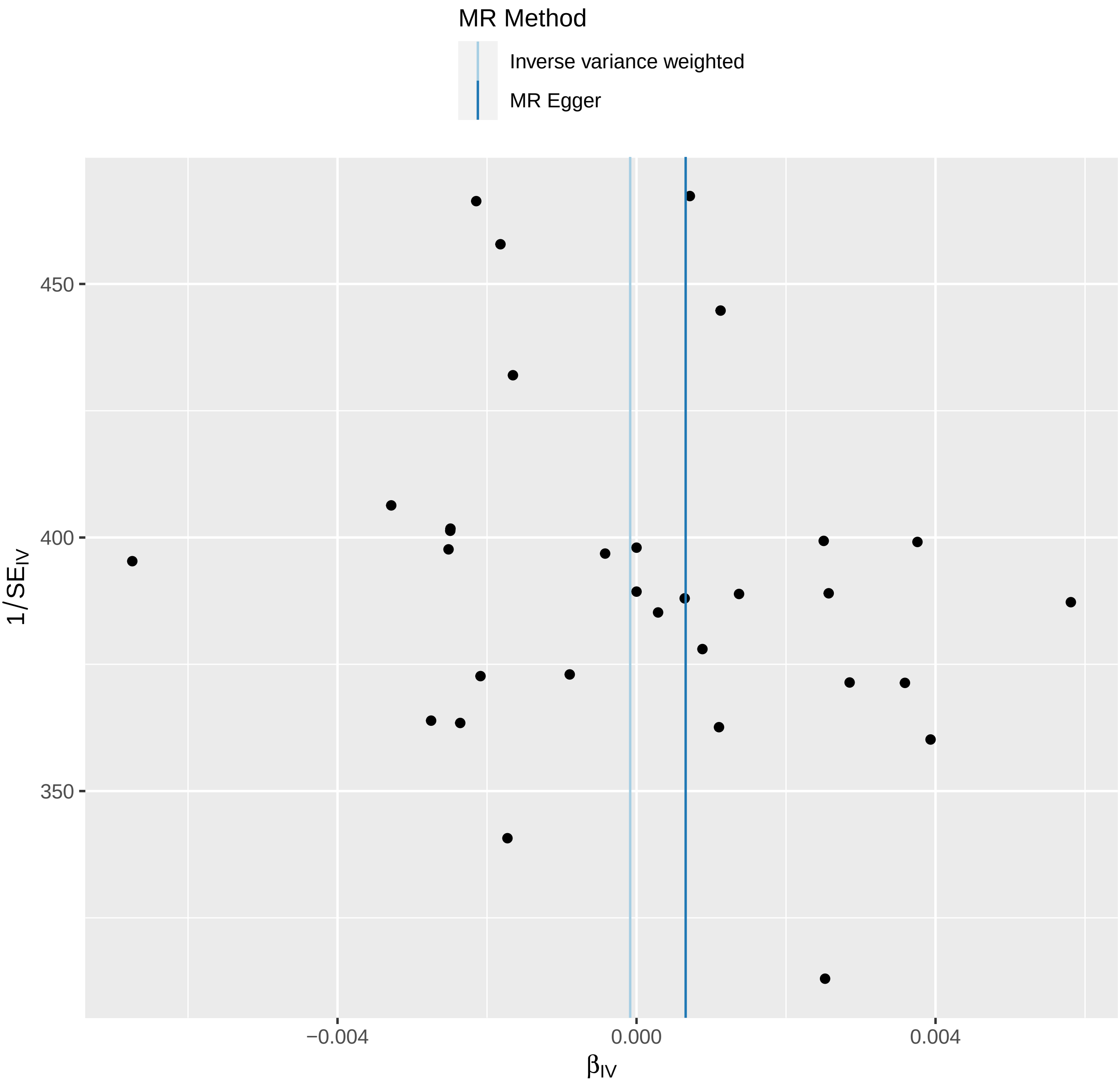

Batch 2−2376 : Prolactin−releasing peptide on eGFR

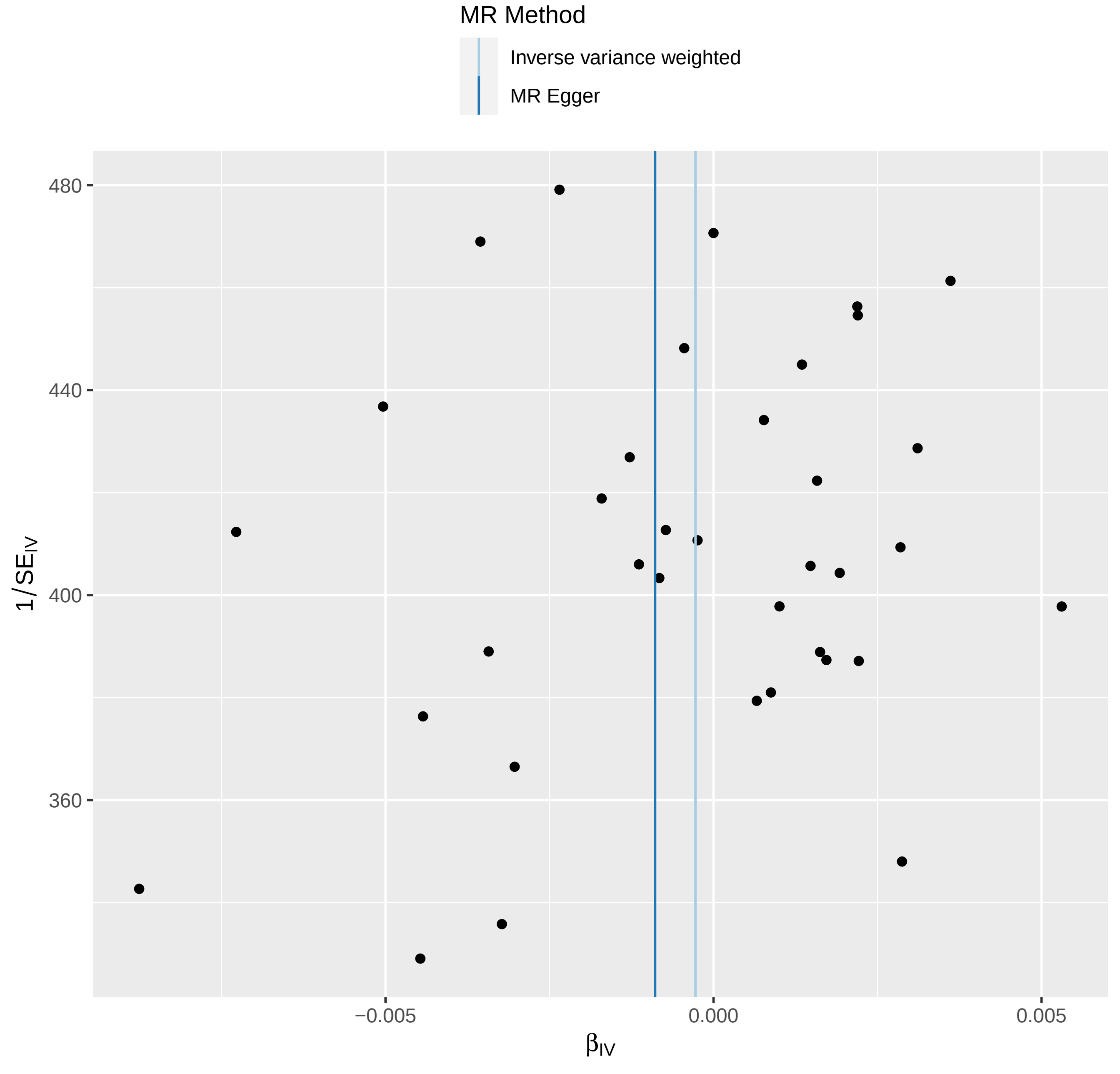

Batch 2−2377 : Prolactin receptor on eGFR

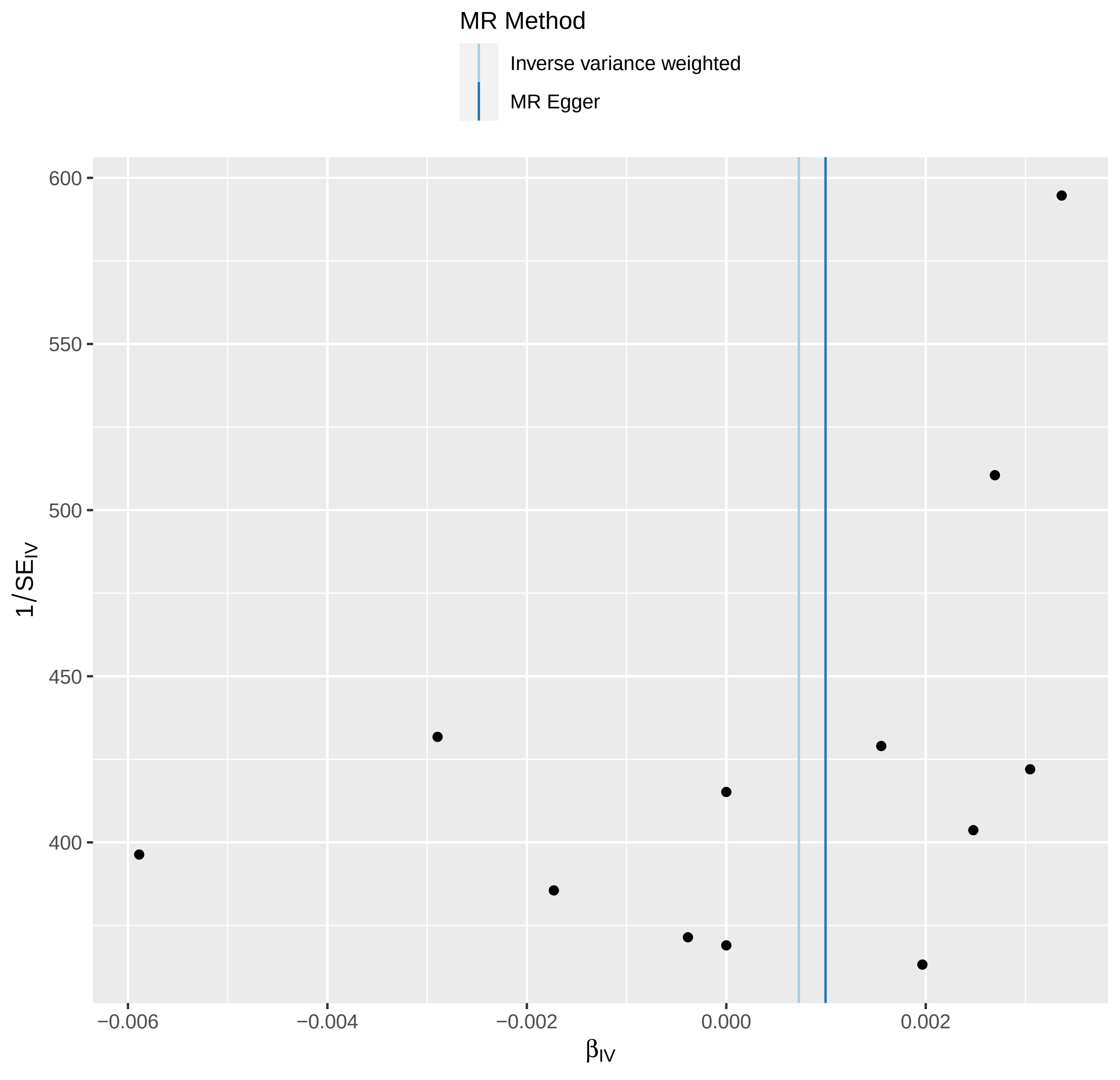

Batch 2−2378 : Protein arginine N−methyltransferase 3 on eGFR

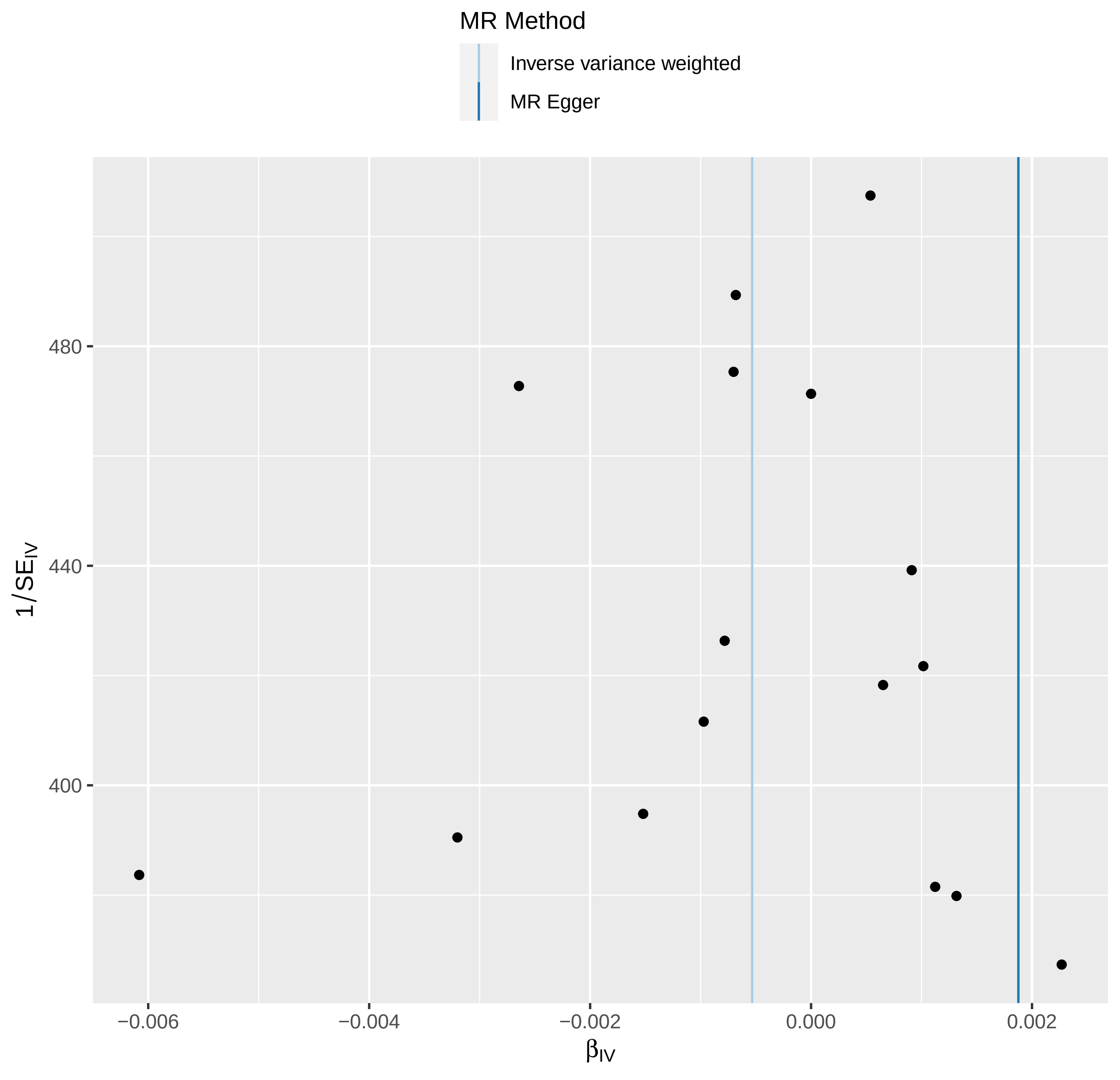

Batch 2−2379 : Major prion protein on eGFR

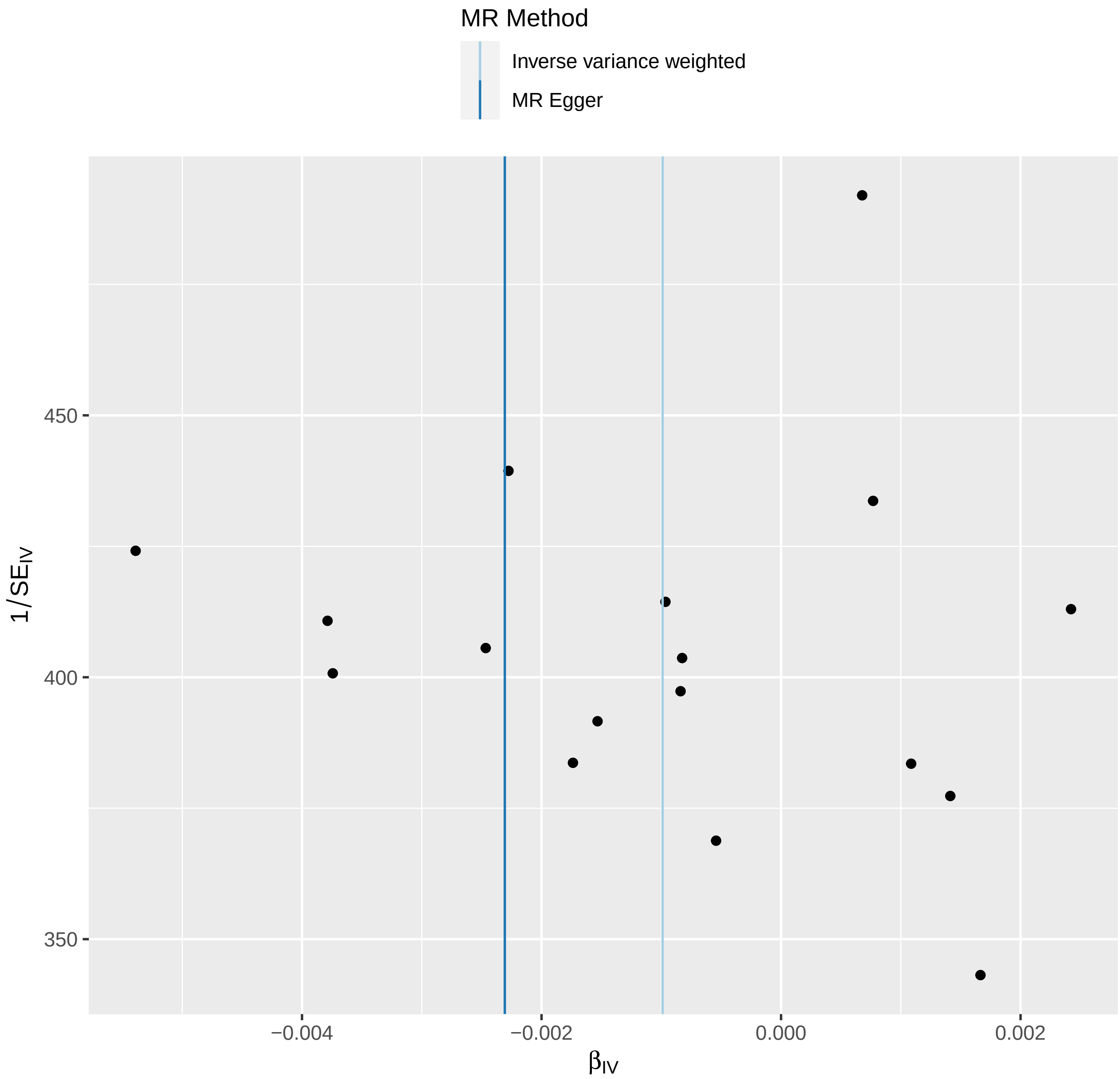

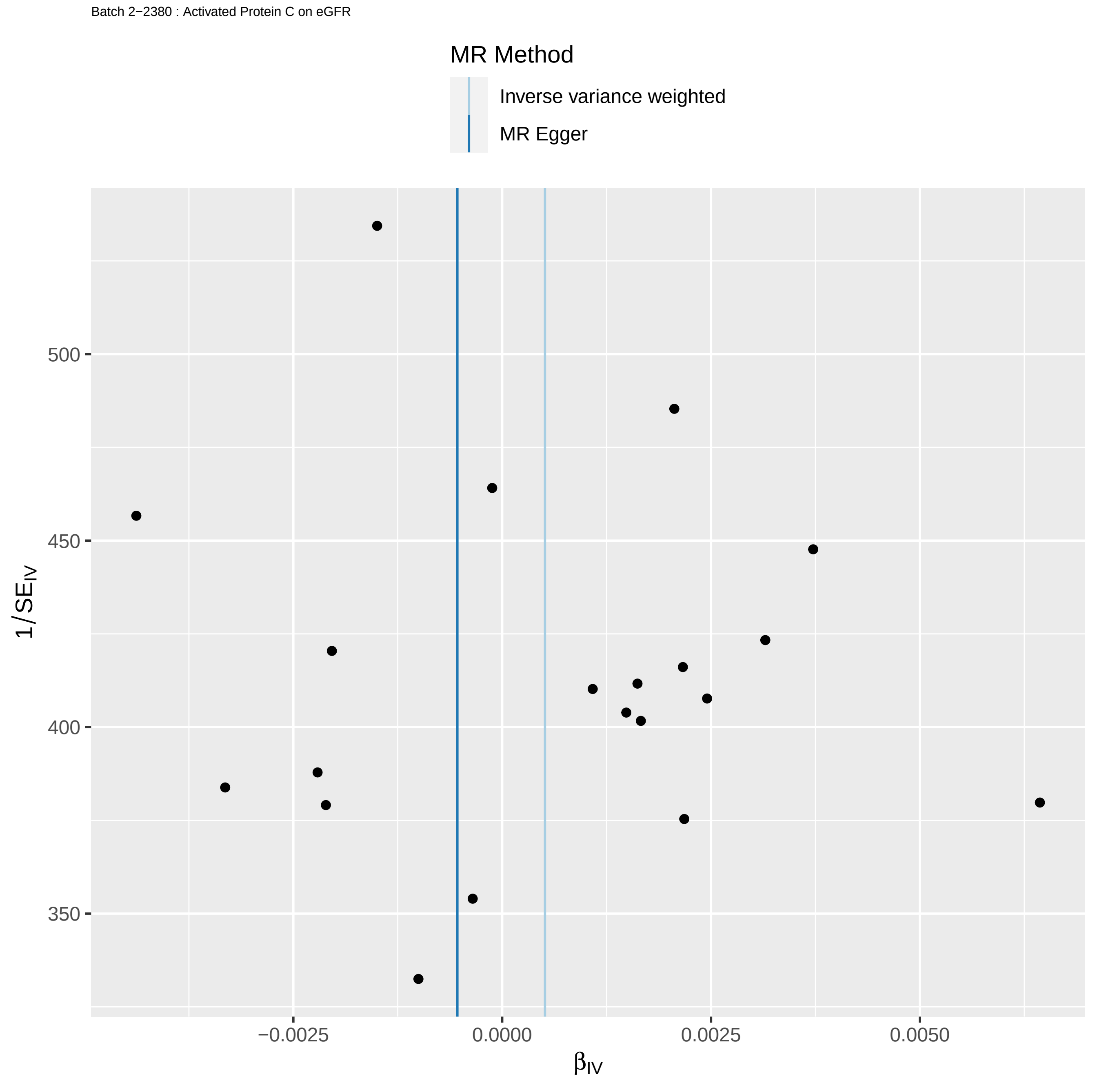
Batch 2−2380 : Activated Protein C on eGFR
MR Method
Inverse variance weighted
MR Egger
500
450
400
350
1/SE_IV
−0.0025
0.0000
0.0025
0.0050
β_IV

Batch 2−2381 : Activated Protein C on eGFR

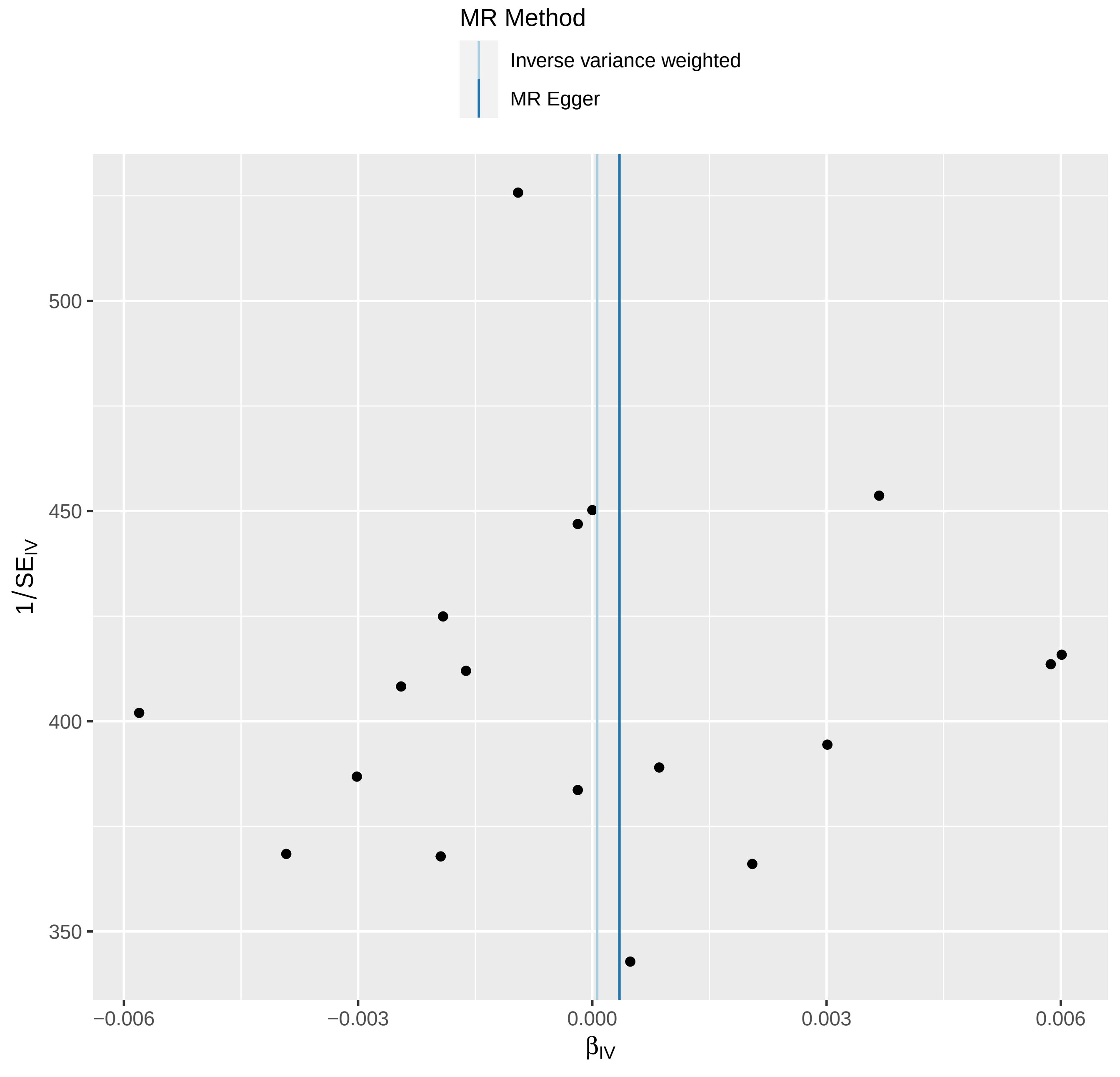

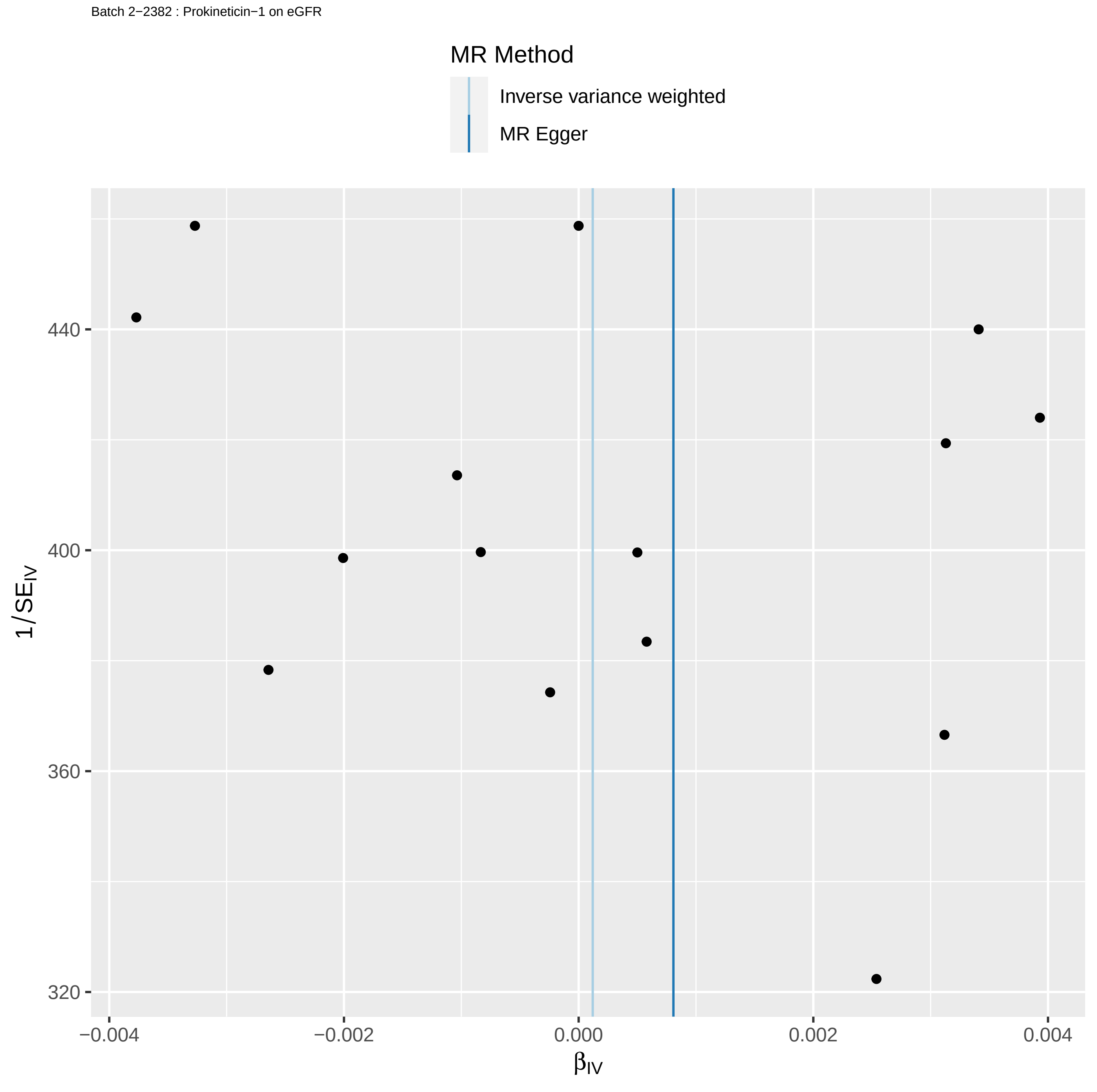
Batch 2−2382 : Prokineticin−1 on eGFR
MR Method
Inverse variance weighted
MR Egger
440
400
360
320
1/SE_IV
−0.004
−0.002
0.000
0.002
0.004
β_IV

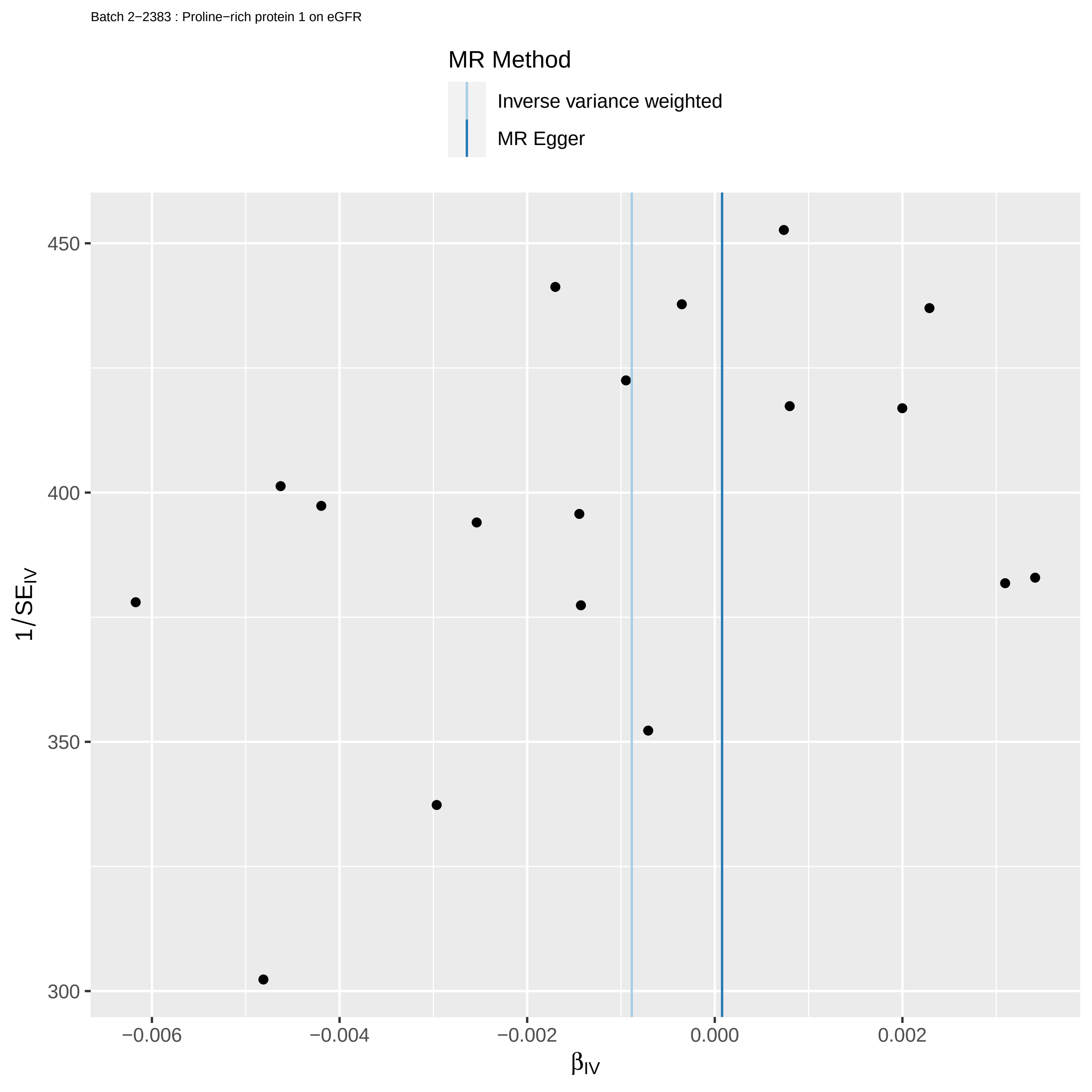

Batch 2−2383 : Proline−rich protein 1 on eGFR
MR Method
Inverse variance weighted
MR Egger
450
400
350
300
1/SE_IV
−0.006
−0.004
−0.002
0.000
0.002
β_IV

Batch 2−2384 : Pre−mRNA−processing factor 6 on eGFR

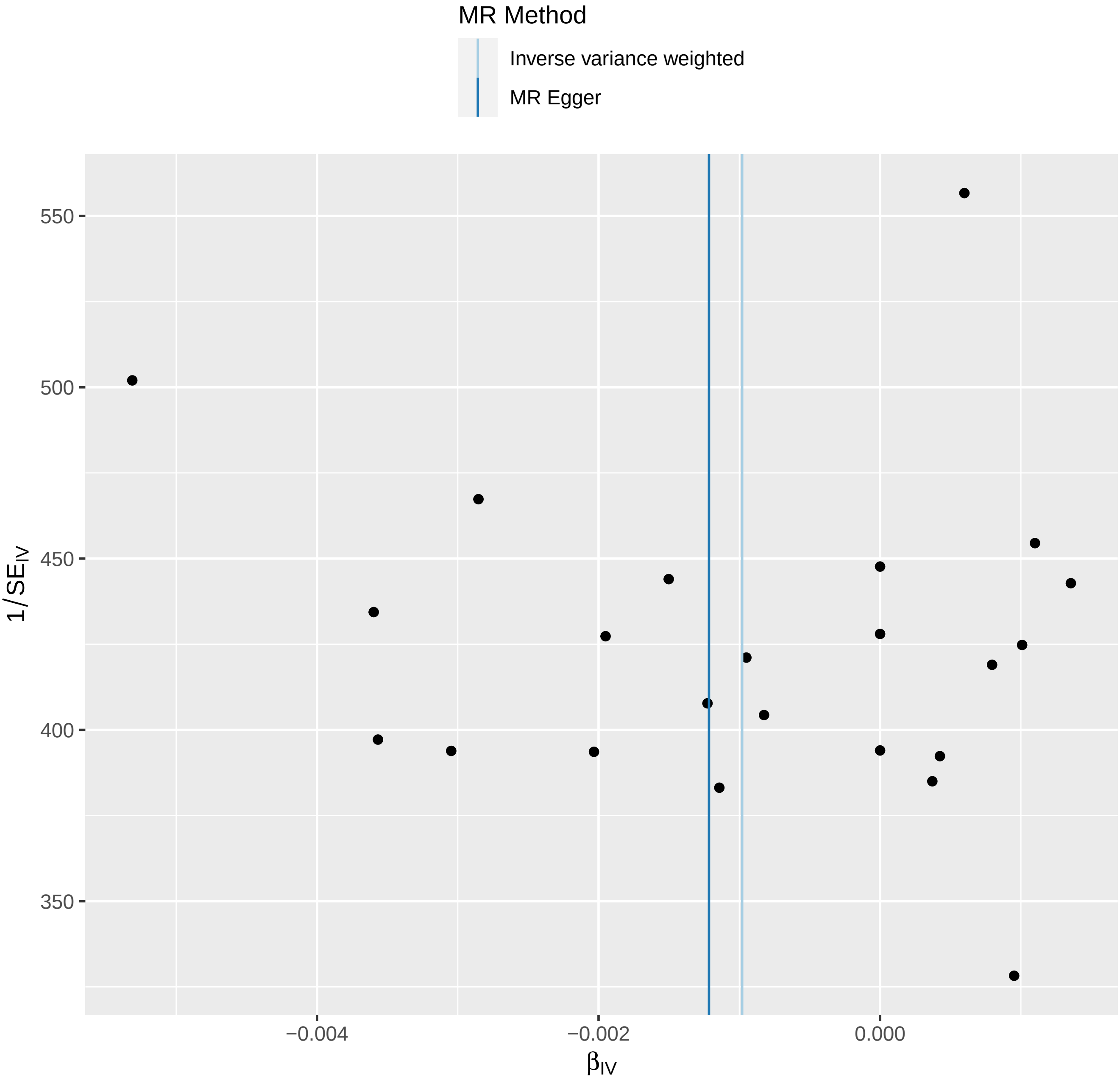

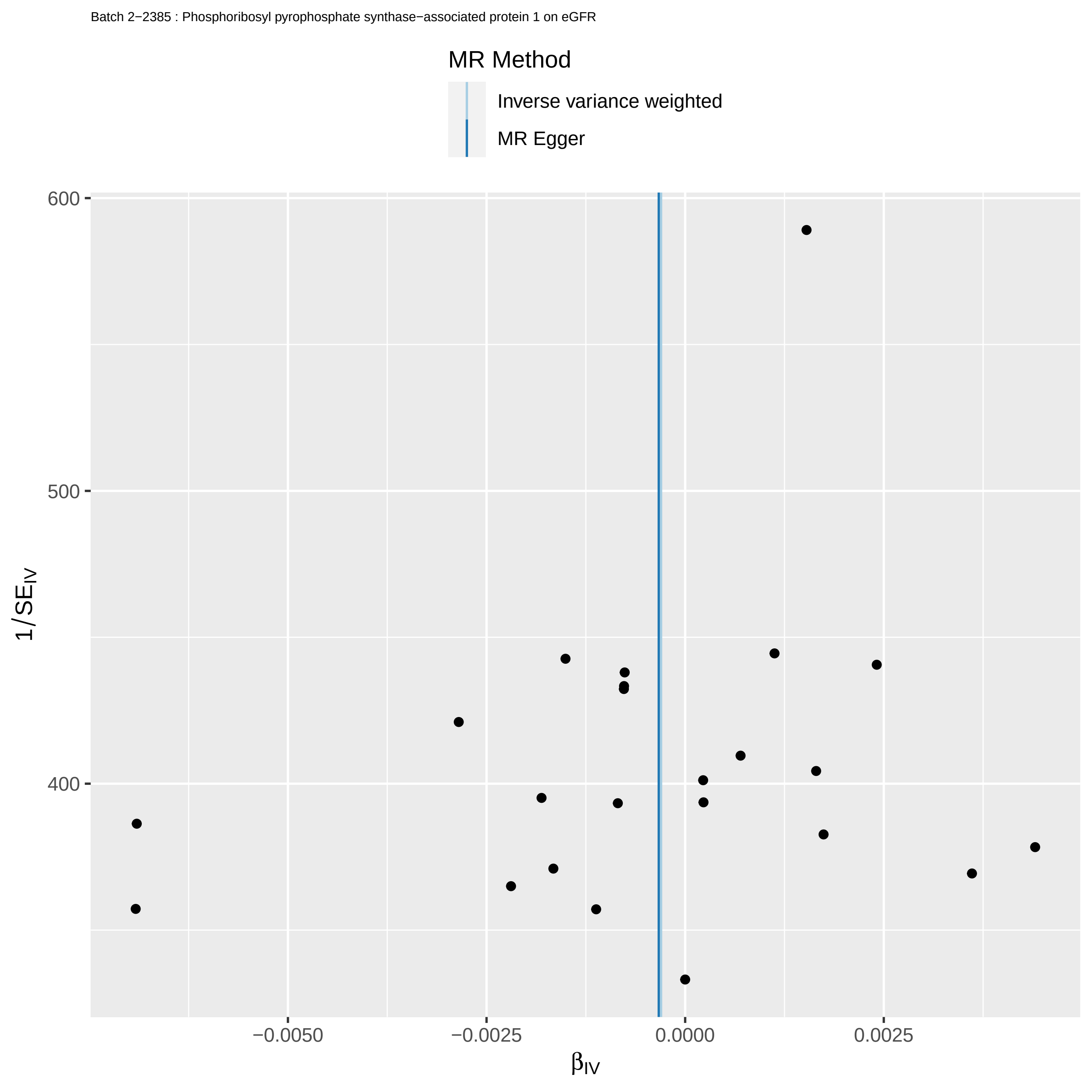

Batch 2−2385 : Phosphoribosyl pyrophosphate synthase−associated protein 1 on eGFR
MR Method
Inverse variance weighted
MR Egger
600
500
400
1/SE_IV
−0.0050
−0.0025
0.0000
0.0025
β_IV

Batch 2−2386 : Protein Largen on eGFR

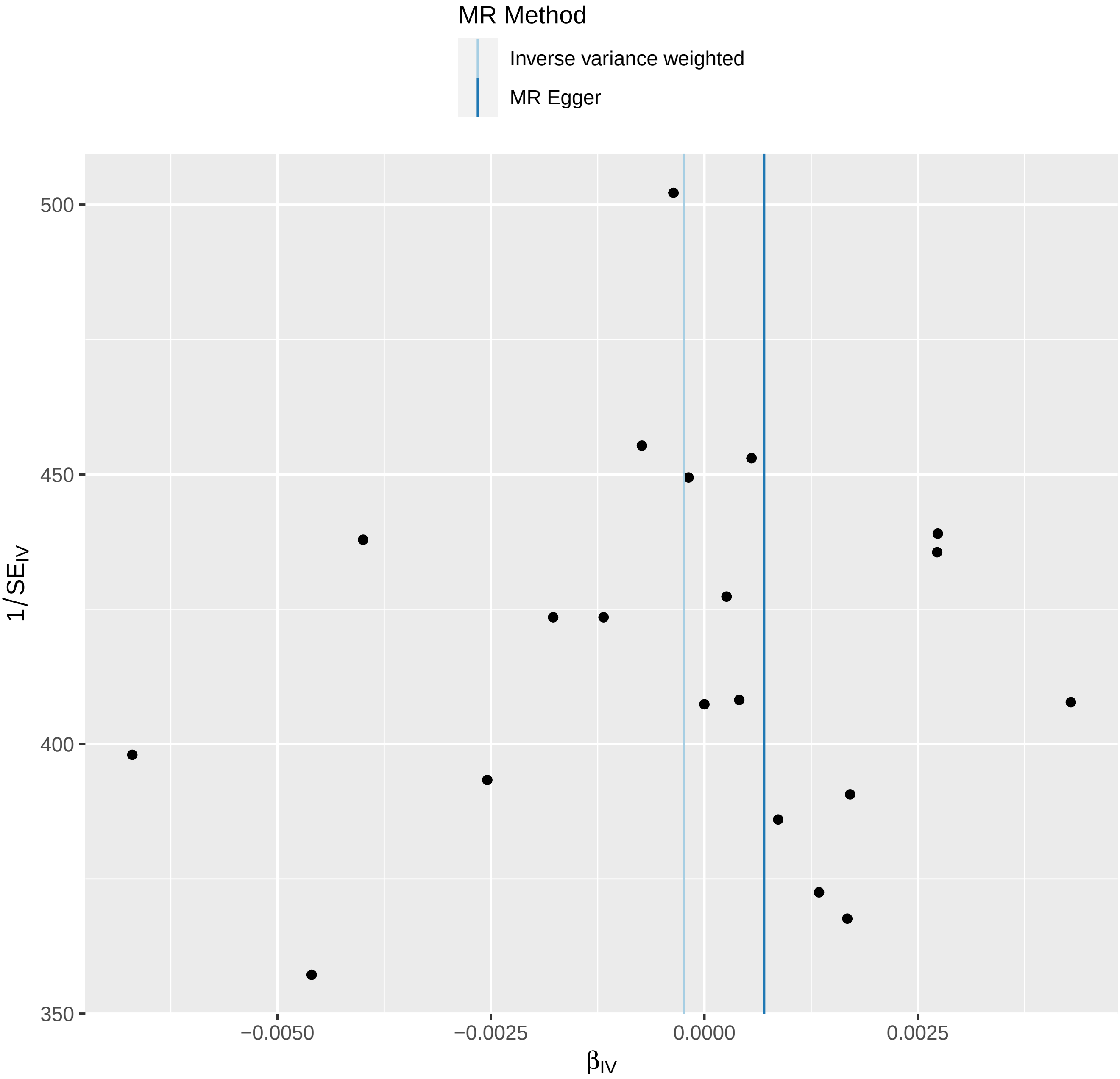

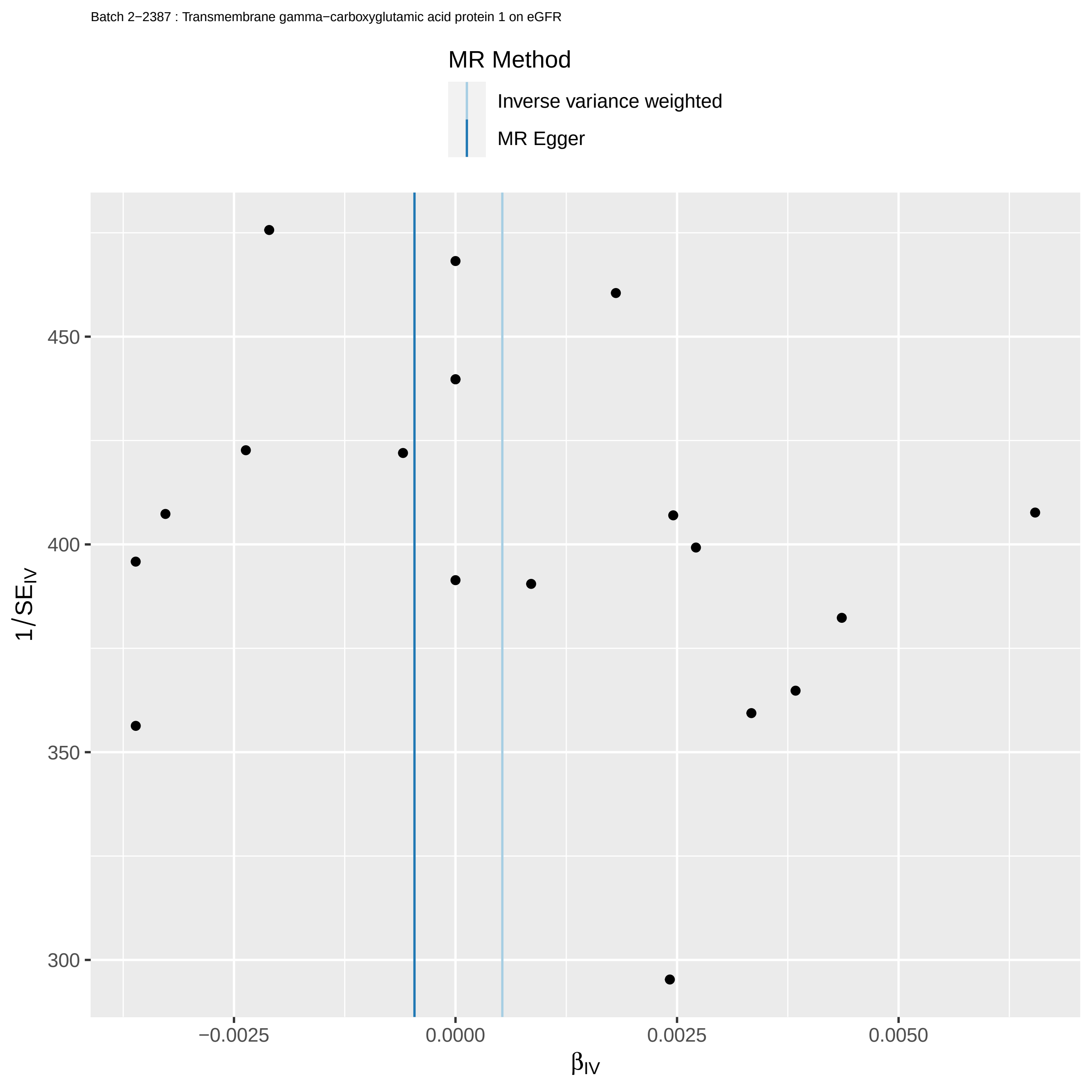
Batch 2−2387 : Transmembrane gamma−carboxyglutamic acid protein 1 on eGFR
MR Method
Inverse variance weighted
MR Egger
450
400
350
300
1/SE_IV
−0.0025
0.0000
0.0025
0.0050
β_IV

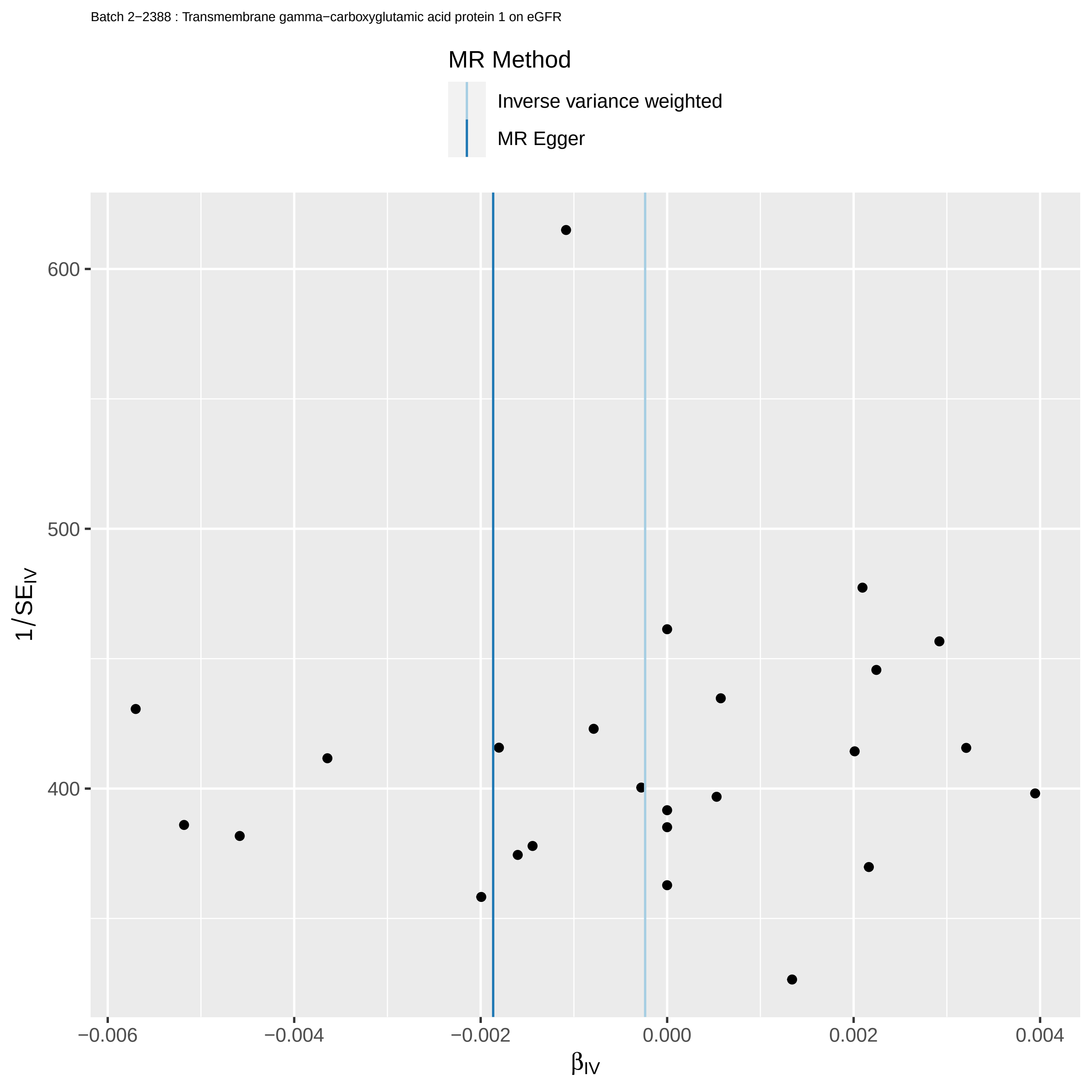
Batch 2−2388 : Transmembrane gamma−carboxyglutamic acid protein 1 on eGFR
MR Method
Inverse variance weighted
MR Egger
600
500
400
1/SE_IV
−0.006
−0.004
−0.002
0.000
0.002
0.004
β_IV

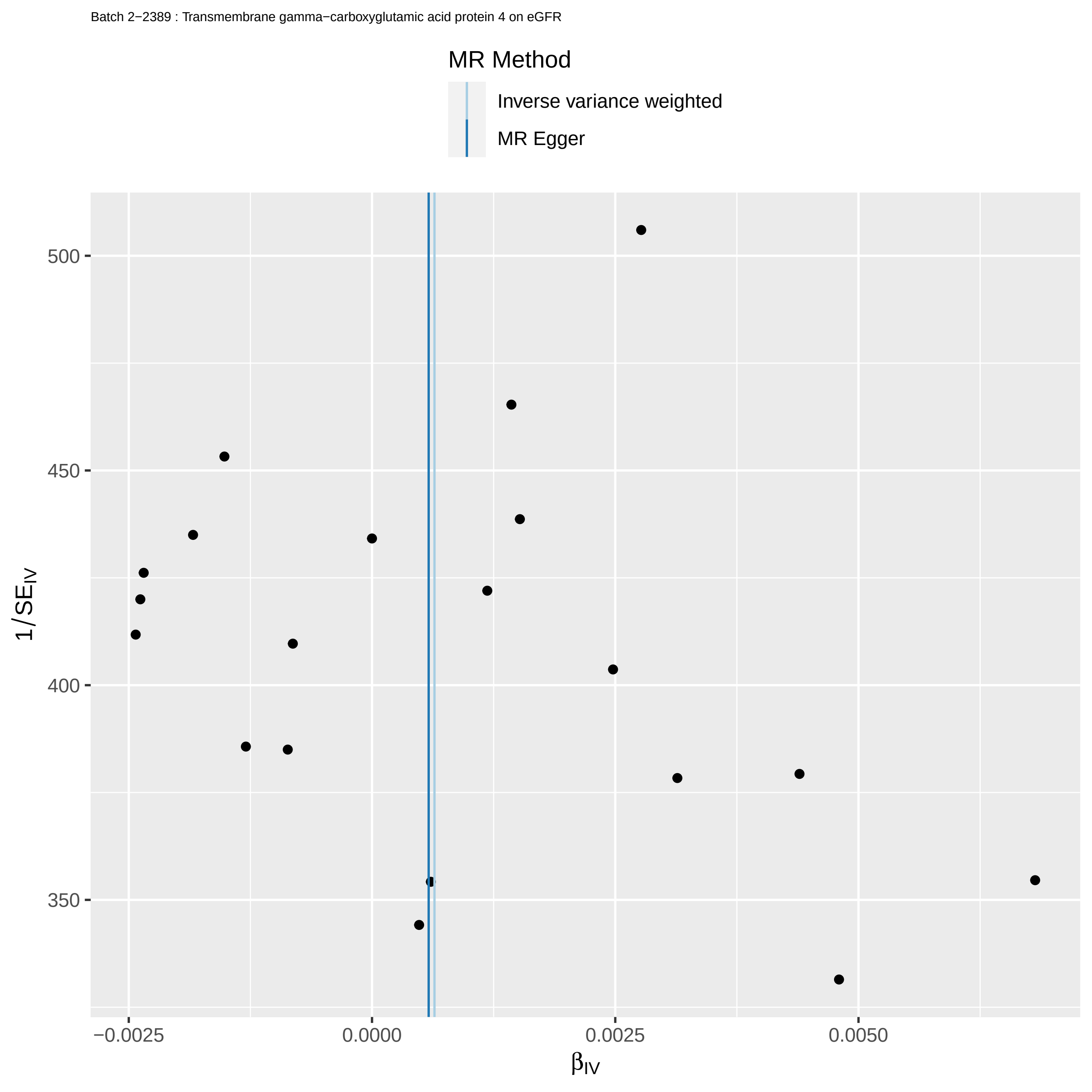

Batch 2−2389 : Transmembrane gamma−carboxyglutamic acid protein 4 on eGFR
MR Method
Inverse variance weighted
MR Egger
500
450
400
350
1/SE_IV
−0.0025
0.0000
0.0025
0.0050
β_IV

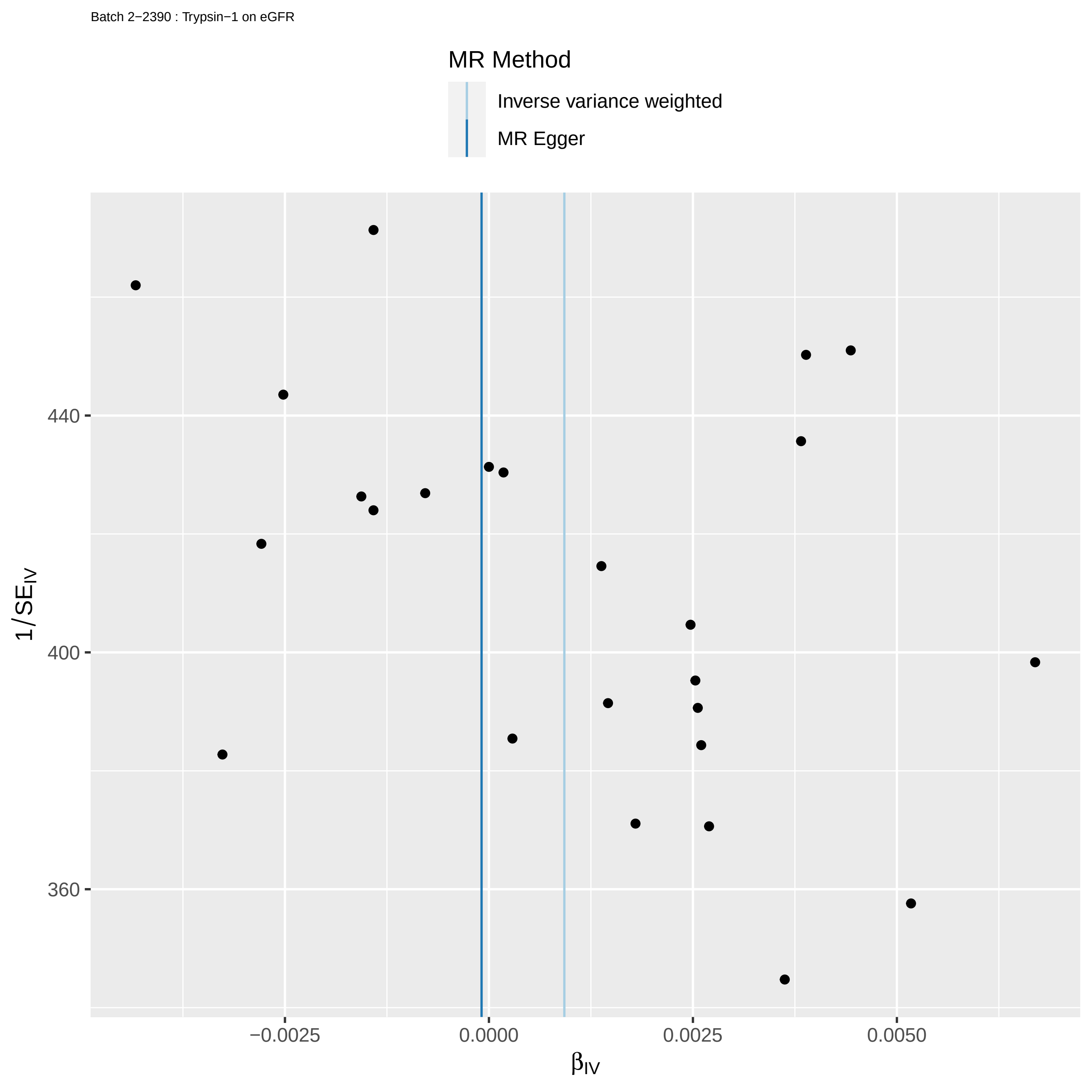

Batch 2−2390 : Trypsin−1 on eGFR
MR Method
Inverse variance weighted
MR Egger
440
400
360
1/SE$_{IV}$
−0.0025
0.0000
0.0025
0.0050
β$_{IV}$

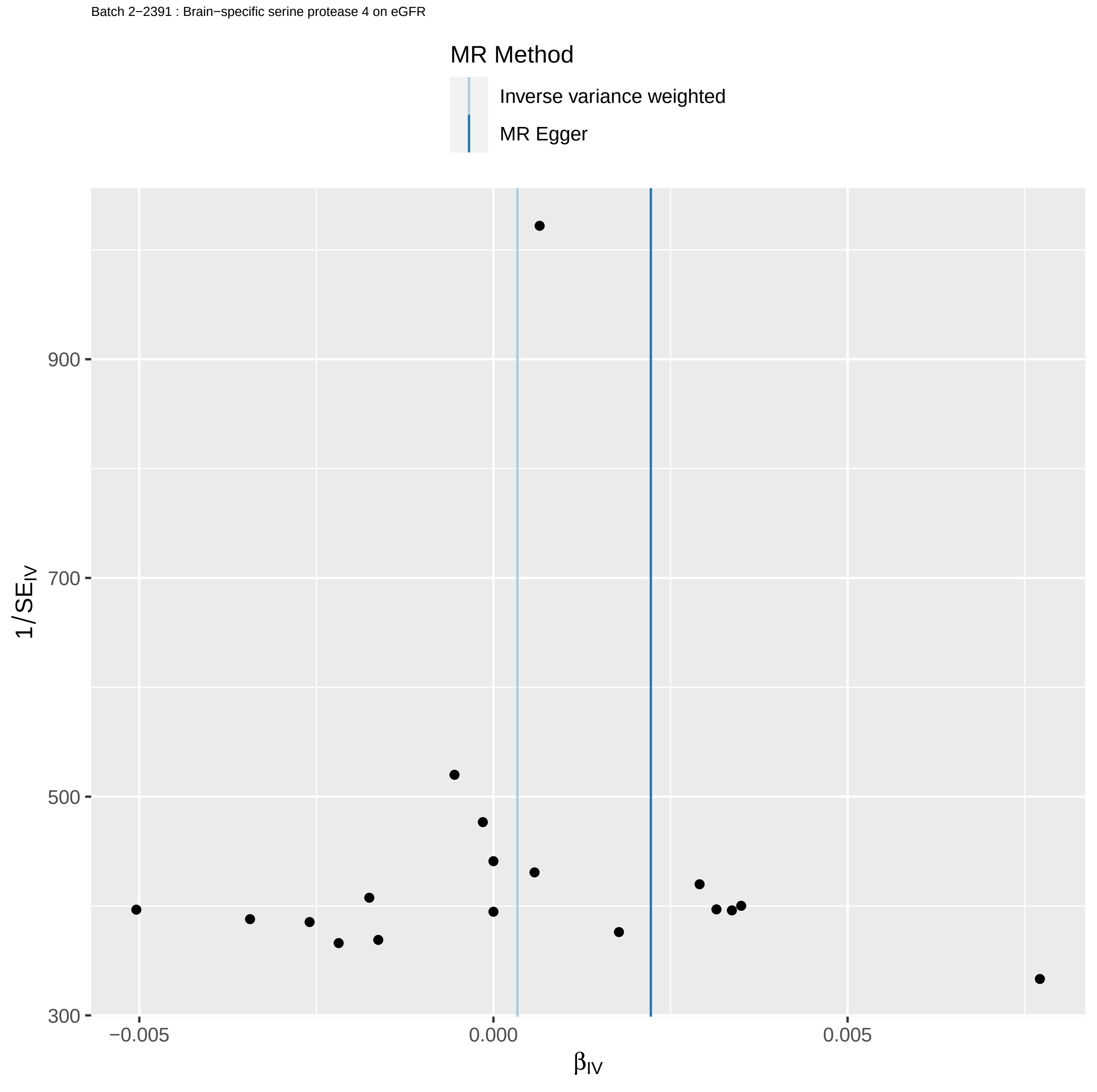
Batch 2−2391 : Brain−specific serine protease 4 on eGFR
MR Method
Inverse variance weighted
MR Egger
1/SE_IV
900
700
500
300
−0.005
0.000
0.005
β_IV

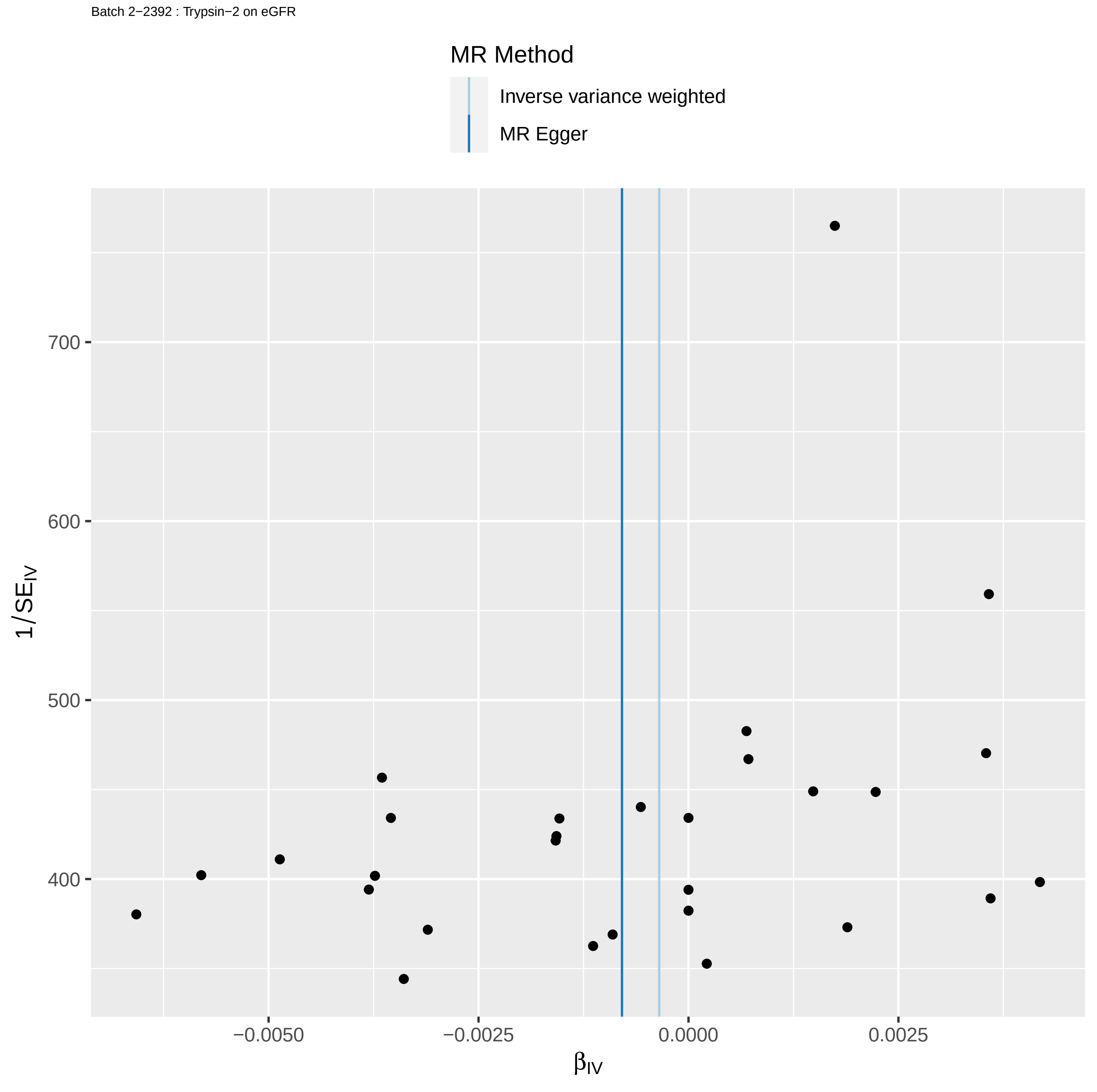

Batch 2−2392 : Trypsin−2 on eGFR
MR Method
Inverse variance weighted
MR Egger
700
600
500
400
$1/SE_{IV}$
−0.0050
−0.0025
0.0000
0.0025
β_{IV}

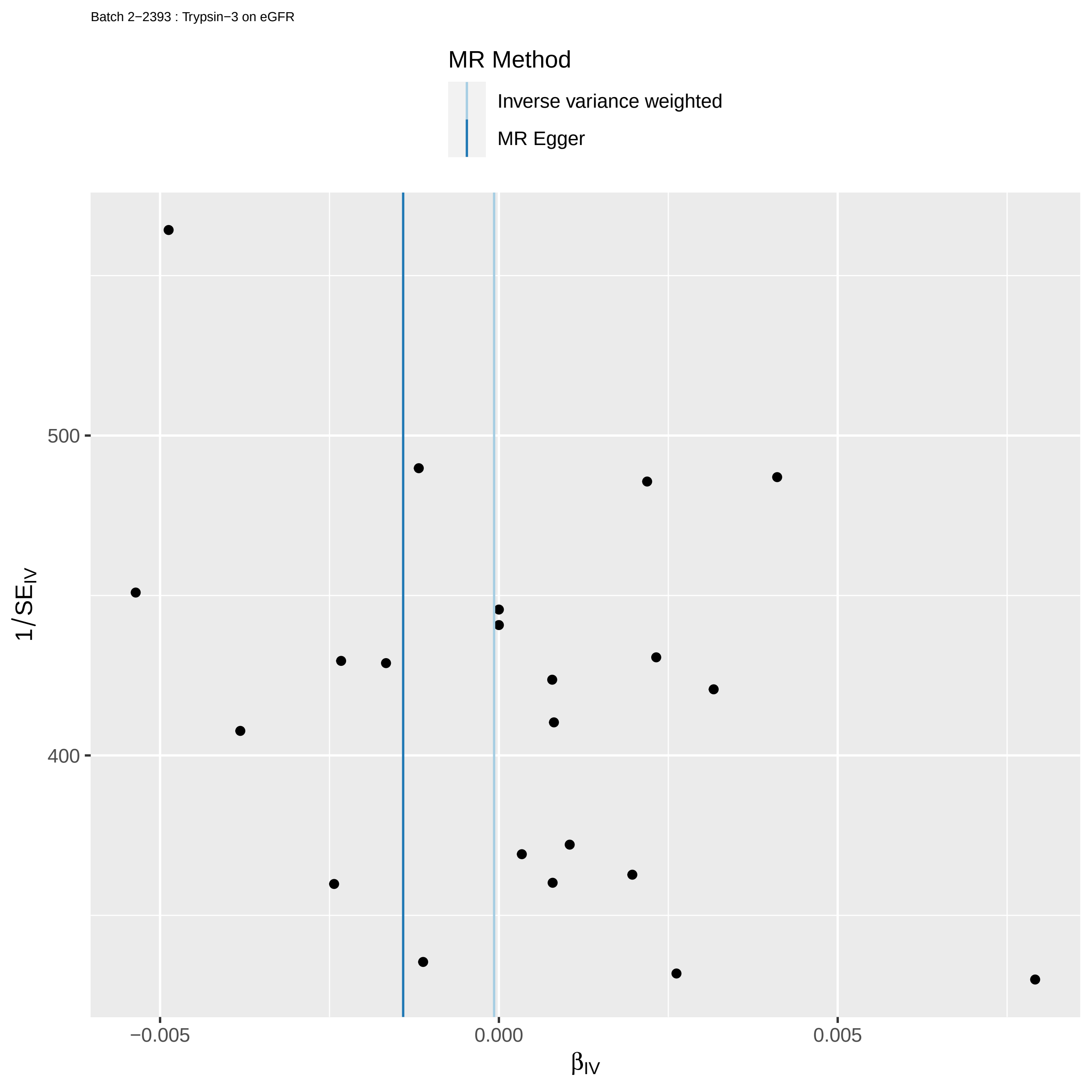
Batch 2−2393 : Trypsin−3 on eGFR
MR Method
Inverse variance weighted
MR Egger
500
400
1/SE_IV
−0.005
0.000
0.005
β_IV

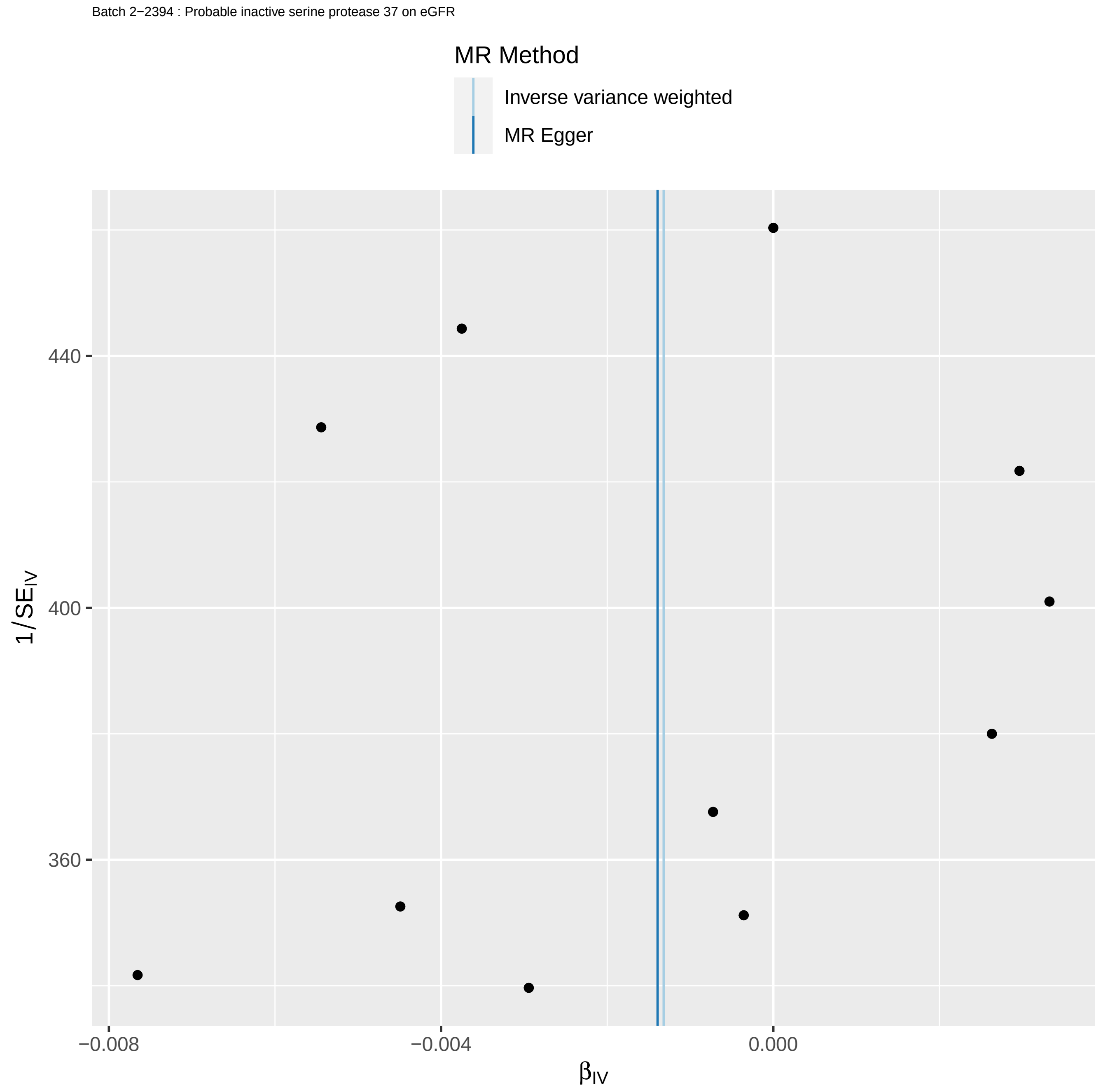

Batch 2−2394 : Probable inactive serine protease 37 on eGFR
MR Method
Inverse variance weighted
MR Egger
440
400
360
1/SE_IV
−0.008
−0.004
0.000
β_IV

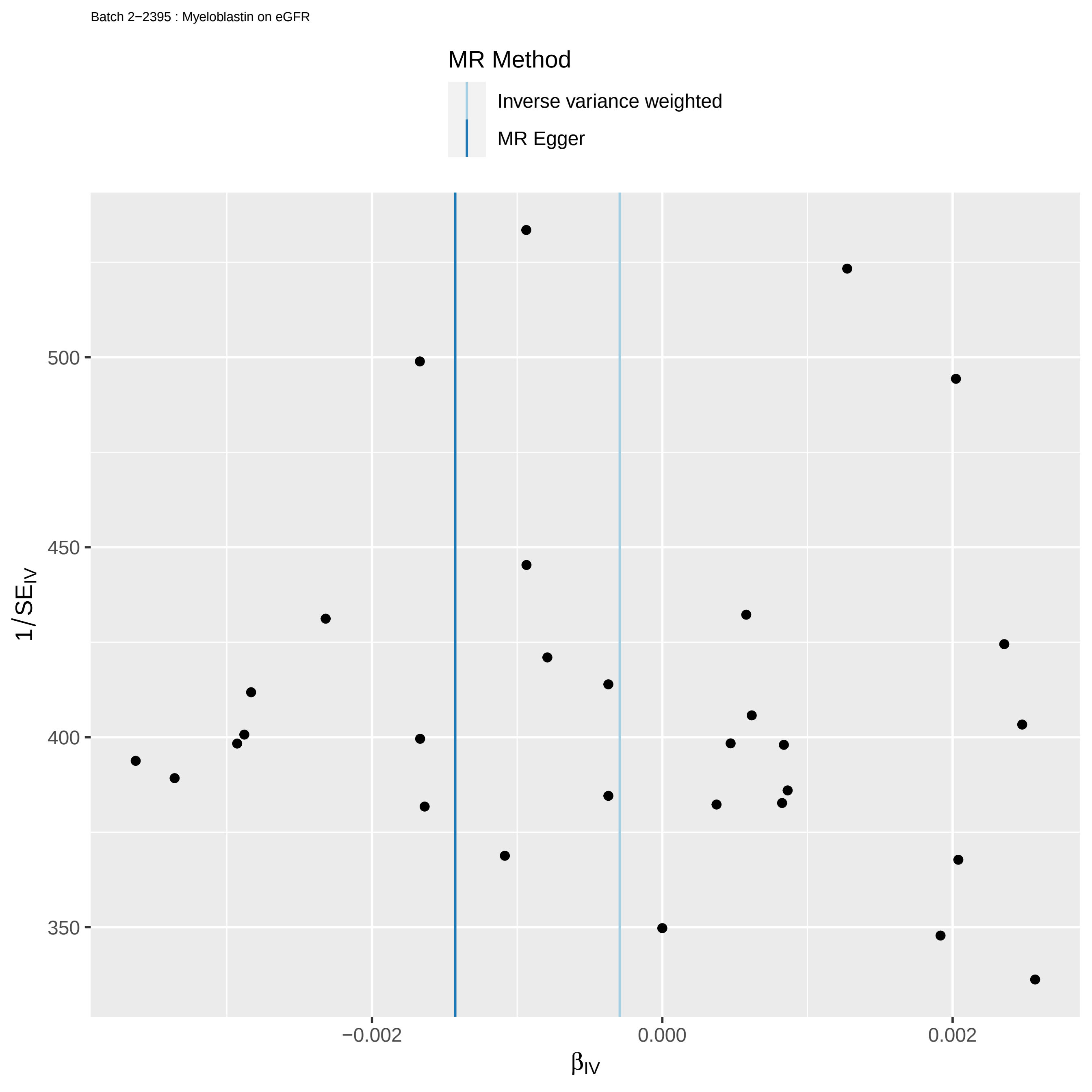

Batch 2−2395 : Myeloblastin on eGFR
MR Method
Inverse variance weighted
MR Egger
500
450
400
350
1/SE$_{IV}$
−0.002
0.000
0.002
β$_{IV}$

Batch 2−2396 : Myeloblastin on eGFR

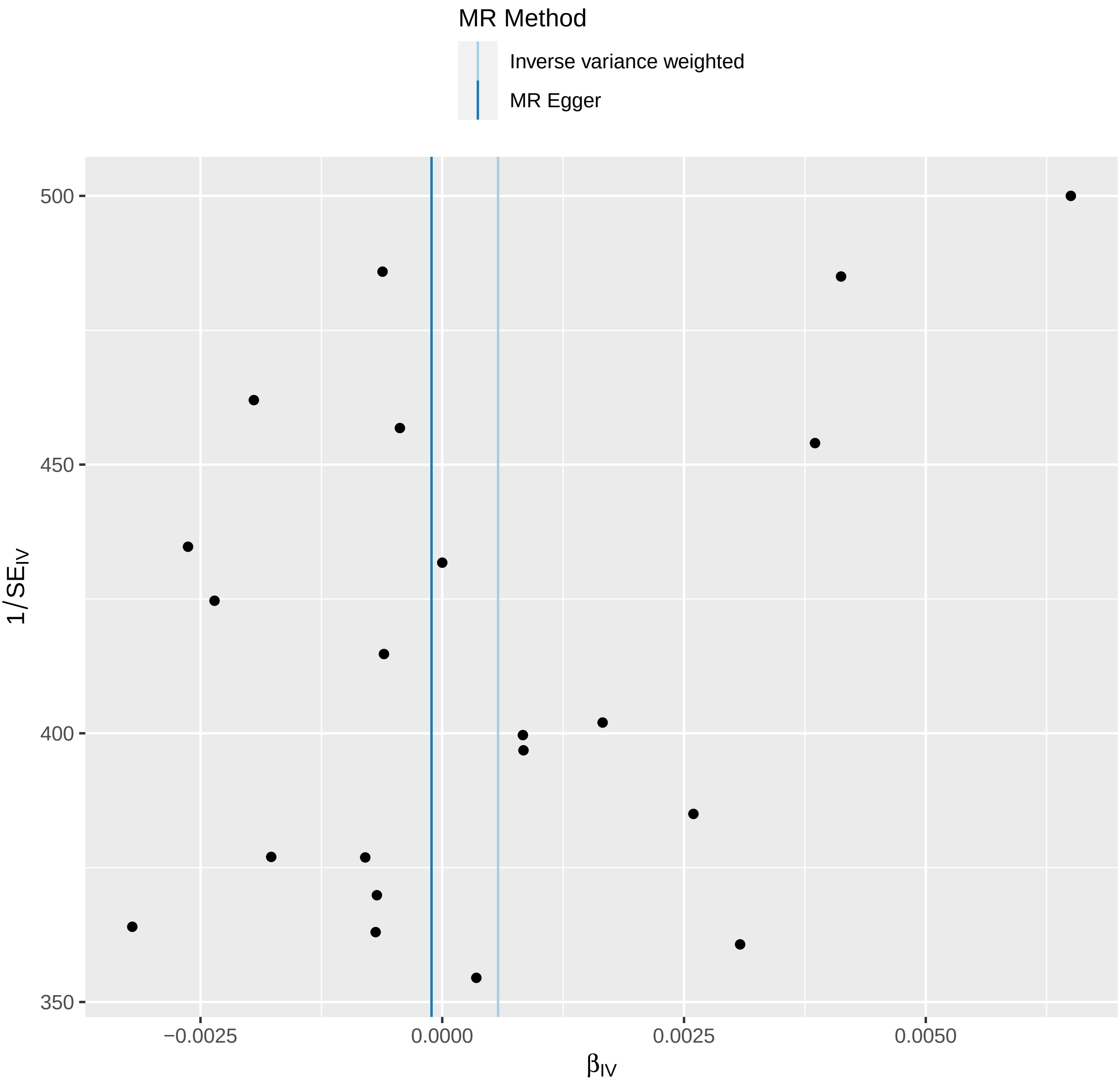

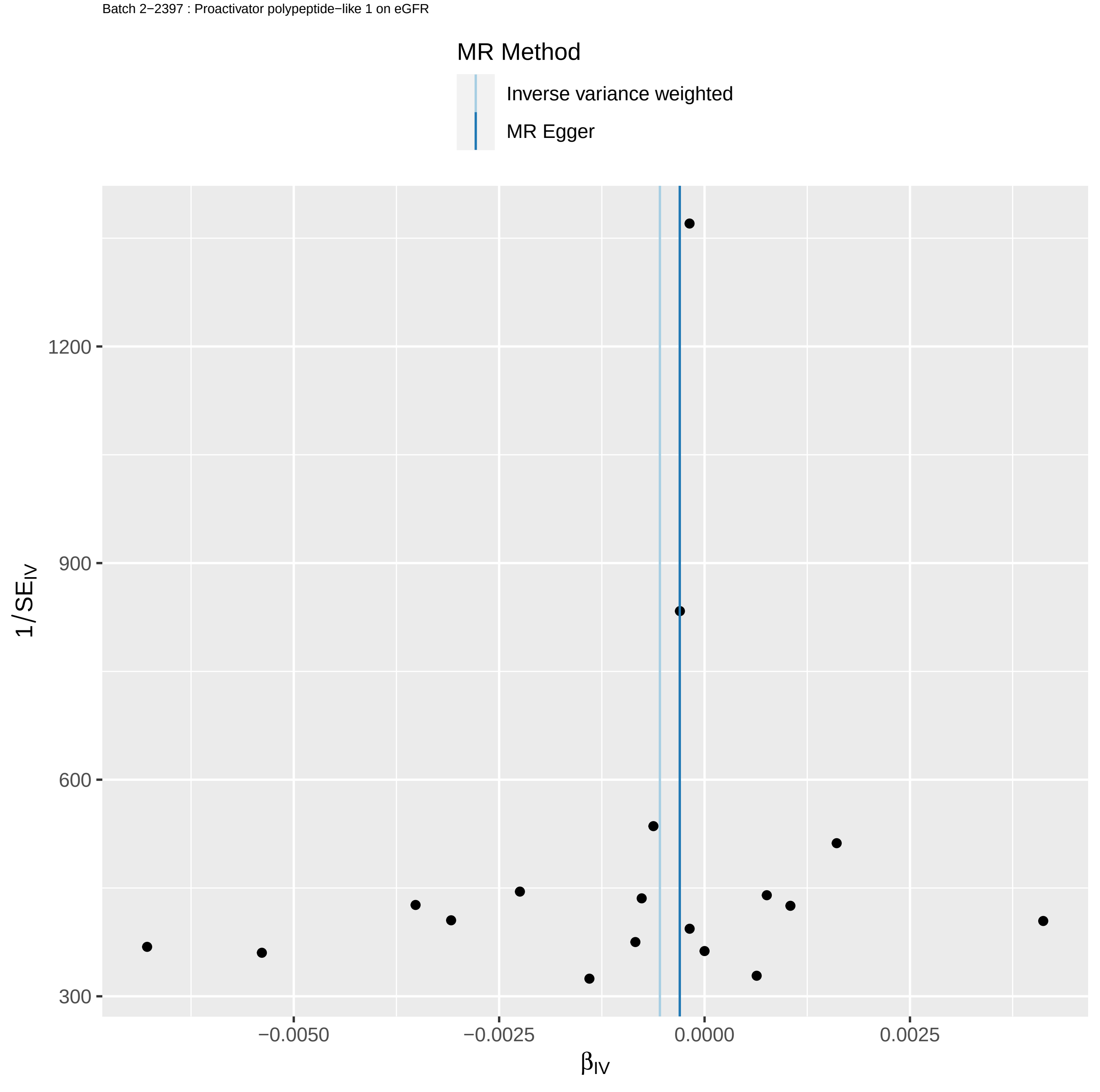
Batch 2−2397 : Proactivator polypeptide−like 1 on eGFR
MR Method
Inverse variance weighted
MR Egger
1200
900
600
300
1/SE_IV
−0.0050
−0.0025
0.0000
0.0025
β_IV

Batch 2−2398 : PH and SEC7 domain−containing protein 1 on eGFR

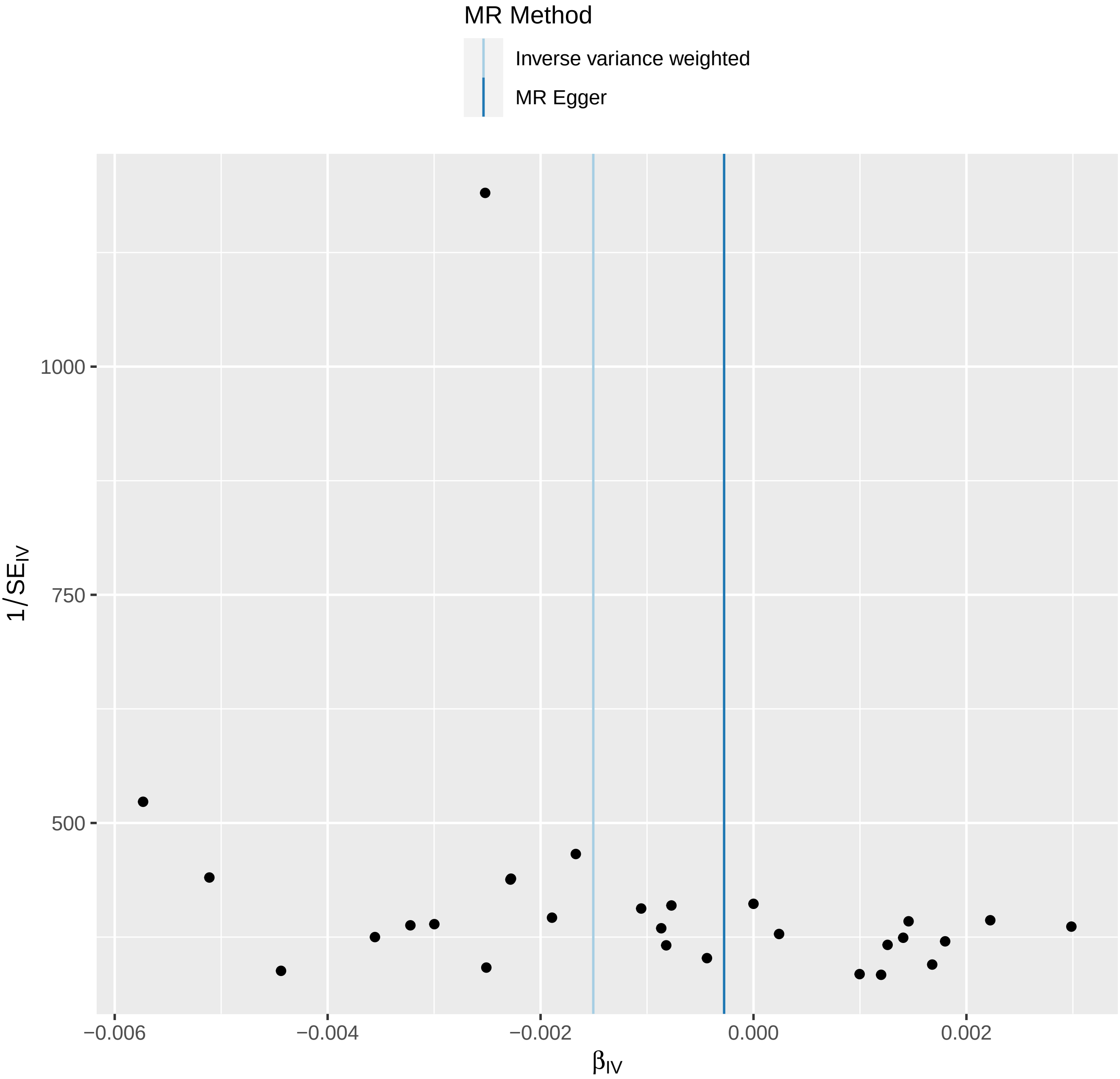

Batch 2−2399 : PH and SEC7 domain−containing protein 2 on eGFR

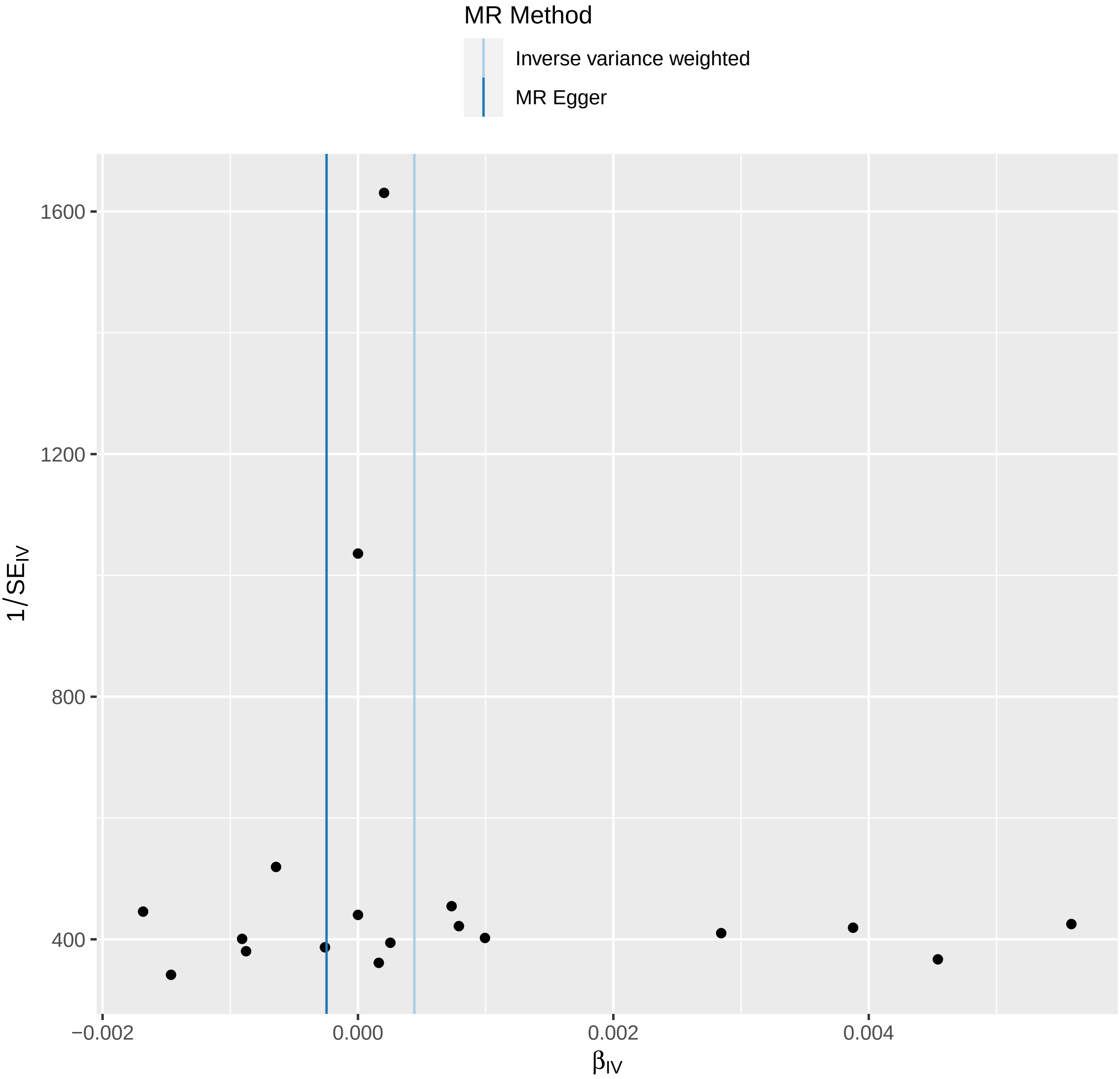

Batch 2−2400 : Pregnancy−specific beta−1−glycoprotein 11 on eGFR

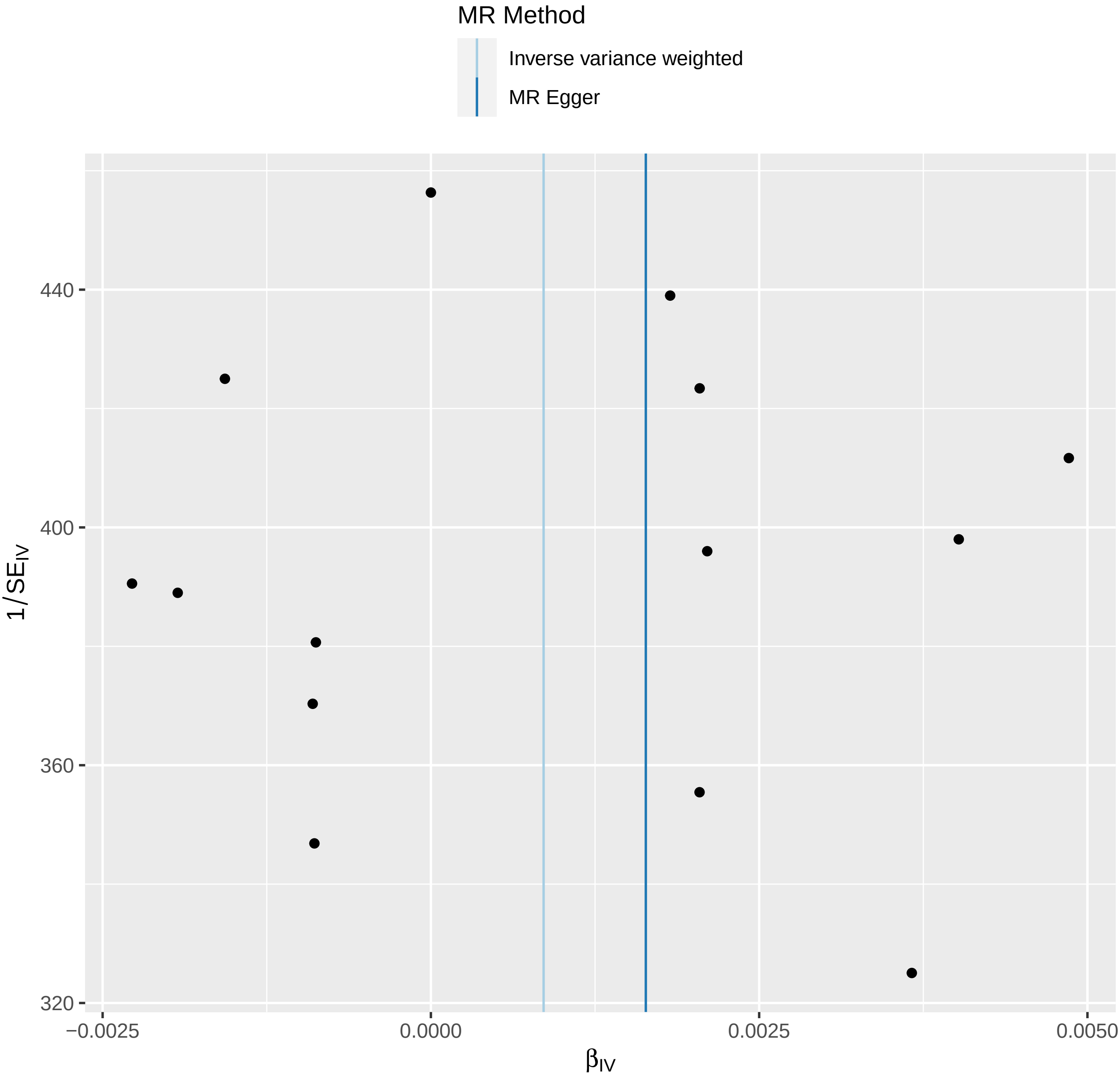

Batch 2−2401 : Pregnancy−specific beta−1−glycoprotein 2 on eGFR

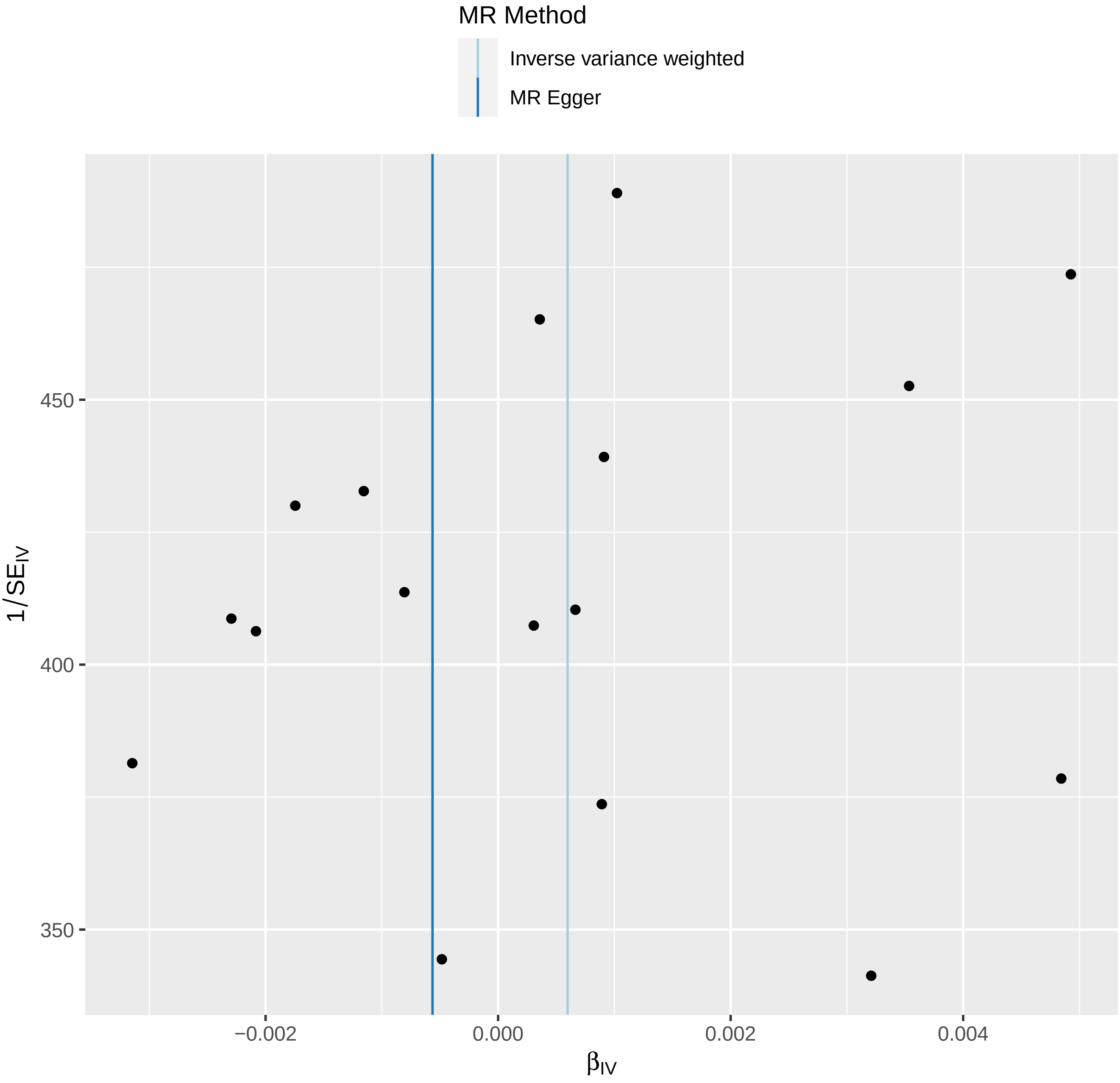

Batch 2−2402 : Pregnancy−specific beta−1−glycoprotein 3 on eGFR

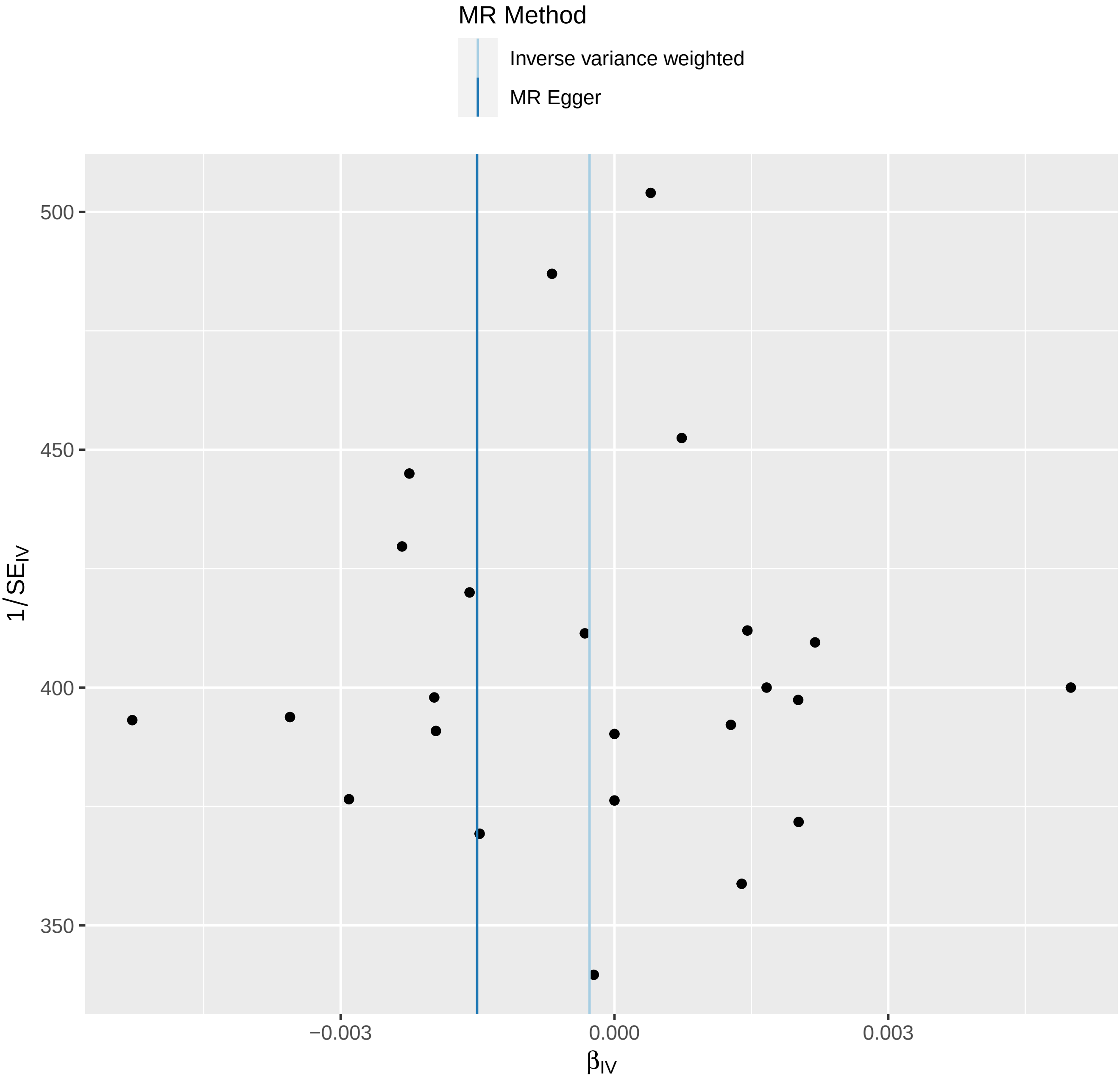

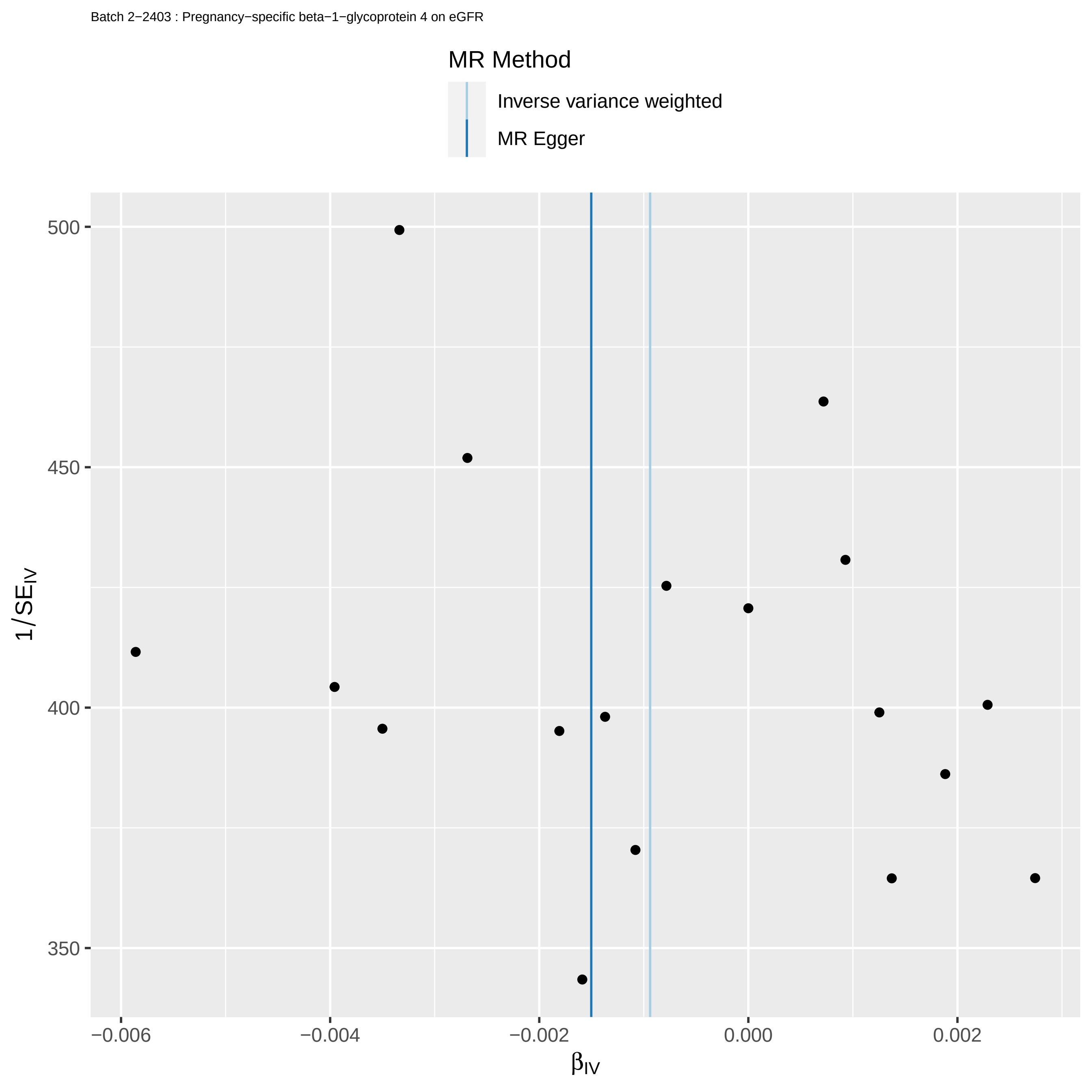

Batch 2−2403 : Pregnancy−specific beta−1−glycoprotein 4 on eGFR
MR Method
Inverse variance weighted
MR Egger
500
450
400
350
1/SE$_{IV}$
−0.006
−0.004
−0.002
0.000
0.002
β$_{IV}$

Batch 2−2404 : Pregnancy−specific beta−1−glycoprotein 5 on eGFR

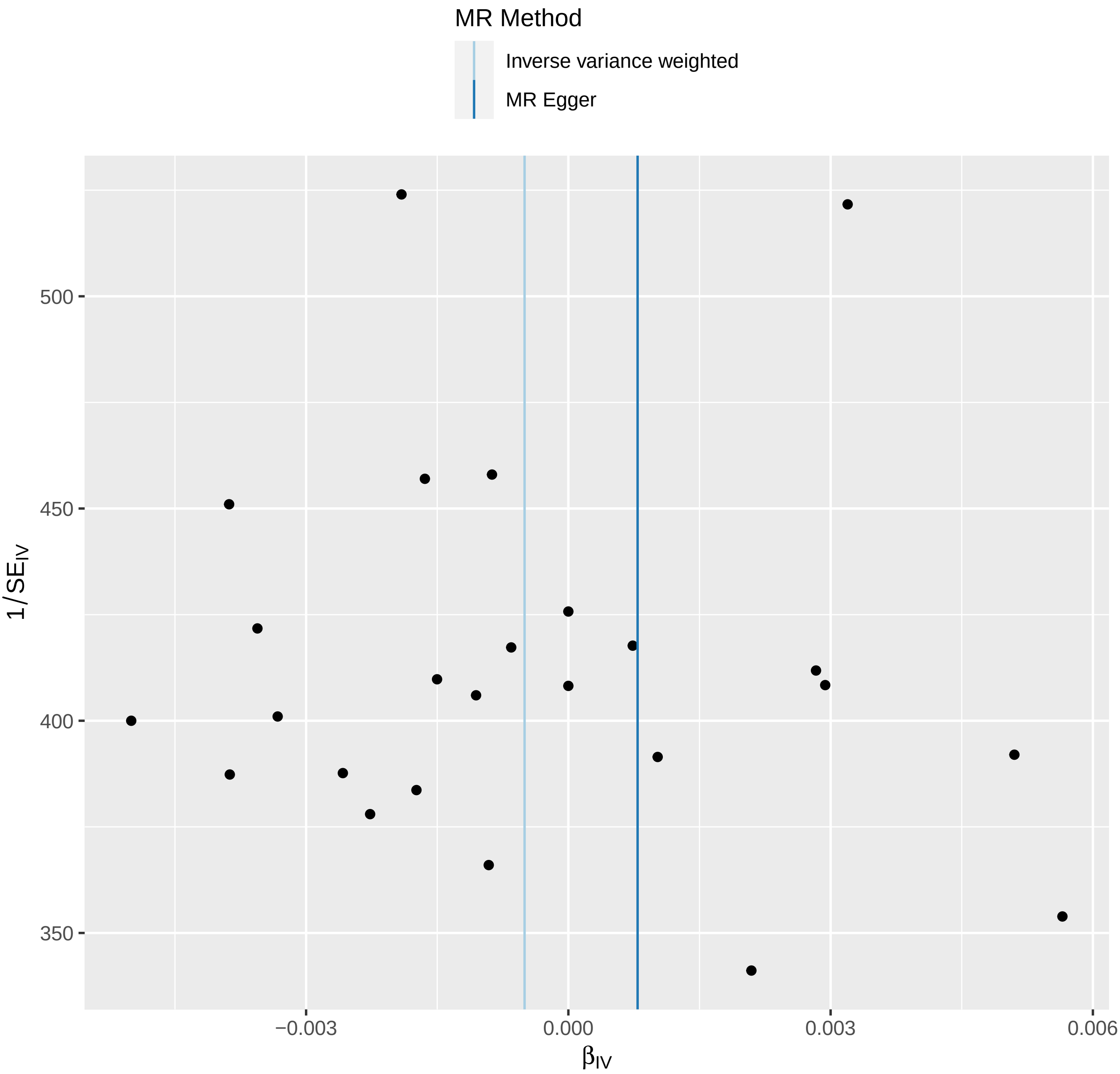

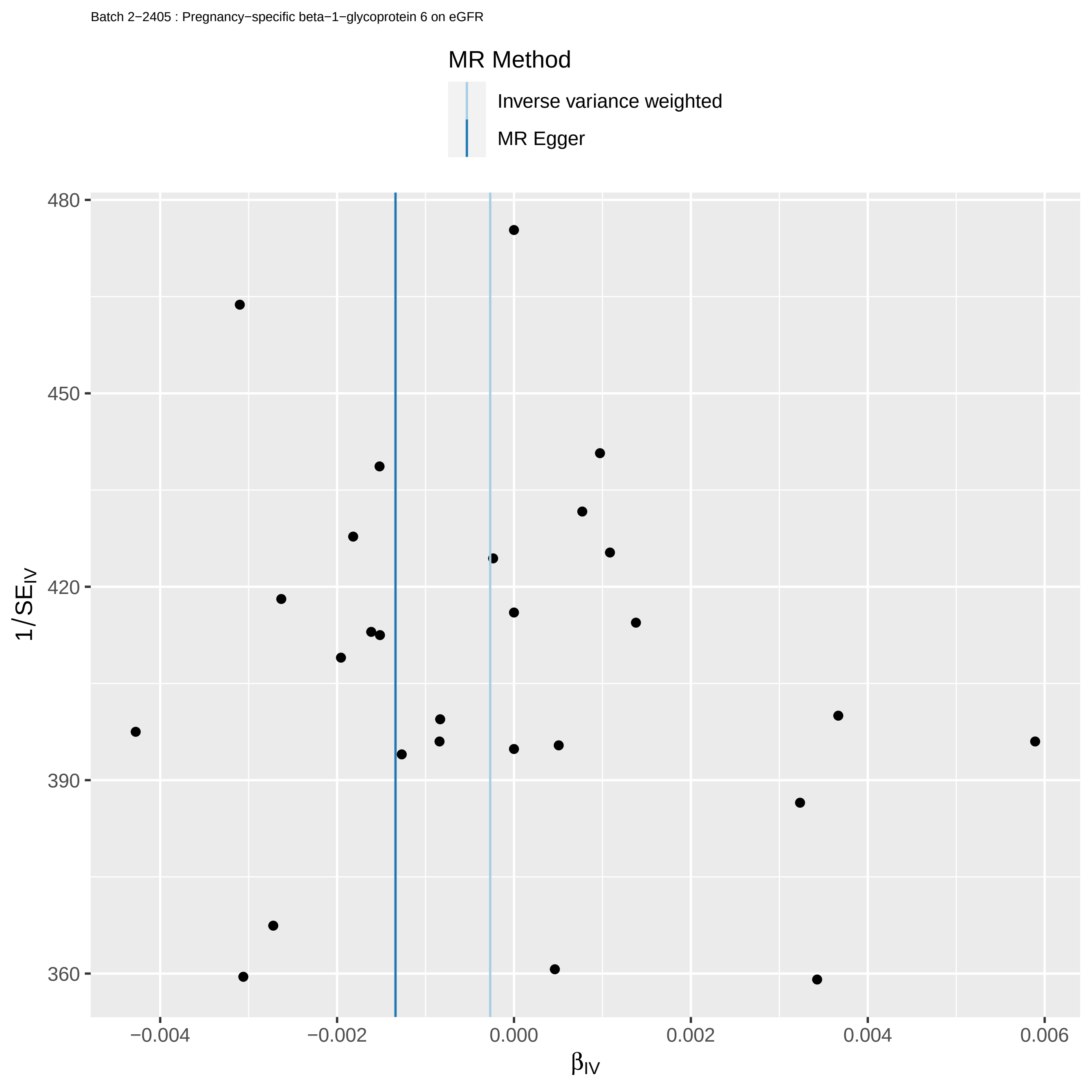

Batch 2−2405 : Pregnancy−specific beta−1−glycoprotein 6 on eGFR
MR Method
Inverse variance weighted
MR Egger
480
450
420
390
360
1/SE$_{IV}$
−0.004
−0.002
0.000
0.002
0.004
0.006
β$_{IV}$

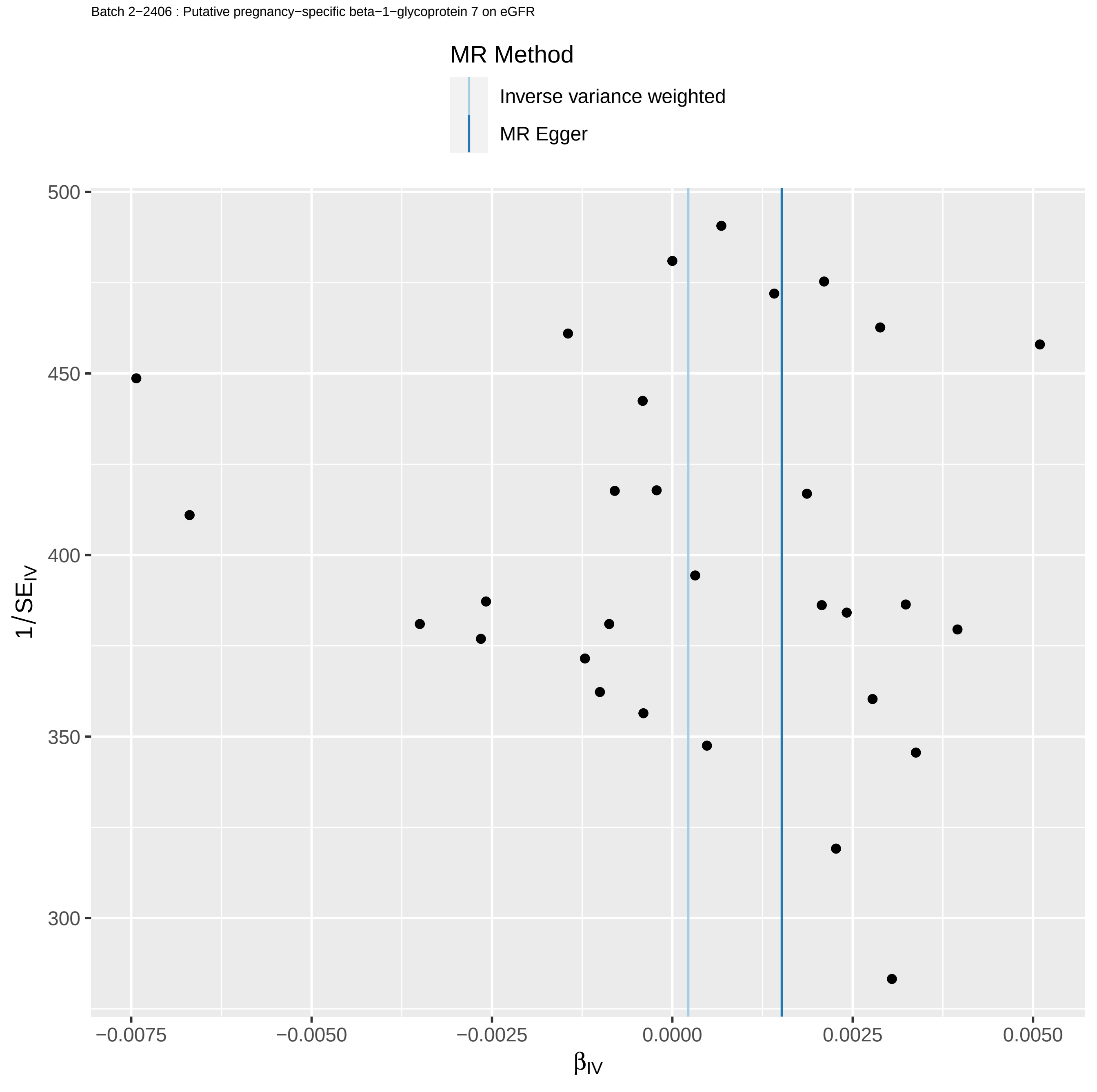
Batch 2−2406 : Putative pregnancy−specific beta−1−glycoprotein 7 on eGFR
MR Method
Inverse variance weighted
MR Egger
500
450
400
350
300
1/SE_IV
−0.0075
−0.0050
−0.0025
0.0000
0.0025
0.0050
β_IV

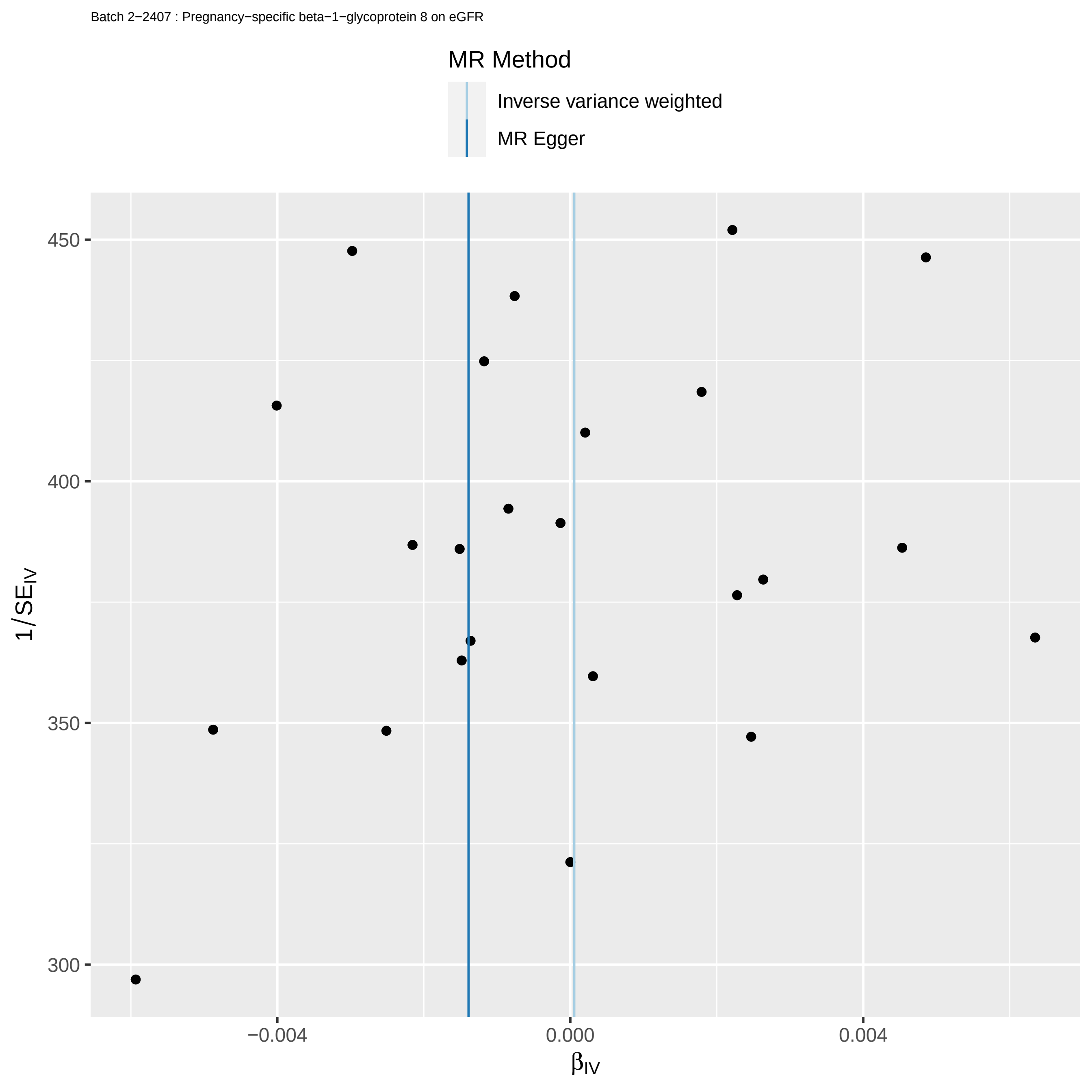

Batch 2−2407 : Pregnancy−specific beta−1−glycoprotein 8 on eGFR
MR Method
Inverse variance weighted
MR Egger
450
400
350
300
1/SE_IV
−0.004
0.000
0.004
β_IV

Batch 2−2408 : Pregnancy−specific beta−1−glycoprotein 9 on eGFR

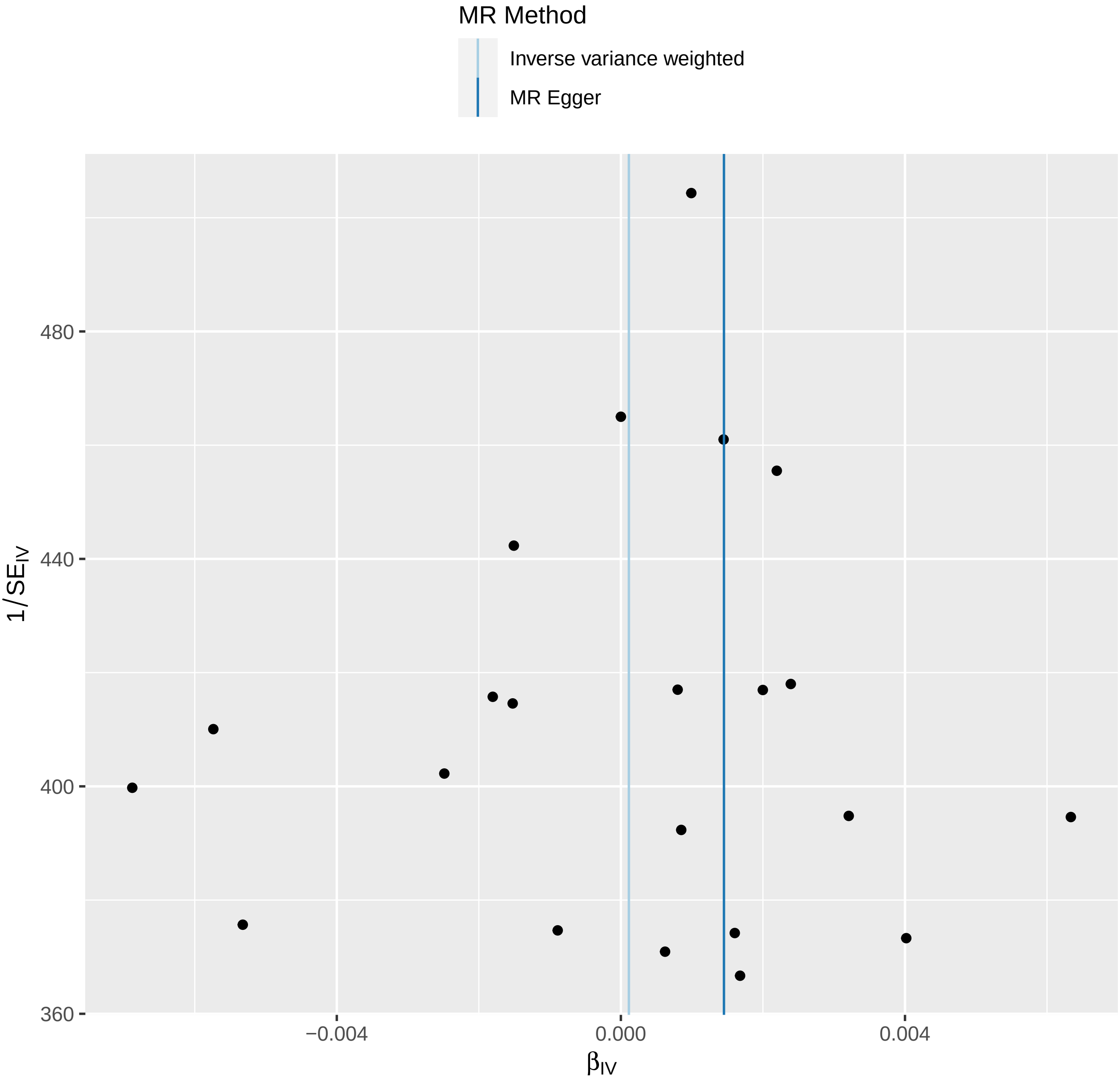

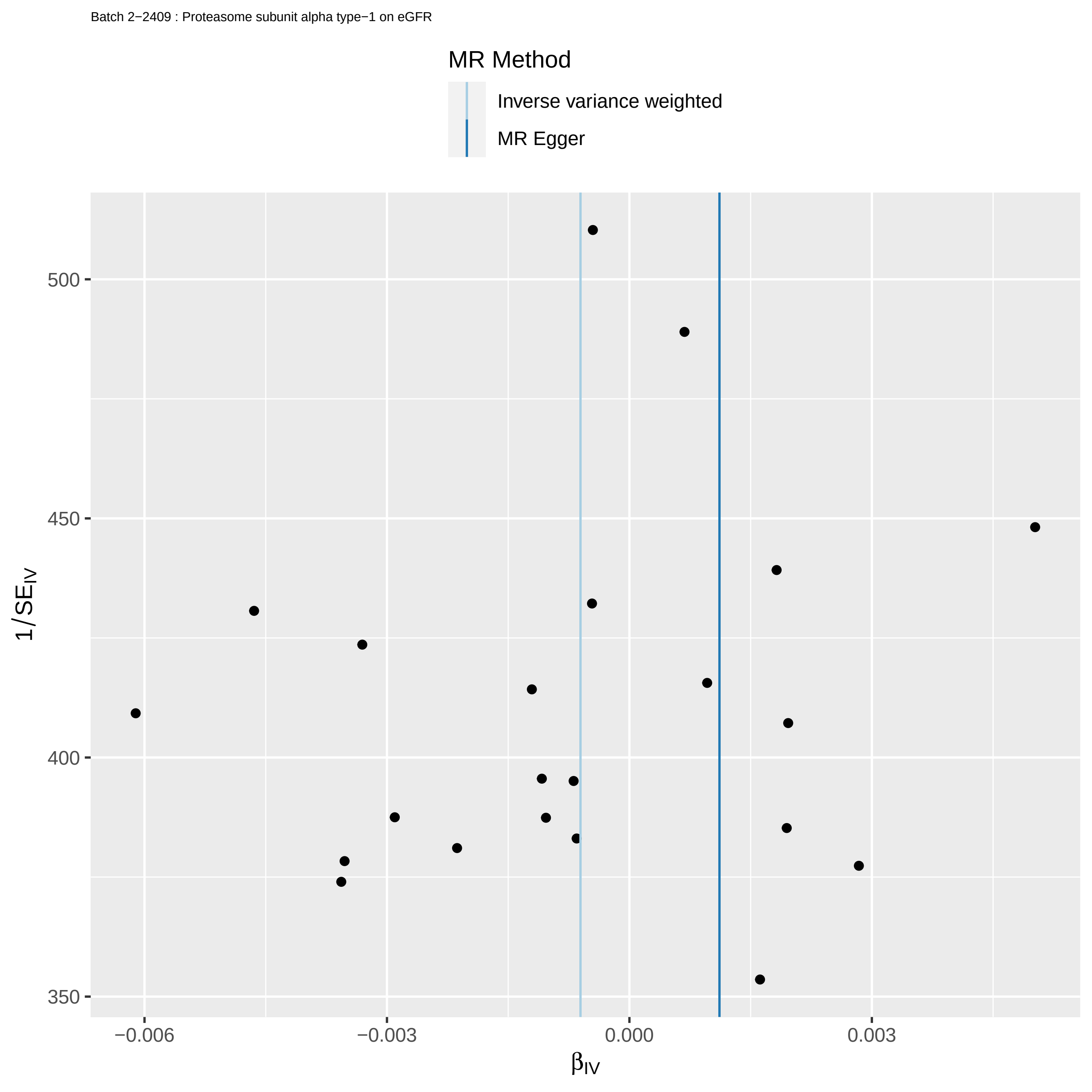

Batch 2−2409 : Proteasome subunit alpha type−1 on eGFR
MR Method
Inverse variance weighted
MR Egger
500
450
400
350
1/SE_IV
−0.006
−0.003
0.000
0.003
β_IV

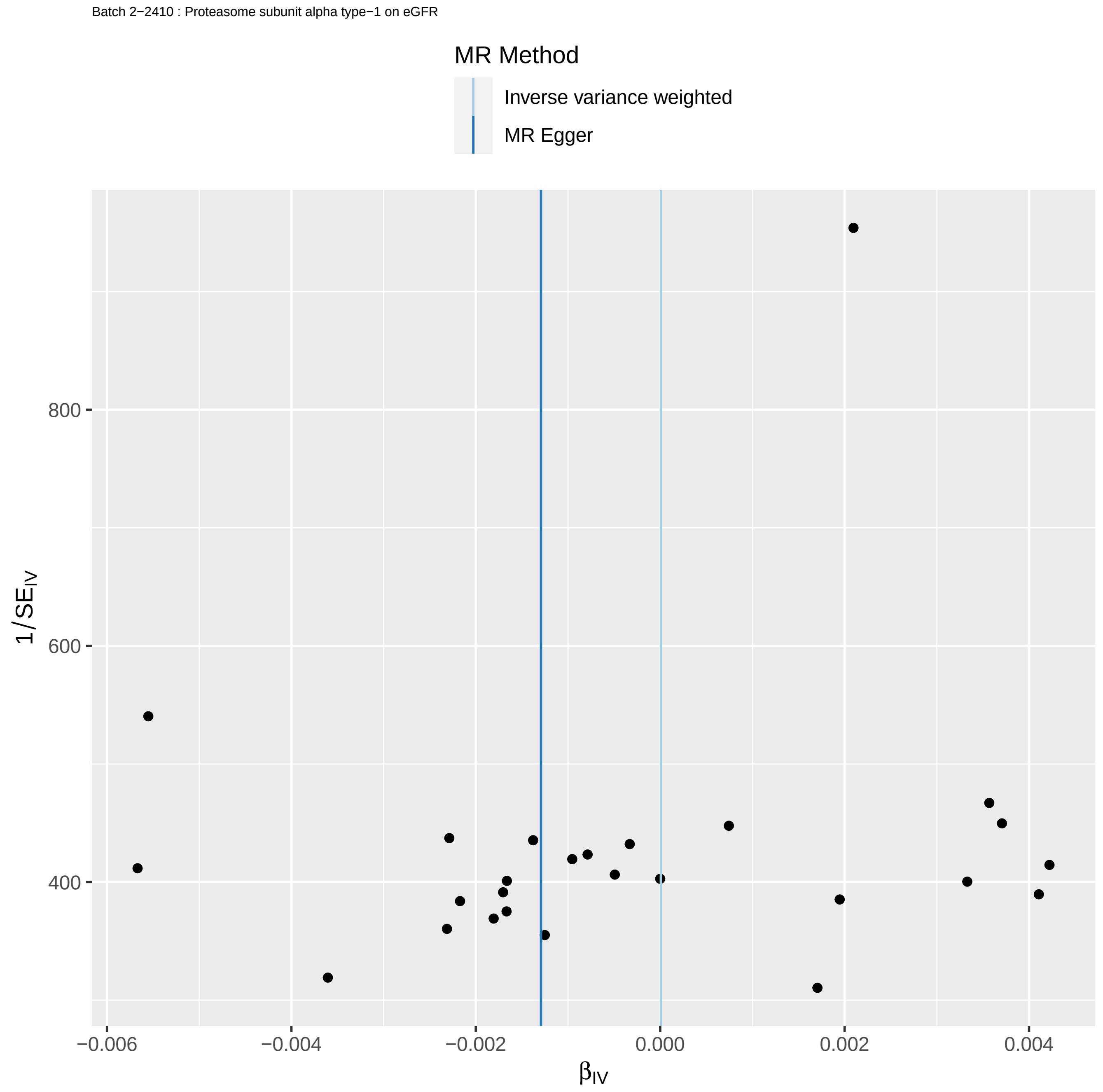
Batch 2−2410 : Proteasome subunit alpha type−1 on eGFR
MR Method
Inverse variance weighted
MR Egger
800
600
400
$1/SE_{IV}$
−0.006
−0.004
−0.002
0.000
0.002
0.004
β_{IV}

Batch 2−2411 : Proteasome subunit alpha type−2 on eGFR

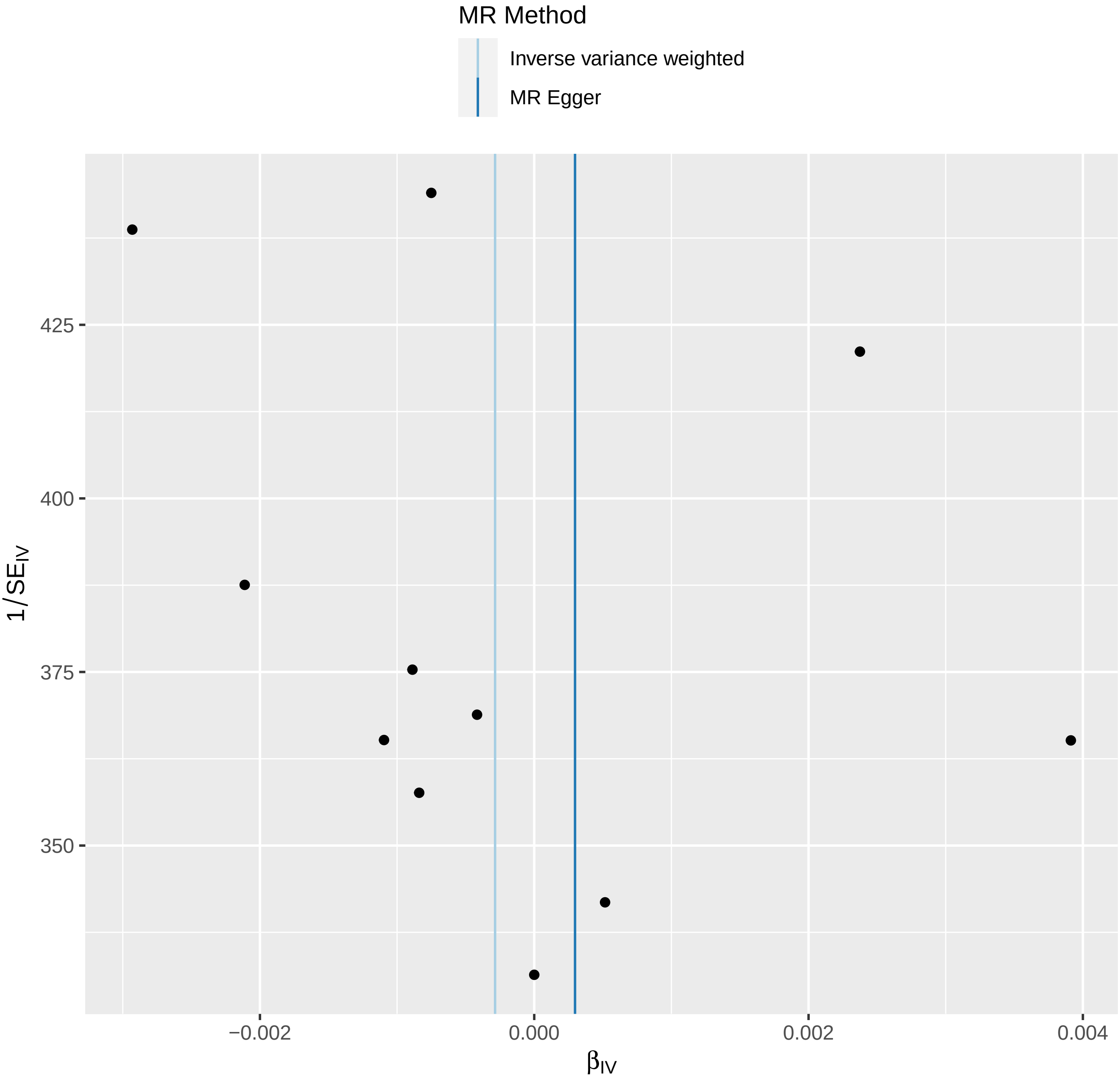

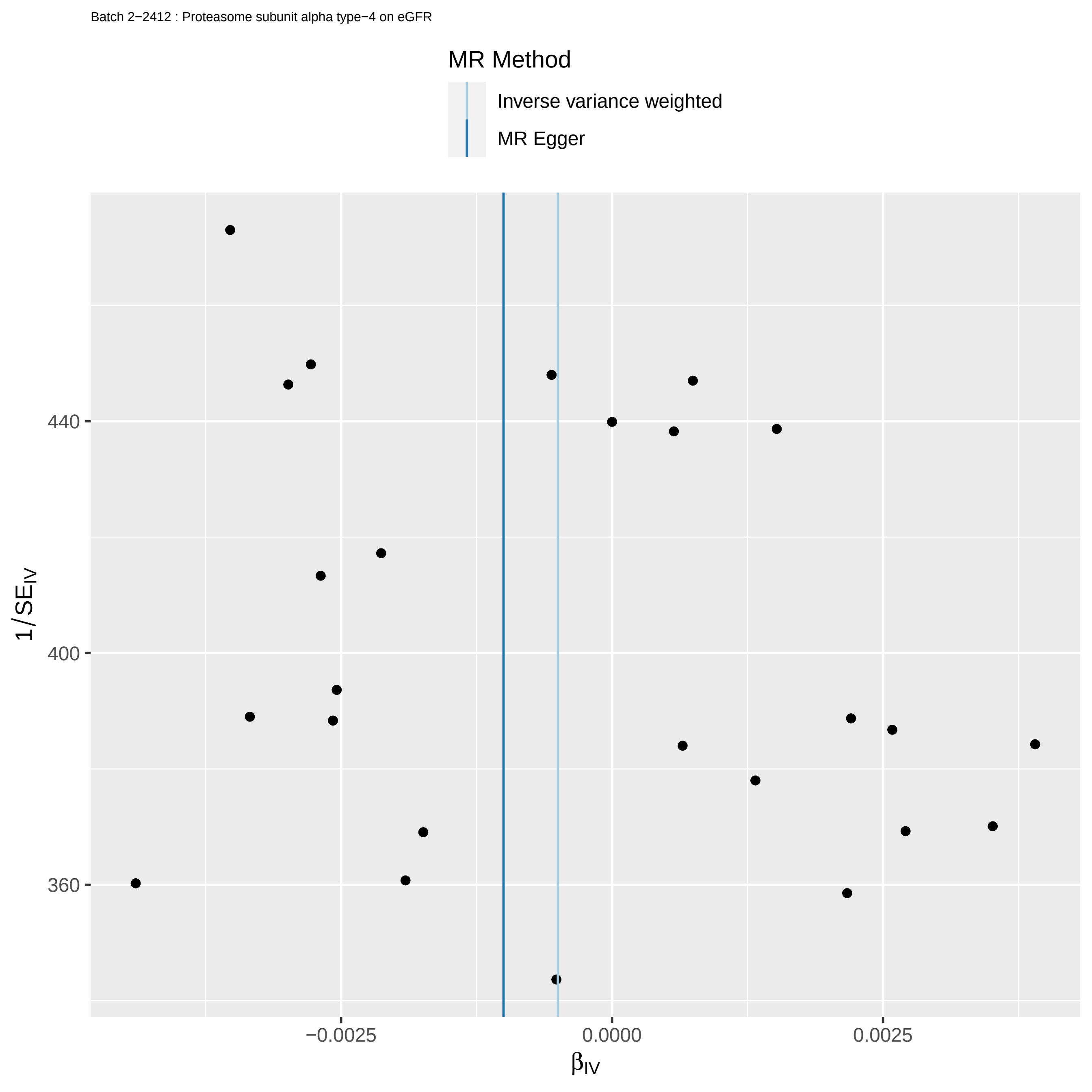

Batch 2−2412 : Proteasome subunit alpha type−4 on eGFR
MR Method
Inverse variance weighted
MR Egger
440
400
360
1/SE_IV
−0.0025
0.0000
0.0025
β_IV

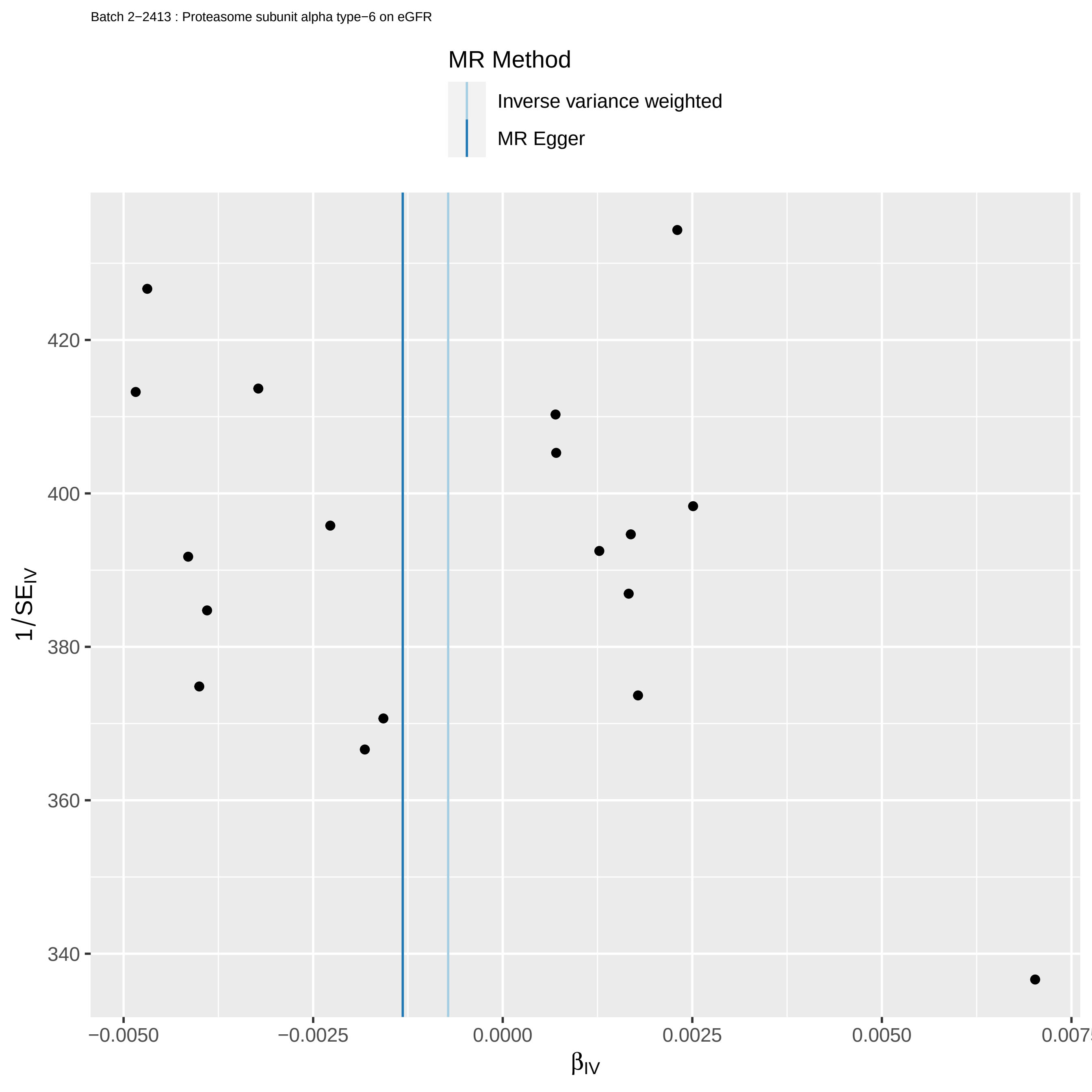
Batch 2−2413 : Proteasome subunit alpha type−6 on eGFR
MR Method
Inverse variance weighted
MR Egger
1/SE$_{IV}$
420
400
380
360
340
−0.0050
−0.0025
0.0000
0.0025
0.0050
β$_{IV}$

Batch 2−2414 : Proteasome subunit beta type−5 on eGFR

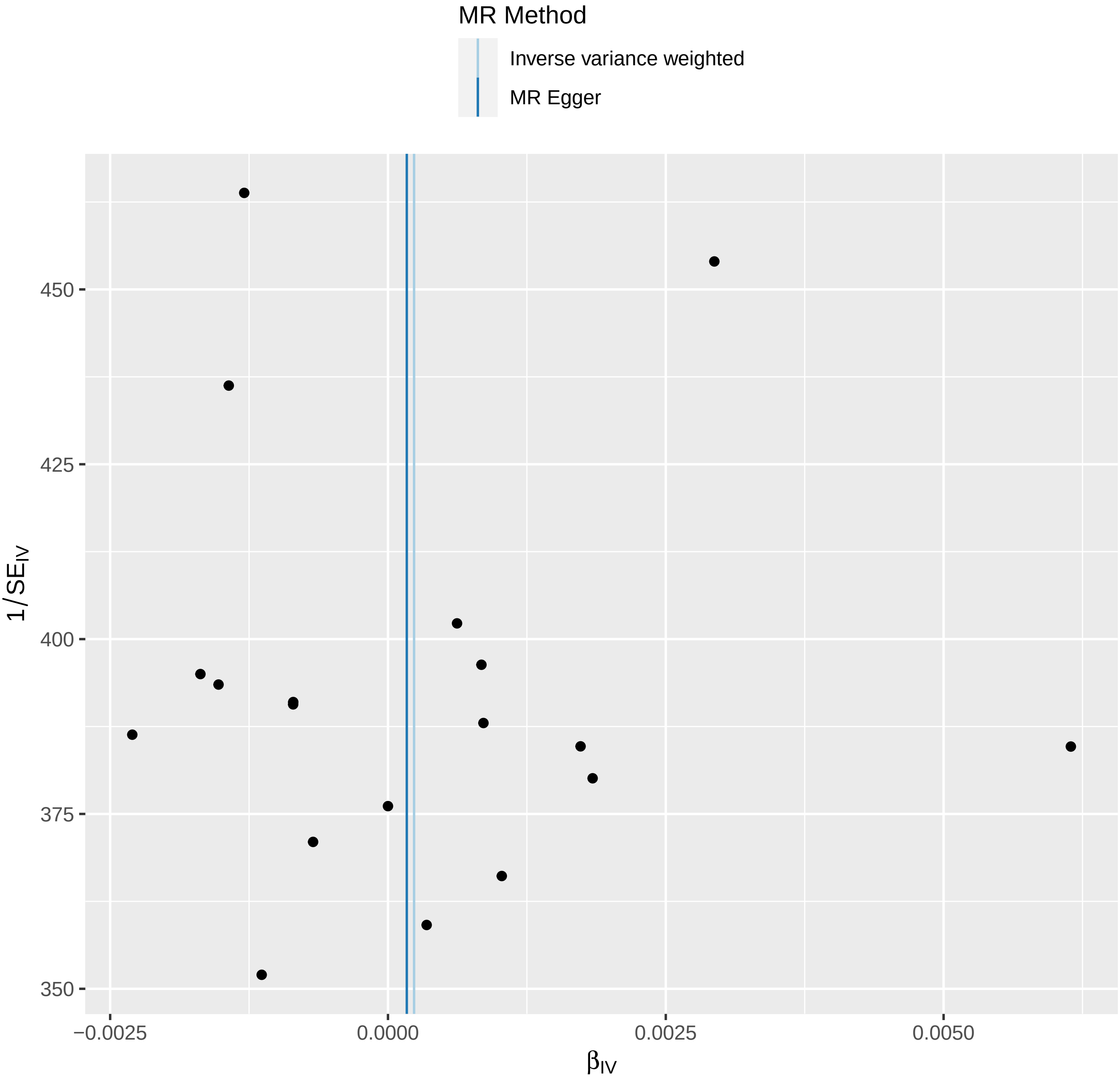

Batch 2−2415 : 26S proteasome non−ATPase regulatory subunit 11 on eGFR

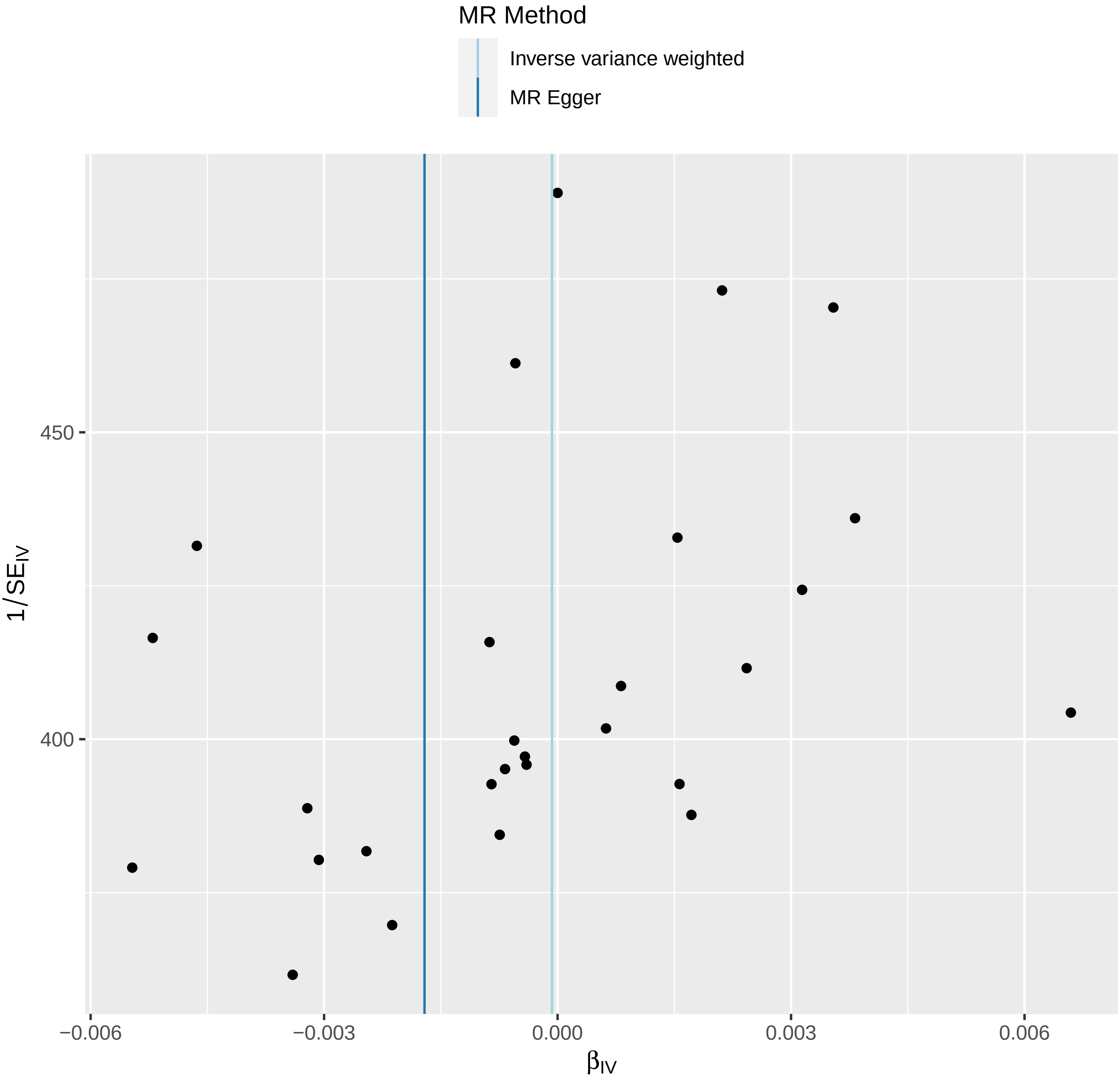

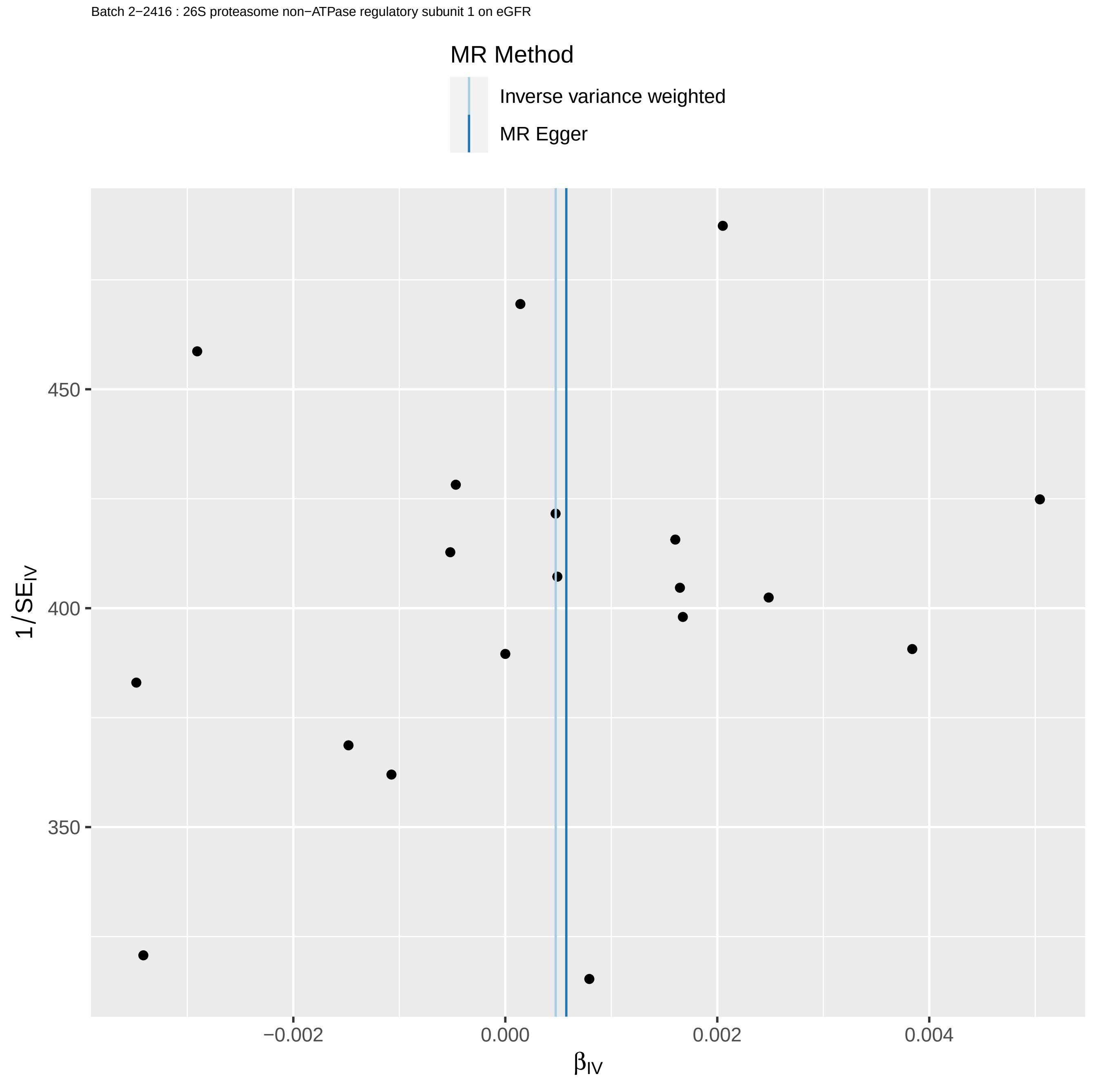

Batch 2−2416 : 26S proteasome non−ATPase regulatory subunit 1 on eGFR
MR Method
Inverse variance weighted
MR Egger
450
400
350
1/SE_IV
−0.002
0.000
0.002
0.004
β_IV

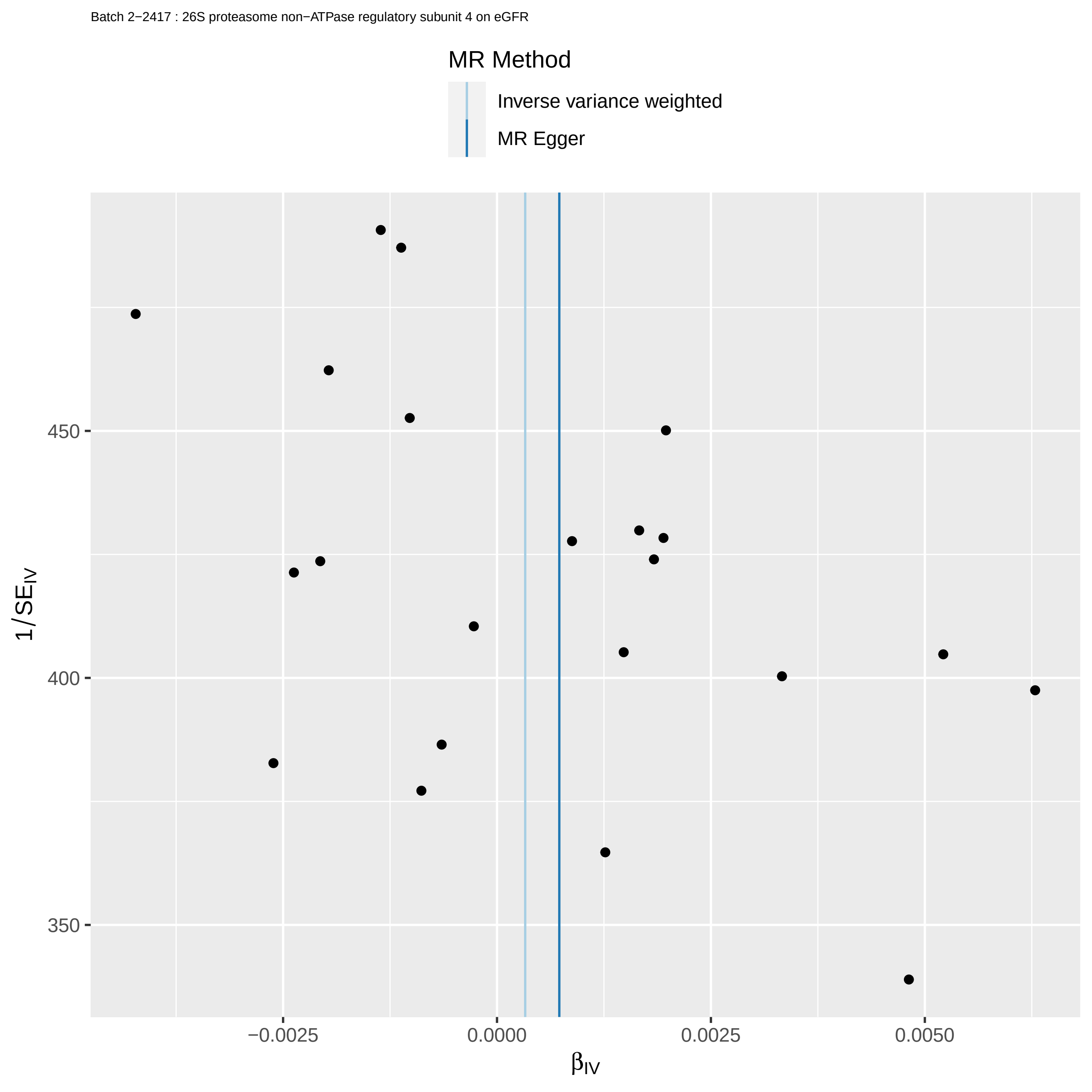

Batch 2−2417 : 26S proteasome non−ATPase regulatory subunit 4 on eGFR
MR Method
Inverse variance weighted
MR Egger
450
400
350
1/SE$_{IV}$
−0.0025
0.0000
0.0025
0.0050
β$_{IV}$

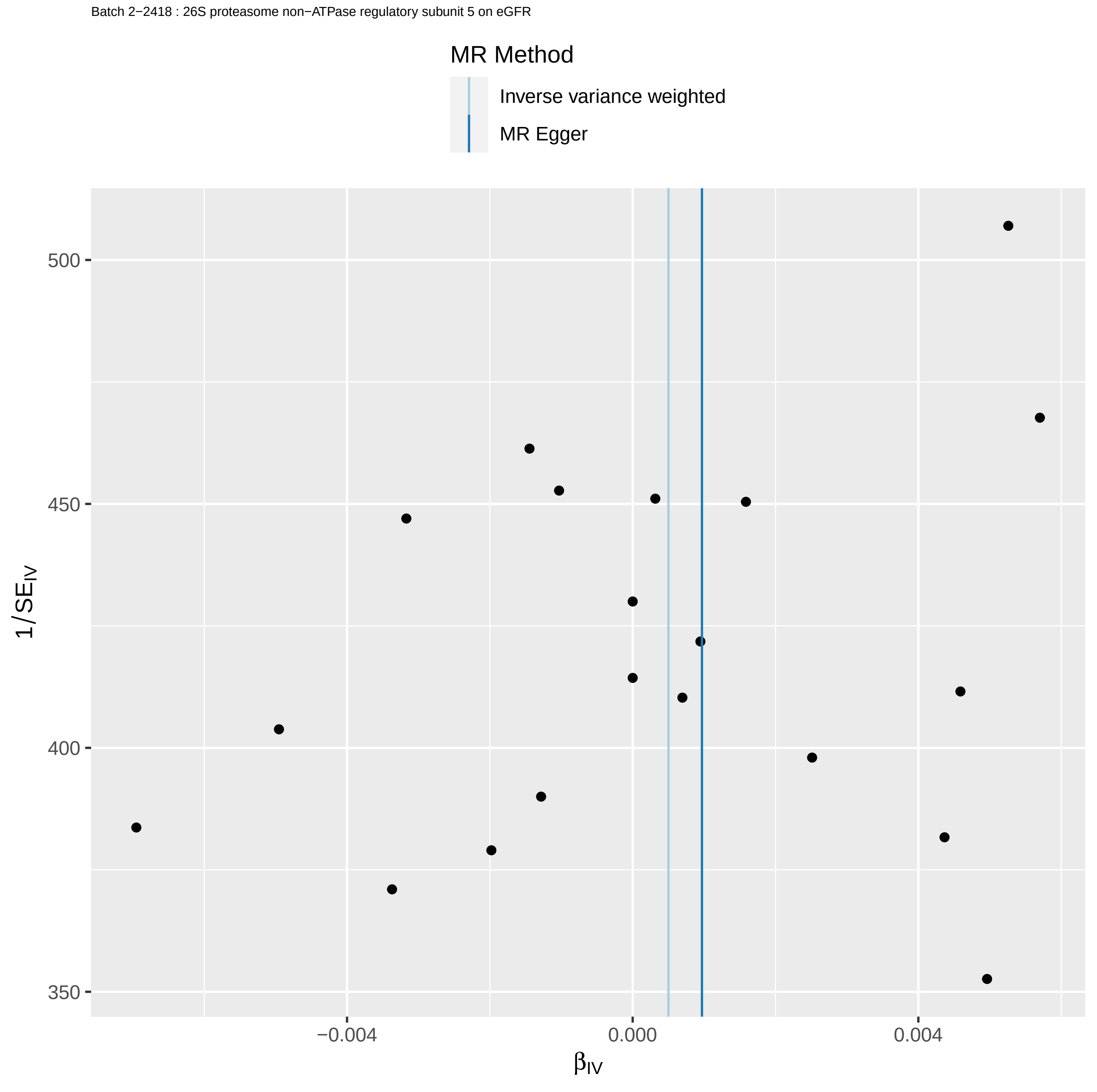
Batch 2−2418 : 26S proteasome non−ATPase regulatory subunit 5 on eGFR
MR Method
Inverse variance weighted
MR Egger
500
450
400
350
1/SE$_{IV}$
−0.004
0.000
0.004
β$_{IV}$

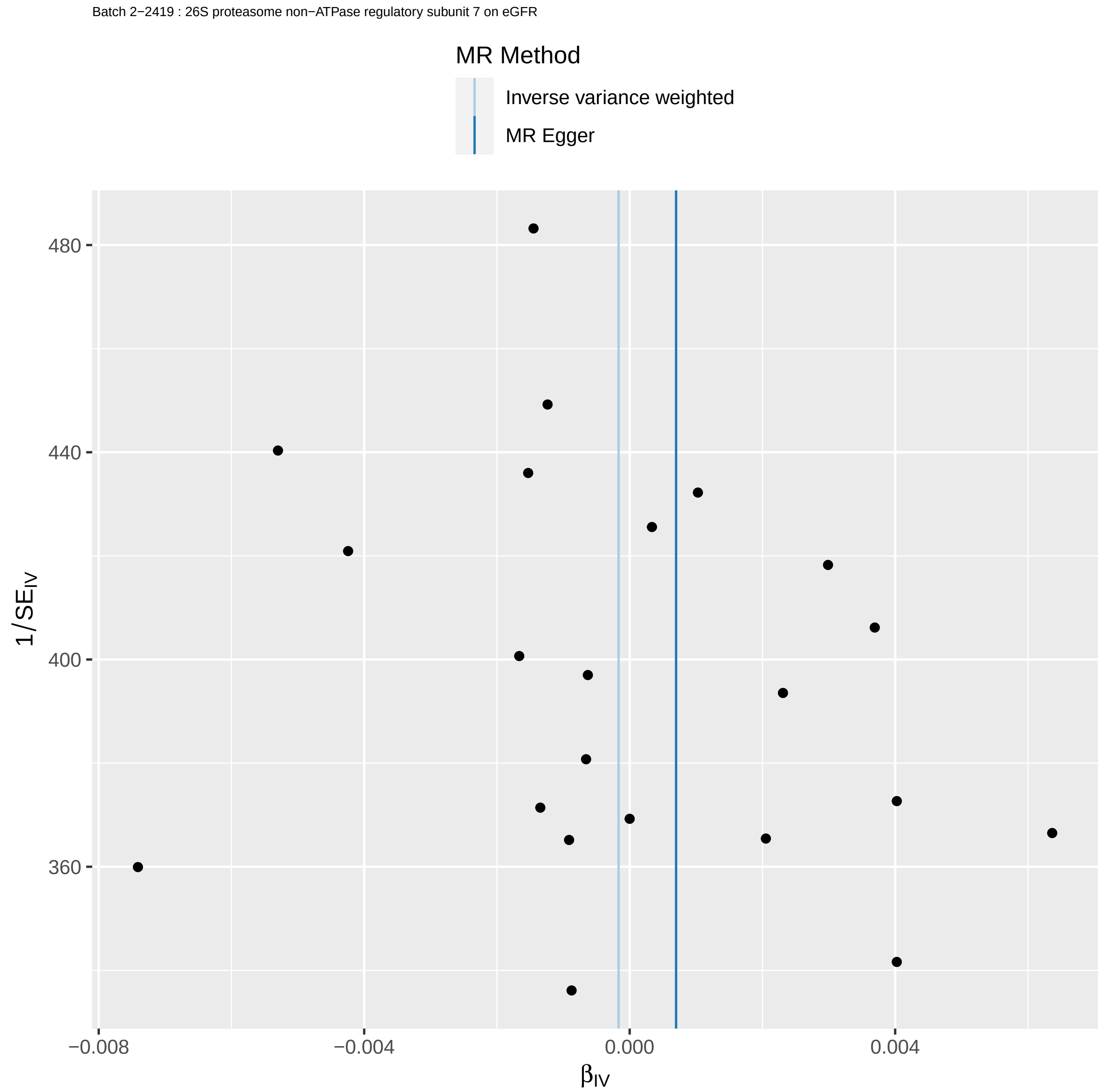

Batch 2−2419 : 26S proteasome non−ATPase regulatory subunit 7 on eGFR
MR Method
Inverse variance weighted
MR Egger
480
440
400
360
1/SE_IV
−0.008
−0.004
0.000
0.004
β_IV

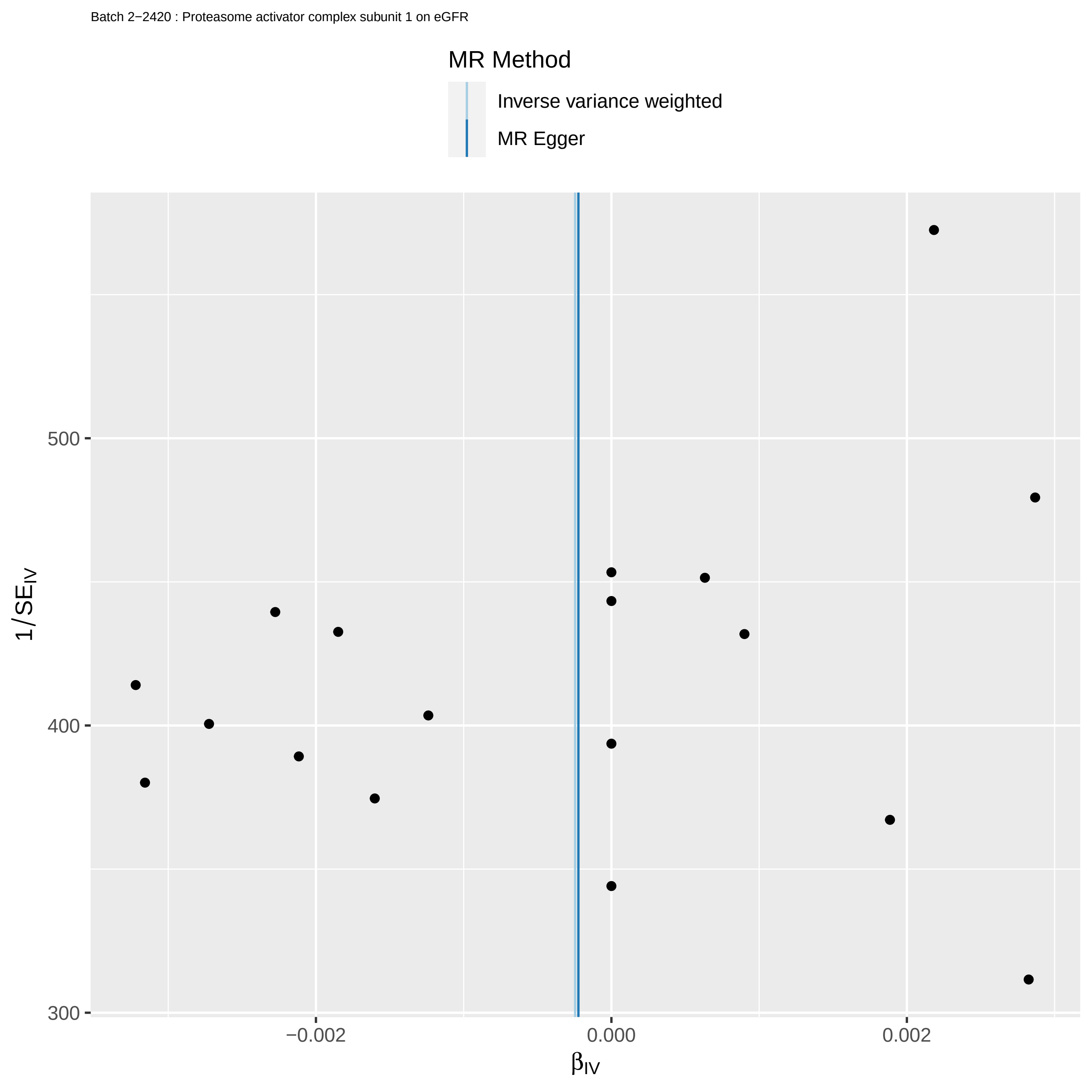
Batch 2−2420 : Proteasome activator complex subunit 1 on eGFR
MR Method
Inverse variance weighted
MR Egger
500
400
300
−0.002
0.000
0.002
1/SE_IV
β_IV

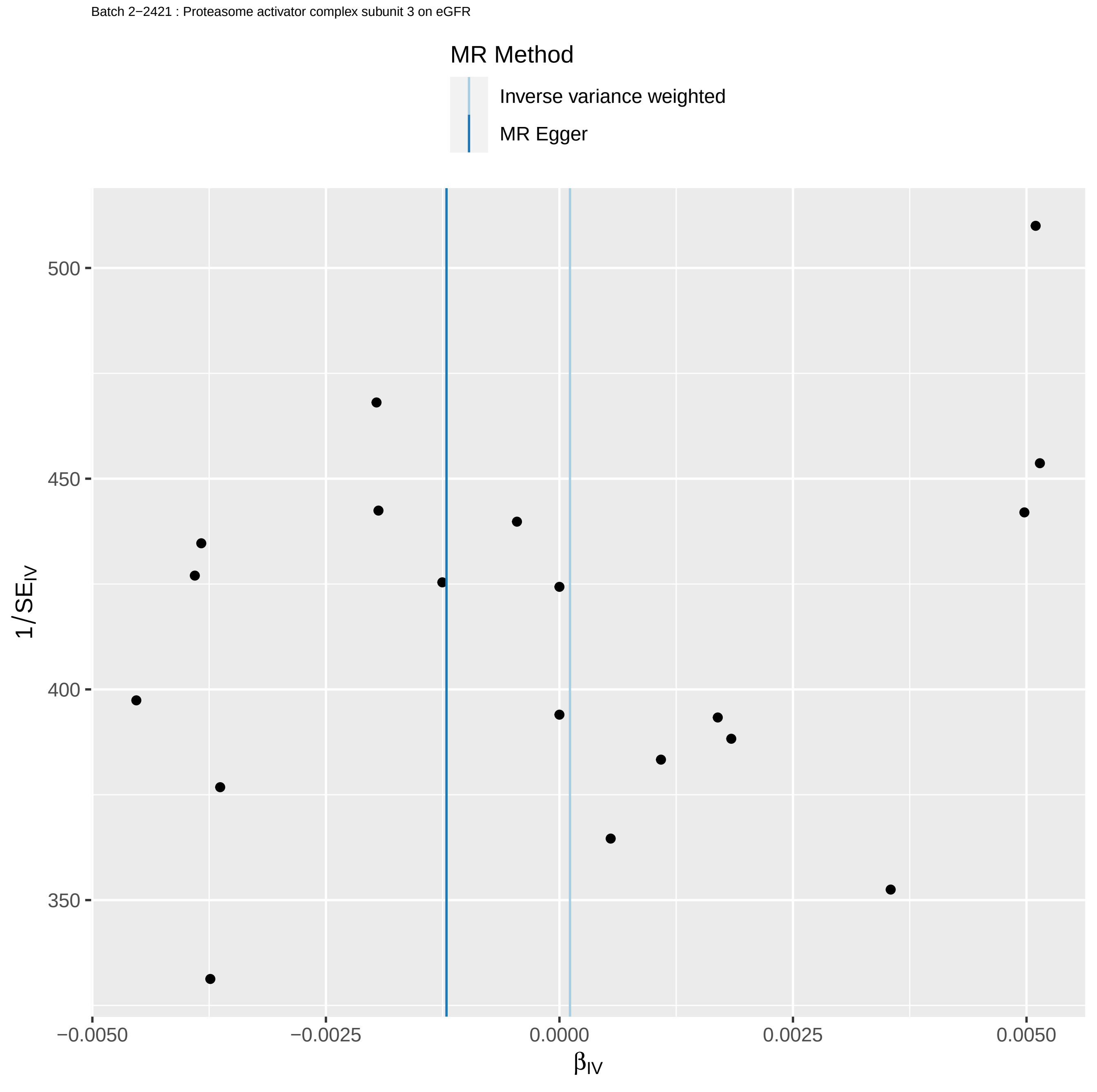
Batch 2−2421 : Proteasome activator complex subunit 3 on eGFR
MR Method
Inverse variance weighted
MR Egger
500
450
400
350
1/SE_IV
−0.0050
−0.0025
0.0000
0.0025
0.0050
β_IV

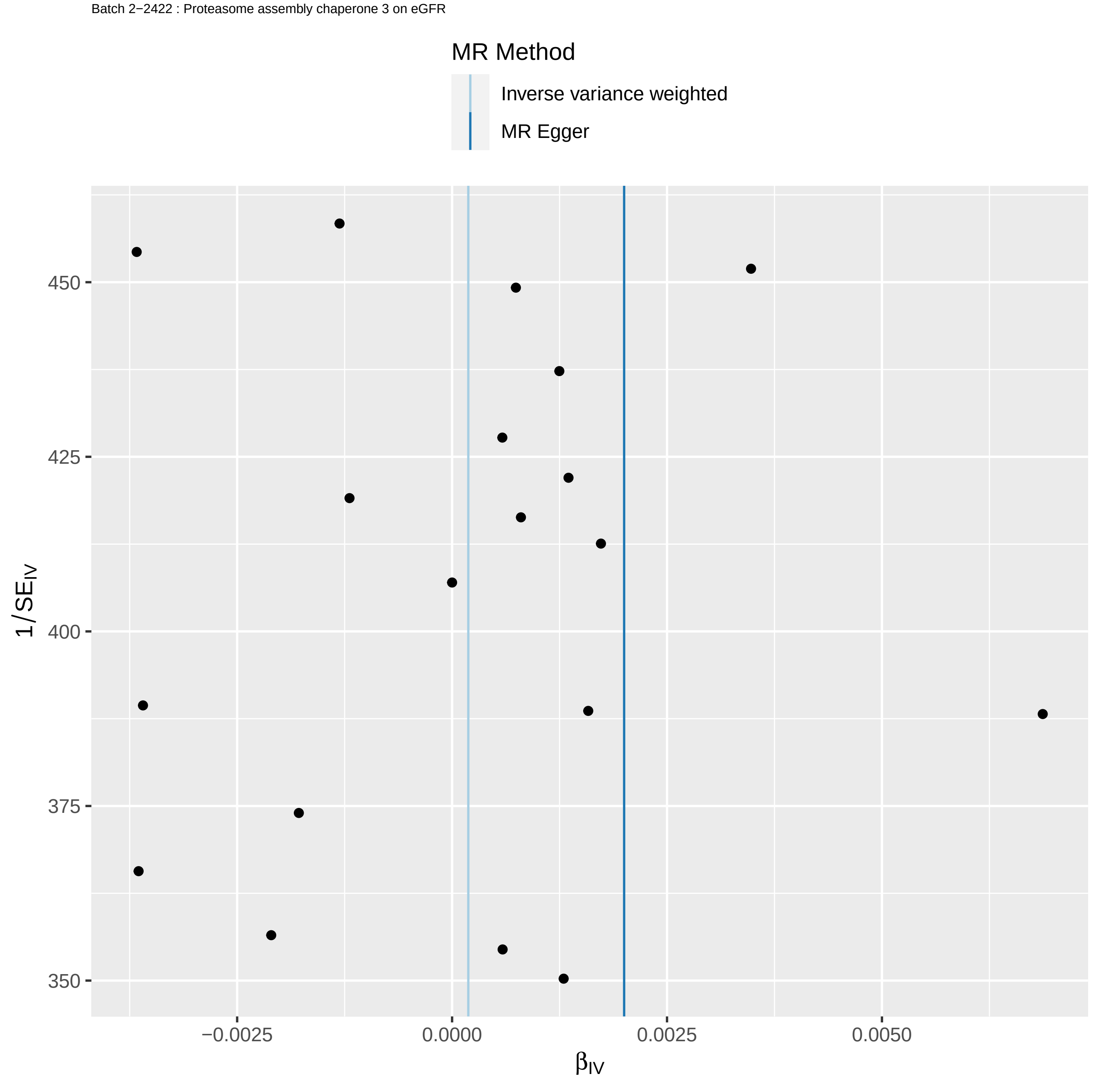
Batch 2−2422 : Proteasome assembly chaperone 3 on eGFR
MR Method
Inverse variance weighted
MR Egger
450
425
400
375
350
1/SE$_{IV}$
−0.0025
0.0000
0.0025
0.0050
β$_{IV}$

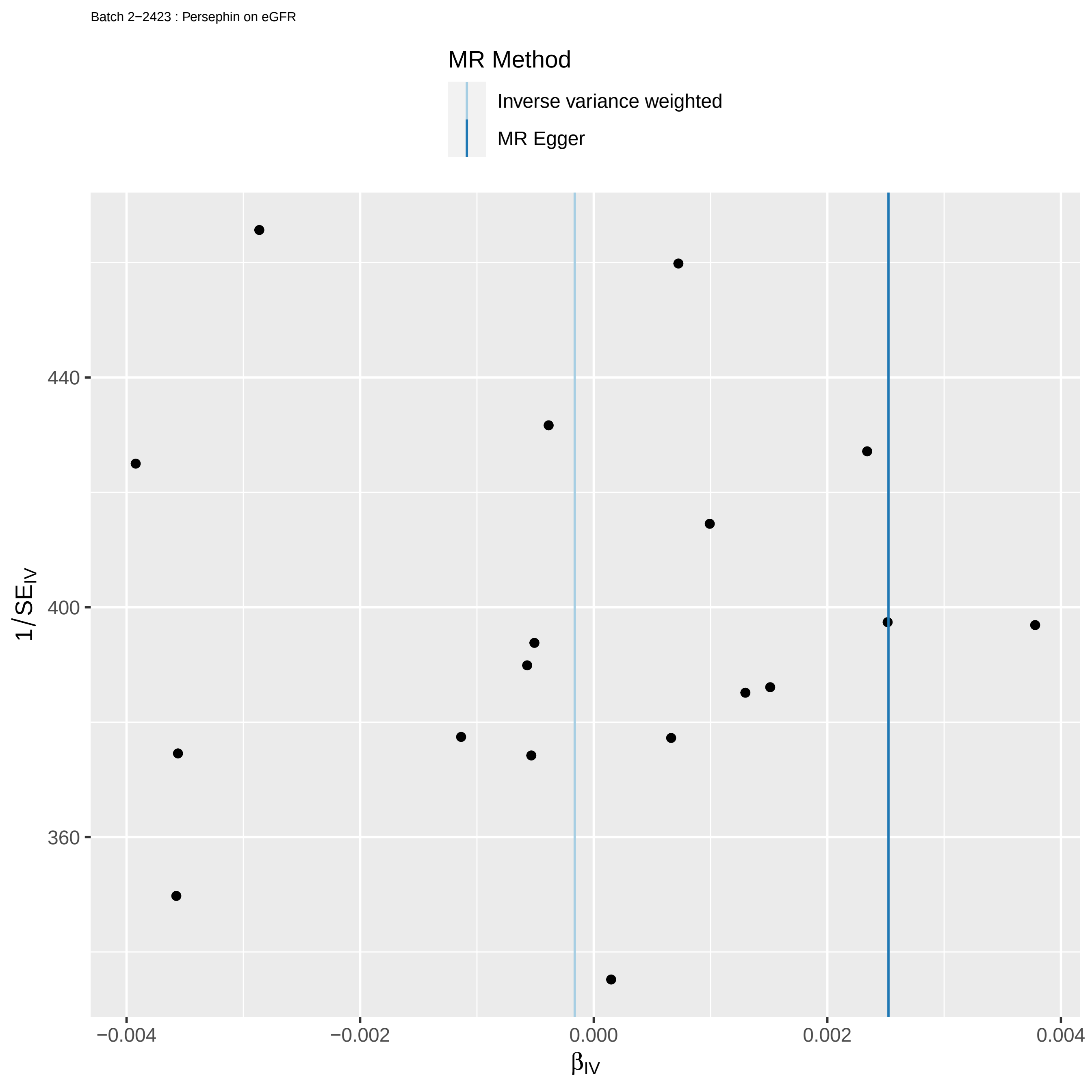
Batch 2−2423 : Persephin on eGFR
MR Method
Inverse variance weighted
MR Egger
440
400
360
1/SE$_{IV}$
−0.004
−0.002
0.000
0.002
0.004
β$_{IV}$

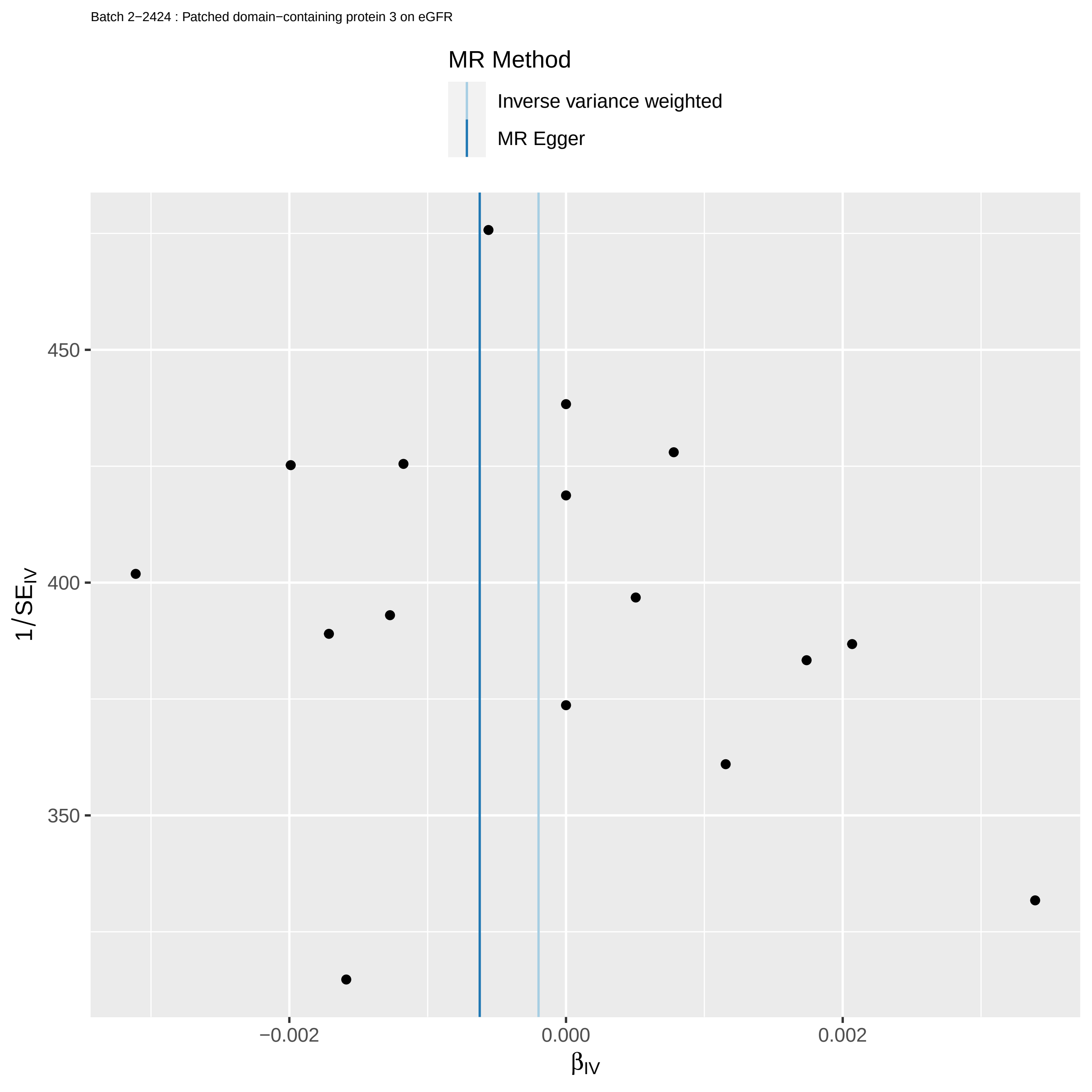

Batch 2−2424 : Patched domain−containing protein 3 on eGFR
MR Method
Inverse variance weighted
MR Egger
450
400
350
1/SE$_{IV}$
−0.002
0.000
0.002
β$_{IV}$

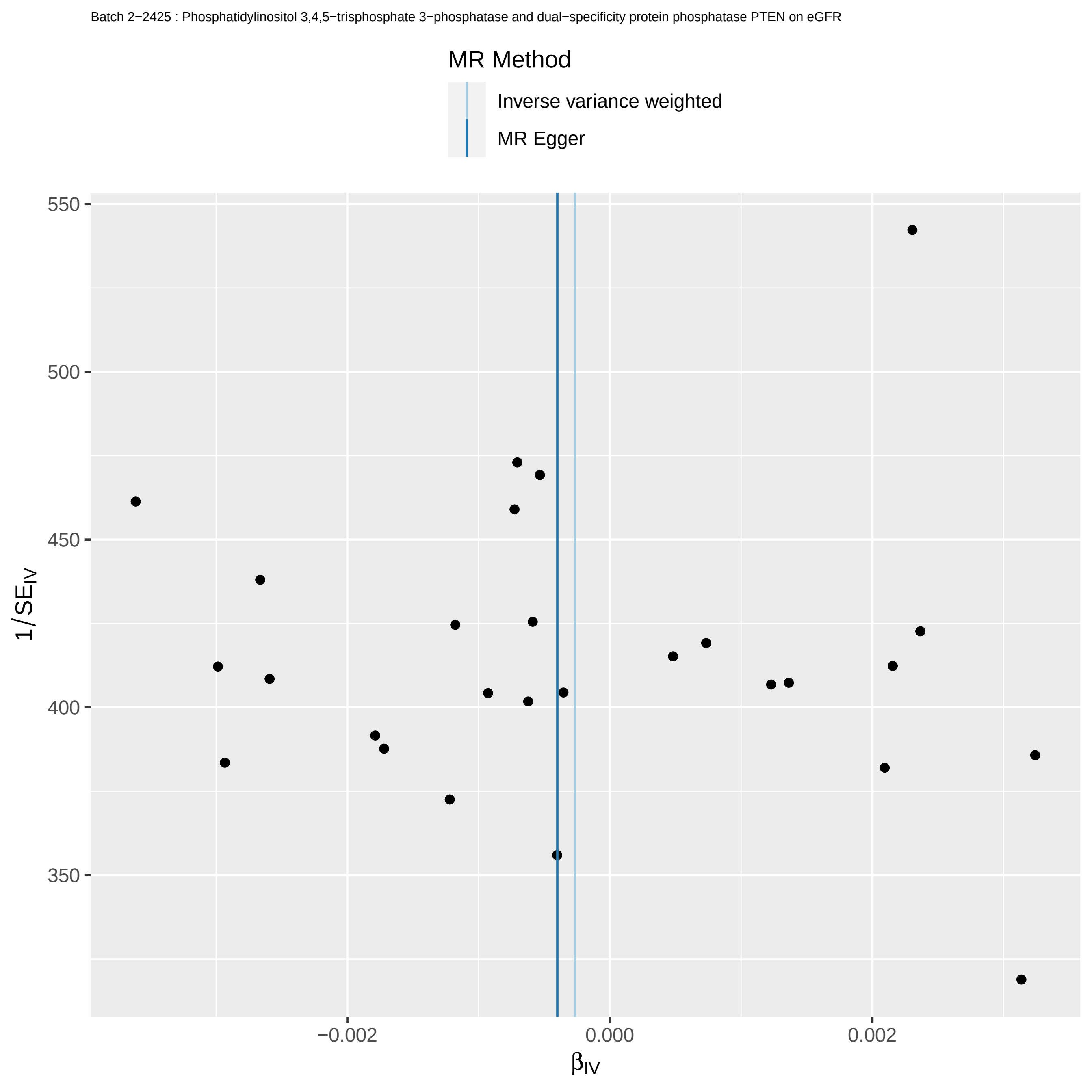
Batch 2−2425 : Phosphatidylinositol 3,4,5−trisphosphate 3−phosphatase and dual−specificity protein phosphatase PTEN on eGFR
MR Method
Inverse variance weighted
MR Egger
550
500
450
400
350
1/SE_IV
−0.002
0.000
0.002
β_IV

Batch 2−2426 : Prostaglandin−H2 D−isomerase on eGFR

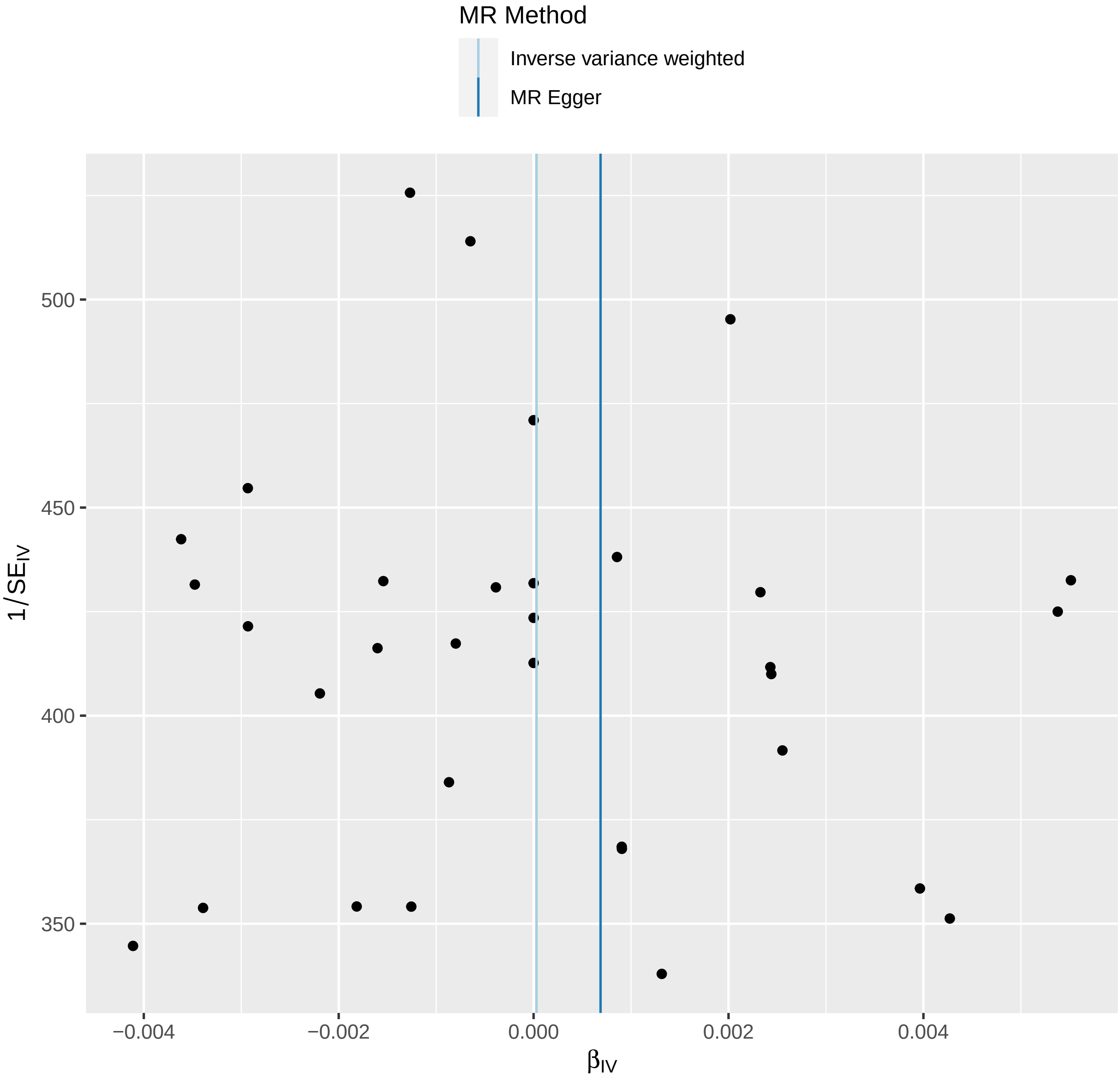

Batch 2−2427 : Prostaglandin F2 receptor negative regulator on eGFR

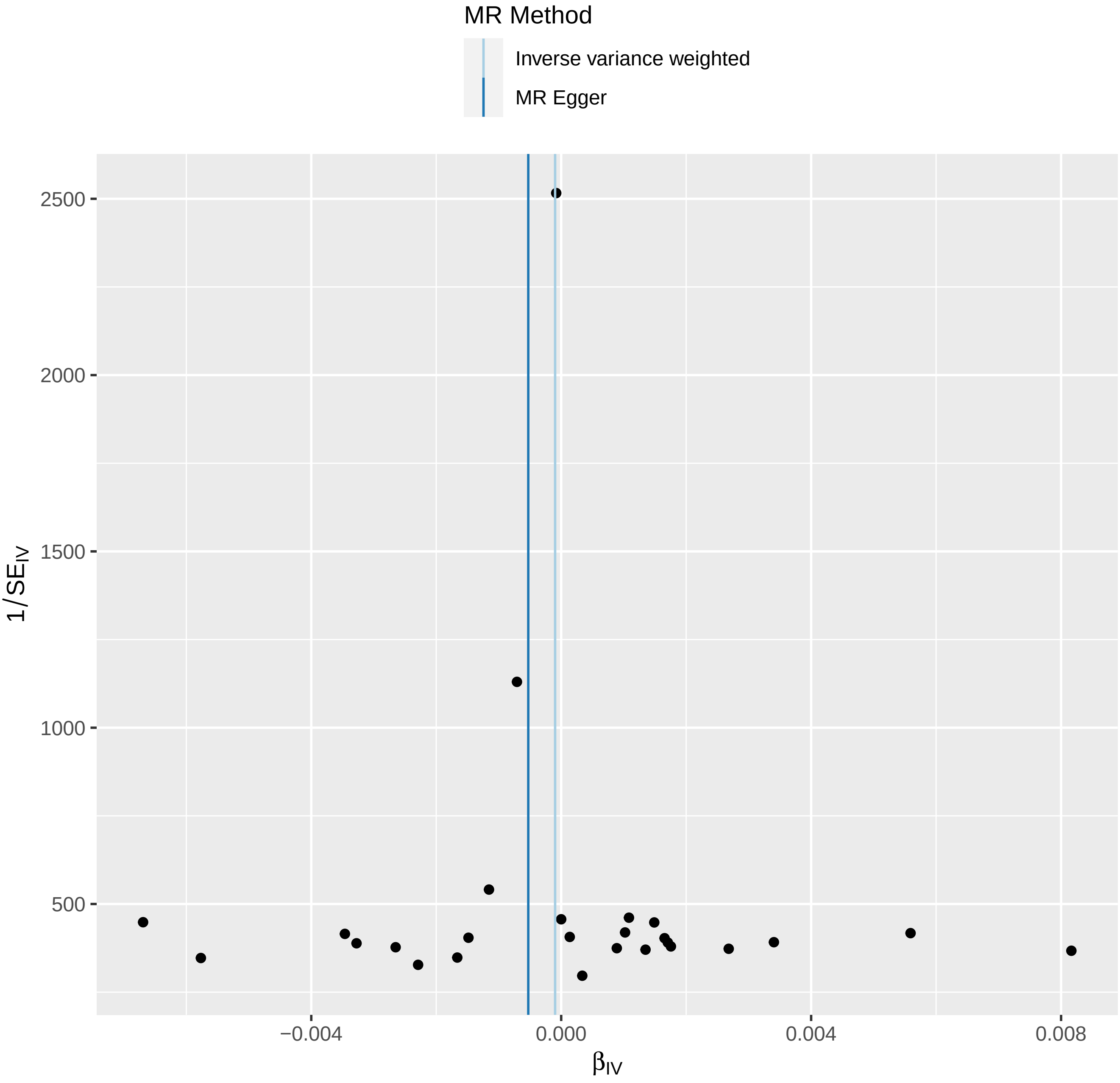

Batch 2−2428 : Prostaglandin reductase 1 on eGFR

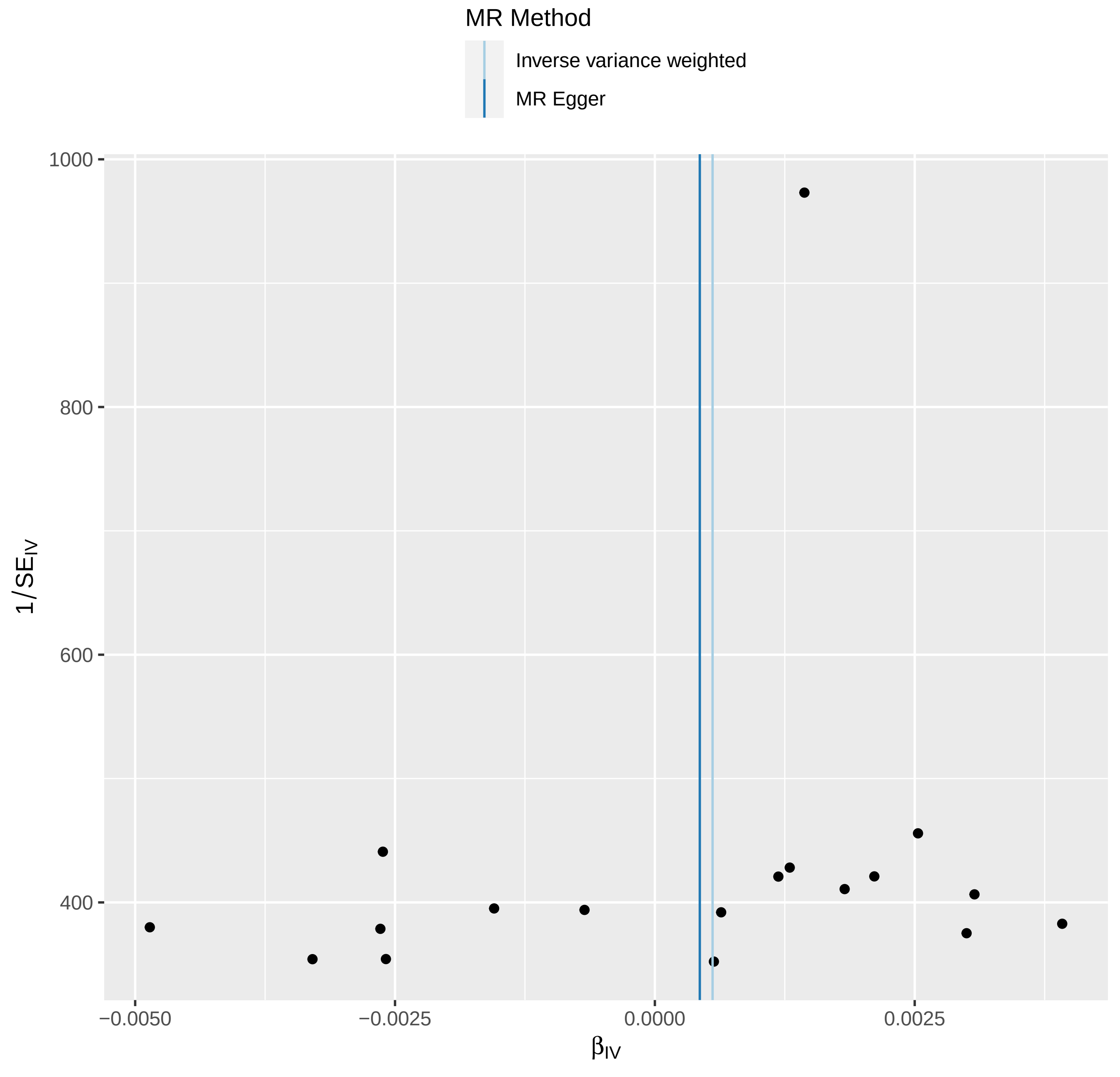

Batch 2−2429 : Parathyroid hormone/parathyroid hormone−related peptide receptor on eGFR

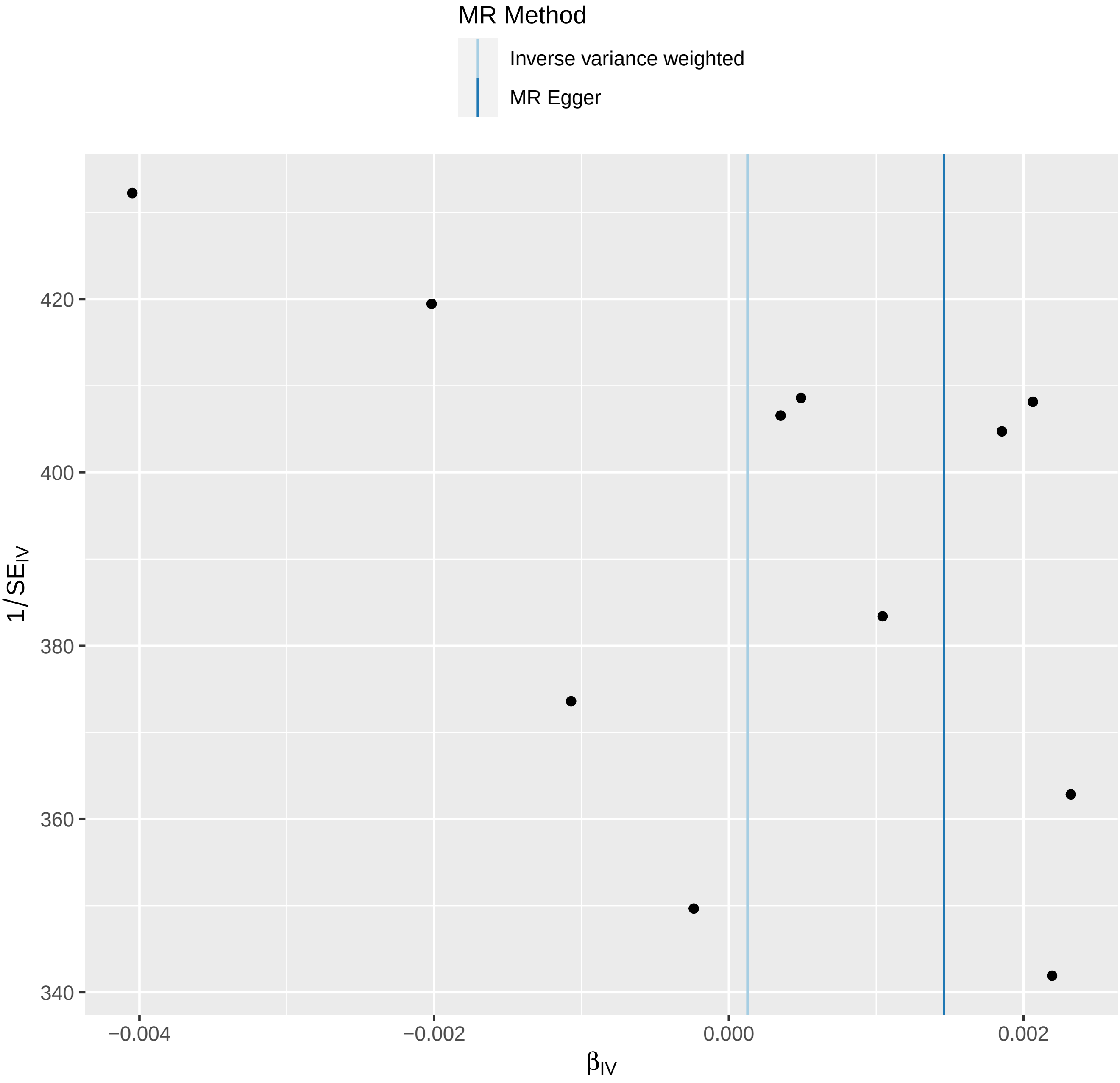

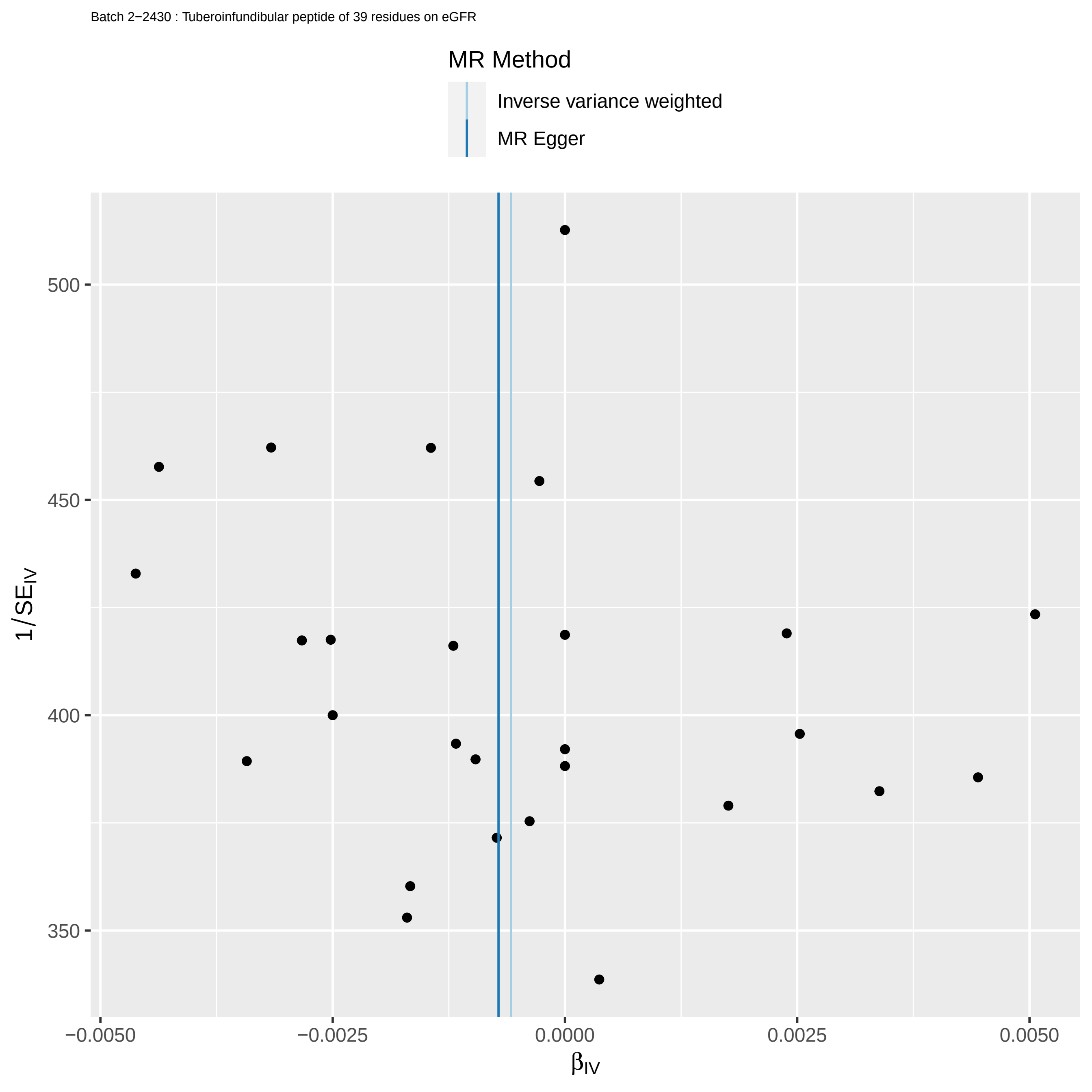

Batch 2−2430 : Tuberoinfundibular peptide of 39 residues on eGFR
MR Method
Inverse variance weighted
MR Egger
500
450
400
350
1/SE_IV
−0.0050
−0.0025
0.0000
0.0025
0.0050
β_IV

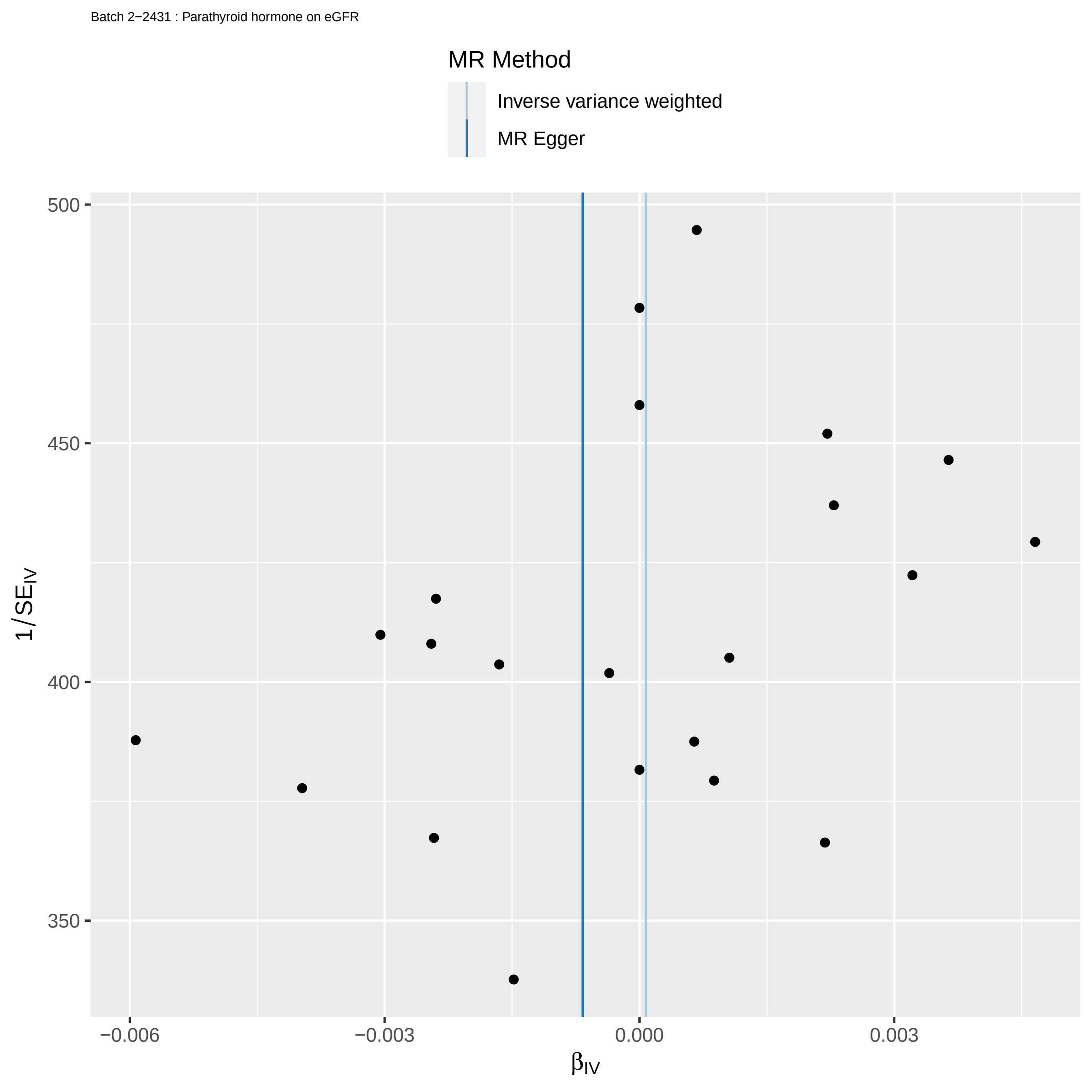

Batch 2−2431 : Parathyroid hormone on eGFR
MR Method
Inverse variance weighted
MR Egger
500
450
400
350
1/SE$_{IV}$
−0.006
−0.003
0.000
0.003
β$_{IV}$

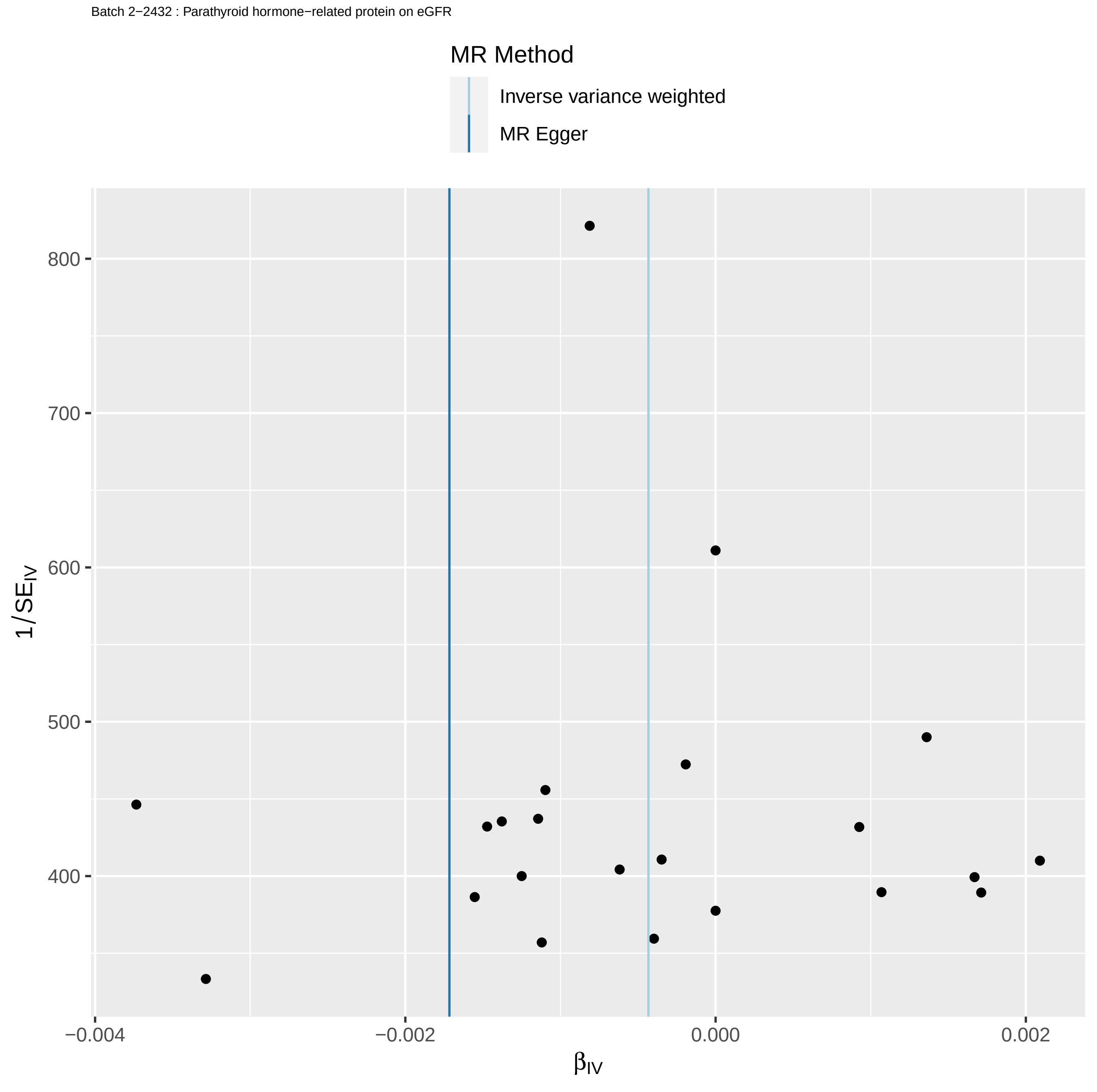
Batch 2−2432 : Parathyroid hormone−related protein on eGFR
MR Method
Inverse variance weighted
MR Egger
800
700
600
500
400
1/SE$_{IV}$
−0.004
−0.002
0.000
0.002
β_{IV}

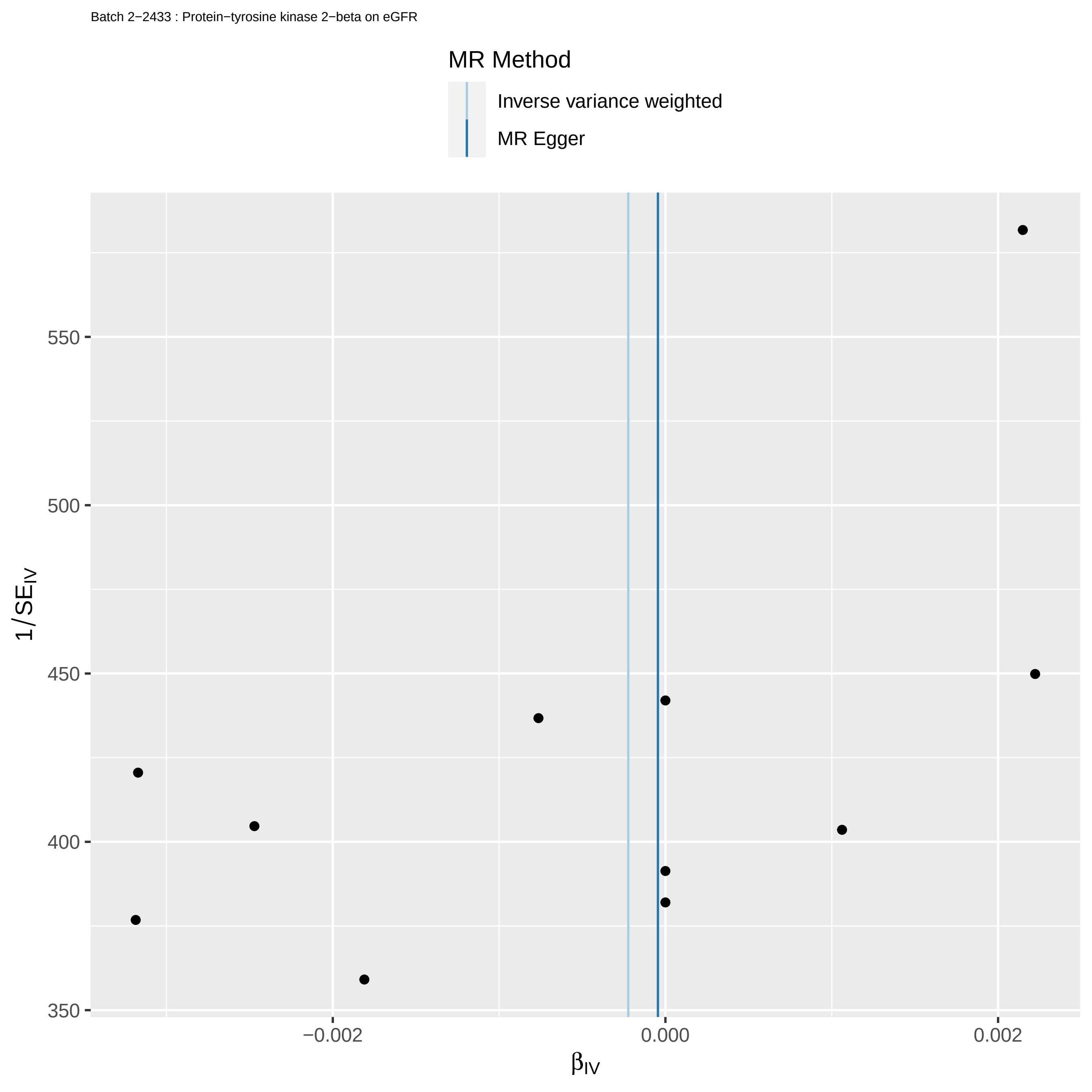

Batch 2−2433 : Protein−tyrosine kinase 2−beta on eGFR
MR Method
Inverse variance weighted
MR Egger
550
500
450
400
350
1/SE_IV
−0.002
0.000
0.002
β_IV

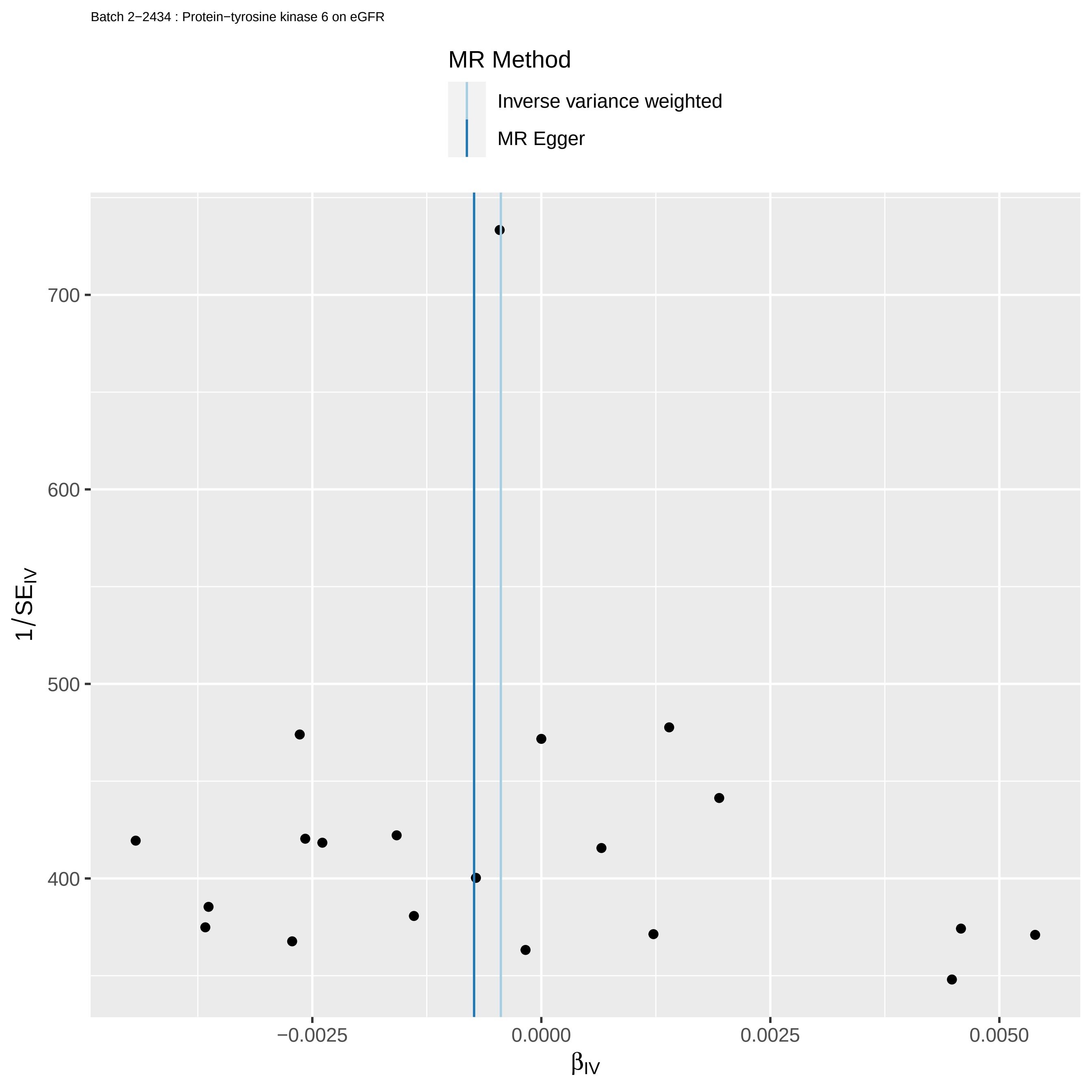
Batch 2−2434 : Protein−tyrosine kinase 6 on eGFR
MR Method
Inverse variance weighted
MR Egger
700
600
500
400
1/SE_IV
−0.0025
0.0000
0.0025
0.0050
β_IV

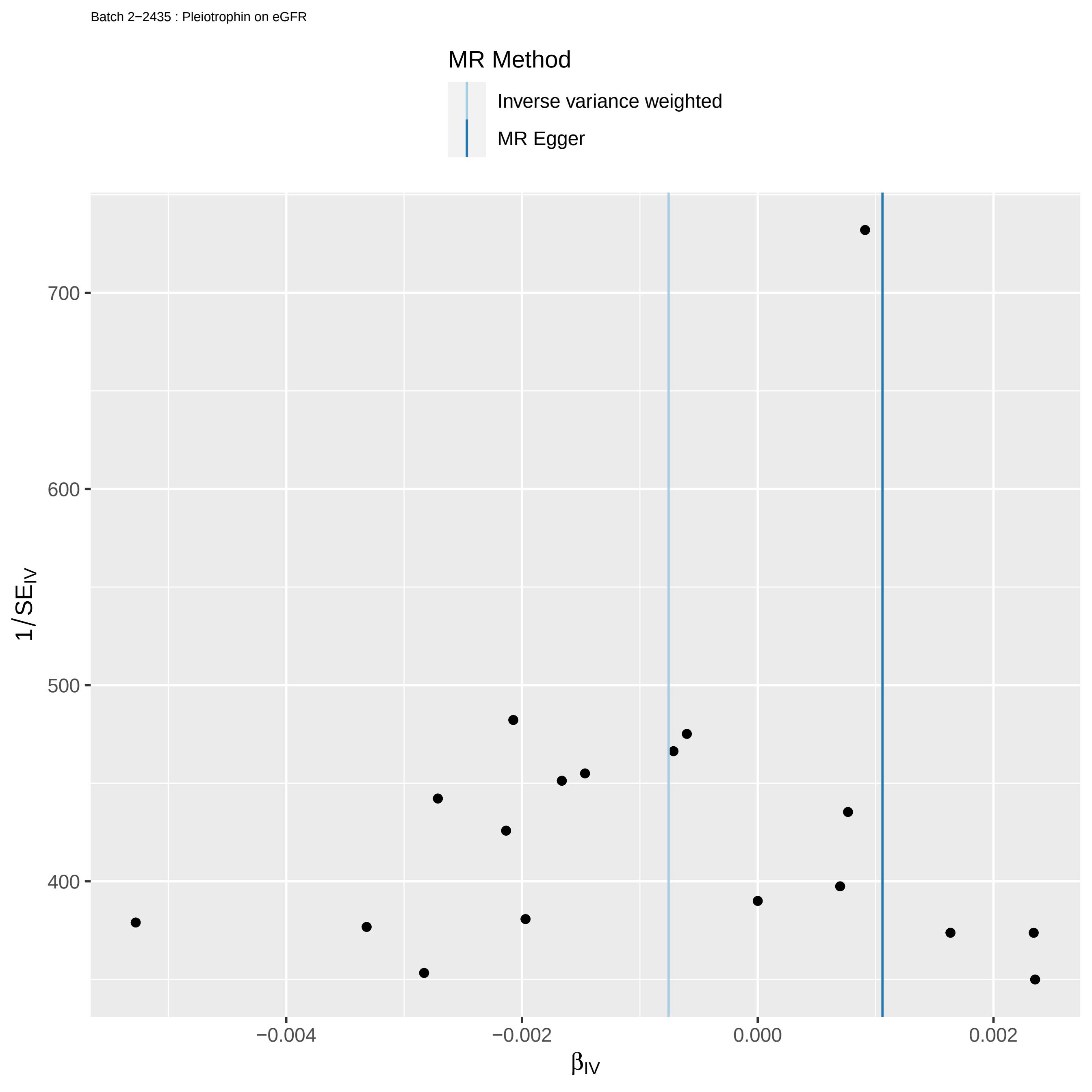
Batch 2−2435 : Pleiotrophin on eGFR
MR Method
Inverse variance weighted
MR Egger
700
600
500
400
1/SE$_{IV}$
−0.004
−0.002
0.000
0.002
β$_{IV}$

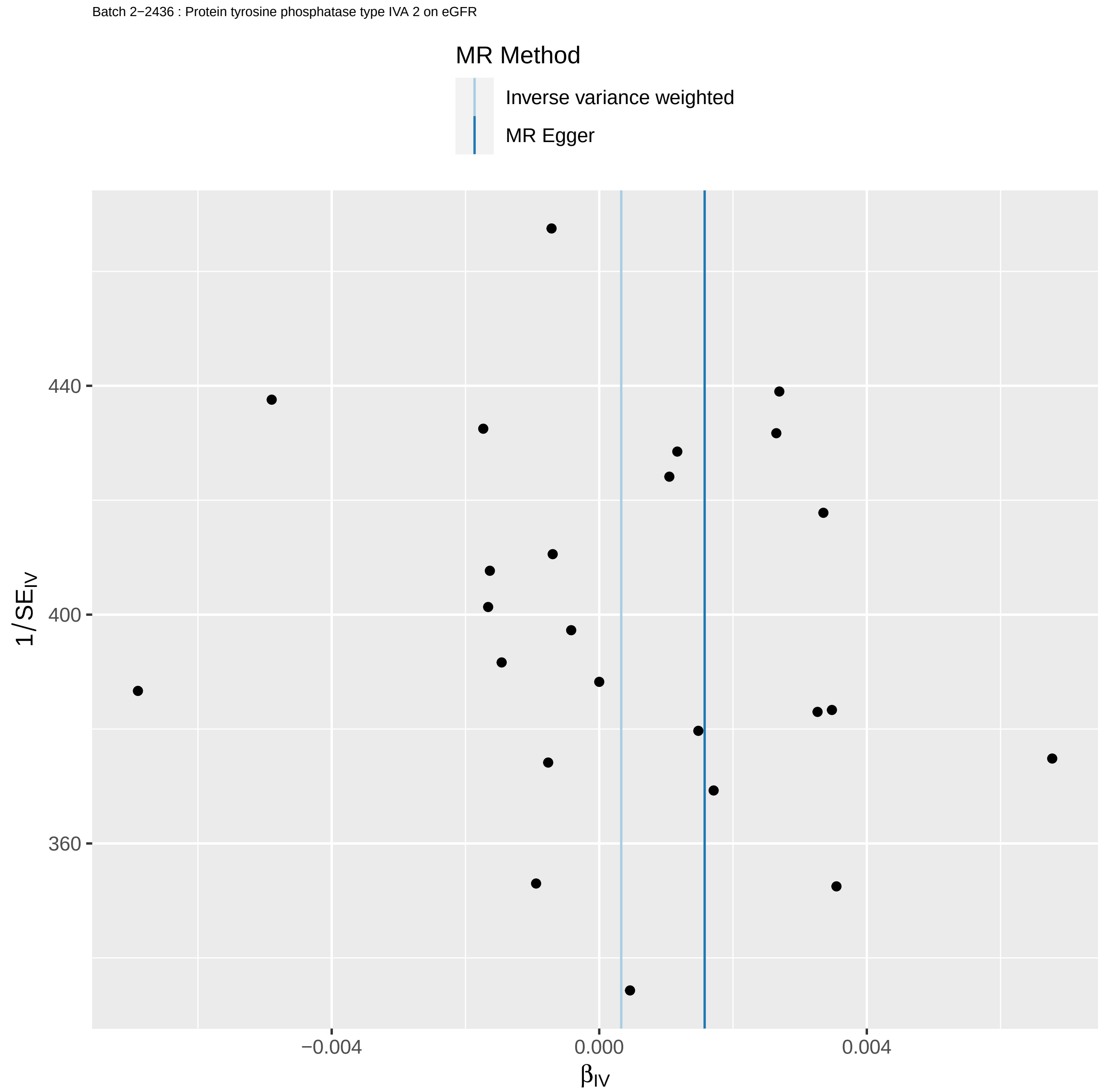
Batch 2−2436 : Protein tyrosine phosphatase type IVA 2 on eGFR
MR Method
Inverse variance weighted
MR Egger
440
400
360
1/SE$_{IV}$
−0.004
0.000
0.004
β$_{IV}$

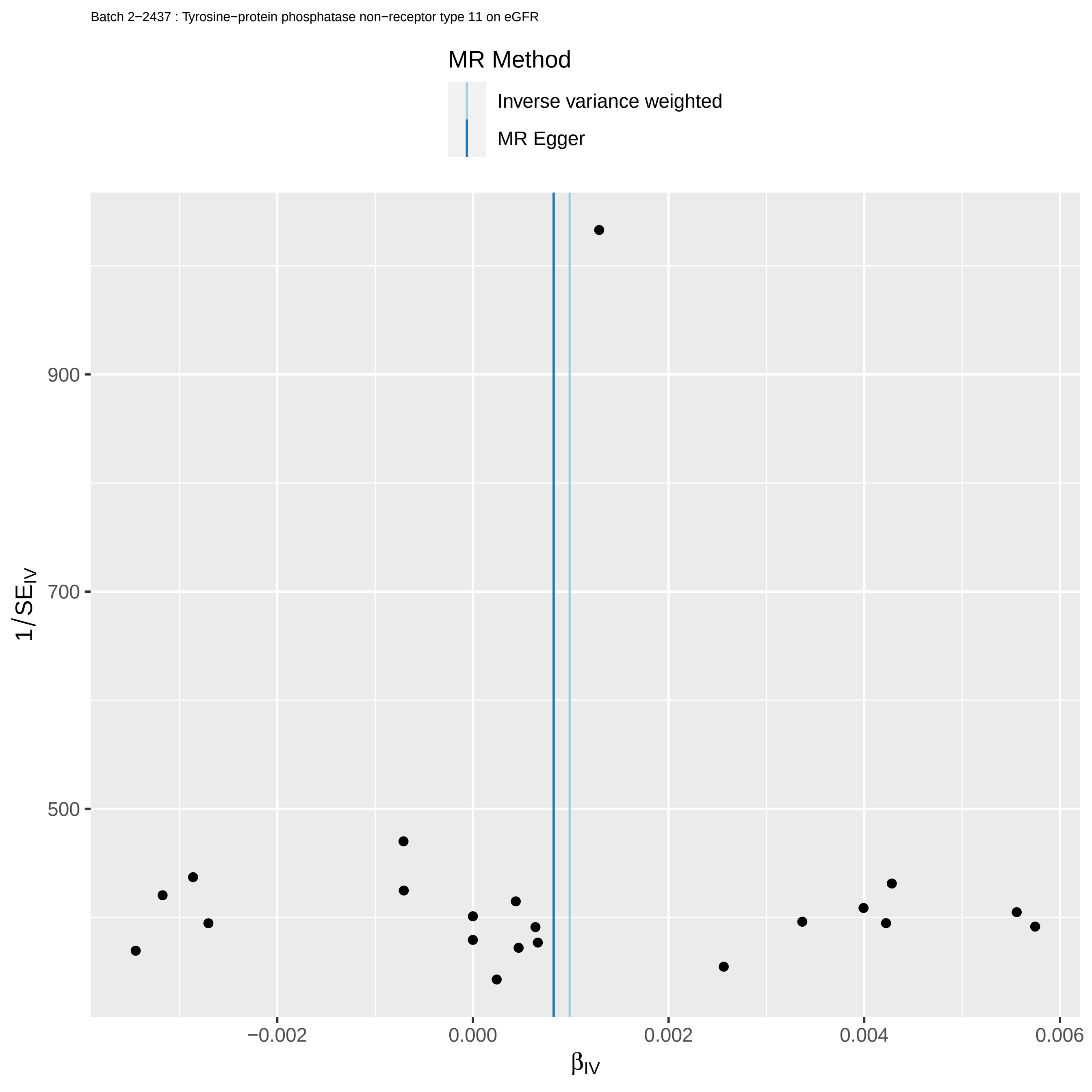
Batch 2−2437 : Tyrosine−protein phosphatase non−receptor type 11 on eGFR
MR Method
Inverse variance weighted
MR Egger
900
700
500
1/SE$_{IV}$
−0.002
0.000
0.002
0.004
0.006
β$_{IV}$

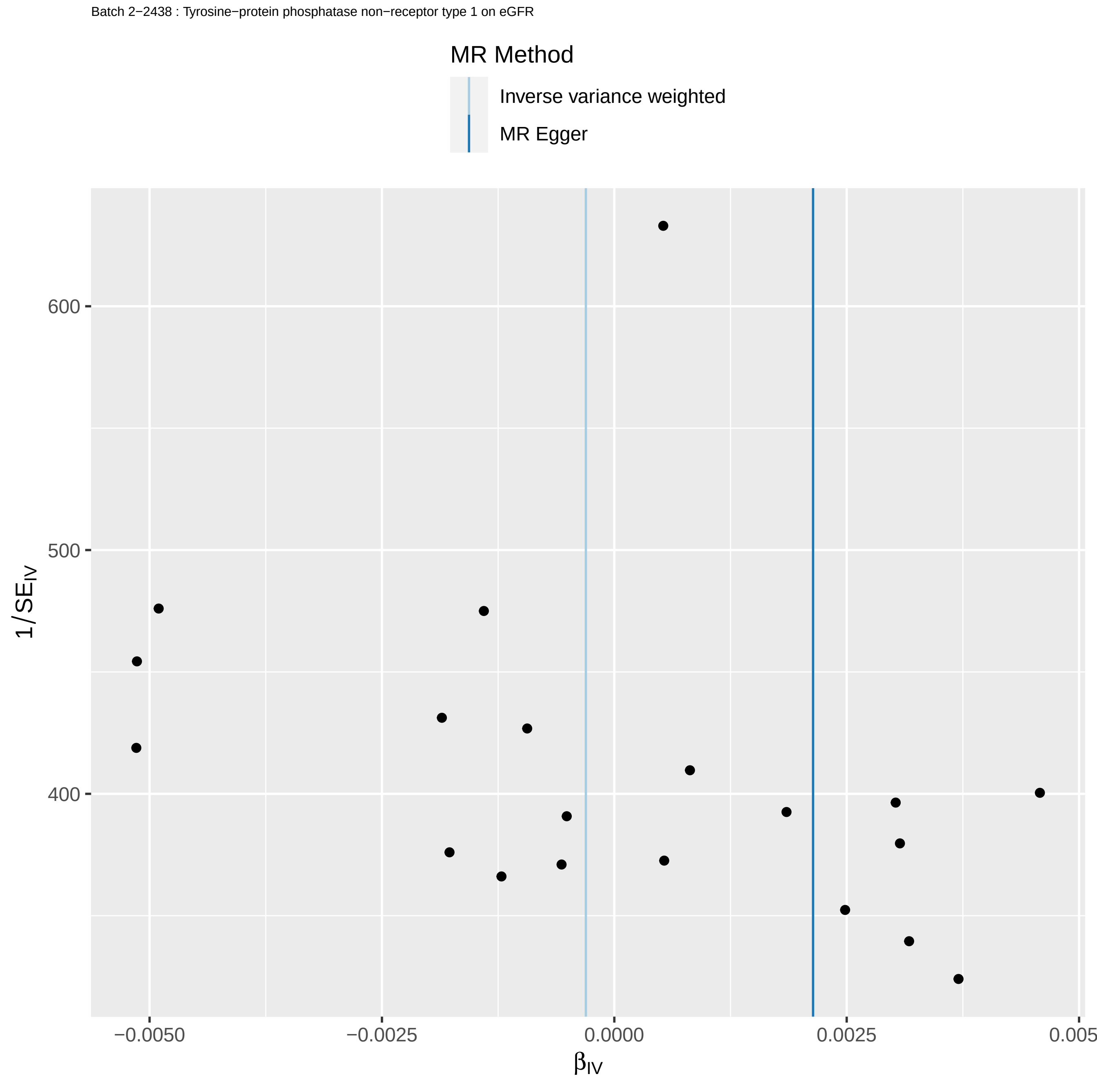
Batch 2−2438 : Tyrosine−protein phosphatase non−receptor type 1 on eGFR
MR Method
Inverse variance weighted
MR Egger
600
500
400
1/SE_IV
−0.0050
−0.0025
0.0000
0.0025
0.005
β_IV

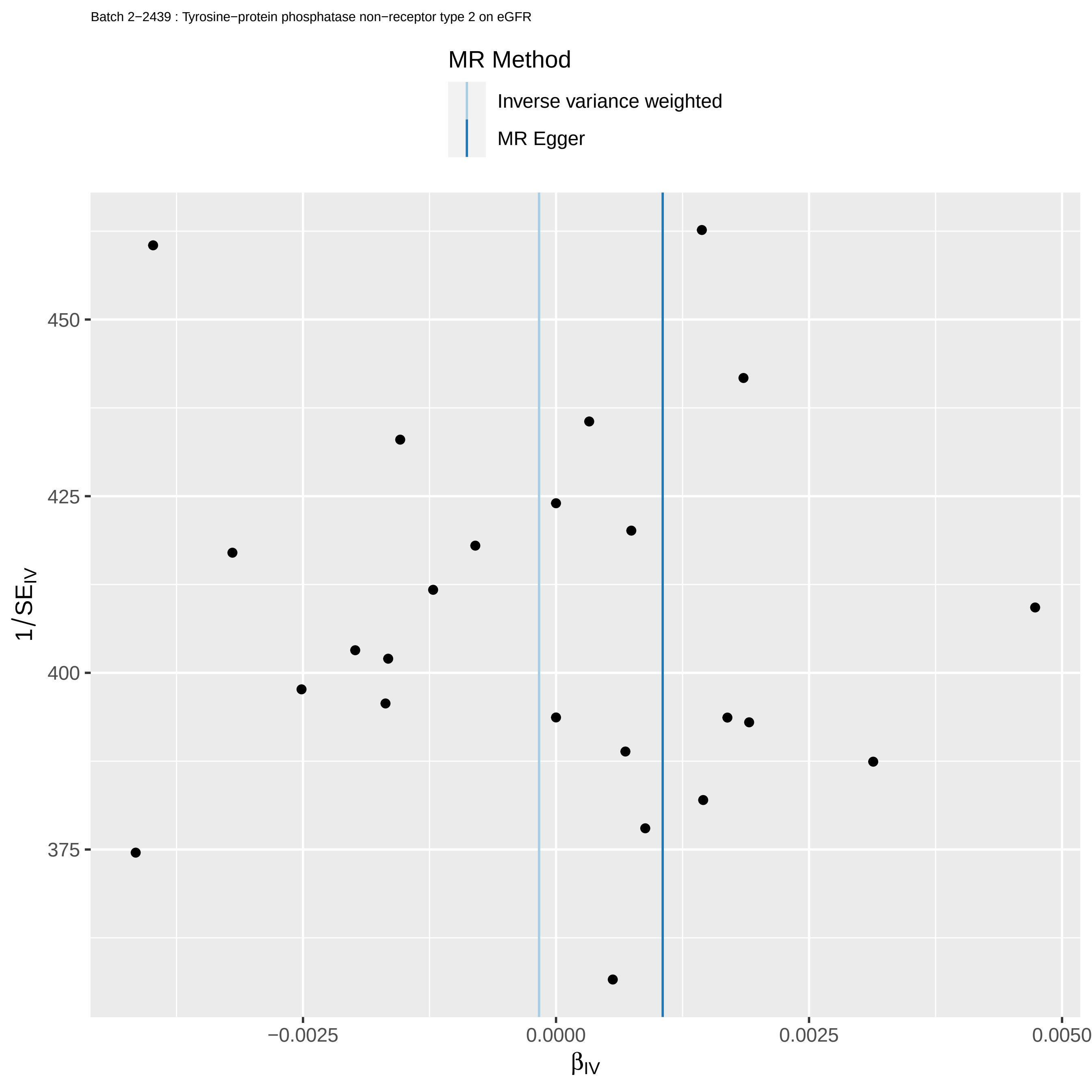
Batch 2−2439 : Tyrosine−protein phosphatase non−receptor type 2 on eGFR
MR Method
Inverse variance weighted
MR Egger
450
425
400
375
1/SE$_{IV}$
−0.0025
0.0000
0.0025
0.0050
β$_{IV}$

Batch 2−2440 : Tyrosine−protein phosphatase non−receptor type 4 on eGFR

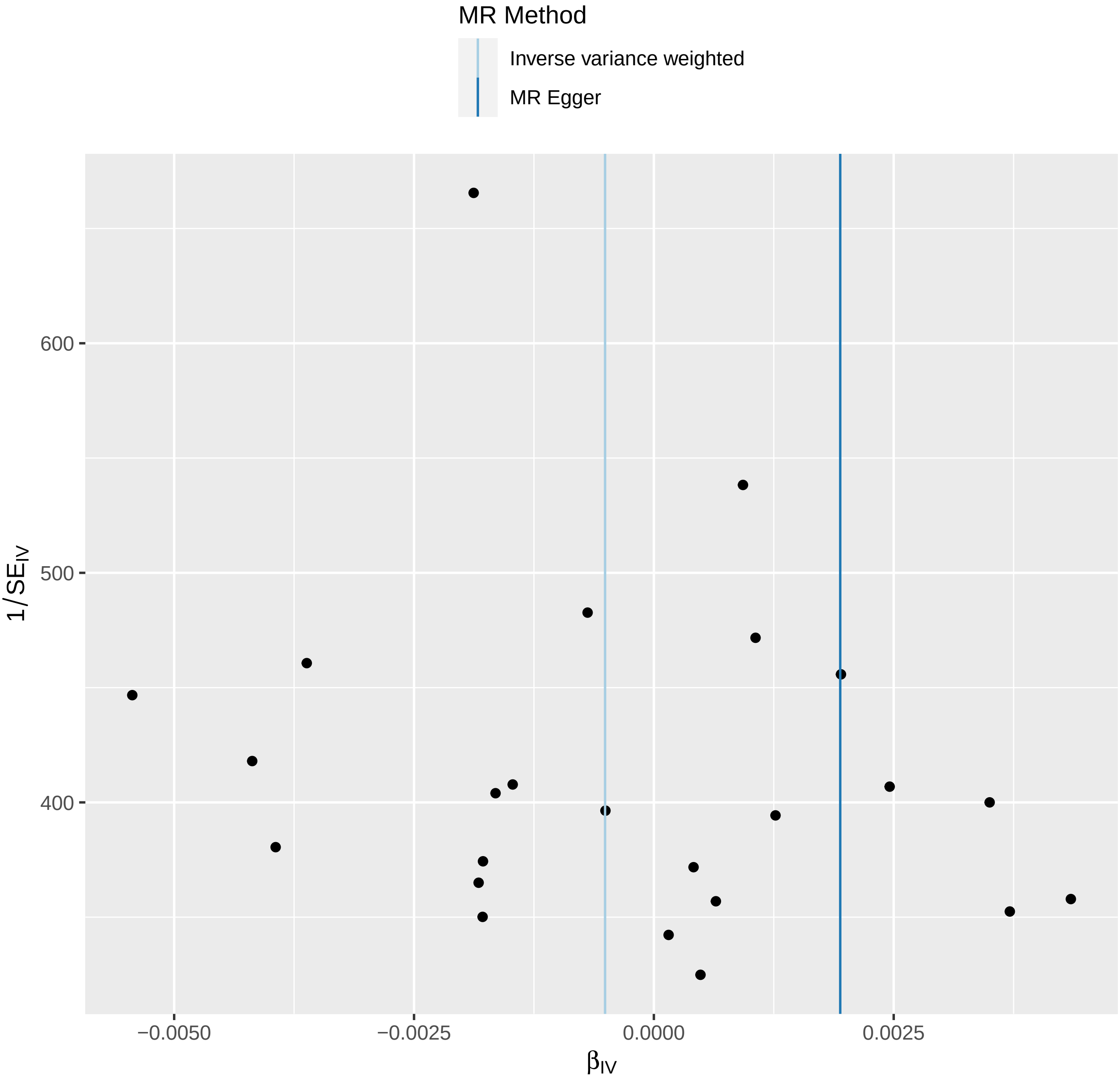

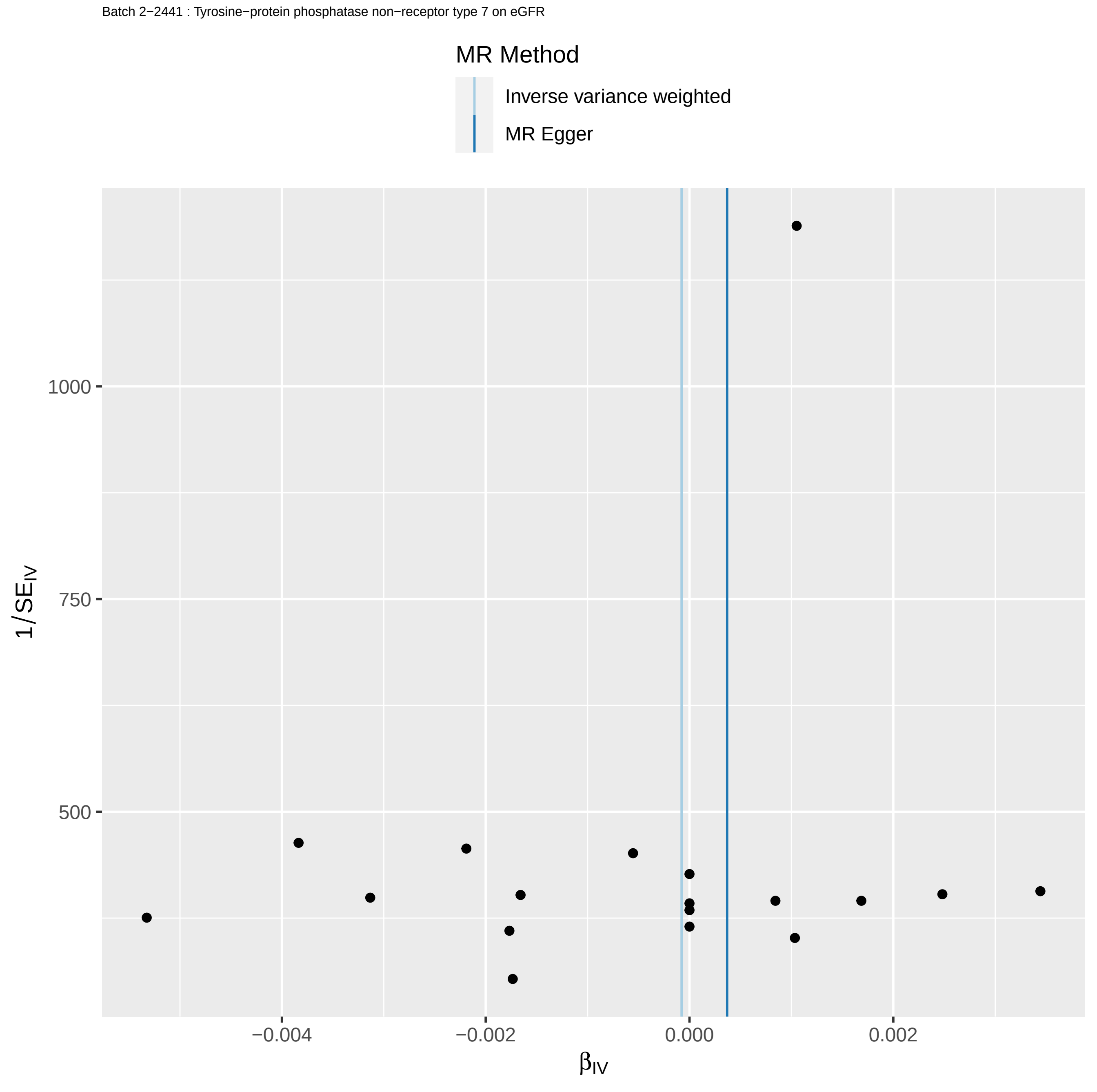
Batch 2−2441 : Tyrosine−protein phosphatase non−receptor type 7 on eGFR
MR Method
Inverse variance weighted
MR Egger
1000
750
500
1/SE$_{IV}$
−0.004
−0.002
0.000
0.002
β$_{IV}$

Batch 2−2442 : Tyrosine−protein phosphatase non−receptor type 9 on eGFR

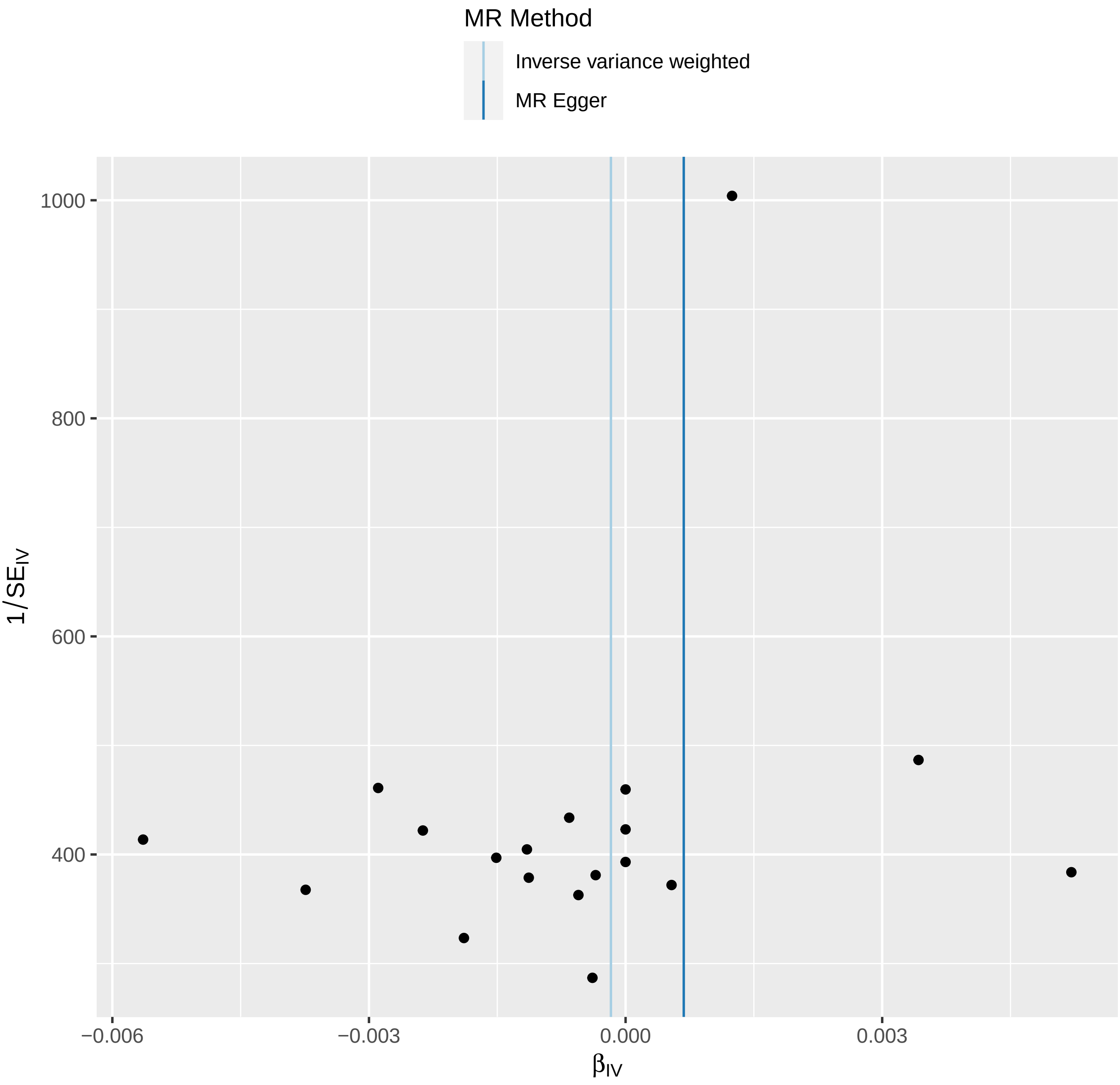

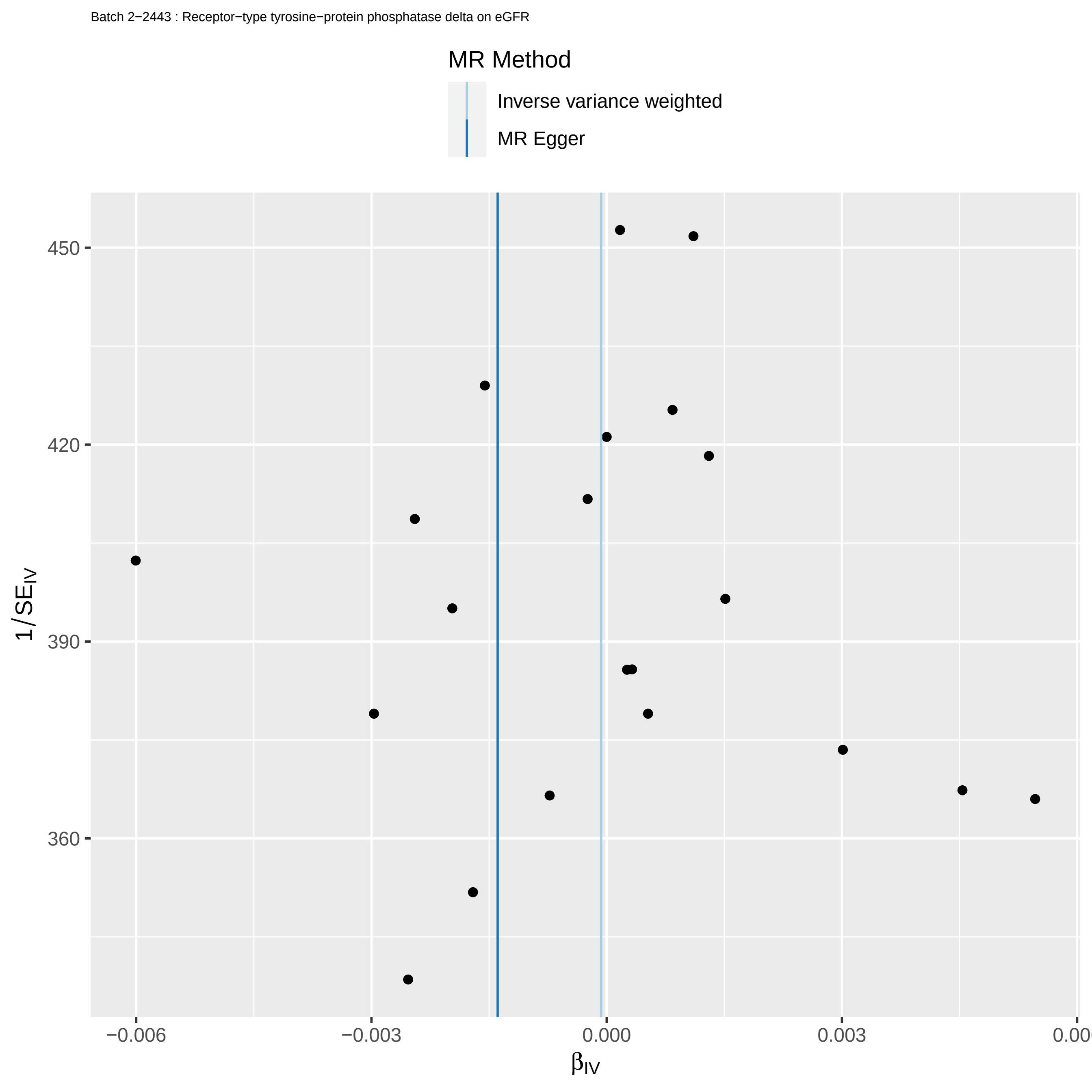

Batch 2−2443 : Receptor−type tyrosine−protein phosphatase delta on eGFR
MR Method
Inverse variance weighted
MR Egger
450
420
390
360
1/SE_IV
−0.006
−0.003
0.000
0.003
β_IV

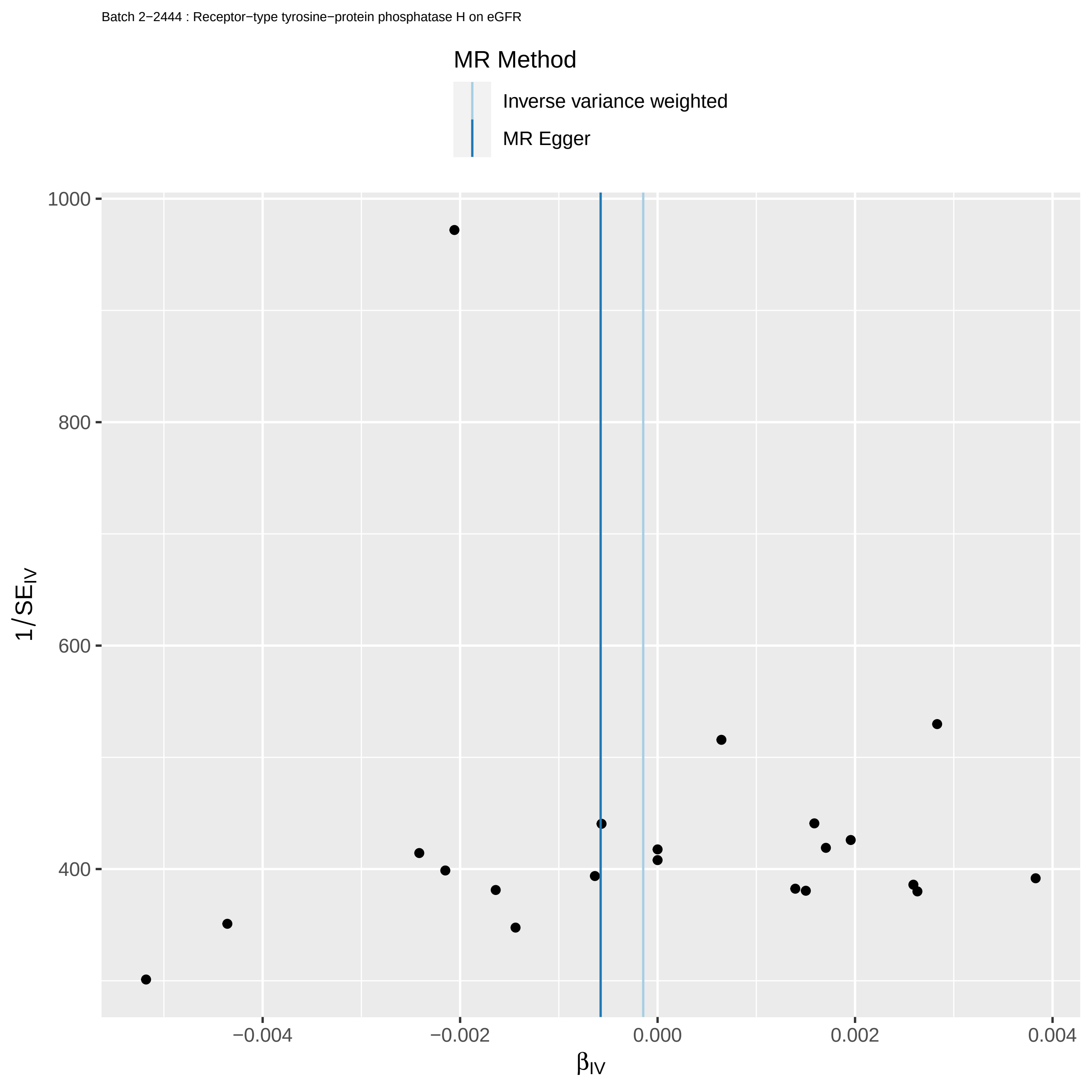

Batch 2−2444 : Receptor−type tyrosine−protein phosphatase H on eGFR
MR Method
Inverse variance weighted
MR Egger
1000
800
600
400
1/SE_IV
−0.004
−0.002
0.000
0.002
0.004
β_IV

Batch 2−2445 : Receptor−type tyrosine−protein phosphatase H on eGFR

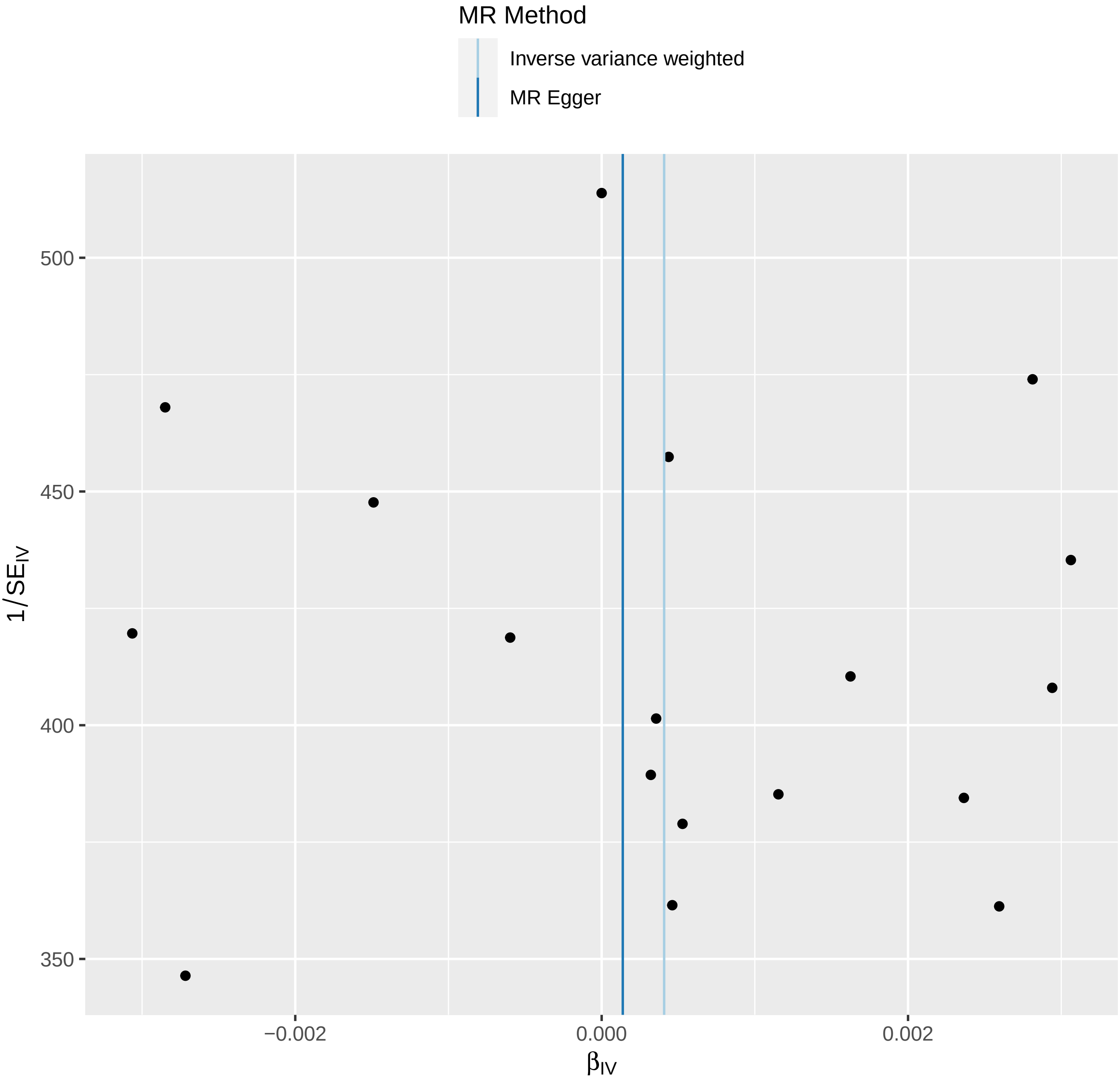

Batch 2−2446 : Receptor−type tyrosine−protein phosphatase eta on eGFR

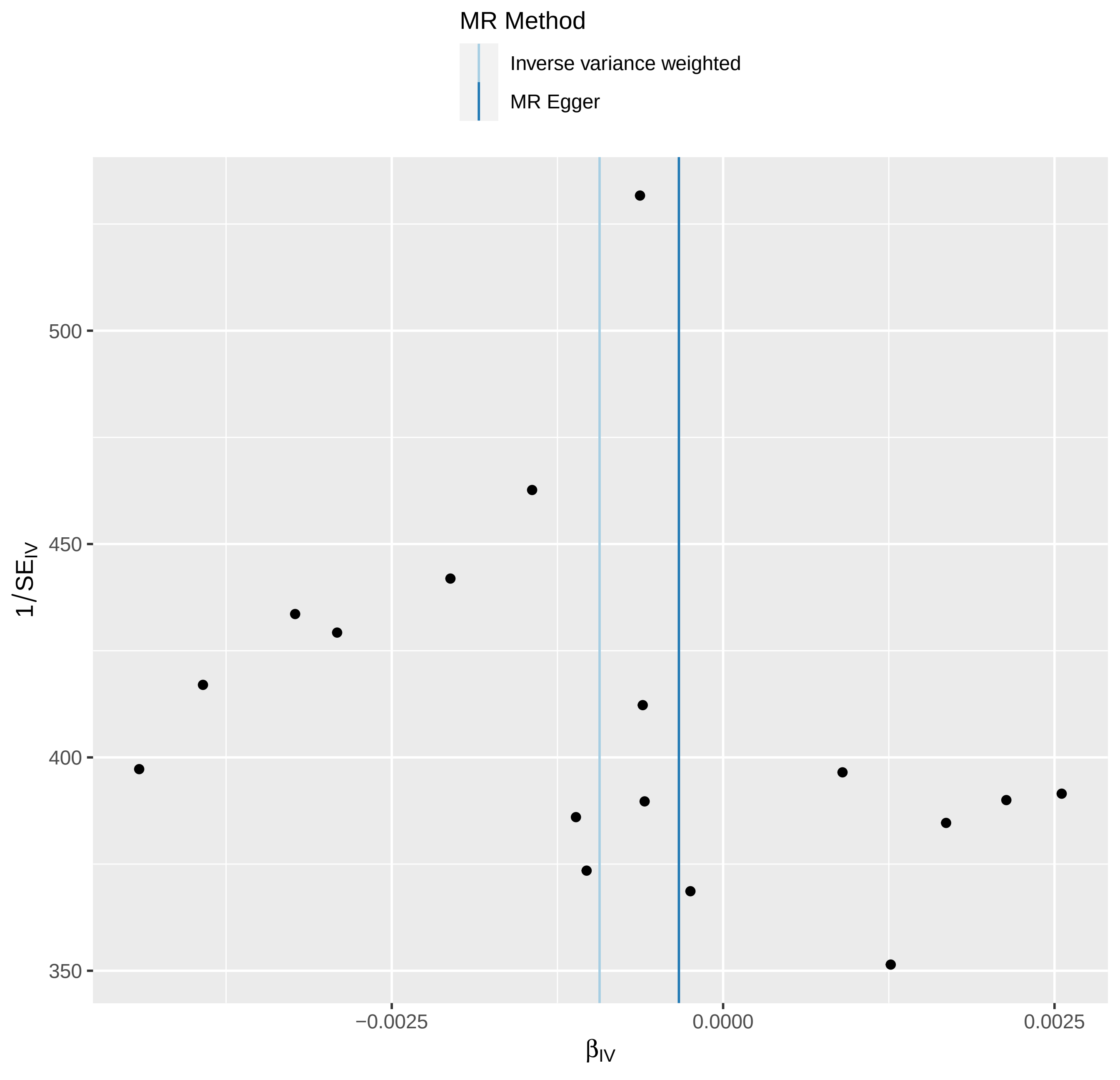

Batch 2−2447 : Receptor−type tyrosine−protein phosphatase R on eGFR

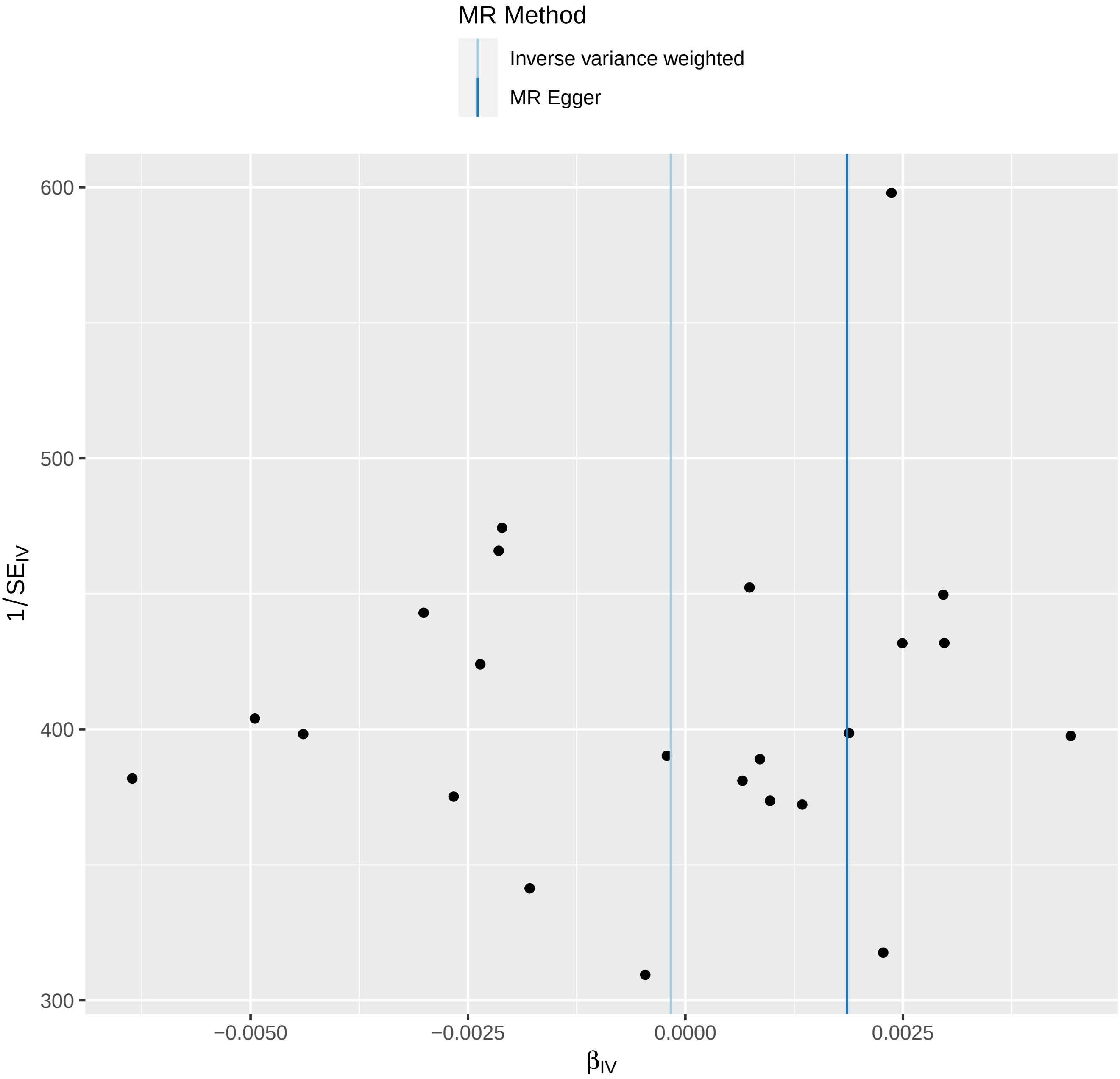

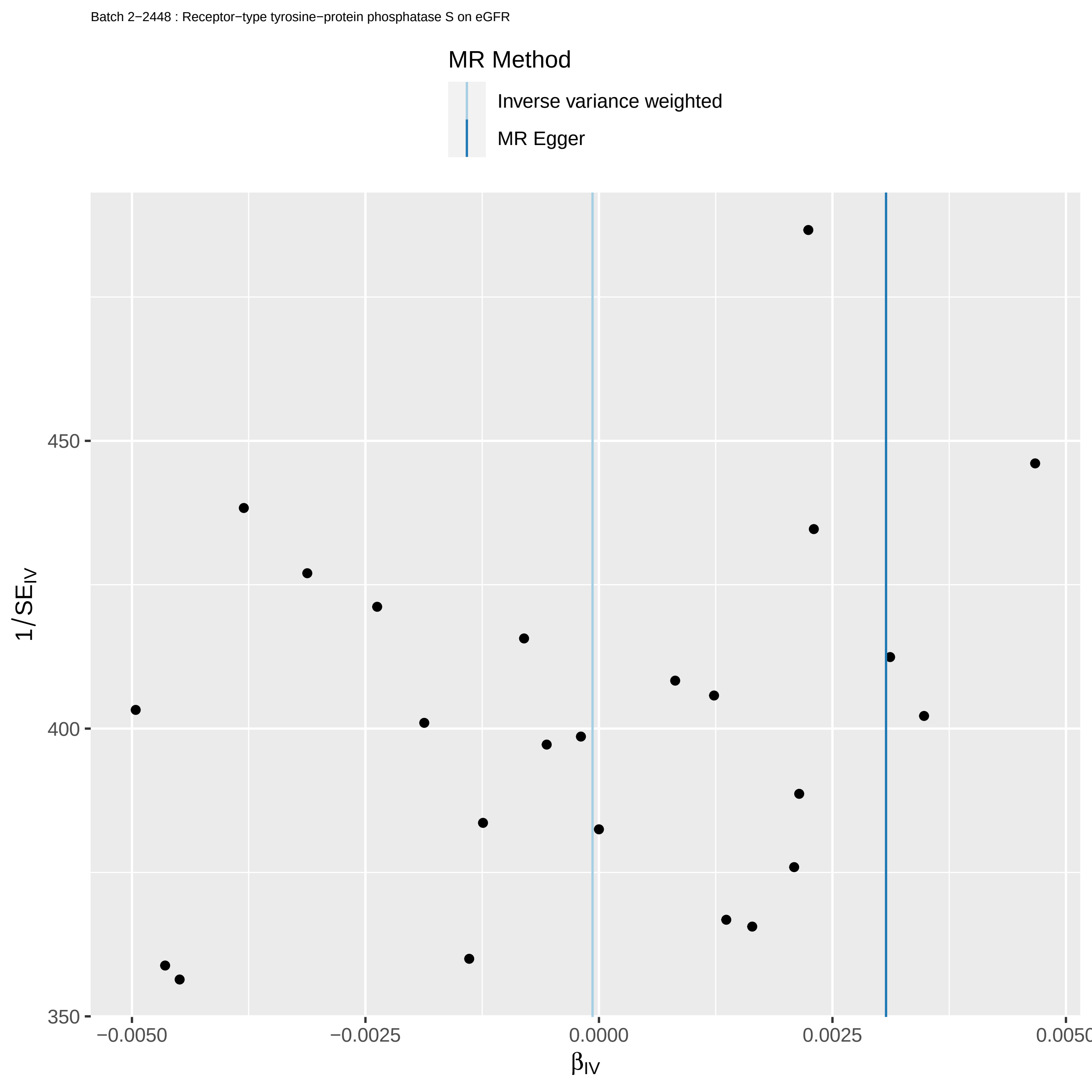
Batch 2−2448 : Receptor−type tyrosine−protein phosphatase S on eGFR
MR Method
Inverse variance weighted
MR Egger
450
400
350
1/SE_IV
−0.0050
−0.0025
0.0000
0.0025
0.0050
β_IV

Batch 2−2449 : Receptor−type tyrosine−protein phosphatase U on eGFR

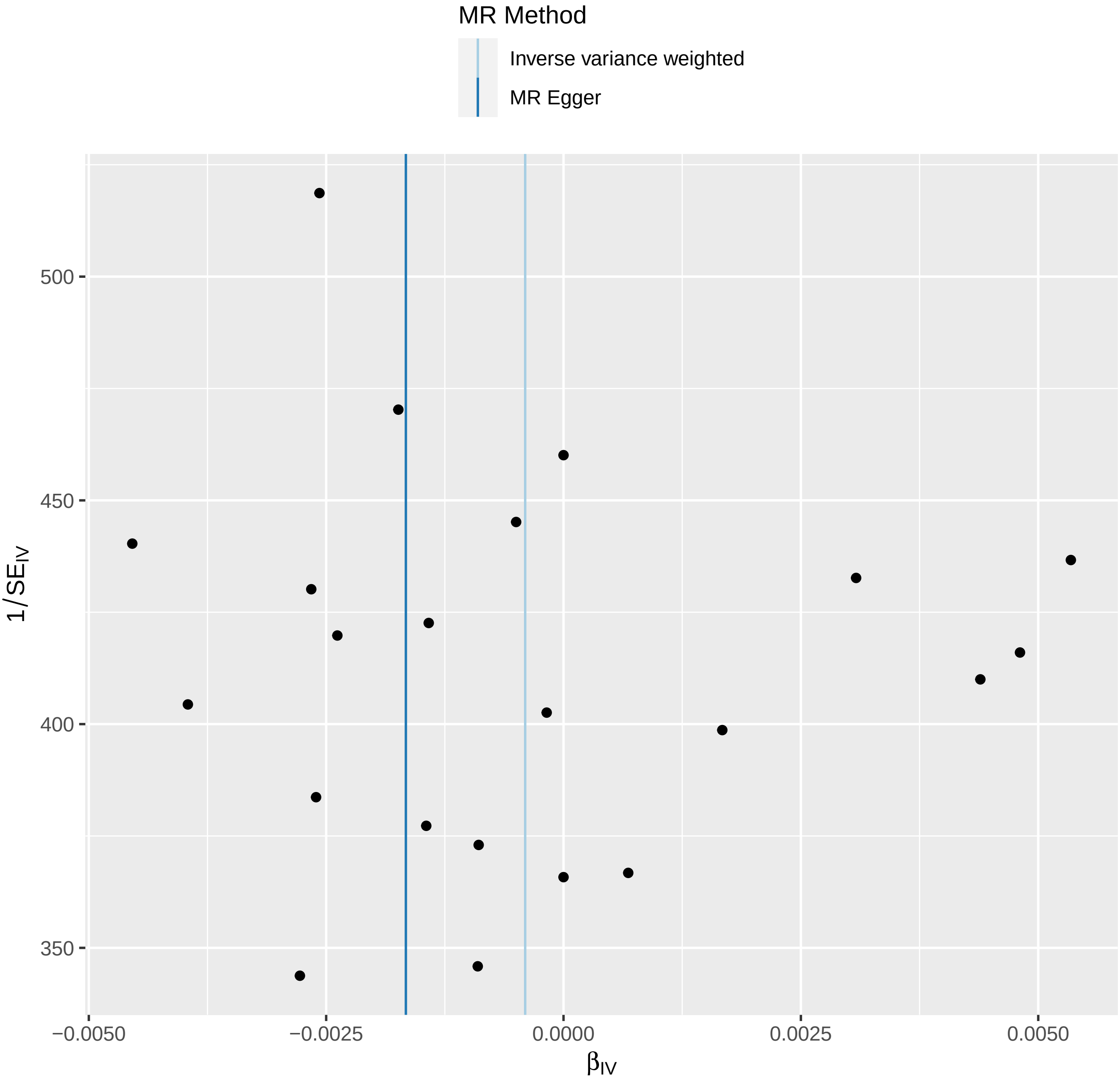

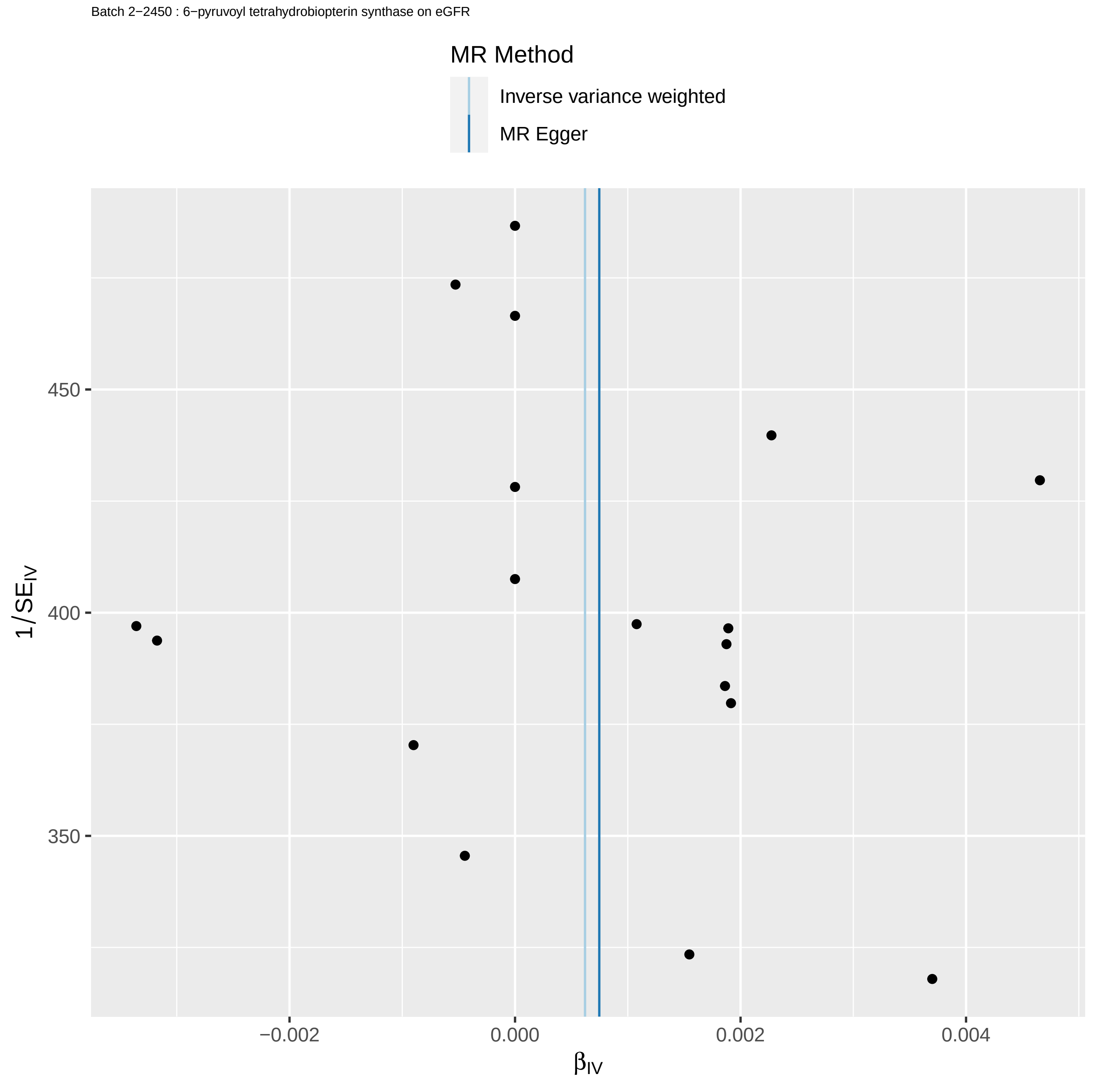

Batch 2−2450 : 6−pyruvoyl tetrahydrobiopterin synthase on eGFR
MR Method
Inverse variance weighted
MR Egger
450
400
350
1/SE$_{IV}$
−0.002
0.000
0.002
0.004
β$_{IV}$

Batch 2−2451 : Pentraxin−related protein PTX3 on eGFR

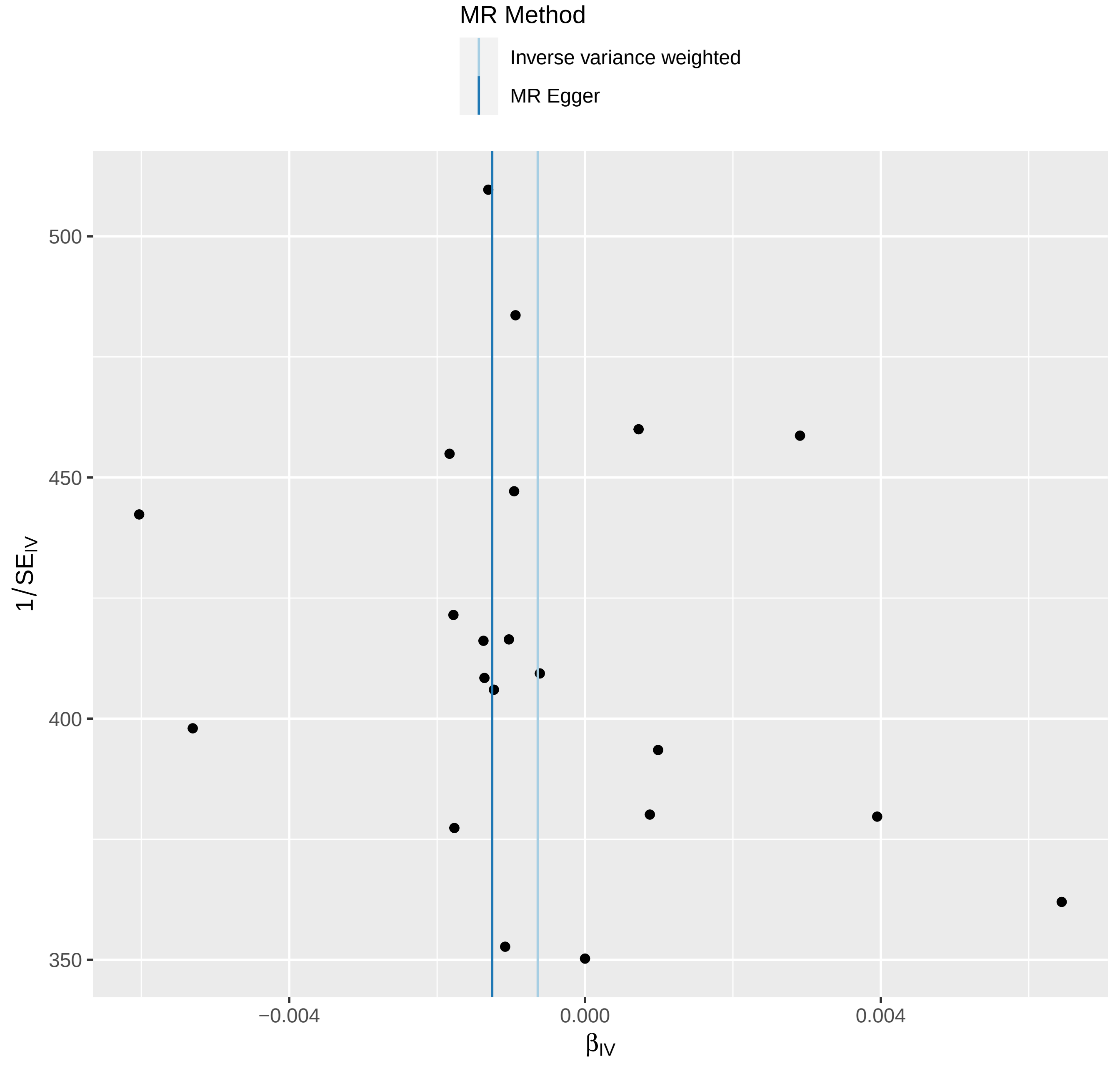

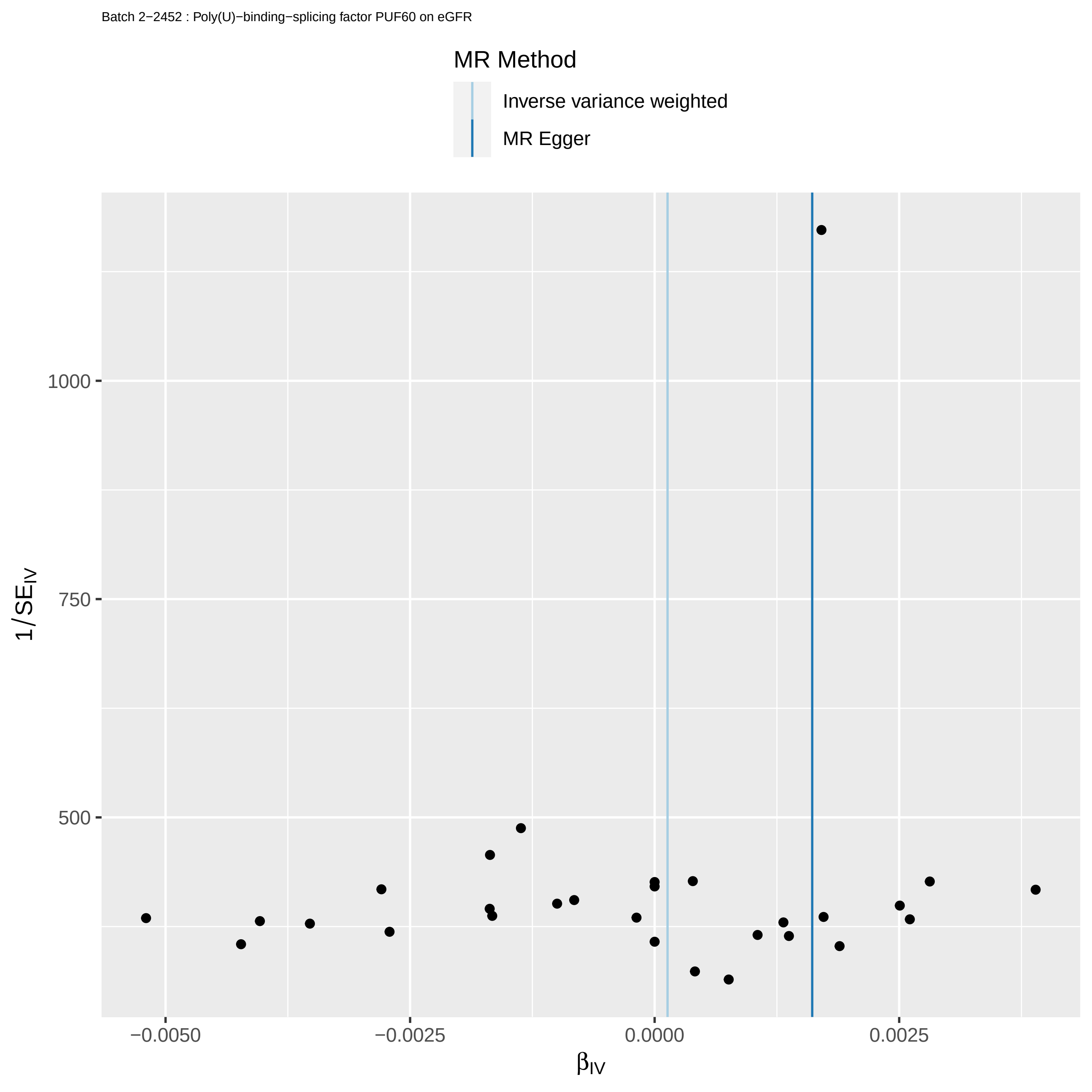
Batch 2−2452 : Poly(U)−binding−splicing factor PUF60 on eGFR
MR Method
Inverse variance weighted
MR Egger
1000
750
500
1/SE_IV
−0.0050
−0.0025
0.0000
0.0025
β_IV

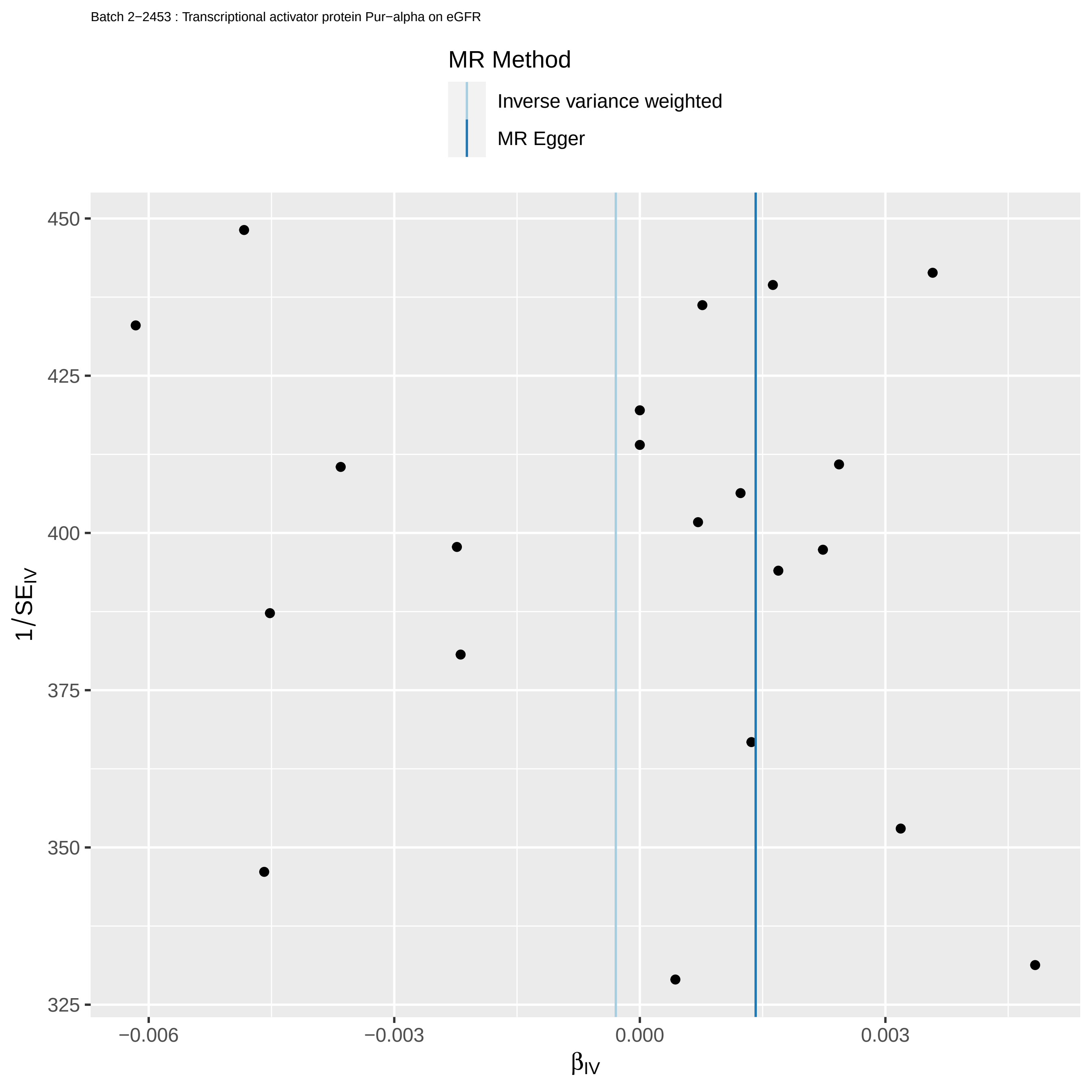

Batch 2−2453 : Transcriptional activator protein Pur−alpha on eGFR
MR Method
Inverse variance weighted
MR Egger
450
425
400
375
350
325
1/SE_IV
−0.006
−0.003
0.000
0.003
β_IV

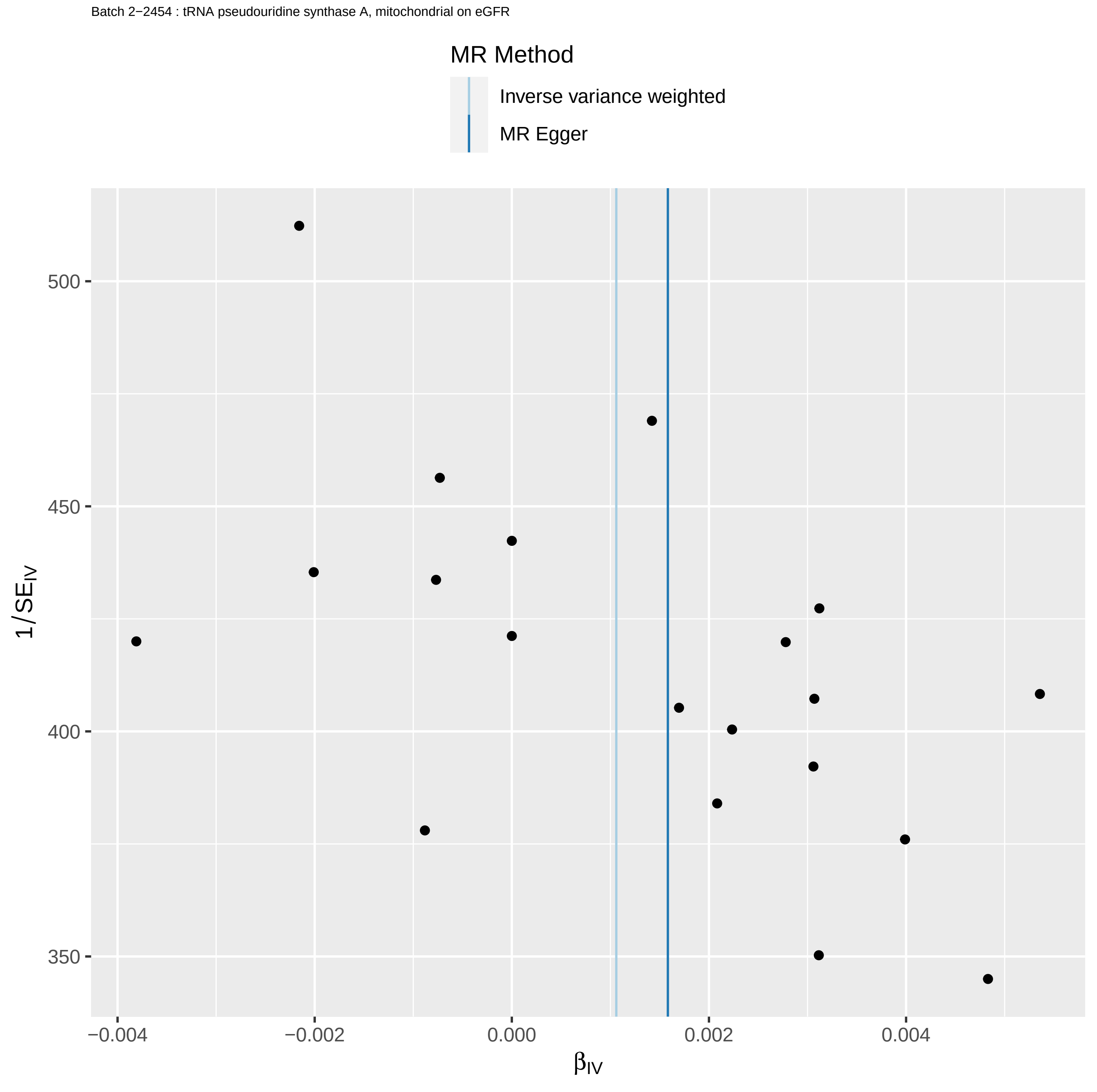

Batch 2−2454 : tRNA pseudouridine synthase A, mitochondrial on eGFR
MR Method
Inverse variance weighted
MR Egger
500
450
400
350
1/SE_IV
−0.004
−0.002
0.000
0.002
0.004
β_IV

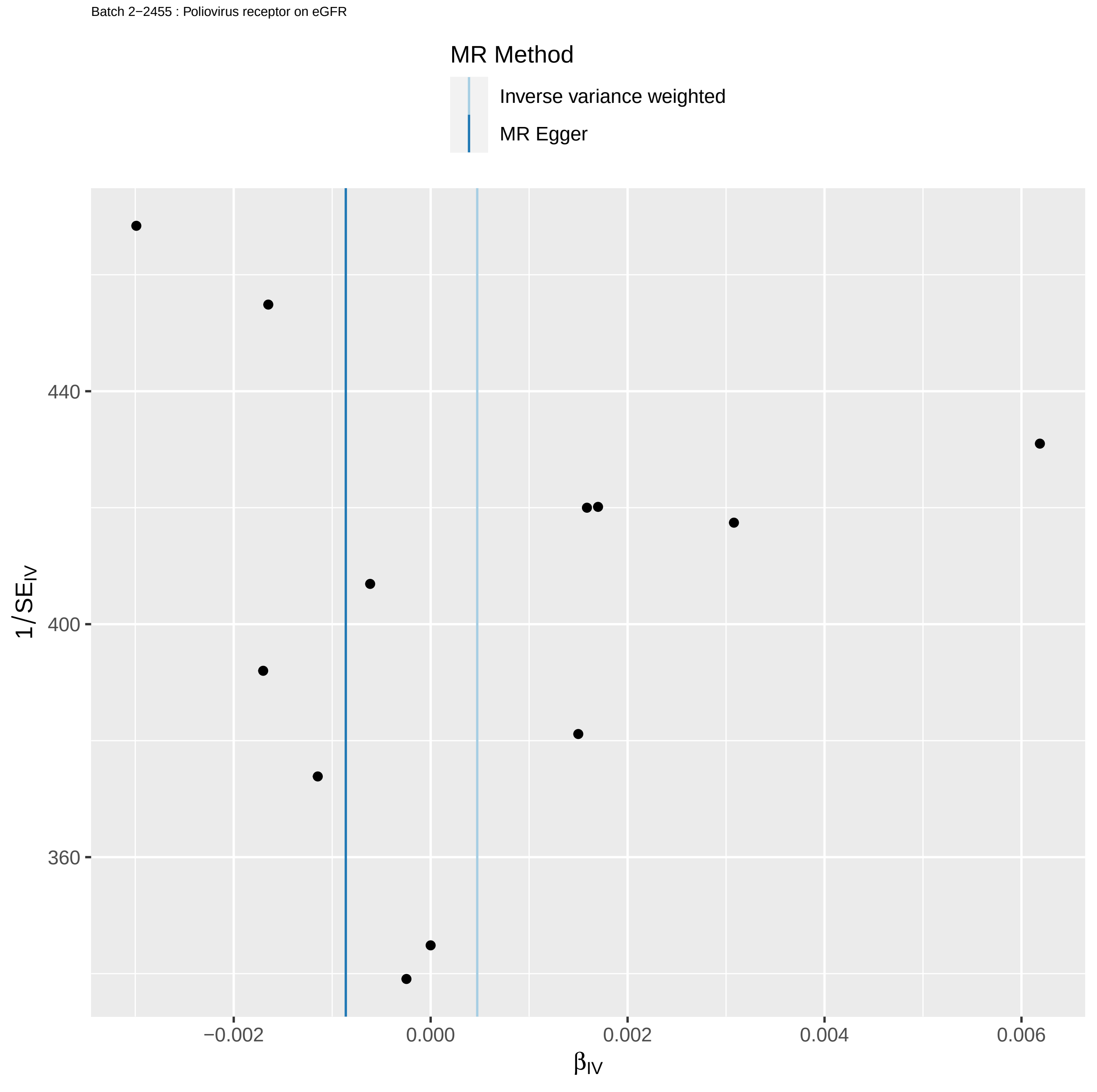

Batch 2−2455 : Poliovirus receptor on eGFR
MR Method
Inverse variance weighted
MR Egger
440
400
360
1/SE$_{IV}$
−0.002
0.000
0.002
0.004
0.006
β$_{IV}$

Batch 2−2456 : Transmembrane protein PVRIG on eGFR

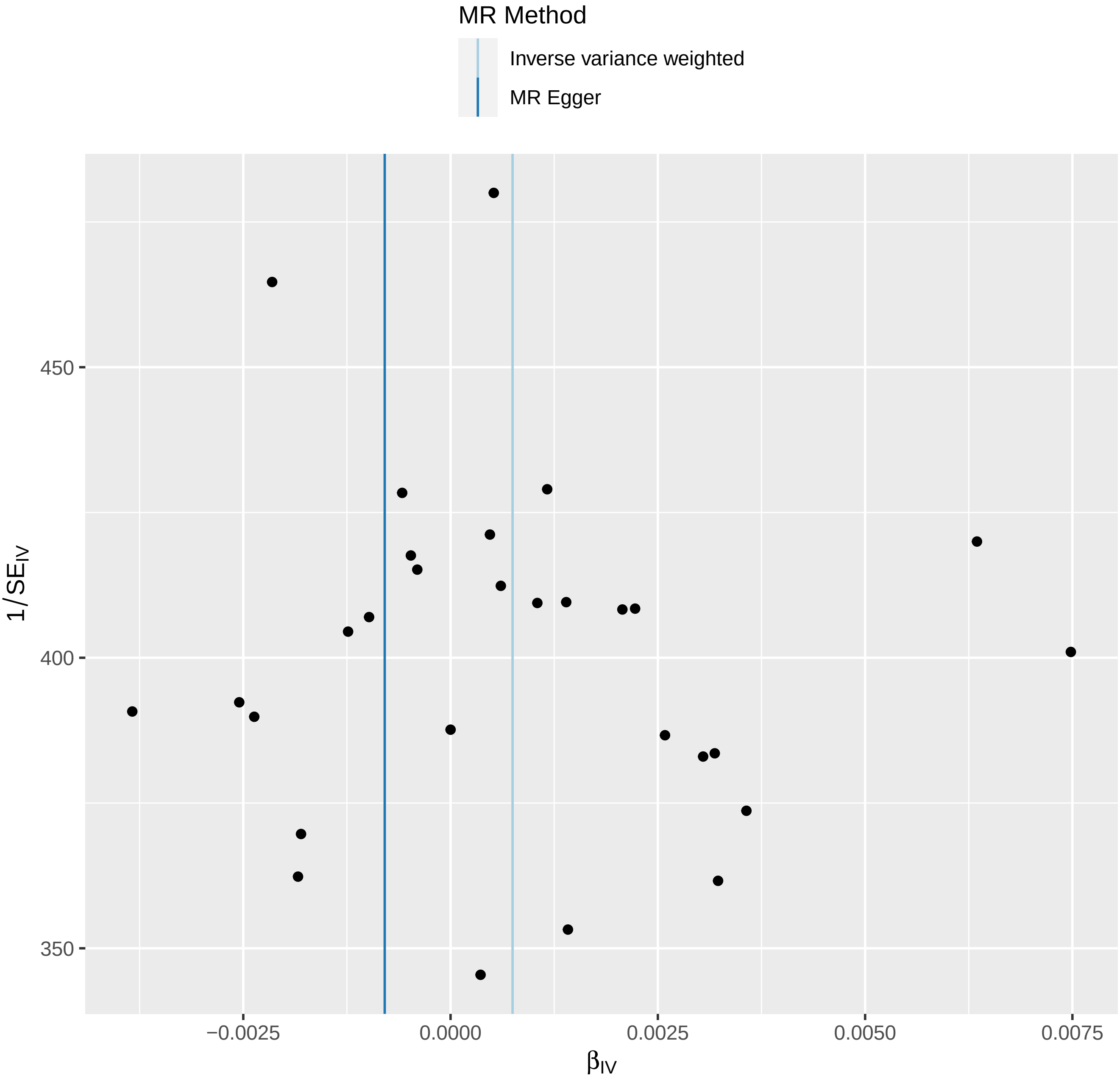

Batch 2−2457 : Nectin−2 on eGFR

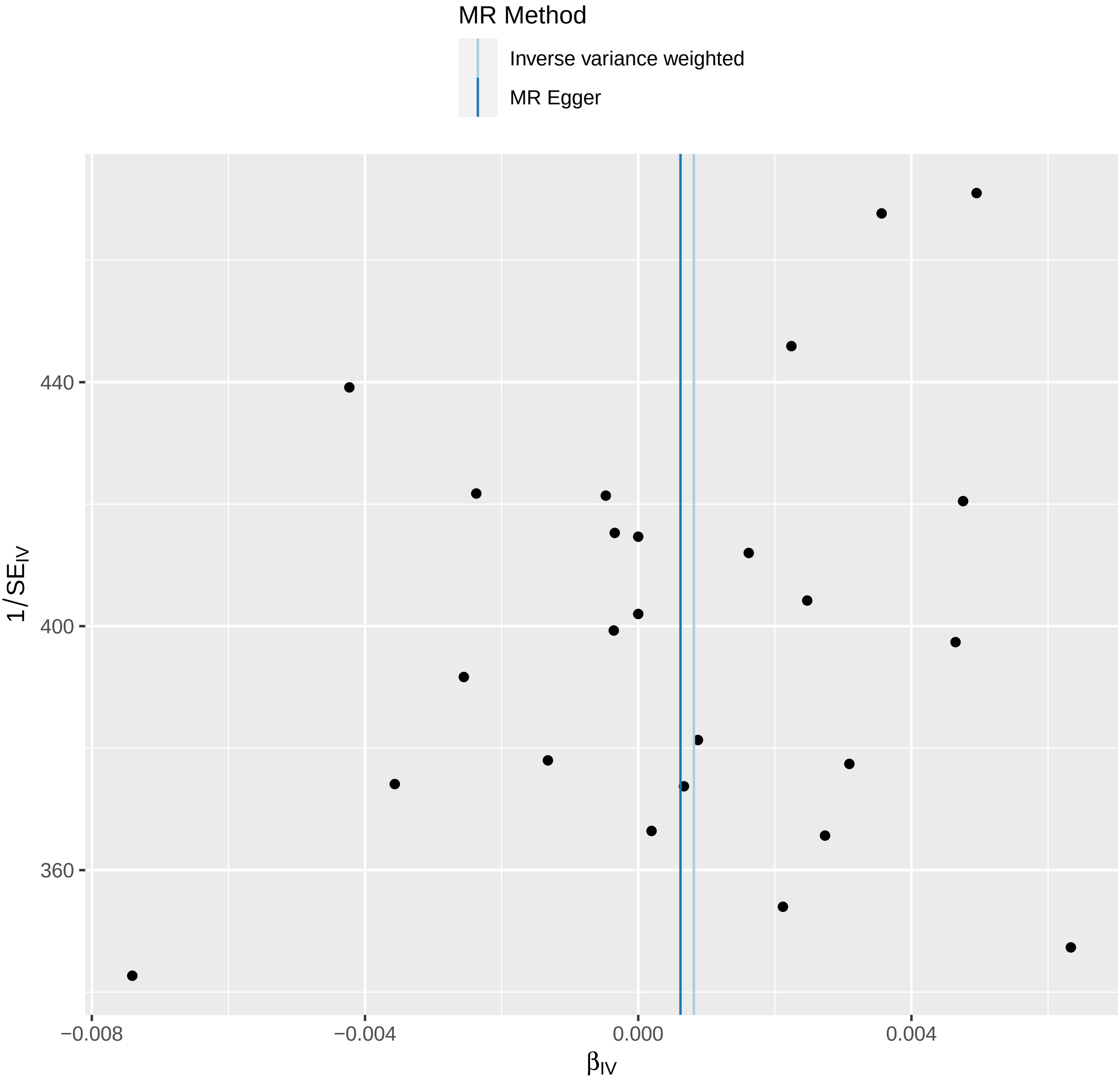

Batch 2−2458 : Nectin−3 on eGFR

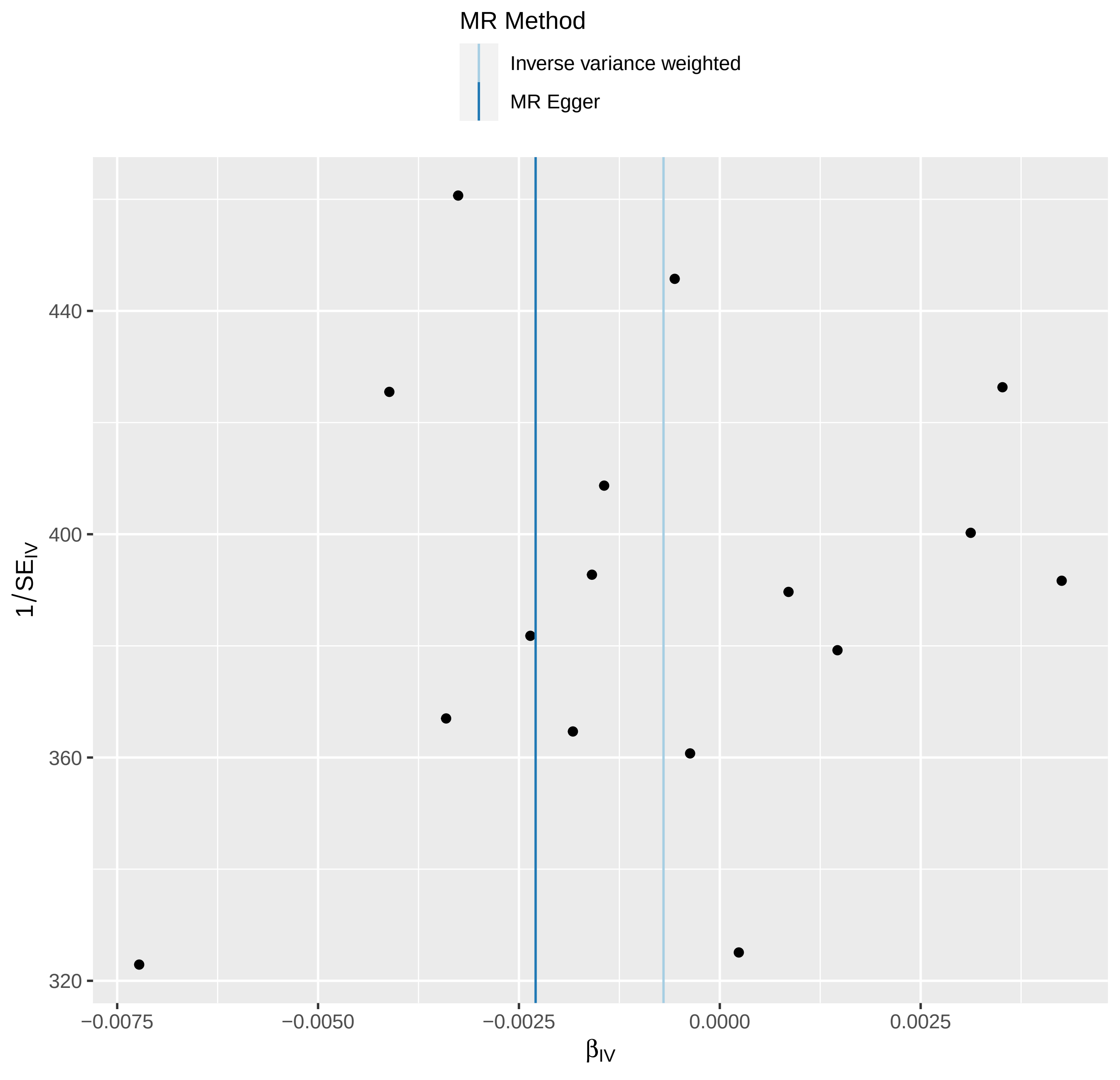

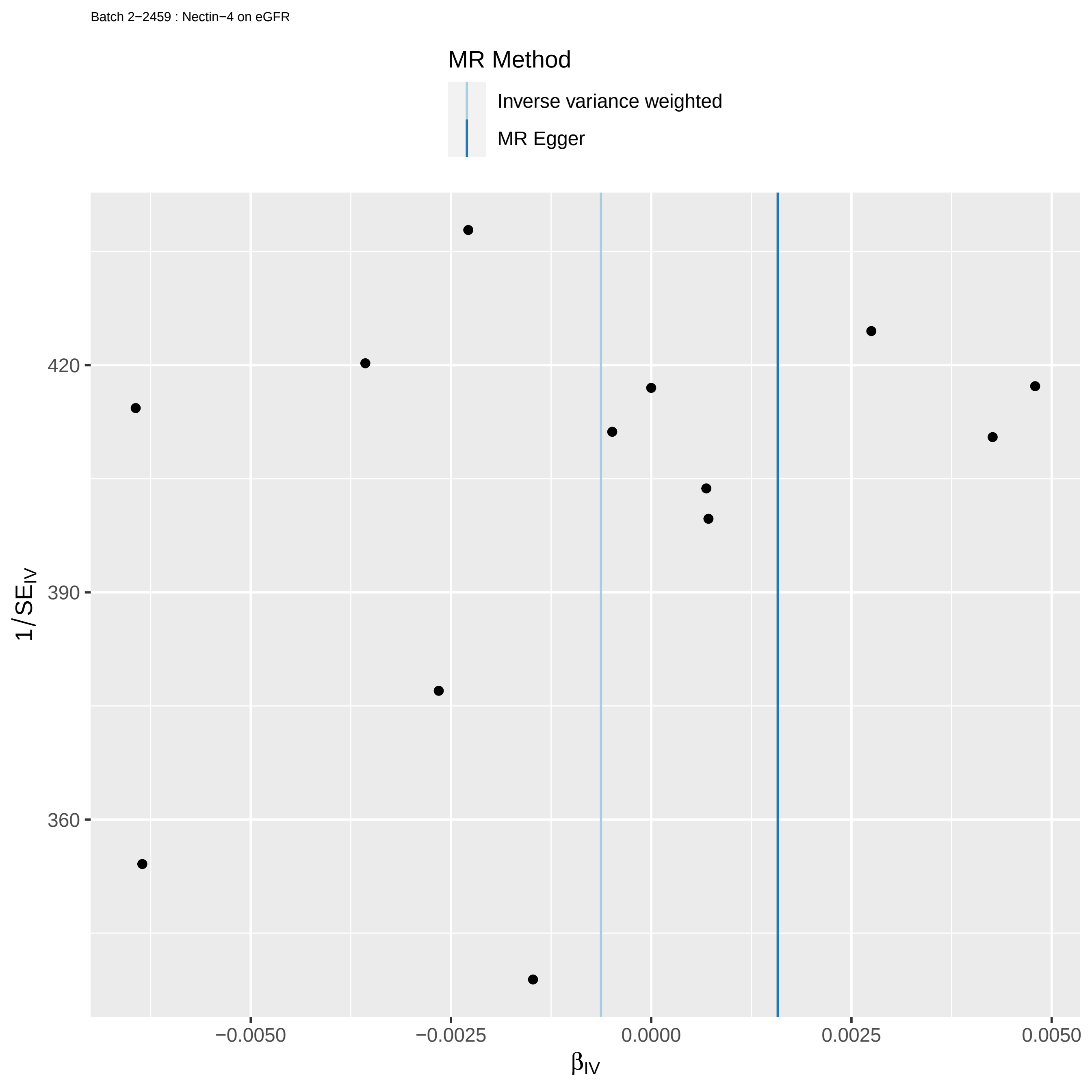
Batch 2−2459 : Nectin−4 on eGFR
MR Method
Inverse variance weighted
MR Egger
420
390
360
1/SE_IV
−0.0050
−0.0025
0.0000
0.0025
0.0050
β_IV

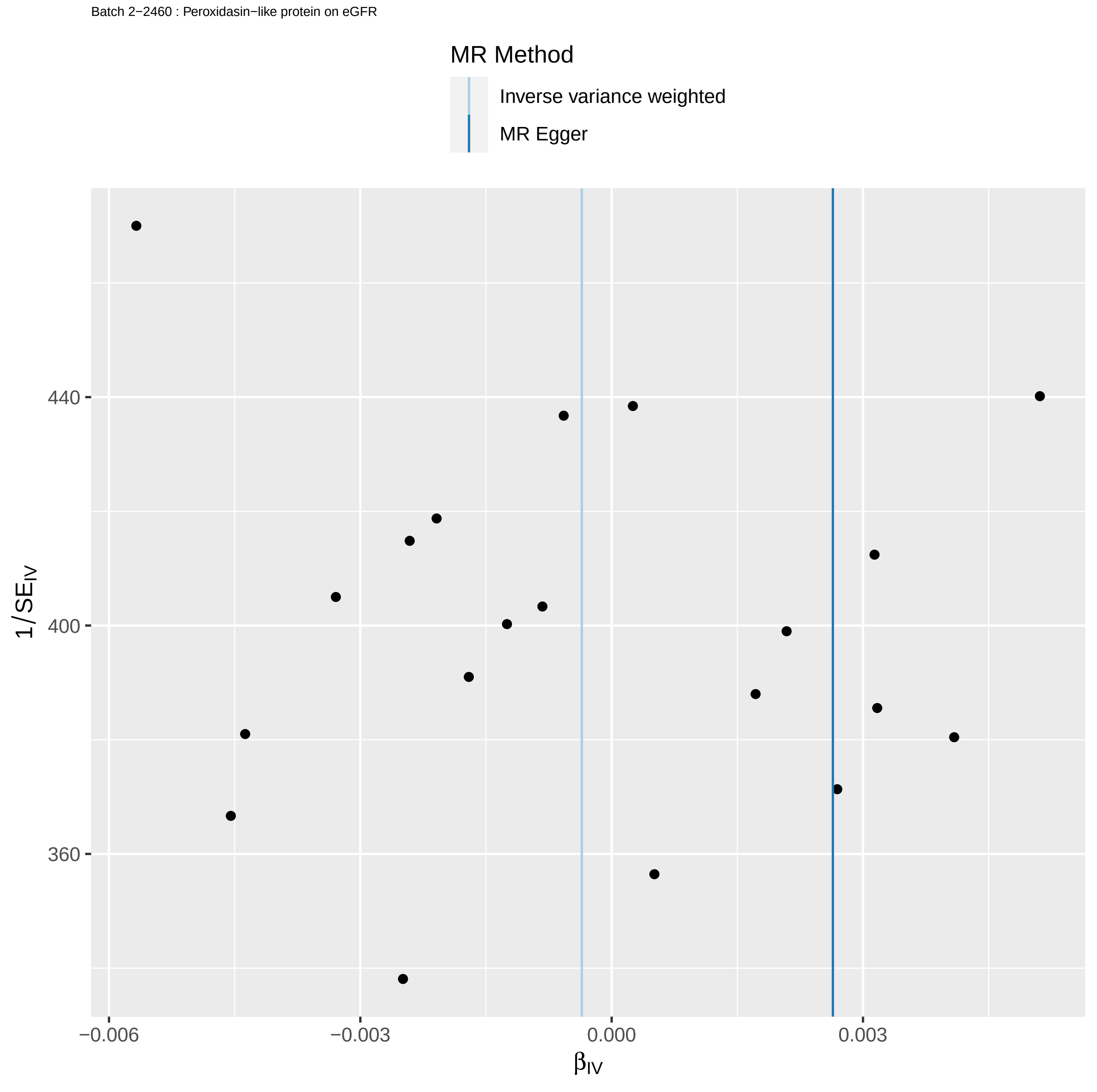
Batch 2−2460 : Peroxidasin−like protein on eGFR
MR Method
Inverse variance weighted
MR Egger
440
400
360
1/SE_IV
−0.006
−0.003
0.000
0.003
β_IV

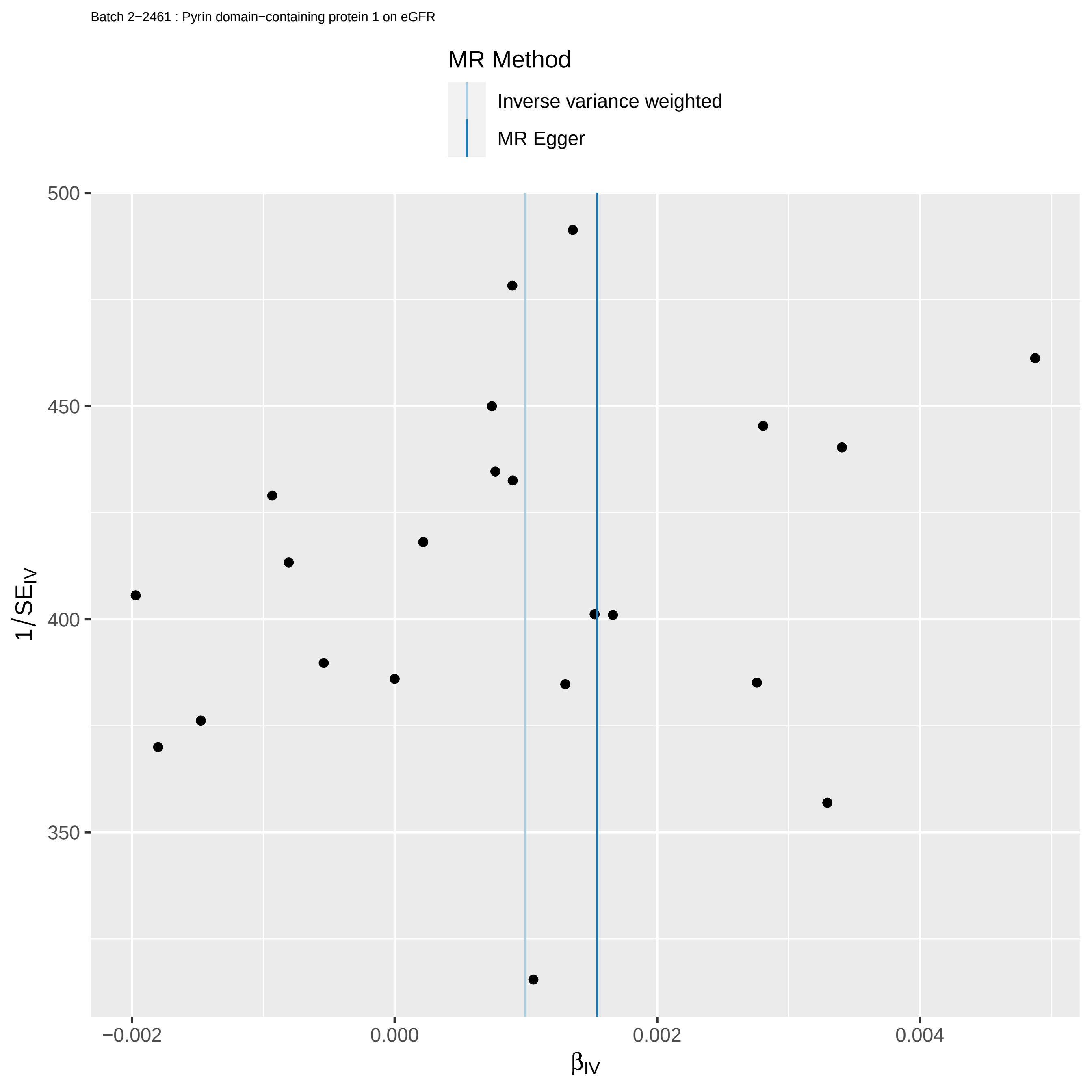

Batch 2−2461 : Pyrin domain−containing protein 1 on eGFR
MR Method
Inverse variance weighted
MR Egger
500
450
400
350
1/SE$_{IV}$
−0.002
0.000
0.002
0.004
β$_{IV}$

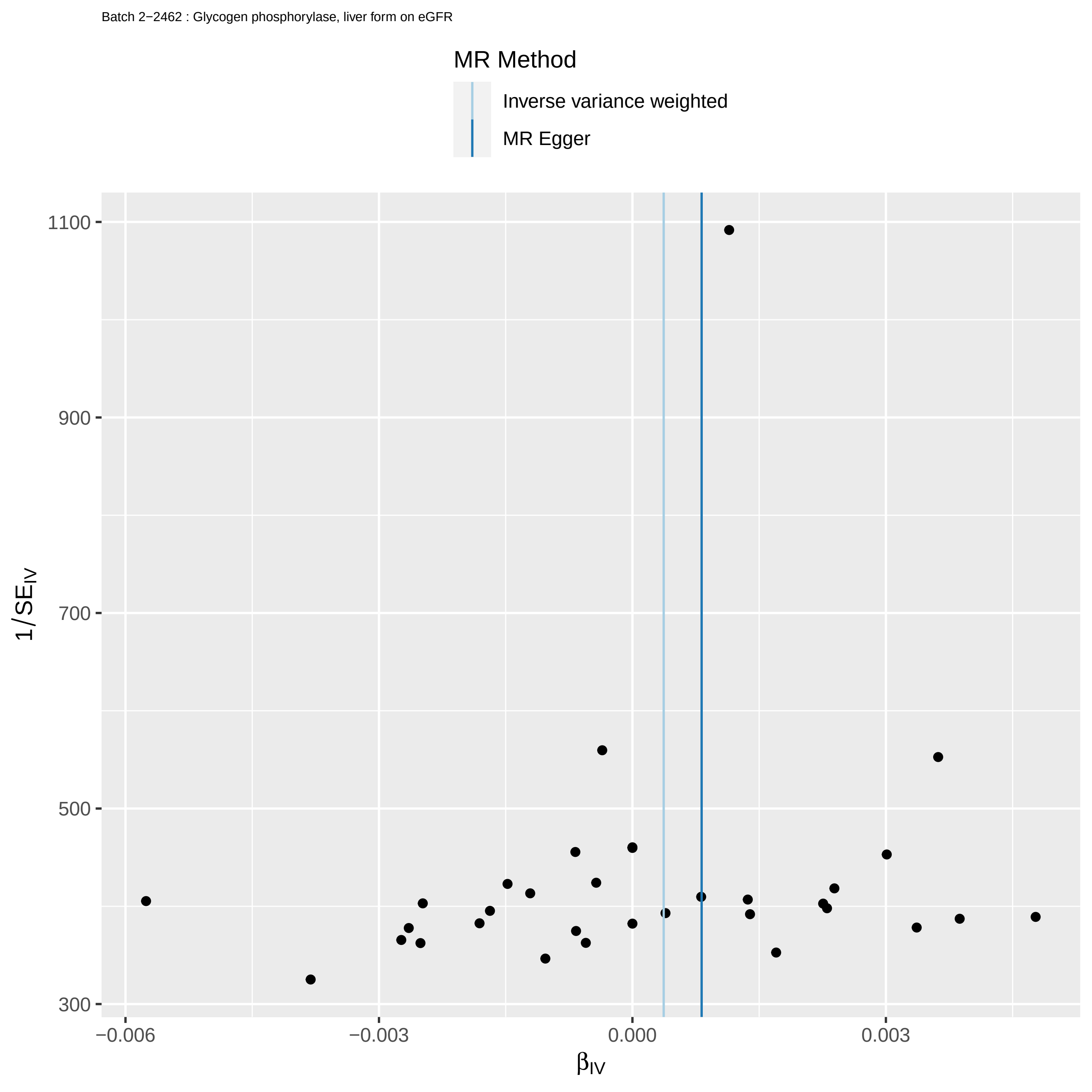
Batch 2−2462 : Glycogen phosphorylase, liver form on eGFR
MR Method
Inverse variance weighted
MR Egger
1100
900
700
500
300
1/SE$_{IV}$
−0.006
−0.003
0.000
0.003
β$_{IV}$

Batch 2−2463 : Peptide YY on eGFR

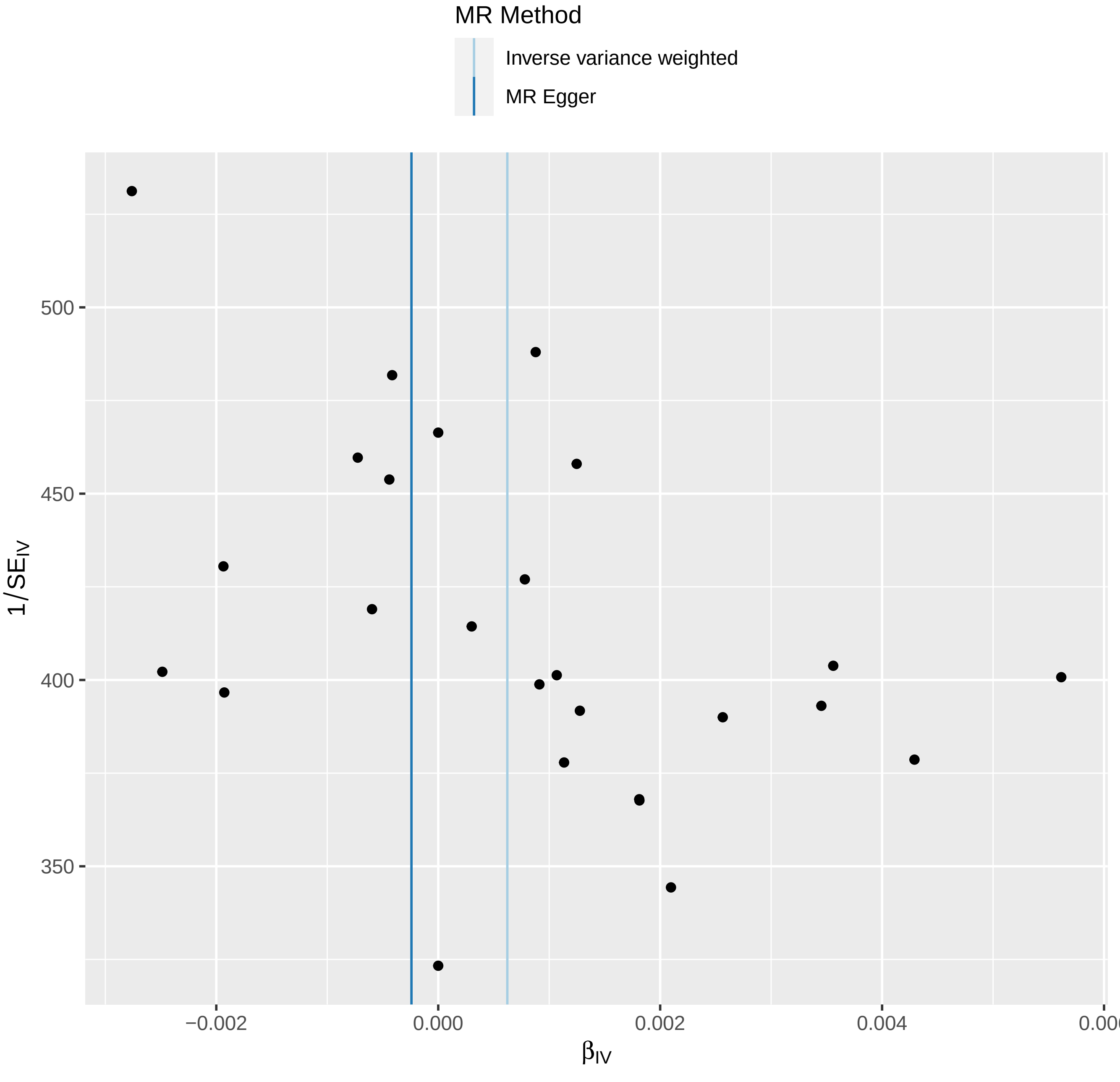

Batch 2−2464 : Dihydropteridine reductase on eGFR

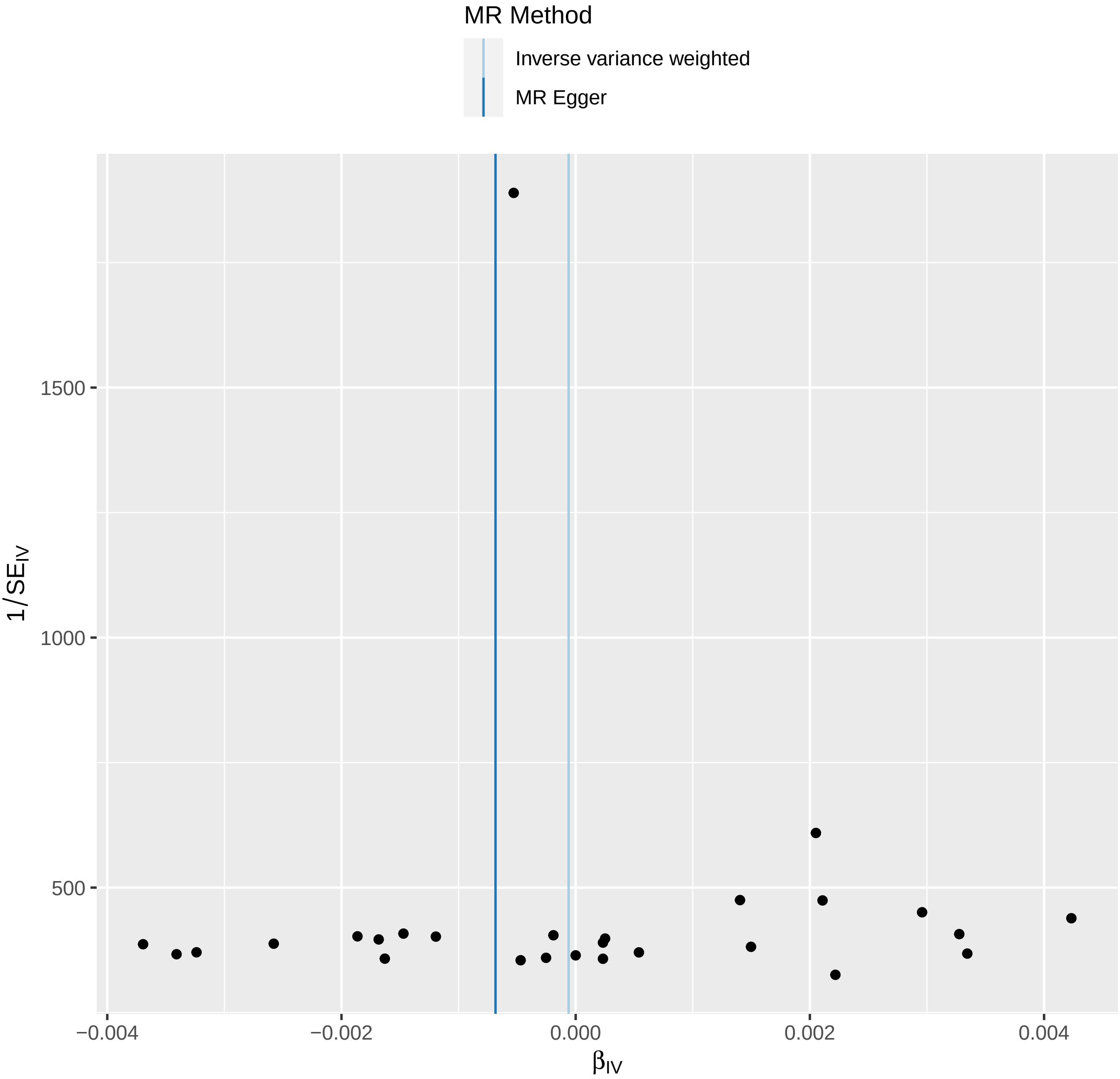

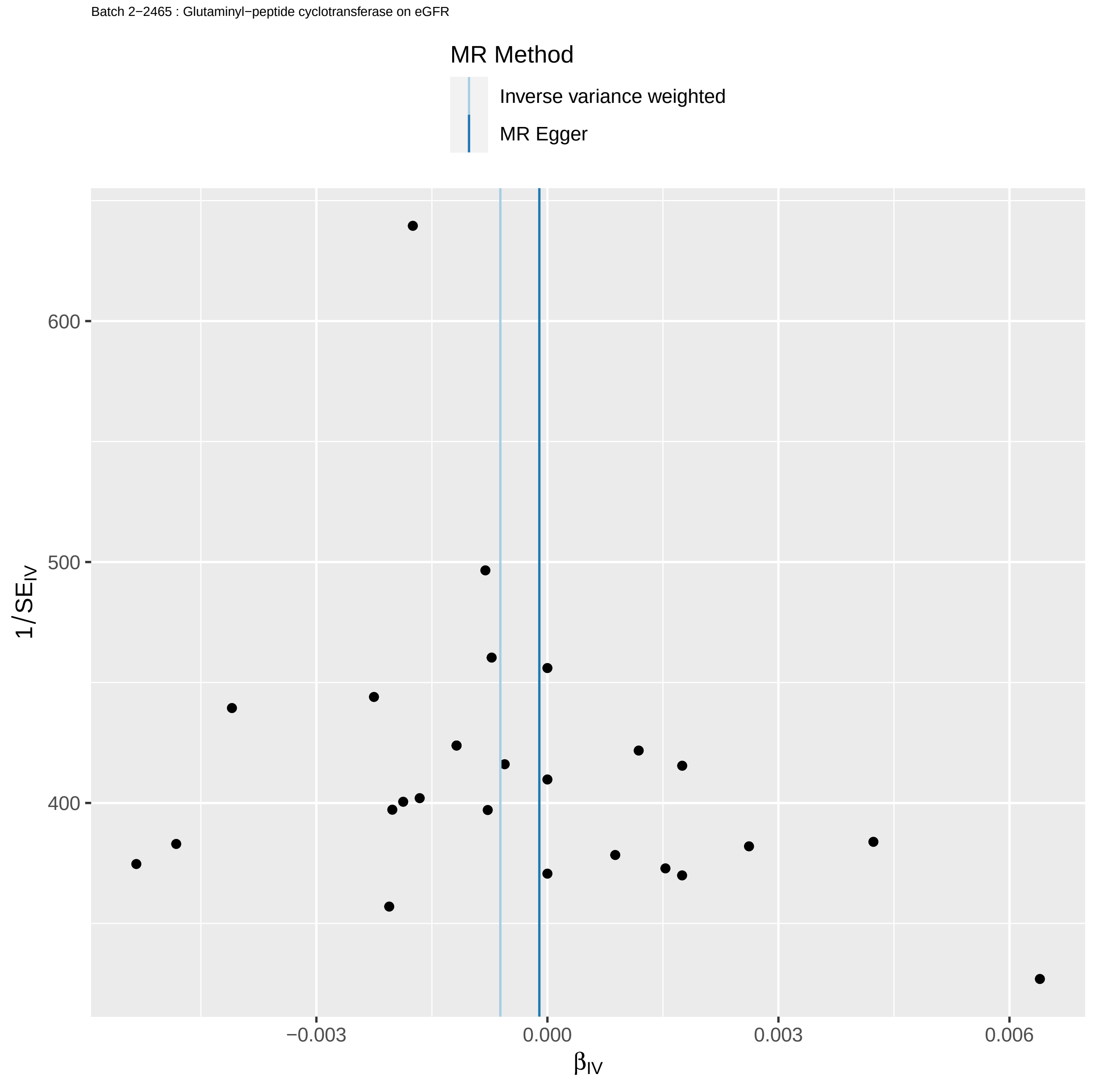

Batch 2−2465 : Glutaminyl−peptide cyclotransferase on eGFR
MR Method
Inverse variance weighted
MR Egger
600
500
400
1/SE_IV
−0.003
0.000
0.003
0.006
β_IV

Batch 2−2466 : Glutaminyl−peptide cyclotransferase−like protein on eGFR

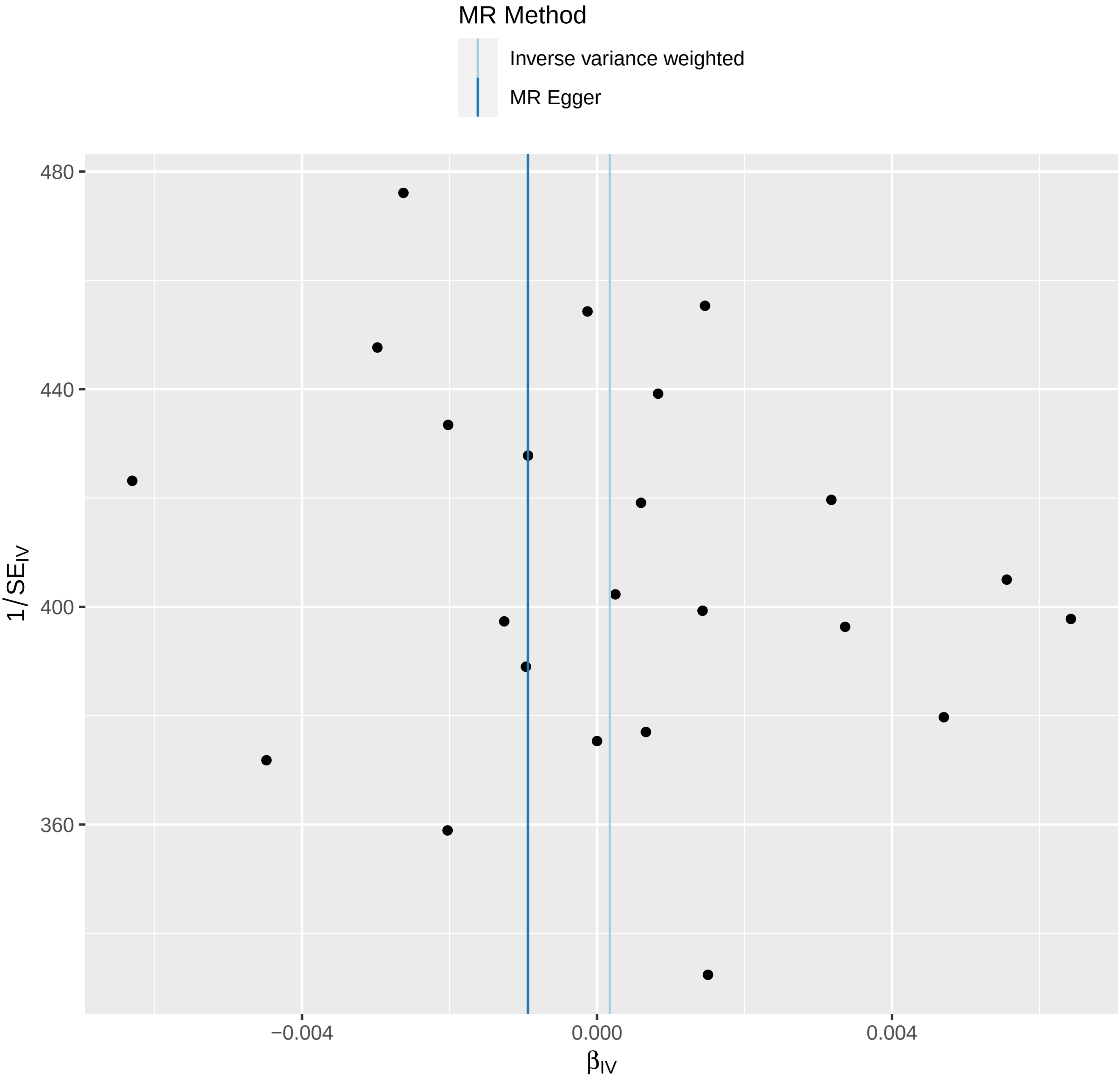

Batch 2−2467 : Orexigenic neuropeptide QRFP on eGFR

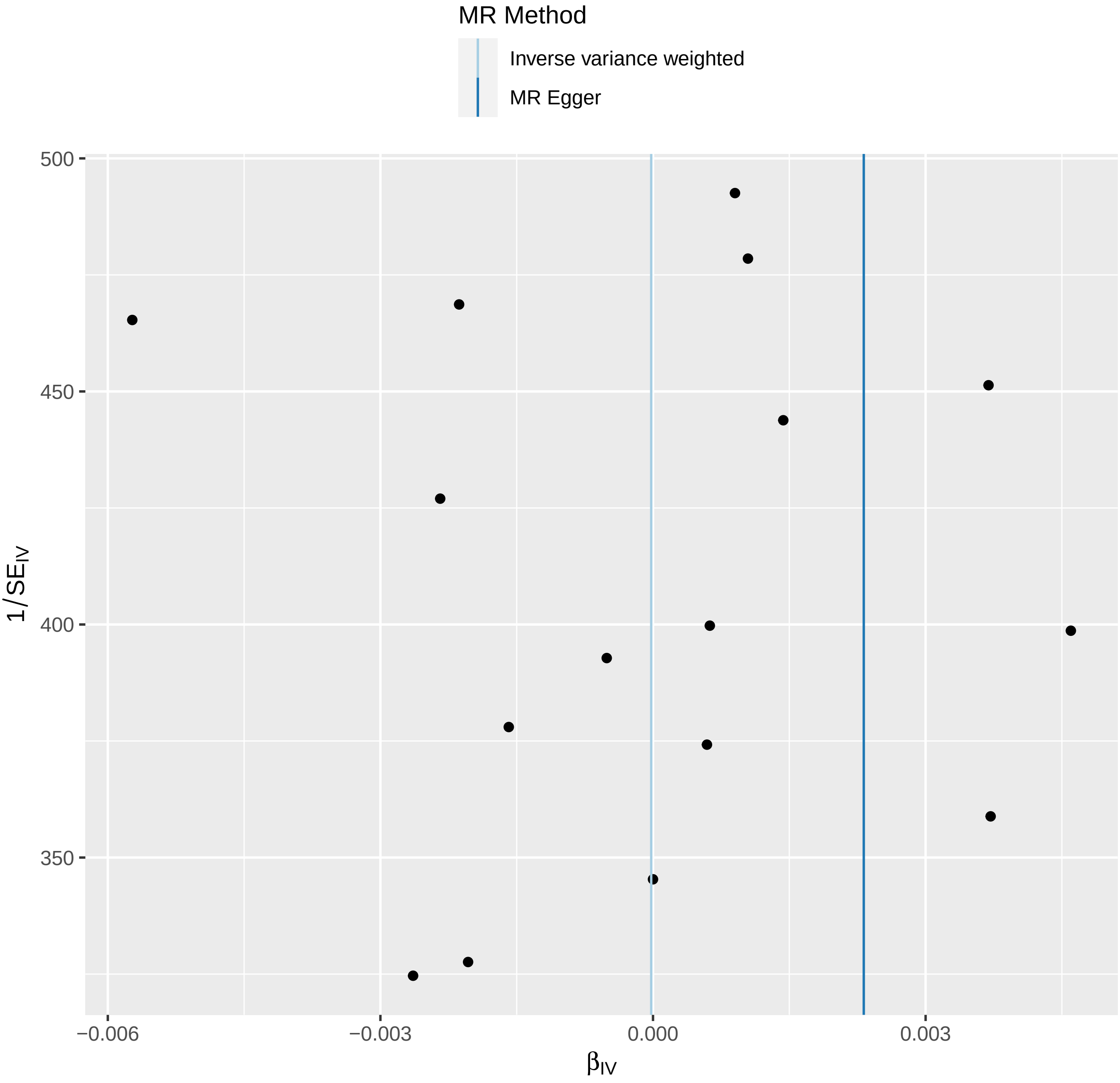

Batch 2−2468 : Sulfhydryl oxidase 1 on eGFR

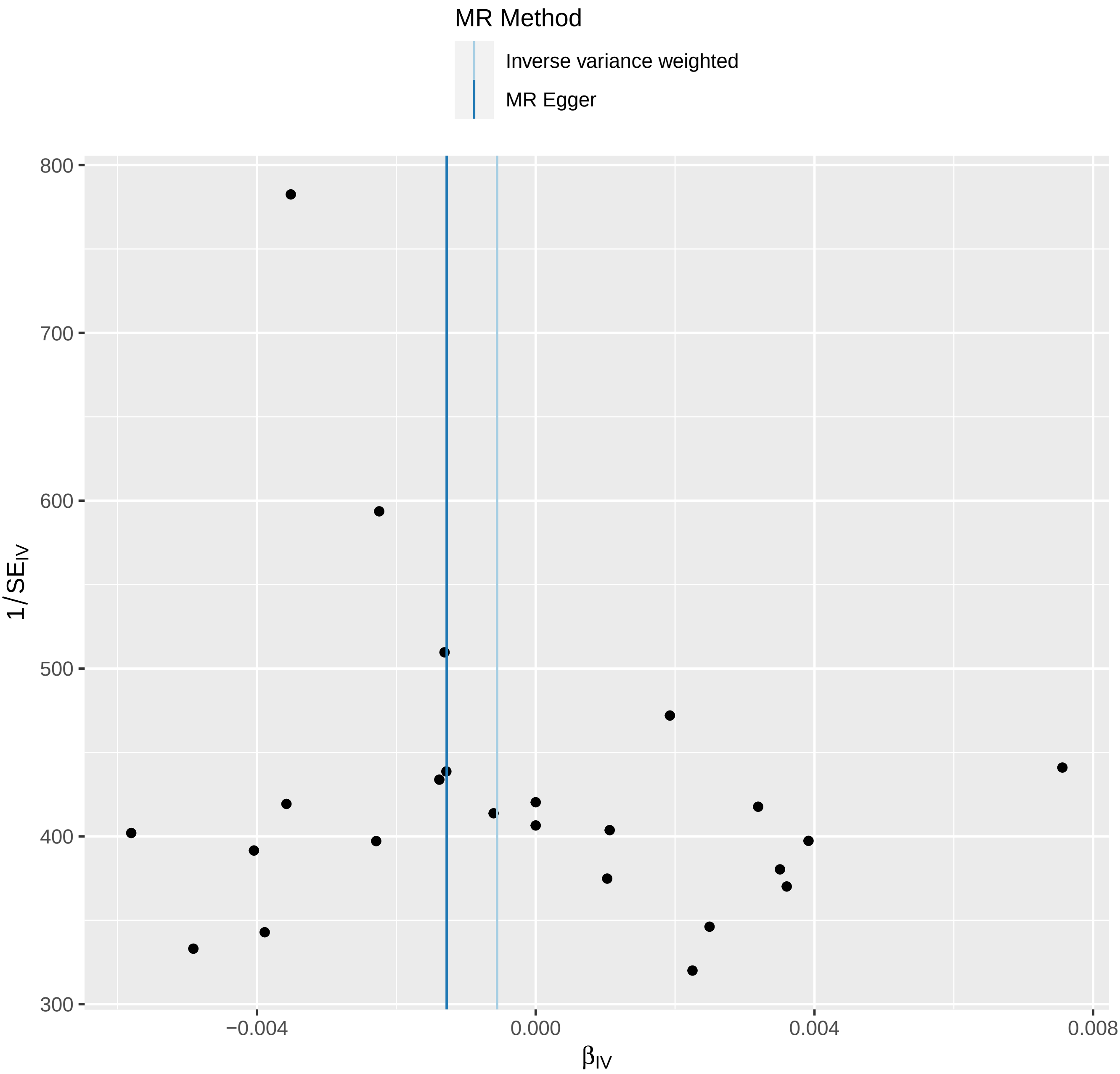

Batch 2−2469 : Sulfhydryl oxidase 1 on eGFR

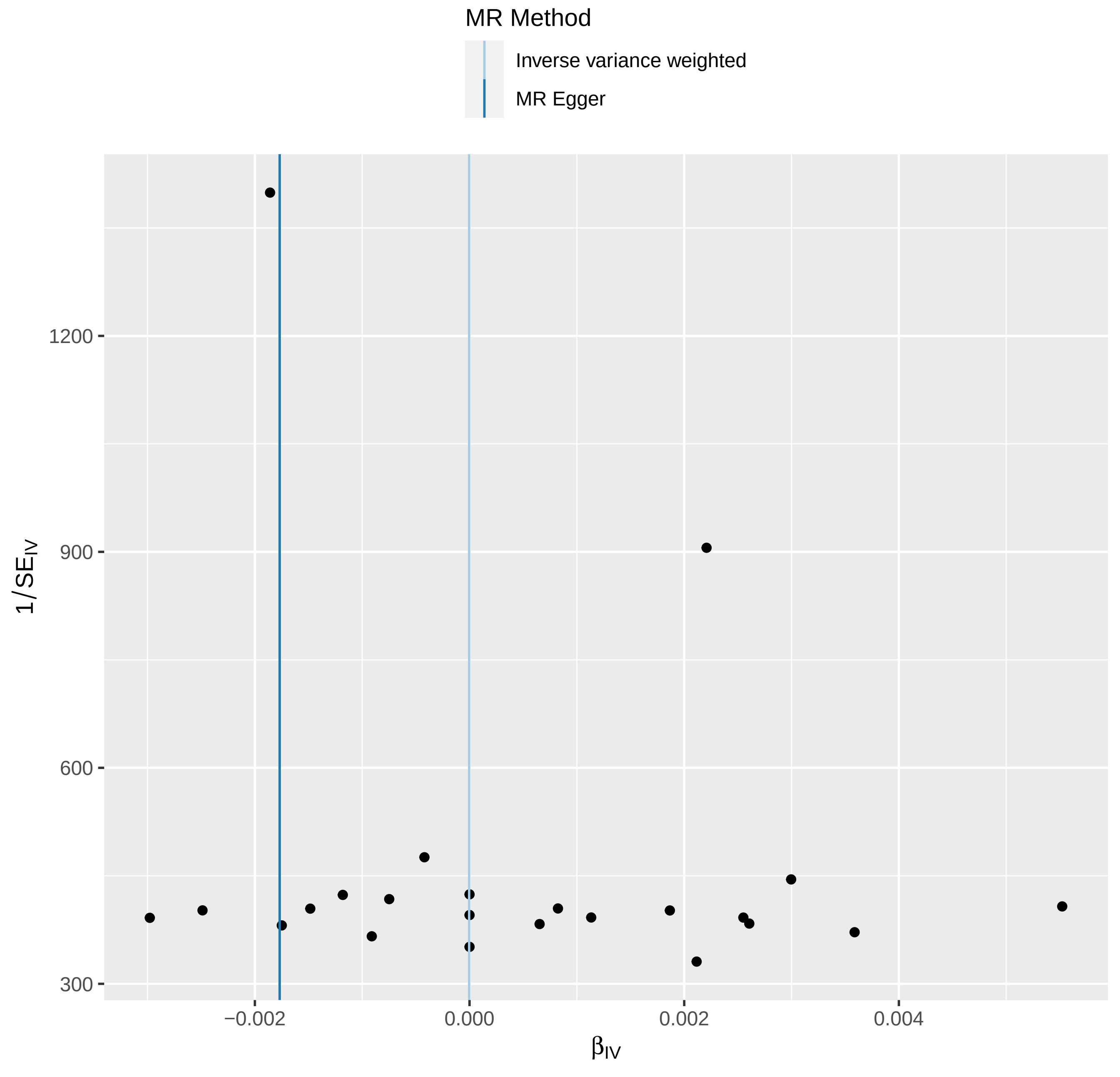

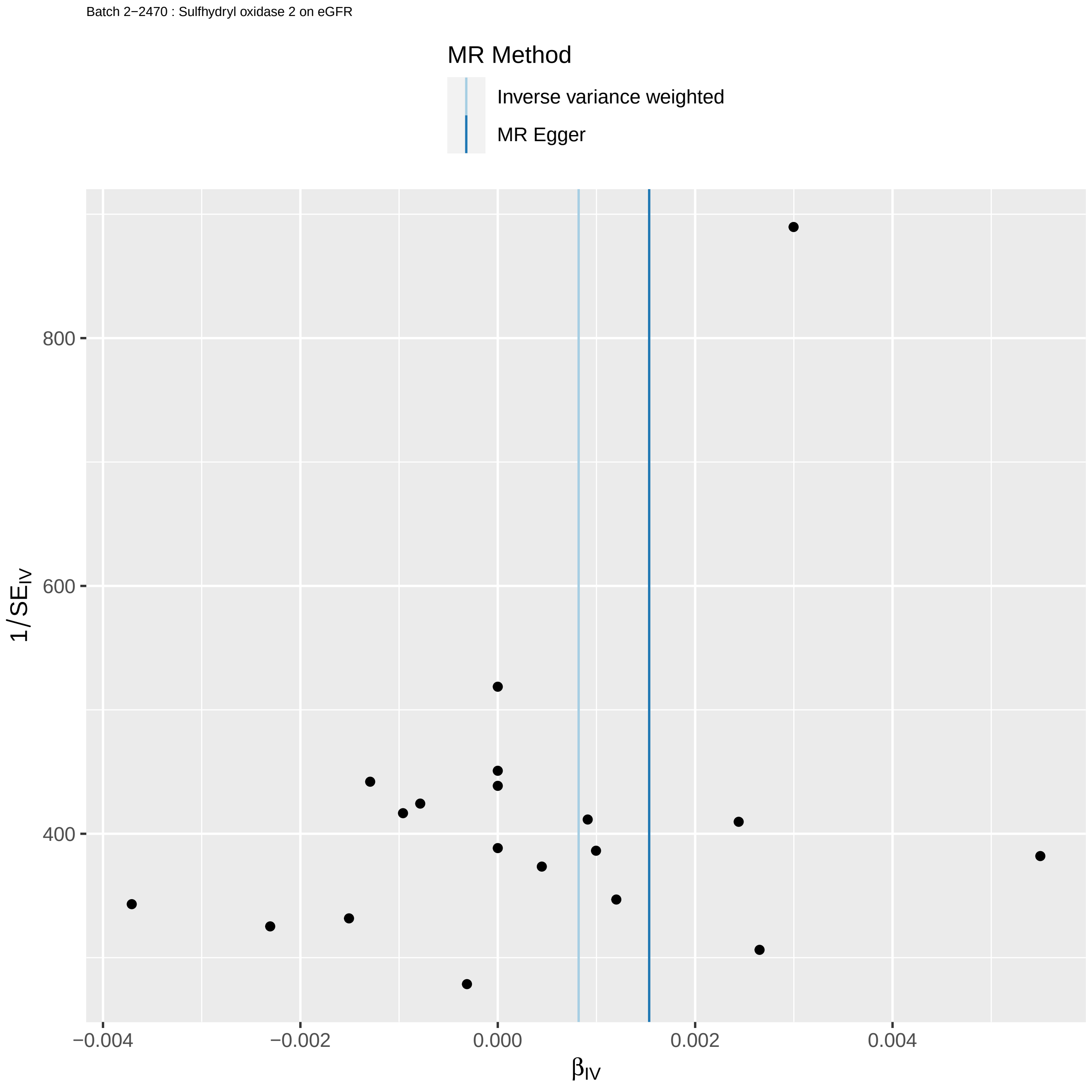

Batch 2−2470 : Sulfhydryl oxidase 2 on eGFR
MR Method
Inverse variance weighted
MR Egger
800
600
400
1/SE$_{IV}$
−0.004
−0.002
0.000
0.002
0.004
β$_{IV}$

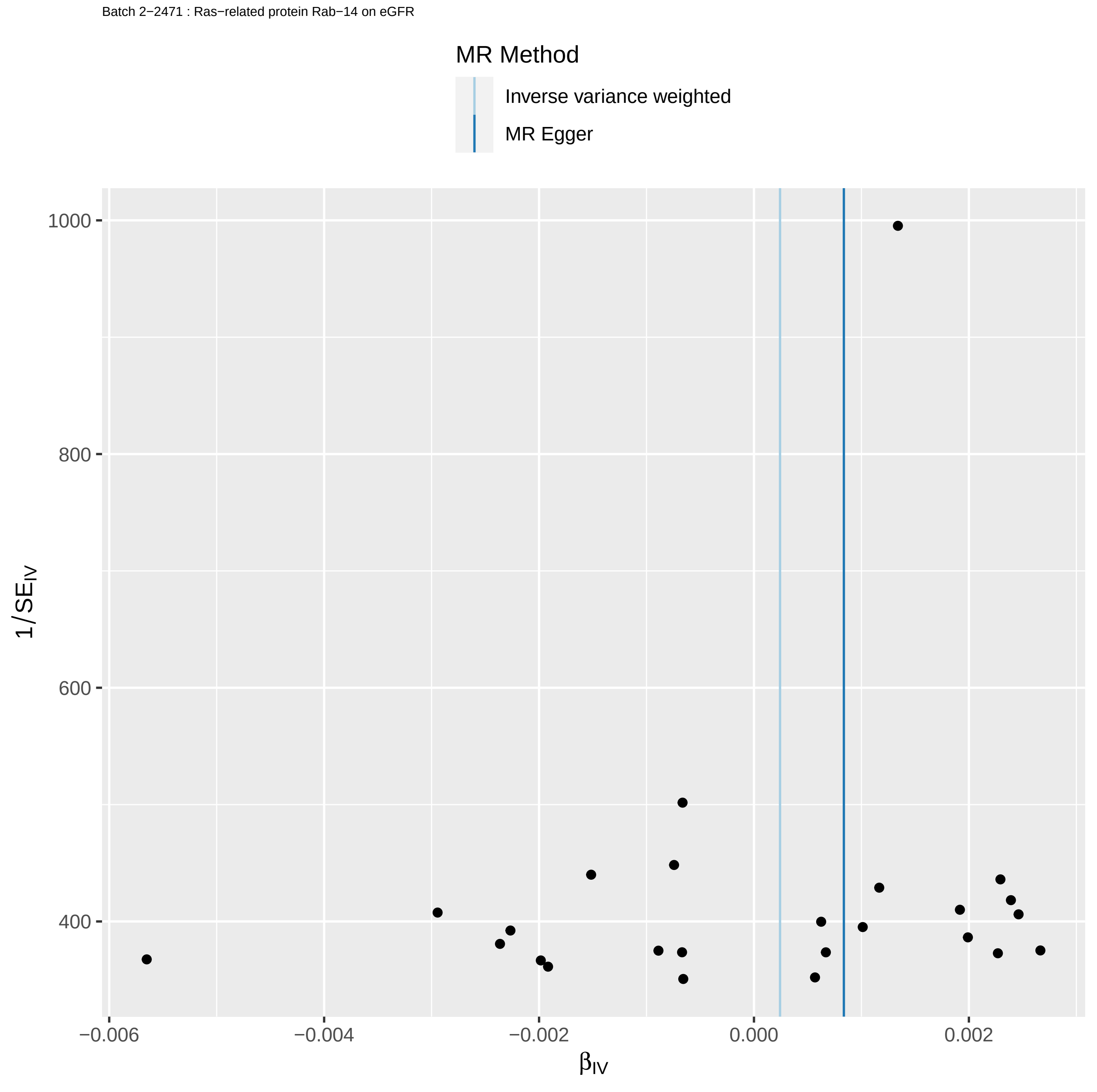

Batch 2−2471 : Ras−related protein Rab−14 on eGFR
MR Method
Inverse variance weighted
MR Egger
1000
800
600
400
1/SE_IV
−0.006
−0.004
−0.002
0.000
0.002
β_IV

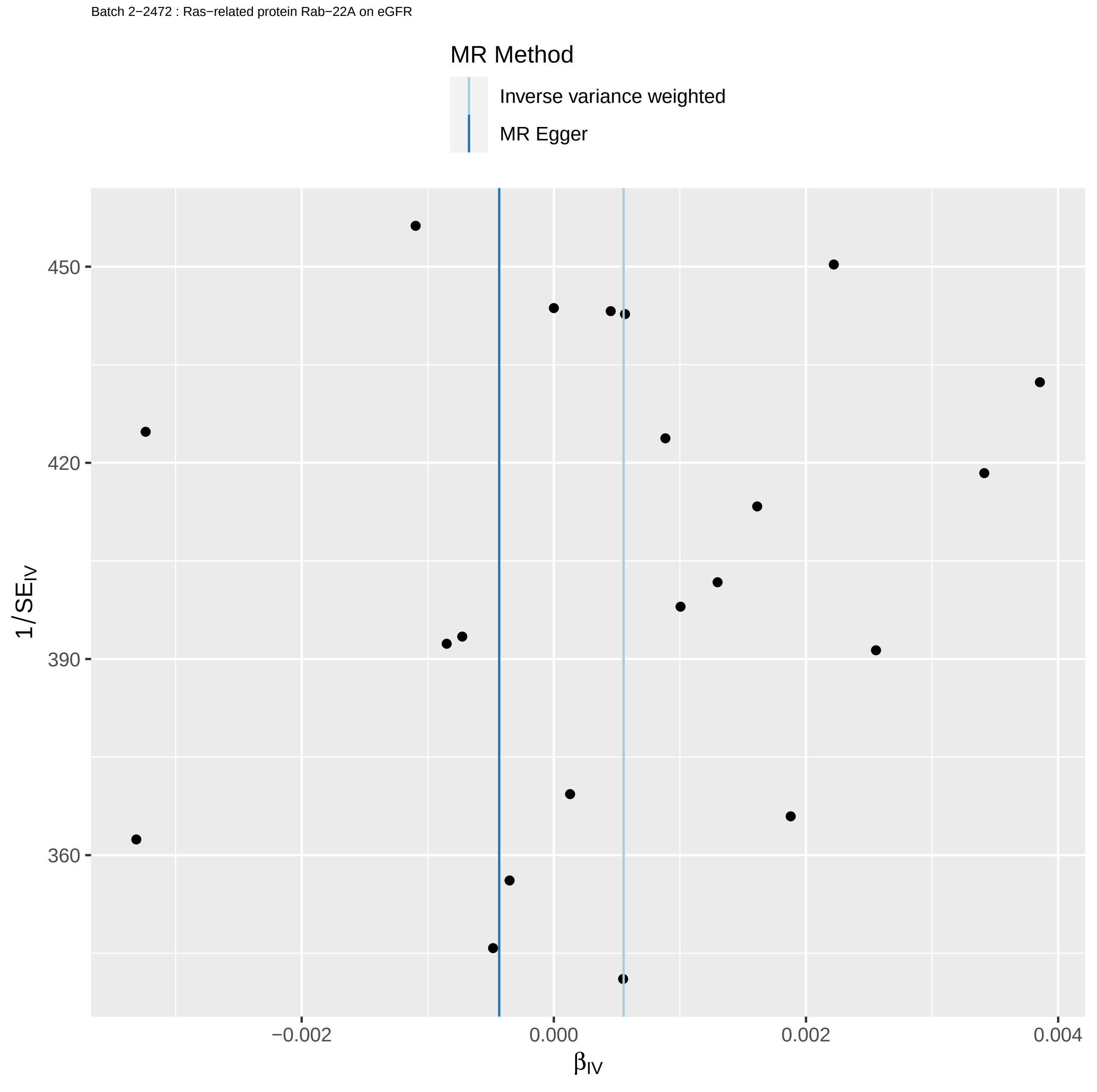

Batch 2−2472 : Ras−related protein Rab−22A on eGFR
MR Method
Inverse variance weighted
MR Egger
450
420
390
360
1/SE_IV
−0.002
0.000
0.002
0.004
β_IV

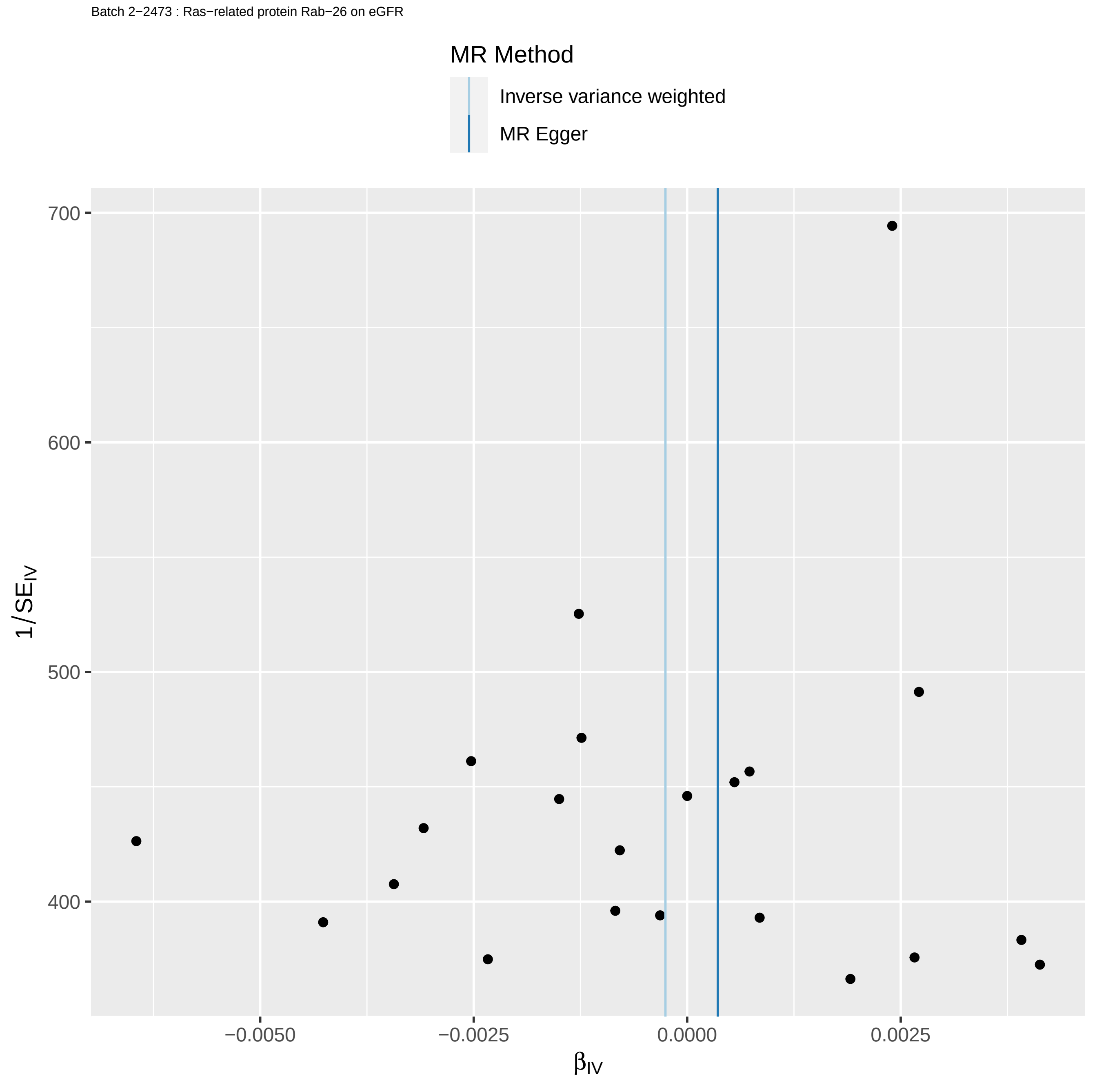

Batch 2−2473 : Ras−related protein Rab−26 on eGFR
MR Method
Inverse variance weighted
MR Egger
700
600
500
400
$1/SE_{IV}$
−0.0050
−0.0025
0.0000
0.0025
β_{IV}

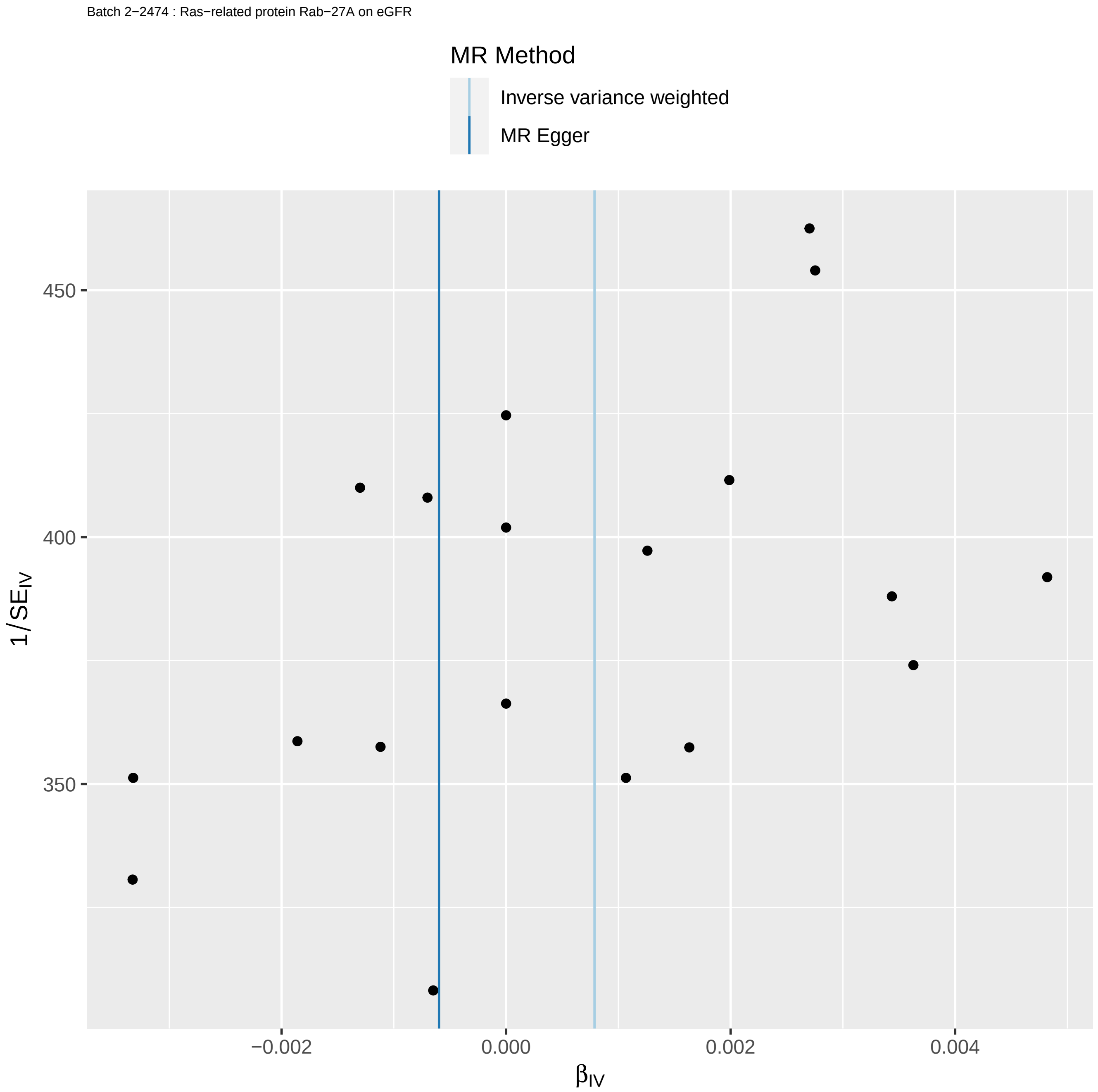

Batch 2−2474 : Ras−related protein Rab−27A on eGFR
MR Method
Inverse variance weighted
MR Egger
450
400
350
1/SE_IV
−0.002
0.000
0.002
0.004
β_IV

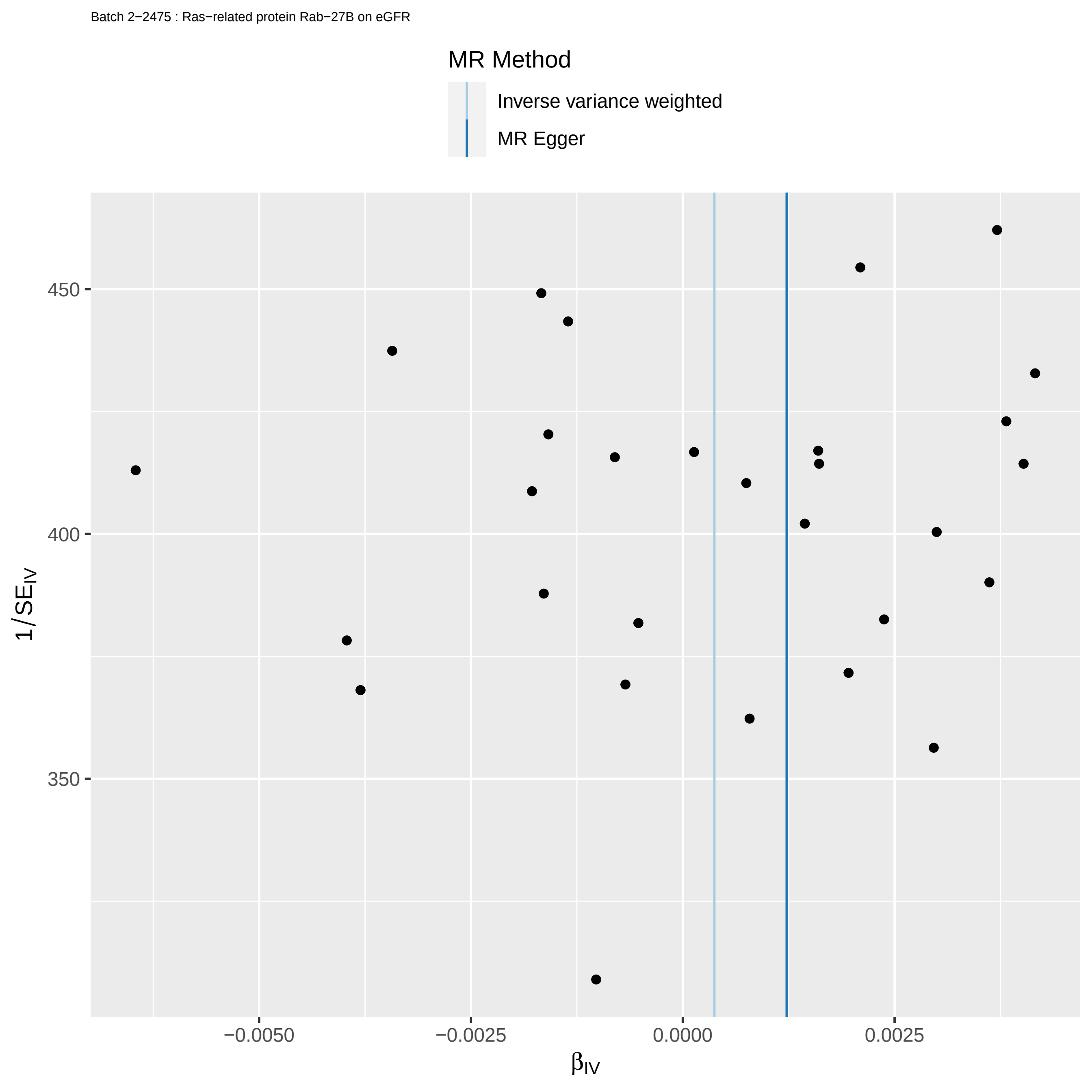

Batch 2−2475 : Ras−related protein Rab−27B on eGFR
MR Method
Inverse variance weighted
MR Egger
450
400
350
1/SE_IV
−0.0050
−0.0025
0.0000
0.0025
β_IV

Batch 2−2476 : Ras−related protein Rab−35 on eGFR

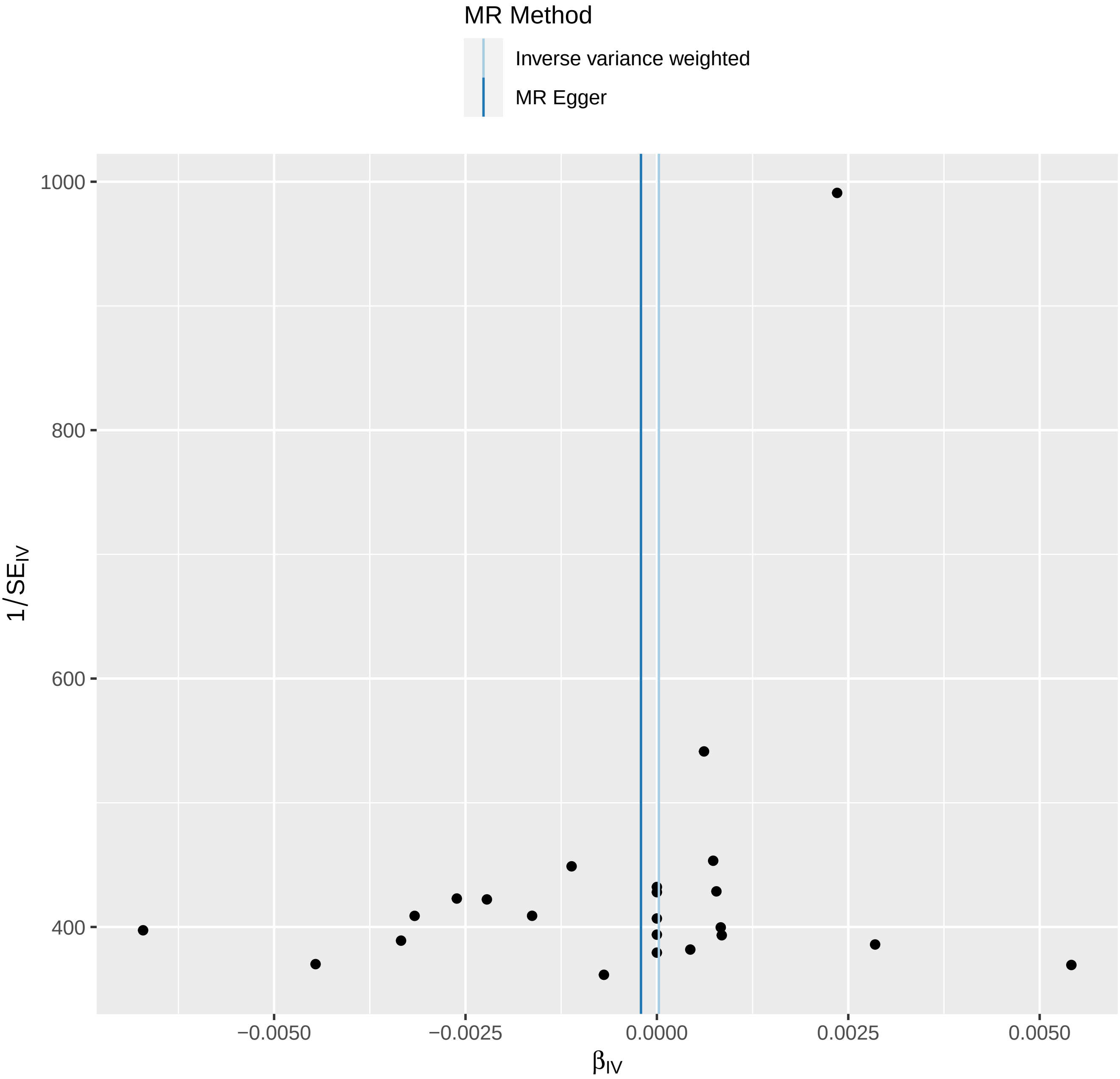

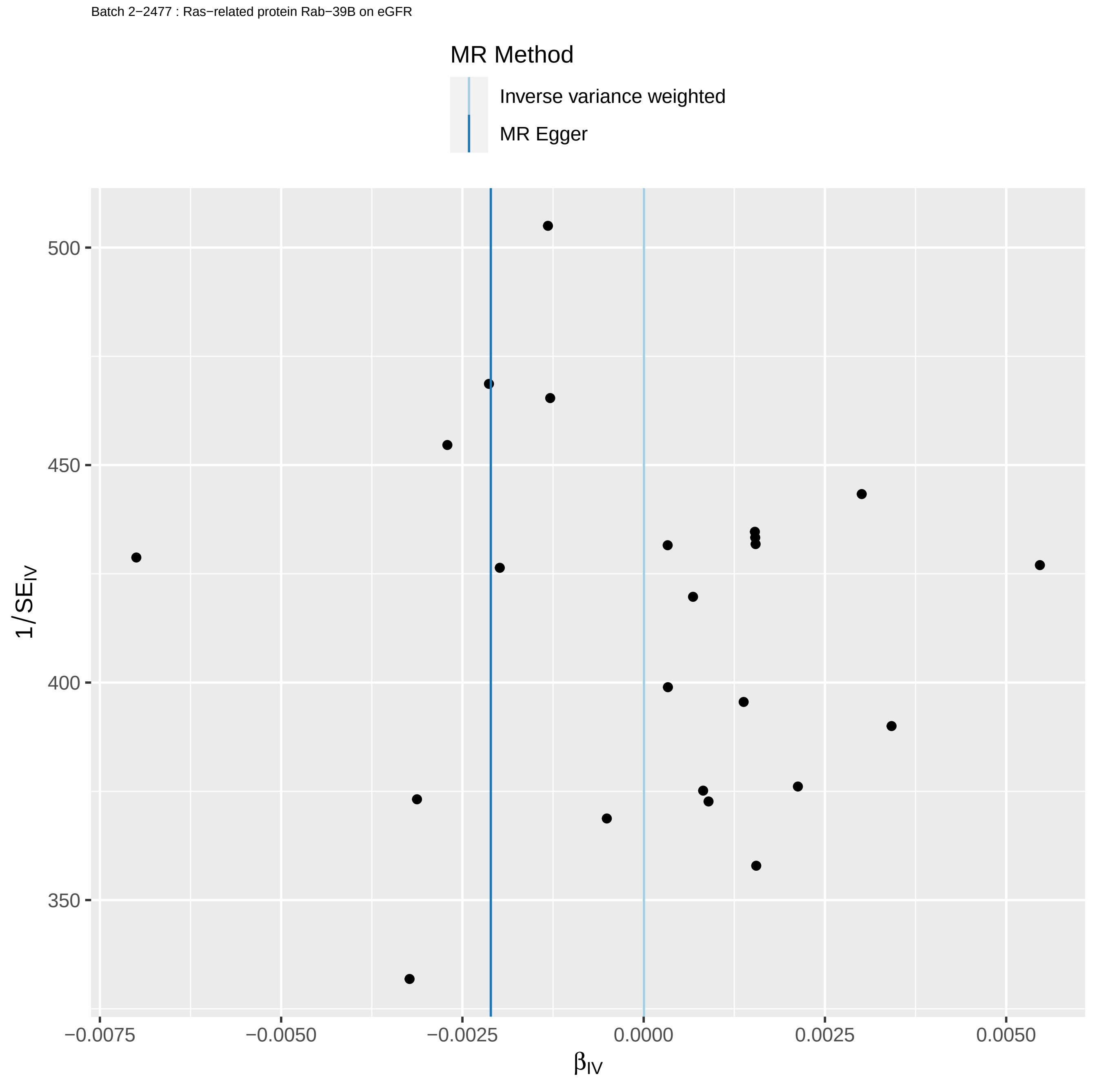

Batch 2−2477 : Ras−related protein Rab−39B on eGFR
MR Method
Inverse variance weighted
MR Egger
500
450
400
350
1/SE_IV
−0.0075
−0.0050
−0.0025
0.0000
0.0025
0.0050
β_IV

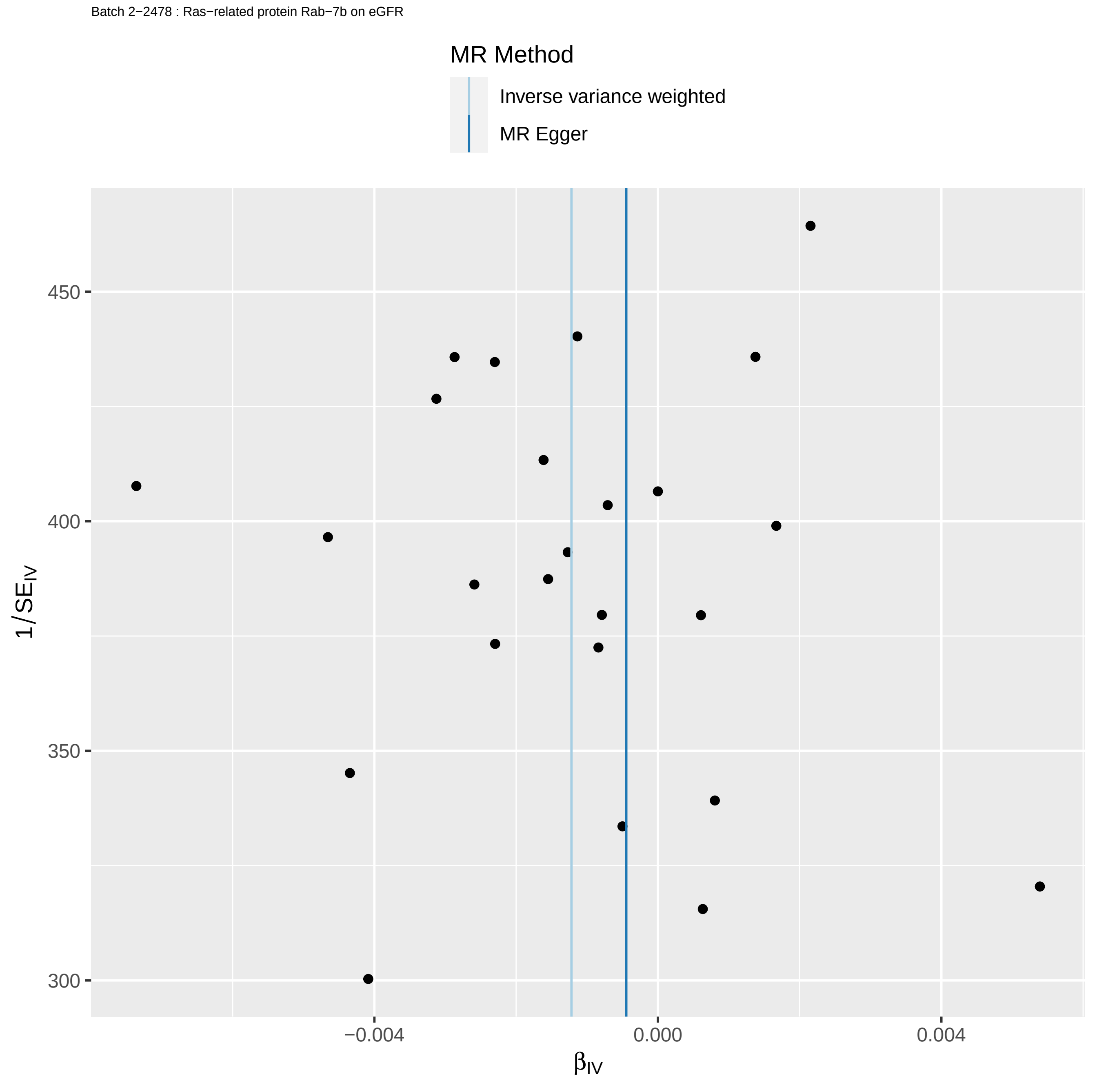

Batch 2−2478 : Ras−related protein Rab−7b on eGFR
MR Method
Inverse variance weighted
MR Egger
450
400
350
300
1/SE$_{IV}$
−0.004
0.000
0.004
β$_{IV}$

Batch 2−2479 : Rab9 effector protein with kelch motifs on eGFR

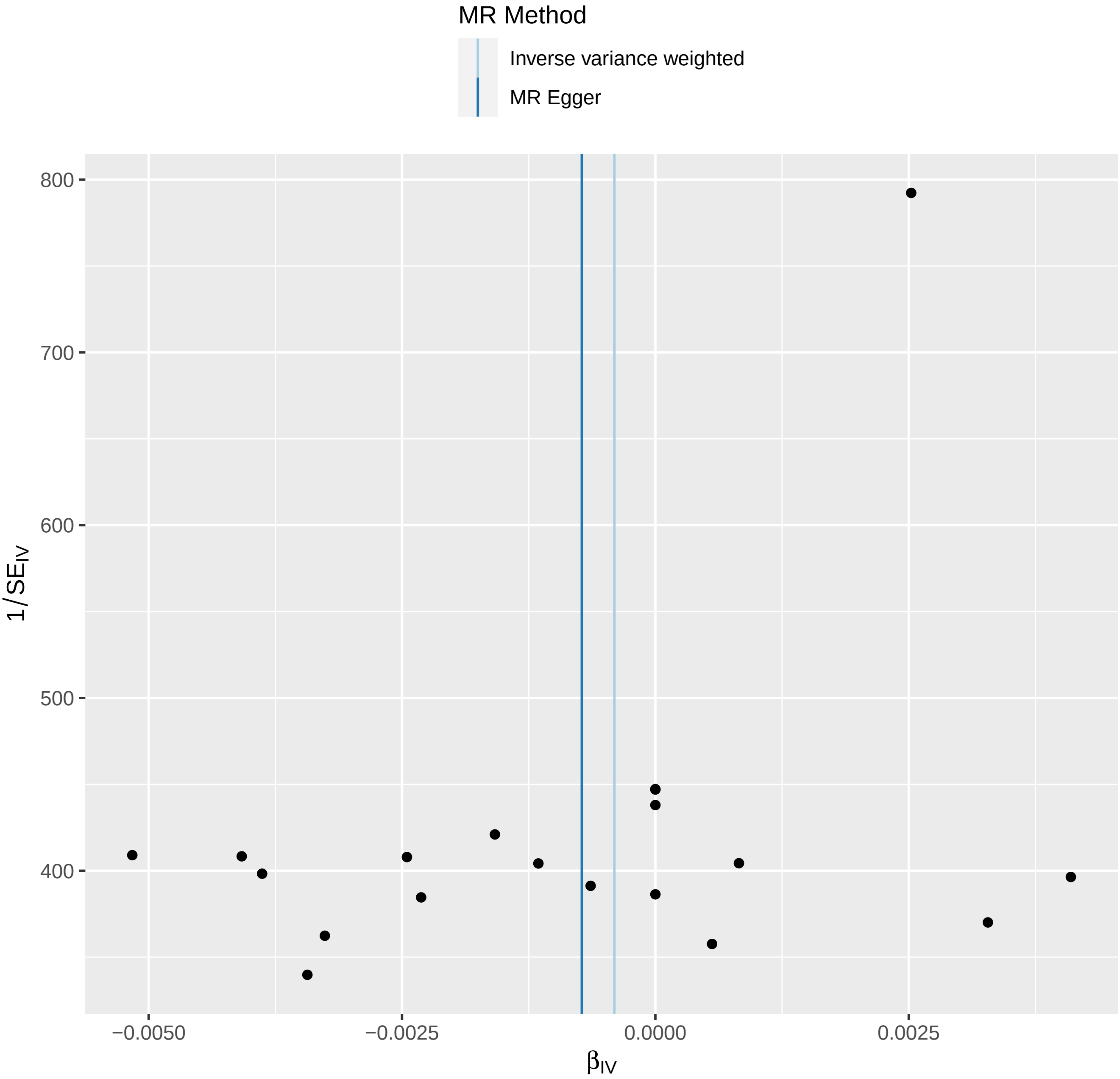

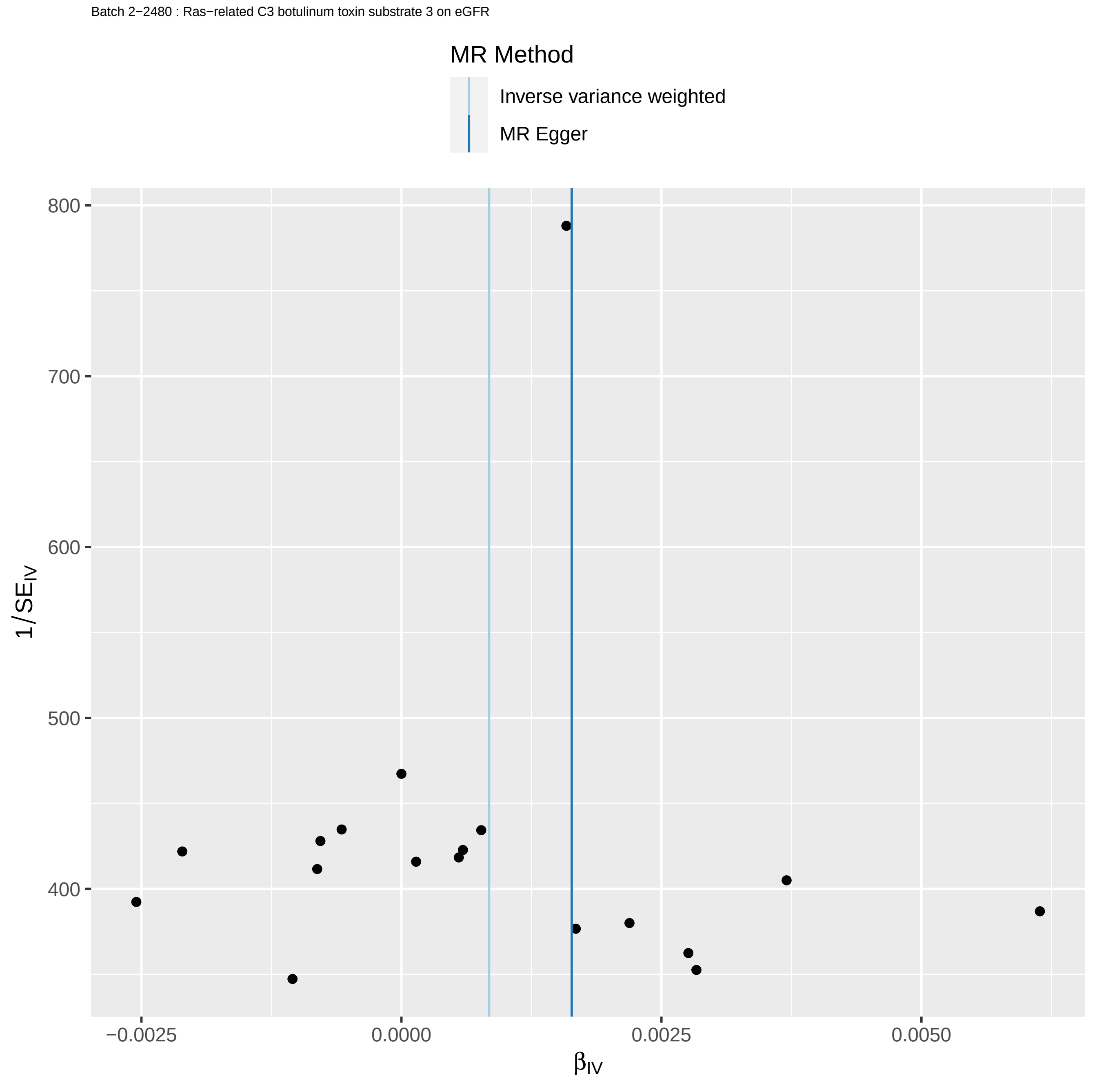
Batch 2−2480 : Ras−related C3 botulinum toxin substrate 3 on eGFR
MR Method
Inverse variance weighted
MR Egger
800
700
600
500
400
1/SE_IV
−0.0025
0.0000
0.0025
0.0050
β_IV

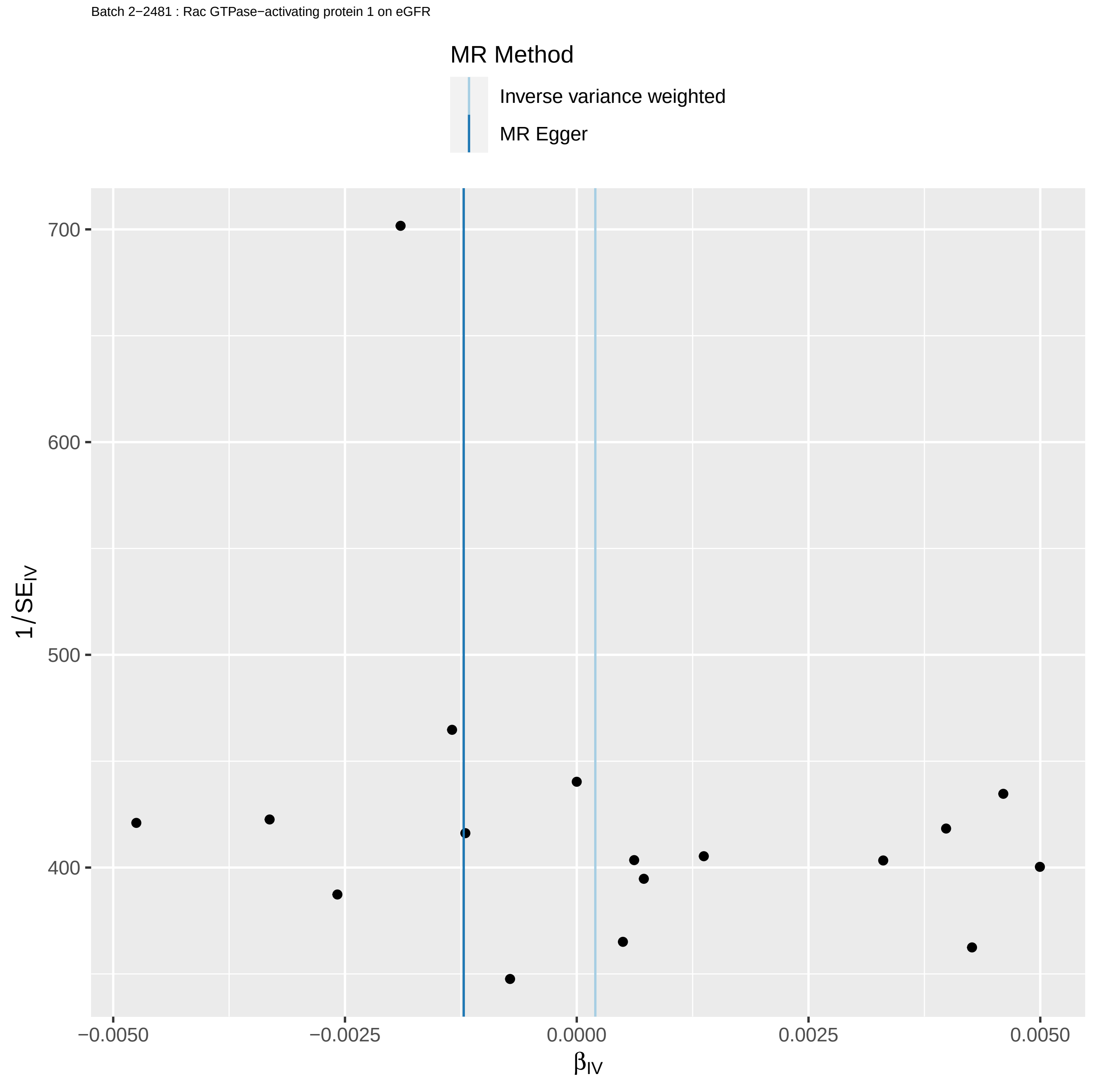

Batch 2−2481 : Rac GTPase−activating protein 1 on eGFR
MR Method
Inverse variance weighted
MR Egger
700
600
500
400
1/SE_IV
−0.0050
−0.0025
0.0000
0.0025
0.0050
β_IV

Batch 2−2482 : Cell cycle checkpoint protein RAD1 on eGFR

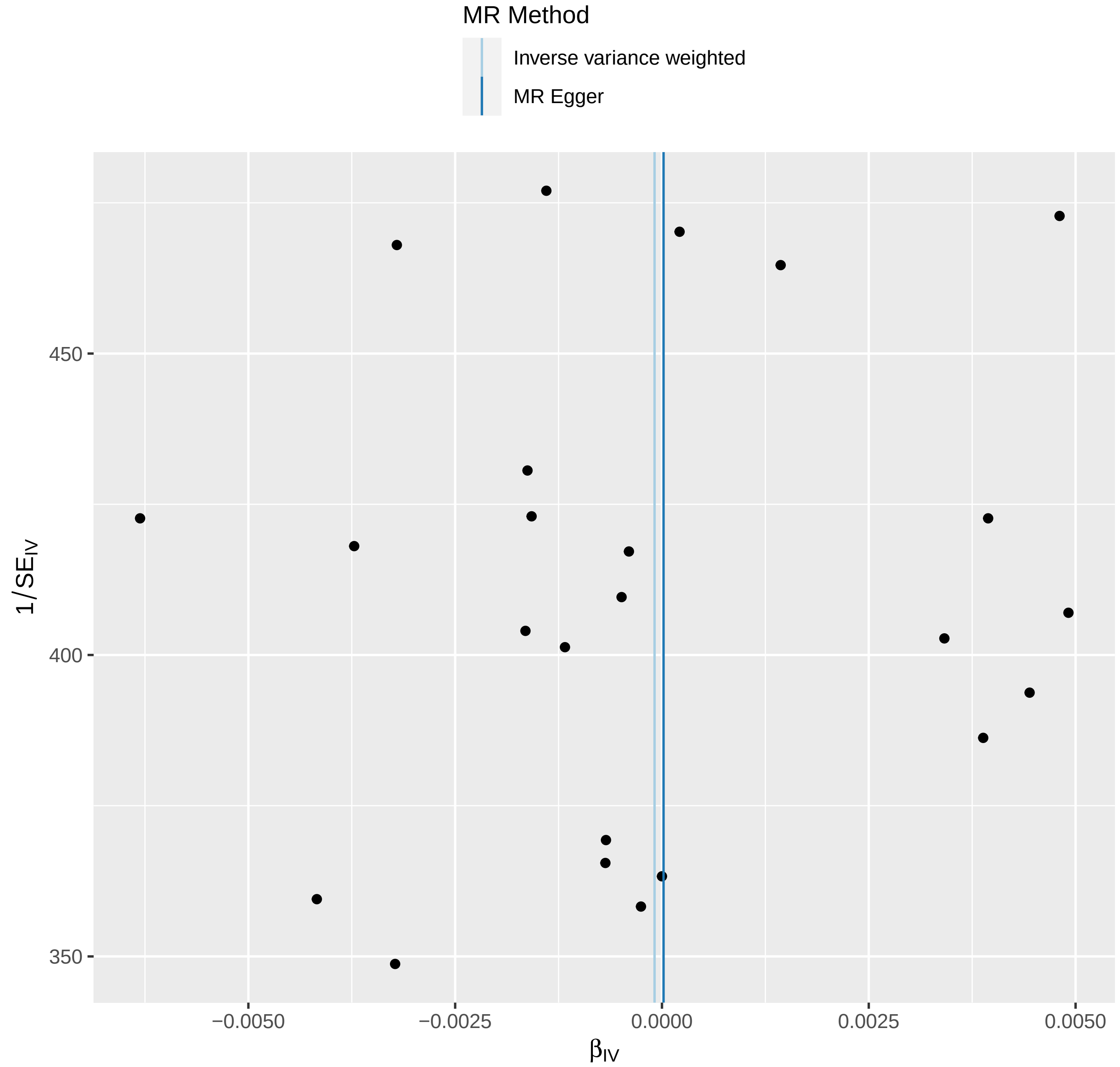

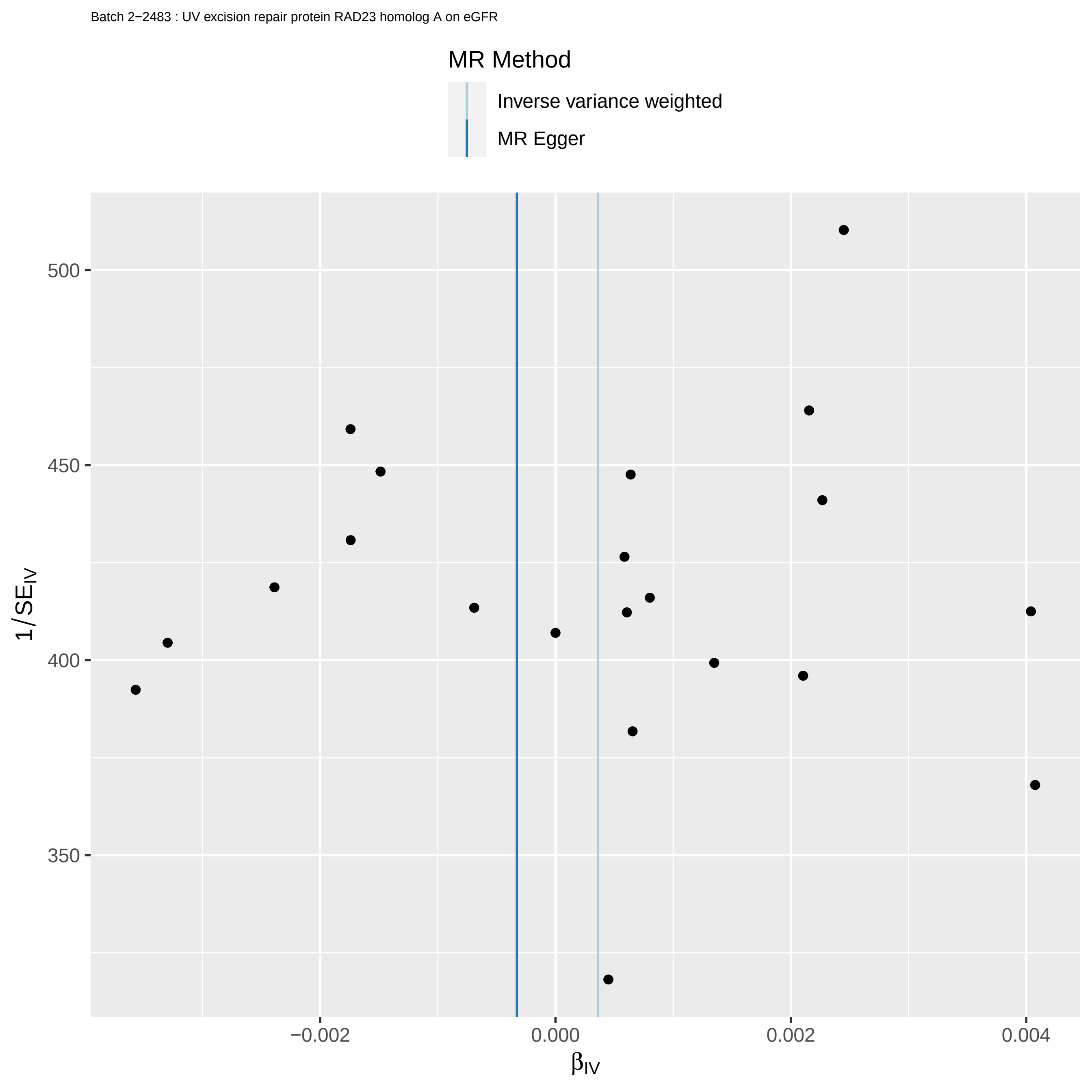

Batch 2−2483 : UV excision repair protein RAD23 homolog A on eGFR
MR Method
Inverse variance weighted
MR Egger
500
450
400
350
−0.002
0.000
0.002
0.004
$1/SE_{IV}$
β_{IV}

Batch 2−2484 : UV excision repair protein RAD23 homolog B on eGFR

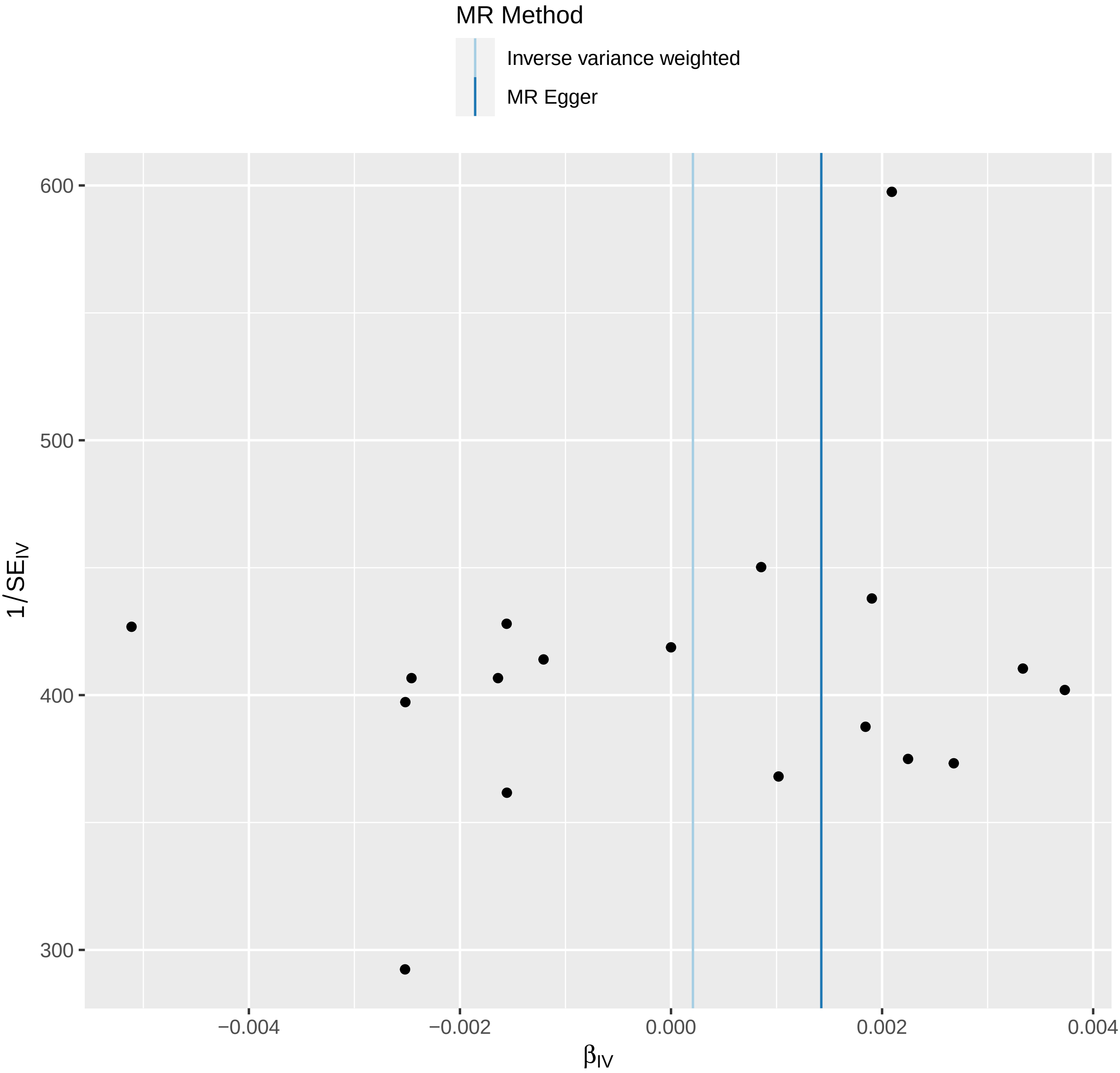

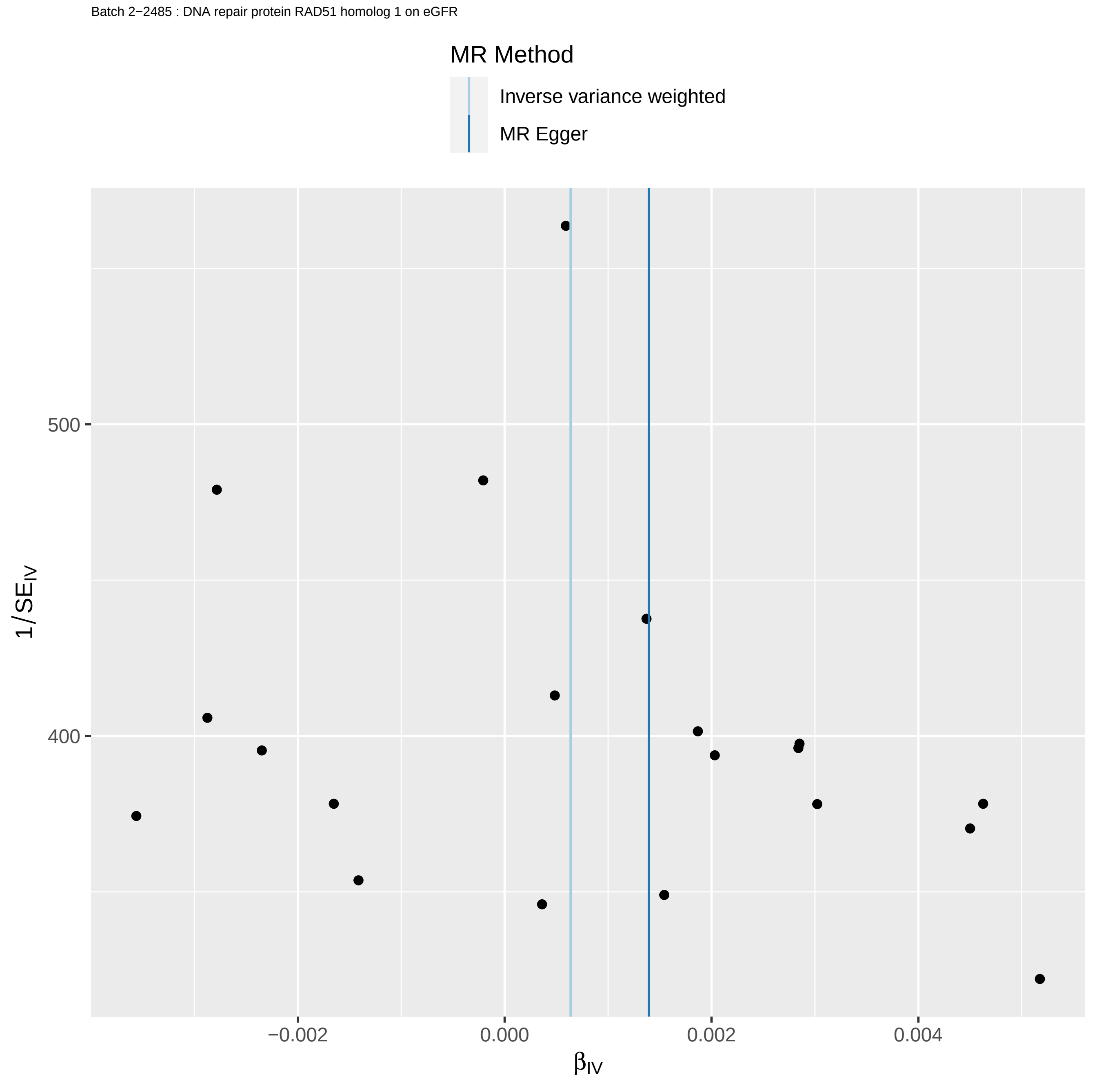

Batch 2−2485 : DNA repair protein RAD51 homolog 1 on eGFR
MR Method
Inverse variance weighted
MR Egger
500
400
1/SE$_{IV}$
−0.002
0.000
0.002
0.004
β$_{IV}$

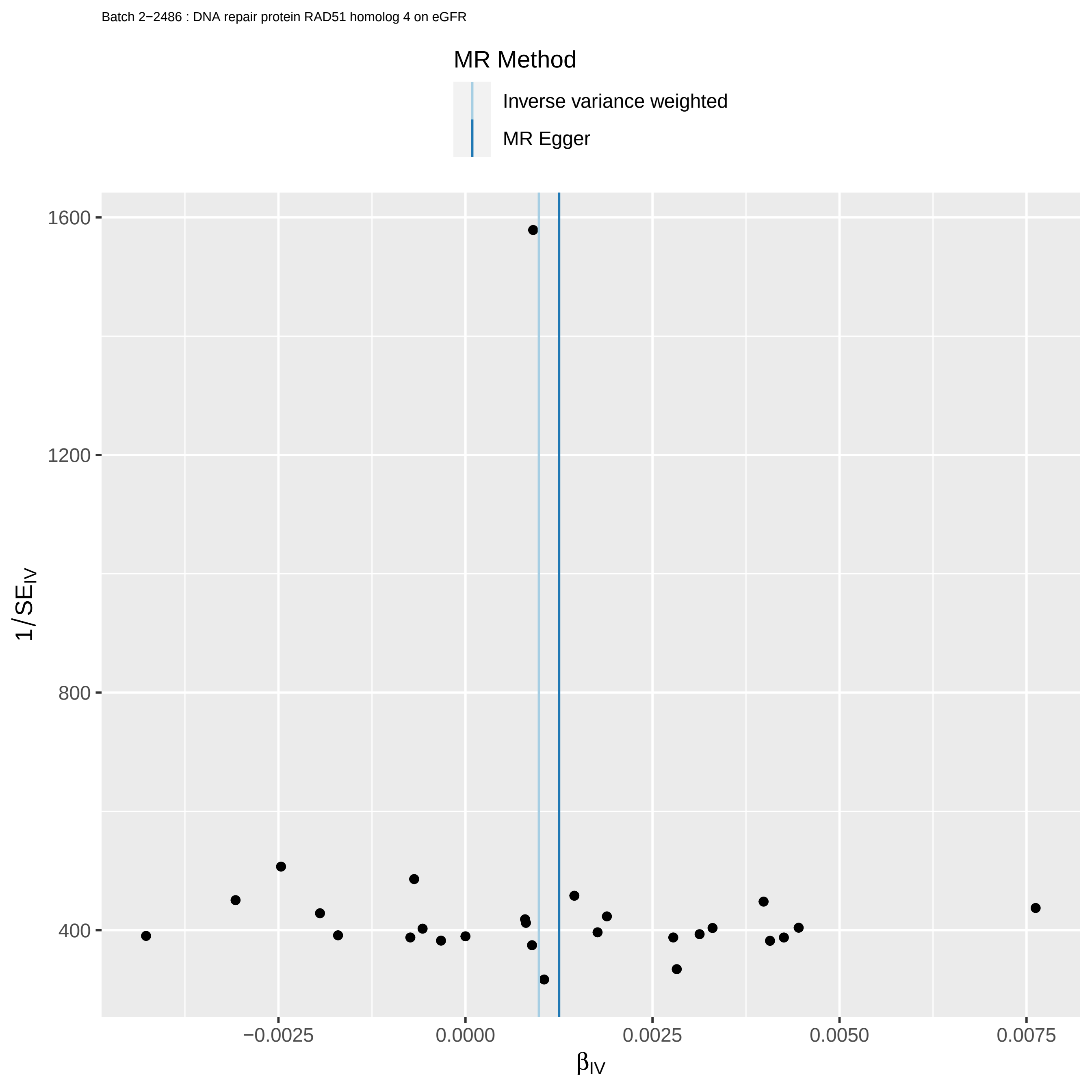

Batch 2−2486 : DNA repair protein RAD51 homolog 4 on eGFR
MR Method
Inverse variance weighted
MR Egger
1600
1200
800
400
1/SE_IV
−0.0025
0.0000
0.0025
0.0050
0.0075
β_IV

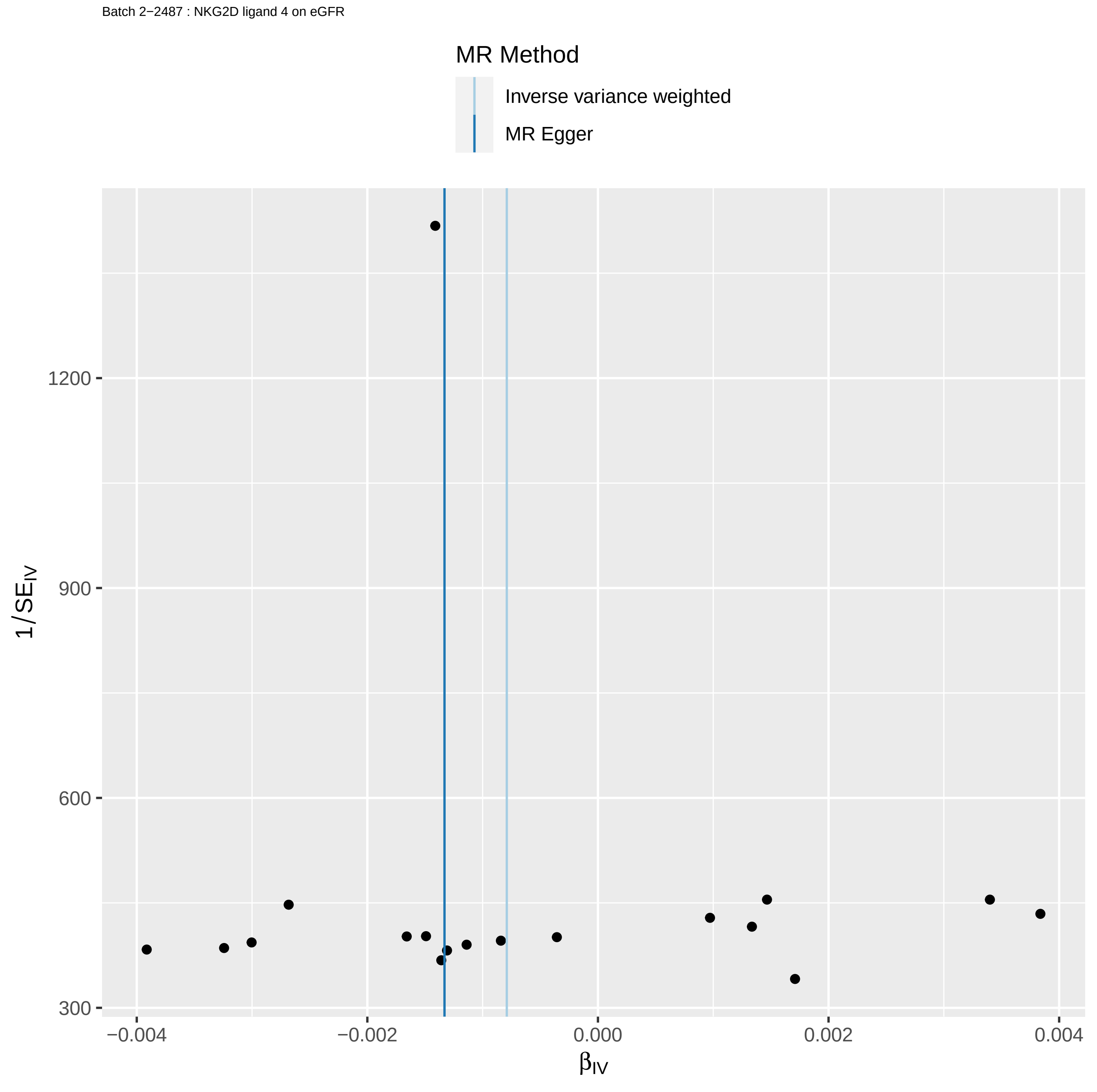

Batch 2−2487 : NKG2D ligand 4 on eGFR
MR Method
Inverse variance weighted
MR Egger
1200
900
600
300
1/SE_IV
−0.004
−0.002
0.000
0.002
0.004
β_IV

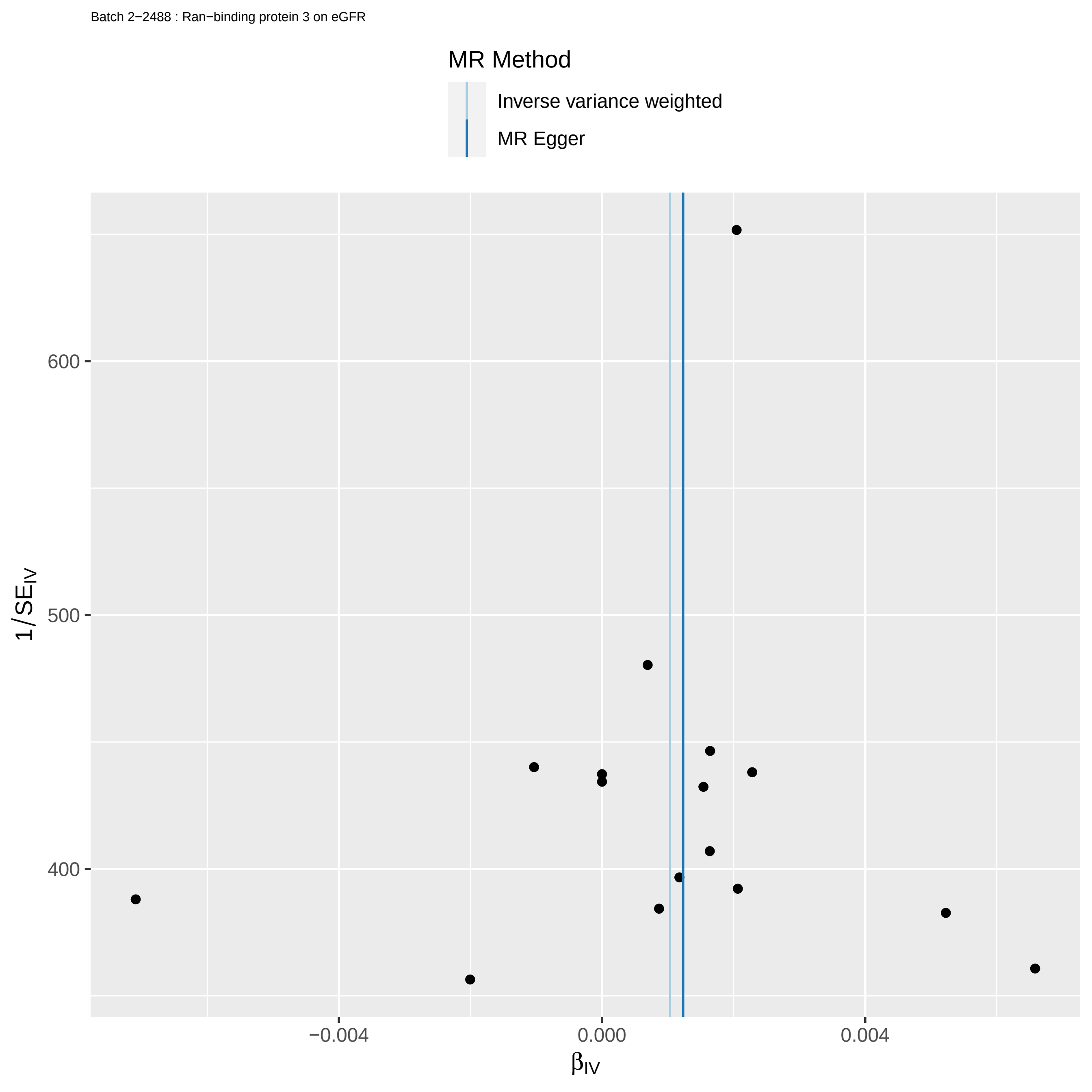

Batch 2−2488 : Ran−binding protein 3 on eGFR
MR Method
Inverse variance weighted
MR Egger
600
500
400
1/SE$_{IV}$
−0.004
0.000
0.004
β$_{IV}$

Batch 2−2489 : Rap1 GTPase−activating protein 1 on eGFR

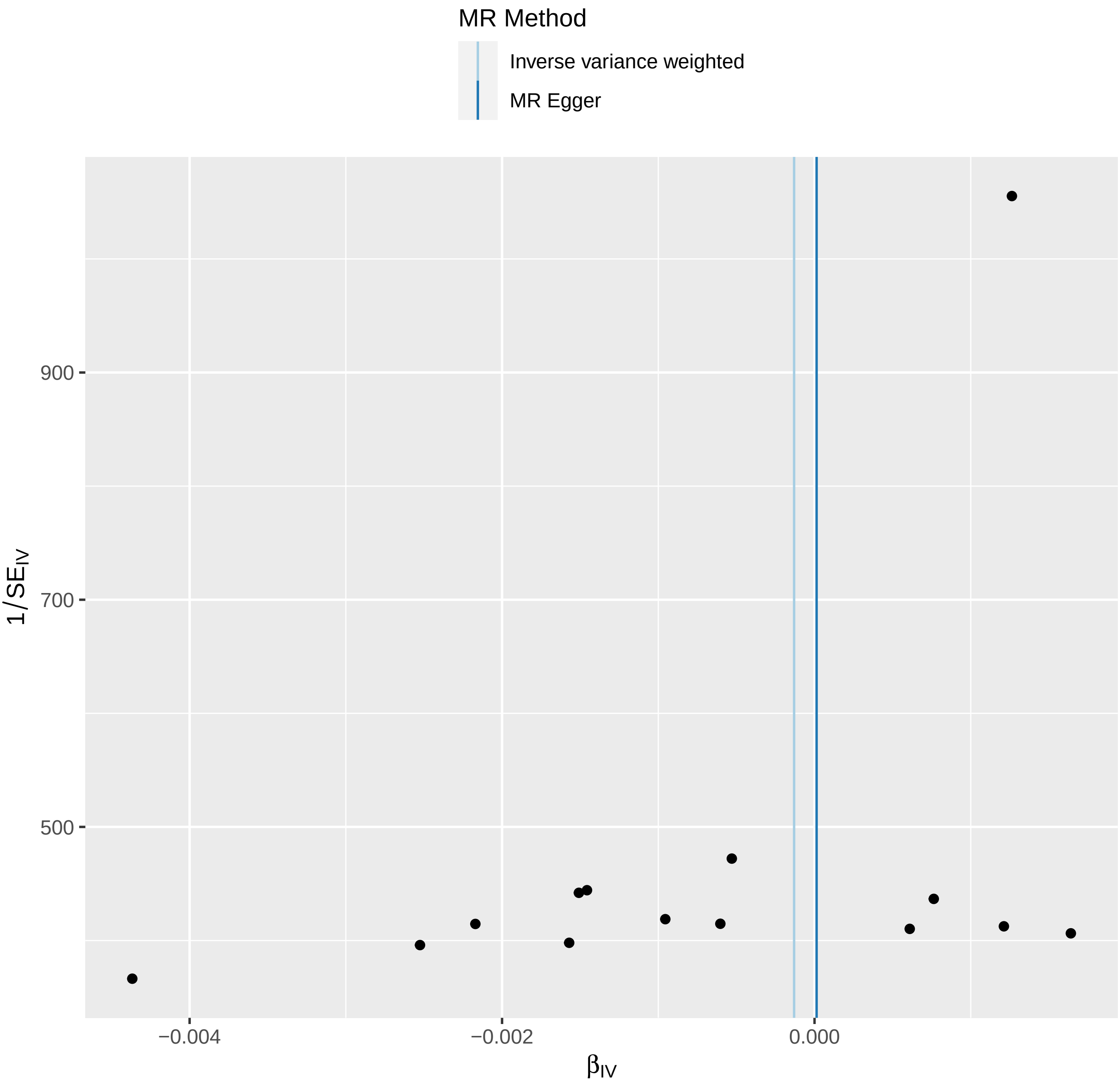

Batch 2−2490 : Rap1 GTPase−GDP dissociation stimulator 1 on eGFR

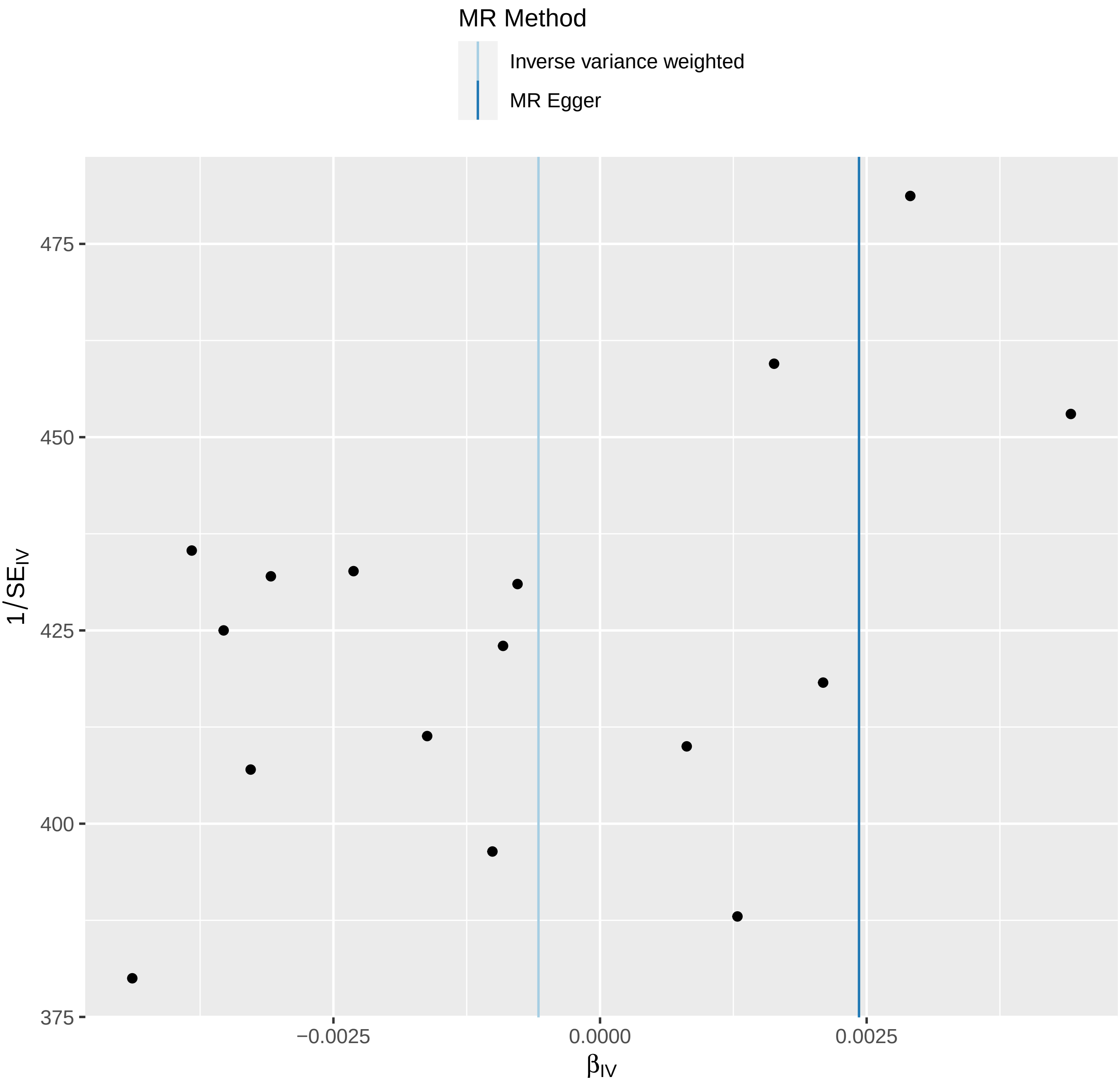

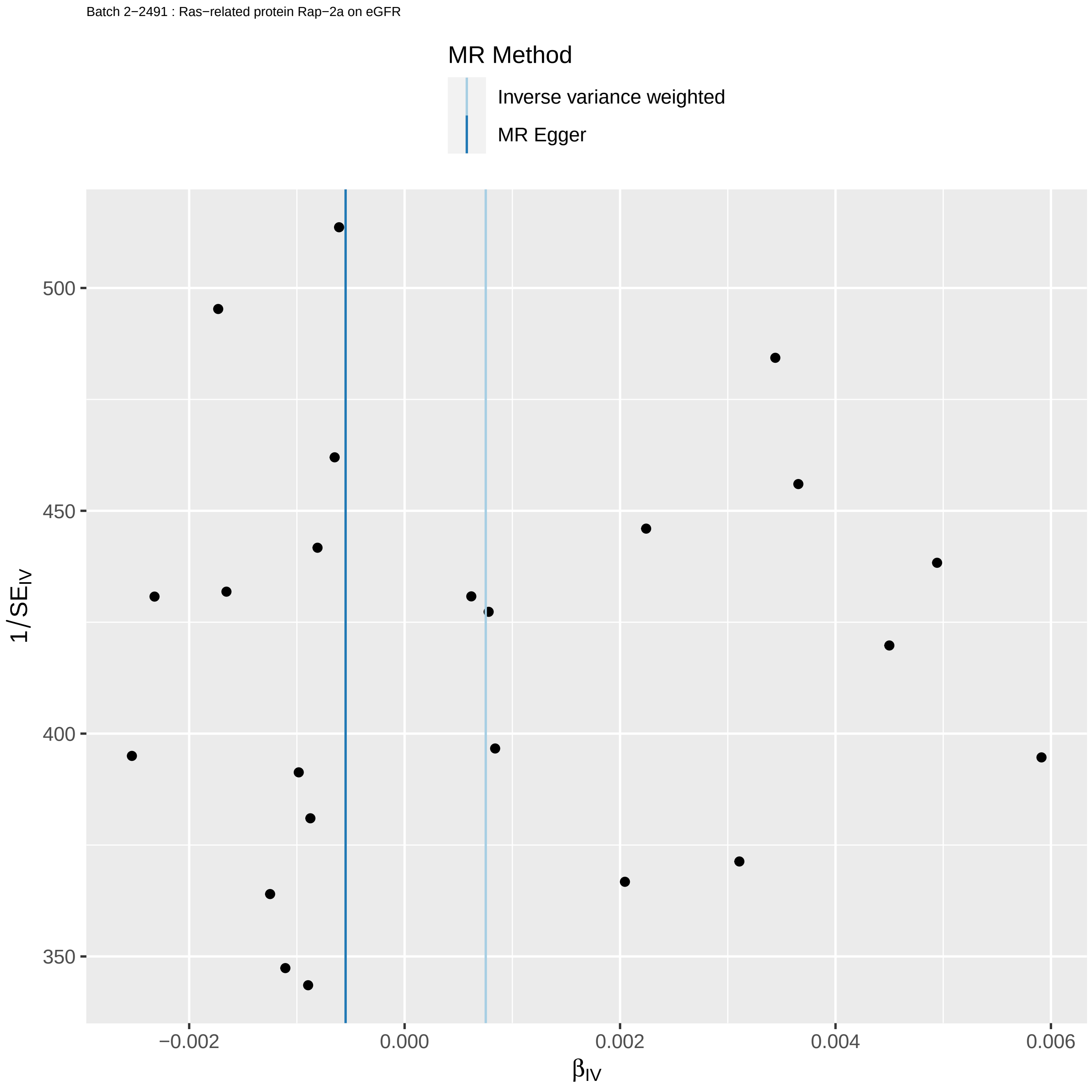
Batch 2−2491 : Ras−related protein Rap−2a on eGFR
MR Method
Inverse variance weighted
MR Egger
500
450
400
350
1/SE_IV
−0.002
0.000
0.002
0.004
0.006
β_IV

Batch 2−2492 : Rap guanine nucleotide exchange factor 5 on eGFR

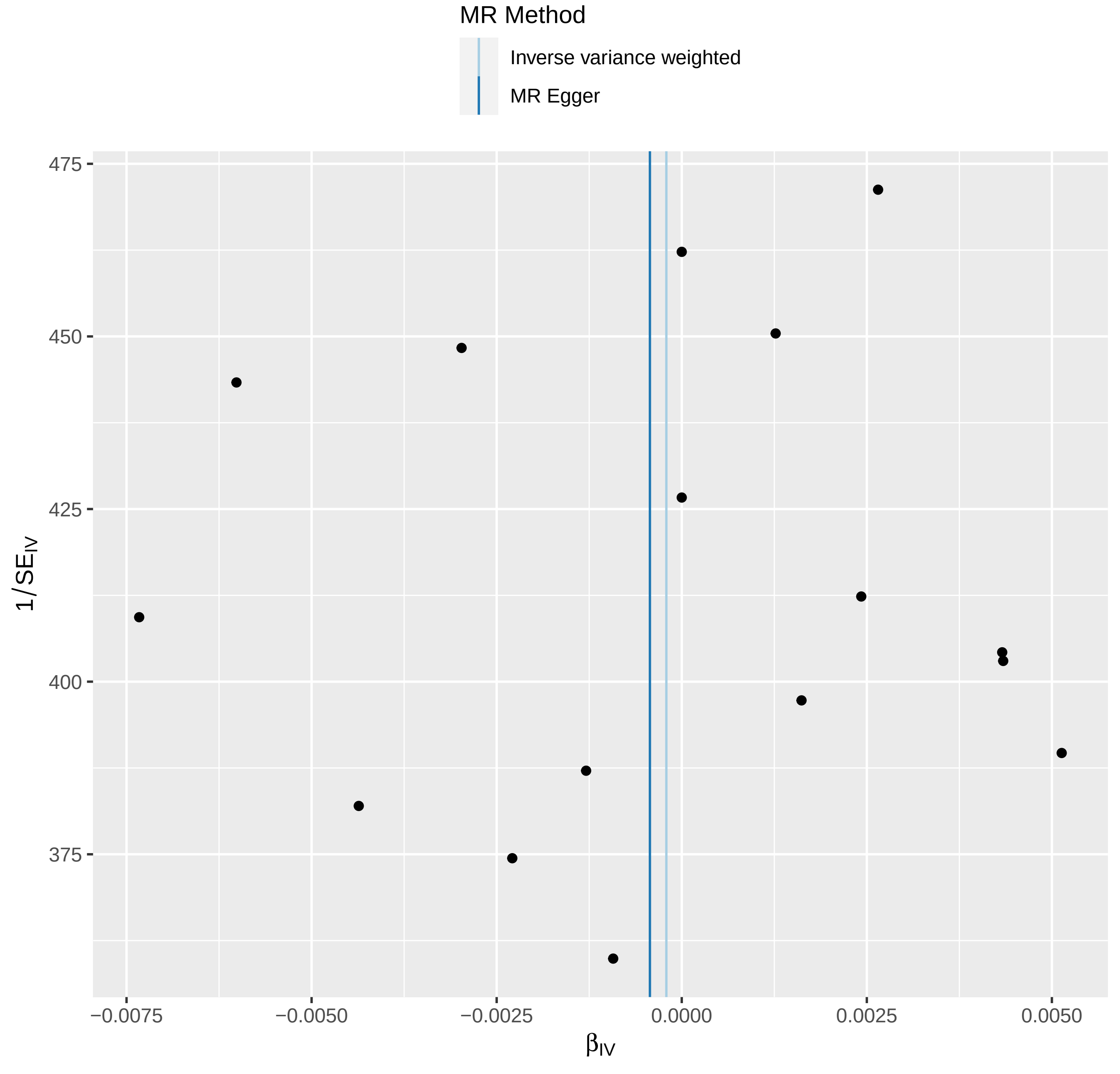

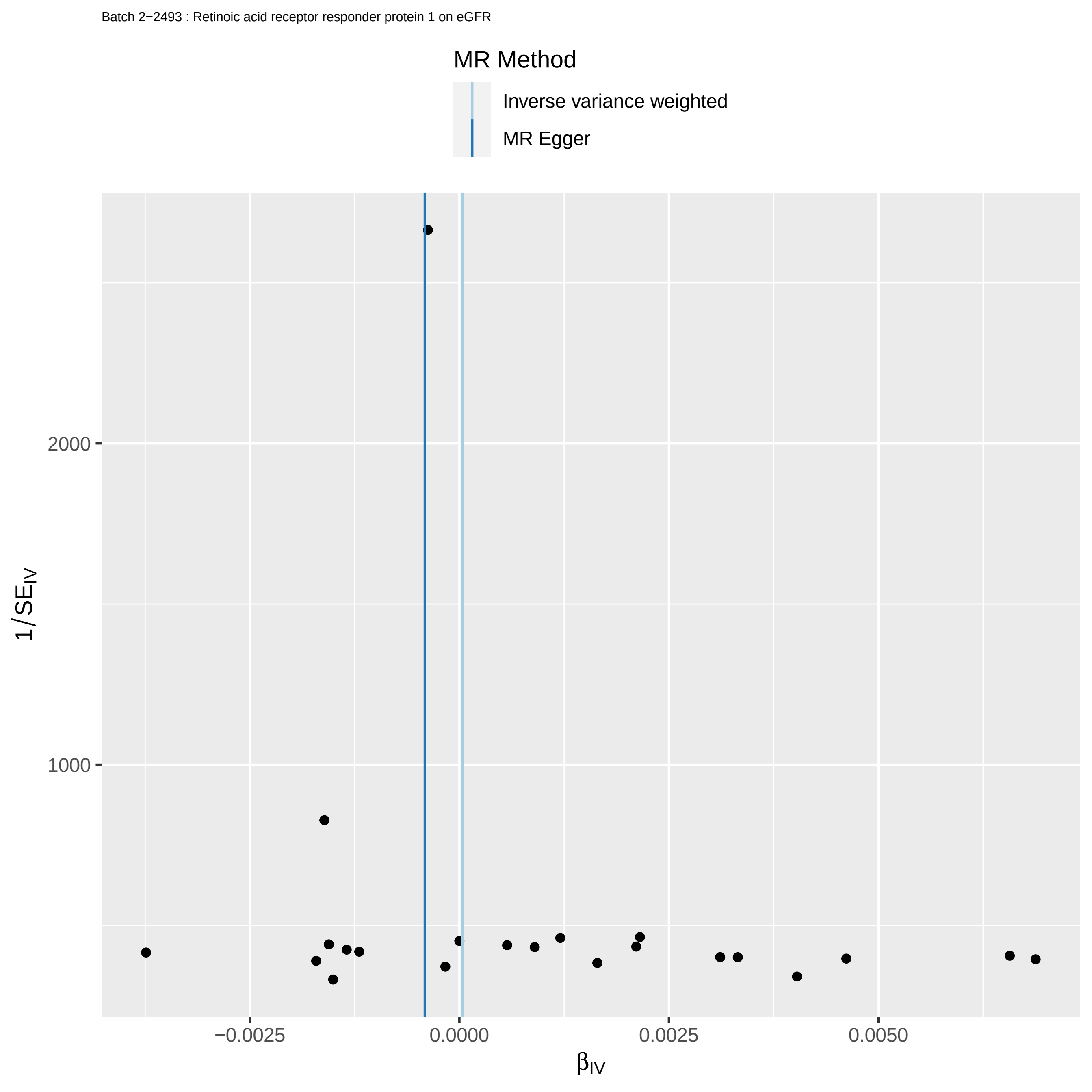

Batch 2−2493 : Retinoic acid receptor responder protein 1 on eGFR
MR Method
Inverse variance weighted
MR Egger
2000
1000
1/SE_IV
−0.0025
0.0000
0.0025
0.0050
β_IV

Batch 2−2494 : Retinoic acid receptor responder protein 3 on eGFR

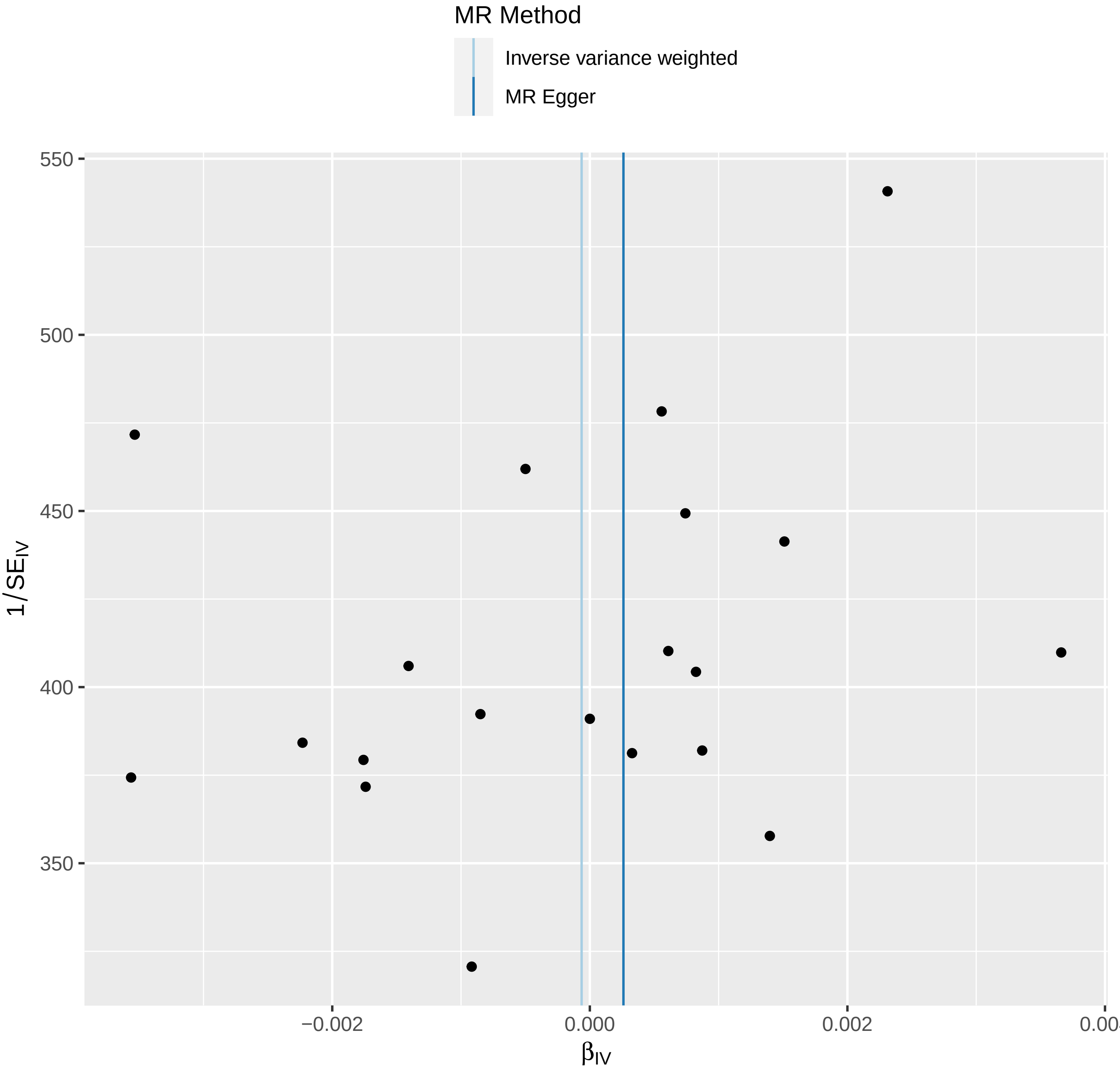

Batch 2−2495 : Ras GTPase−activating protein 1 on eGFR

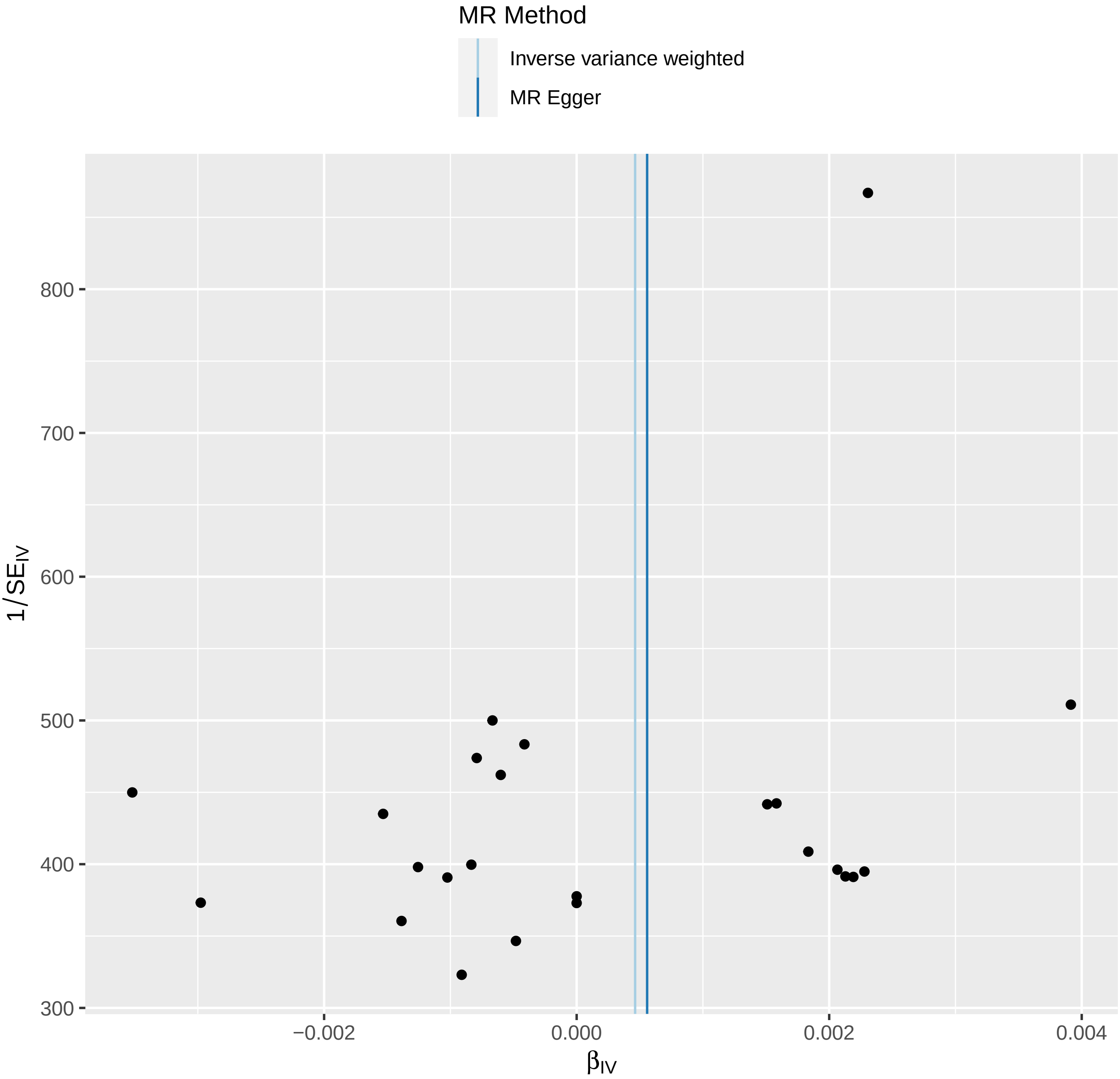

Batch 2−2496 : Retinoblastoma−associated protein on eGFR

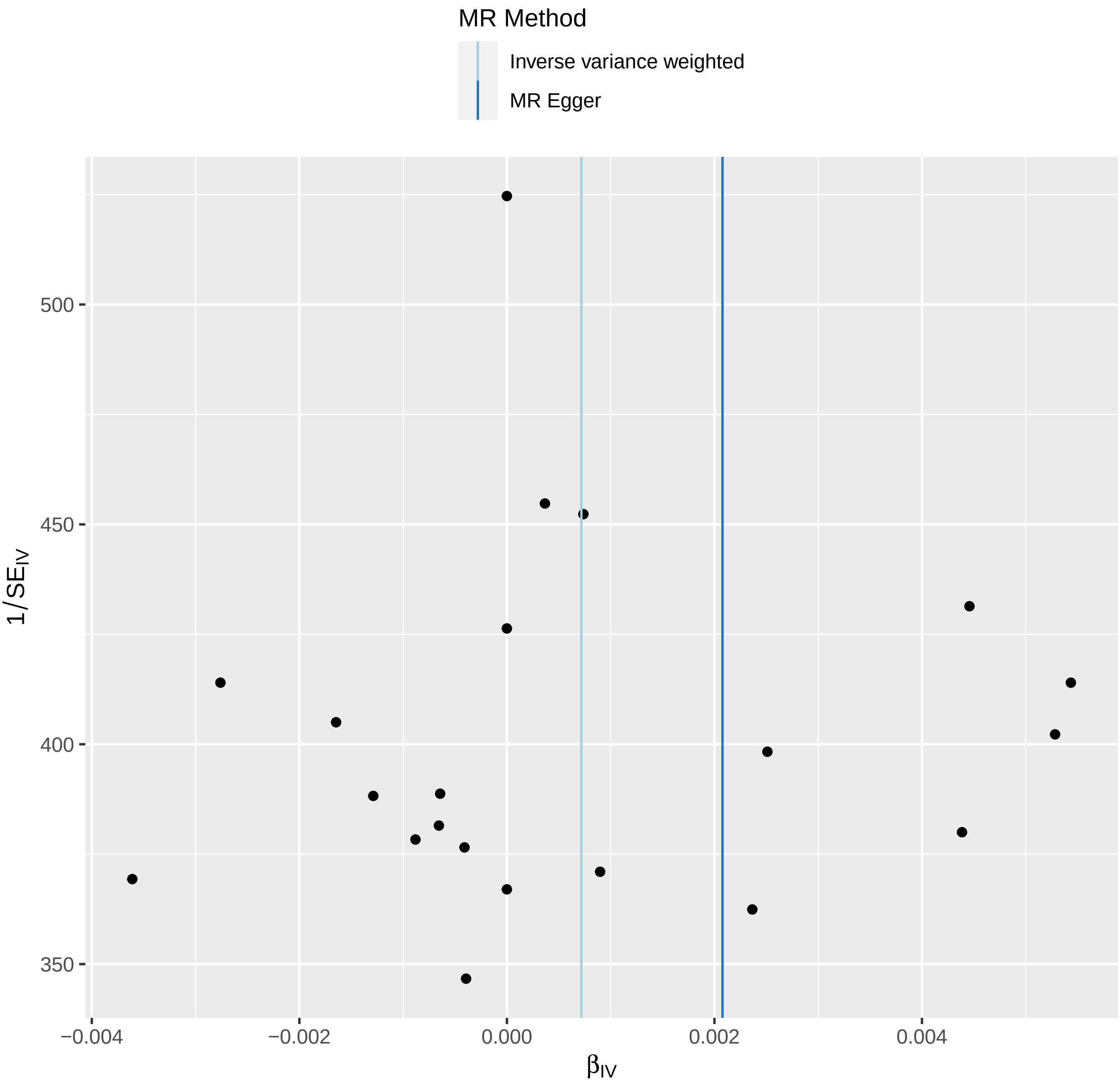

Batch 2−2497 : Retinoblastoma−binding protein 5 on eGFR

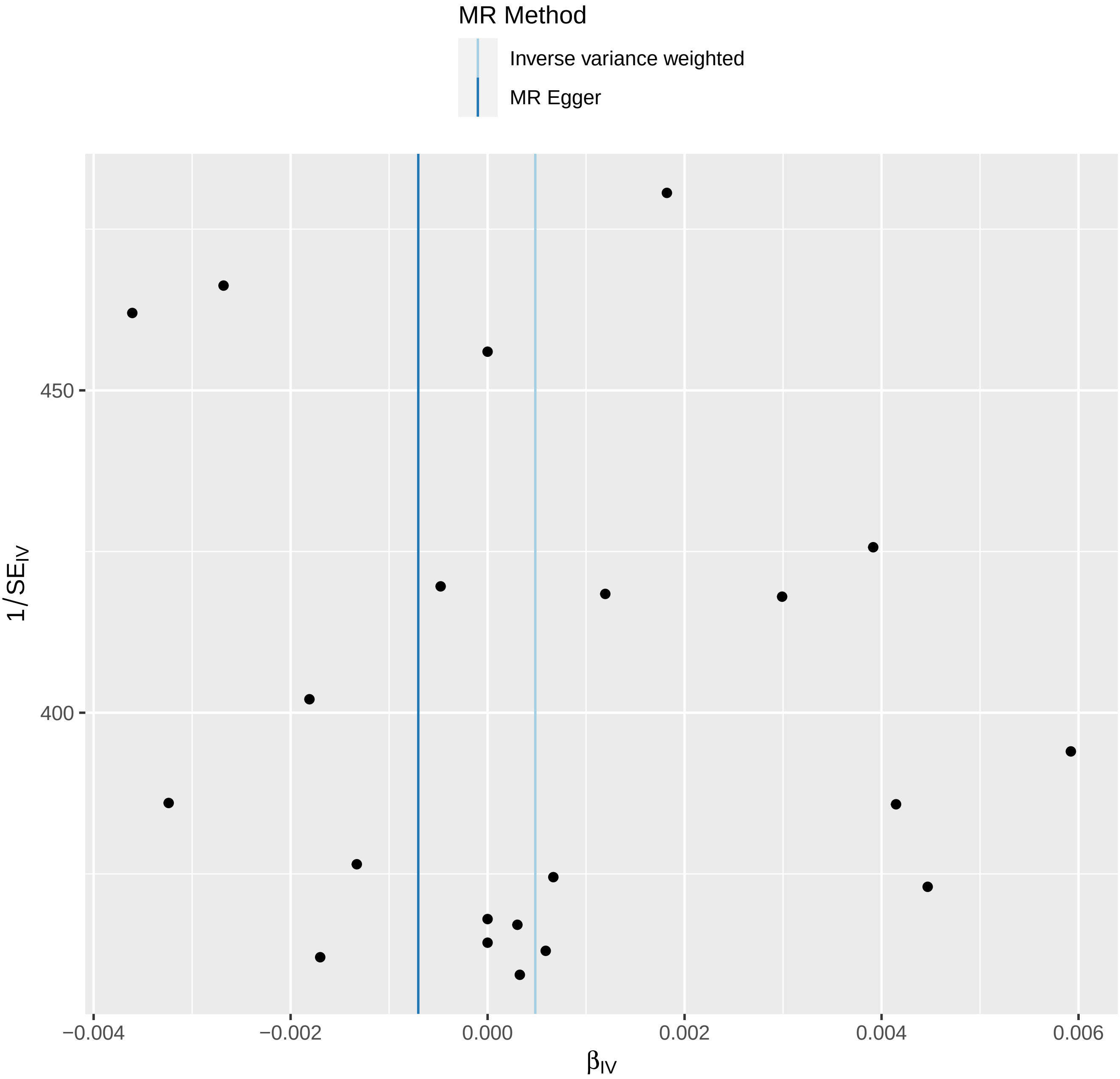

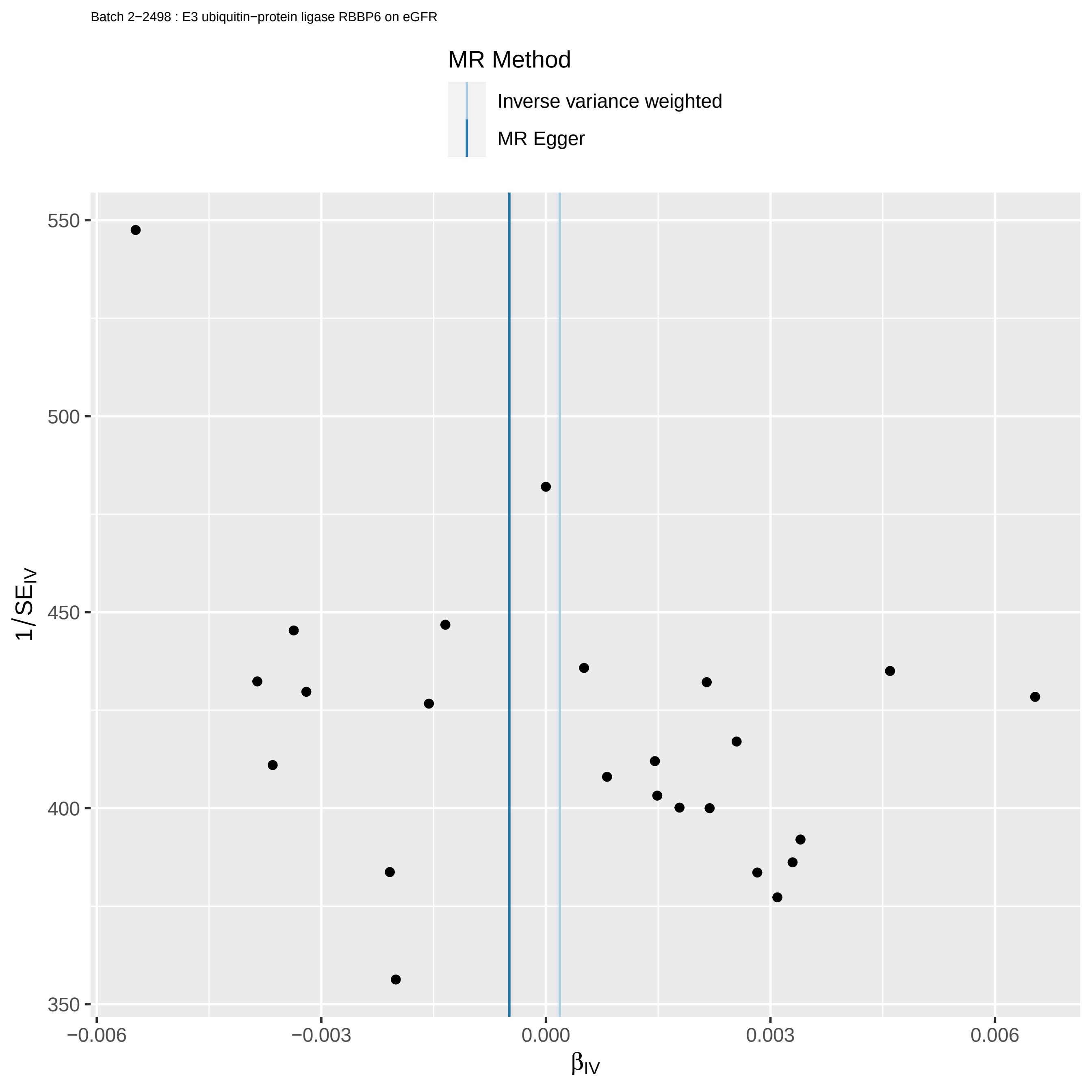

Batch 2−2498 : E3 ubiquitin−protein ligase RBBP6 on eGFR
MR Method
Inverse variance weighted
MR Egger
1/SE_IV
550
500
450
400
350
−0.006
−0.003
0.000
0.003
0.006
β_IV

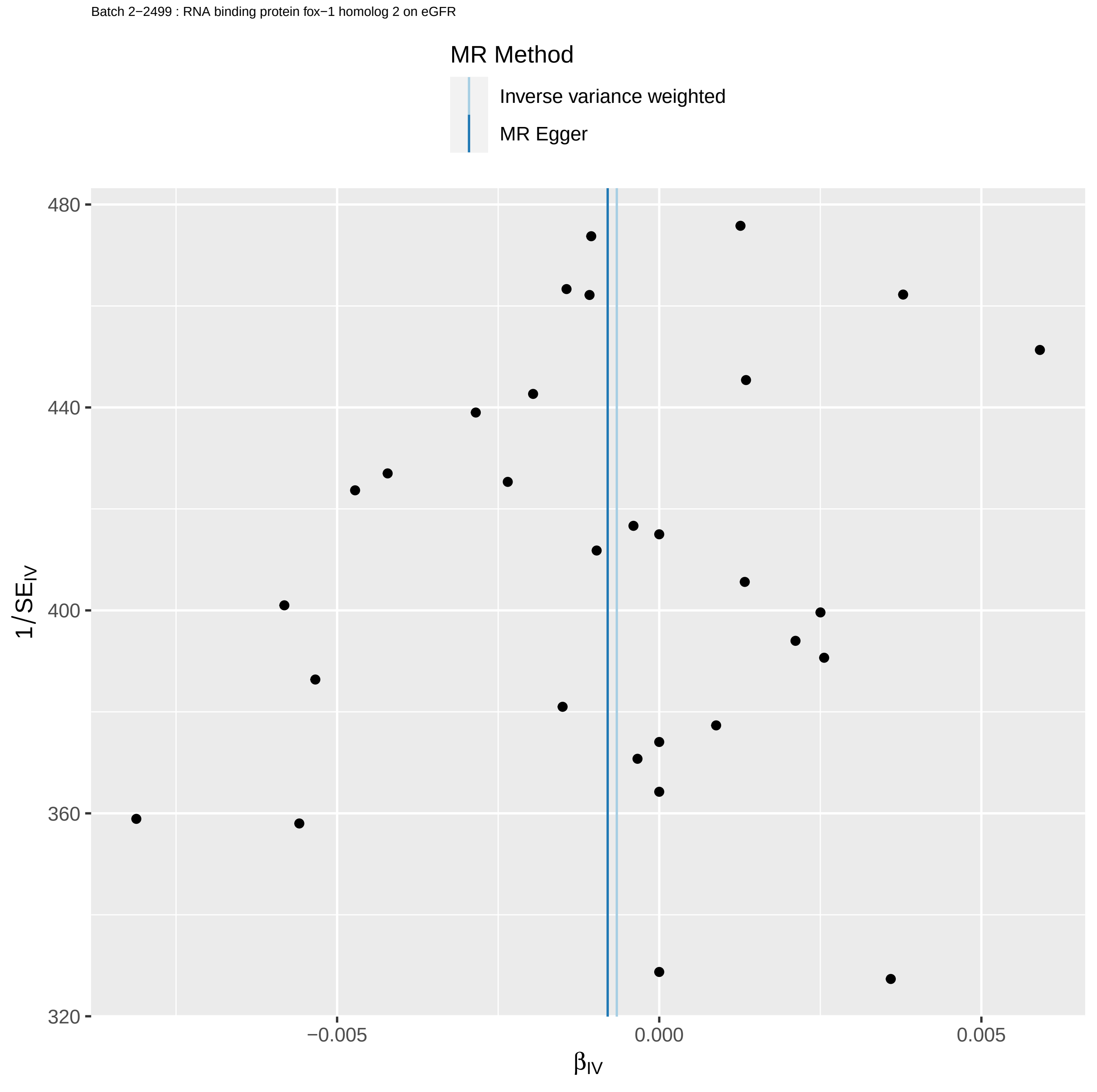
Batch 2−2499 : RNA binding protein fox−1 homolog 2 on eGFR
MR Method
Inverse variance weighted
MR Egger
480
440
400
360
320
1/SE_IV
−0.005
0.000
0.005
β_IV

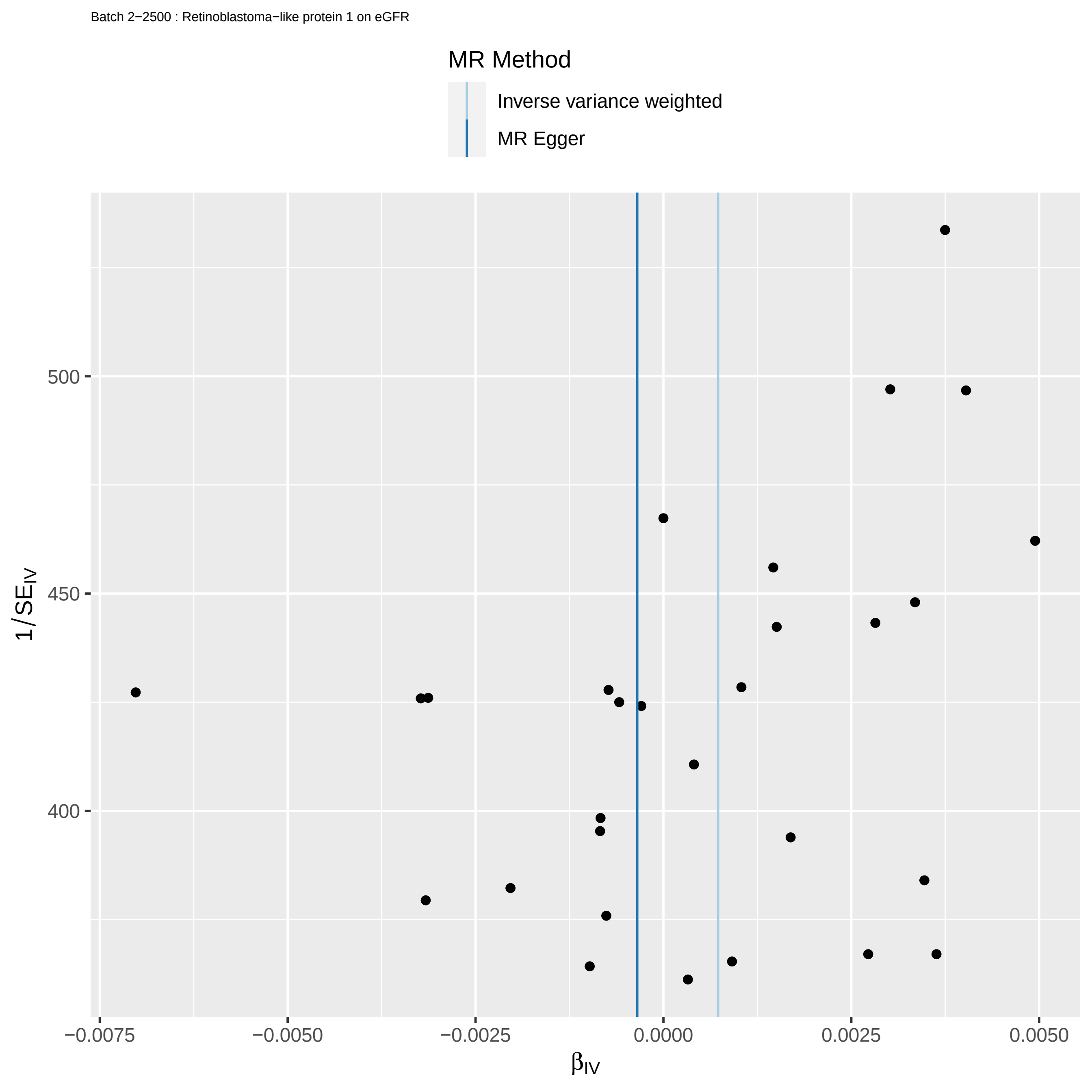

Batch 2−2500 : Retinoblastoma−like protein 1 on eGFR
MR Method
Inverse variance weighted
MR Egger
500
450
400
1/SE_IV
−0.0075
−0.0050
−0.0025
0.0000
0.0025
0.0050
β_IV

Batch 2−2501 : Probable RNA−binding protein 19 on eGFR

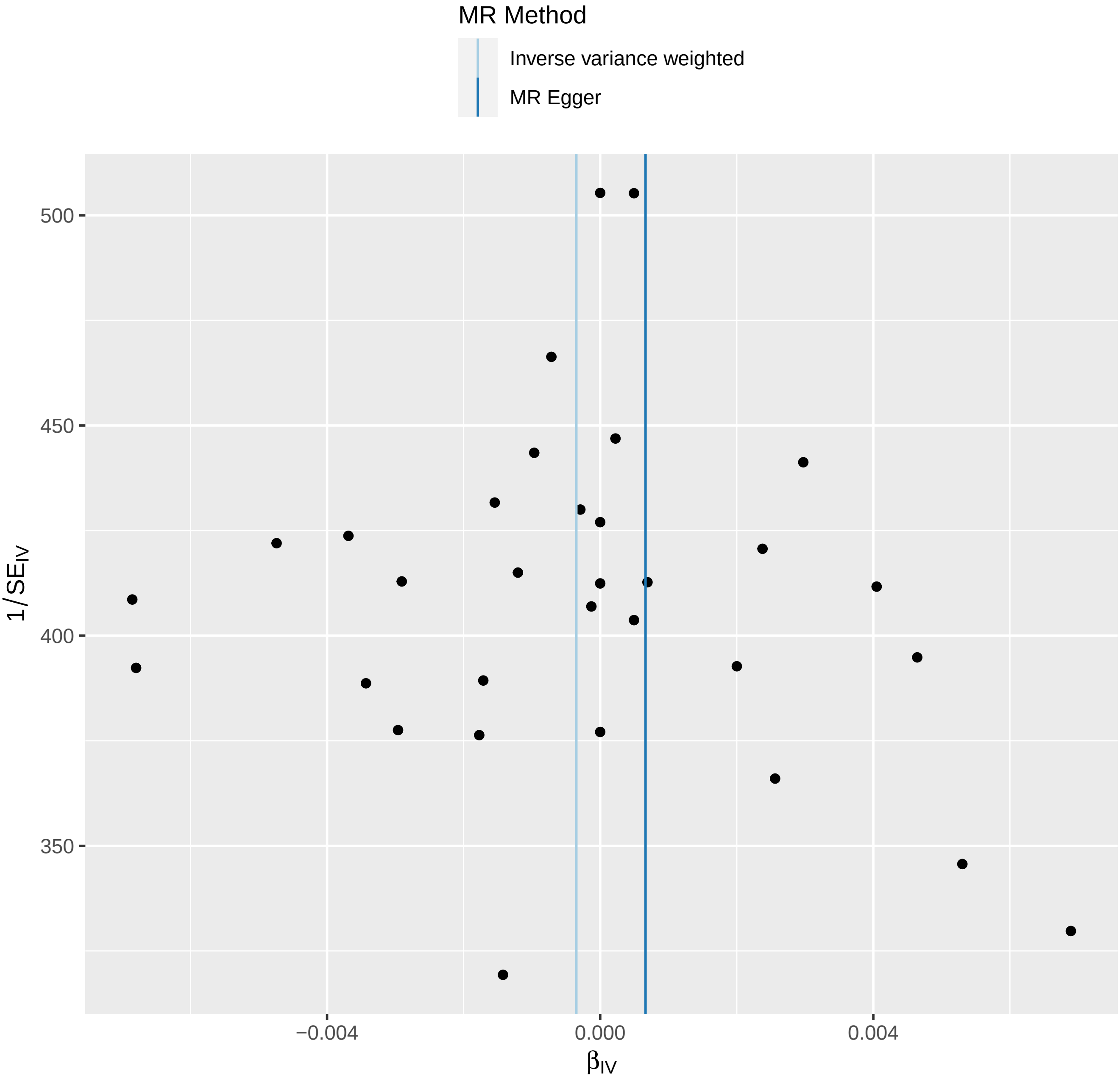

Batch 2−2502 : Probable RNA−binding protein 23 on eGFR

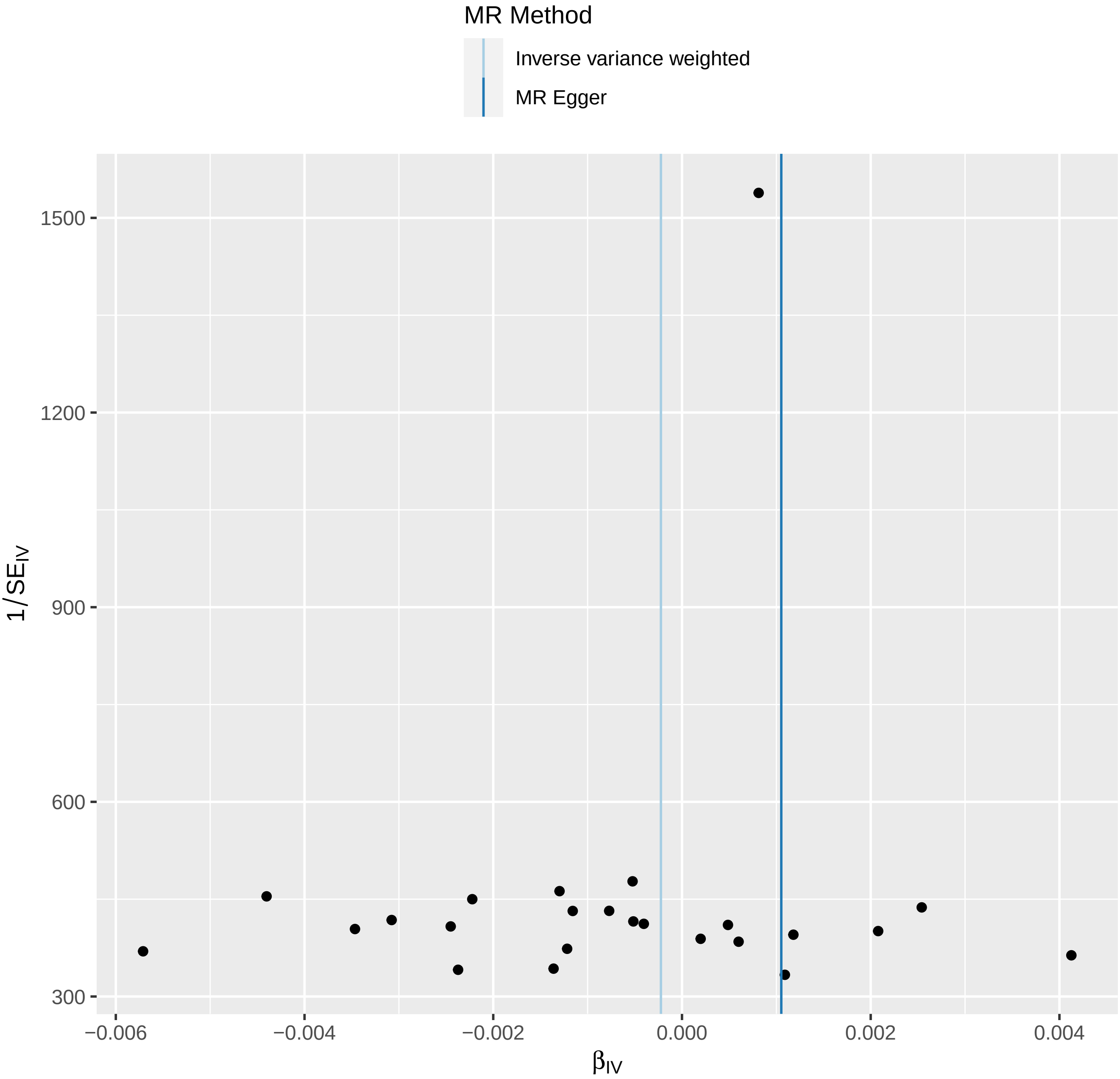

Batch 2−2503 : RNA−binding protein 24 on eGFR

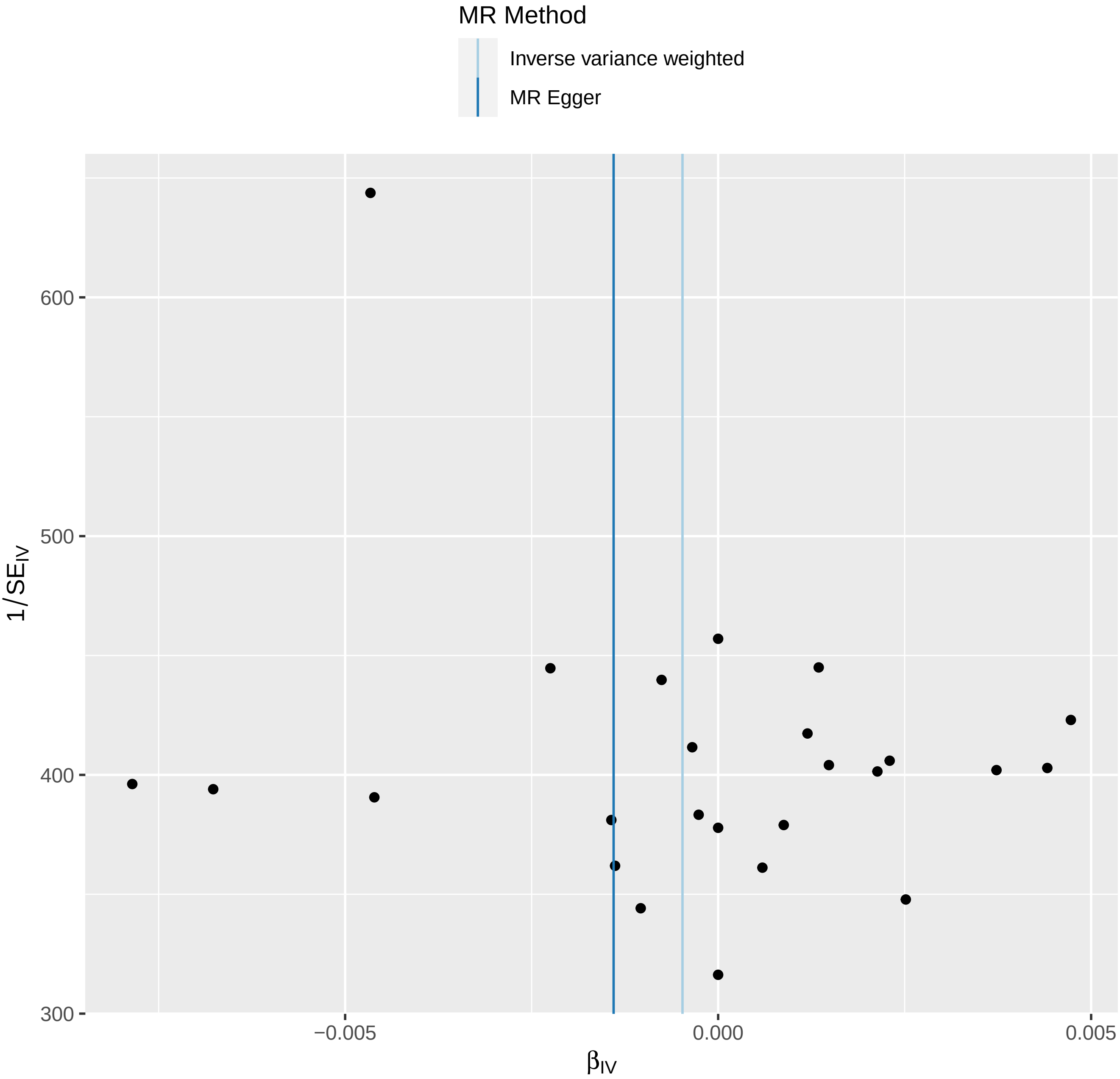

Batch 2−2504 : RNA−binding protein 28 on eGFR

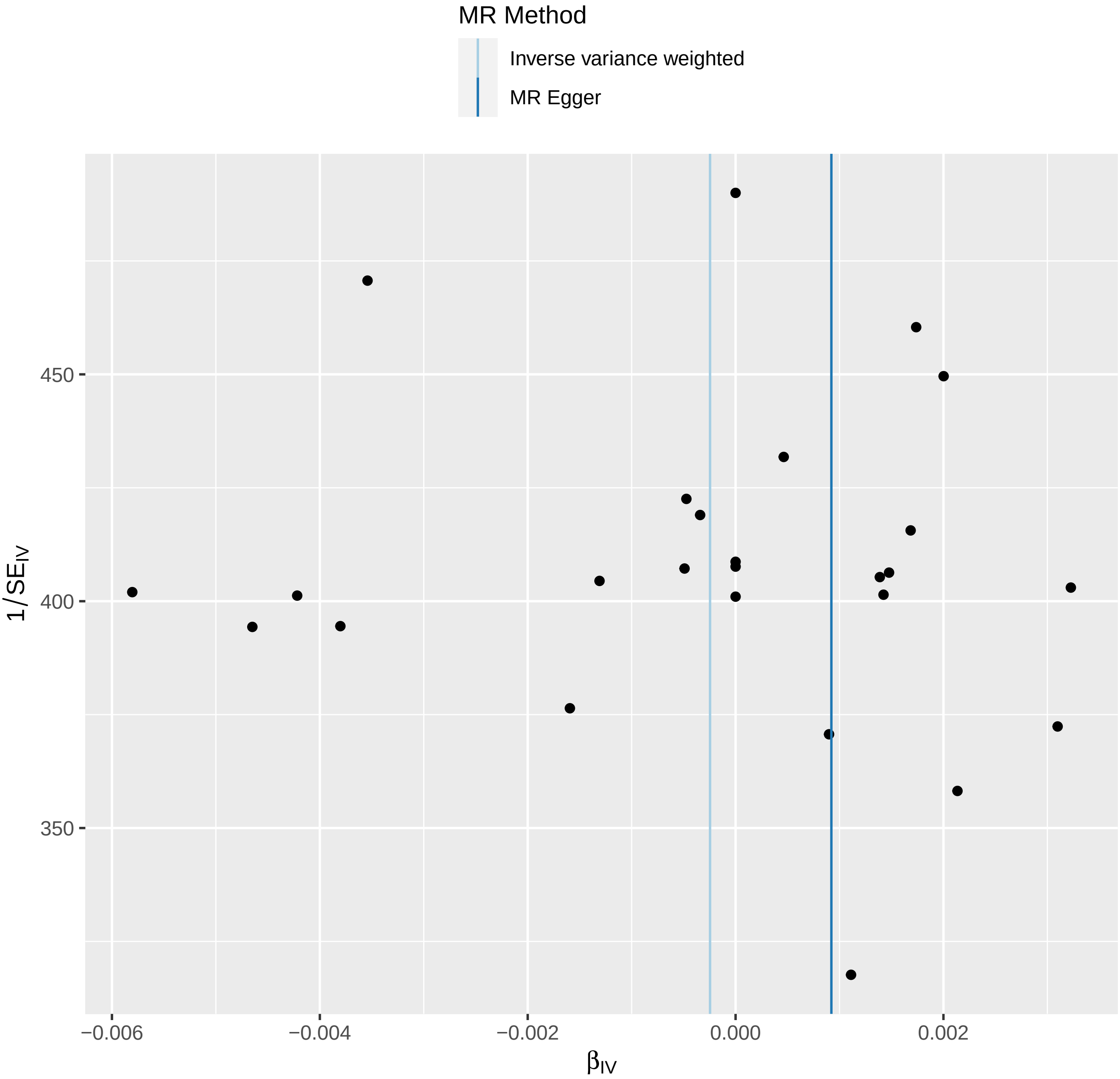

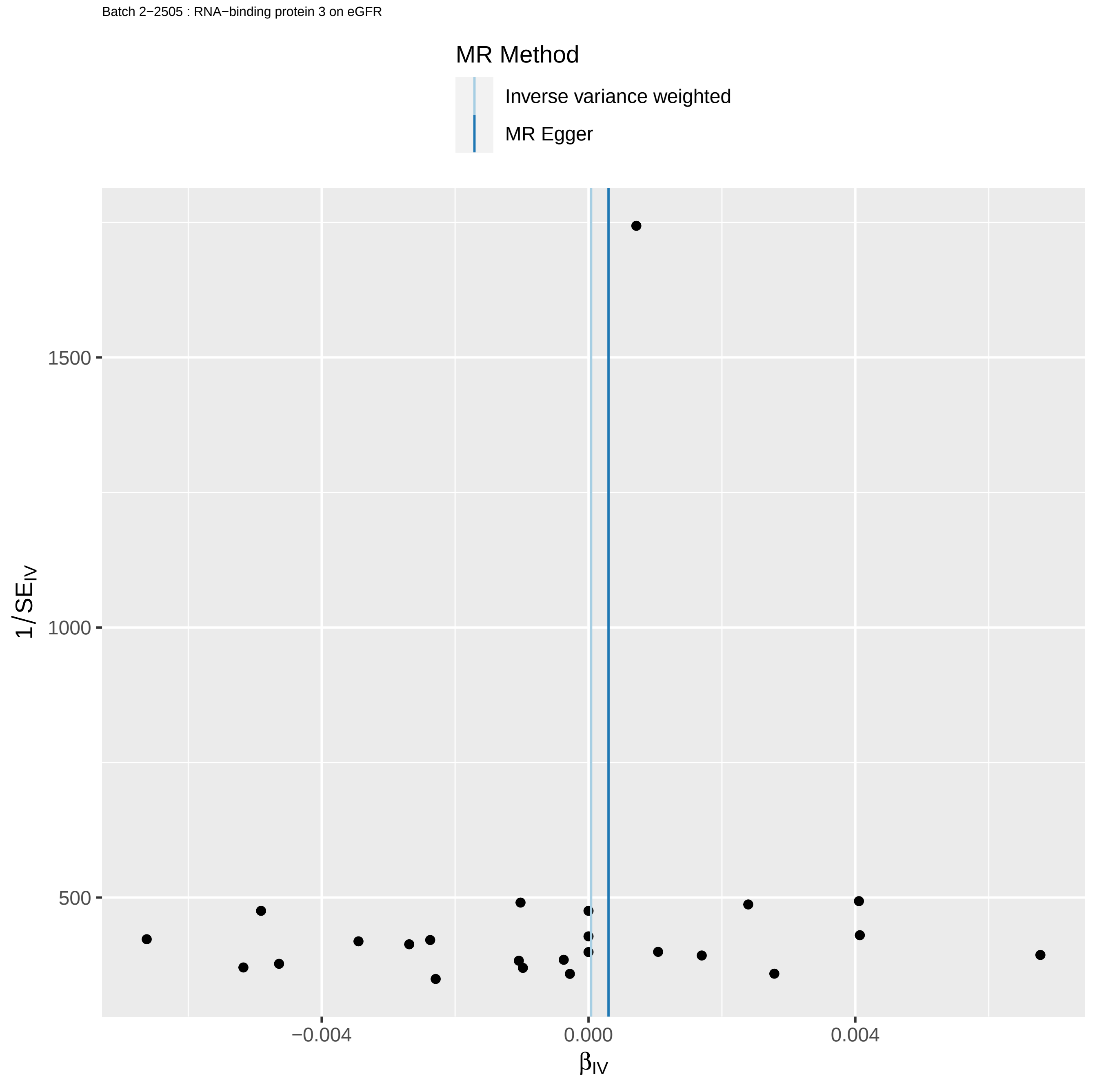

Batch 2−2505 : RNA−binding protein 3 on eGFR
MR Method
Inverse variance weighted
MR Egger
1500
1000
500
1/SE$_{IV}$
−0.004
0.000
0.004
β$_{IV}$

Batch 2−2506 : RNA−binding protein 39 on eGFR

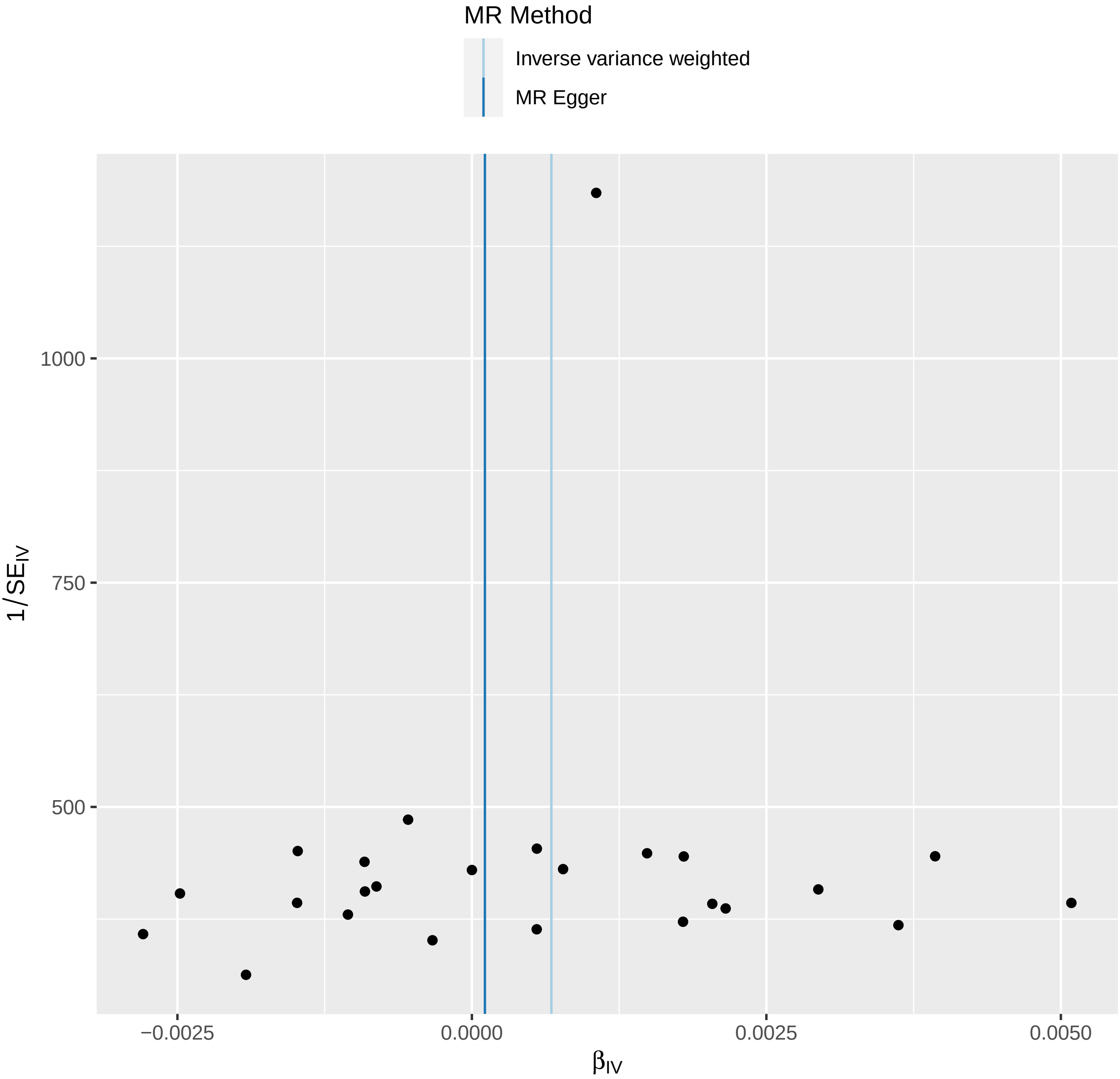

Batch 2−2507 : Retinol−binding protein 4 on eGFR

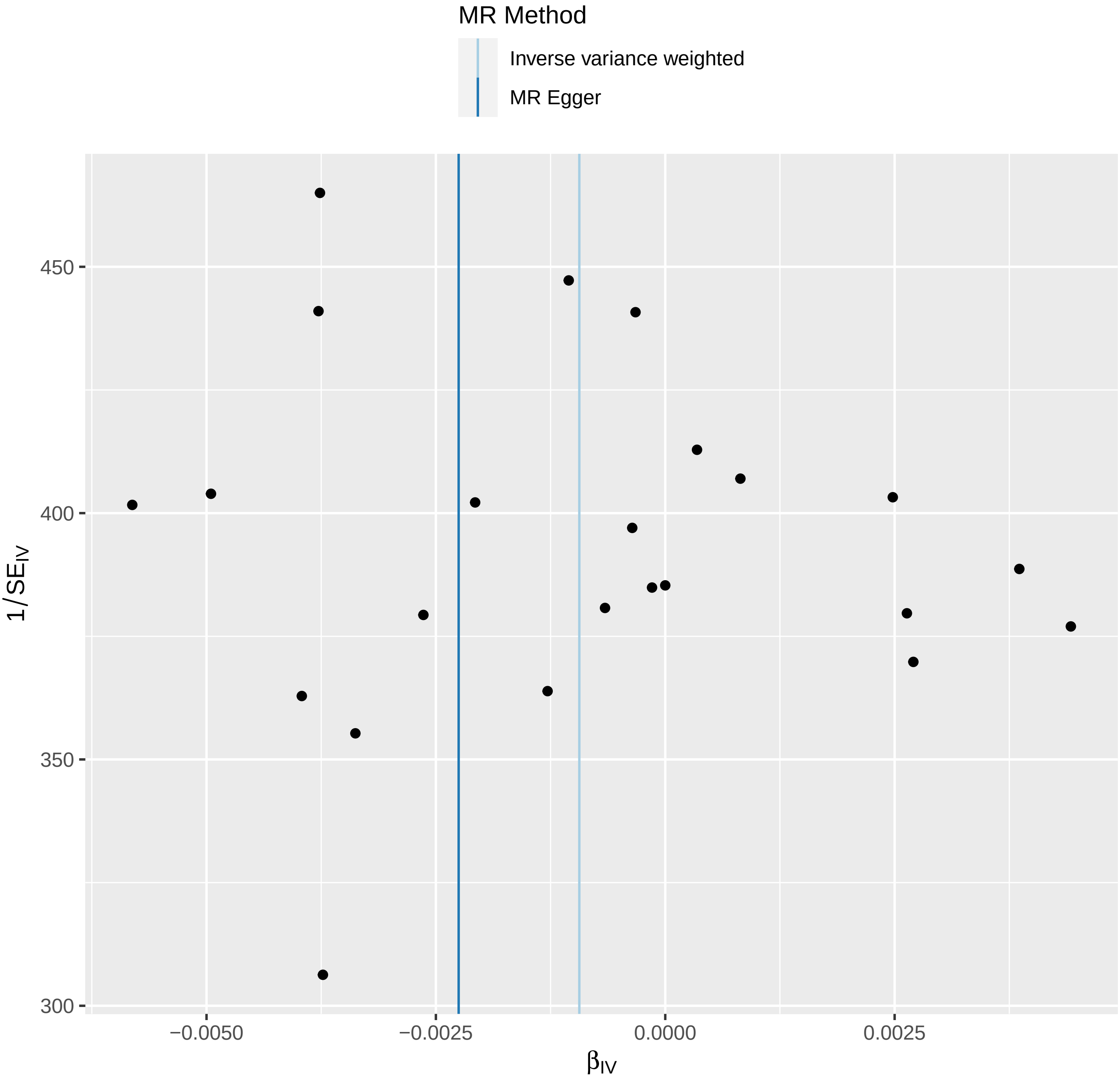

Batch 2−2508 : Retinoid−binding protein 7 on eGFR

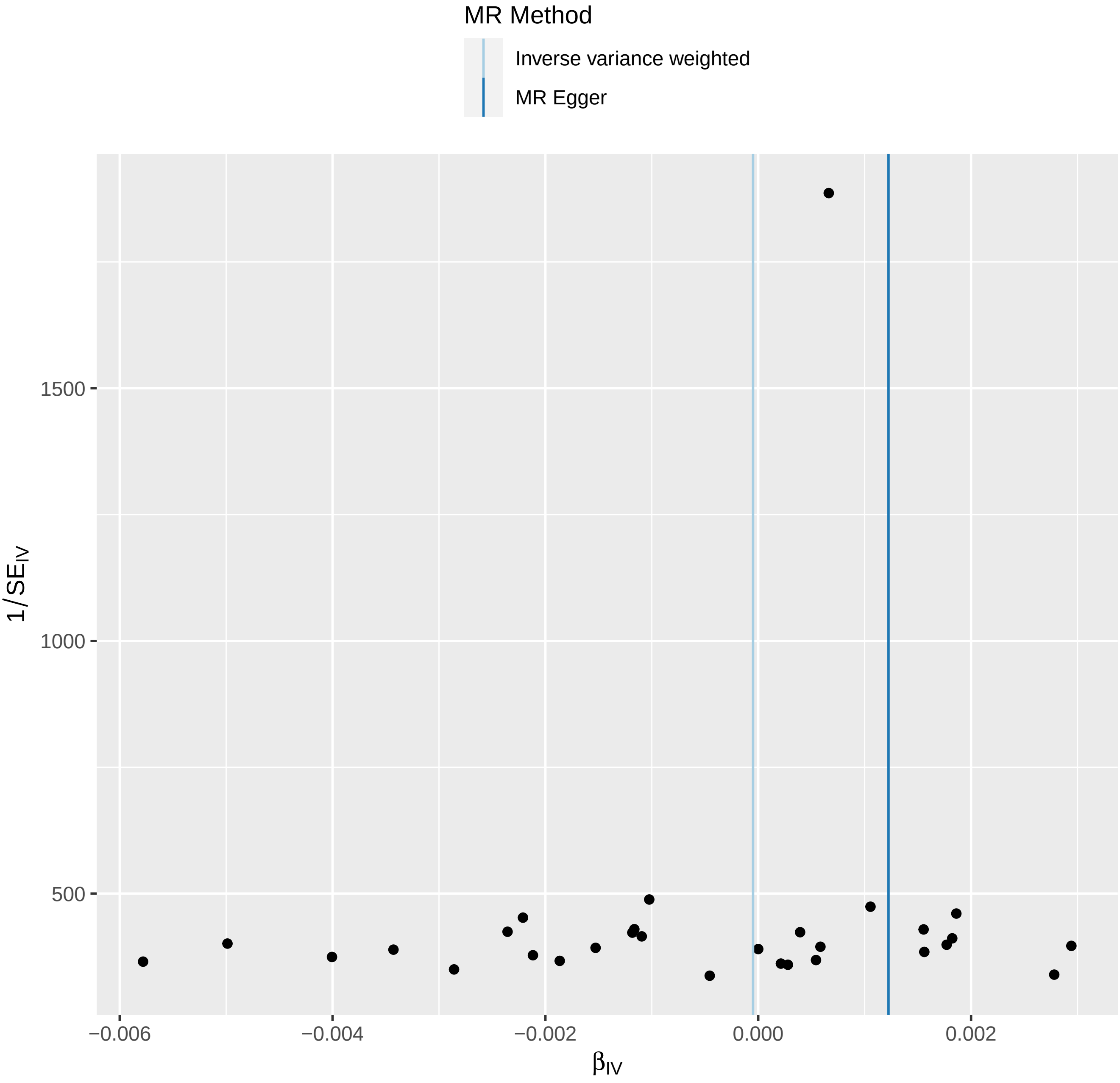

Batch 2−2509 : Calcipressin−1 on eGFR

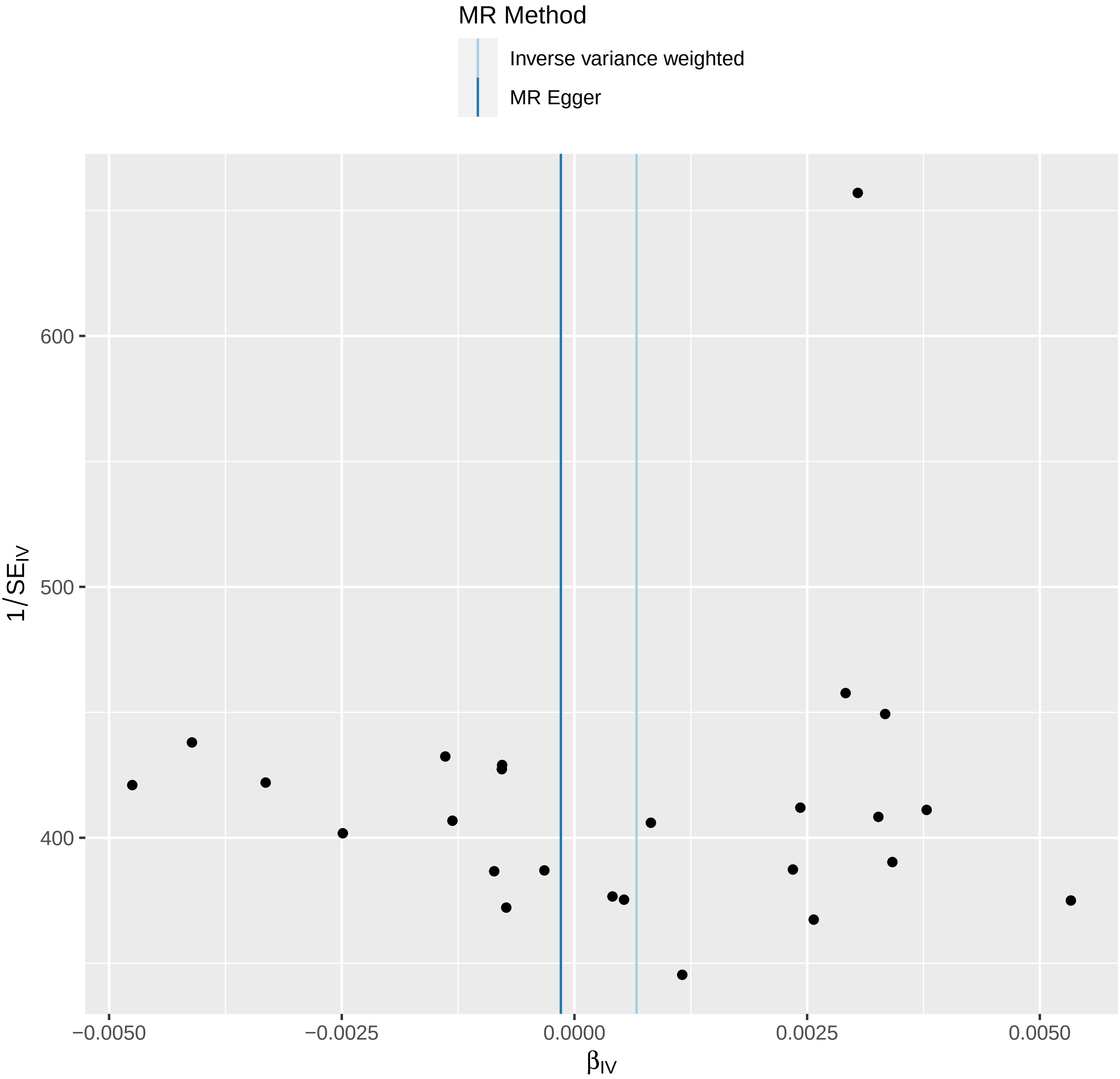

Batch 2−2510 : Reticulocalbin−2 on eGFR

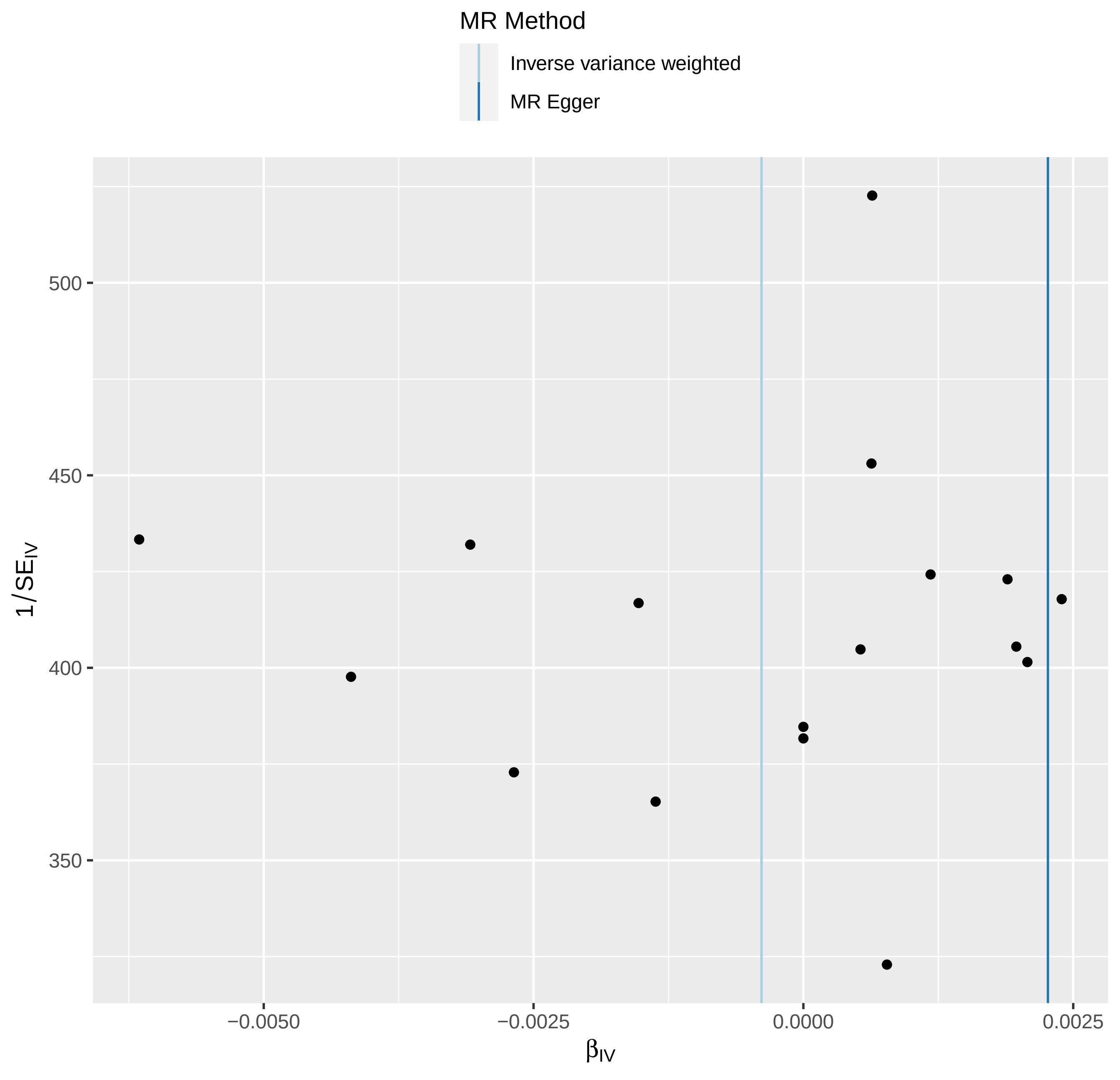

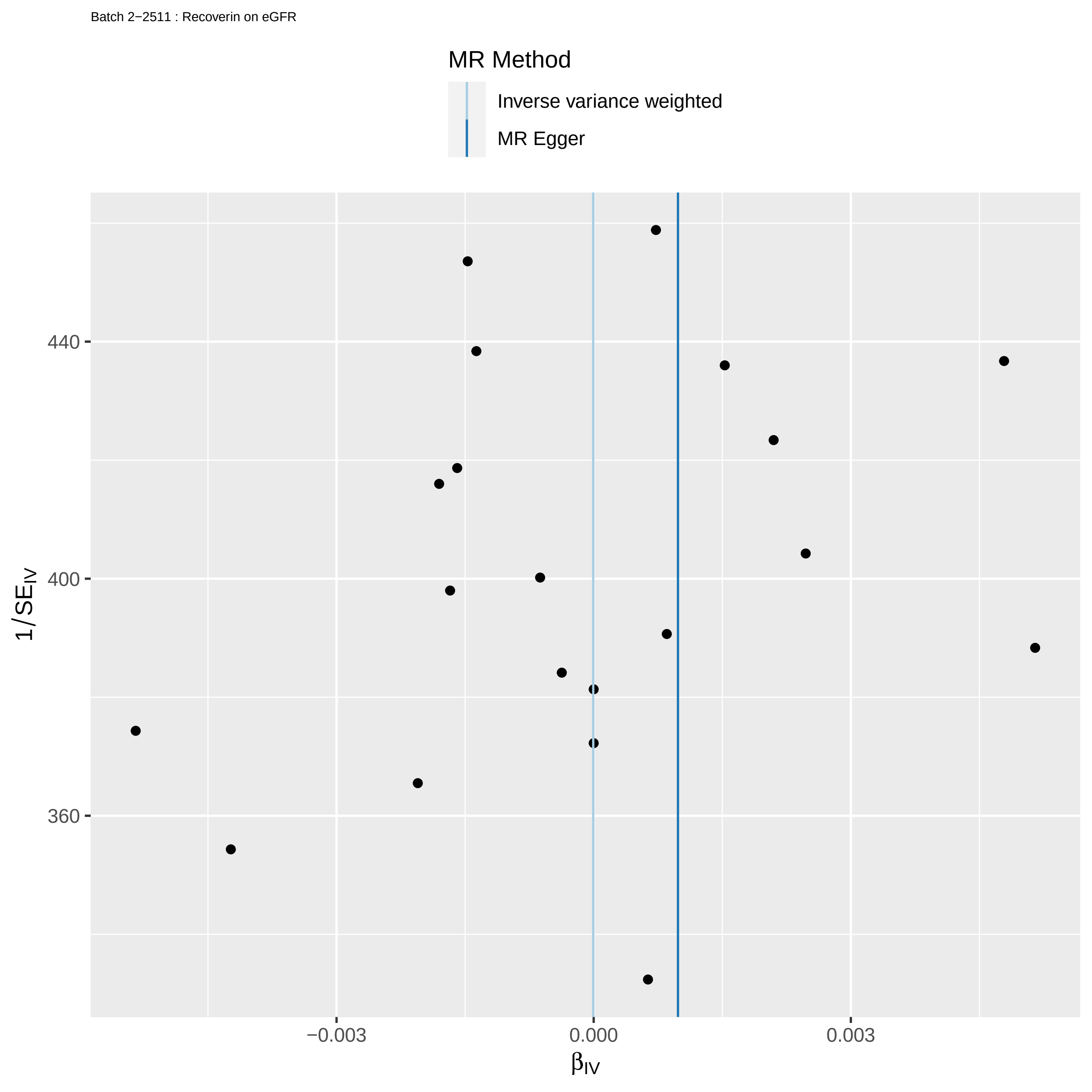

Batch 2−2511 : Recoverin on eGFR
MR Method
Inverse variance weighted
MR Egger
440
400
360
1/SE_IV
−0.003
0.000
0.003
β_IV

Batch 2−2512 : Retinol dehydrogenase 16 on eGFR

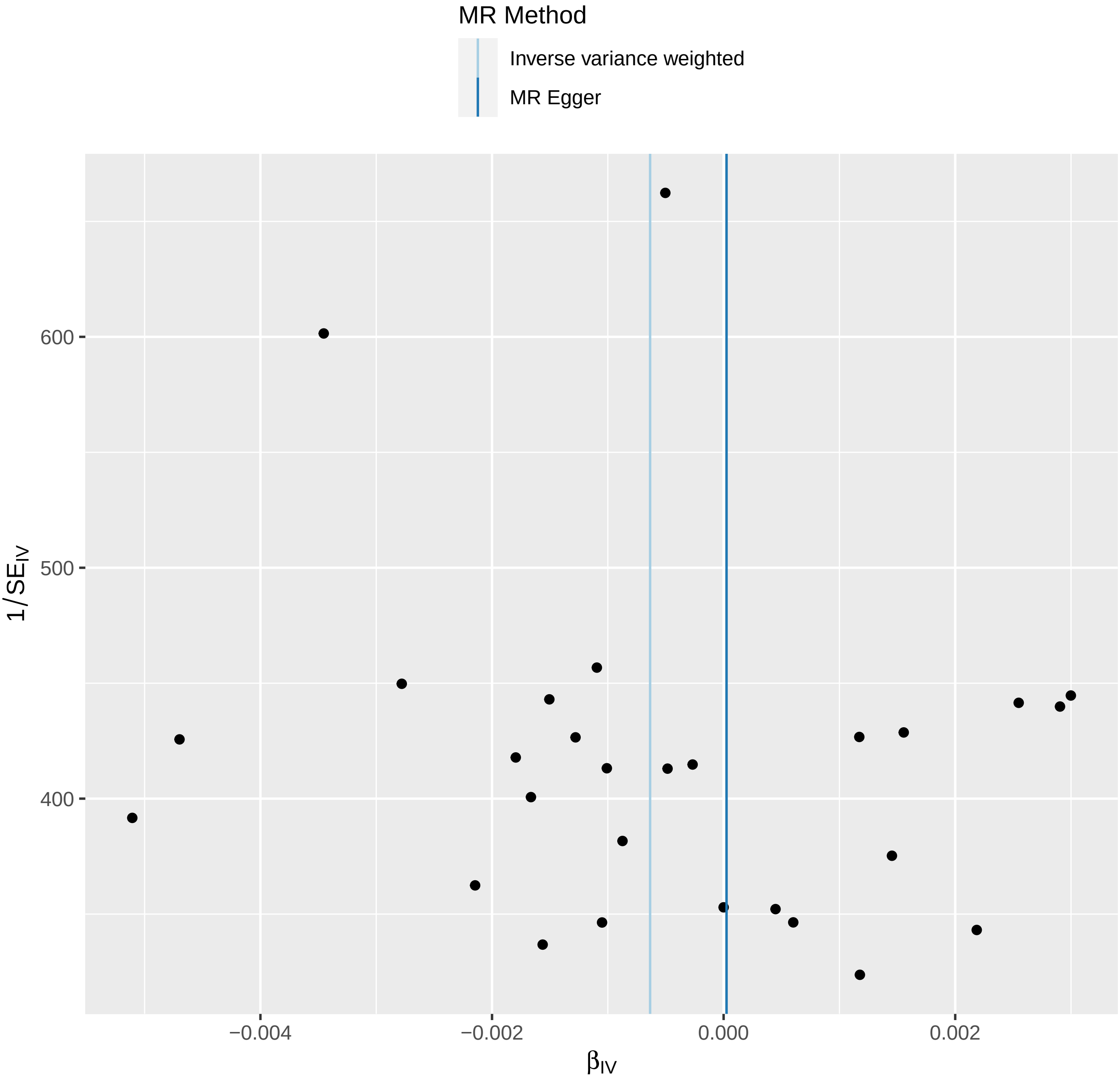

Batch 2−2513 : ATP−dependent DNA helicase Q1 on eGFR

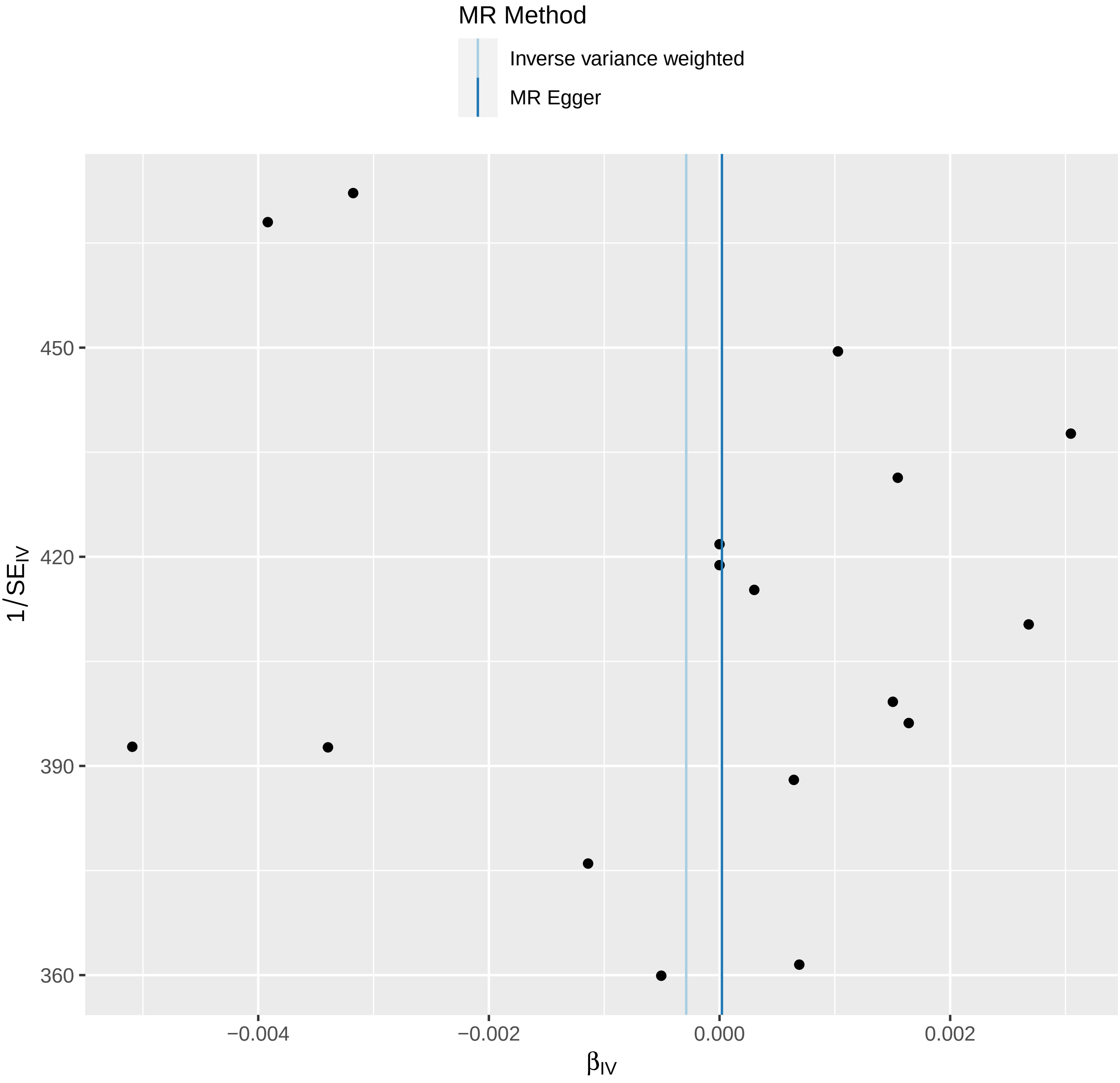

Batch 2−2514 : Regenerating islet−derived protein 3−alpha on eGFR

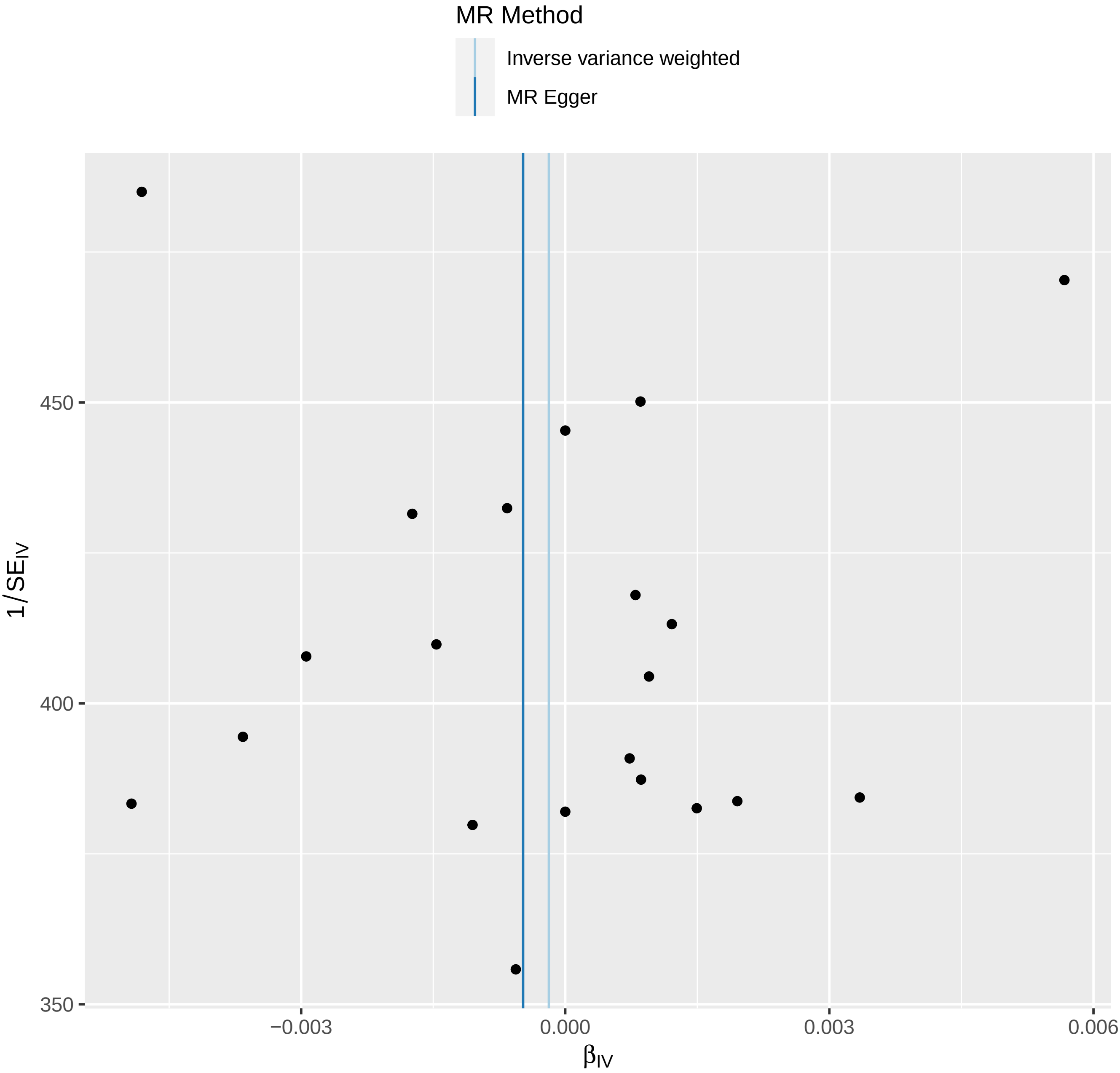

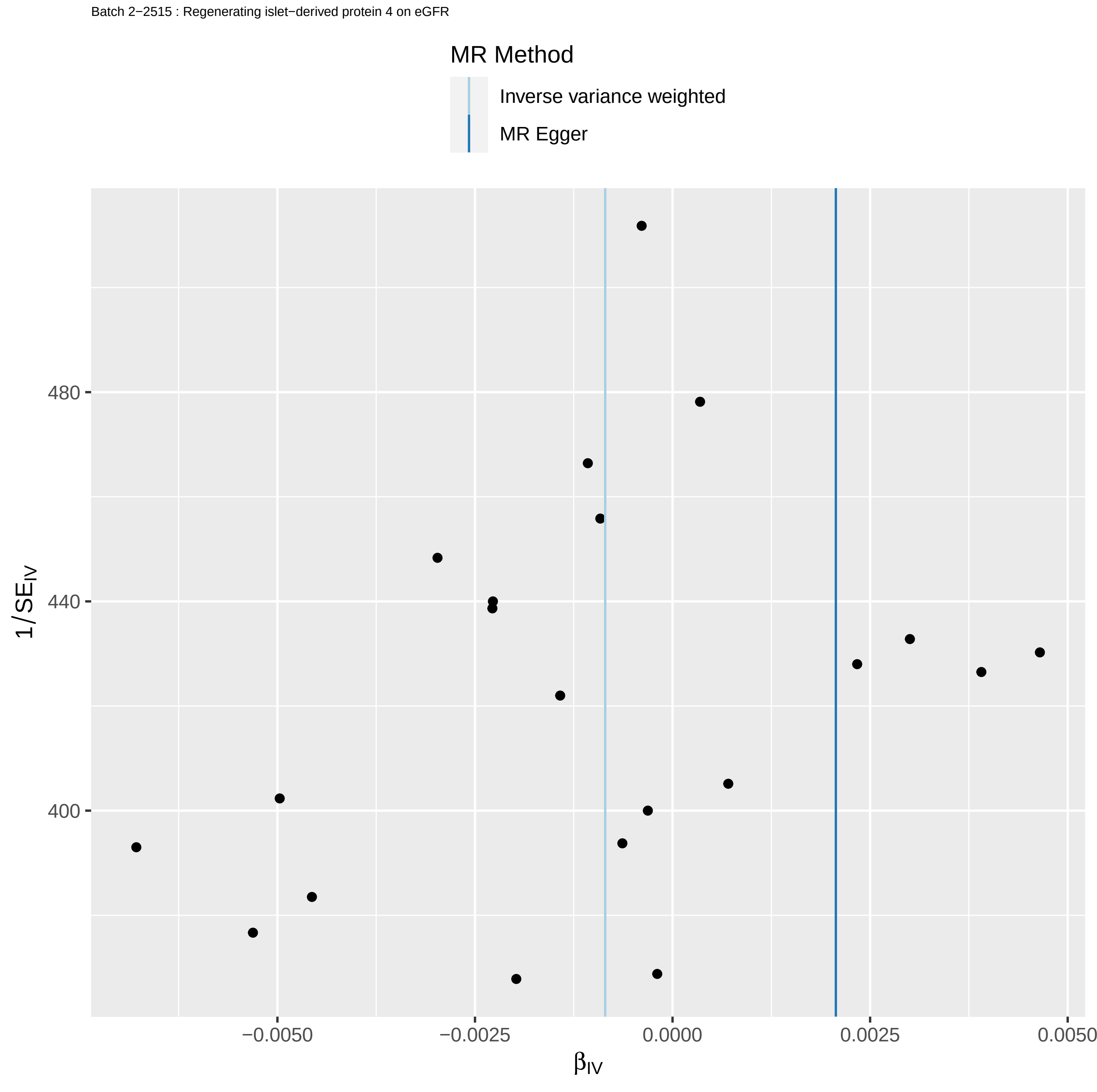
Batch 2−2515 : Regenerating islet−derived protein 4 on eGFR
MR Method
Inverse variance weighted
MR Egger
480
440
400
1/SE$_{IV}$
−0.0050
−0.0025
0.0000
0.0025
0.0050
β_{IV}

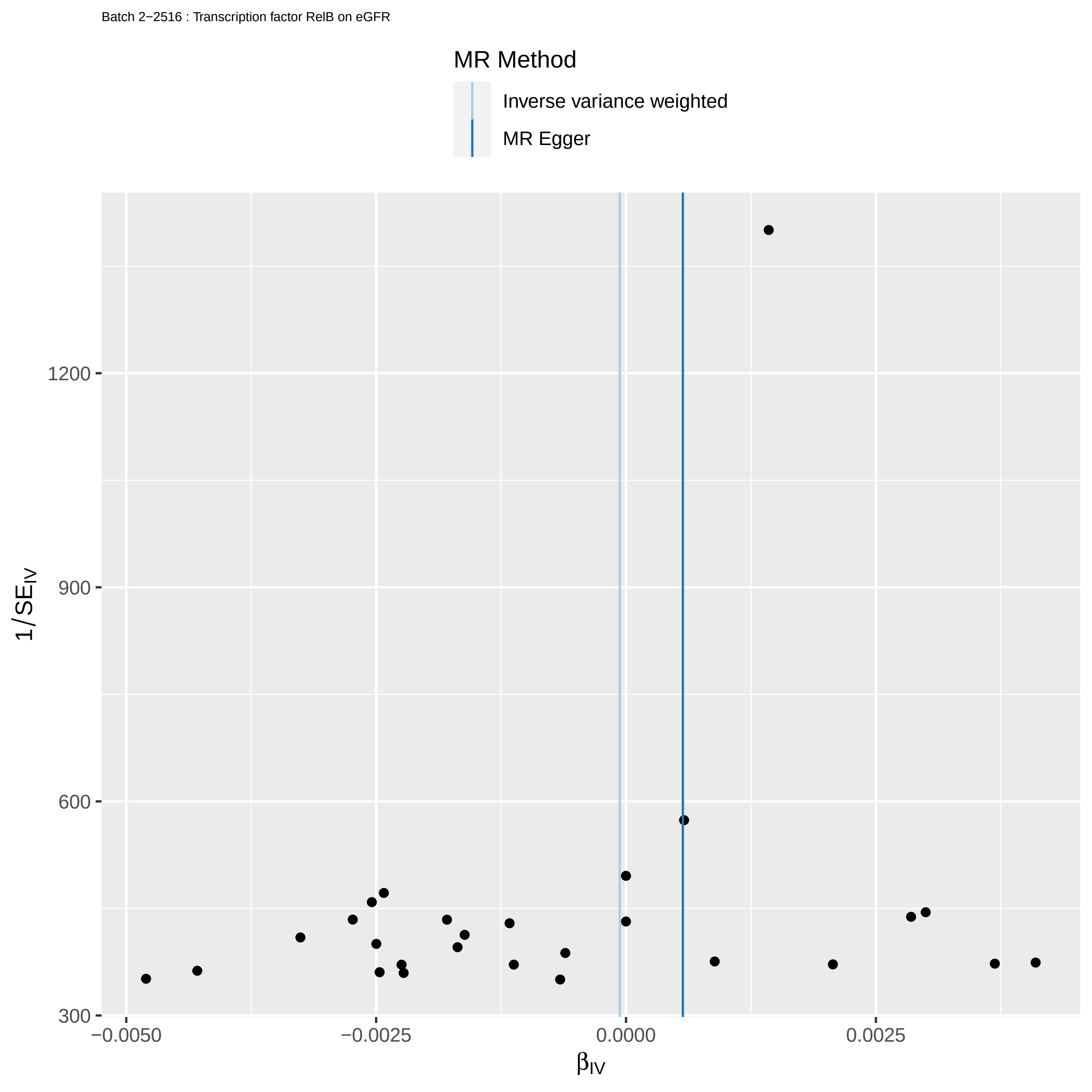

Batch 2−2516 : Transcription factor RelB on eGFR
MR Method
Inverse variance weighted
MR Egger
1200
900
600
300
1/SE_IV
−0.0050
−0.0025
0.0000
0.0025
β_IV

Batch 2−2517 : RELT−like protein 1 on eGFR

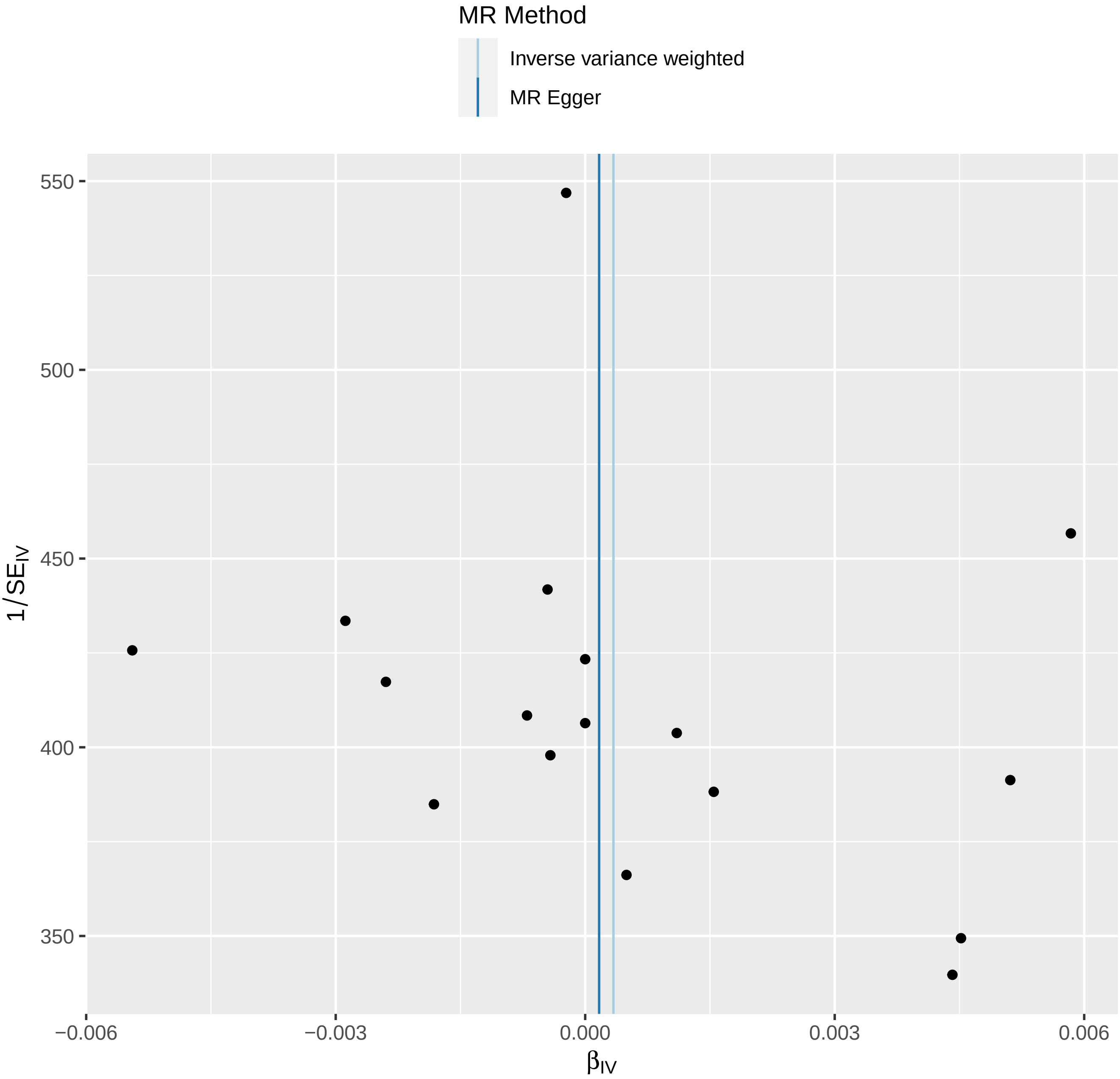

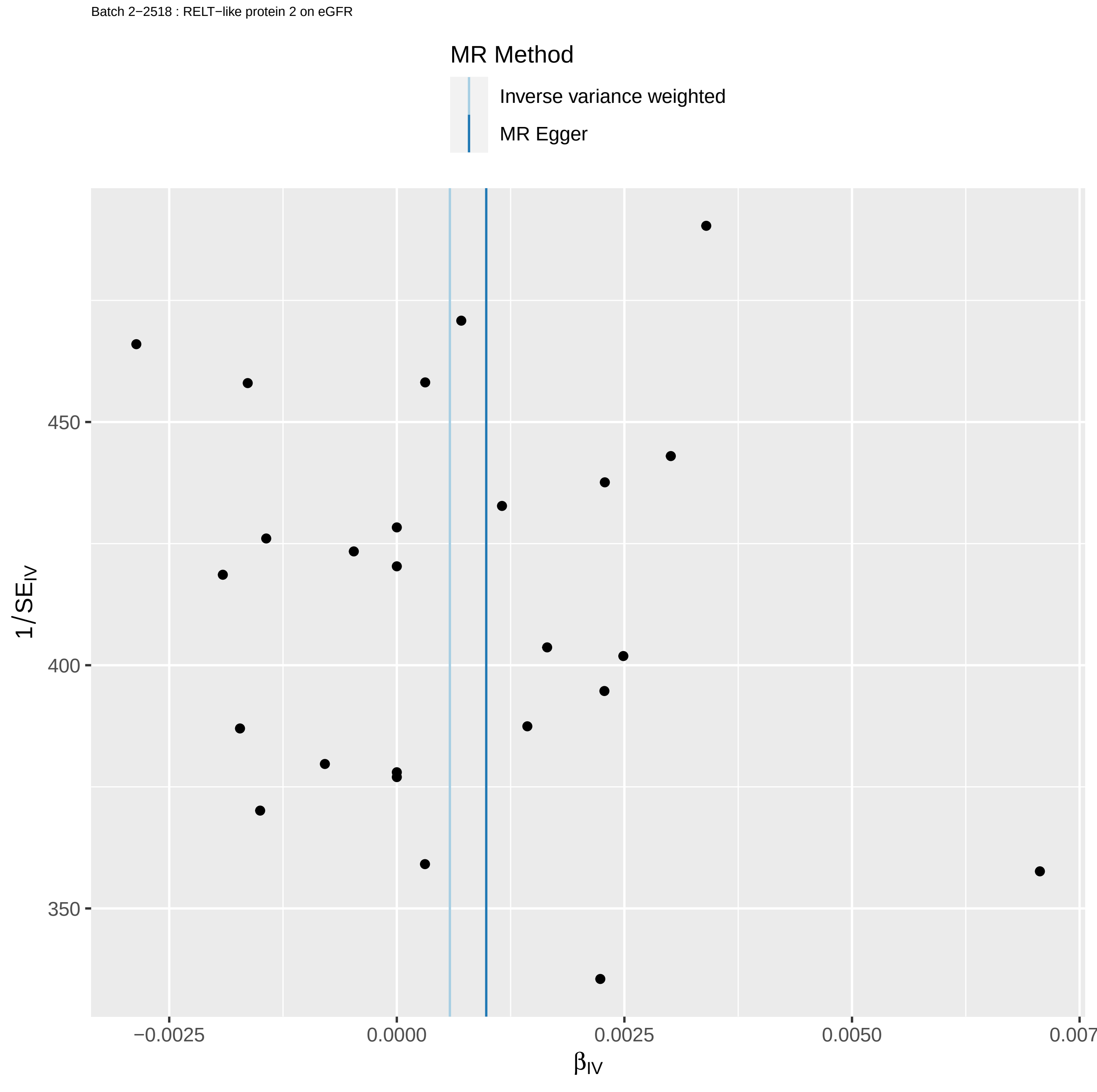

Batch 2−2518 : RELT−like protein 2 on eGFR
MR Method
Inverse variance weighted
MR Egger
450
400
350
1/SE$_{IV}$
−0.0025
0.0000
0.0025
0.0050
0.007
β$_{IV}$

Batch 2−2519 : Tumor necrosis factor receptor superfamily member 19L on eGFR

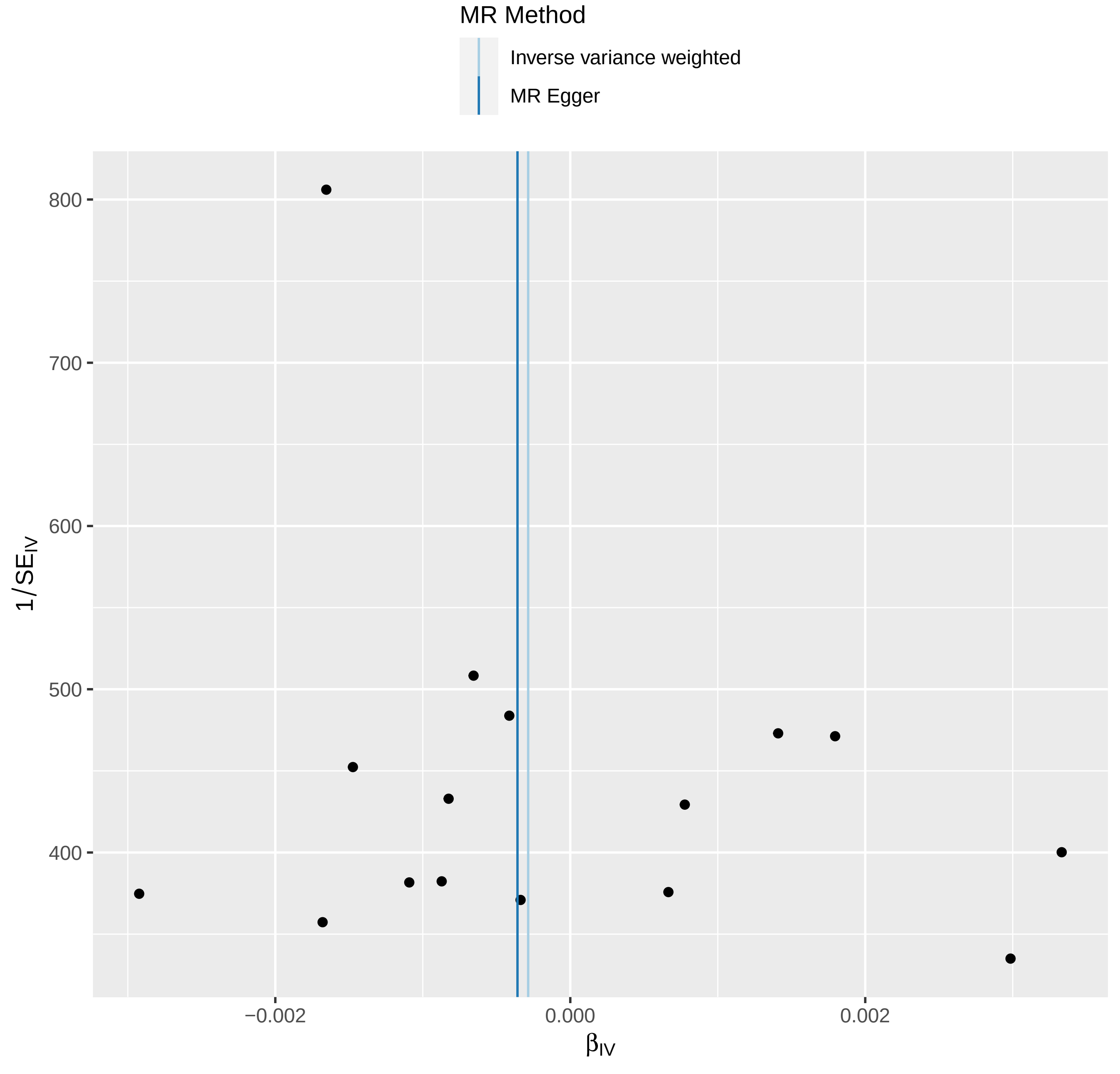

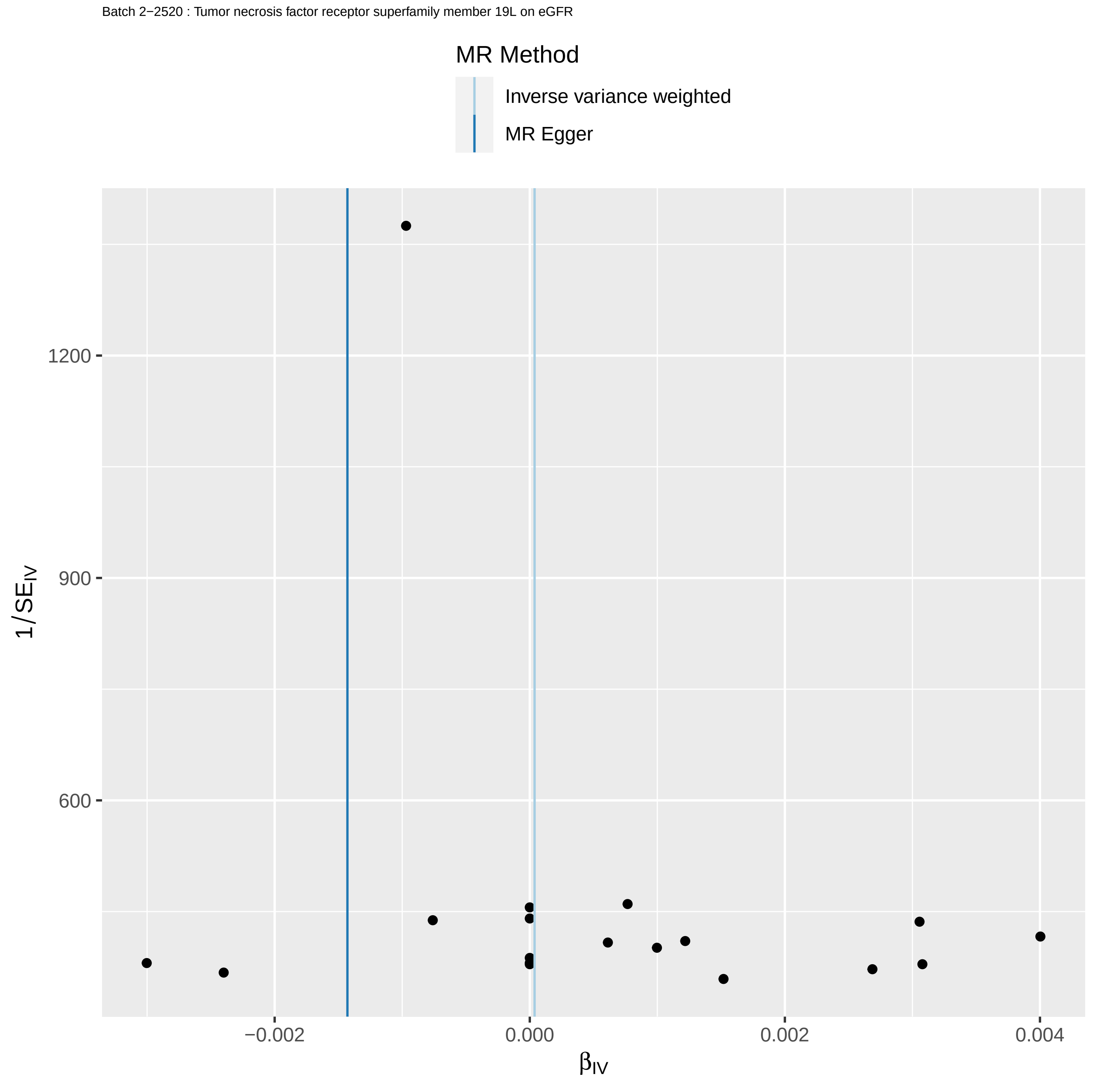

Batch 2−2520 : Tumor necrosis factor receptor superfamily member 19L on eGFR
MR Method
Inverse variance weighted
MR Egger
1200
900
600
1/SE_IV
−0.002
0.000
0.002
0.004
β_IV

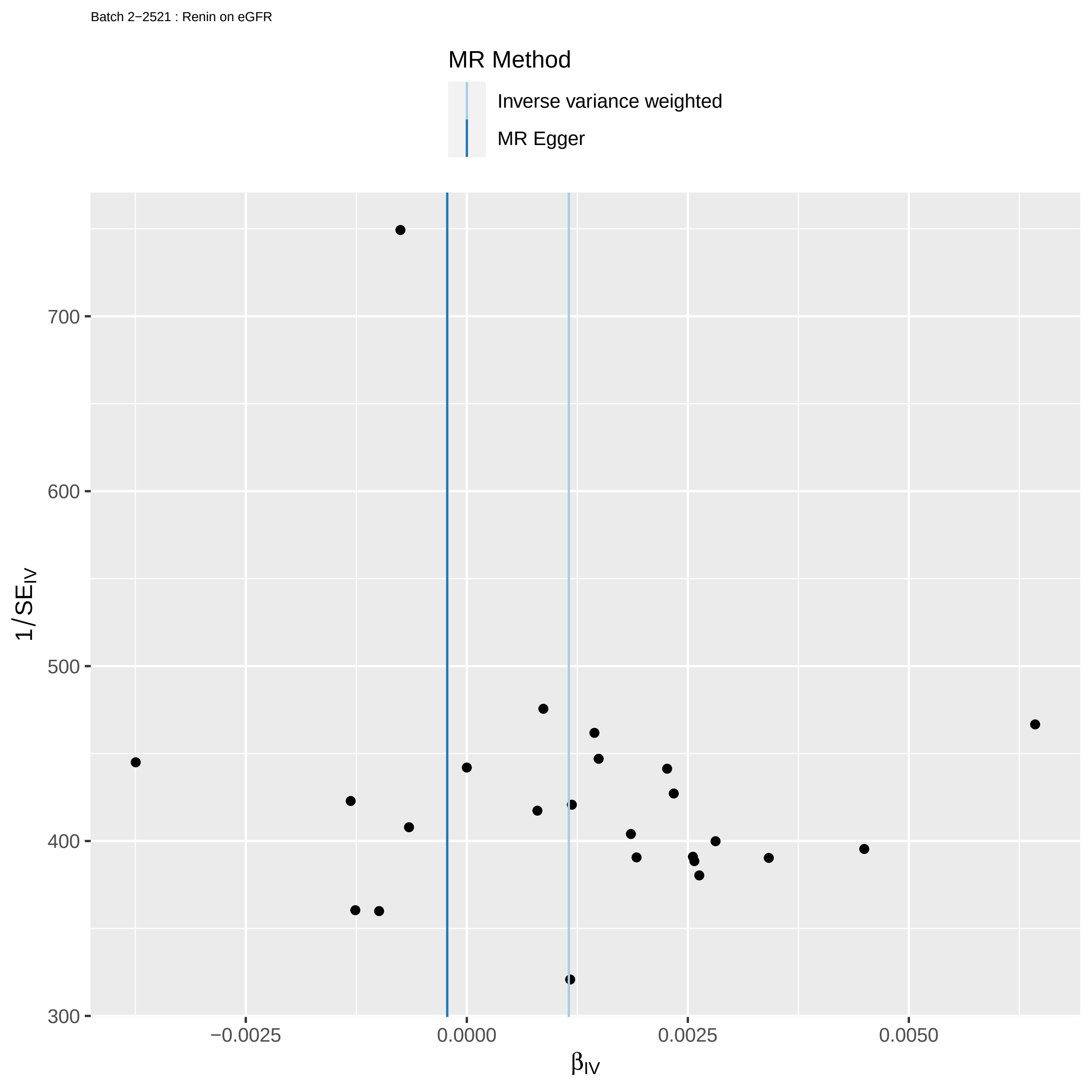
Batch 2−2521 : Renin on eGFR
MR Method
Inverse variance weighted
MR Egger
1/SE_IV
β_IV
700
600
500
400
300
−0.0025
0.0000
0.0025
0.0050

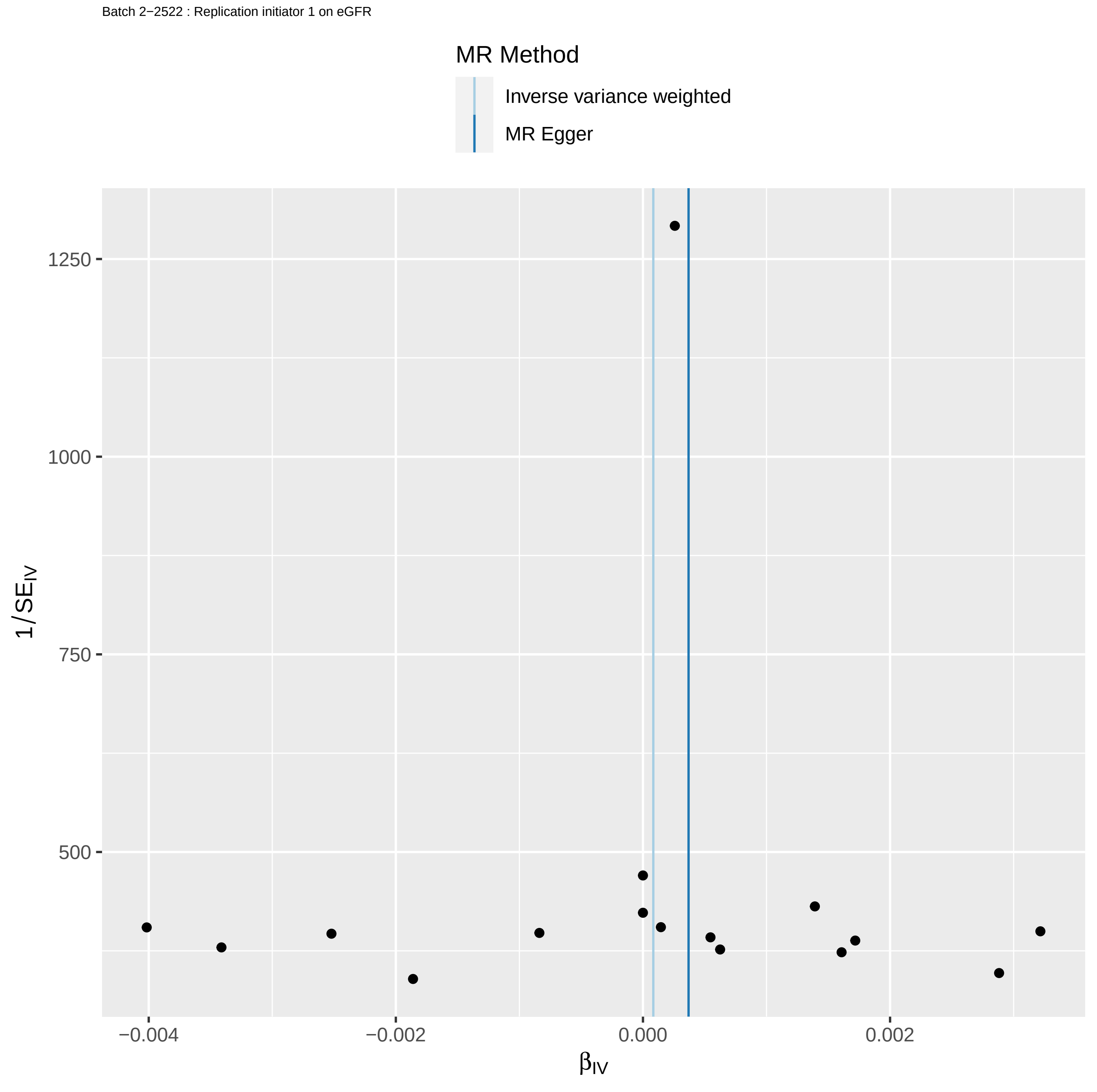
Batch 2−2522 : Replication initiator 1 on eGFR
MR Method
Inverse variance weighted
MR Egger
1250
1000
750
500
1/SE$_{IV}$
−0.004
−0.002
0.000
0.002
β$_{IV}$

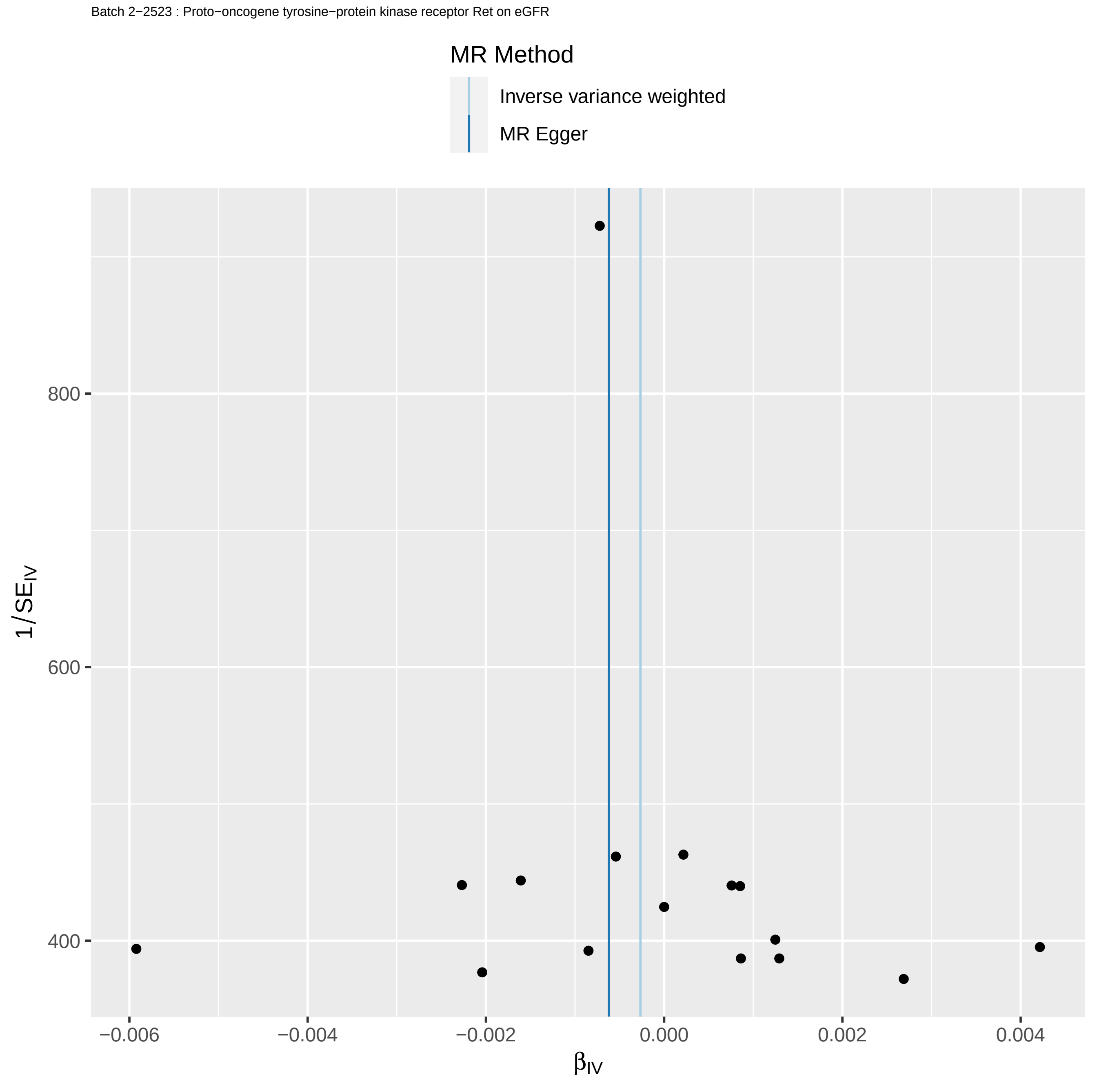

Batch 2−2523 : Proto−oncogene tyrosine−protein kinase receptor Ret on eGFR
MR Method
Inverse variance weighted
MR Egger
800
600
400
1/SE$_{IV}$
−0.006
−0.004
−0.002
0.000
0.002
0.004
β_{IV}

Batch 2−2524 : Resistin on eGFR

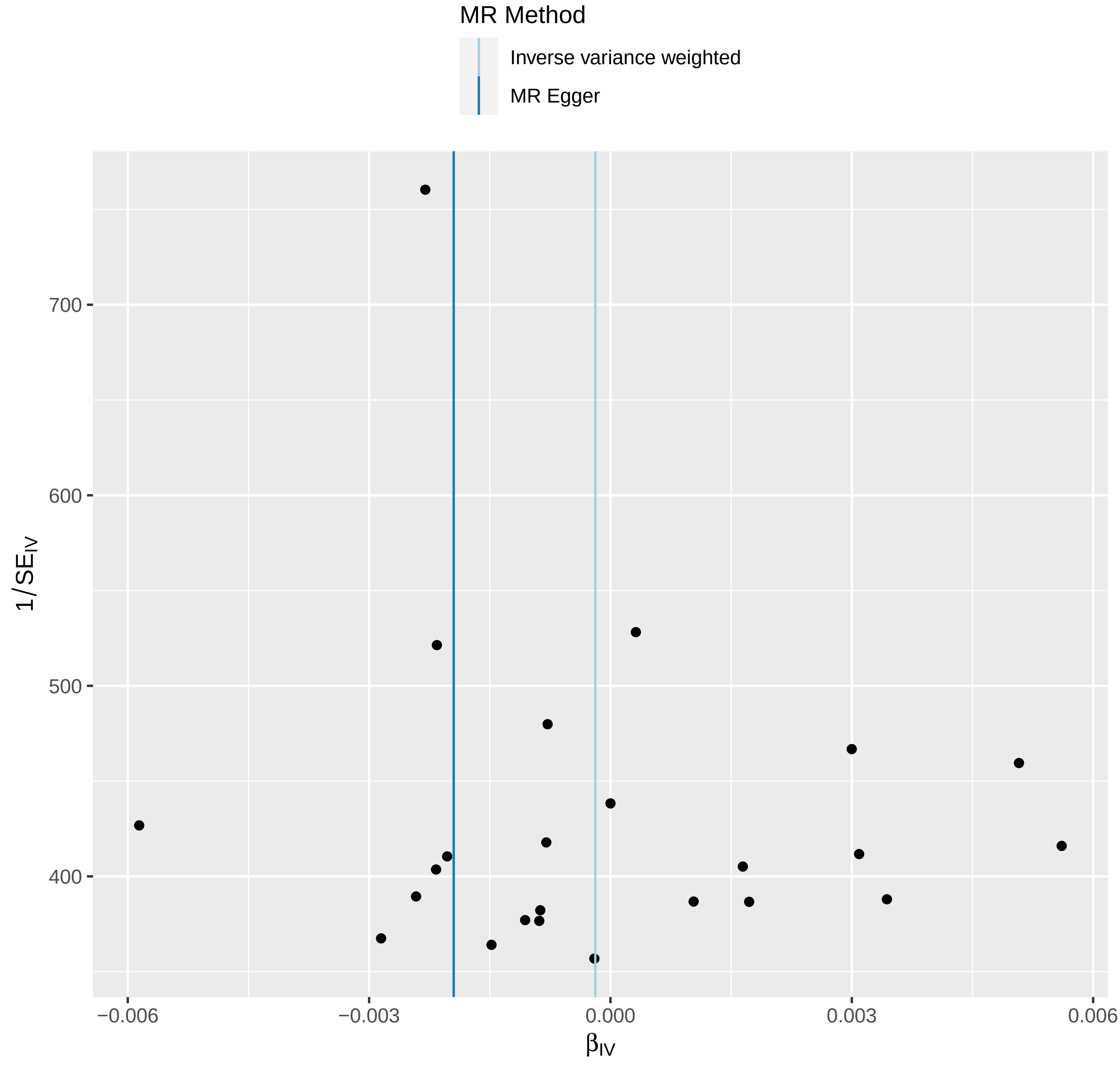

Batch 2−2525 : All−trans−retinol 13,14−reductase on eGFR

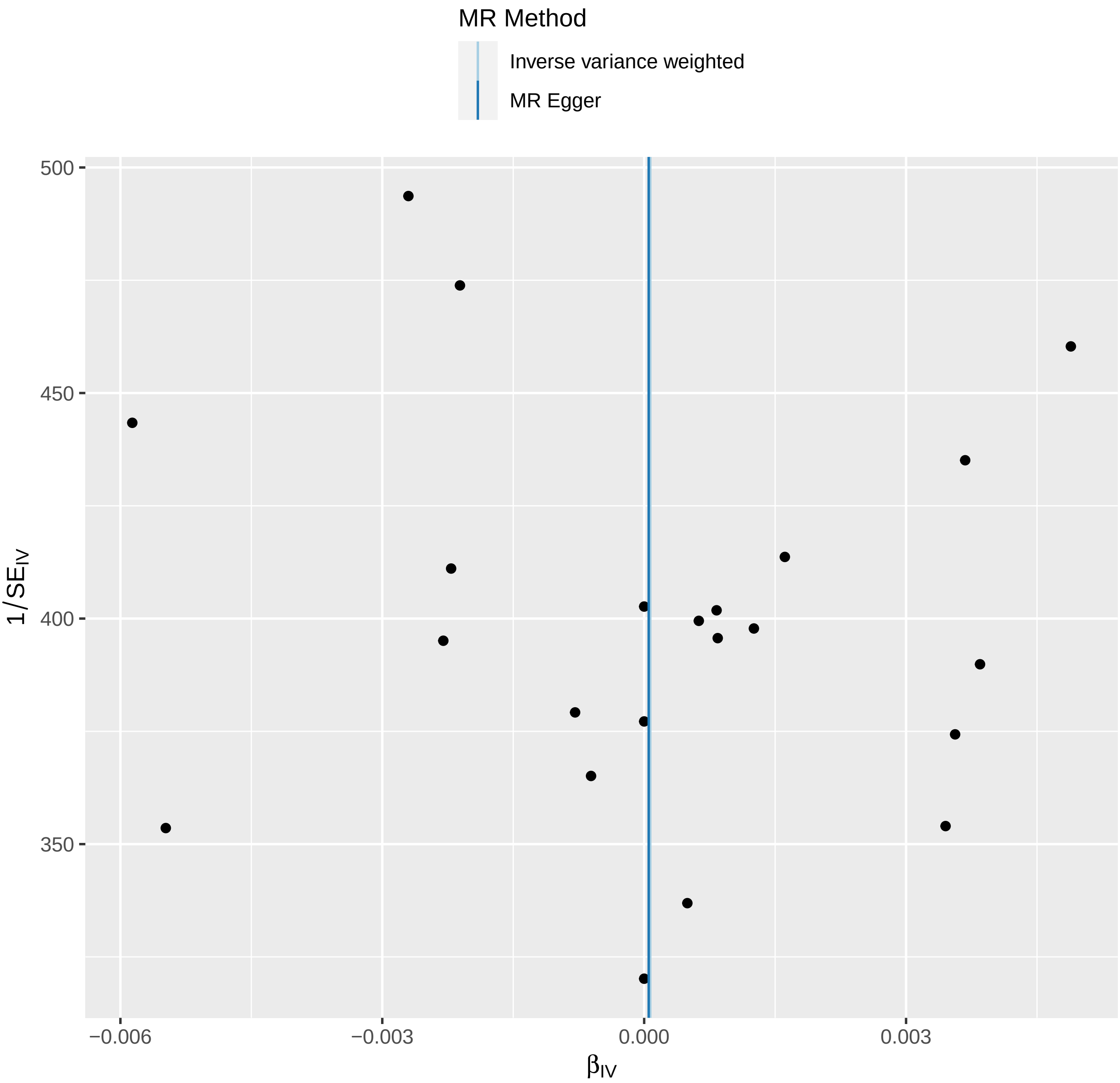

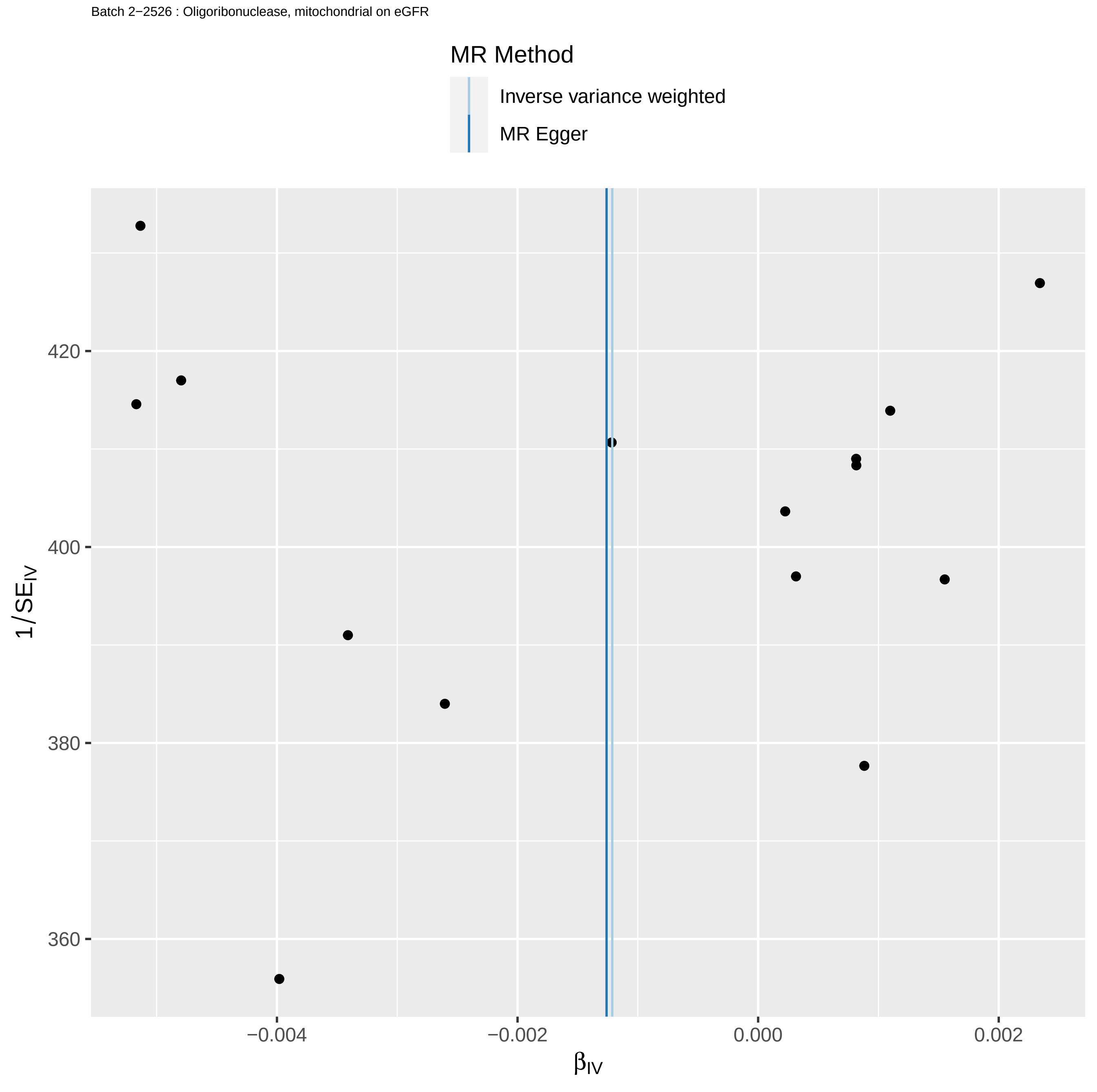
Batch 2−2526 : Oligoribonuclease, mitochondrial on eGFR
MR Method
Inverse variance weighted
MR Egger
420
400
380
360
−0.004
−0.002
0.000
0.002
$1/SE_{IV}$
β_{IV}

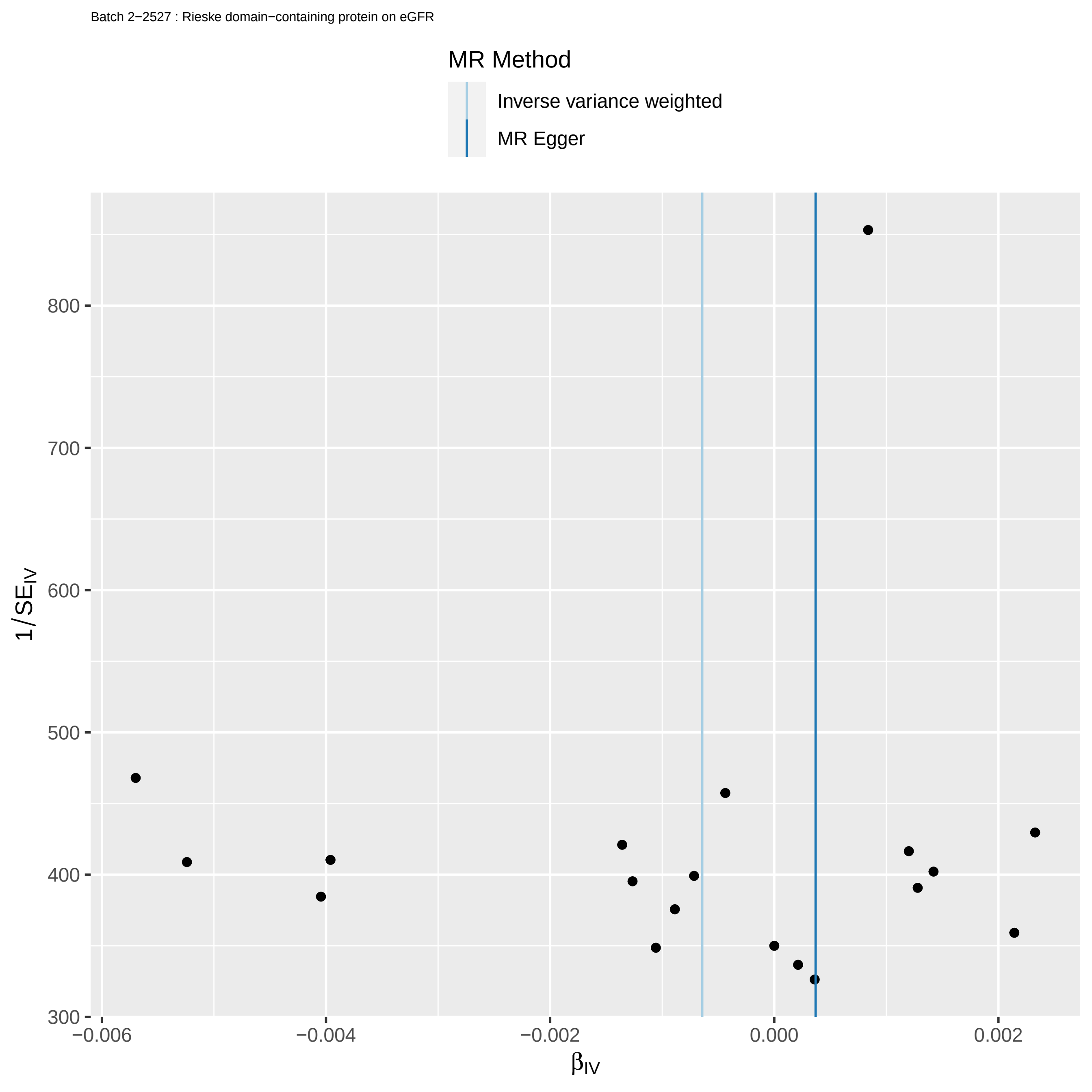

Batch 2−2527 : Rieske domain−containing protein on eGFR
MR Method
Inverse variance weighted
MR Egger
800
700
600
500
400
300
1/SE_IV
−0.006
−0.004
−0.002
0.000
0.002
β_IV

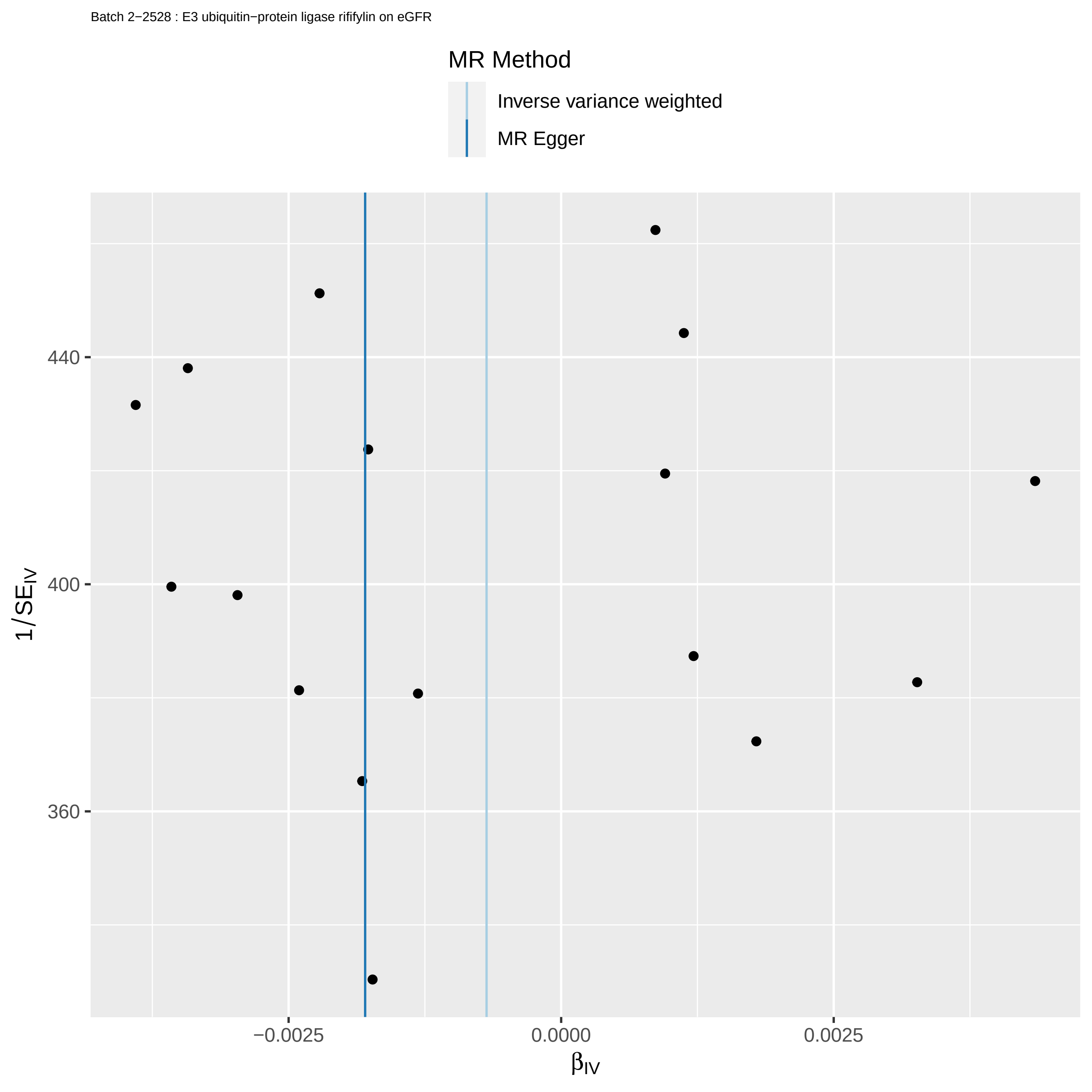

Batch 2−2528 : E3 ubiquitin−protein ligase rififylin on eGFR
MR Method
Inverse variance weighted
MR Egger
440
400
360
1/SE_IV
−0.0025
0.0000
0.0025
β_IV

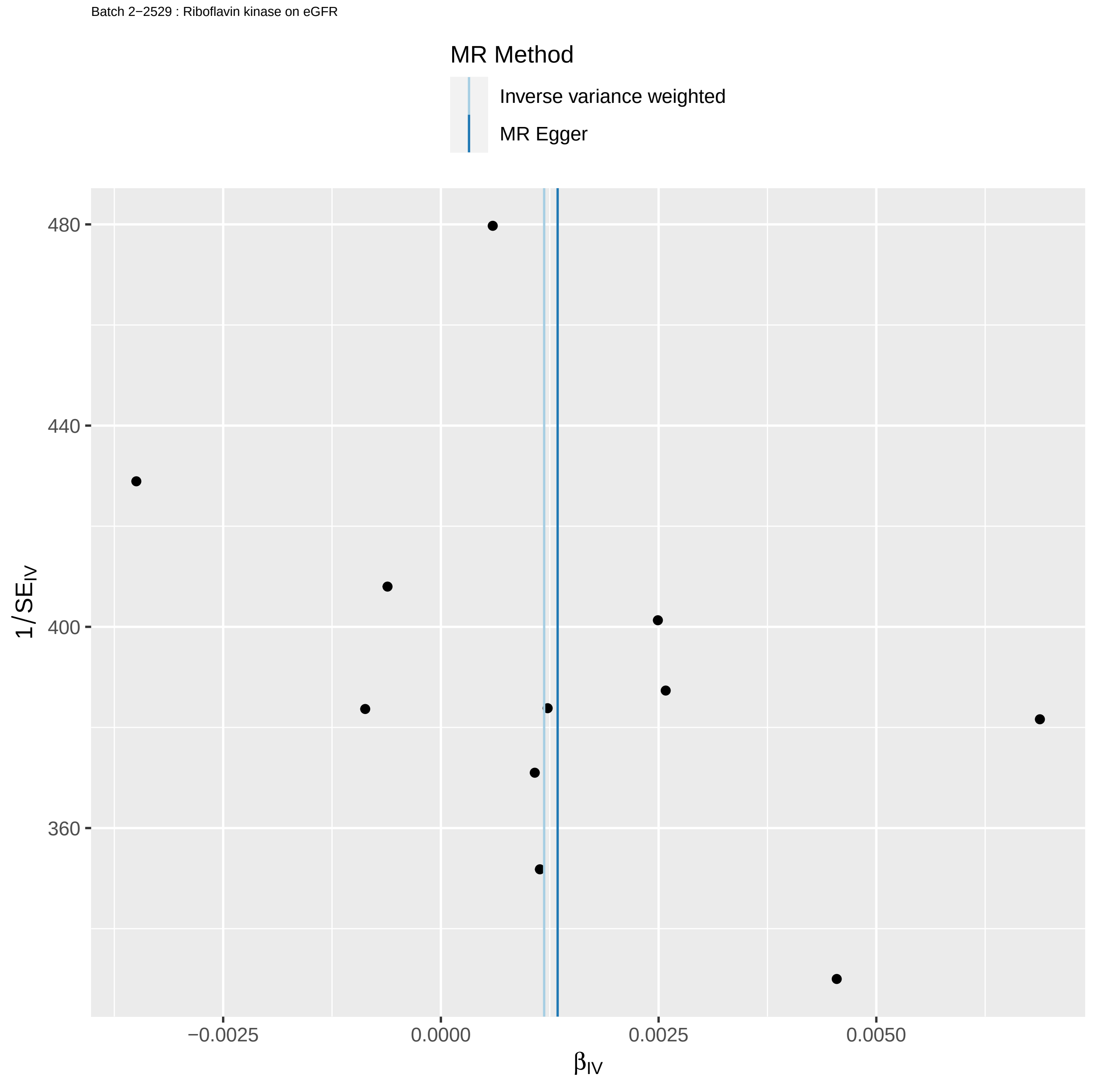

Batch 2−2529 : Riboflavin kinase on eGFR
MR Method
Inverse variance weighted
MR Egger
480
440
400
360
1/SE_IV
−0.0025
0.0000
0.0025
0.0050
β_IV

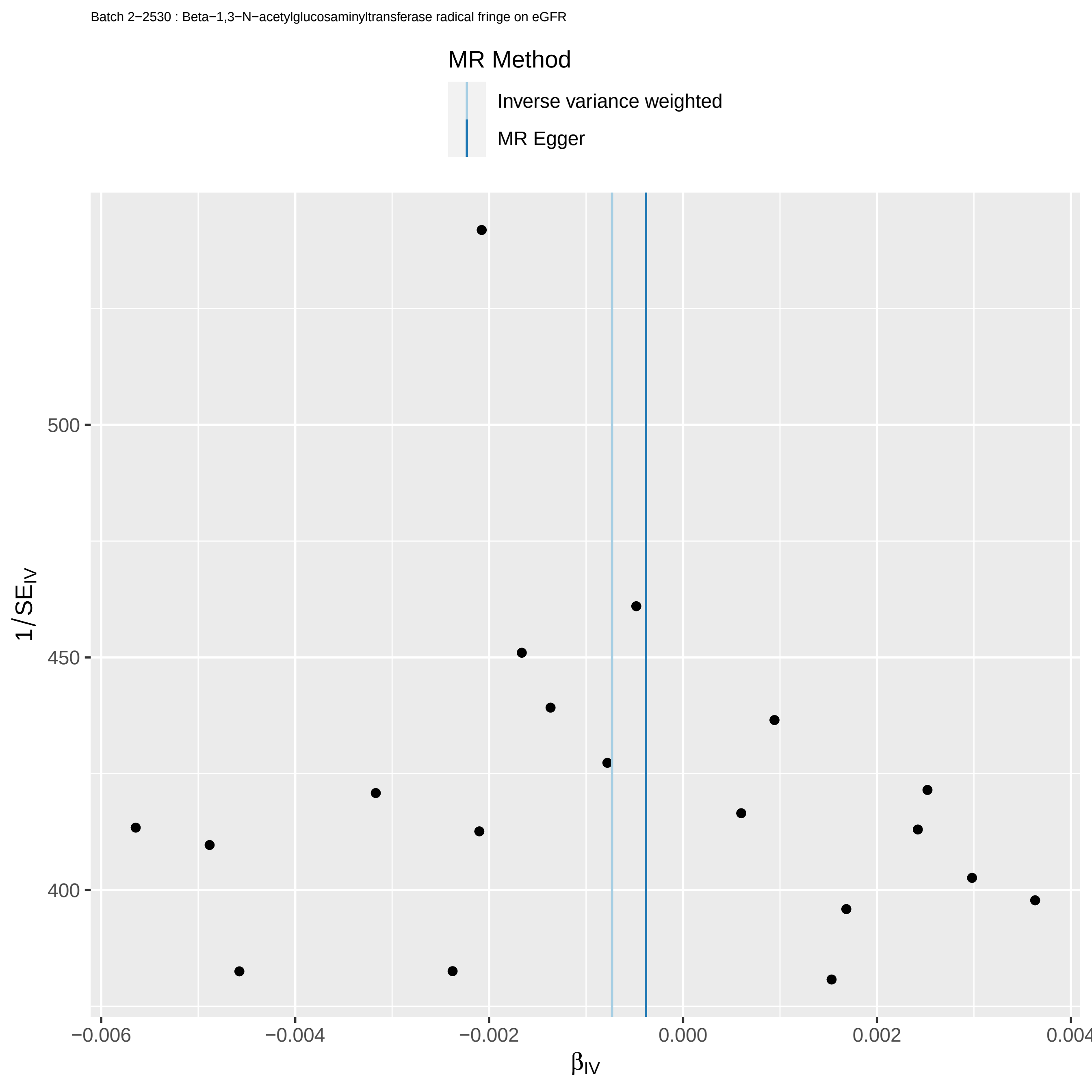

Batch 2−2530 : Beta−1,3−N−acetylglucosaminyltransferase radical fringe on eGFR
MR Method
Inverse variance weighted
MR Egger
500
450
400
1/SE_IV
−0.006
−0.004
−0.002
0.000
0.002
0.004
β_IV

Batch 2−2531 : Regulatory factor X−associated protein on eGFR

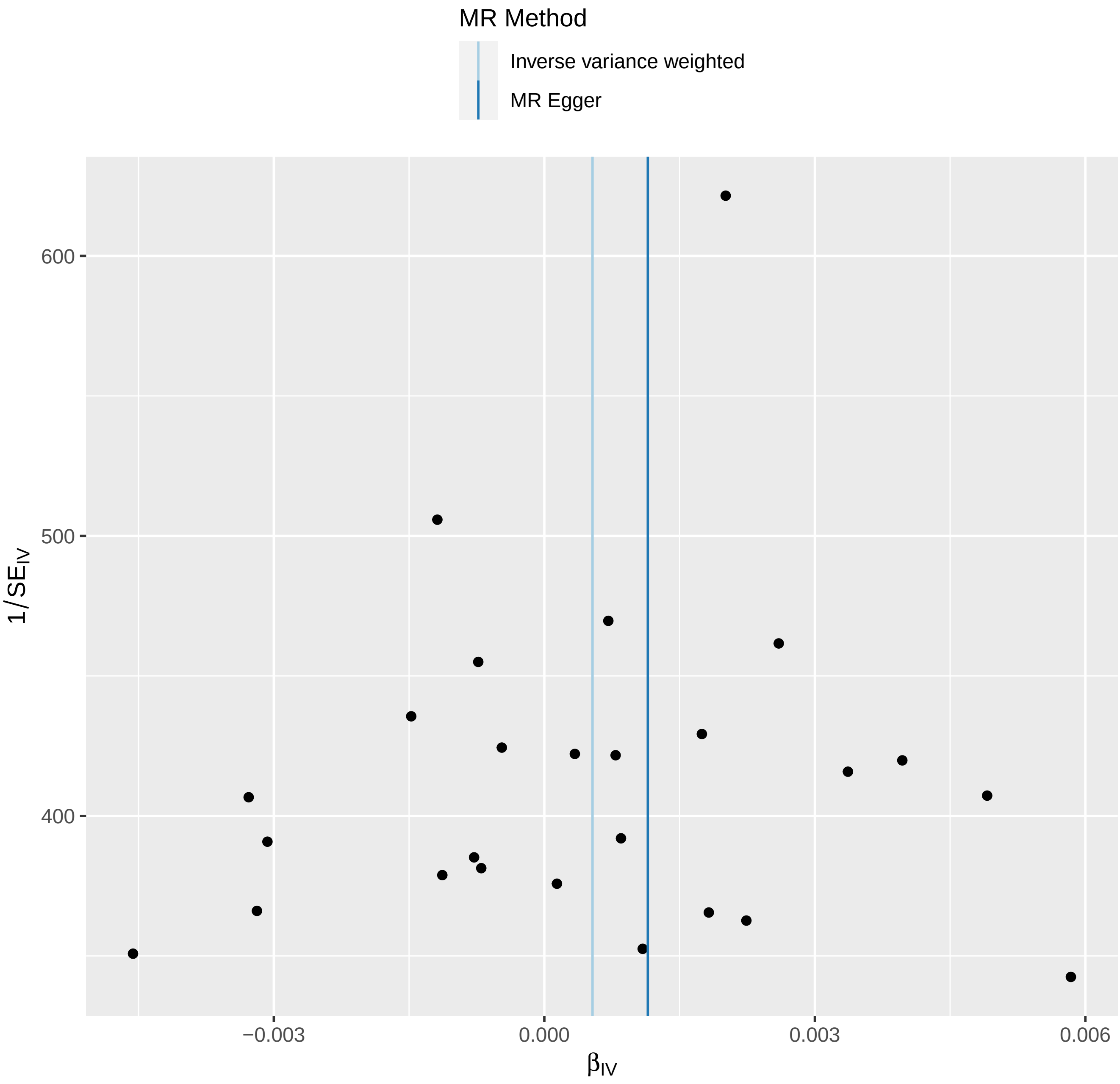

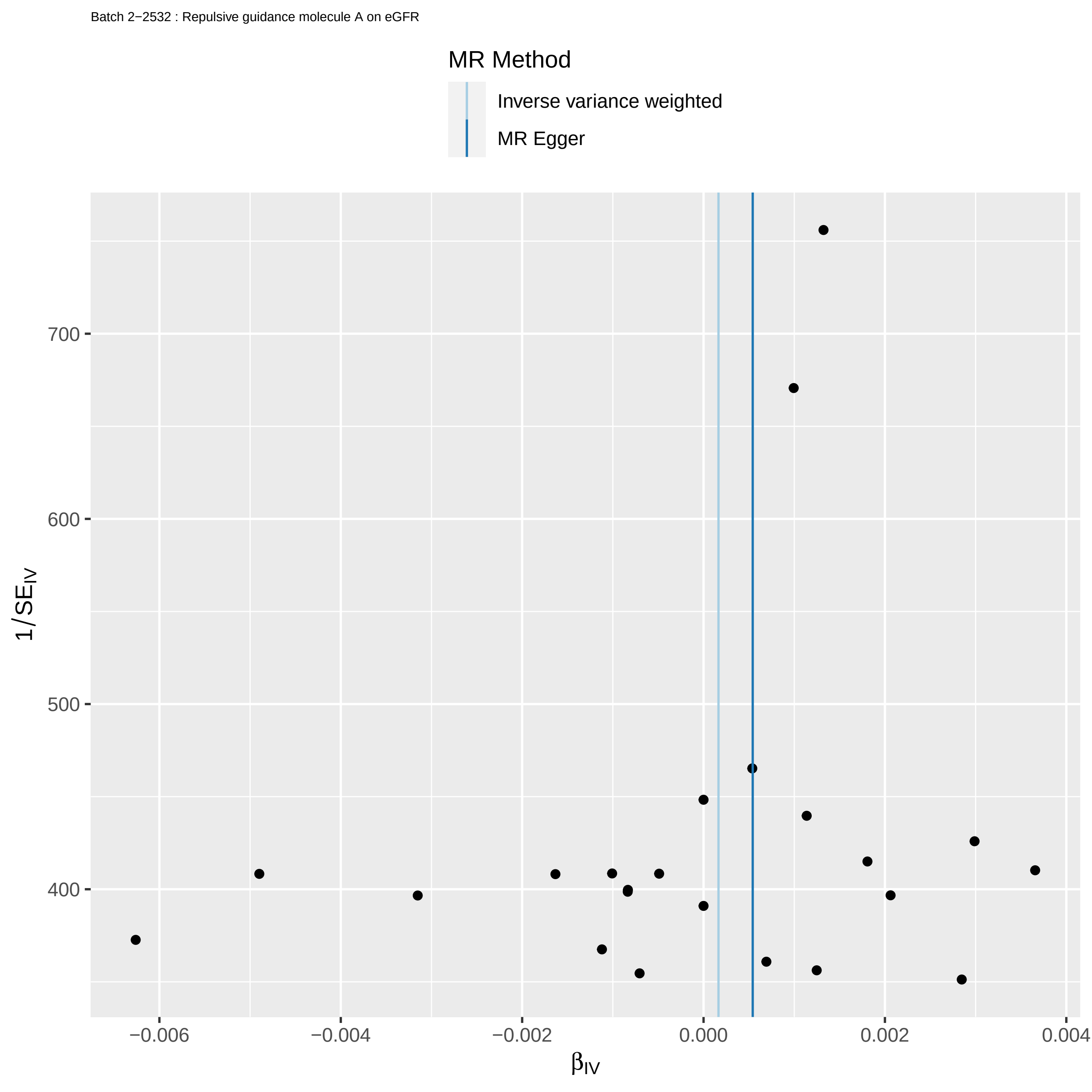
Batch 2−2532 : Repulsive guidance molecule A on eGFR
MR Method
Inverse variance weighted
MR Egger
700
600
500
400
1/SE_IV
−0.006
−0.004
−0.002
0.000
0.002
0.004
β_IV

Batch 2−2533 : RGM domain family member B on eGFR

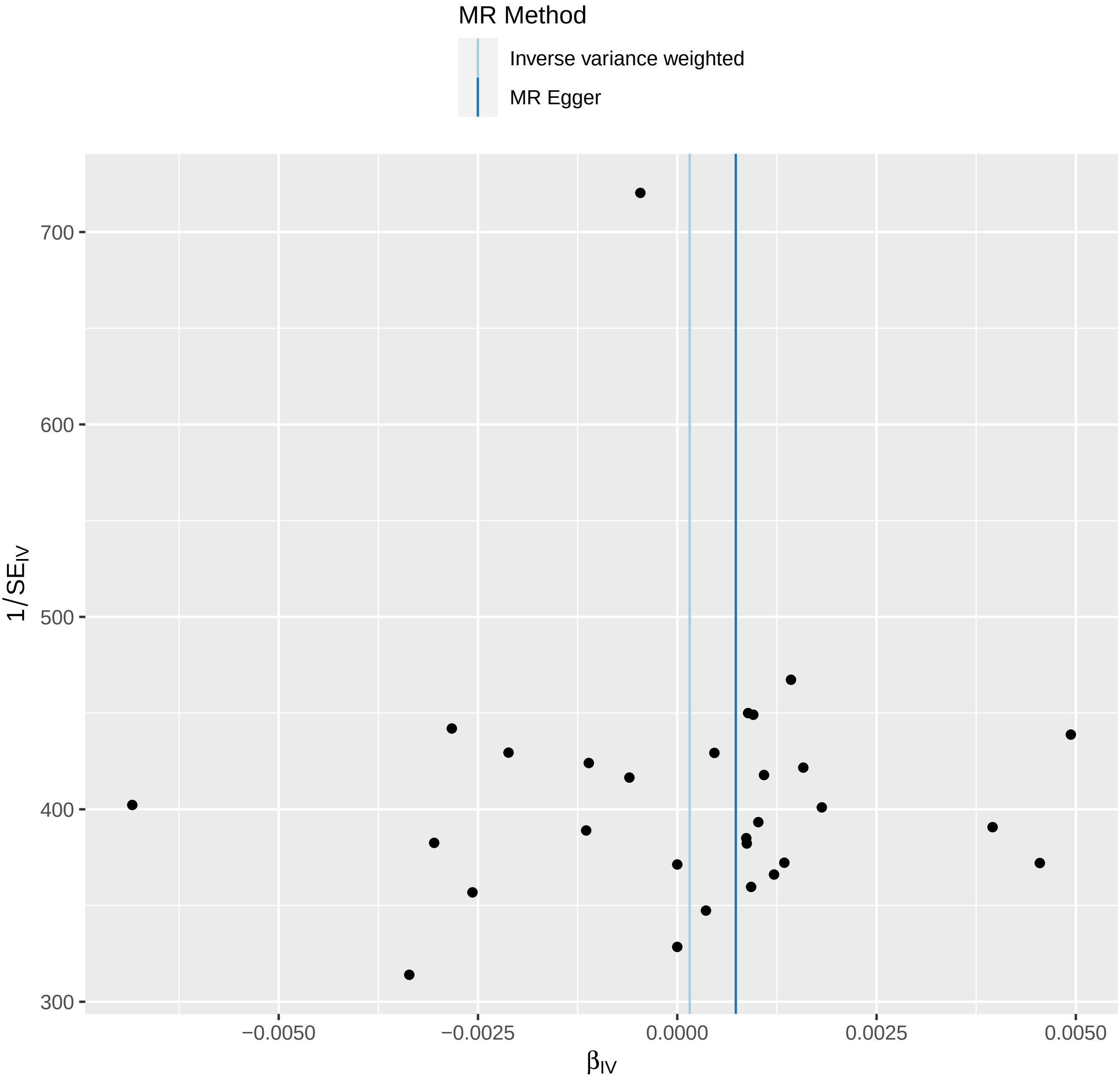

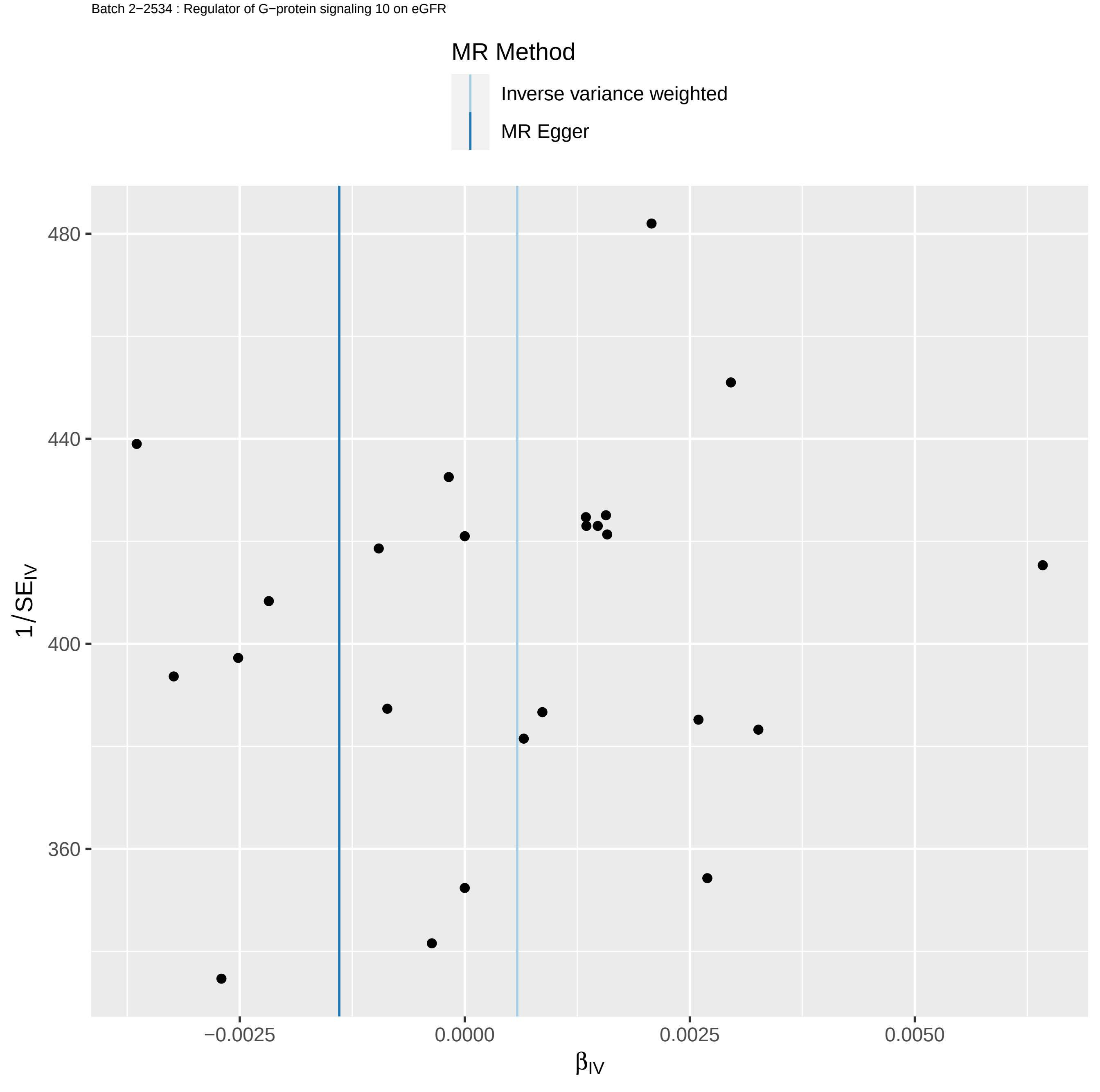

Batch 2−2534 : Regulator of G−protein signaling 10 on eGFR
MR Method
Inverse variance weighted
MR Egger
480
440
400
360
1/SE_IV
−0.0025
0.0000
0.0025
0.0050
β_IV

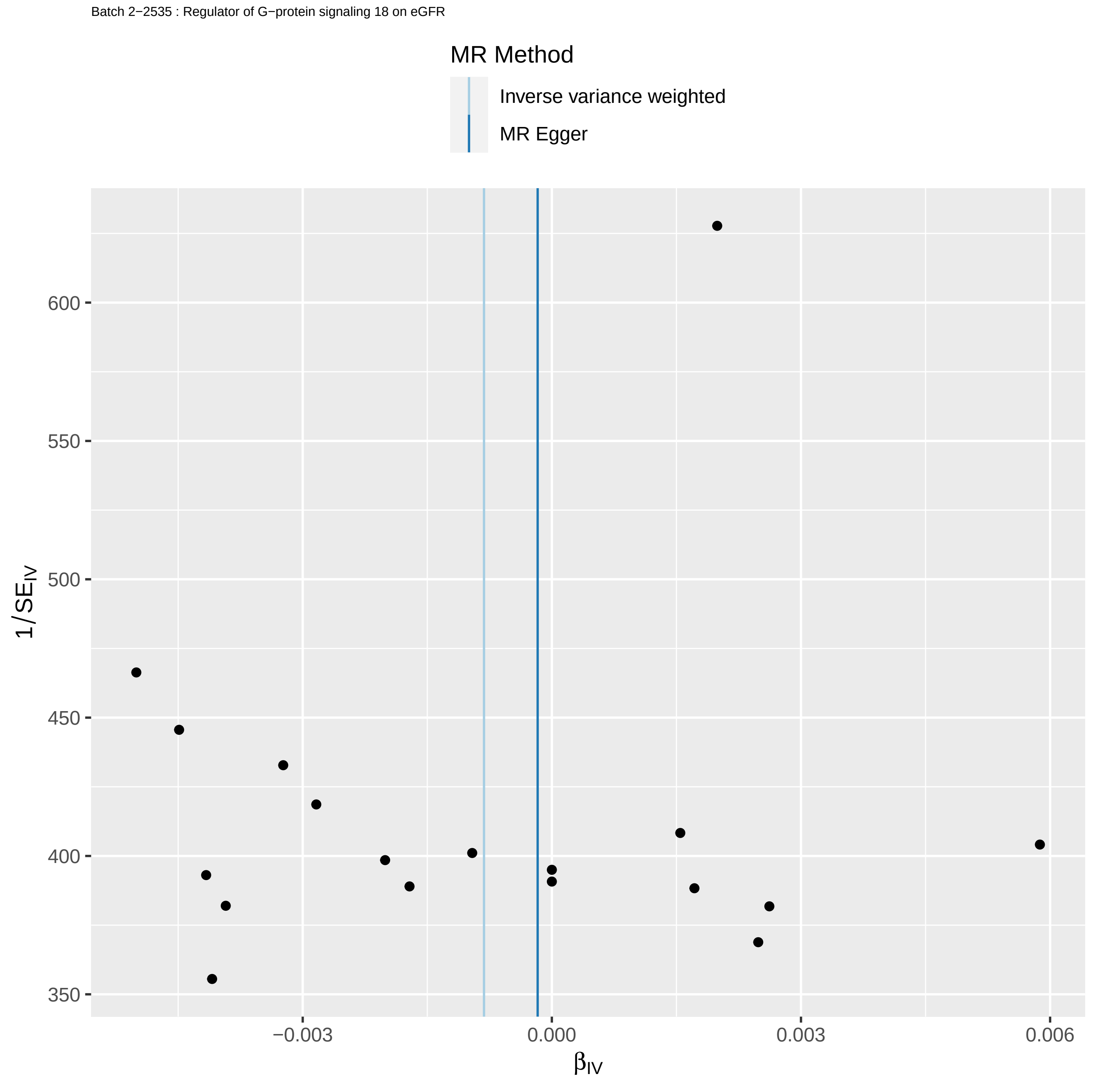

Batch 2−2535 : Regulator of G−protein signaling 18 on eGFR
MR Method
Inverse variance weighted
MR Egger
600
550
500
450
400
350
1/SE_IV
−0.003
0.000
0.003
0.006
β_IV

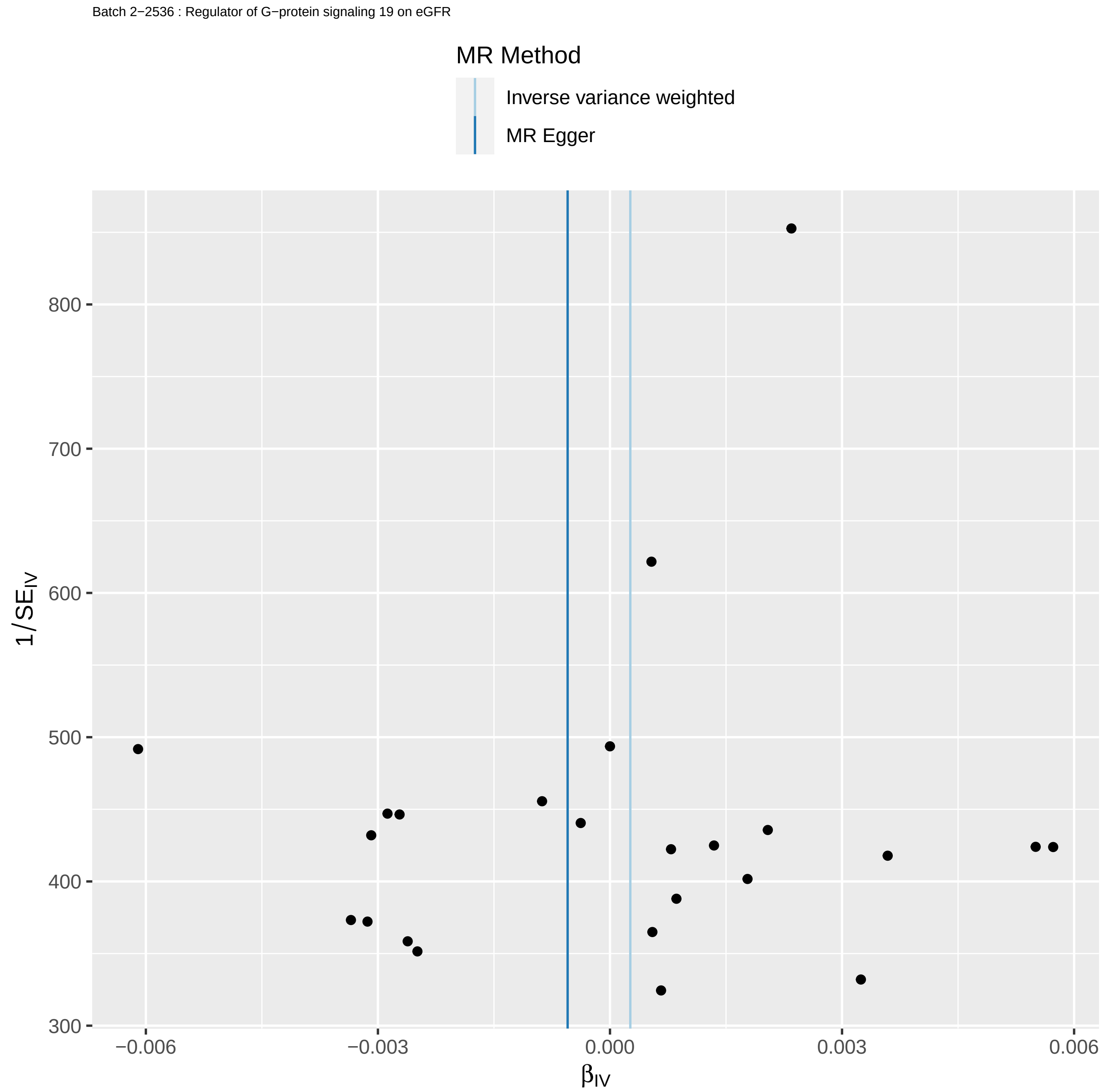

Batch 2−2536 : Regulator of G−protein signaling 19 on eGFR
MR Method
Inverse variance weighted
MR Egger
1/SE_IV
800
700
600
500
400
300
−0.006
−0.003
0.000
0.003
0.006
β_IV

Batch 2−2537 : Regulator of G−protein signaling 3 on eGFR

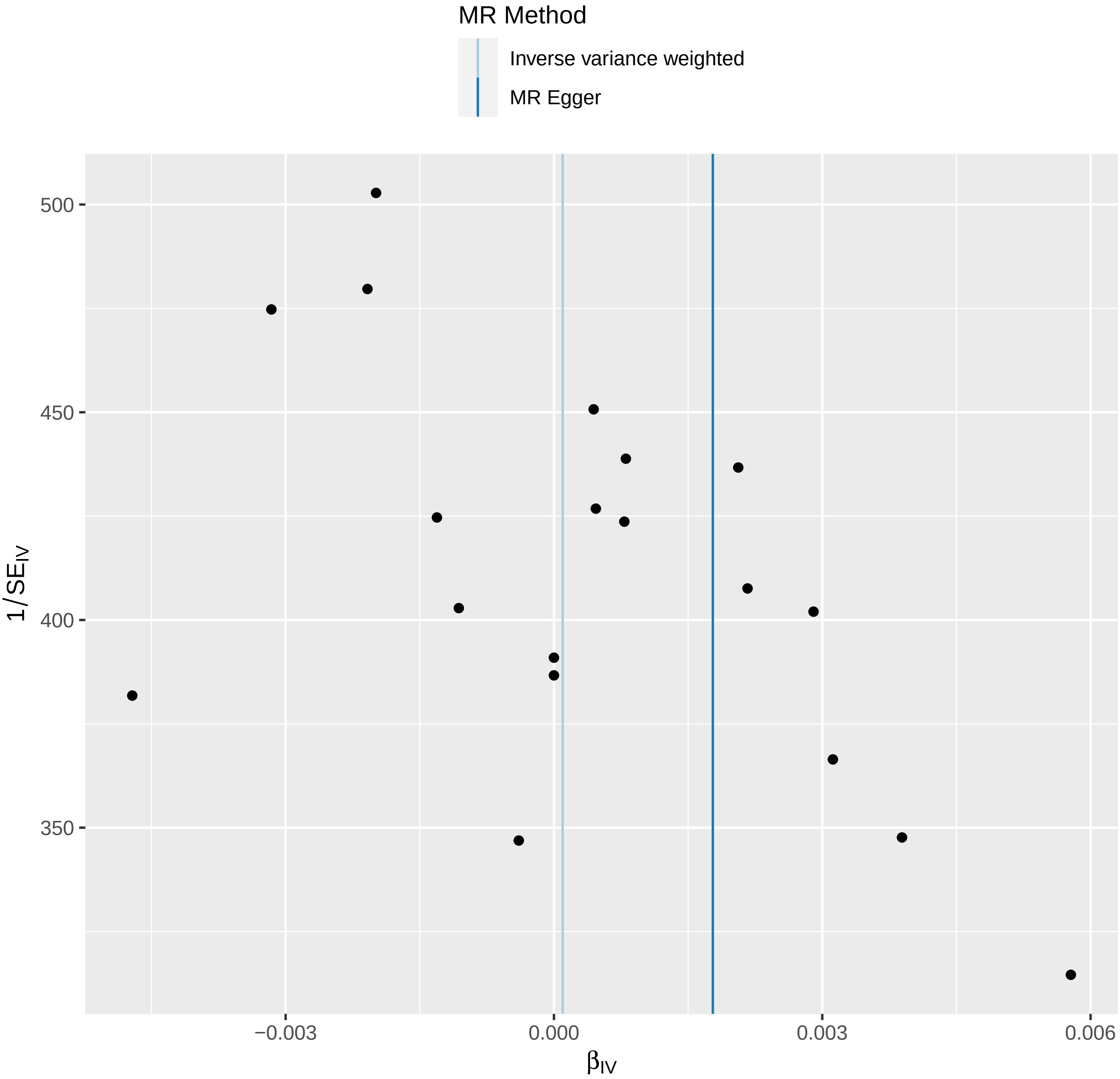

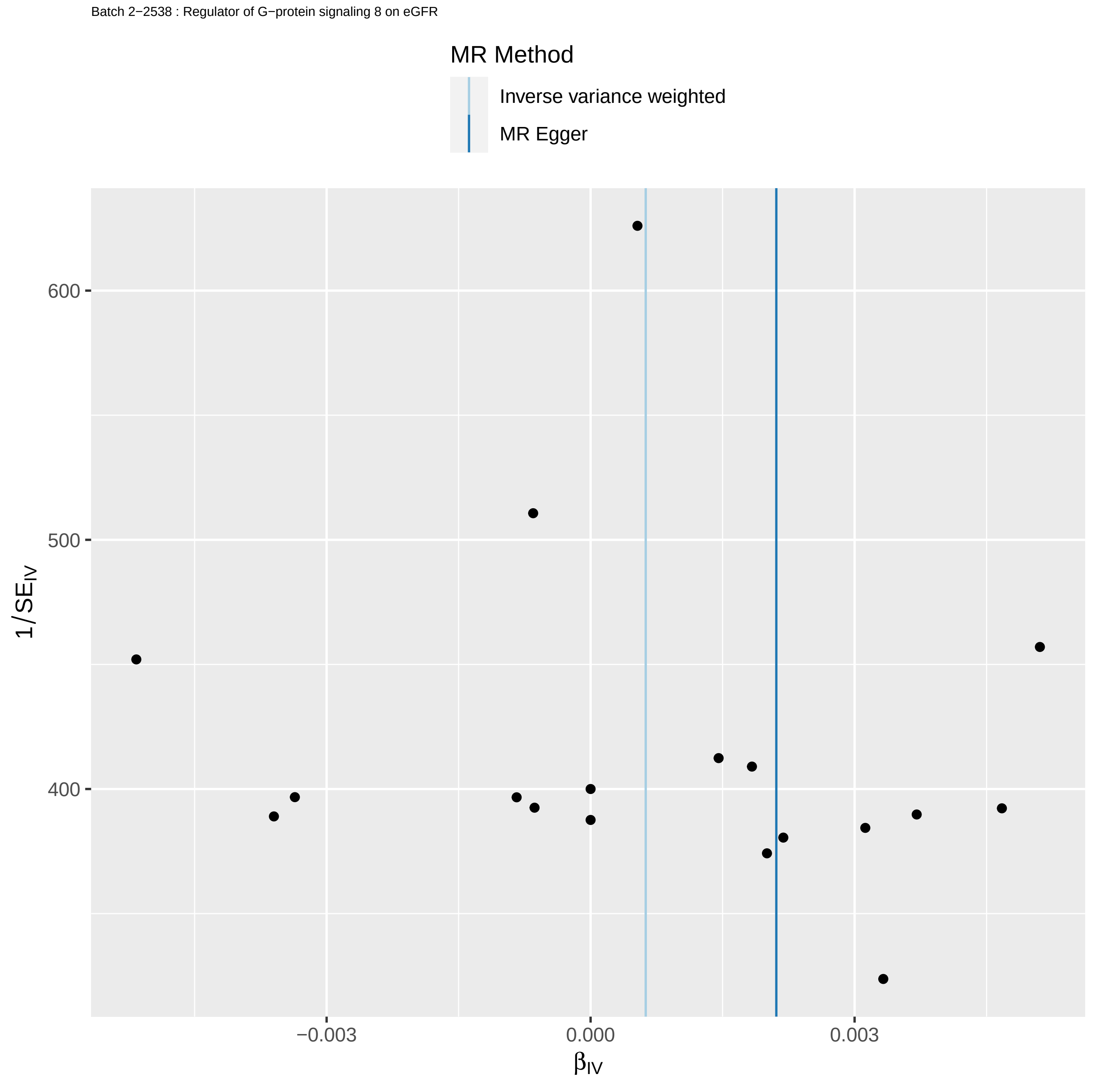

Batch 2−2538 : Regulator of G−protein signaling 8 on eGFR
MR Method
Inverse variance weighted
MR Egger
600
500
400
1/SE_IV
−0.003
0.000
0.003
β_IV

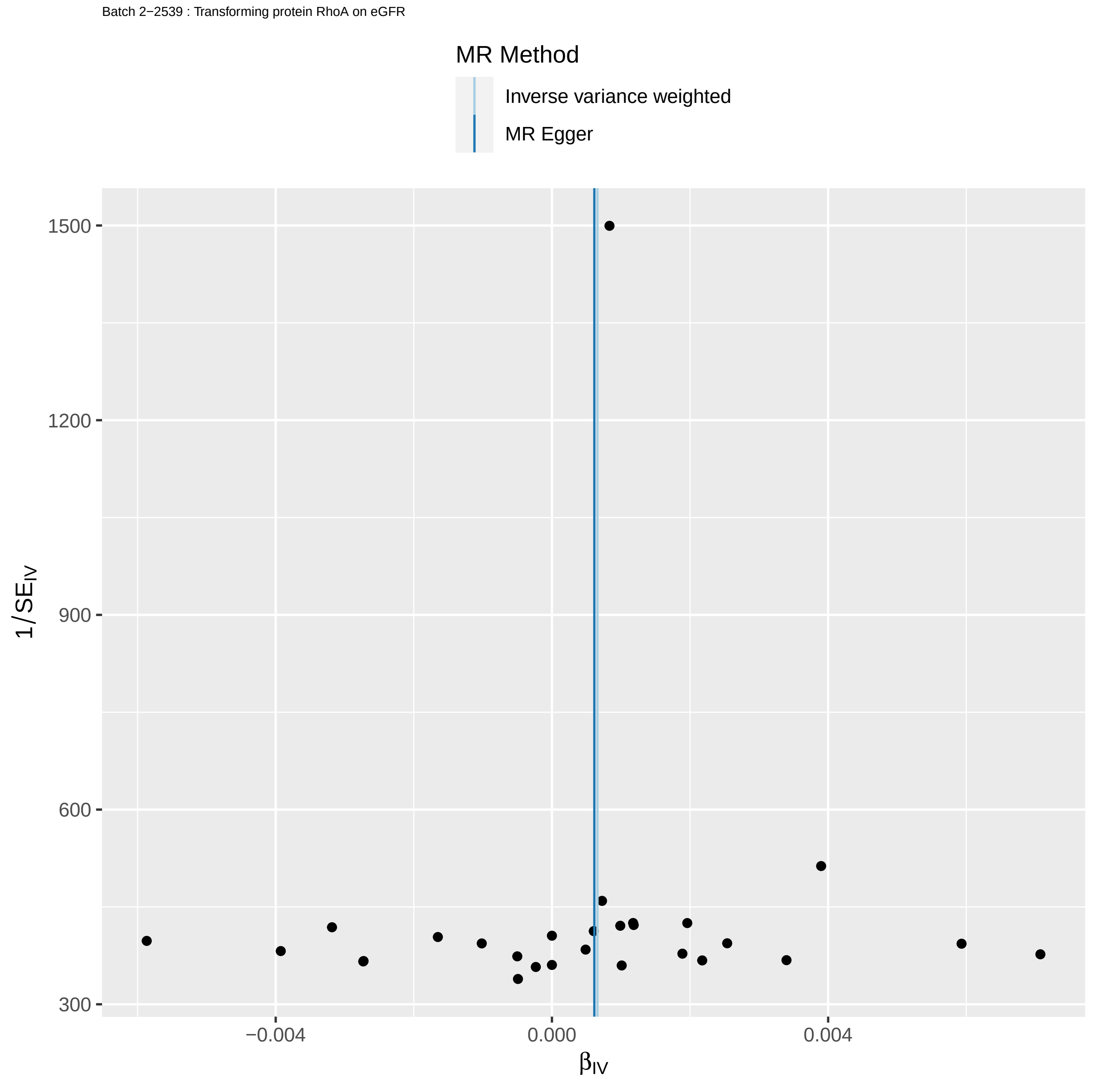
Batch 2−2539 : Transforming protein RhoA on eGFR
MR Method
Inverse variance weighted
MR Egger
1500
1200
900
600
300
1/SE_IV
−0.004
0.000
0.004
β_IV

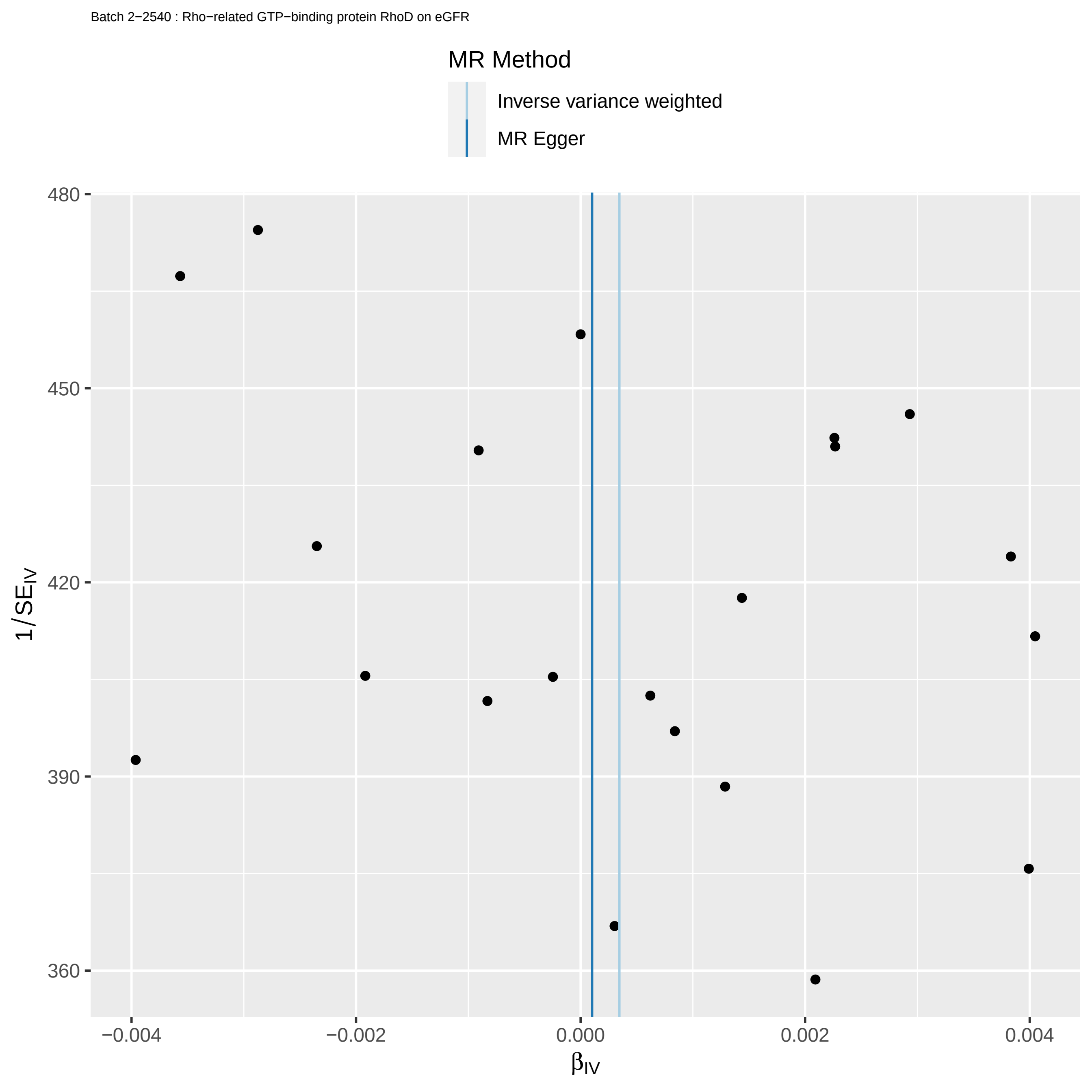

Batch 2−2540 : Rho−related GTP−binding protein RhoD on eGFR
MR Method
Inverse variance weighted
MR Egger
480
450
420
390
360
1/SE_IV
−0.004
−0.002
0.000
0.002
0.004
β_IV

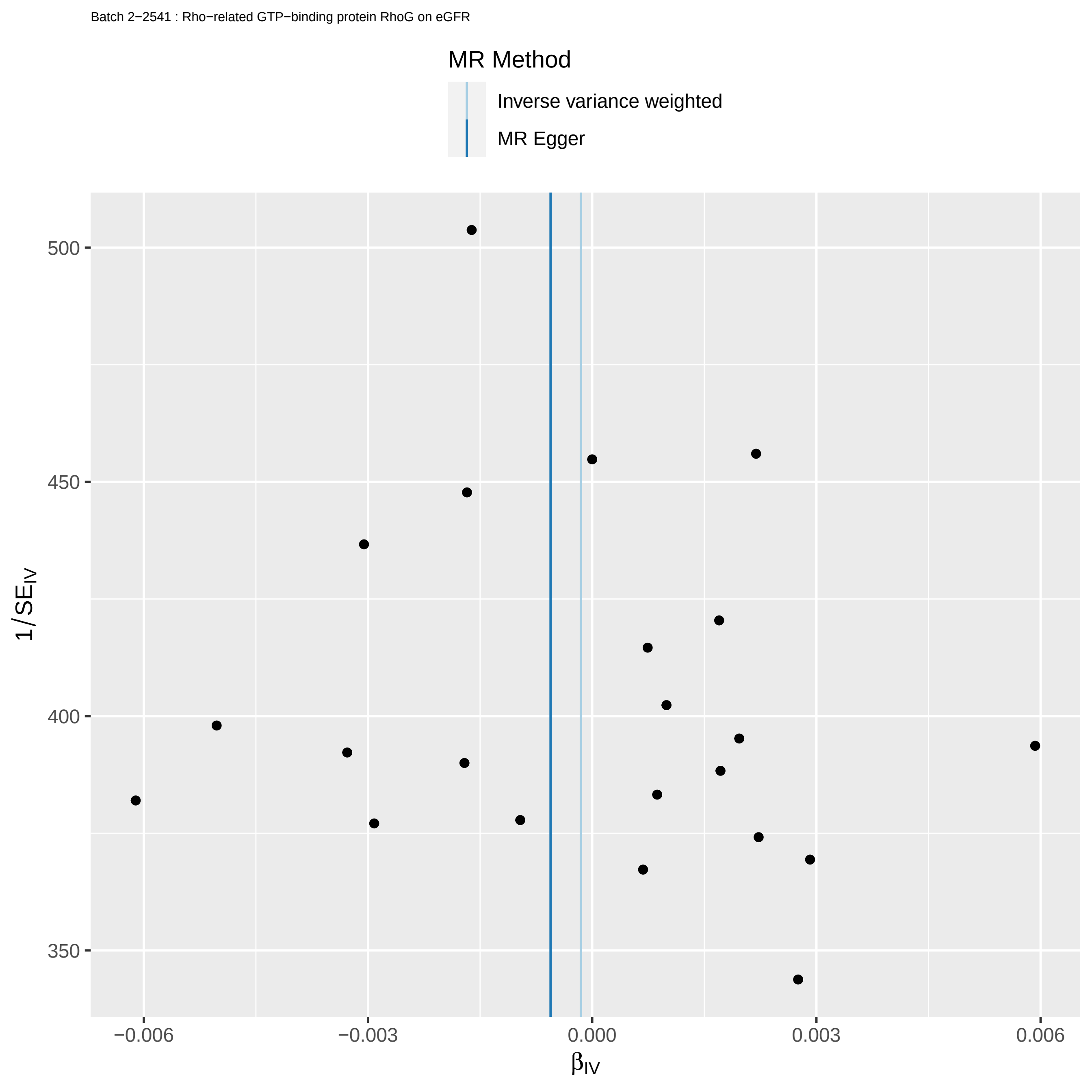

Batch 2−2541 : Rho−related GTP−binding protein RhoG on eGFR
MR Method
Inverse variance weighted
MR Egger
500
450
400
350
1/SE$_{IV}$
−0.006
−0.003
0.000
0.003
0.006
β$_{IV}$

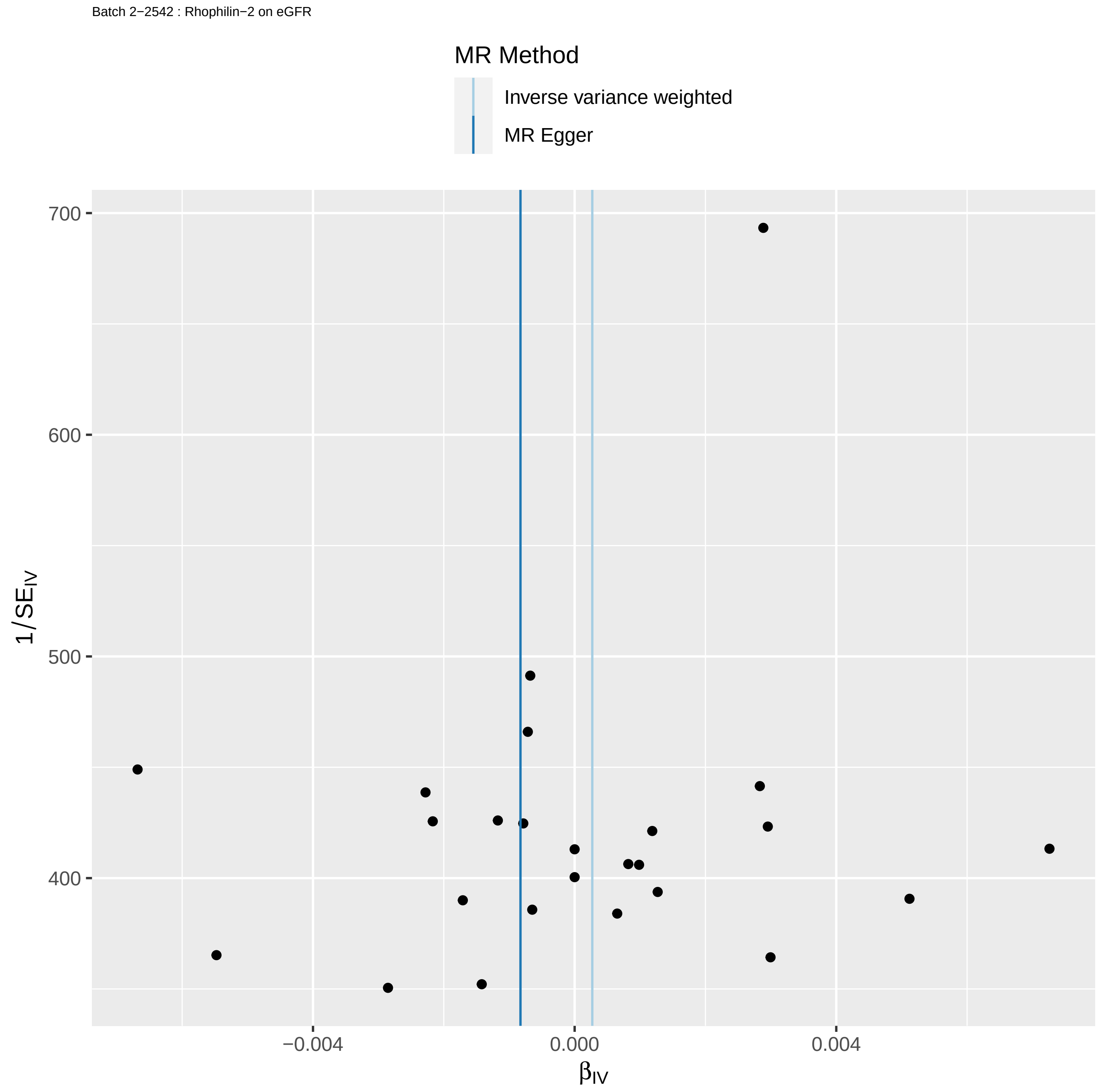
Batch 2−2542 : Rhophilin−2 on eGFR
MR Method
Inverse variance weighted
MR Egger
700
600
500
400
1/SE_IV
−0.004
0.000
0.004
β_IV

Batch 2−2543 : Protein RIC−3 on eGFR

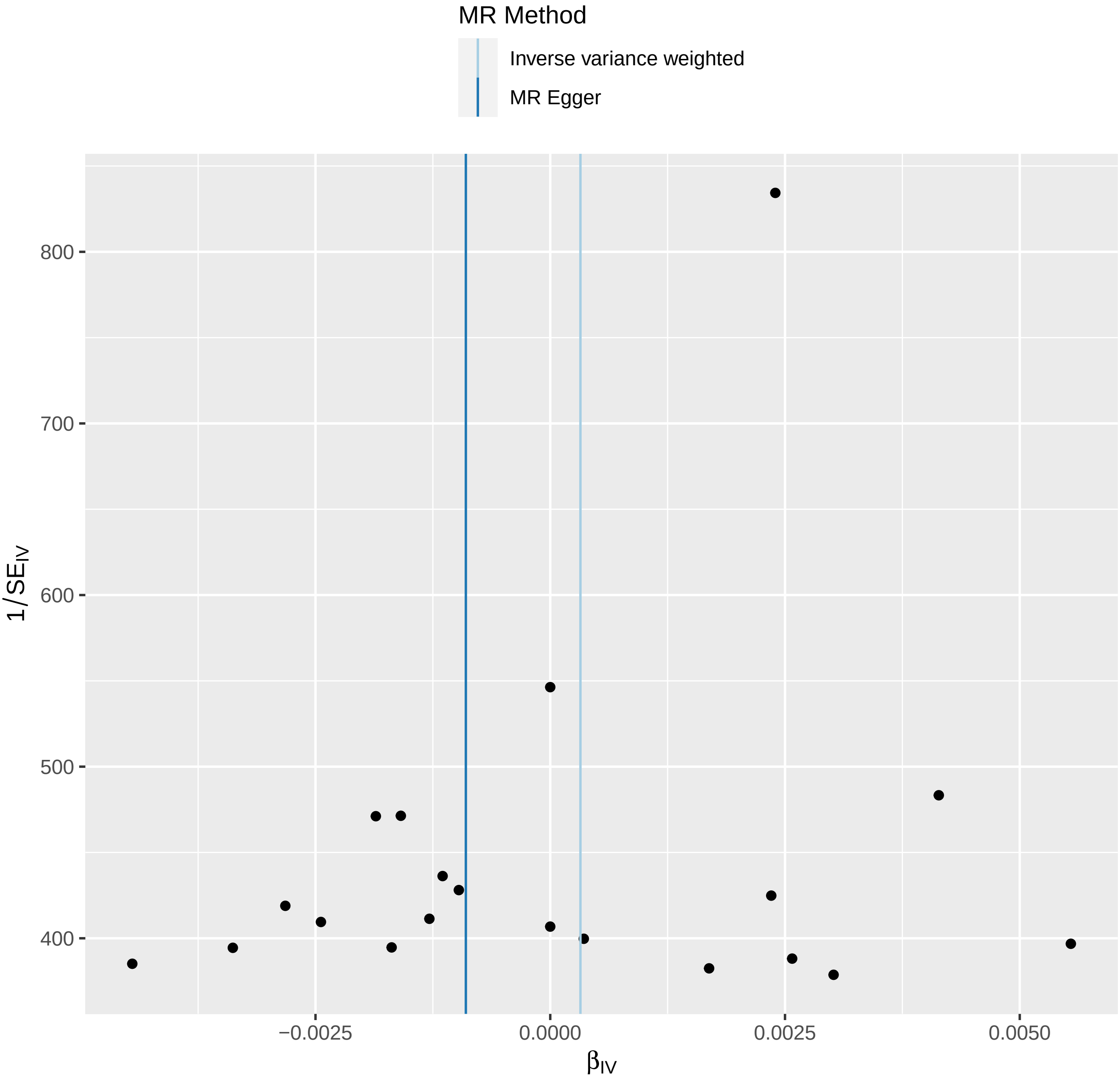

Batch 2−2544 : Protein RIC−3 on eGFR

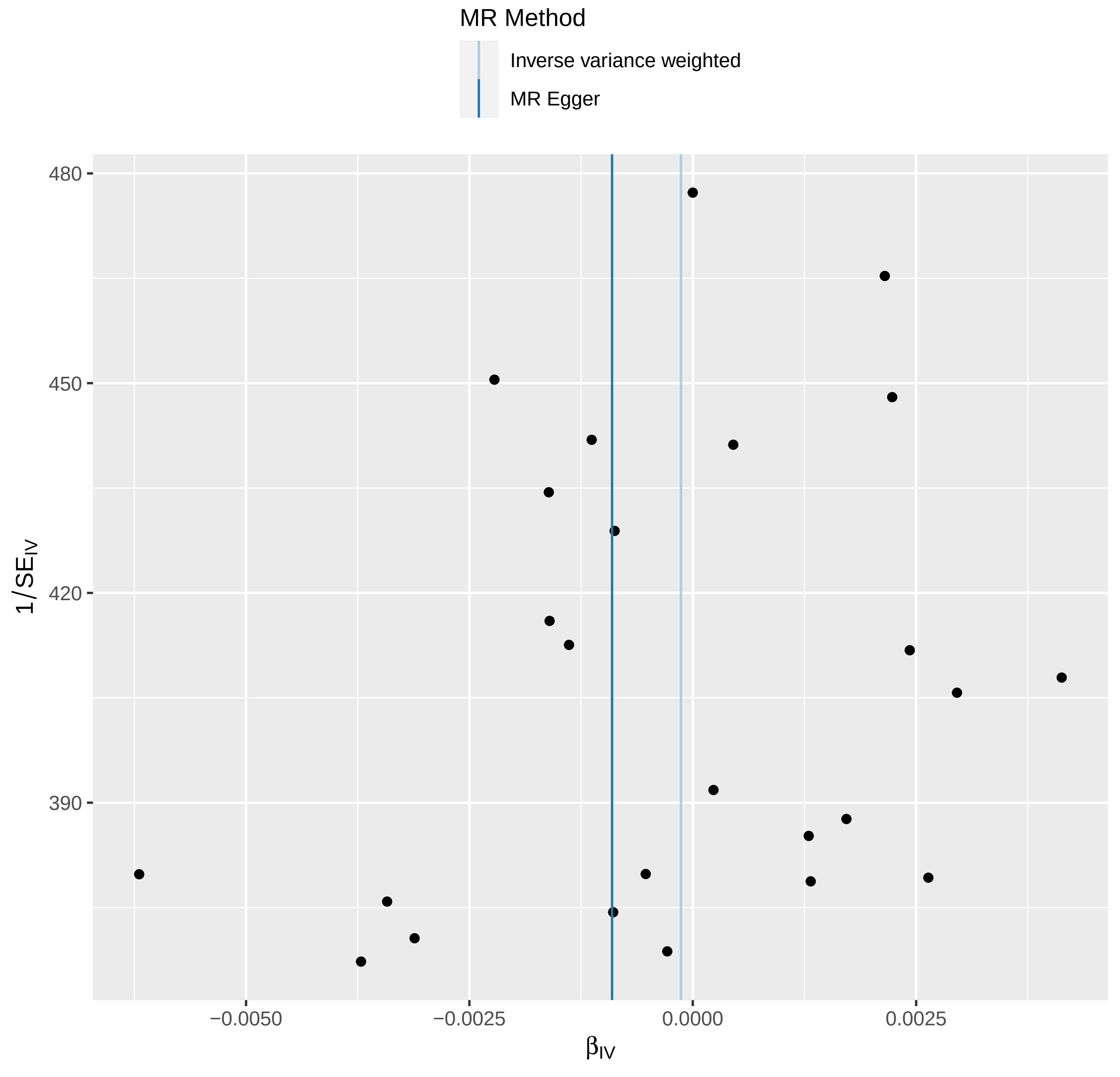

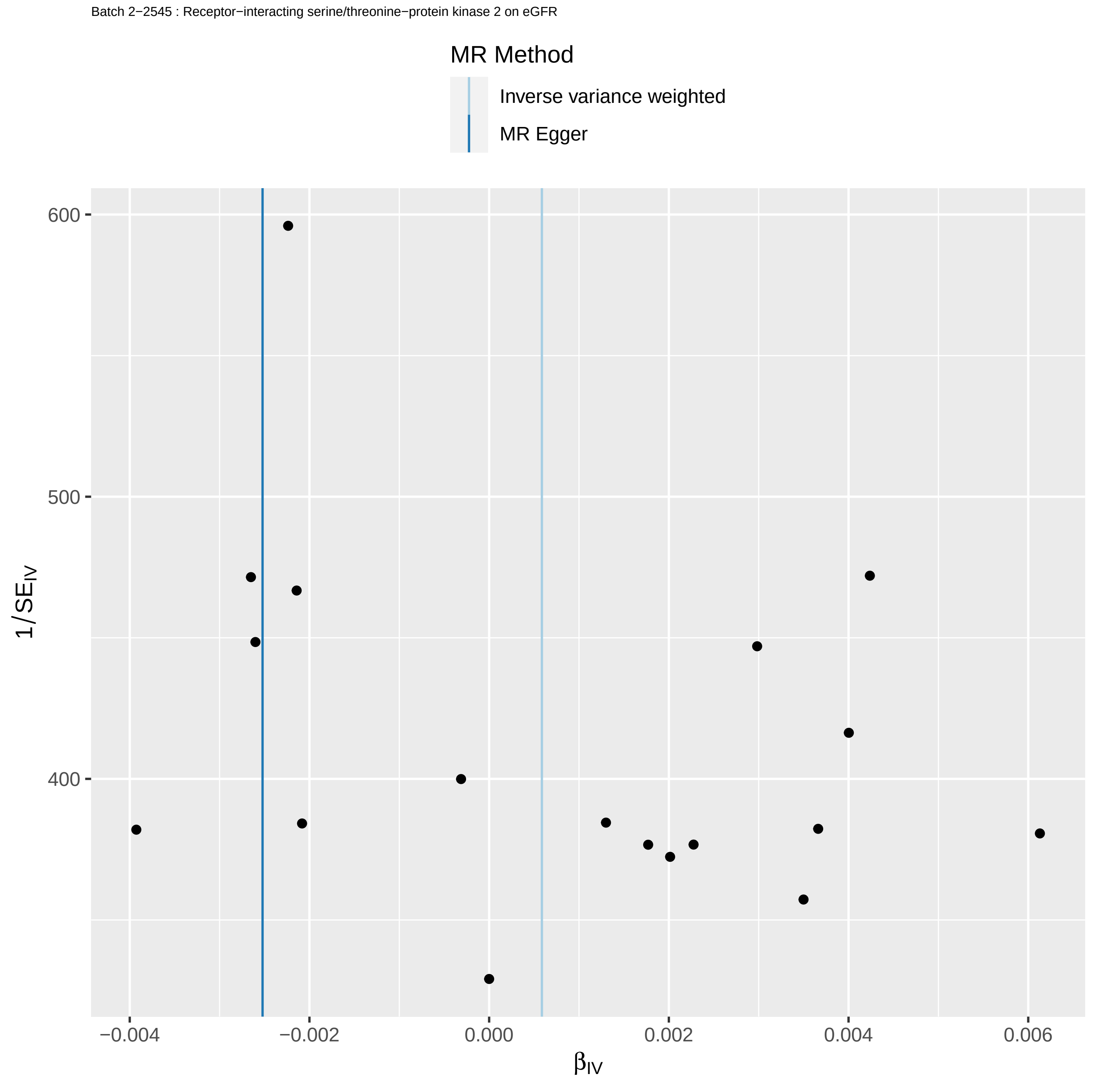

Batch 2−2545 : Receptor−interacting serine/threonine−protein kinase 2 on eGFR
MR Method
Inverse variance weighted
MR Egger
600
500
400
1/SE$_{IV}$
−0.004
−0.002
0.000
0.002
0.004
0.006
β$_{IV}$

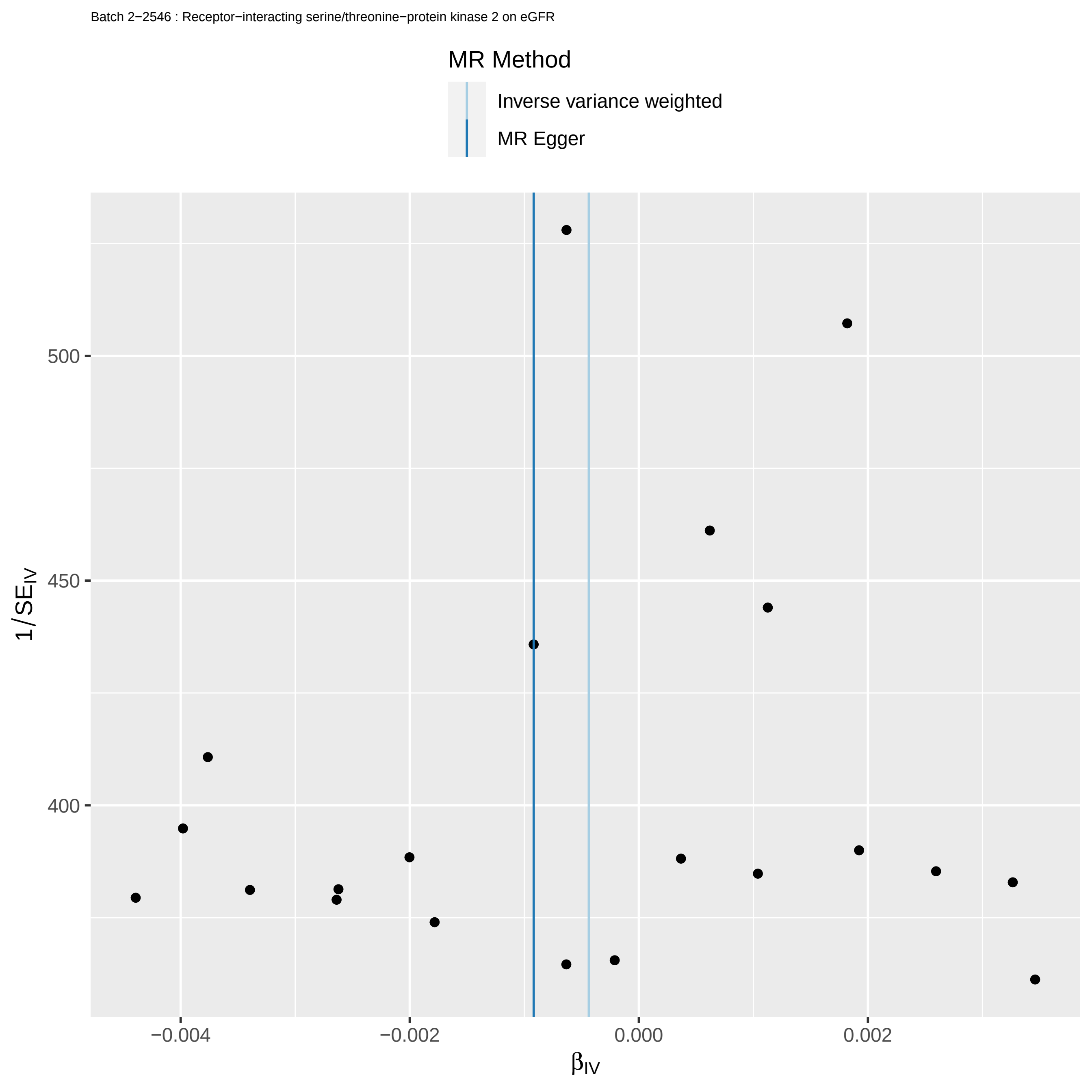
Batch 2−2546 : Receptor−interacting serine/threonine−protein kinase 2 on eGFR
MR Method
Inverse variance weighted
MR Egger
500
450
400
1/SE_IV
−0.004
−0.002
0.000
0.002
β_IV

Batch 2−2547 : Protein ripply1 on eGFR

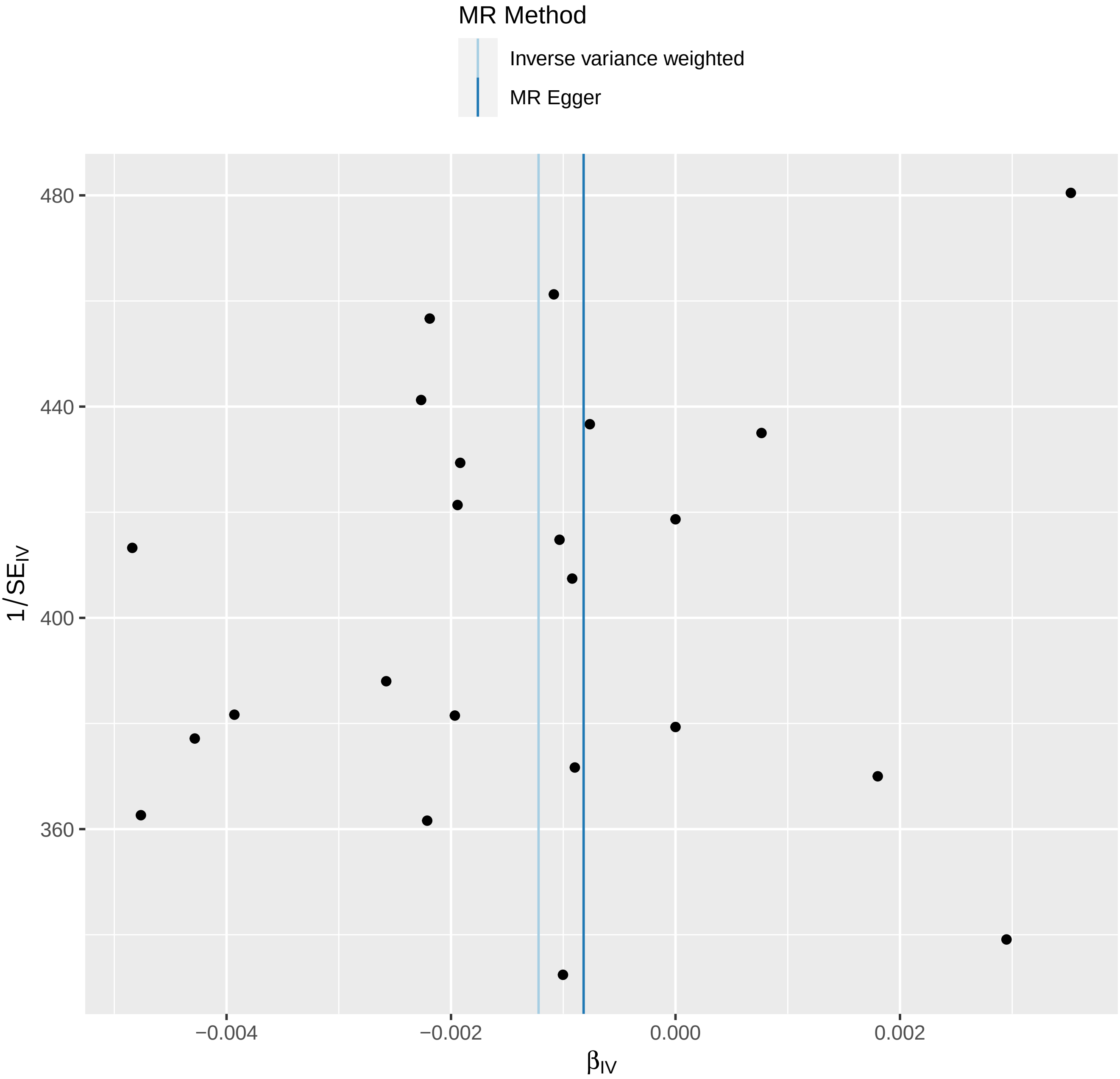

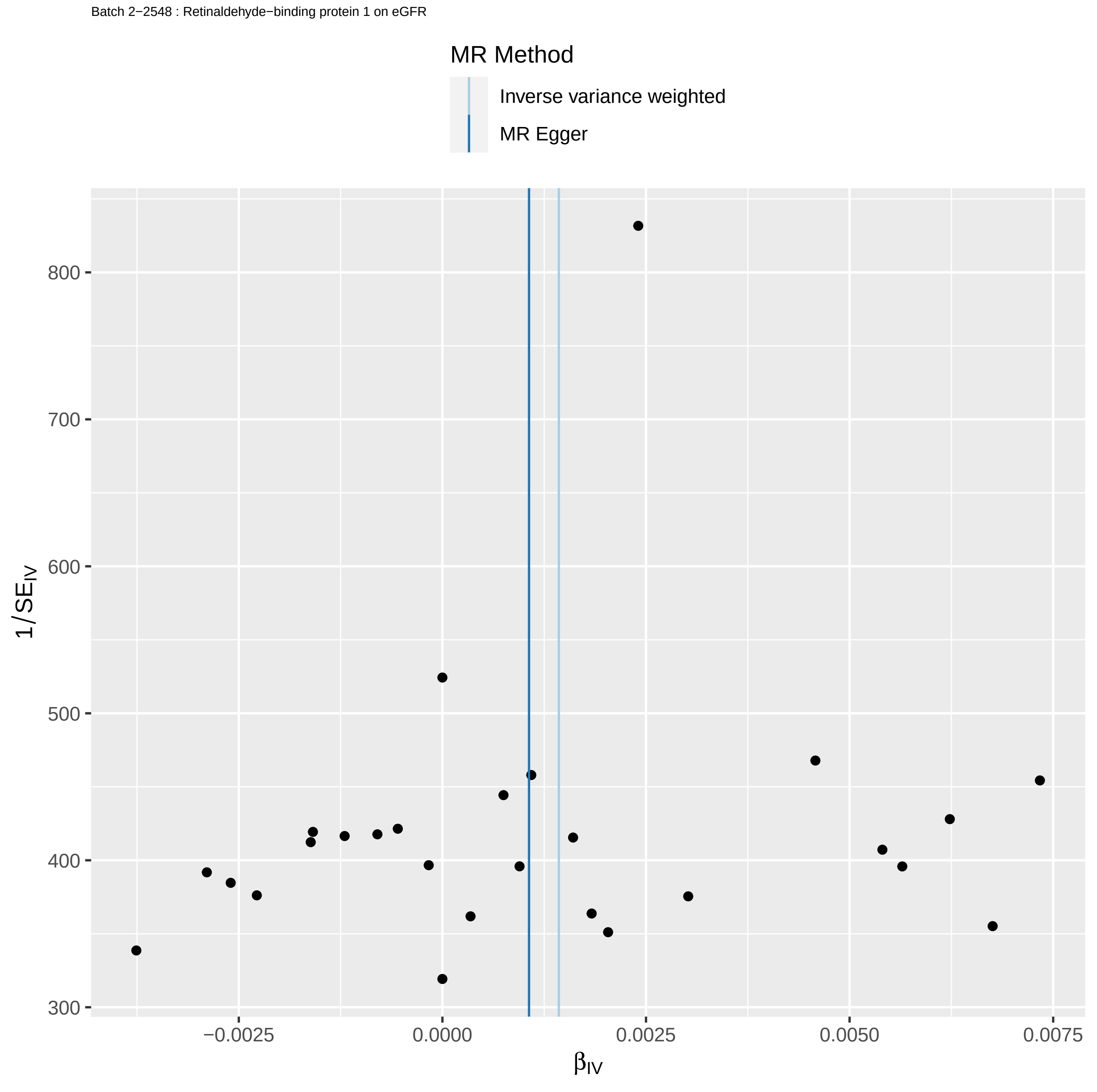

Batch 2−2548 : Retinaldehyde−binding protein 1 on eGFR
MR Method
Inverse variance weighted
MR Egger
800
700
600
500
400
300
1/SE$_{IV}$
−0.0025
0.0000
0.0025
0.0050
0.0075
β$_{IV}$

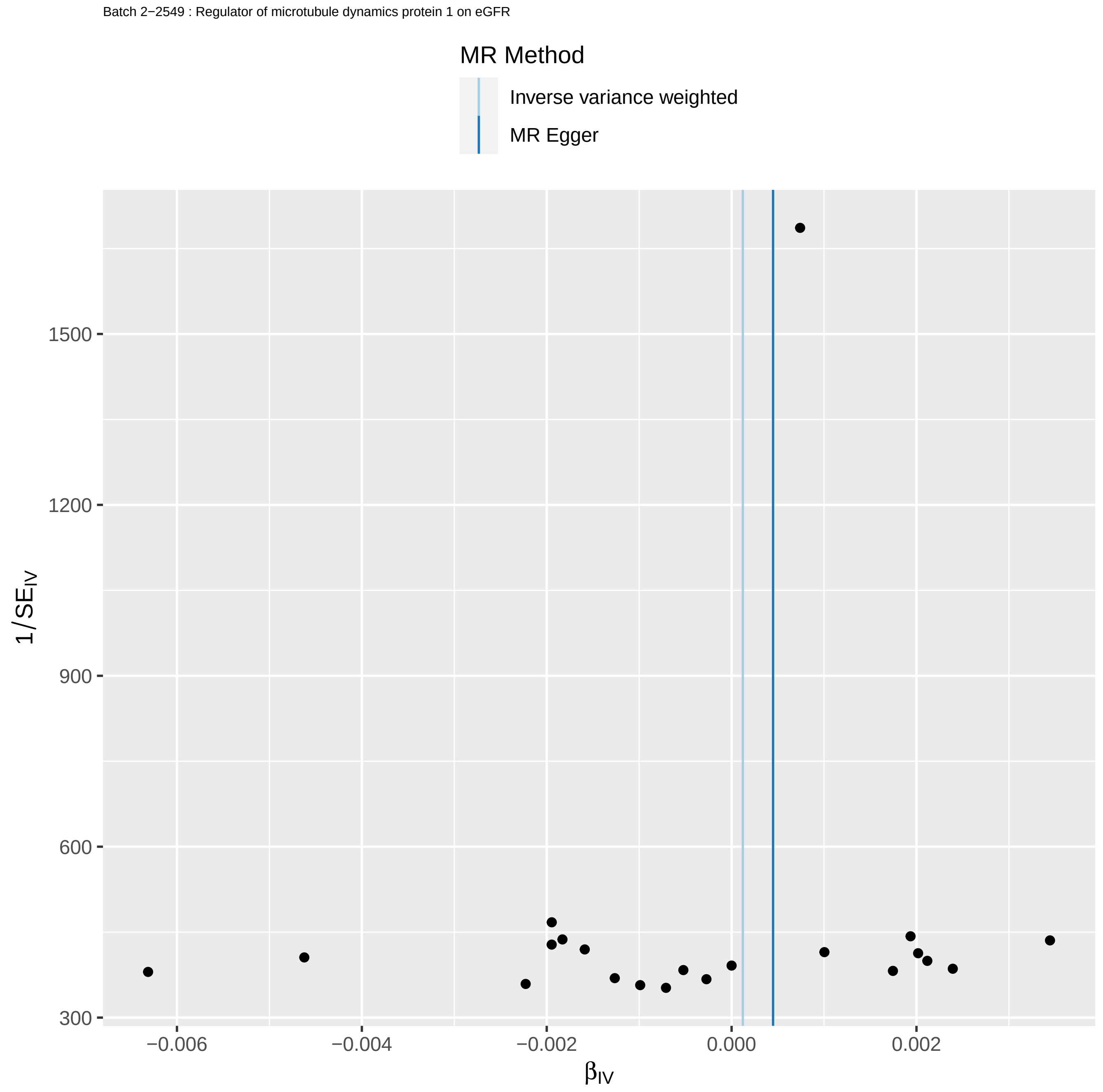
Batch 2−2549 : Regulator of microtubule dynamics protein 1 on eGFR
MR Method
Inverse variance weighted
MR Egger
1500
1200
900
600
300
1/SE$_{IV}$
−0.006
−0.004
−0.002
0.000
0.002
β$_{IV}$

Batch 2−2550 : Regulator of microtubule dynamics protein 3 on eGFR

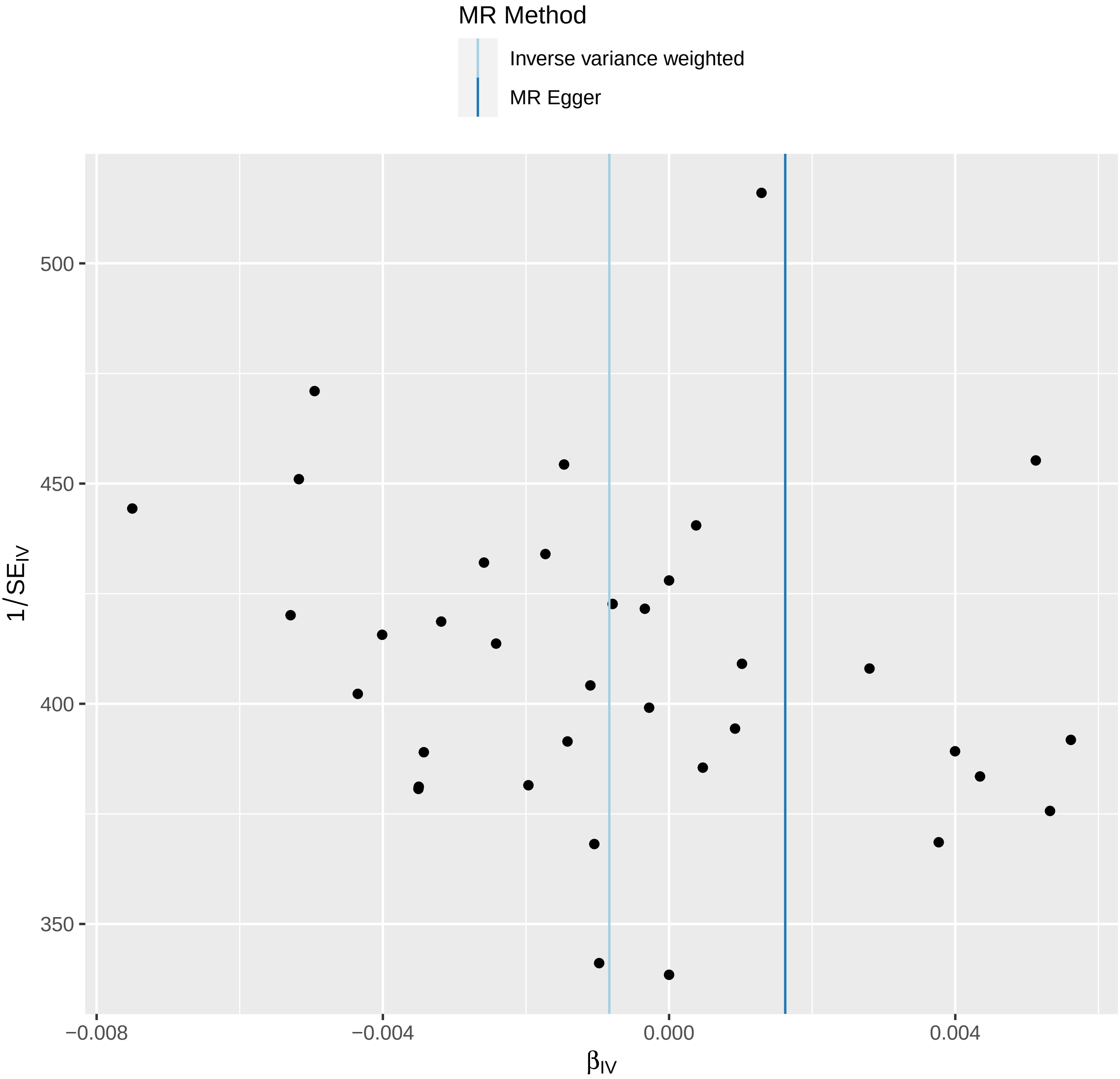

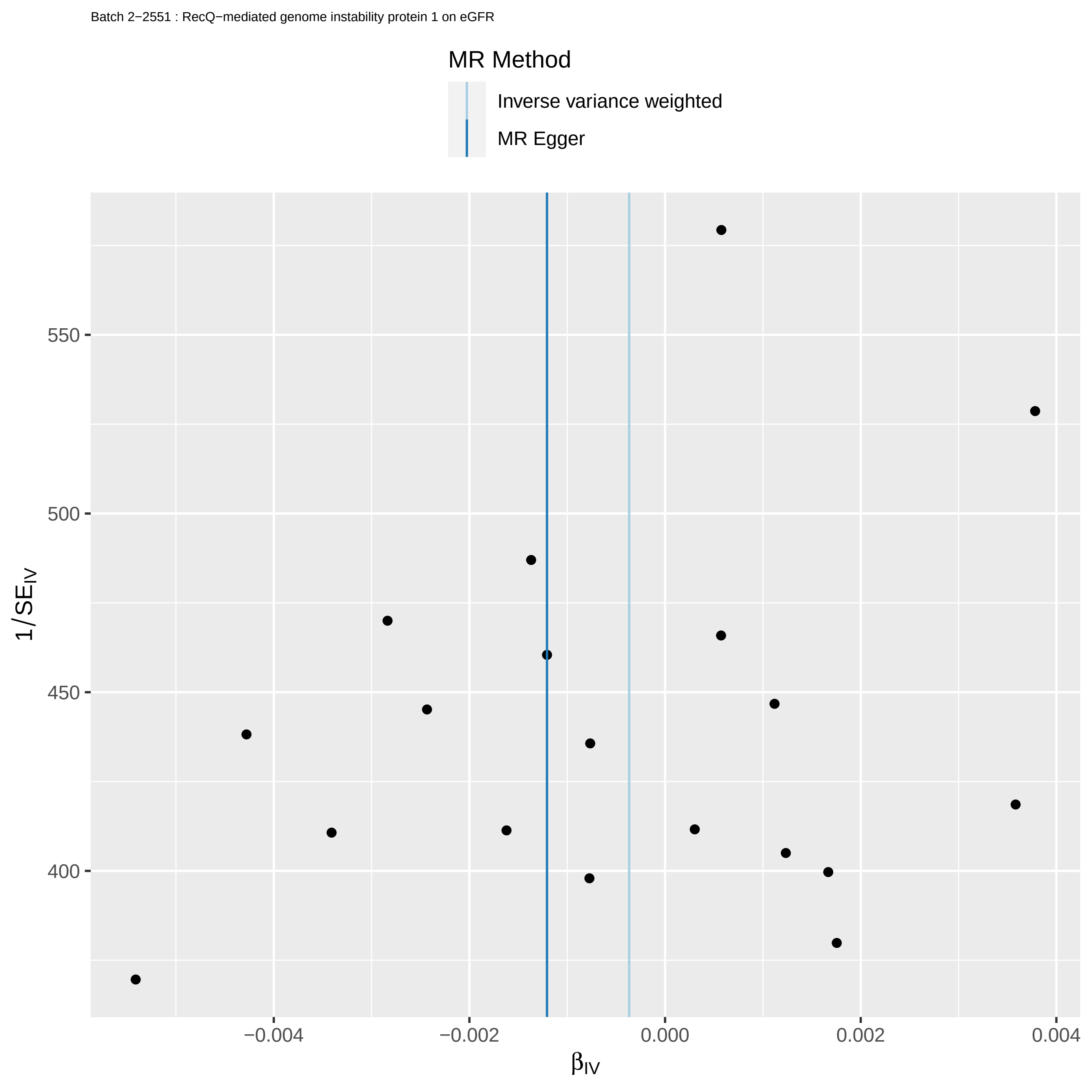

Batch 2−2551 : RecQ−mediated genome instability protein 1 on eGFR
MR Method
Inverse variance weighted
MR Egger
550
500
450
400
1/SE$_{IV}$
−0.004
−0.002
0.000
0.002
0.004
β$_{IV}$

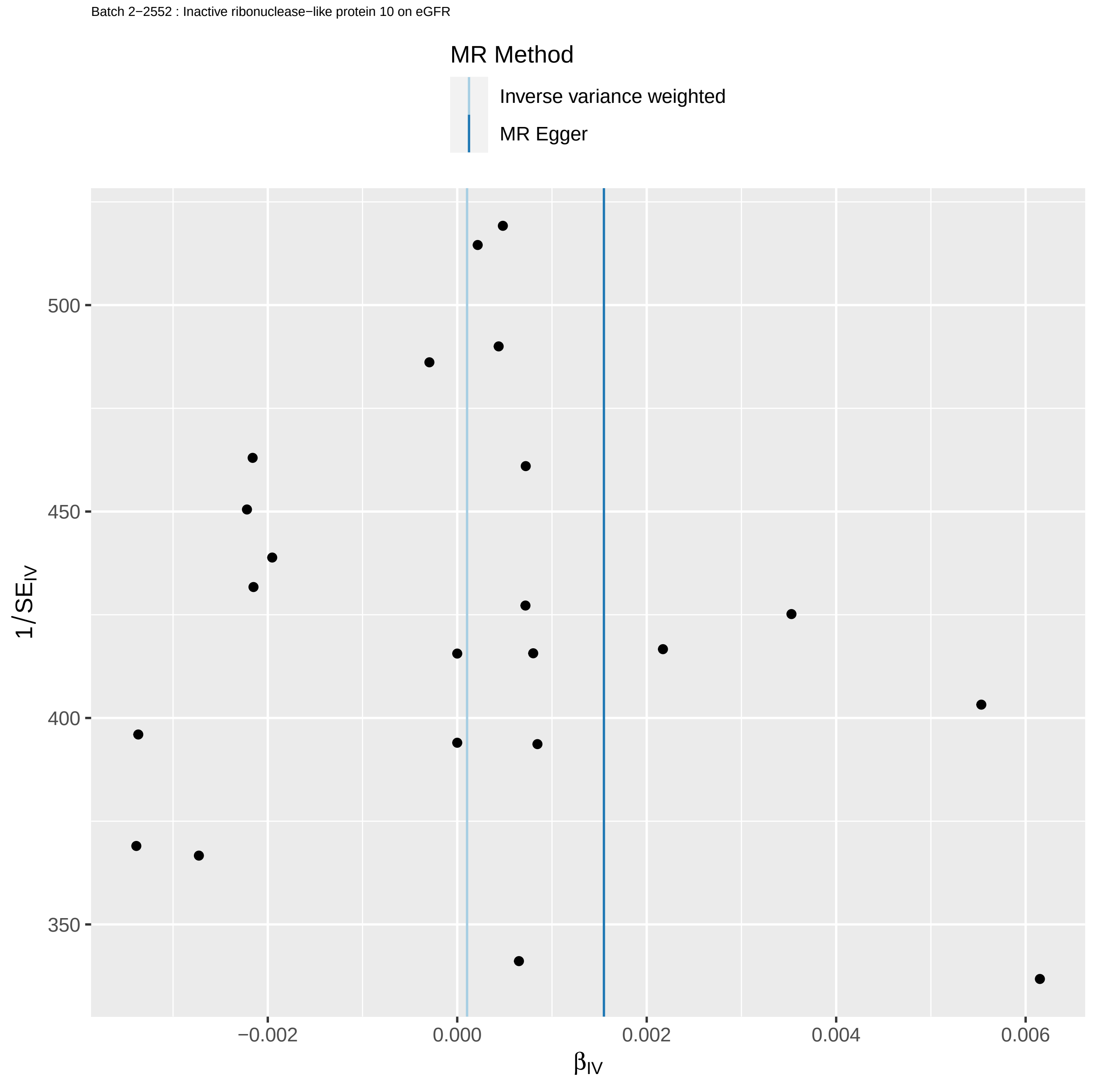

Batch 2−2552 : Inactive ribonuclease−like protein 10 on eGFR
MR Method
Inverse variance weighted
MR Egger
500
450
400
350
$1/SE_{IV}$
−0.002
0.000
0.002
0.004
0.006
β_{IV}

Batch 2−2553 : Probable inactive ribonuclease−like protein 13 on eGFR

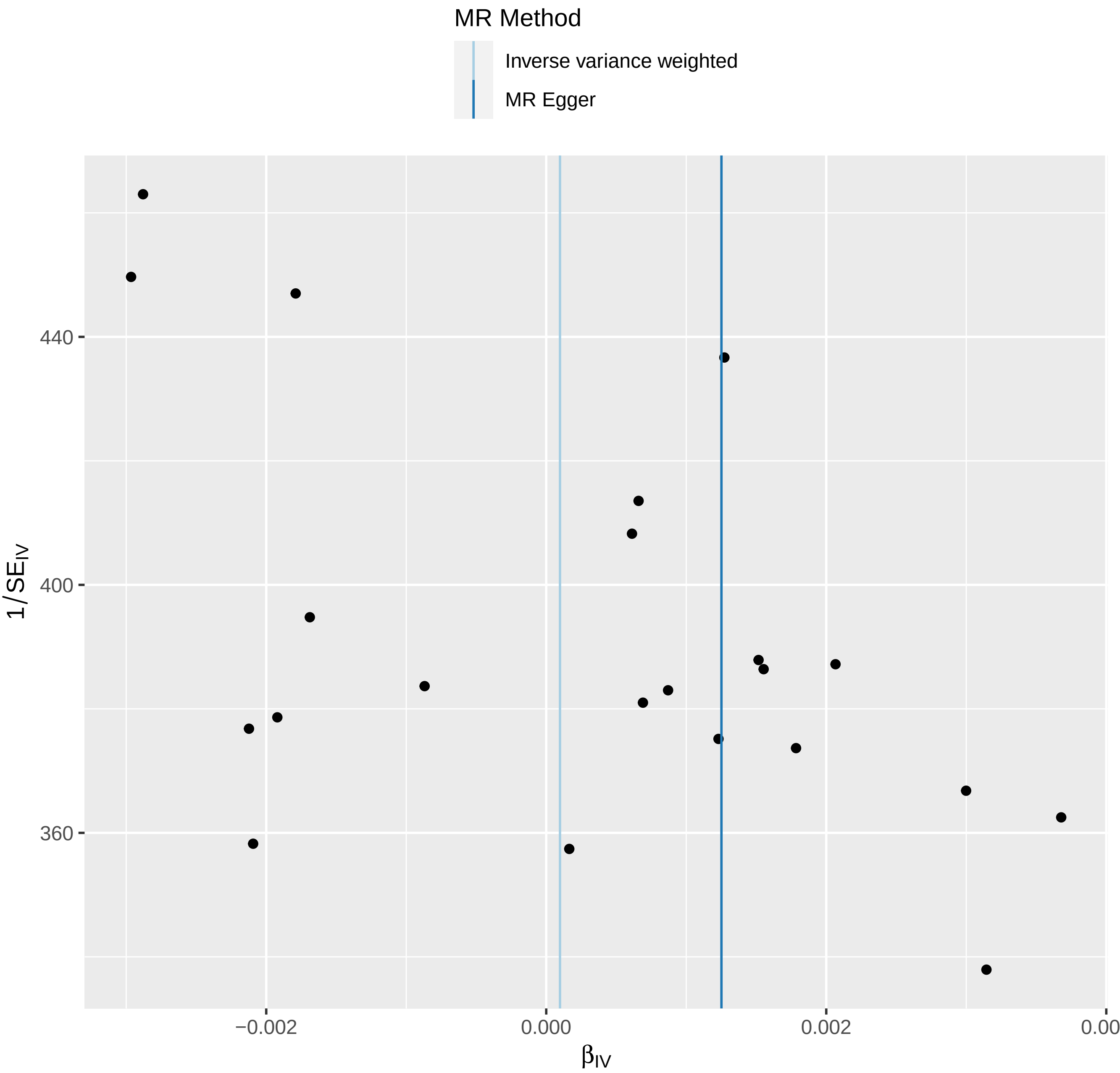

Batch 2−2554 : Ribonuclease pancreatic on eGFR

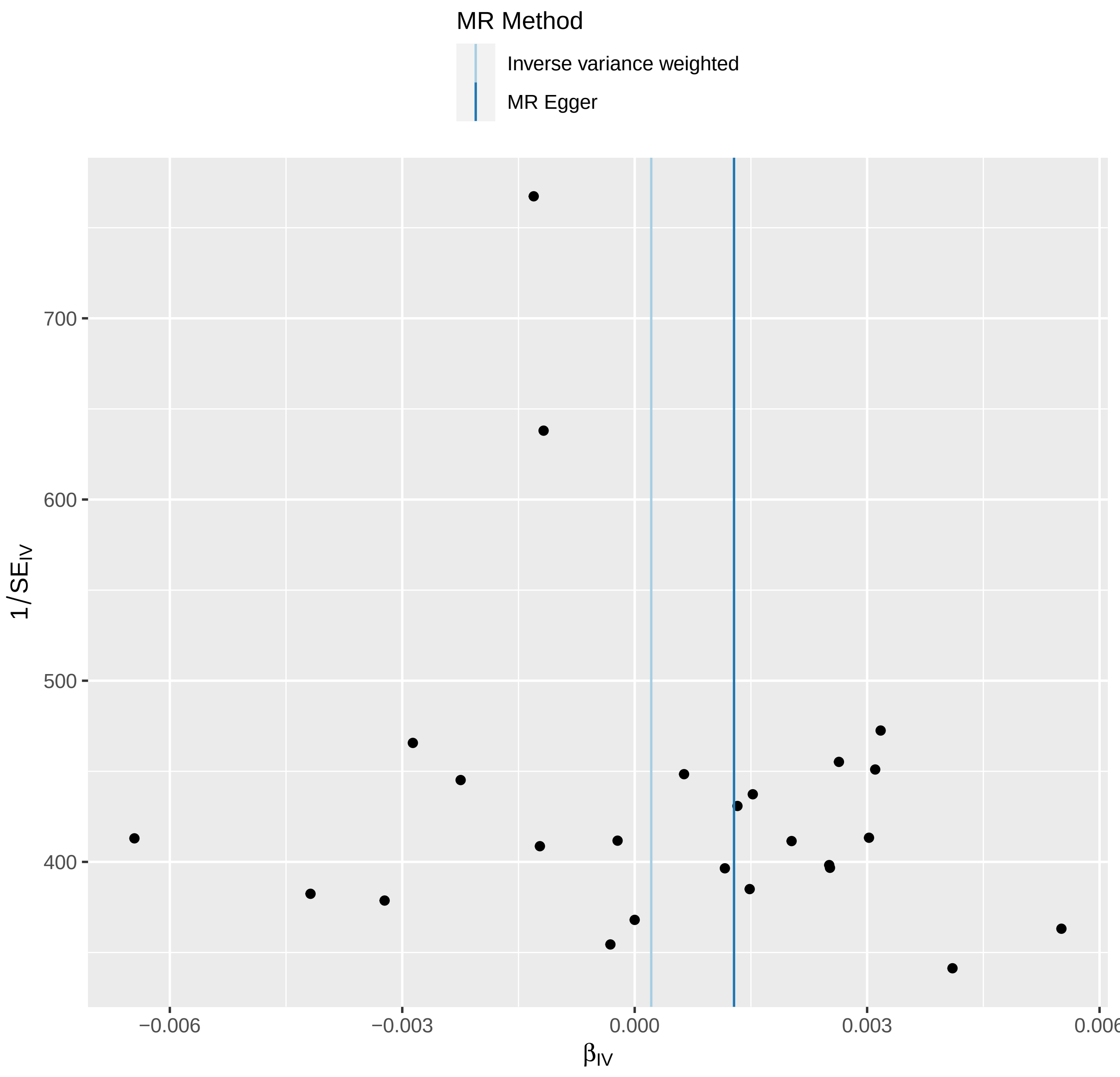

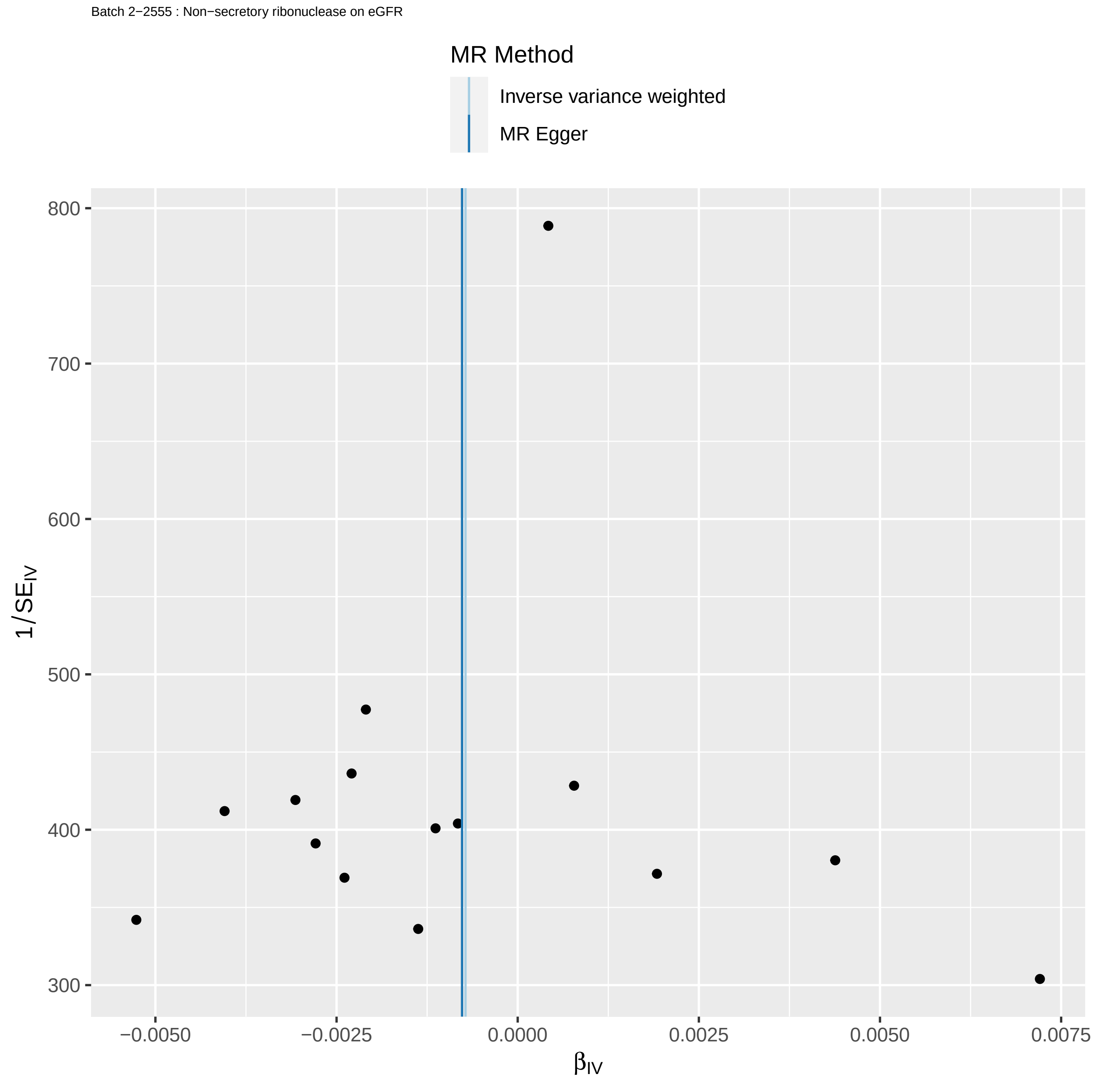

Batch 2−2555 : Non−secretory ribonuclease on eGFR
MR Method
Inverse variance weighted
MR Egger
800
700
600
500
400
300
1/SE$_{IV}$
−0.0050
−0.0025
0.0000
0.0025
0.0050
0.0075
β$_{IV}$

Batch 2−2556 : Ribonuclease 4 on eGFR

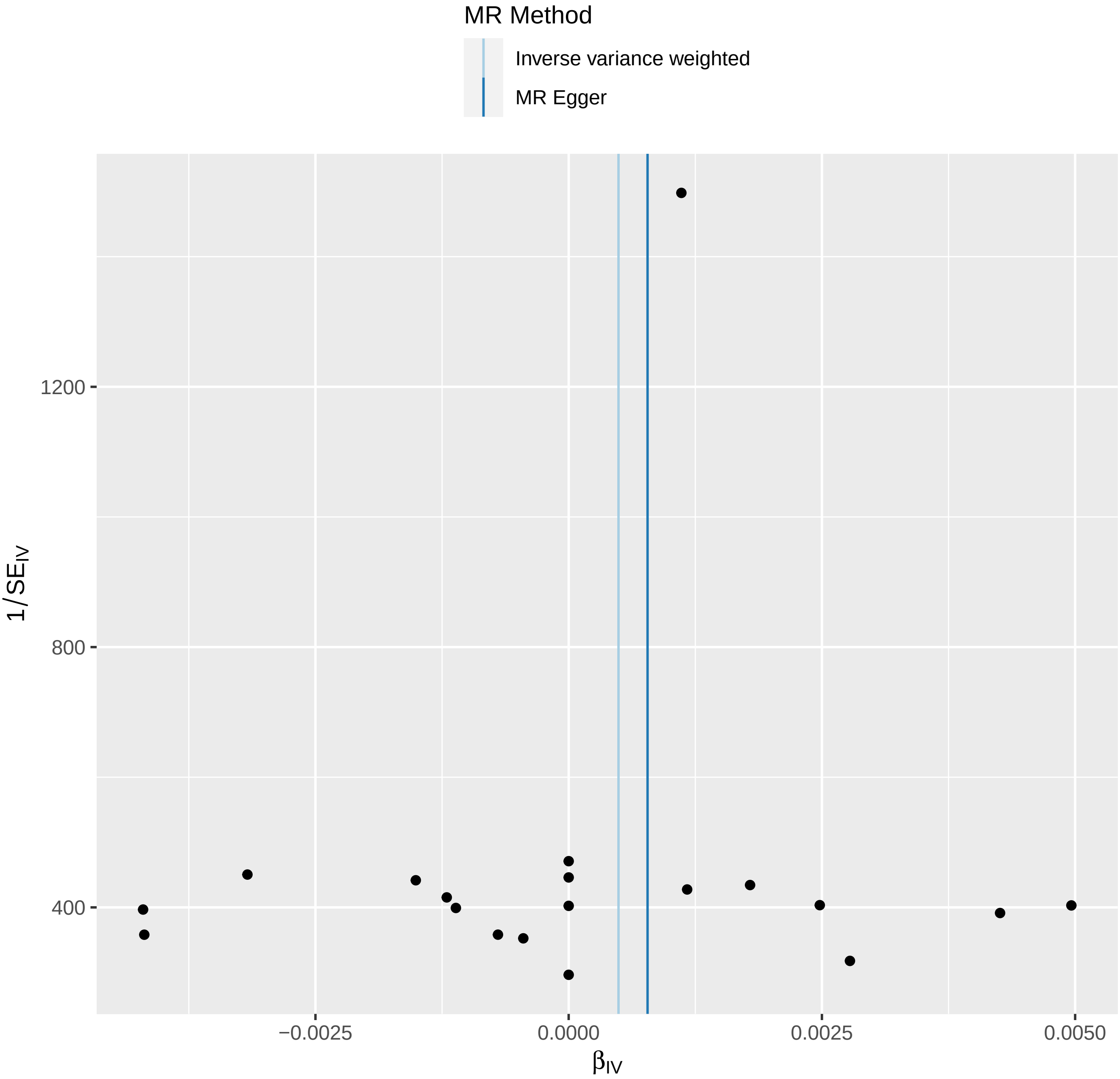

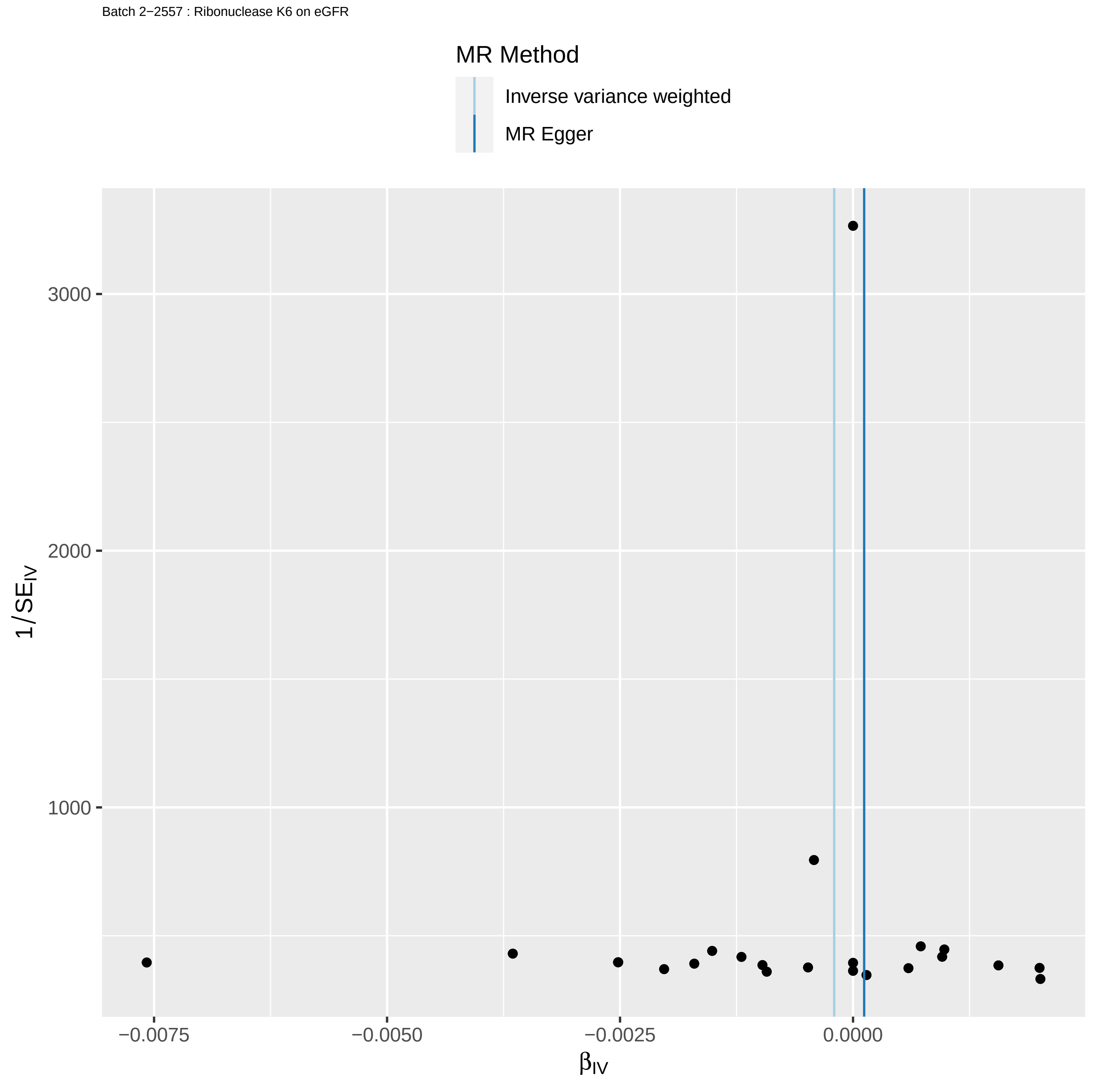

Batch 2−2557 : Ribonuclease K6 on eGFR
MR Method
Inverse variance weighted
MR Egger
3000
2000
1000
−0.0075
−0.0050
−0.0025
0.0000
$1/SE_{IV}$
β_{IV}

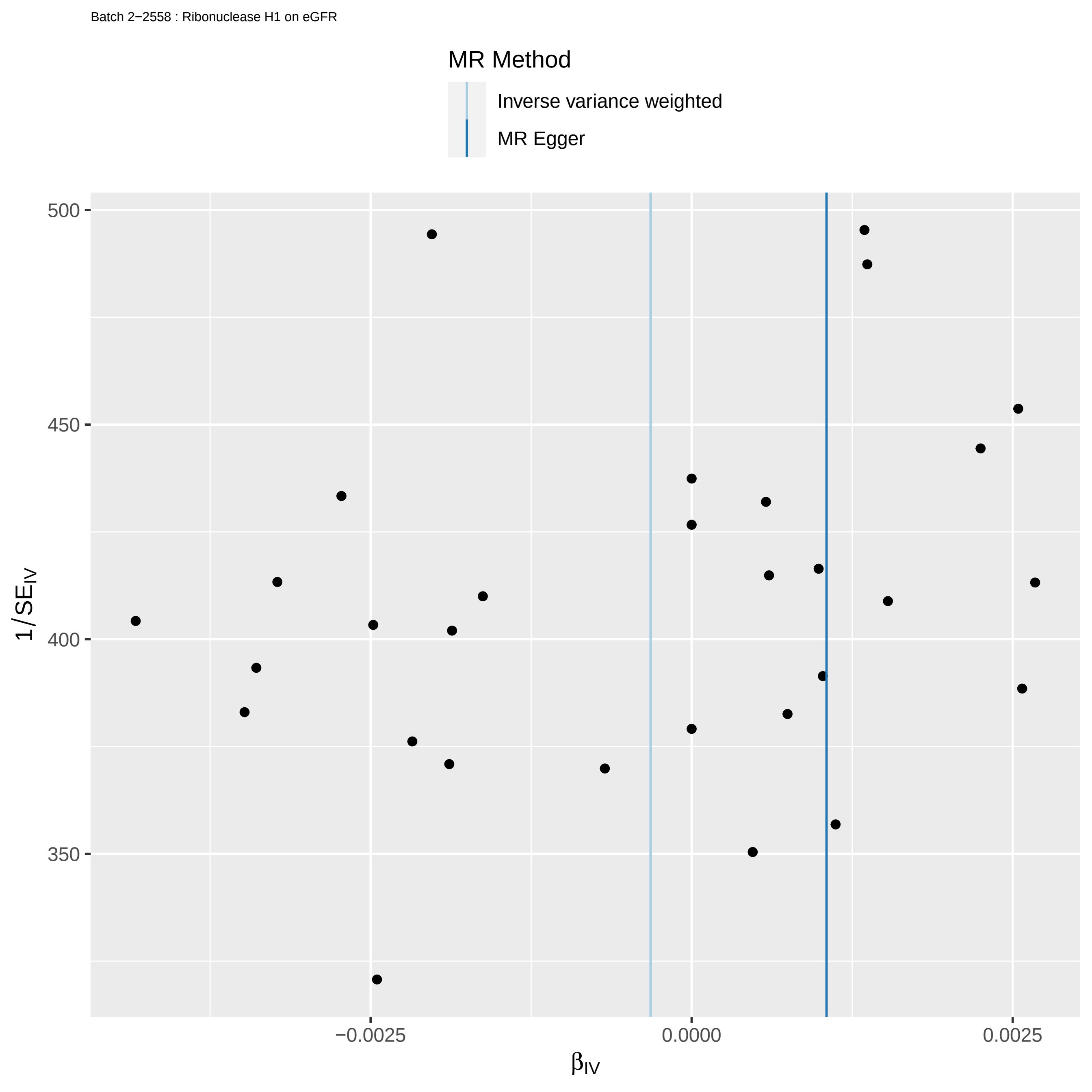
Batch 2−2558 : Ribonuclease H1 on eGFR
MR Method
Inverse variance weighted
MR Egger
500
450
400
350
1/SE_IV
−0.0025
0.0000
0.0025
β_IV

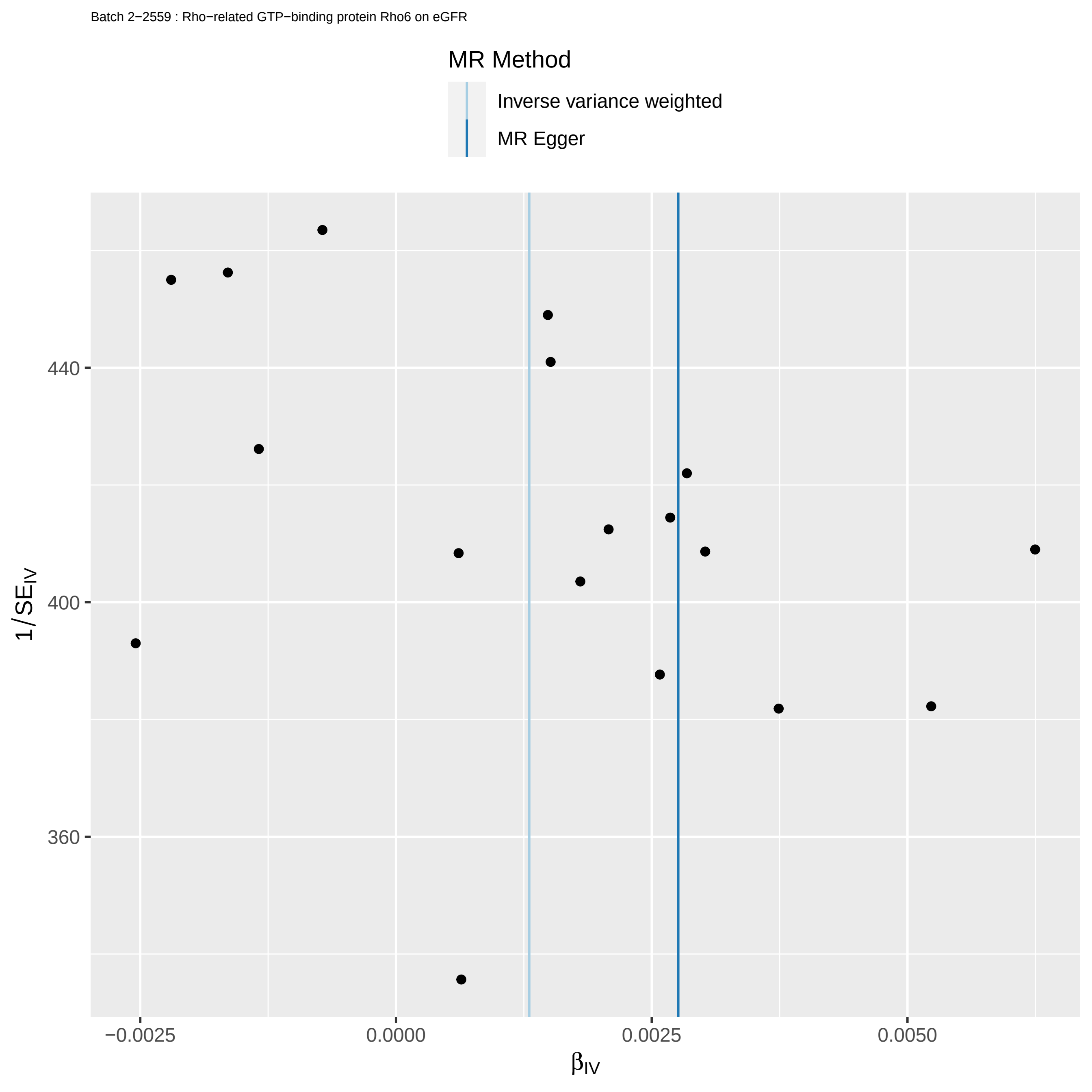

Batch 2−2559 : Rho−related GTP−binding protein Rho6 on eGFR
MR Method
Inverse variance weighted
MR Egger
440
400
360
1/SE_IV
−0.0025
0.0000
0.0025
0.0050
β_IV

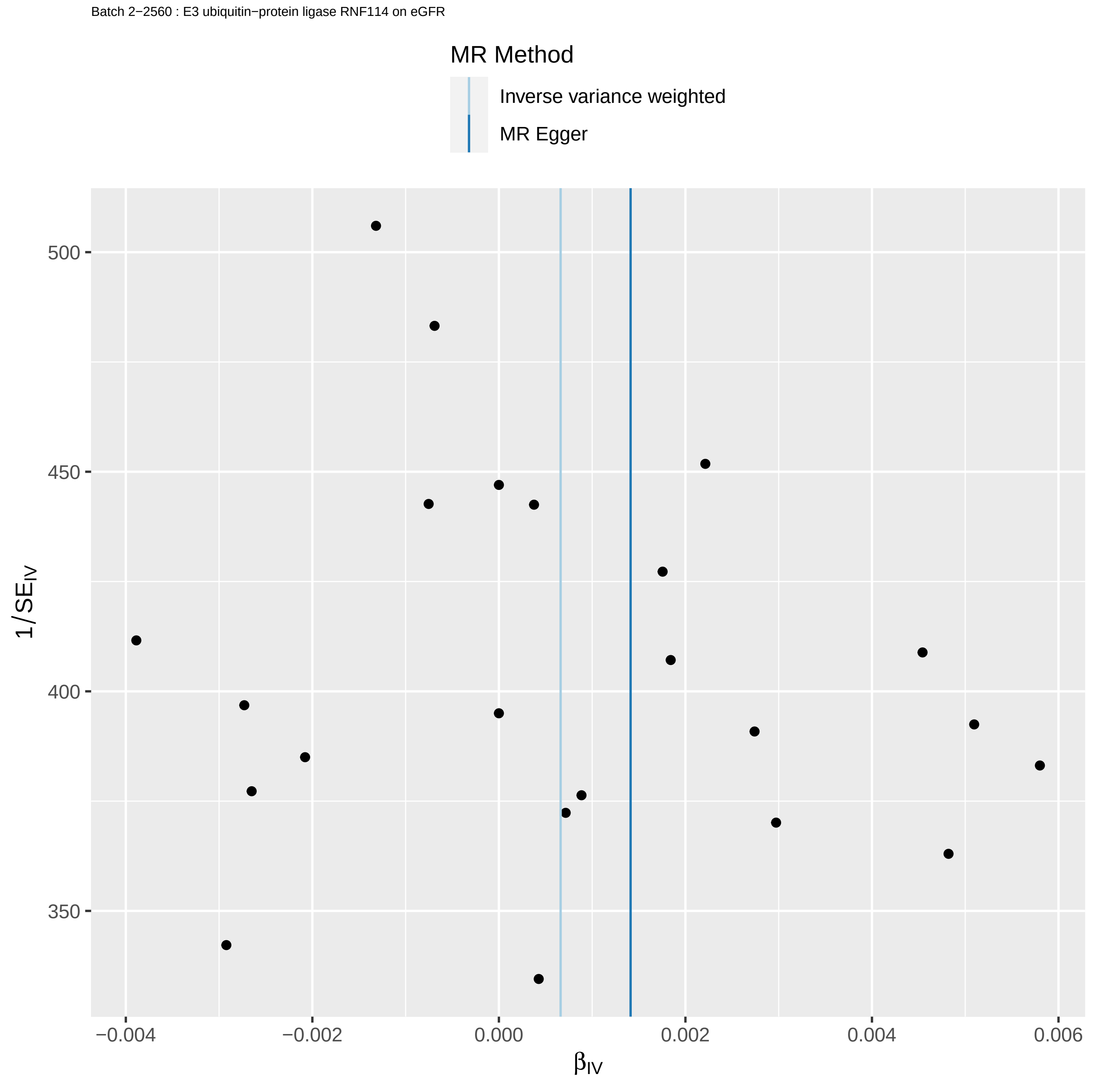

Batch 2−2560 : E3 ubiquitin−protein ligase RNF114 on eGFR
MR Method
Inverse variance weighted
MR Egger
500
450
400
350
1/SE_IV
−0.004
−0.002
0.000
0.002
0.004
0.006
β_IV

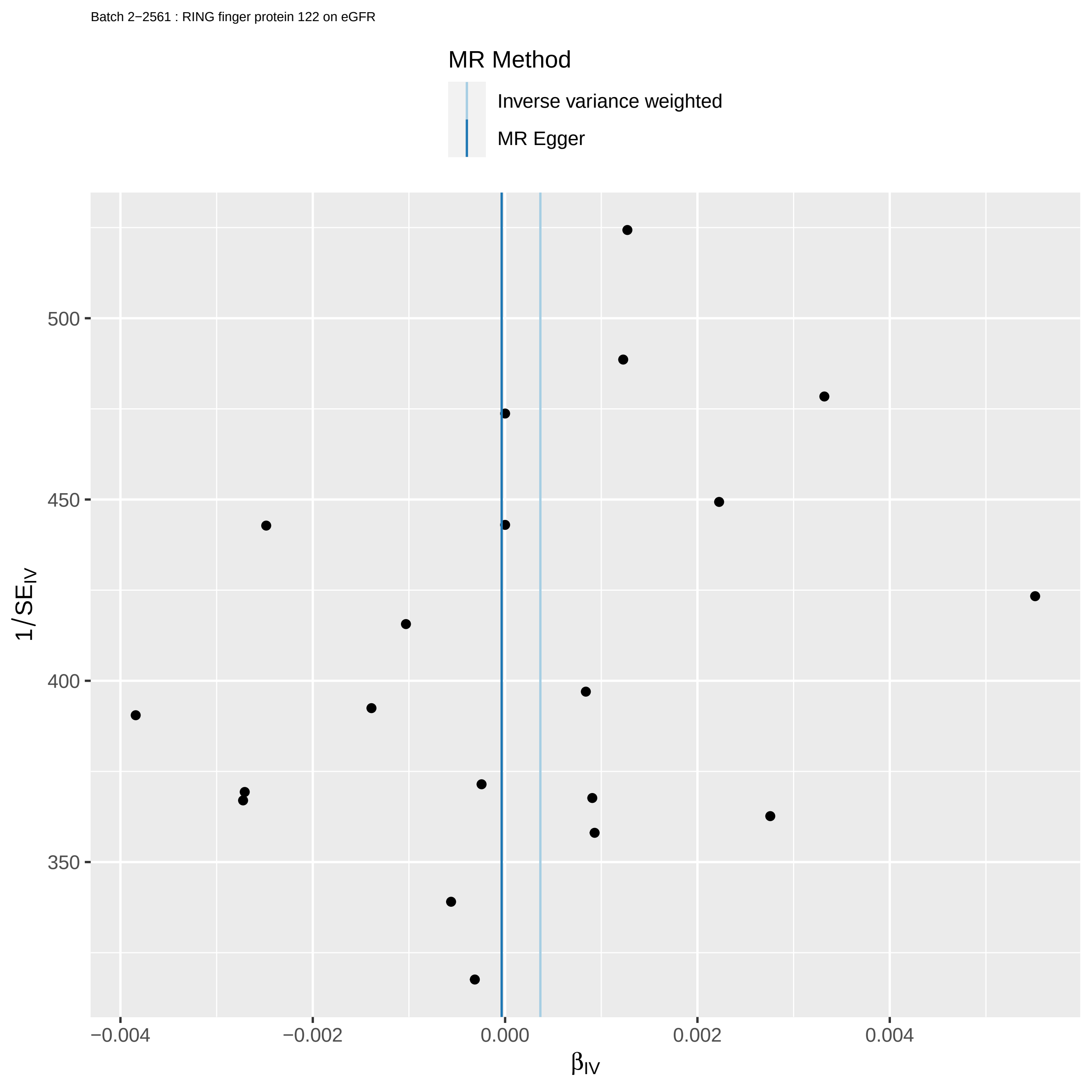
Batch 2−2561 : RING finger protein 122 on eGFR
MR Method
Inverse variance weighted
MR Egger
500
450
400
350
1/SE_IV
−0.004
−0.002
0.000
0.002
0.004
β_IV

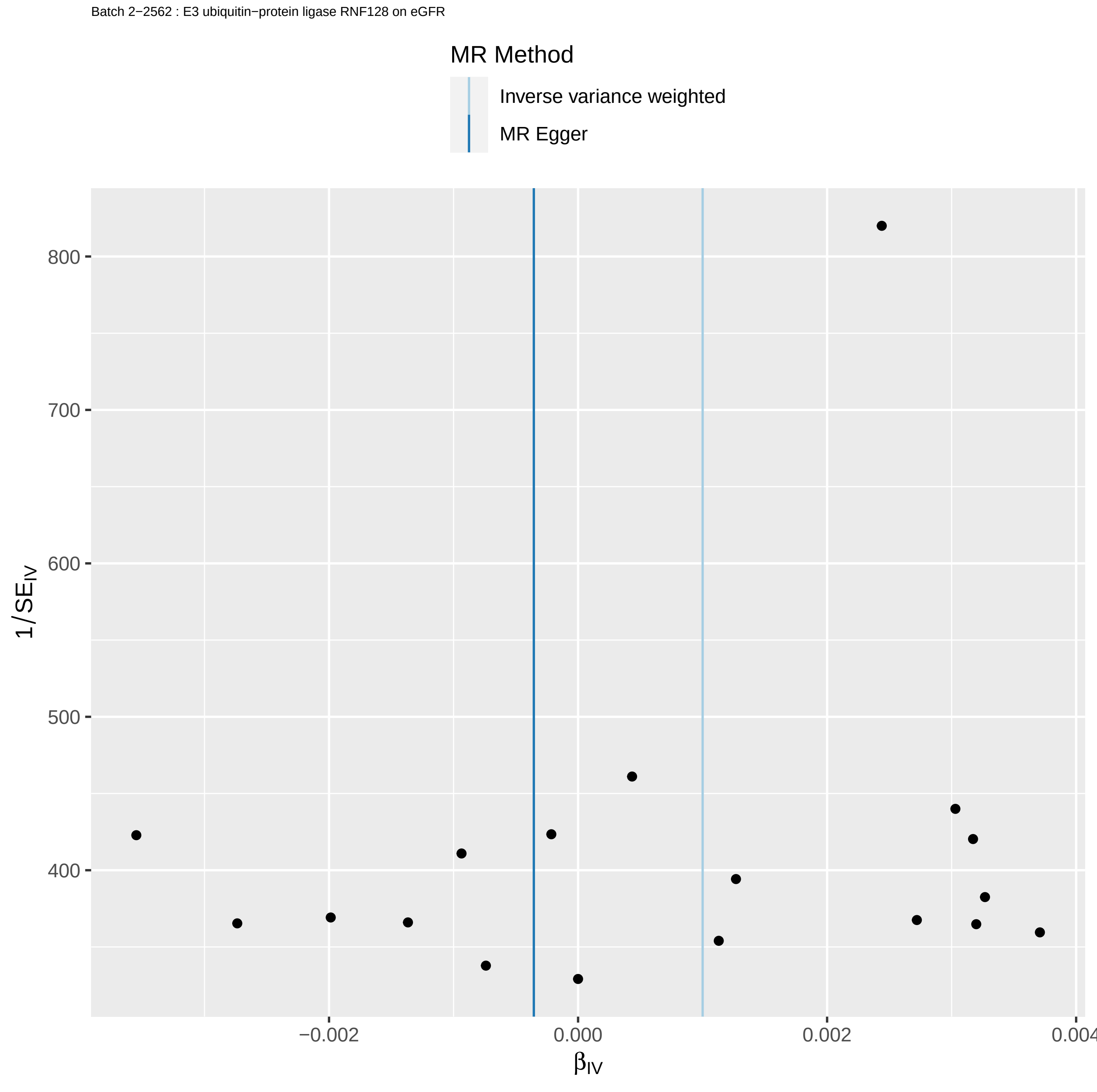

Batch 2−2562 : E3 ubiquitin−protein ligase RNF128 on eGFR
MR Method
Inverse variance weighted
MR Egger
800
700
600
500
400
1/SE_IV
−0.002
0.000
0.002
0.004
β_IV

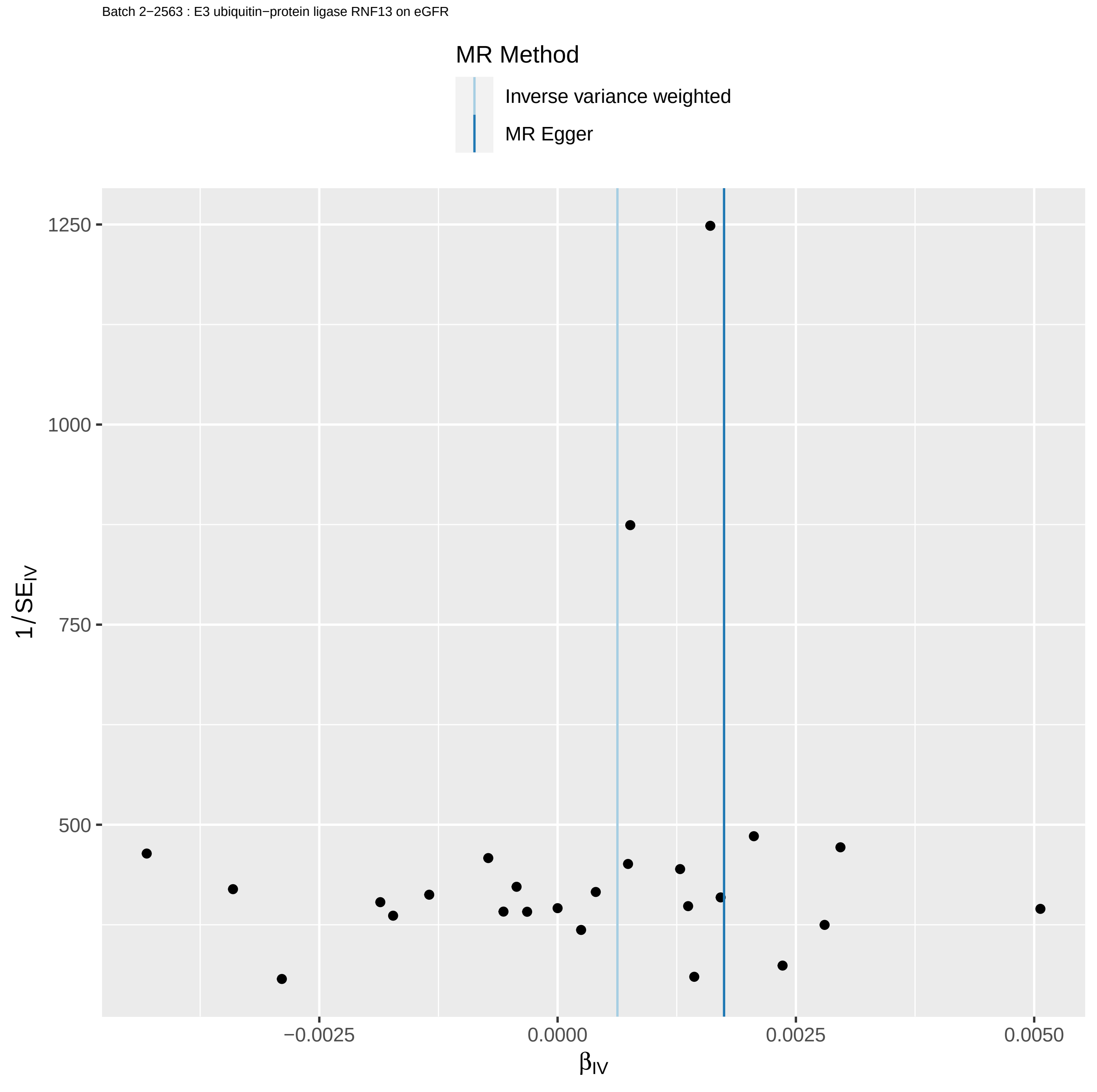

Batch 2−2563 : E3 ubiquitin−protein ligase RNF13 on eGFR
MR Method
Inverse variance weighted
MR Egger
1250
1000
750
500
1/SE_IV
−0.0025
0.0000
0.0025
0.0050
β_IV

Batch 2−2564 : RING finger protein 148 on eGFR

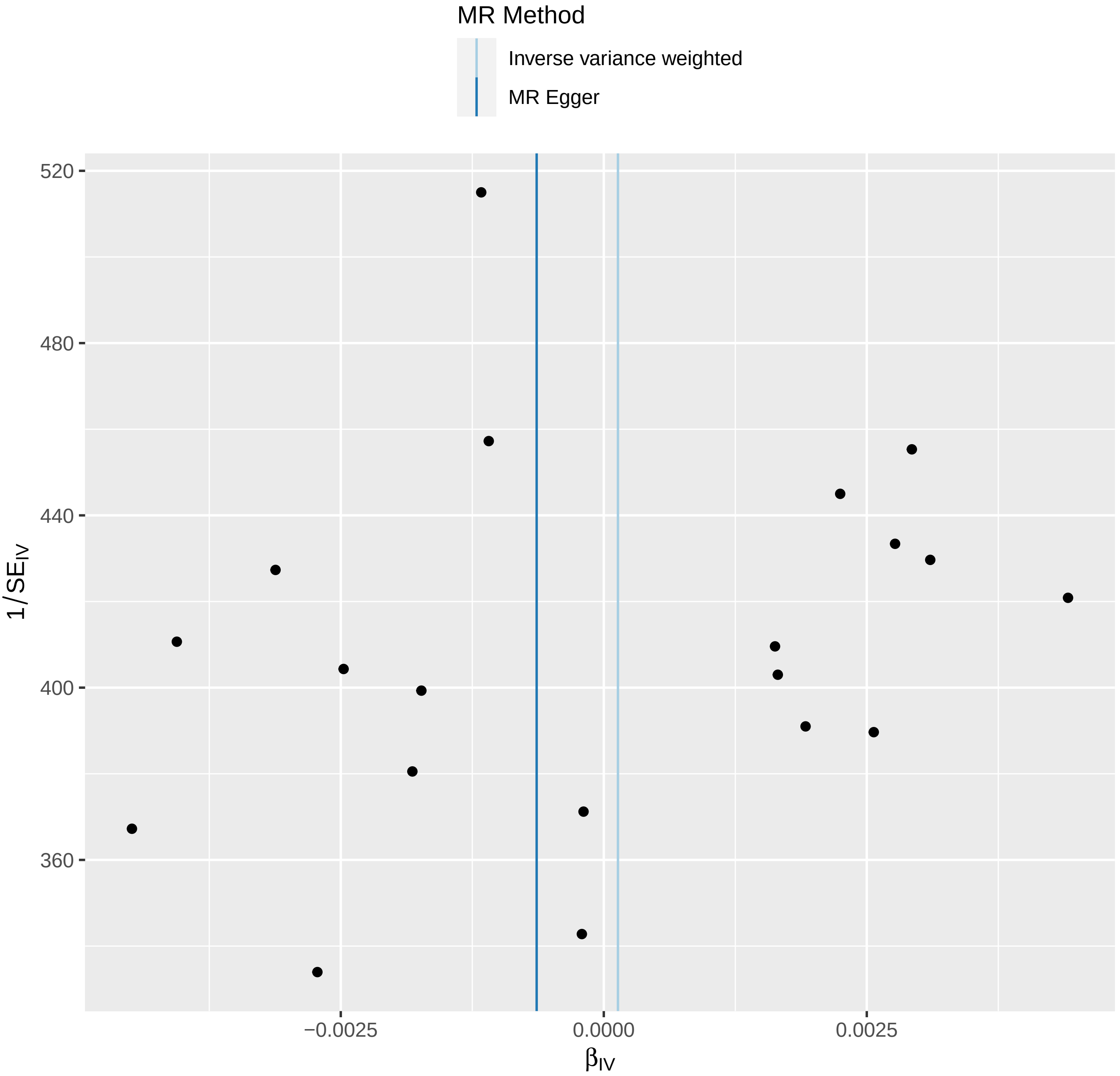

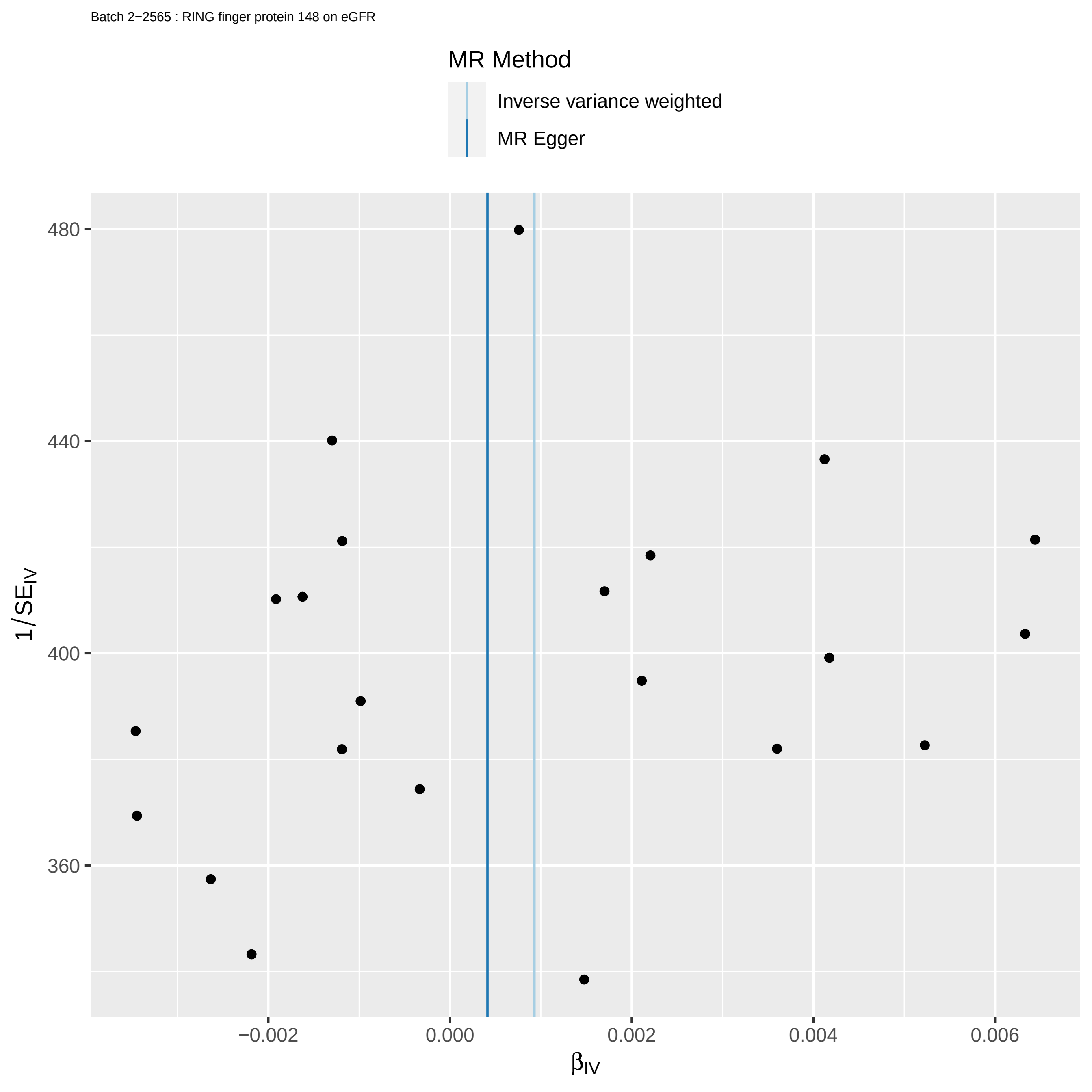
Batch 2−2565 : RING finger protein 148 on eGFR
MR Method
Inverse variance weighted
MR Egger
480
440
400
360
1/SE$_{IV}$
−0.002
0.000
0.002
0.004
0.006
β$_{IV}$

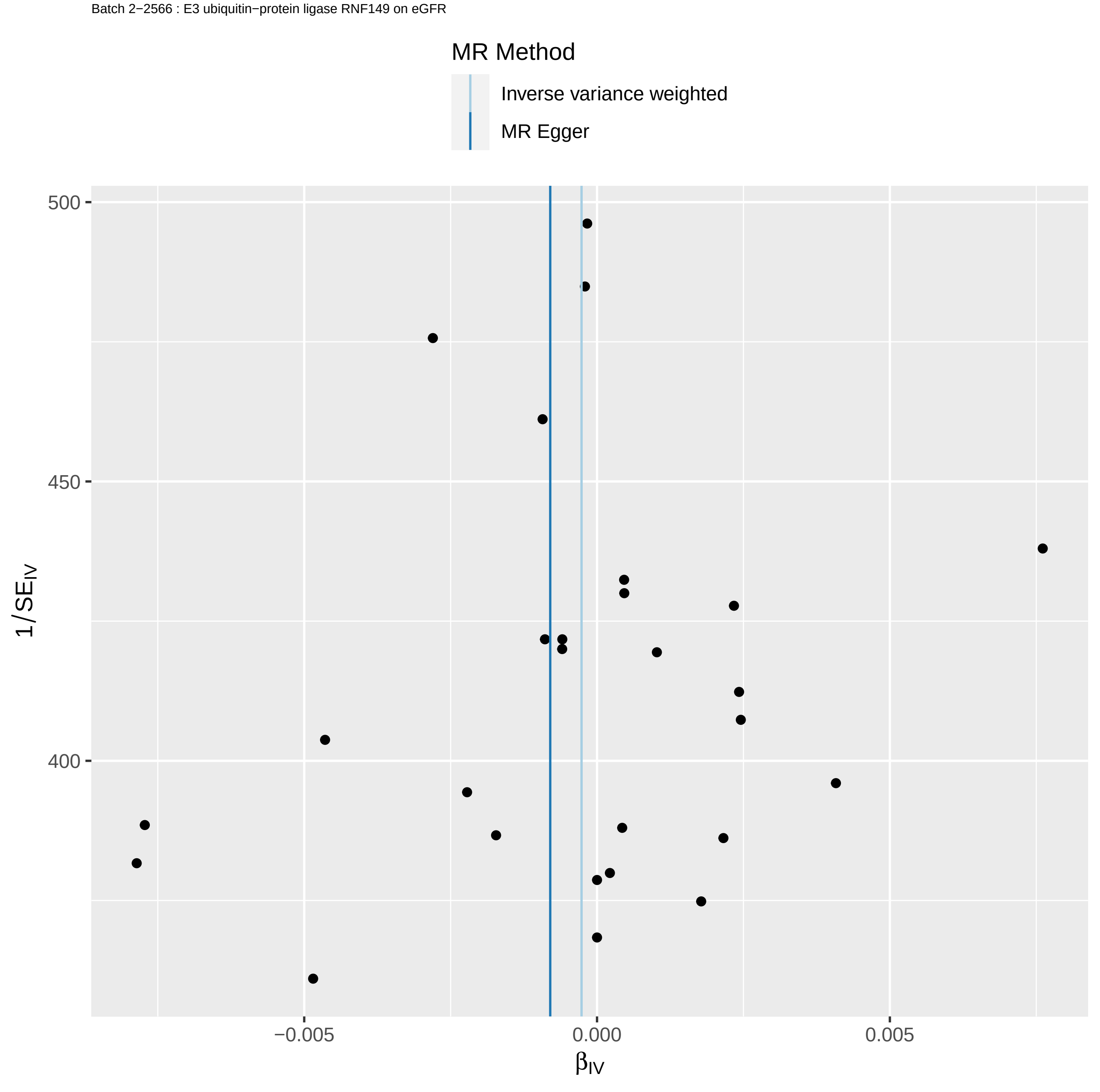

Batch 2−2566 : E3 ubiquitin−protein ligase RNF149 on eGFR
MR Method
Inverse variance weighted
MR Egger
500
450
400
1/SE$_{IV}$
−0.005
0.000
0.005
β$_{IV}$

Batch 2−2567 : RING finger protein 150 on eGFR

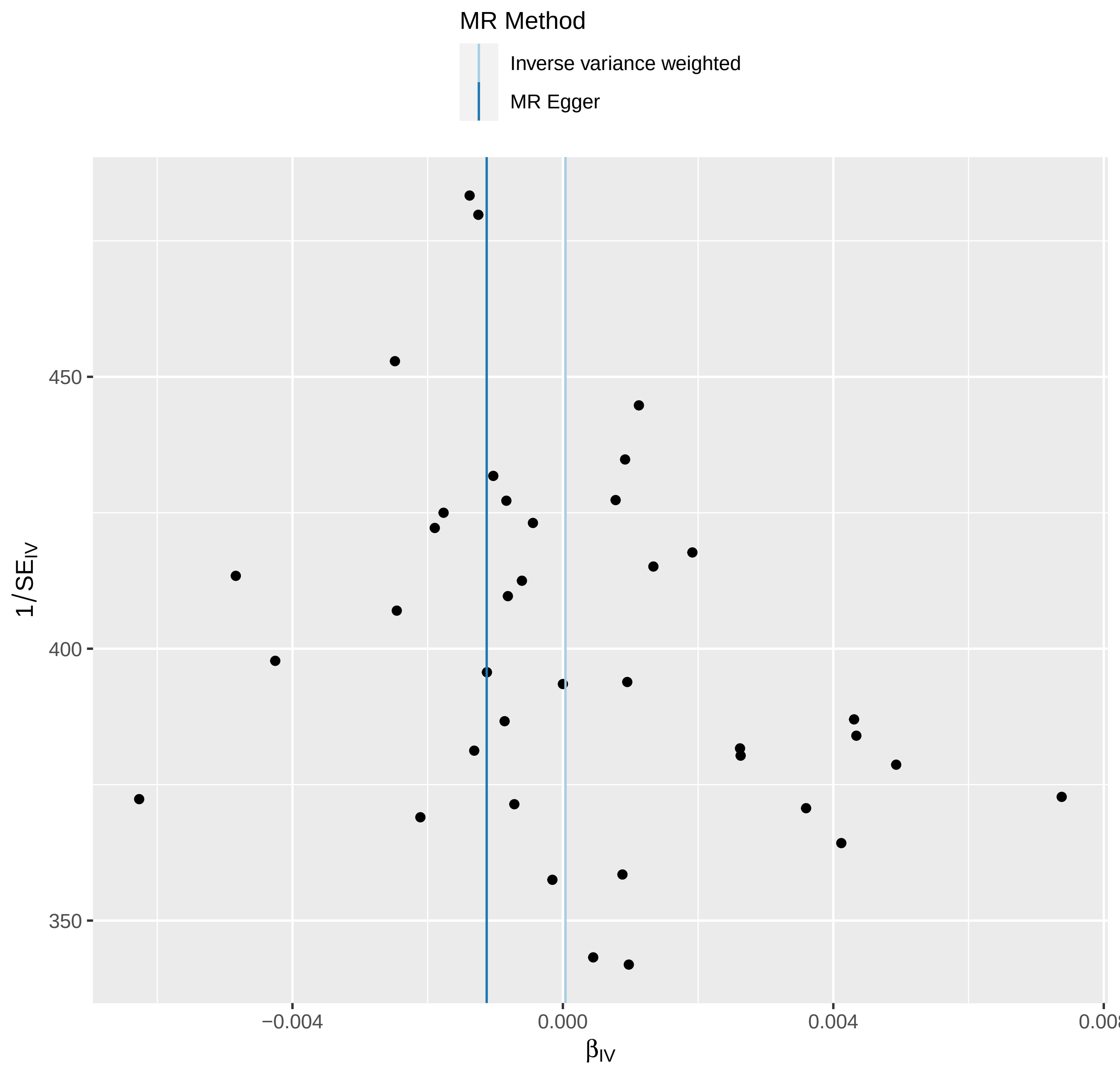

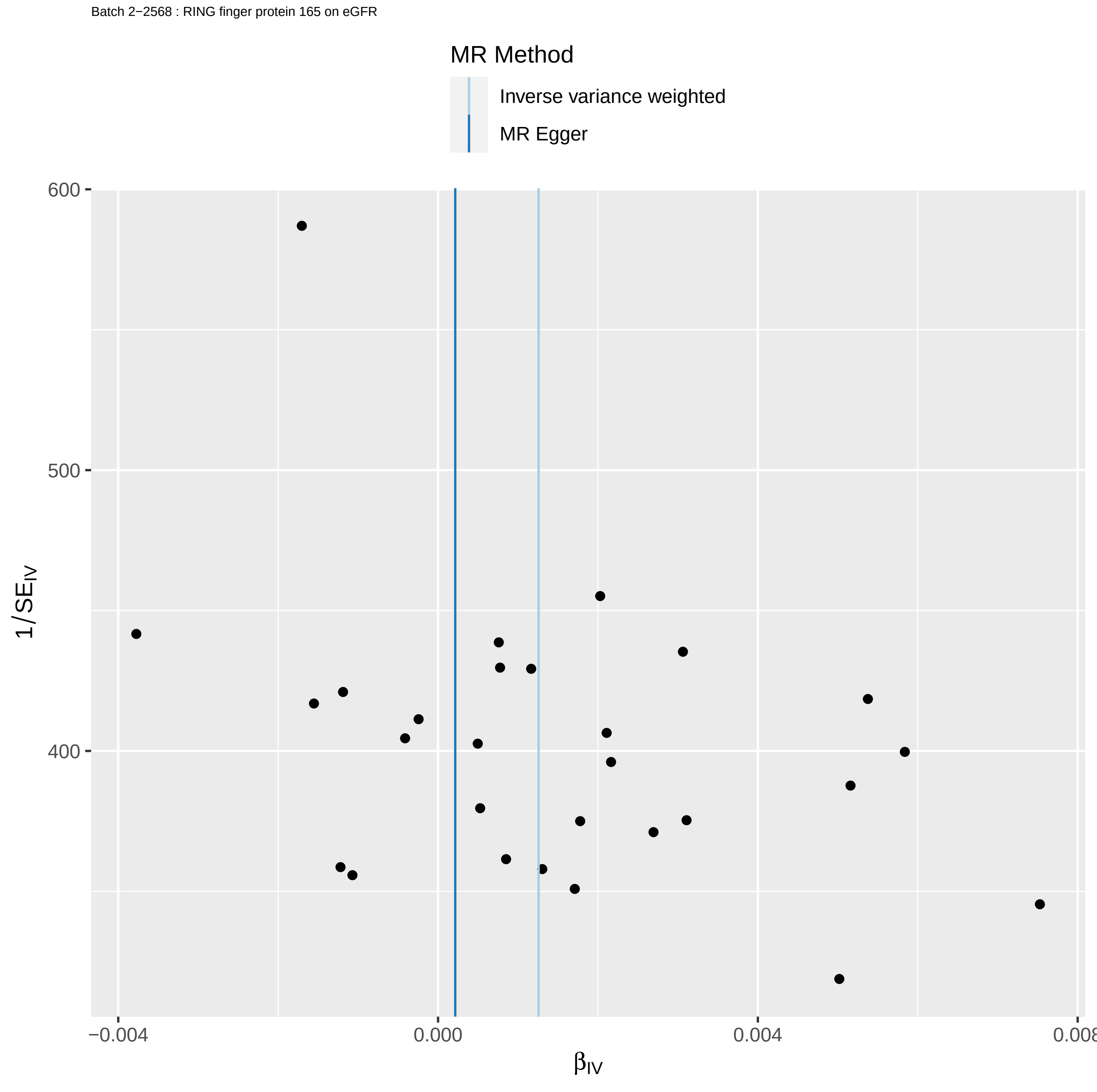
Batch 2−2568 : RING finger protein 165 on eGFR
MR Method
Inverse variance weighted
MR Egger
600
500
400
1/SE_IV
−0.004
0.000
0.004
0.008
β_IV

Batch 2−2569 : RING finger protein 215 on eGFR

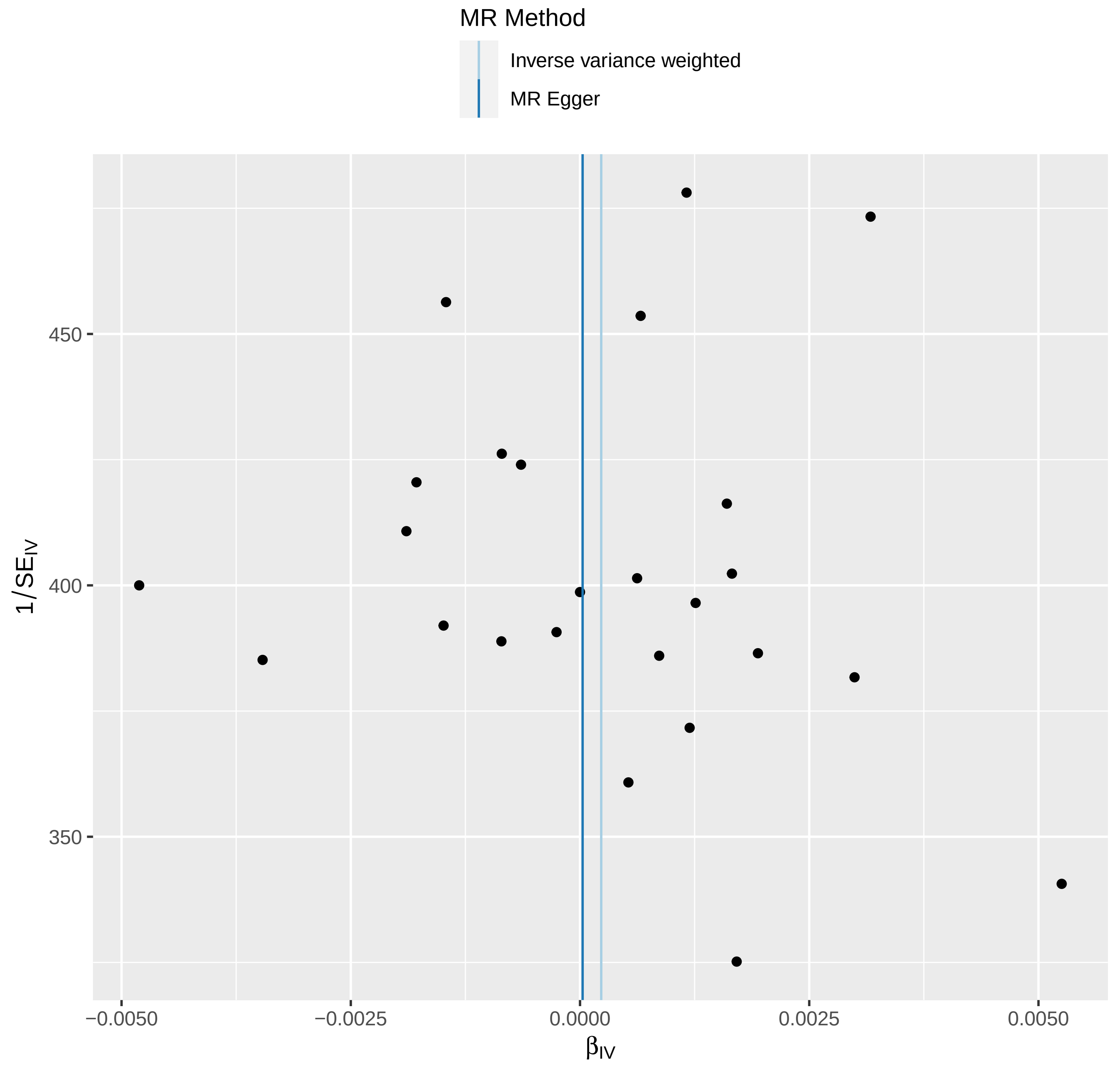

Batch 2−2570 : RING finger protein 219 on eGFR

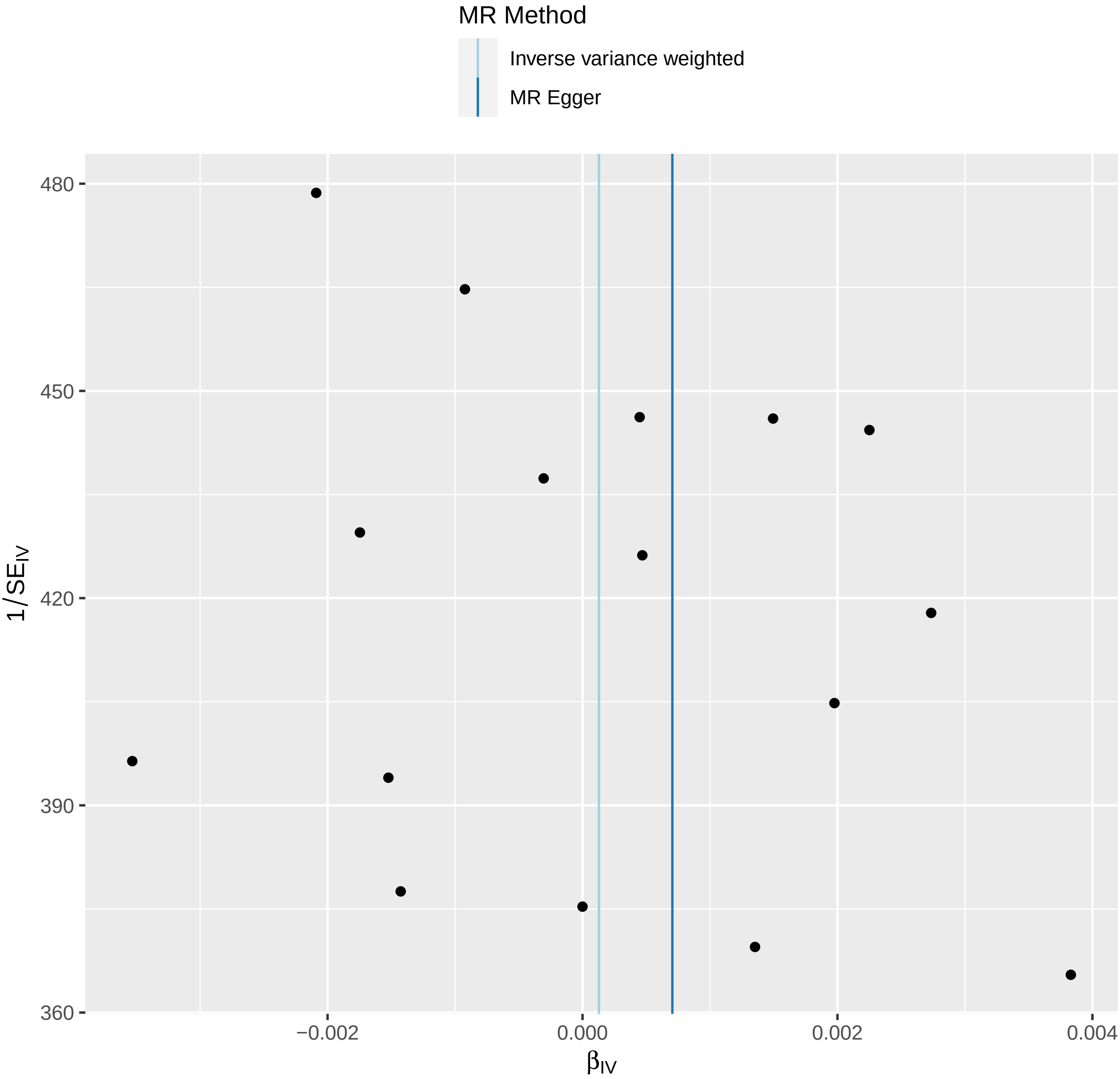

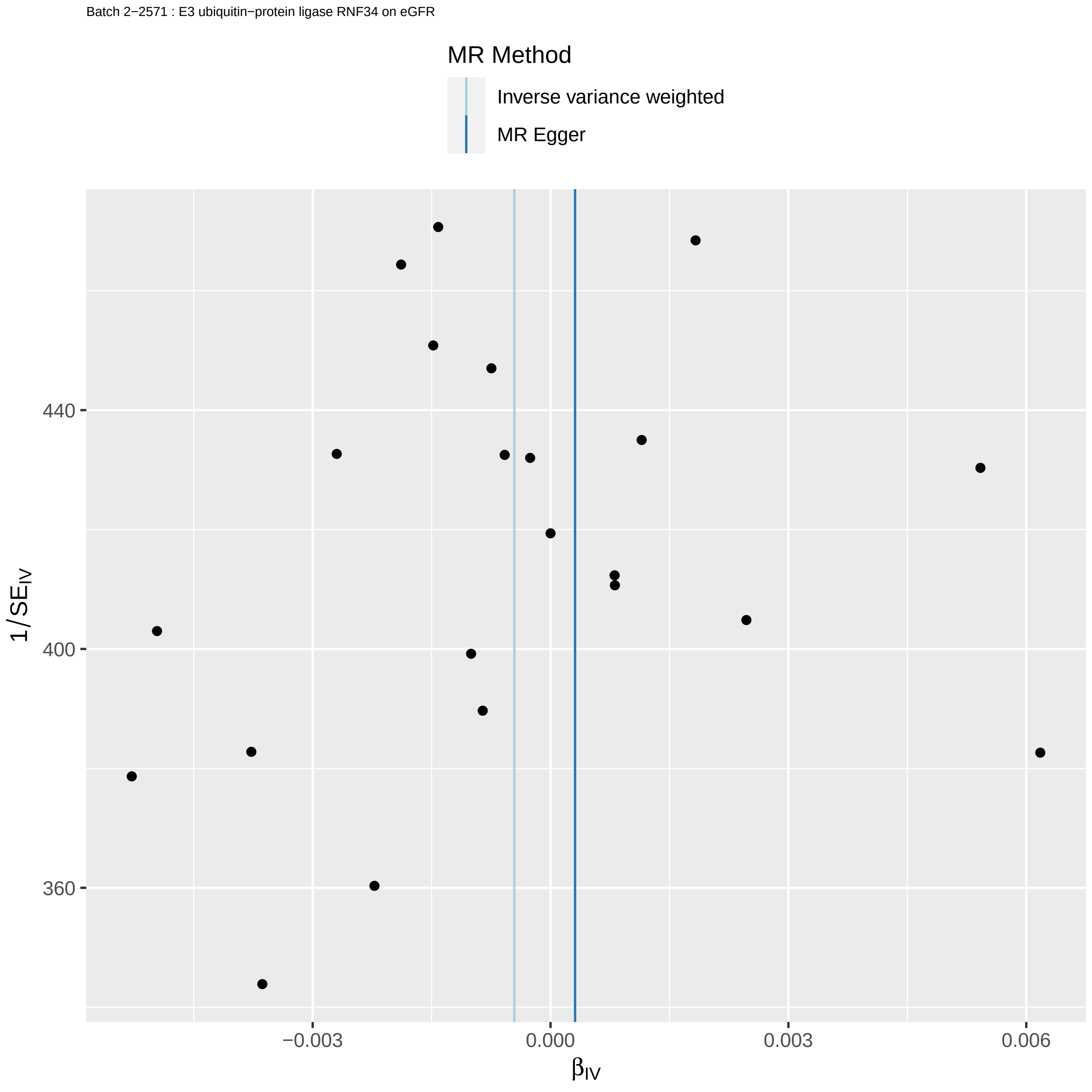
Batch 2−2571 : E3 ubiquitin−protein ligase RNF34 on eGFR
MR Method
Inverse variance weighted
MR Egger
440
400
360
$1/SE_{IV}$
−0.003
0.000
0.003
0.006
β_{IV}

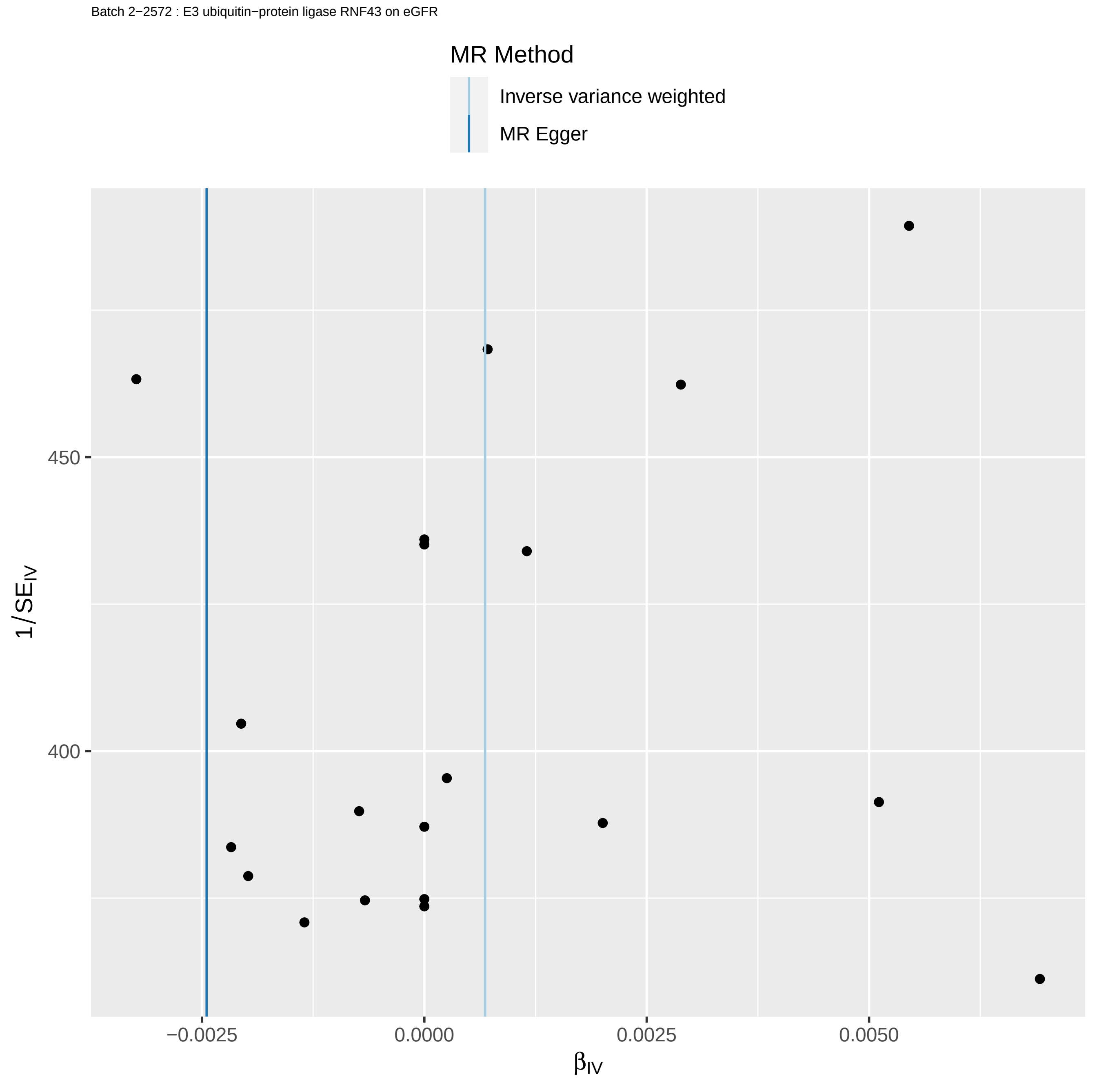

Batch 2−2572 : E3 ubiquitin−protein ligase RNF43 on eGFR
MR Method
Inverse variance weighted
MR Egger
450
400
1/SE_IV
−0.0025
0.0000
0.0025
0.0050
β_IV

Batch 2−2573 : E3 ubiquitin−protein ligase RNF8 on eGFR

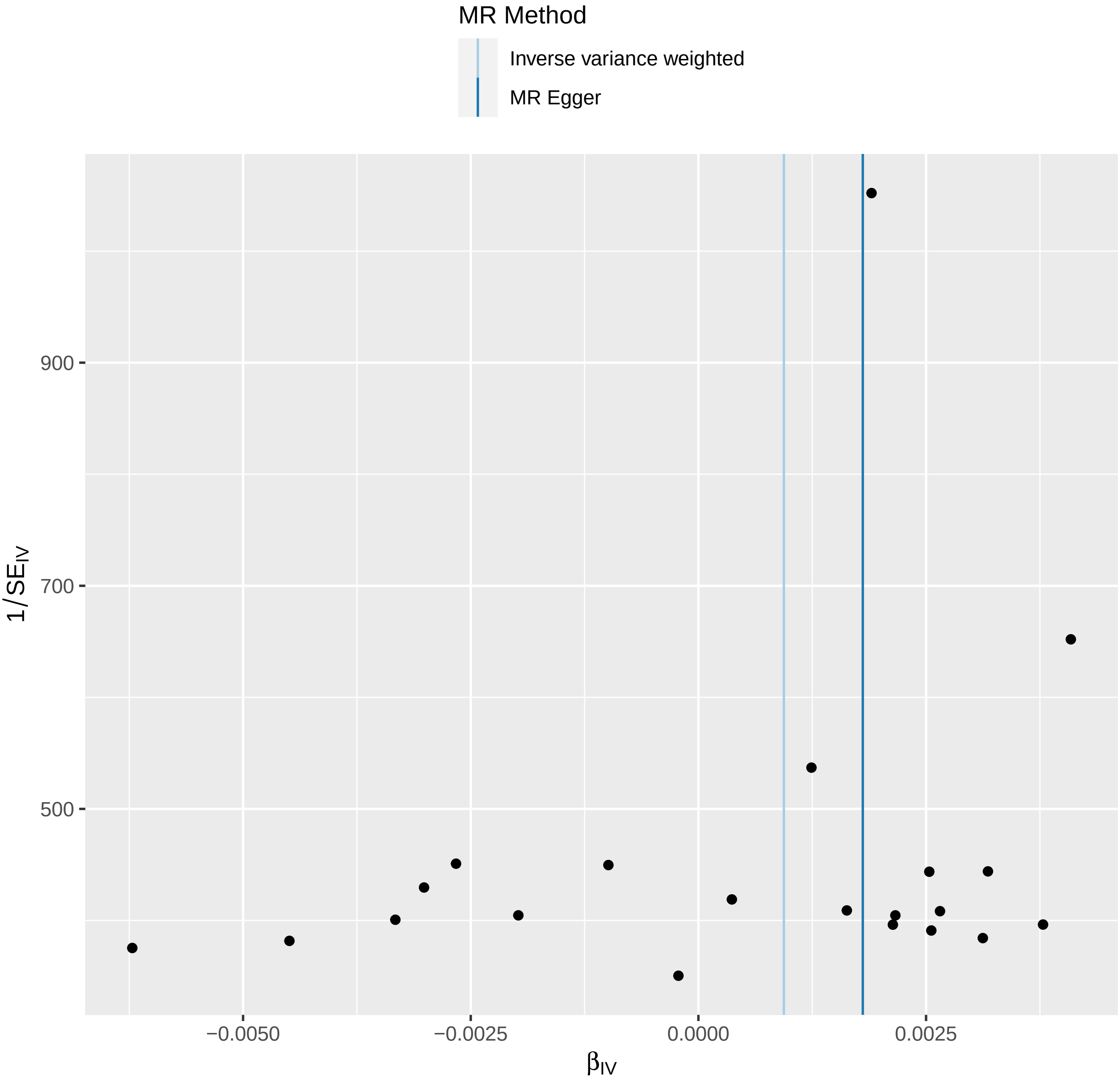

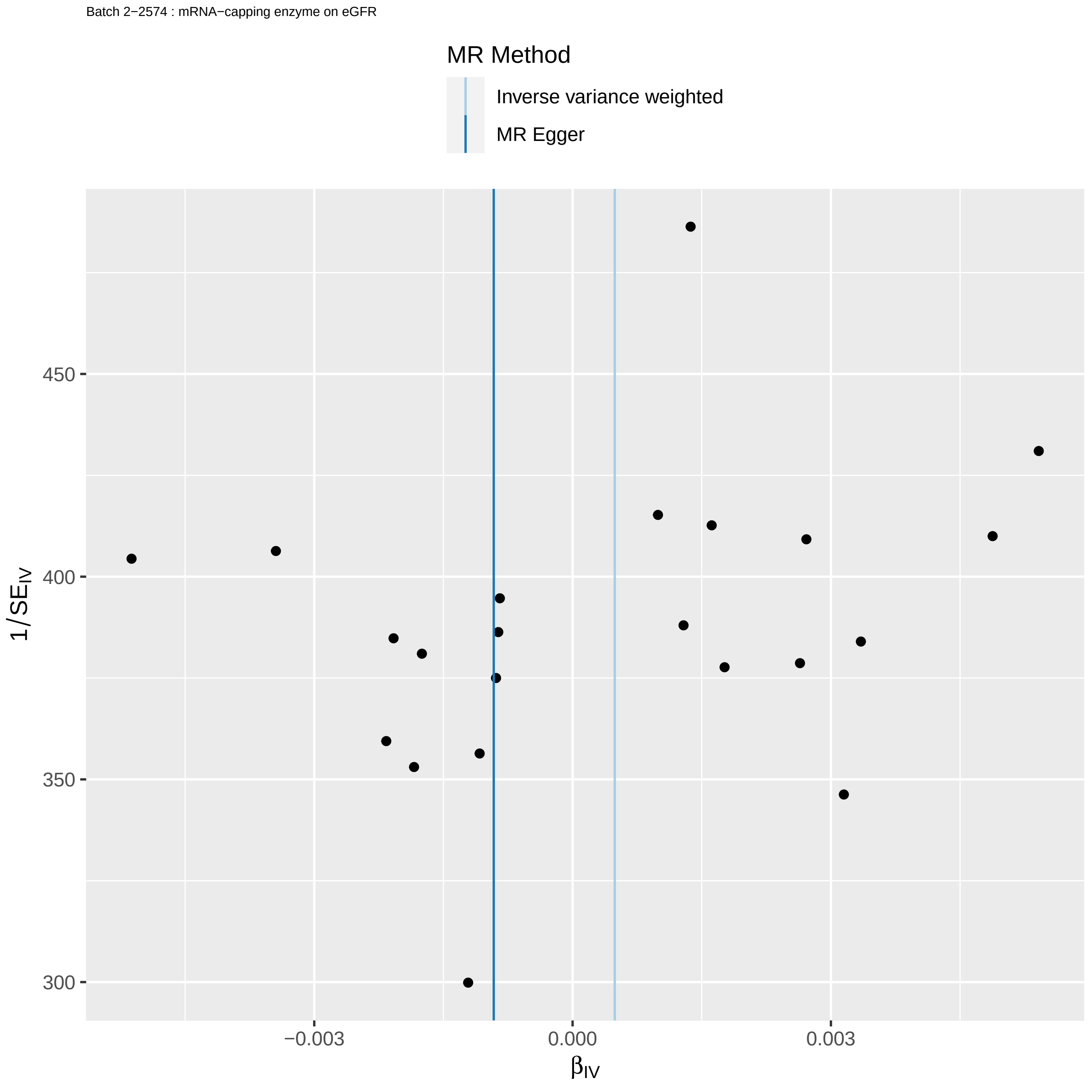

Batch 2−2574 : mRNA−capping enzyme on eGFR
MR Method
Inverse variance weighted
MR Egger
450
400
350
300
1/SE$_{IV}$
−0.003
0.000
0.003
β$_{IV}$

Batch 2−2575 : rRNA methyltransferase 3, mitochondrial on eGFR

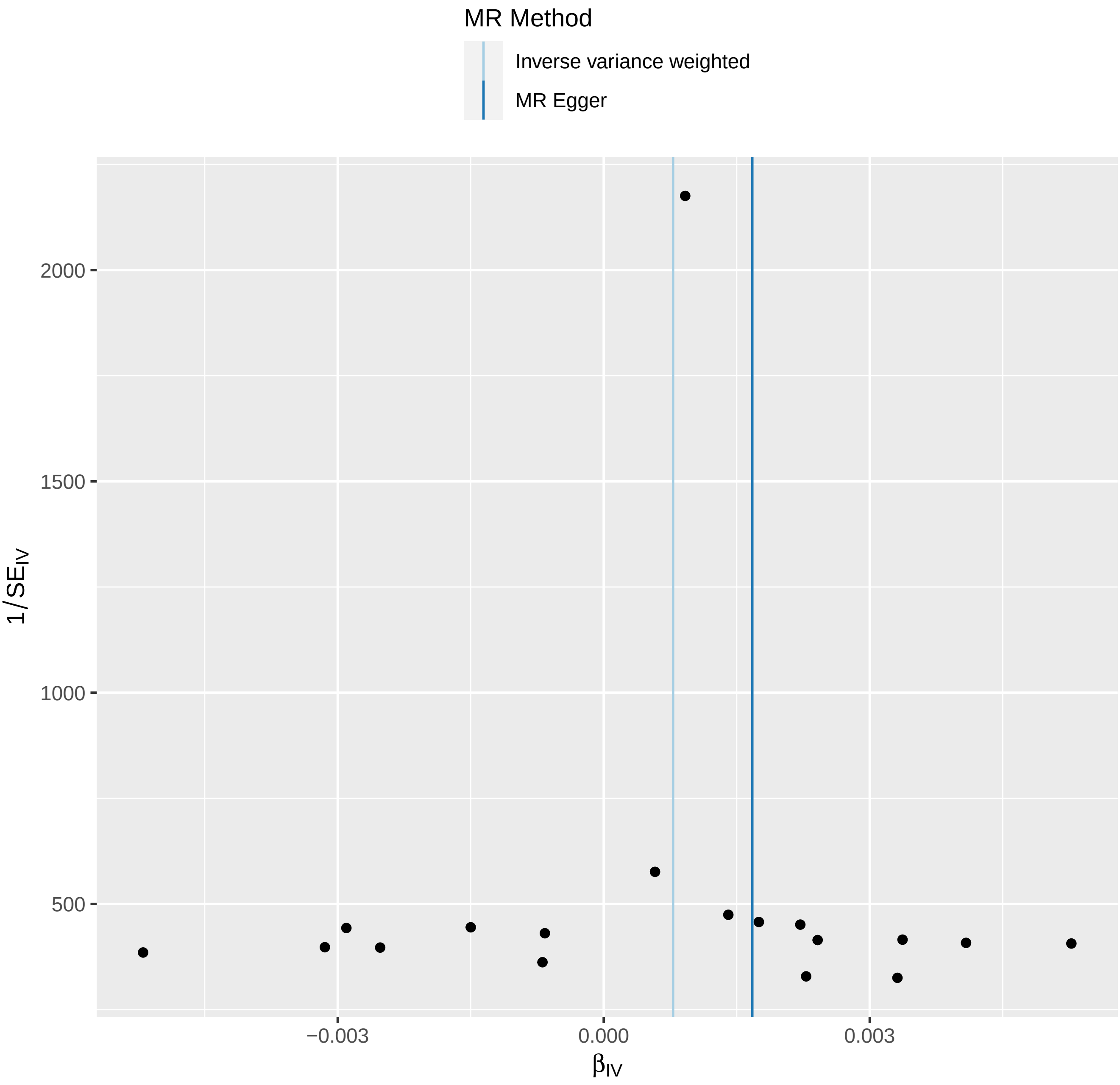

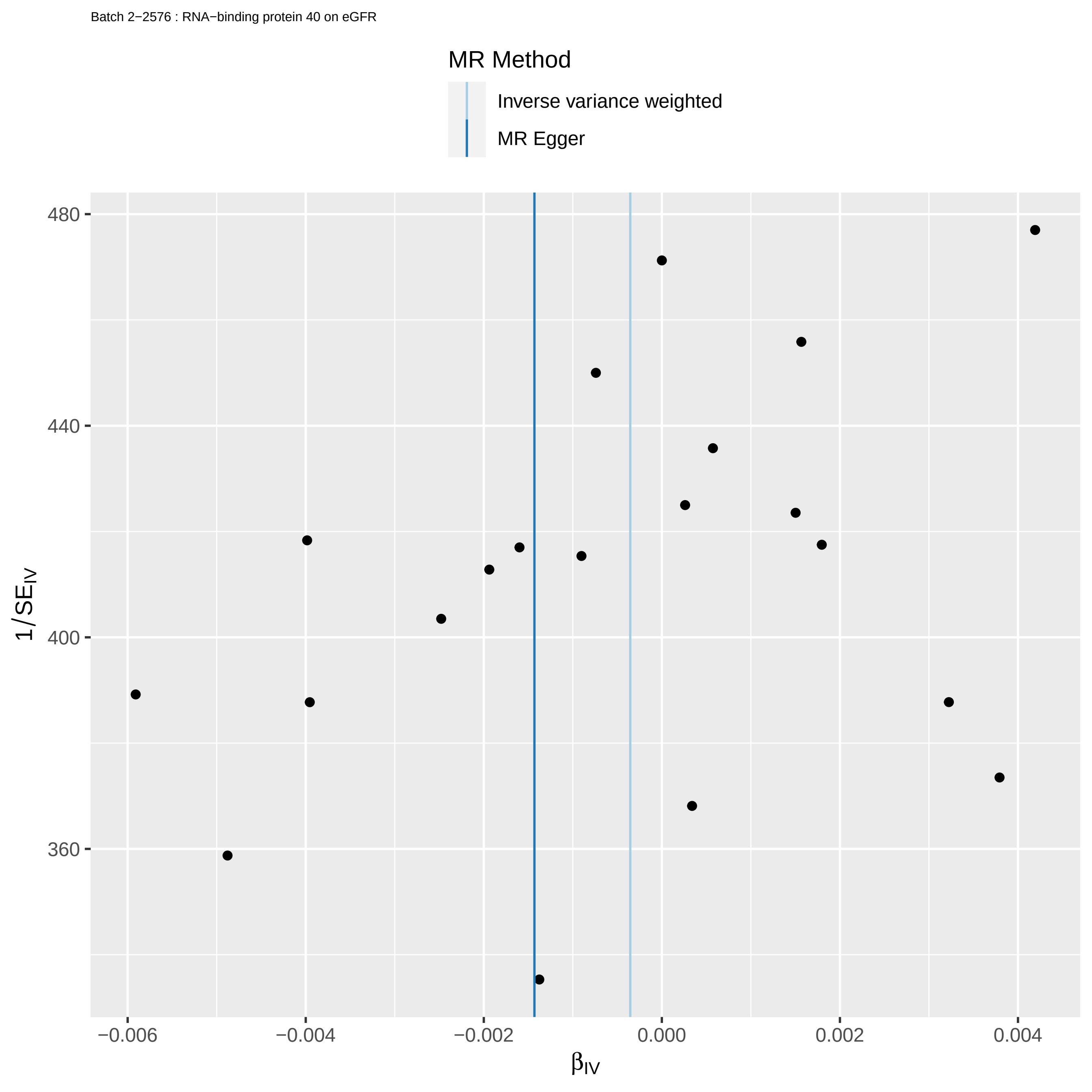

Batch 2−2576 : RNA−binding protein 40 on eGFR
MR Method
Inverse variance weighted
MR Egger
480
440
400
360
1/SE$_{IV}$
−0.006
−0.004
−0.002
0.000
0.002
0.004
β$_{IV}$

Batch 2−2577 : Roundabout homolog 1 on eGFR

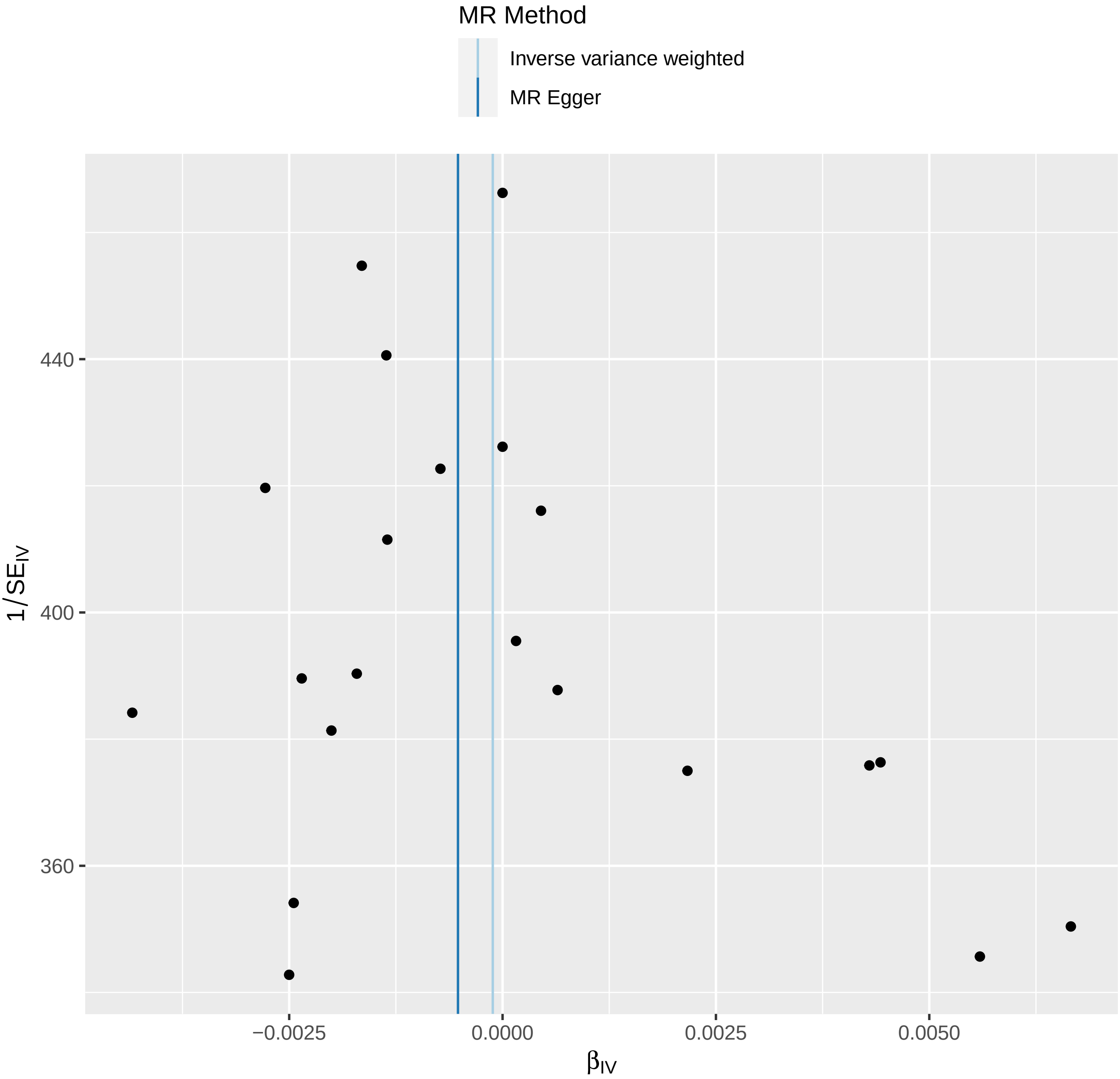

Batch 2−2578 : Roundabout homolog 2 on eGFR

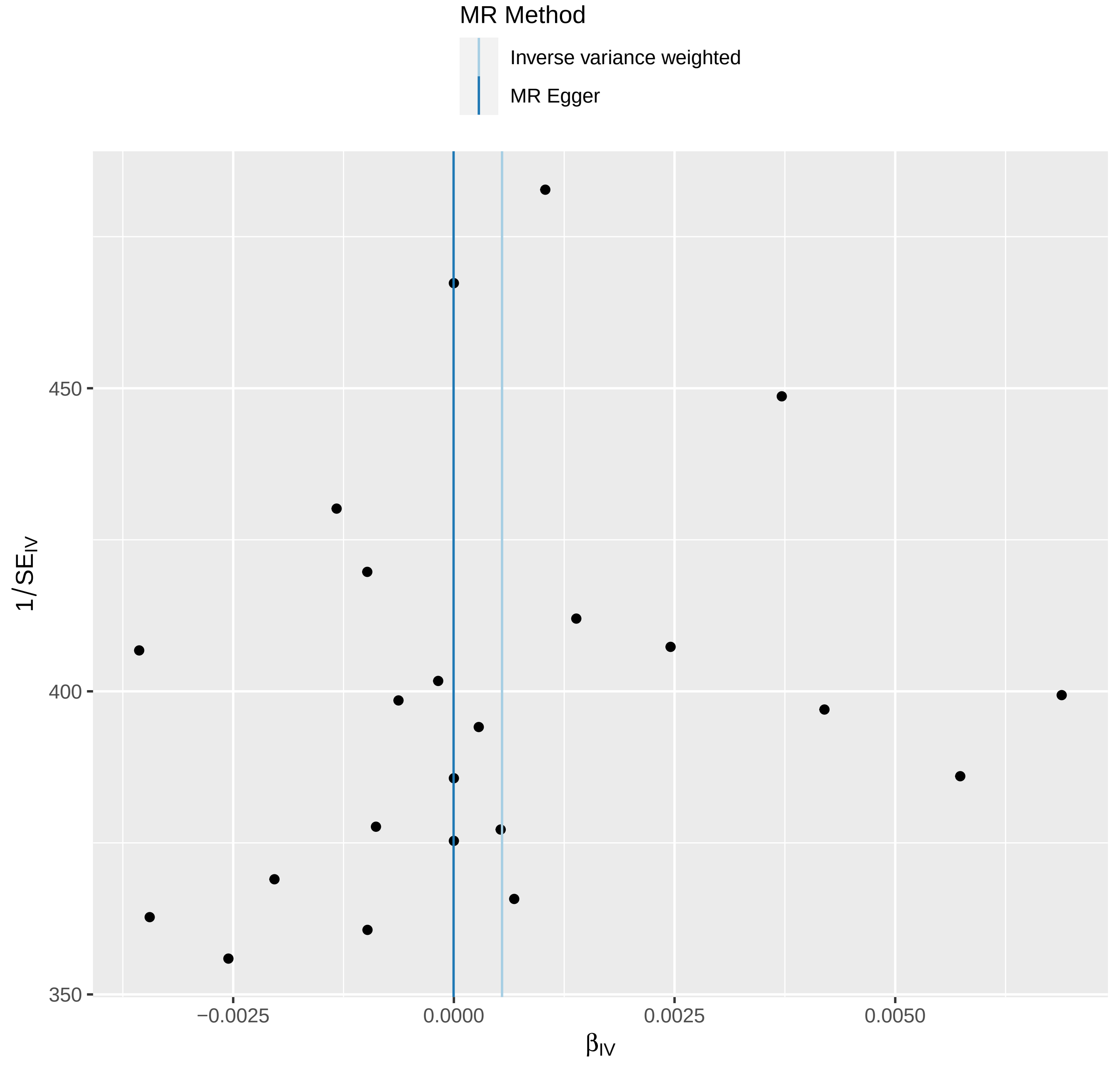

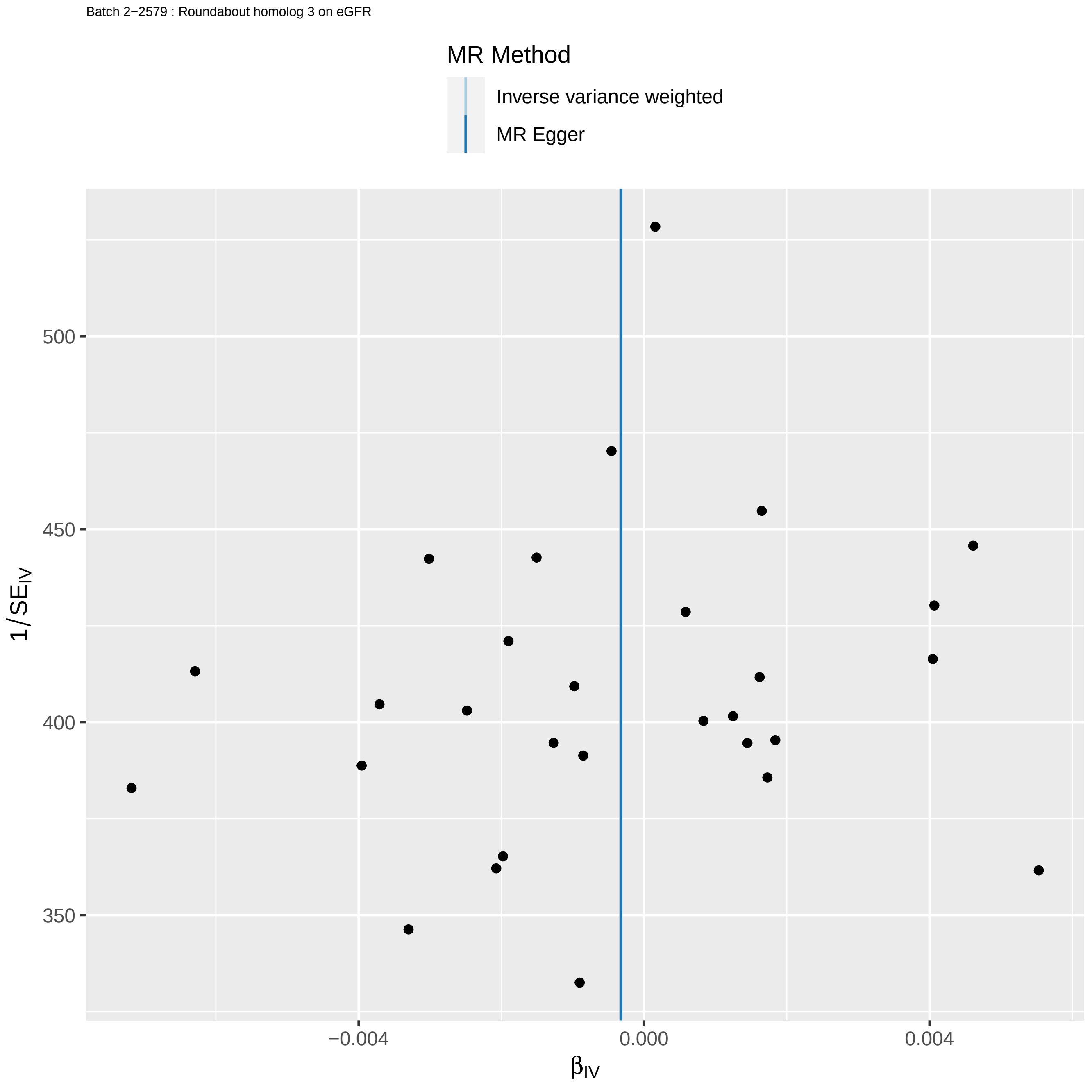
Batch 2−2579 : Roundabout homolog 3 on eGFR
MR Method
Inverse variance weighted
MR Egger
500
450
400
350
1/SE_IV
−0.004
0.000
0.004
β_IV

Batch 2−2580 : Rho−associated protein kinase 2 on eGFR

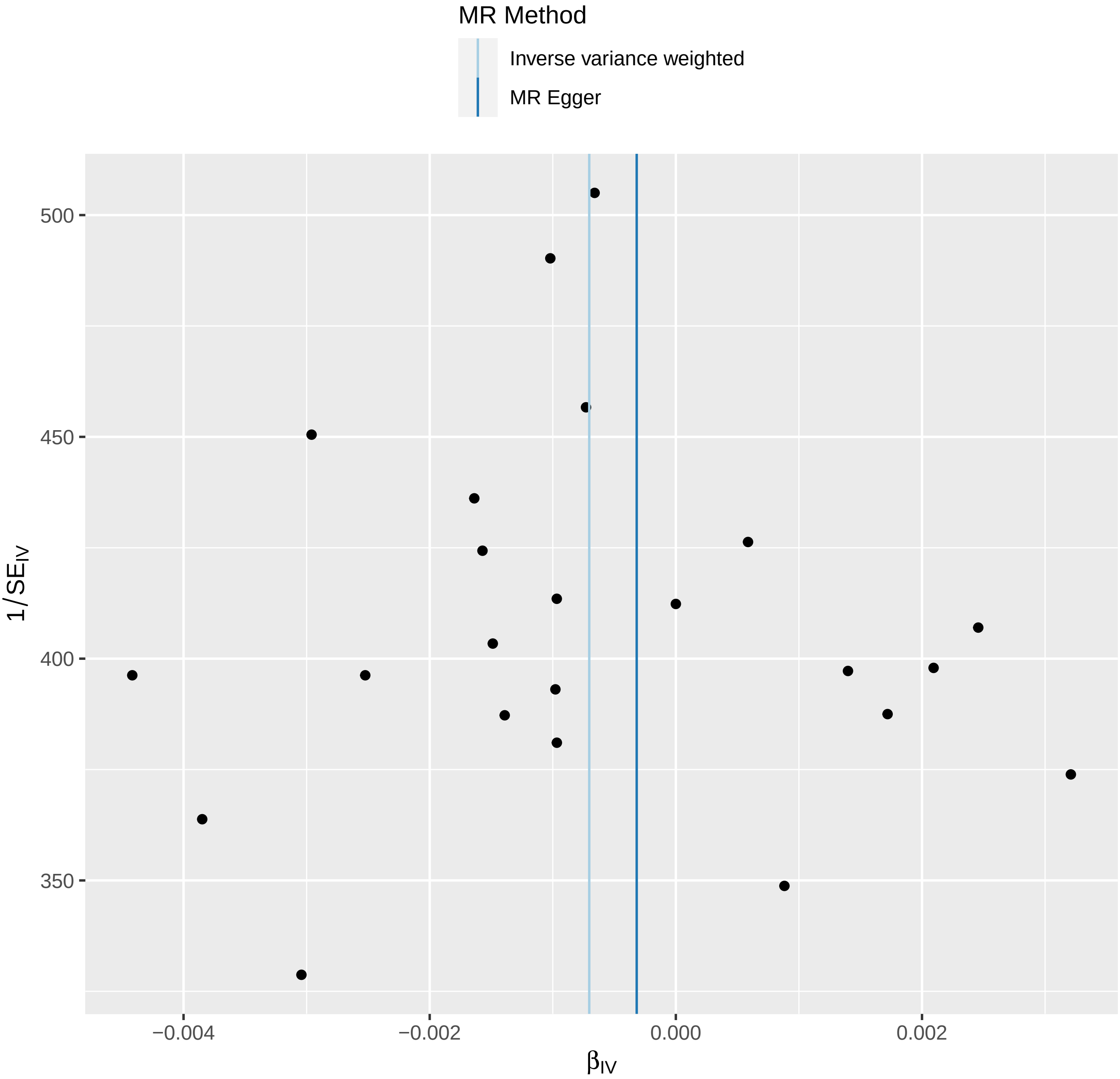

Batch 2−2581 : Tyrosine−protein kinase transmembrane receptor ROR1 on eGFR

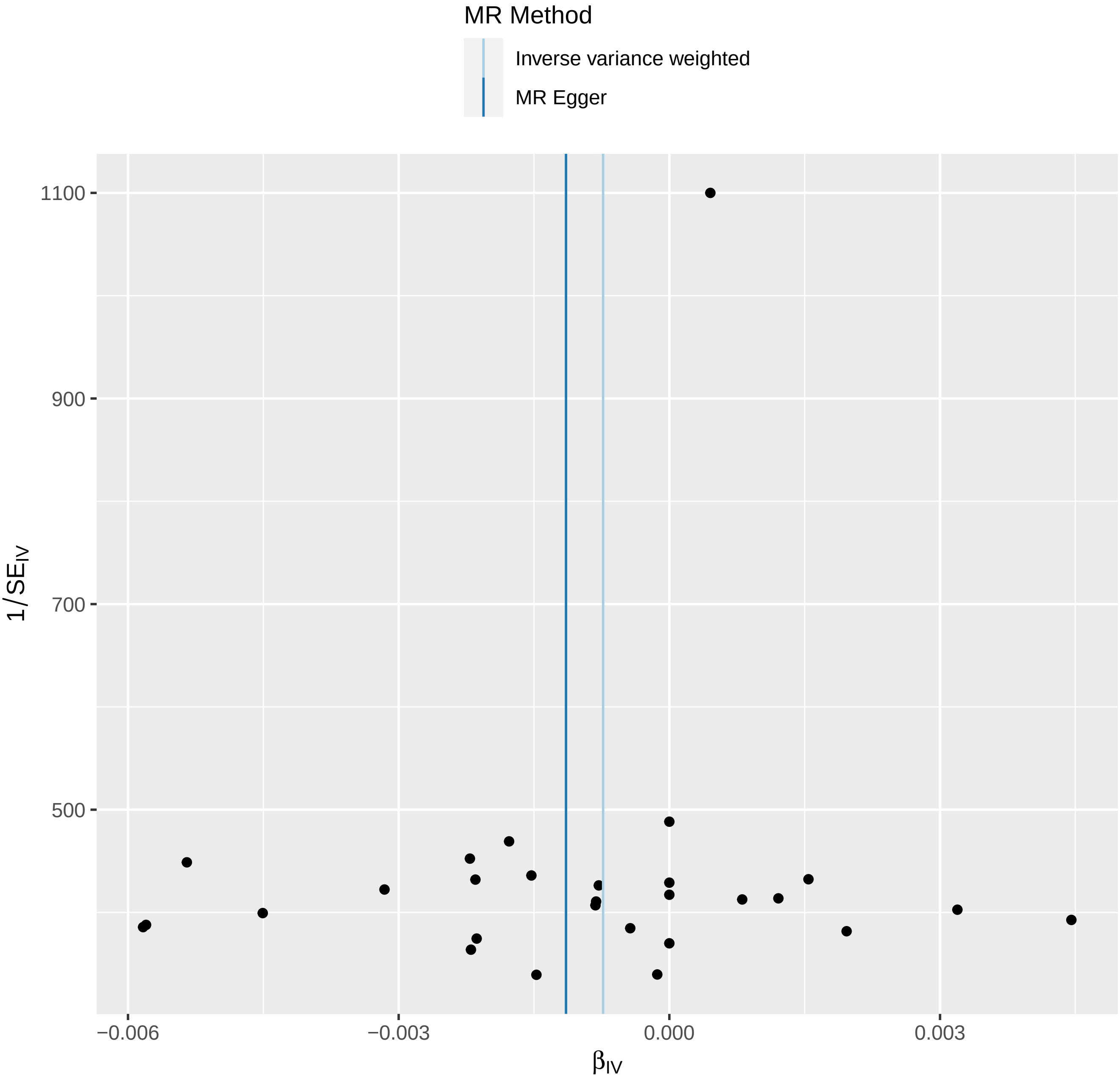

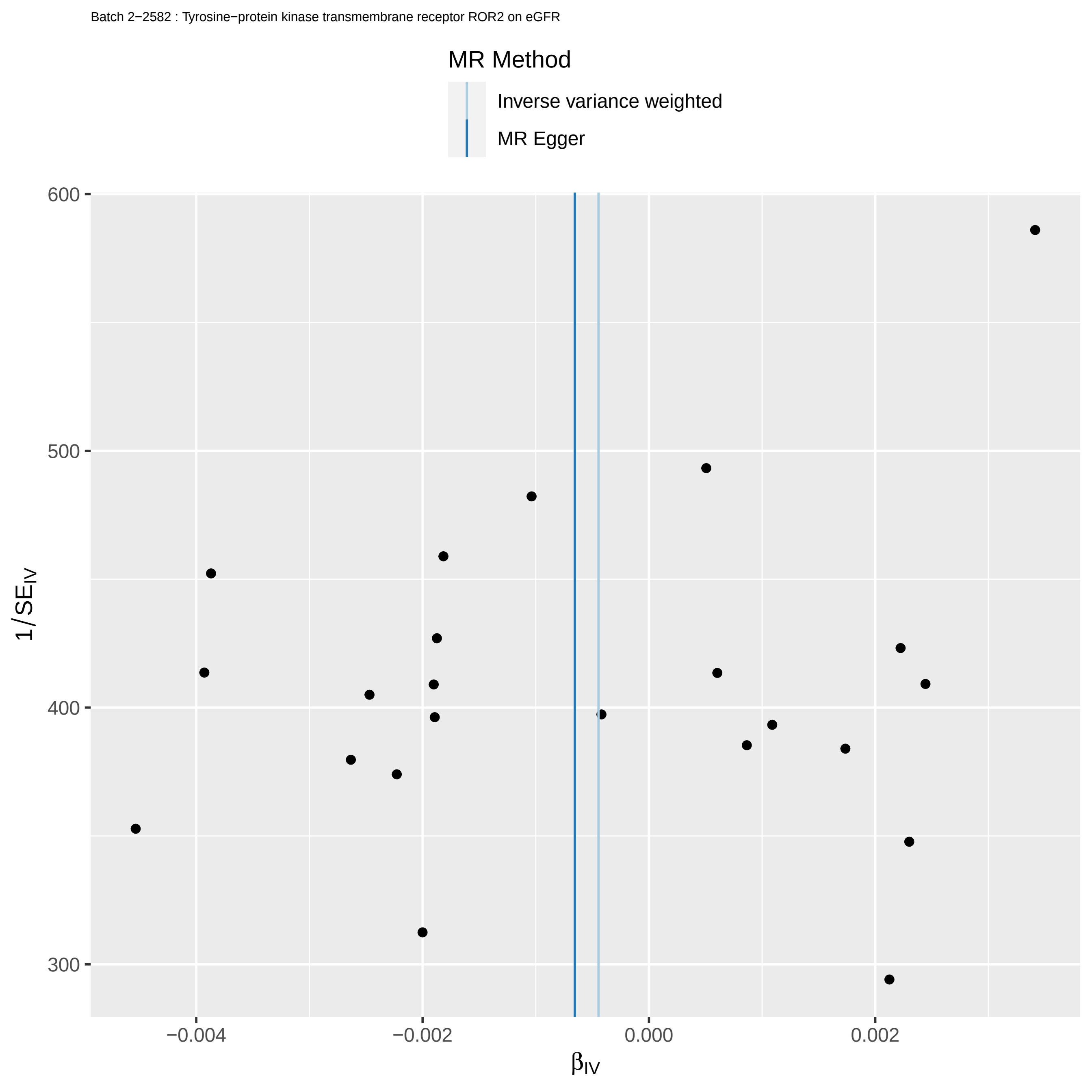
Batch 2−2582 : Tyrosine−protein kinase transmembrane receptor ROR2 on eGFR
MR Method
Inverse variance weighted
MR Egger
600
500
400
300
1/SE_IV
−0.004
−0.002
0.000
0.002
β_IV

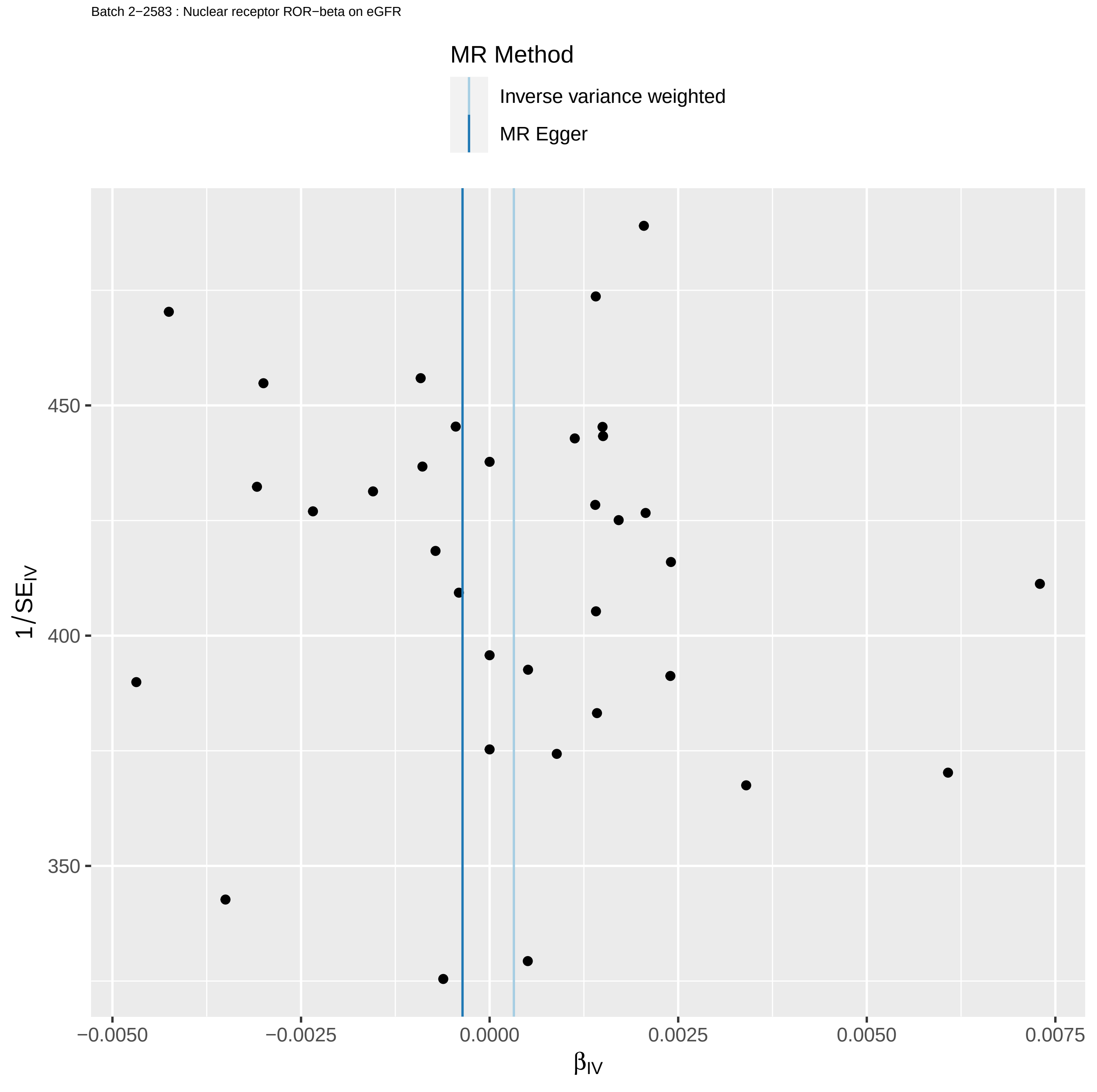

Batch 2−2583 : Nuclear receptor ROR−beta on eGFR
MR Method
Inverse variance weighted
MR Egger
450
400
350
1/SE$_{IV}$
−0.0050
−0.0025
0.0000
0.0025
0.0050
0.0075
β$_{IV}$

Batch 2−2584 : Ribulose−phosphate 3−epimerase on eGFR

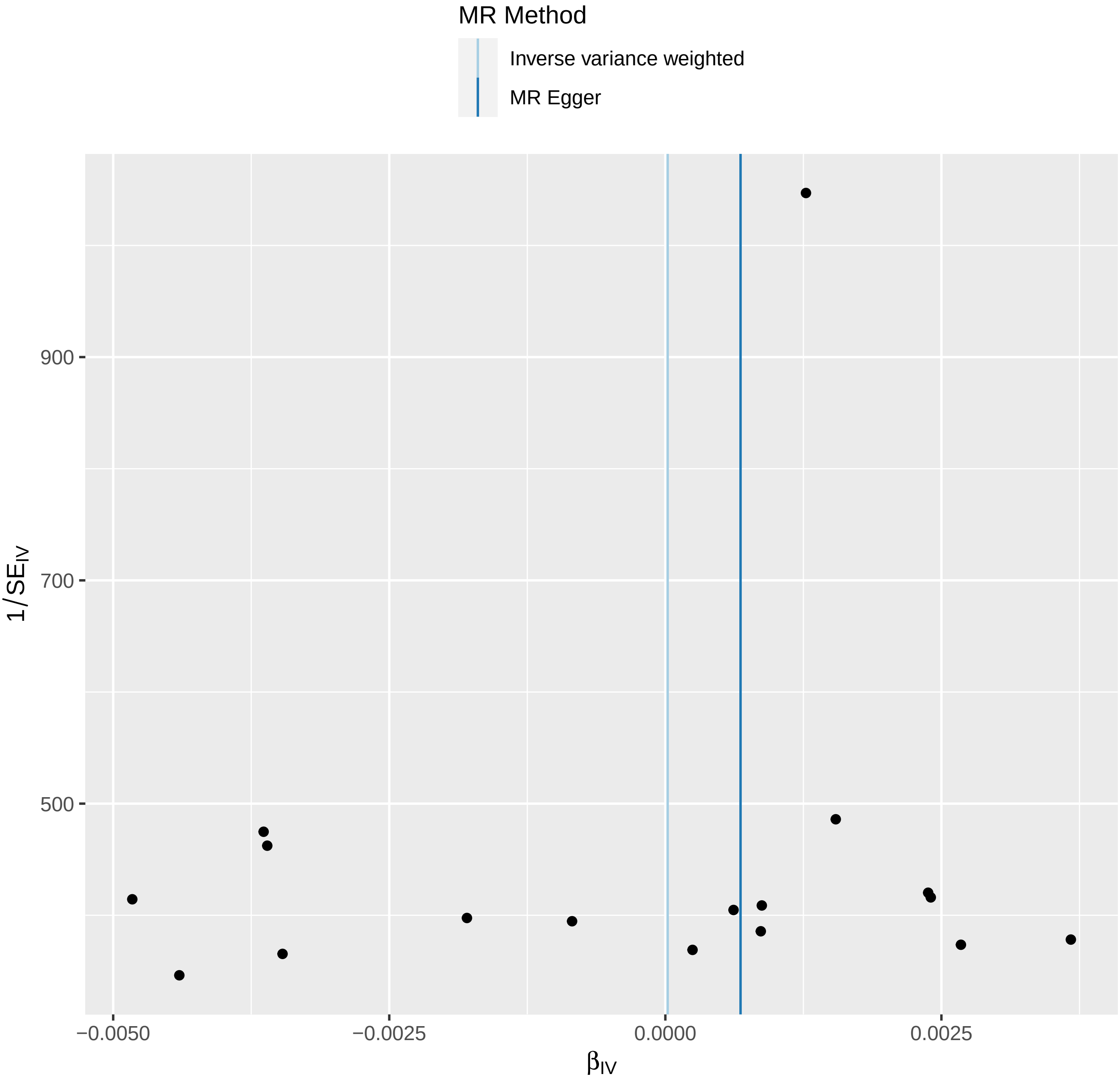

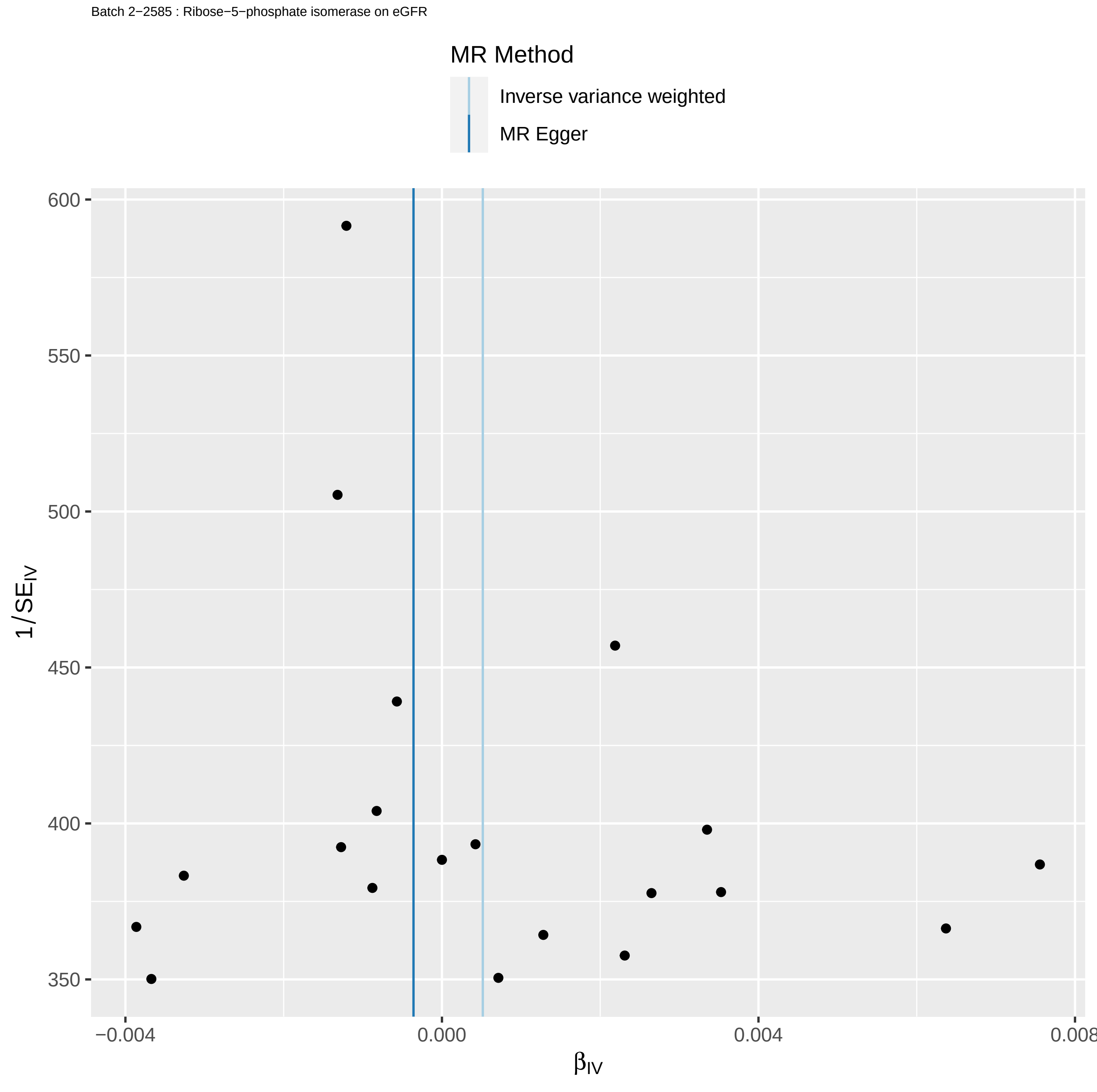
Batch 2−2585 : Ribose−5−phosphate isomerase on eGFR
MR Method
Inverse variance weighted
MR Egger
600
550
500
450
400
350
1/SE$_{IV}$
−0.004
0.000
0.004
0.008
β$_{IV}$

Batch 2−2586 : 60S ribosomal protein L30 on eGFR

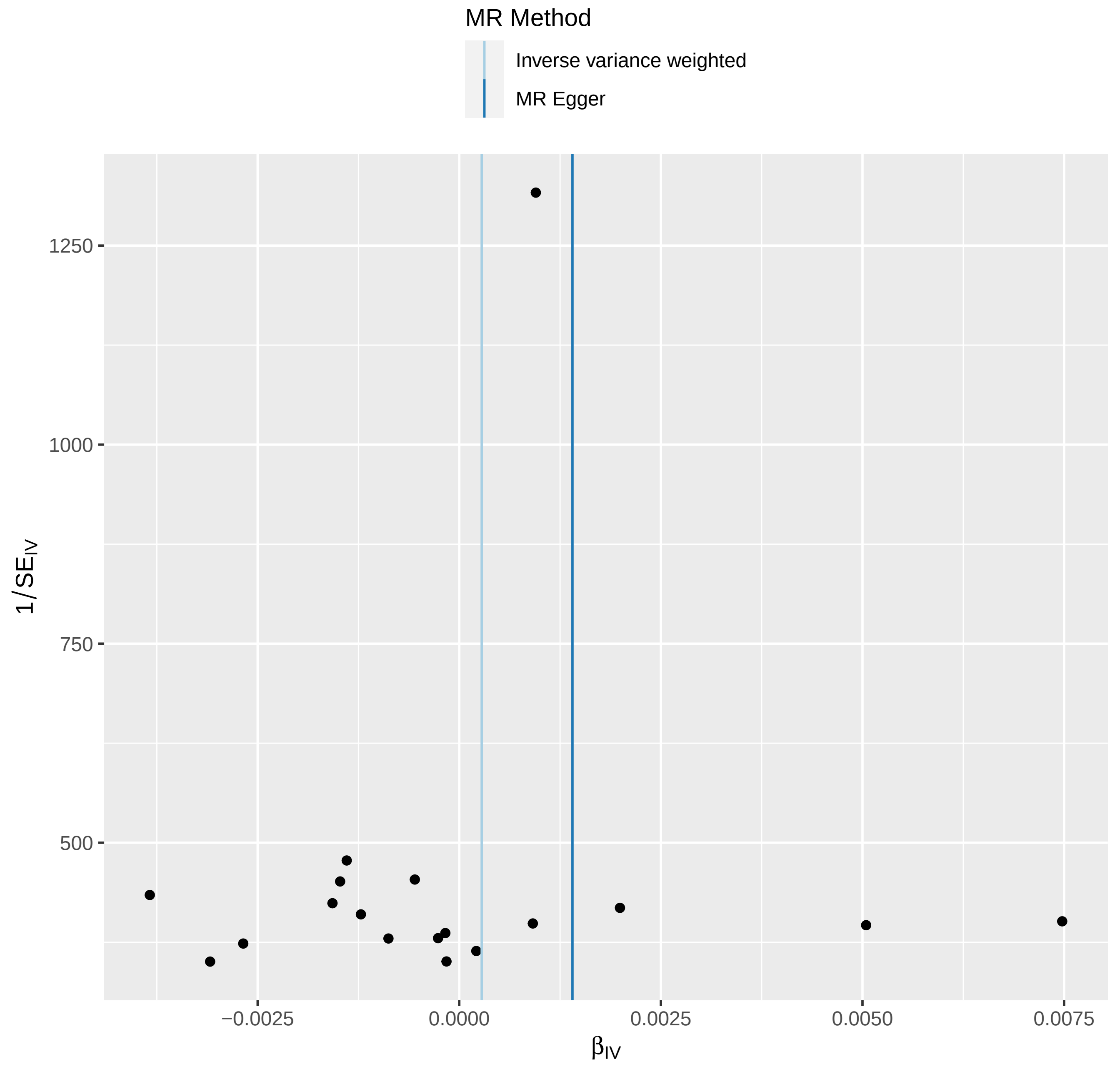

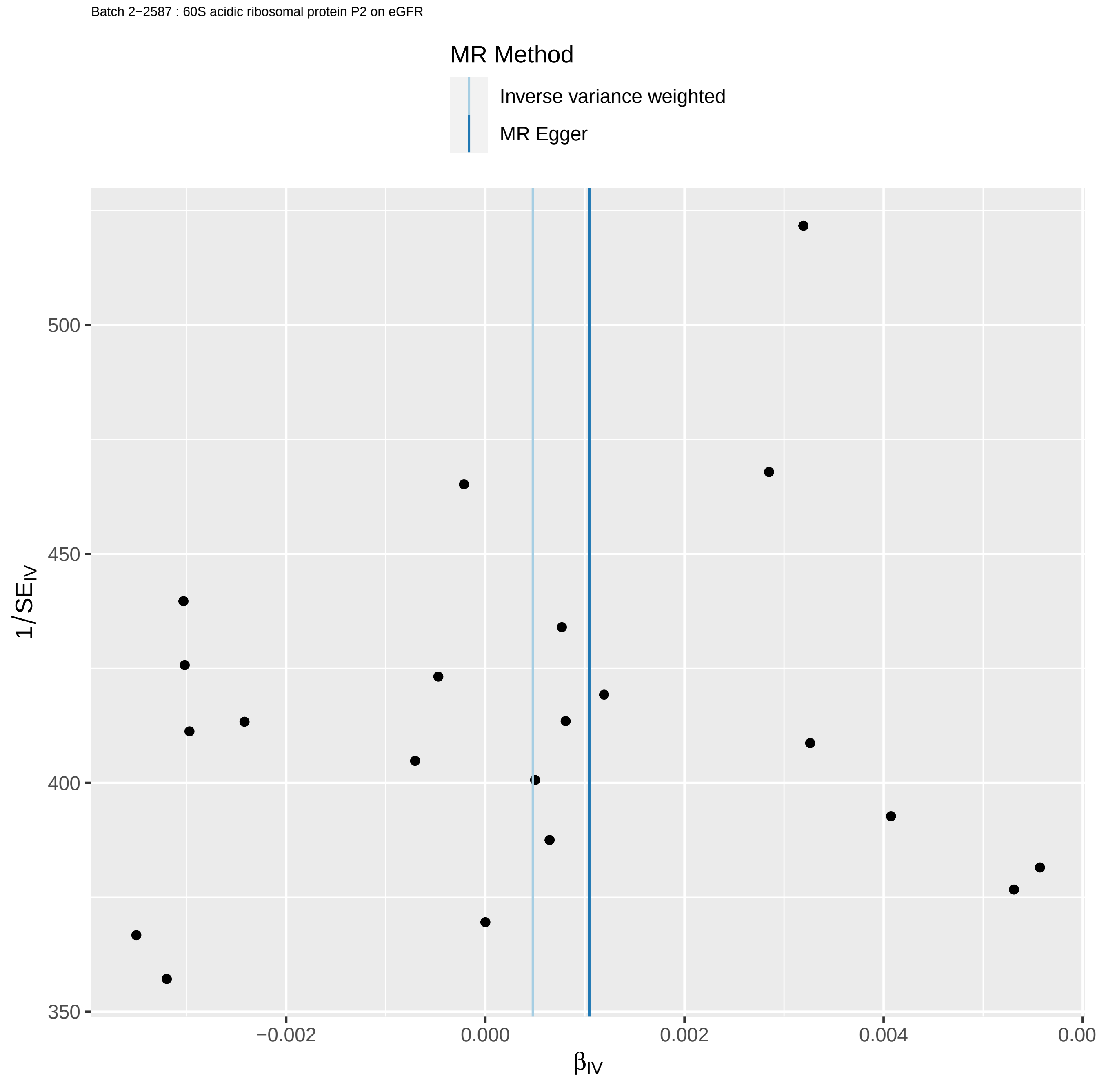

Batch 2−2587 : 60S acidic ribosomal protein P2 on eGFR
MR Method
Inverse variance weighted
MR Egger
500
450
400
350
1/SE_IV
−0.002
0.000
0.002
0.004
0.00
β_IV

Batch 2−2588 : Dolichyl−diphosphooligosaccharide−−protein glycosyltransferase subunit 1 on eGFR

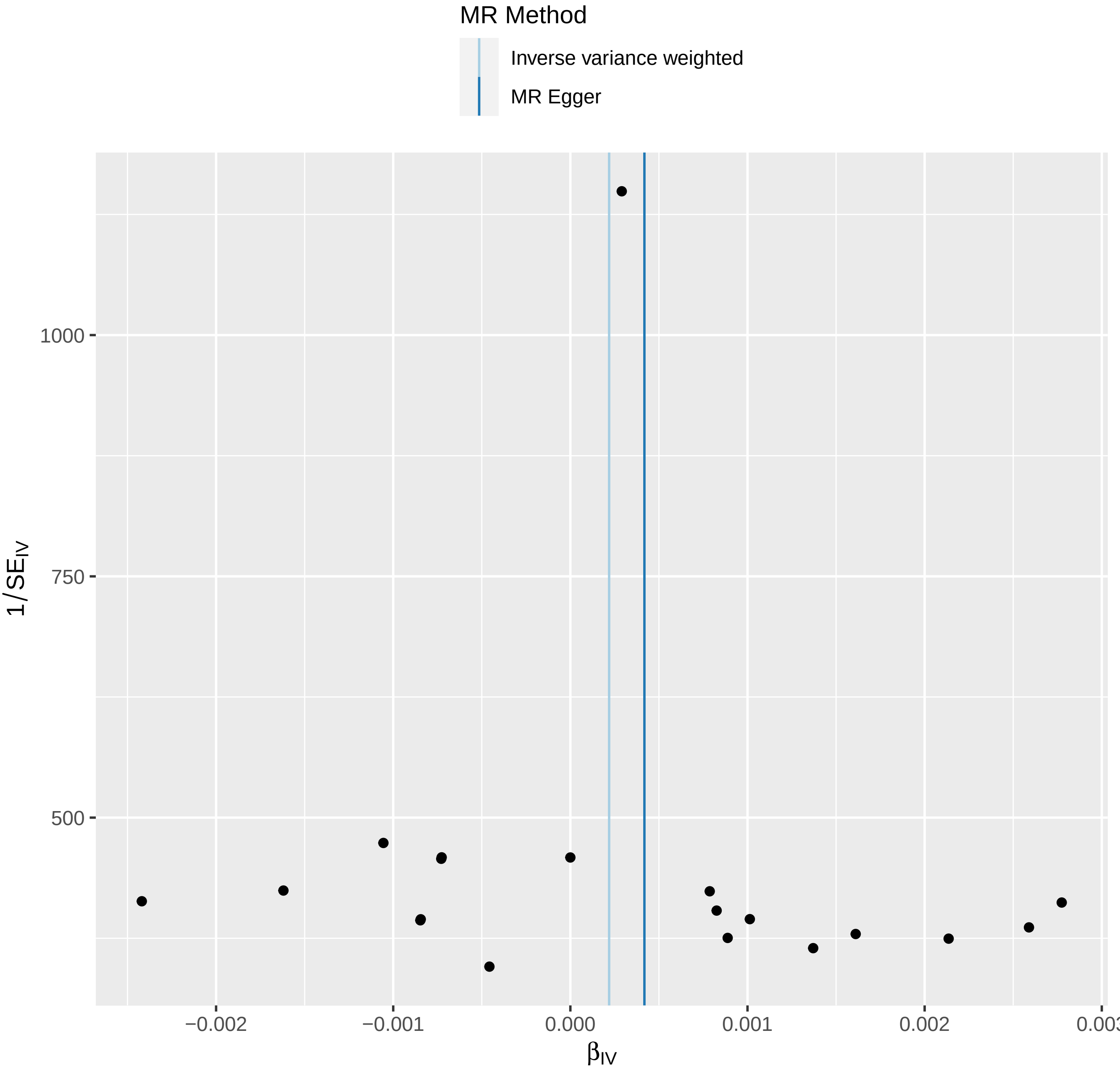

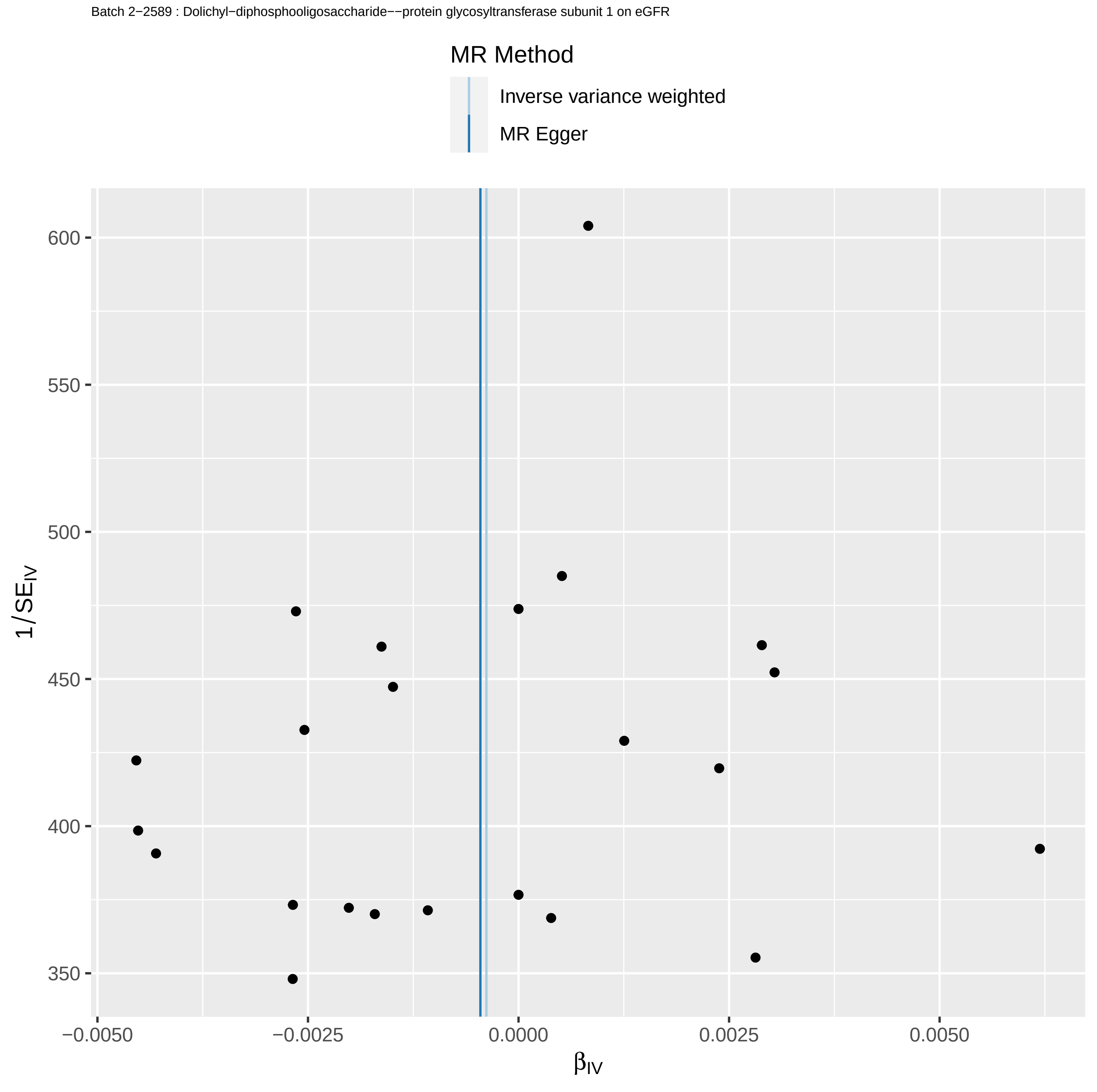

Batch 2−2589 : Dolichyl−diphosphooligosaccharide−−protein glycosyltransferase subunit 1 on eGFR
MR Method
Inverse variance weighted
MR Egger
600
550
500
450
400
350
1/SE_IV
−0.0050
−0.0025
0.0000
0.0025
0.0050
β_IV

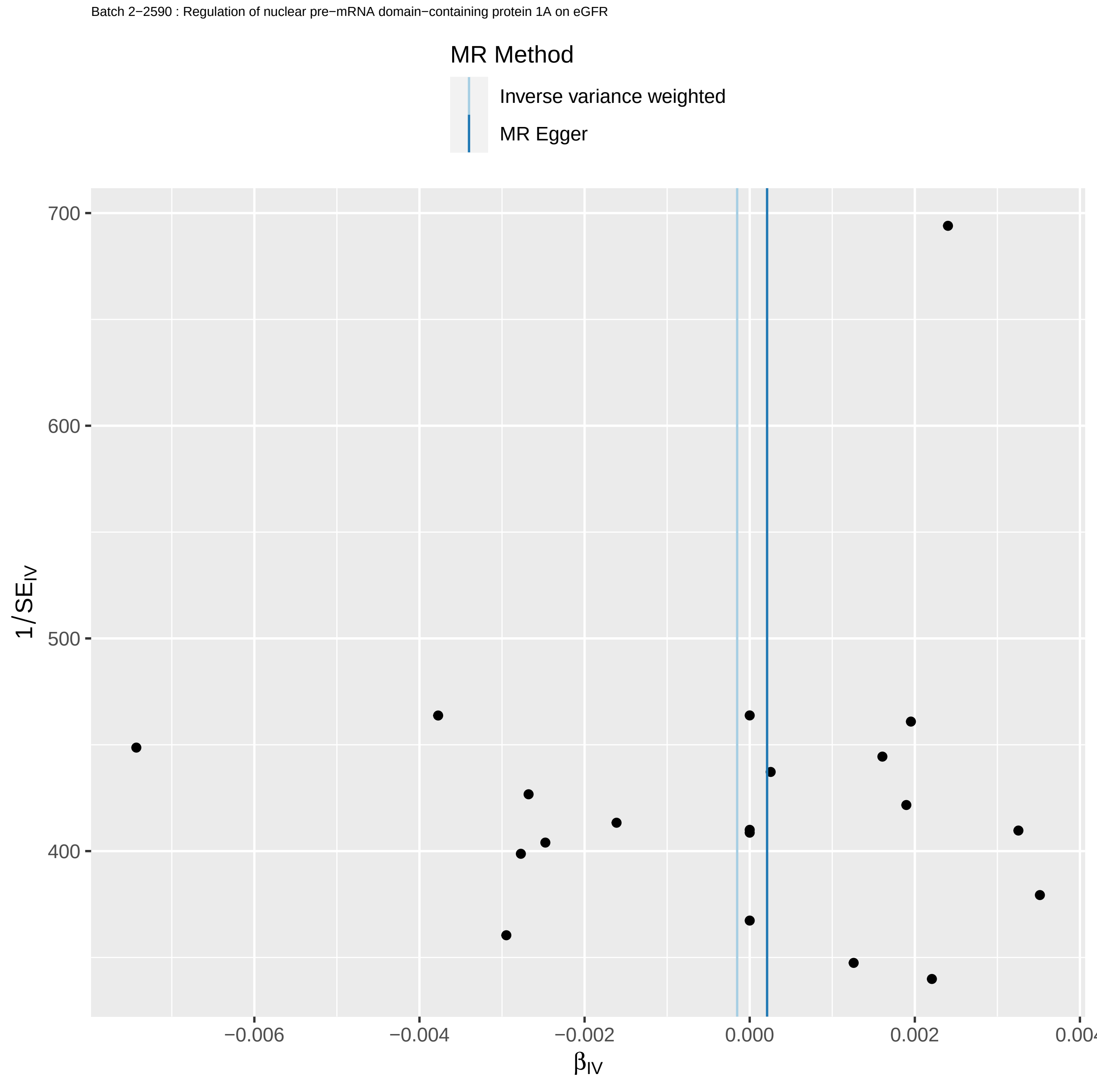

Batch 2−2590 : Regulation of nuclear pre−mRNA domain−containing protein 1A on eGFR
MR Method
Inverse variance weighted
MR Egger
700
600
500
400
1/SE$_{IV}$
−0.006
−0.004
−0.002
0.000
0.002
0.004
β$_{IV}$

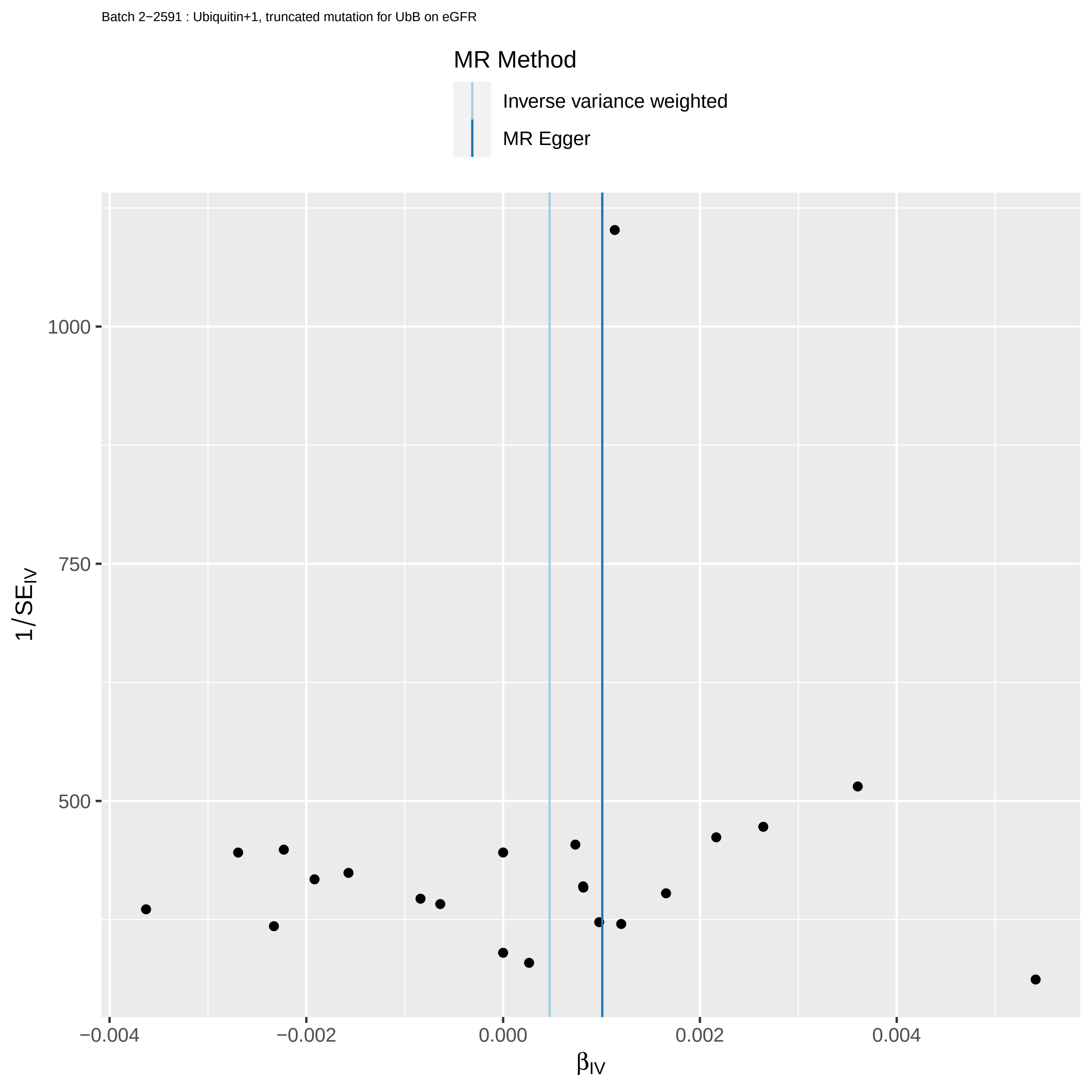

Batch 2−2591 : Ubiquitin+1, truncated mutation for UbB on eGFR
MR Method
Inverse variance weighted
MR Egger
1000
750
500
1/SE_IV
−0.004
−0.002
0.000
0.002
0.004
β_IV

Batch 2−2592 : Ubiquitin on eGFR

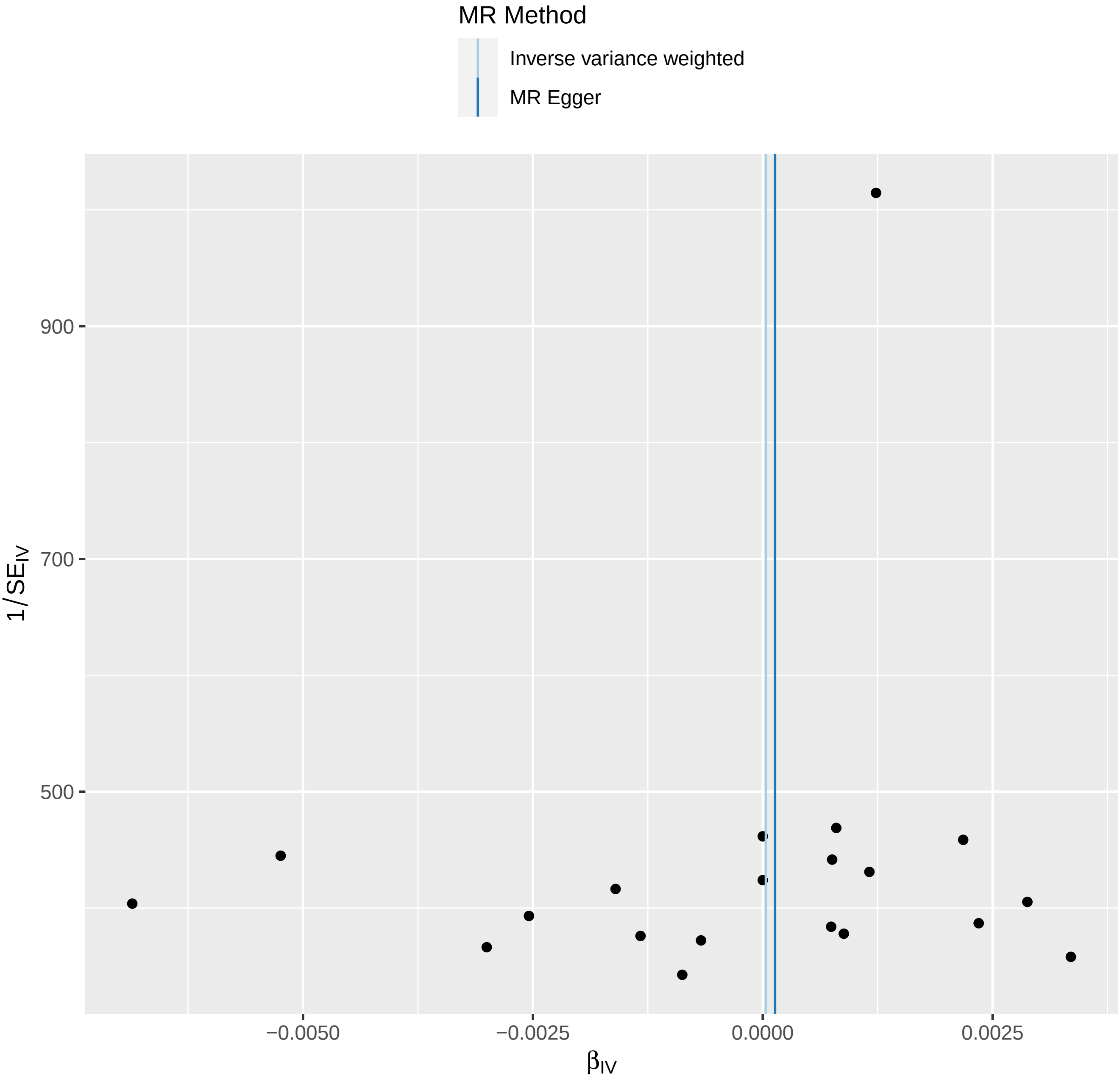

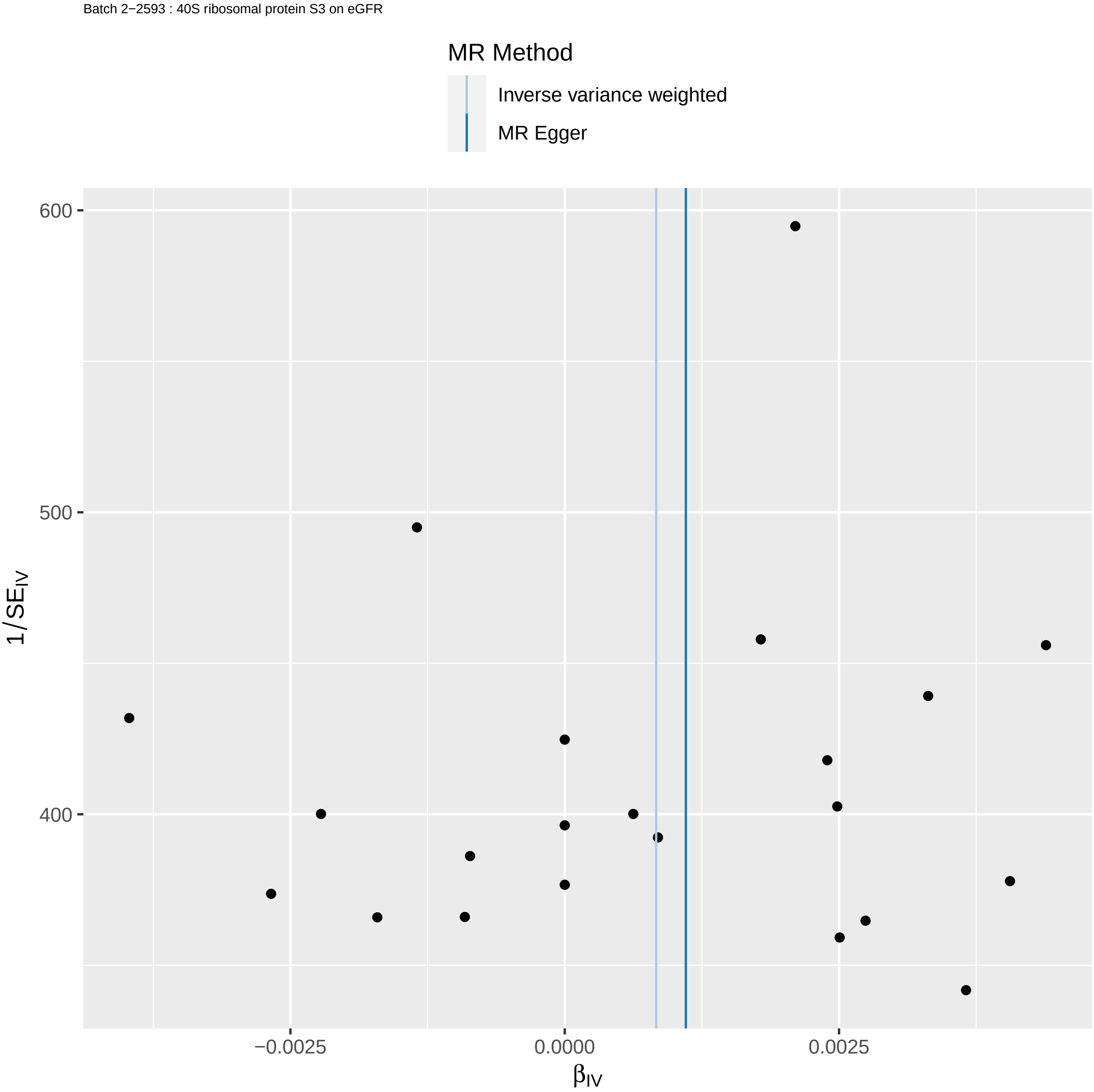
Batch 2−2593 : 40S ribosomal protein S3 on eGFR
MR Method
Inverse variance weighted
MR Egger
600
500
400
1/SE_IV
−0.0025
0.0000
0.0025
β_IV

Batch 2−2594 : 40S ribosomal protein S3a on eGFR

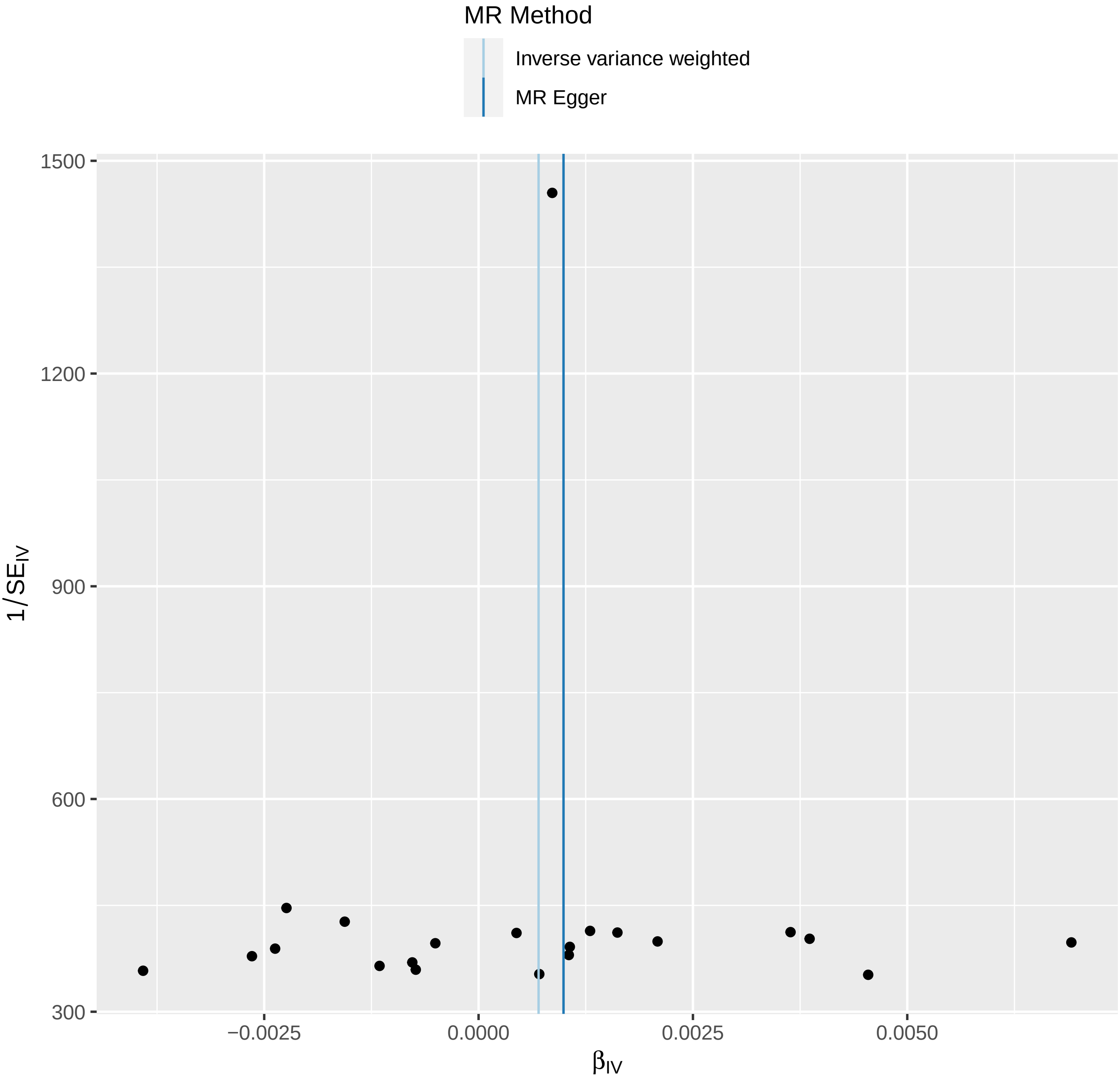

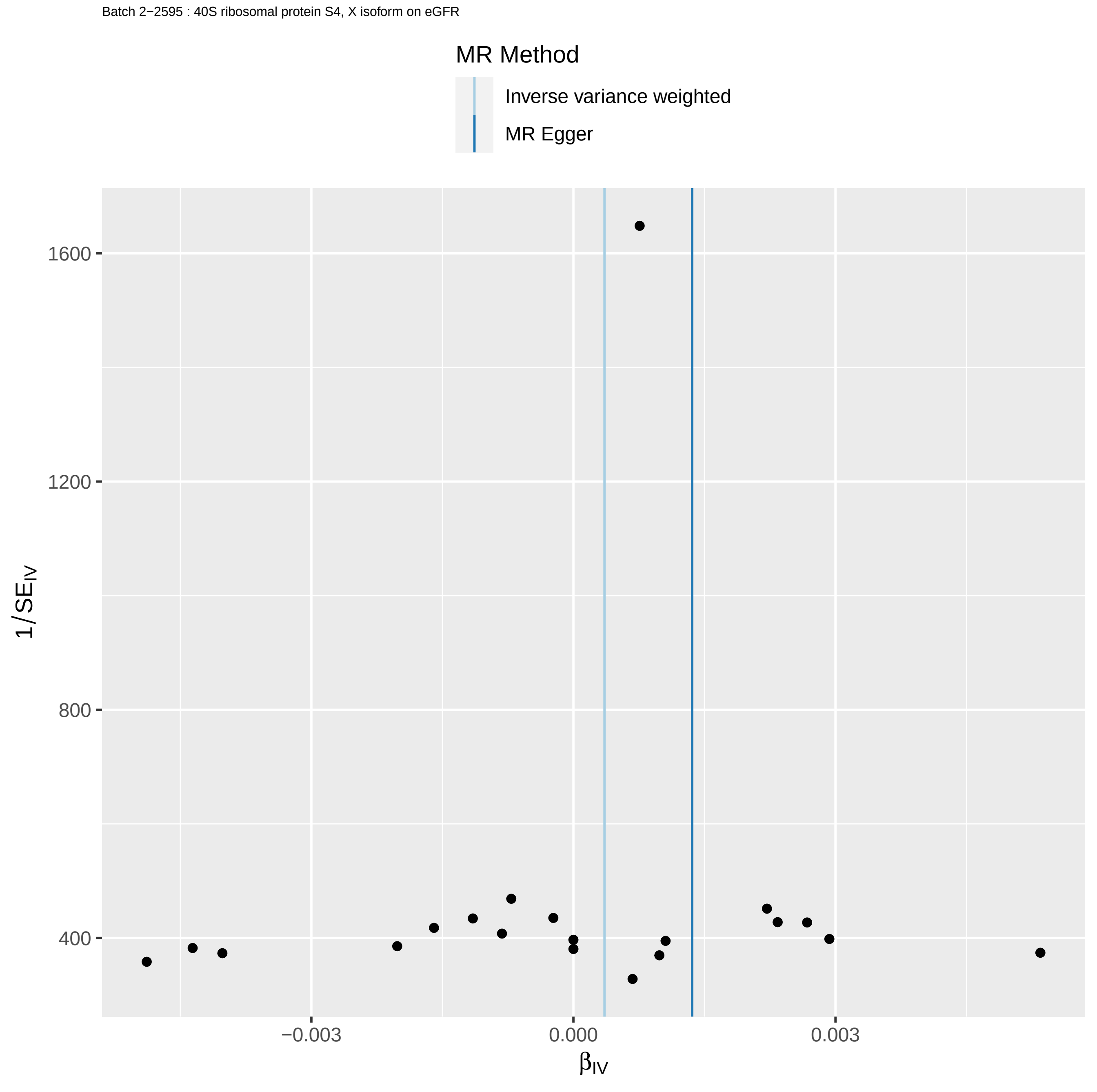

Batch 2−2595 : 40S ribosomal protein S4, X isoform on eGFR
MR Method
Inverse variance weighted
MR Egger
1600
1200
800
400
1/SE$_{IV}$
−0.003
0.000
0.003
β$_{IV}$

Batch 2−2596 : Ribosomal protein S6 kinase alpha−6 on eGFR

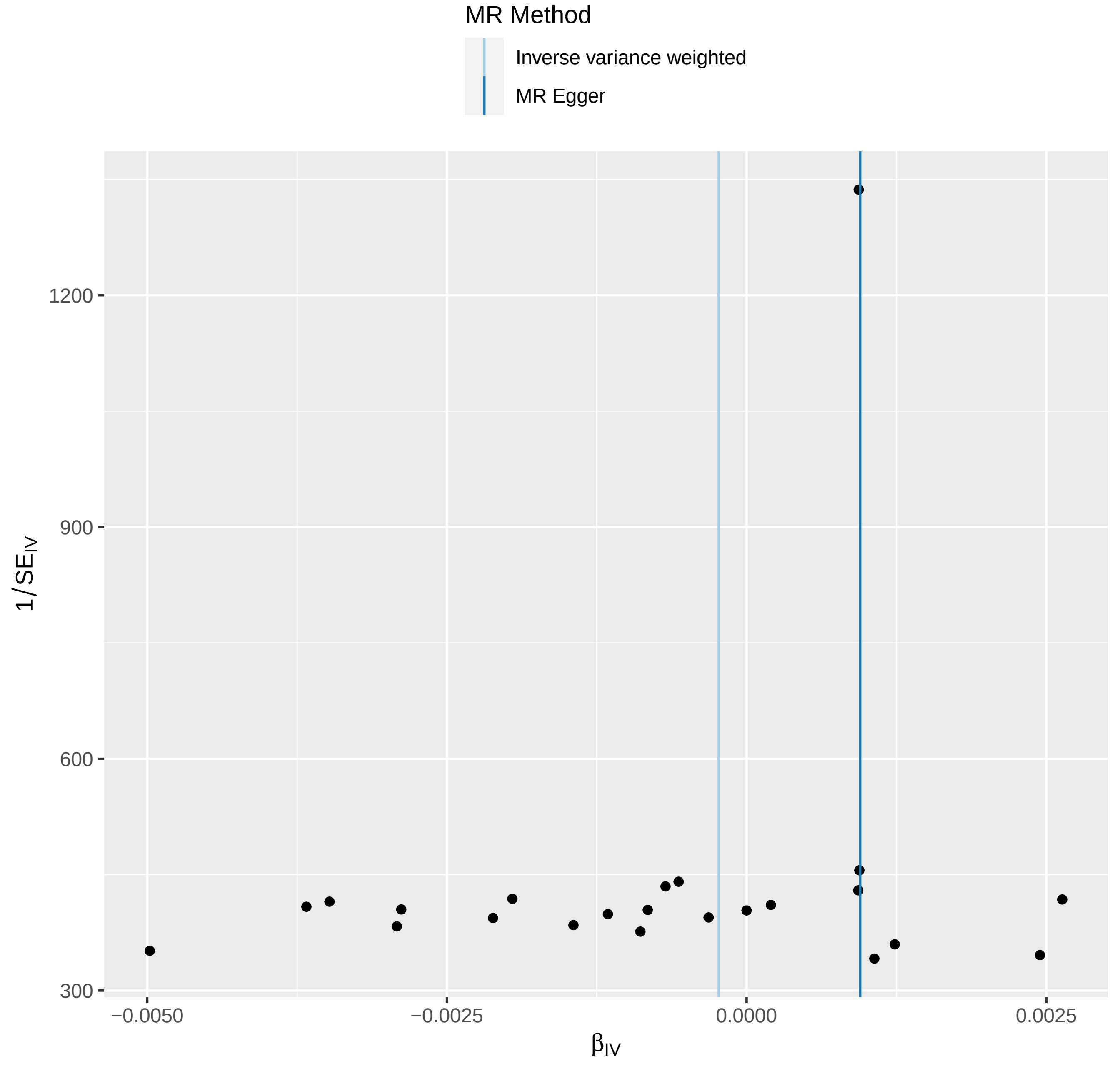

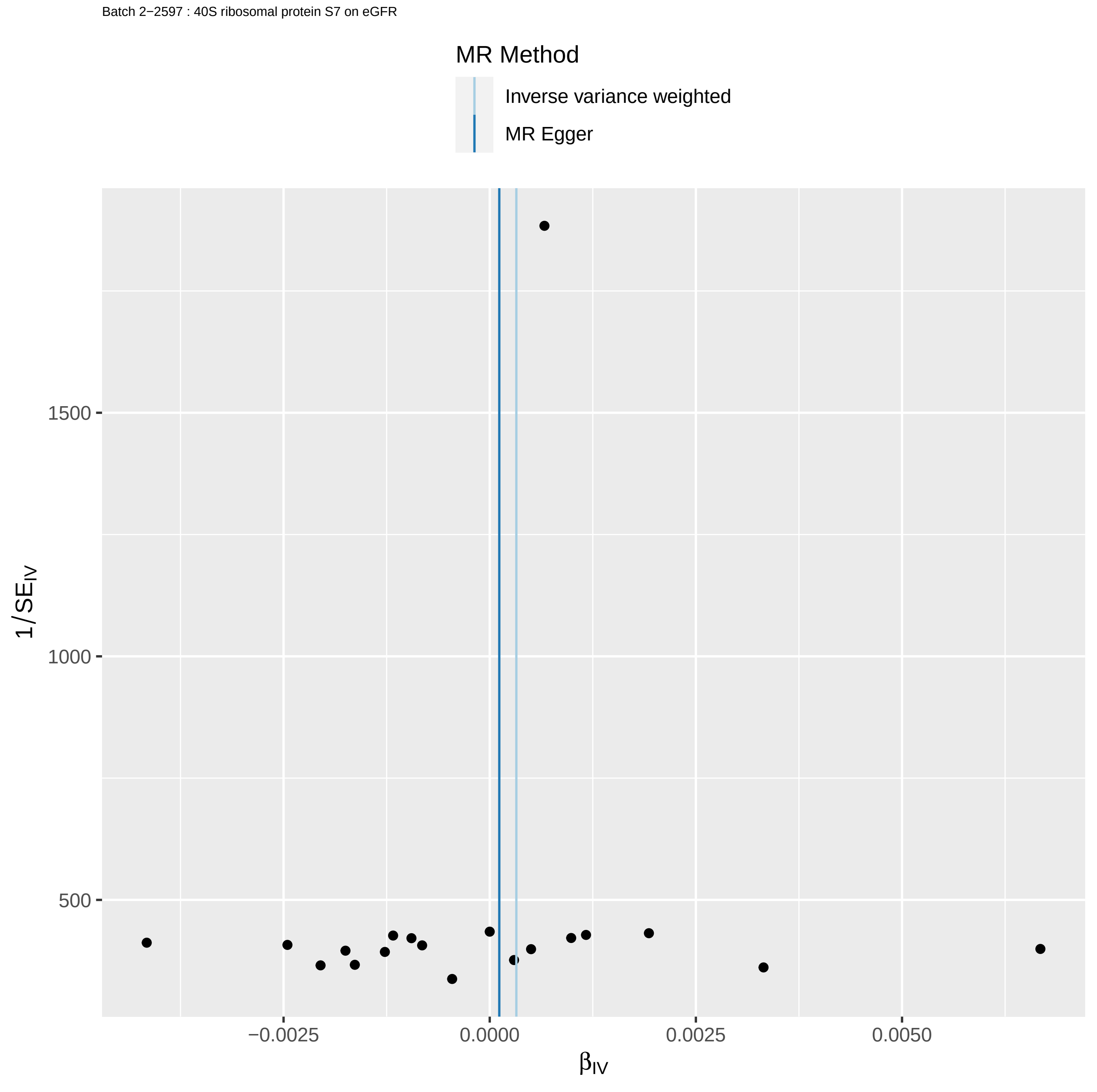
Batch 2−2597 : 40S ribosomal protein S7 on eGFR
MR Method
Inverse variance weighted
MR Egger
1500
1000
500
1/SE_IV
−0.0025
0.0000
0.0025
0.0050
β_IV

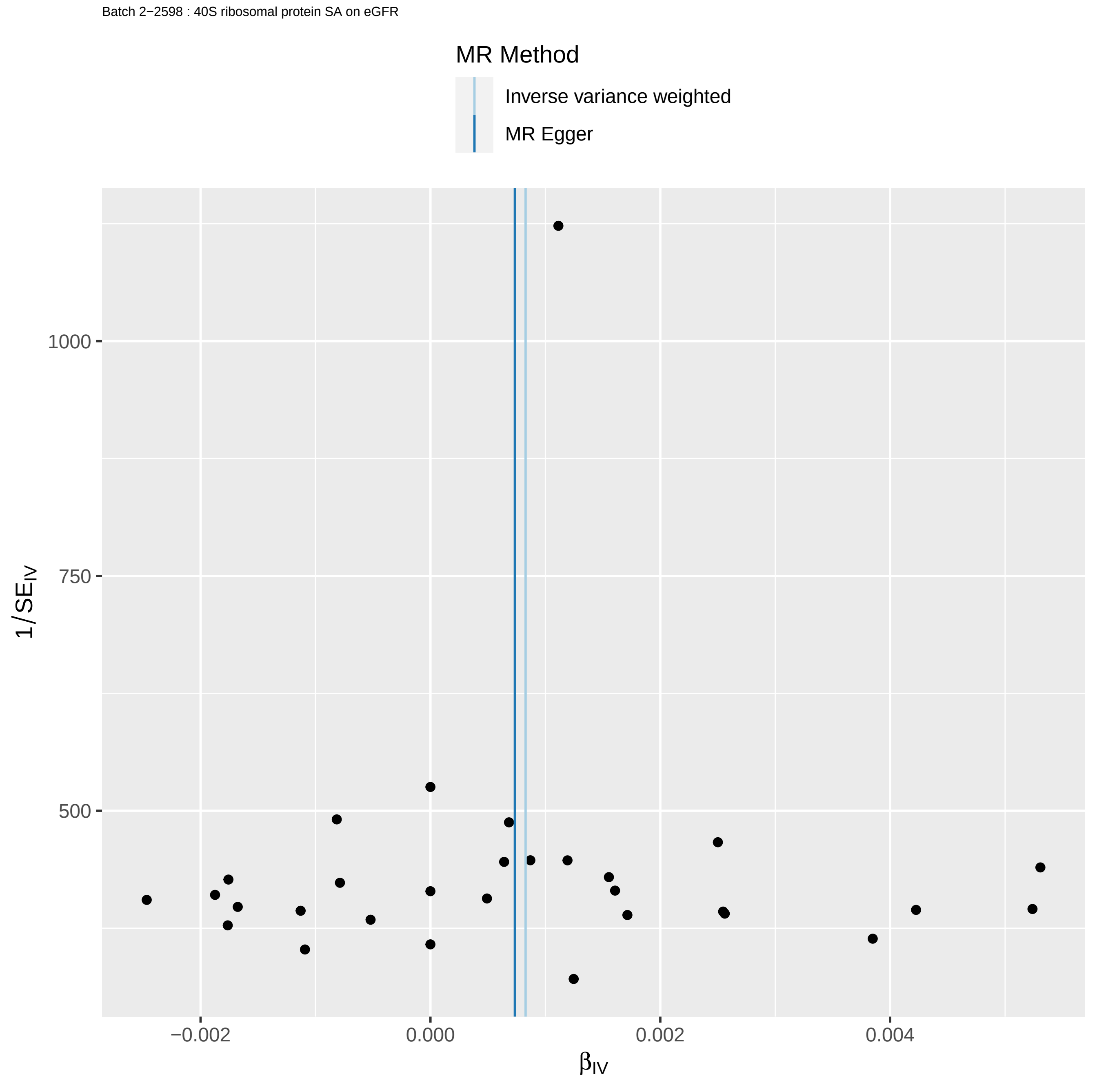
Batch 2−2598 : 40S ribosomal protein SA on eGFR
MR Method
Inverse variance weighted
MR Egger
1000
750
500
1/SE$_{IV}$
−0.002
0.000
0.002
0.004
β$_{IV}$

Batch 2−2599 : Cell differentiation protein RCD1 homolog on eGFR

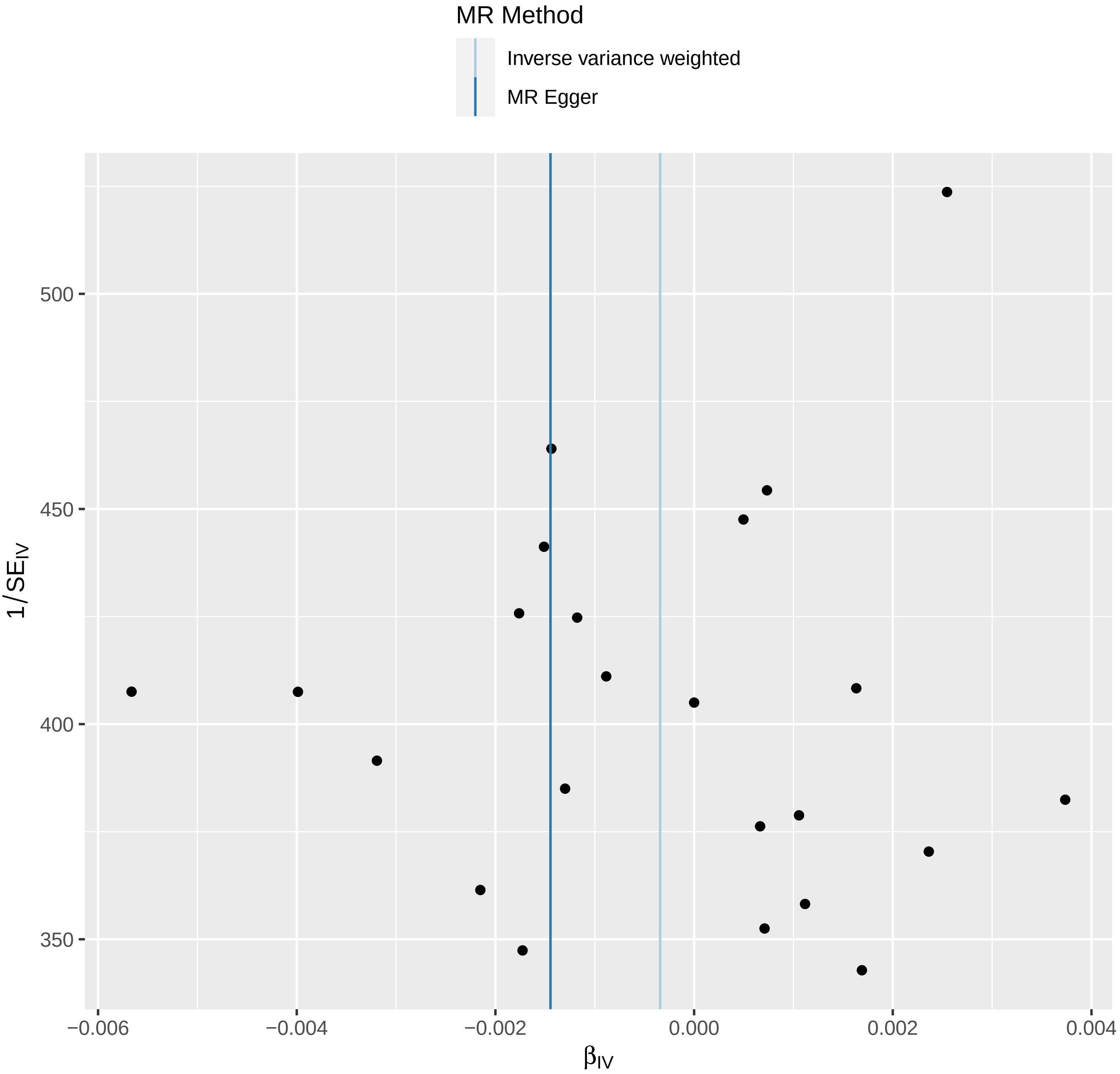

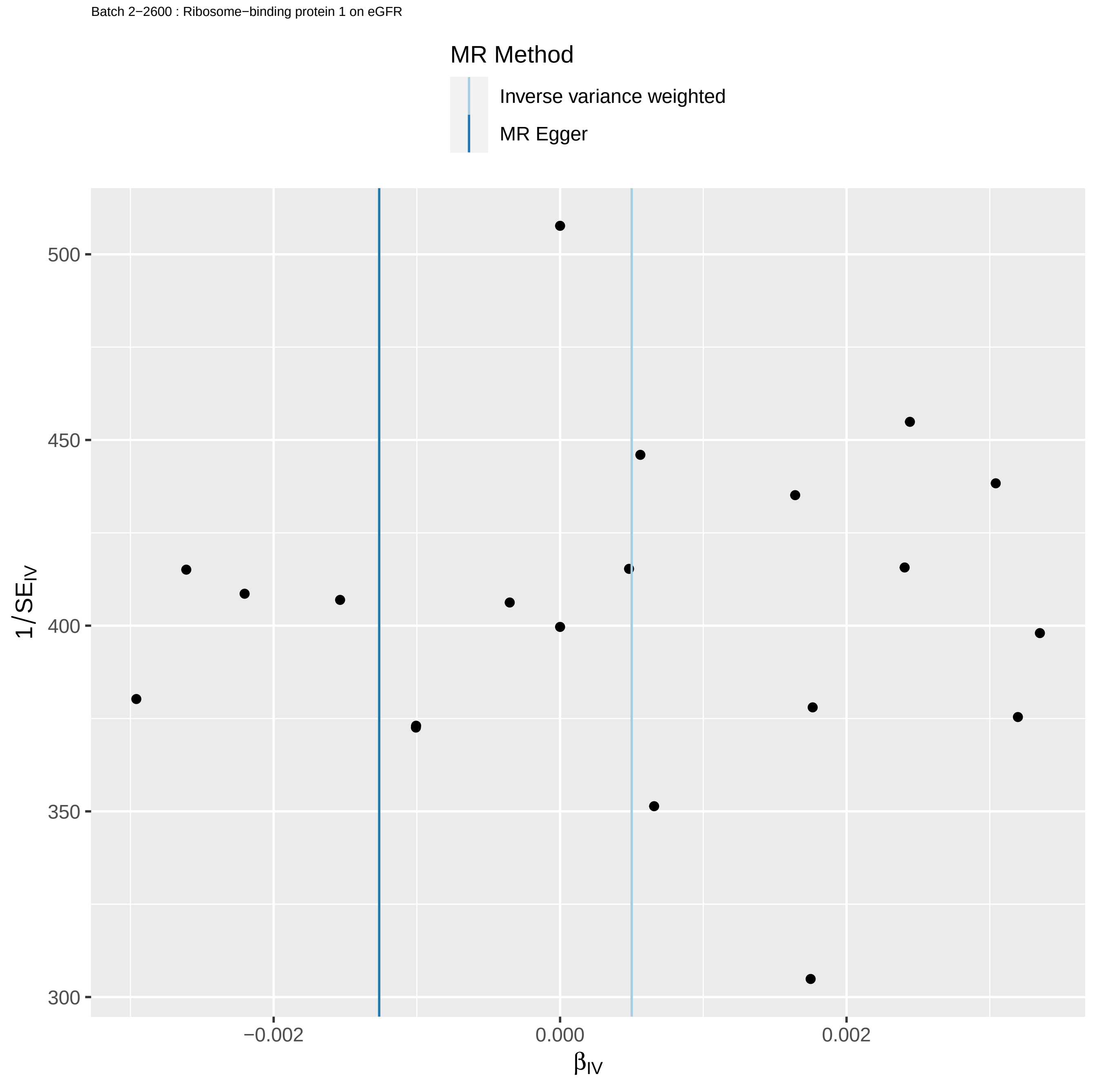

Batch 2−2600 : Ribosome−binding protein 1 on eGFR
MR Method
Inverse variance weighted
MR Egger
500
450
400
350
300
1/SE$_{IV}$
−0.002
0.000
0.002
β_{IV}

Batch 2−2601 : Ribonucleoside−diphosphate reductase large subunit on eGFR

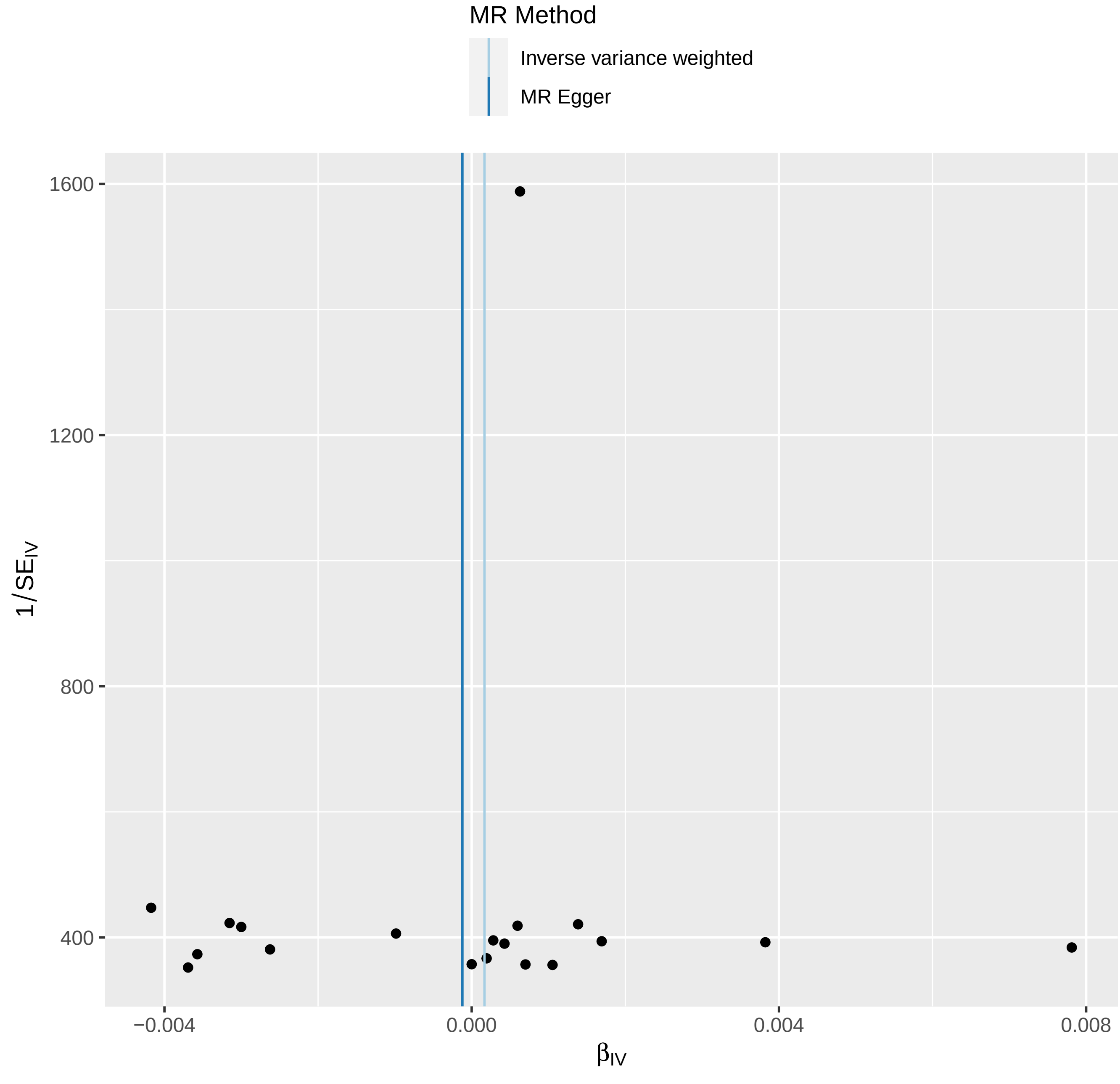

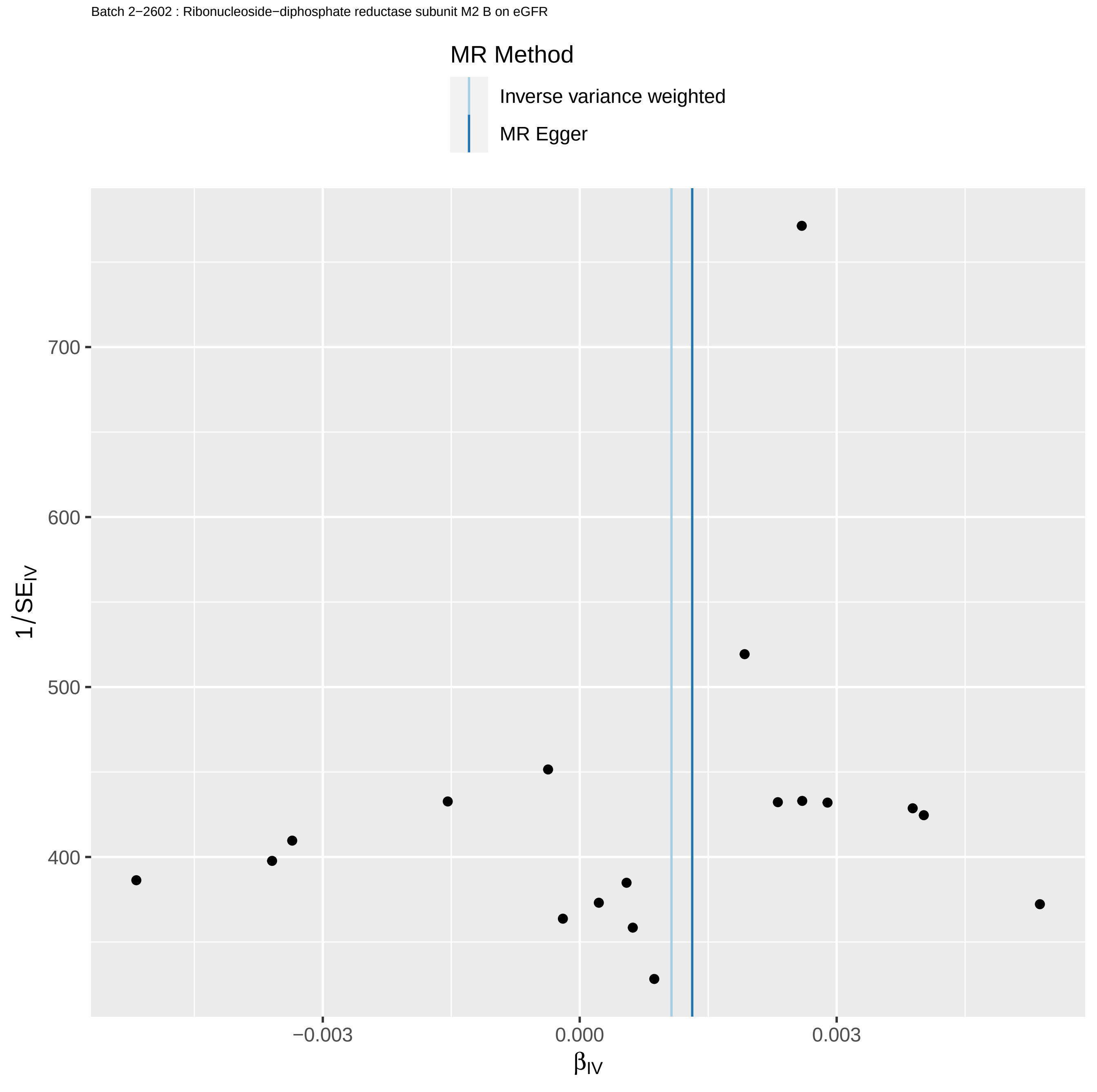

Batch 2−2602 : Ribonucleoside−diphosphate reductase subunit M2 B on eGFR
MR Method
Inverse variance weighted
MR Egger
700
600
500
400
1/SE$_{IV}$
−0.003
0.000
0.003
β$_{IV}$

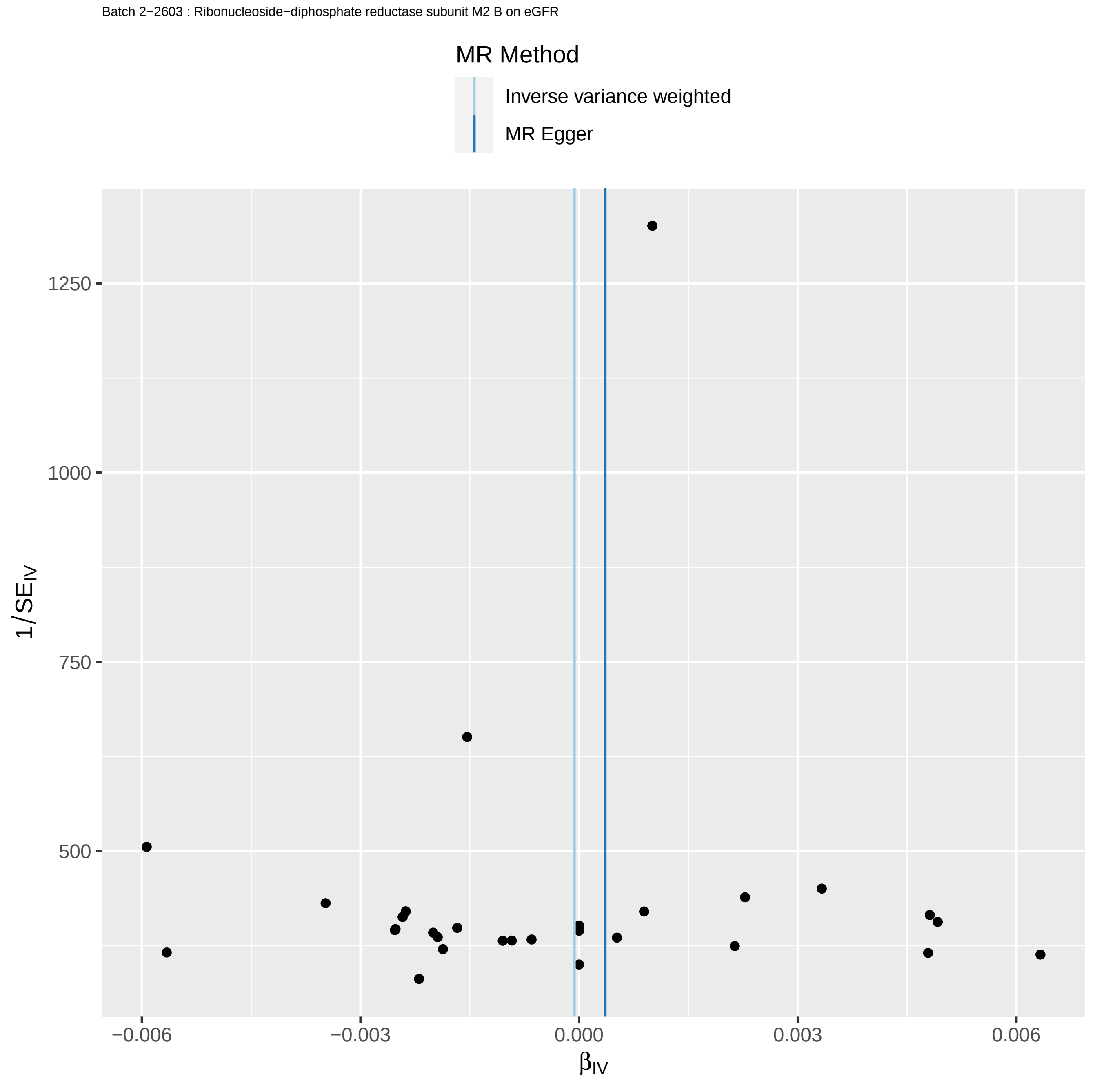
Batch 2−2603 : Ribonucleoside−diphosphate reductase subunit M2 B on eGFR
MR Method
Inverse variance weighted
MR Egger
1250
1000
750
500
1/SE_IV
−0.006
−0.003
0.000
0.003
0.006
β_IV

Batch 2−2604 : Retinoschisin on eGFR

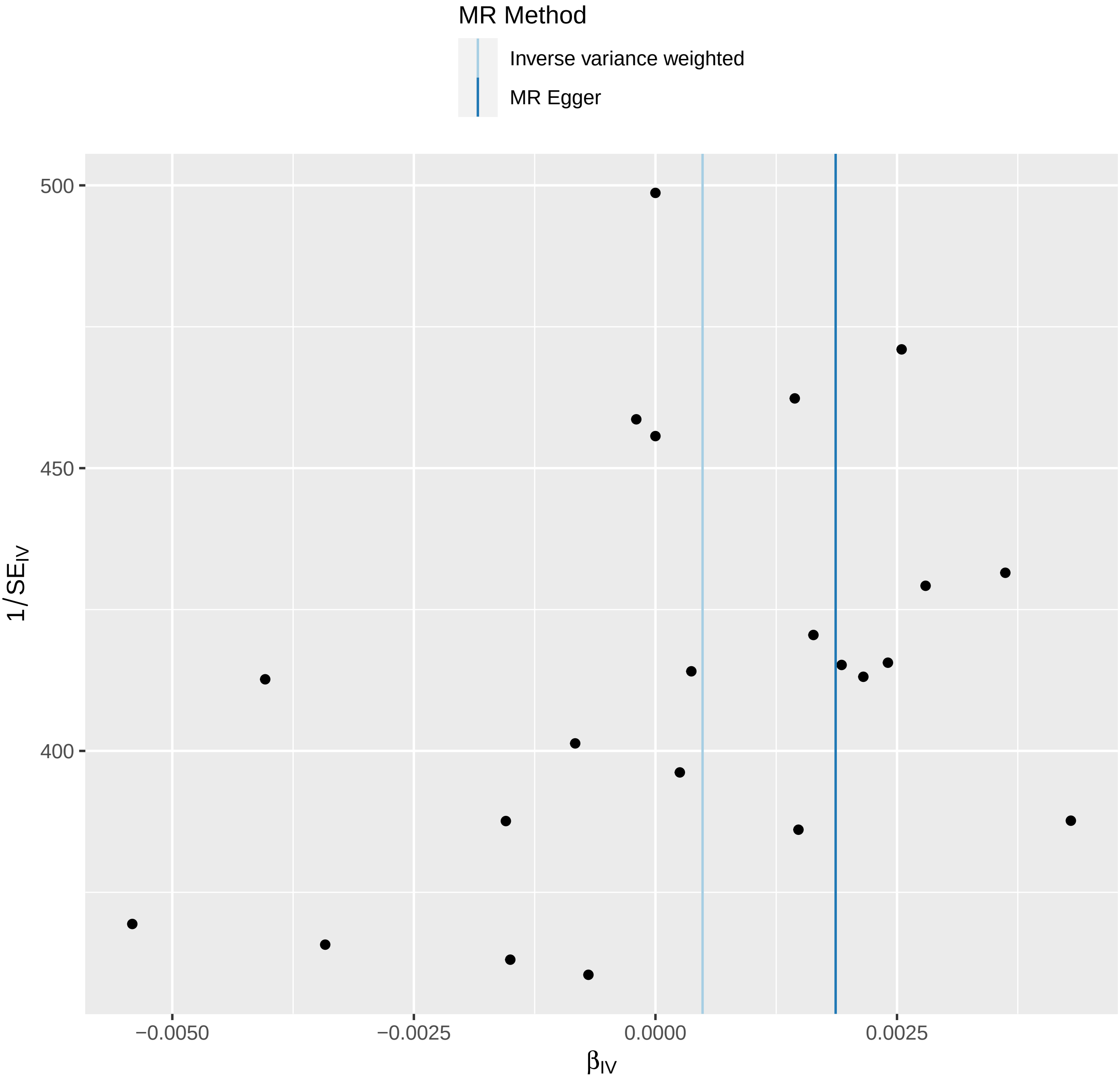

Batch 2−2605 : R−spondin−3 on eGFR

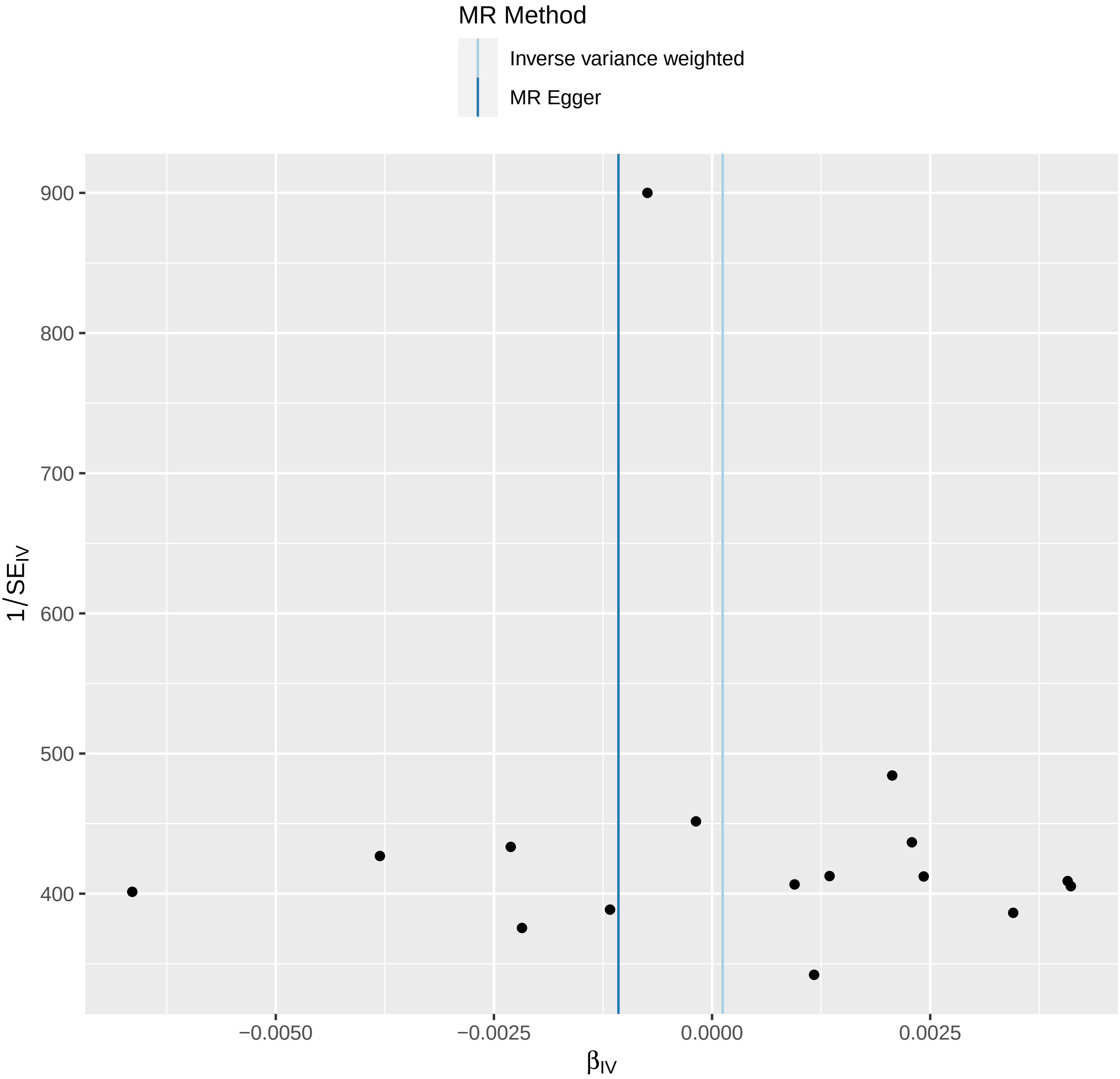

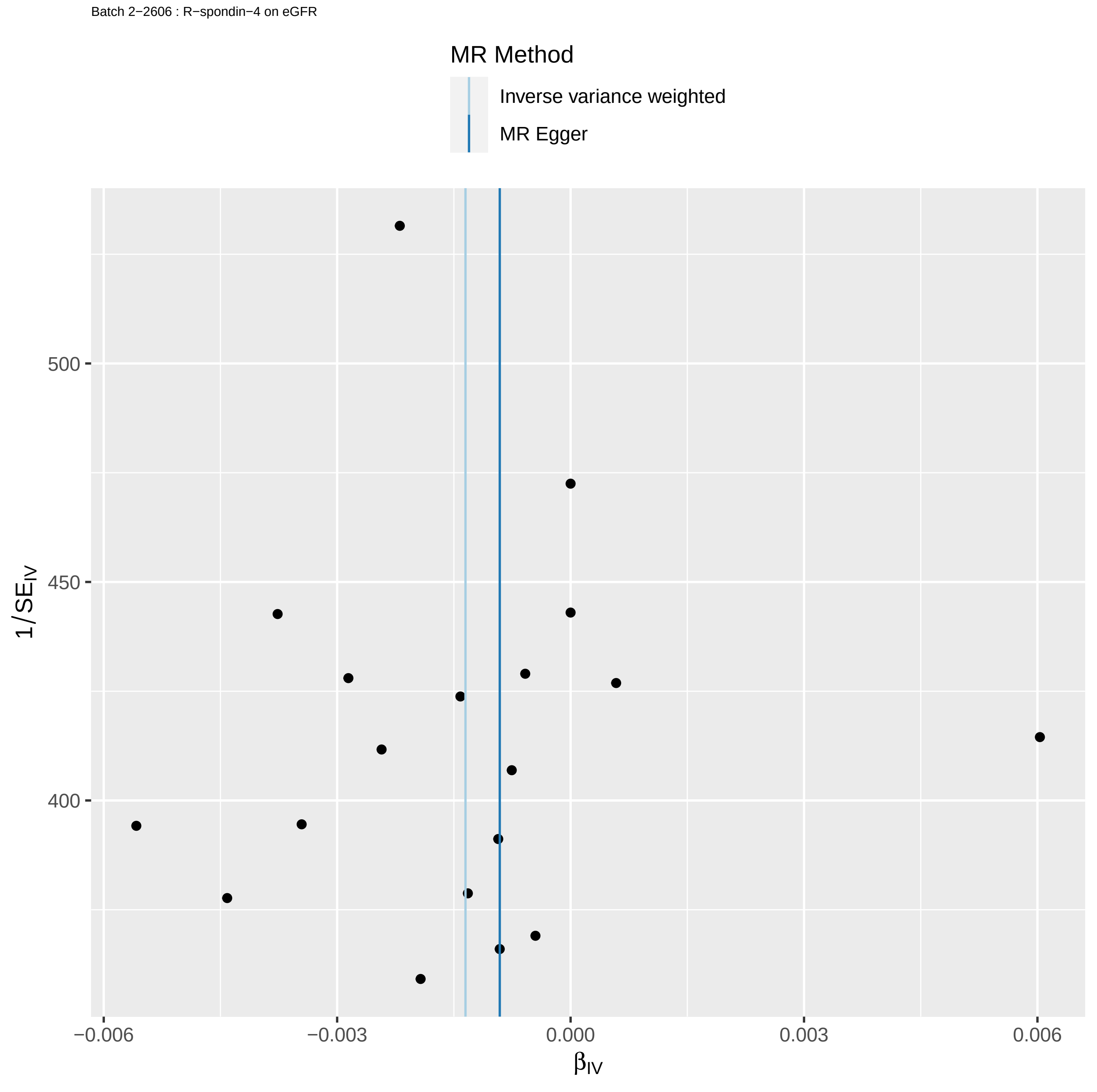

Batch 2−2606 : R−spondin−4 on eGFR
MR Method
Inverse variance weighted
MR Egger
500
450
400
1/SE$_{IV}$
−0.006
−0.003
0.000
0.003
0.006
β$_{IV}$

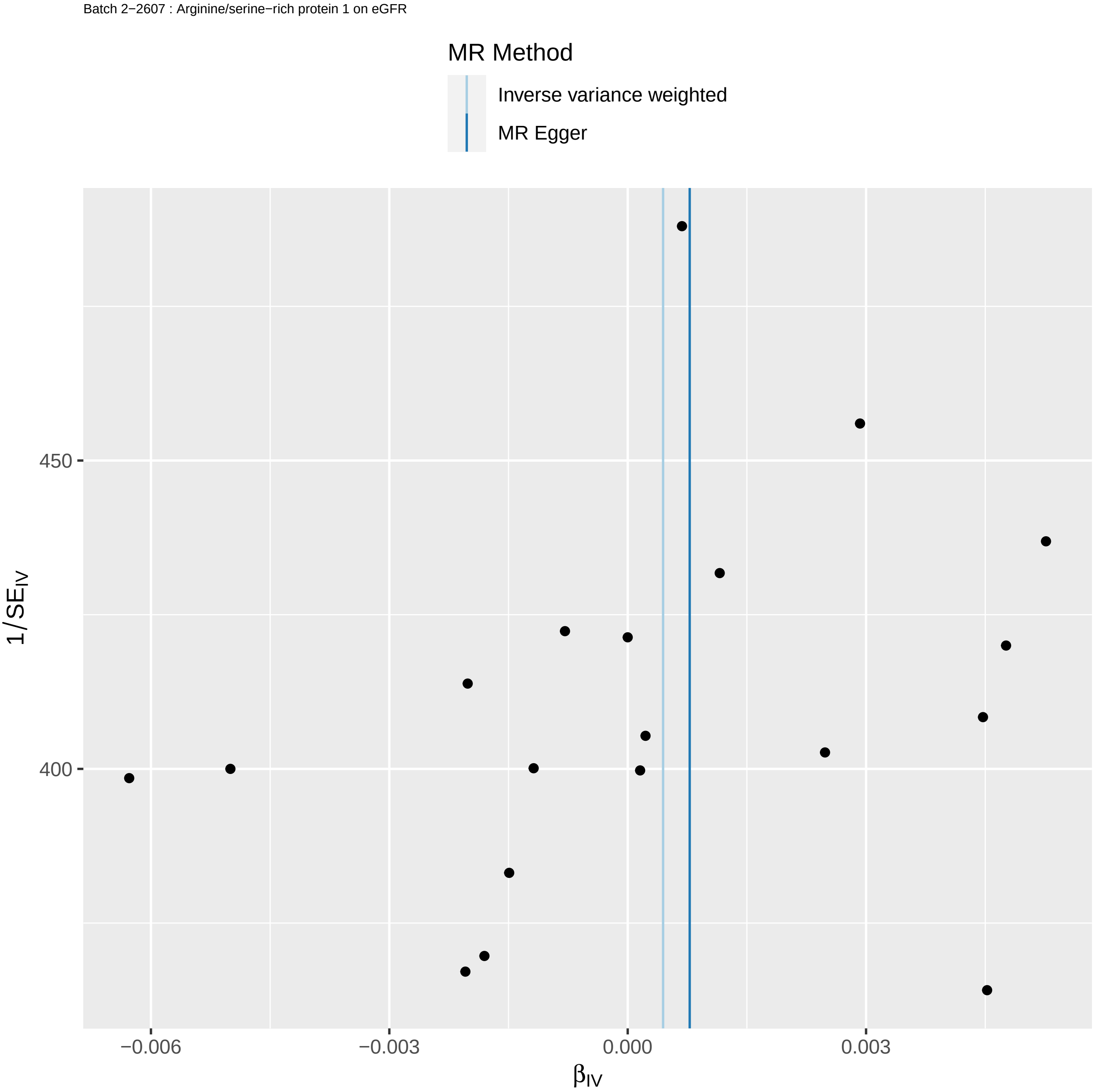

Batch 2−2607 : Arginine/serine−rich protein 1 on eGFR
MR Method
Inverse variance weighted
MR Egger
450
400
1/SE$_{IV}$
−0.006
−0.003
0.000
0.003
β$_{IV}$

Batch 2−2608 : Retbindin on eGFR

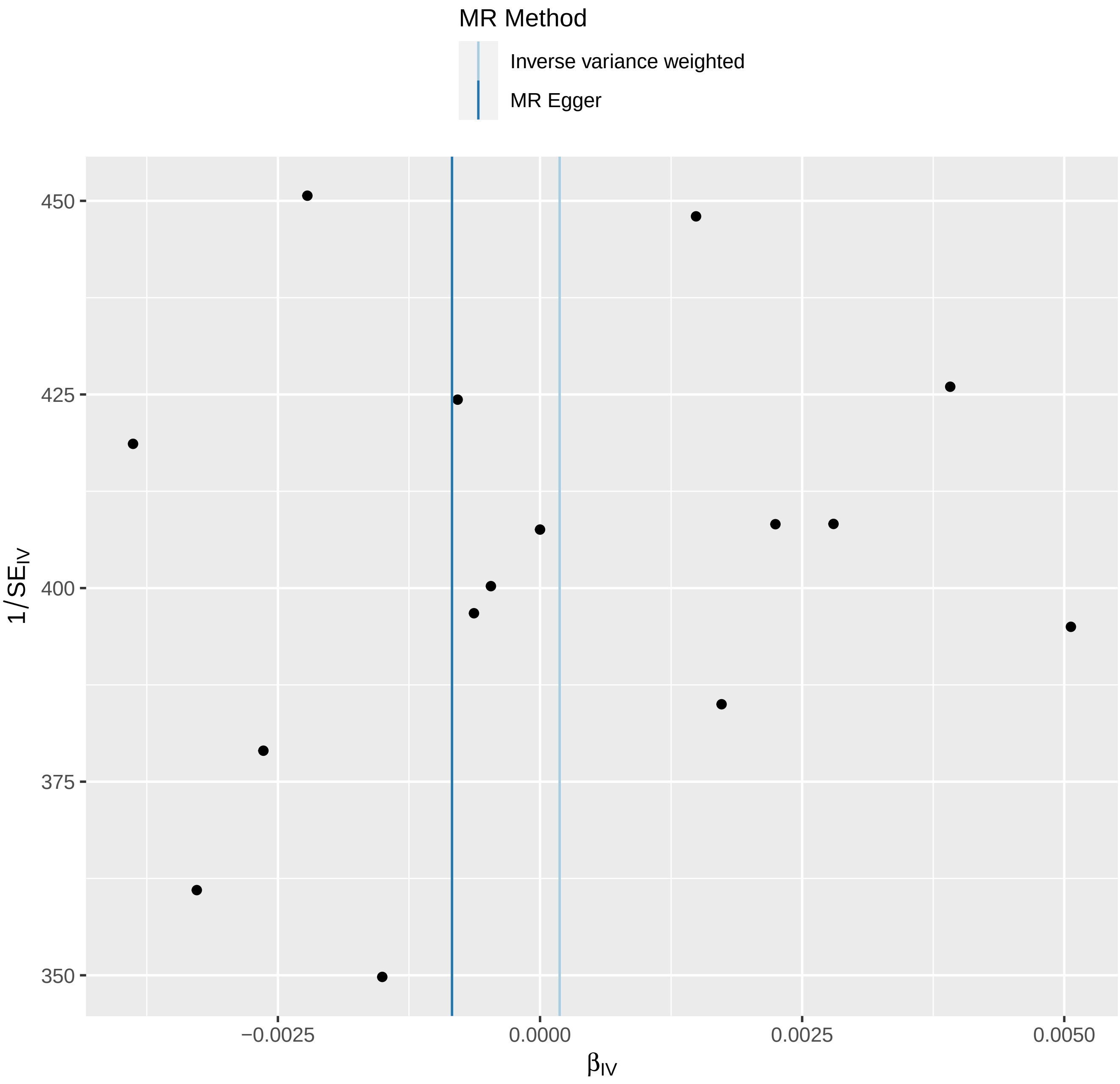

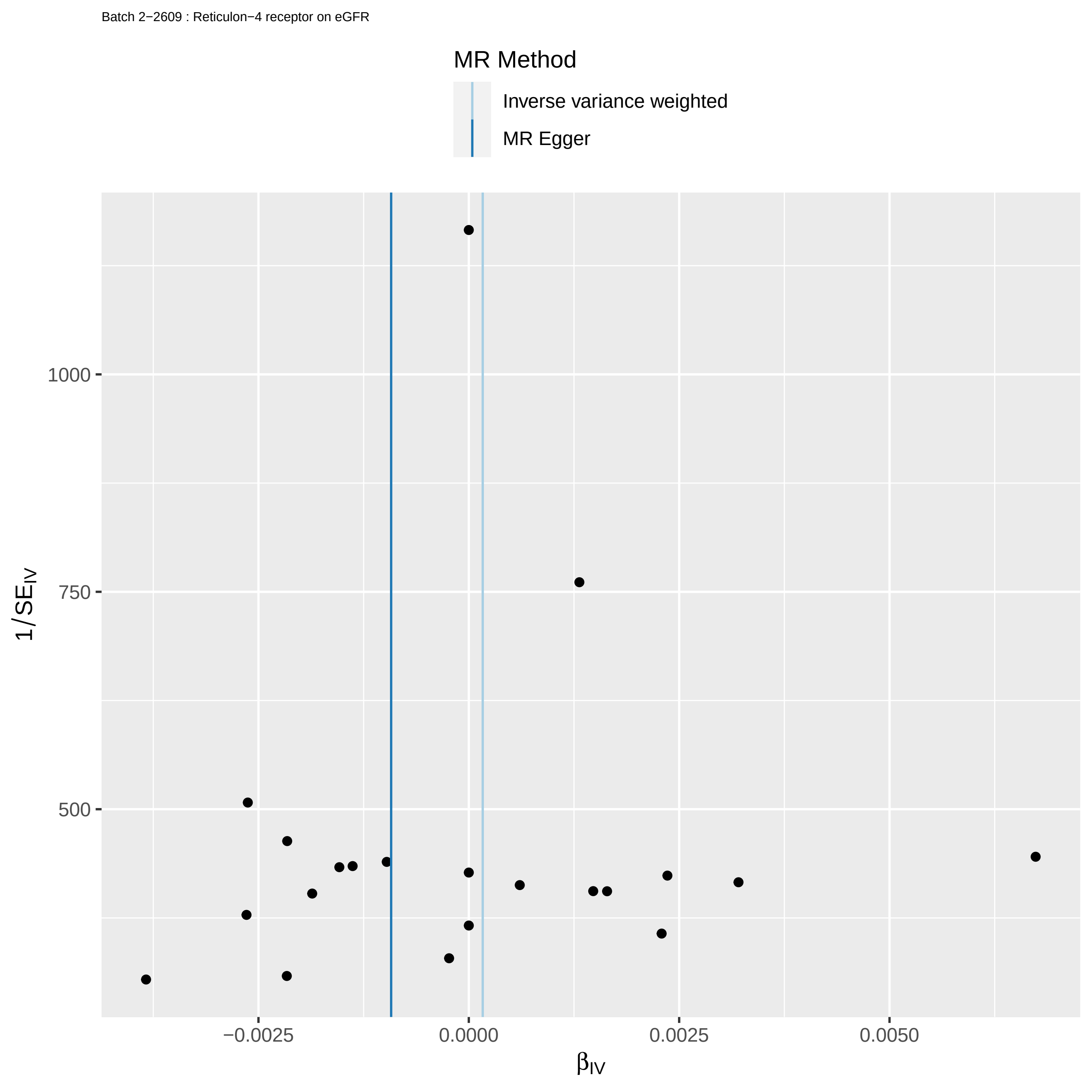

Batch 2−2609 : Reticulon−4 receptor on eGFR
MR Method
Inverse variance weighted
MR Egger
1000
750
500
$1/SE_{IV}$
−0.0025
0.0000
0.0025
0.0050
β_{IV}

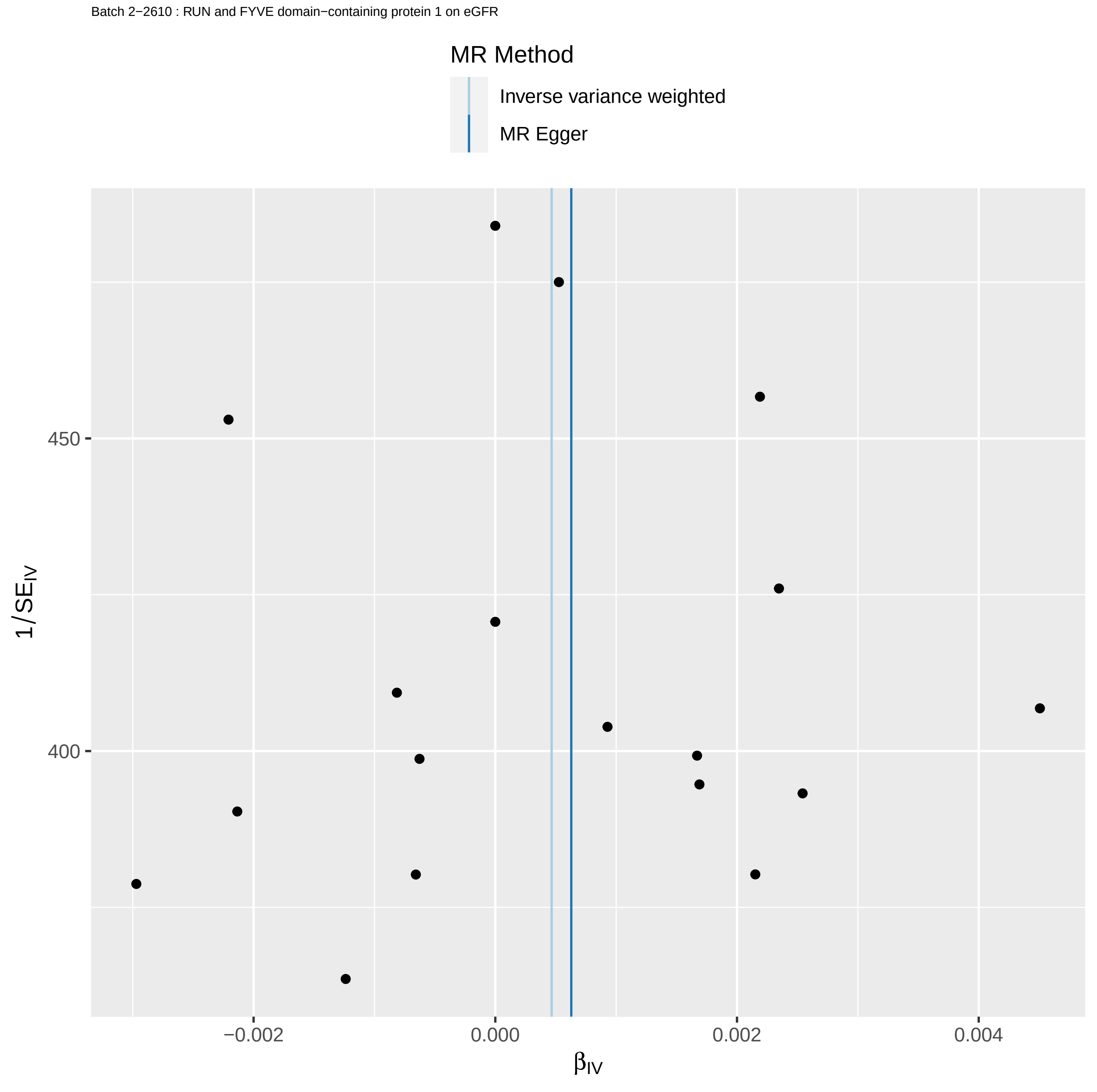

Batch 2−2610 : RUN and FYVE domain−containing protein 1 on eGFR
MR Method
Inverse variance weighted
MR Egger
450
400
1/SE$_{IV}$
−0.002
0.000
0.002
0.004
β$_{IV}$

Batch 2−2611 : Runt−related transcription factor 3 on eGFR

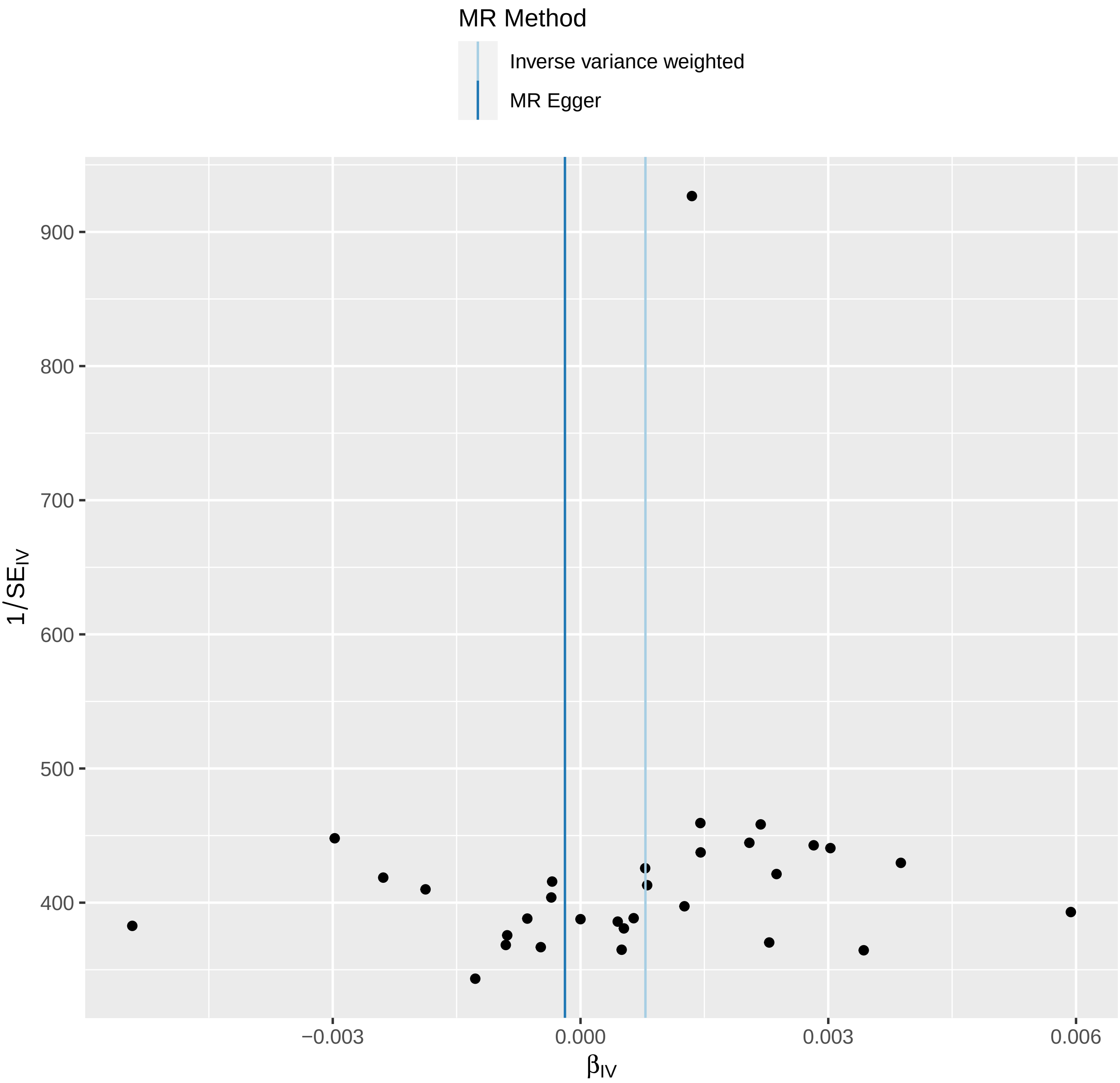

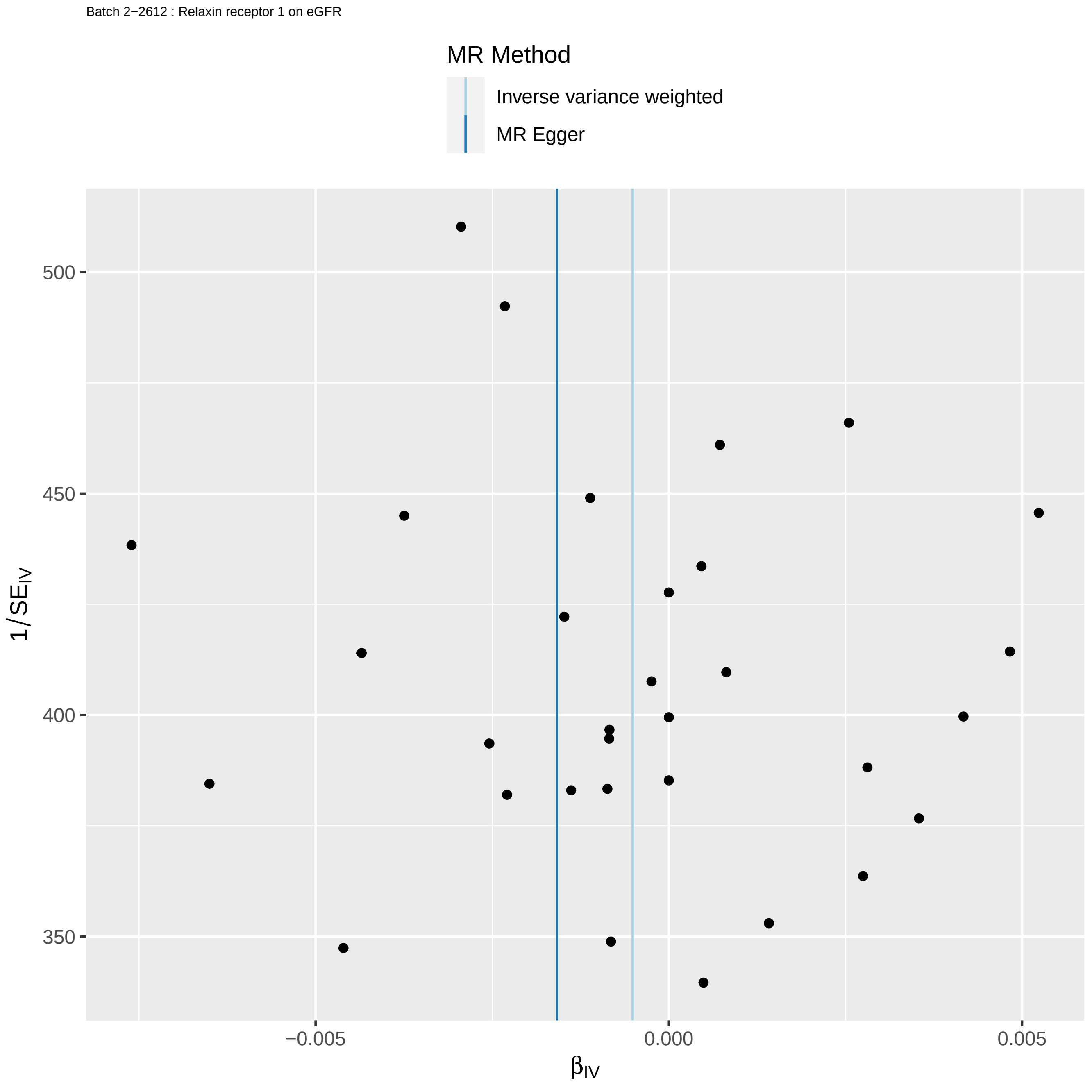
Batch 2−2612 : Relaxin receptor 1 on eGFR
MR Method
Inverse variance weighted
MR Egger
500
450
400
350
1/SE_IV
−0.005
0.000
0.005
β_IV

Batch 2−2613 : Tyrosine−protein kinase RYK on eGFR

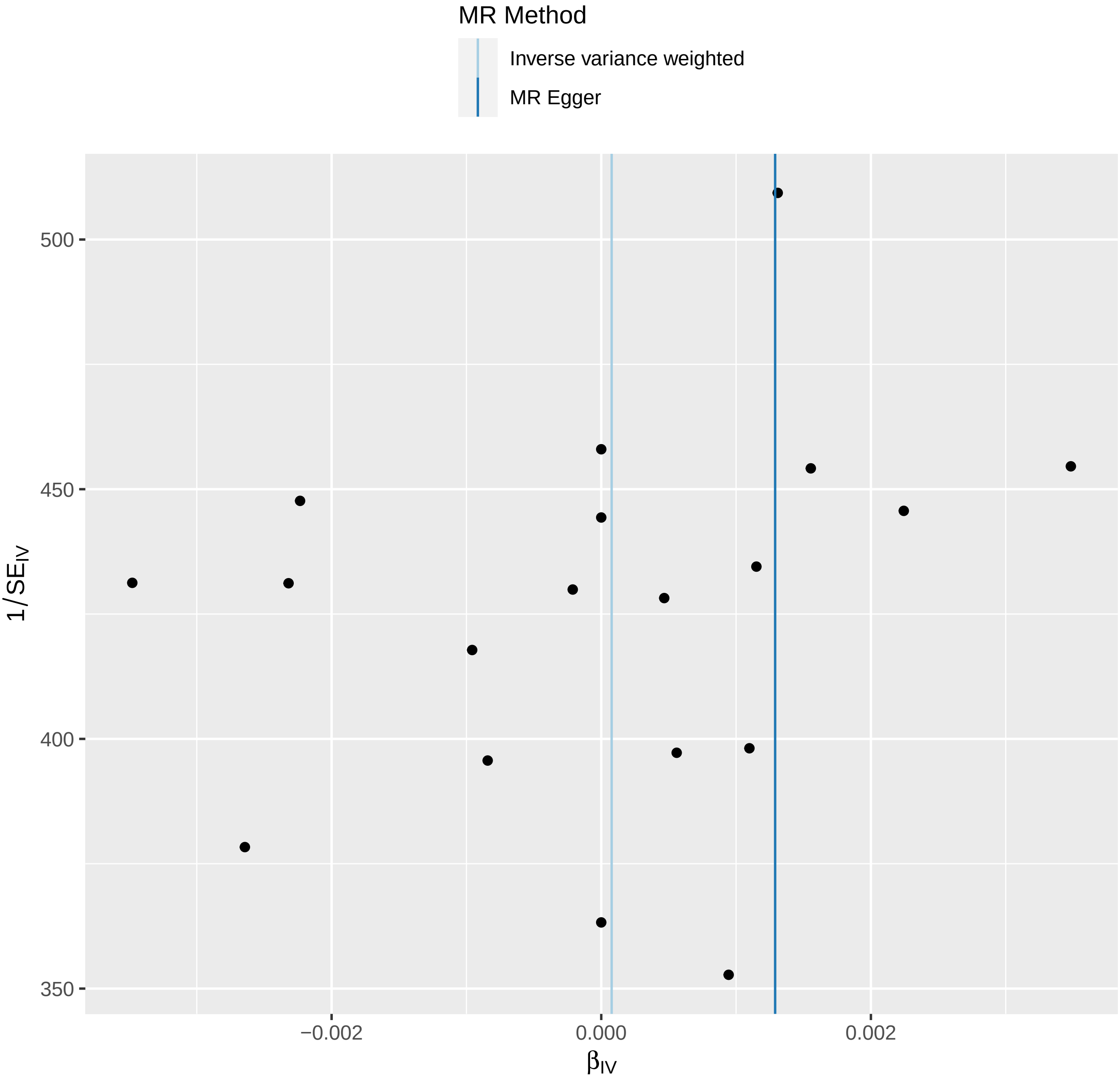

Batch 2−2614 : Protein S100−A11 on eGFR

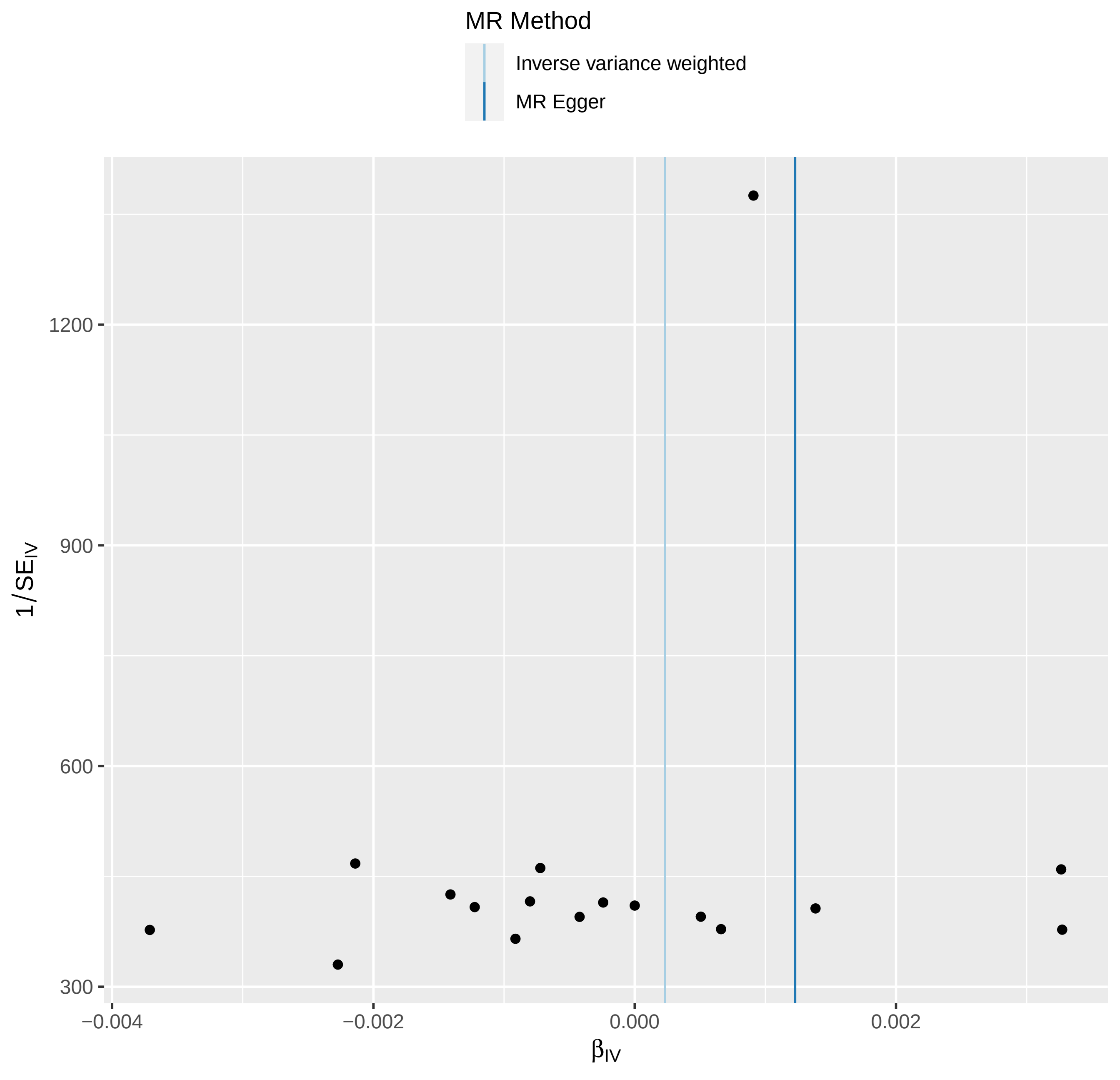

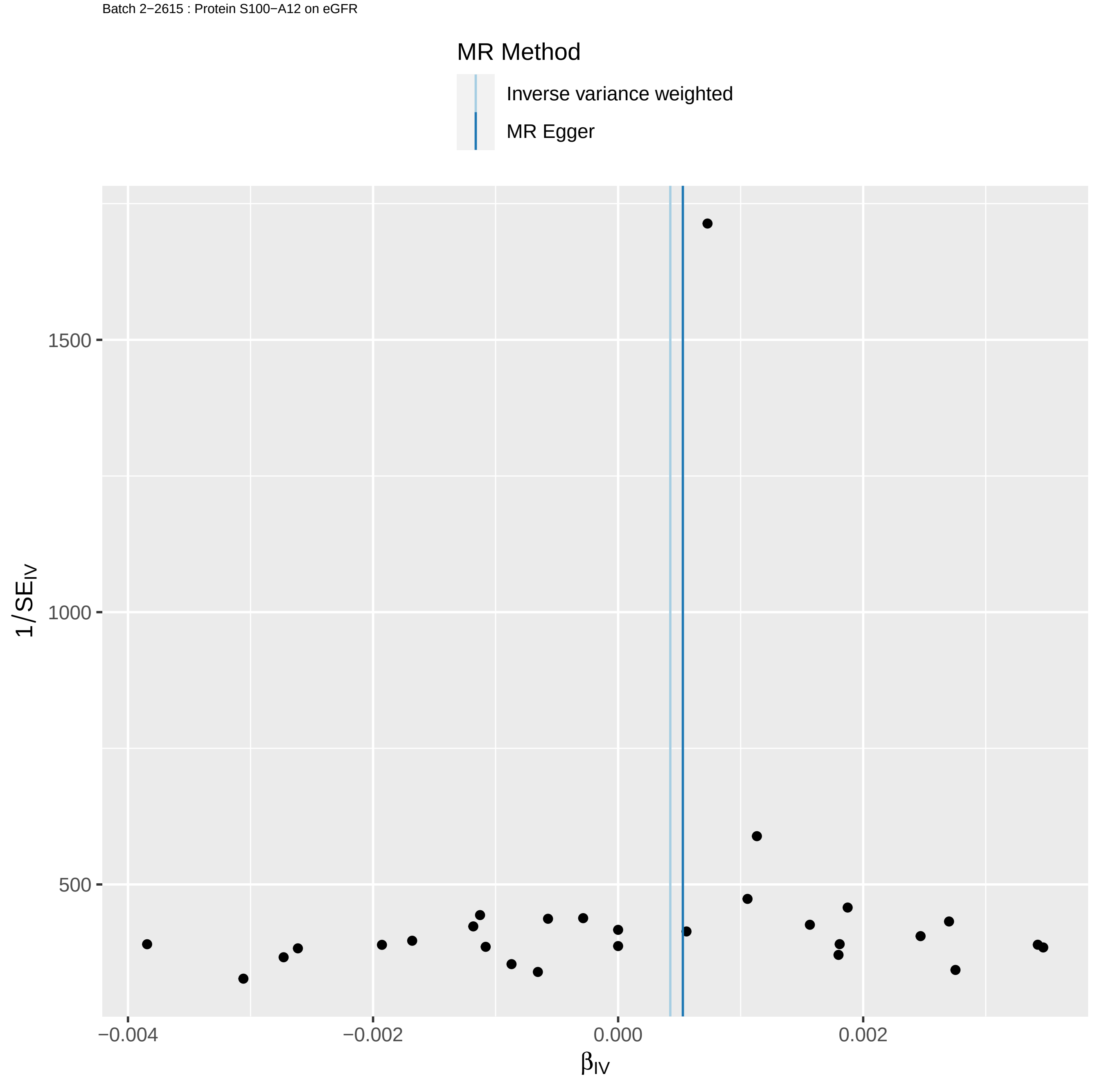

Batch 2−2615 : Protein S100−A12 on eGFR
MR Method
Inverse variance weighted
MR Egger
1500
1000
500
1/SE$_{IV}$
−0.004
−0.002
0.000
0.002
β$_{IV}$

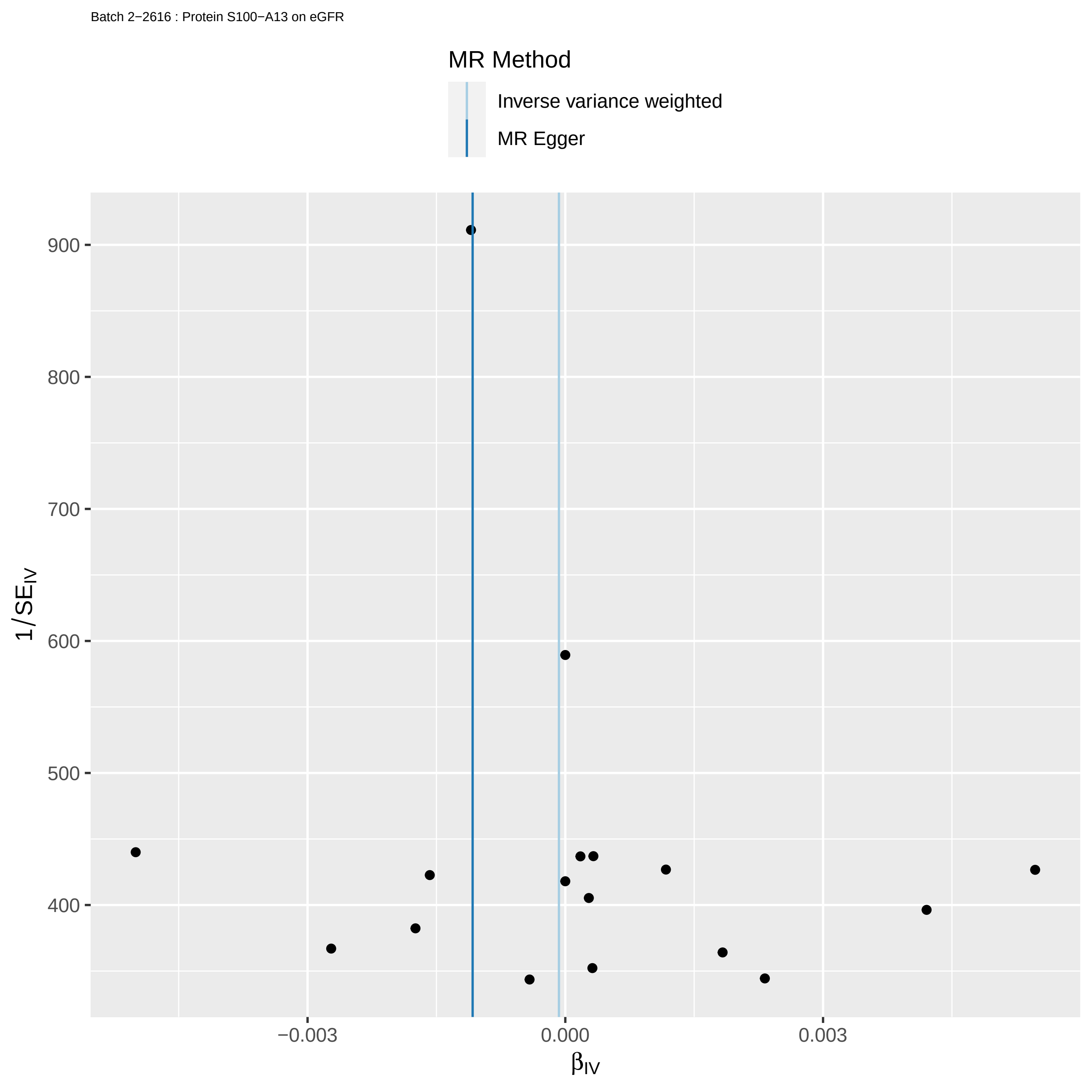

Batch 2−2616 : Protein S100−A13 on eGFR
MR Method
Inverse variance weighted
MR Egger
900
800
700
600
500
400
1/SE$_{IV}$
−0.003
0.000
0.003
β$_{IV}$

Batch 2−2617 : Protein S100−A2 on eGFR

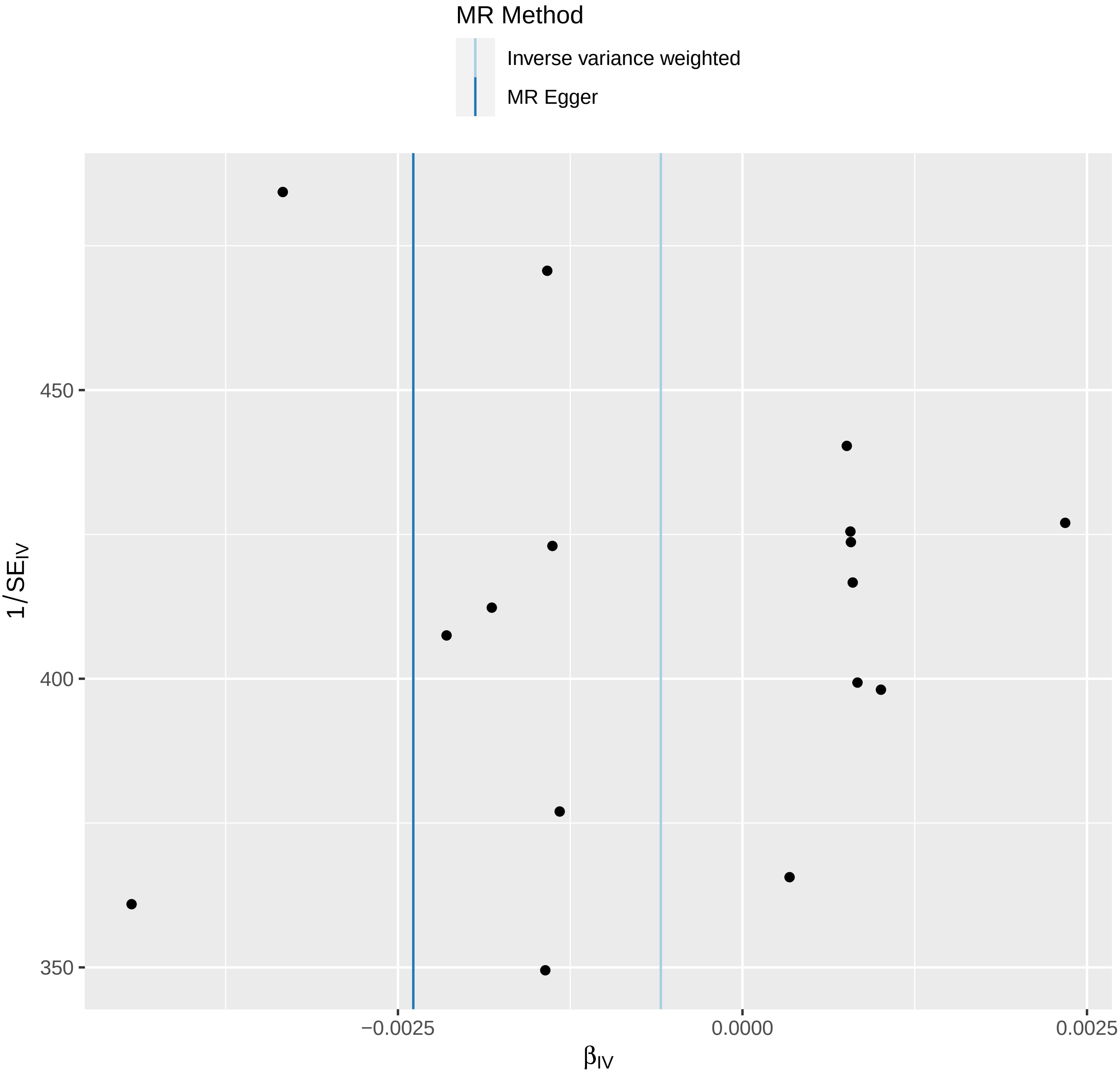

Batch 2−2618 : Protein S100−A4 on eGFR

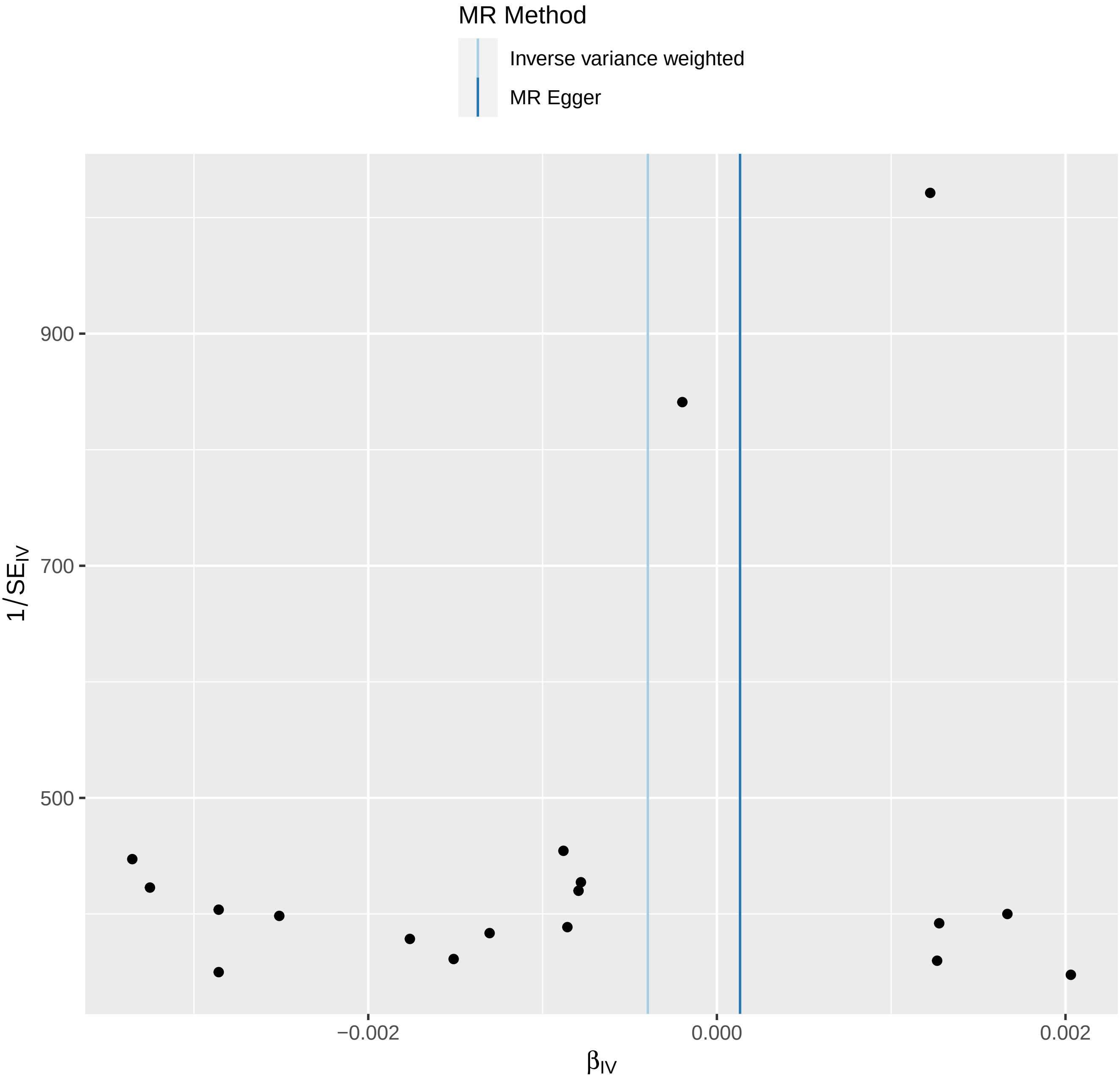

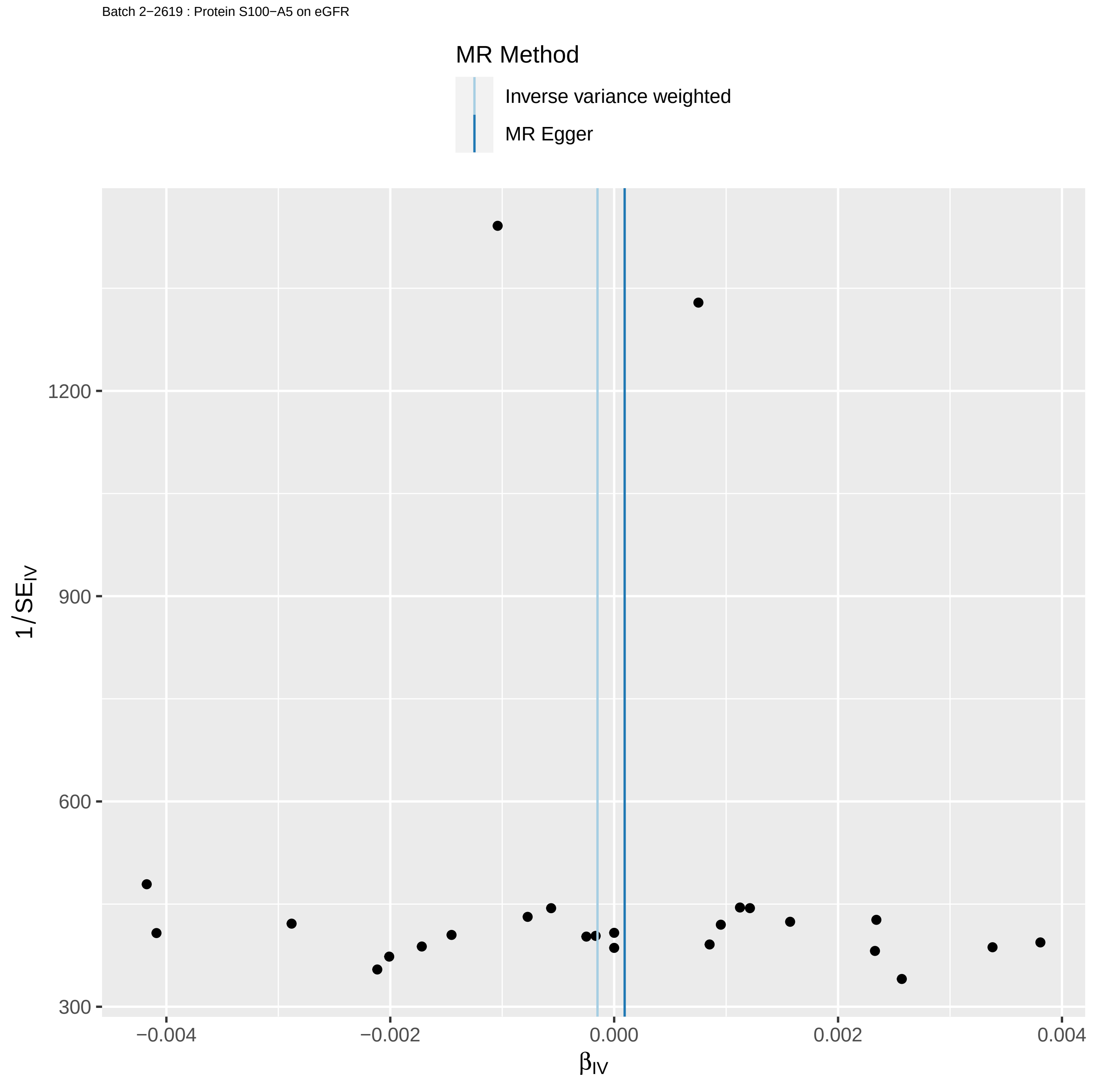

Batch 2−2619 : Protein S100−A5 on eGFR
MR Method
Inverse variance weighted
MR Egger
1200
900
600
300
1/SE_IV
−0.004
−0.002
0.000
0.002
0.004
β_IV

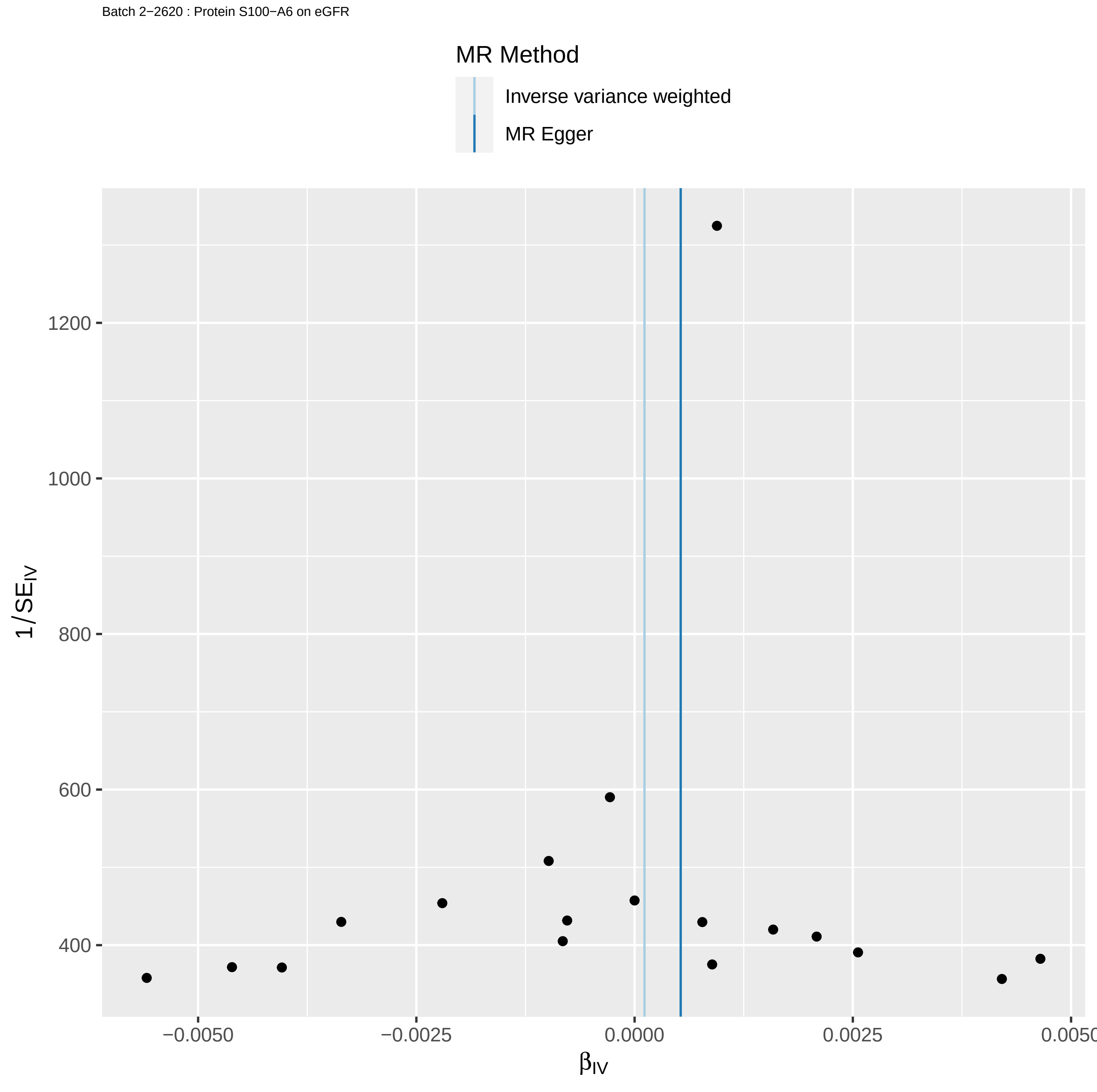

Batch 2−2620 : Protein S100−A6 on eGFR
MR Method
Inverse variance weighted
MR Egger
1200
1000
800
600
400
1/SE_IV
−0.0050
−0.0025
0.0000
0.0025
0.0050
β_IV

Batch 2−2621 : Protein S100−A7 on eGFR

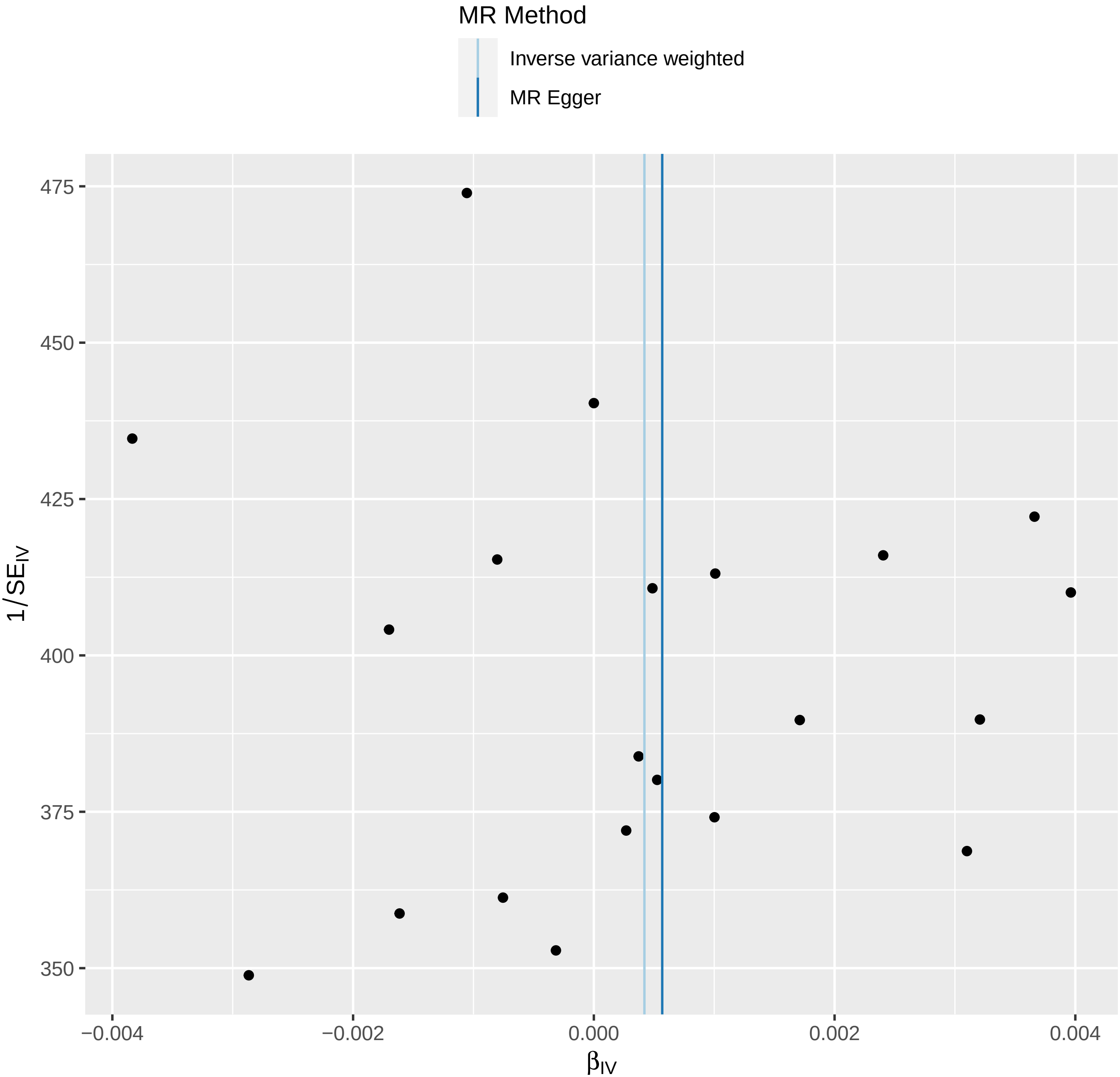

Batch 2−2622 : Protein S100−A9 on eGFR

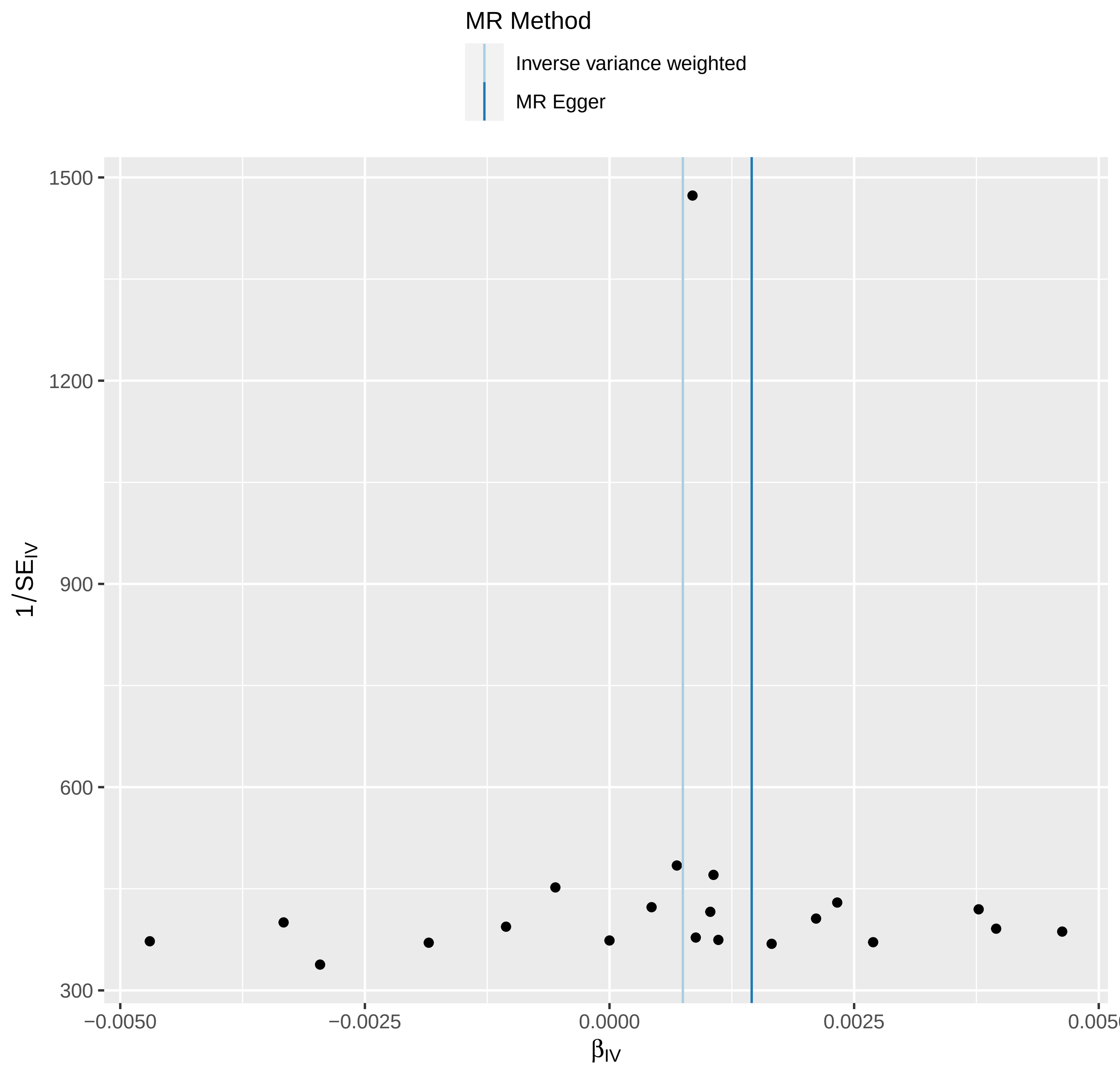

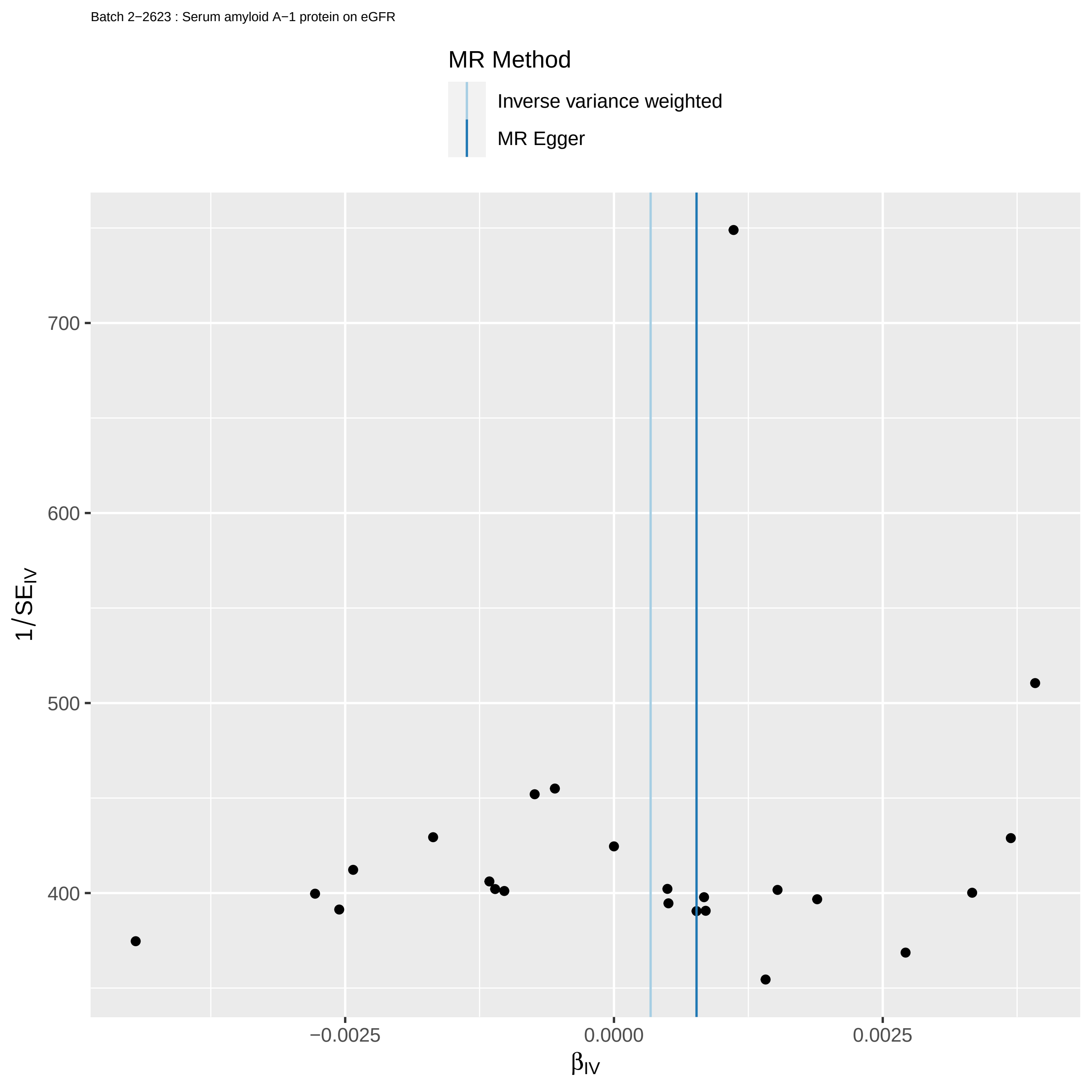

Batch 2−2623 : Serum amyloid A−1 protein on eGFR
MR Method
Inverse variance weighted
MR Egger
700
600
500
400
1/SE$_{IV}$
−0.0025
0.0000
0.0025
β$_{IV}$

Batch 2−2624 : Deoxynucleoside triphosphate triphosphohydrolase SAMHD1 on eGFR

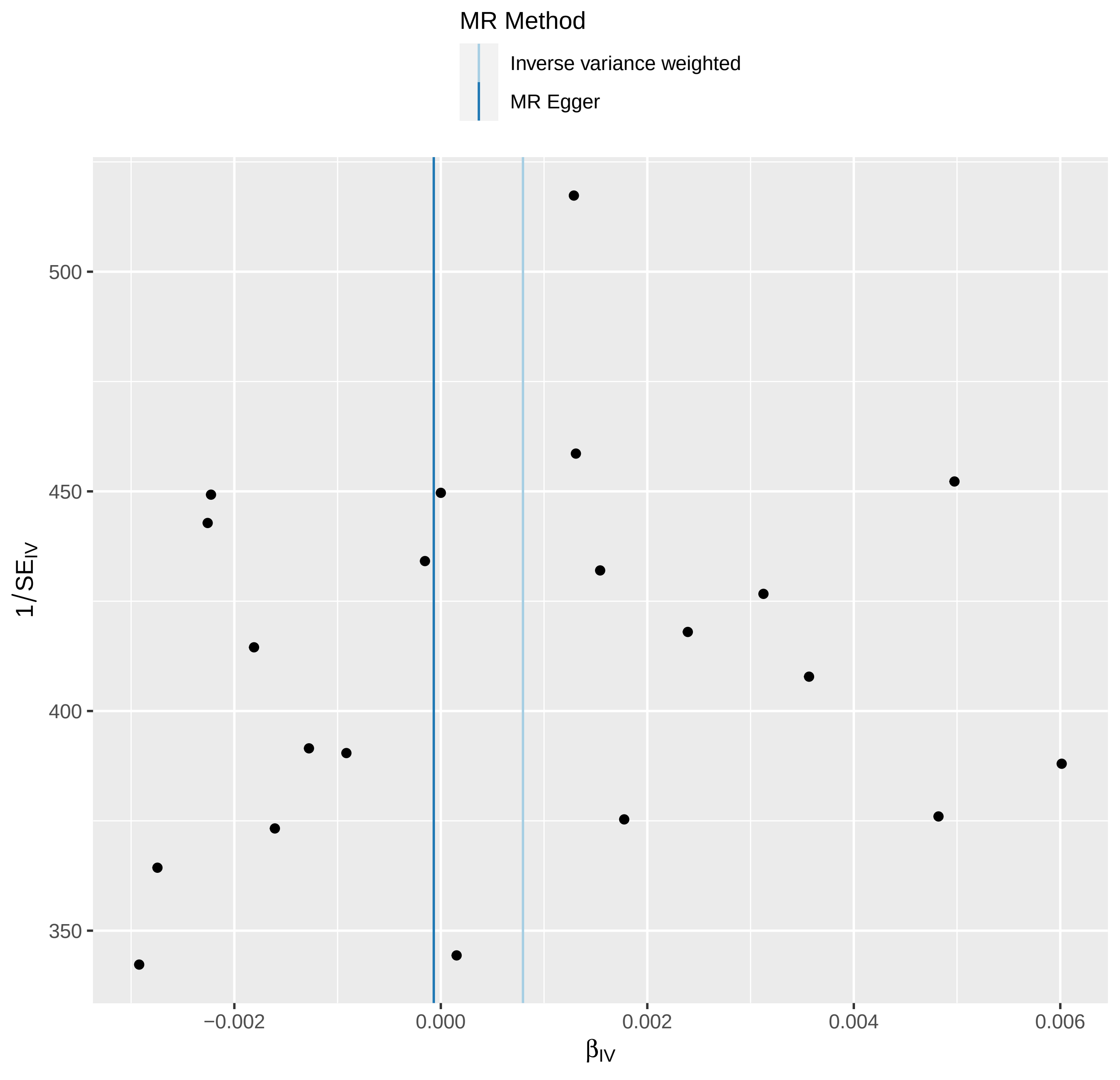

Batch 2−2625 : Histone deacetylase complex subunit SAP18 on eGFR

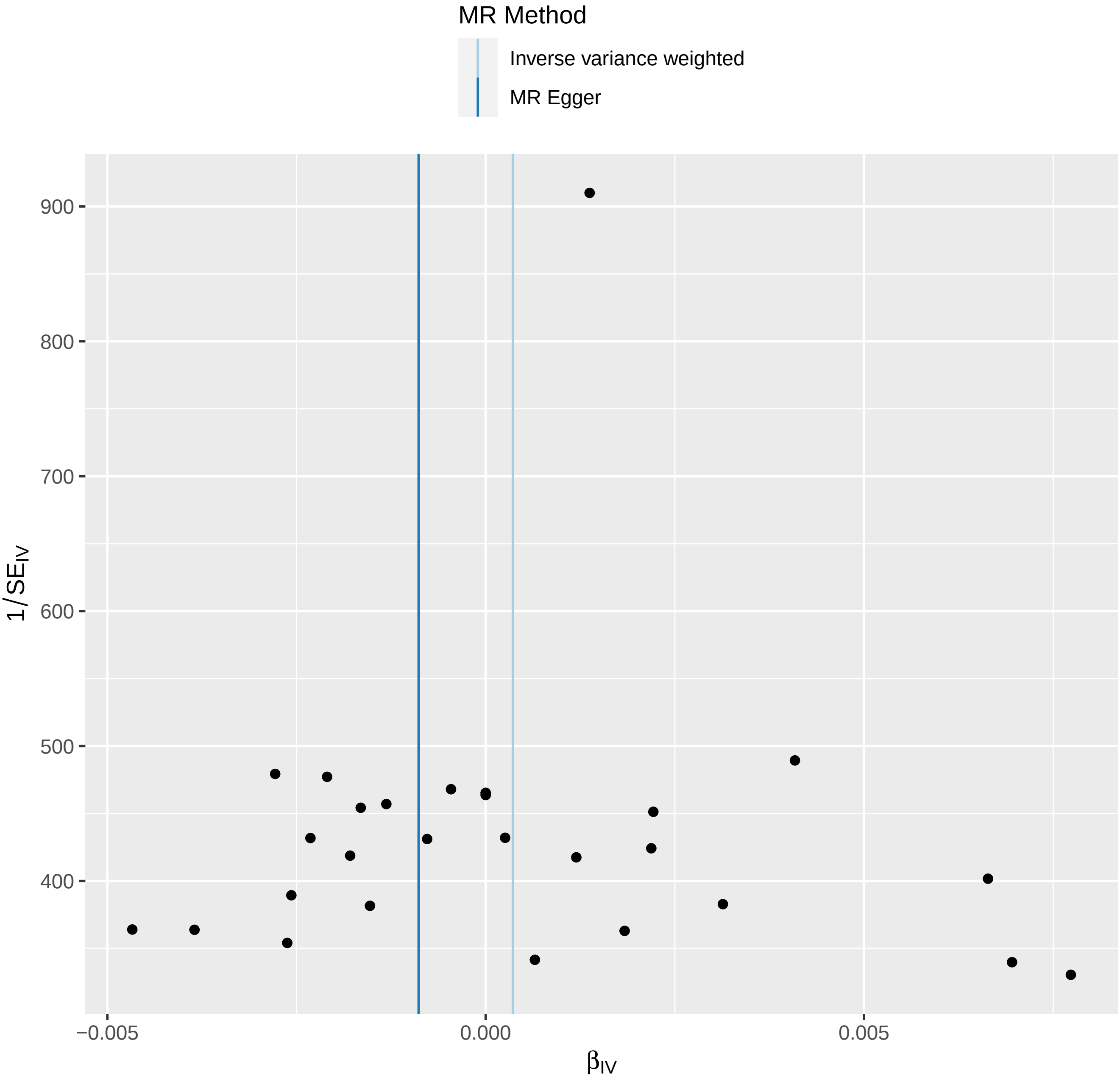

Batch 2−2626 : Histone deacetylase complex subunit SAP30 on eGFR

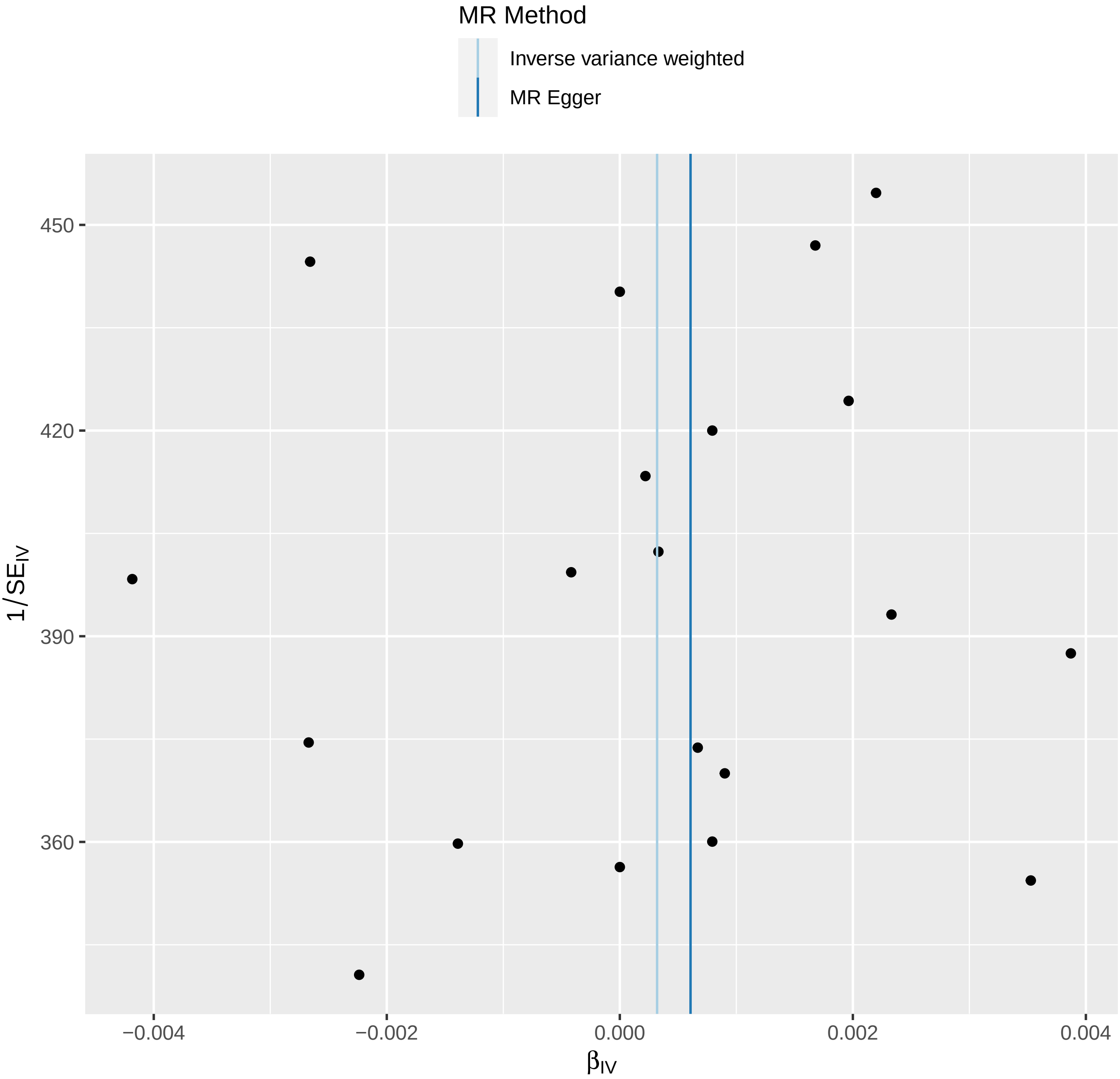

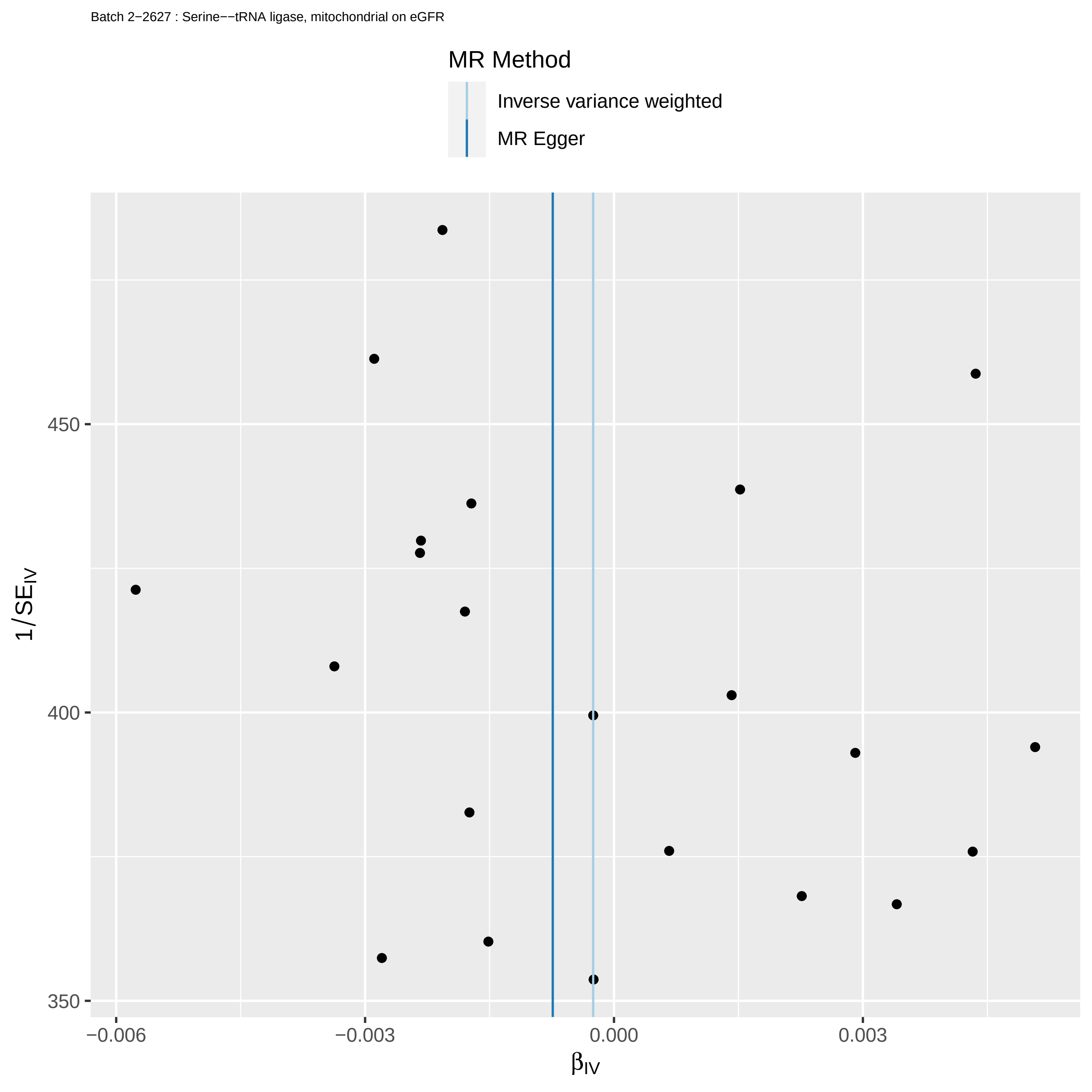
Batch 2−2627 : Serine−−tRNA ligase, mitochondrial on eGFR
MR Method
Inverse variance weighted
MR Egger
450
400
350
1/SE_IV
−0.006
−0.003
0.000
0.003
β_IV

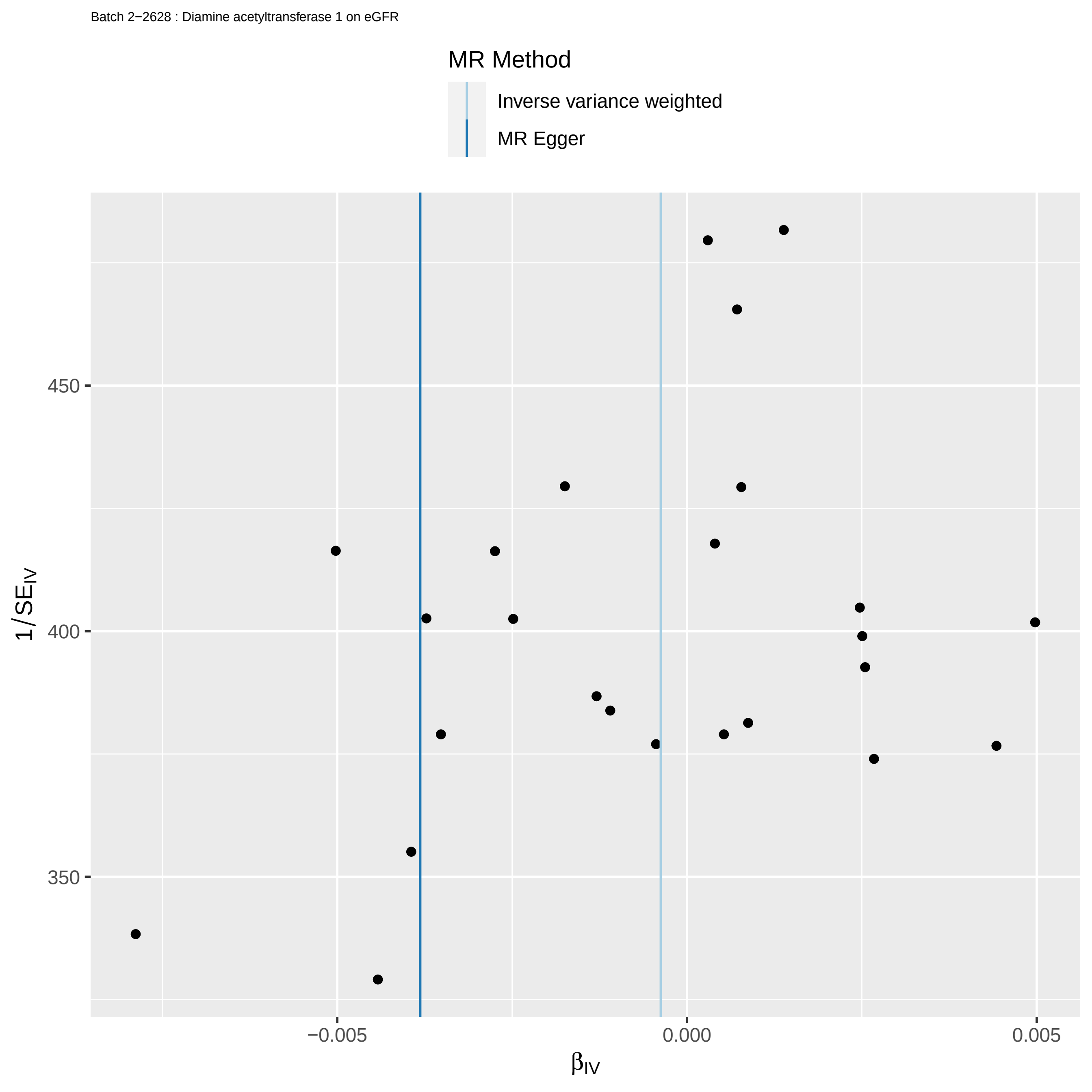

Batch 2−2628 : Diamine acetyltransferase 1 on eGFR
MR Method
Inverse variance weighted
MR Egger
450
400
350
1/SE$_{IV}$
−0.005
0.000
0.005
β$_{IV}$

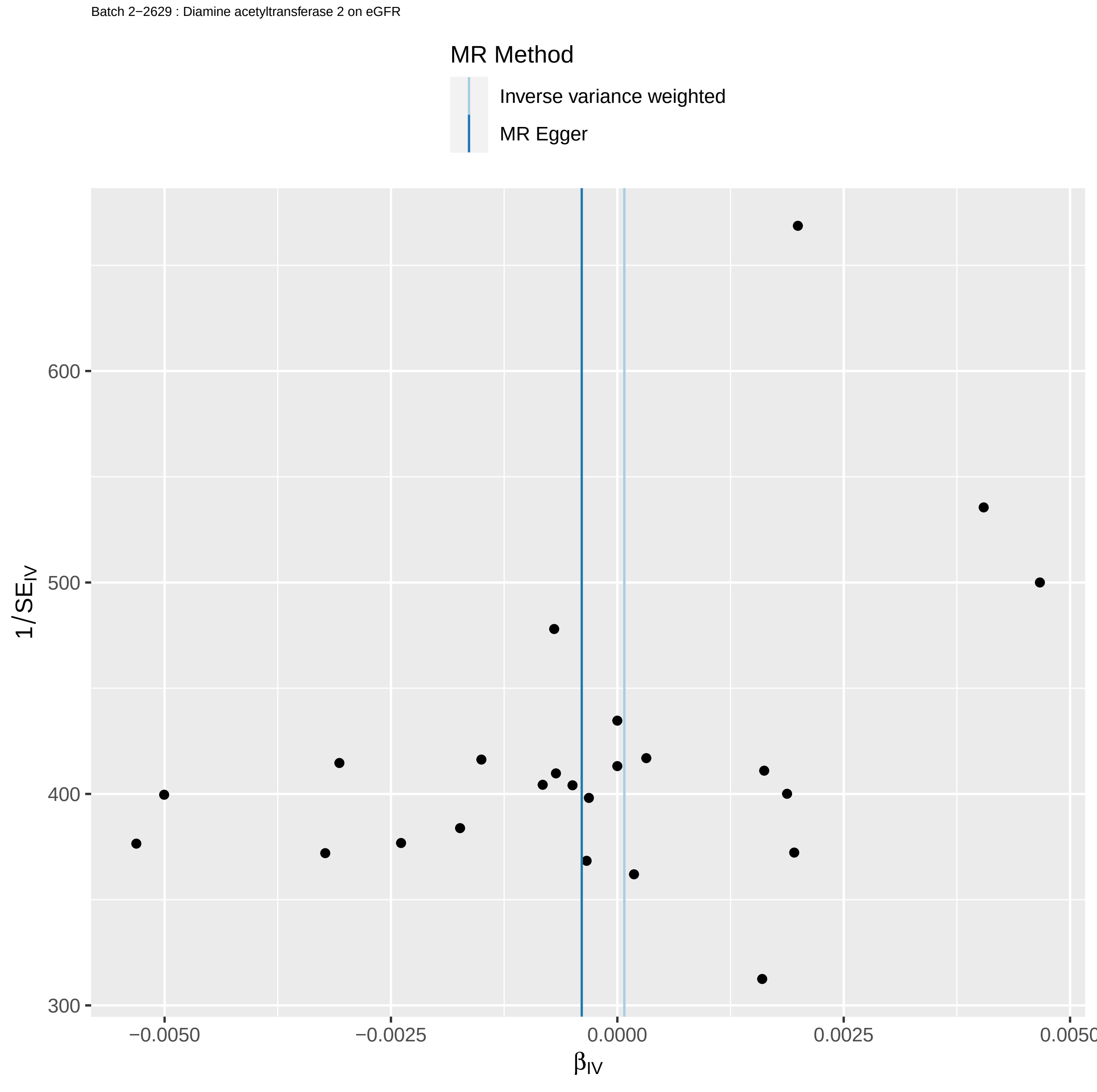

Batch 2−2629 : Diamine acetyltransferase 2 on eGFR
MR Method
Inverse variance weighted
MR Egger
600
500
400
300
1/SE_IV
−0.0050
−0.0025
0.0000
0.0025
0.0050
β_IV

Batch 2−2630 : DNA−binding protein SATB1 on eGFR

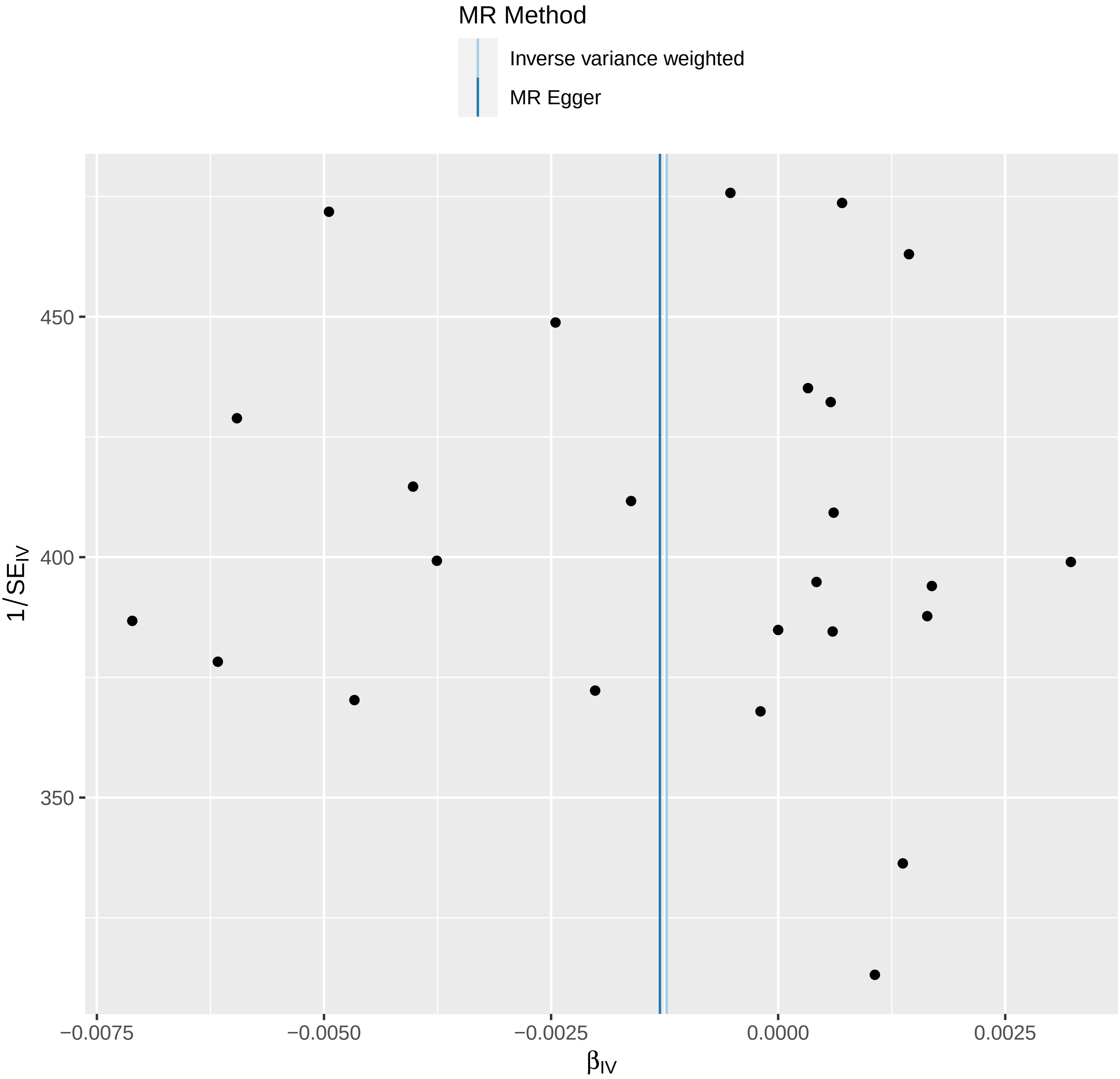

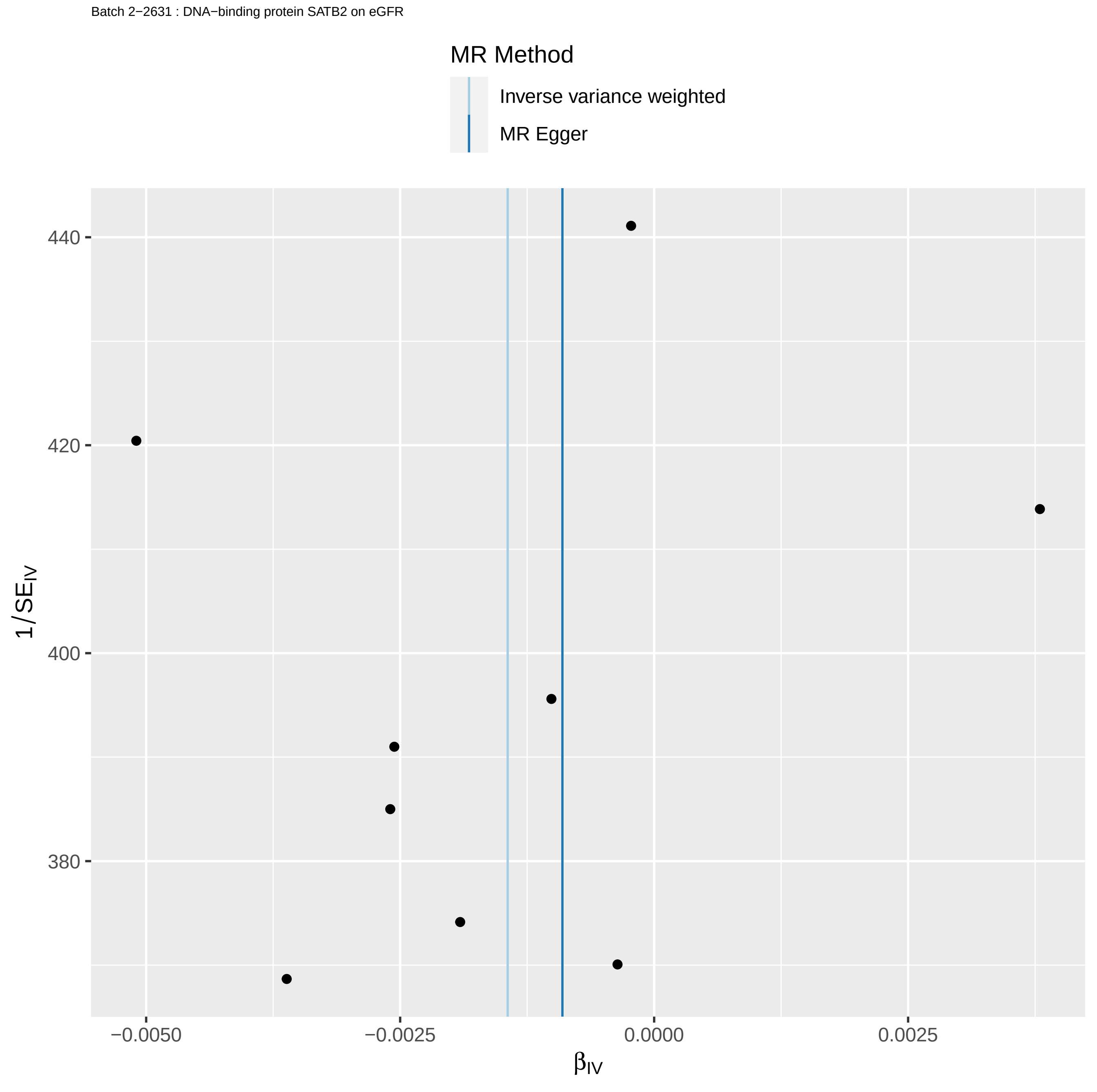
Batch 2−2631 : DNA−binding protein SATB2 on eGFR
MR Method
Inverse variance weighted
MR Egger
440
420
400
380
1/SE$_{IV}$
−0.0050
−0.0025
0.0000
0.0025
β$_{IV}$

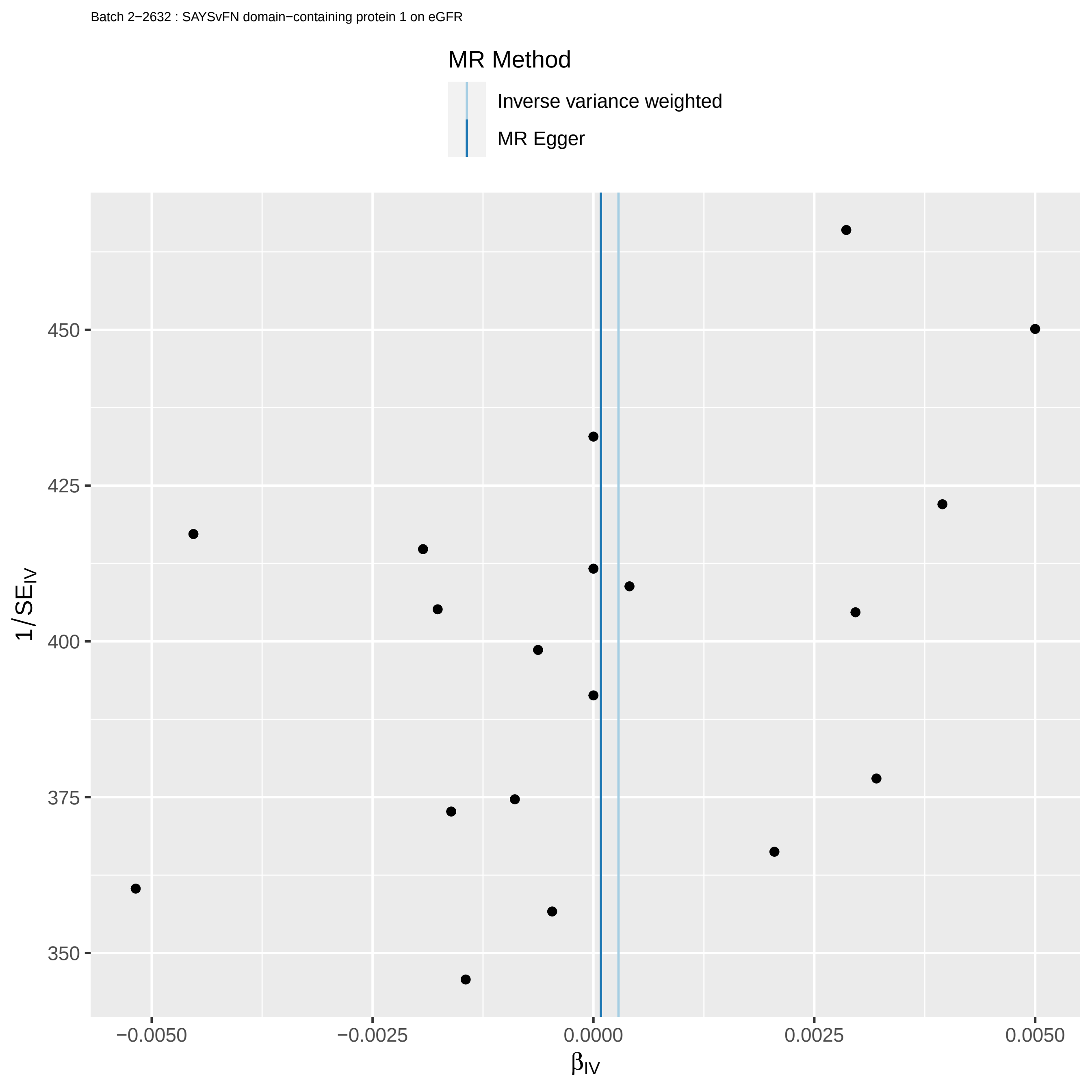
Batch 2−2632 : SAYSvFN domain−containing protein 1 on eGFR
MR Method
Inverse variance weighted
MR Egger
450
425
400
375
350
1/SE$_{IV}$
−0.0050
−0.0025
0.0000
0.0025
0.0050
β$_{IV}$

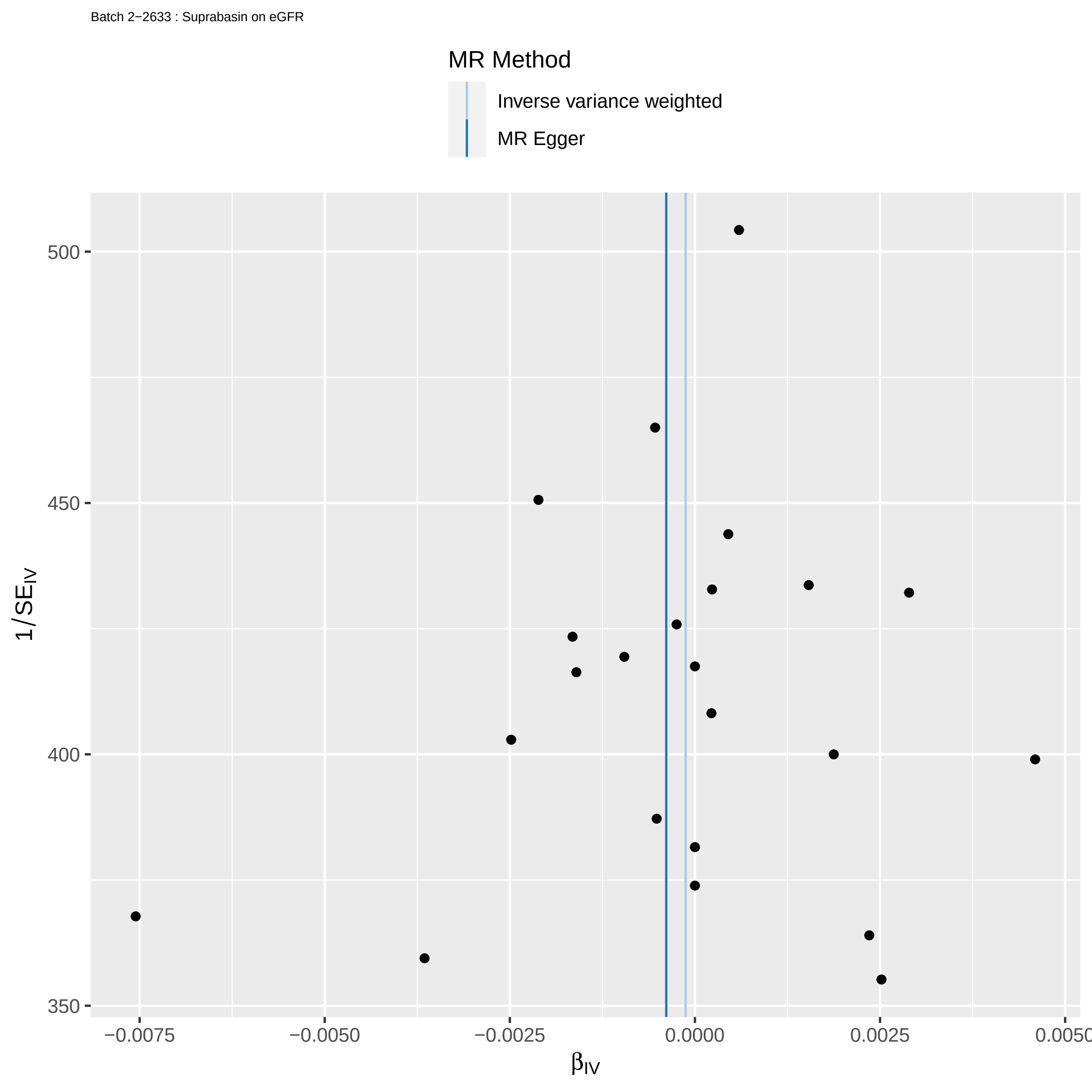
Batch 2−2633 : Suprabasin on eGFR
MR Method
Inverse variance weighted
MR Egger
1/SE_IV
β_IV
500
450
400
350
−0.0075
−0.0050
−0.0025
0.0000
0.0025
0.0050

Batch 2−2634 : Secretory carrier−associated membrane protein 5 on eGFR

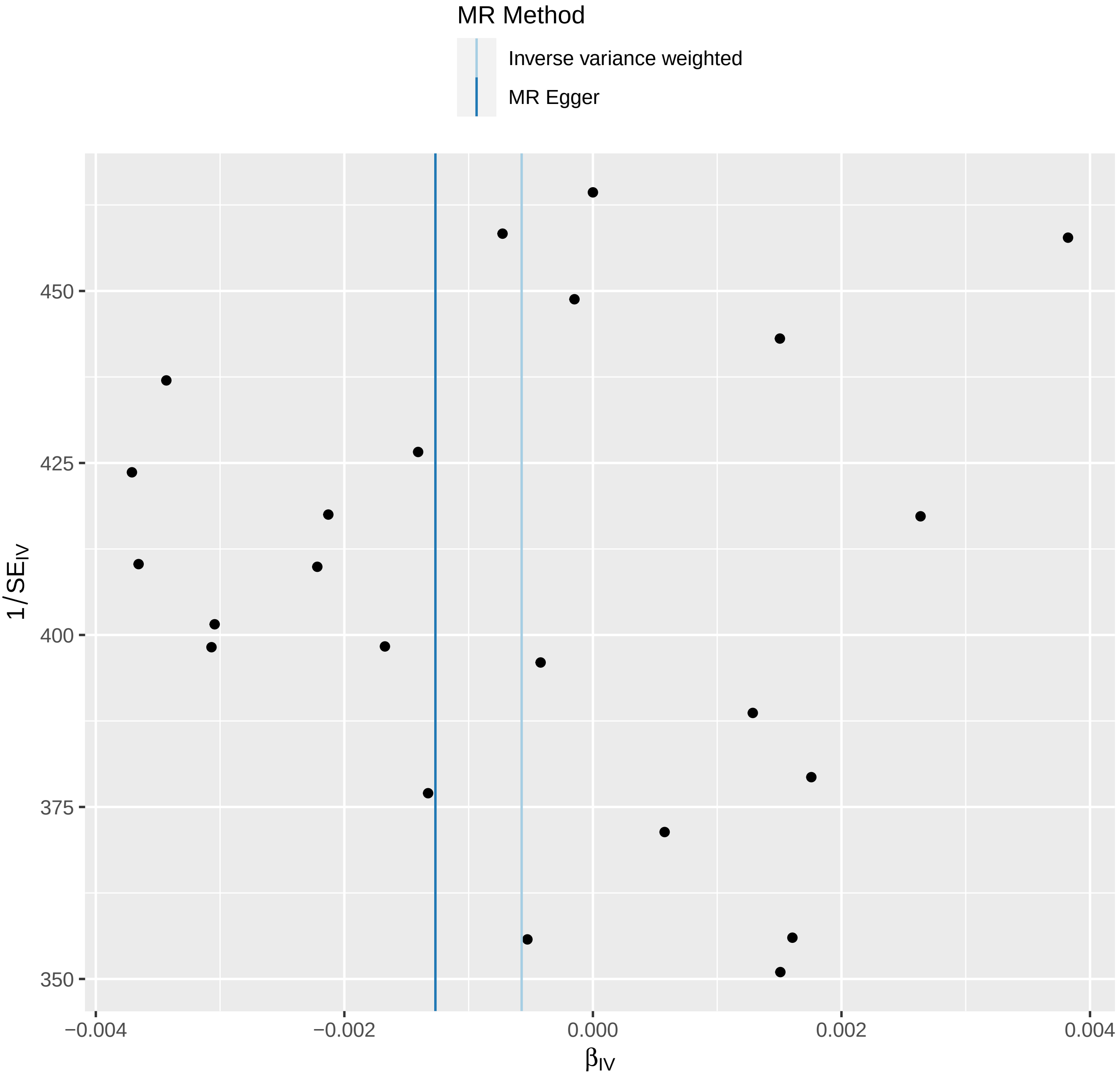

Batch 2−2635 : Scavenger receptor class A member 3 on eGFR

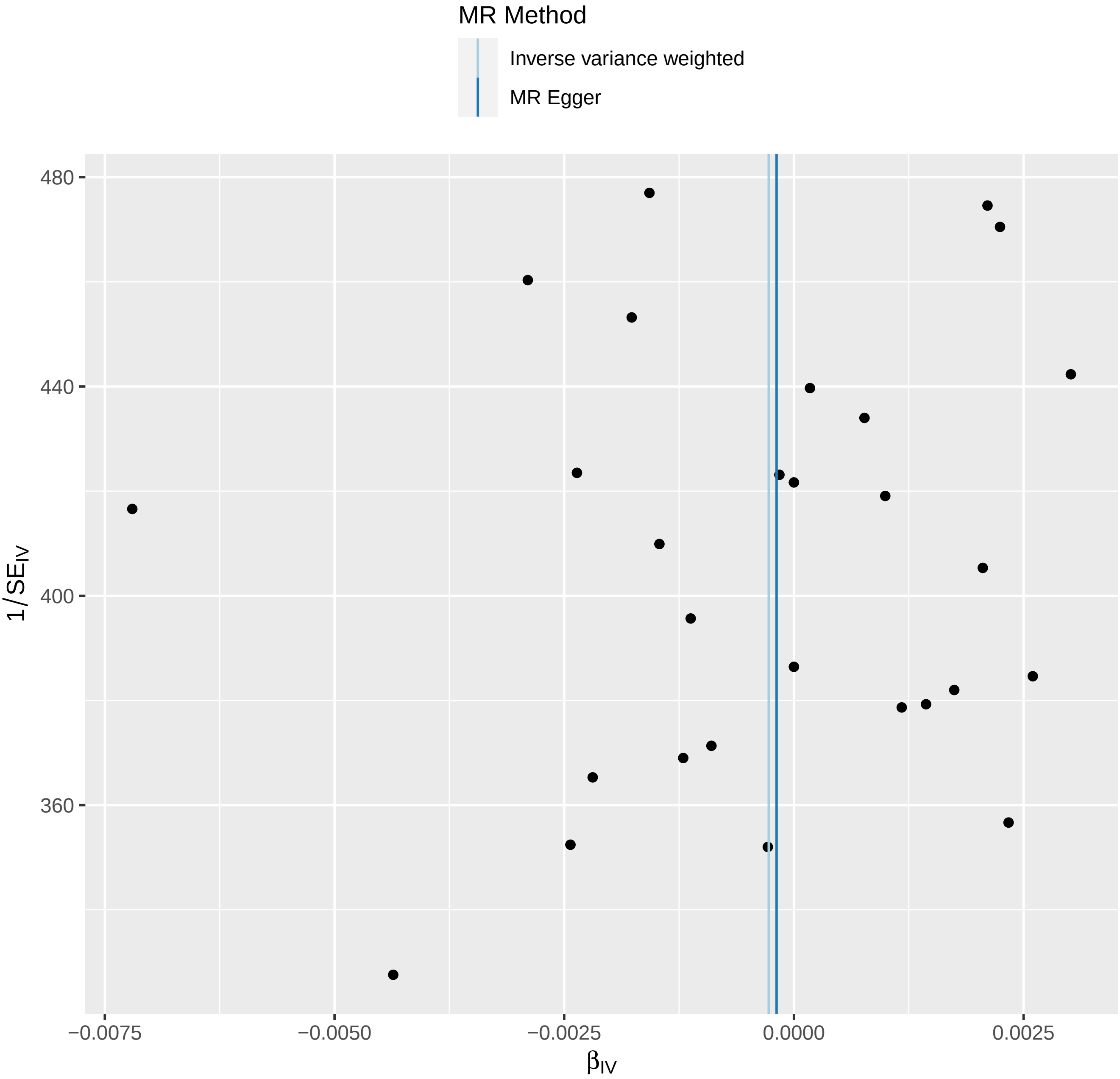

Batch 2−2636 : Scavenger receptor class A member 3 on eGFR

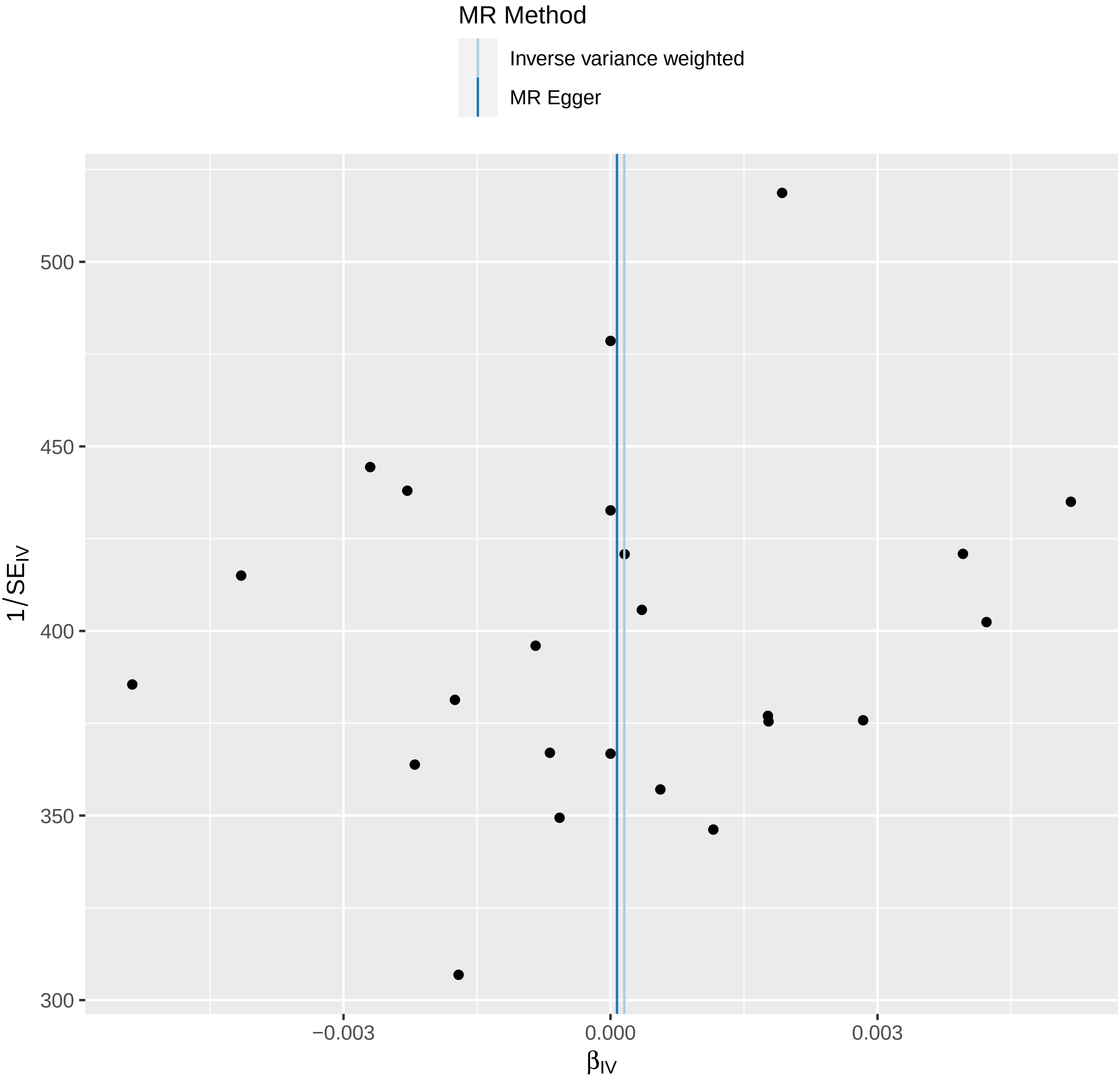

Batch 2−2637 : Scavenger receptor class A member 3 on eGFR

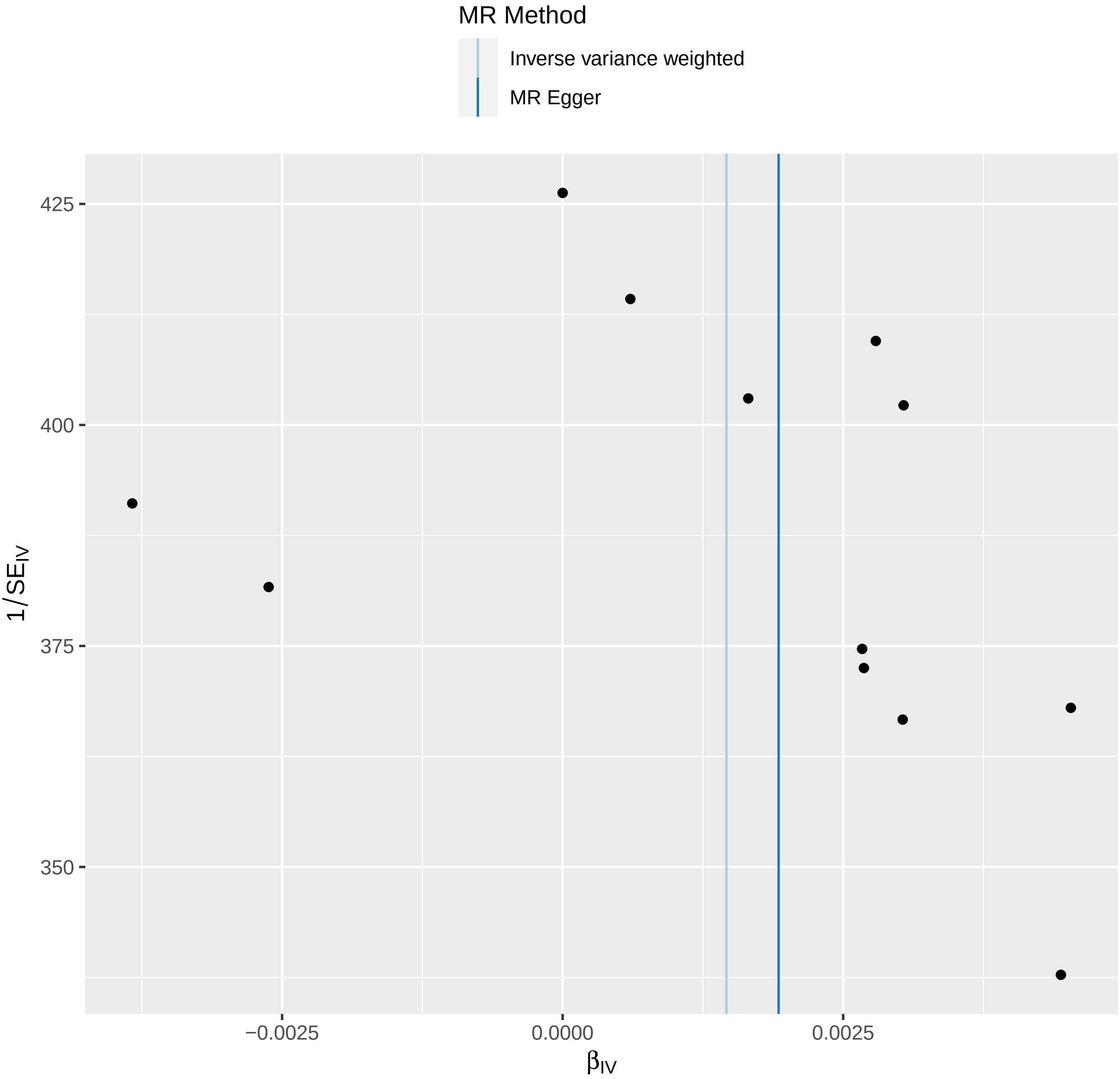

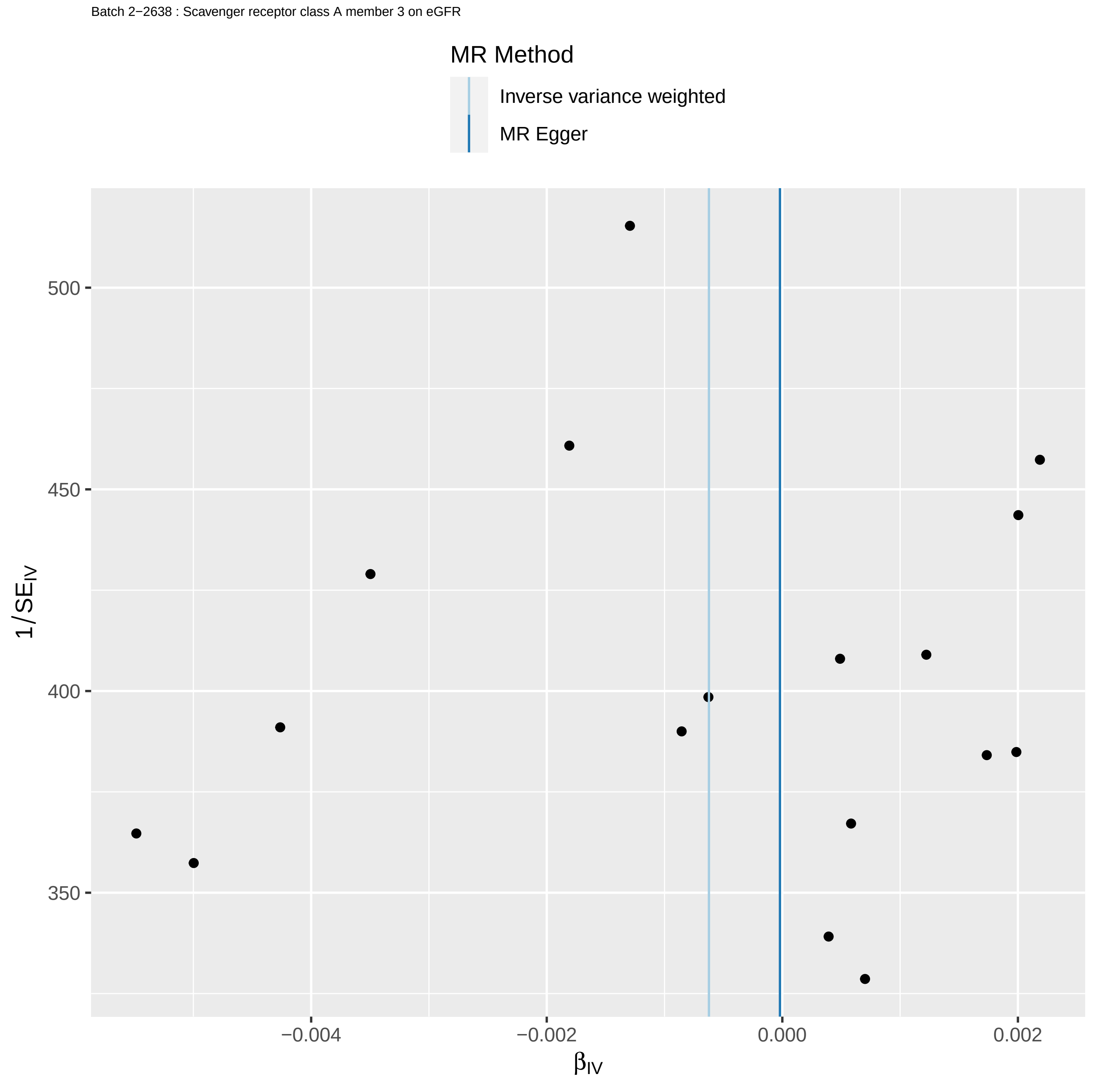
Batch 2−2638 : Scavenger receptor class A member 3 on eGFR
MR Method
Inverse variance weighted
MR Egger
500
450
400
350
1/SE$_{IV}$
−0.004
−0.002
0.000
0.002
β_{IV}

Batch 2−2639 : Scavenger receptor class A member 5 on eGFR

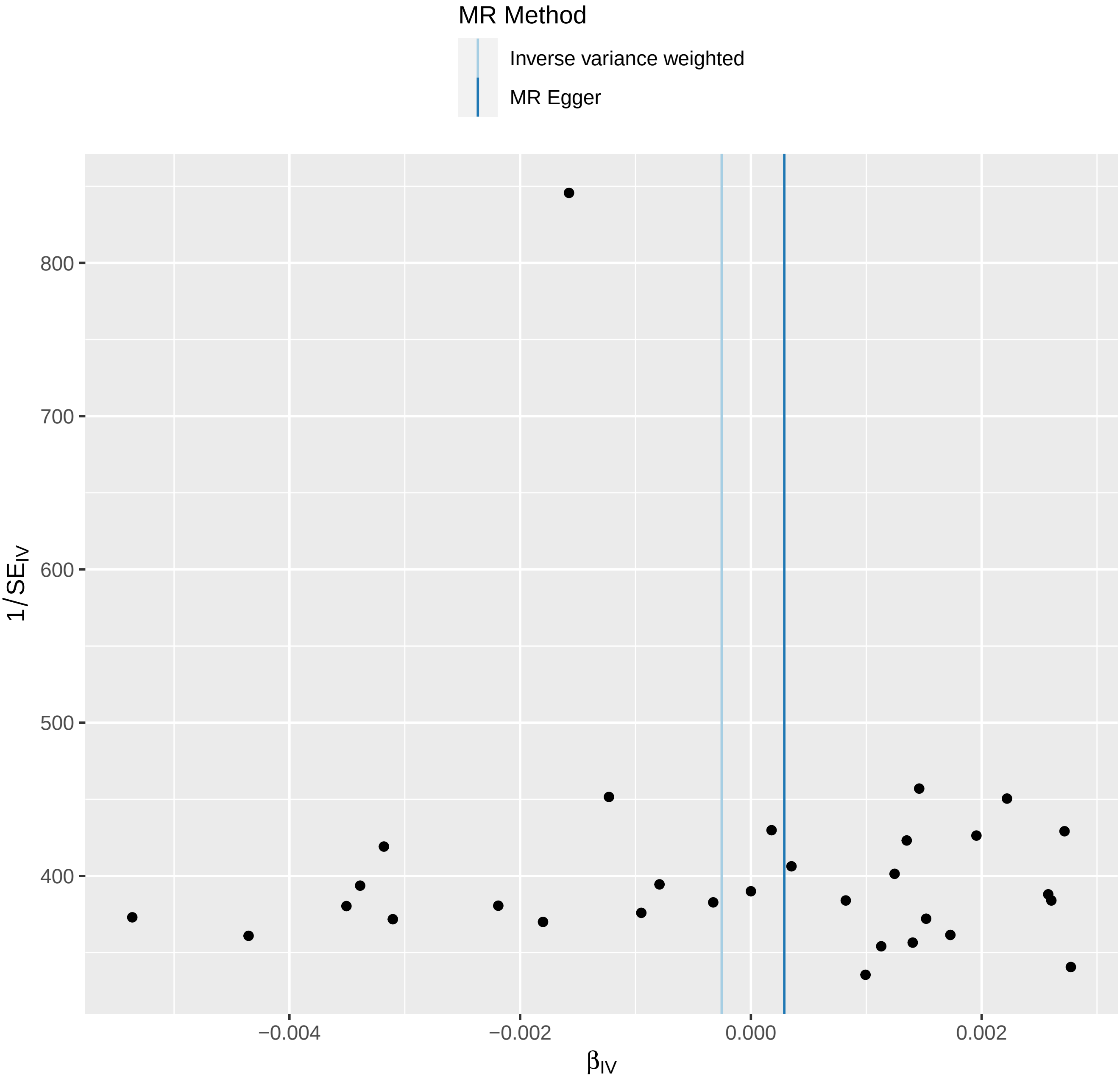

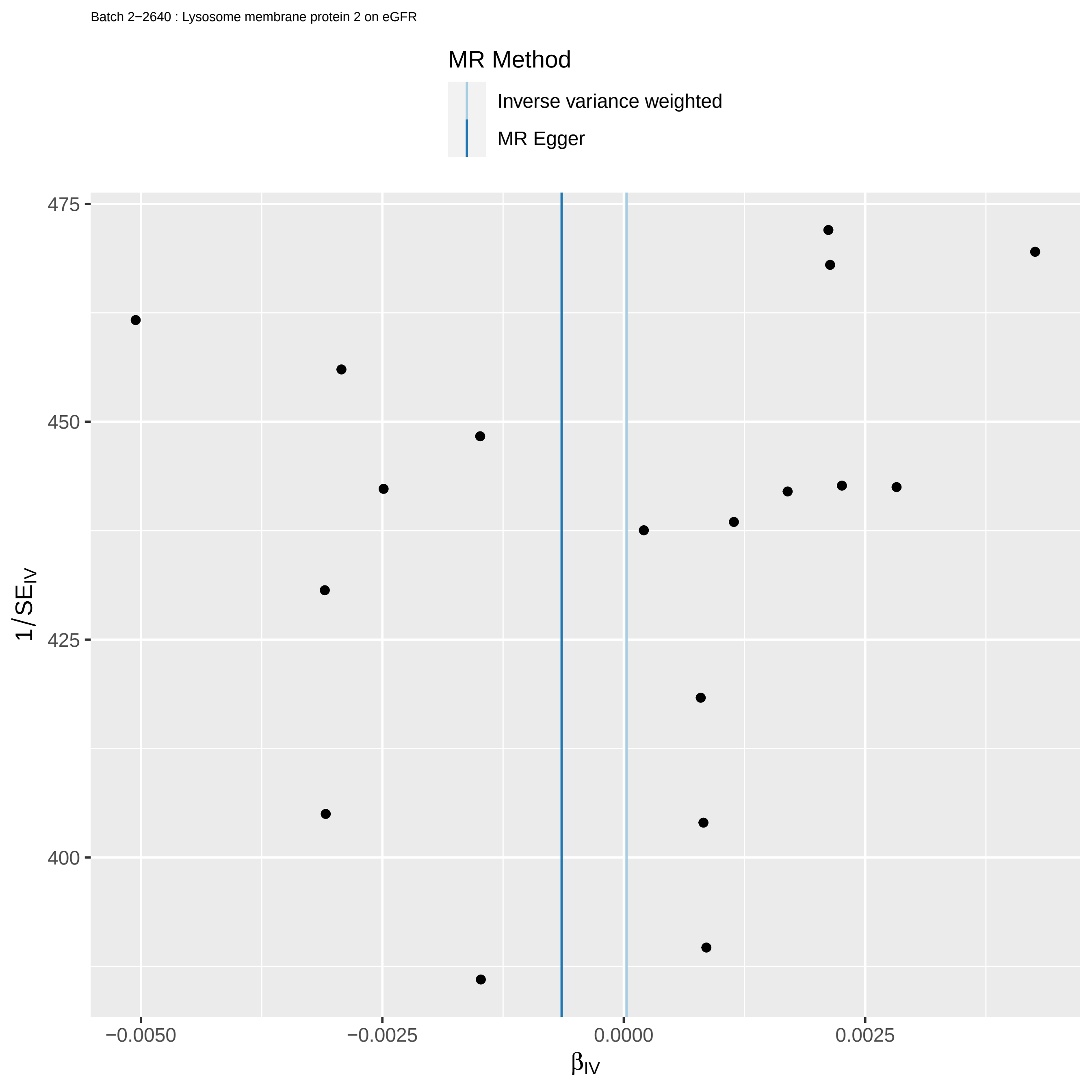

Batch 2−2640 : Lysosome membrane protein 2 on eGFR
MR Method
Inverse variance weighted
MR Egger
475
450
425
400
1/SE_IV
−0.0050
−0.0025
0.0000
0.0025
β_IV

Batch 2−2641 : Scavenger receptor class F member 2 on eGFR

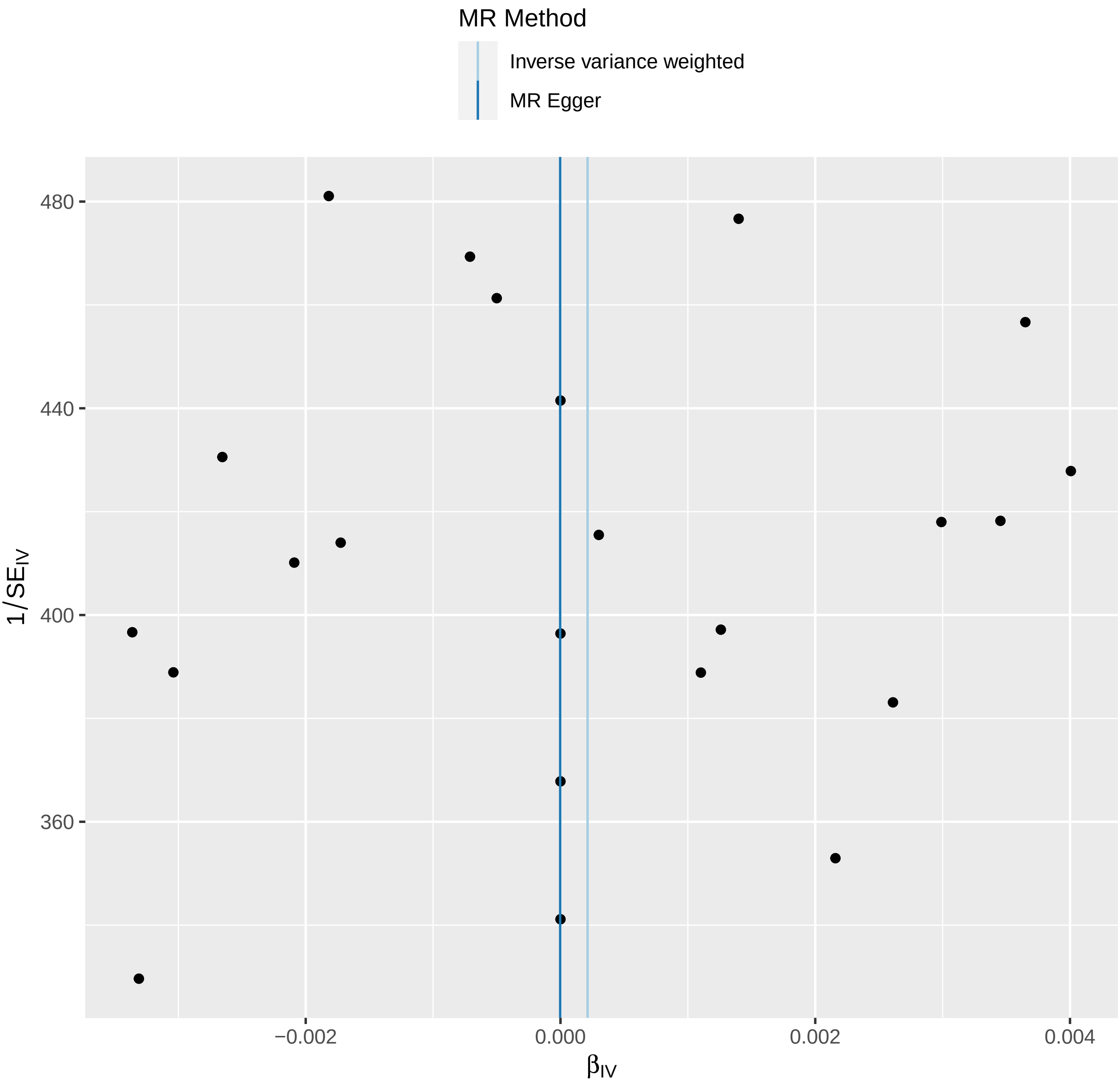

Batch 2−2642 : Scavenger receptor class F member 2 on eGFR

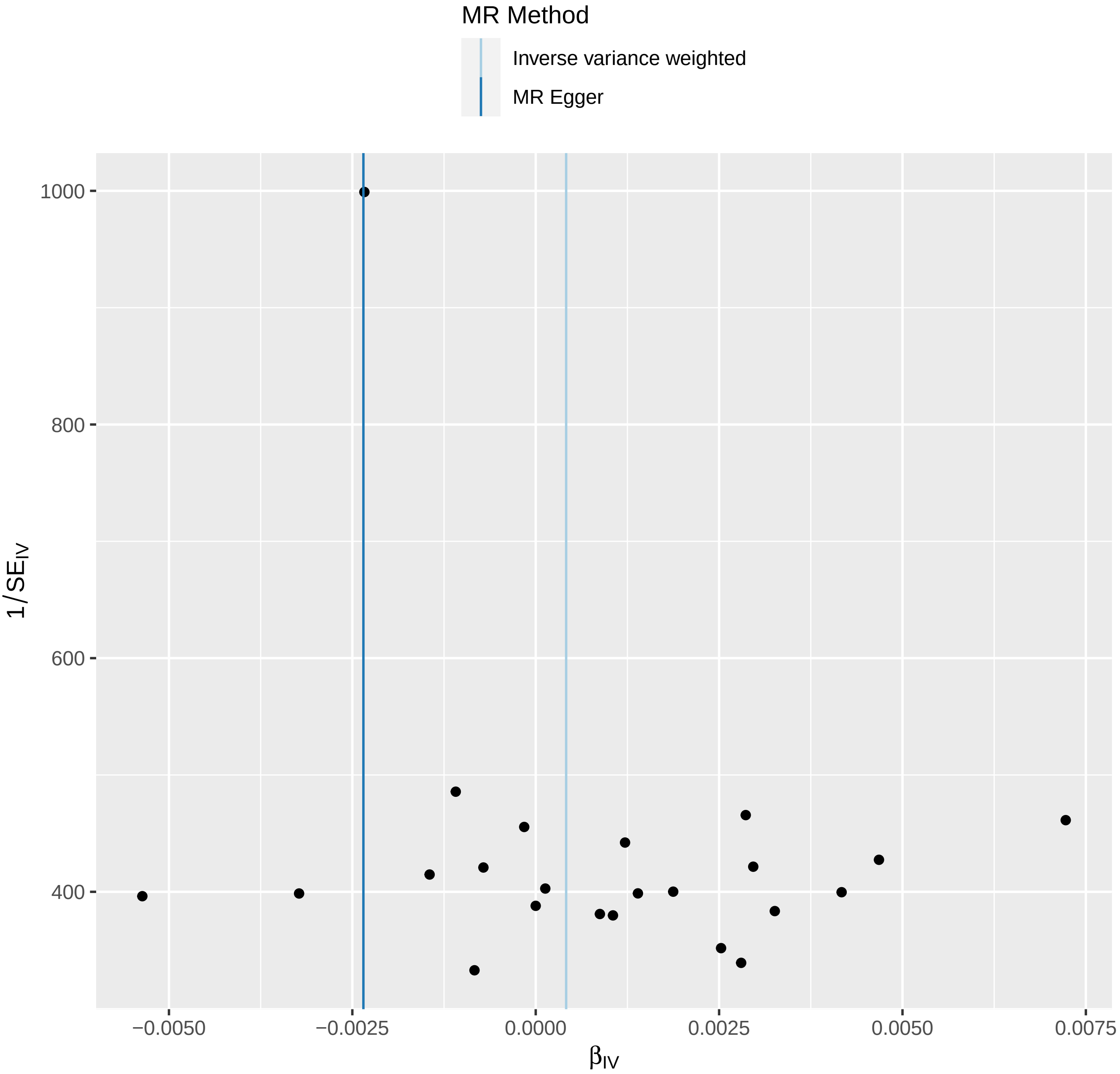

Batch 2−2643 : Scavenger receptor class F member 2 on eGFR

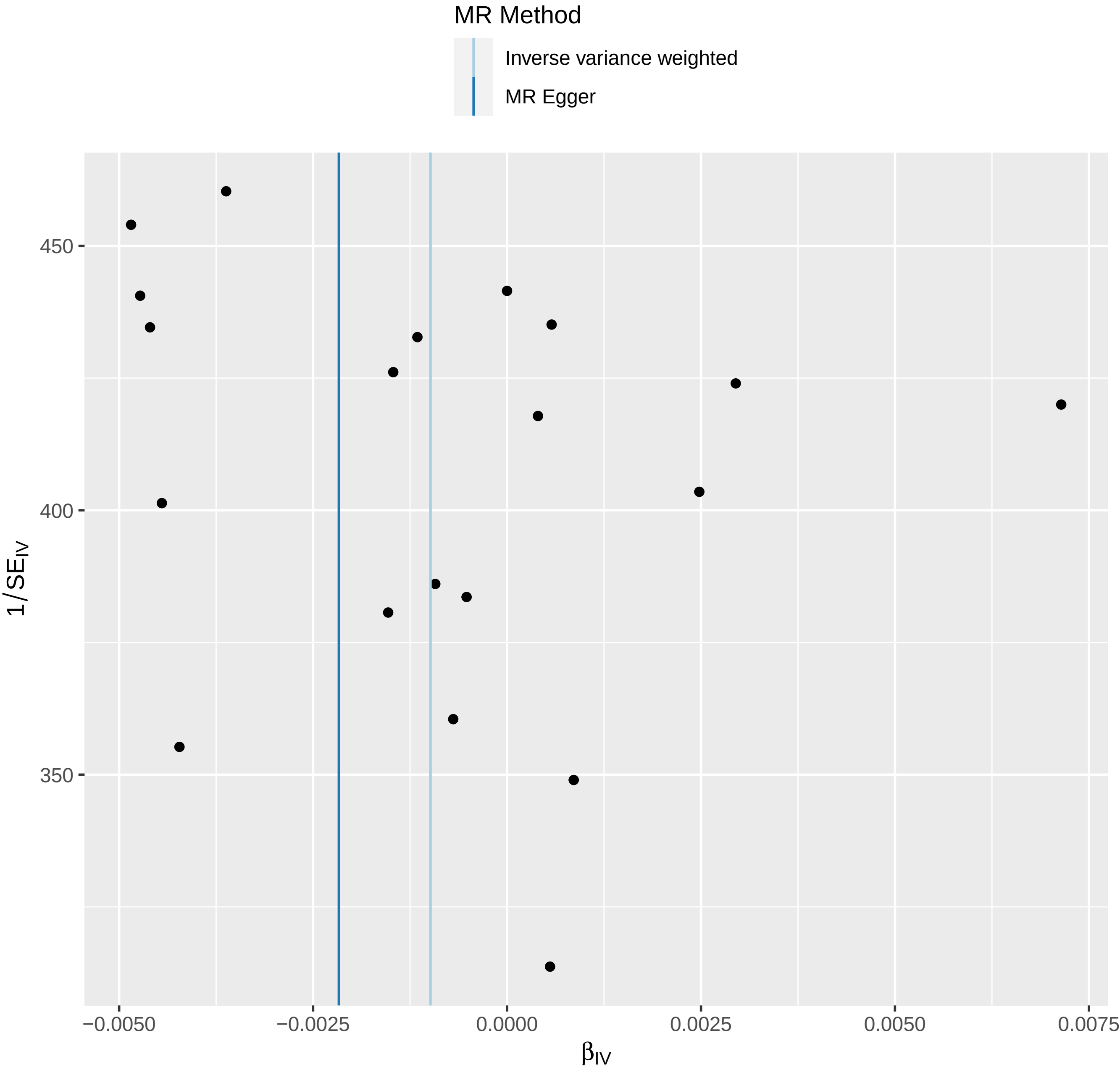

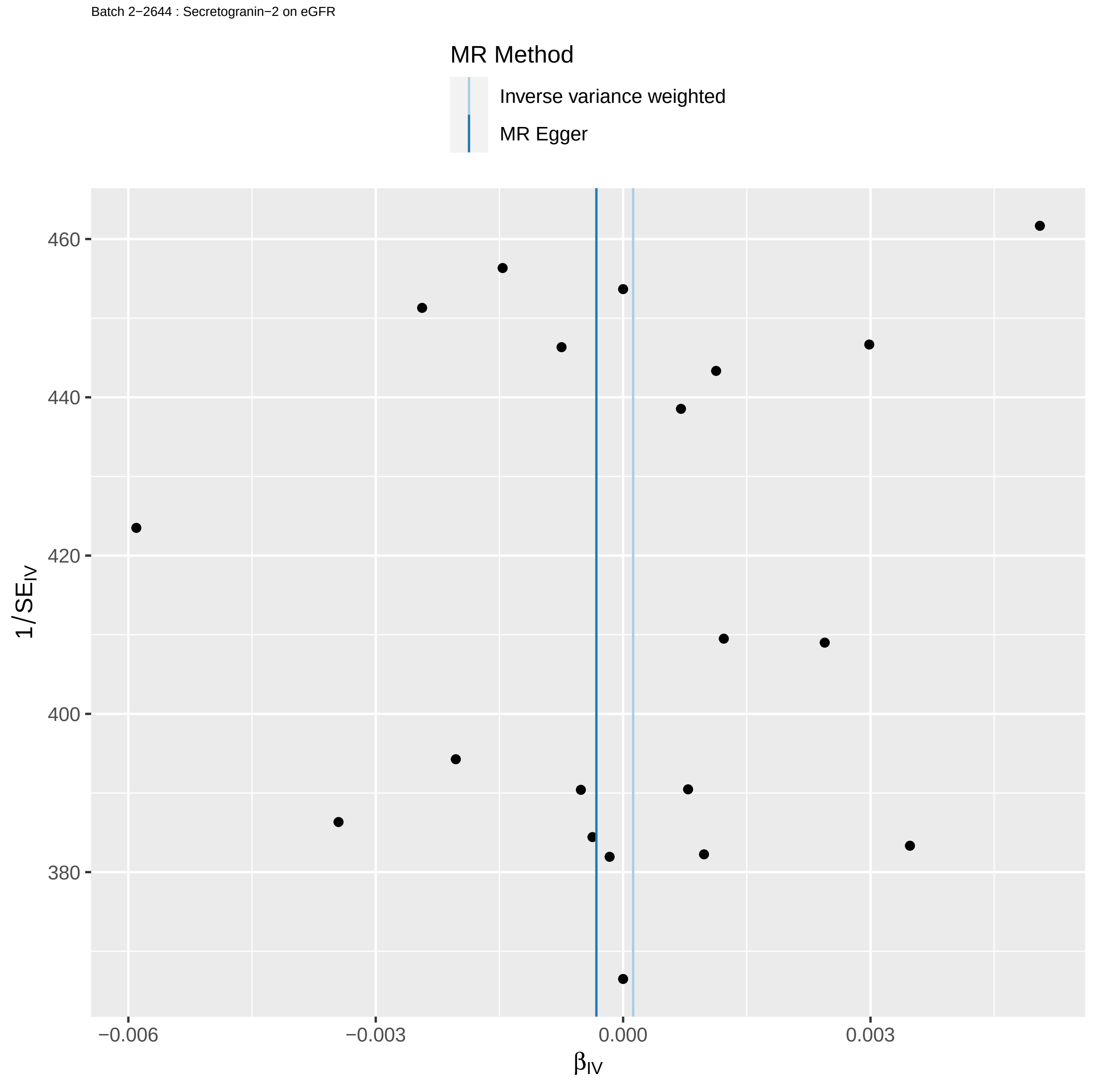

Batch 2−2644 : Secretogranin−2 on eGFR
MR Method
Inverse variance weighted
MR Egger
460
440
420
400
380
1/SE_IV
−0.006
−0.003
0.000
0.003
β_IV

Batch 2−2645 : Secretogranin−3 on eGFR

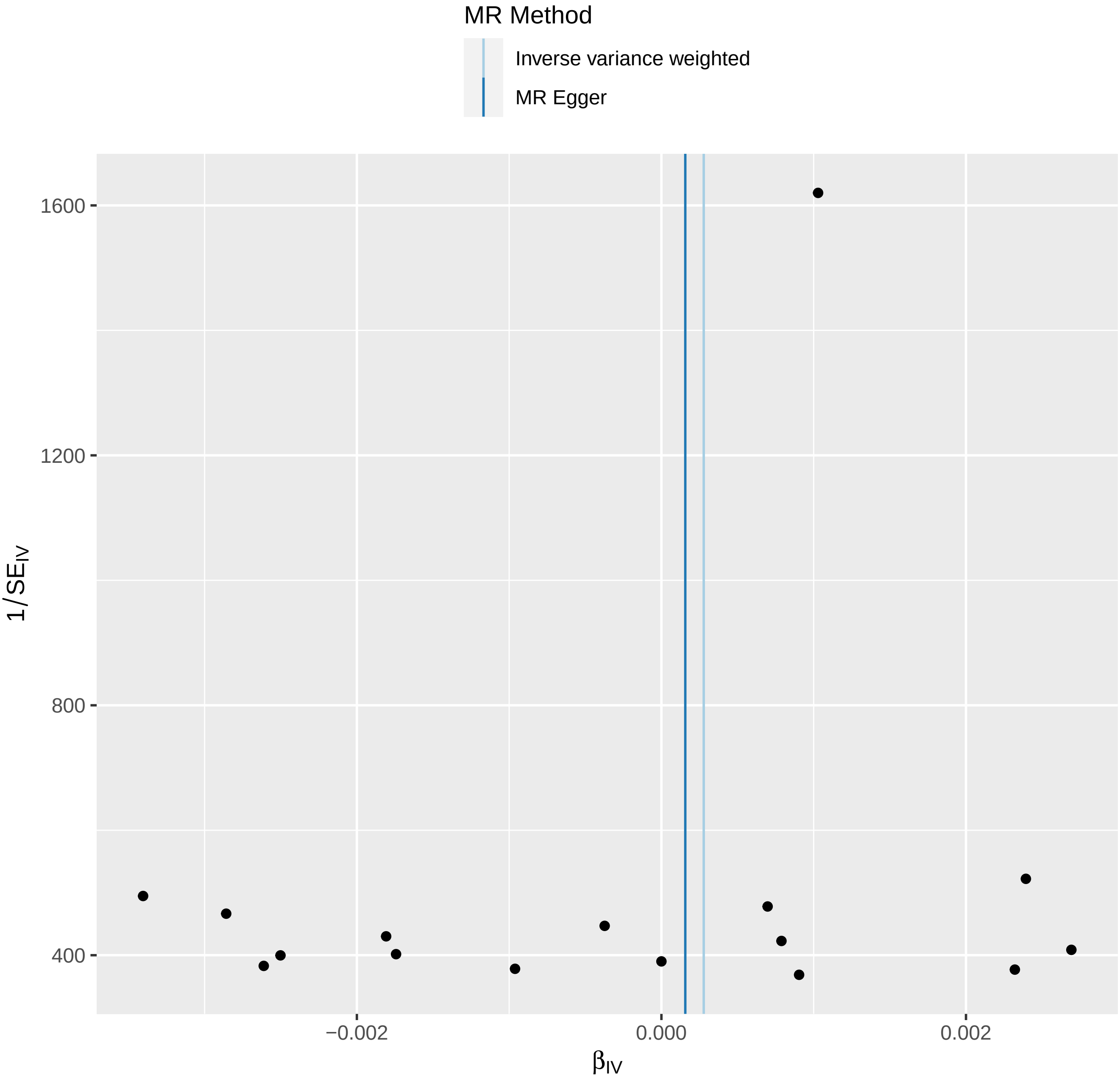

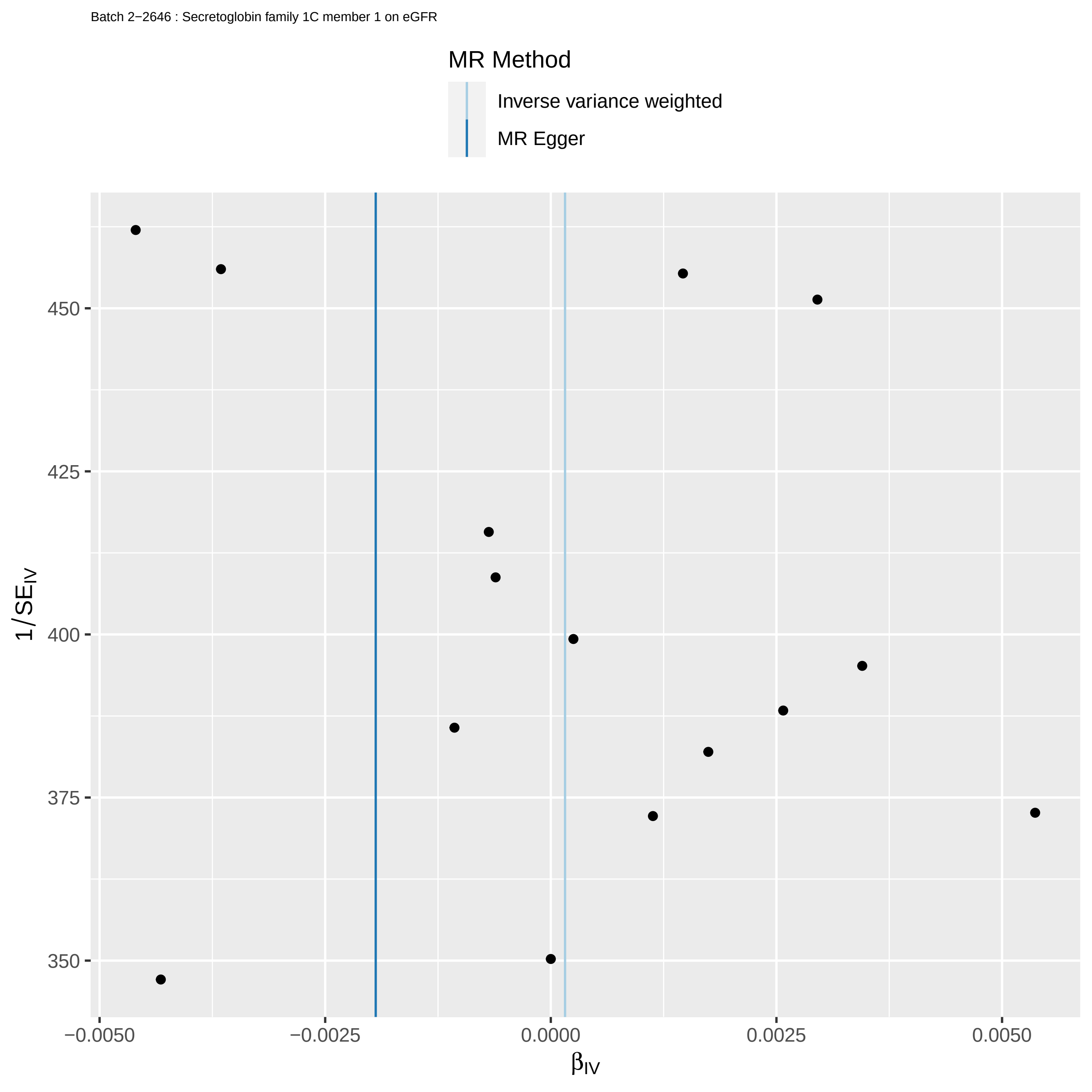

Batch 2−2646 : Secretoglobin family 1C member 1 on eGFR
MR Method
Inverse variance weighted
MR Egger
450
425
400
375
350
1/SE_IV
−0.0050
−0.0025
0.0000
0.0025
0.0050
β_IV

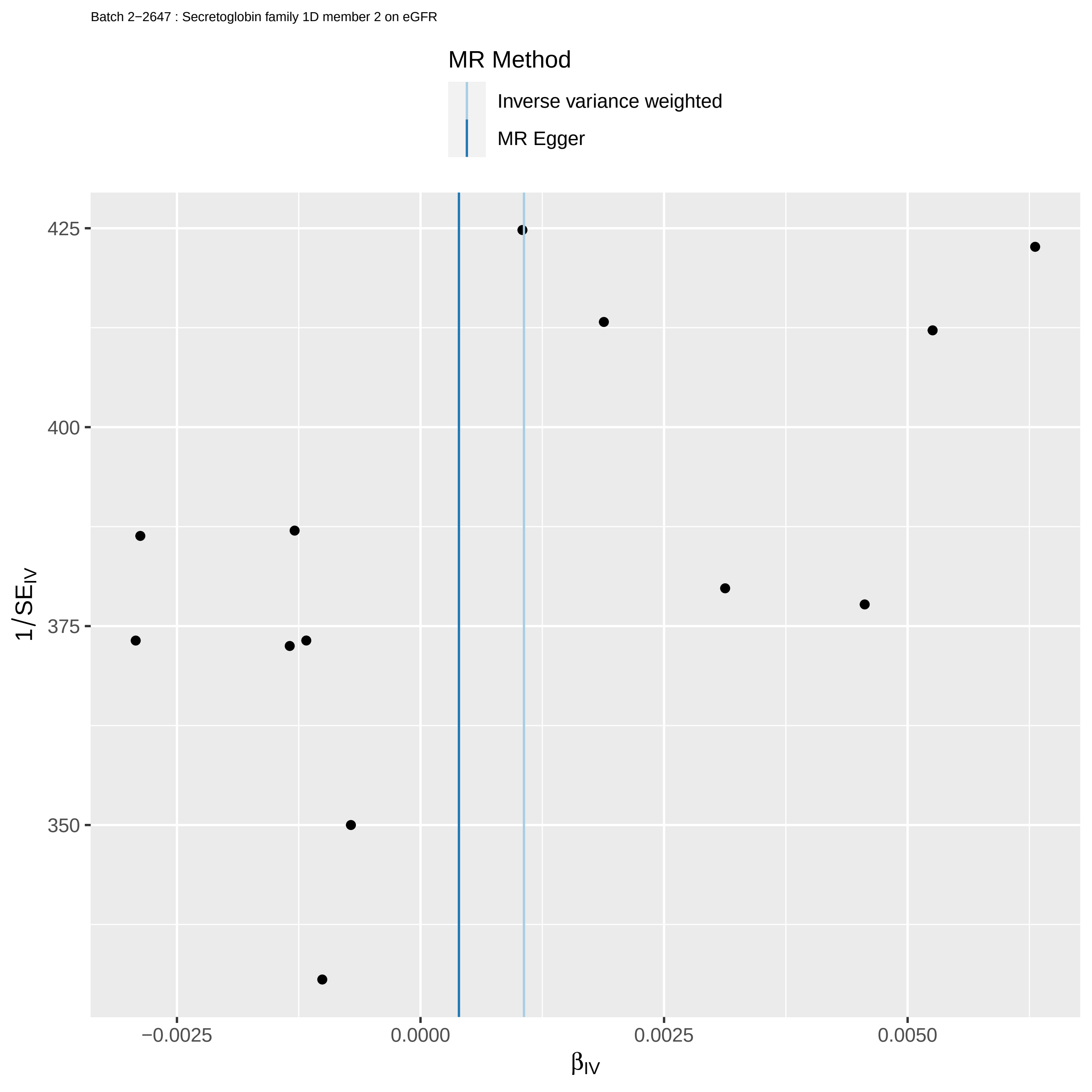
Batch 2−2647 : Secretoglobin family 1D member 2 on eGFR
MR Method
Inverse variance weighted
MR Egger
425
400
375
350
1/SE_IV
−0.0025
0.0000
0.0025
0.0050
β_IV

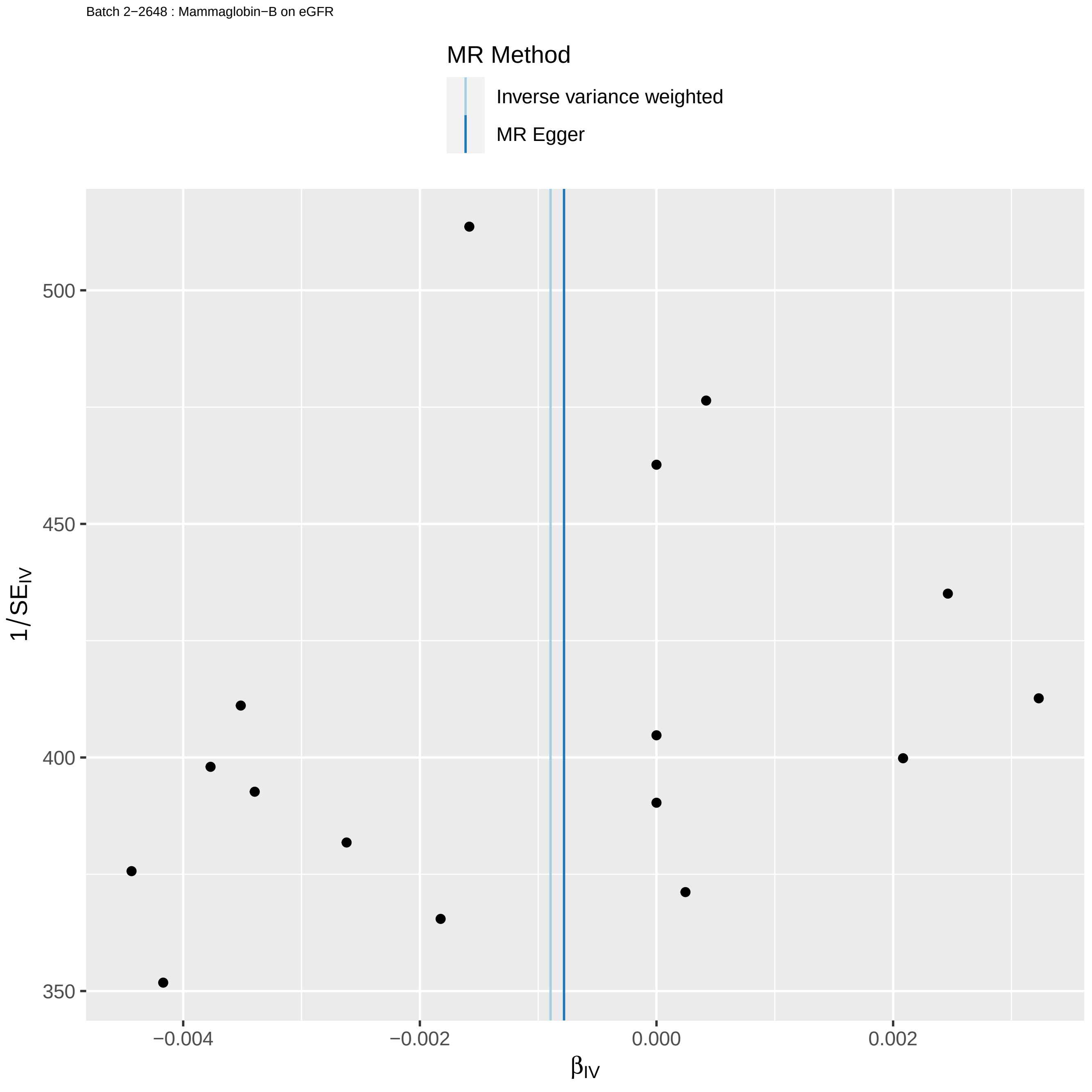
Batch 2−2648 : Mammaglobin−B on eGFR
MR Method
Inverse variance weighted
MR Egger
500
450
400
350
1/SE_IV
−0.004
−0.002
0.000
0.002
β_IV

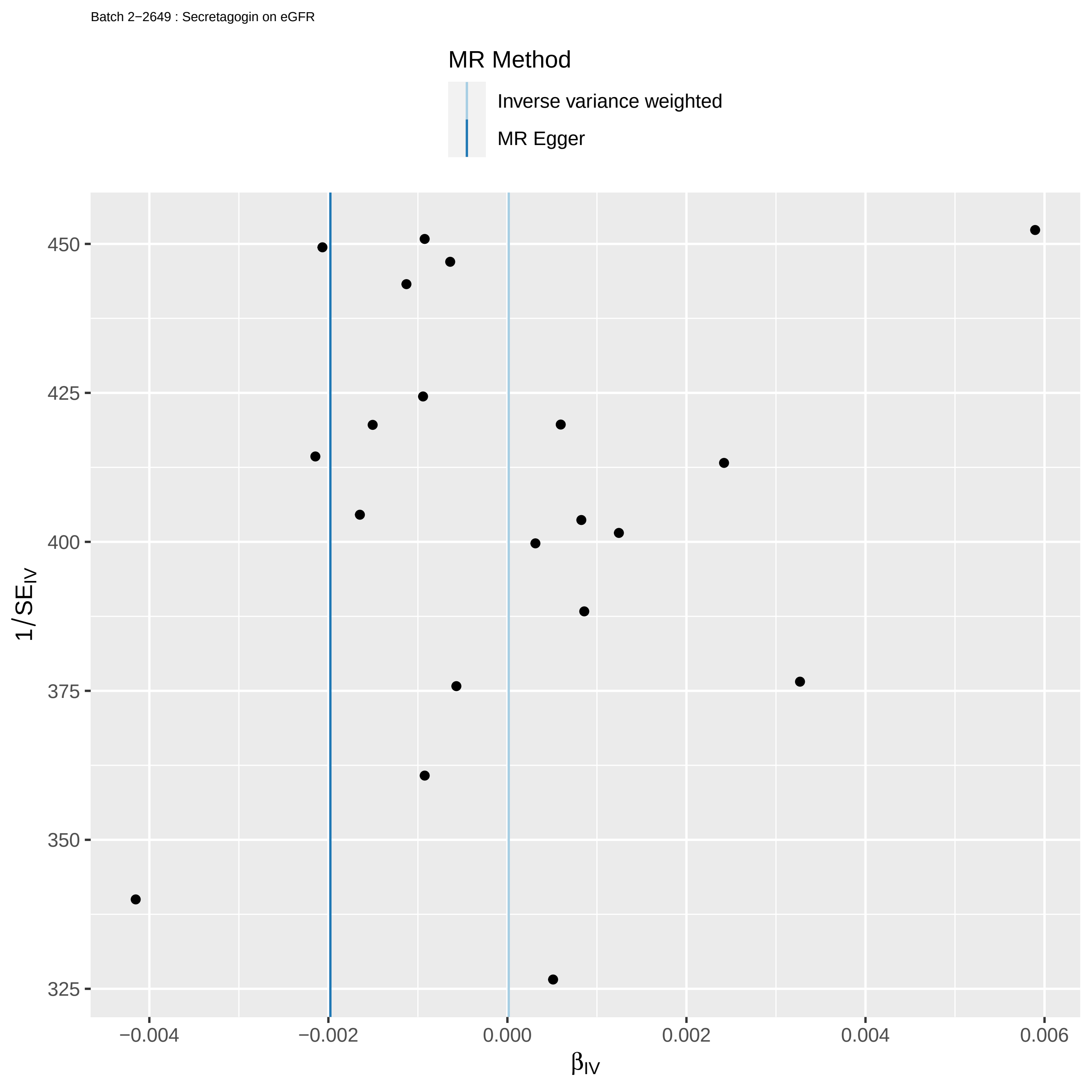
Batch 2−2649 : Secretagogin on eGFR
MR Method
Inverse variance weighted
MR Egger
1/SE_IV
450
425
400
375
350
325
−0.004
−0.002
0.000
0.002
0.004
0.006
β_IV

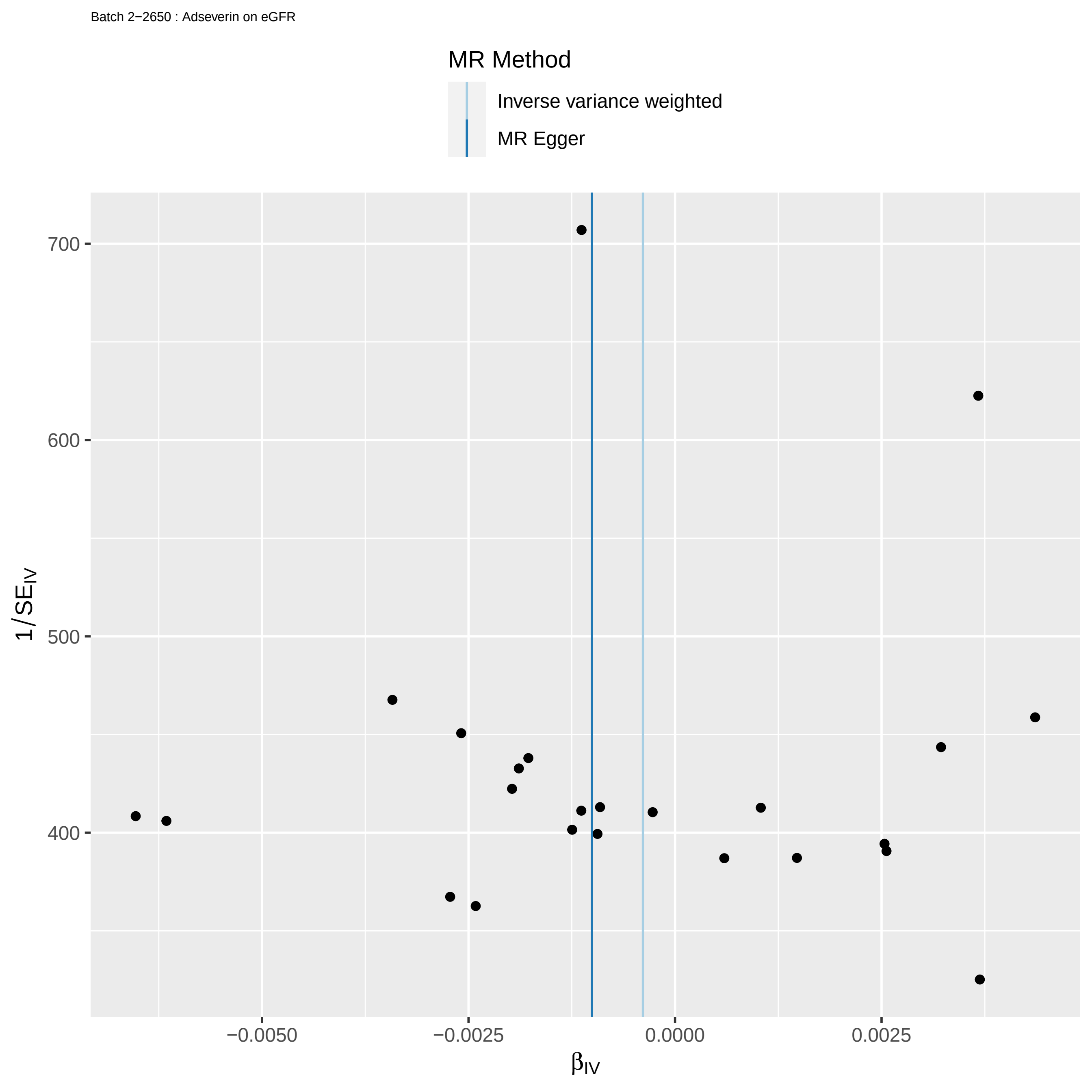

Batch 2−2650 : Adseverin on eGFR
MR Method
Inverse variance weighted
MR Egger
700
600
500
400
1/SE$_{IV}$
−0.0050
−0.0025
0.0000
0.0025
β$_{IV}$

Batch 2−2651 : Polycomb protein SCMH1 on eGFR

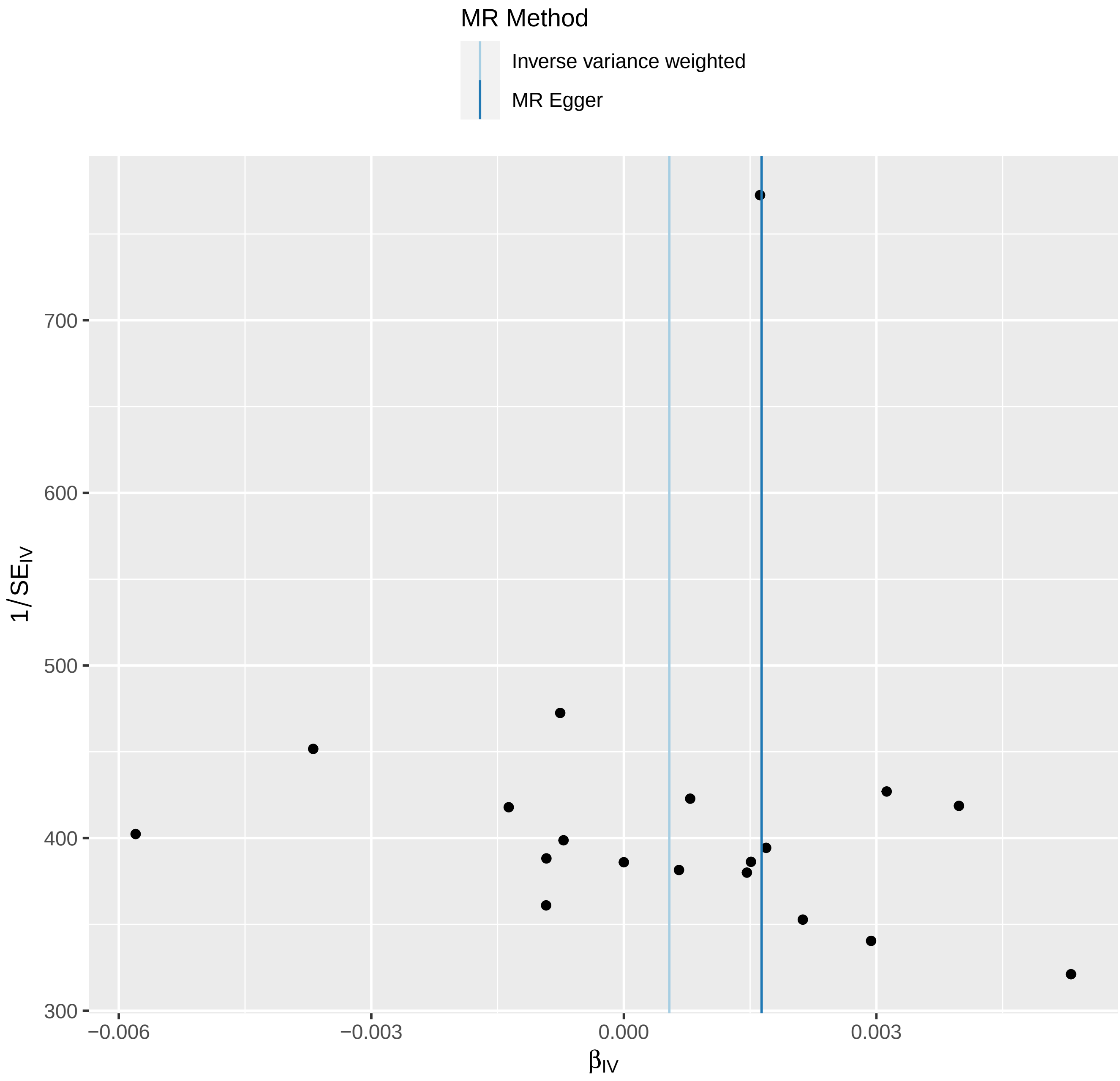

Batch 2−2652 : Sodium channel subunit beta−2 on eGFR

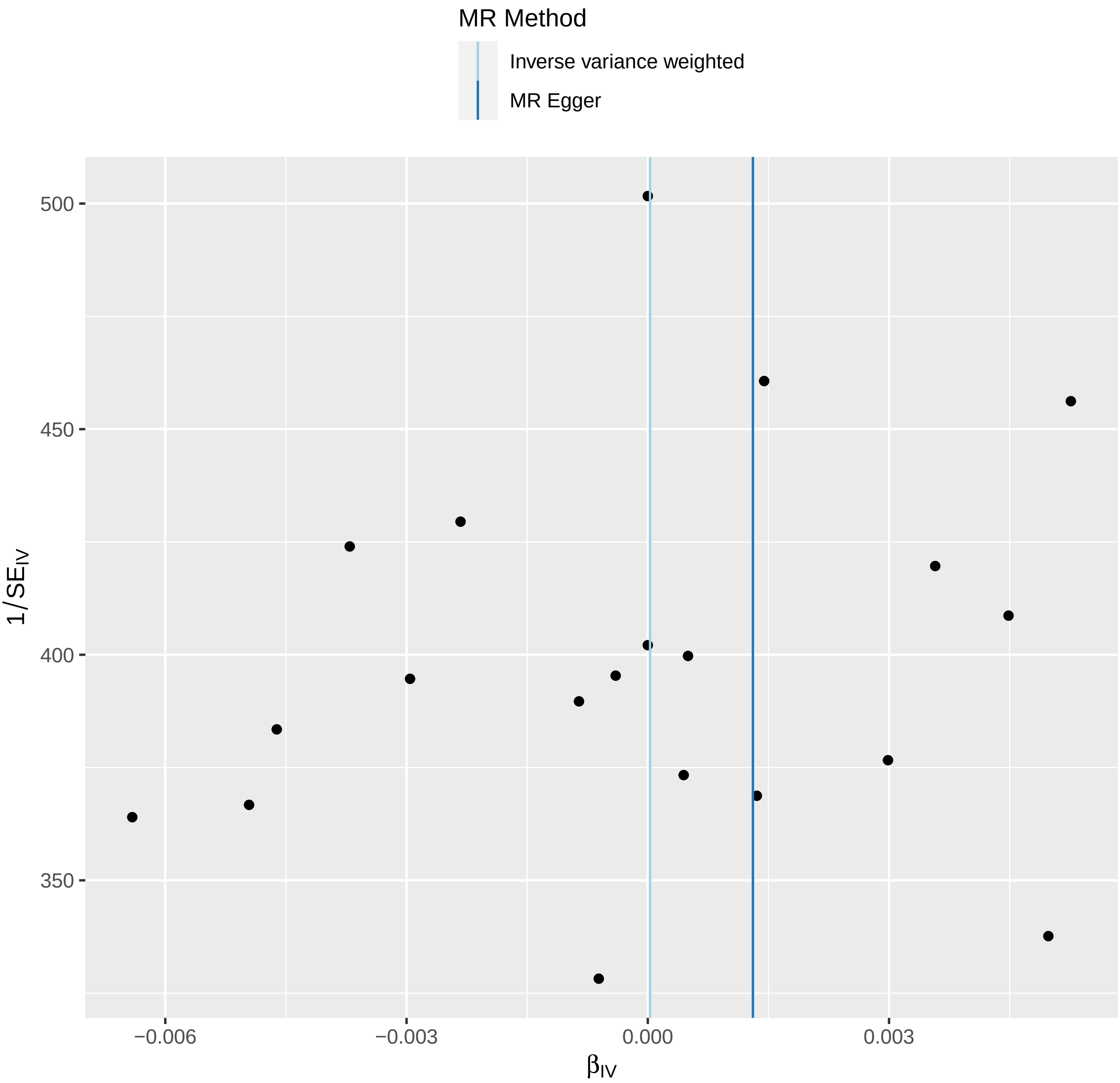

Batch 2−2653 : Protein SCO1 homolog, mitochondrial on eGFR

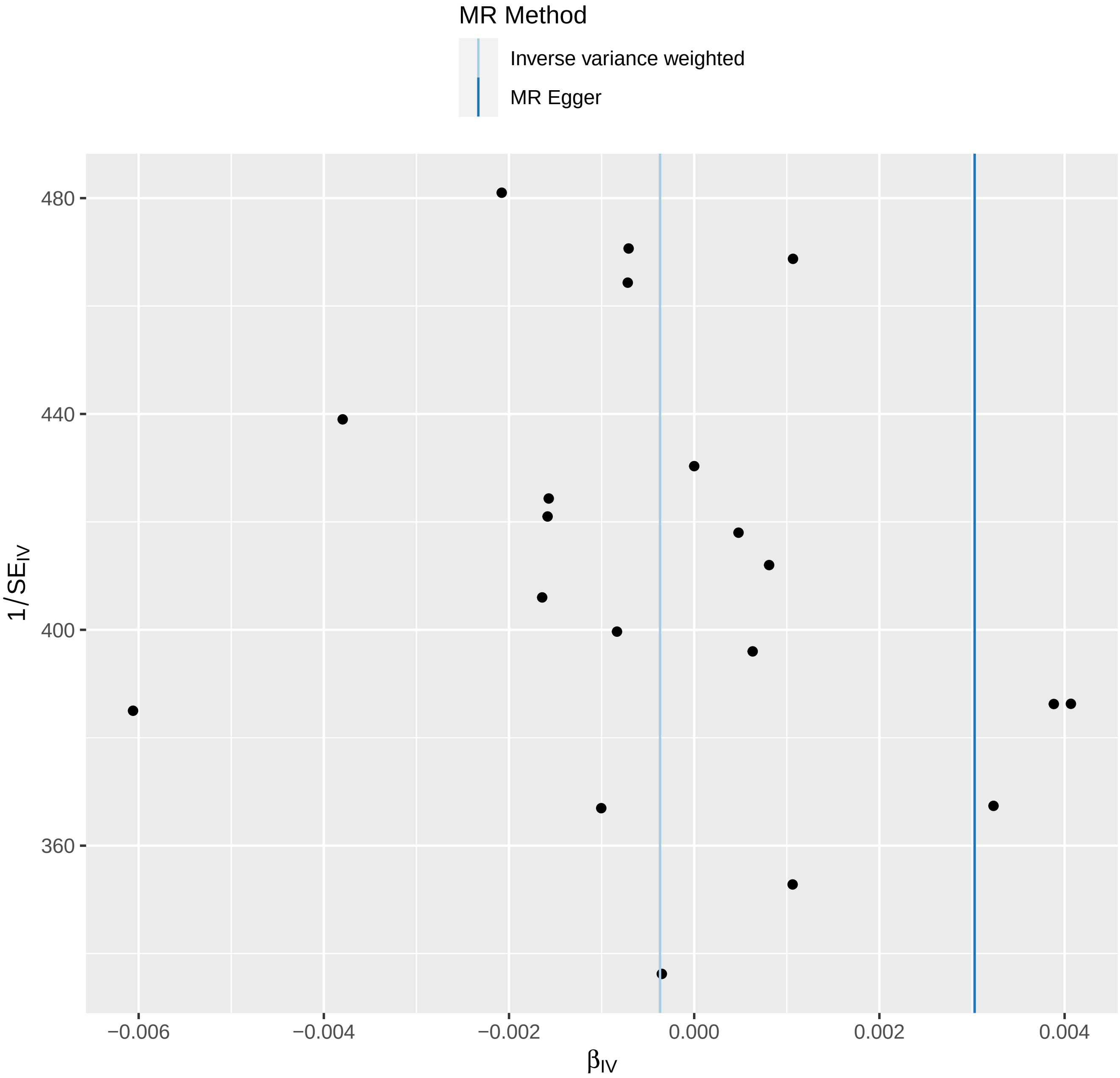

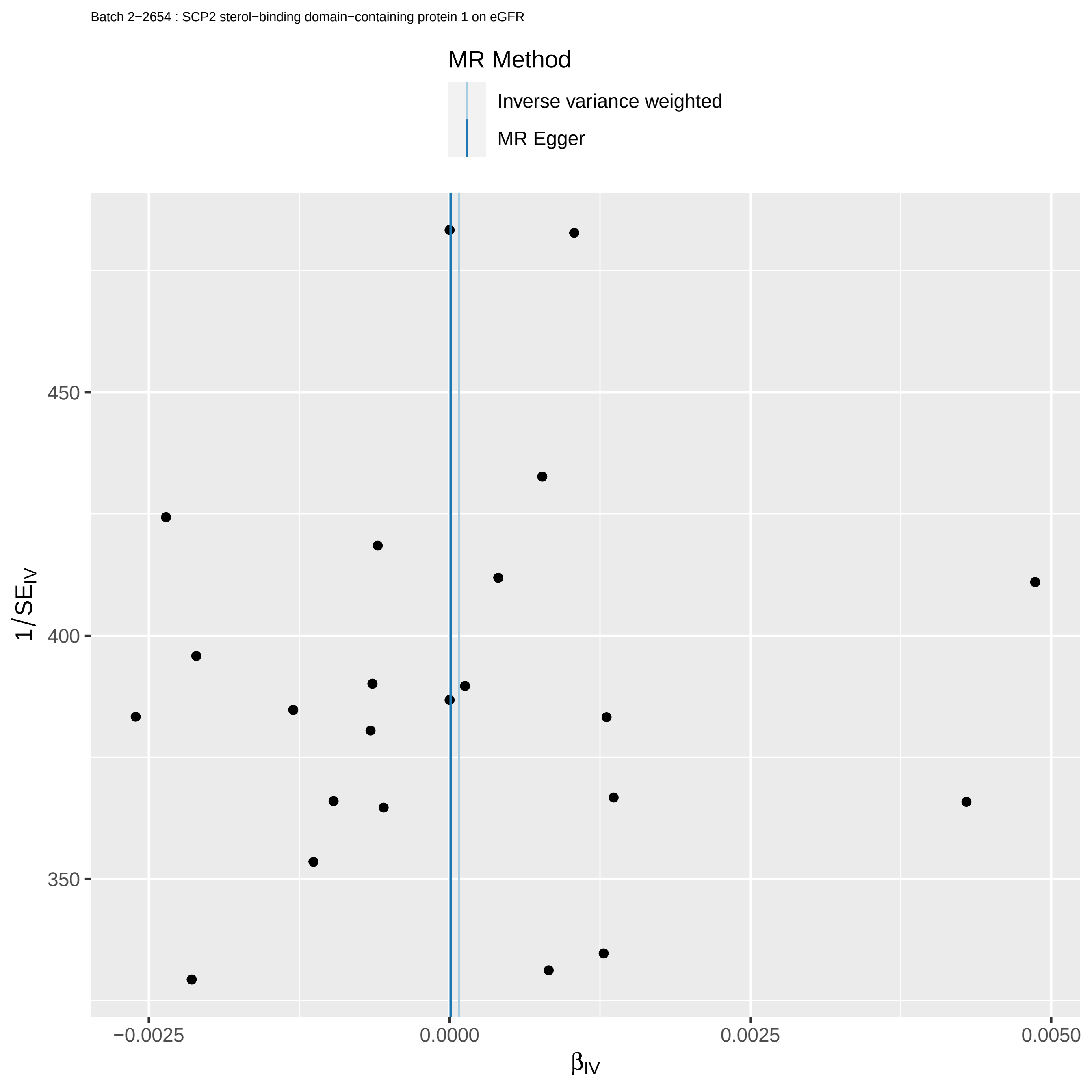
Batch 2−2654 : SCP2 sterol−binding domain−containing protein 1 on eGFR
MR Method
Inverse variance weighted
MR Egger
450
400
350
1/SE_IV
−0.0025
0.0000
0.0025
0.0050
β_IV

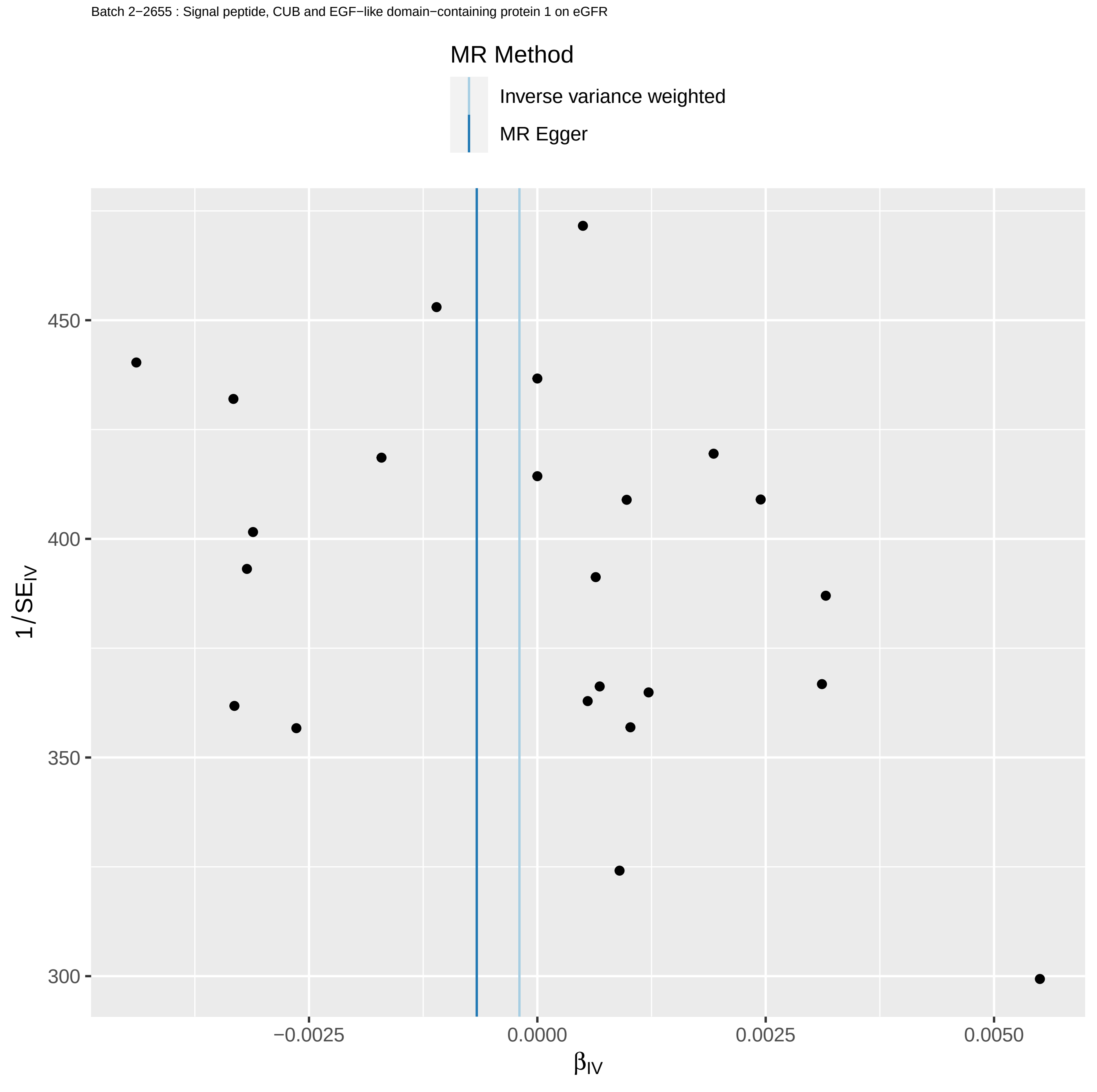

Batch 2−2655 : Signal peptide, CUB and EGF−like domain−containing protein 1 on eGFR
MR Method
Inverse variance weighted
MR Egger
450
400
350
300
1/SE_IV
−0.0025
0.0000
0.0025
0.0050
β_IV

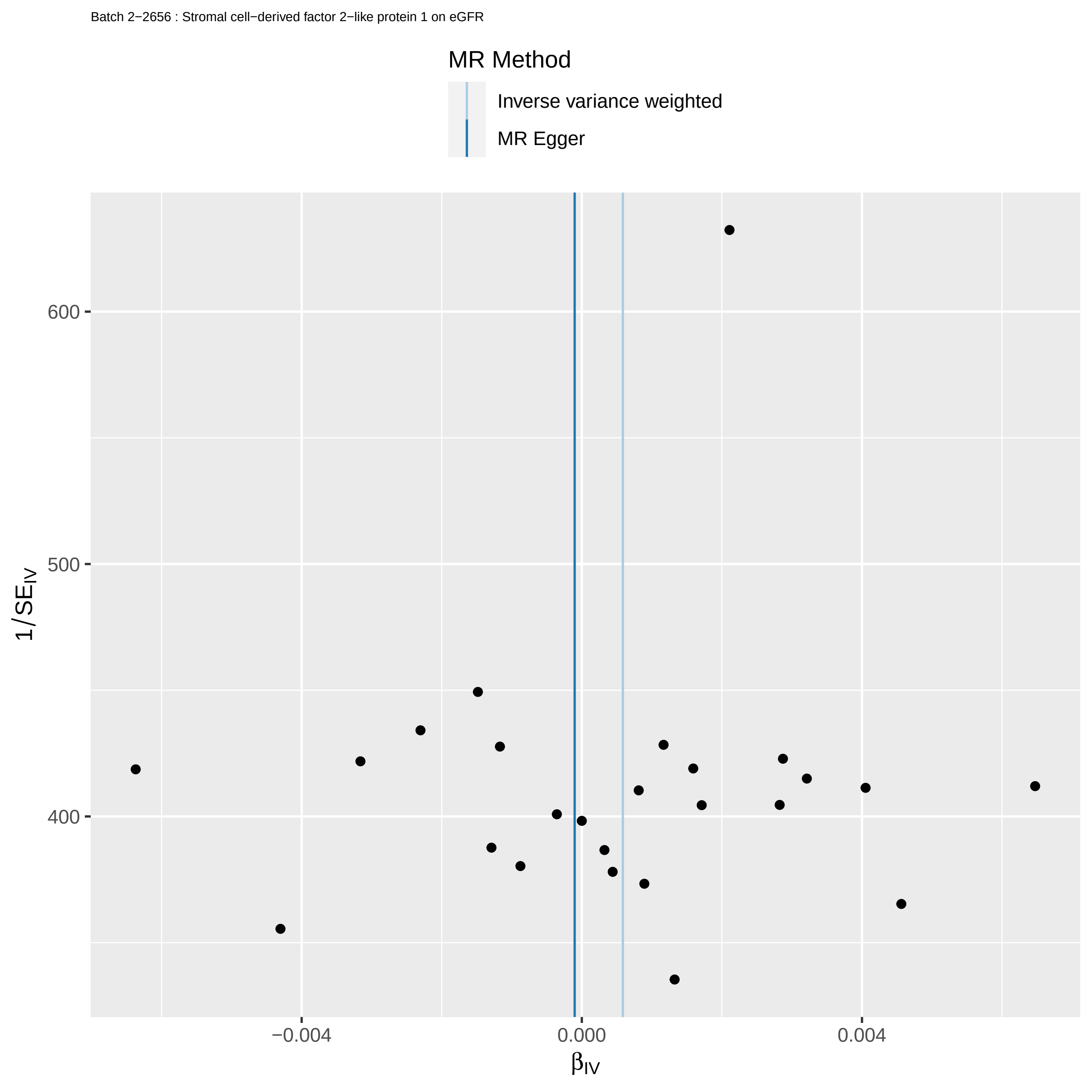

Batch 2−2656 : Stromal cell−derived factor 2−like protein 1 on eGFR
MR Method
Inverse variance weighted
MR Egger
600
500
400
1/SE$_{IV}$
−0.004
0.000
0.004
β_{IV}

Batch 2−2657 : Succinate dehydrogenase assembly factor 2, mitochondrial on eGFR

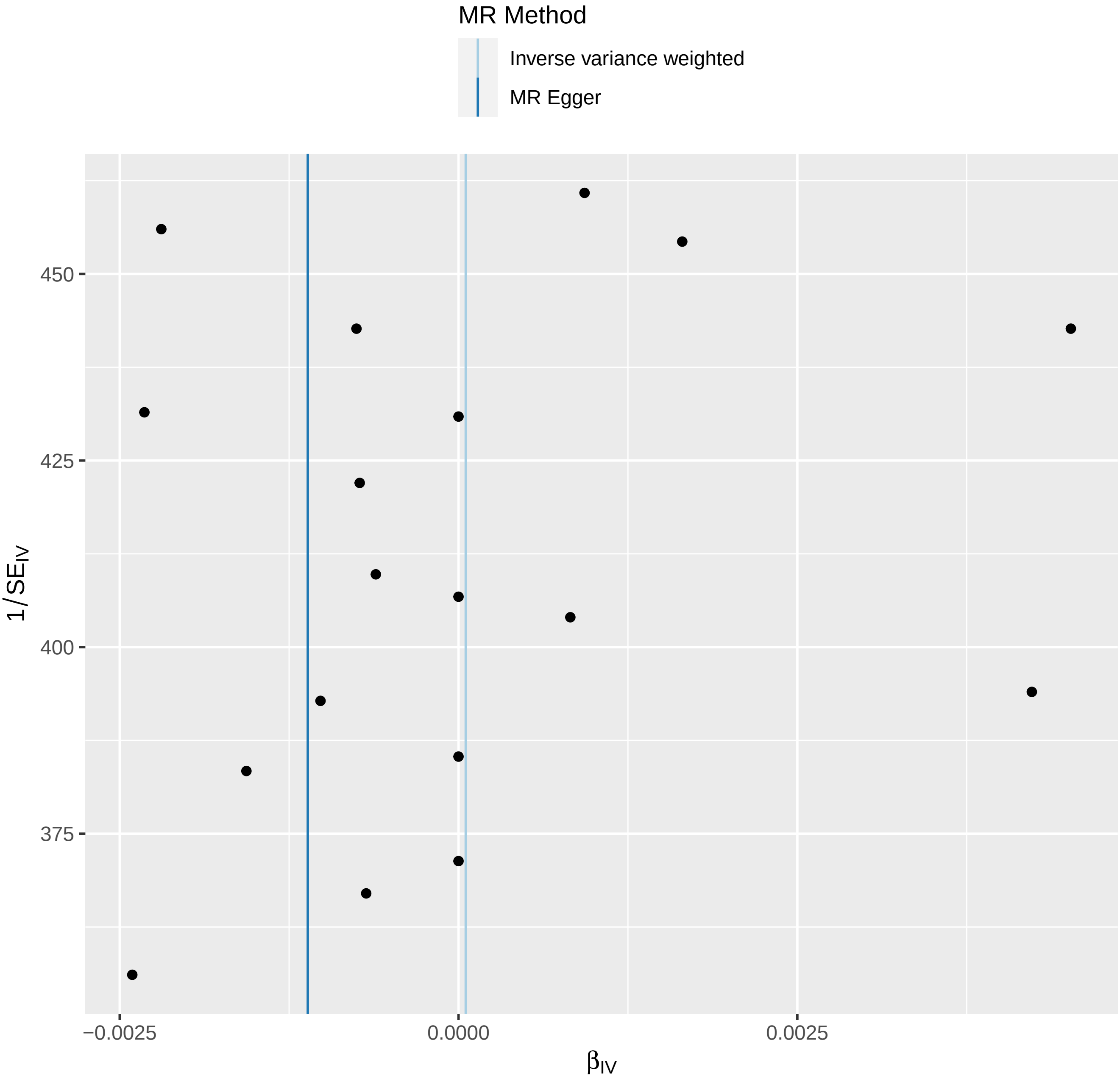

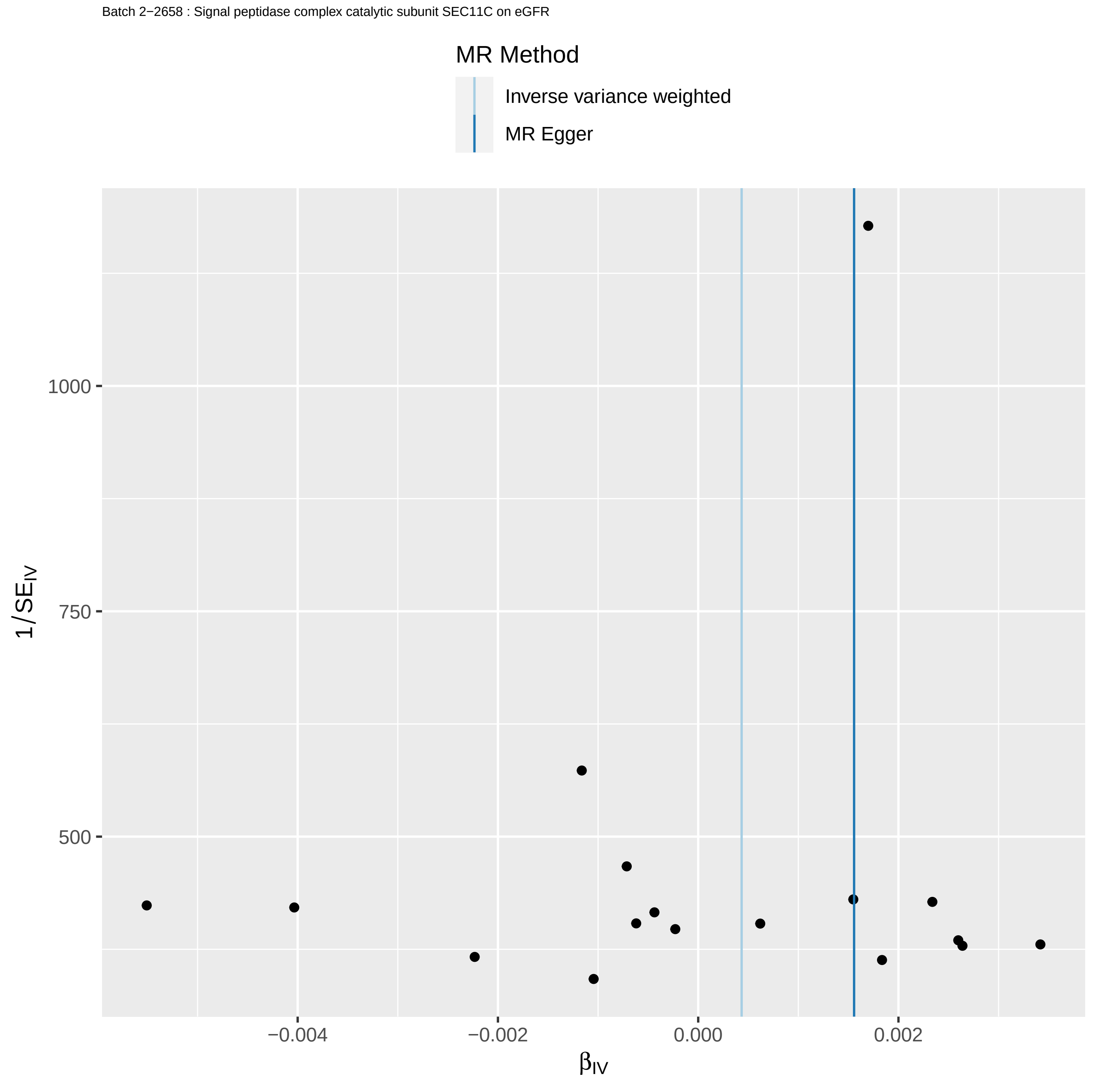
Batch 2−2658 : Signal peptidase complex catalytic subunit SEC11C on eGFR
MR Method
Inverse variance weighted
MR Egger
1000
750
500
1/SE$_{IV}$
−0.004
−0.002
0.000
0.002
β$_{IV}$

Batch 2−2659 : Protein SEC13 homolog on eGFR

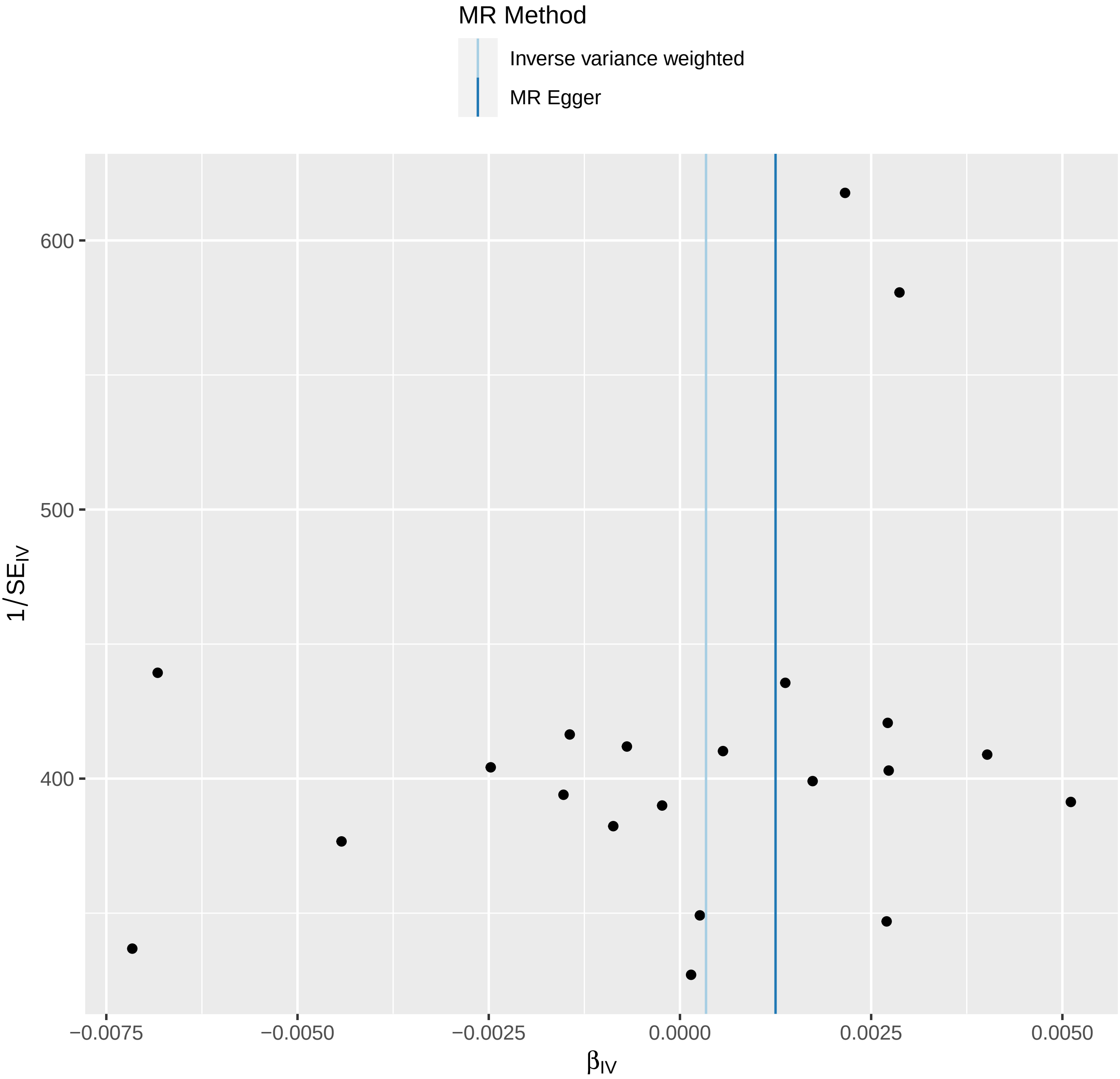

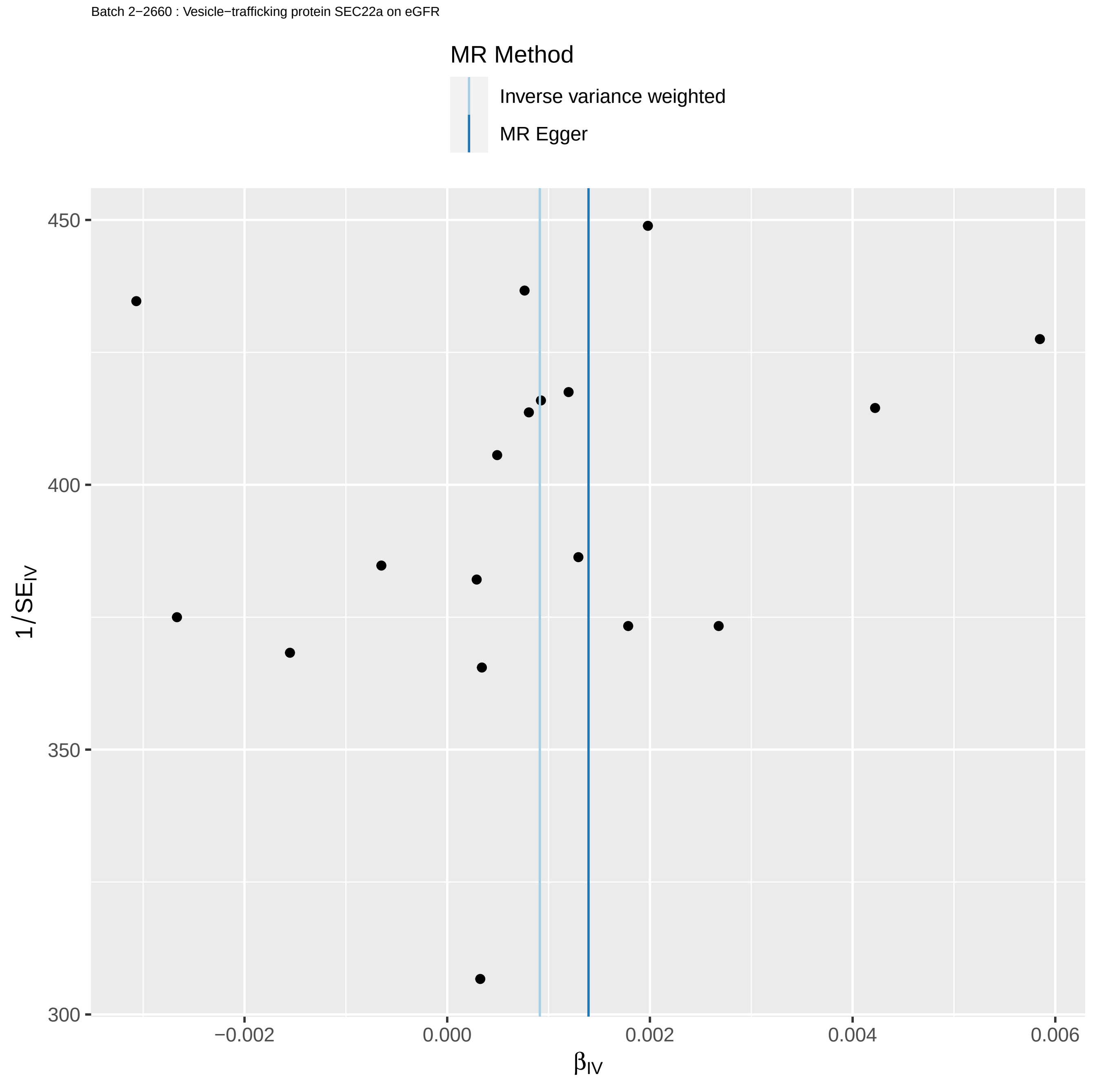
Batch 2−2660 : Vesicle−trafficking protein SEC22a on eGFR
MR Method
Inverse variance weighted
MR Egger
450
400
350
300
−0.002
0.000
0.002
0.004
0.006
$1/SE_{IV}$
β_{IV}

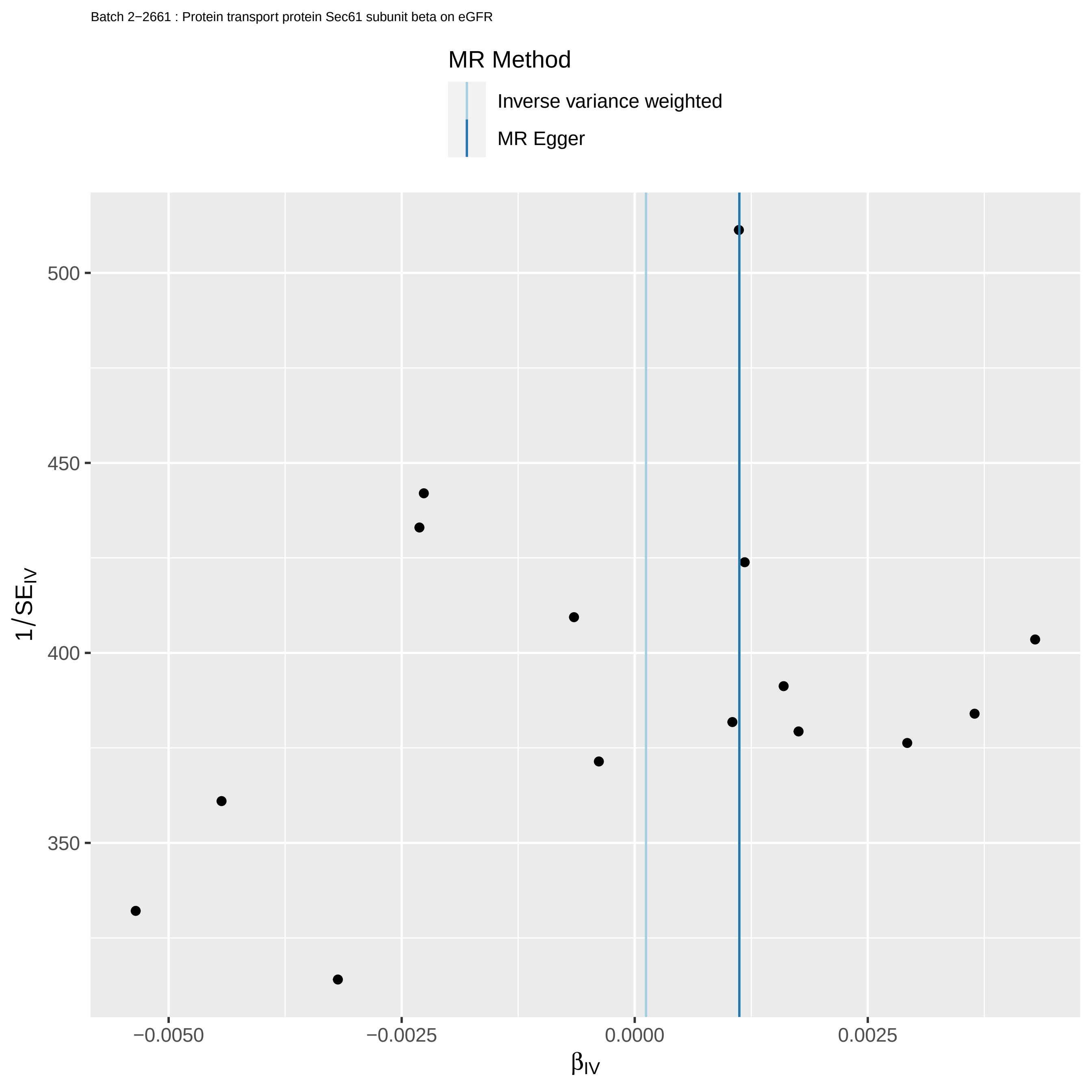
Batch 2−2661 : Protein transport protein Sec61 subunit beta on eGFR
MR Method
Inverse variance weighted
MR Egger
500
450
400
350
1/SE_IV
−0.0050
−0.0025
0.0000
0.0025
β_IV

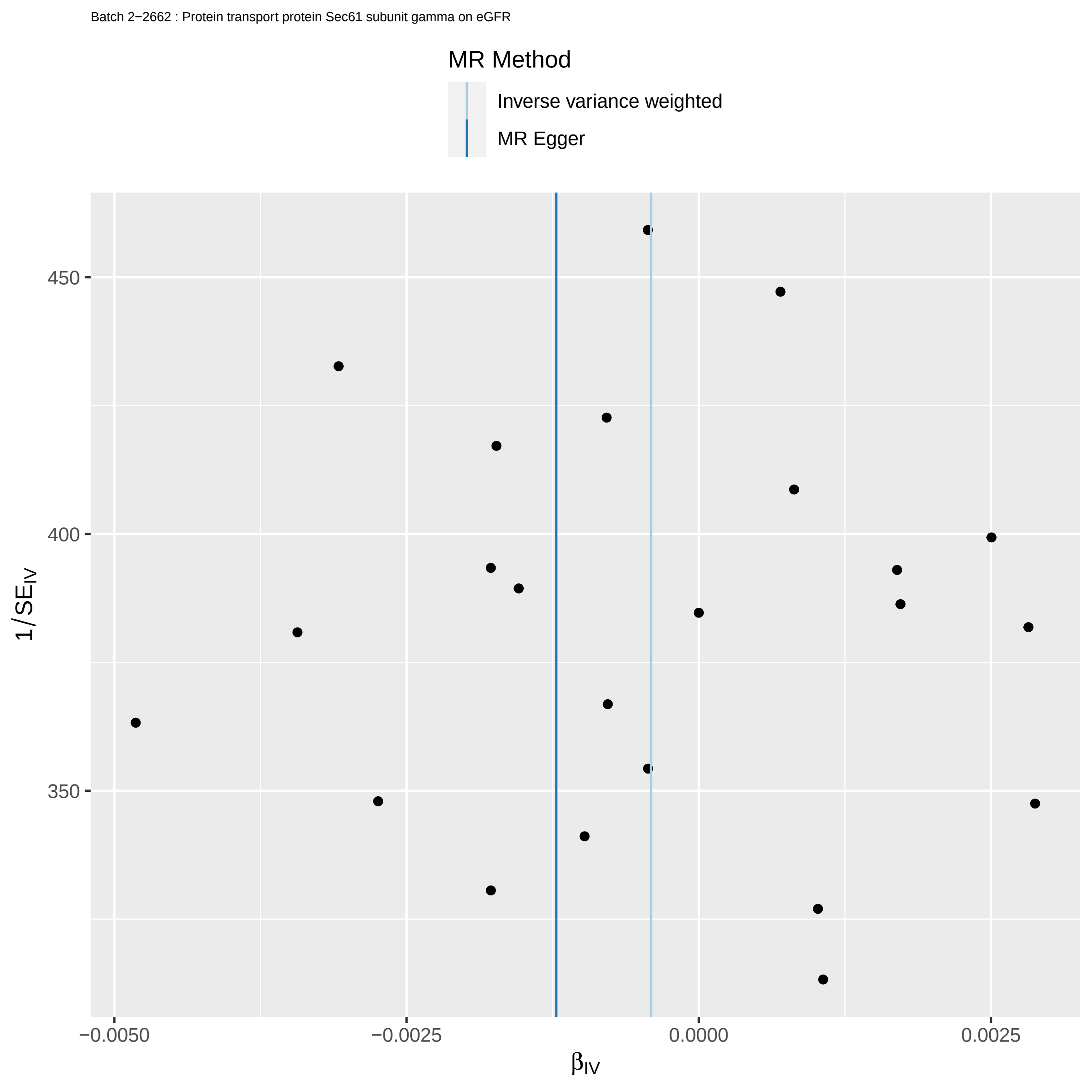

Batch 2−2662 : Protein transport protein Sec61 subunit gamma on eGFR
MR Method
Inverse variance weighted
MR Egger
450
400
350
1/SE_IV
−0.0050
−0.0025
0.0000
0.0025
β_IV

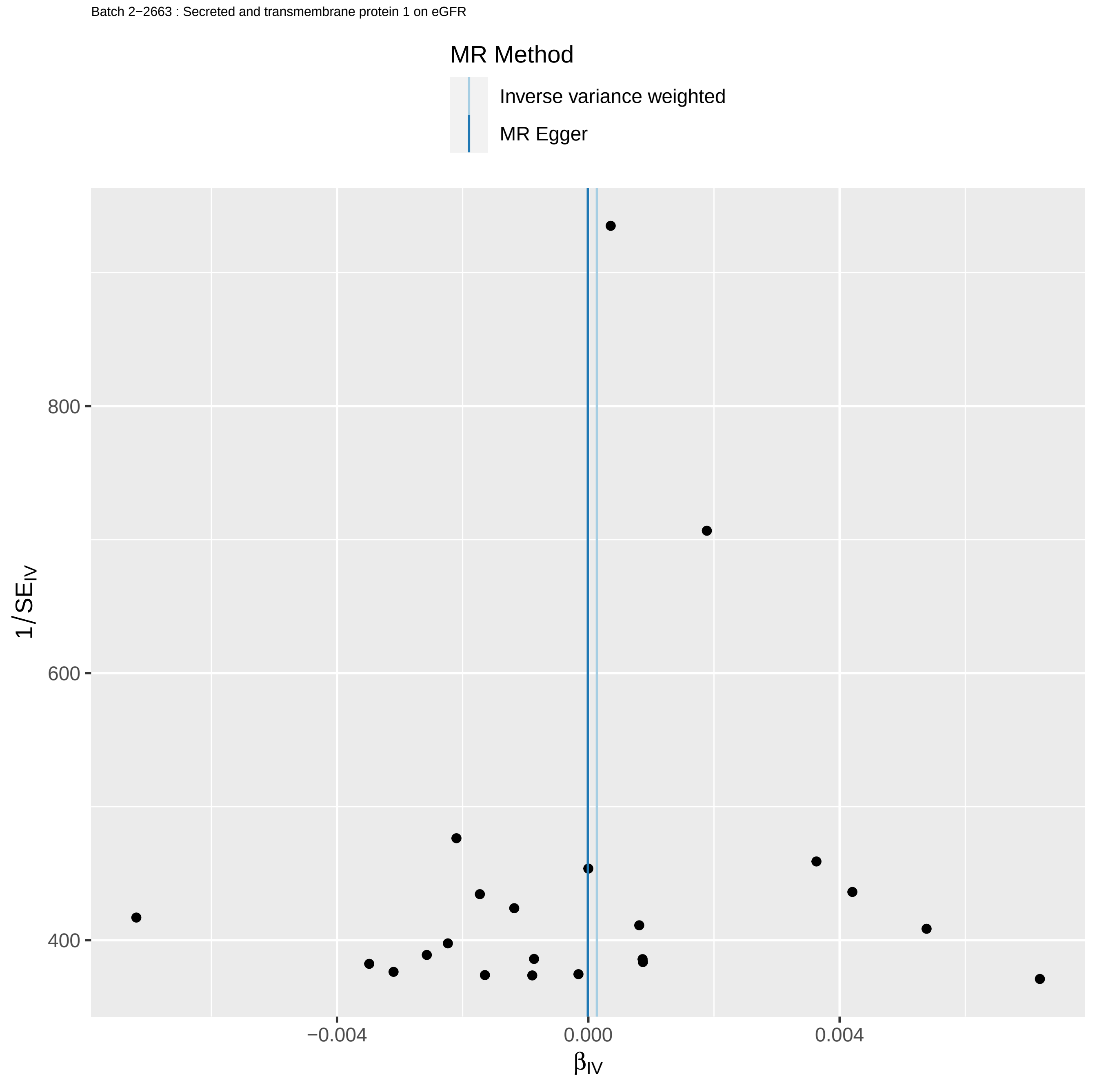

Batch 2−2663 : Secreted and transmembrane protein 1 on eGFR
MR Method
Inverse variance weighted
MR Egger
800
600
400
1/SE_IV
−0.004
0.000
0.004
β_IV

Batch 2−2664 : Protein sel−1 homolog 2 on eGFR

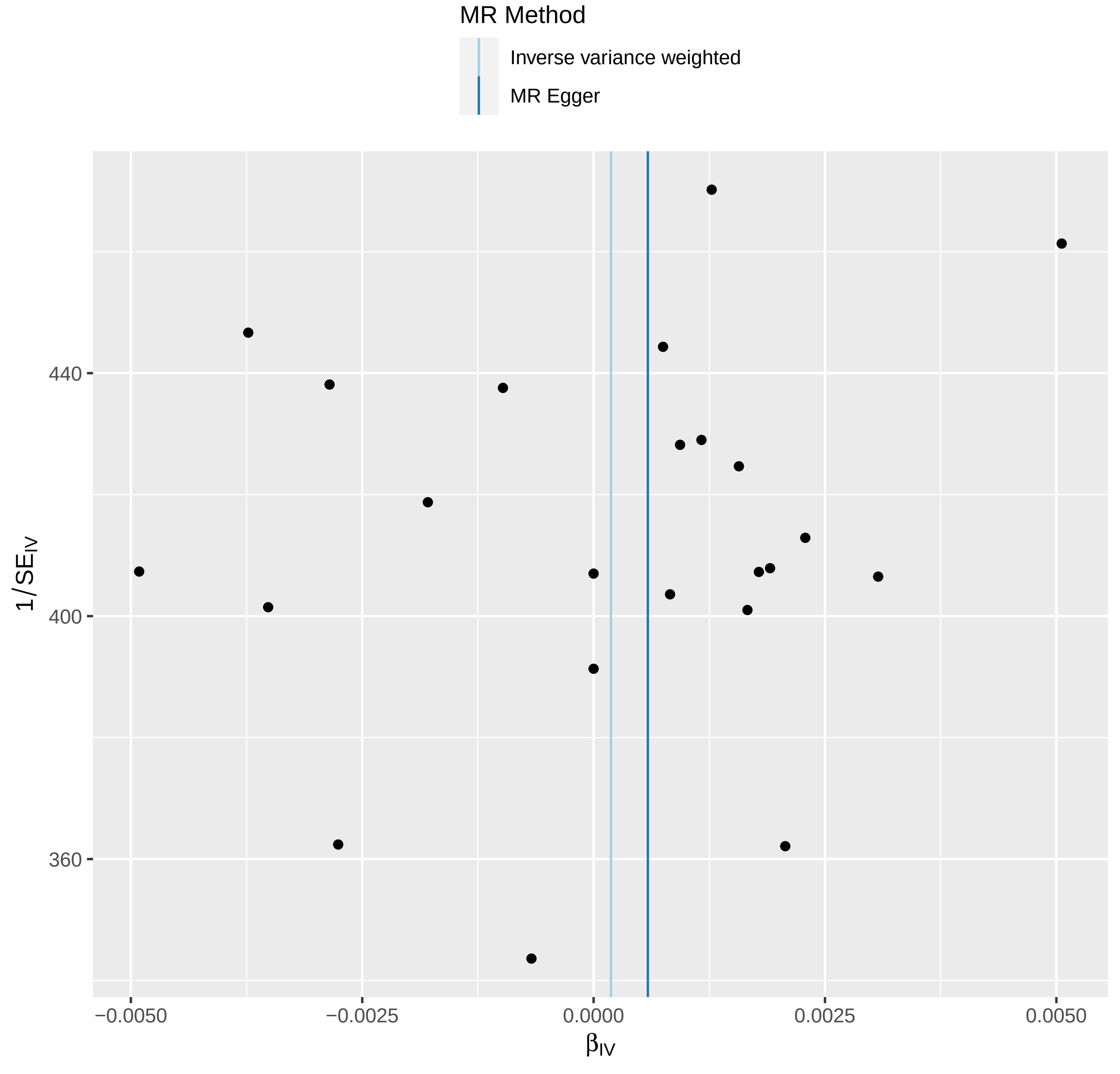

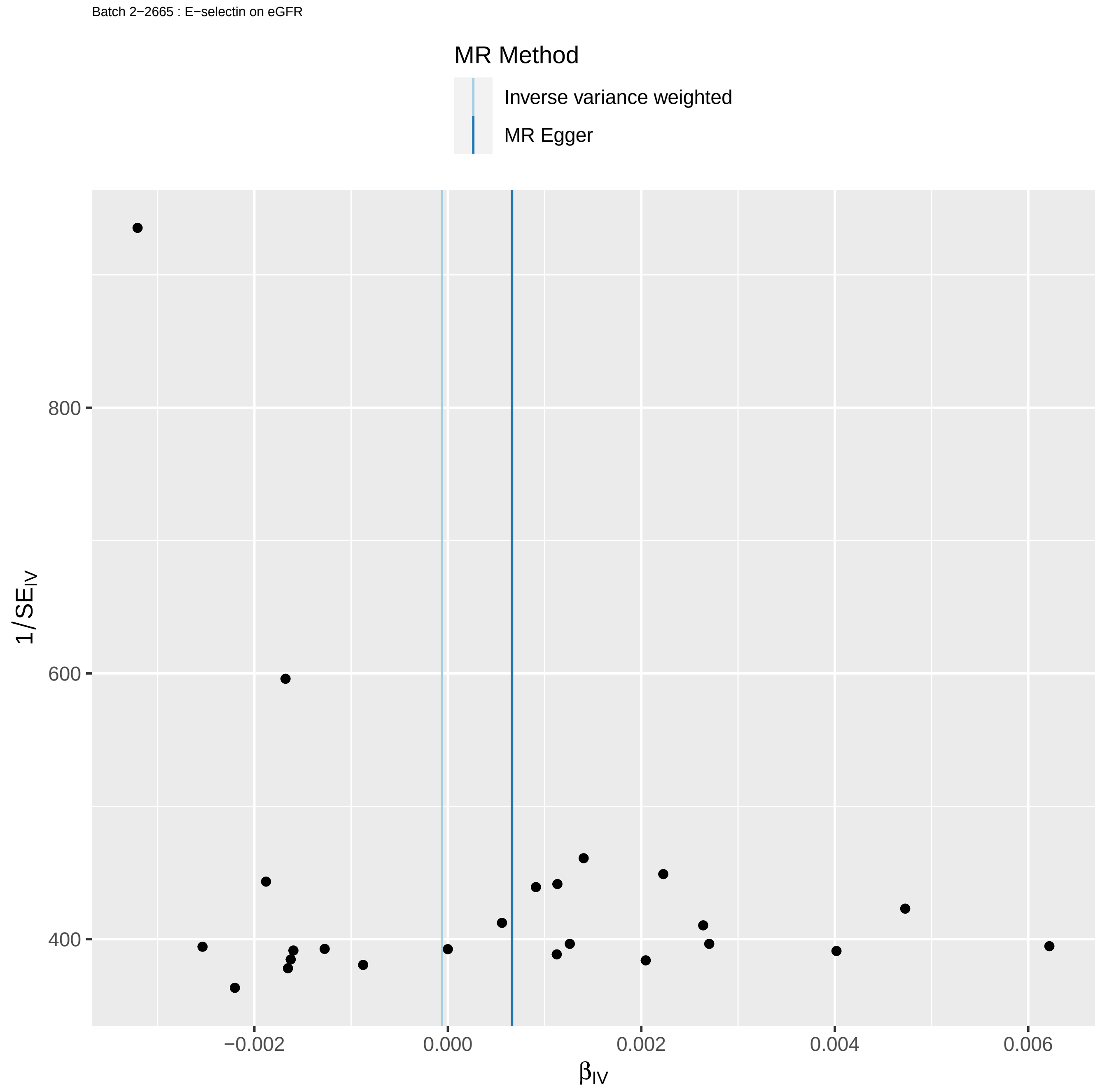
Batch 2−2665 : E−selectin on eGFR
MR Method
Inverse variance weighted
MR Egger
800
600
400
1/SE$_{IV}$
−0.002
0.000
0.002
0.004
0.006
β$_{IV}$

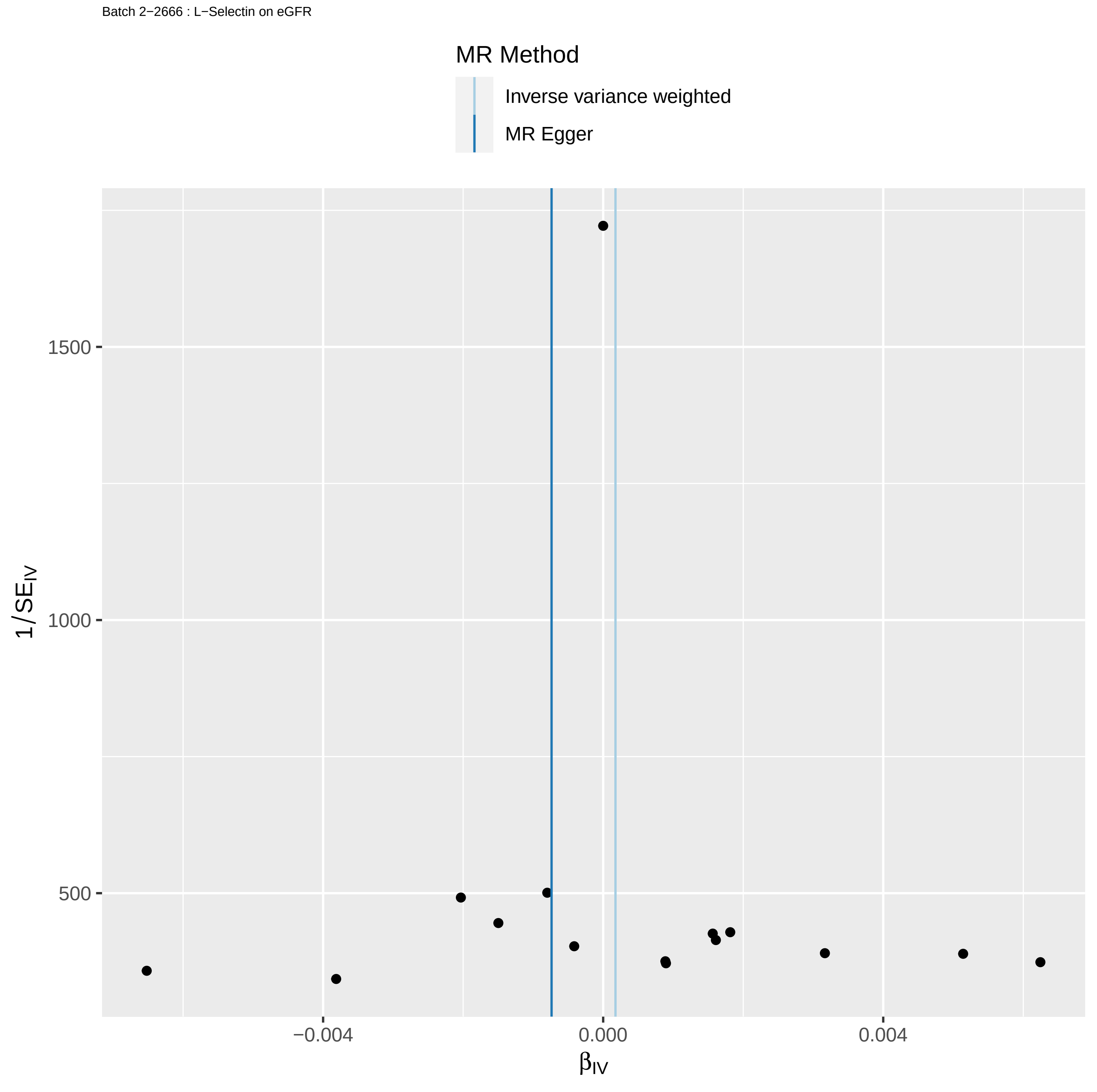

Batch 2−2666 : L−Selectin on eGFR
MR Method
Inverse variance weighted
MR Egger
1500
1000
500
1/SE$_{IV}$
−0.004
0.000
0.004
β$_{IV}$

Batch 2−2667 : P−selectin on eGFR

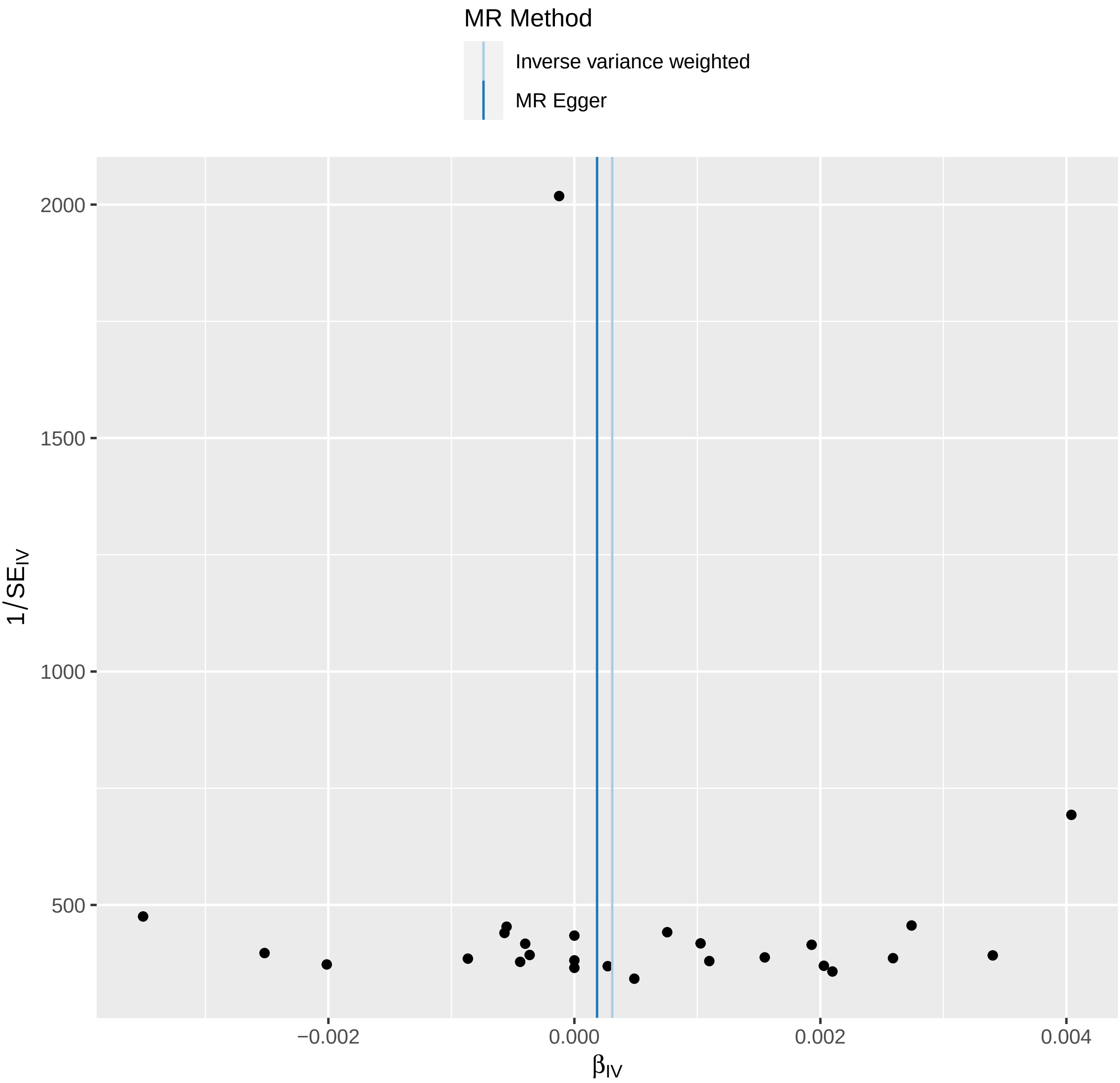

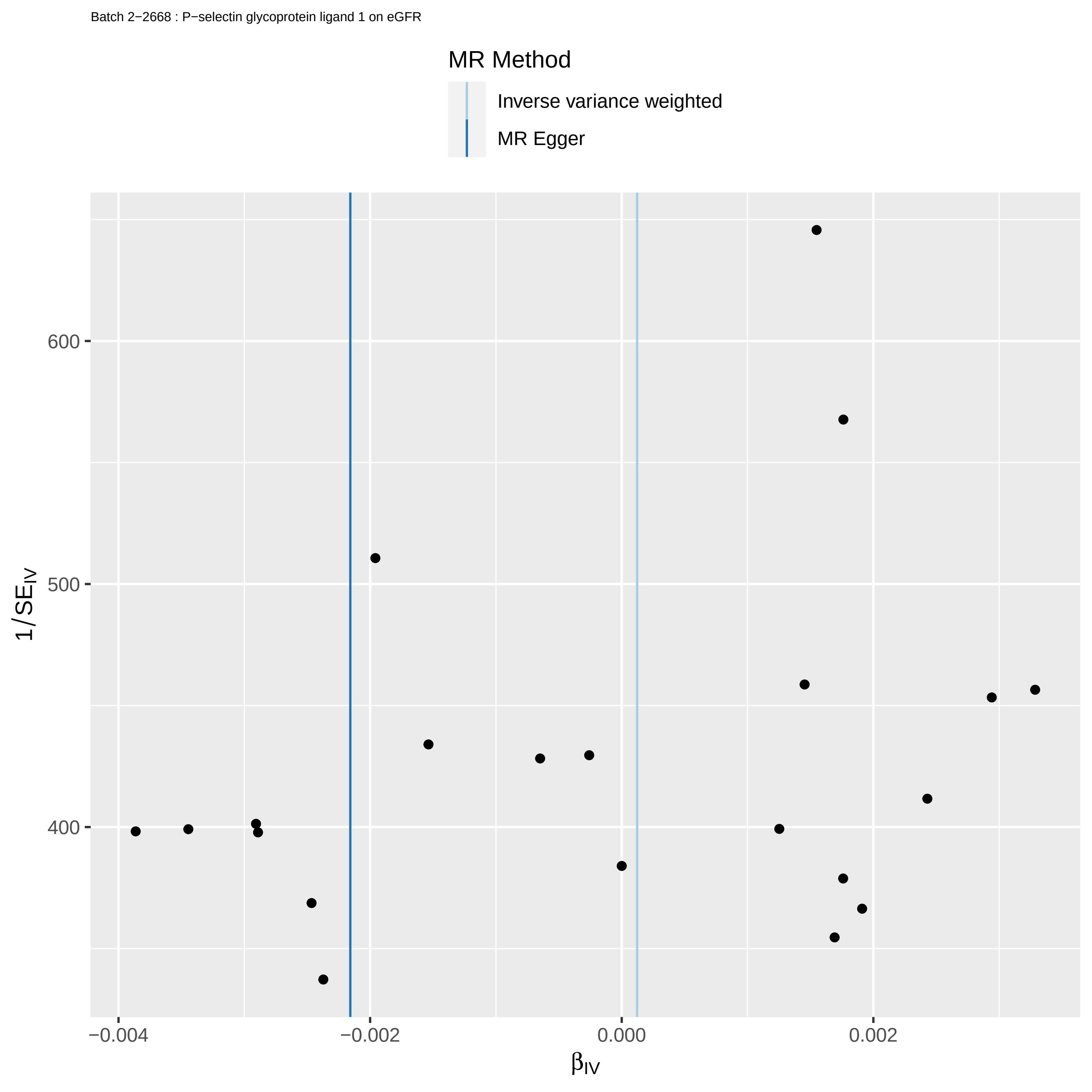

Batch 2−2668 : P−selectin glycoprotein ligand 1 on eGFR
MR Method
Inverse variance weighted
MR Egger
600
500
400
1/SE$_{IV}$
−0.004
−0.002
0.000
0.002
β$_{IV}$

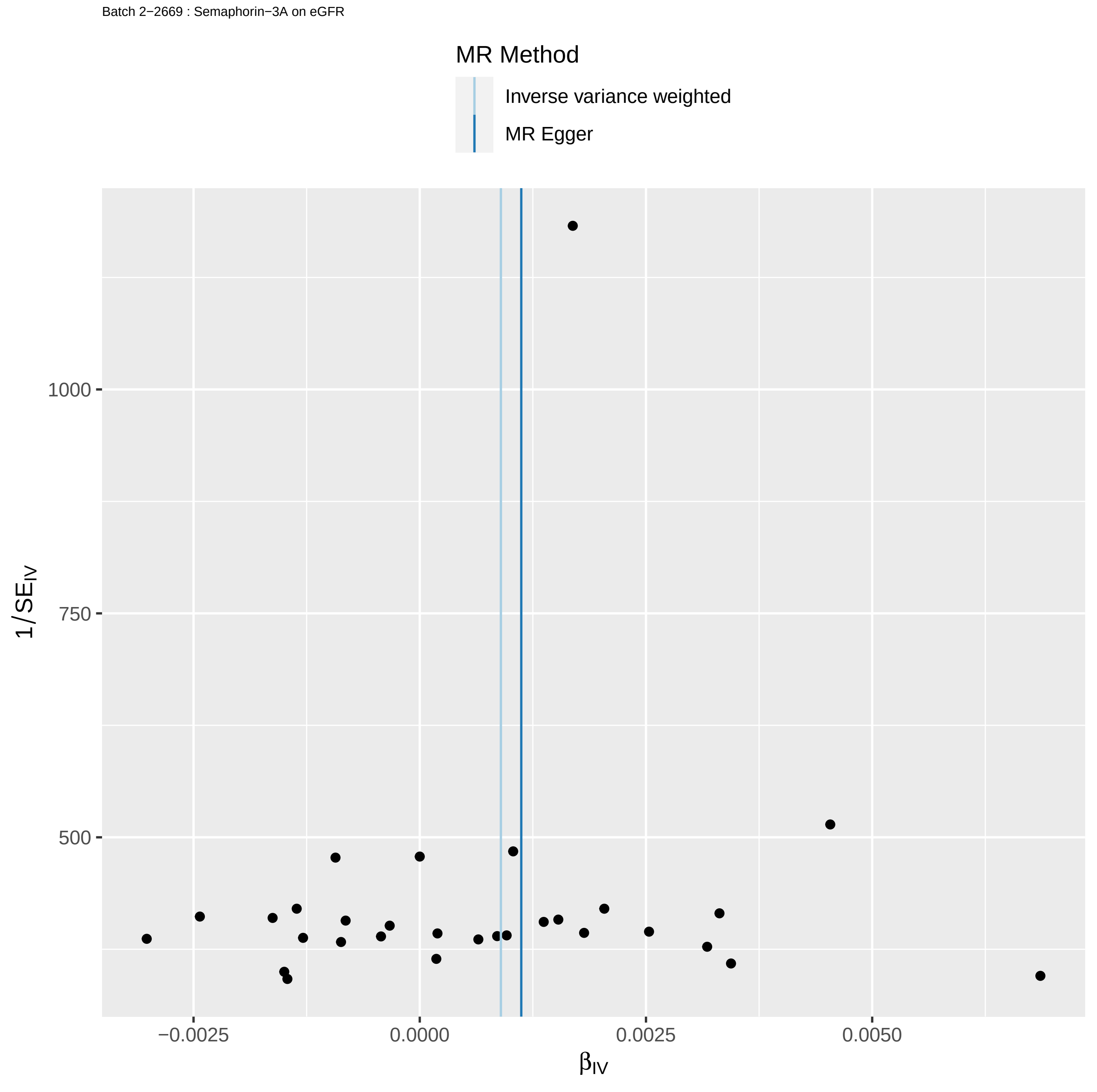

Batch 2−2669 : Semaphorin−3A on eGFR
MR Method
Inverse variance weighted
MR Egger
1000
750
500
1/SE_IV
−0.0025
0.0000
0.0025
0.0050
β_IV

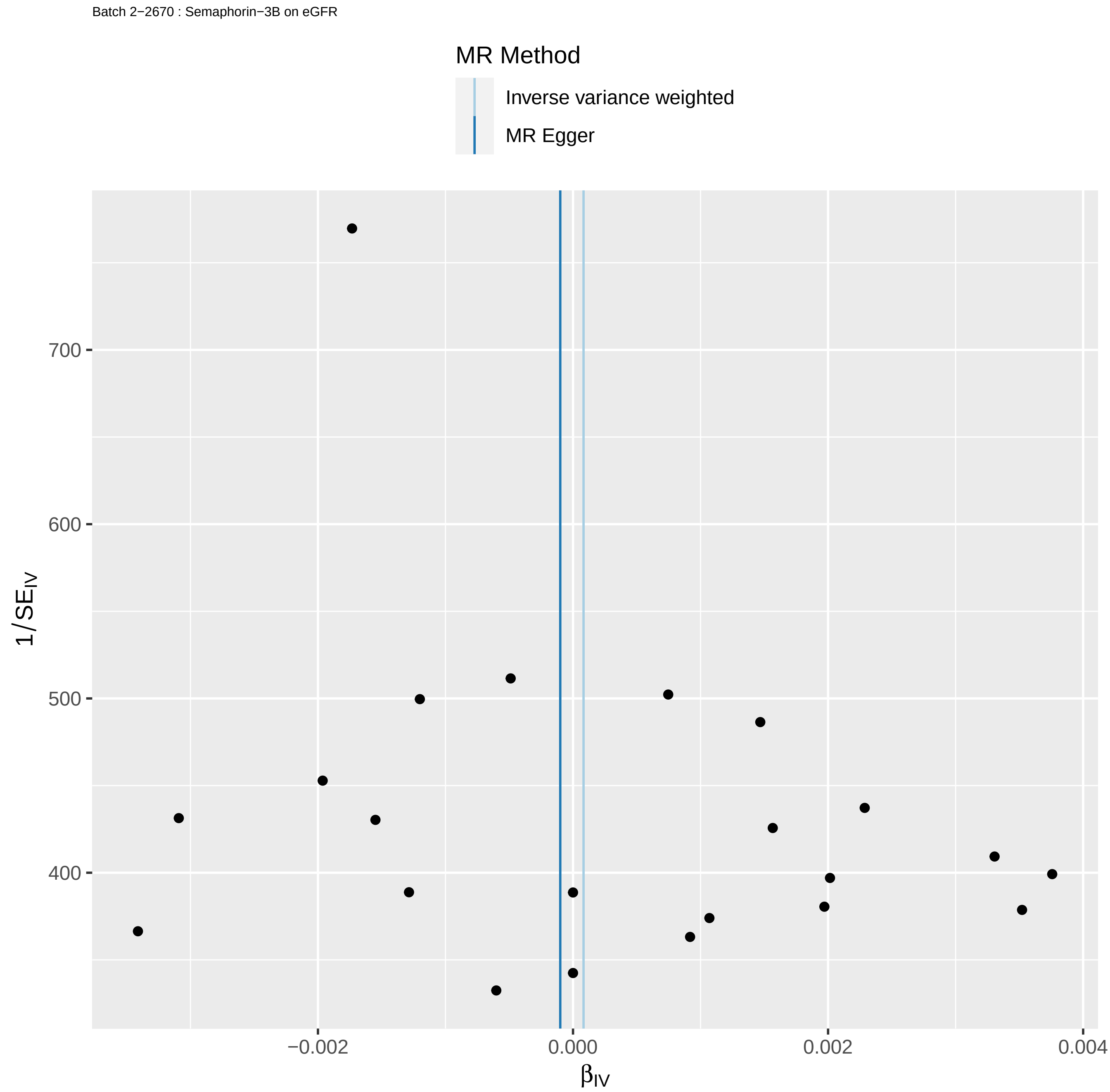

Batch 2−2670 : Semaphorin−3B on eGFR
MR Method
Inverse variance weighted
MR Egger
700
600
500
400
1/SE$_{IV}$
−0.002
0.000
0.002
0.004
β$_{IV}$

Batch 2−2671 : Semaphorin−3C on eGFR

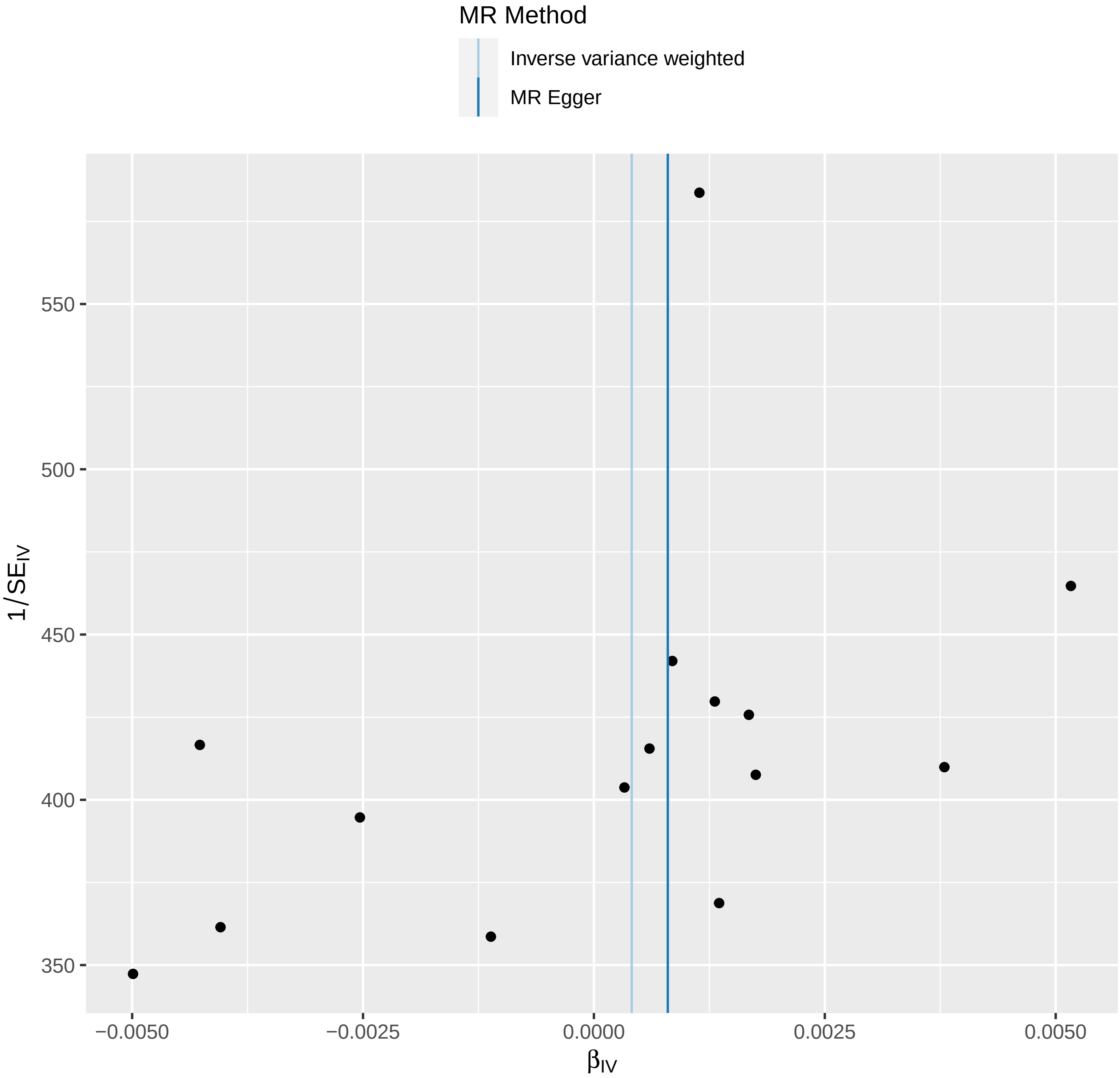

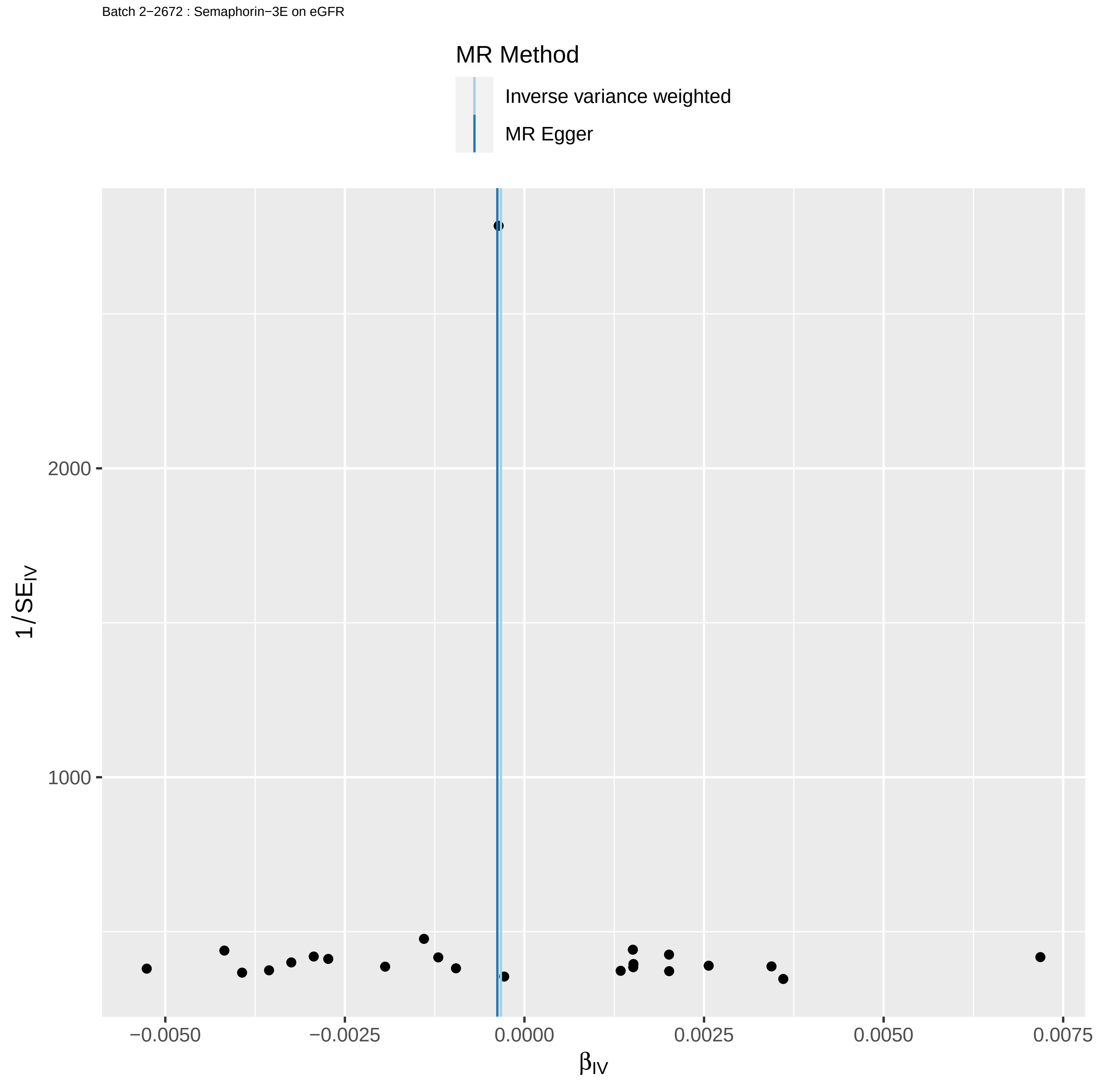

Batch 2−2672 : Semaphorin−3E on eGFR
MR Method
Inverse variance weighted
MR Egger
2000
1000
1/SE_IV
−0.0050
−0.0025
0.0000
0.0025
0.0050
0.0075
β_IV

Batch 2−2673 : Semaphorin−3G on eGFR

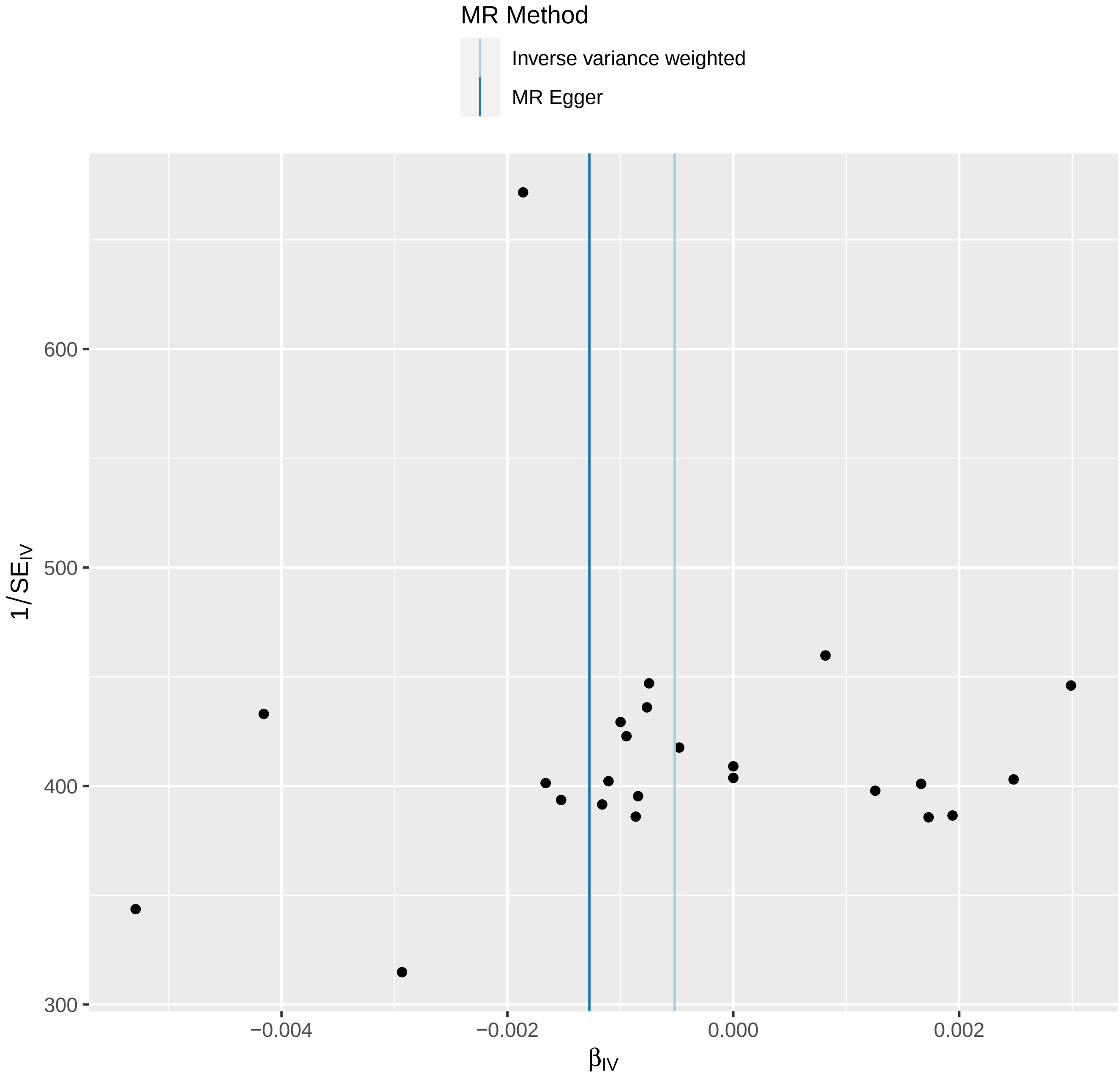

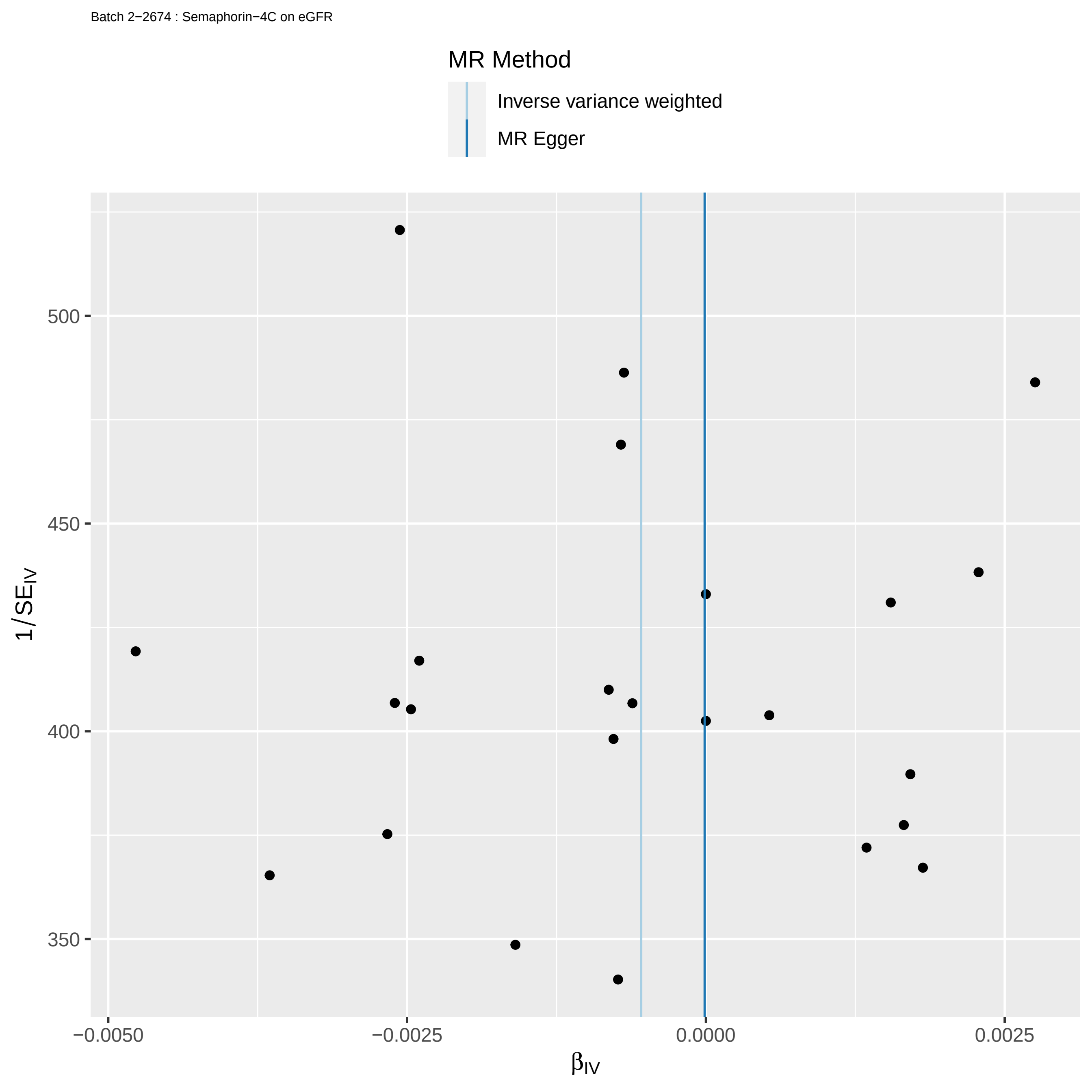
Batch 2−2674 : Semaphorin−4C on eGFR
MR Method
Inverse variance weighted
MR Egger
1/SE_IV
500
450
400
350
−0.0050
−0.0025
0.0000
0.0025
β_IV

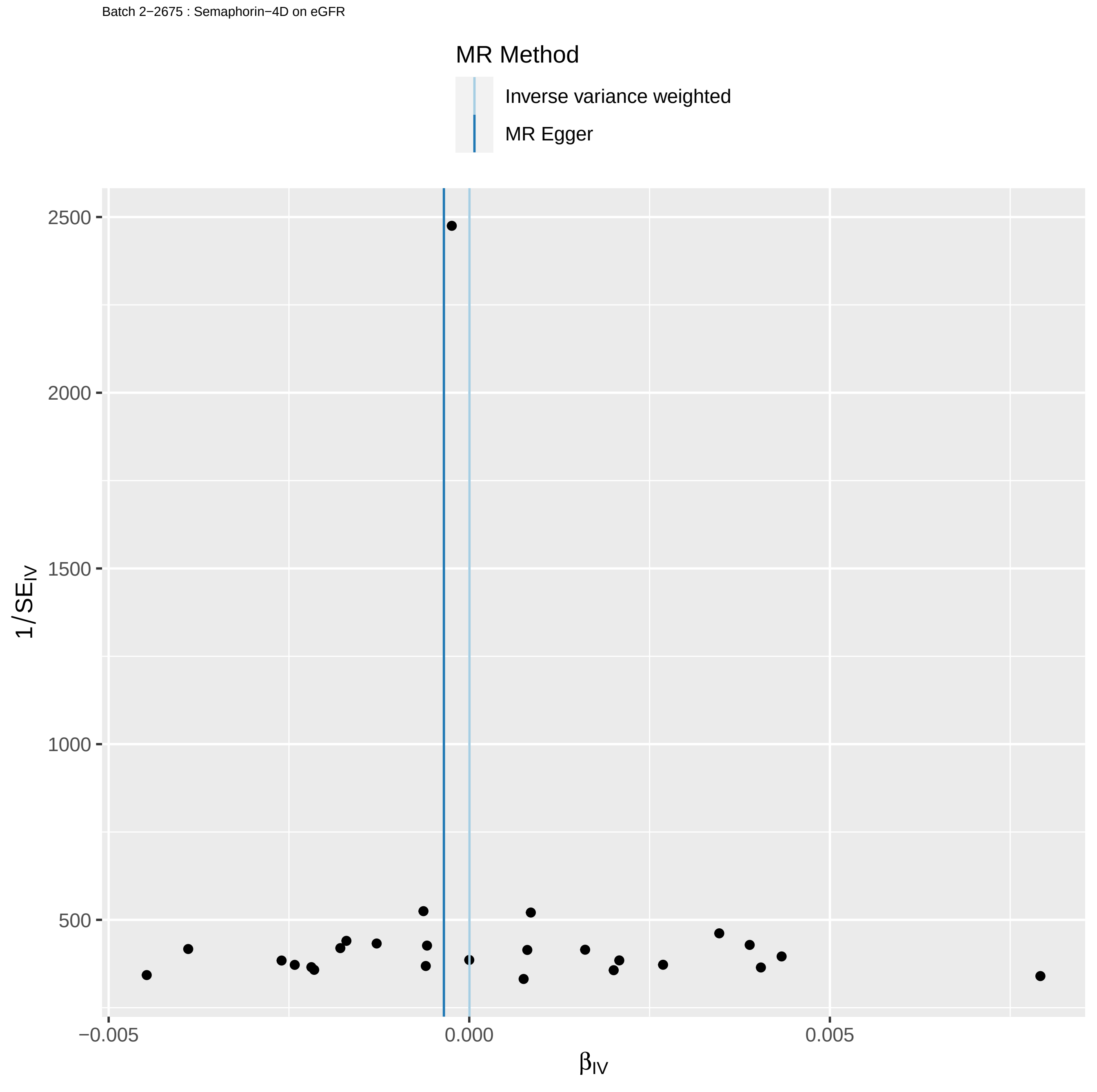

Batch 2−2675 : Semaphorin−4D on eGFR
MR Method
Inverse variance weighted
MR Egger
2500
2000
1500
1000
500
1/SE_IV
−0.005
0.000
0.005
β_IV

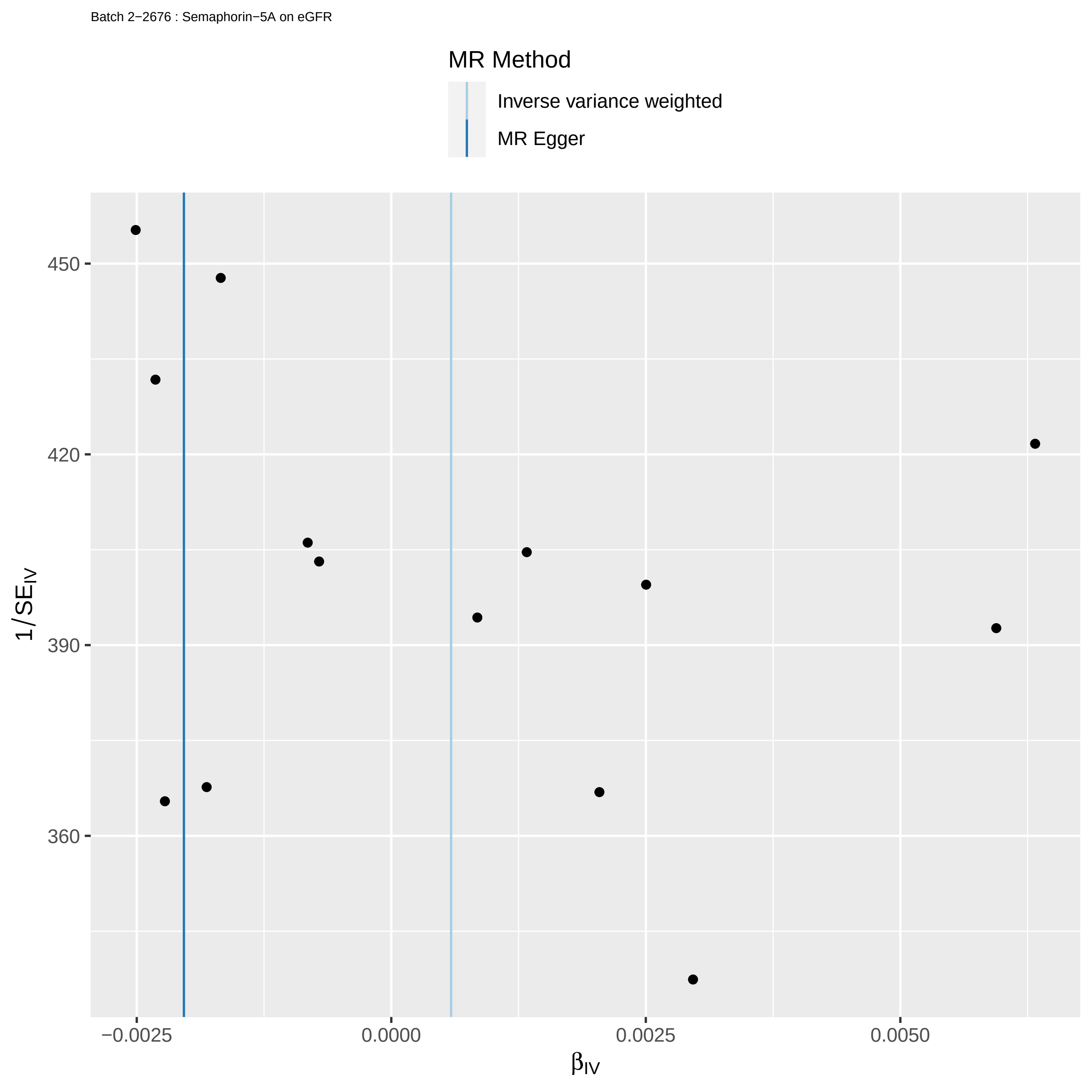
Batch 2−2676 : Semaphorin−5A on eGFR
MR Method
Inverse variance weighted
MR Egger
450
420
390
360
1/SE_IV
−0.0025
0.0000
0.0025
0.0050
β_IV

Batch 2−2677 : Semaphorin−6A on eGFR

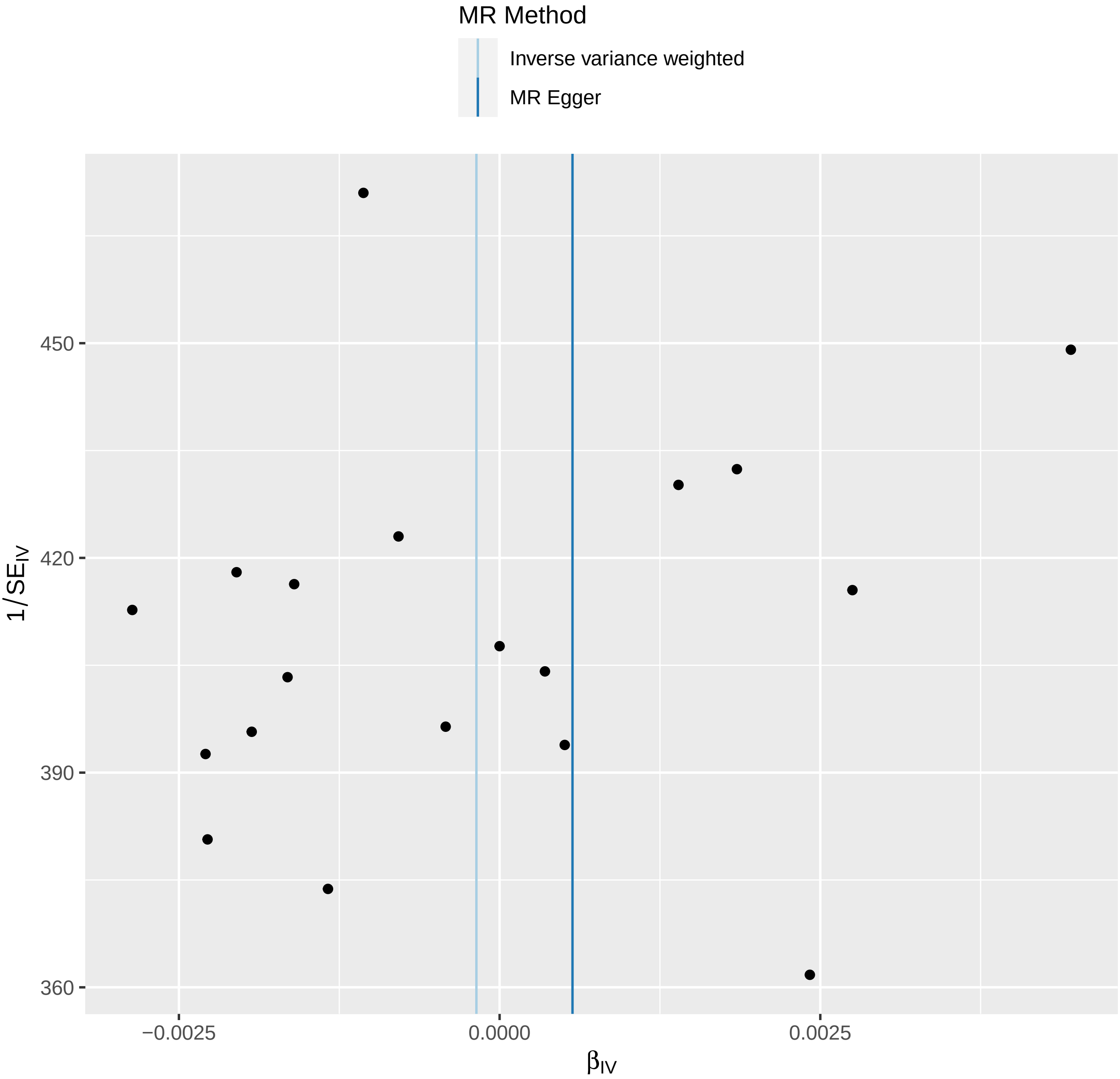

Batch 2−2678 : Semaphorin−6A on eGFR

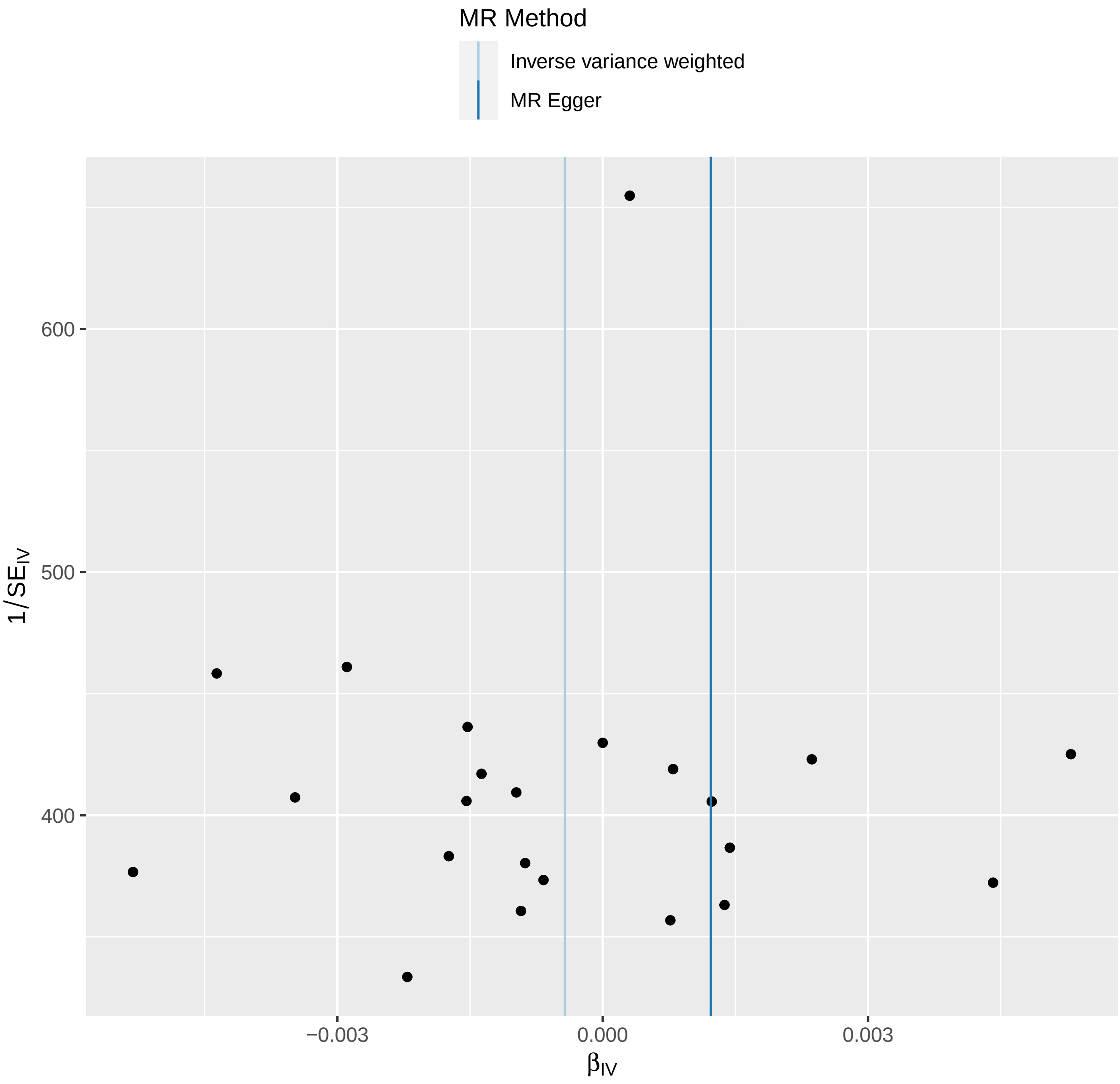

Batch 2−2679 : Semaphorin−6C on eGFR

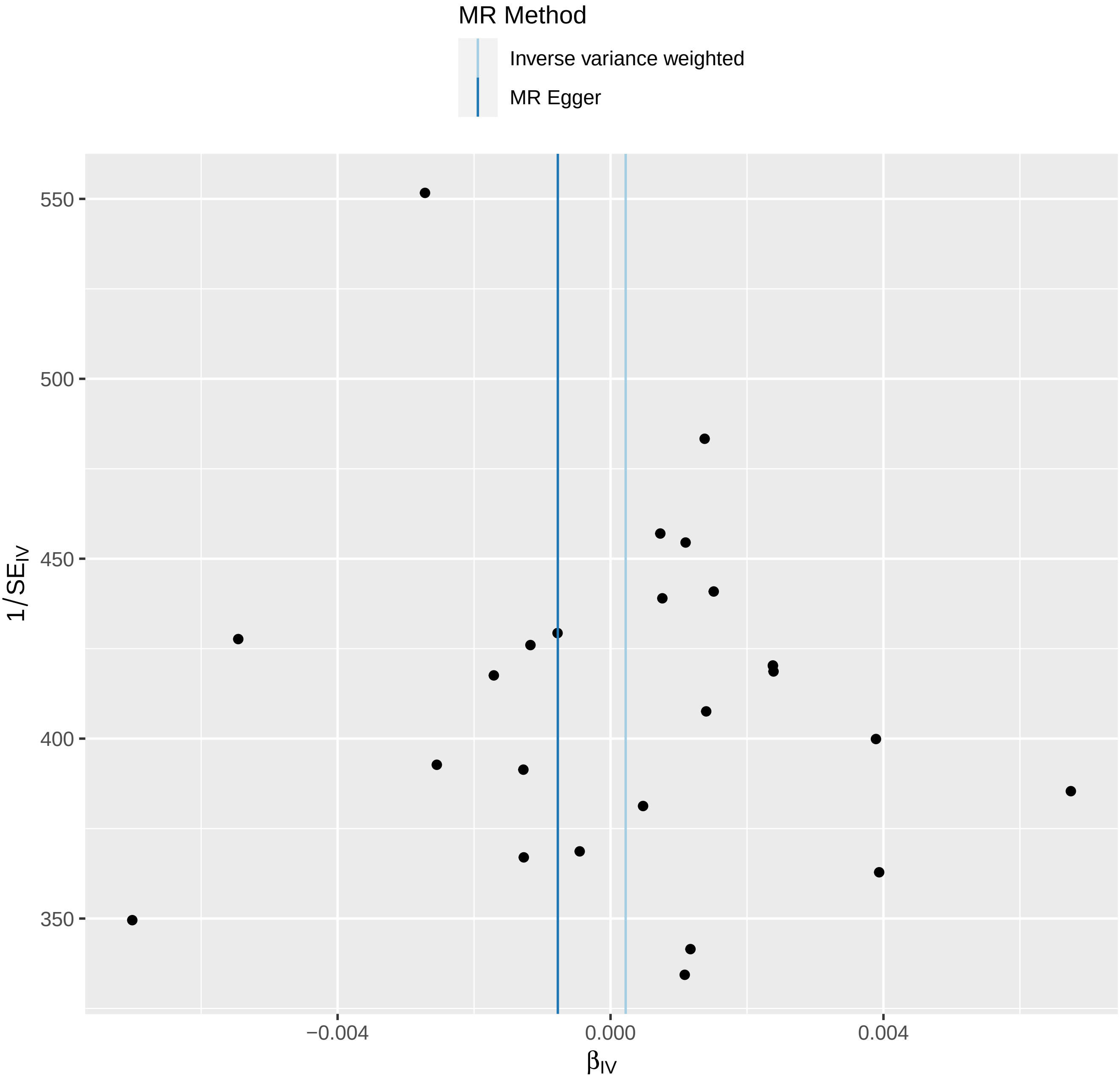

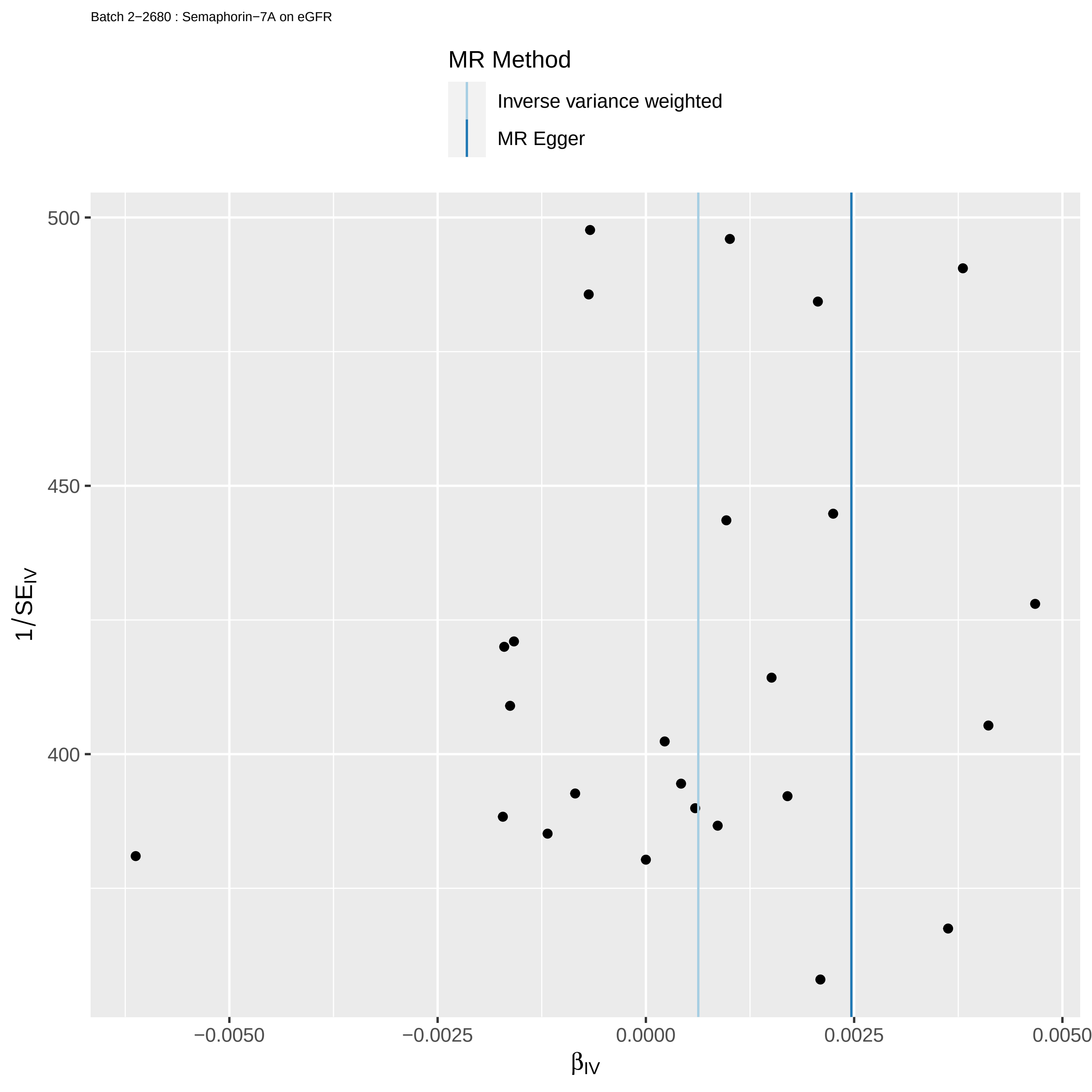
Batch 2−2680 : Semaphorin−7A on eGFR
MR Method
Inverse variance weighted
MR Egger
500
450
400
1/SE$_{IV}$
−0.0050
−0.0025
0.0000
0.0025
0.0050
β$_{IV}$

Batch 2−2681 : Semenogelin−1 on eGFR

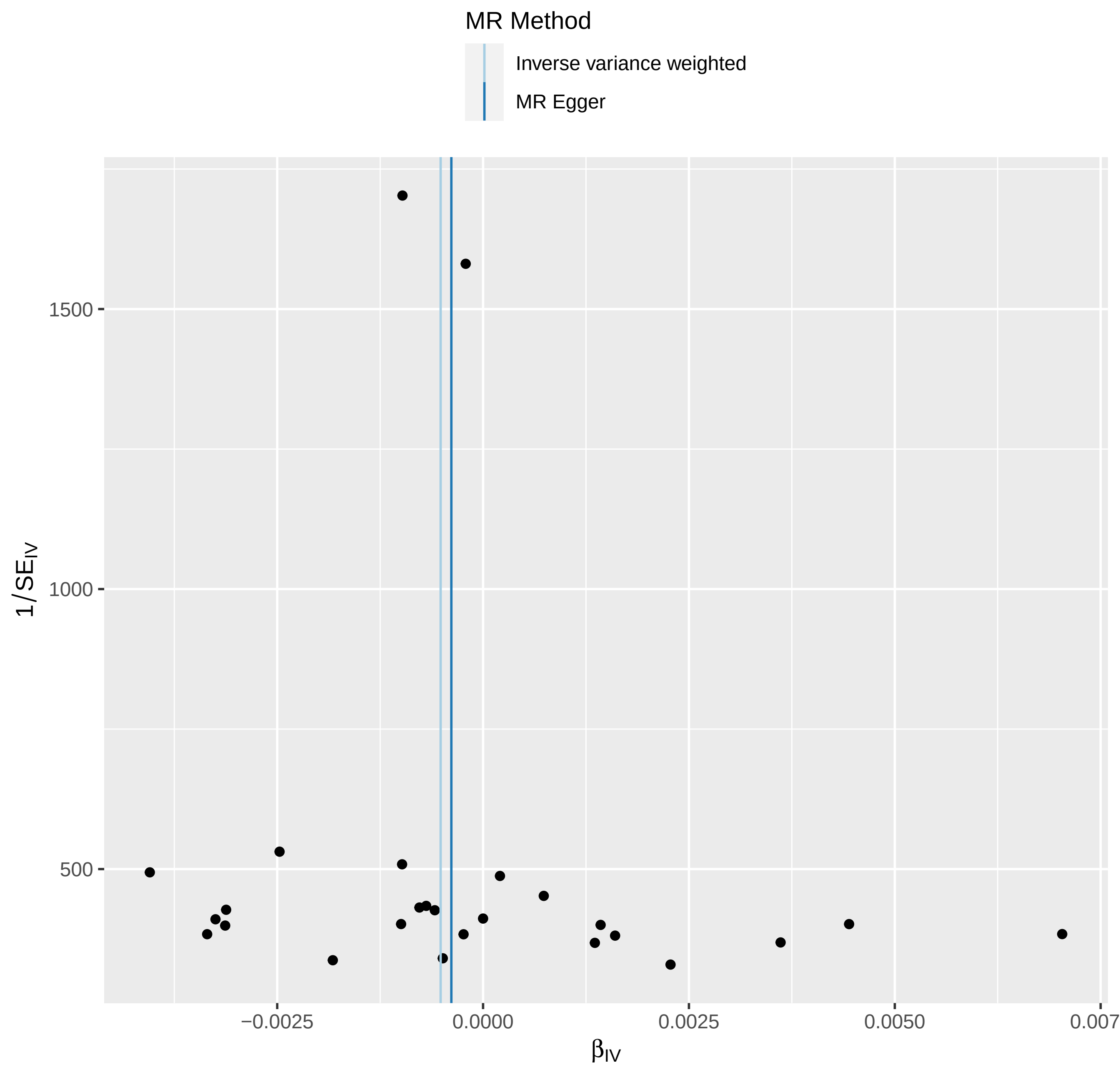

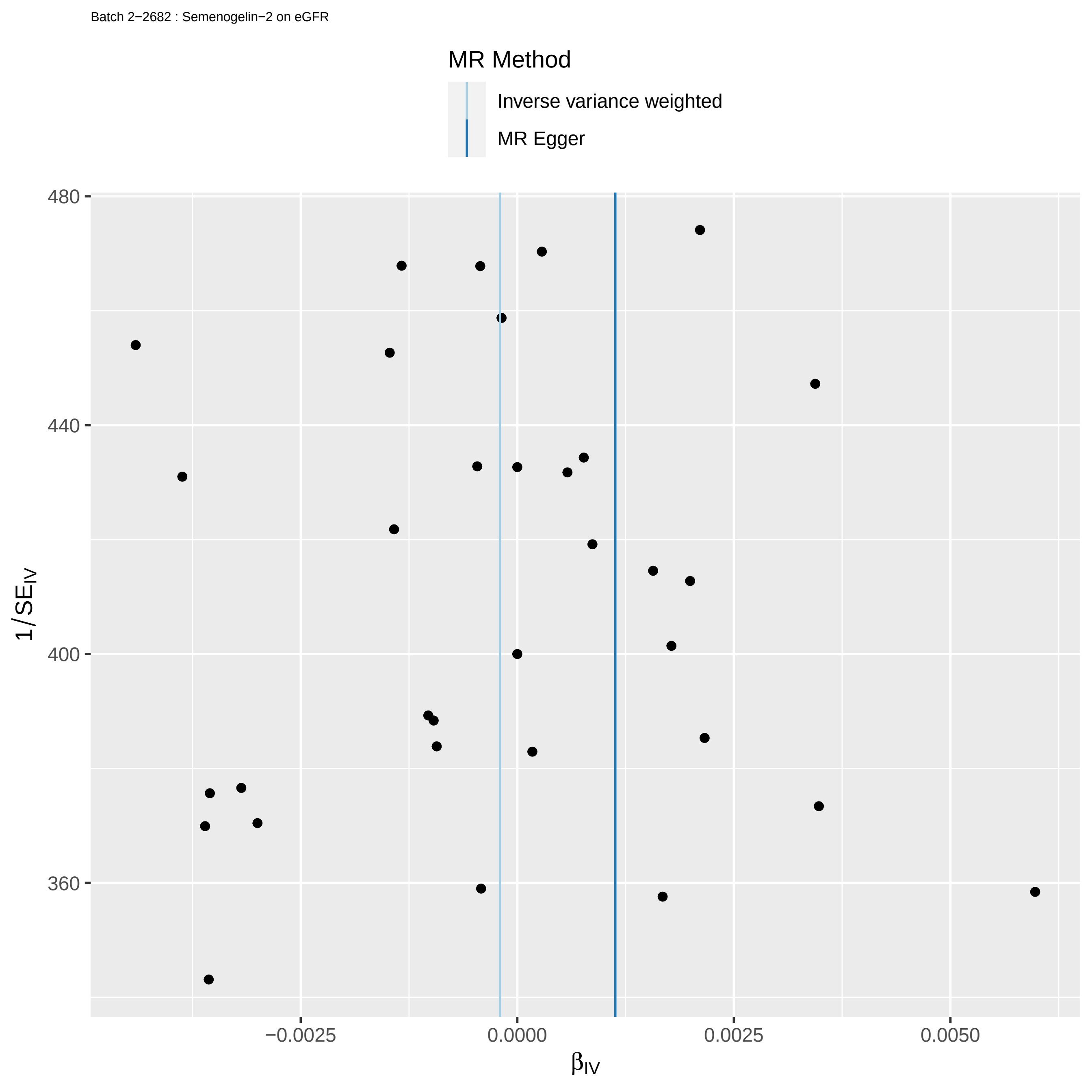
Batch 2−2682 : Semenogelin−2 on eGFR
MR Method
Inverse variance weighted
MR Egger
480
440
400
360
1/SE$_{IV}$
−0.0025
0.0000
0.0025
0.0050
β$_{IV}$

Batch 2−2683 : Sentrin−specific protease 7 on eGFR

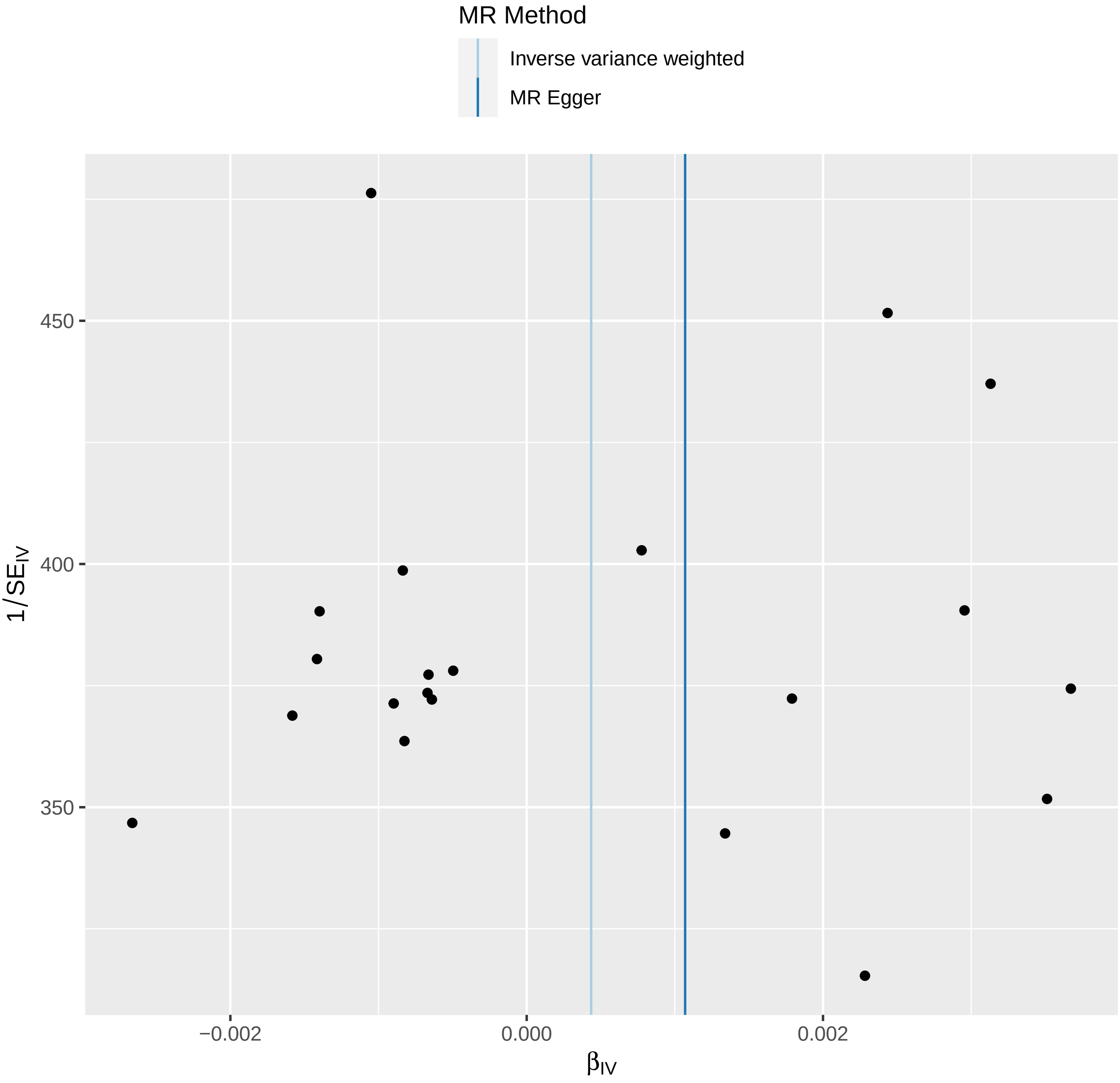

Batch 2−2684 : Selenide, water dikinase 1 on eGFR

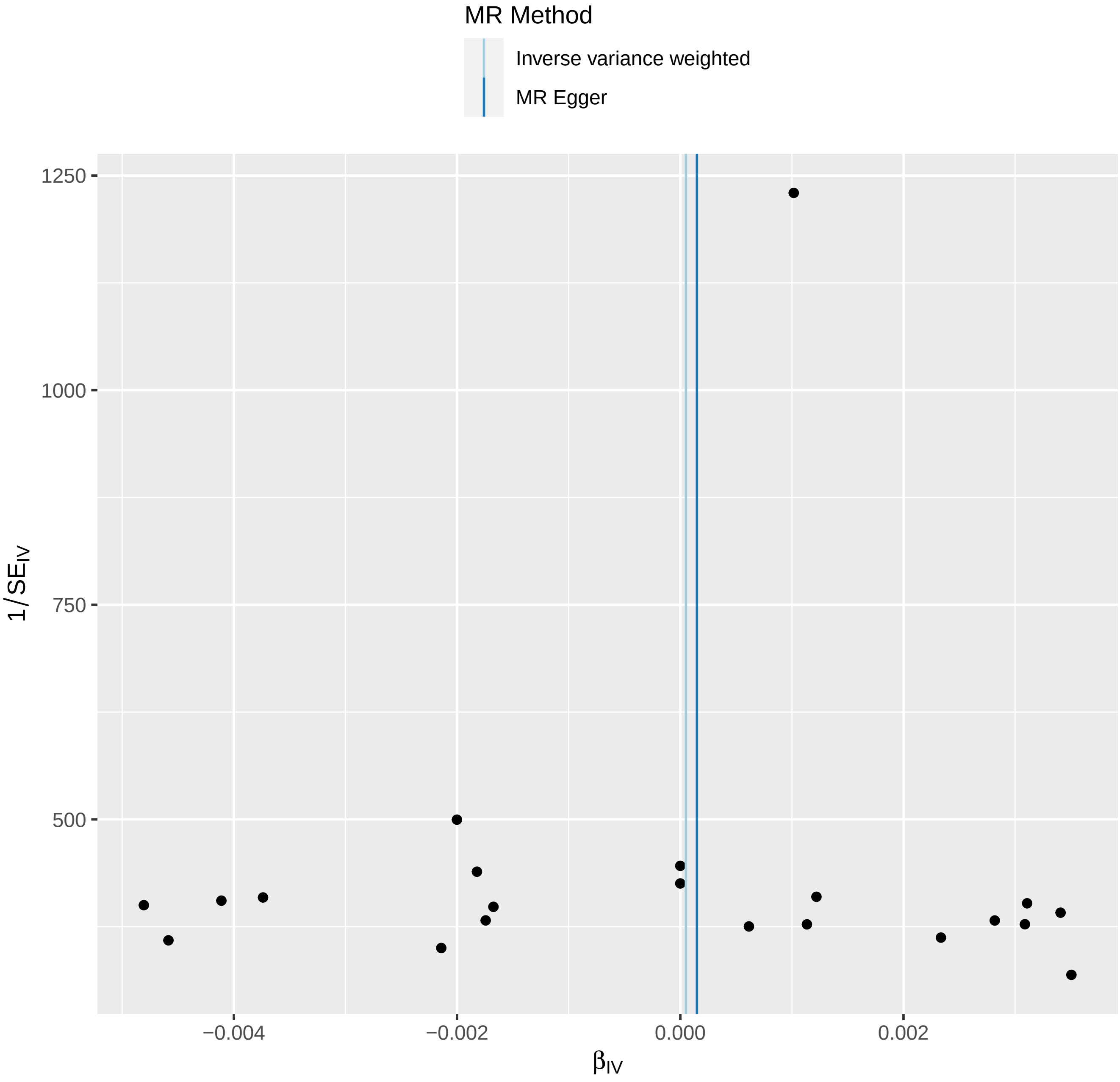

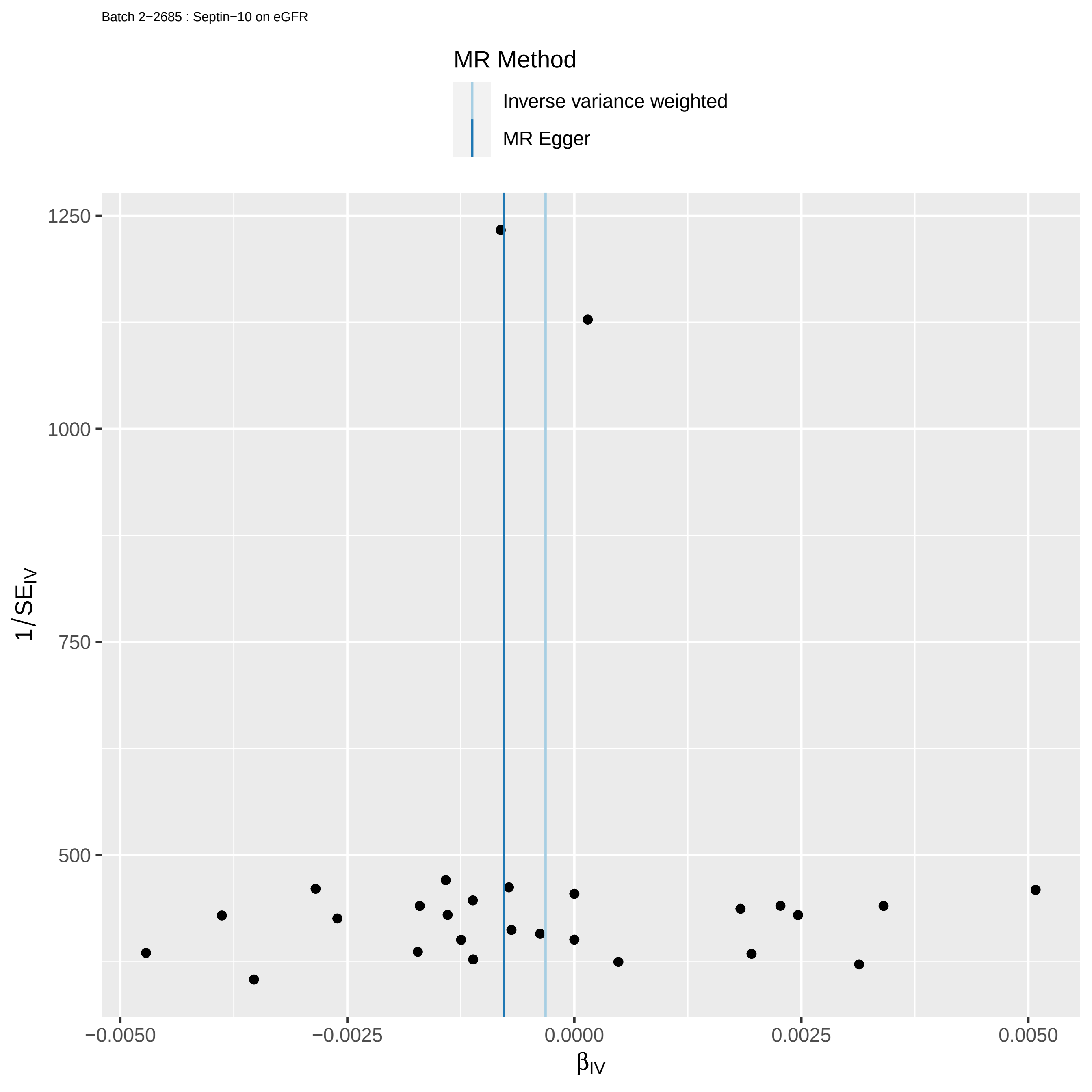

Batch 2−2685 : Septin−10 on eGFR
MR Method
Inverse variance weighted
MR Egger
1250
1000
750
500
1/SE_IV
−0.0050
−0.0025
0.0000
0.0025
0.0050
β_IV

Batch 2−2686 : Protein SERAC1 on eGFR

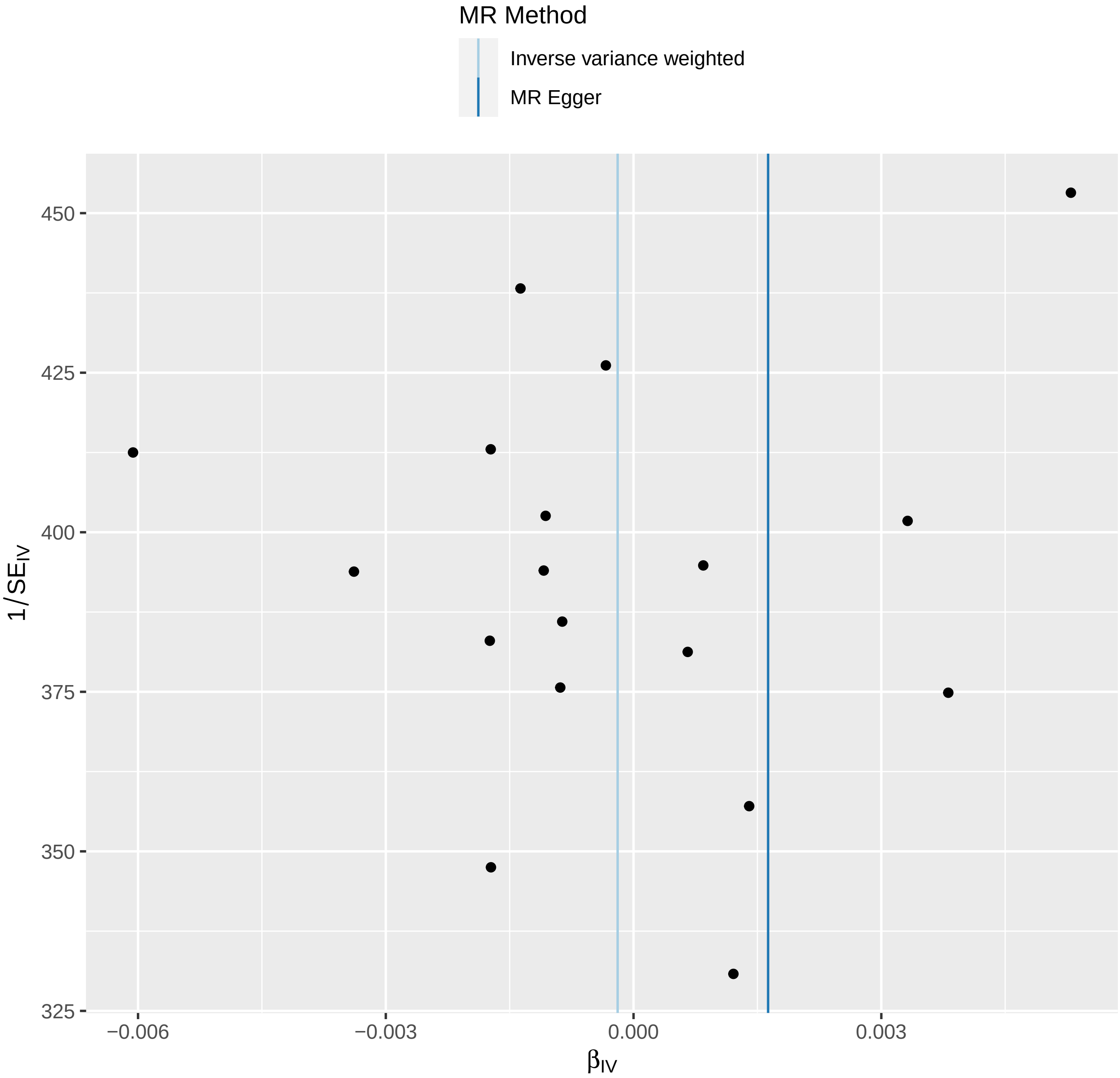

Batch 2−2687 : Small EDRK−rich factor 1 on eGFR

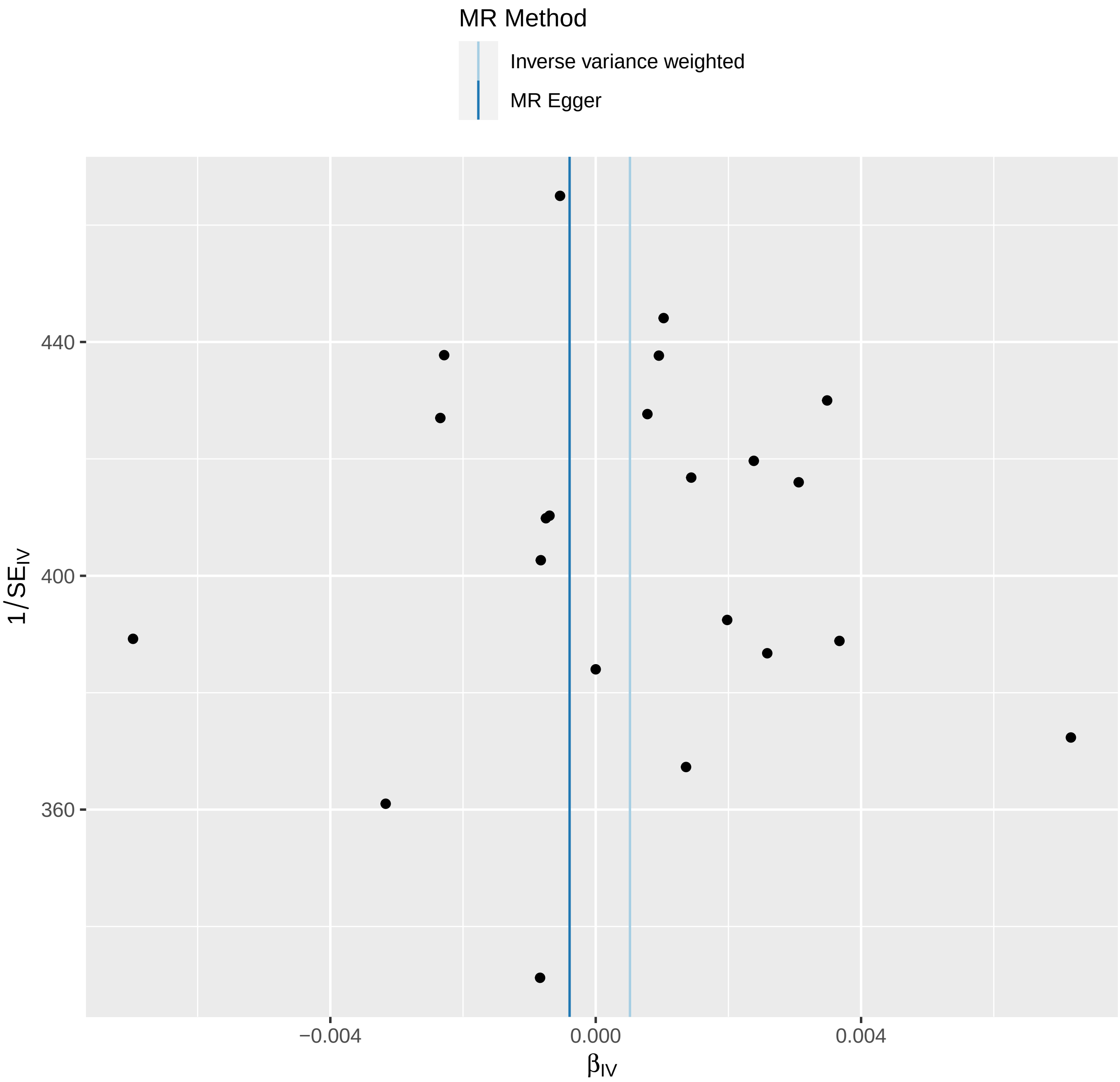

Batch 2−2688 : Protein Z−dependent protease inhibitor on eGFR

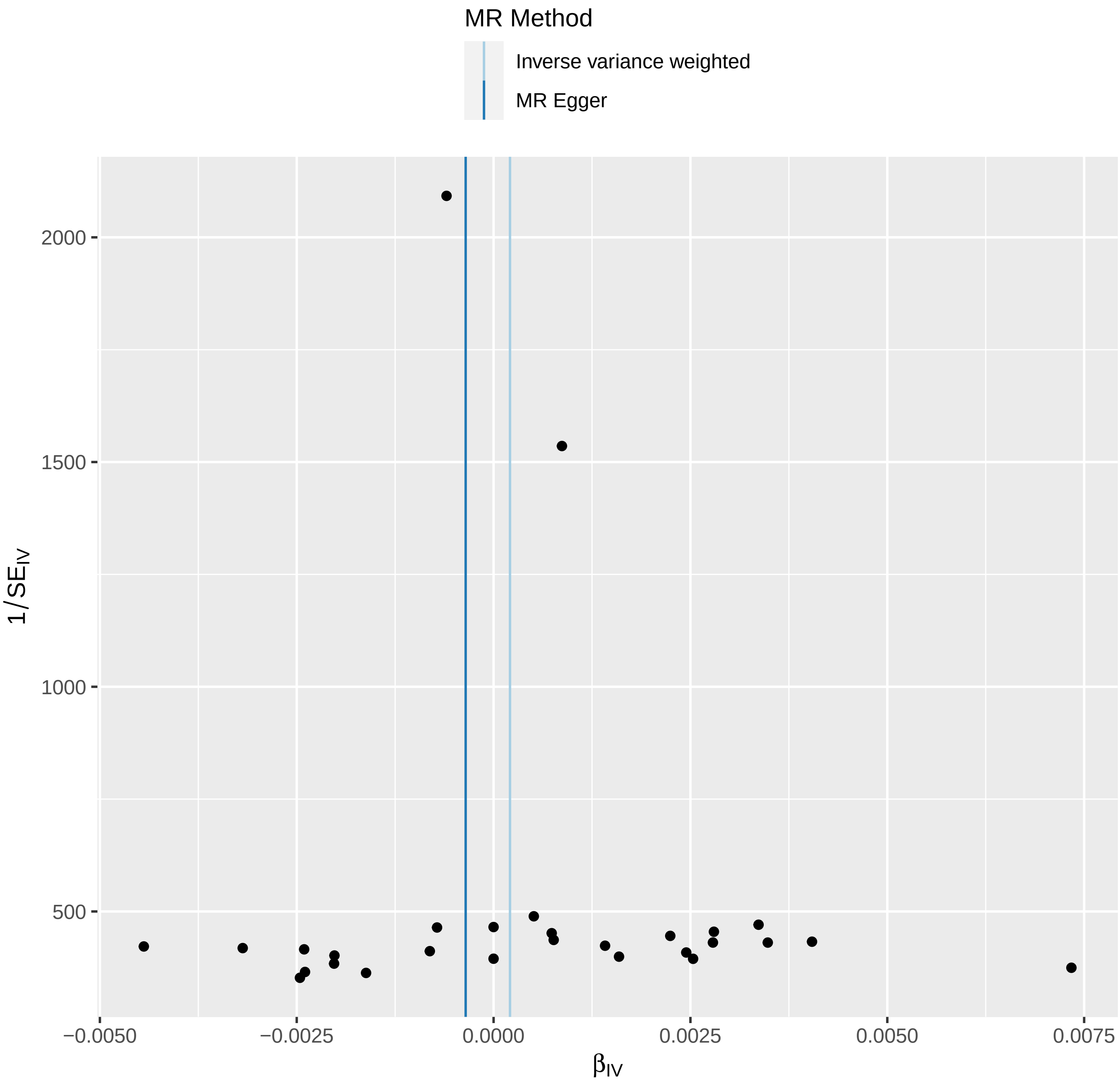

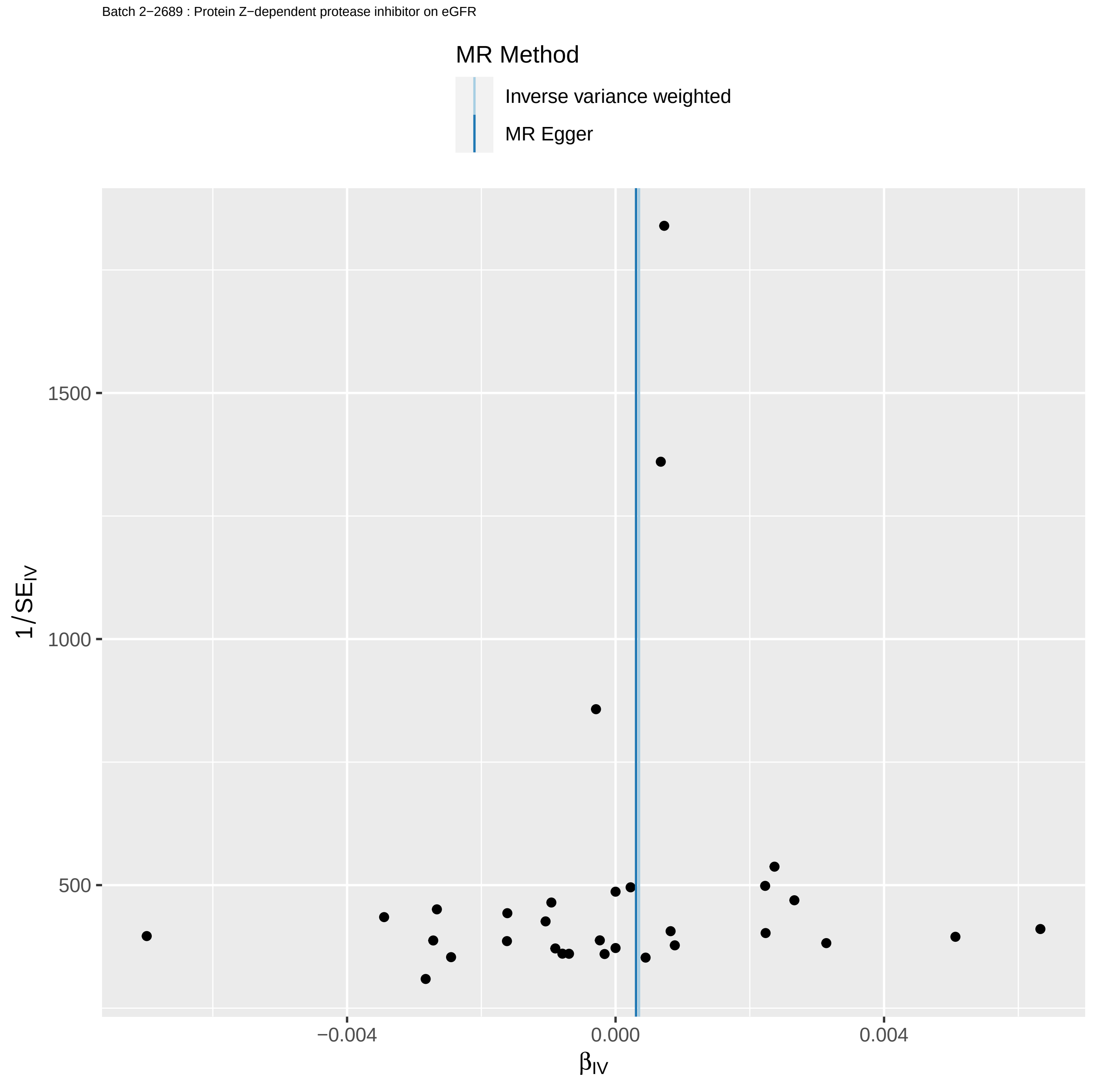

Batch 2−2689 : Protein Z−dependent protease inhibitor on eGFR
MR Method
Inverse variance weighted
MR Egger
1500
1000
500
1/SE$_{IV}$
−0.004
0.000
0.004
β$_{IV}$

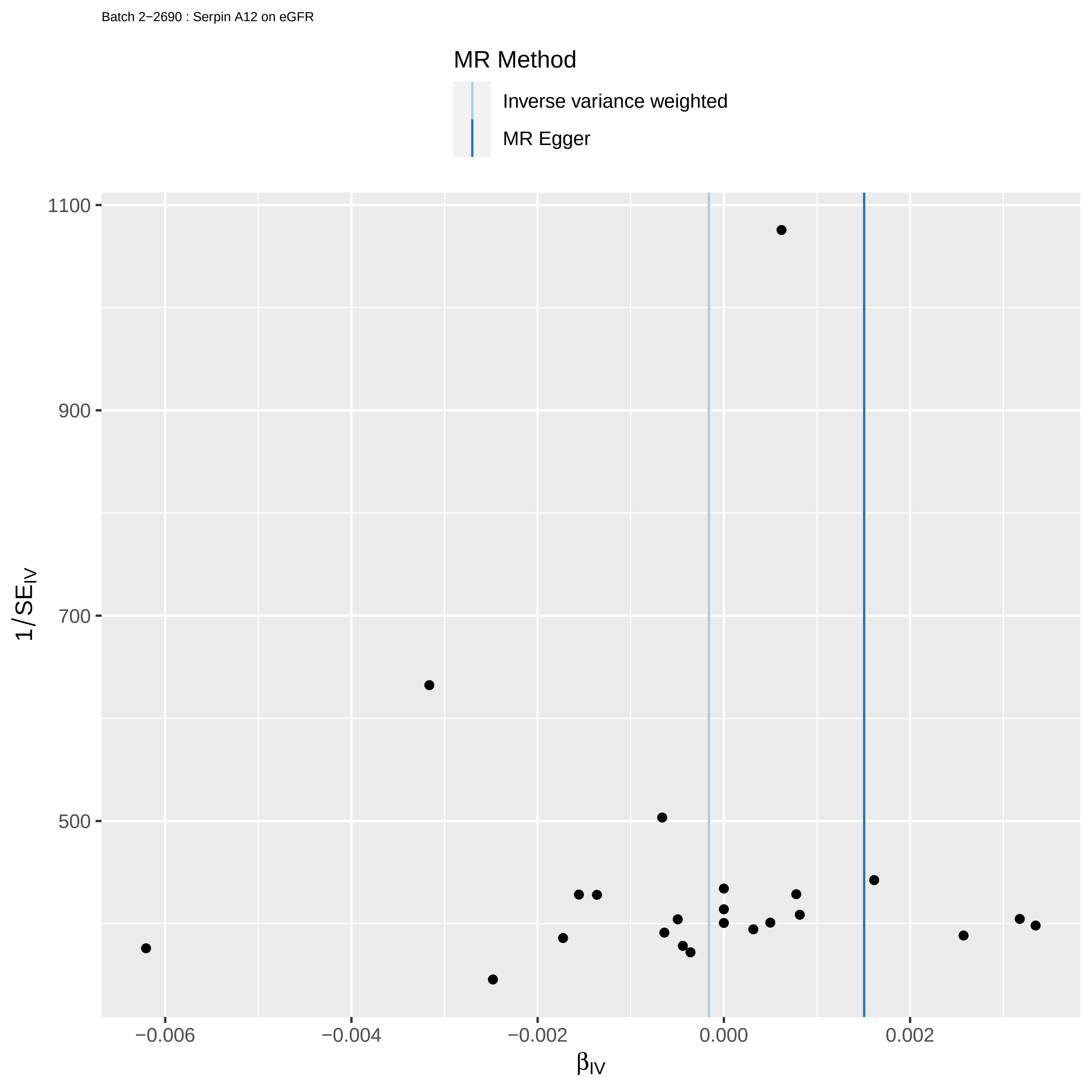
Batch 2−2690 : Serpin A12 on eGFR
MR Method
Inverse variance weighted
MR Egger
1100
900
700
500
1/SE$_{IV}$
−0.006
−0.004
−0.002
0.000
0.002
β$_{IV}$

Batch 2−2691 : Kallistatin on eGFR

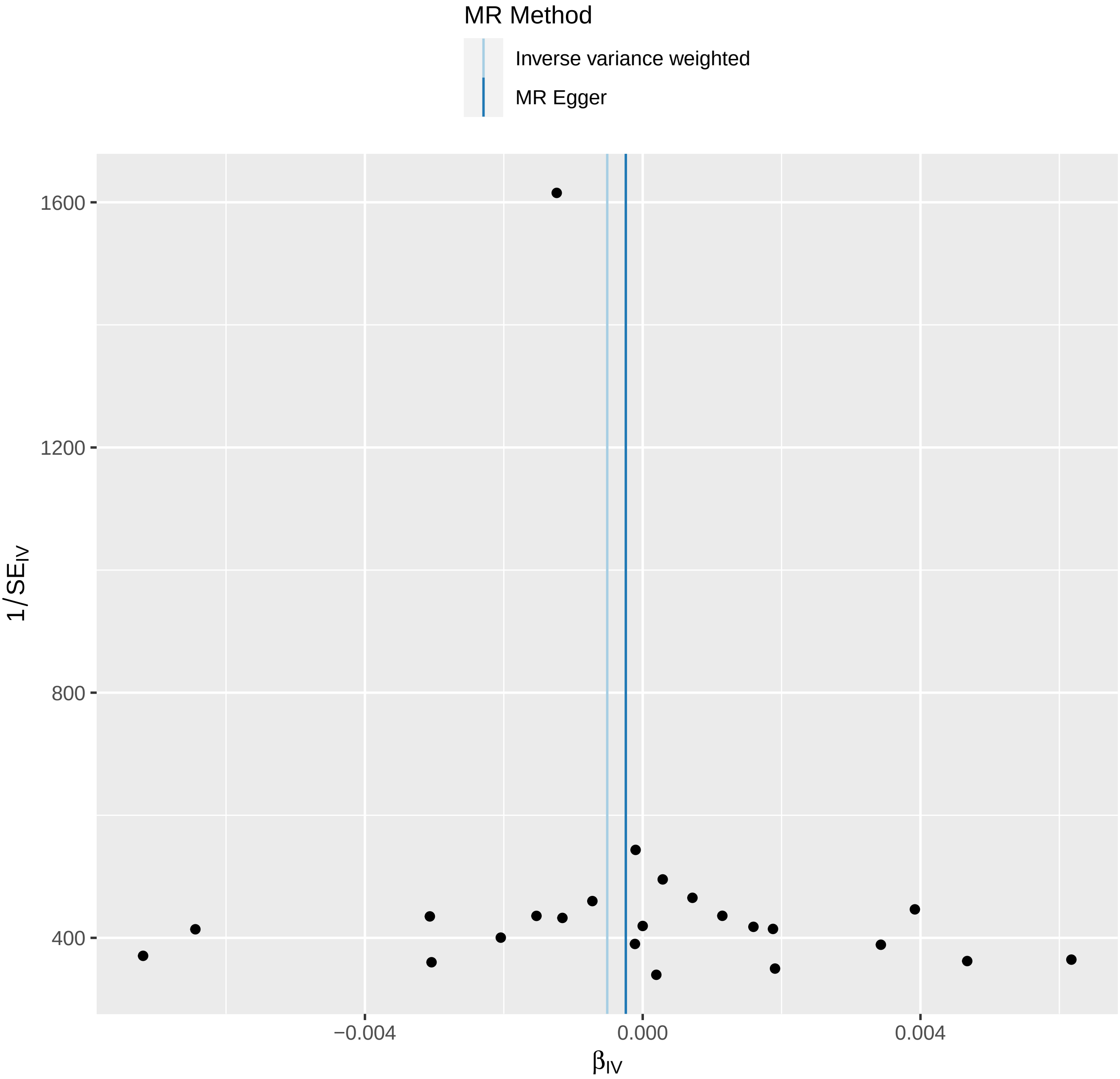

Batch 2−2692 : Kallistatin on eGFR

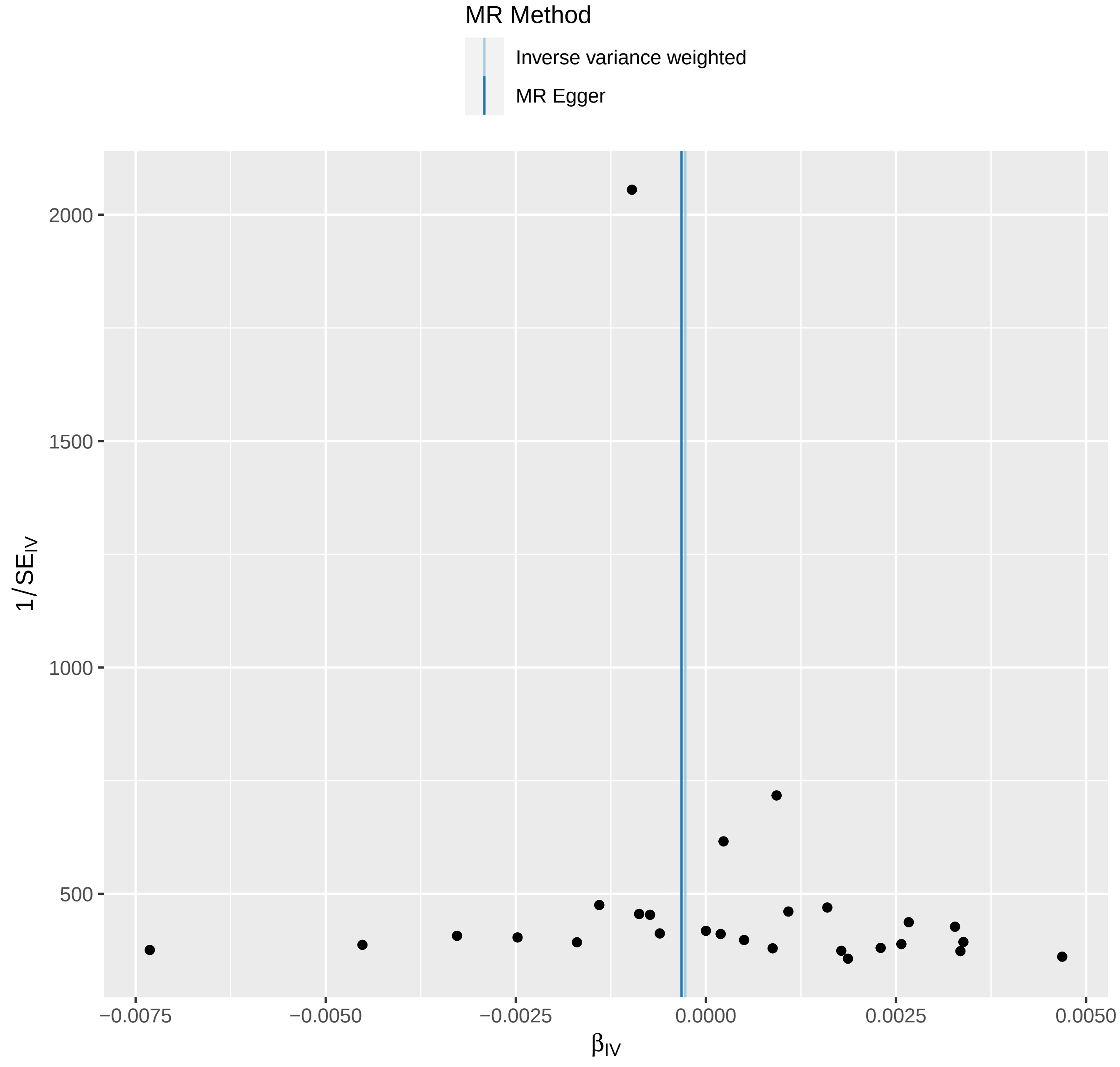

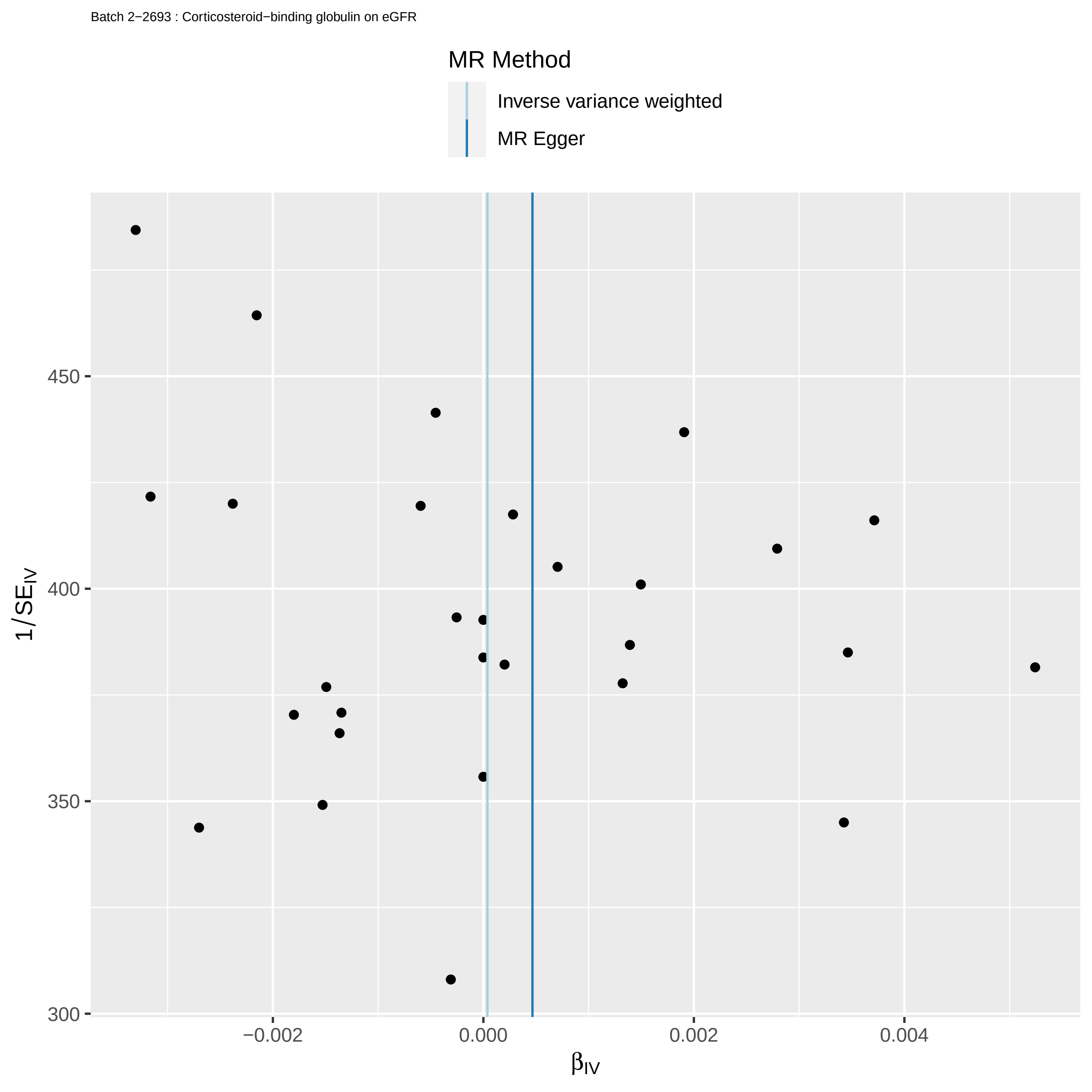

Batch 2−2693 : Corticosteroid−binding globulin on eGFR
MR Method
Inverse variance weighted
MR Egger
450
400
350
300
−0.002
0.000
0.002
0.004
1/SE_IV
β_IV

Batch 2−2694 : Serpin A9 on eGFR

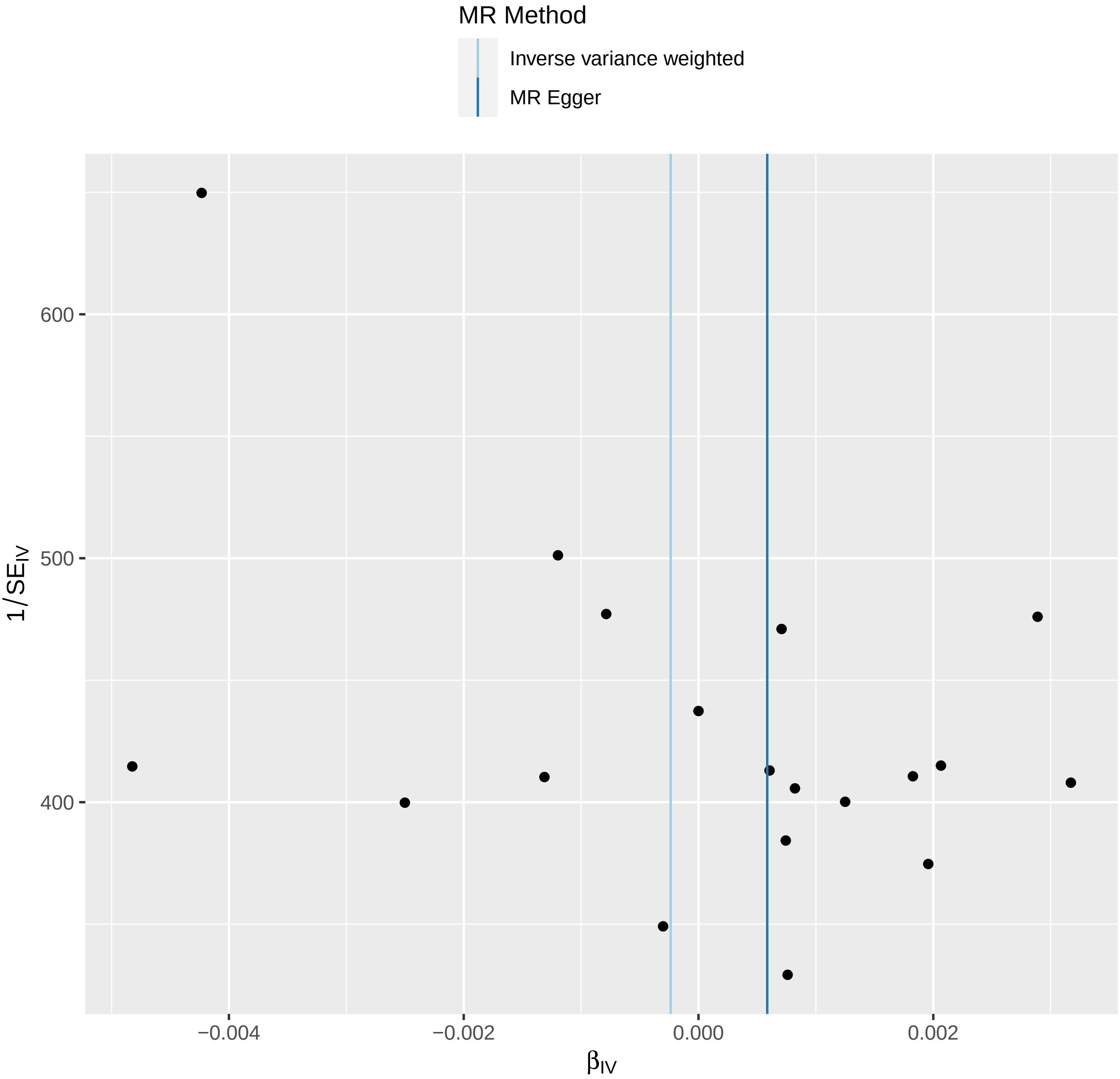

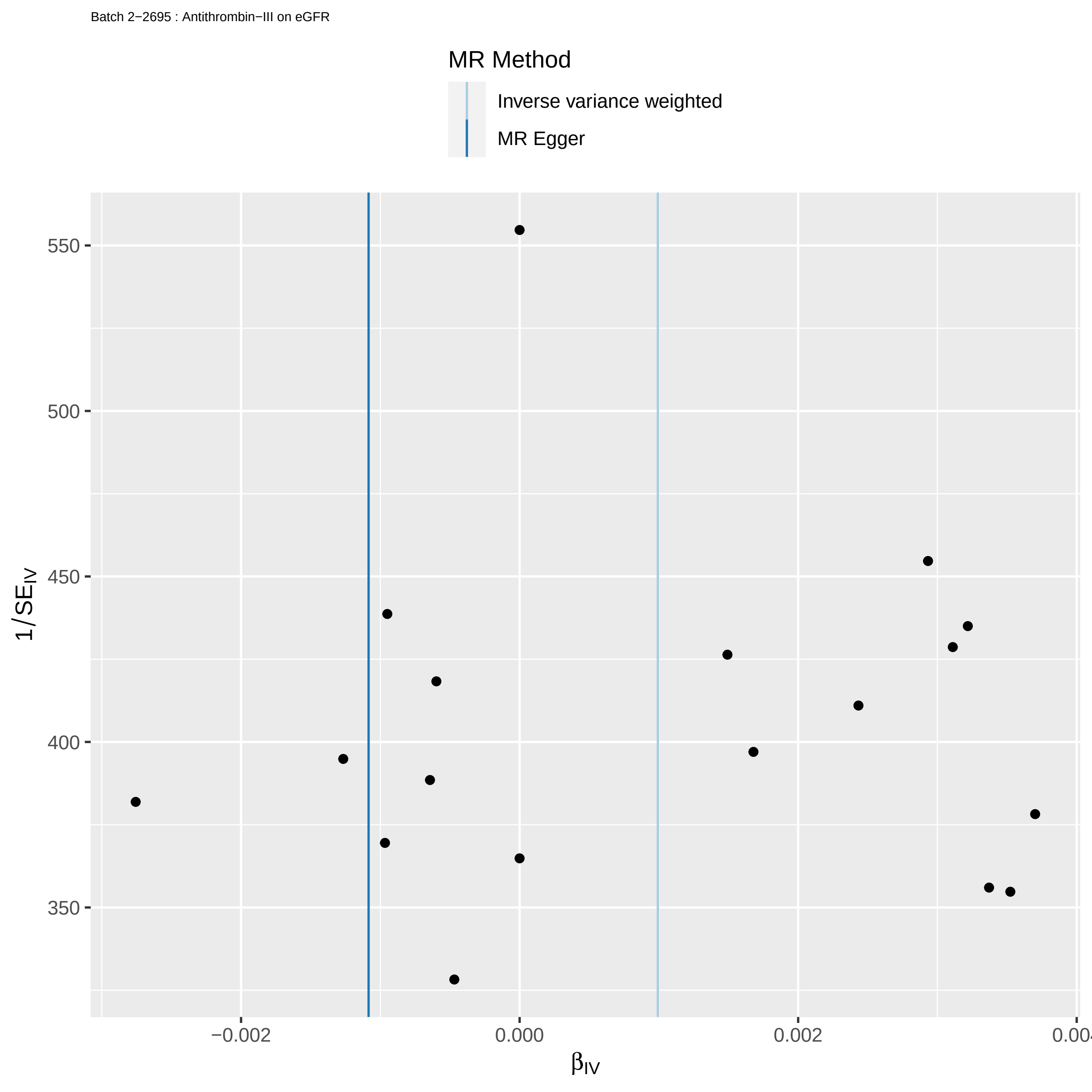

Batch 2−2695 : Antithrombin−III on eGFR
MR Method
Inverse variance weighted
MR Egger
550
500
450
400
350
1/SE$_{IV}$
−0.002
0.000
0.002
β$_{IV}$

Batch 2−2696 : Plasminogen activator inhibitor 1 on eGFR

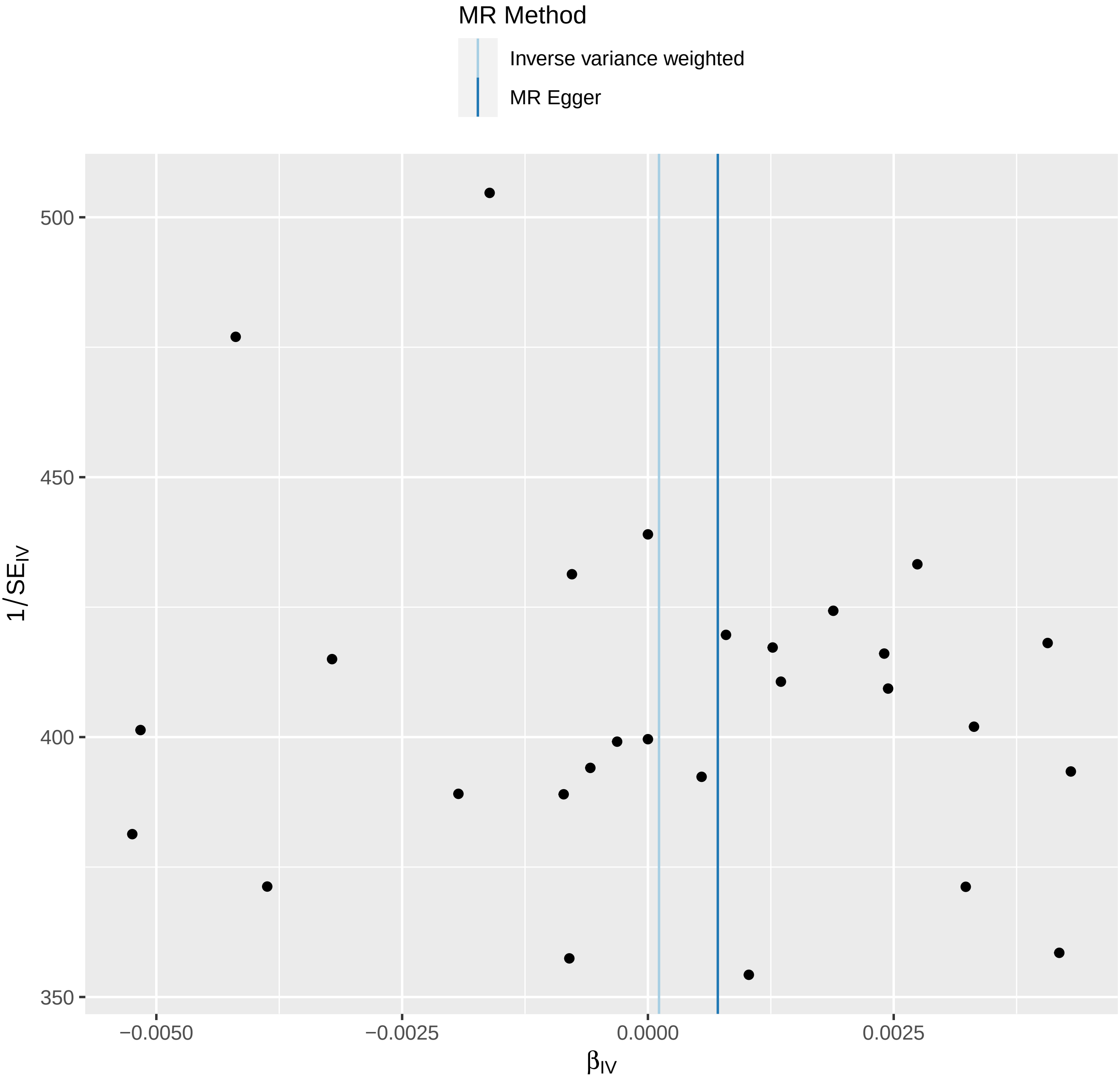

Batch 2−2697 : Glia−derived nexin on eGFR

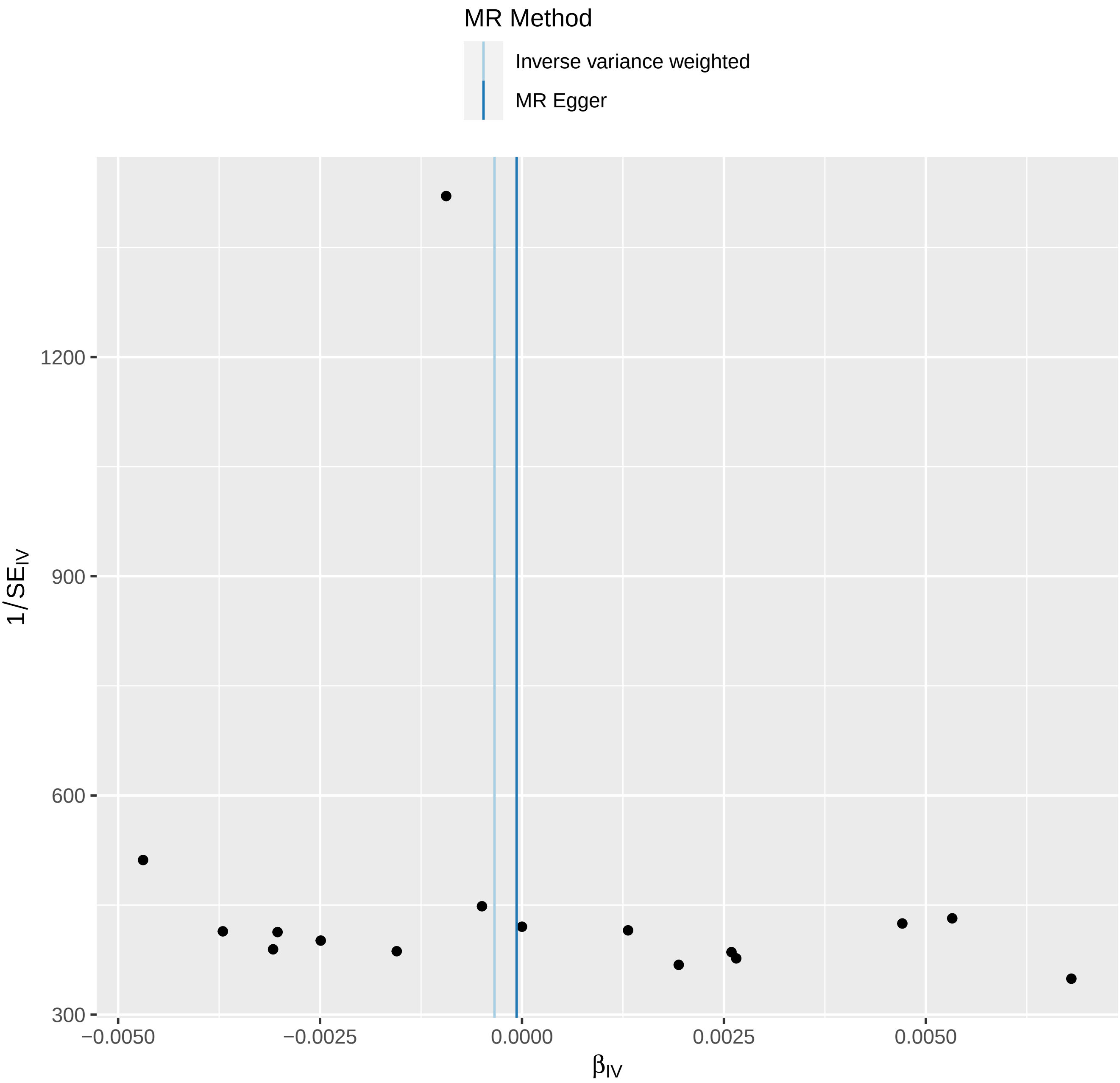

Batch 2−2698 : Pigment epithelium−derived factor on eGFR

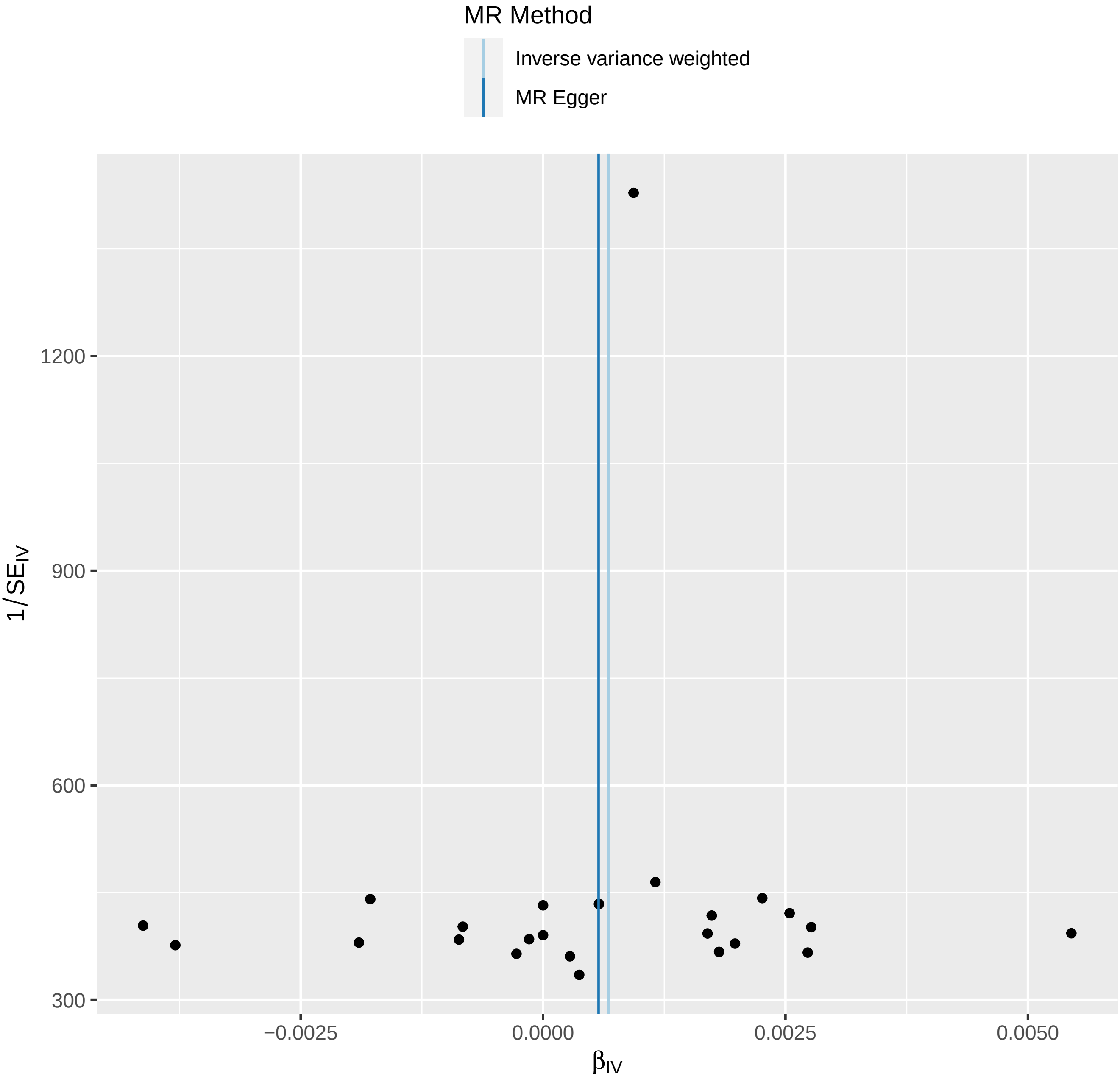

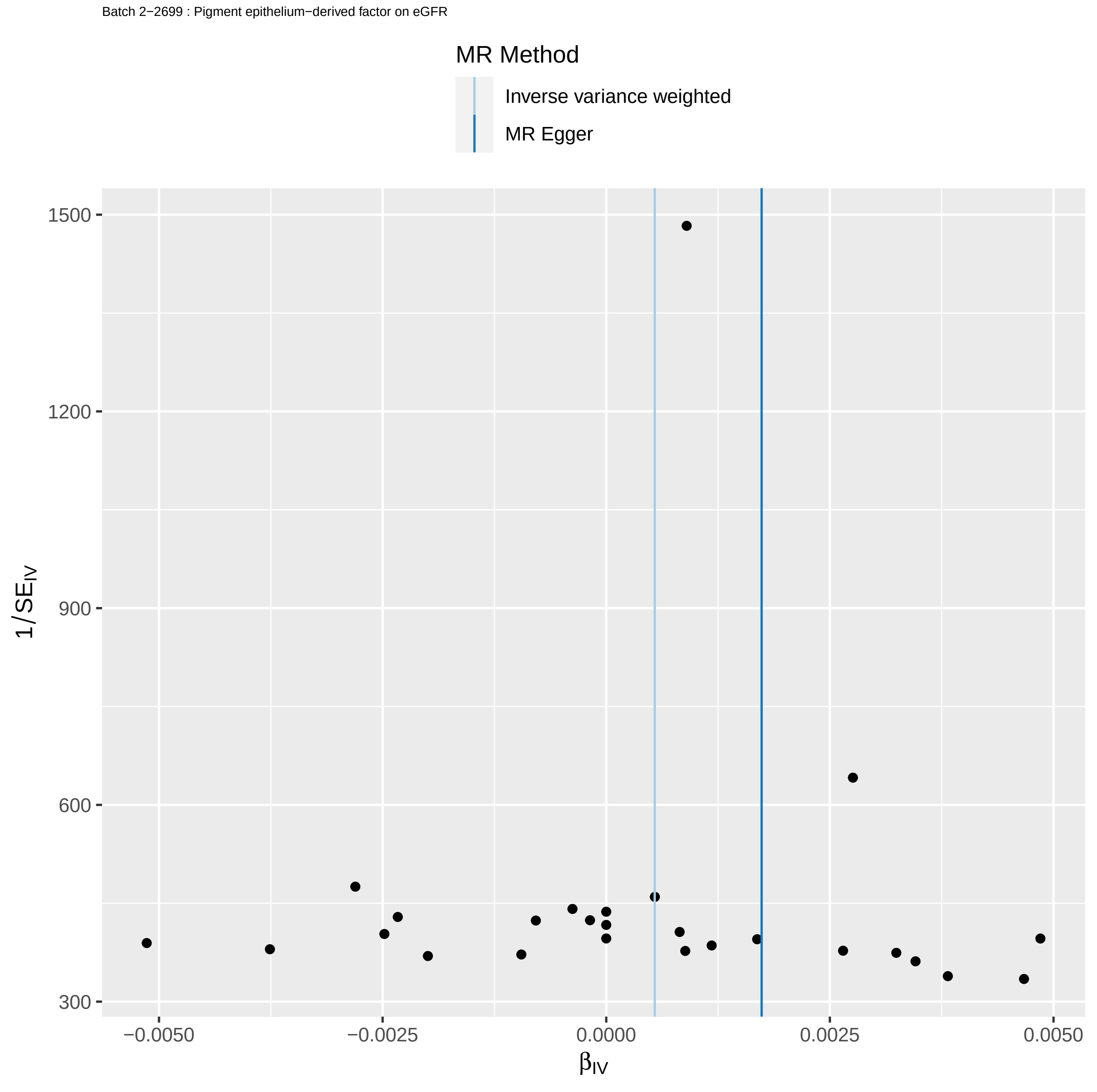

Batch 2−2699 : Pigment epithelium−derived factor on eGFR
MR Method
Inverse variance weighted
MR Egger
1500
1200
900
600
300
1/SE_IV
−0.0050
−0.0025
0.0000
0.0025
0.0050
β_IV

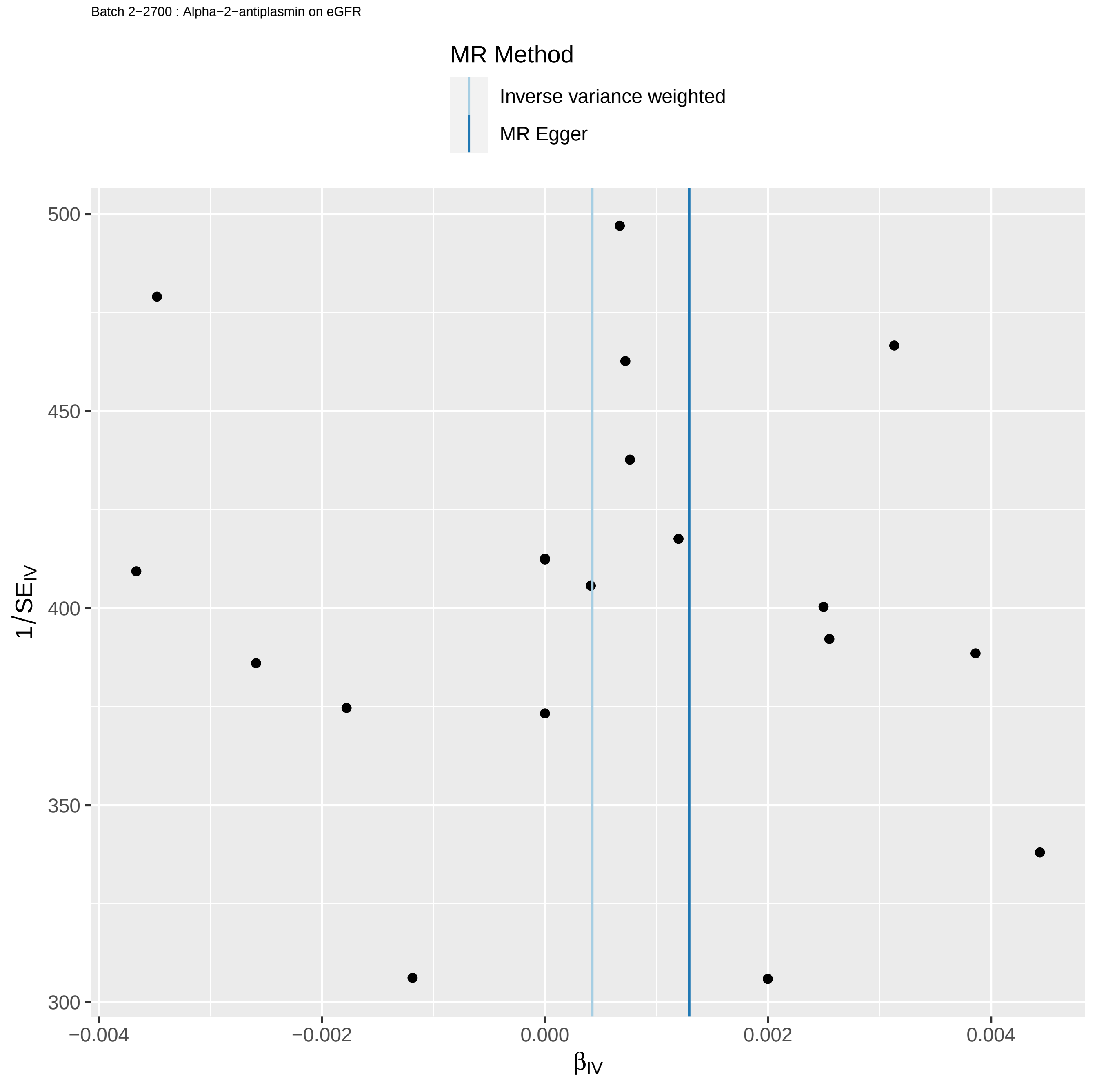
Batch 2−2700 : Alpha−2−antiplasmin on eGFR
MR Method
Inverse variance weighted
MR Egger
500
450
400
350
300
1/SE_IV
−0.004
−0.002
0.000
0.002
0.004
β_IV

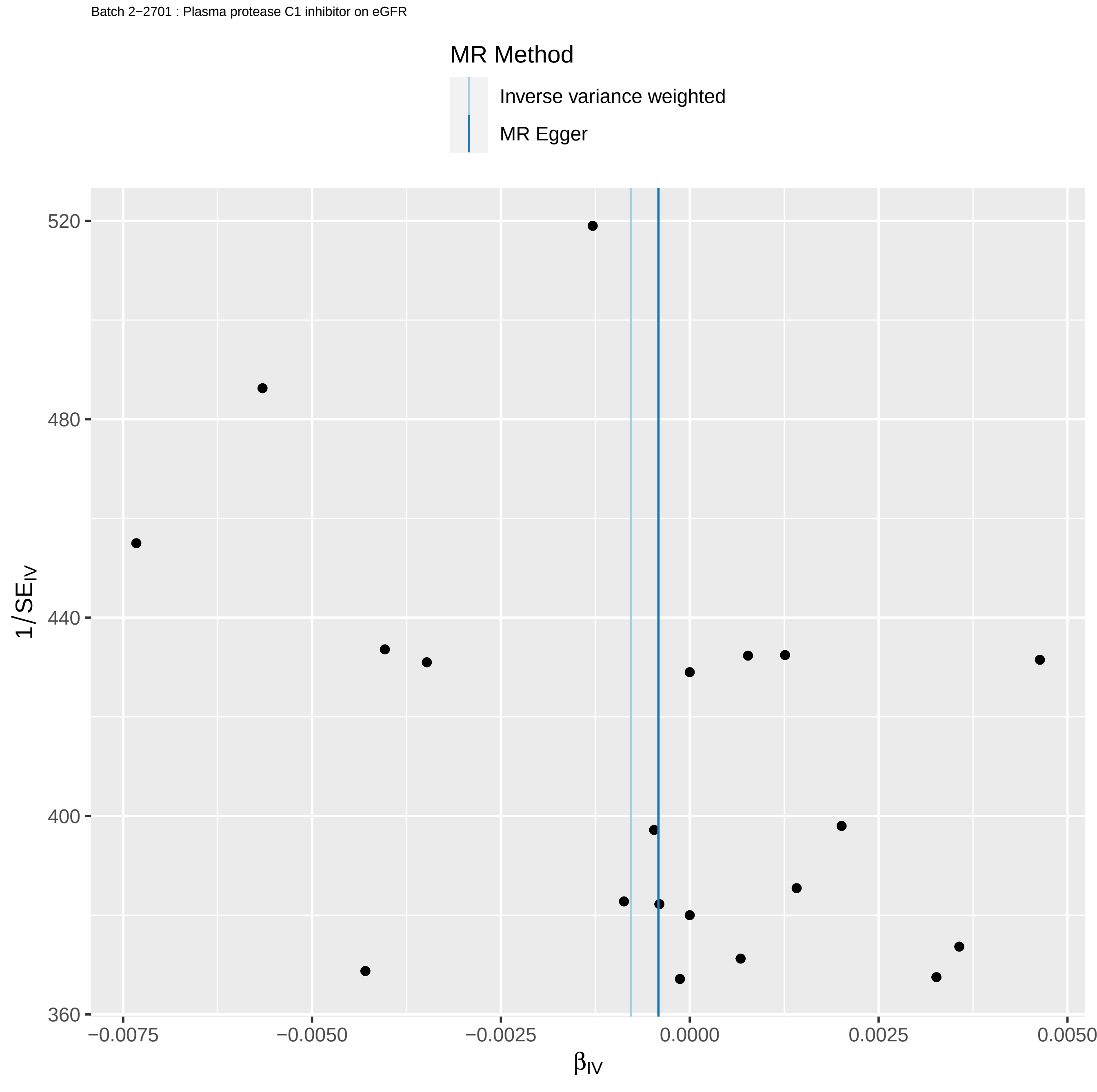
Batch 2−2701 : Plasma protease C1 inhibitor on eGFR
MR Method
Inverse variance weighted
MR Egger
1/SE_IV
520
480
440
400
360
−0.0075
−0.0050
−0.0025
0.0000
0.0025
0.0050
β_IV

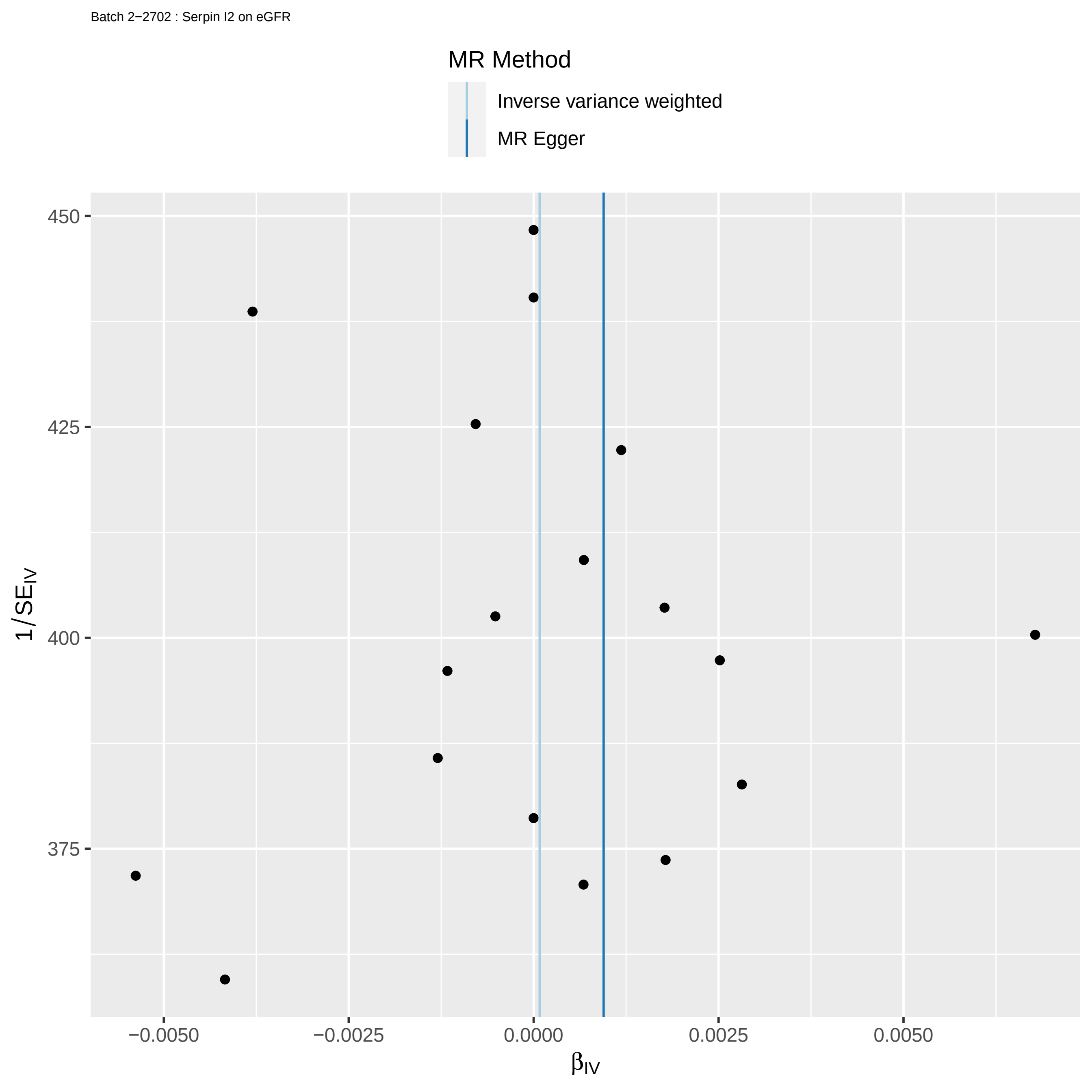

Batch 2−2702 : Serpin I2 on eGFR
MR Method
Inverse variance weighted
MR Egger
450
425
400
375
1/SE_IV
−0.0050
−0.0025
0.0000
0.0025
0.0050
β_IV

Batch 2−2703 : Histone−lysine N−methyltransferase SETD2 on eGFR

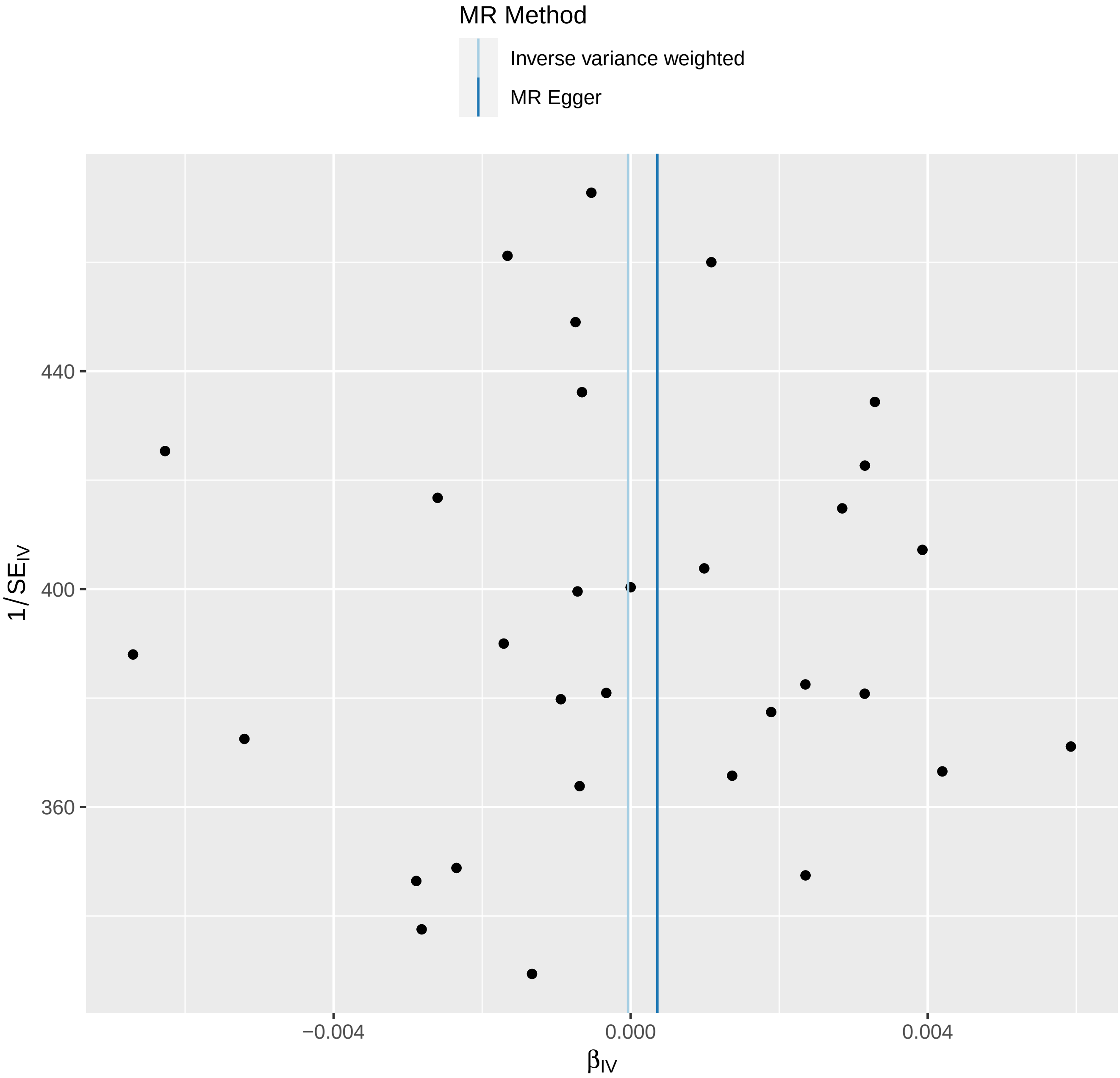

Batch 2−2704 : Histone−lysine N−methyltransferase SETMAR on eGFR

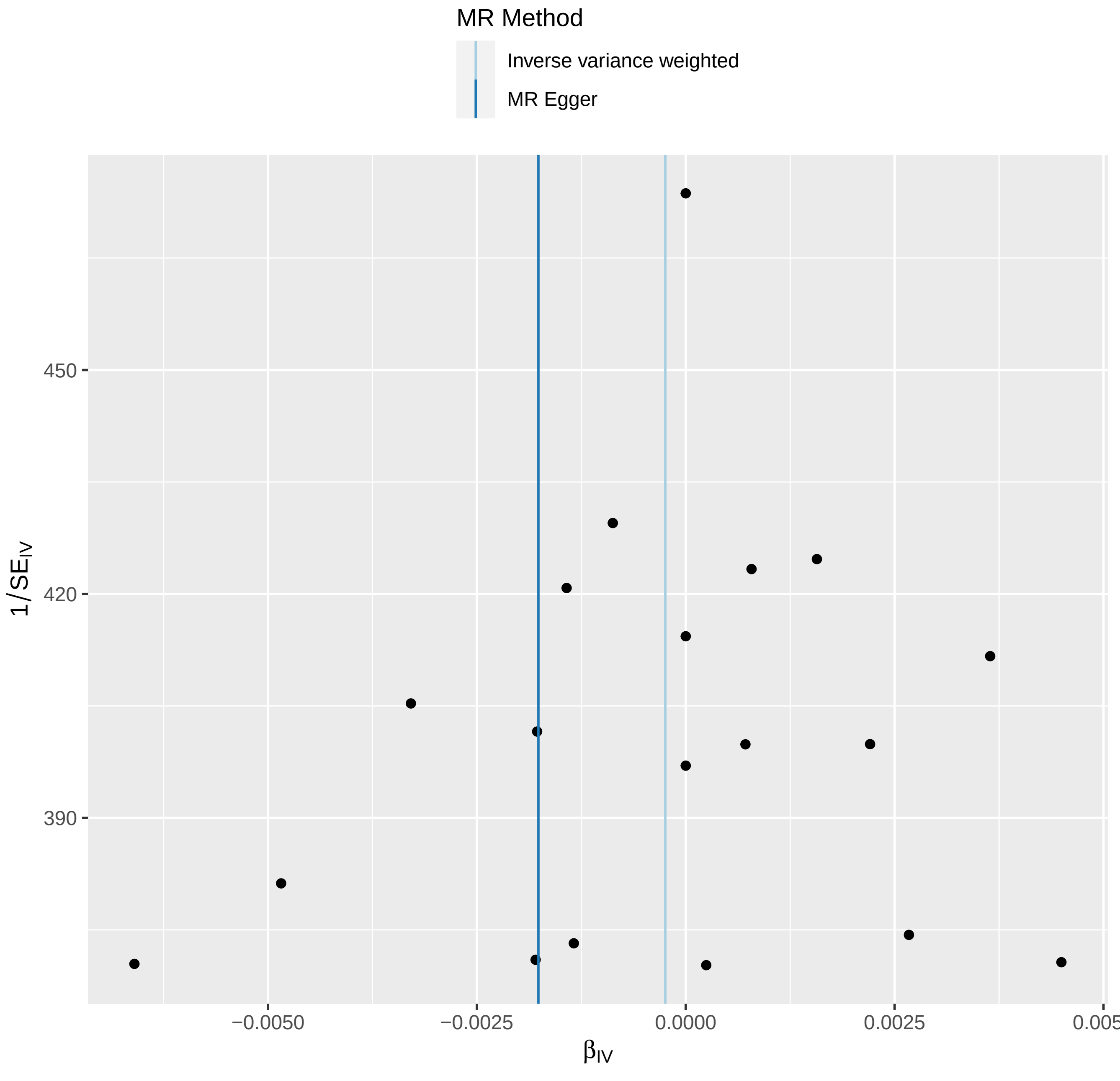

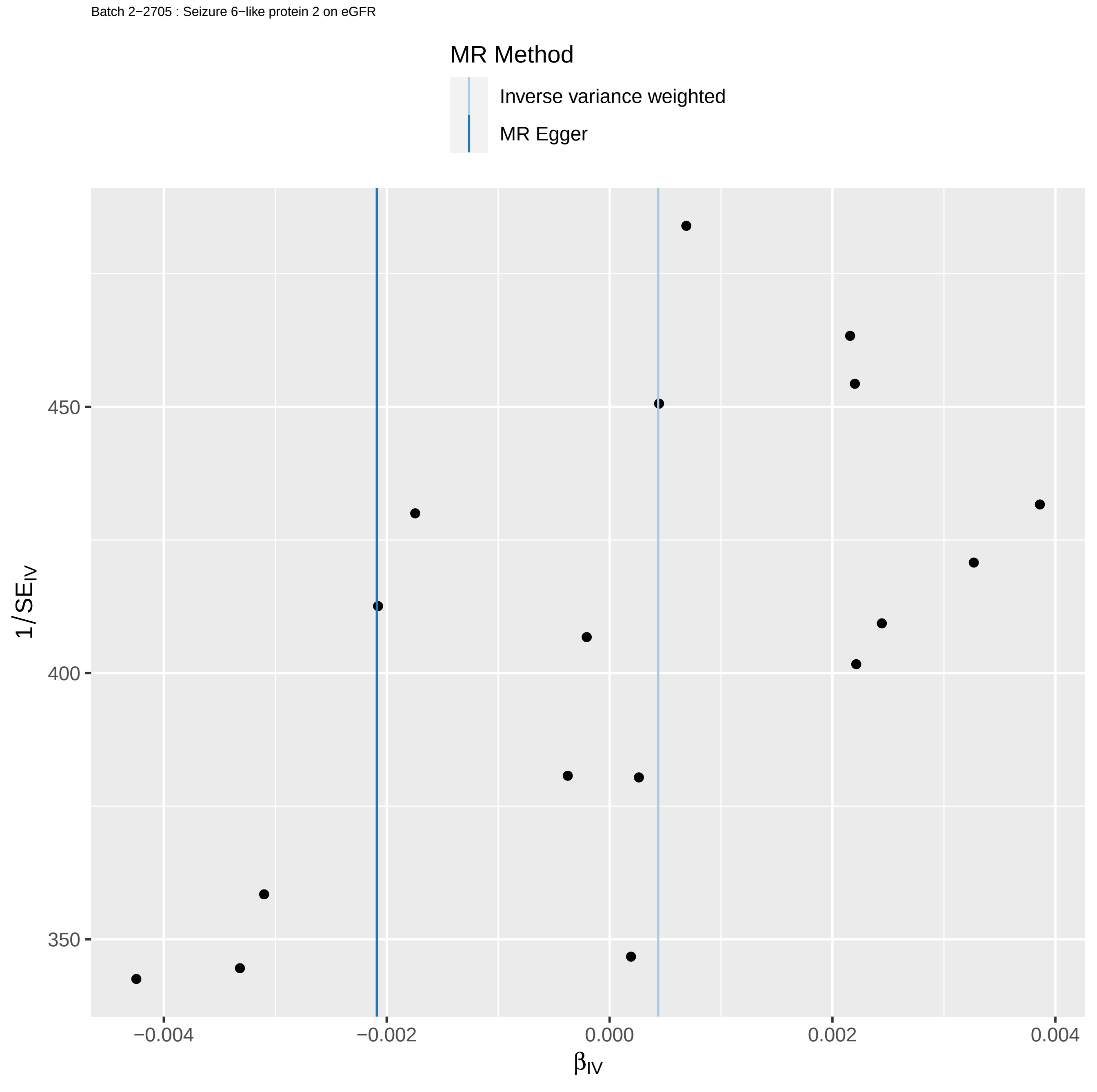

Batch 2−2705 : Seizure 6−like protein 2 on eGFR
MR Method
Inverse variance weighted
MR Egger
450
400
350
1/SE$_{IV}$
−0.004
−0.002
0.000
0.002
0.004
β$_{IV}$

Batch 2−2706 : Splicing factor 1 on eGFR

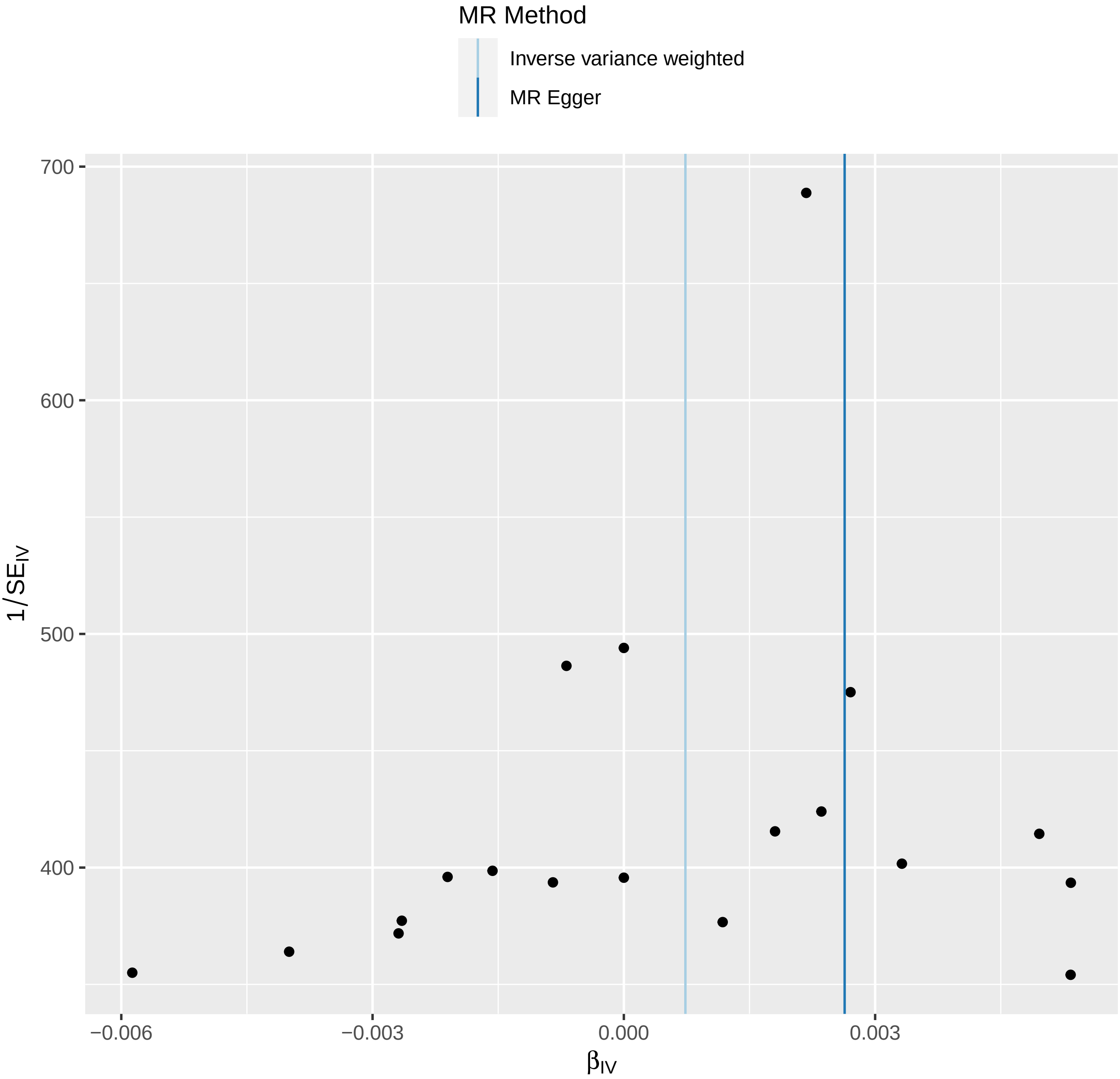

Batch 2−2707 : Splicing factor 3B subunit 4 on eGFR

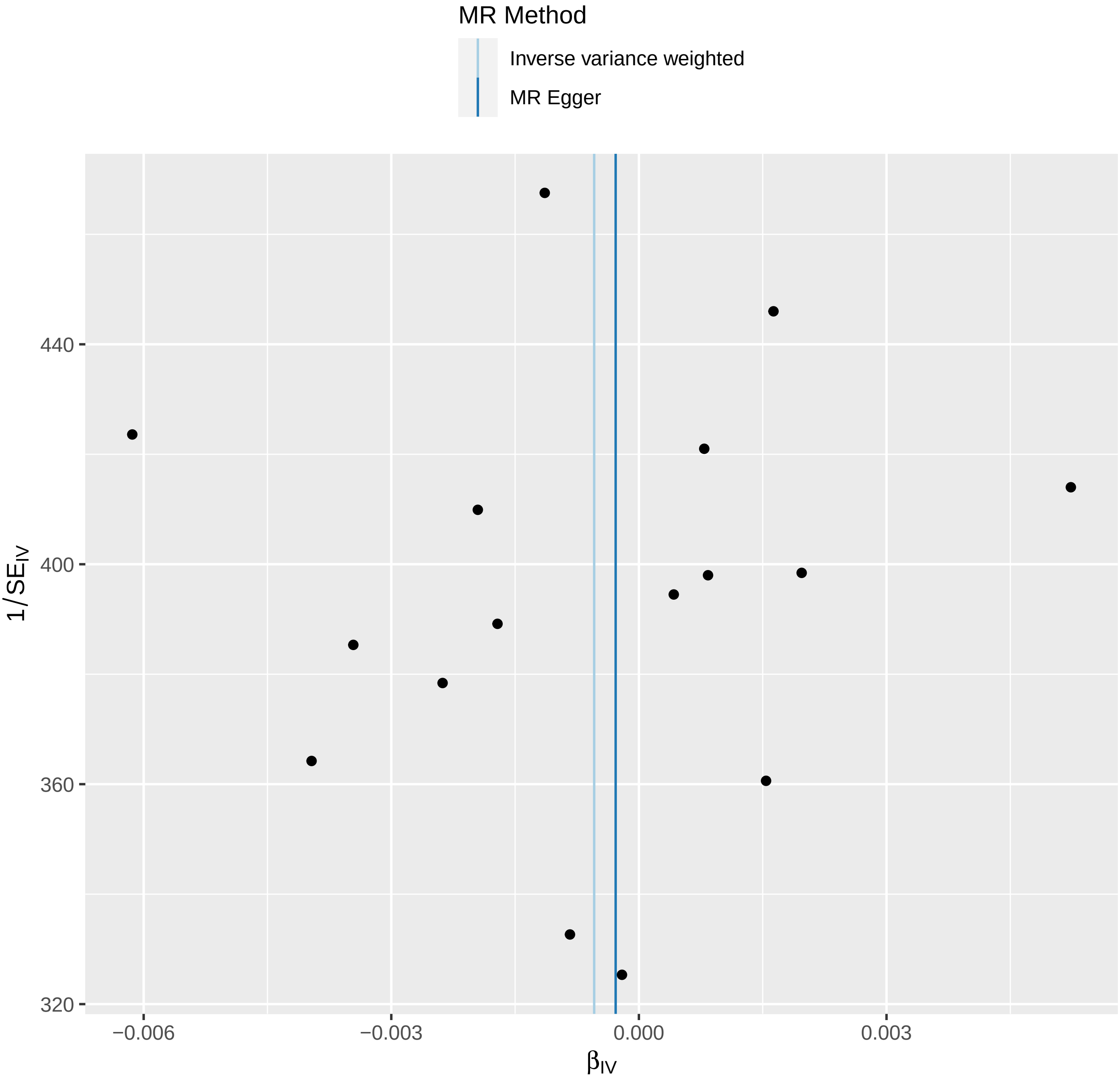

Batch 2−2708 : 14−3−3 protein sigma on eGFR

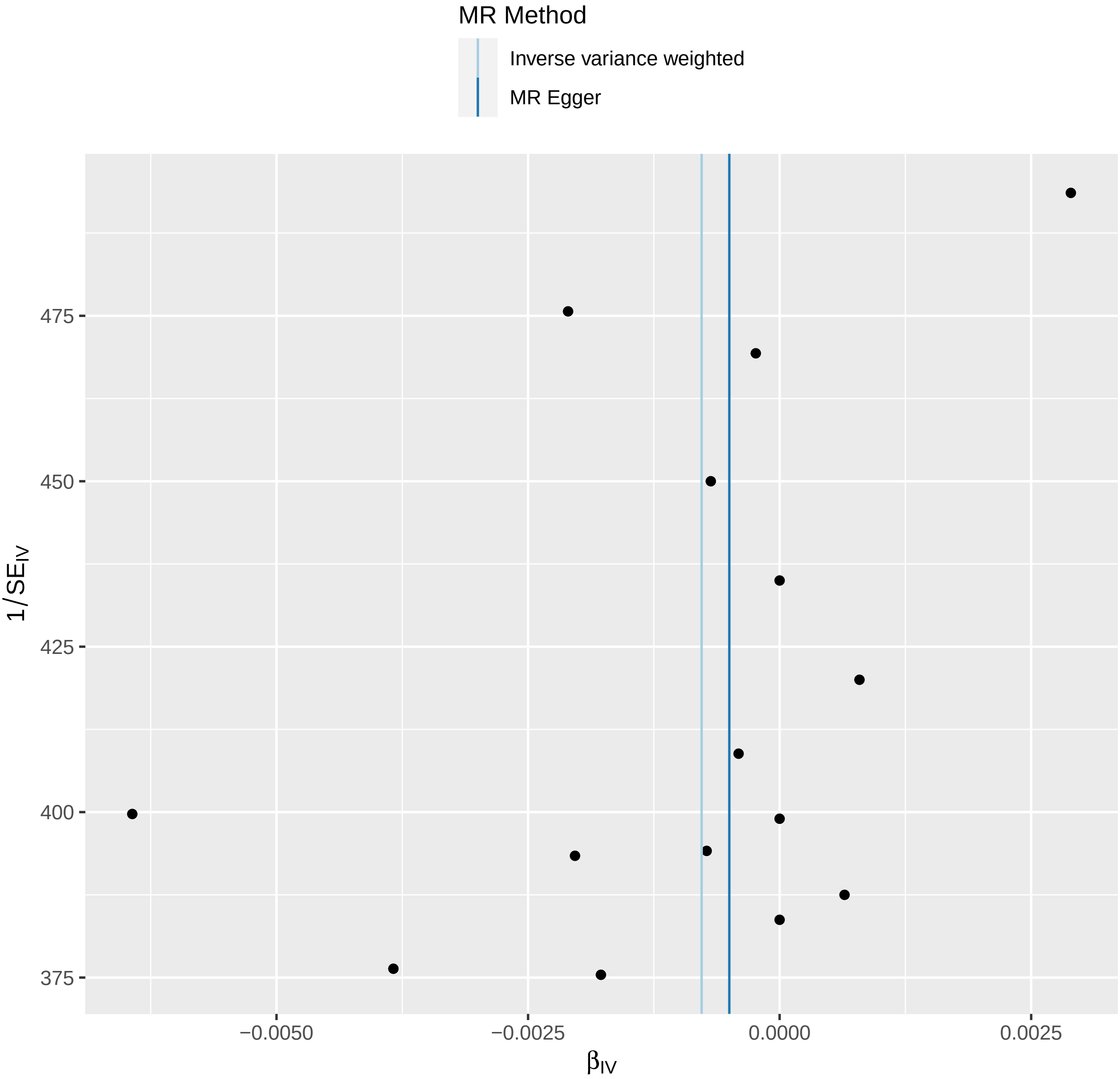

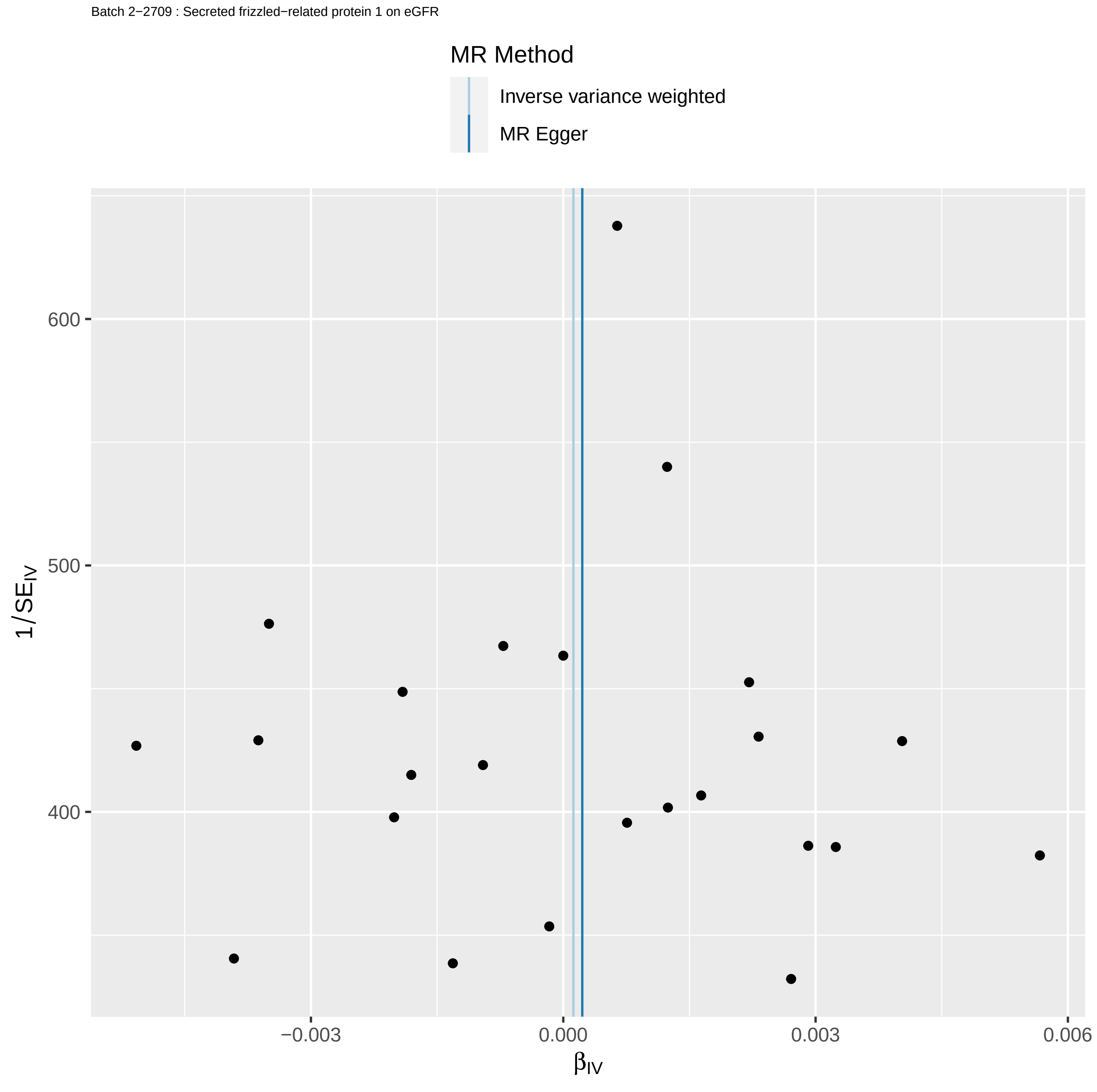

Batch 2−2709 : Secreted frizzled−related protein 1 on eGFR
MR Method
Inverse variance weighted
MR Egger
600
500
400
1/SE$_{IV}$
−0.003
0.000
0.003
0.006
β$_{IV}$

Batch 2−2710 : Secreted frizzled−related protein 2 on eGFR

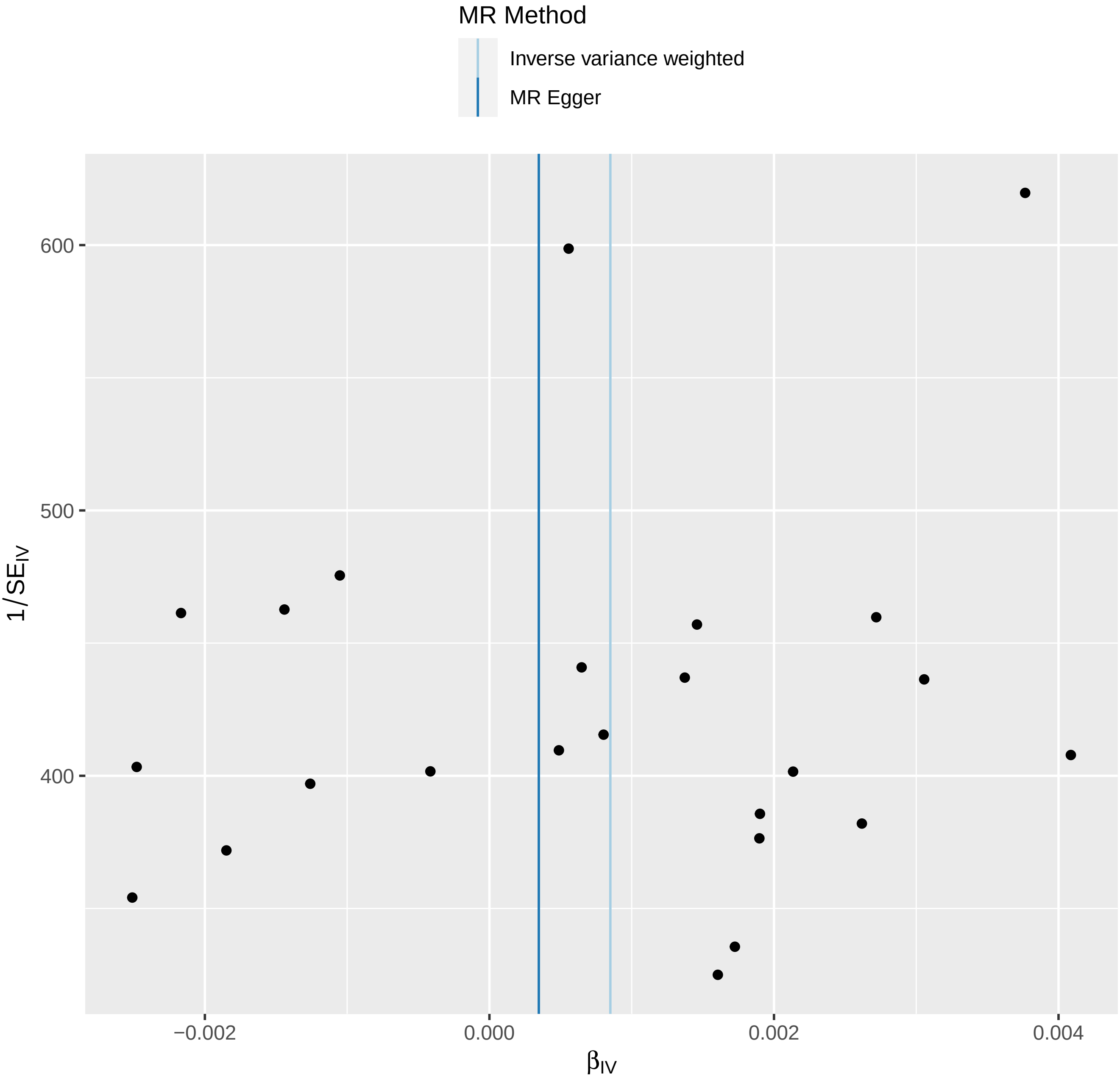

Batch 2−2711 : Pulmonary surfactant−associated protein C on eGFR

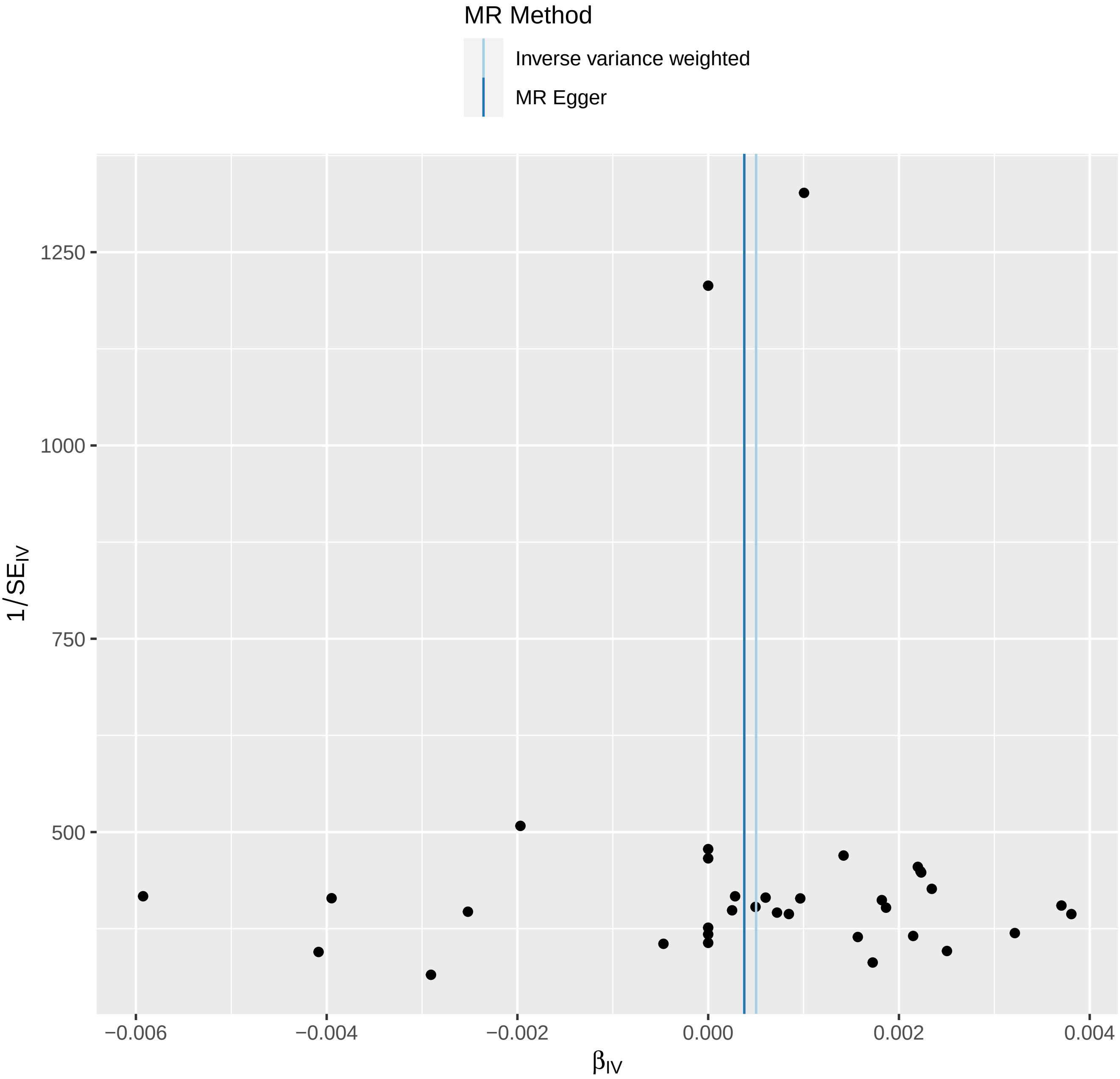

Batch 2−2712 : Pulmonary surfactant−associated protein D on eGFR

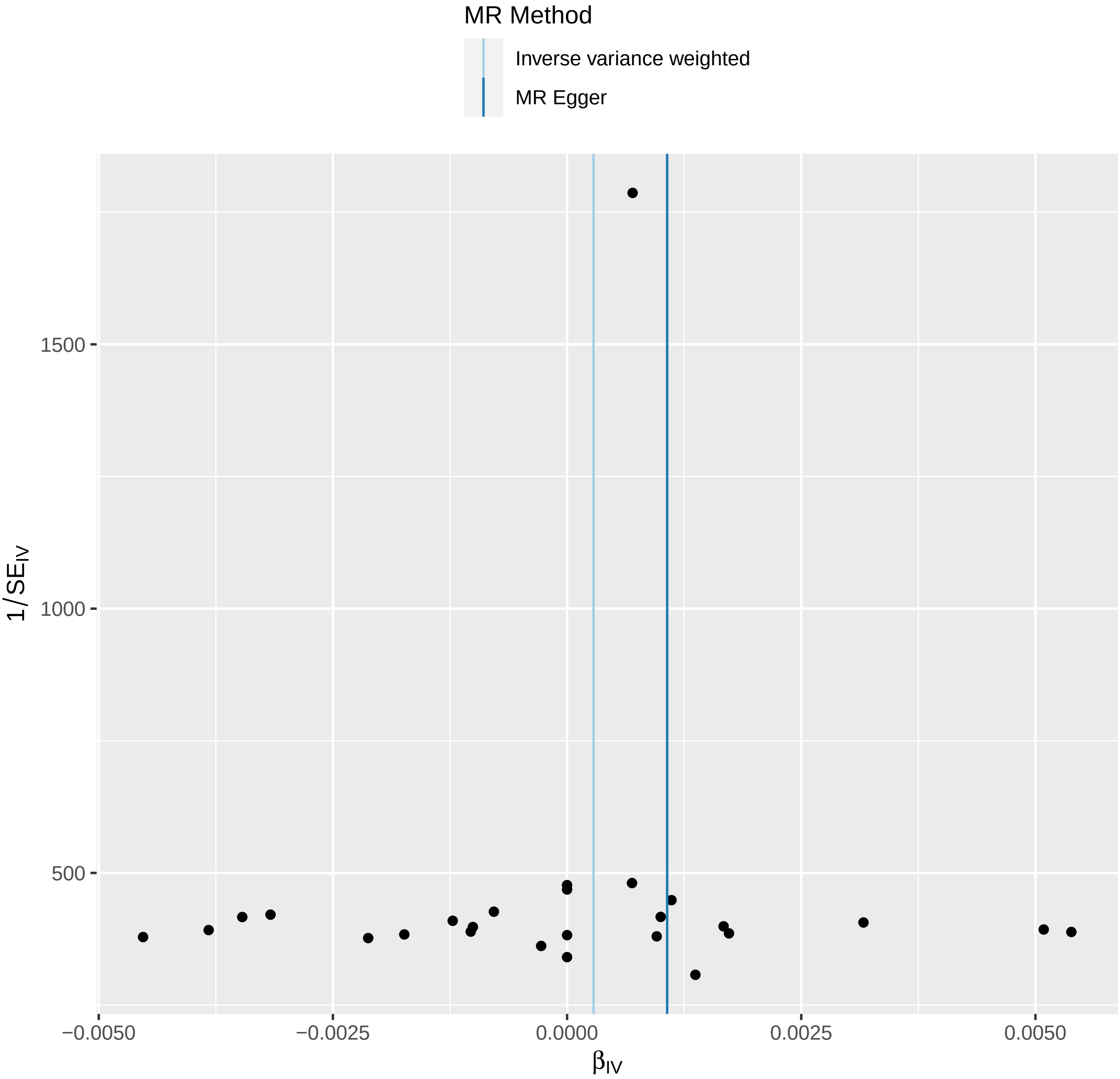

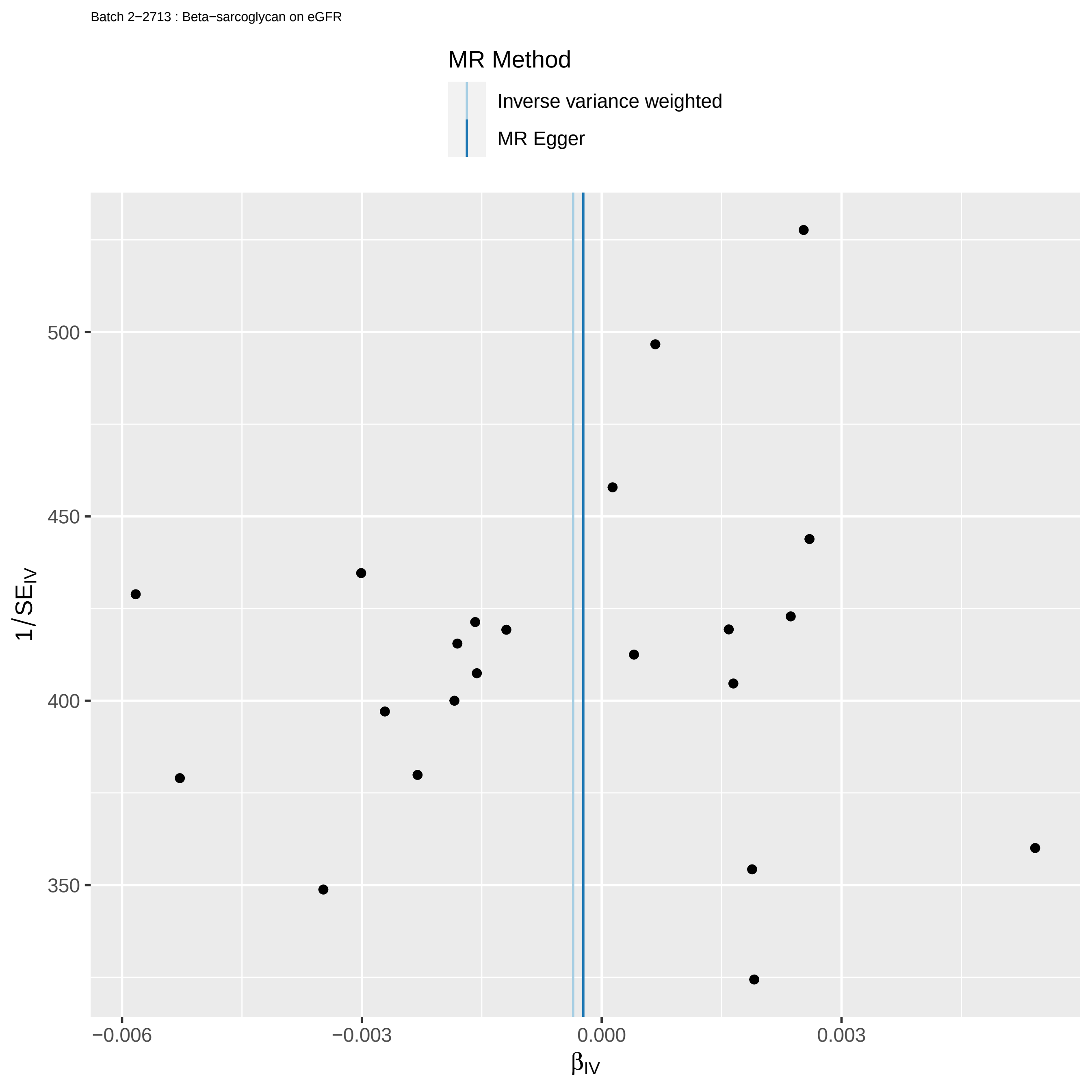

Batch 2−2713 : Beta−sarcoglycan on eGFR
MR Method
Inverse variance weighted
MR Egger
500
450
400
350
1/SE_IV
−0.006
−0.003
0.000
0.003
β_IV

Batch 2−2714 : Small glutamine−rich tetratricopeptide repeat−containing protein alpha on eGFR

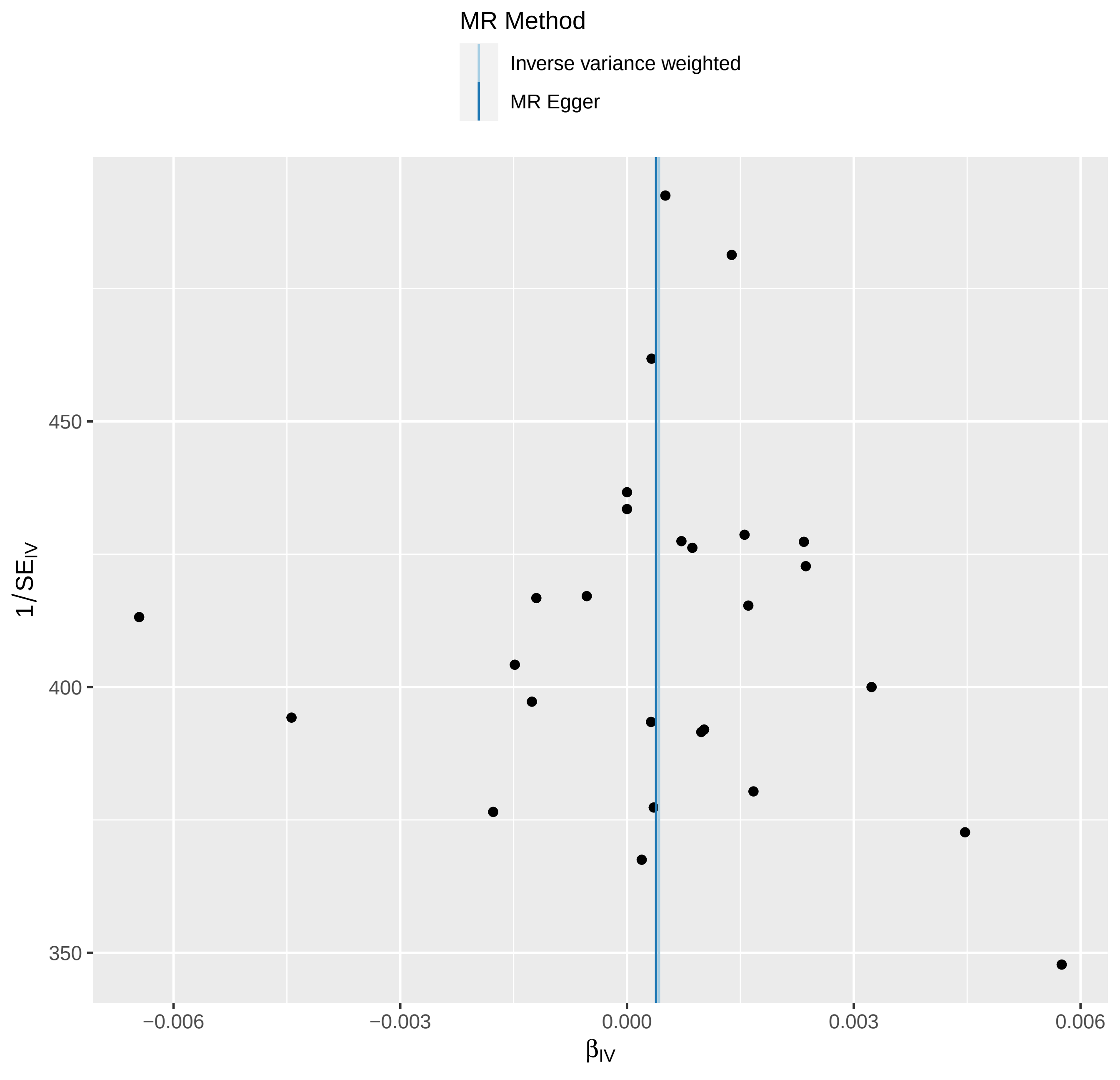

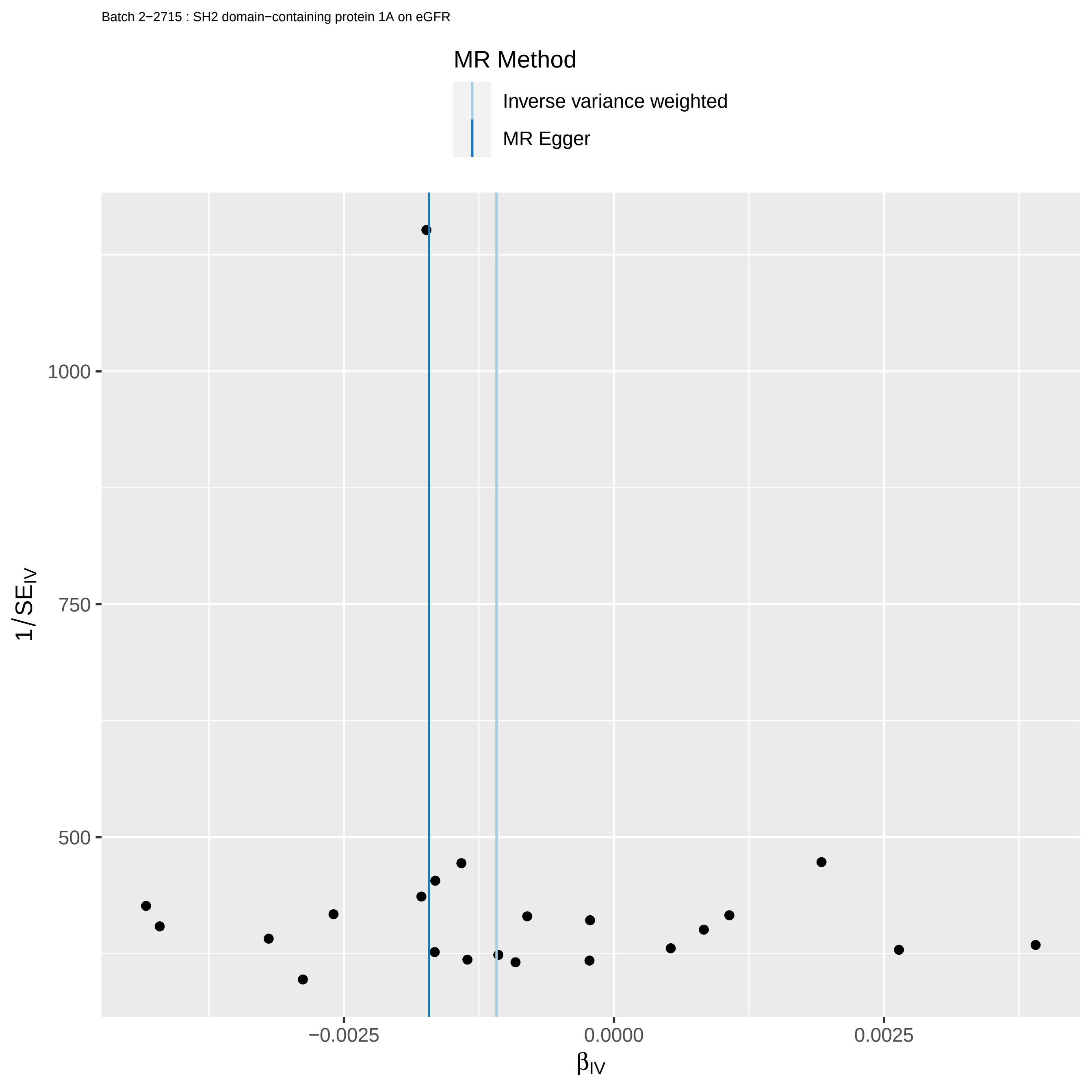

Batch 2−2715 : SH2 domain−containing protein 1A on eGFR
MR Method
Inverse variance weighted
MR Egger
1000
750
500
1/SE$_{IV}$
−0.0025
0.0000
0.0025
β$_{IV}$

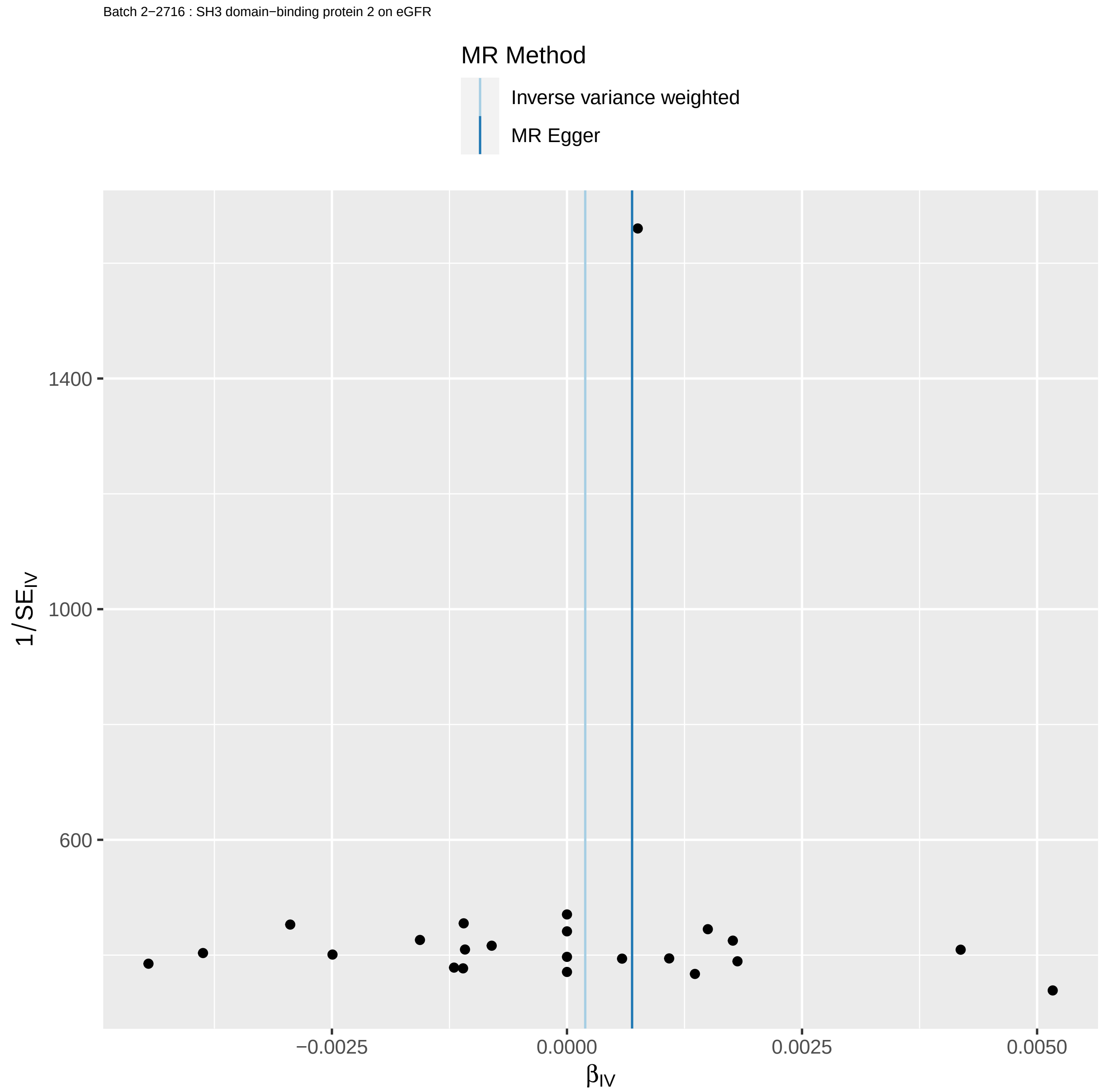

Batch 2−2716 : SH3 domain−binding protein 2 on eGFR
MR Method
Inverse variance weighted
MR Egger
1400
1000
600
1/SE$_{IV}$
−0.0025
0.0000
0.0025
0.0050
β_{IV}

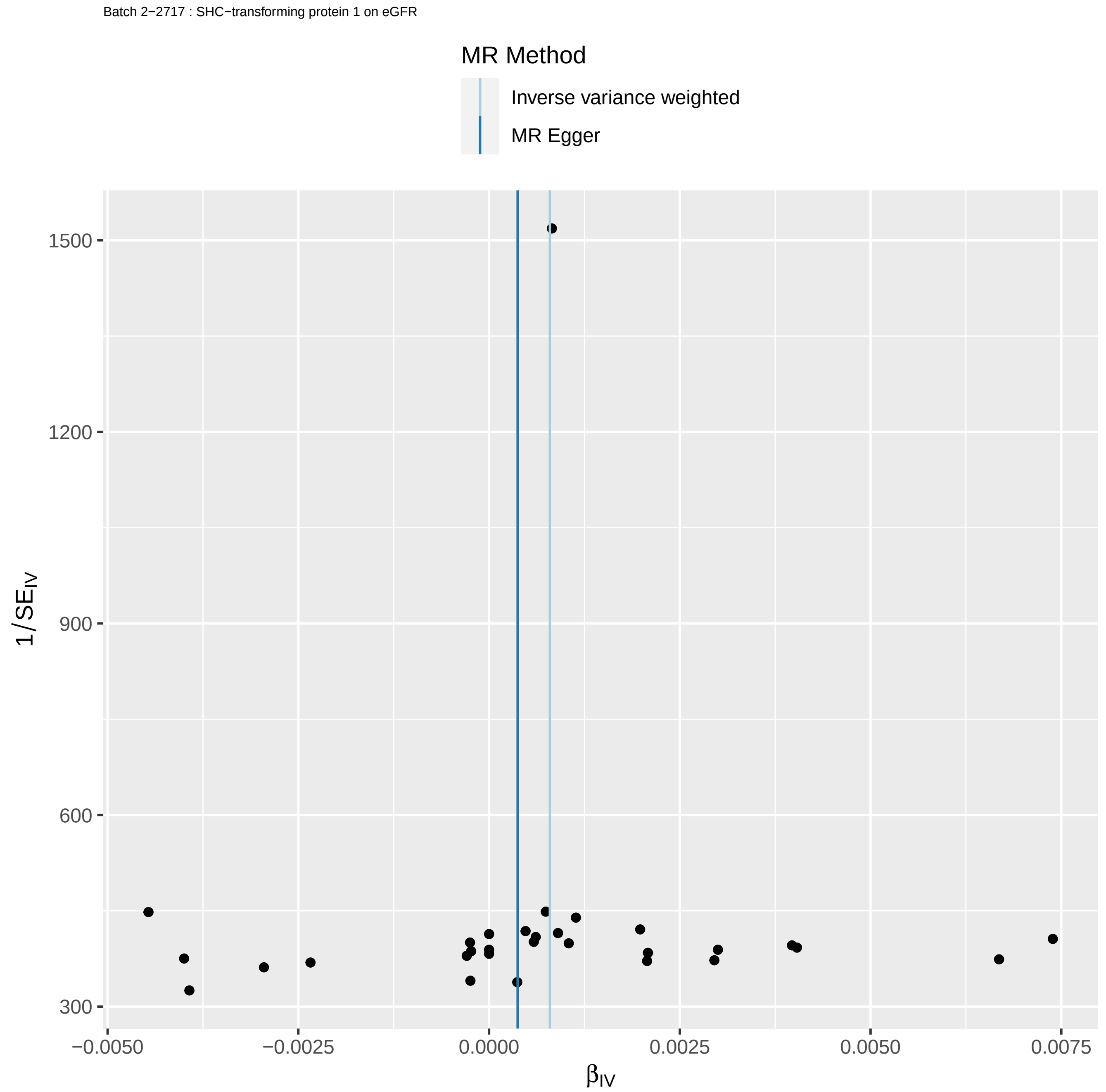
Batch 2−2717 : SHC−transforming protein 1 on eGFR
MR Method
Inverse variance weighted
MR Egger
1500
1200
900
600
300
1/SE$_{IV}$
−0.0050
−0.0025
0.0000
0.0025
0.0050
0.0075
β$_{IV}$

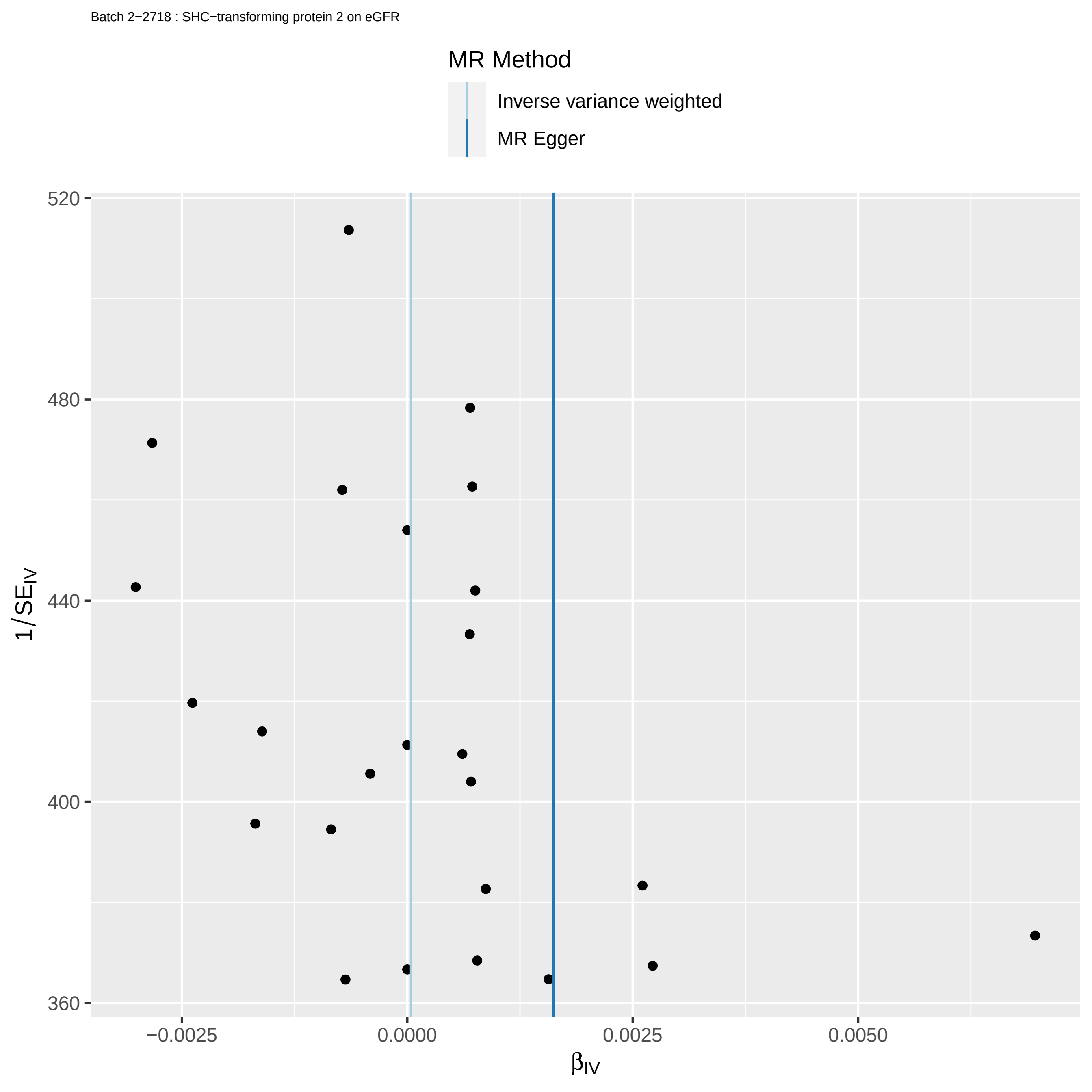
Batch 2−2718 : SHC−transforming protein 2 on eGFR
MR Method
Inverse variance weighted
MR Egger
520
480
440
400
360
1/SE$_{IV}$
−0.0025
0.0000
0.0025
0.0050
β$_{IV}$

Batch 2−2719 : SHC−transforming protein 4 on eGFR

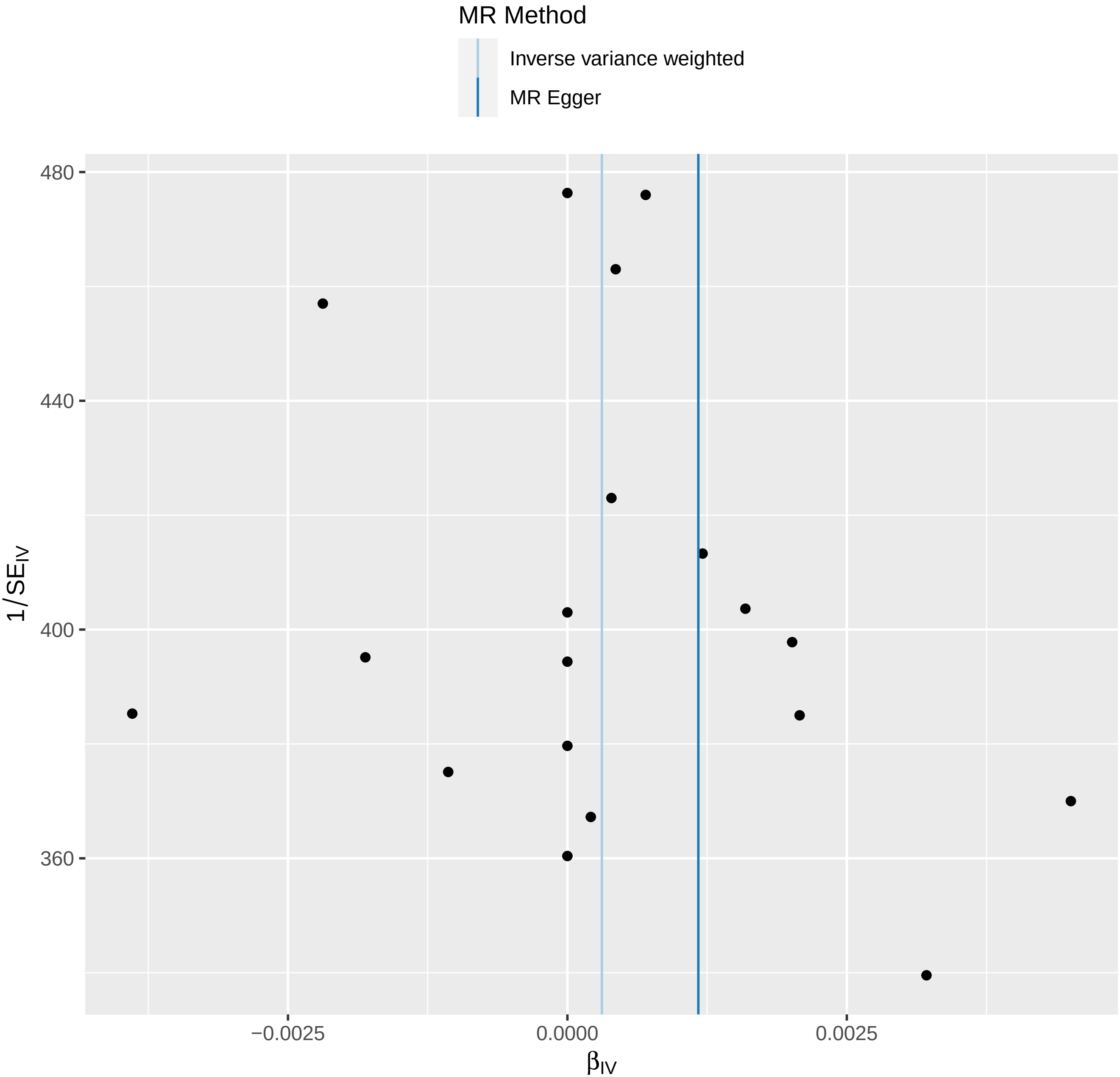

Batch 2−2720 : Protein shisa−3 homolog on eGFR

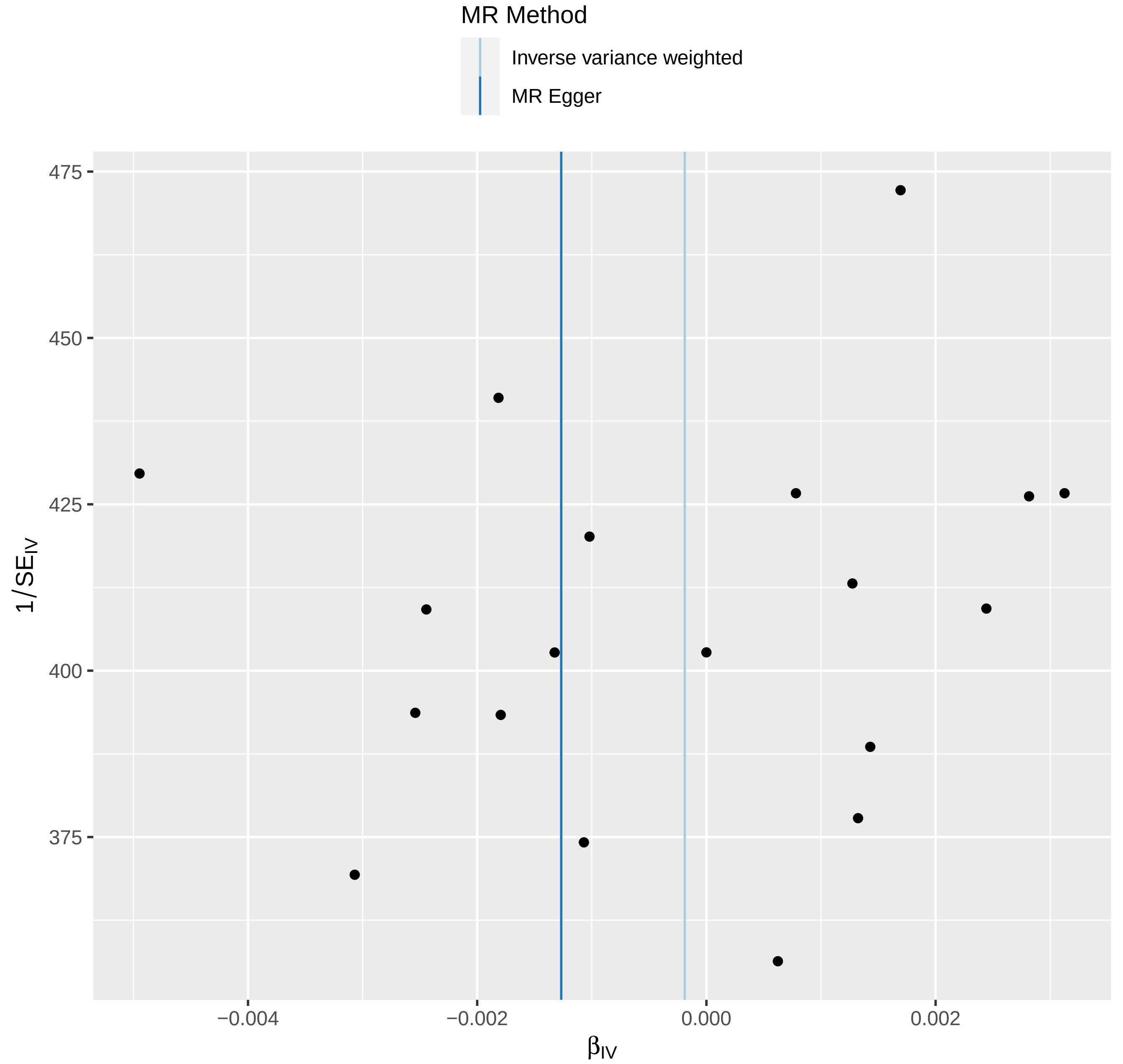

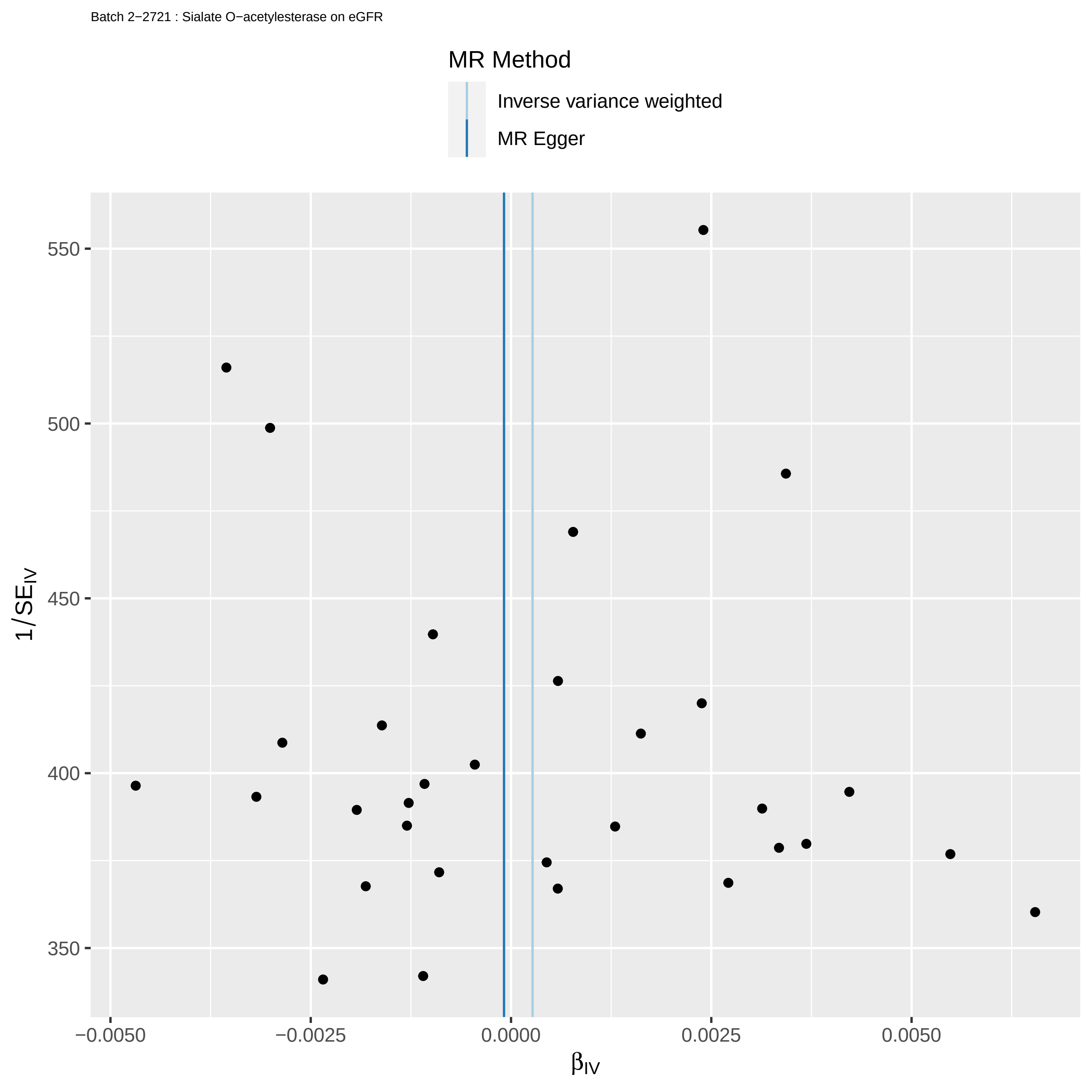

Batch 2−2721 : Sialate O−acetylesterase on eGFR
MR Method
Inverse variance weighted
MR Egger
550
500
450
400
350
1/SE$_{IV}$
−0.0050
−0.0025
0.0000
0.0025
0.0050
β_{IV}

Batch 2−2722 : Single Ig IL−1−related receptor on eGFR

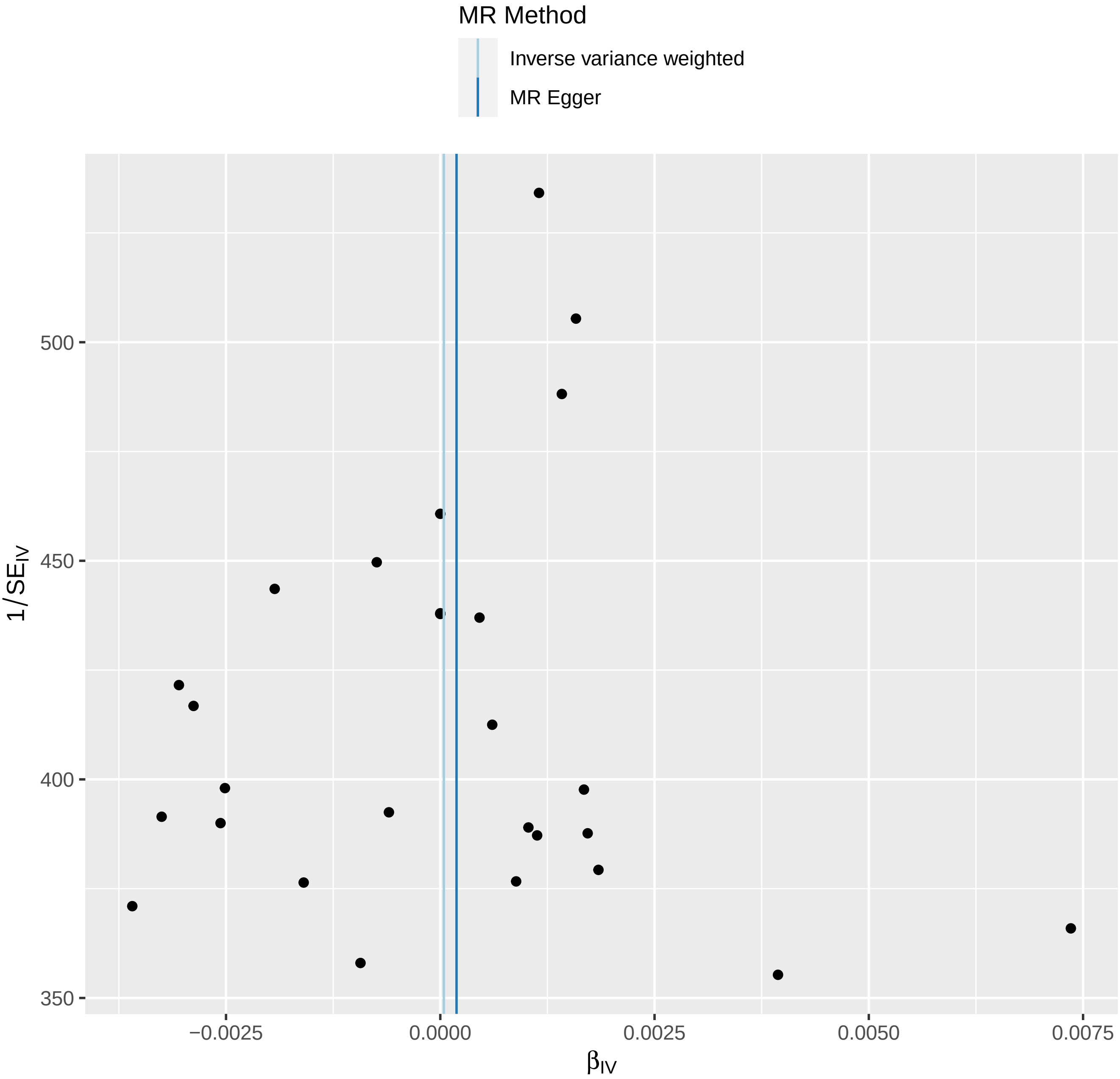

Batch 2−2723 : Sialic acid−binding Ig−like lectin 10 on eGFR

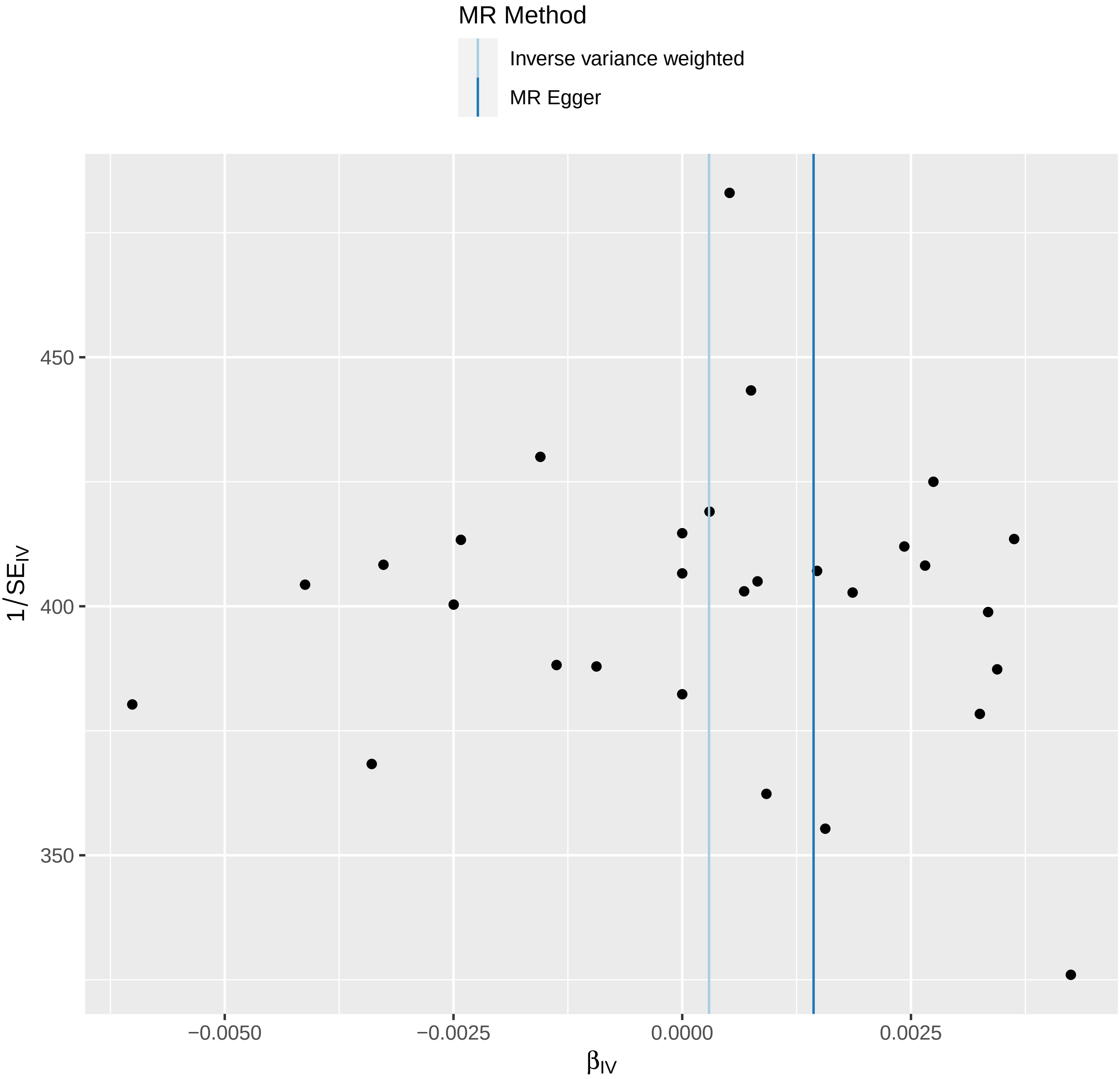

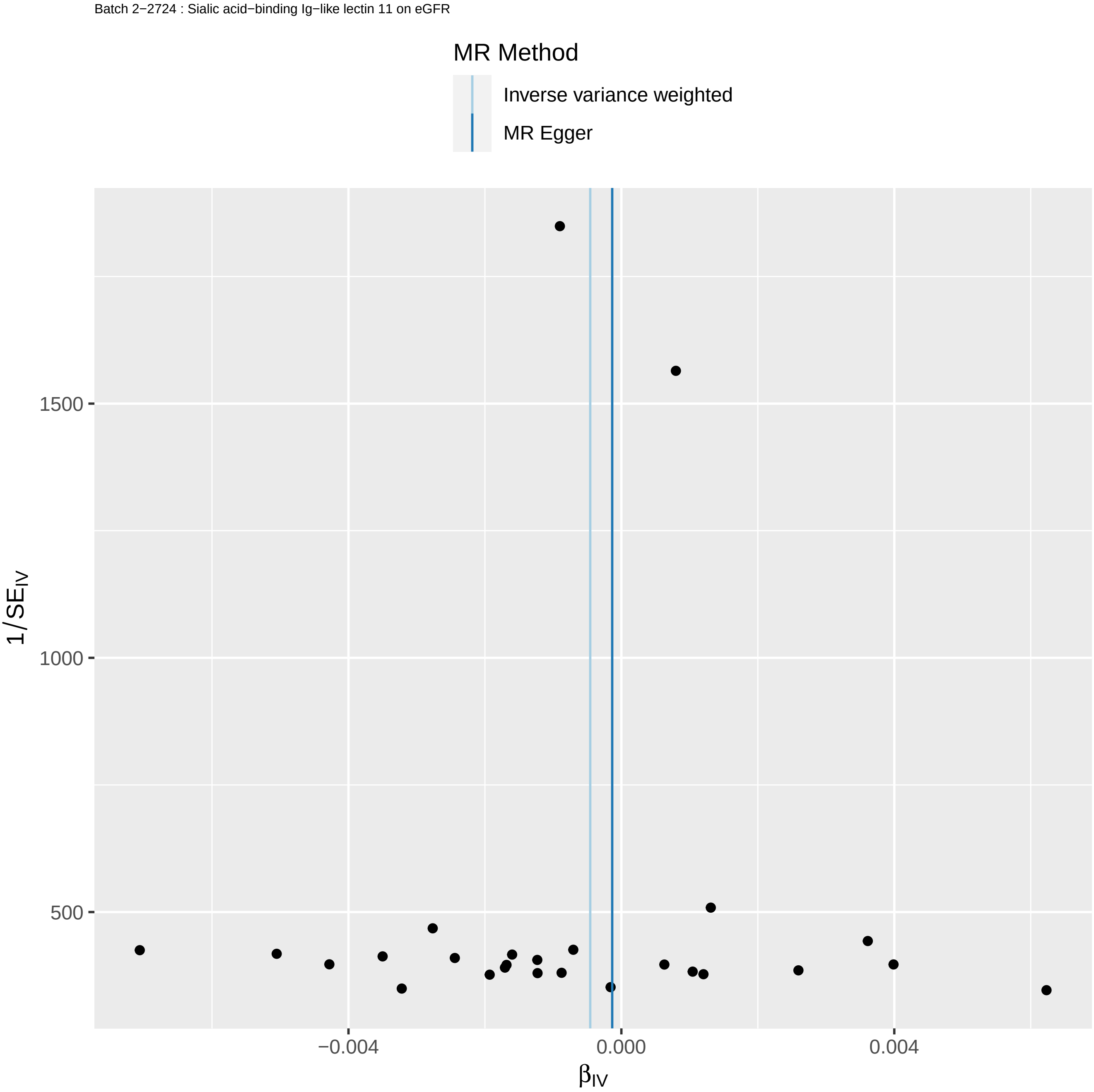

Batch 2−2724 : Sialic acid−binding Ig−like lectin 11 on eGFR
MR Method
Inverse variance weighted
MR Egger
1500
1000
500
1/SE$_{IV}$
−0.004
0.000
0.004
β$_{IV}$

Batch 2−2725 : Sialic acid−binding Ig−like lectin 12 on eGFR

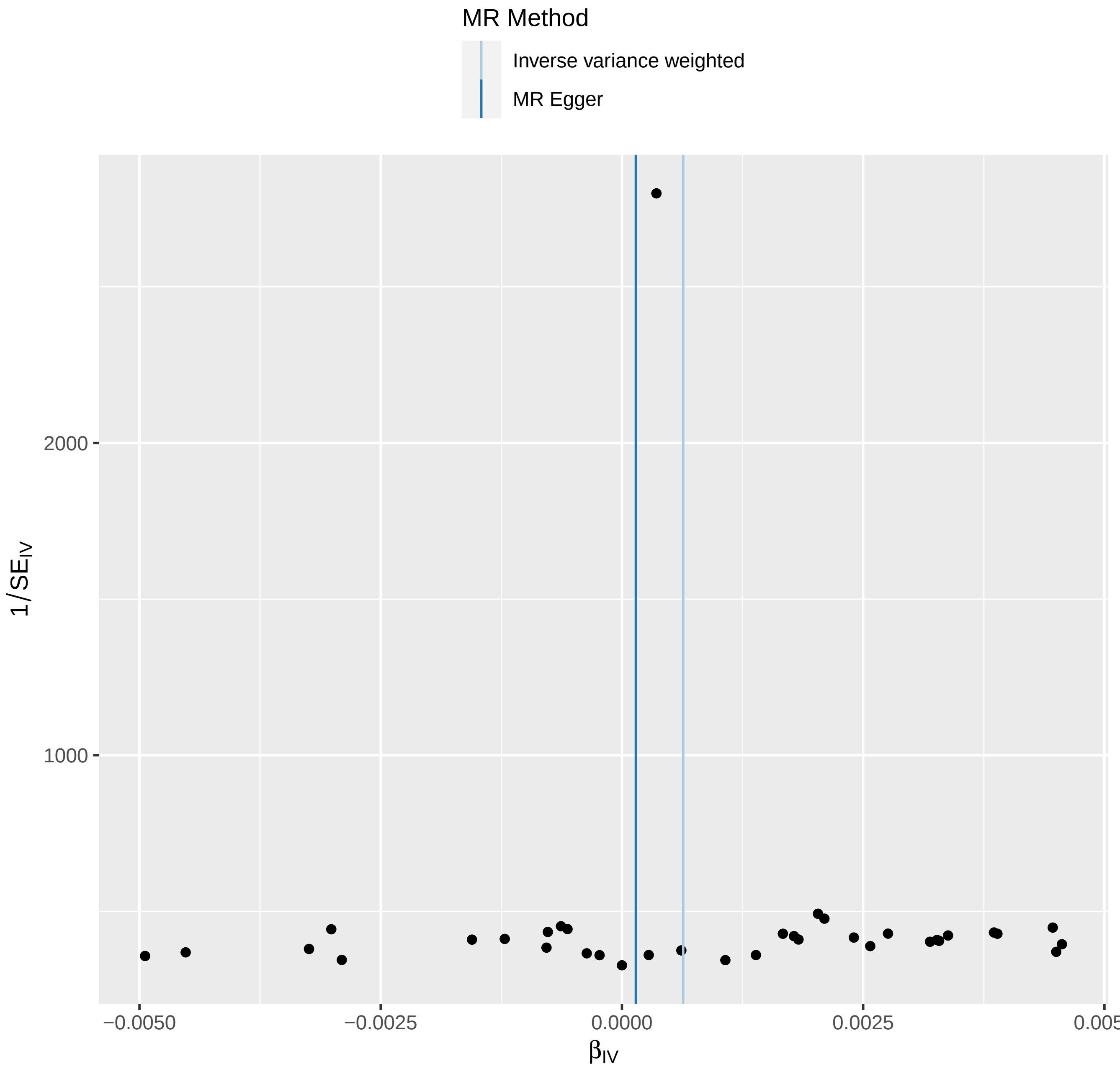

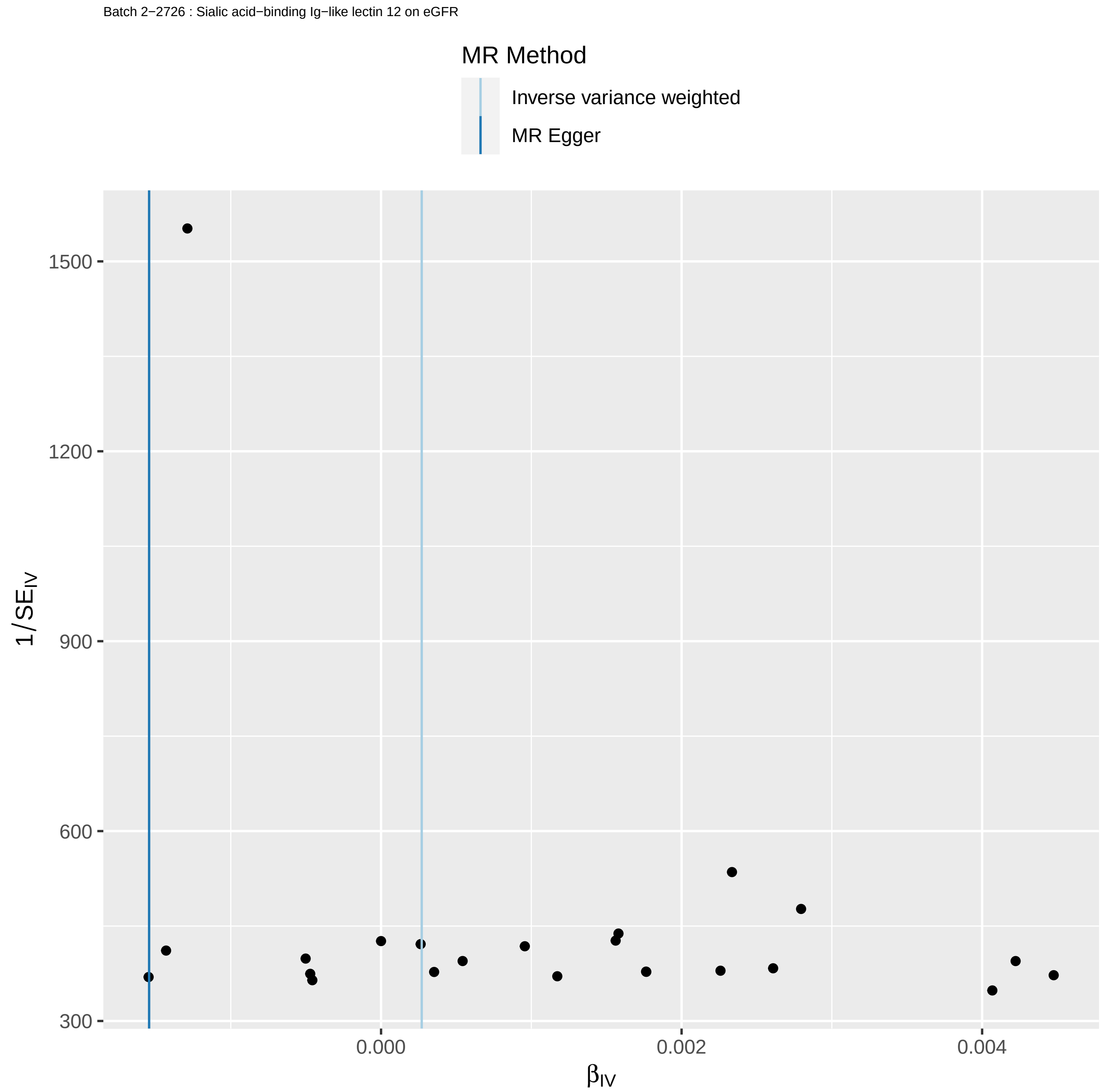

Batch 2−2726 : Sialic acid−binding Ig−like lectin 12 on eGFR
MR Method
Inverse variance weighted
MR Egger
1500
1200
900
600
300
0.000
0.002
0.004
$1/SE_{IV}$
β_{IV}

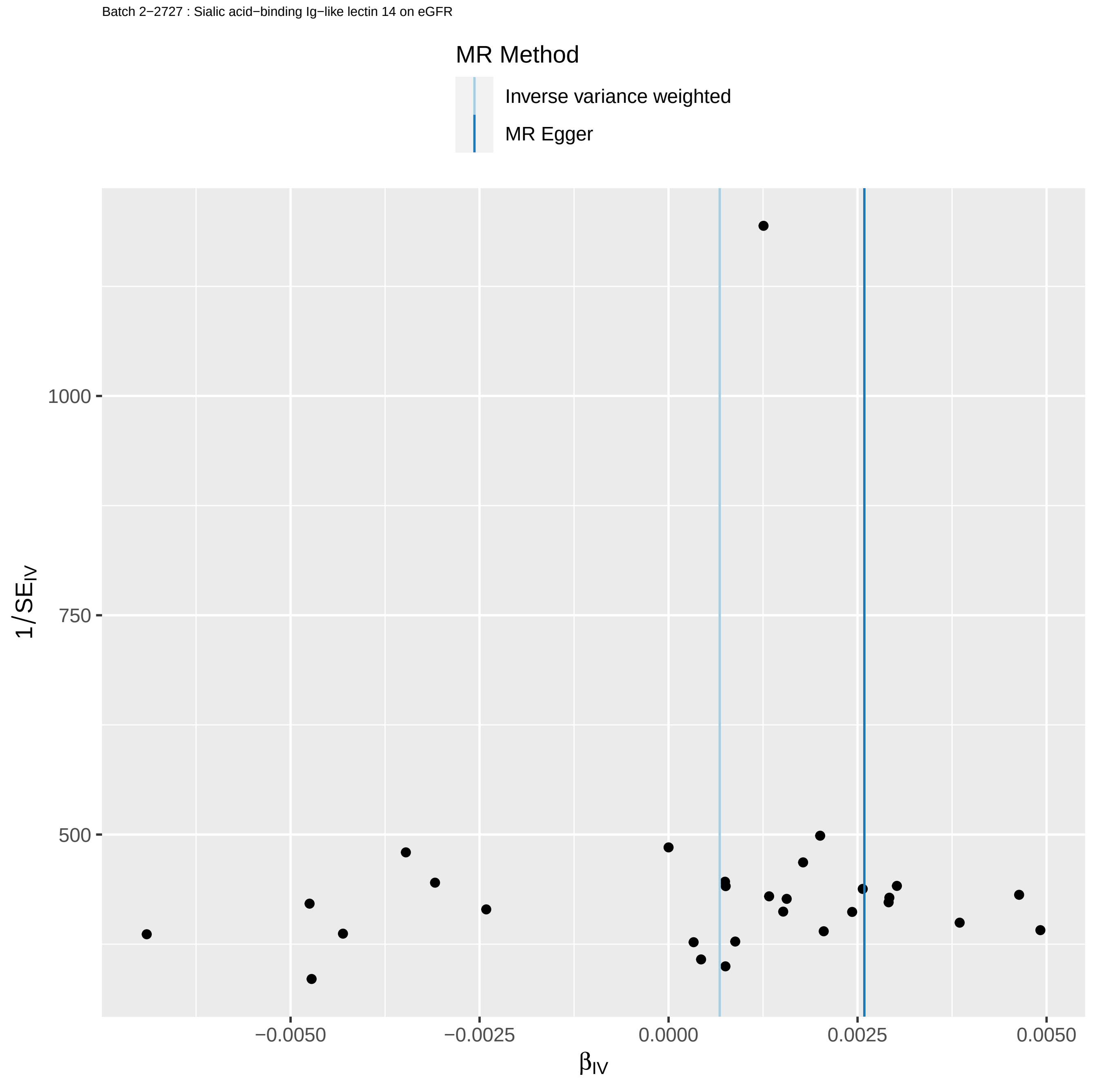
Batch 2−2727 : Sialic acid−binding Ig−like lectin 14 on eGFR
MR Method
Inverse variance weighted
MR Egger
1000
750
500
1/SE$_{IV}$
−0.0050
−0.0025
0.0000
0.0025
0.0050
β$_{IV}$

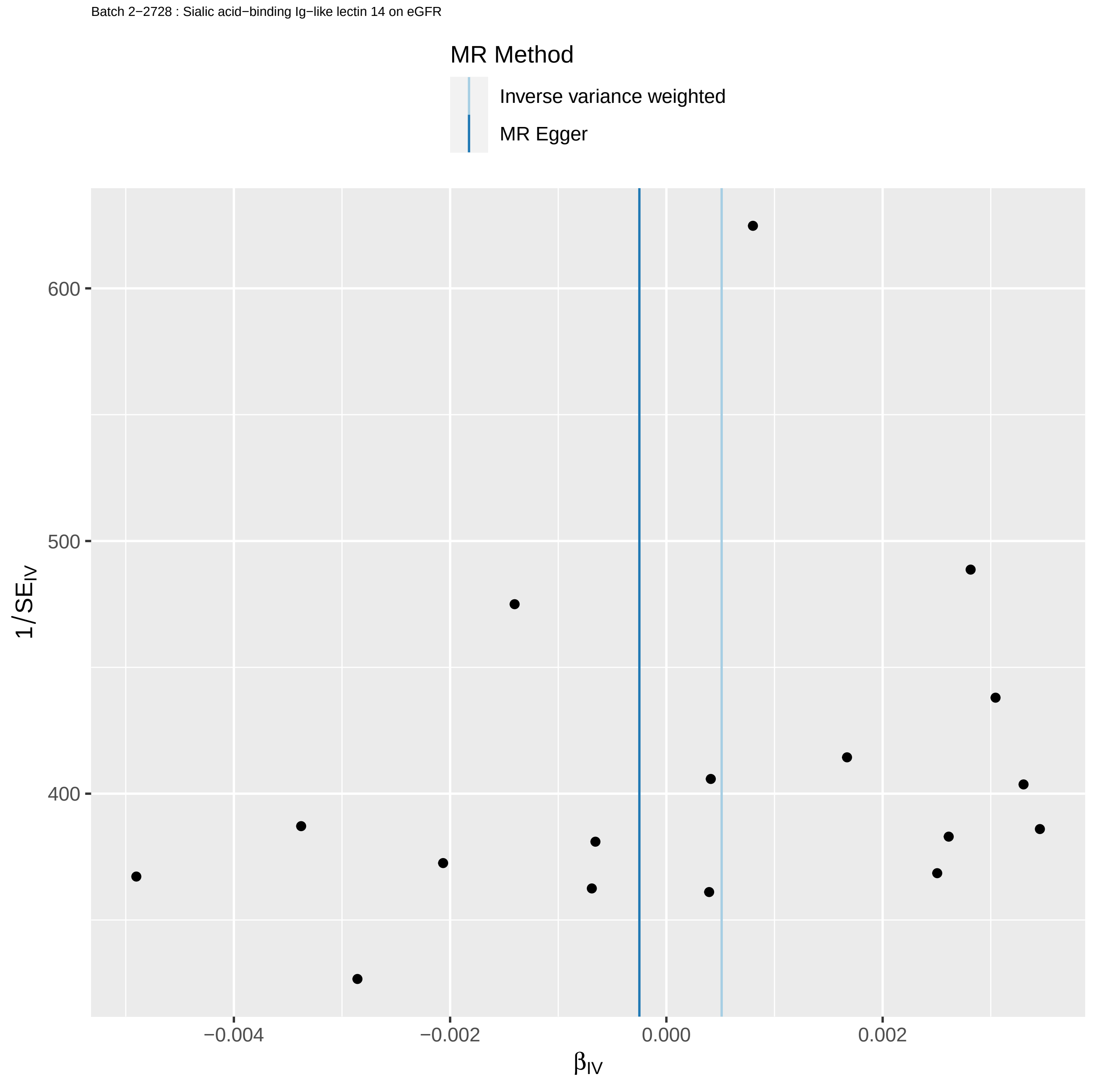
Batch 2−2728 : Sialic acid−binding Ig−like lectin 14 on eGFR
MR Method
Inverse variance weighted
MR Egger
600
500
400
1/SE_IV
−0.004
−0.002
0.000
0.002
β_IV

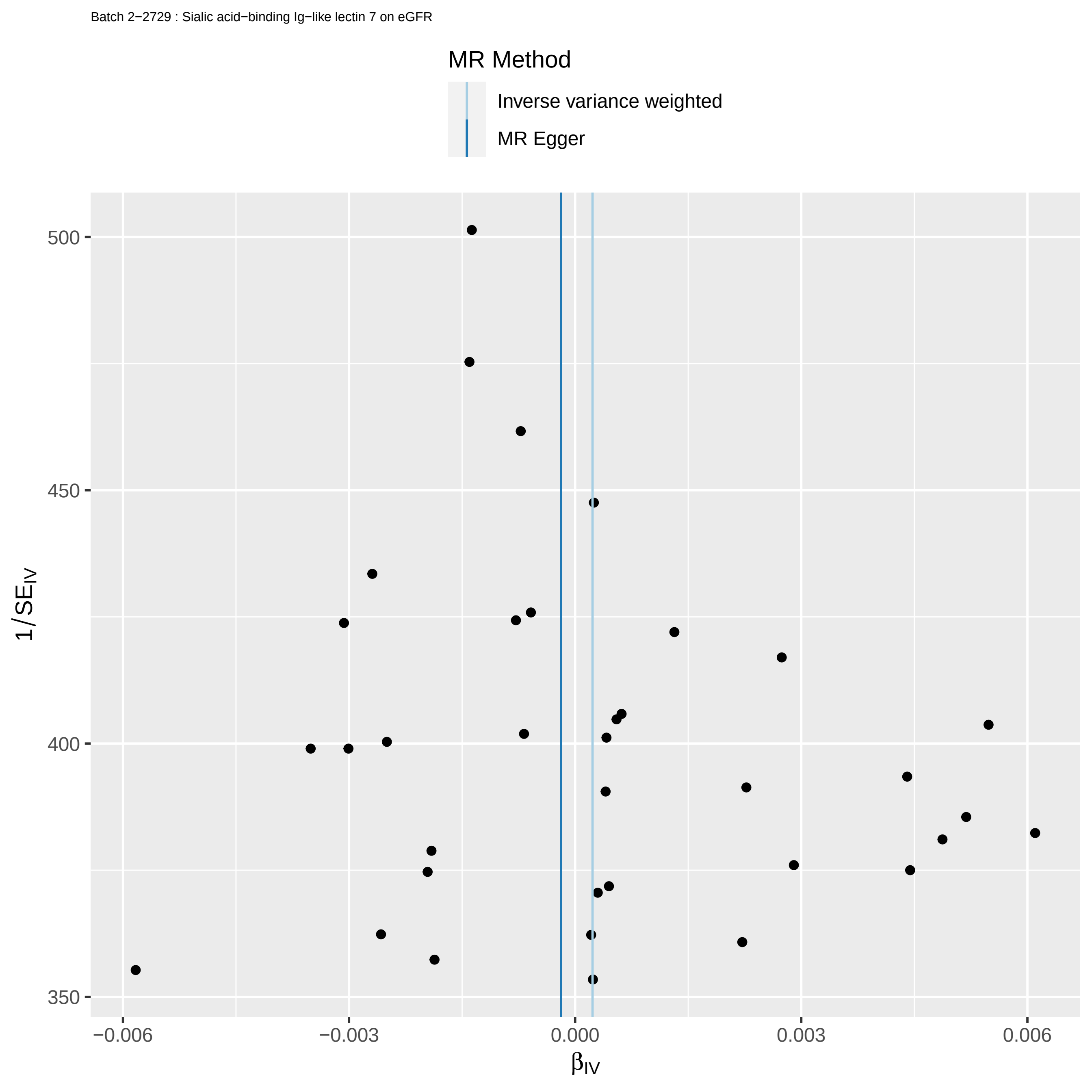
Batch 2−2729 : Sialic acid−binding Ig−like lectin 7 on eGFR
MR Method
Inverse variance weighted
MR Egger
1/SE_IV
500
450
400
350
−0.006
−0.003
0.000
0.003
0.006
β_IV

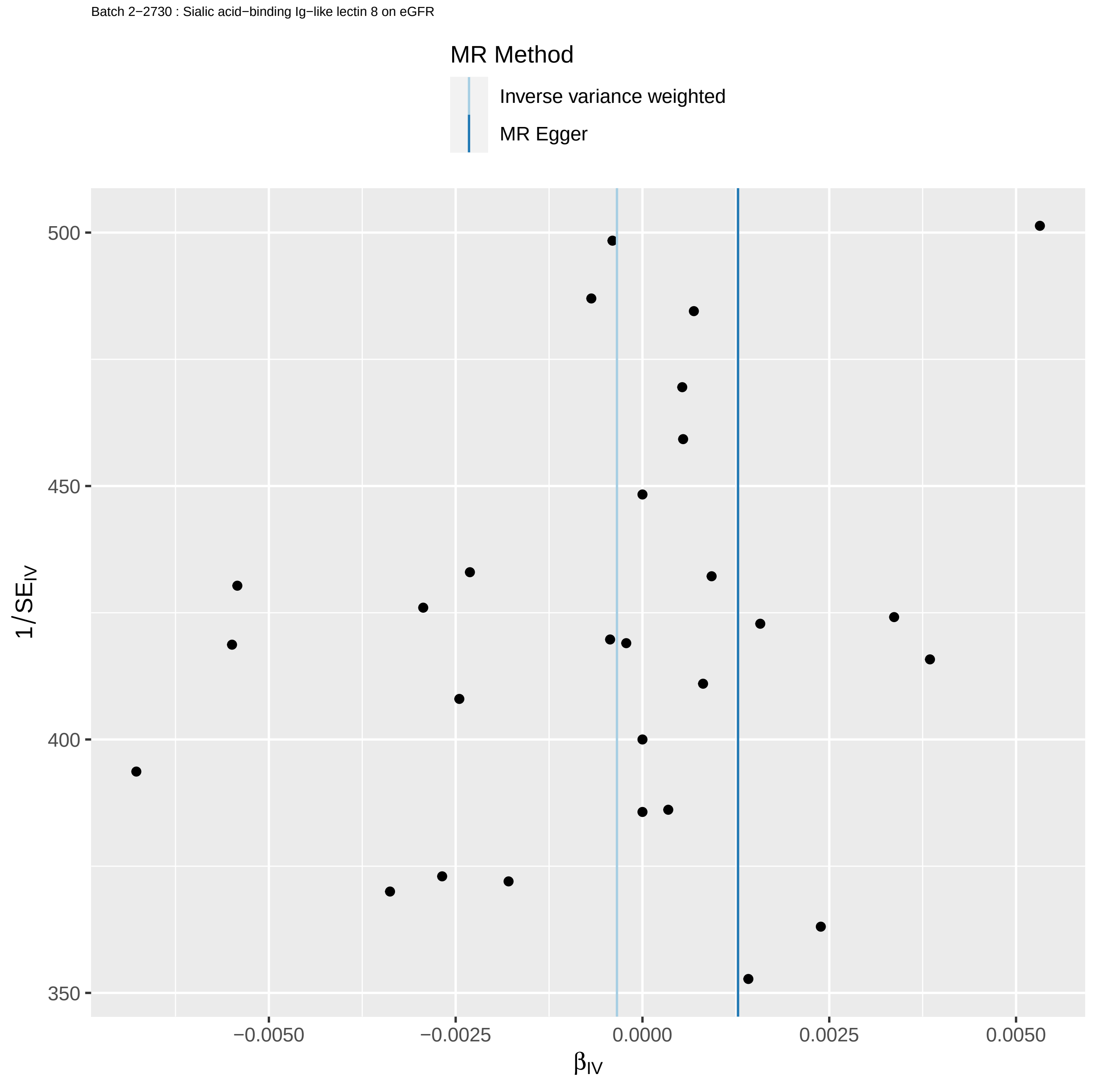
Batch 2−2730 : Sialic acid−binding Ig−like lectin 8 on eGFR
MR Method
Inverse variance weighted
MR Egger
500
450
400
350
1/SE$_{IV}$
−0.0050
−0.0025
0.0000
0.0025
0.0050
β$_{IV}$

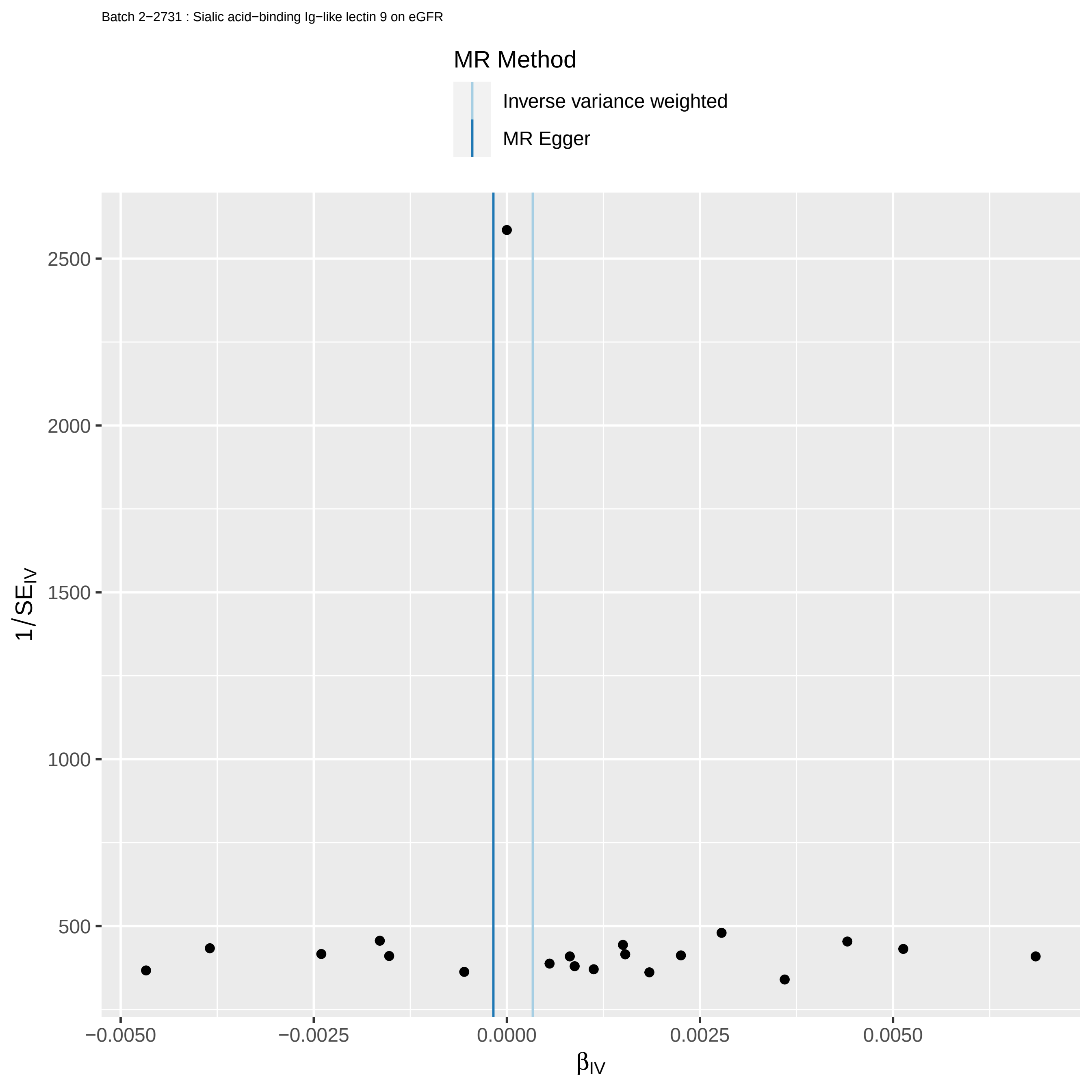
Batch 2−2731 : Sialic acid−binding Ig−like lectin 9 on eGFR
MR Method
Inverse variance weighted
MR Egger
2500
2000
1500
1000
500
$1/SE_{IV}$
−0.0050
−0.0025
0.0000
0.0025
0.0050
β_{IV}

Batch 2−2732 : Tyrosine−protein phosphatase non−receptor type substrate 1 on eGFR

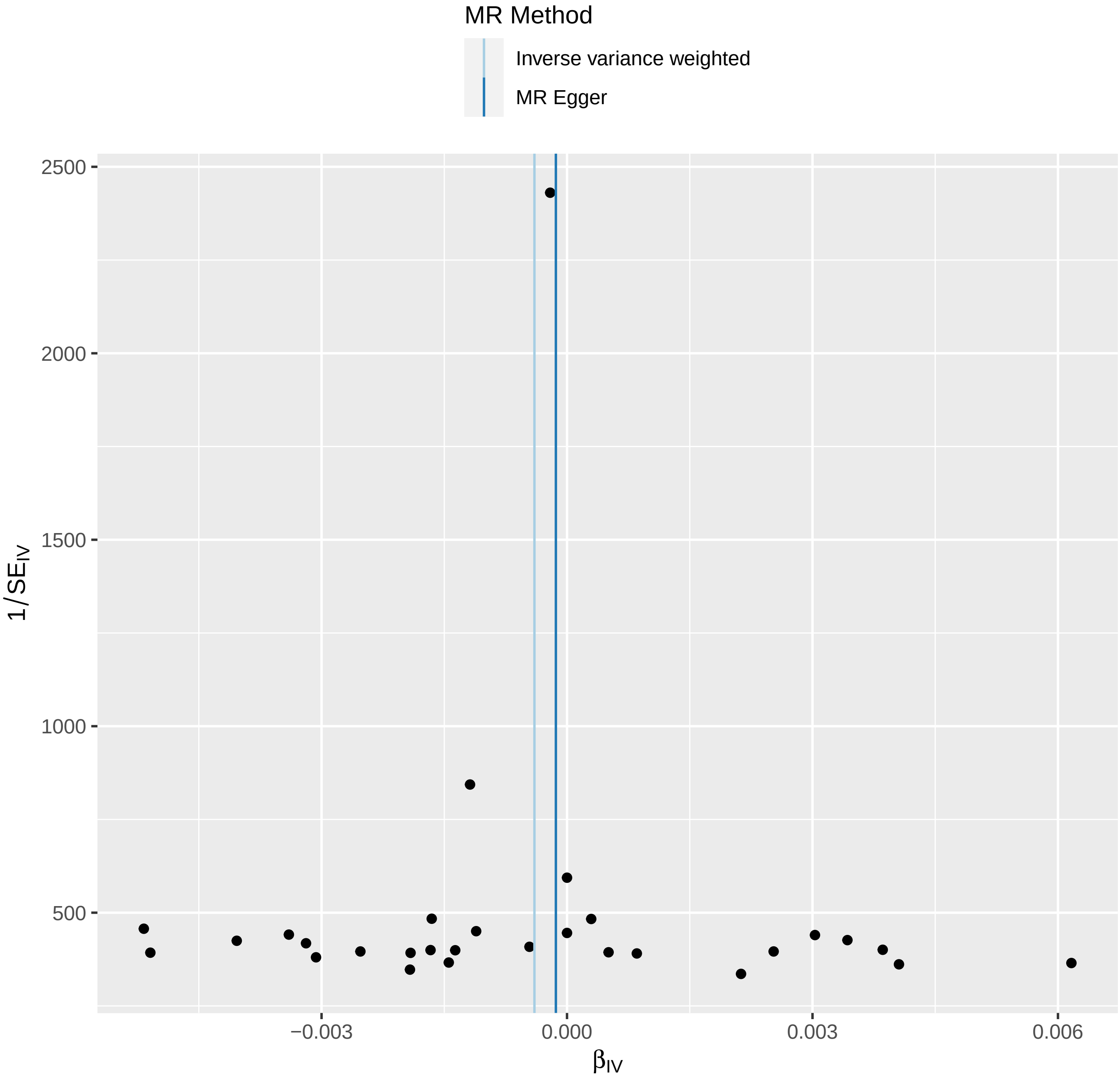

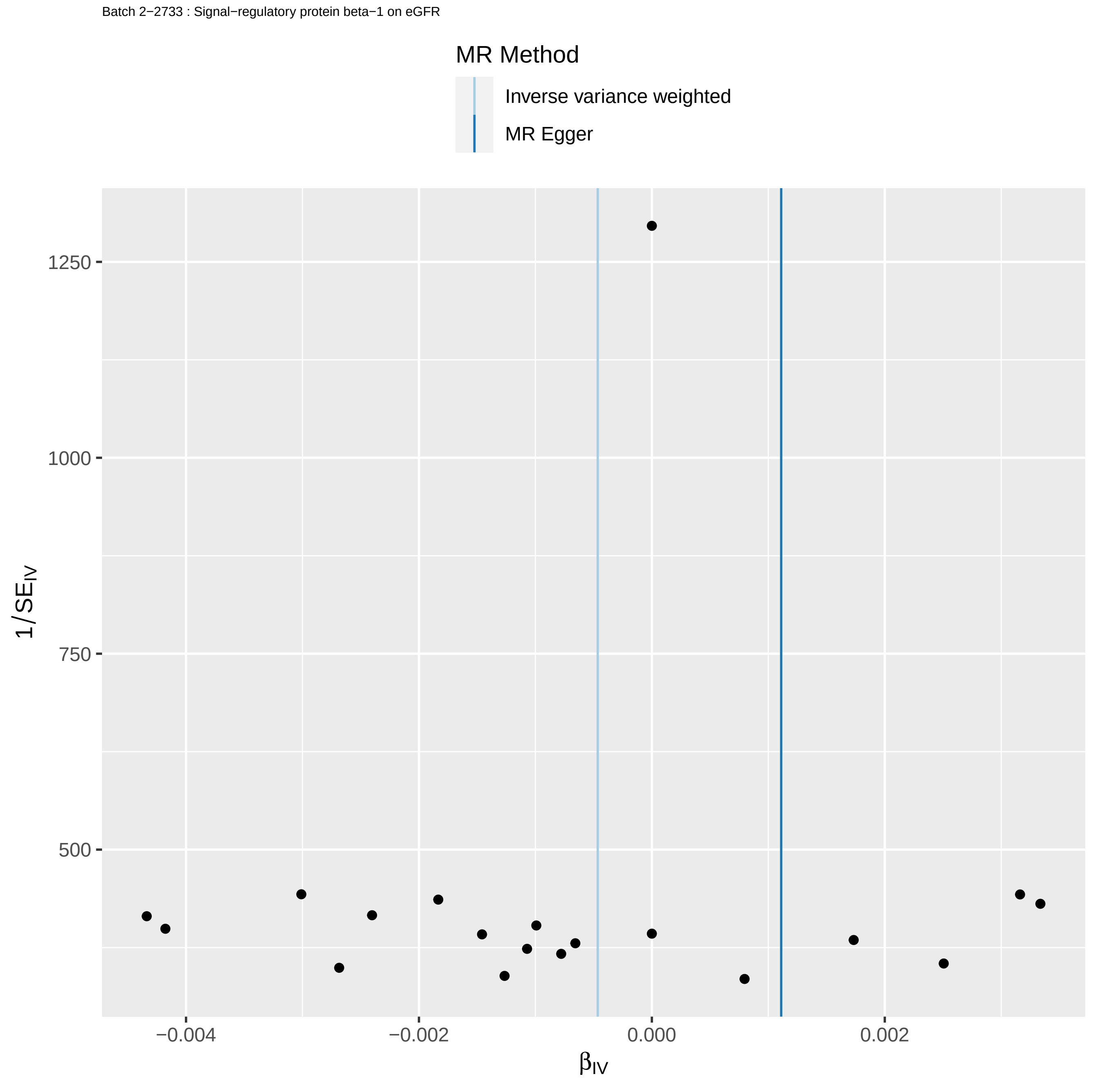

Batch 2−2733 : Signal−regulatory protein beta−1 on eGFR
MR Method
Inverse variance weighted
MR Egger
1250
1000
750
500
$1/SE_{IV}$
−0.004
−0.002
0.000
0.002
β_{IV}

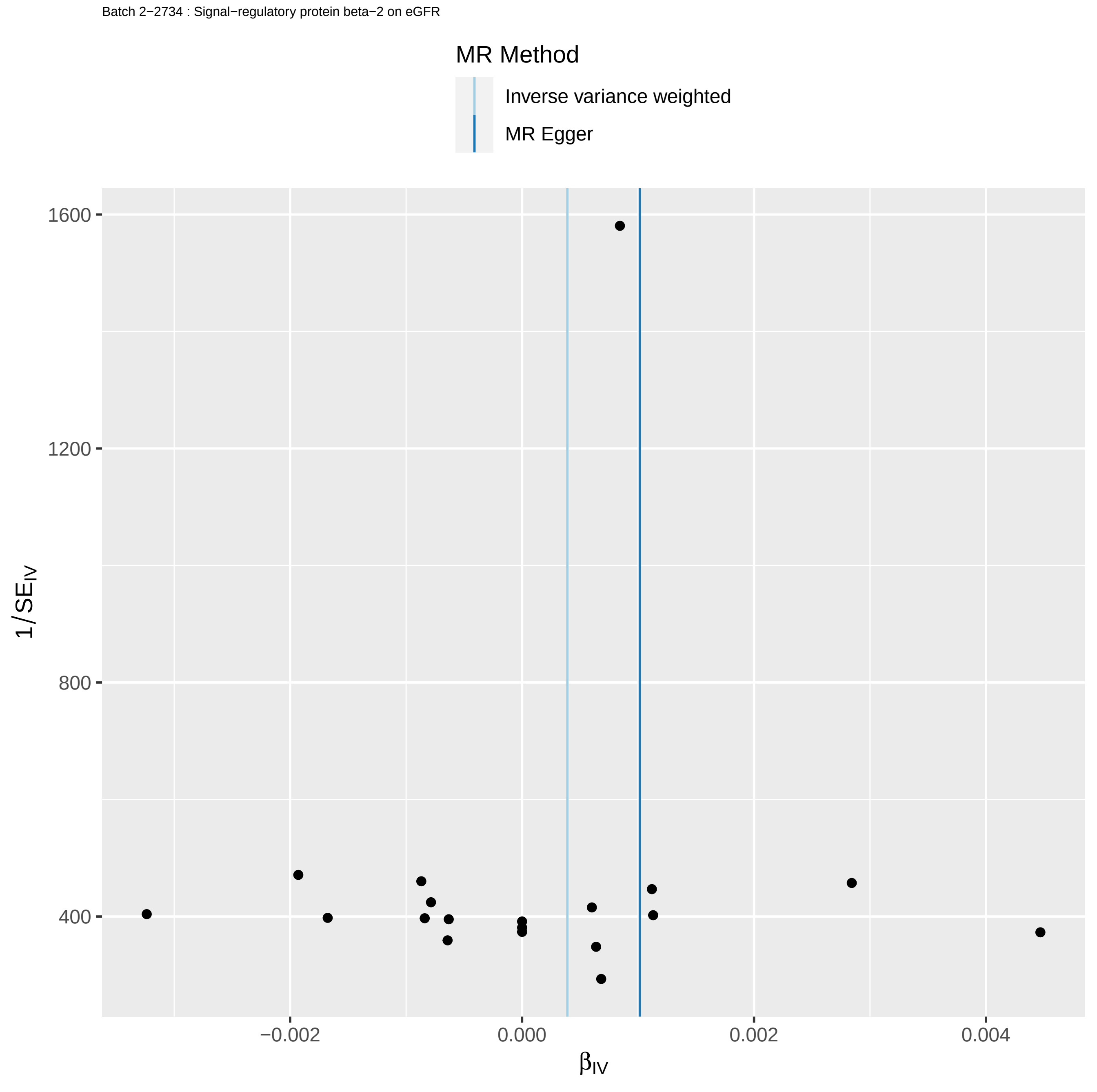

Batch 2−2734 : Signal−regulatory protein beta−2 on eGFR
MR Method
Inverse variance weighted
MR Egger
1600
1200
800
400
$1/SE_{IV}$
−0.002
0.000
0.002
0.004
β_{IV}

Batch 2−2735 : Signal−regulatory protein gamma on eGFR

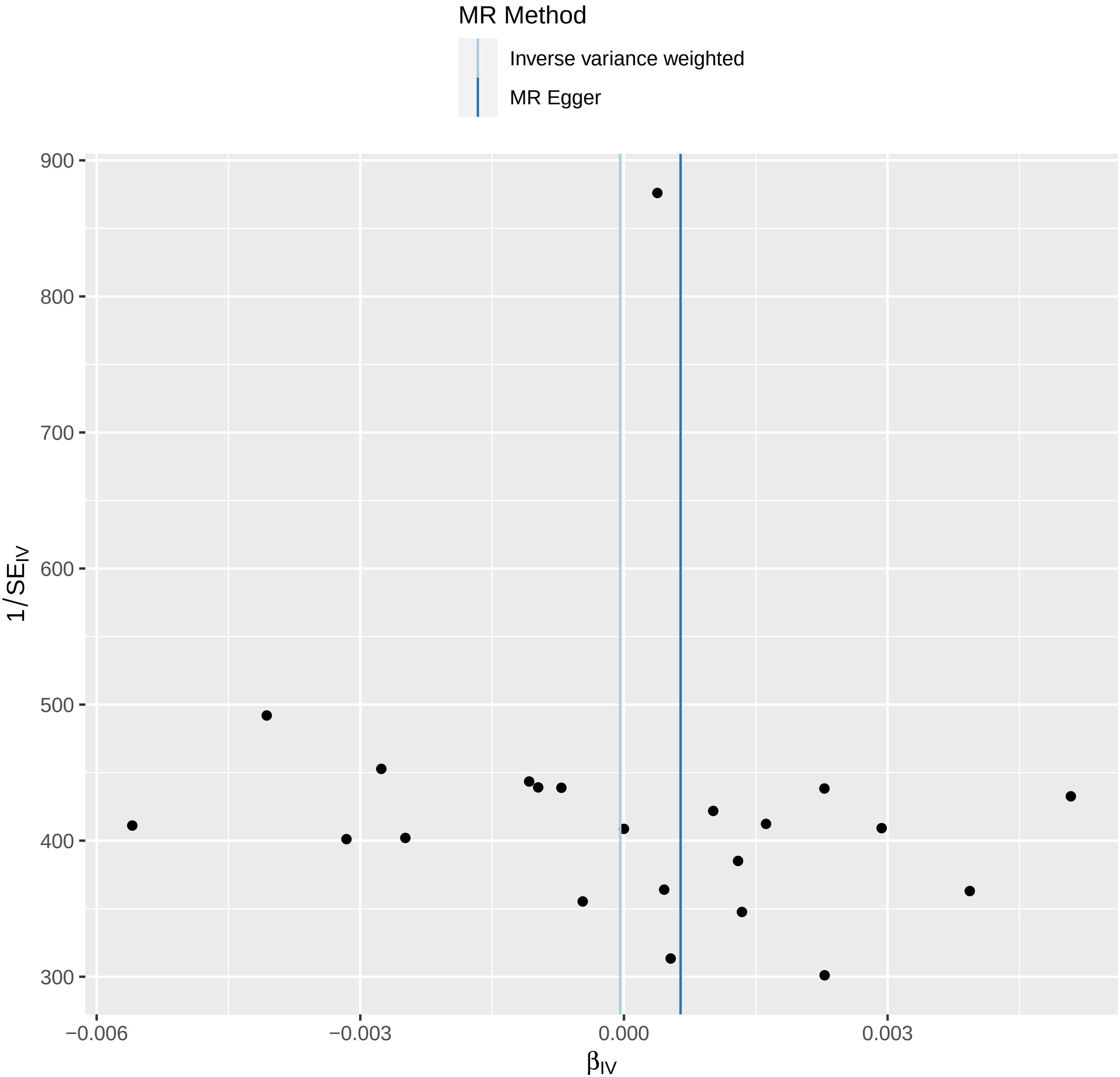

Batch 2−2736 : NAD−dependent protein deacetylase sirtuin−2 on eGFR

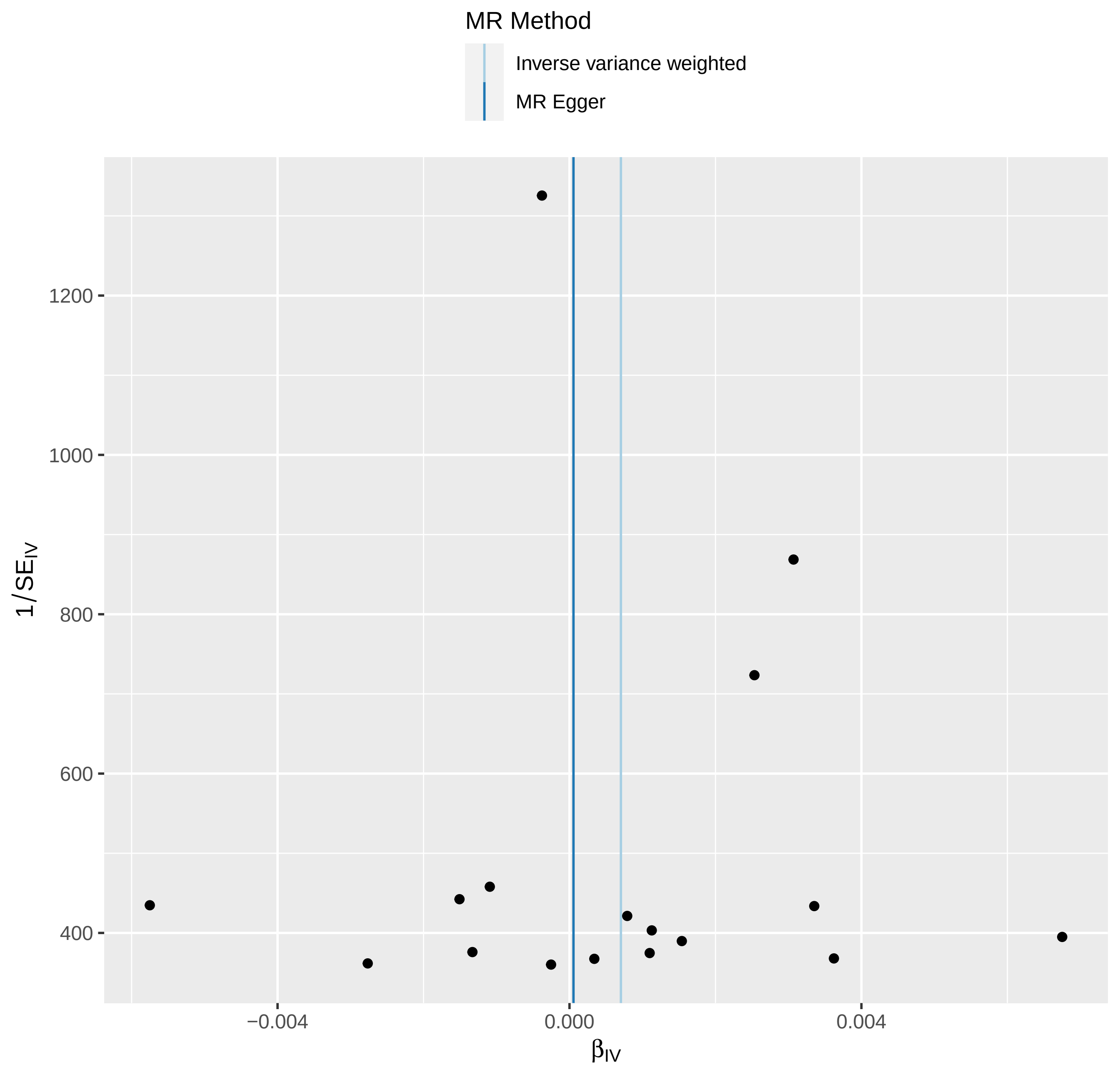

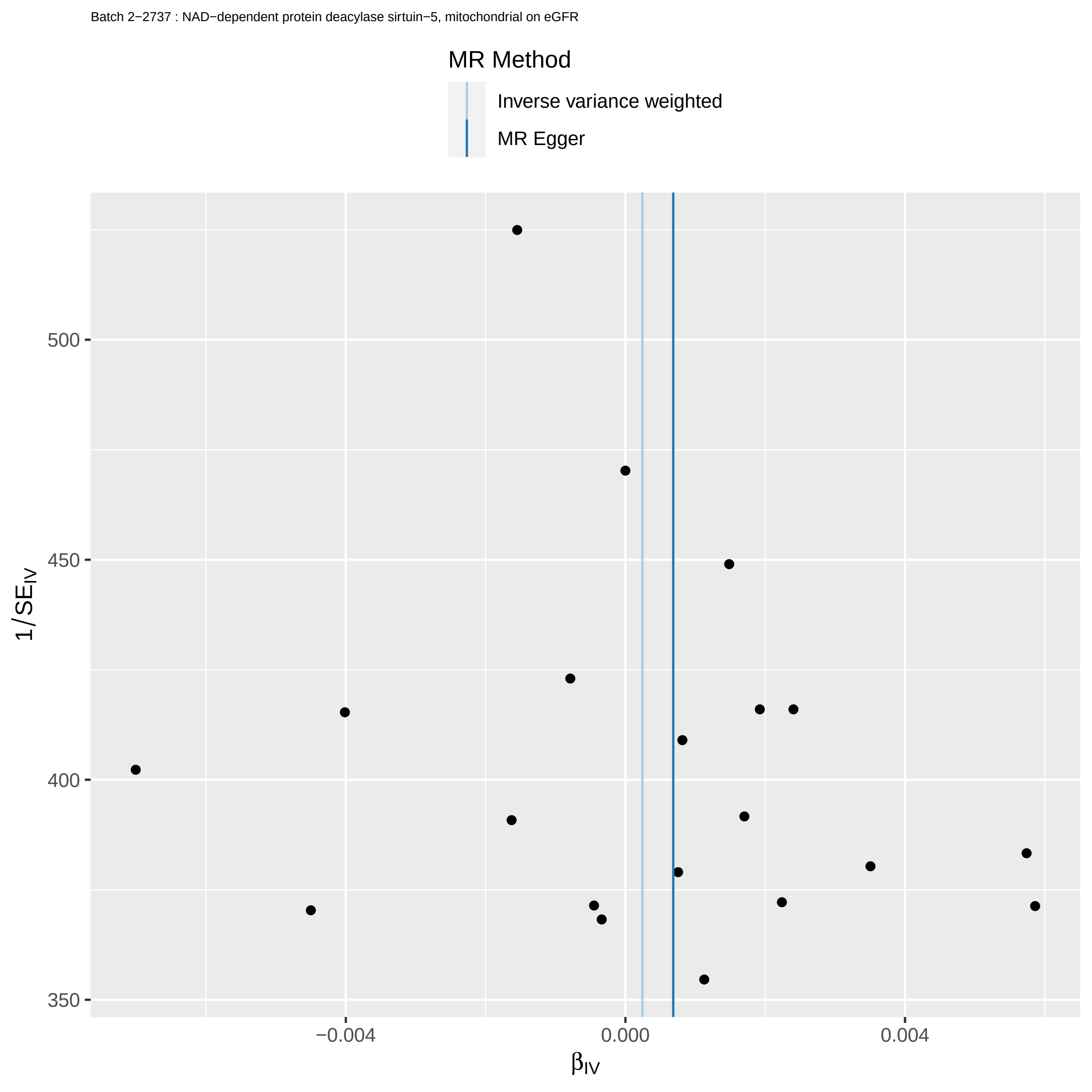
Batch 2−2737 : NAD−dependent protein deacylase sirtuin−5, mitochondrial on eGFR
MR Method
Inverse variance weighted
MR Egger
500
450
400
350
1/SE$_{IV}$
−0.004
0.000
0.004
β_{IV}

Batch 2−2738 : Signaling threshold−regulating transmembrane adapter 1 on eGFR

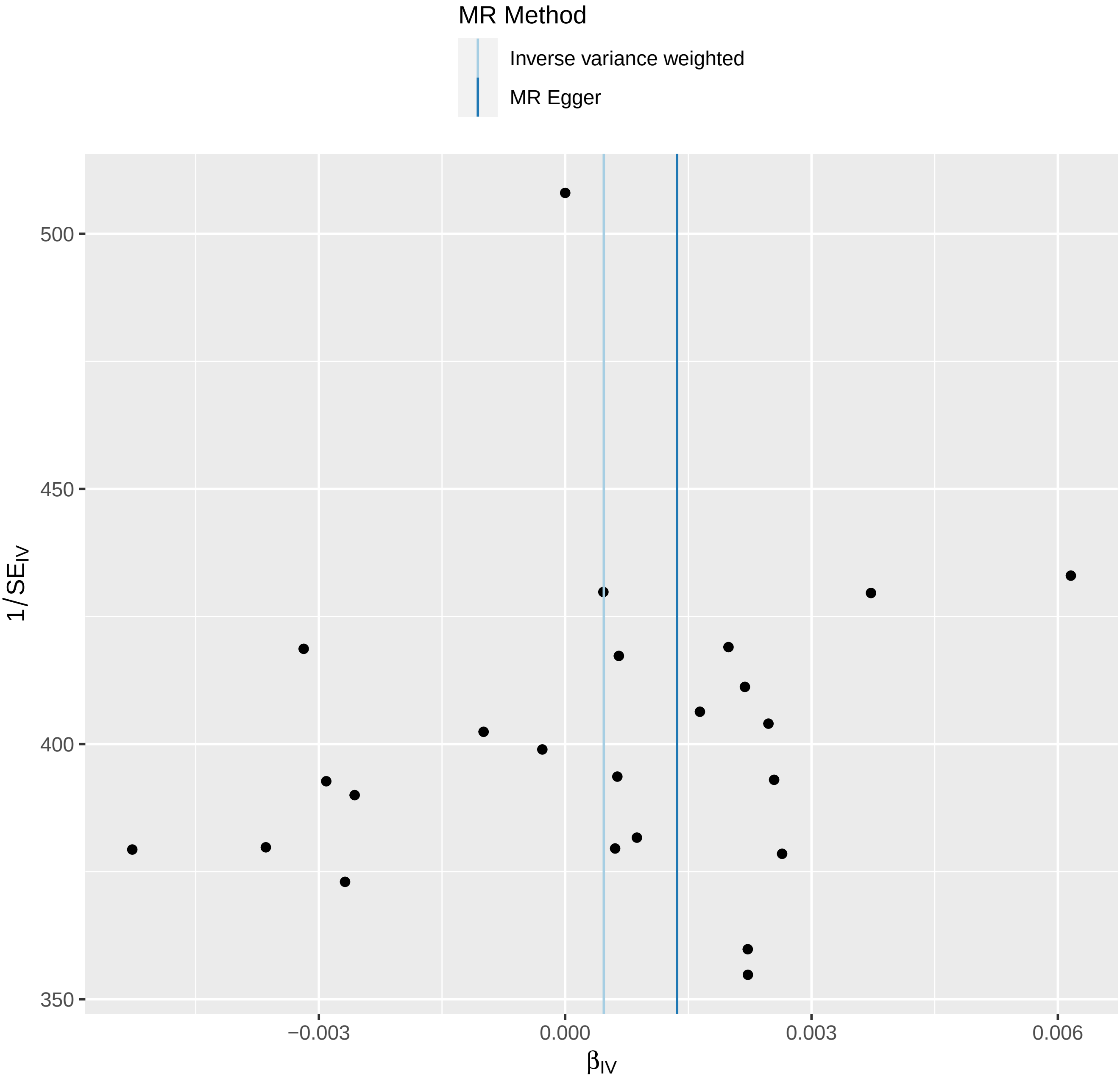

Batch 2−2739 : Ski−like protein on eGFR

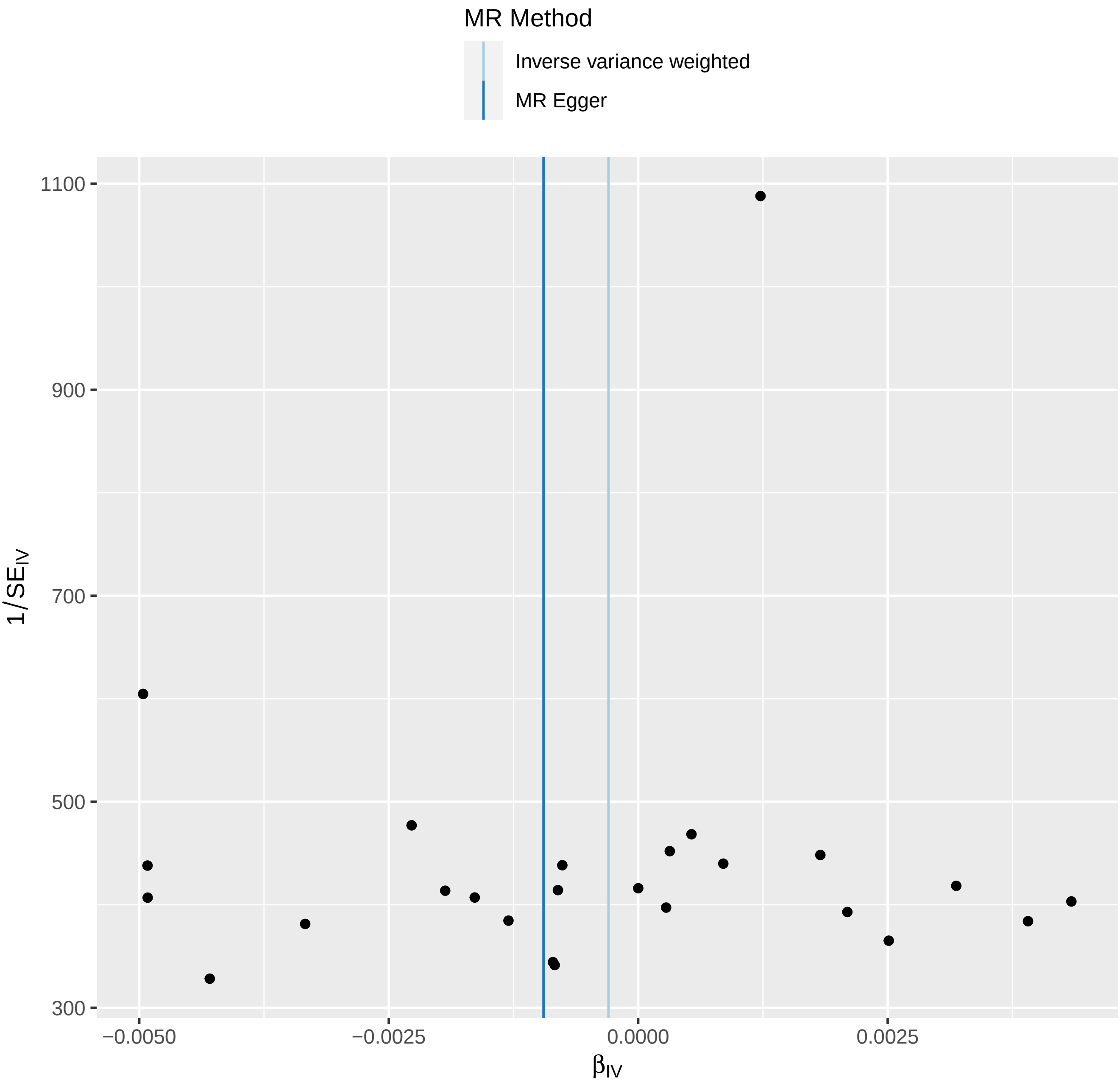

Batch 2−2740 : S−phase kinase−associated protein 1 on eGFR

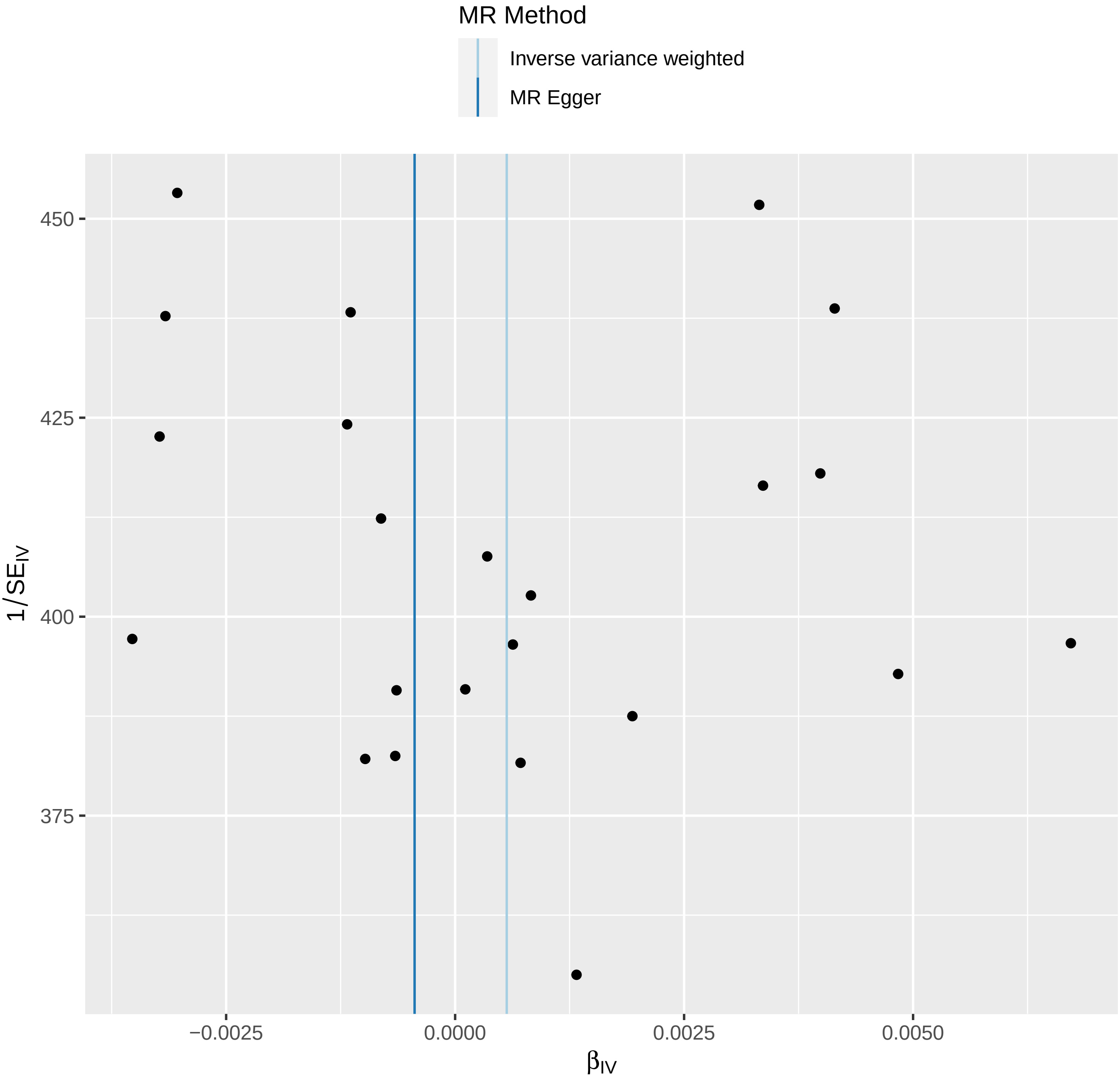

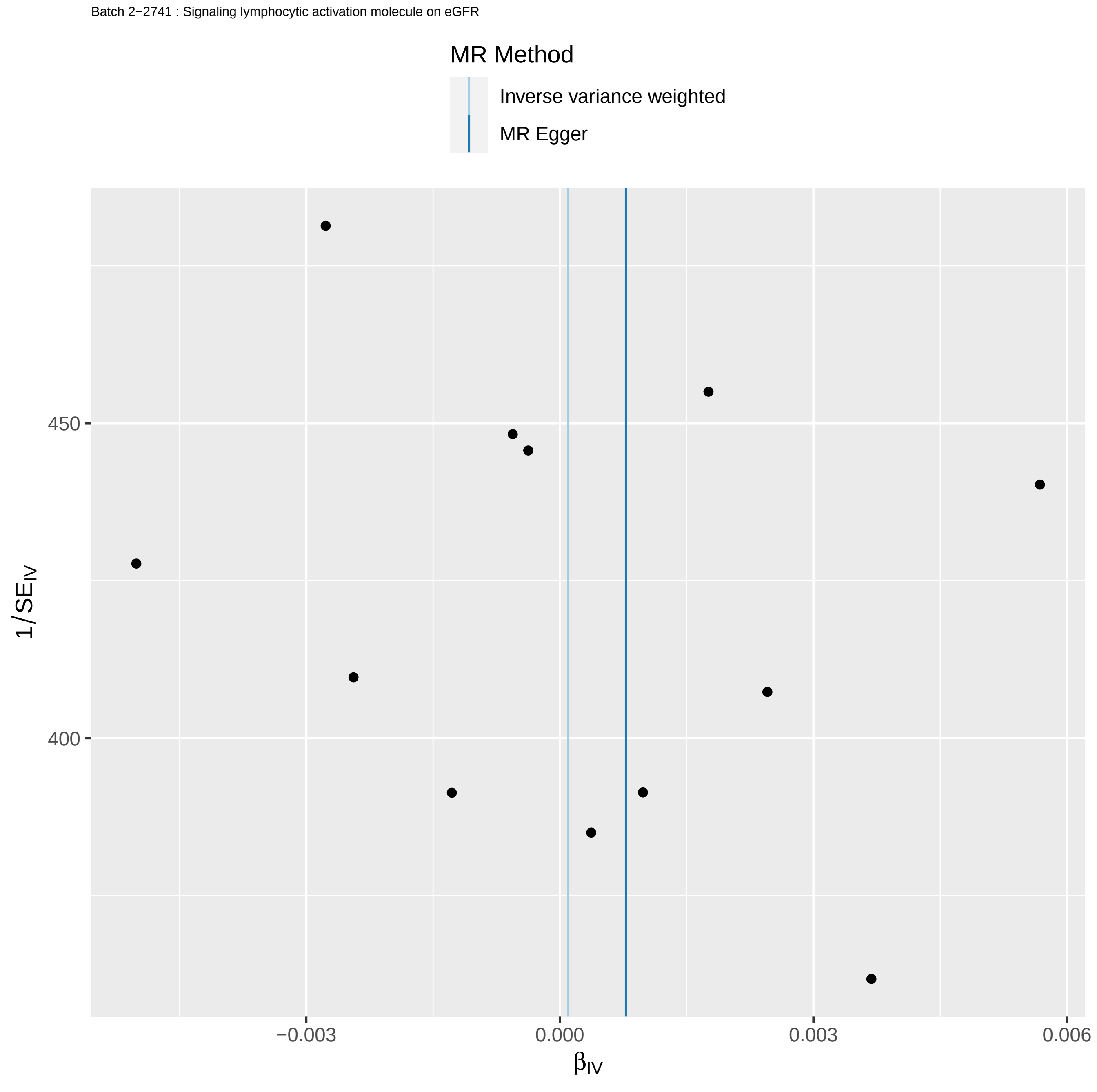

Batch 2−2741 : Signaling lymphocytic activation molecule on eGFR
MR Method
Inverse variance weighted
MR Egger
450
400
1/SE$_{IV}$
−0.003
0.000
0.003
0.006
β$_{IV}$

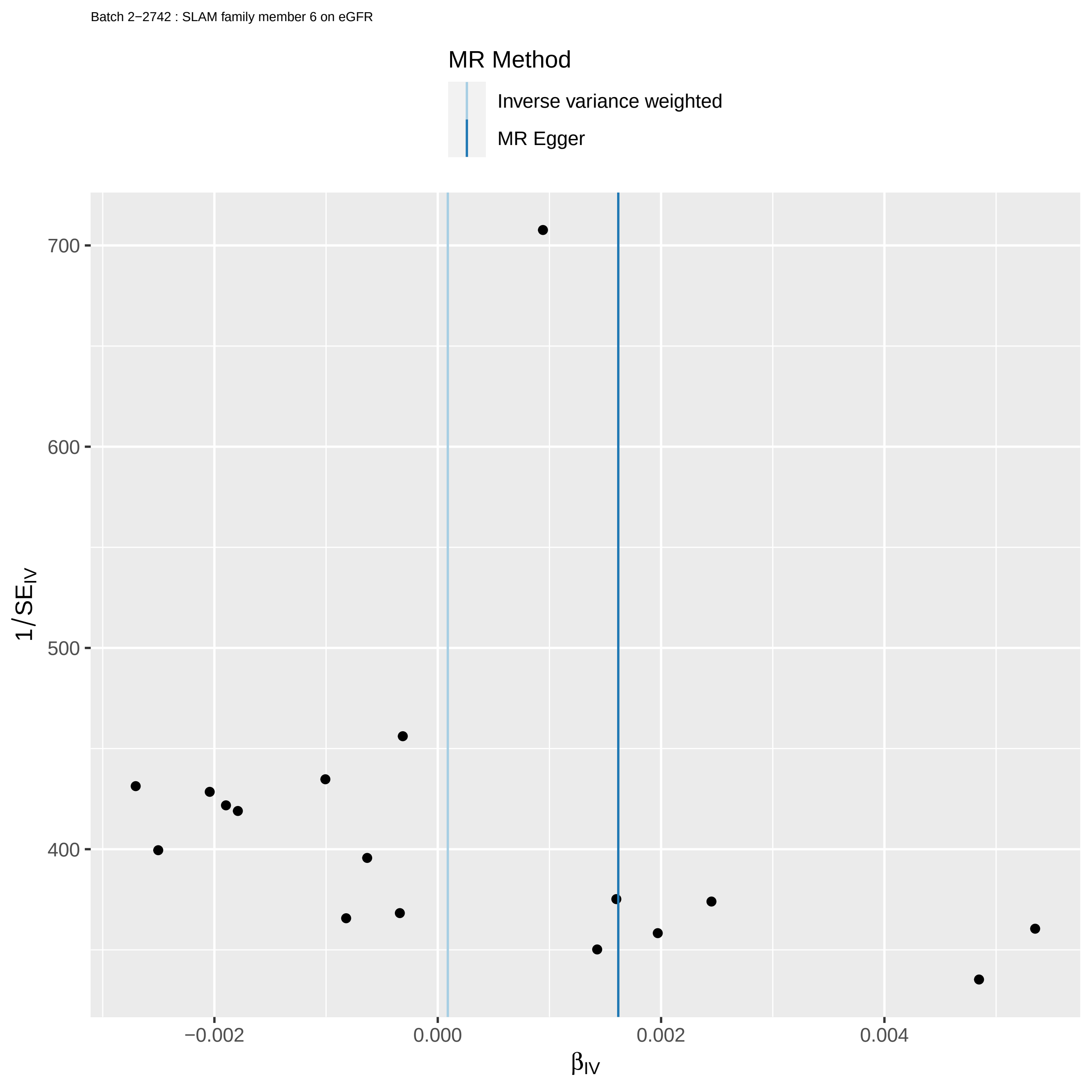

Batch 2−2742 : SLAM family member 6 on eGFR
MR Method
Inverse variance weighted
MR Egger
700
600
500
400
1/SE$_{IV}$
−0.002
0.000
0.002
0.004
β$_{IV}$

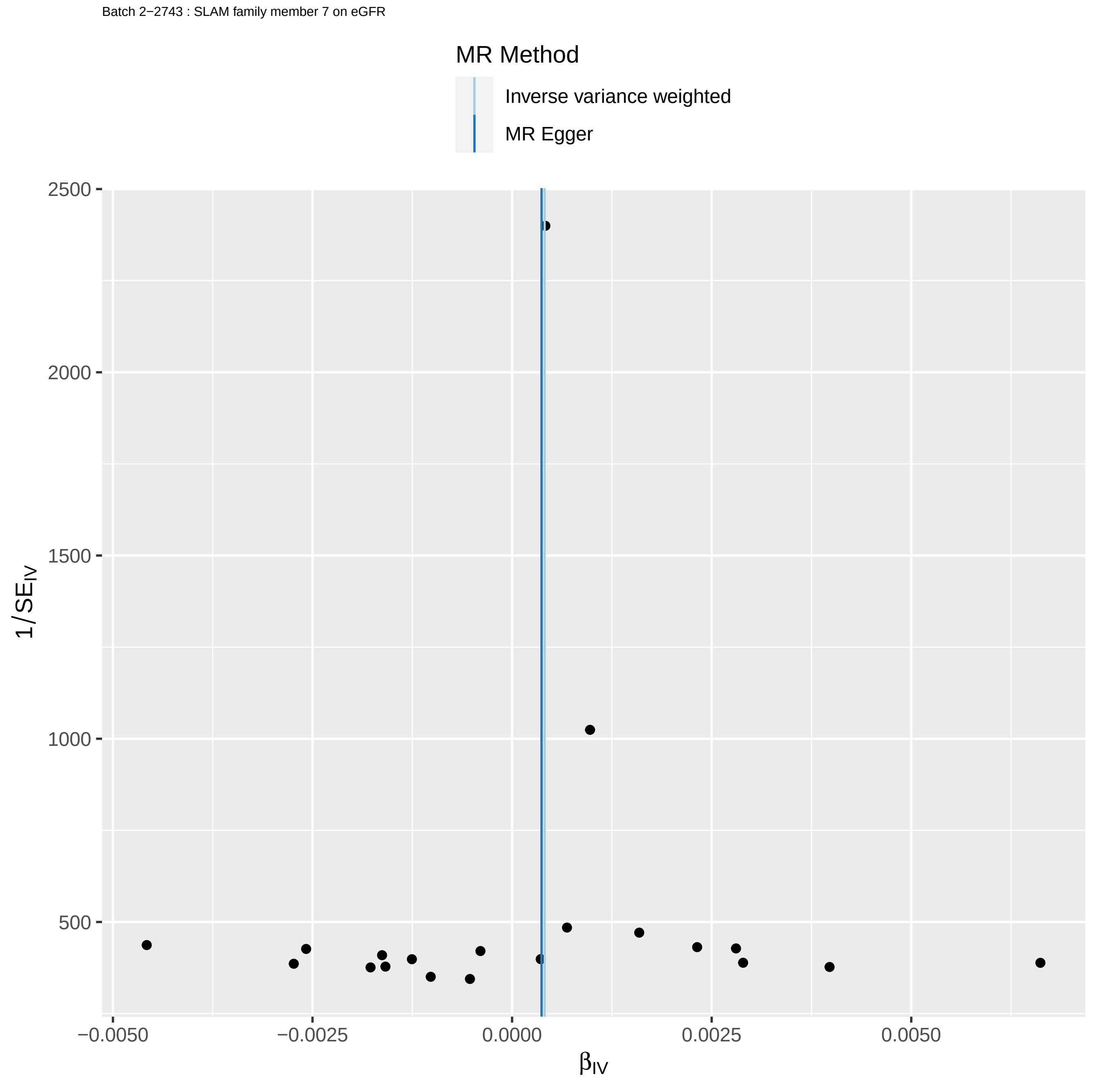

Batch 2−2743 : SLAM family member 7 on eGFR
MR Method
Inverse variance weighted
MR Egger
2500
2000
1500
1000
500
1/SE$_{IV}$
−0.0050
−0.0025
0.0000
0.0025
0.0050
β$_{IV}$

Batch 2−2744 : SLAM family member 7 on eGFR

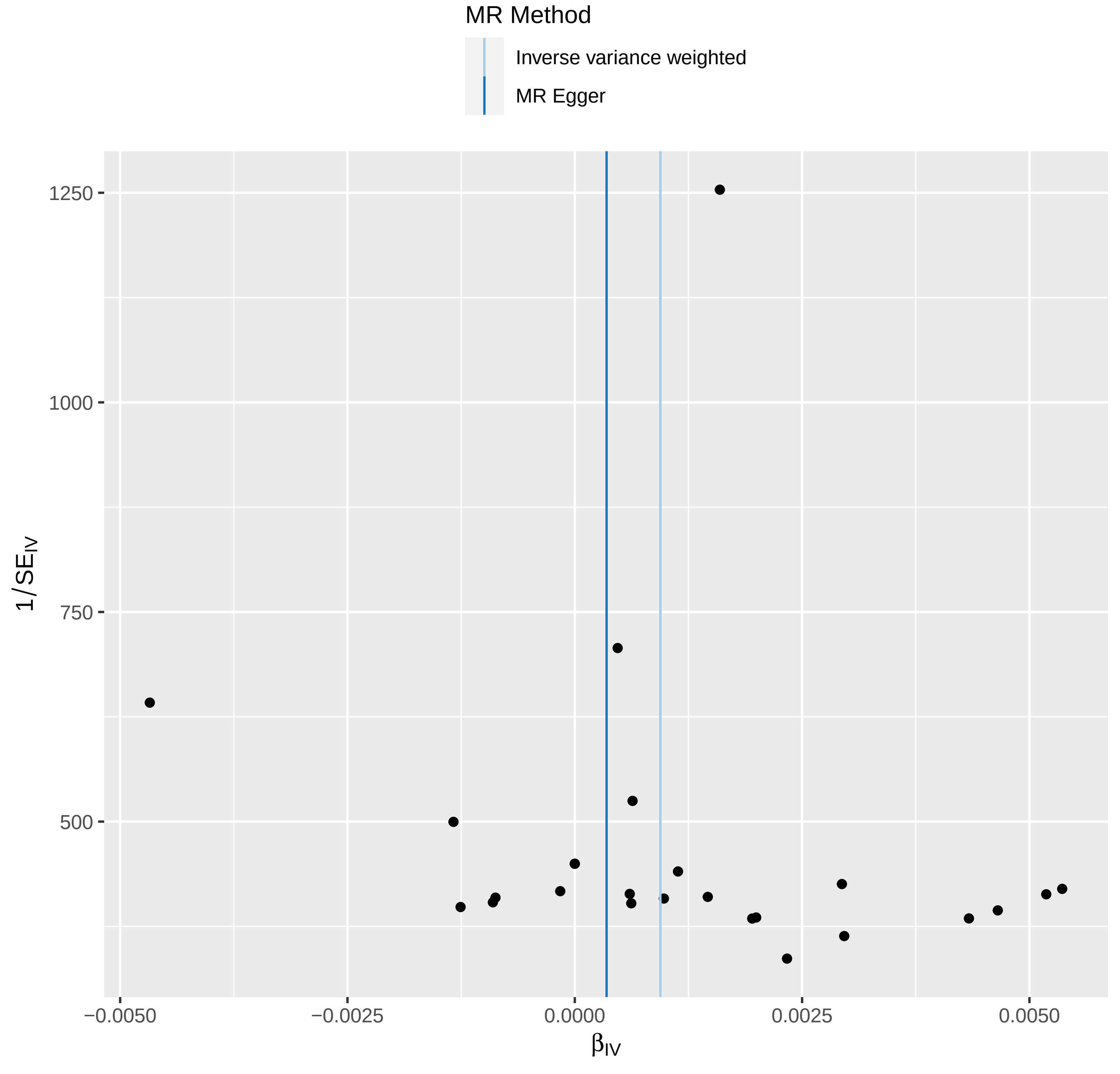

Batch 2−2745 : SLAM family member 8 on eGFR

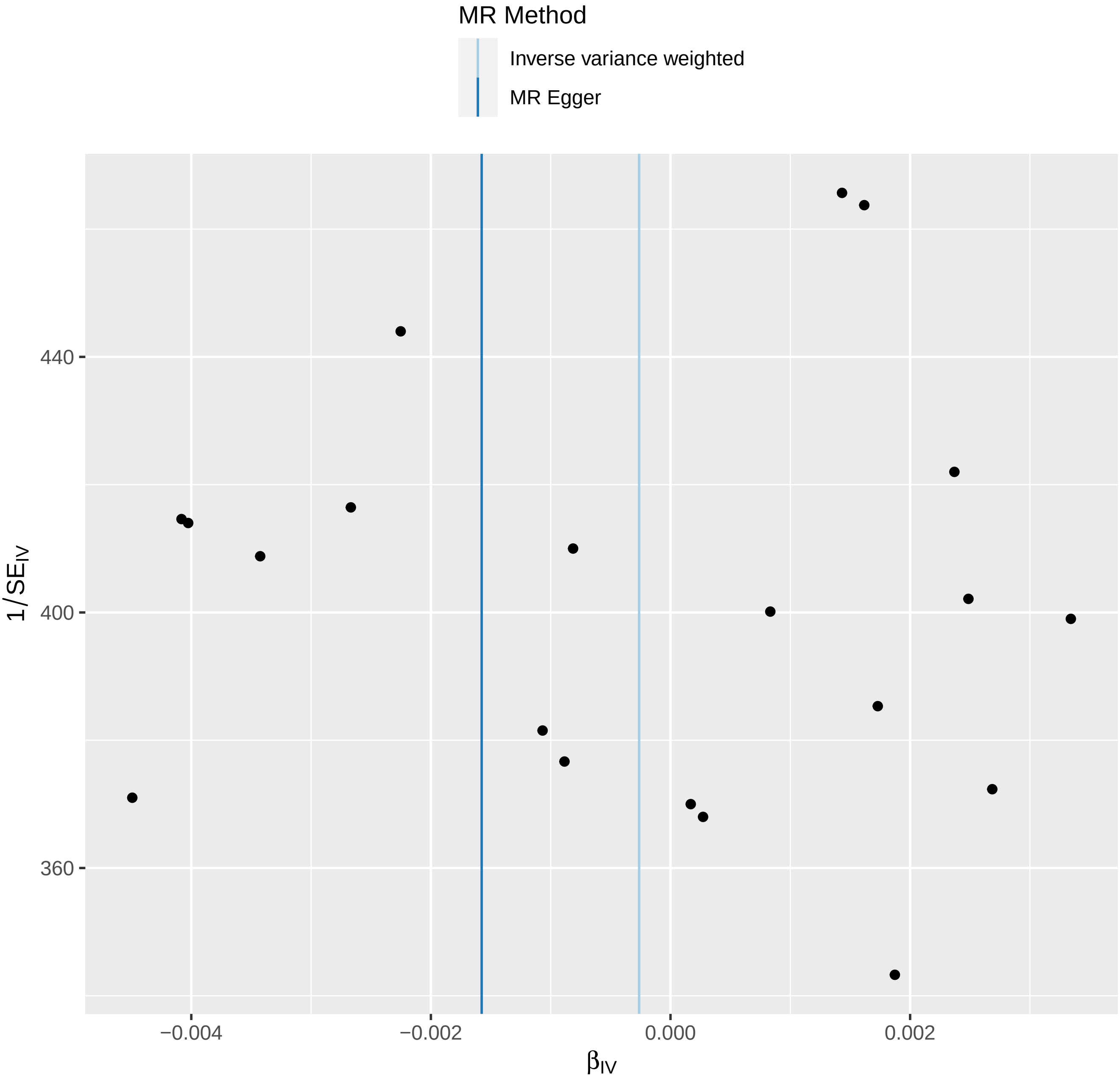

Batch 2−2746 : Urea transporter 1 on eGFR

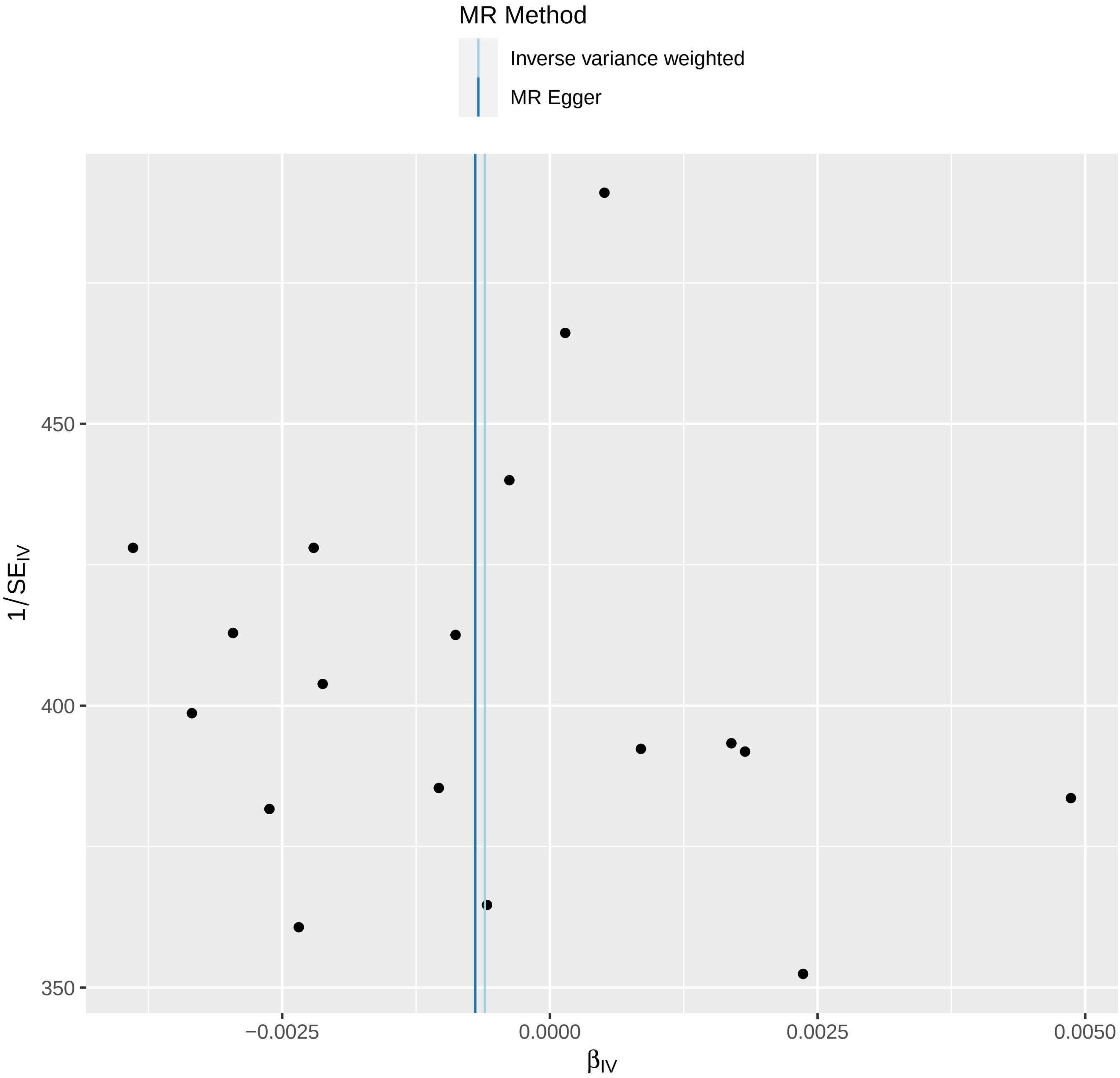

Batch 2−2747 : Urea transporter 2 on eGFR

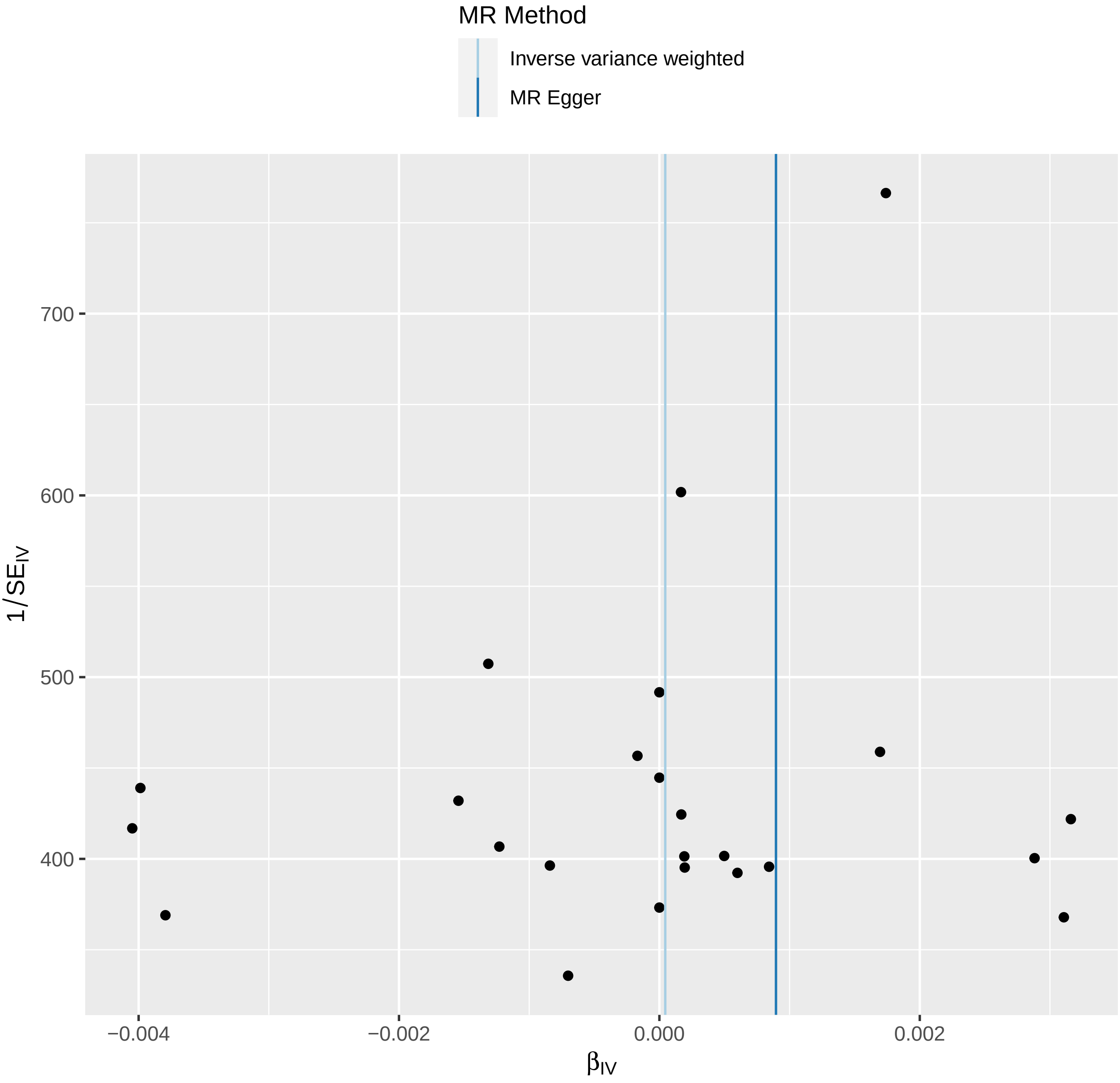

Batch 2−2748 : Solute carrier family 22 member 16 on eGFR

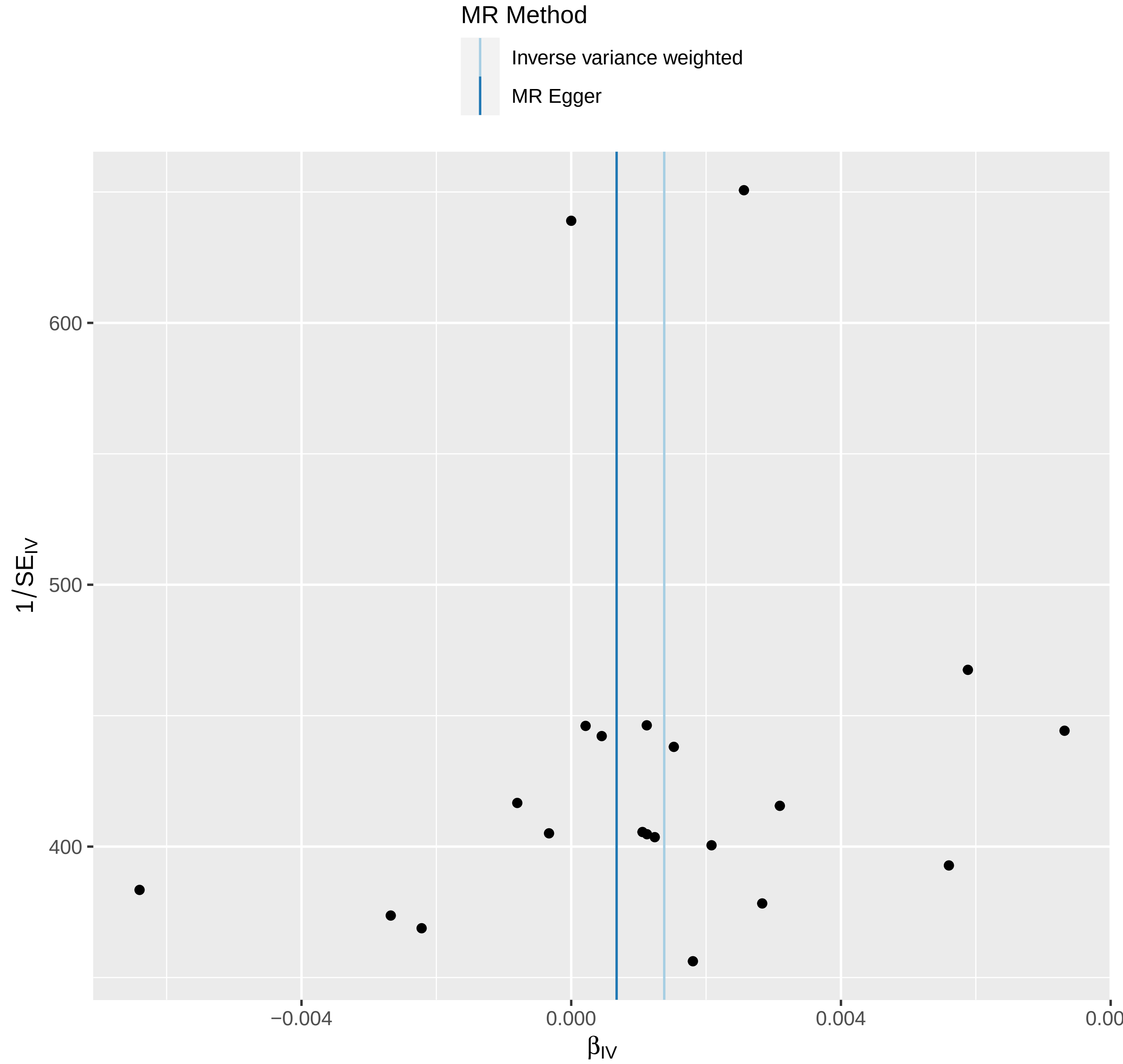

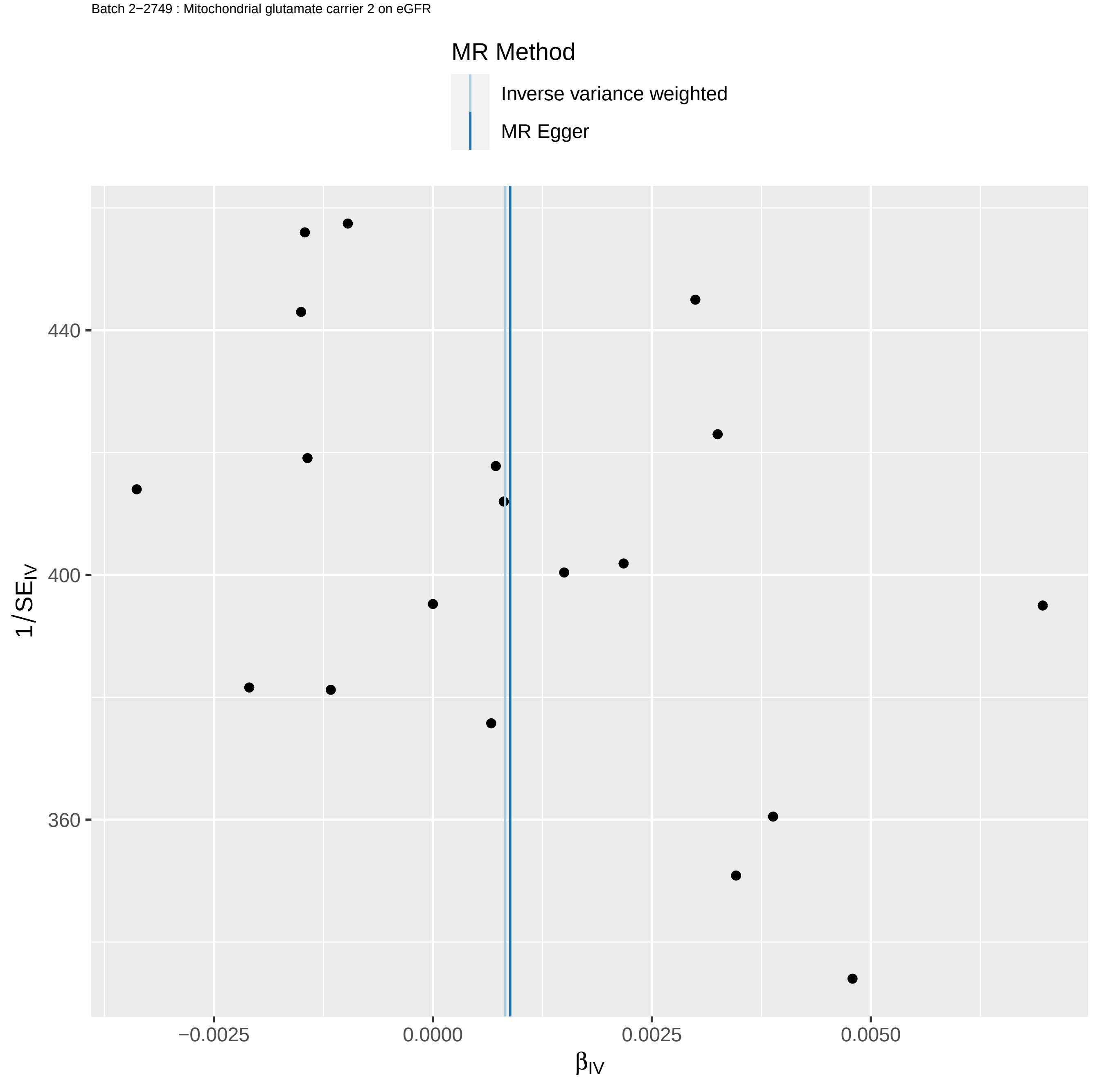
Batch 2−2749 : Mitochondrial glutamate carrier 2 on eGFR
MR Method
Inverse variance weighted
MR Egger
440
400
360
1/SE$_{IV}$
−0.0025
0.0000
0.0025
0.0050
β$_{IV}$

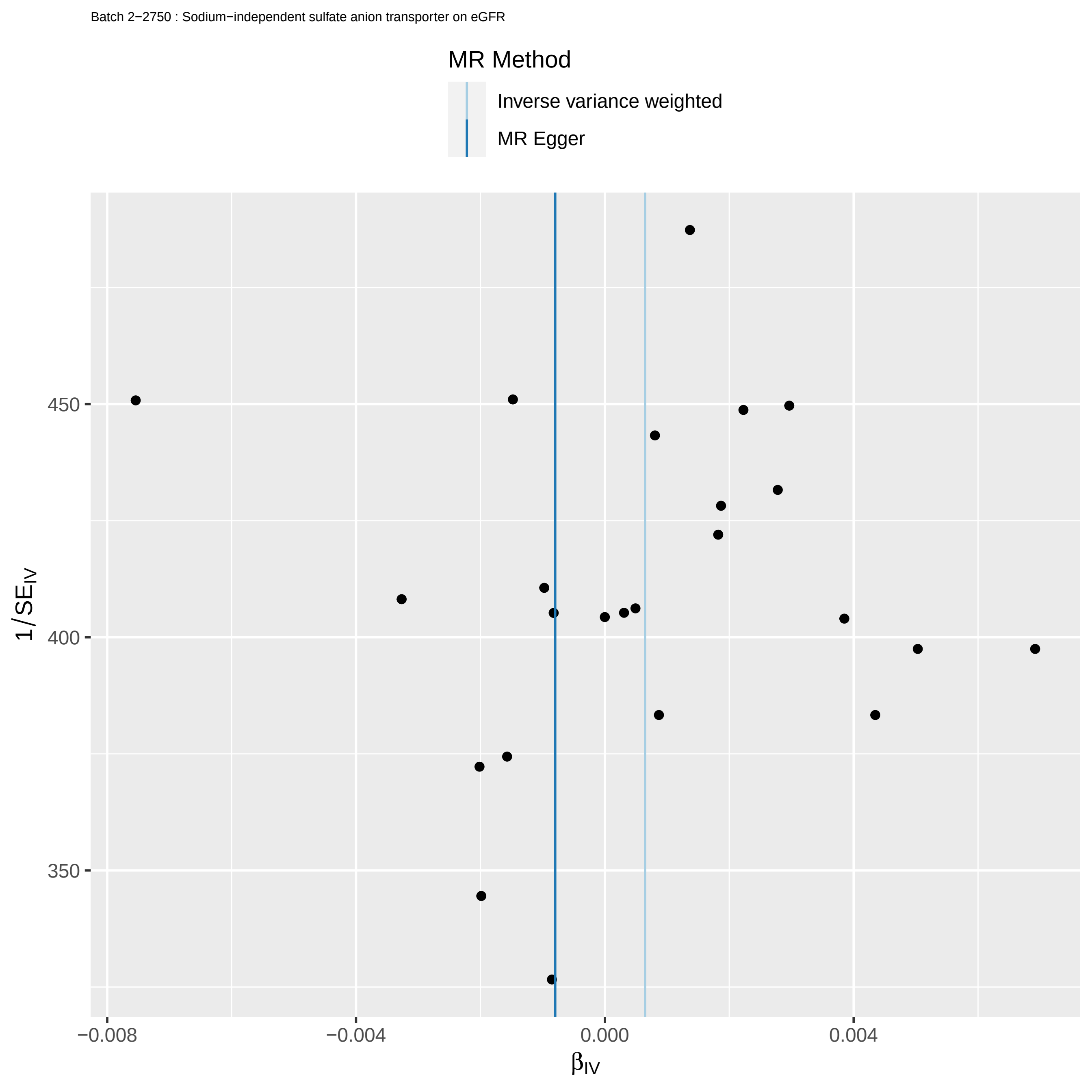

Batch 2−2750 : Sodium−independent sulfate anion transporter on eGFR
MR Method
Inverse variance weighted
MR Egger
450
400
350
$1/SE_{IV}$
−0.008
−0.004
0.000
0.004
β_{IV}

Batch 2−2751 : Prestin on eGFR

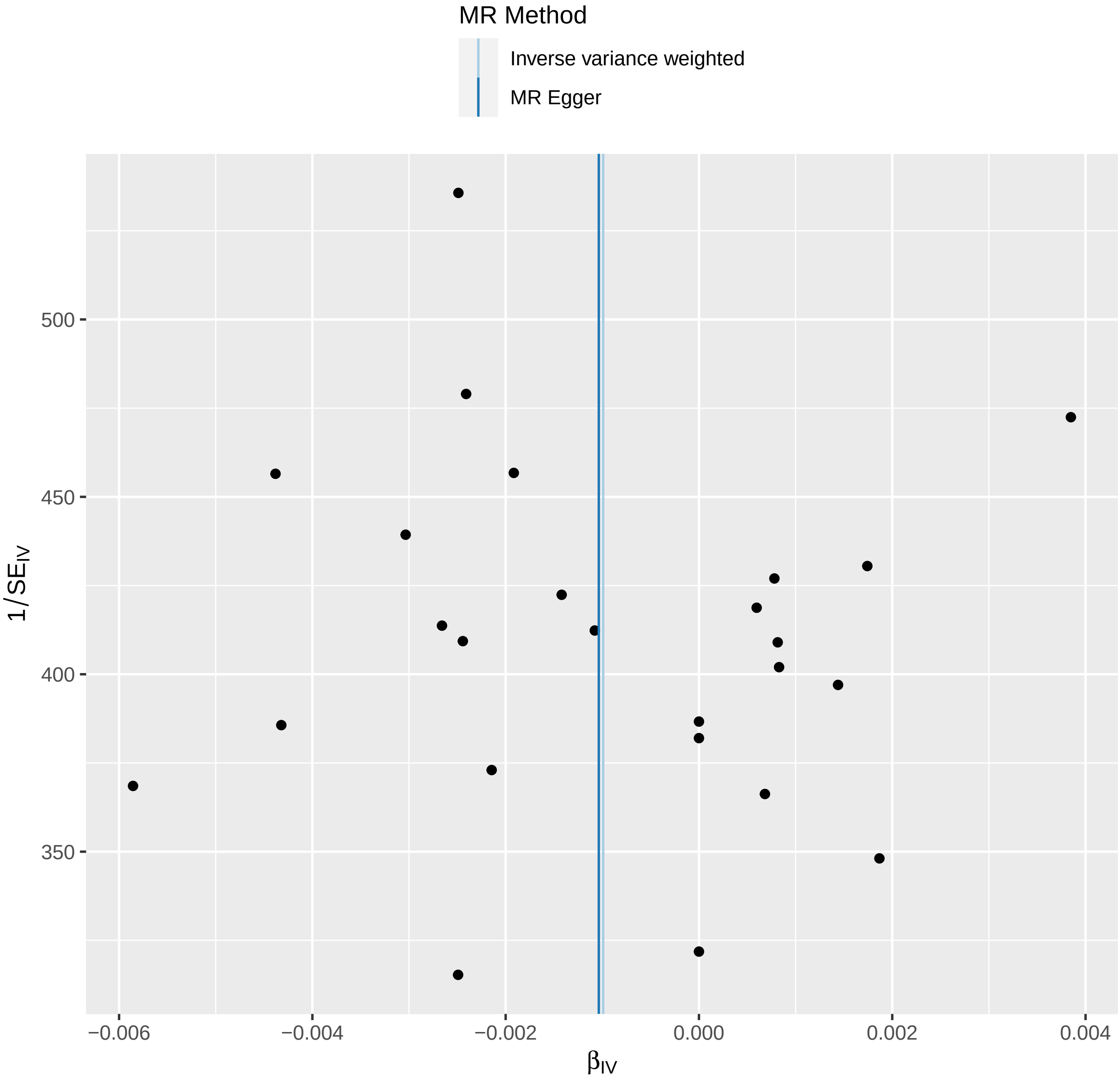

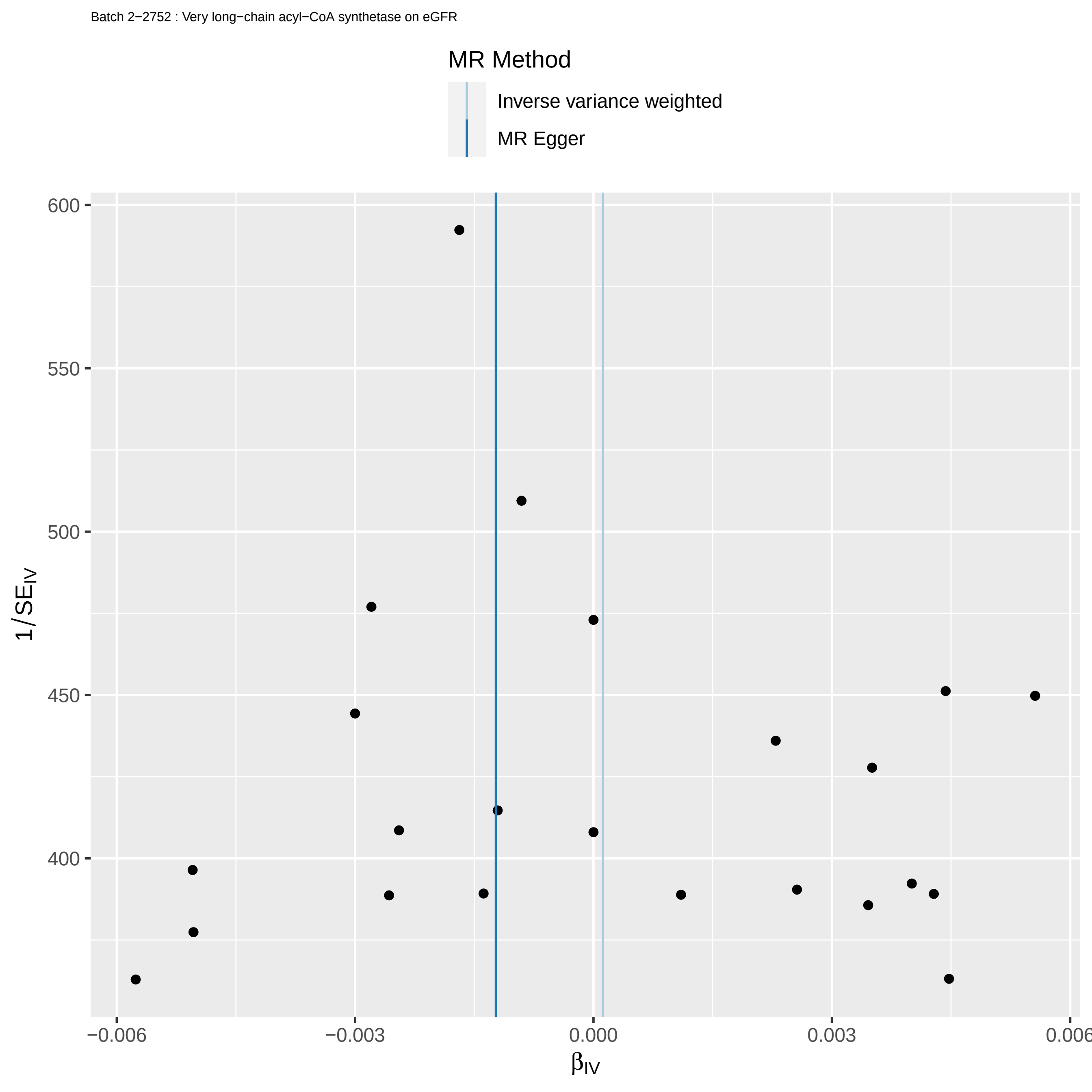

Batch 2−2752 : Very long−chain acyl−CoA synthetase on eGFR
MR Method
Inverse variance weighted
MR Egger
600
550
500
450
400
1/SE_IV
−0.006
−0.003
0.000
0.003
0.006
β_IV

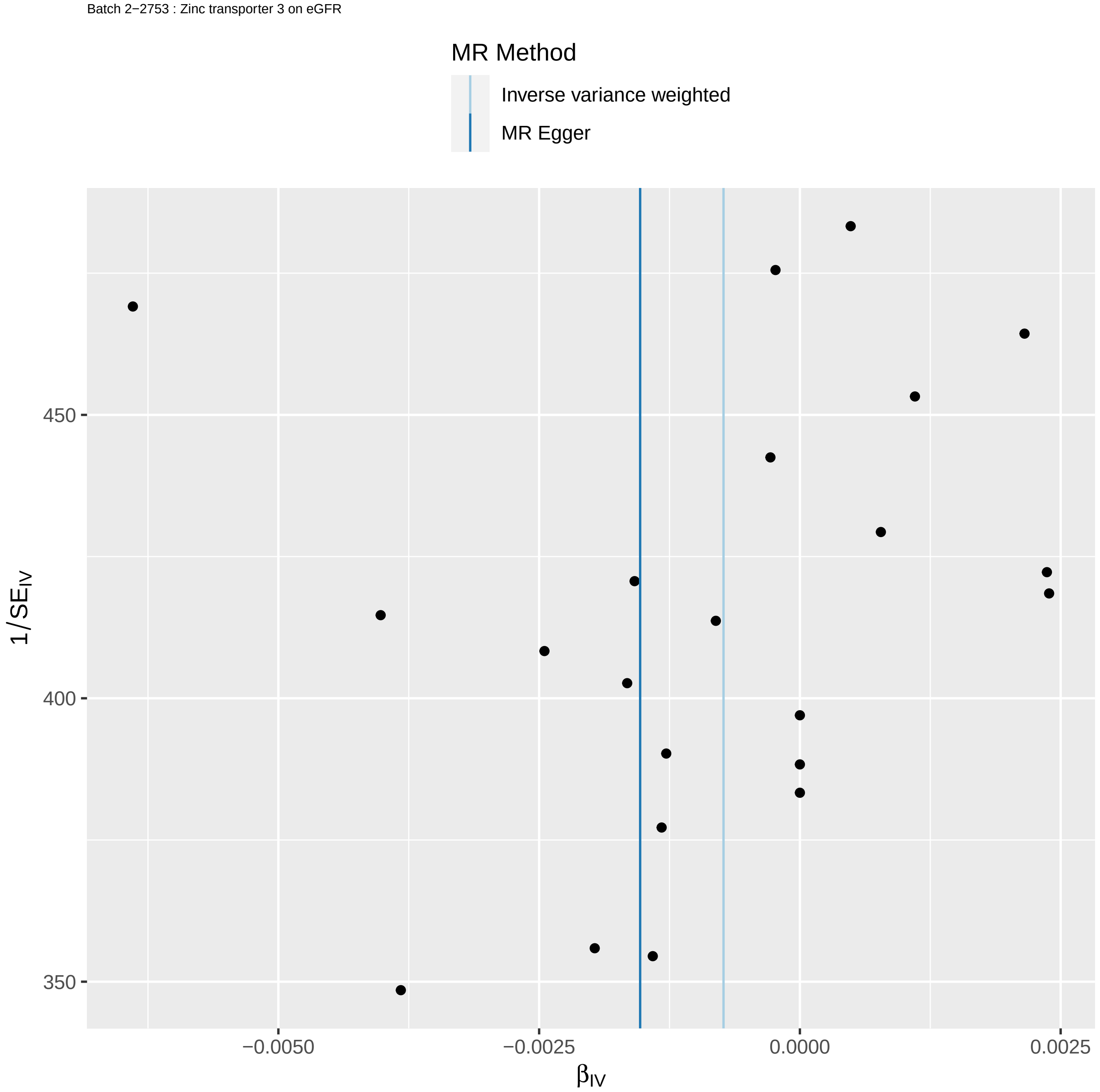

Batch 2−2753 : Zinc transporter 3 on eGFR
MR Method
Inverse variance weighted
MR Egger
450
400
350
1/SE_IV
−0.0050
−0.0025
0.0000
0.0025
β_IV

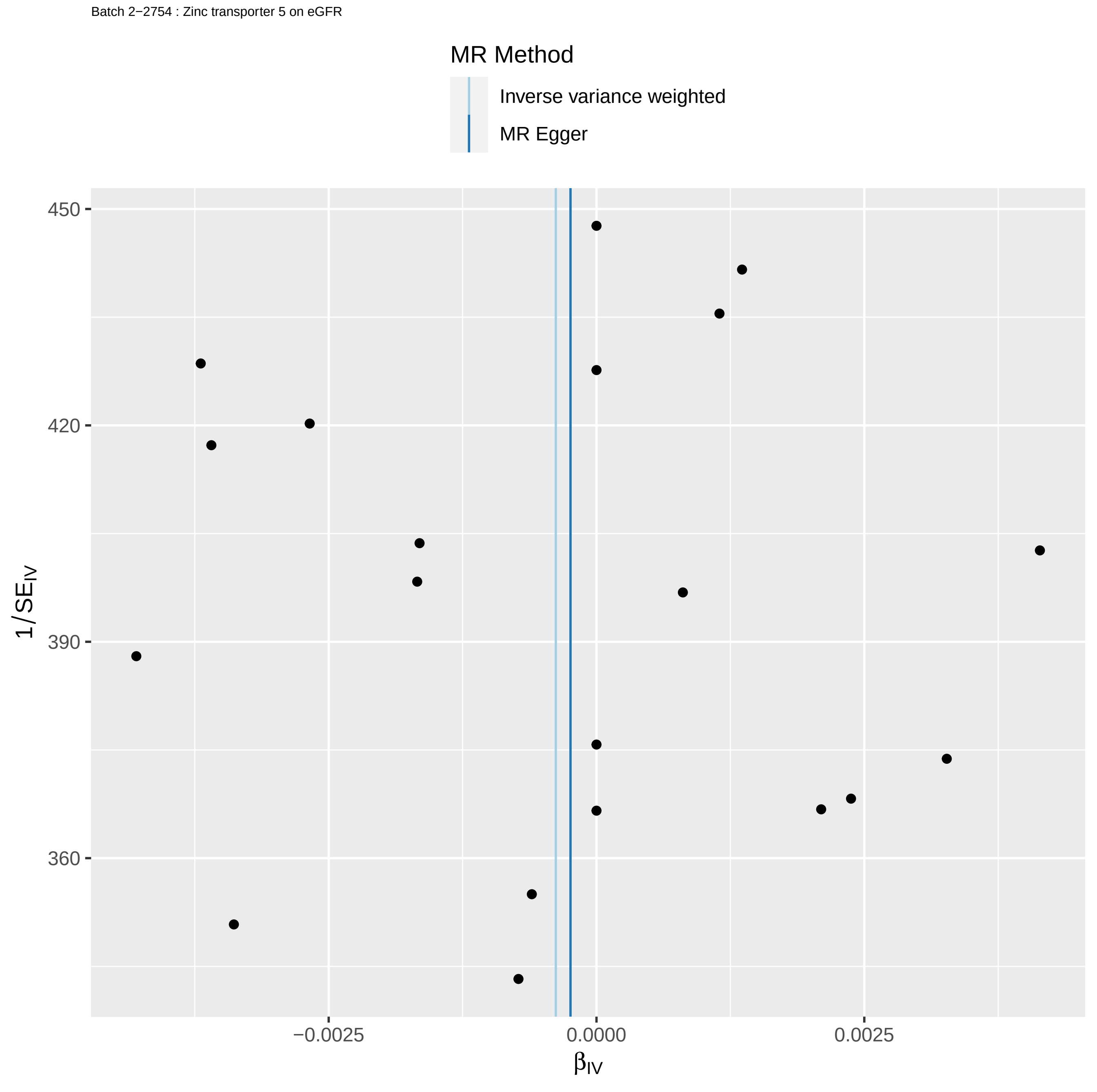

Batch 2−2754 : Zinc transporter 5 on eGFR
MR Method
Inverse variance weighted
MR Egger
450
420
390
360
1/SE_IV
−0.0025
0.0000
0.0025
β_IV

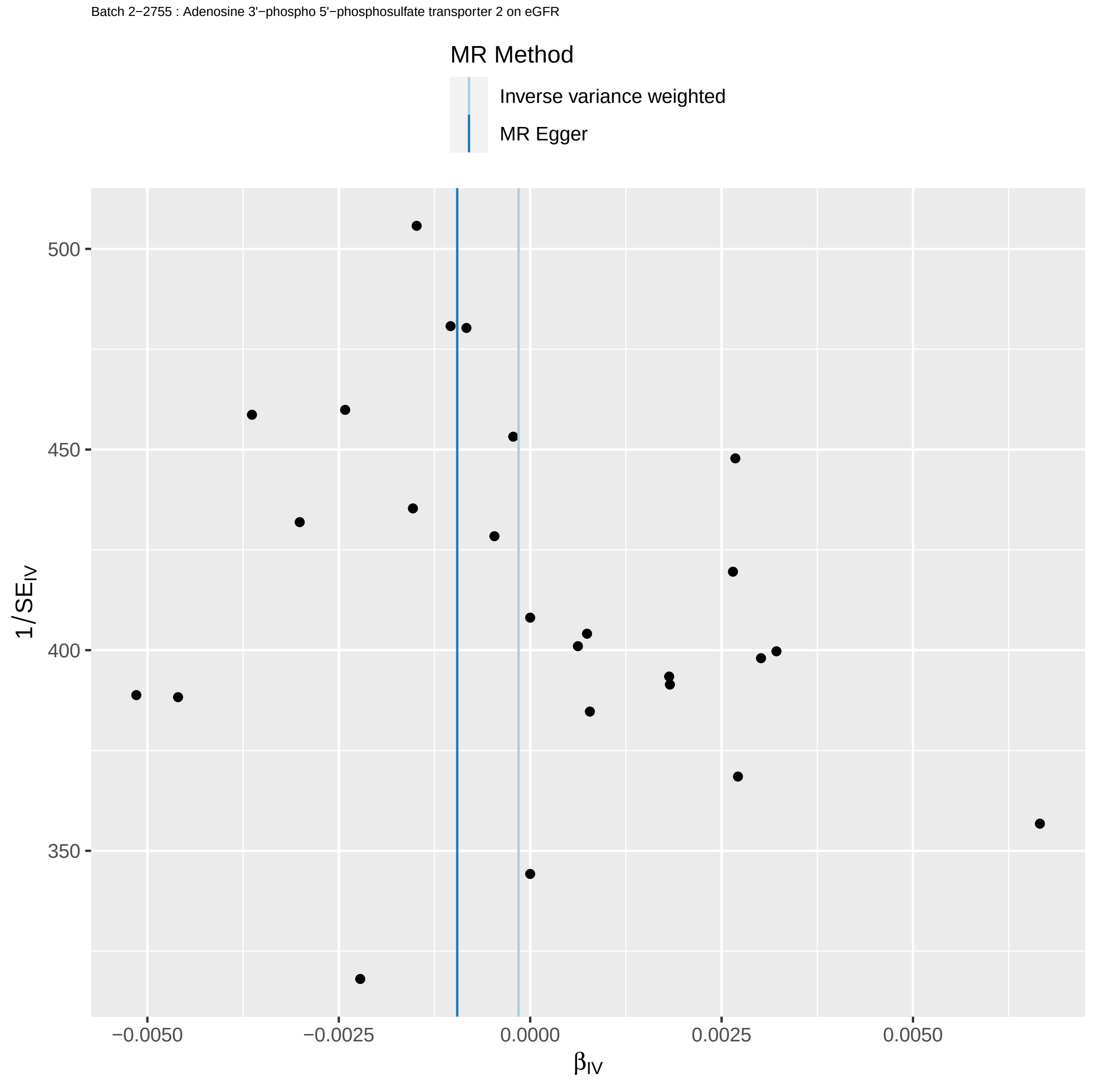

Batch 2−2755 : Adenosine 3'−phospho 5'−phosphosulfate transporter 2 on eGFR
MR Method
Inverse variance weighted
MR Egger
500
450
400
350
1/SE_IV
−0.0050
−0.0025
0.0000
0.0025
0.0050
β_IV

Batch 2−2756 : Solute carrier family 35 member G2 on eGFR

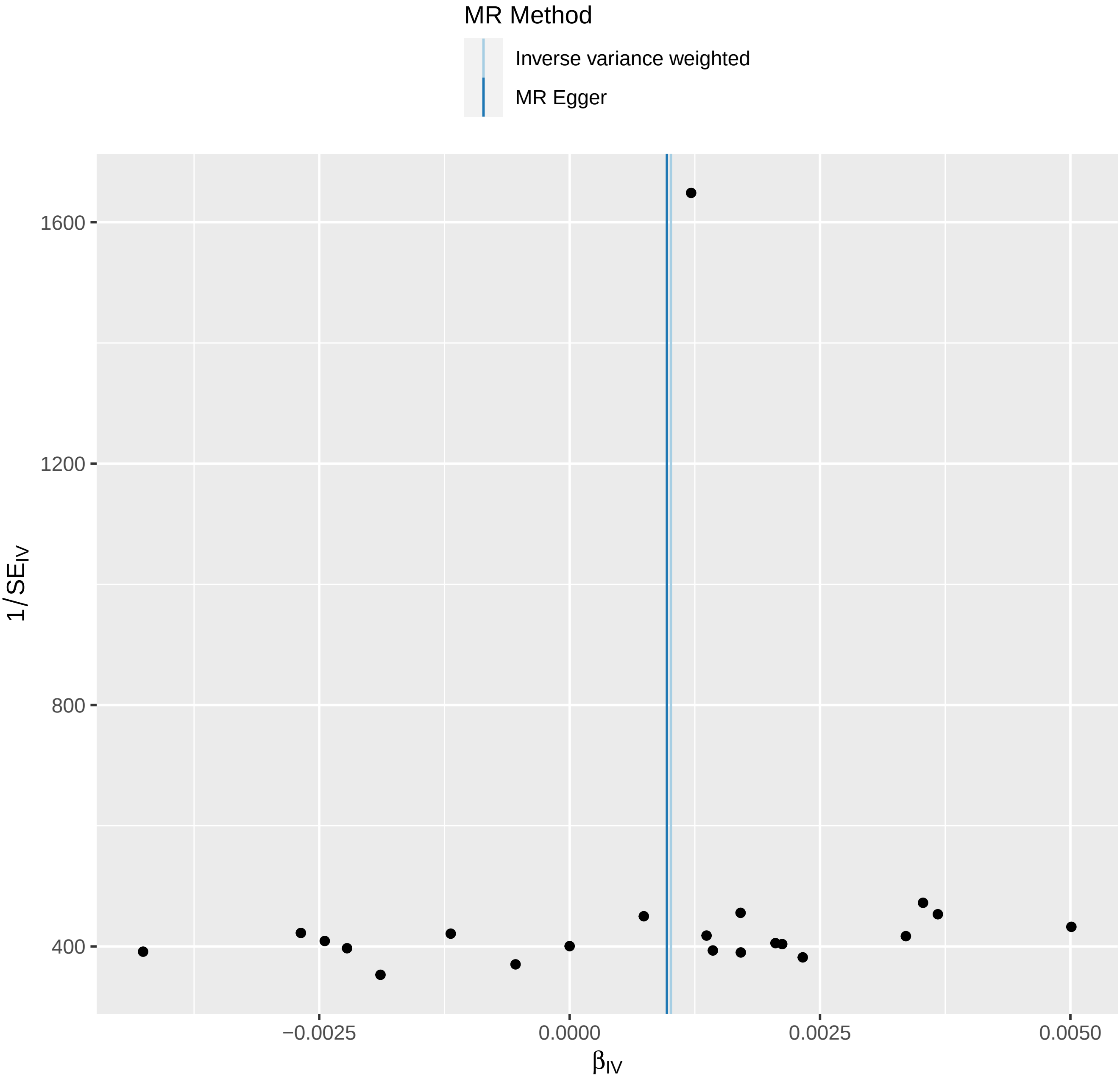

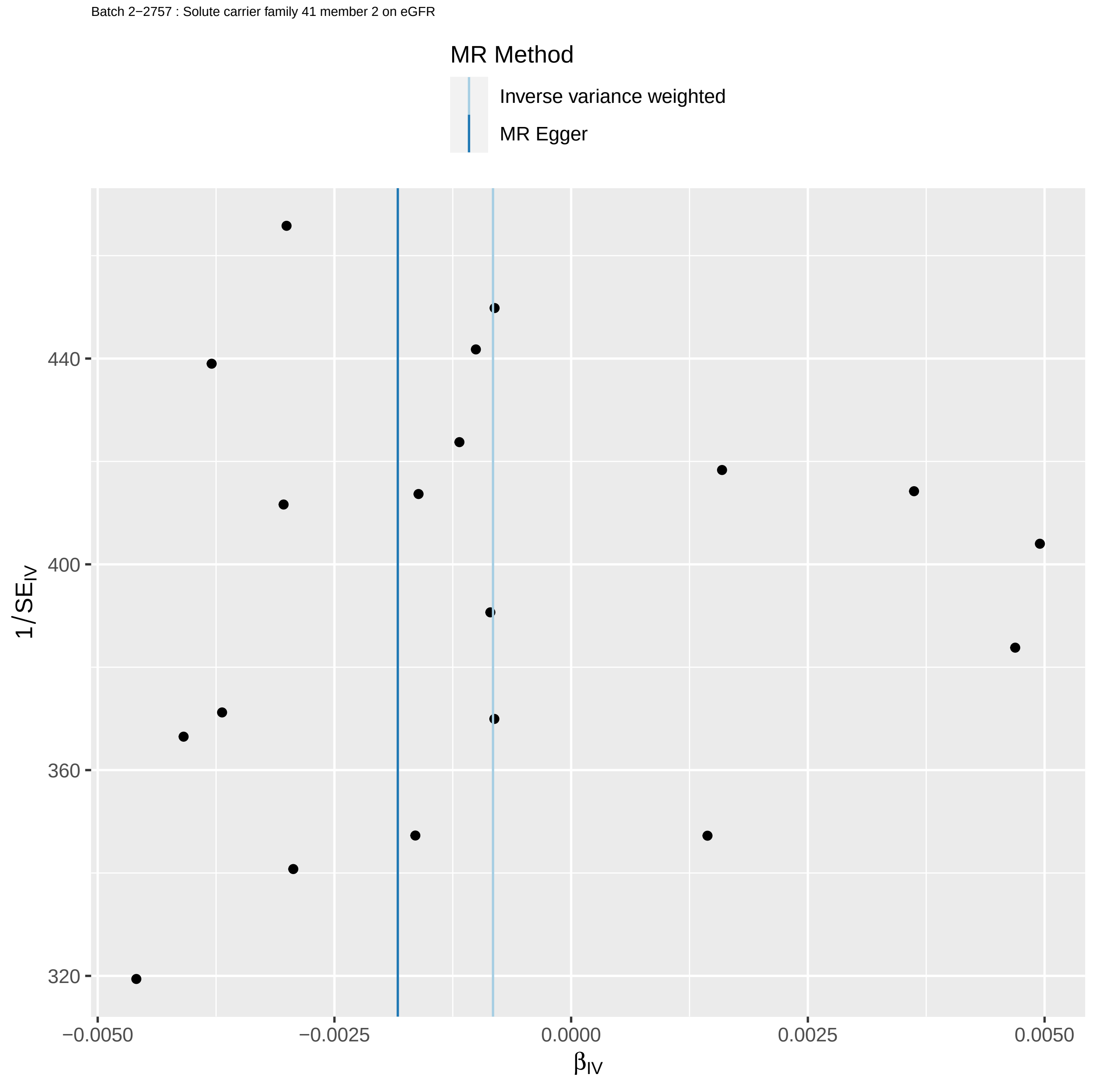

Batch 2−2757 : Solute carrier family 41 member 2 on eGFR
MR Method
Inverse variance weighted
MR Egger
440
400
360
320
1/SE_IV
−0.0050
−0.0025
0.0000
0.0025
0.0050
β_IV

Batch 2−2758 : Electroneutral sodium bicarbonate exchanger 1 on eGFR

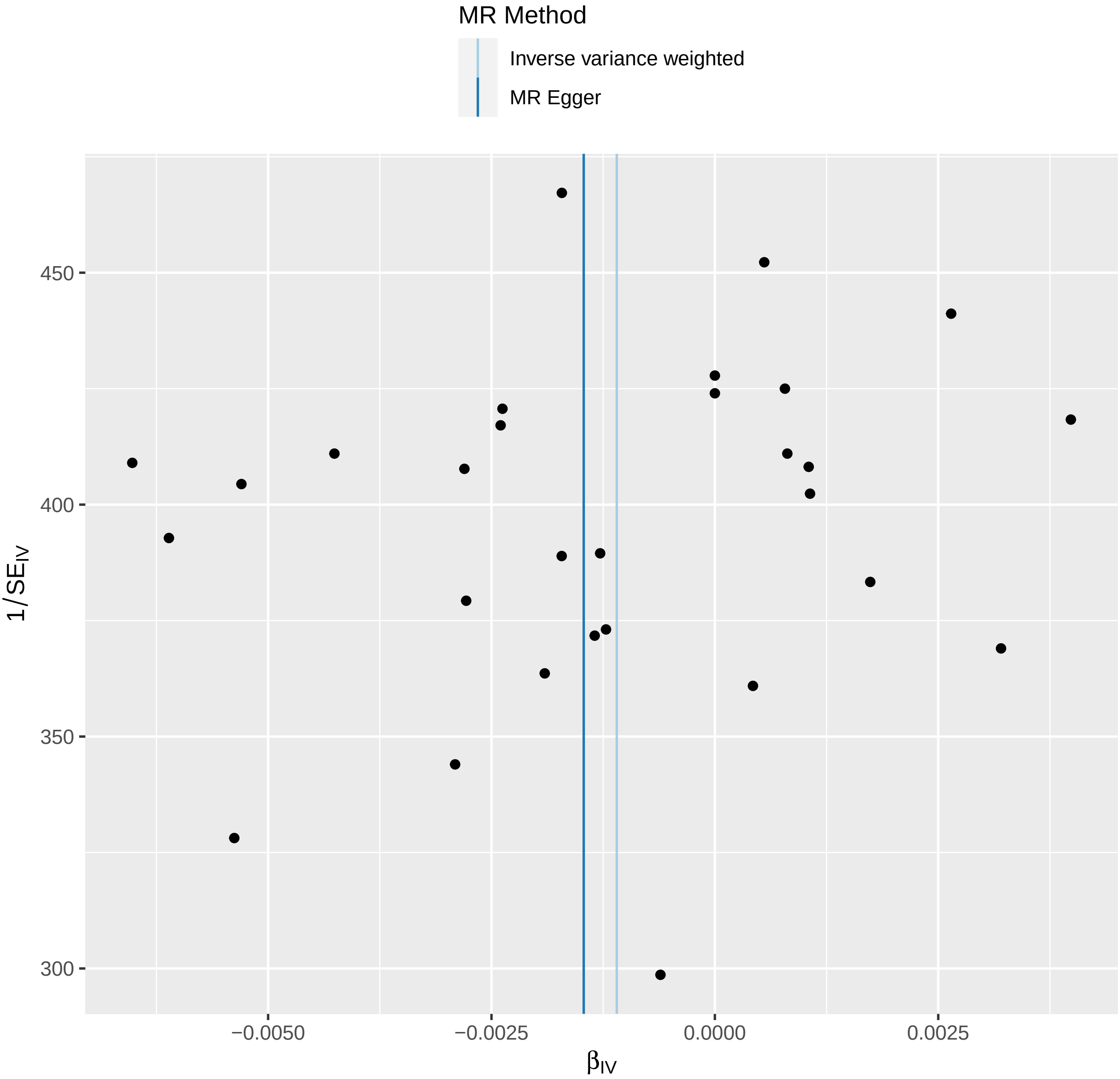

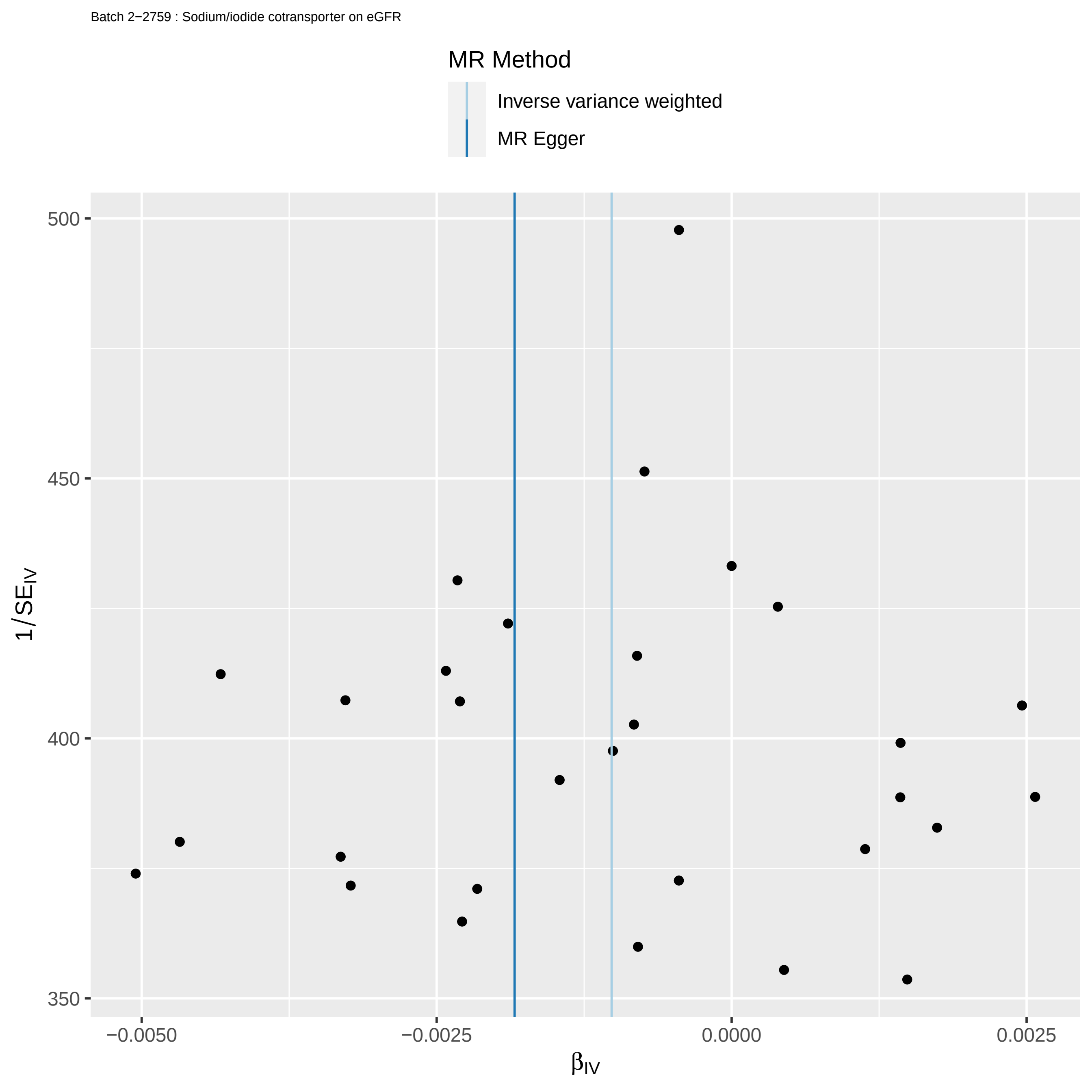

Batch 2−2759 : Sodium/iodide cotransporter on eGFR
MR Method
Inverse variance weighted
MR Egger
500
450
400
350
1/SE_IV
−0.0050
−0.0025
0.0000
0.0025
β_IV

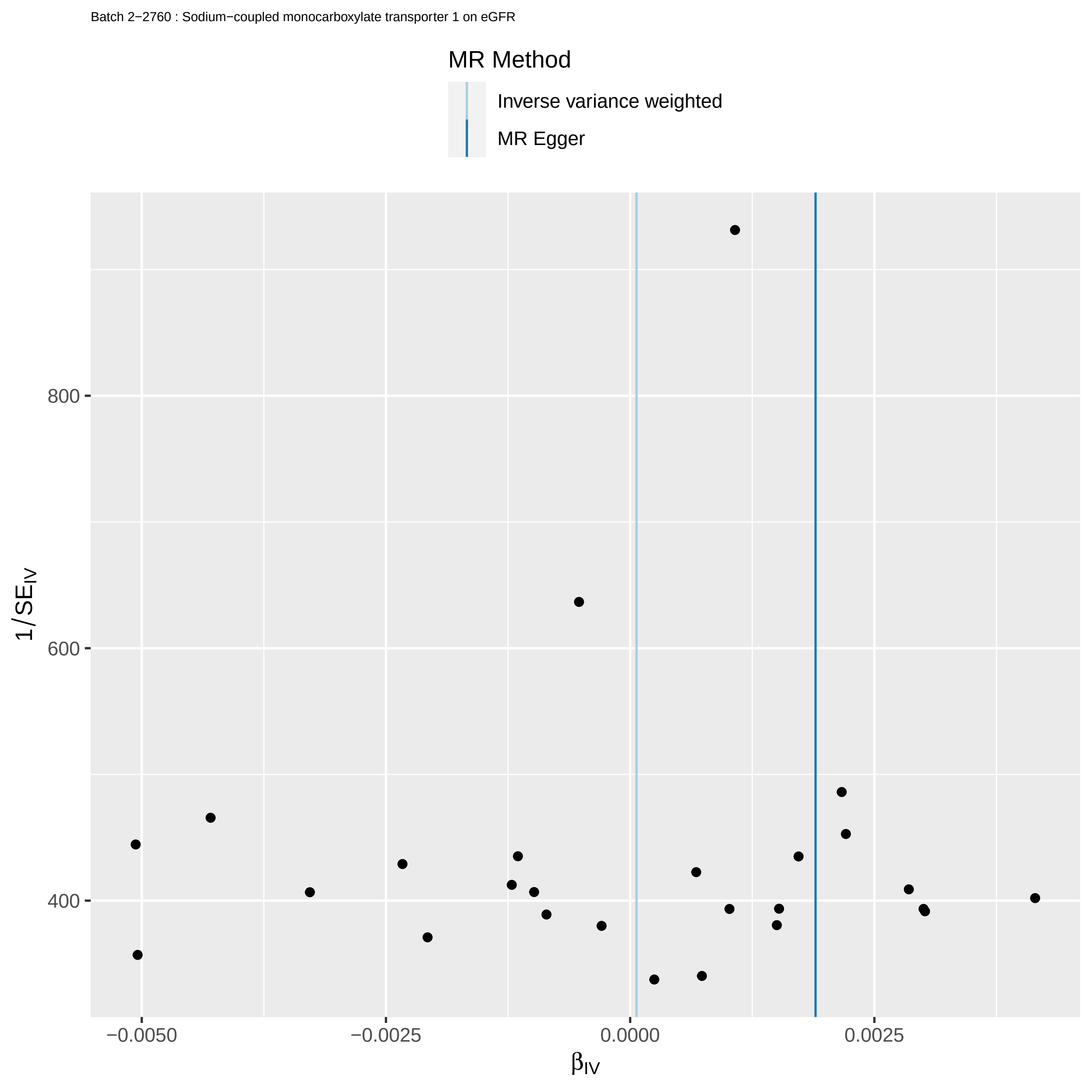
Batch 2−2760 : Sodium−coupled monocarboxylate transporter 1 on eGFR
MR Method
Inverse variance weighted
MR Egger
800
600
400
1/SE_IV
−0.0050
−0.0025
0.0000
0.0025
β_IV

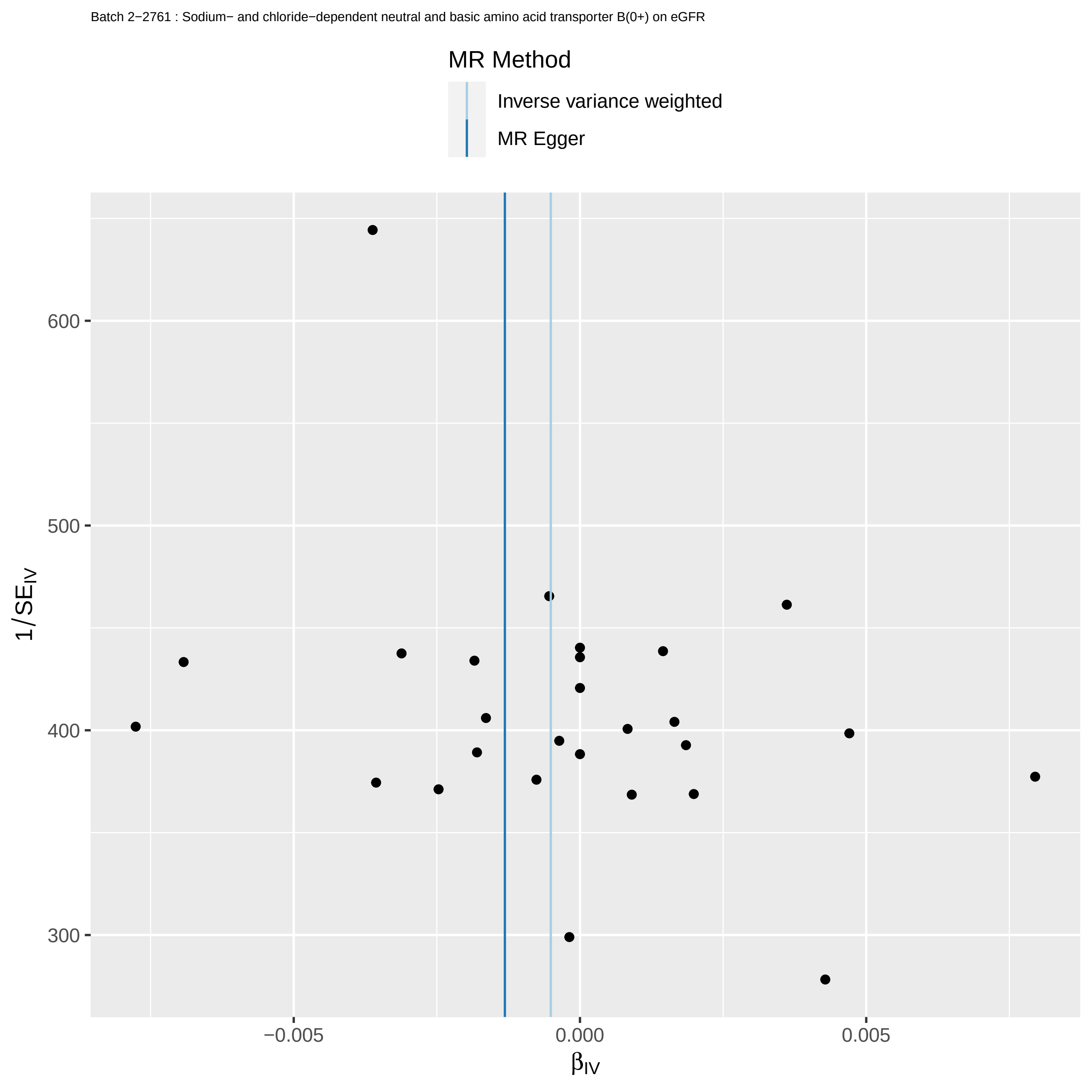
Batch 2−2761 : Sodium− and chloride−dependent neutral and basic amino acid transporter B(0+) on eGFR
MR Method
Inverse variance weighted
MR Egger
600
500
400
300
−0.005
0.000
0.005
1/SE_IV
β_IV

Batch 2−2762 : Orphan sodium− and chloride−dependent neurotransmitter transporter NTT5 on eGFR

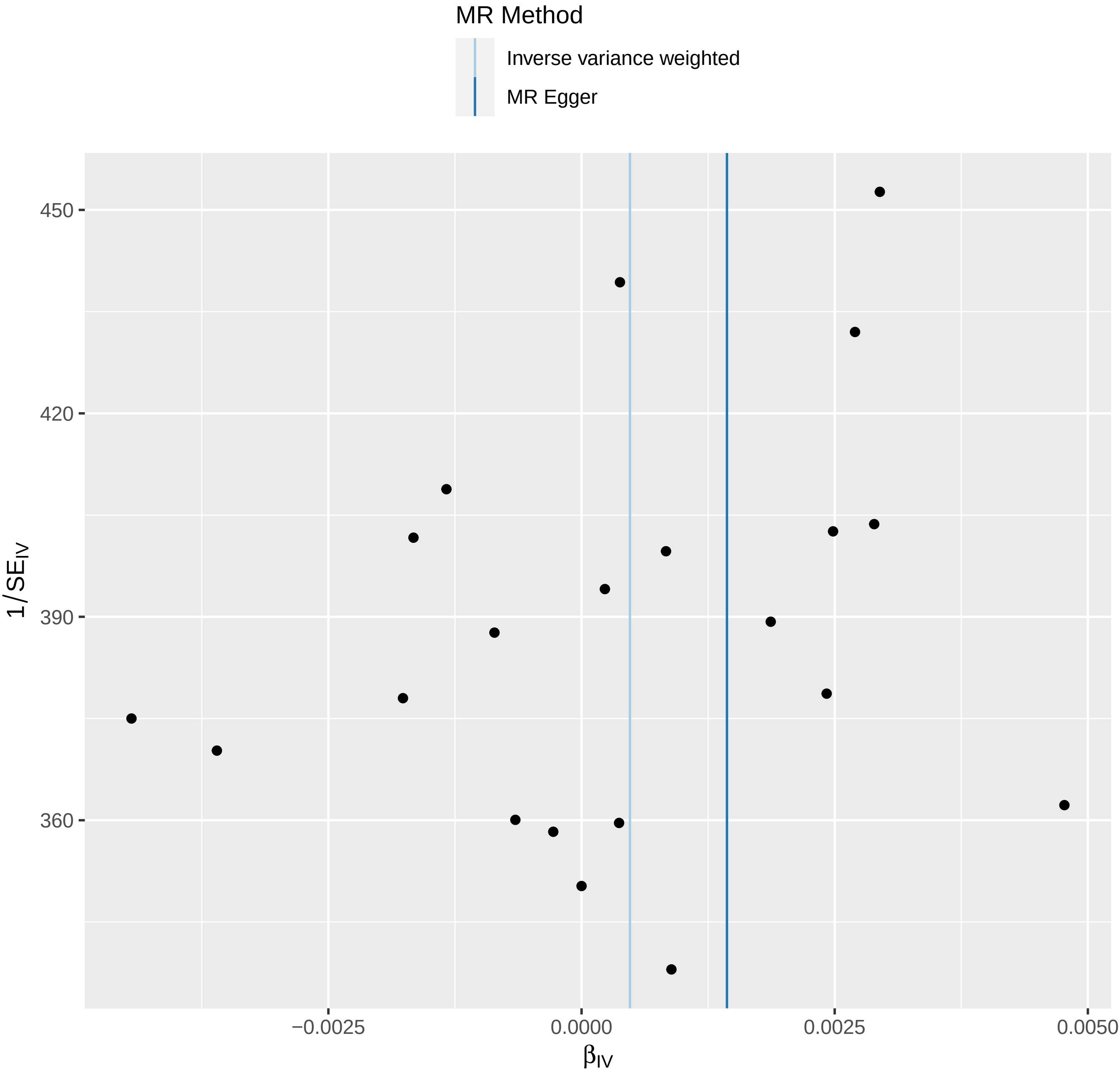

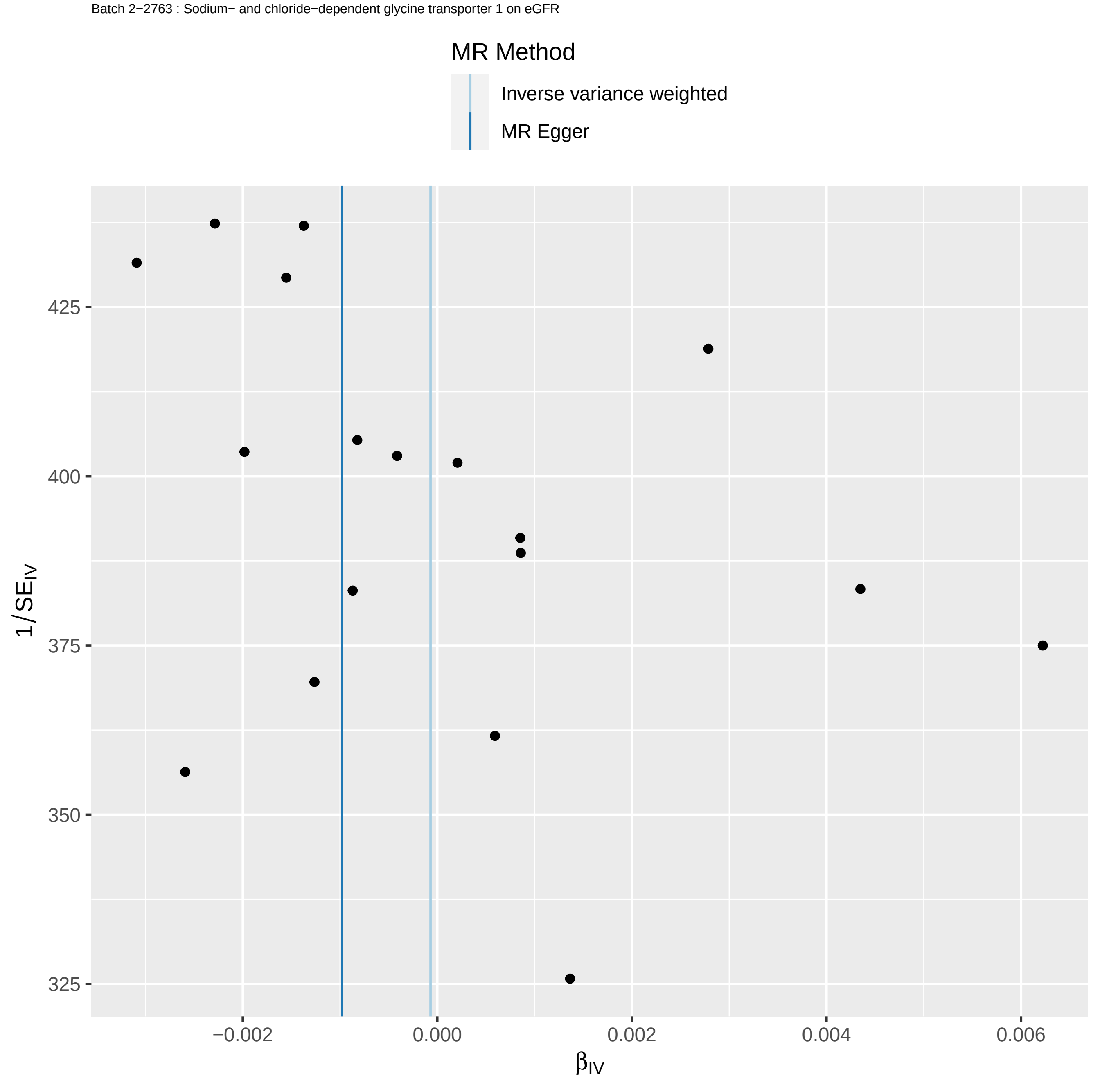
Batch 2−2763 : Sodium− and chloride−dependent glycine transporter 1 on eGFR
MR Method
Inverse variance weighted
MR Egger
425
400
375
350
325
1/SE$_{IV}$
−0.002
0.000
0.002
0.004
0.006
β$_{IV}$

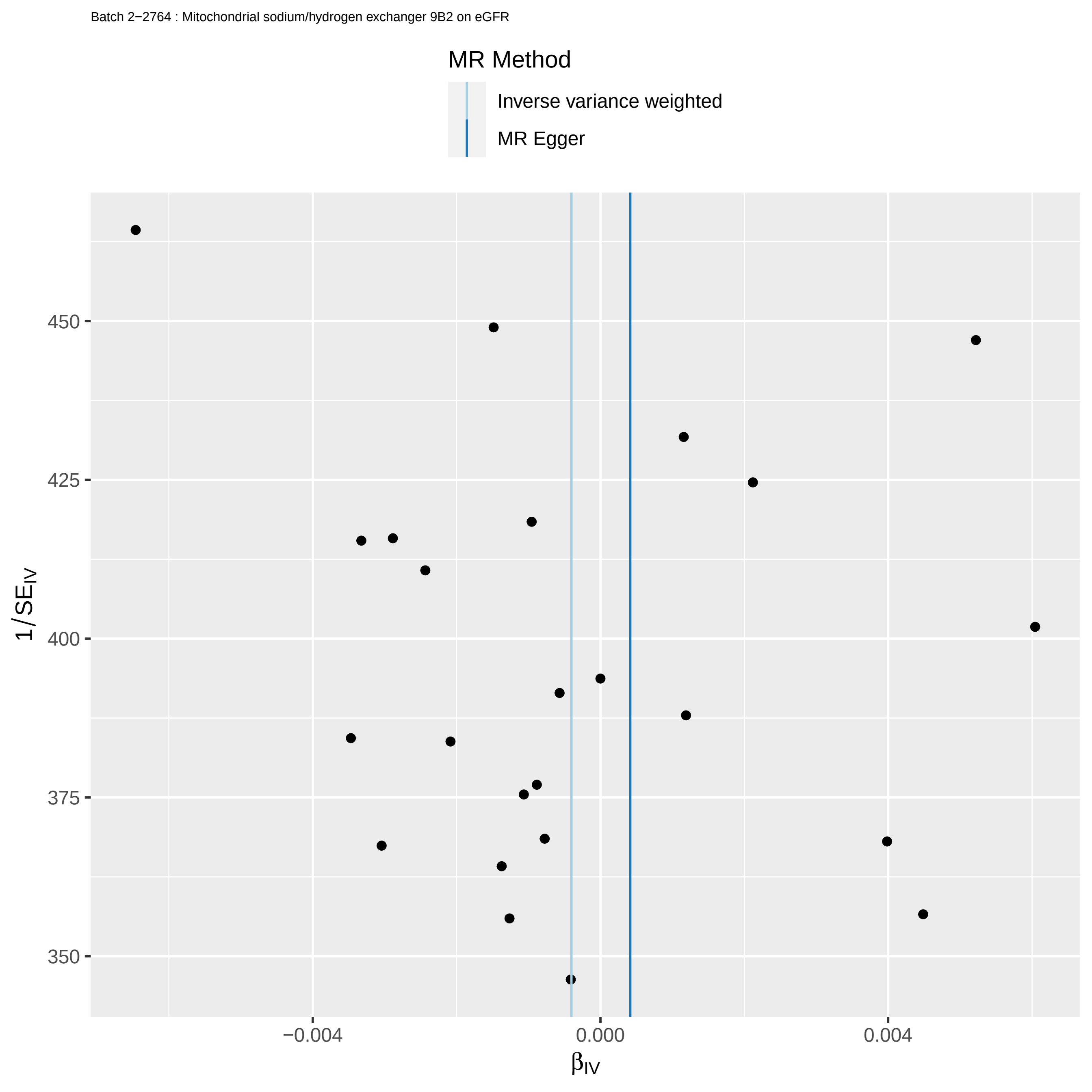

Batch 2−2764 : Mitochondrial sodium/hydrogen exchanger 9B2 on eGFR
MR Method
Inverse variance weighted
MR Egger
450
425
400
375
350
1/SE_IV
−0.004
0.000
0.004
β_IV

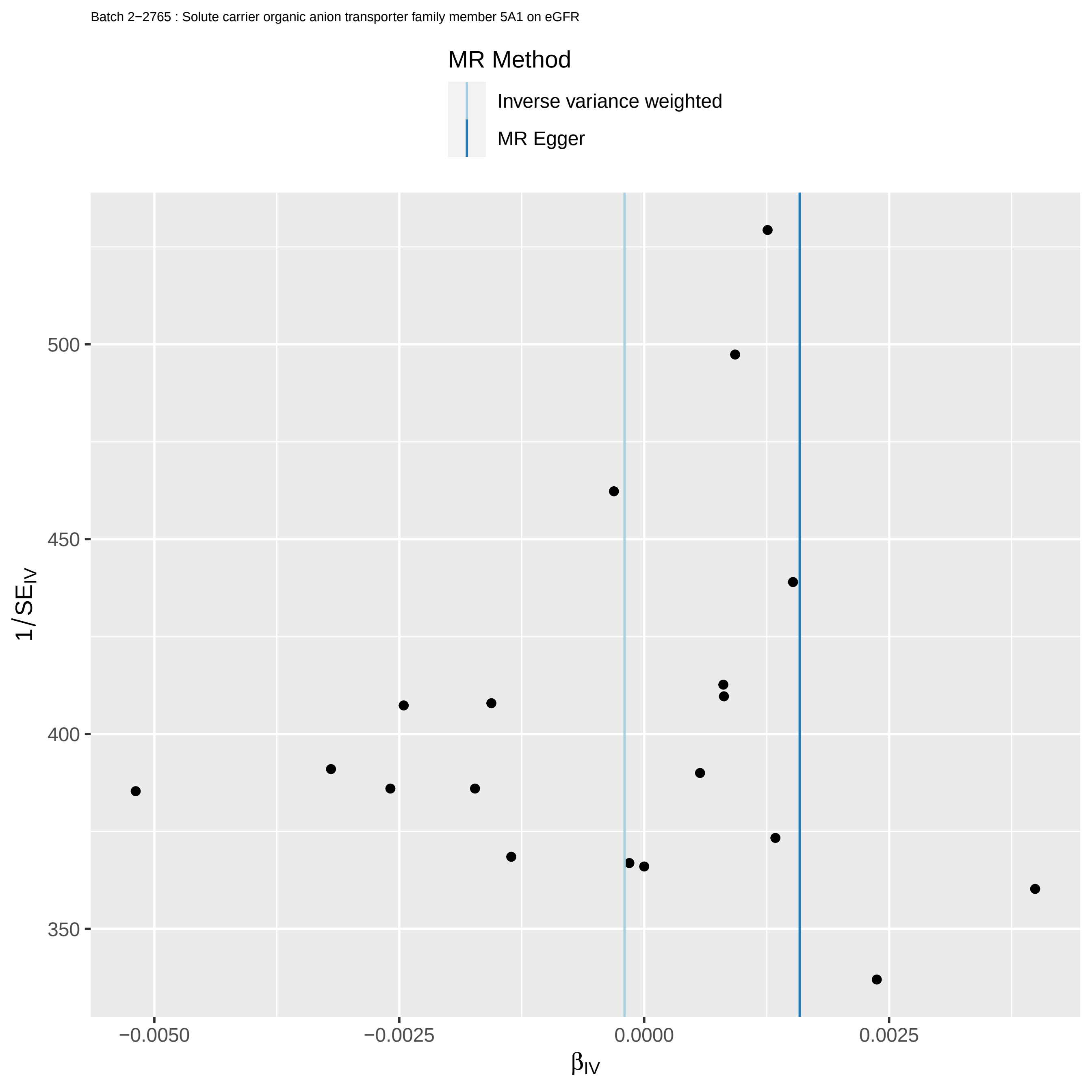
Batch 2−2765 : Solute carrier organic anion transporter family member 5A1 on eGFR
MR Method
Inverse variance weighted
MR Egger
500
450
400
350
1/SE_IV
−0.0050
−0.0025
0.0000
0.0025
β_IV

Batch 2−2766 : SLIT and NTRK−like protein 1 on eGFR

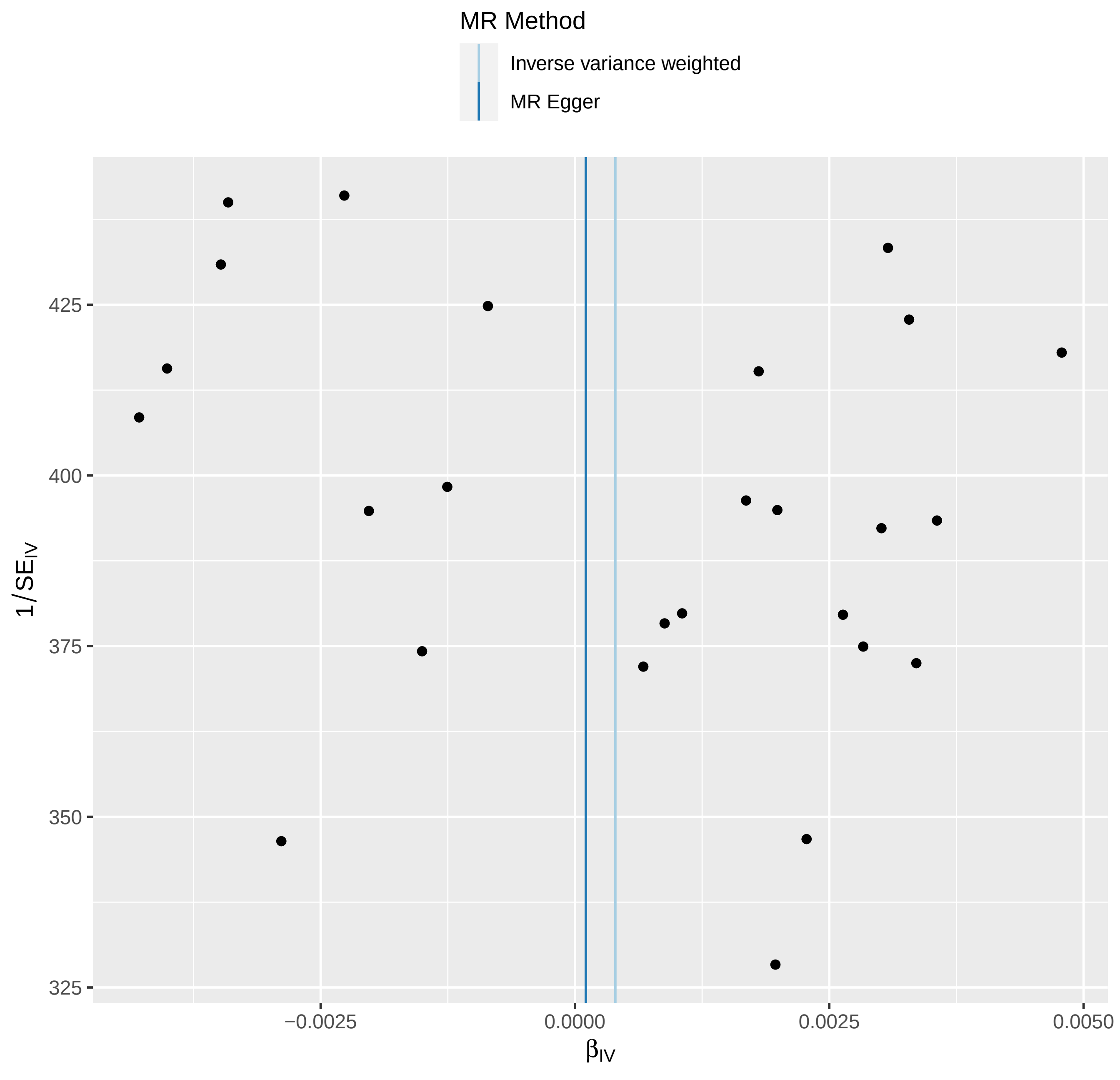

Batch 2−2767 : SLIT and NTRK−like protein 3 on eGFR

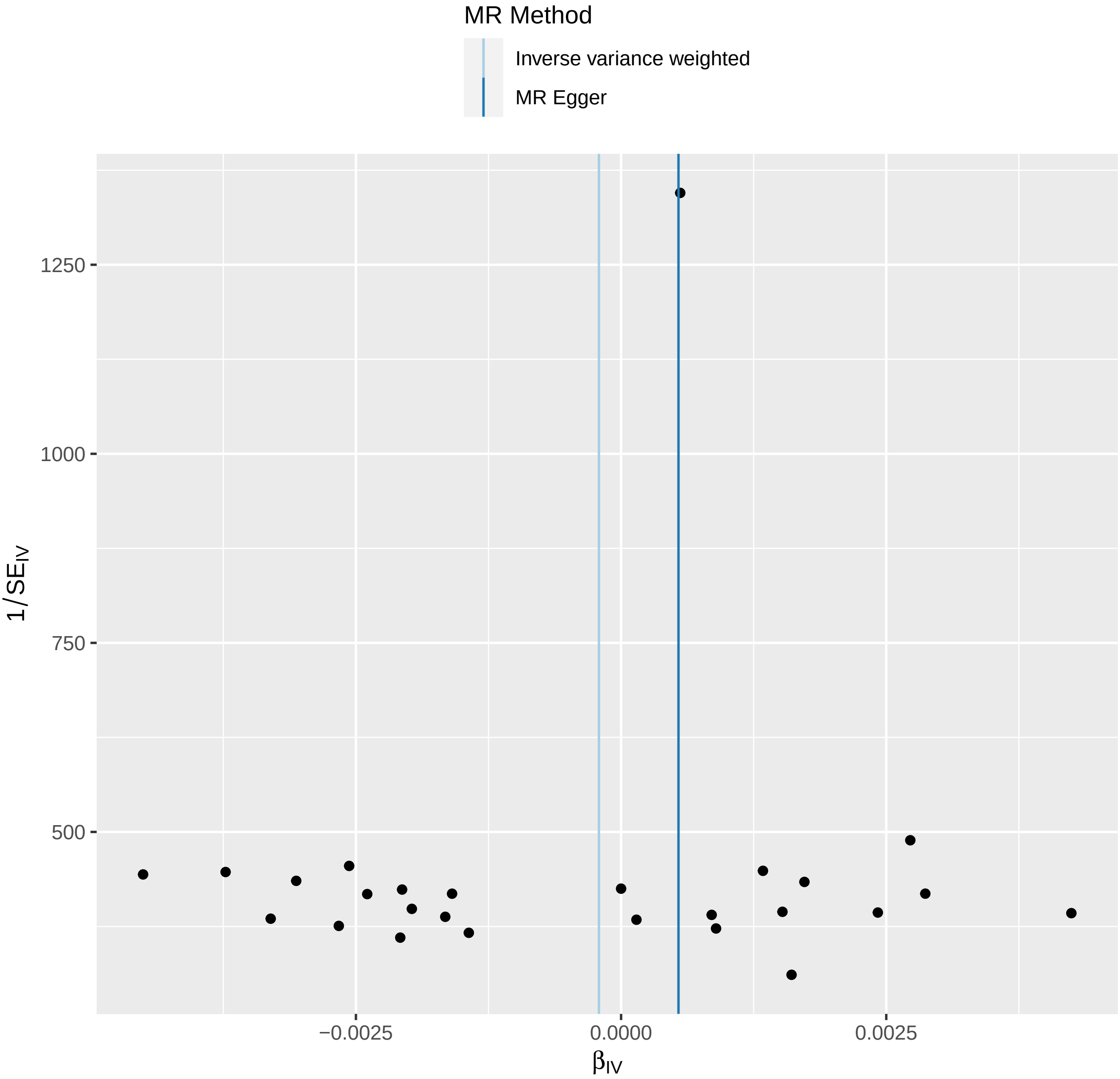

Batch 2−2768 : SLIT and NTRK−like protein 4 on eGFR

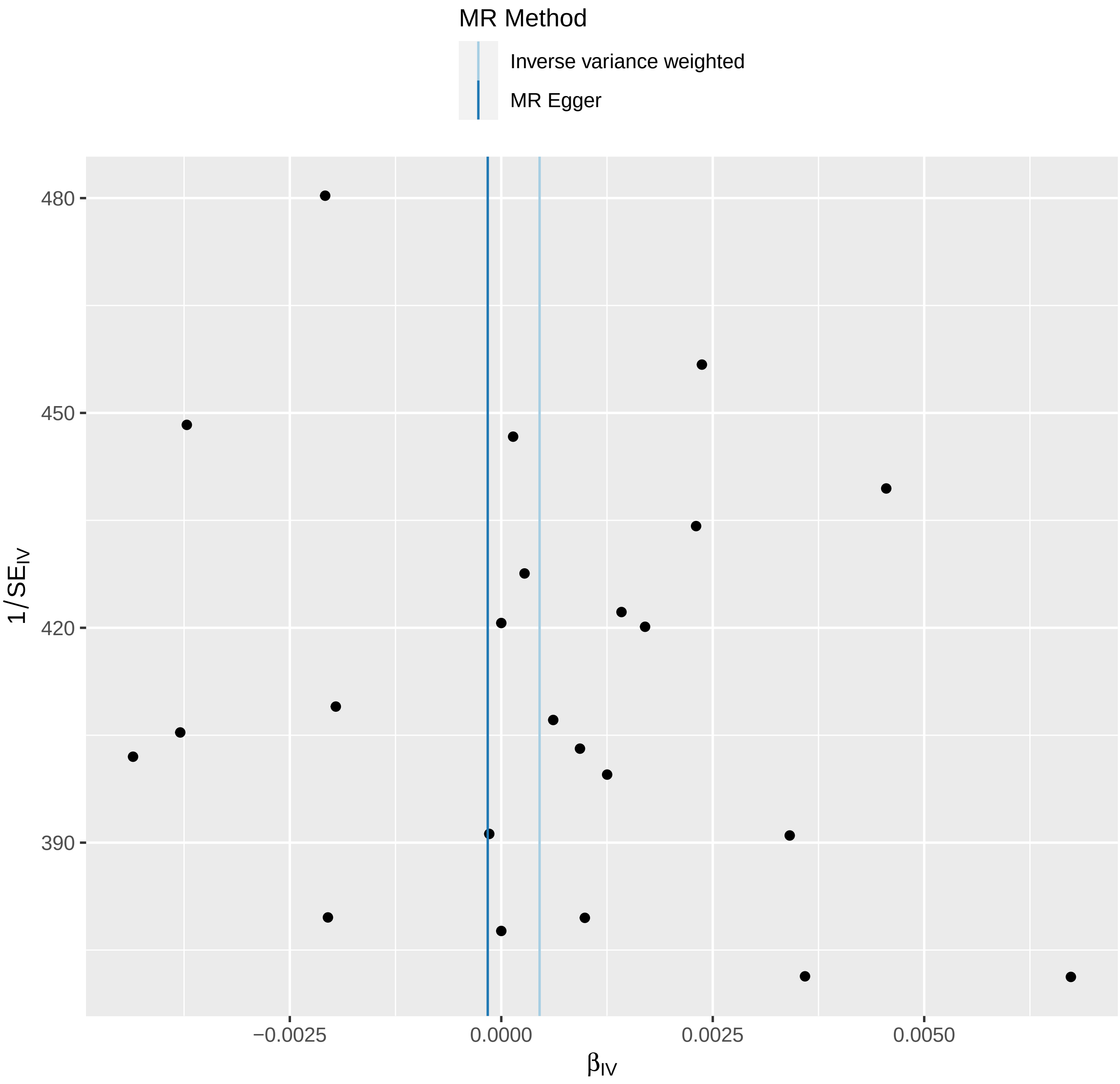

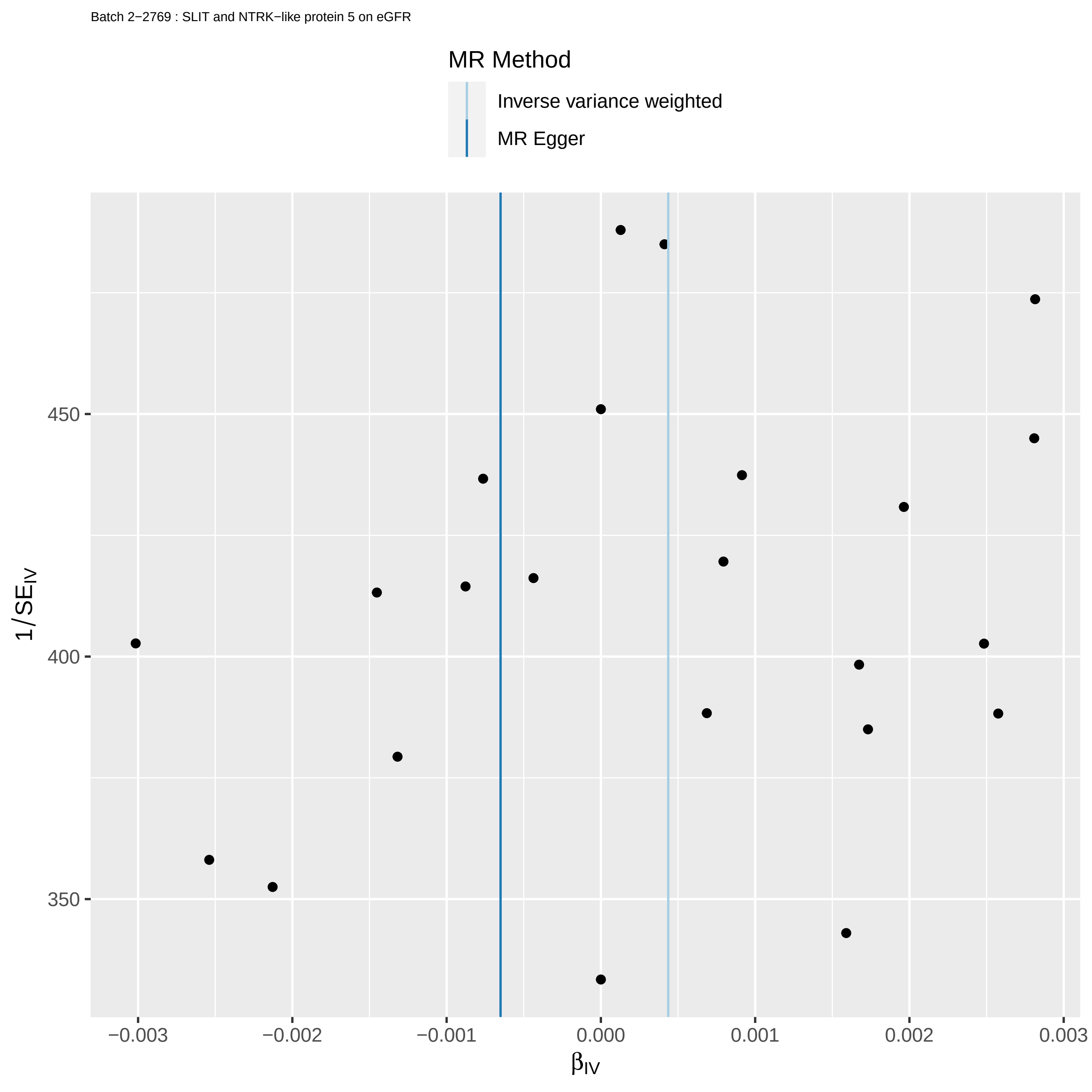

Batch 2−2769 : SLIT and NTRK−like protein 5 on eGFR
MR Method
Inverse variance weighted
MR Egger
1/SE_IV
450
400
350
β_IV
−0.003
−0.002
−0.001
0.000
0.001
0.002
0.003

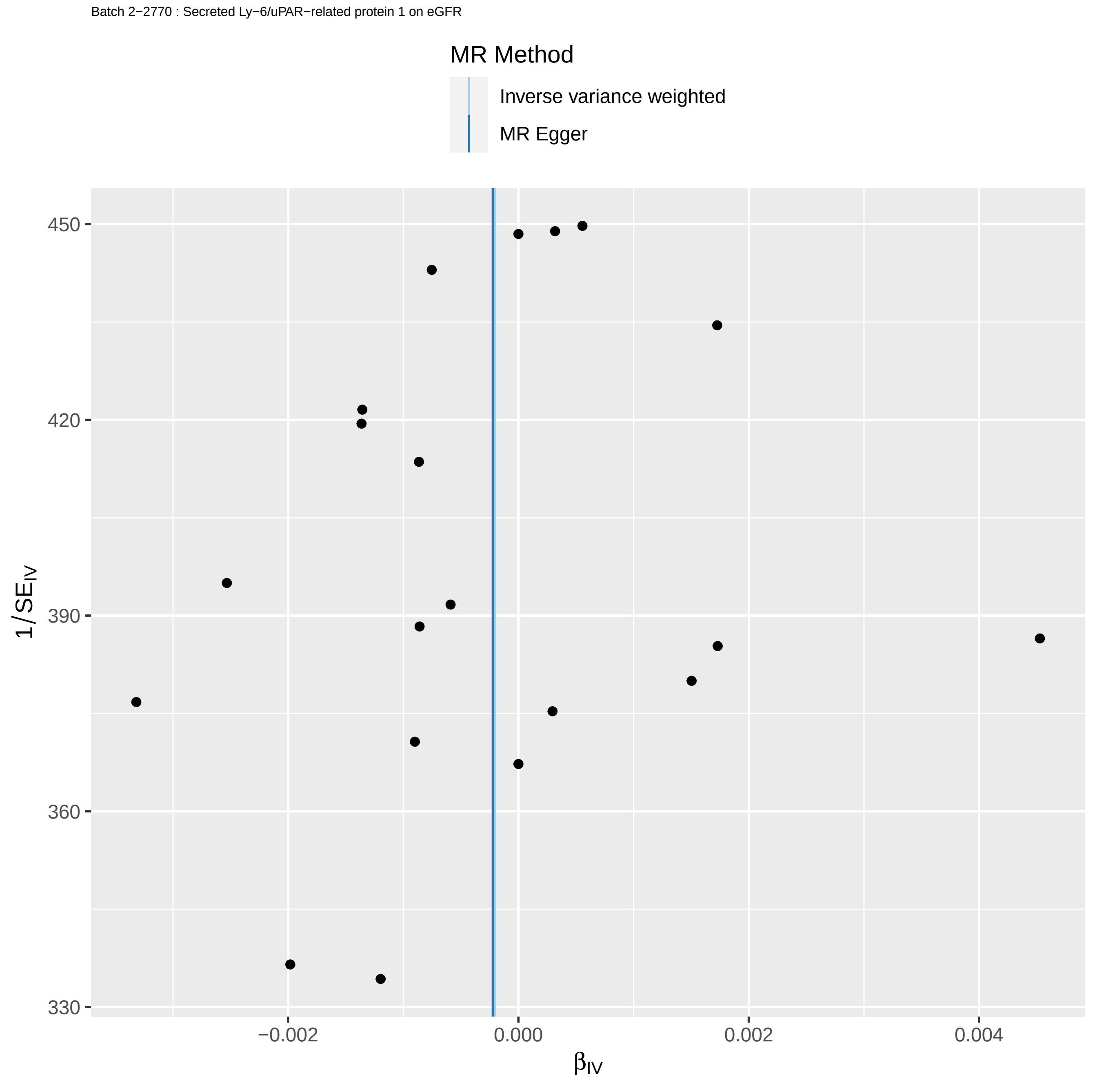
Batch 2−2770 : Secreted Ly−6/uPAR−related protein 1 on eGFR
MR Method
Inverse variance weighted
MR Egger
450
420
390
360
330
1/SE_IV
−0.002
0.000
0.002
0.004
β_IV

Batch 2−2771 : Mothers against decapentaplegic homolog 2 on eGFR

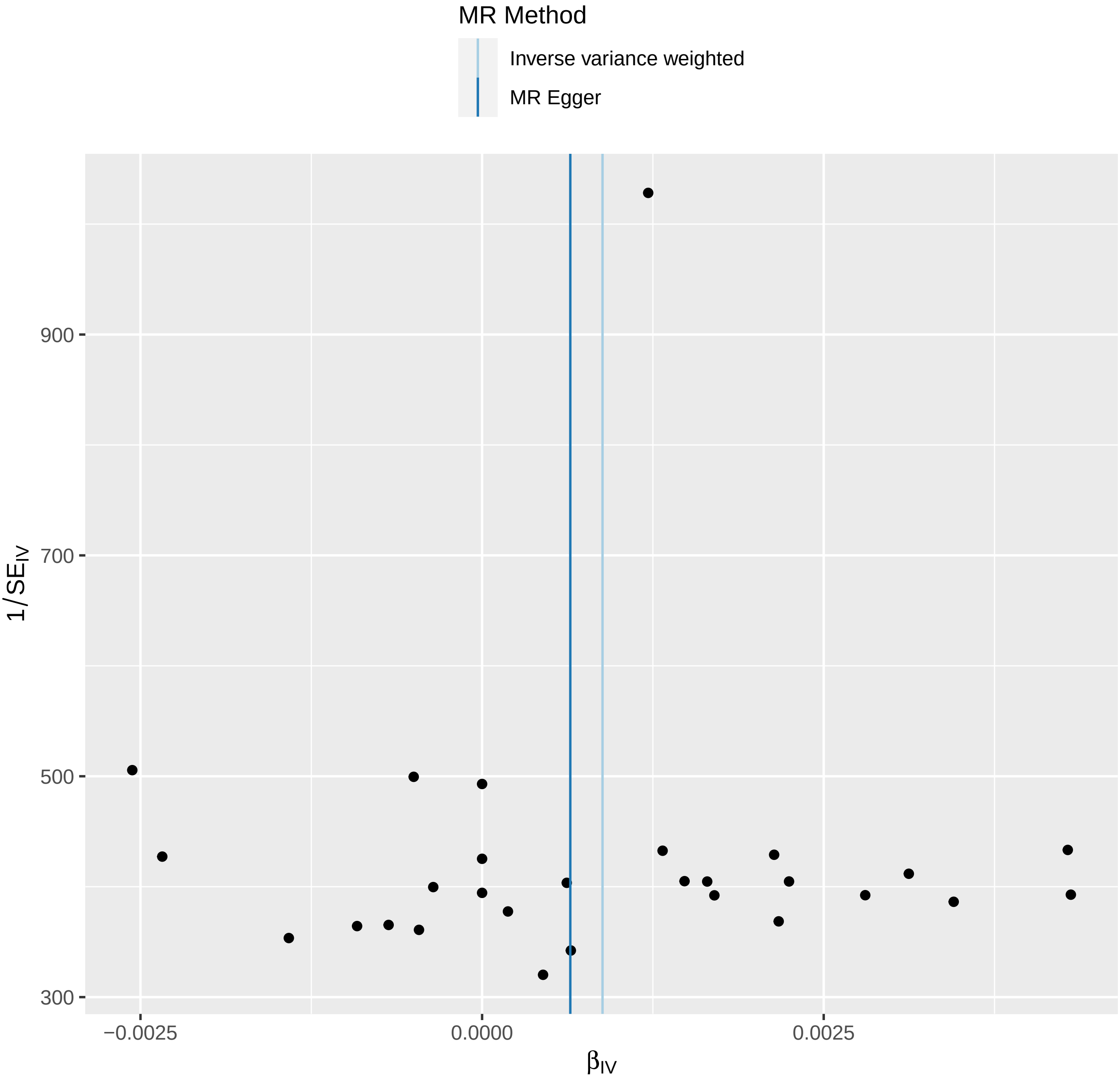

Batch 2−2772 : Mothers against decapentaplegic homolog 4 on eGFR

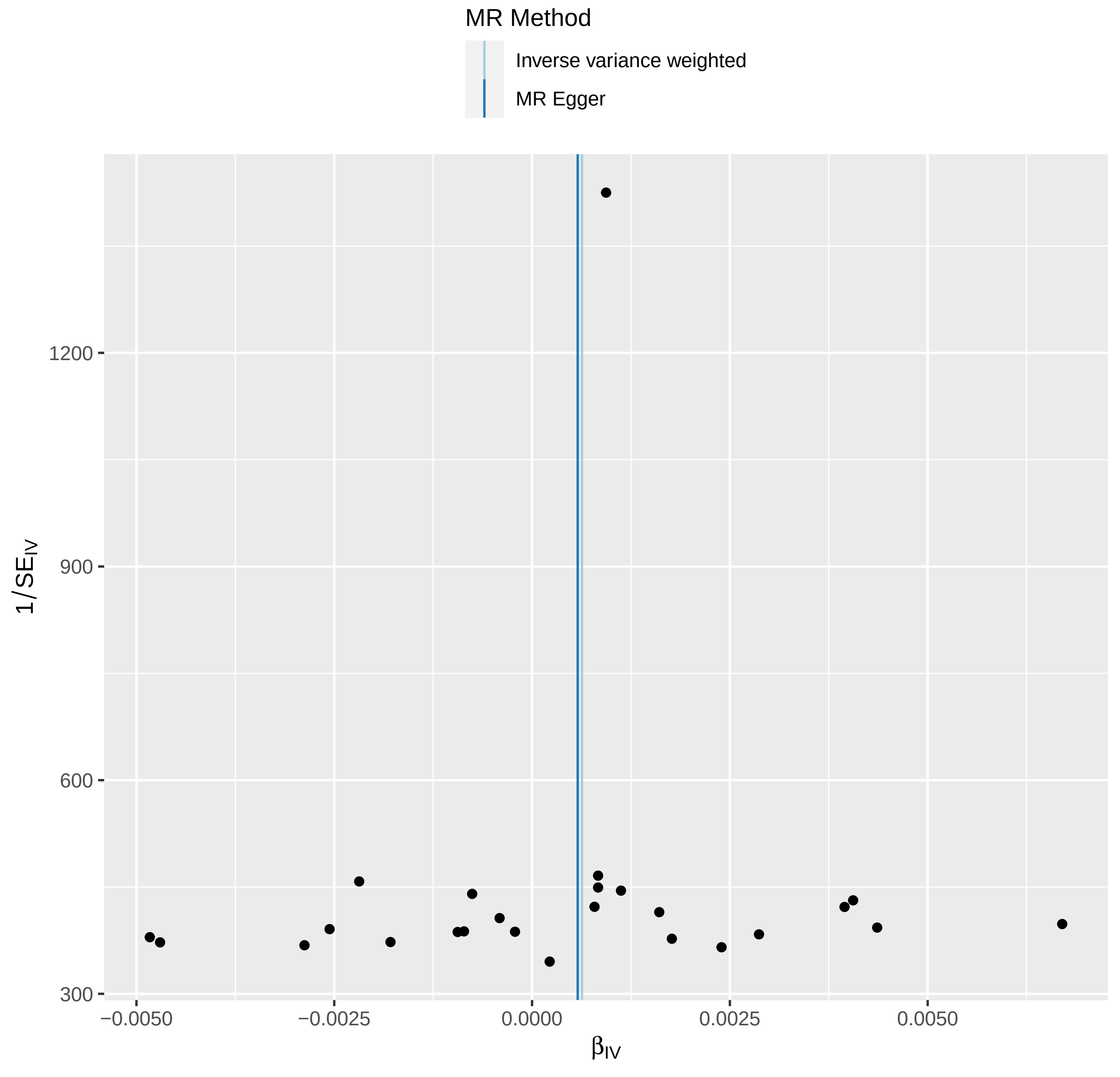

Batch 2−2773 : Stromal membrane−associated protein 1 on eGFR

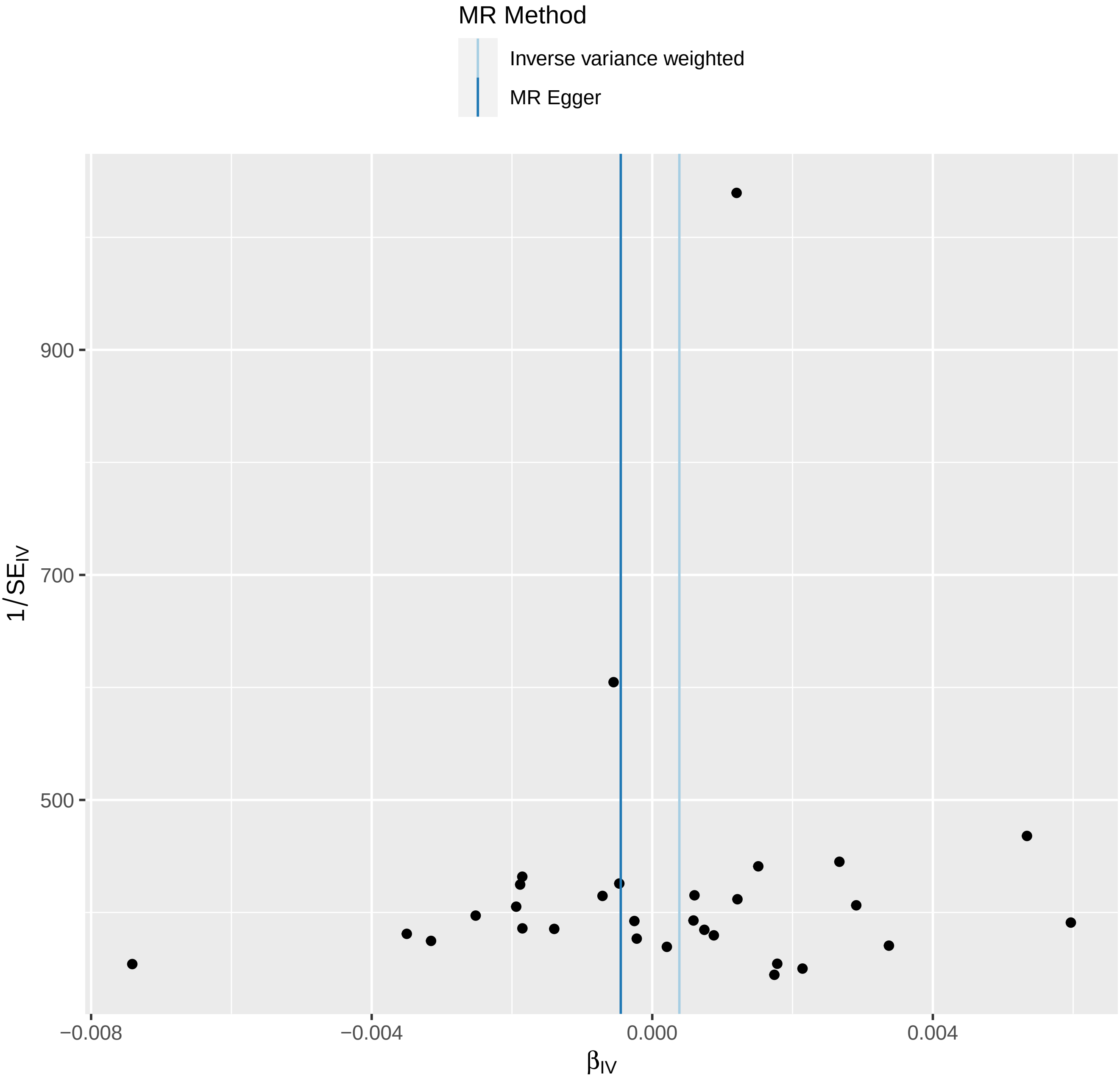

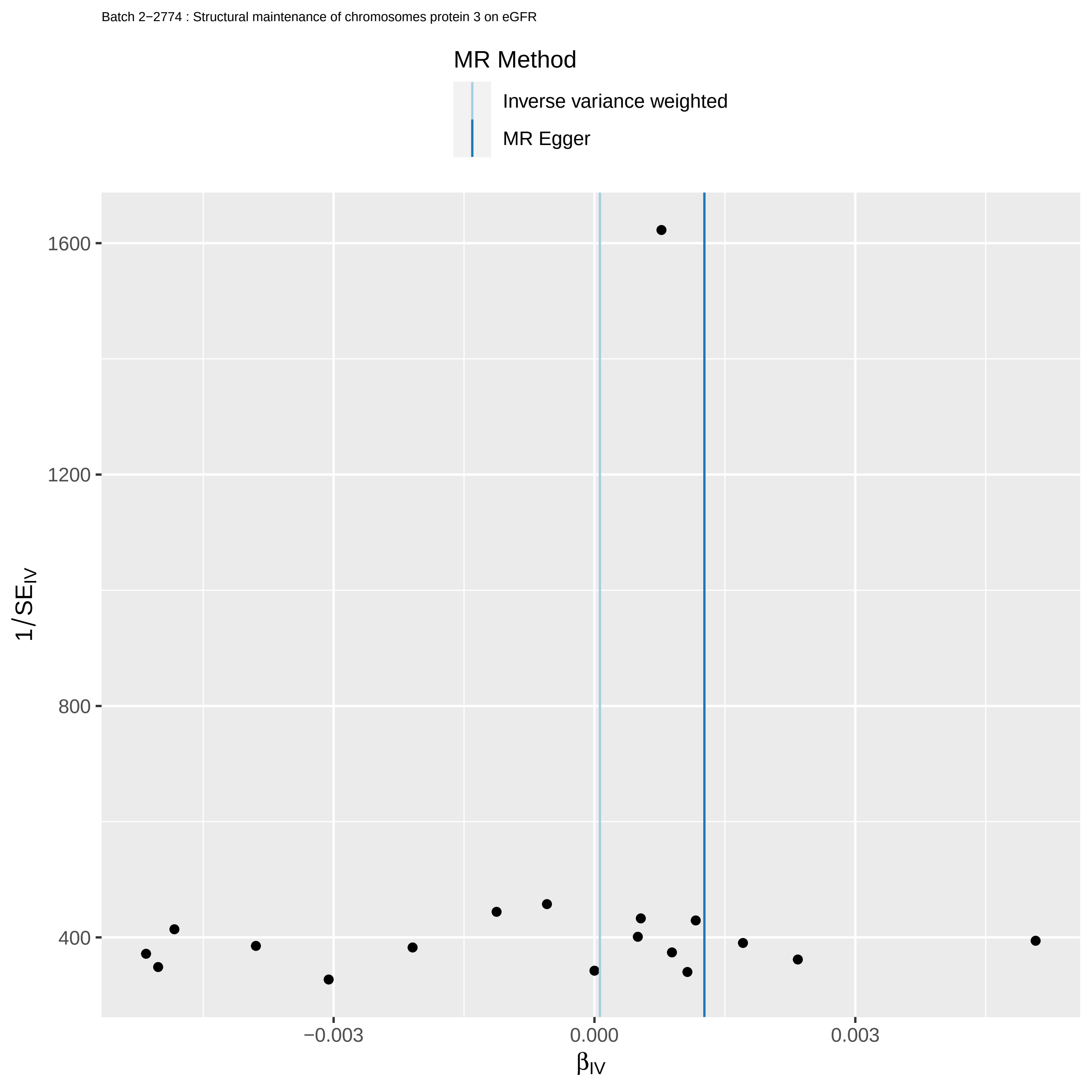

Batch 2−2774 : Structural maintenance of chromosomes protein 3 on eGFR
MR Method
Inverse variance weighted
MR Egger
1600
1200
800
400
−0.003
0.000
0.003
β_{IV}
$1/SE_{IV}$

Batch 2−2775 : Single−pass membrane and coiled−coil domain−containing protein 2 on eGFR

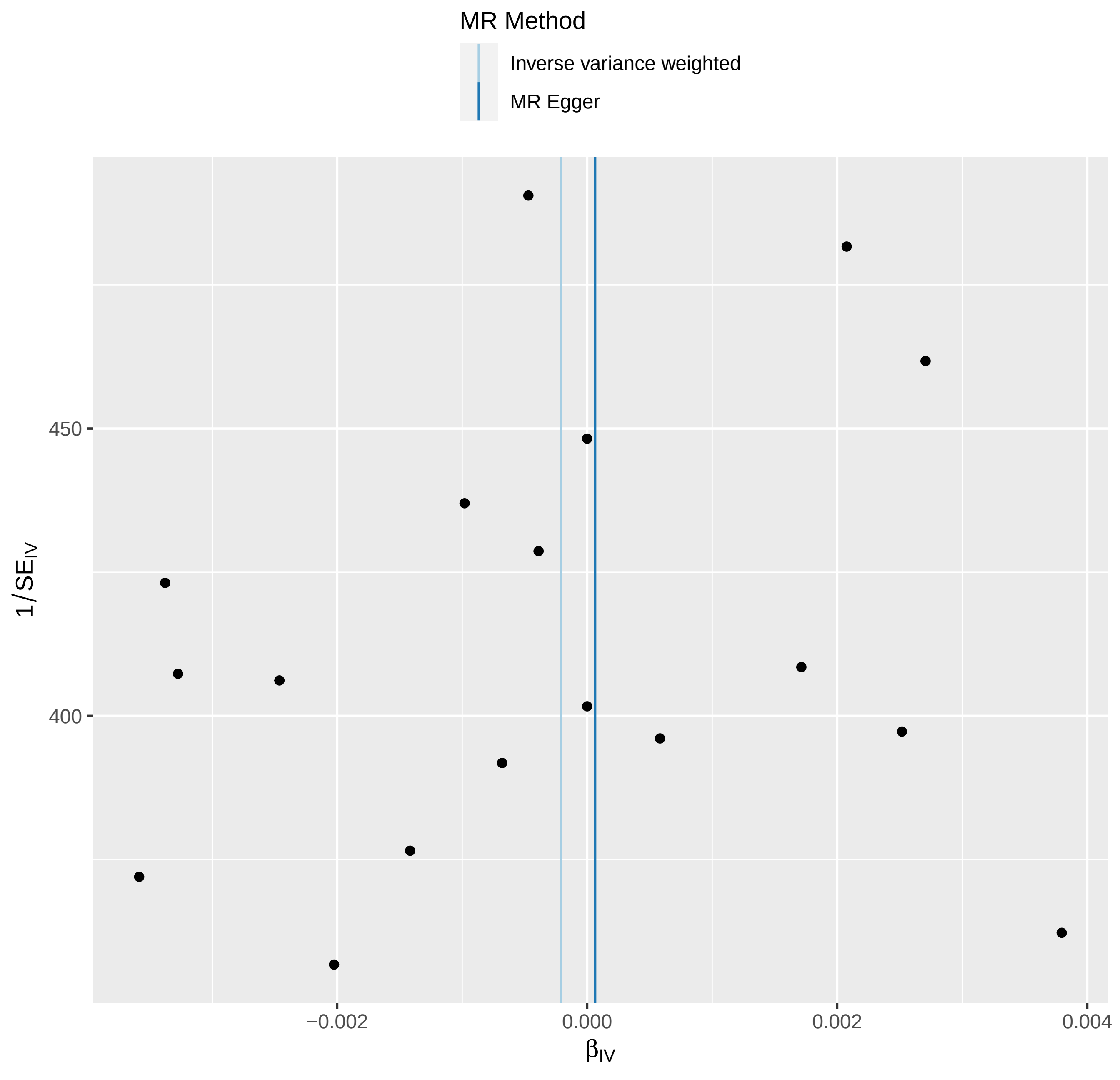

Batch 2−2776 : Essential MCU regulator, mitochondrial on eGFR

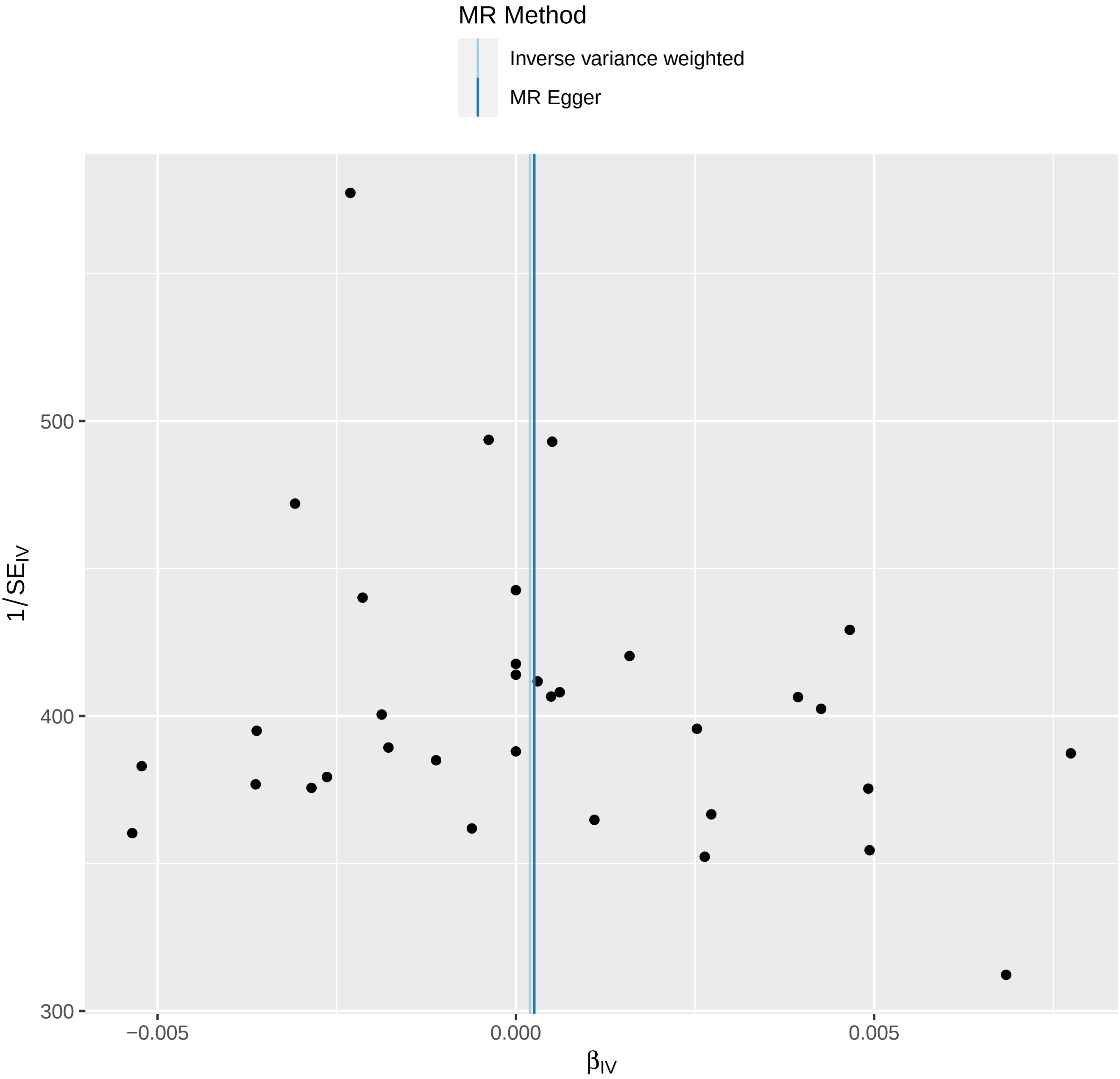

Batch 2−2777 : Small integral membrane protein 10 on eGFR

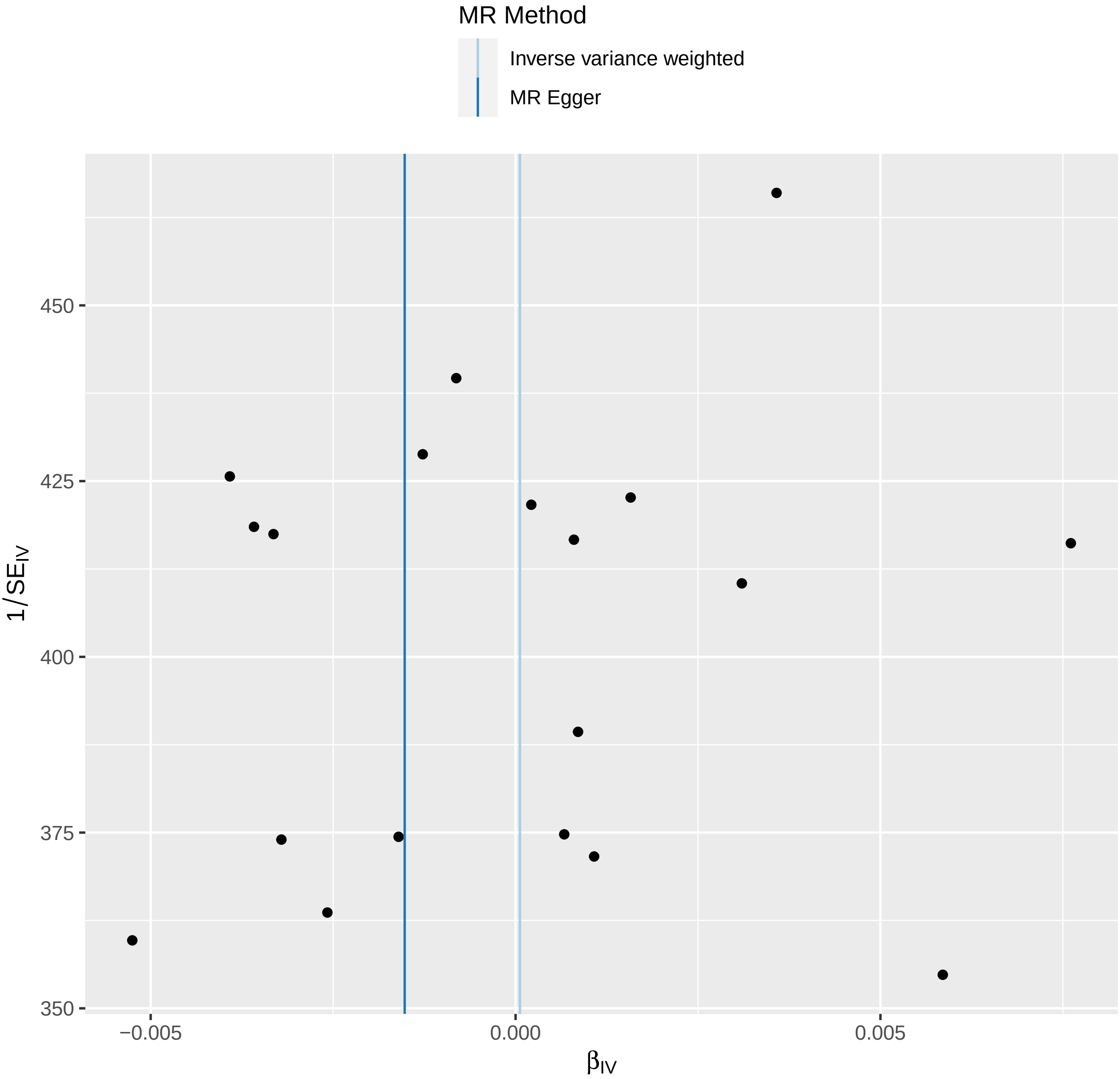

Batch 2−2778 : Small integral membrane protein 9 on eGFR

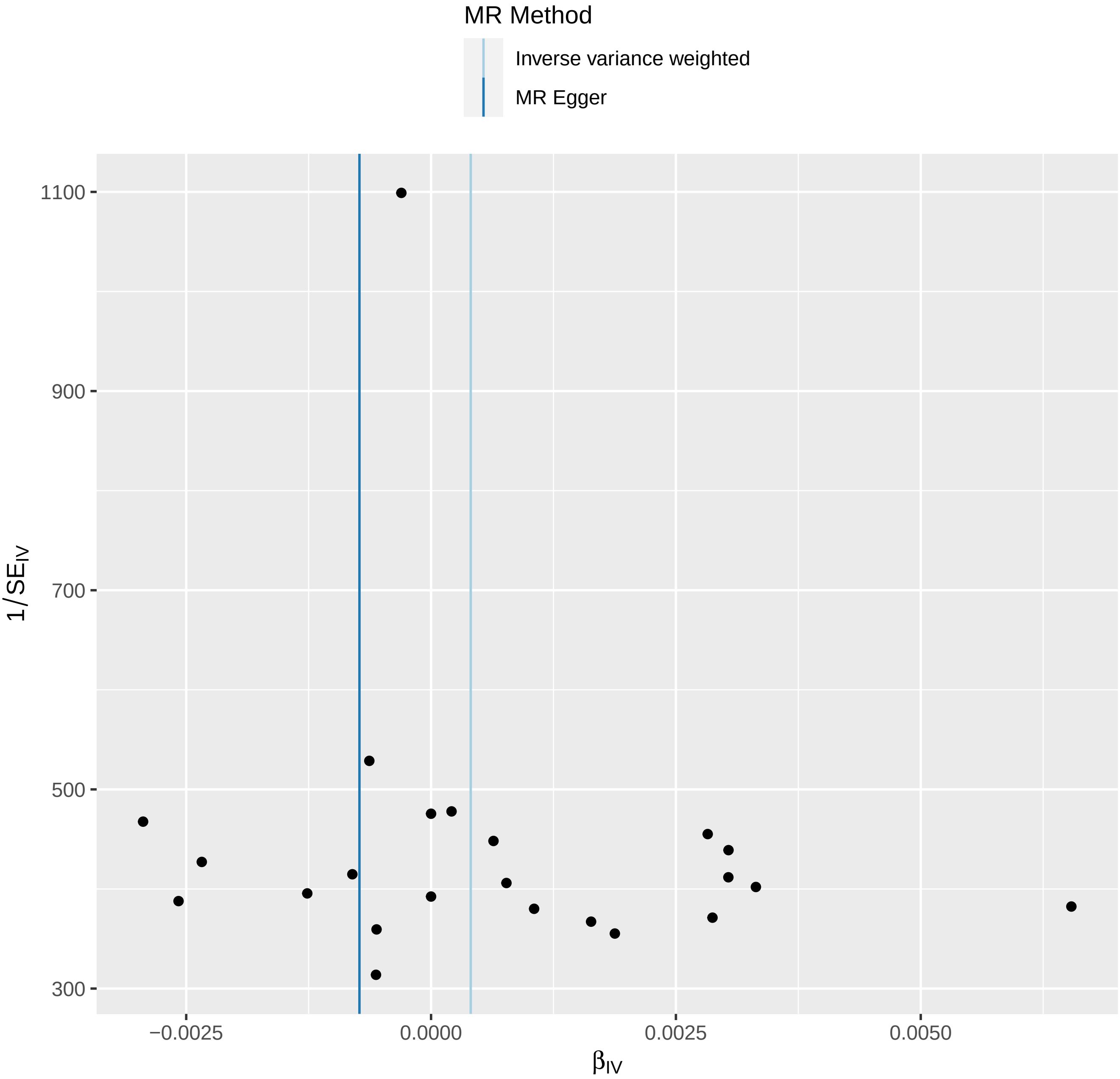

Batch 2−2779 : SPARC−related modular calcium−binding protein 1 on eGFR

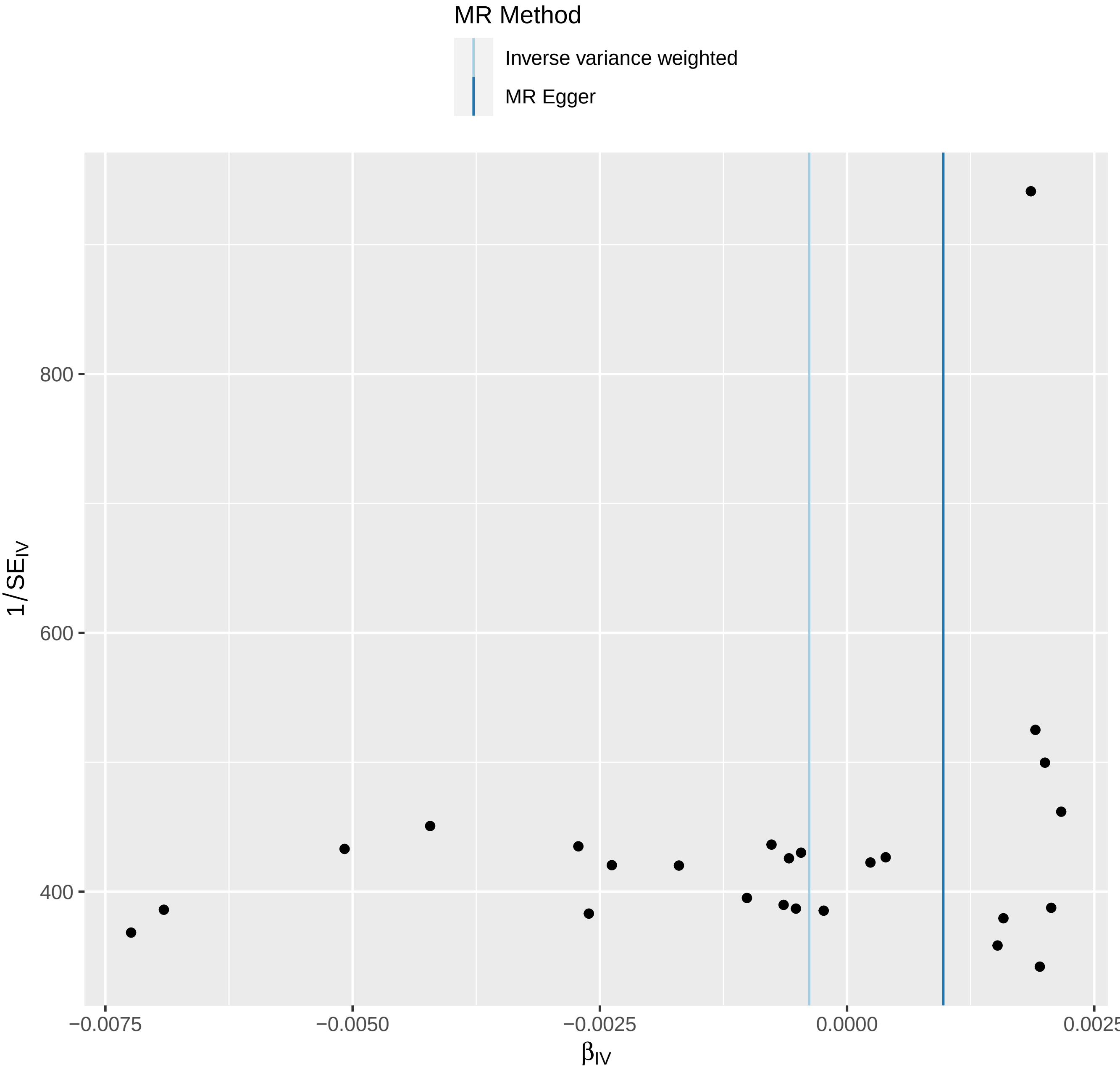

Batch 2−2780 : Acid sphingomyelinase−like phosphodiesterase 3a on eGFR

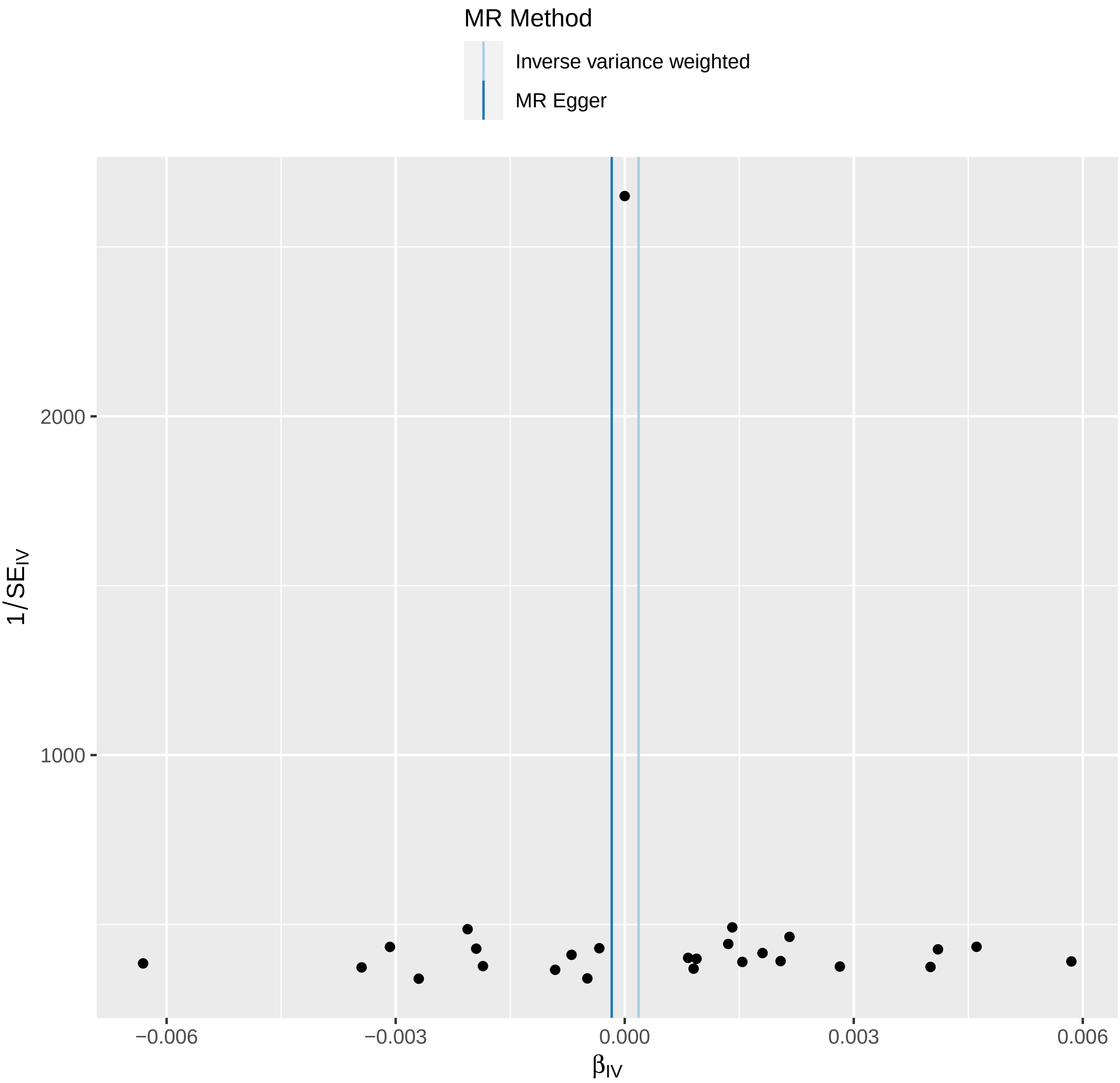

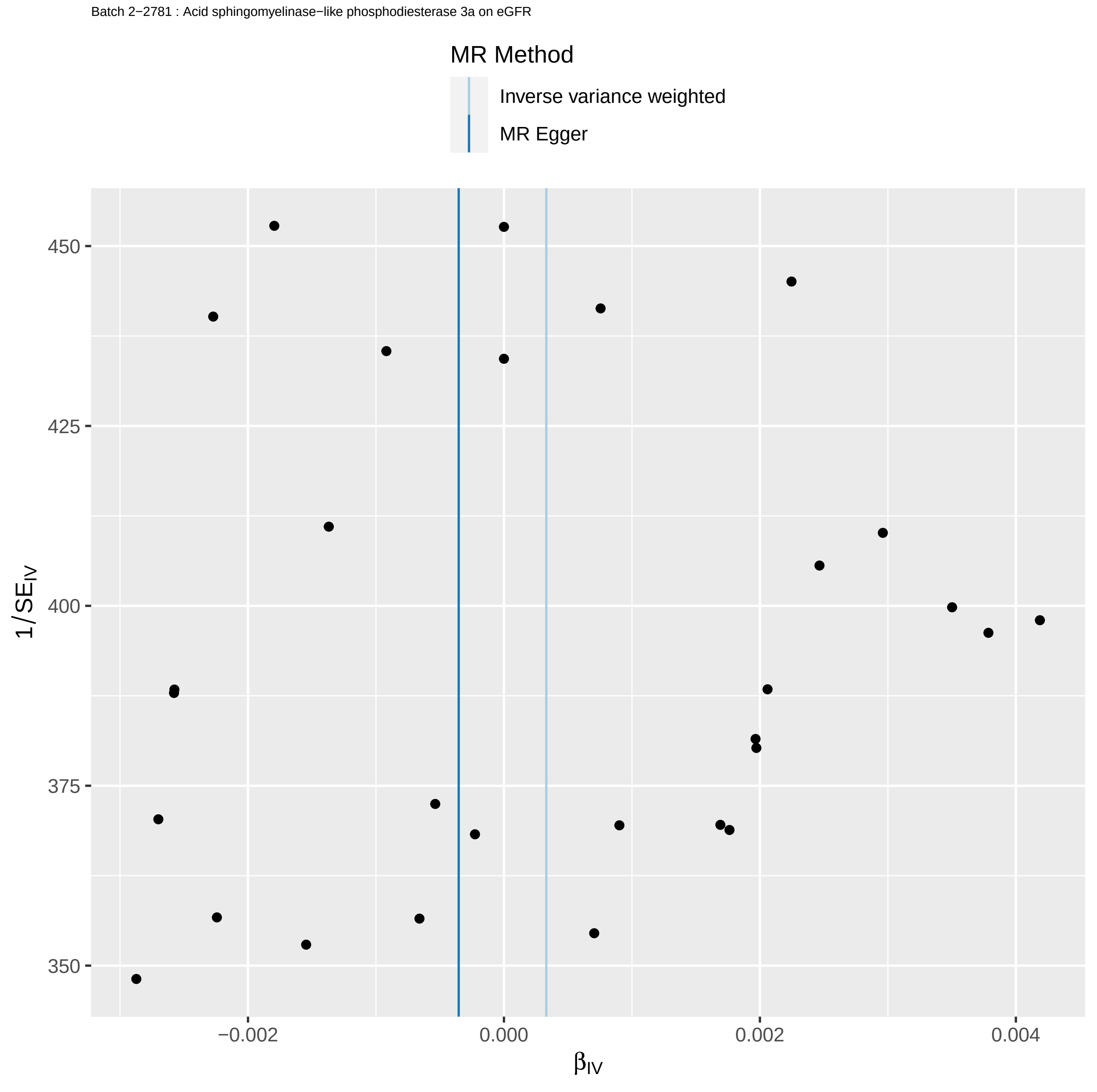

Batch 2−2781 : Acid sphingomyelinase−like phosphodiesterase 3a on eGFR
MR Method
Inverse variance weighted
MR Egger
450
425
400
375
350
1/SE$_{IV}$
−0.002
0.000
0.002
0.004
β$_{IV}$

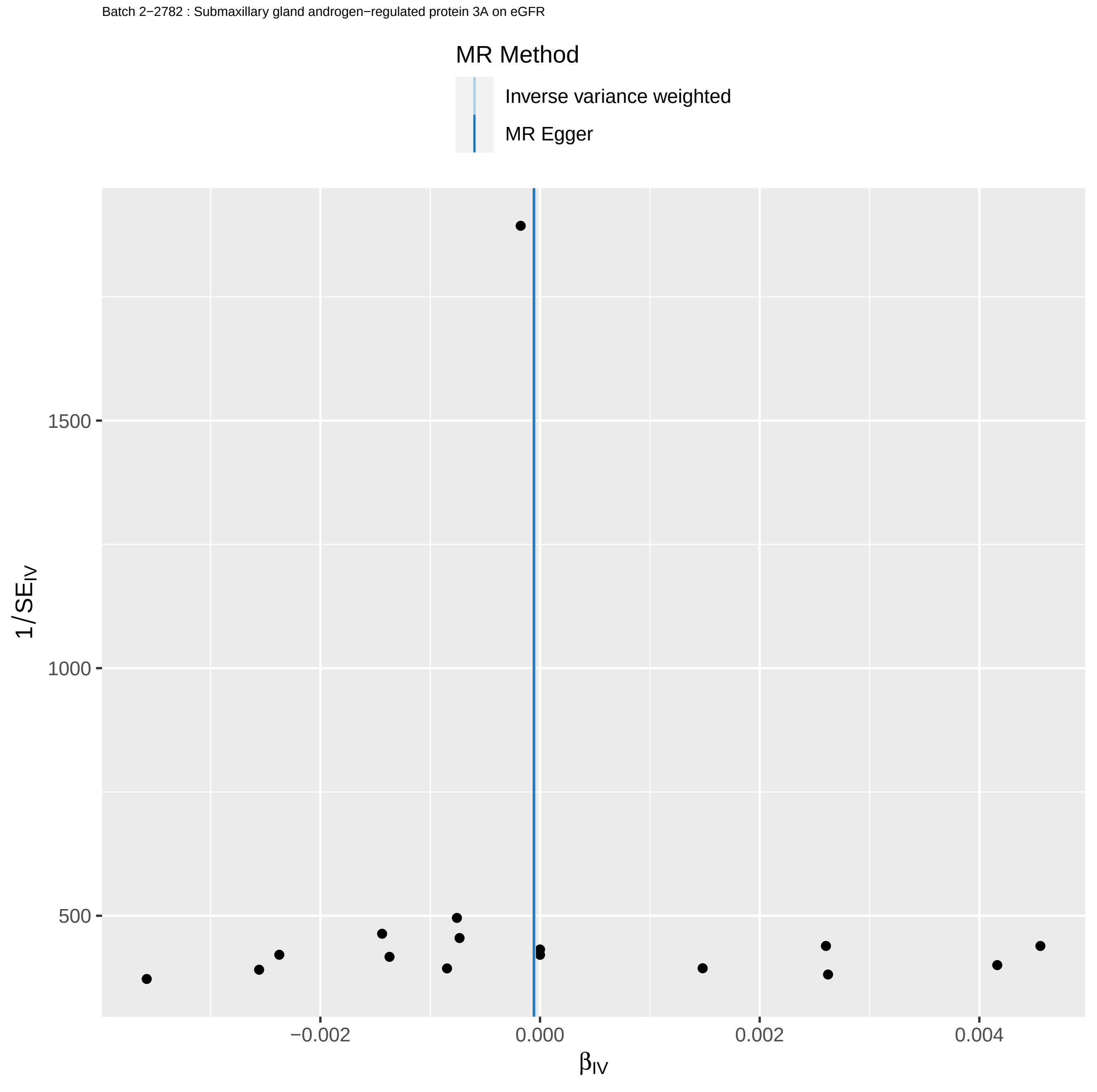
Batch 2−2782 : Submaxillary gland androgen−regulated protein 3A on eGFR
MR Method
Inverse variance weighted
MR Egger
1500
1000
500
1/SE_IV
−0.002
0.000
0.002
0.004
β_IV

Batch 2−2783 : Submaxillary gland androgen−regulated protein 3B on eGFR

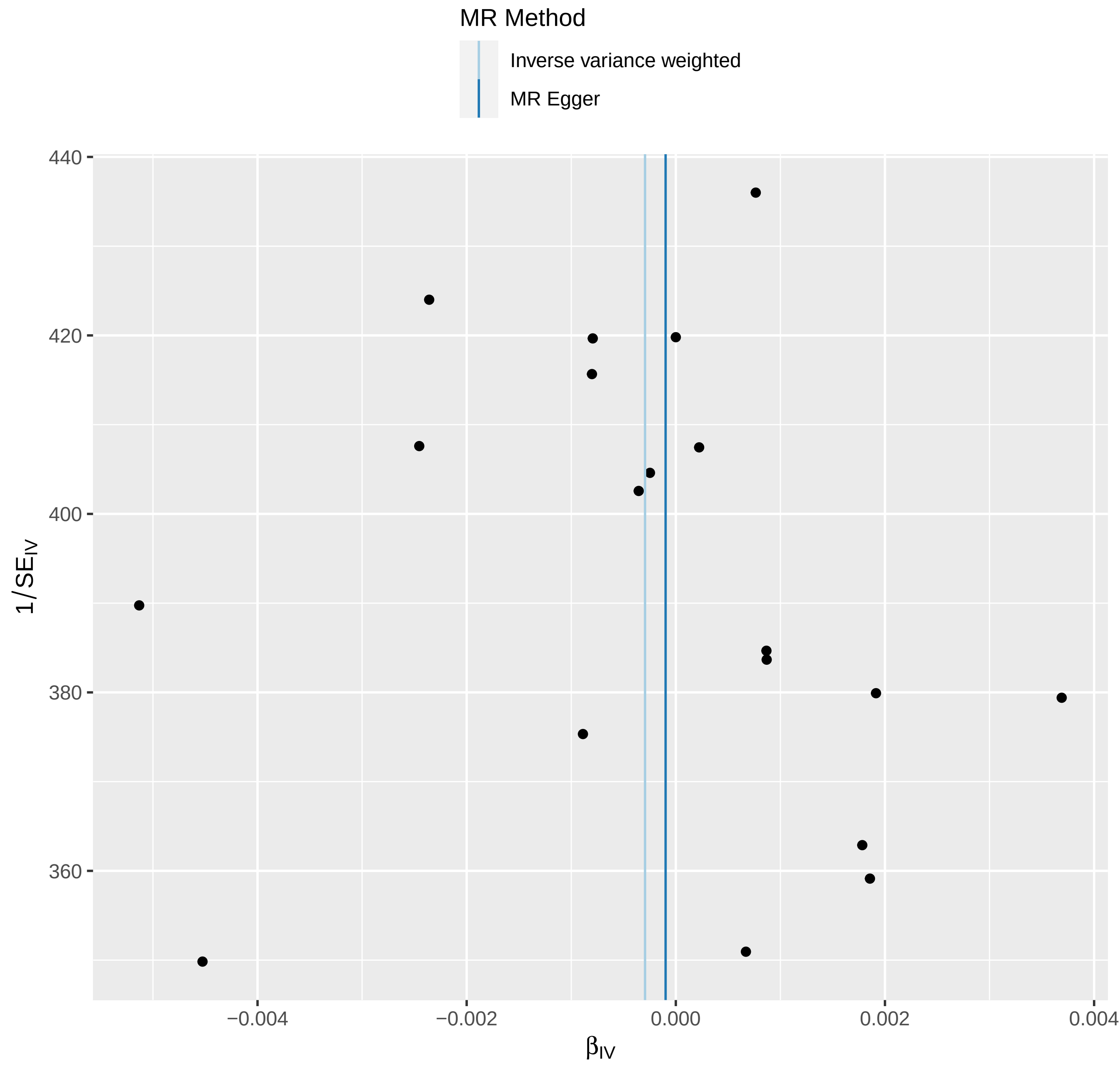

Batch 2−2784 : N−lysine methyltransferase SMYD2 on eGFR

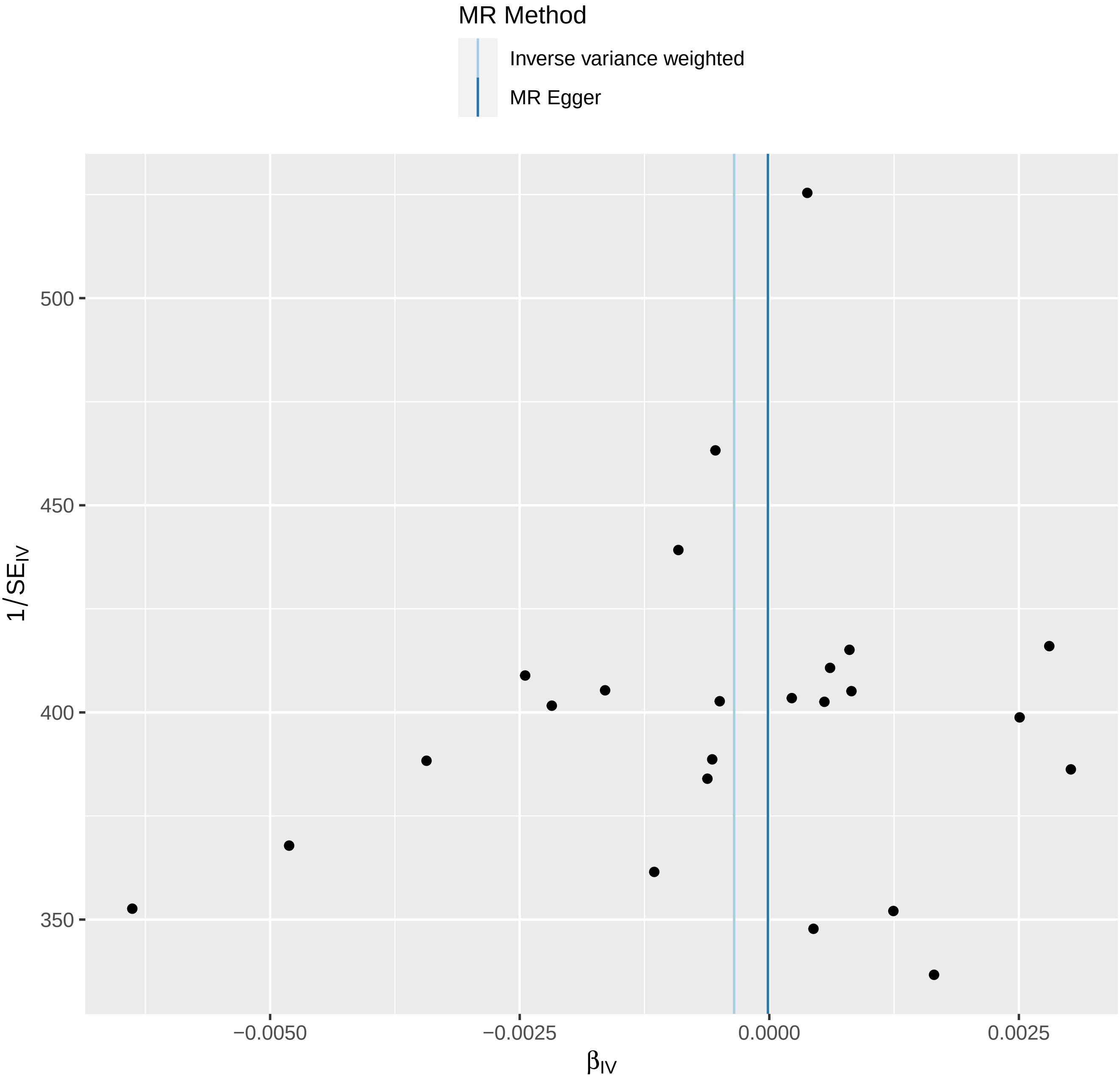

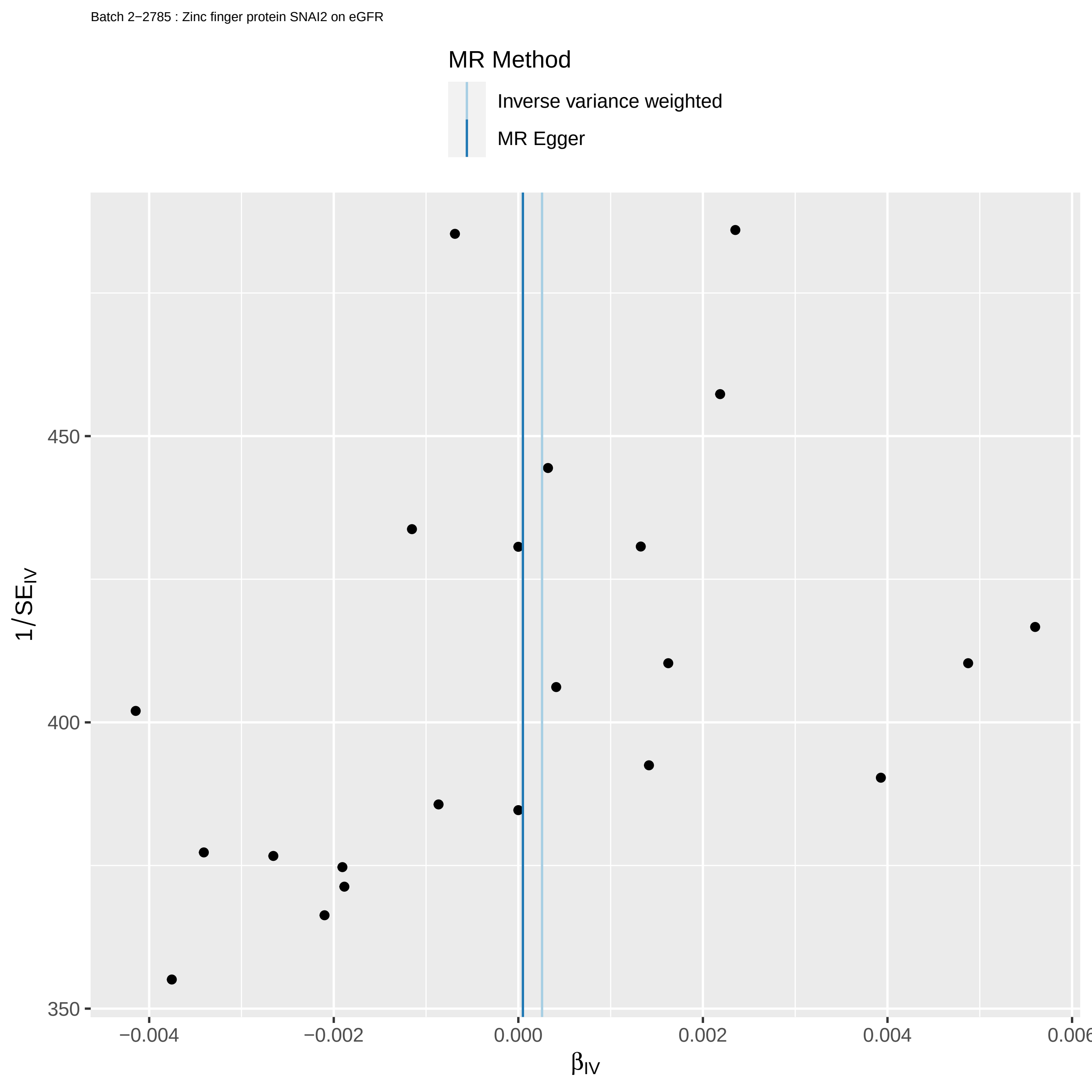
Batch 2−2785 : Zinc finger protein SNAI2 on eGFR
MR Method
Inverse variance weighted
MR Egger
450
400
350
1/SE_IV
−0.004
−0.002
0.000
0.002
0.004
0.006
β_IV

Batch 2−2786 : Synaptosomal−associated protein 25 on eGFR

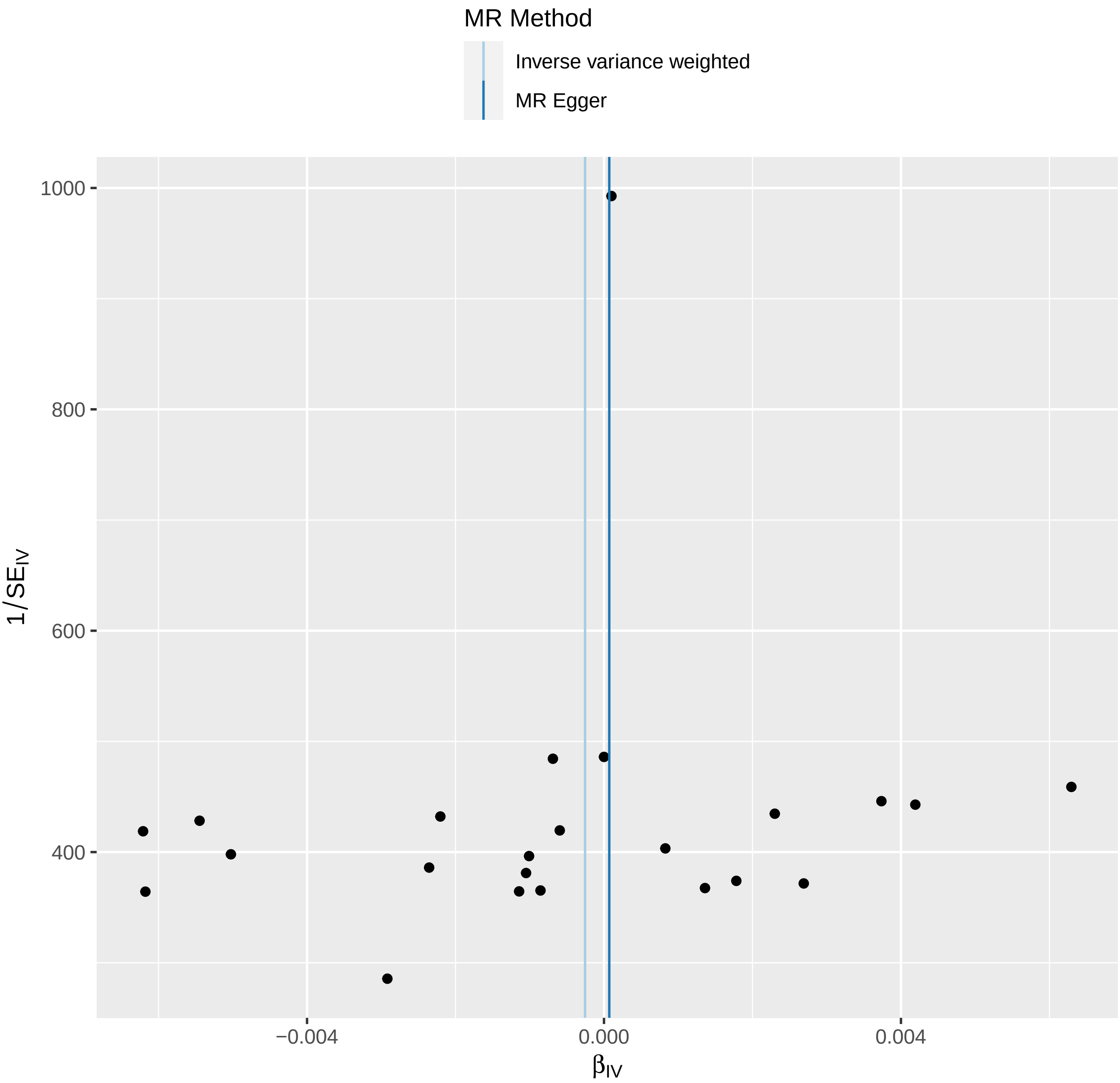

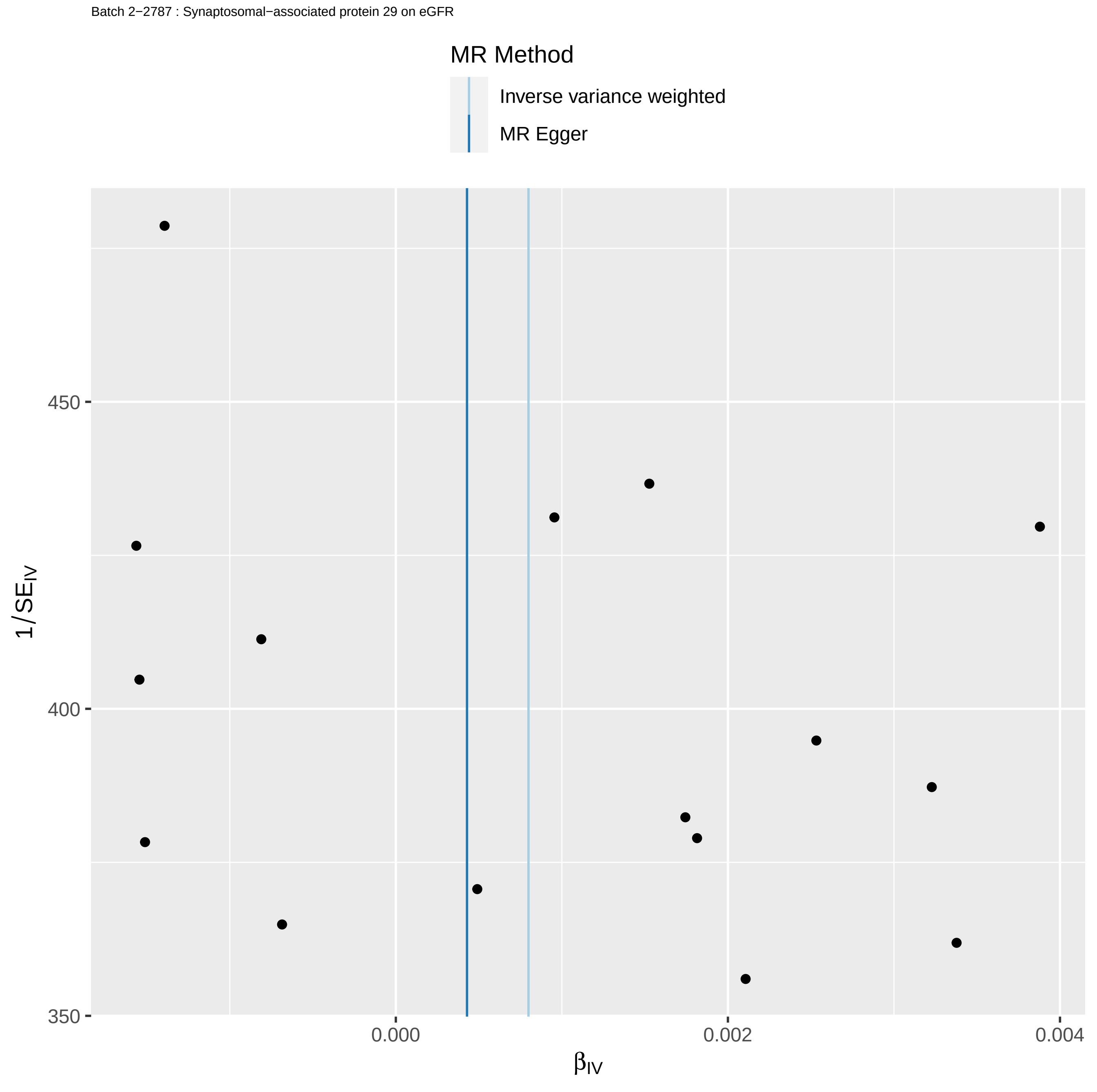
Batch 2−2787 : Synaptosomal−associated protein 29 on eGFR
MR Method
Inverse variance weighted
MR Egger
450
400
350
1/SE_IV
0.000
0.002
0.004
β_IV

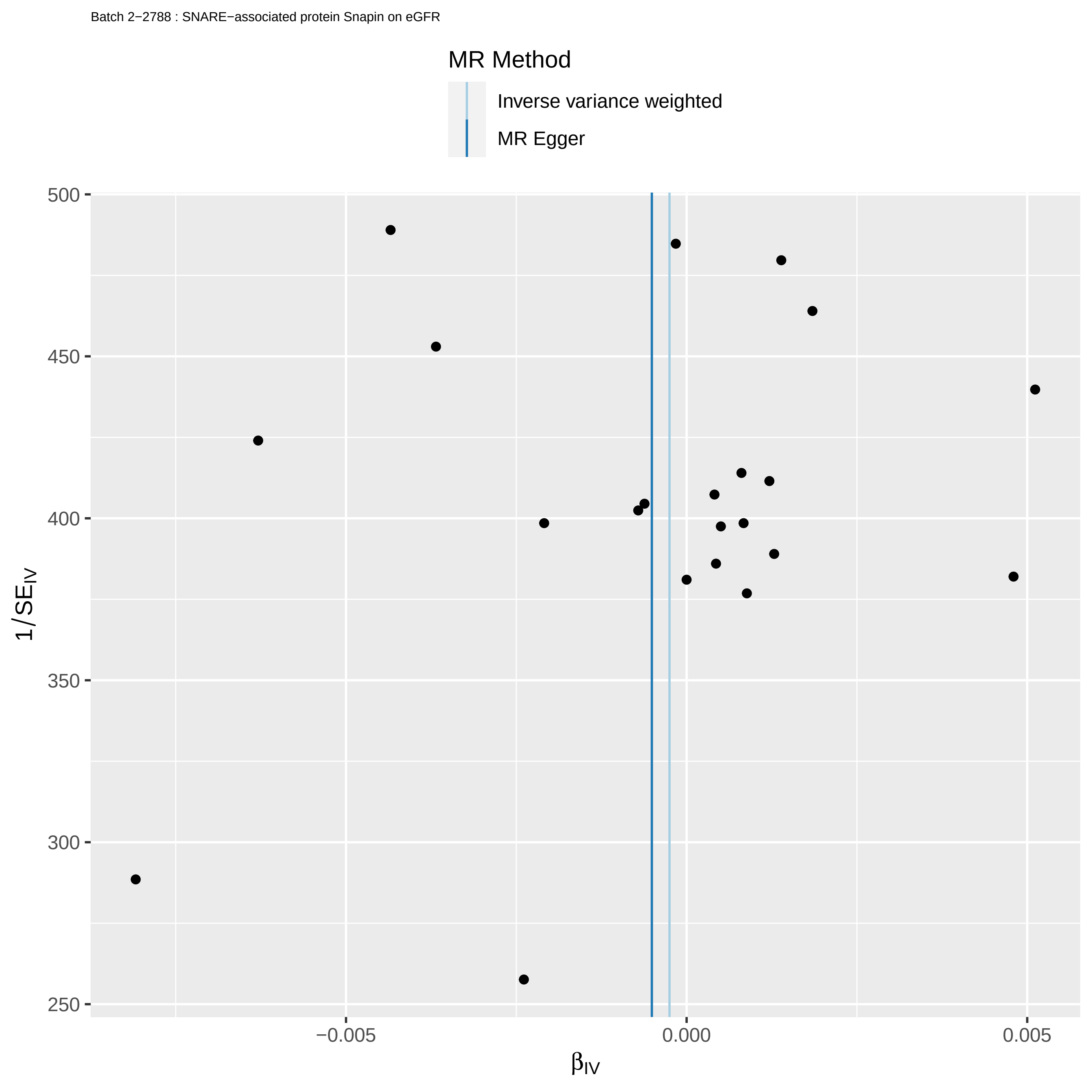
Batch 2−2788 : SNARE−associated protein Snapin on eGFR
MR Method
Inverse variance weighted
MR Egger
500
450
400
350
300
250
1/SE_IV
−0.005
0.000
0.005
β_IV

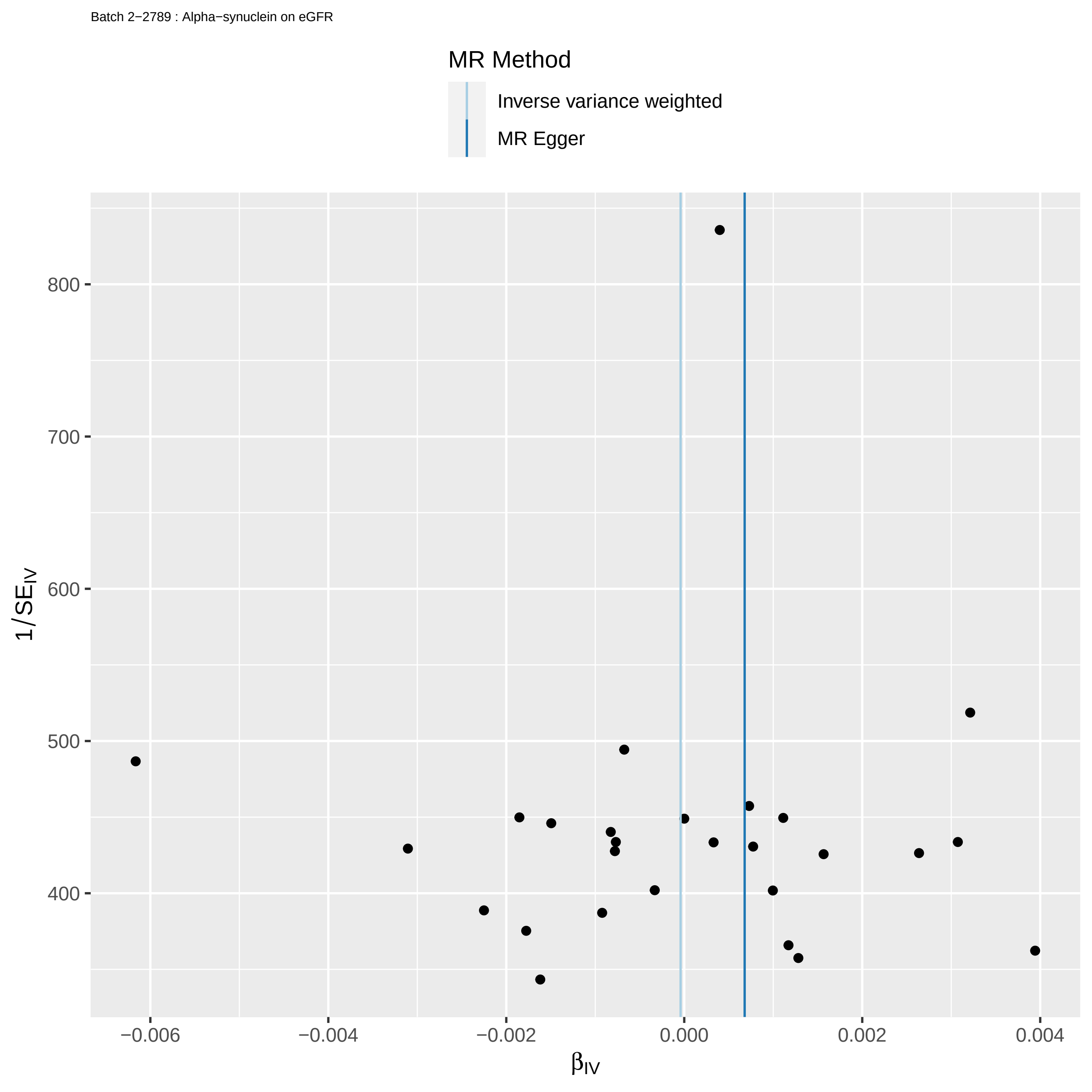

Batch 2−2789 : Alpha−synuclein on eGFR
MR Method
Inverse variance weighted
MR Egger
800
700
600
500
400
1/SE_IV
−0.006
−0.004
−0.002
0.000
0.002
0.004
β_IV

Batch 2−2790 : Alpha−synuclein on eGFR

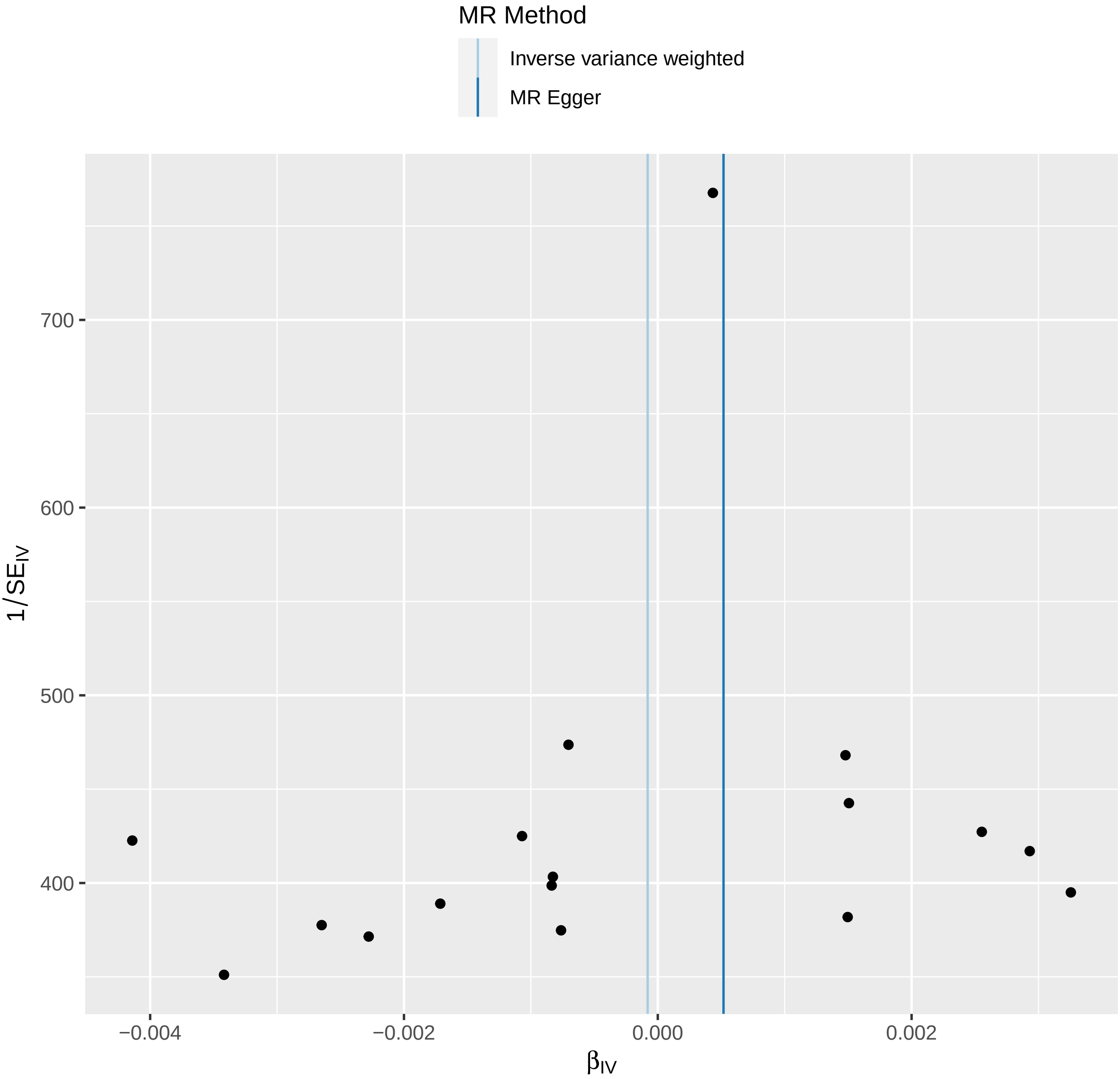

Batch 2−2791 : U1 small nuclear ribonucleoprotein A on eGFR

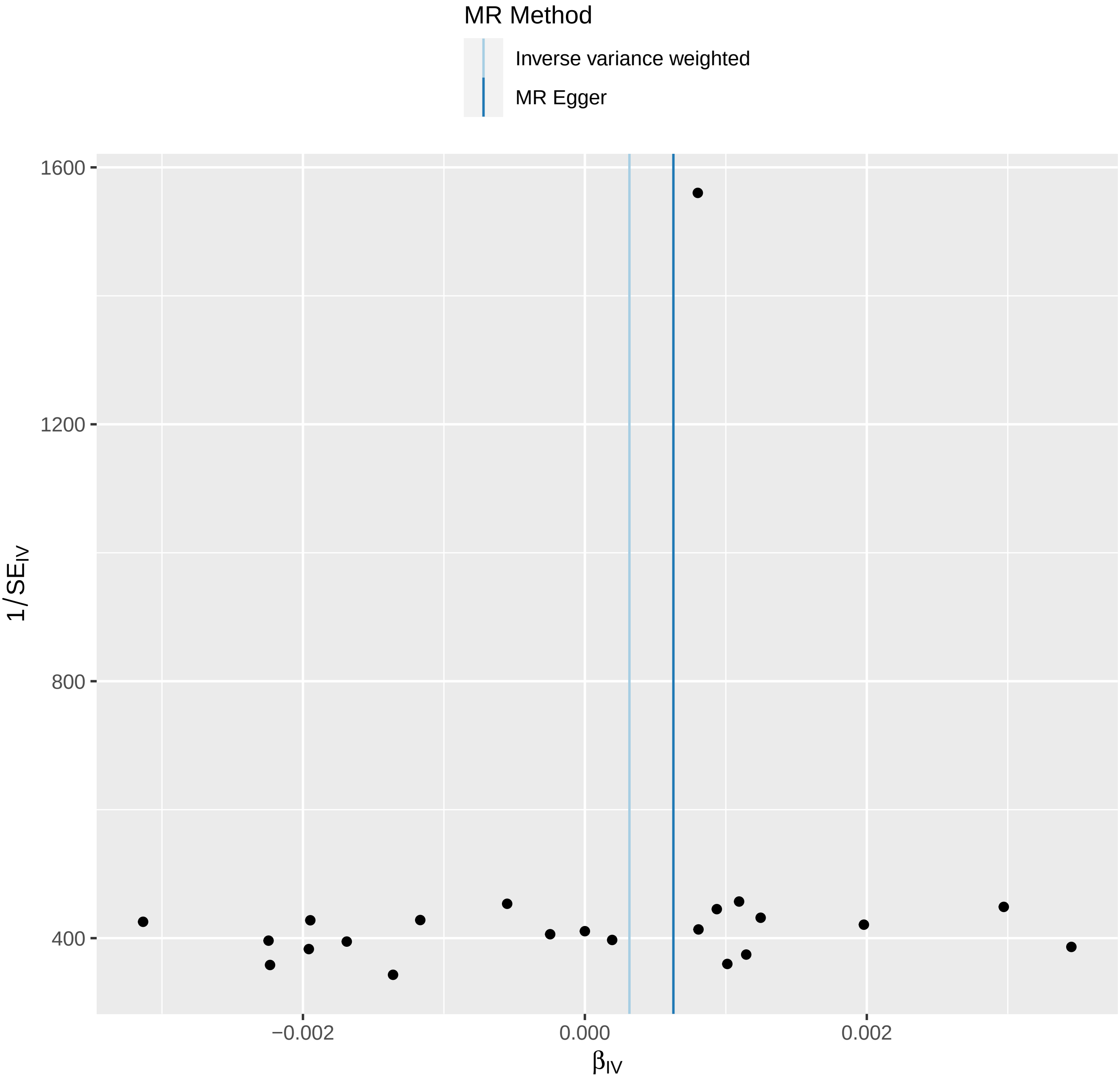

Batch 2−2792 : Sorting nexin−17 on eGFR

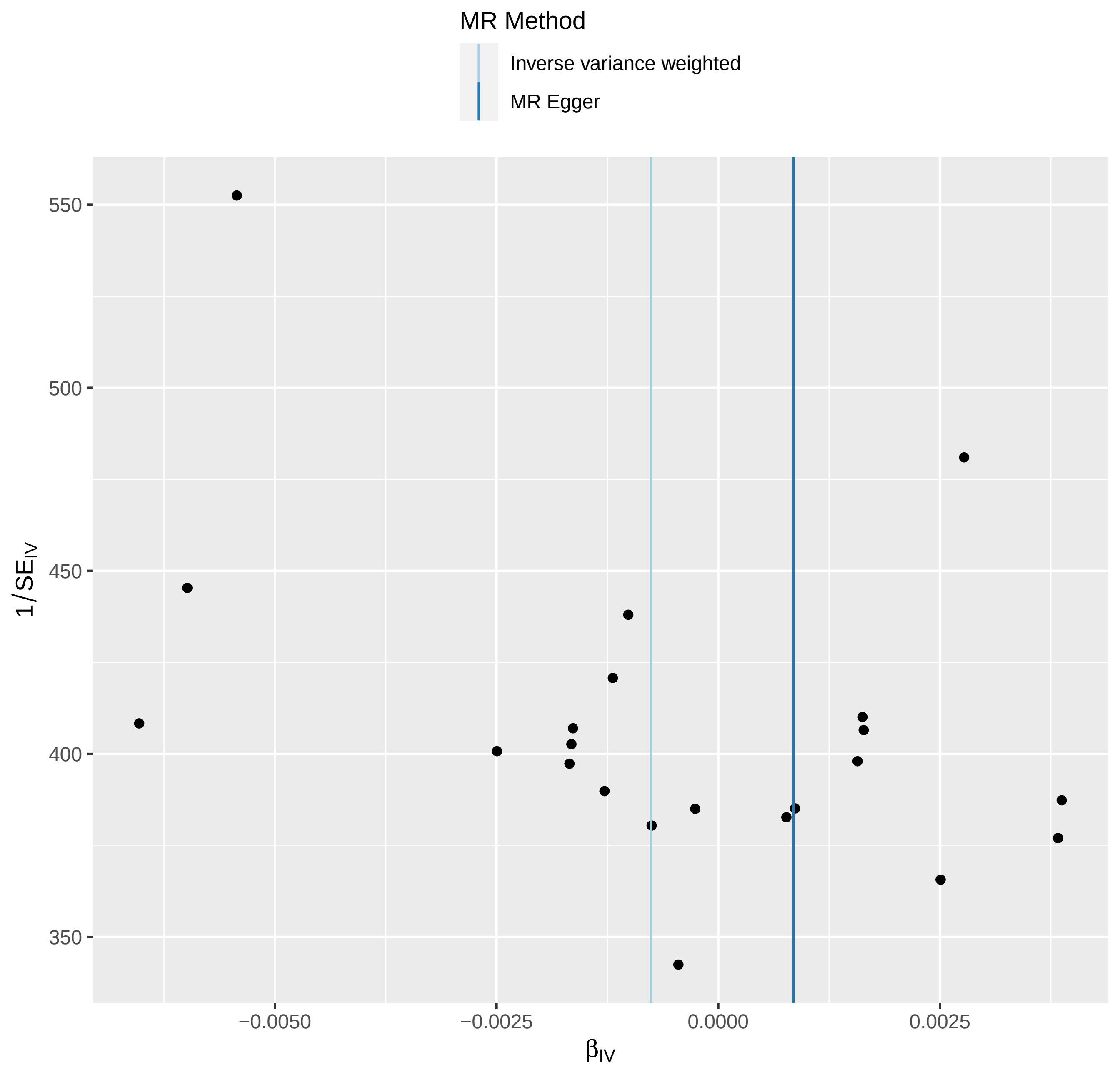

Batch 2−2793 : Sorting nexin−1 on eGFR

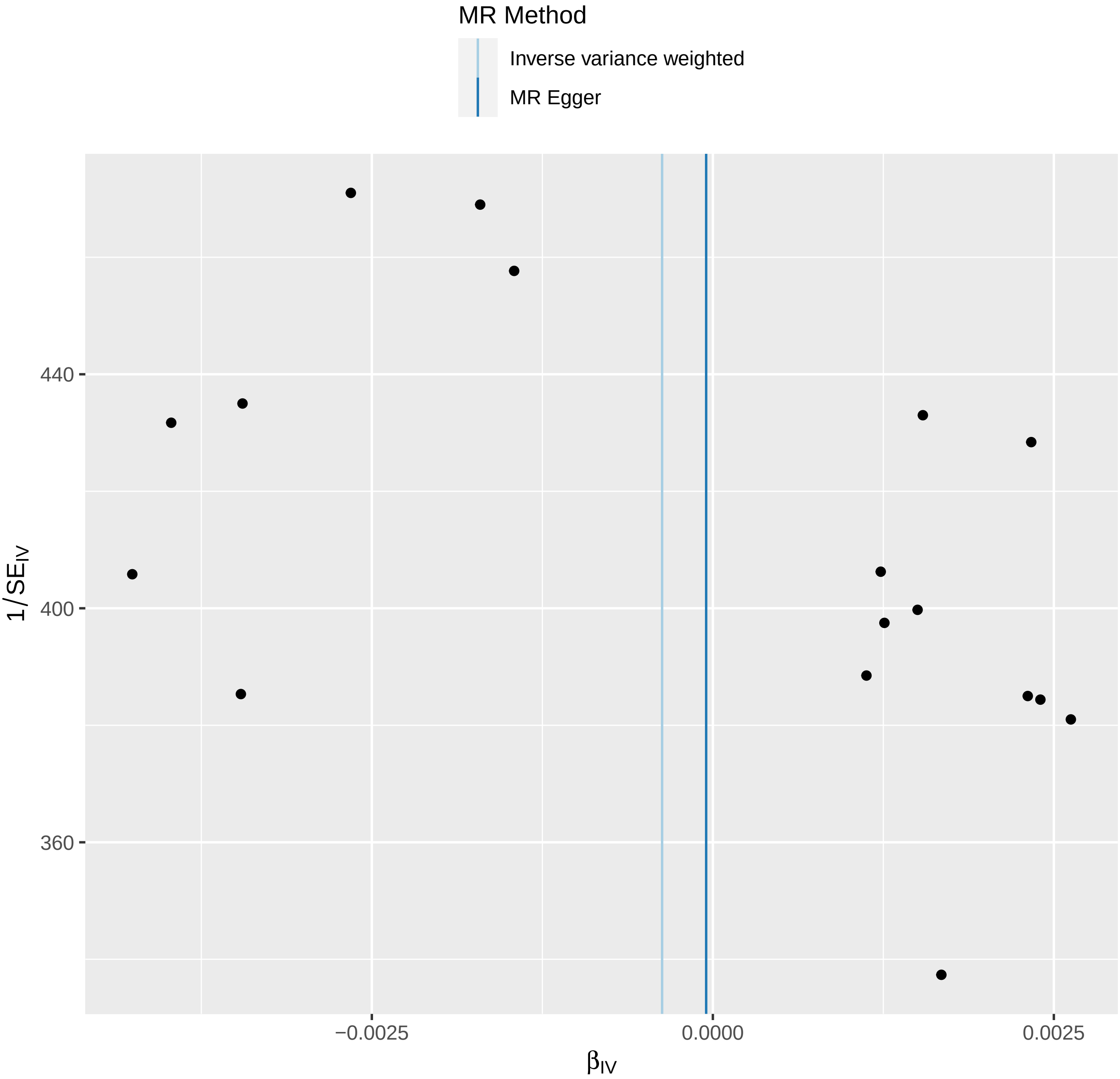

Batch 2−2794 : Sorting nexin−4 on eGFR

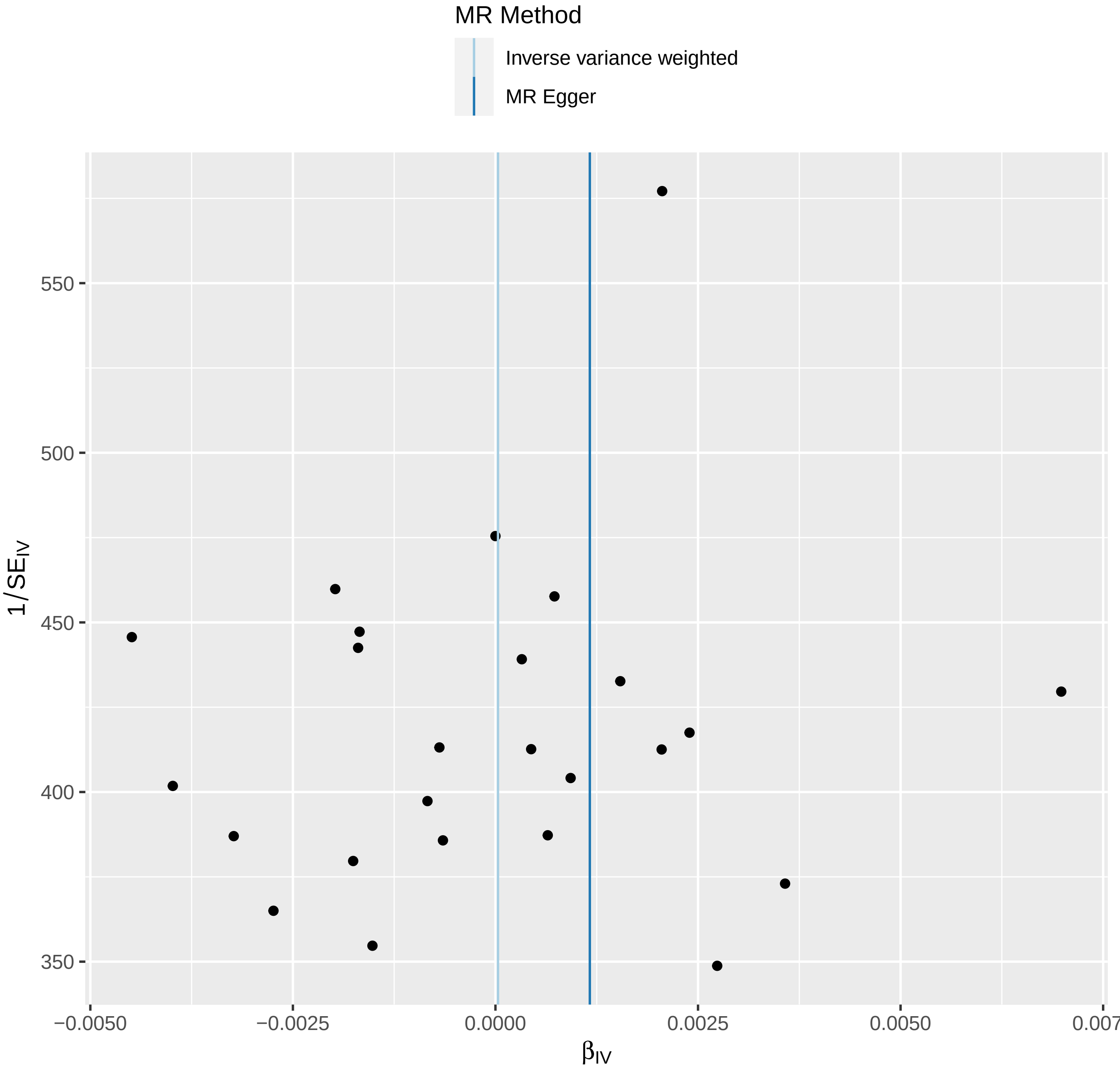

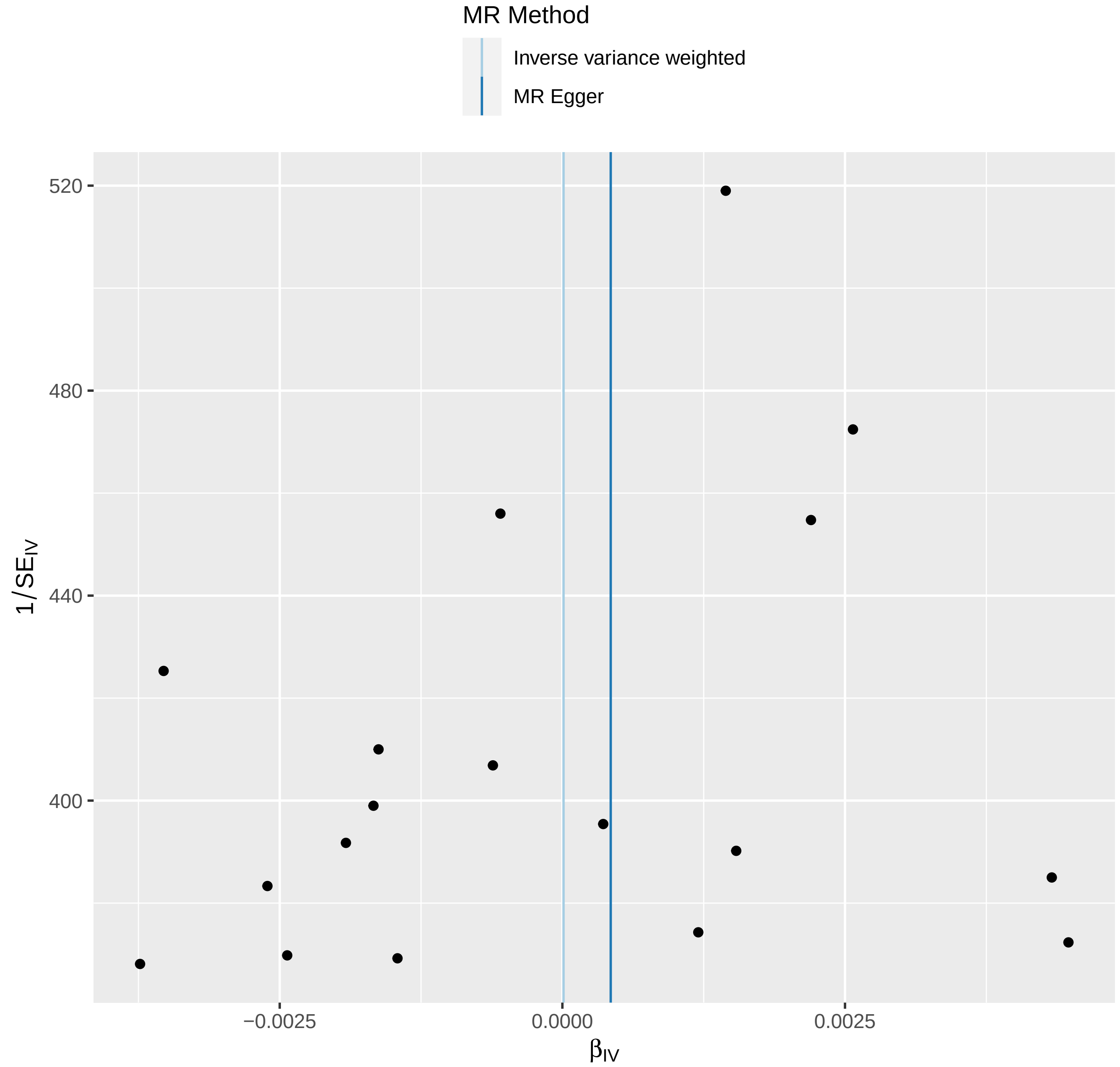
MR Method
Inverse variance weighted
MR Egger
520
480
440
400
1/SE$_{IV}$
−0.0025
0.0000
0.0025
β$_{IV}$

Batch 2−2796 : Sorting nexin−8 on eGFR

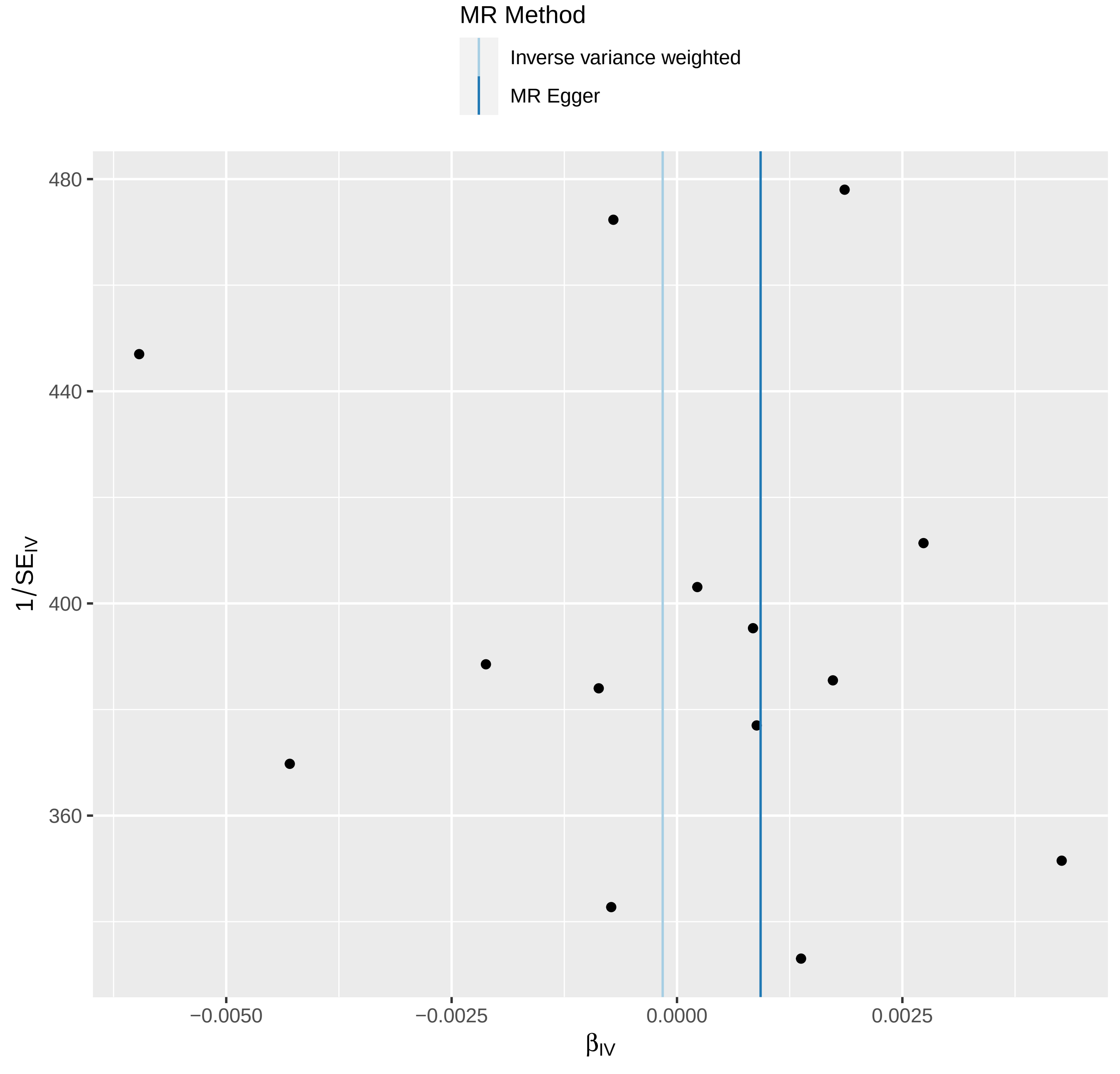

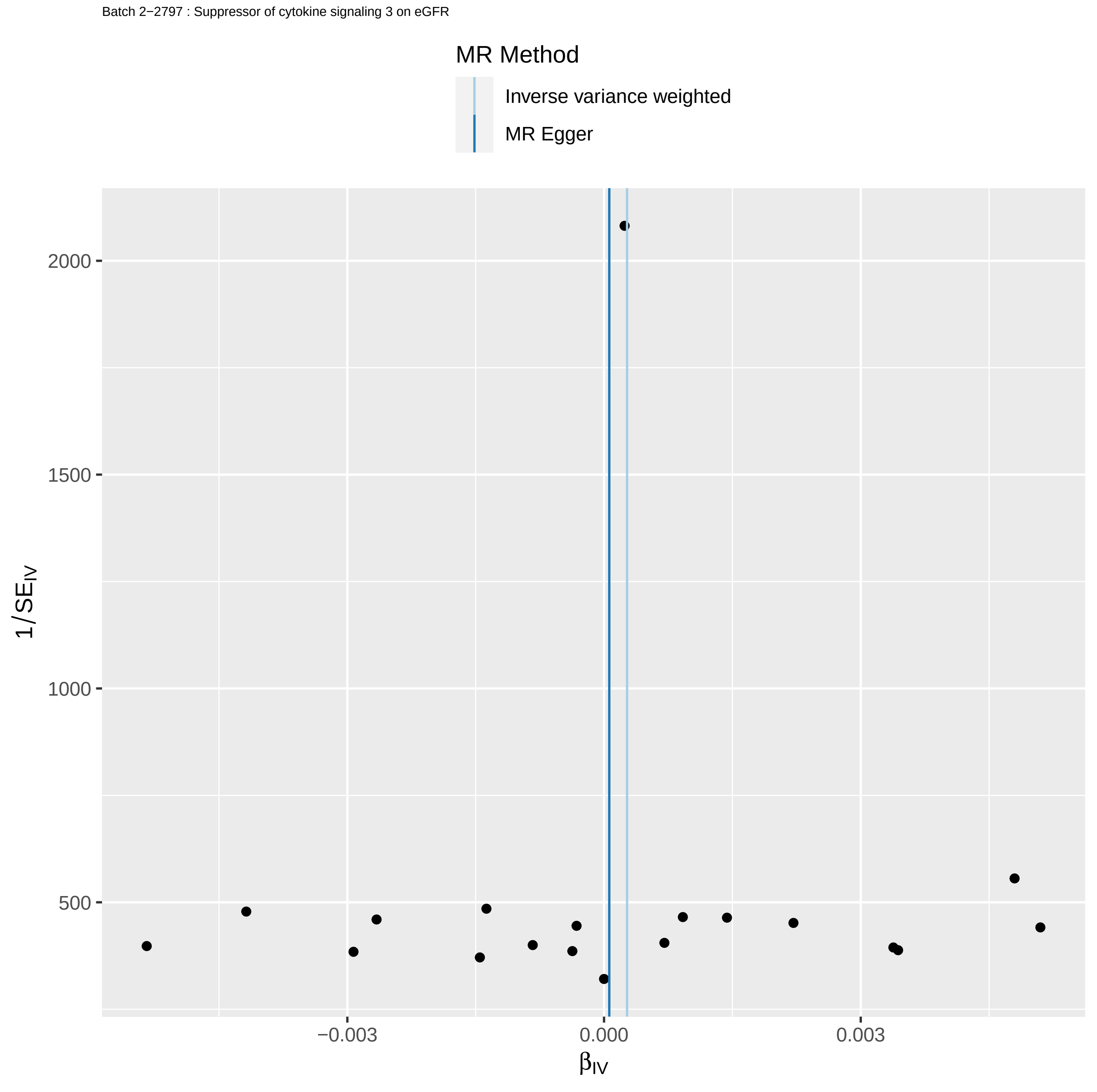

Batch 2−2797 : Suppressor of cytokine signaling 3 on eGFR
MR Method
Inverse variance weighted
MR Egger
2000
1500
1000
500
1/SE_IV
−0.003
0.000
0.003
β_IV

Batch 2−2798 : Suppressor of cytokine signaling 7 on eGFR

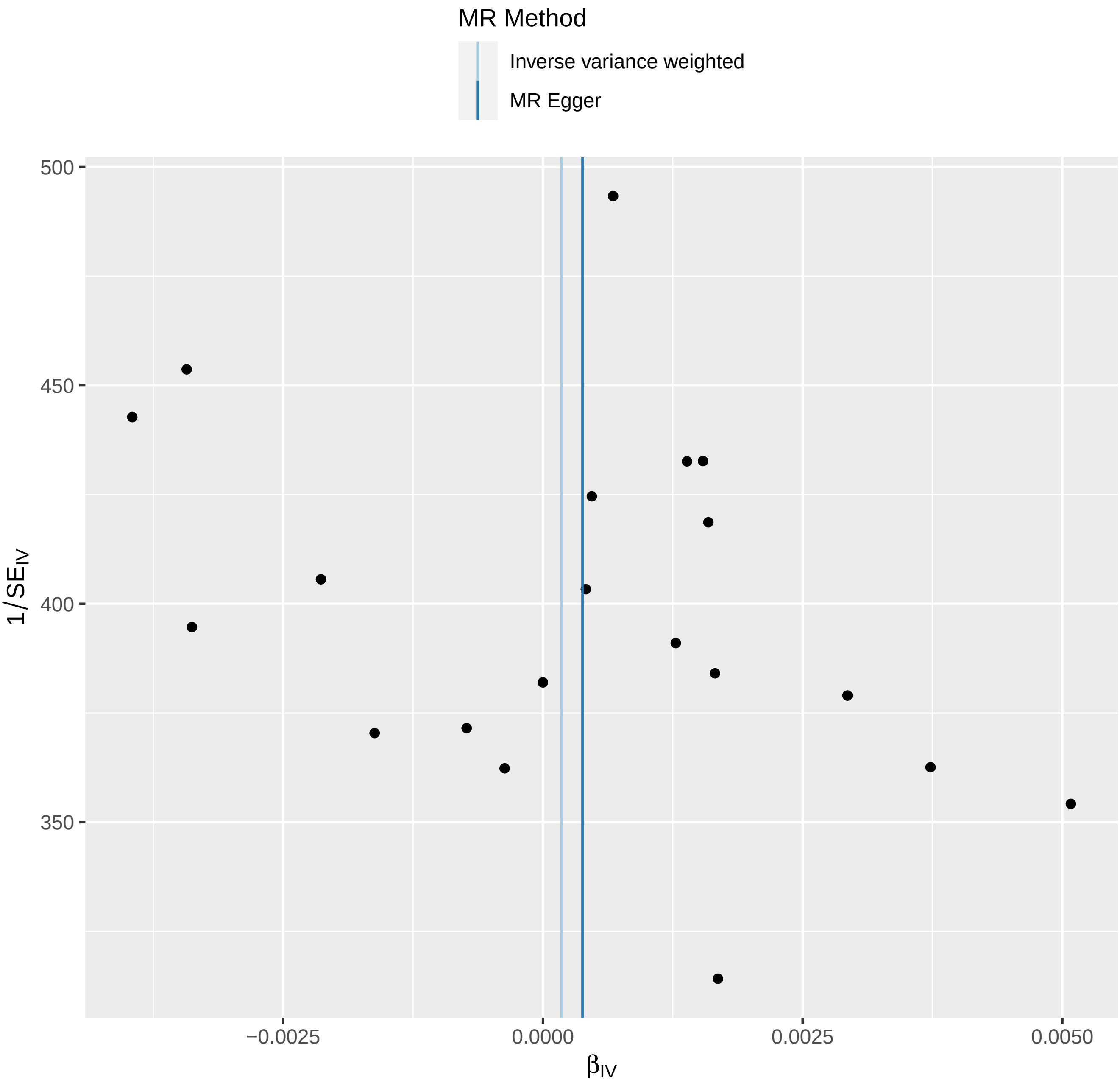

Batch 2−2799 : Superoxide dismutase [Mn], mitochondrial on eGFR

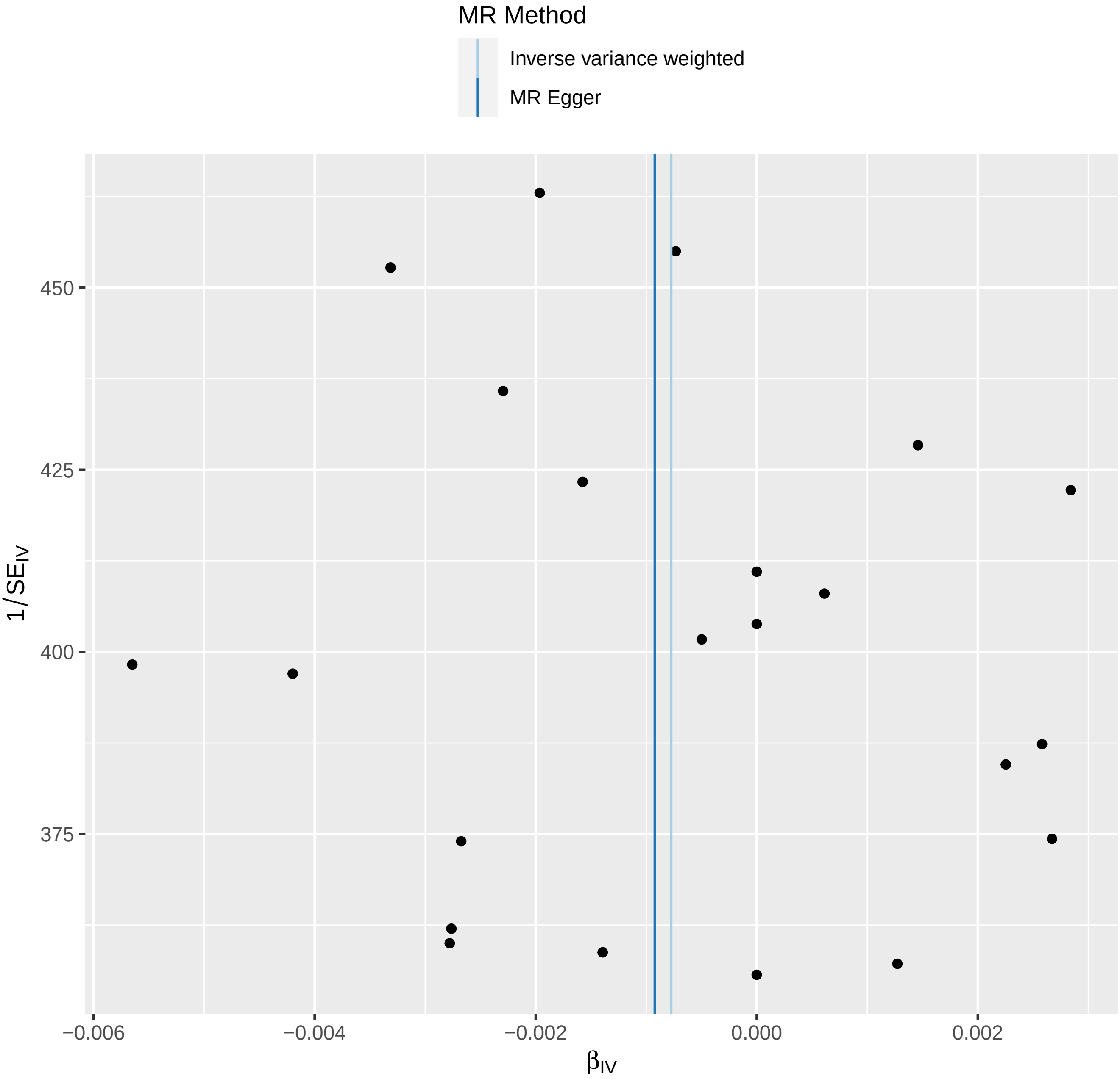

Batch 2−2800 : Extracellular superoxide dismutase [Cu−Zn] on eGFR

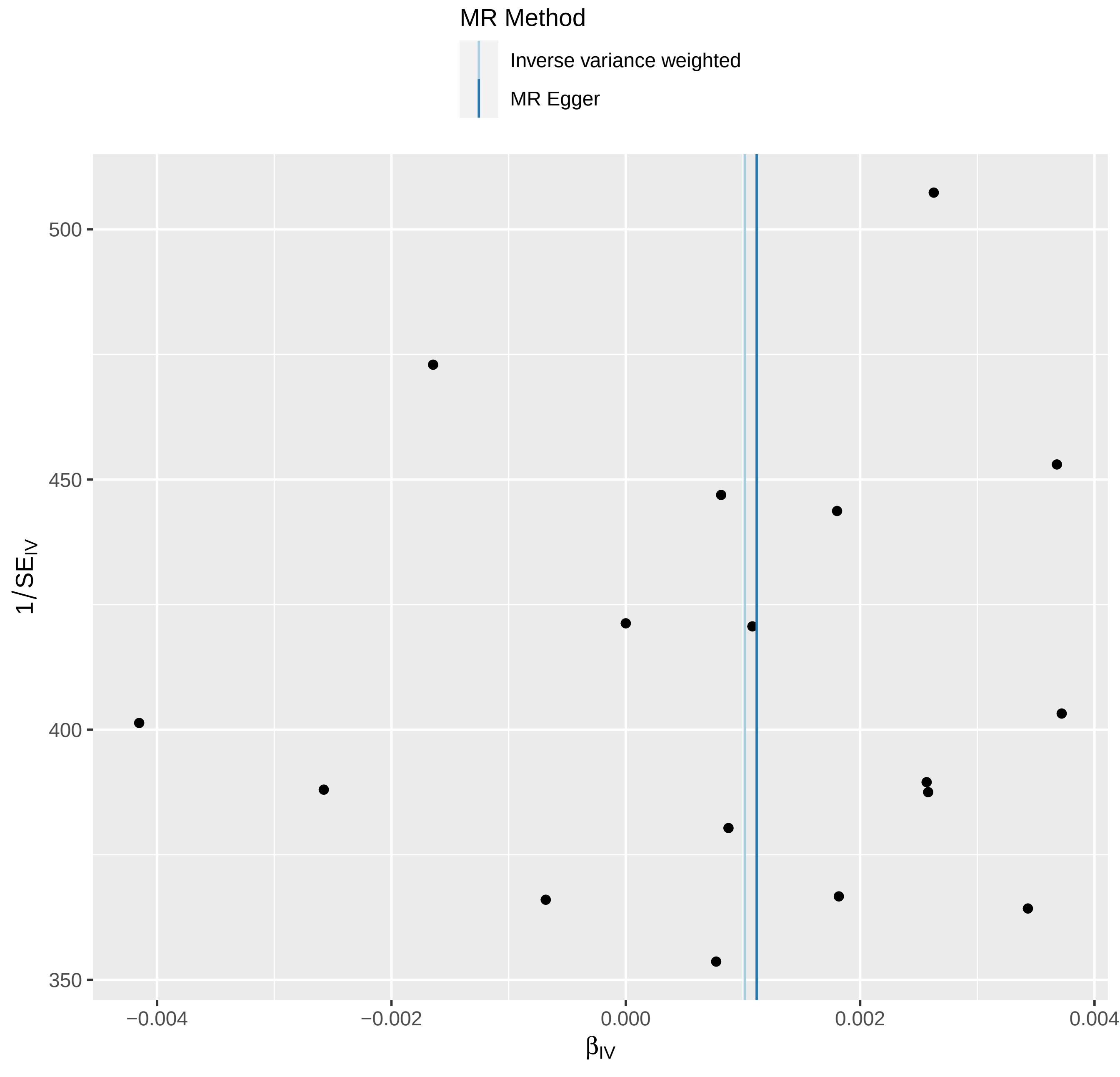

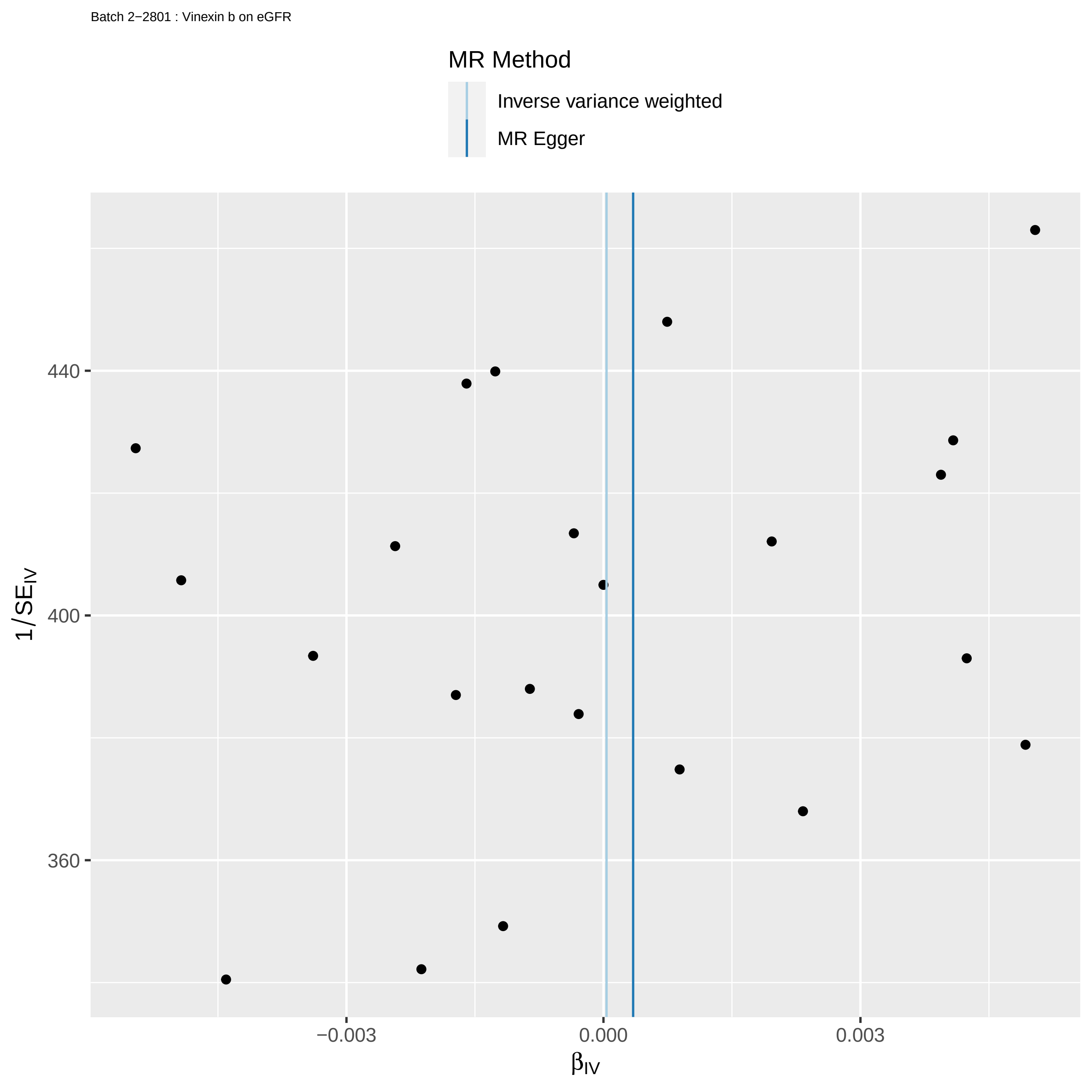
Batch 2−2801 : Vinexin b on eGFR
MR Method
Inverse variance weighted
MR Egger
440
400
360
1/SE$_{IV}$
−0.003
0.000
0.003
β$_{IV}$

Batch 2−2802 : Sortilin on eGFR

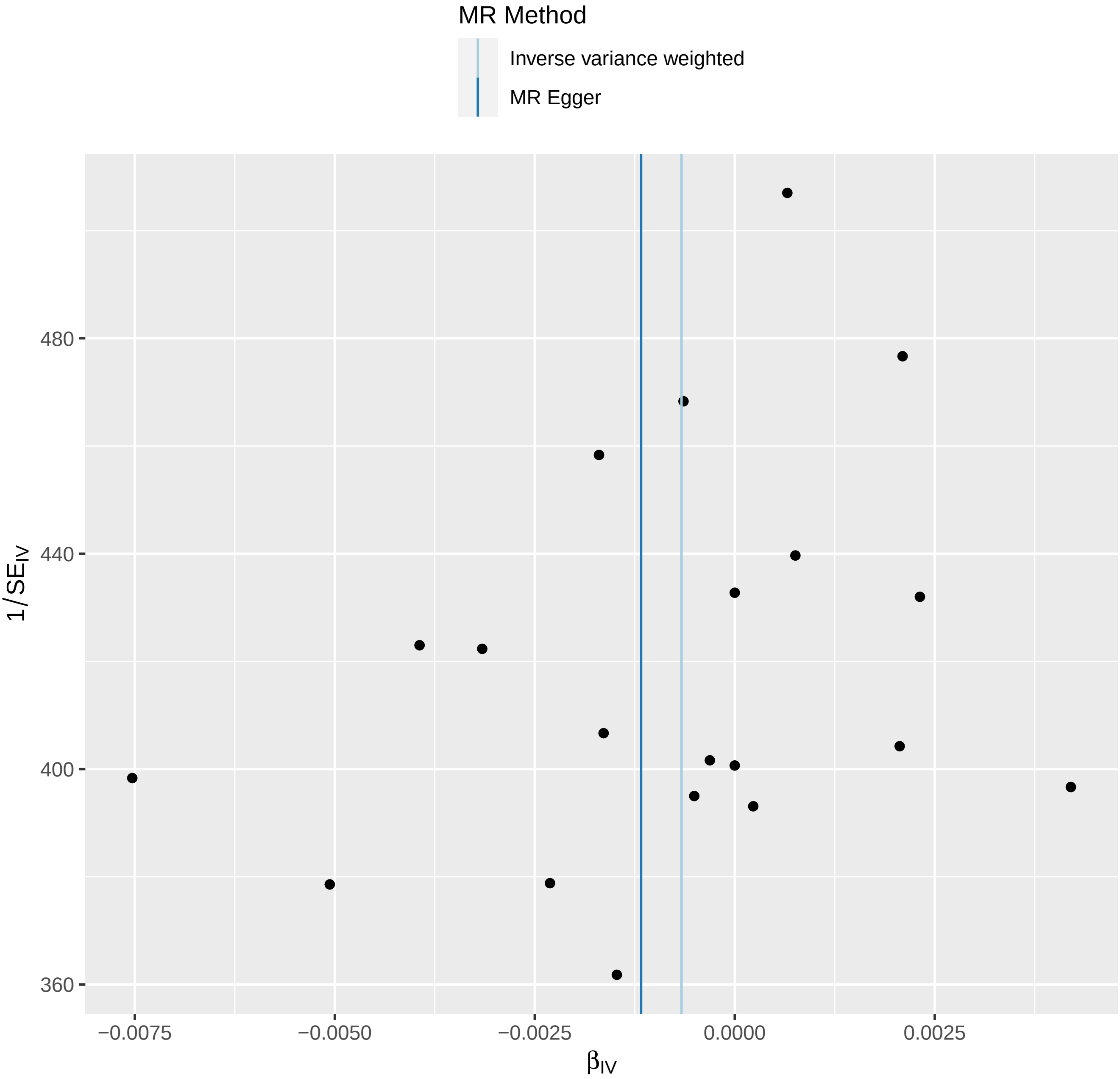

Batch 2−2803 : Sclerostin on eGFR

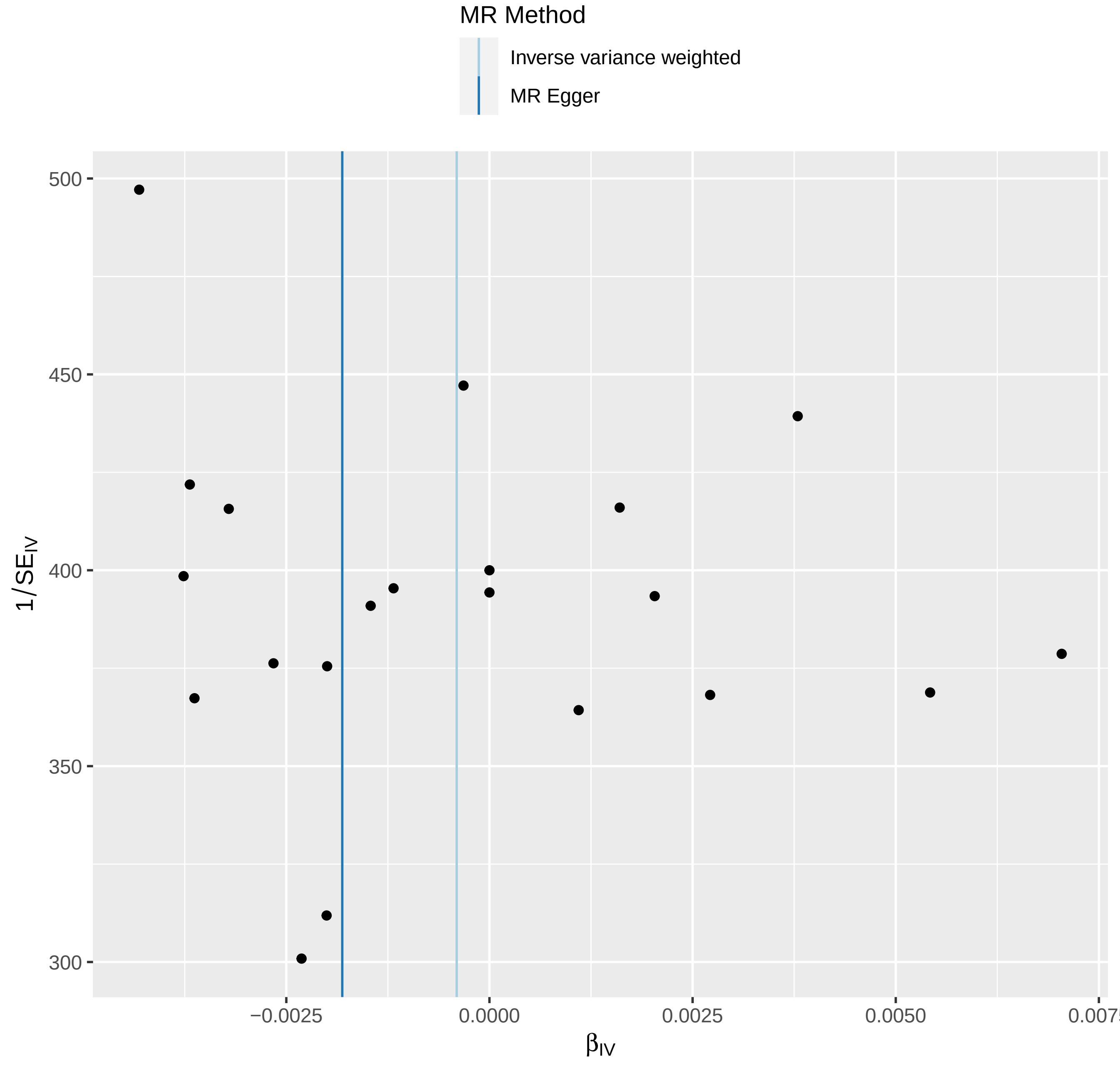

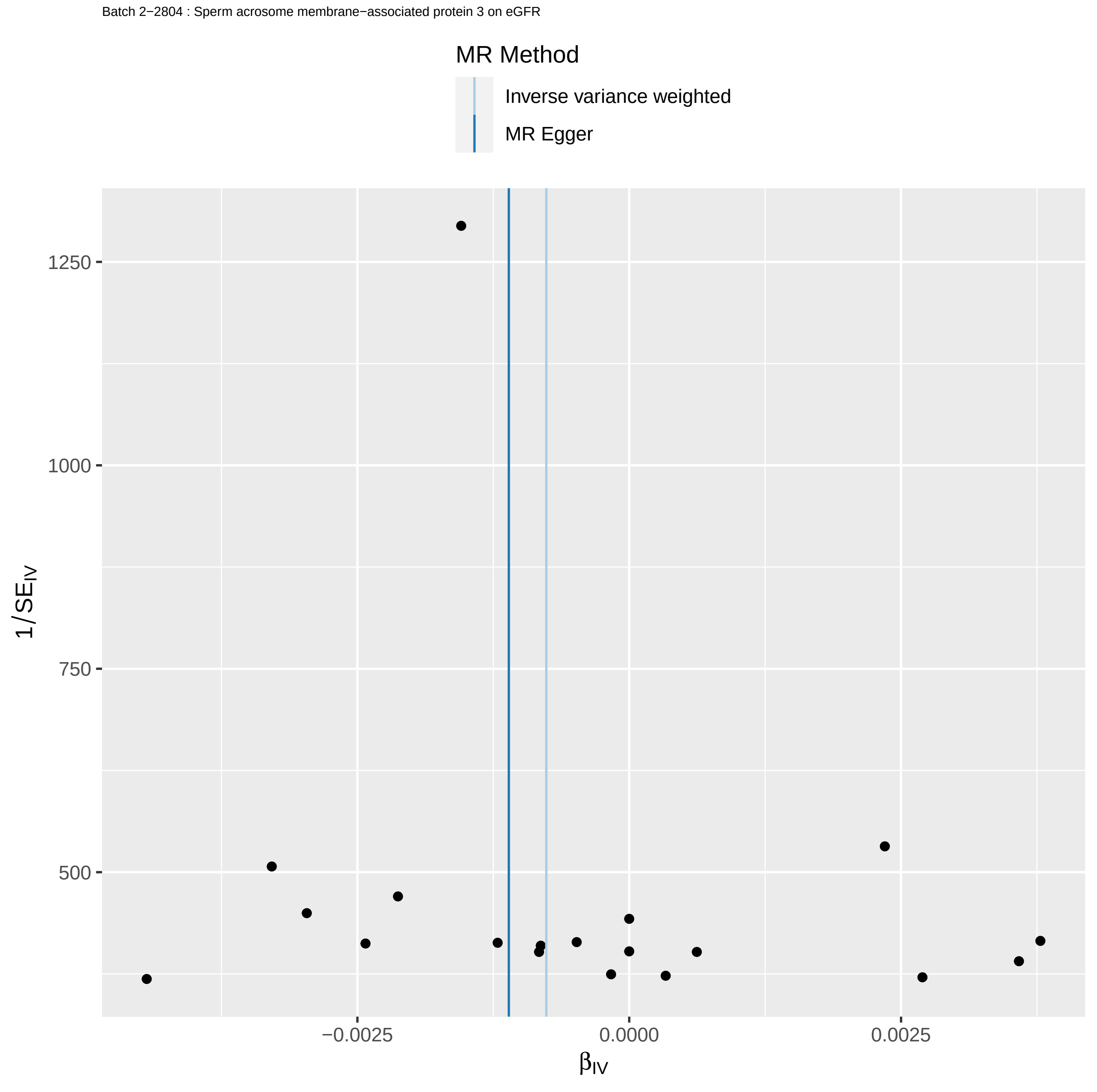

Batch 2−2804 : Sperm acrosome membrane−associated protein 3 on eGFR
MR Method
Inverse variance weighted
MR Egger
1250
1000
750
500
1/SE_IV
−0.0025
0.0000
0.0025
β_IV

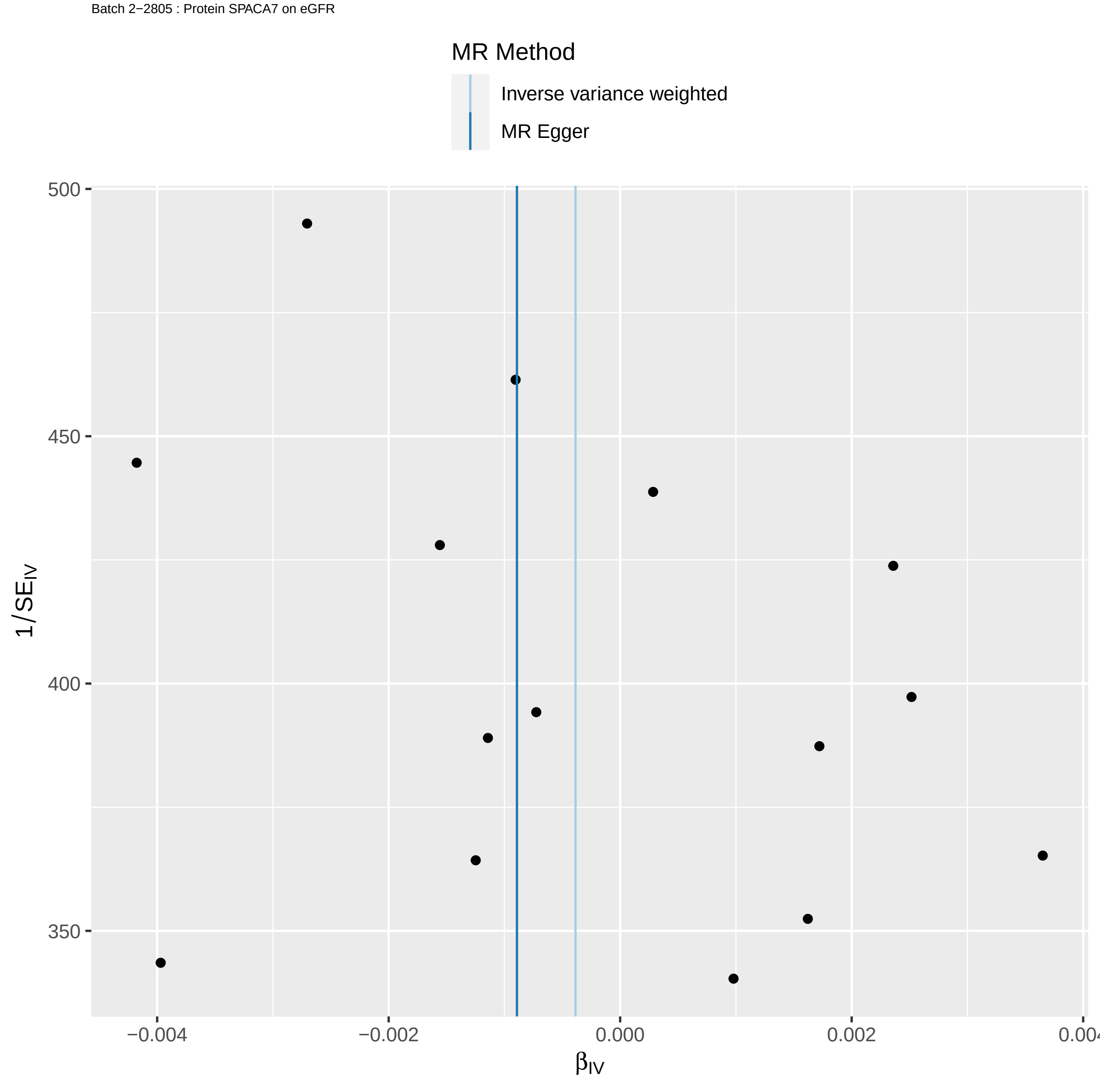
Batch 2−2805 : Protein SPACA7 on eGFR
MR Method
Inverse variance weighted
MR Egger
500
450
400
350
1/SE_IV
−0.004
−0.002
0.000
0.002
0.004
β_IV

Batch 2−2806 : Sperm−associated antigen 11A on eGFR

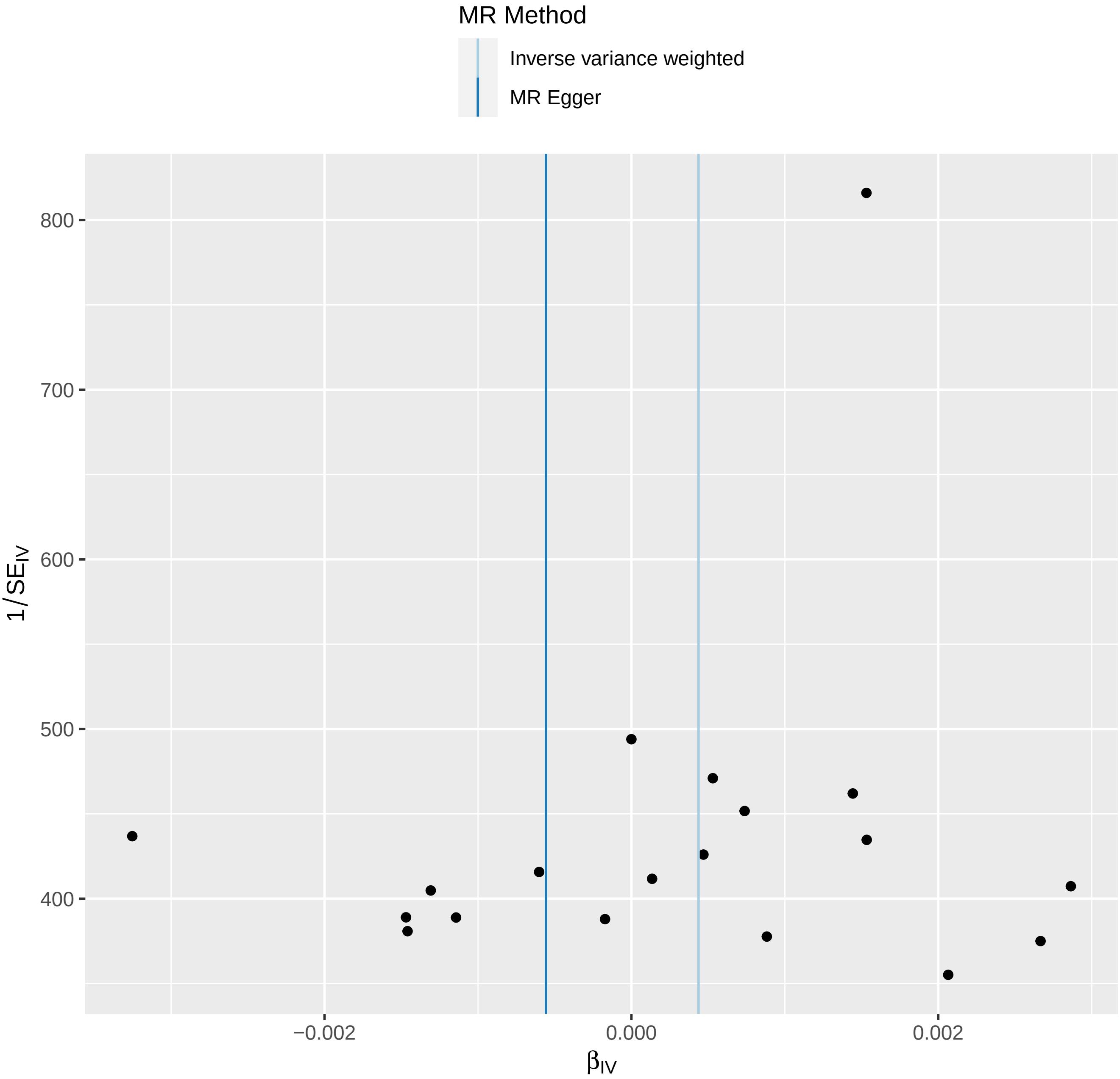

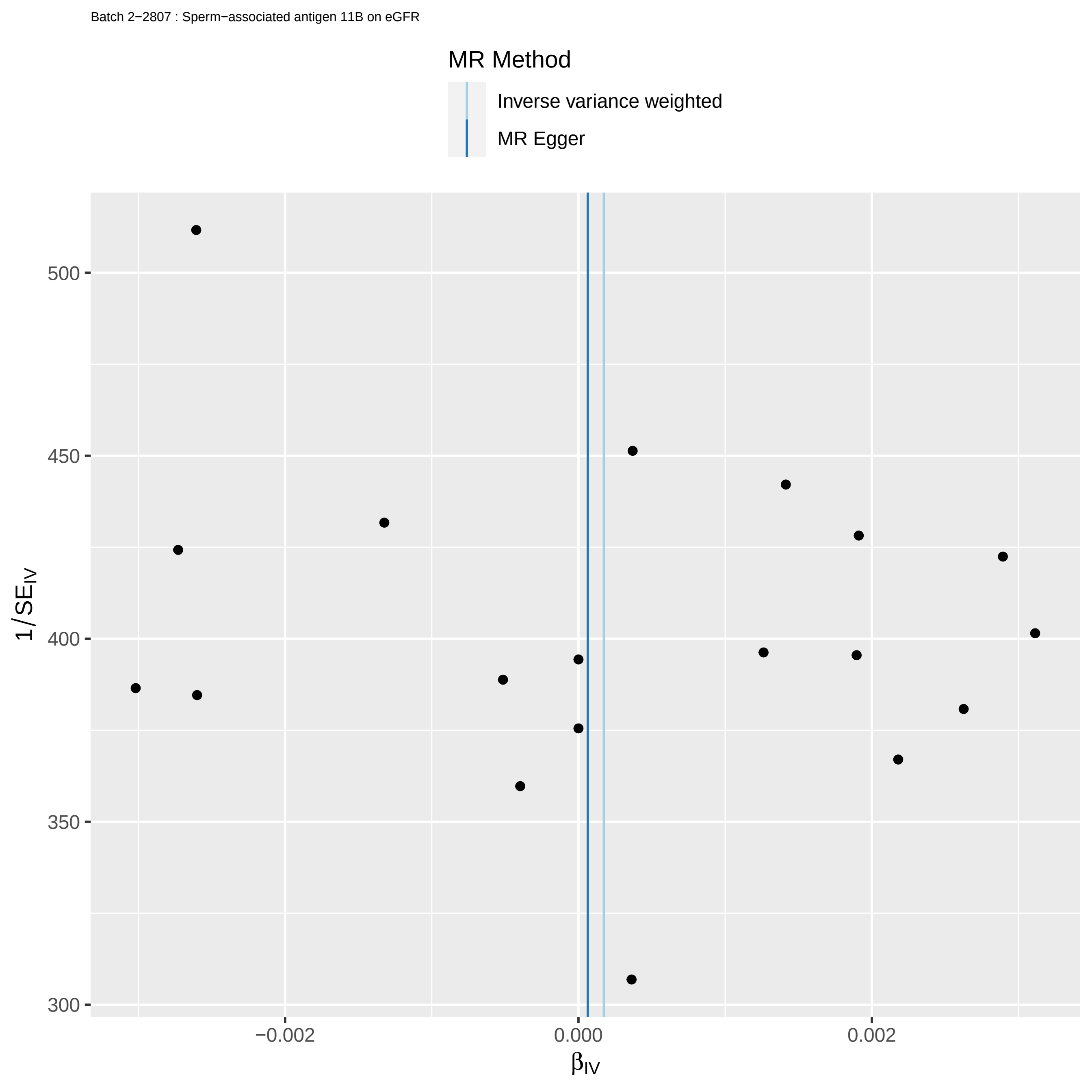
Batch 2−2807 : Sperm−associated antigen 11B on eGFR
MR Method
Inverse variance weighted
MR Egger
500
450
400
350
300
−0.002
0.000
0.002
$1/SE_{IV}$
β_{IV}

Batch 2−2808 : SPARC on eGFR

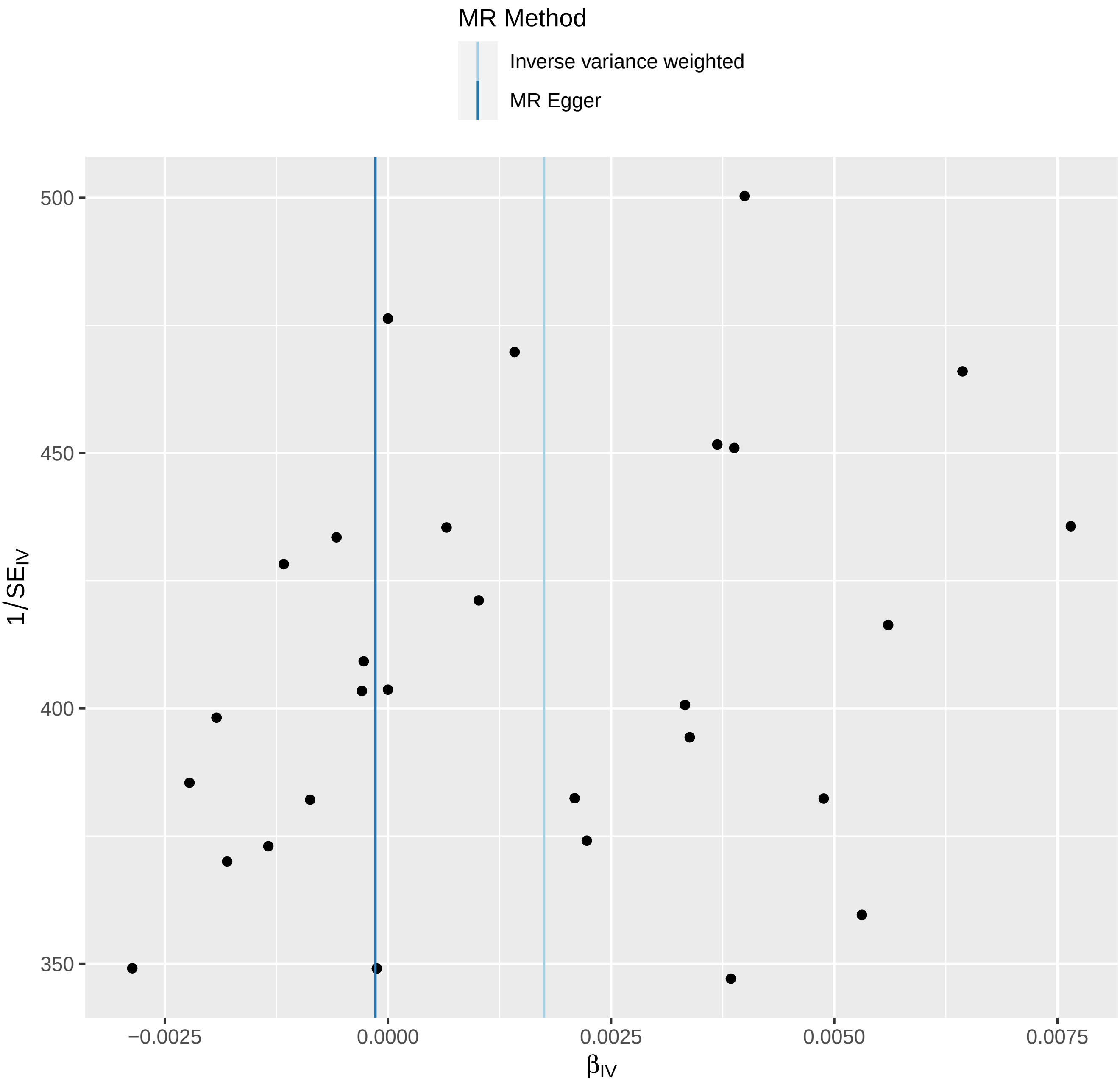

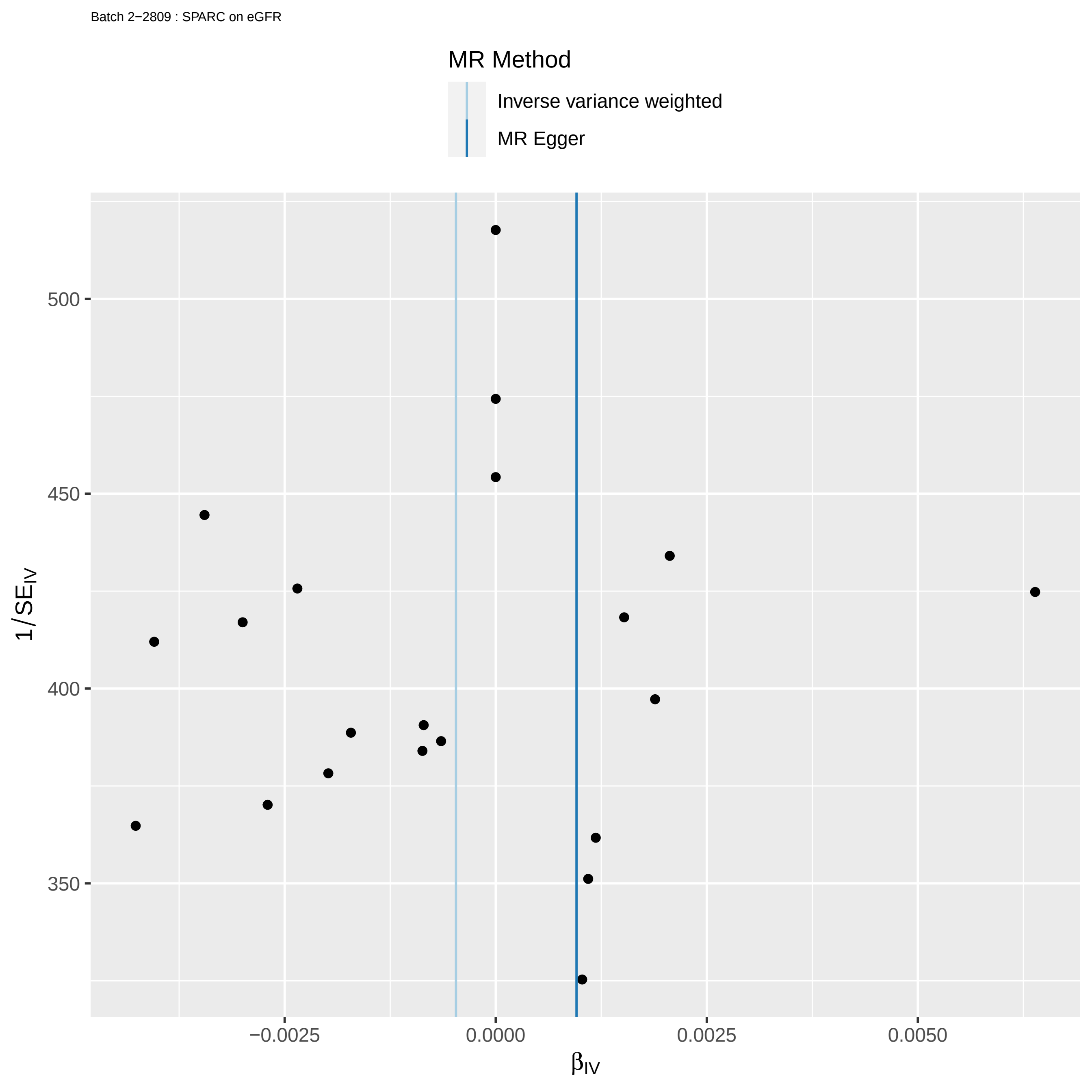
Batch 2−2809 : SPARC on eGFR
MR Method
Inverse variance weighted
MR Egger
500
450
400
350
1/SE_IV
−0.0025
0.0000
0.0025
0.0050
β_IV

Batch 2−2810 : SPARC−like protein 1 on eGFR

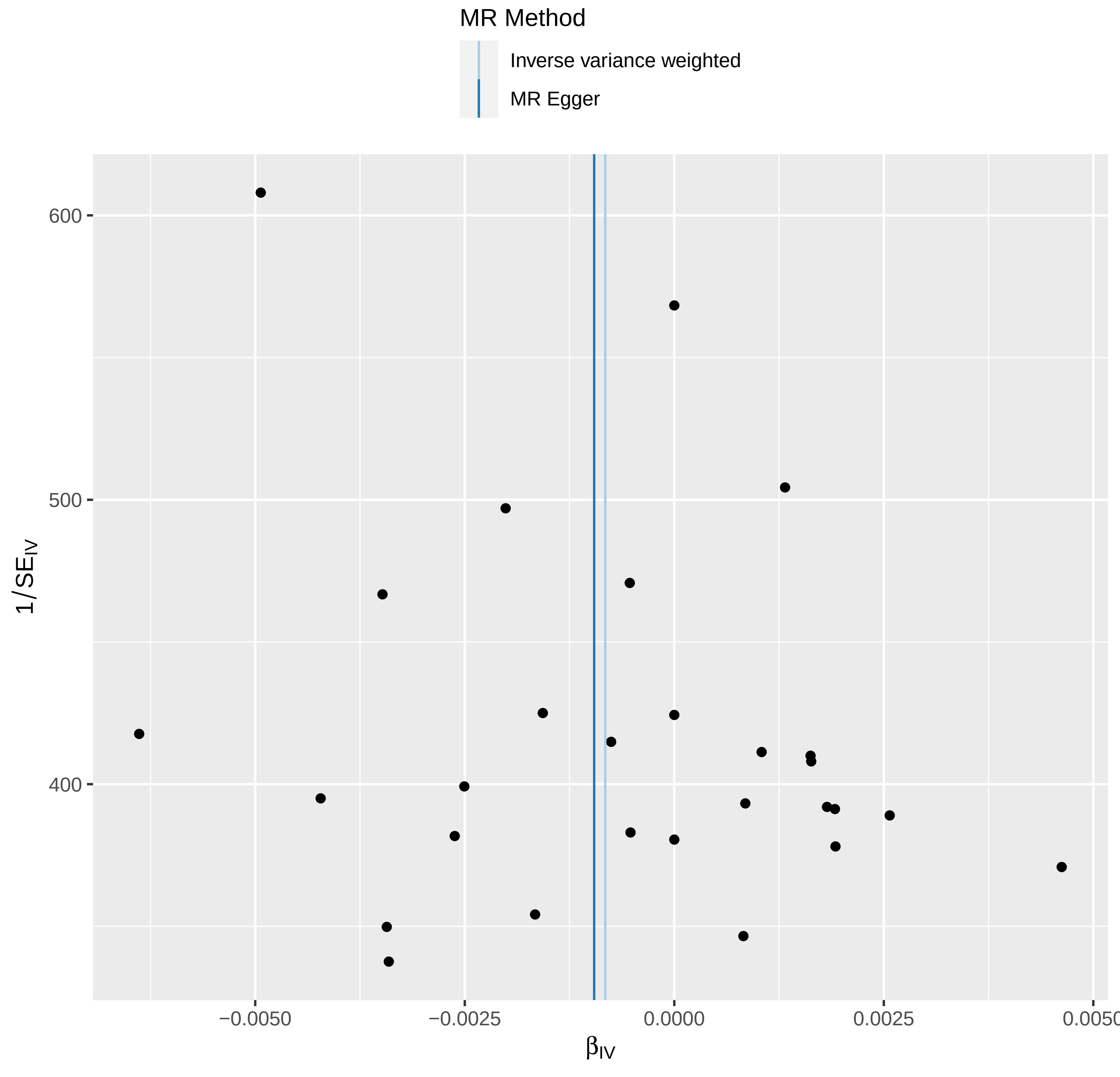

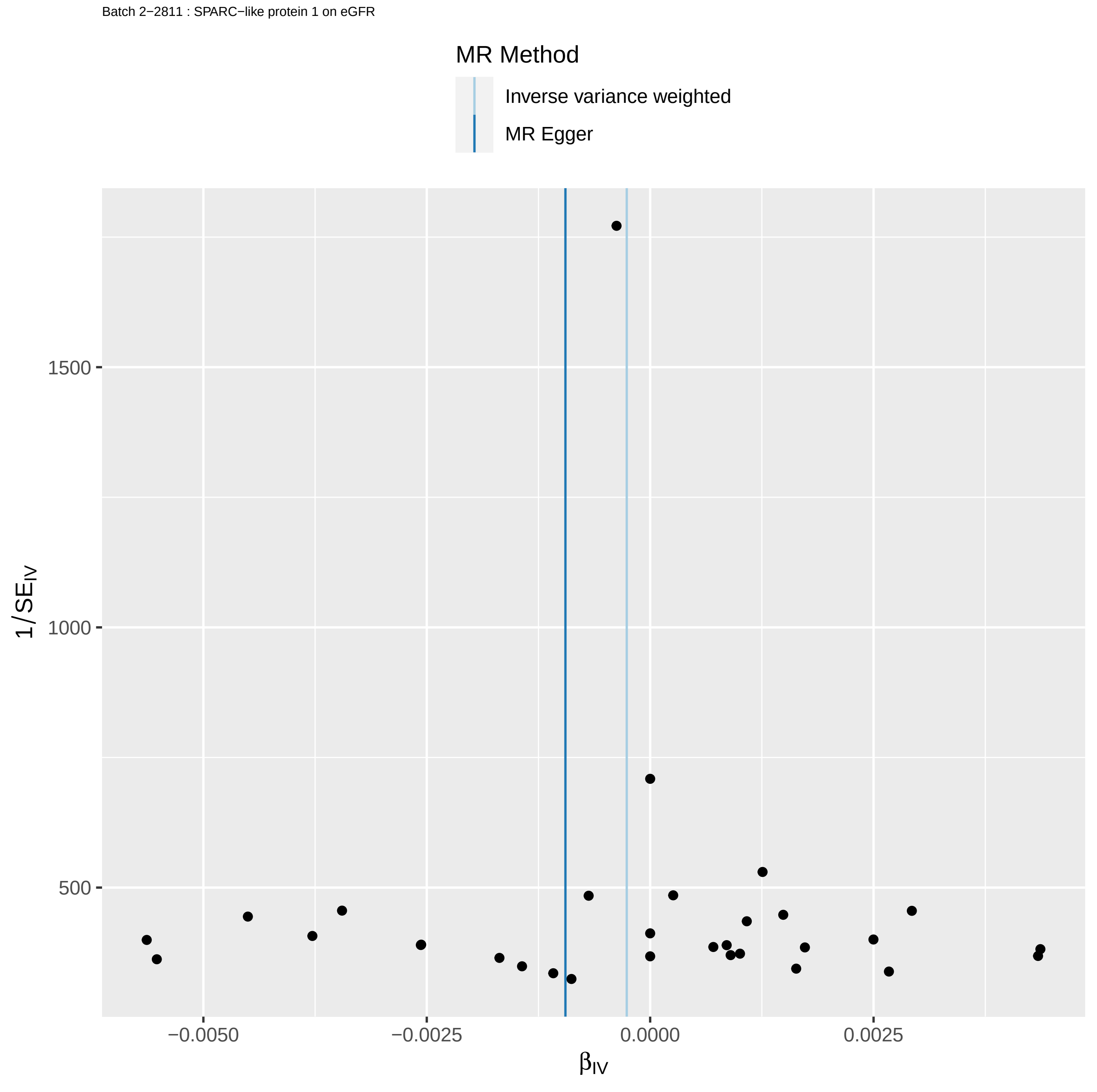

Batch 2−2811 : SPARC−like protein 1 on eGFR
MR Method
Inverse variance weighted
MR Egger
1500
1000
500
1/SE$_{IV}$
−0.0050
−0.0025
0.0000
0.0025
β$_{IV}$

Batch 2−2812 : Spastin on eGFR

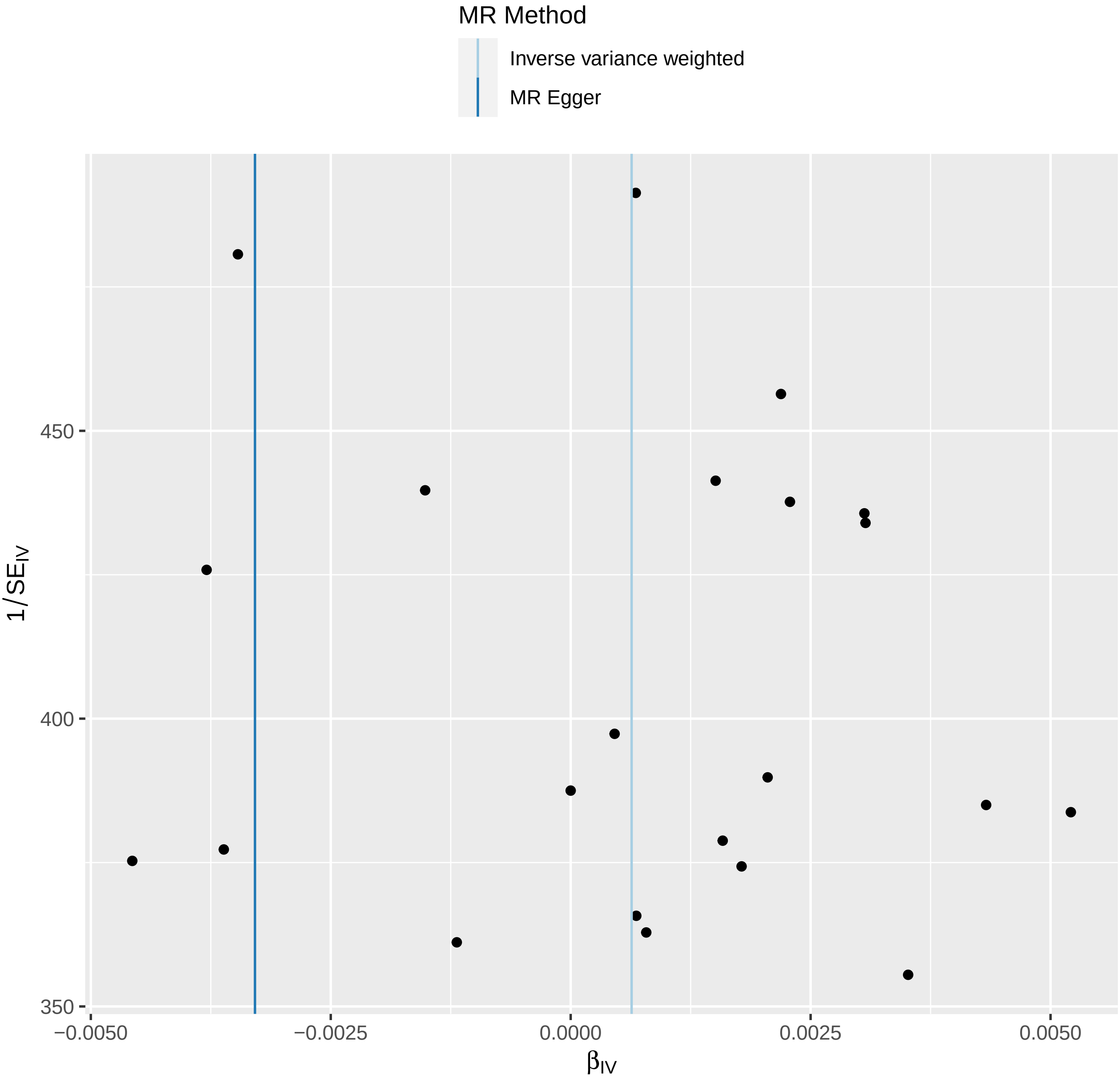

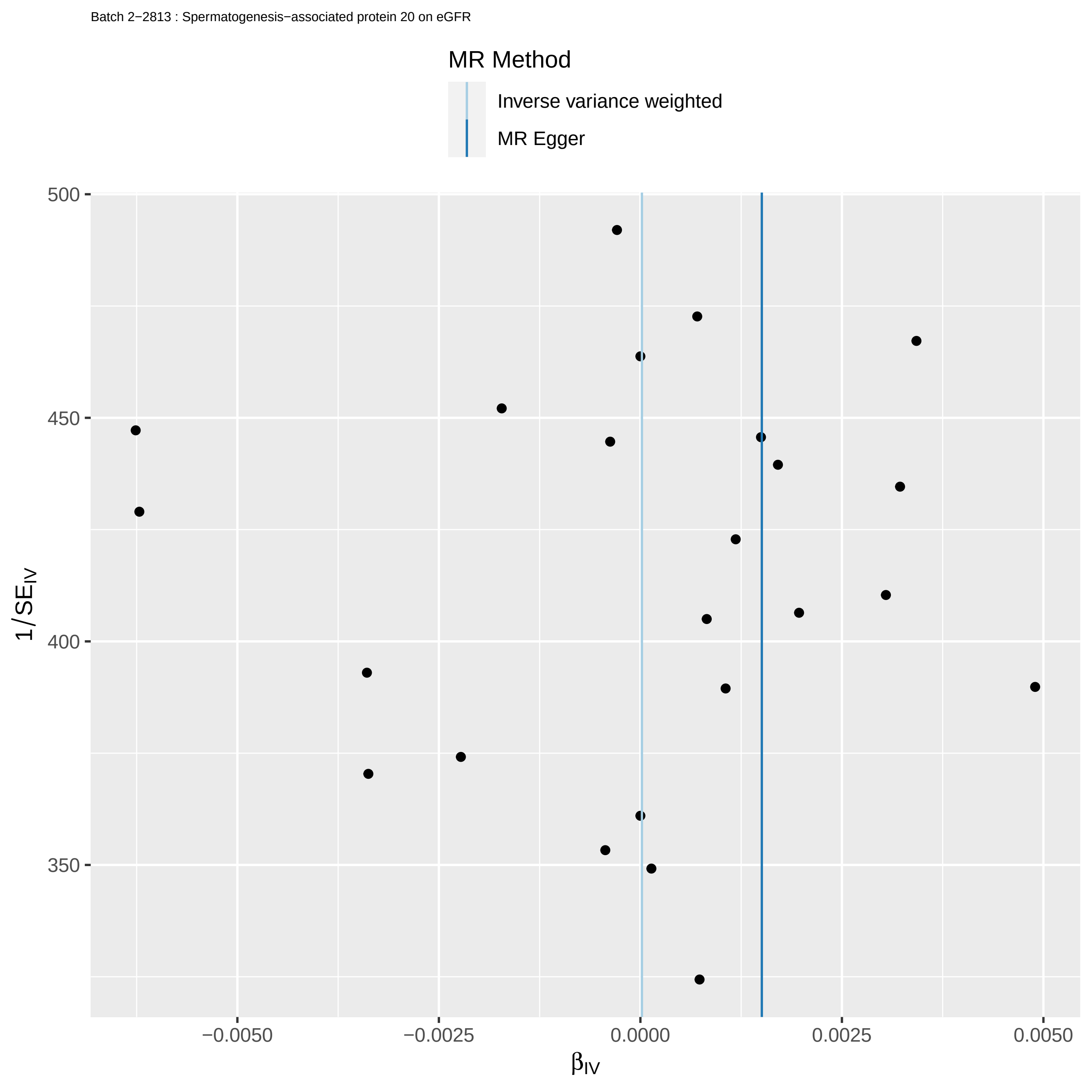

Batch 2−2813 : Spermatogenesis−associated protein 20 on eGFR
MR Method
Inverse variance weighted
MR Egger
500
450
400
350
1/SE_IV
−0.0050
−0.0025
0.0000
0.0025
0.0050
β_IV

Batch 2−2814 : Spermatogenesis−associated protein 9 on eGFR

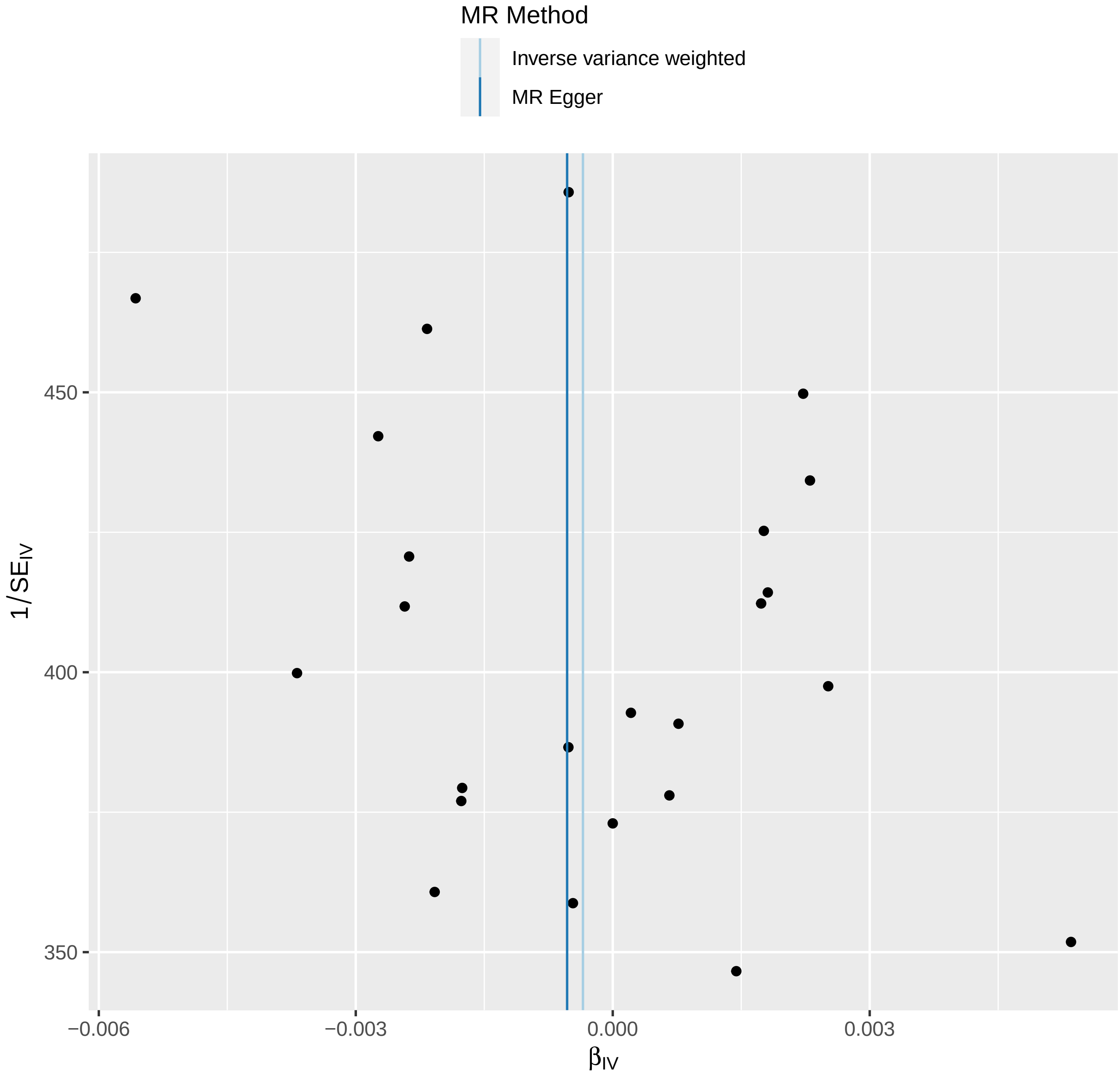

Batch 2−2815 : Maspardin on eGFR

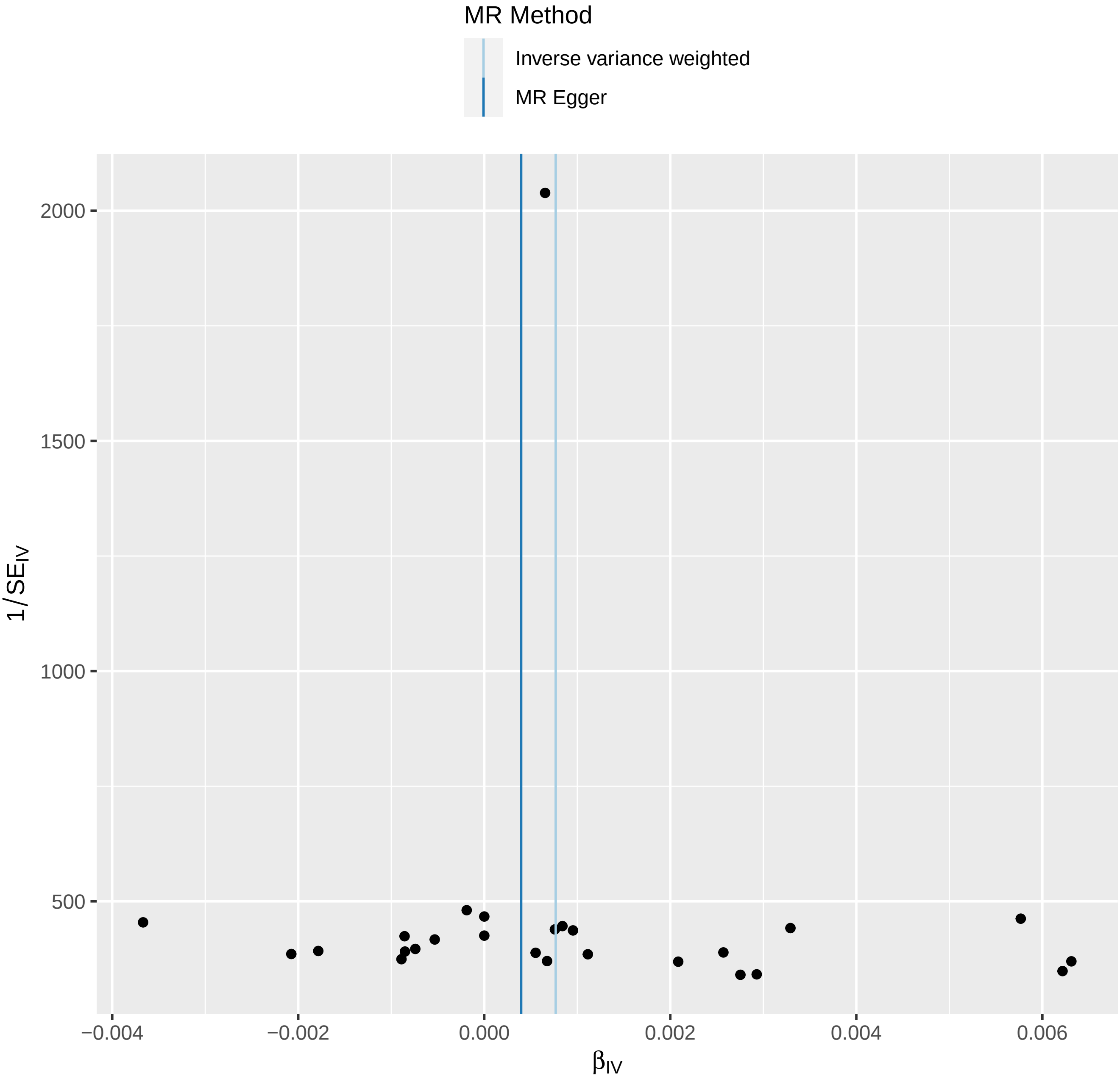

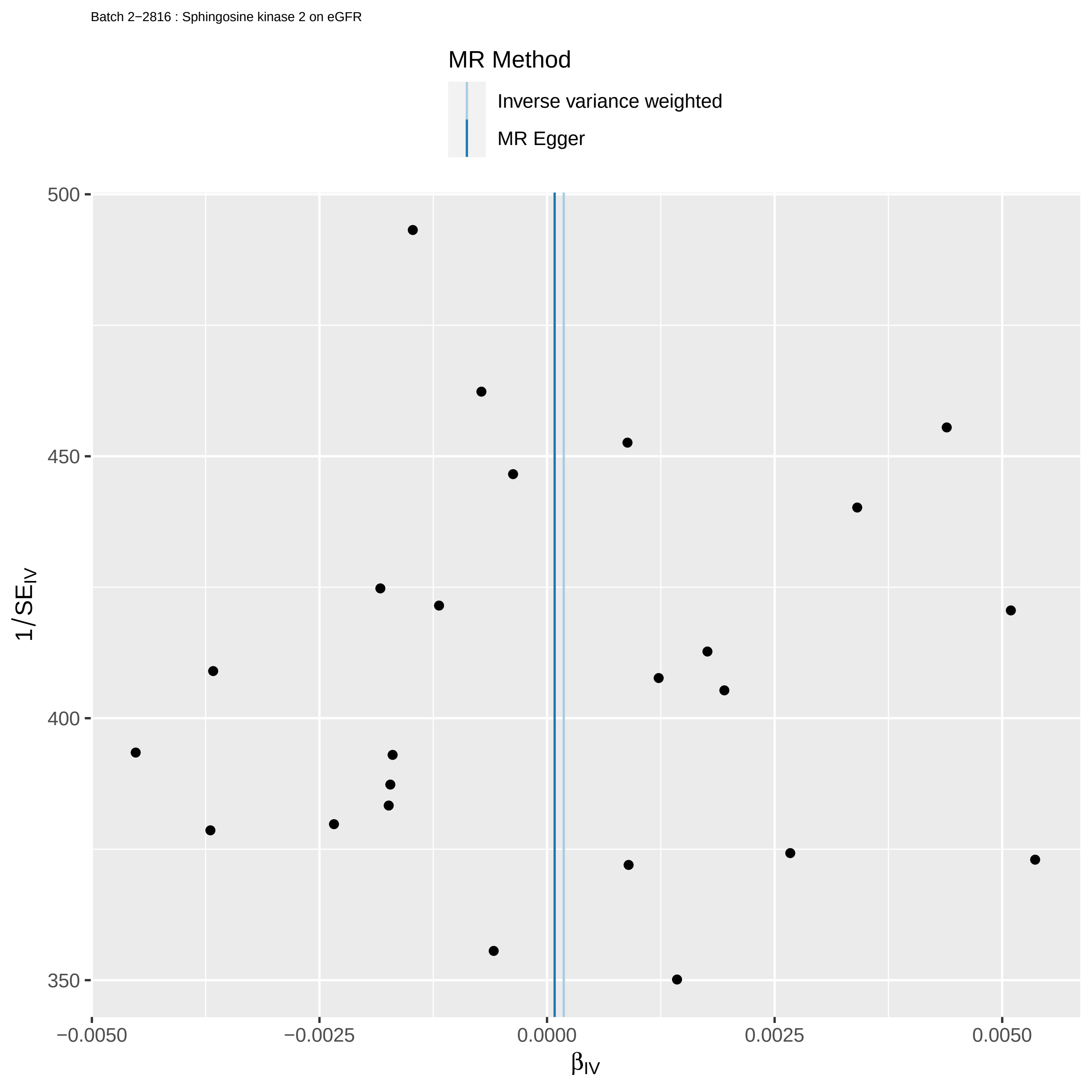
Batch 2−2816 : Sphingosine kinase 2 on eGFR
MR Method
Inverse variance weighted
MR Egger
500
450
400
350
1/SE_IV
−0.0050
−0.0025
0.0000
0.0025
0.0050
β_IV

Batch 2−2817 : Serine protease inhibitor Kazal−type 13 on eGFR

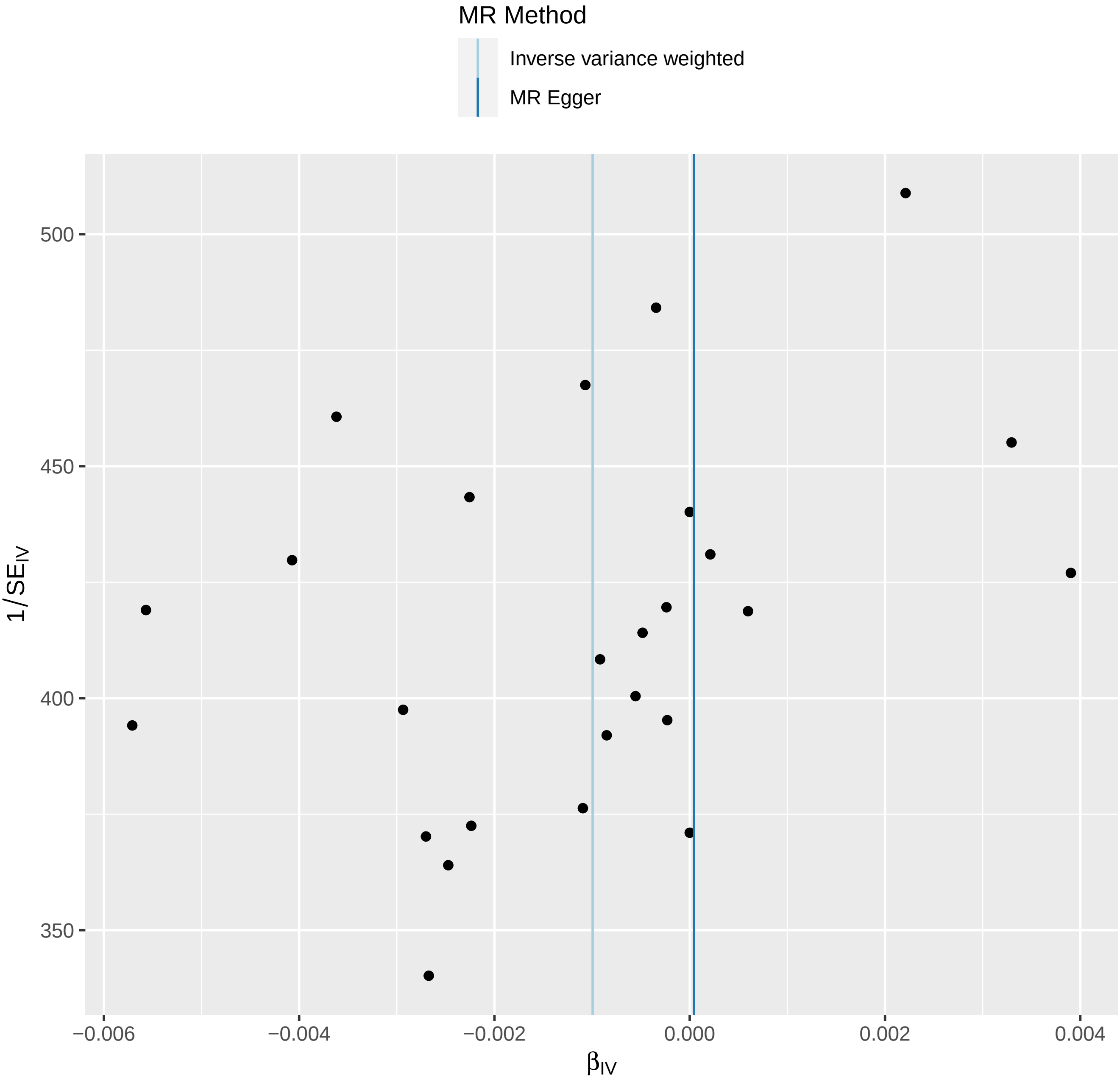

Batch 2−2818 : Serine protease inhibitor Kazal−type 2 on eGFR

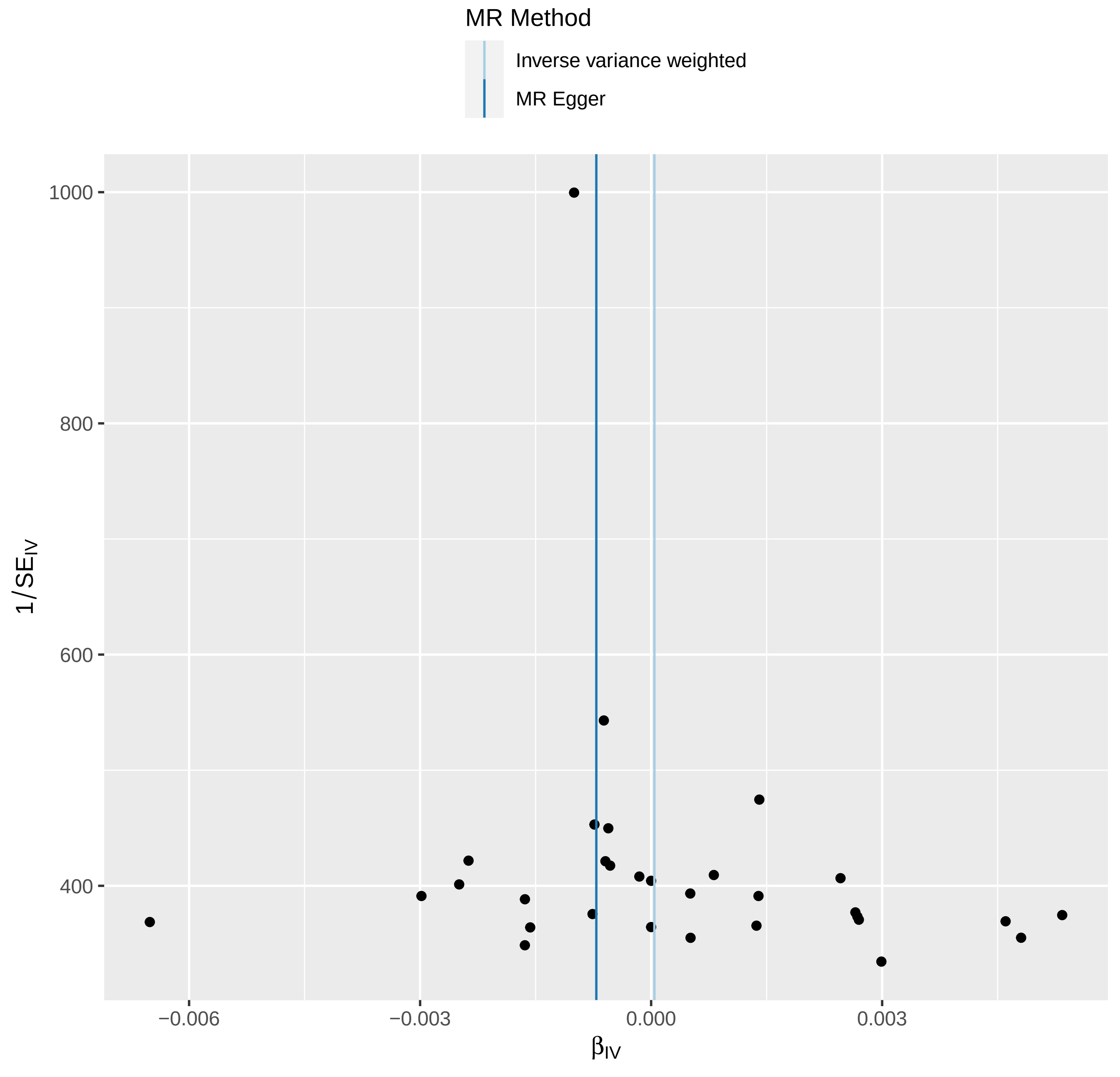

Batch 2−2819 : Serine protease inhibitor Kazal−type 5 on eGFR

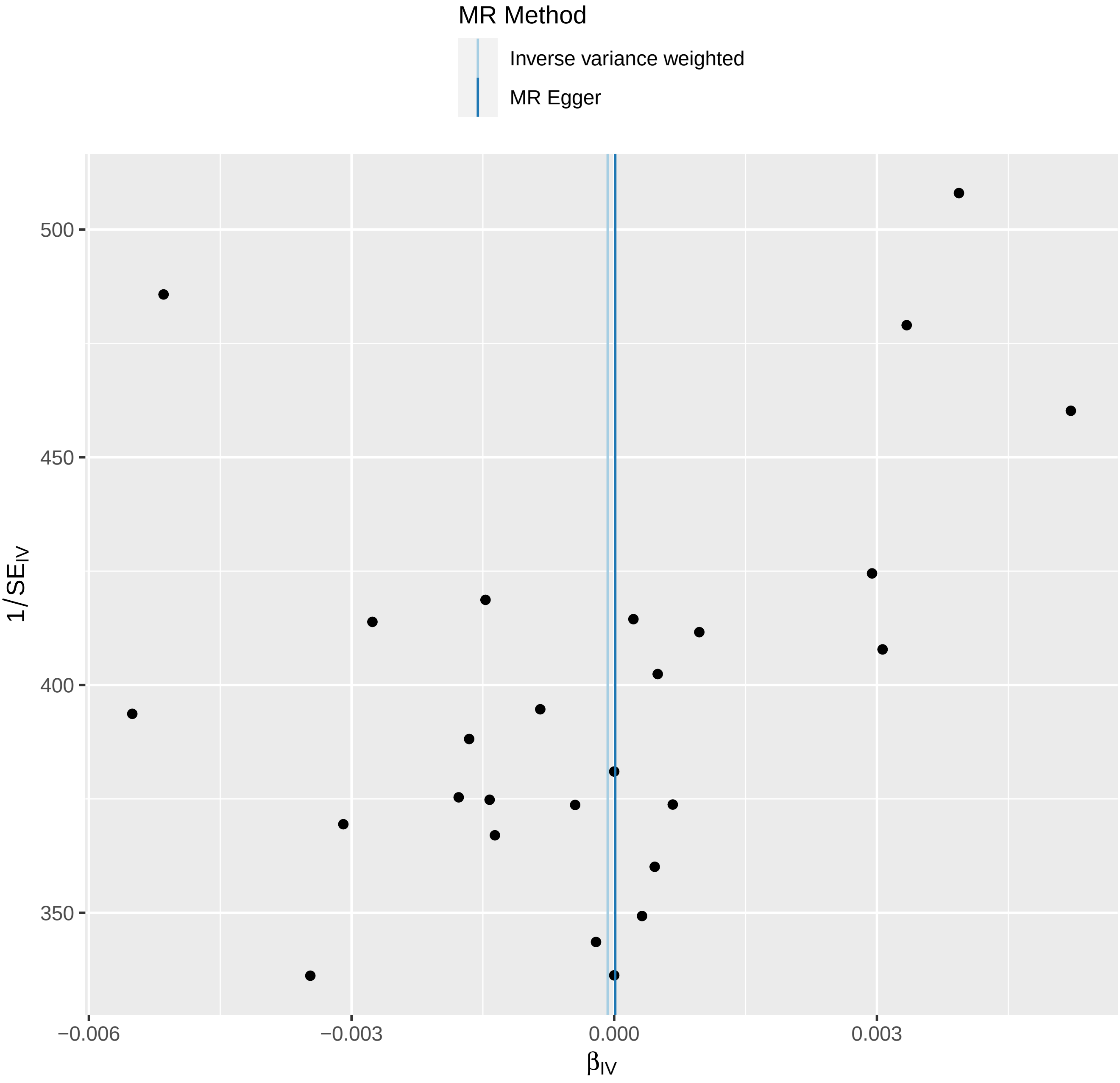

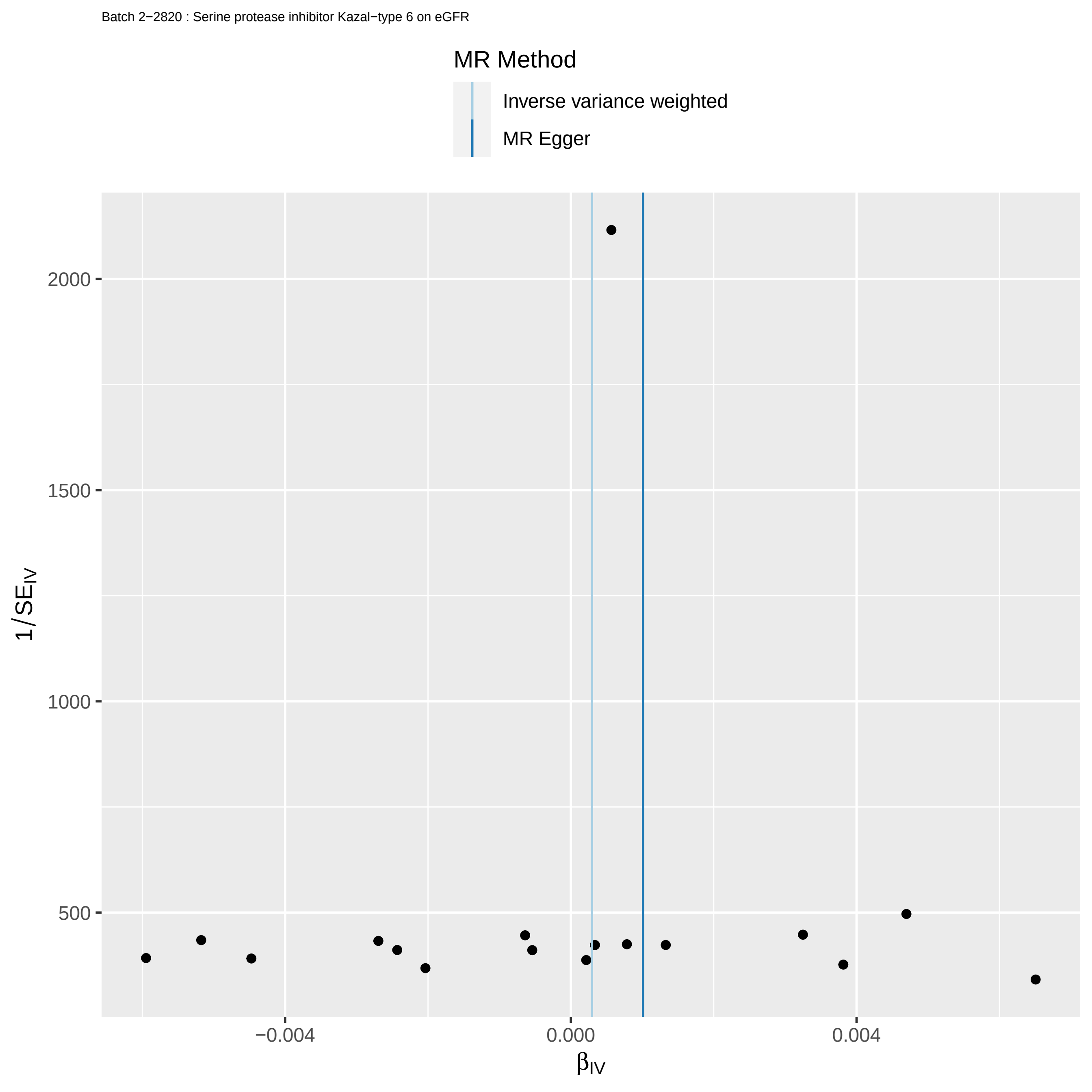

Batch 2−2820 : Serine protease inhibitor Kazal−type 6 on eGFR
MR Method
Inverse variance weighted
MR Egger
2000
1500
1000
500
1/SE_IV
−0.004
0.000
0.004
β_IV

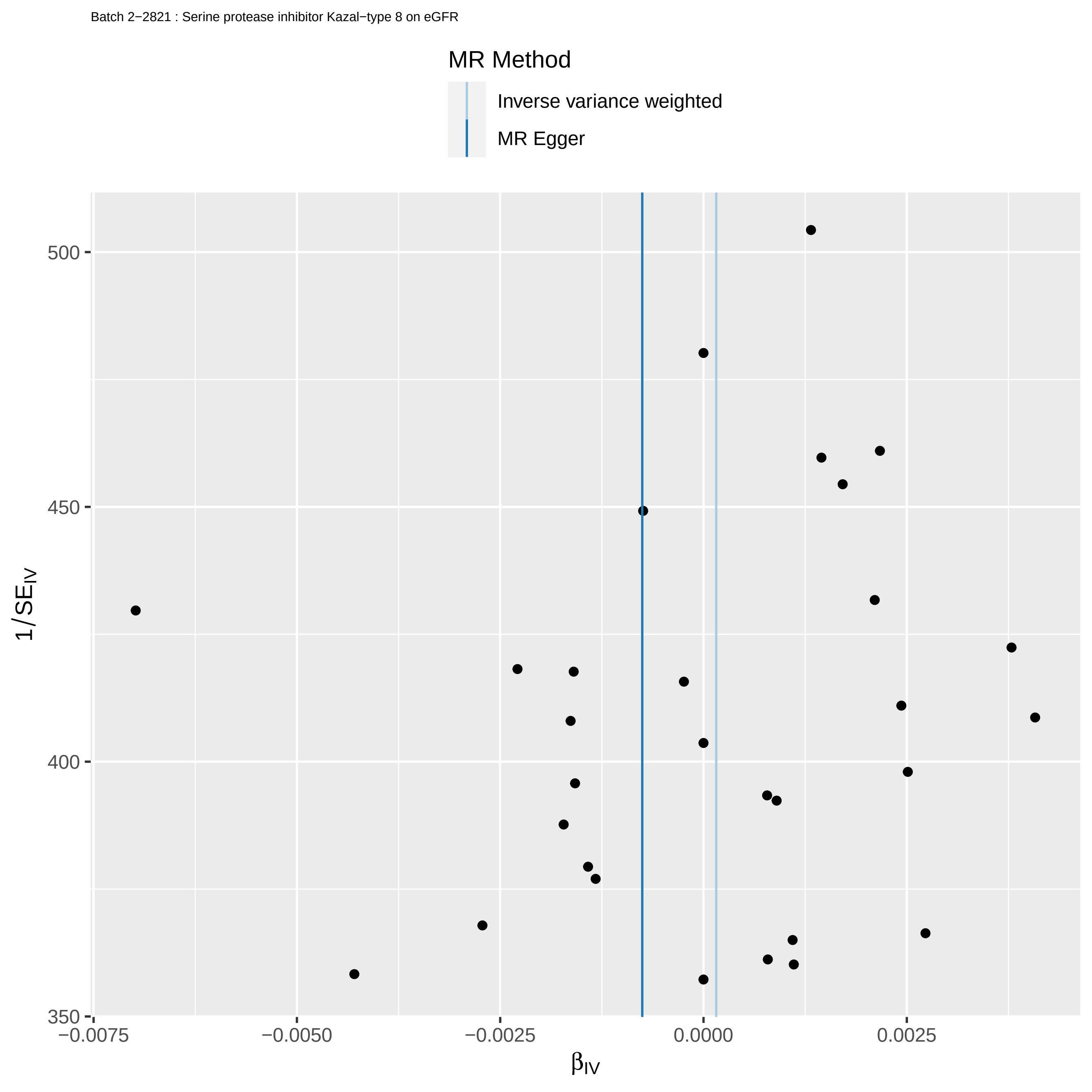

Batch 2−2821 : Serine protease inhibitor Kazal−type 8 on eGFR
MR Method
Inverse variance weighted
MR Egger
500
450
400
350
1/SE_IV
−0.0075
−0.0050
−0.0025
0.0000
0.0025
β_IV

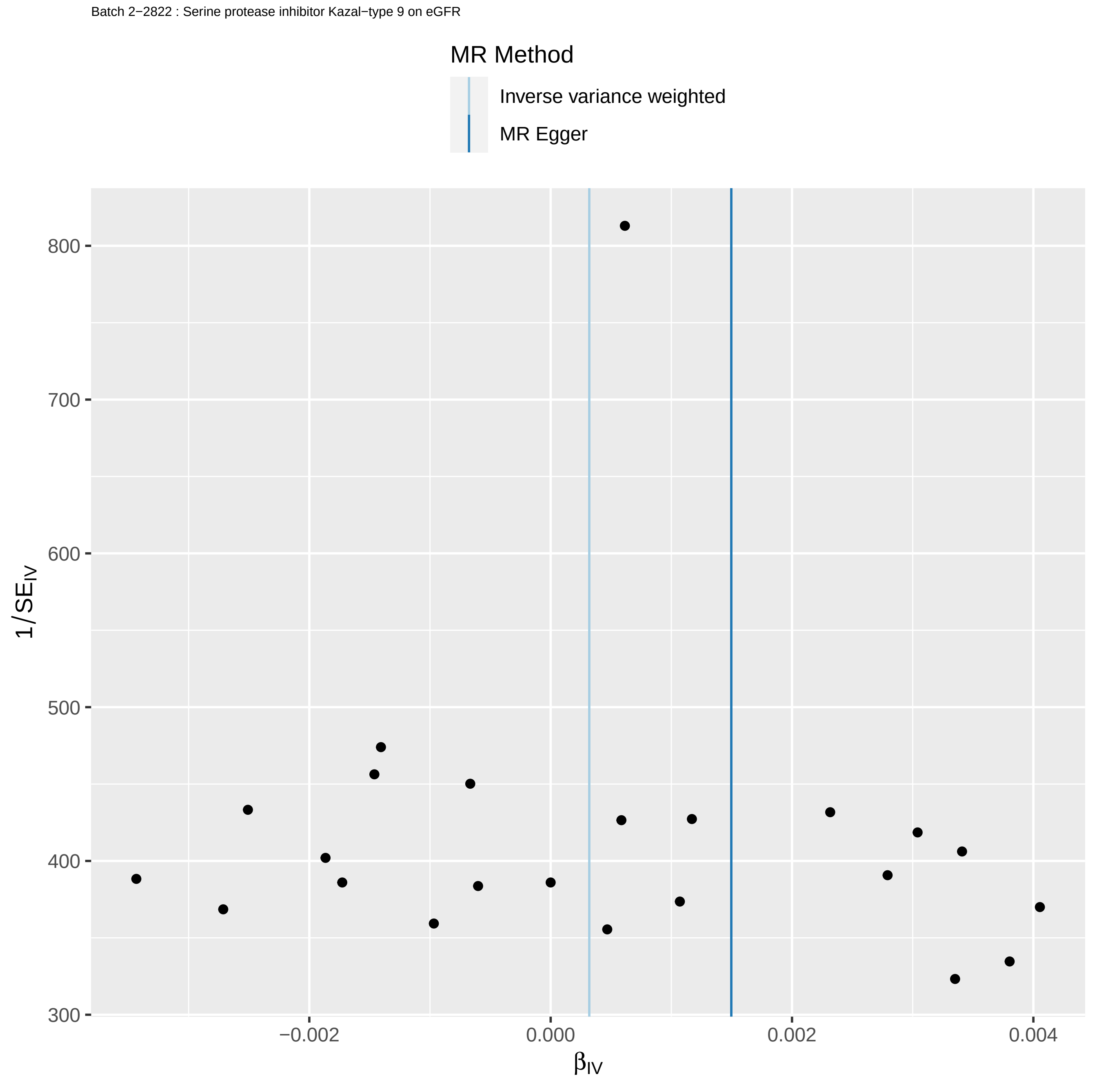

Batch 2−2822 : Serine protease inhibitor Kazal−type 9 on eGFR
MR Method
Inverse variance weighted
MR Egger
800
700
600
500
400
300
1/SE_IV
−0.002
0.000
0.002
0.004
β_IV

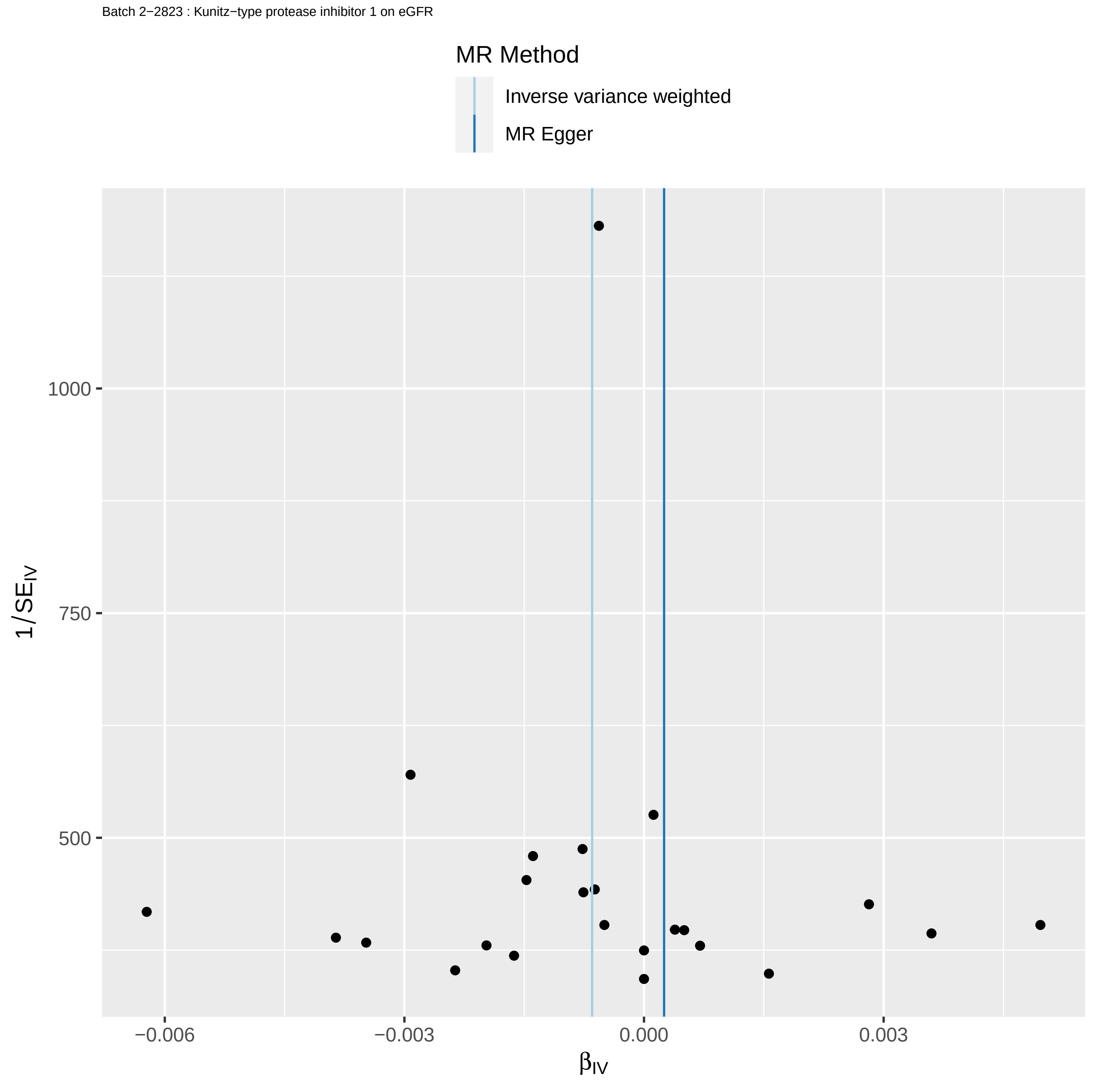

Batch 2−2823 : Kunitz−type protease inhibitor 1 on eGFR
MR Method
Inverse variance weighted
MR Egger
1000
750
500
1/SE$_{IV}$
−0.006
−0.003
0.000
0.003
β_{IV}

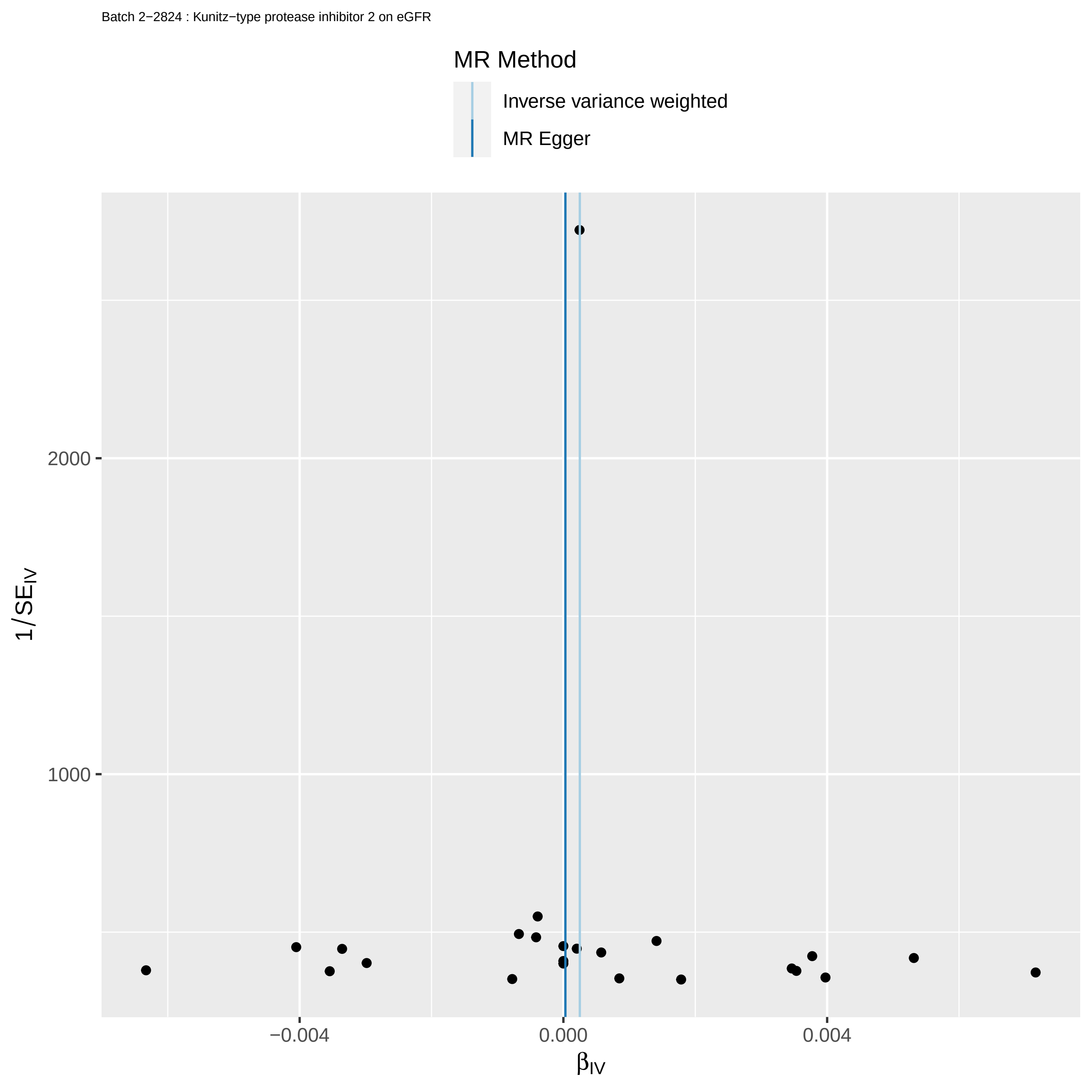
Batch 2−2824 : Kunitz−type protease inhibitor 2 on eGFR
MR Method
Inverse variance weighted
MR Egger
2000
1000
1/SE_IV
−0.004
0.000
0.004
β_IV

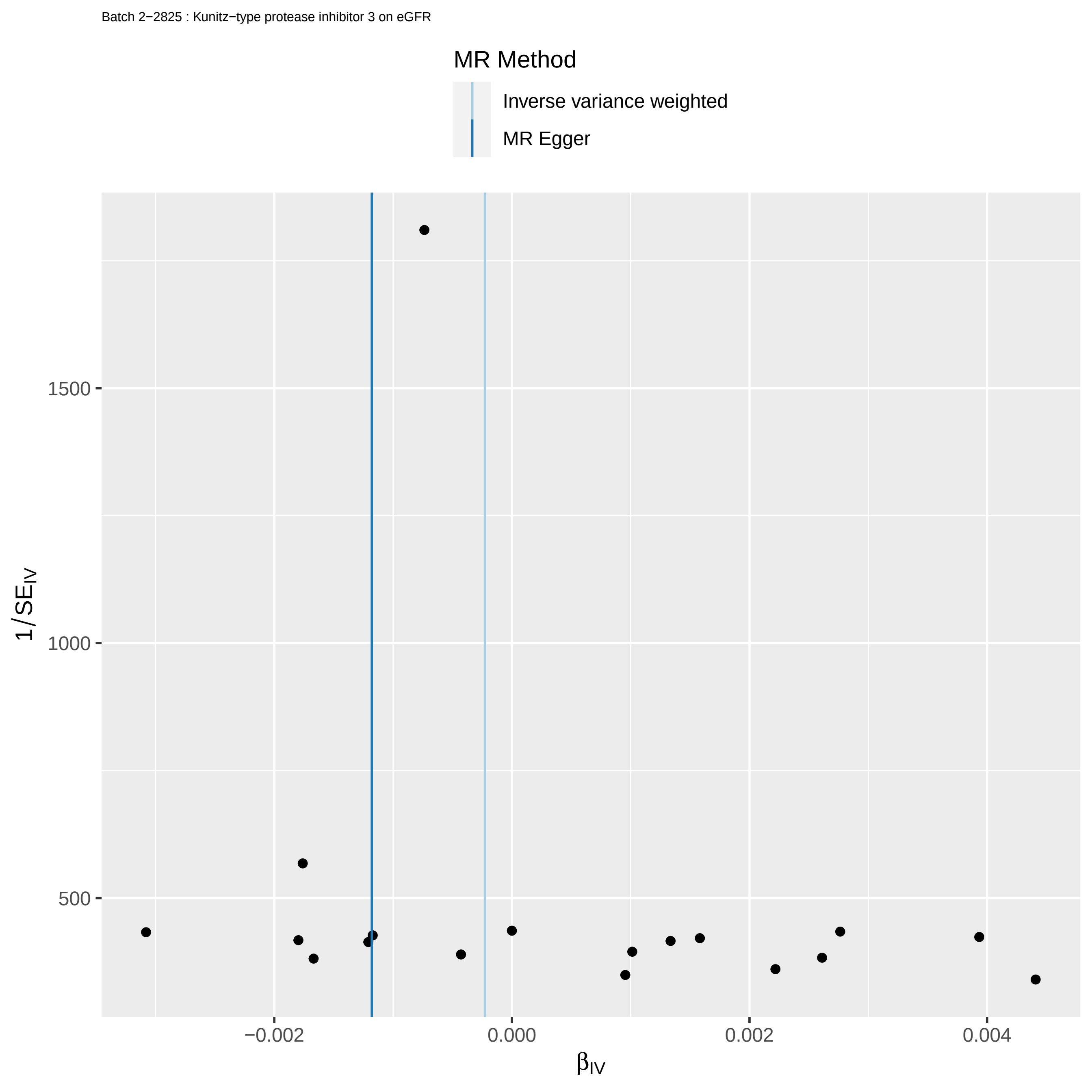

Batch 2−2825 : Kunitz−type protease inhibitor 3 on eGFR
MR Method
Inverse variance weighted
MR Egger
1500
1000
500
1/SE_IV
−0.002
0.000
0.002
0.004
β_IV

Batch 2−2826 : Testican−1 on eGFR

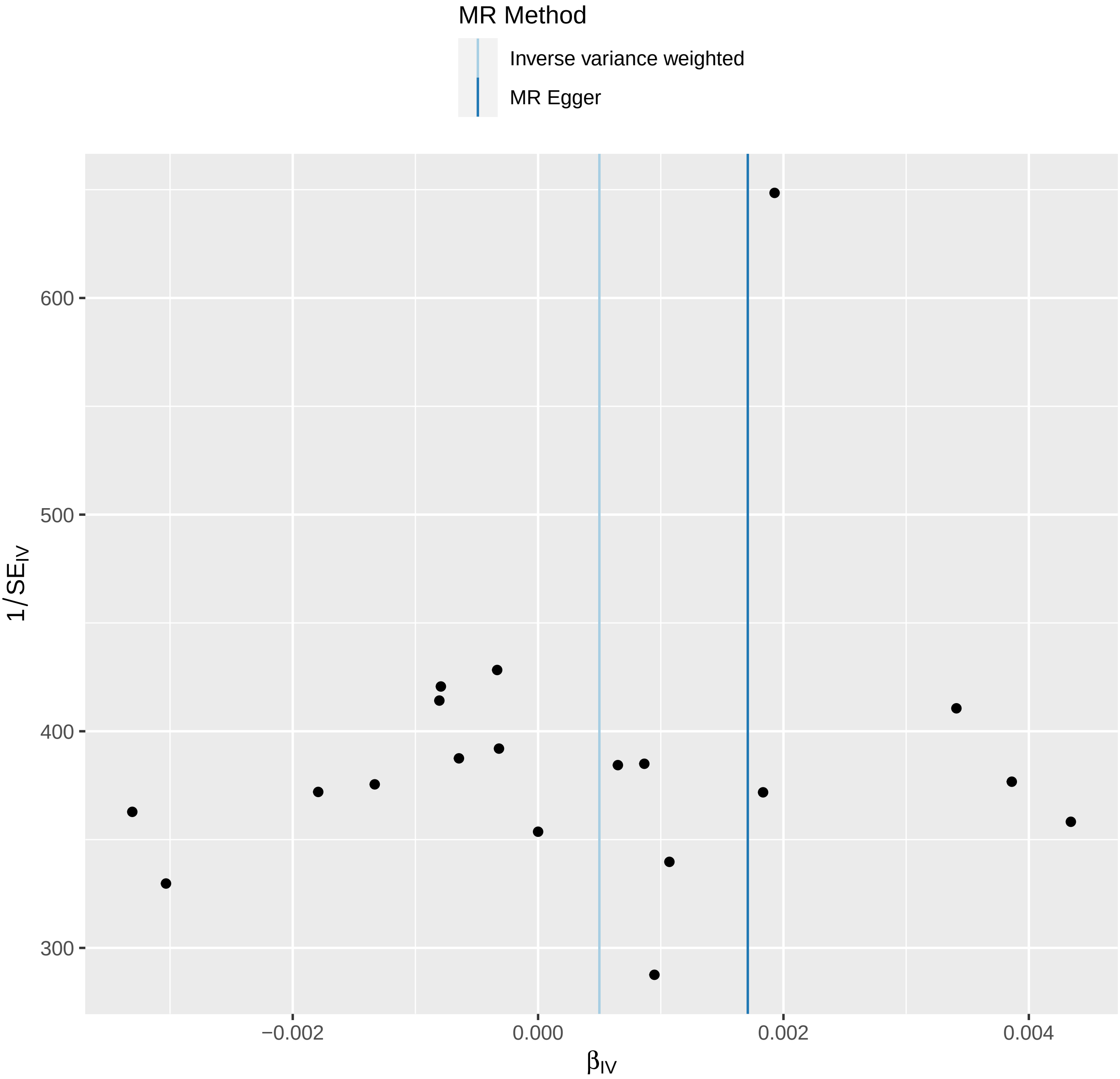

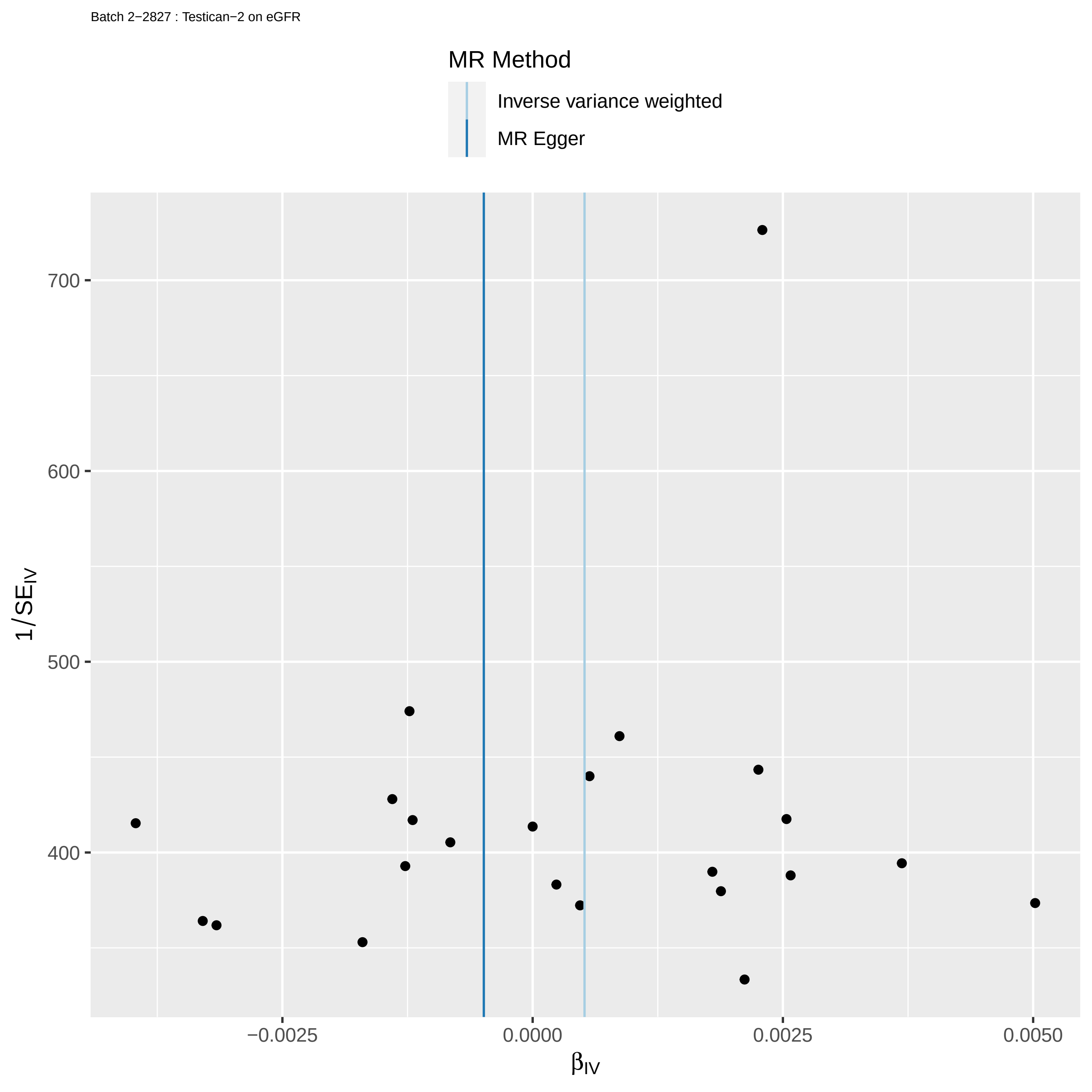
Batch 2−2827 : Testican−2 on eGFR
MR Method
Inverse variance weighted
MR Egger
700
600
500
400
1/SE$_{IV}$
−0.0025
0.0000
0.0025
0.0050
β$_{IV}$

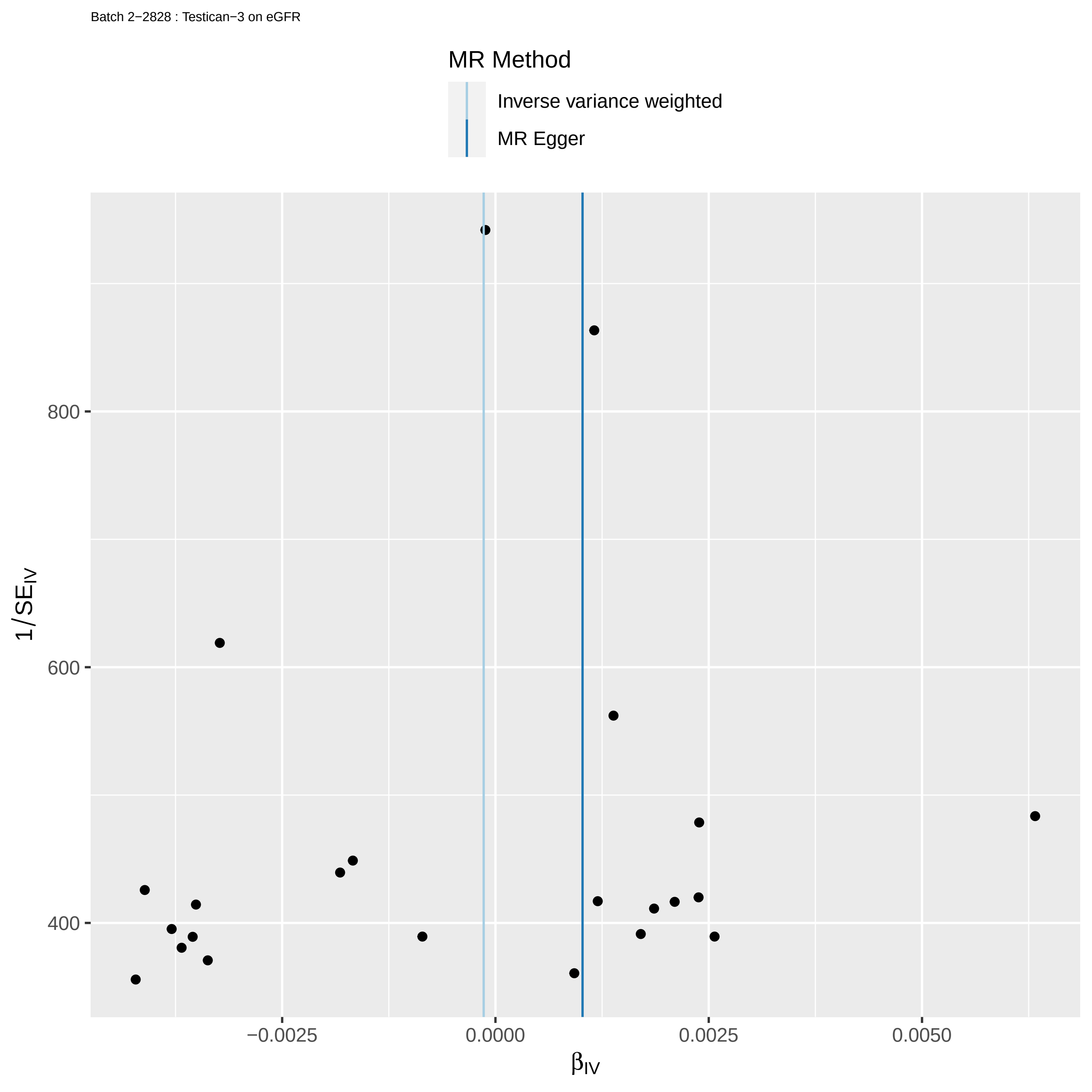

Batch 2−2828 : Testican−3 on eGFR
MR Method
Inverse variance weighted
MR Egger
800
600
400
1/SE$_{IV}$
−0.0025
0.0000
0.0025
0.0050
β$_{IV}$

Batch 2−2829 : Spondin−1 on eGFR

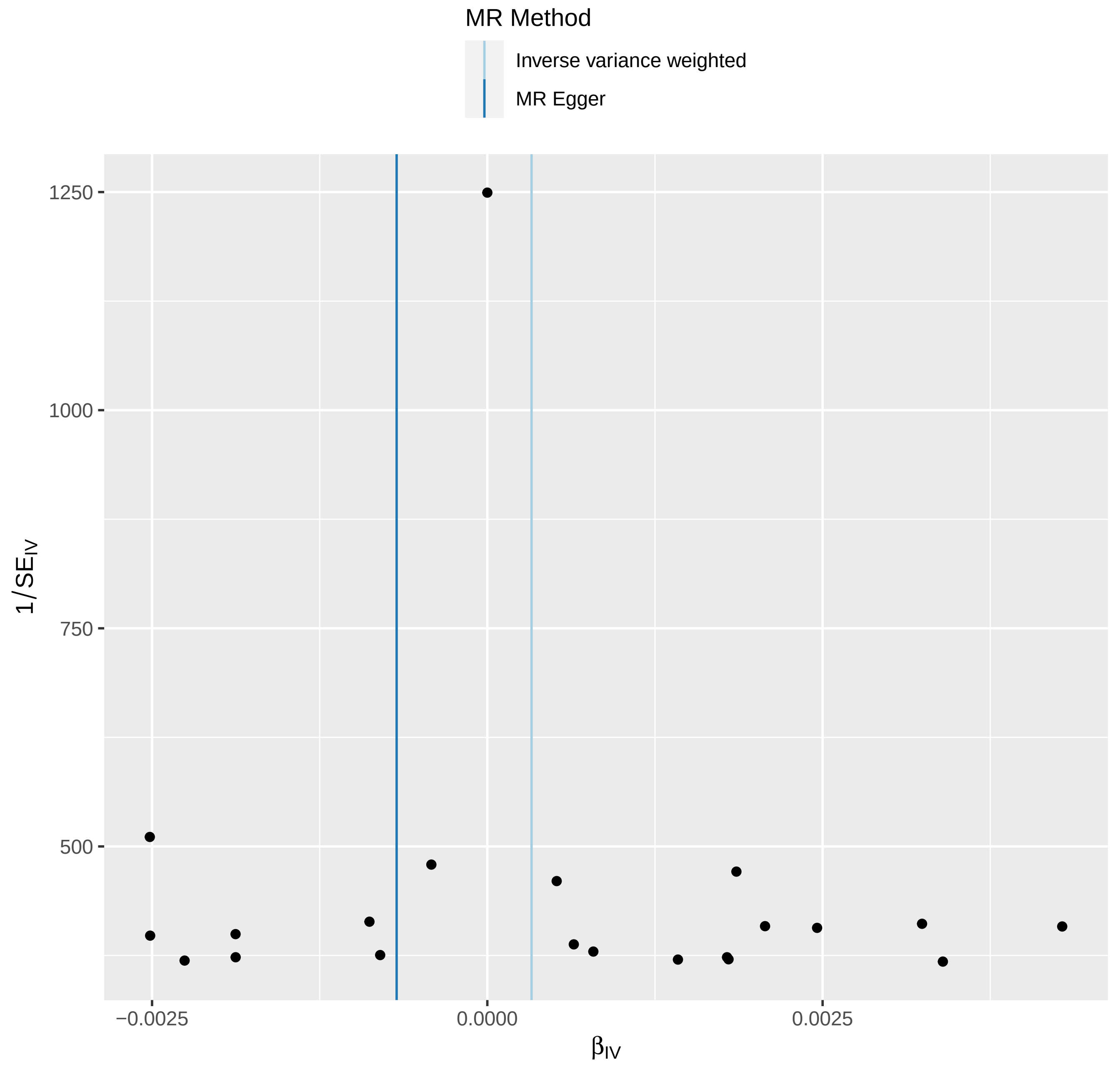

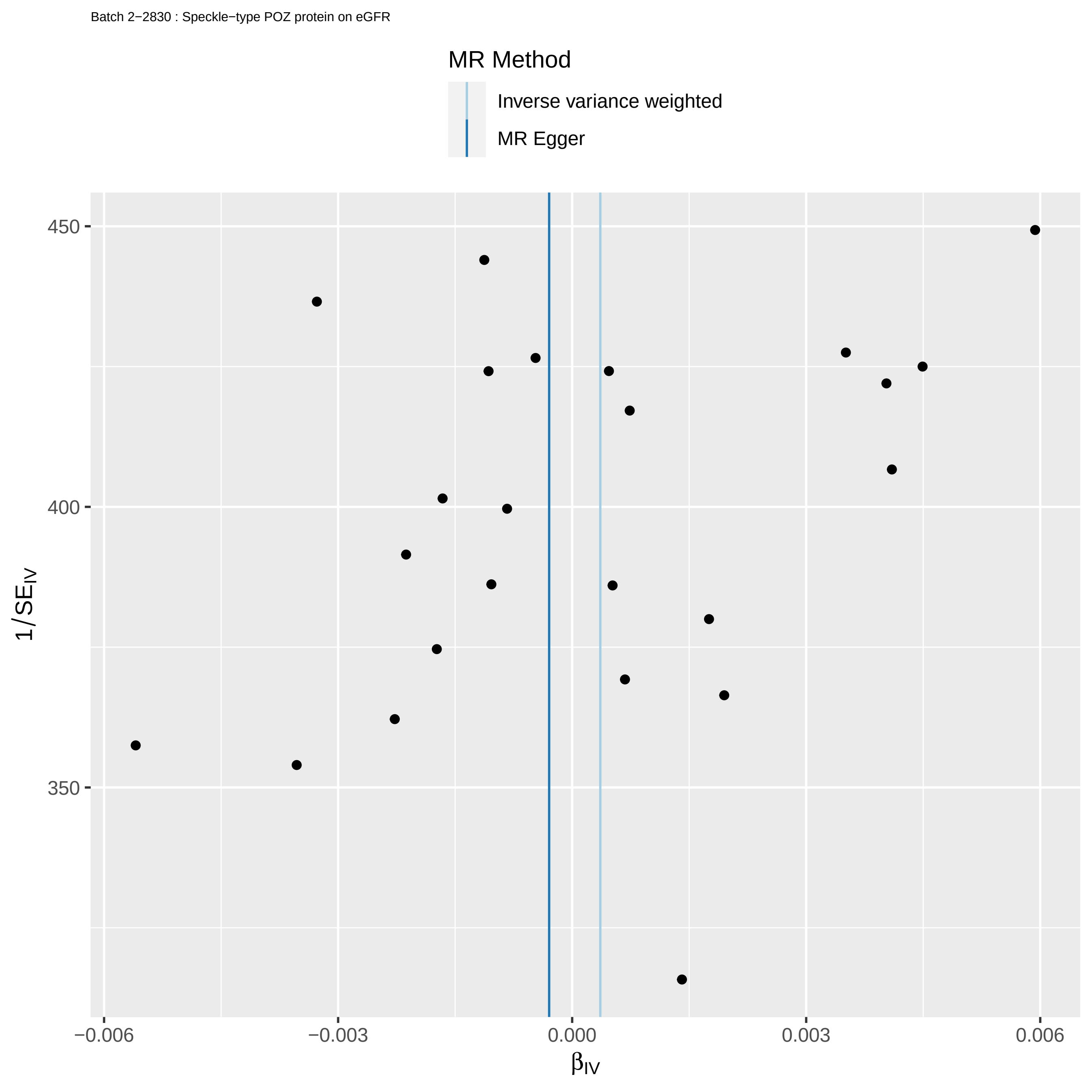

Batch 2−2830 : Speckle−type POZ protein on eGFR
MR Method
Inverse variance weighted
MR Egger
450
400
350
$1/SE_{IV}$
−0.006
−0.003
0.000
0.003
0.006
β_{IV}

Batch 2−2831 : Sepiapterin reductase on eGFR

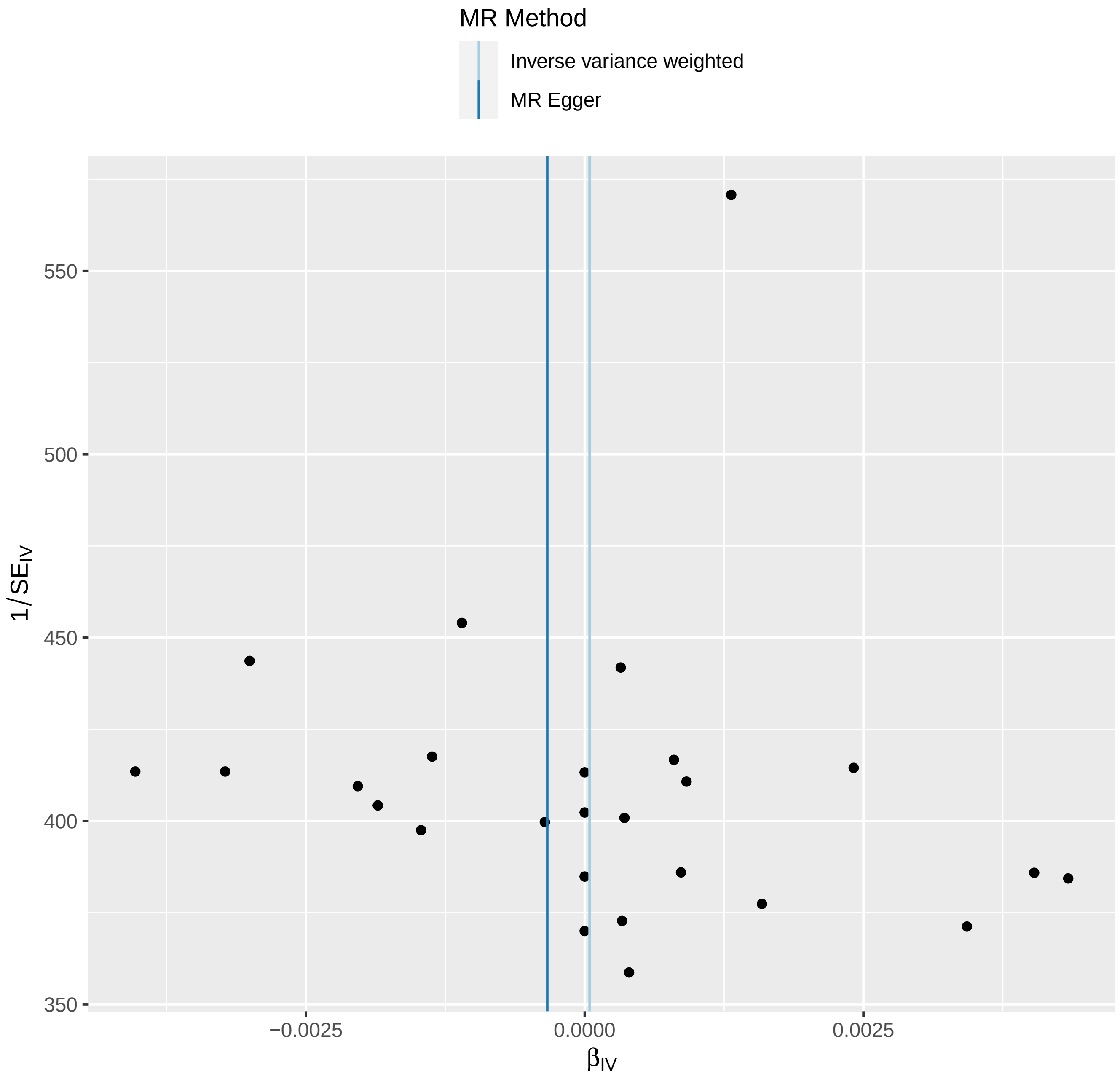

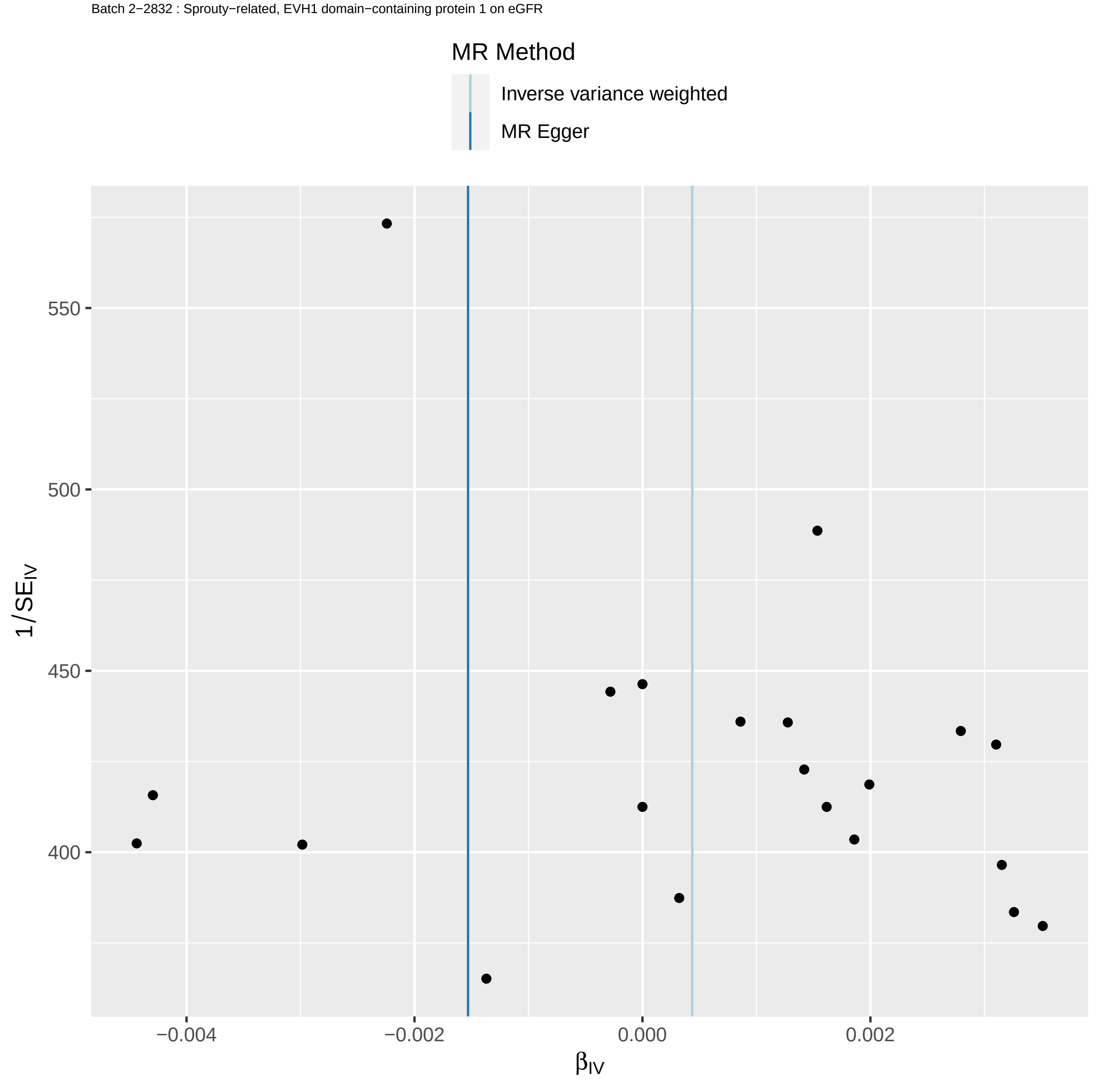

Batch 2−2832 : Sprouty−related, EVH1 domain−containing protein 1 on eGFR
MR Method
Inverse variance weighted
MR Egger
550
500
450
400
1/SE_IV
−0.004
−0.002
0.000
0.002
β_IV

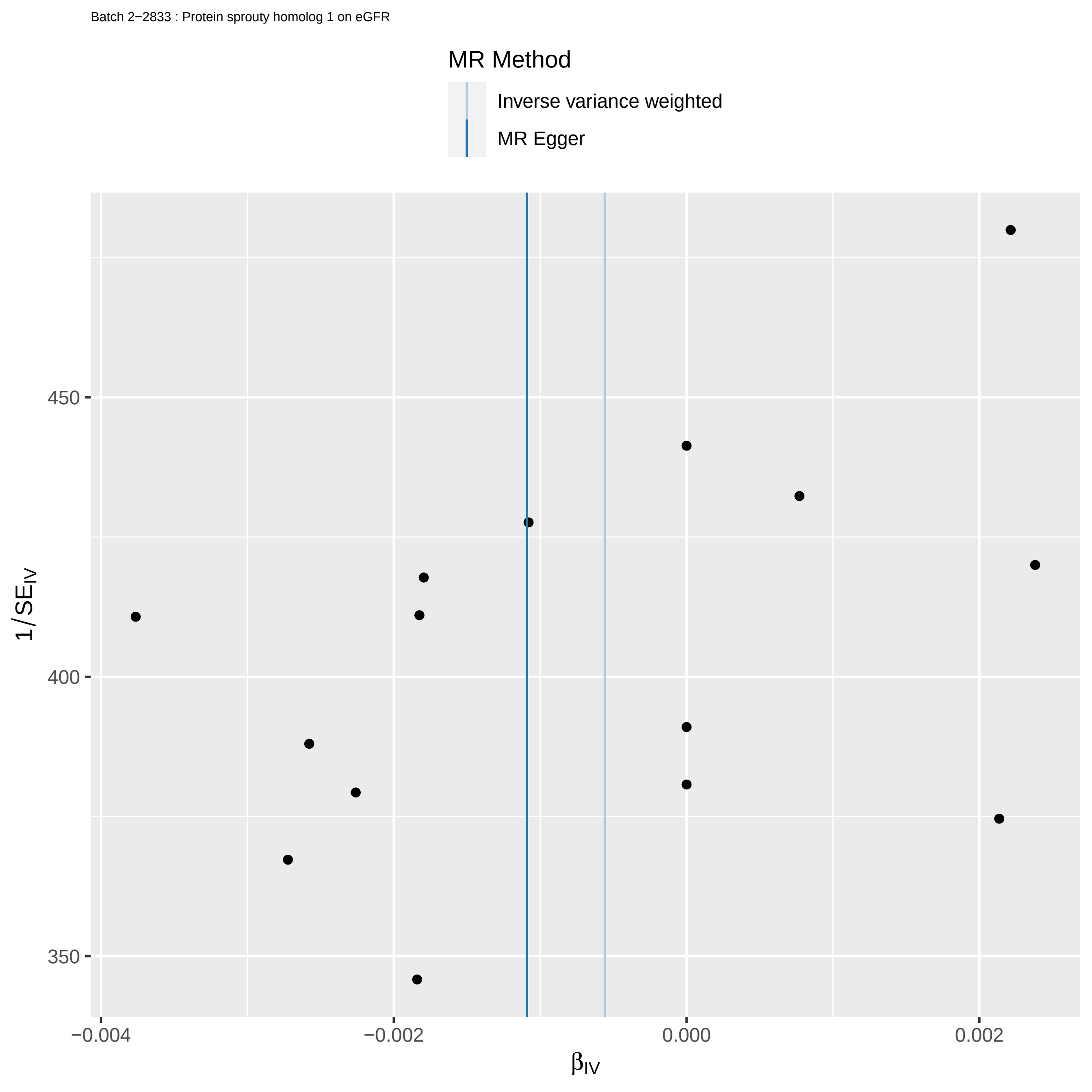

Batch 2−2833 : Protein sprouty homolog 1 on eGFR
MR Method
Inverse variance weighted
MR Egger
450
400
350
1/SE_IV
−0.004
−0.002
0.000
0.002
β_IV

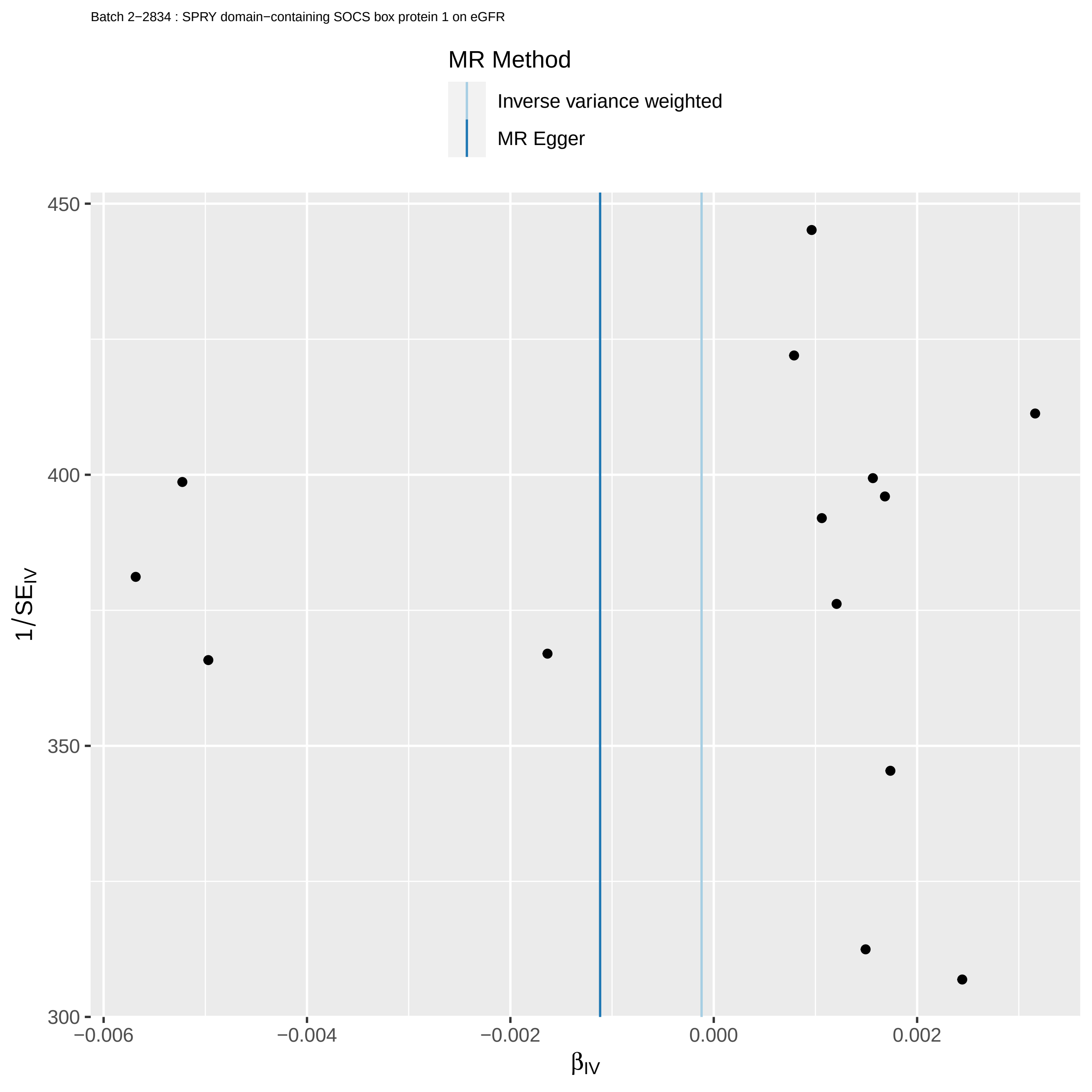

Batch 2−2834 : SPRY domain−containing SOCS box protein 1 on eGFR
MR Method
Inverse variance weighted
MR Egger
450
400
350
300
1/SE$_{IV}$
−0.006
−0.004
−0.002
0.000
0.002
β$_{IV}$

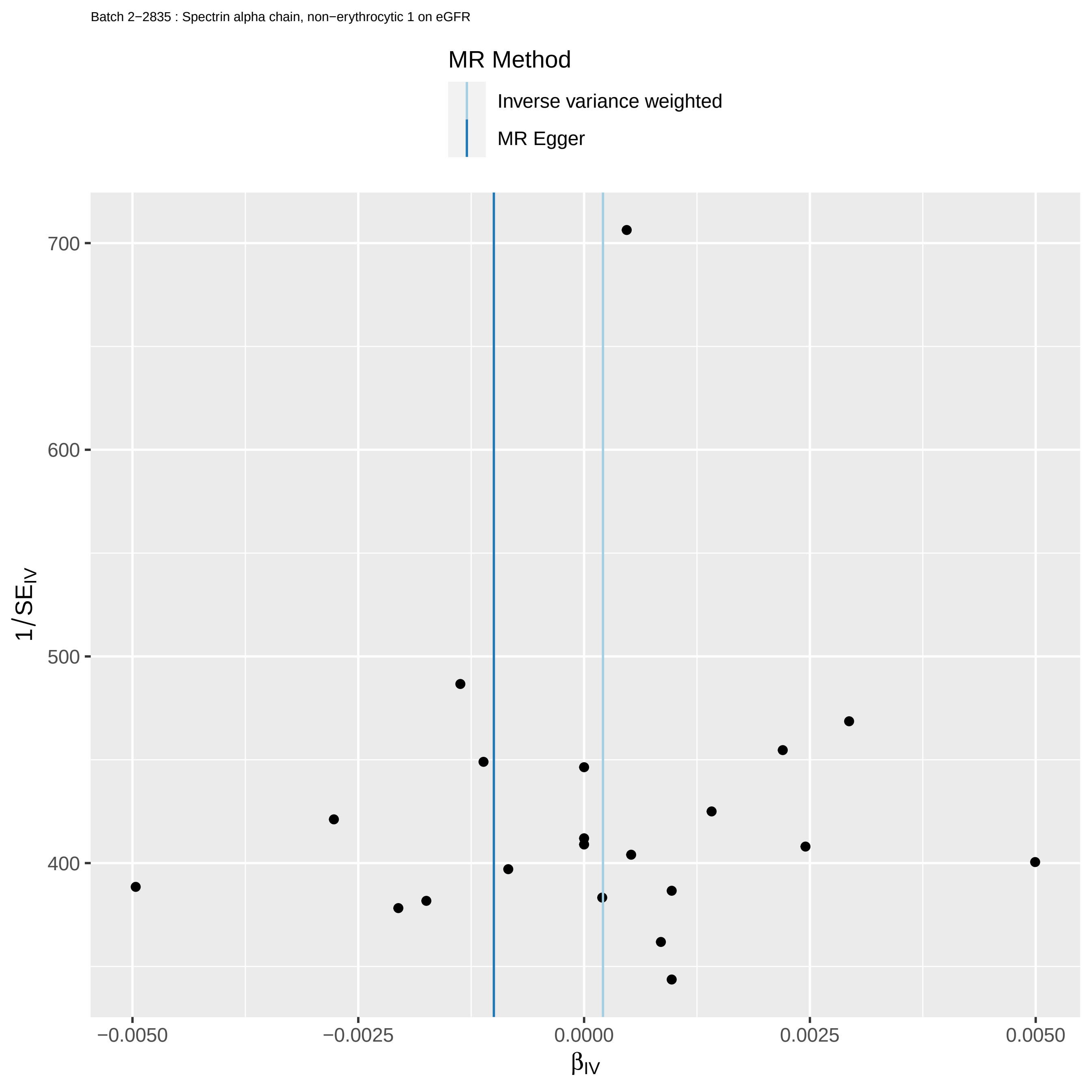

Batch 2−2835 : Spectrin alpha chain, non−erythrocytic 1 on eGFR
MR Method
Inverse variance weighted
MR Egger
700
600
500
400
1/SE_IV
−0.0050
−0.0025
0.0000
0.0025
0.0050
β_IV

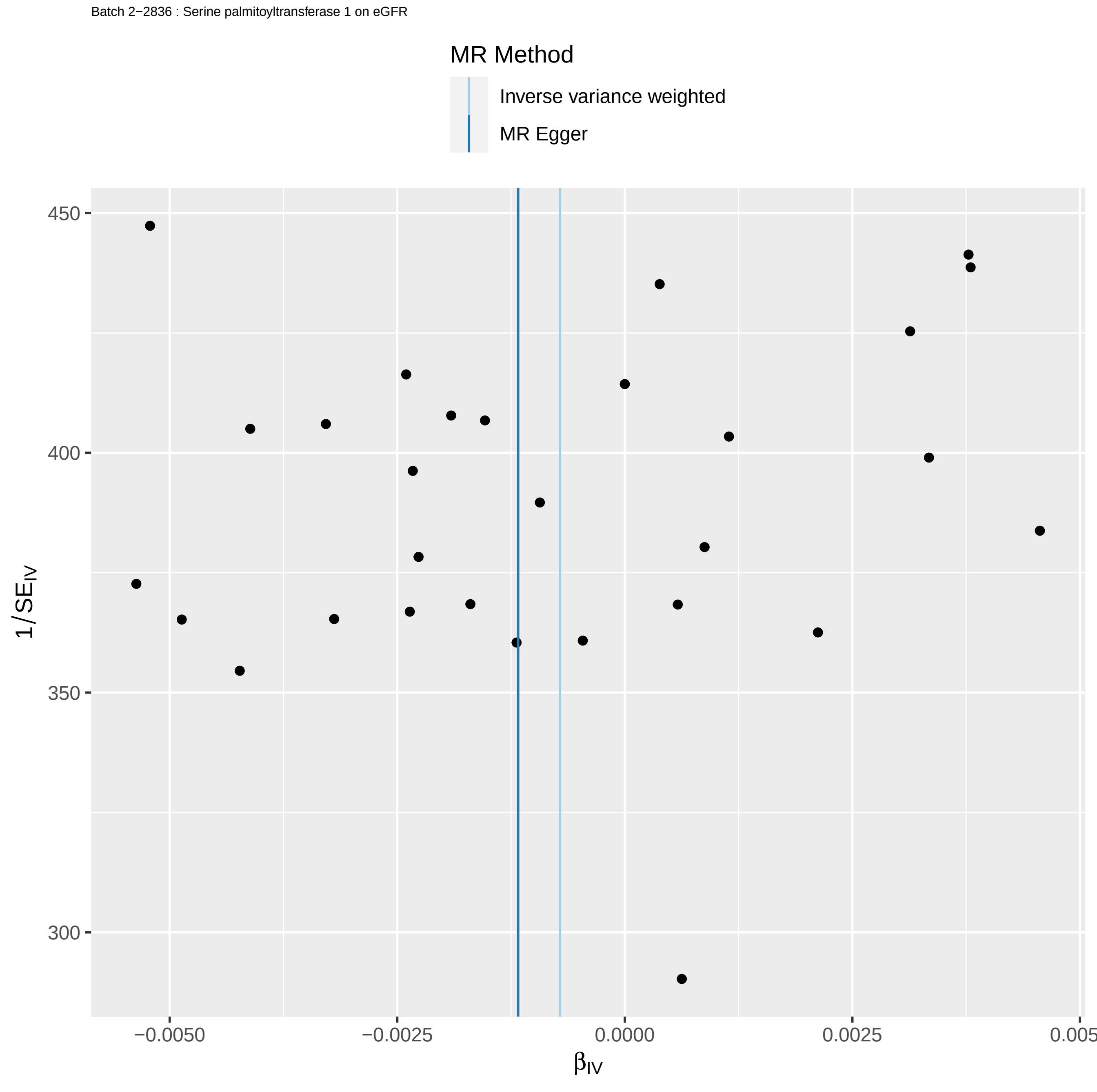

Batch 2−2836 : Serine palmitoyltransferase 1 on eGFR
MR Method
Inverse variance weighted
MR Egger
450
400
350
300
1/SE_IV
−0.0050
−0.0025
0.0000
0.0025
0.005
β_IV

Batch 2−2837 : Serine palmitoyltransferase 2 on eGFR

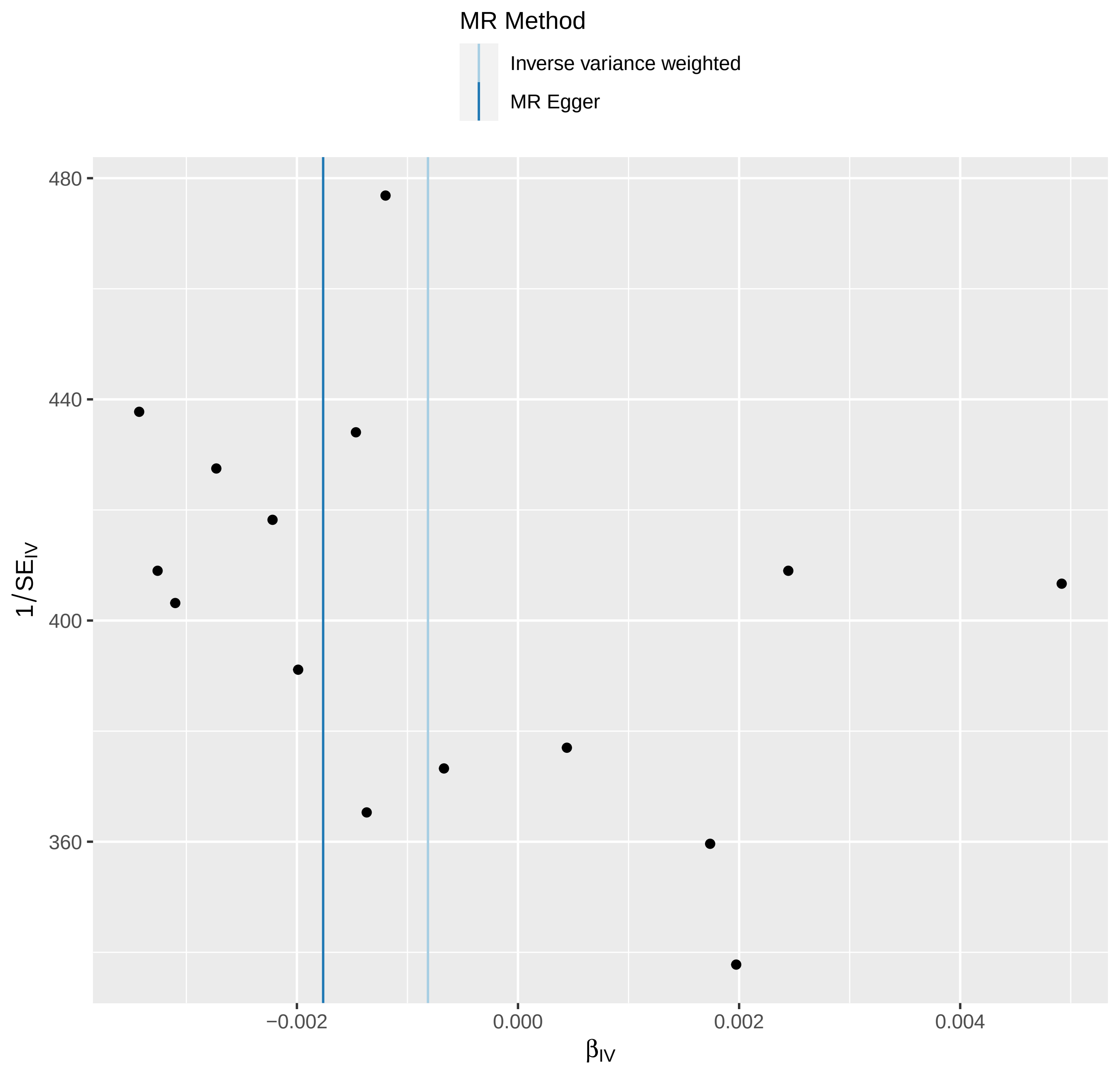

Batch 2−2838 : Serine/arginine−rich splicing factor 6 on eGFR

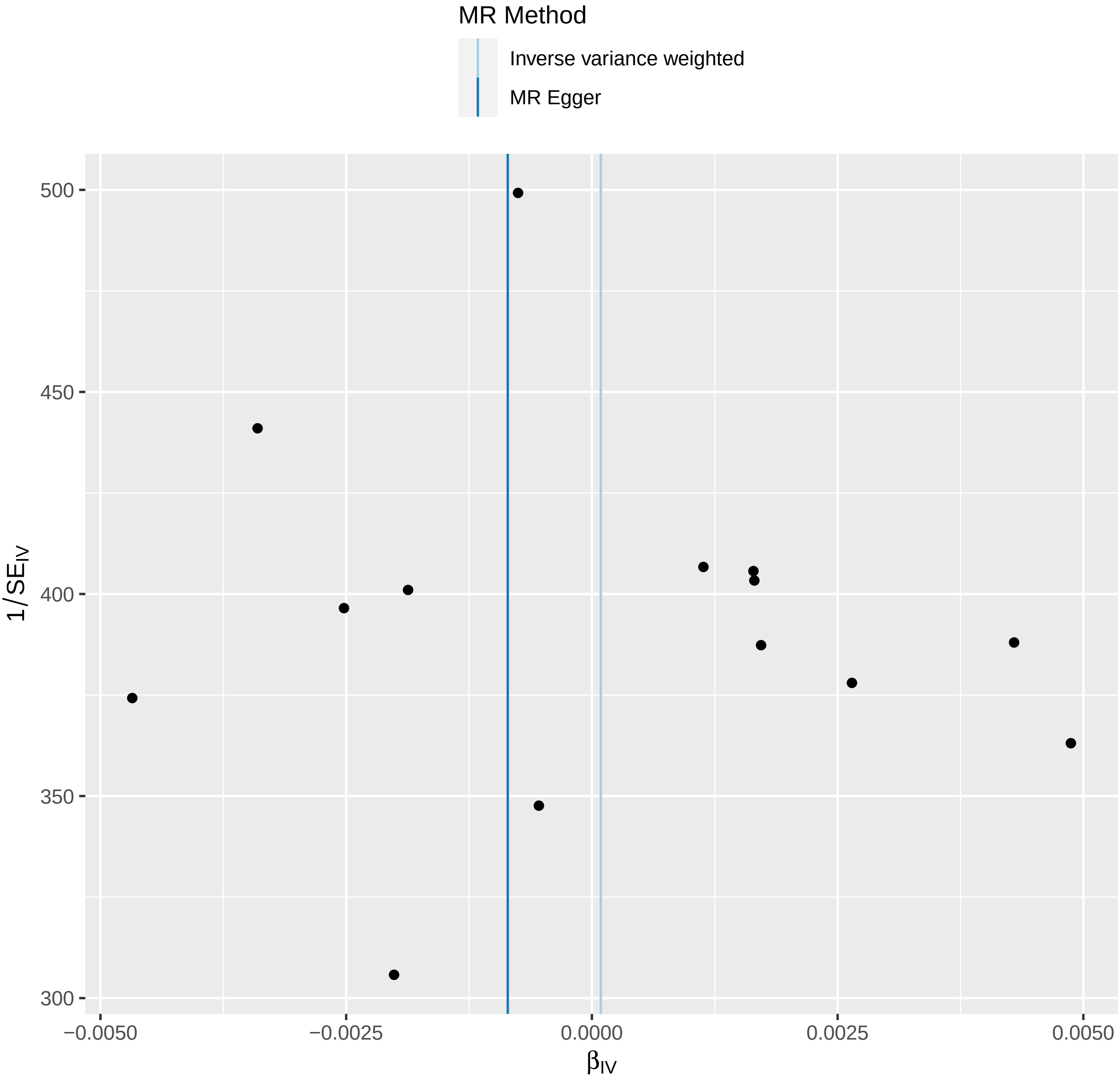

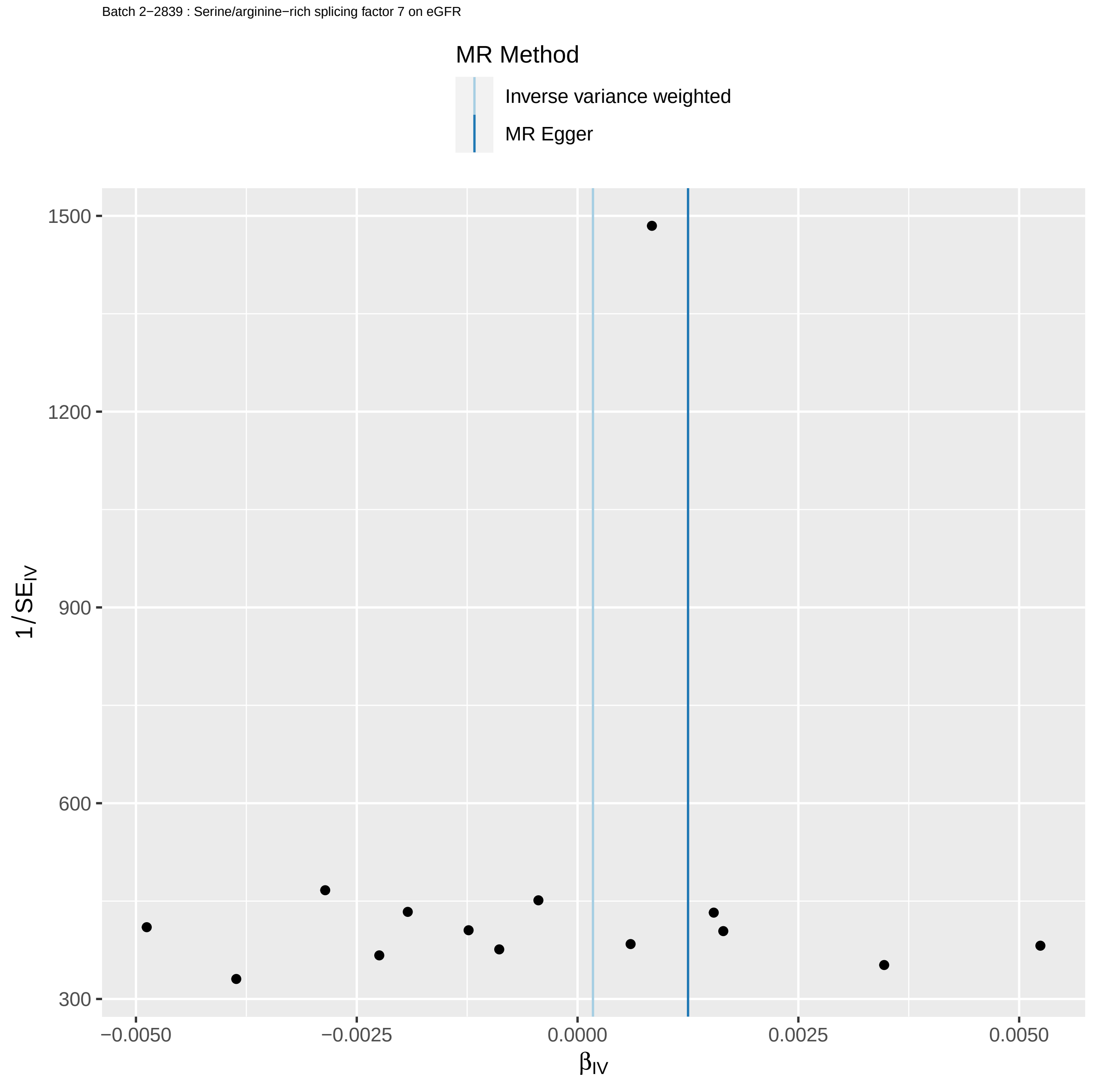

Batch 2−2839 : Serine/arginine−rich splicing factor 7 on eGFR
MR Method
Inverse variance weighted
MR Egger
1500
1200
900
600
300
$1/SE_{IV}$
−0.0050
−0.0025
0.0000
0.0025
0.0050
β_{IV}

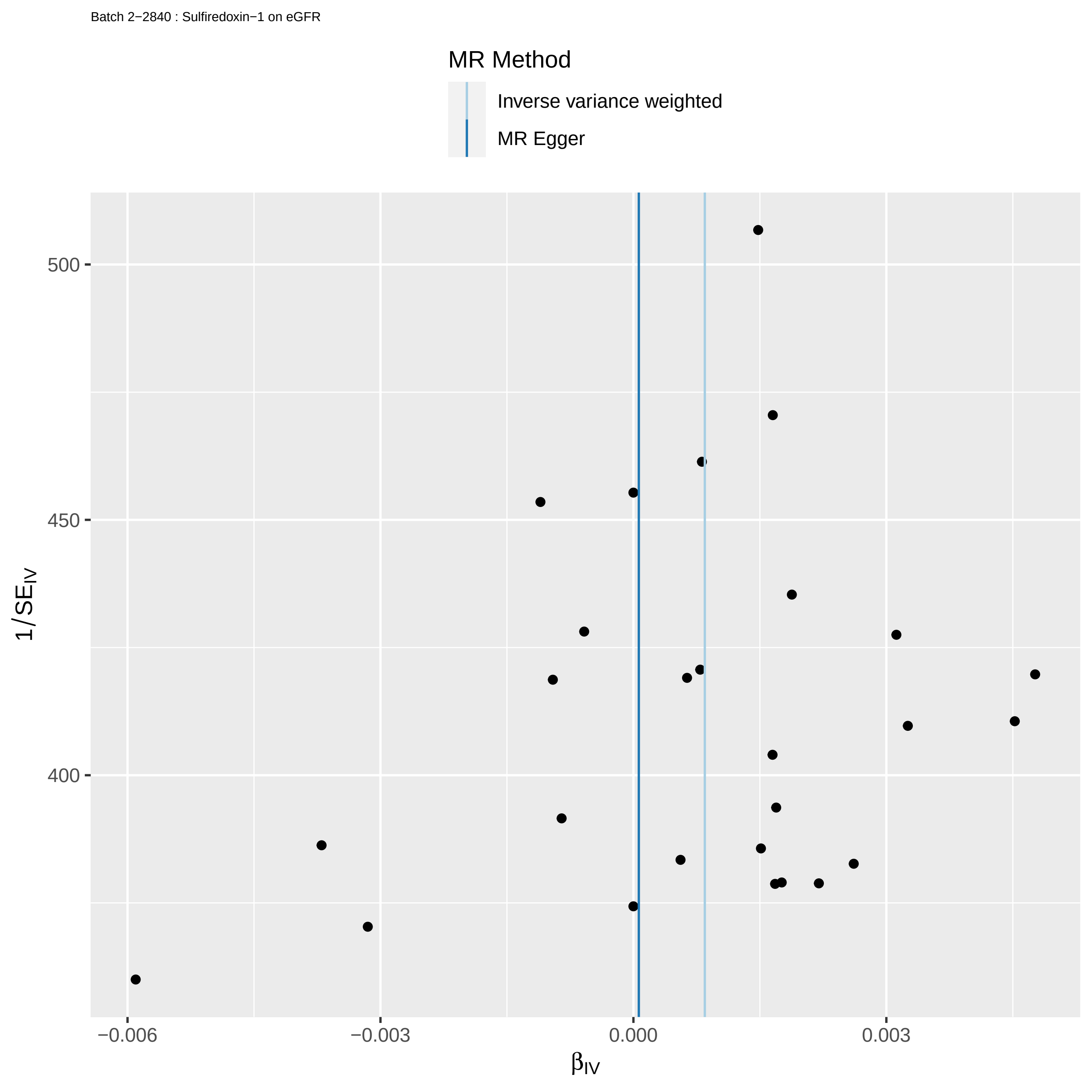

Batch 2−2840 : Sulfiredoxin−1 on eGFR
MR Method
Inverse variance weighted
MR Egger
500
450
400
1/SE_IV
−0.006
−0.003
0.000
0.003
β_IV

Batch 2−2841 : Lupus La protein on eGFR

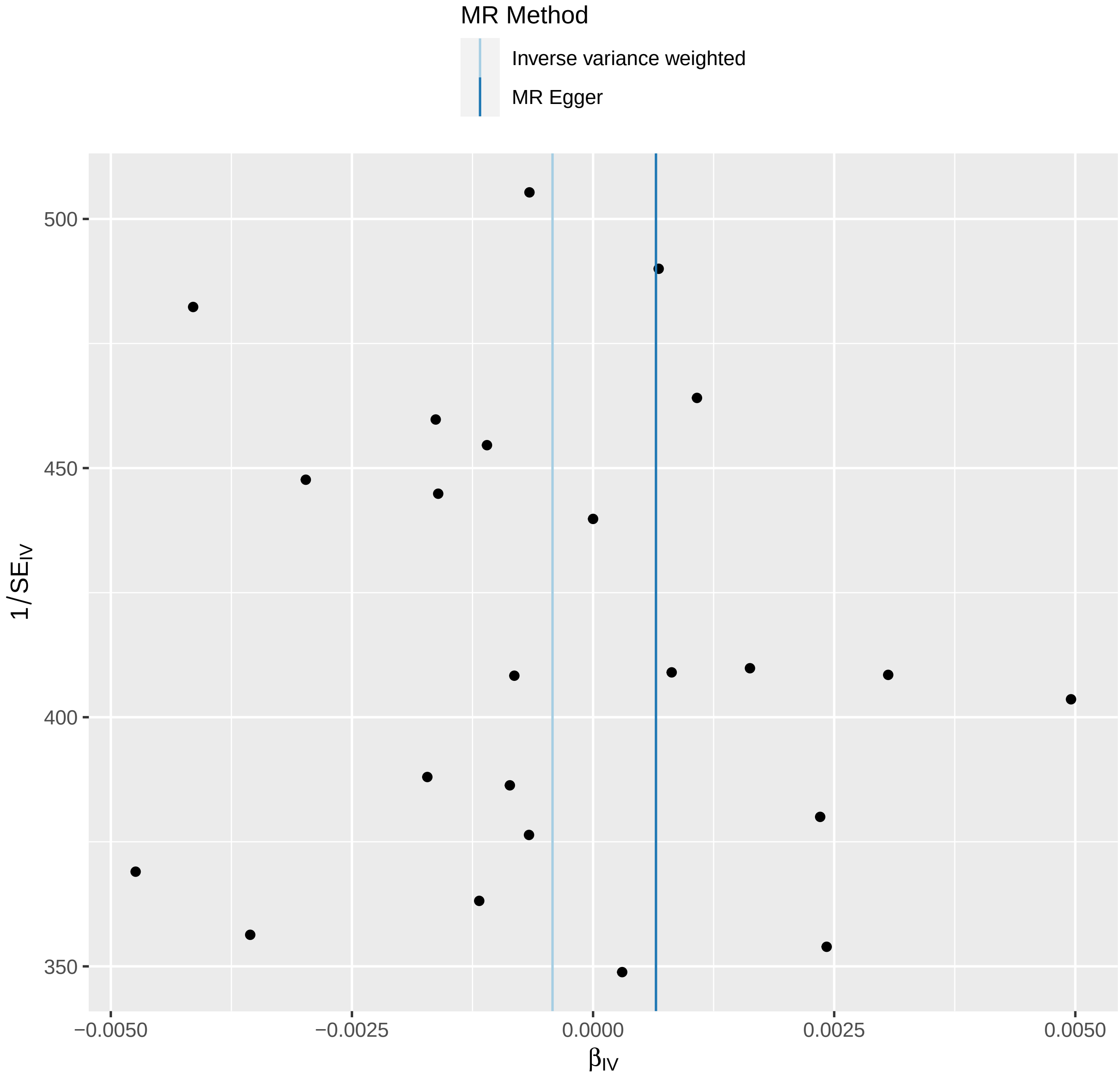

Batch 2−2842 : Lupus La protein on eGFR

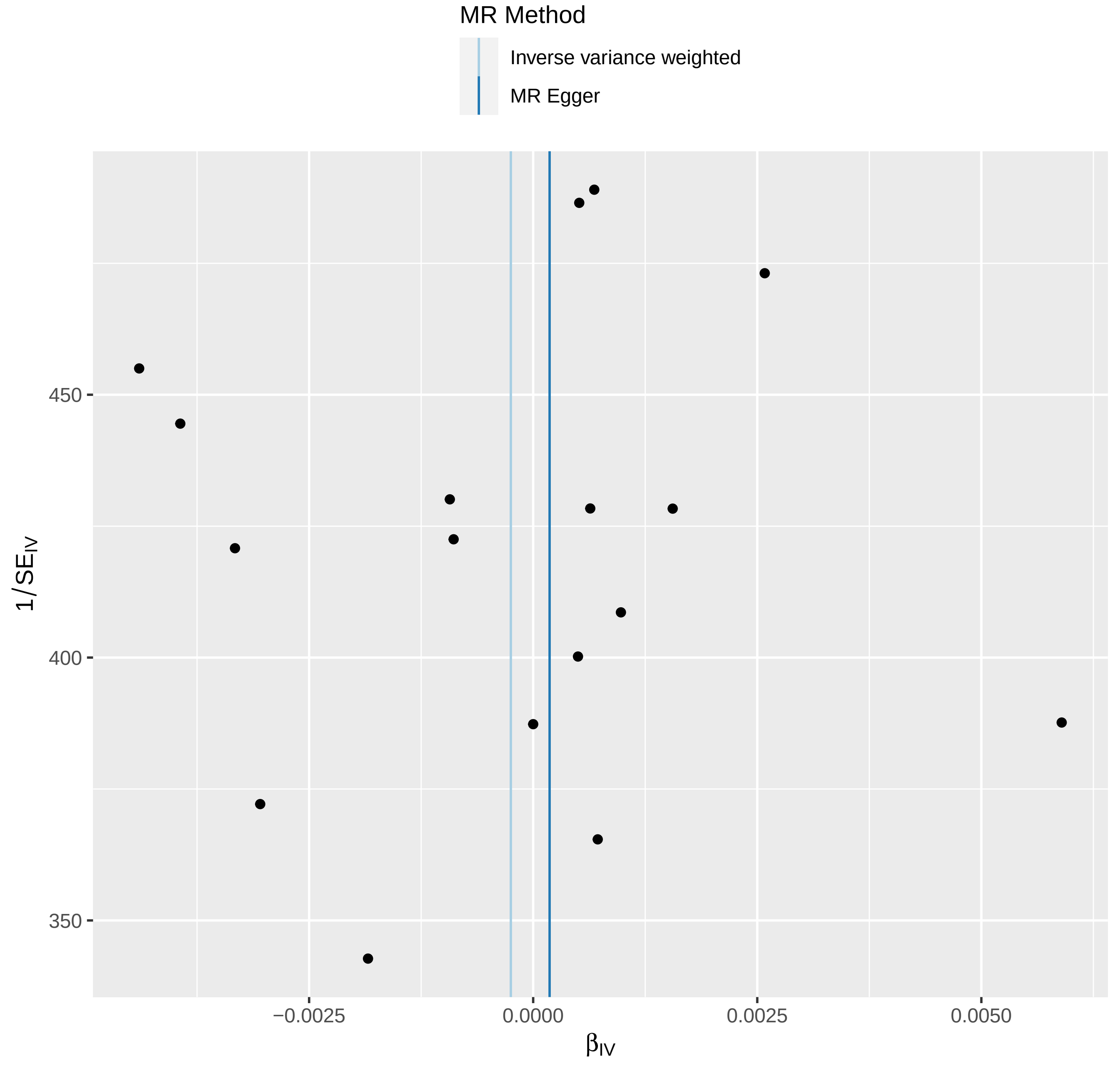

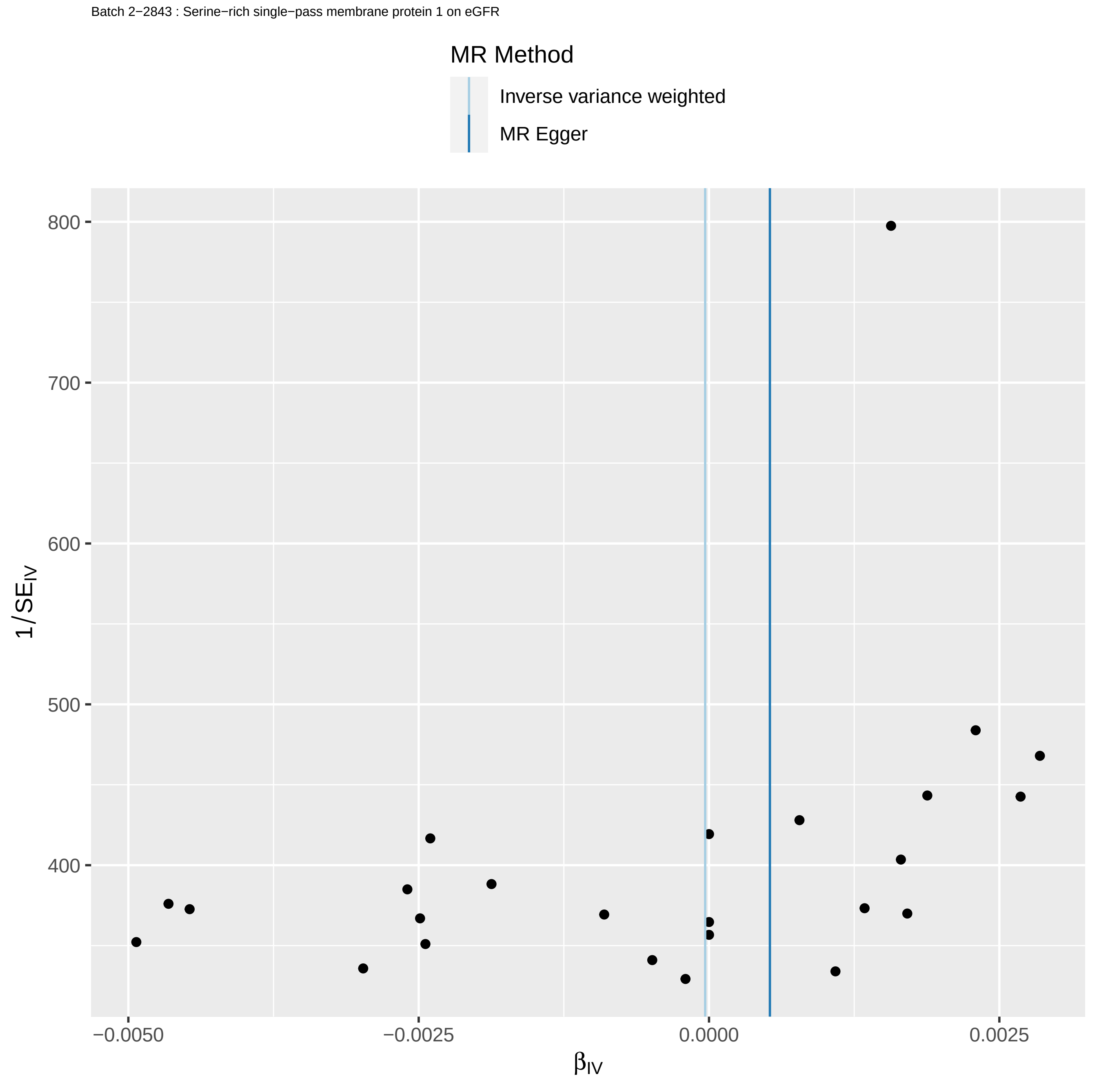
Batch 2−2843 : Serine−rich single−pass membrane protein 1 on eGFR
MR Method
Inverse variance weighted
MR Egger
800
700
600
500
400
1/SE$_{IV}$
−0.0050
−0.0025
0.0000
0.0025
β$_{IV}$

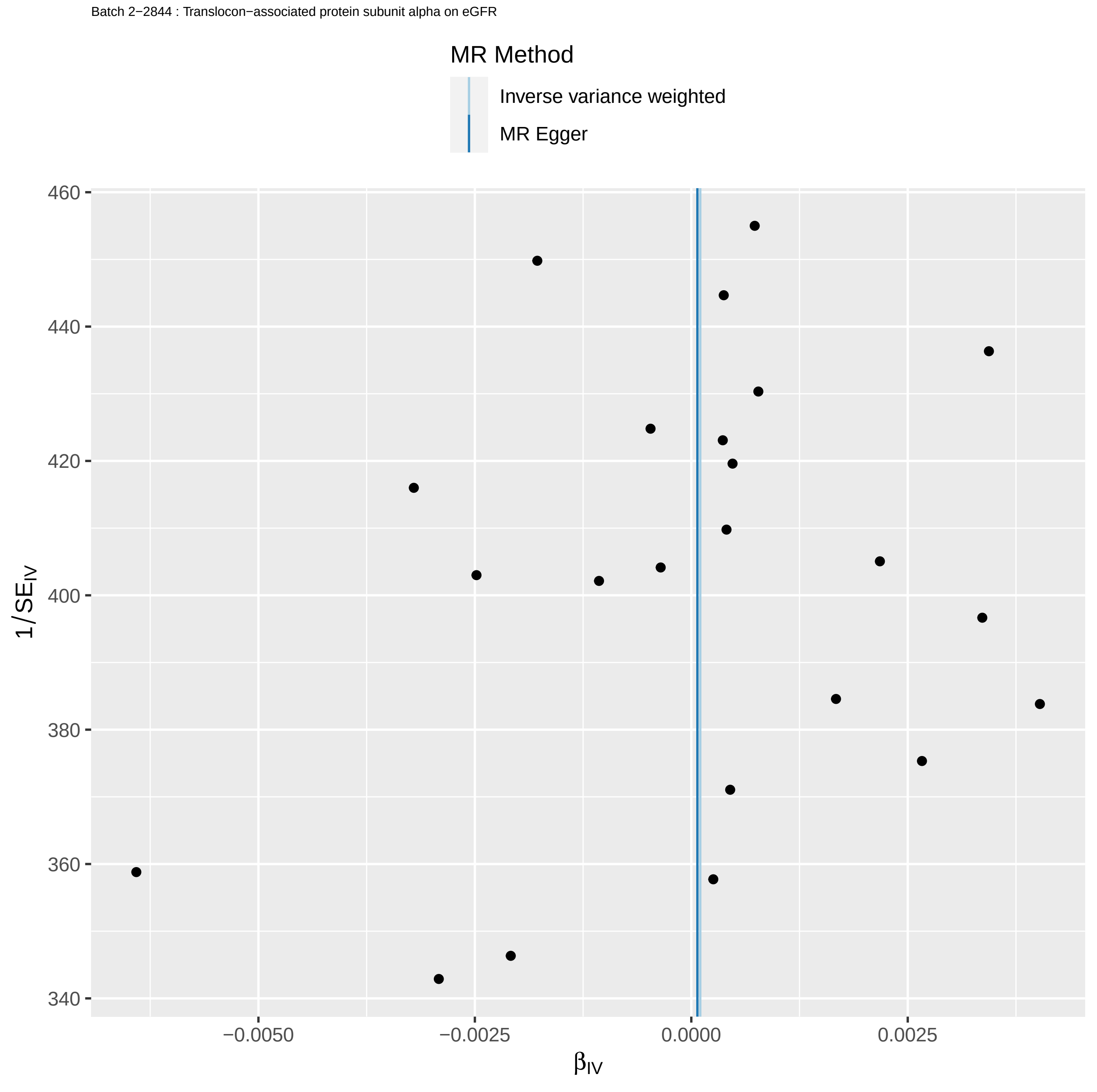
Batch 2−2844 : Translocon−associated protein subunit alpha on eGFR
MR Method
Inverse variance weighted
MR Egger
460
440
420
400
380
360
340
1/SE$_{IV}$
−0.0050
−0.0025
0.0000
0.0025
β_{IV}

Batch 2−2845 : FACT complex subunit SSRP1 on eGFR

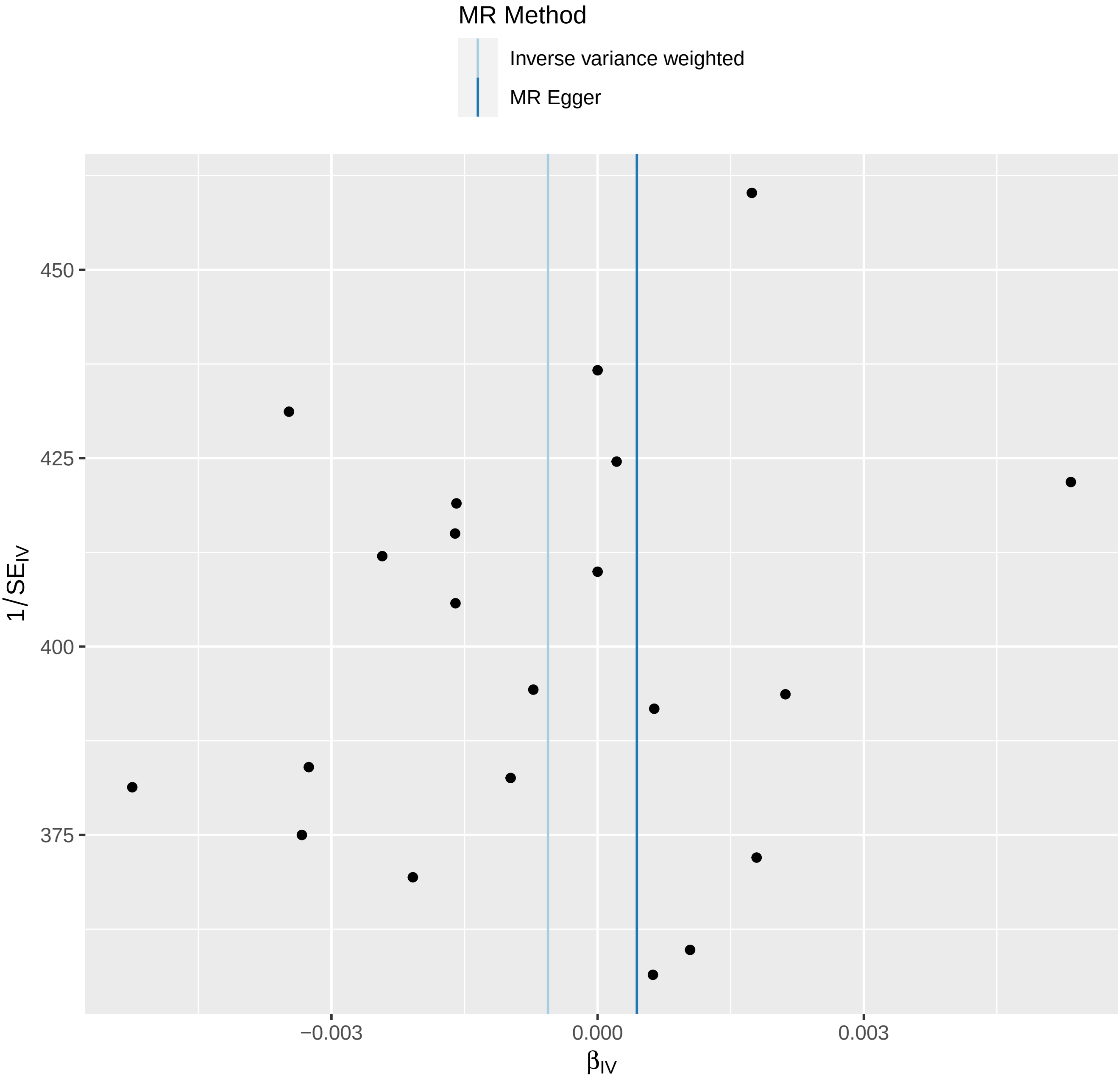

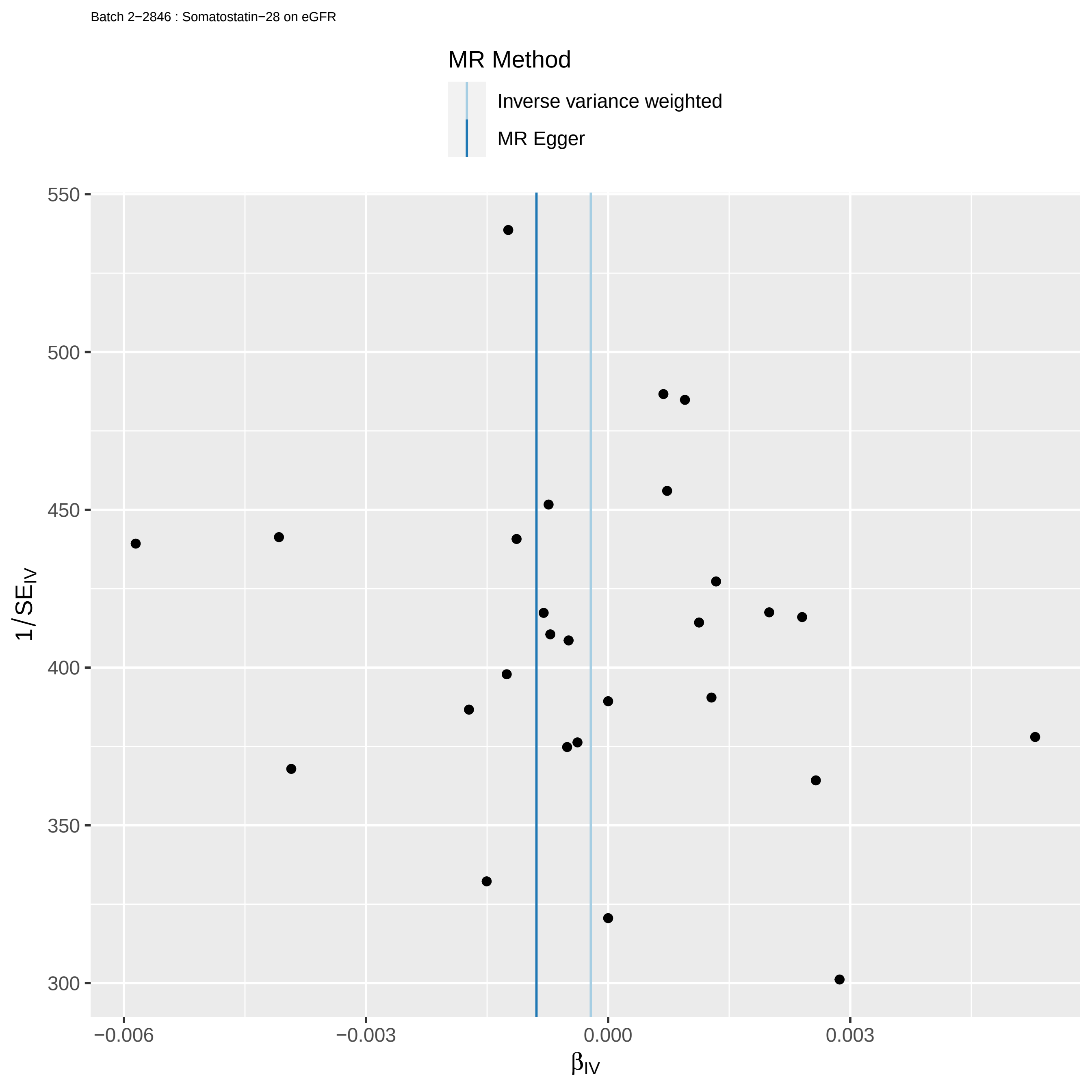
Batch 2−2846 : Somatostatin−28 on eGFR
MR Method
Inverse variance weighted
MR Egger
550
500
450
400
350
300
1/SE_IV
−0.006
−0.003
0.000
0.003
β_IV

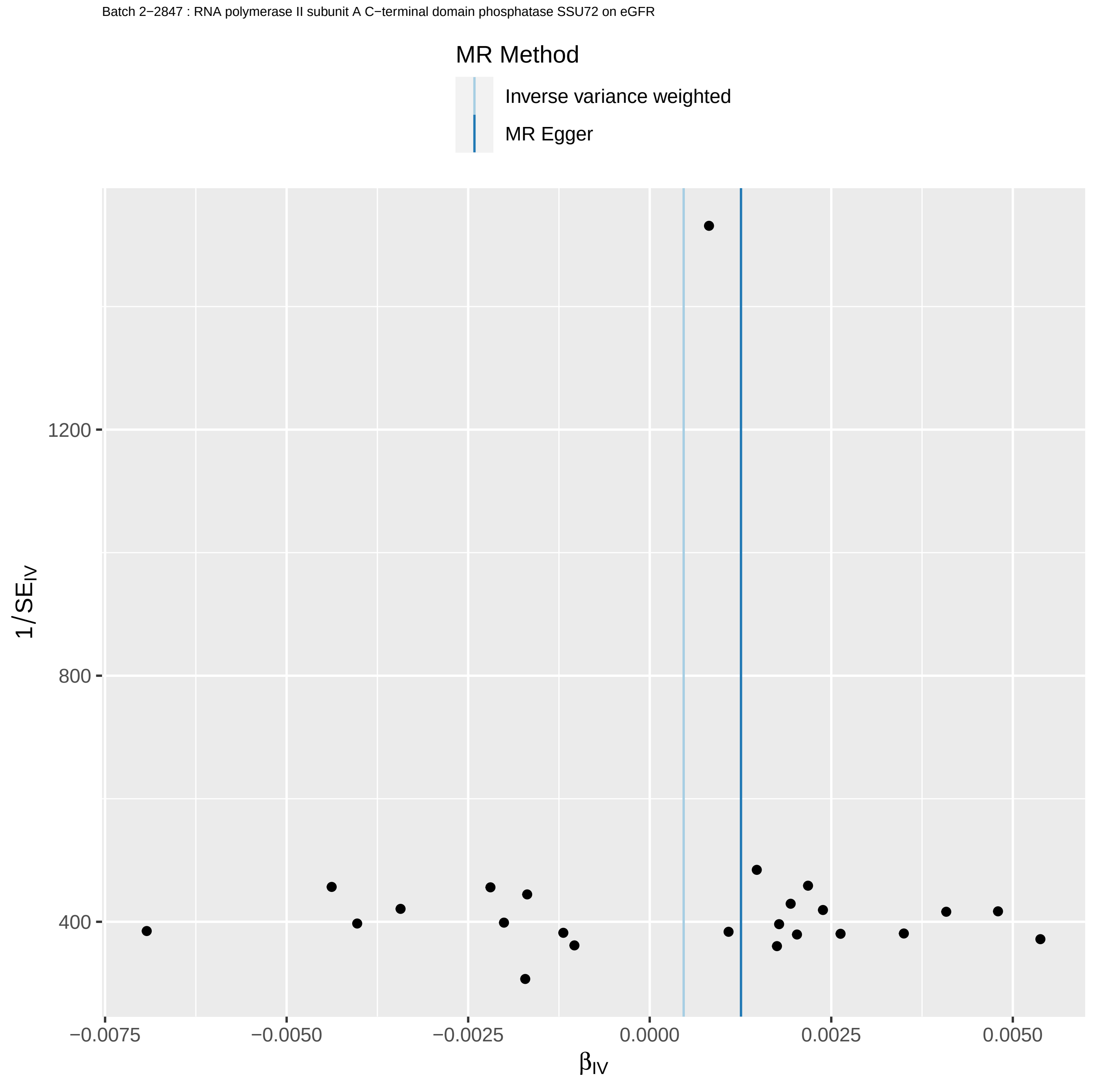

Batch 2−2847 : RNA polymerase II subunit A C−terminal domain phosphatase SSU72 on eGFR
MR Method
Inverse variance weighted
MR Egger
1200
800
400
1/SE_IV
−0.0075
−0.0050
−0.0025
0.0000
0.0025
0.0050
β_IV

Batch 2−2848 : CMP−N−acetylneuraminate−beta−galactosamide−alpha−2,3−sialyltransferase 1 on eGFR

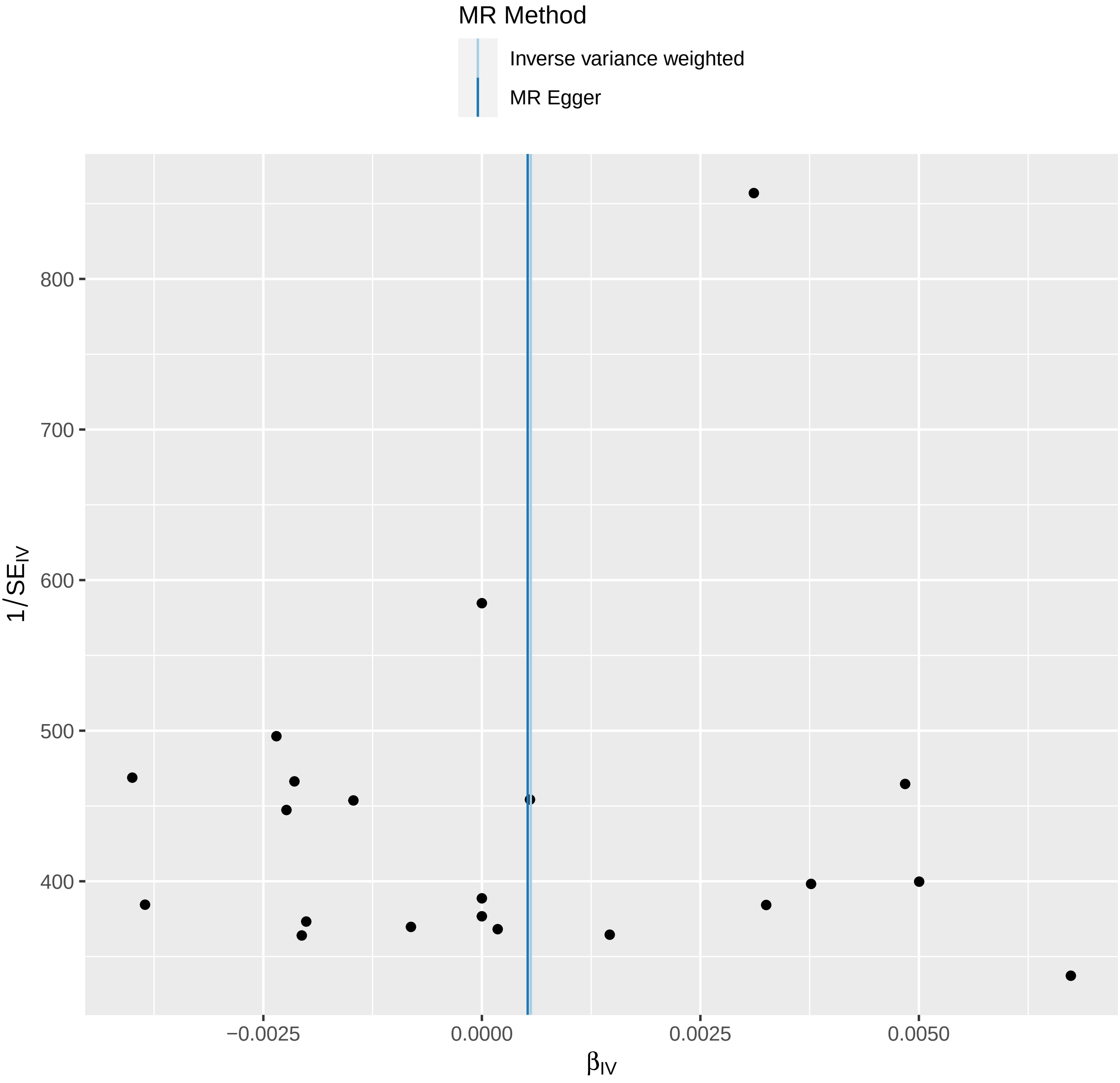

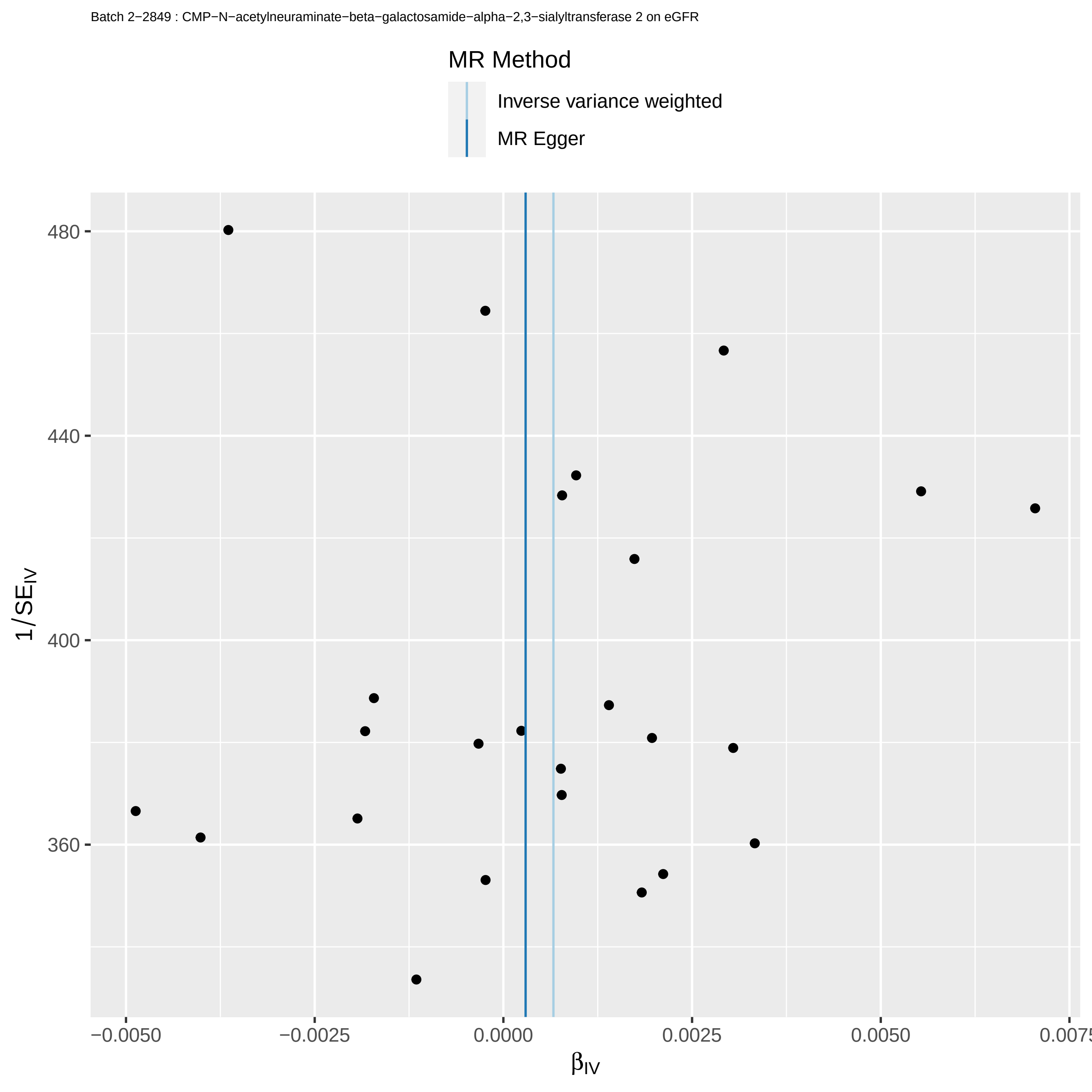

Batch 2−2849 : CMP−N−acetylneuraminate−beta−galactosamide−alpha−2,3−sialyltransferase 2 on eGFR
MR Method
Inverse variance weighted
MR Egger
480
440
400
360
1/SE_IV
−0.0050
−0.0025
0.0000
0.0025
0.0050
0.0075
β_IV

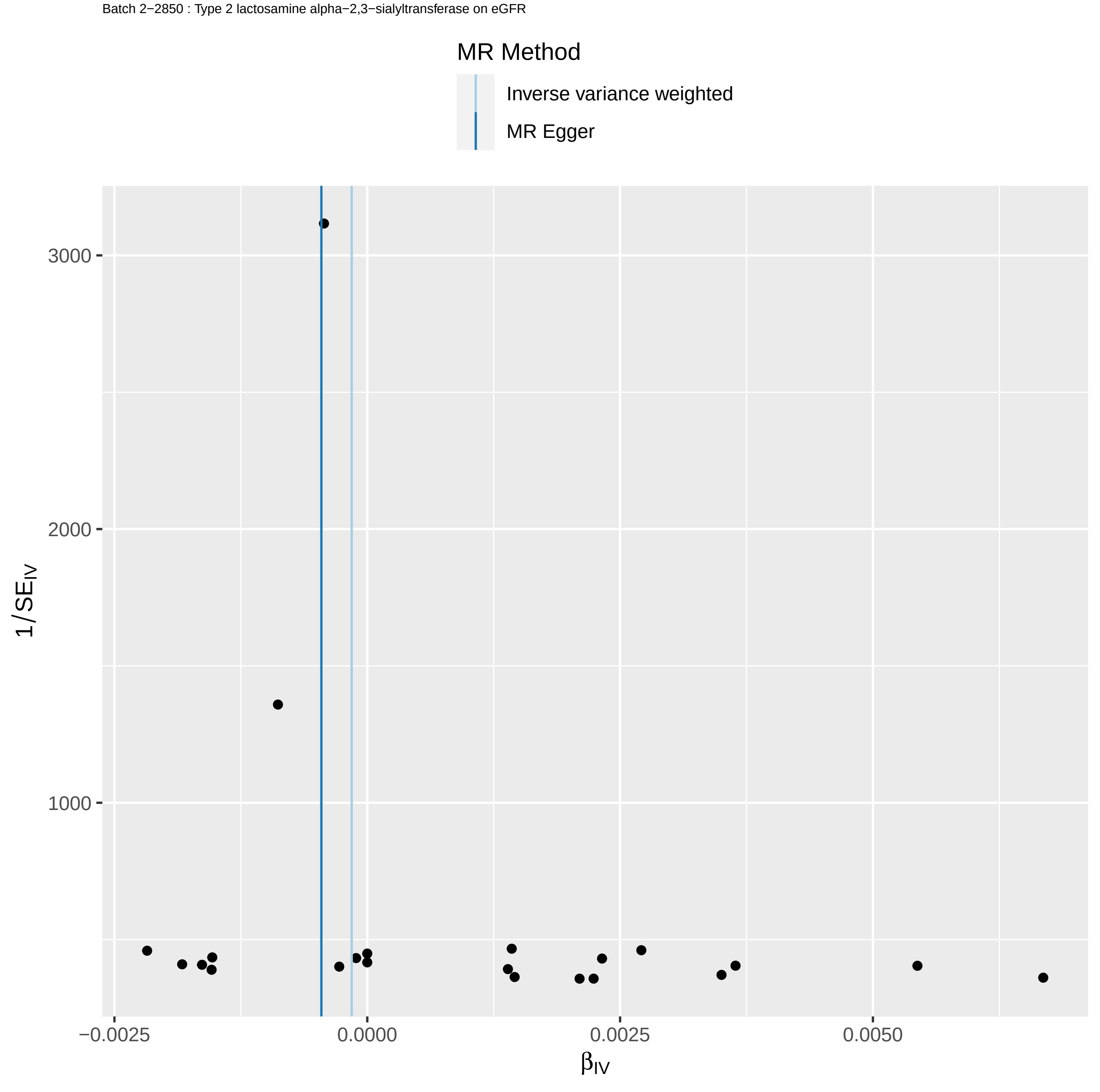

Batch 2−2850 : Type 2 lactosamine alpha−2,3−sialyltransferase on eGFR
MR Method
Inverse variance weighted
MR Egger
3000
2000
1000
1/SE_IV
−0.0025
0.0000
0.0025
0.0050
β_IV

Batch 2−2851 : Beta−galactoside alpha−2,6−sialyltransferase 1 on eGFR

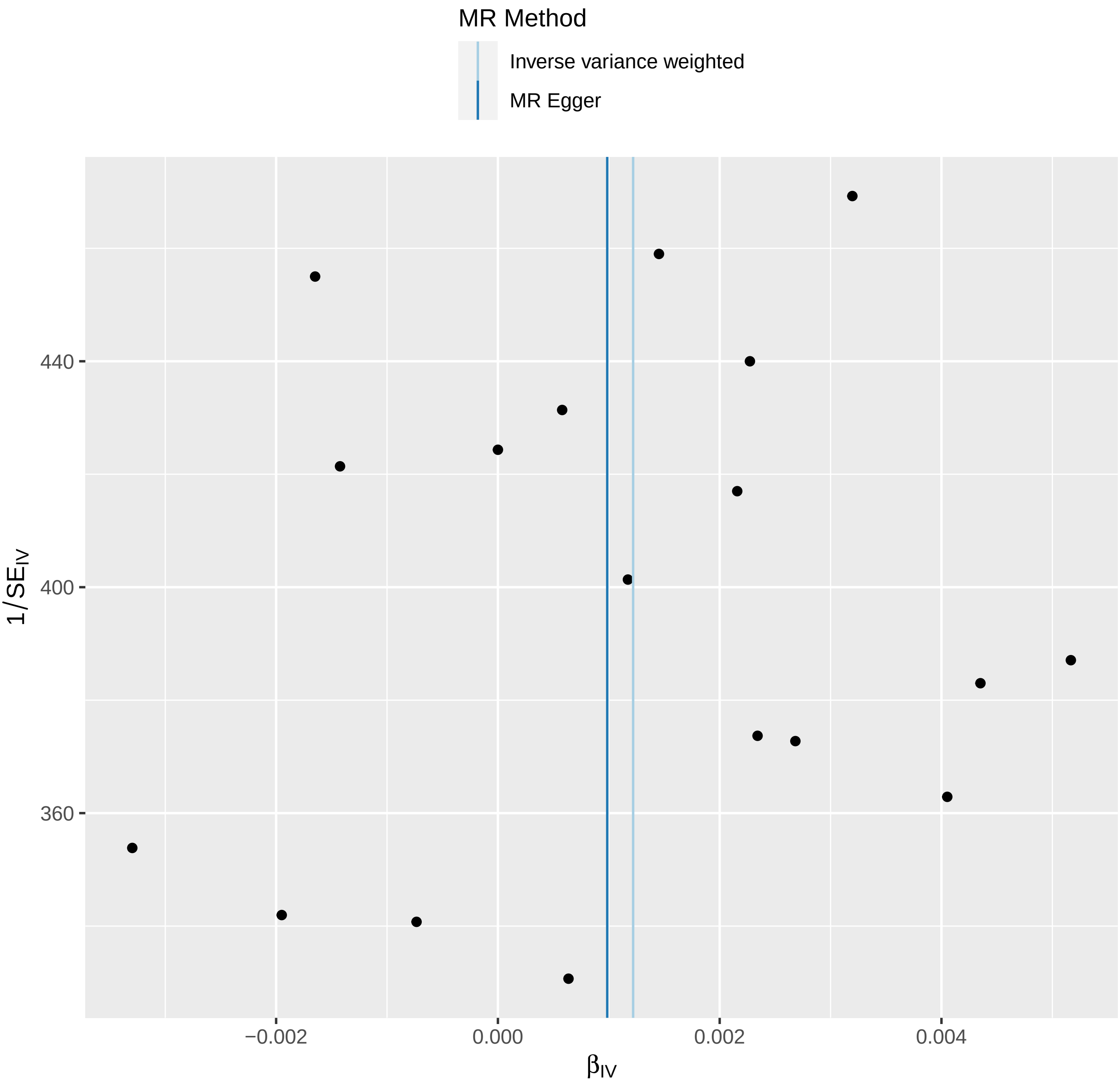

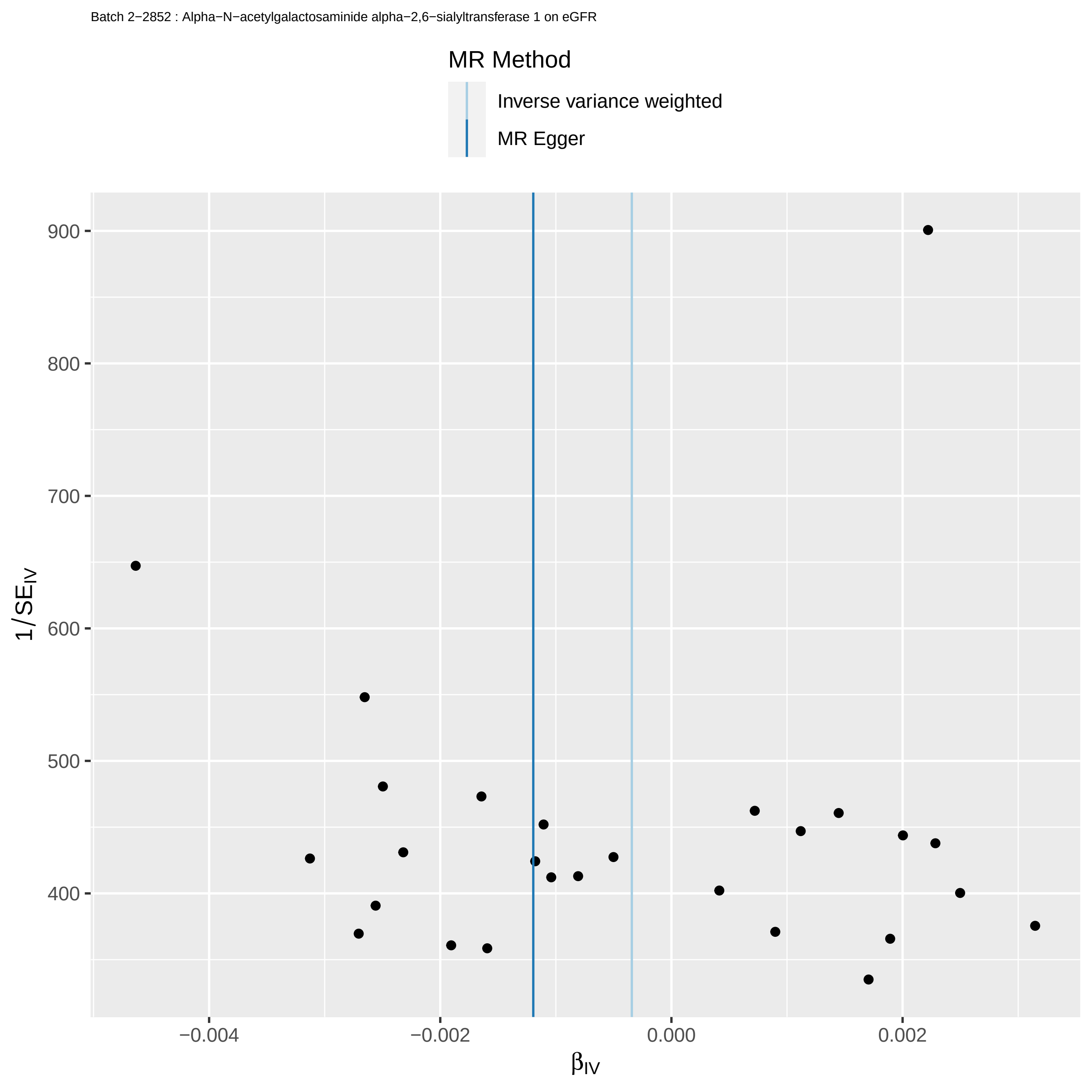
Batch 2−2852 : Alpha−N−acetylgalactosaminide alpha−2,6−sialyltransferase 1 on eGFR
MR Method
Inverse variance weighted
MR Egger
900
800
700
600
500
400
1/SE$_{IV}$
−0.004
−0.002
0.000
0.002
β$_{IV}$

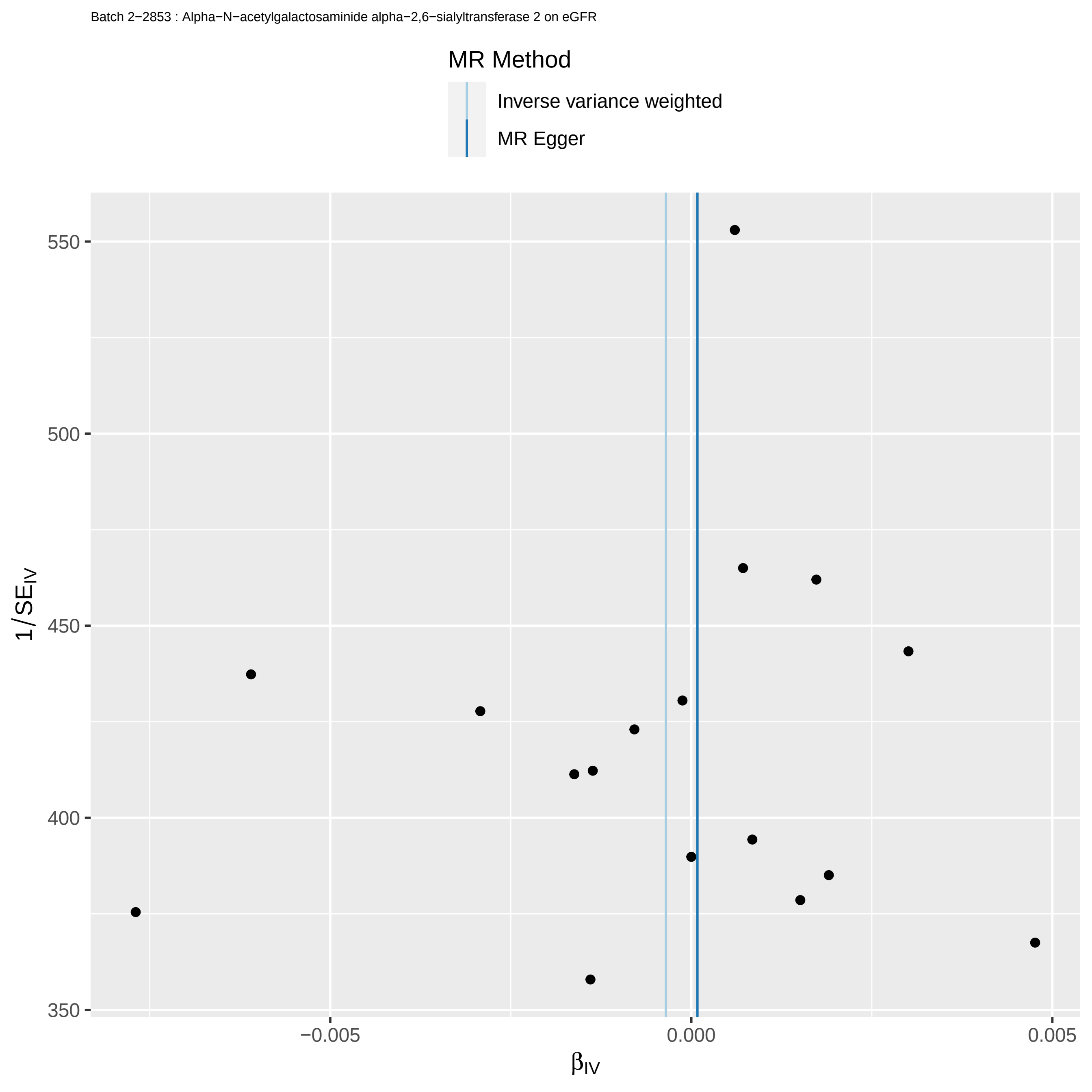
Batch 2−2853 : Alpha−N−acetylgalactosaminide alpha−2,6−sialyltransferase 2 on eGFR
MR Method
Inverse variance weighted
MR Egger
550
500
450
400
350
1/SE_IV
−0.005
0.000
0.005
β_IV

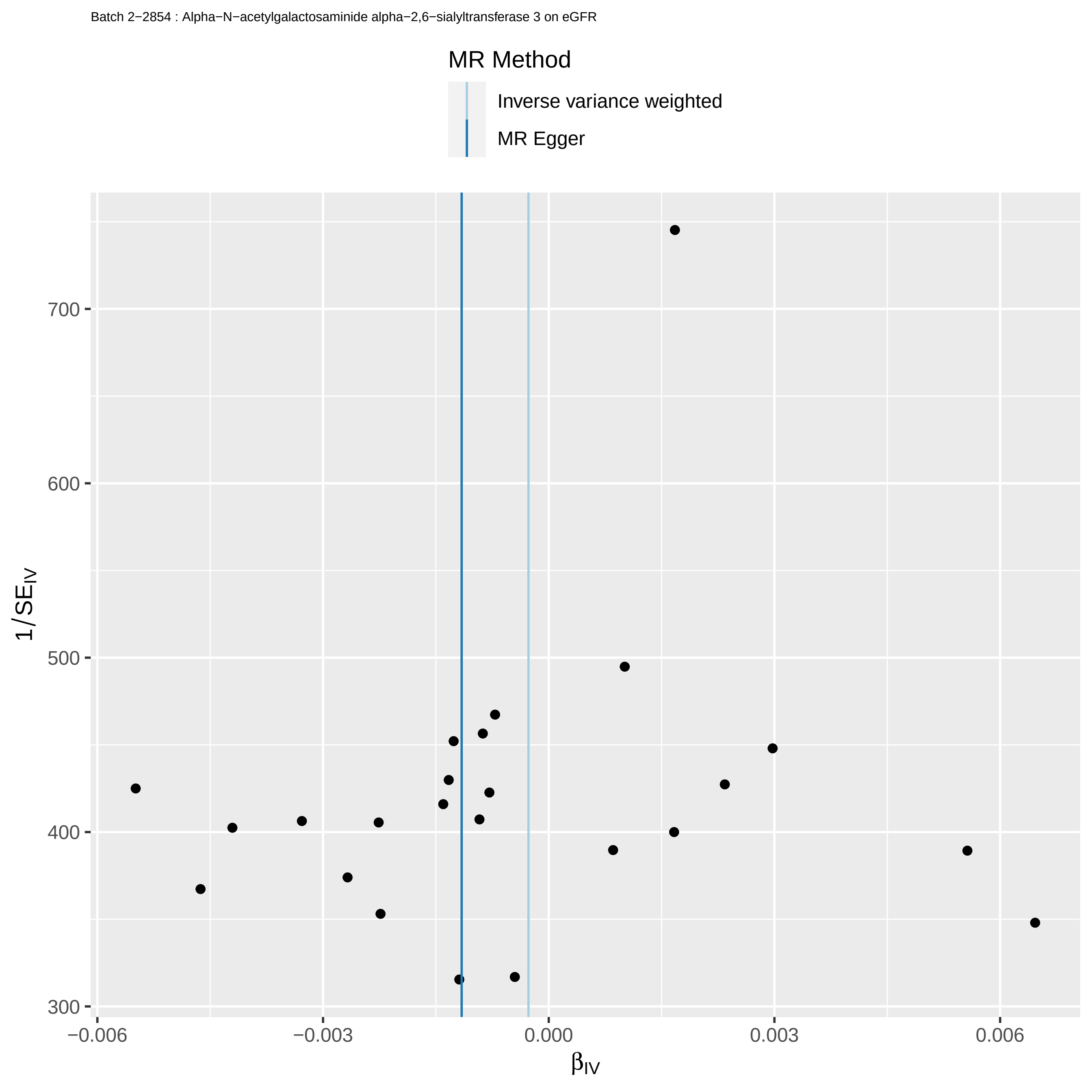
Batch 2−2854 : Alpha−N−acetylgalactosaminide alpha−2,6−sialyltransferase 3 on eGFR
MR Method
Inverse variance weighted
MR Egger
700
600
500
400
300
$1/SE_{IV}$
−0.006
−0.003
0.000
0.003
0.006
β_{IV}

Batch 2−2855 : Alpha−N−acetylgalactosaminide alpha−2,6−sialyltransferase 3 on eGFR

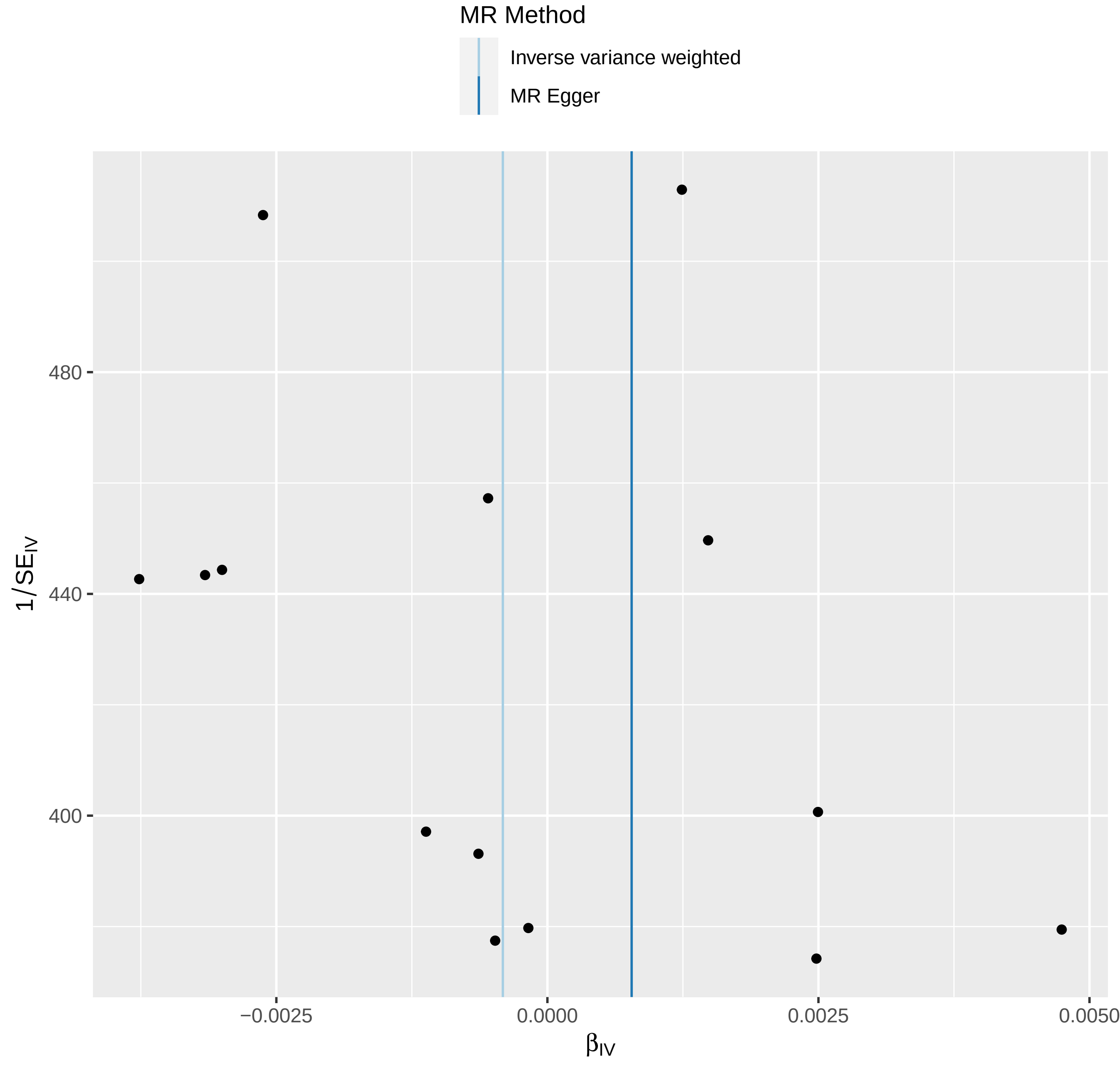

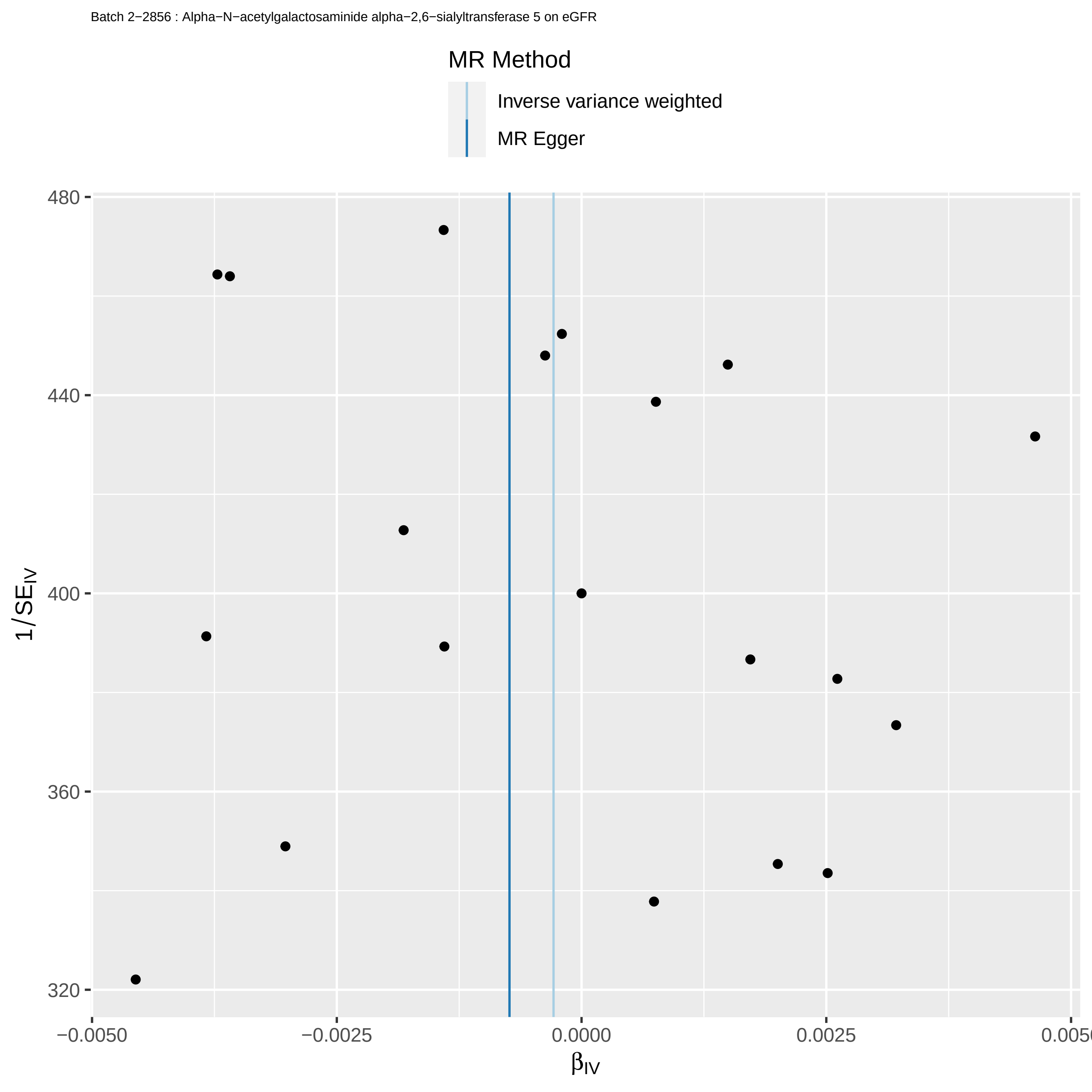

Batch 2−2856 : Alpha−N−acetylgalactosaminide alpha−2,6−sialyltransferase 5 on eGFR
MR Method
Inverse variance weighted
MR Egger
480
440
400
360
320
1/SE$_{IV}$
−0.0050
−0.0025
0.0000
0.0025
0.0050
β$_{IV}$

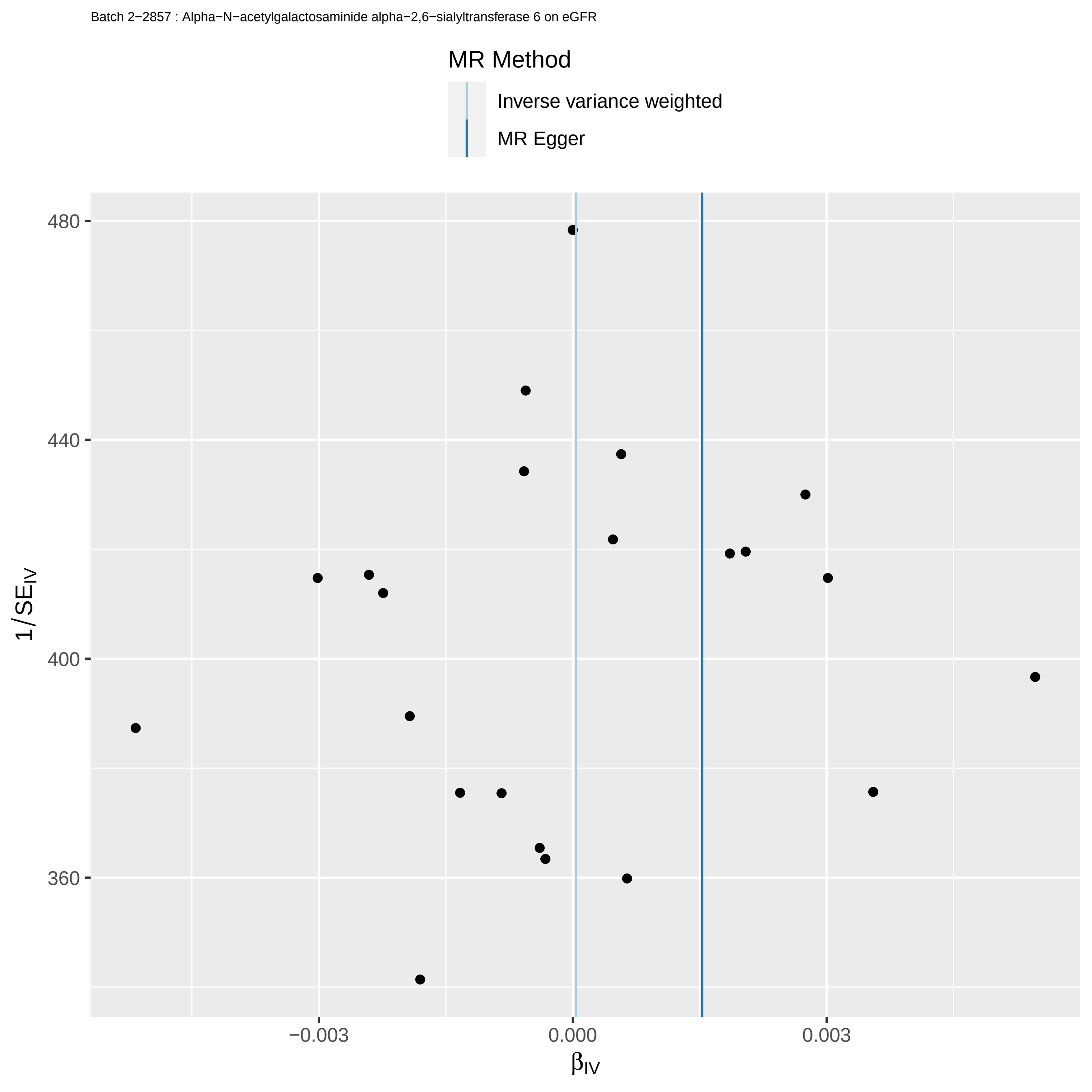
Batch 2−2857 : Alpha−N−acetylgalactosaminide alpha−2,6−sialyltransferase 6 on eGFR
MR Method
Inverse variance weighted
MR Egger
480
440
400
360
$1/SE_{IV}$
−0.003
0.000
0.003
β_{IV}

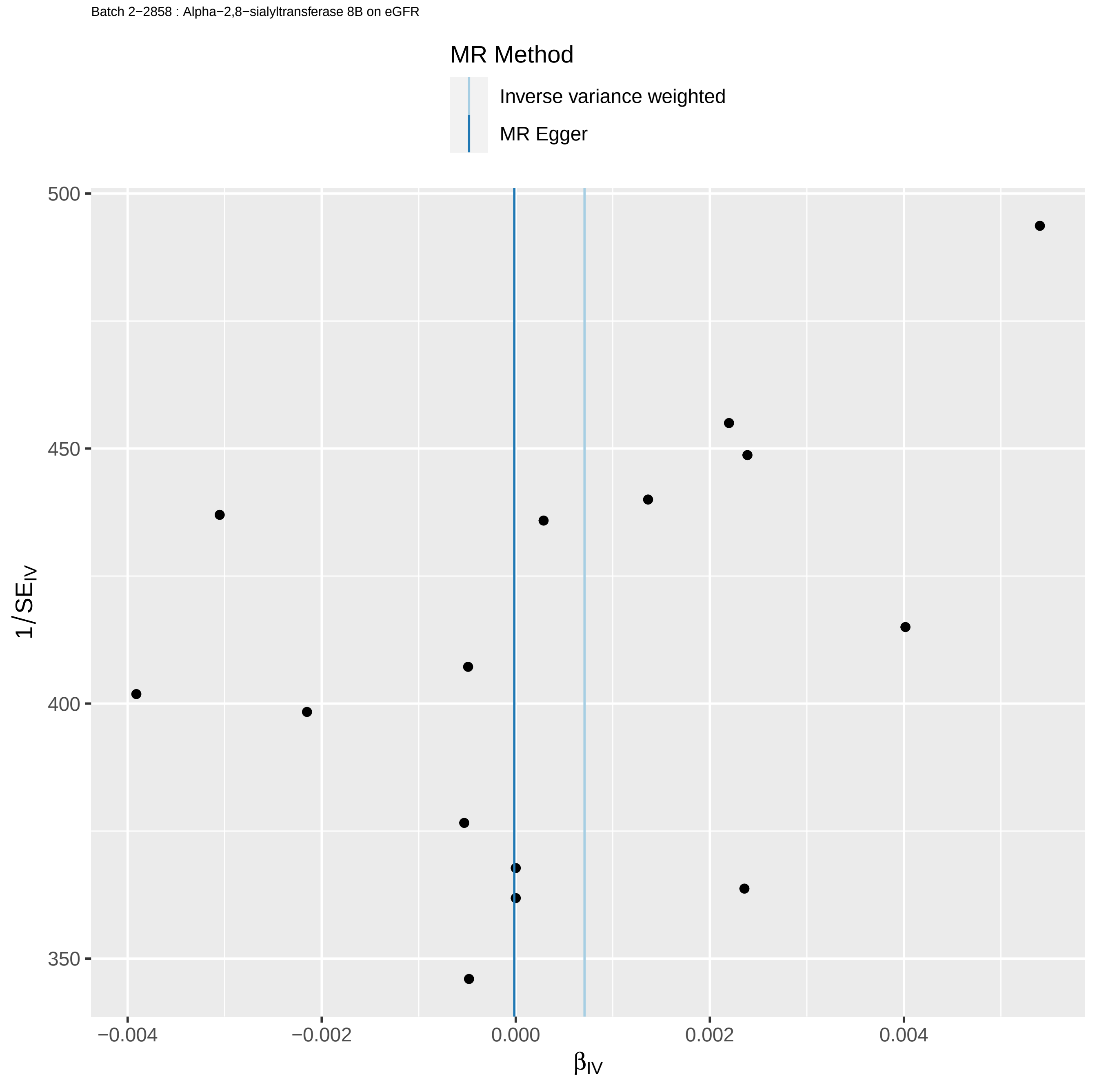

Batch 2−2858 : Alpha−2,8−sialyltransferase 8B on eGFR
MR Method
Inverse variance weighted
MR Egger
500
450
400
350
1/SE_IV
−0.004
−0.002
0.000
0.002
0.004
β_IV

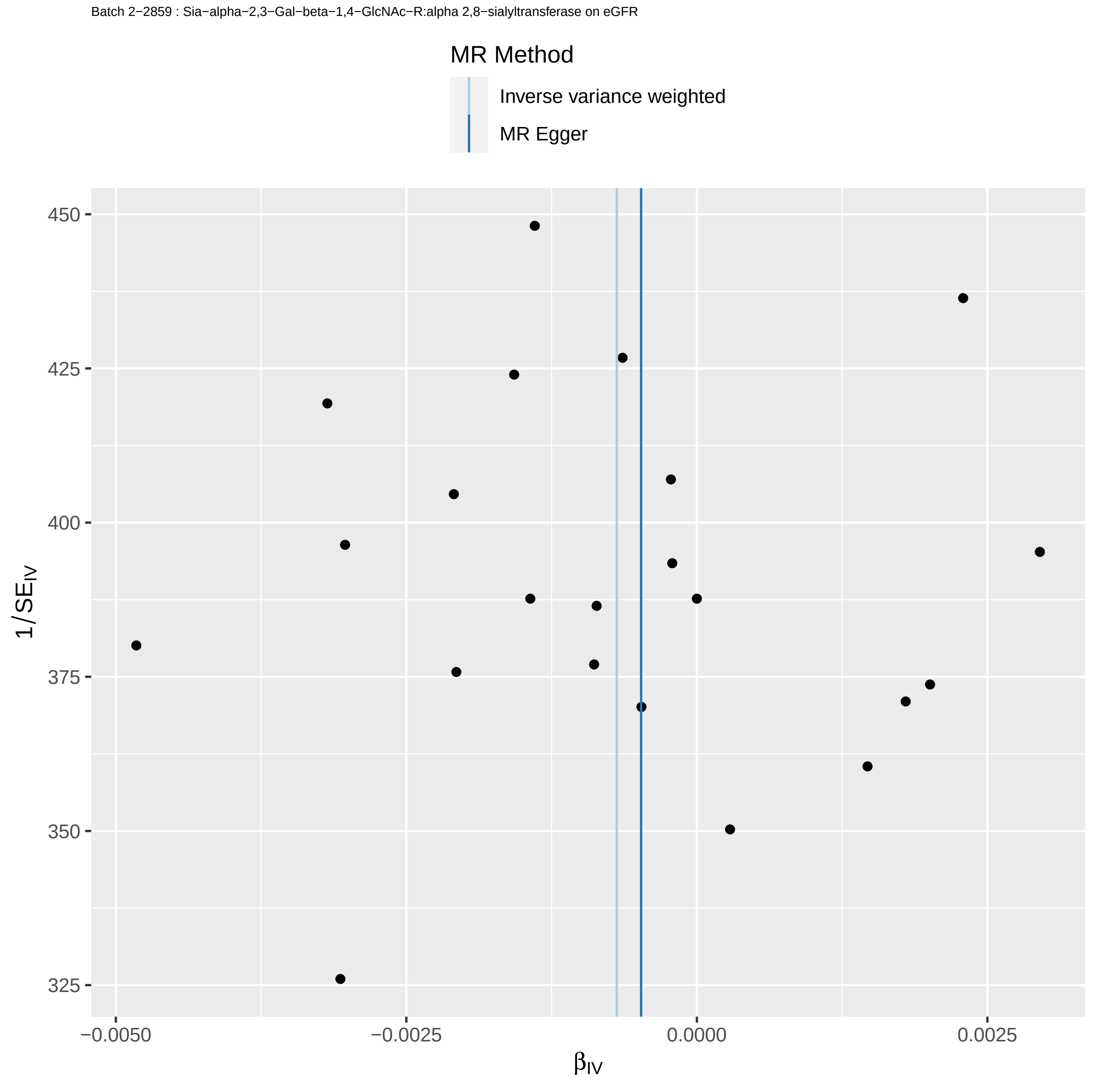
Batch 2−2859 : Sia−alpha−2,3−Gal−beta−1,4−GlcNAc−R:alpha 2,8−sialyltransferase on eGFR
MR Method
Inverse variance weighted
MR Egger
450
425
400
375
350
325
1/SE_IV
−0.0050
−0.0025
0.0000
0.0025
β_IV

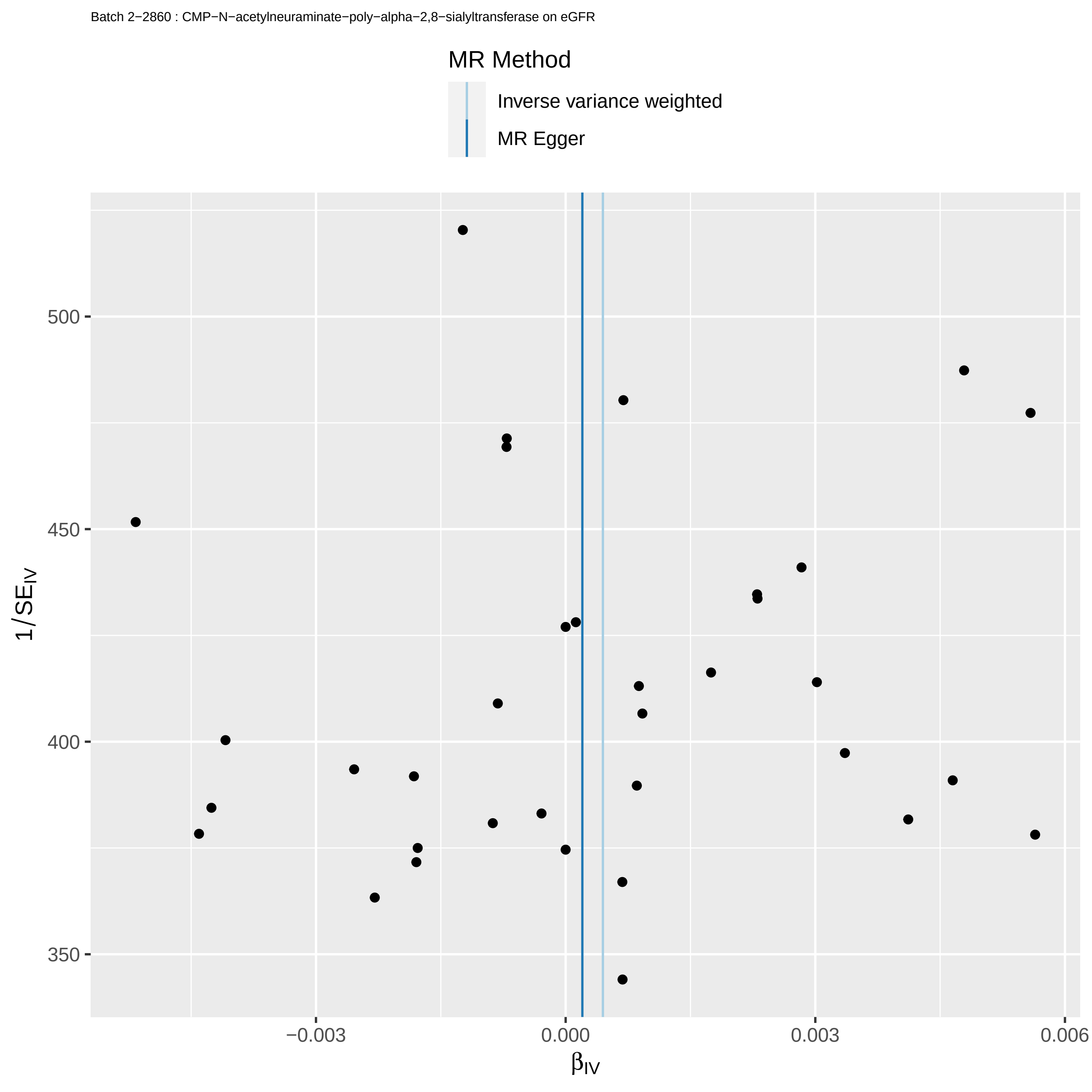
Batch 2−2860 : CMP−N−acetylneuraminate−poly−alpha−2,8−sialyltransferase on eGFR
MR Method
Inverse variance weighted
MR Egger
500
450
400
350
1/SE_IV
−0.003
0.000
0.003
0.006
β_IV

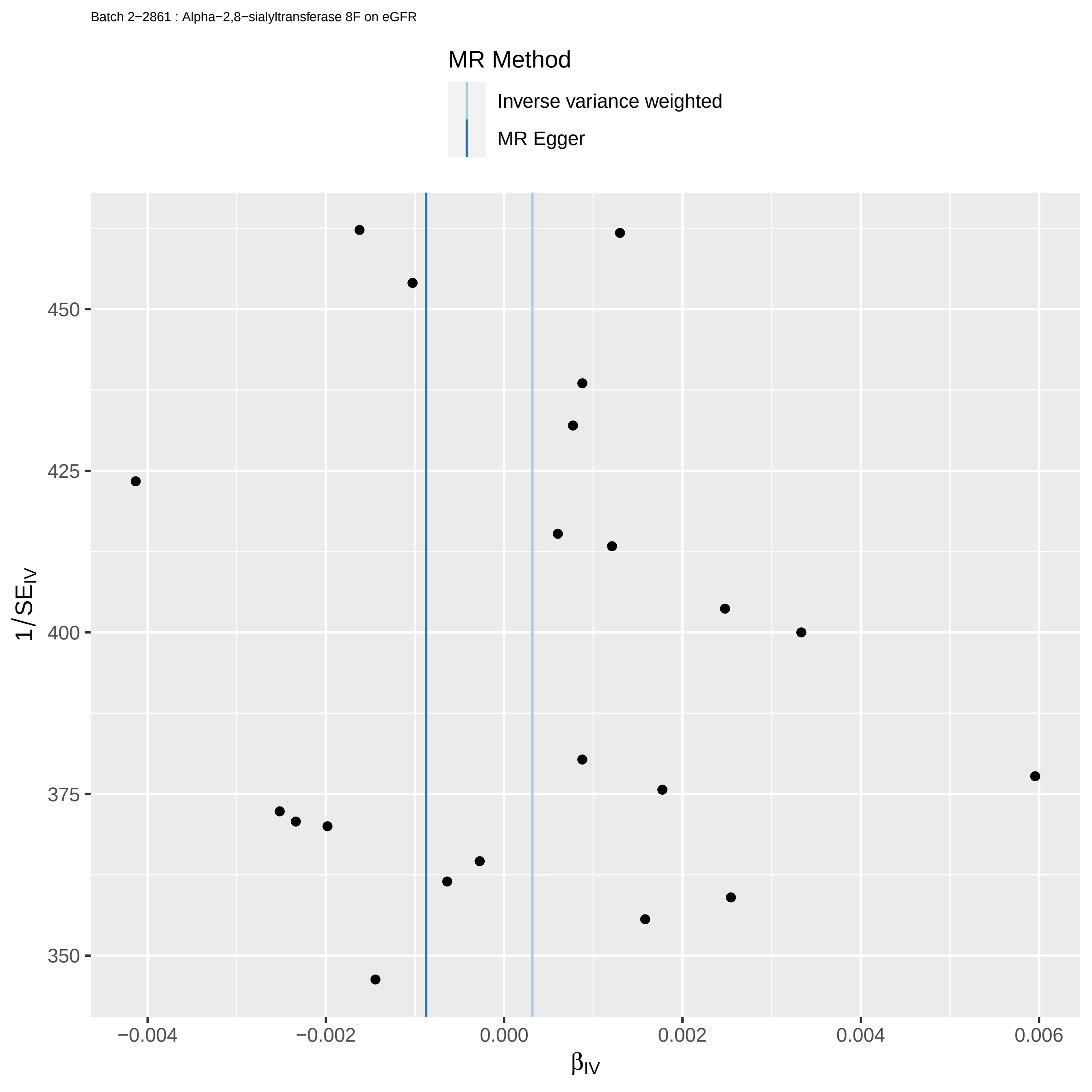
Batch 2−2861 : Alpha−2,8−sialyltransferase 8F on eGFR
MR Method
Inverse variance weighted
MR Egger
450
425
400
375
350
1/SE_IV
−0.004
−0.002
0.000
0.002
0.004
0.006
β_IV

Batch 2−2862 : Stabilin−1 on eGFR

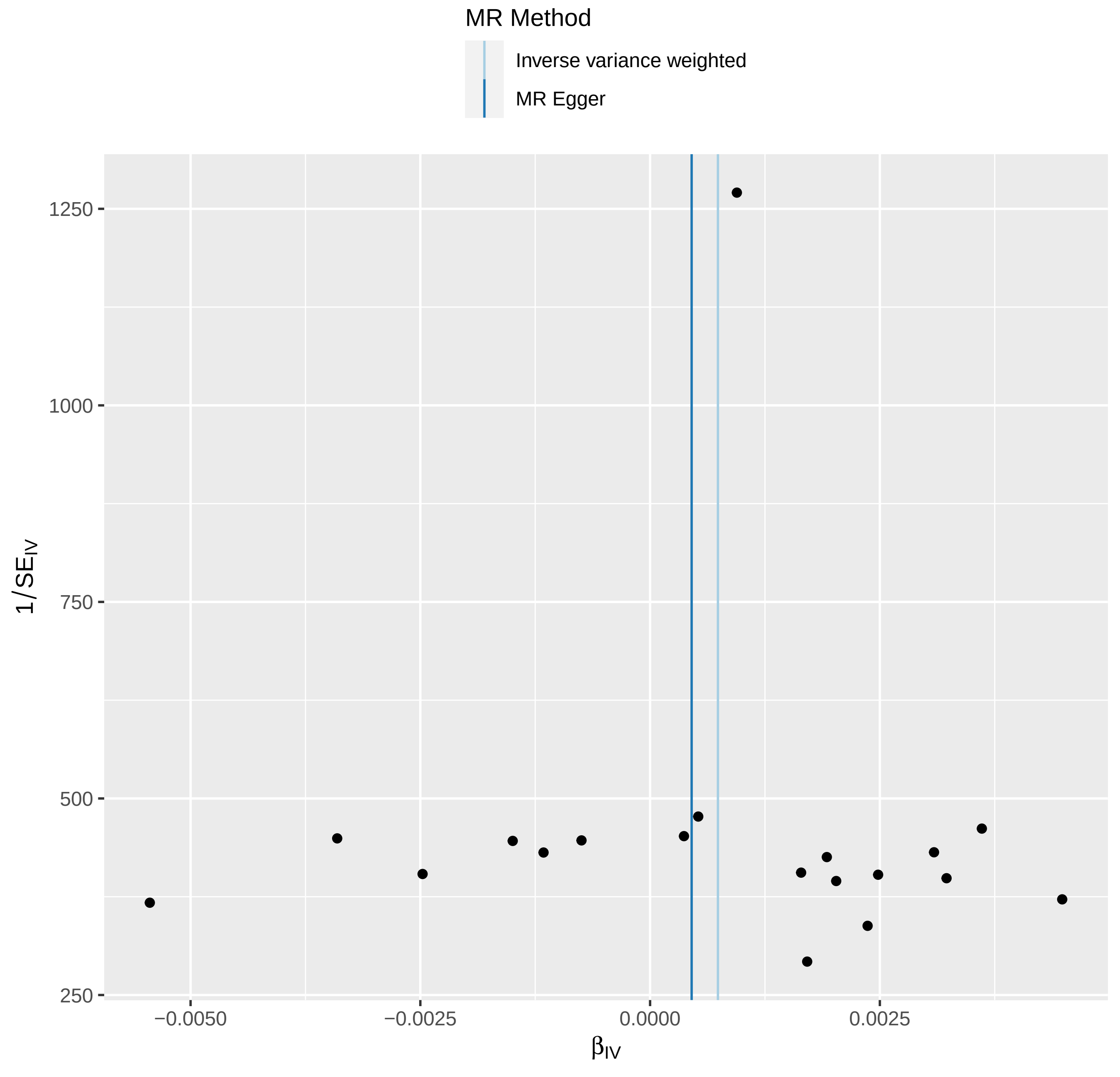

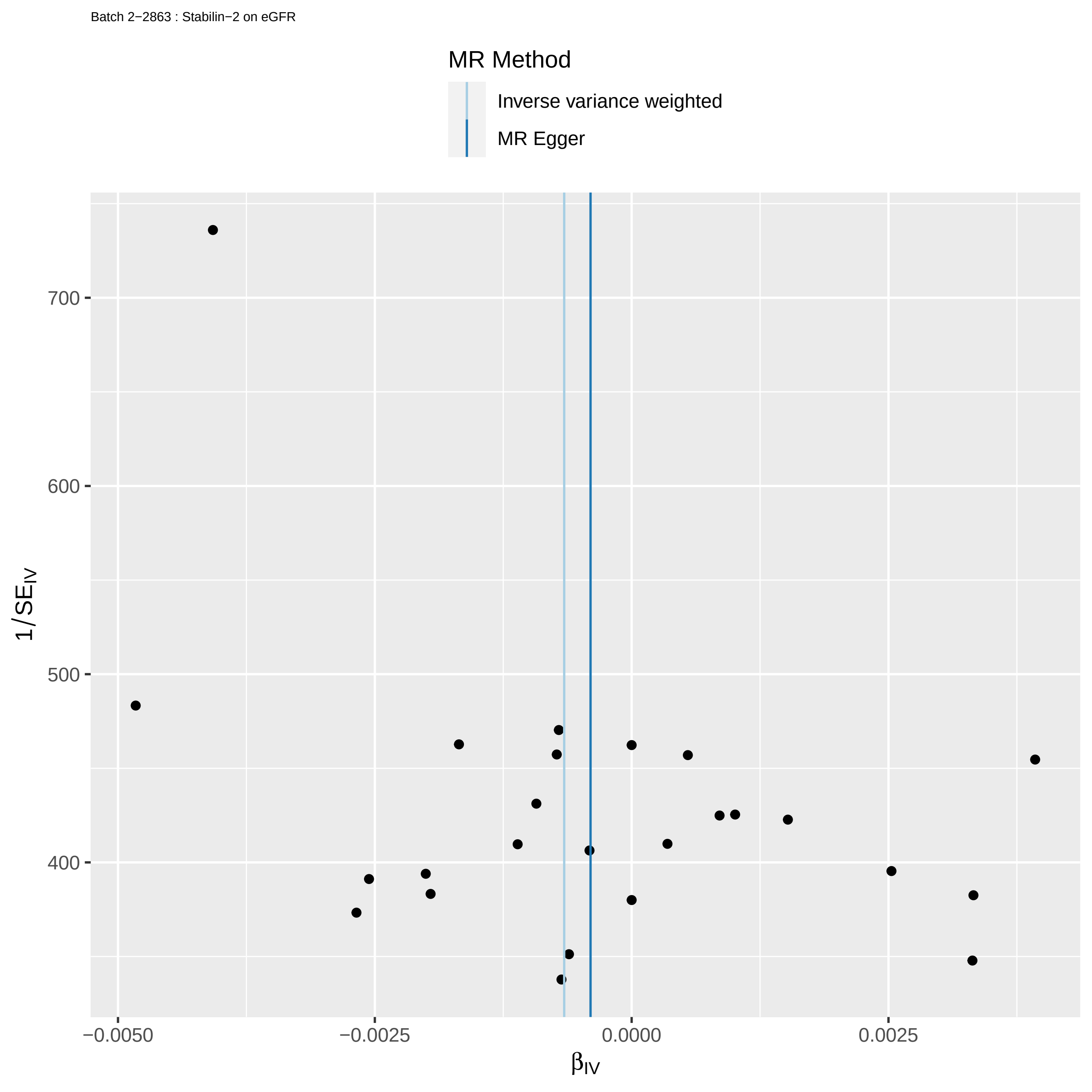

Batch 2−2863 : Stabilin−2 on eGFR
MR Method
Inverse variance weighted
MR Egger
700
600
500
400
1/SE_IV
−0.0050
−0.0025
0.0000
0.0025
β_IV

Batch 2−2864 : AMSH−like protease on eGFR

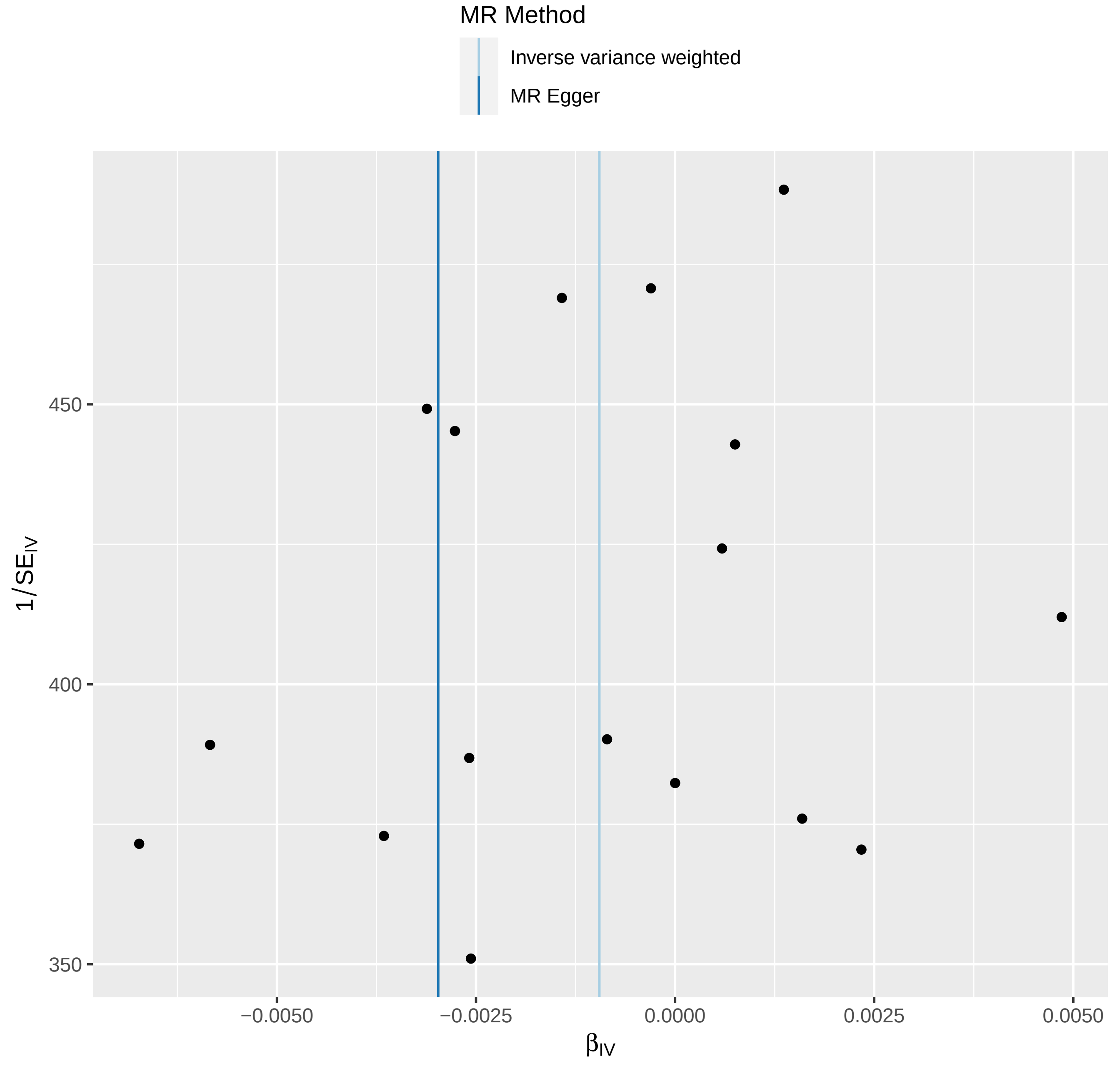

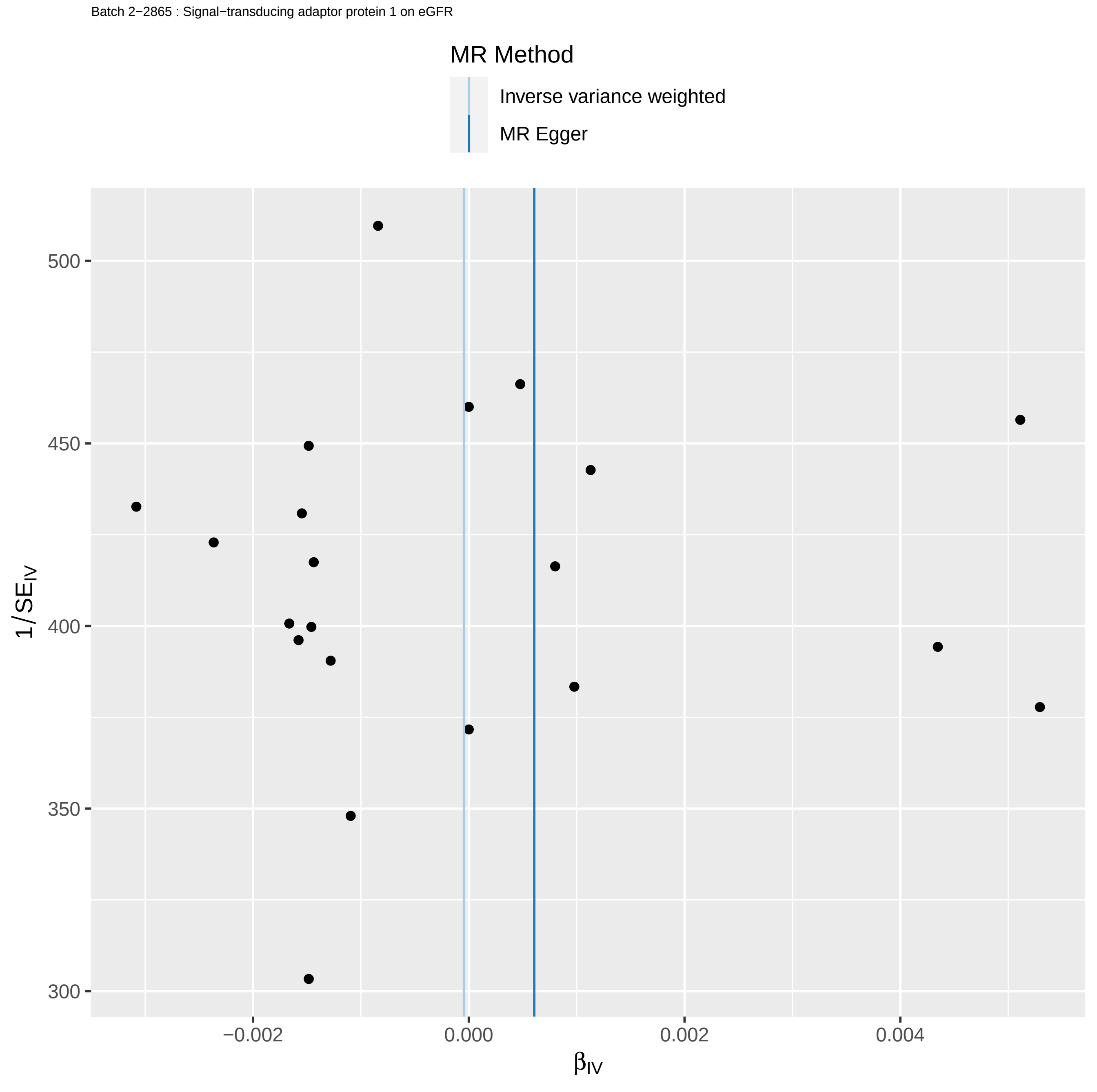
Batch 2−2865 : Signal−transducing adaptor protein 1 on eGFR
MR Method
Inverse variance weighted
MR Egger
500
450
400
350
300
1/SE_IV
−0.002
0.000
0.002
0.004
β_IV

Batch 2−2866 : Steroidogenic acute regulatory protein, mitochondrial on eGFR

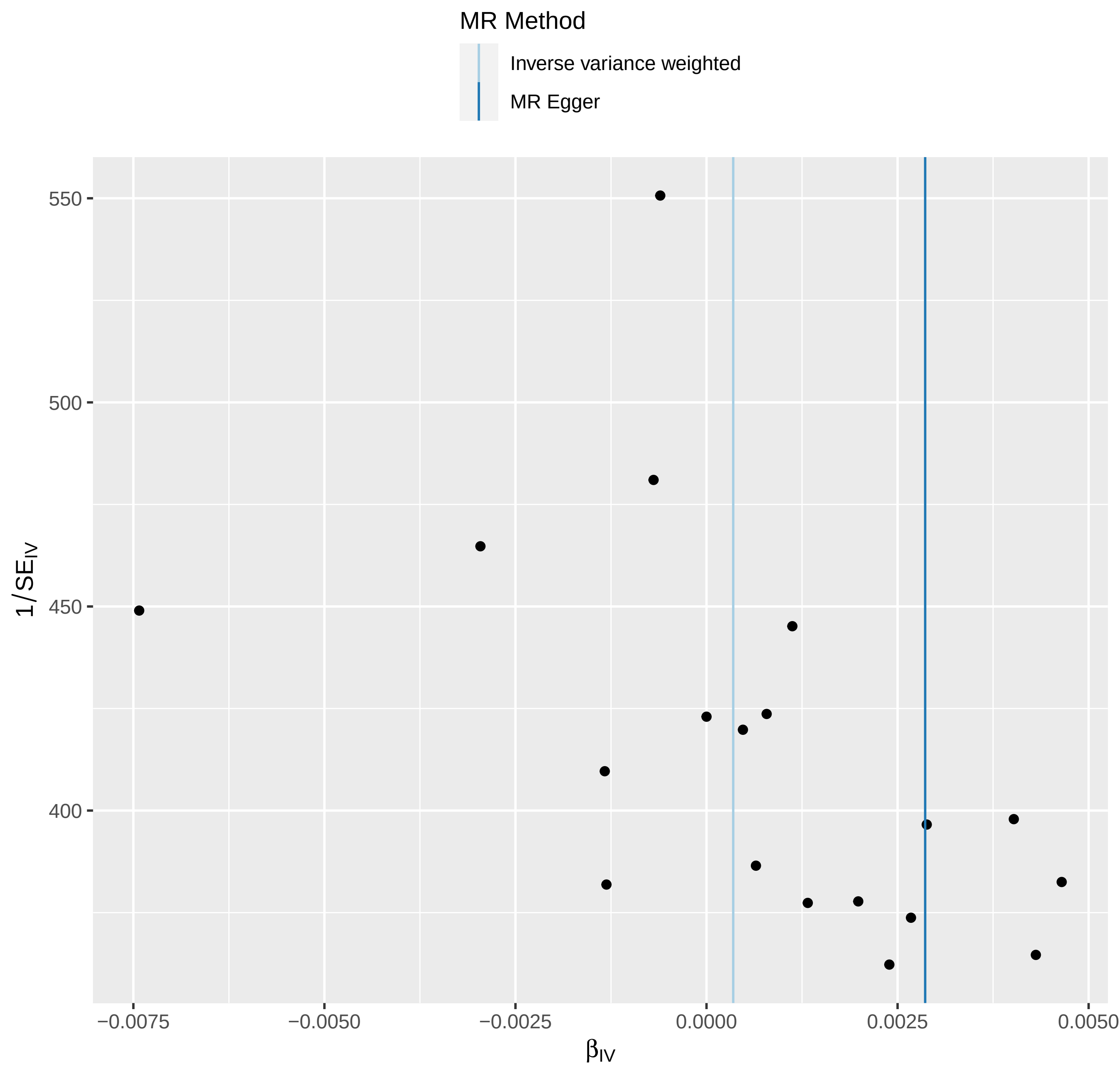

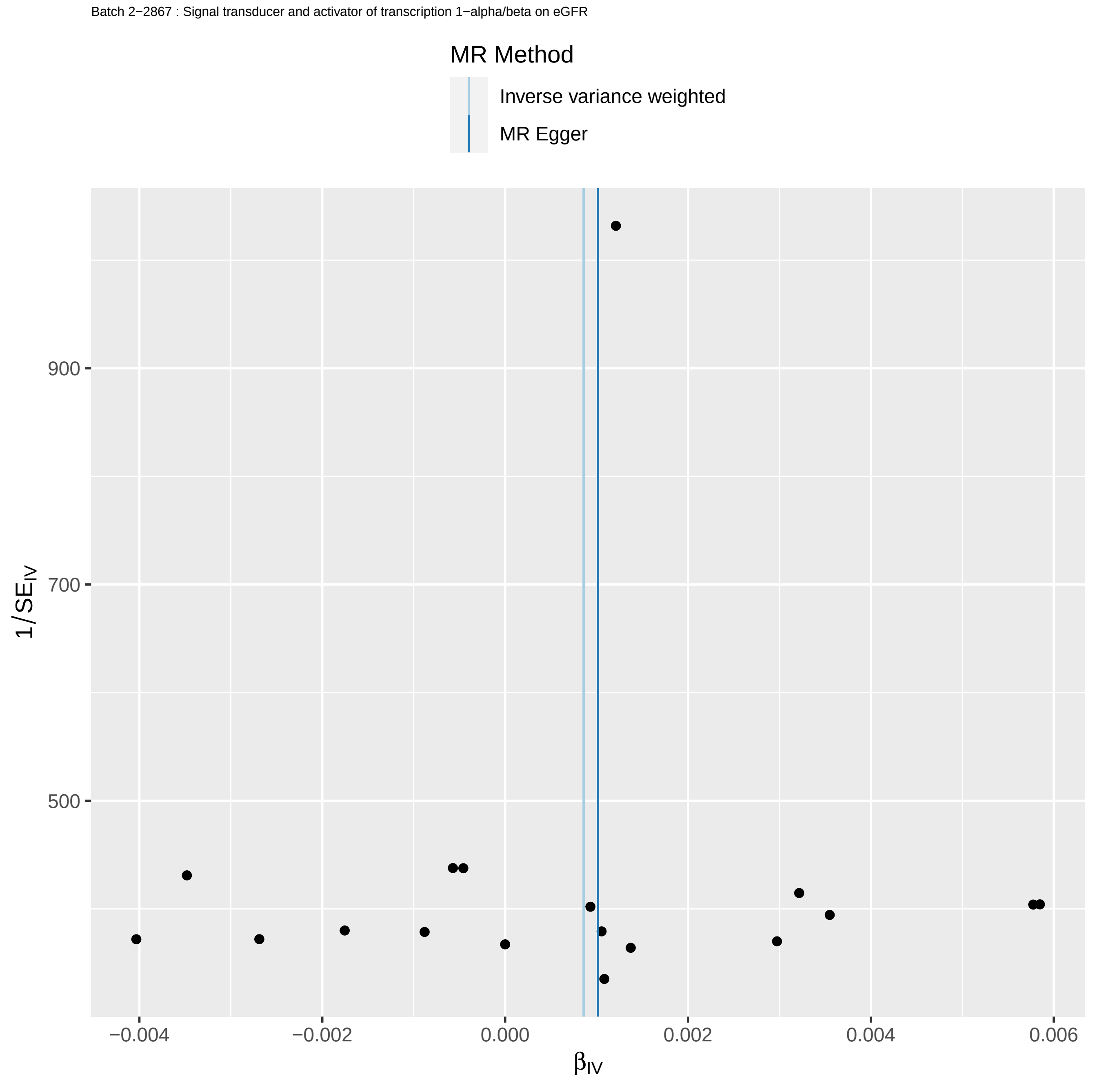

Batch 2−2867 : Signal transducer and activator of transcription 1−alpha/beta on eGFR
MR Method
Inverse variance weighted
MR Egger
900
700
500
$1/SE_{IV}$
−0.004
−0.002
0.000
0.002
0.004
0.006
β_{IV}

Batch 2−2868 : Signal transducer and activator of transcription 1−alpha/beta on eGFR

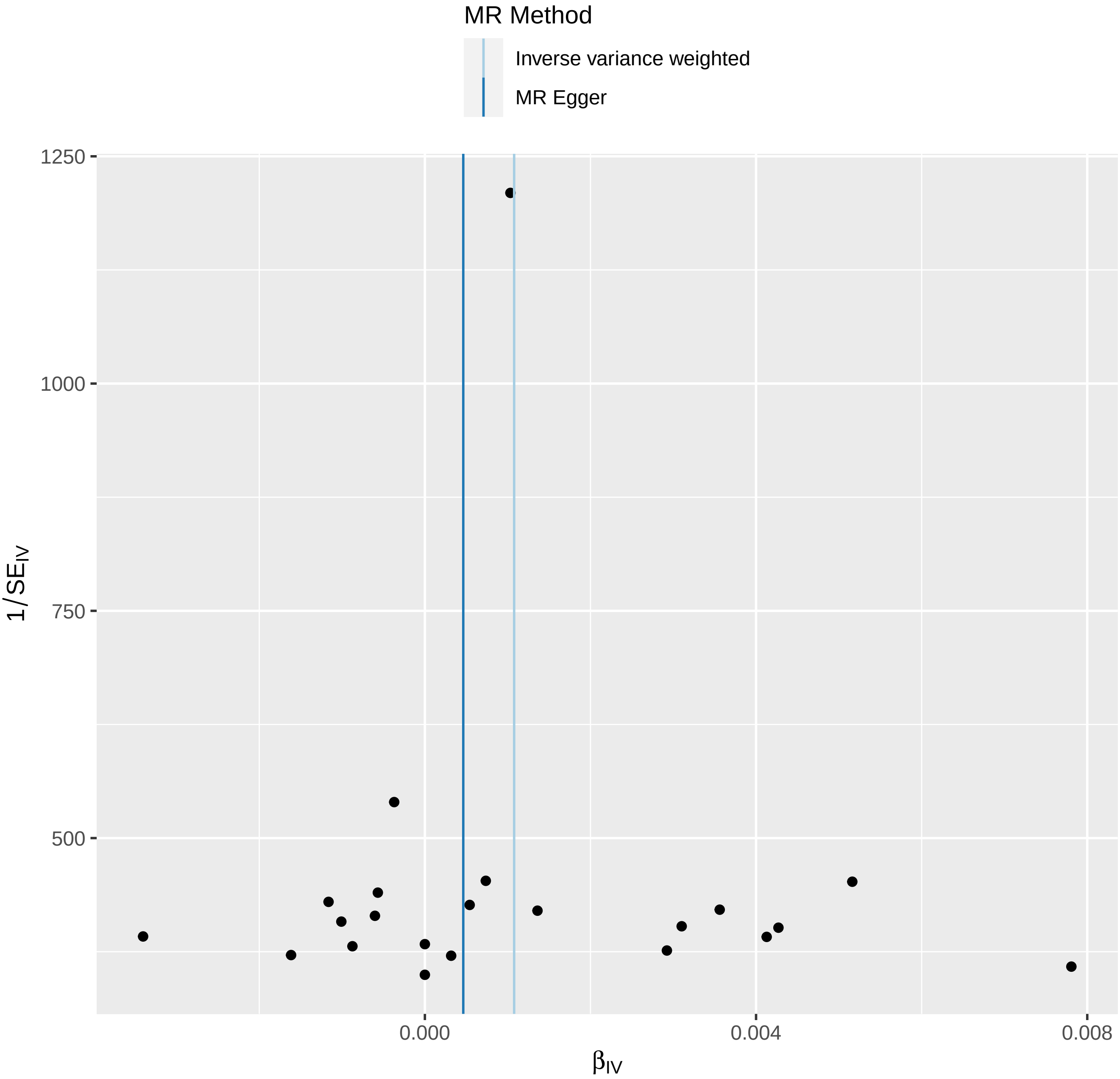

Batch 2−2869 : Signal transducer and activator of transcription 3 on eGFR

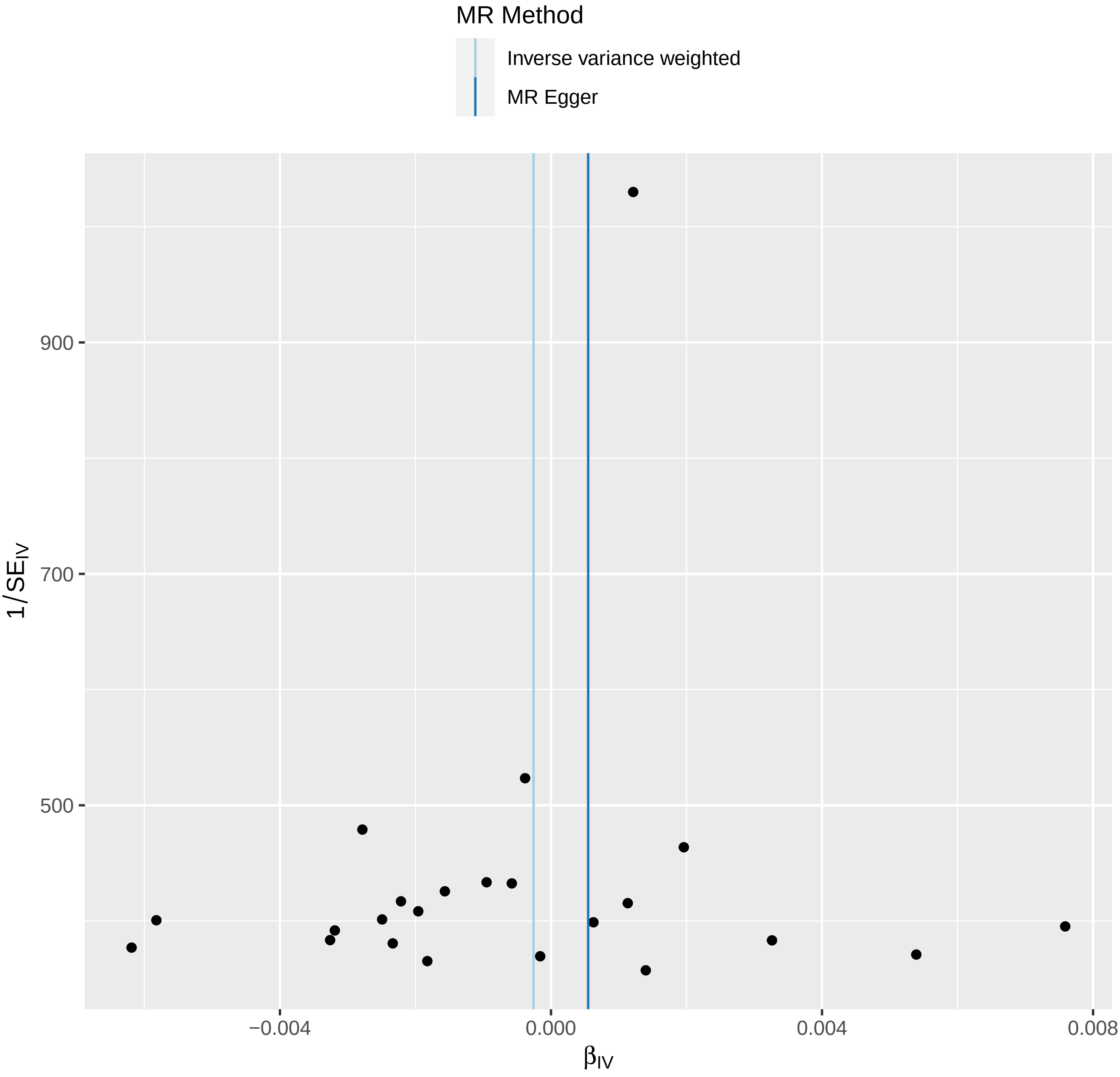

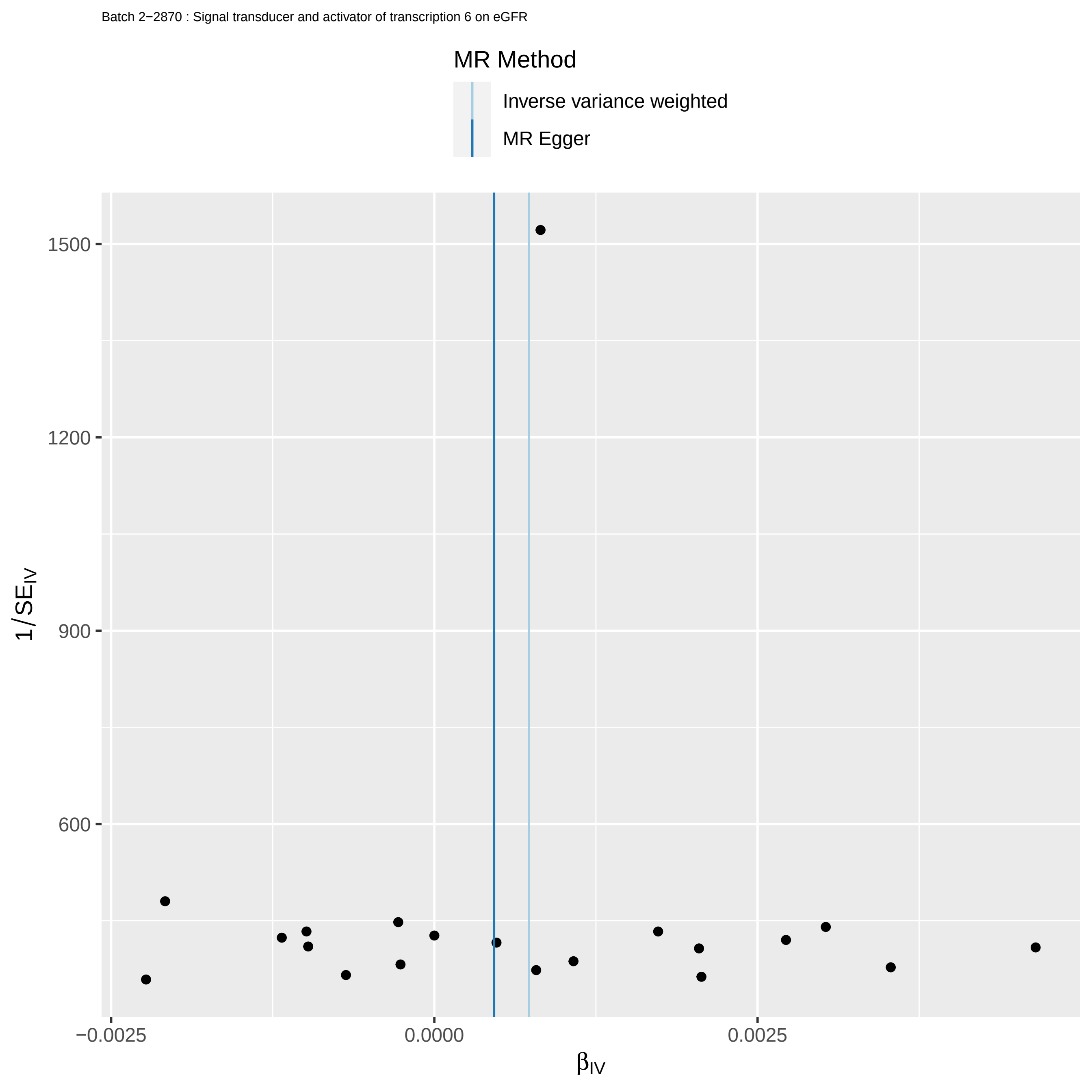

Batch 2−2870 : Signal transducer and activator of transcription 6 on eGFR
MR Method
Inverse variance weighted
MR Egger
1500
1200
900
600
1/SE_IV
−0.0025
0.0000
0.0025
β_IV

Batch 2−2871 : Double−stranded RNA−binding protein Staufen homolog 1 on eGFR

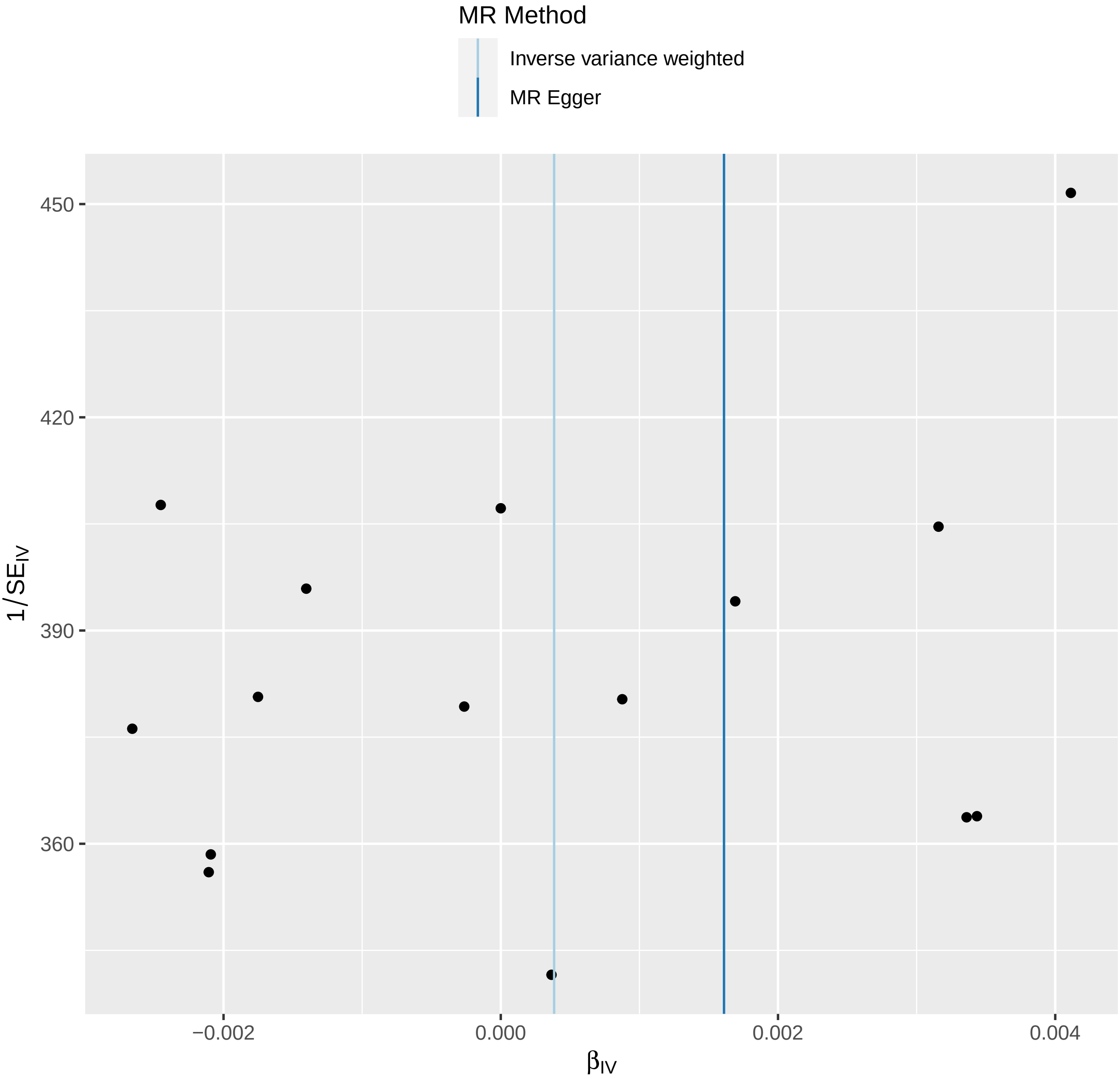

Batch 2−2872 : Double−stranded RNA−binding protein Staufen homolog 2 on eGFR

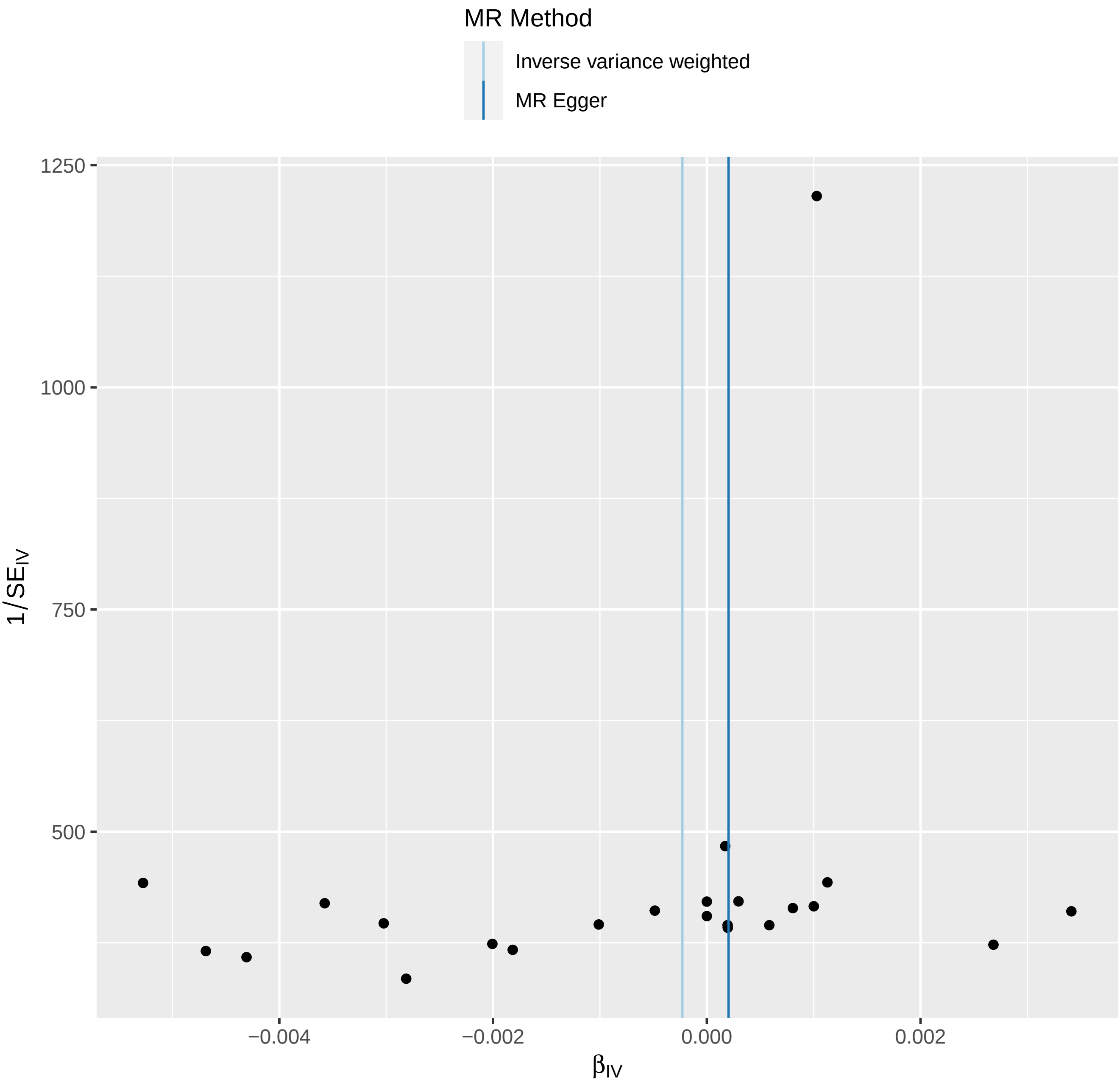

Batch 2−2873 : Stanniocalcin−1 on eGFR

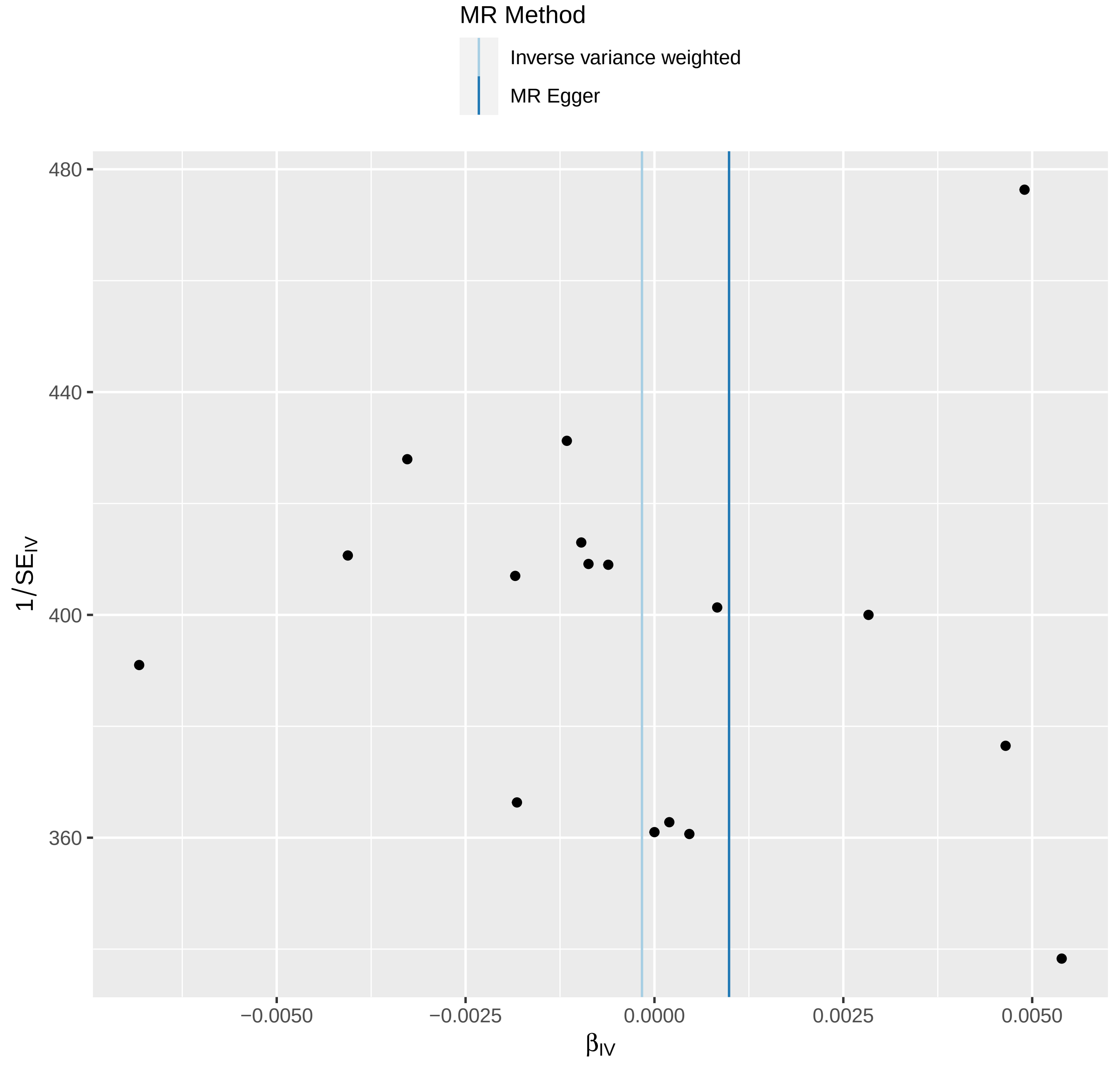

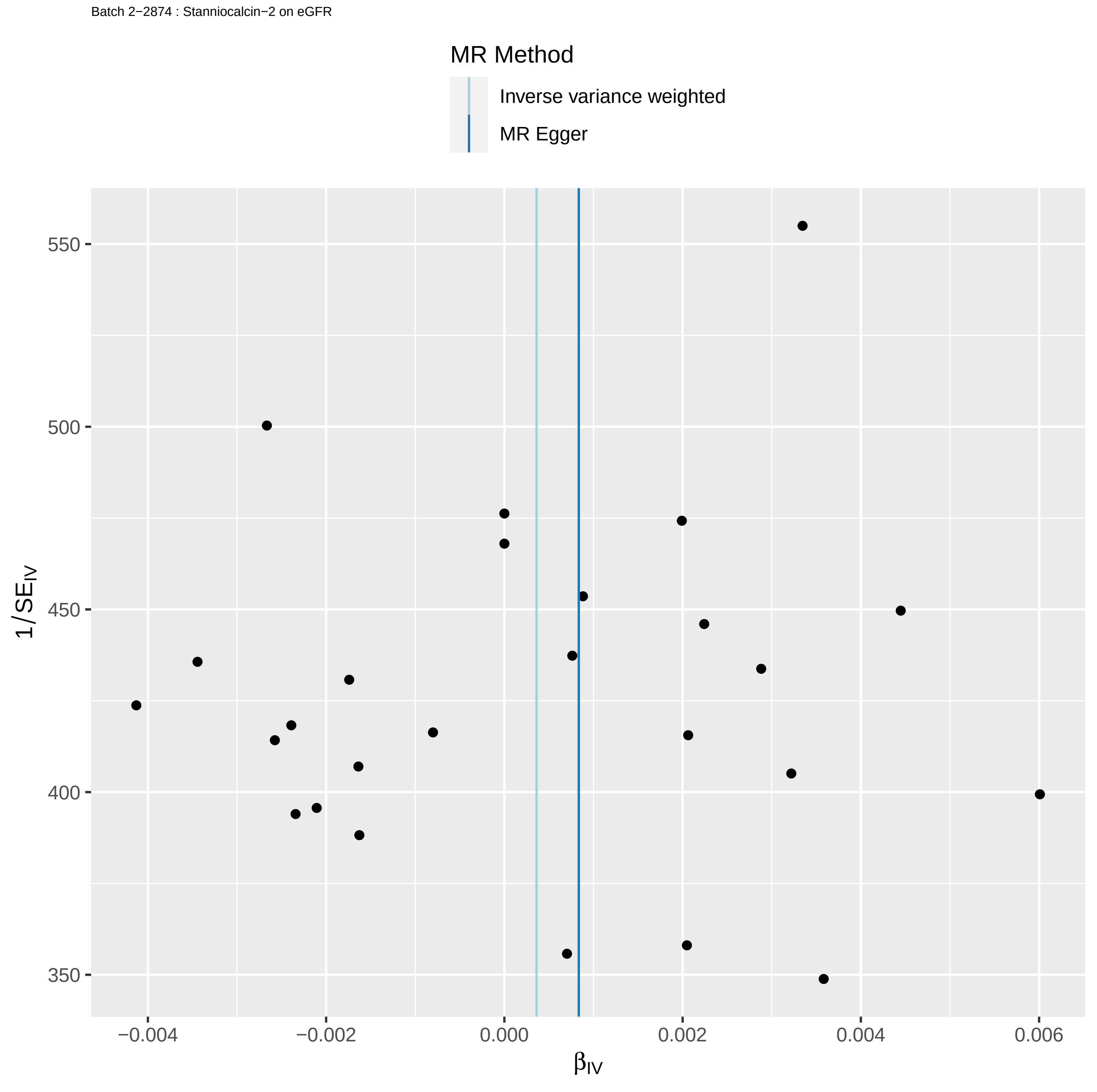

Batch 2−2874 : Stanniocalcin−2 on eGFR
MR Method
Inverse variance weighted
MR Egger
550
500
450
400
350
1/SE$_{IV}$
−0.004
−0.002
0.000
0.002
0.004
0.006
β_{IV}

Batch 2−2875 : Stromal interaction molecule 1 on eGFR

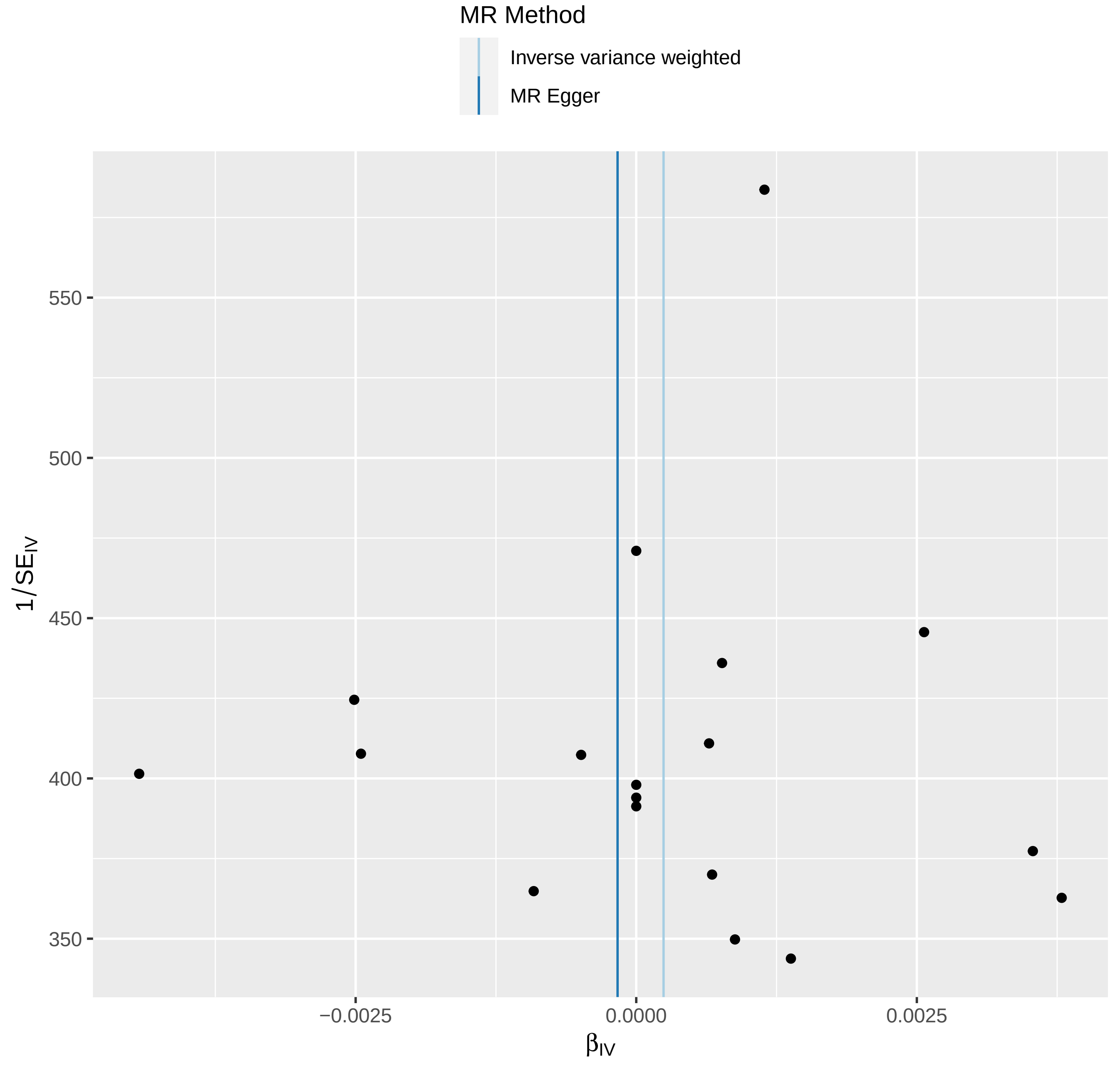

Batch 2−2876 : Stress−induced−phosphoprotein 1 on eGFR

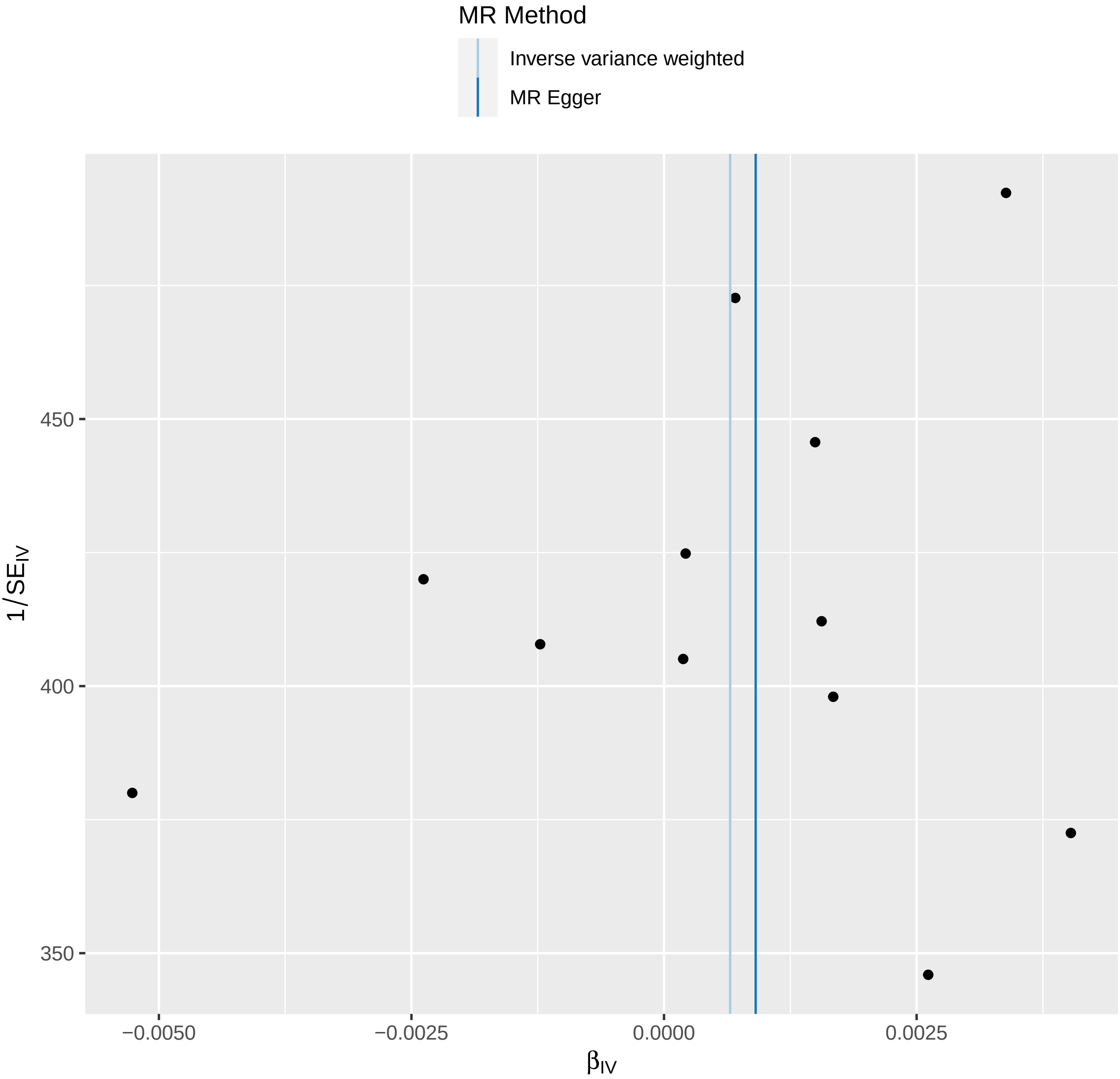

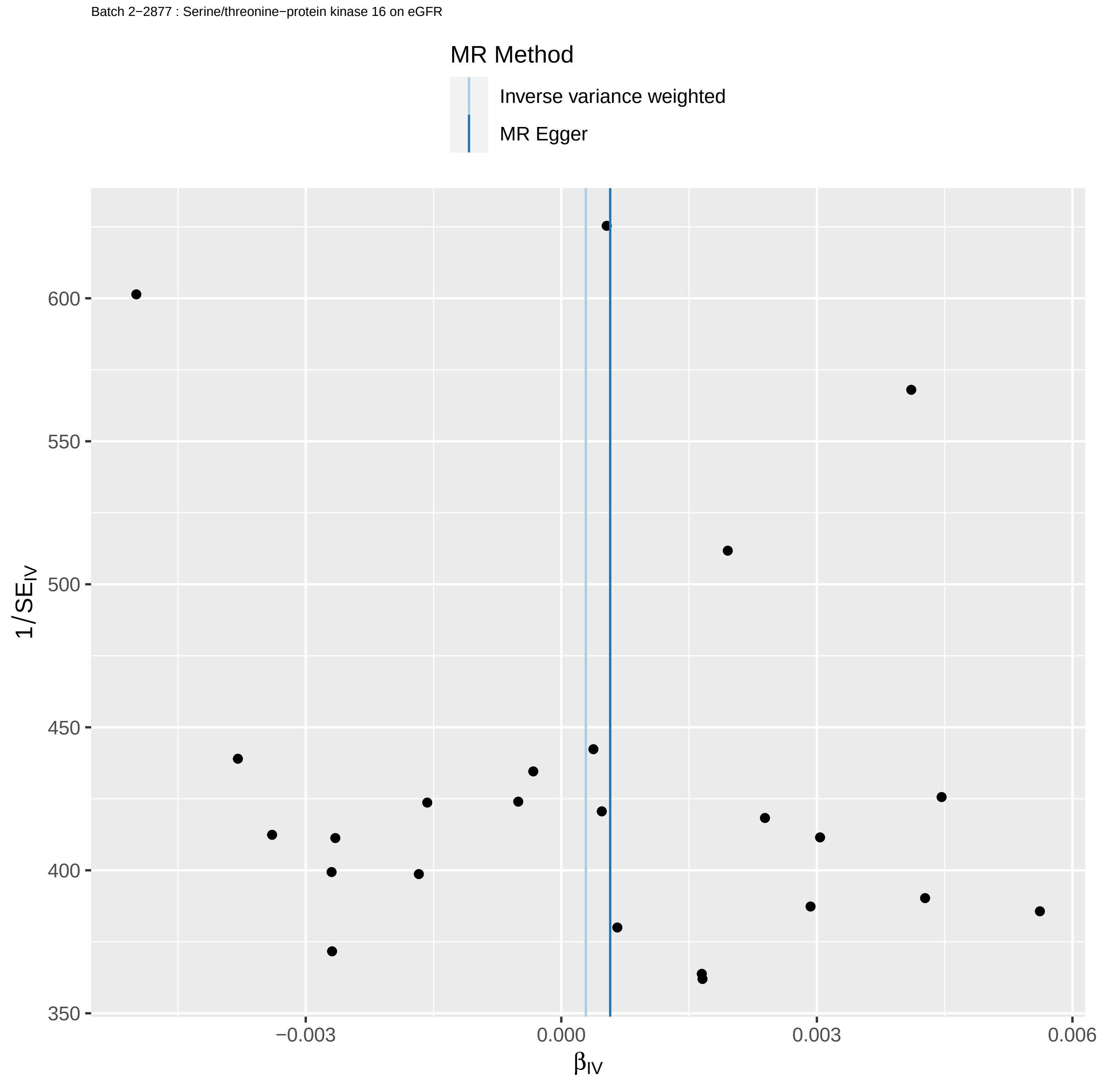

Batch 2−2877 : Serine/threonine−protein kinase 16 on eGFR
MR Method
Inverse variance weighted
MR Egger
600
550
500
450
400
350
1/SE_IV
−0.003
0.000
0.003
0.006
β_IV

Batch 2−2878 : Serine/threonine−protein kinase 17B on eGFR

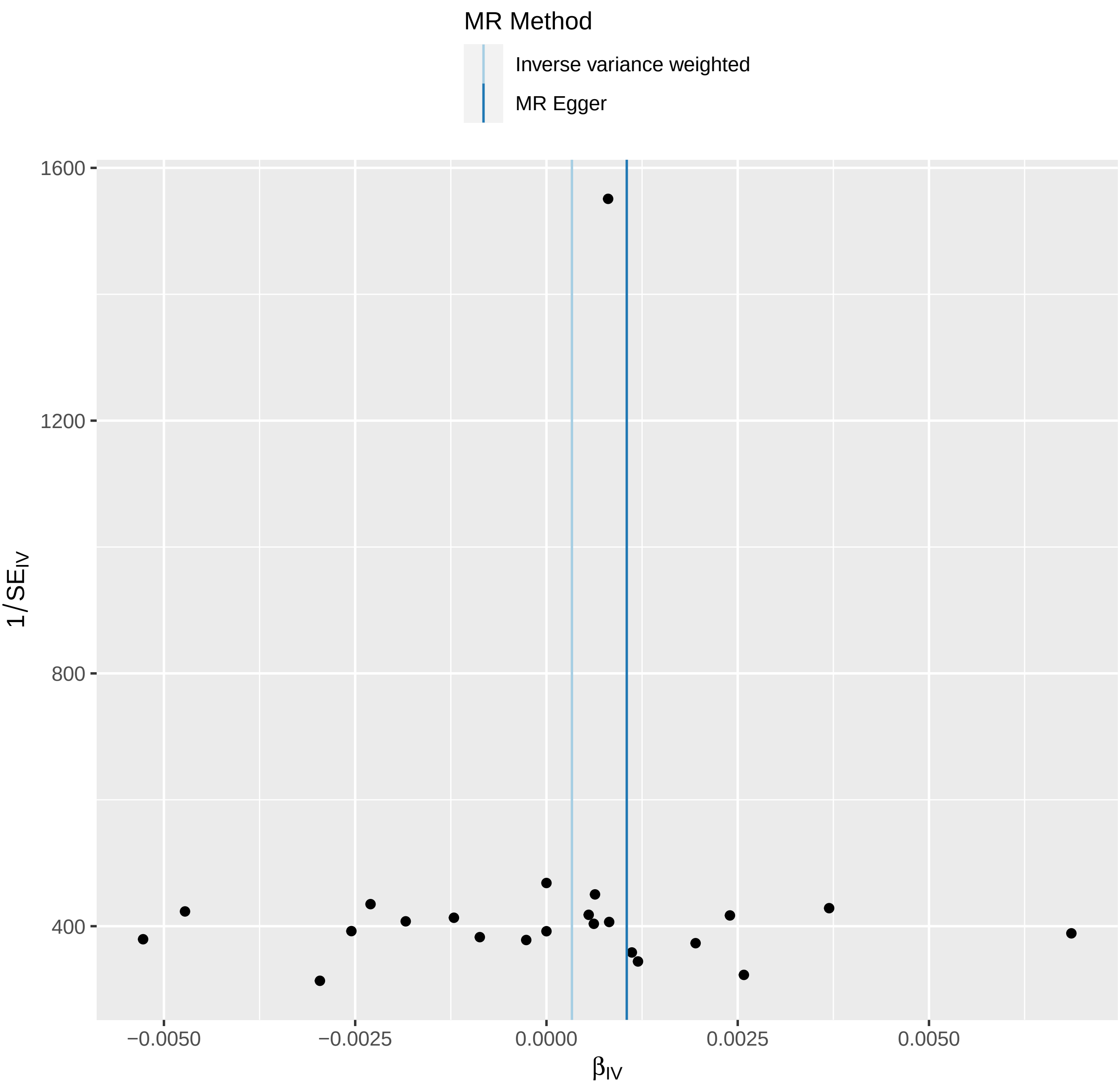

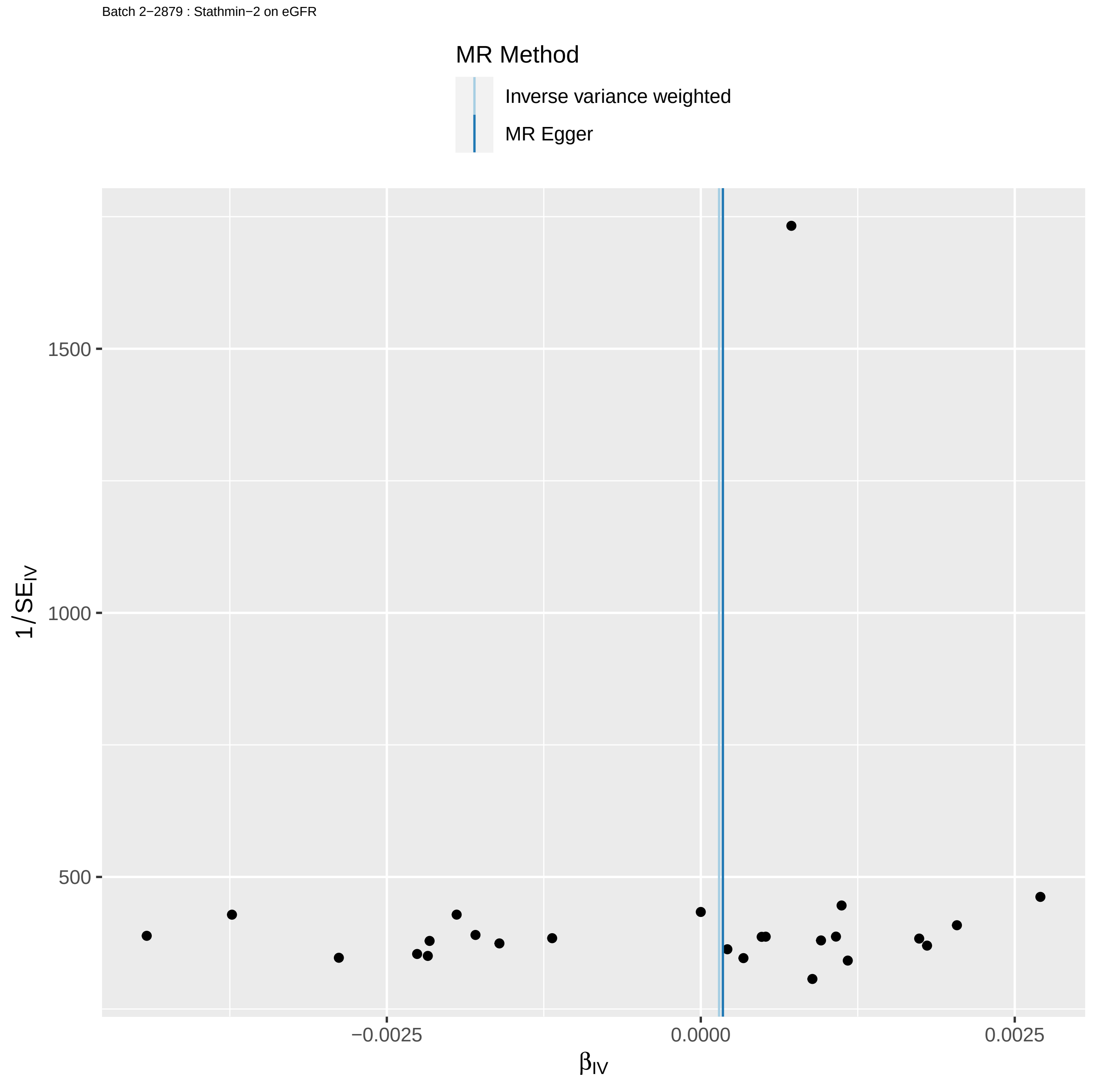
Batch 2−2879 : Stathmin−2 on eGFR
MR Method
Inverse variance weighted
MR Egger
1500
1000
500
1/SE$_{IV}$
−0.0025
0.0000
0.0025
β$_{IV}$

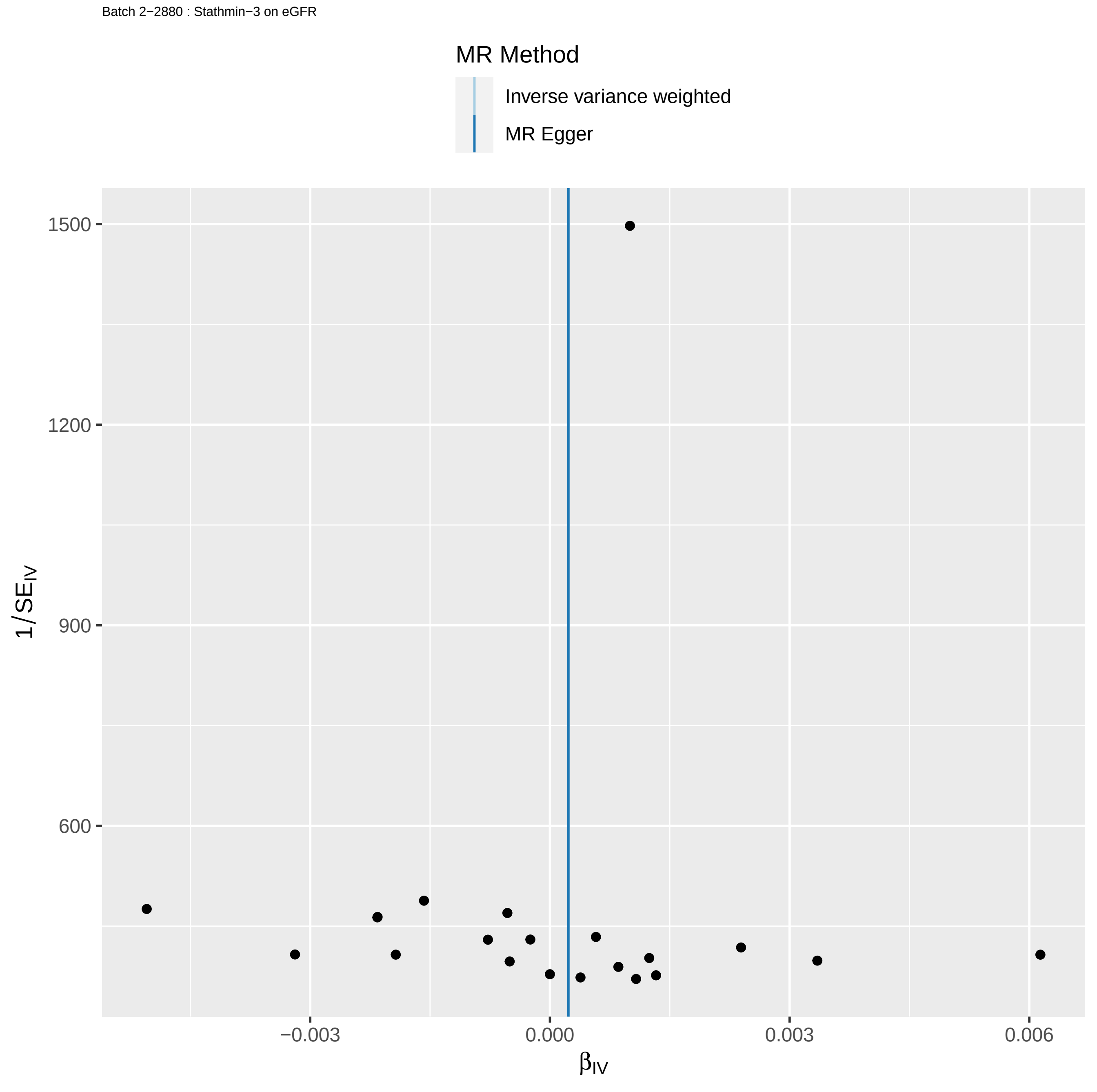

Batch 2−2880 : Stathmin−3 on eGFR
MR Method
Inverse variance weighted
MR Egger
1500
1200
900
600
1/SE_IV
−0.003
0.000
0.003
0.006
β_IV

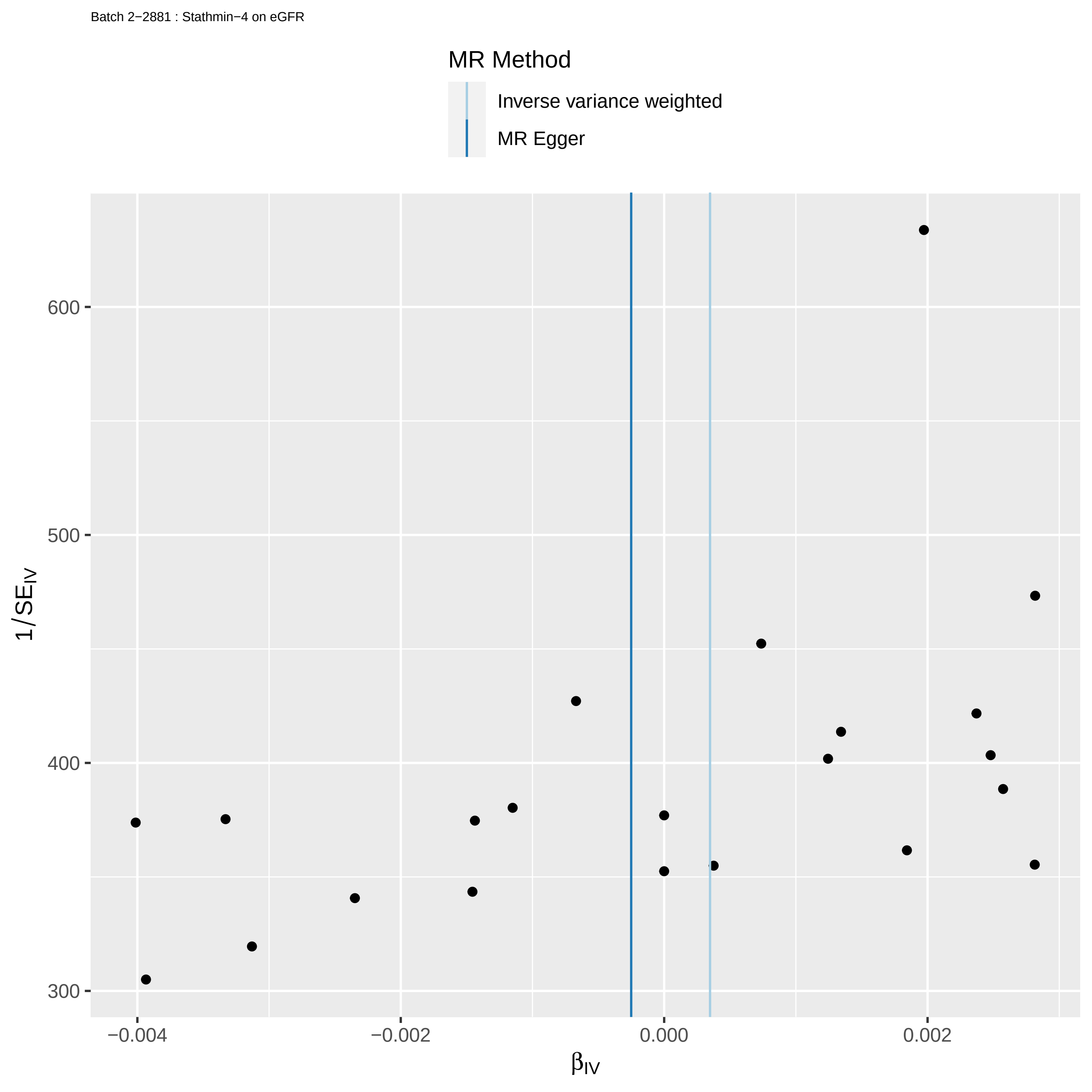

Batch 2−2881 : Stathmin−4 on eGFR
MR Method
Inverse variance weighted
MR Egger
600
500
400
300
1/SE_IV
−0.004
−0.002
0.000
0.002
β_IV

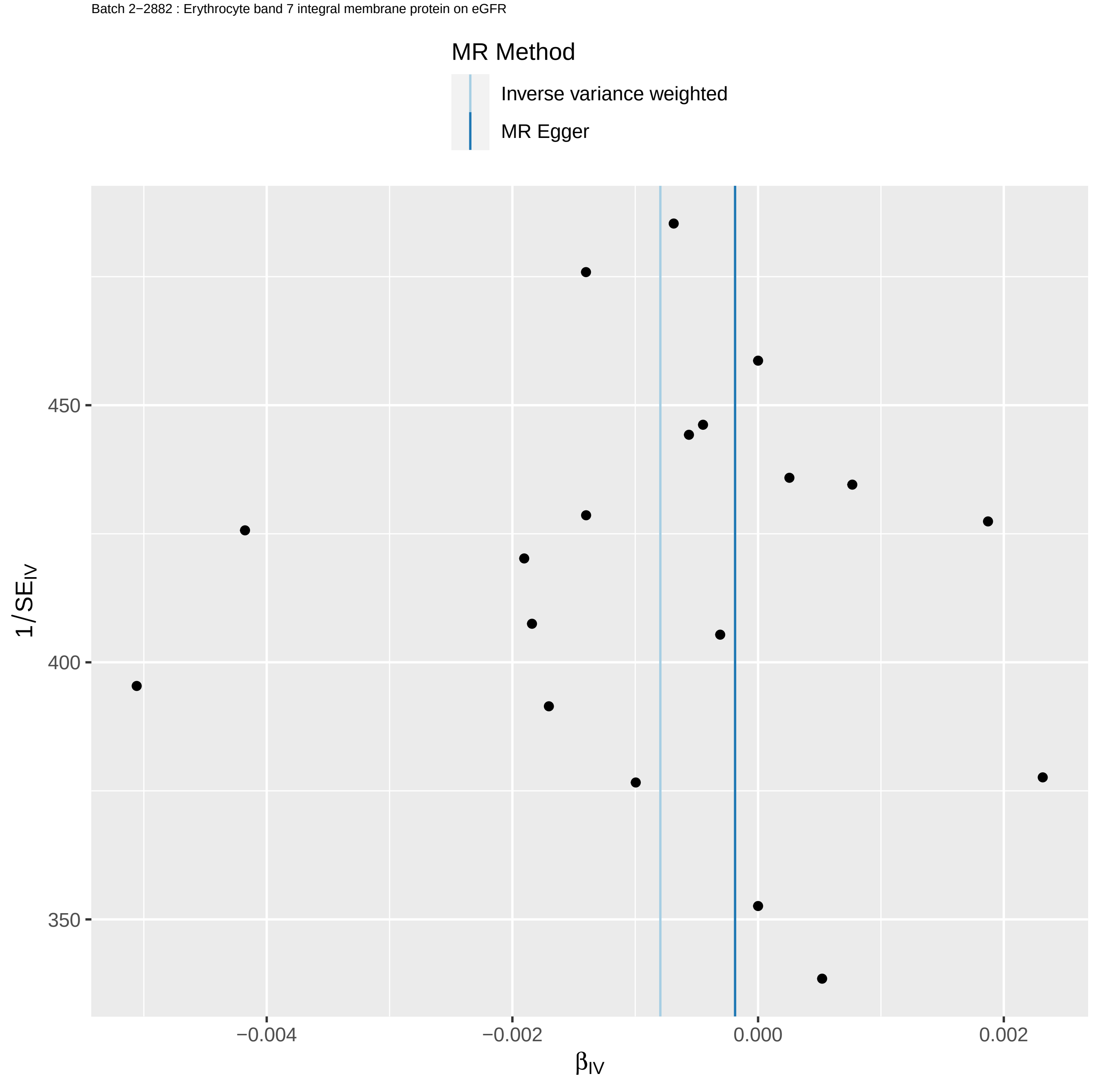

Batch 2−2882 : Erythrocyte band 7 integral membrane protein on eGFR
MR Method
Inverse variance weighted
MR Egger
450
400
350
1/SE$_{IV}$
−0.004
−0.002
0.000
0.002
β_{IV}

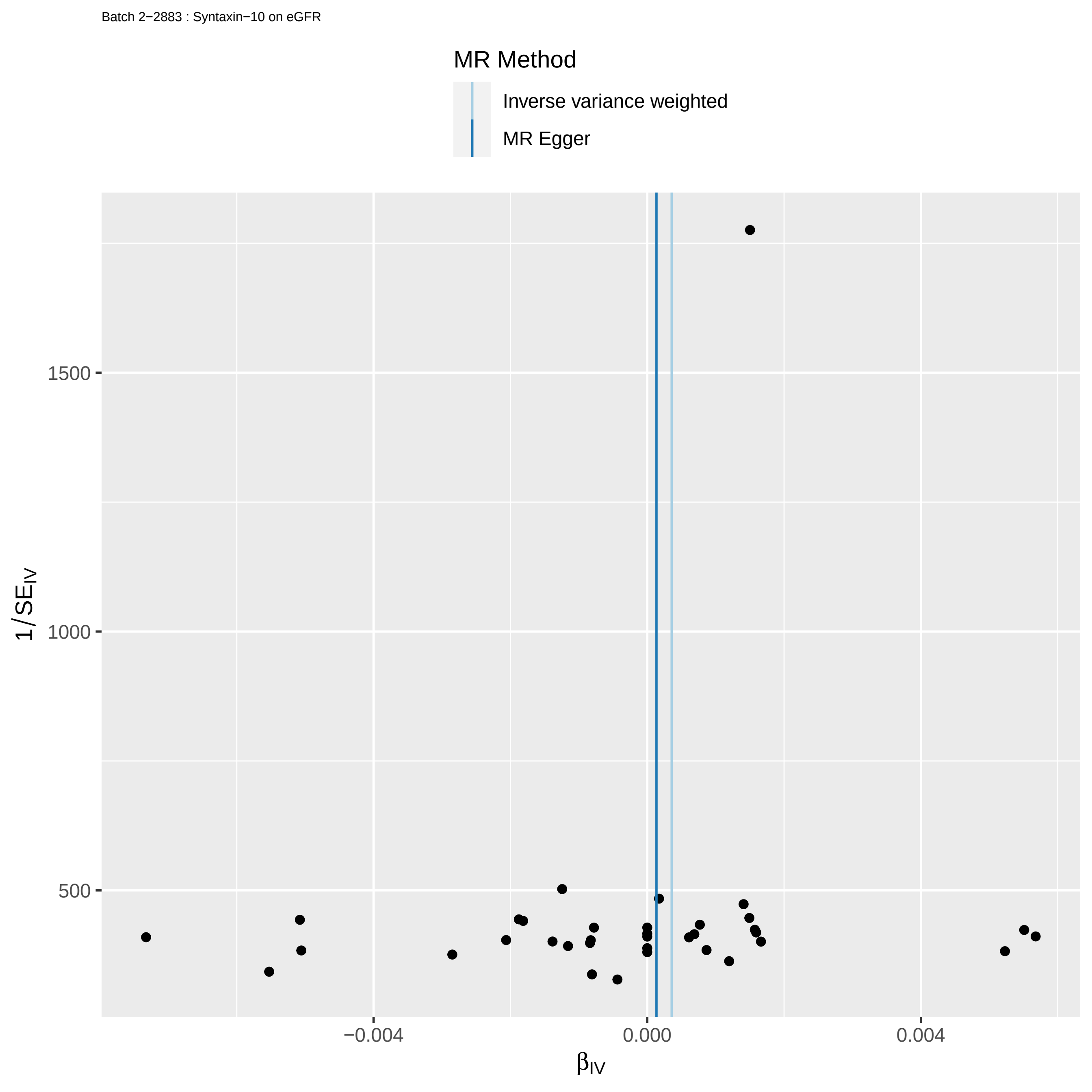

Batch 2−2883 : Syntaxin−10 on eGFR
MR Method
Inverse variance weighted
MR Egger
1500
1000
500
1/SE_IV
−0.004
0.000
0.004
β_IV

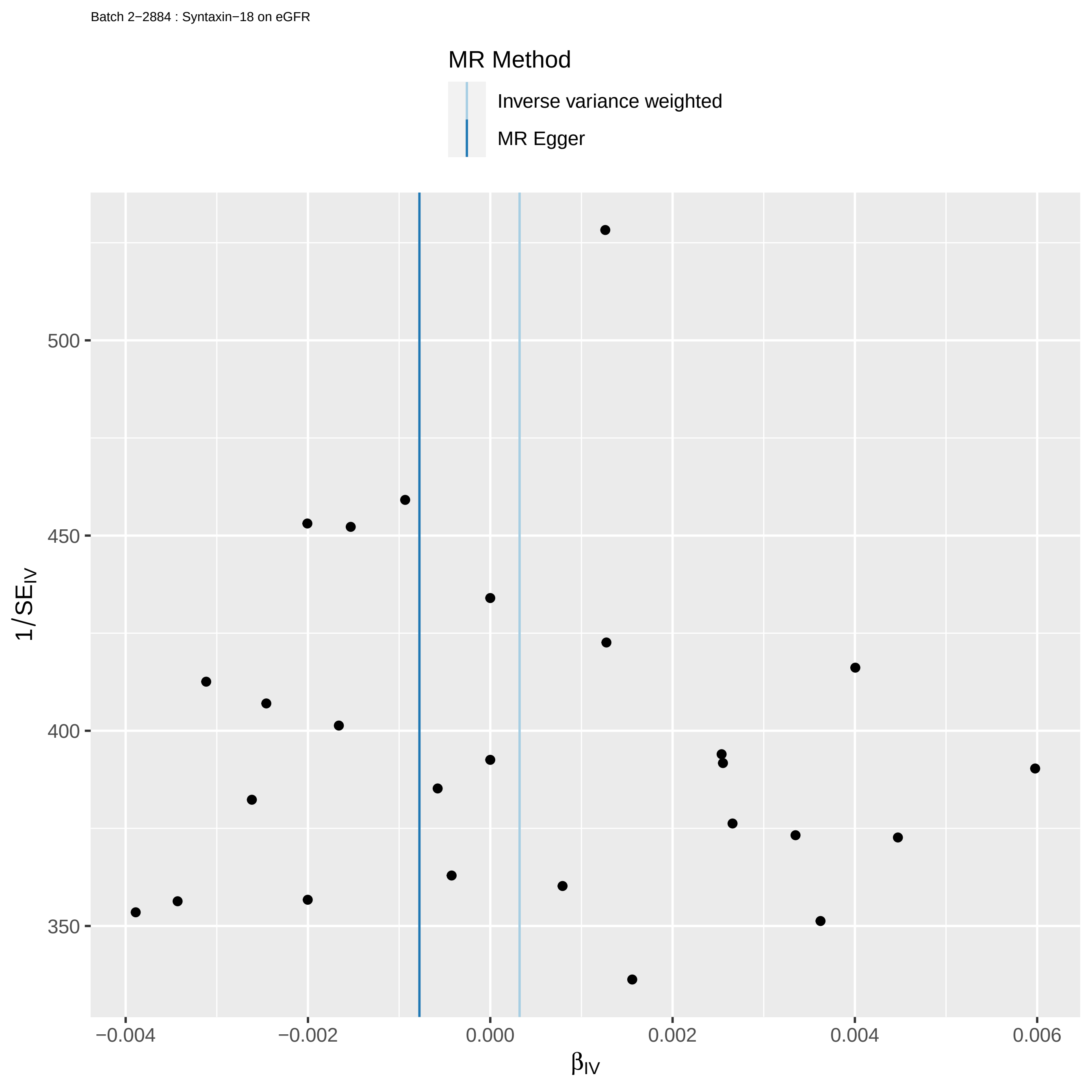

Batch 2−2884 : Syntaxin−18 on eGFR
MR Method
Inverse variance weighted
MR Egger
500
450
400
350
1/SE_IV
−0.004
−0.002
0.000
0.002
0.004
0.006
β_IV

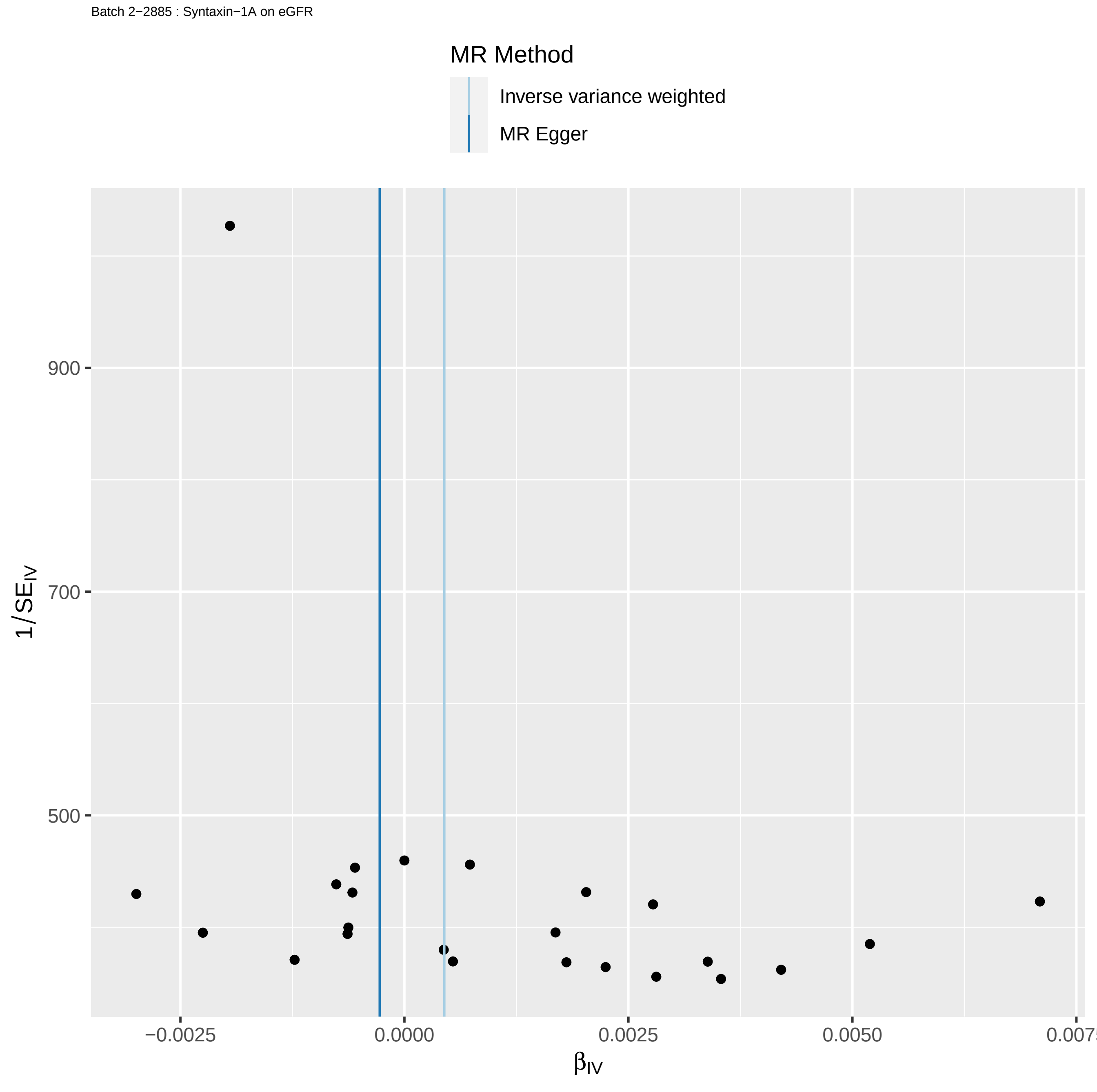

Batch 2−2885 : Syntaxin−1A on eGFR
MR Method
Inverse variance weighted
MR Egger
900
700
500
1/SE$_{IV}$
−0.0025
0.0000
0.0025
0.0050
0.0075
β$_{IV}$

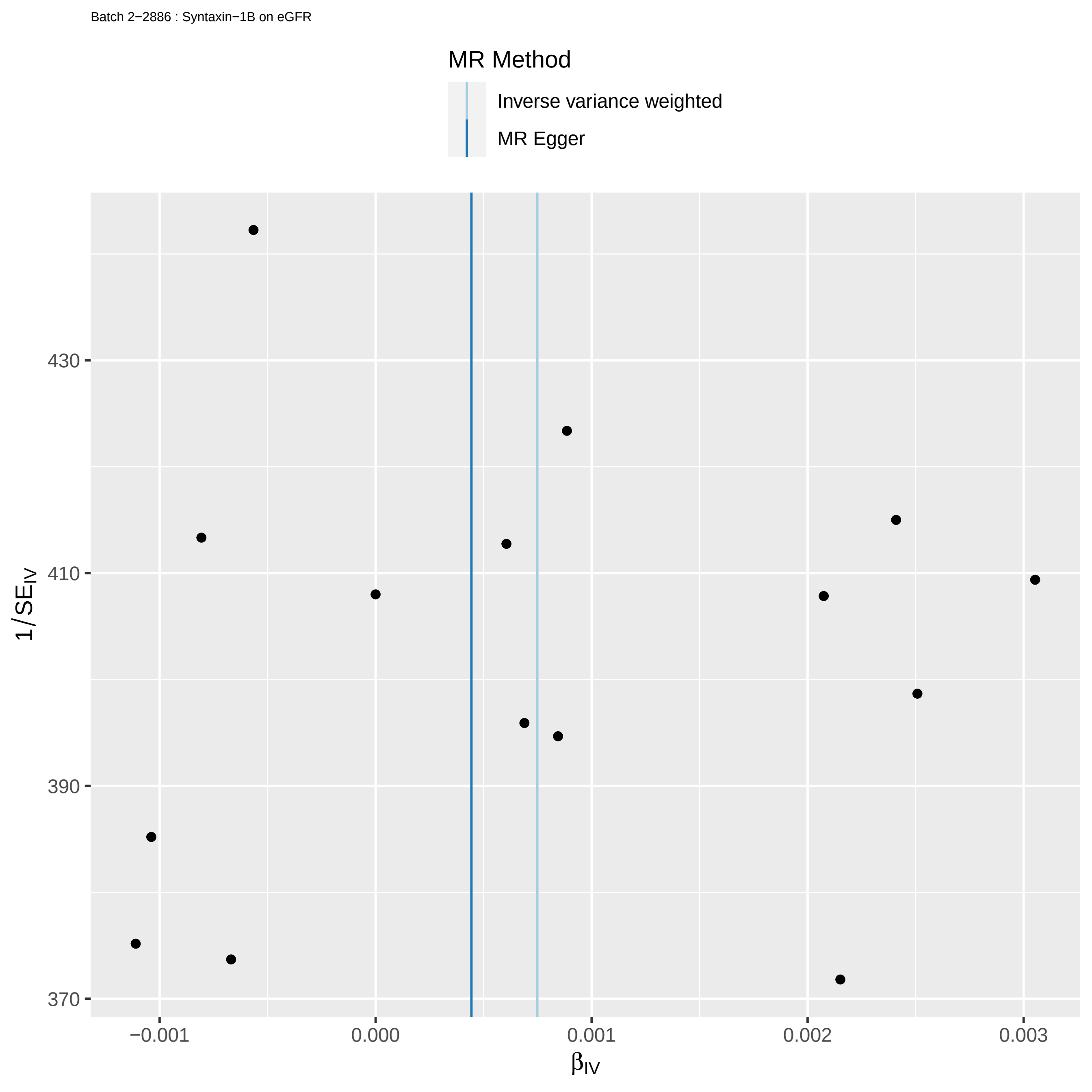
Batch 2−2886 : Syntaxin−1B on eGFR
MR Method
Inverse variance weighted
MR Egger
430
410
390
370
1/SE$_{IV}$
−0.001
0.000
0.001
0.002
0.003
β$_{IV}$

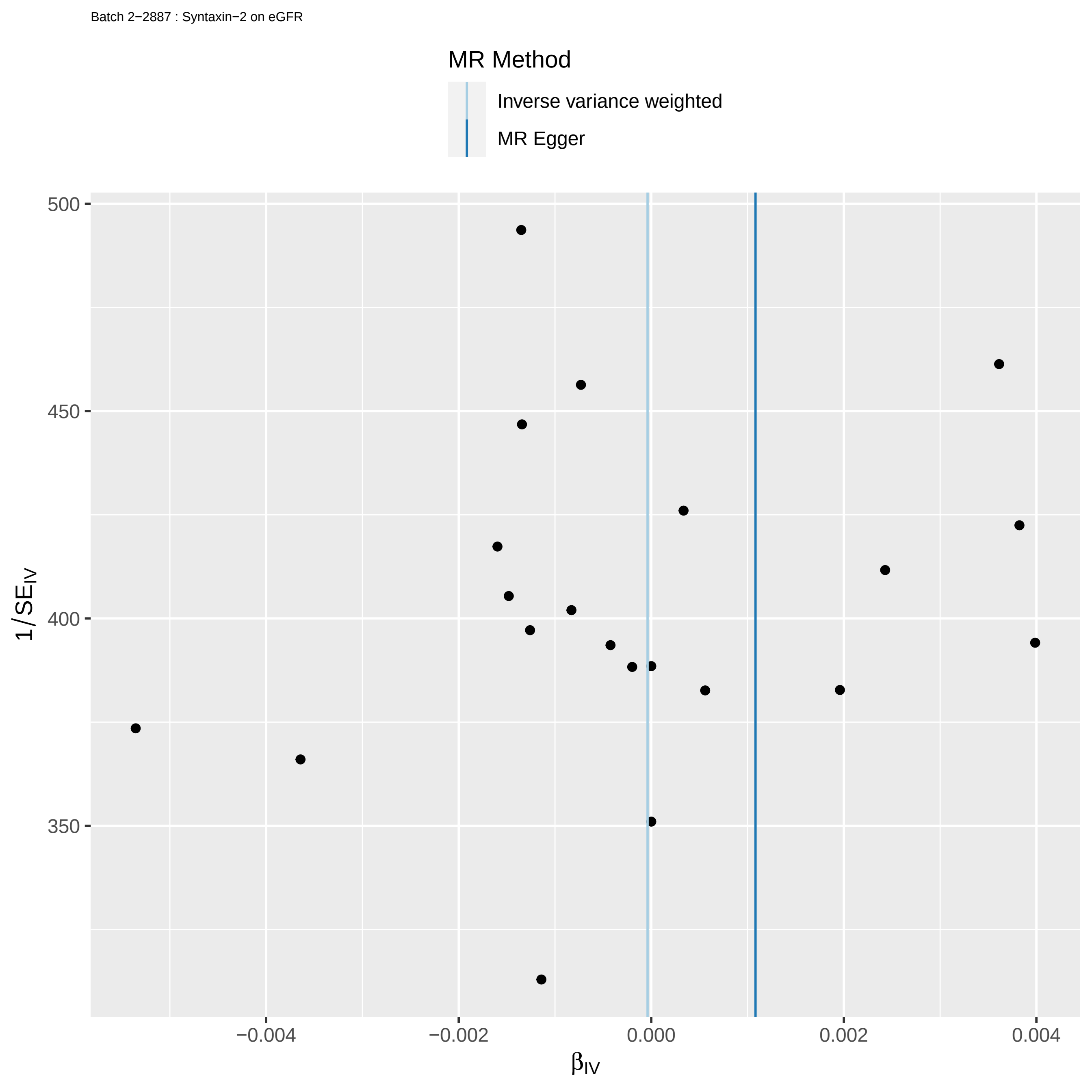

Batch 2−2887 : Syntaxin−2 on eGFR
MR Method
Inverse variance weighted
MR Egger
500
450
400
350
1/SE_IV
−0.004
−0.002
0.000
0.002
0.004
β_IV

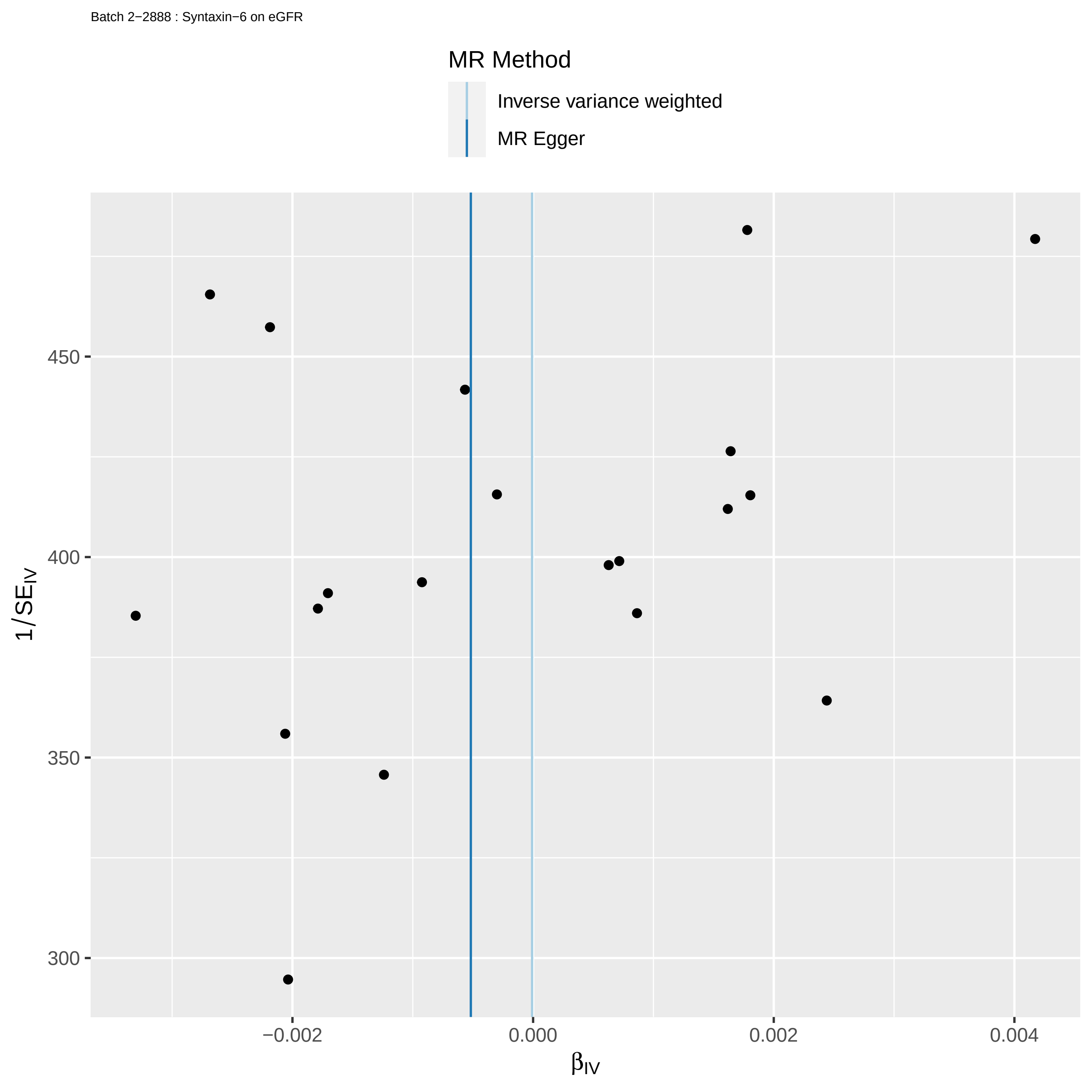

Batch 2−2888 : Syntaxin−6 on eGFR
MR Method
Inverse variance weighted
MR Egger
450
400
350
300
1/SE$_{IV}$
−0.002
0.000
0.002
0.004
β$_{IV}$

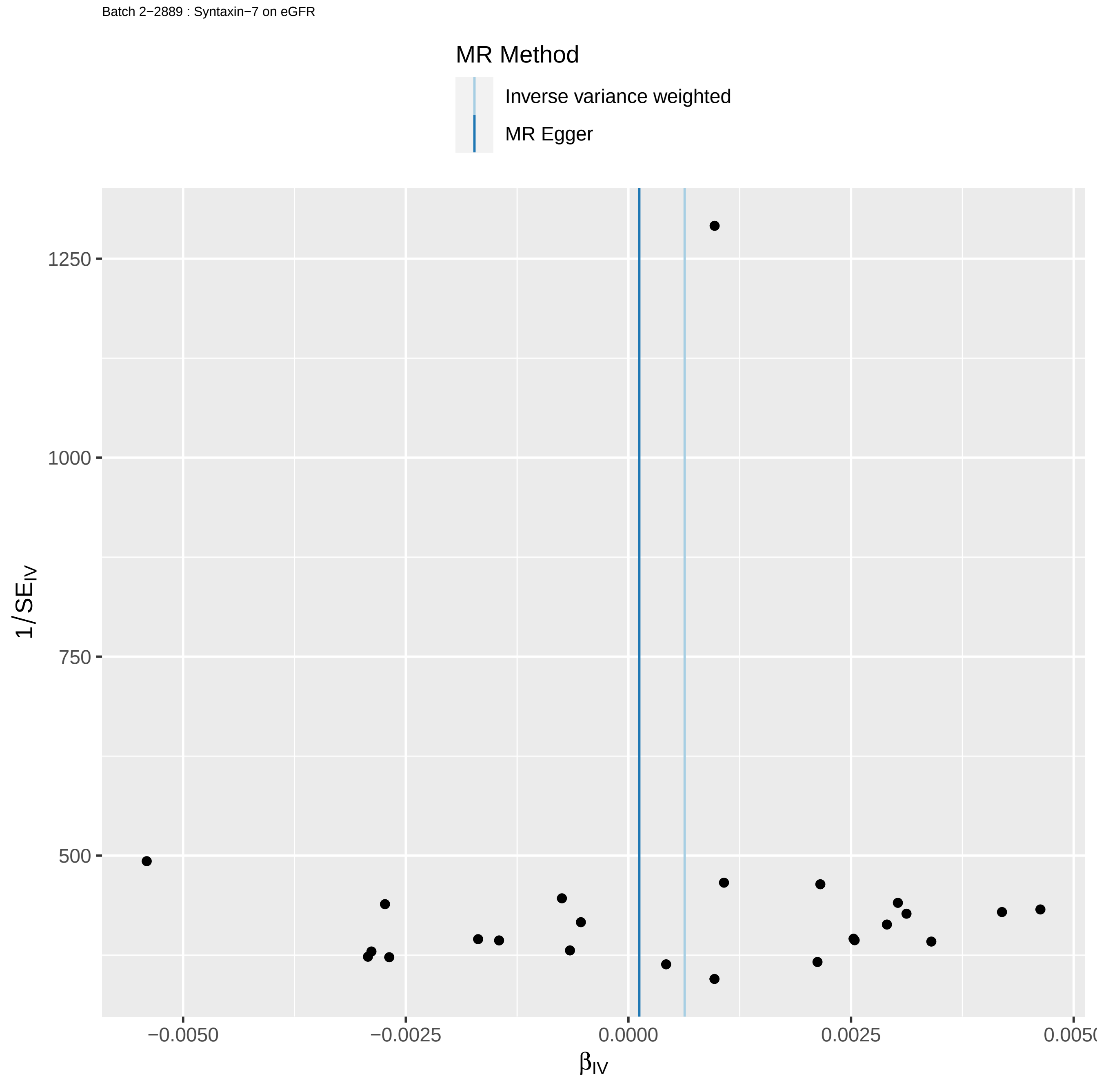

Batch 2−2889 : Syntaxin−7 on eGFR
MR Method
Inverse variance weighted
MR Egger
1250
1000
750
500
1/SE$_{IV}$
−0.0050
−0.0025
0.0000
0.0025
0.0050
β$_{IV}$

Batch 2−2890 : Syntaxin−8 on eGFR

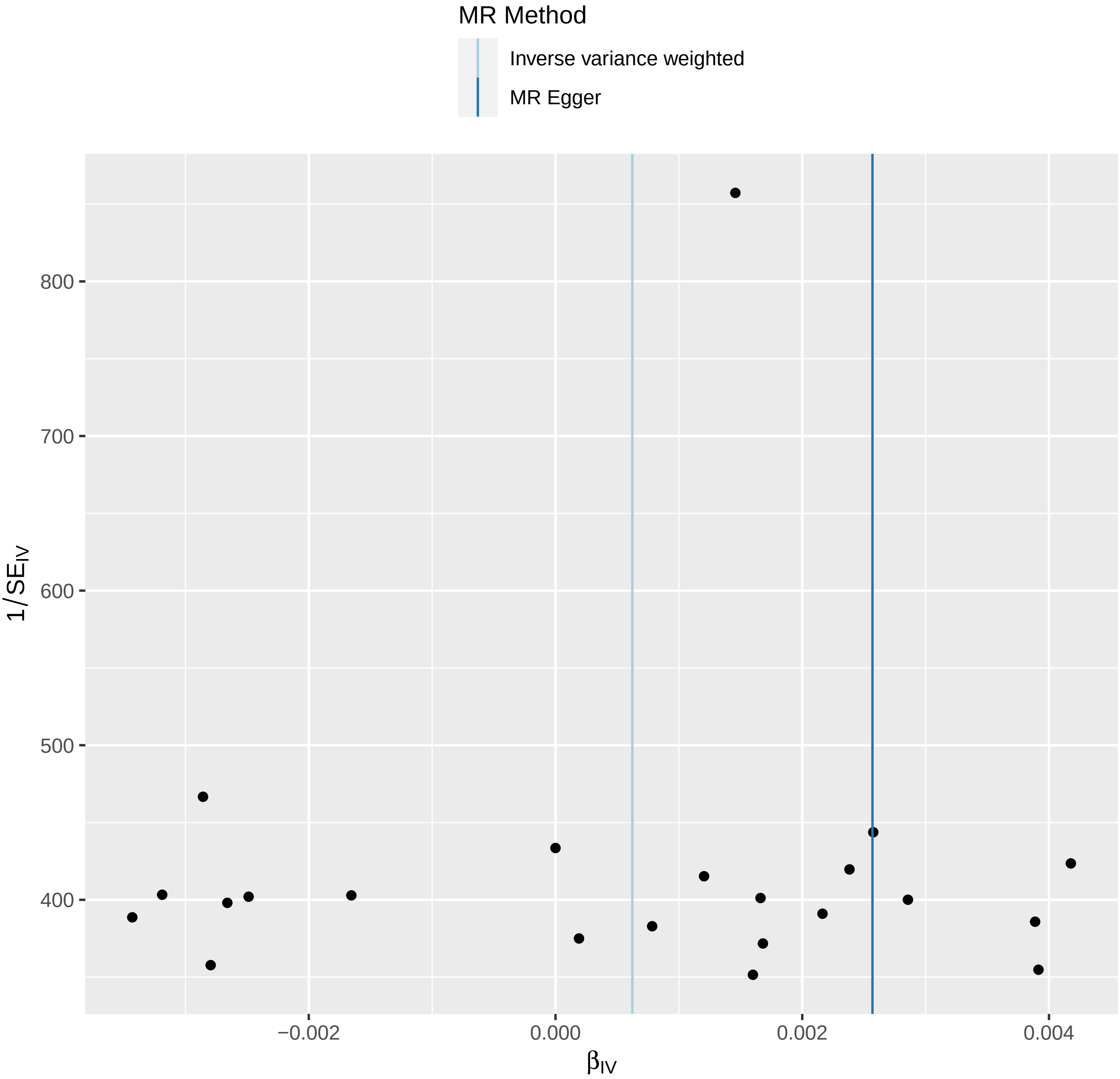

Batch 2−2891 : Extracellular sulfatase Sulf−2 on eGFR

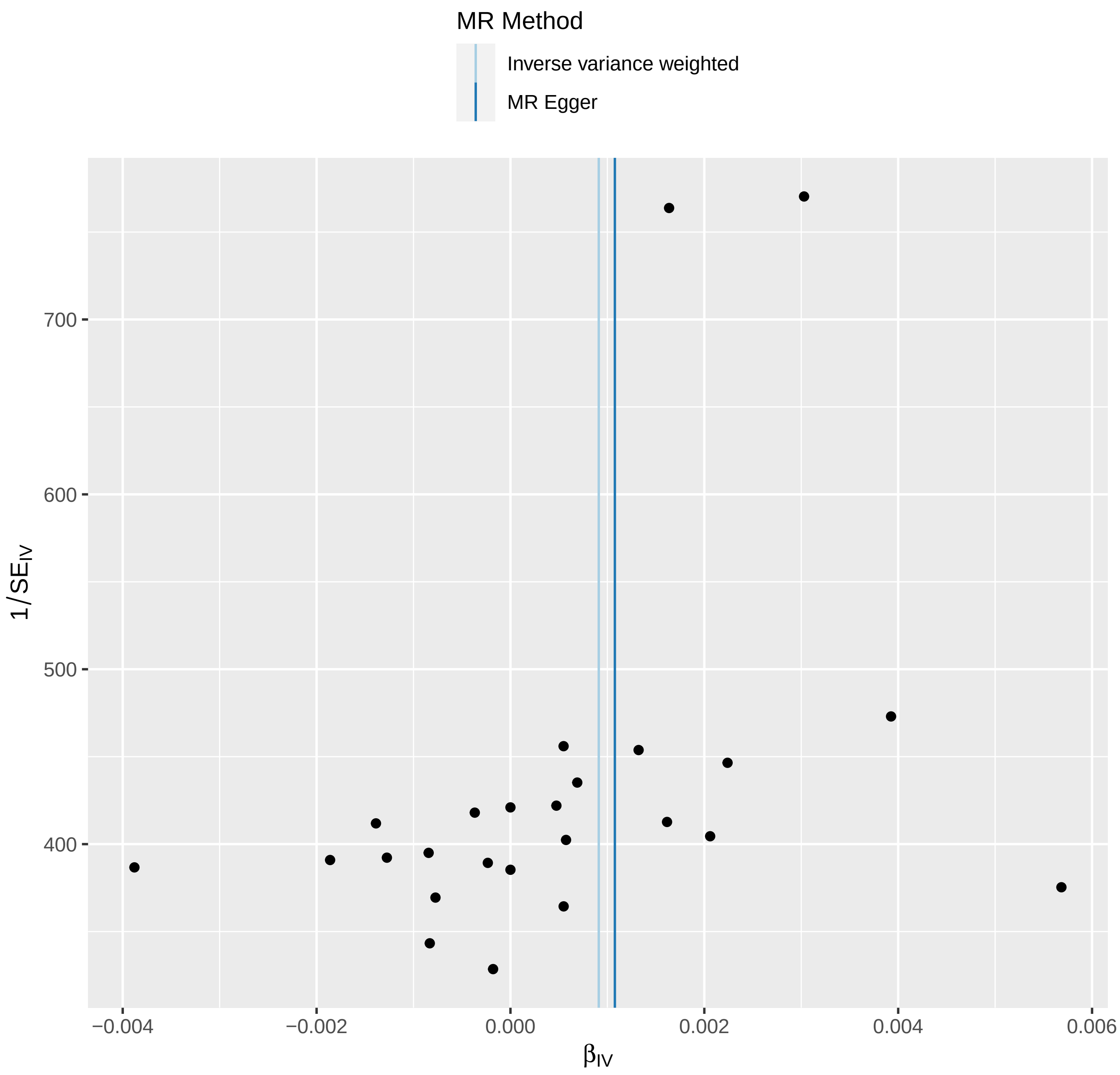

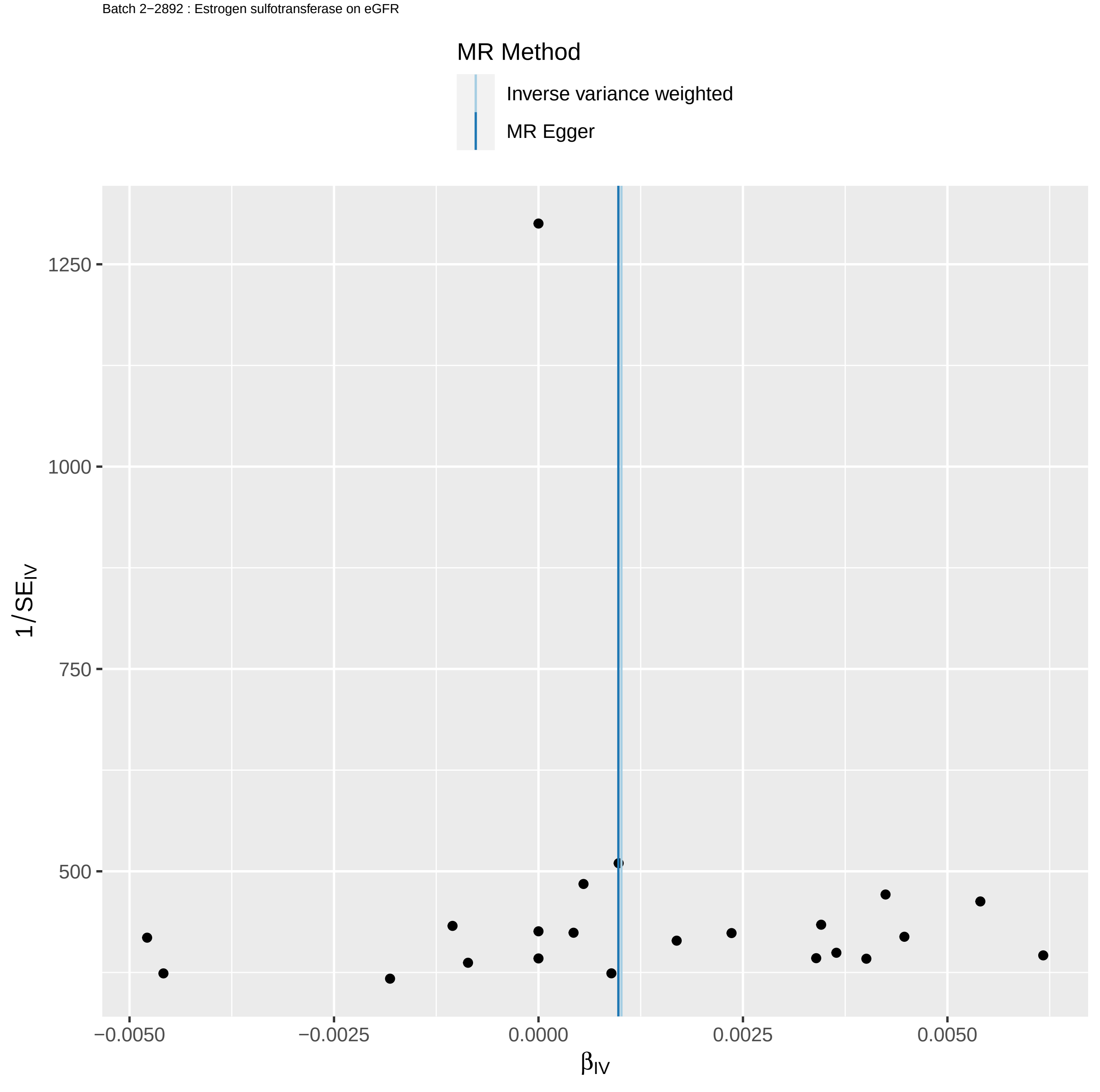
Batch 2−2892 : Estrogen sulfotransferase on eGFR
MR Method
Inverse variance weighted
MR Egger
1250
1000
750
500
1/SE$_{IV}$
−0.0050
−0.0025
0.0000
0.0025
0.0050
β$_{IV}$

Batch 2−2893 : Bile salt sulfotransferase on eGFR

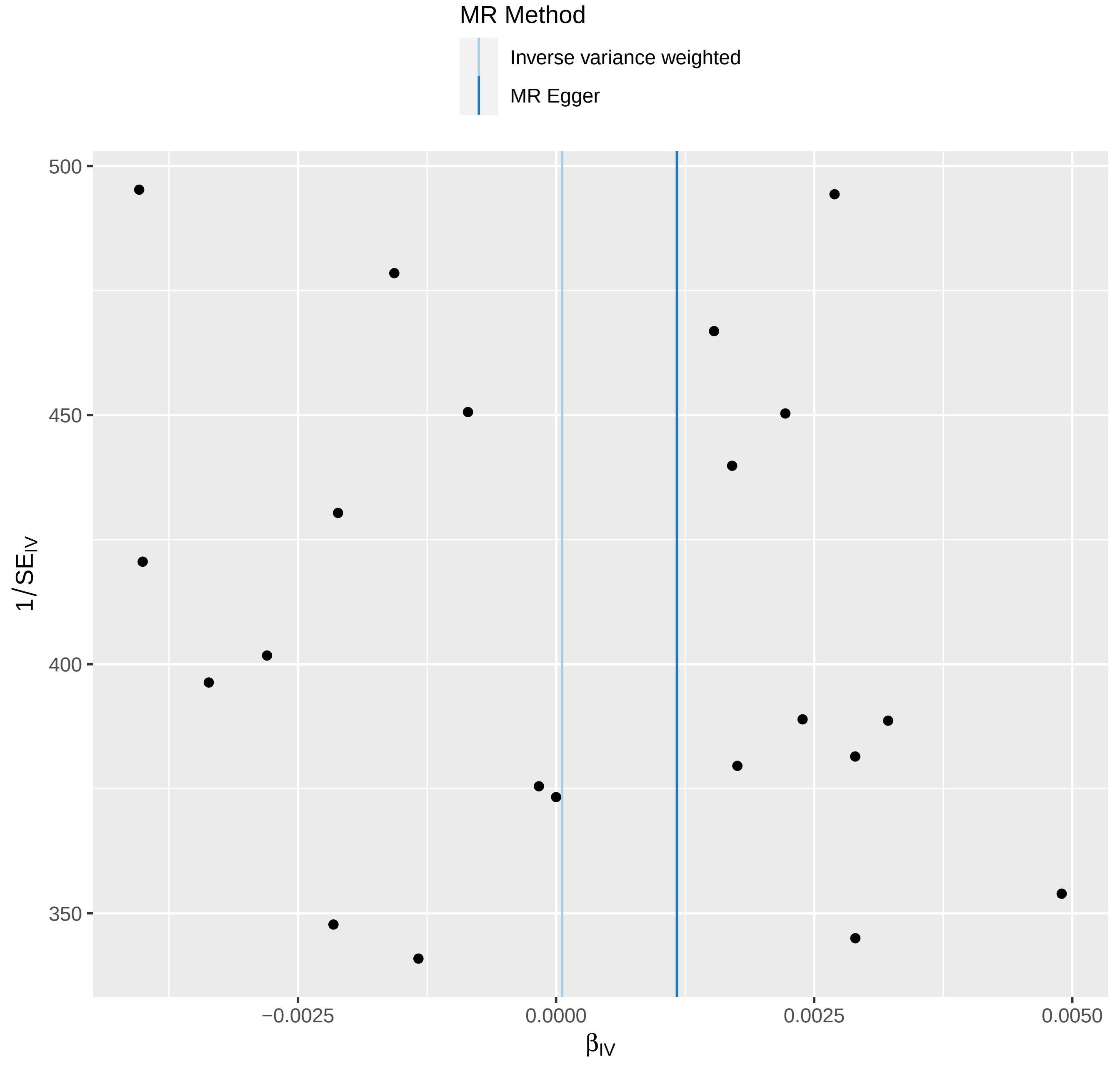

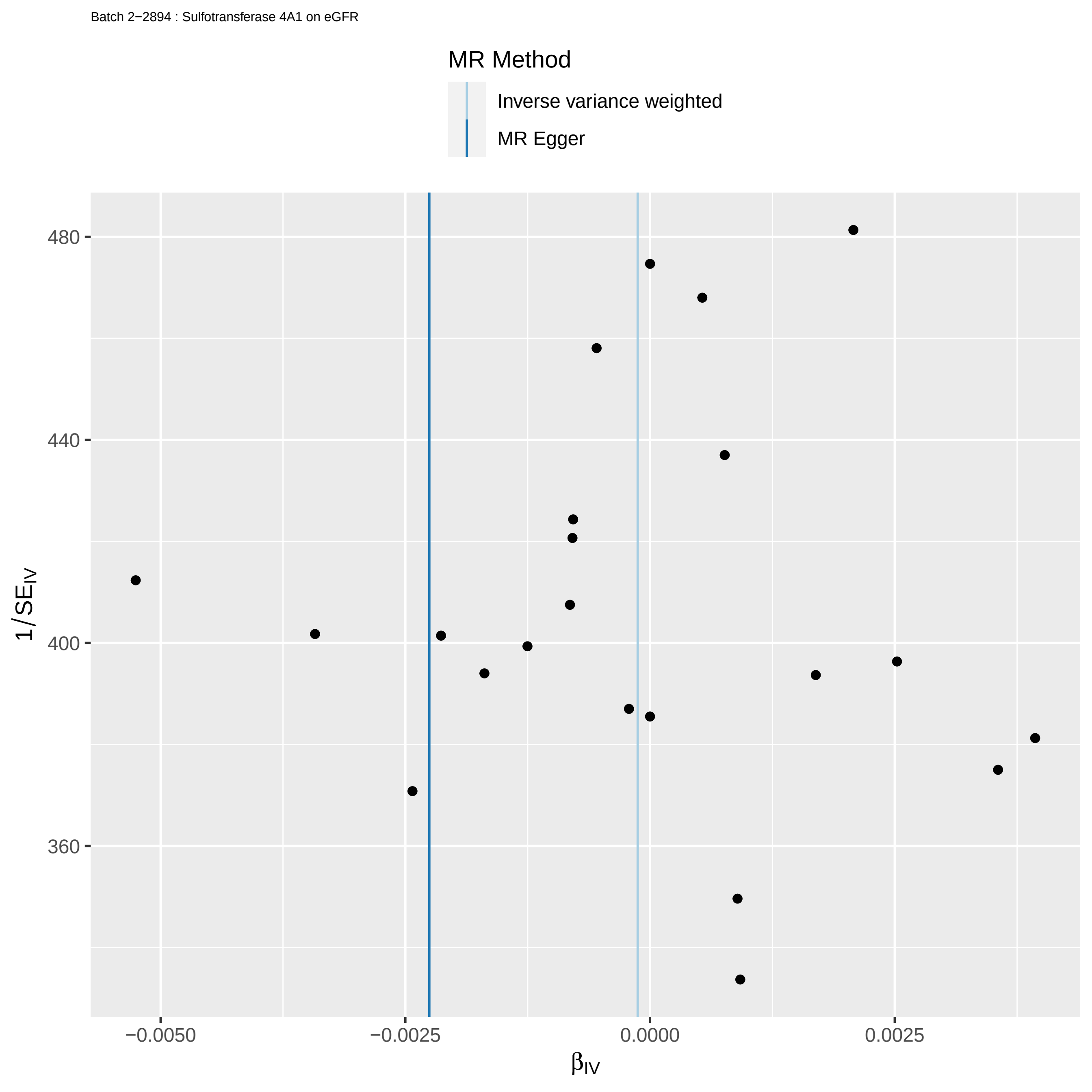

Batch 2−2894 : Sulfotransferase 4A1 on eGFR
MR Method
Inverse variance weighted
MR Egger
480
440
400
360
1/SE$_{IV}$
−0.0050
−0.0025
0.0000
0.0025
β$_{IV}$

Batch 2−2895 : Sulfatase−modifying factor 1 on eGFR

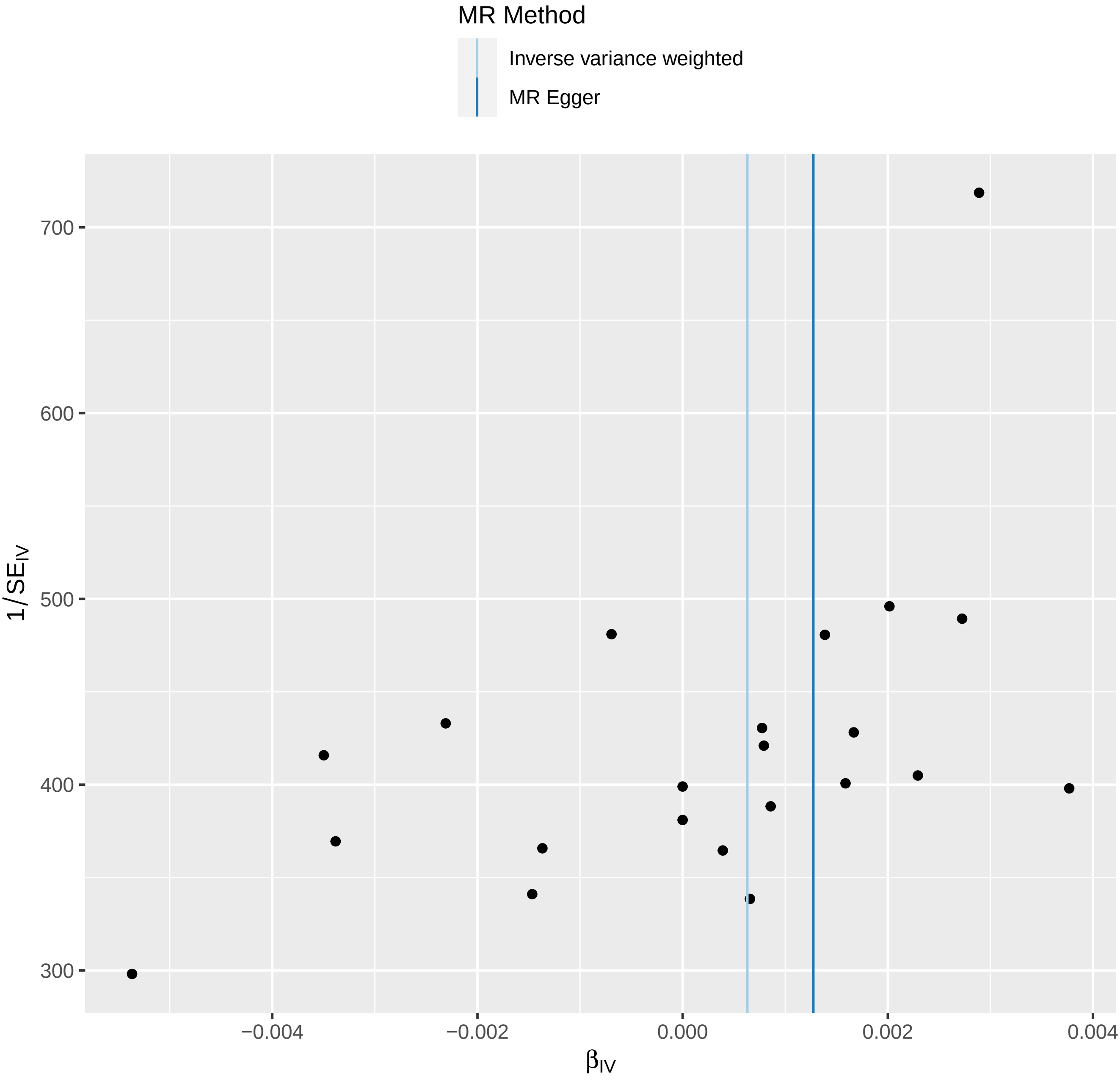

Batch 2−2896 : Small ubiquitin−related modifier 3 on eGFR

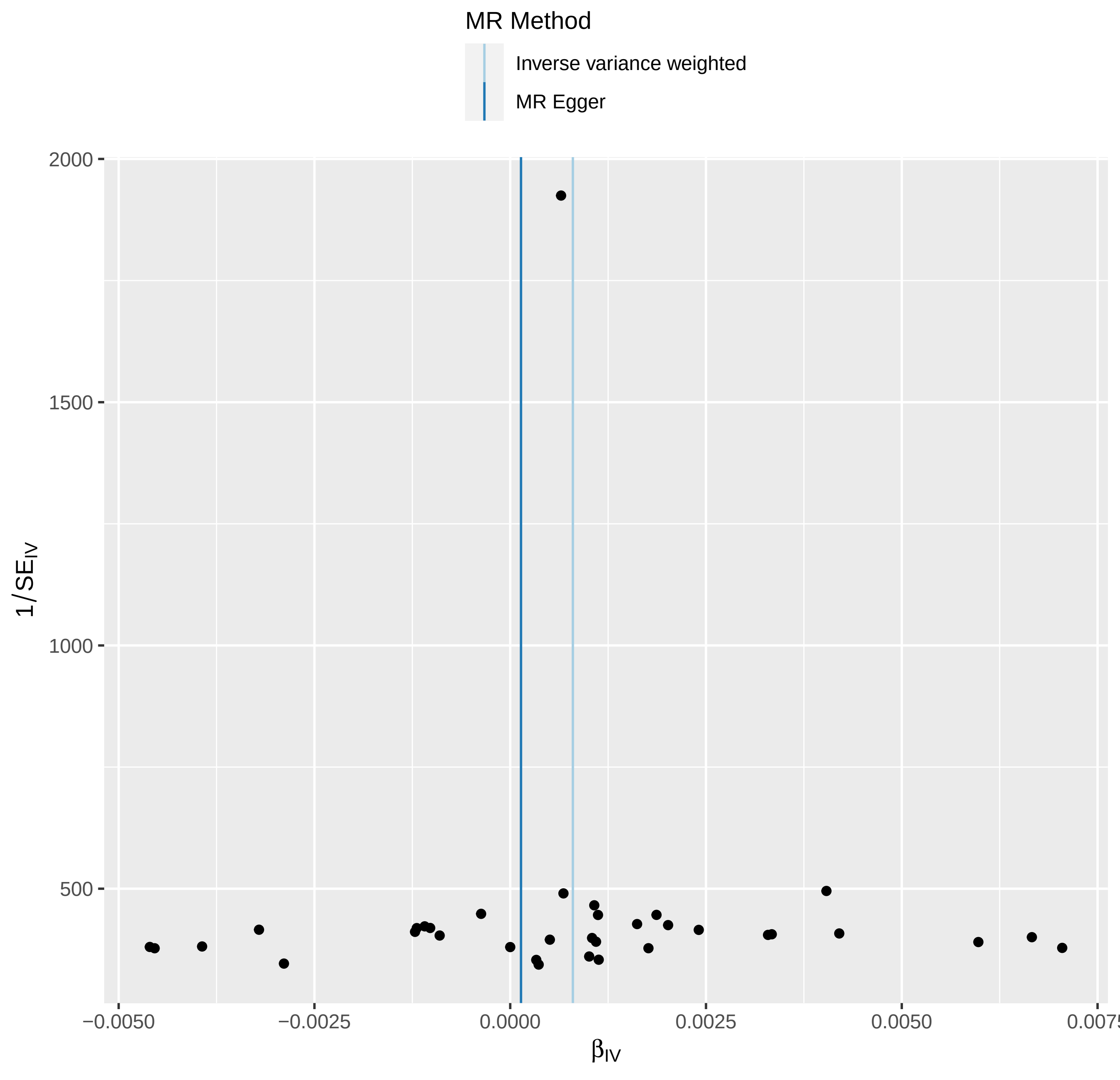

Batch 2−2897 : Small ubiquitin−related modifier 3 on eGFR

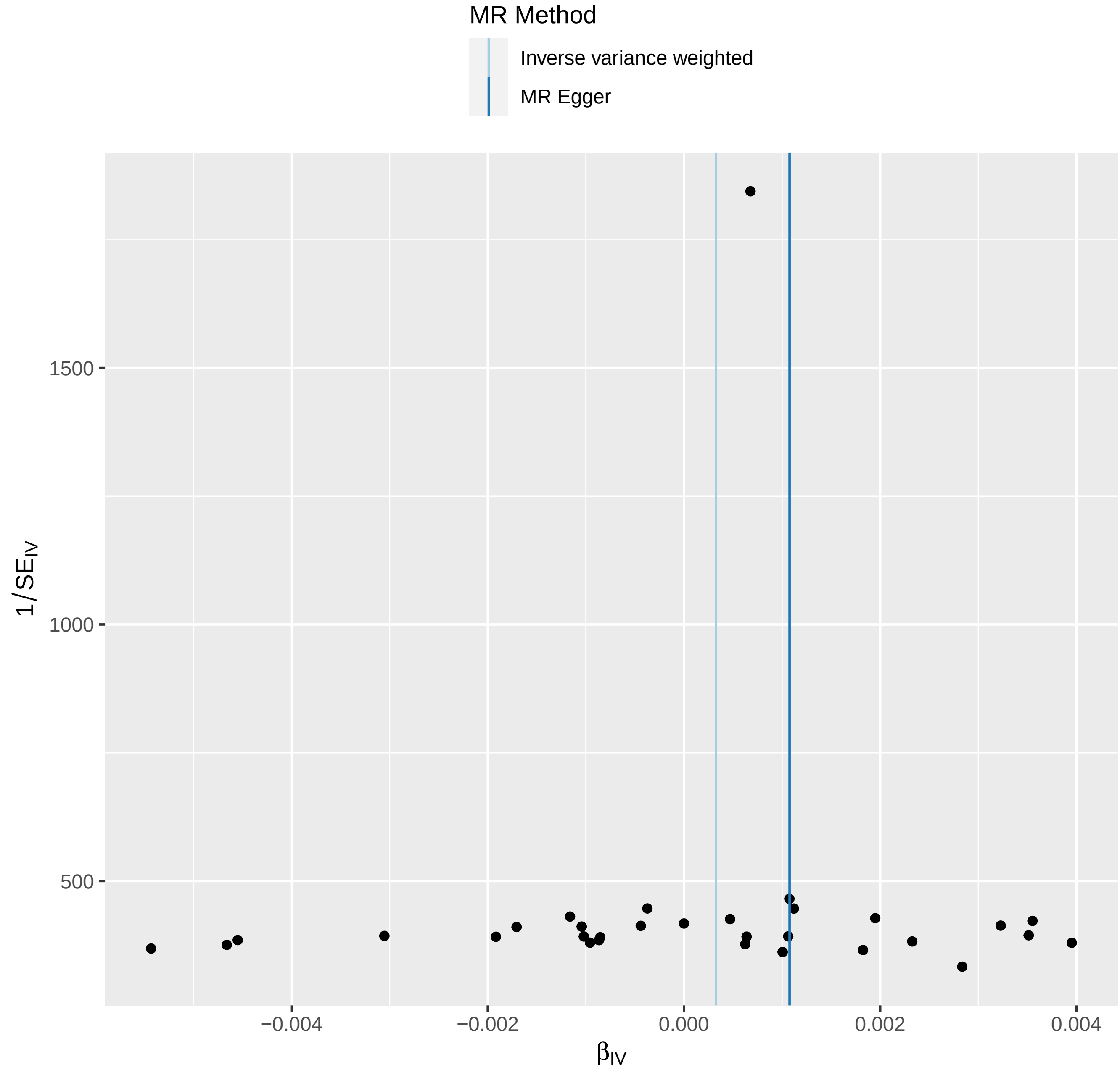

Batch 2−2898 : SUN domain−containing protein 3 on eGFR

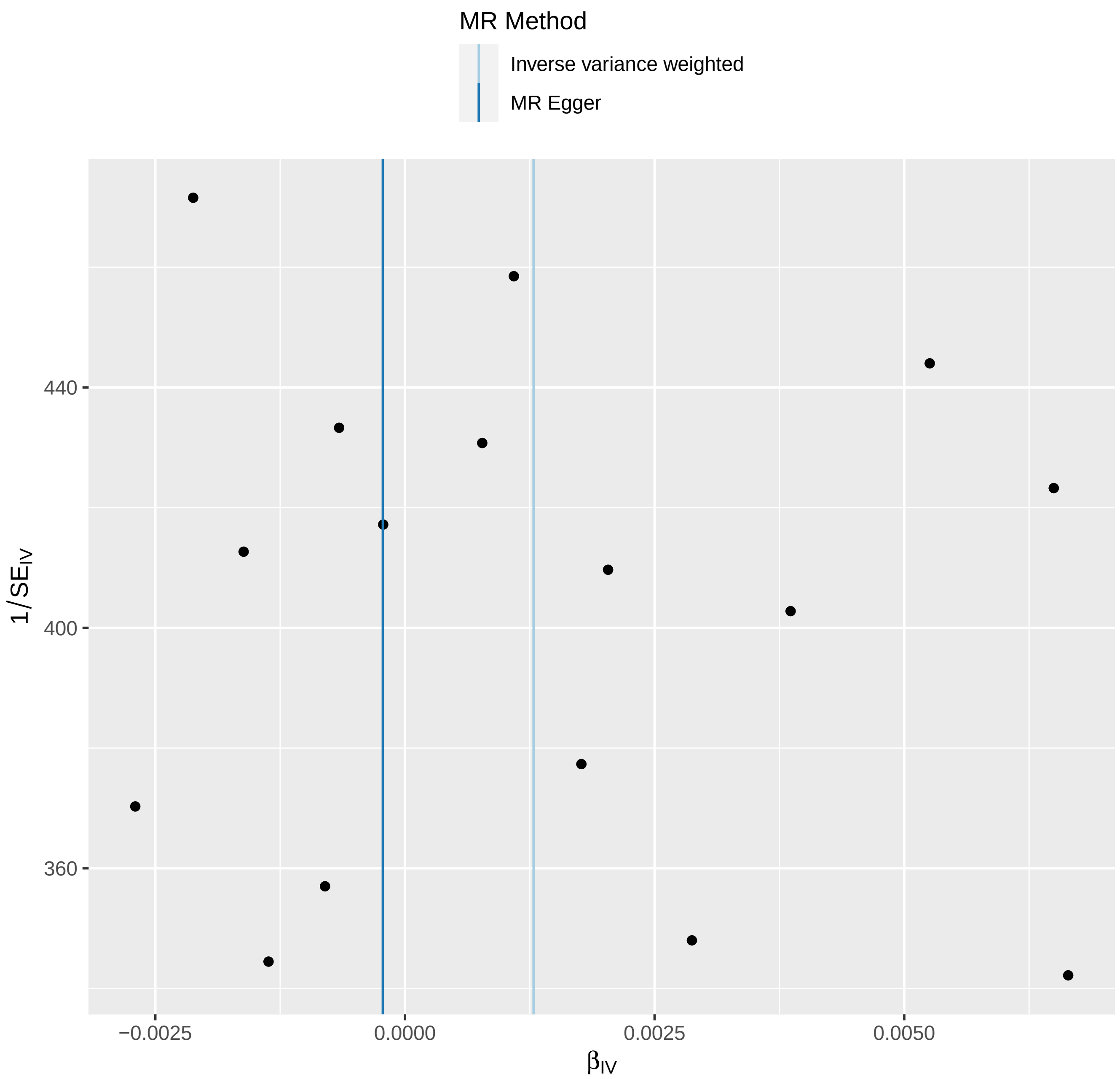

Batch 2−2899 : SUN domain−containing protein 5 on eGFR

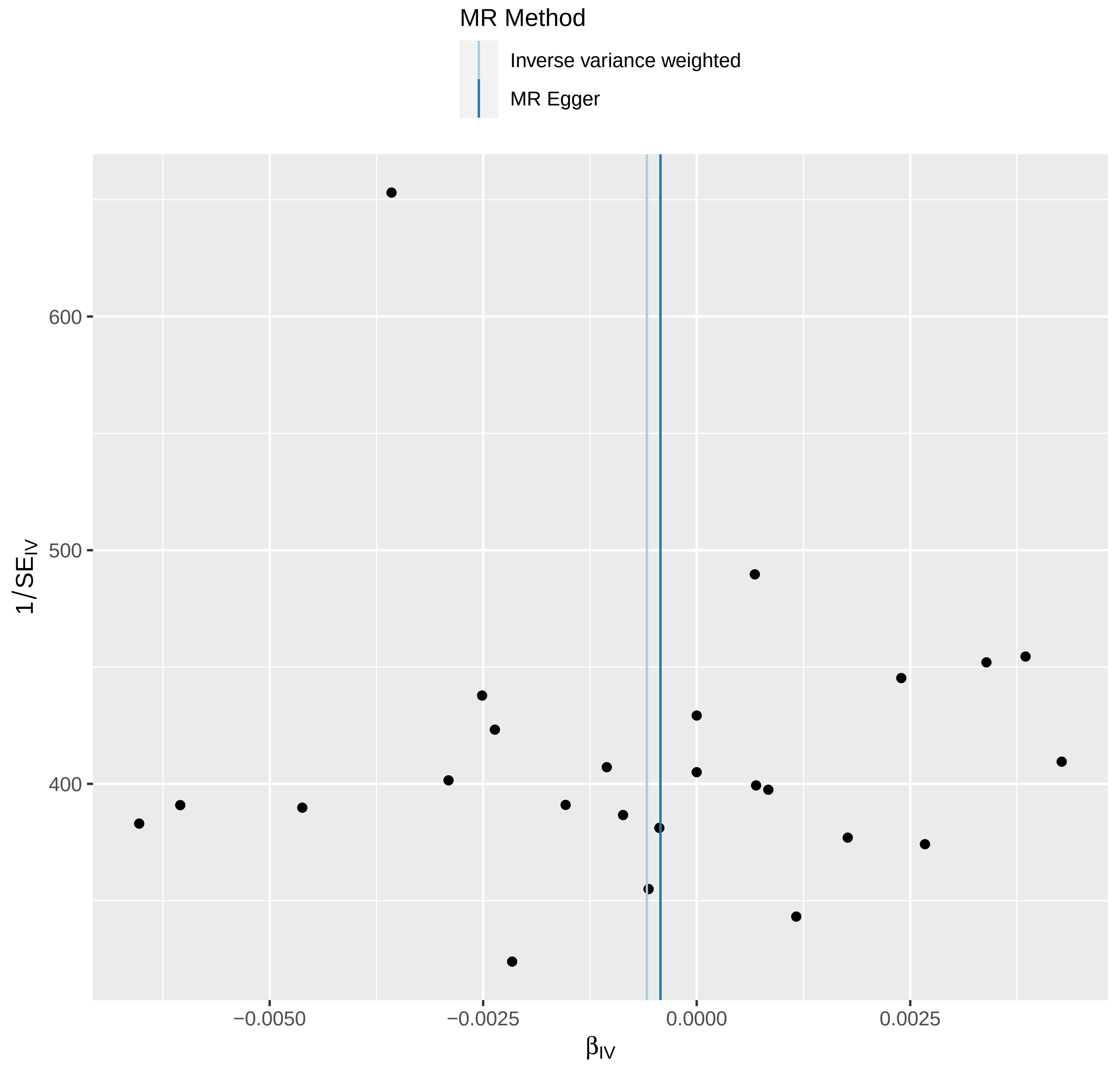

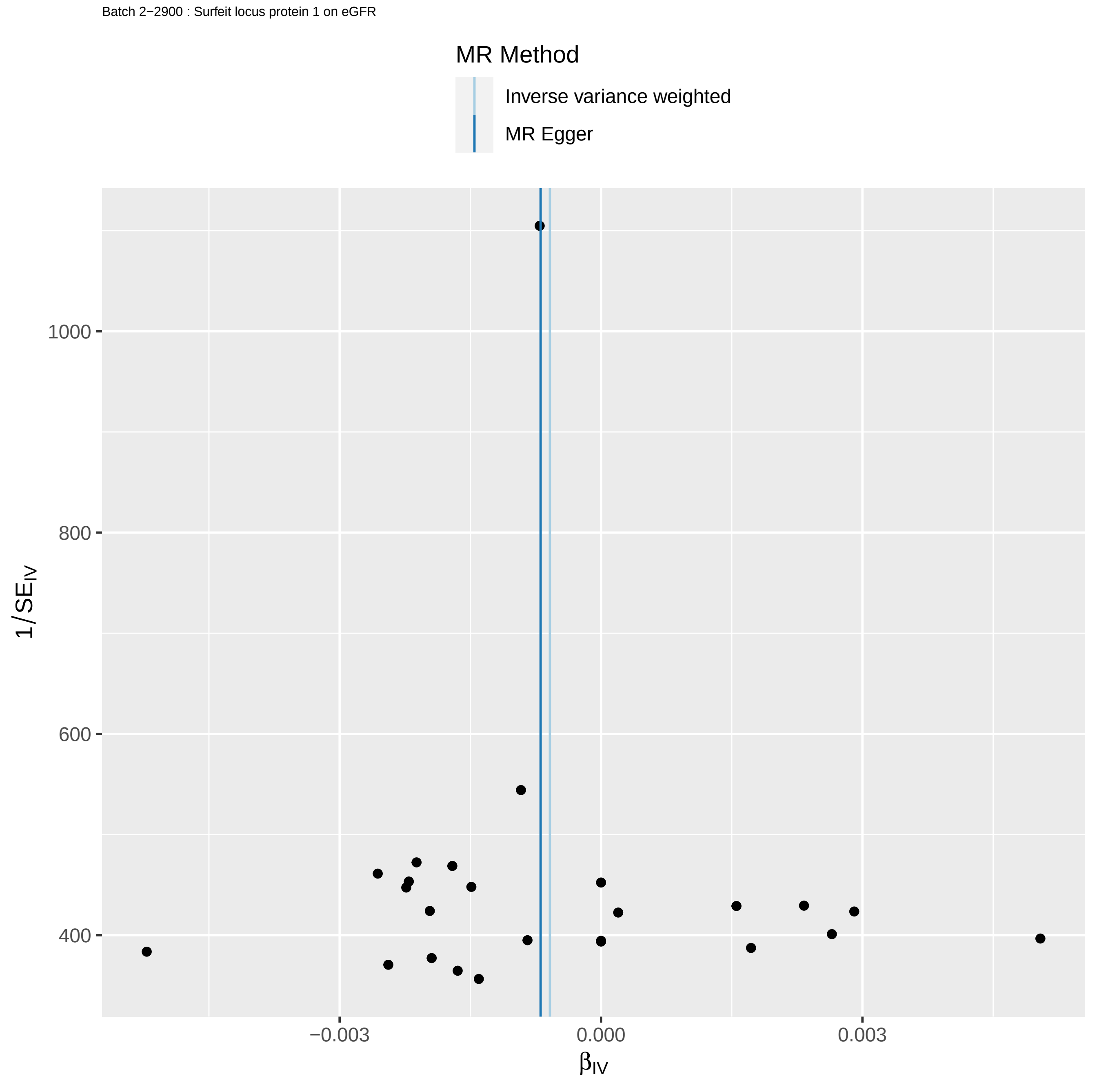

Batch 2−2900 : Surfeit locus protein 1 on eGFR
MR Method
Inverse variance weighted
MR Egger
1000
800
600
400
1/SE$_{IV}$
−0.003
0.000
0.003
β$_{IV}$

Batch 2−2901 : Sushi domain−containing protein 1 on eGFR

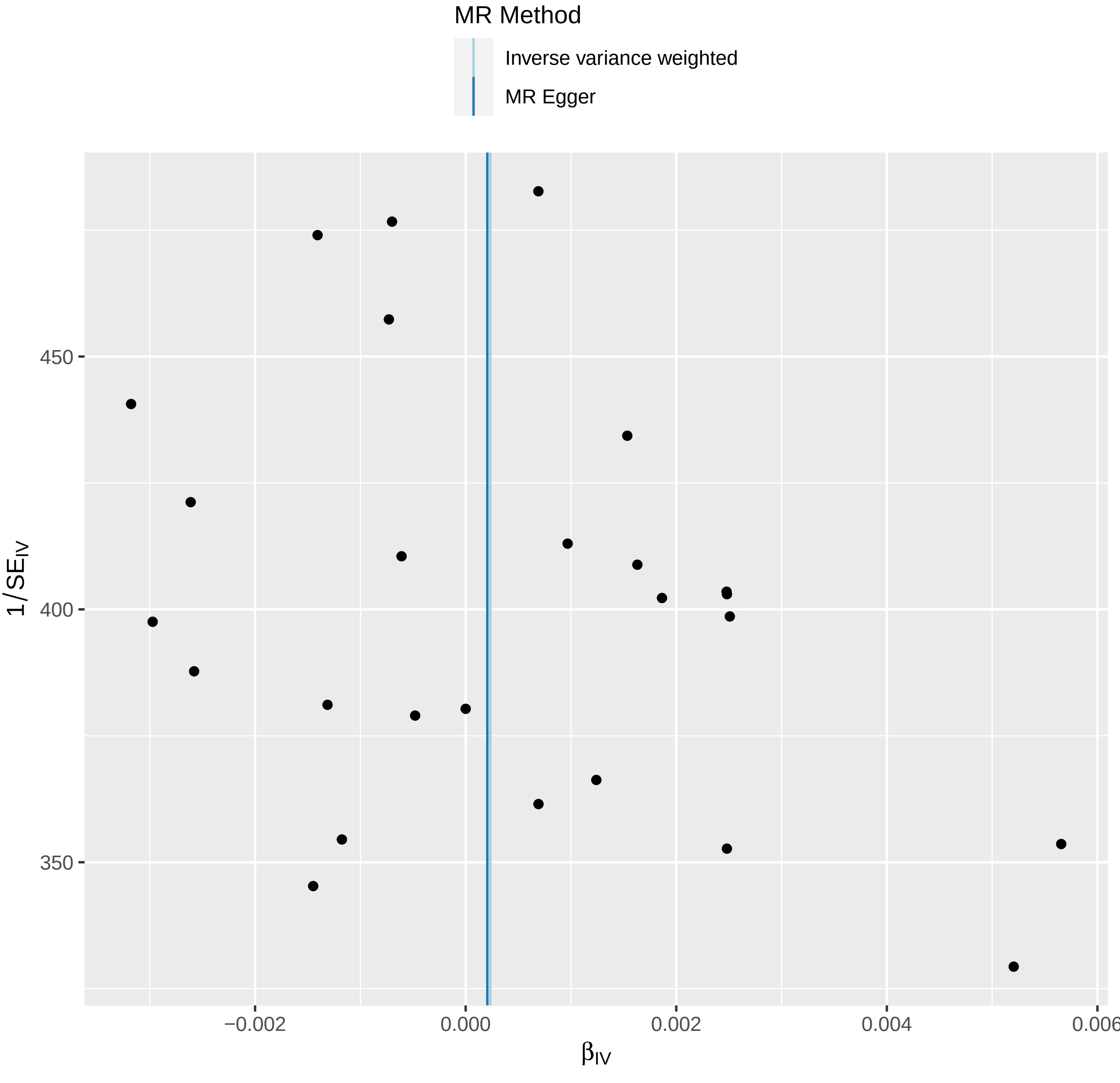

Batch 2−2902 : Sushi domain−containing protein 2 on eGFR

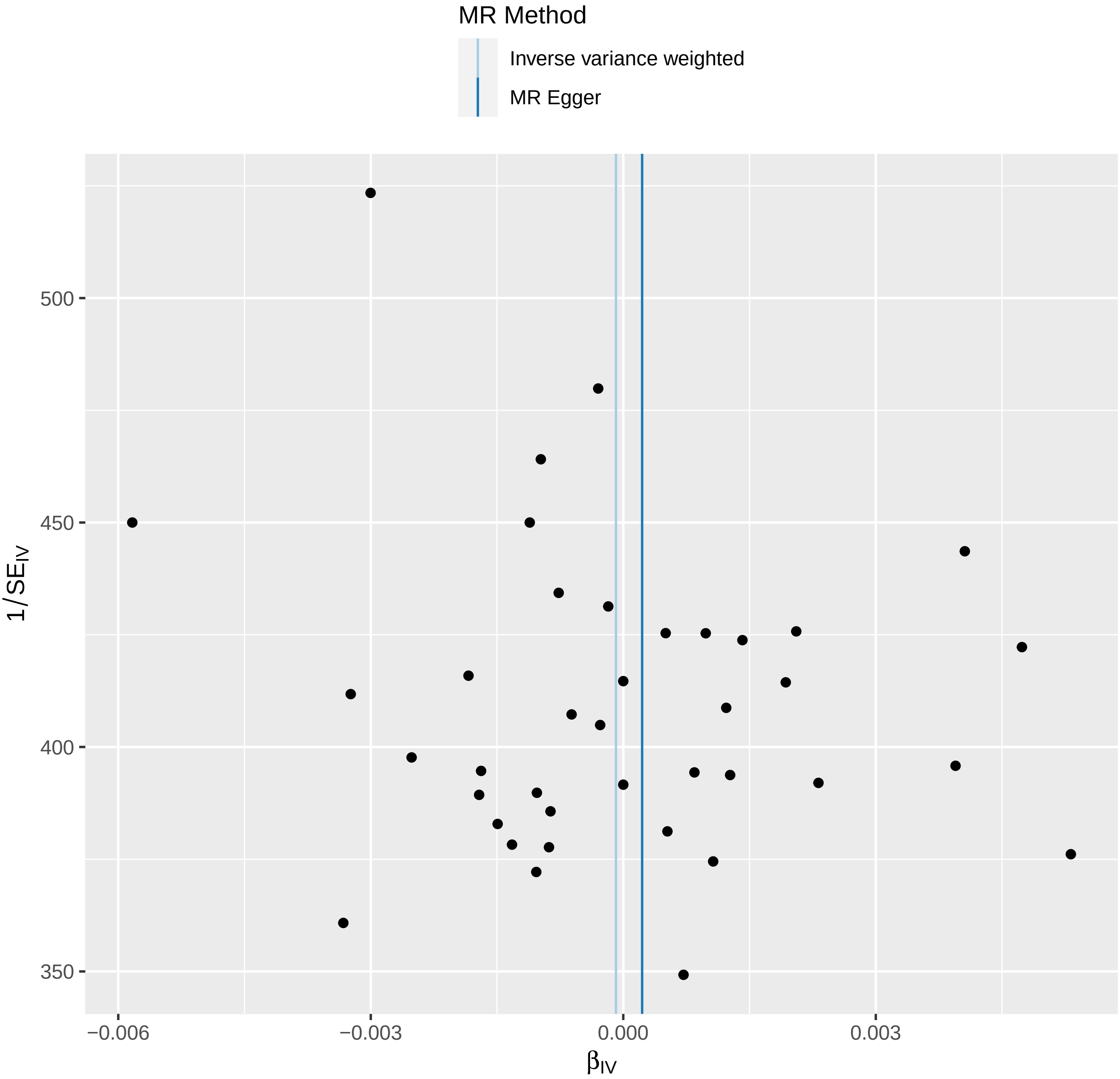

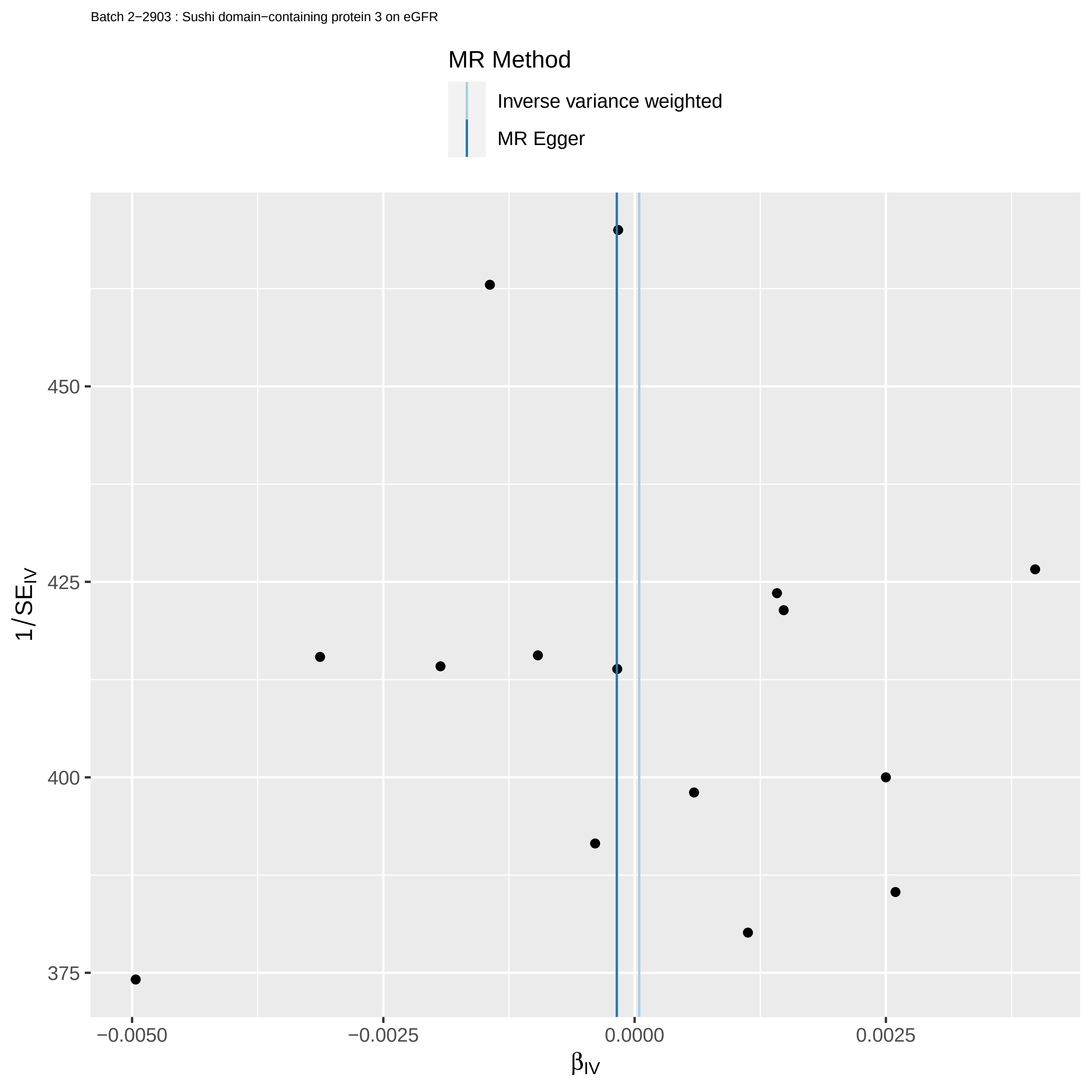
Batch 2−2903 : Sushi domain−containing protein 3 on eGFR
MR Method
Inverse variance weighted
MR Egger
450
425
400
375
1/SE_IV
−0.0050
−0.0025
0.0000
0.0025
β_IV

Batch 2−2904 : Histone−lysine N−methyltransferase SUV420H2 on eGFR

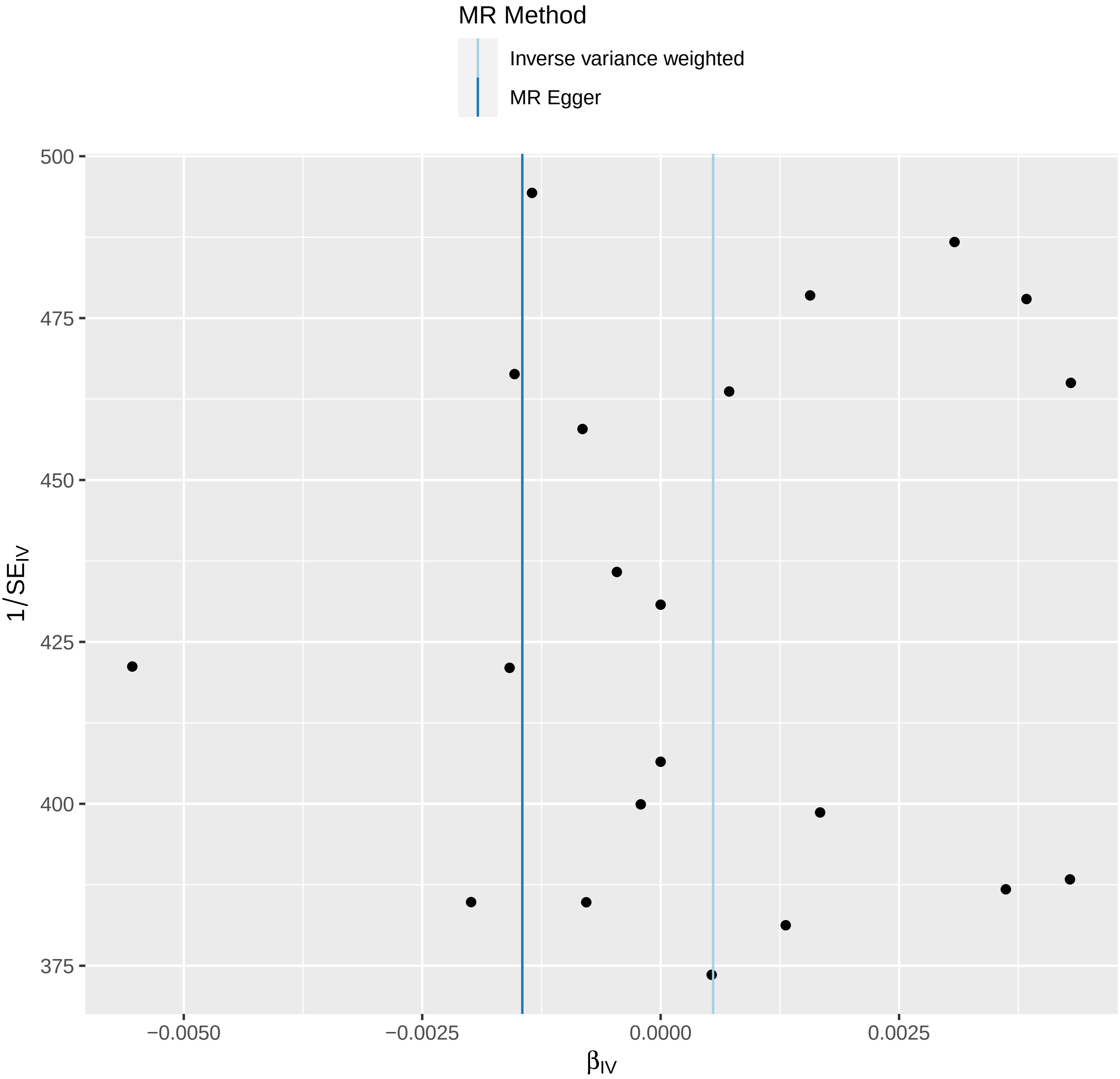

Batch 2−2905 : Synaptic vesicle glycoprotein 2A on eGFR

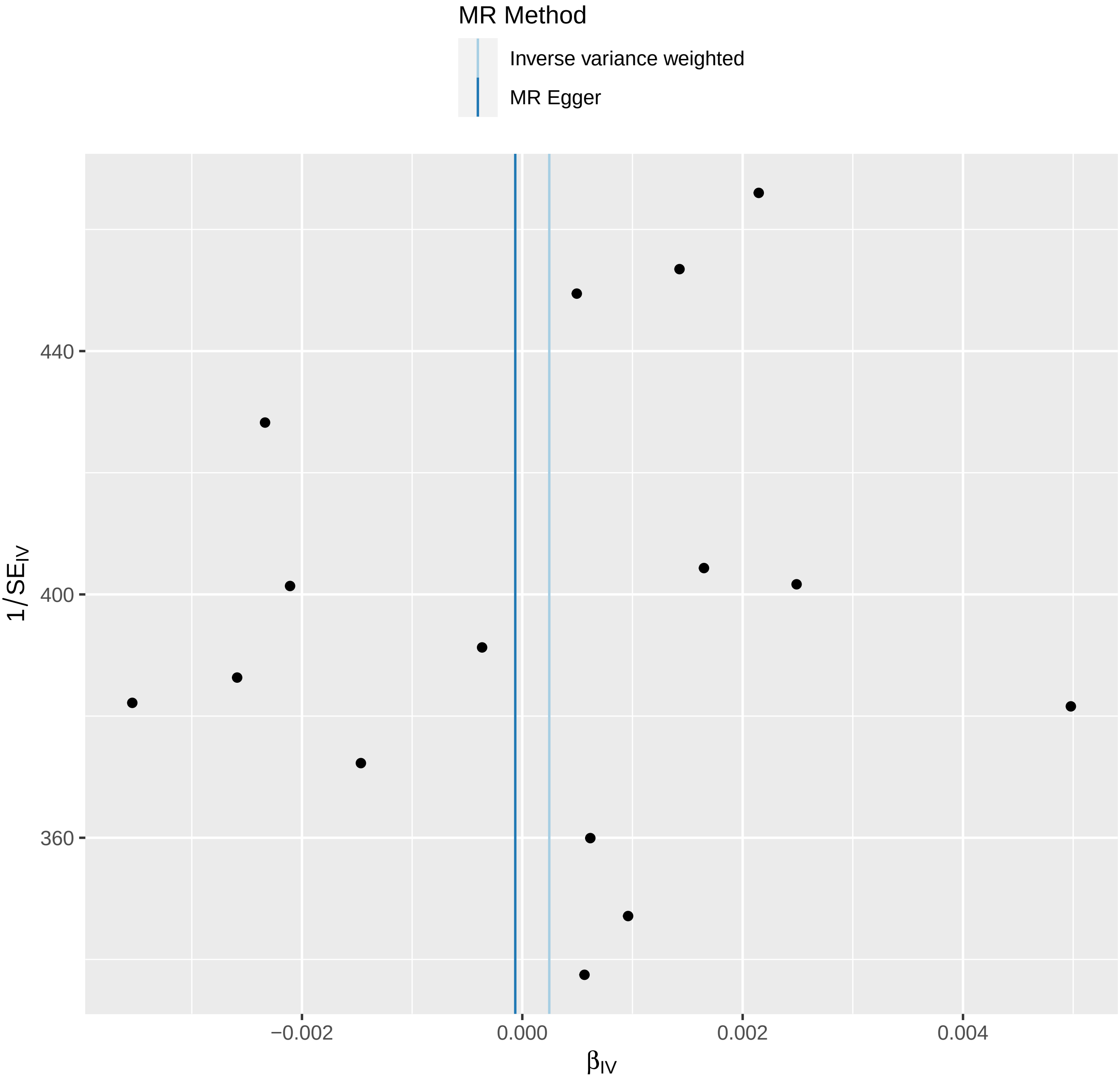

Batch 2−2906 : Sushi, von Willebrand factor type A, EGF and pentraxin domain−containing protein 1 on eGFR

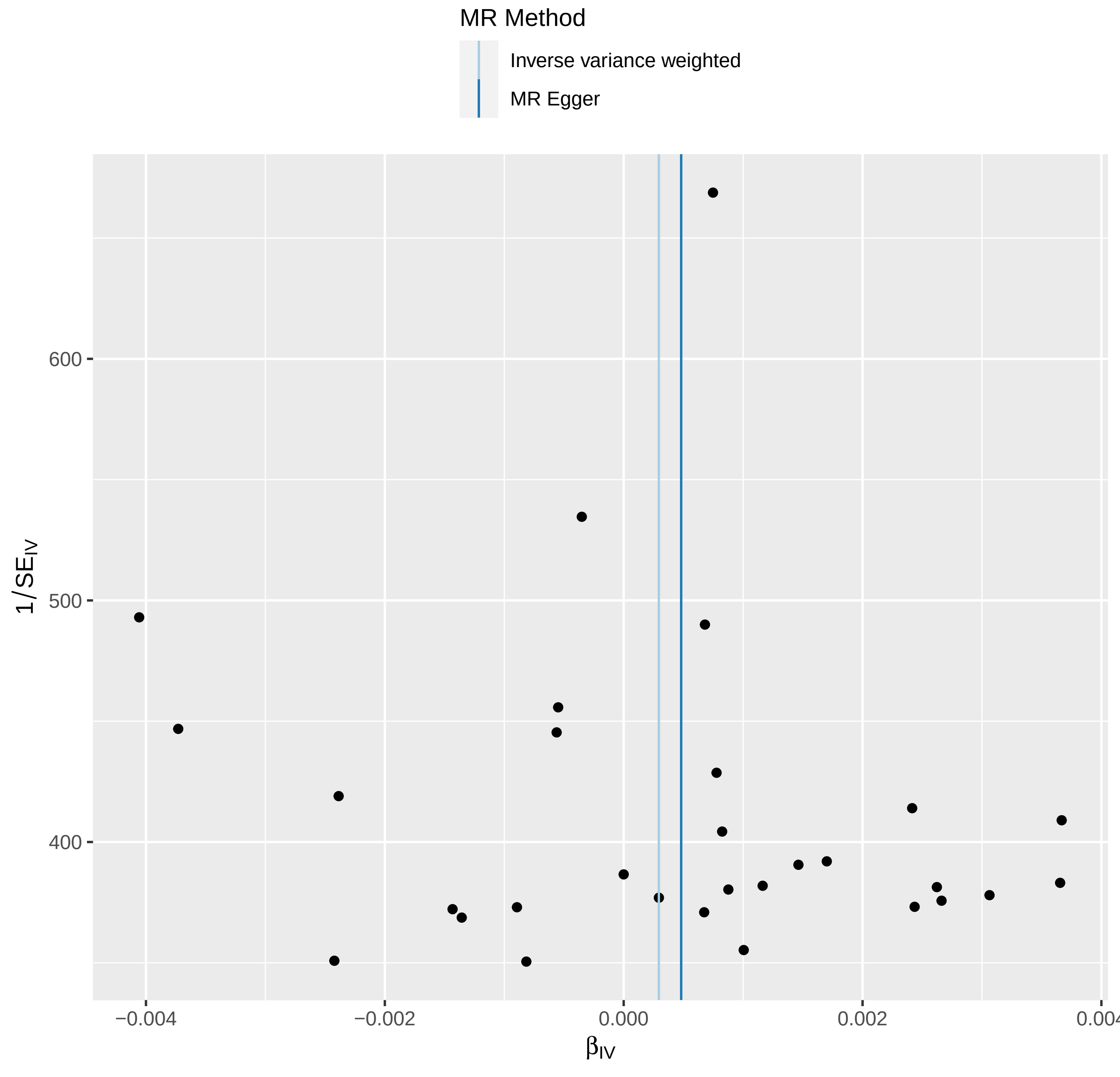

Batch 2−2907 : Sushi, von Willebrand factor type A, EGF and pentraxin domain−containing protein 1 on eGFR

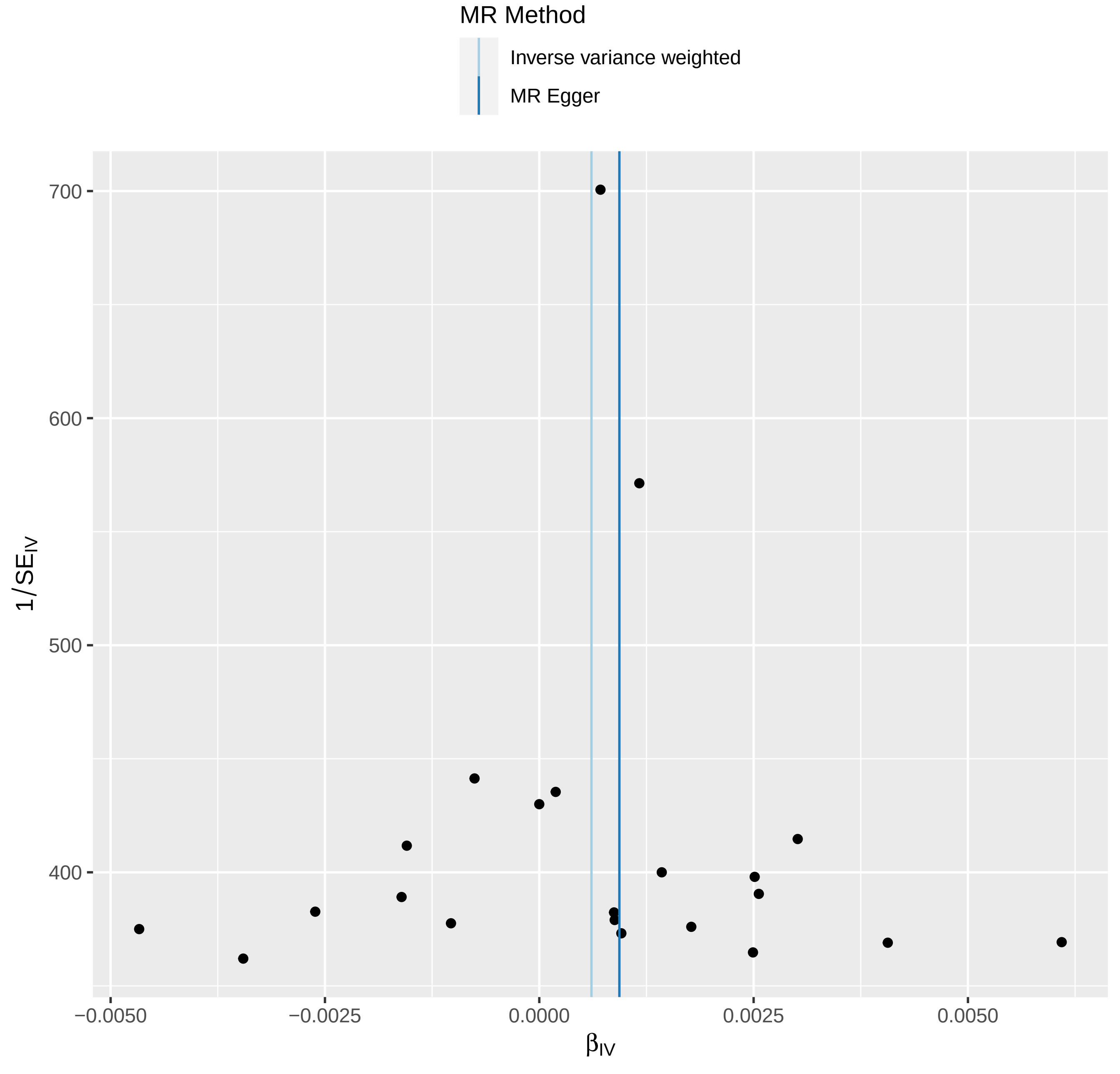

Batch 2−2908 : Switch−associated protein 70 on eGFR

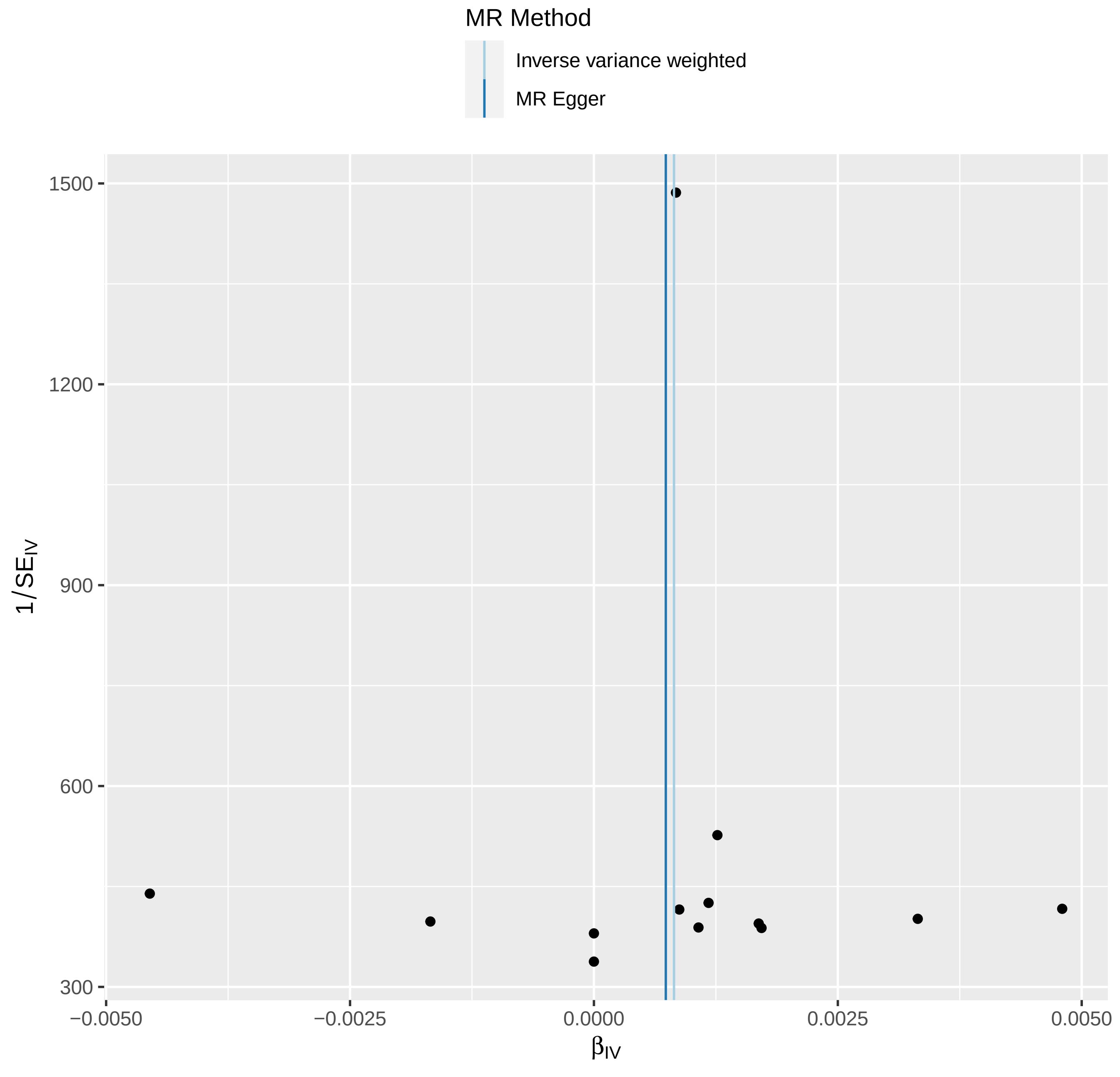

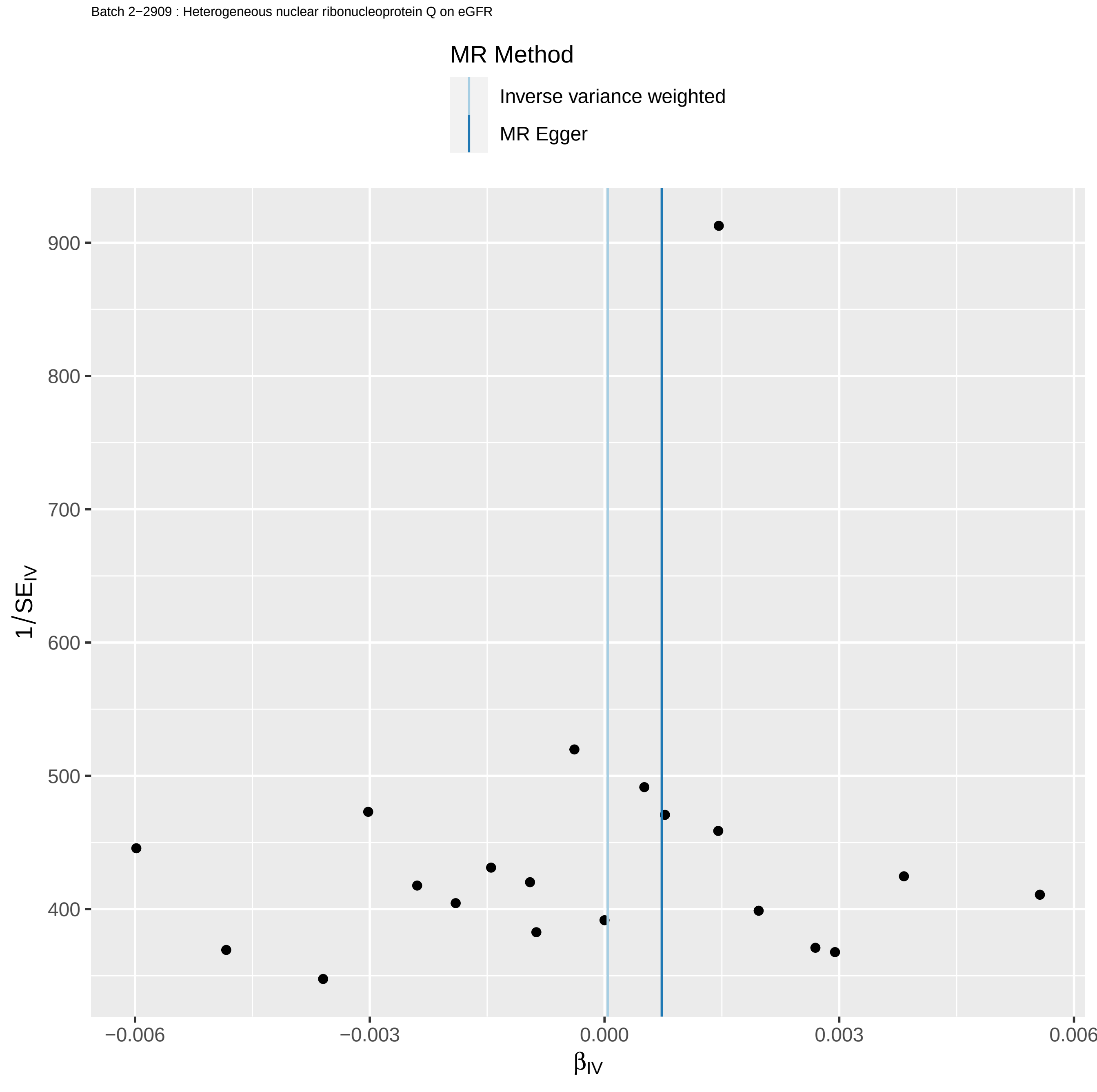

Batch 2−2909 : Heterogeneous nuclear ribonucleoprotein Q on eGFR
MR Method
Inverse variance weighted
MR Egger
900
800
700
600
500
400
1/SE_IV
−0.006
−0.003
0.000
0.003
0.006
β_IV

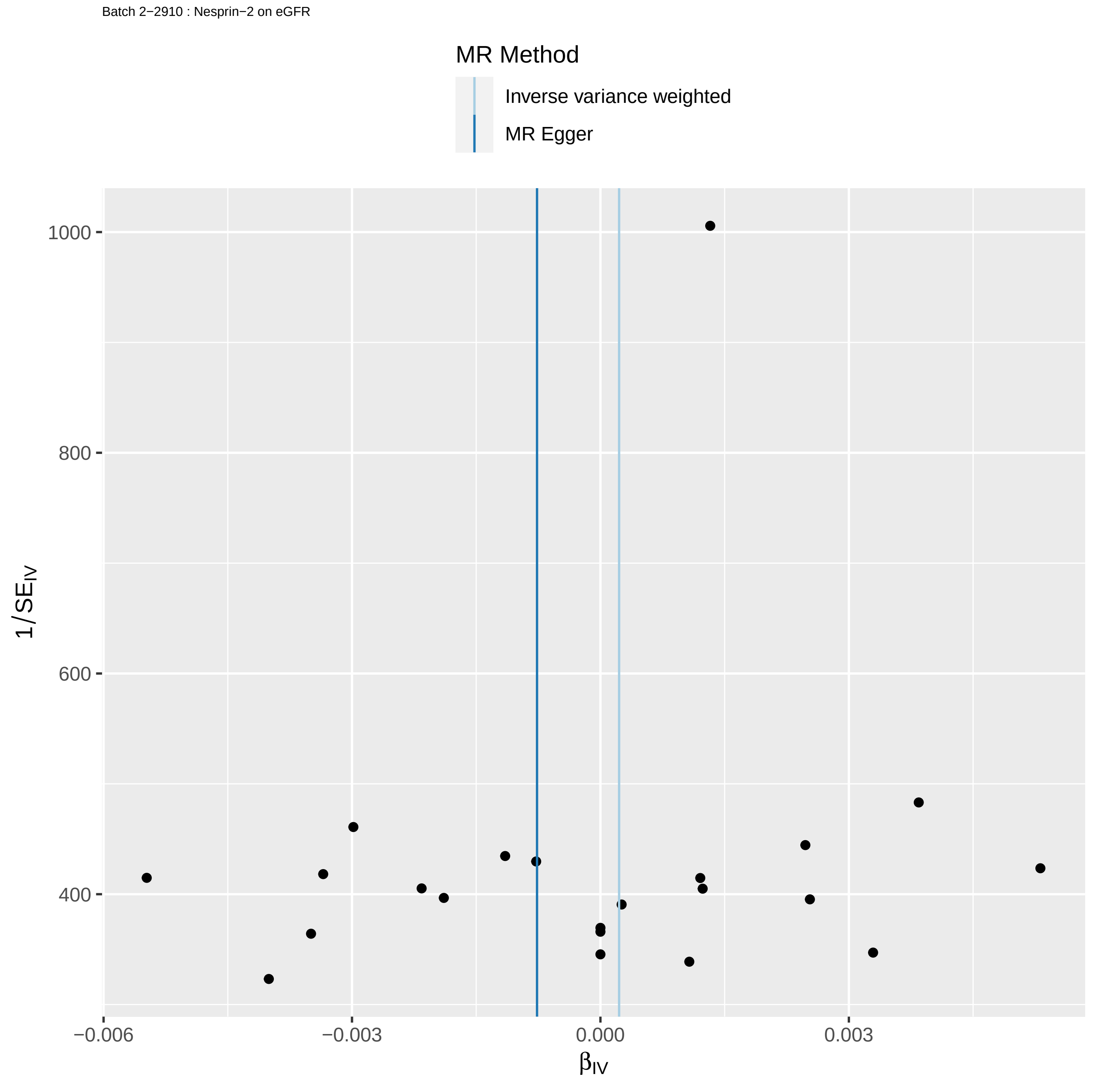

Batch 2−2910 : Nesprin−2 on eGFR
MR Method
Inverse variance weighted
MR Egger
1000
800
600
400
1/SE_IV
−0.006
−0.003
0.000
0.003
β_IV

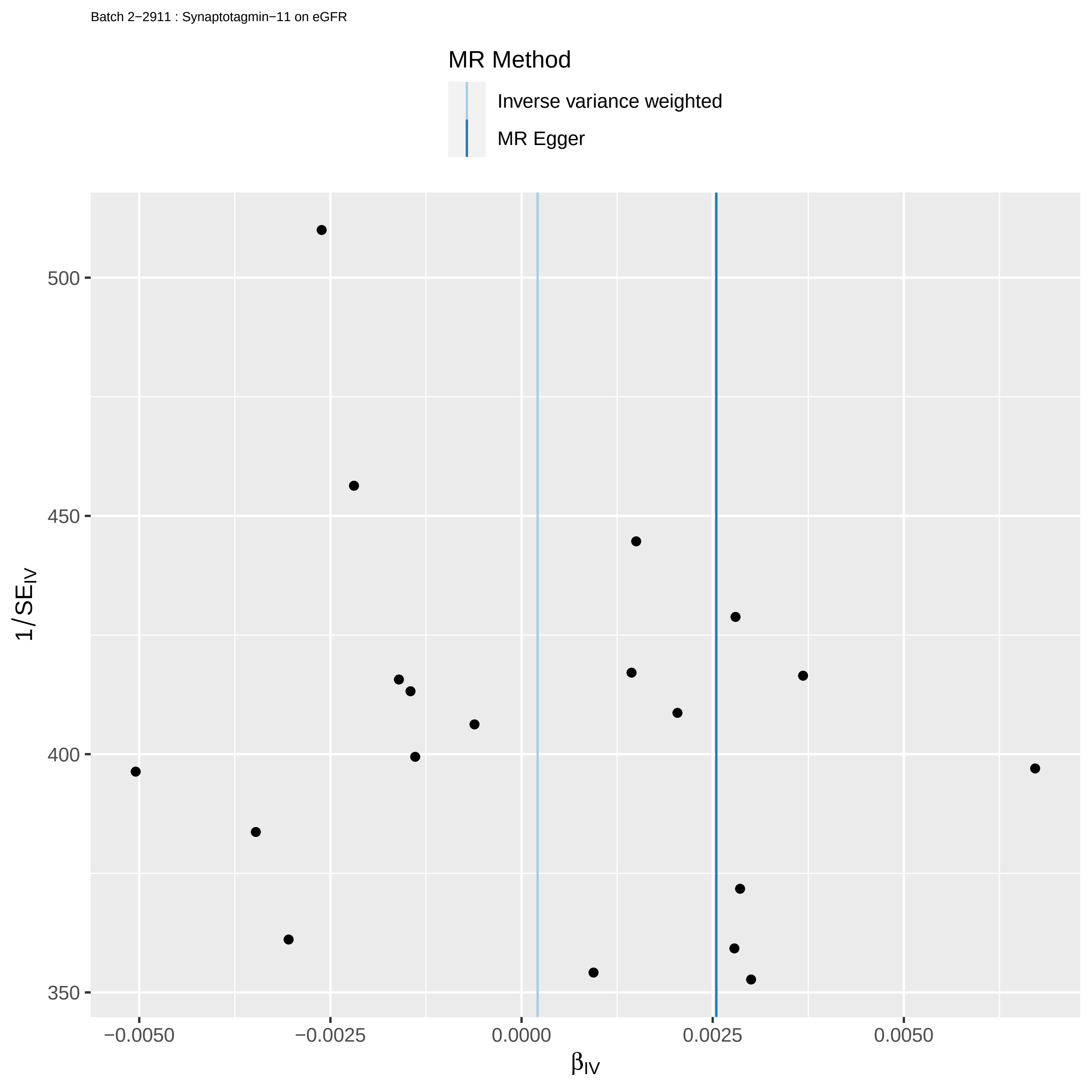

Batch 2−2911 : Synaptotagmin−11 on eGFR
MR Method
Inverse variance weighted
MR Egger
500
450
400
350
1/SE_IV
−0.0050
−0.0025
0.0000
0.0025
0.0050
β_IV

Batch 2−2912 : Synaptotagmin−17 on eGFR

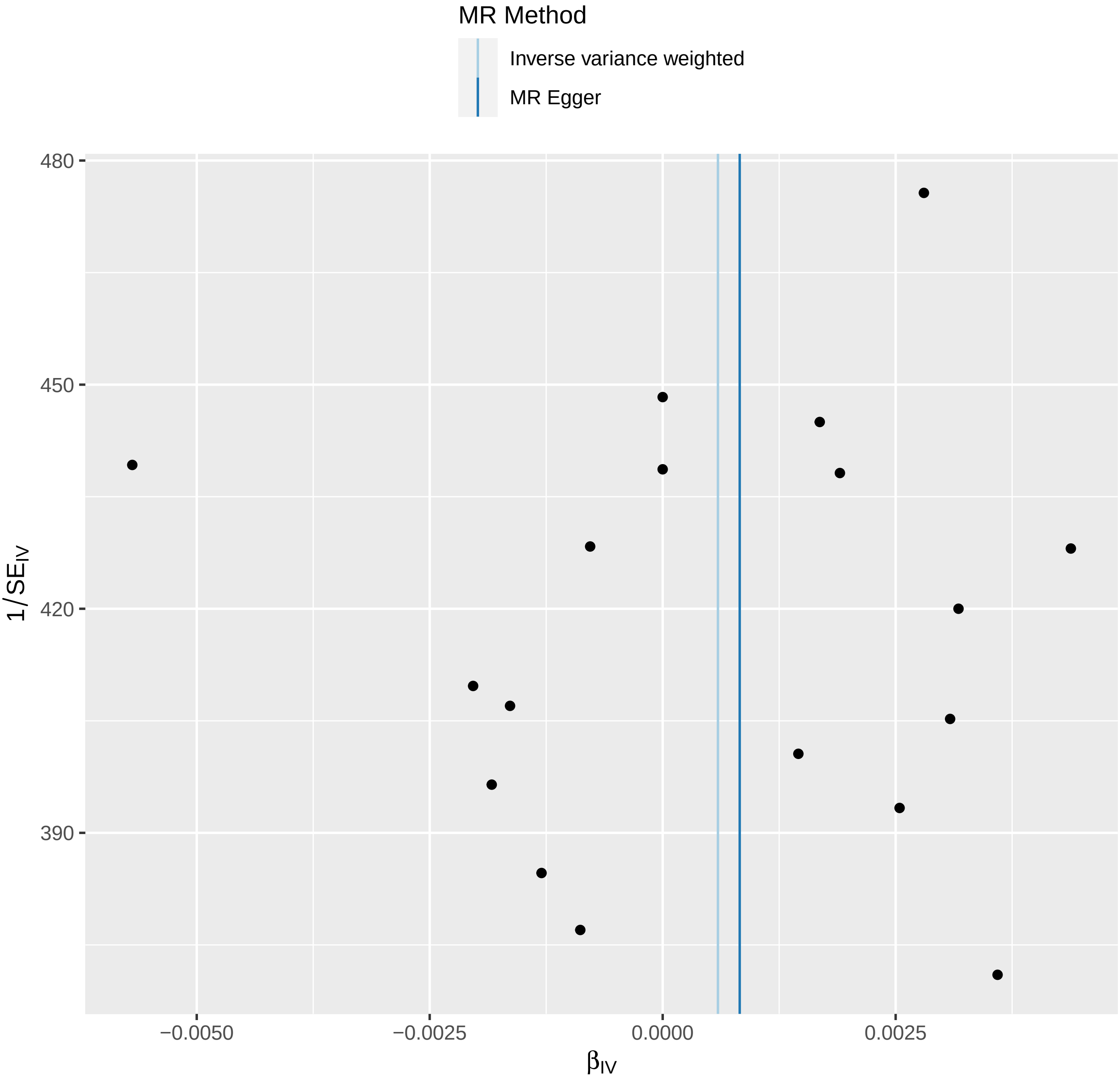

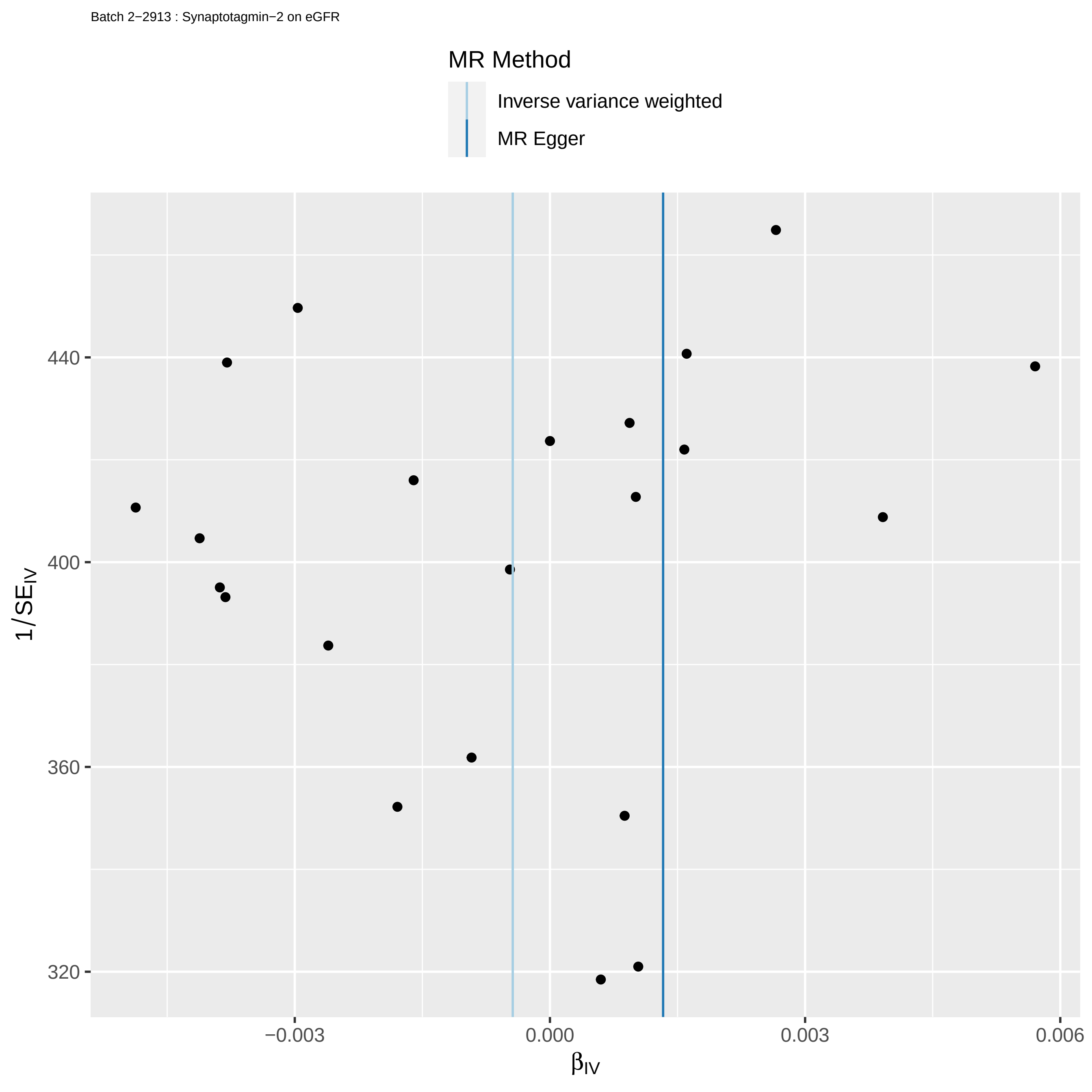

Batch 2−2913 : Synaptotagmin−2 on eGFR
MR Method
Inverse variance weighted
MR Egger
440
400
360
320
1/SE_IV
−0.003
0.000
0.003
0.006
β_IV

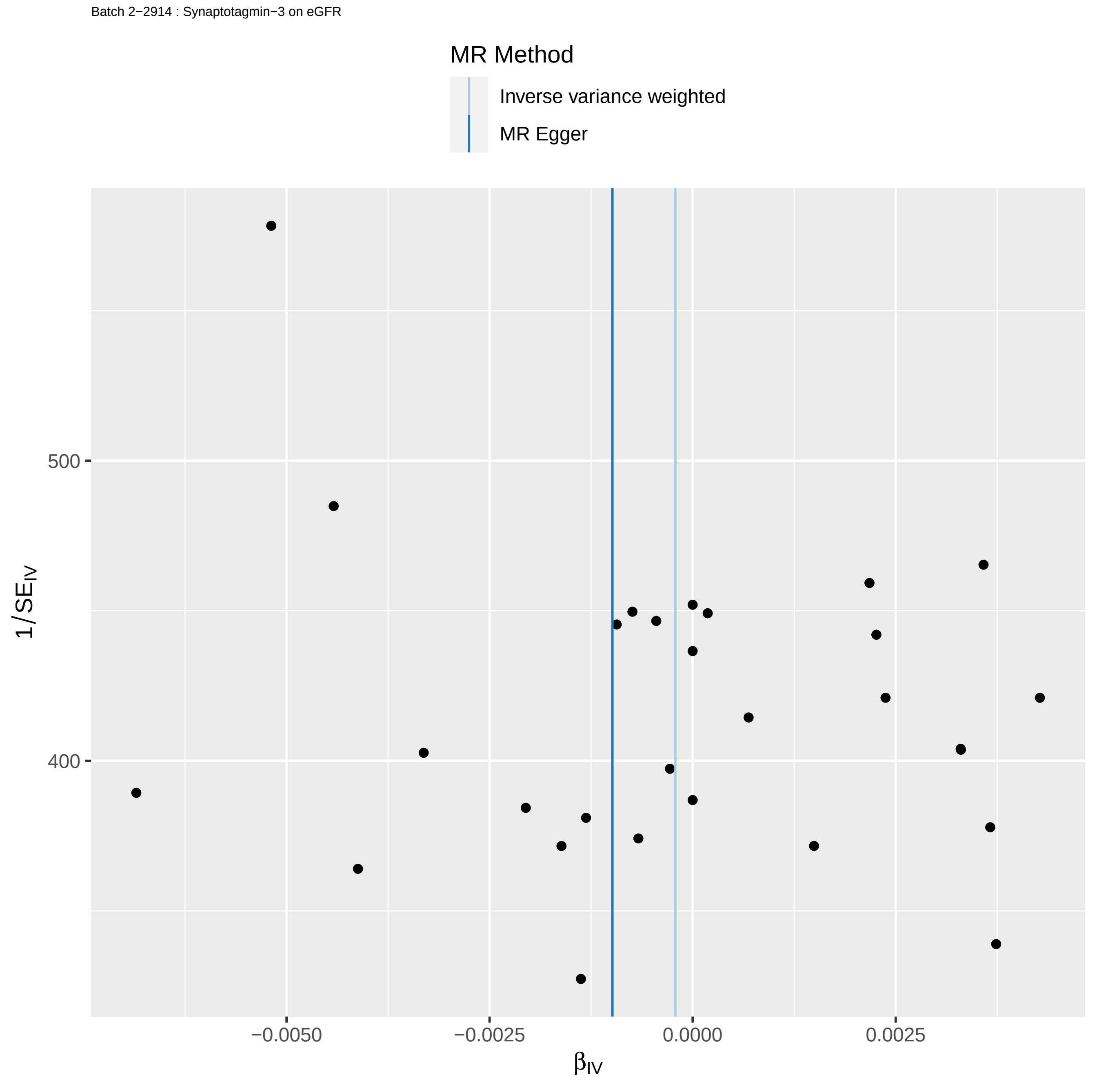
Batch 2−2914 : Synaptotagmin−3 on eGFR
MR Method
Inverse variance weighted
MR Egger
500
400
1/SE_IV
−0.0050
−0.0025
0.0000
0.0025
β_IV

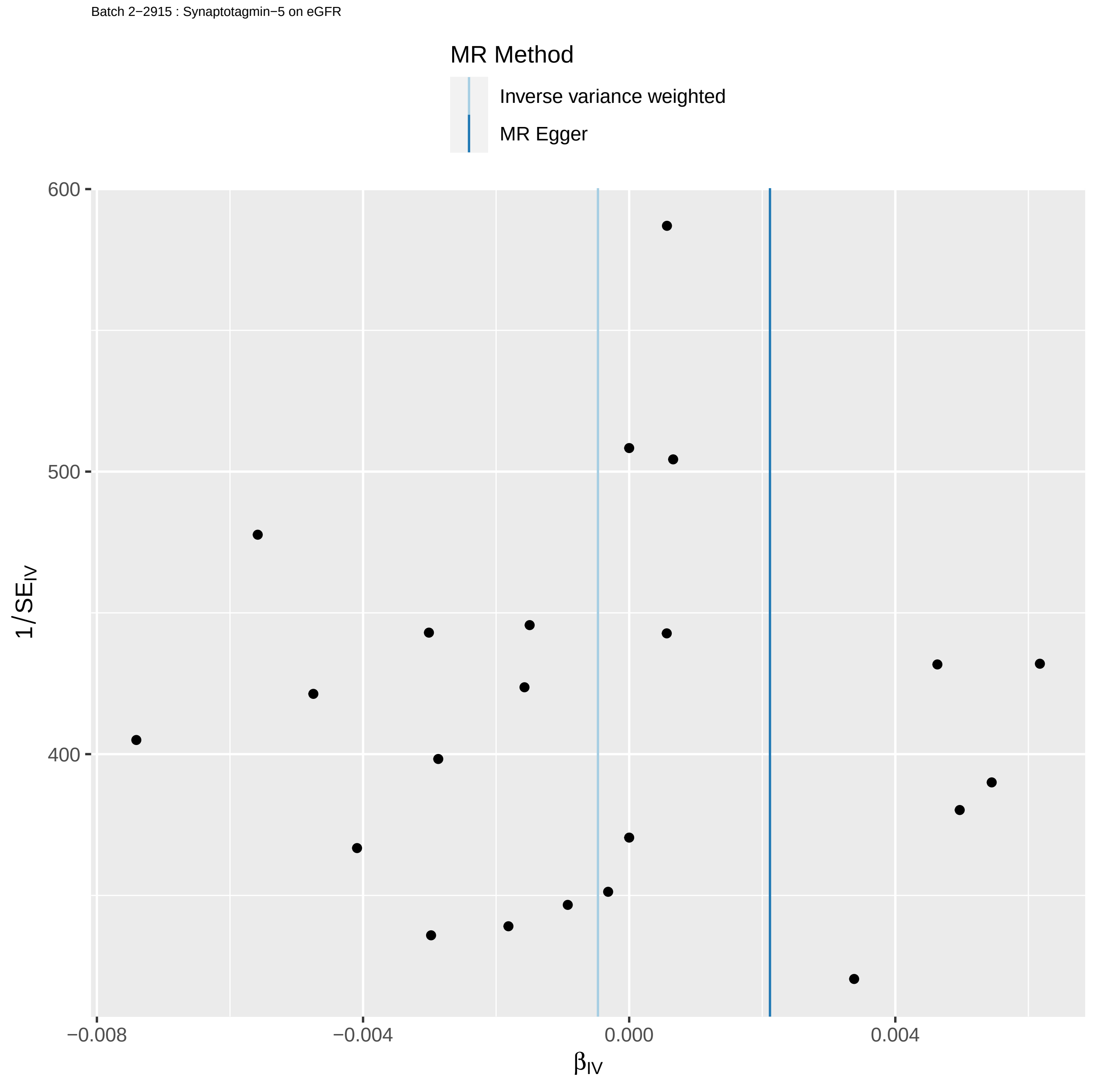
Batch 2−2915 : Synaptotagmin−5 on eGFR
MR Method
Inverse variance weighted
MR Egger
600
500
400
1/SE$_{IV}$
−0.008
−0.004
0.000
0.004
β$_{IV}$

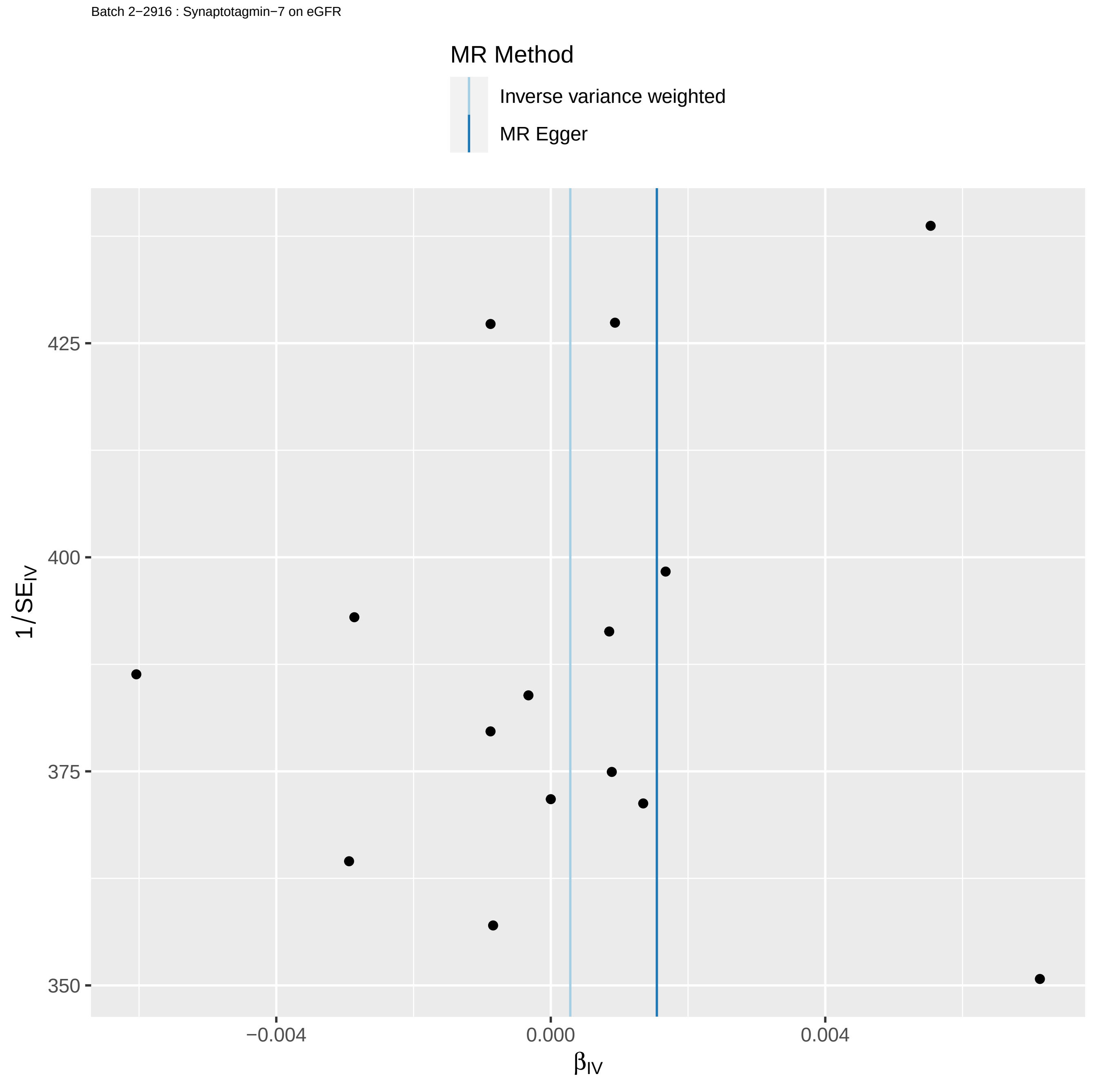
Batch 2−2916 : Synaptotagmin−7 on eGFR
MR Method
Inverse variance weighted
MR Egger
425
400
375
350
1/SE_IV
−0.004
0.000
0.004
β_IV

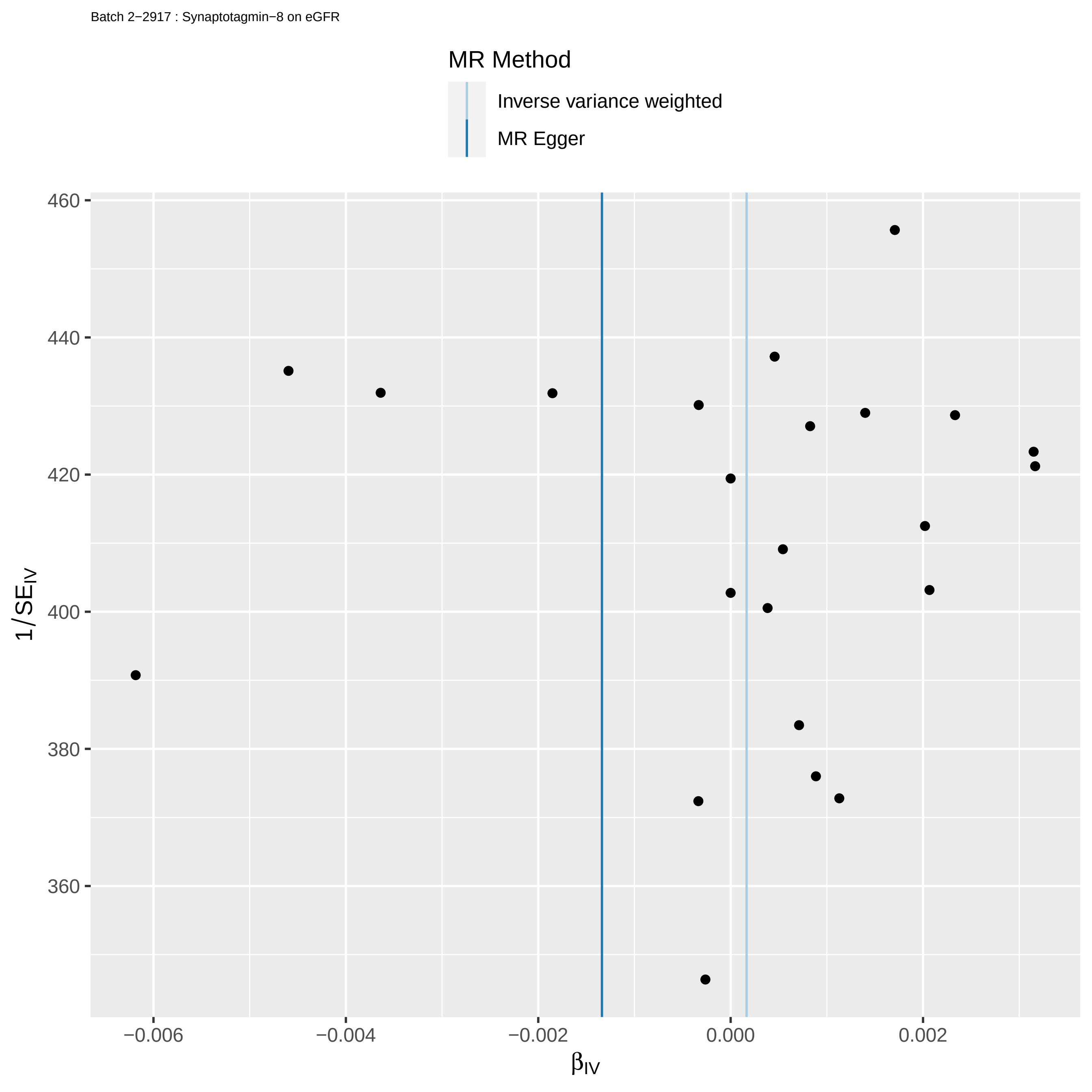
Batch 2−2917 : Synaptotagmin−8 on eGFR
MR Method
Inverse variance weighted
MR Egger
460
440
420
400
380
360
1/SE_IV
−0.006
−0.004
−0.002
0.000
0.002
β_IV

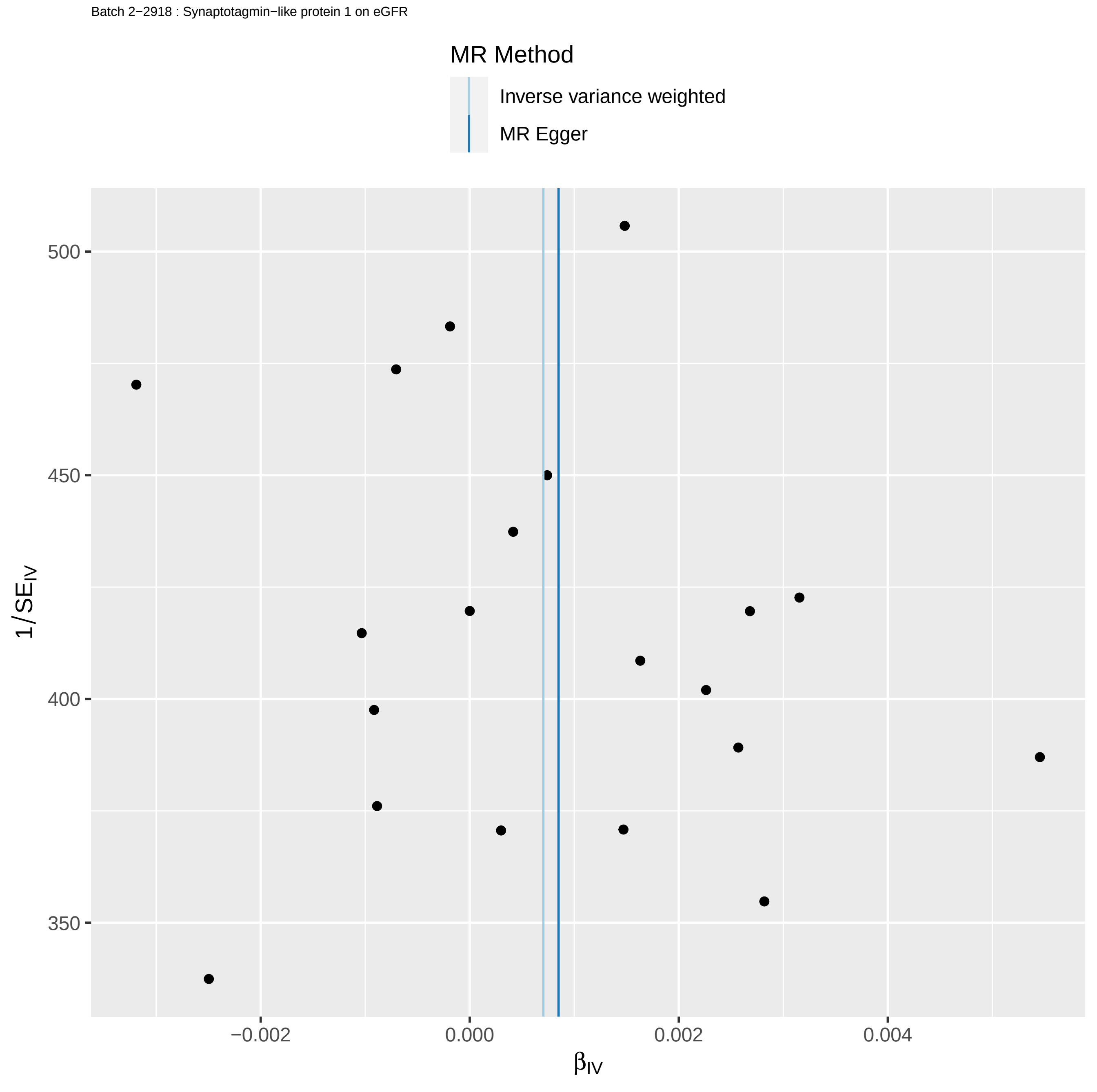
Batch 2−2918 : Synaptotagmin−like protein 1 on eGFR
MR Method
Inverse variance weighted
MR Egger
500
450
400
350
−0.002
0.000
0.002
0.004
1/SE_IV
β_IV

Batch 2−2919 : Synaptotagmin−like protein 4 on eGFR

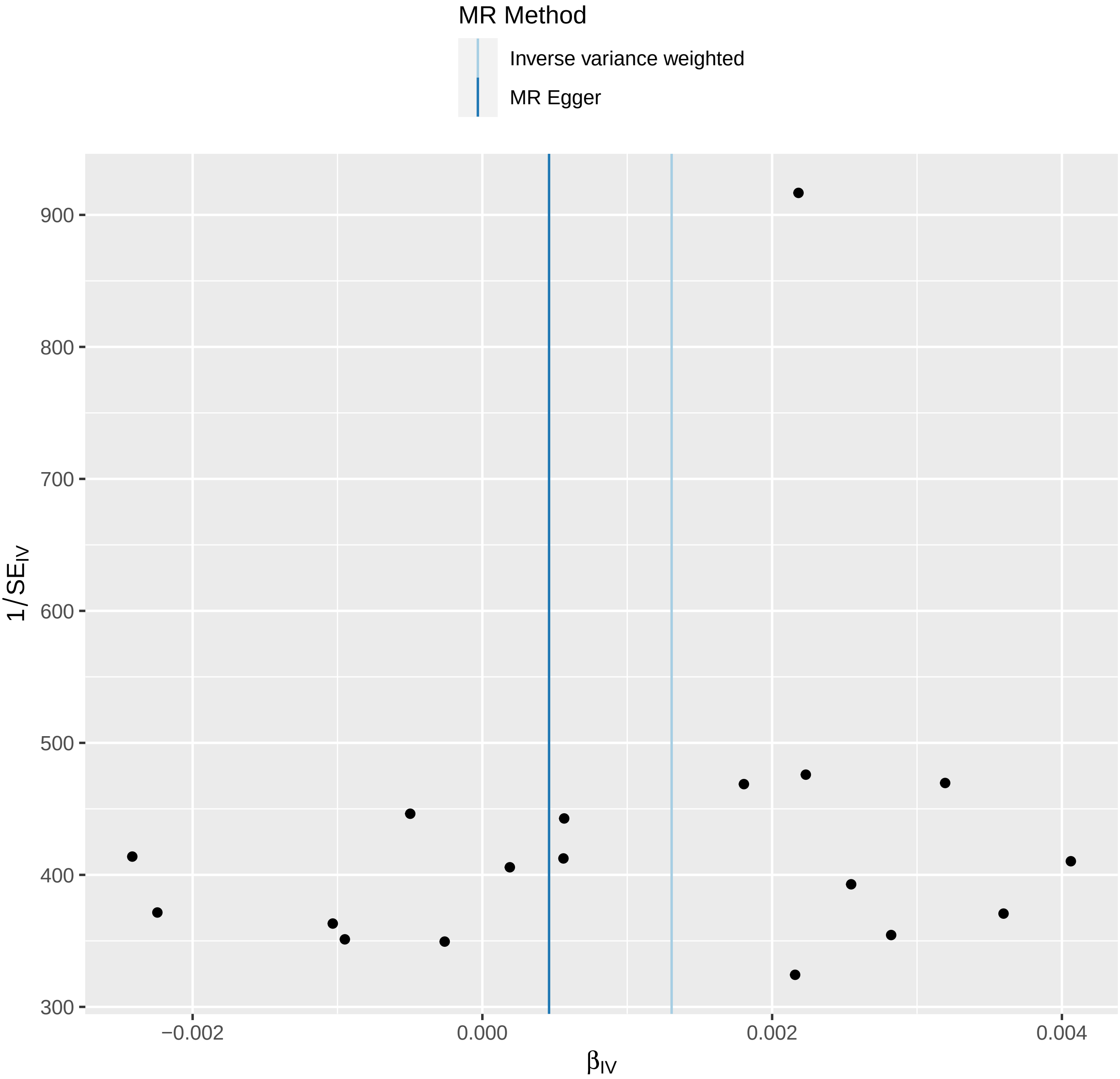

Batch 2−2920 : Protachykinin−1 on eGFR

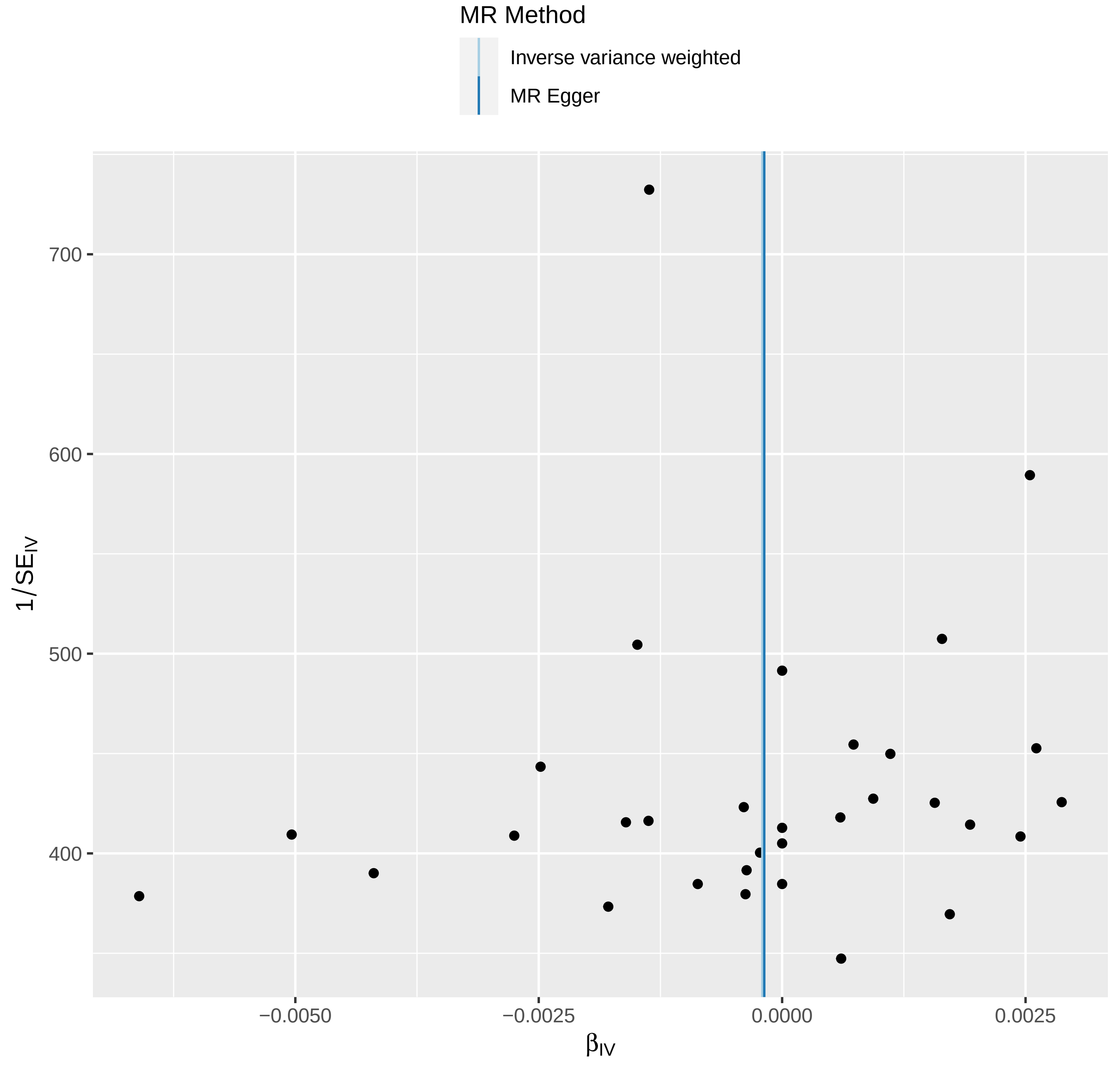

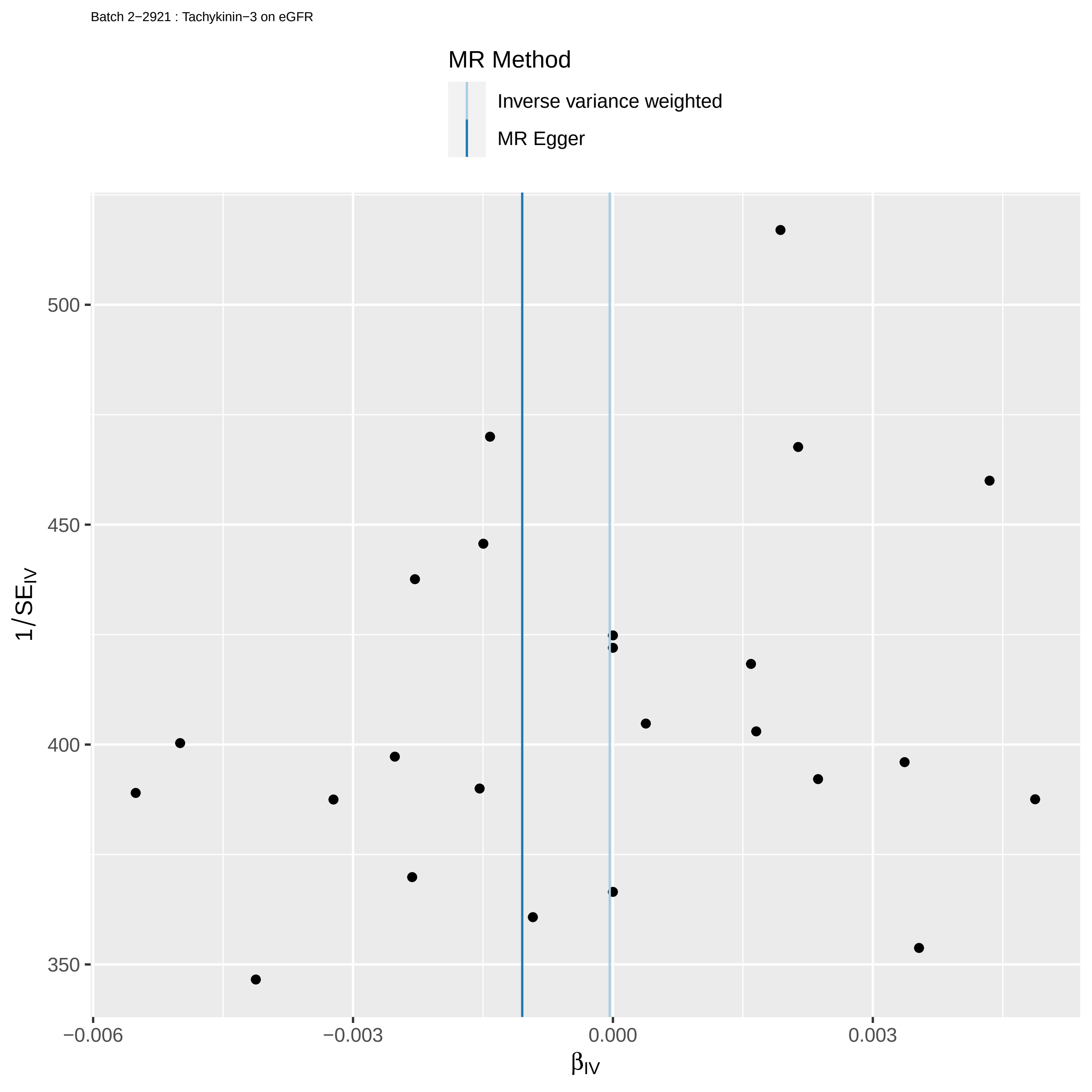
Batch 2−2921 : Tachykinin−3 on eGFR
MR Method
Inverse variance weighted
MR Egger
500
450
400
350
1/SE$_{IV}$
−0.006
−0.003
0.000
0.003
β$_{IV}$

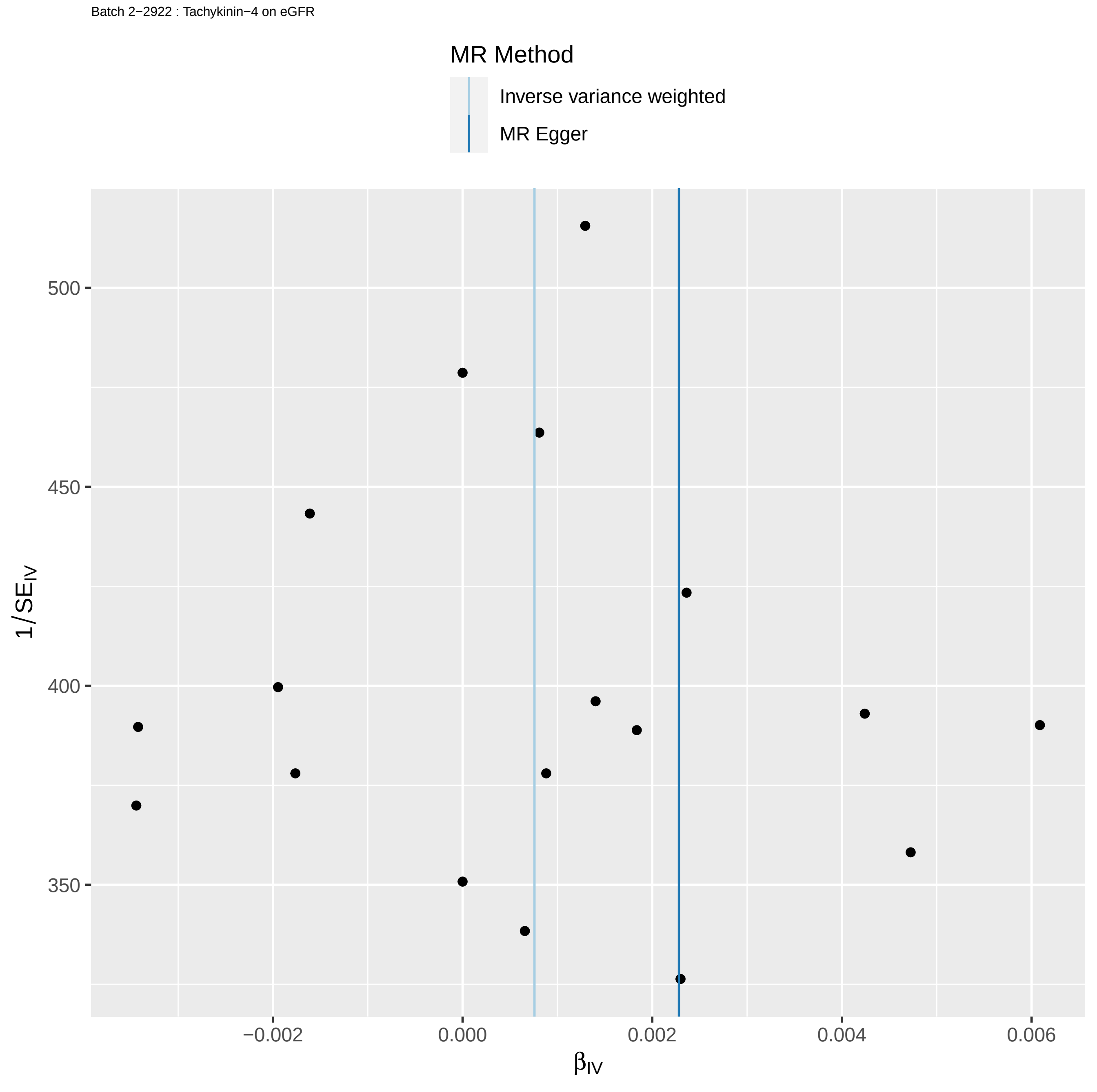

Batch 2−2922 : Tachykinin−4 on eGFR
MR Method
Inverse variance weighted
MR Egger
500
450
400
350
1/SE_IV
−0.002
0.000
0.002
0.004
0.006
β_IV

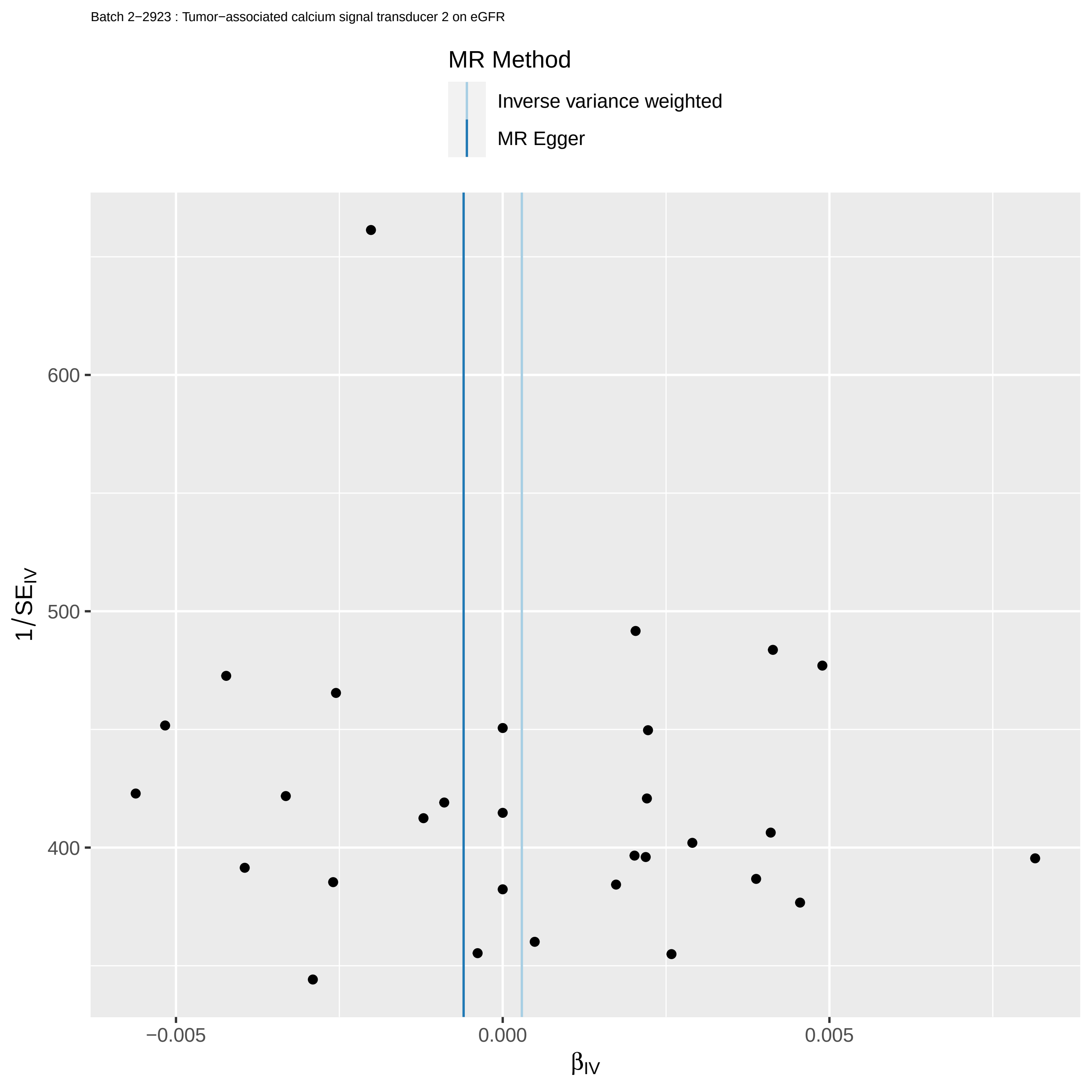

Batch 2−2923 : Tumor−associated calcium signal transducer 2 on eGFR
MR Method
Inverse variance weighted
MR Egger
600
500
400
1/SE_IV
−0.005
0.000
0.005
β_IV

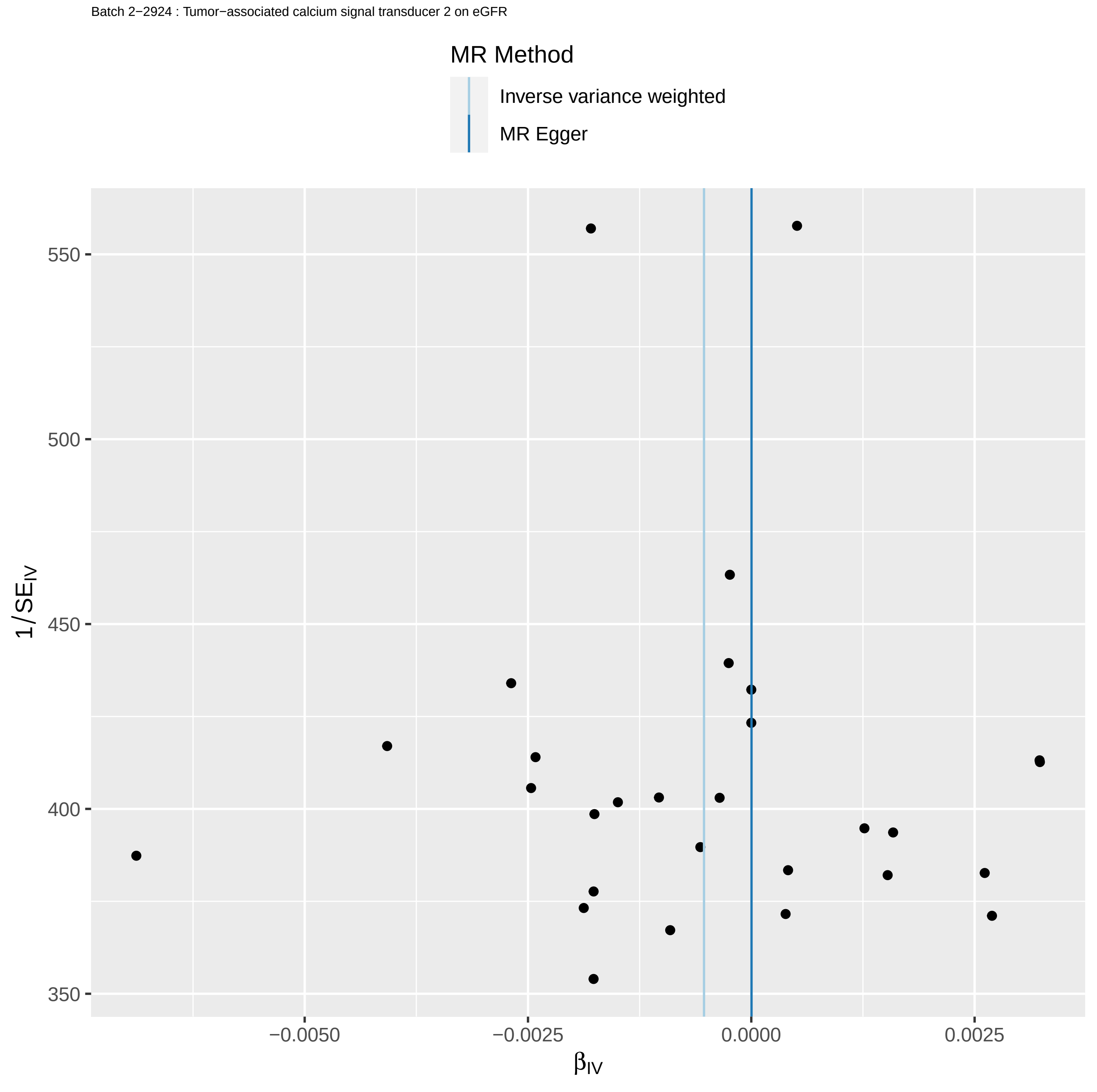

Batch 2−2924 : Tumor−associated calcium signal transducer 2 on eGFR
MR Method
Inverse variance weighted
MR Egger
550
500
450
400
350
1/SE$_{IV}$
−0.0050
−0.0025
0.0000
0.0025
β$_{IV}$

Batch 2−2925 : Transgelin on eGFR

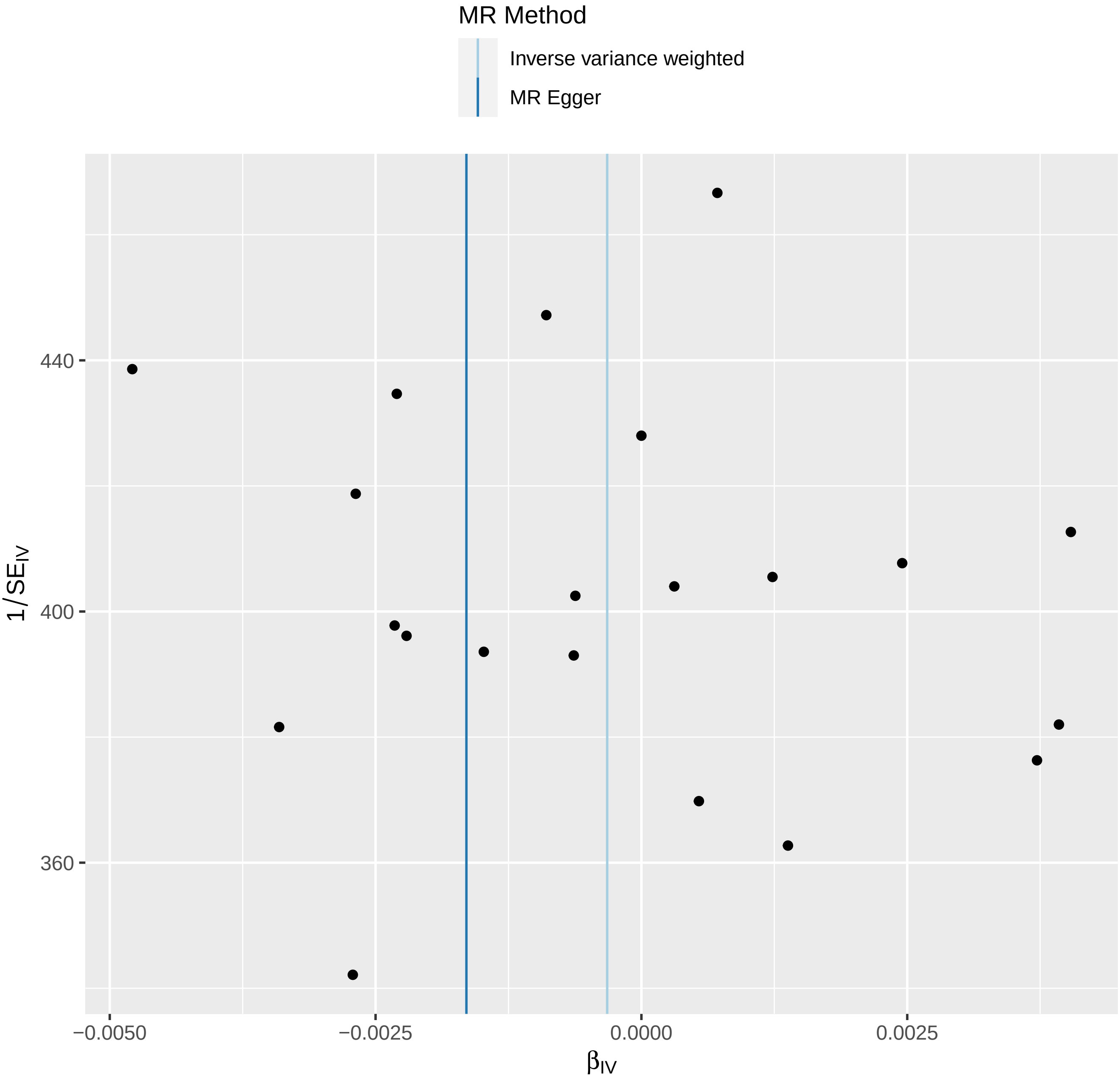

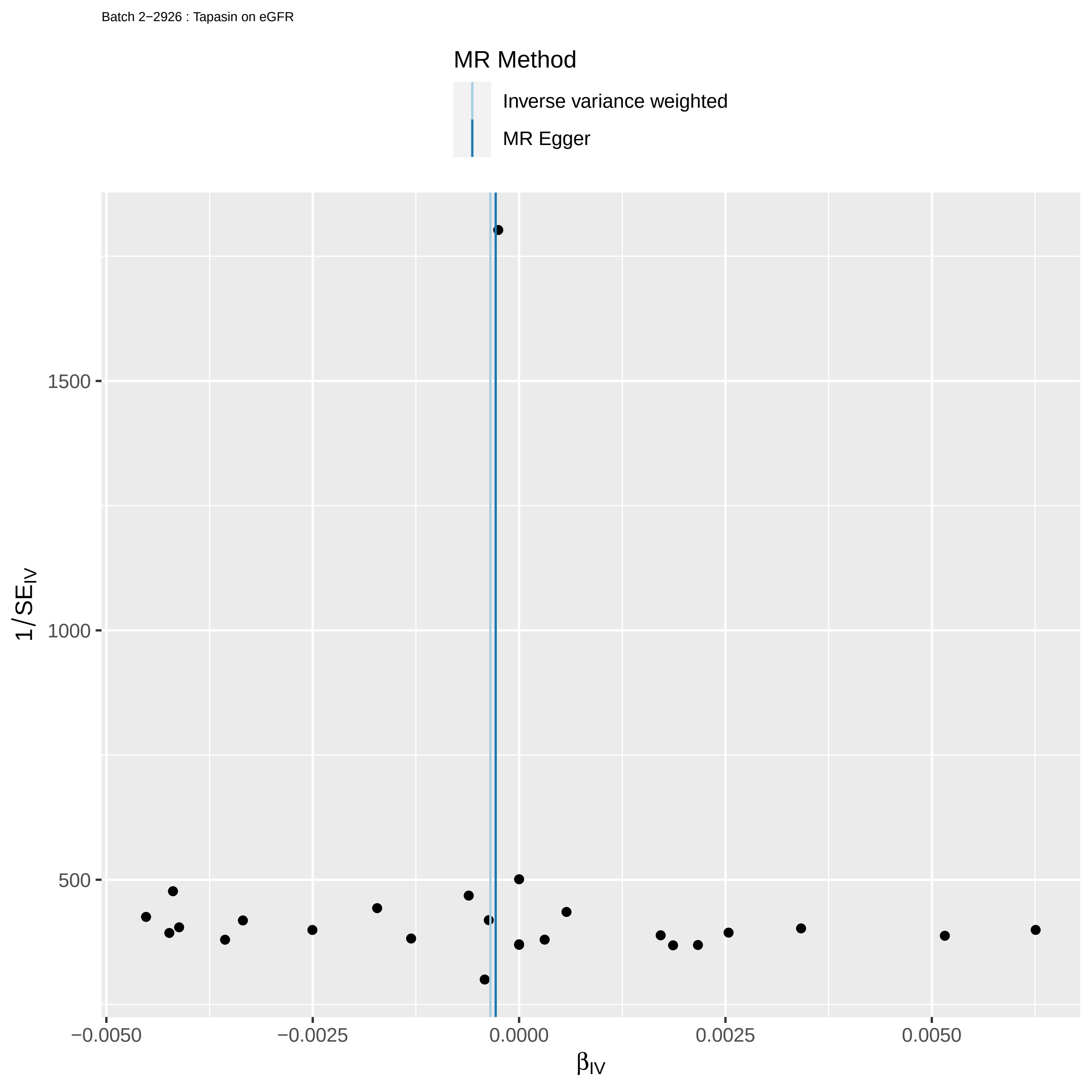

Batch 2−2926 : Tapasin on eGFR
MR Method
Inverse variance weighted
MR Egger
1500
1000
500
1/SE_IV
−0.0050
−0.0025
0.0000
0.0025
0.0050
β_IV

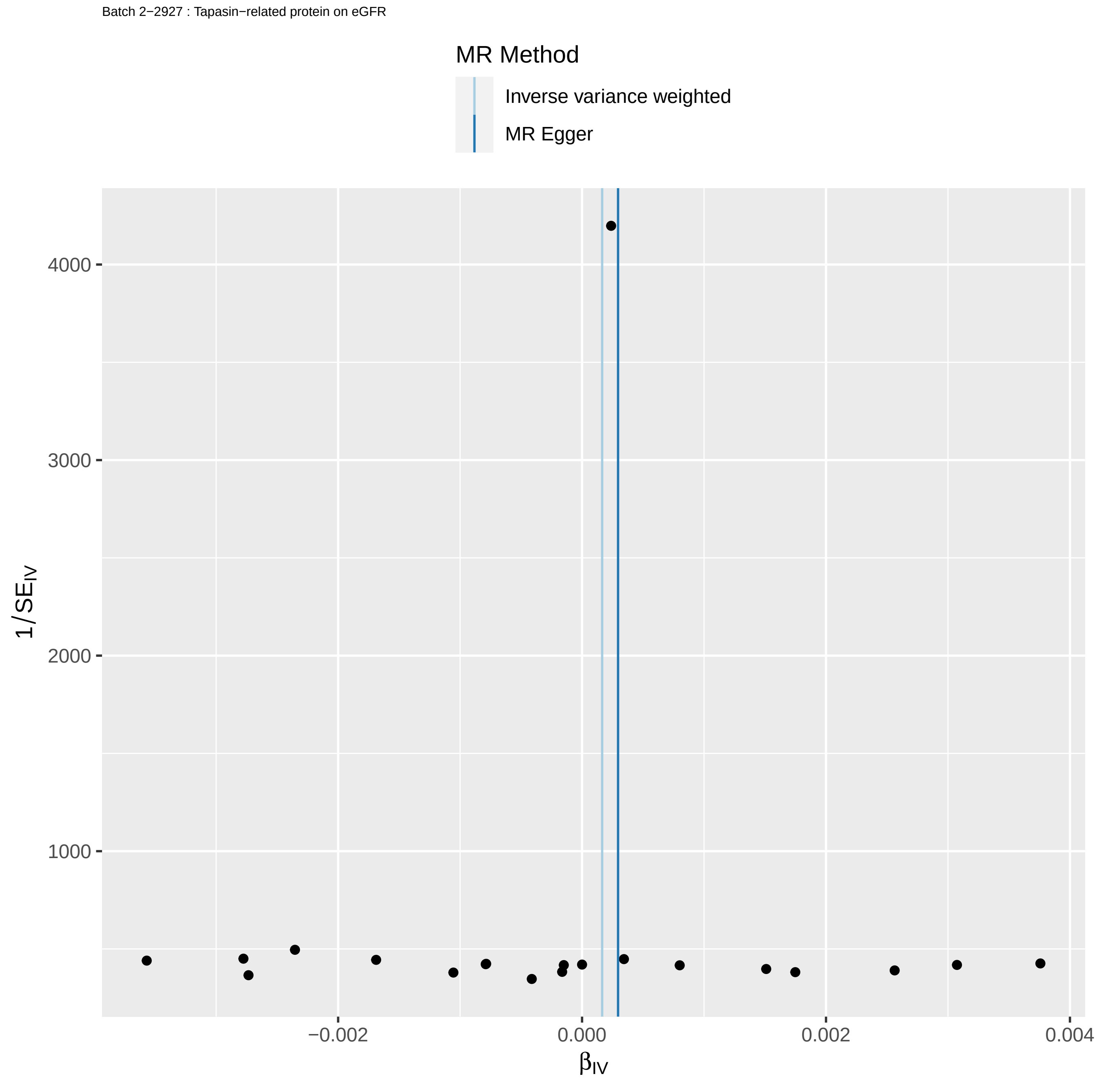

Batch 2−2927 : Tapasin−related protein on eGFR
MR Method
Inverse variance weighted
MR Egger
4000
3000
2000
1000
1/SE_IV
−0.002
0.000
0.002
0.004
β_IV

Batch 2−2928 : Tax1−binding protein 1 on eGFR

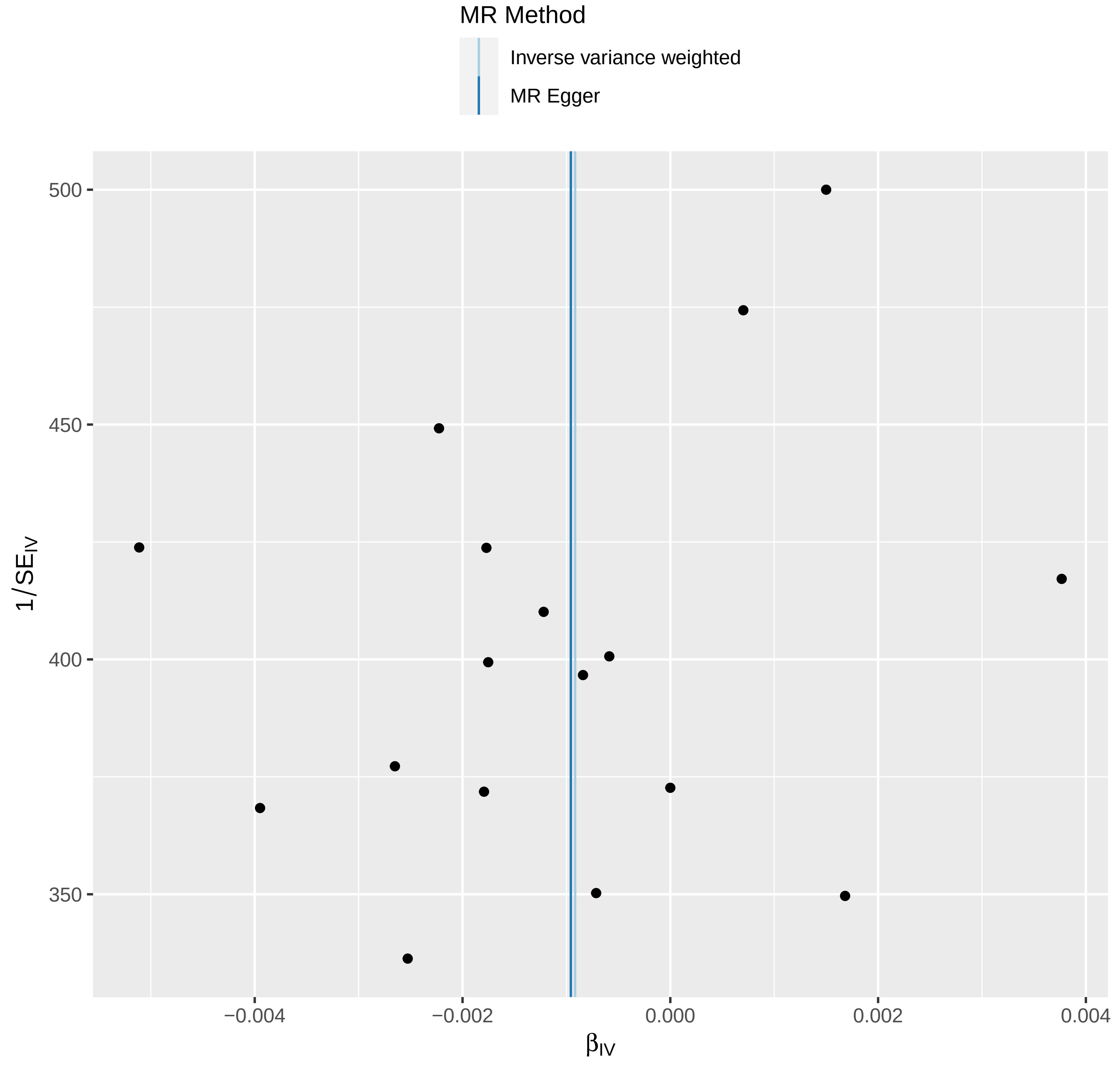

Batch 2−2929 : Tax1−binding protein 3 on eGFR

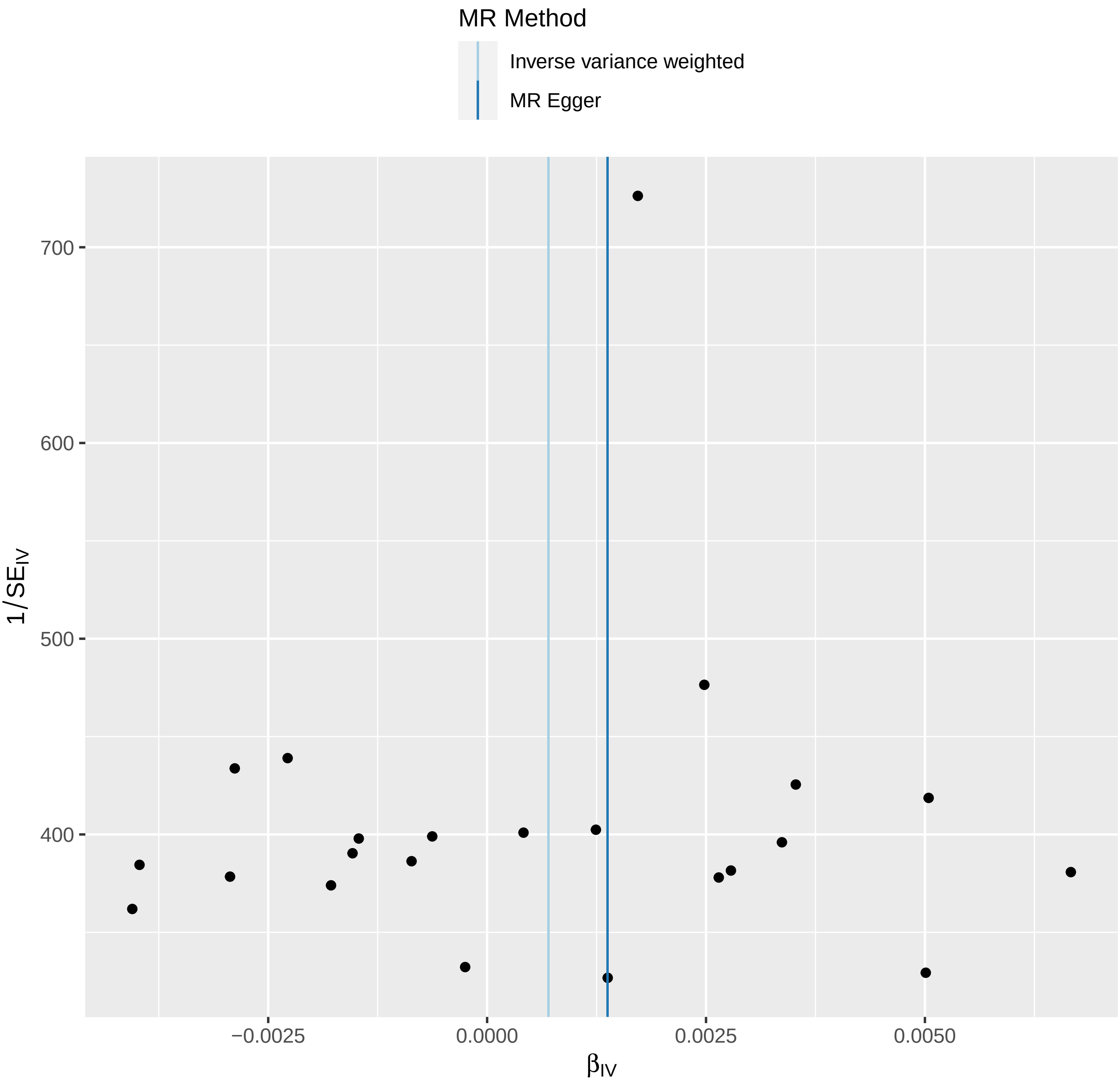

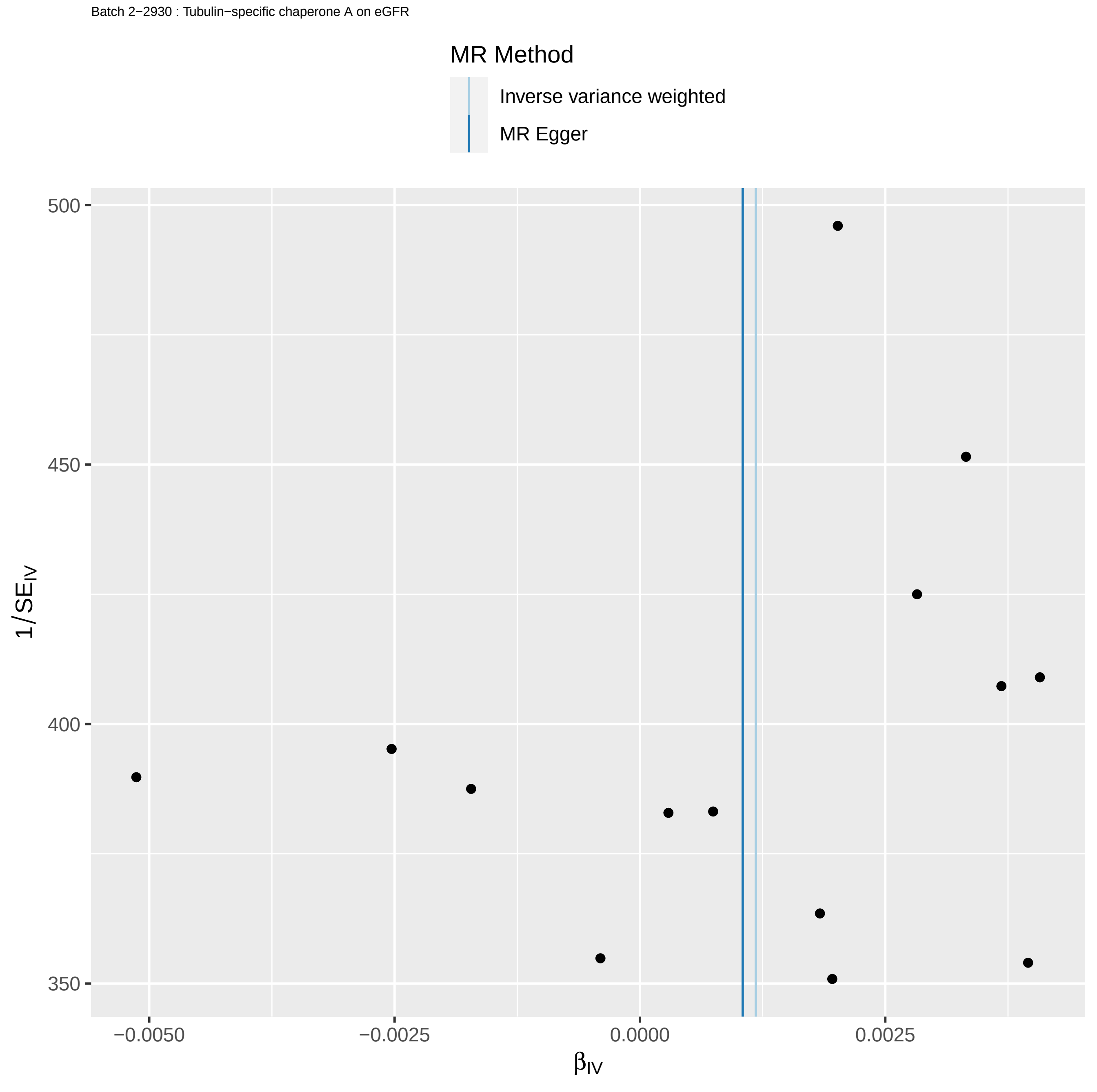
Batch 2−2930 : Tubulin−specific chaperone A on eGFR
MR Method
Inverse variance weighted
MR Egger
500
450
400
350
1/SE_IV
−0.0050
−0.0025
0.0000
0.0025
β_IV

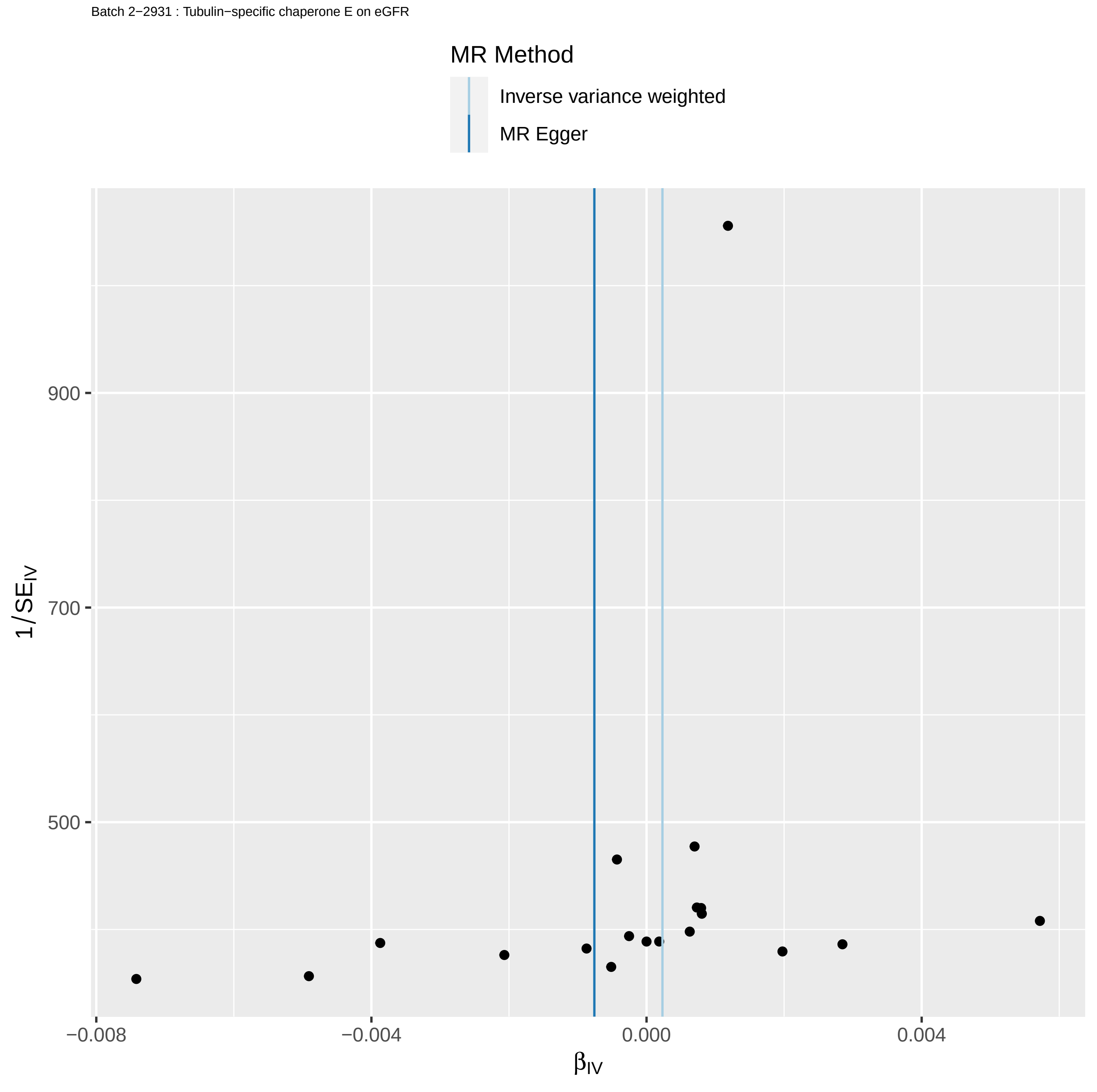
Batch 2−2931 : Tubulin−specific chaperone E on eGFR
MR Method
Inverse variance weighted
MR Egger
900
700
500
1/SE$_{IV}$
−0.008
−0.004
0.000
0.004
β_{IV}

Batch 2−2932 : Serine/threonine−protein kinase TBK1 on eGFR

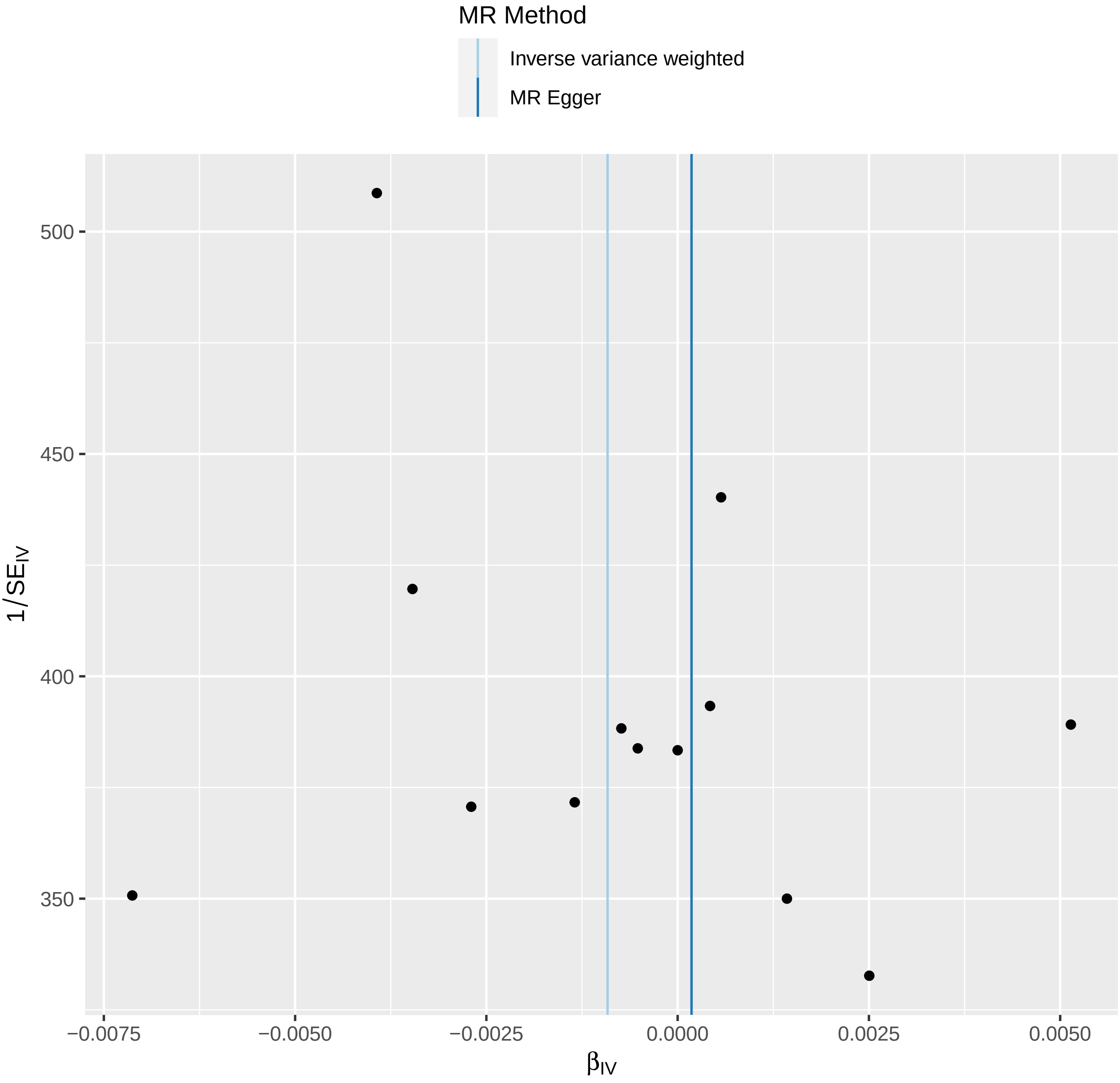

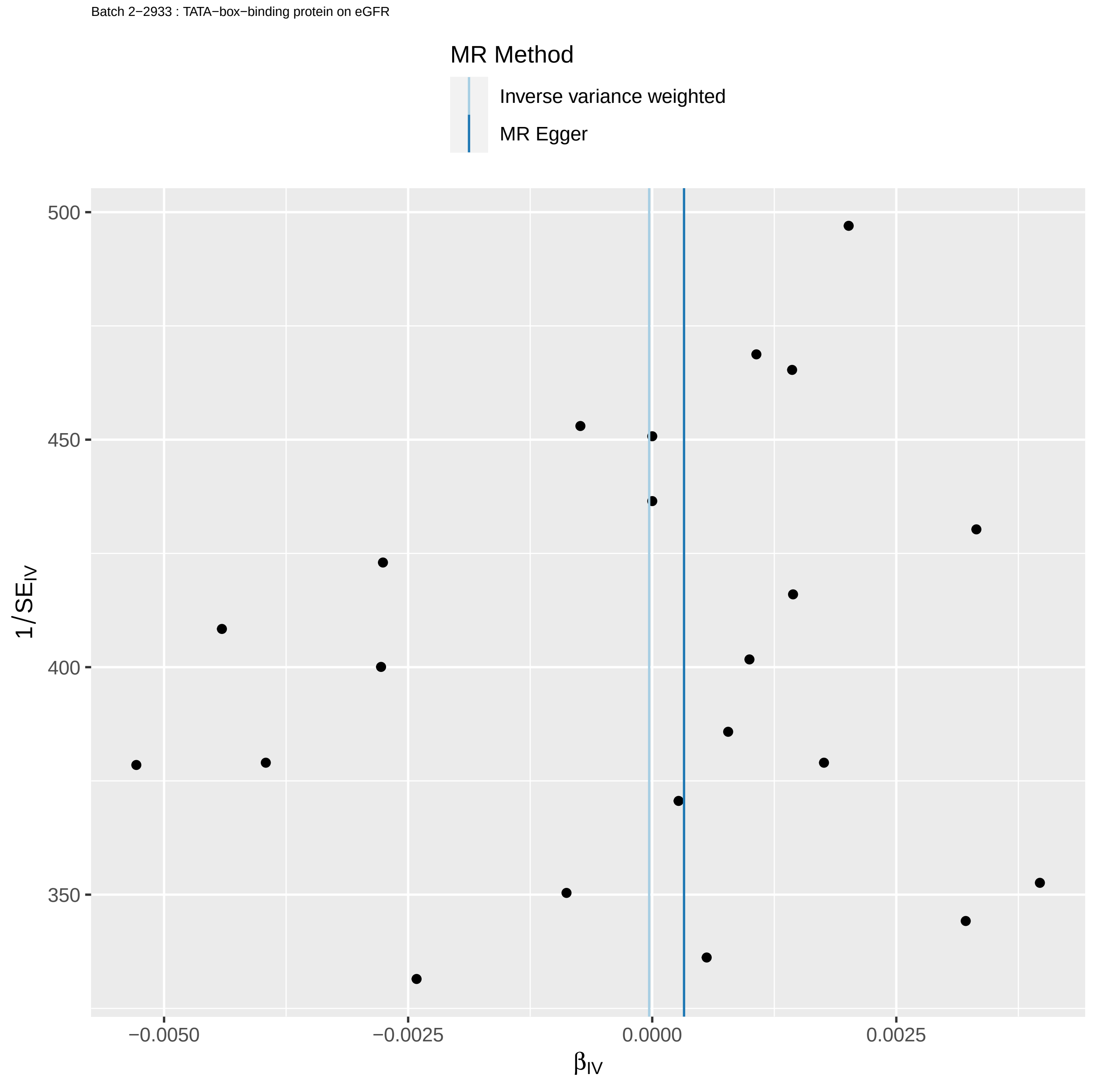

Batch 2−2933 : TATA−box−binding protein on eGFR
MR Method
Inverse variance weighted
MR Egger
500
450
400
350
1/SE_IV
−0.0050
−0.0025
0.0000
0.0025
β_IV

Batch 2−2934 : T−box transcription factor TBX22 on eGFR

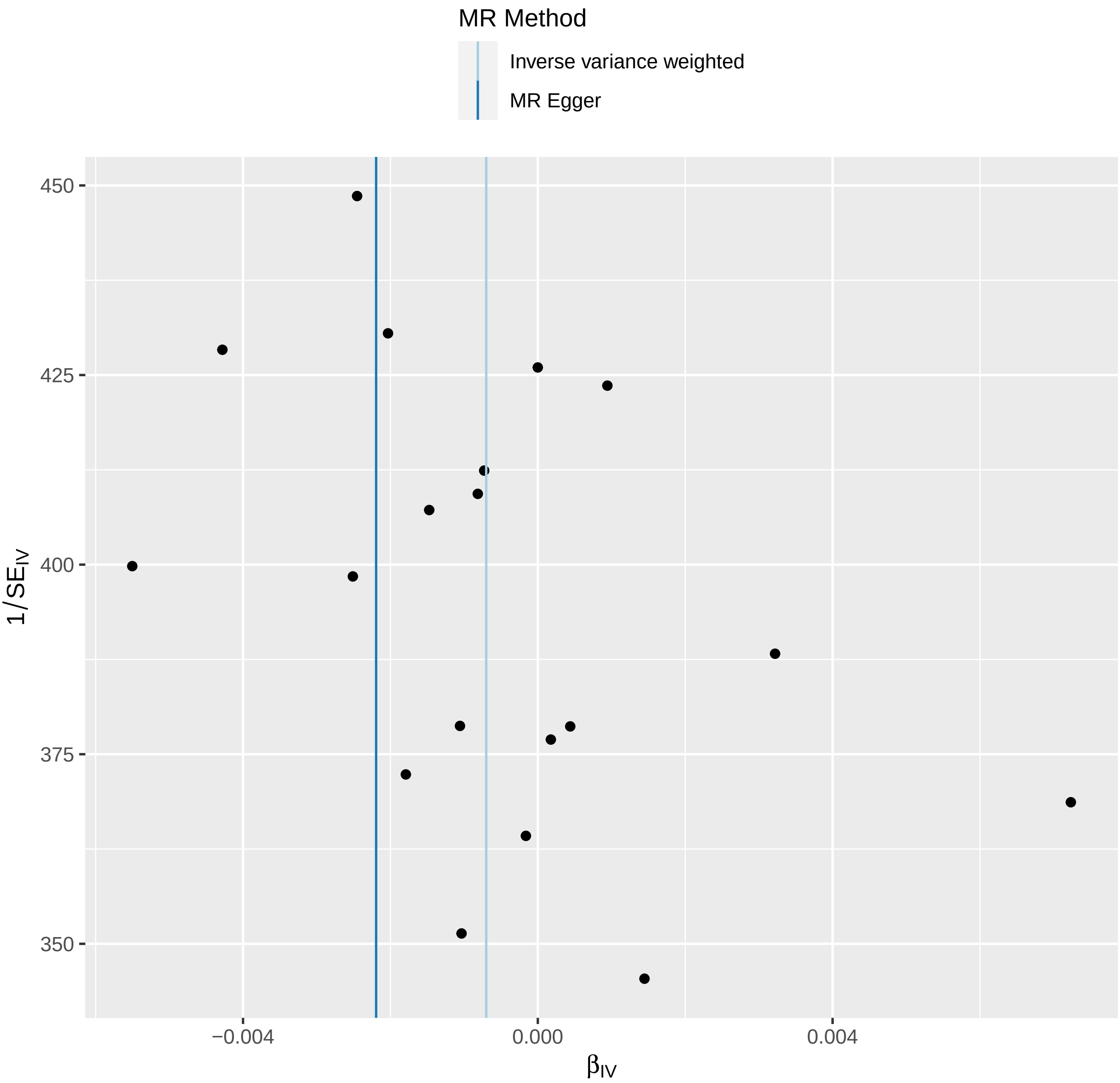

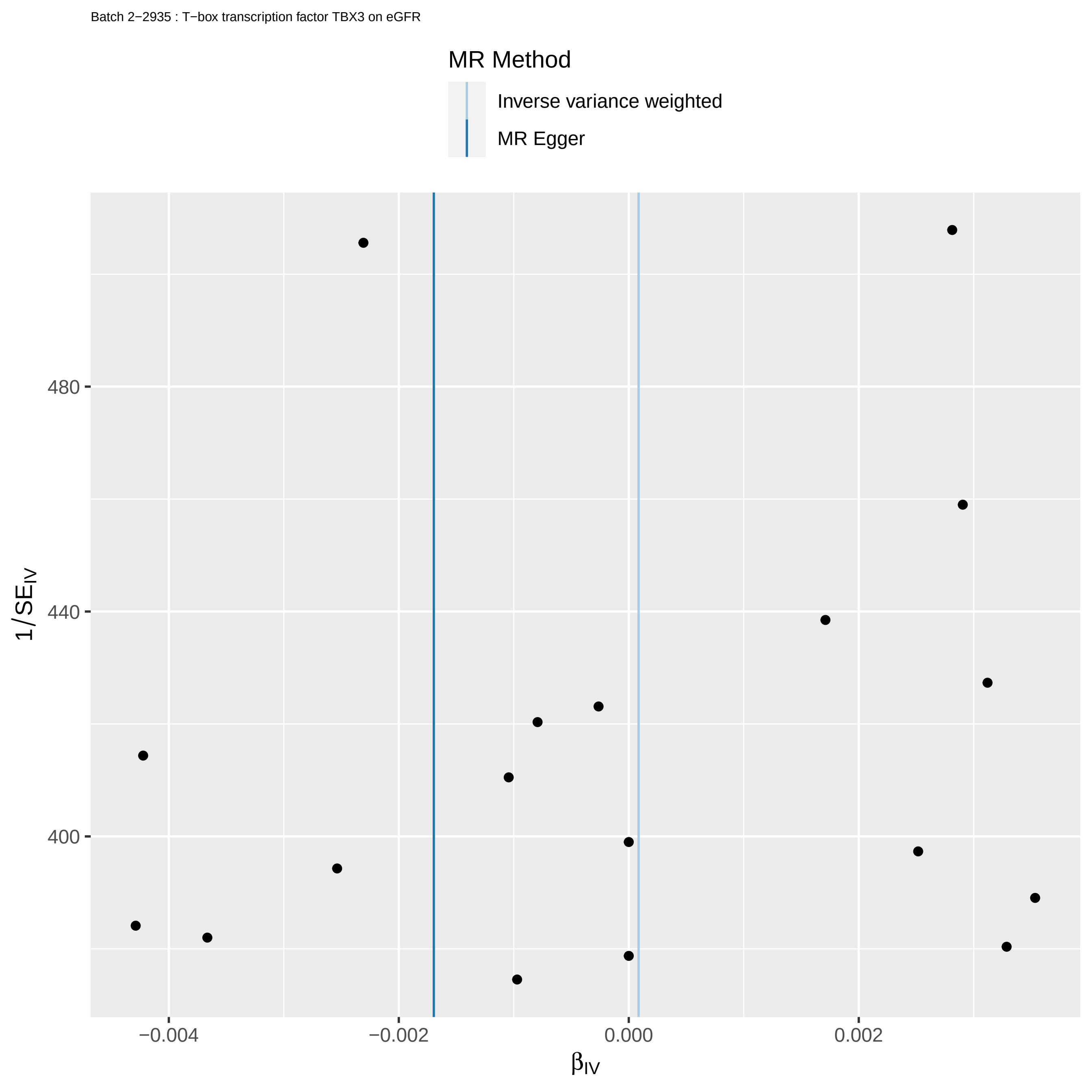

Batch 2−2935 : T−box transcription factor TBX3 on eGFR
MR Method
Inverse variance weighted
MR Egger
480
440
400
1/SE_IV
−0.004
−0.002
0.000
0.002
β_IV

Batch 2−2936 : T−box transcription factor TBX5 on eGFR

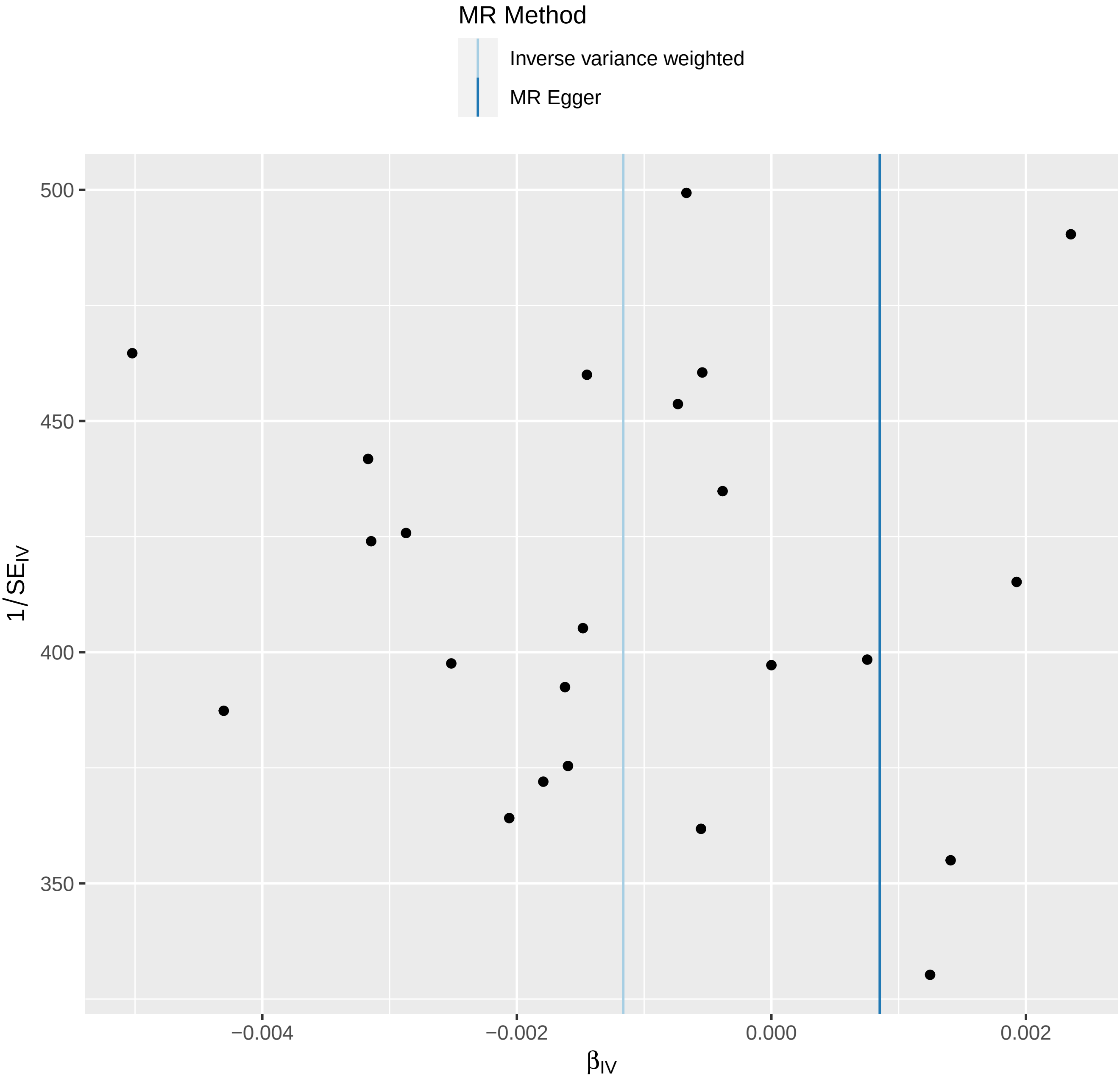

Batch 2−2937 : Thromboxane−A synthase on eGFR

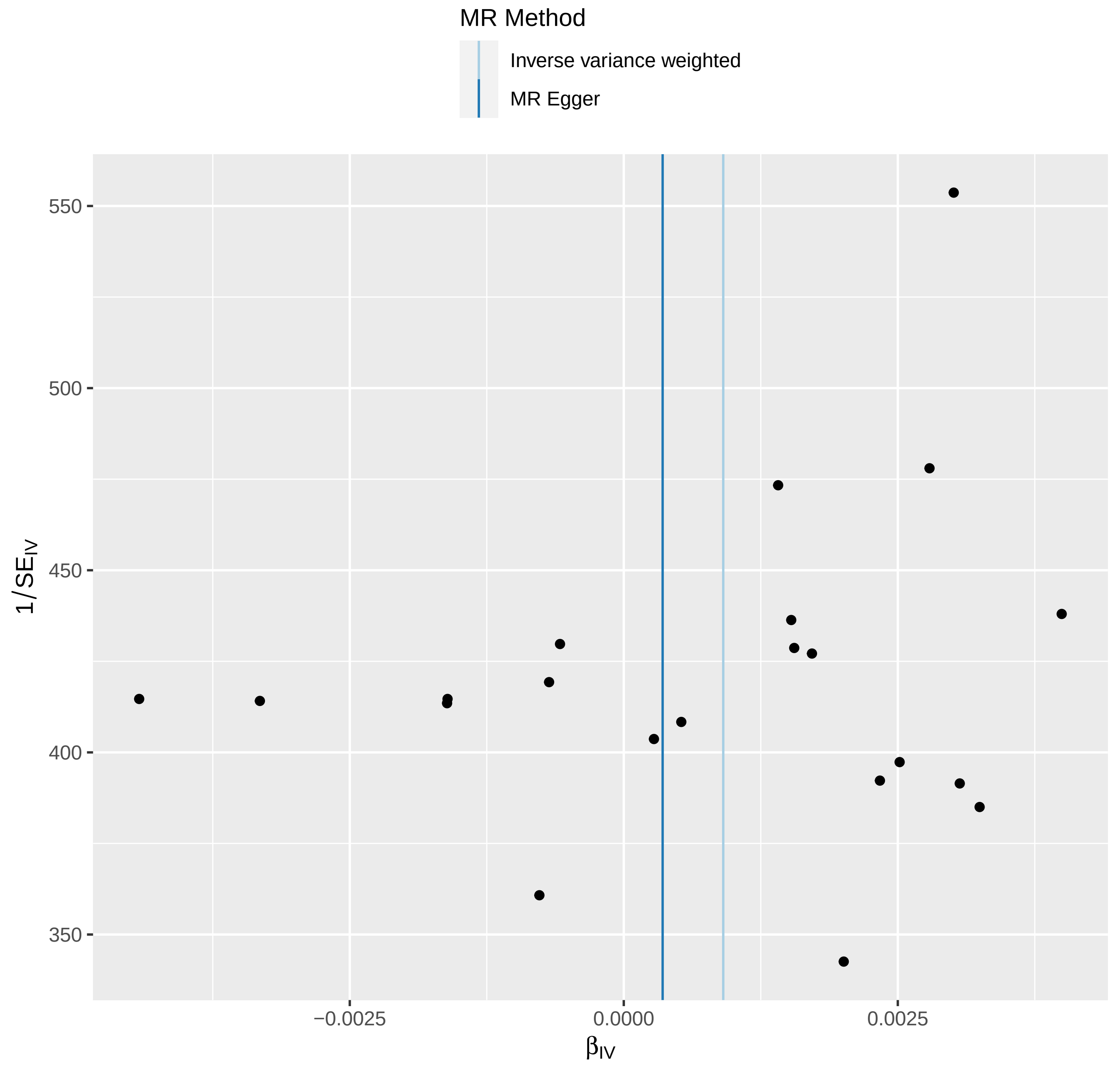

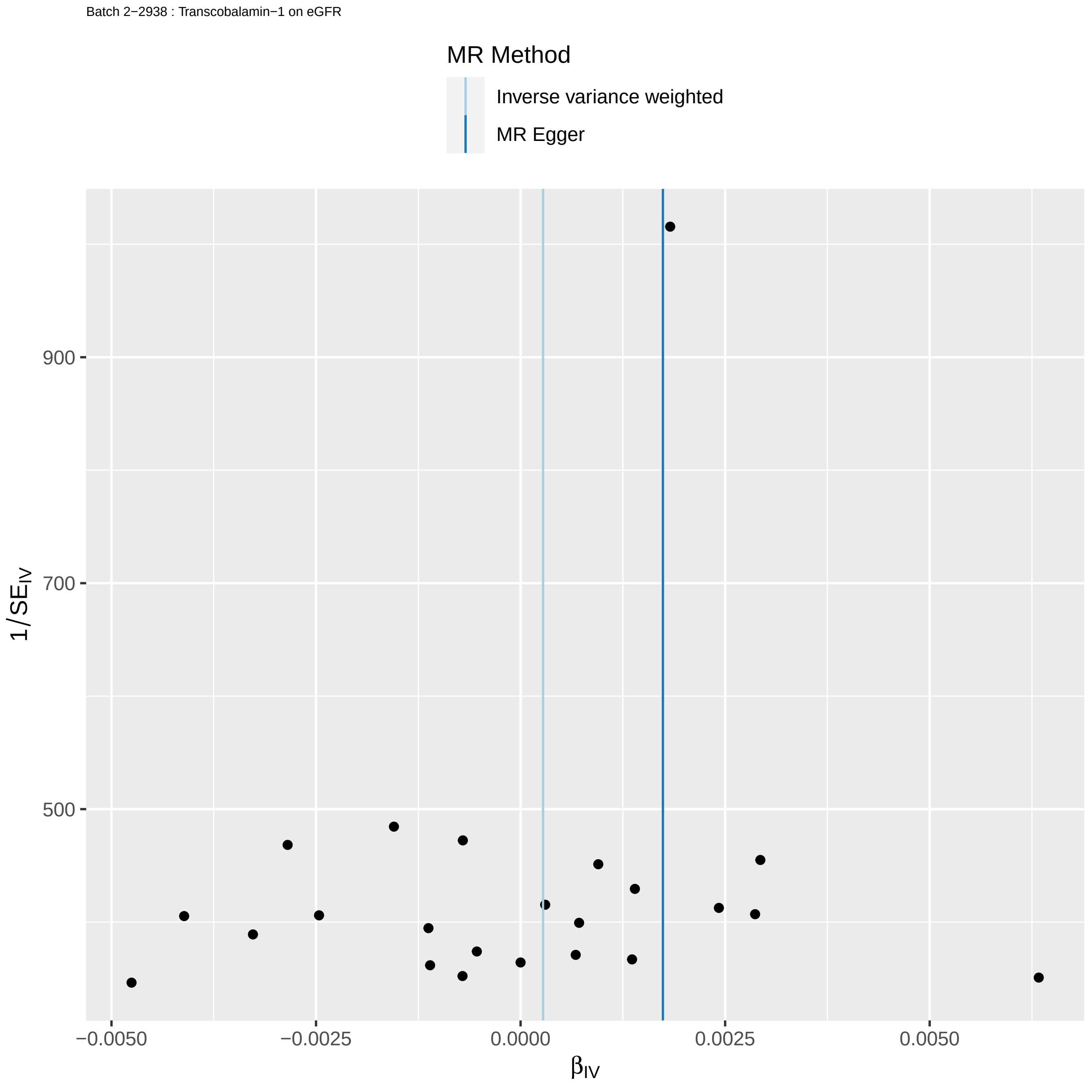
Batch 2−2938 : Transcobalamin−1 on eGFR
MR Method
Inverse variance weighted
MR Egger
900
700
500
1/SE$_{IV}$
−0.0050
−0.0025
0.0000
0.0025
0.0050
β$_{IV}$

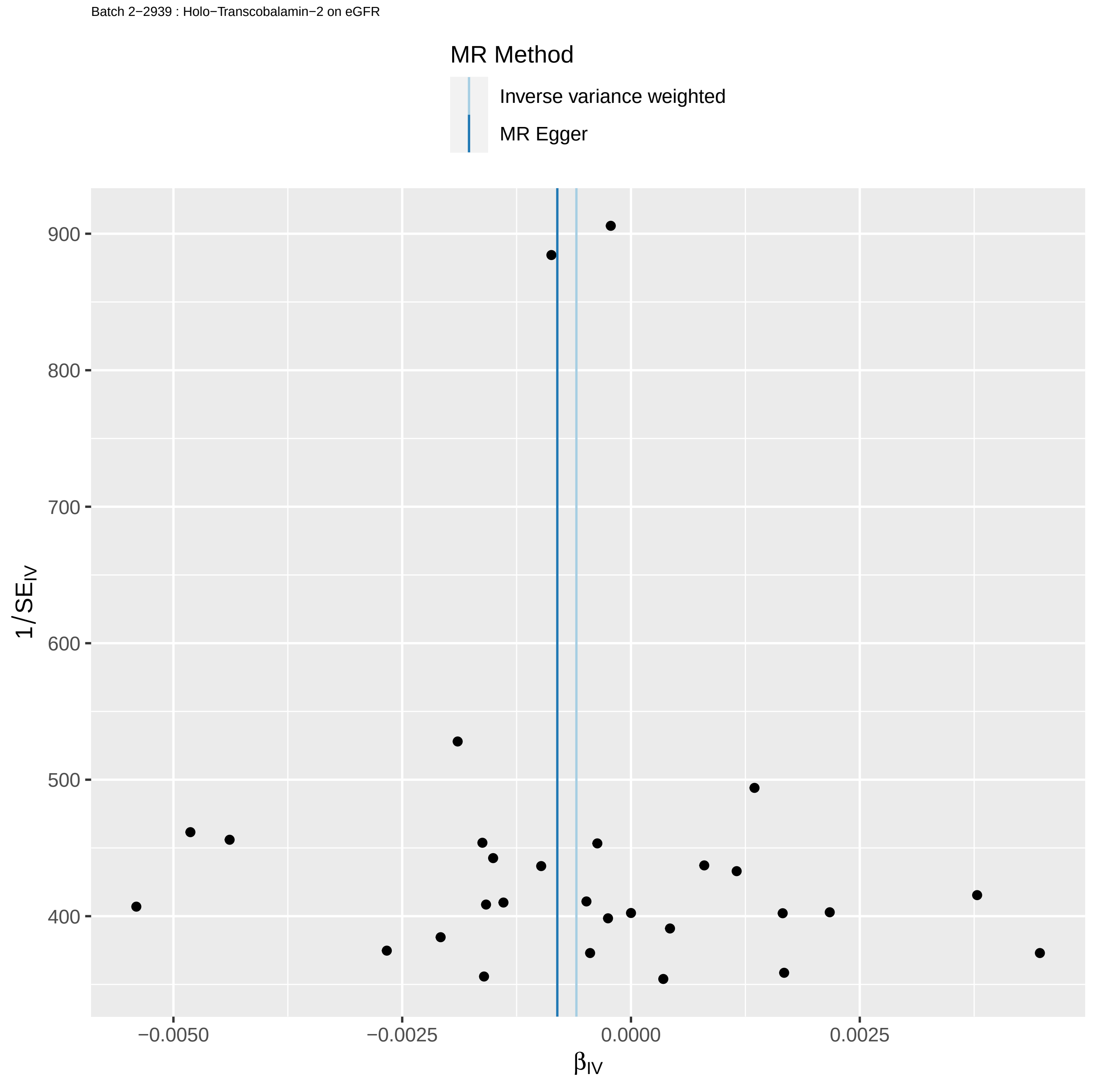

Batch 2−2939 : Holo−Transcobalamin−2 on eGFR
MR Method
Inverse variance weighted
MR Egger
900
800
700
600
500
400
1/SE_IV
−0.0050
−0.0025
0.0000
0.0025
β_IV

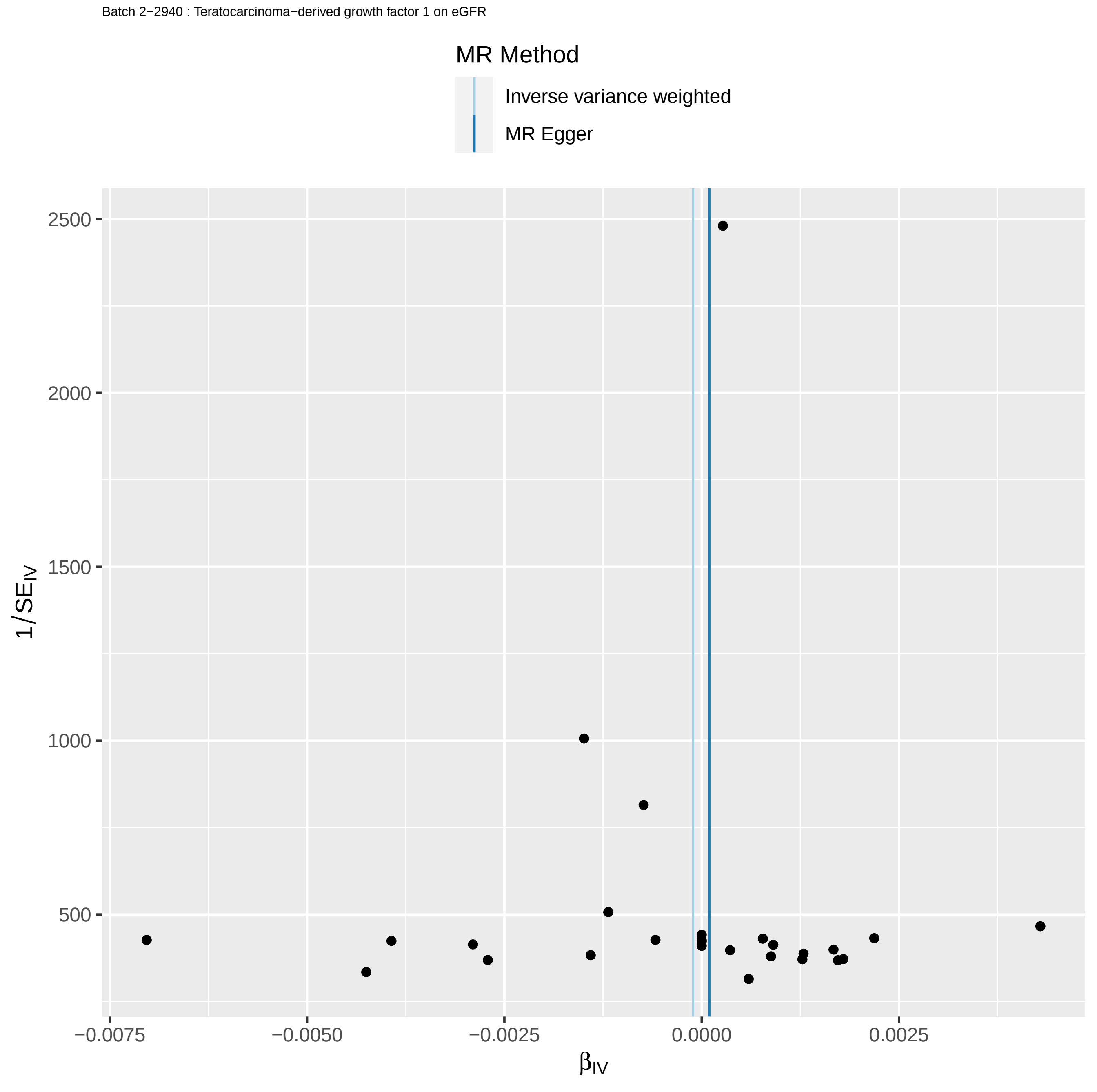

Batch 2−2940 : Teratocarcinoma−derived growth factor 1 on eGFR
MR Method
Inverse variance weighted
MR Egger
2500
2000
1500
1000
500
1/SE$_{IV}$
−0.0075
−0.0050
−0.0025
0.0000
0.0025
β$_{IV}$

Batch 2−2941 : Tyrosyl−DNA phosphodiesterase 1 on eGFR

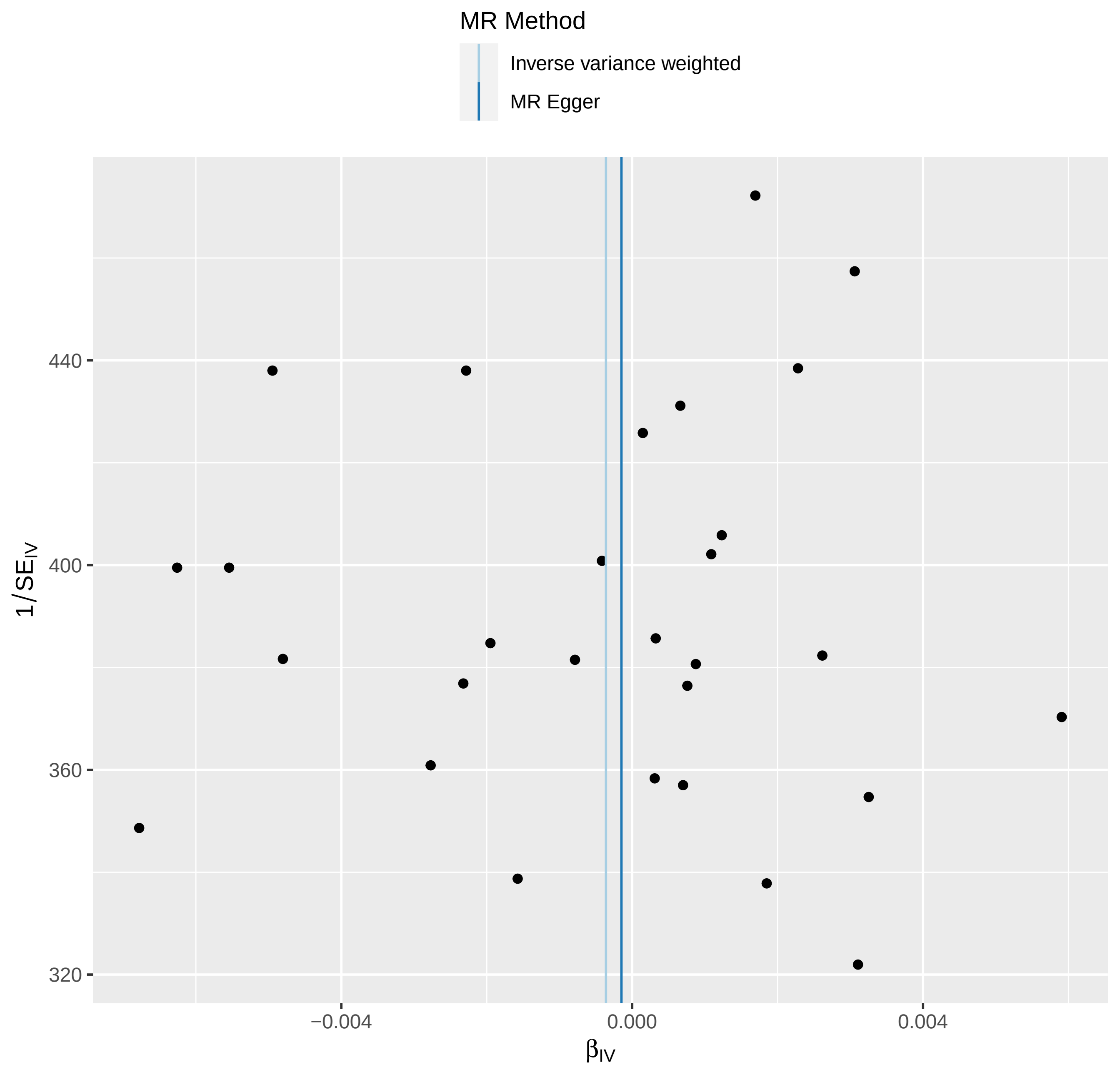

Batch 2−2942 : Transcriptional enhancer factor TEF−5 on eGFR

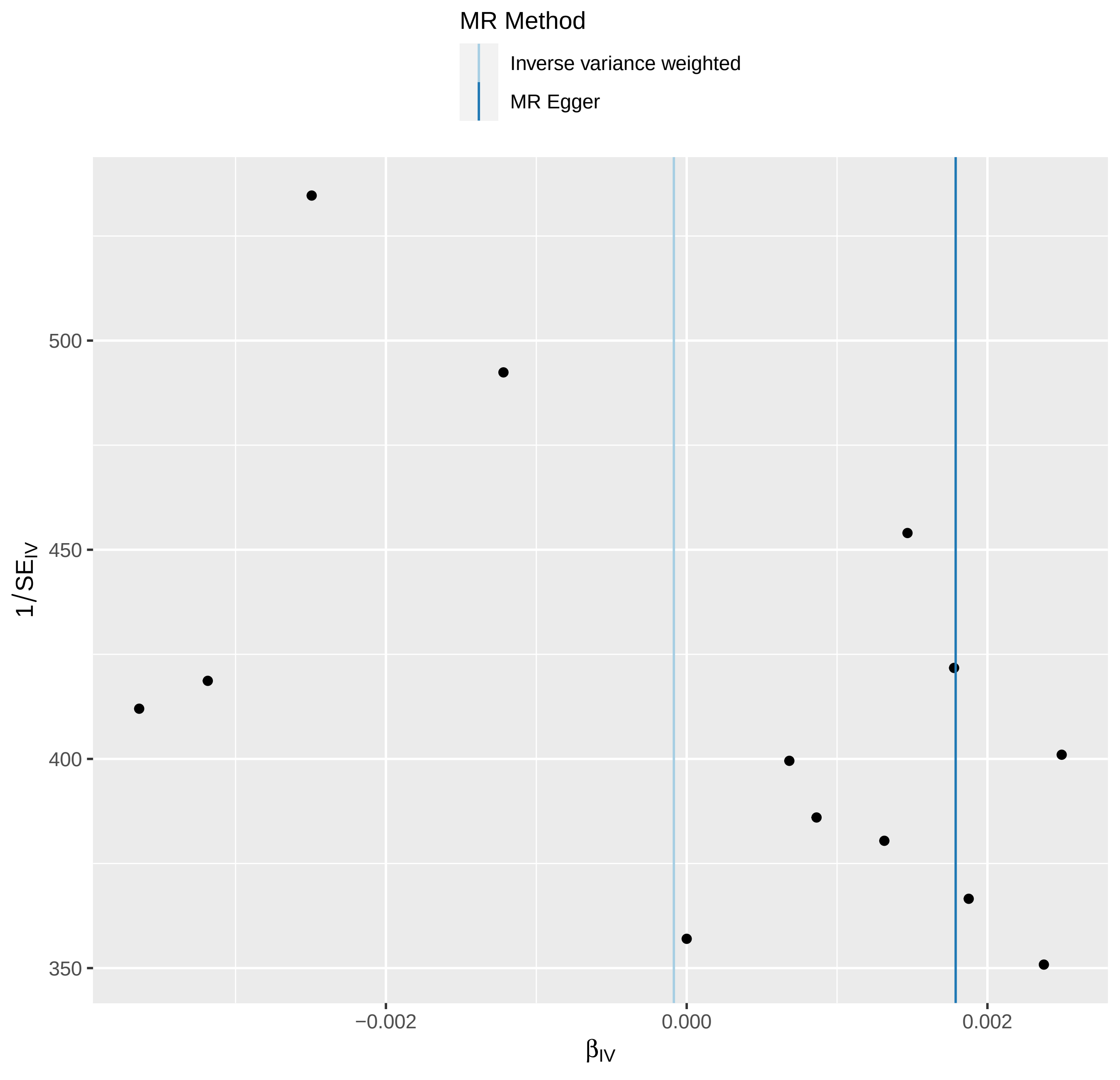

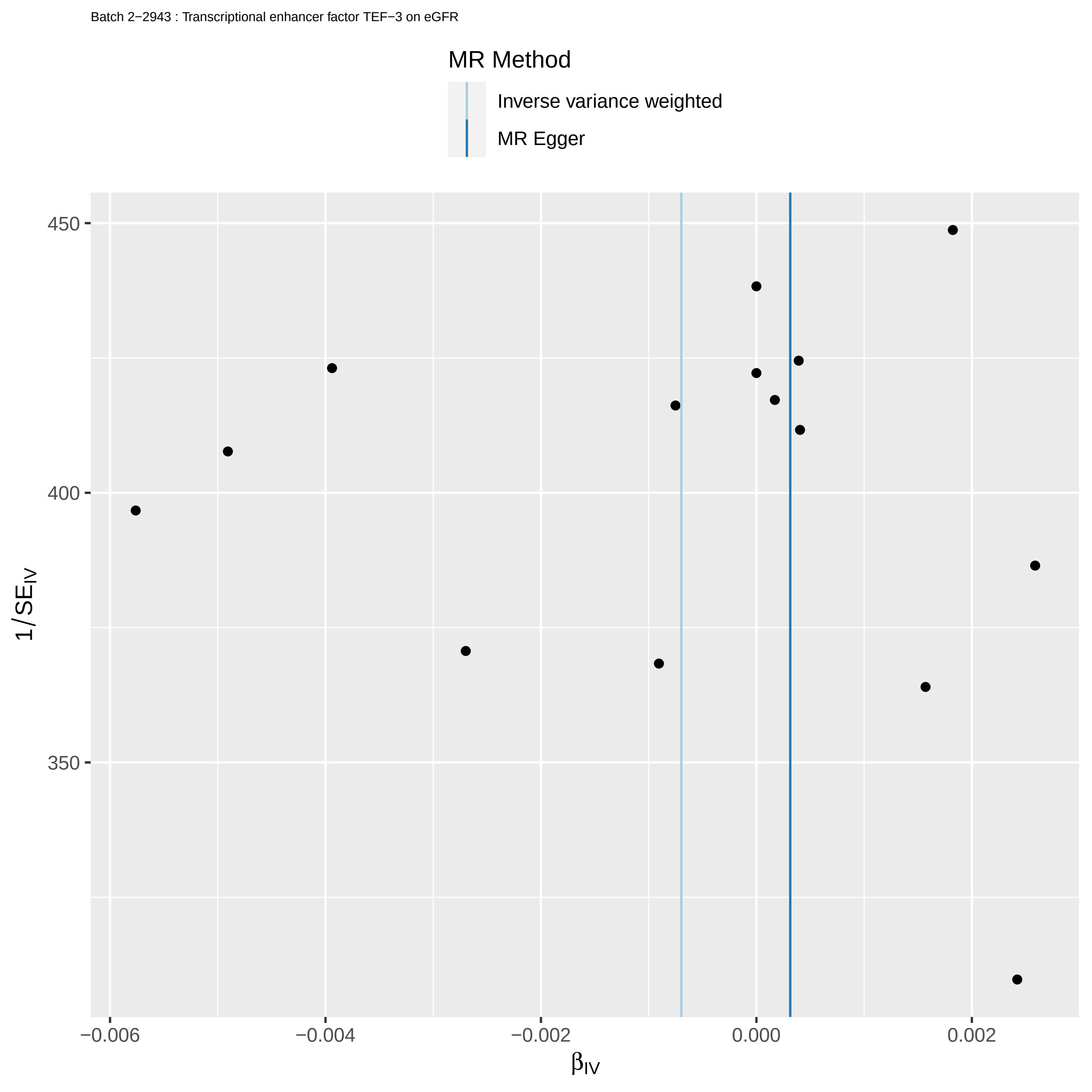
Batch 2−2943 : Transcriptional enhancer factor TEF−3 on eGFR
MR Method
Inverse variance weighted
MR Egger
450
400
350
1/SE_IV
−0.006
−0.004
−0.002
0.000
0.002
β_IV

Batch 2−2944 : Angiopoietin−1 receptor, soluble on eGFR

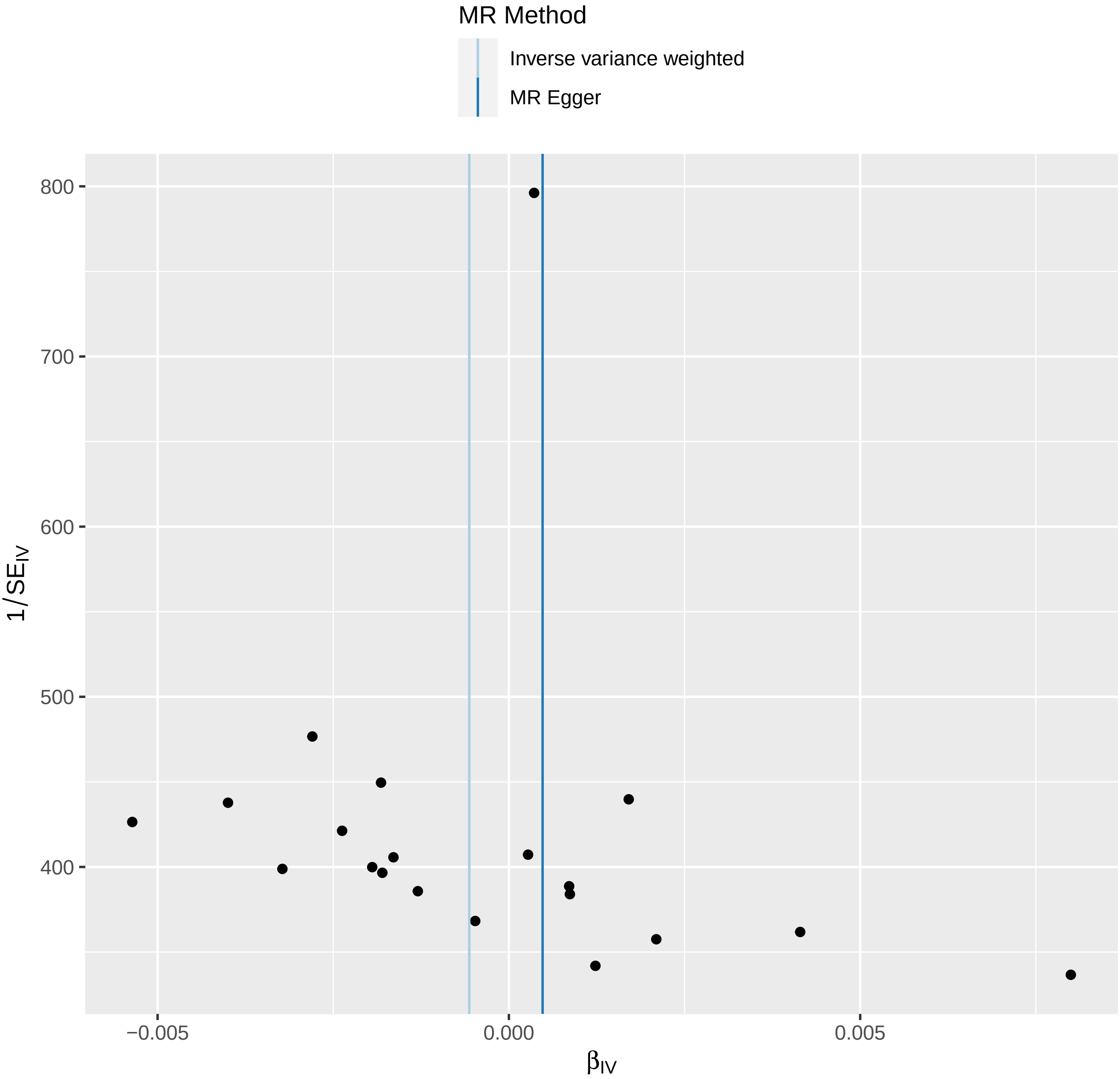

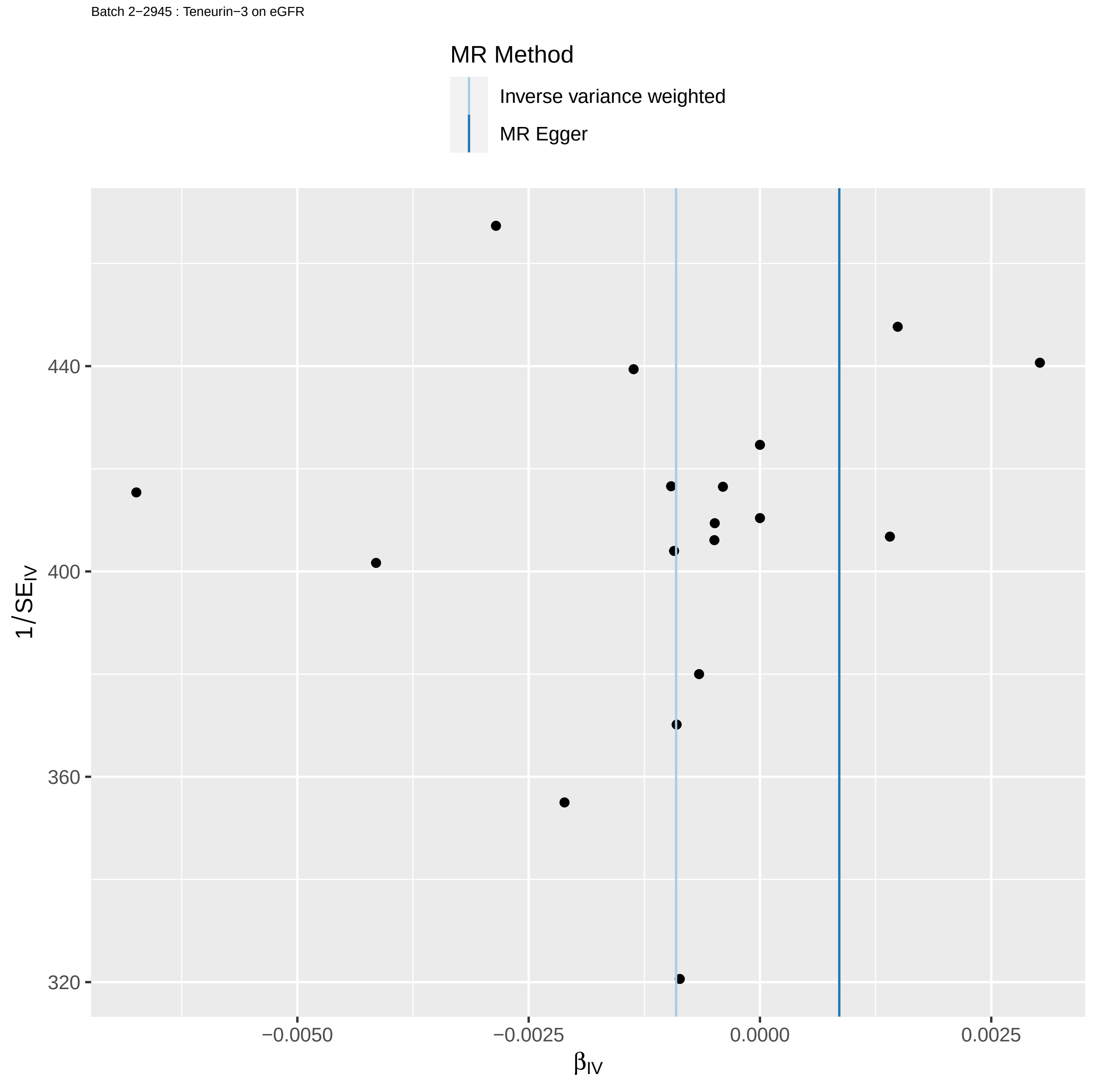
Batch 2−2945 : Teneurin−3 on eGFR
MR Method
Inverse variance weighted
MR Egger
440
400
360
320
1/SE$_{IV}$
−0.0050
−0.0025
0.0000
0.0025
β_{IV}

Batch 2−2946 : Teneurin−4 on eGFR

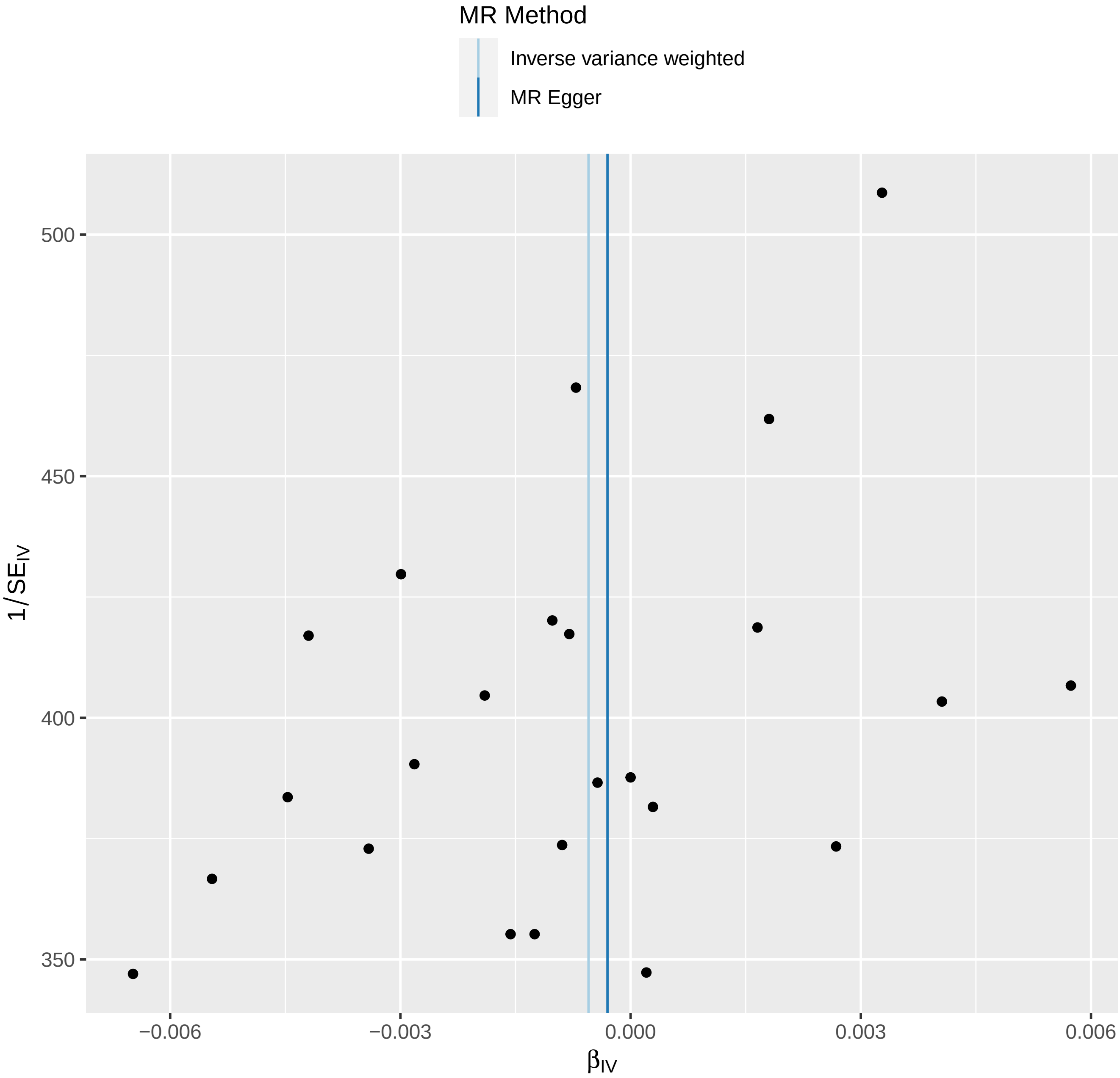

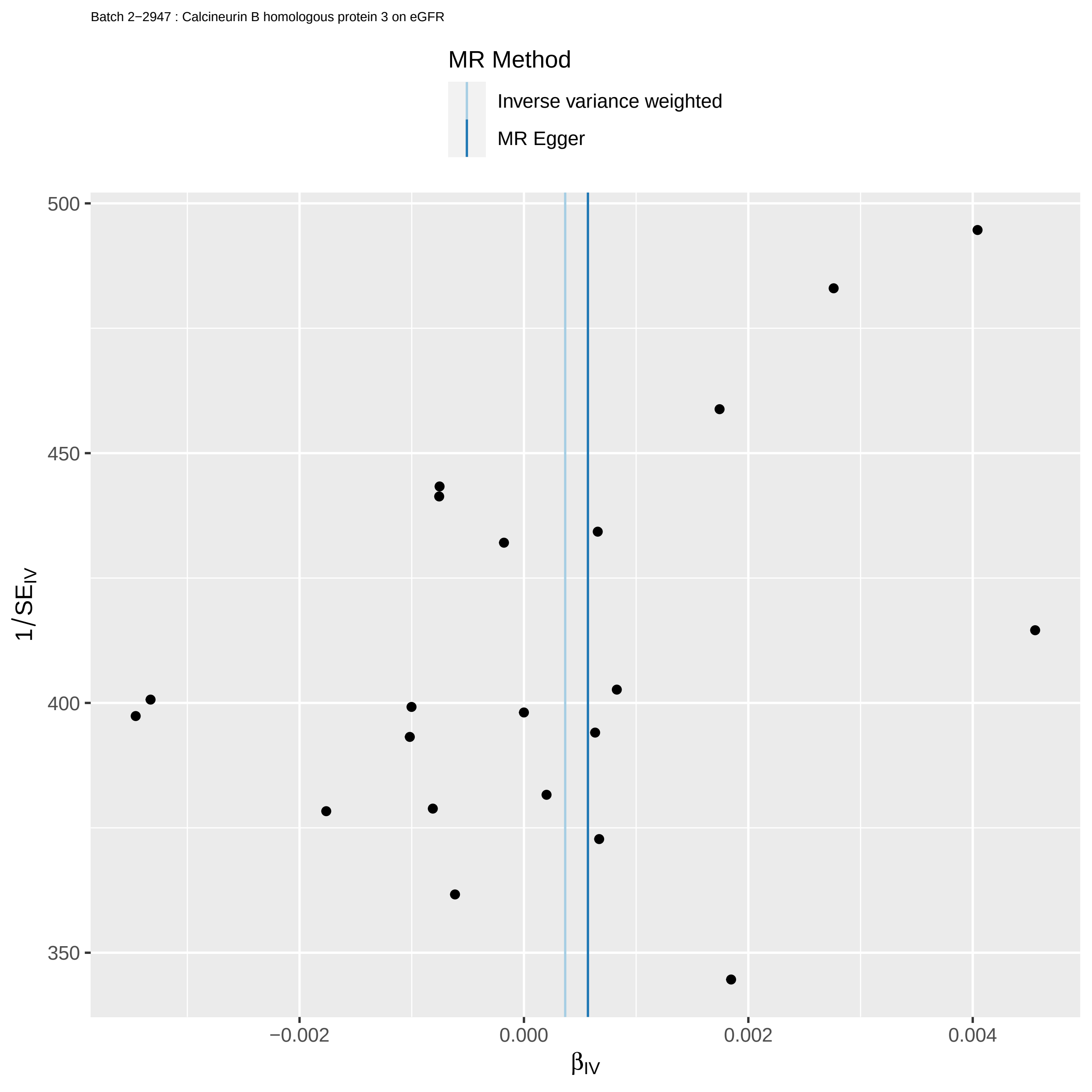

Batch 2−2947 : Calcineurin B homologous protein 3 on eGFR
MR Method
Inverse variance weighted
MR Egger
500
450
400
350
1/SE_IV
−0.002
0.000
0.002
0.004
β_IV

Batch 2−2948 : Testis−expressed sequence 29 protein on eGFR

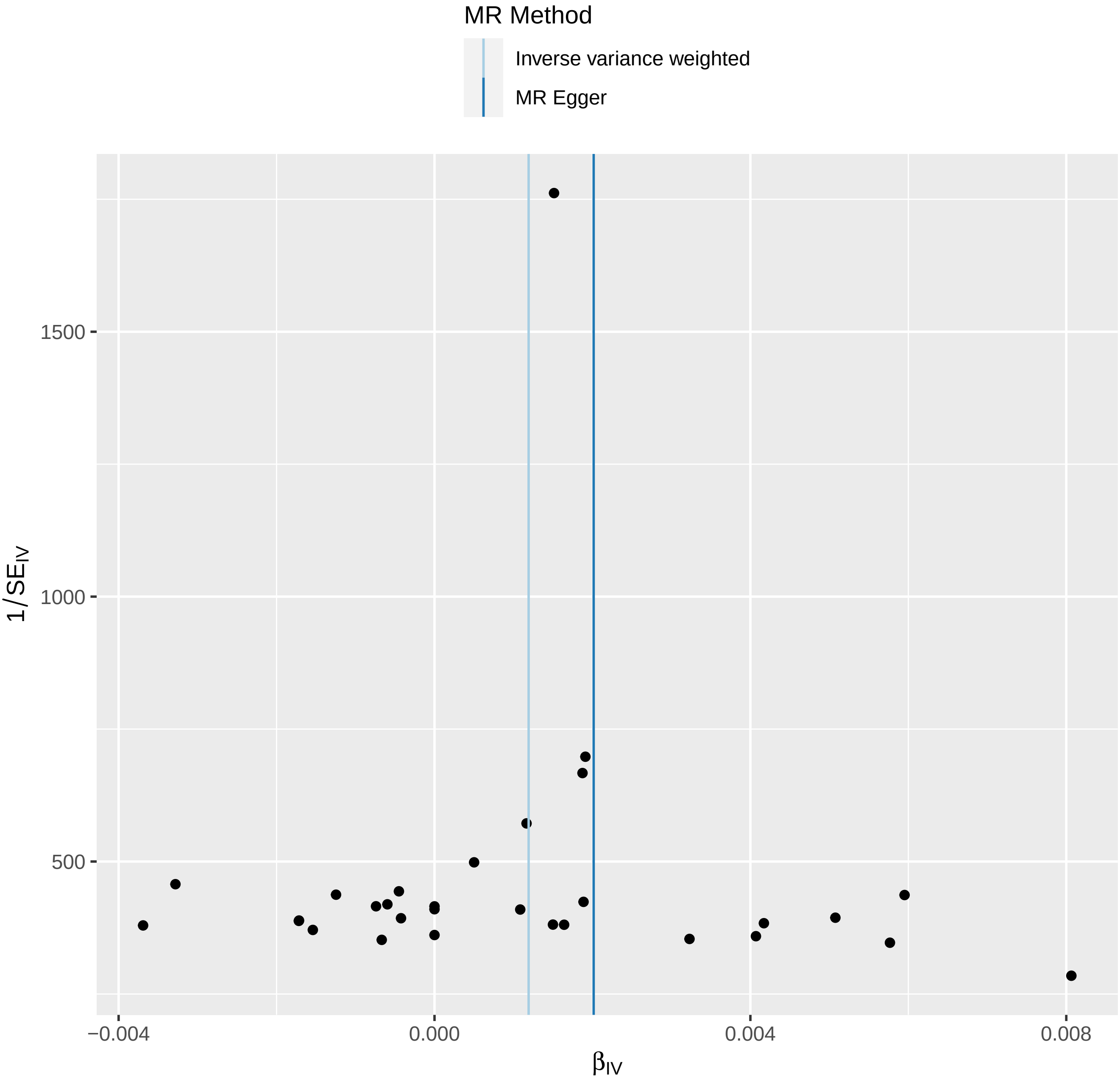

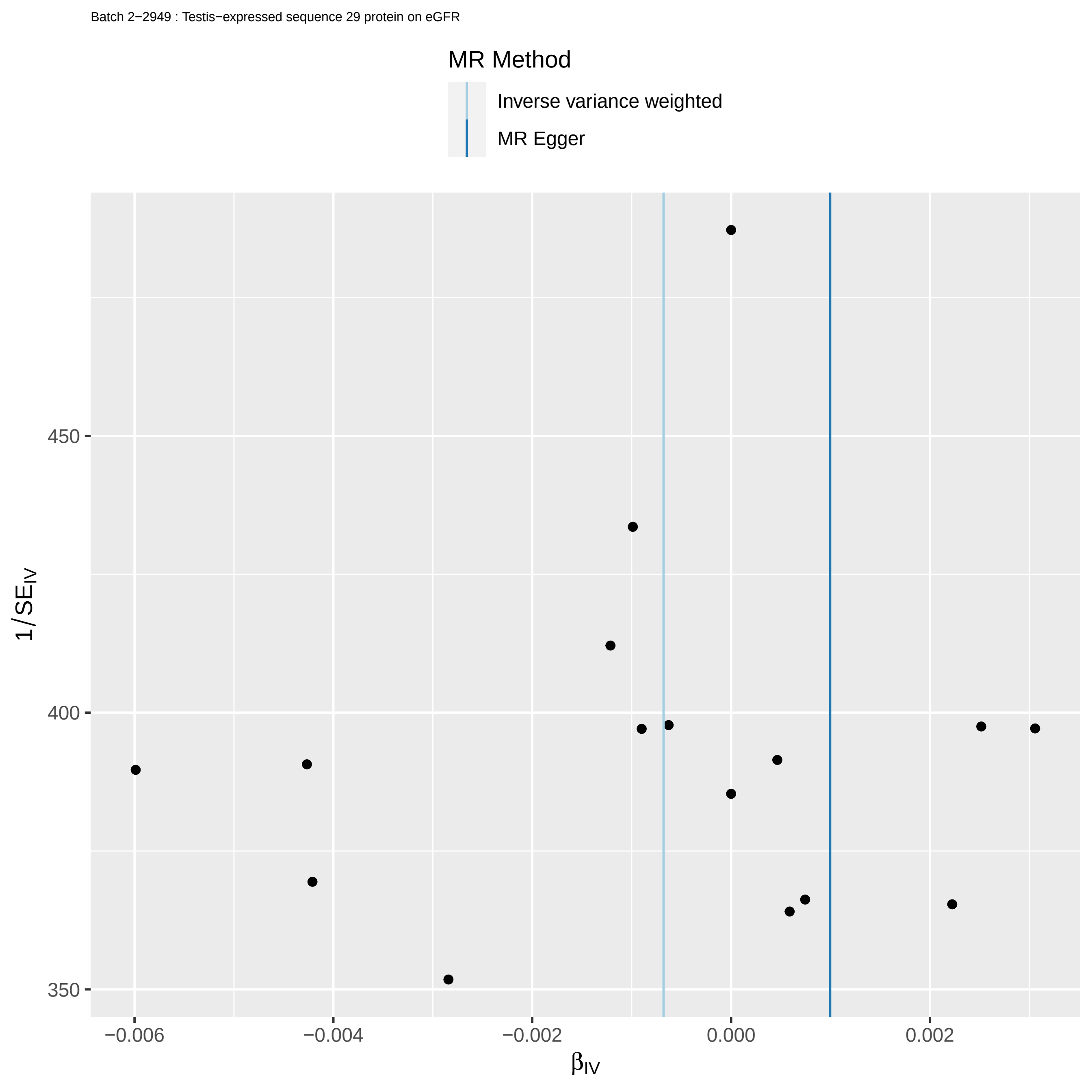
Batch 2−2949 : Testis−expressed sequence 29 protein on eGFR
MR Method
Inverse variance weighted
MR Egger
450
400
350
1/SE$_{IV}$
−0.006
−0.004
−0.002
0.000
0.002
β$_{IV}$

Batch 2−2950 : Serotransferrin on eGFR

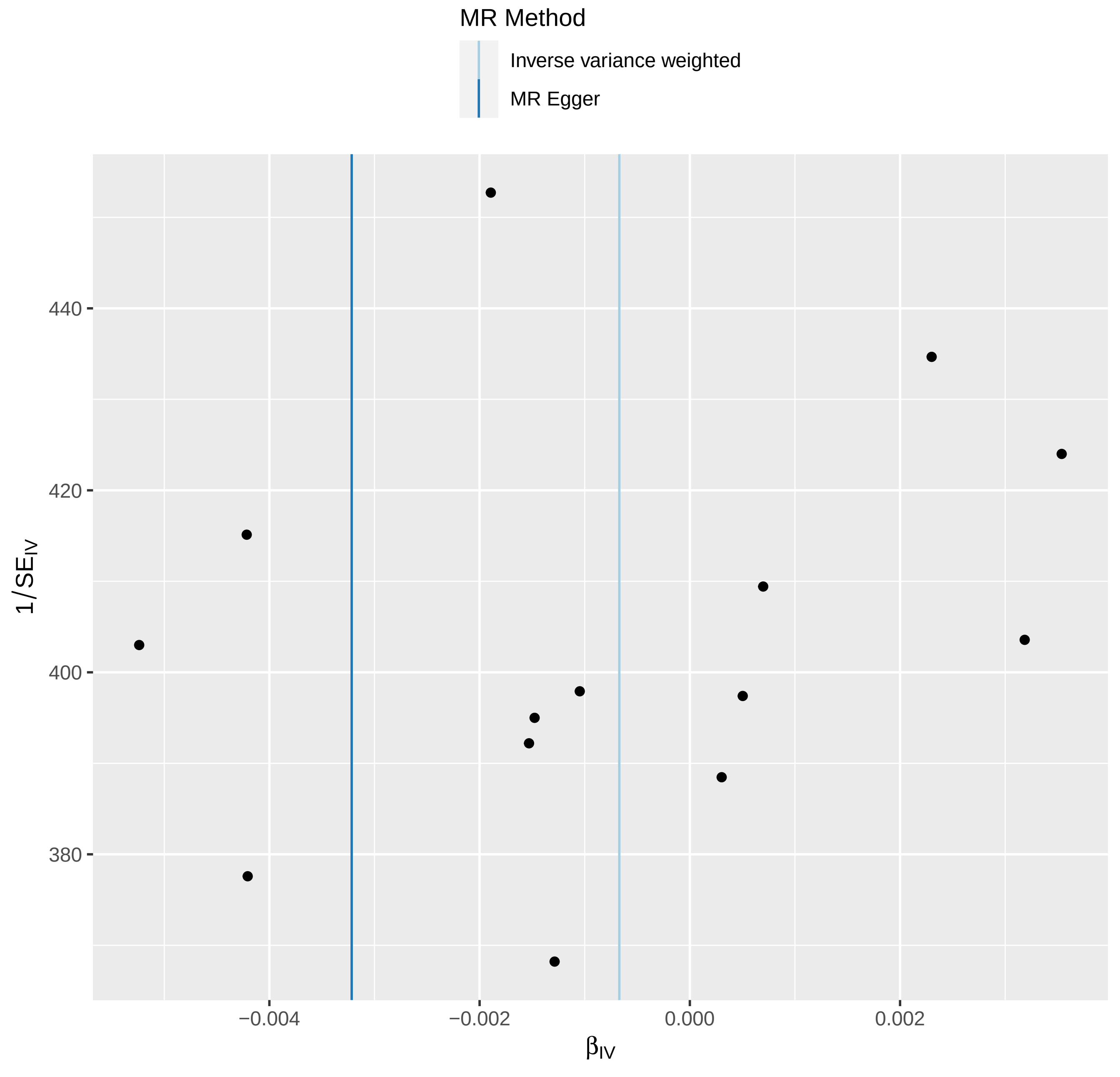

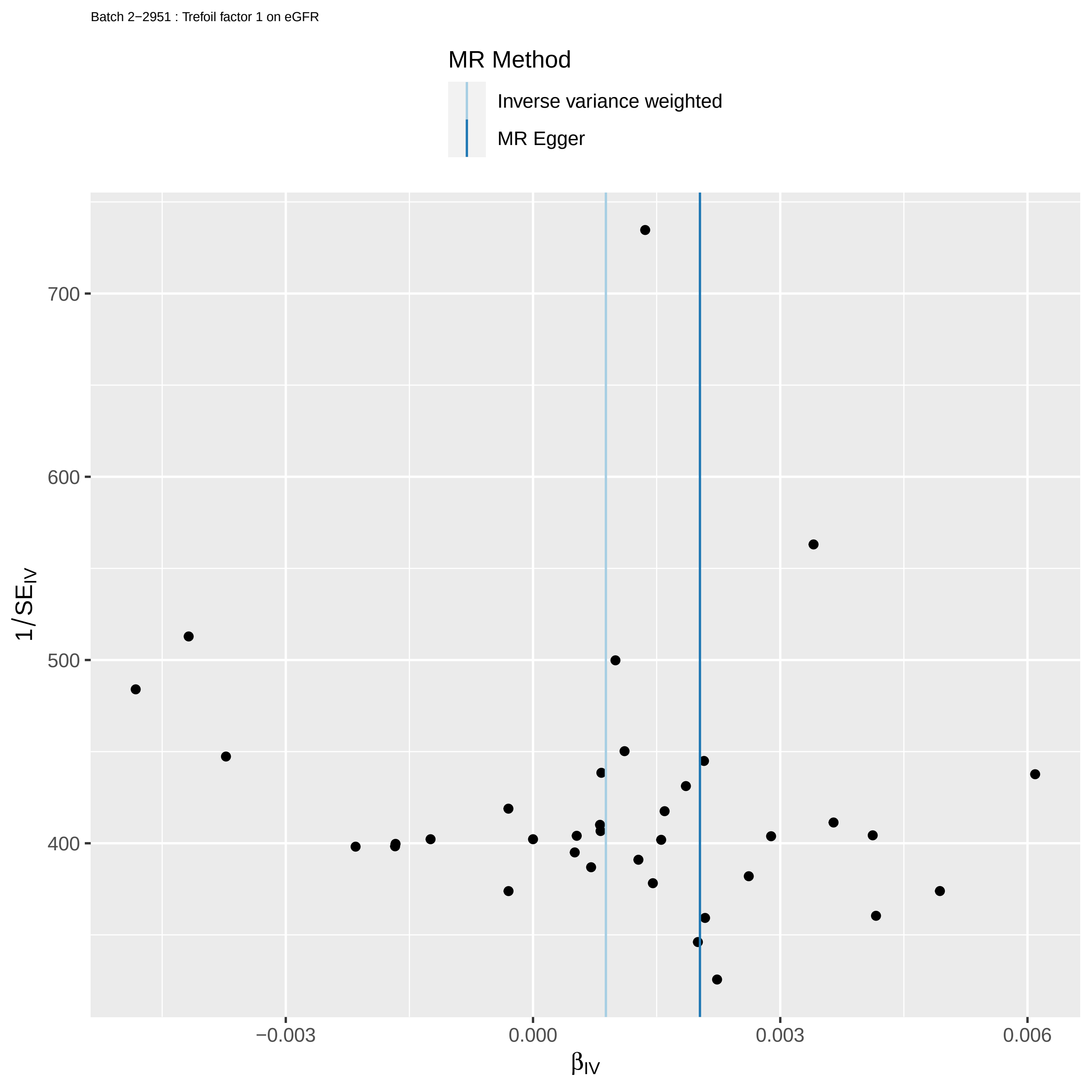

Batch 2−2951 : Trefoil factor 1 on eGFR
MR Method
Inverse variance weighted
MR Egger
700
600
500
400
1/SE$_{IV}$
−0.003
0.000
0.003
0.006
β$_{IV}$

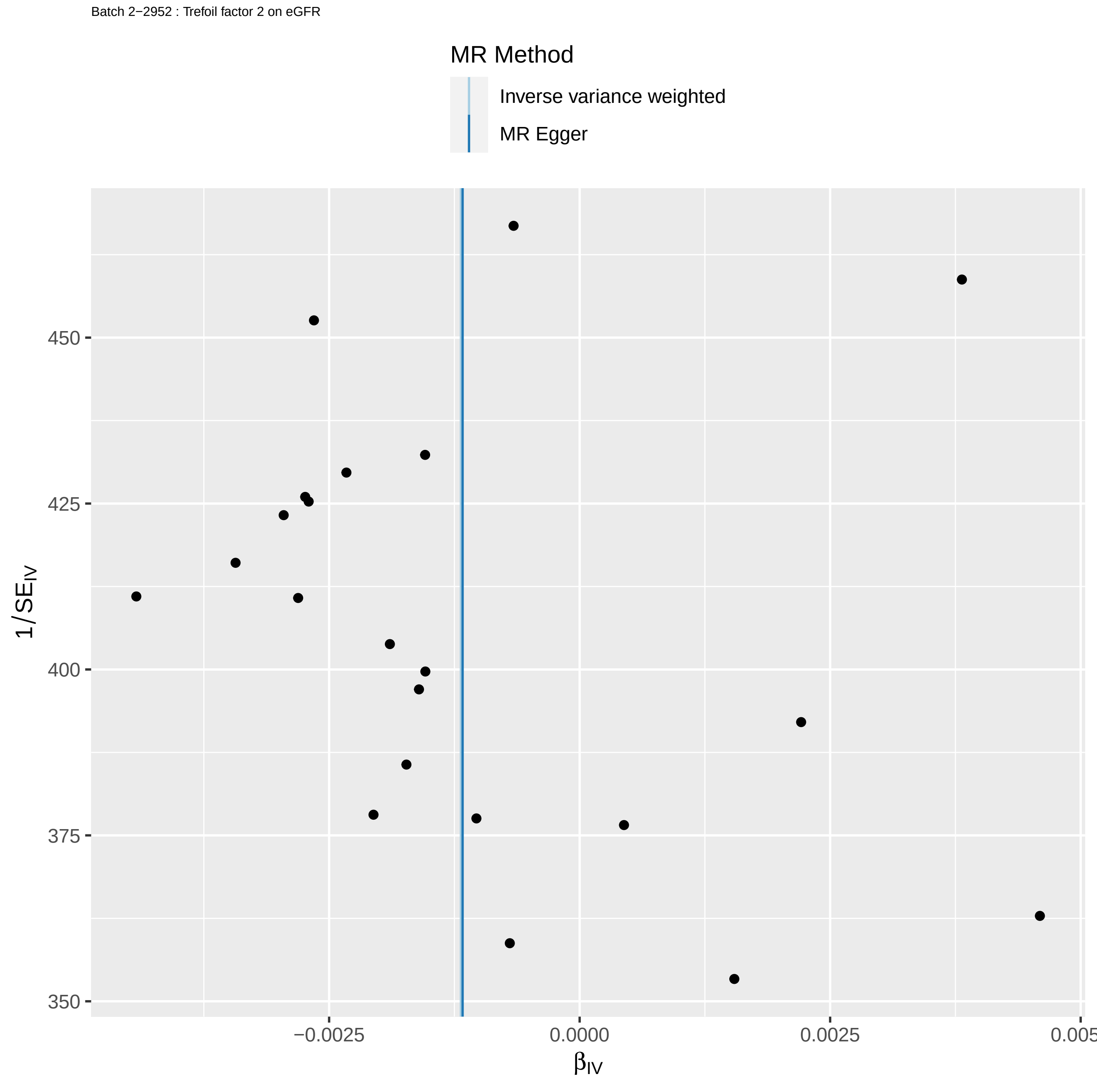

Batch 2−2952 : Trefoil factor 2 on eGFR
MR Method
Inverse variance weighted
MR Egger
450
425
400
375
350
1/SE_IV
−0.0025
0.0000
0.0025
0.005
β_IV

Batch 2−2953 : Trefoil factor 3 on eGFR

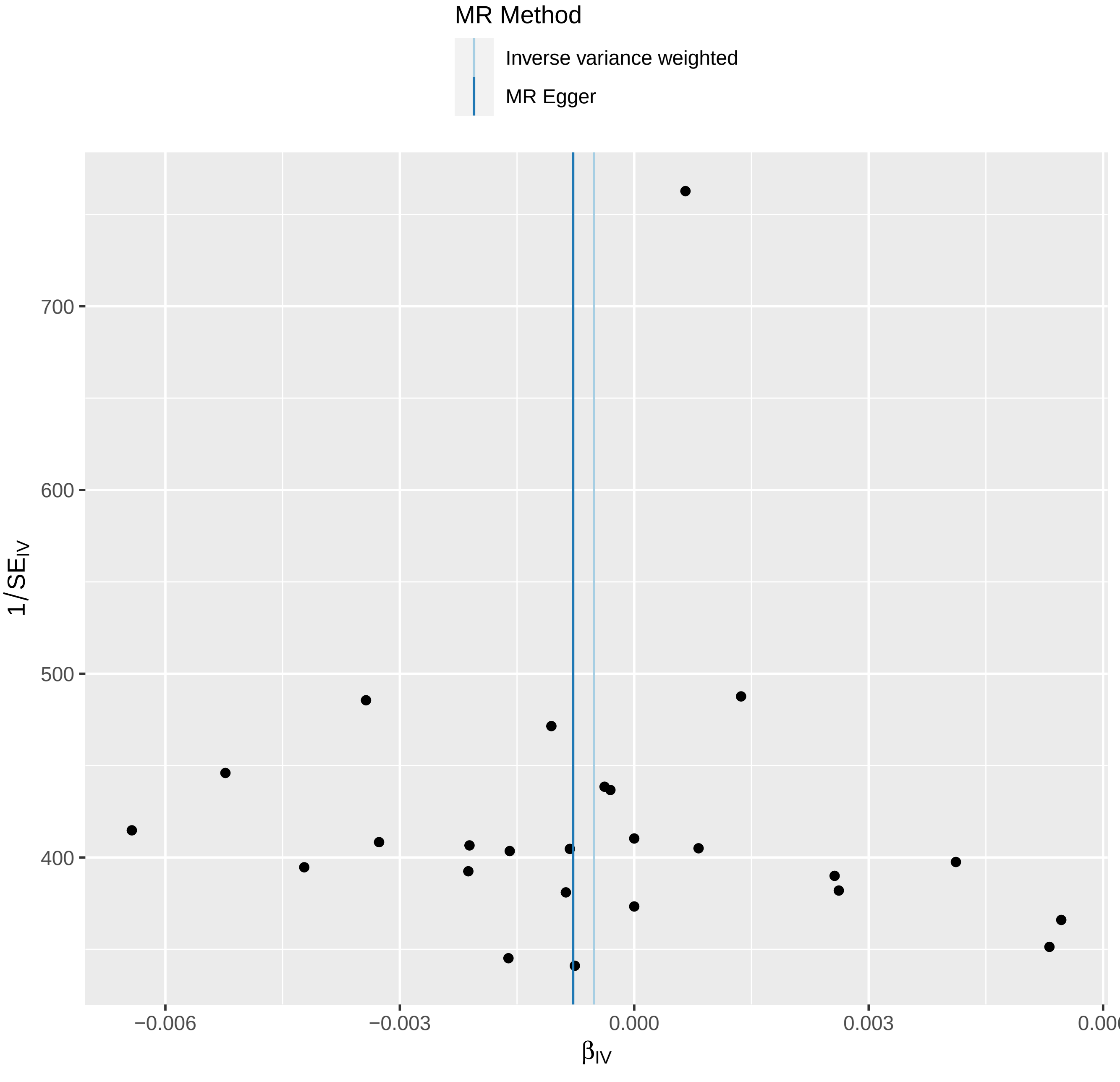

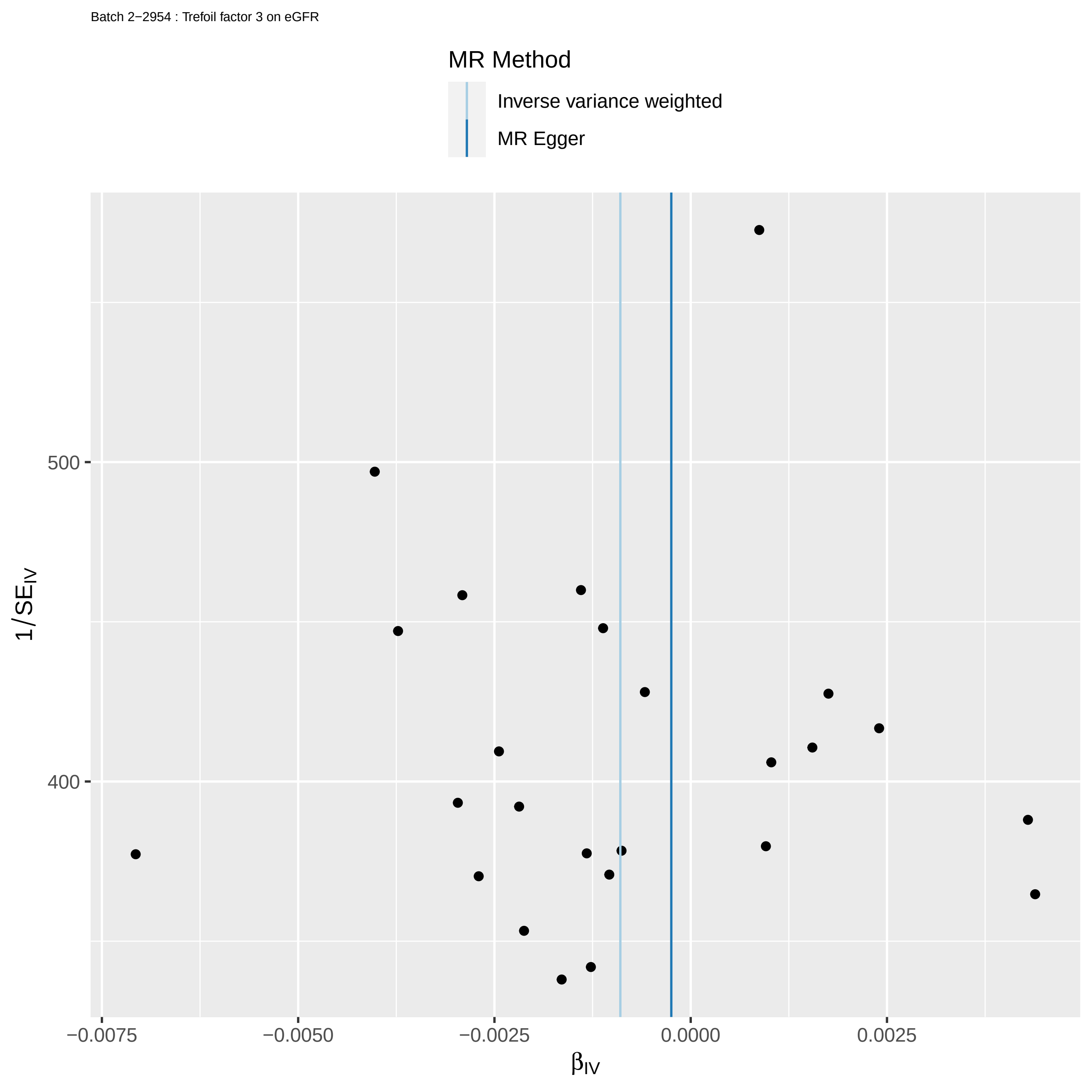

Batch 2−2954 : Trefoil factor 3 on eGFR
MR Method
Inverse variance weighted
MR Egger
500
400
1/SE_IV
−0.0075
−0.0050
−0.0025
0.0000
0.0025
β_IV

Batch 2−2955 : Tuftelin−interacting protein 11 on eGFR

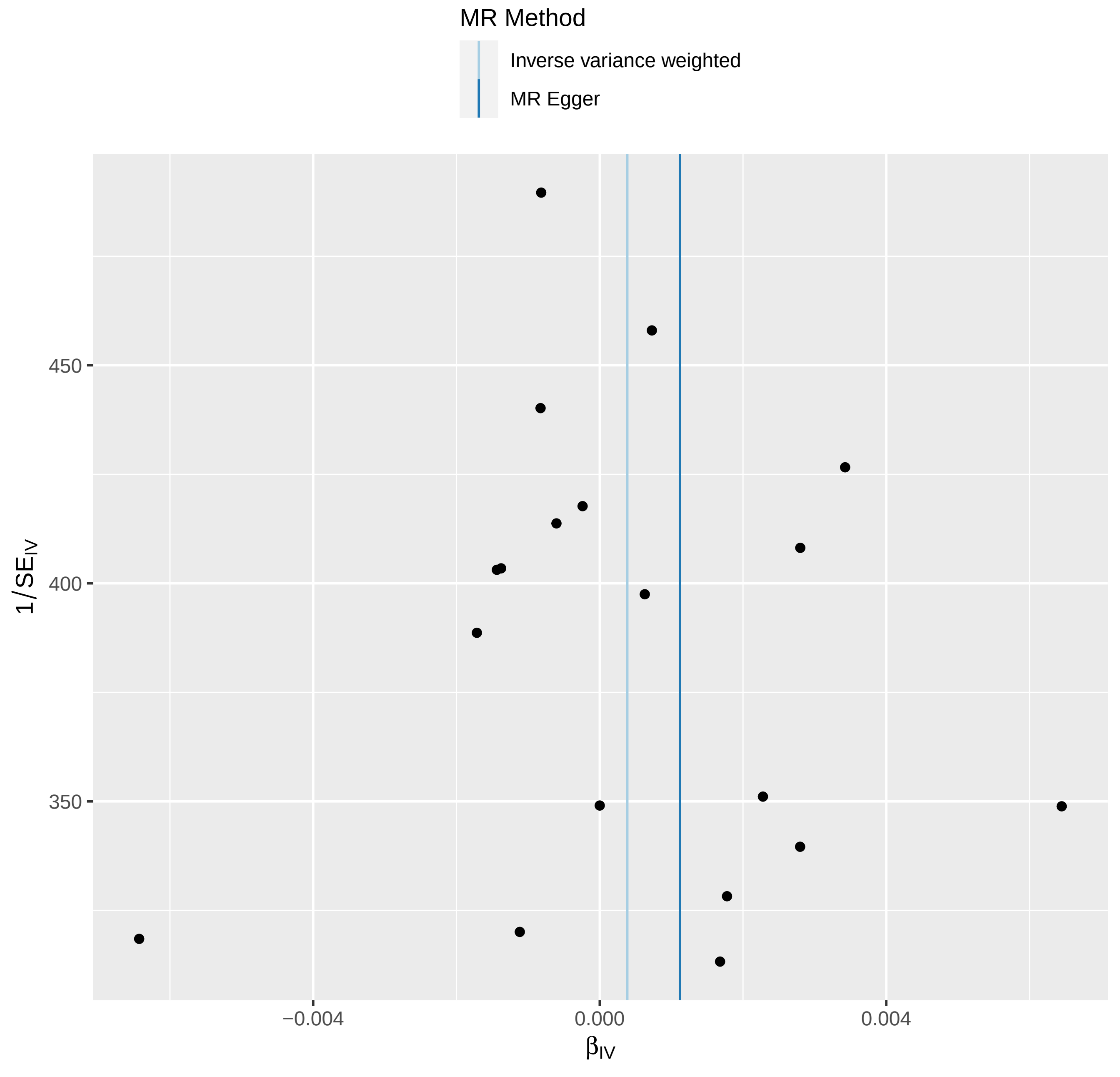

Batch 2−2956 : Tissue factor pathway inhibitor 2 on eGFR

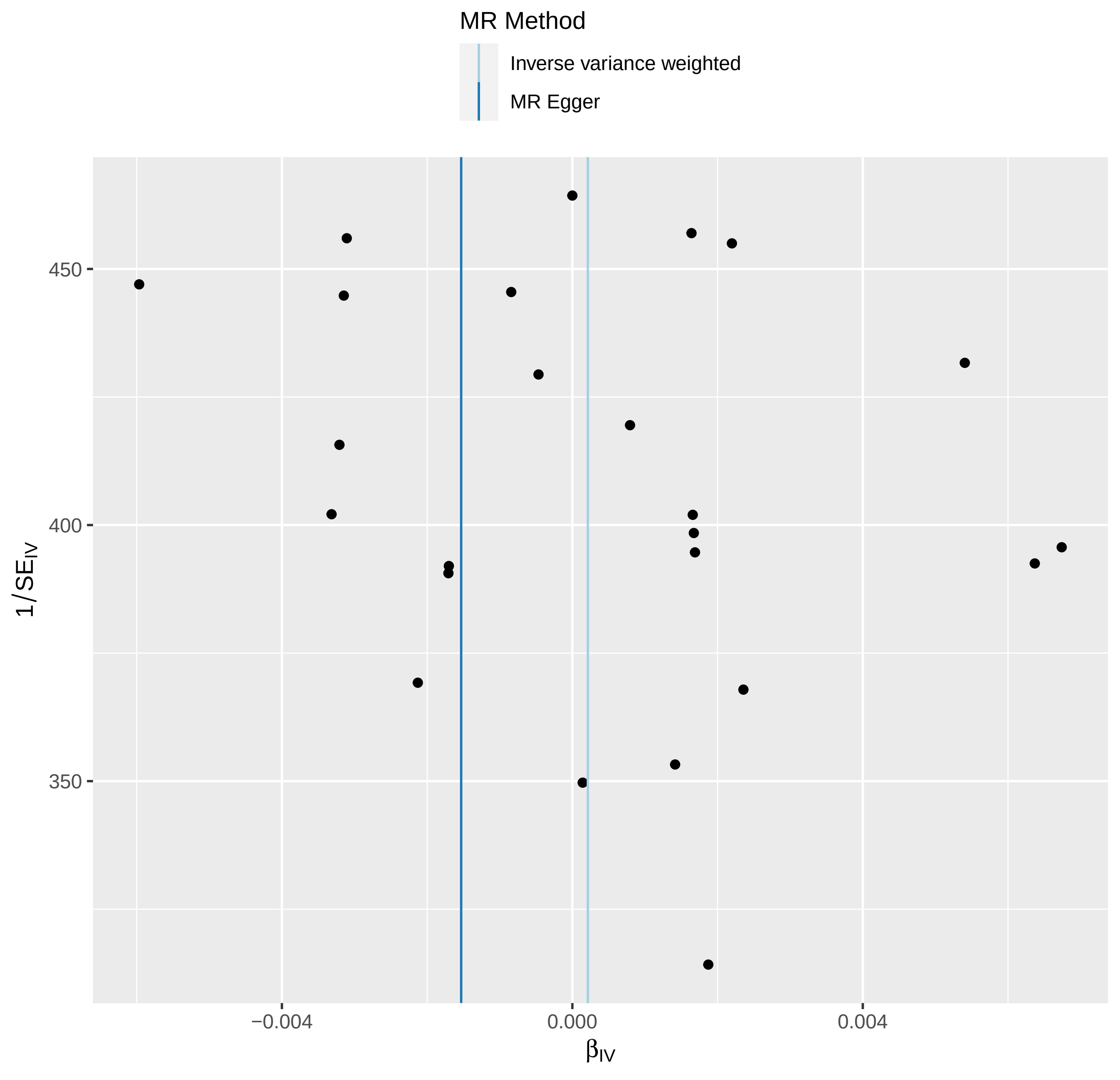

Batch 2−2957 : Tissue factor pathway inhibitor on eGFR

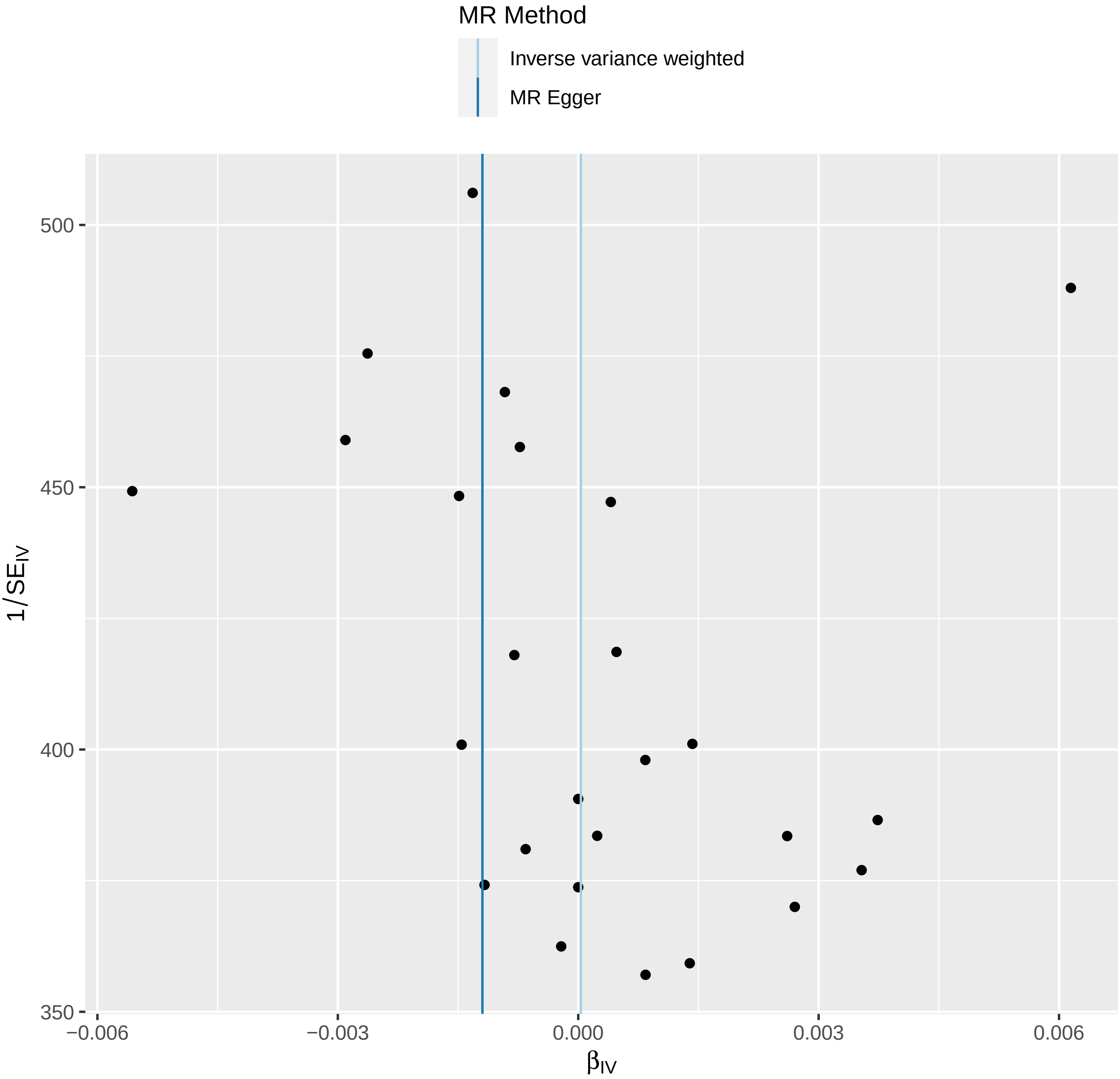

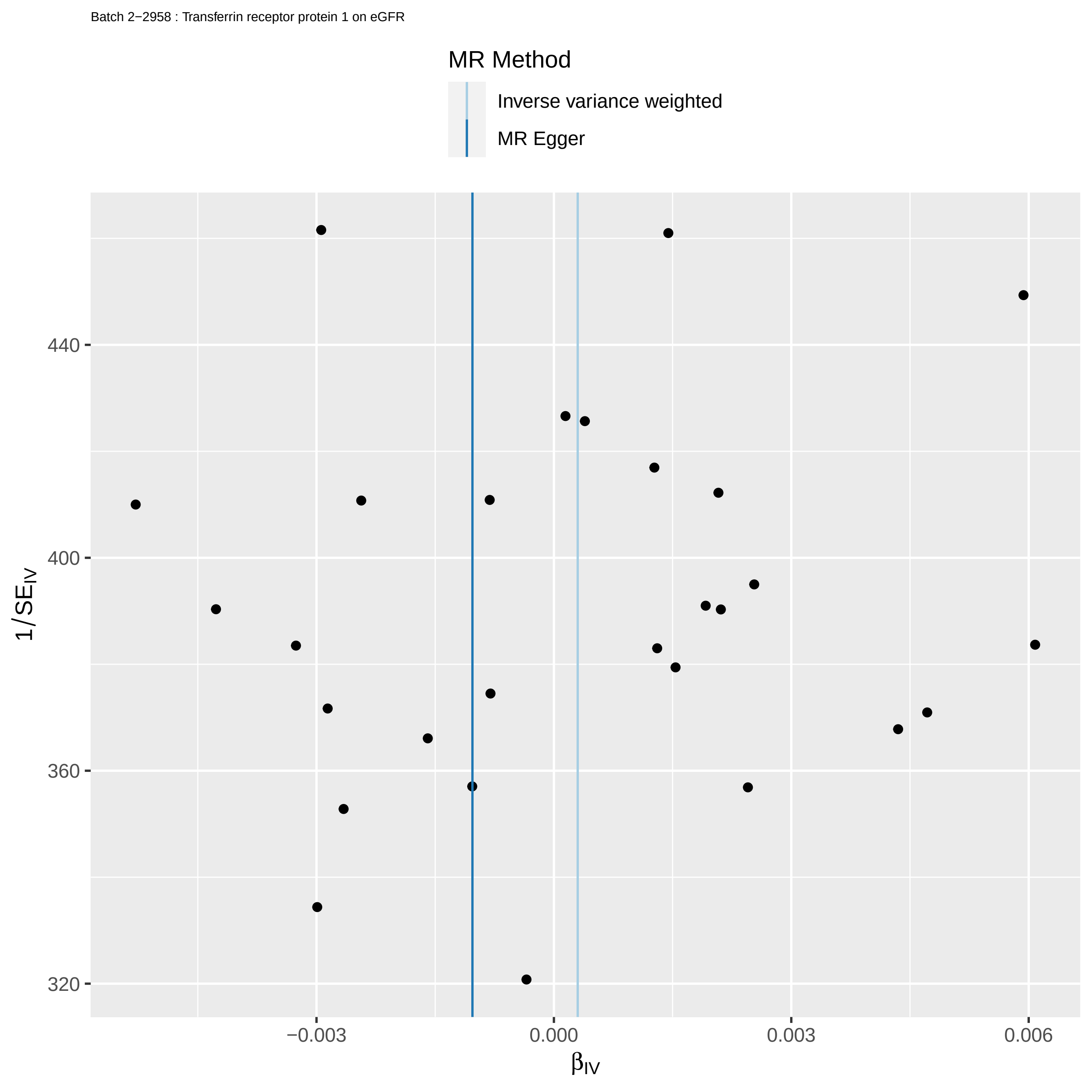

Batch 2−2958 : Transferrin receptor protein 1 on eGFR
MR Method
Inverse variance weighted
MR Egger
440
400
360
320
1/SE$_{IV}$
−0.003
0.000
0.003
0.006
β$_{IV}$

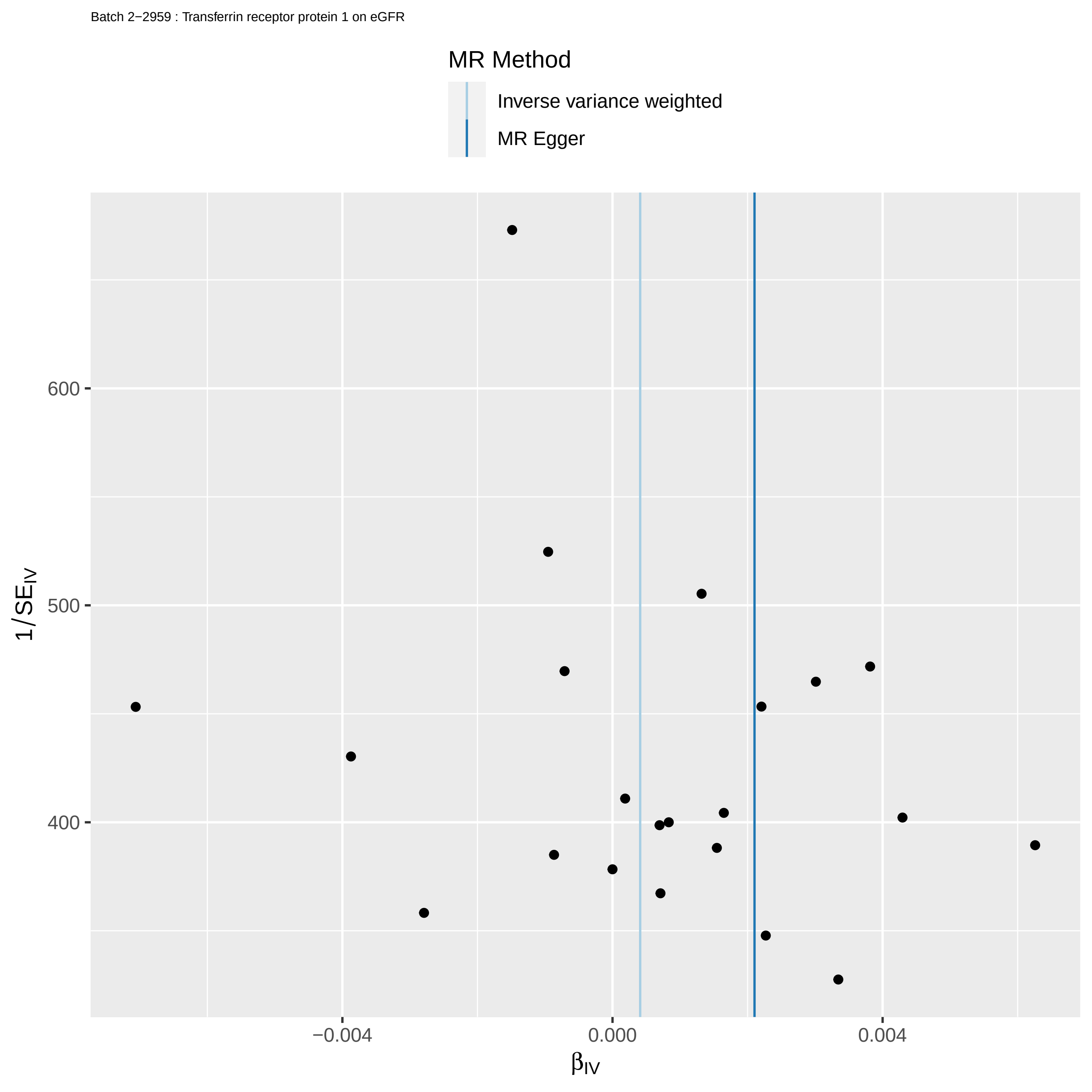
Batch 2−2959 : Transferrin receptor protein 1 on eGFR
MR Method
Inverse variance weighted
MR Egger
600
500
400
1/SE_IV
−0.004
0.000
0.004
β_IV

Batch 2−2960 : Thyroglobulin on eGFR

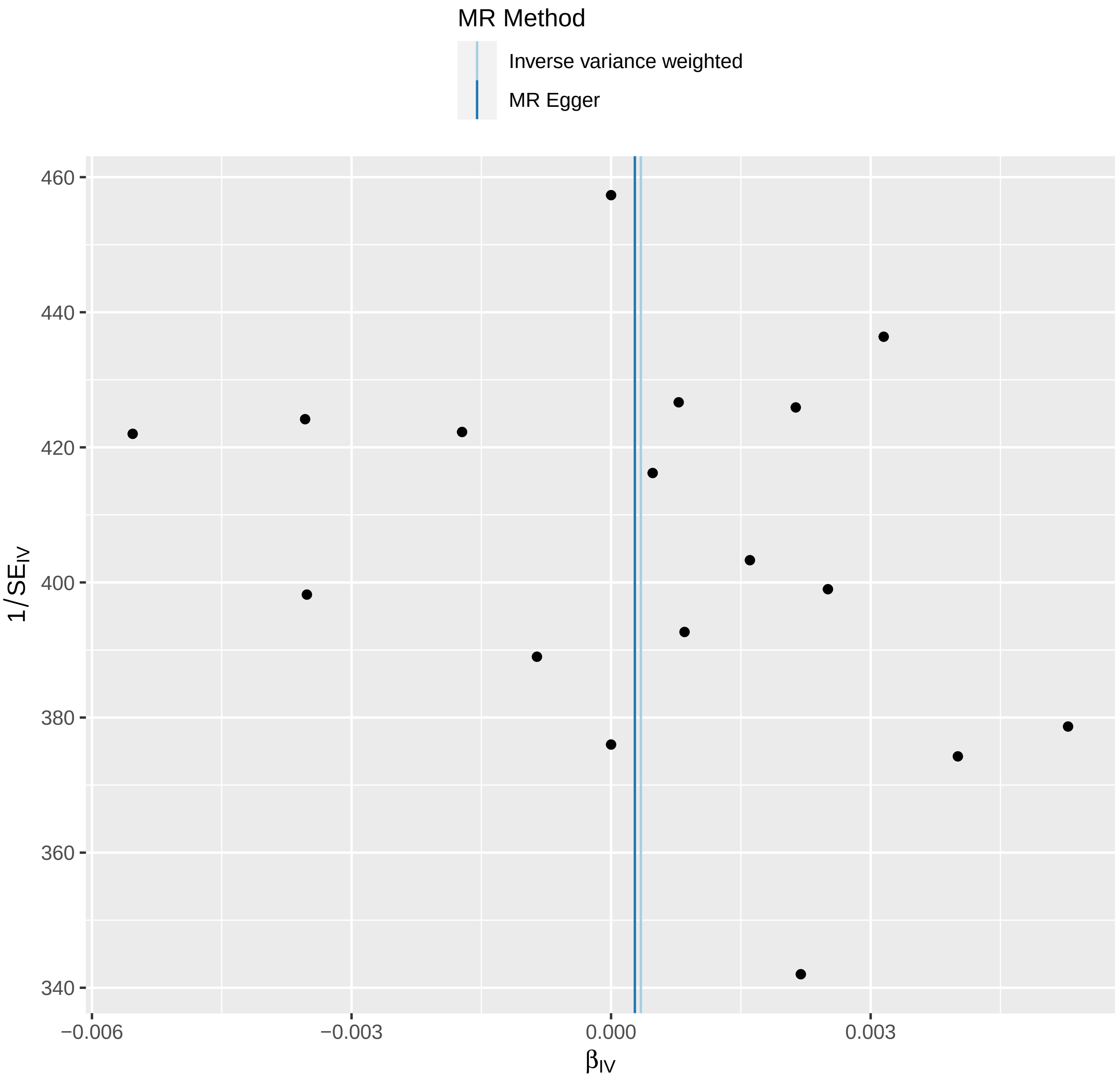

Batch 2−2961 : Transforming growth factor alpha on eGFR

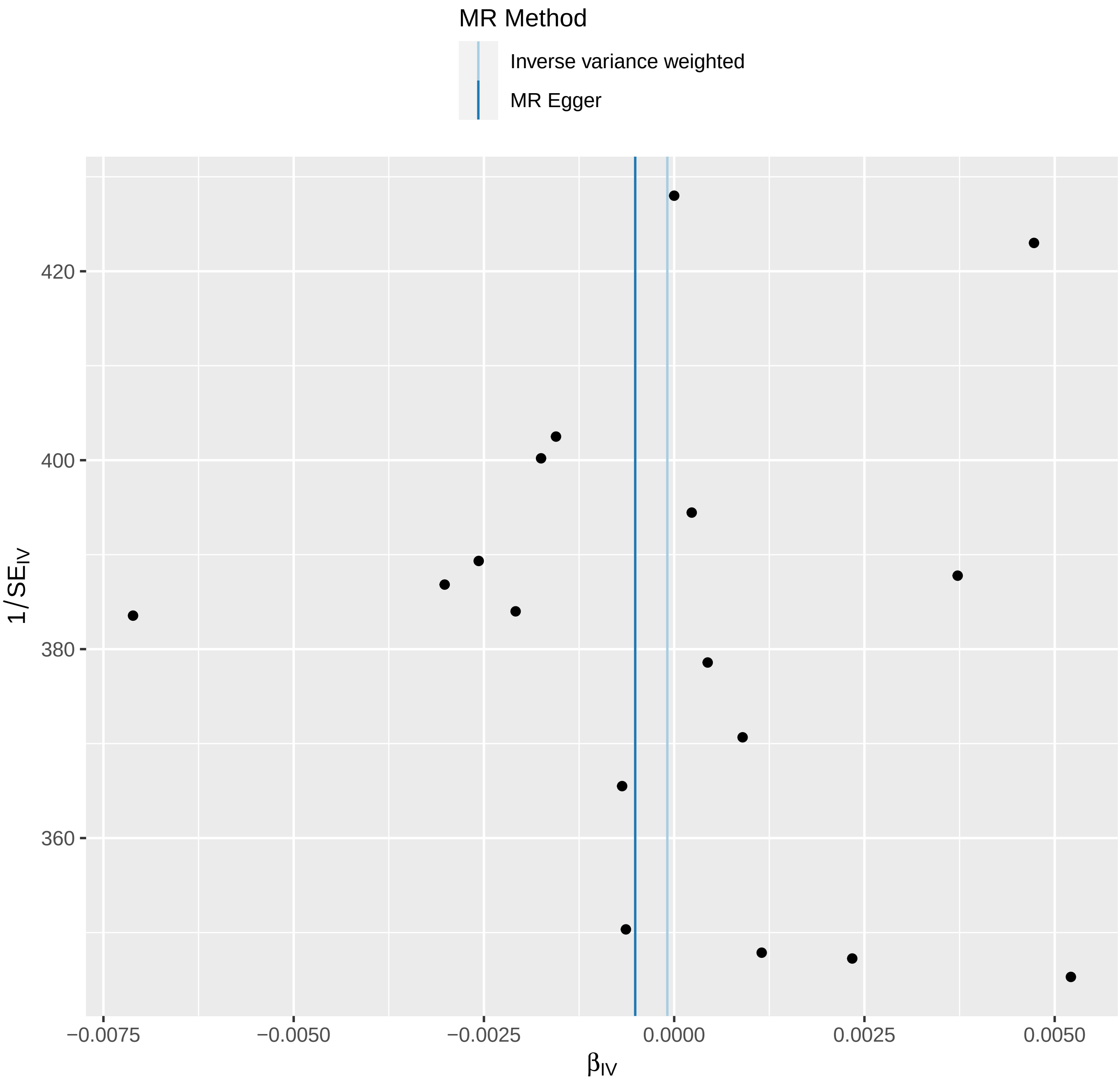

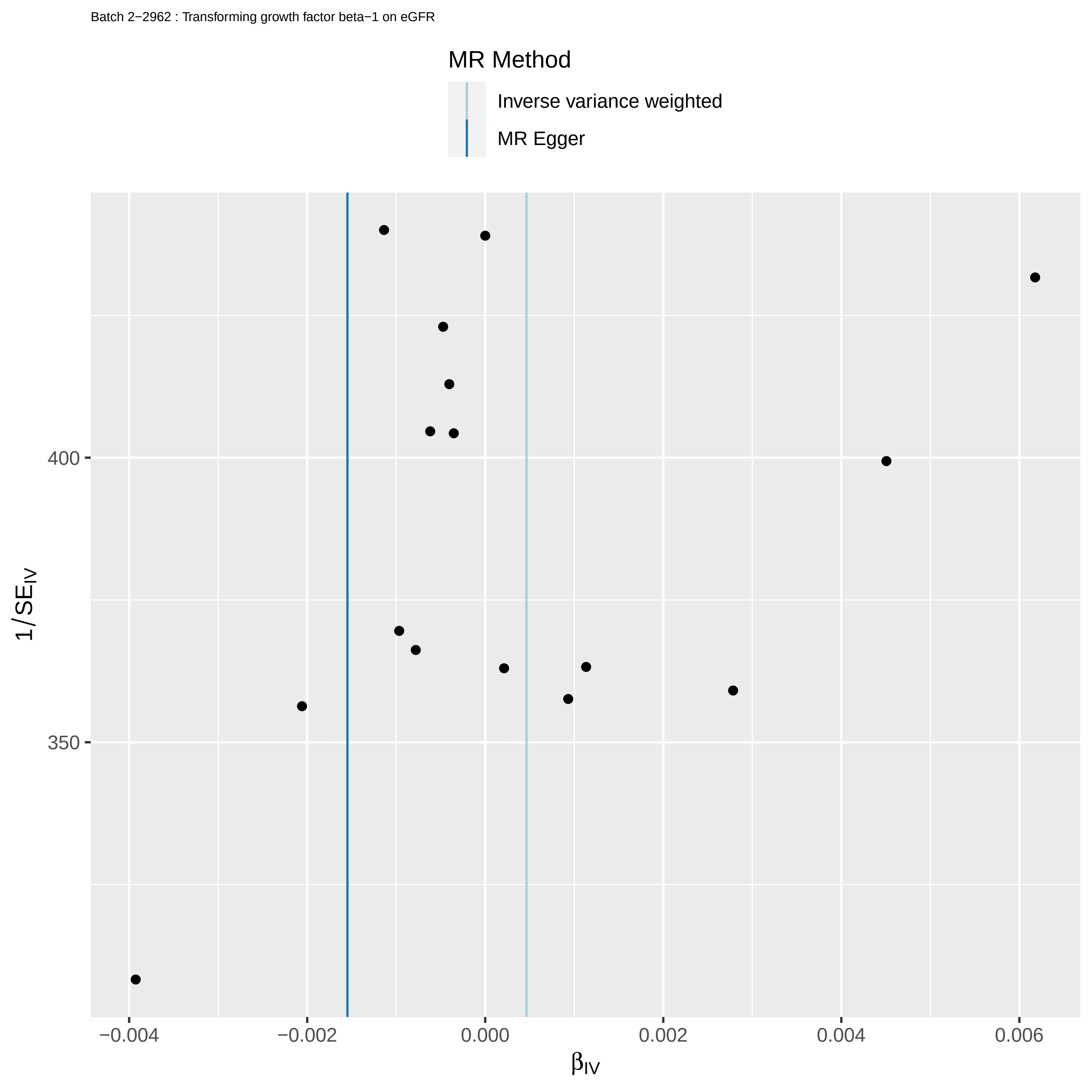
Batch 2−2962 : Transforming growth factor beta−1 on eGFR
MR Method
Inverse variance weighted
MR Egger
400
350
1/SE$_{IV}$
−0.004
−0.002
0.000
0.002
0.004
0.006
β$_{IV}$

Batch 2−2963 : Transforming growth factor beta−1−induced transcript 1 protein on eGFR

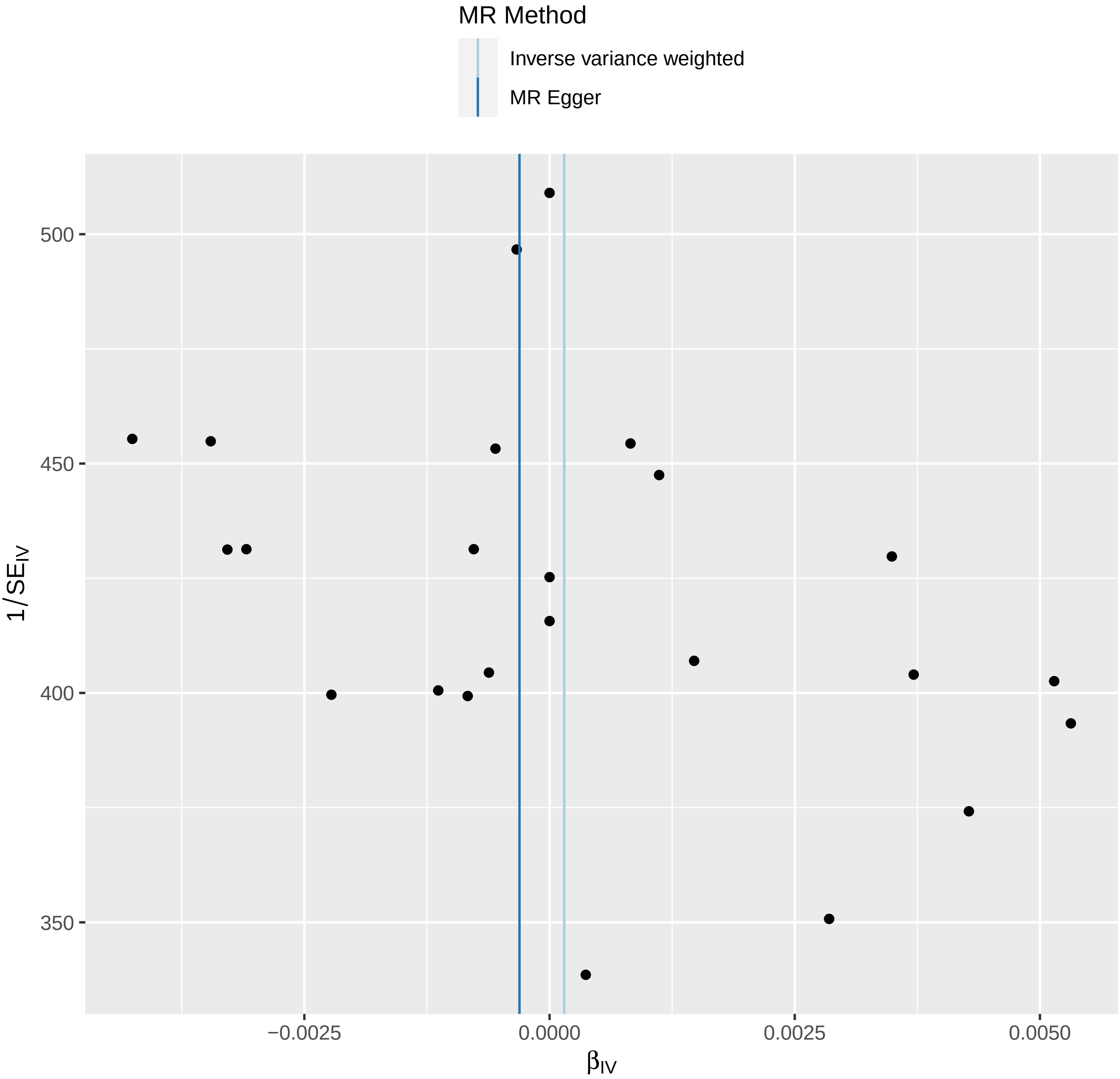

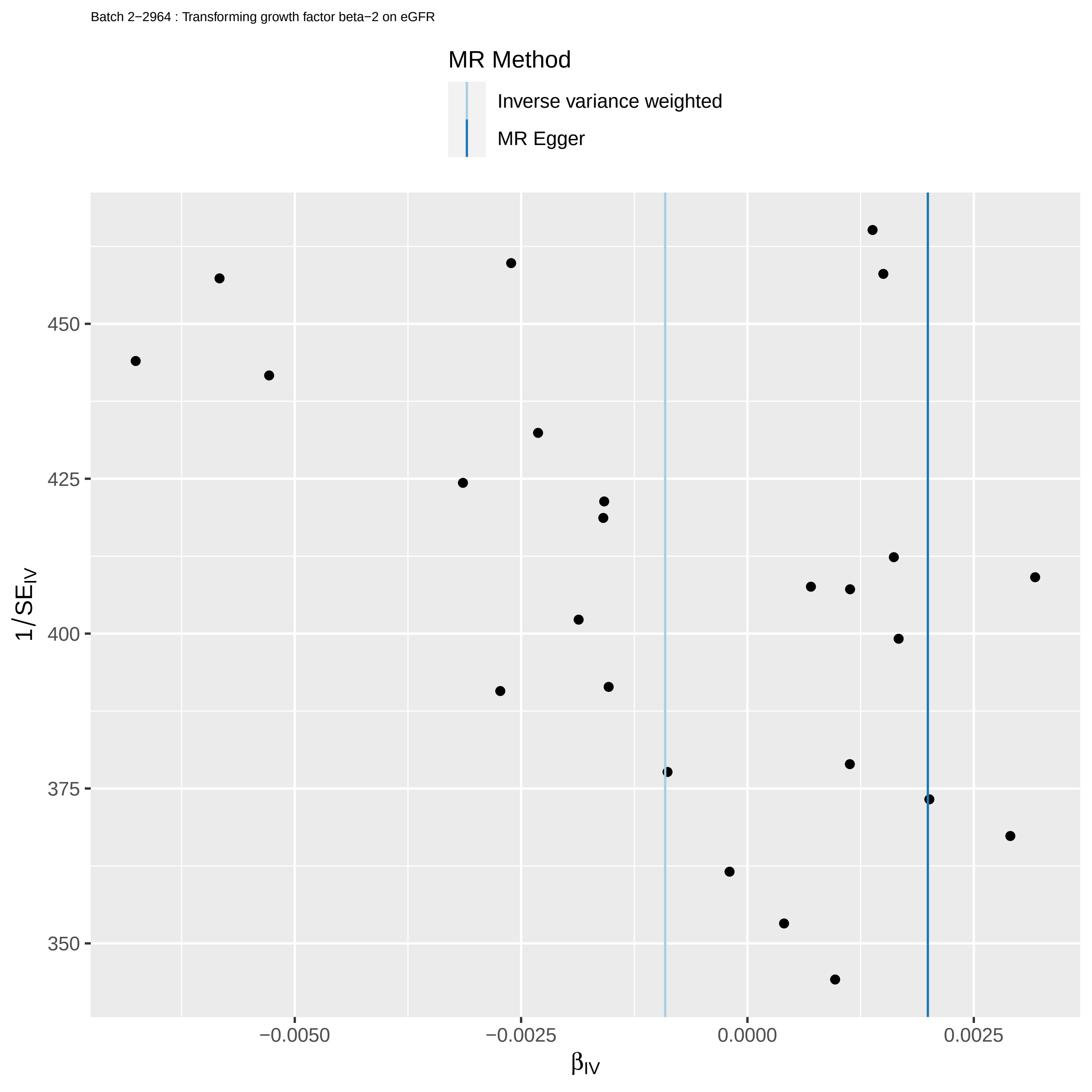

Batch 2−2964 : Transforming growth factor beta−2 on eGFR
MR Method
Inverse variance weighted
MR Egger
450
425
400
375
350
1/SE_IV
−0.0050
−0.0025
0.0000
0.0025
β_IV

Batch 2−2965 : Transforming growth factor beta−3 on eGFR

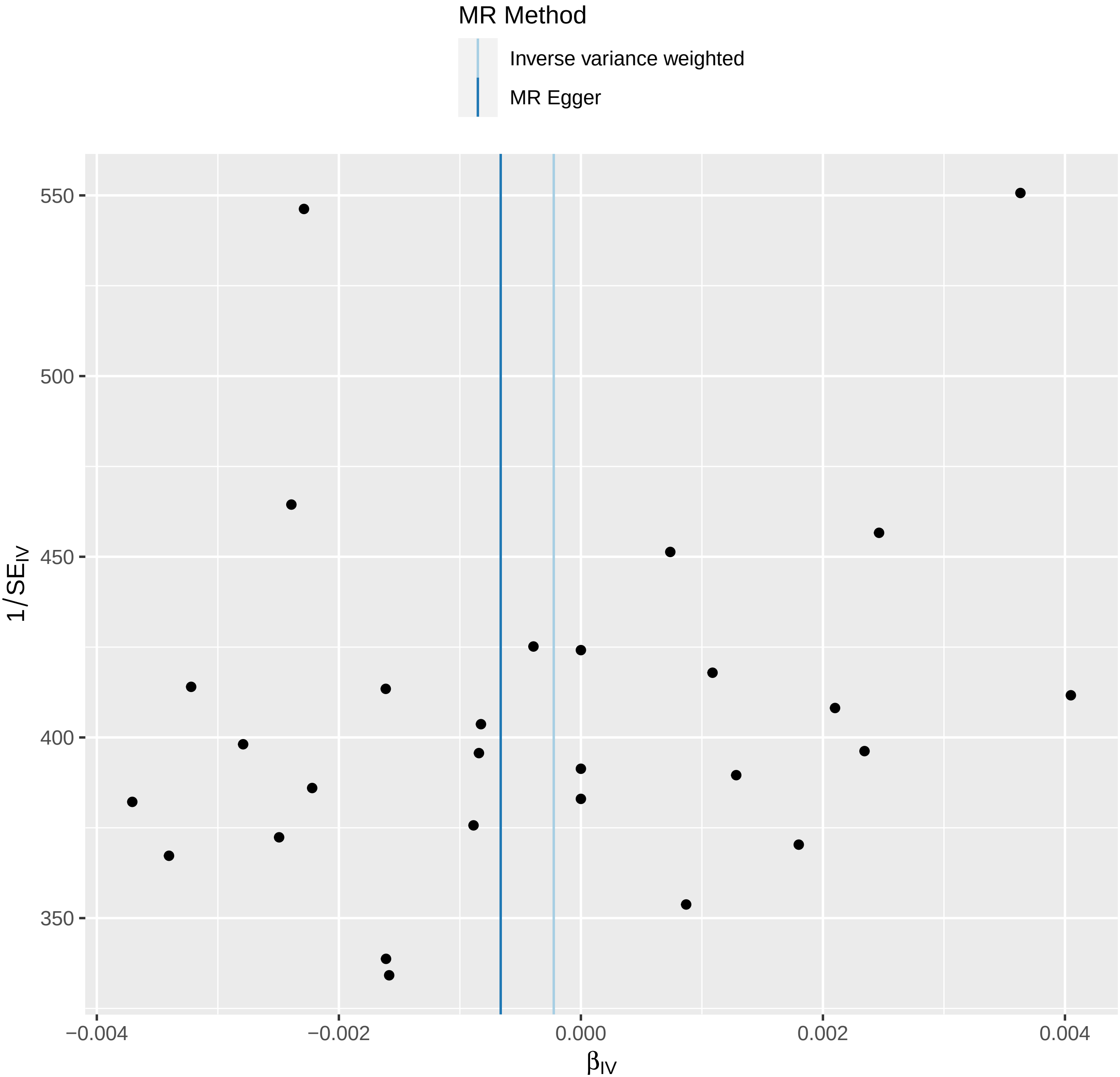

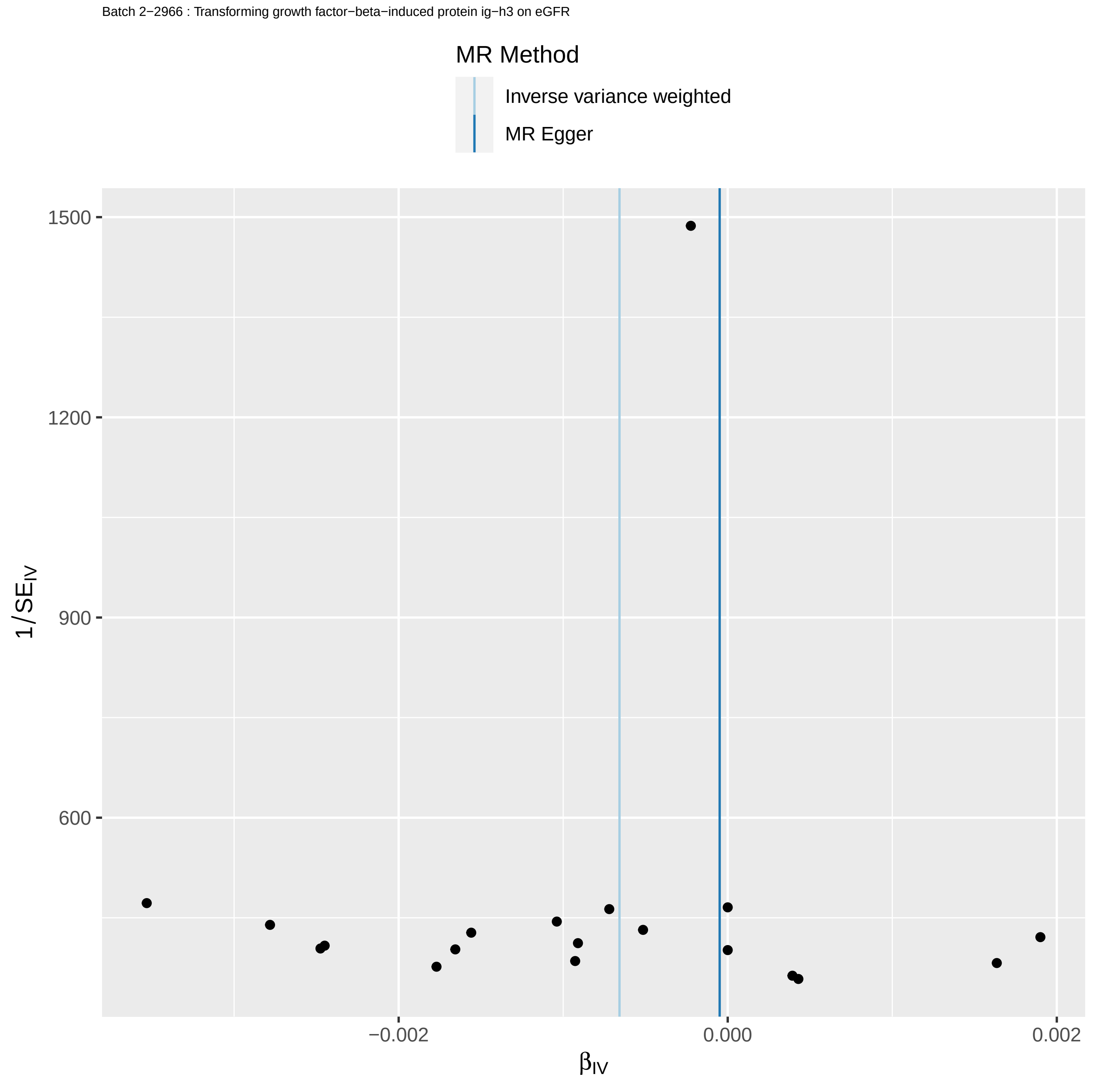
Batch 2−2966 : Transforming growth factor−beta−induced protein ig−h3 on eGFR
MR Method
Inverse variance weighted
MR Egger
1500
1200
900
600
1/SE$_{IV}$
−0.002
0.000
0.002
β$_{IV}$

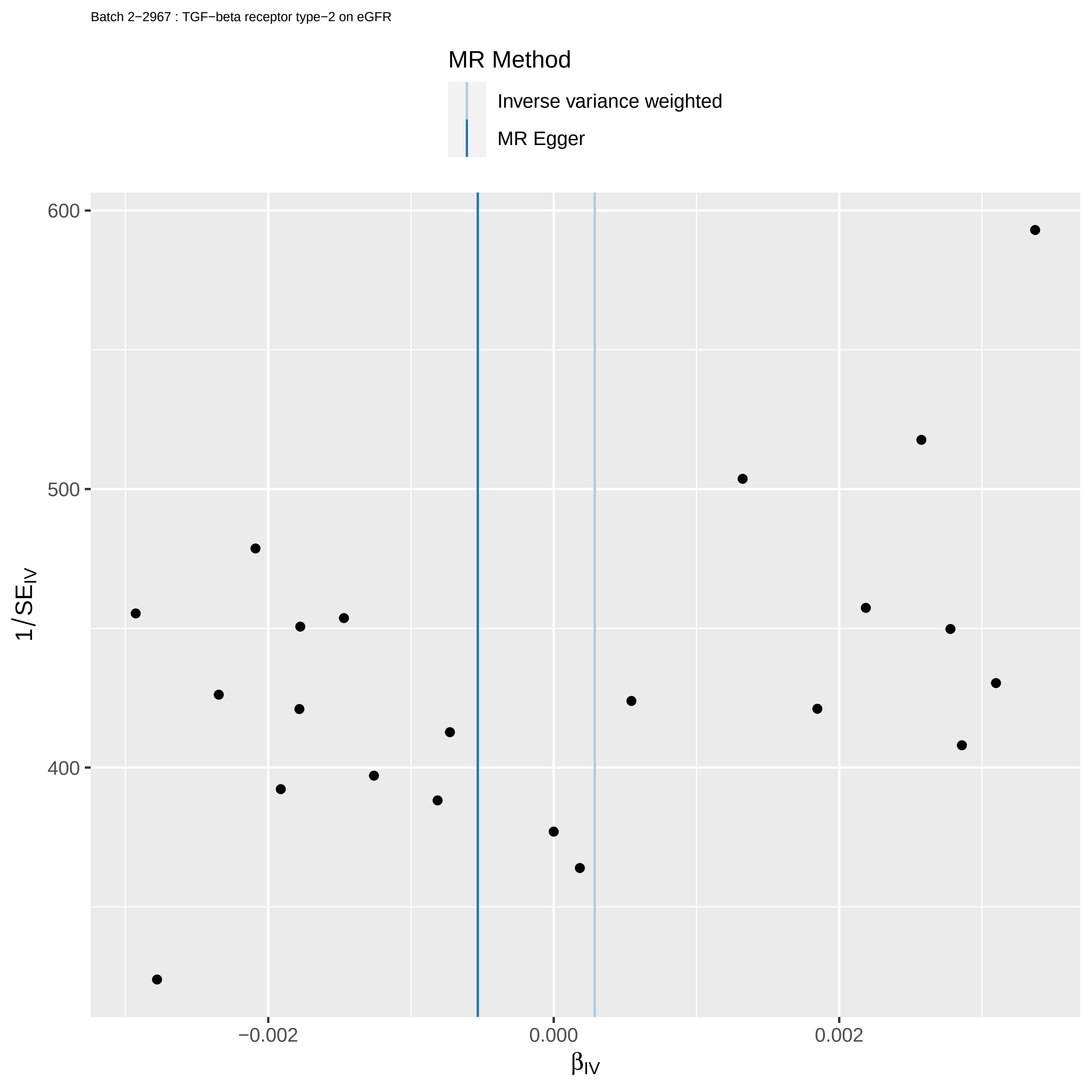
Batch 2−2967 : TGF−beta receptor type−2 on eGFR
MR Method
Inverse variance weighted
MR Egger
600
500
400
1/SE_IV
−0.002
0.000
0.002
β_IV

Batch 2−2968 : Transforming growth factor beta receptor type 3 on eGFR

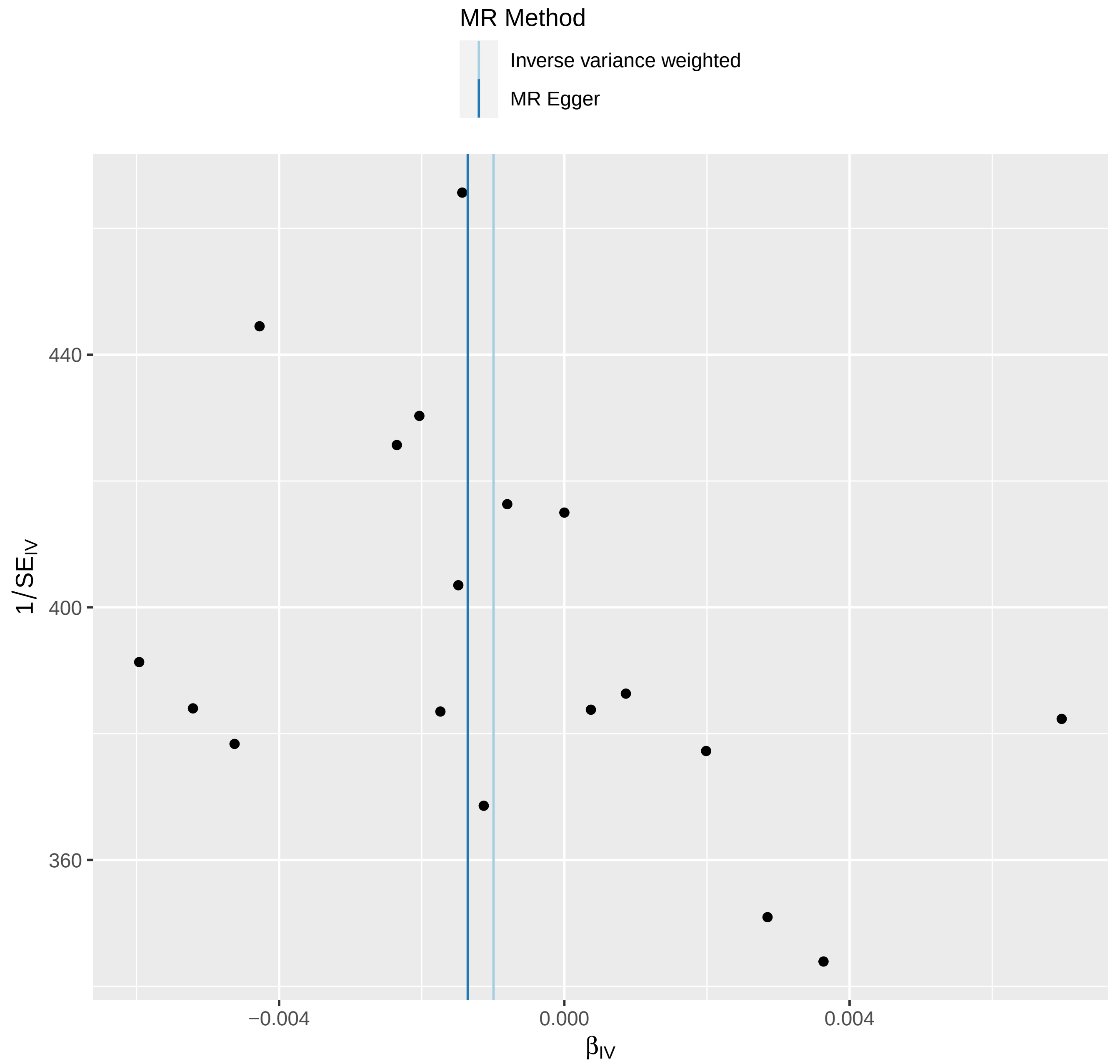

Batch 2−2969 : Homeobox protein TGIF2 on eGFR

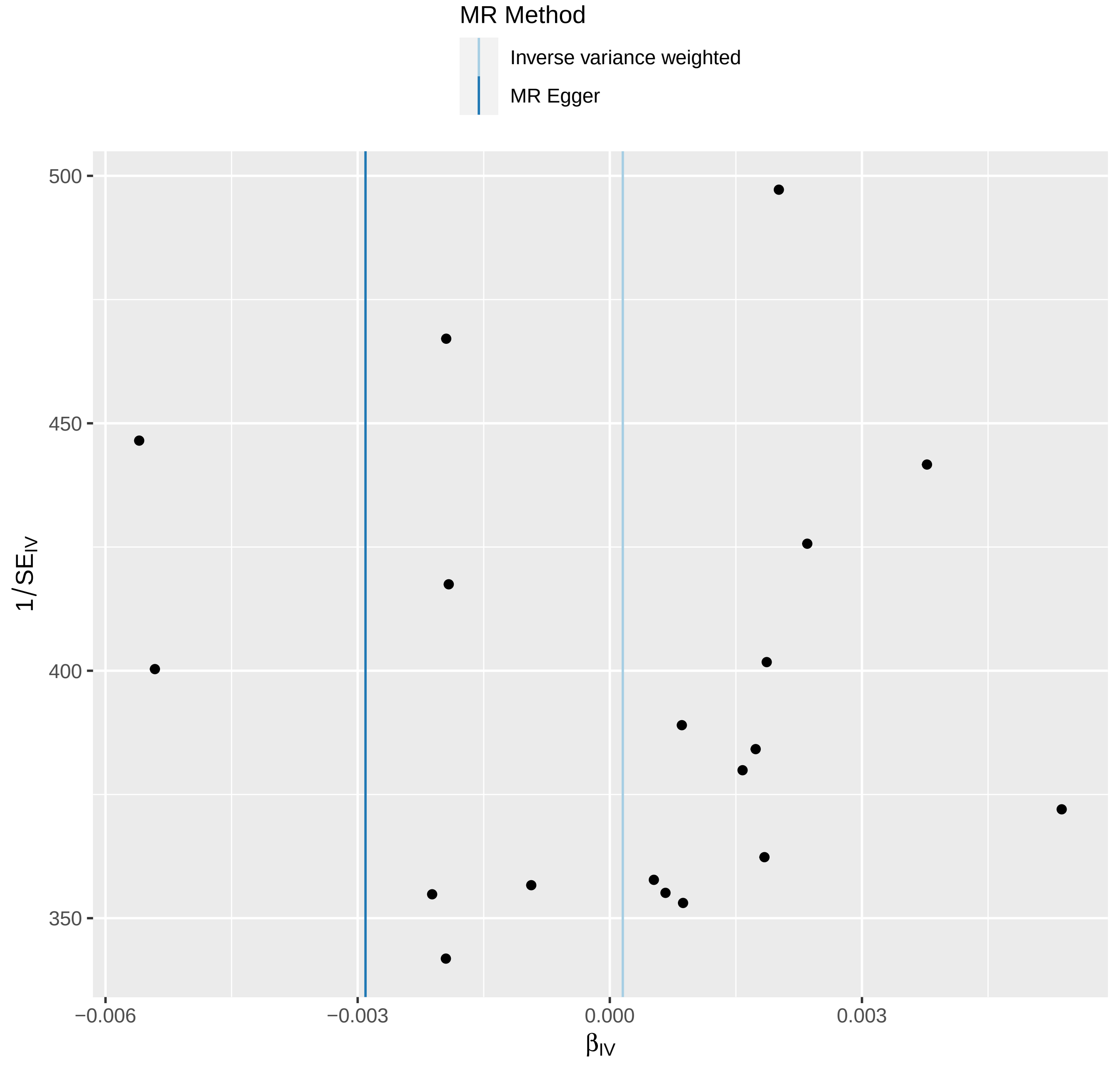

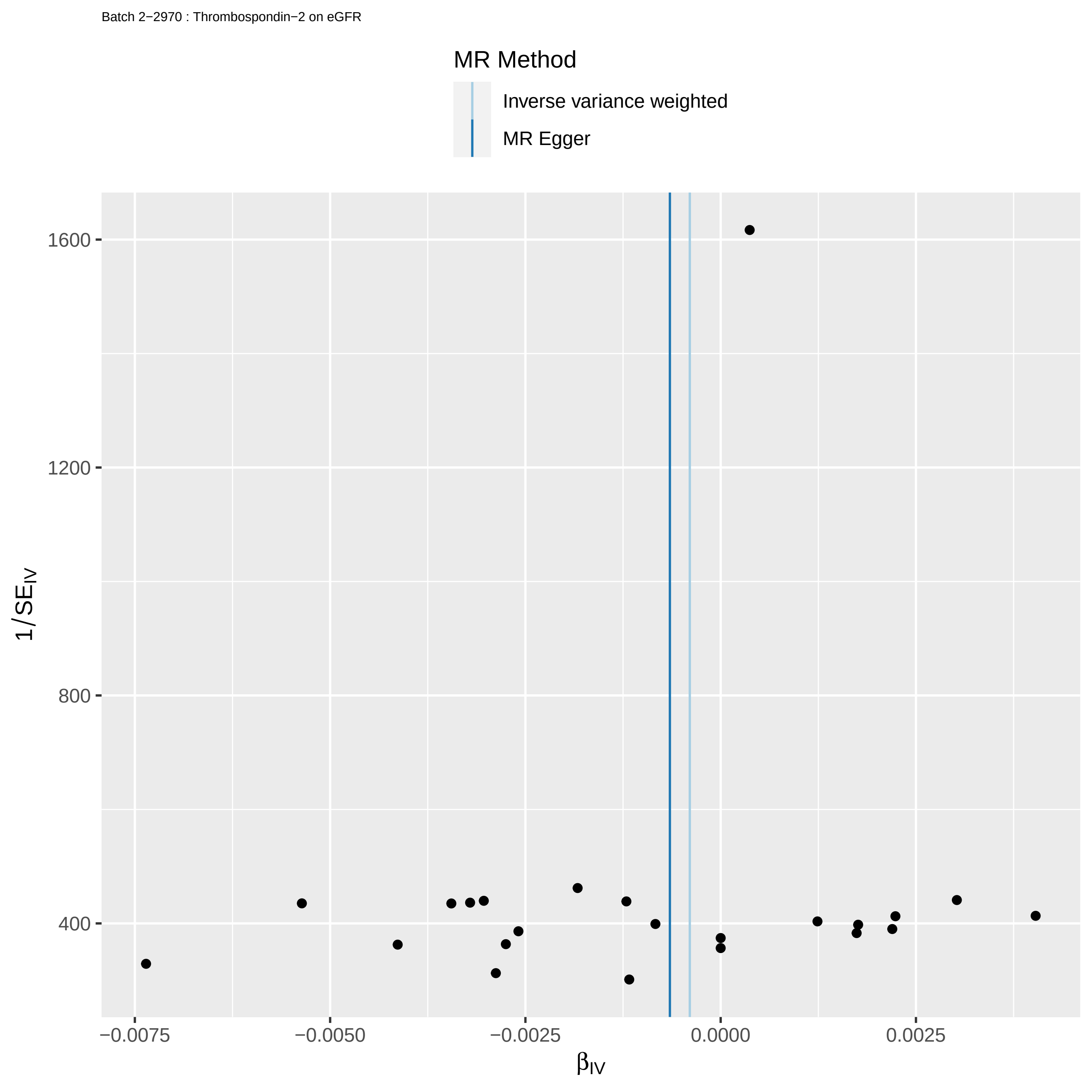

Batch 2−2970 : Thrombospondin−2 on eGFR
MR Method
Inverse variance weighted
MR Egger
1600
1200
800
400
1/SE_IV
−0.0075
−0.0050
−0.0025
0.0000
0.0025
β_IV

Batch 2−2971 : Thrombospondin−3 on eGFR

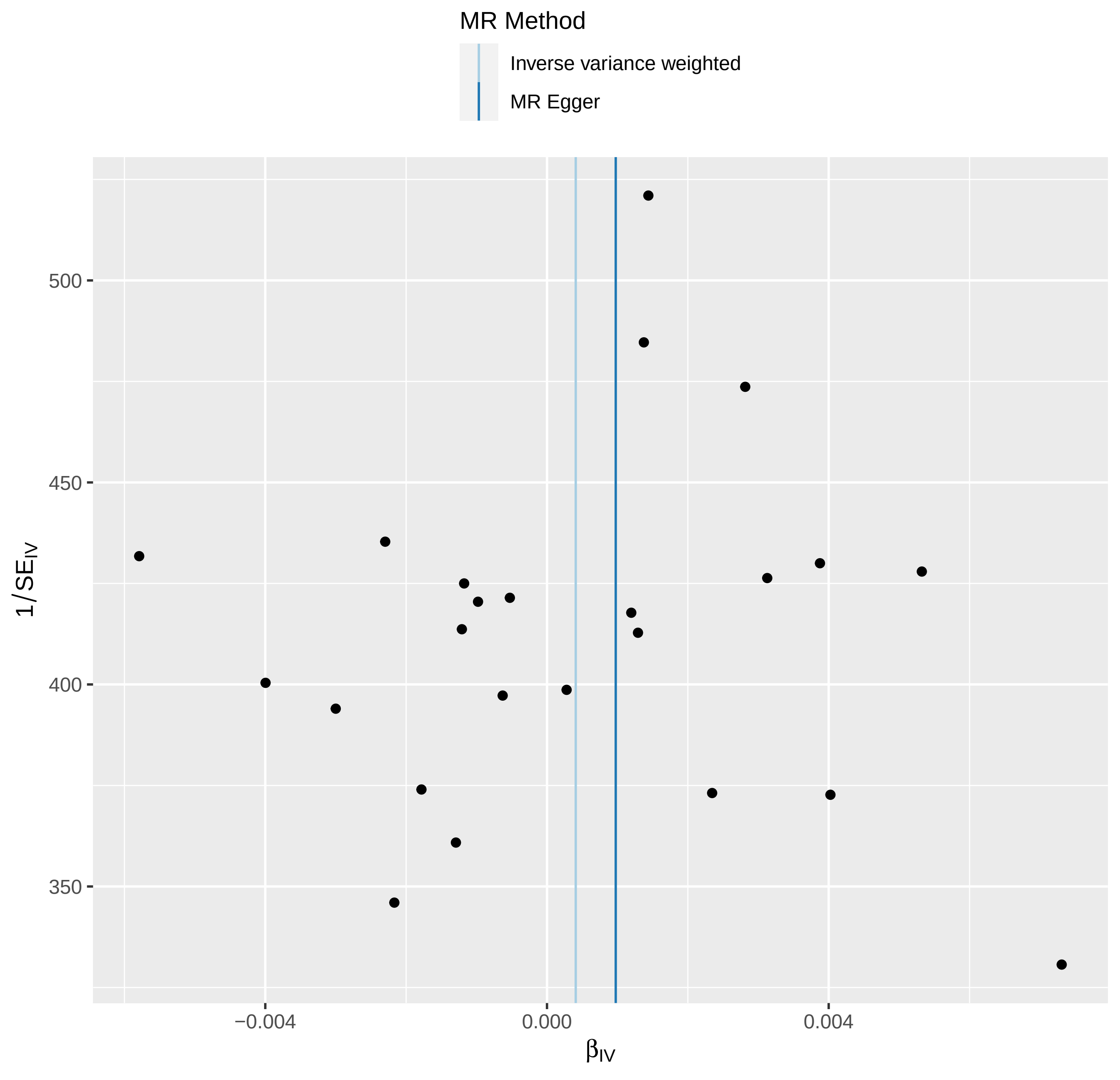

Batch 2−2972 : Thrombopoietin on eGFR

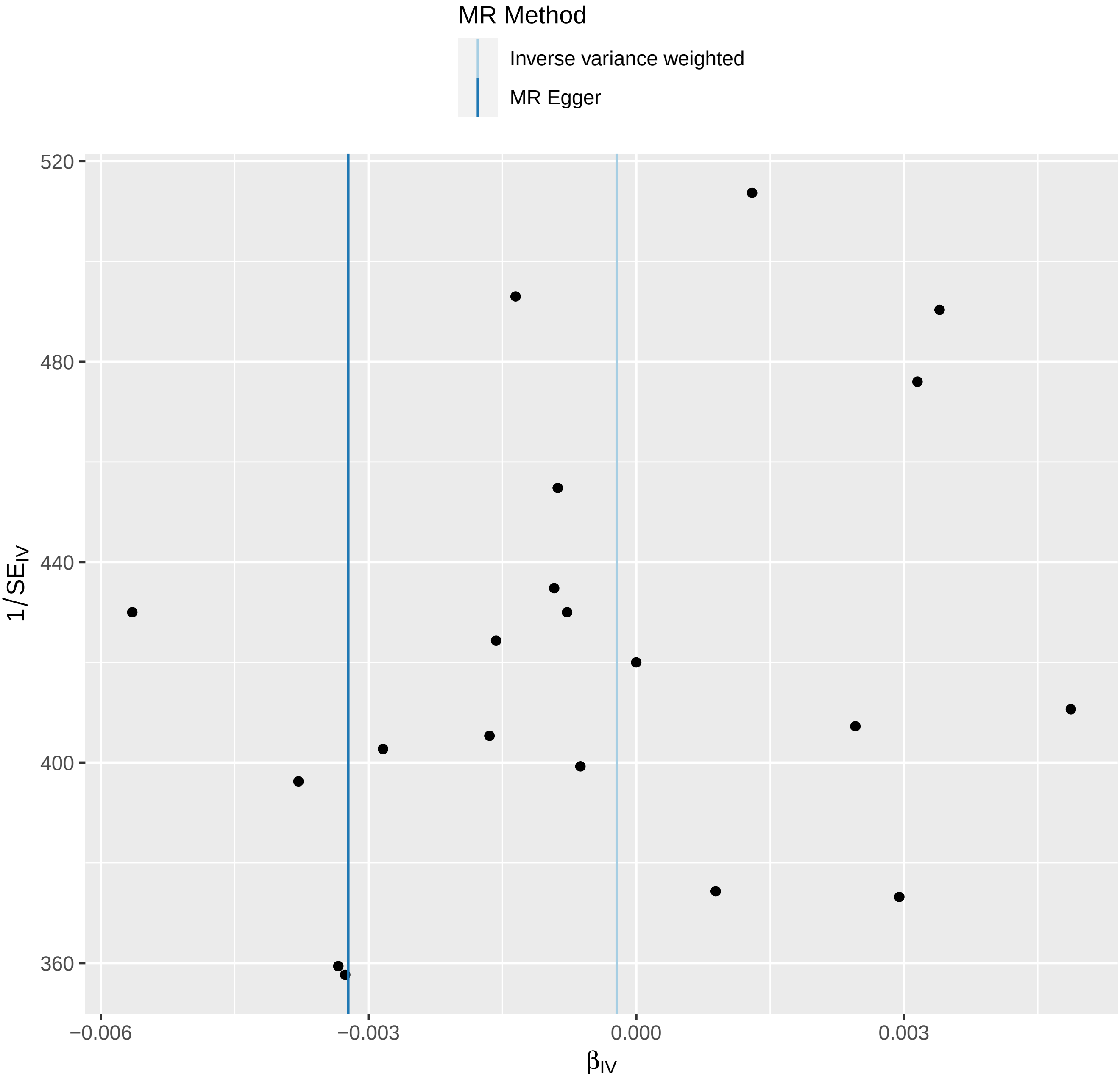

Batch 2−2973 : Thrombopoietin on eGFR

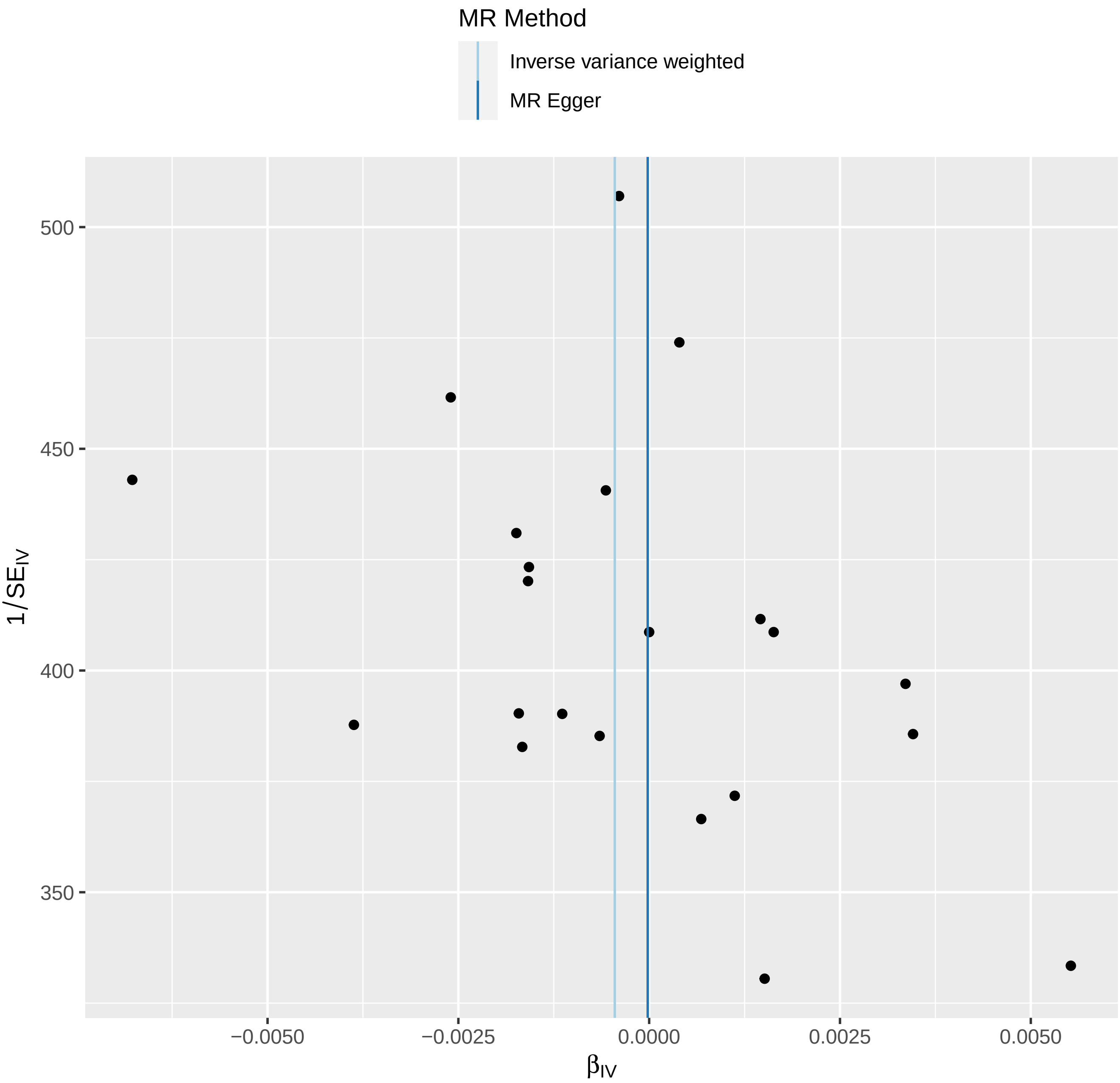

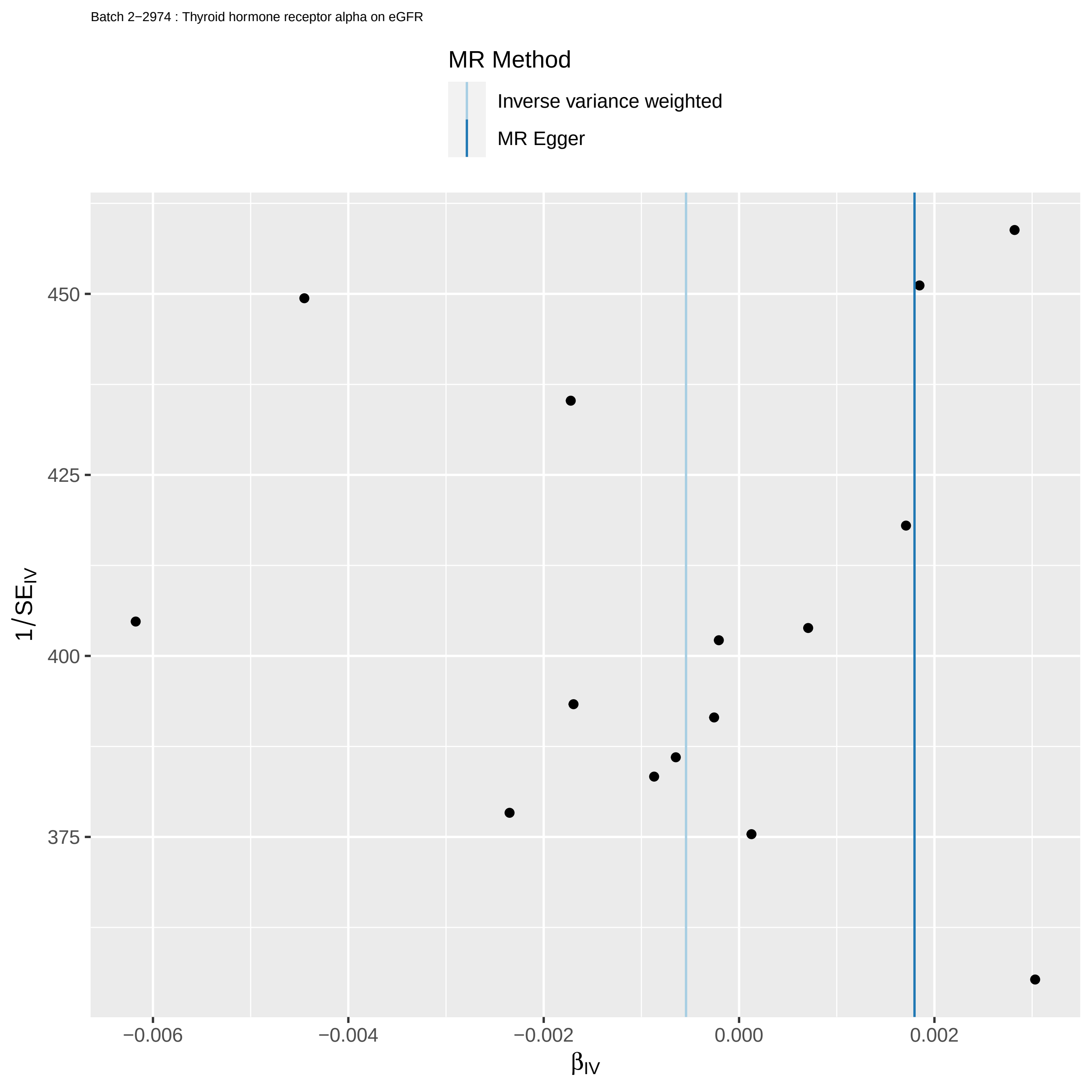
Batch 2−2974 : Thyroid hormone receptor alpha on eGFR
MR Method
Inverse variance weighted
MR Egger
450
425
400
375
1/SE_IV
−0.006
−0.004
−0.002
0.000
0.002
β_IV

Batch 2−2975 : Thrombospondin type−1 domain−containing protein 1 on eGFR

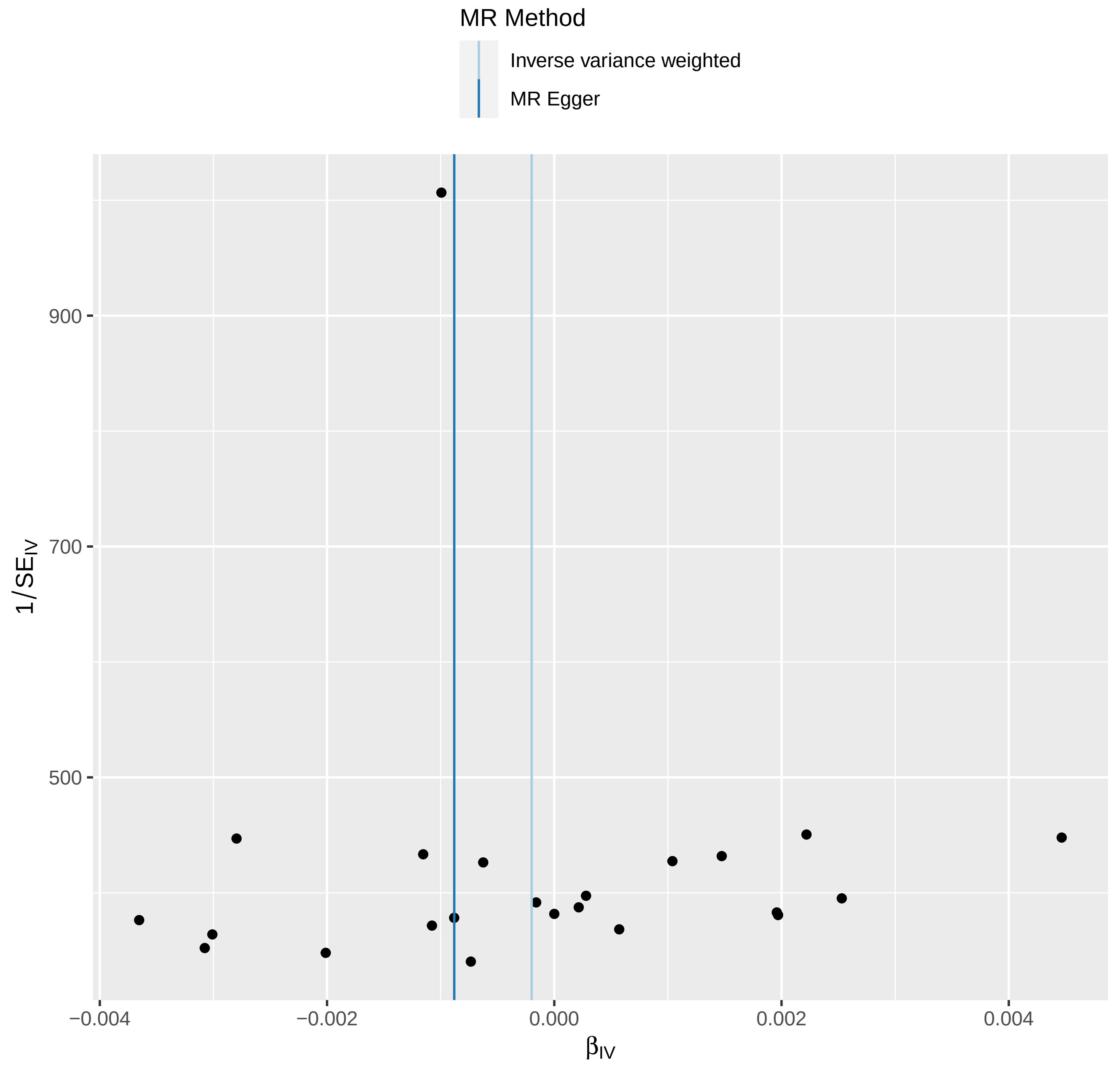

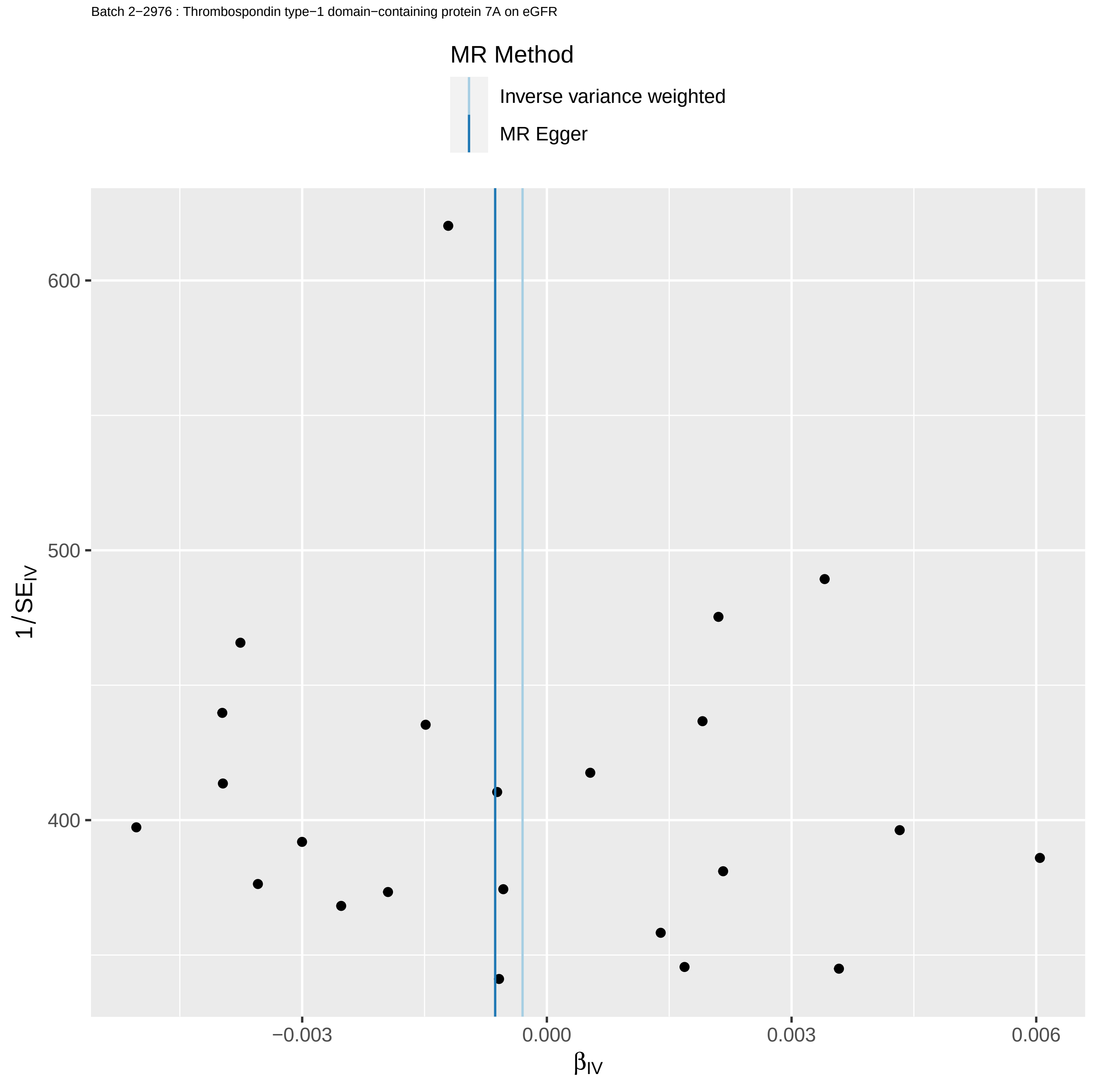
Batch 2−2976 : Thrombospondin type−1 domain−containing protein 7A on eGFR
MR Method
Inverse variance weighted
MR Egger
600
500
400
1/SE_IV
−0.003
0.000
0.003
0.006
β_IV

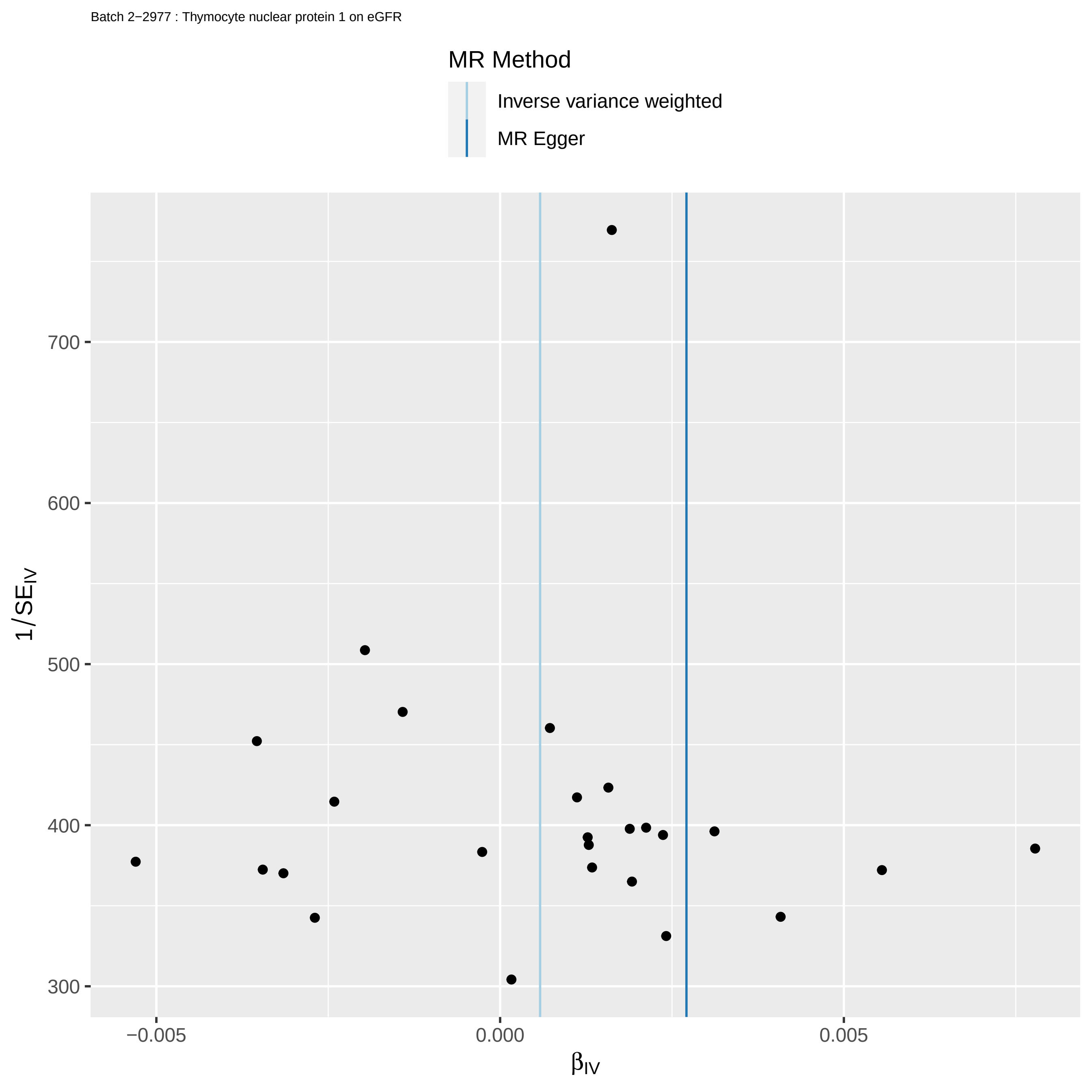

Batch 2−2977 : Thymocyte nuclear protein 1 on eGFR
MR Method
Inverse variance weighted
MR Egger
700
600
500
400
300
1/SE$_{IV}$
−0.005
0.000
0.005
β$_{IV}$

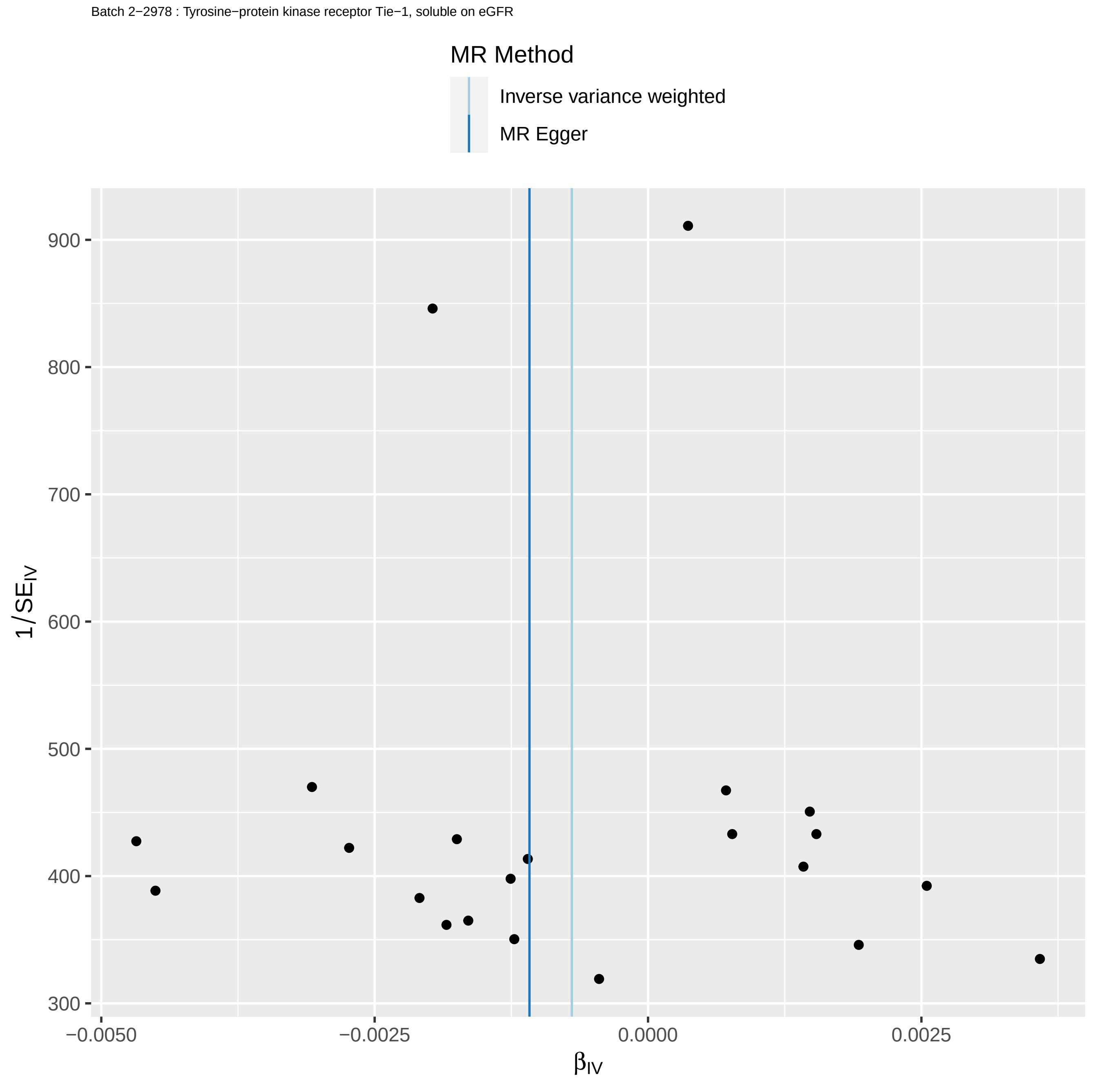

Batch 2−2978 : Tyrosine−protein kinase receptor Tie−1, soluble on eGFR
MR Method
Inverse variance weighted
MR Egger
900
800
700
600
500
400
300
$1/SE_{IV}$
−0.0050
−0.0025
0.0000
0.0025
β_{IV}

Batch 2−2979 : Fructose−2,6−bisphosphatase TIGAR on eGFR

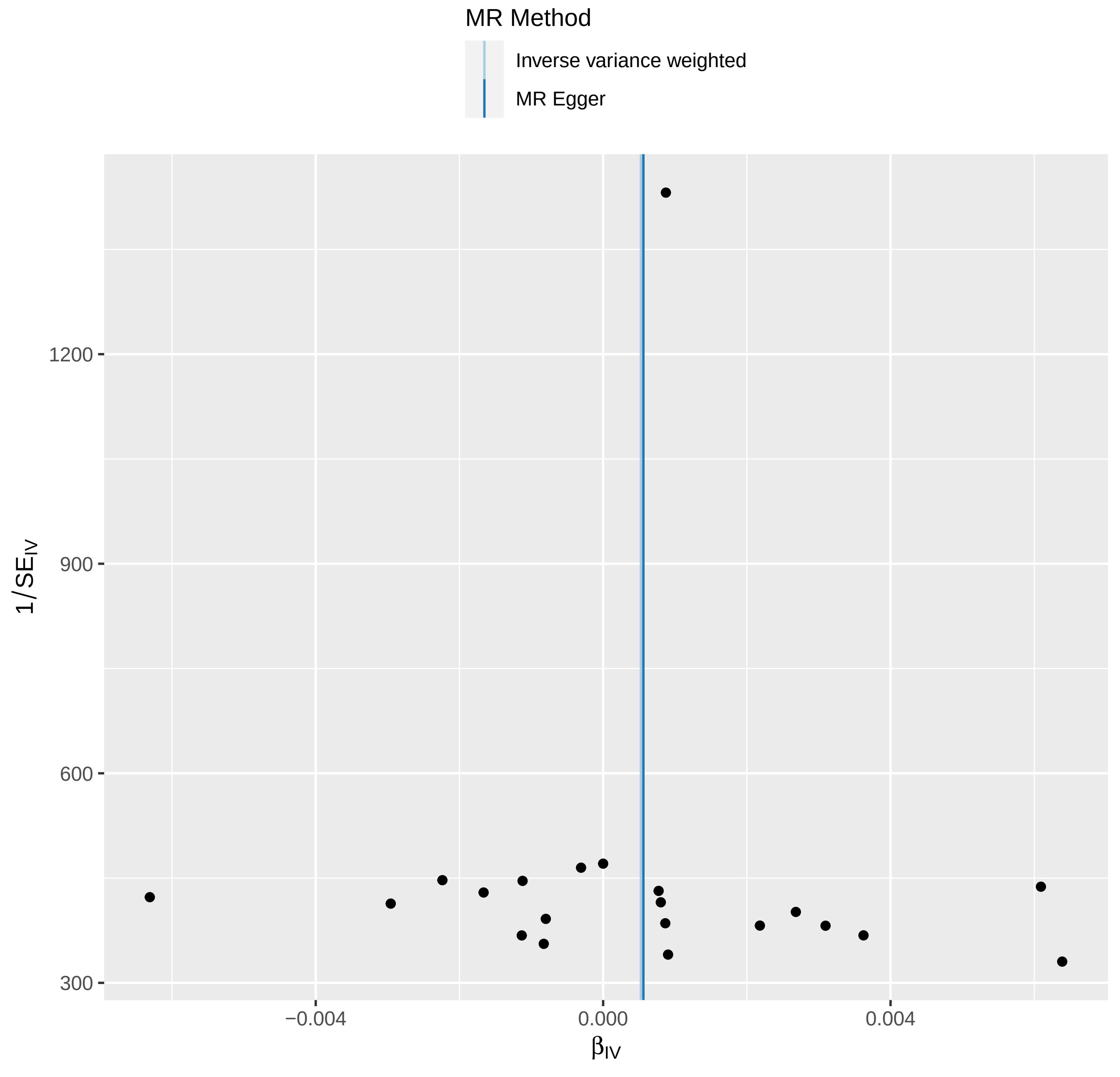

Batch 2−2980 : Metalloproteinase inhibitor 4 on eGFR

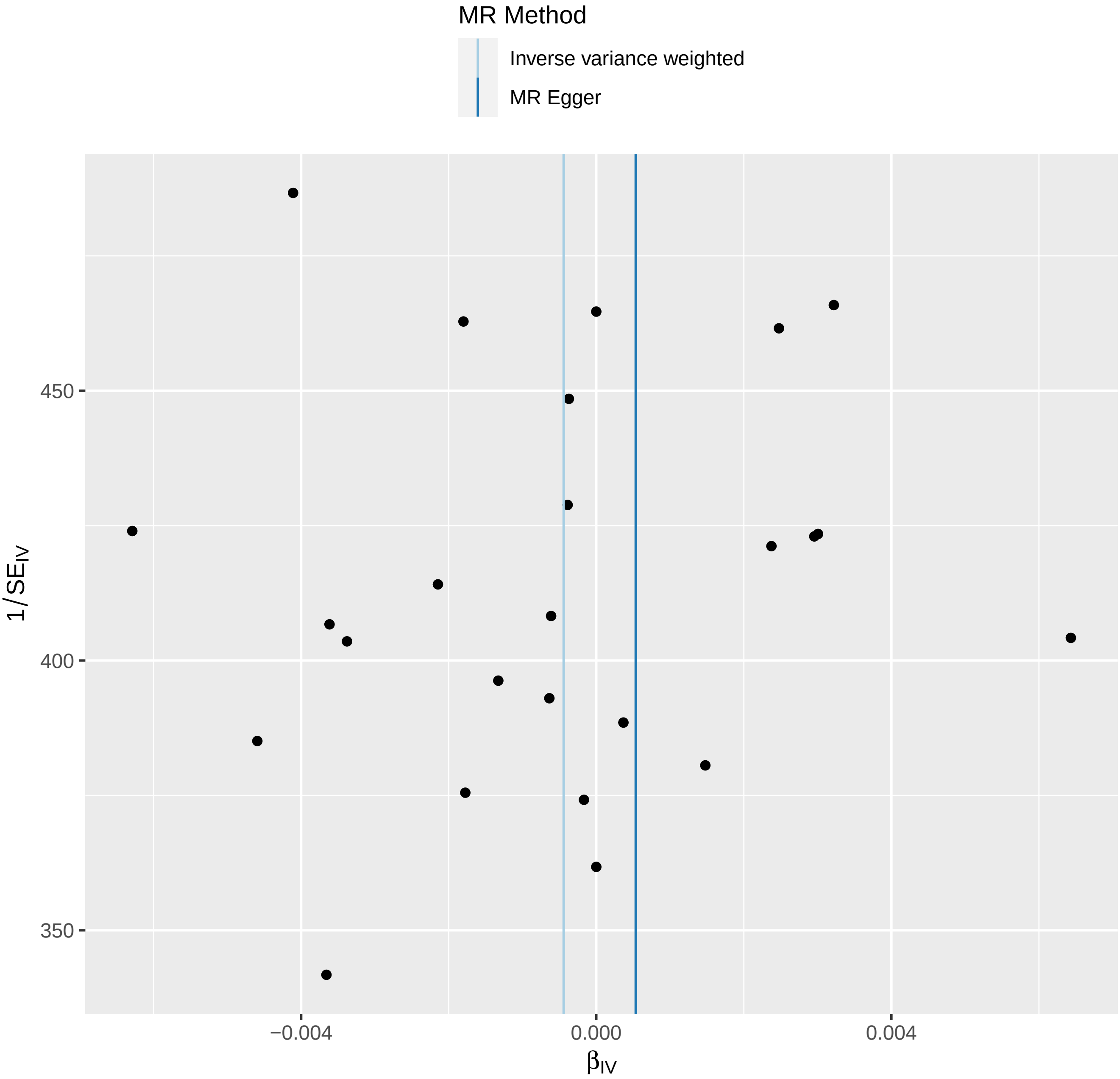

Batch 2−2981 : Tubulointerstitial nephritis antigen−like on eGFR

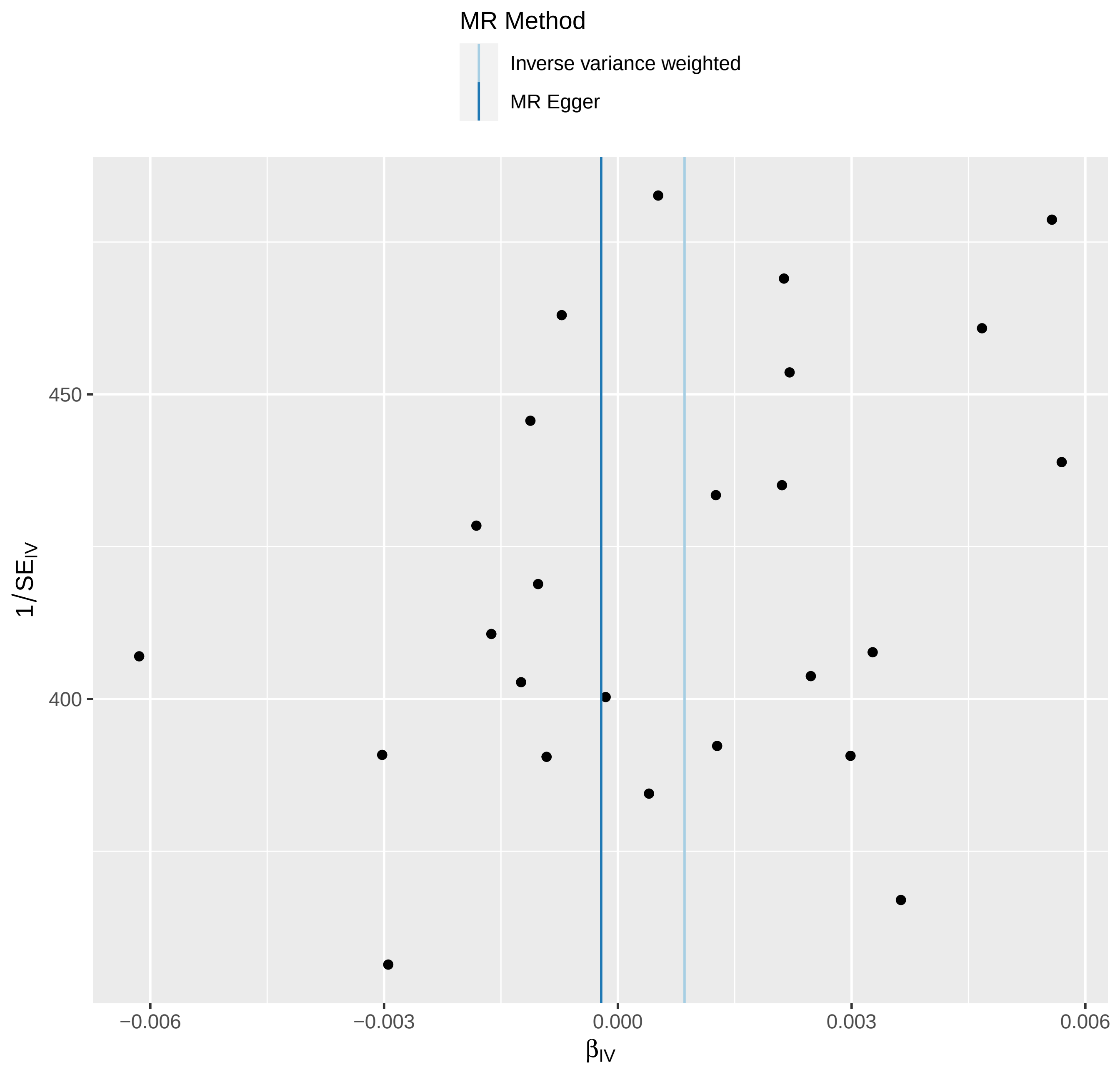

Batch 2−2982 : Toll/interleukin−1 receptor domain−containing adapter protein on eGFR

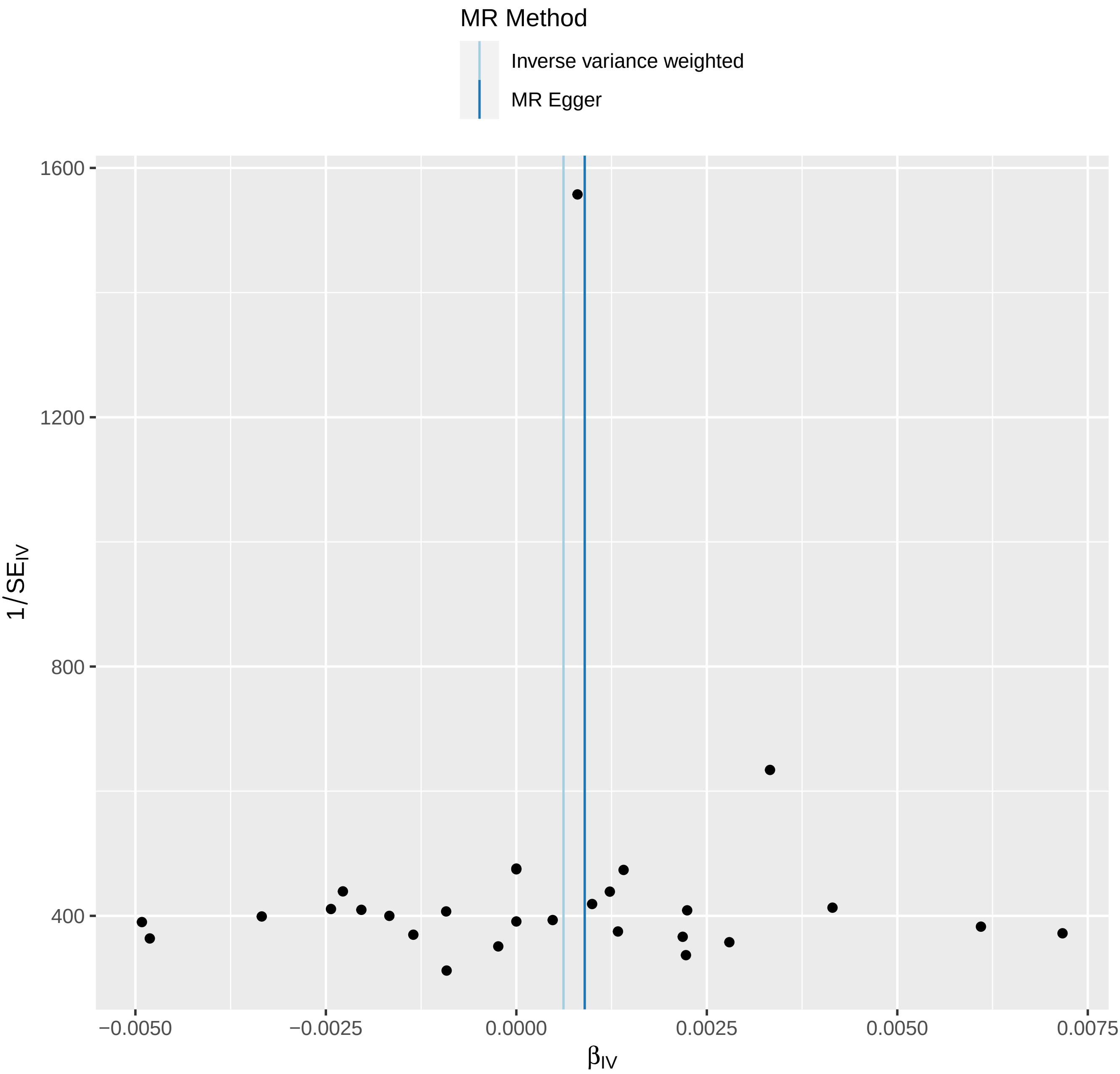

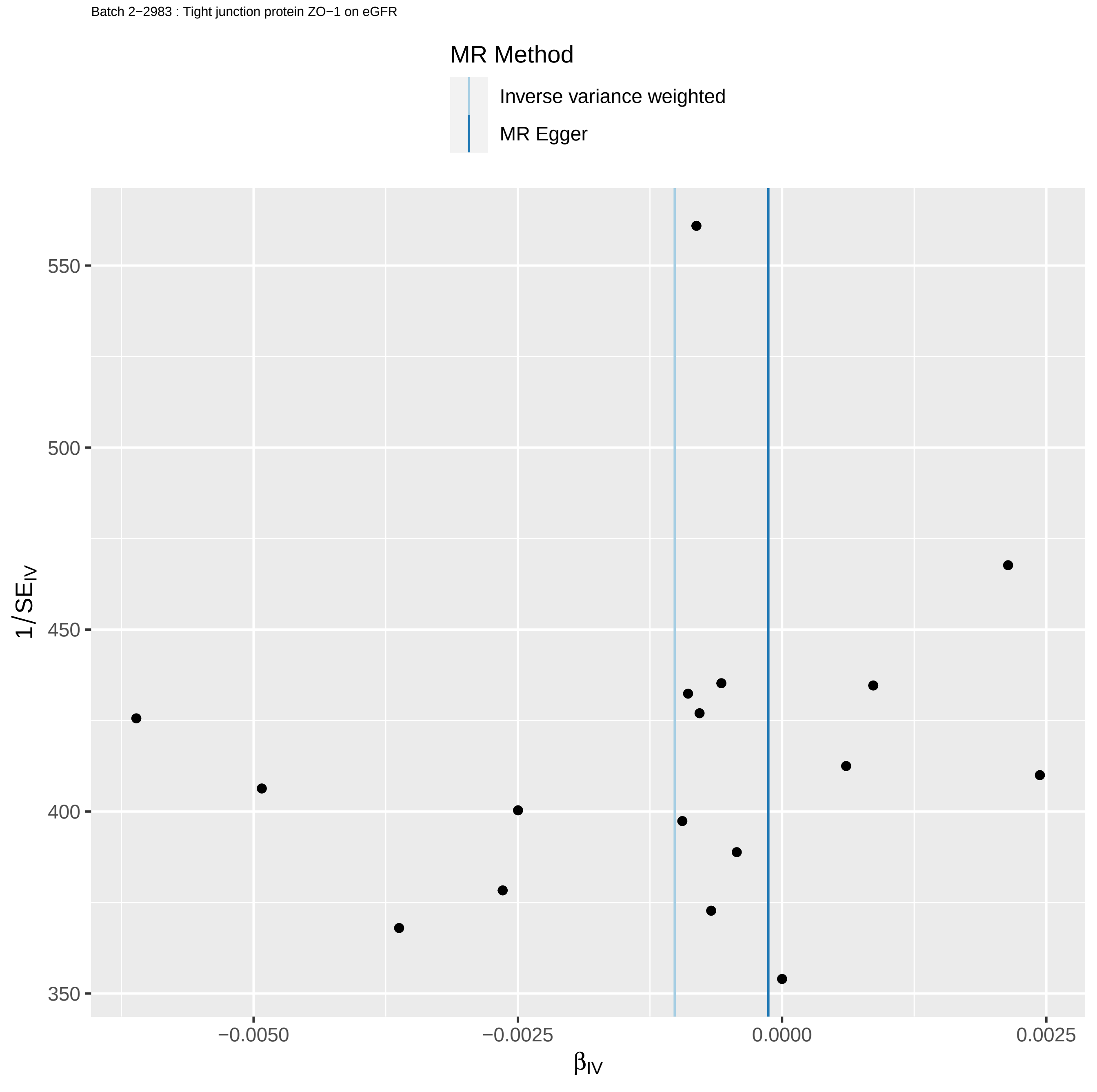
Batch 2−2983 : Tight junction protein ZO−1 on eGFR
MR Method
Inverse variance weighted
MR Egger
550
500
450
400
350
1/SE_IV
−0.0050
−0.0025
0.0000
0.0025
β_IV

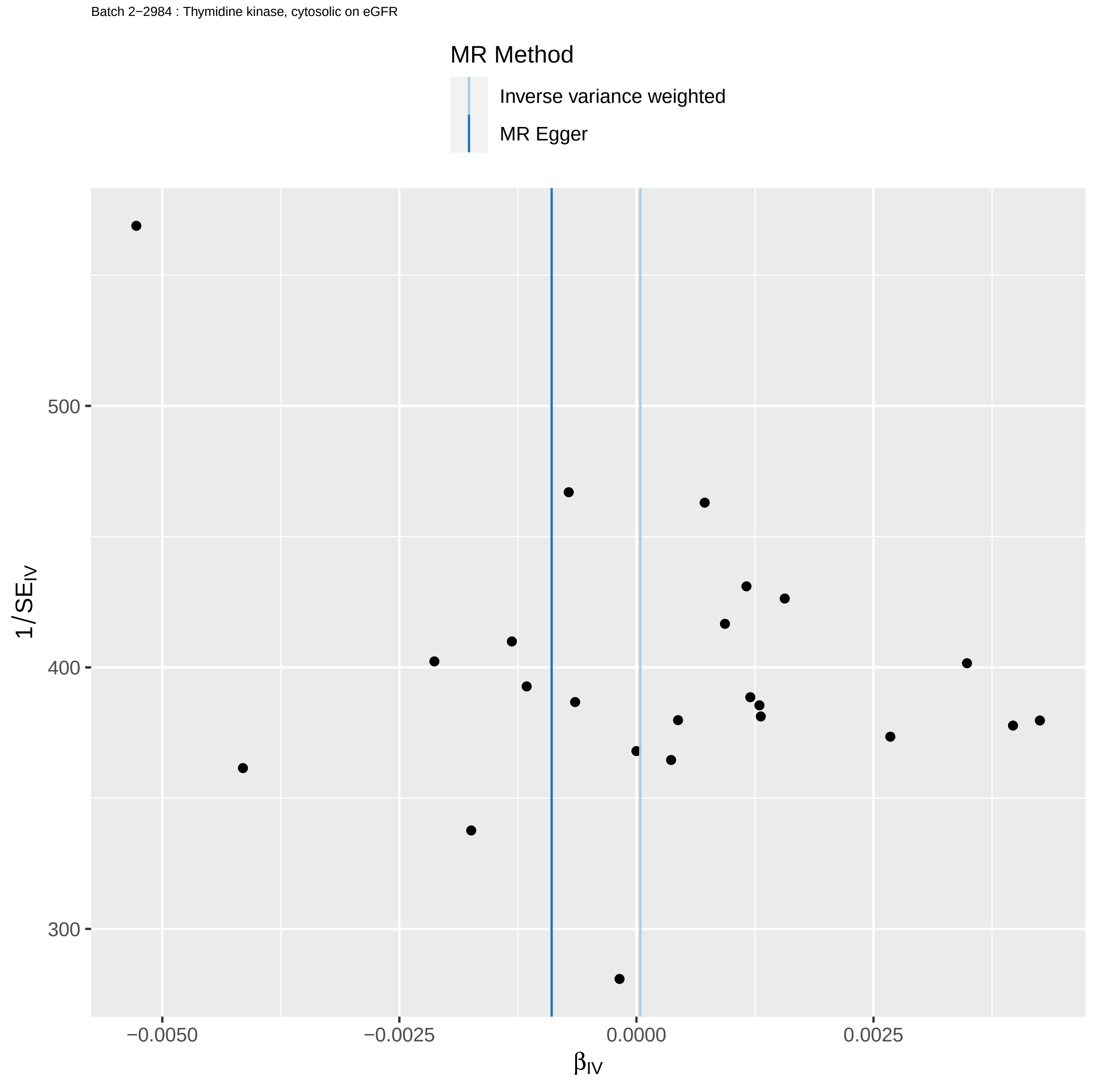
Batch 2−2984 : Thymidine kinase, cytosolic on eGFR
MR Method
Inverse variance weighted
MR Egger
500
400
300
1/SE_IV
−0.0050
−0.0025
0.0000
0.0025
β_IV

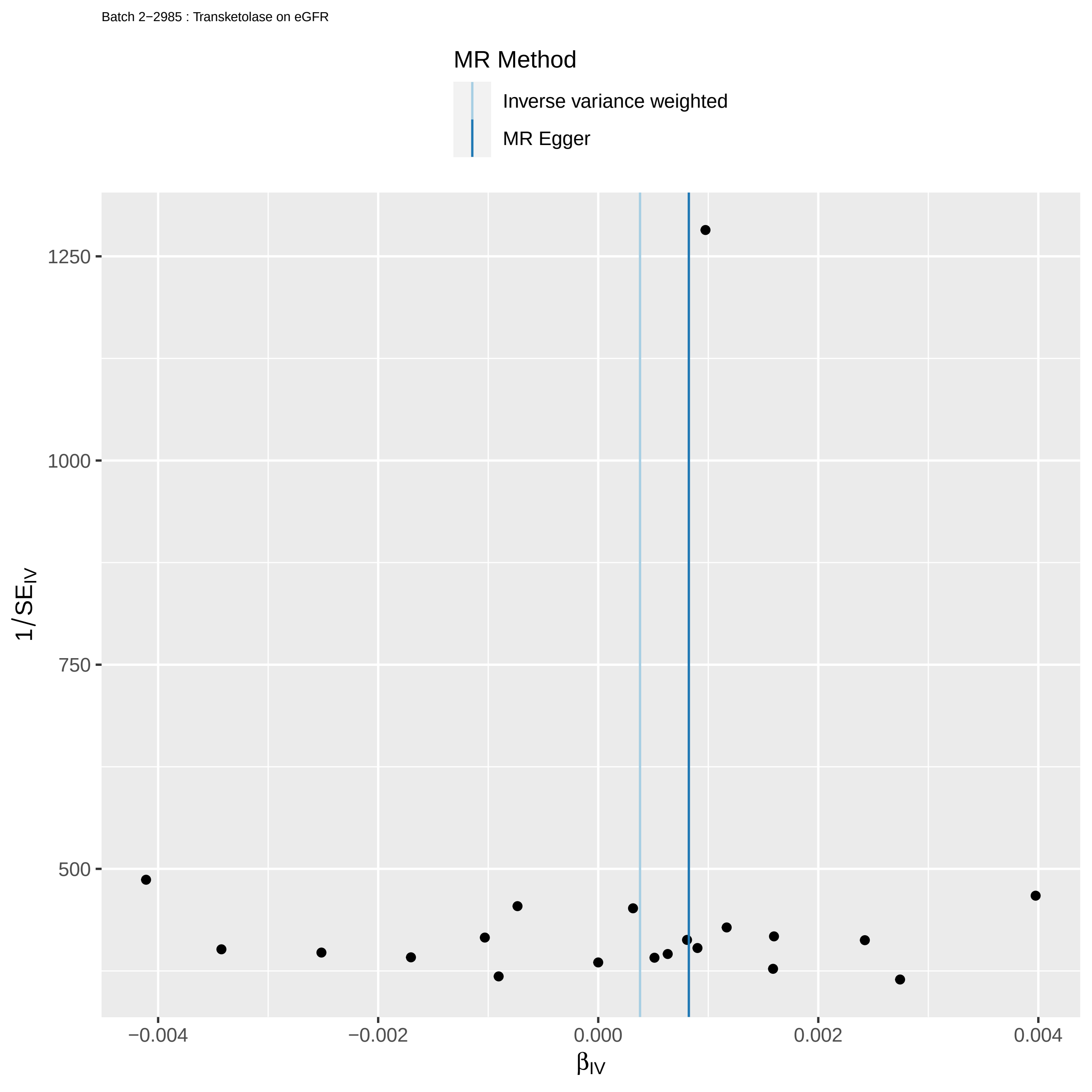

Batch 2−2985 : Transketolase on eGFR
MR Method
Inverse variance weighted
MR Egger
1250
1000
750
500
1/SE$_{IV}$
−0.004
−0.002
0.000
0.002
0.004
β$_{IV}$

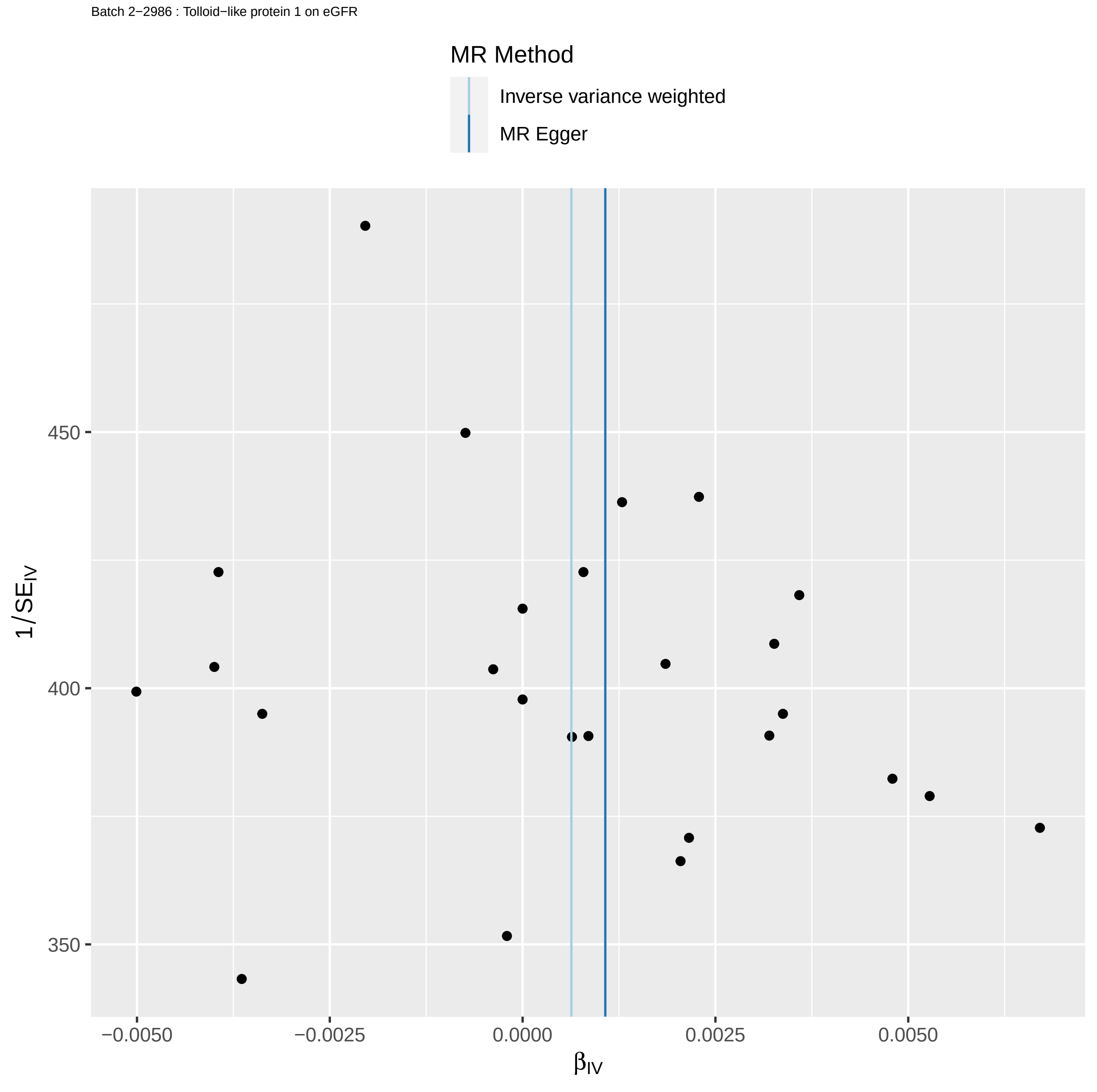
Batch 2−2986 : Tolloid−like protein 1 on eGFR
MR Method
Inverse variance weighted
MR Egger
1/SE_IV
450
400
350
−0.0050
−0.0025
0.0000
0.0025
0.0050
β_IV

Batch 2−2987 : Talin−2 on eGFR

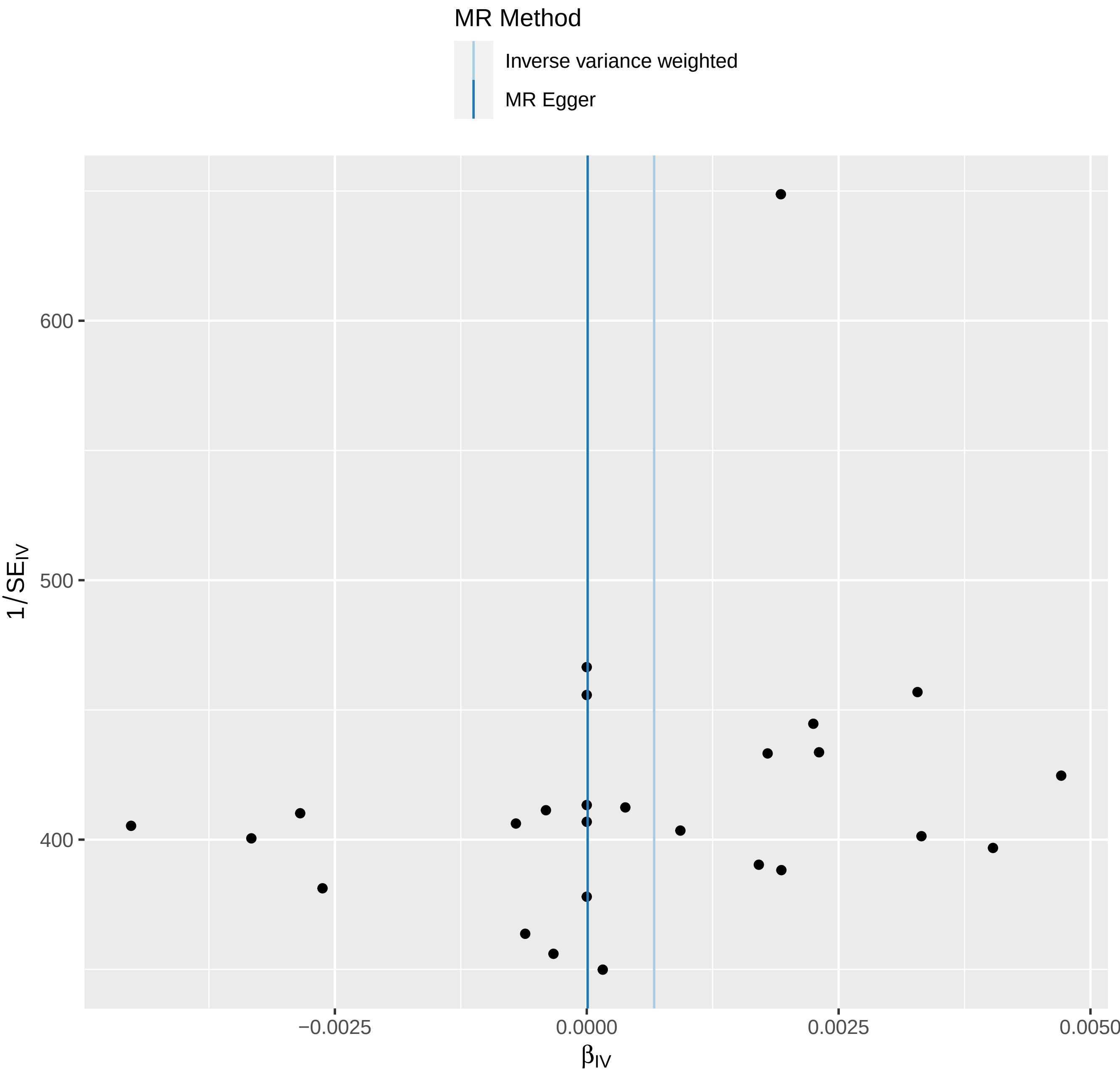

Batch 2−2988 : Toll−like receptor 1 on eGFR

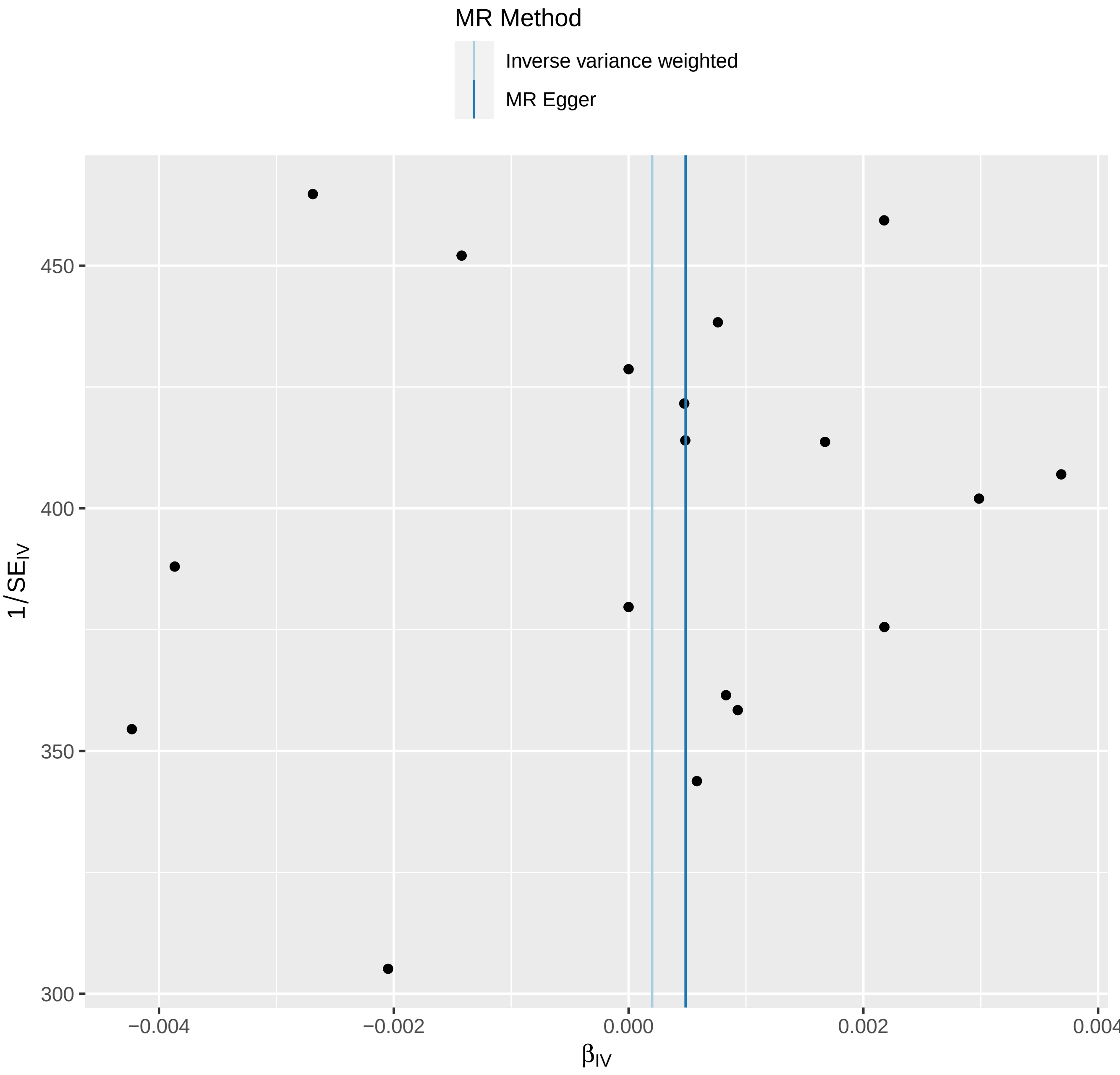

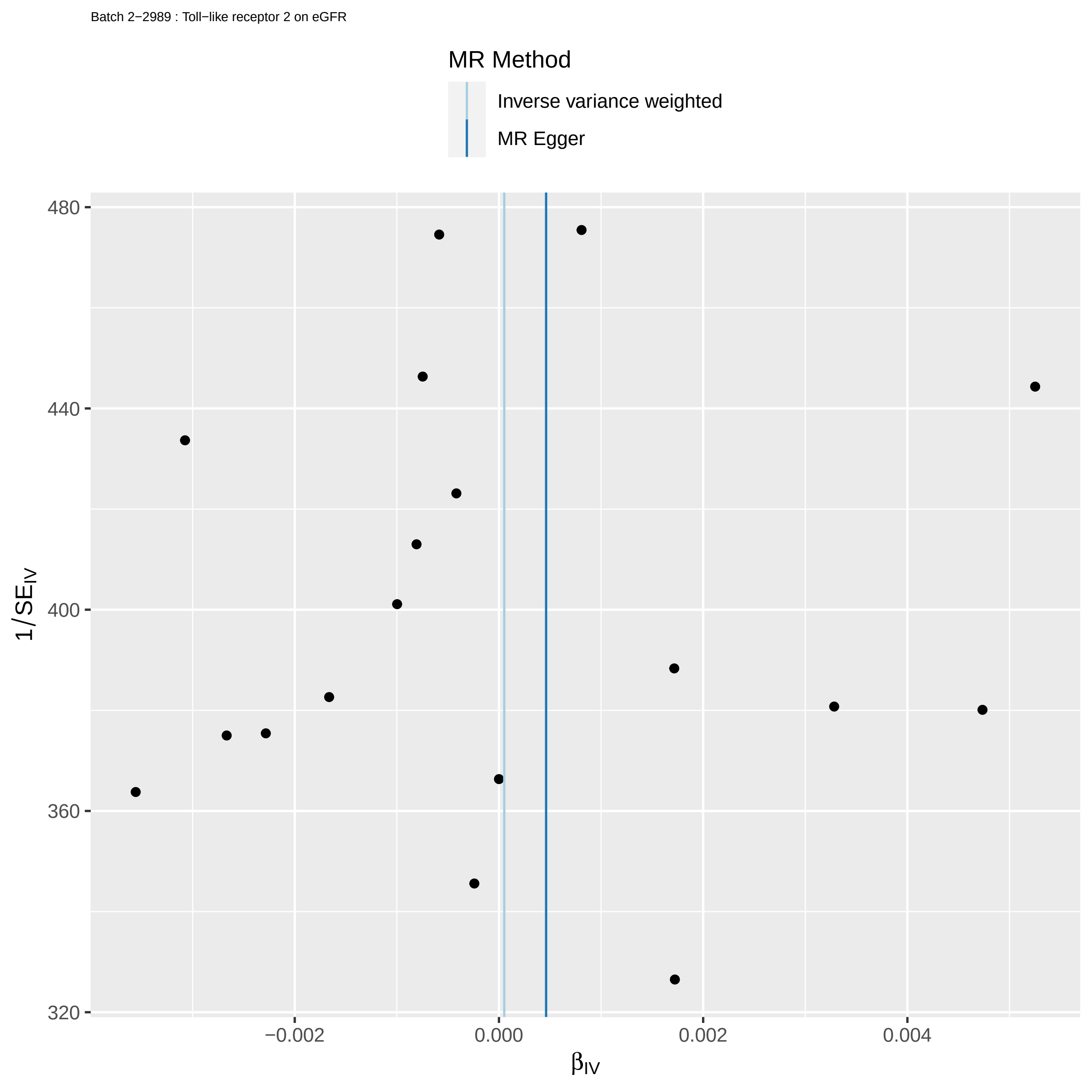
Batch 2−2989 : Toll−like receptor 2 on eGFR
MR Method
Inverse variance weighted
MR Egger
480
440
400
360
320
1/SE_IV
−0.002
0.000
0.002
0.004
β_IV

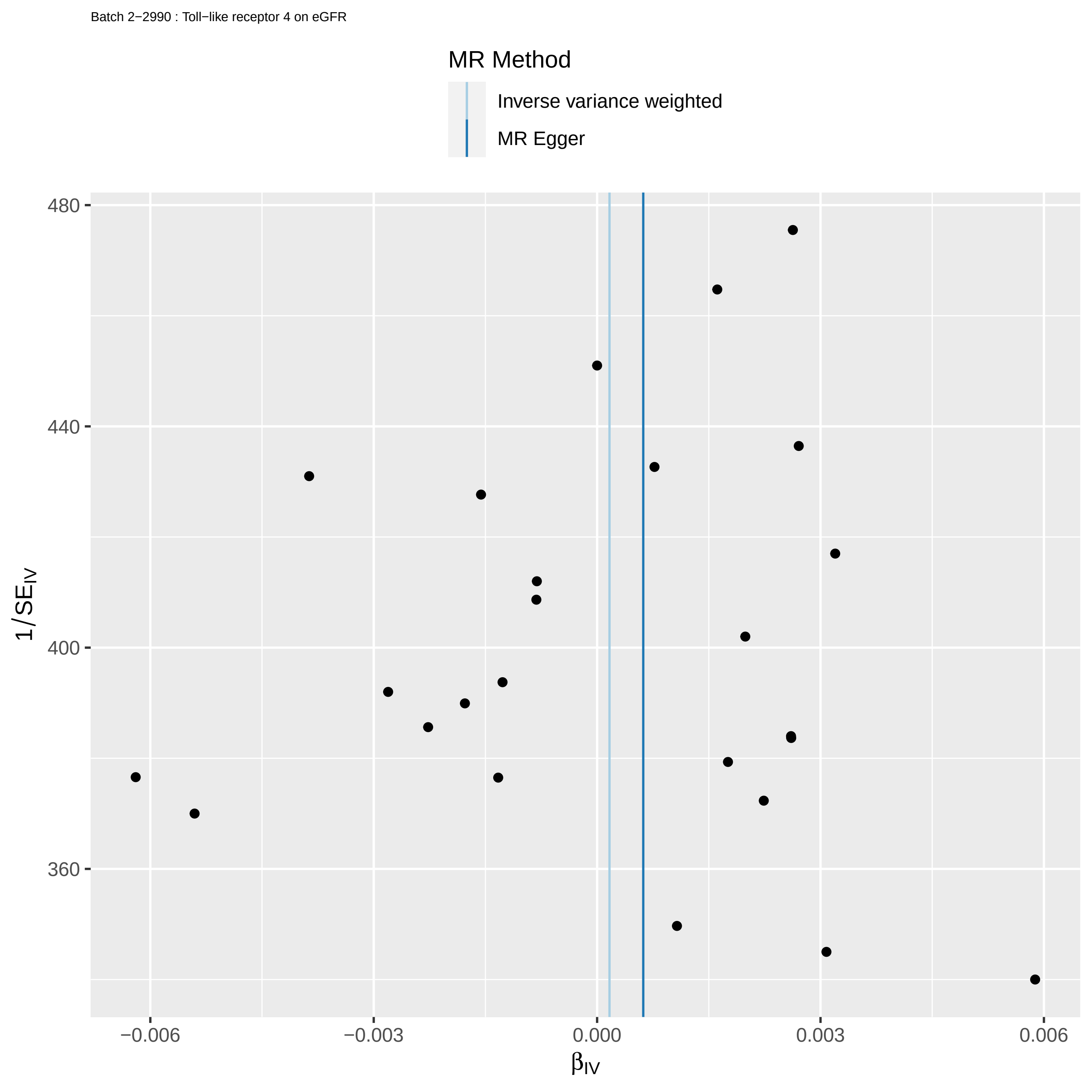

Batch 2−2990 : Toll−like receptor 4 on eGFR
MR Method
Inverse variance weighted
MR Egger
480
440
400
360
1/SE_IV
−0.006
−0.003
0.000
0.003
0.006
β_IV

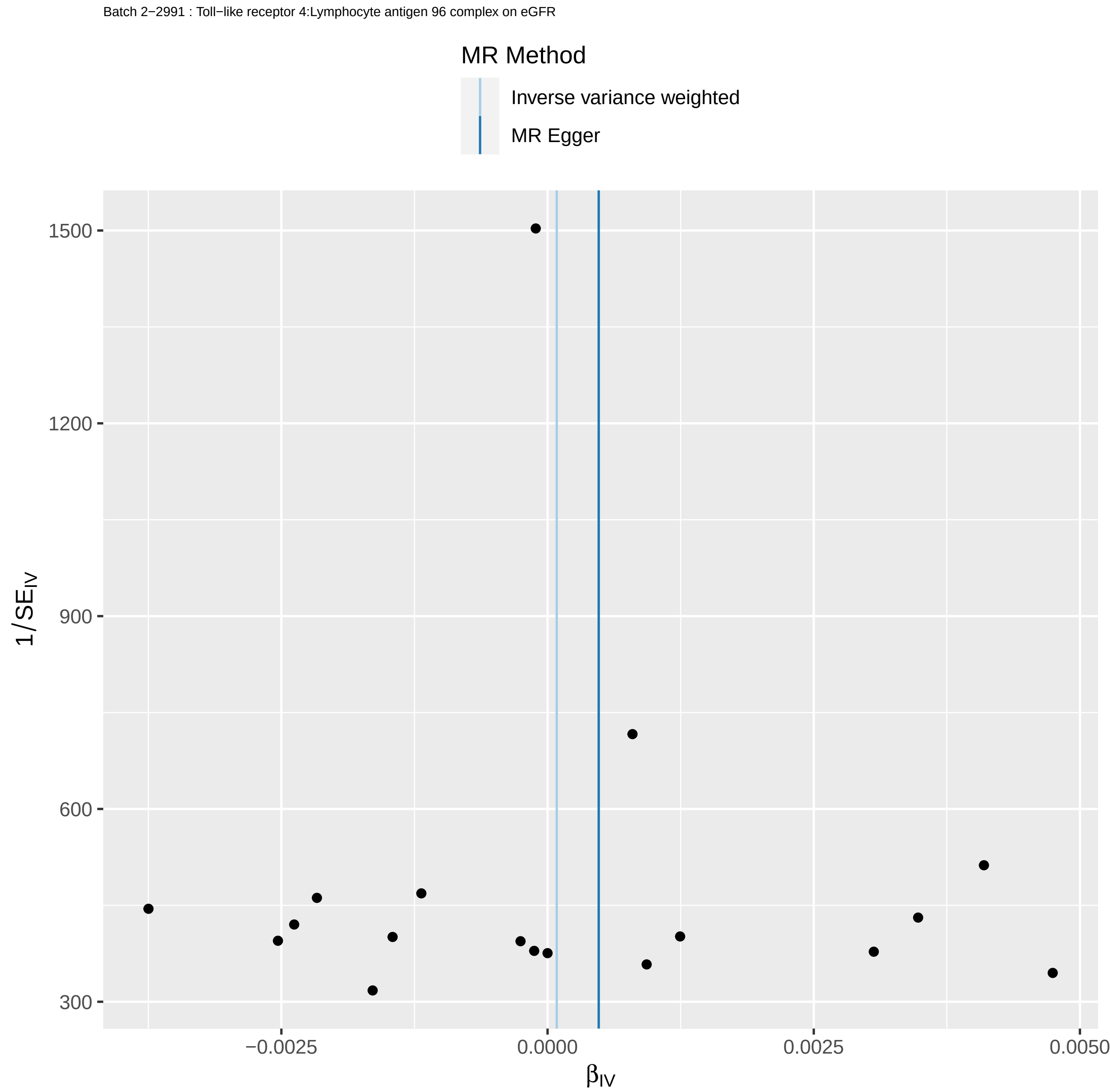
Batch 2−2991 : Toll−like receptor 4:Lymphocyte antigen 96 complex on eGFR
MR Method
Inverse variance weighted
MR Egger
1500
1200
900
600
300
1/SE_IV
−0.0025
0.0000
0.0025
0.0050
β_IV

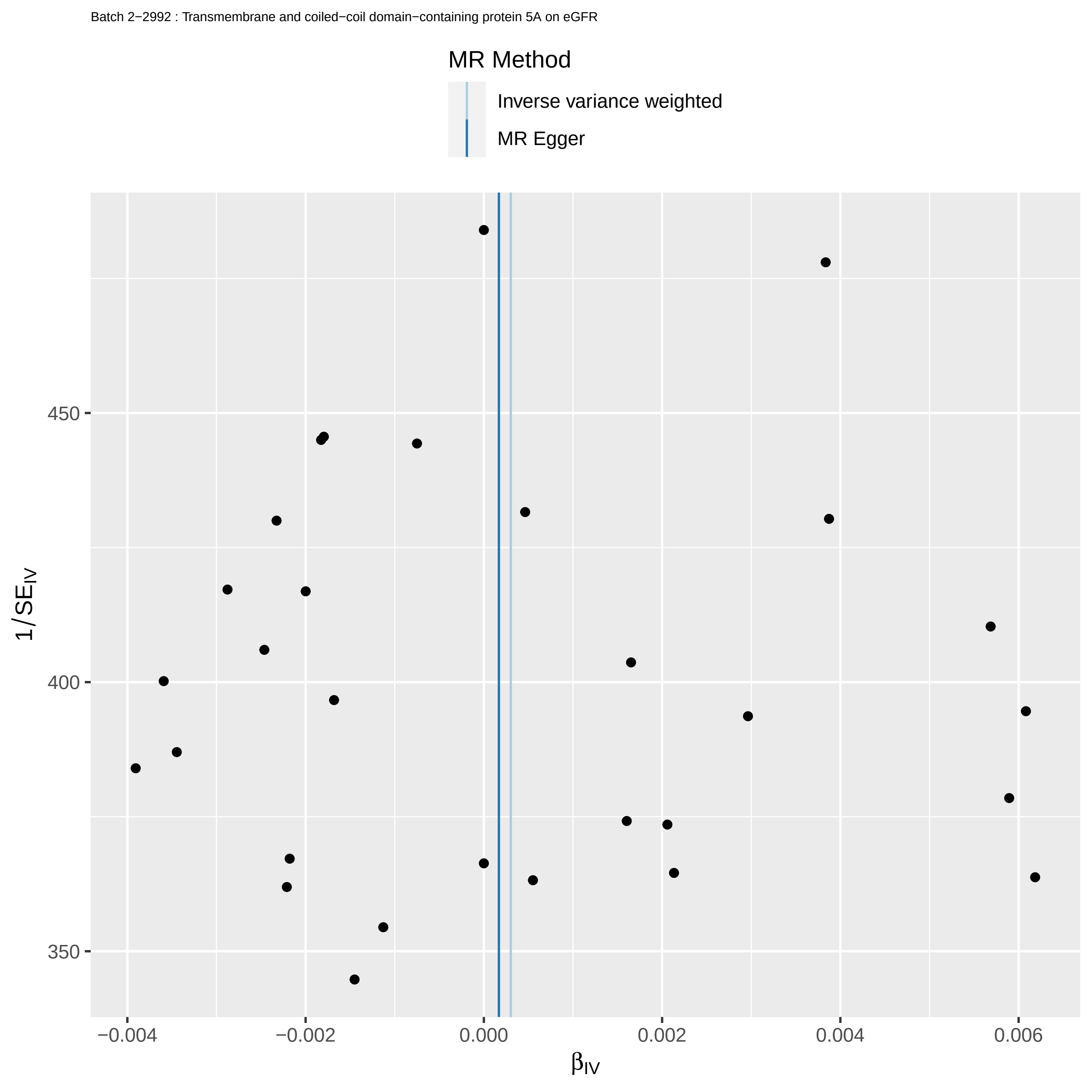

Batch 2−2992 : Transmembrane and coiled−coil domain−containing protein 5A on eGFR
MR Method
Inverse variance weighted
MR Egger
450
400
350
1/SE_IV
−0.004
−0.002
0.000
0.002
0.004
0.006
β_IV

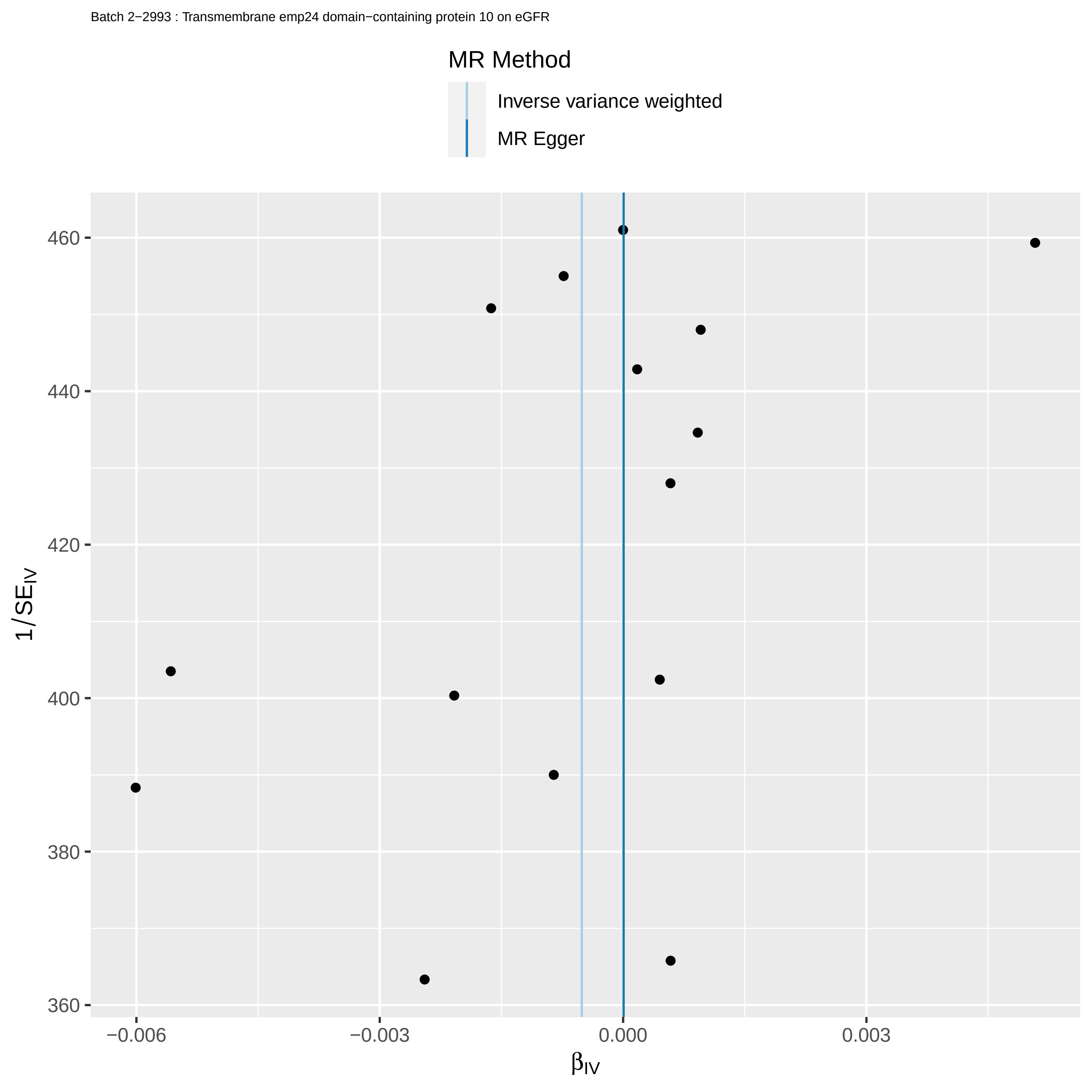

Batch 2−2993 : Transmembrane emp24 domain−containing protein 10 on eGFR
MR Method
Inverse variance weighted
MR Egger
460
440
420
400
380
360
1/SE$_{IV}$
−0.006
−0.003
0.000
0.003
β$_{IV}$

Batch 2−2994 : Transmembrane emp24 domain−containing protein 2 on eGFR

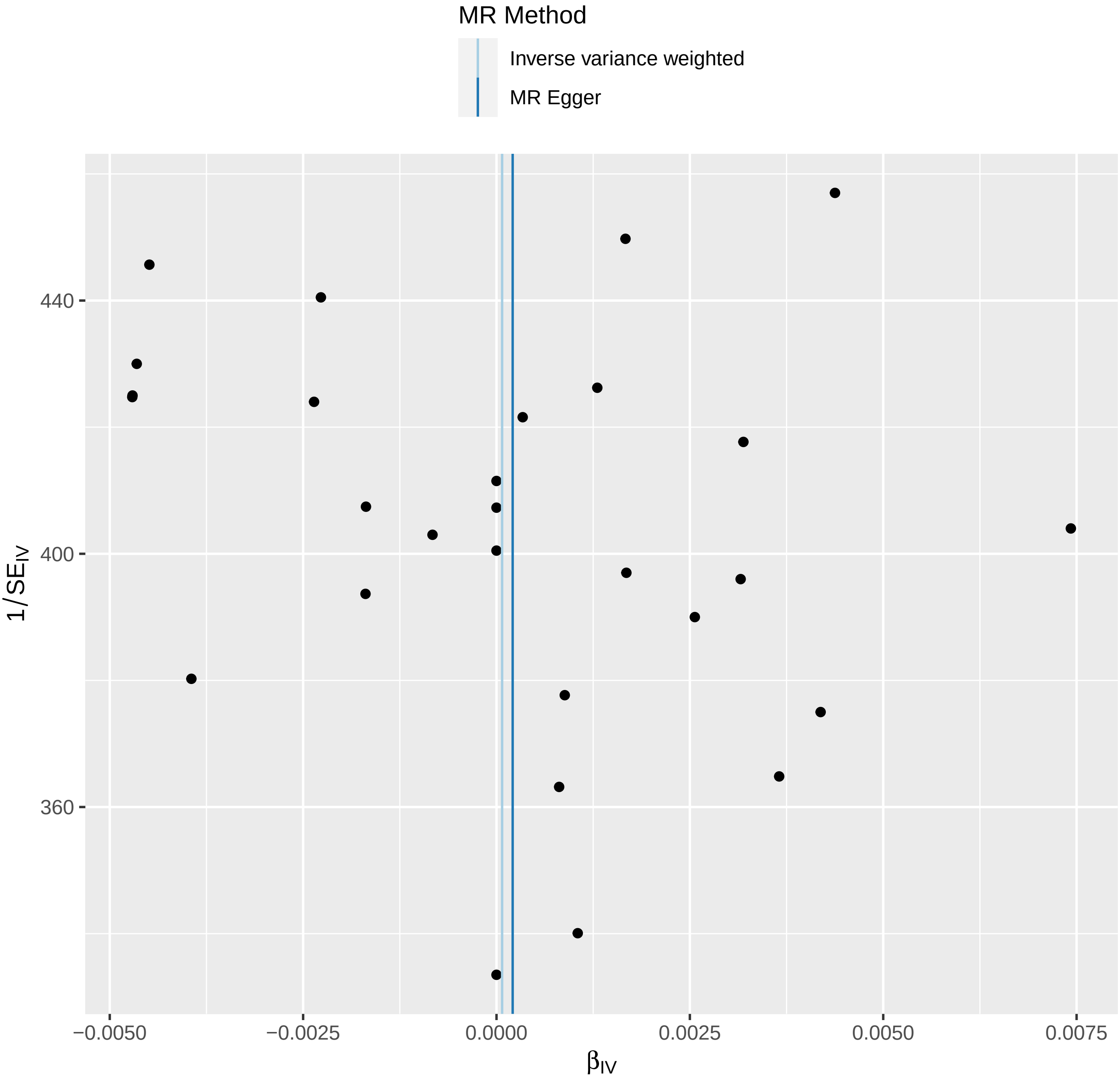

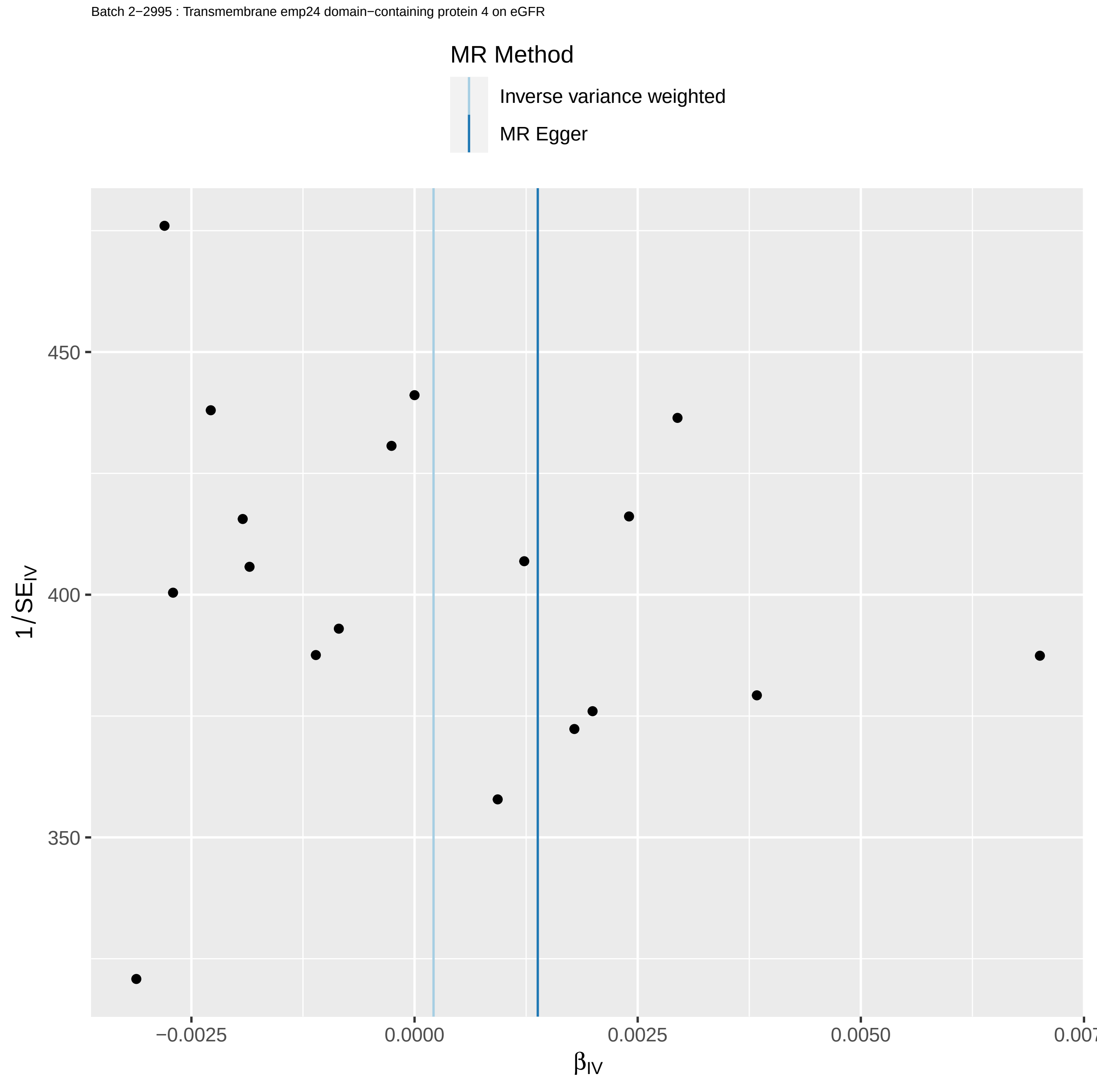

Batch 2−2995 : Transmembrane emp24 domain−containing protein 4 on eGFR
MR Method
Inverse variance weighted
MR Egger
450
400
350
−0.0025
0.0000
0.0025
0.0050
0.007
1/SE_IV
β_IV

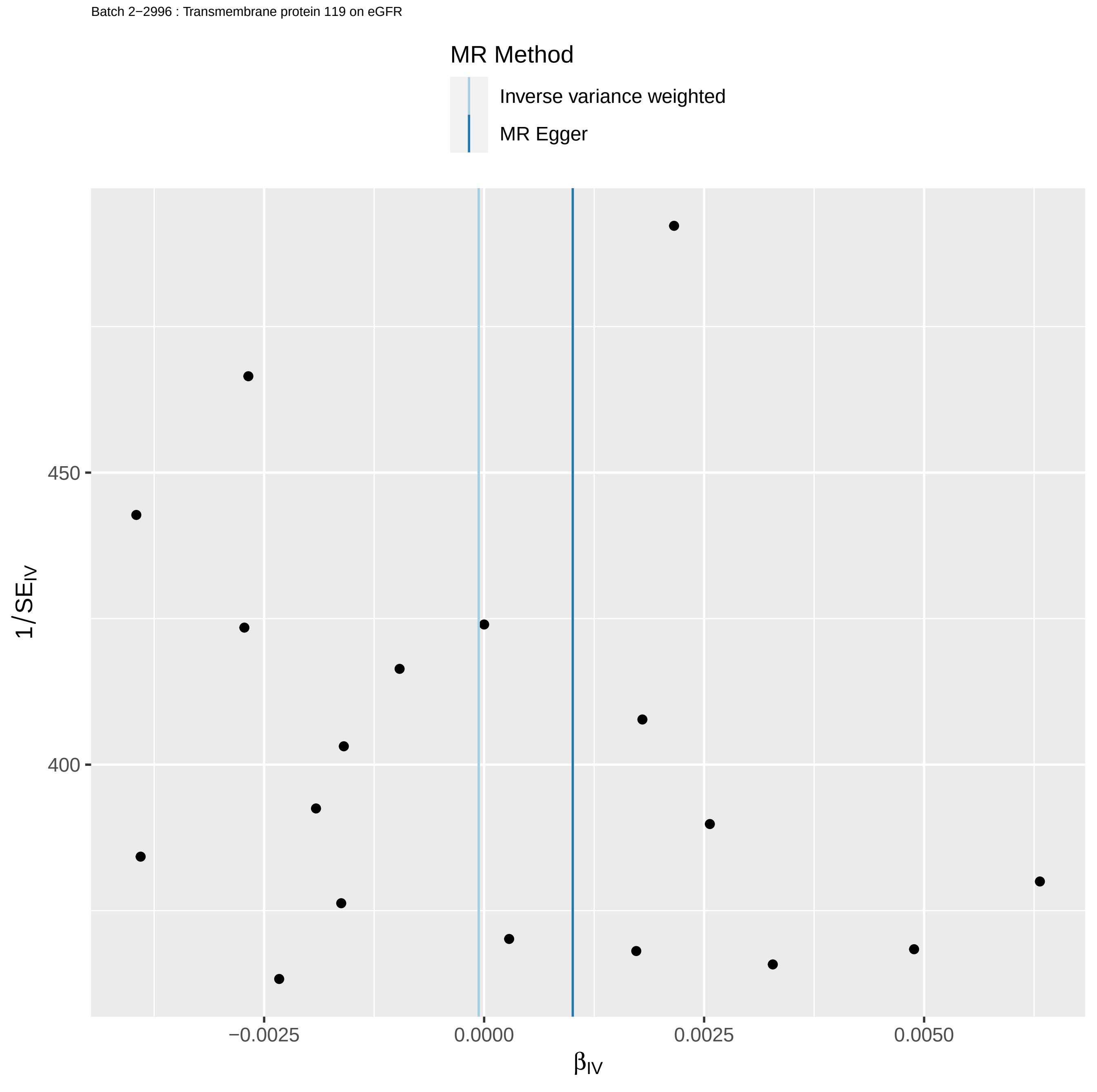

Batch 2−2996 : Transmembrane protein 119 on eGFR
MR Method
Inverse variance weighted
MR Egger
450
400
1/SE_IV
−0.0025
0.0000
0.0025
0.0050
β_IV

Batch 2−2997 : Transmembrane protein 132A on eGFR

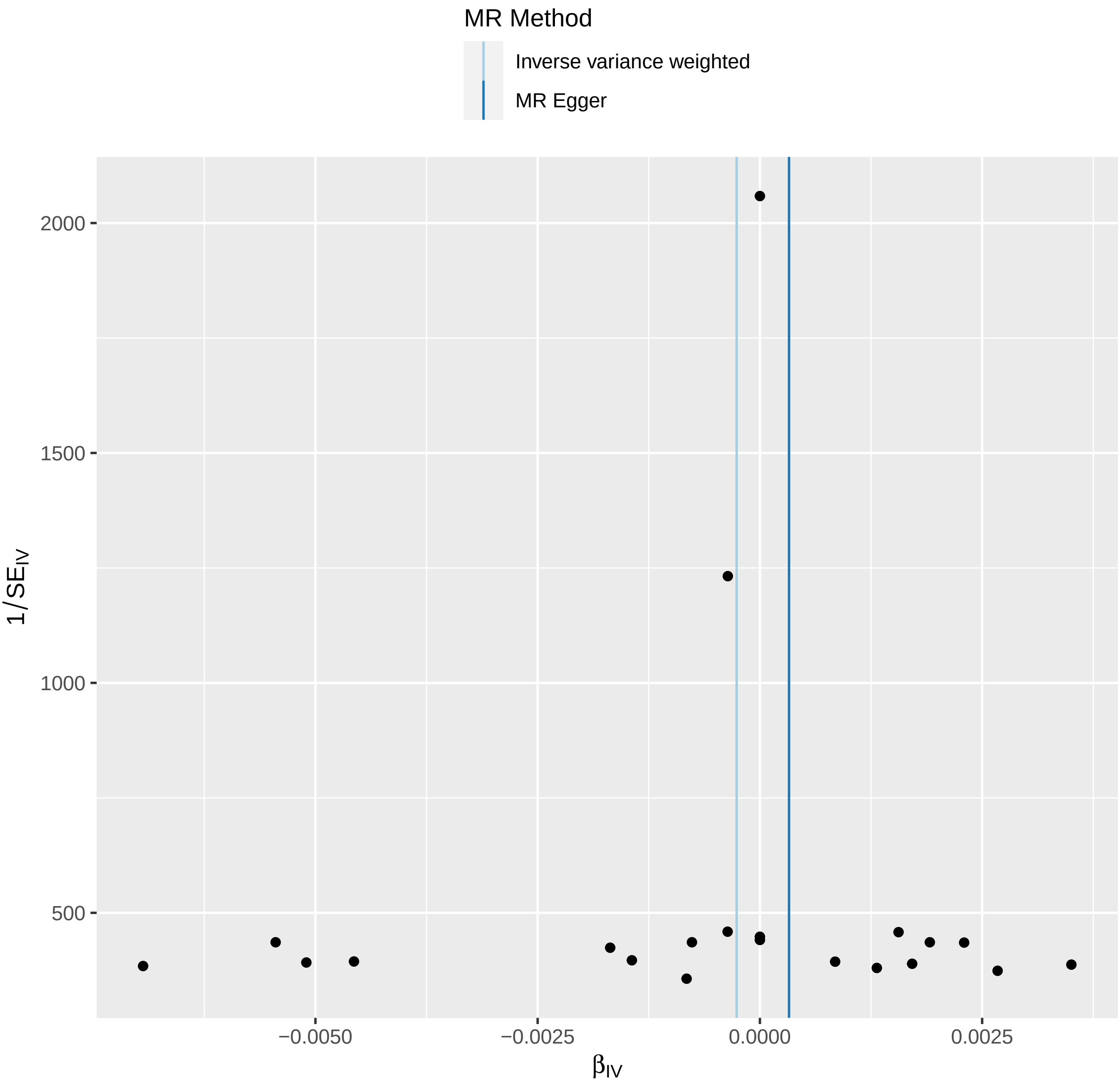

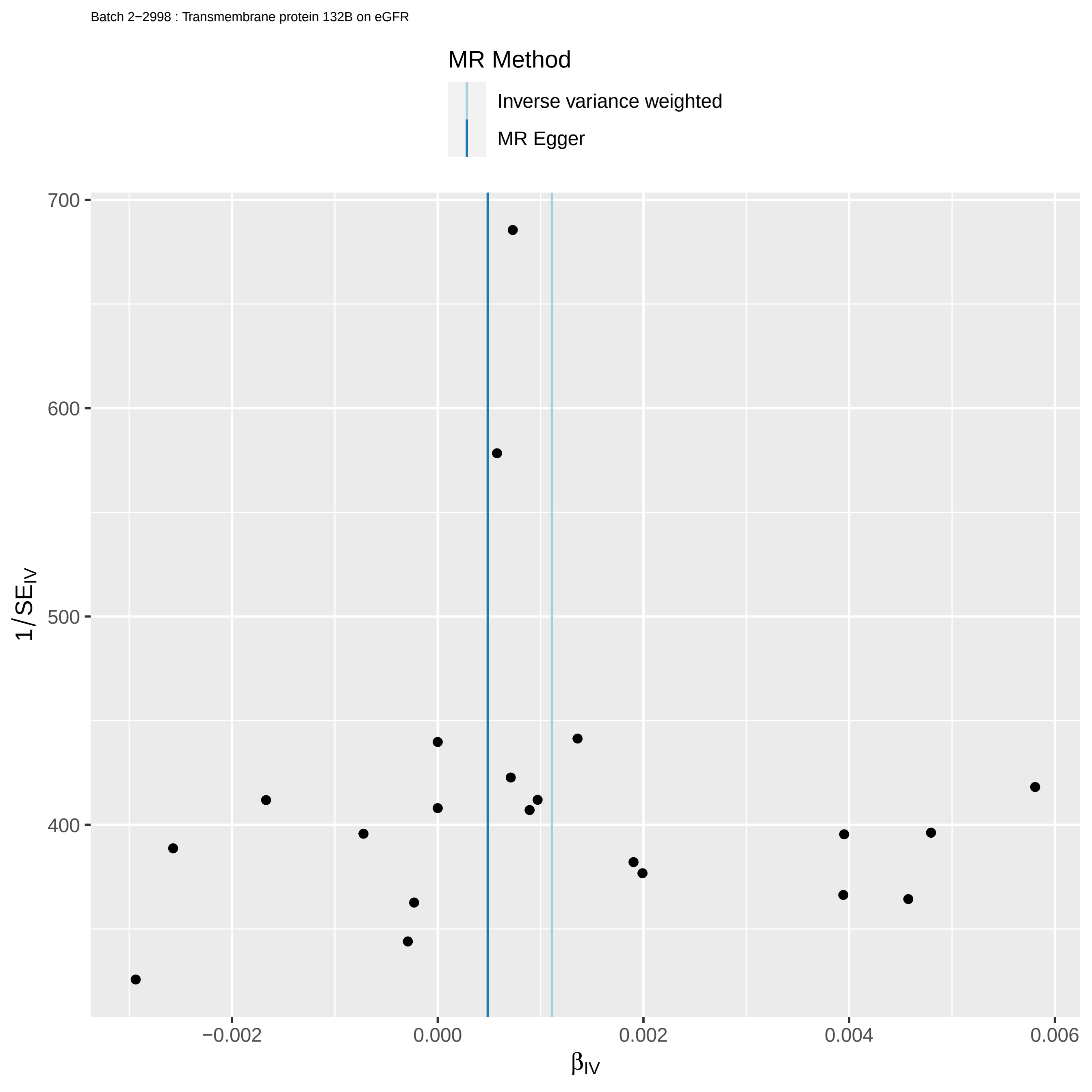
Batch 2−2998 : Transmembrane protein 132B on eGFR
MR Method
Inverse variance weighted
MR Egger
700
600
500
400
1/SE_IV
−0.002
0.000
0.002
0.004
0.006
β_IV

Batch 2−2999 : Transmembrane protein 132C on eGFR

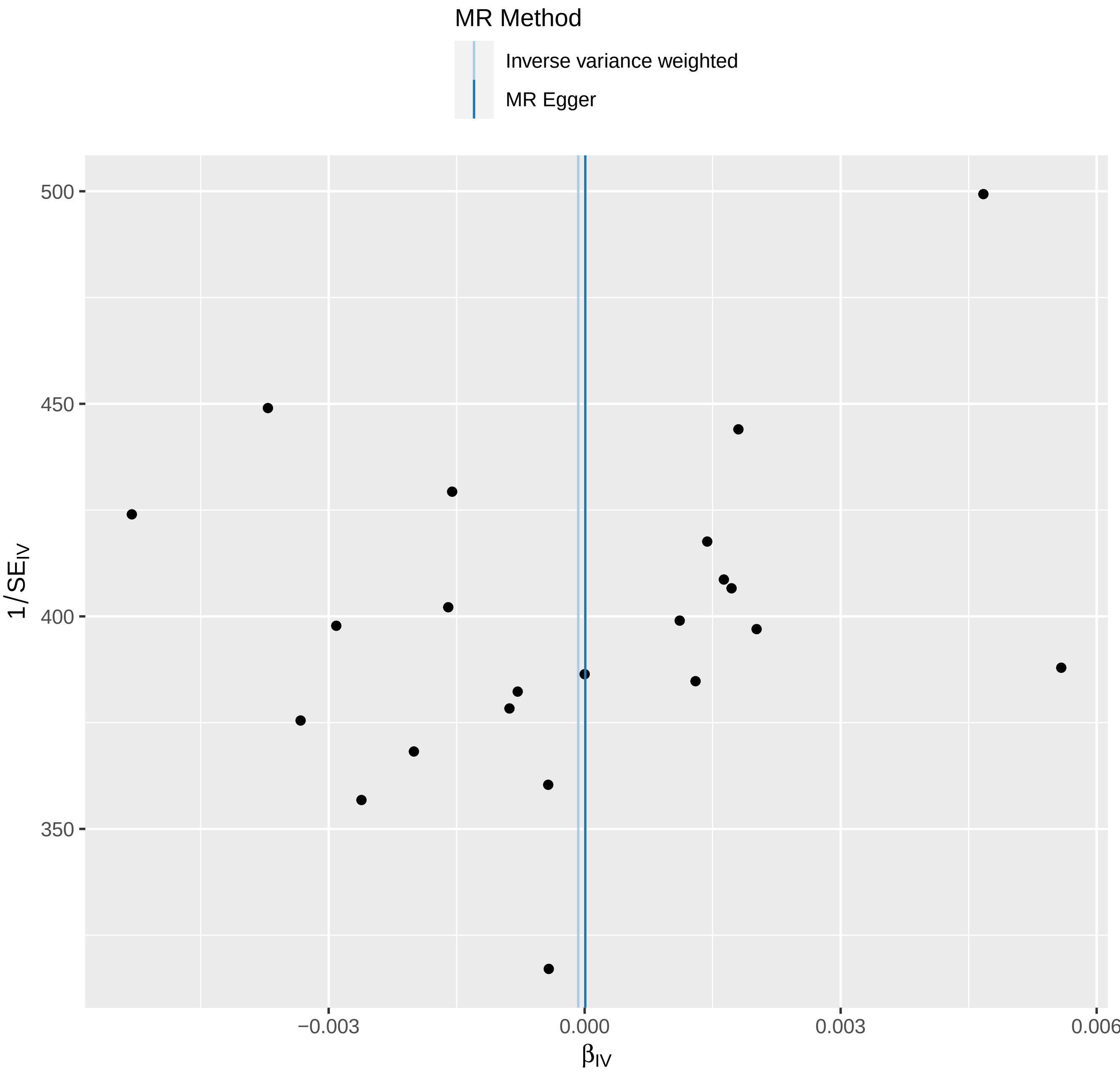

Batch 2−3000 : Transmembrane protein 132C on eGFR

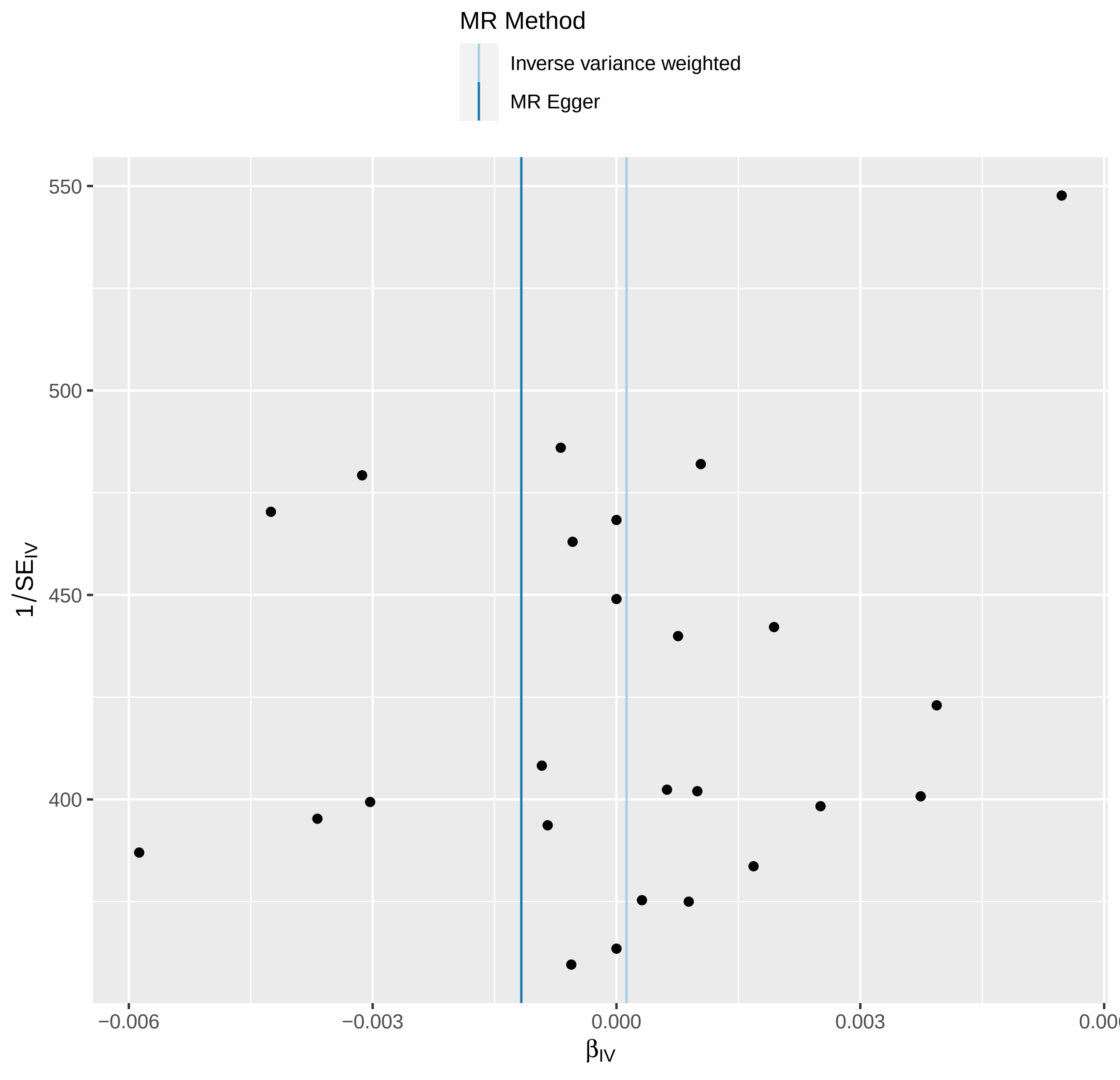

Batch 2−3001 : Transmembrane protein 132D on eGFR

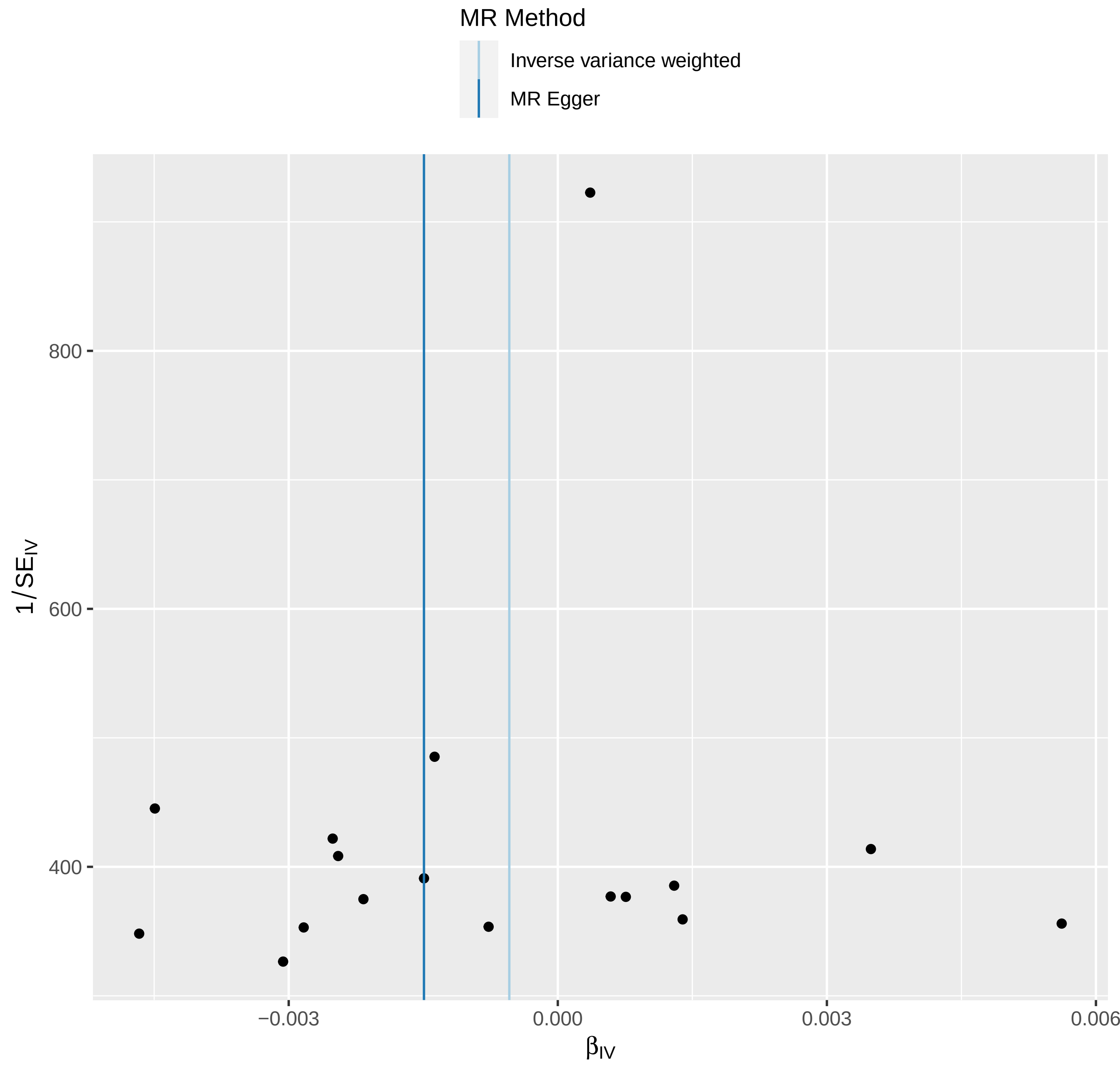

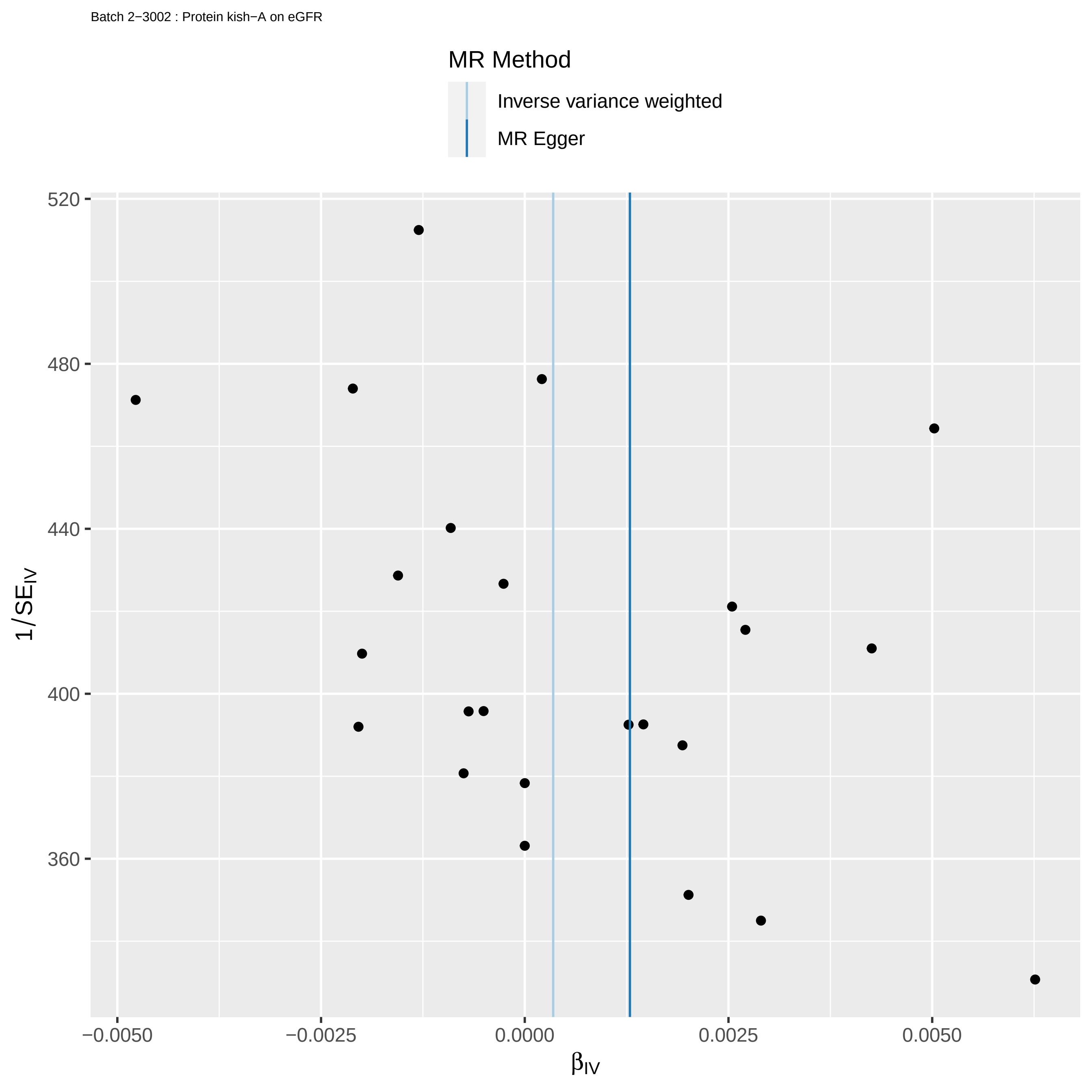
Batch 2−3002 : Protein kish−A on eGFR
MR Method
Inverse variance weighted
MR Egger
520
480
440
400
360
1/SE_IV
−0.0050
−0.0025
0.0000
0.0025
0.0050
β_IV

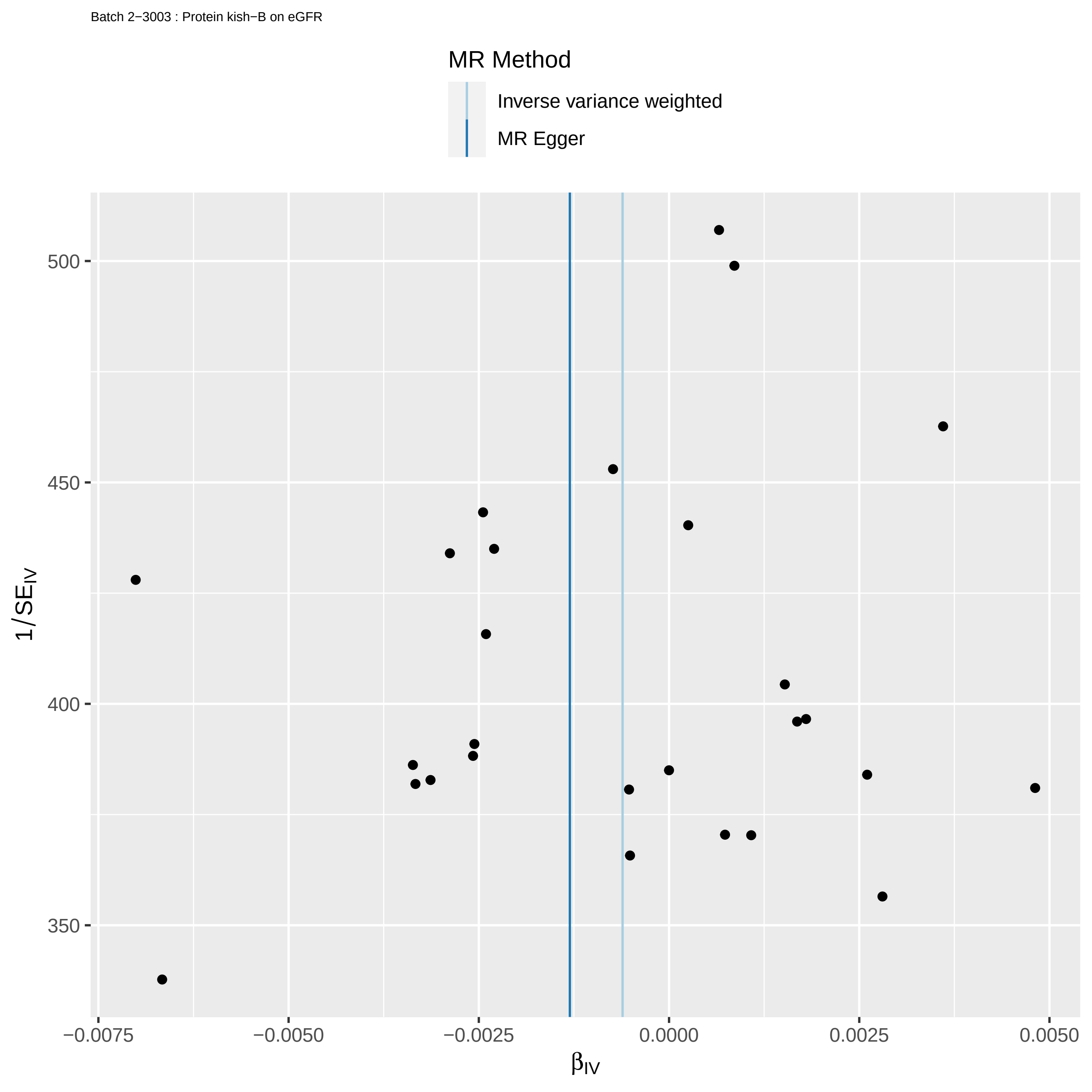

Batch 2−3003 : Protein kish−B on eGFR
MR Method
Inverse variance weighted
MR Egger
500
450
400
350
1/SE_IV
−0.0075
−0.0050
−0.0025
0.0000
0.0025
0.0050
β_IV

Batch 2−3004 : Transmembrane protein 190 on eGFR

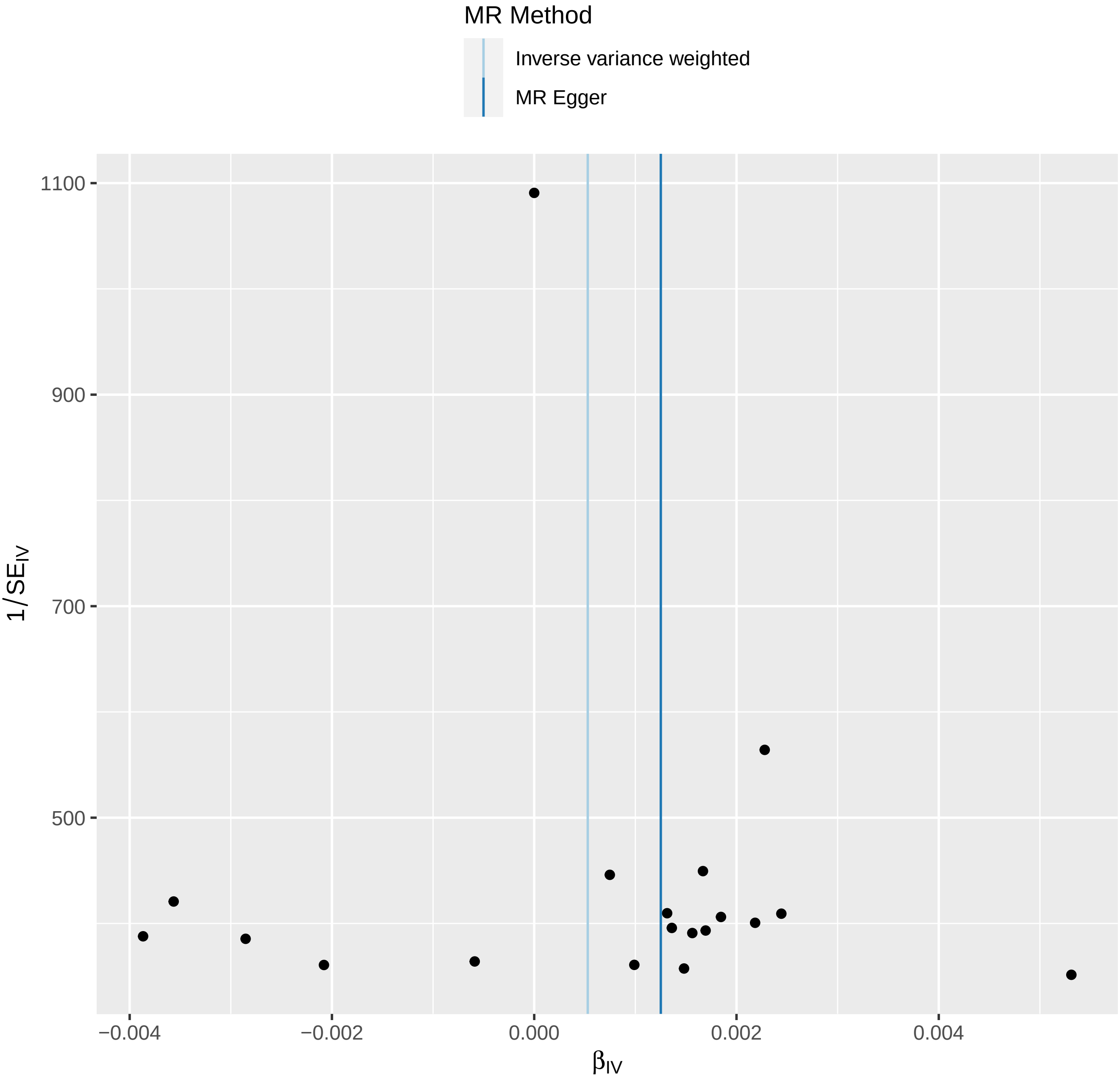

Batch 2−3005 : Transmembrane protein 230 on eGFR

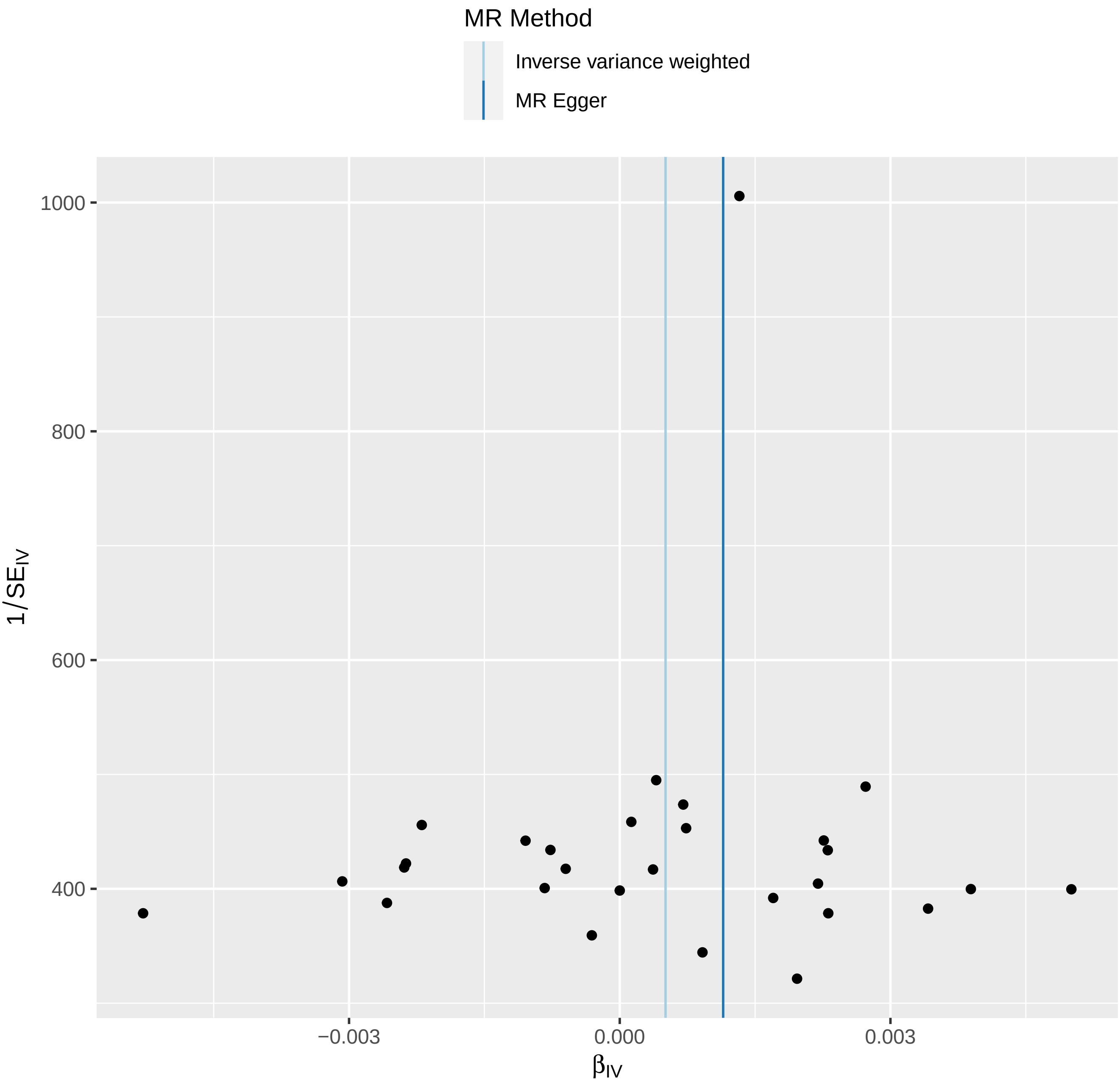

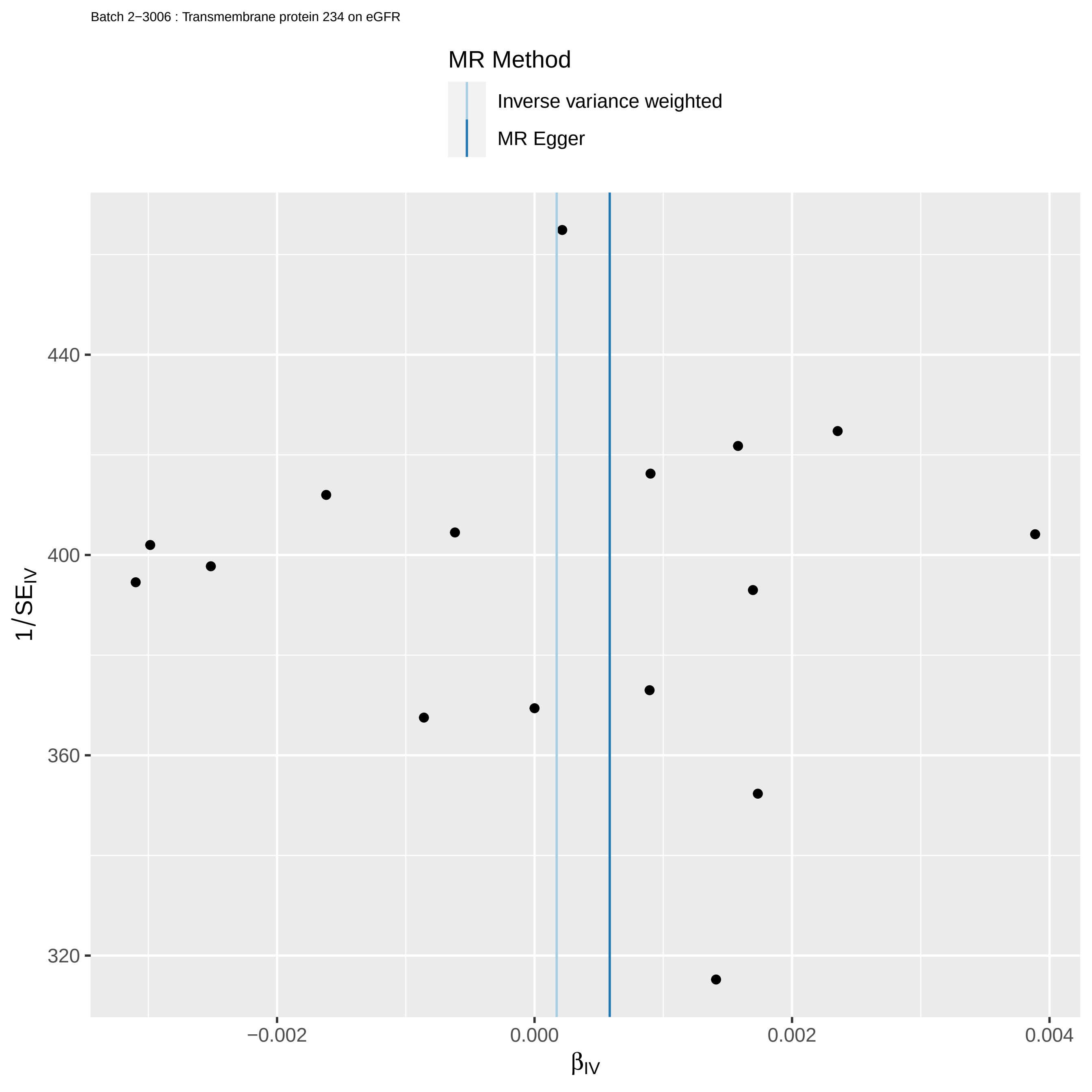

Batch 2−3006 : Transmembrane protein 234 on eGFR
MR Method
Inverse variance weighted
MR Egger
440
400
360
320
1/SE$_{IV}$
−0.002
0.000
0.002
0.004
β_{IV}

Batch 2−3007 : Transmembrane protein 237 on eGFR

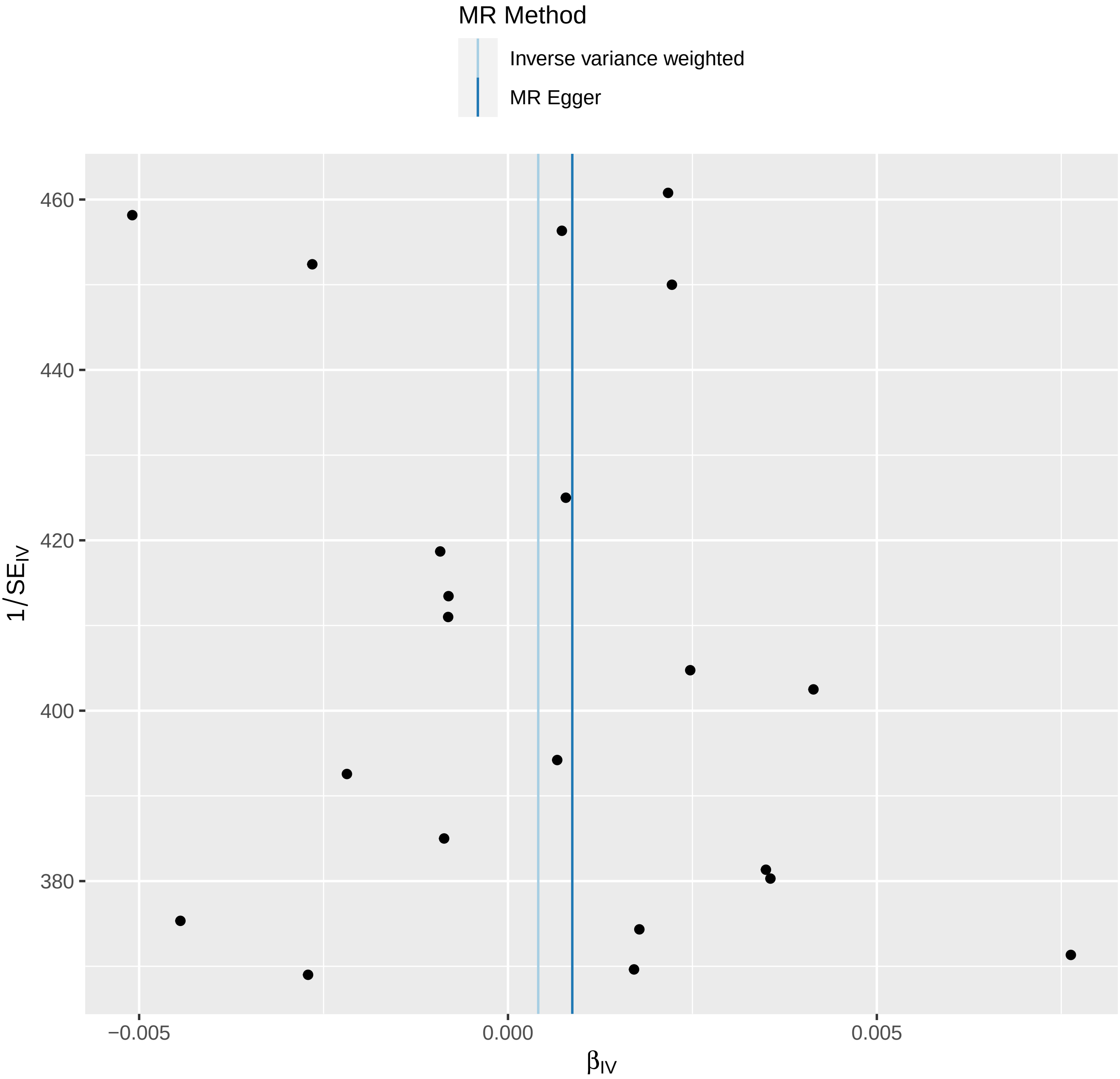

Batch 2−3008 : Collectrin on eGFR

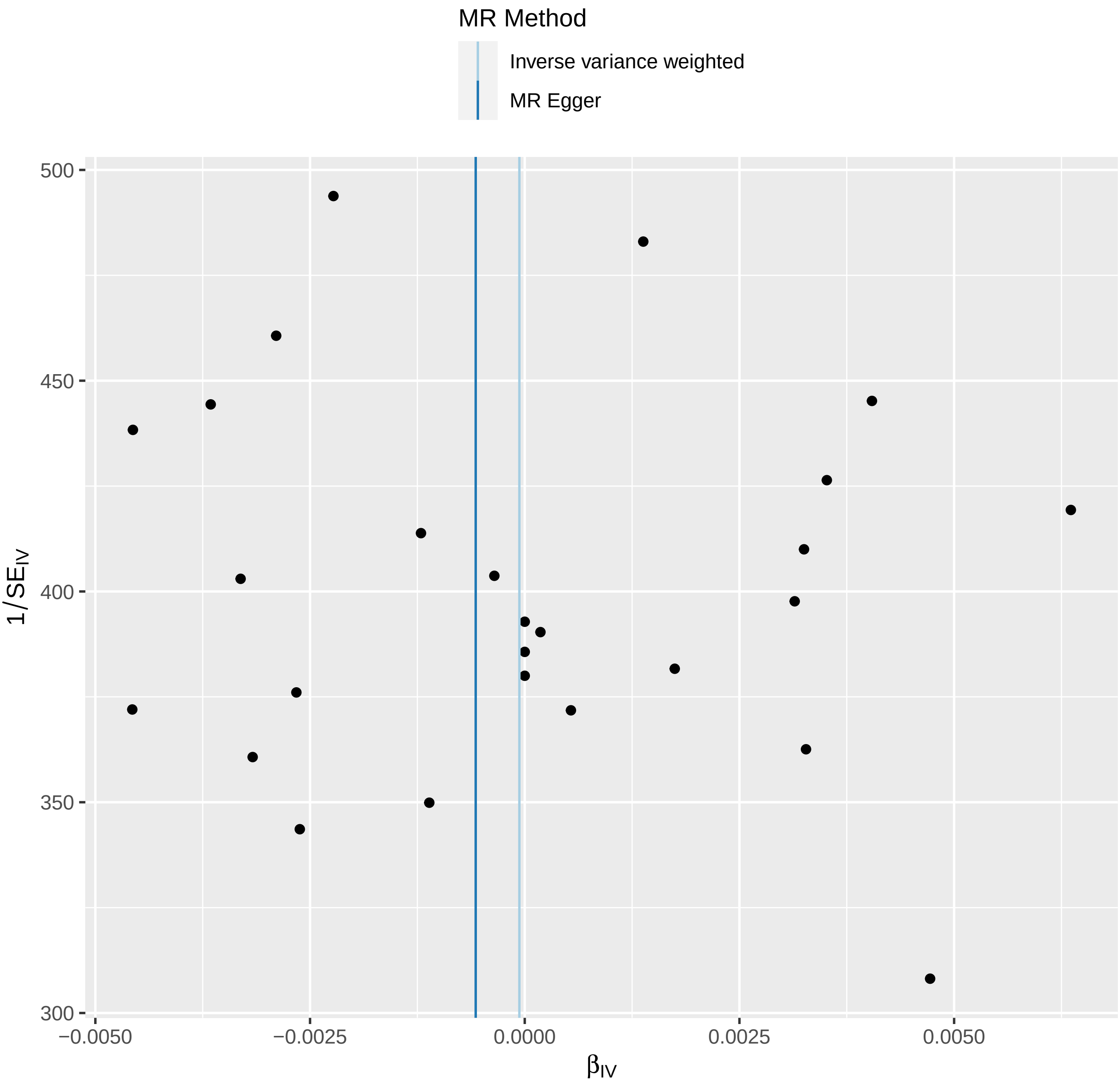

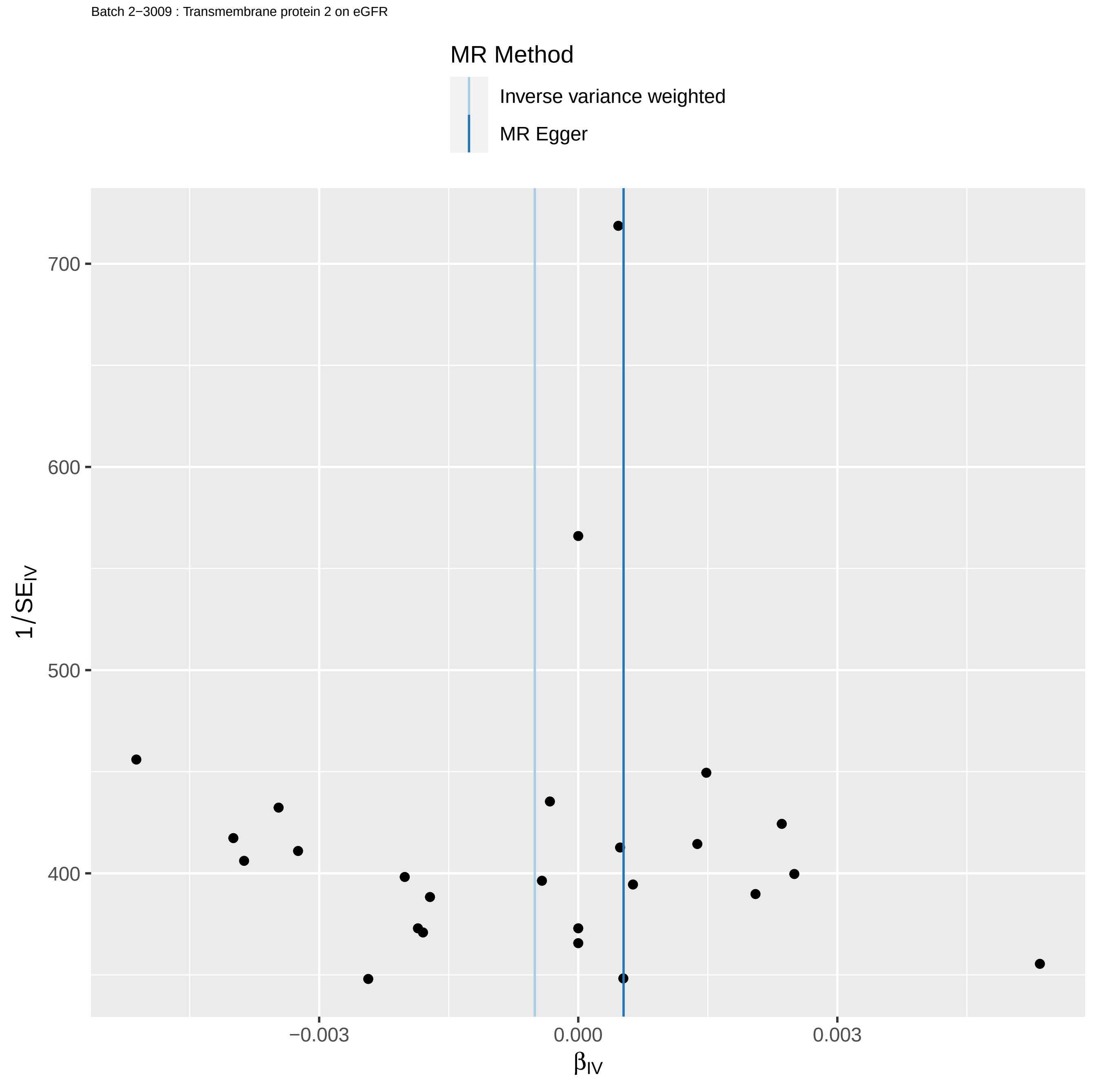

Batch 2−3009 : Transmembrane protein 2 on eGFR
MR Method
Inverse variance weighted
MR Egger
700
600
500
400
1/SE_IV
−0.003
0.000
0.003
β_IV

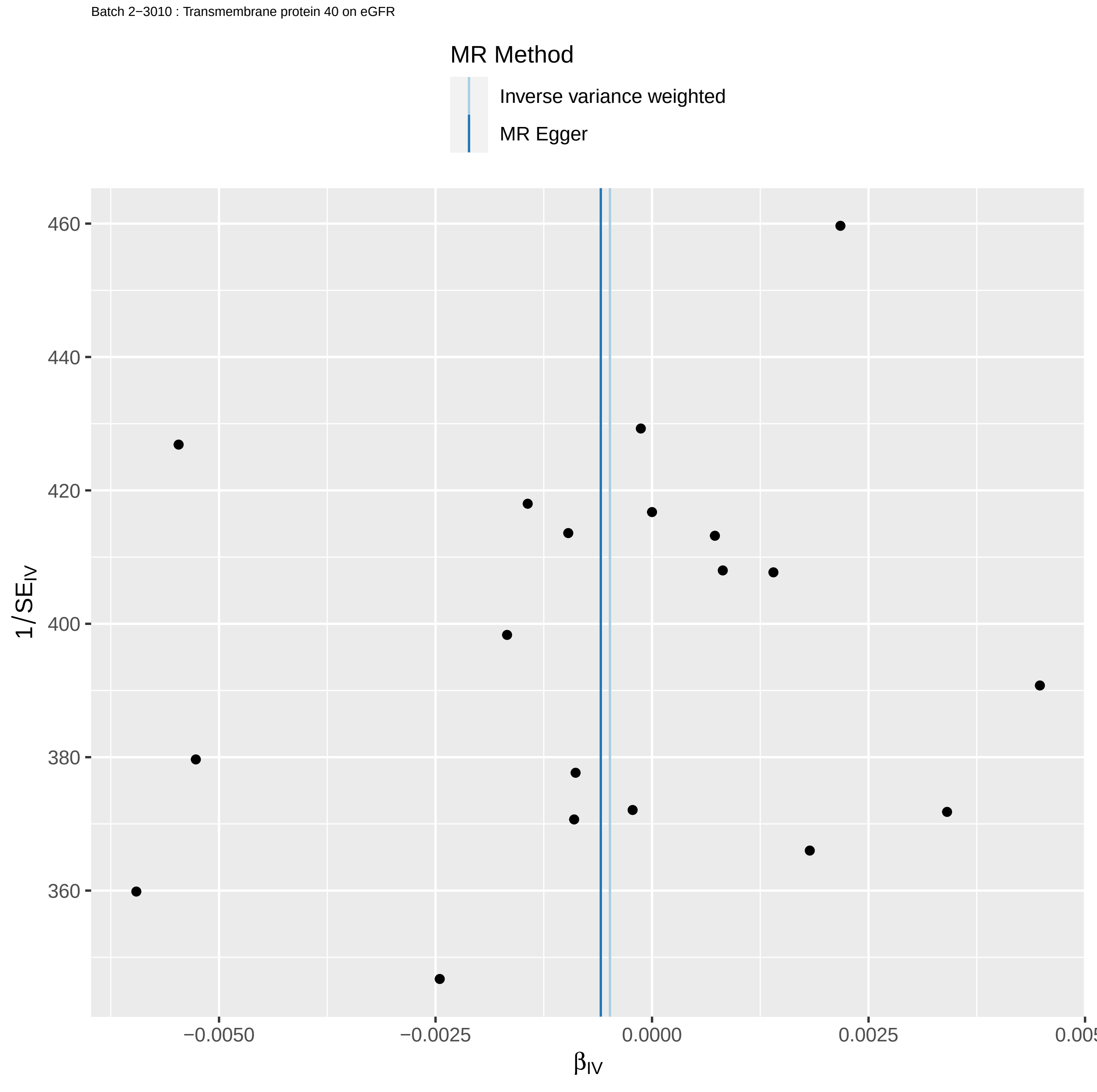
Batch 2−3010 : Transmembrane protein 40 on eGFR
MR Method
Inverse variance weighted
MR Egger
460
440
420
400
380
360
1/SE_IV
−0.0050
−0.0025
0.0000
0.0025
0.005
β_IV

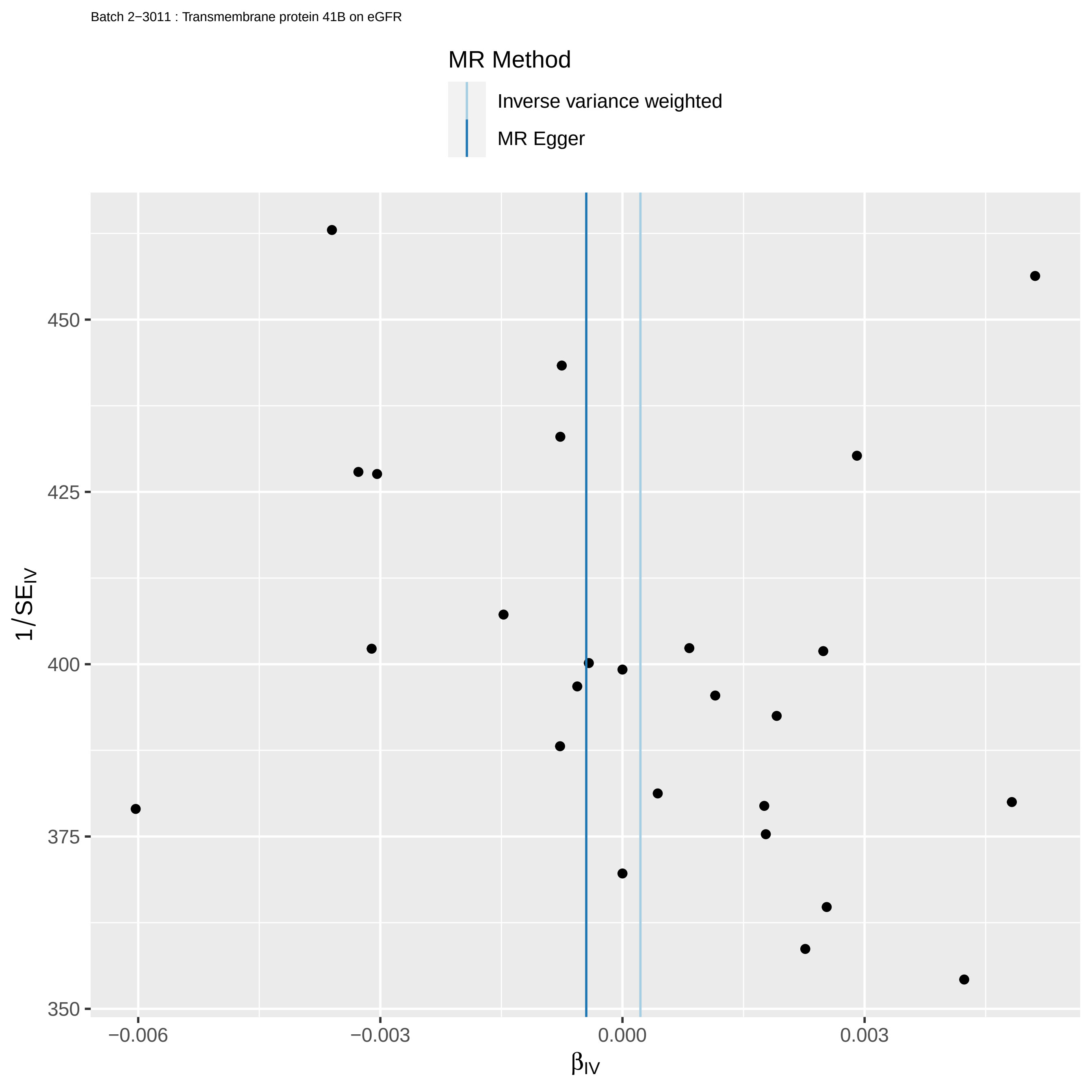

Batch 2−3011 : Transmembrane protein 41B on eGFR
MR Method
Inverse variance weighted
MR Egger
450
425
400
375
350
1/SE_IV
−0.006
−0.003
0.000
0.003
β_IV

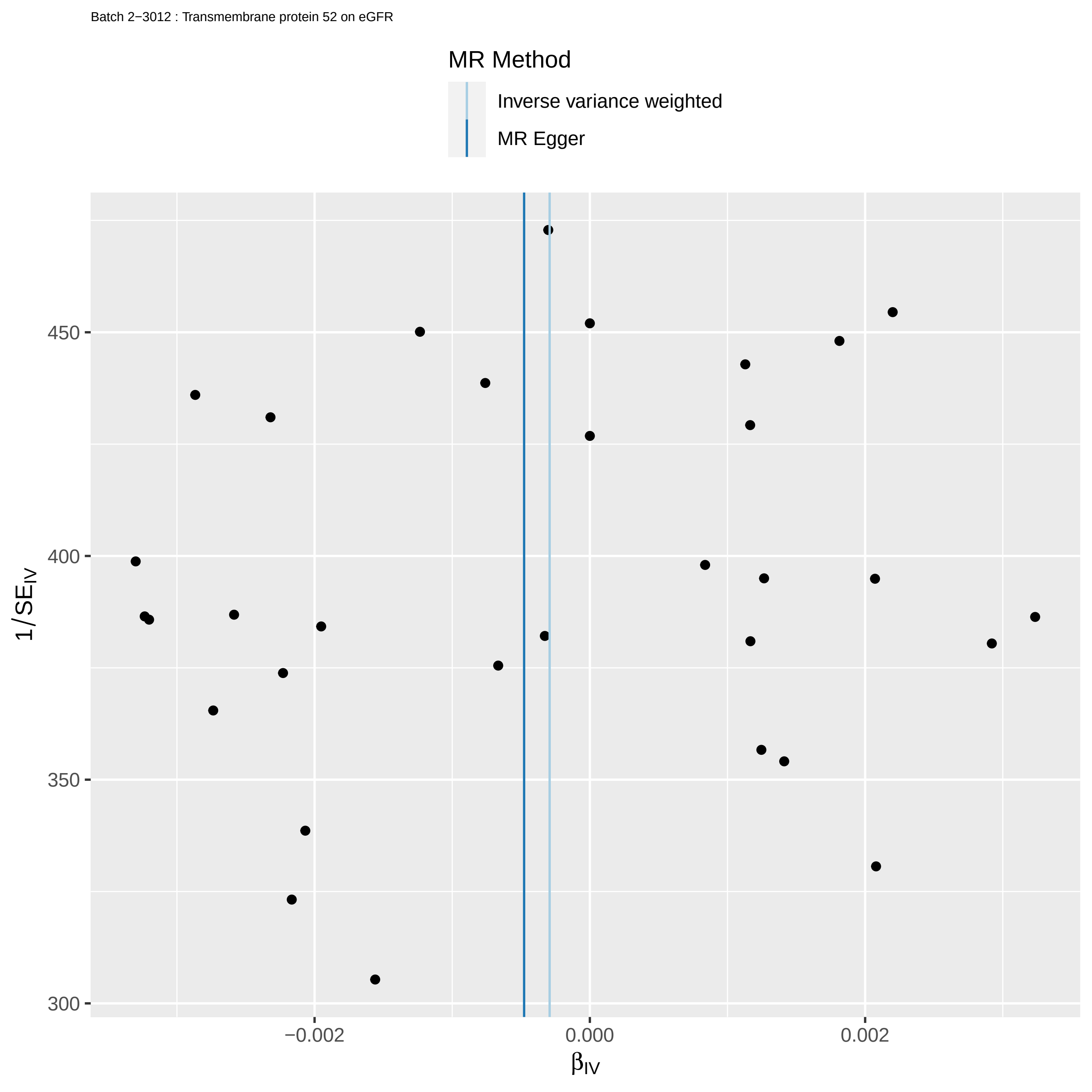

Batch 2−3012 : Transmembrane protein 52 on eGFR
MR Method
Inverse variance weighted
MR Egger
450
400
350
300
1/SE$_{IV}$
−0.002
0.000
0.002
β$_{IV}$

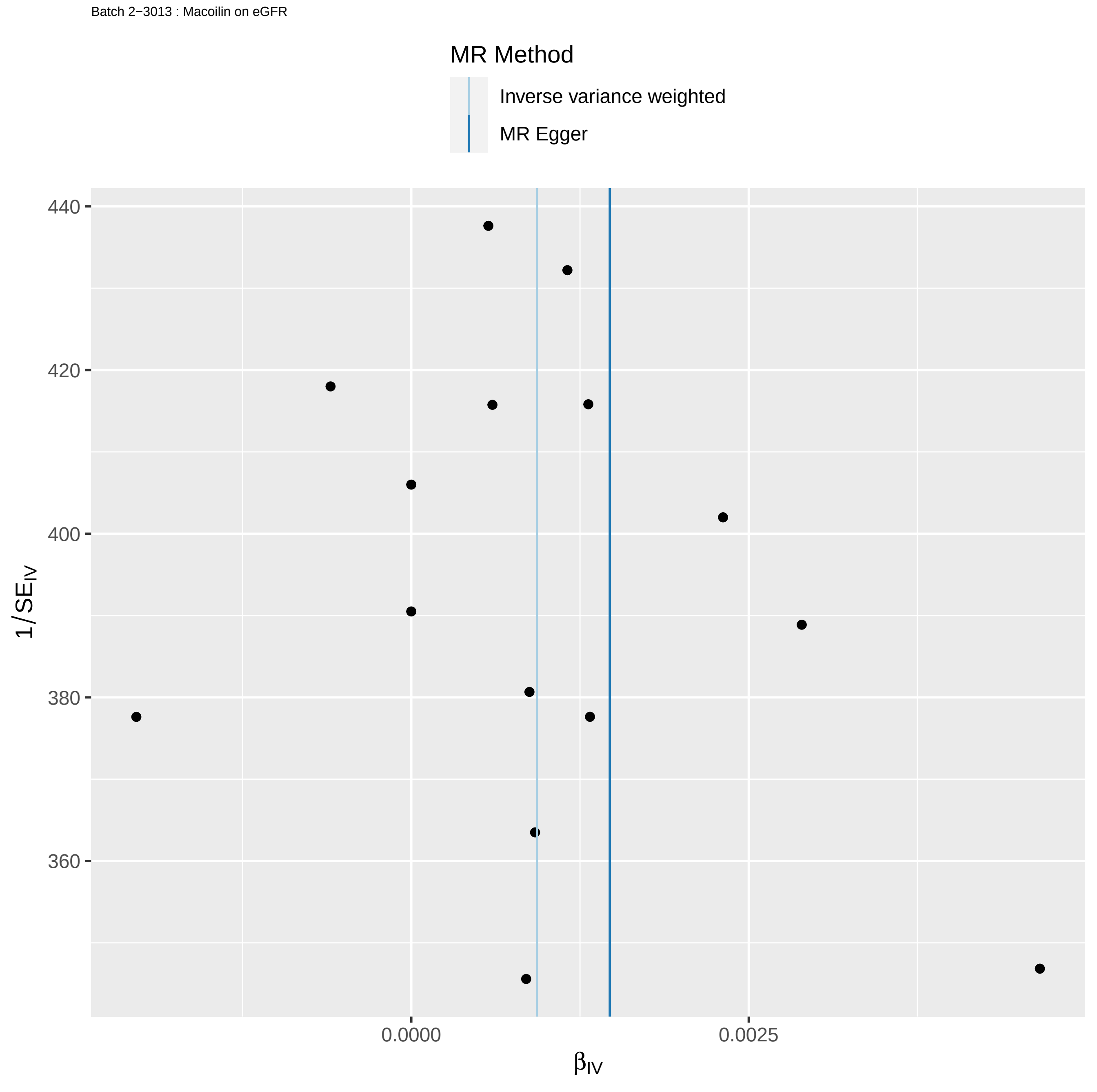

Batch 2−3013 : Macoilin on eGFR
MR Method
Inverse variance weighted
MR Egger
440
420
400
380
360
1/SE_IV
0.0000
0.0025
β_IV

Batch 2−3014 : Transmembrane protein 59−like on eGFR

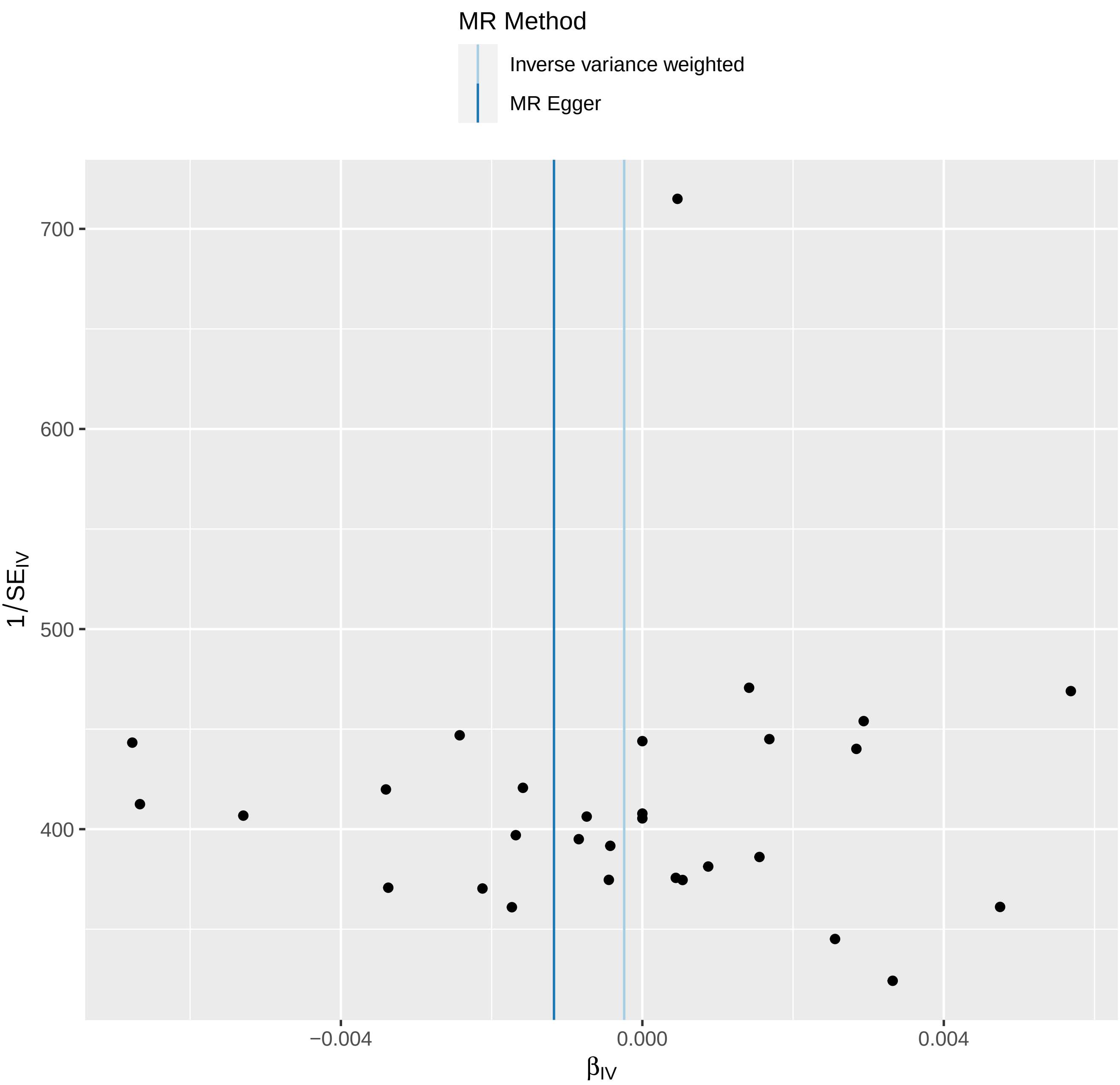

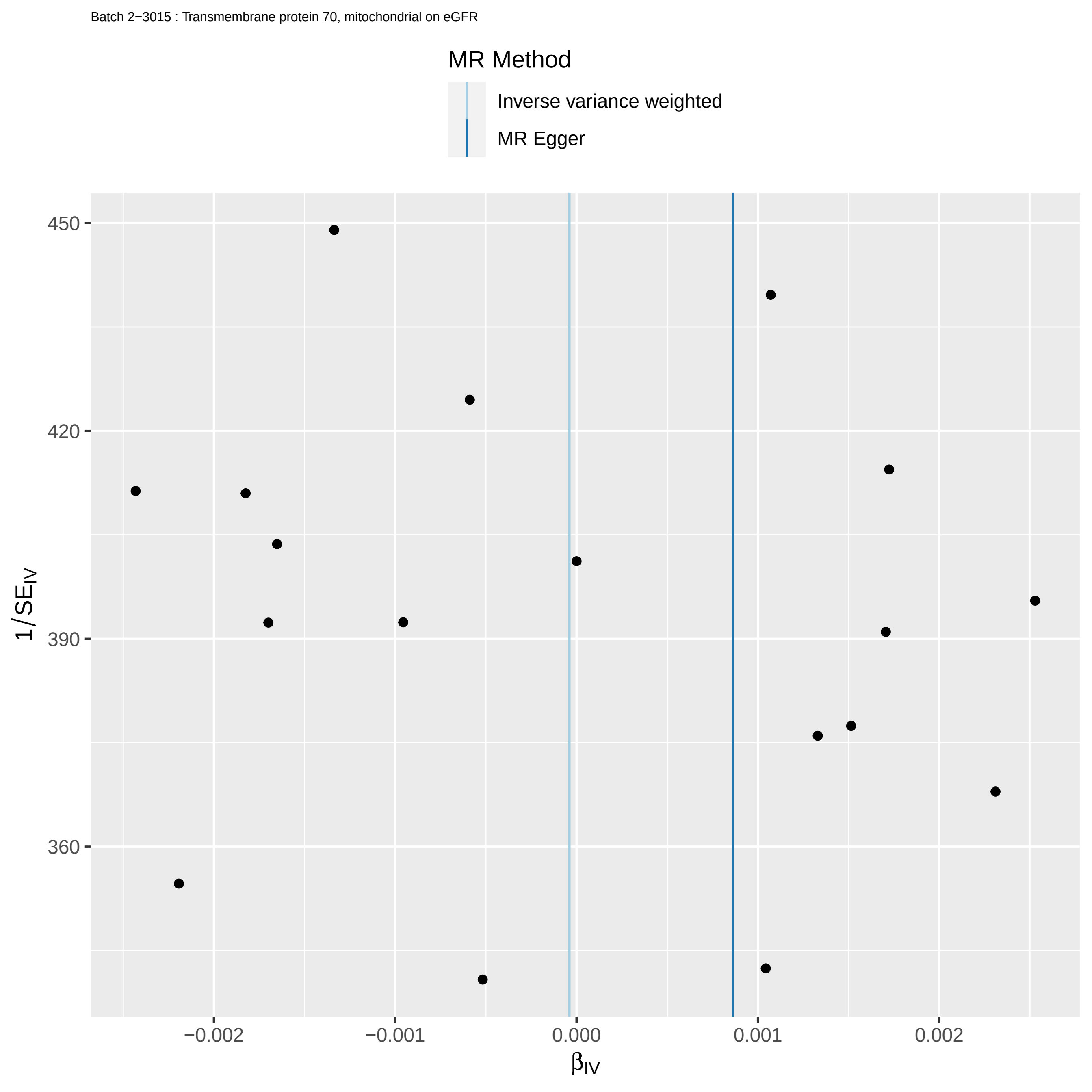

Batch 2−3015 : Transmembrane protein 70, mitochondrial on eGFR
MR Method
Inverse variance weighted
MR Egger
450
420
390
360
1/SE_IV
−0.002
−0.001
0.000
0.001
0.002
β_IV

Batch 2−3016 : Transmembrane protein 87B on eGFR

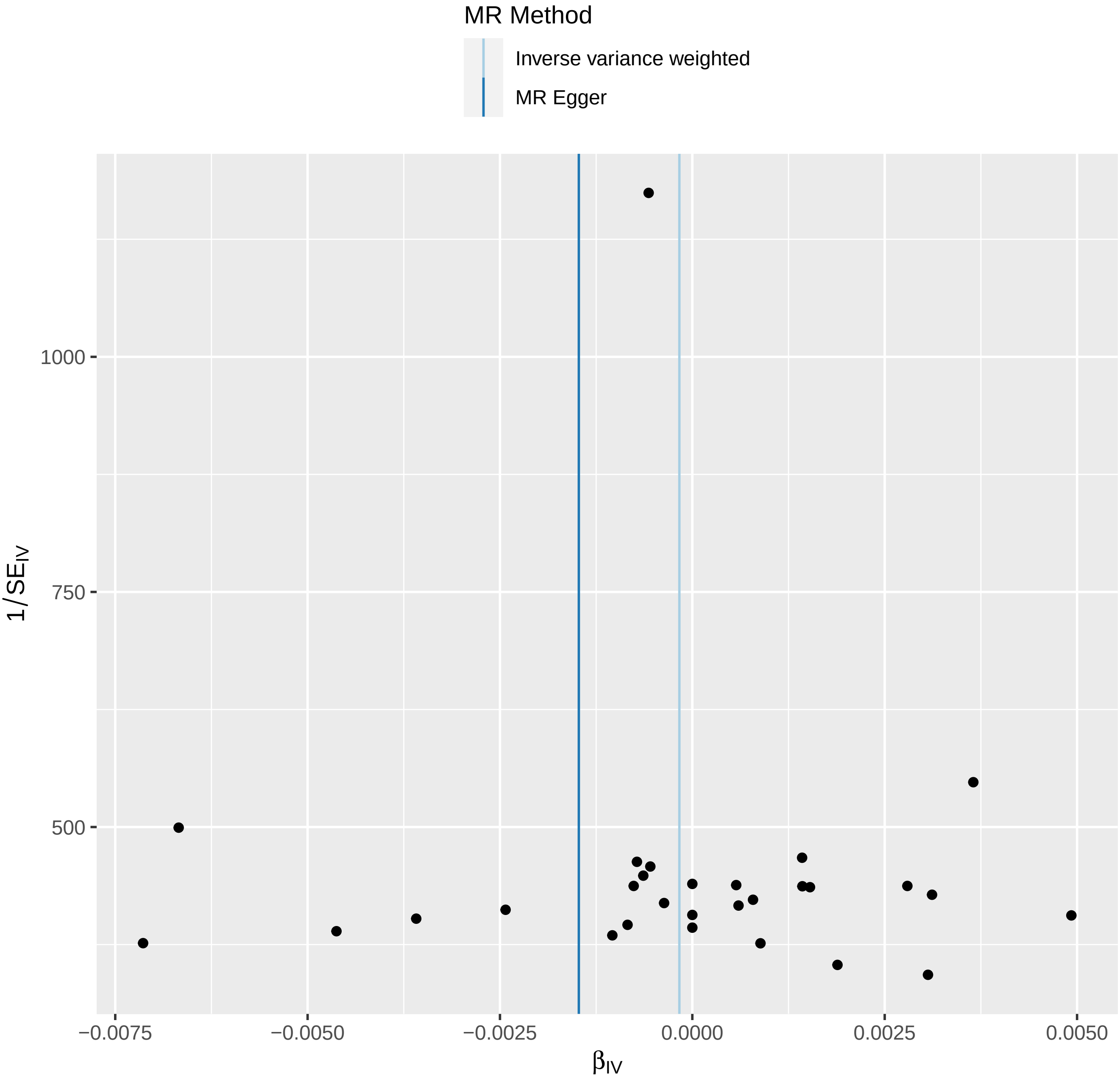

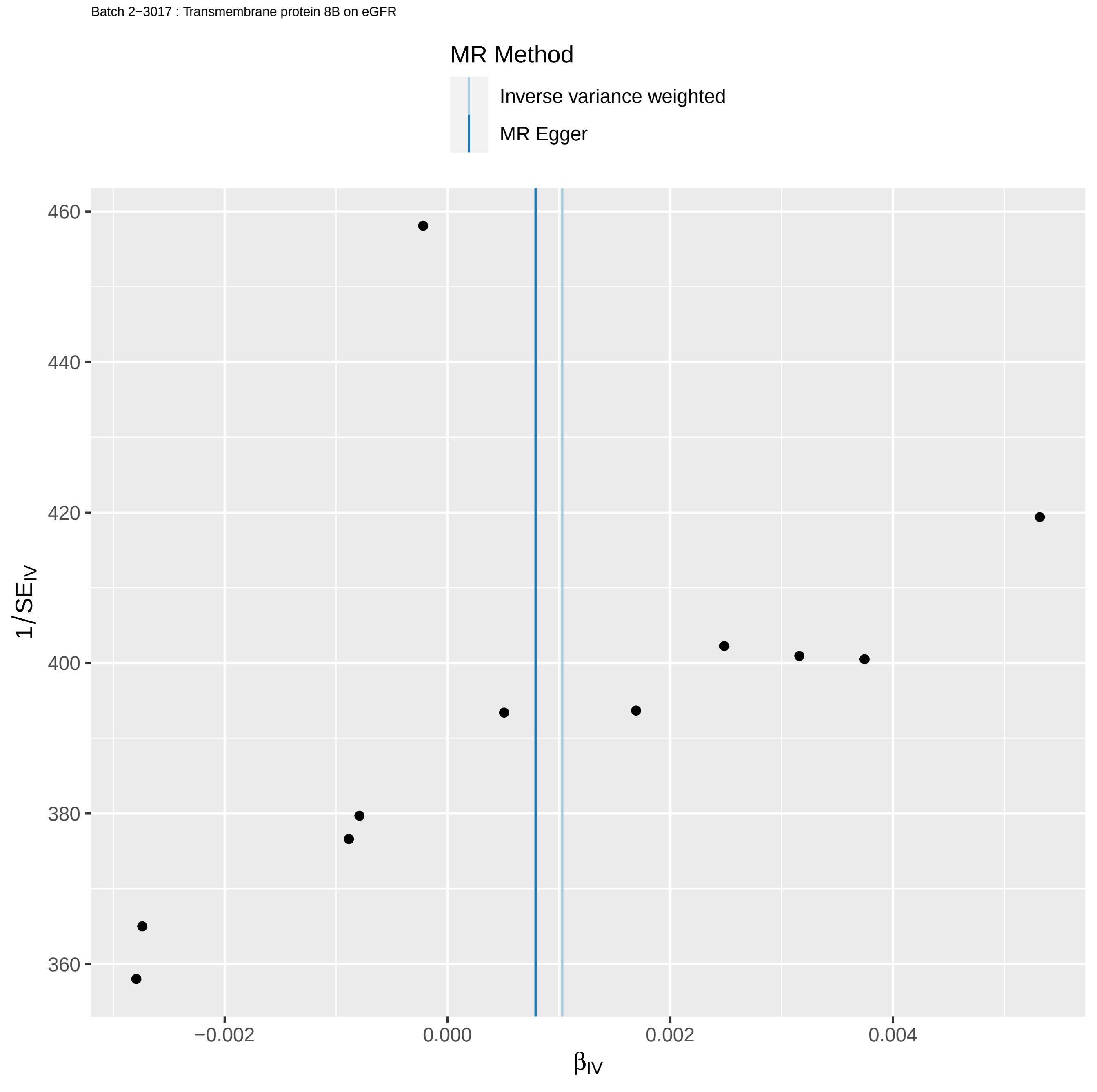
Batch 2−3017 : Transmembrane protein 8B on eGFR
MR Method
Inverse variance weighted
MR Egger
460
440
420
400
380
360
1/SE_IV
−0.002
0.000
0.002
0.004
β_IV

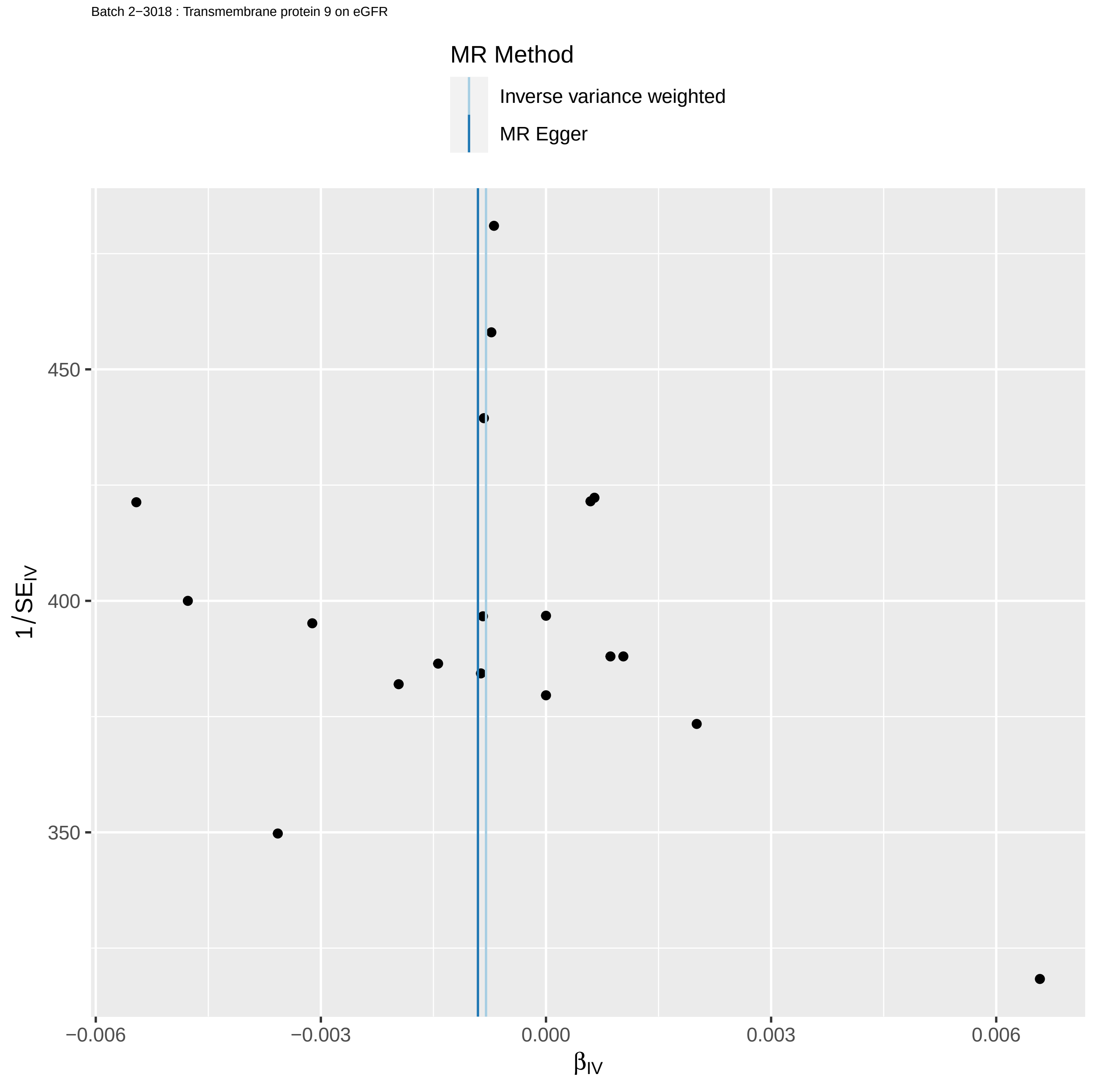
Batch 2−3018 : Transmembrane protein 9 on eGFR
MR Method
Inverse variance weighted
MR Egger
450
400
350
1/SE$_{IV}$
−0.006
−0.003
0.000
0.003
0.006
β_{IV}

Batch 2−3019 : Transmembrane inner ear expressed protein on eGFR

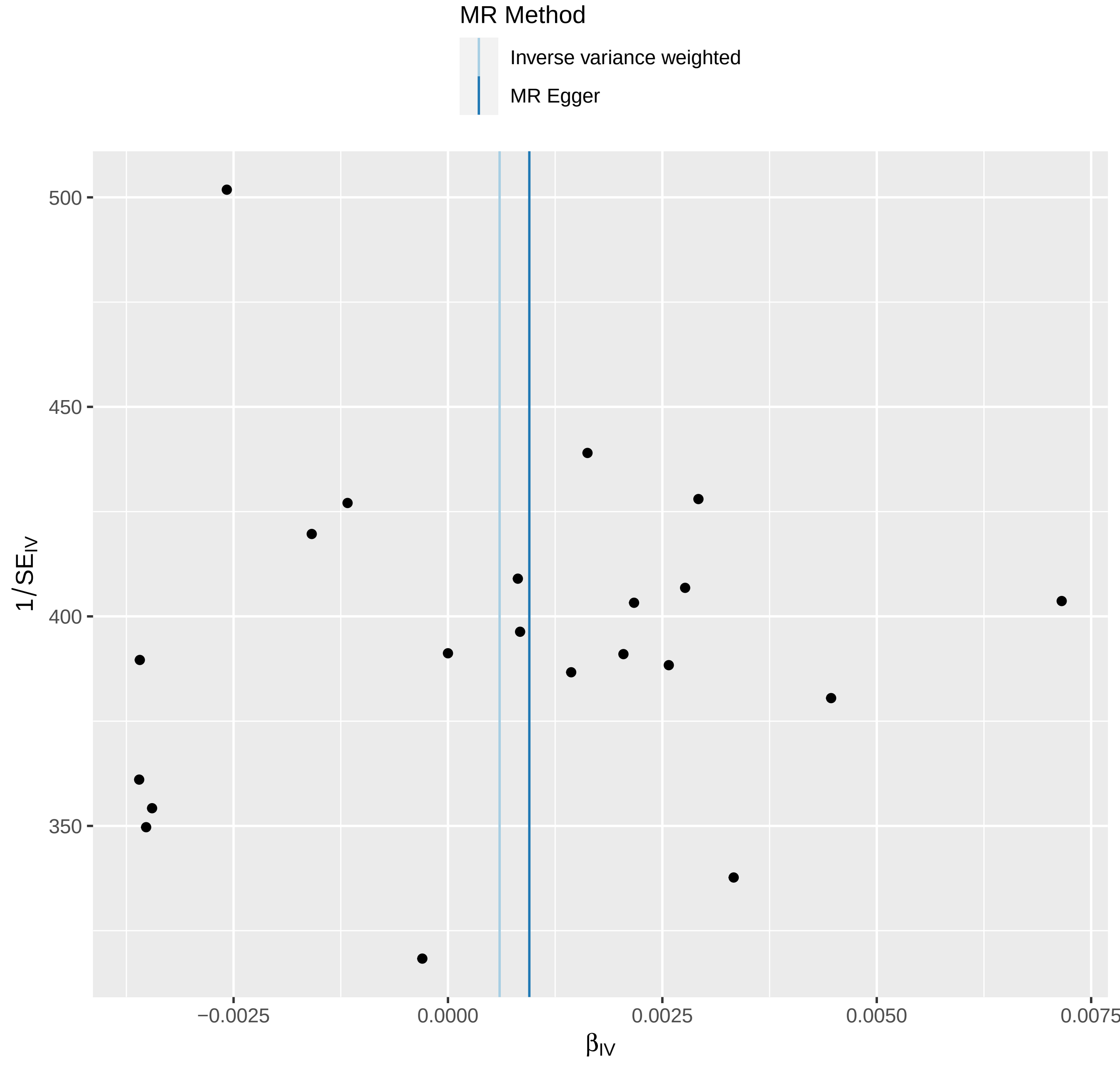

Batch 2−3020 : Tropomodulin−1 on eGFR

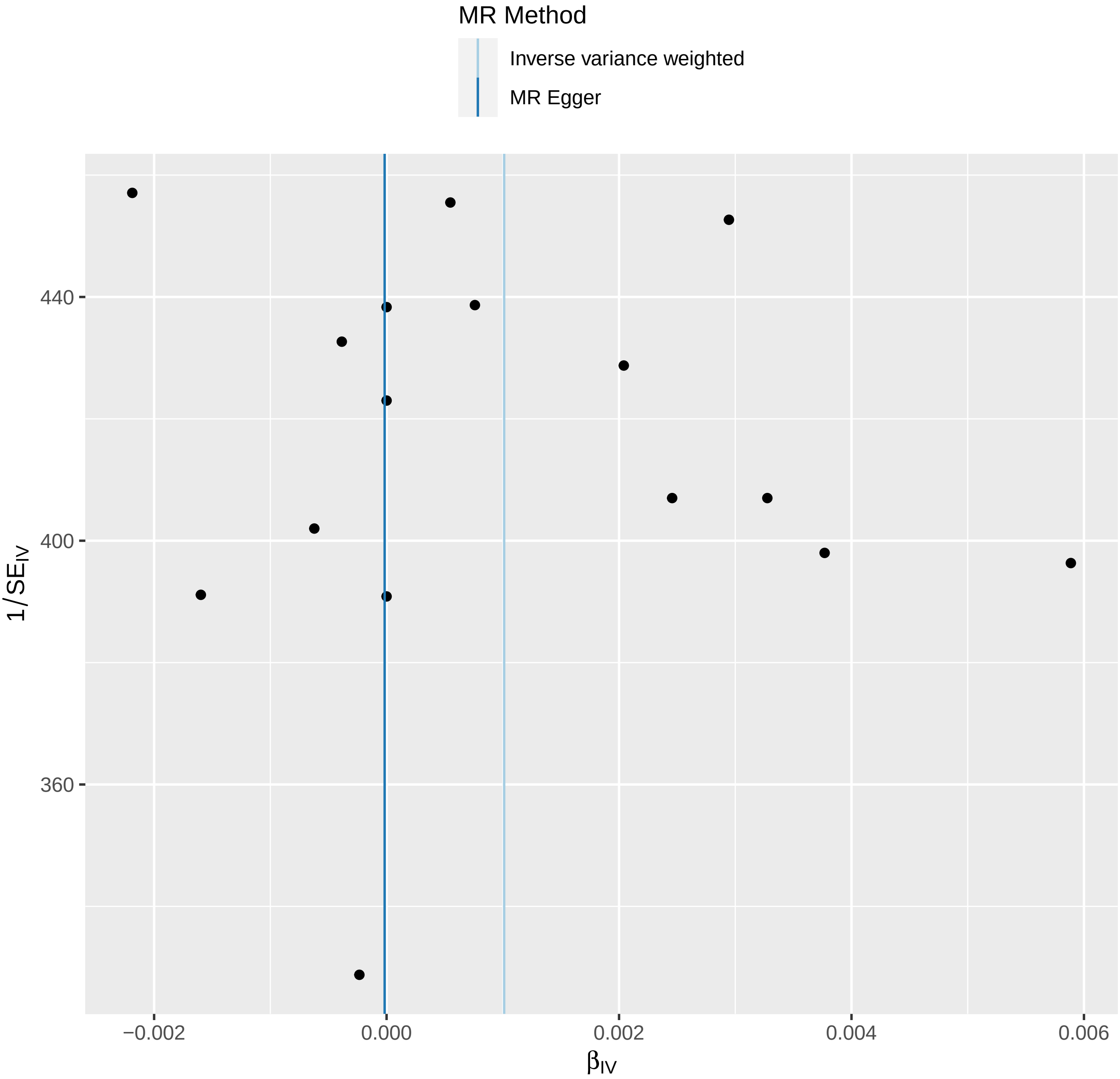

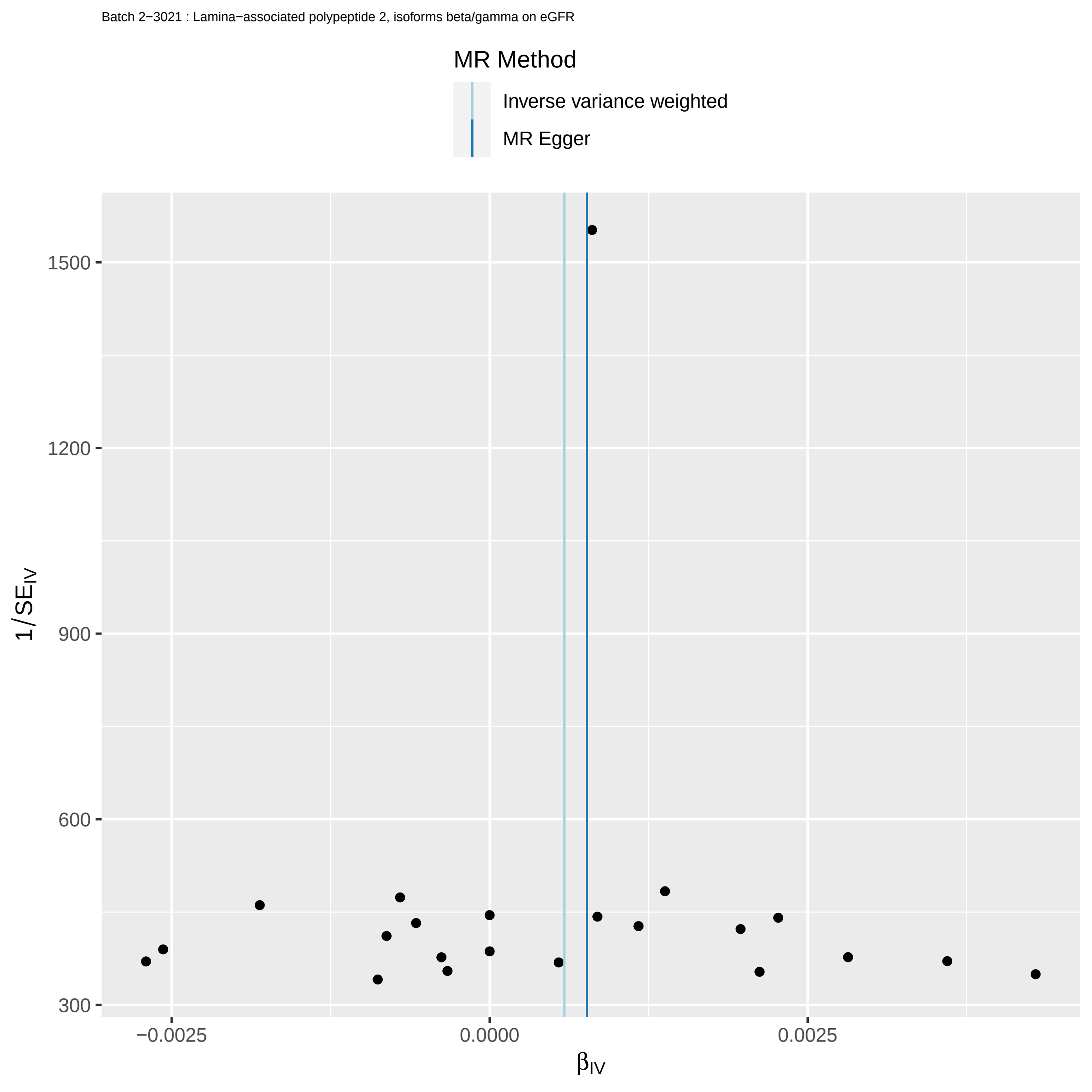
Batch 2−3021 : Lamina−associated polypeptide 2, isoforms beta/gamma on eGFR
MR Method
Inverse variance weighted
MR Egger
1500
1200
900
600
300
−0.0025
0.0000
0.0025
$1/SE_{IV}$
β_{IV}

Batch 2−3022 : Transmembrane protease serine 11A on eGFR

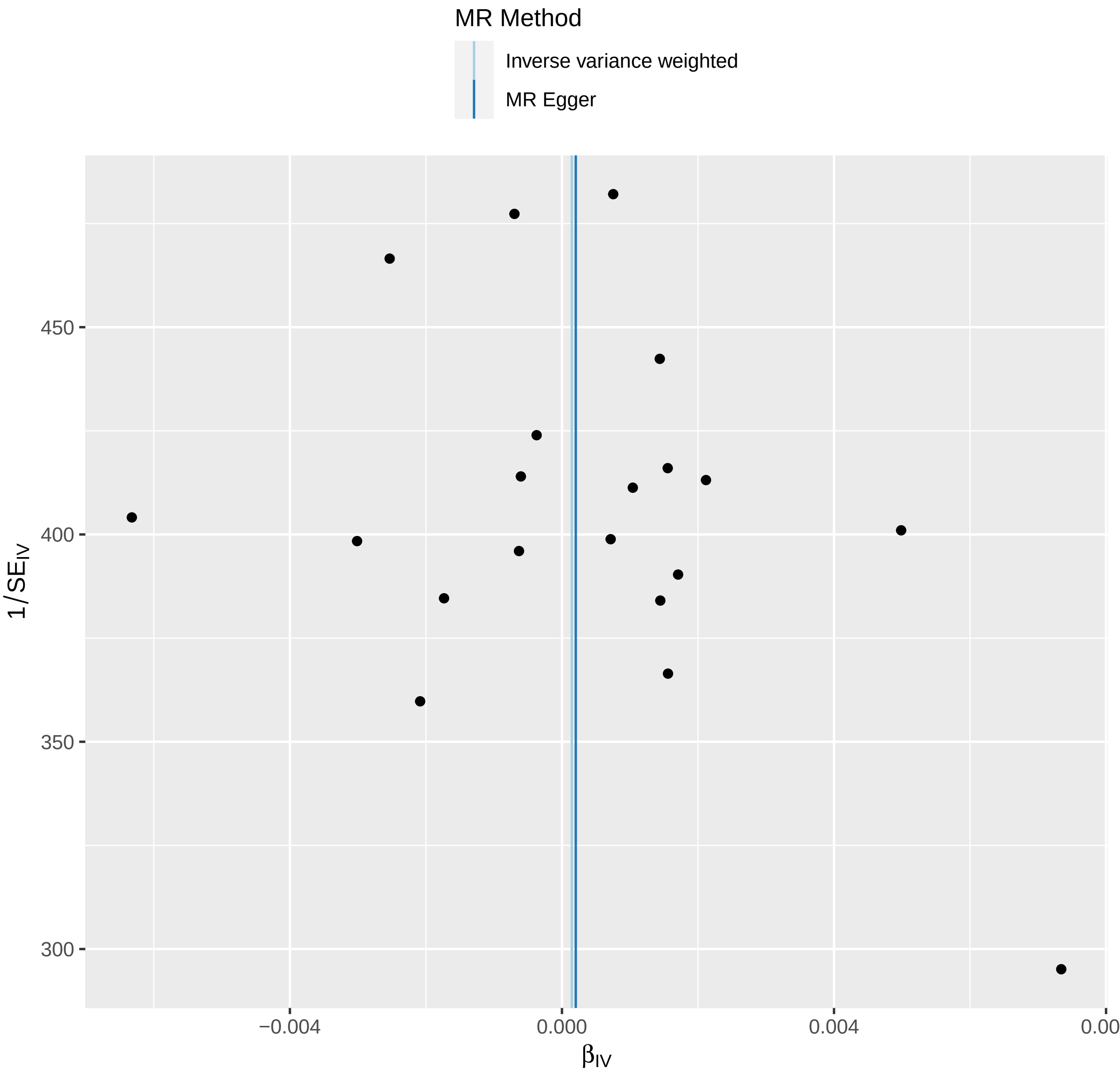

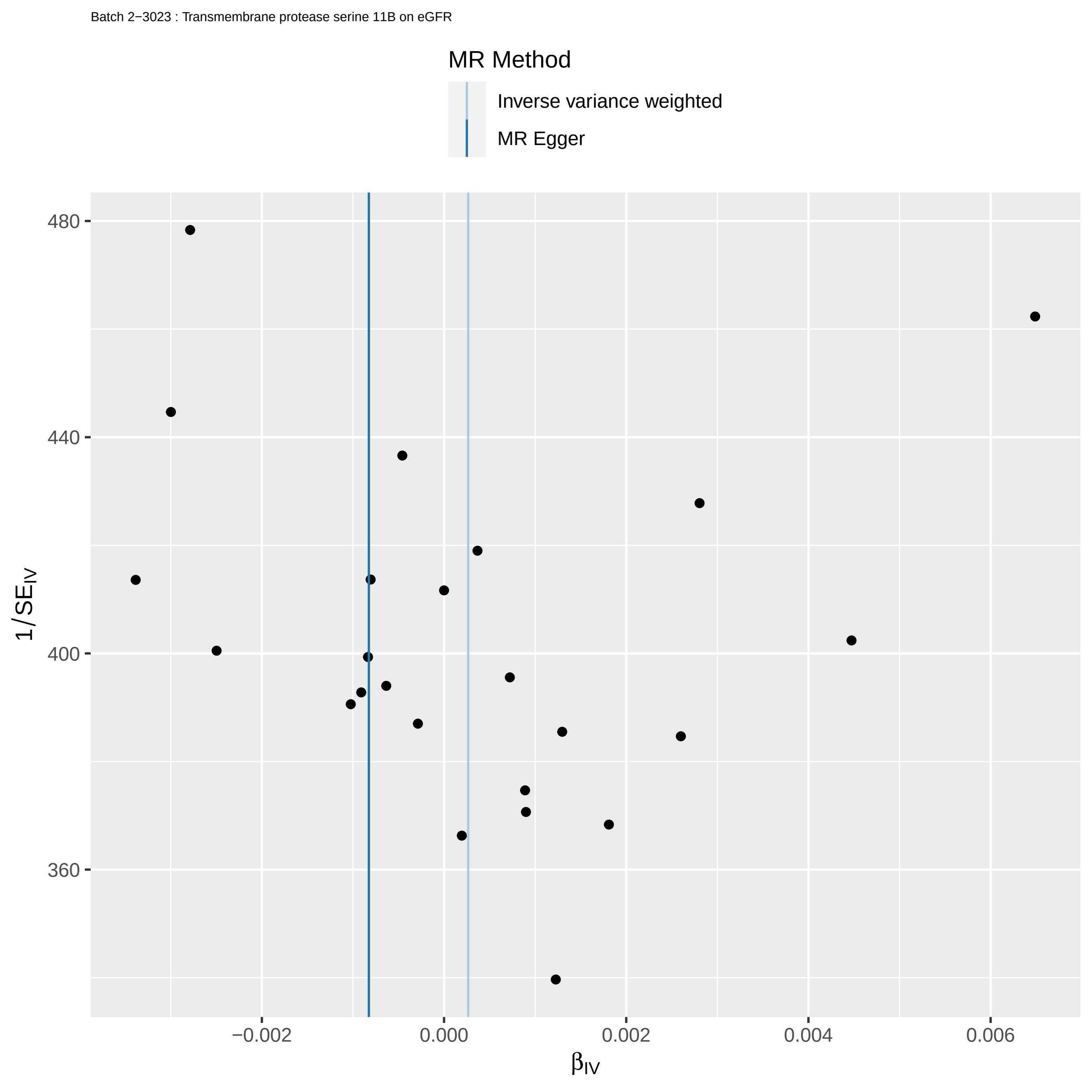
Batch 2−3023 : Transmembrane protease serine 11B on eGFR
MR Method
Inverse variance weighted
MR Egger
480
440
400
360
1/SE$_{IV}$
−0.002
0.000
0.002
0.004
0.006
β$_{IV}$

Batch 2−3024 : Transmembrane protease serine 11D on eGFR

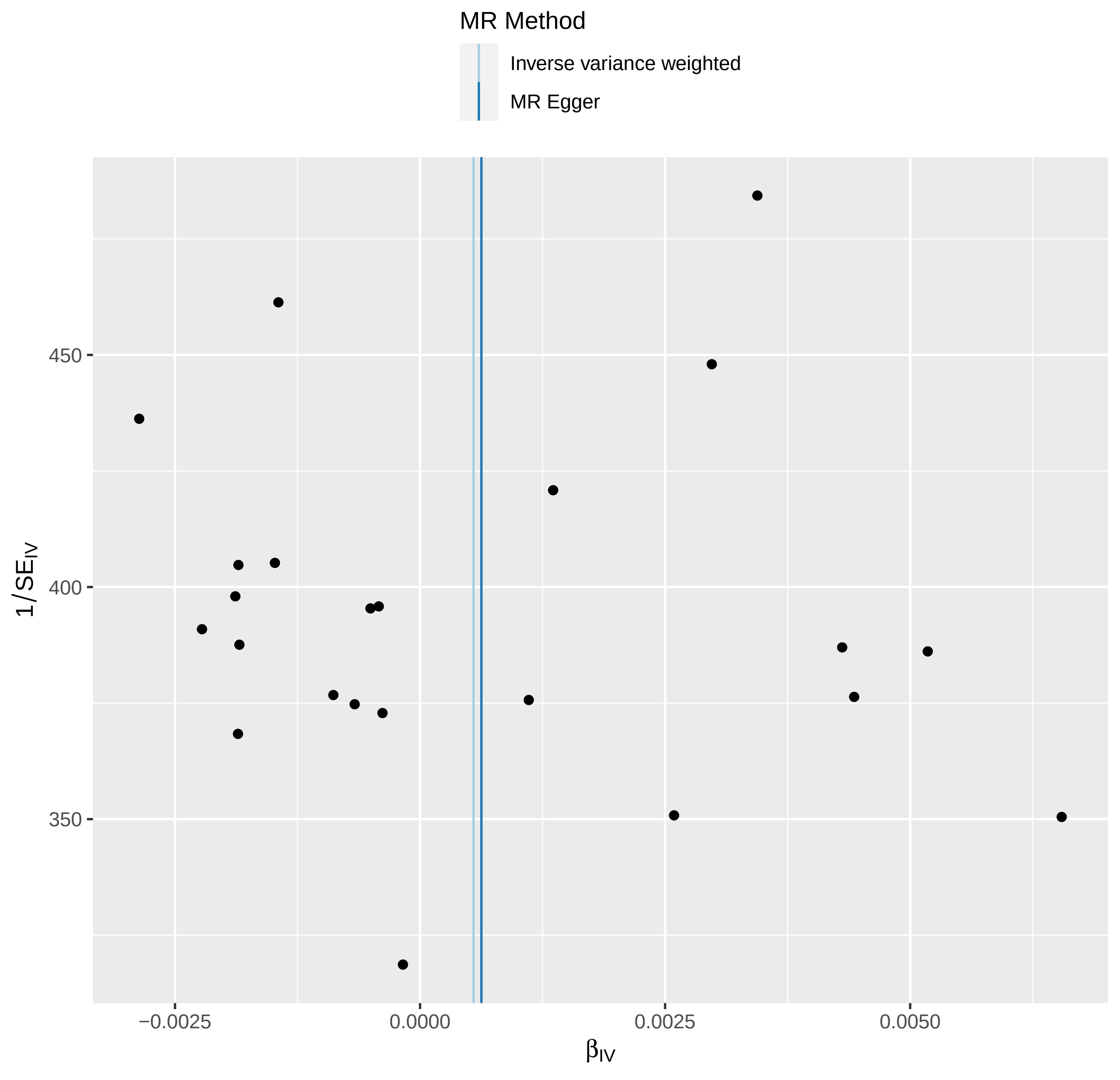

Batch 2−3025 : Enteropeptidase on eGFR

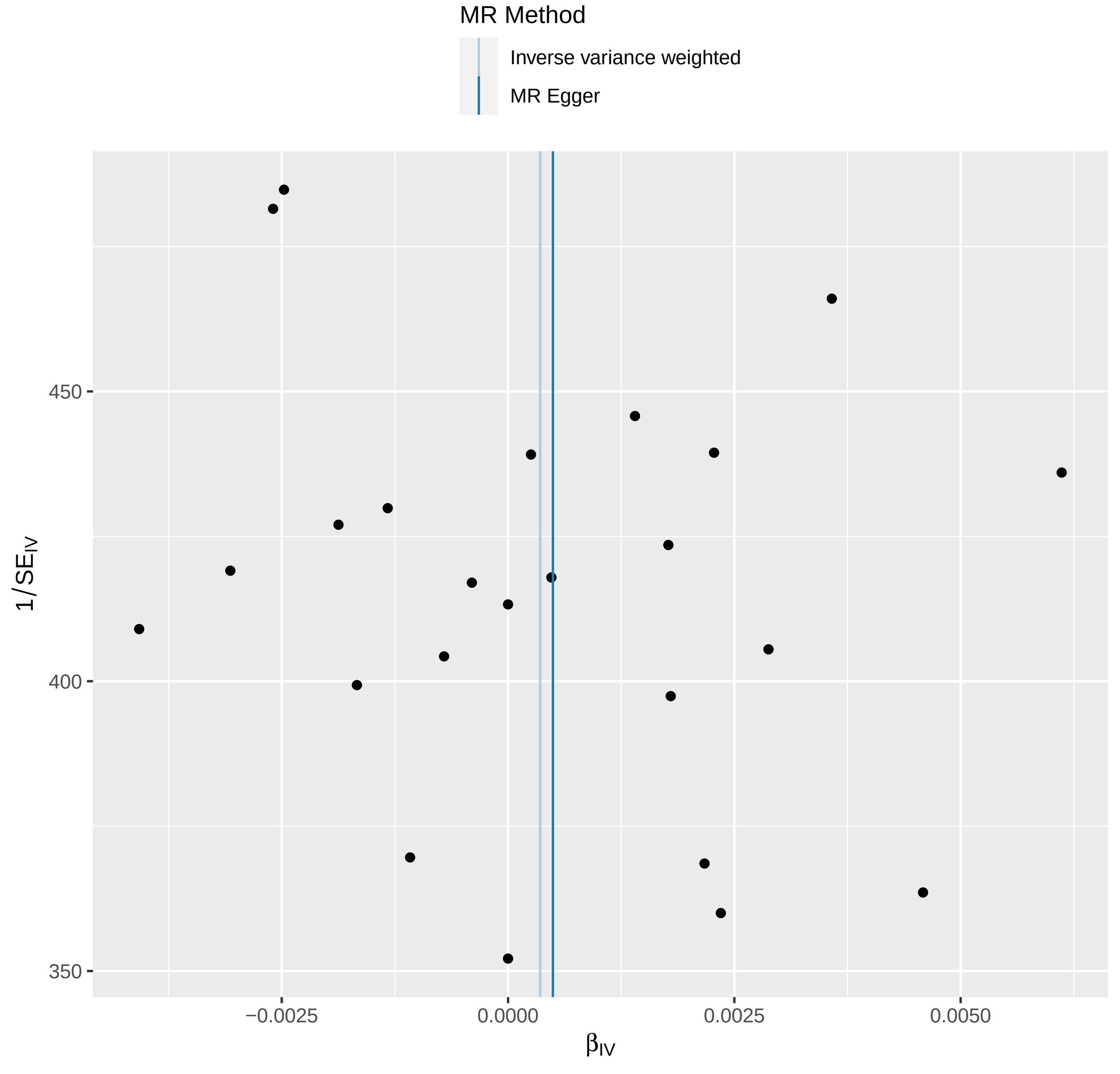

Batch 2−3026 : Transmembrane protease serine 5 on eGFR

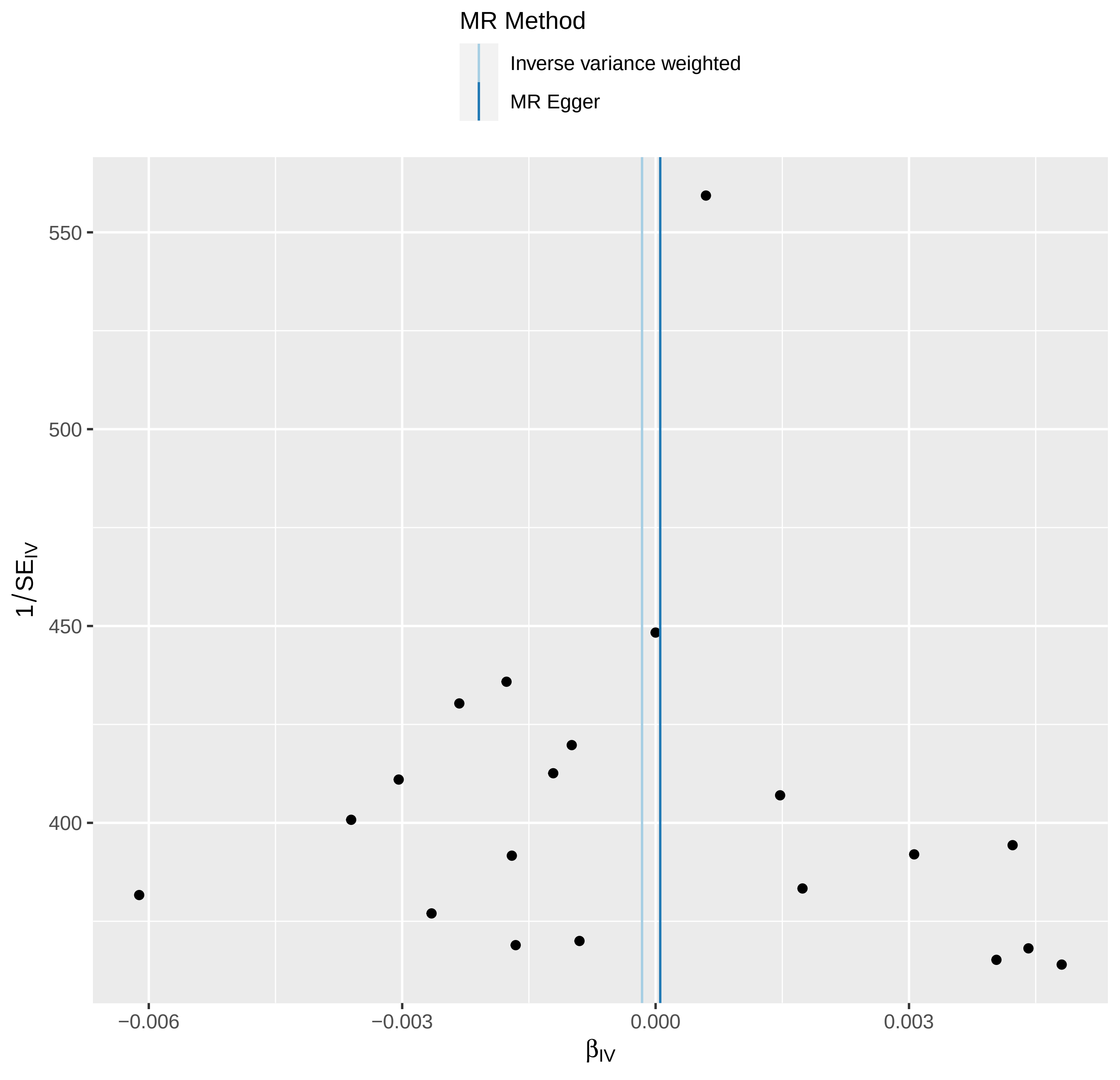

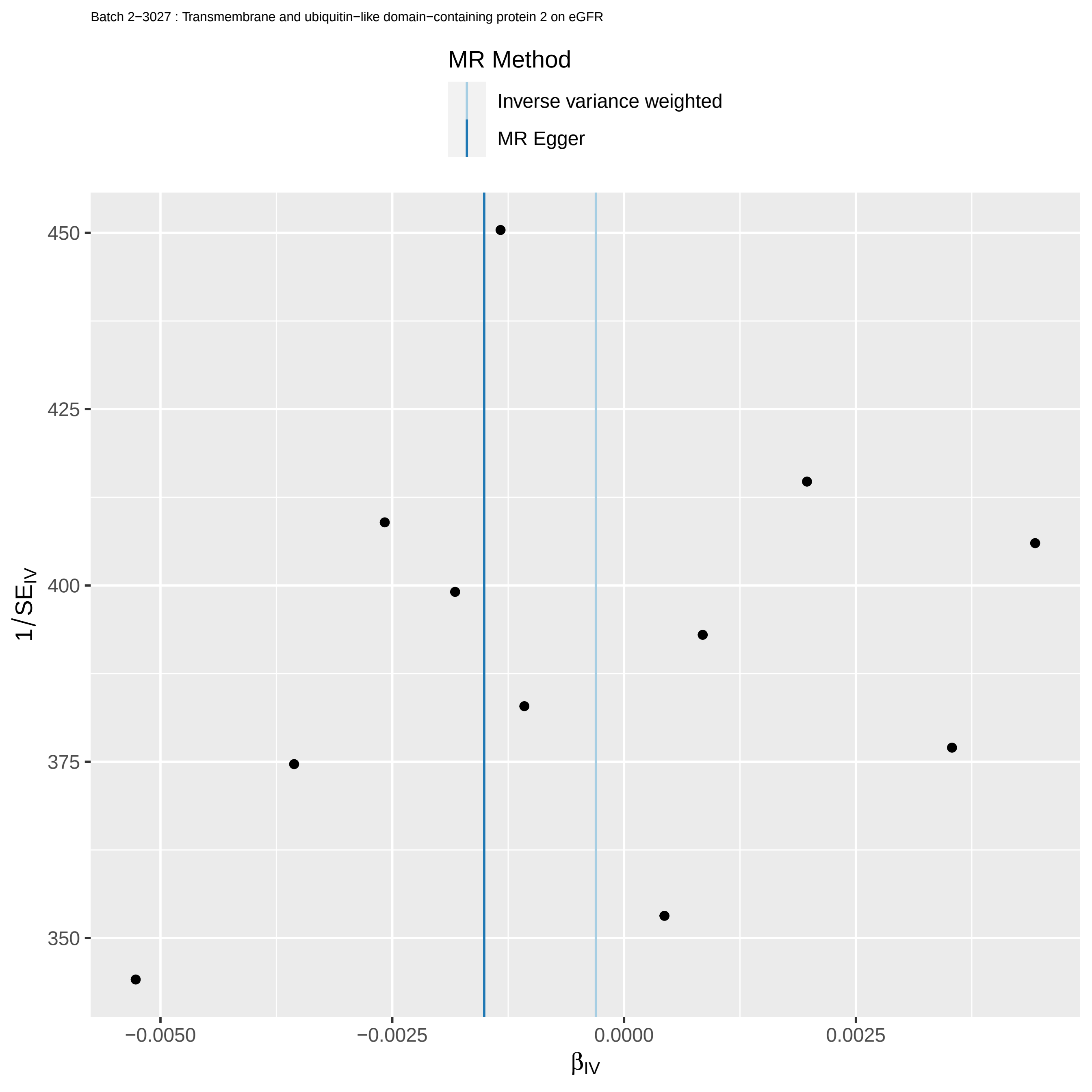
Batch 2−3027 : Transmembrane and ubiquitin−like domain−containing protein 2 on eGFR
MR Method
Inverse variance weighted
MR Egger
450
425
400
375
350
1/SE$_{IV}$
−0.0050
−0.0025
0.0000
0.0025
β$_{IV}$

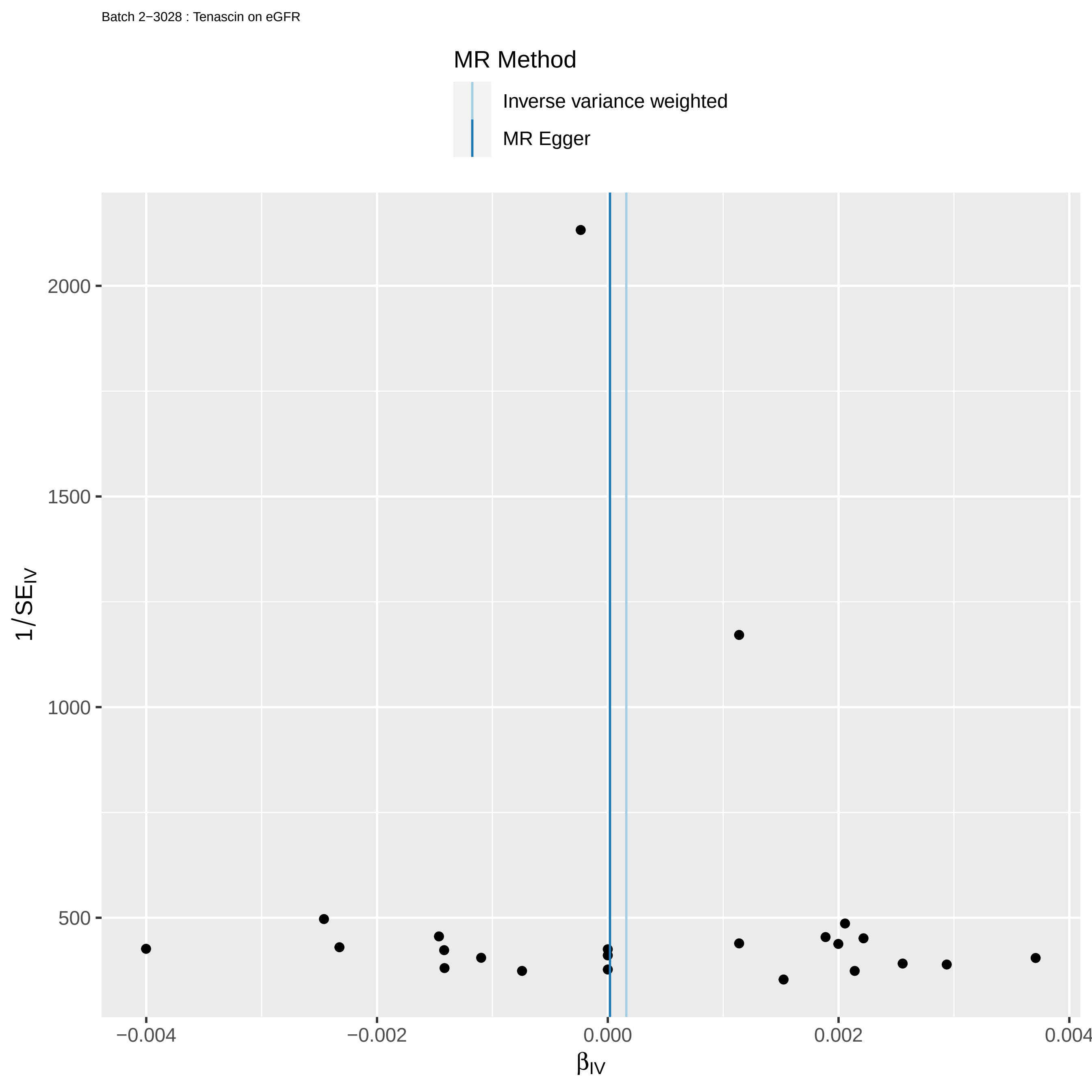

Batch 2−3028 : Tenascin on eGFR
MR Method
Inverse variance weighted
MR Egger
2000
1500
1000
500
1/SE$_{IV}$
−0.004
−0.002
0.000
0.002
0.004
β$_{IV}$

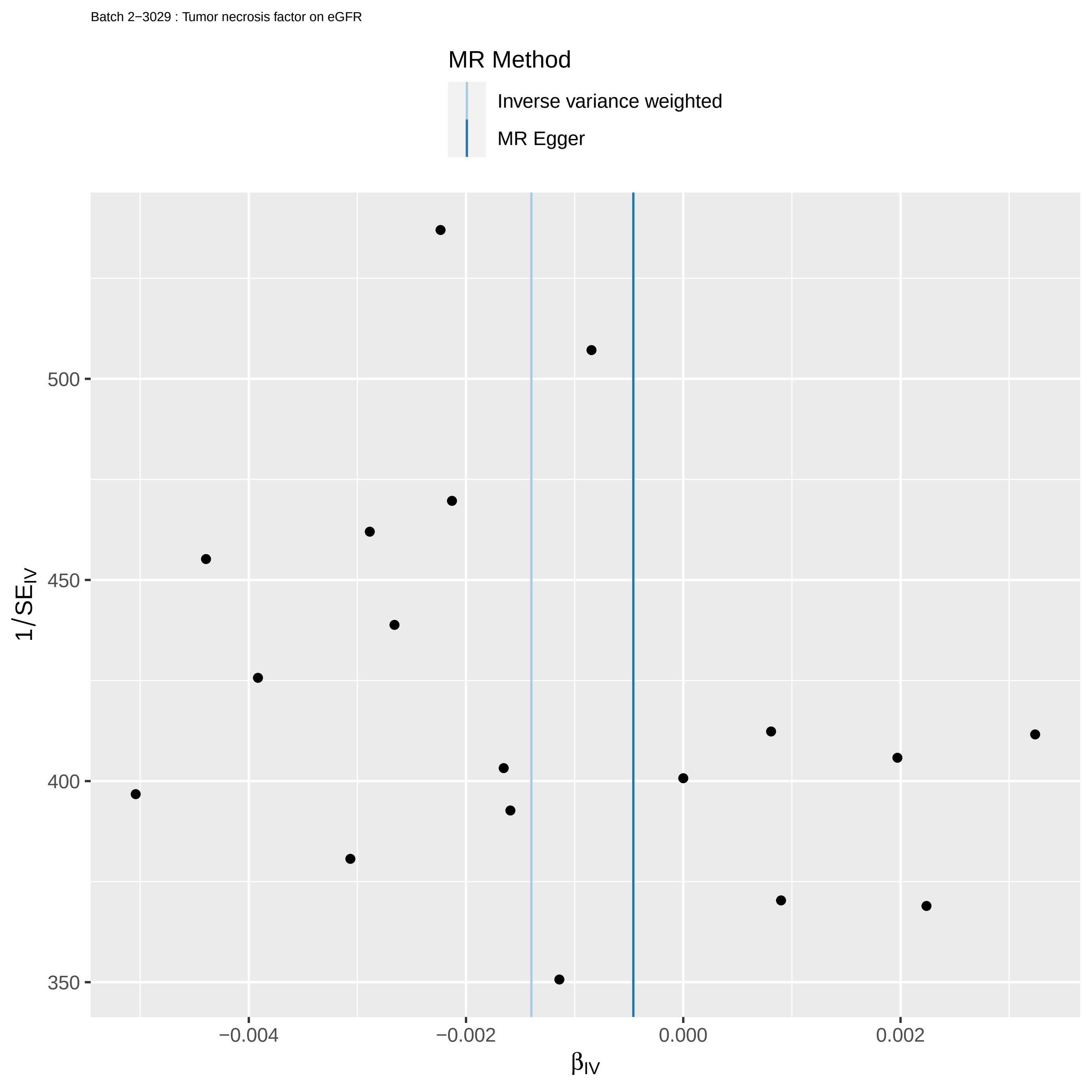

Batch 2−3029 : Tumor necrosis factor on eGFR
MR Method
Inverse variance weighted
MR Egger
500
450
400
350
1/SE$_{IV}$
−0.004
−0.002
0.000
0.002
β$_{IV}$

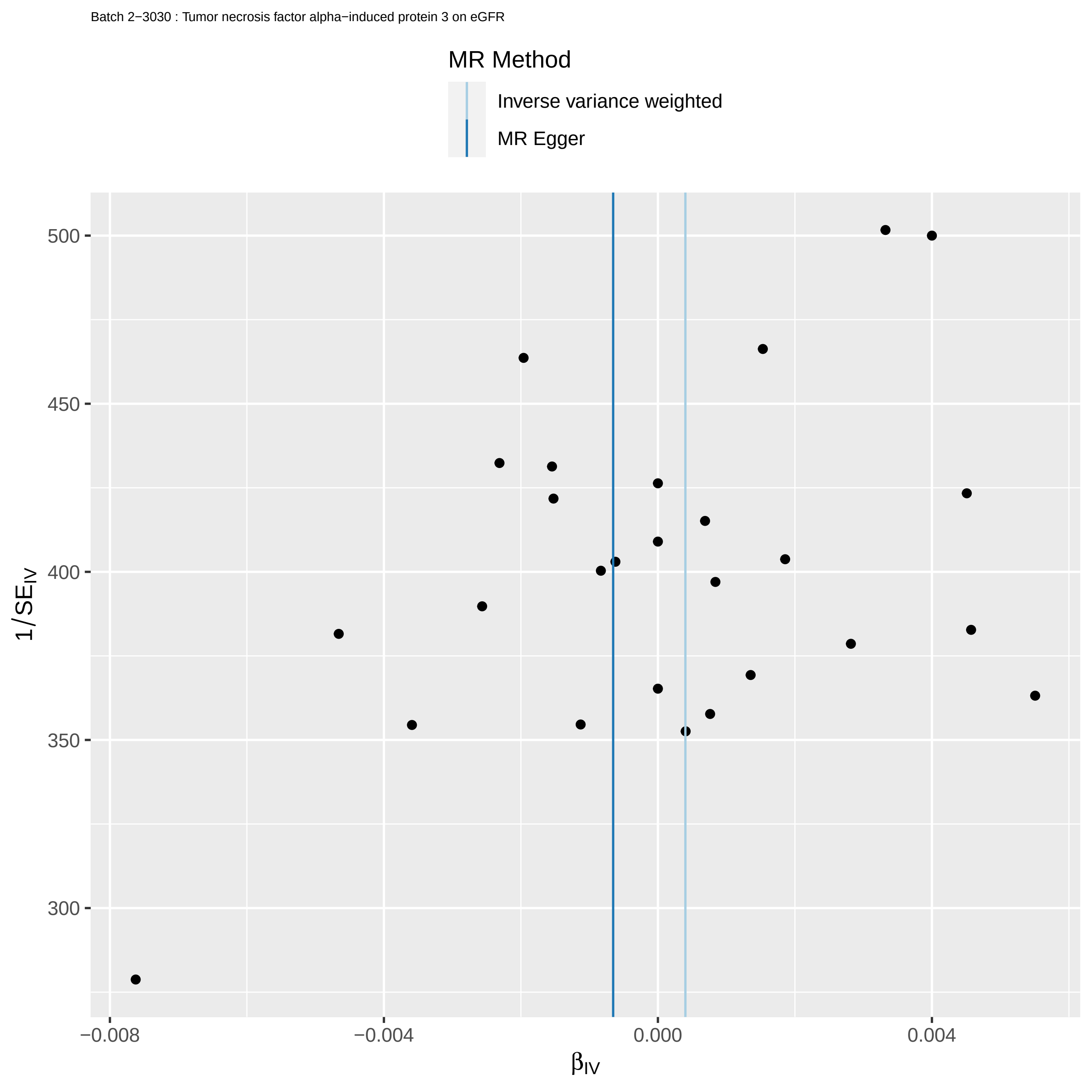
Batch 2−3030 : Tumor necrosis factor alpha−induced protein 3 on eGFR
MR Method
Inverse variance weighted
MR Egger
500
450
400
350
300
1/SE_IV
−0.008
−0.004
0.000
0.004
β_IV

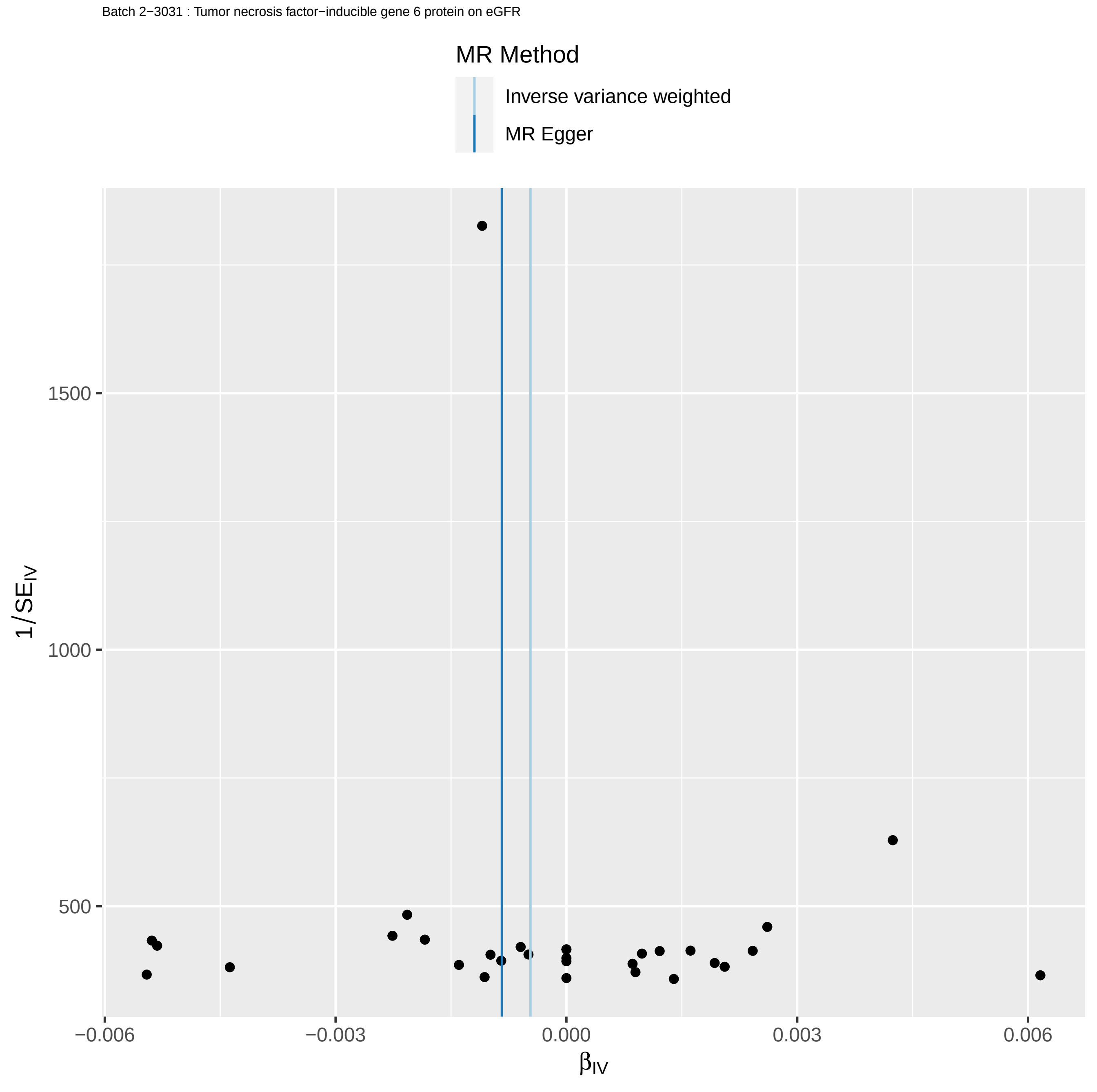

Batch 2−3031 : Tumor necrosis factor−inducible gene 6 protein on eGFR
MR Method
Inverse variance weighted
MR Egger
1500
1000
500
$1/SE_{IV}$
−0.006
−0.003
0.000
0.003
0.006
β_{IV}

Batch 2−3032 : Tumor necrosis factor receptor superfamily member 10A on eGFR

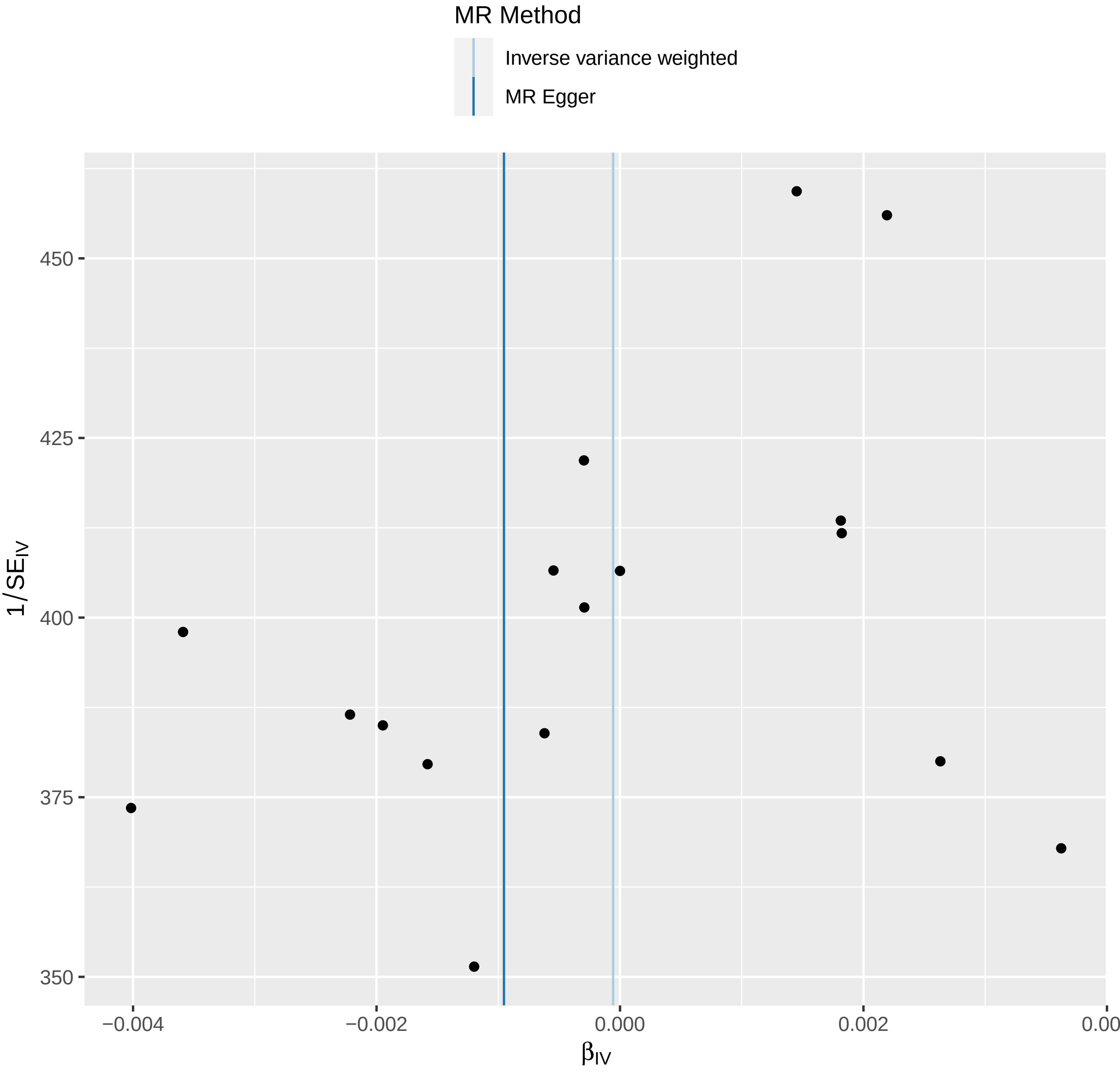

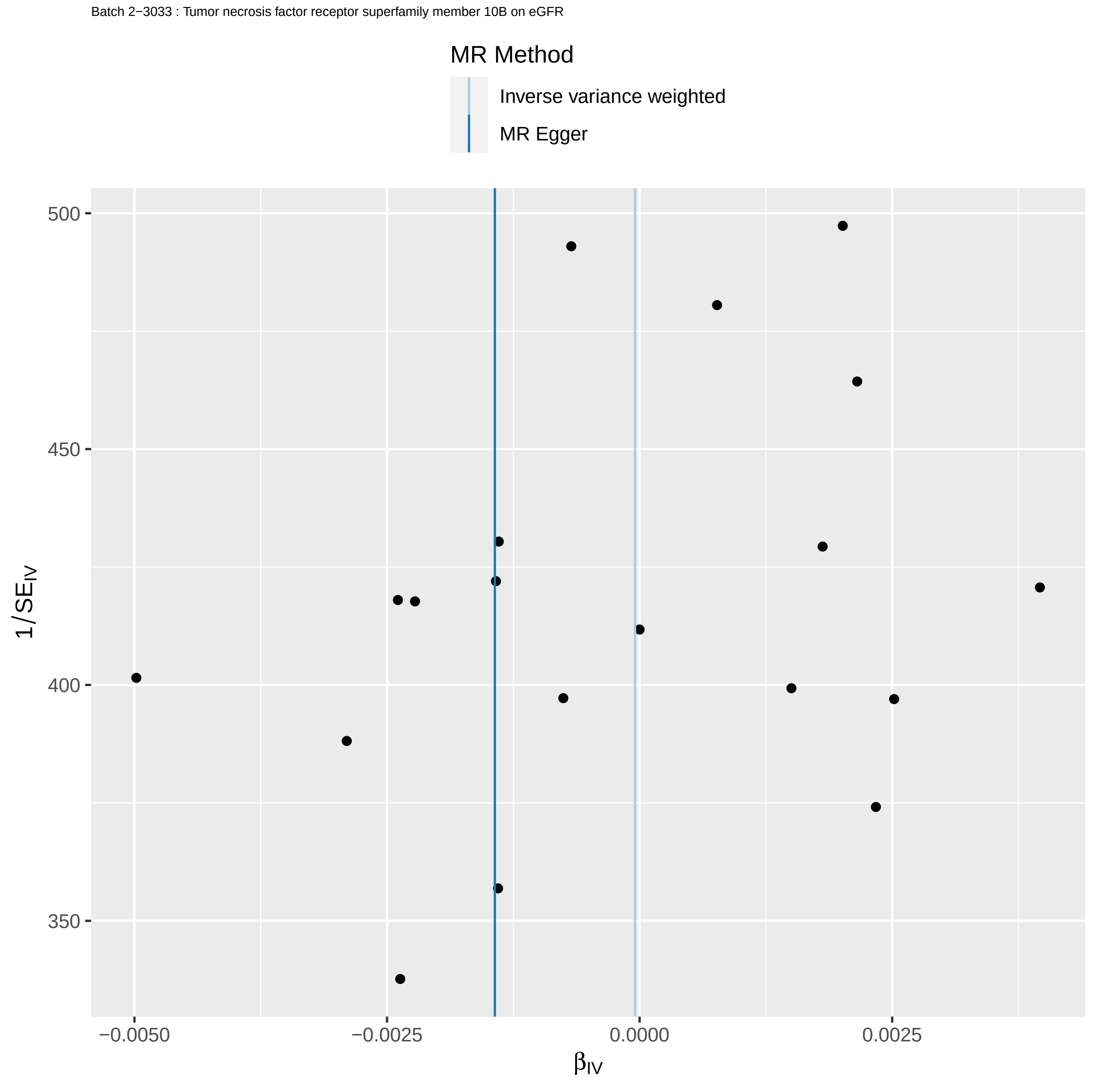

Batch 2−3033 : Tumor necrosis factor receptor superfamily member 10B on eGFR
MR Method
Inverse variance weighted
MR Egger
500
450
400
350
1/SE_IV
−0.0050
−0.0025
0.0000
0.0025
β_IV

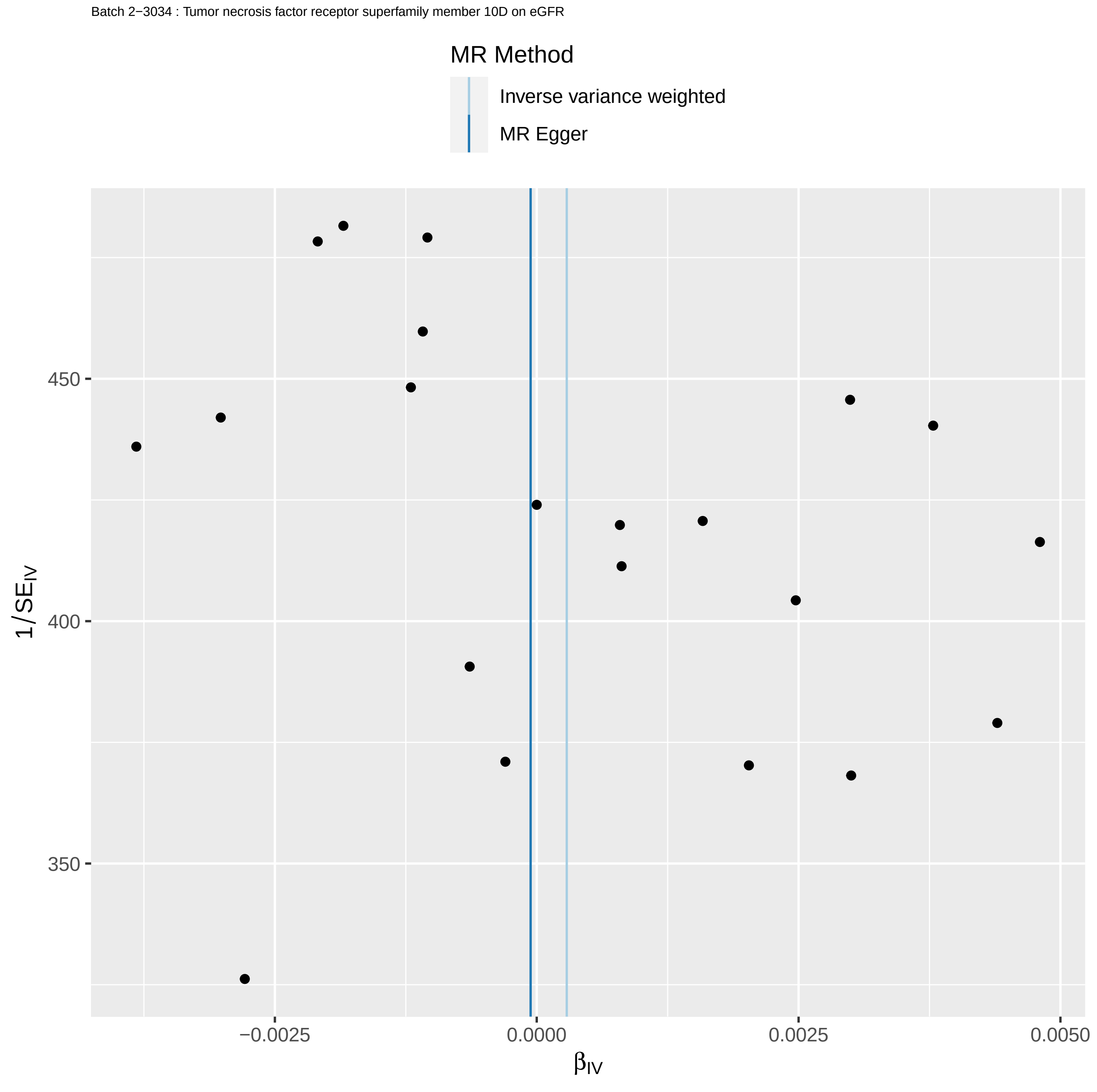

Batch 2−3034 : Tumor necrosis factor receptor superfamily member 10D on eGFR
MR Method
Inverse variance weighted
MR Egger
450
400
350
1/SE$_{IV}$
−0.0025
0.0000
0.0025
0.0050
β_{IV}

Batch 2−3035 : Tumor necrosis factor receptor superfamily member 11A on eGFR

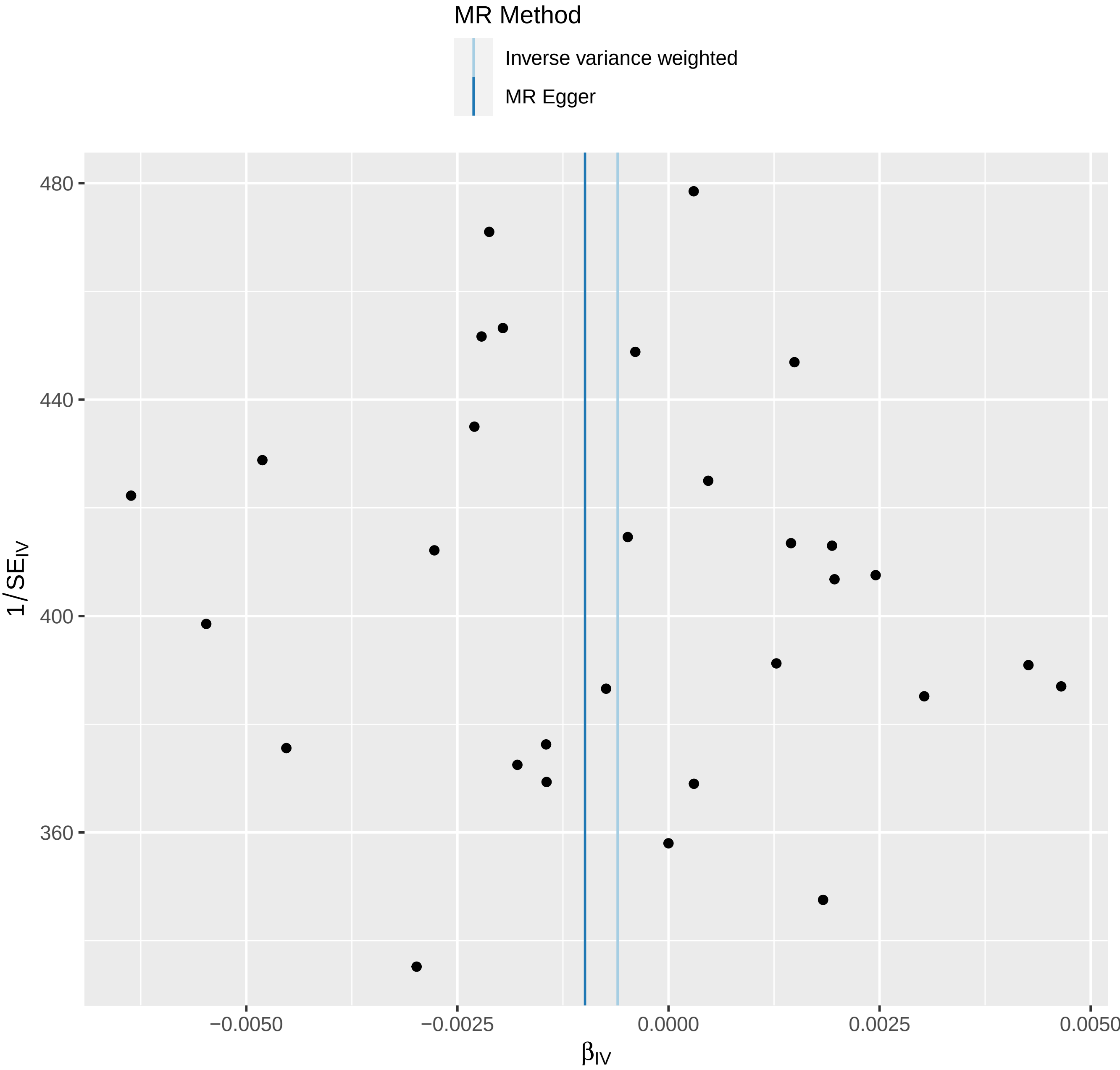

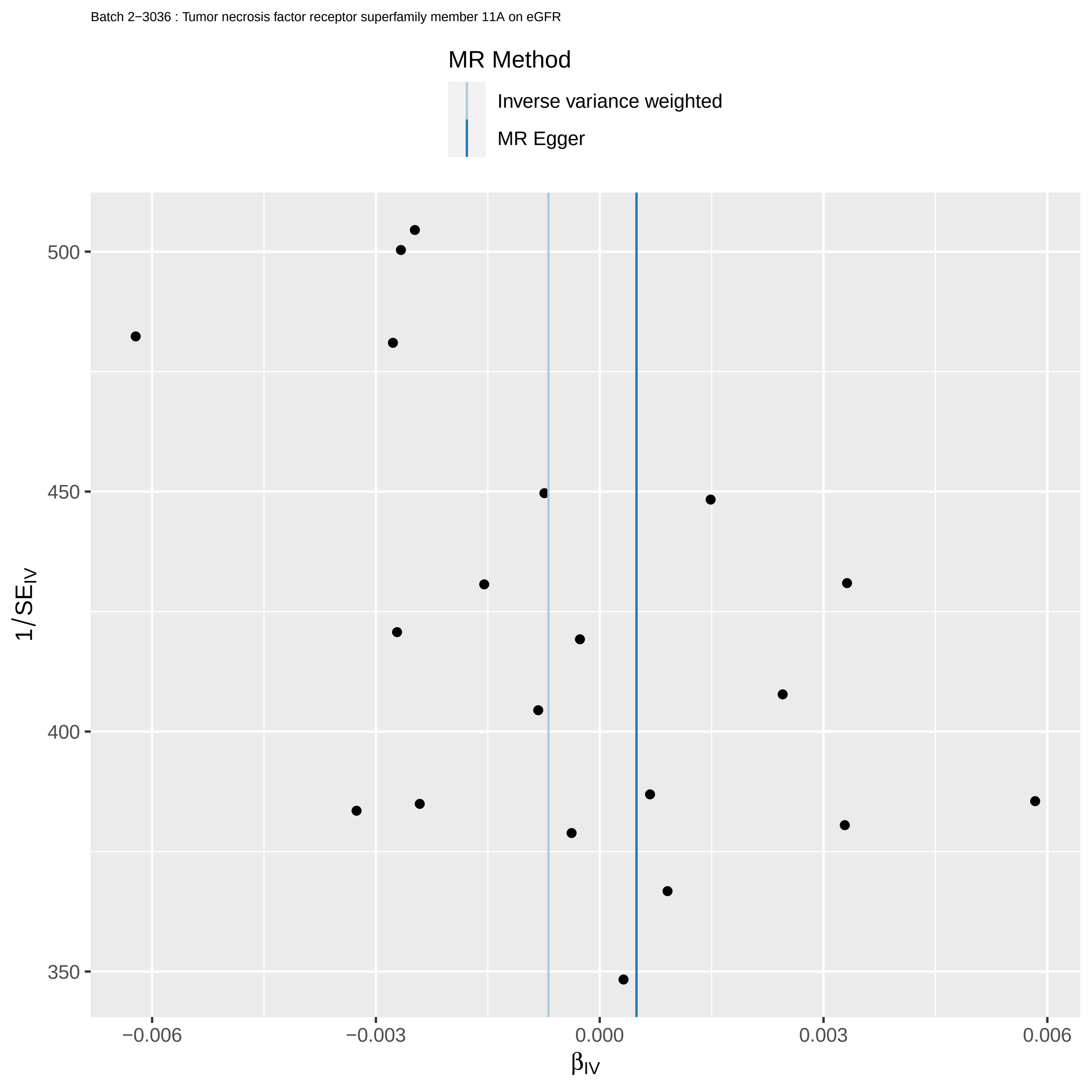

Batch 2−3036 : Tumor necrosis factor receptor superfamily member 11A on eGFR
MR Method
Inverse variance weighted
MR Egger
500
450
400
350
1/SE$_{IV}$
−0.006
−0.003
0.000
0.003
0.006
β$_{IV}$

Batch 2−3037 : Tumor necrosis factor receptor superfamily member 11A on eGFR

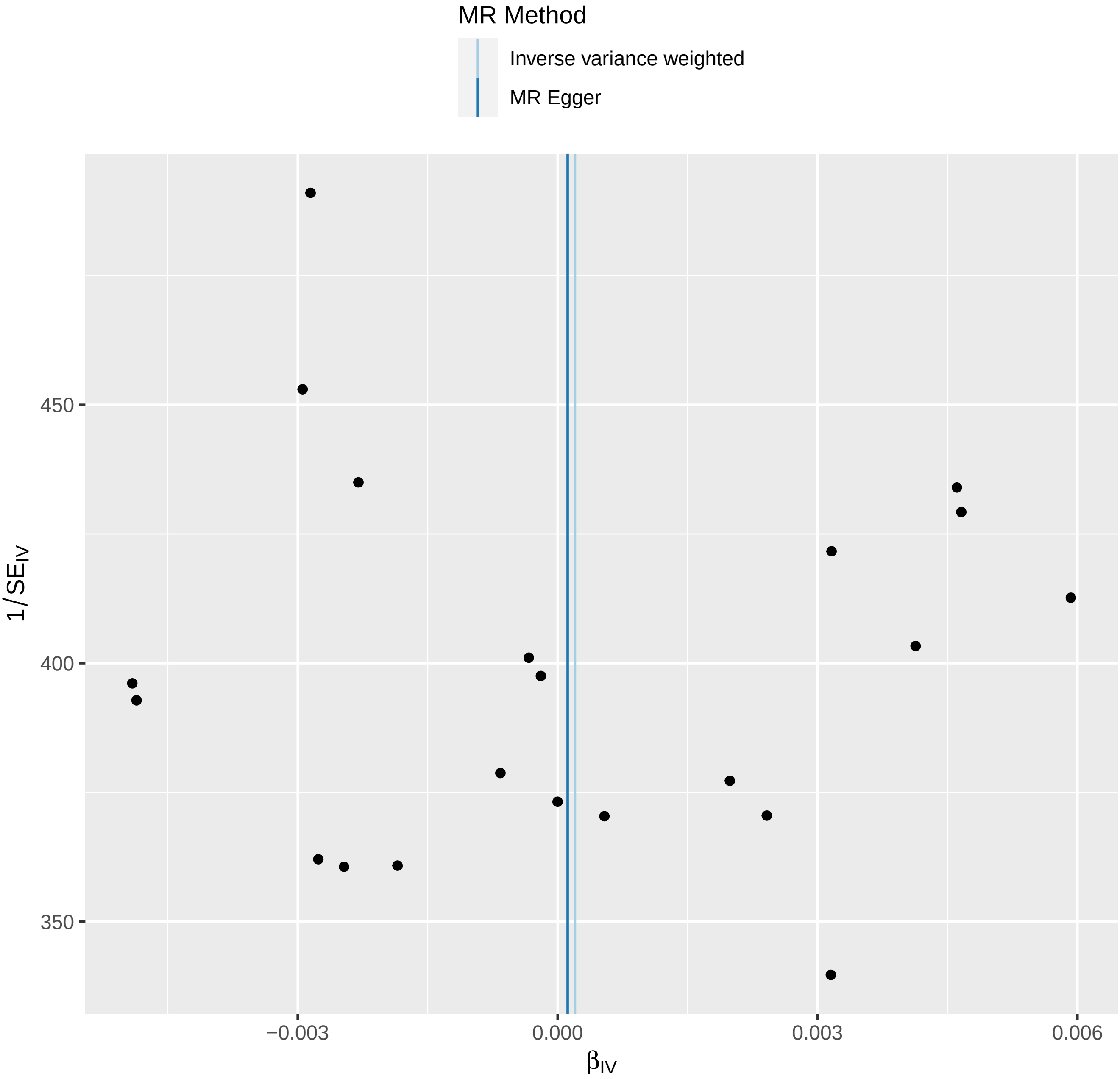

Batch 2−3038 : Tumor necrosis factor receptor superfamily member 11B on eGFR

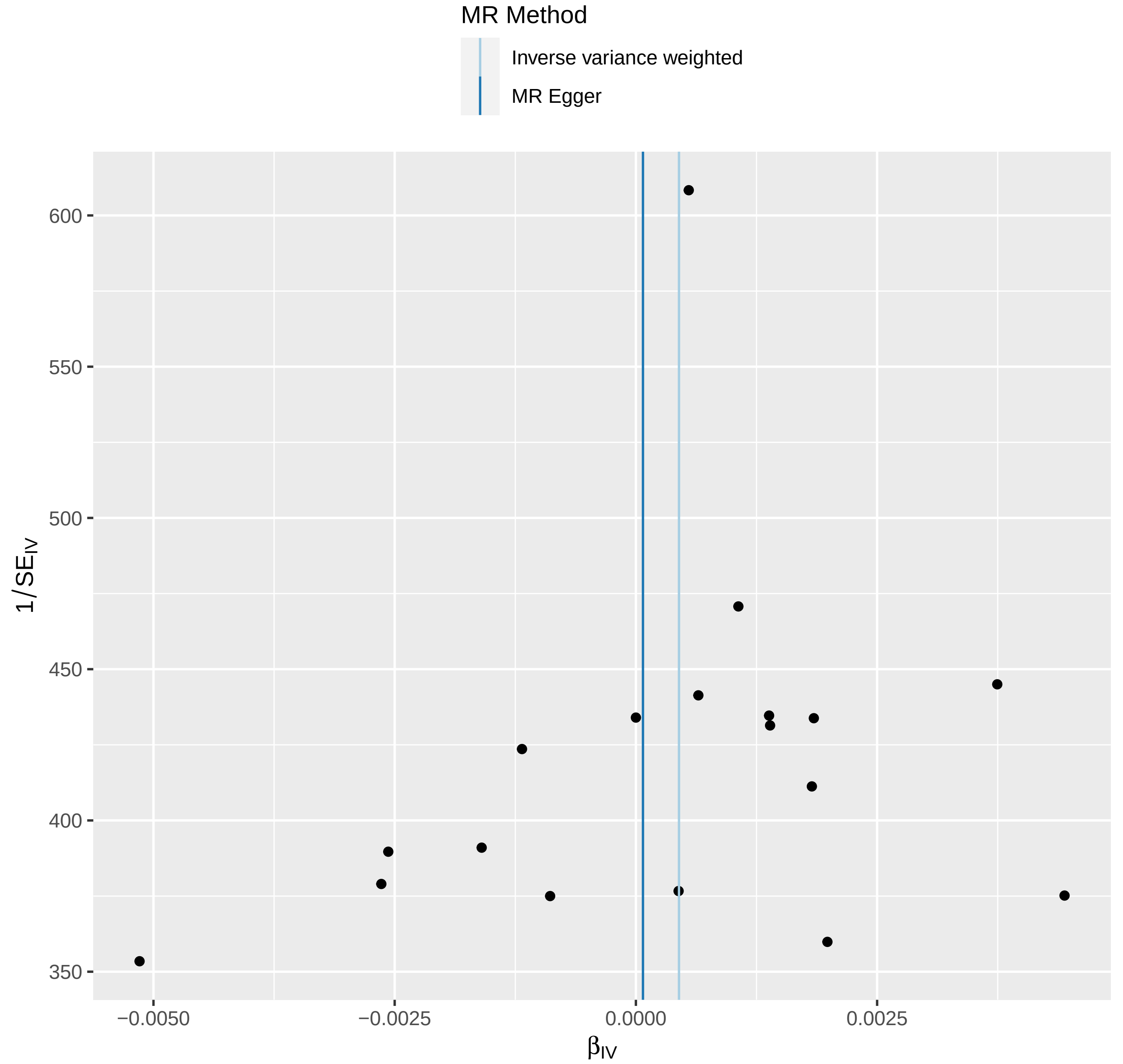

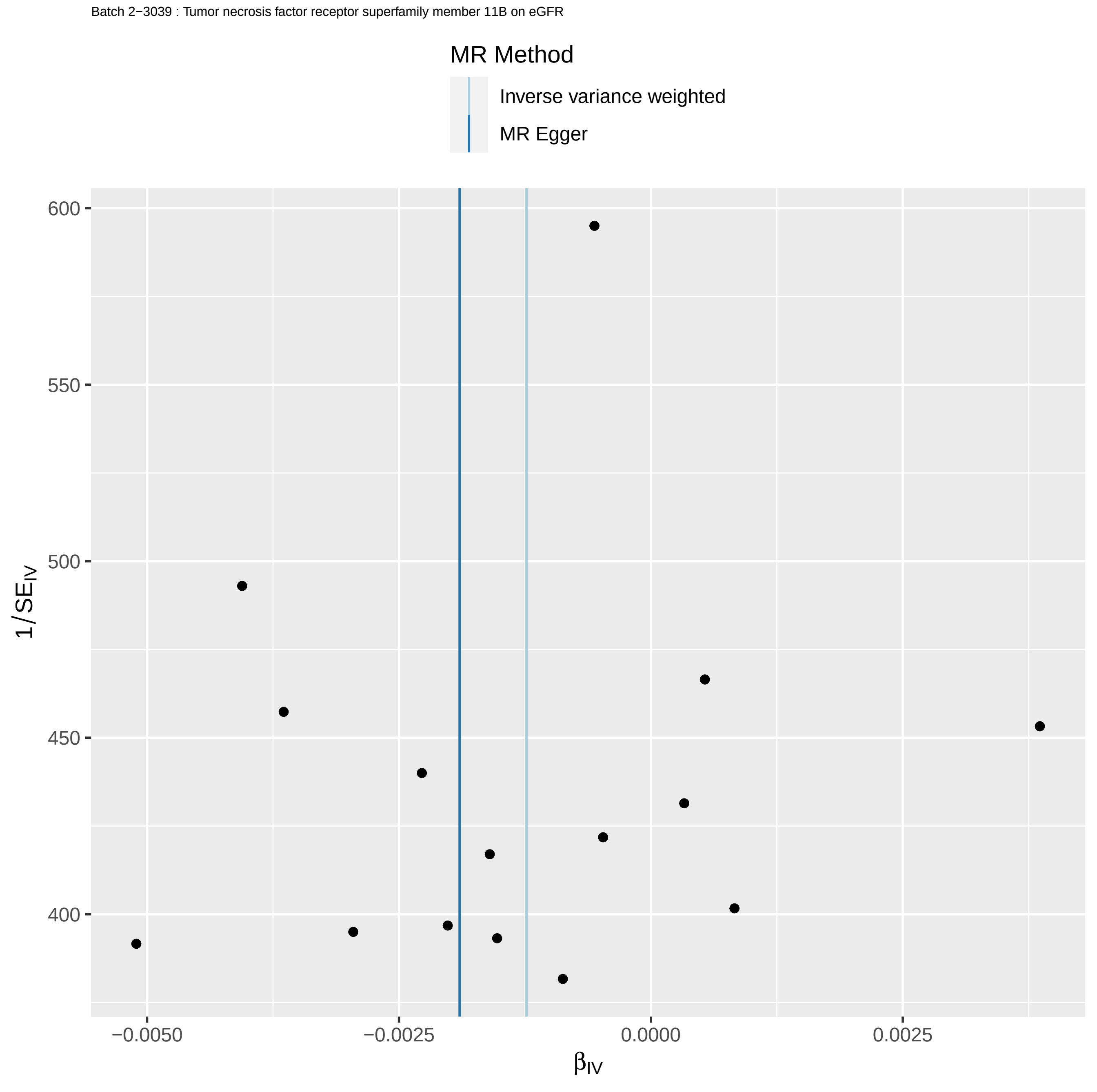
Batch 2−3039 : Tumor necrosis factor receptor superfamily member 11B on eGFR
MR Method
Inverse variance weighted
MR Egger
600
550
500
450
400
1/SE_IV
−0.0050
−0.0025
0.0000
0.0025
β_IV

Batch 2−3040 : Tumor necrosis factor receptor superfamily member 12A on eGFR

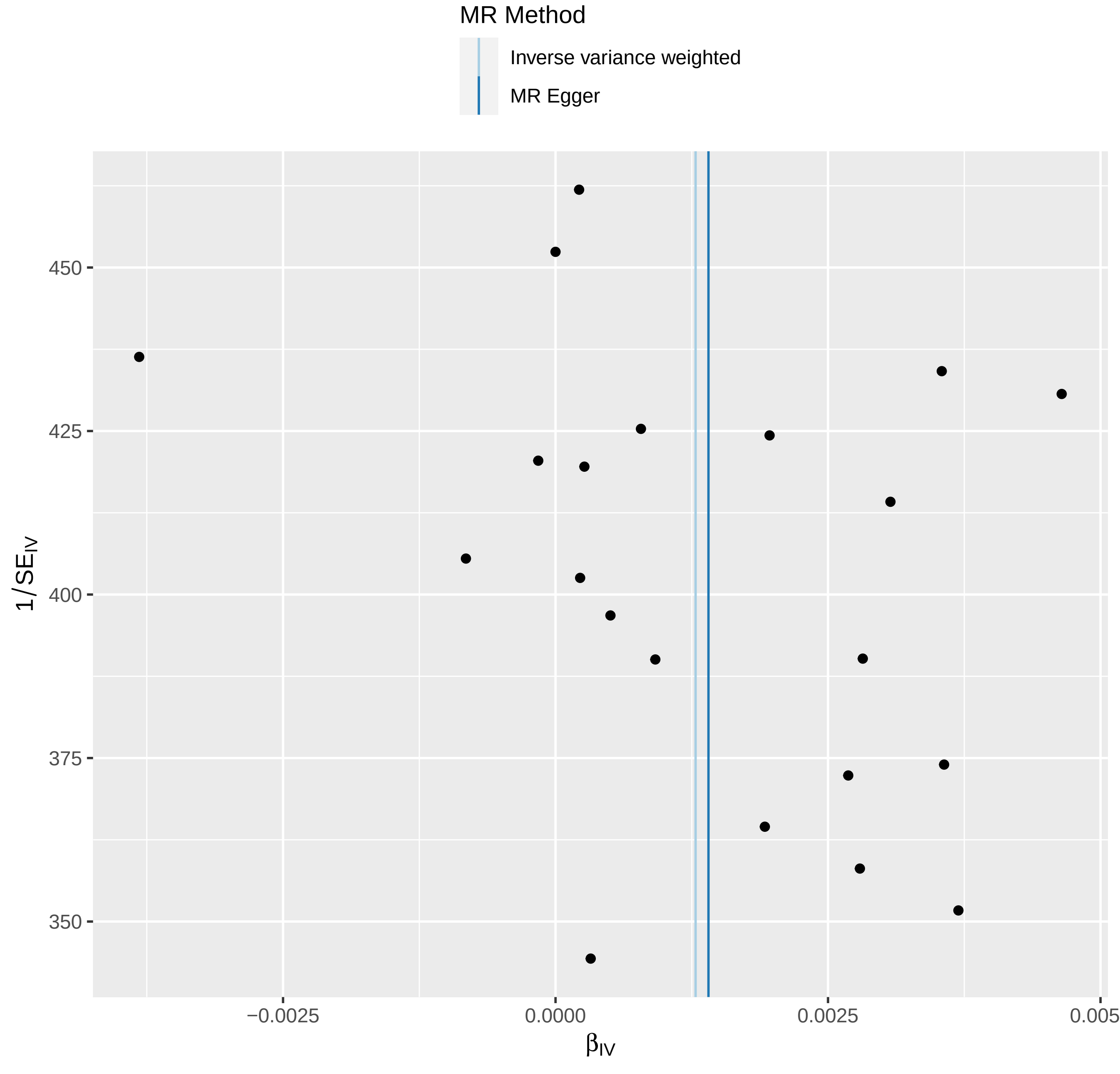

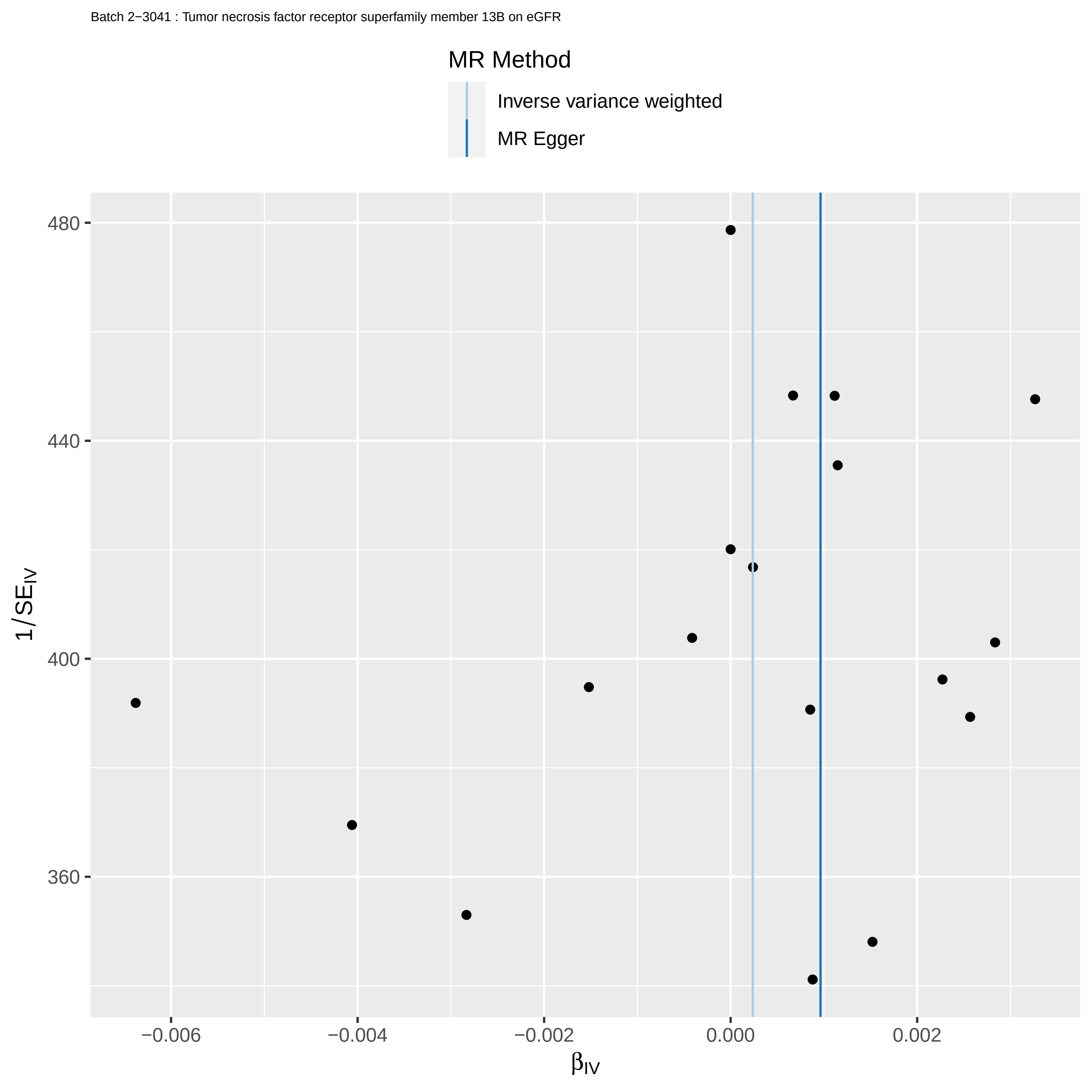

Batch 2−3041 : Tumor necrosis factor receptor superfamily member 13B on eGFR
MR Method
Inverse variance weighted
MR Egger
480
440
400
360
1/SE$_{IV}$
−0.006
−0.004
−0.002
0.000
0.002
β$_{IV}$

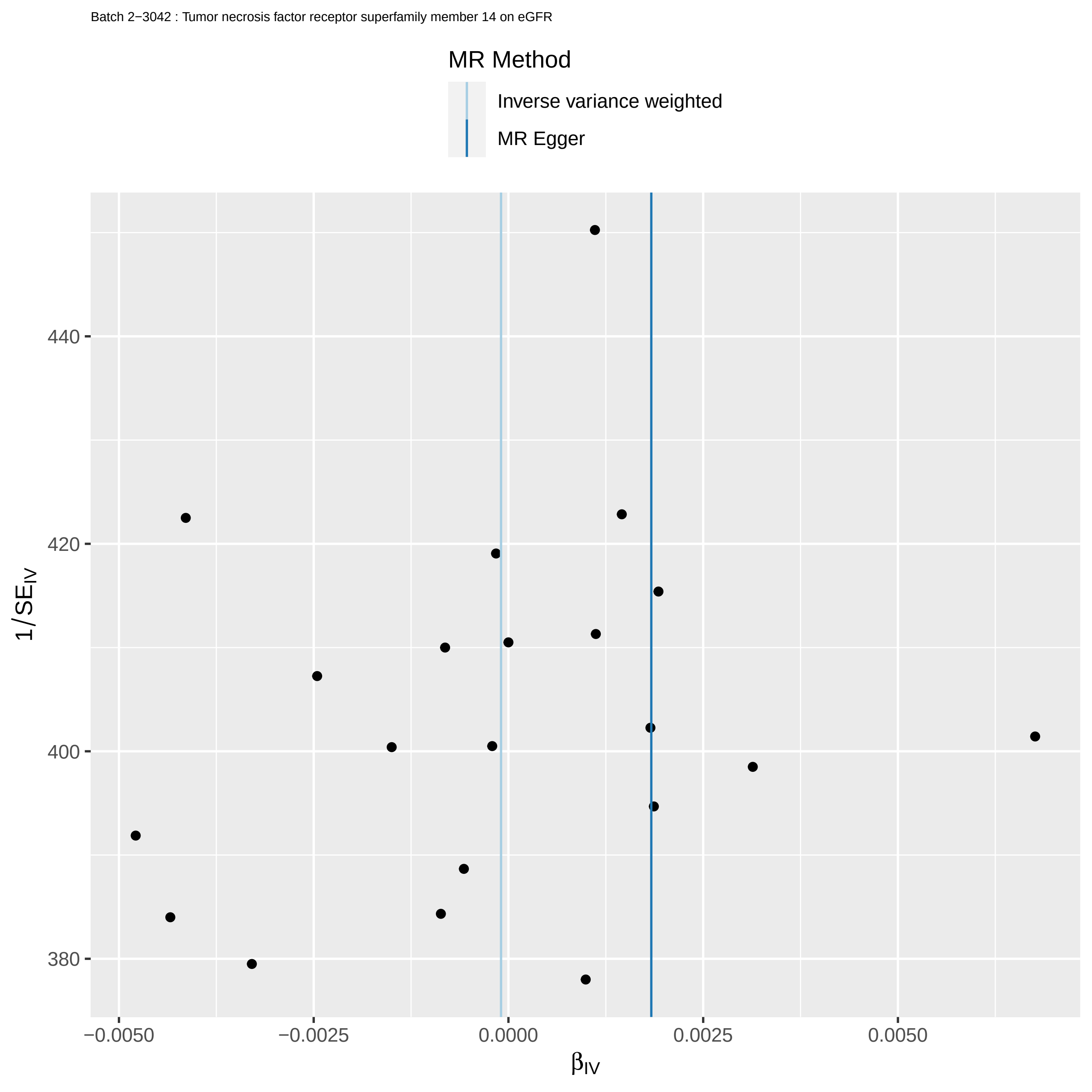
Batch 2−3042 : Tumor necrosis factor receptor superfamily member 14 on eGFR
MR Method
Inverse variance weighted
MR Egger
440
420
400
380
1/SE$_{IV}$
−0.0050
−0.0025
0.0000
0.0025
0.0050
β$_{IV}$

Batch 2−3043 : Tumor necrosis factor receptor superfamily member 17 on eGFR

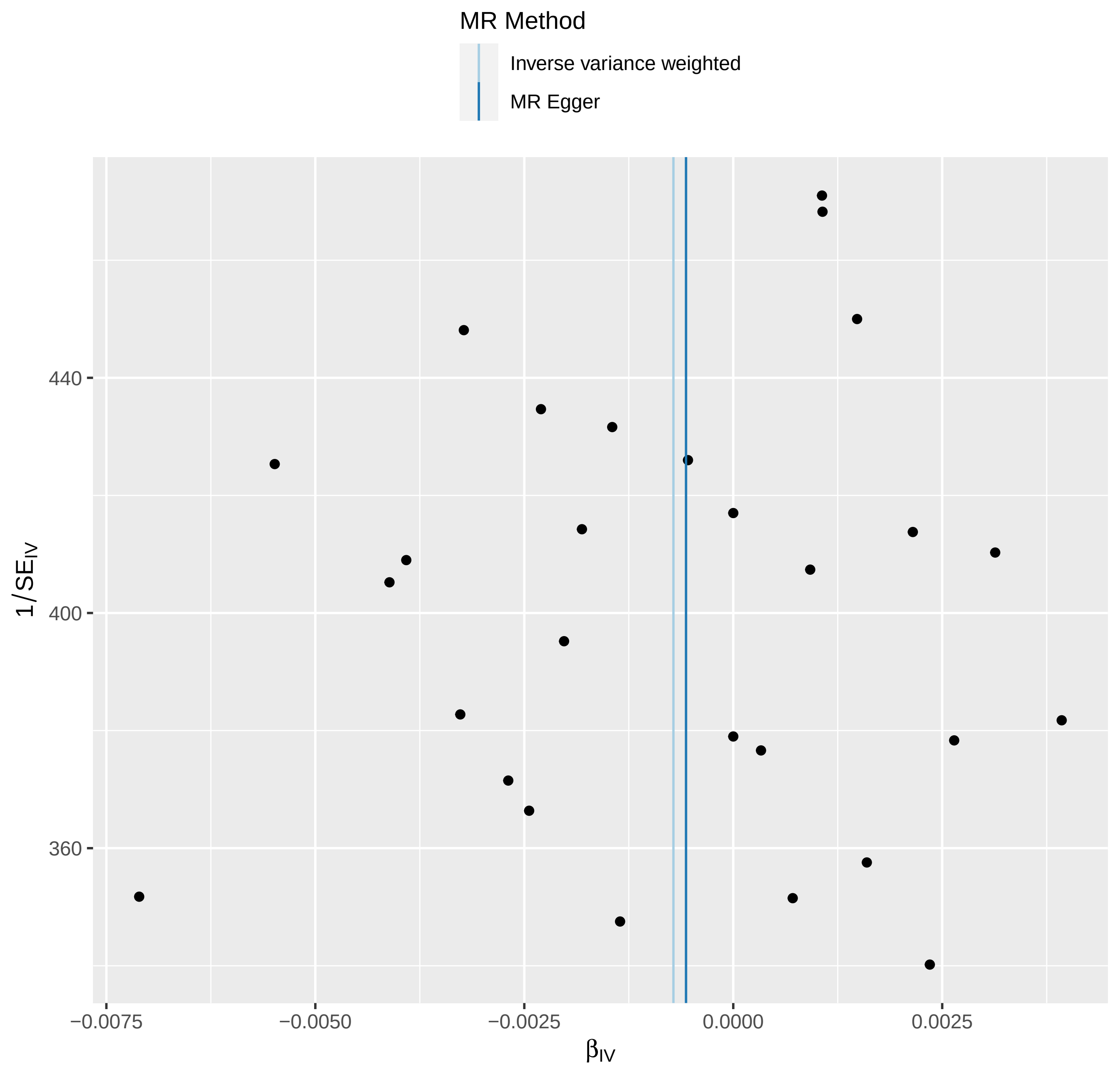

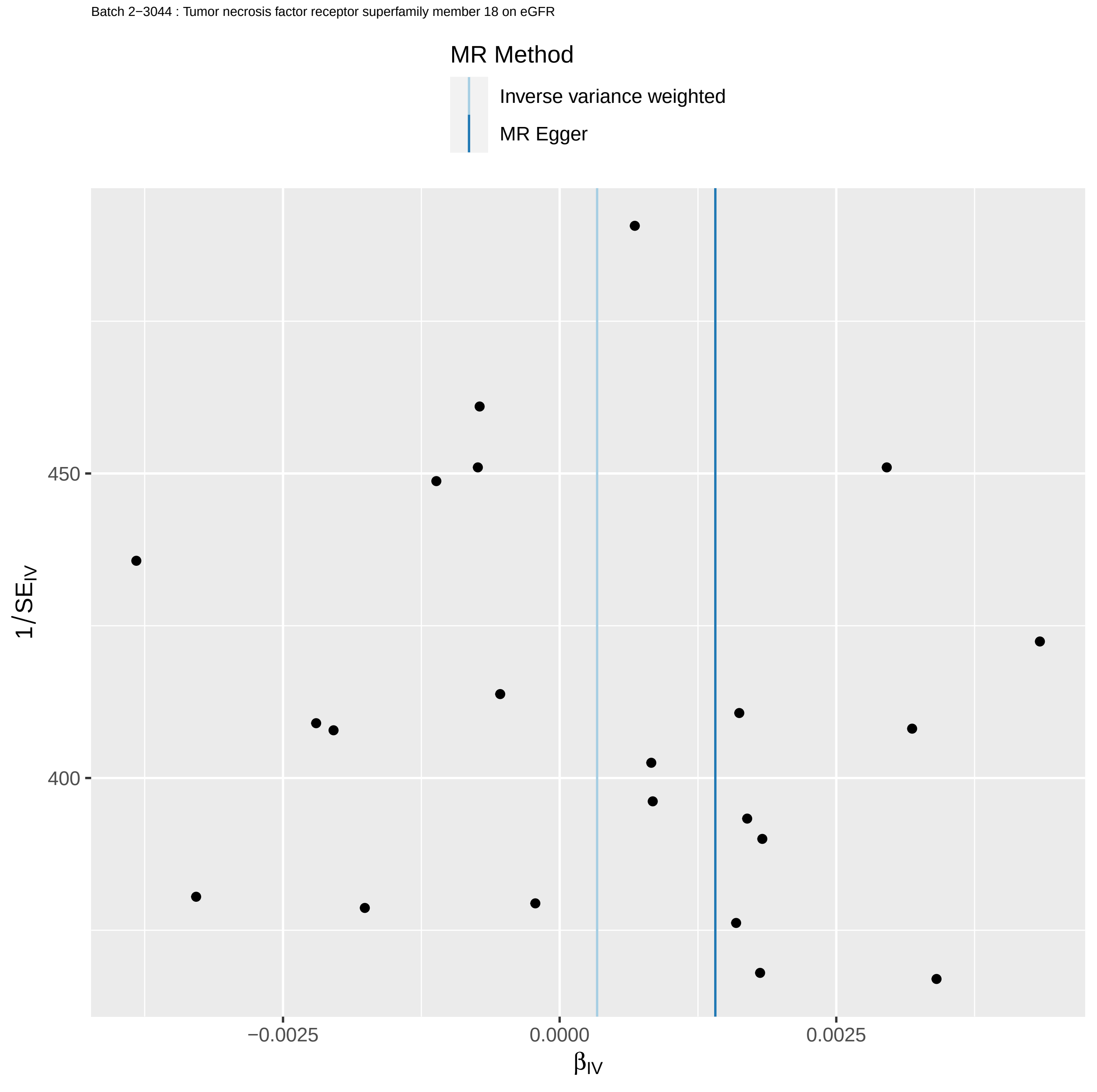
Batch 2−3044 : Tumor necrosis factor receptor superfamily member 18 on eGFR
MR Method
Inverse variance weighted
MR Egger
450
400
1/SE_IV
−0.0025
0.0000
0.0025
β_IV

Batch 2−3045 : Tumor necrosis factor receptor superfamily member 19 on eGFR

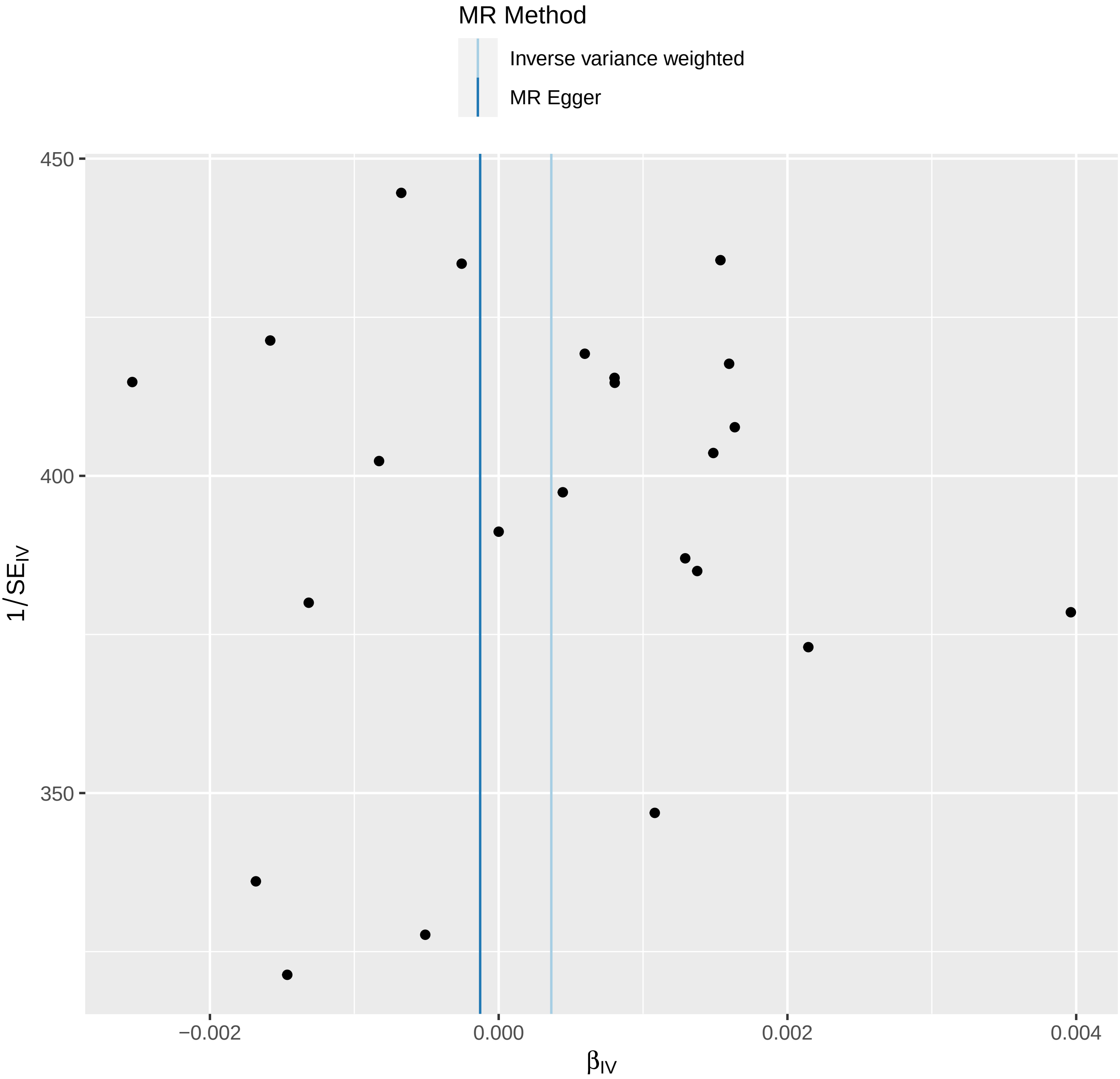

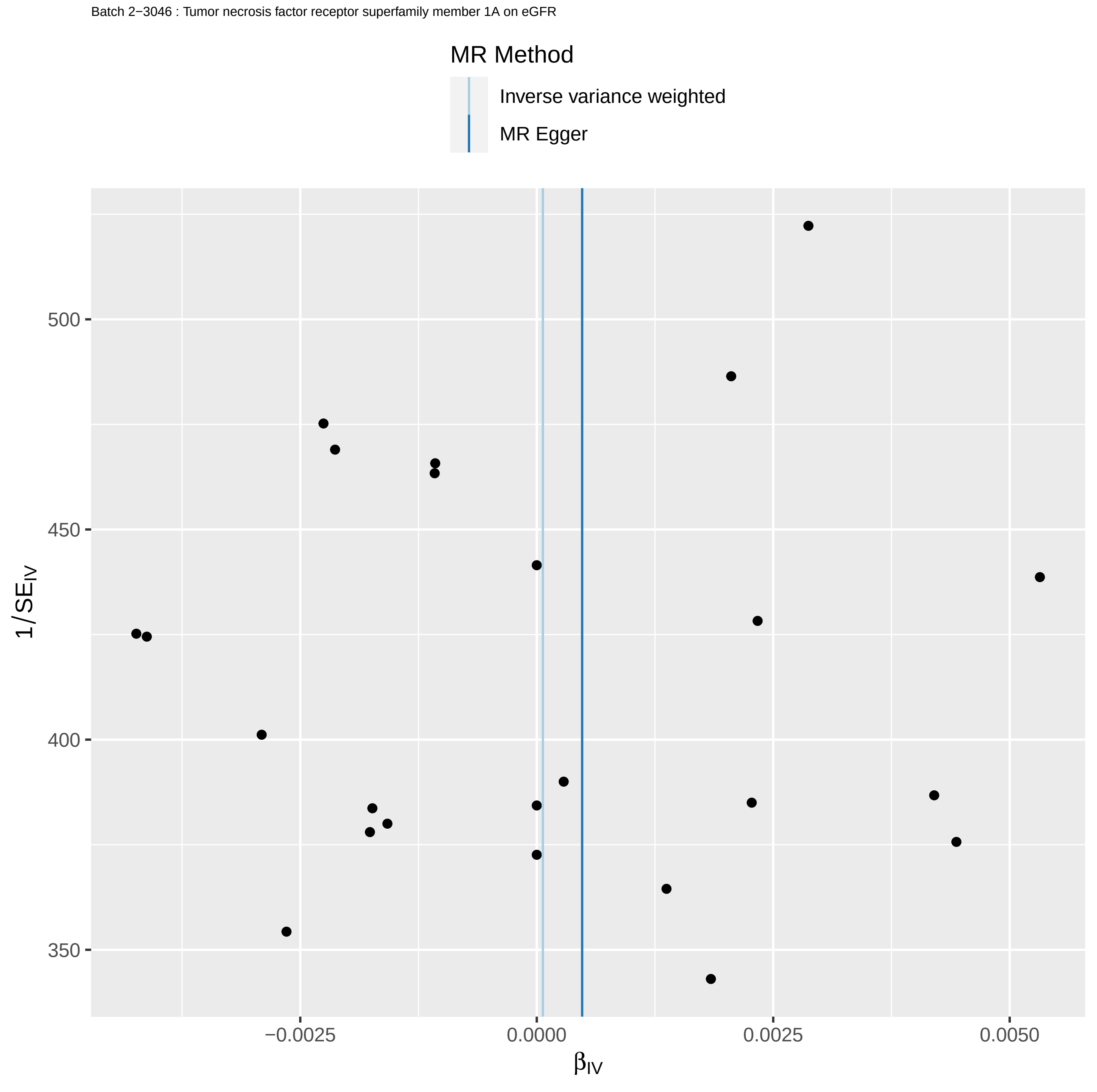
Batch 2−3046 : Tumor necrosis factor receptor superfamily member 1A on eGFR
MR Method
Inverse variance weighted
MR Egger
500
450
400
350
1/SE_IV
−0.0025
0.0000
0.0025
0.0050
β_IV

Batch 2−3047 : Tumor necrosis factor receptor superfamily member 1B on eGFR

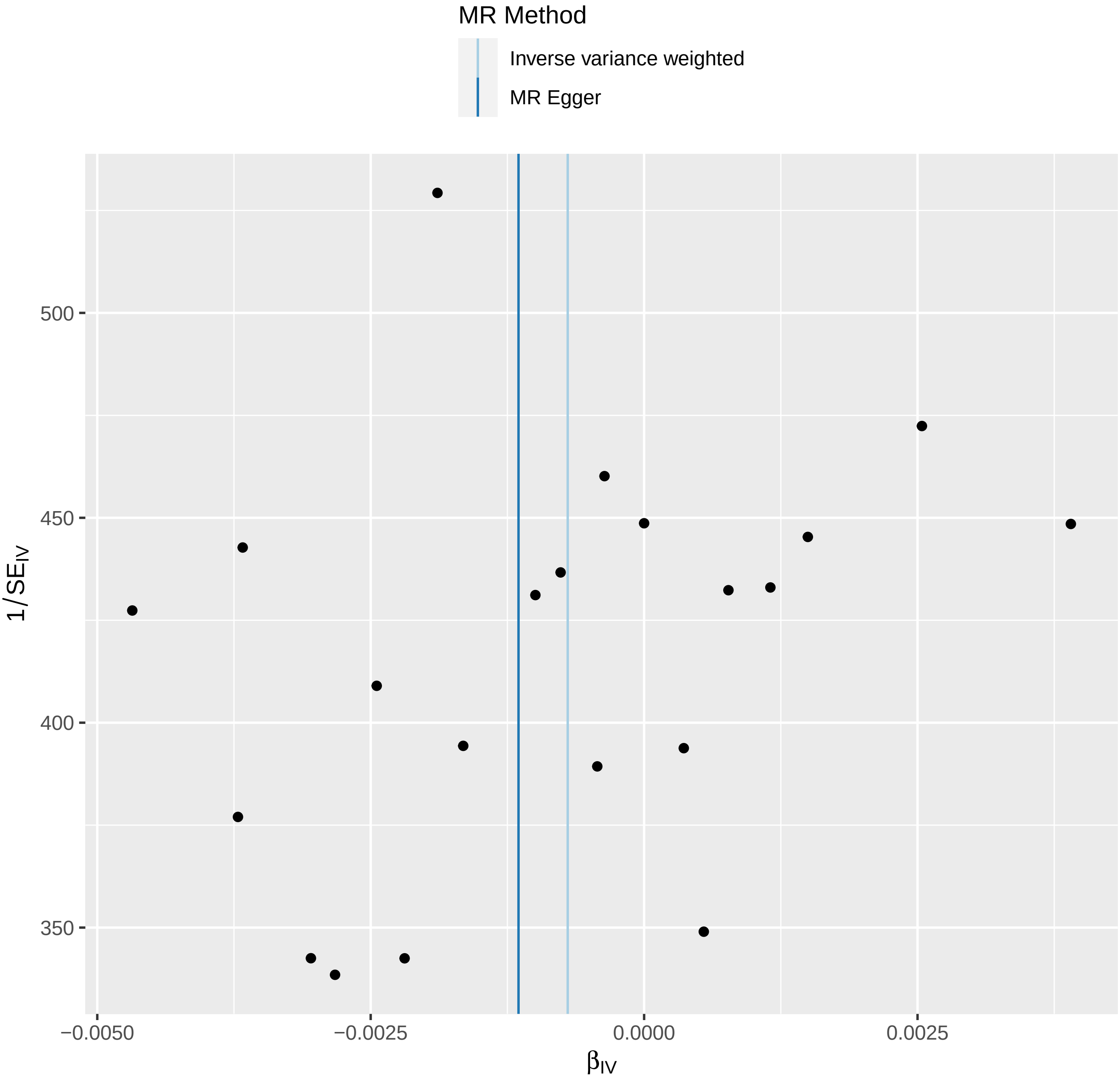

Batch 2−3048 : Tumor necrosis factor receptor superfamily member 1B on eGFR

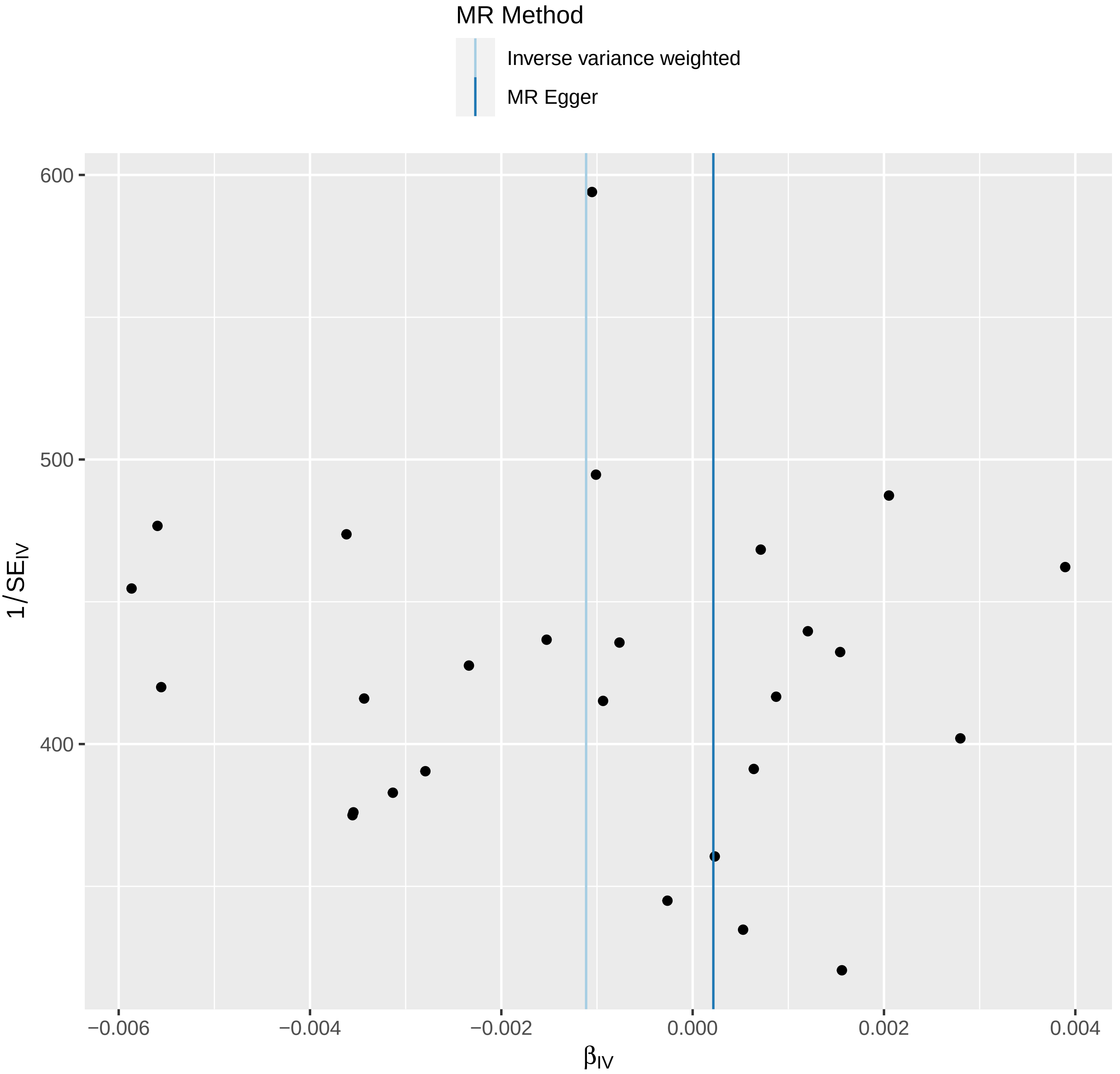

Batch 2−3049 : Tumor necrosis factor receptor superfamily member 21 on eGFR

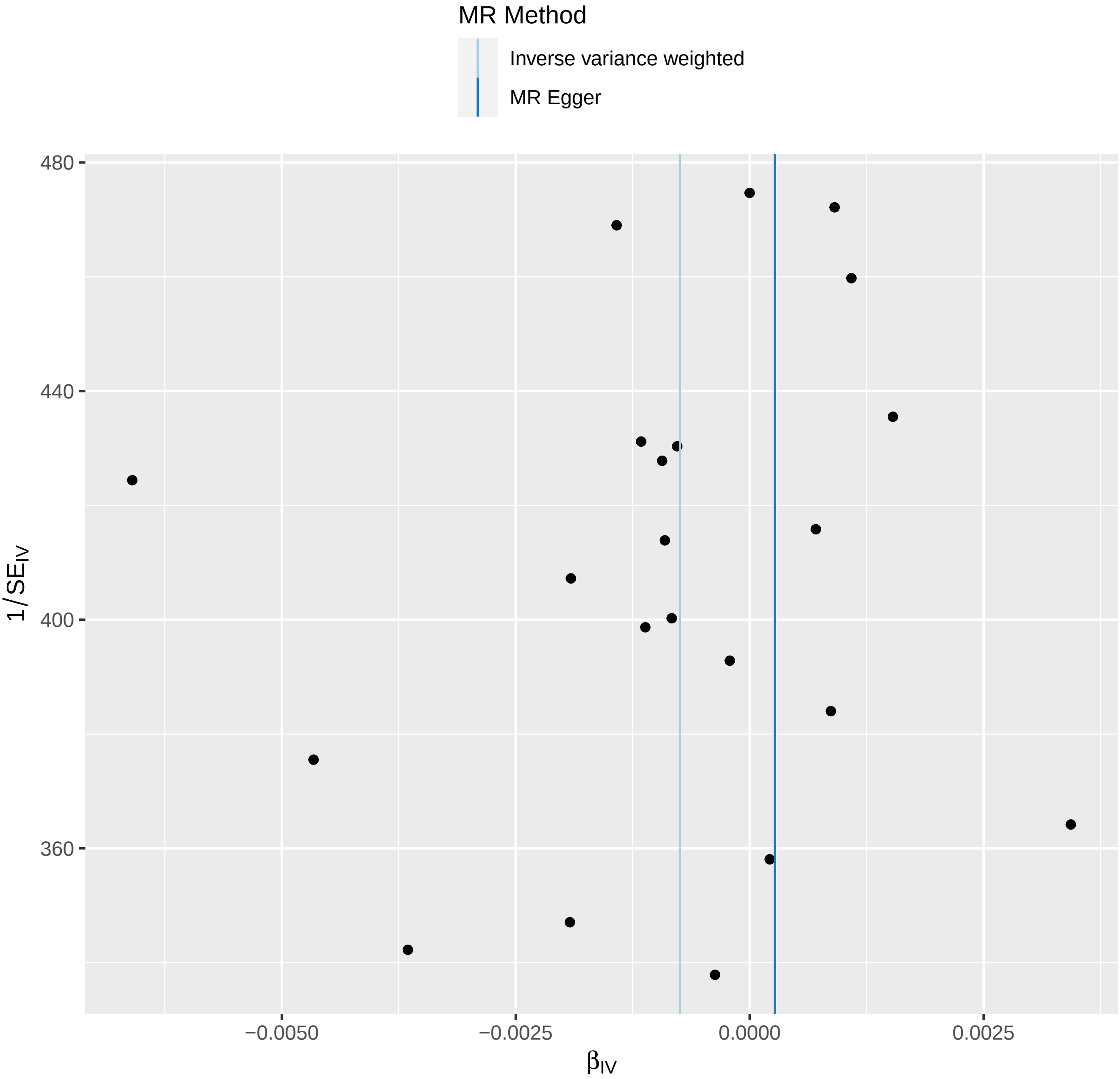

Batch 2−3050 : Tumor necrosis factor receptor superfamily member 21 on eGFR

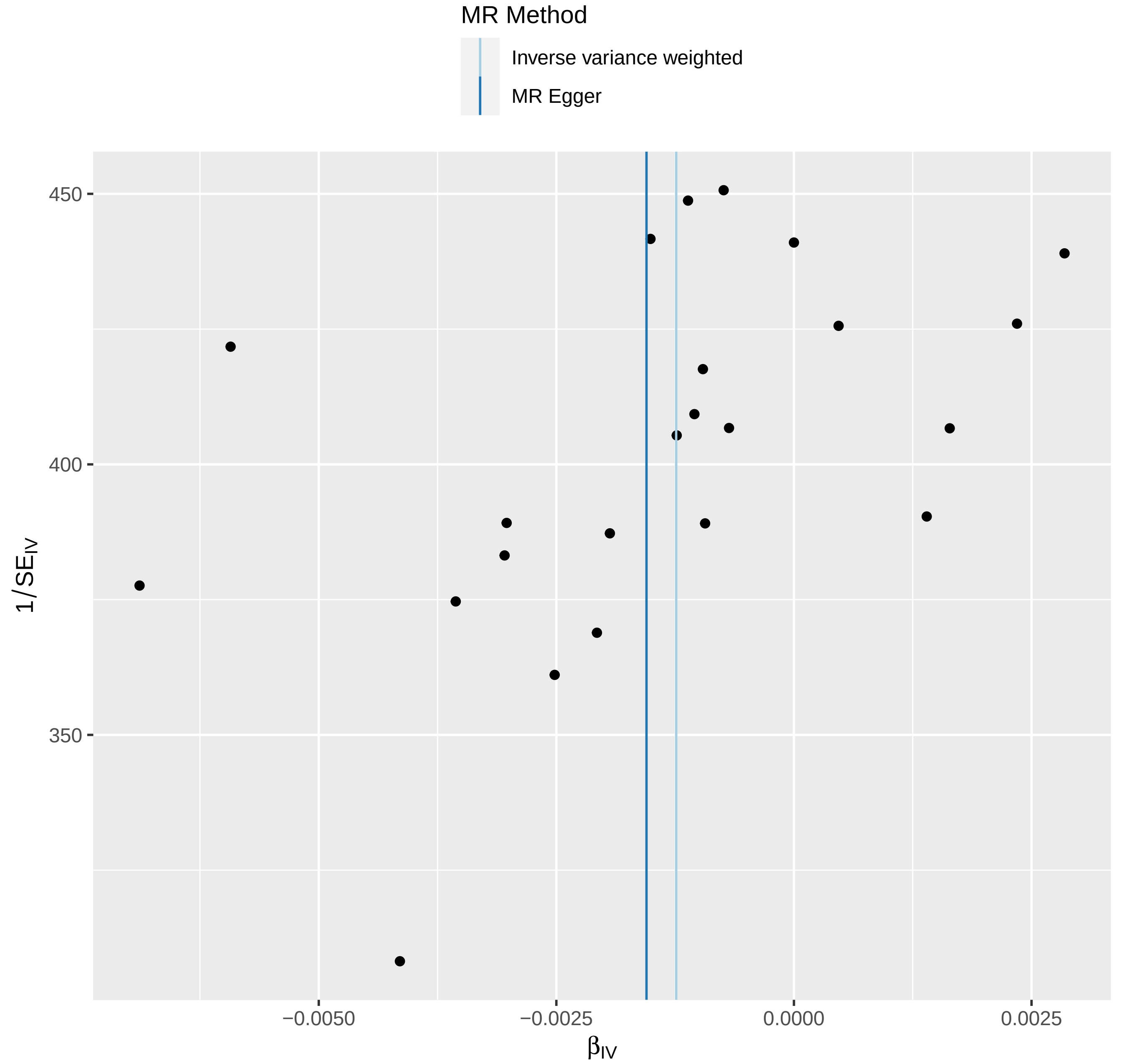

Batch 2−3051 : Tumor necrosis factor receptor superfamily member 4 on eGFR

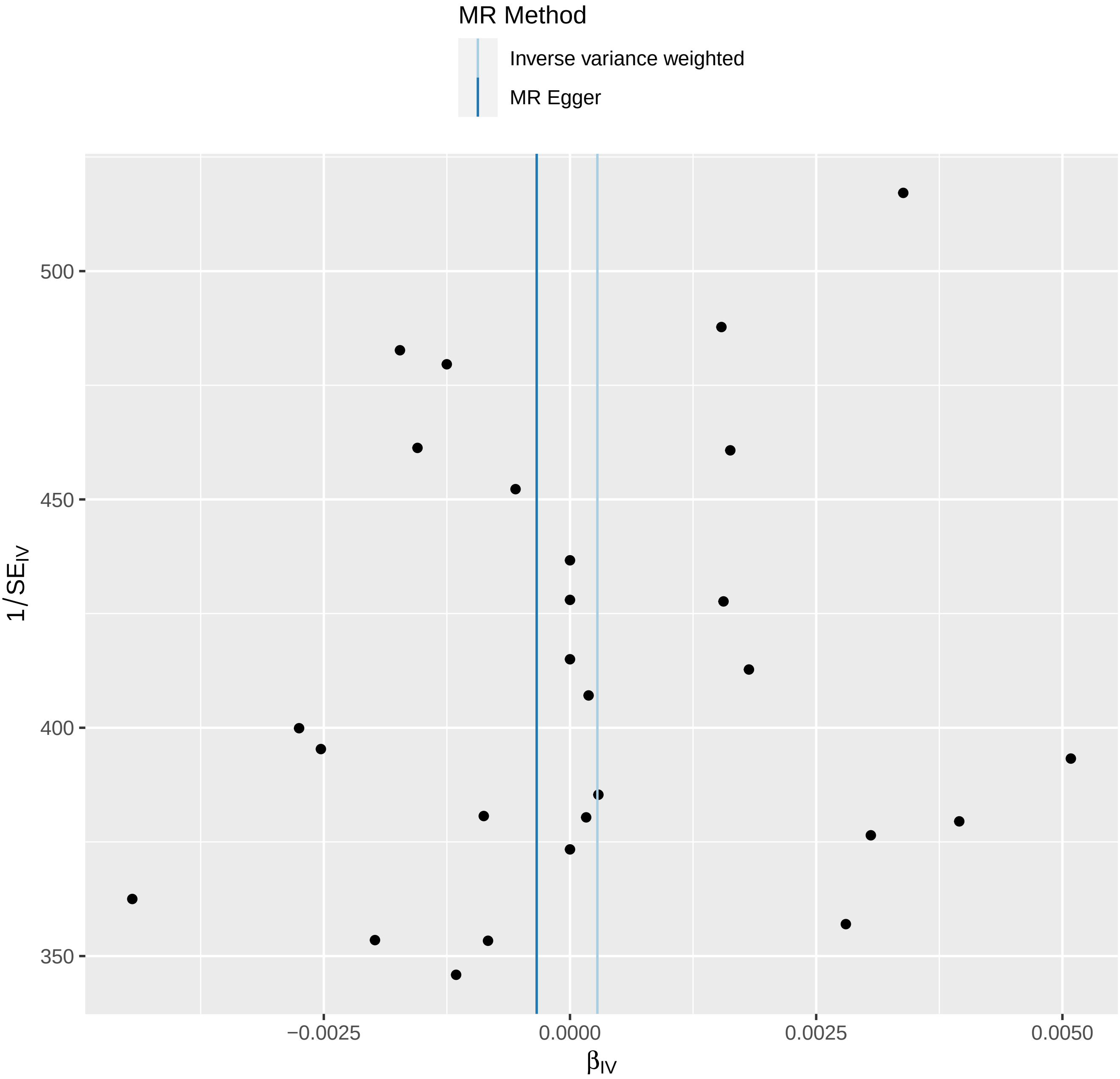

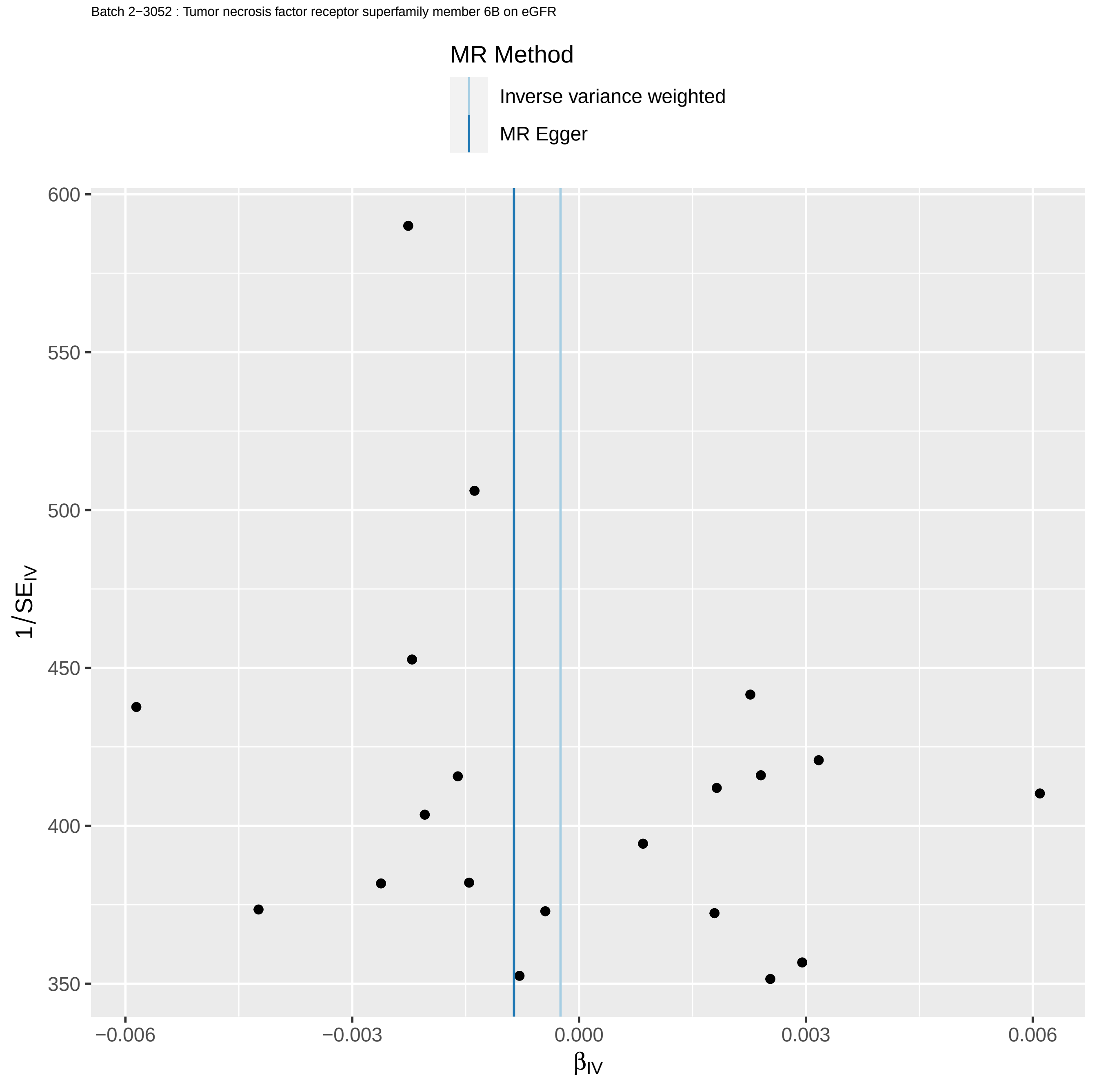
Batch 2−3052 : Tumor necrosis factor receptor superfamily member 6B on eGFR
MR Method
Inverse variance weighted
MR Egger
600
550
500
450
400
350
1/SE_IV
−0.006
−0.003
0.000
0.003
0.006
β_IV

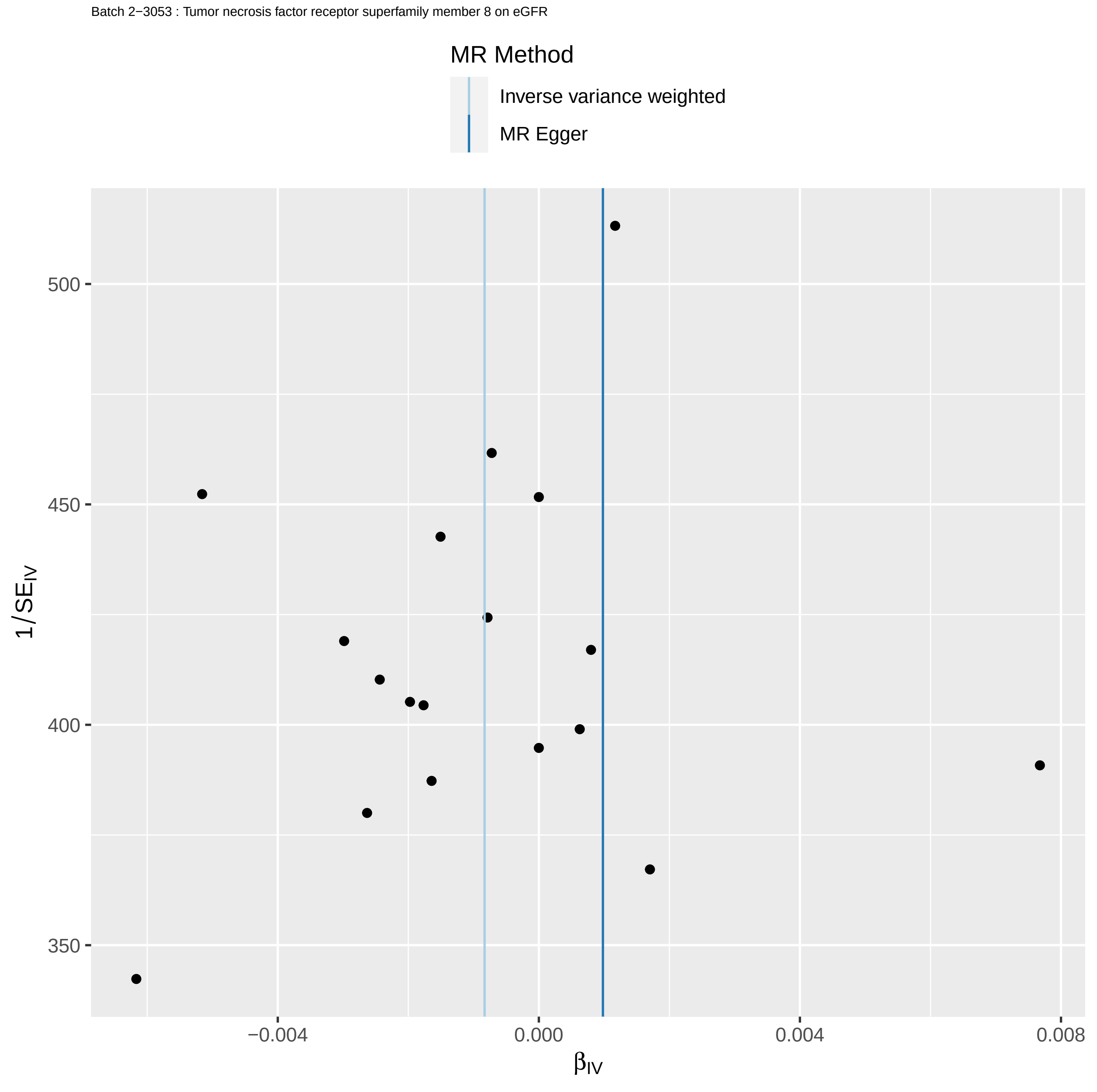
Batch 2−3053 : Tumor necrosis factor receptor superfamily member 8 on eGFR
MR Method
Inverse variance weighted
MR Egger
500
450
400
350
1/SE_IV
−0.004
0.000
0.004
0.008
β_IV

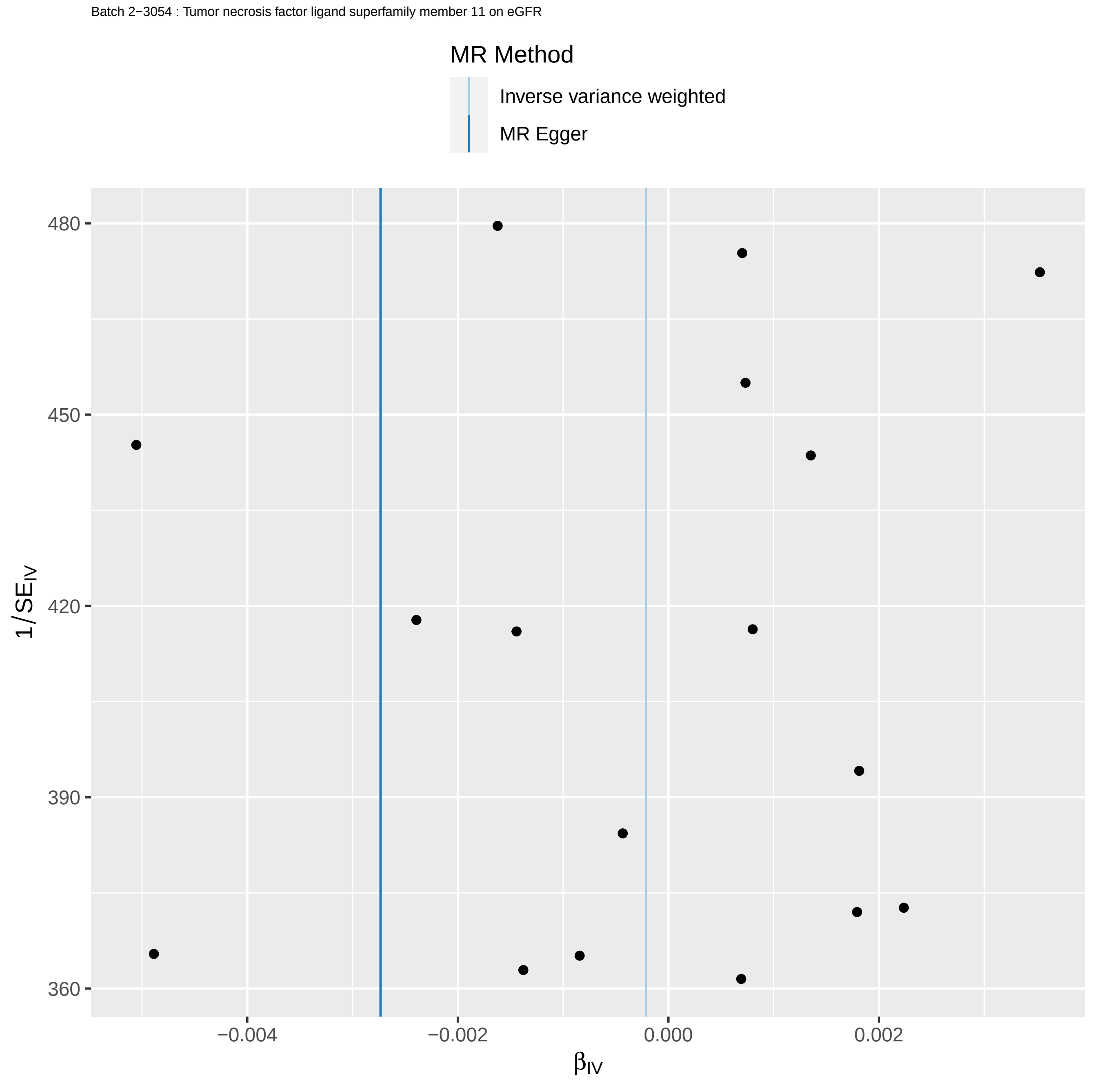
Batch 2−3054 : Tumor necrosis factor ligand superfamily member 11 on eGFR
MR Method
Inverse variance weighted
MR Egger
480
450
420
390
360
1/SE_IV
−0.004
−0.002
0.000
0.002
β_IV

Batch 2−3055 : Tumor necrosis factor ligand superfamily member 12 on eGFR

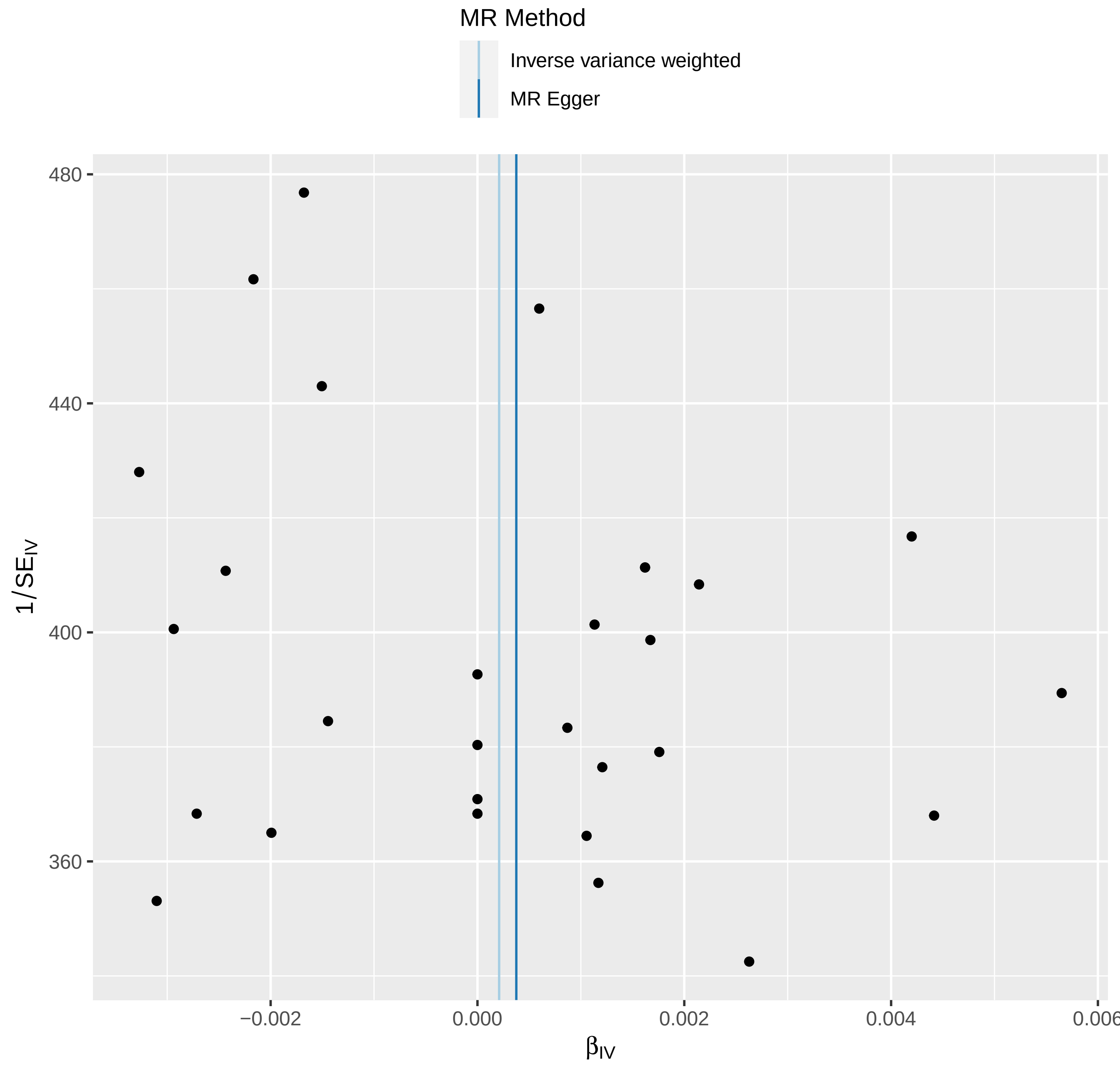

Batch 2−3056 : Tumor necrosis factor ligand superfamily member 13B on eGFR

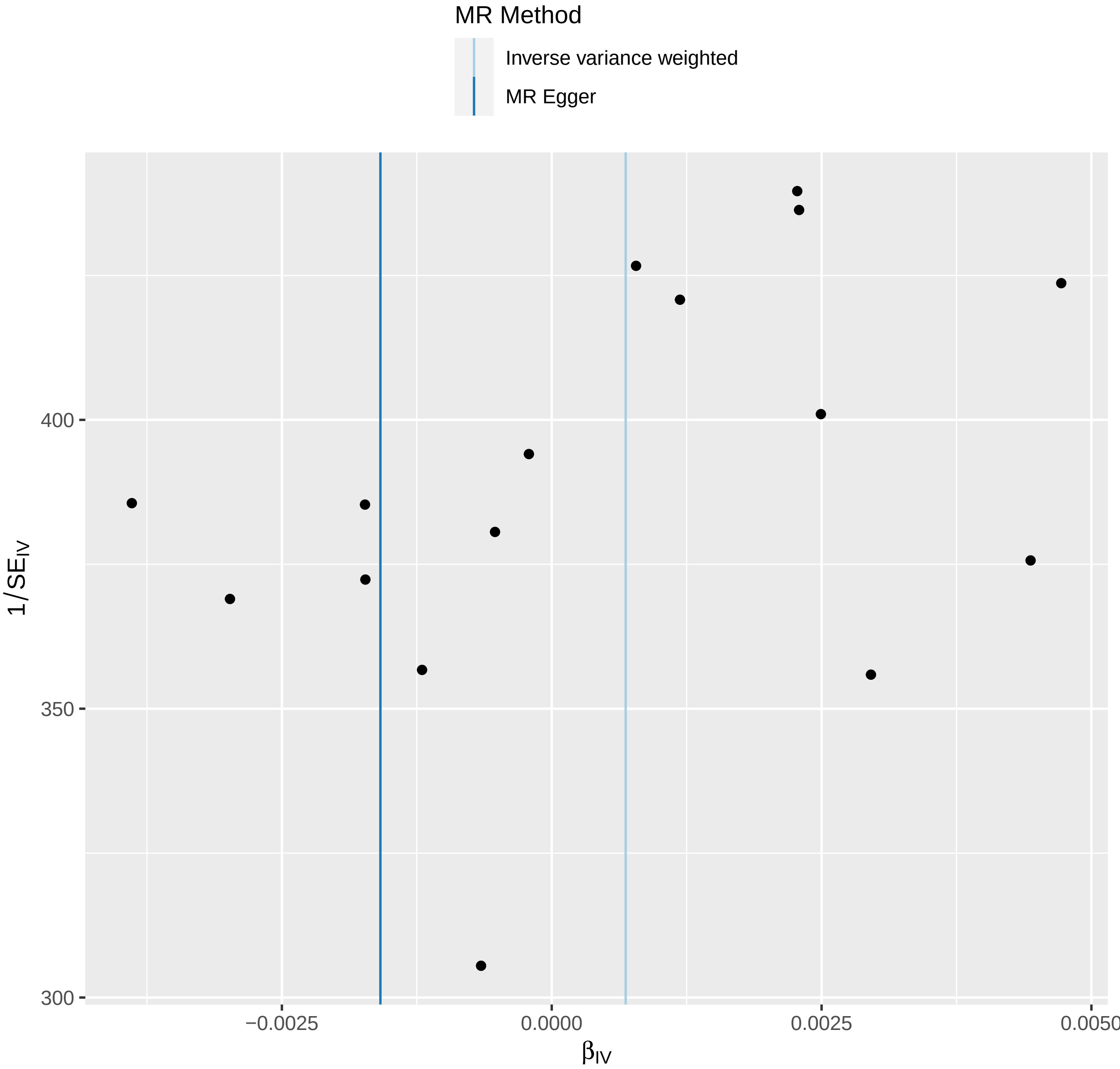

Batch 2−3057 : Tumor necrosis factor ligand superfamily member 14 on eGFR

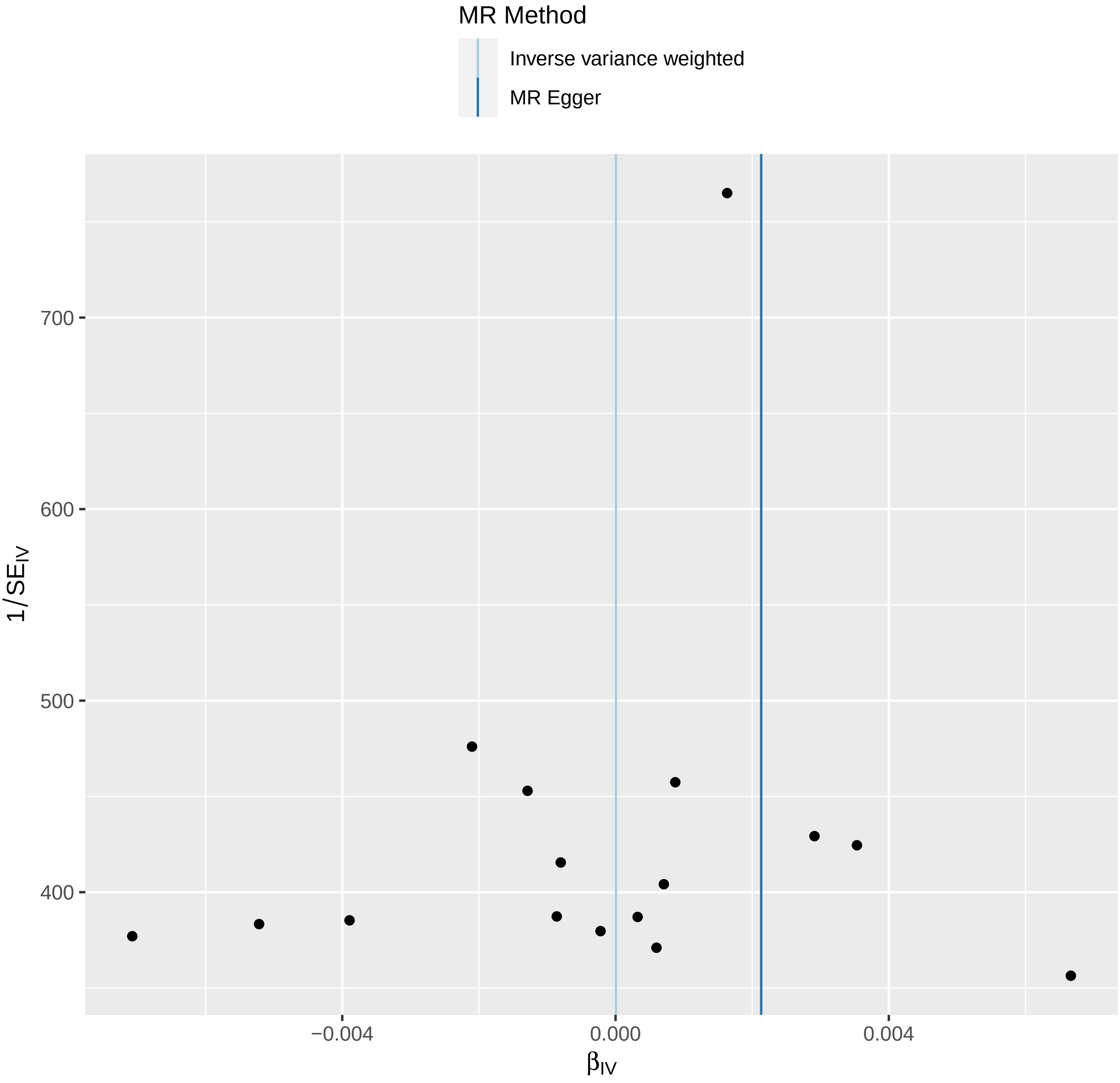

Batch 2−3058 : Tumor necrosis factor ligand superfamily member 15 on eGFR

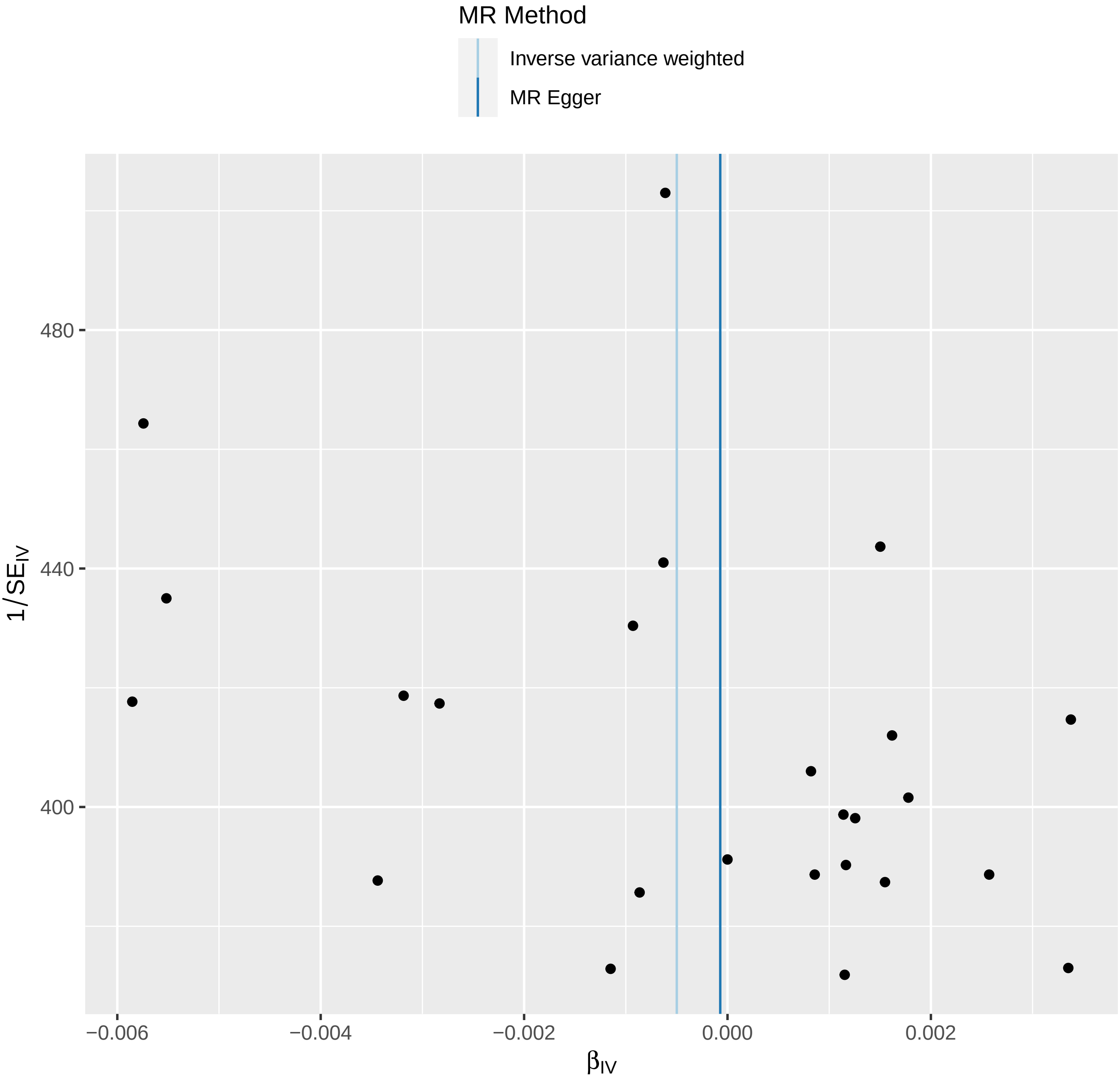

Batch 2−3059 : Tumor necrosis factor ligand superfamily member 15 on eGFR

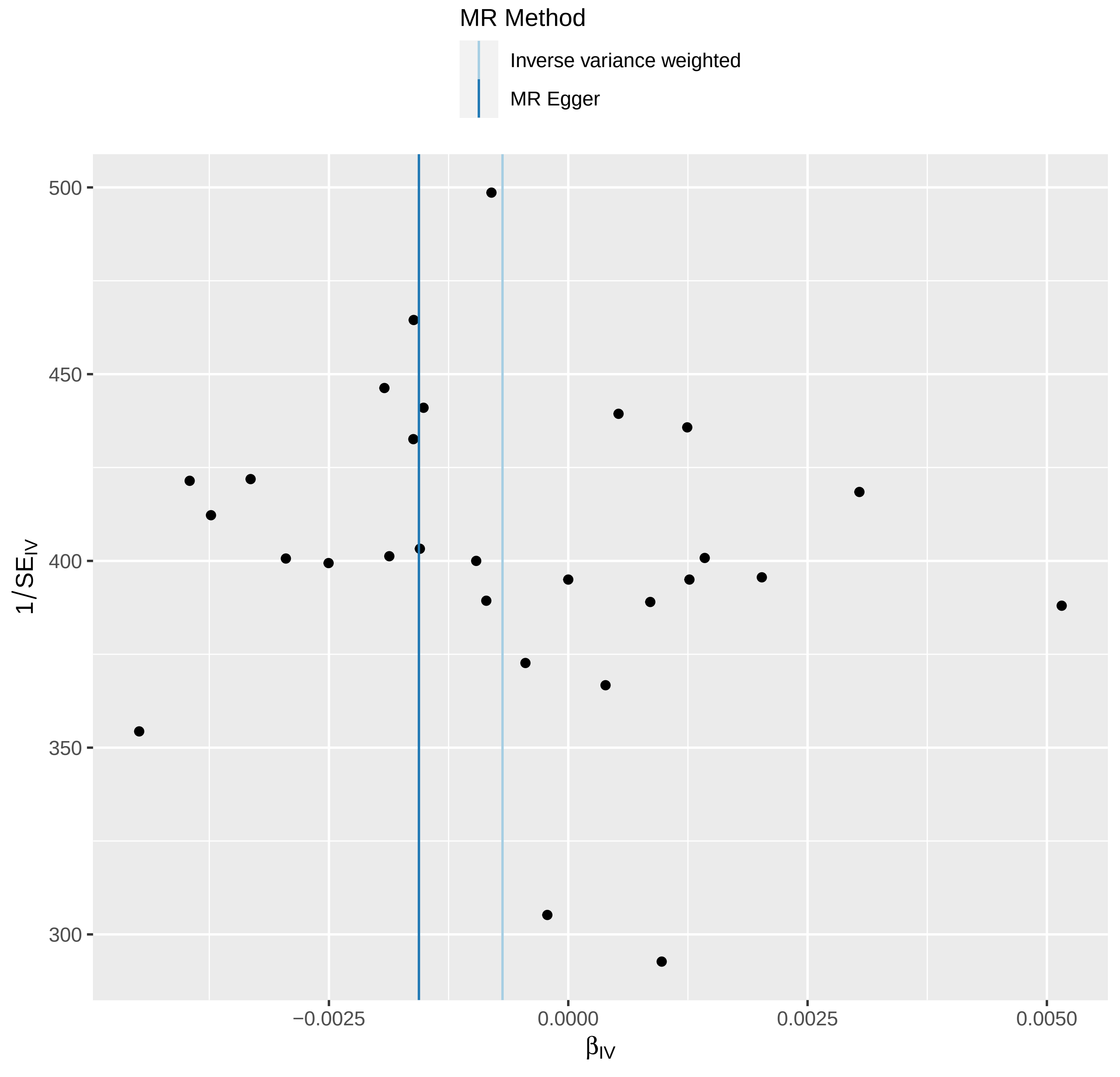

Batch 2−3060 : Tumor necrosis factor ligand superfamily member 18 on eGFR

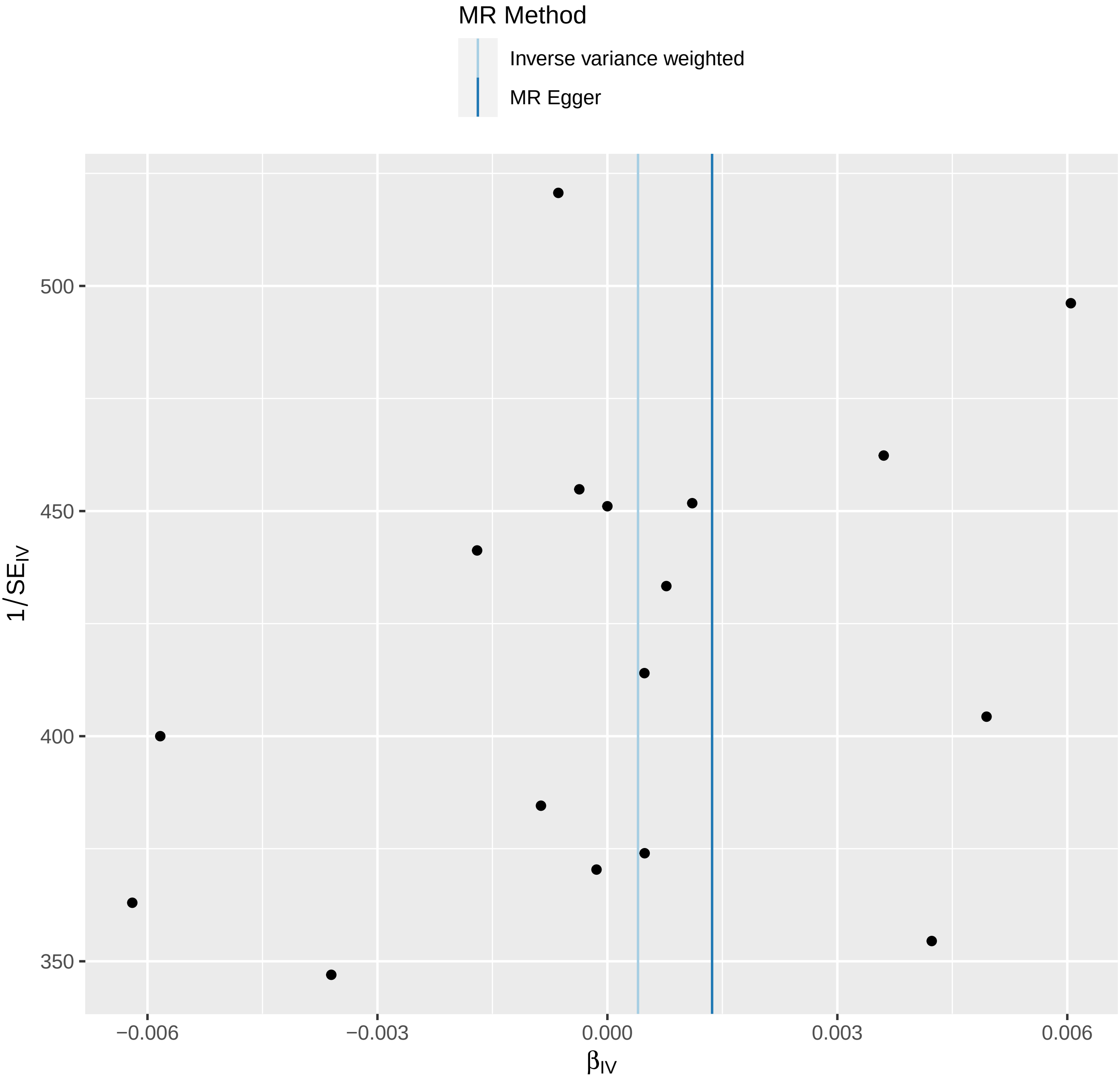

Batch 2−3061 : Tumor necrosis factor ligand superfamily member 4 on eGFR

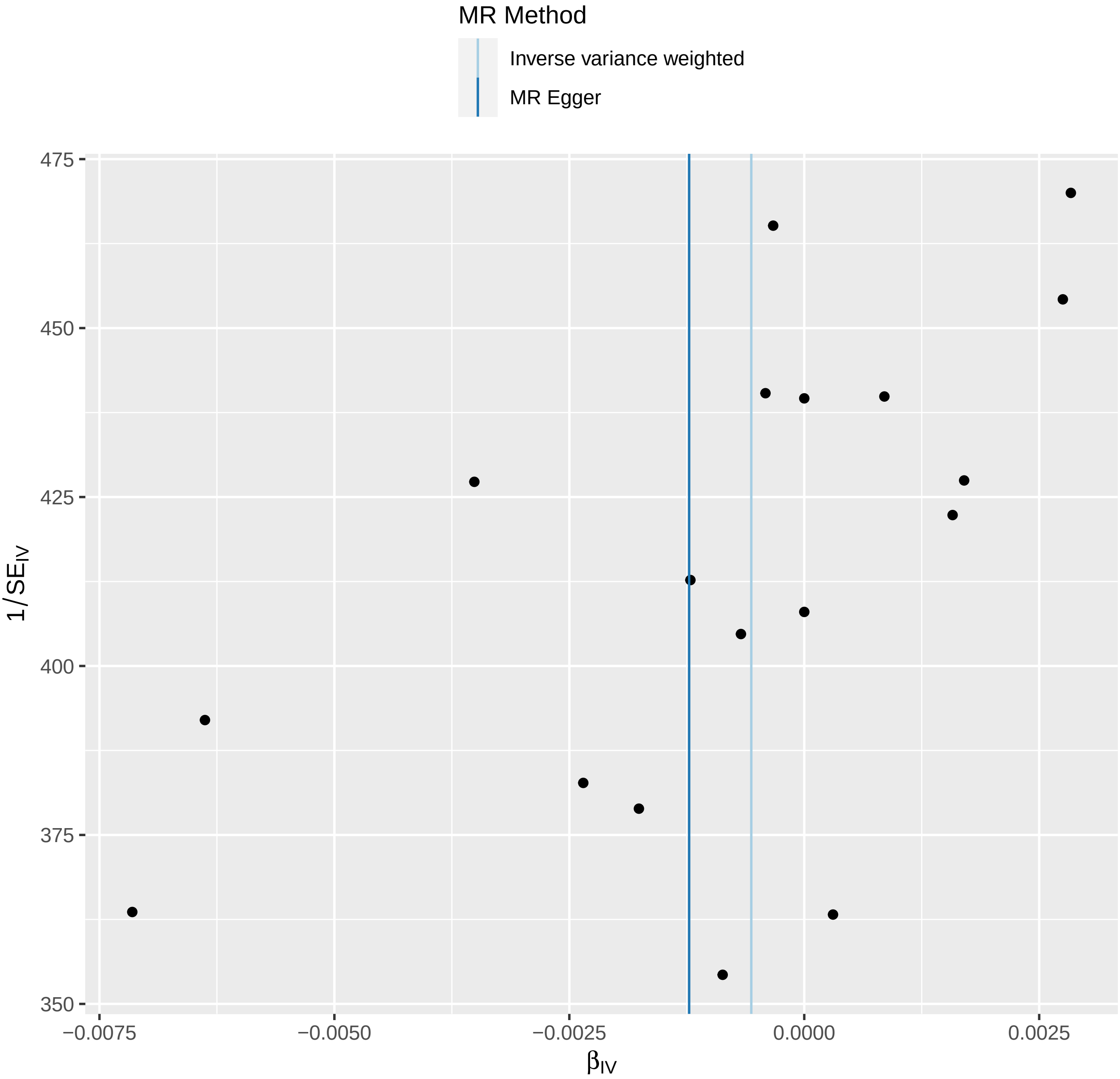

Batch 2−3062 : Tumor necrosis factor ligand superfamily member 4 on eGFR

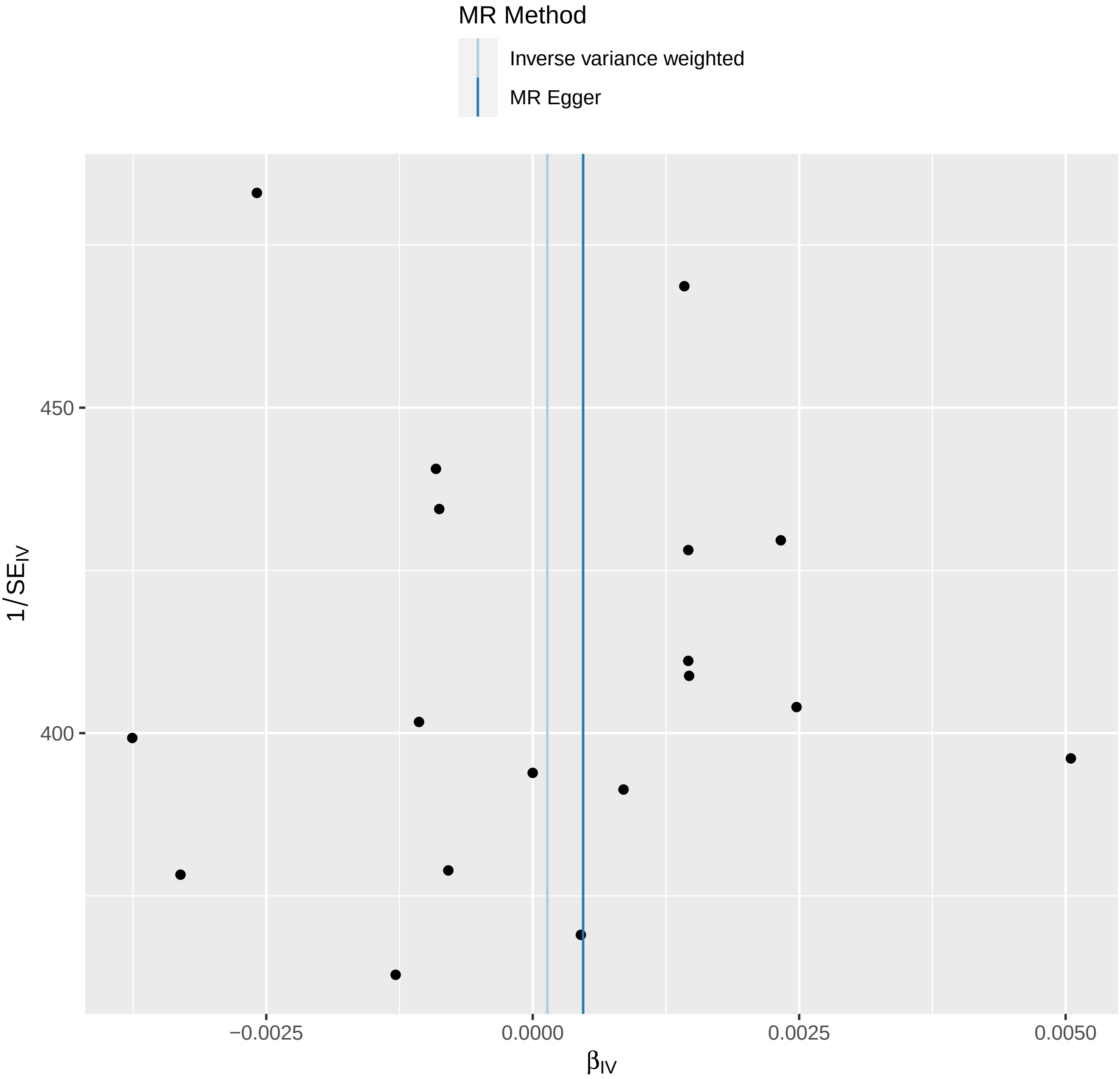

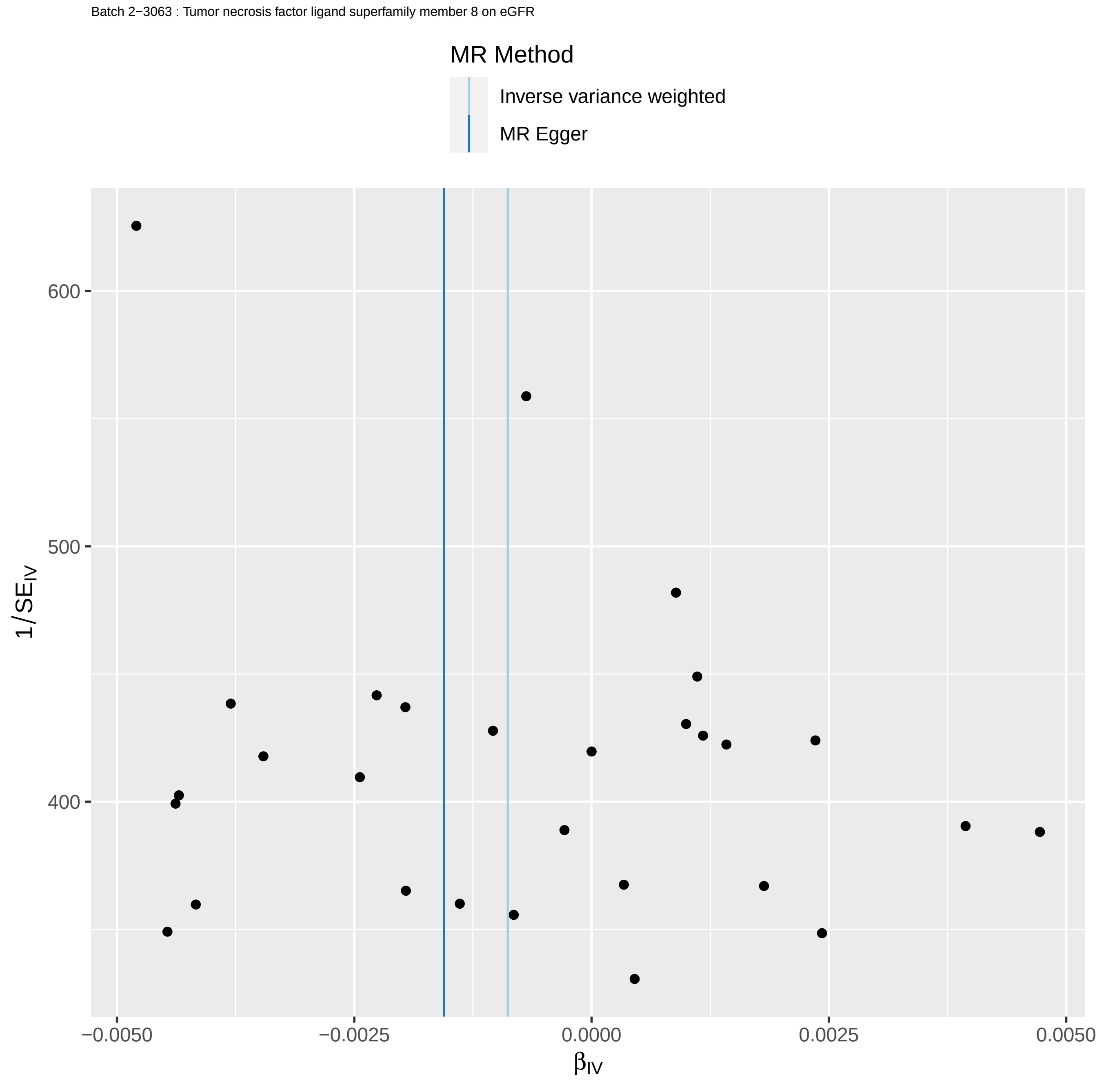
Batch 2−3063 : Tumor necrosis factor ligand superfamily member 8 on eGFR
MR Method
Inverse variance weighted
MR Egger
600
500
400
1/SE$_{IV}$
−0.0050
−0.0025
0.0000
0.0025
0.0050
β_{IV}

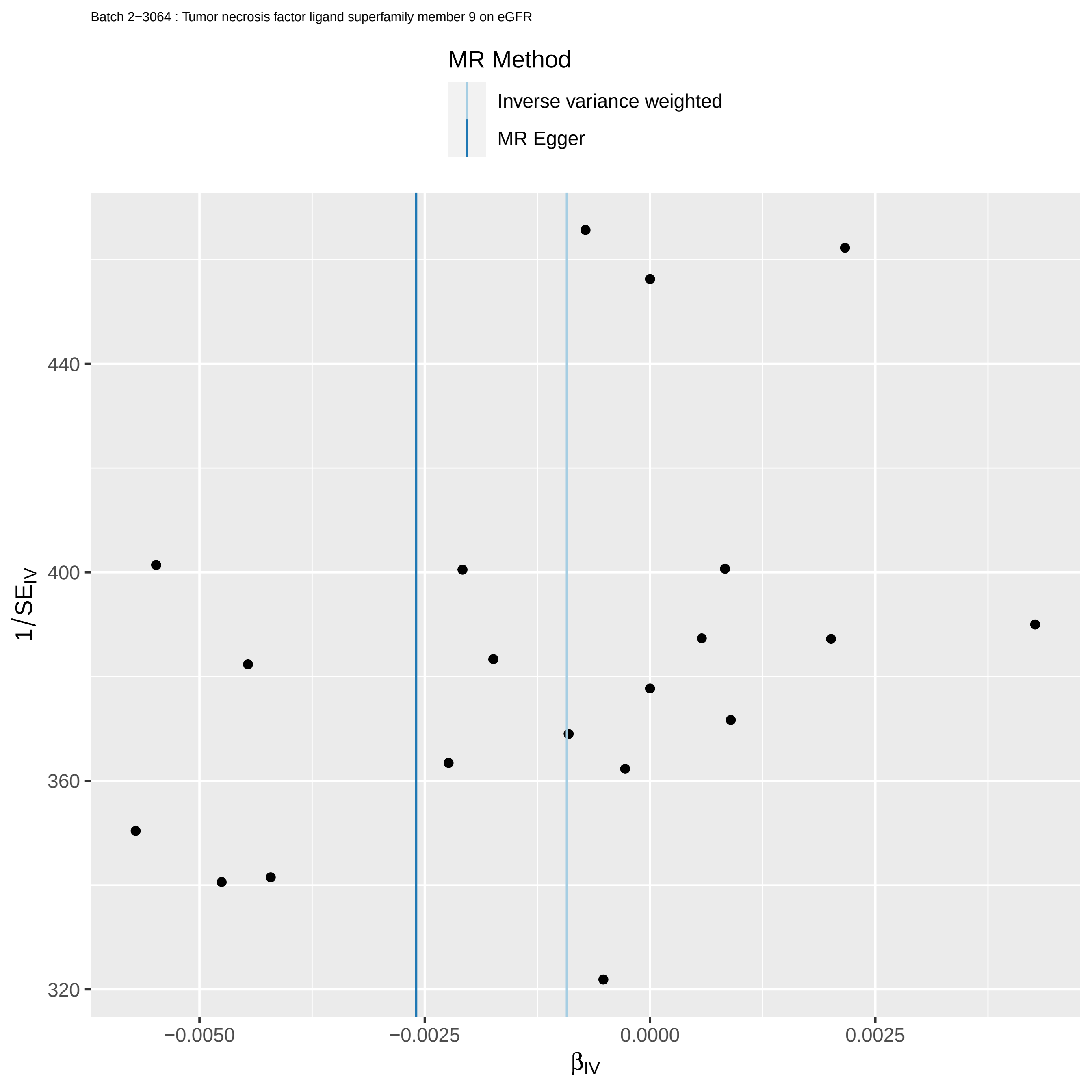
Batch 2−3064 : Tumor necrosis factor ligand superfamily member 9 on eGFR
MR Method
Inverse variance weighted
MR Egger
440
400
360
320
1/SE_IV
−0.0050
−0.0025
0.0000
0.0025
β_IV

Batch 2−3065 : Tenomodulin on eGFR

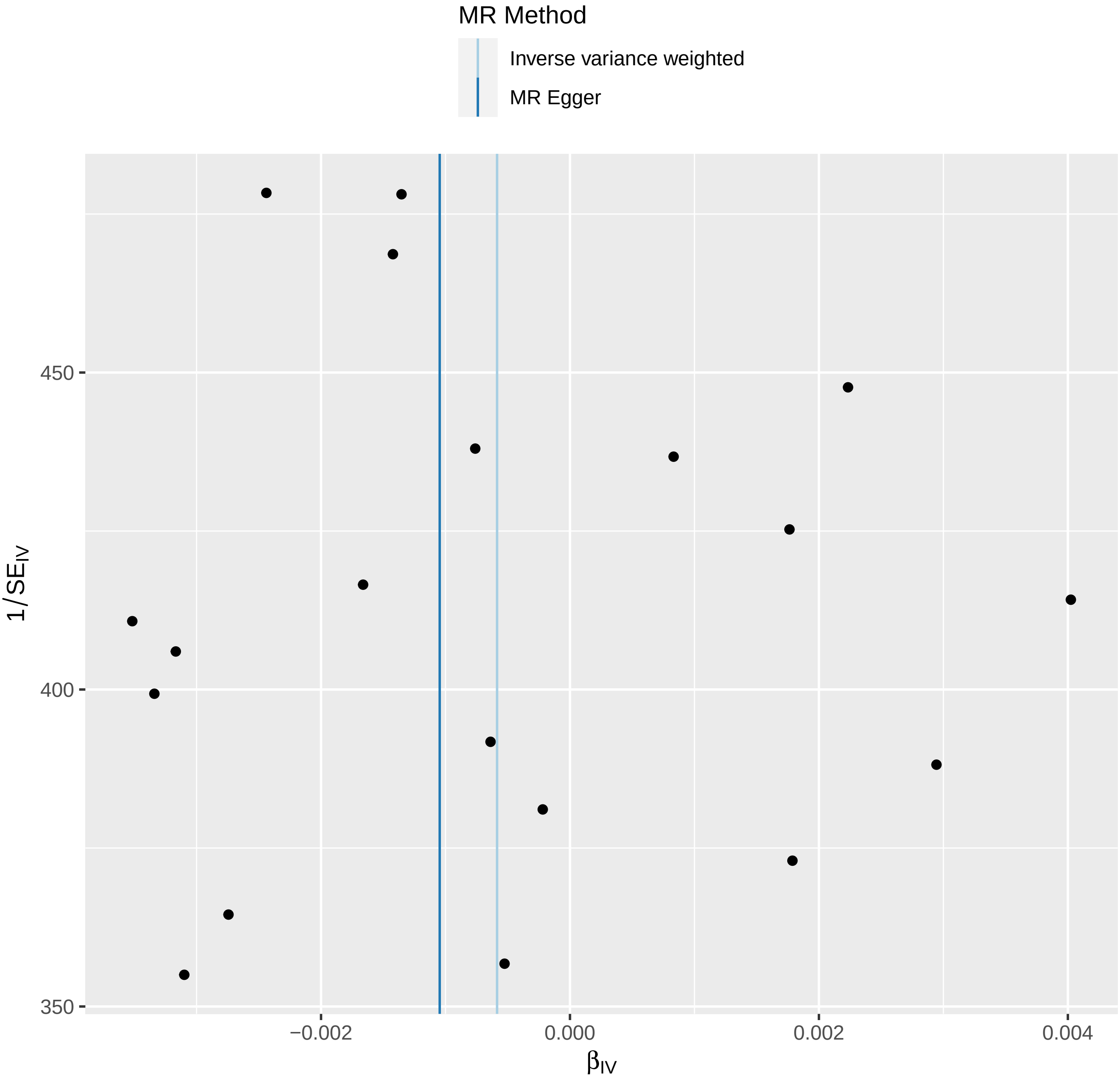

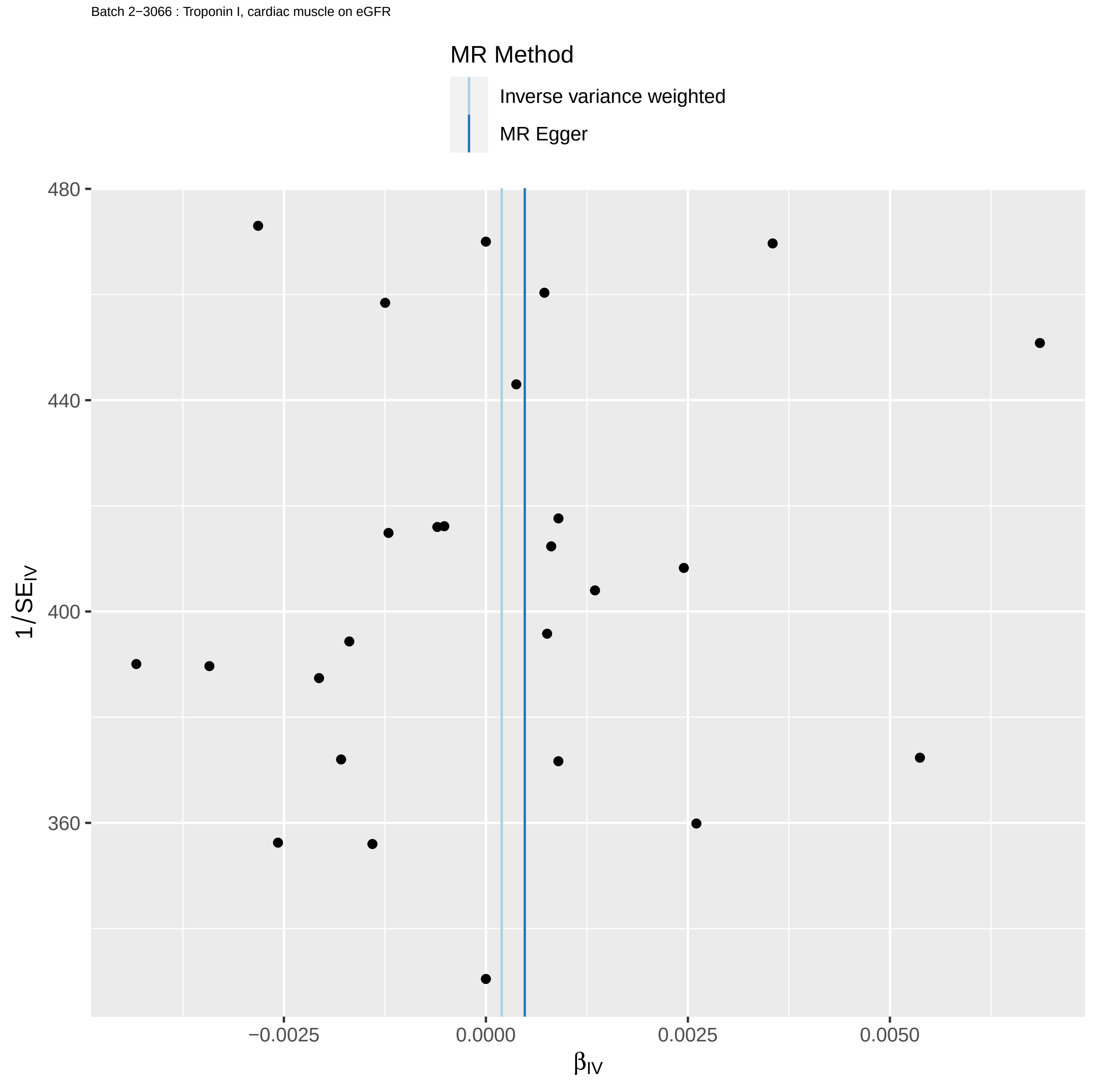

Batch 2−3066 : Troponin I, cardiac muscle on eGFR
MR Method
Inverse variance weighted
MR Egger
480
440
400
360
1/SE$_{IV}$
−0.0025
0.0000
0.0025
0.0050
β$_{IV}$

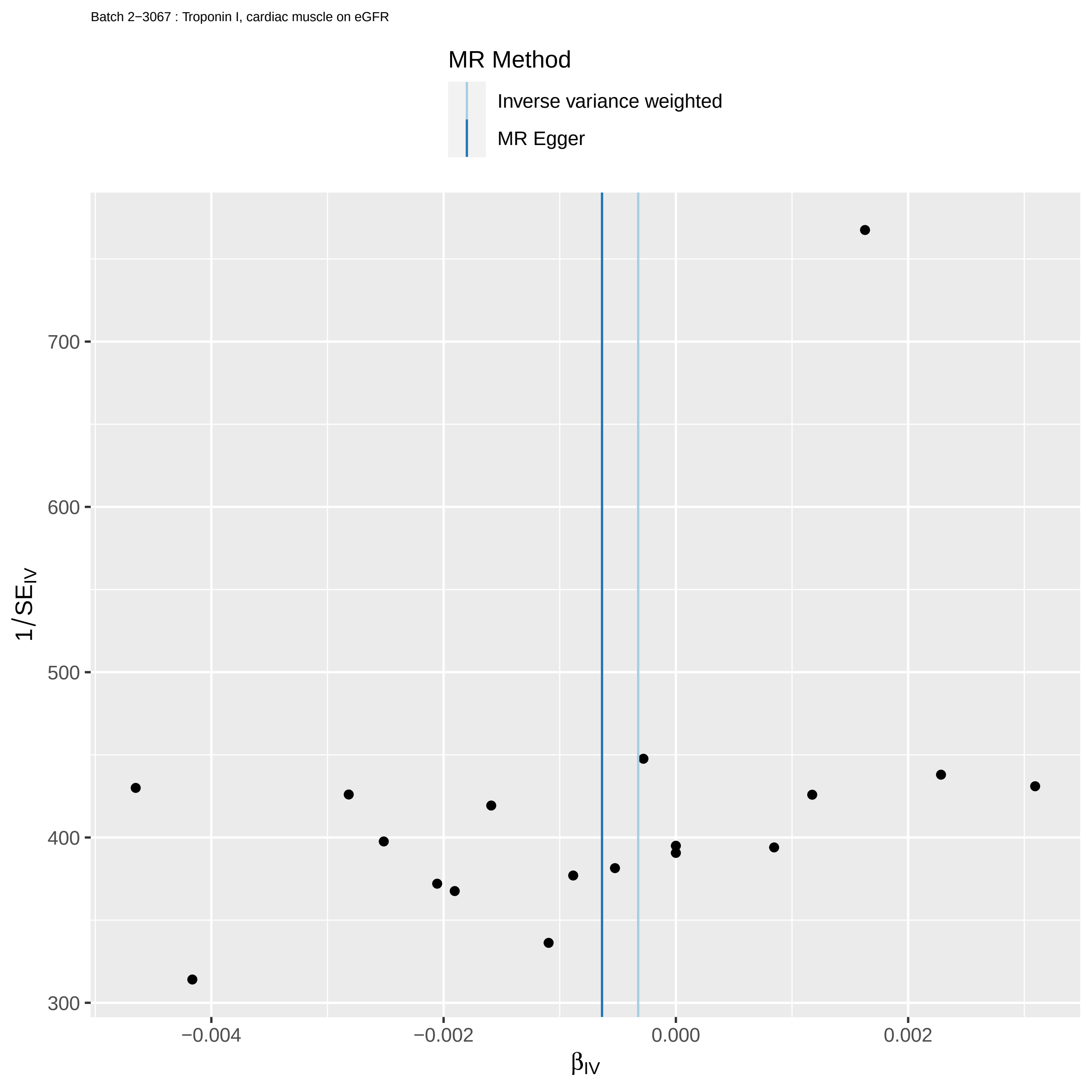
Batch 2−3067 : Troponin I, cardiac muscle on eGFR
MR Method
Inverse variance weighted
MR Egger
1/SE_IV
700
600
500
400
300
β_IV
−0.004
−0.002
0.000
0.002

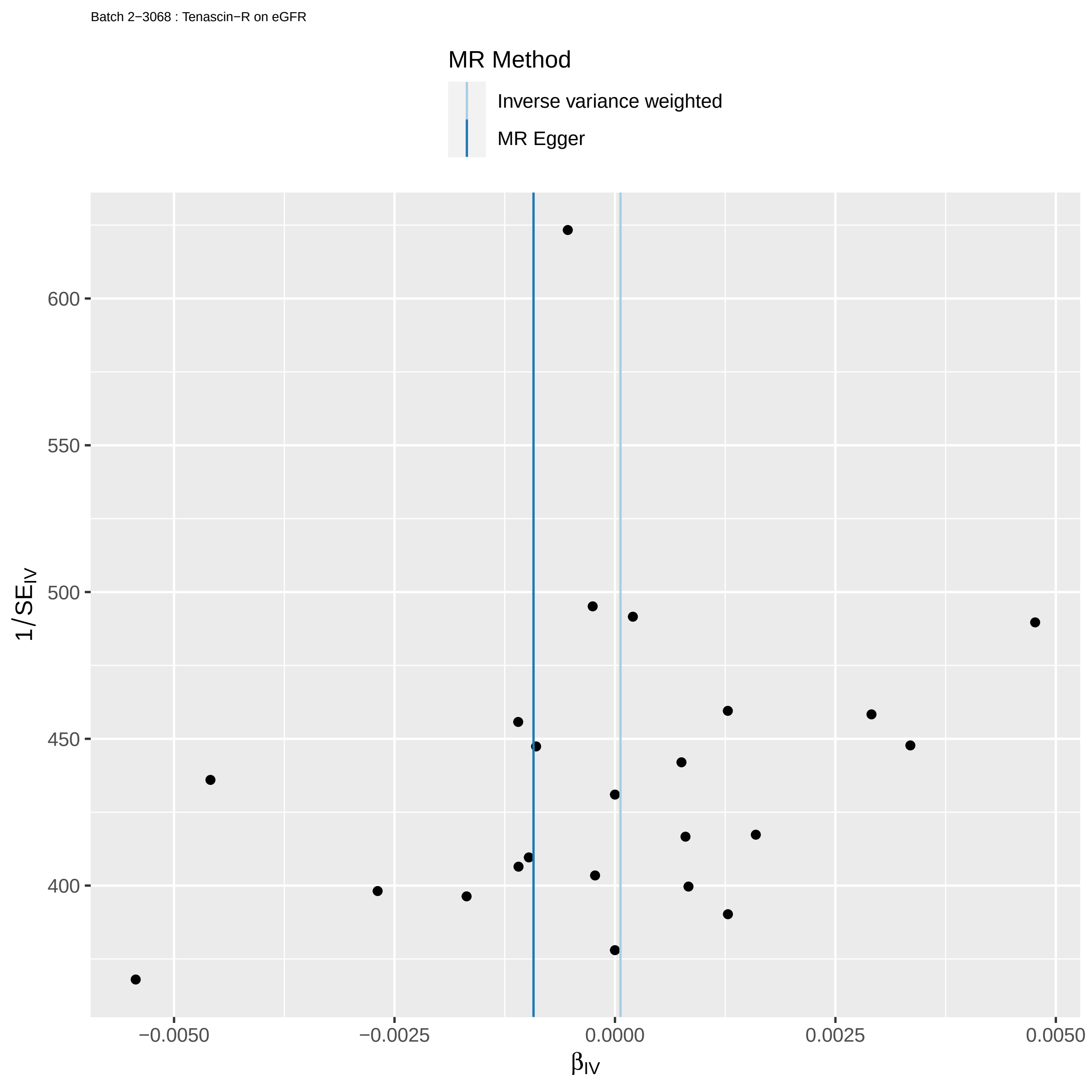

Batch 2−3068 : Tenascin−R on eGFR
MR Method
Inverse variance weighted
MR Egger
600
550
500
450
400
1/SE_IV
−0.0050
−0.0025
0.0000
0.0025
0.0050
β_IV

Batch 2−3069 : Tensin−2 on eGFR

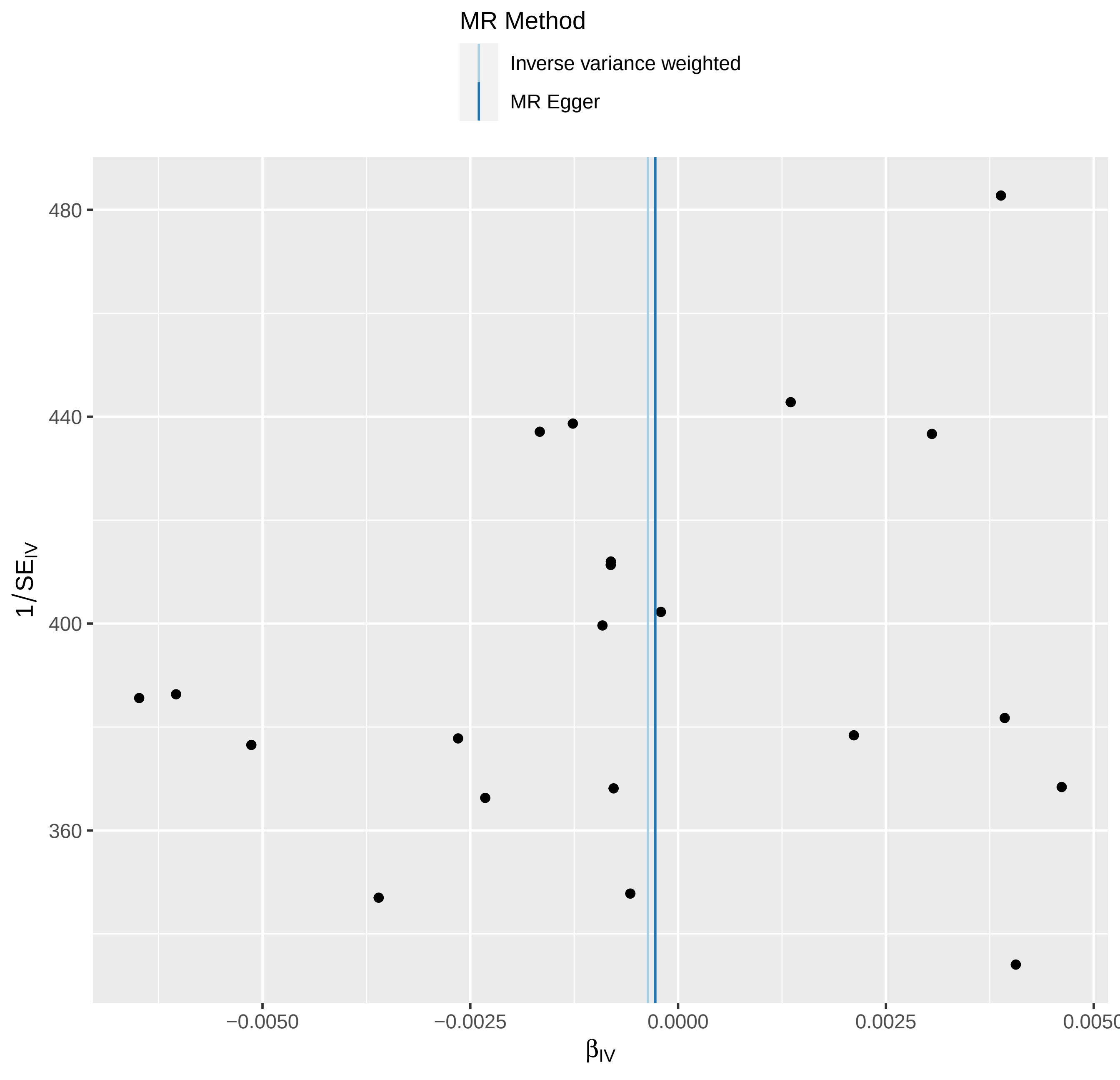

Batch 2−3070 : Tensin−4 on eGFR

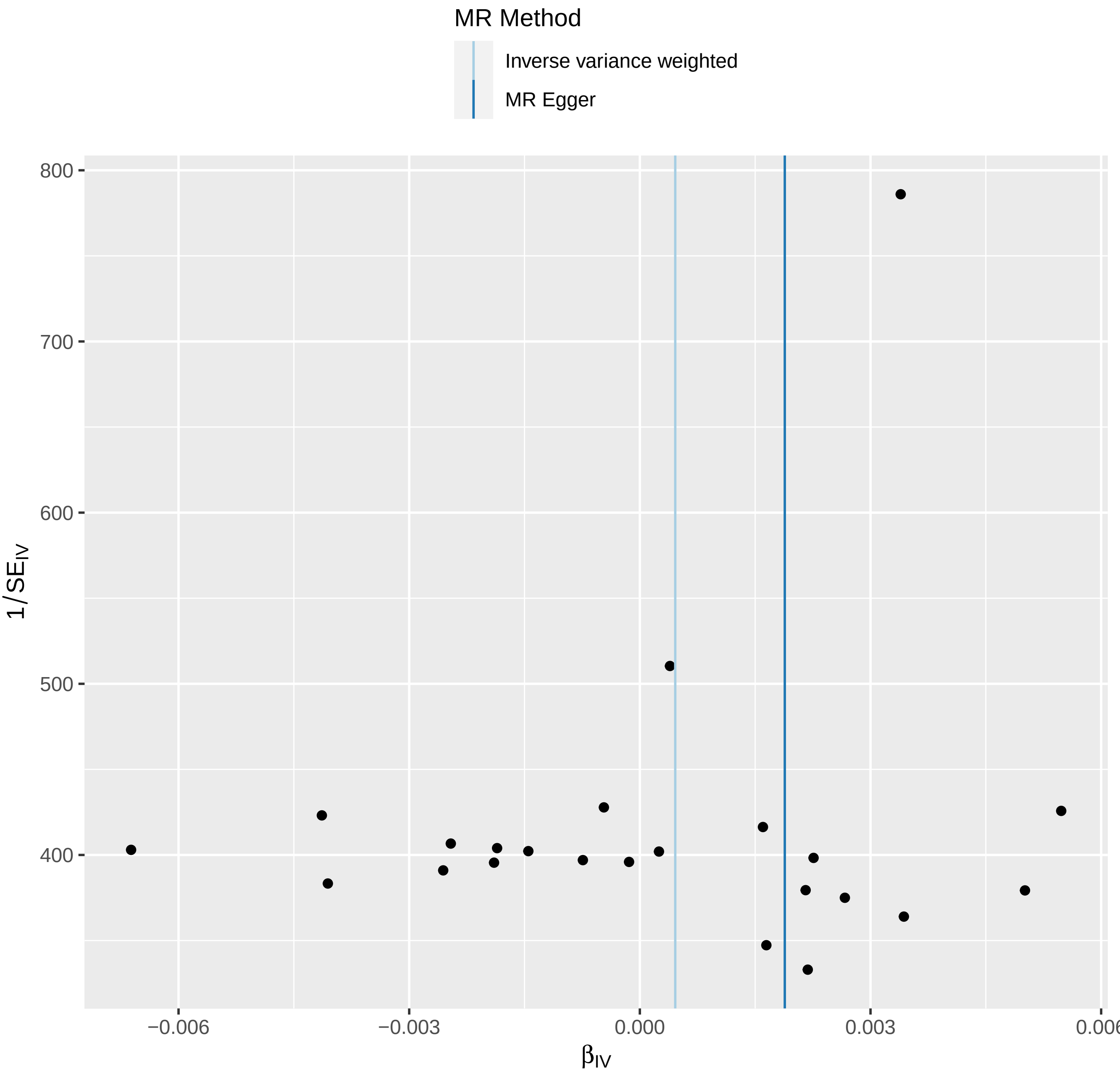

Batch 2−3071 : Tenascin−X on eGFR

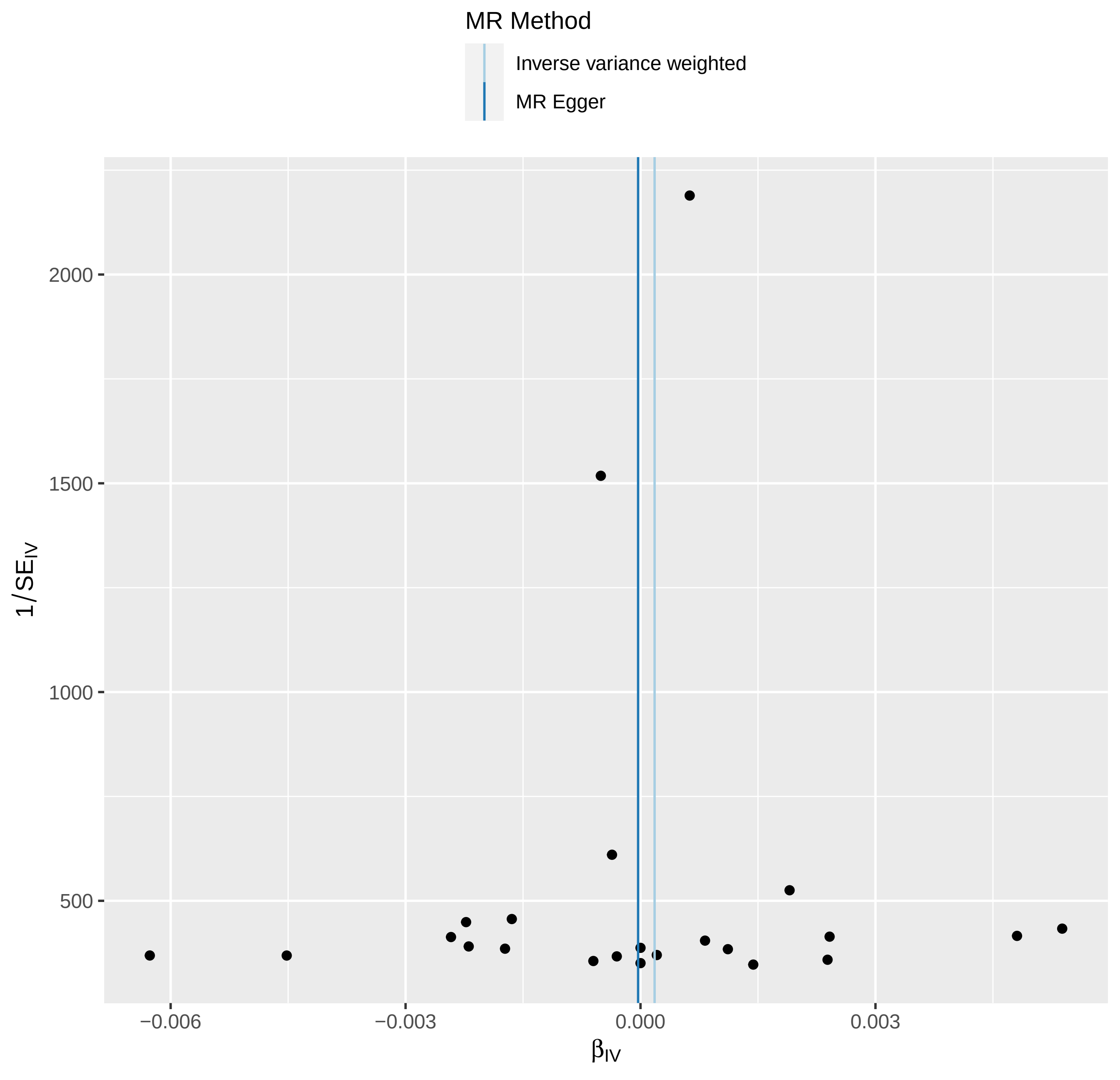

Batch 2−3072 : Toll−interacting protein on eGFR

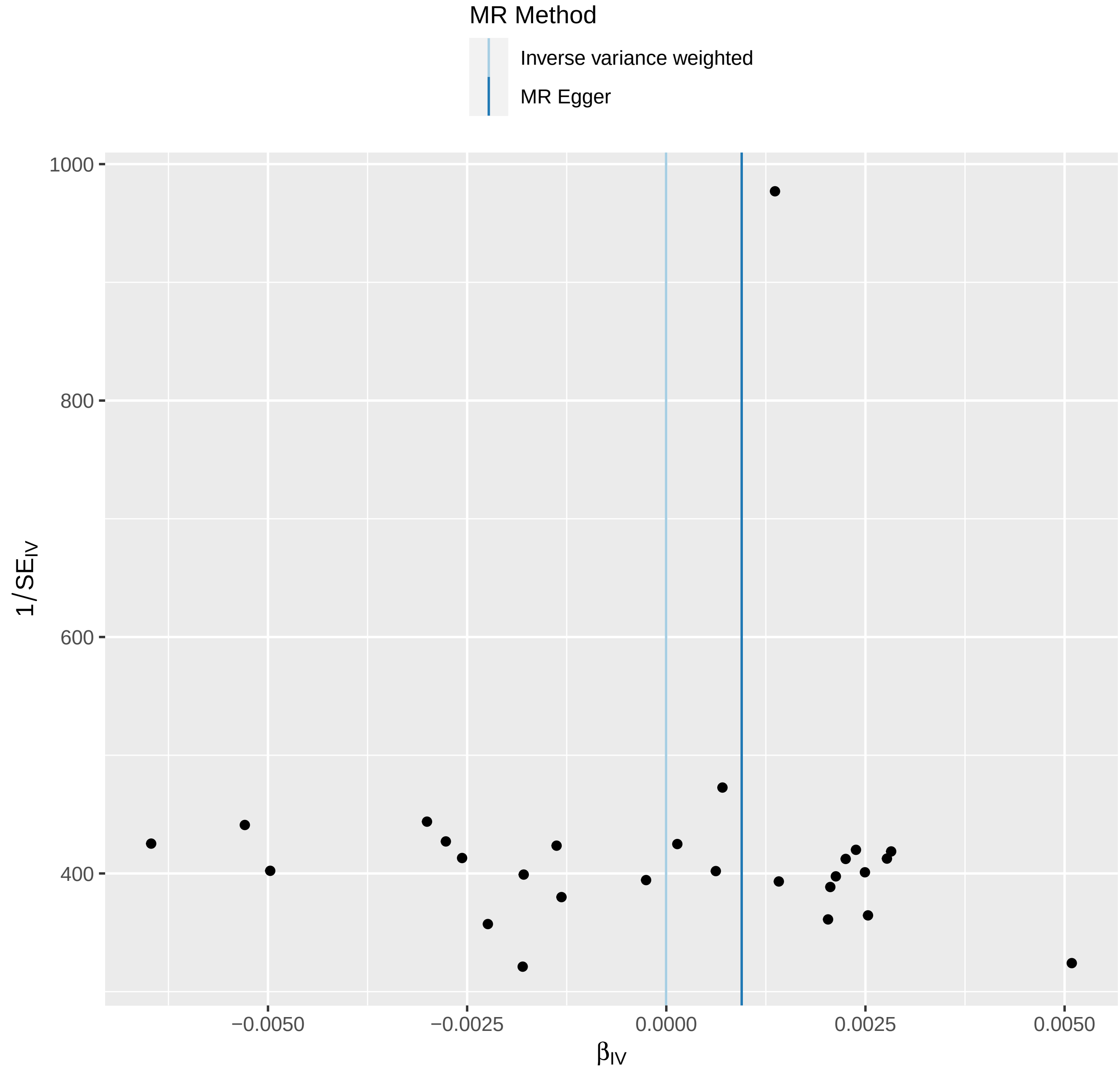

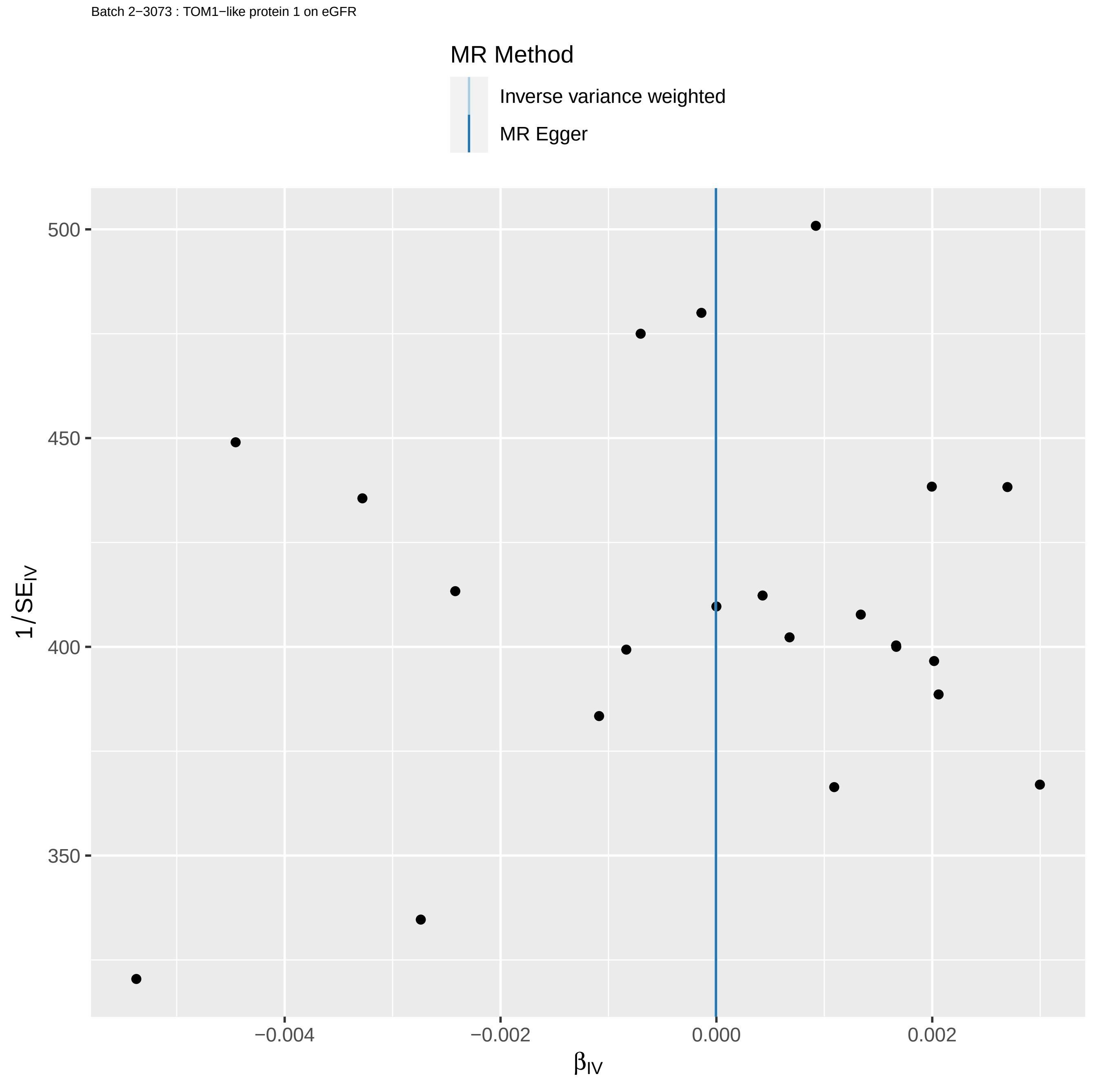

Batch 2−3073 : TOM1−like protein 1 on eGFR
MR Method
Inverse variance weighted
MR Egger
500
450
400
350
1/SE_IV
−0.004
−0.002
0.000
0.002
β_IV

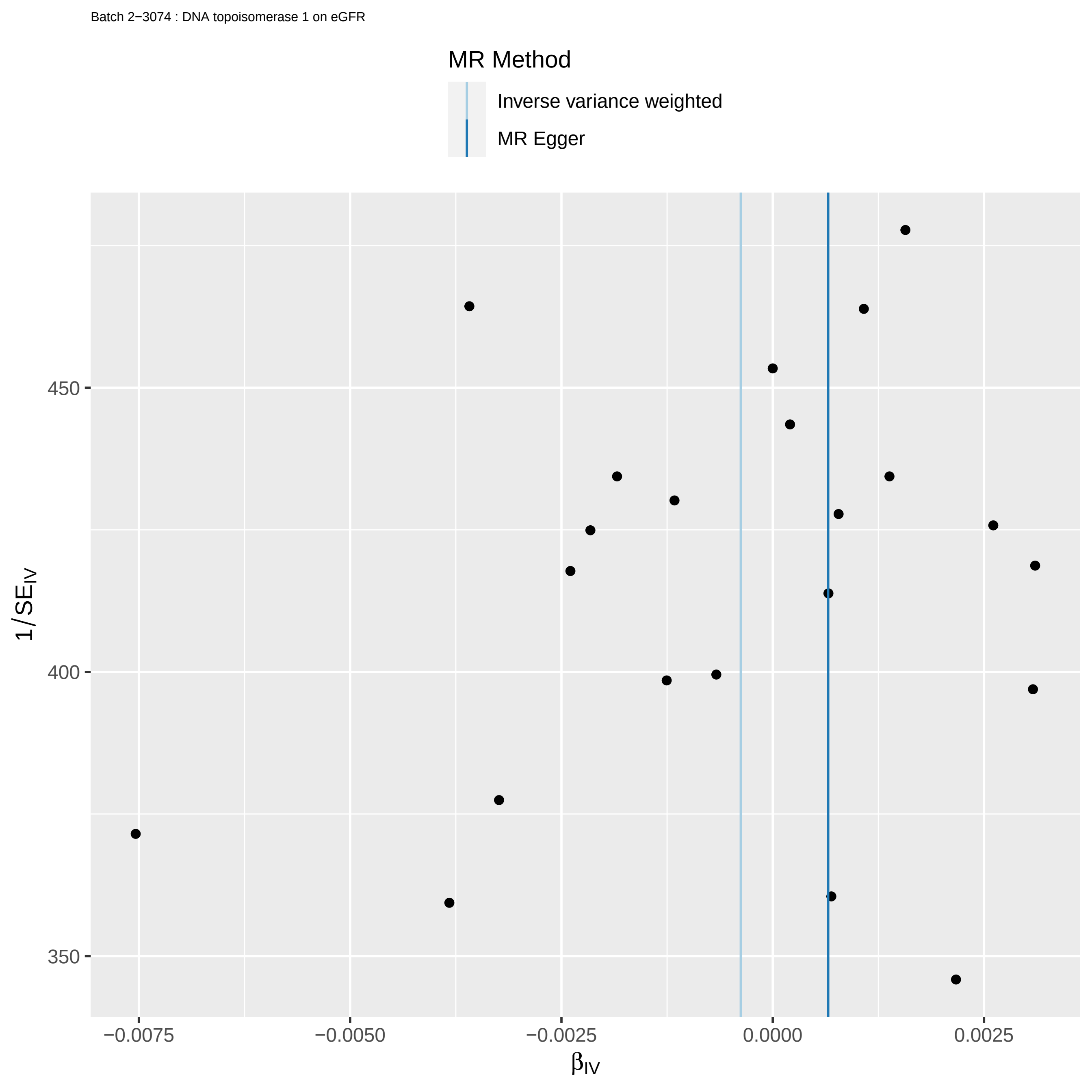
Batch 2−3074 : DNA topoisomerase 1 on eGFR
MR Method
Inverse variance weighted
MR Egger
450
400
350
1/SE_IV
−0.0075
−0.0050
−0.0025
0.0000
0.0025
β_IV

Batch 2−3075 : DNA topoisomerase 2−binding protein 1 on eGFR

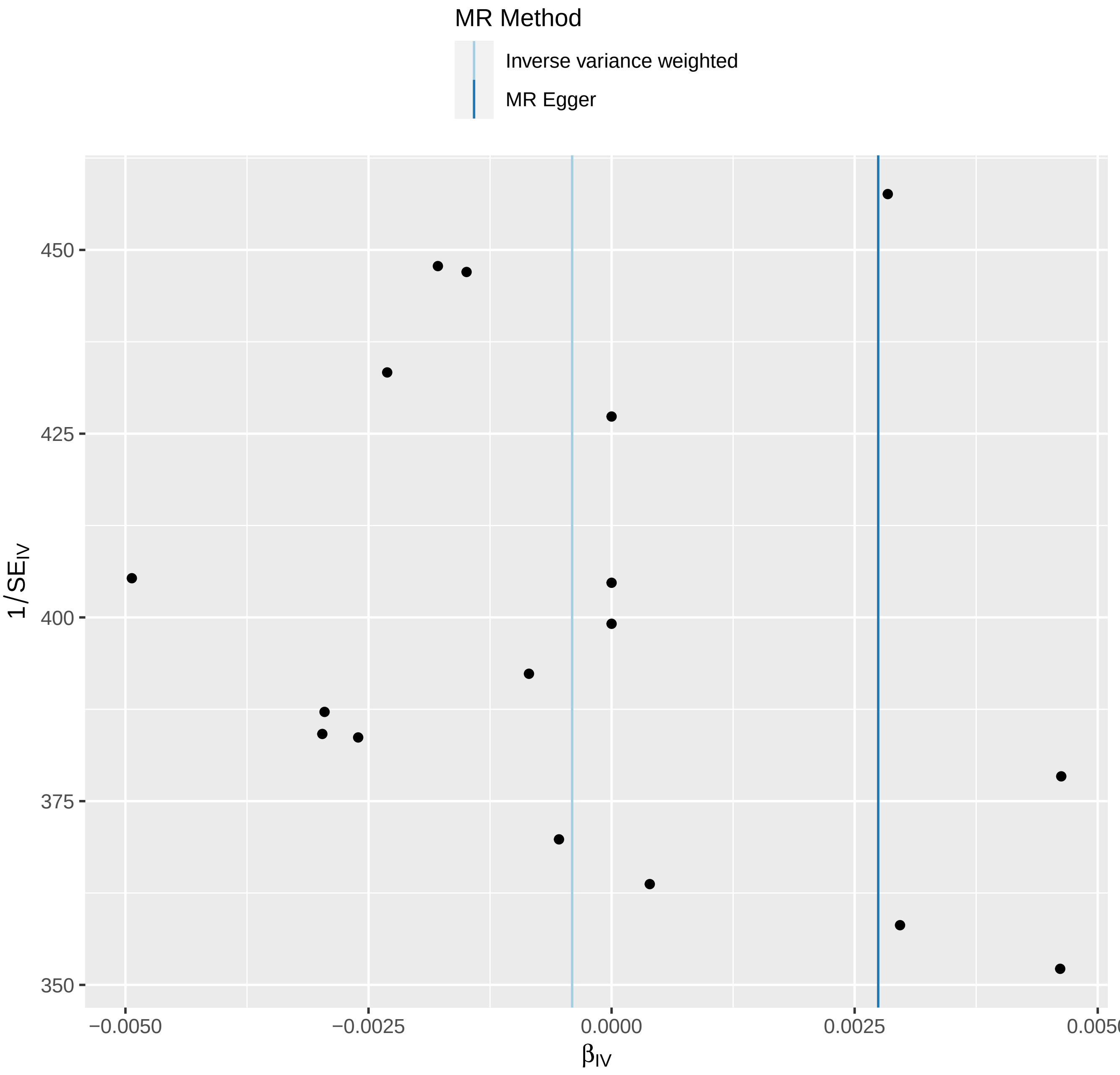

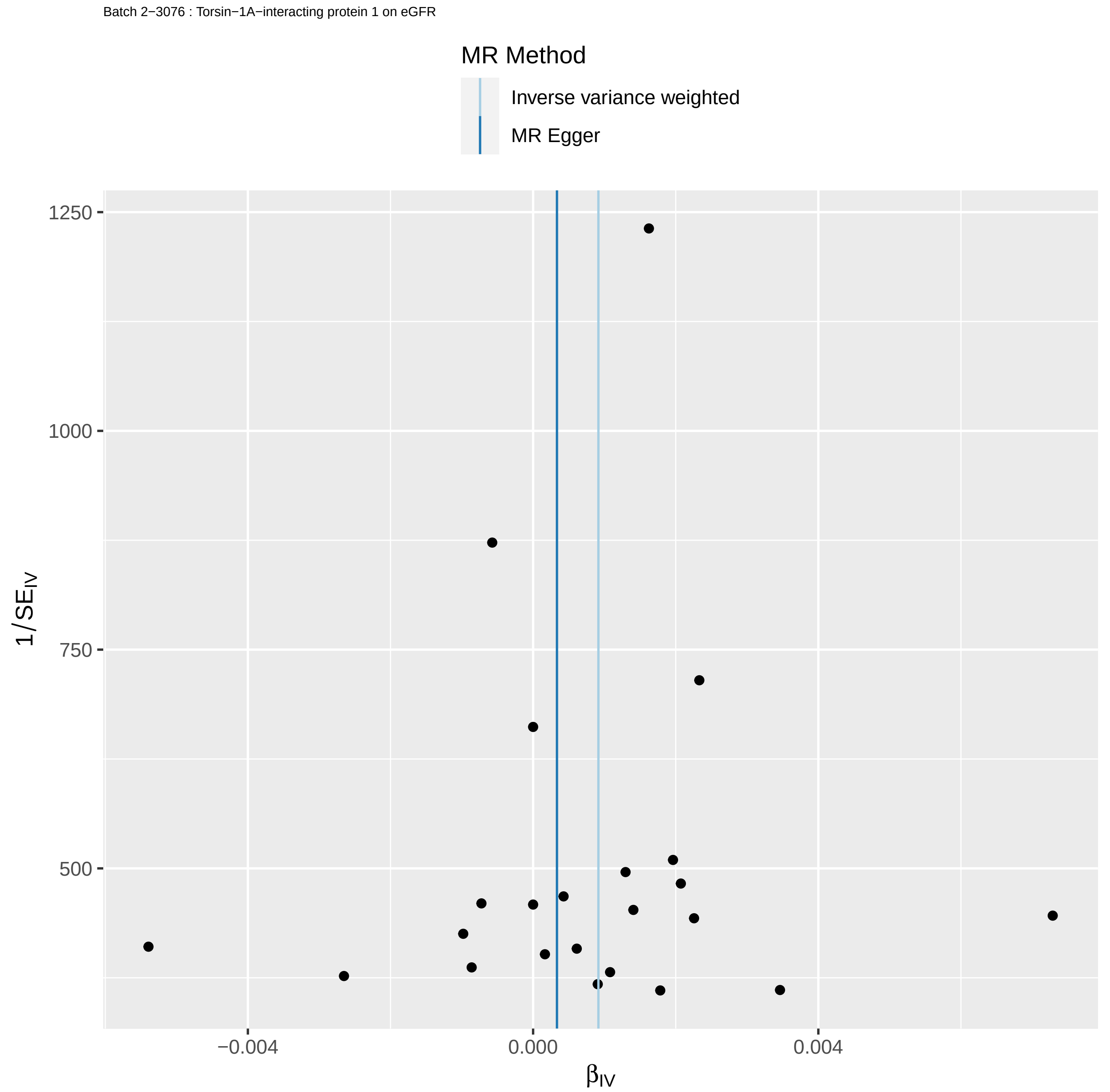

Batch 2−3076 : Torsin−1A−interacting protein 1 on eGFR
MR Method
Inverse variance weighted
MR Egger
1250
1000
750
500
1/SE_IV
−0.004
0.000
0.004
β_IV

Batch 2−3077 : Torsin−1A−interacting protein 2 on eGFR

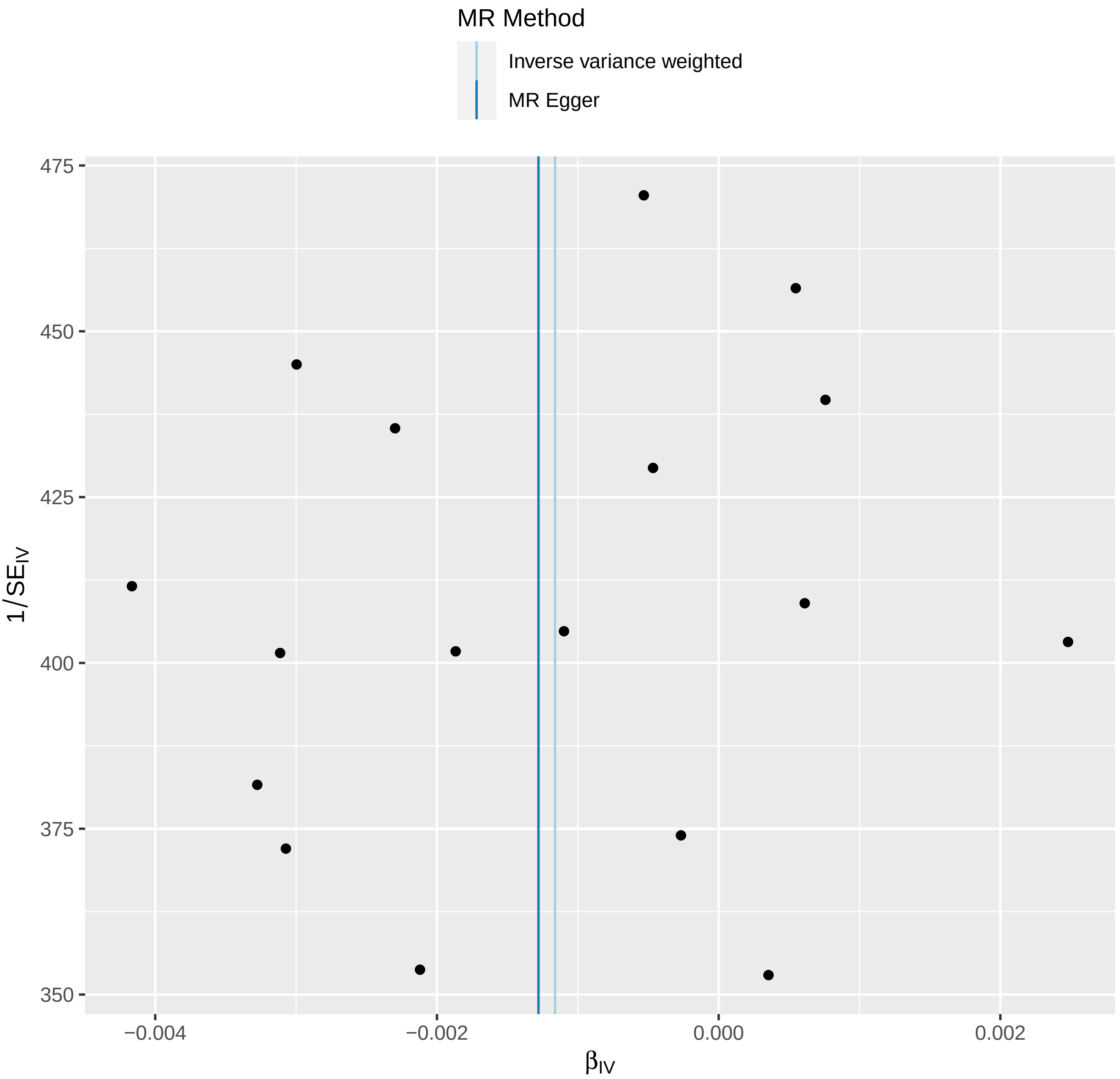

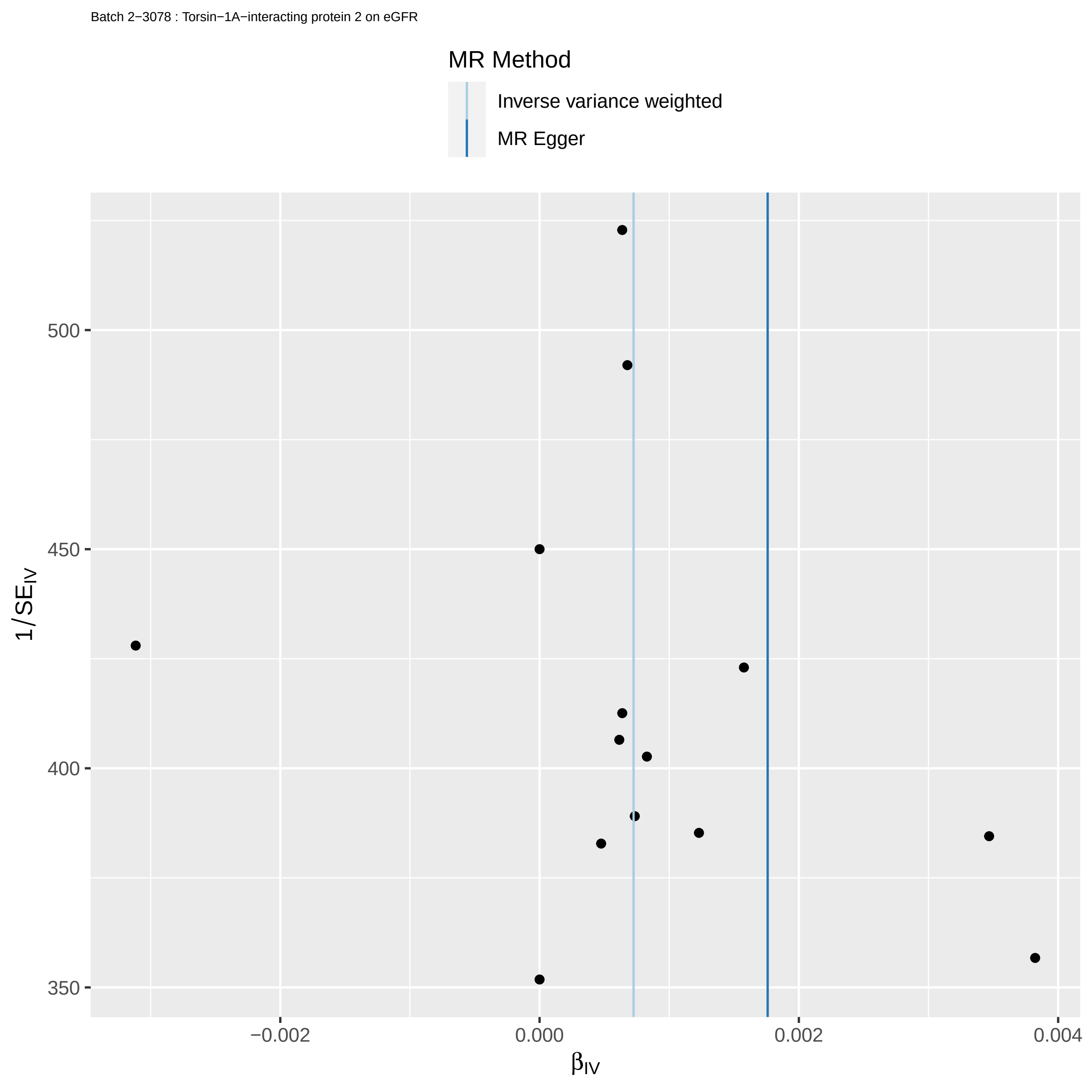

Batch 2−3078 : Torsin−1A−interacting protein 2 on eGFR
MR Method
Inverse variance weighted
MR Egger
500
450
400
350
1/SE$_{IV}$
−0.002
0.000
0.002
0.004
β$_{IV}$

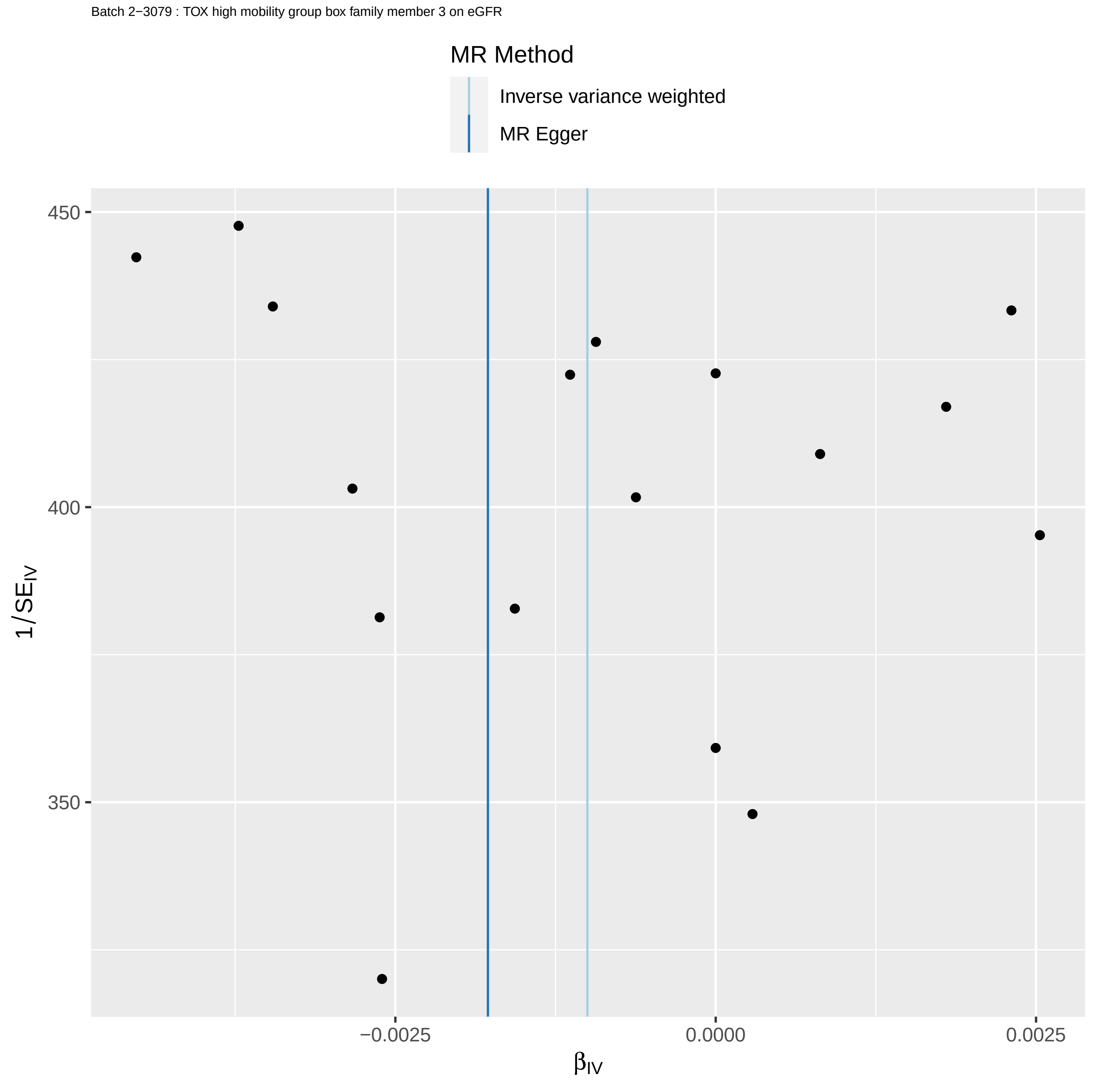

Batch 2−3079 : TOX high mobility group box family member 3 on eGFR
MR Method
Inverse variance weighted
MR Egger
450
400
350
$1/SE_{IV}$
−0.0025
0.0000
0.0025
β_{IV}

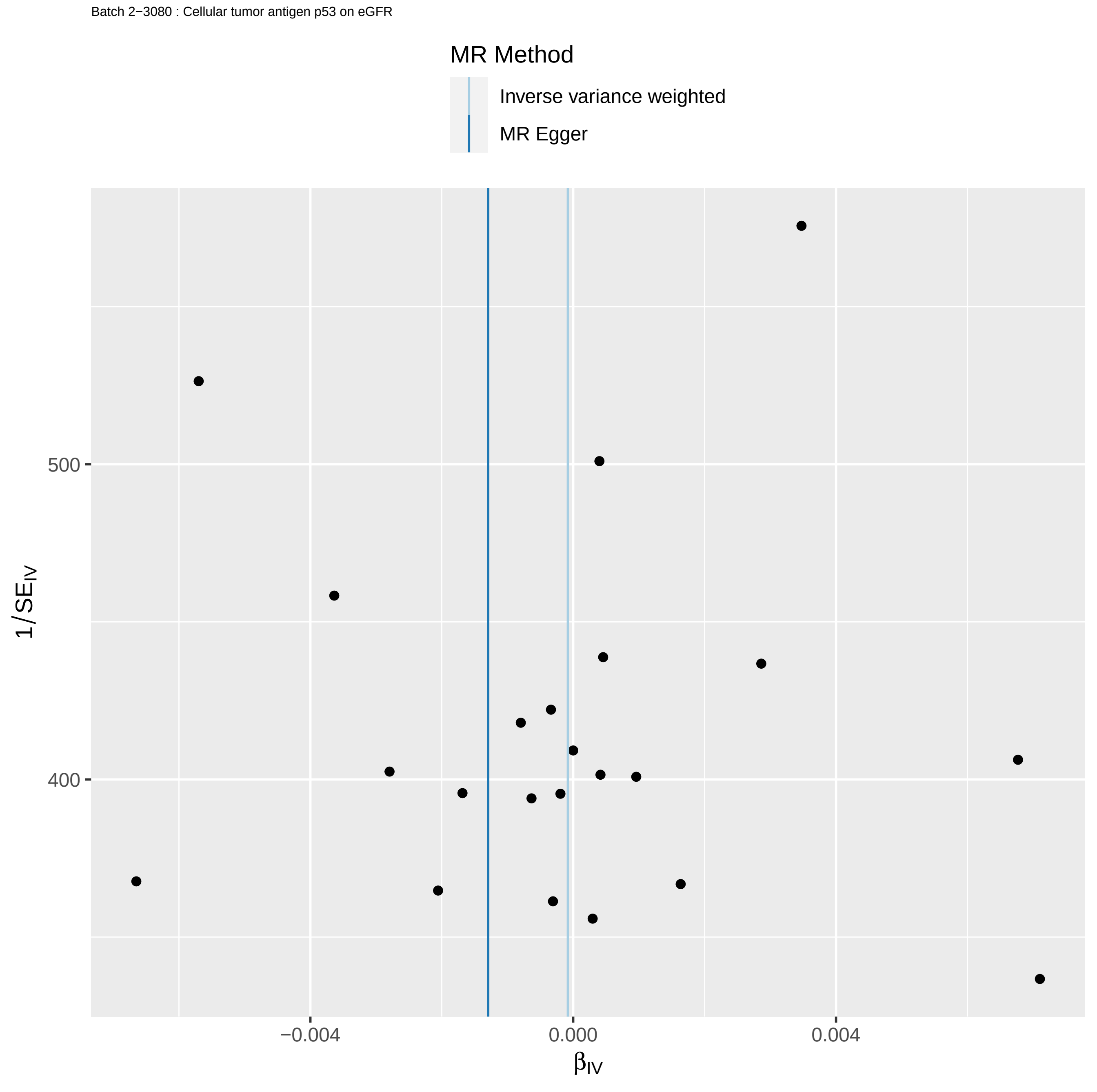

Batch 2−3080 : Cellular tumor antigen p53 on eGFR
MR Method
Inverse variance weighted
MR Egger
500
400
1/SE$_{IV}$
−0.004
0.000
0.004
β_{IV}

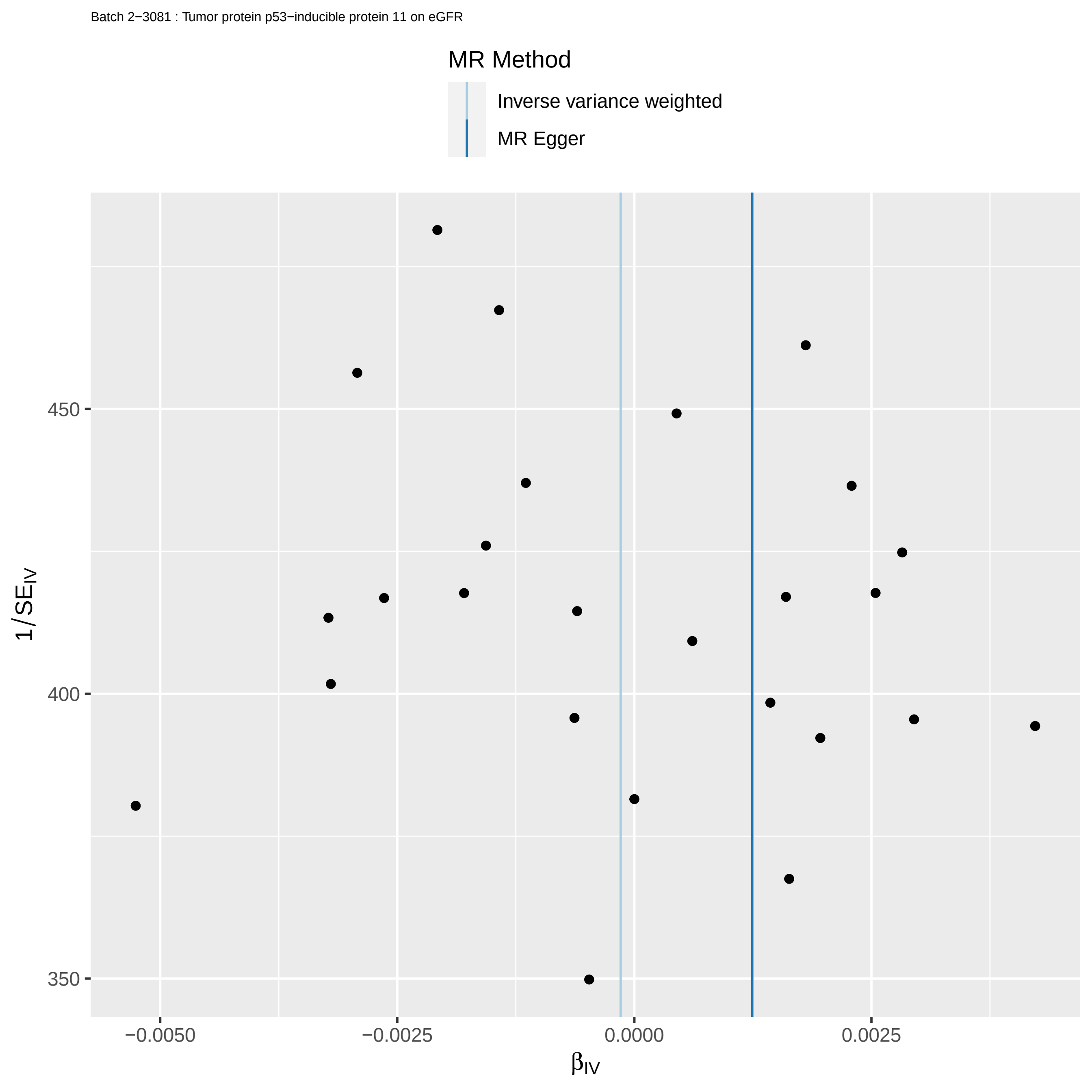
Batch 2−3081 : Tumor protein p53−inducible protein 11 on eGFR
MR Method
Inverse variance weighted
MR Egger
1/SE_IV
450
400
350
−0.0050
−0.0025
0.0000
0.0025
β_IV

Batch 2−3082 : Tumor protein 63 on eGFR

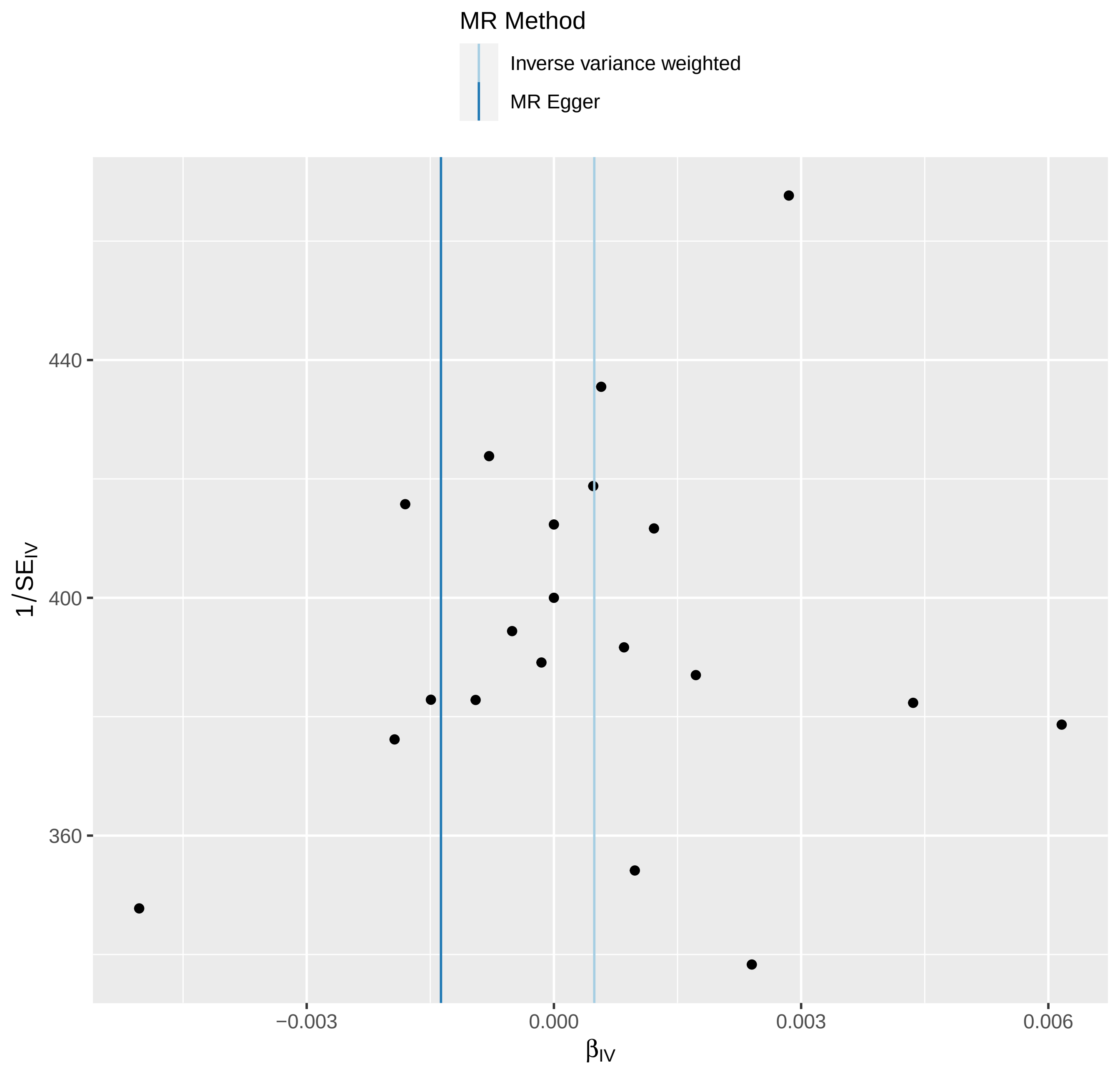

Batch 2−3083 : Triosephosphate isomerase on eGFR

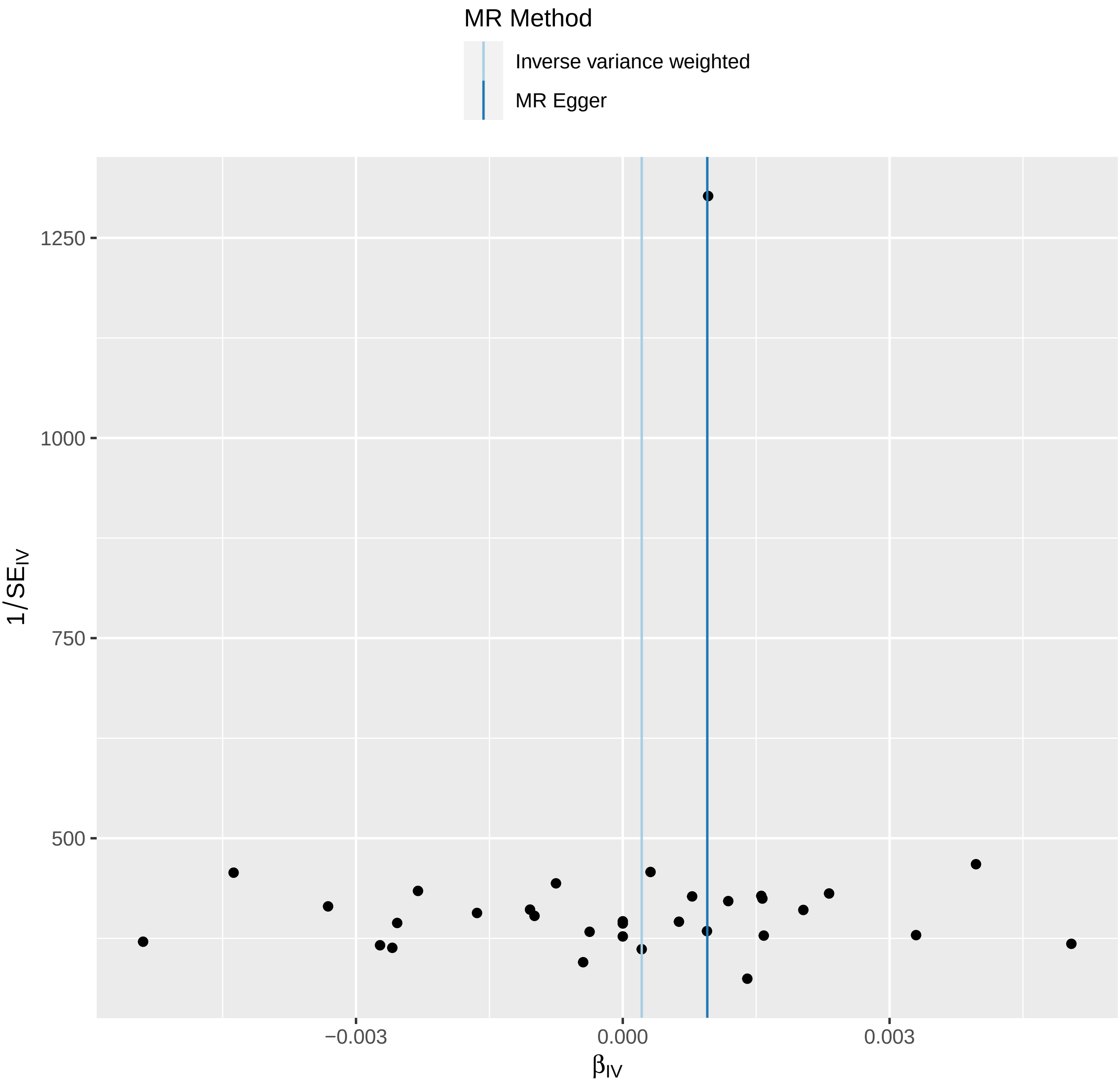

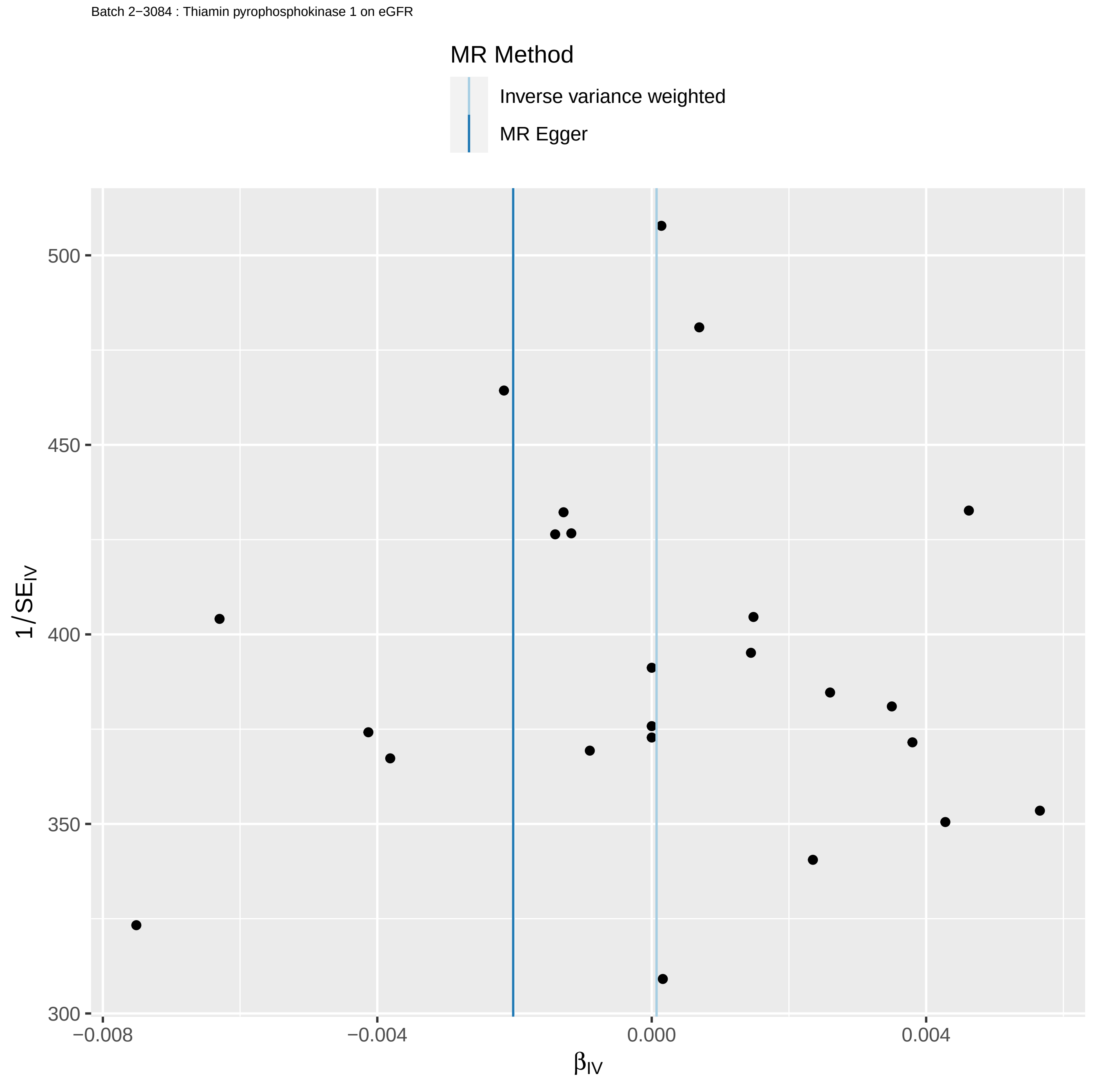
Batch 2−3084 : Thiamin pyrophosphokinase 1 on eGFR
MR Method
Inverse variance weighted
MR Egger
500
450
400
350
300
1/SE_IV
−0.008
−0.004
0.000
0.004
β_IV

Batch 2−3085 : Tropomyosin alpha−1 chain on eGFR

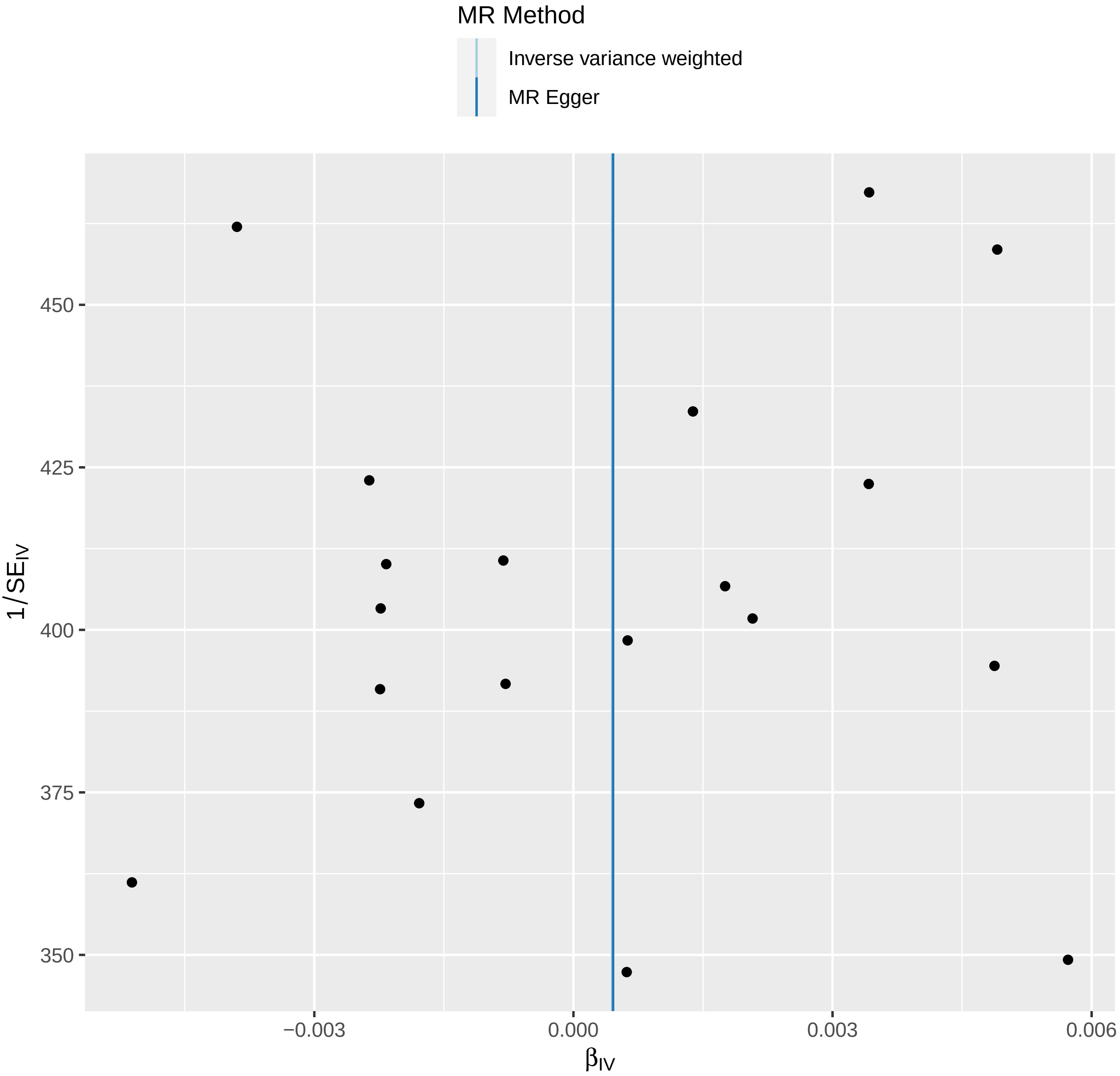

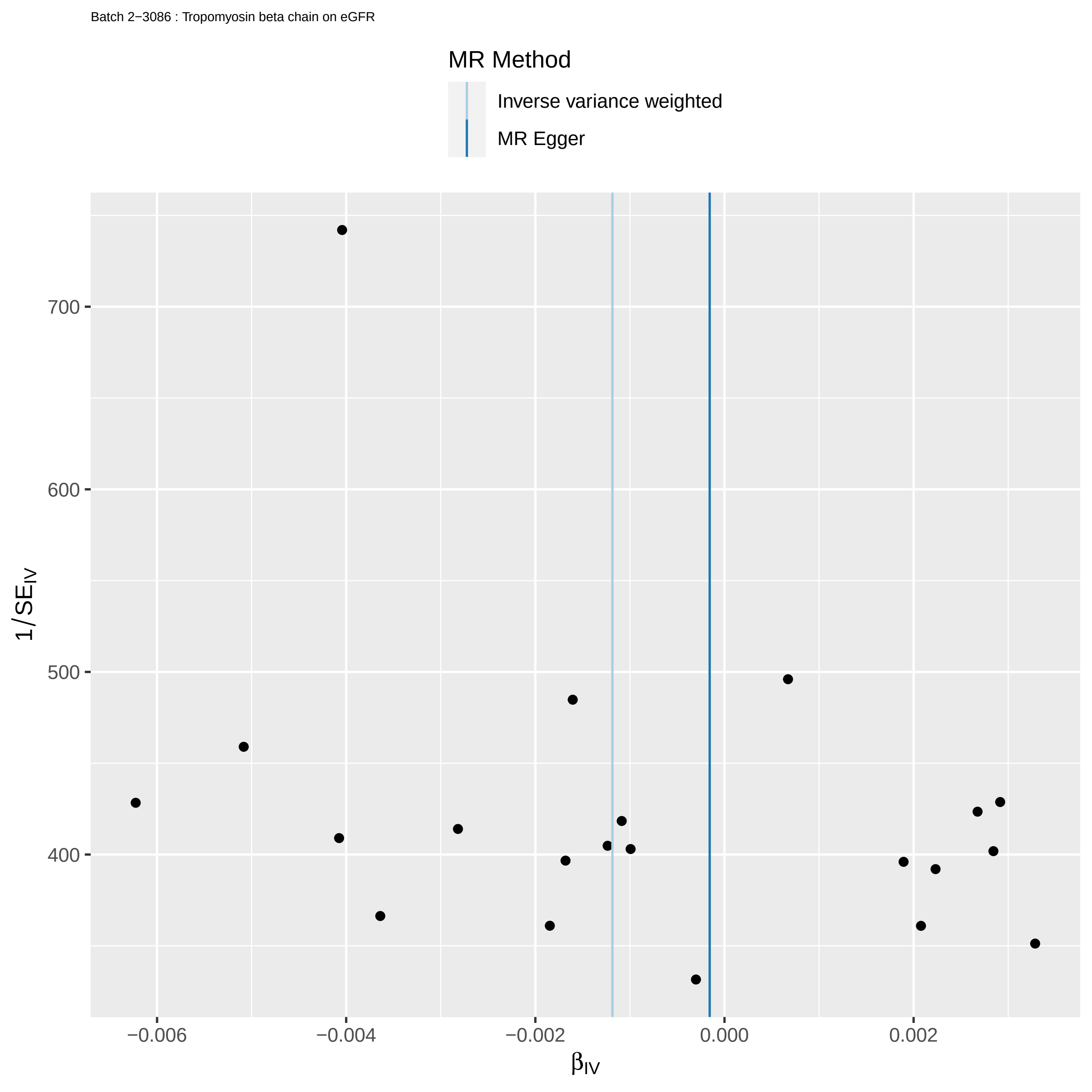
Batch 2−3086 : Tropomyosin beta chain on eGFR
MR Method
Inverse variance weighted
MR Egger
700
600
500
400
1/SE$_{IV}$
−0.006
−0.004
−0.002
0.000
0.002
β$_{IV}$

Batch 2−3087 : Tropomyosin alpha−3 chain on eGFR

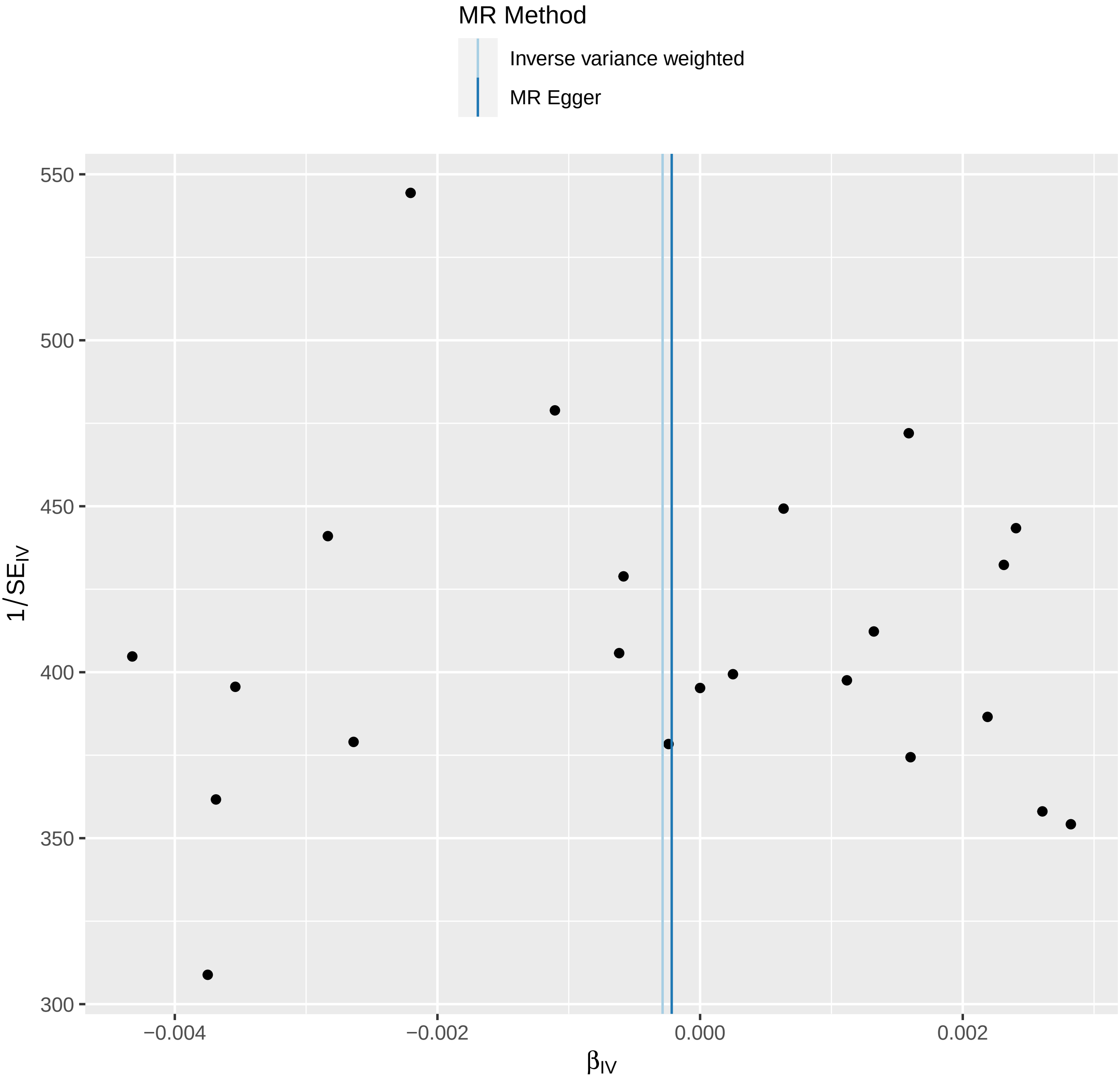

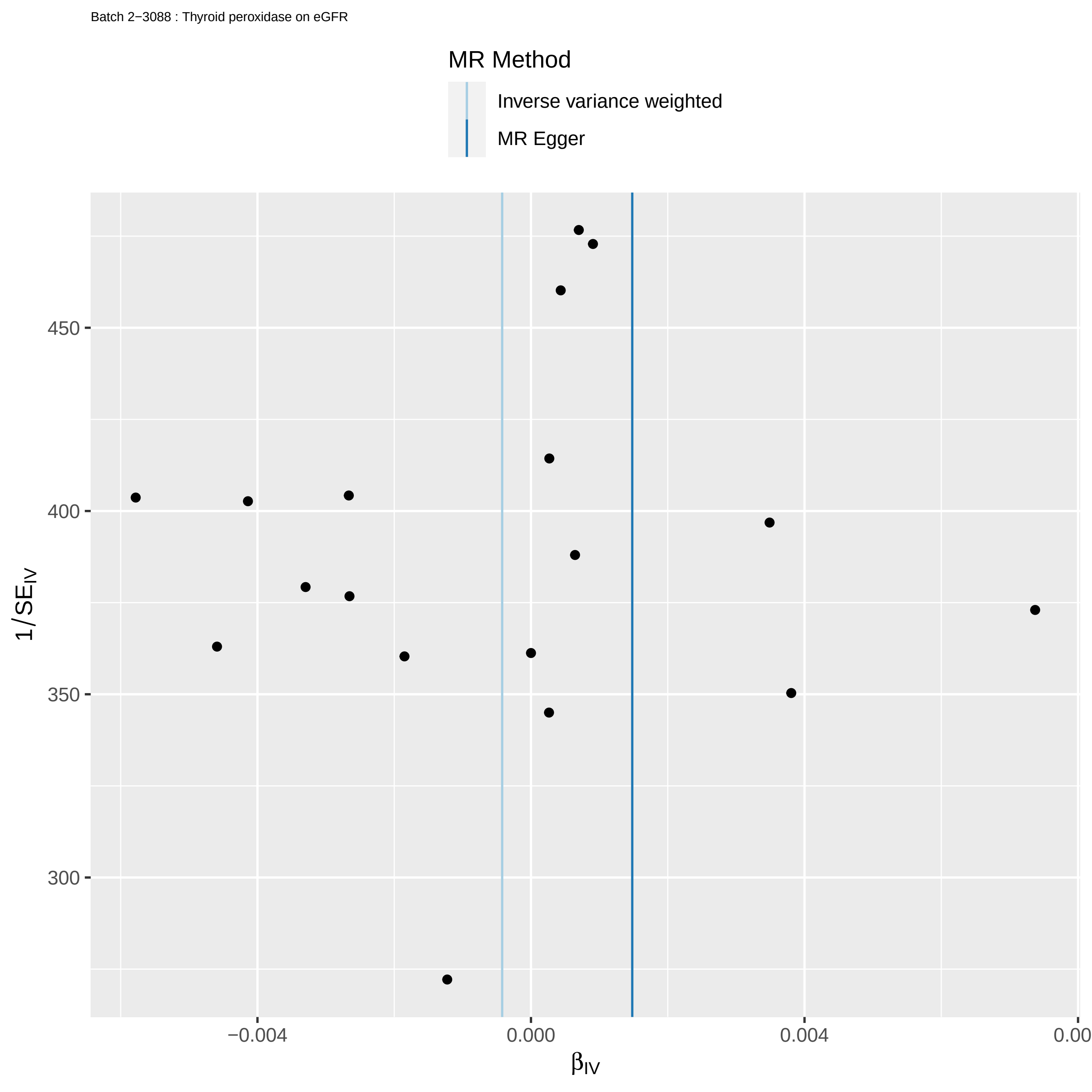

Batch 2−3088 : Thyroid peroxidase on eGFR
MR Method
Inverse variance weighted
MR Egger
450
400
350
300
1/SE_IV
−0.004
0.000
0.004
0.00
β_IV

Batch 2−3089 : Tubulin polymerization−promoting protein family member 2 on eGFR

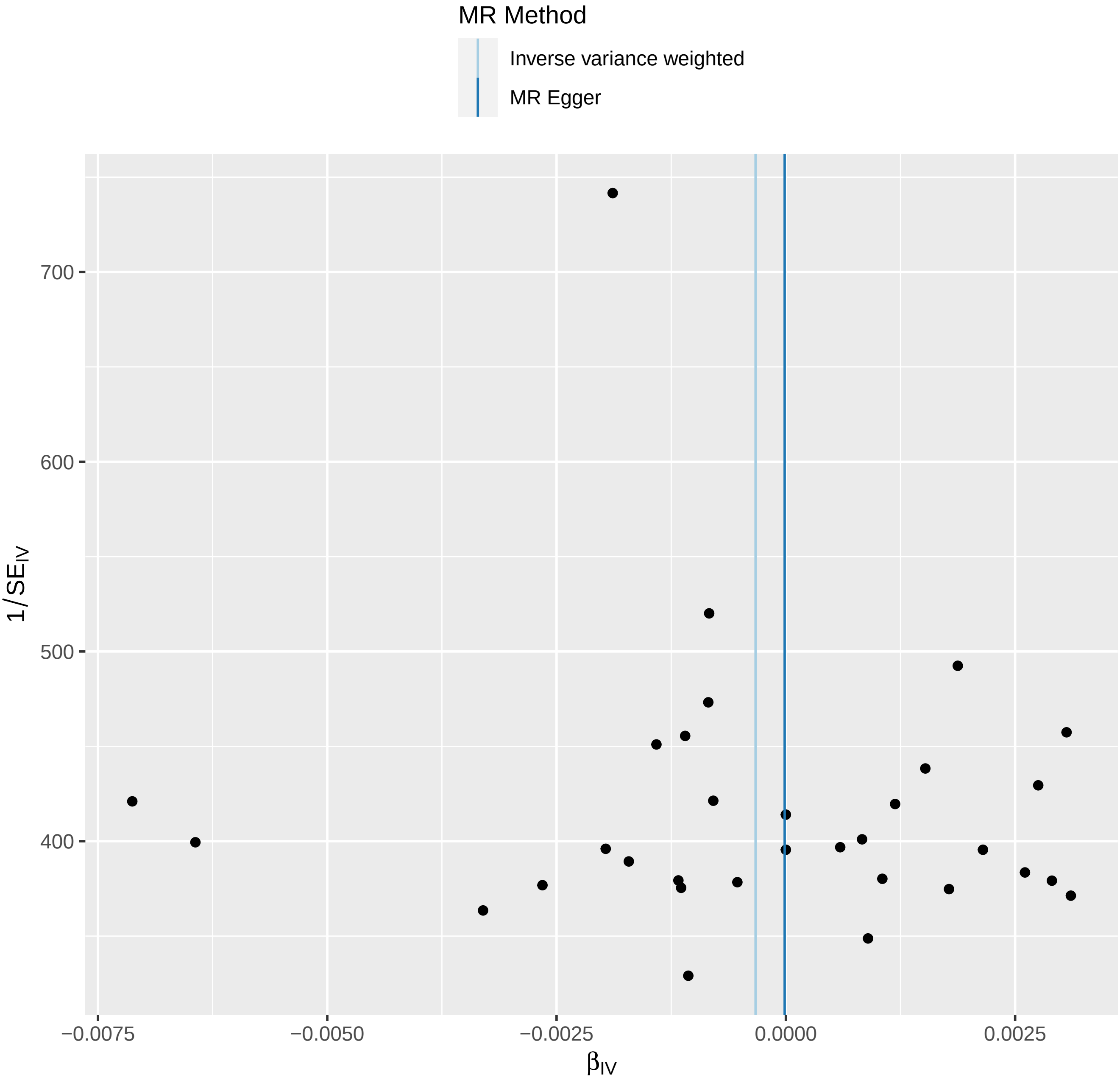

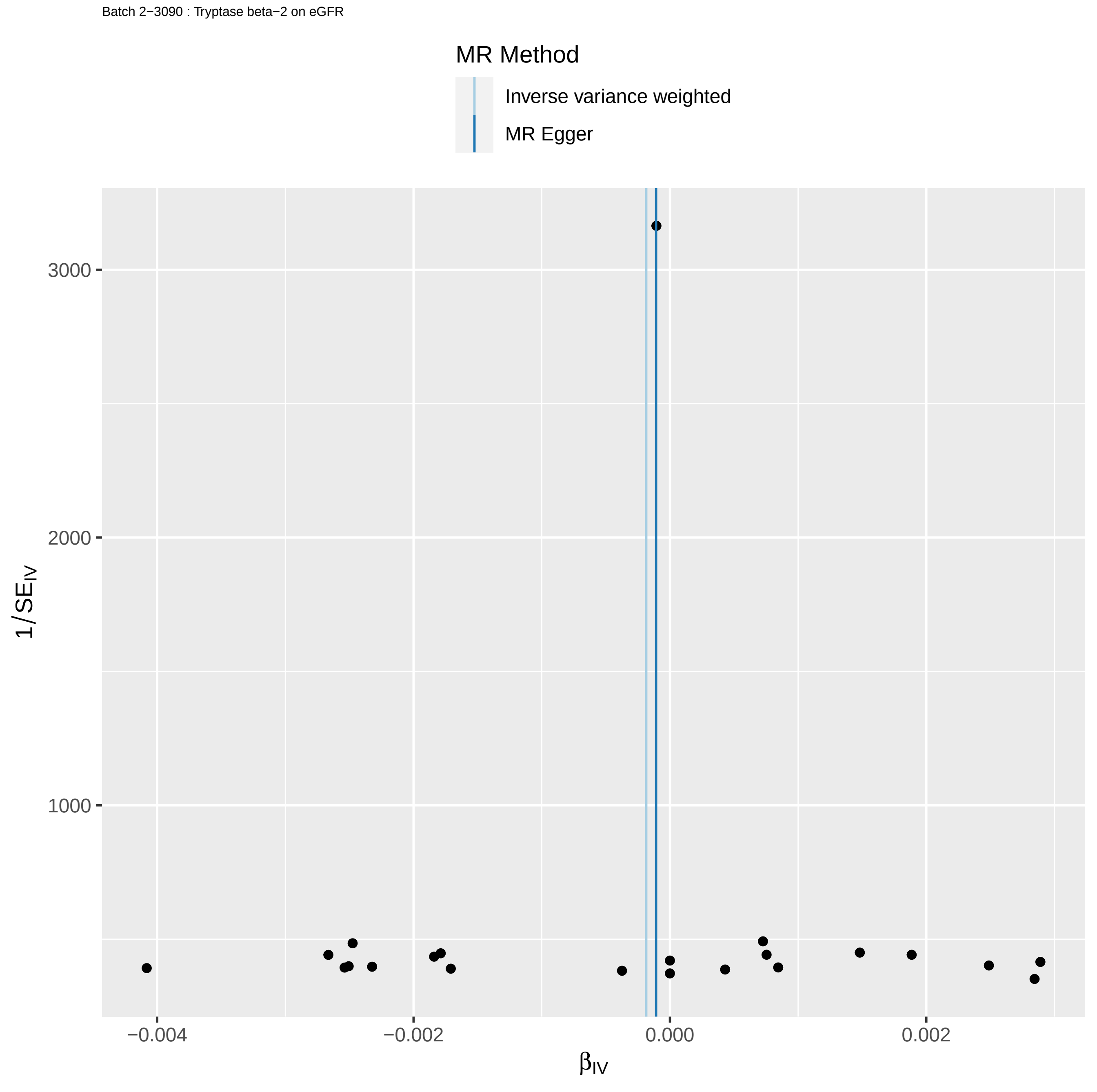

Batch 2−3090 : Tryptase beta−2 on eGFR
MR Method
Inverse variance weighted
MR Egger
3000
2000
1000
1/SE$_{IV}$
−0.004
−0.002
0.000
0.002
β$_{IV}$

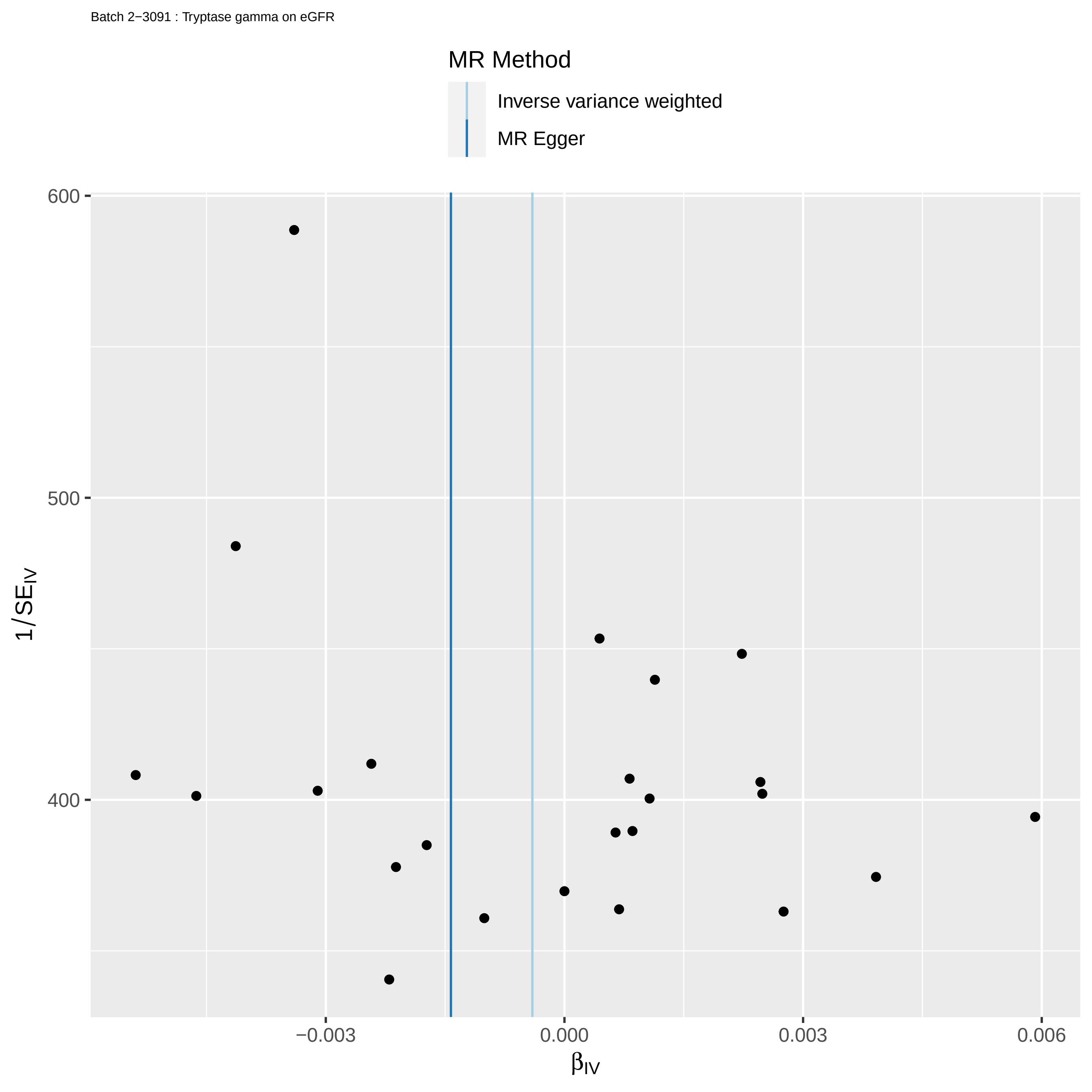

Batch 2−3091 : Tryptase gamma on eGFR
MR Method
Inverse variance weighted
MR Egger
600
500
400
1/SE$_{IV}$
−0.003
0.000
0.003
0.006
β$_{IV}$

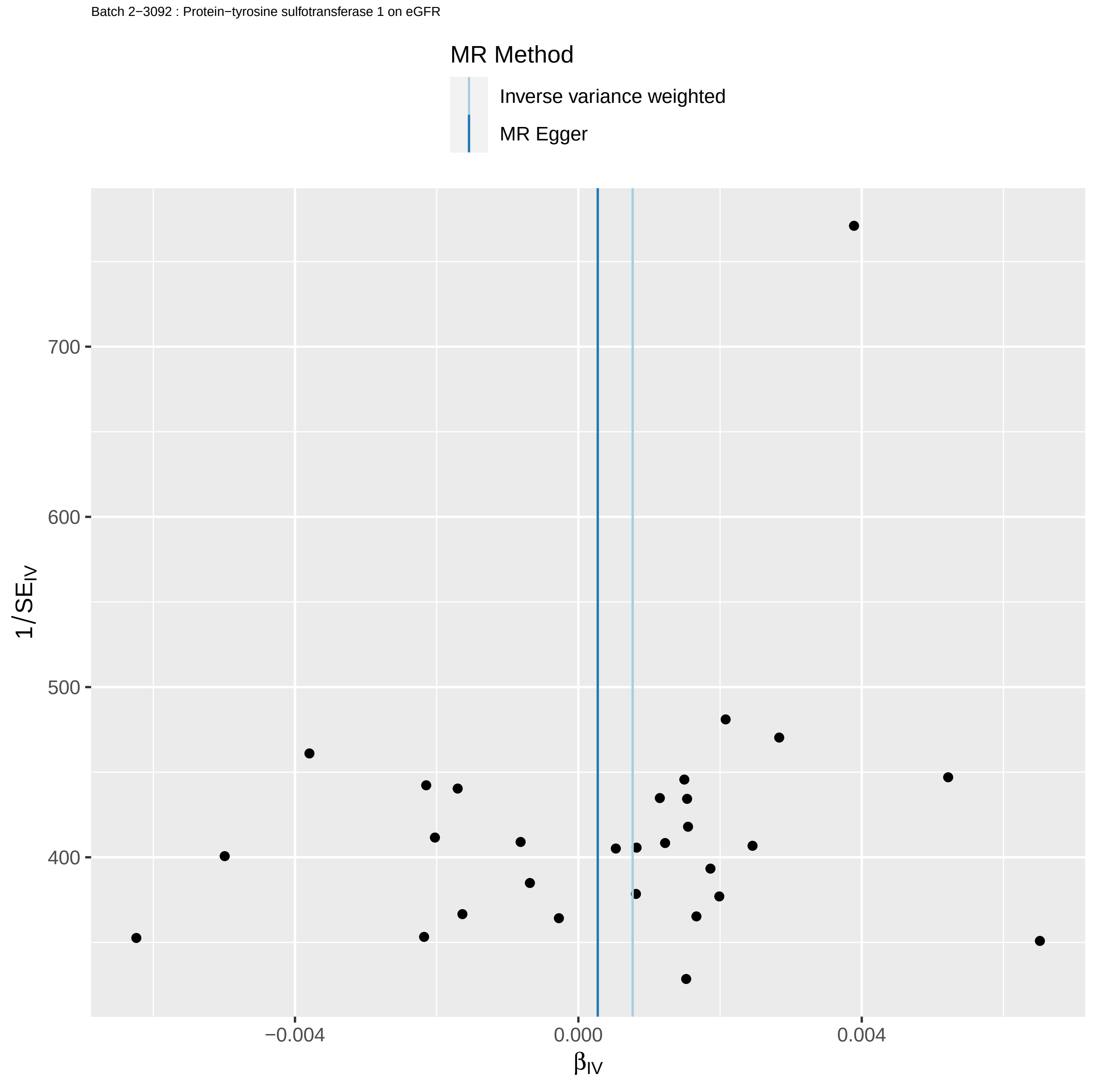

Batch 2−3092 : Protein−tyrosine sulfotransferase 1 on eGFR
MR Method
Inverse variance weighted
MR Egger
700
600
500
400
1/SE_IV
−0.004
0.000
0.004
β_IV

Batch 2−3093 : Protein−tyrosine sulfotransferase 2 on eGFR

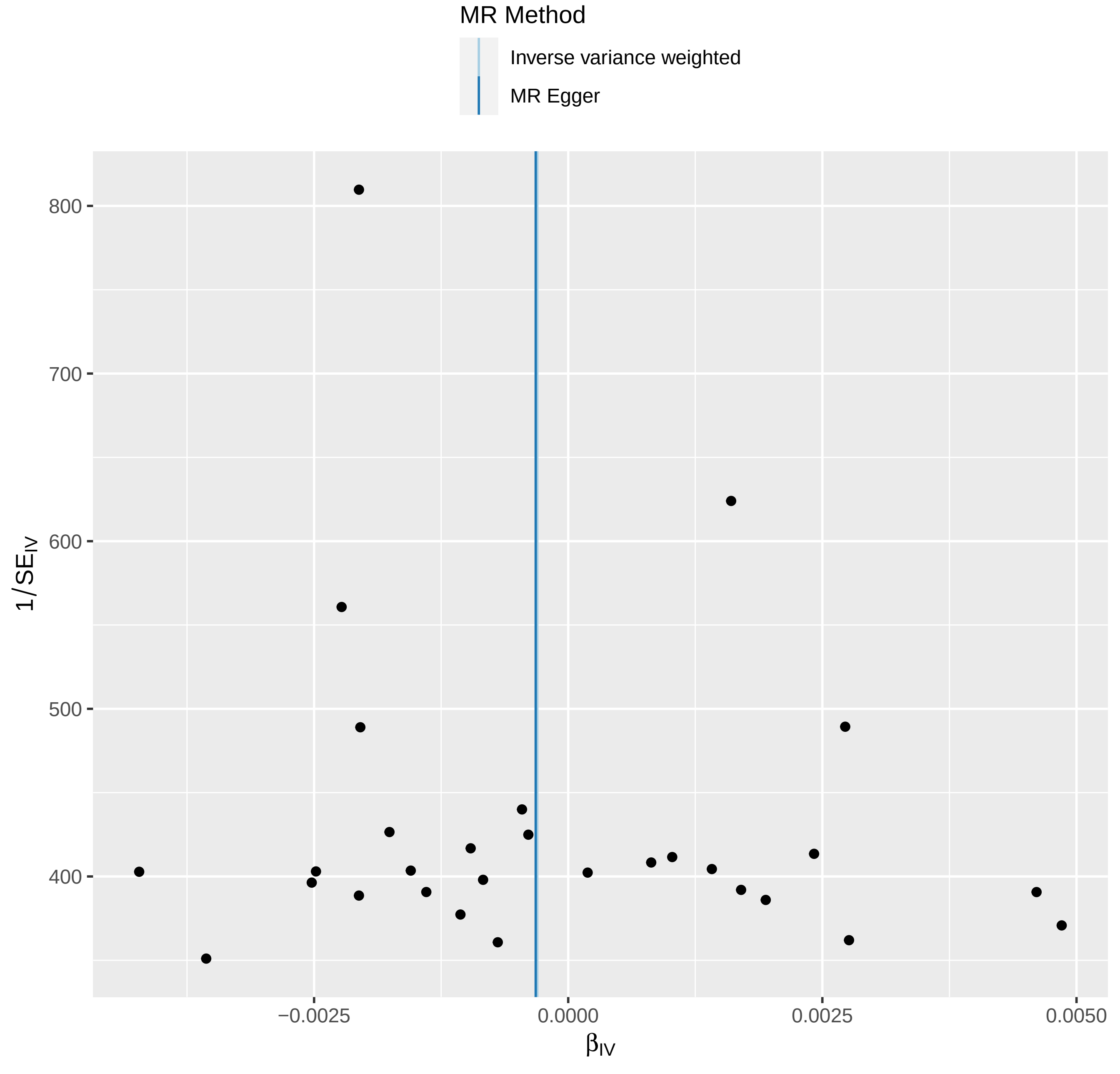

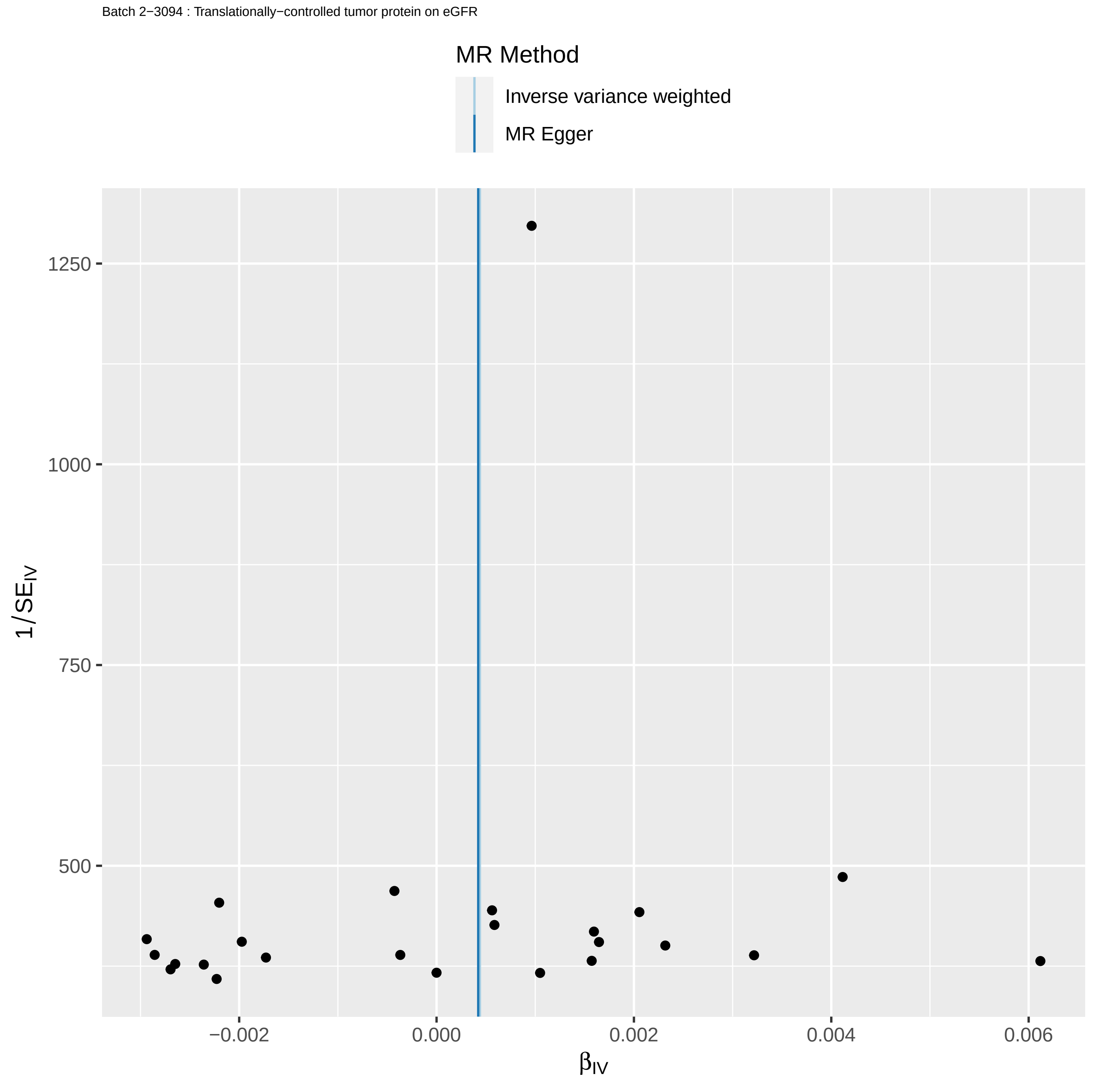

Batch 2−3094 : Translationally−controlled tumor protein on eGFR
MR Method
Inverse variance weighted
MR Egger
1250
1000
750
500
−0.002
0.000
0.002
0.004
0.006
β_{IV}
$1/SE_{IV}$

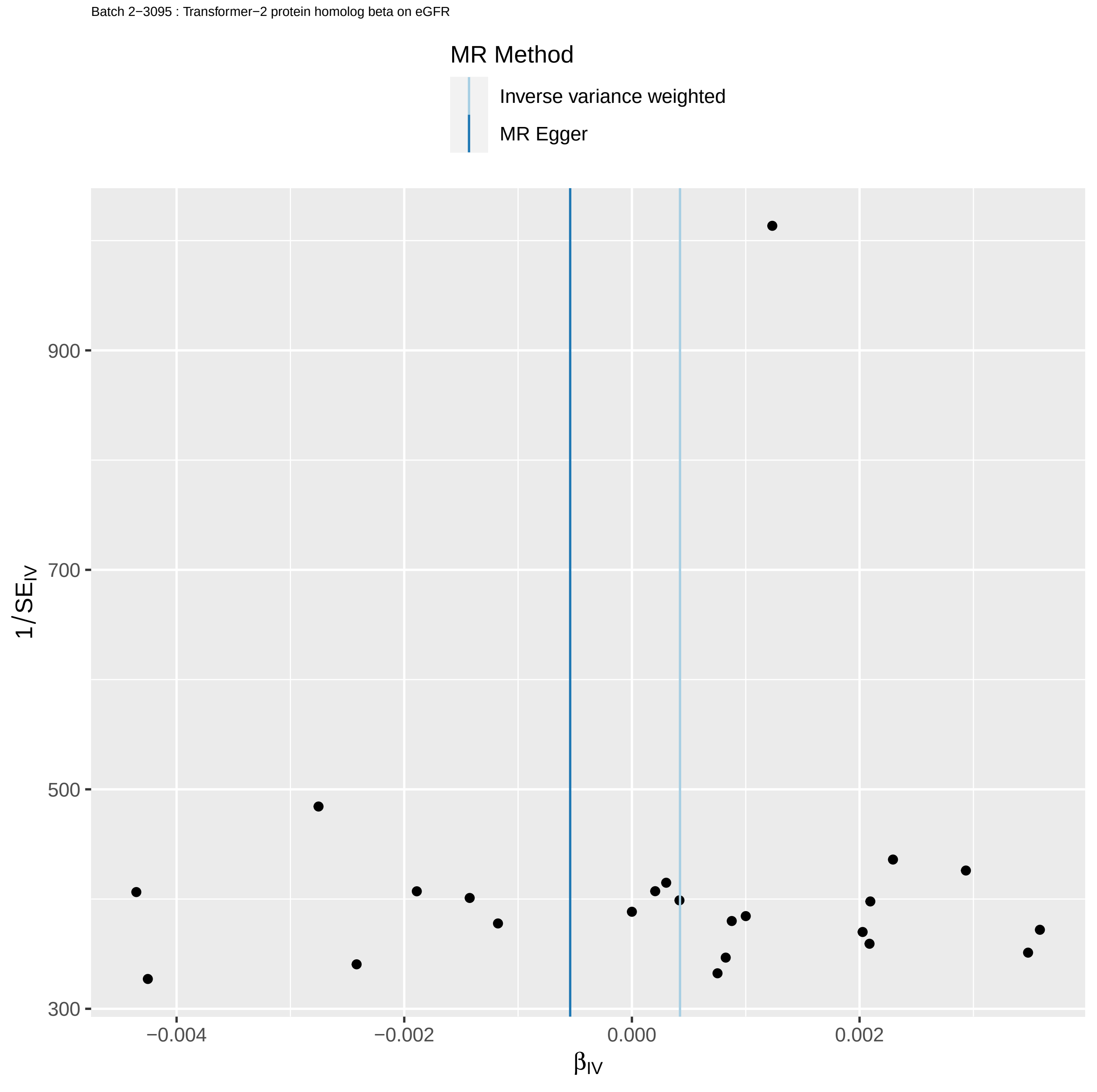
Batch 2−3095 : Transformer−2 protein homolog beta on eGFR
MR Method
Inverse variance weighted
MR Egger
900
700
500
300
1/SE_IV
−0.004
−0.002
0.000
0.002
β_IV

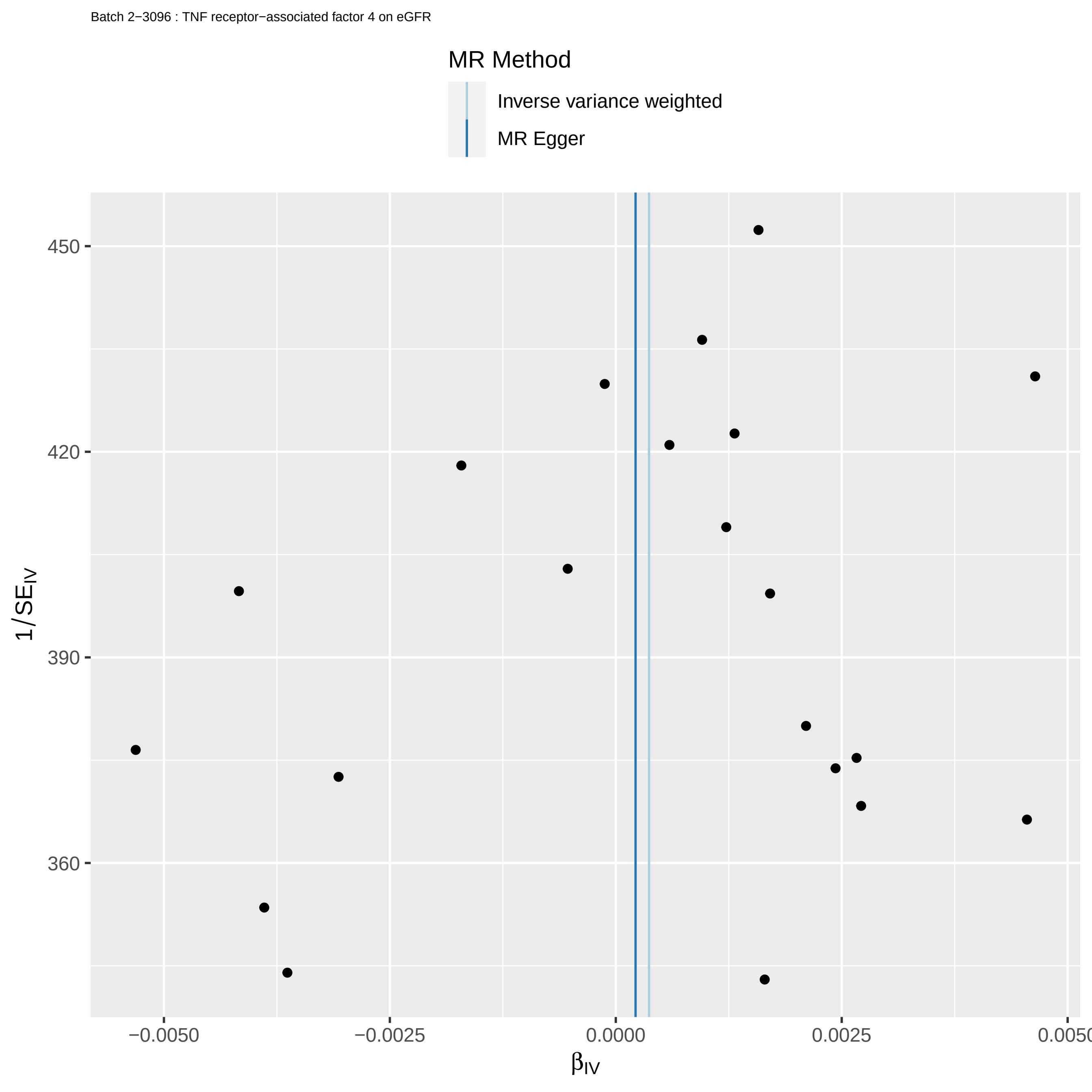

Batch 2−3096 : TNF receptor−associated factor 4 on eGFR
MR Method
Inverse variance weighted
MR Egger
450
420
390
360
1/SE_IV
−0.0050
−0.0025
0.0000
0.0025
0.0050
β_IV

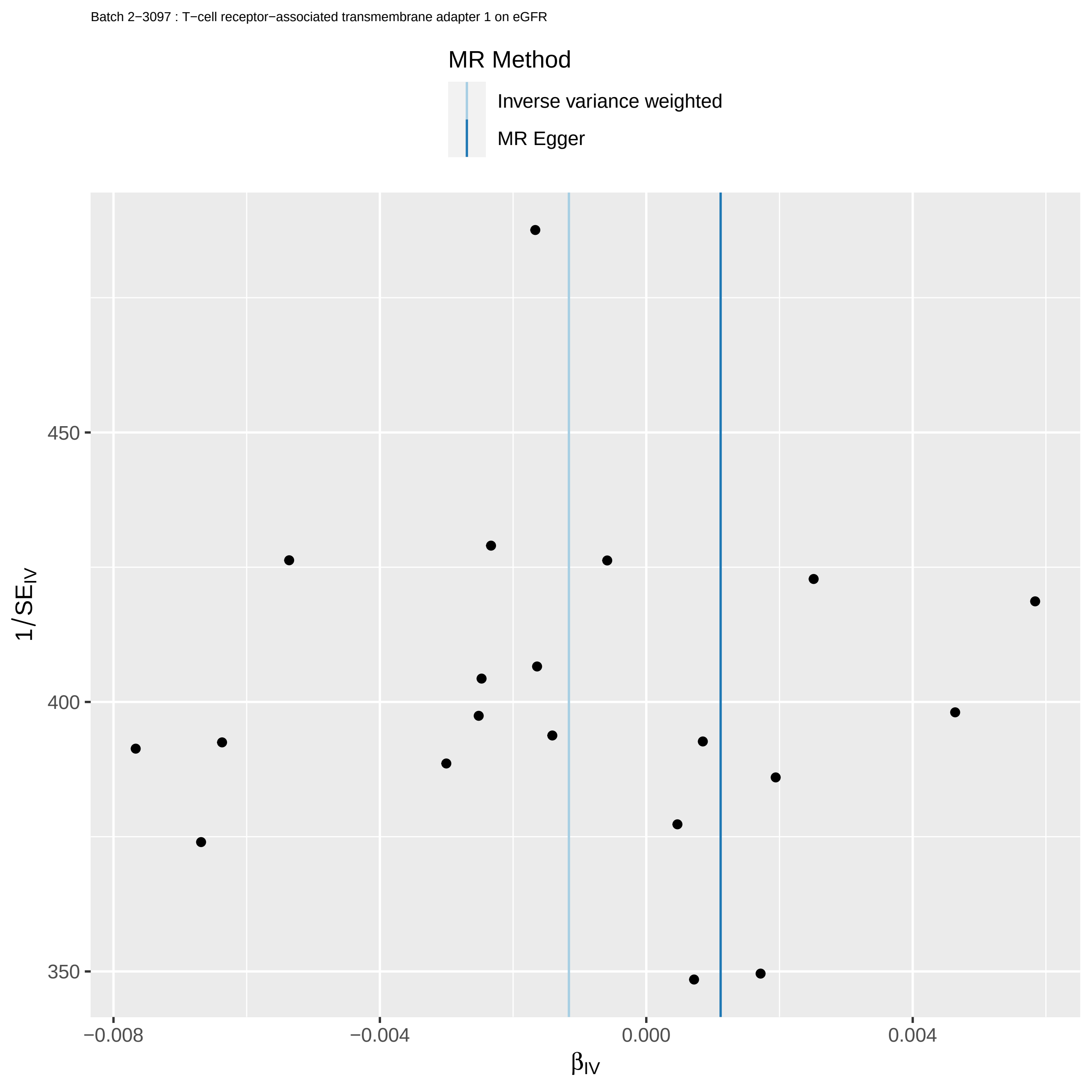
Batch 2−3097 : T−cell receptor−associated transmembrane adapter 1 on eGFR
MR Method
Inverse variance weighted
MR Egger
450
400
350
$1/SE_{IV}$
−0.008
−0.004
0.000
0.004
β_{IV}

Batch 2−3098 : Triggering receptor expressed on myeloid cells 1 on eGFR

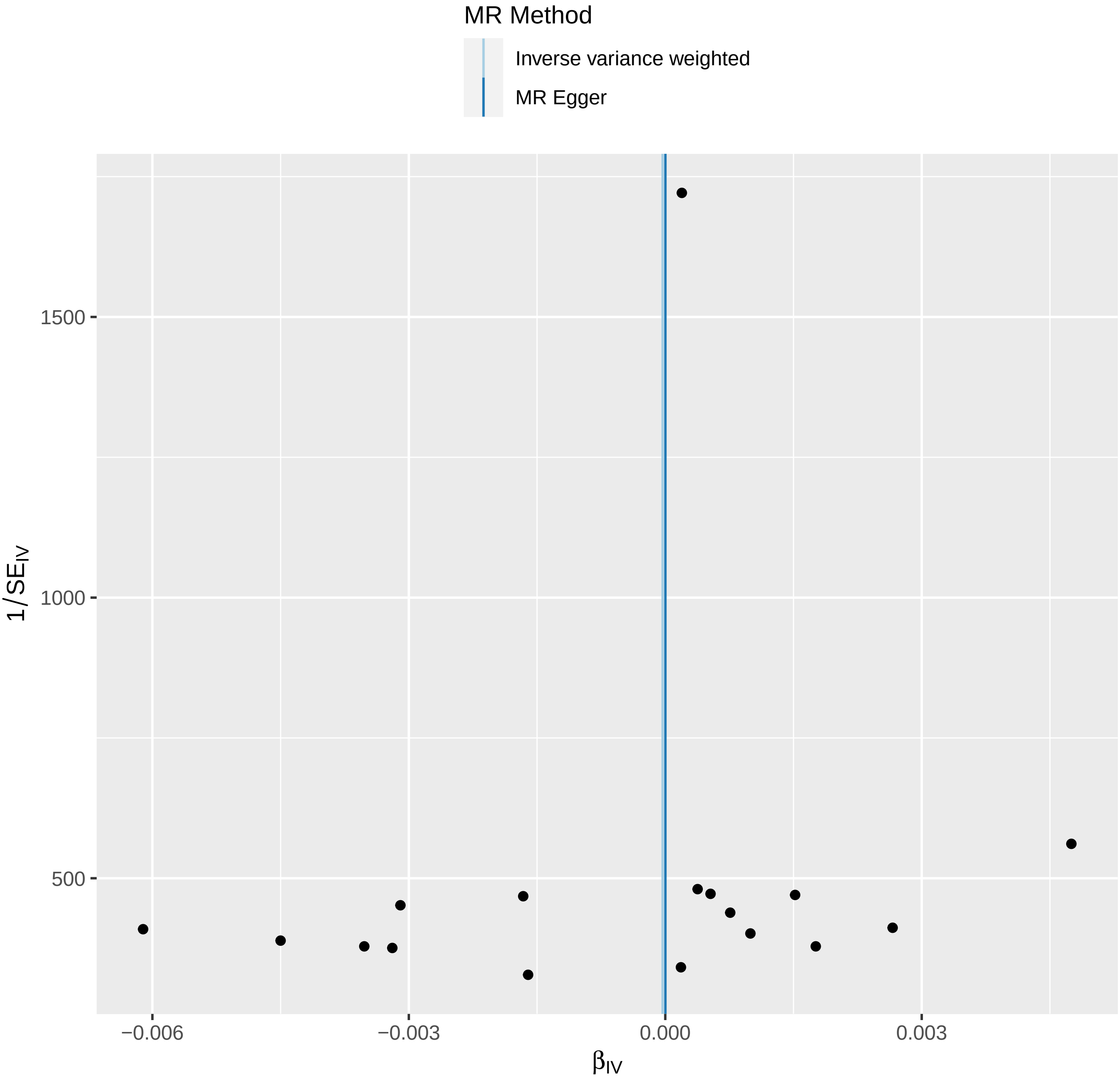

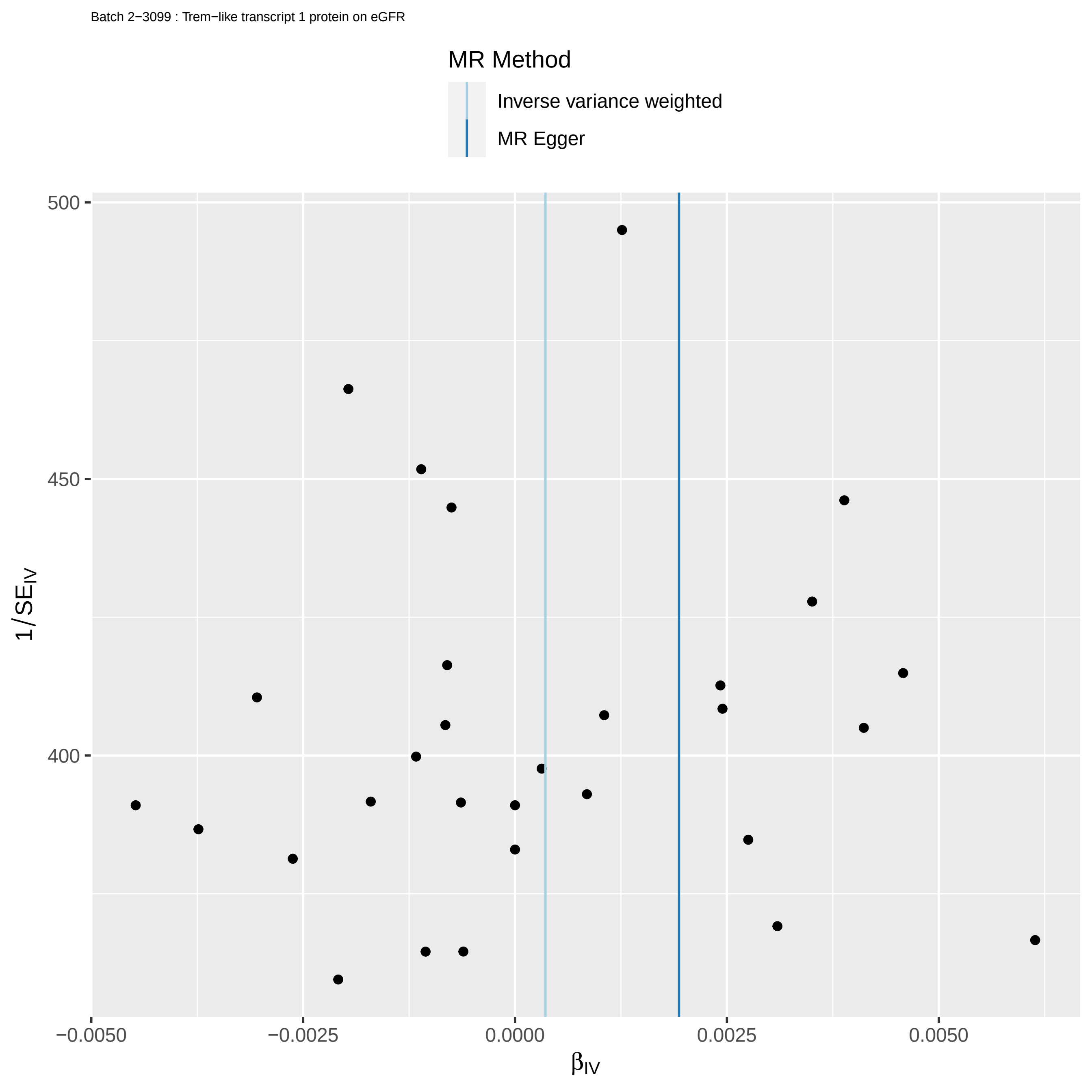

Batch 2−3099 : Trem−like transcript 1 protein on eGFR
MR Method
Inverse variance weighted
MR Egger
500
450
400
1/SE$_{IV}$
−0.0050
−0.0025
0.0000
0.0025
0.0050
β$_{IV}$

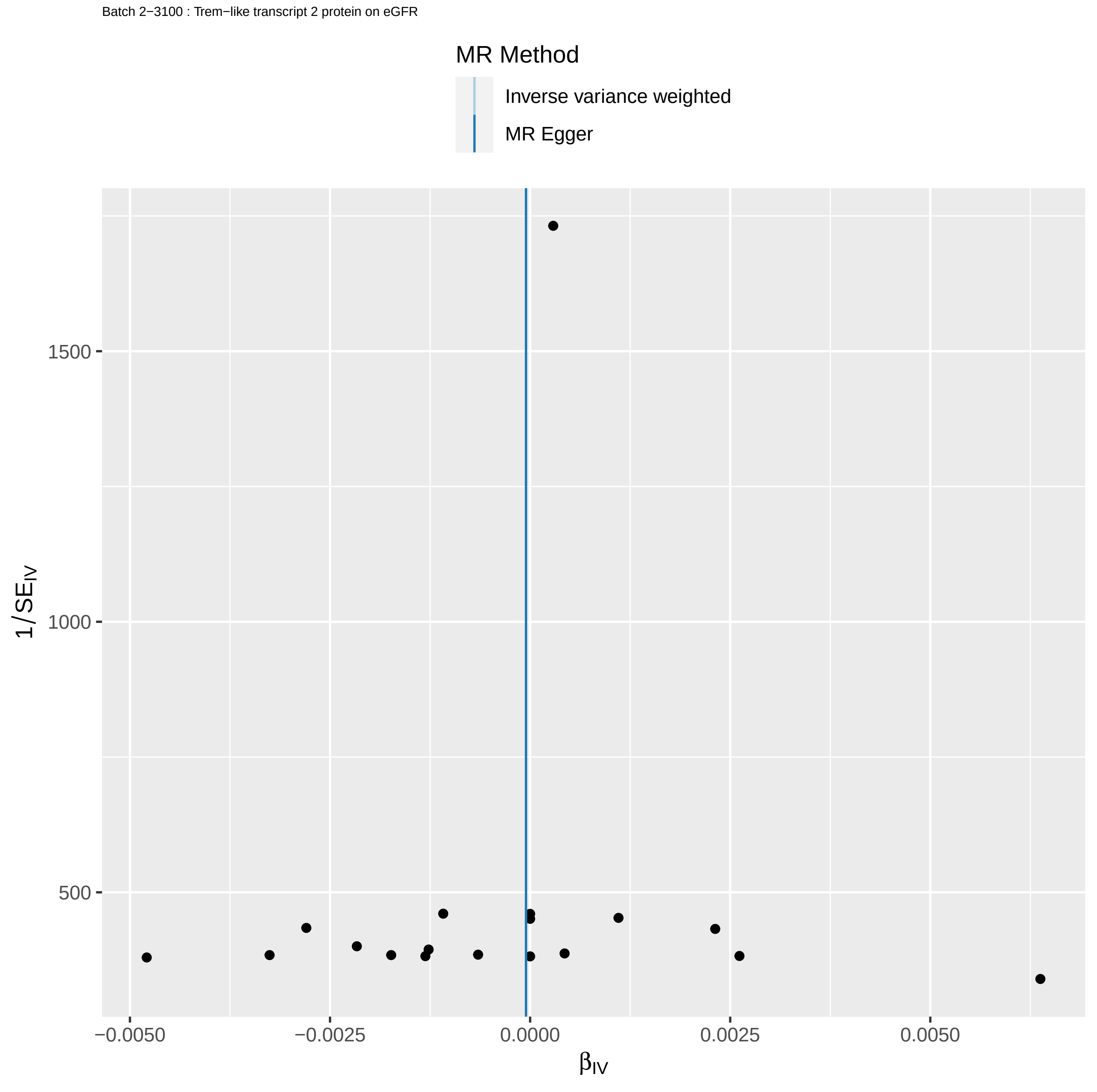

Batch 2−3100 : Trem−like transcript 2 protein on eGFR
MR Method
Inverse variance weighted
MR Egger
1500
1000
500
1/SE$_{IV}$
−0.0050
−0.0025
0.0000
0.0025
0.0050
β$_{IV}$

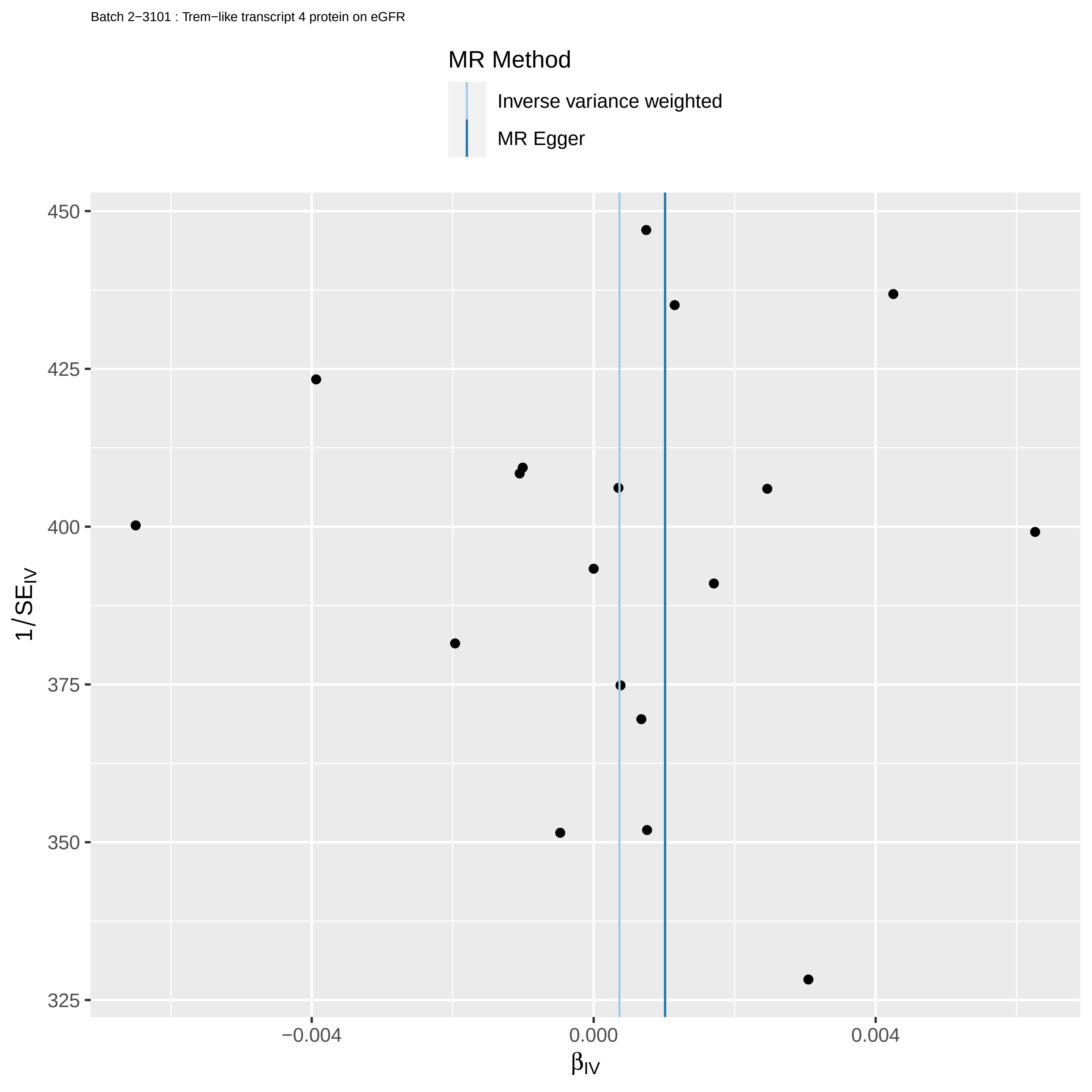
Batch 2−3101 : Trem−like transcript 4 protein on eGFR
MR Method
Inverse variance weighted
MR Egger
450
425
400
375
350
325
1/SE_IV
−0.004
0.000
0.004
β_IV

Batch 2−3102 : Thyrotropin−releasing hormone on eGFR

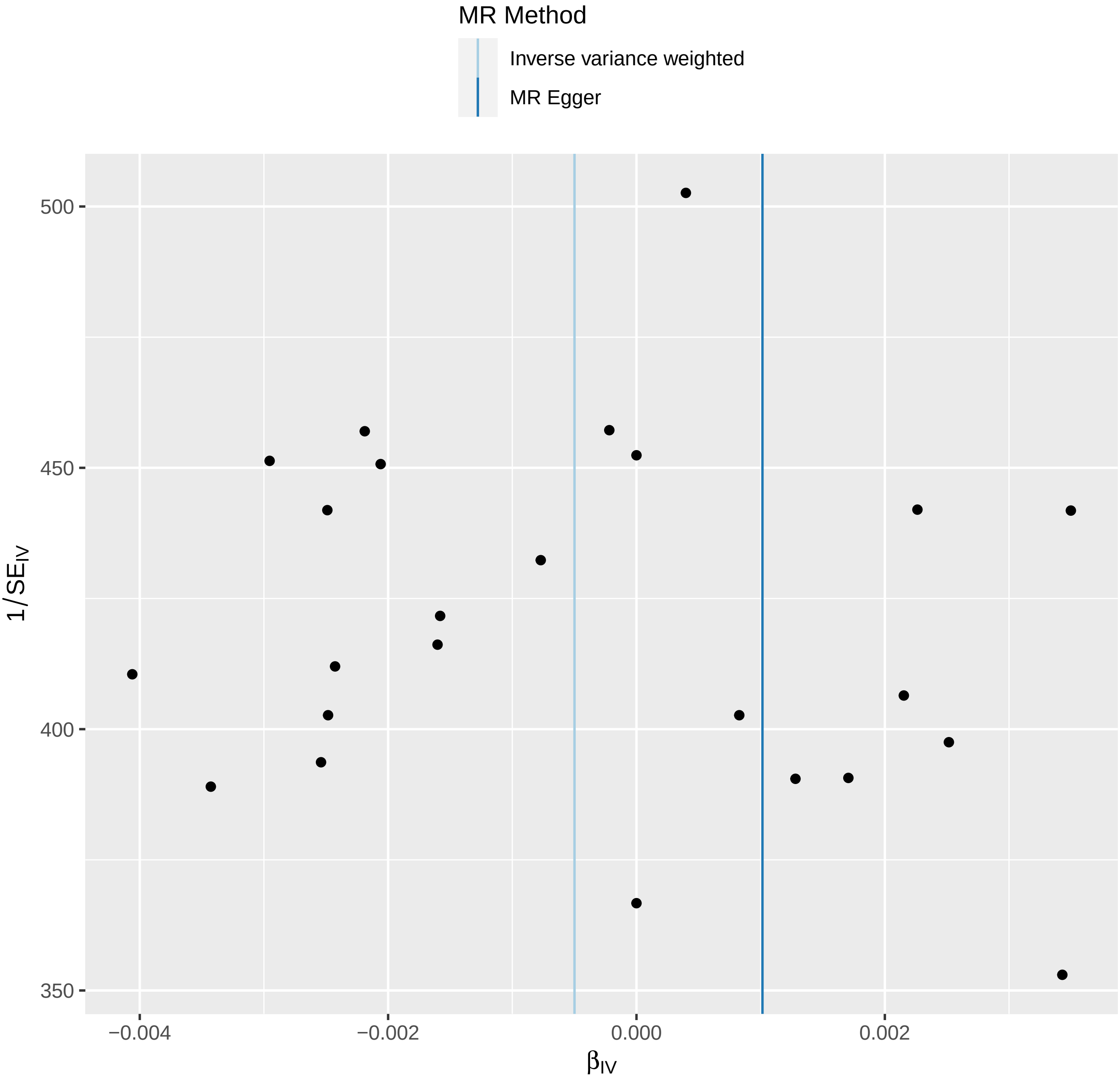

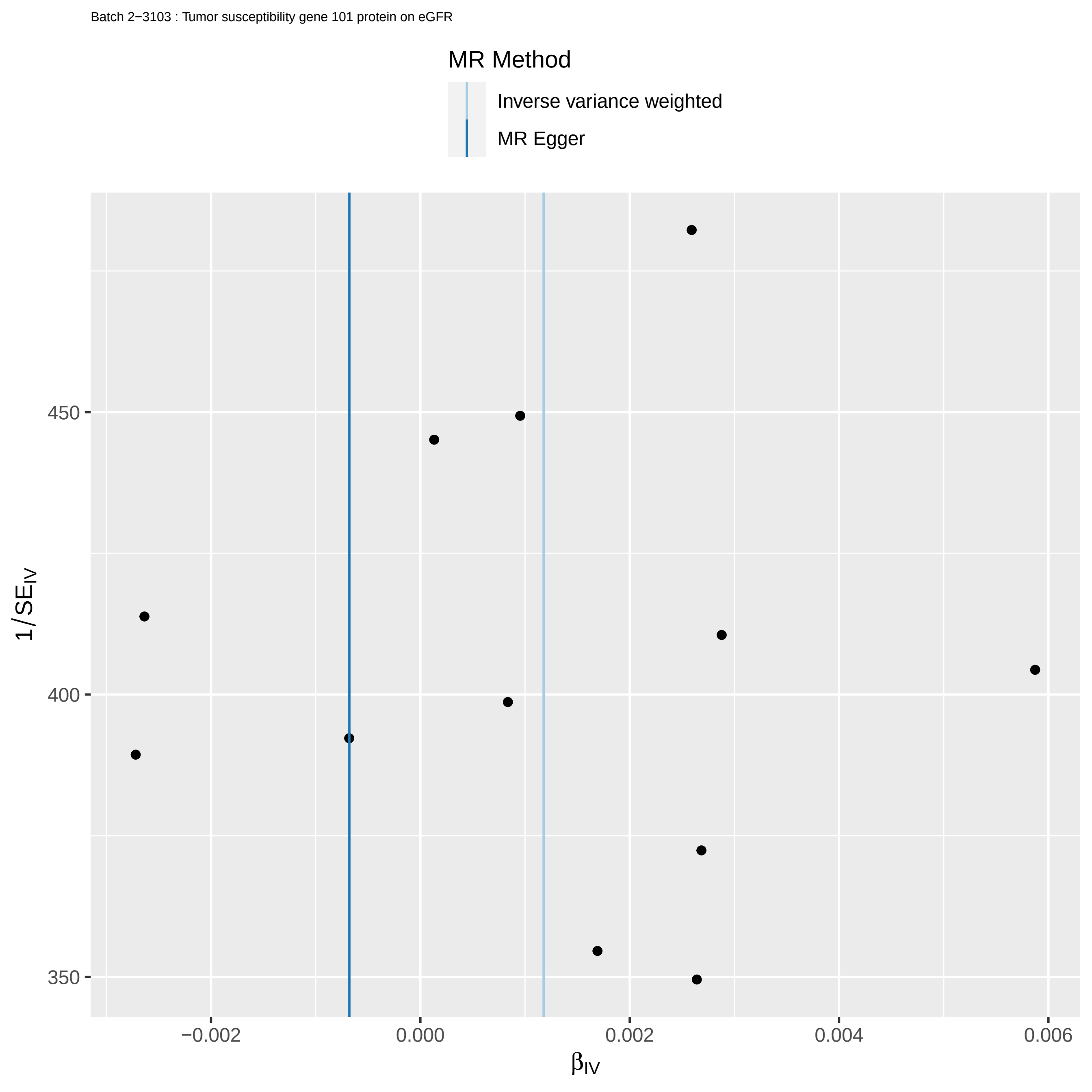

Batch 2−3103 : Tumor susceptibility gene 101 protein on eGFR
MR Method
Inverse variance weighted
MR Egger
450
400
350
1/SE$_{IV}$
−0.002
0.000
0.002
0.004
0.006
β$_{IV}$

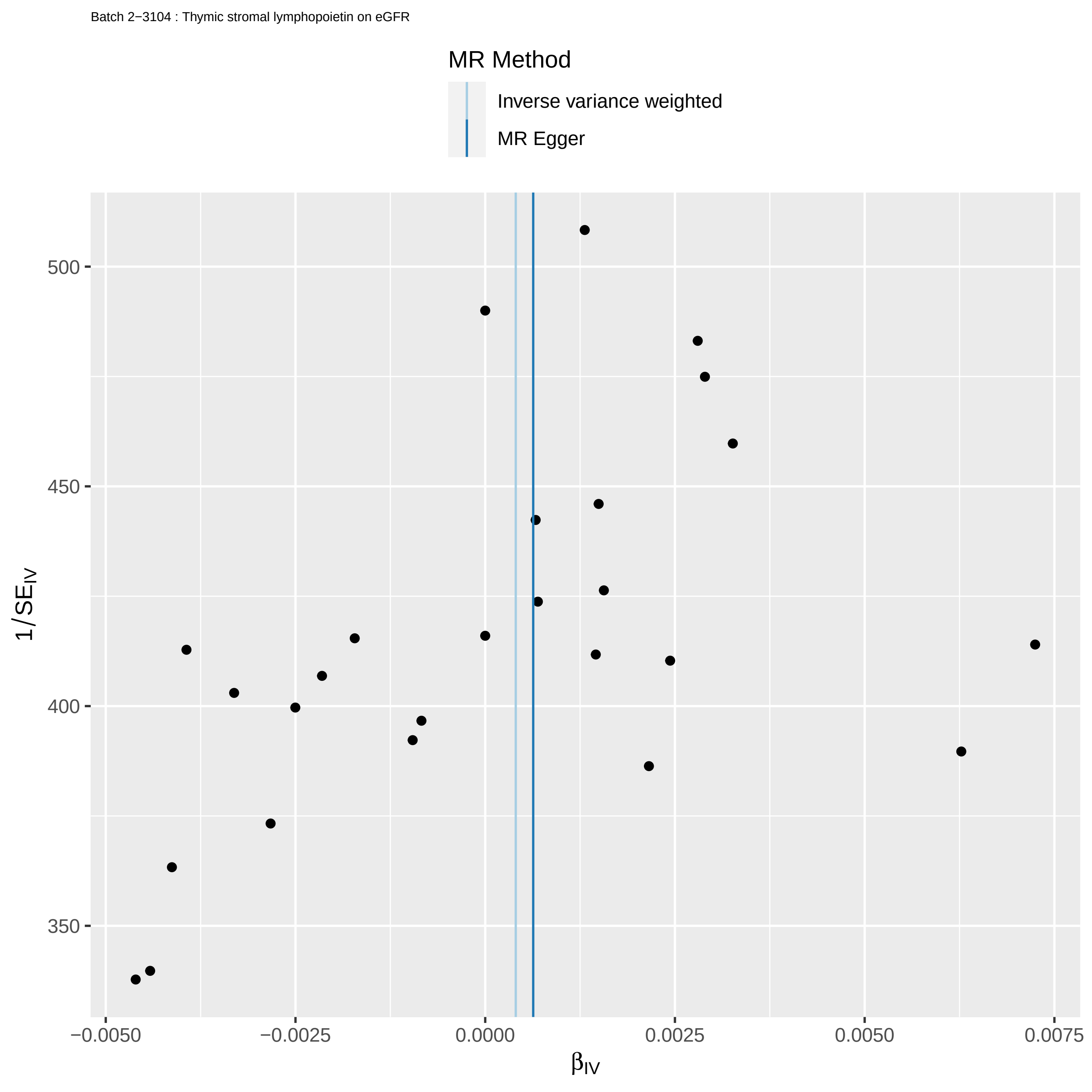

Batch 2−3104 : Thymic stromal lymphopoietin on eGFR
MR Method
Inverse variance weighted
MR Egger
500
450
400
350
1/SE$_{IV}$
−0.0050
−0.0025
0.0000
0.0025
0.0050
0.0075
β$_{IV}$

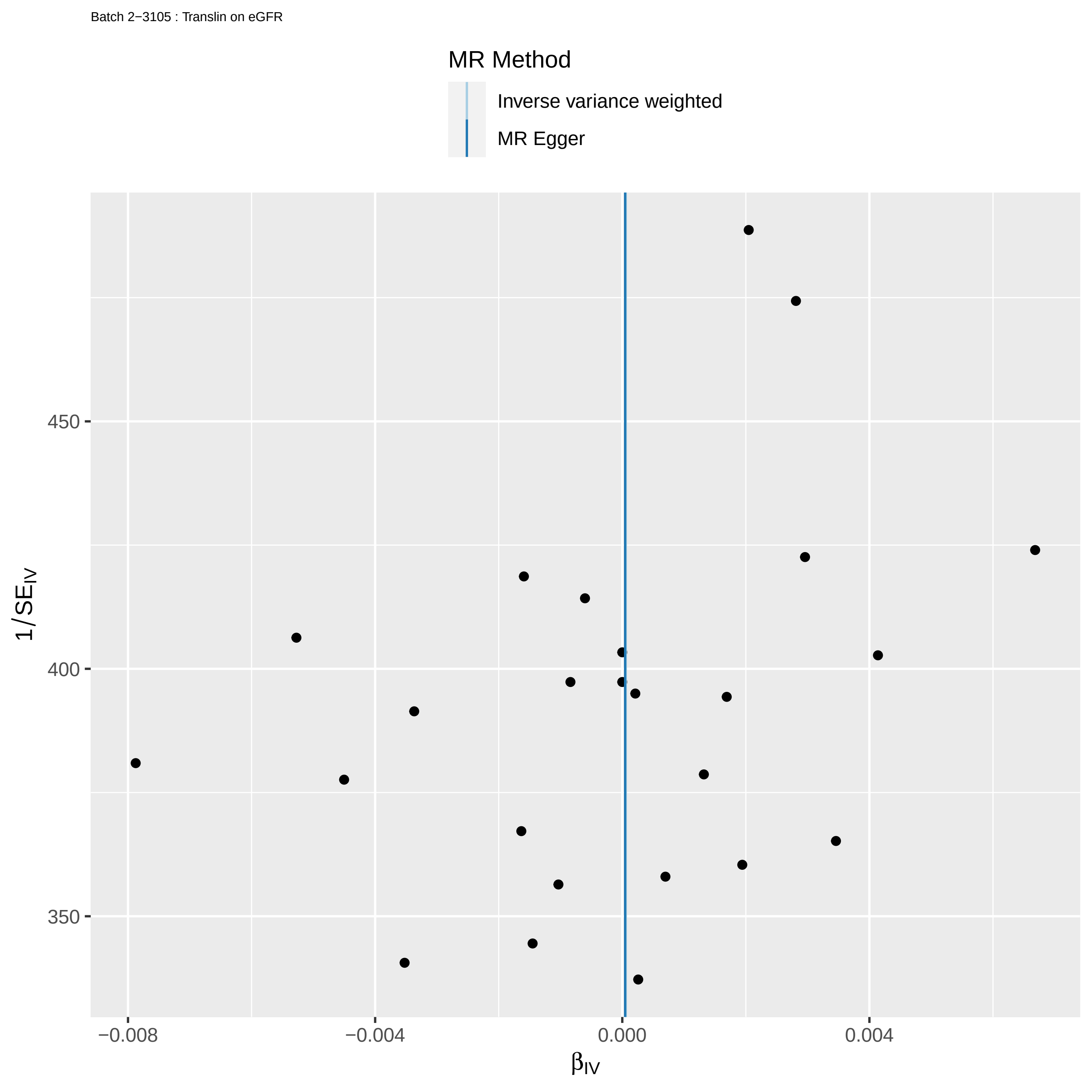
Batch 2−3105 : Translin on eGFR
MR Method
Inverse variance weighted
MR Egger
450
400
350
1/SE_IV
−0.008
−0.004
0.000
0.004
β_IV

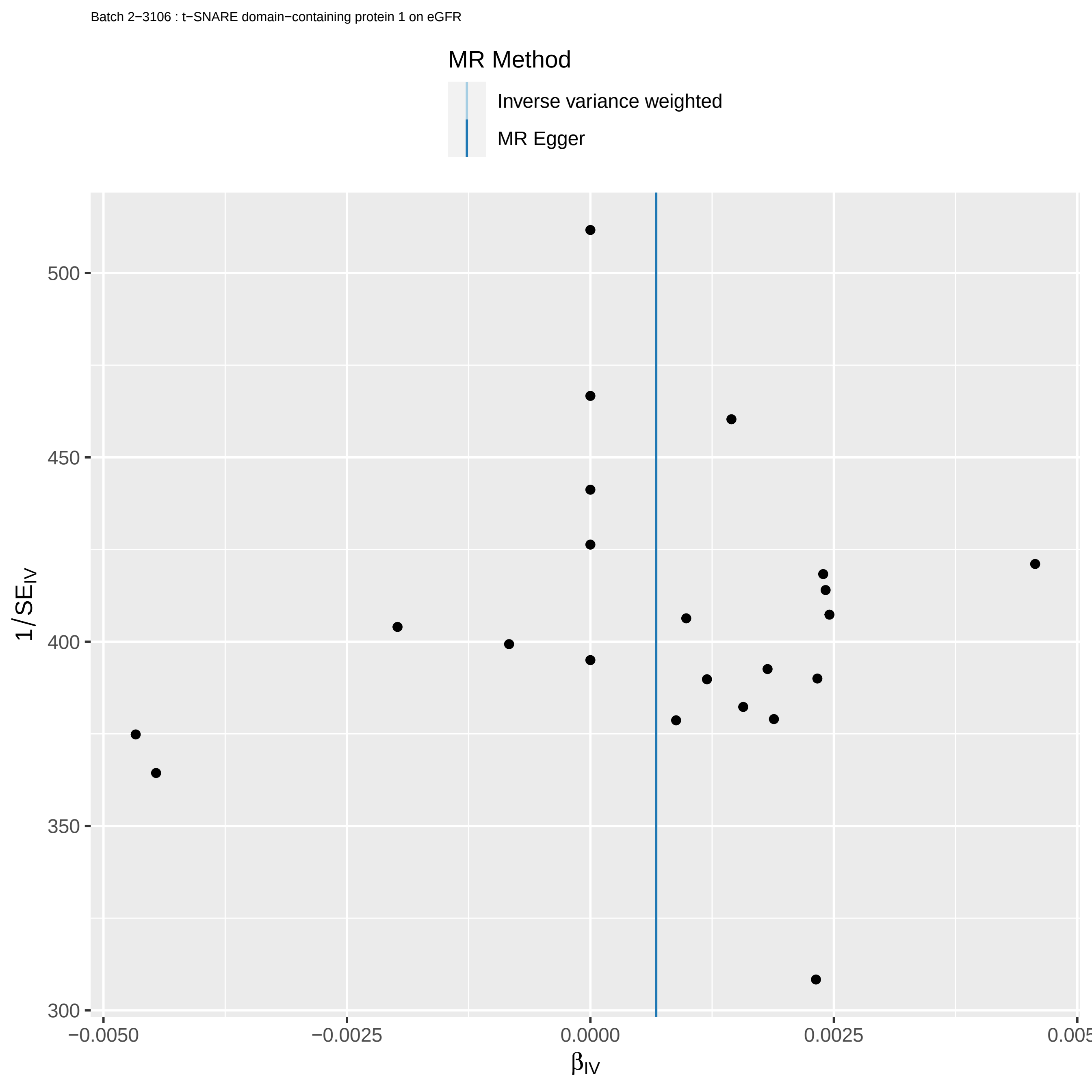
Batch 2−3106 : t−SNARE domain−containing protein 1 on eGFR
MR Method
Inverse variance weighted
MR Egger
500
450
400
350
300
1/SE_IV
−0.0050
−0.0025
0.0000
0.0025
0.005
β_IV

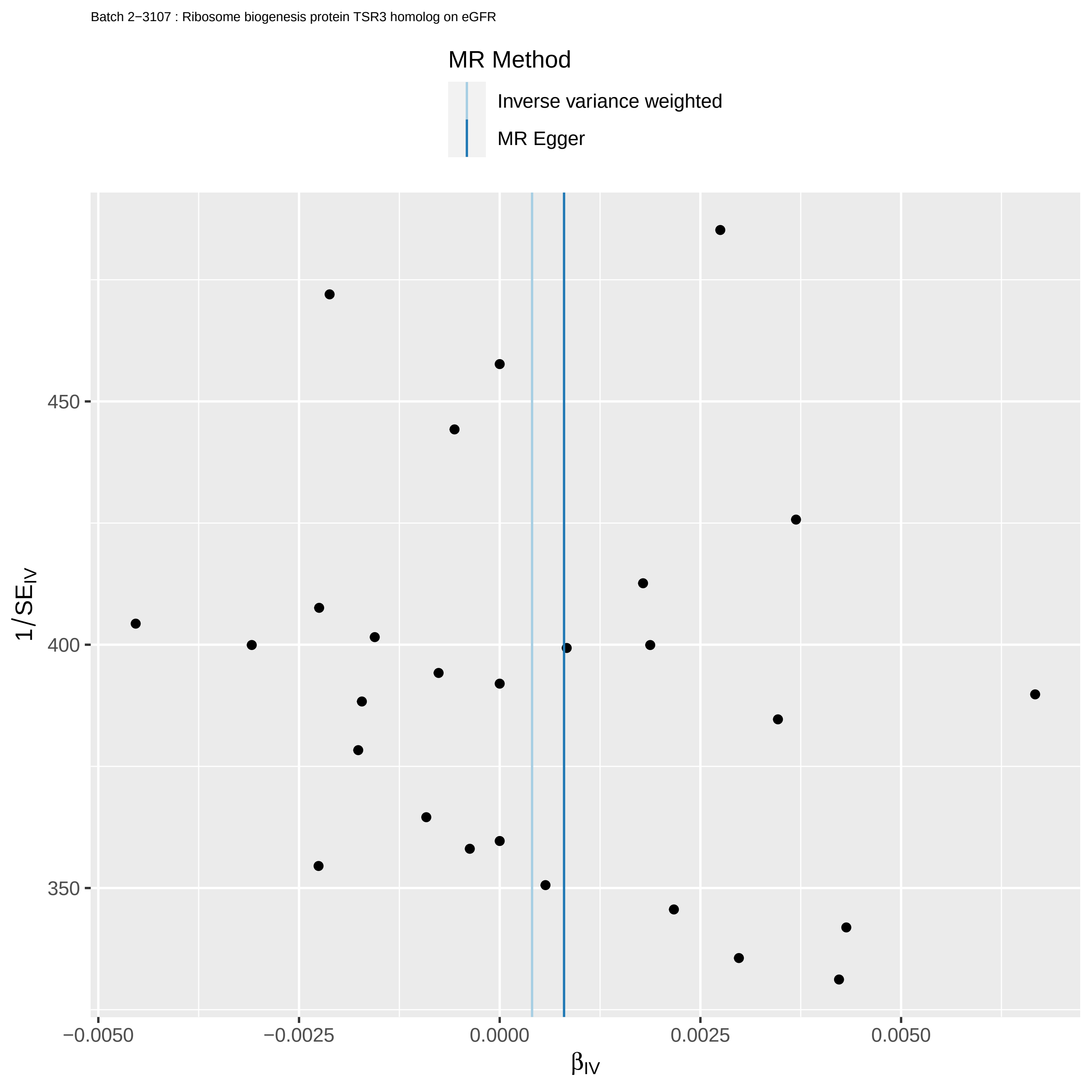

Batch 2−3107 : Ribosome biogenesis protein TSR3 homolog on eGFR
MR Method
Inverse variance weighted
MR Egger
450
400
350
1/SE_IV
−0.0050
−0.0025
0.0000
0.0025
0.0050
β_IV

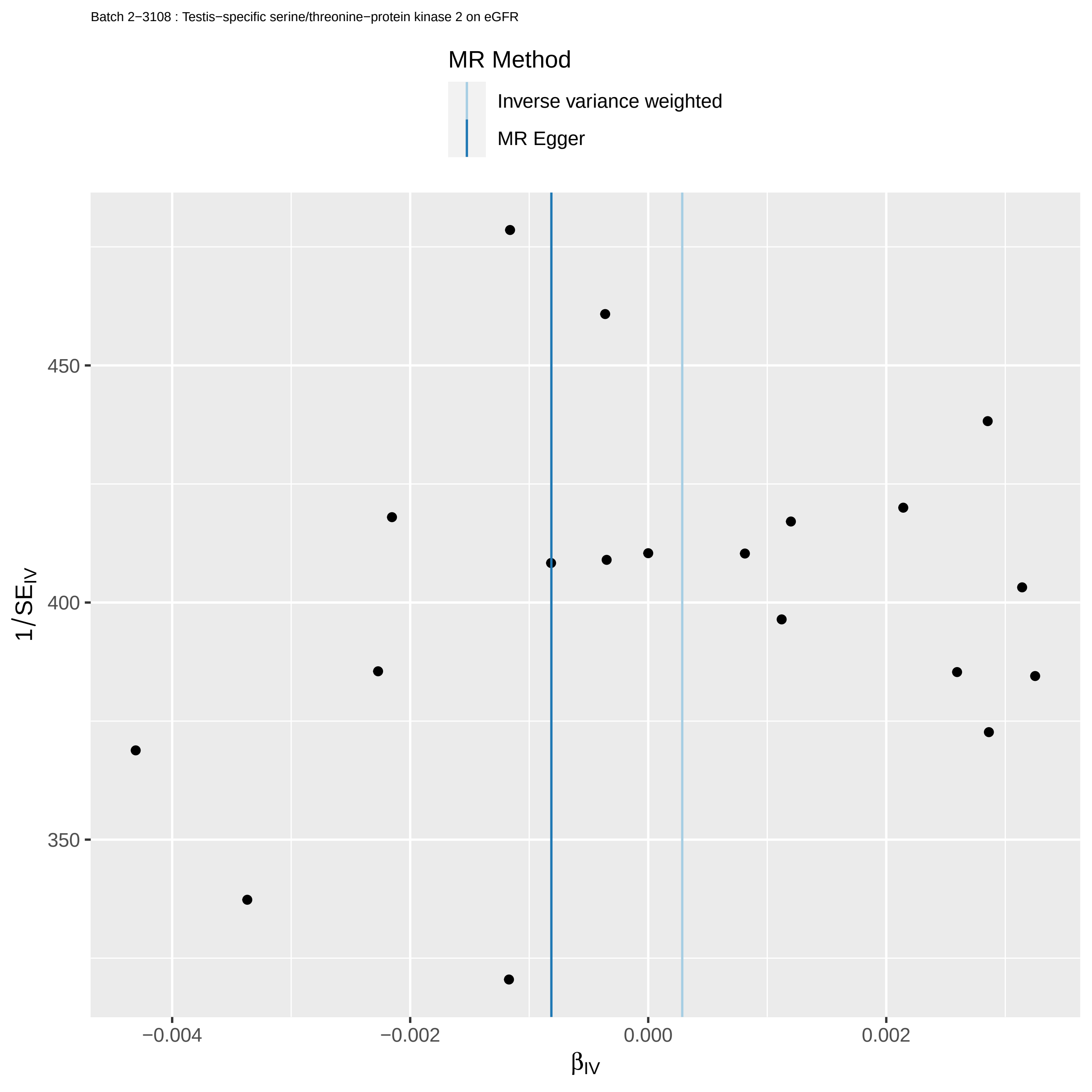
Batch 2−3108 : Testis−specific serine/threonine−protein kinase 2 on eGFR
MR Method
Inverse variance weighted
MR Egger
1/SE_IV
450
400
350
−0.004
−0.002
0.000
0.002
β_IV

Batch 2−3109 : Thiosulfate sulfurtransferase on eGFR

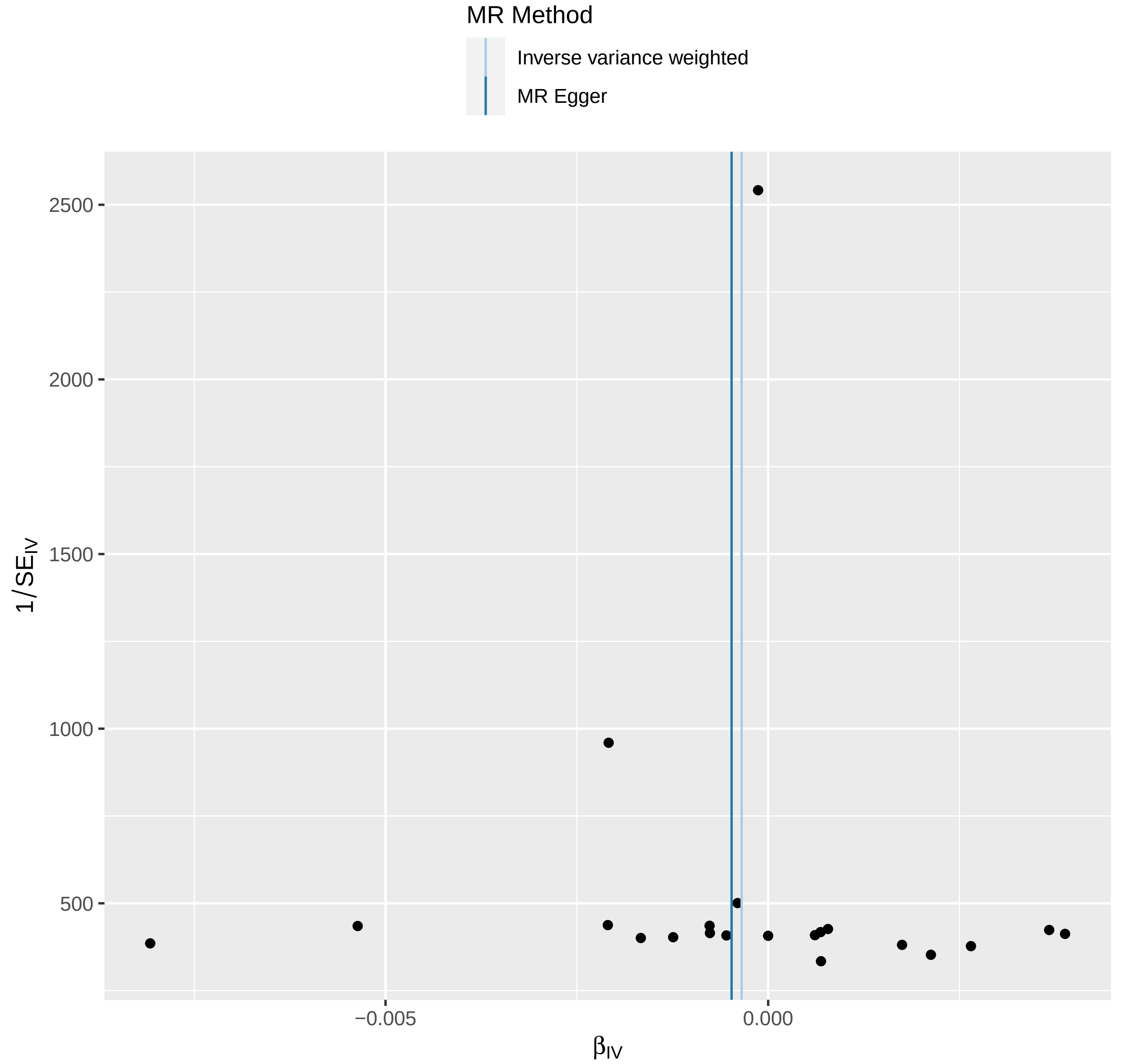

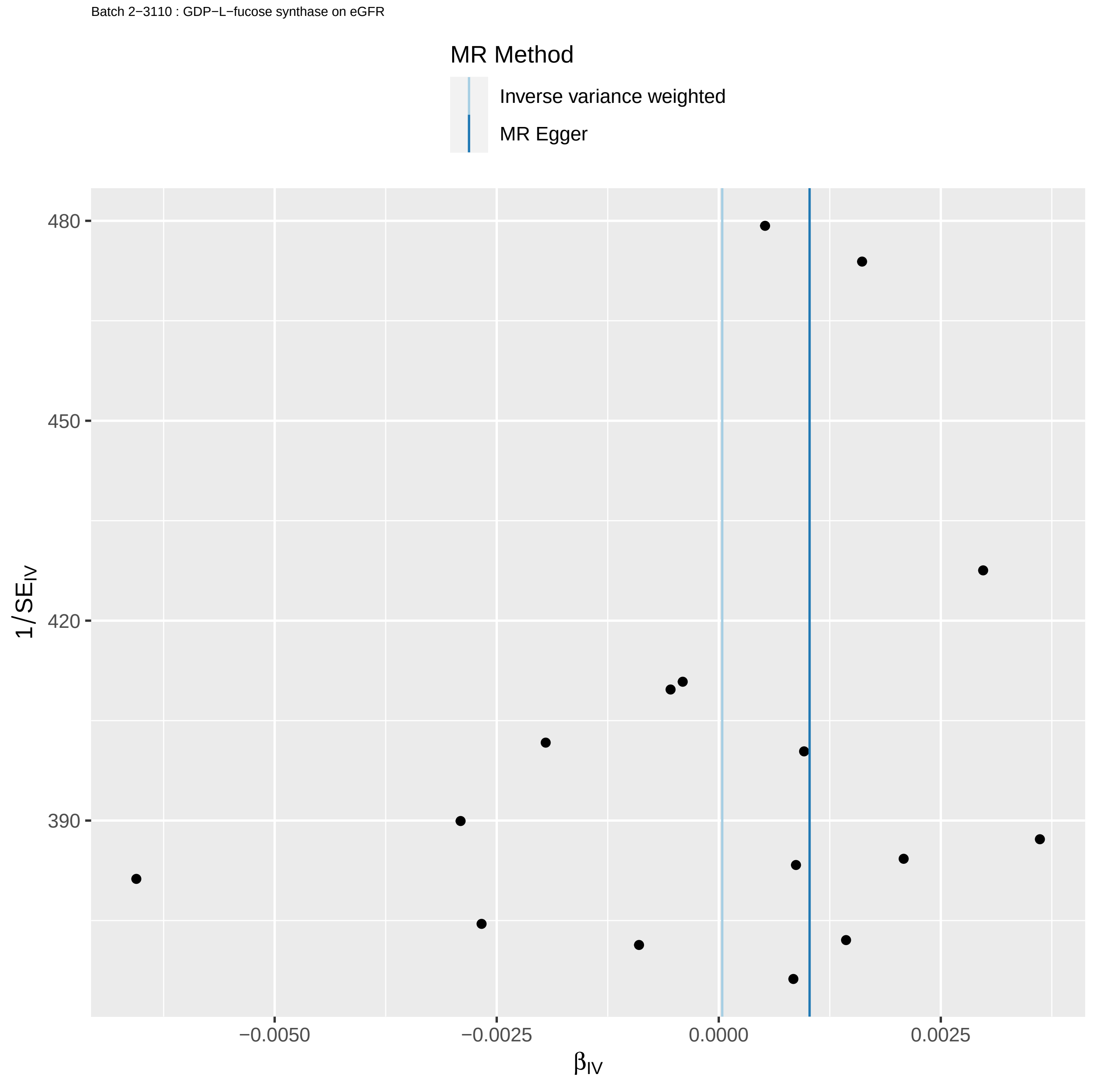

Batch 2−3110 : GDP−L−fucose synthase on eGFR
MR Method
Inverse variance weighted
MR Egger
480
450
420
390
1/SE_IV
−0.0050
−0.0025
0.0000
0.0025
β_IV

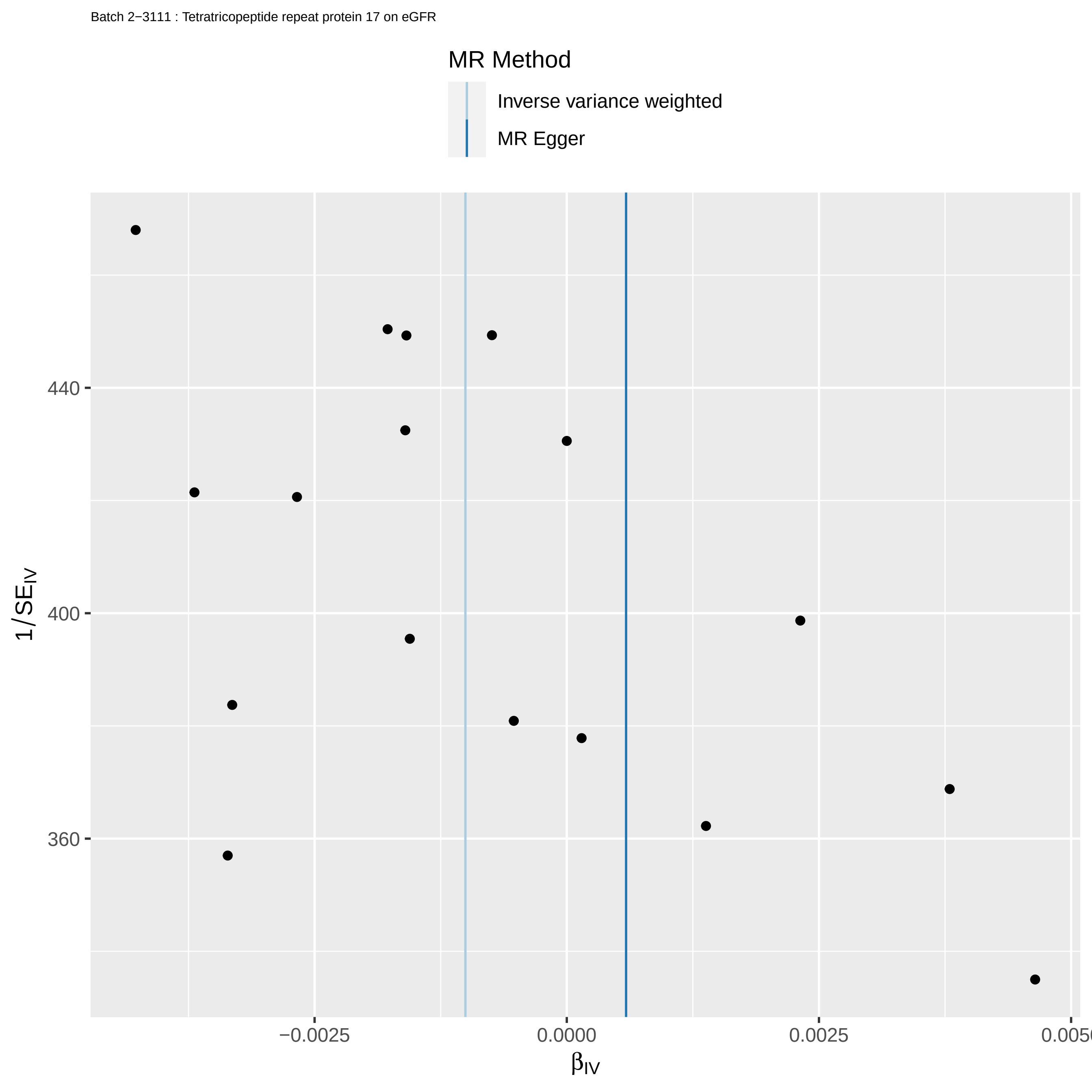
Batch 2−3111 : Tetratricopeptide repeat protein 17 on eGFR
MR Method
Inverse variance weighted
MR Egger
440
400
360
1/SE_IV
−0.0025
0.0000
0.0025
0.005
β_IV

Batch 2−3112 : Tetratricopeptide repeat protein 17 on eGFR

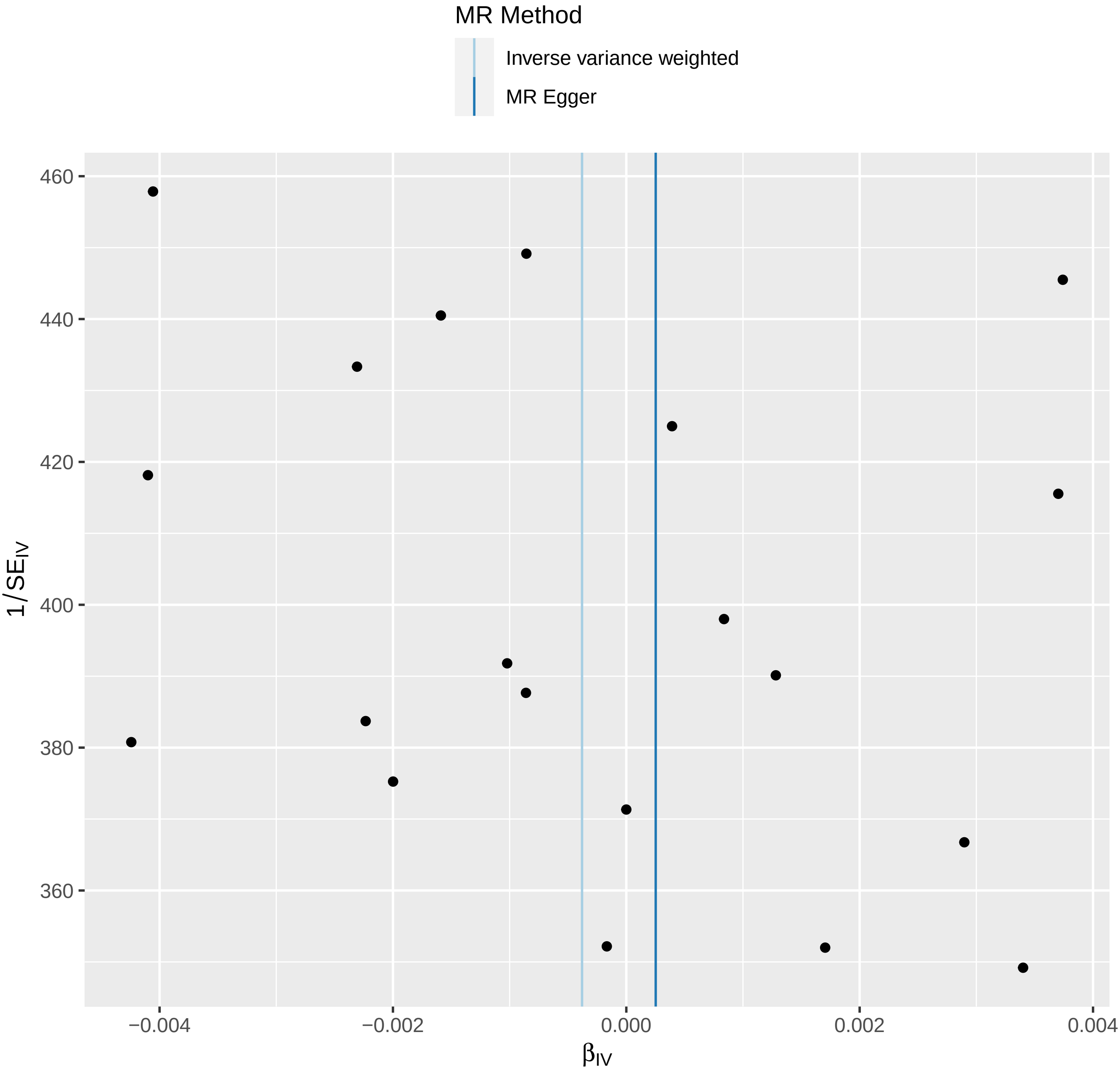

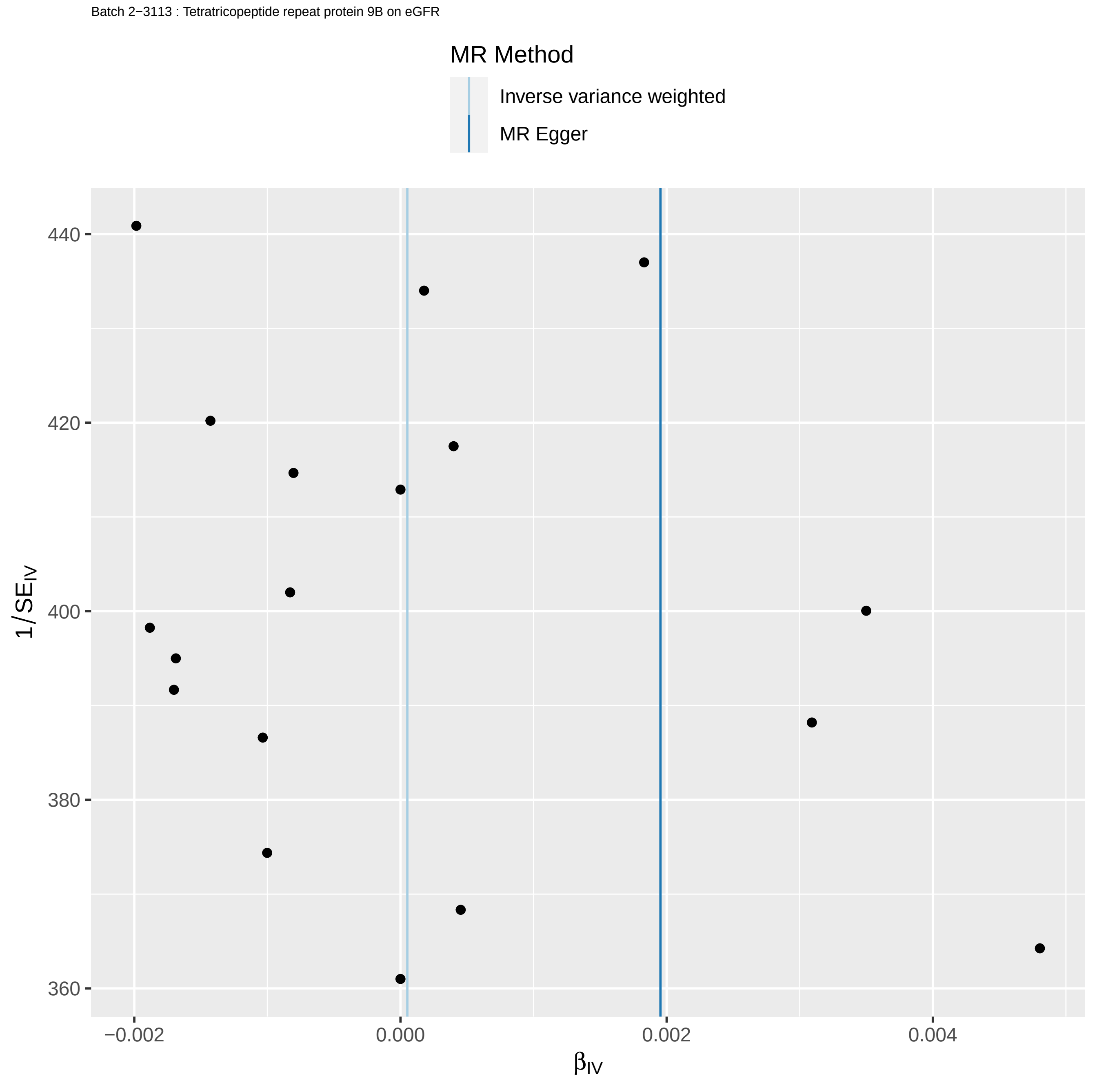

Batch 2−3113 : Tetratricopeptide repeat protein 9B on eGFR
MR Method
Inverse variance weighted
MR Egger
440
420
400
380
360
1/SE_IV
−0.002
0.000
0.002
0.004
β_IV

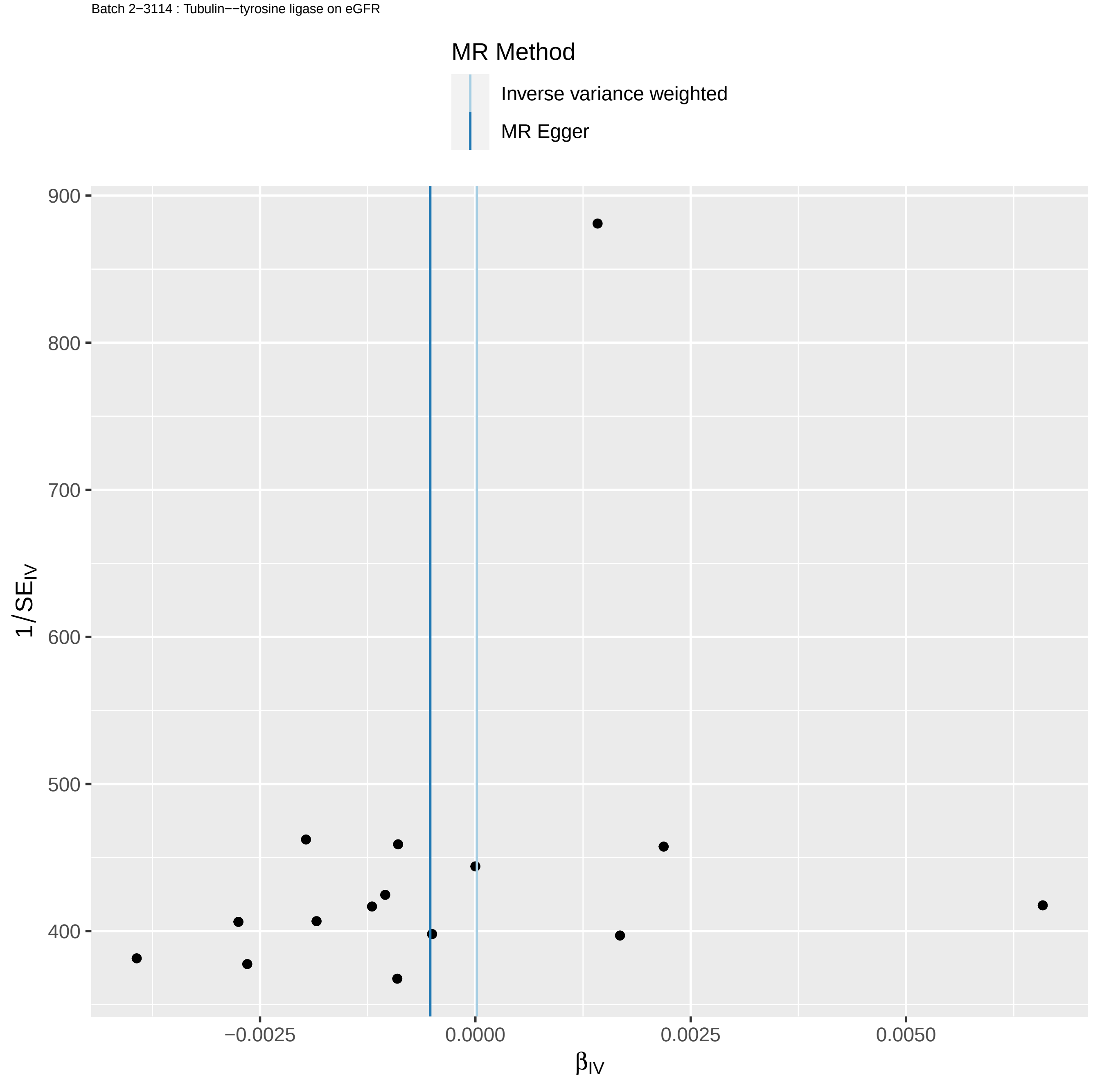
Batch 2−3114 : Tubulin−−tyrosine ligase on eGFR
MR Method
Inverse variance weighted
MR Egger
900
800
700
600
500
400
1/SE_IV
−0.0025
0.0000
0.0025
0.0050
β_IV

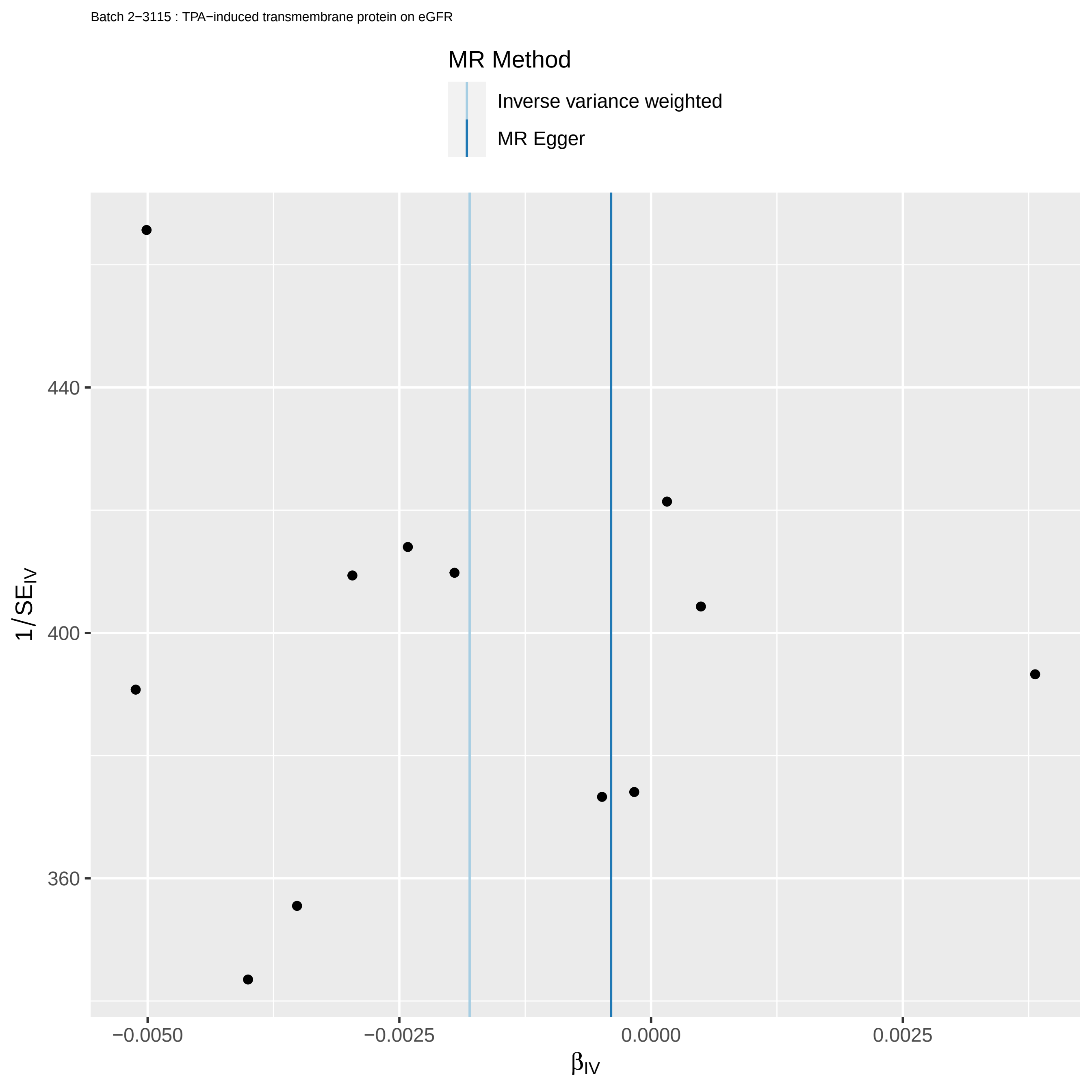

Batch 2−3115 : TPA−induced transmembrane protein on eGFR
MR Method
Inverse variance weighted
MR Egger
440
400
360
1/SE_IV
−0.0050
−0.0025
0.0000
0.0025
β_IV

Batch 2−3116 : Titin on eGFR

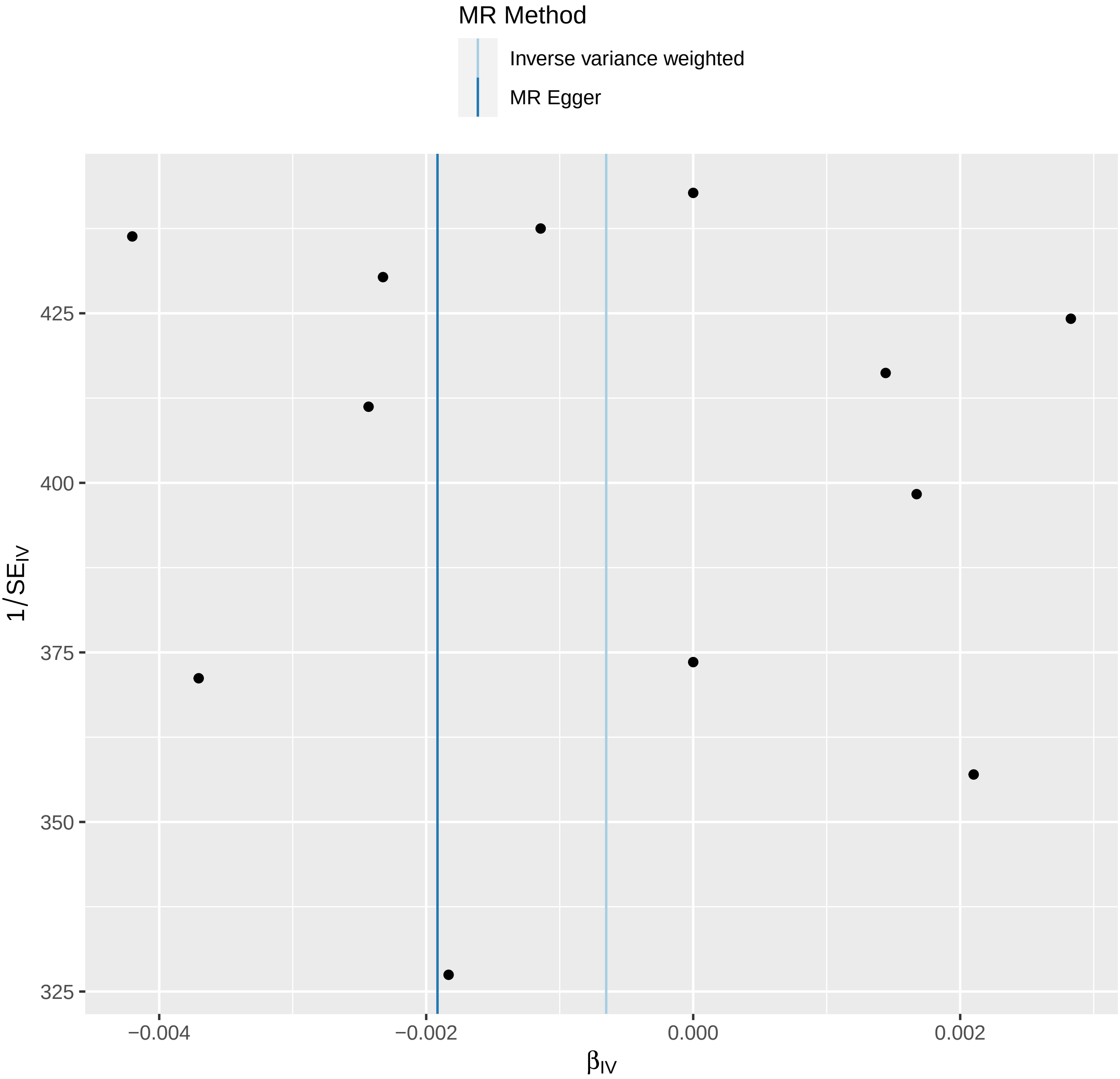

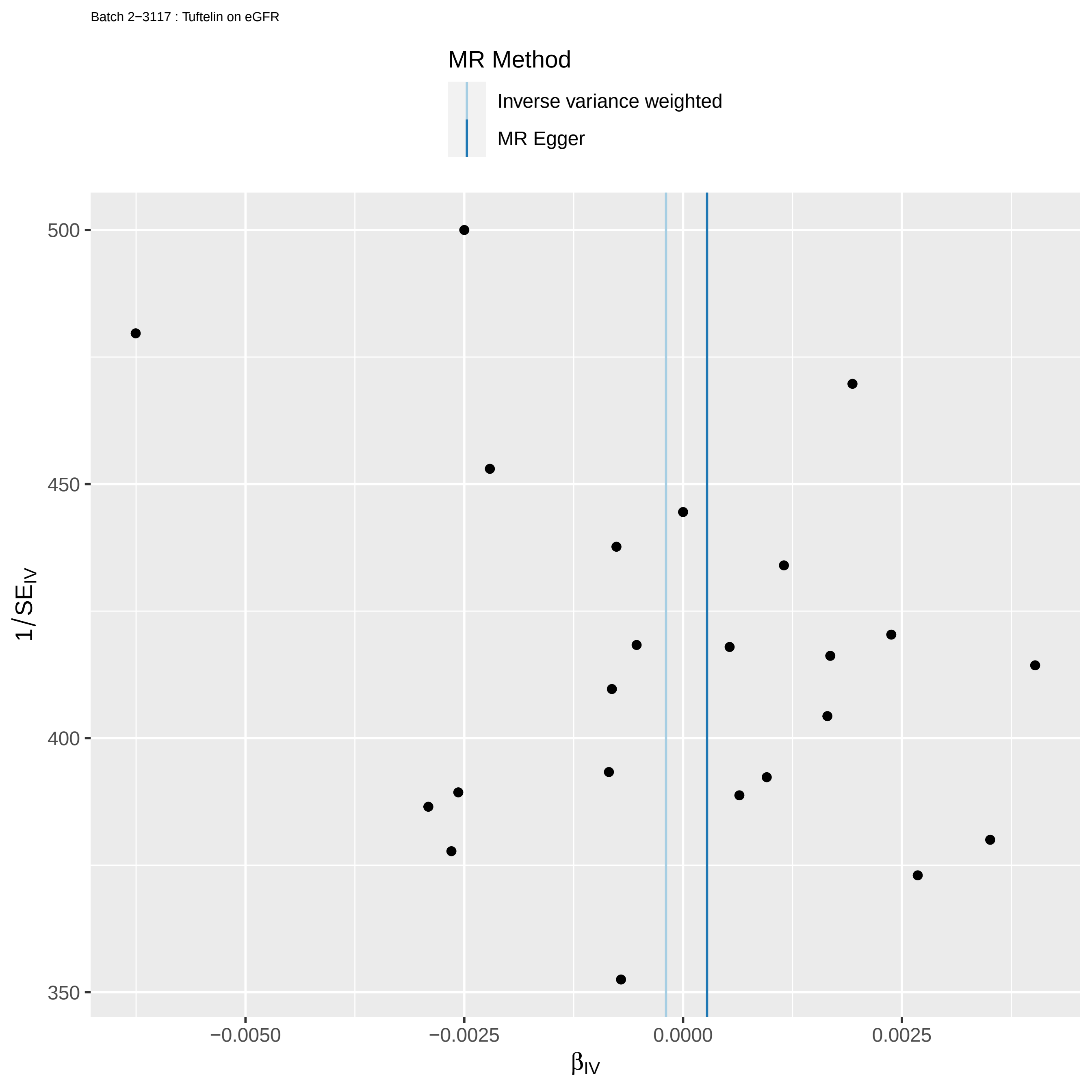

Batch 2−3117 : Tuftelin on eGFR
MR Method
Inverse variance weighted
MR Egger
500
450
400
350
−0.0050
−0.0025
0.0000
0.0025
$1/SE_{IV}$
β_{IV}

Batch 2−3118 : Twinfilin−1 on eGFR

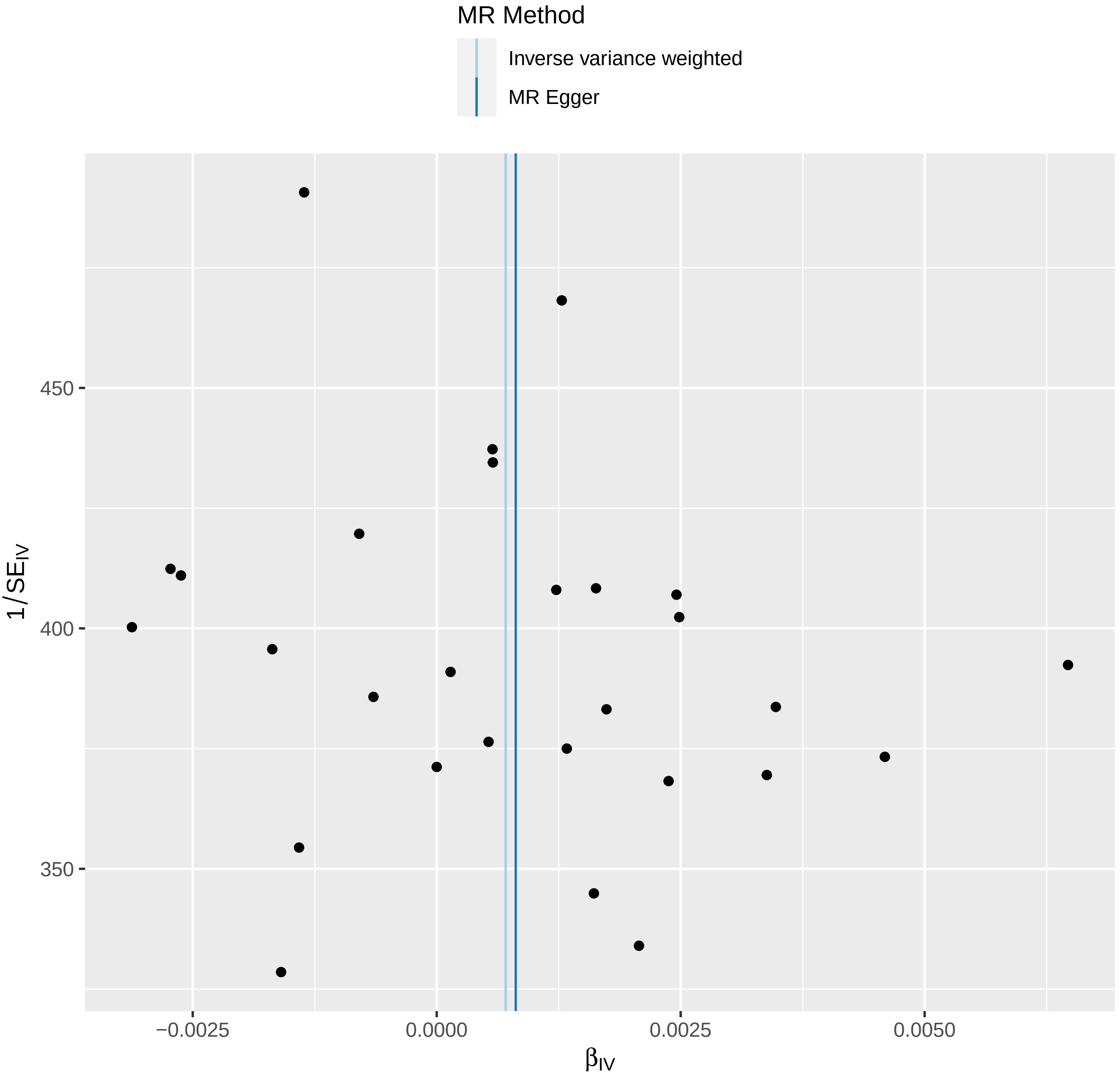

Batch 2−3119 : Twisted gastrulation protein homolog 1 on eGFR

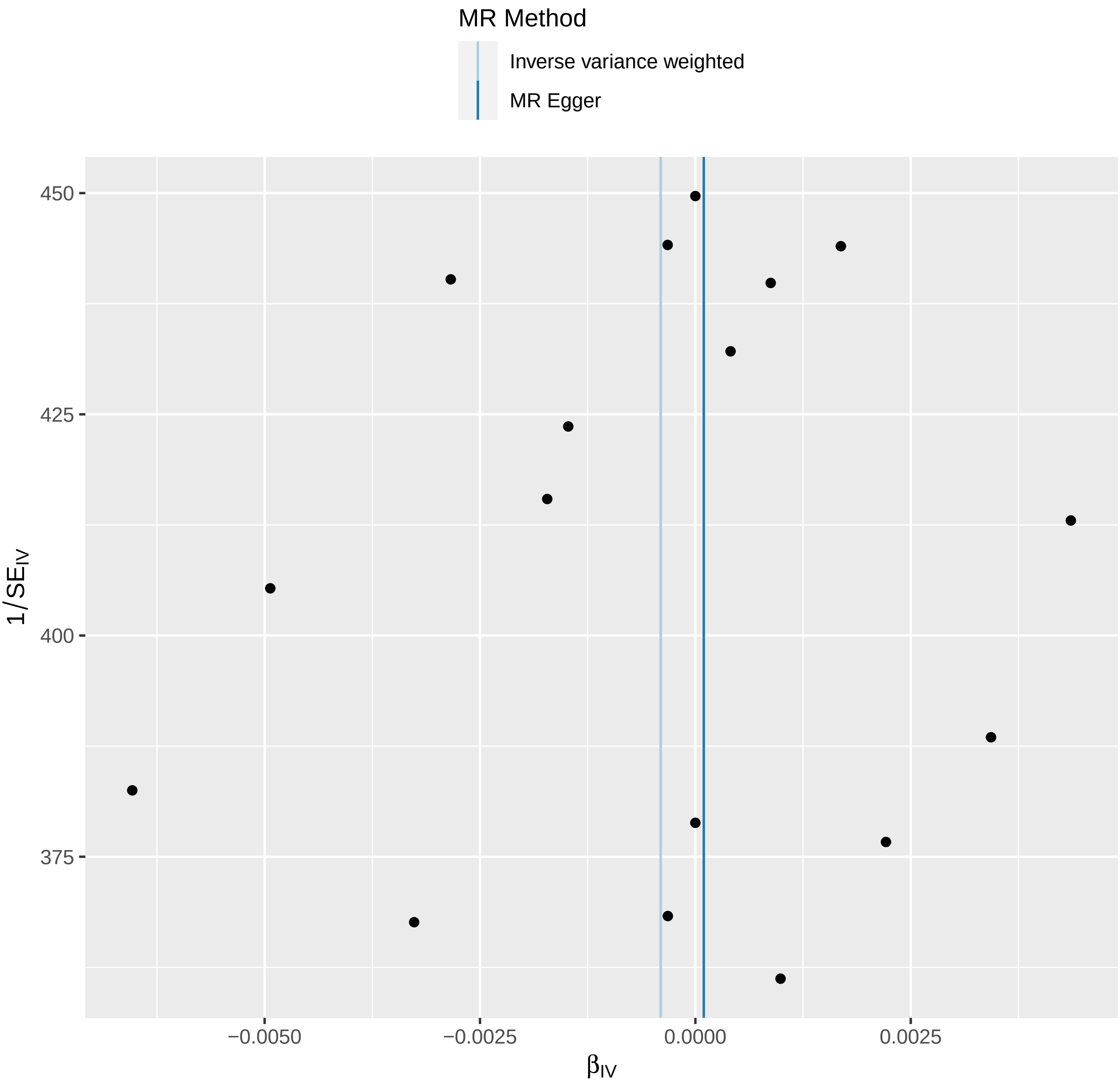

Batch 2−3120 : Alpha−taxilin on eGFR

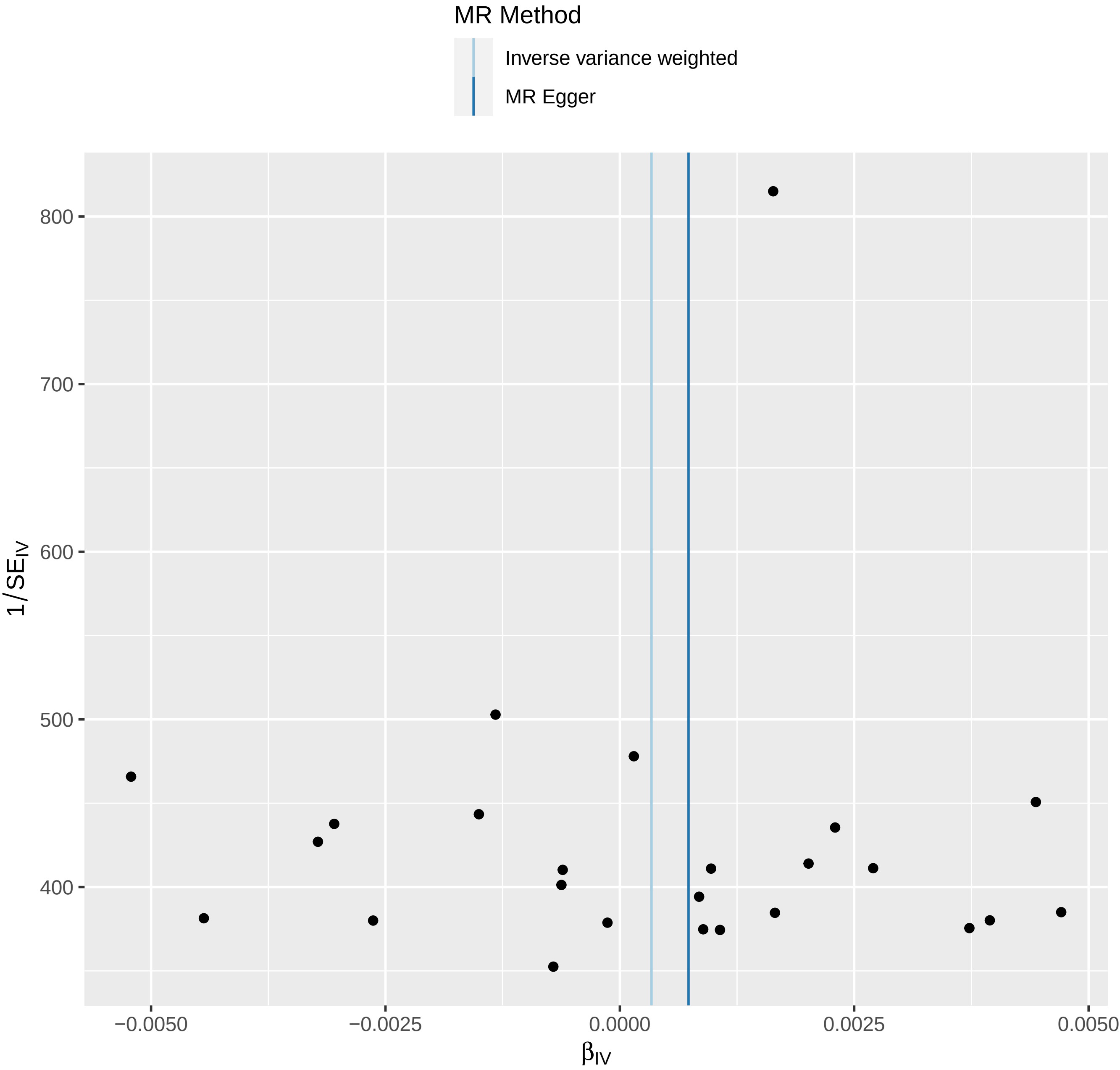

Batch 2−3121 : Thioredoxin on eGFR

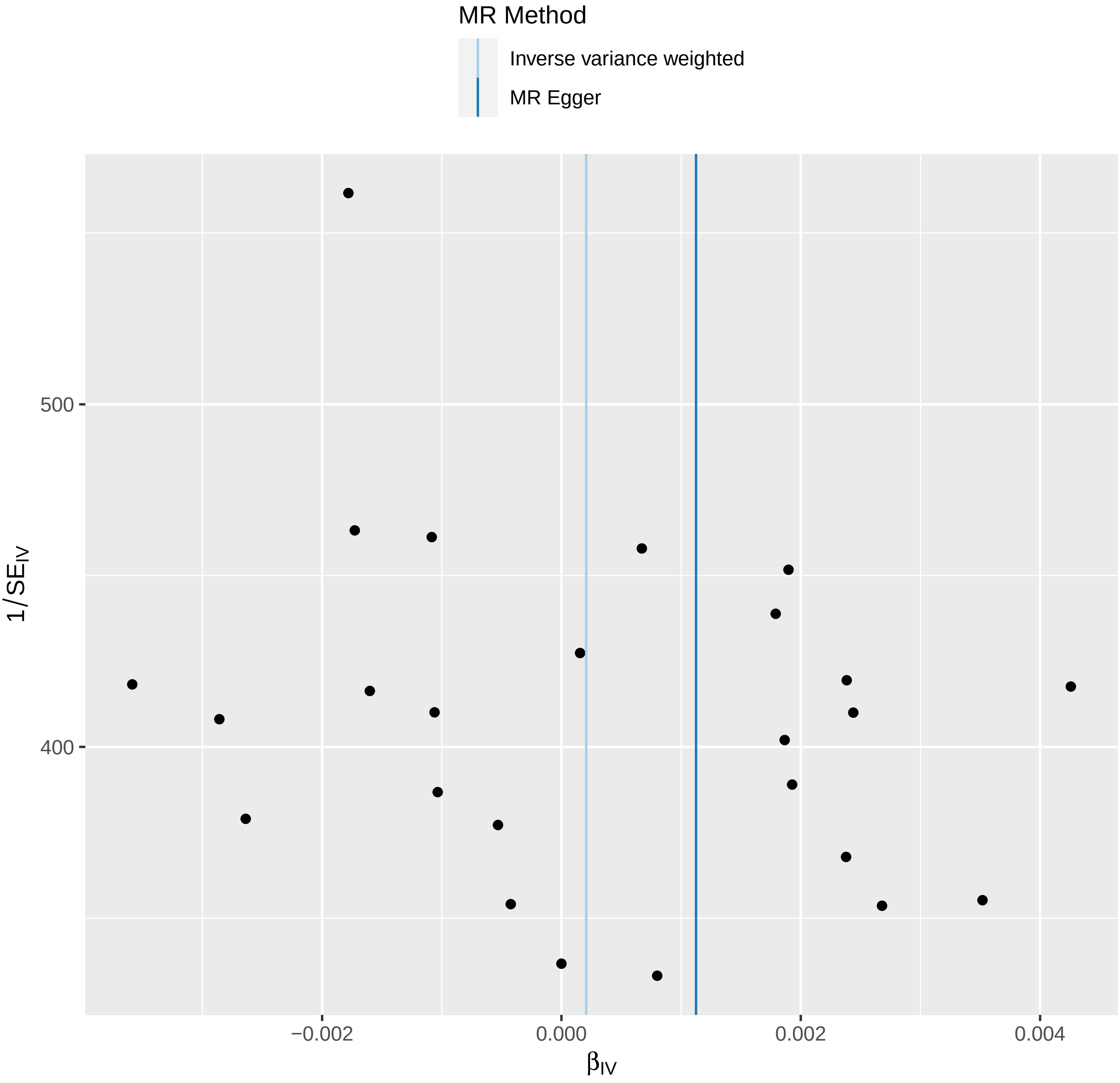

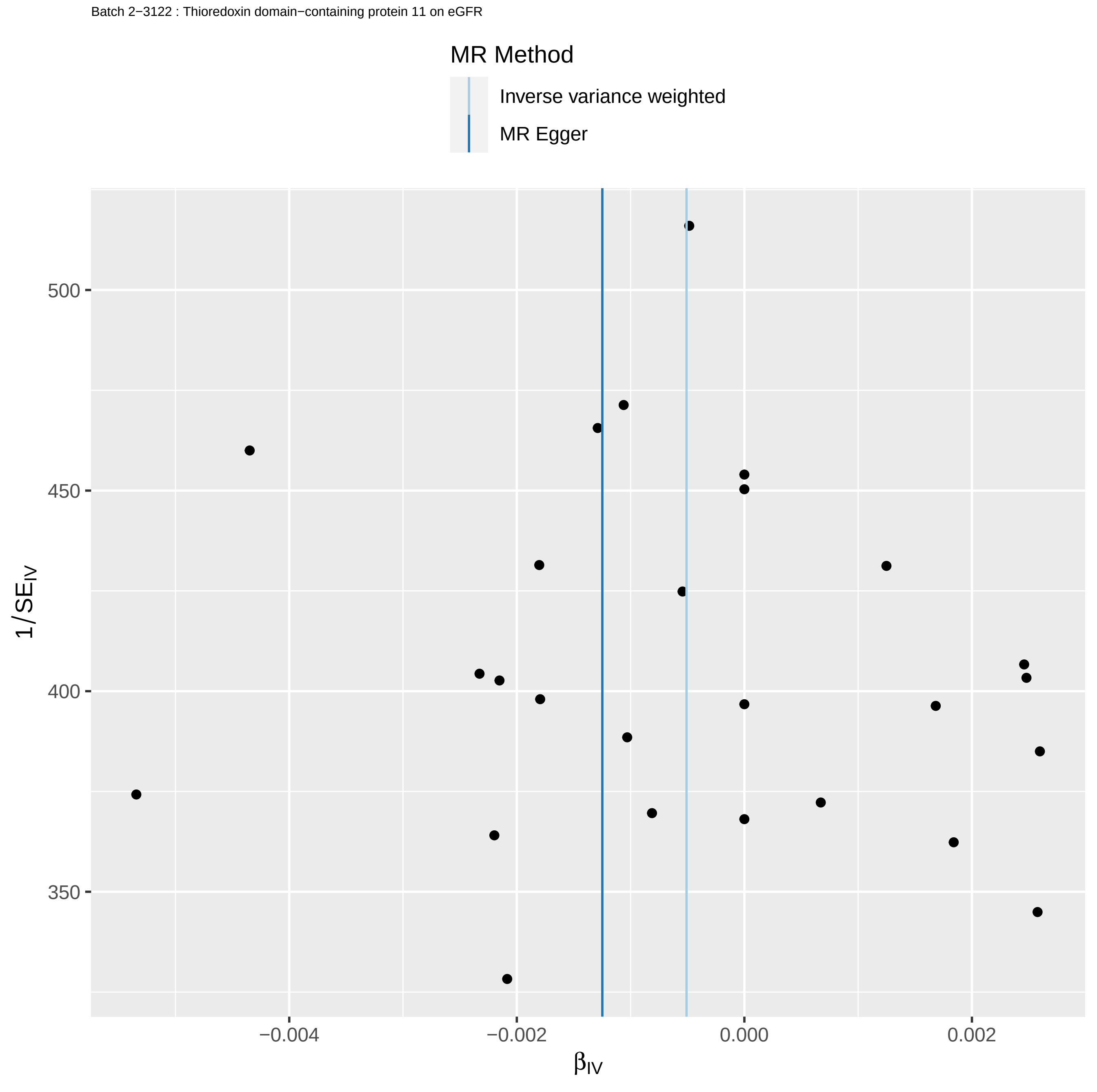
Batch 2−3122 : Thioredoxin domain−containing protein 11 on eGFR
MR Method
Inverse variance weighted
MR Egger
500
450
400
350
$1/SE_{IV}$
−0.004
−0.002
0.000
0.002
β_{IV}

Batch 2−3123 : Thioredoxin domain−containing protein 12 on eGFR

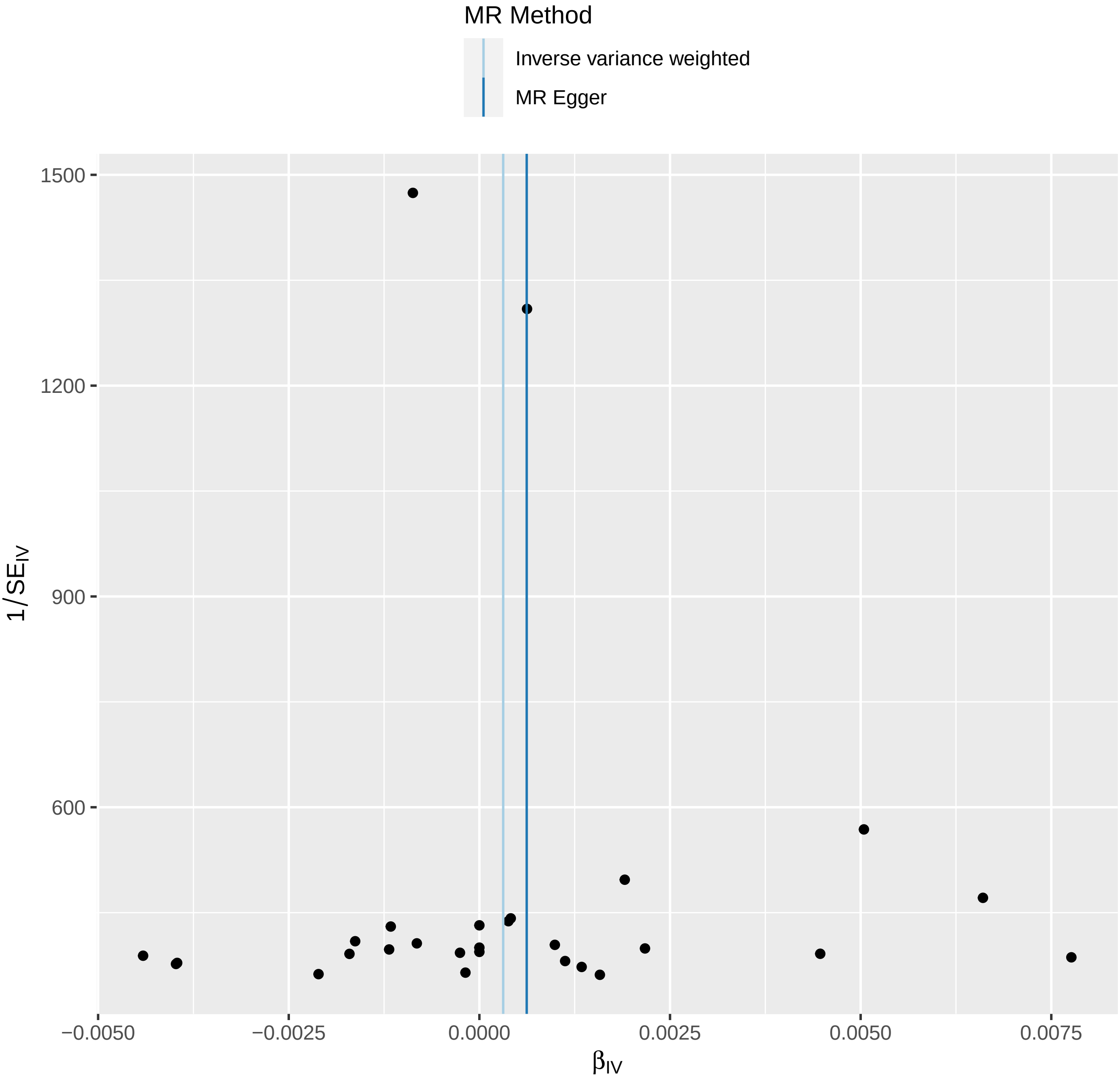

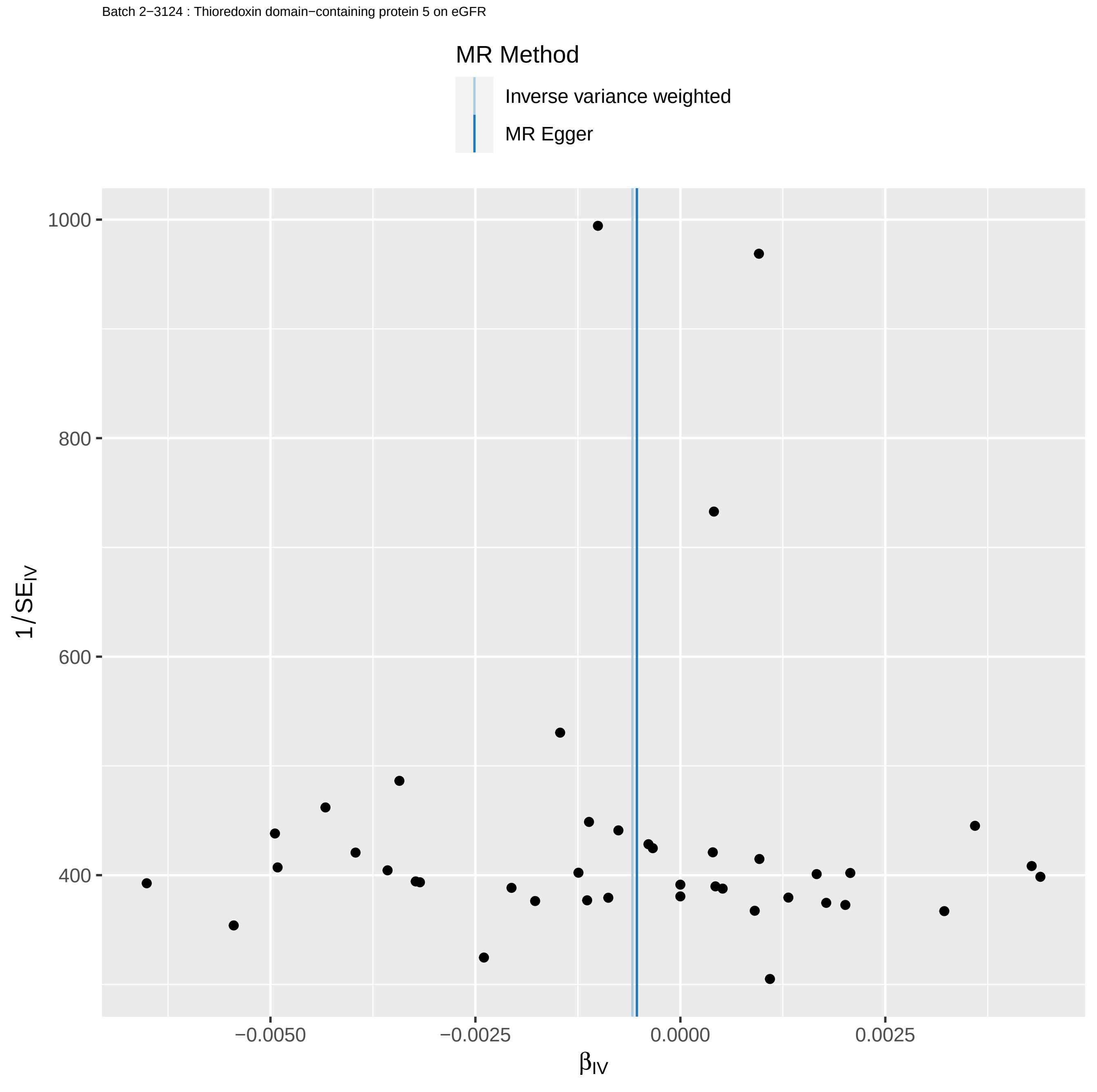
Batch 2−3124 : Thioredoxin domain−containing protein 5 on eGFR
MR Method
Inverse variance weighted
MR Egger
1000
800
600
400
1/SE_IV
−0.0050
−0.0025
0.0000
0.0025
β_IV

Batch 2−3125 : Thioredoxin−interacting protein on eGFR

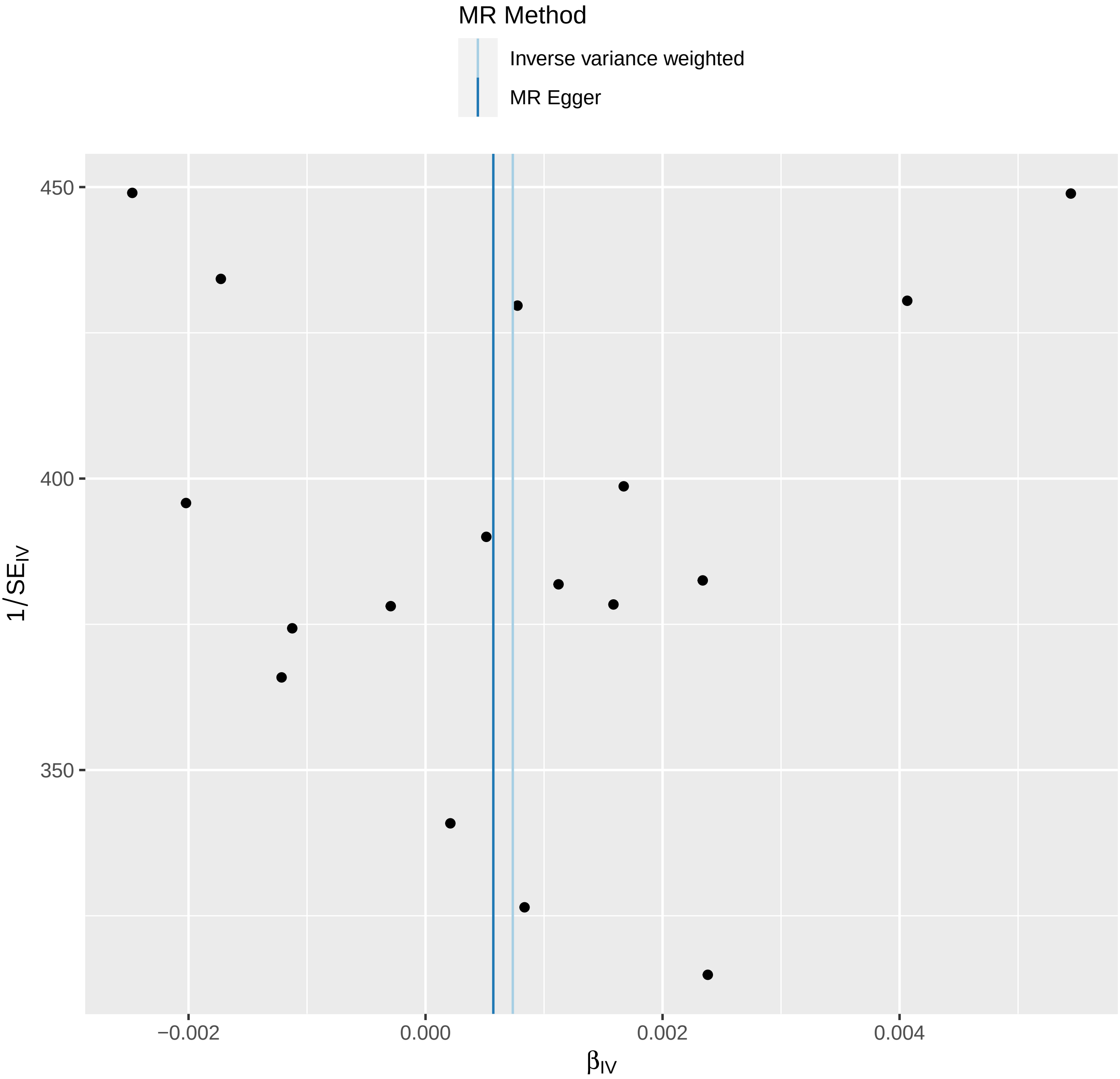

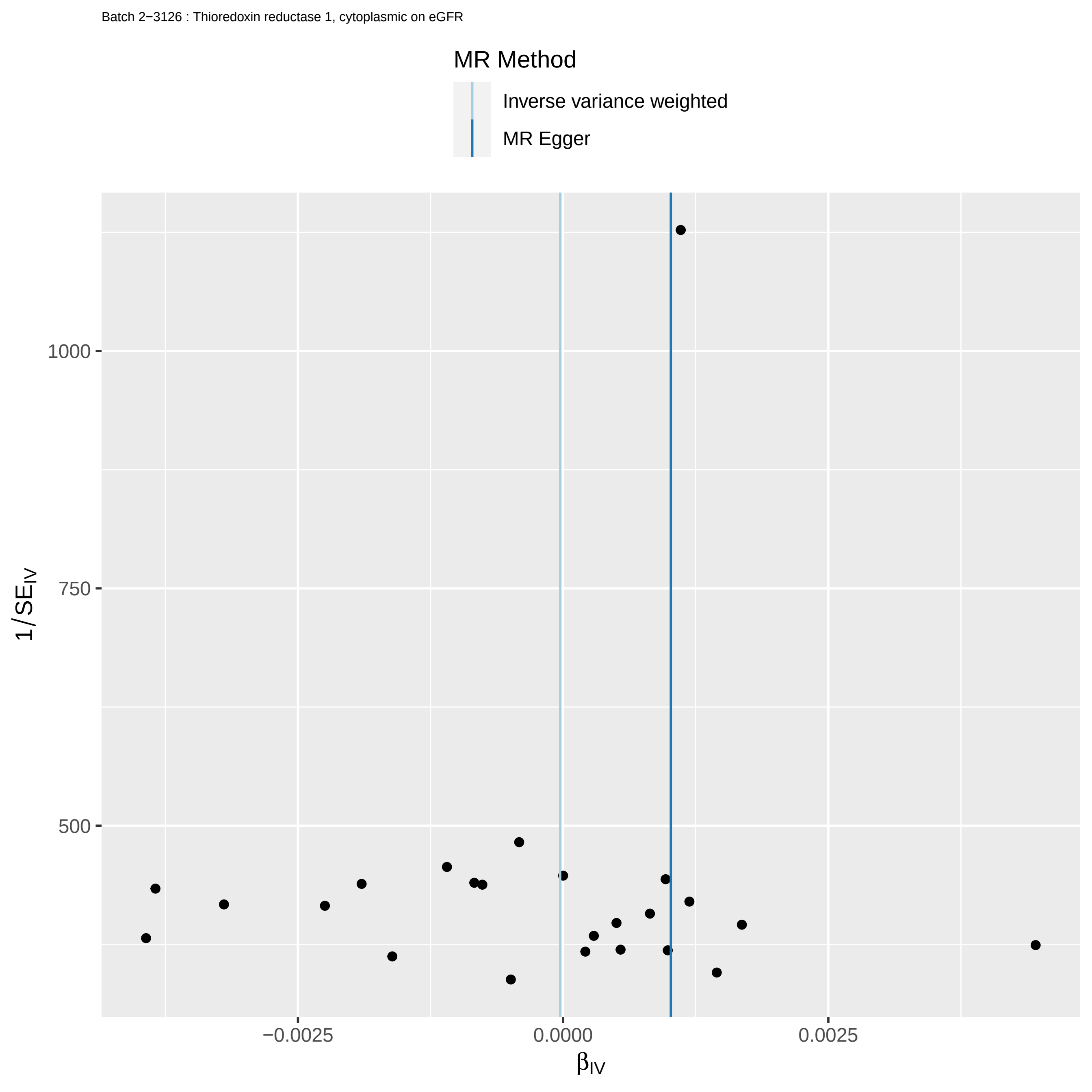

Batch 2−3126 : Thioredoxin reductase 1, cytoplasmic on eGFR
MR Method
Inverse variance weighted
MR Egger
1000
750
500
−0.0025
0.0000
0.0025
$1/SE_{IV}$
β_{IV}

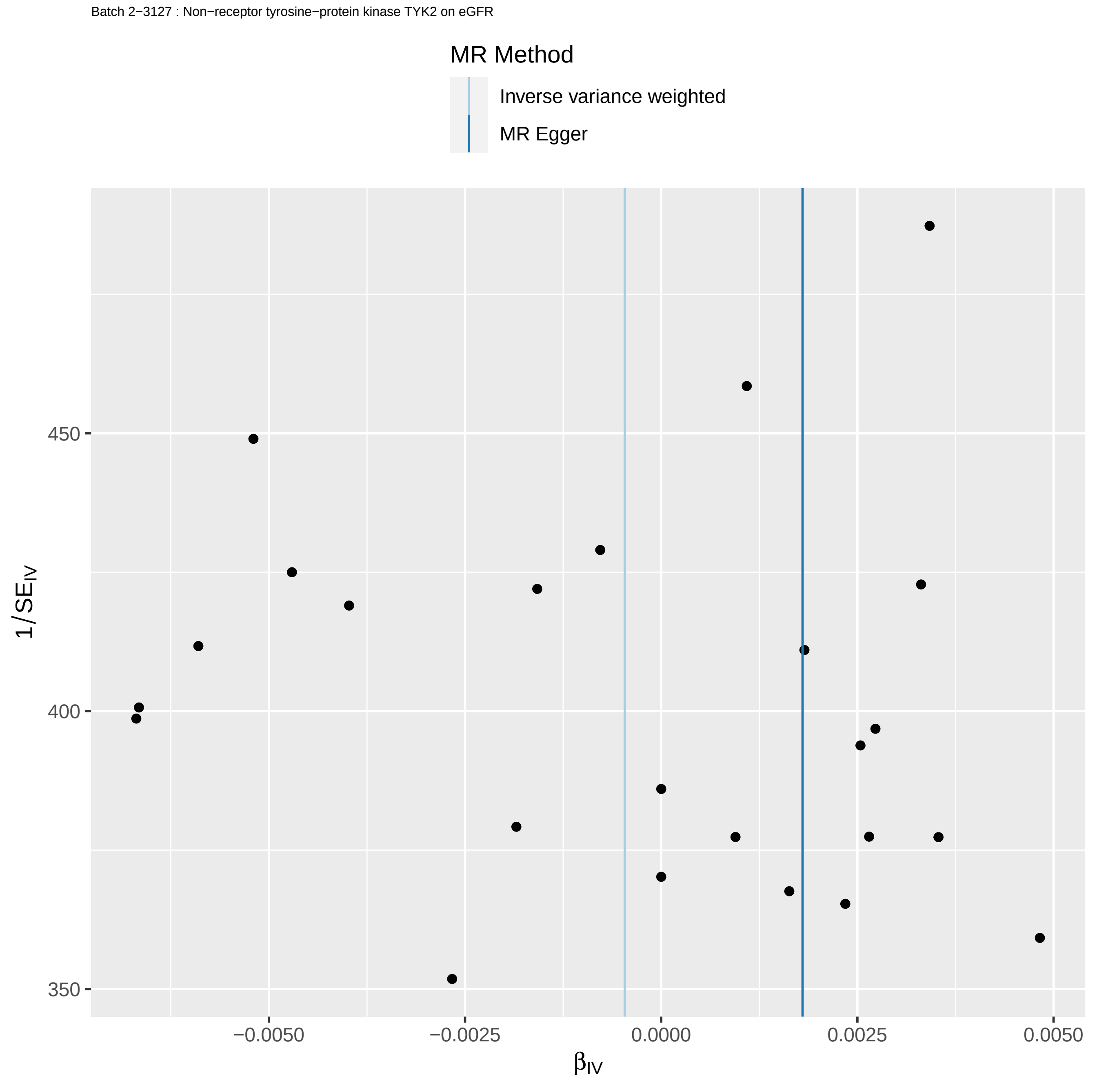

Batch 2−3127 : Non−receptor tyrosine−protein kinase TYK2 on eGFR
MR Method
Inverse variance weighted
MR Egger
1/SE$_{IV}$
450
400
350
−0.0050
−0.0025
0.0000
0.0025
0.0050
β$_{IV}$

Batch 2−3128 : TYMS opposite strand protein on eGFR

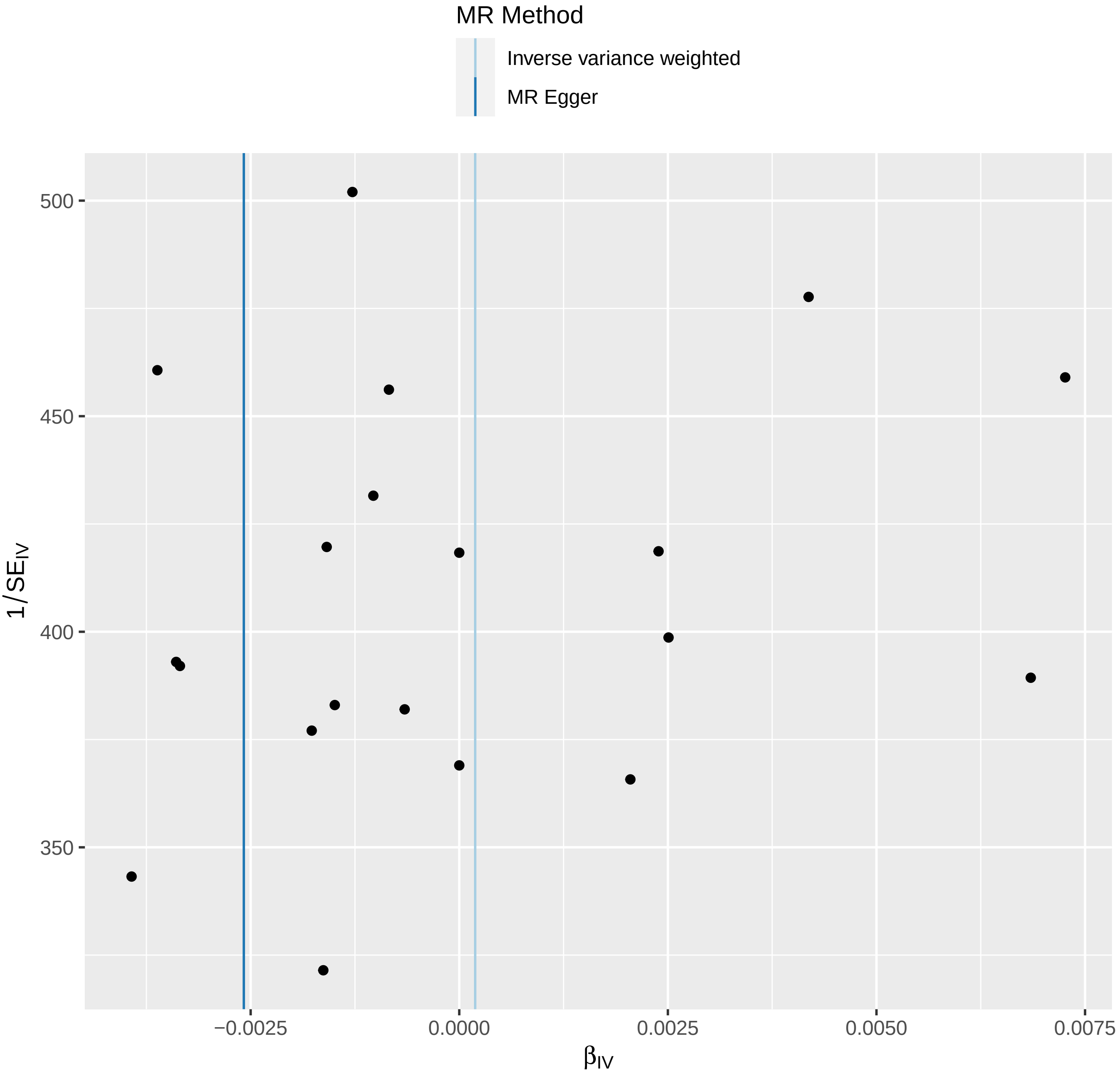

Batch 2−3129 : Tyrosine−protein kinase receptor TYRO3 on eGFR

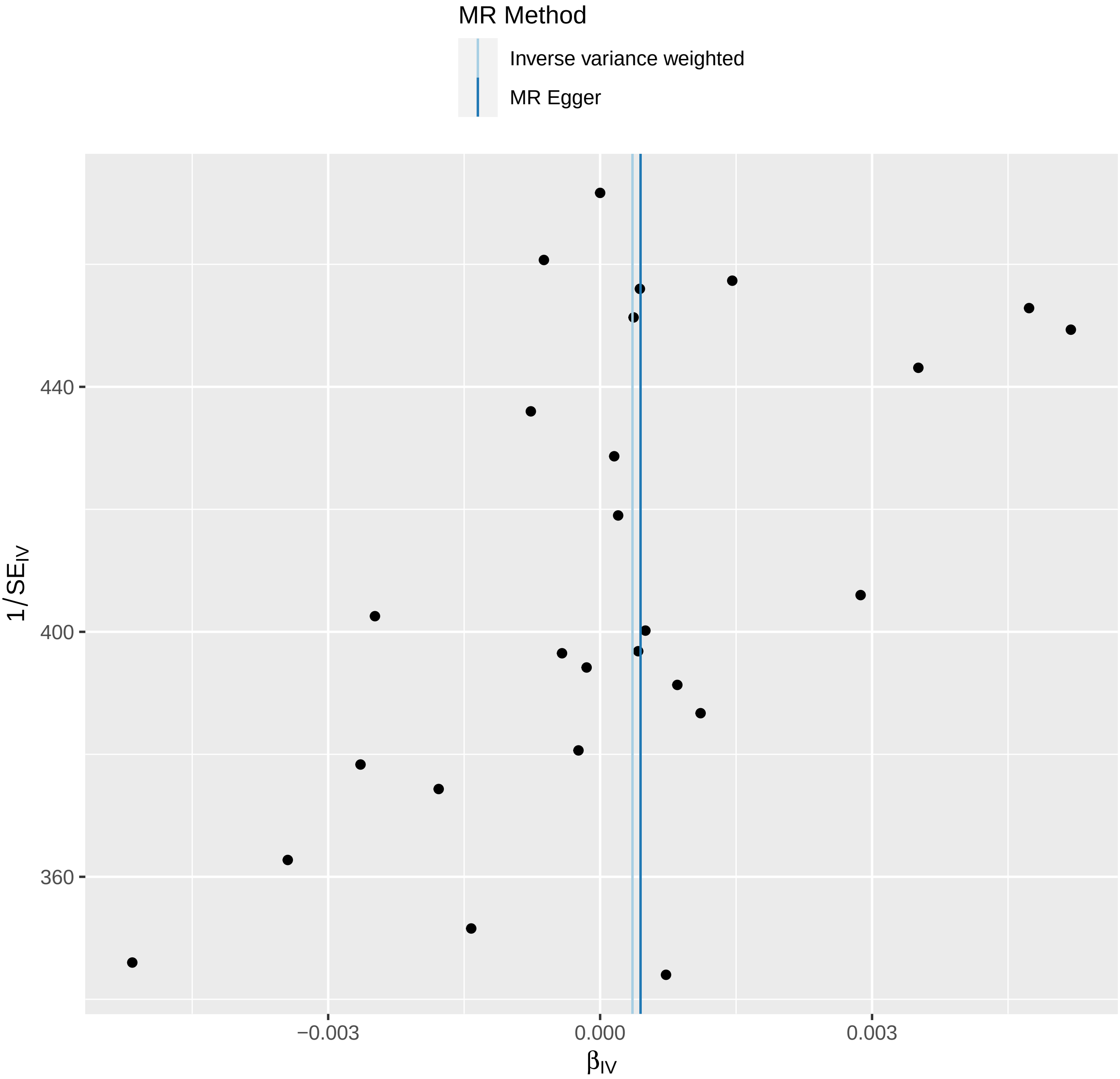

Batch 2−3130 : Splicing factor U2AF 65 kDa subunit on eGFR

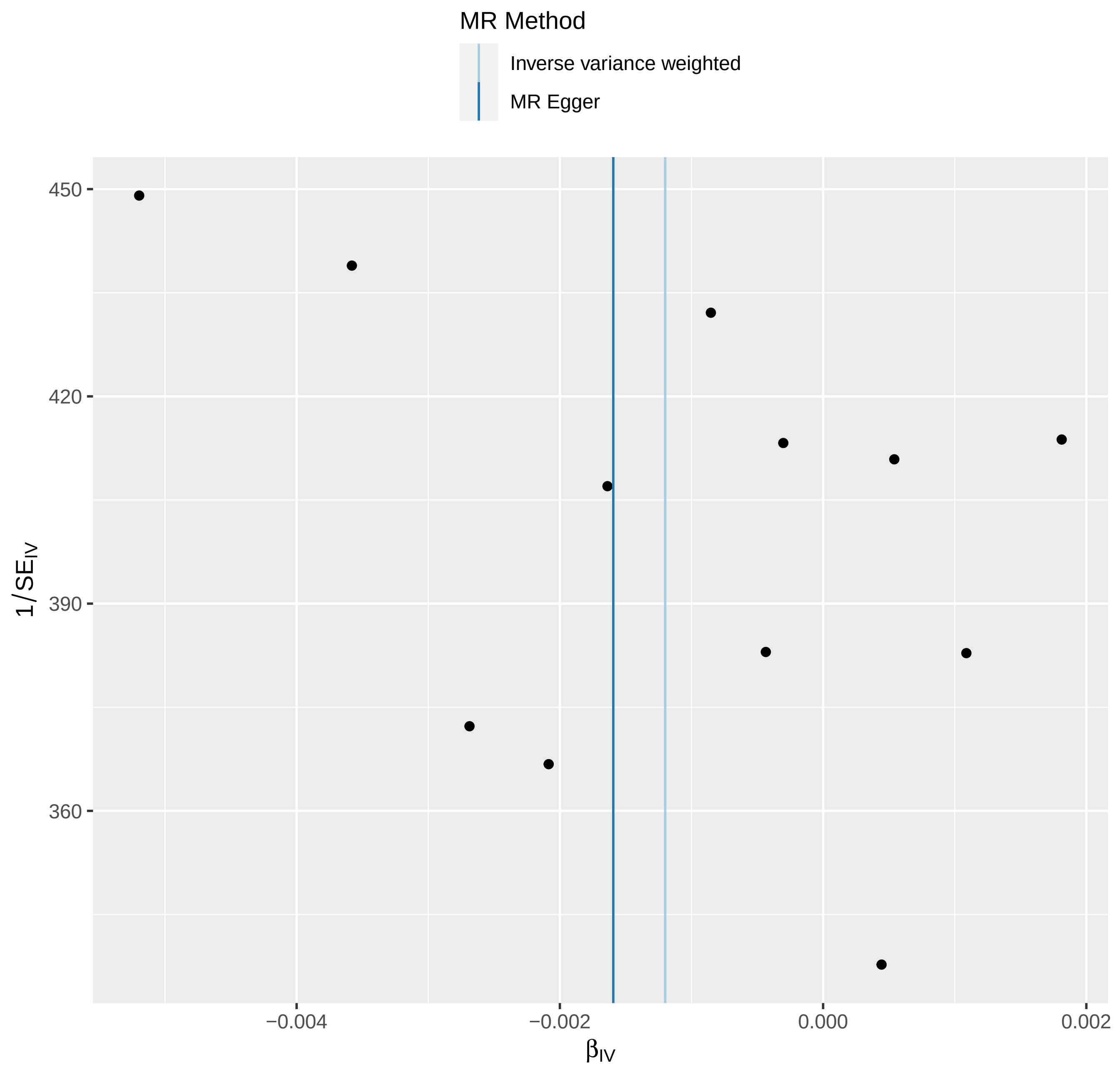

Batch 2−3131 : UDP−N−acetylhexosamine pyrophosphorylase on eGFR

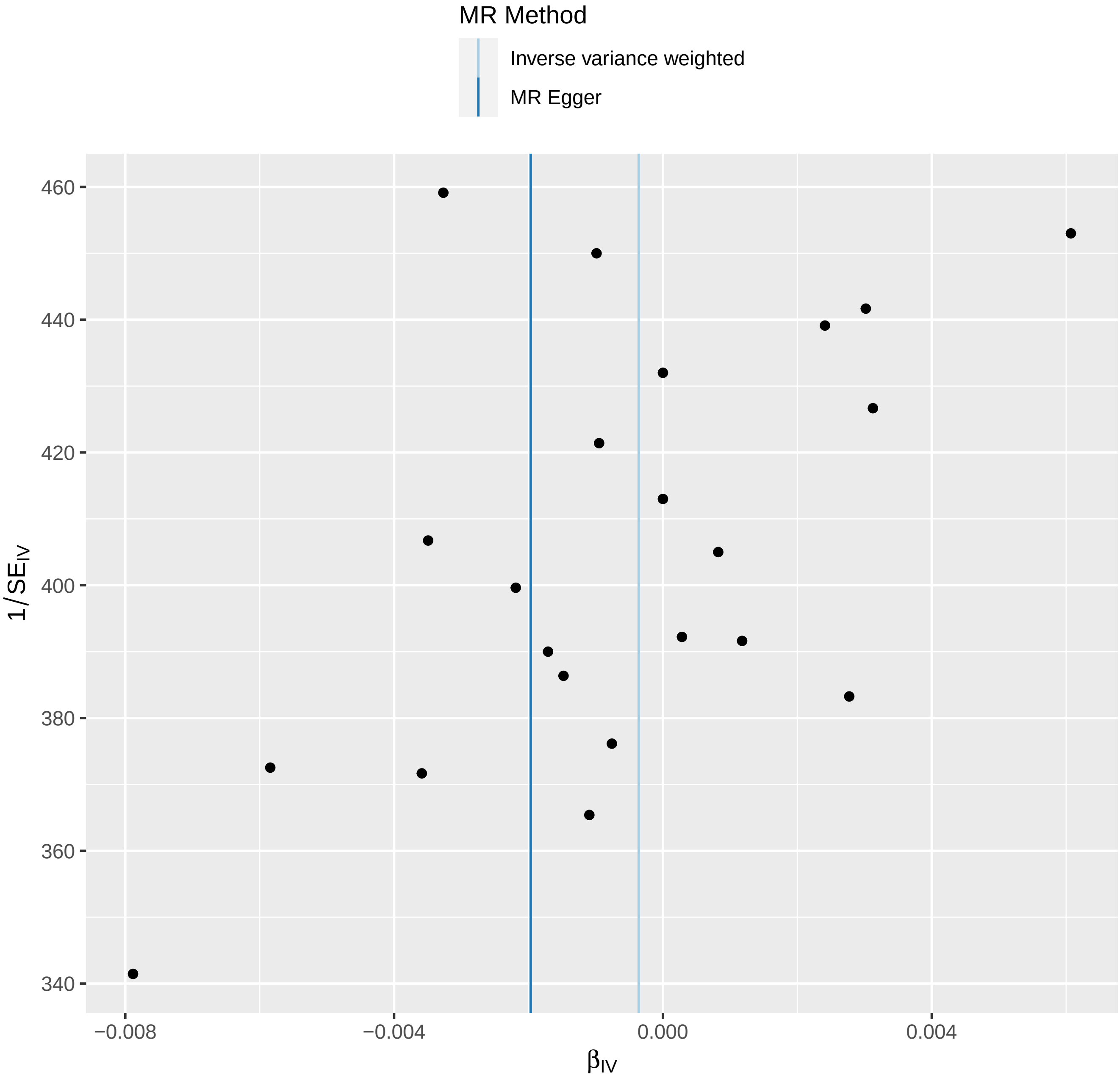

Batch 2−3132 : PolyUbiquitin K48−linked on eGFR

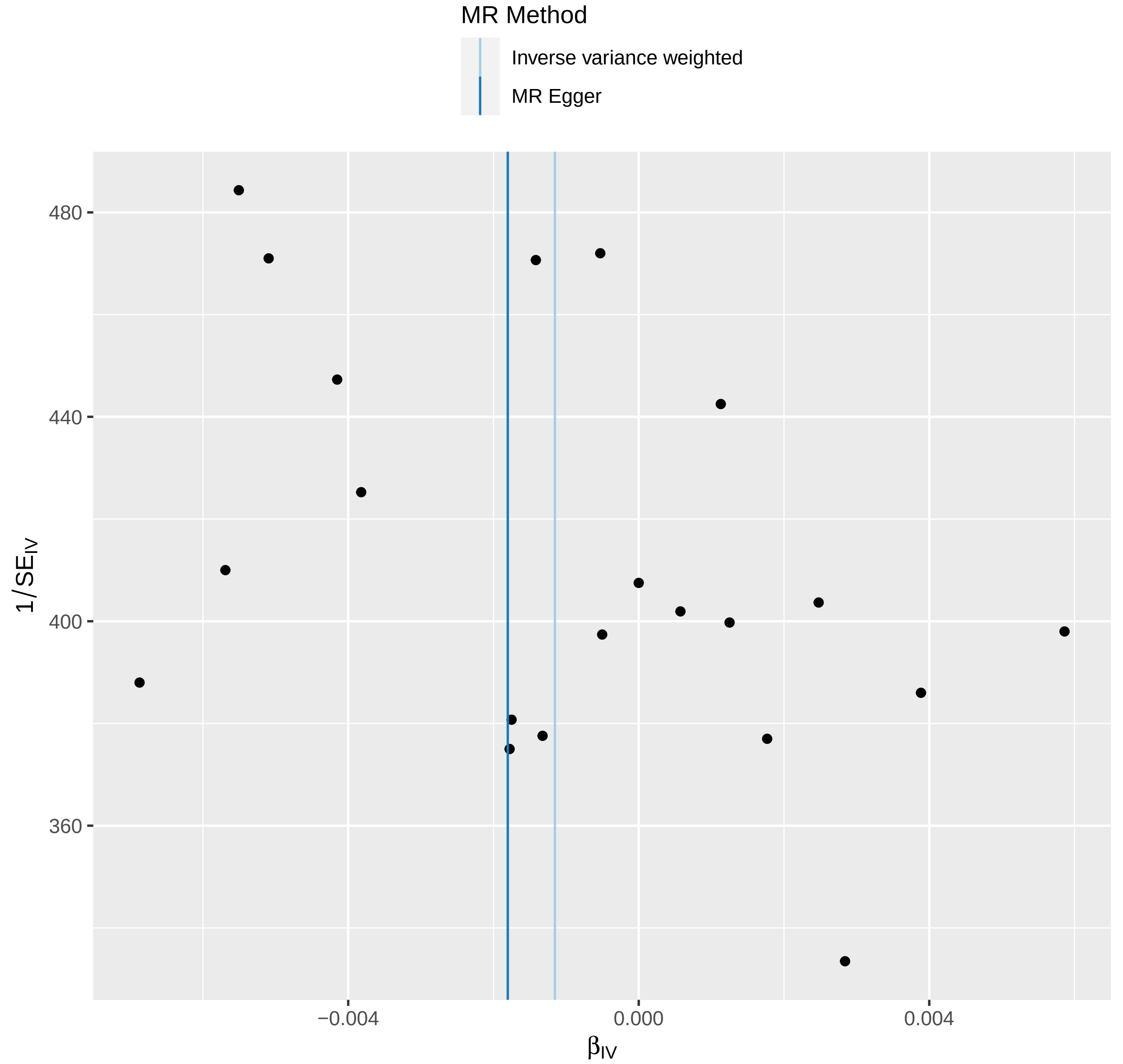

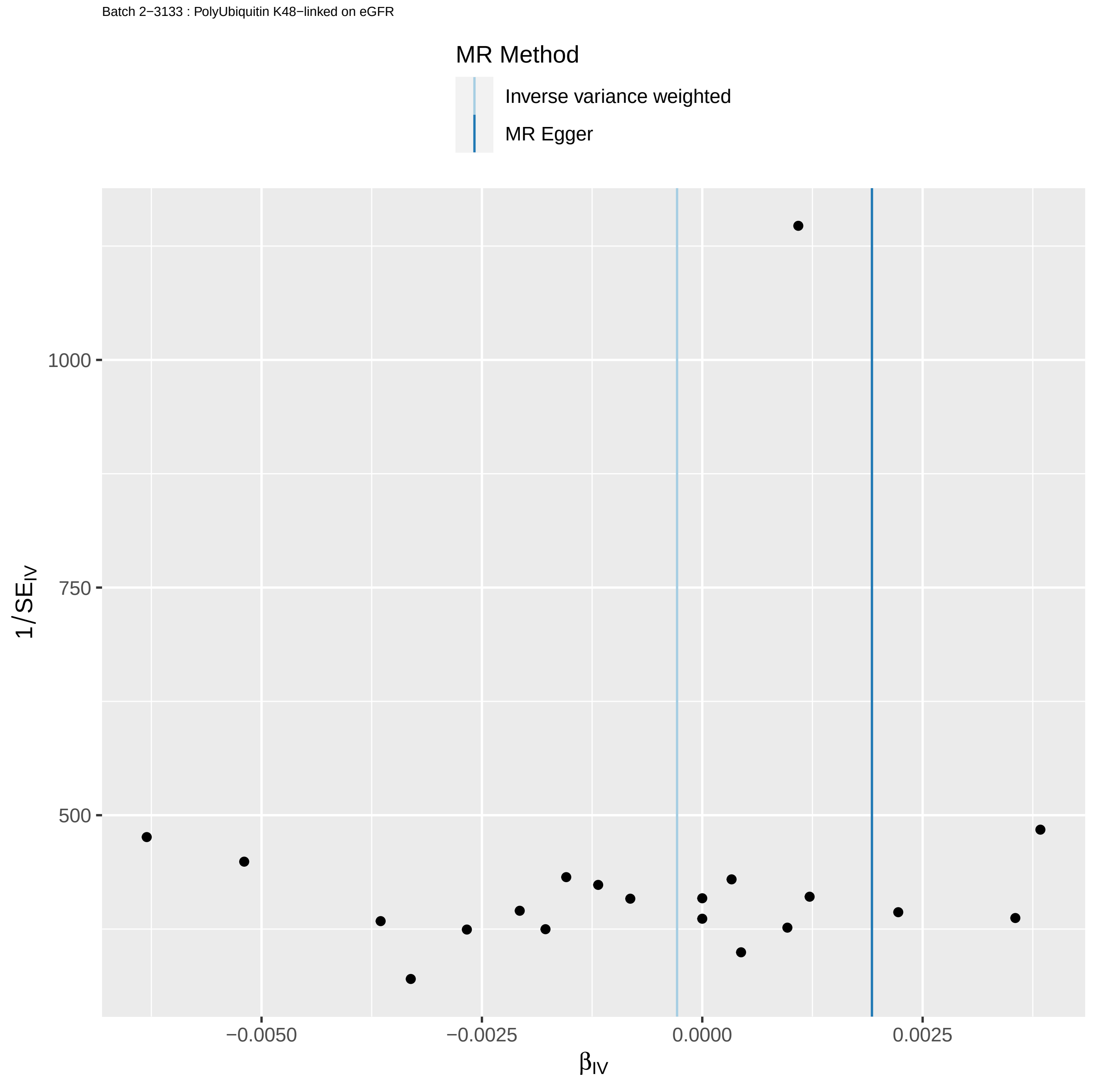

Batch 2−3133 : PolyUbiquitin K48−linked on eGFR
MR Method
Inverse variance weighted
MR Egger
1000
750
500
1/SE$_{IV}$
−0.0050
−0.0025
0.0000
0.0025
β$_{IV}$

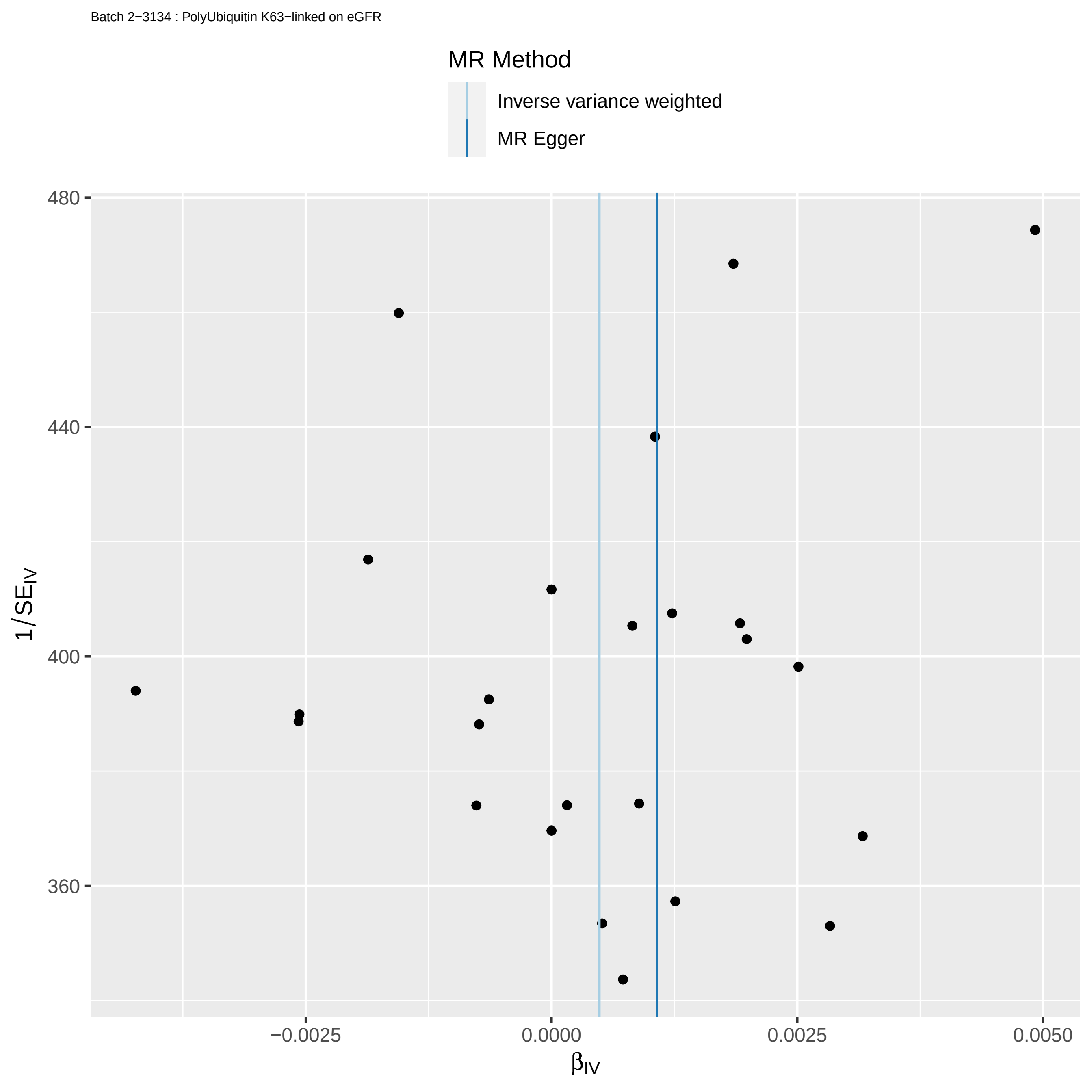
Batch 2−3134 : PolyUbiquitin K63−linked on eGFR
MR Method
Inverse variance weighted
MR Egger
480
440
400
360
1/SE_IV
−0.0025
0.0000
0.0025
0.0050
β_IV

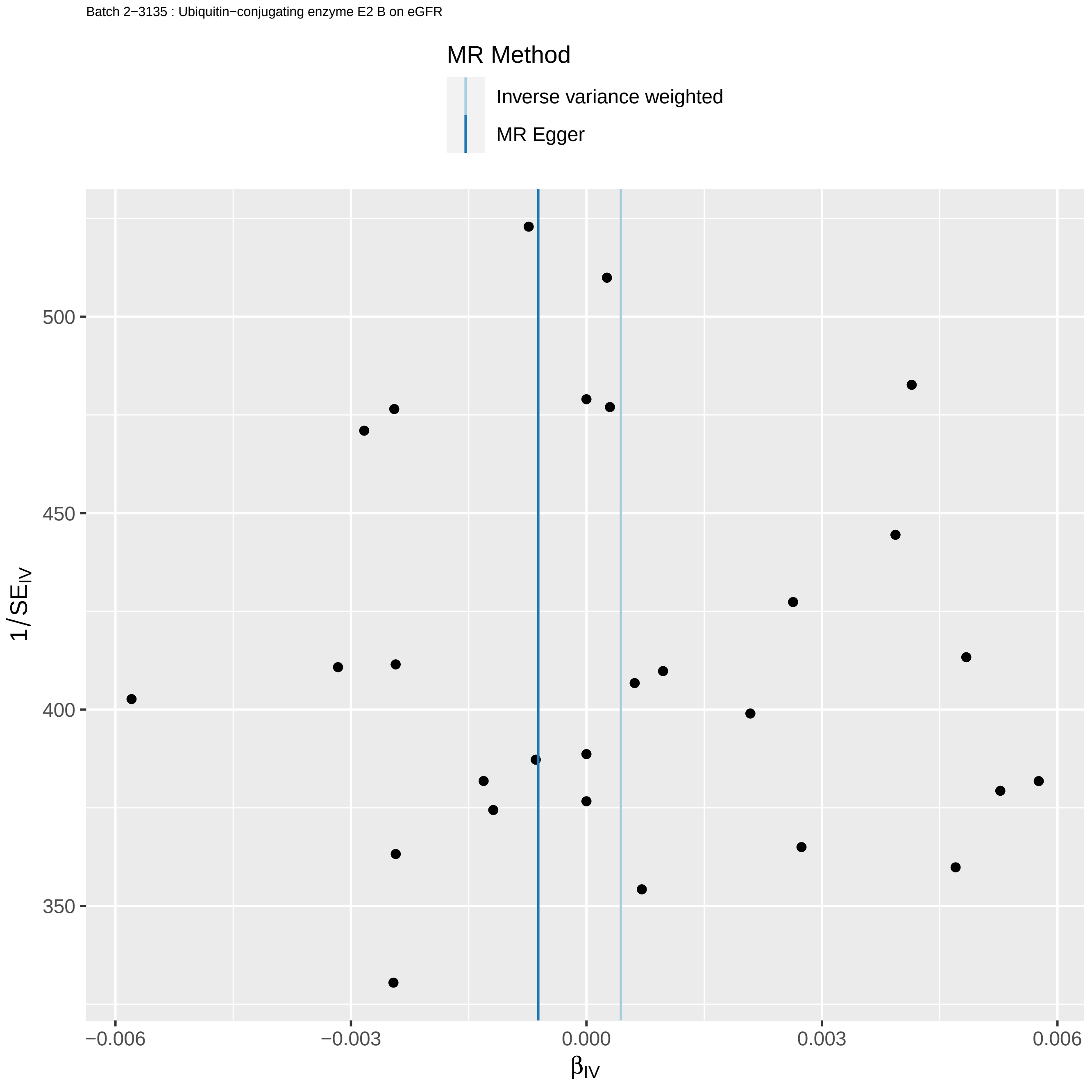

Batch 2−3135 : Ubiquitin−conjugating enzyme E2 B on eGFR
MR Method
Inverse variance weighted
MR Egger
500
450
400
350
1/SE$_{IV}$
−0.006
−0.003
0.000
0.003
0.006
β$_{IV}$

Batch 2−3136 : Ubiquitin−conjugating enzyme E2 D4 on eGFR

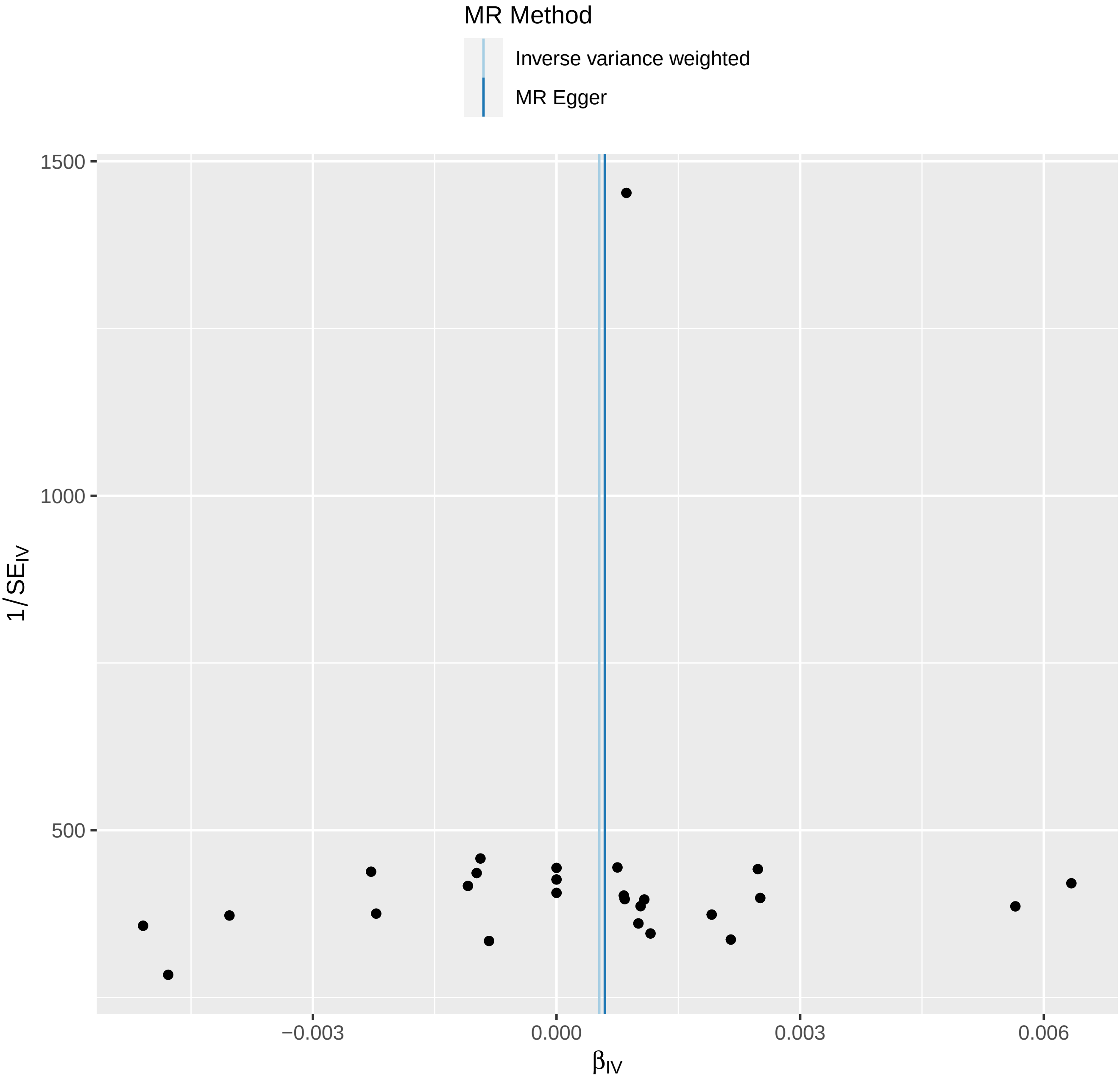

Batch 2−3137 : Ubiquitin−conjugating enzyme E2 G2 on eGFR

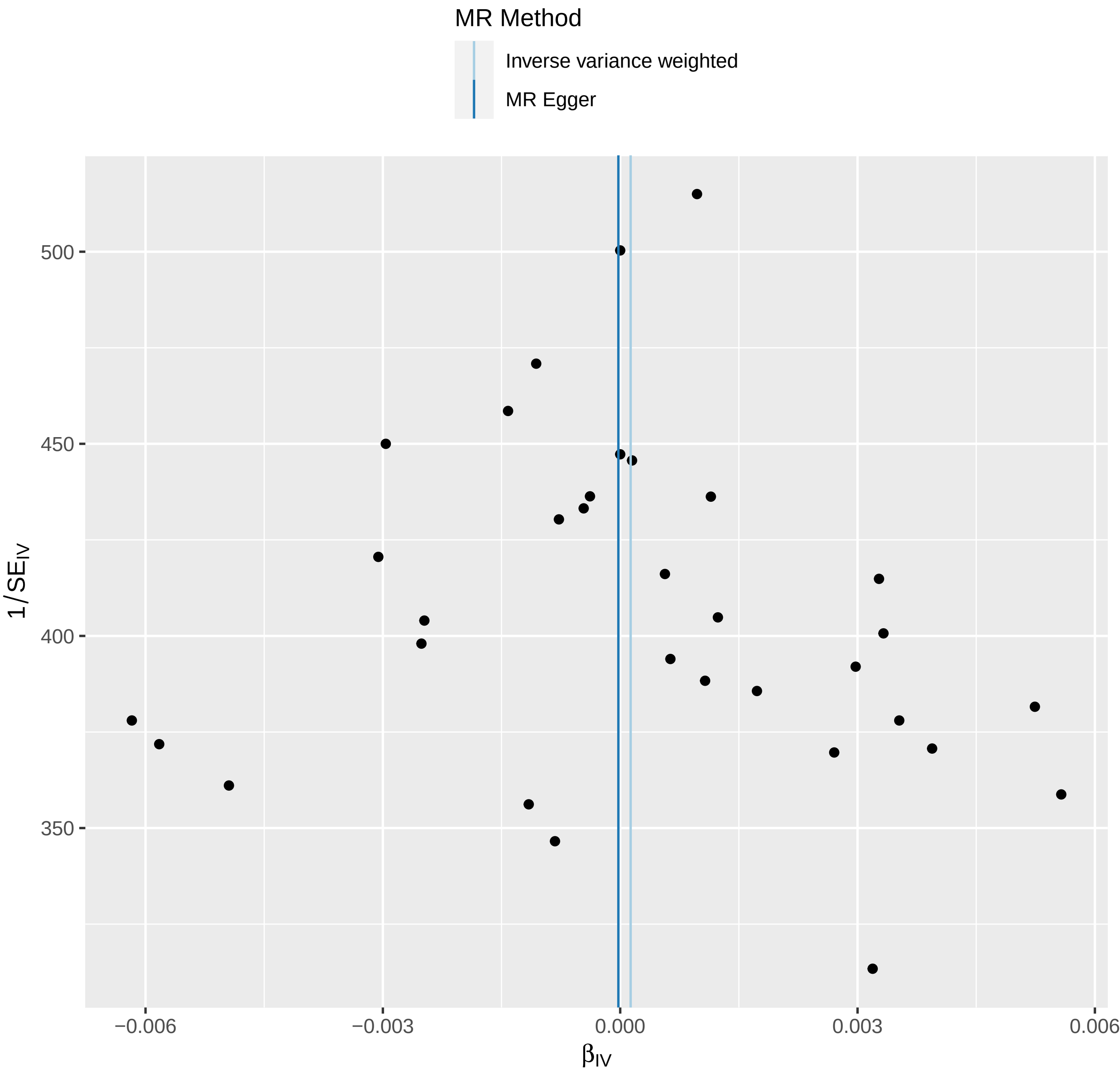

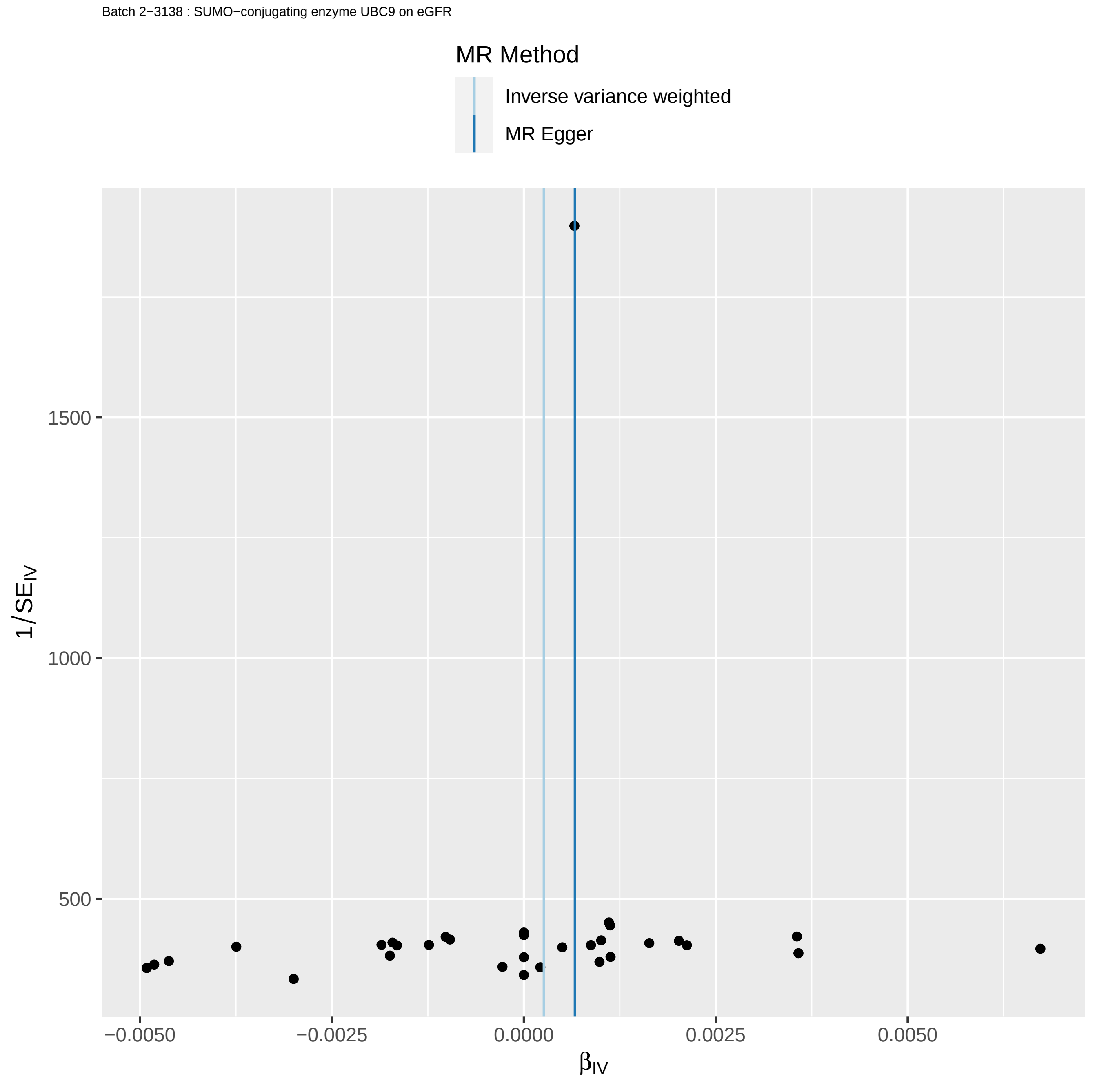
Batch 2−3138 : SUMO−conjugating enzyme UBC9 on eGFR
MR Method
Inverse variance weighted
MR Egger
1/SE_IV
1500
1000
500
−0.0050
−0.0025
0.0000
0.0025
0.0050
β_IV

Batch 2−3139 : Ubiquitin−conjugating enzyme E2 J1 on eGFR

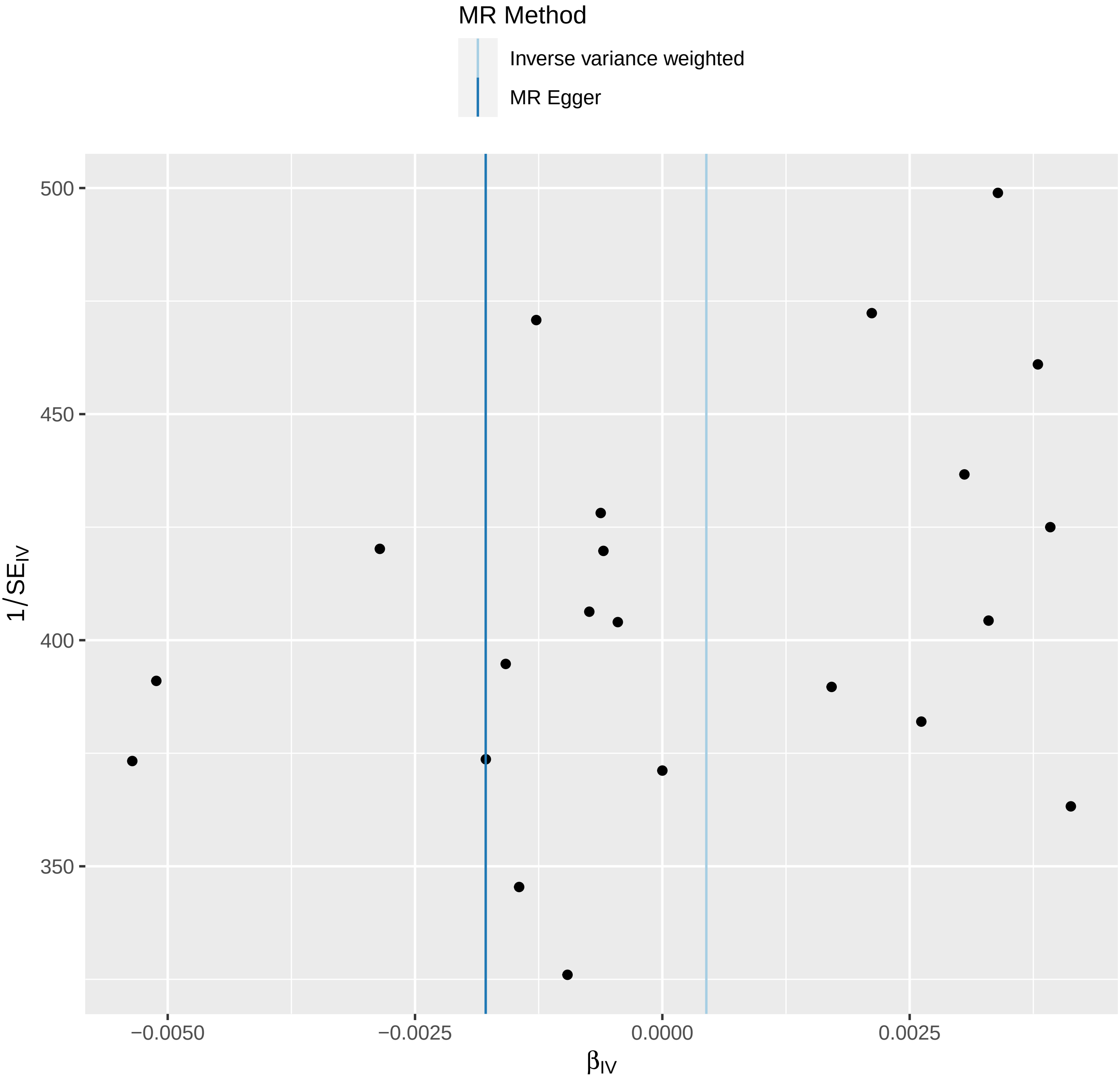

Batch 2−3140 : Ubiquitin−conjugating enzyme E2 J2 on eGFR

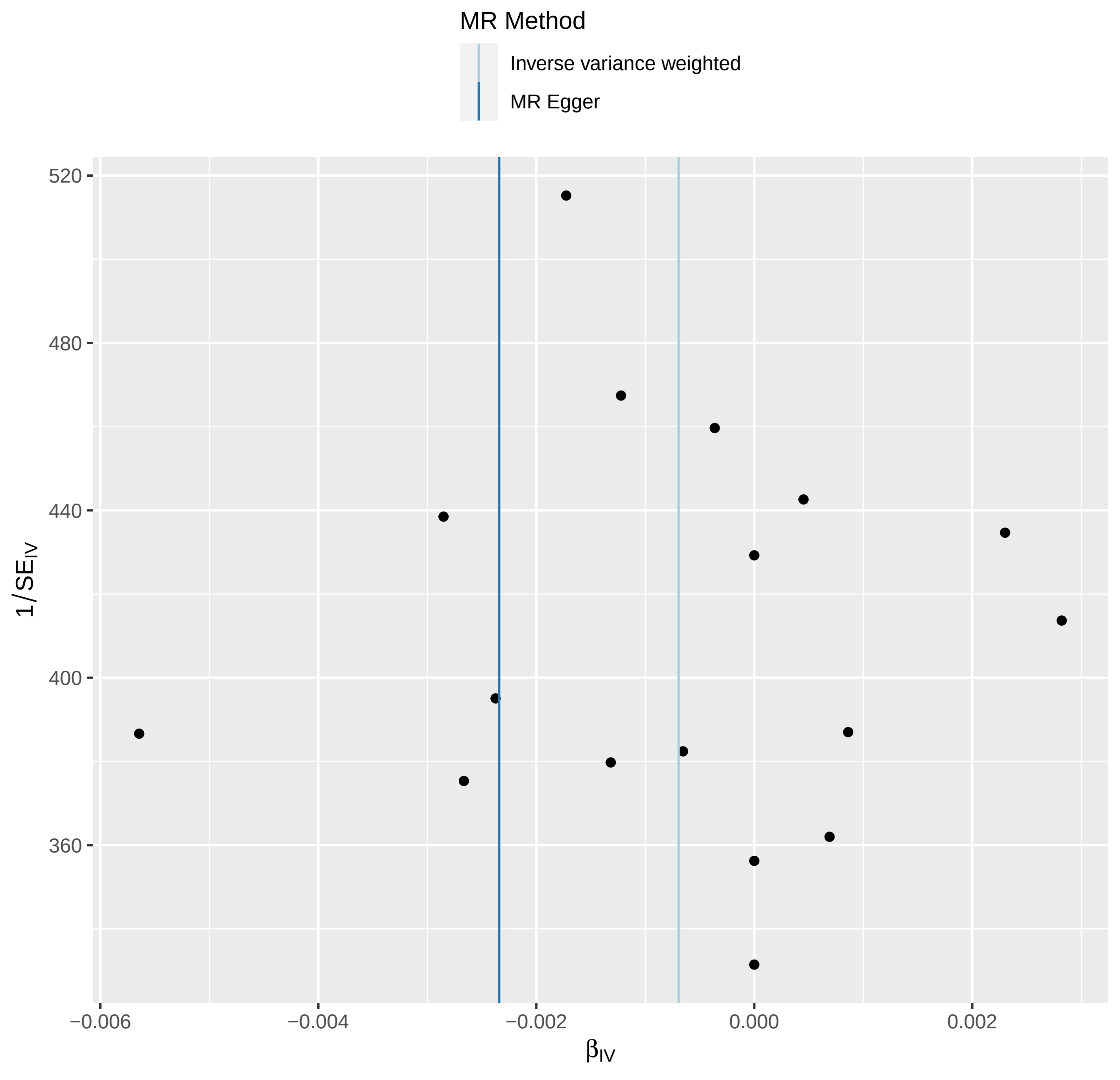

Batch 2−3141 : Ubiquitin−conjugating enzyme E2 J2 on eGFR

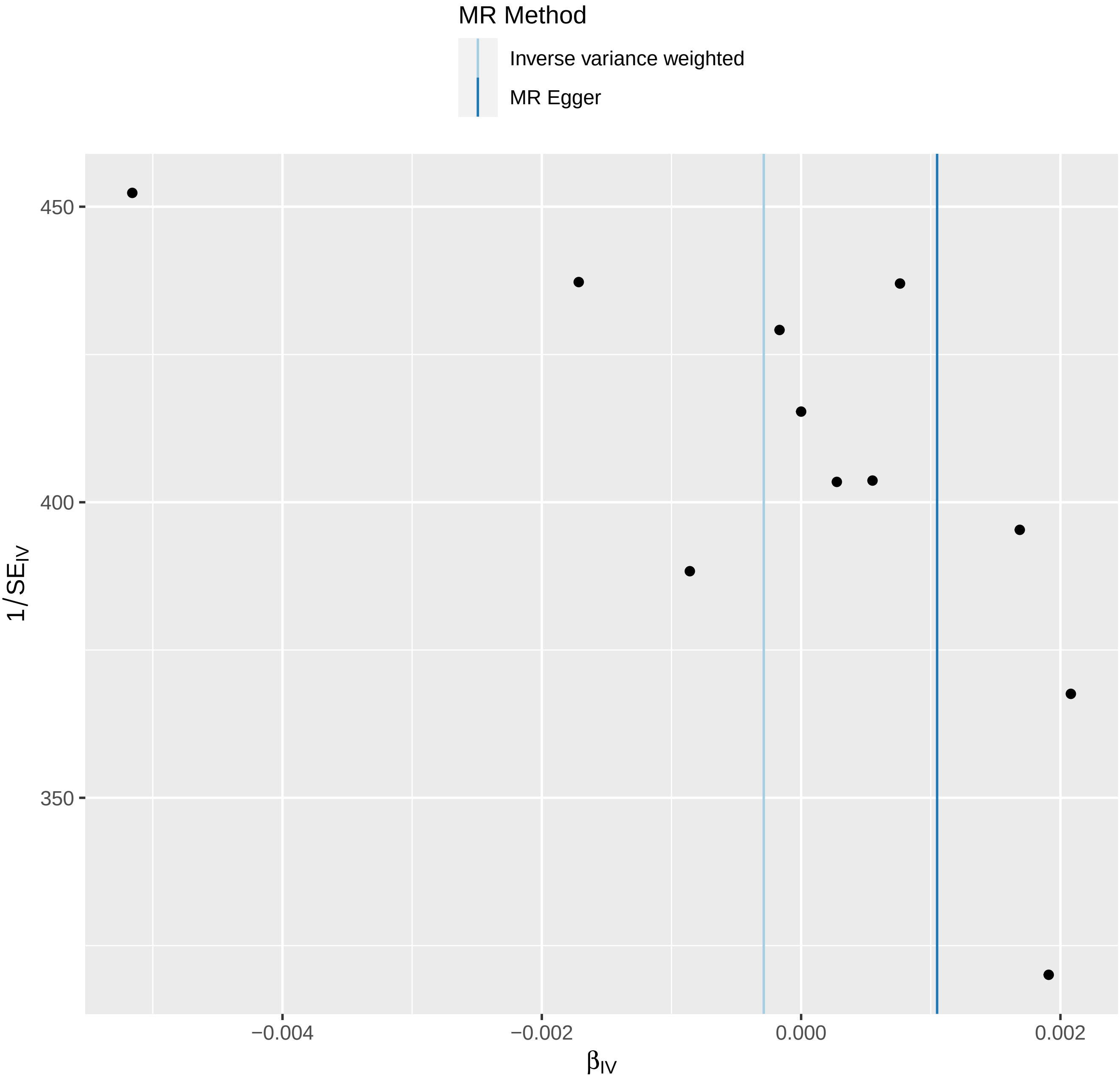

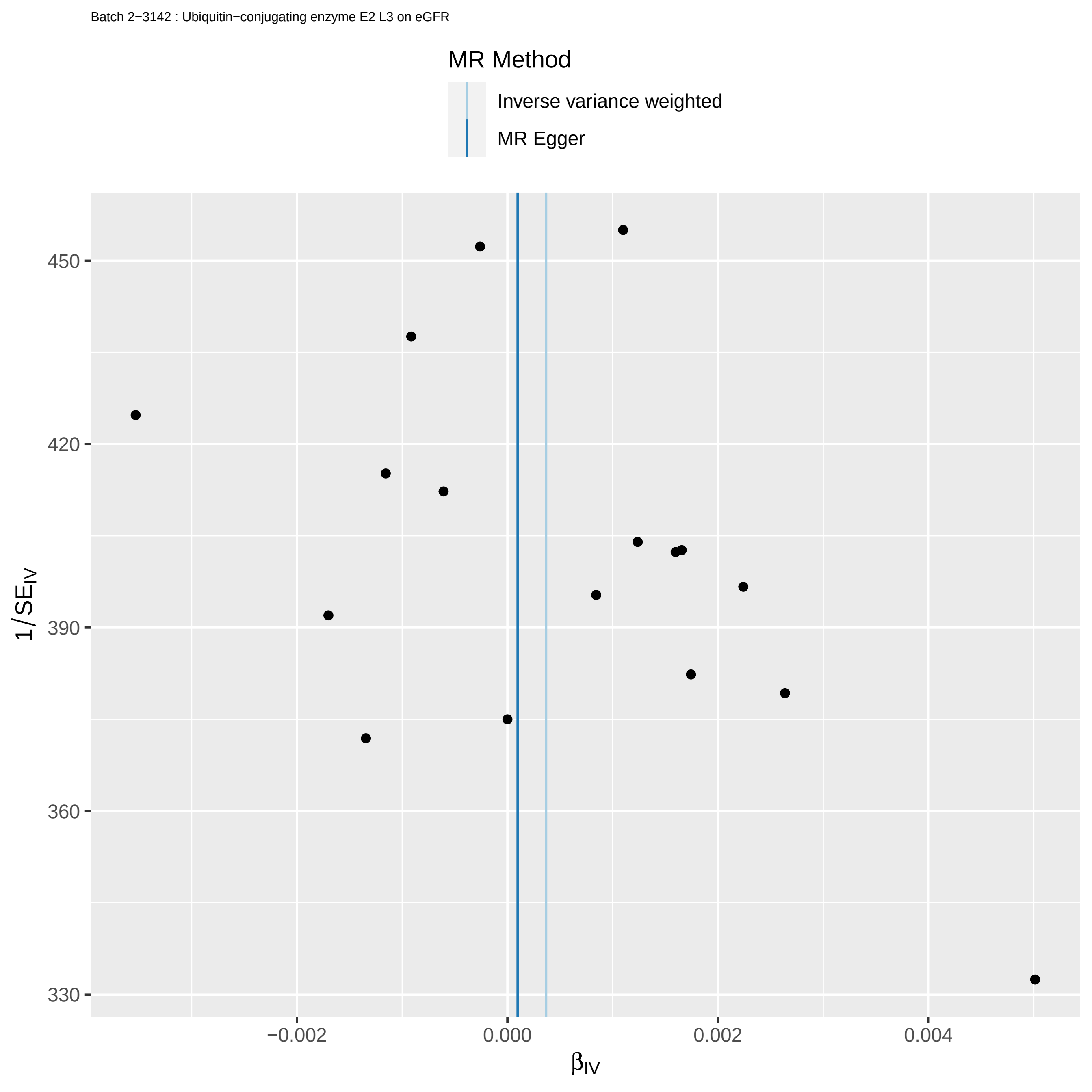
Batch 2−3142 : Ubiquitin−conjugating enzyme E2 L3 on eGFR
MR Method
Inverse variance weighted
MR Egger
450
420
390
360
330
1/SE_IV
−0.002
0.000
0.002
0.004
β_IV

Batch 2−3143 : Ubiquitin−conjugating enzyme E2 N on eGFR

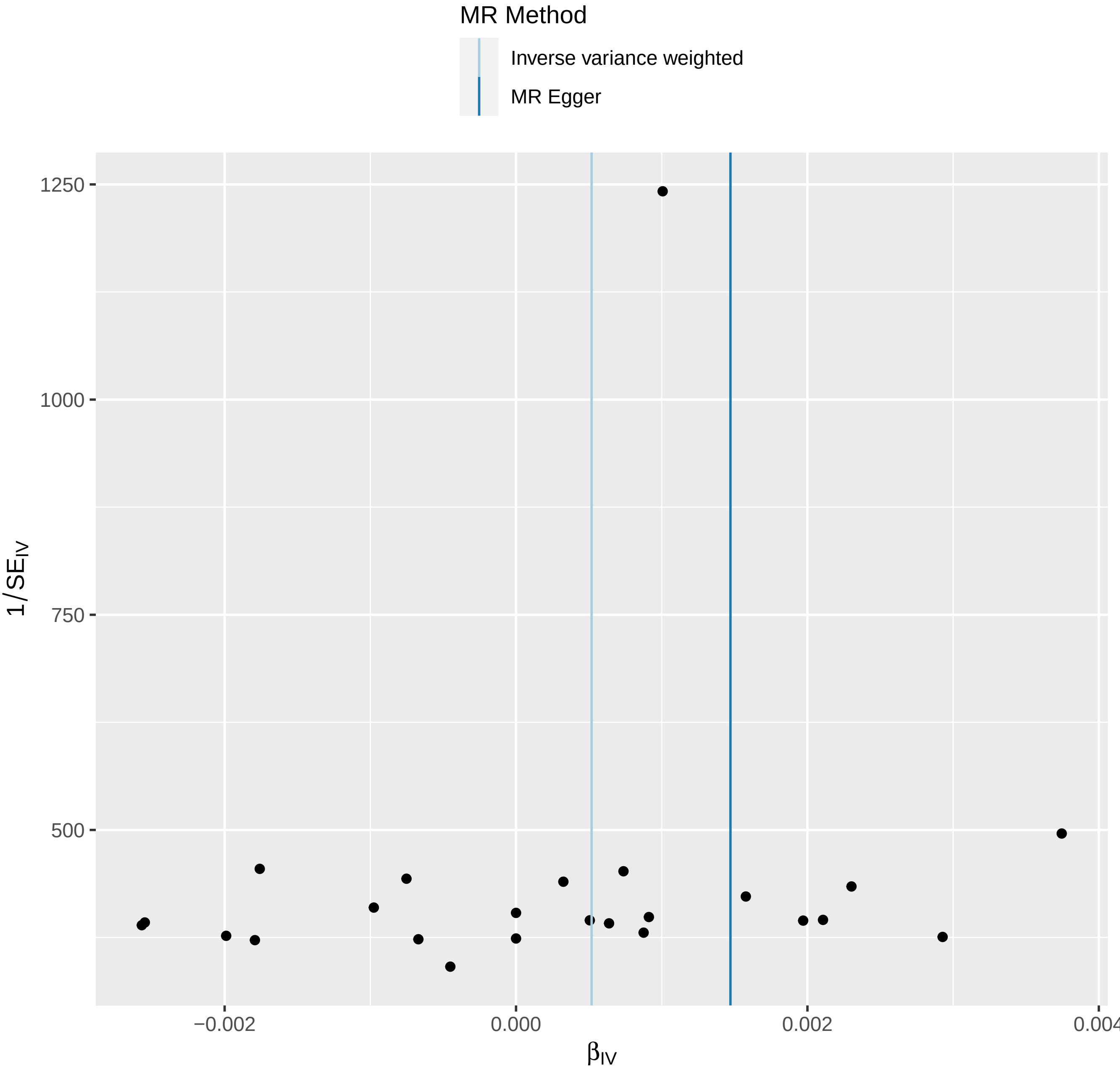

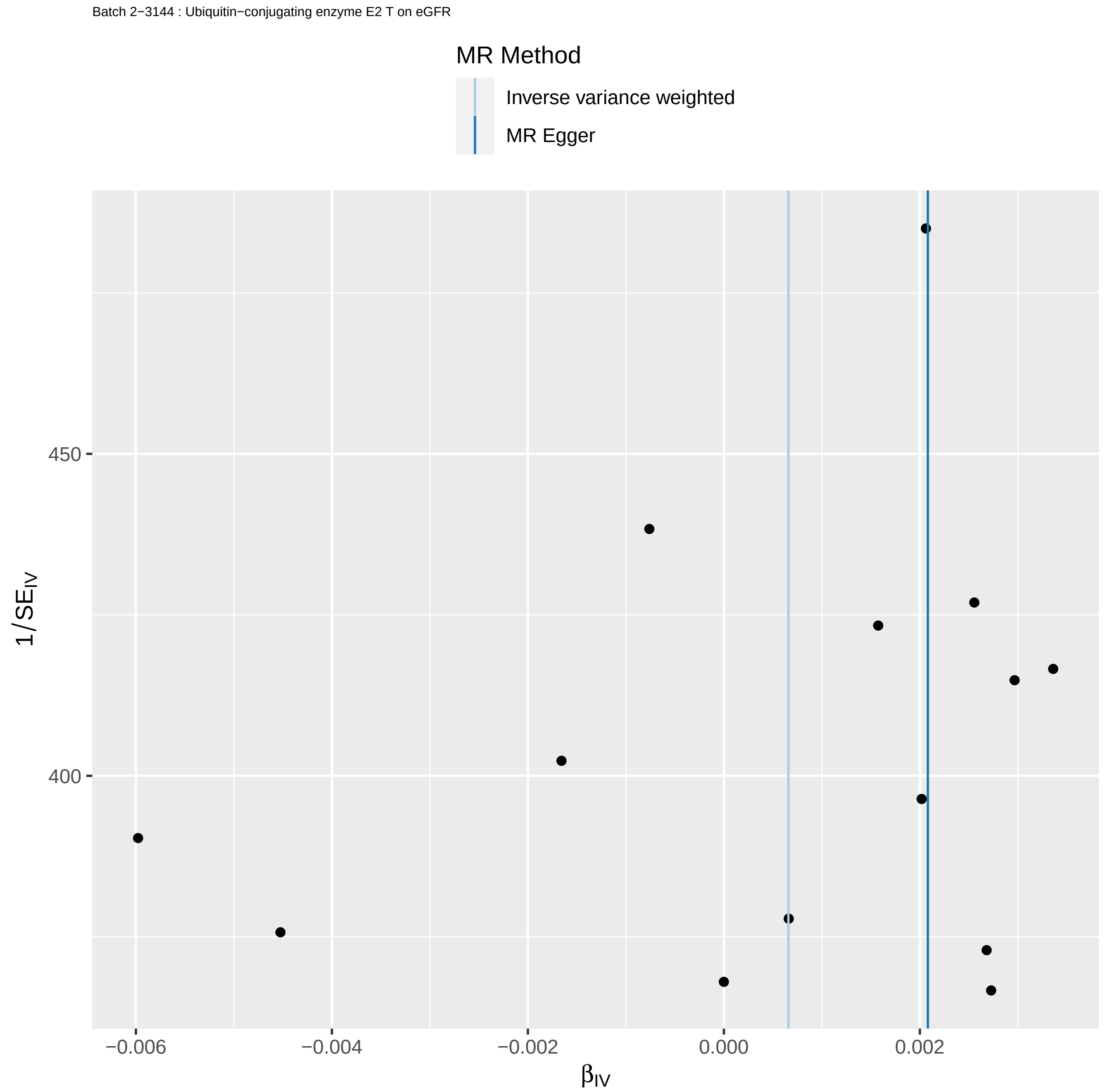
Batch 2−3144 : Ubiquitin−conjugating enzyme E2 T on eGFR
MR Method
Inverse variance weighted
MR Egger
450
400
1/SE$_{IV}$
−0.006
−0.004
−0.002
0.000
0.002
β$_{IV}$

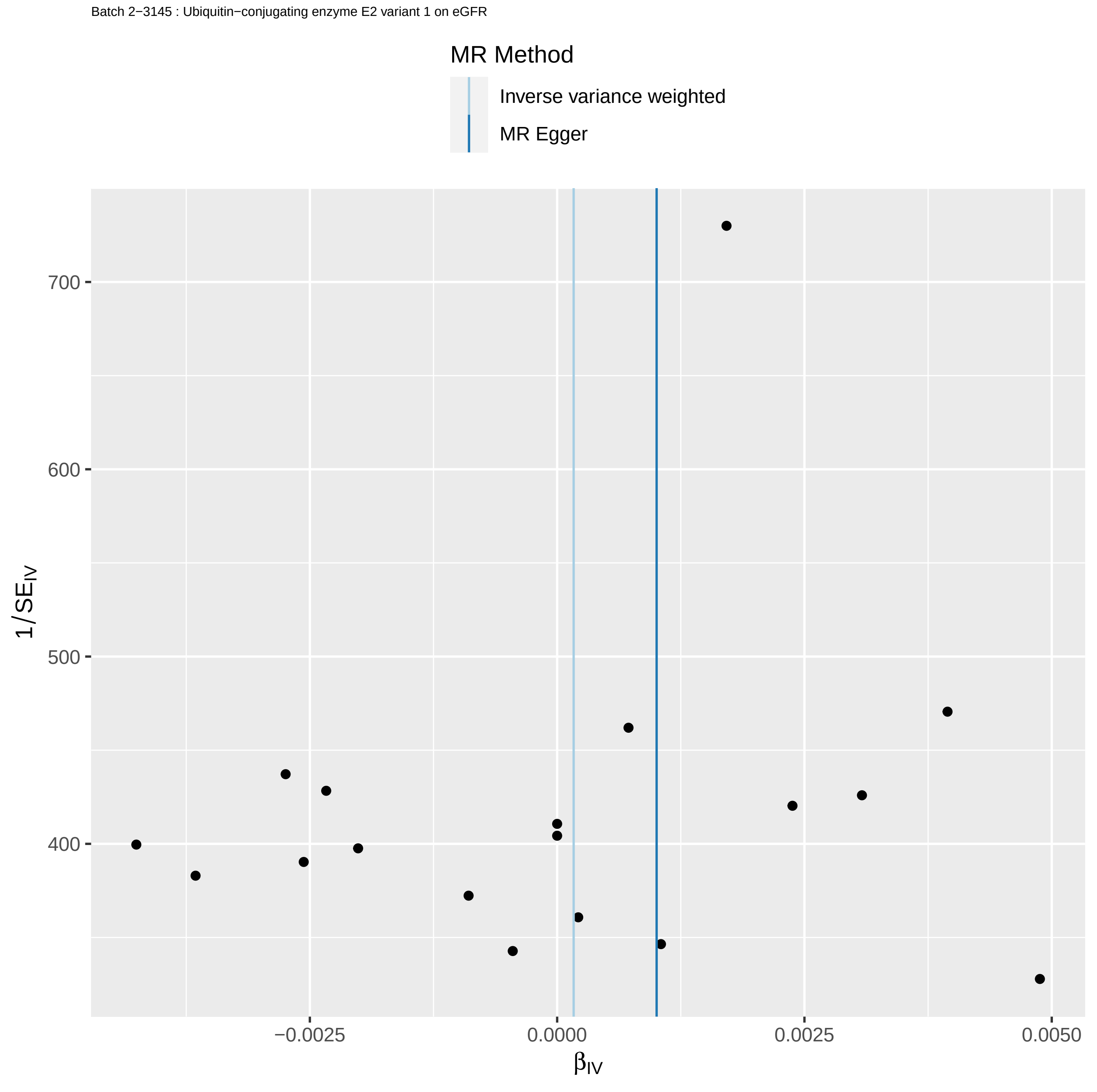
Batch 2−3145 : Ubiquitin−conjugating enzyme E2 variant 1 on eGFR
MR Method
Inverse variance weighted
MR Egger
700
600
500
400
1/SE_IV
−0.0025
0.0000
0.0025
0.0050
β_IV

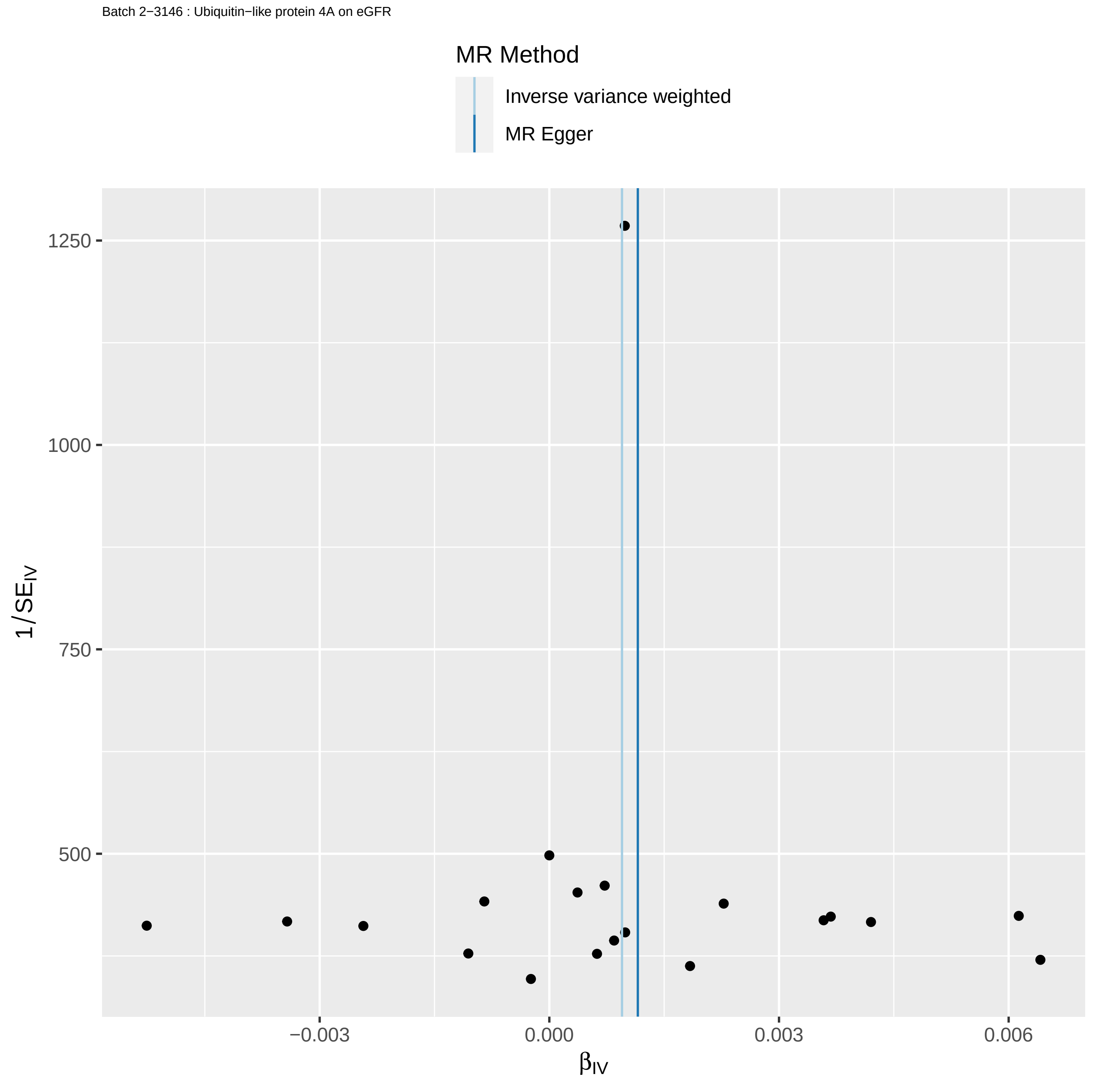
Batch 2−3146 : Ubiquitin−like protein 4A on eGFR
MR Method
Inverse variance weighted
MR Egger
1250
1000
750
500
1/SE_IV
−0.003
0.000
0.003
0.006
β_IV

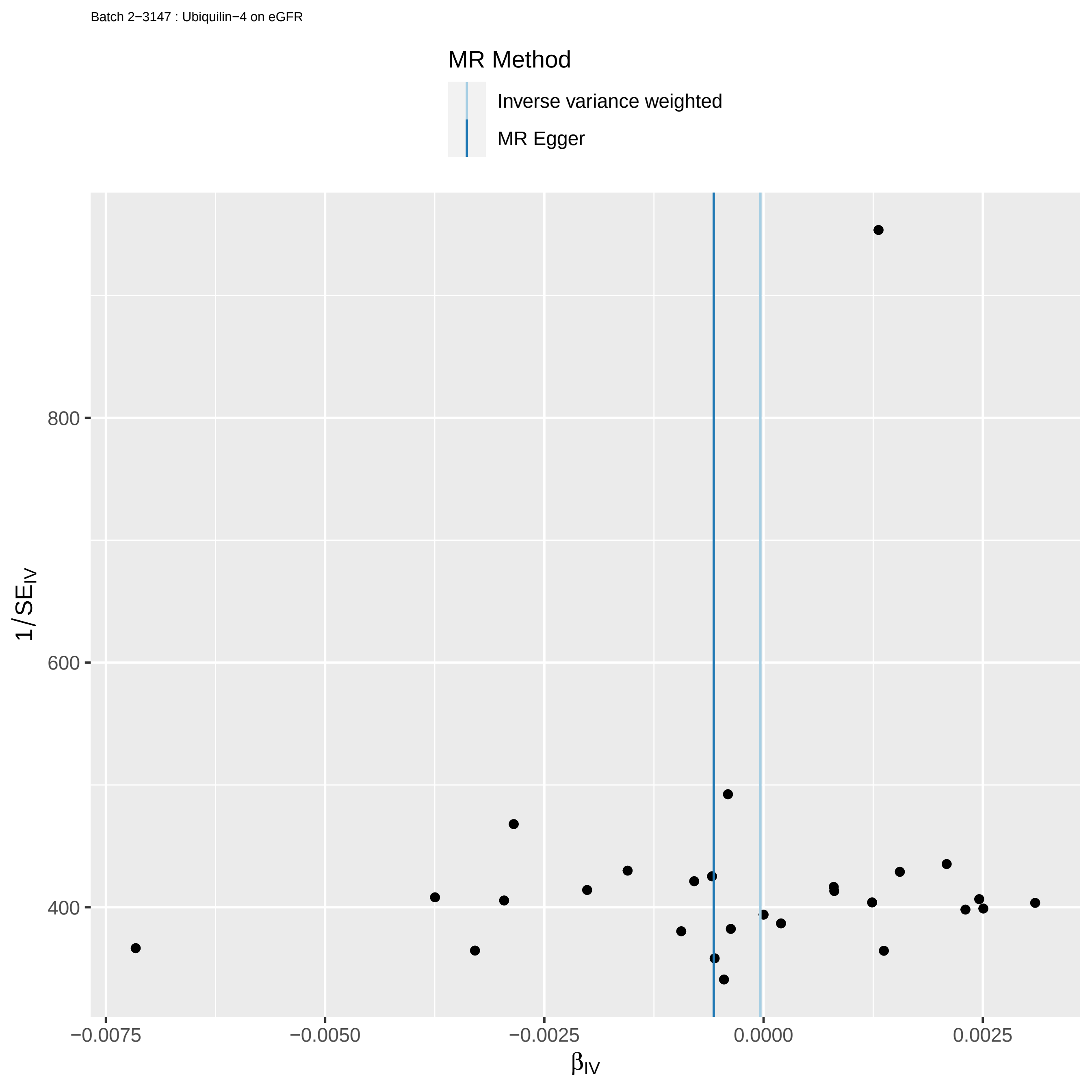
Batch 2−3147 : Ubiquilin−4 on eGFR
MR Method
Inverse variance weighted
MR Egger
800
600
400
1/SE$_{IV}$
−0.0075
−0.0050
−0.0025
0.0000
0.0025
β$_{IV}$

Batch 2−3148 : Ubiquitin domain−containing protein 2 on eGFR

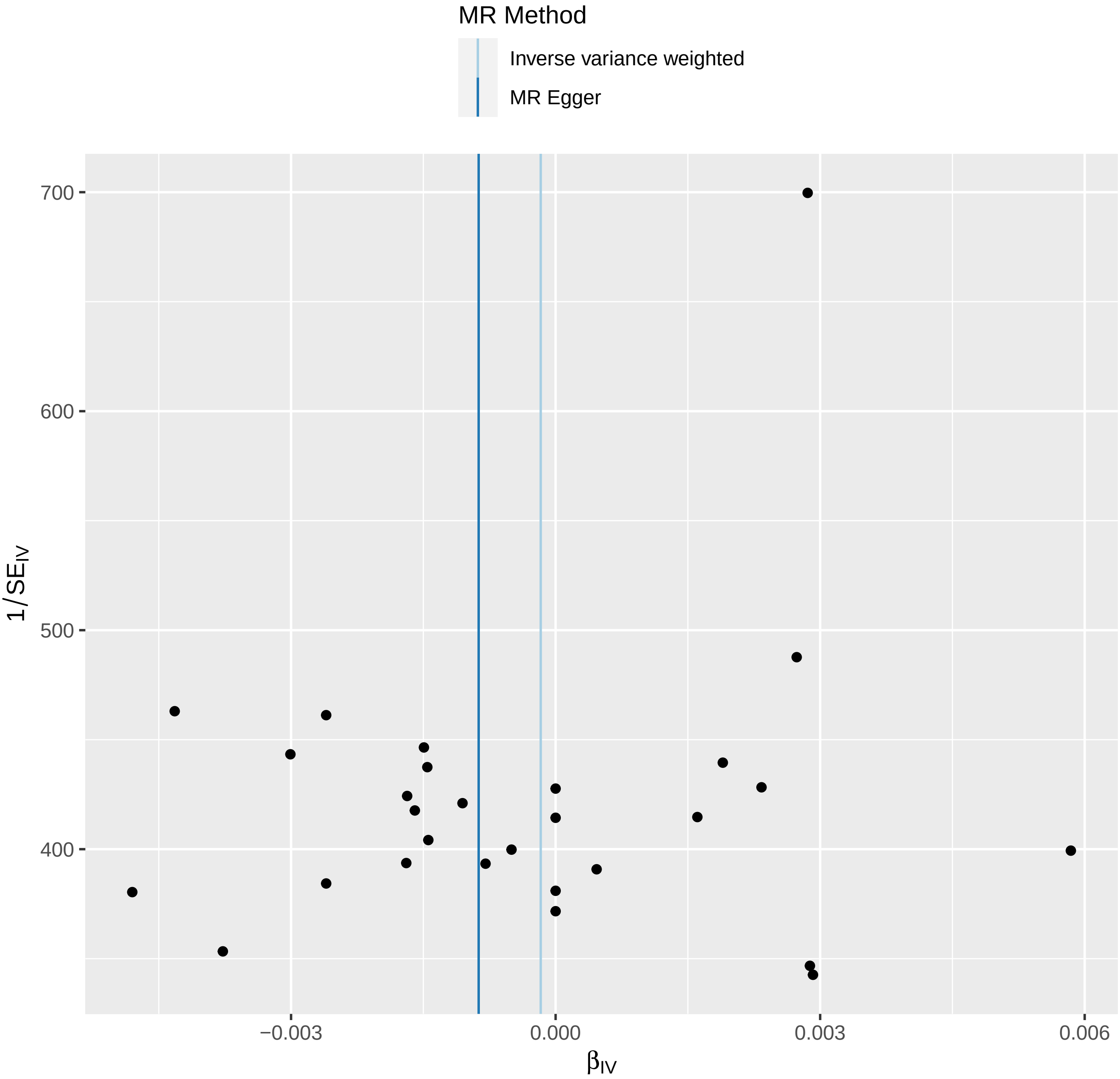

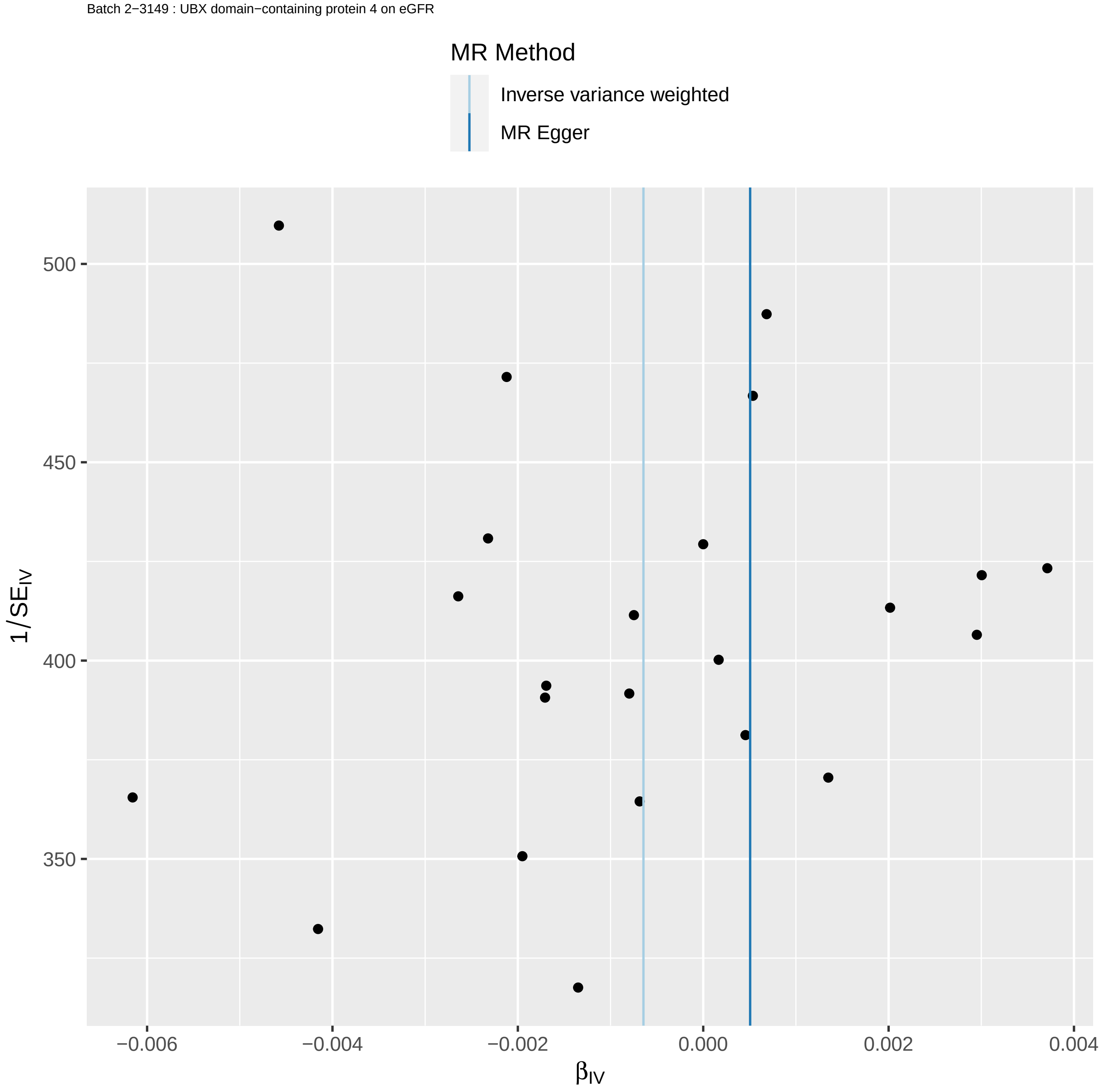
Batch 2−3149 : UBX domain−containing protein 4 on eGFR
MR Method
Inverse variance weighted
MR Egger
500
450
400
350
1/SE$_{IV}$
−0.006
−0.004
−0.002
0.000
0.002
0.004
β$_{IV}$

Batch 2−3150 : UBX domain−containing protein 4 on eGFR

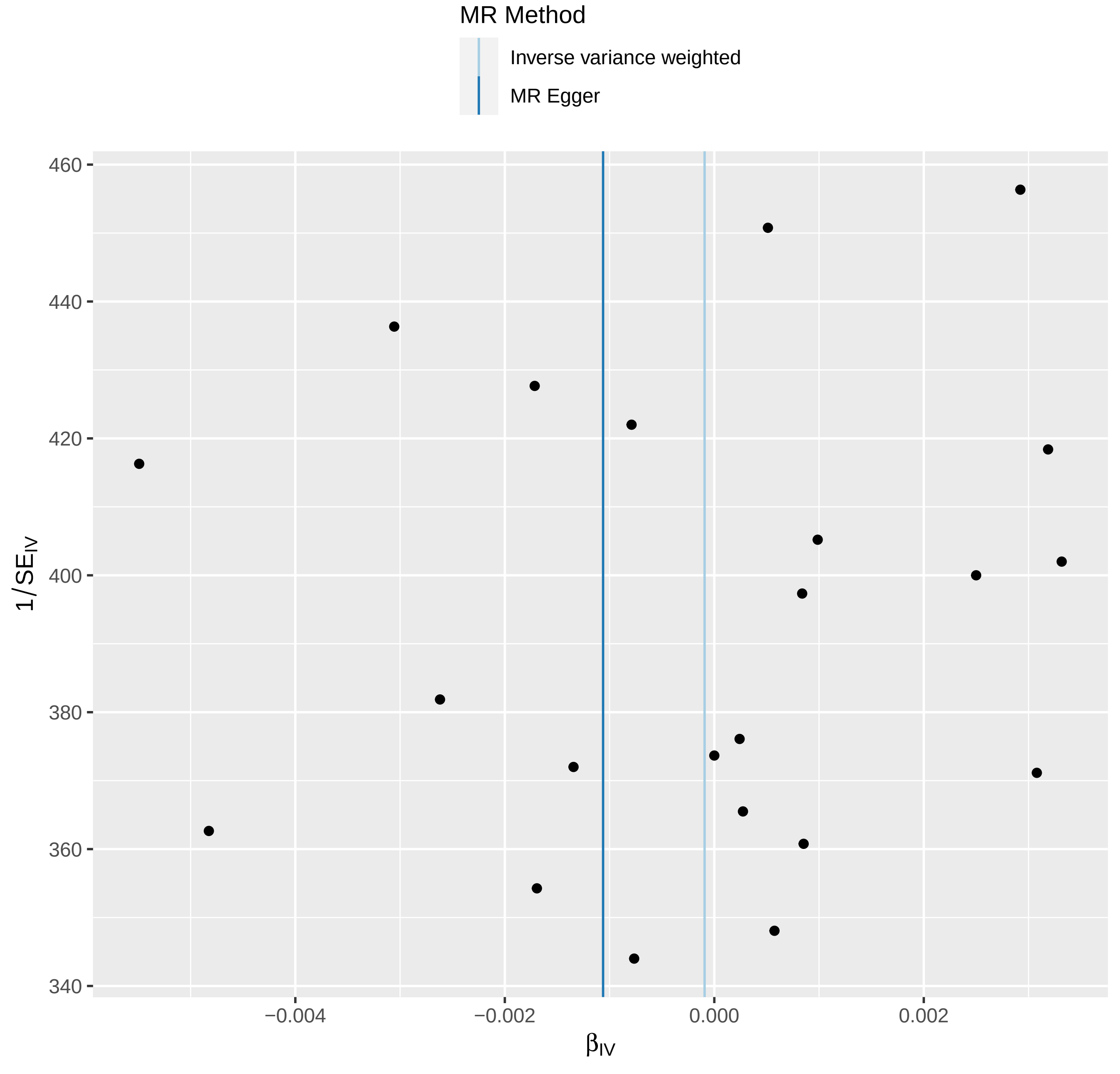

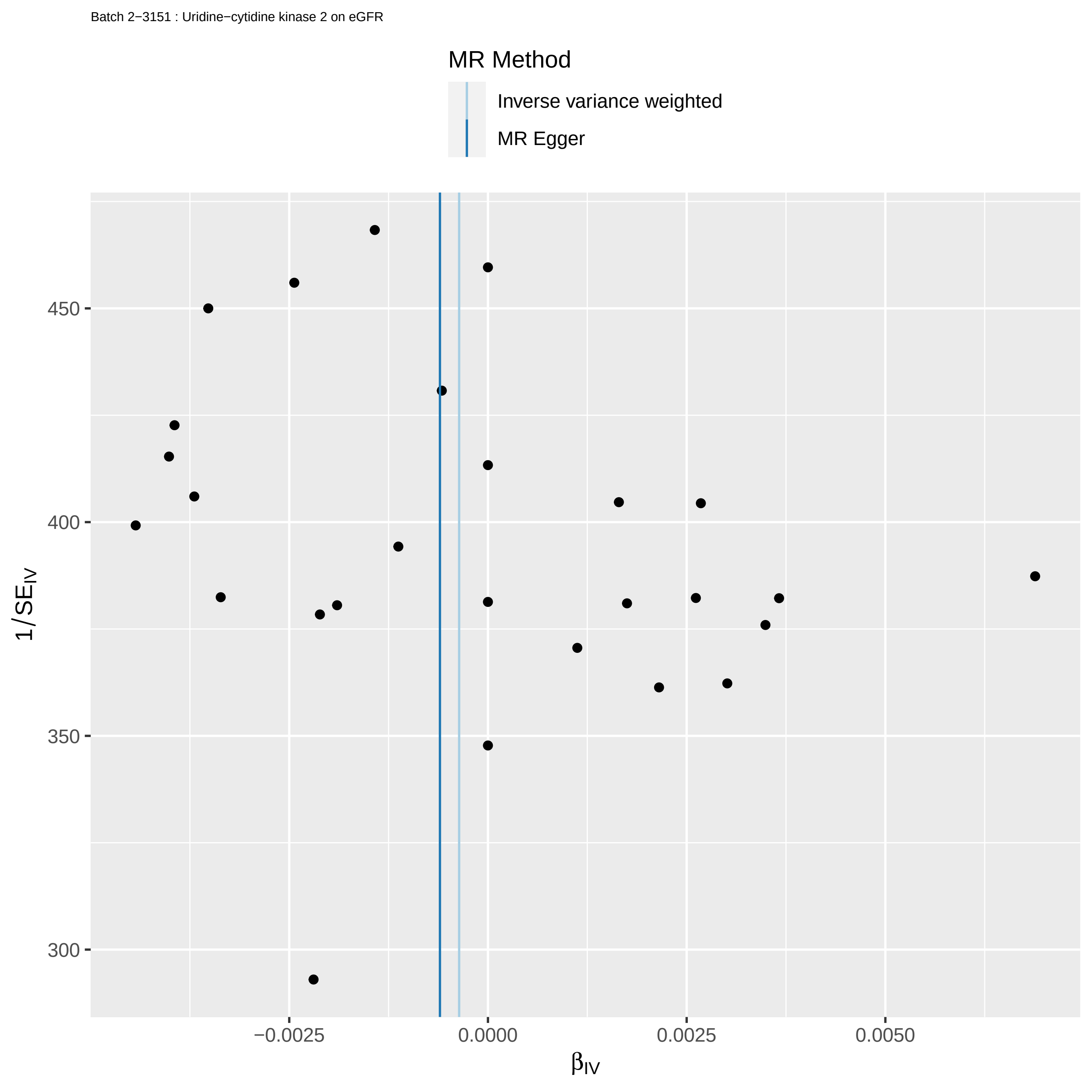
Batch 2−3151 : Uridine−cytidine kinase 2 on eGFR
MR Method
Inverse variance weighted
MR Egger
450
400
350
300
1/SE_IV
−0.0025
0.0000
0.0025
0.0050
β_IV

Batch 2−3152 : Unique cartilage matrix−associated protein on eGFR

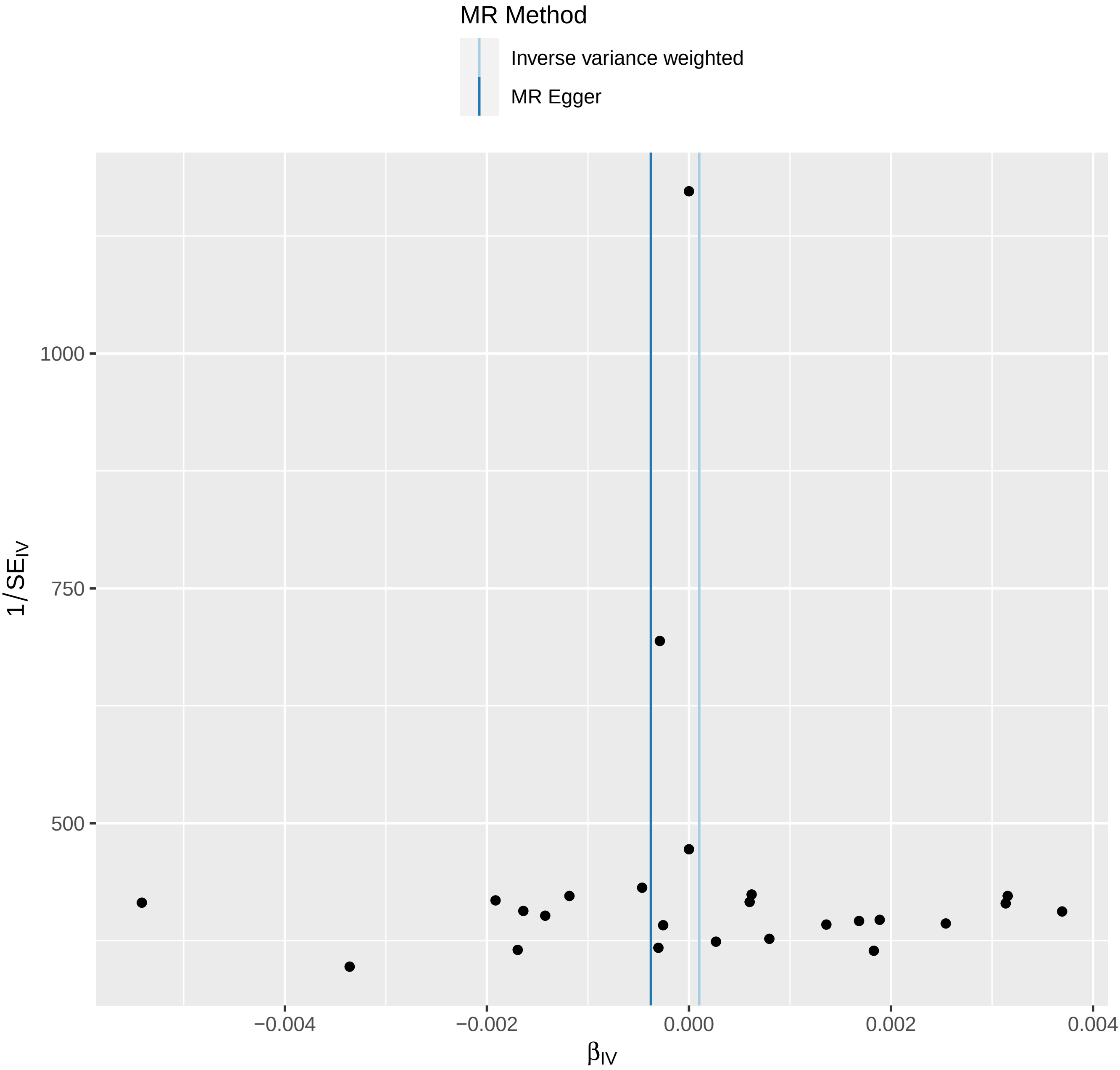

Batch 2−3153 : Ubiquitin−fold modifier−conjugating enzyme 1 on eGFR

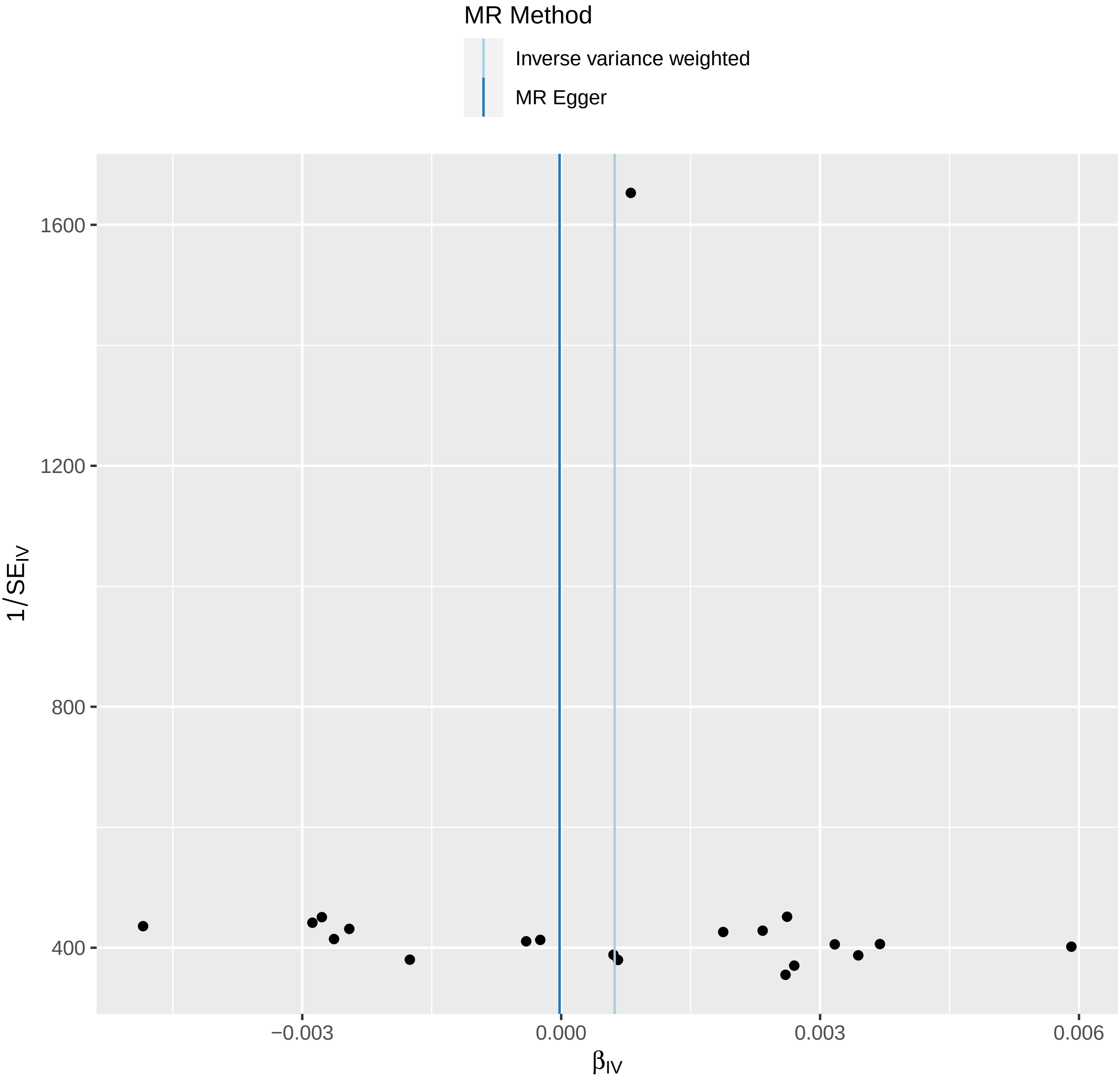

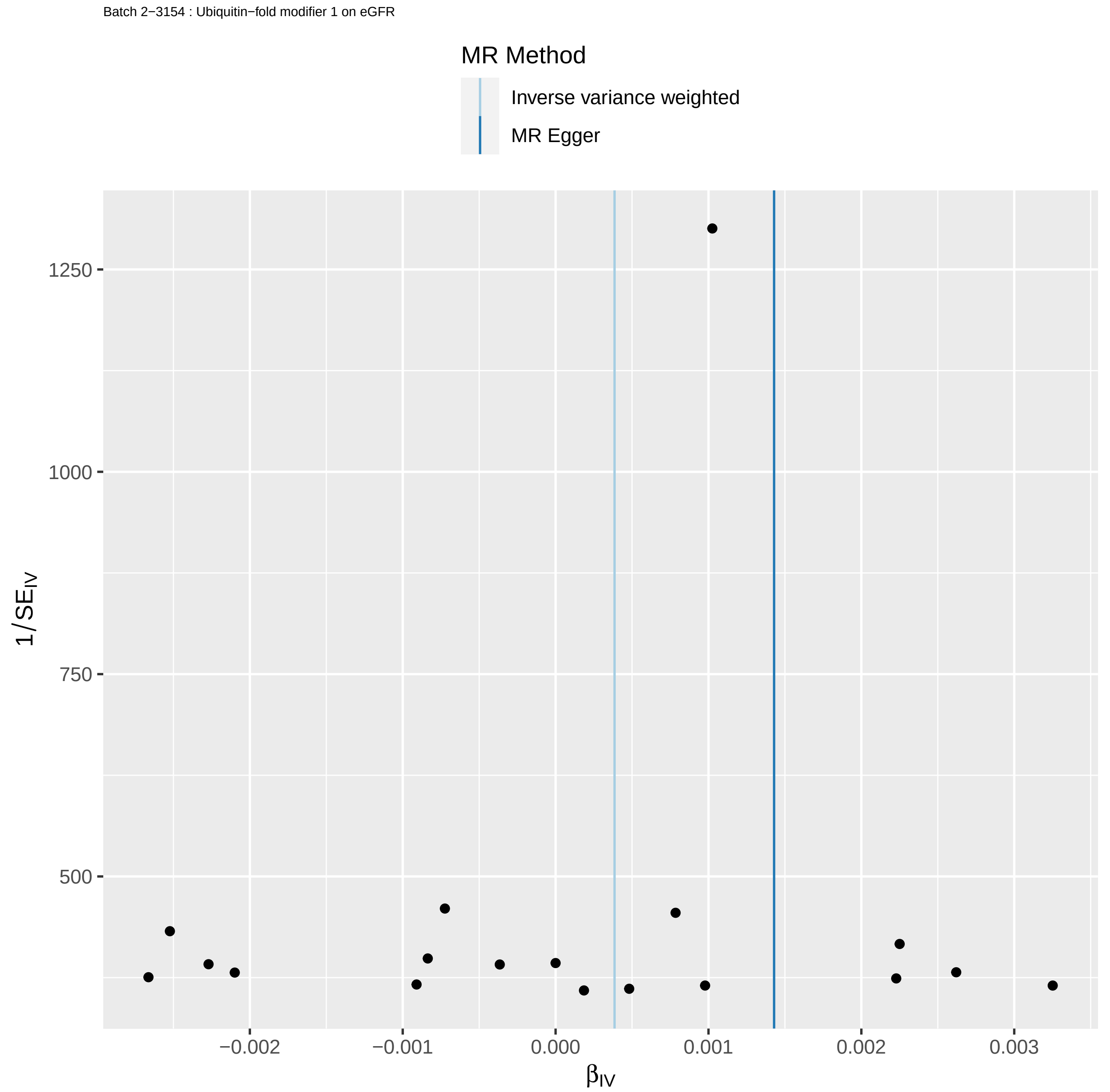

Batch 2−3154 : Ubiquitin−fold modifier 1 on eGFR
MR Method
Inverse variance weighted
MR Egger
1250
1000
750
500
1/SE_IV
−0.002
−0.001
0.000
0.001
0.002
0.003
β_IV

Batch 2−3155 : UTP−−glucose−1−phosphate uridylyltransferase on eGFR

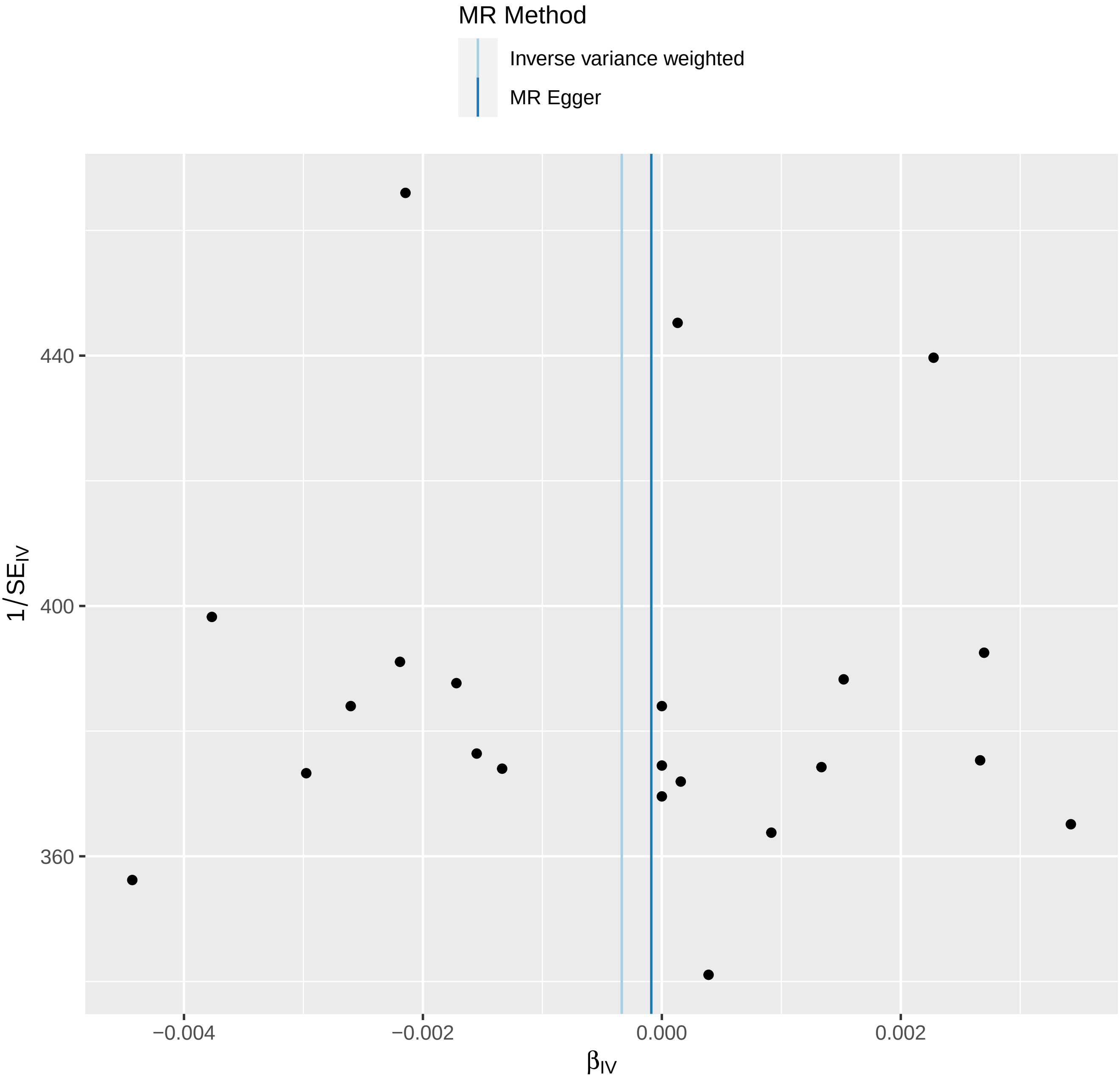

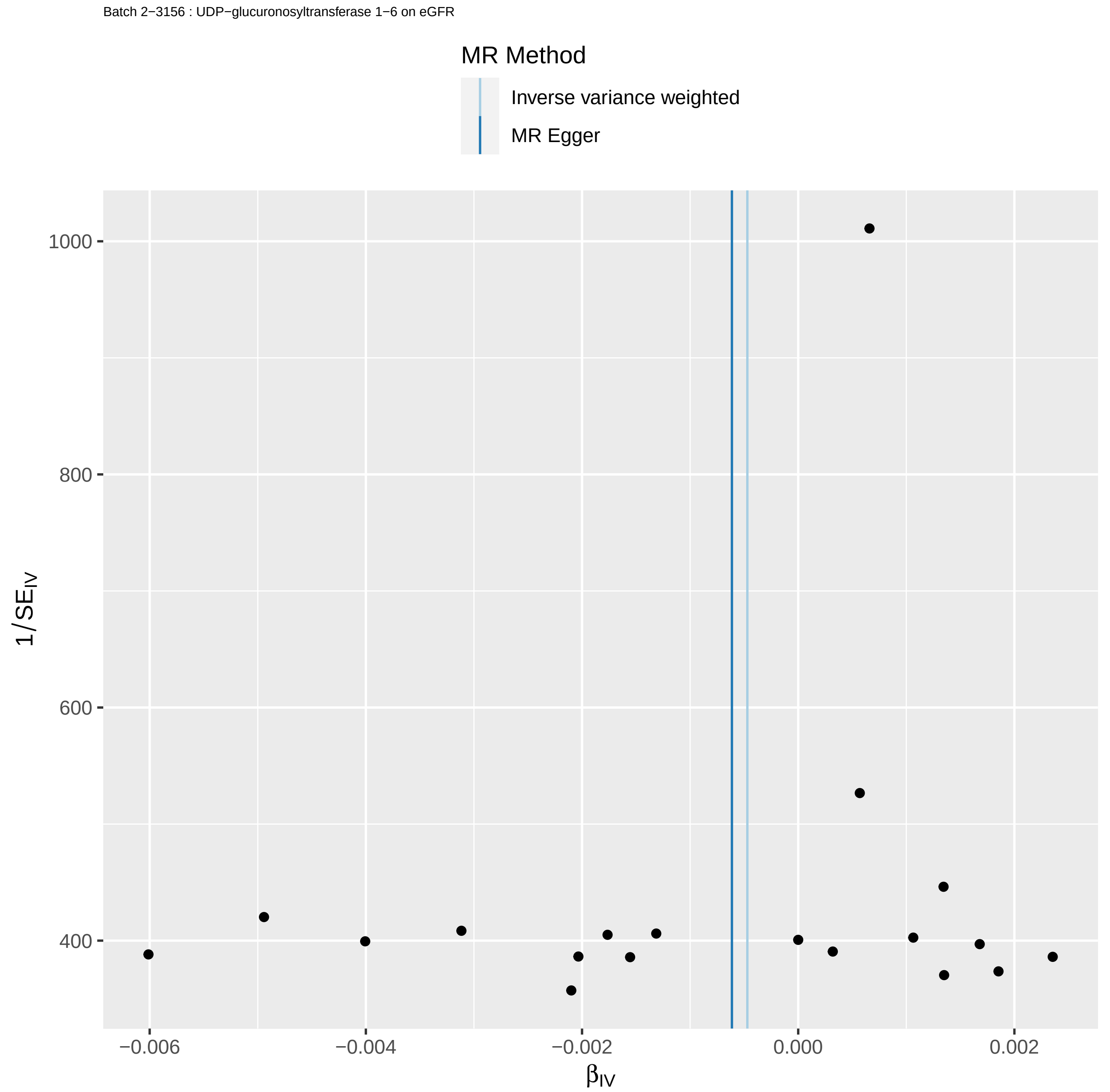
Batch 2−3156 : UDP−glucuronosyltransferase 1−6 on eGFR
MR Method
Inverse variance weighted
MR Egger
1000
800
600
400
1/SE$_{IV}$
−0.006
−0.004
−0.002
0.000
0.002
β$_{IV}$

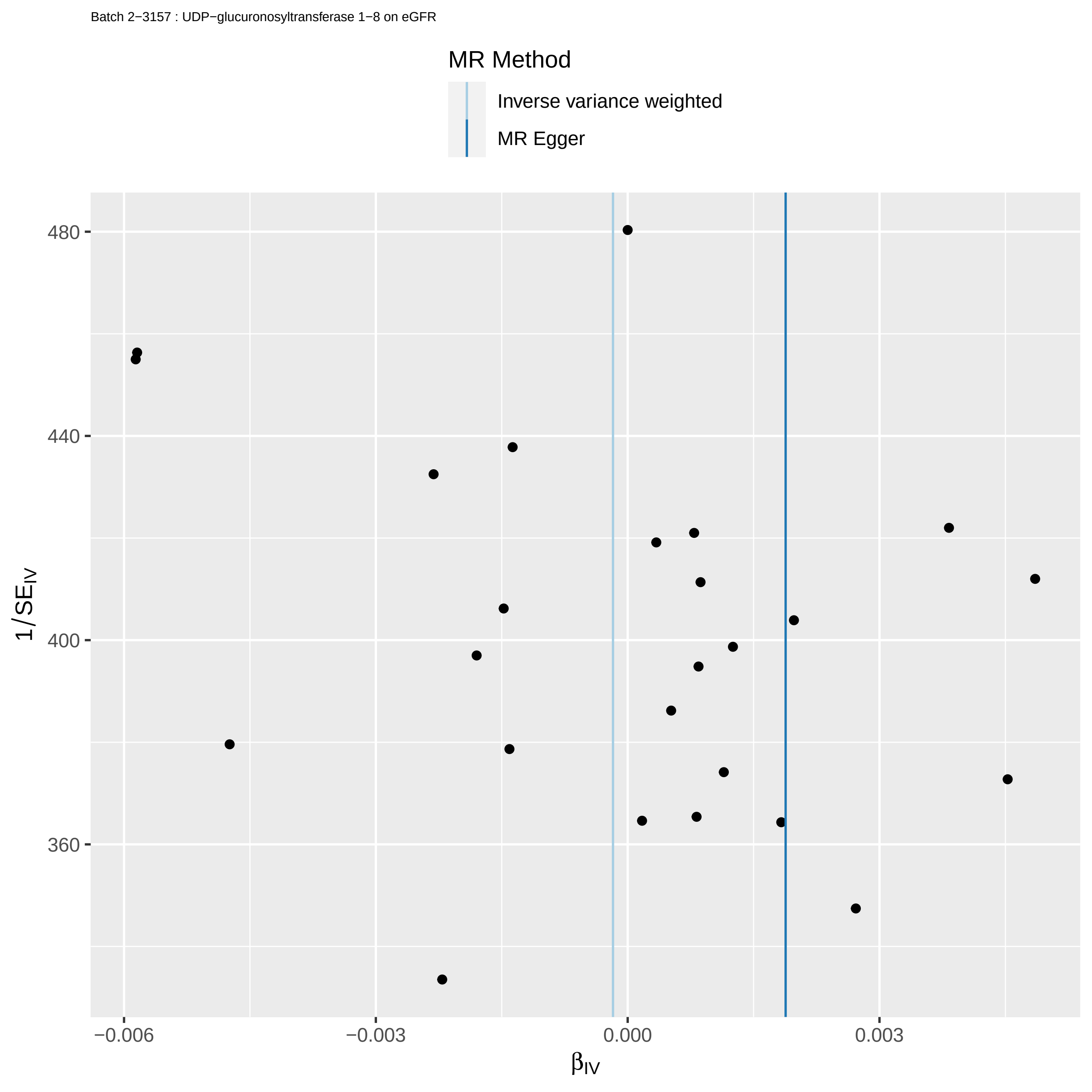
Batch 2−3157 : UDP−glucuronosyltransferase 1−8 on eGFR
MR Method
Inverse variance weighted
MR Egger
480
440
400
360
1/SE$_{IV}$
−0.006
−0.003
0.000
0.003
β$_{IV}$

Batch 2−3158 : UDP−glucuronosyltransferase 2A1 on eGFR

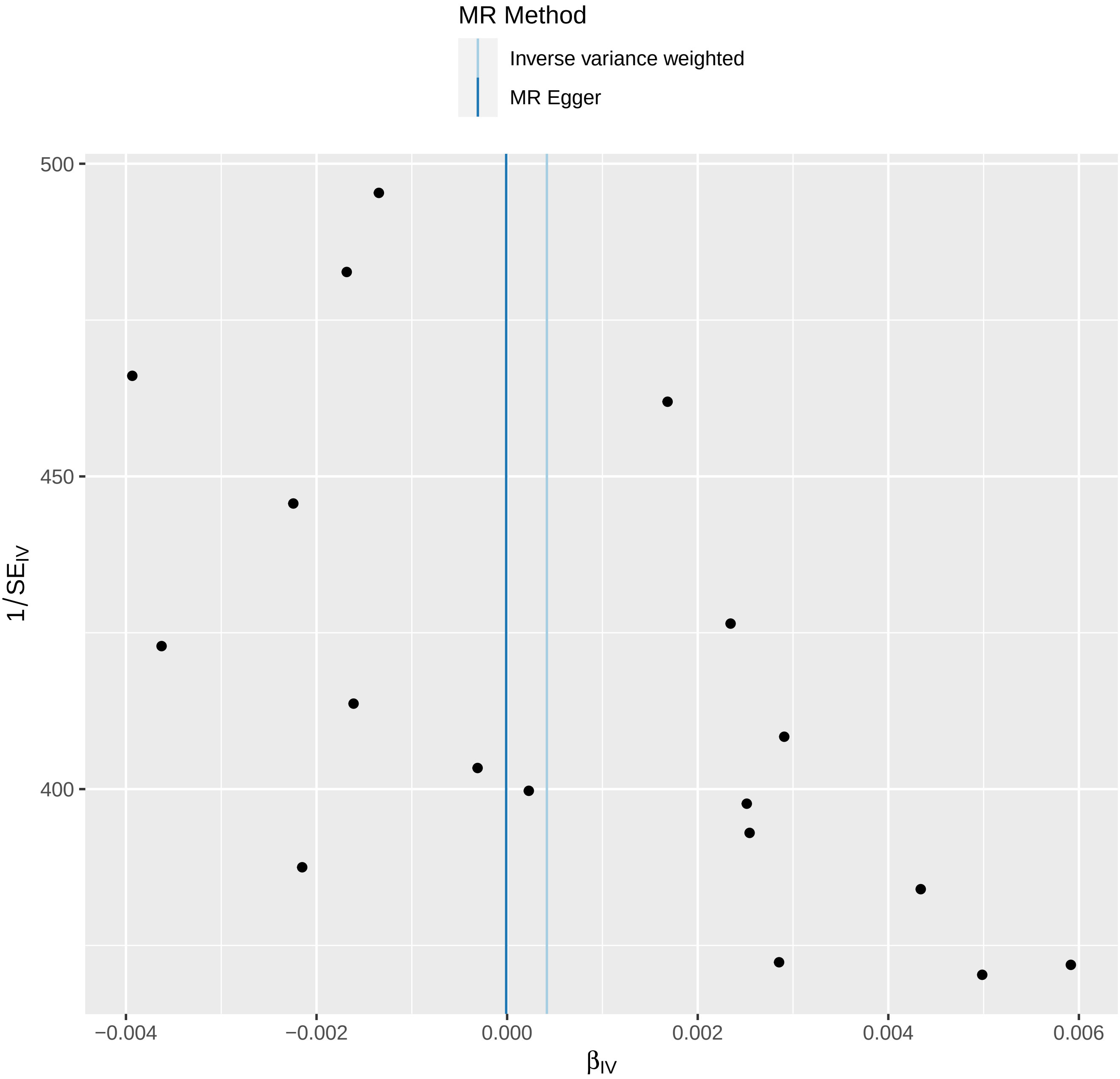

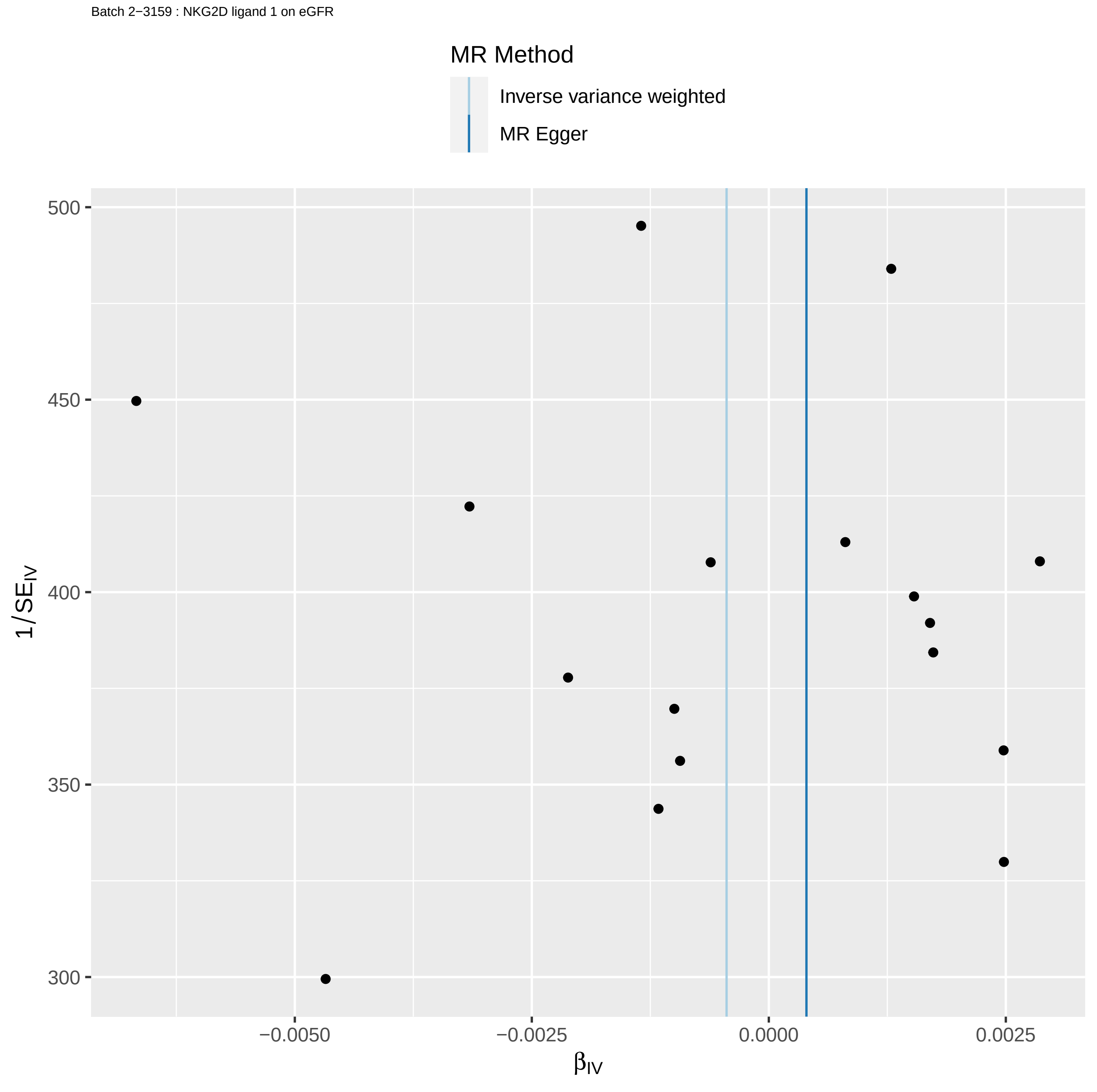

Batch 2−3159 : NKG2D ligand 1 on eGFR
MR Method
Inverse variance weighted
MR Egger
500
450
400
350
300
1/SE$_{IV}$
−0.0050
−0.0025
0.0000
0.0025
β$_{IV}$

Batch 2−3160 : NKG2D ligand 1 on eGFR

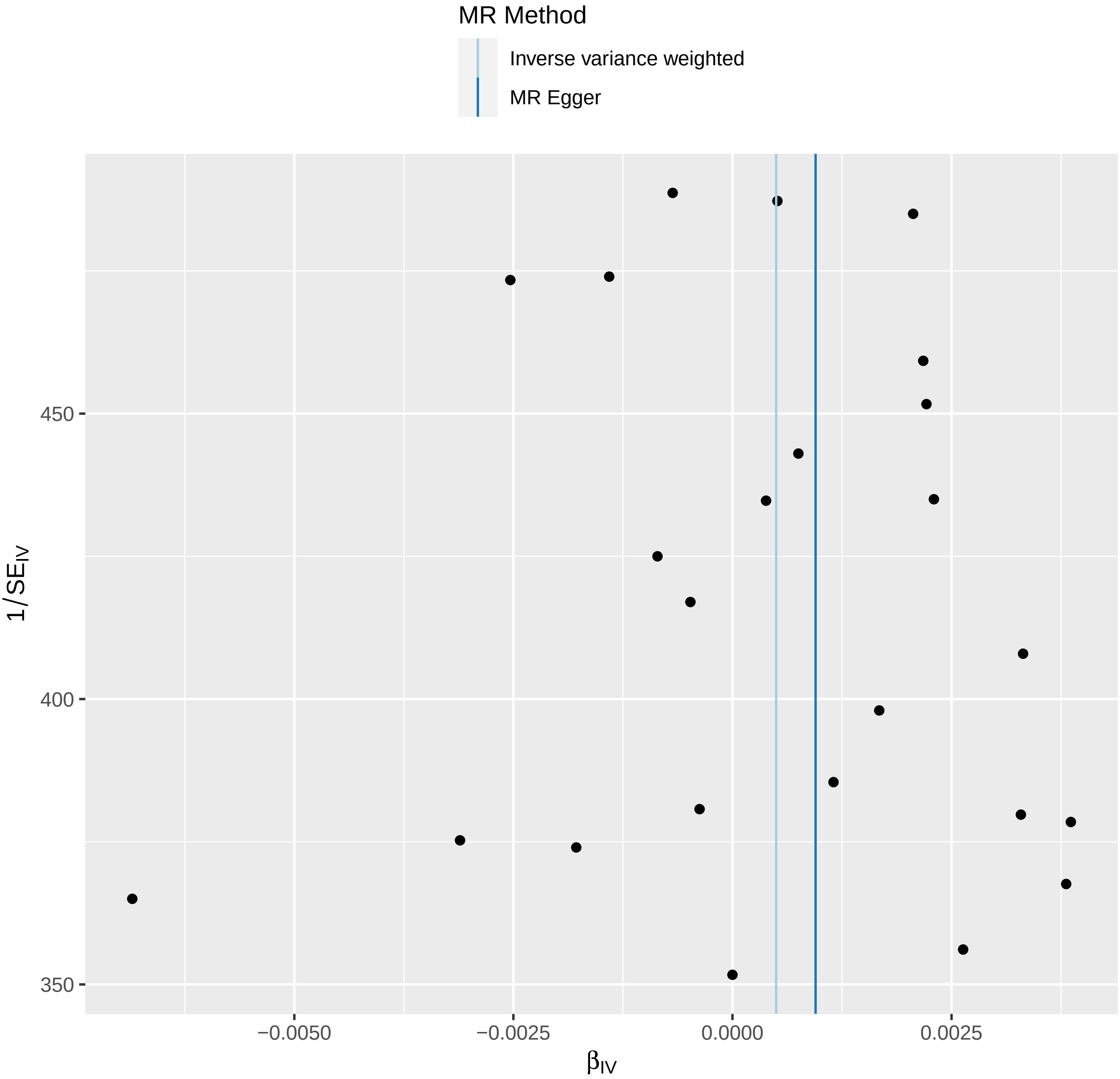

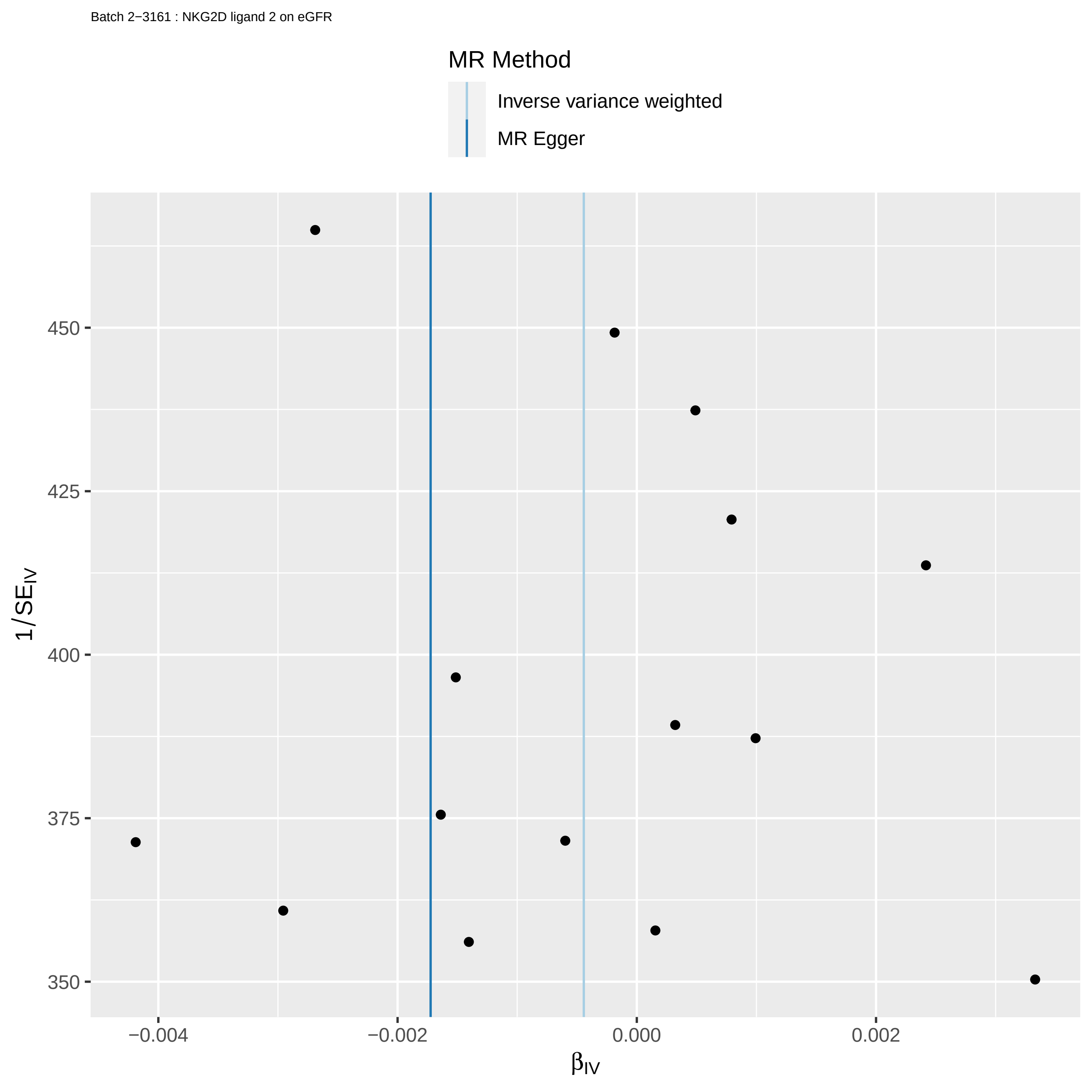
Batch 2−3161 : NKG2D ligand 2 on eGFR
MR Method
Inverse variance weighted
MR Egger
450
425
400
375
350
1/SE_IV
−0.004
−0.002
0.000
0.002
β_IV

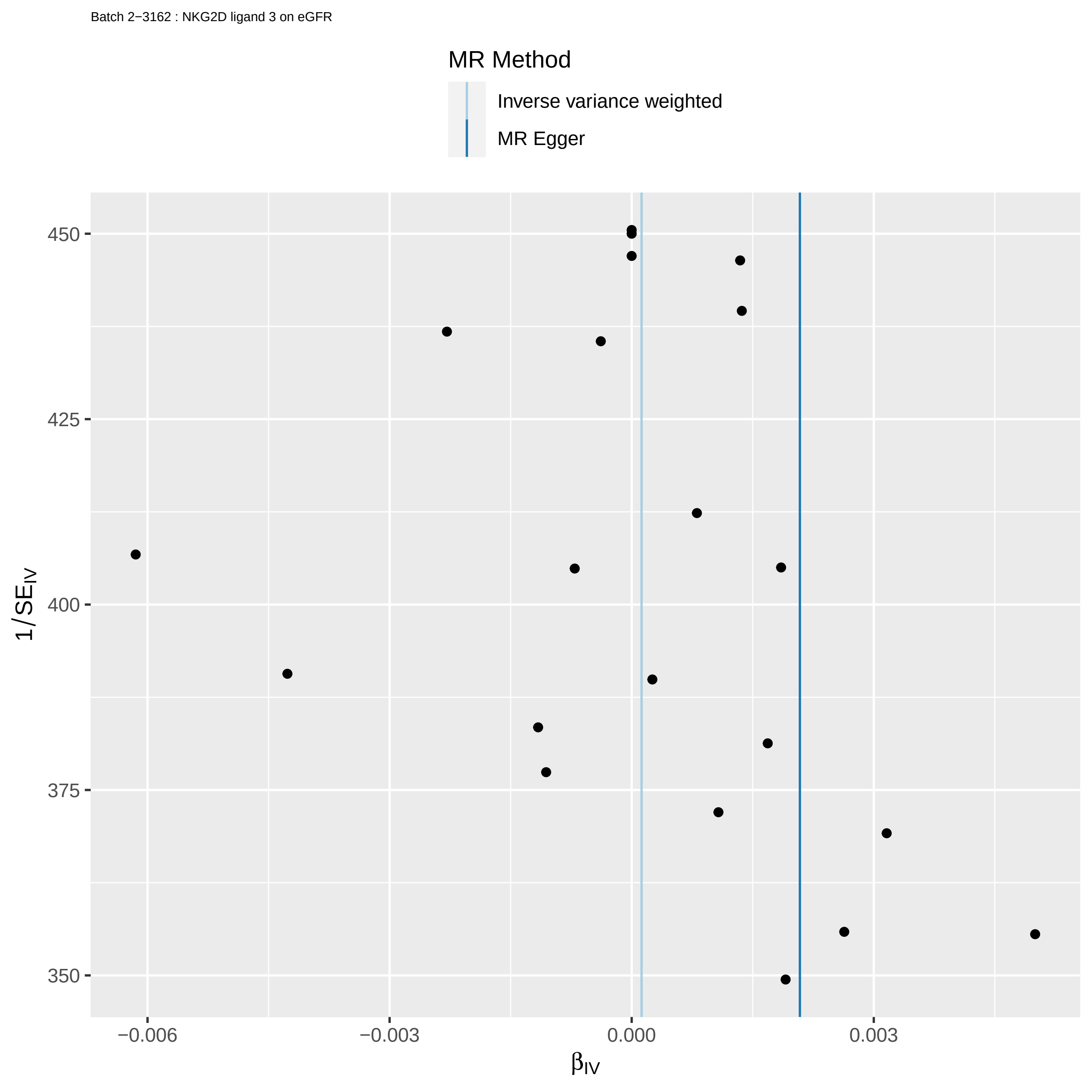
Batch 2−3162 : NKG2D ligand 3 on eGFR
MR Method
Inverse variance weighted
MR Egger
450
425
400
375
350
1/SE_IV
−0.006
−0.003
0.000
0.003
β_IV

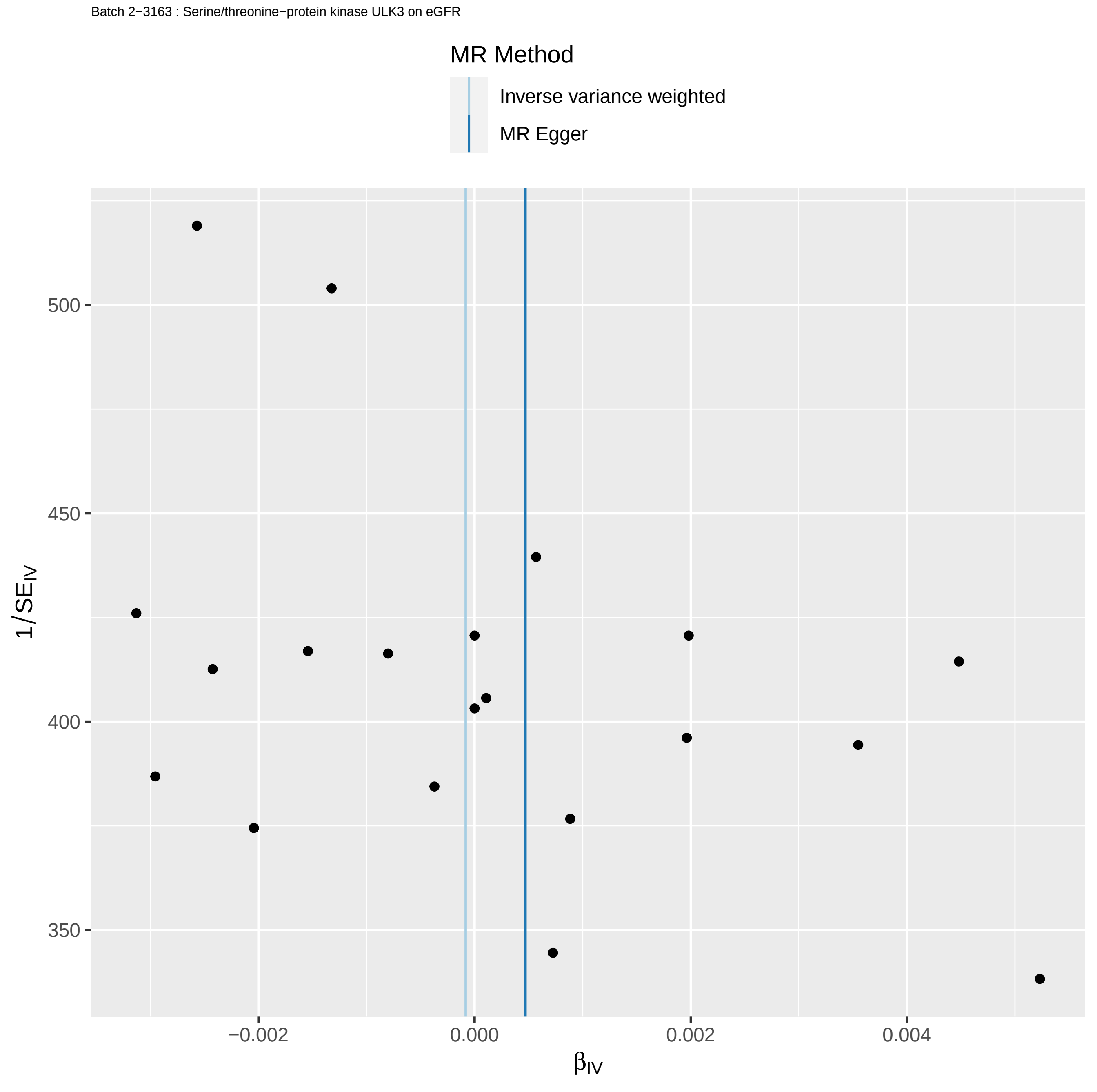

Batch 2−3163 : Serine/threonine−protein kinase ULK3 on eGFR
MR Method
Inverse variance weighted
MR Egger
500
450
400
350
1/SE_IV
−0.002
0.000
0.002
0.004
β_IV

Batch 2−3164 : Uromodulin−like 1 on eGFR

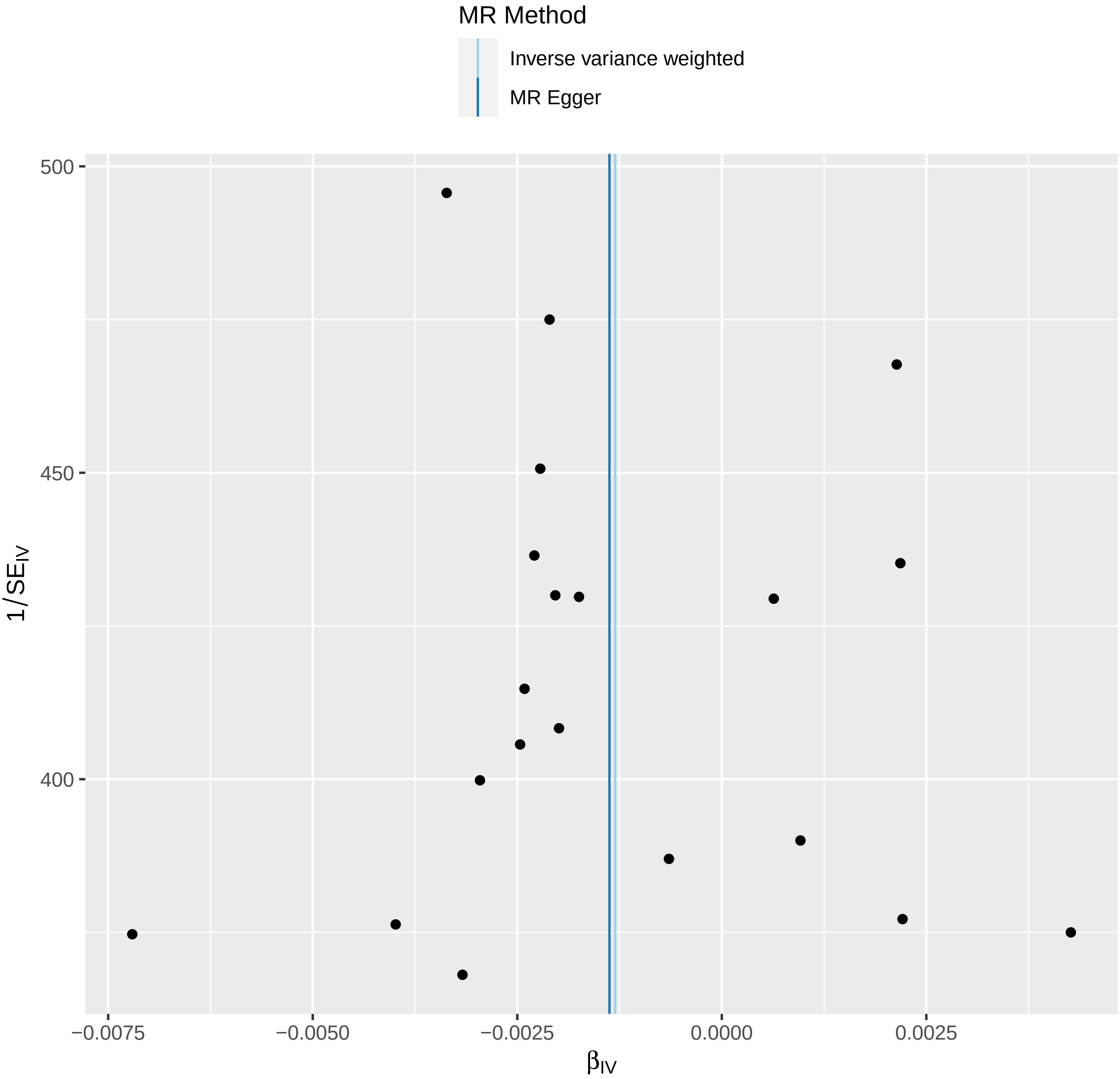

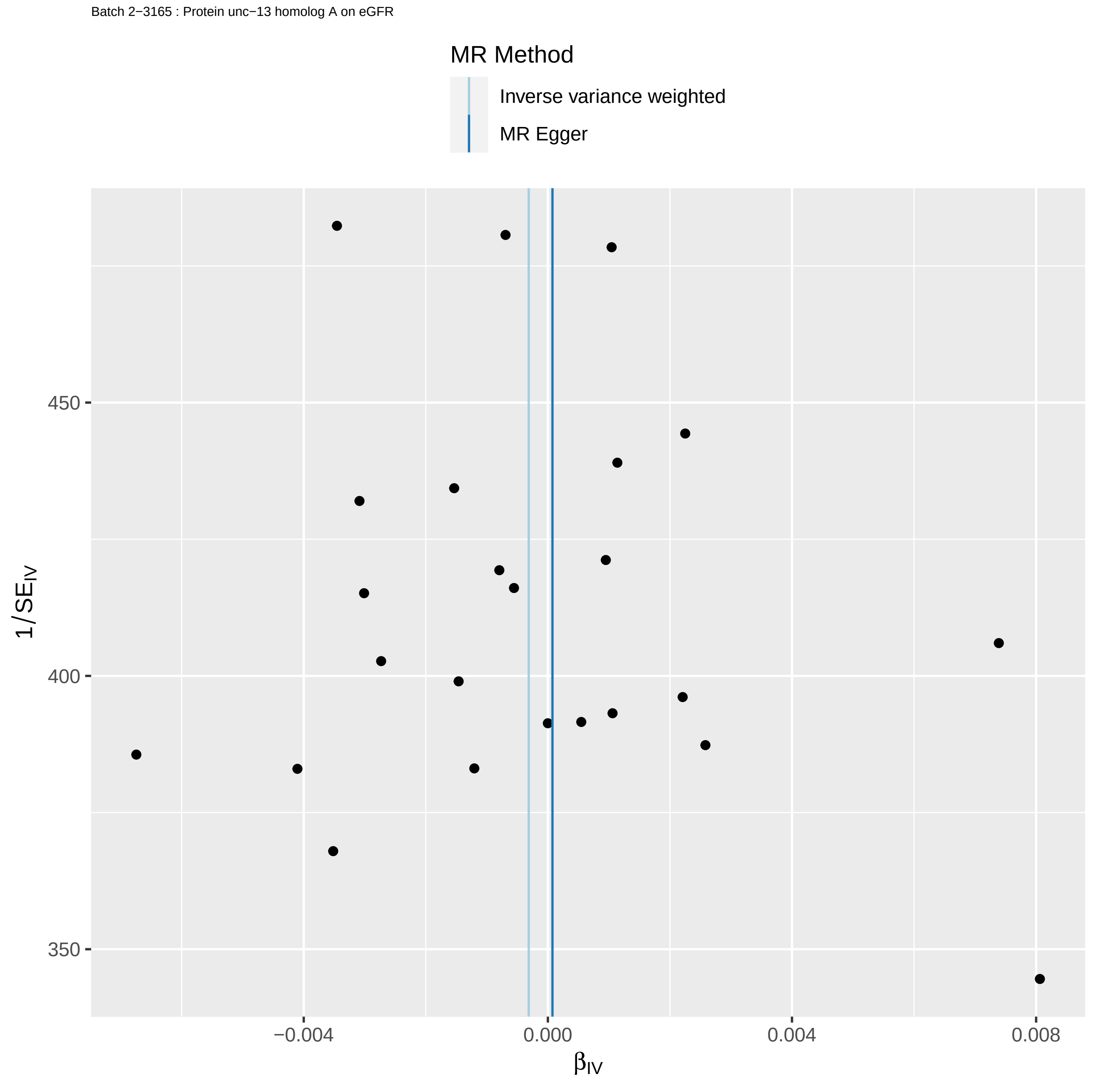
Batch 2−3165 : Protein unc−13 homolog A on eGFR
MR Method
Inverse variance weighted
MR Egger
450
400
350
1/SE_IV
−0.004
0.000
0.004
0.008
β_IV

Batch 2−3166 : Netrin receptor UNC5A on eGFR

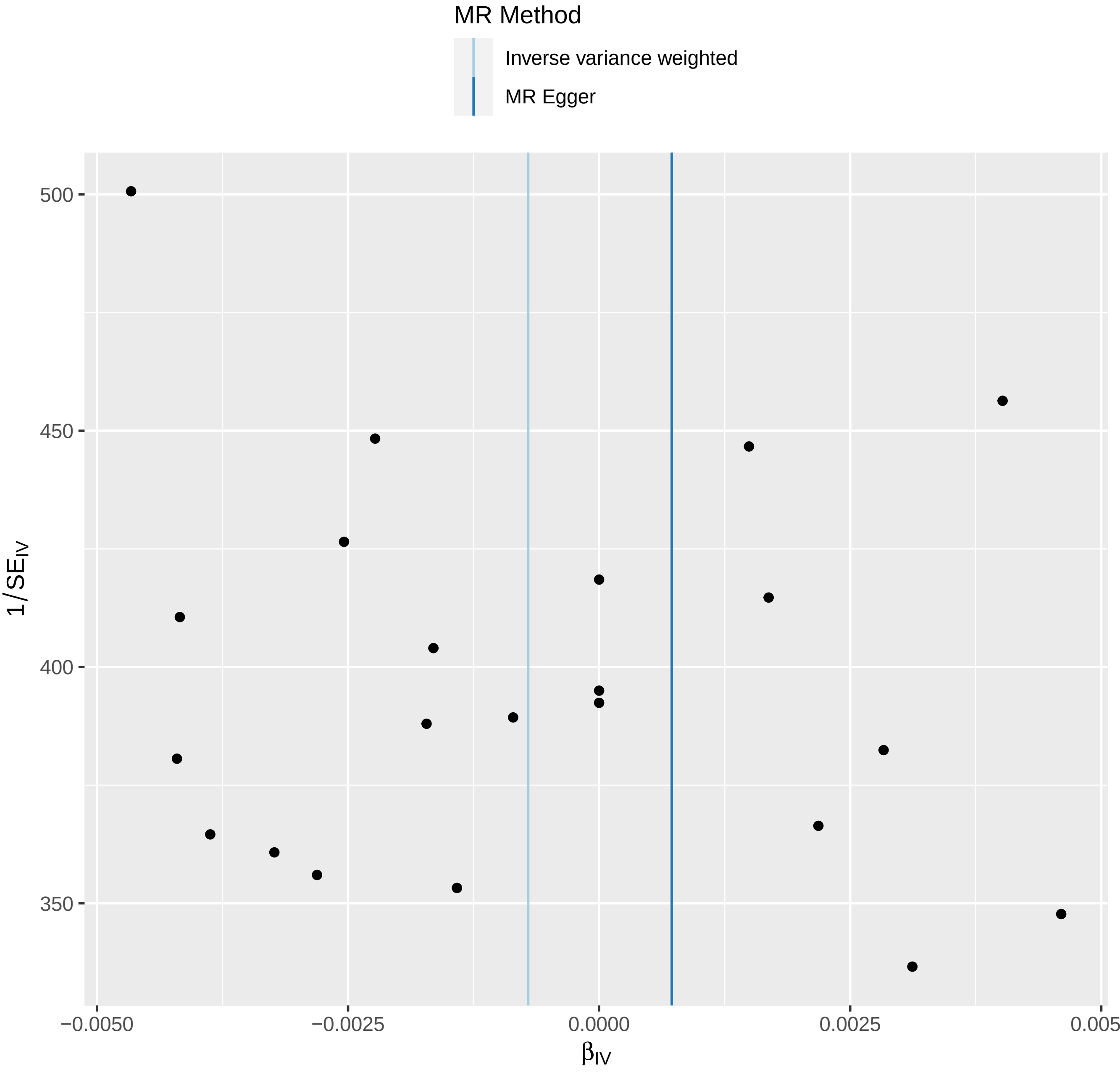

Batch 2−3167 : Netrin receptor UNC5B on eGFR

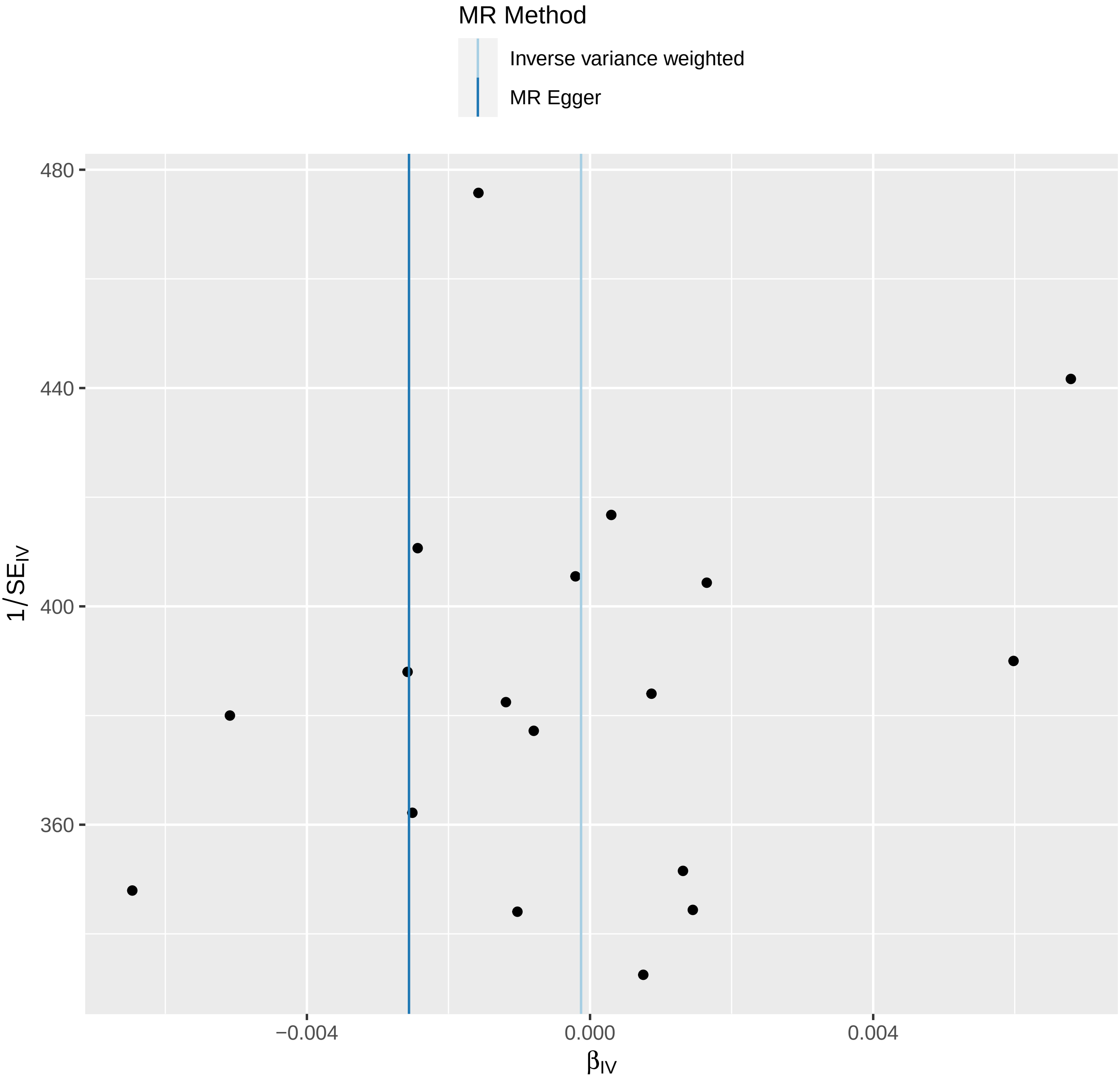

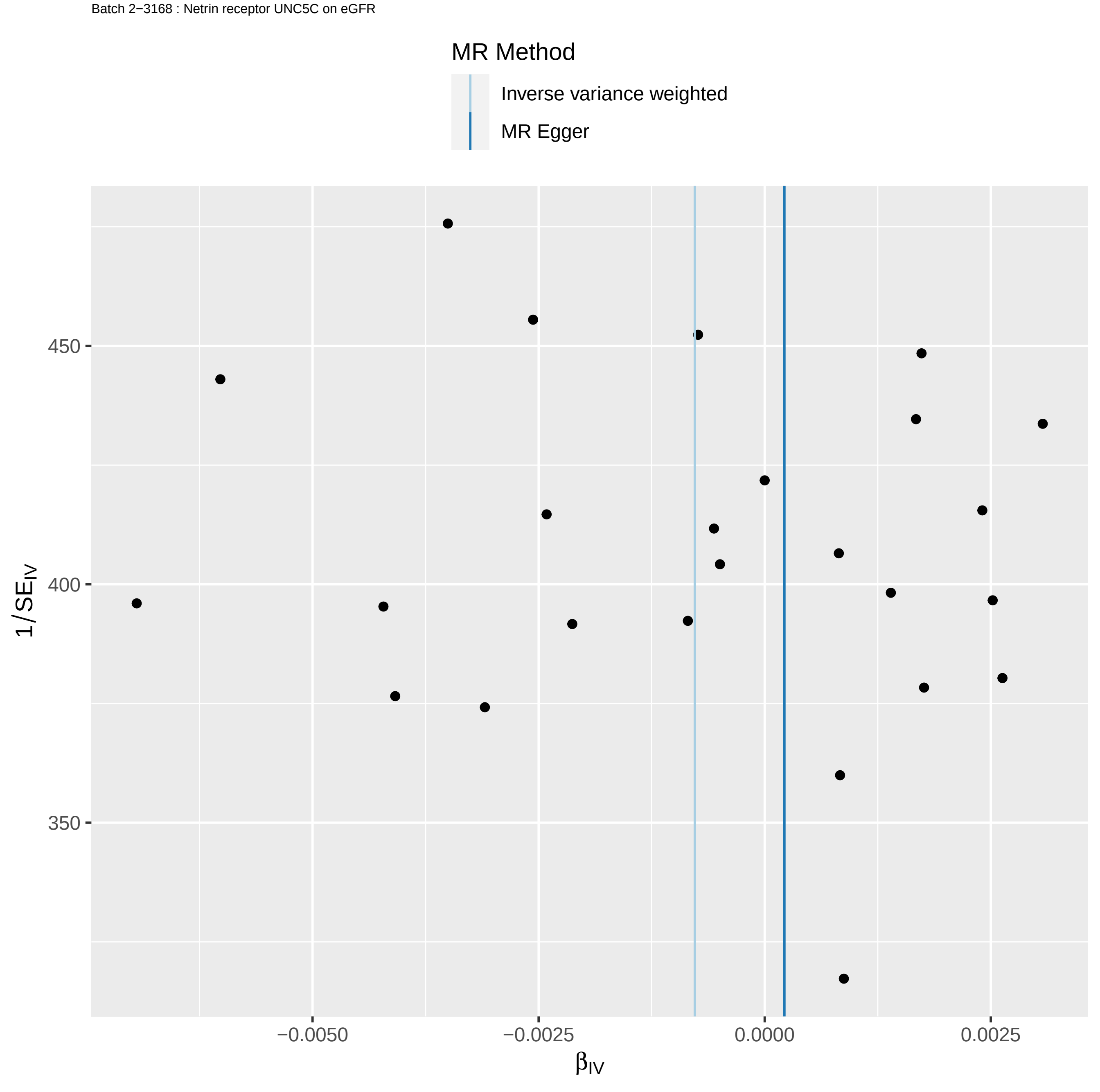

Batch 2−3168 : Netrin receptor UNC5C on eGFR
MR Method
Inverse variance weighted
MR Egger
450
400
350
−0.0050
−0.0025
0.0000
0.0025
$1/SE_{IV}$
β_{IV}

Batch 2−3169 : Netrin receptor UNC5D on eGFR

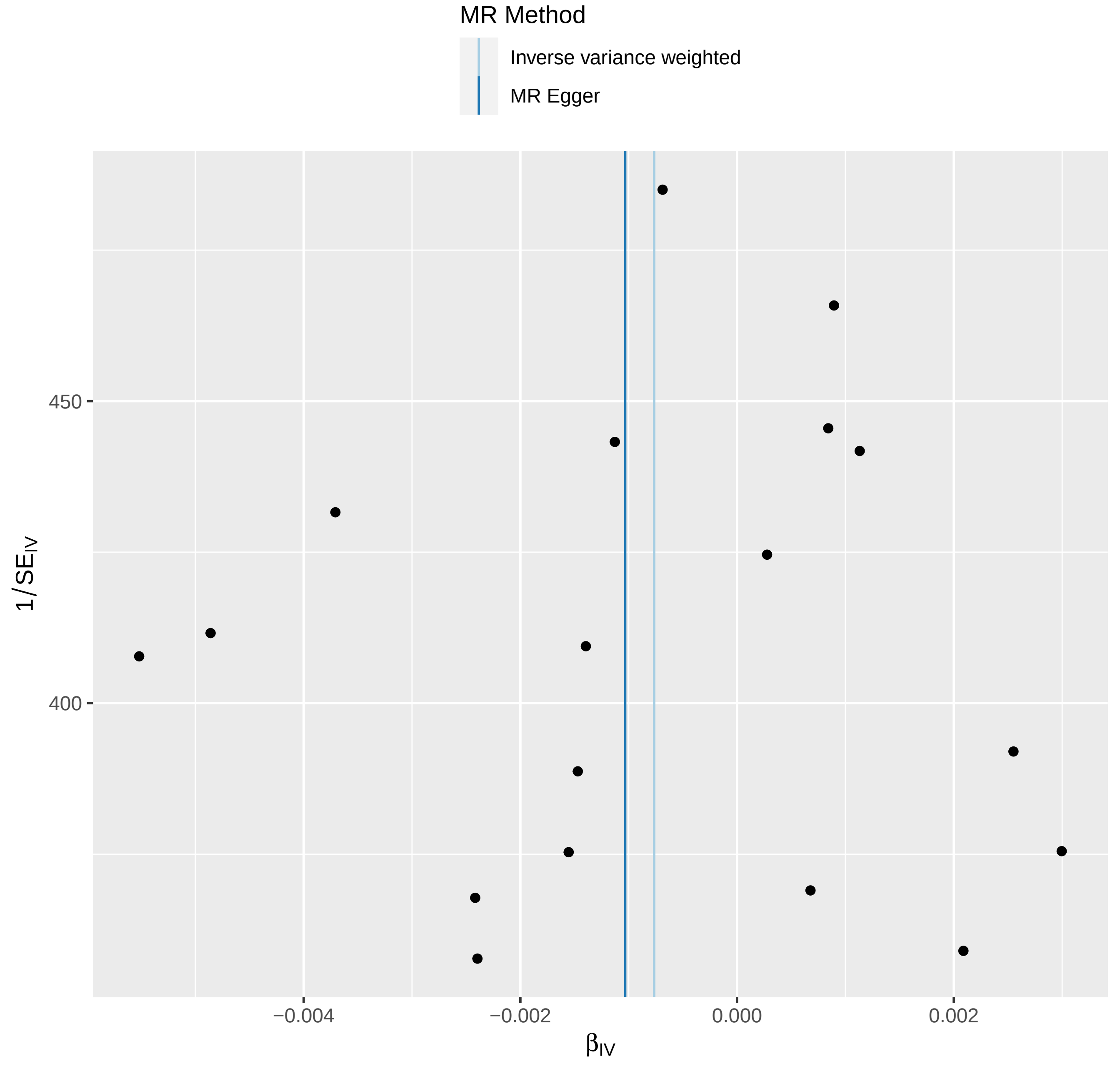

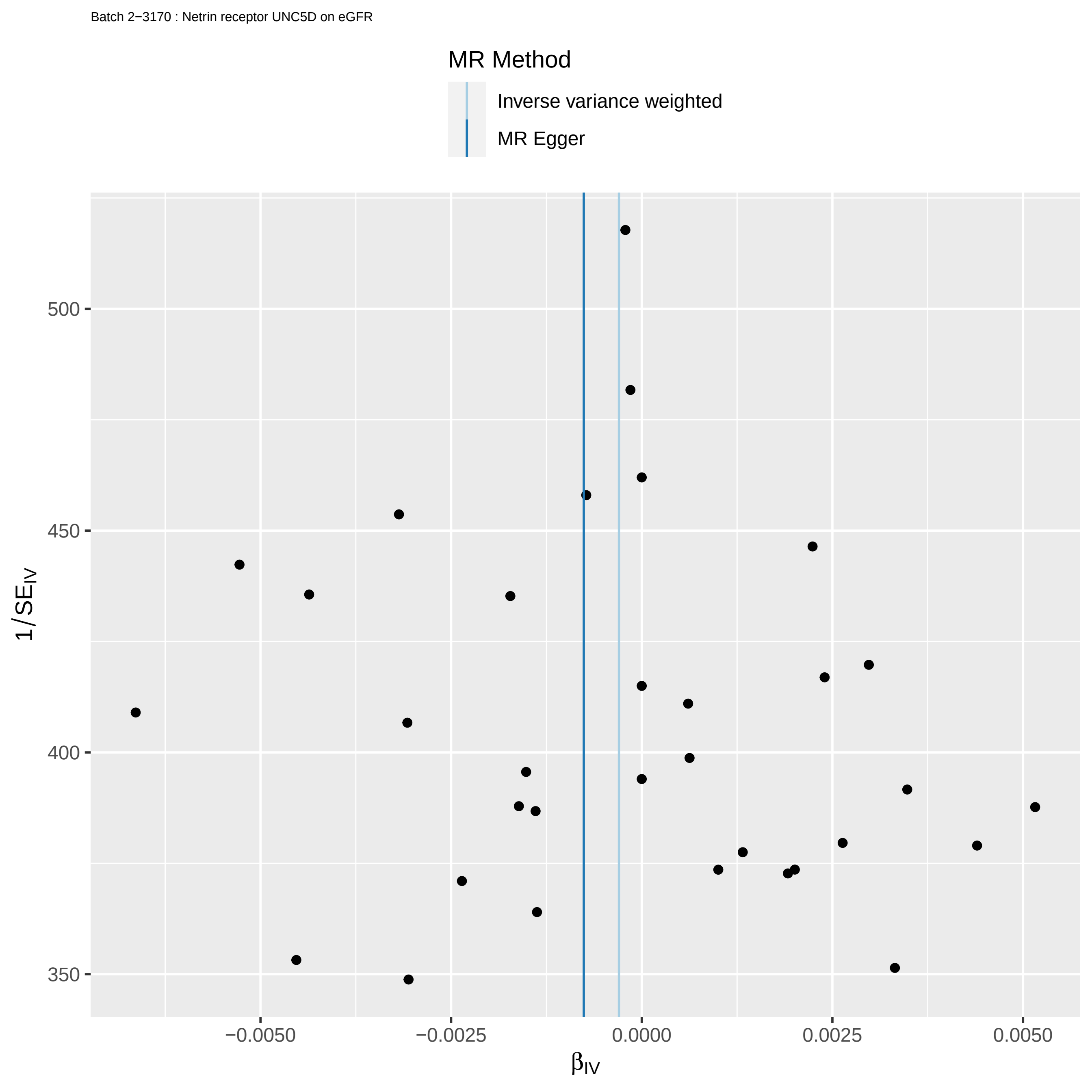

Batch 2−3170 : Netrin receptor UNC5D on eGFR
MR Method
Inverse variance weighted
MR Egger
500
450
400
350
1/SE_IV
−0.0050
−0.0025
0.0000
0.0025
0.0050
β_IV

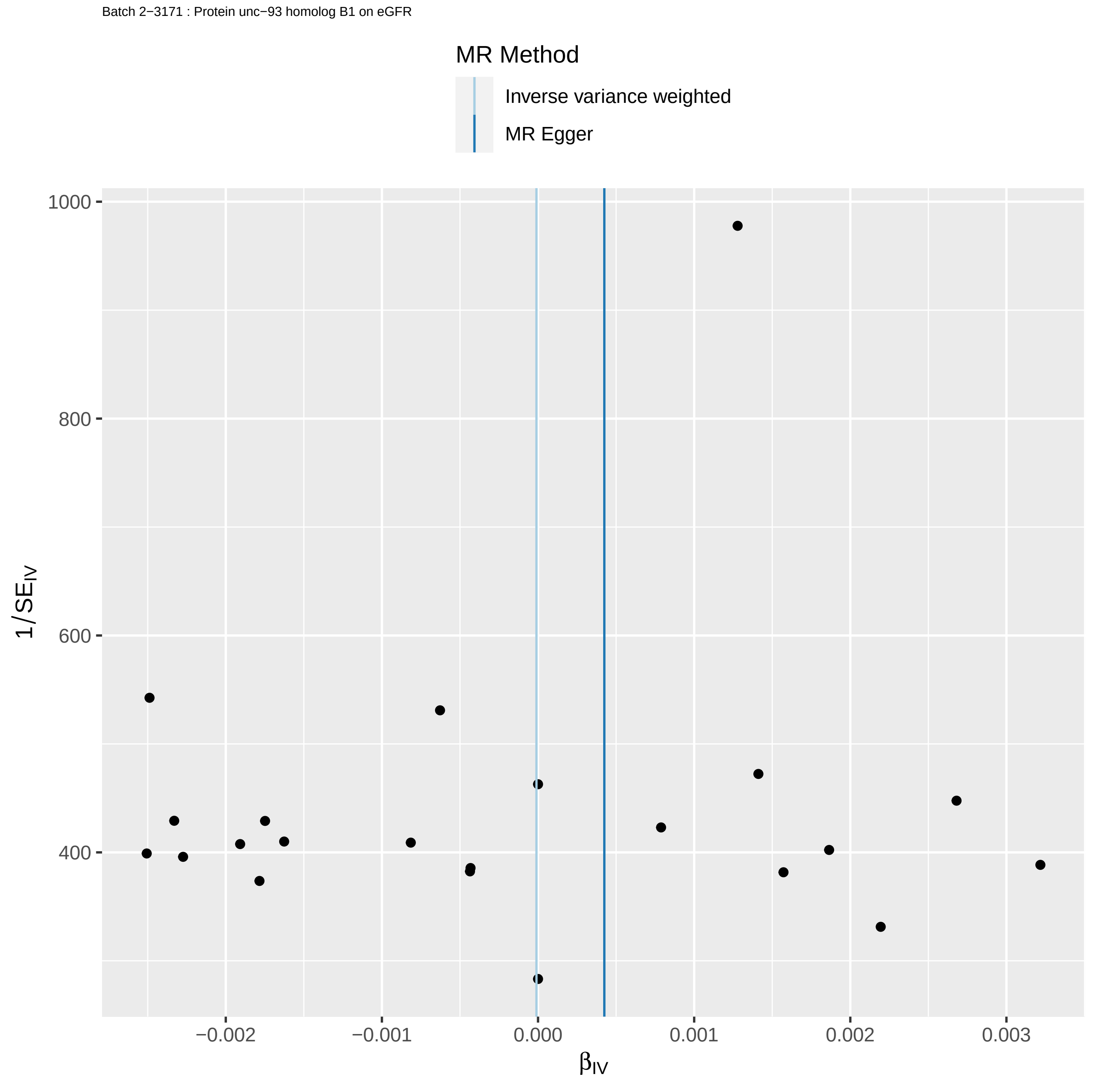

Batch 2−3171 : Protein unc−93 homolog B1 on eGFR
MR Method
Inverse variance weighted
MR Egger
1000
800
600
400
1/SE$_{IV}$
−0.002
−0.001
0.000
0.001
0.002
0.003
β$_{IV}$

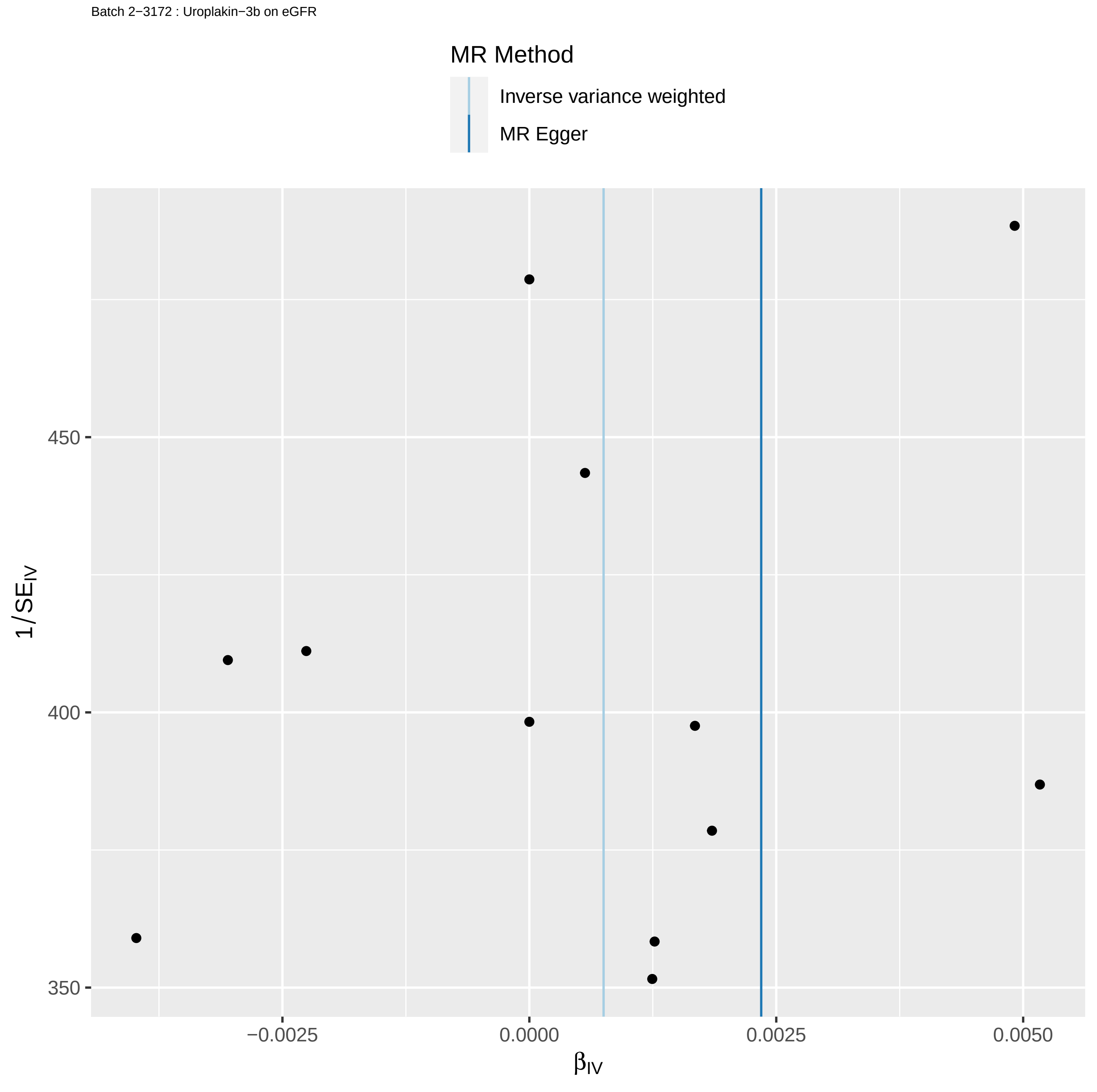
Batch 2−3172 : Uroplakin−3b on eGFR
MR Method
Inverse variance weighted
MR Egger
450
400
350
1/SE_IV
−0.0025
0.0000
0.0025
0.0050
β_IV

Batch 2−3173 : Uroplakin−3b−like protein on eGFR

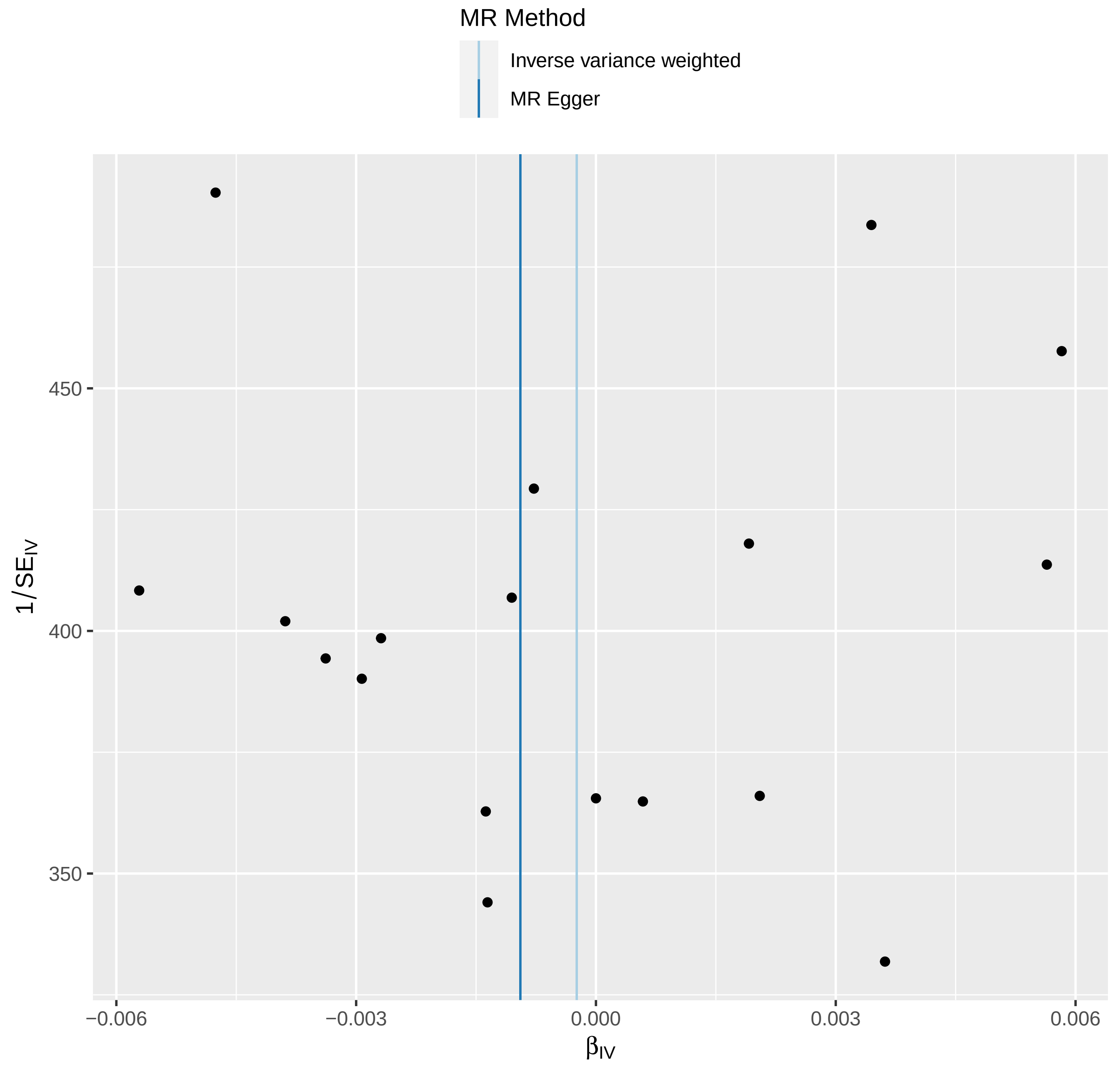

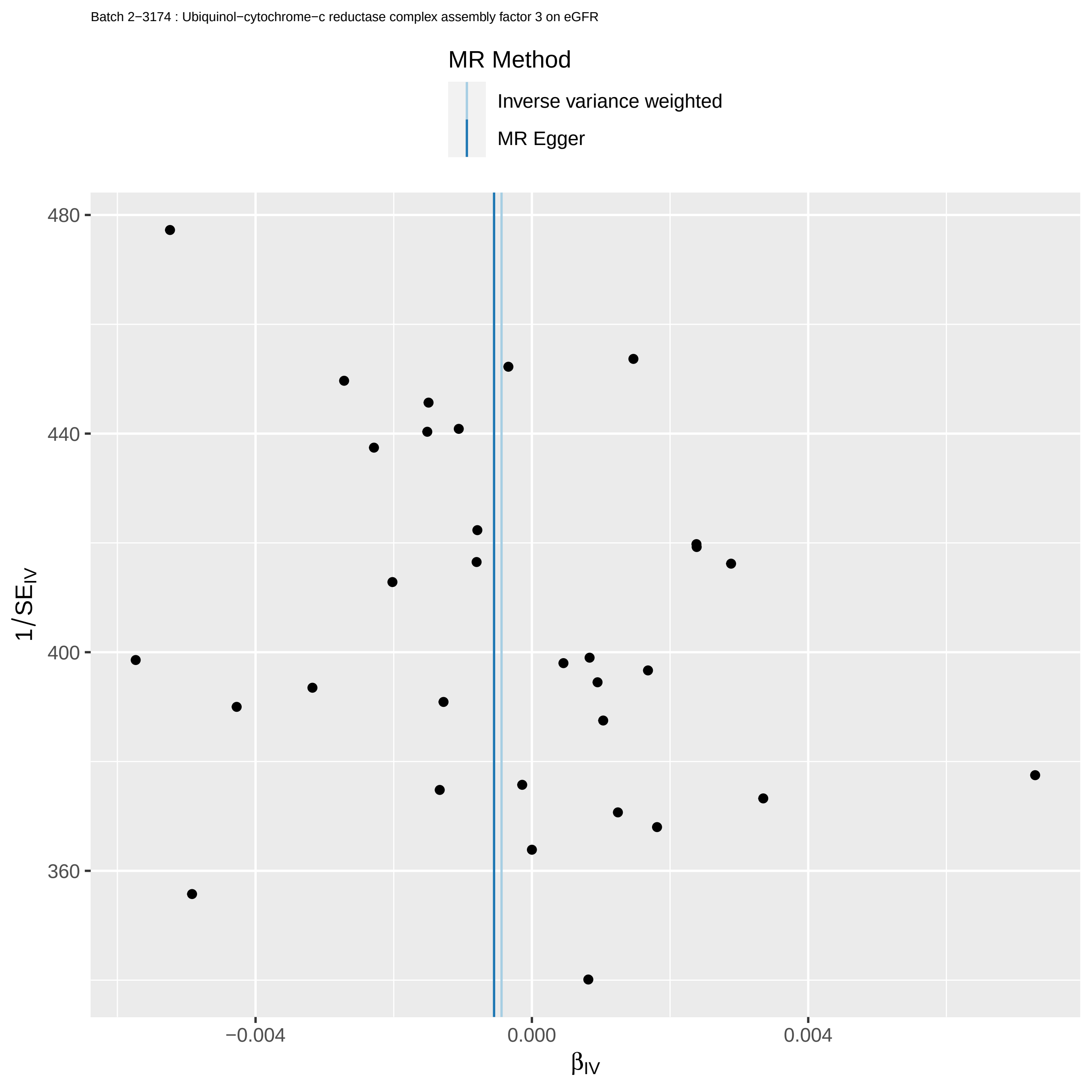
Batch 2−3174 : Ubiquinol−cytochrome−c reductase complex assembly factor 3 on eGFR
MR Method
Inverse variance weighted
MR Egger
480
440
400
360
1/SE_IV
−0.004
0.000
0.004
β_IV

Batch 2−3175 : Cytochrome b−c1 complex subunit 7 on eGFR

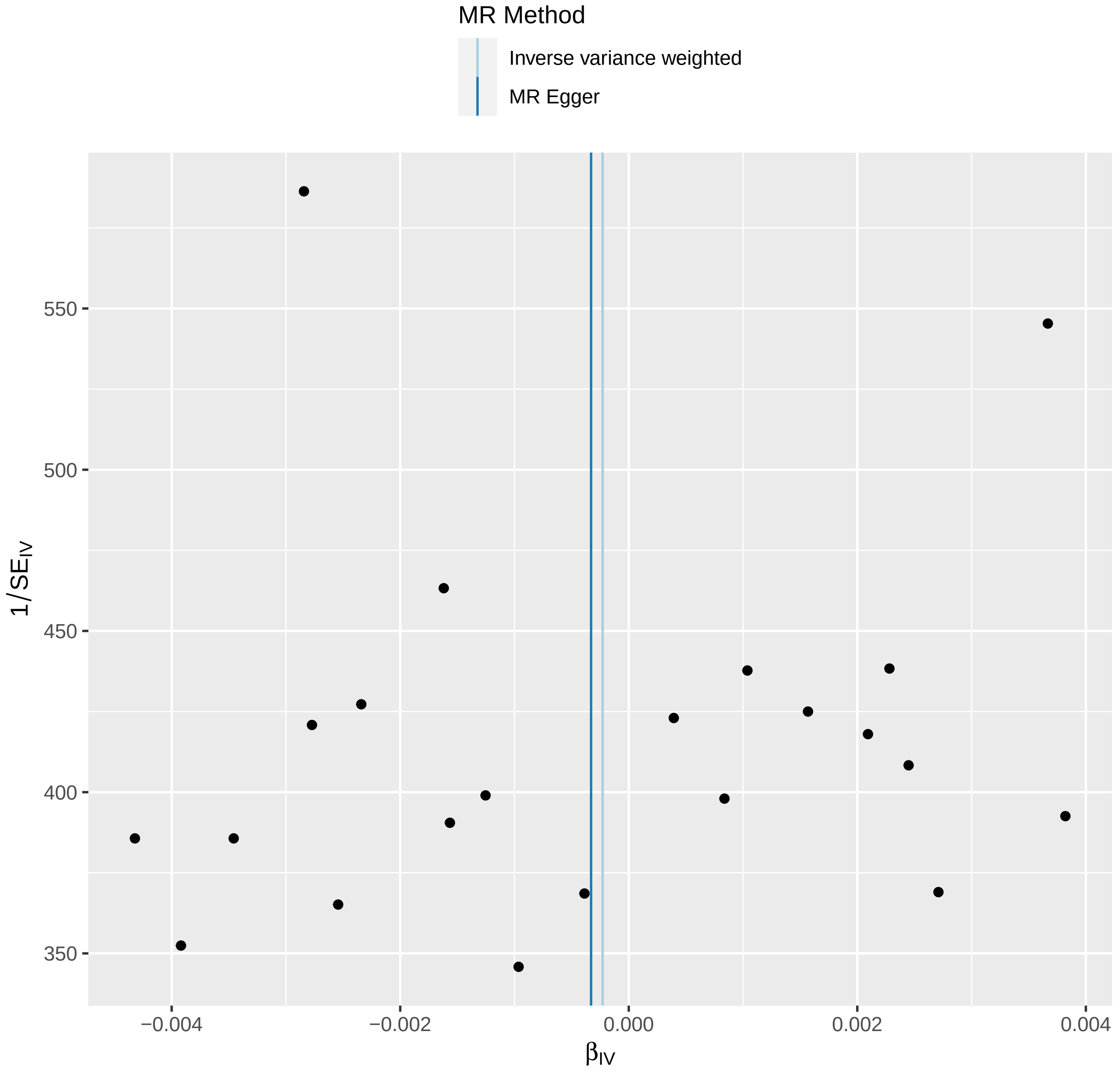

Batch 2−3176 : Uroporphyrinogen−III synthase on eGFR

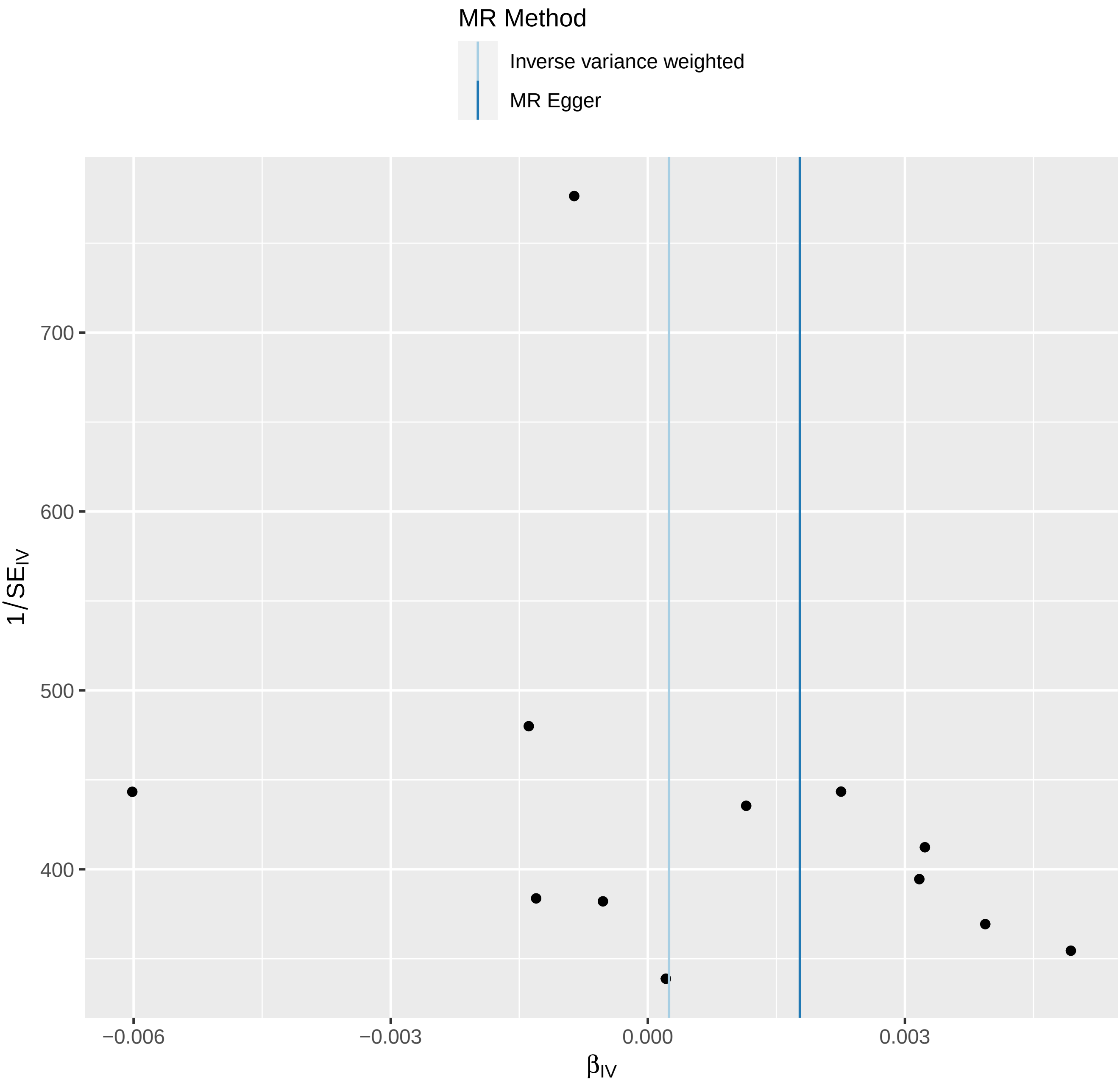

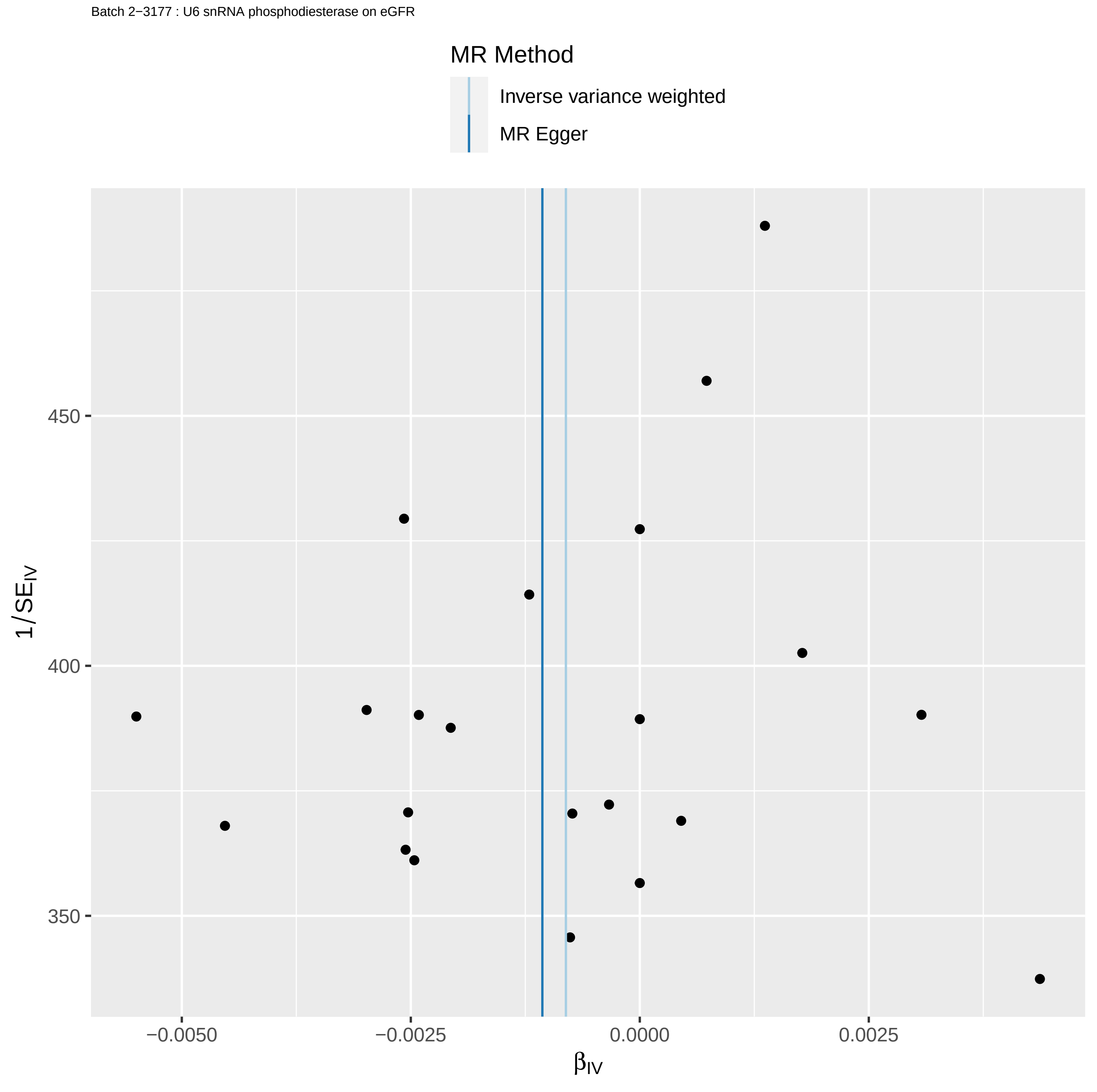
Batch 2−3177 : U6 snRNA phosphodiesterase on eGFR
MR Method
Inverse variance weighted
MR Egger
1/SE_IV
450
400
350
−0.0050
−0.0025
0.0000
0.0025
β_IV

Batch 2−3178 : Vesicle transport protein USE1 on eGFR

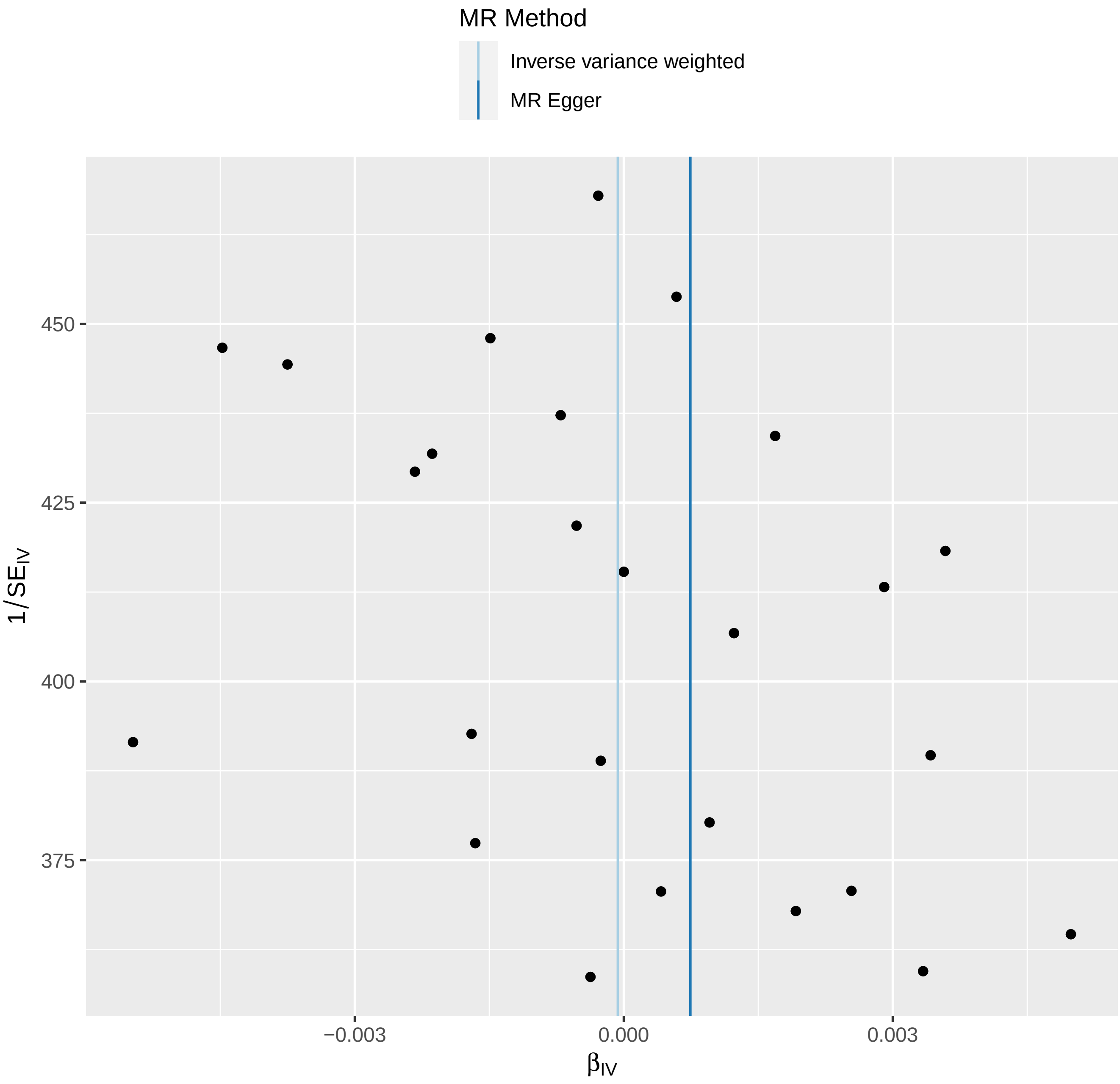

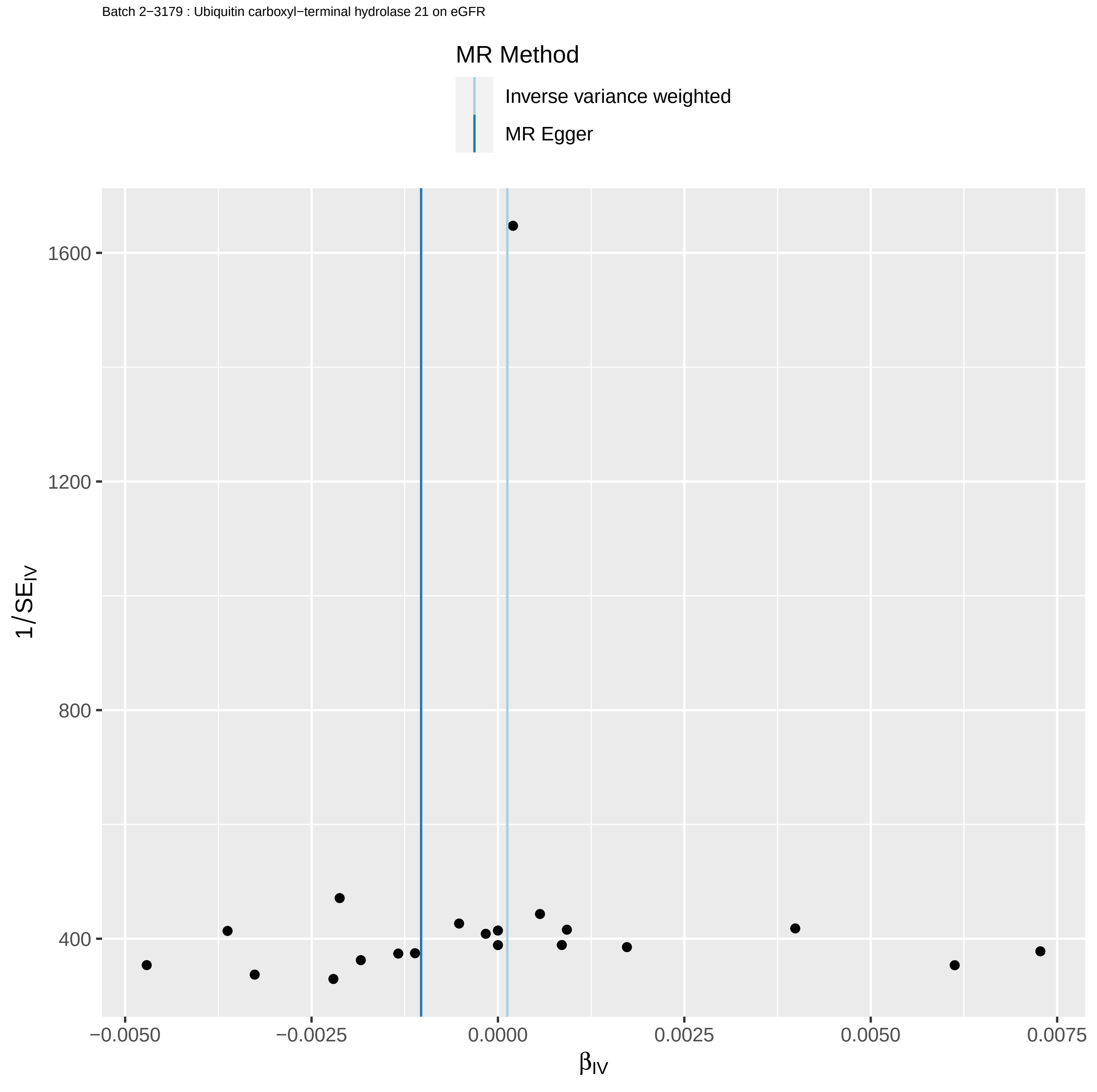
Batch 2−3179 : Ubiquitin carboxyl−terminal hydrolase 21 on eGFR
MR Method
Inverse variance weighted
MR Egger
1600
1200
800
400
−0.0050
−0.0025
0.0000
0.0025
0.0050
0.0075
β_{IV}
$1/SE_{IV}$

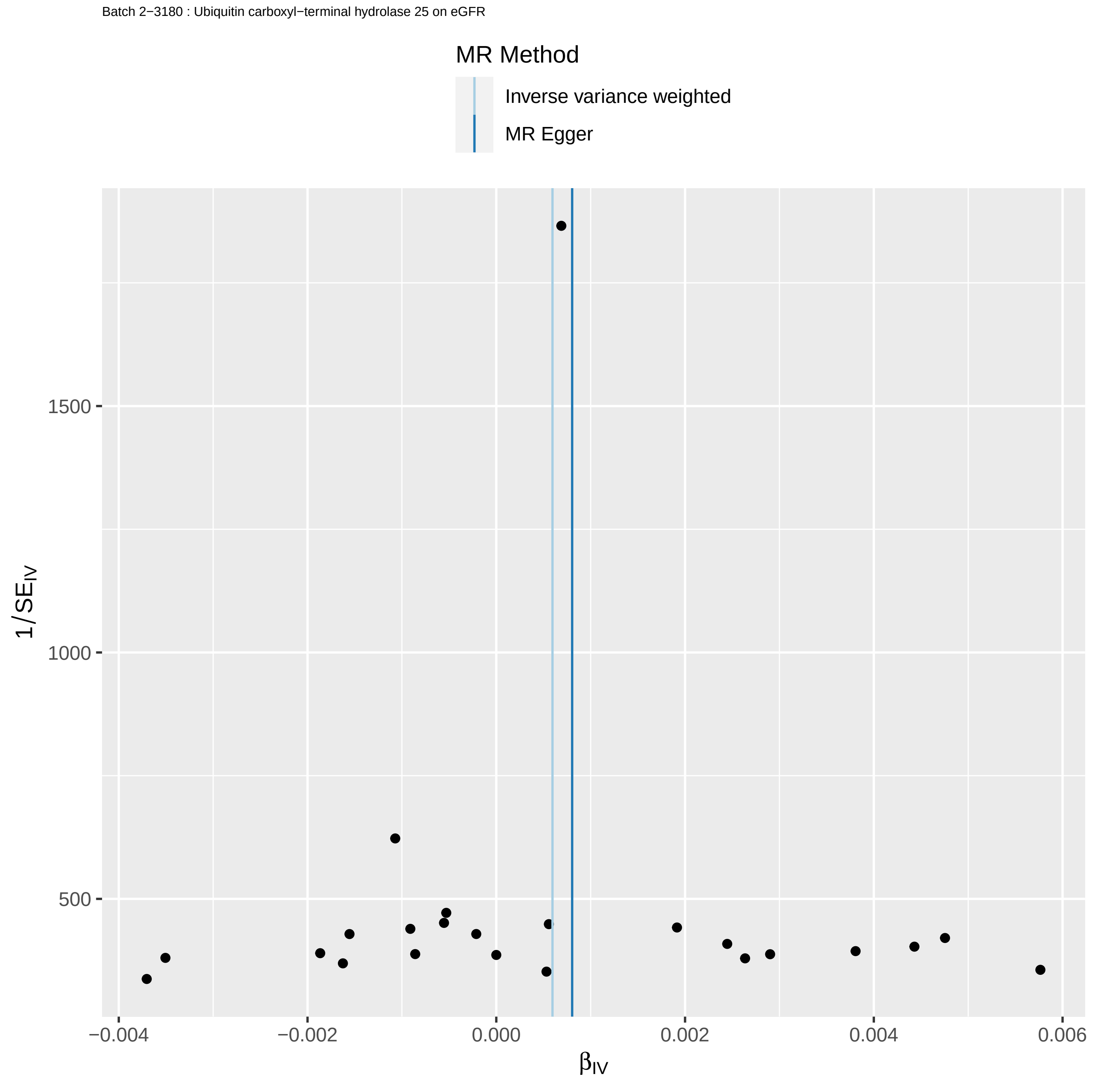

Batch 2−3180 : Ubiquitin carboxyl−terminal hydrolase 25 on eGFR
MR Method
Inverse variance weighted
MR Egger
1500
1000
500
1/SE$_{IV}$
−0.004
−0.002
0.000
0.002
0.004
0.006
β$_{IV}$

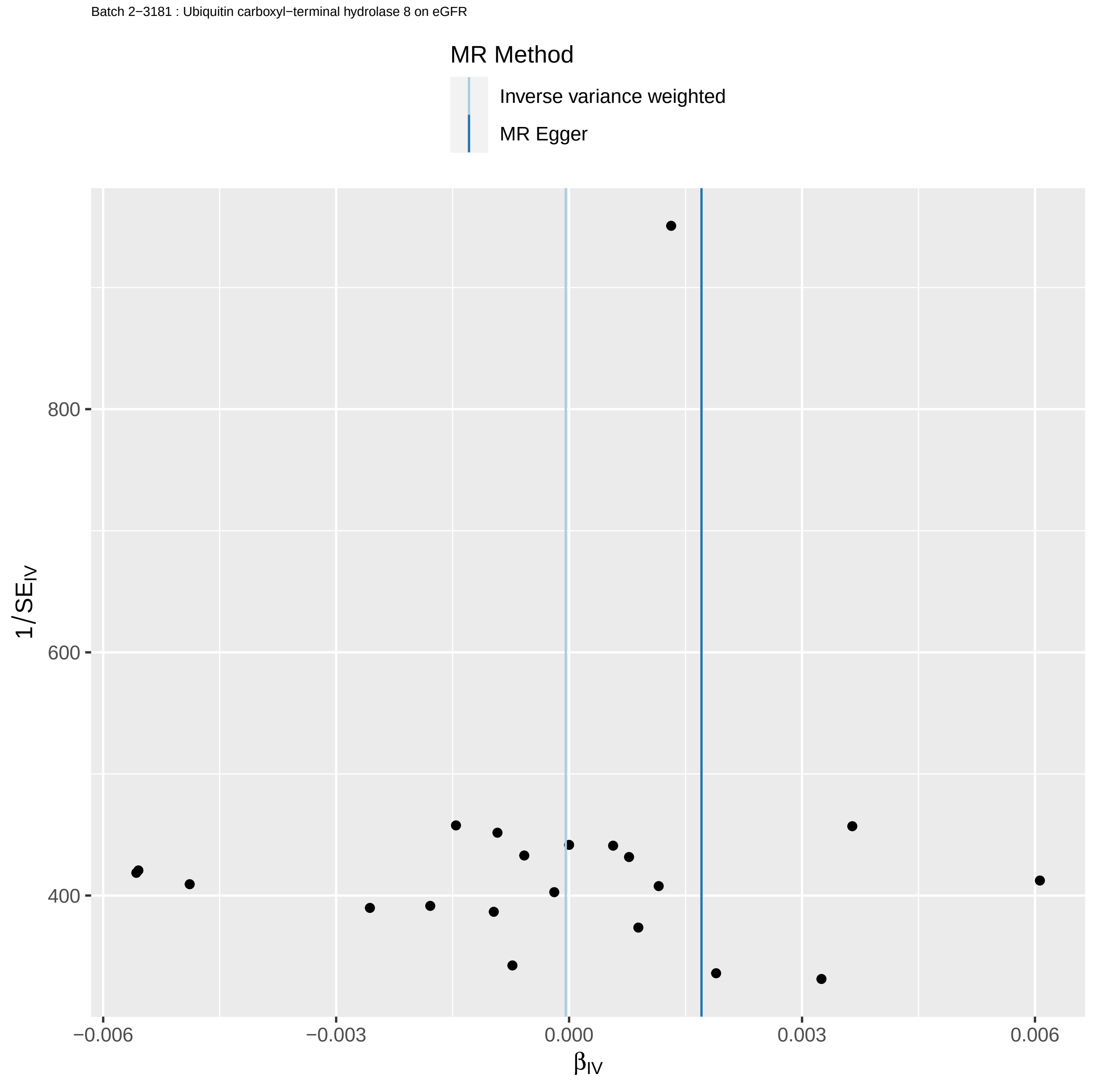

Batch 2−3181 : Ubiquitin carboxyl−terminal hydrolase 8 on eGFR
MR Method
Inverse variance weighted
MR Egger
800
600
400
1/SE$_{IV}$
−0.006
−0.003
0.000
0.003
0.006
β$_{IV}$

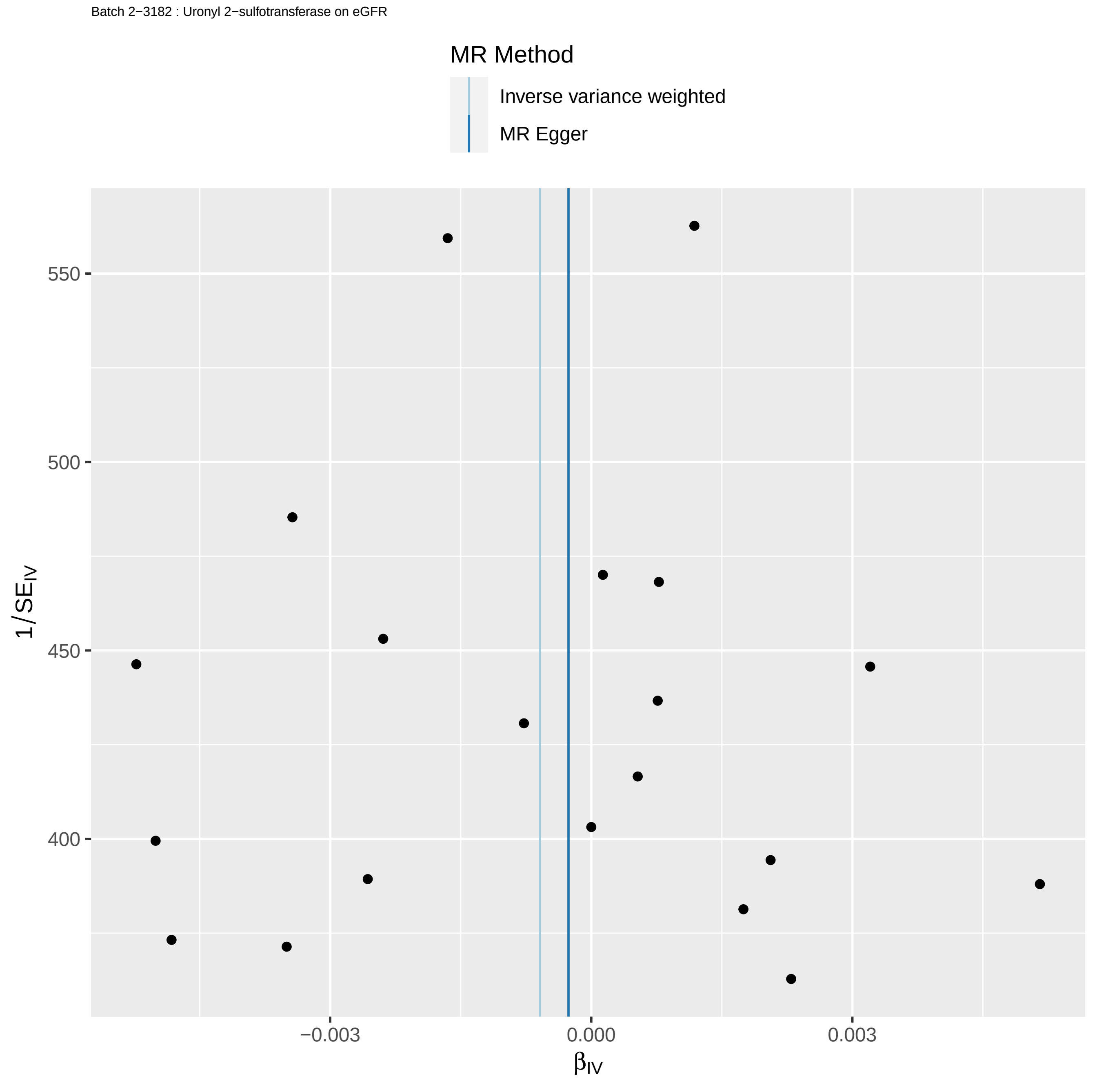

Batch 2−3182 : Uronyl 2−sulfotransferase on eGFR
MR Method
Inverse variance weighted
MR Egger
550
500
450
400
1/SE$_{IV}$
−0.003
0.000
0.003
β$_{IV}$

Batch 2−3183 : Urotensin−2 receptor on eGFR

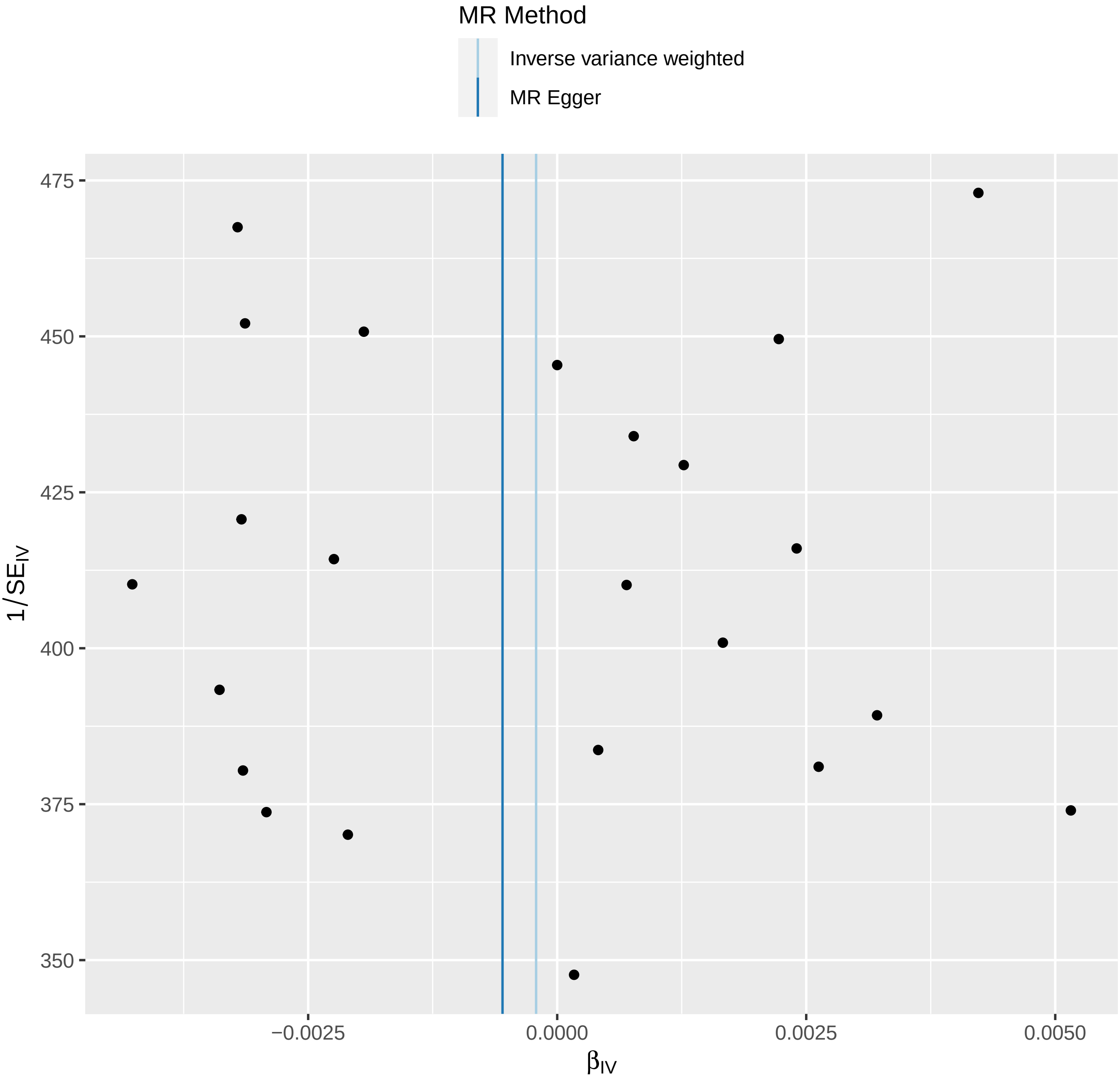

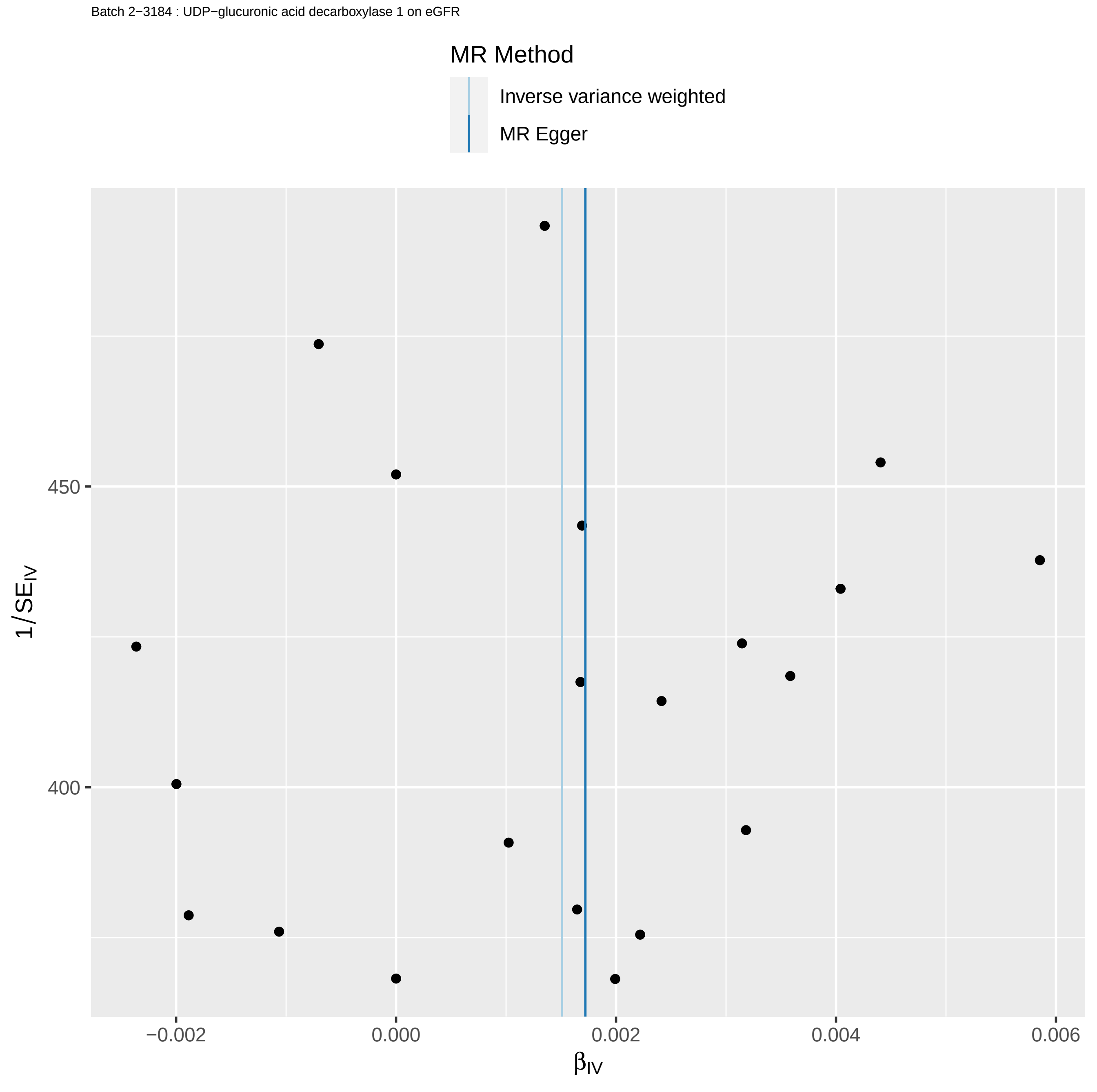

Batch 2−3184 : UDP−glucuronic acid decarboxylase 1 on eGFR
MR Method
Inverse variance weighted
MR Egger
450
400
1/SE_IV
−0.002
0.000
0.002
0.004
0.006
β_IV

Batch 2−3185 : Vesicle−associated membrane protein 3 on eGFR

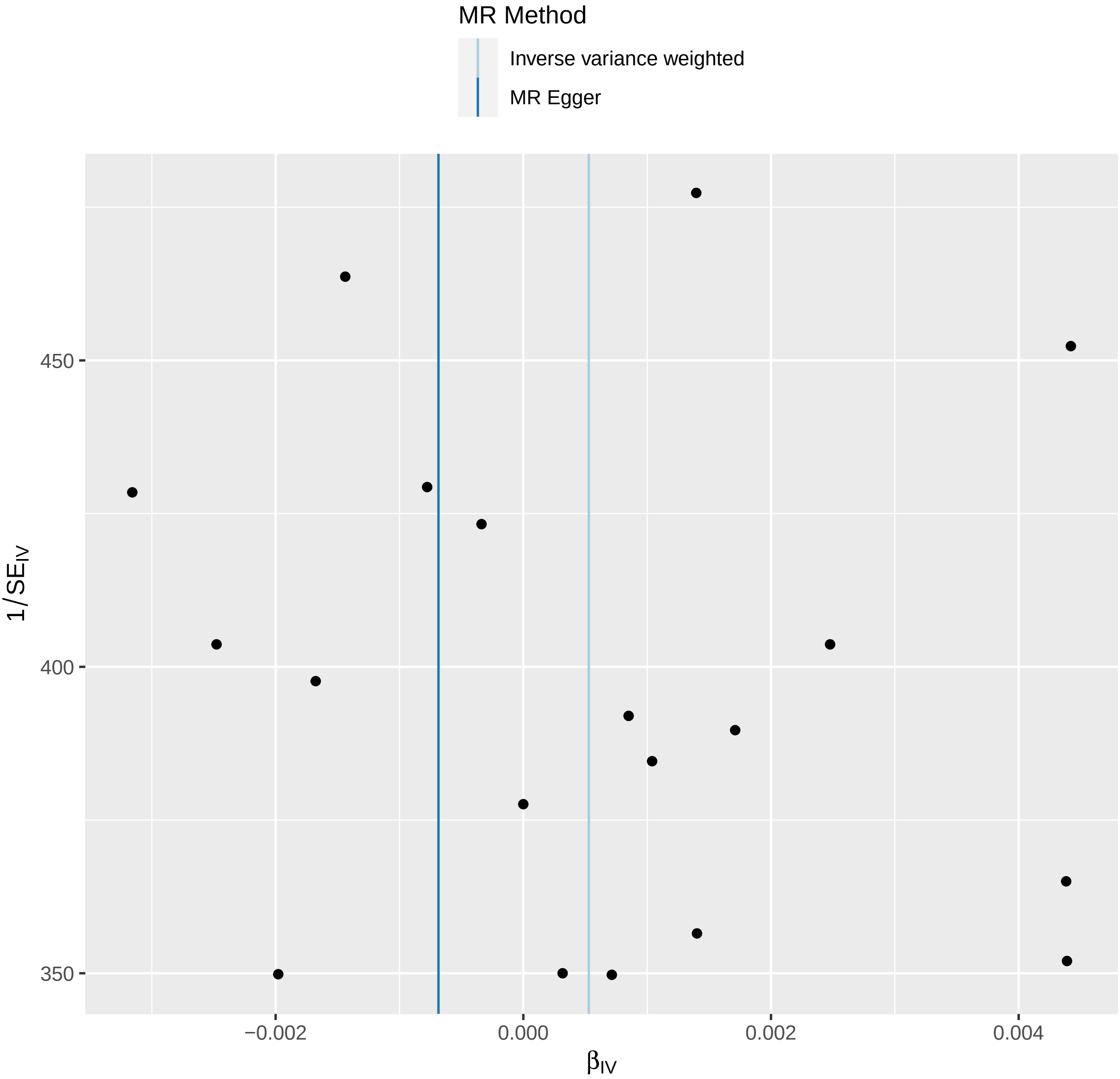

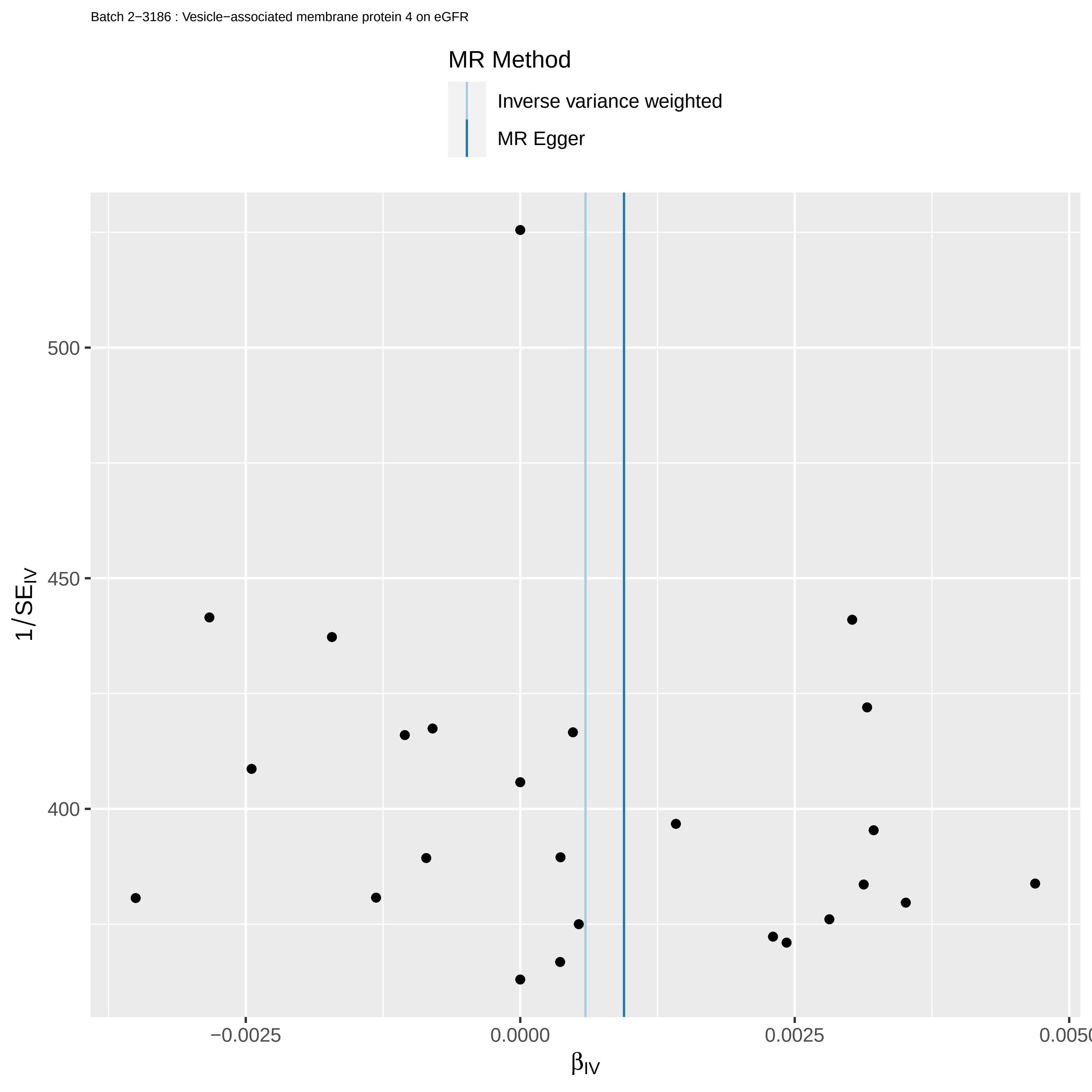

Batch 2−3186 : Vesicle−associated membrane protein 4 on eGFR
MR Method
Inverse variance weighted
MR Egger
500
450
400
1/SE$_{IV}$
−0.0025
0.0000
0.0025
0.0050
β$_{IV}$

Batch 2−3187 : Vesicle−associated membrane protein 5 on eGFR

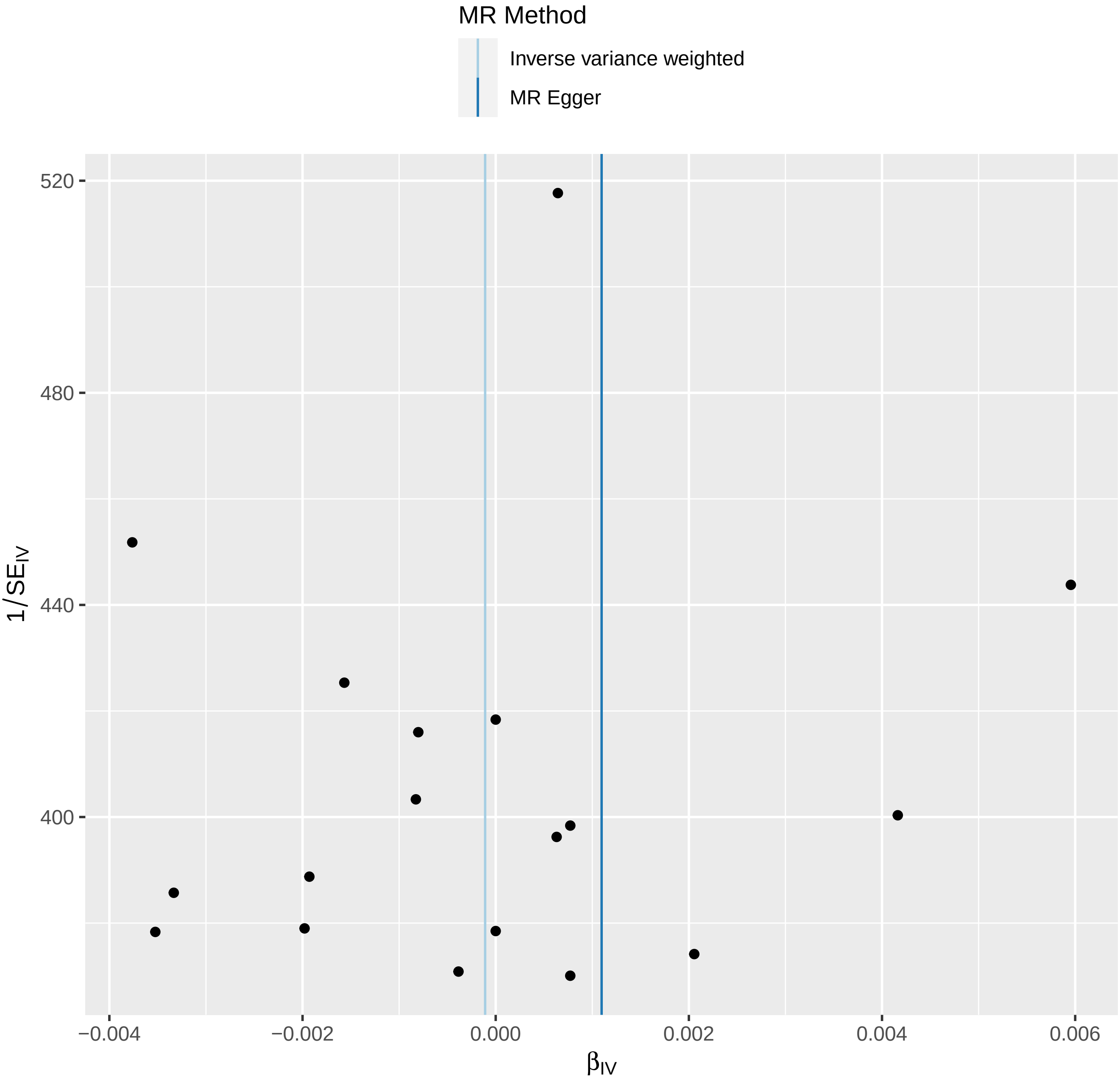

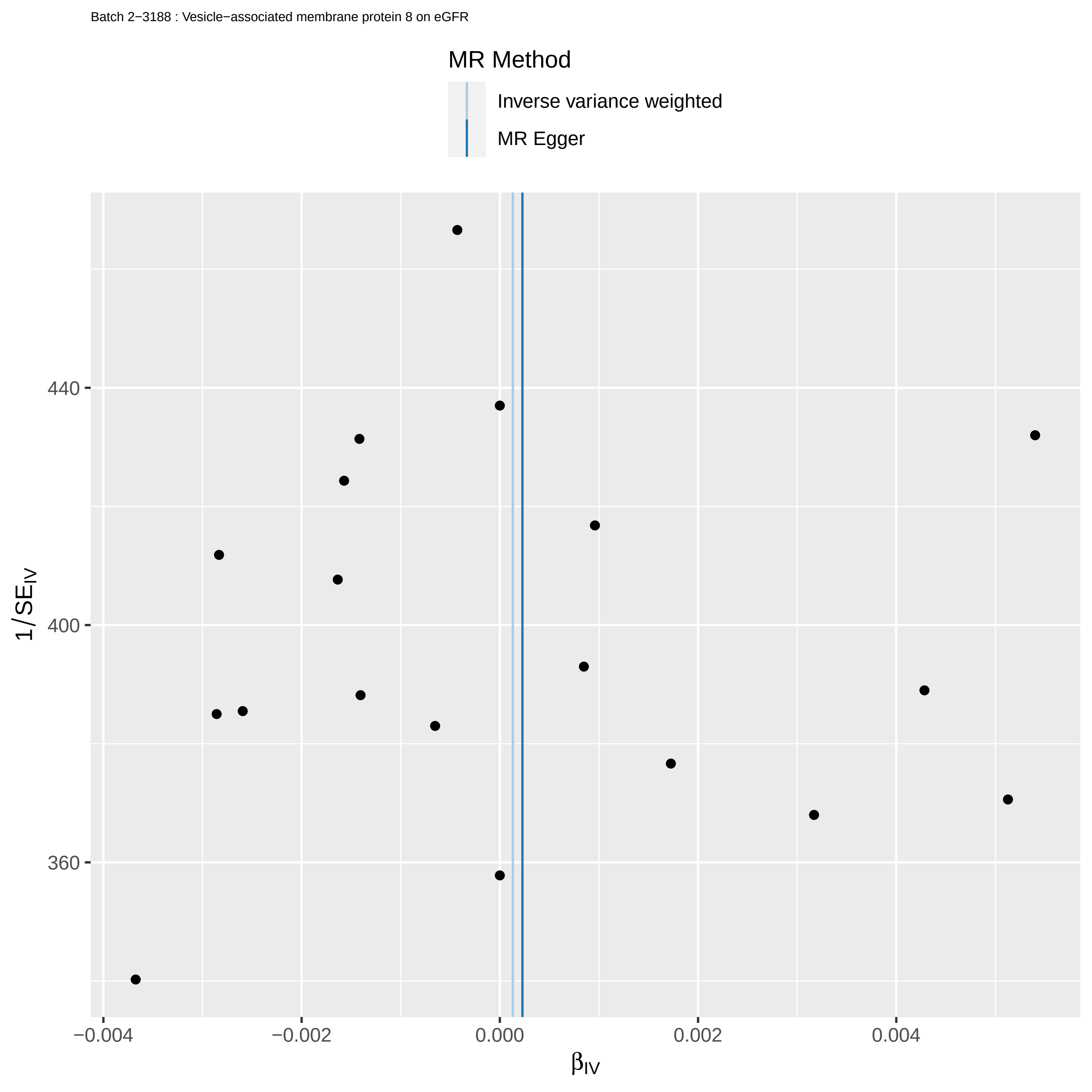
Batch 2−3188 : Vesicle−associated membrane protein 8 on eGFR
MR Method
Inverse variance weighted
MR Egger
440
400
360
−0.004
−0.002
0.000
0.002
0.004
$1/SE_{IV}$
β_{IV}

Batch 2−3189 : Vesicle−associated membrane protein−associated protein A on eGFR

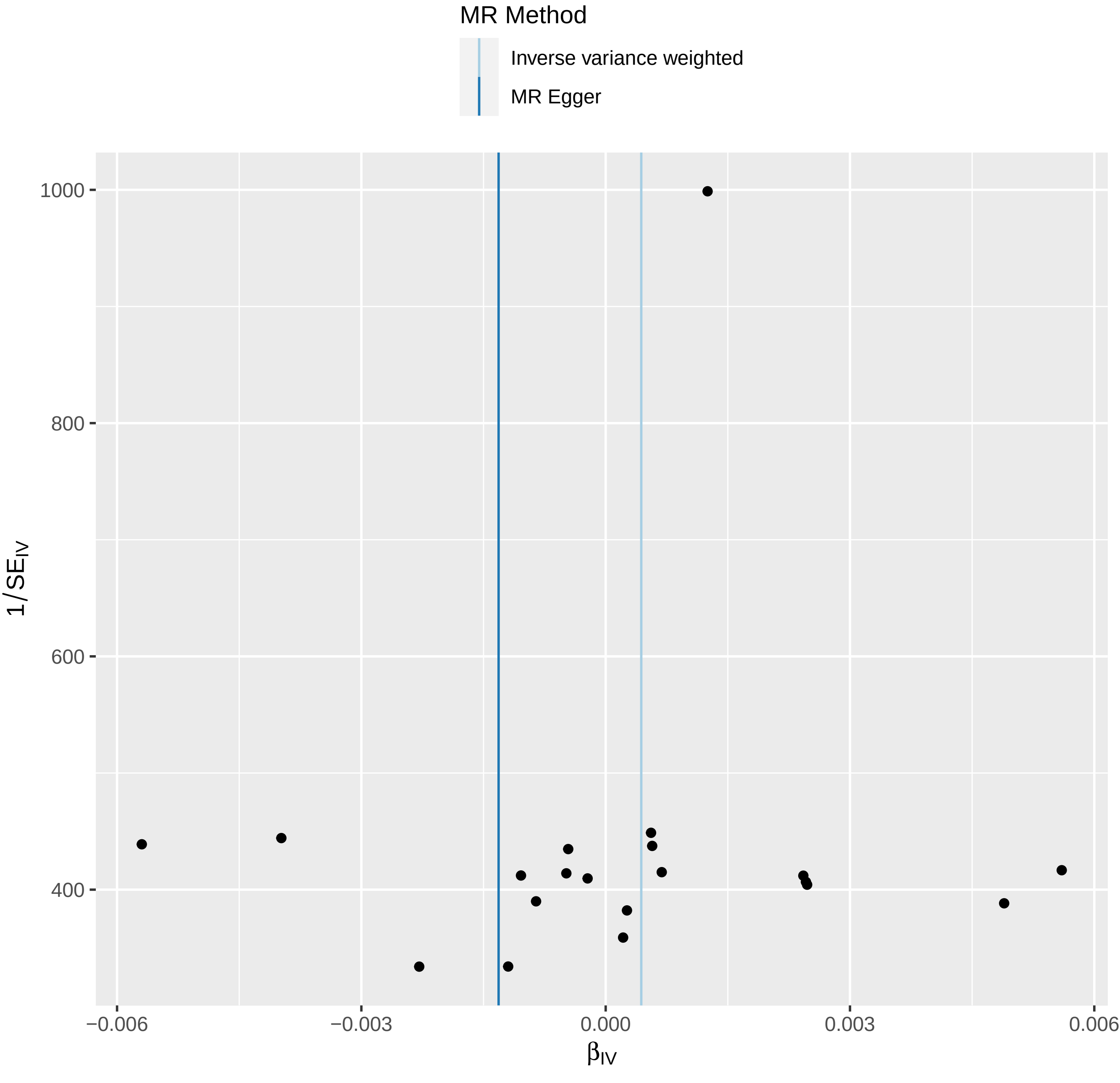

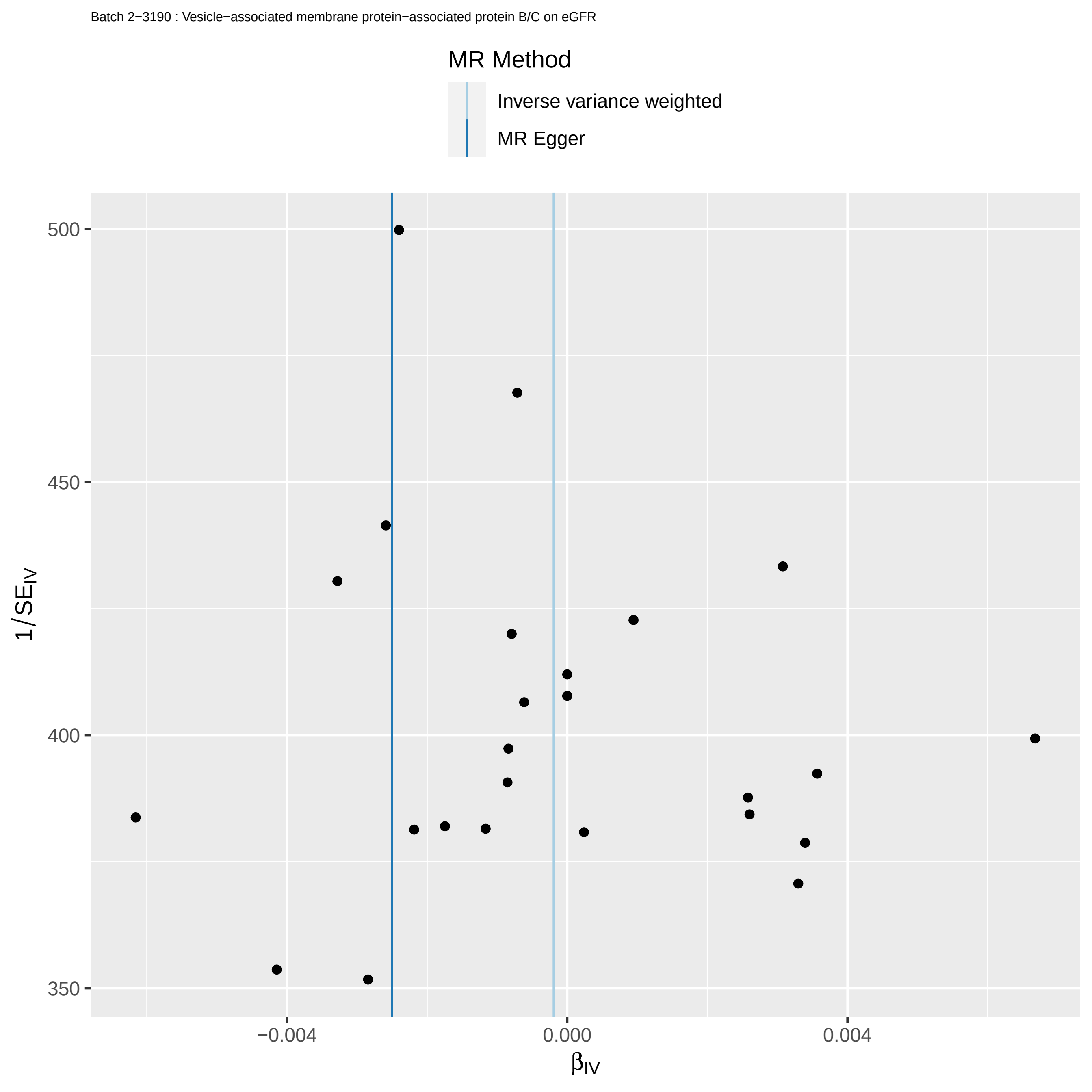

Batch 2−3190 : Vesicle−associated membrane protein−associated protein B/C on eGFR
MR Method
Inverse variance weighted
MR Egger
500
450
400
350
−0.004
0.000
0.004
$1/SE_{IV}$
β_{IV}

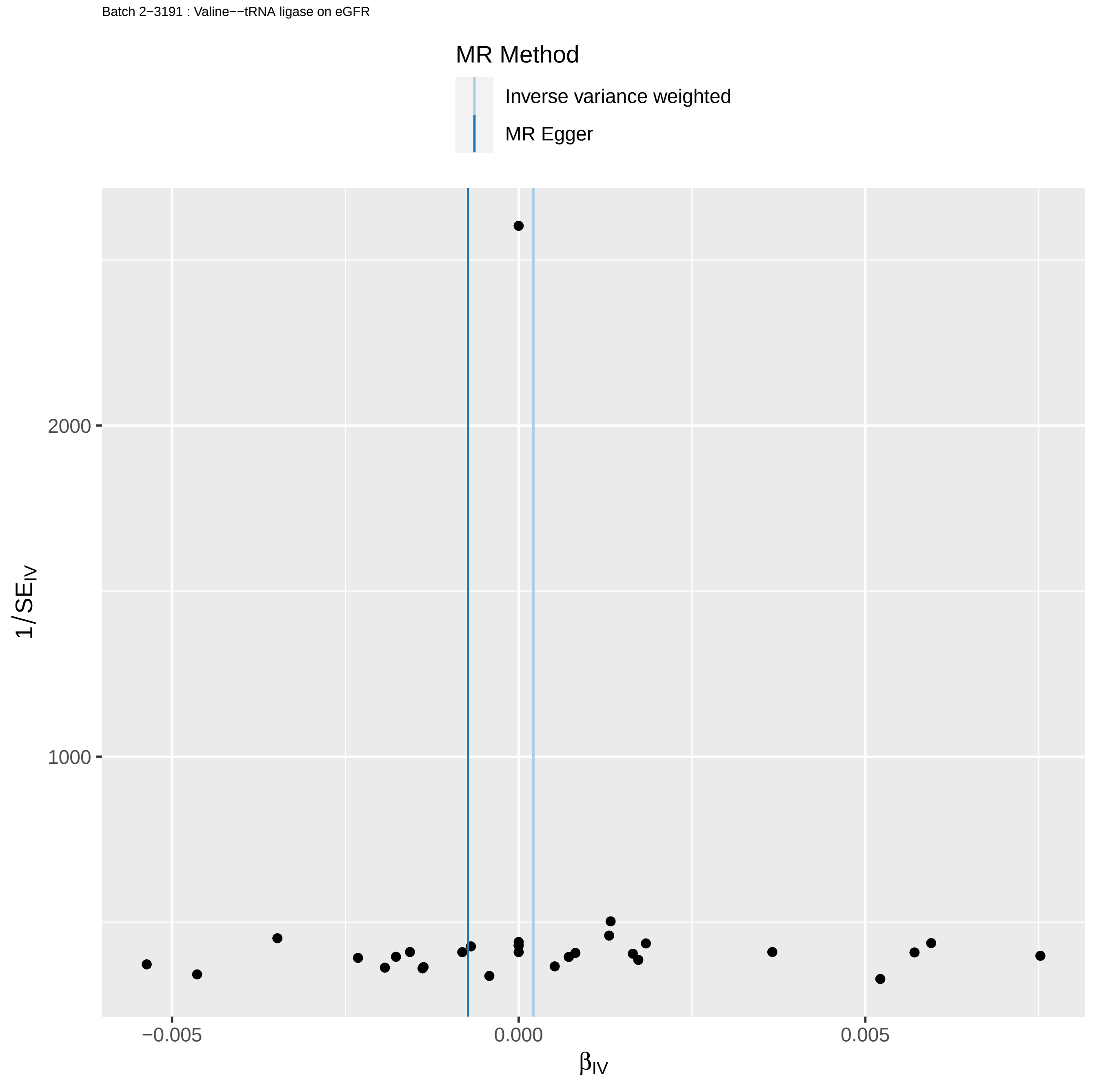

Batch 2−3191 : Valine−−tRNA ligase on eGFR
MR Method
Inverse variance weighted
MR Egger
2000
1000
1/SE$_{IV}$
−0.005
0.000
0.005
β$_{IV}$

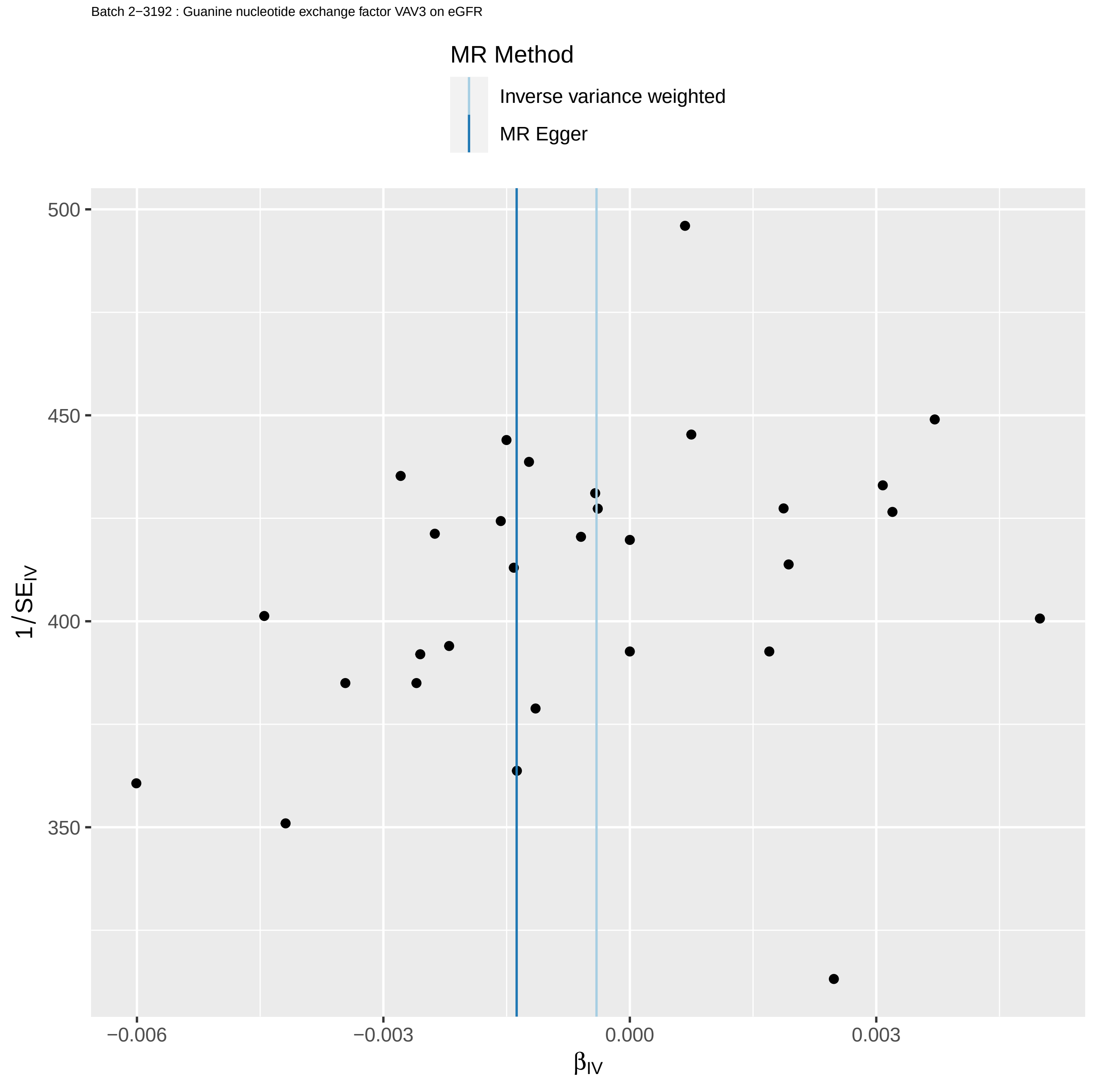
Batch 2−3192 : Guanine nucleotide exchange factor VAV3 on eGFR
MR Method
Inverse variance weighted
MR Egger
500
450
400
350
1/SE_IV
−0.006
−0.003
0.000
0.003
β_IV

Batch 2−3193 : Vascular cell adhesion protein 1 on eGFR

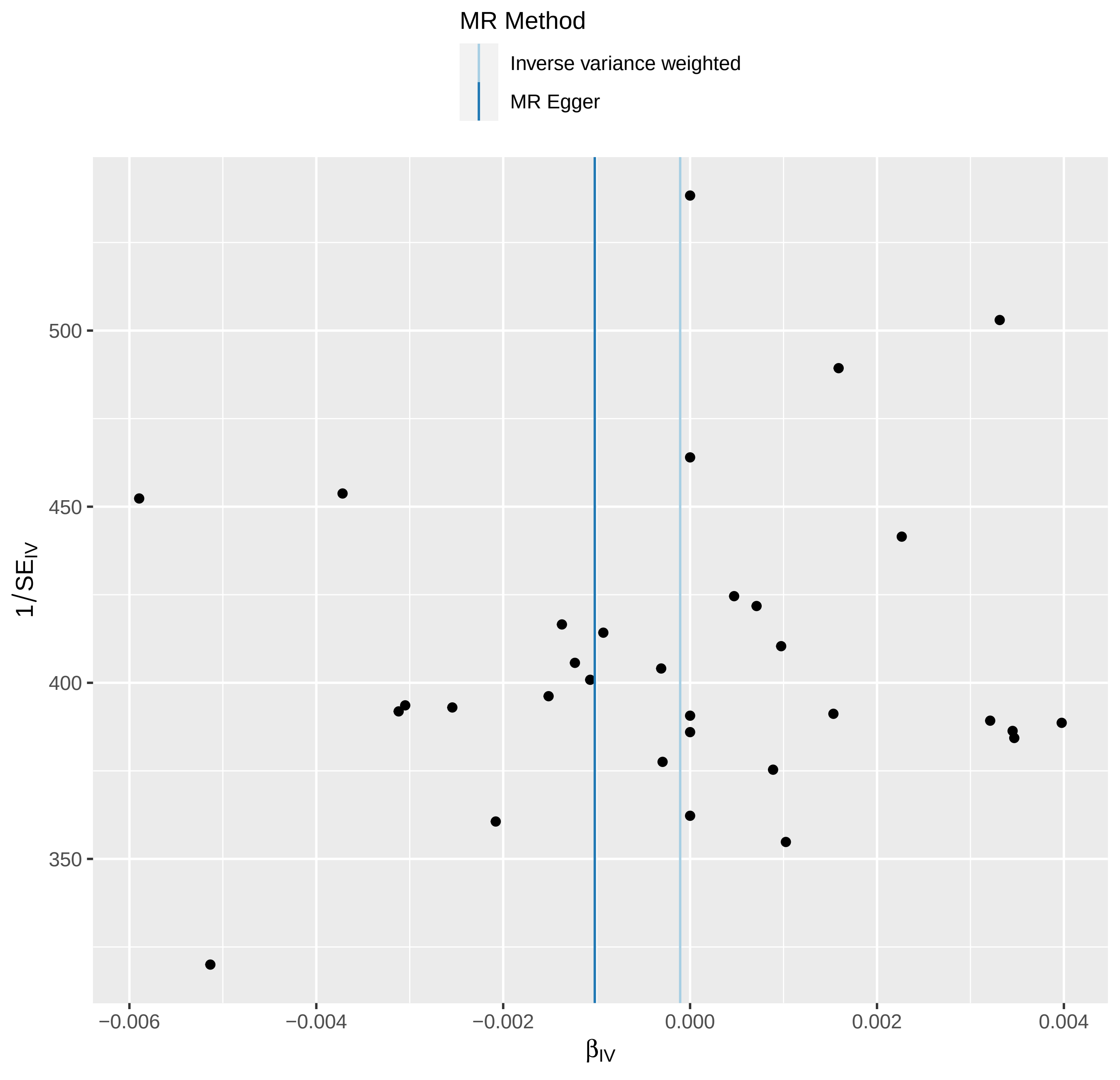

Batch 2−3194 : Versican core protein on eGFR

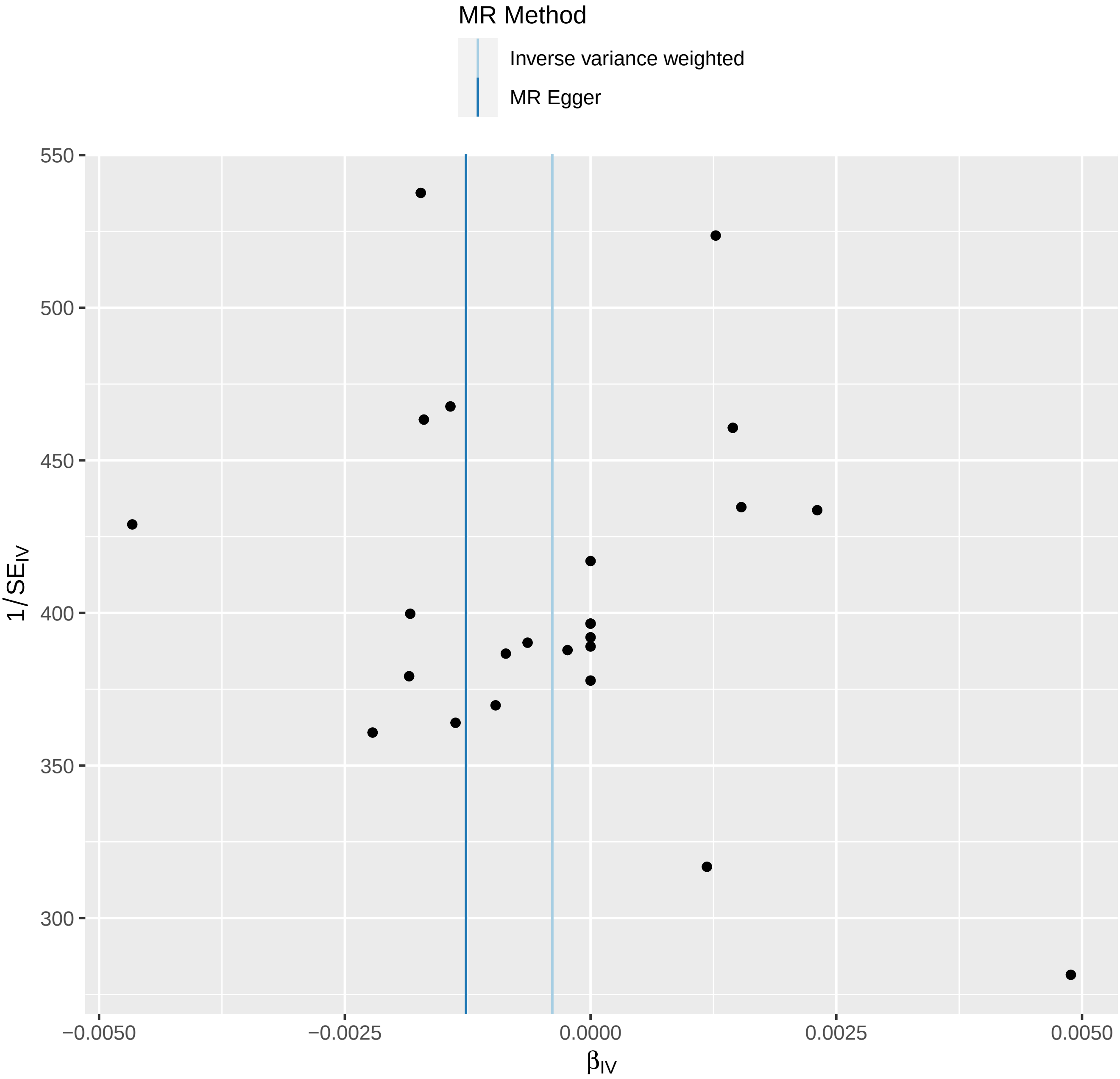

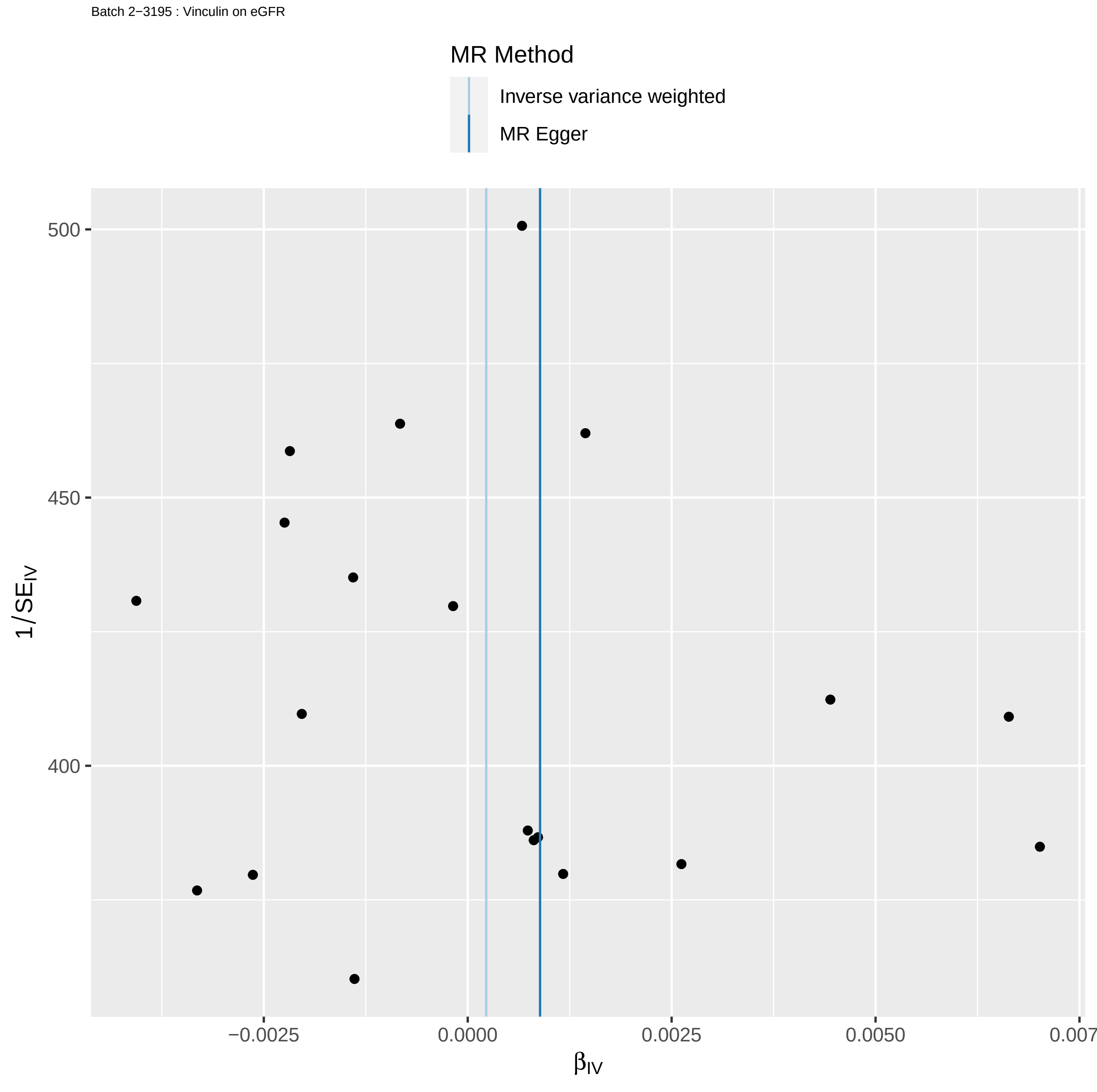

Batch 2−3195 : Vinculin on eGFR
MR Method
Inverse variance weighted
MR Egger
500
450
400
1/SE_IV
−0.0025
0.0000
0.0025
0.0050
0.007
β_IV

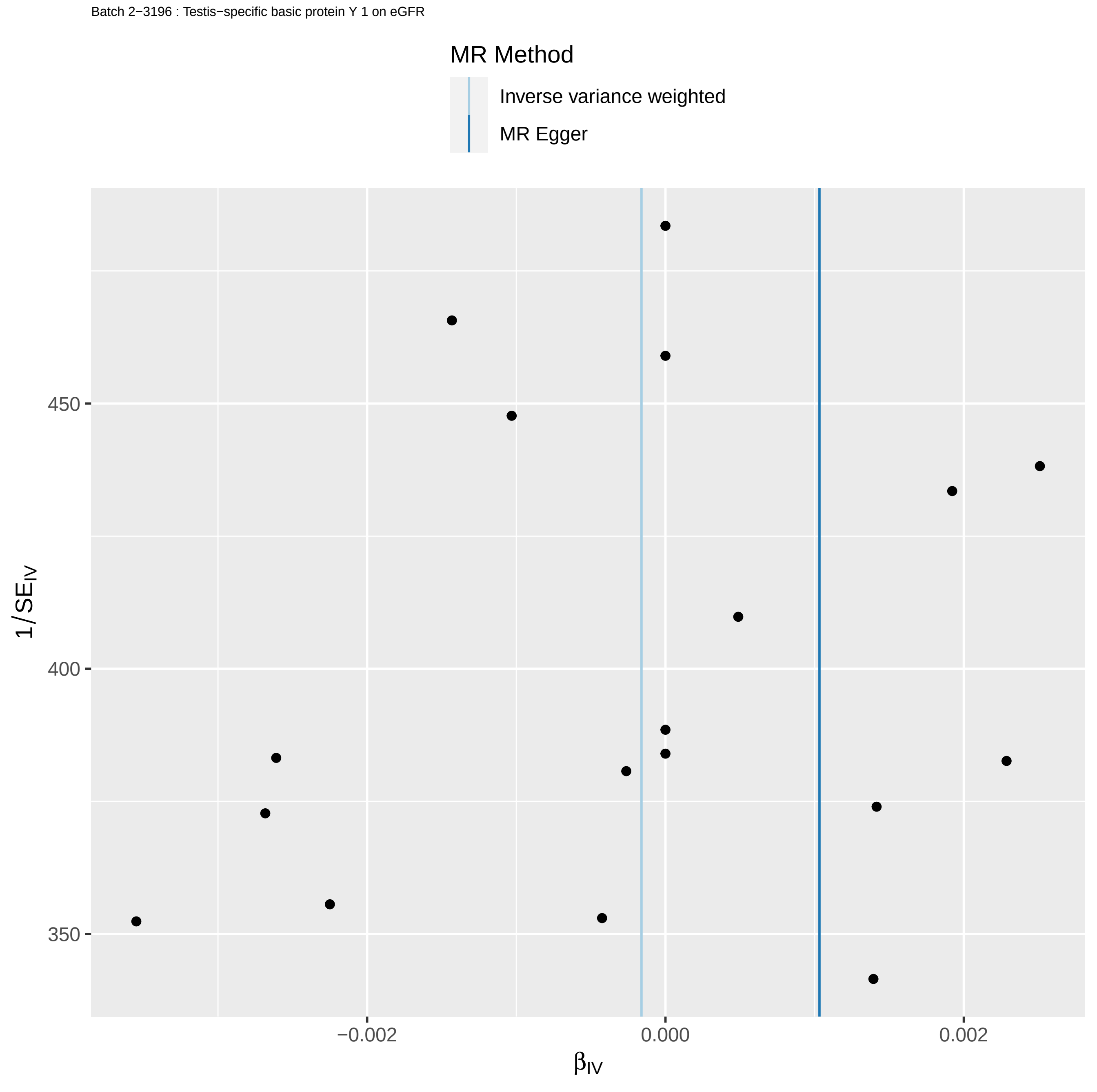
Batch 2−3196 : Testis−specific basic protein Y 1 on eGFR
MR Method
Inverse variance weighted
MR Egger
450
400
350
1/SE$_{IV}$
−0.002
0.000
0.002
β$_{IV}$

Batch 2−3197 : Vascular endothelial growth factor A, isoform 121 on eGFR

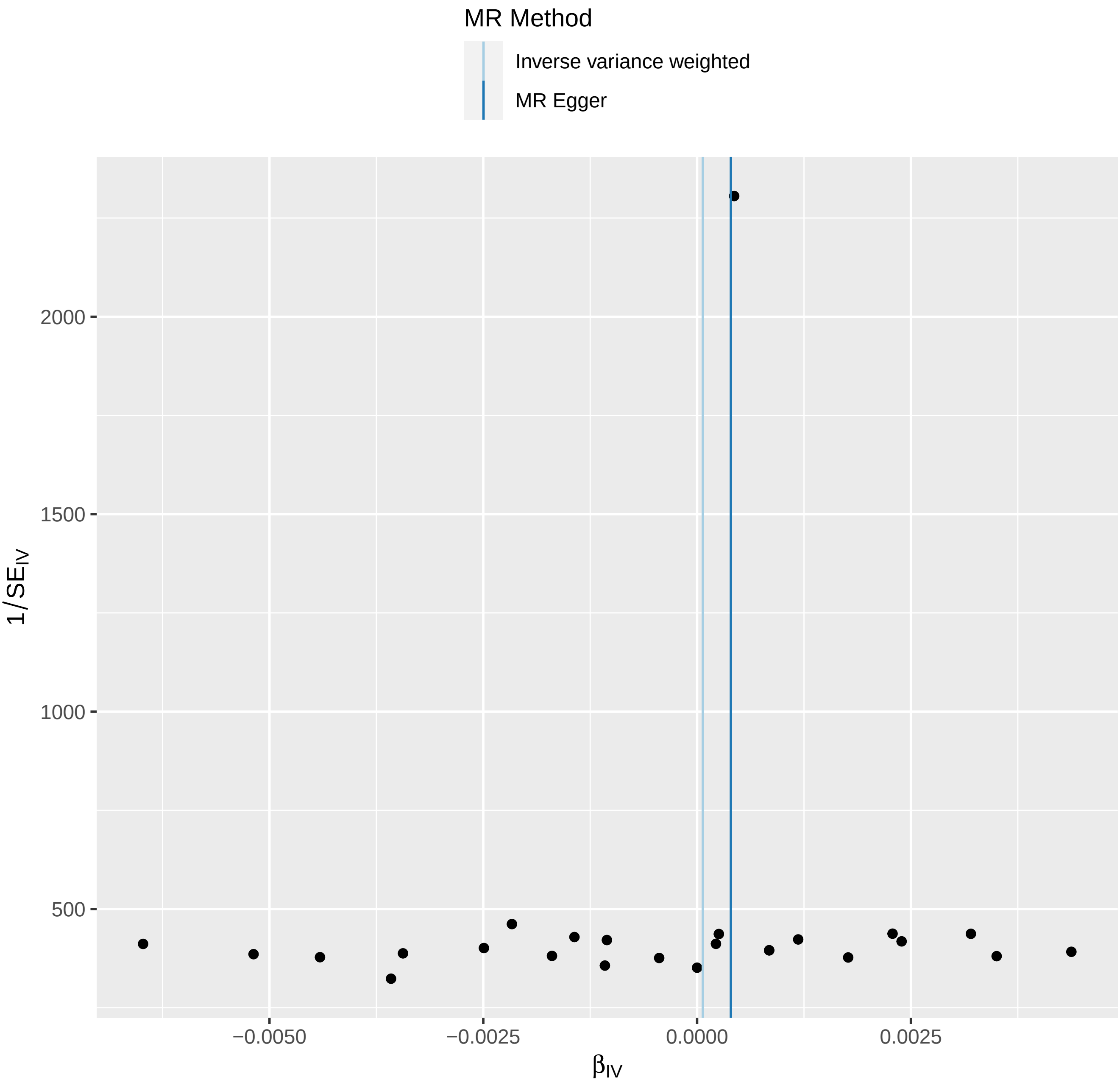

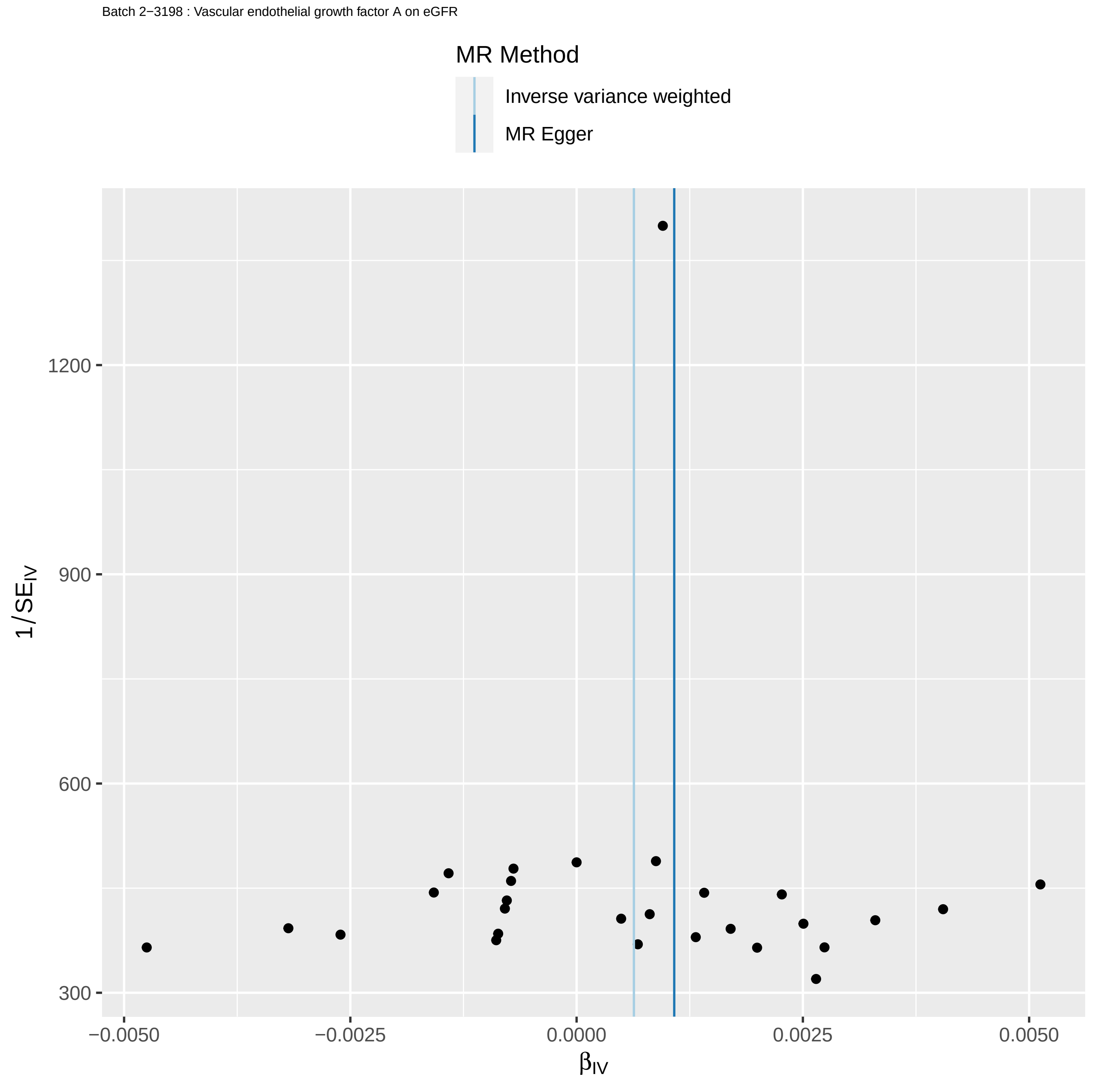
Batch 2−3198 : Vascular endothelial growth factor A on eGFR
MR Method
Inverse variance weighted
MR Egger
1200
900
600
300
1/SE_IV
−0.0050
−0.0025
0.0000
0.0025
0.0050
β_IV

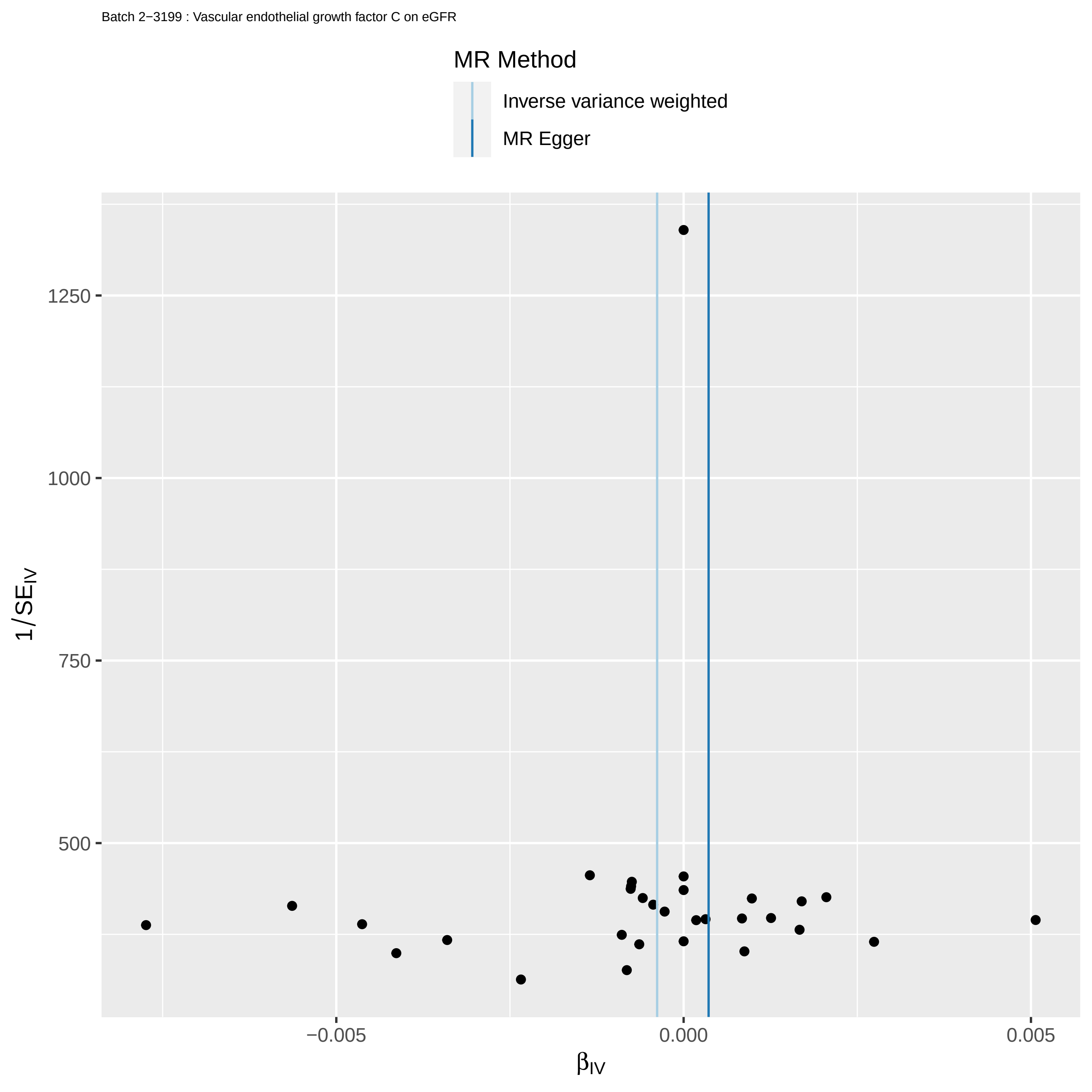

Batch 2−3199 : Vascular endothelial growth factor C on eGFR
MR Method
Inverse variance weighted
MR Egger
1250
1000
750
500
1/SE$_{IV}$
−0.005
0.000
0.005
β$_{IV}$

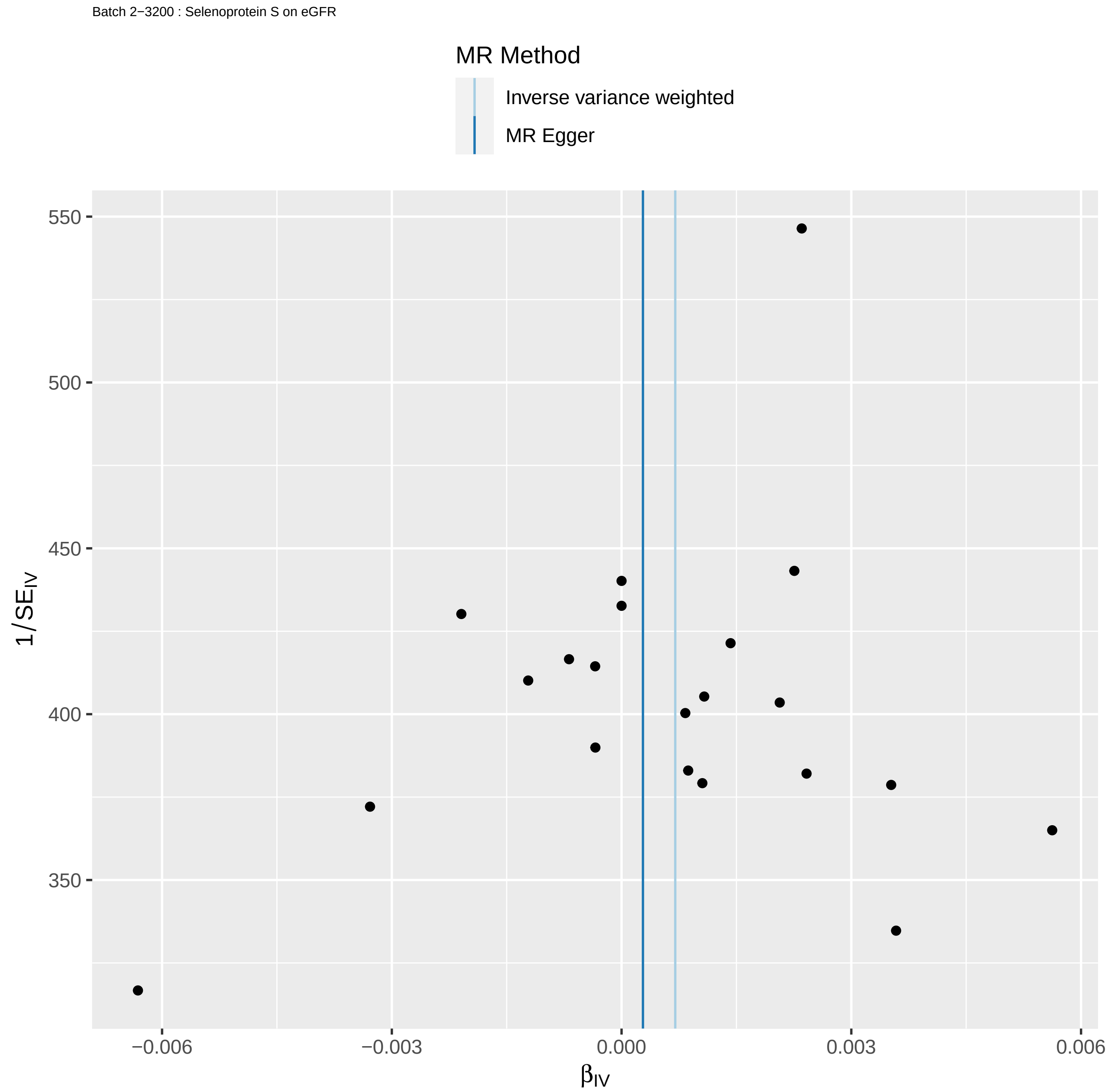

Batch 2−3200 : Selenoprotein S on eGFR
MR Method
Inverse variance weighted
MR Egger
550
500
450
400
350
1/SE_IV
−0.006
−0.003
0.000
0.003
0.006
β_IV

Batch 2−3201 : Vitrin on eGFR

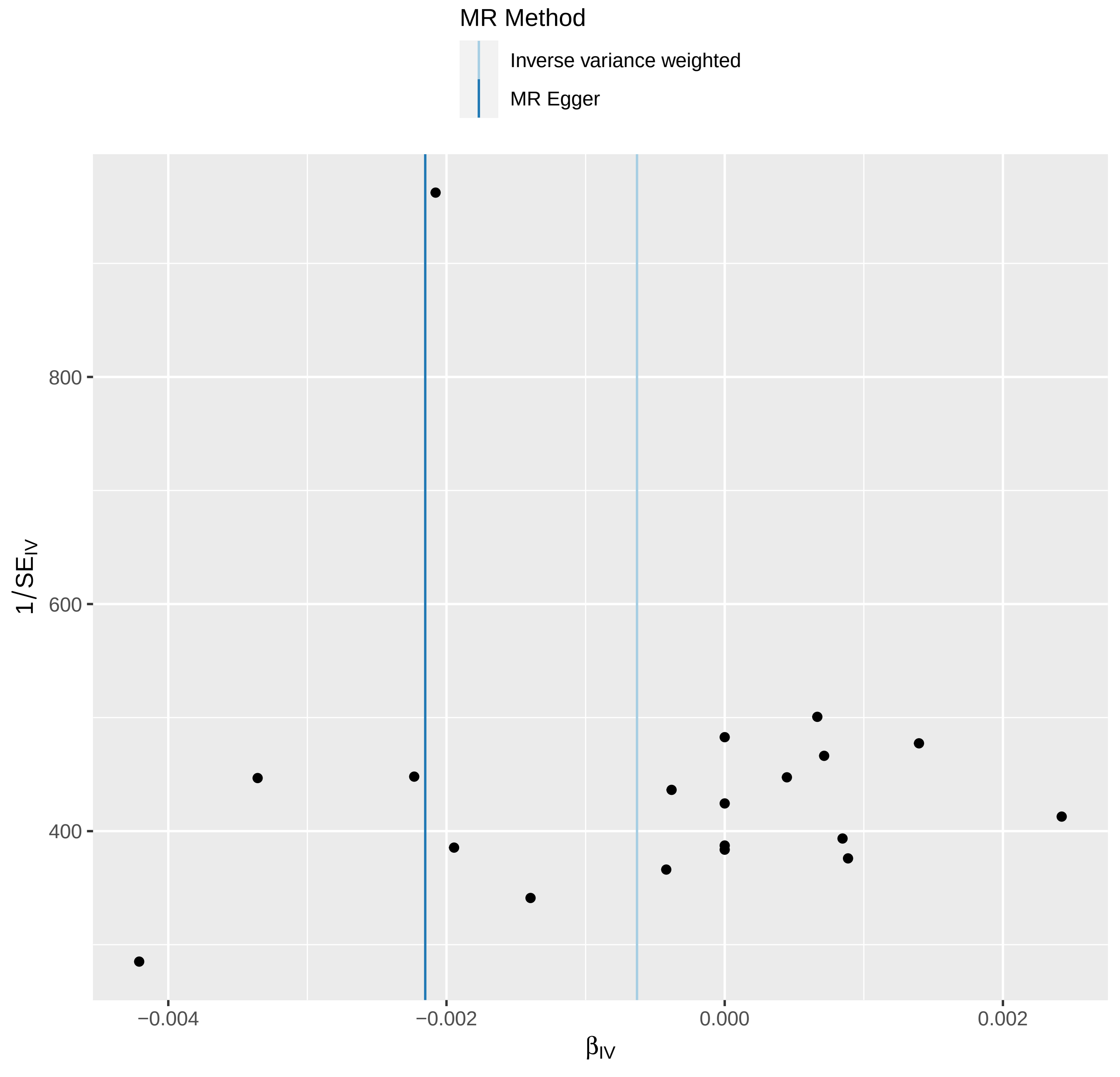

Batch 2−3202 : Charged multivesicular body protein 3 on eGFR

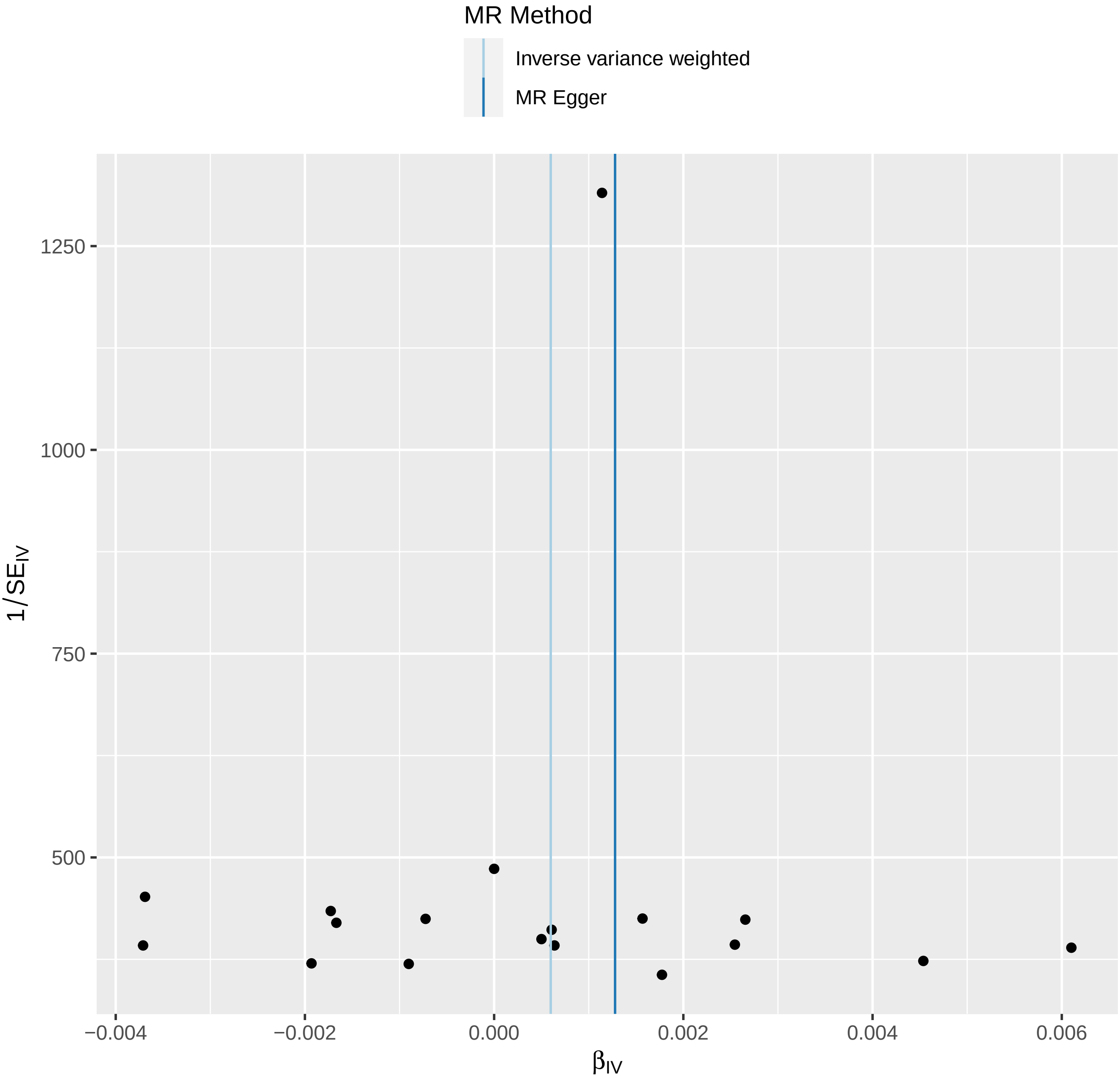

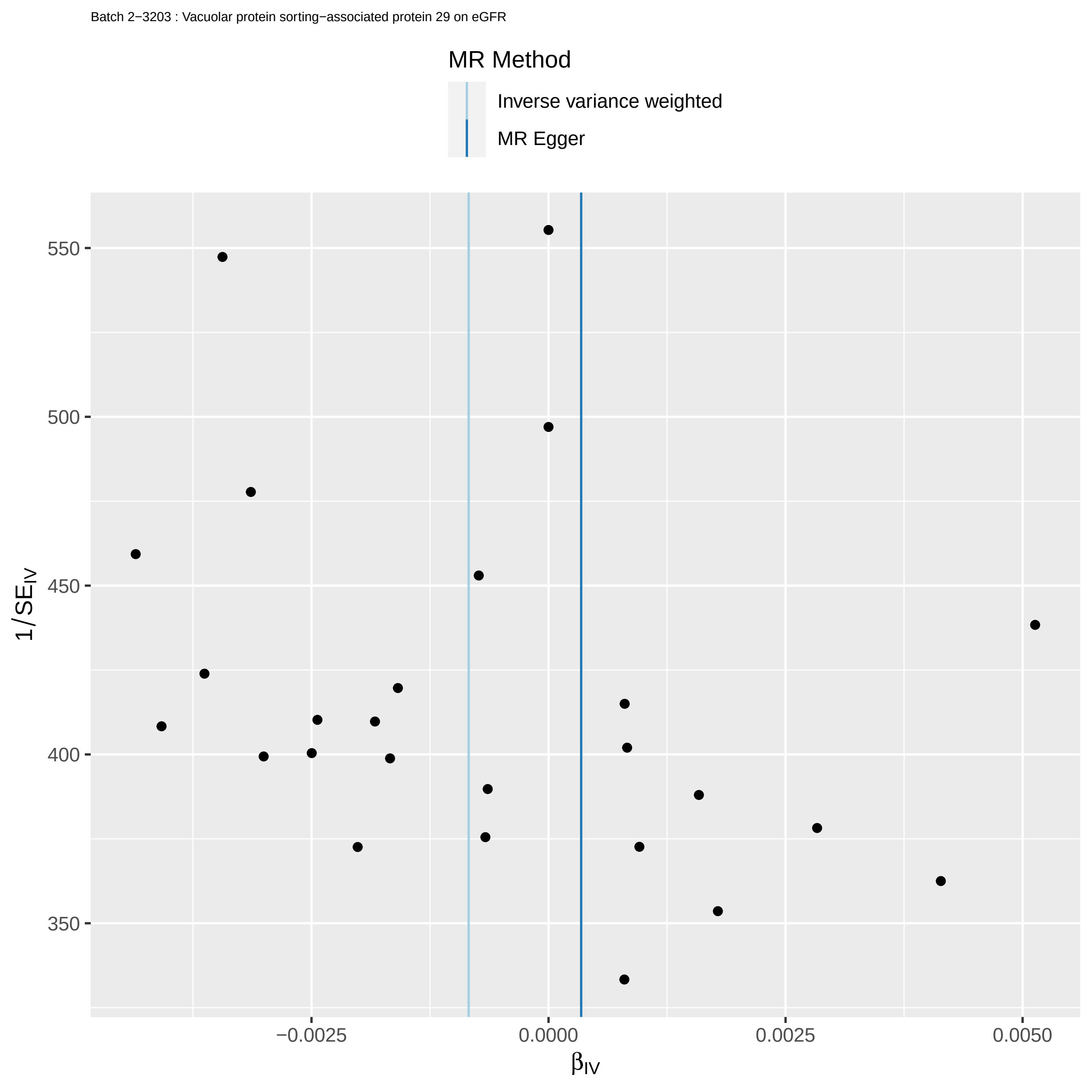
Batch 2−3203 : Vacuolar protein sorting−associated protein 29 on eGFR
MR Method
Inverse variance weighted
MR Egger
550
500
450
400
350
1/SE_IV
−0.0025
0.0000
0.0025
0.0050
β_IV

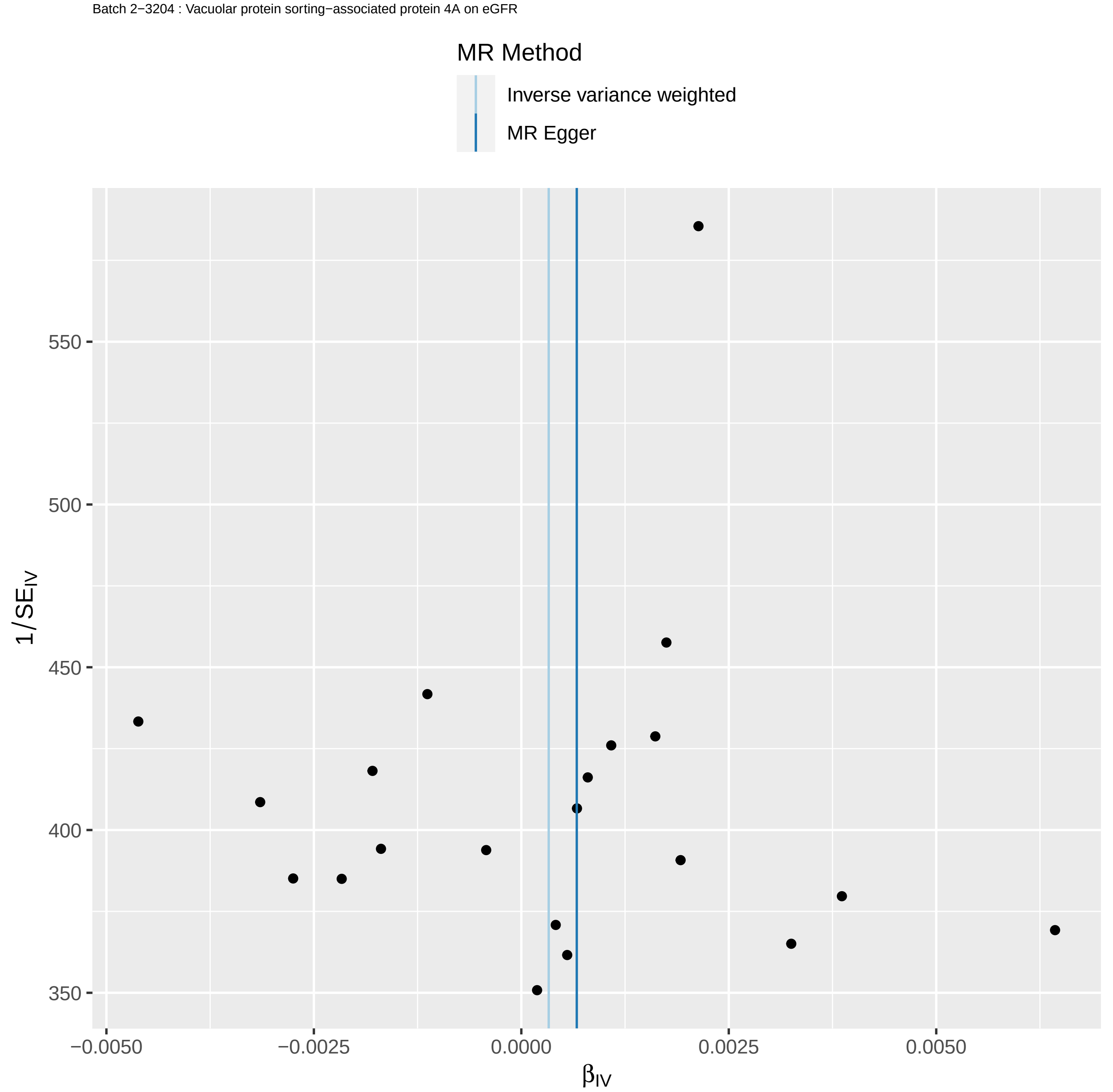

Batch 2−3204 : Vacuolar protein sorting−associated protein 4A on eGFR
MR Method
Inverse variance weighted
MR Egger
550
500
450
400
350
1/SE_IV
−0.0050
−0.0025
0.0000
0.0025
0.0050
β_IV

Batch 2−3205 : Vacuolar protein sorting−associated protein 4B on eGFR

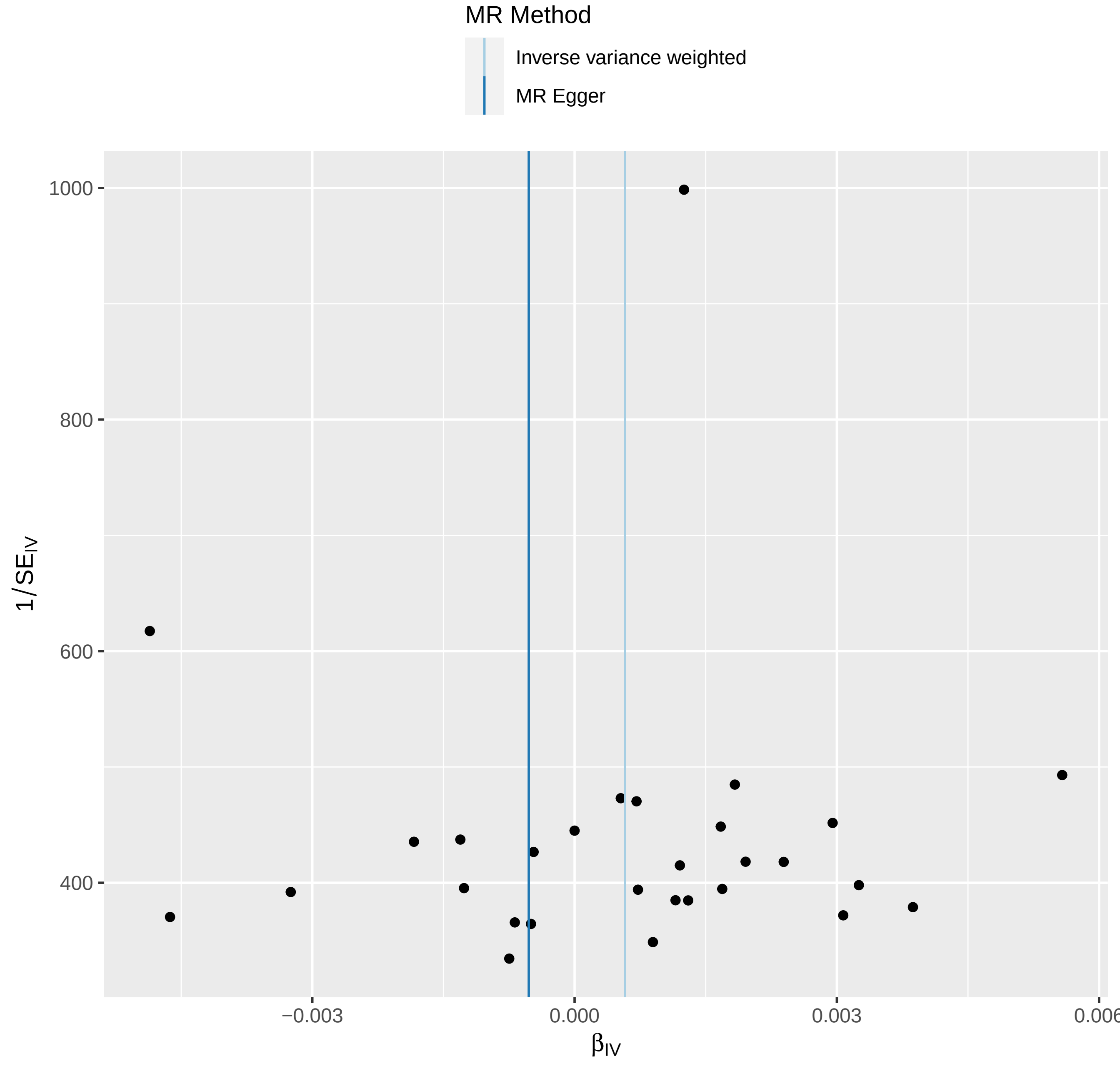

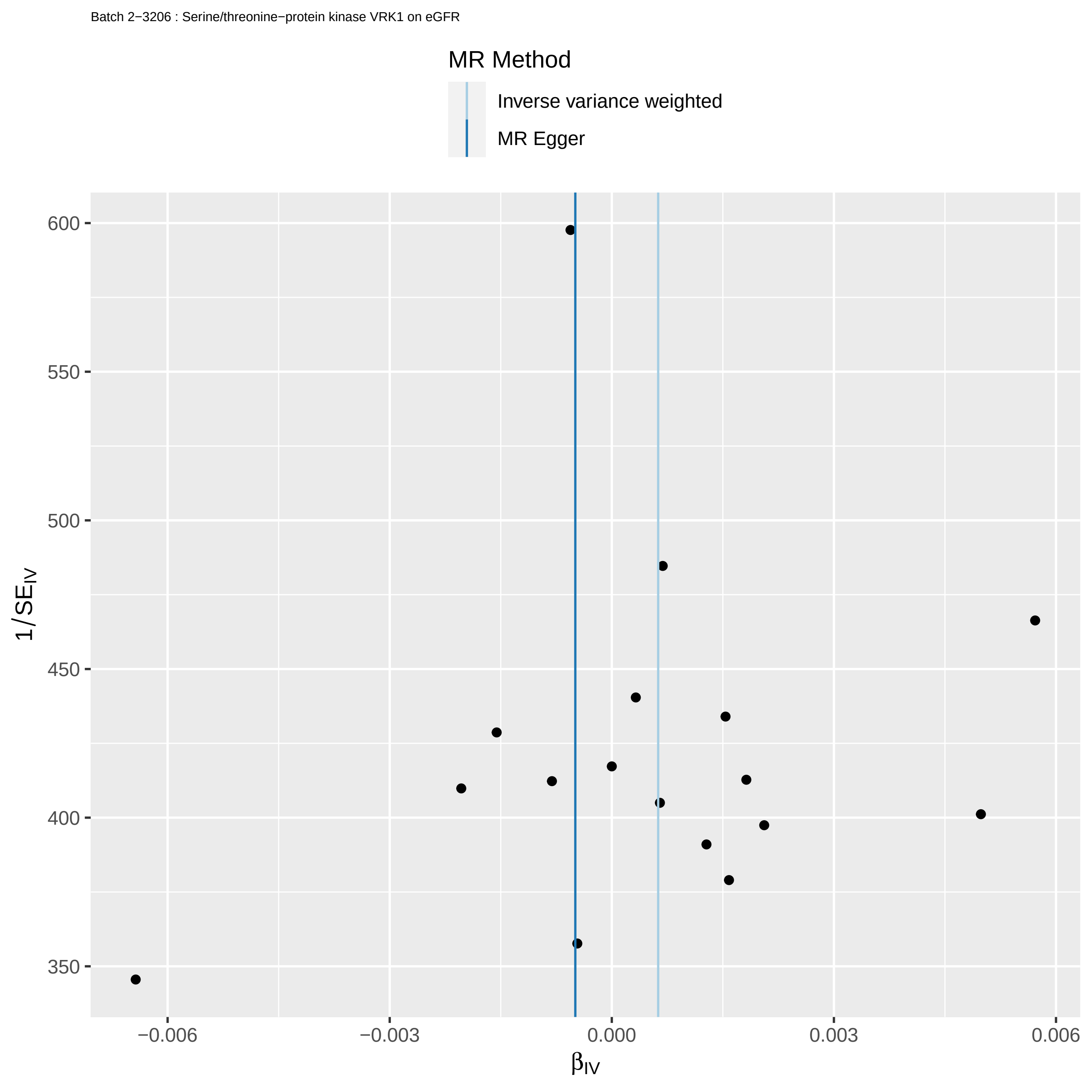

Batch 2−3206 : Serine/threonine−protein kinase VRK1 on eGFR
MR Method
Inverse variance weighted
MR Egger
600
550
500
450
400
350
1/SE_IV
−0.006
−0.003
0.000
0.003
0.006
β_IV

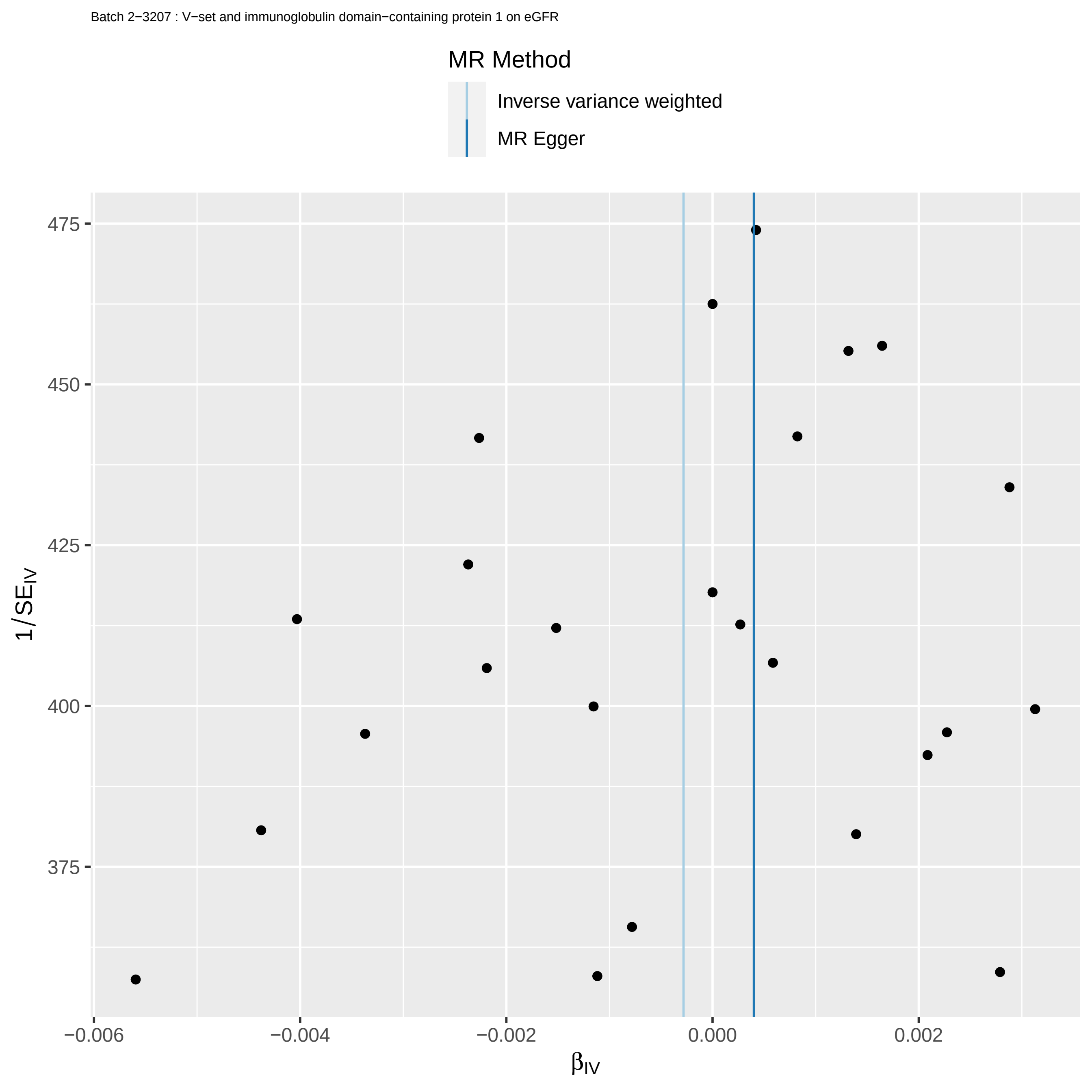

Batch 2−3207 : V−set and immunoglobulin domain−containing protein 1 on eGFR
MR Method
Inverse variance weighted
MR Egger
475
450
425
400
375
1/SE_IV
−0.006
−0.004
−0.002
0.000
0.002
β_IV

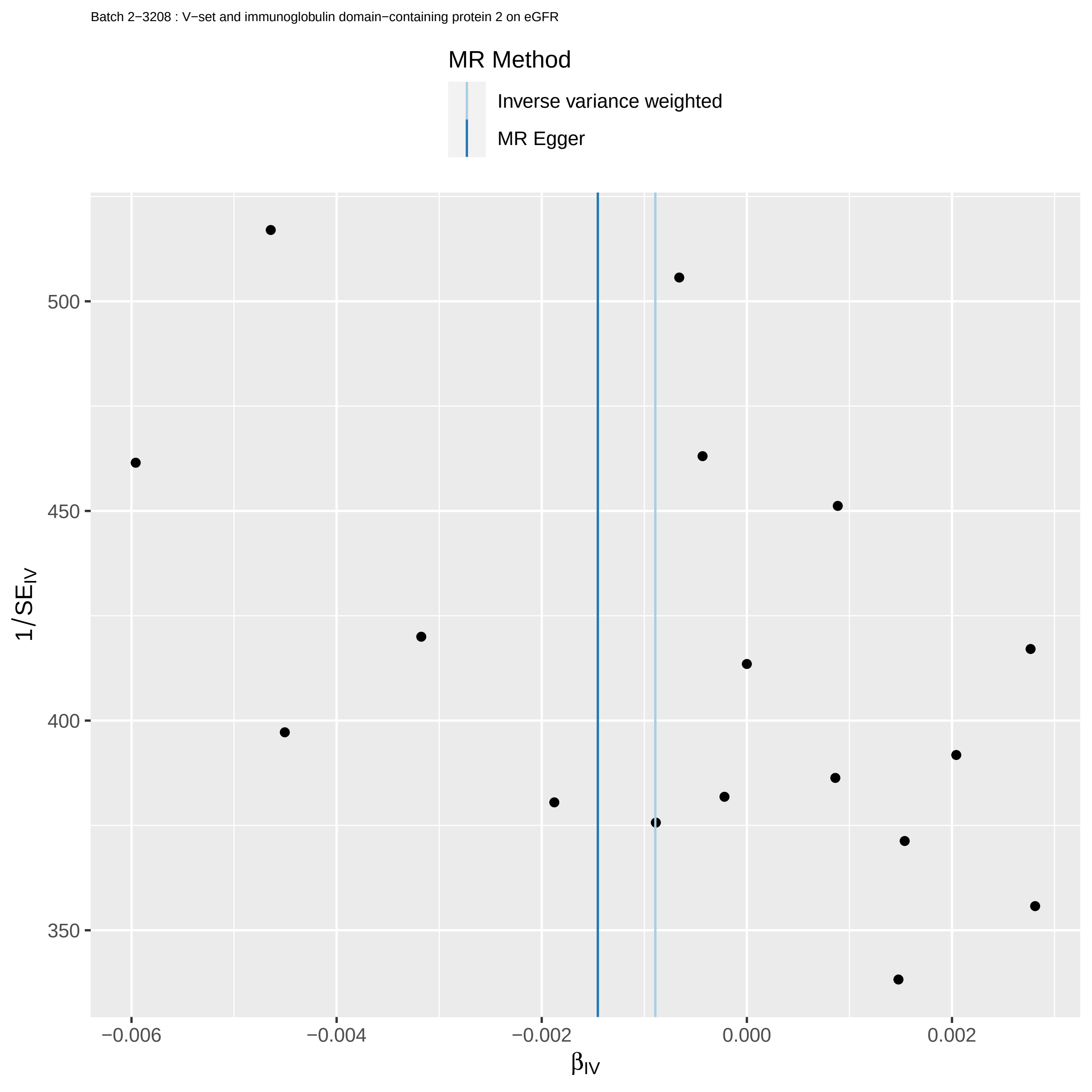

Batch 2−3208 : V−set and immunoglobulin domain−containing protein 2 on eGFR
MR Method
Inverse variance weighted
MR Egger
500
450
400
350
1/SE_IV
−0.006
−0.004
−0.002
0.000
0.002
β_IV

Batch 2−3209 : V−set and transmembrane domain−containing protein 2−like protein on eGFR

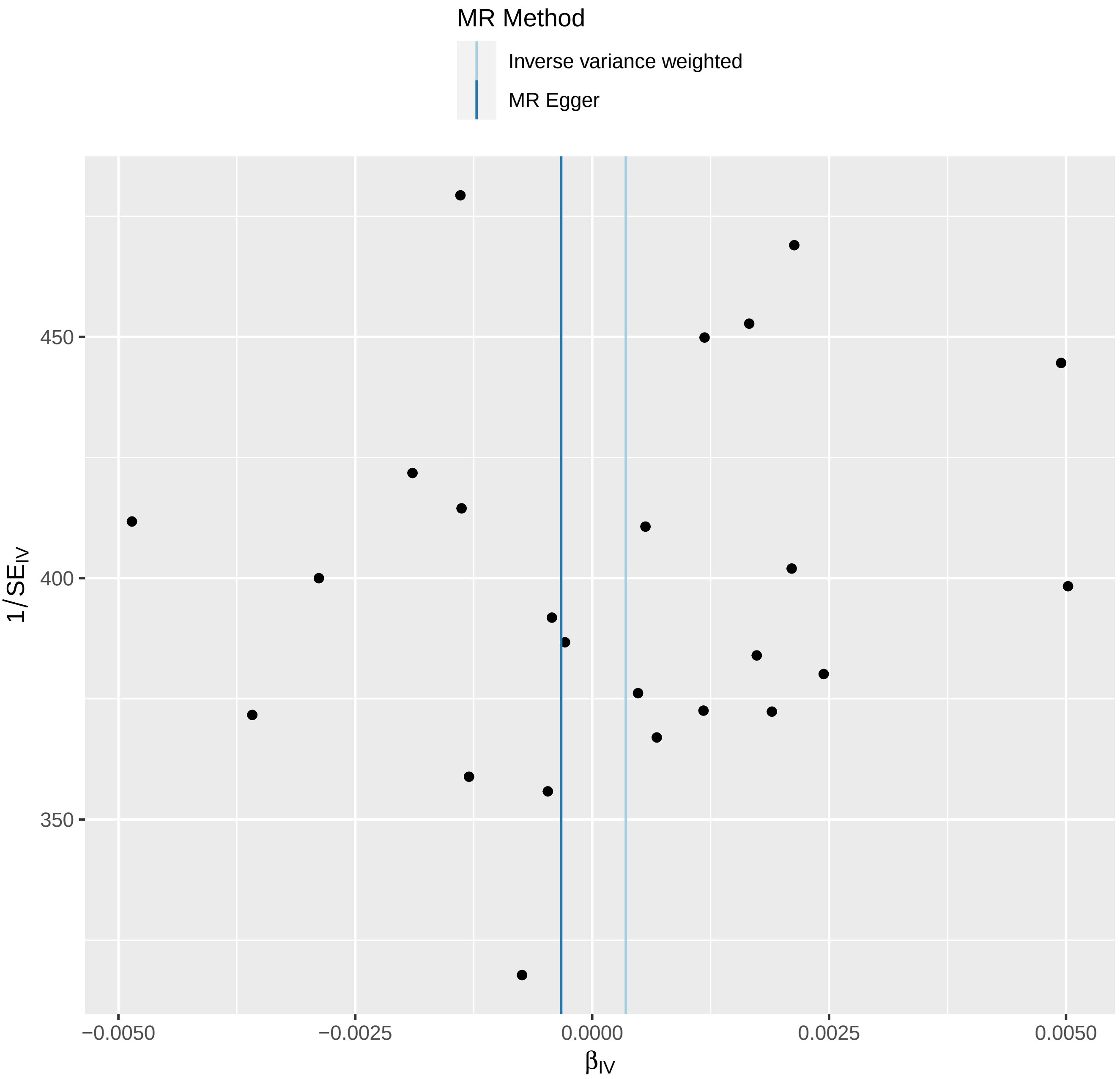

Batch 2−3210 : V−set and transmembrane domain−containing protein 4 on eGFR

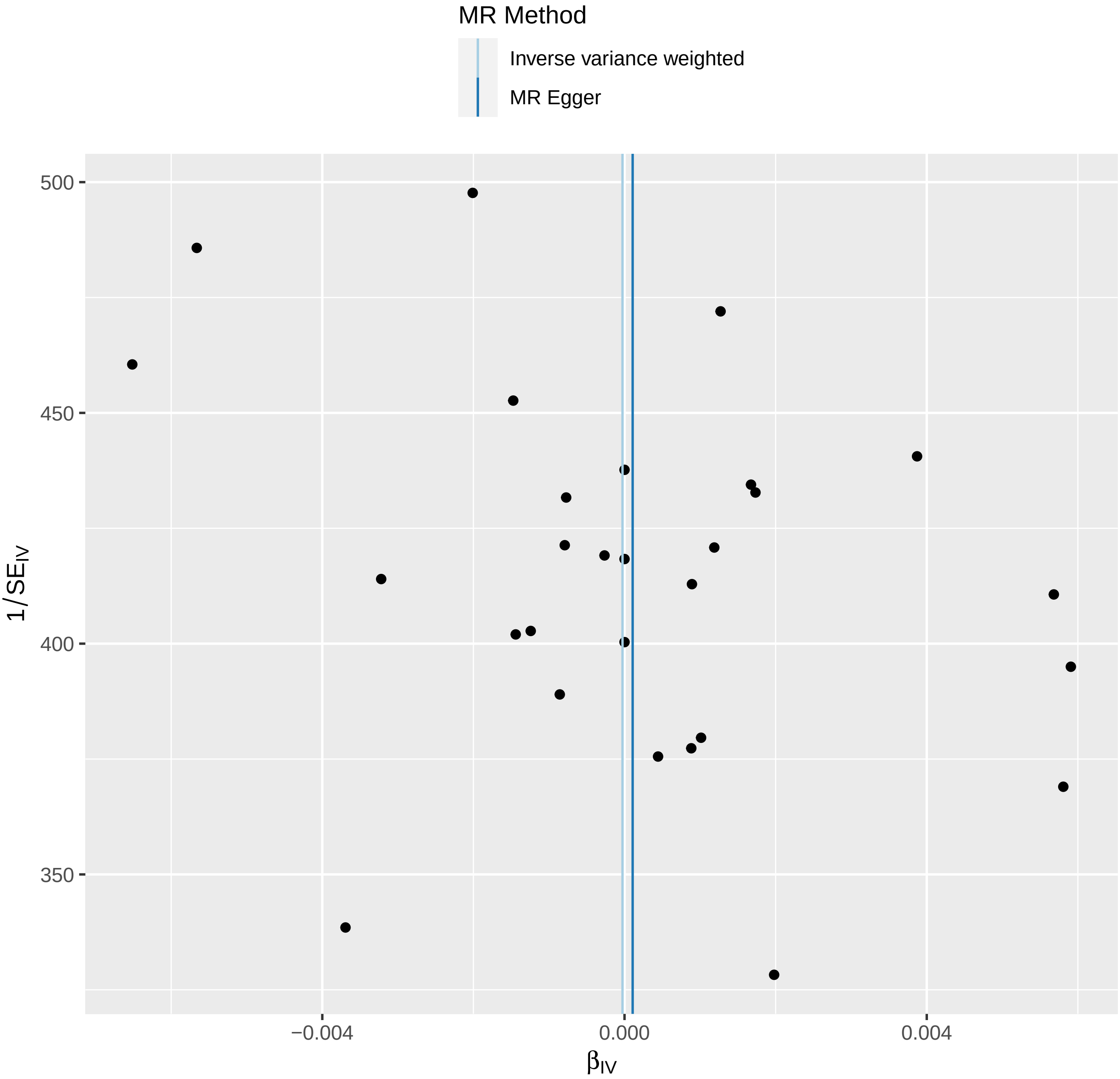

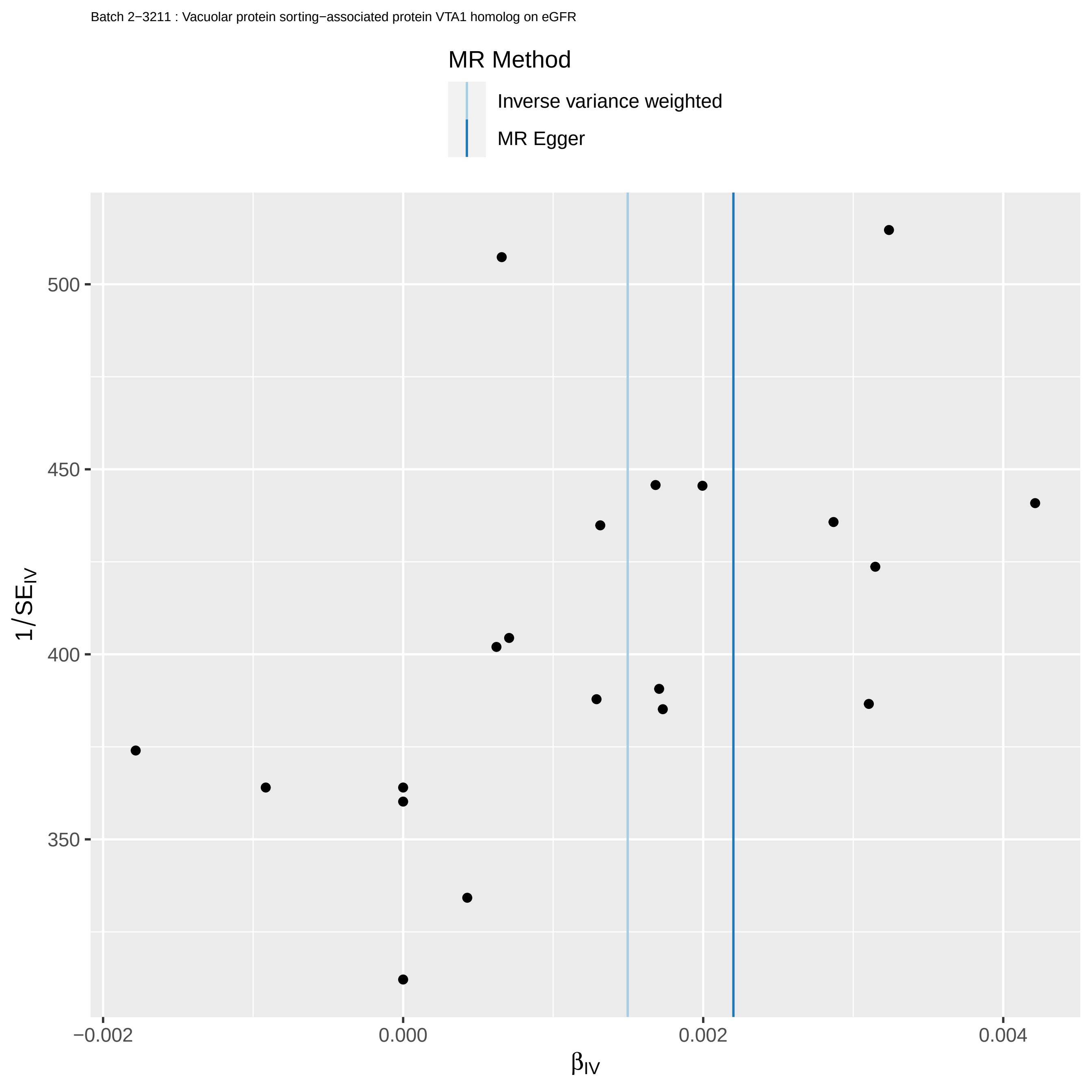
Batch 2−3211 : Vacuolar protein sorting−associated protein VTA1 homolog on eGFR
MR Method
Inverse variance weighted
MR Egger
500
450
400
350
1/SE_IV
−0.002
0.000
0.002
0.004
β_IV

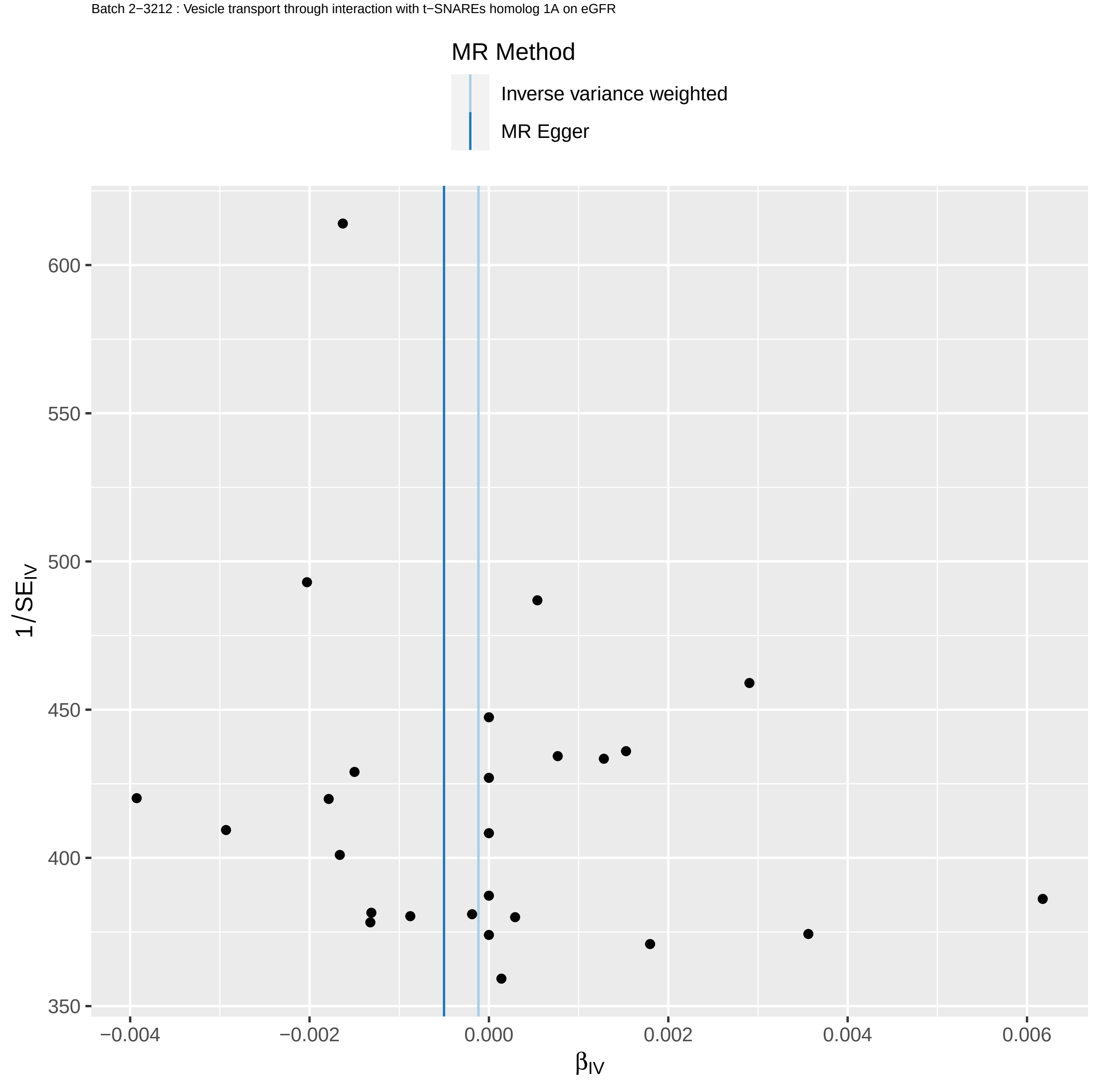
Batch 2−3212 : Vesicle transport through interaction with t−SNAREs homolog 1A on eGFR
MR Method
Inverse variance weighted
MR Egger
600
550
500
450
400
350
1/SE$_{IV}$
−0.004
−0.002
0.000
0.002
0.004
0.006
β$_{IV}$

Batch 2−3213 : Vesicle transport through interaction with t−SNAREs homolog 1B on eGFR

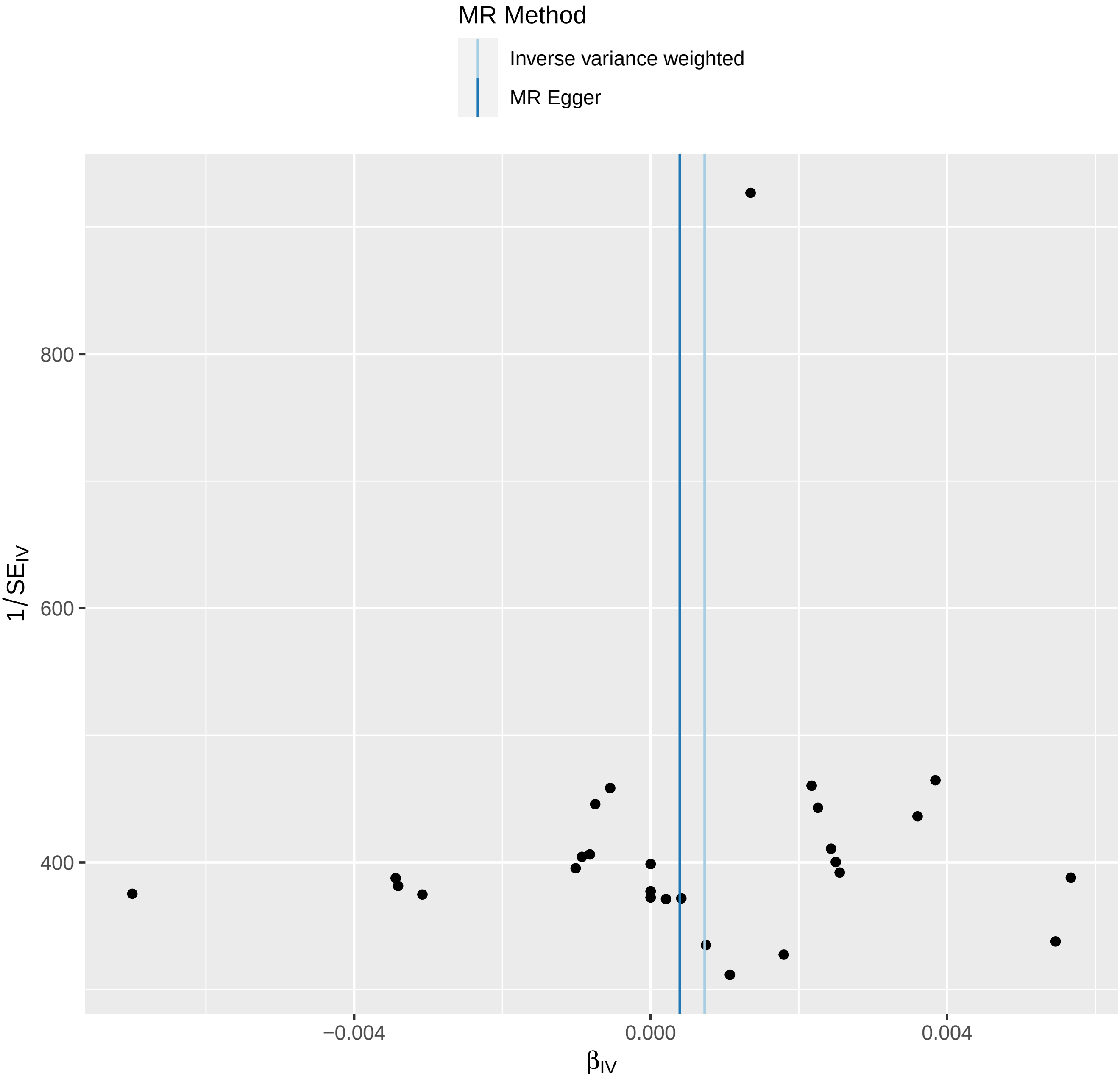

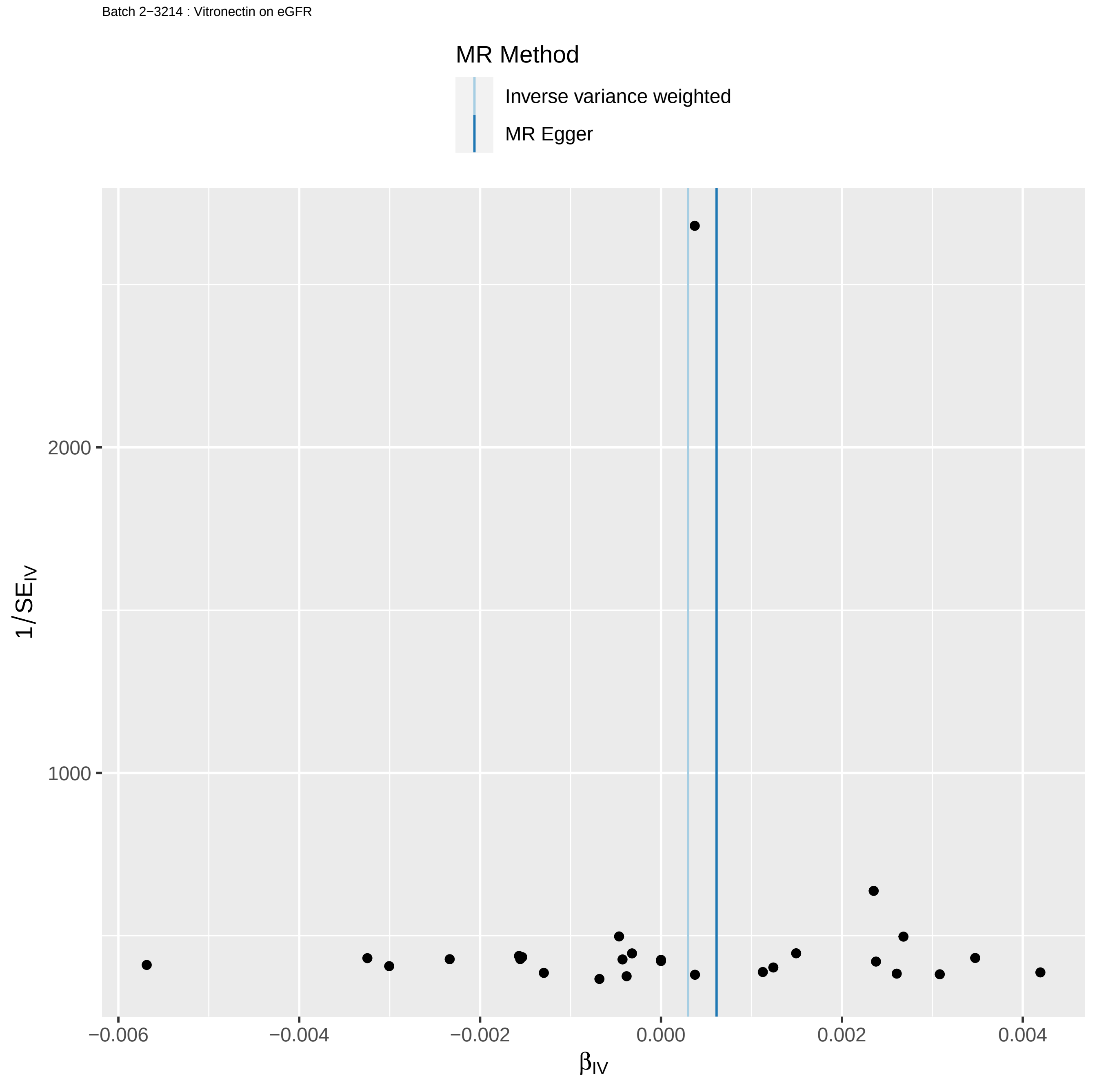

Batch 2−3214 : Vitronectin on eGFR
MR Method
Inverse variance weighted
MR Egger
2000
1000
1/SE$_{IV}$
−0.006
−0.004
−0.002
0.000
0.002
0.004
β$_{IV}$

Batch 2−3215 : Vitronectin on eGFR

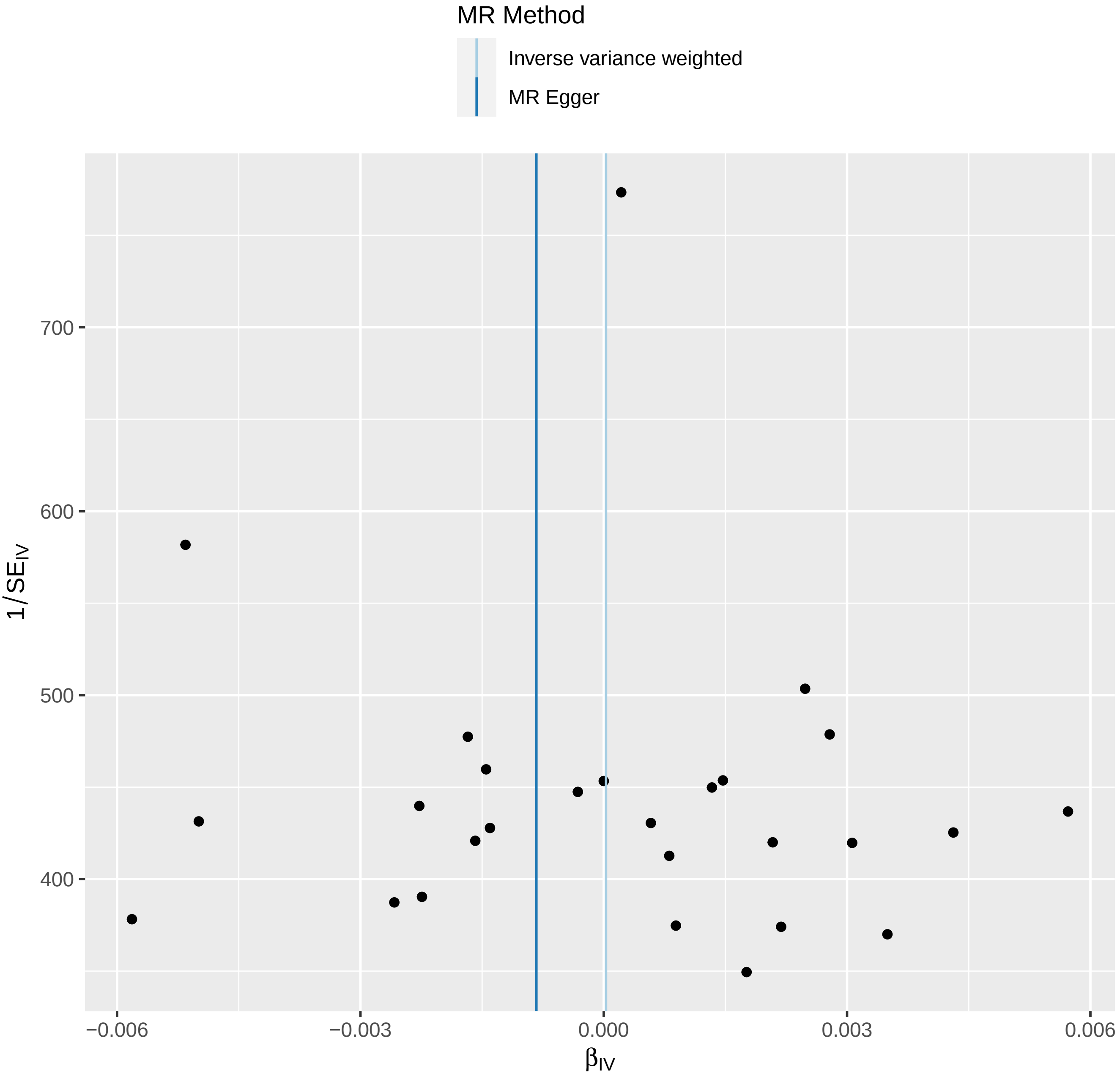

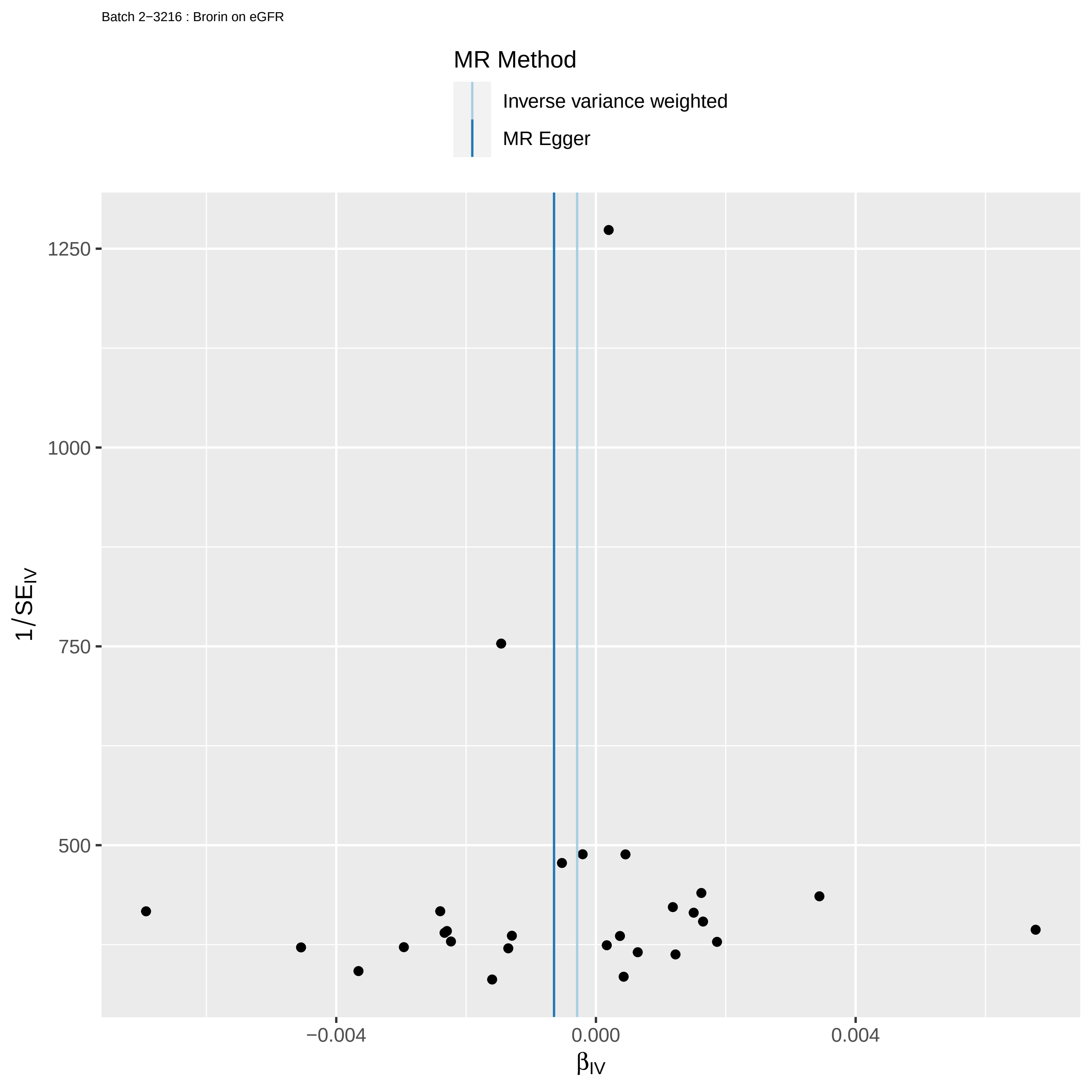
Batch 2−3216 : Brorin on eGFR
MR Method
Inverse variance weighted
MR Egger
1250
1000
750
500
1/SE_IV
−0.004
0.000
0.004
β_IV

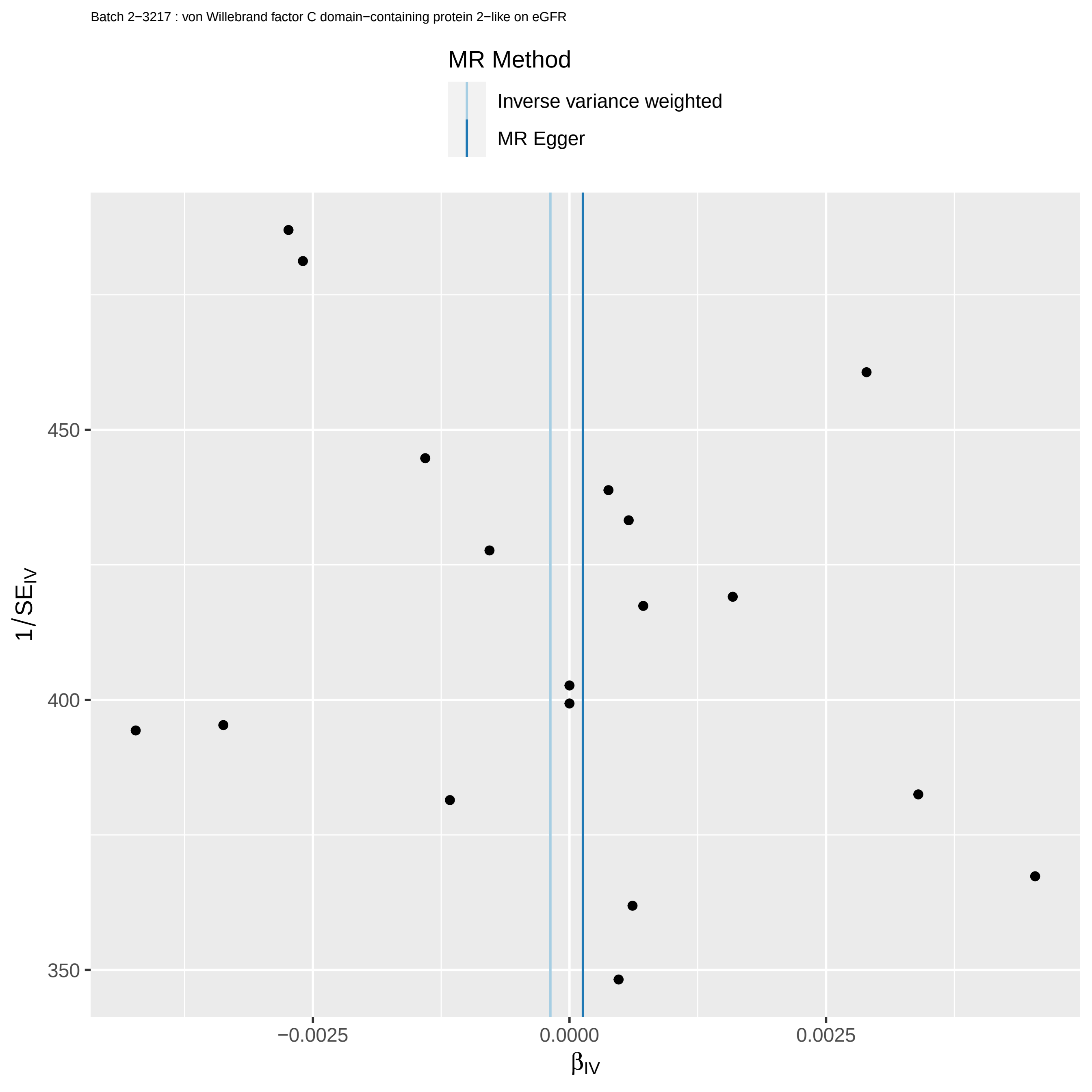
Batch 2−3217 : von Willebrand factor C domain−containing protein 2−like on eGFR
MR Method
Inverse variance weighted
MR Egger
450
400
350
1/SE$_{IV}$
−0.0025
0.0000
0.0025
β$_{IV}$

Batch 2−3218 : WW domain−binding protein 1 on eGFR

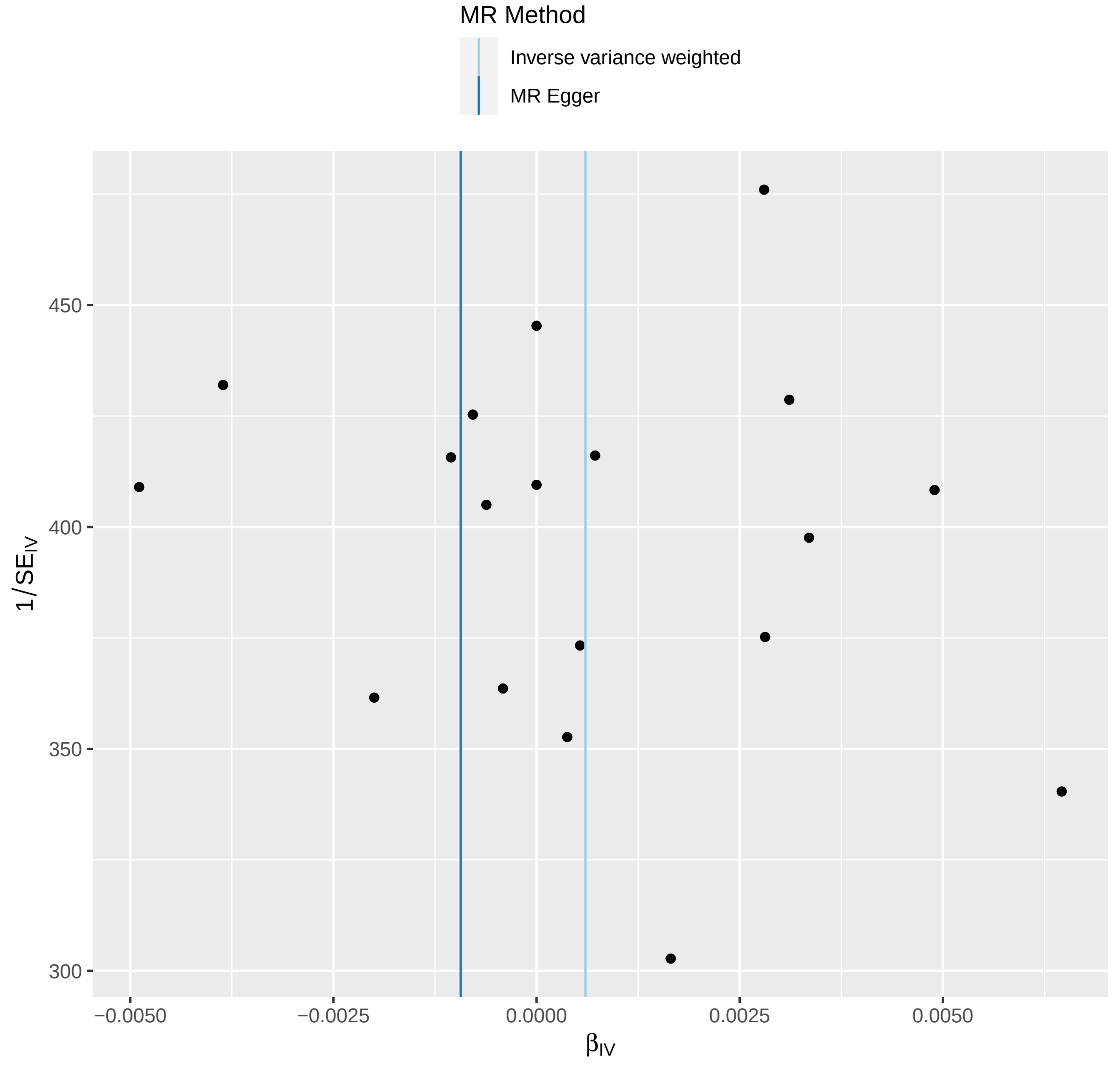

Batch 2−3219 : WW domain binding protein 1−like on eGFR

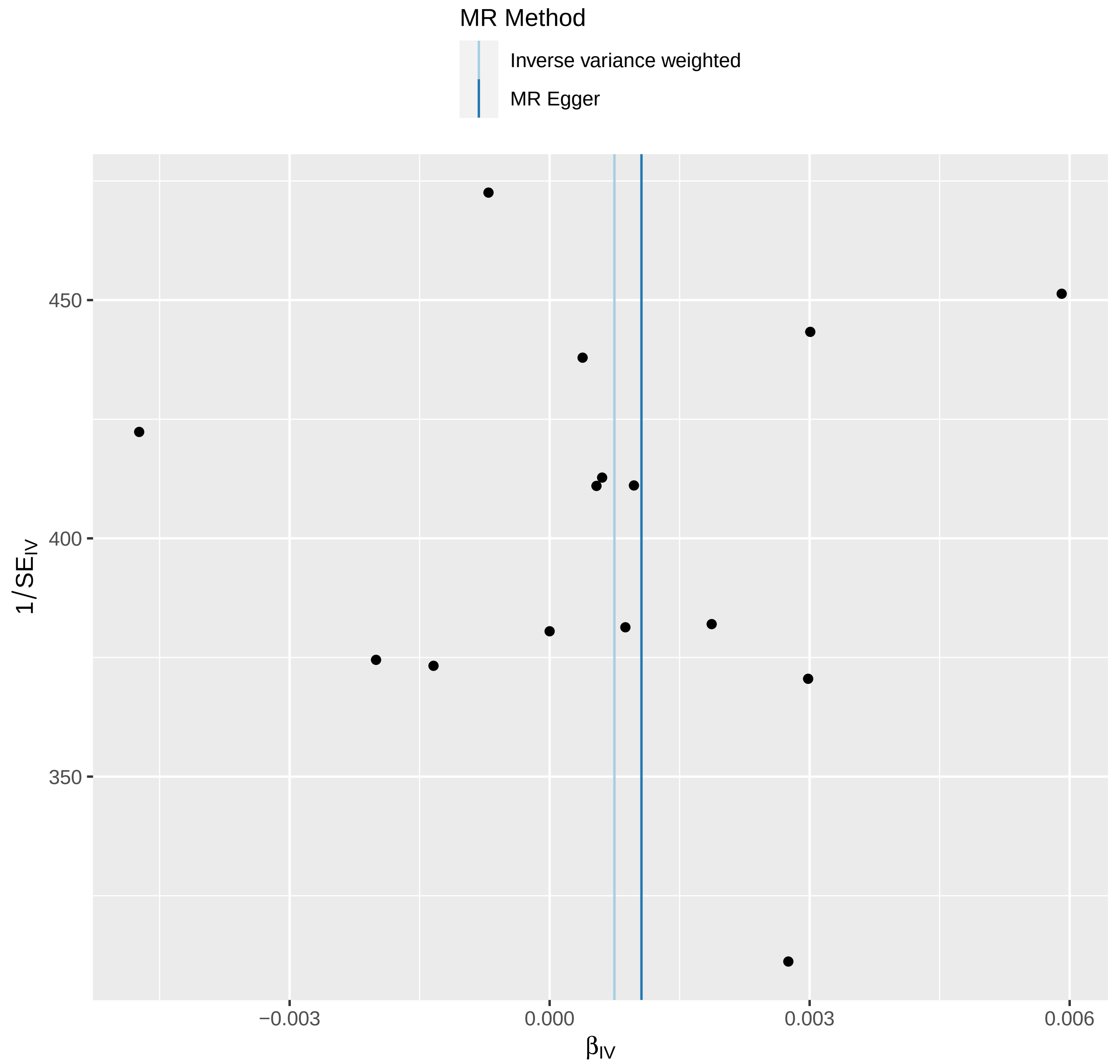

Batch 2−3220 : WD repeat−containing protein 1 on eGFR

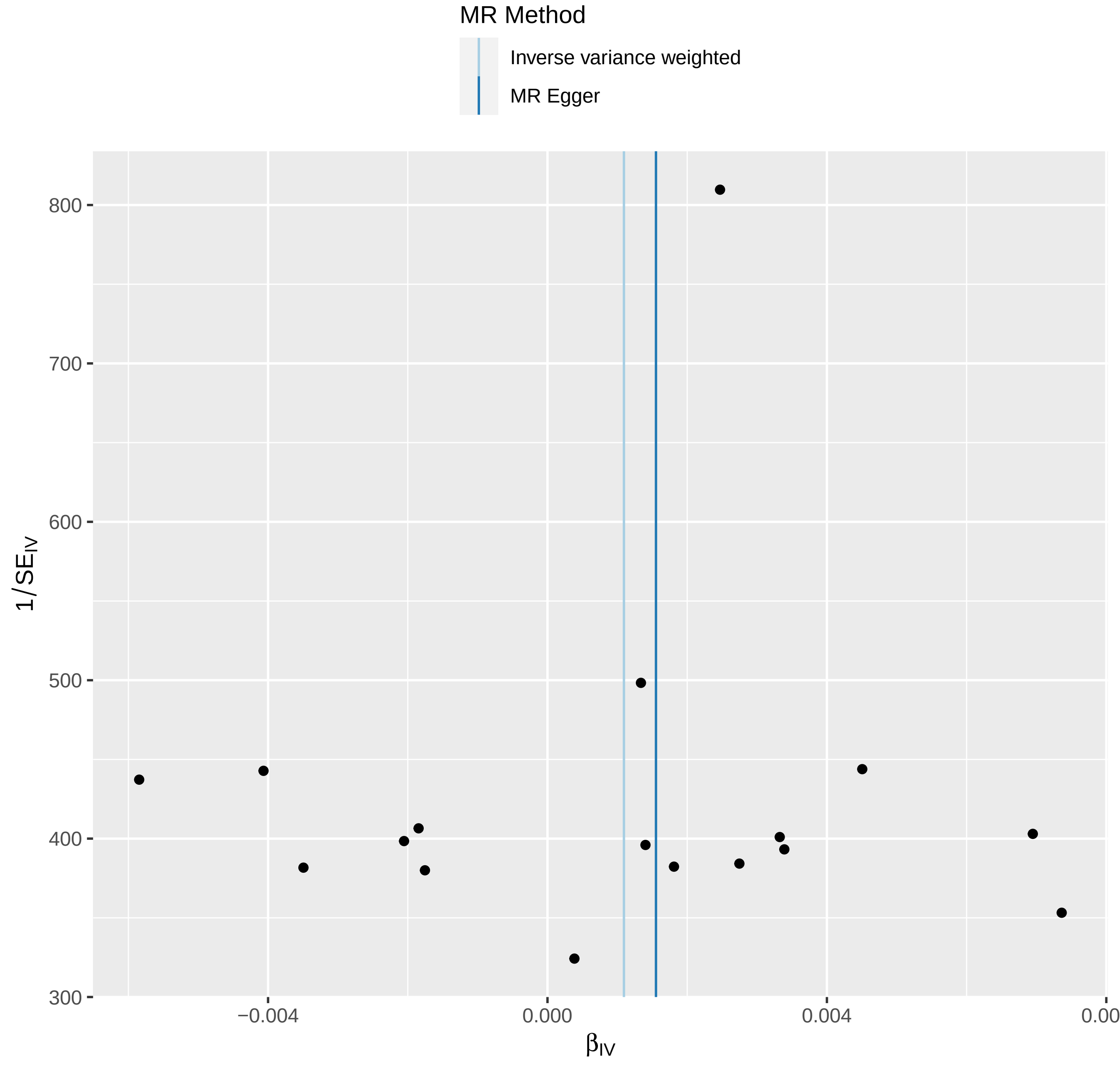

Batch 2−3221 : WAP four−disulfide core domain protein 10A on eGFR

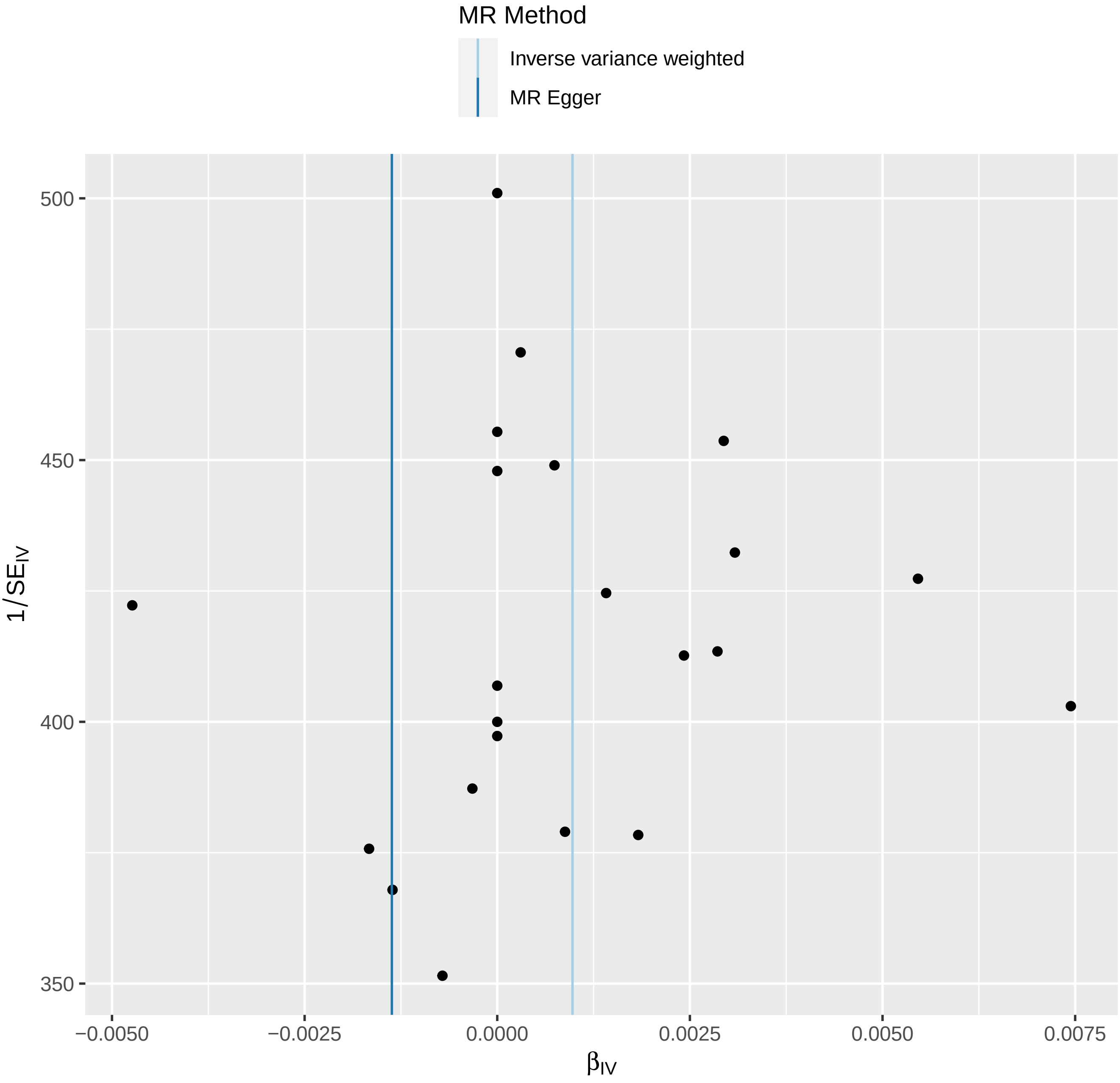

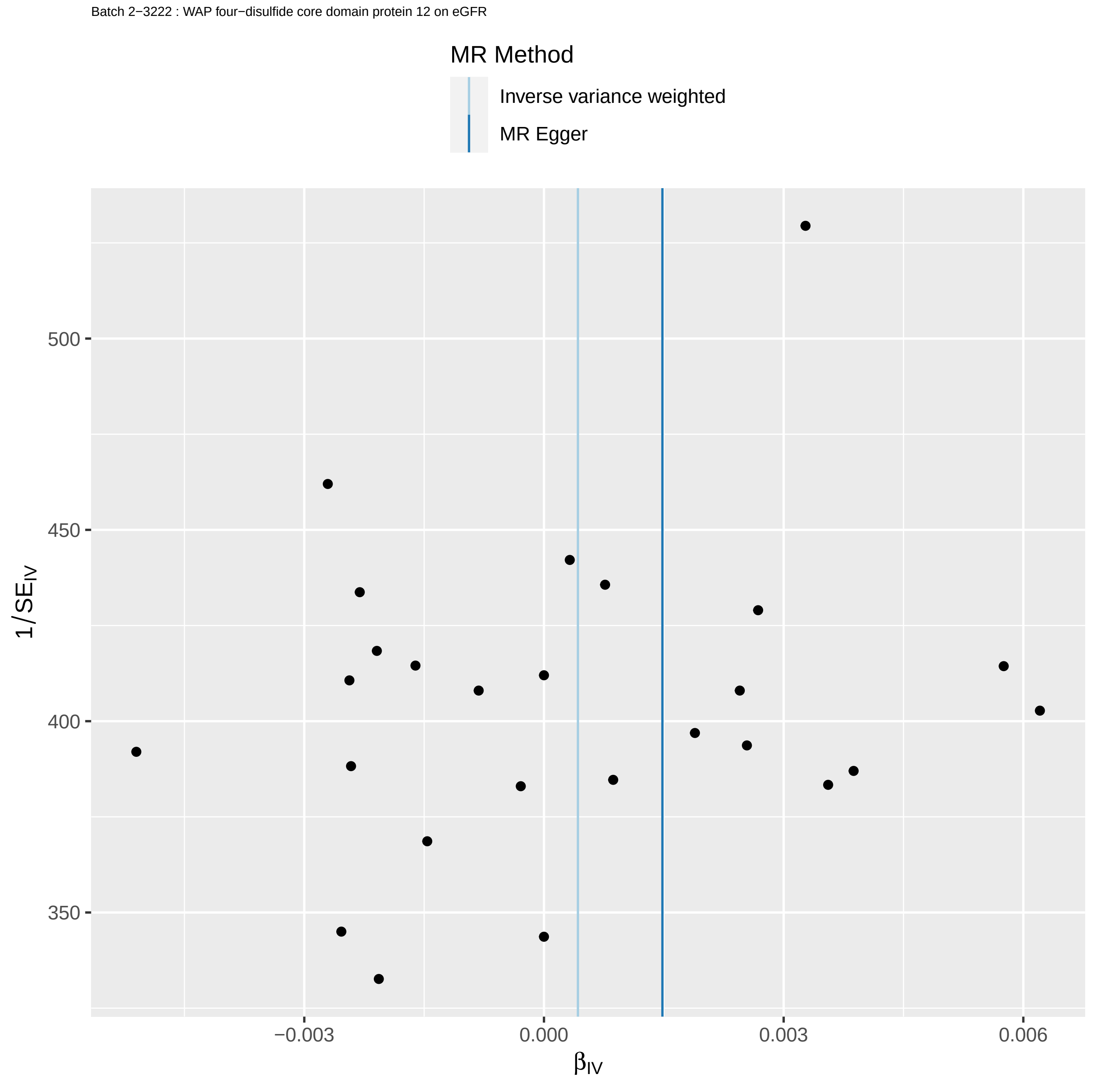

Batch 2−3222 : WAP four−disulfide core domain protein 12 on eGFR
MR Method
Inverse variance weighted
MR Egger
500
450
400
350
1/SE_IV
−0.003
0.000
0.003
0.006
β_IV

Batch 2−3223 : WAP four−disulfide core domain protein 13 on eGFR

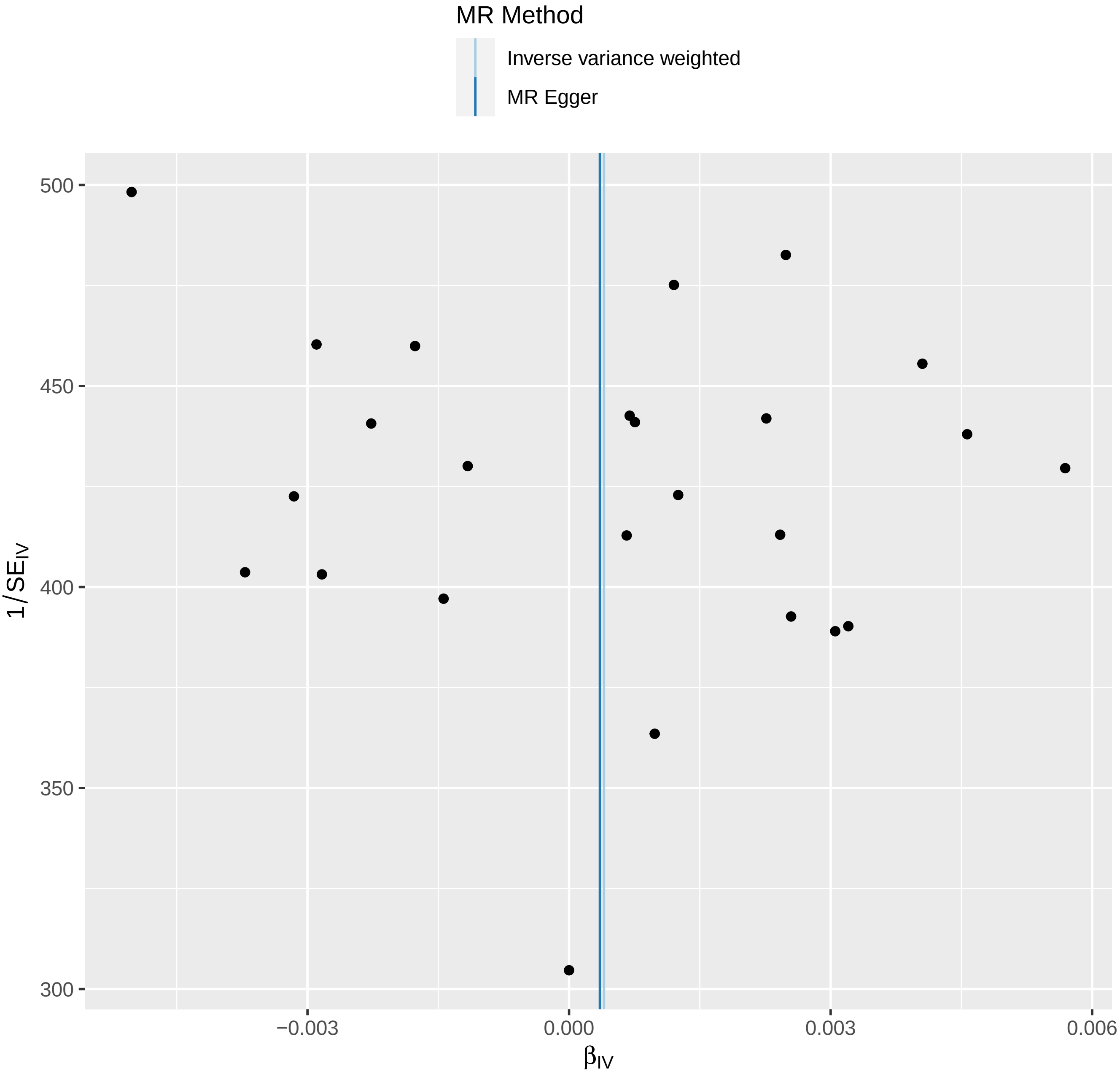

Batch 2−3224 : WAP four−disulfide core domain protein 2 on eGFR

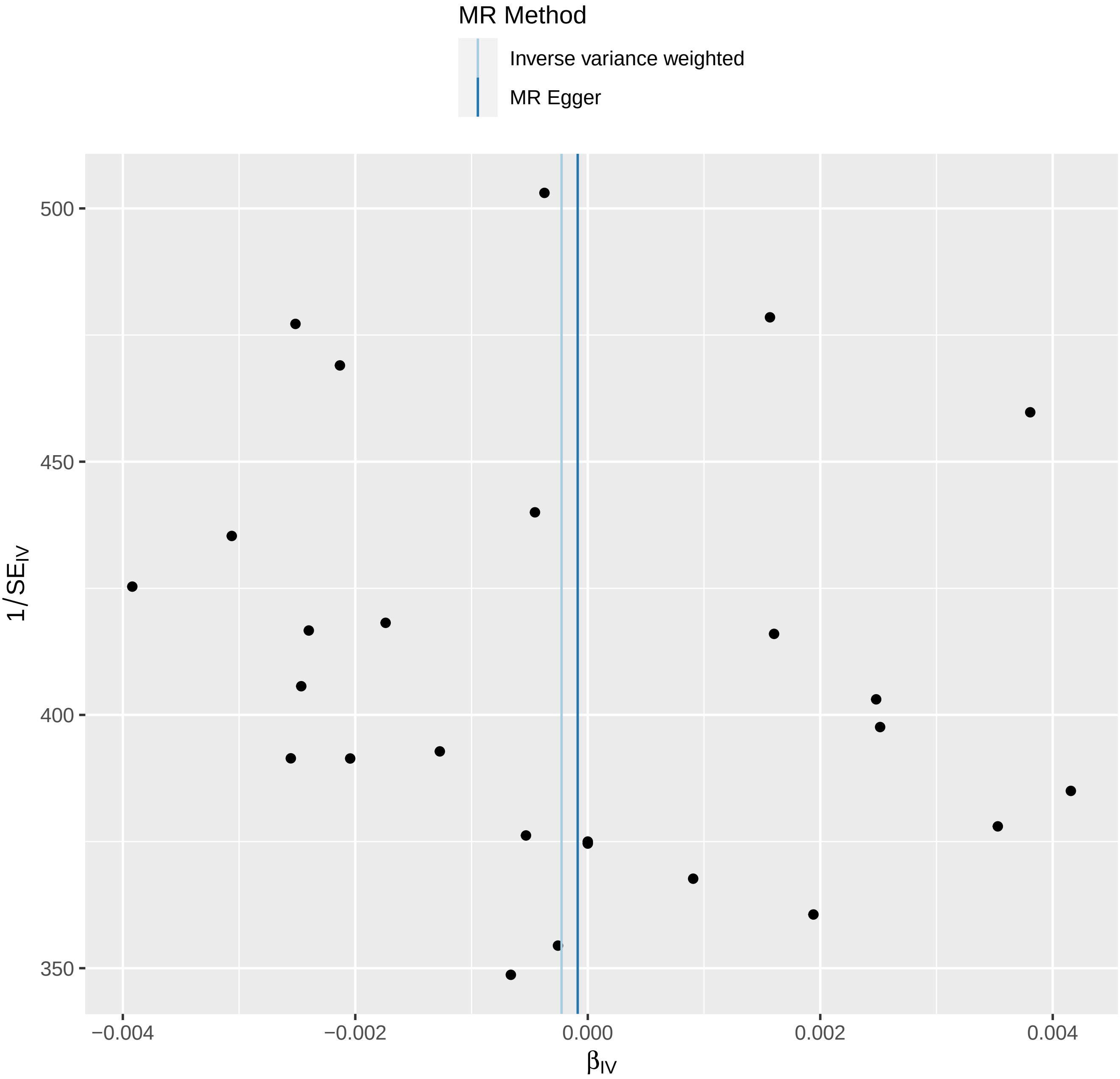

Batch 2−3225 : WAP four−disulfide core domain protein 3 on eGFR

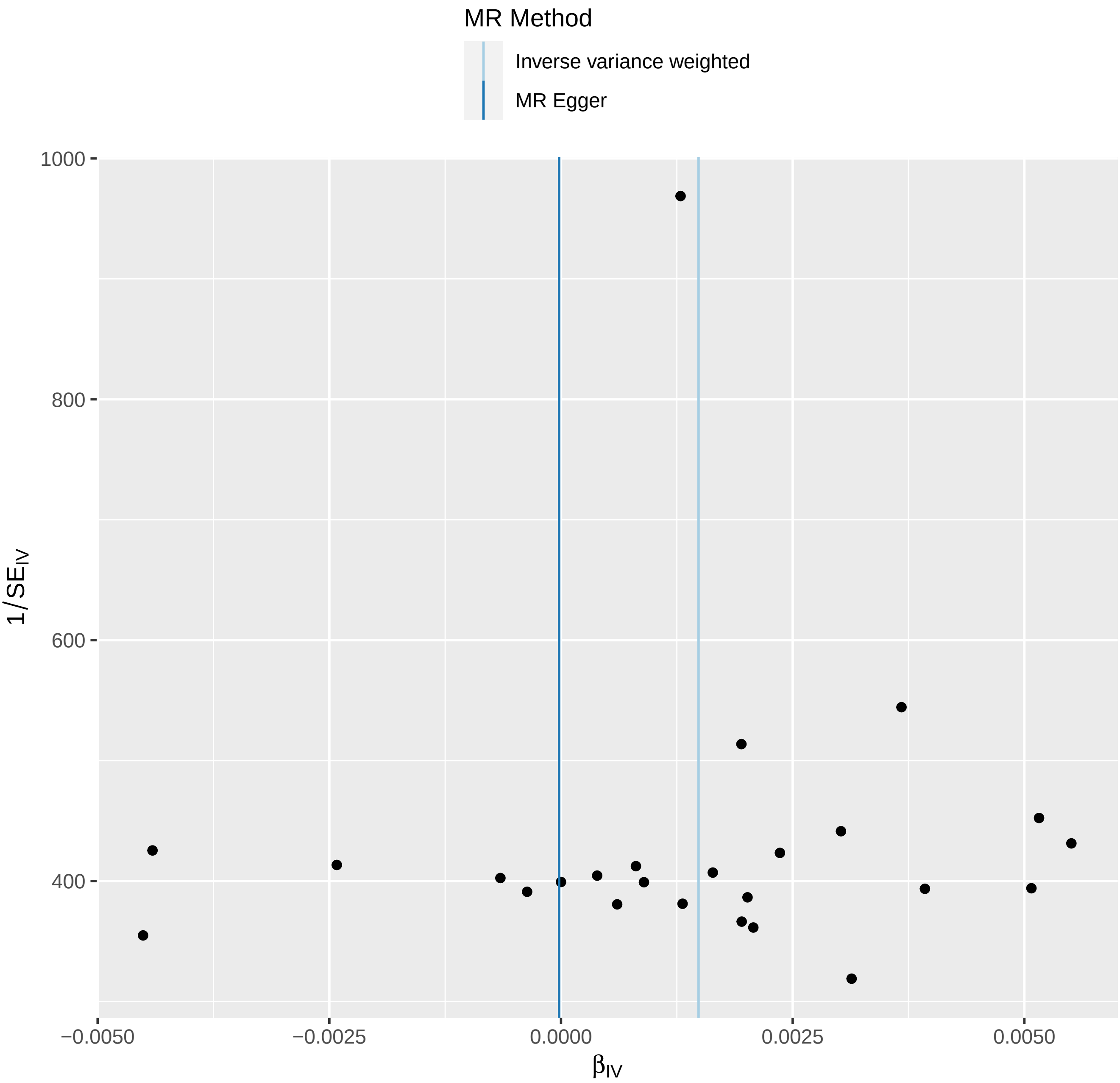

Batch 2−3226 : WAP four−disulfide core domain protein 5 on eGFR

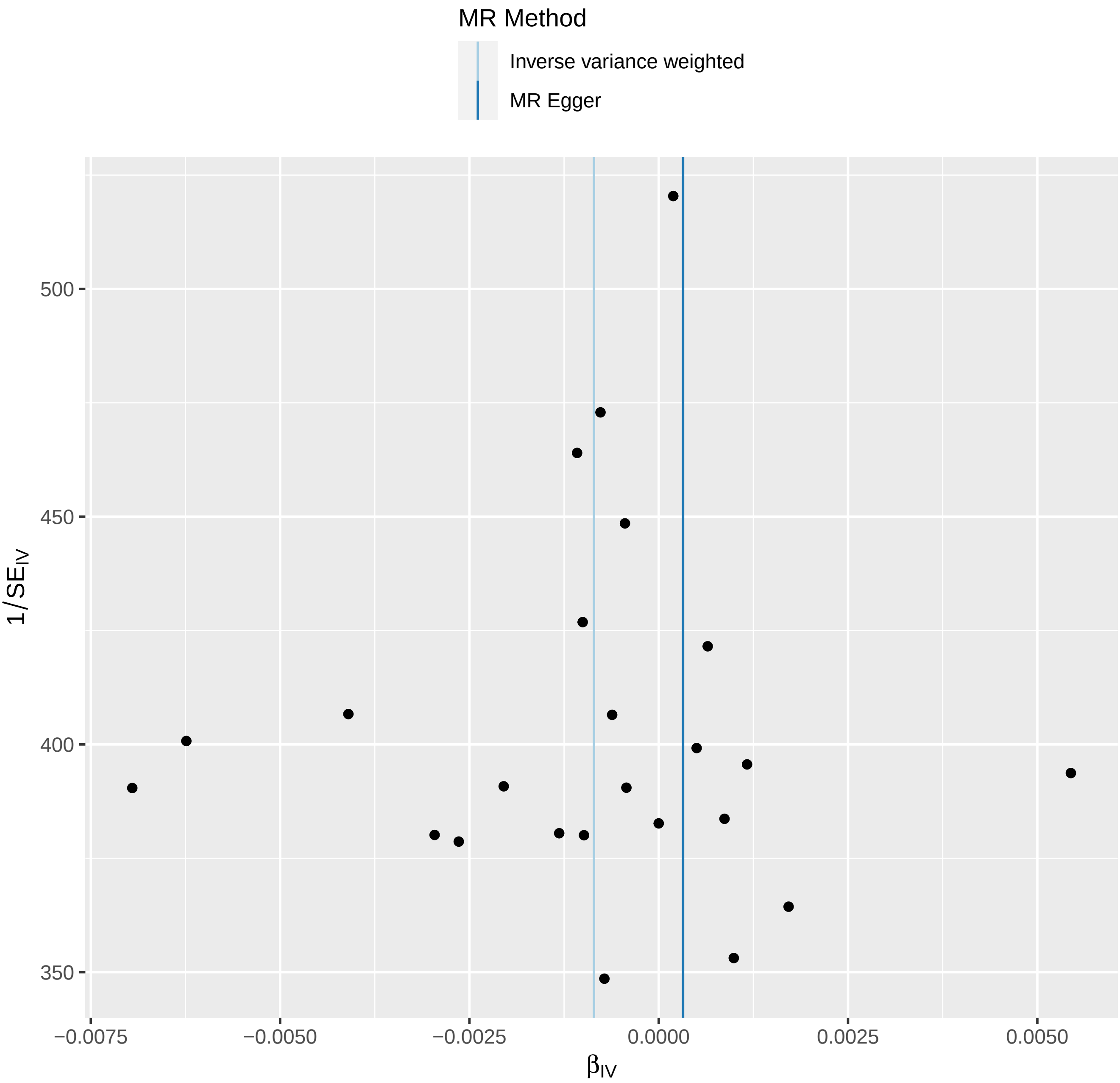

Batch 2−3227 : WAP, kazal, immunoglobulin, kunitz and NTR domain−containing protein 1 on eGFR

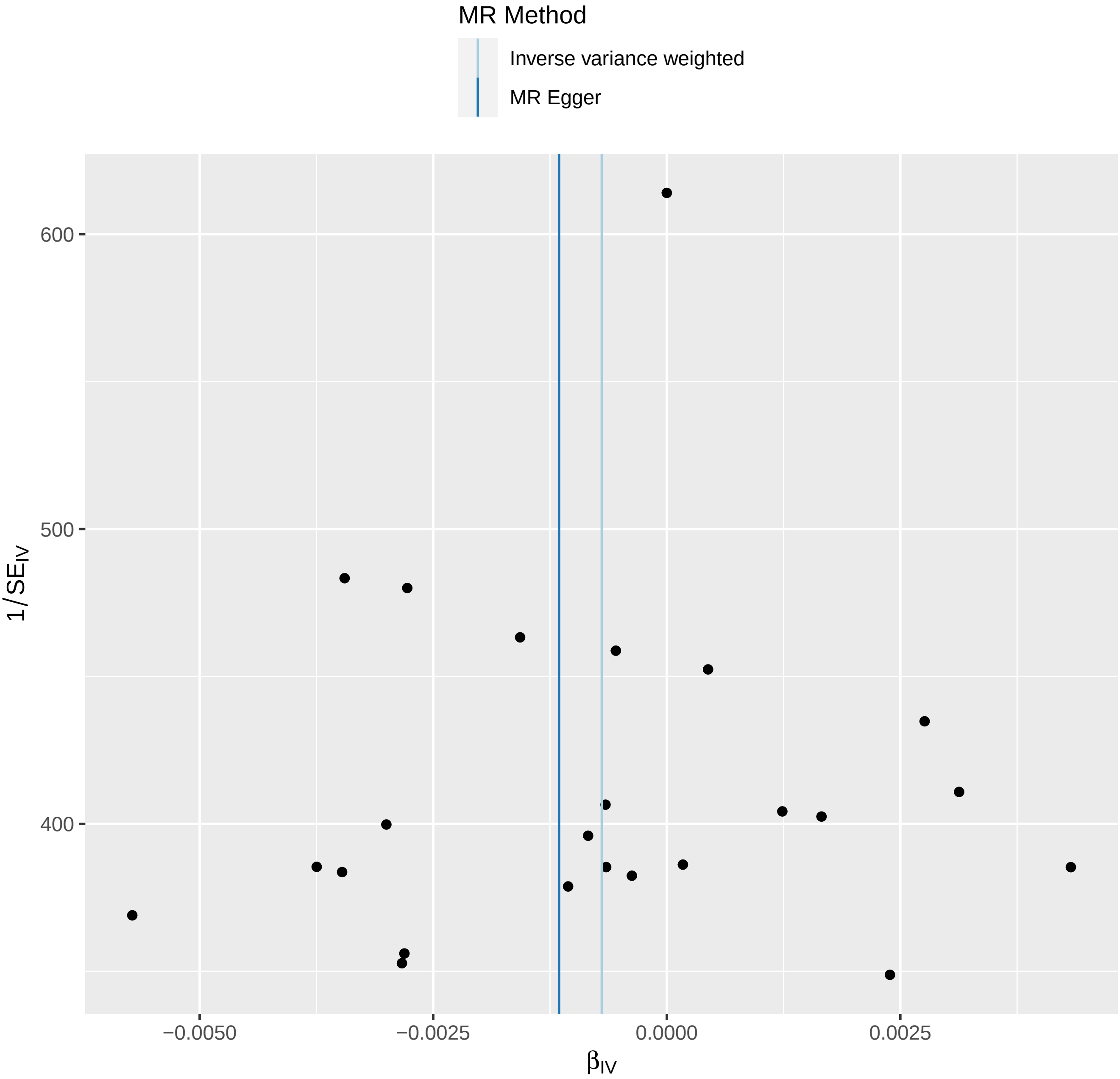

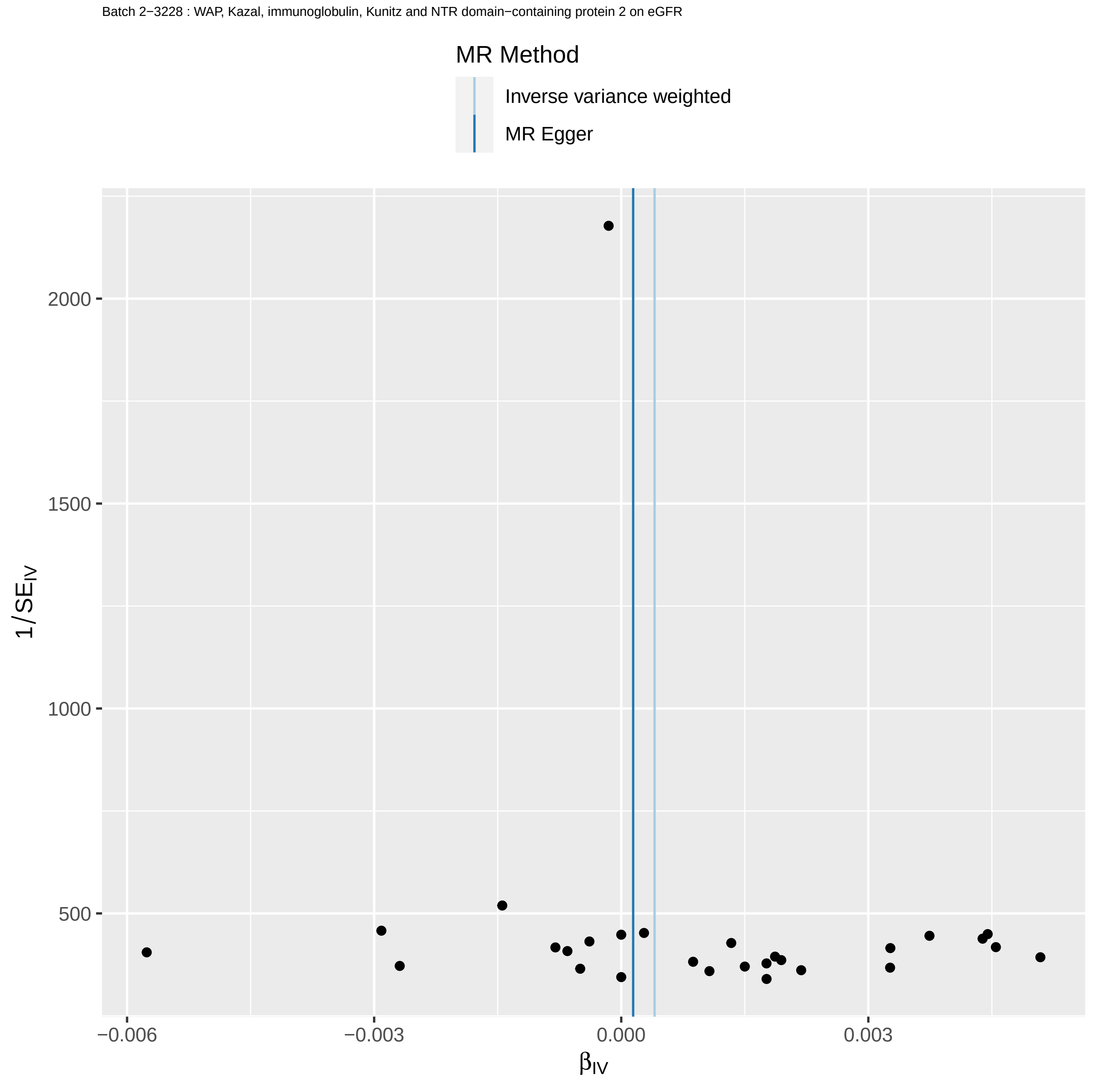

Batch 2−3228 : WAP, Kazal, immunoglobulin, Kunitz and NTR domain−containing protein 2 on eGFR
MR Method
Inverse variance weighted
MR Egger
2000
1500
1000
500
1/SE$_{IV}$
−0.006
−0.003
0.000
0.003
β$_{IV}$

Batch 2−3229 : WAP, Kazal, immunoglobulin, Kunitz and NTR domain−containing protein 2 on eGFR

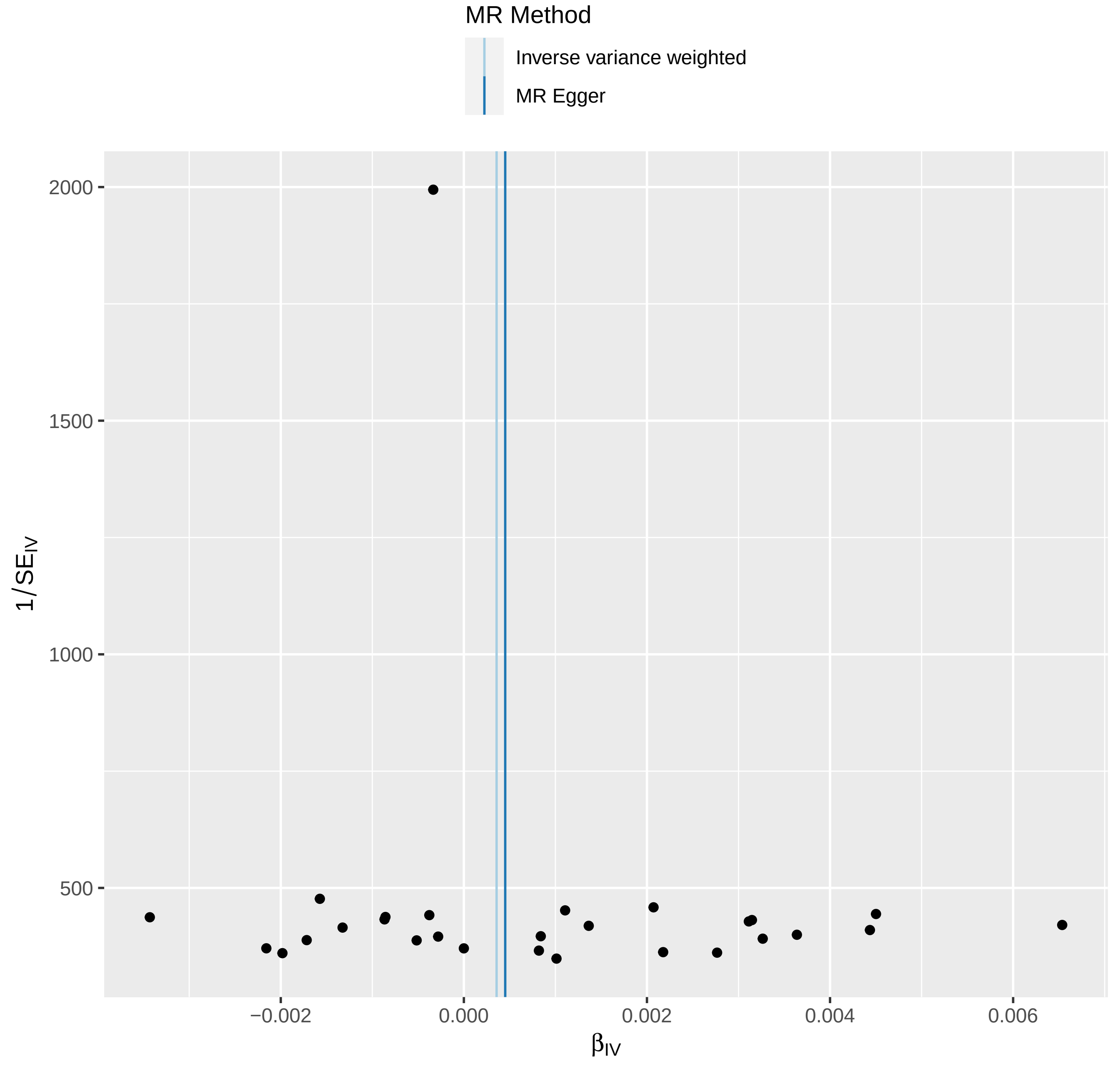

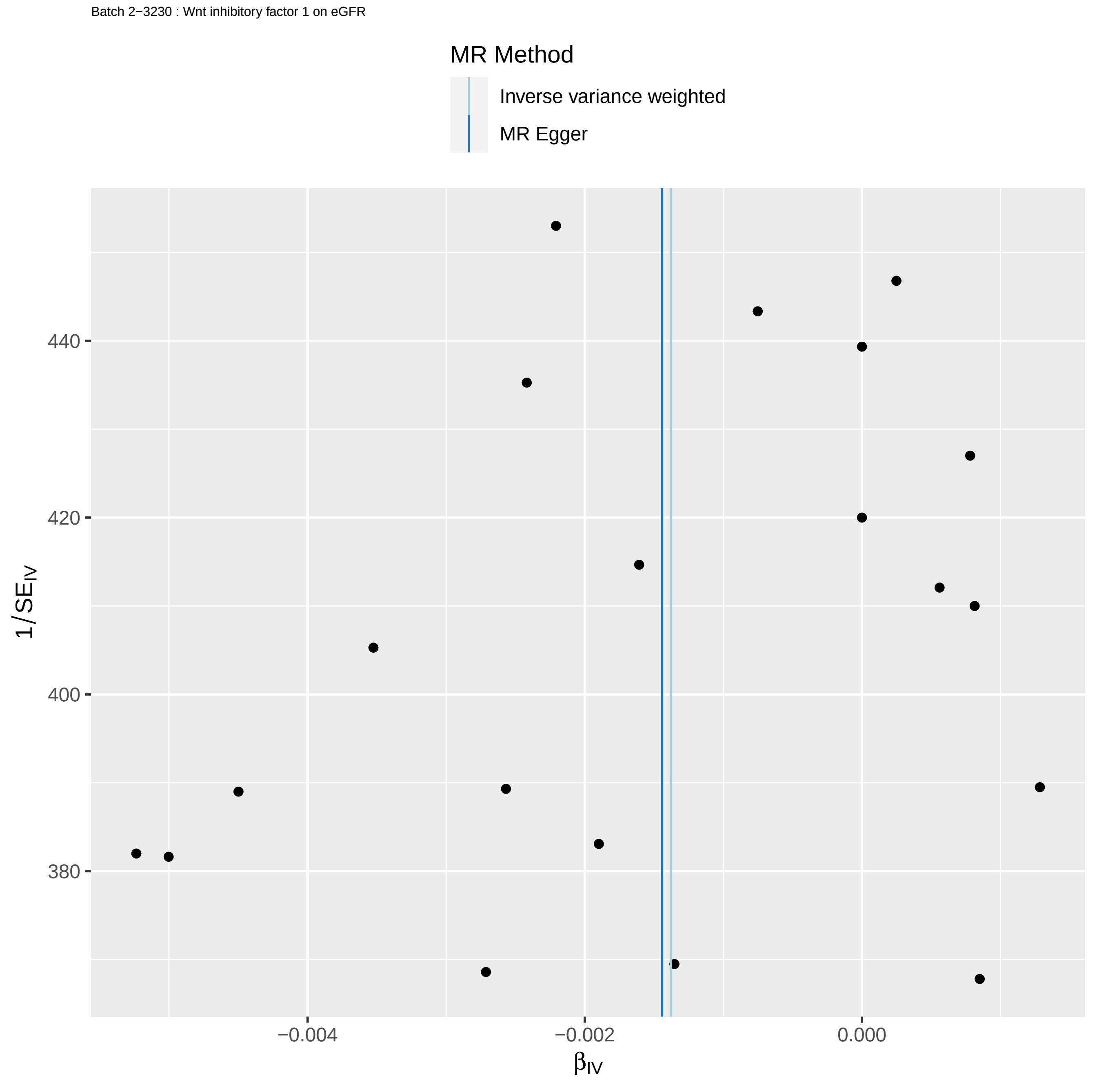
Batch 2−3230 : Wnt inhibitory factor 1 on eGFR
MR Method
Inverse variance weighted
MR Egger
440
420
400
380
1/SE_IV
−0.004
−0.002
0.000
β_IV

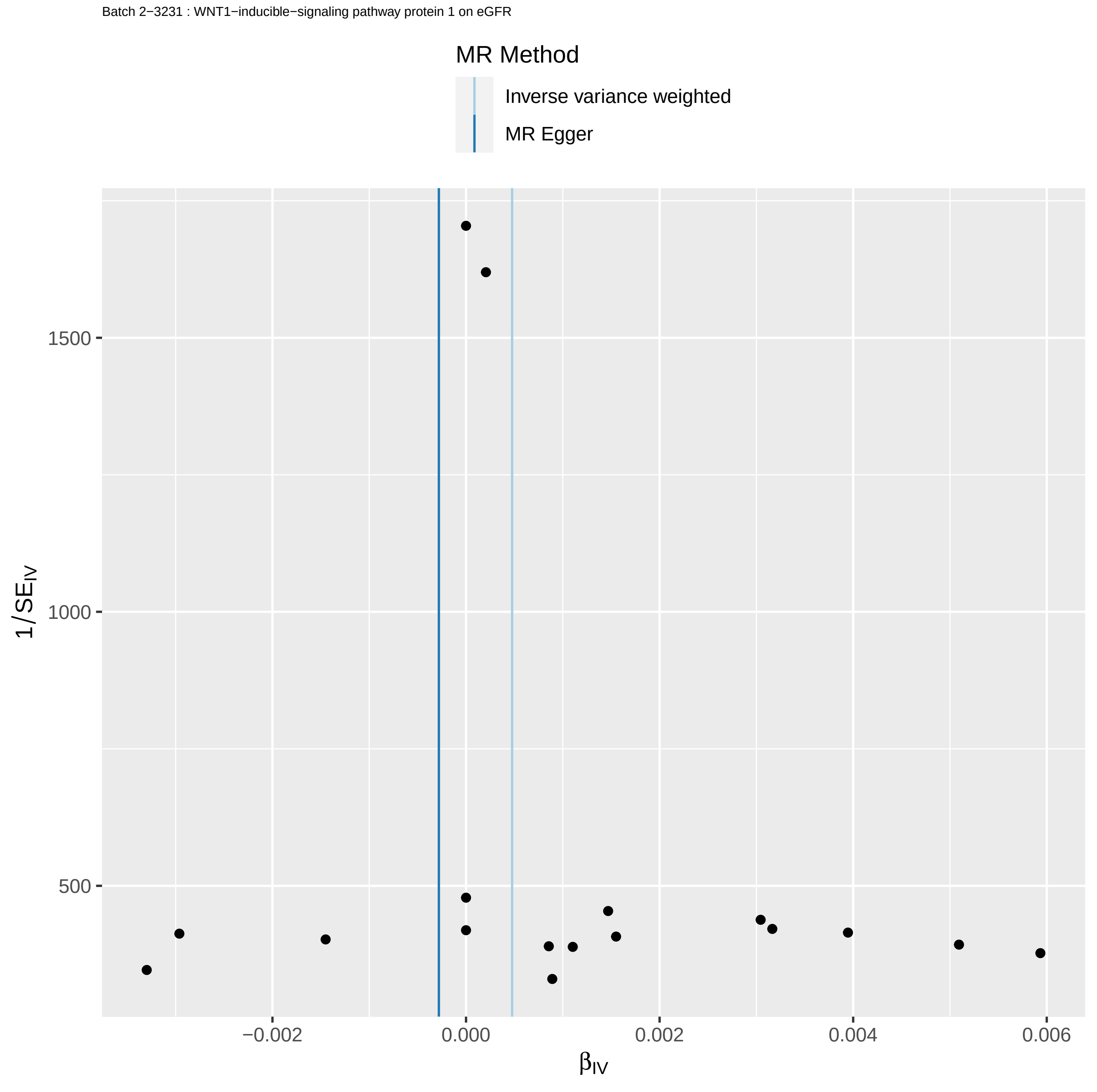
Batch 2−3231 : WNT1−inducible−signaling pathway protein 1 on eGFR
MR Method
Inverse variance weighted
MR Egger
1500
1000
500
1/SE_IV
−0.002
0.000
0.002
0.004
0.006
β_IV

Batch 2−3232 : WNT1−inducible−signaling pathway protein 3 on eGFR

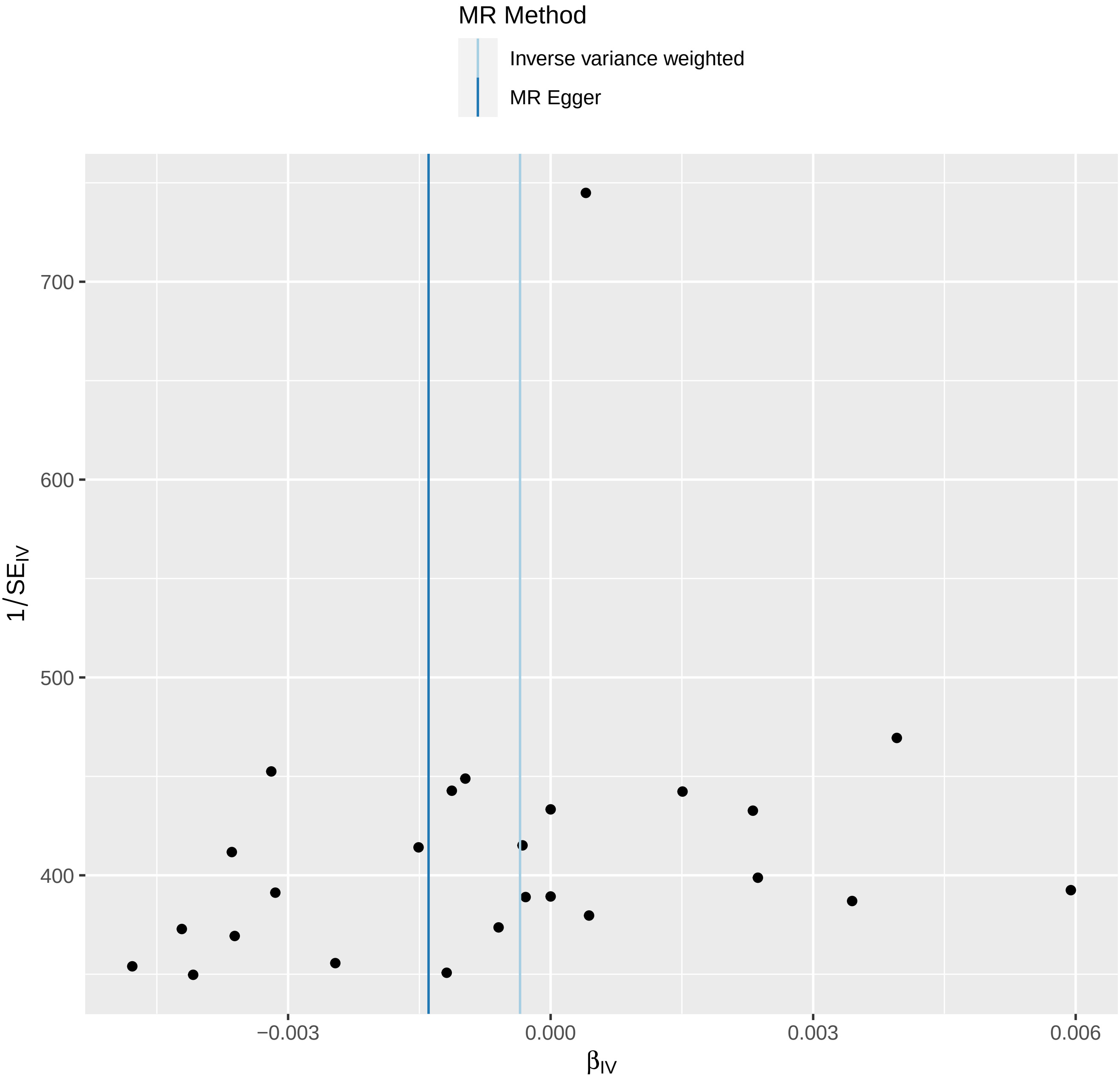

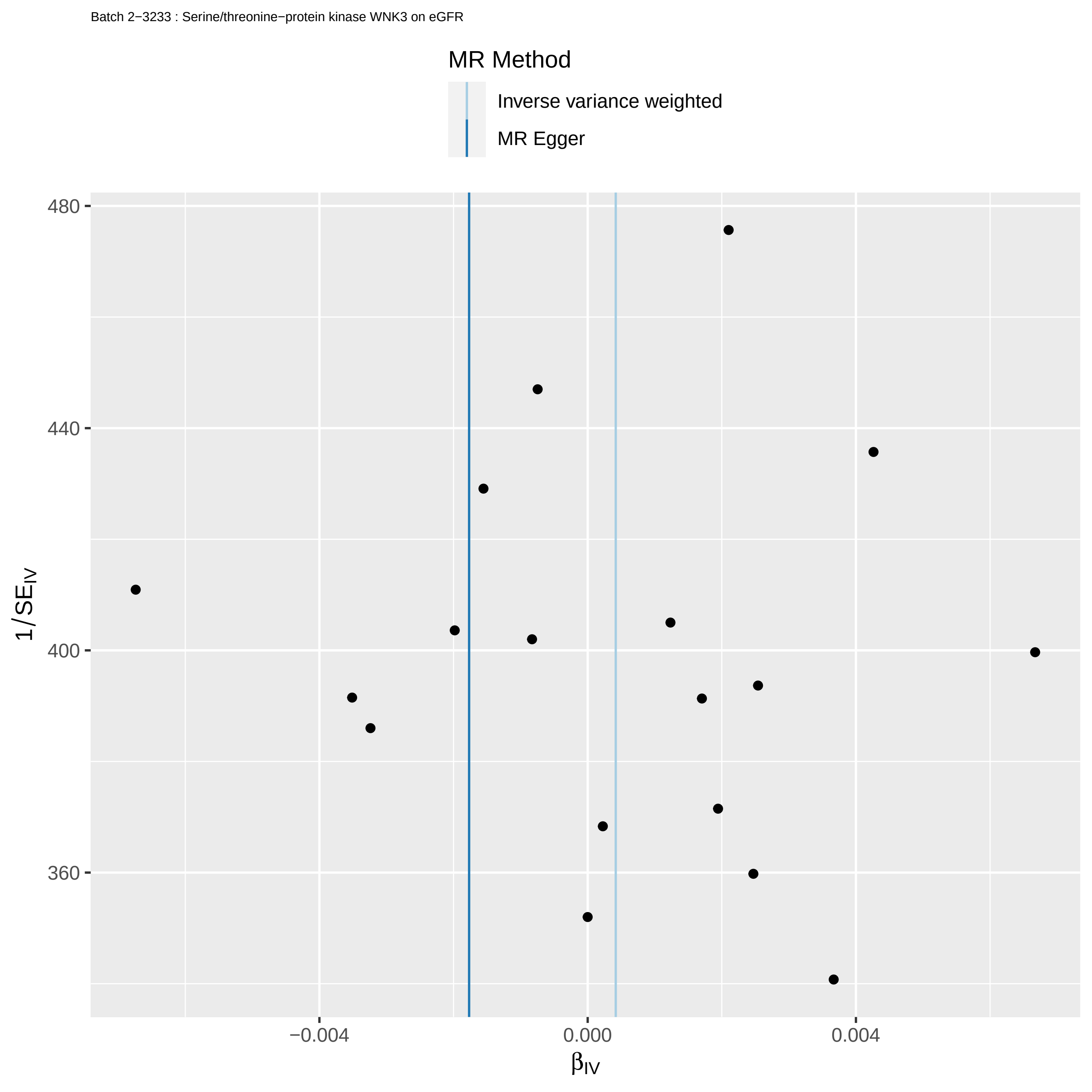
Batch 2−3233 : Serine/threonine−protein kinase WNK3 on eGFR
MR Method
Inverse variance weighted
MR Egger
480
440
400
360
1/SE_IV
−0.004
0.000
0.004
β_IV

Batch 2−3234 : Protein Wnt−7a on eGFR

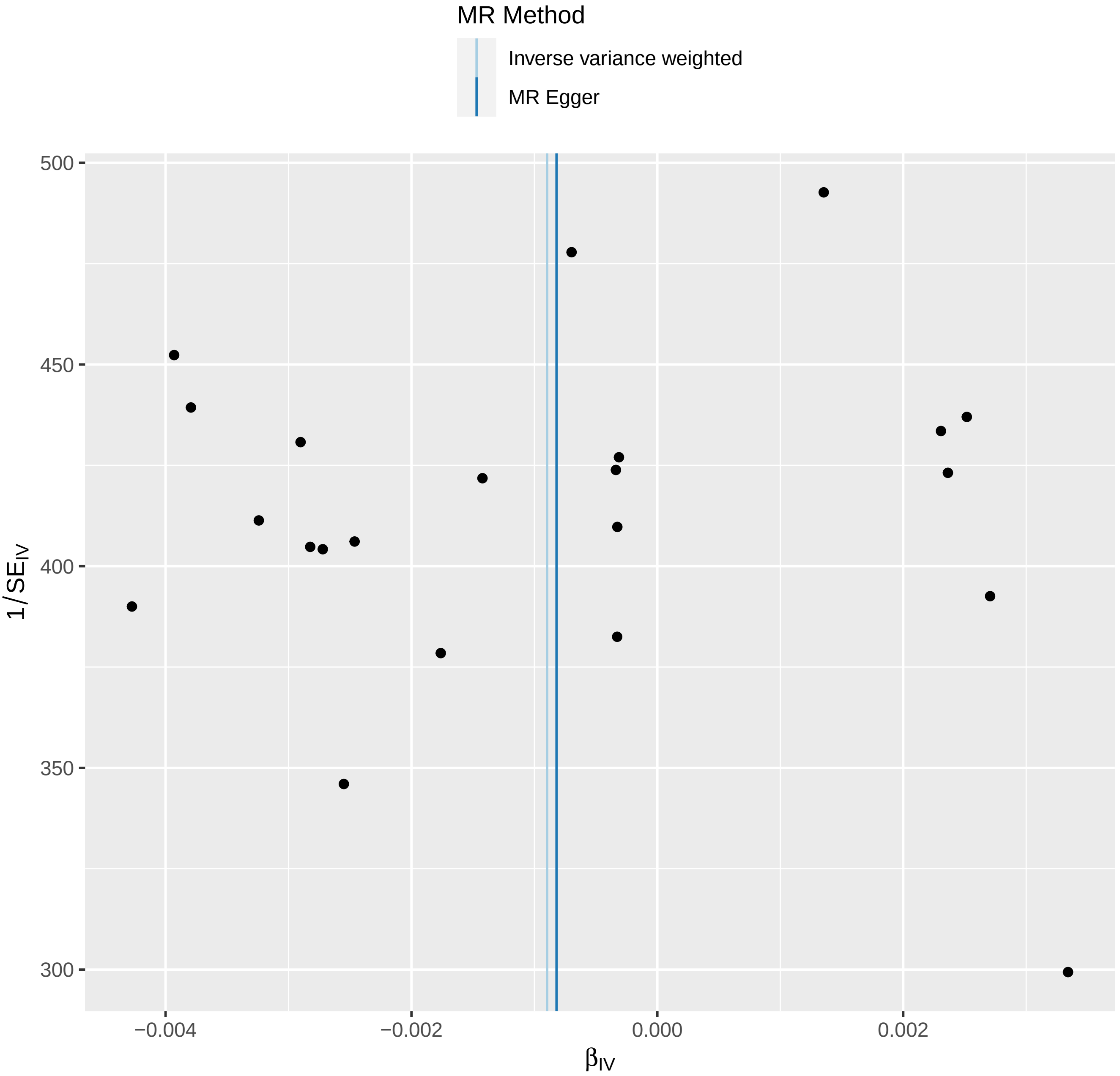

Batch 2−3235 : WSC domain−containing protein 2 on eGFR

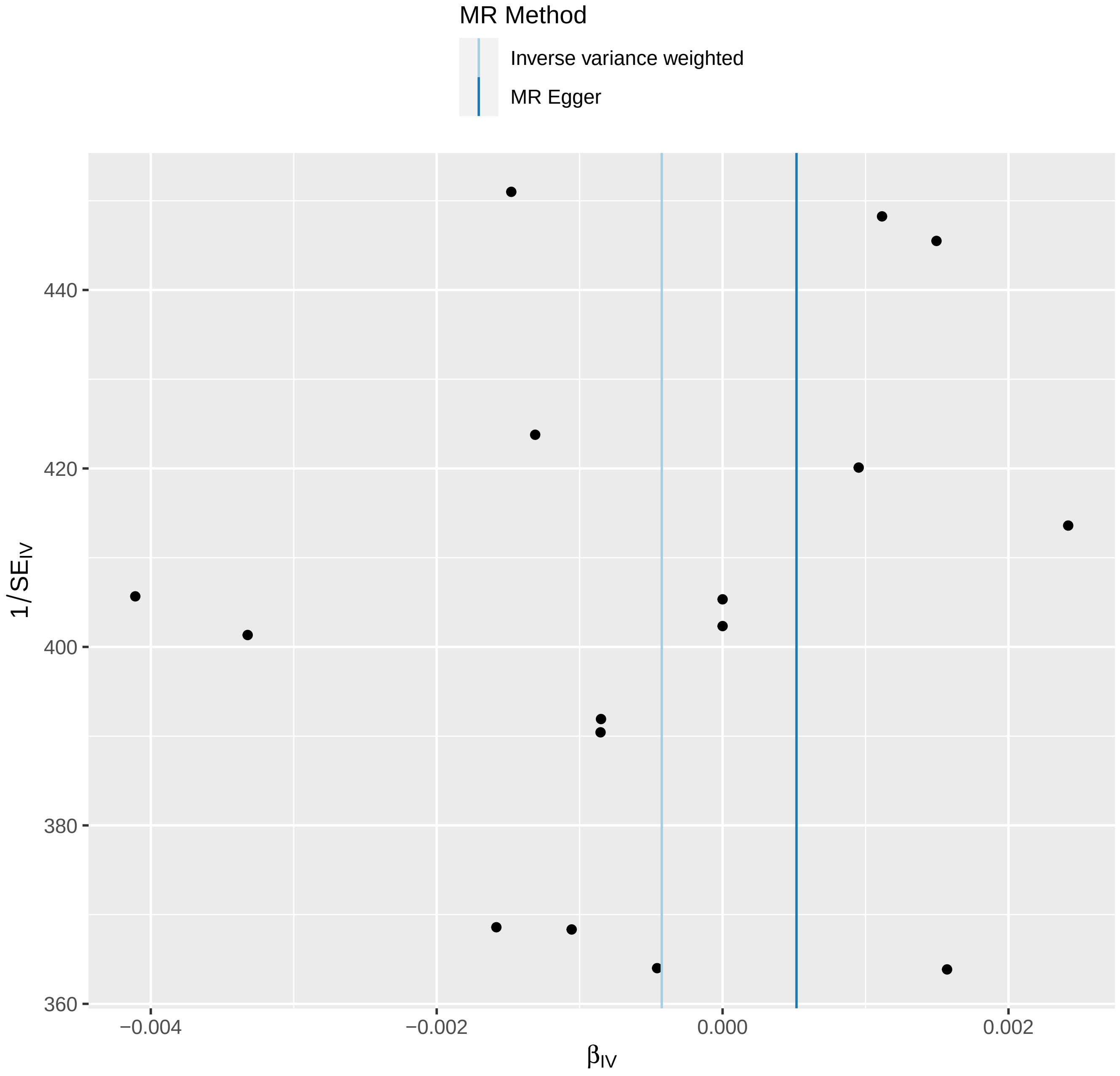

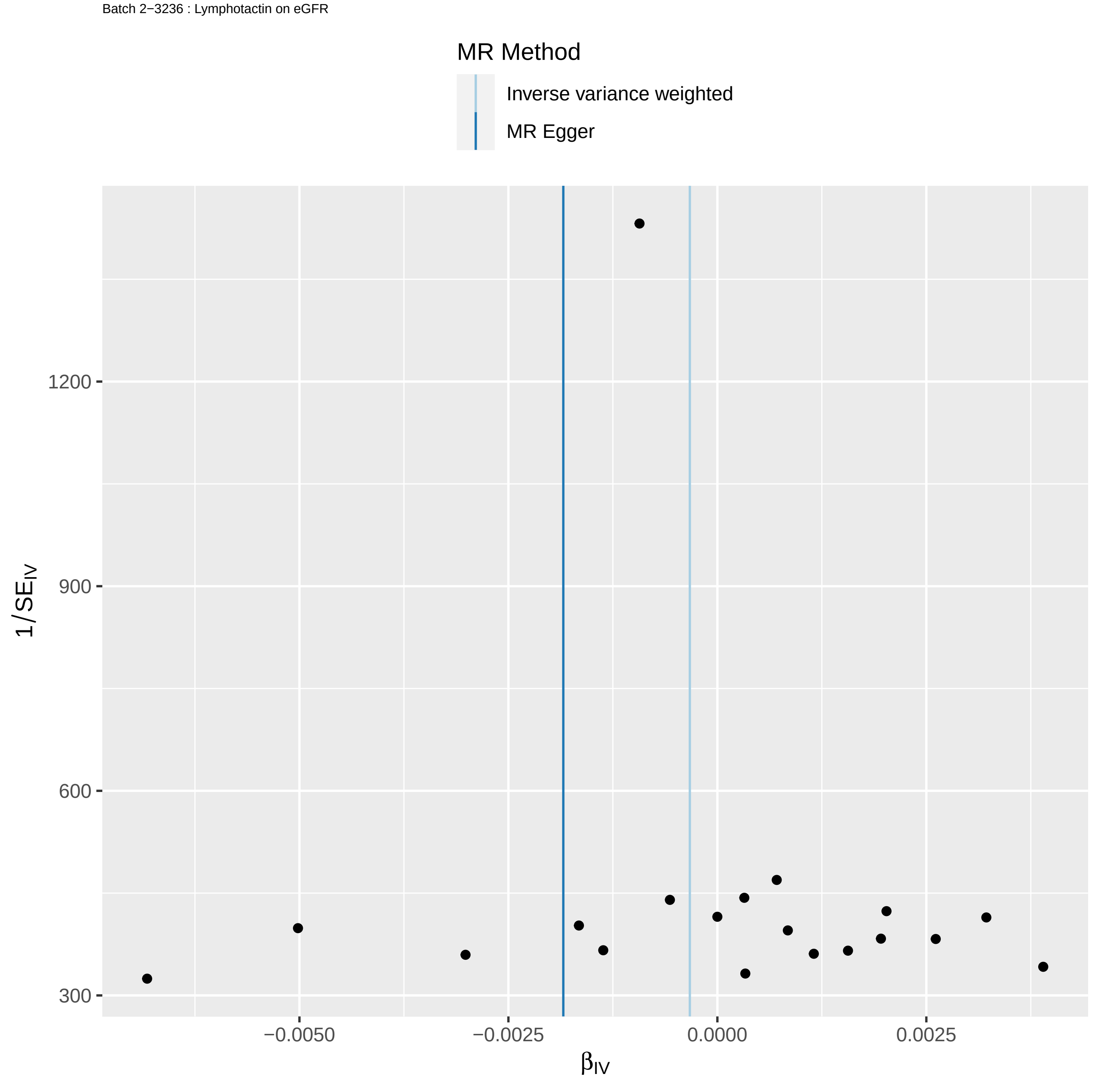

Batch 2−3236 : Lymphotactin on eGFR
MR Method
Inverse variance weighted
MR Egger
1200
900
600
300
1/SE$_{IV}$
−0.0050
−0.0025
0.0000
0.0025
β_{IV}

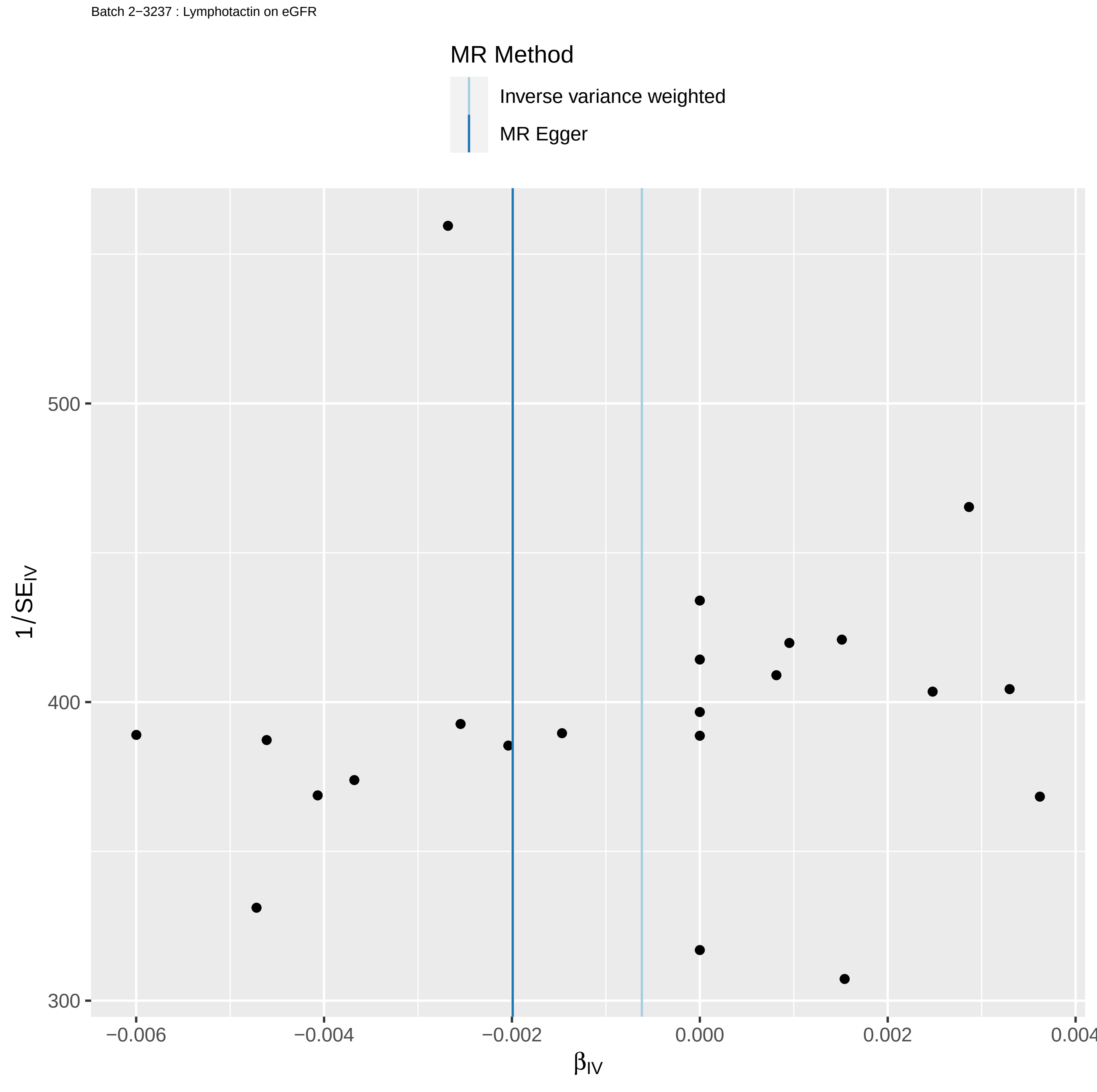
Batch 2−3237 : Lymphotactin on eGFR
MR Method
Inverse variance weighted
MR Egger
500
400
300
1/SE$_{IV}$
−0.006
−0.004
−0.002
0.000
0.002
0.004
β$_{IV}$

Batch 2−3238 : Xanthine dehydrogenase/oxidase on eGFR

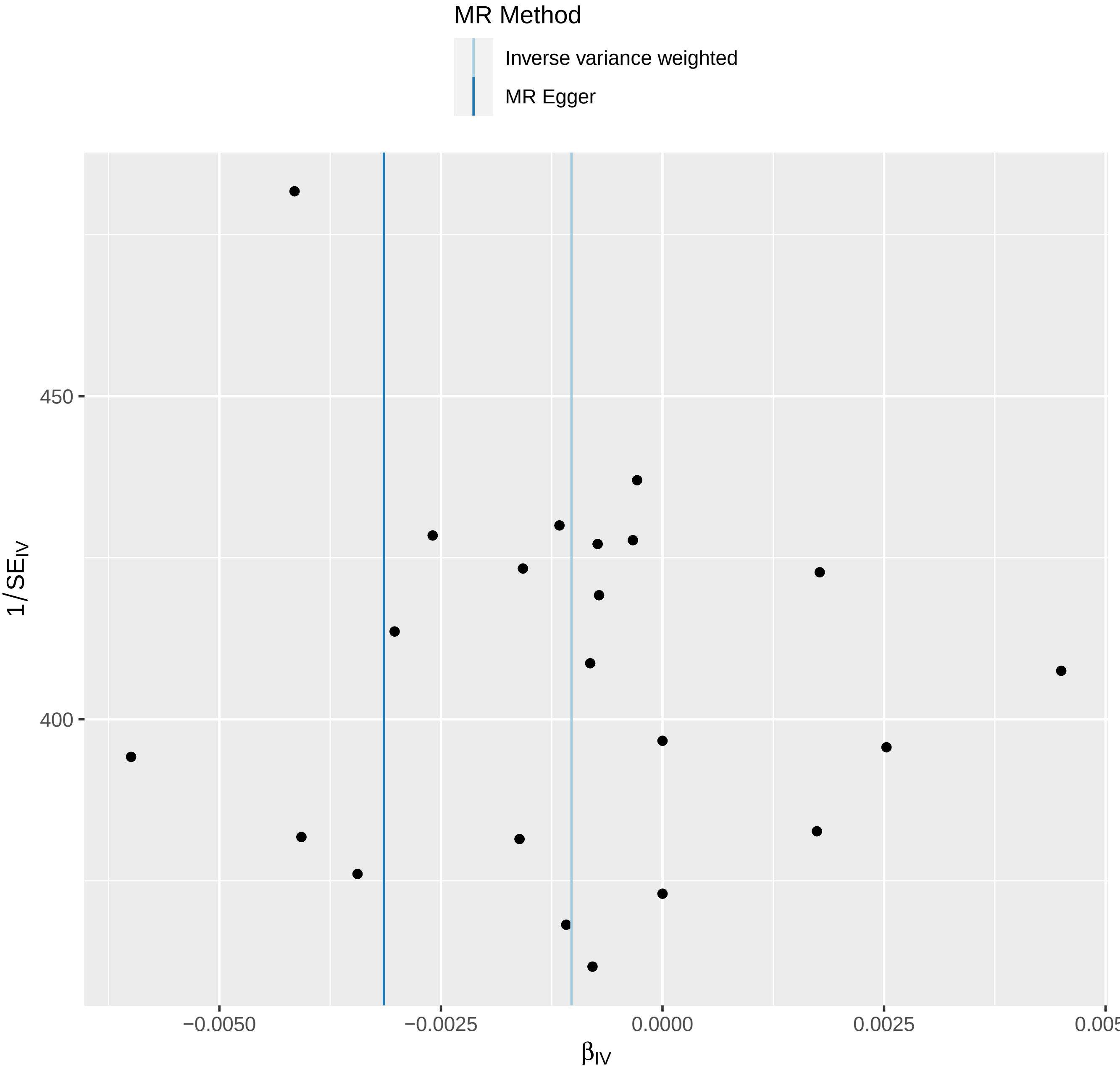

Batch 2−3239 : Glycoprotein Xg on eGFR

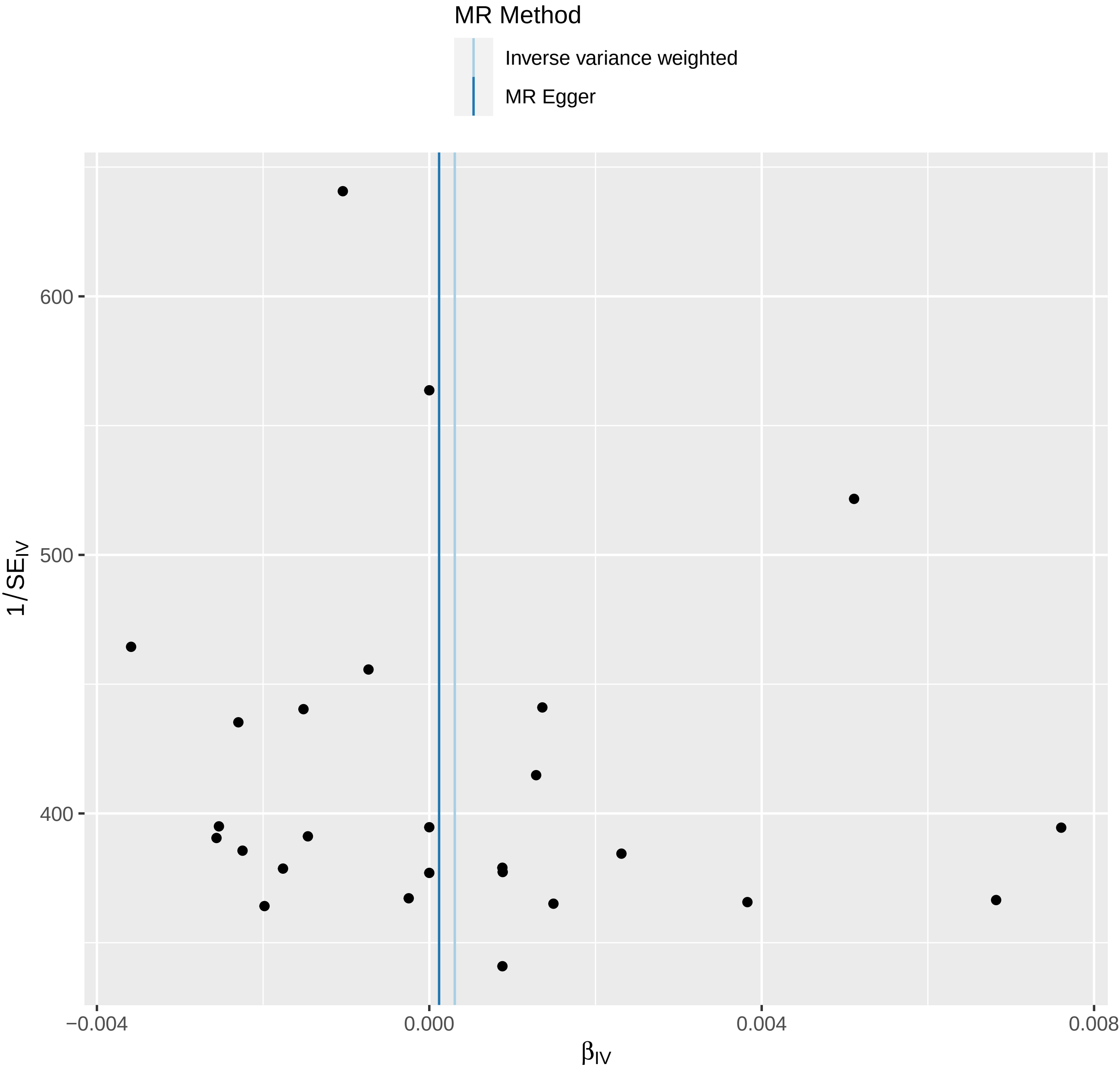

Batch 2−3240 : Xaa−Pro aminopeptidase 1 on eGFR

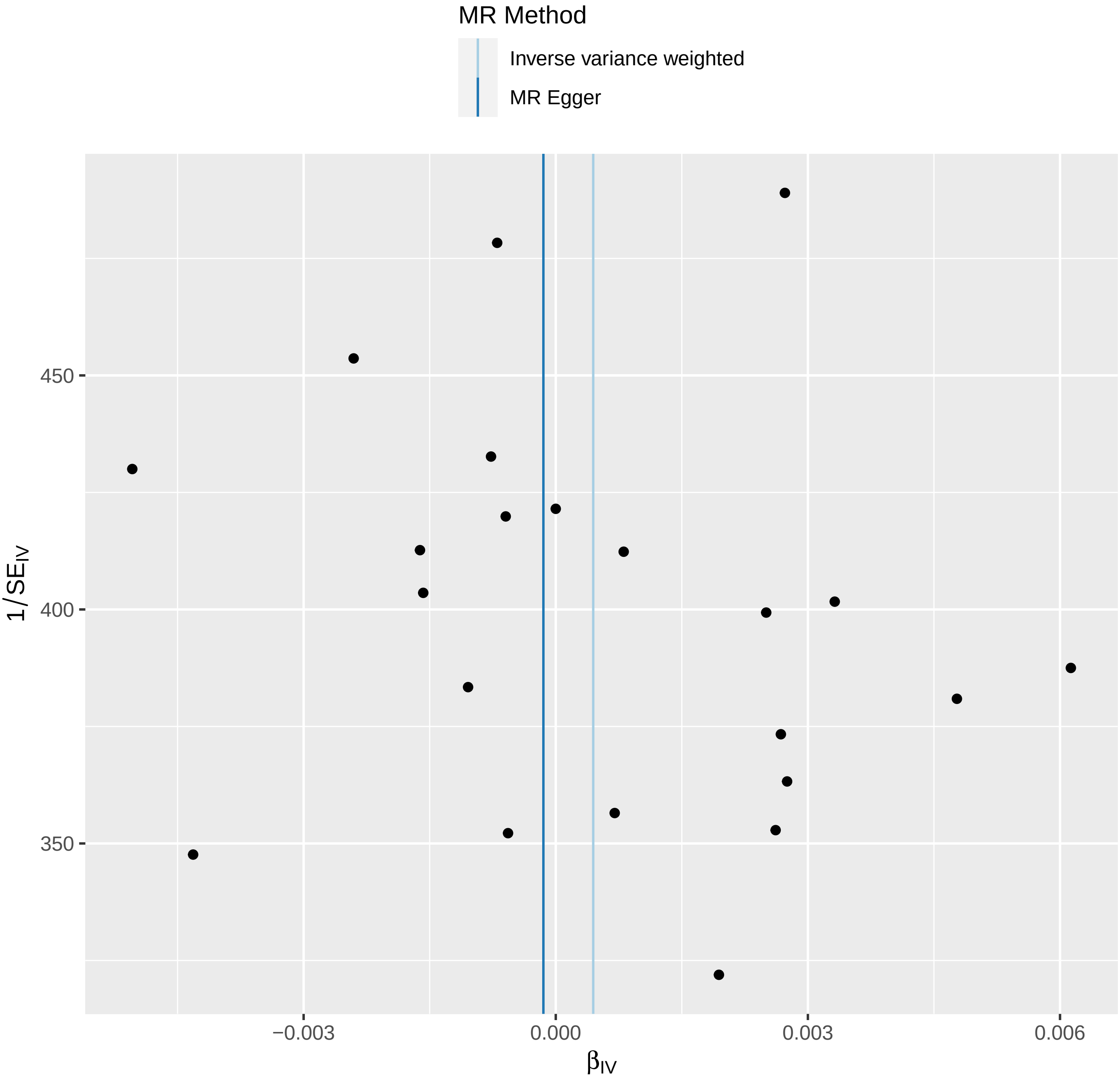

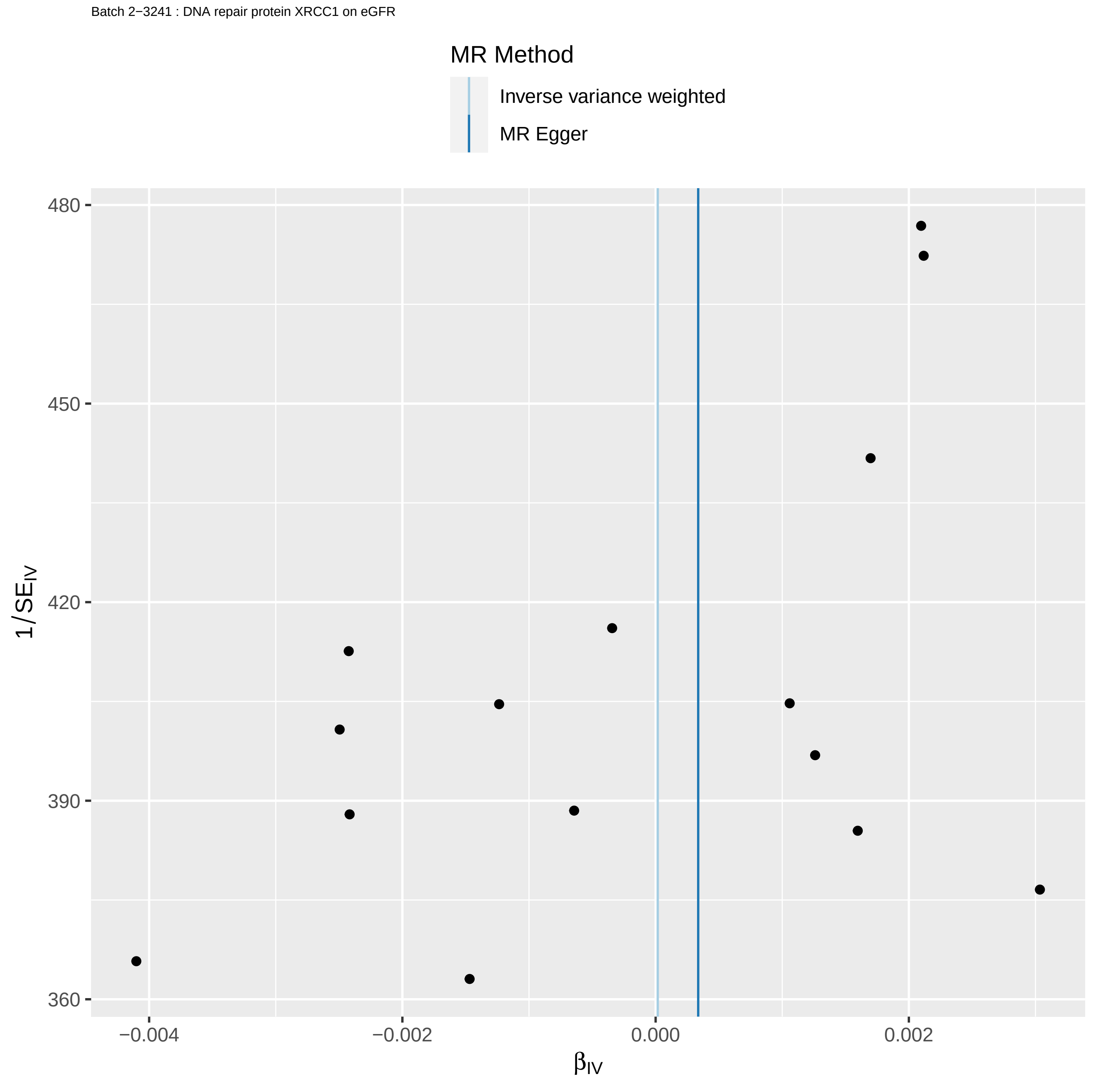

Batch 2−3241 : DNA repair protein XRCC1 on eGFR
MR Method
Inverse variance weighted
MR Egger
480
450
420
390
360
1/SE_IV
−0.004
−0.002
0.000
0.002
β_IV

Batch 2−3242 : DNA repair protein XRCC4 on eGFR

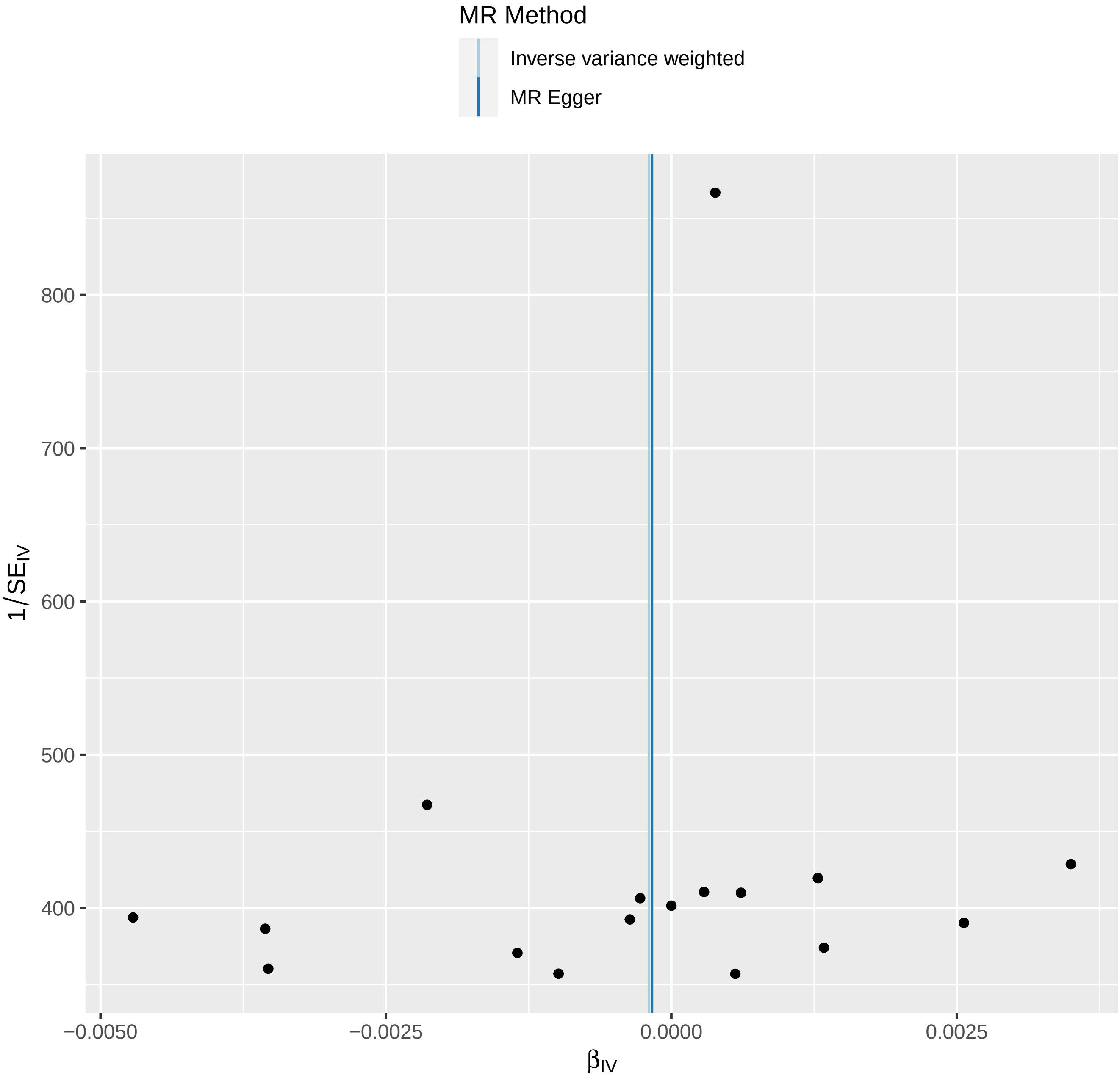

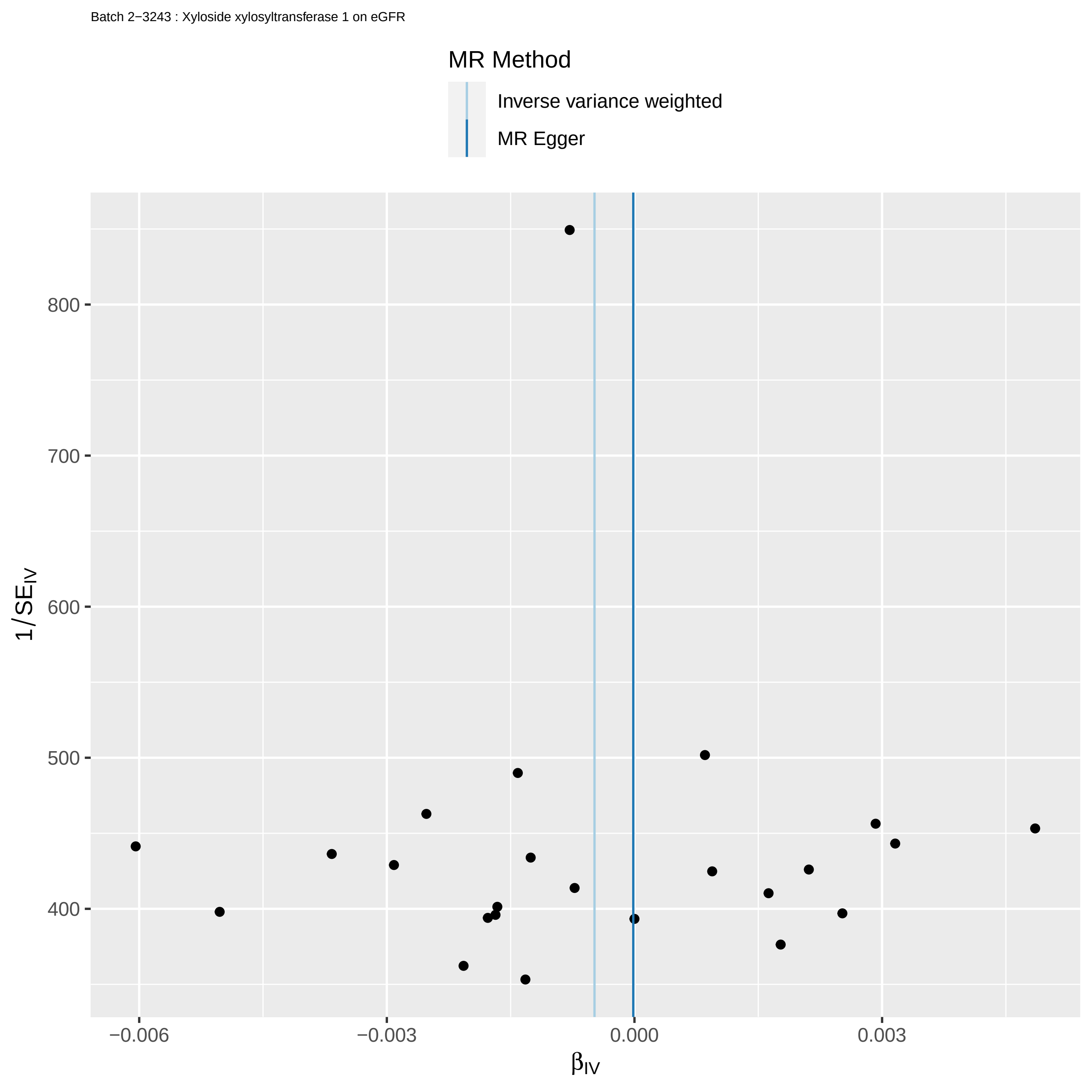

Batch 2−3243 : Xyloside xylosyltransferase 1 on eGFR
MR Method
Inverse variance weighted
MR Egger
800
700
600
500
400
1/SE_IV
−0.006
−0.003
0.000
0.003
β_IV

Batch 2−3244 : Y−box−binding protein 2 on eGFR

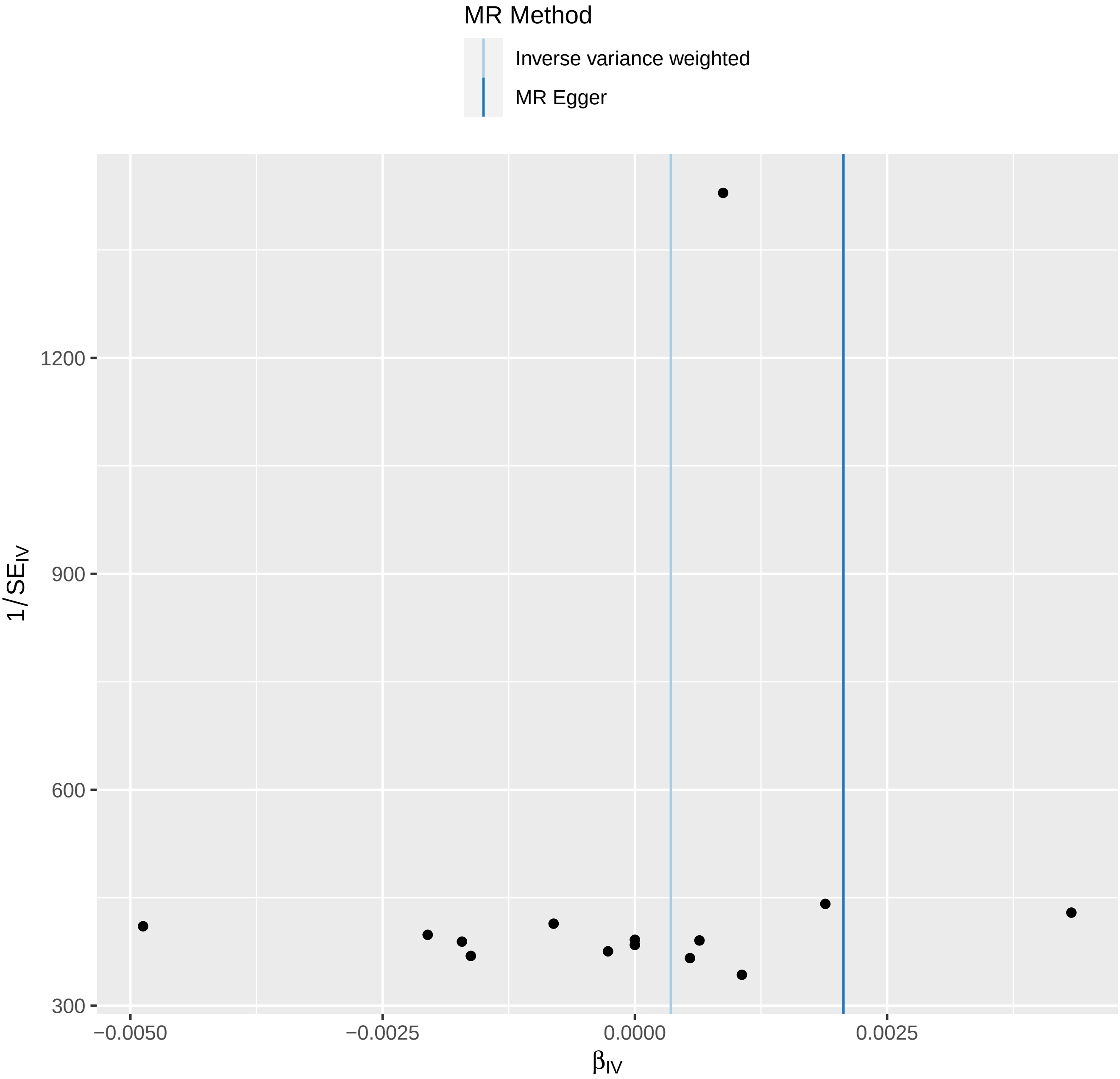

Batch 2−3245 : Tyrosine−protein kinase Yes on eGFR

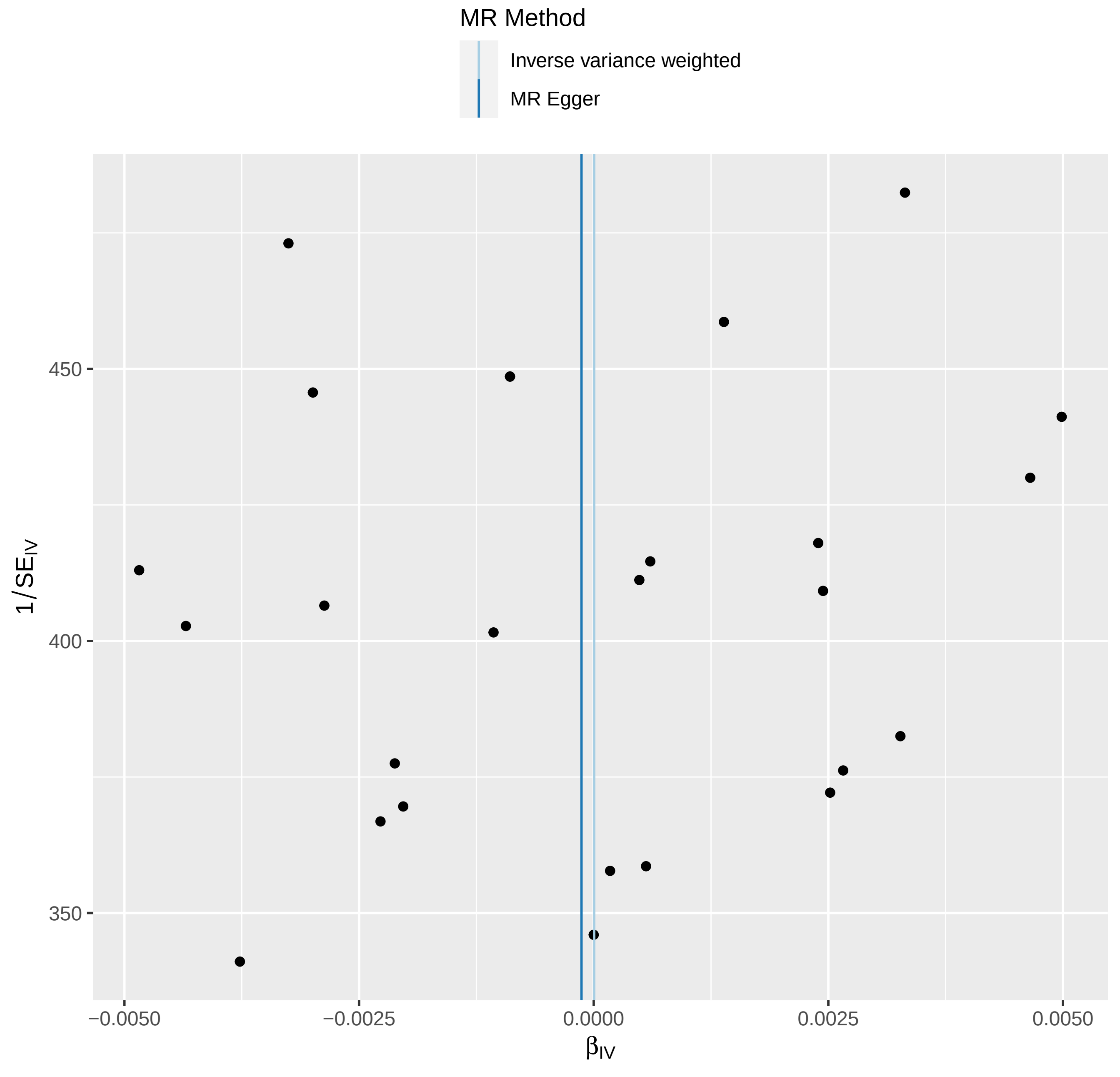

Batch 2−3246 : Protein YIPF6 on eGFR

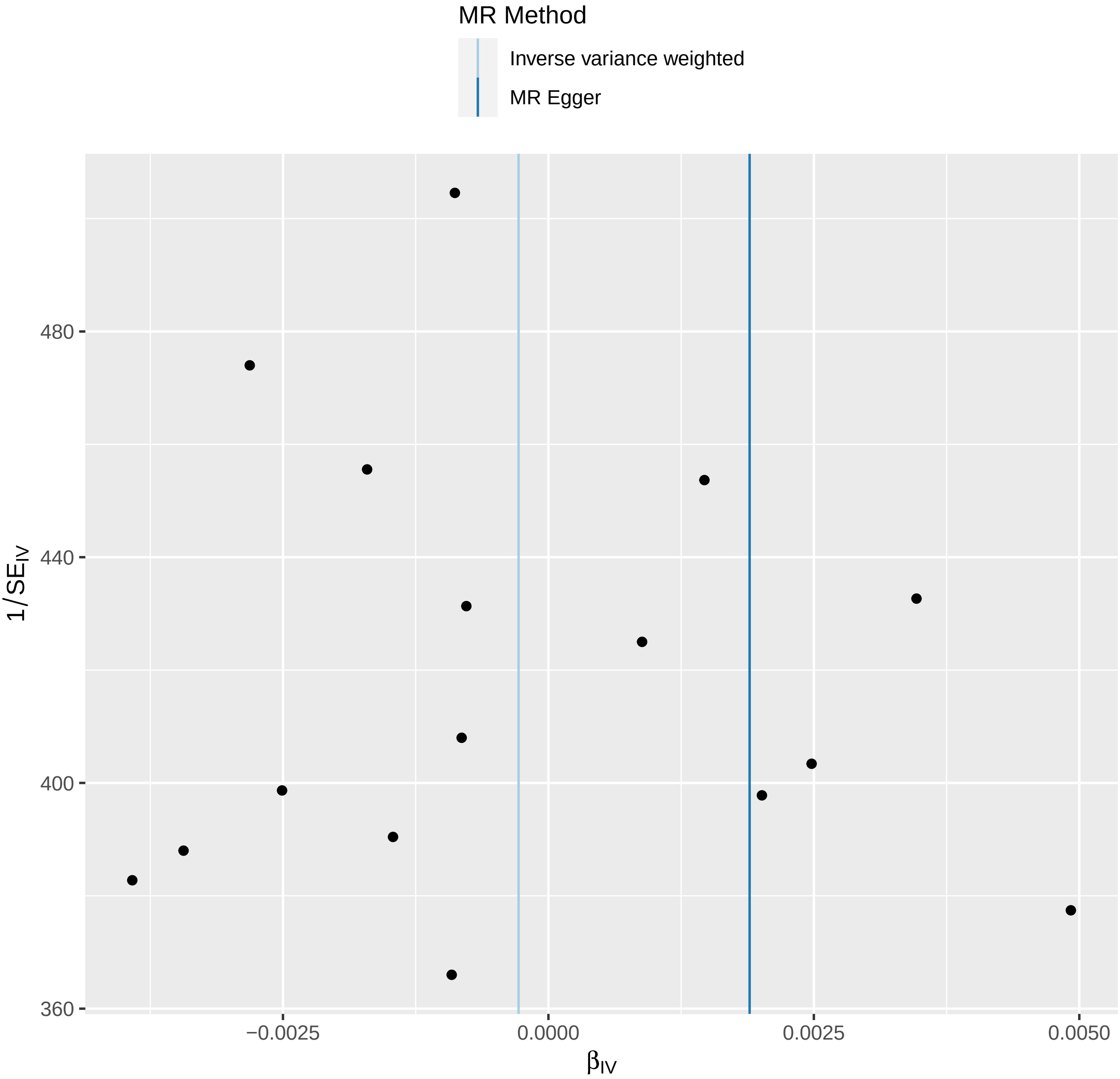

Batch 2−3247 : YTH domain−containing protein 1 on eGFR

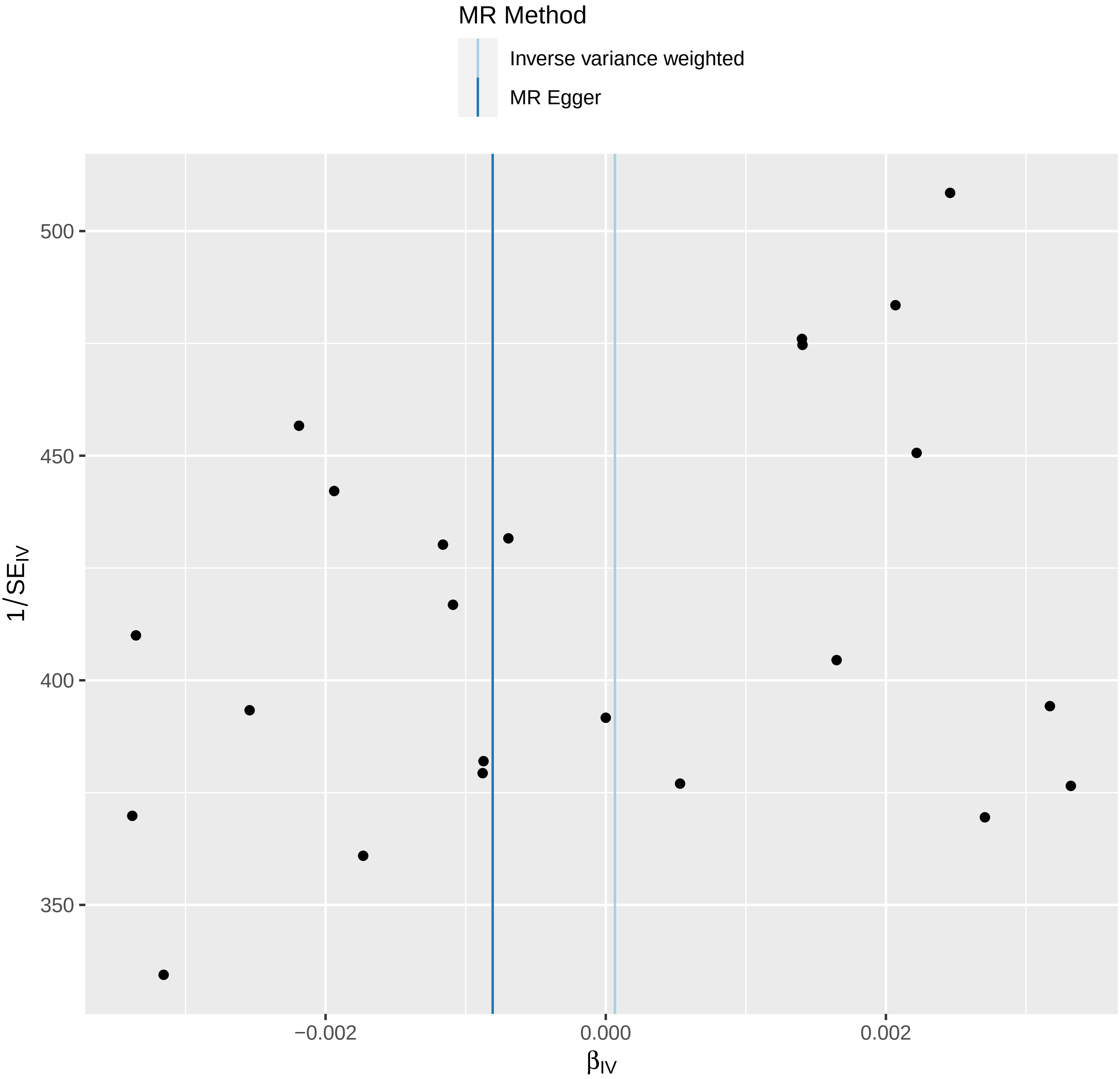

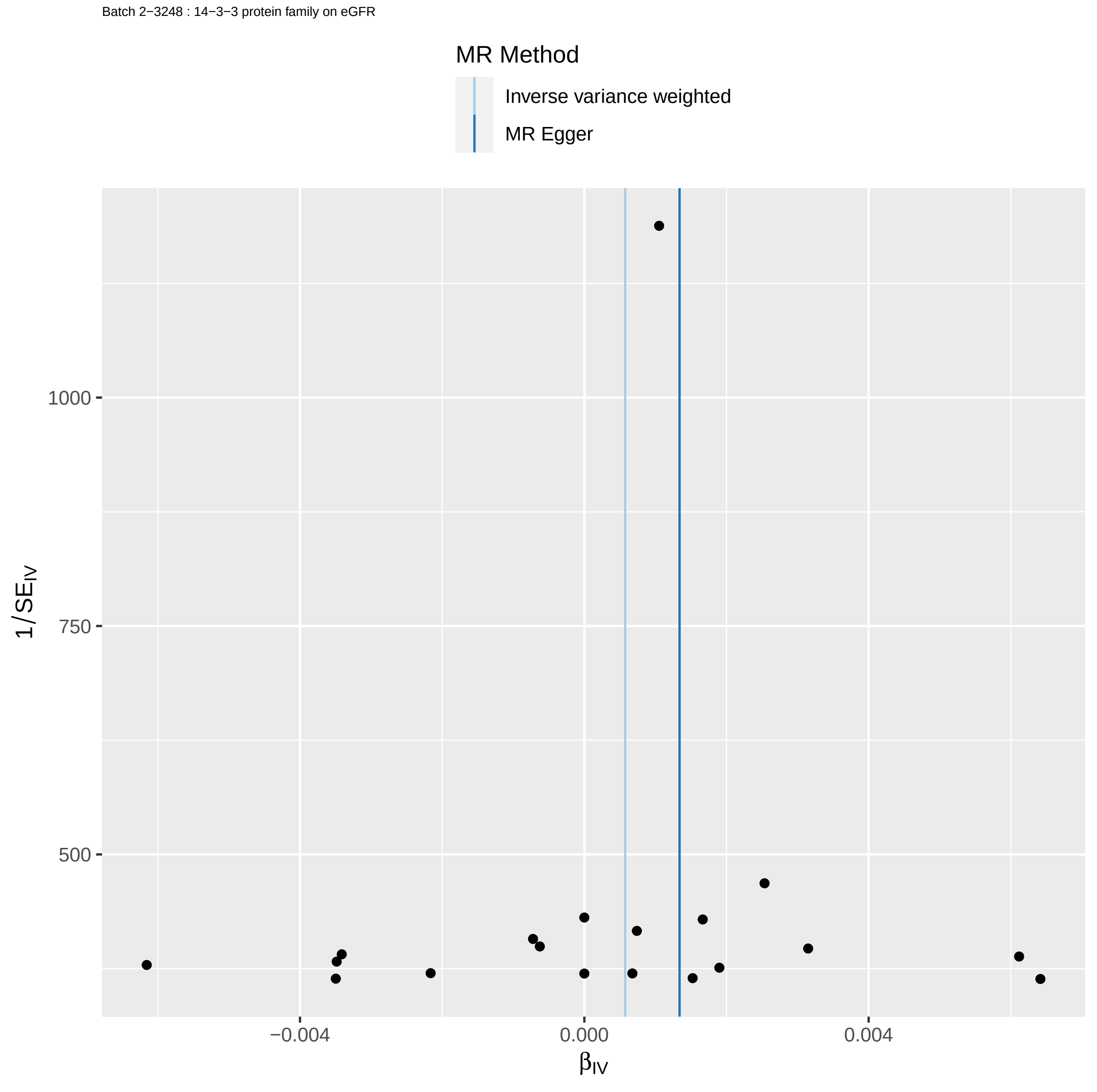

Batch 2−3248 : 14−3−3 protein family on eGFR
MR Method
Inverse variance weighted
MR Egger
1000
750
500
1/SE_IV
−0.004
0.000
0.004
β_IV

Batch 2−3249 : 14−3−3 protein epsilon on eGFR

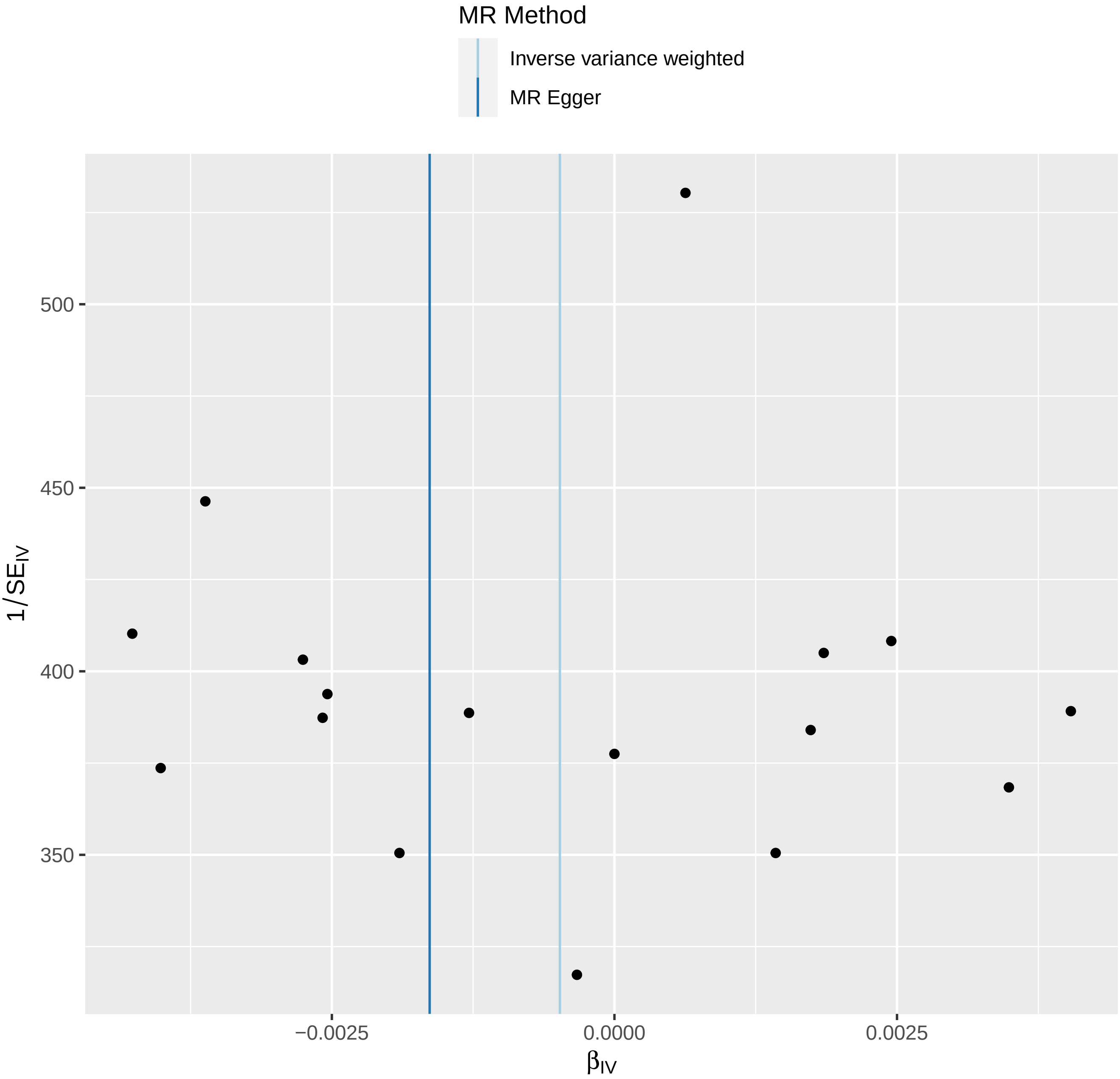

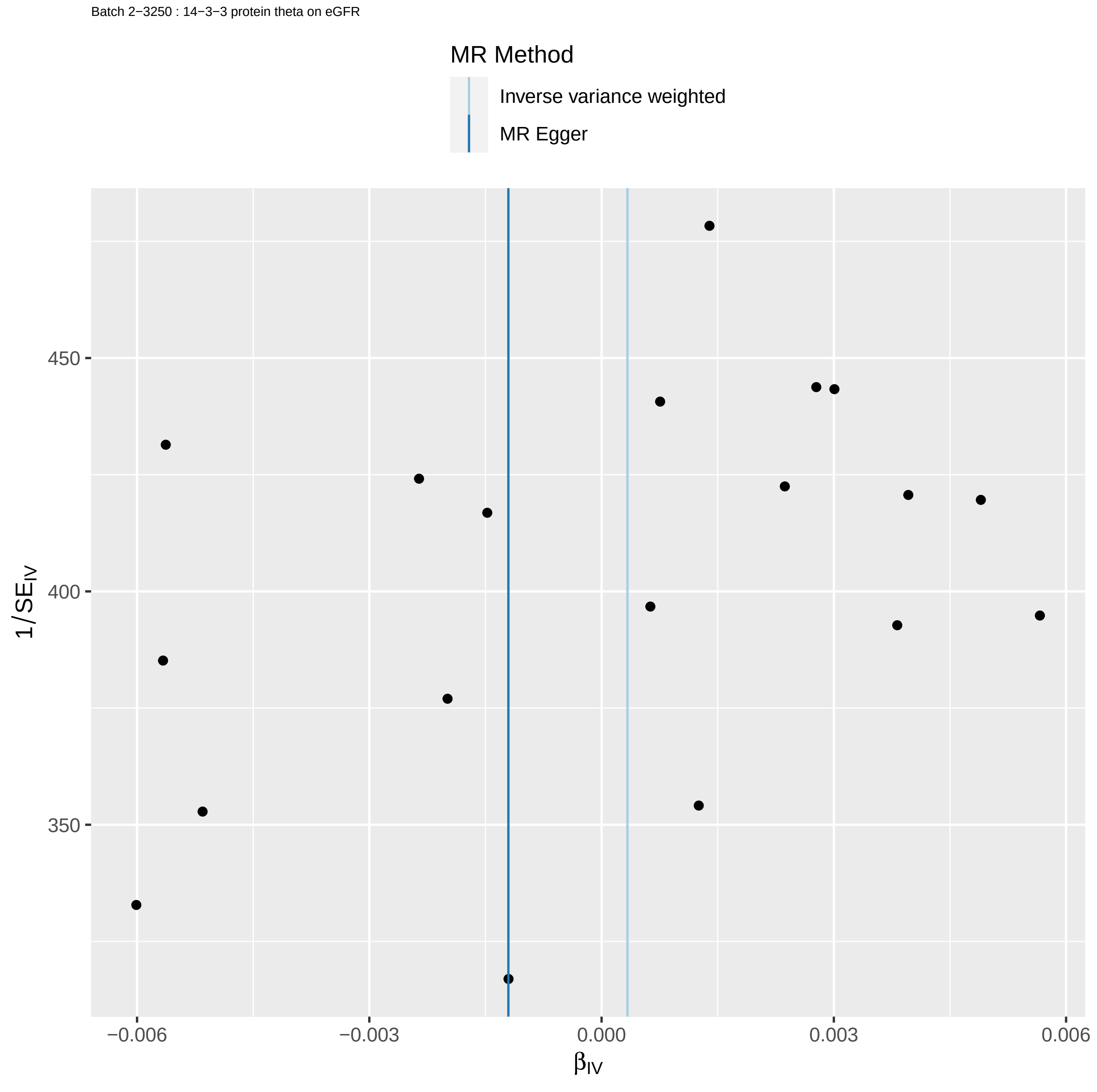

Batch 2−3250 : 14−3−3 protein theta on eGFR
MR Method
Inverse variance weighted
MR Egger
450
400
350
1/SE$_{IV}$
−0.006
−0.003
0.000
0.003
0.006
β$_{IV}$

Batch 2−3251 : 14−3−3 protein zeta/delta on eGFR

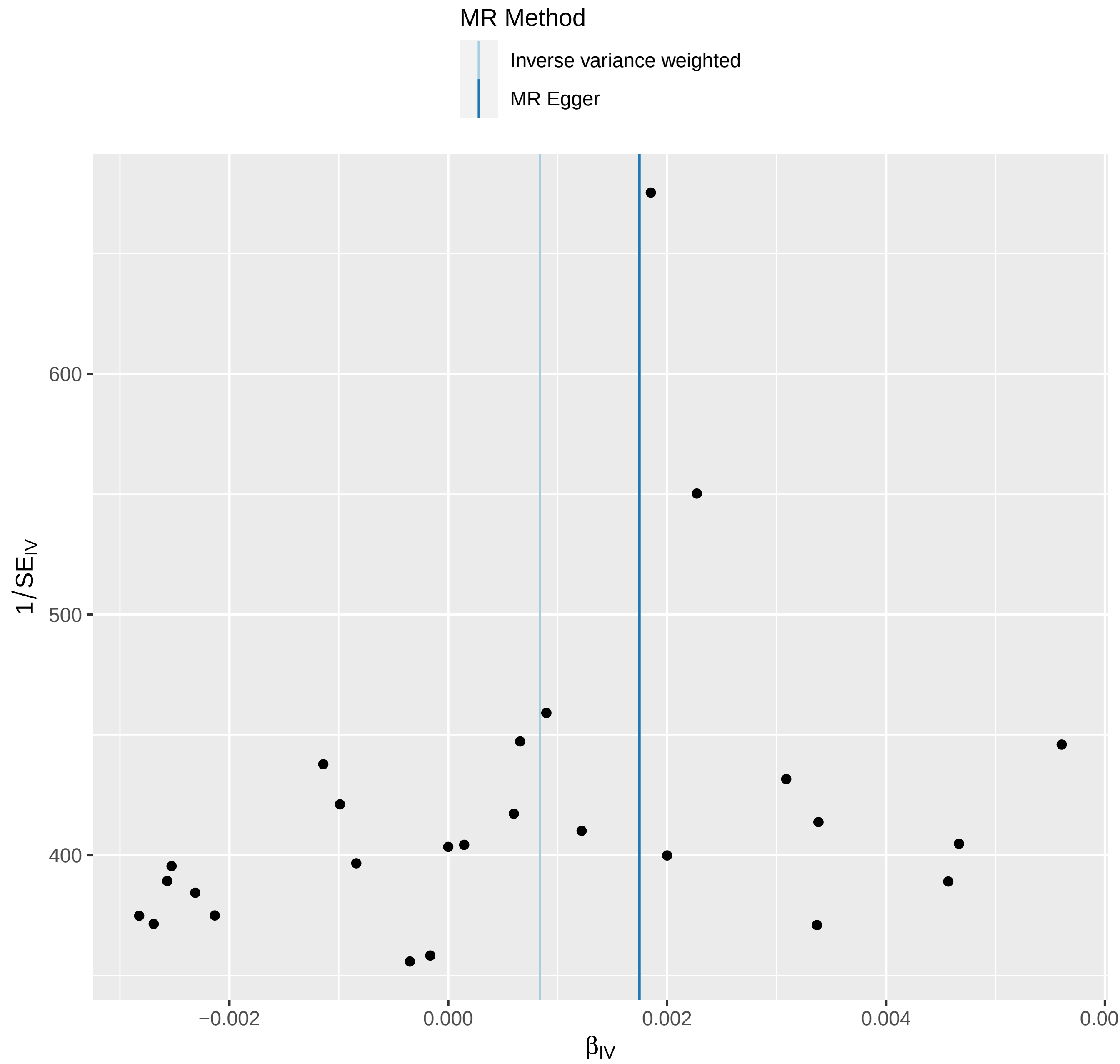

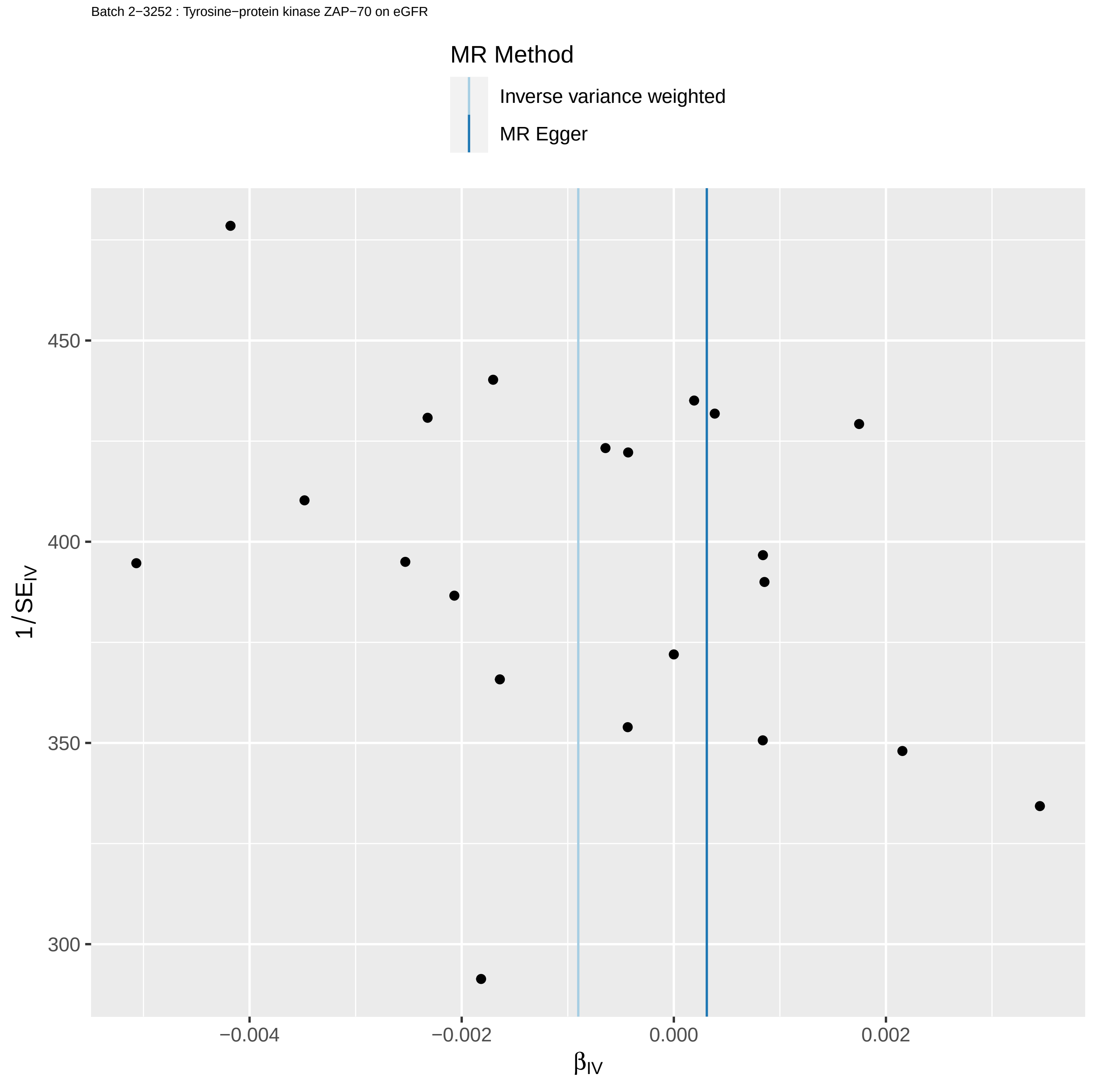
Batch 2−3252 : Tyrosine−protein kinase ZAP−70 on eGFR
MR Method
Inverse variance weighted
MR Egger
450
400
350
300
1/SE_IV
−0.004
−0.002
0.000
0.002
β_IV

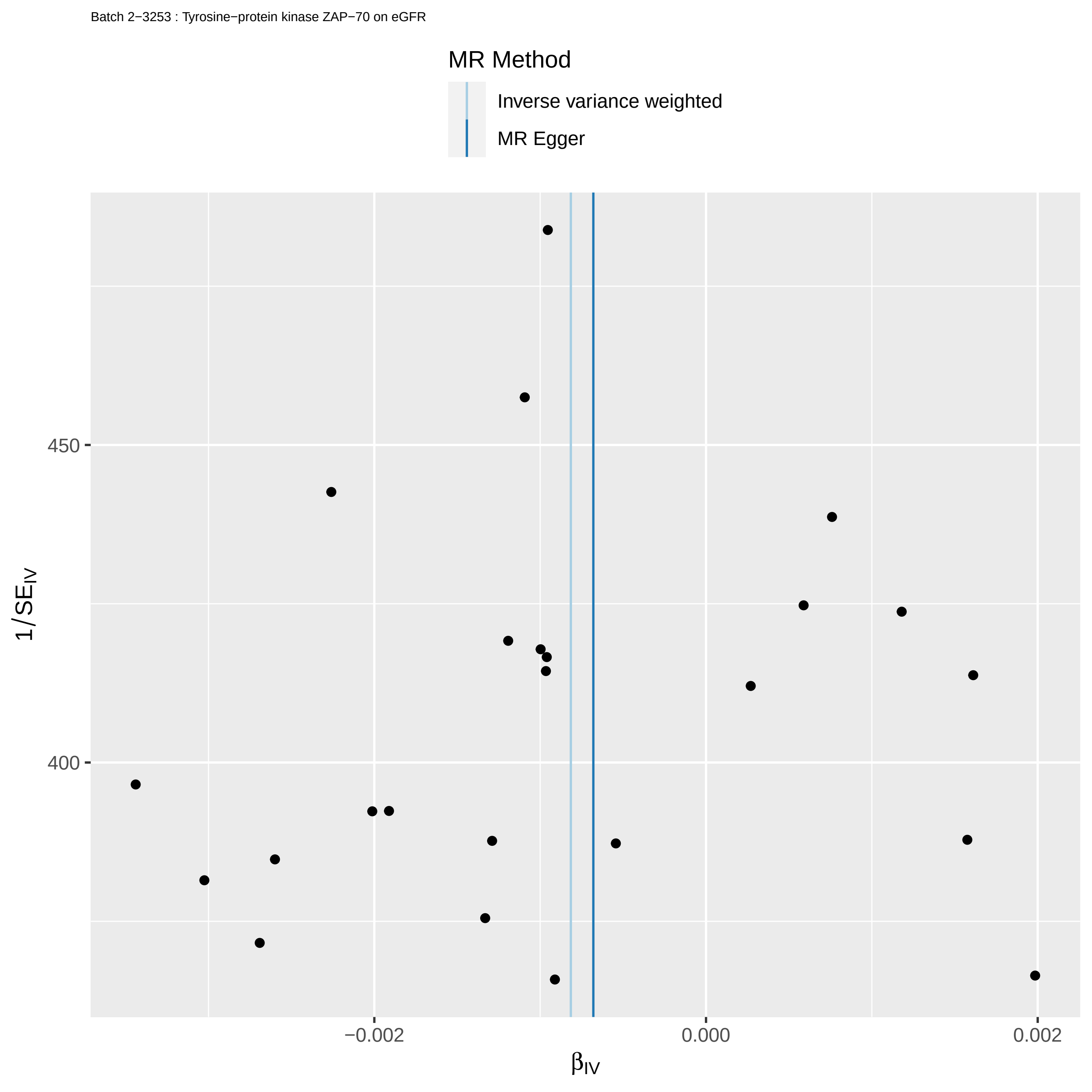

Batch 2−3253 : Tyrosine−protein kinase ZAP−70 on eGFR
MR Method
Inverse variance weighted
MR Egger
450
400
1/SE_IV
−0.002
0.000
0.002
β_IV

Batch 2−3254 : Tyrosine−protein kinase ZAP−70 on eGFR

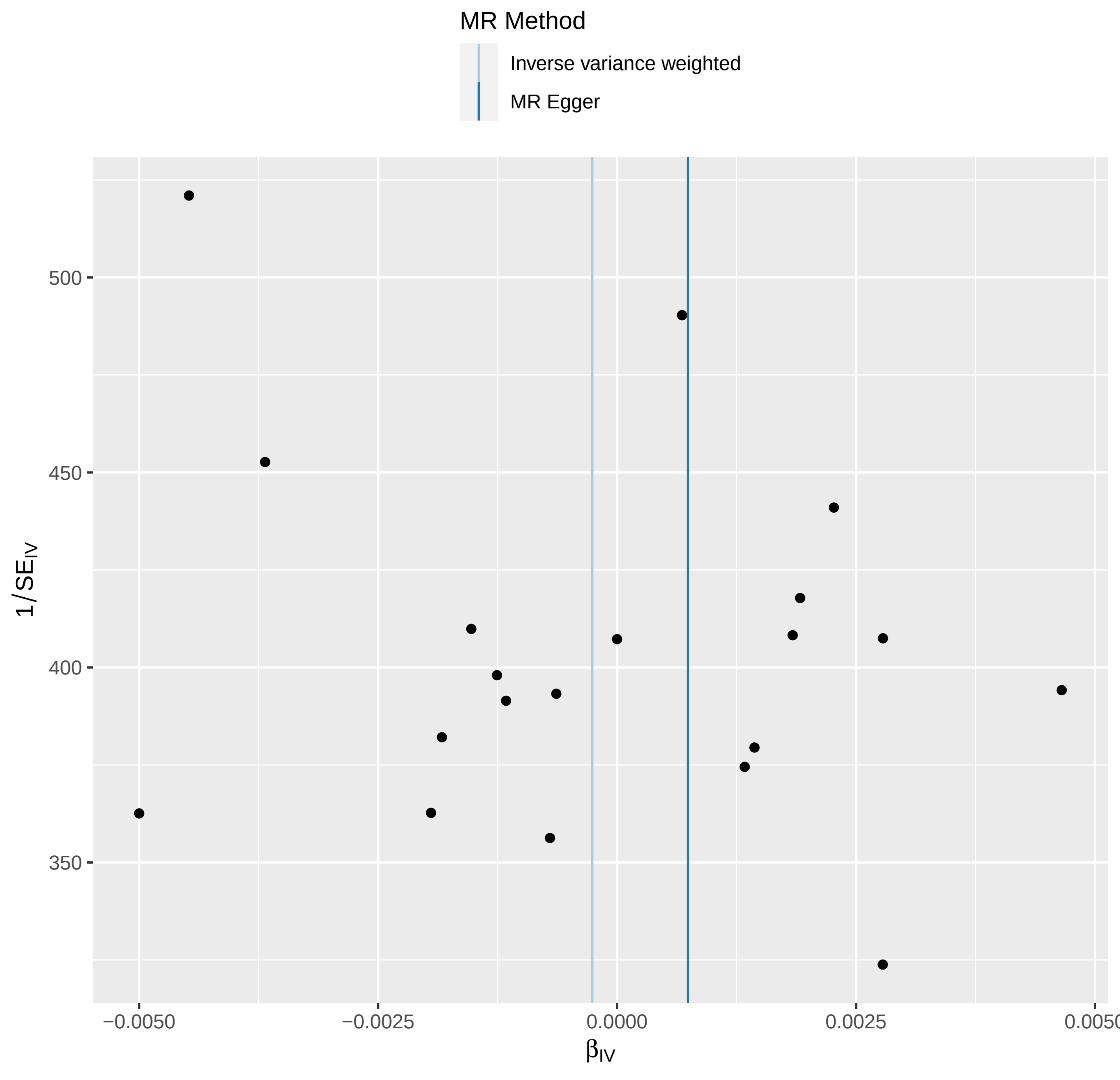

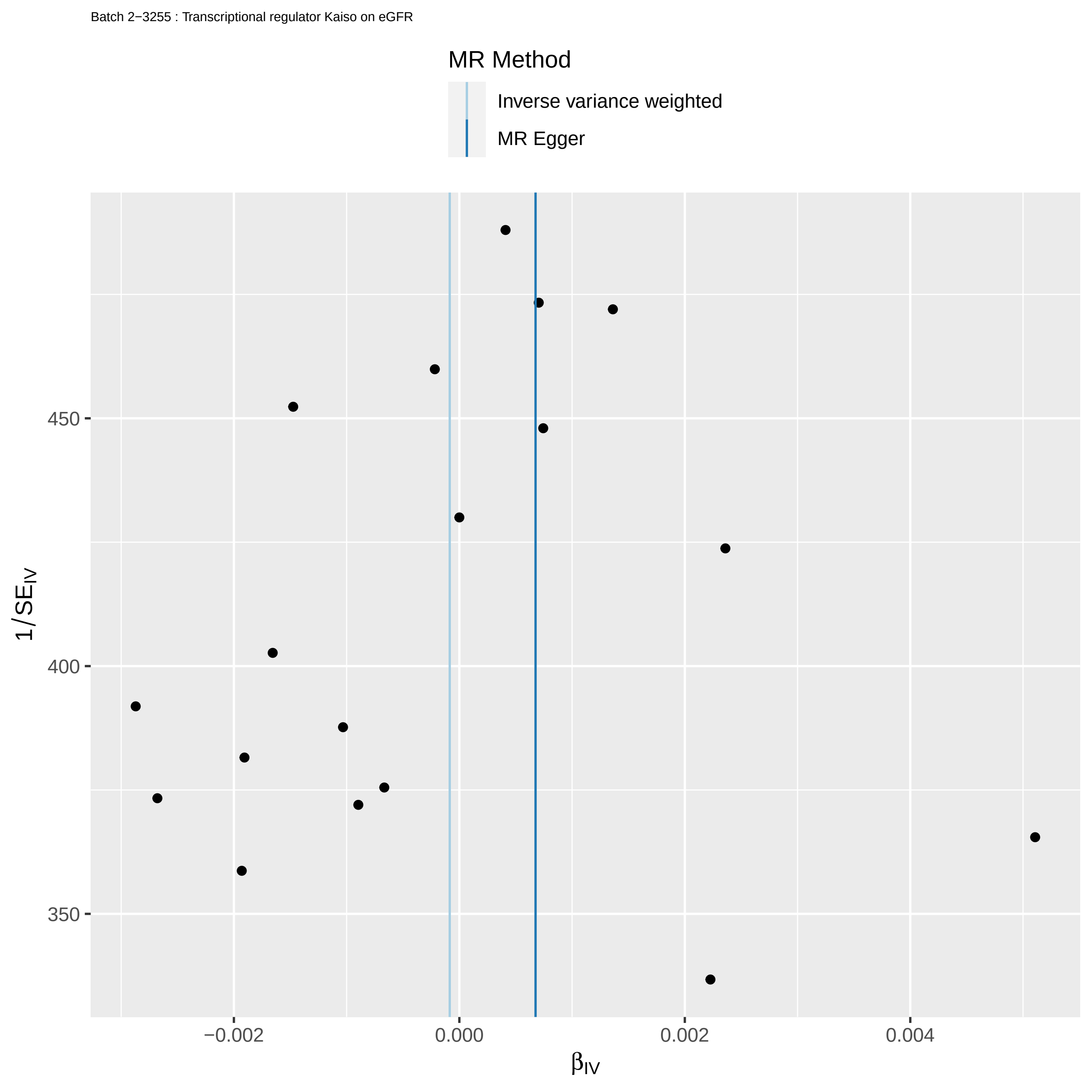
Batch 2−3255 : Transcriptional regulator Kaiso on eGFR
MR Method
Inverse variance weighted
MR Egger
1/SE_IV
450
400
350
−0.002
0.000
0.002
0.004
β_IV

Batch 2−3256 : E3 ubiquitin−protein ligase ZFP91 on eGFR

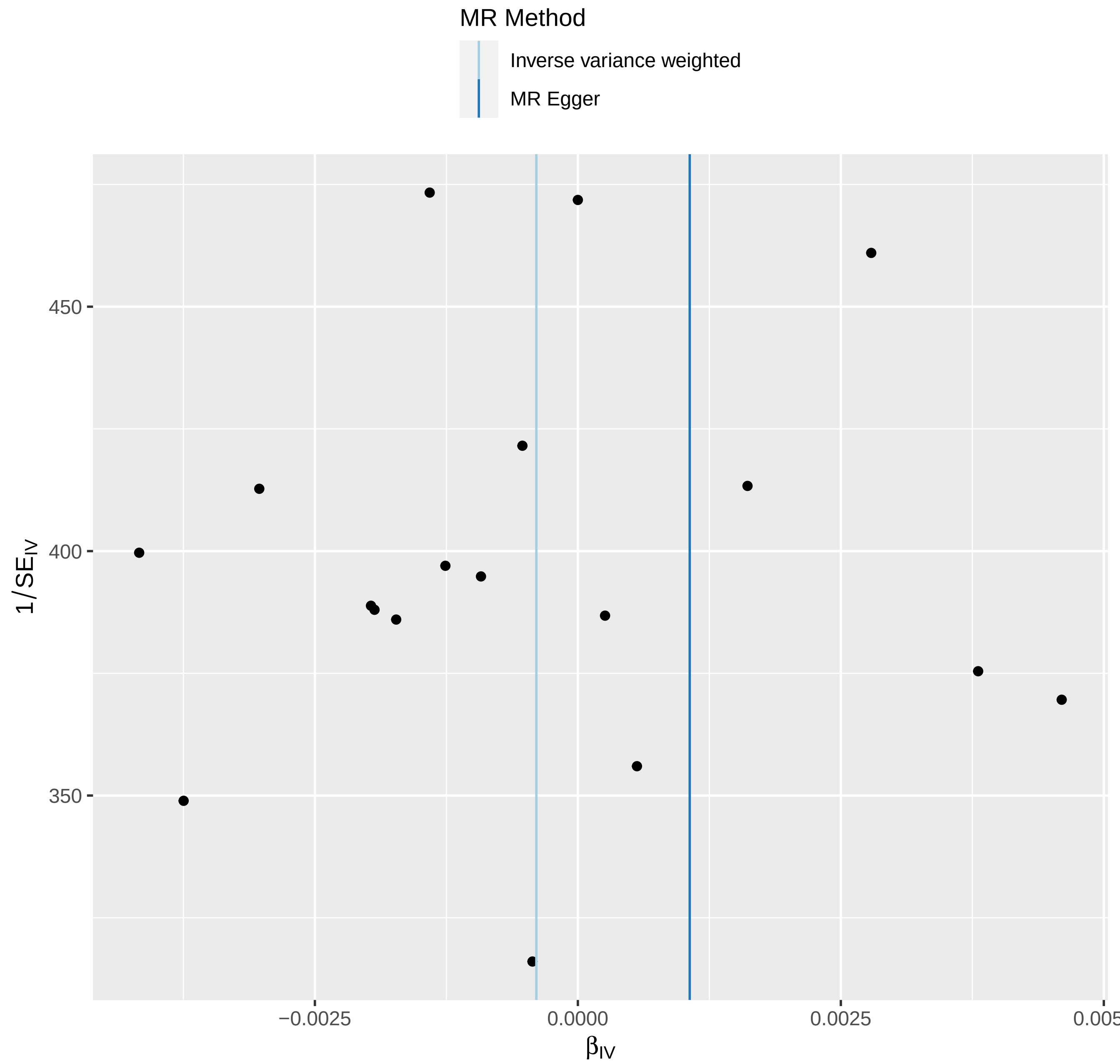

Batch 2−3257 : Protrudin on eGFR

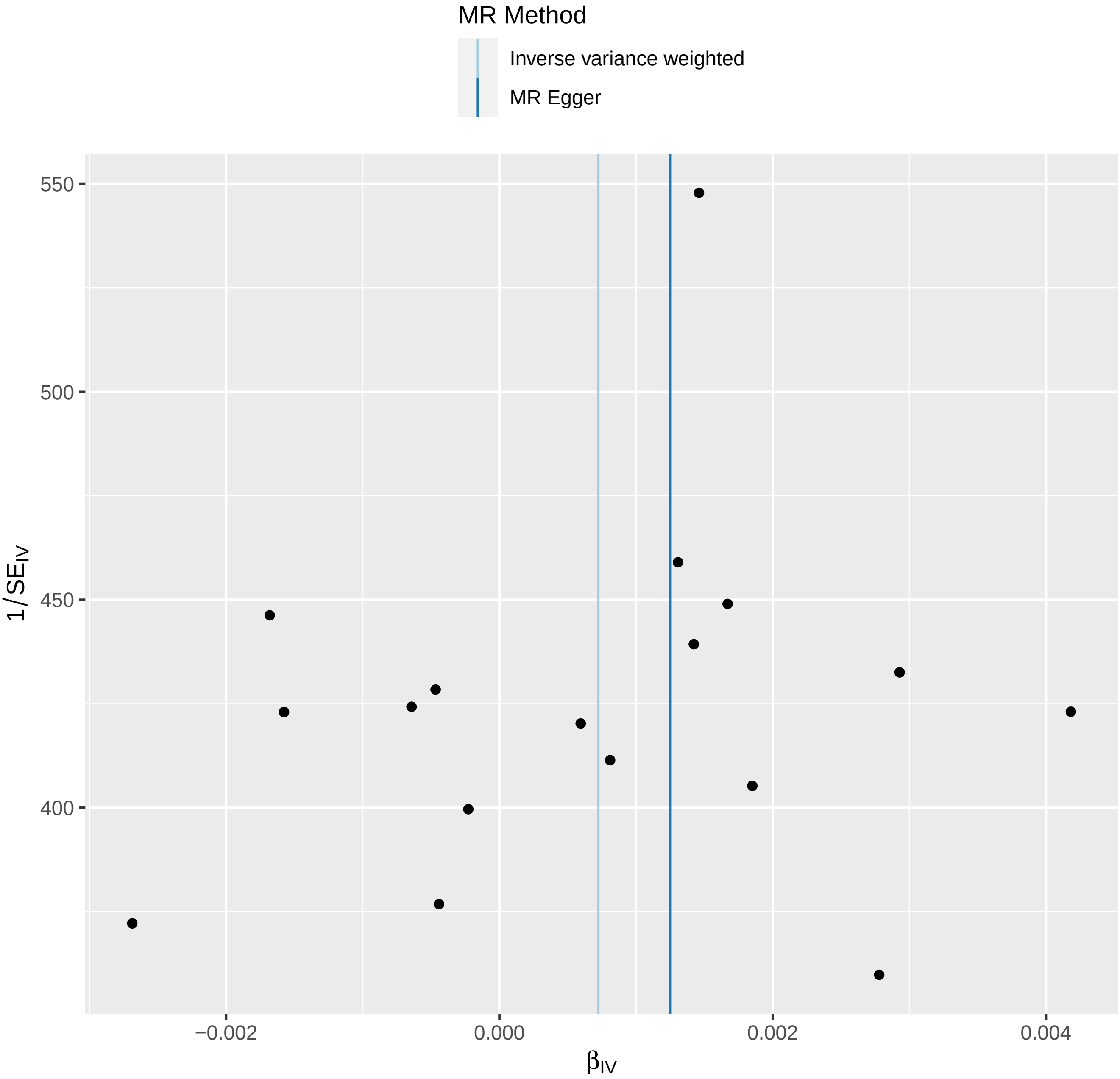

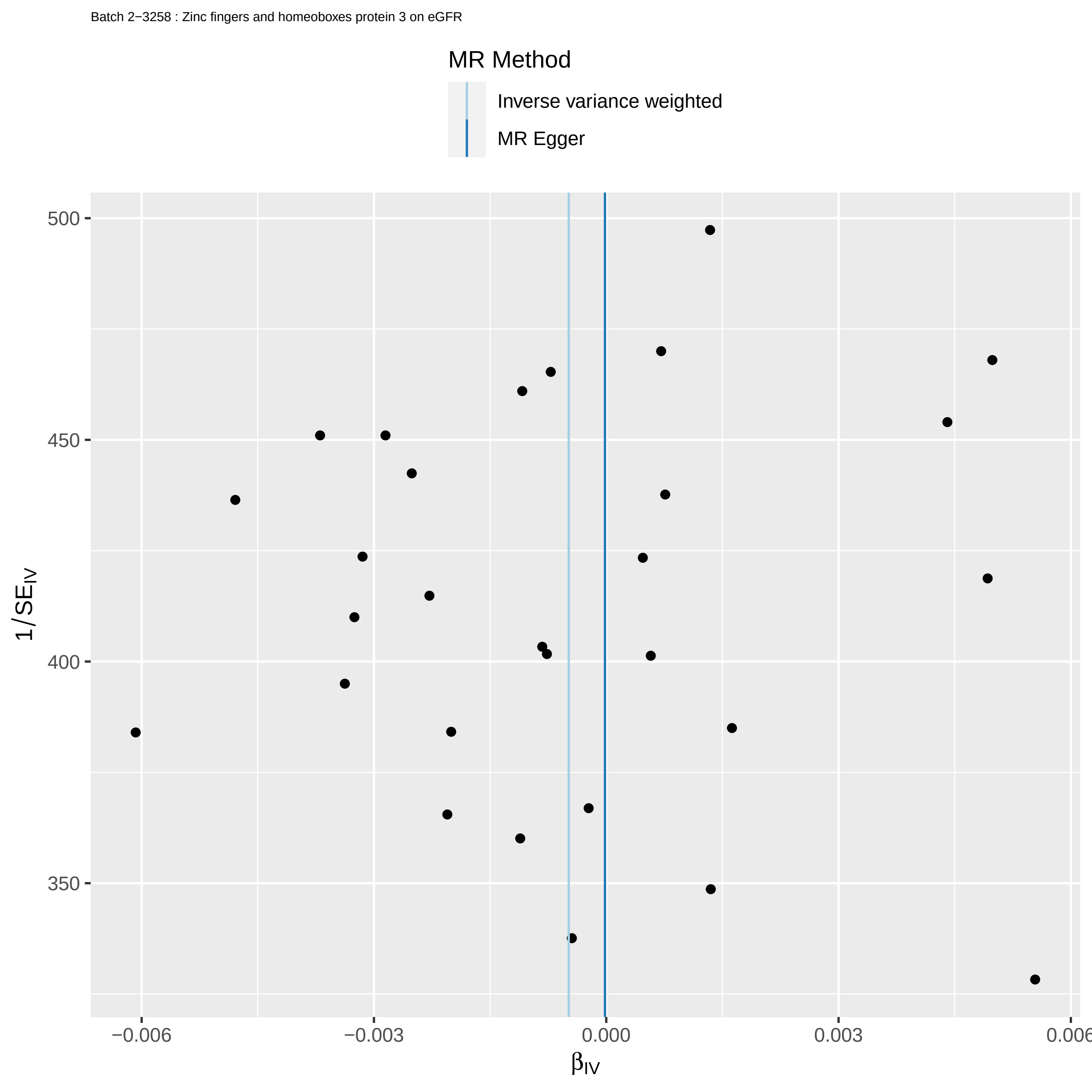

Batch 2−3258 : Zinc fingers and homeoboxes protein 3 on eGFR
MR Method
Inverse variance weighted
MR Egger
500
450
400
350
1/SE$_{IV}$
−0.006
−0.003
0.000
0.003
0.006
β$_{IV}$

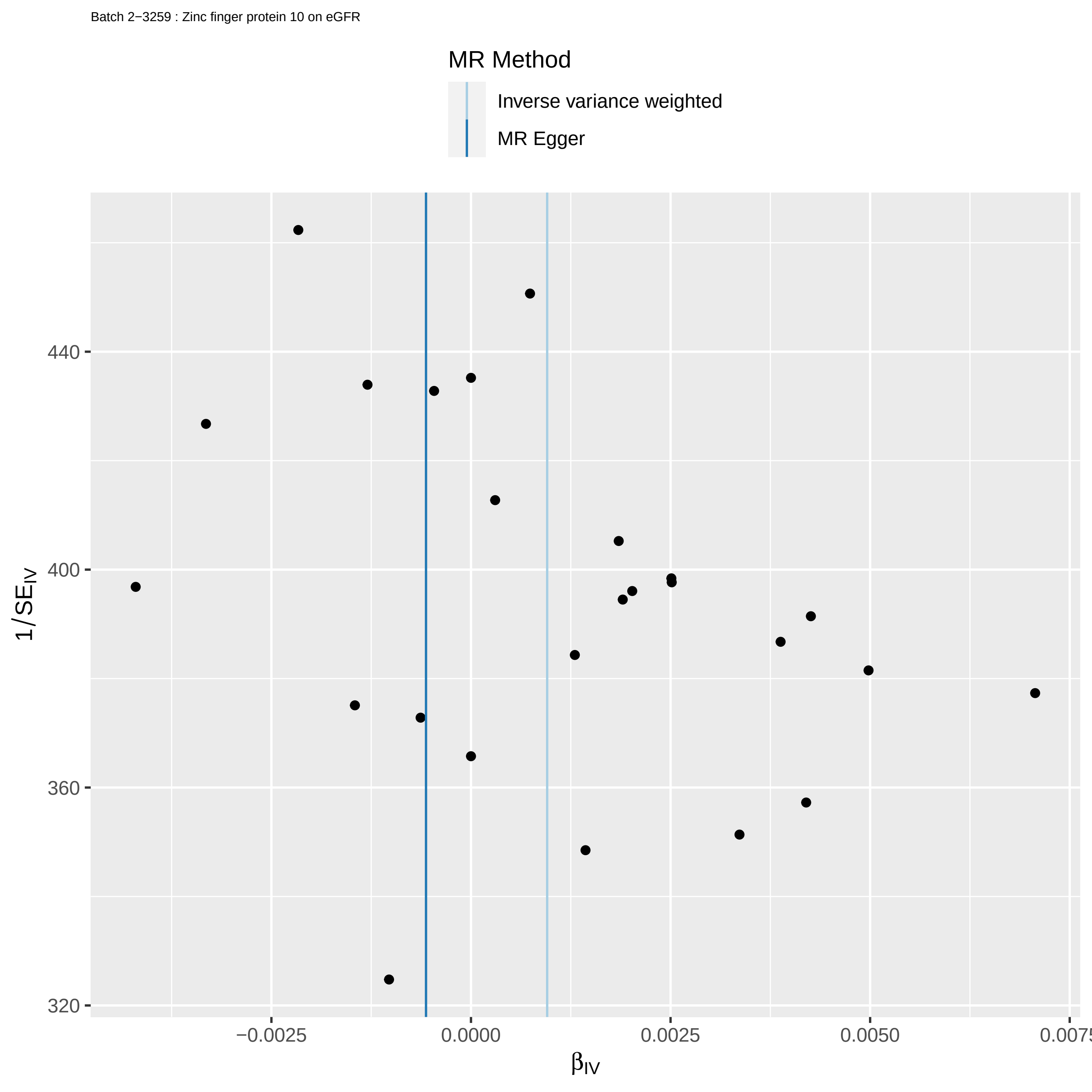
Batch 2−3259 : Zinc finger protein 10 on eGFR
MR Method
Inverse variance weighted
MR Egger
440
400
360
320
1/SE$_{IV}$
−0.0025
0.0000
0.0025
0.0050
0.0075
β$_{IV}$

Batch 2−3260 : Zinc finger protein 134 on eGFR

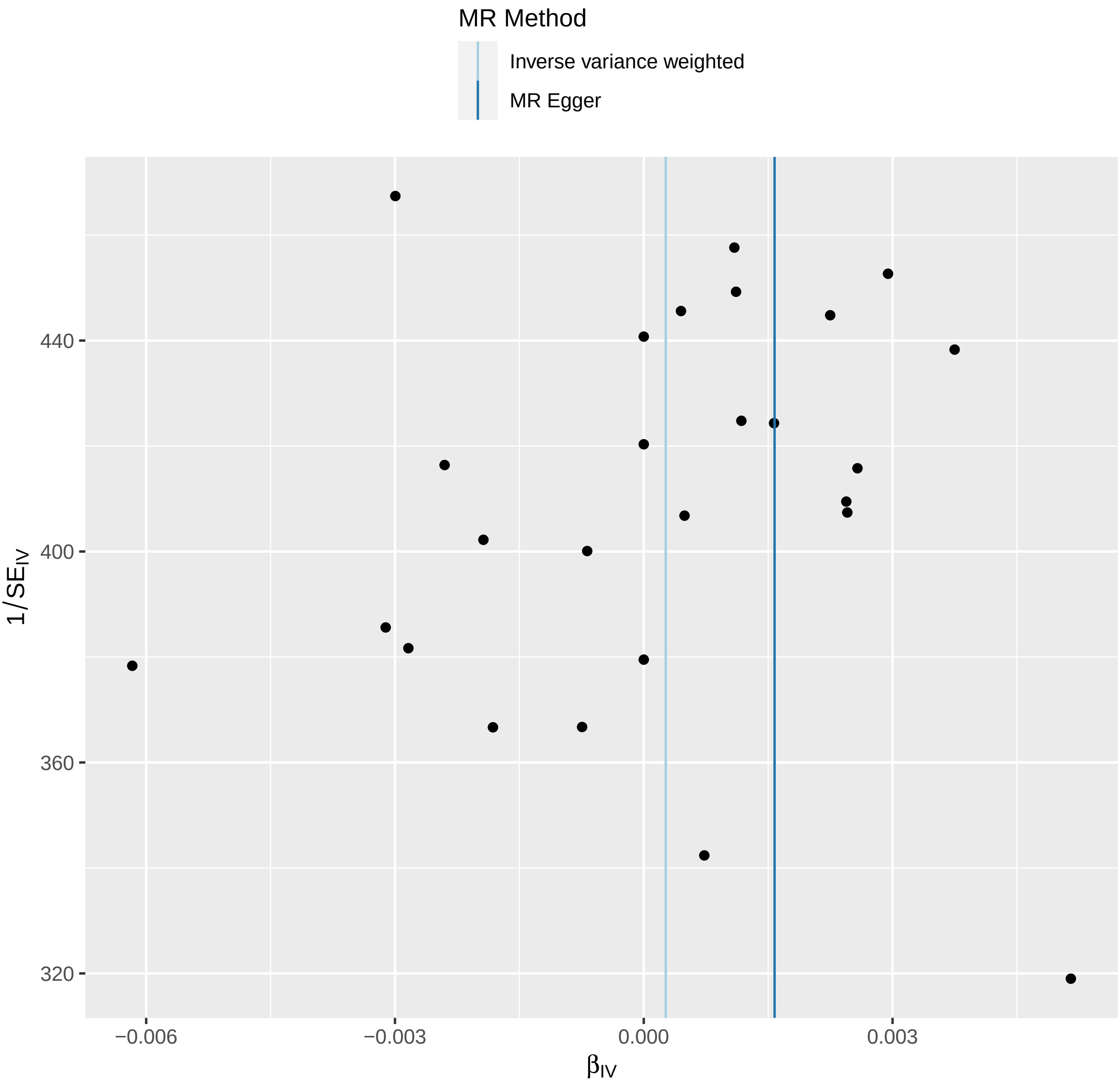

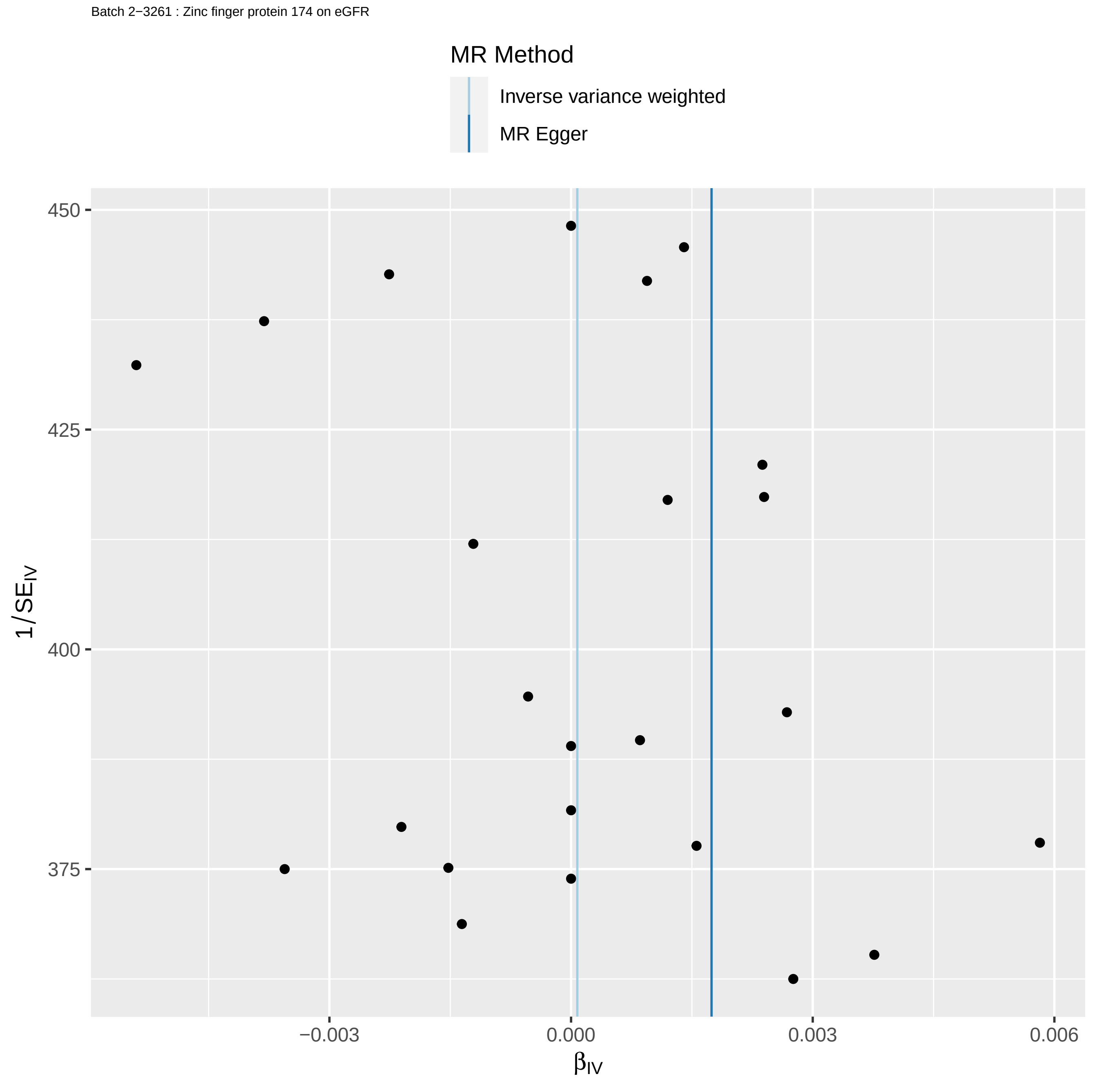
Batch 2−3261 : Zinc finger protein 174 on eGFR
MR Method
Inverse variance weighted
MR Egger
450
425
400
375
1/SE_IV
−0.003
0.000
0.003
0.006
β_IV

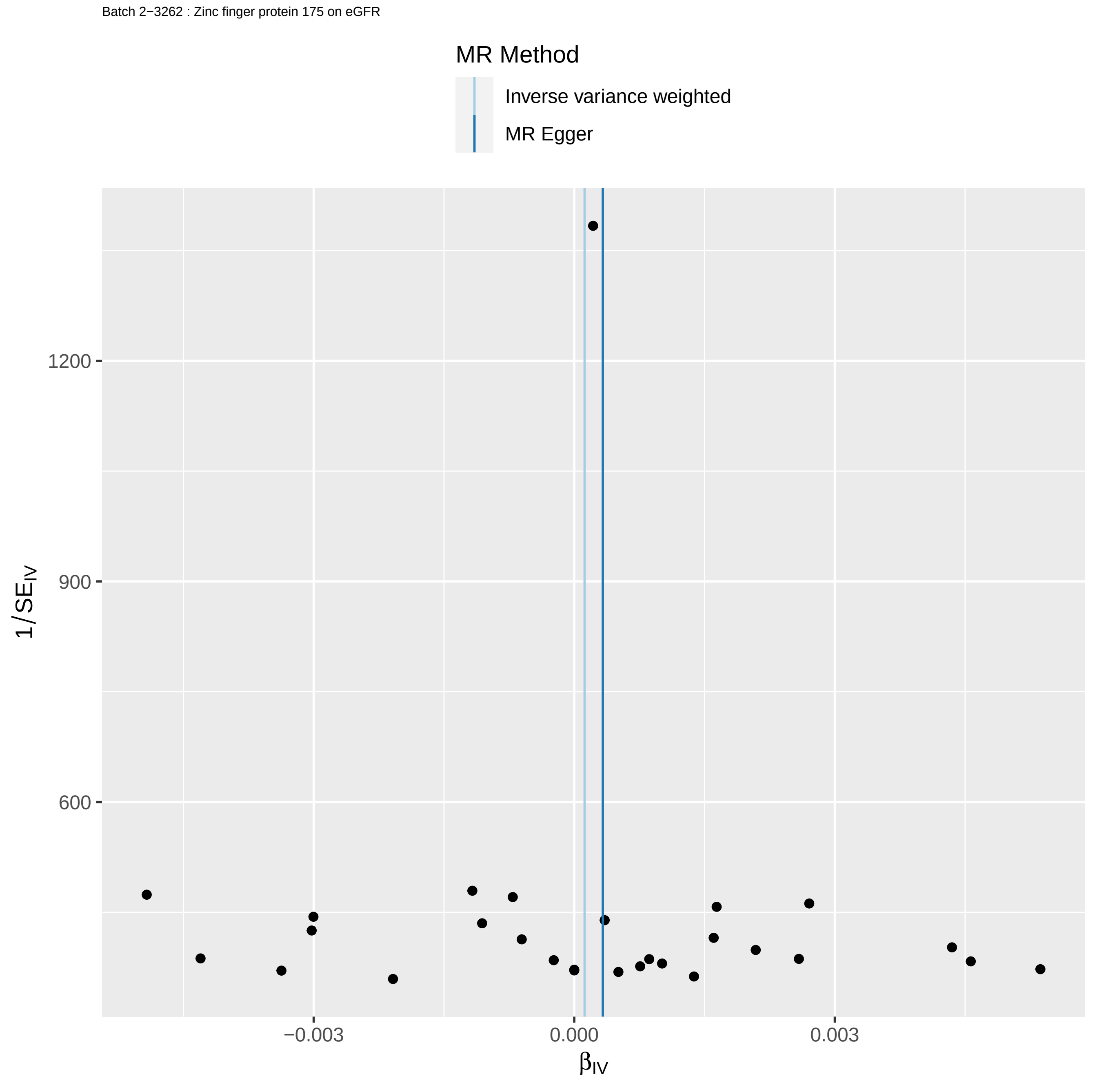
Batch 2−3262 : Zinc finger protein 175 on eGFR
MR Method
Inverse variance weighted
MR Egger
1200
900
600
1/SE$_{IV}$
−0.003
0.000
0.003
β_{IV}

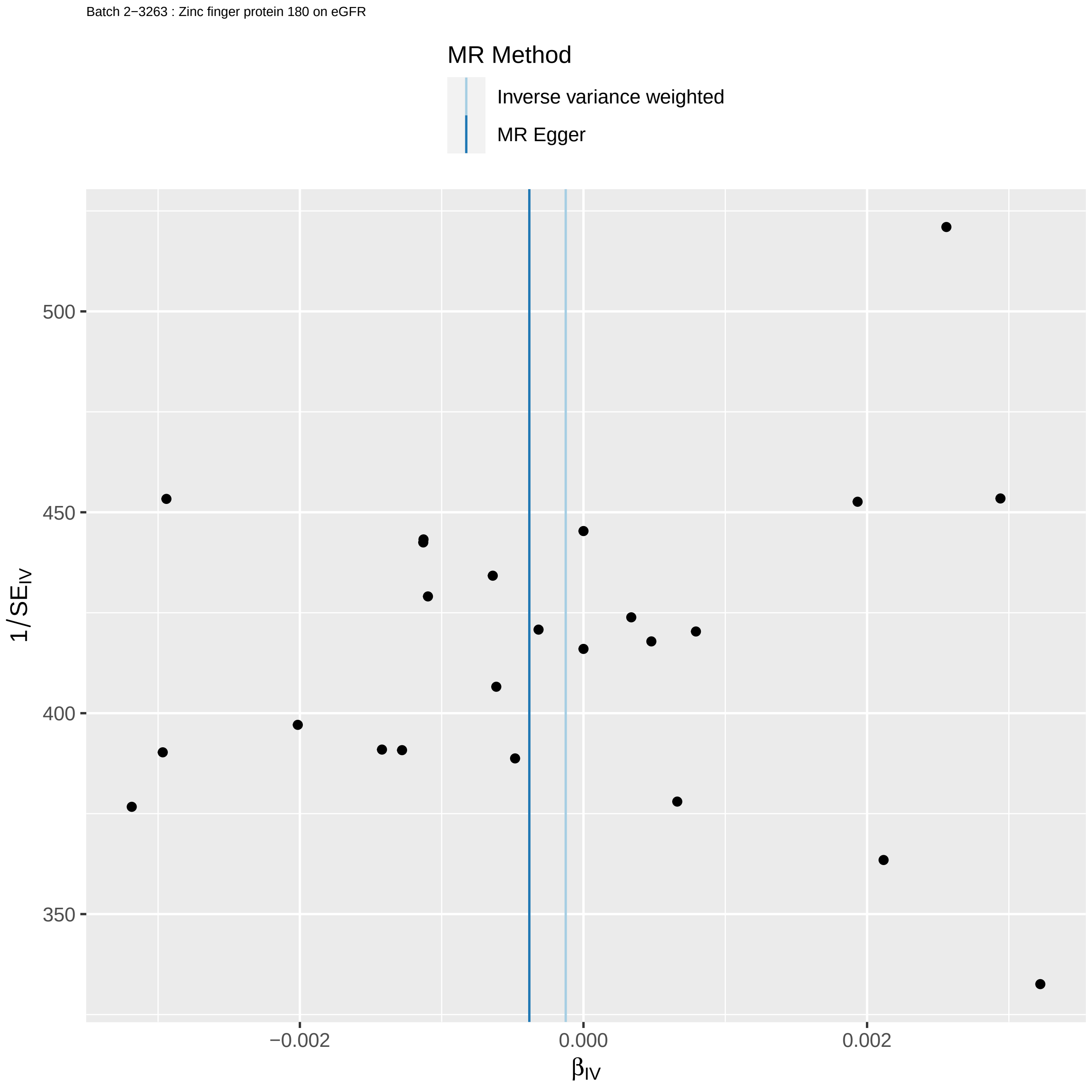

Batch 2−3263 : Zinc finger protein 180 on eGFR
MR Method
Inverse variance weighted
MR Egger
500
450
400
350
1/SE_IV
−0.002
0.000
0.002
β_IV

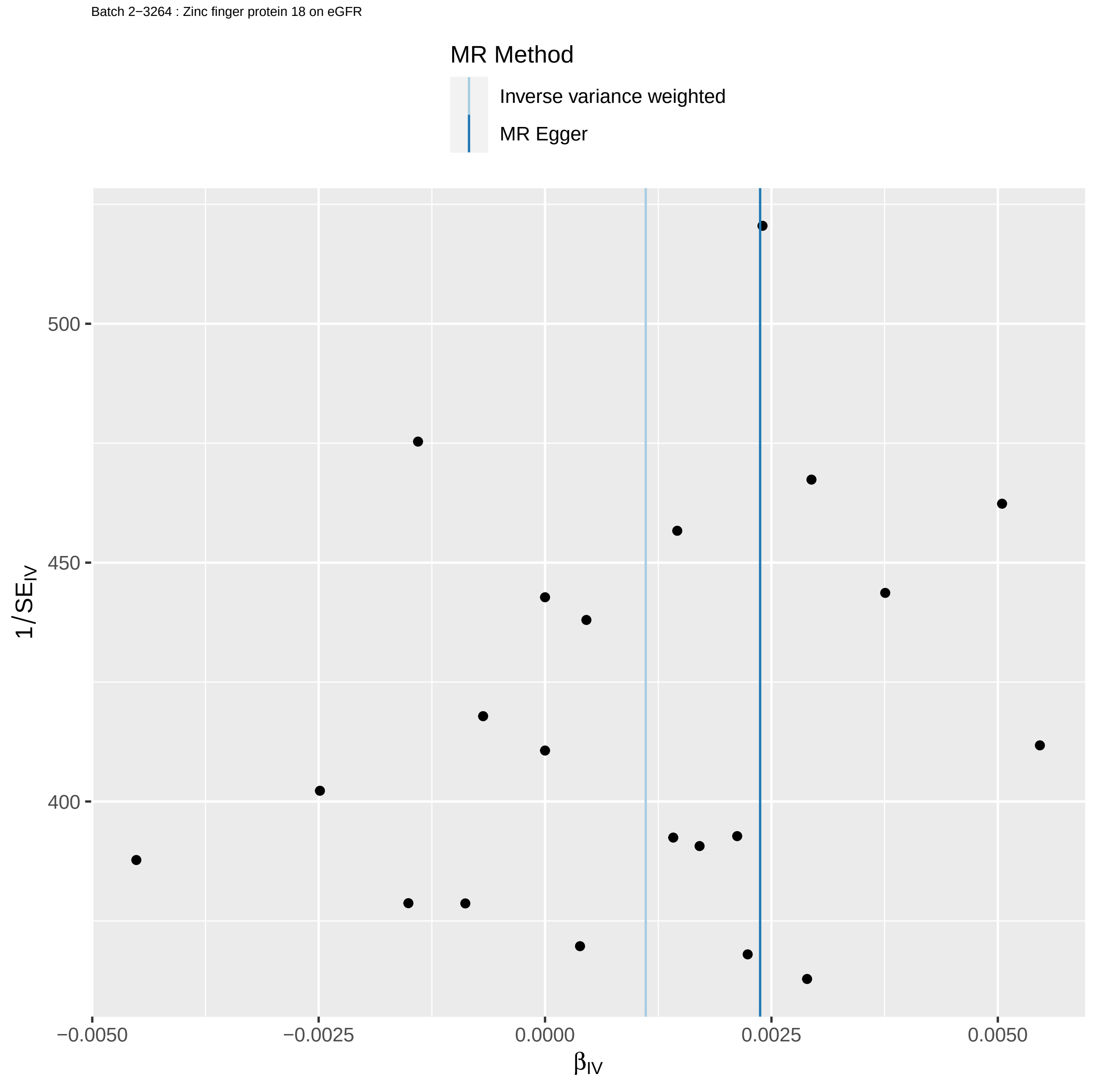

Batch 2−3264 : Zinc finger protein 18 on eGFR
MR Method
Inverse variance weighted
MR Egger
500
450
400
1/SE$_{IV}$
−0.0050
−0.0025
0.0000
0.0025
0.0050
β$_{IV}$

Batch 2−3265 : Zinc finger protein 23 on eGFR

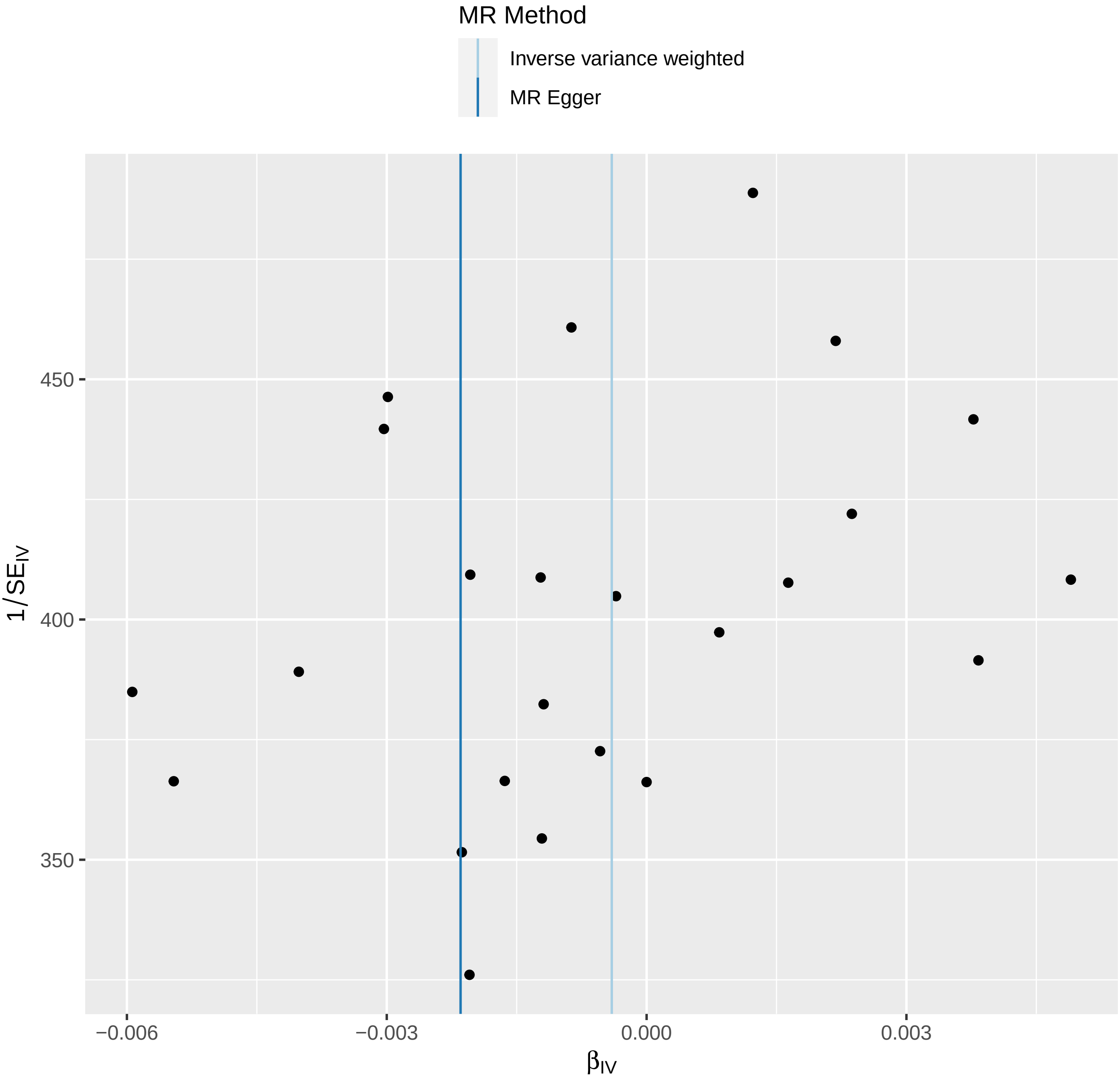

Batch 2−3266 : Zinc finger protein 264 on eGFR

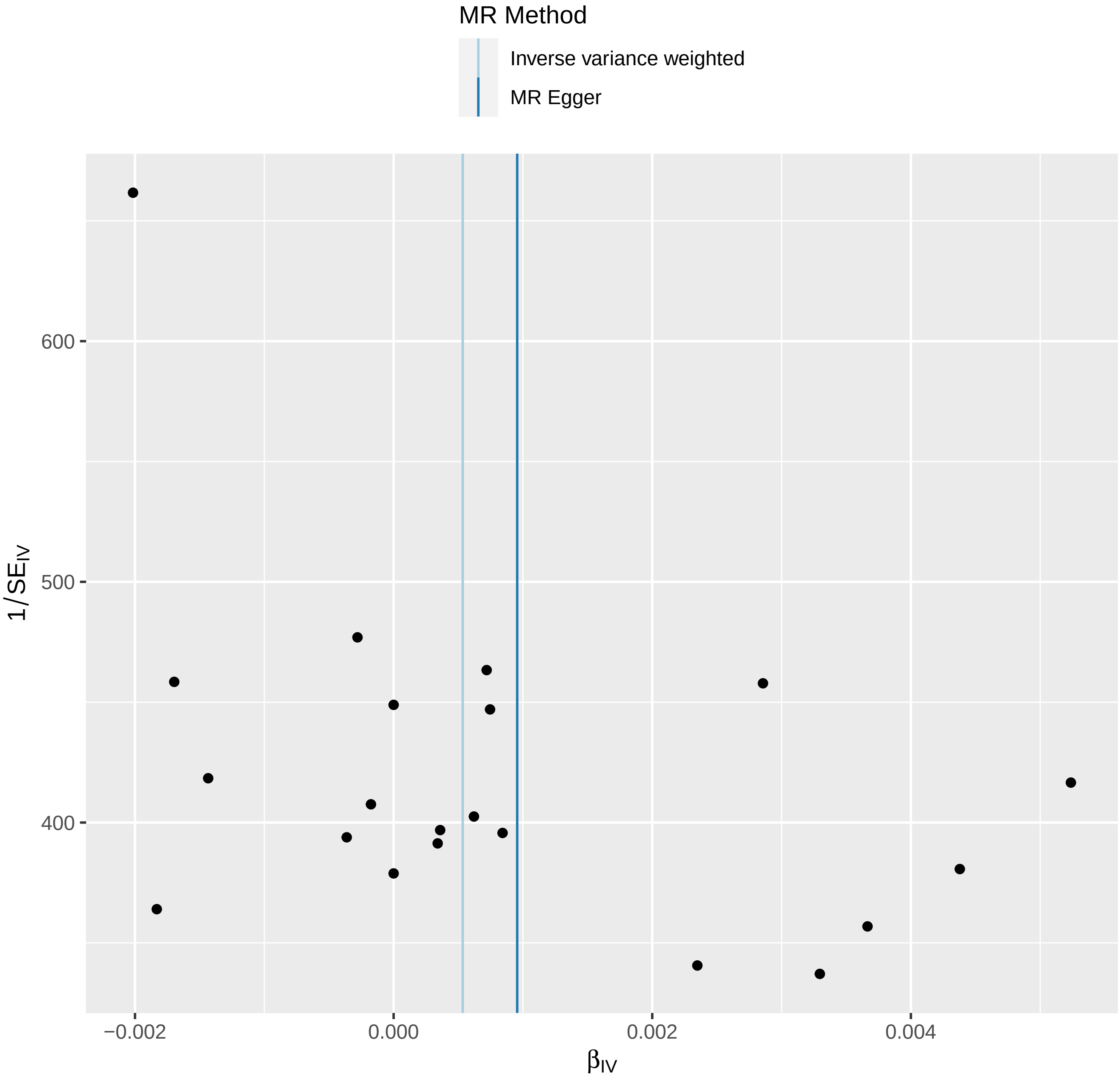

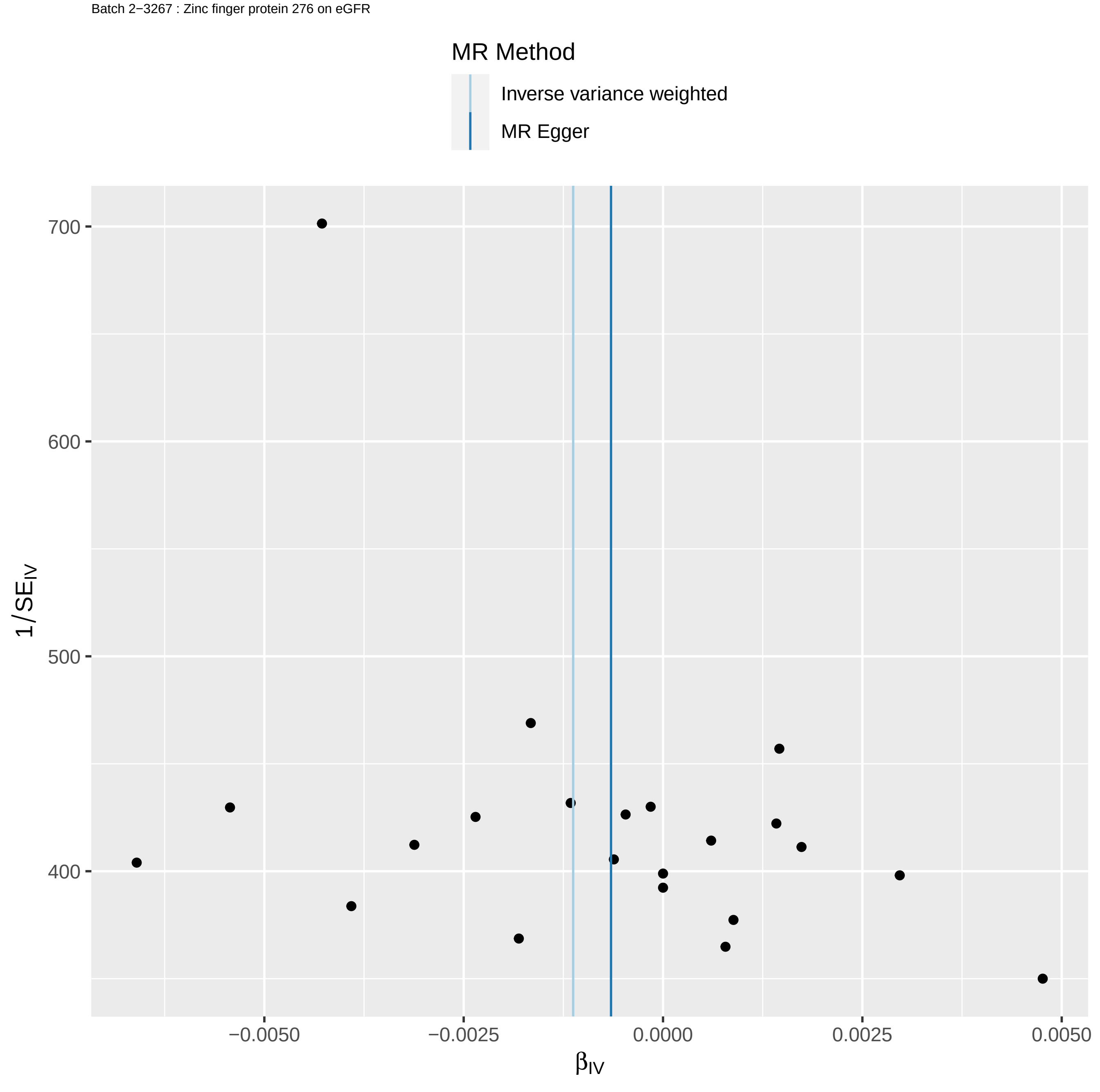

Batch 2−3267 : Zinc finger protein 276 on eGFR
MR Method
Inverse variance weighted
MR Egger
700
600
500
400
1/SE_IV
−0.0050
−0.0025
0.0000
0.0025
0.0050
β_IV

Batch 2−3268 : Zinc finger protein 329 on eGFR

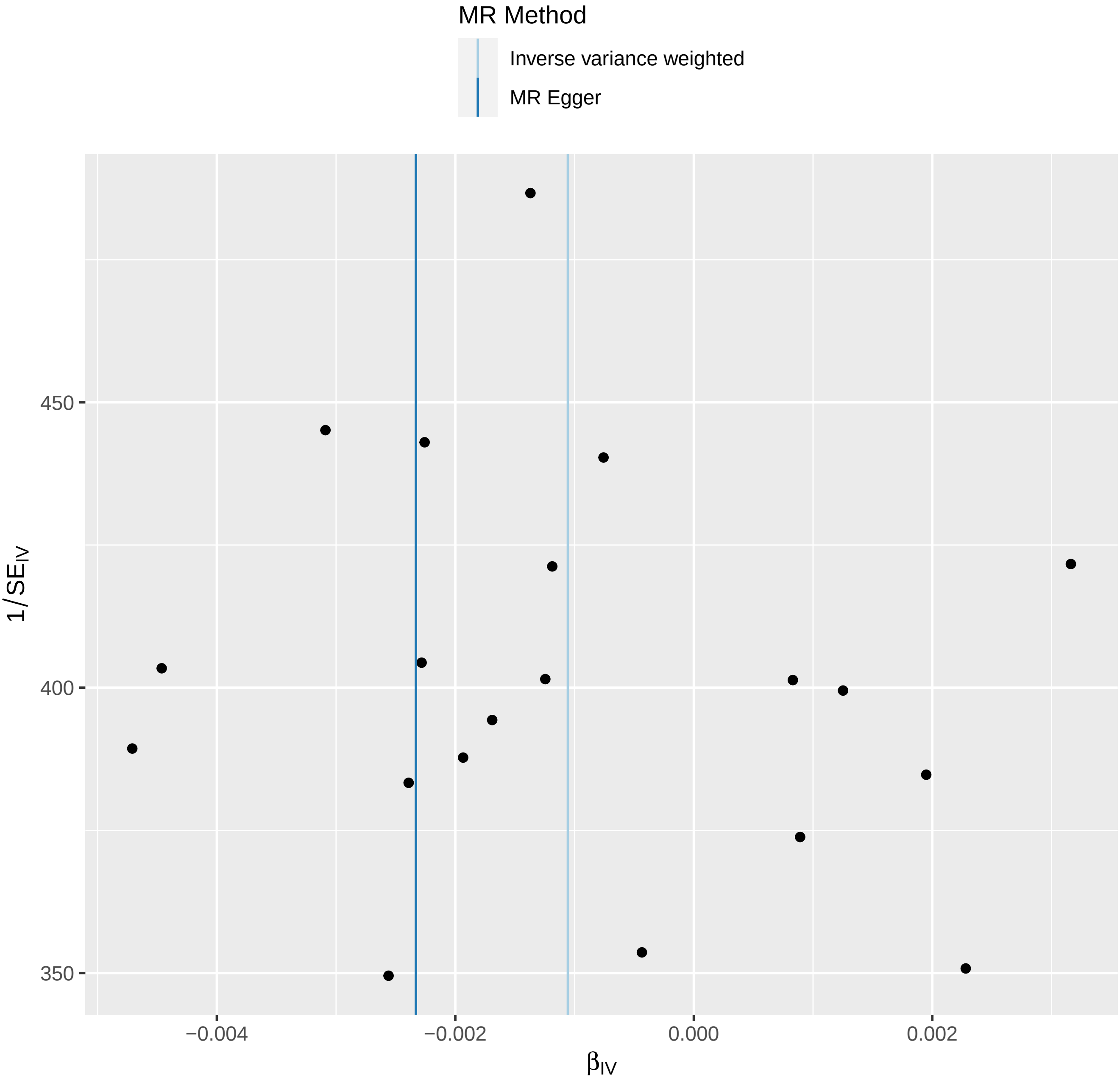

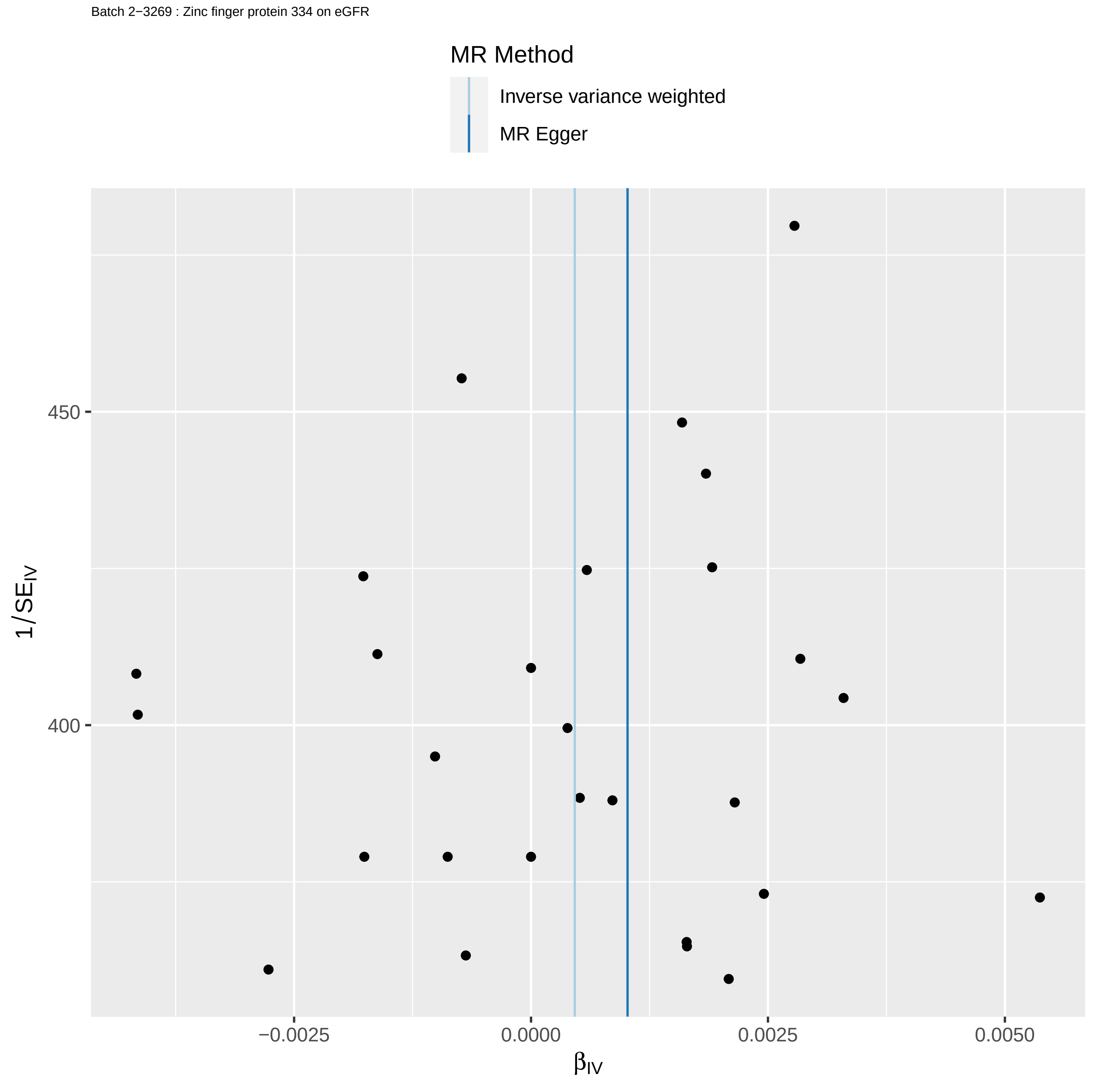

Batch 2−3269 : Zinc finger protein 334 on eGFR
MR Method
Inverse variance weighted
MR Egger
450
400
1/SE$_{IV}$
−0.0025
0.0000
0.0025
0.0050
β$_{IV}$

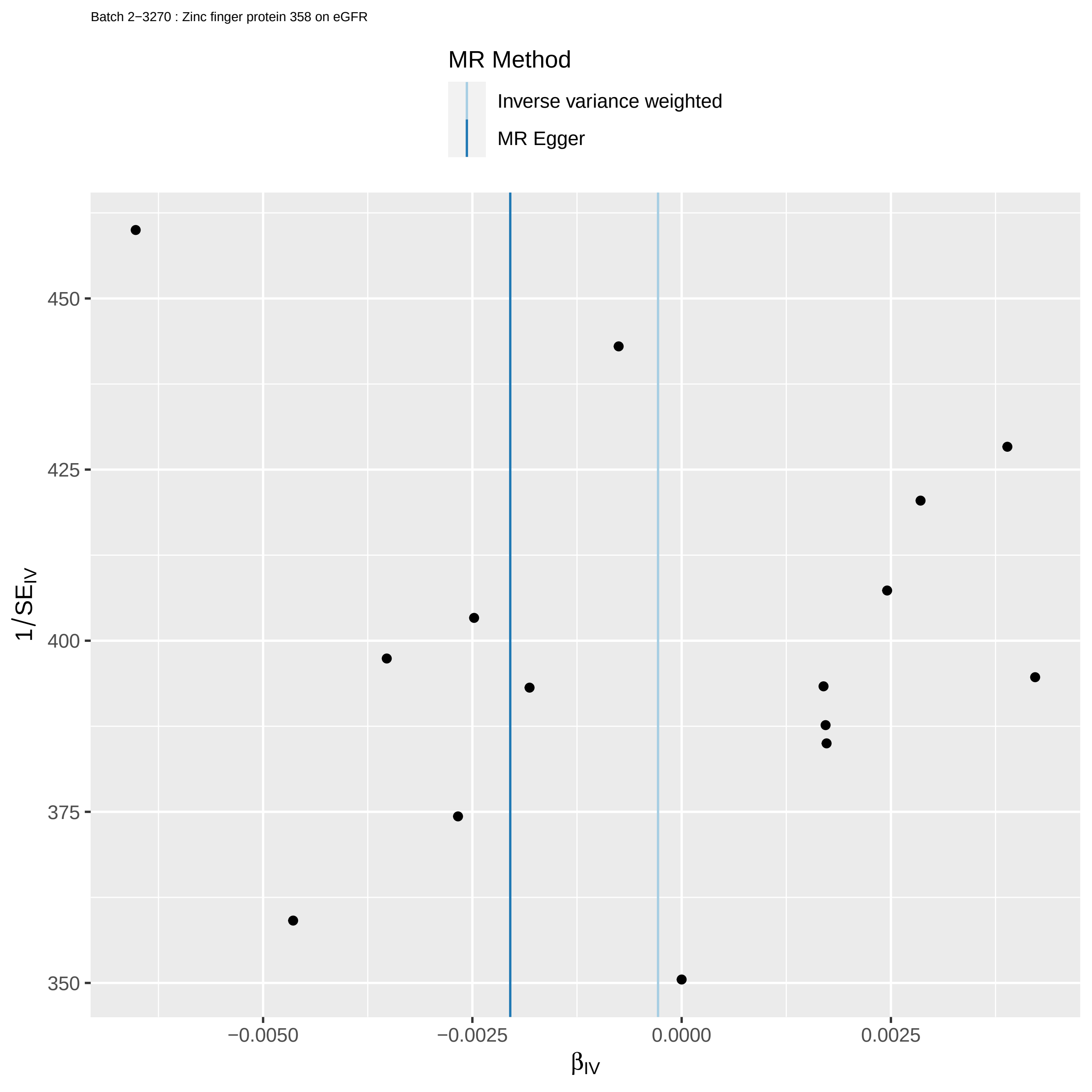

Batch 2−3270 : Zinc finger protein 358 on eGFR
MR Method
Inverse variance weighted
MR Egger
450
425
400
375
350
1/SE$_{IV}$
−0.0050
−0.0025
0.0000
0.0025
β$_{IV}$

Batch 2−3271 : Zinc finger protein 382 on eGFR

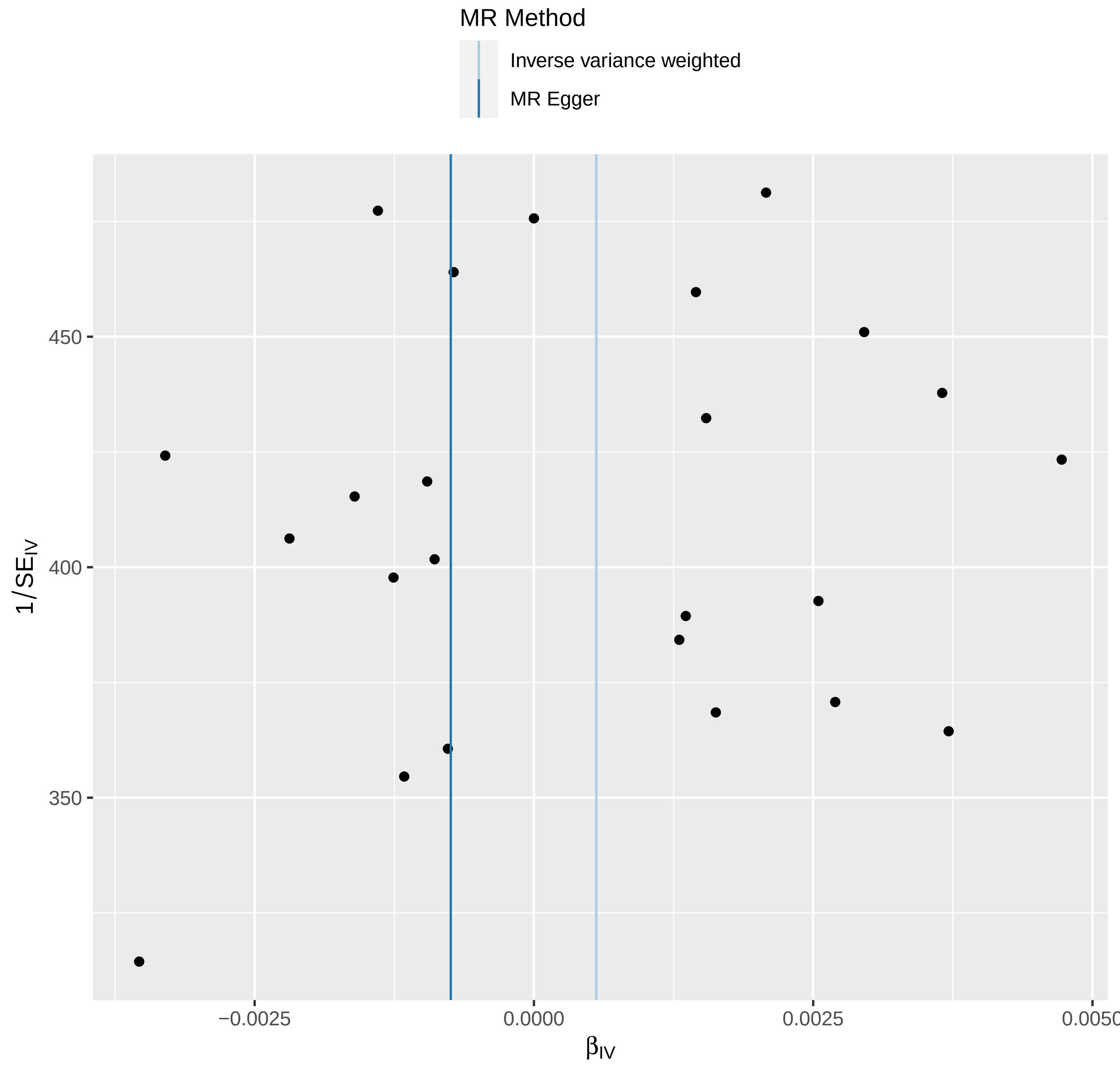

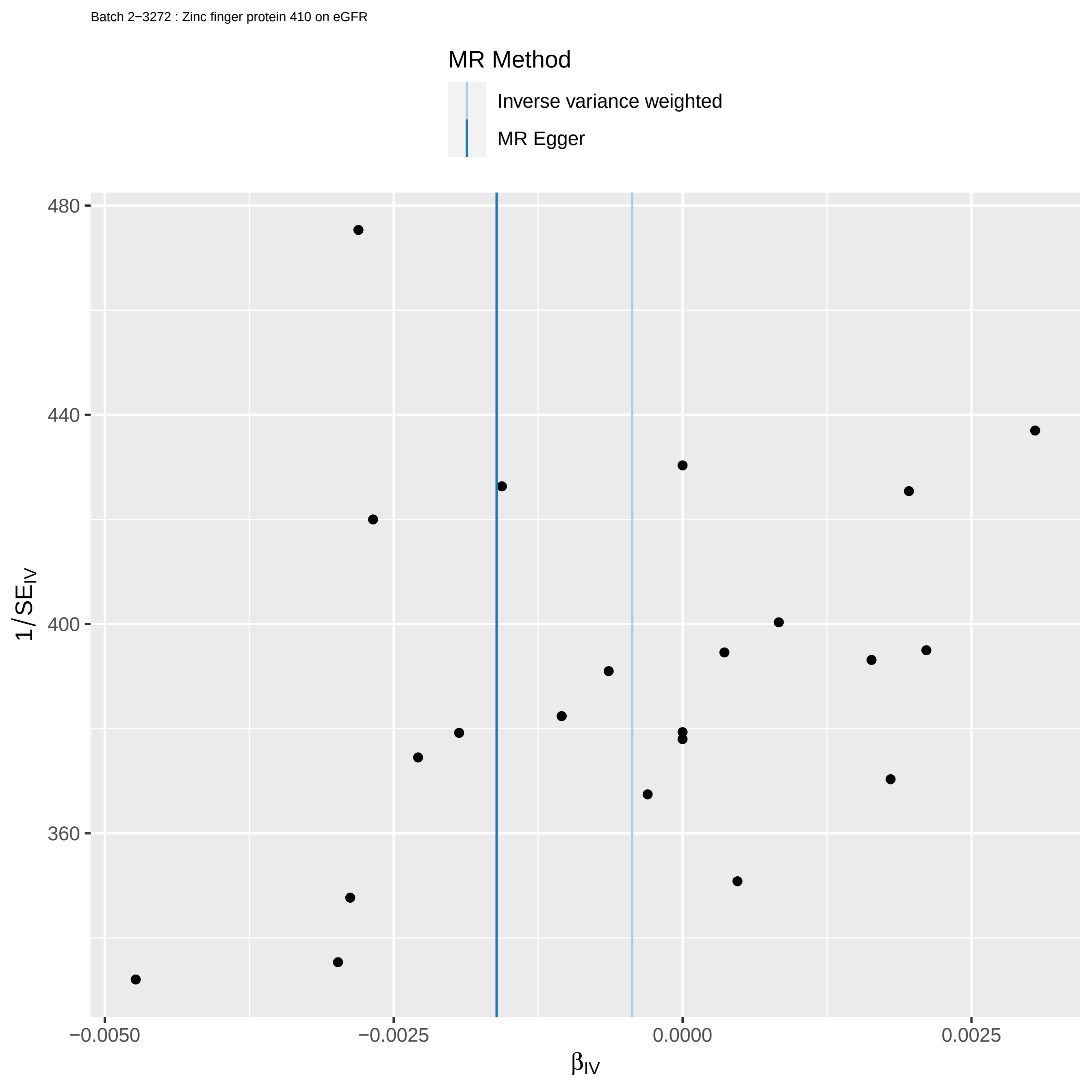
Batch 2−3272 : Zinc finger protein 410 on eGFR
MR Method
Inverse variance weighted
MR Egger
480
440
400
360
1/SE$_{IV}$
−0.0050
−0.0025
0.0000
0.0025
β$_{IV}$

Batch 2−3273 : Zinc finger protein 415 on eGFR

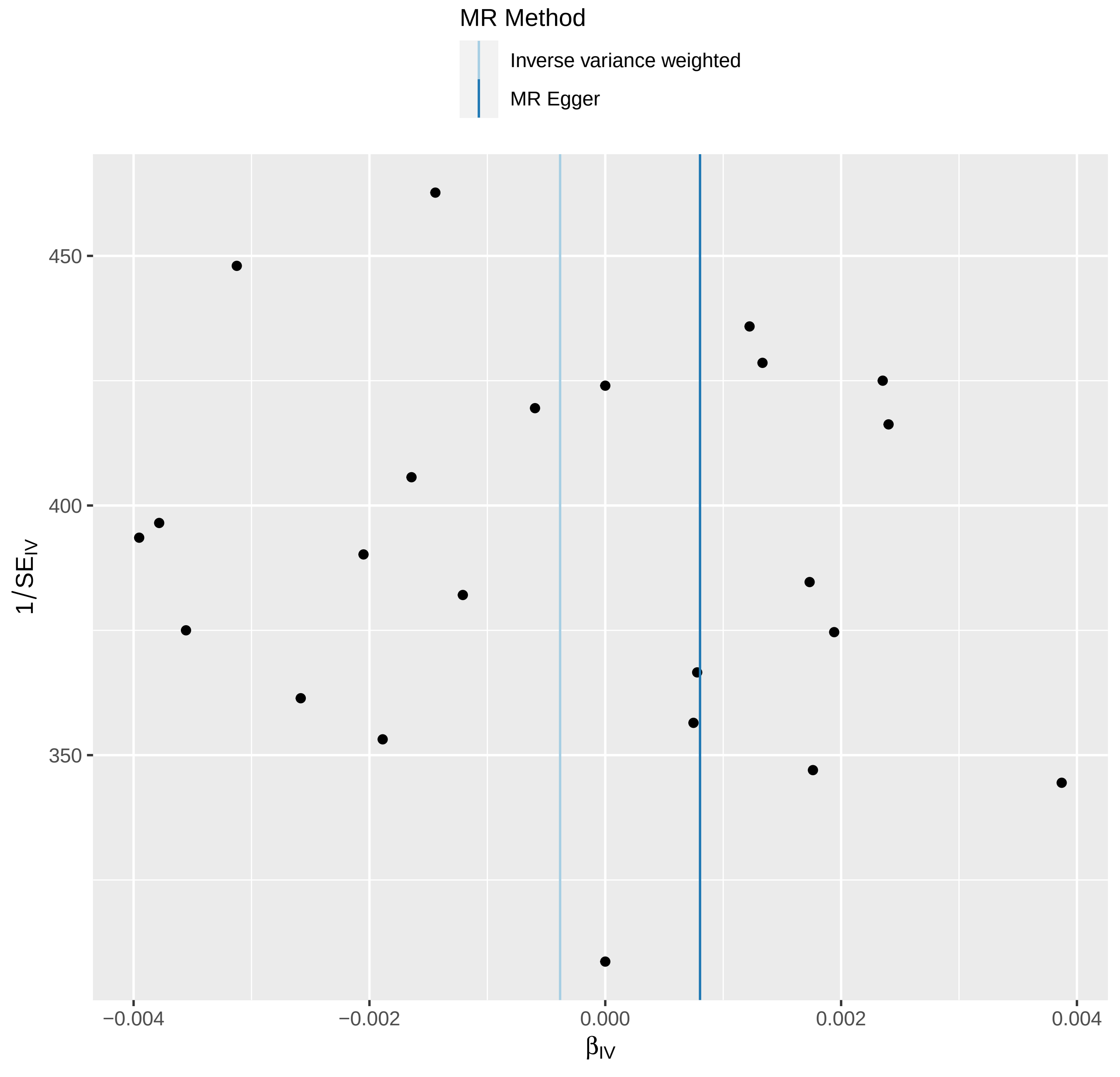

Batch 2−3274 : Zinc finger protein 566 on eGFR

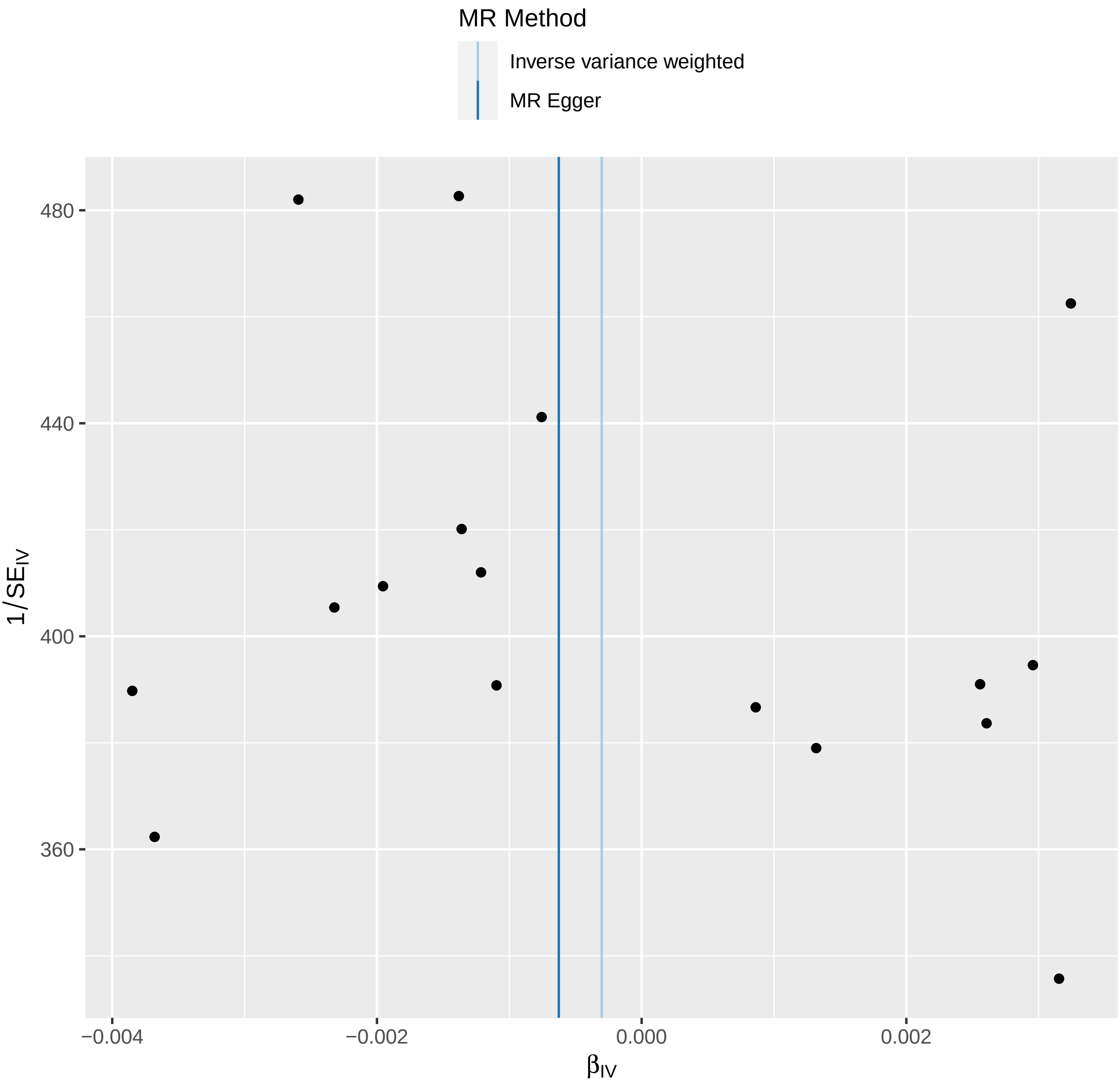

Batch 2−3275 : Zinc finger protein 75D on eGFR

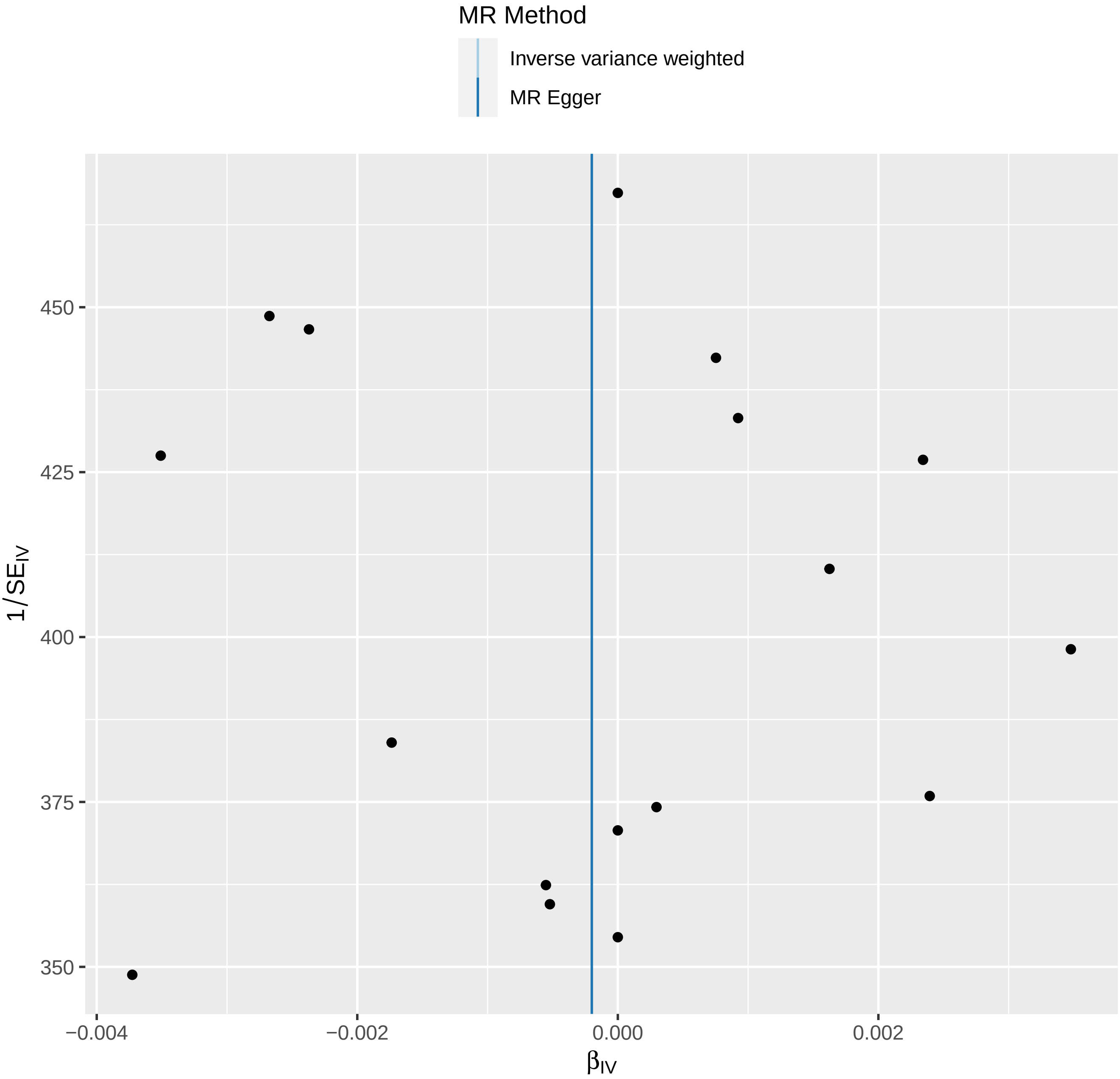

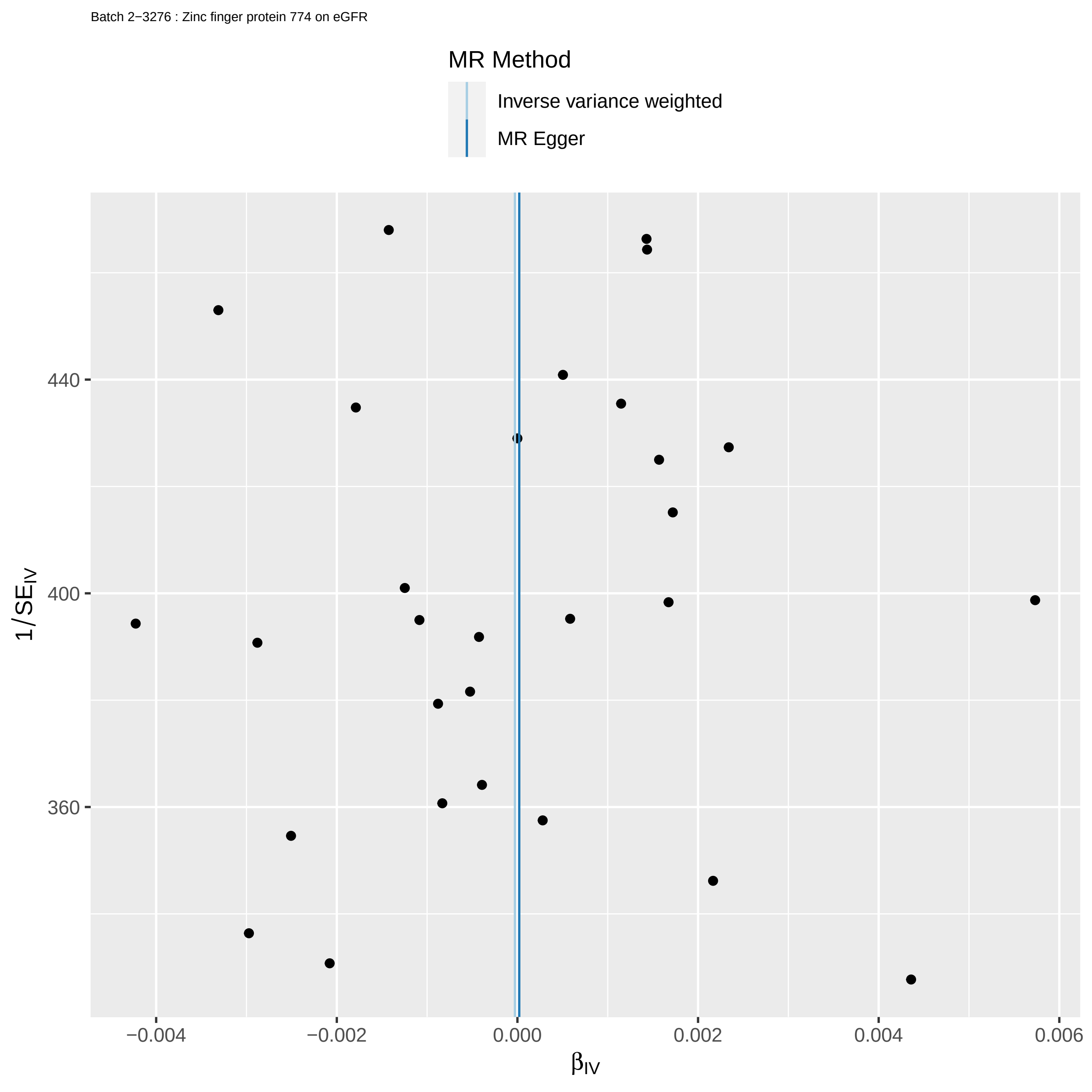
Batch 2−3276 : Zinc finger protein 774 on eGFR
MR Method
Inverse variance weighted
MR Egger
440
400
360
1/SE_IV
−0.004
−0.002
0.000
0.002
0.004
0.006
β_IV

Batch 2−3277 : Zinc finger protein 843 on eGFR

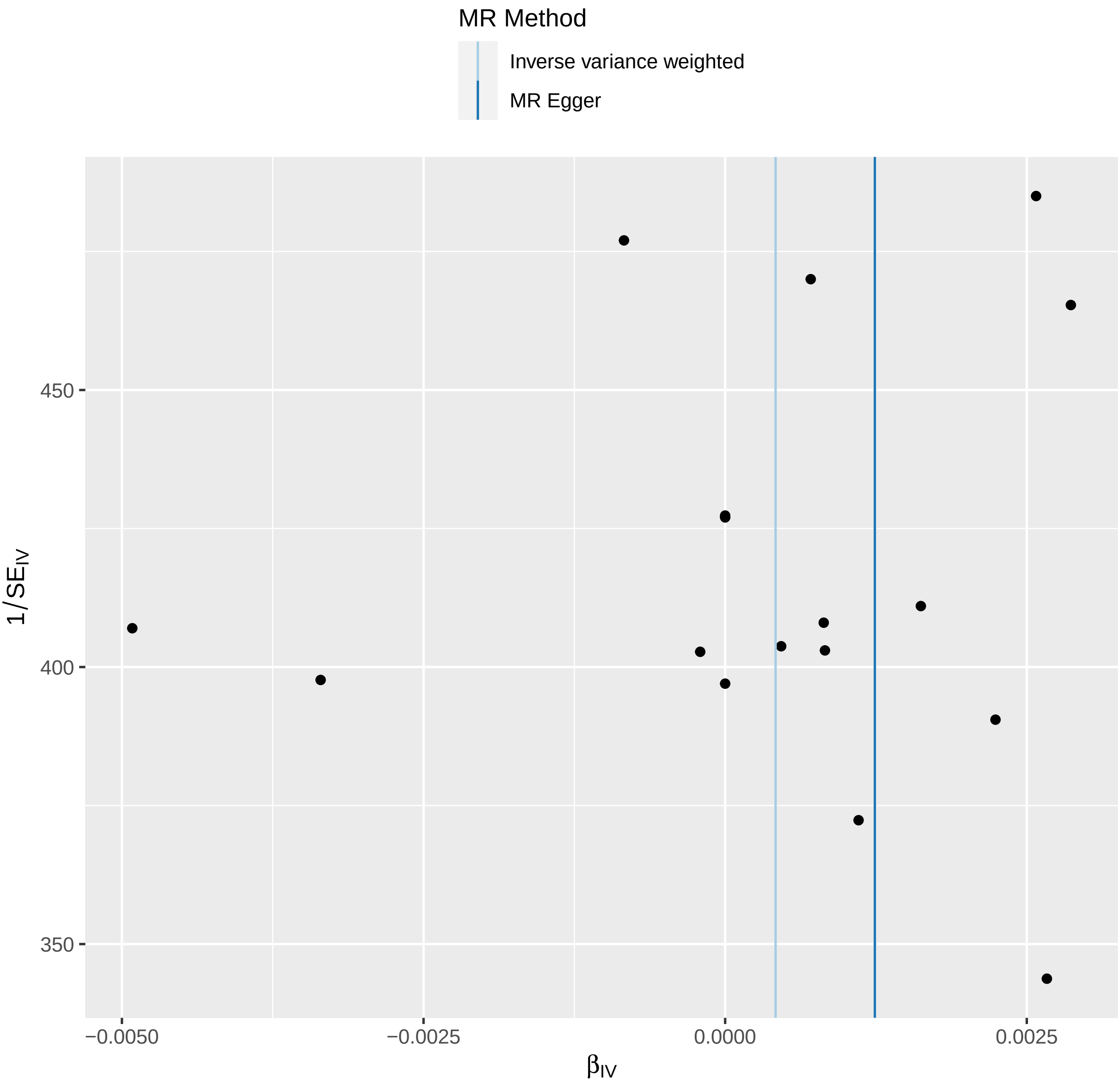

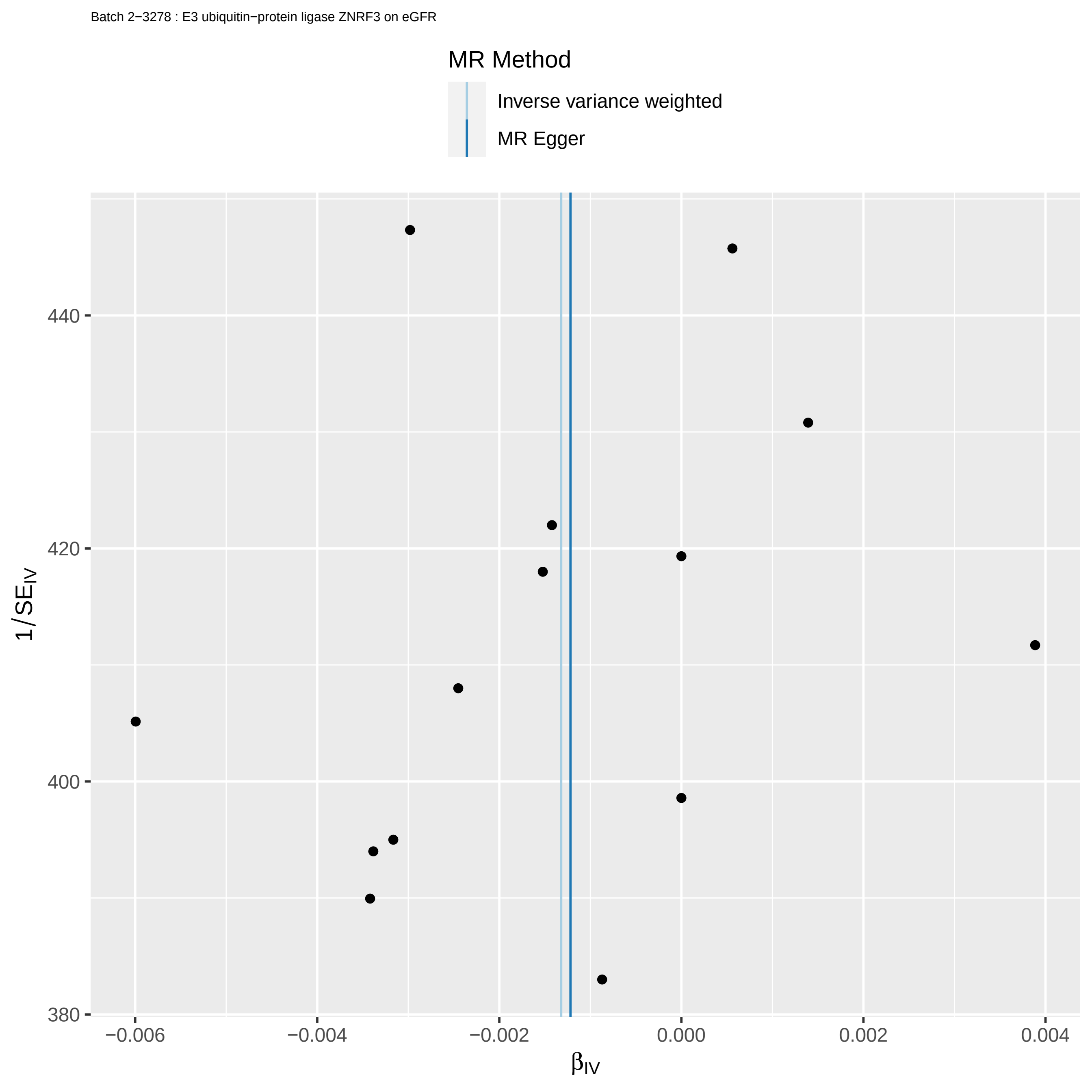
Batch 2−3278 : E3 ubiquitin−protein ligase ZNRF3 on eGFR
MR Method
Inverse variance weighted
MR Egger
440
420
400
380
1/SE_IV
−0.006
−0.004
−0.002
0.000
0.002
0.004
β_IV

Batch 2−3279 : E3 ubiquitin−protein ligase ZNRF3 on eGFR

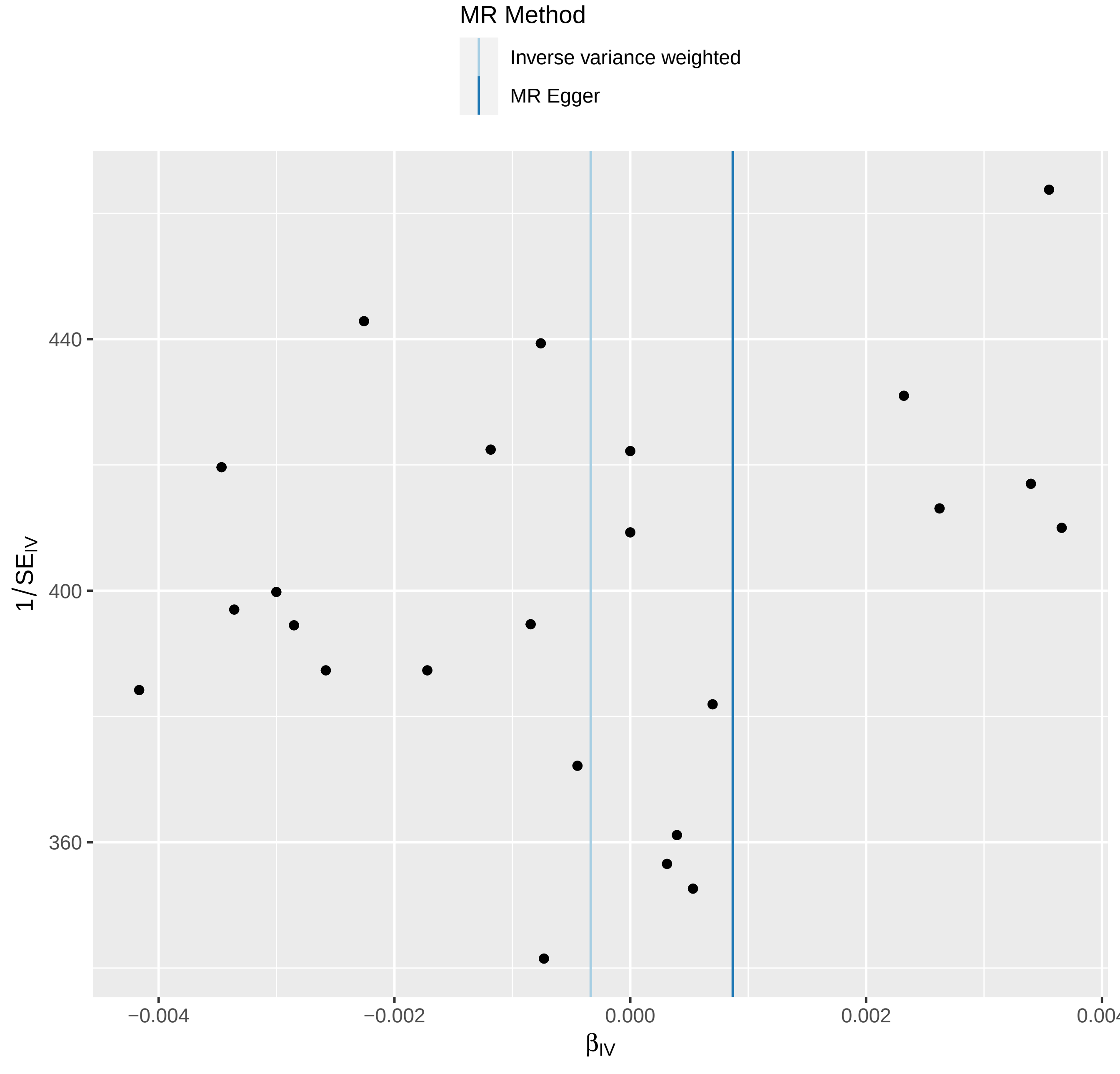

Batch 2−3280 : Zinc/RING finger protein 4 on eGFR

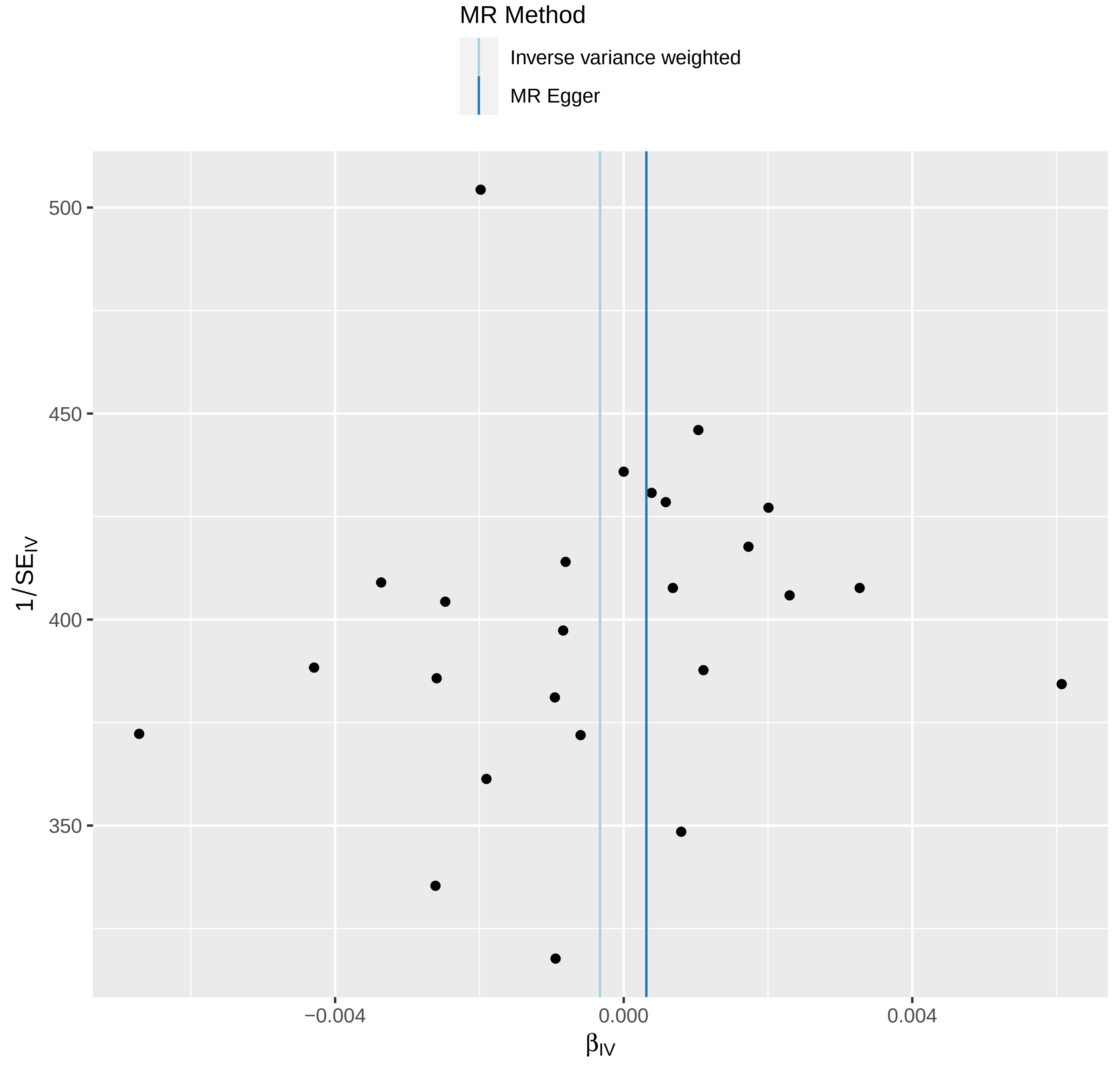

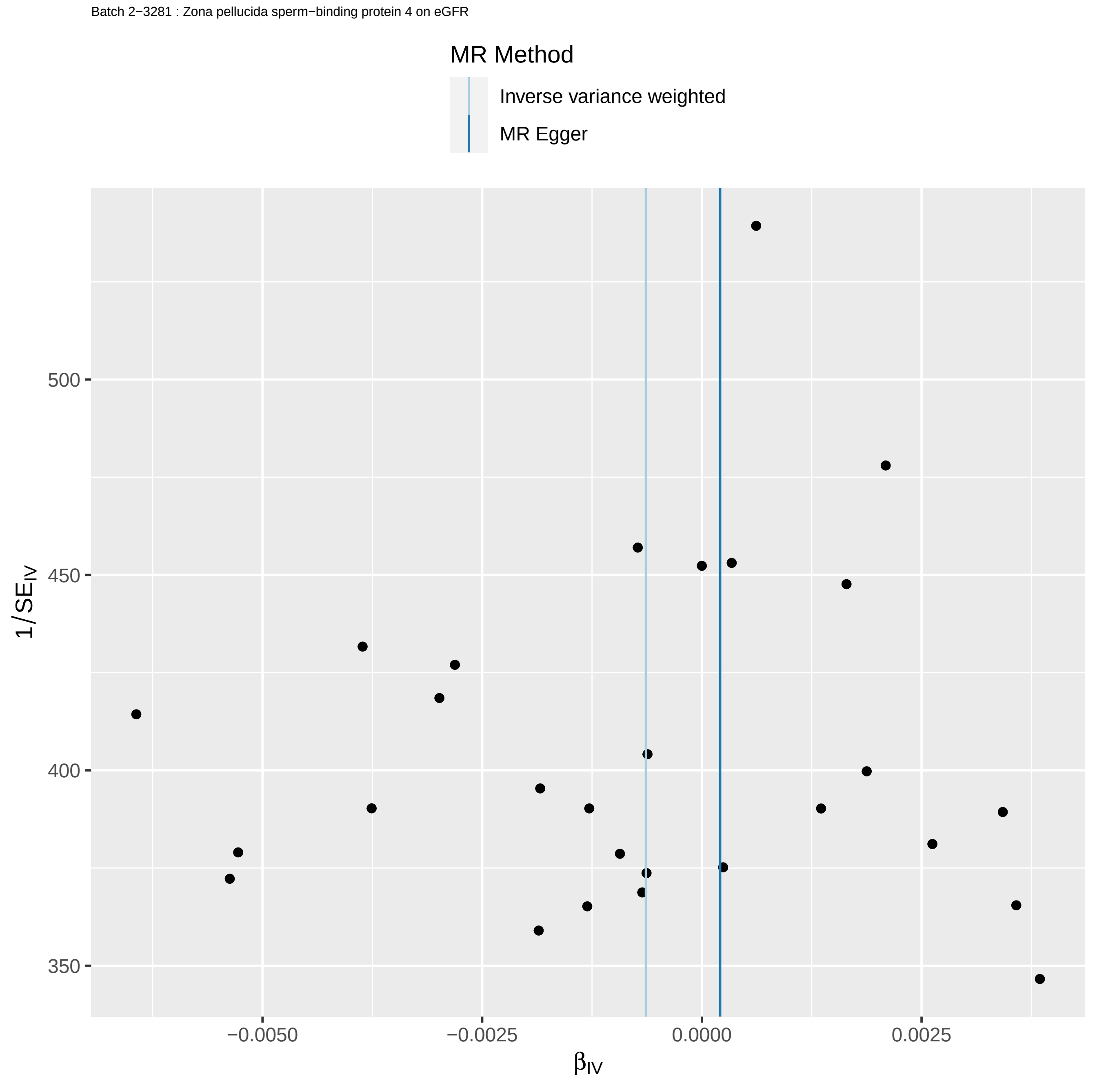

Batch 2−3281 : Zona pellucida sperm−binding protein 4 on eGFR
MR Method
Inverse variance weighted
MR Egger
500
450
400
350
1/SE$_{IV}$
−0.0050
−0.0025
0.0000
0.0025
β$_{IV}$

Batch 2−3282 : Zona pellucida−like domain−containing protein 1 on eGFR

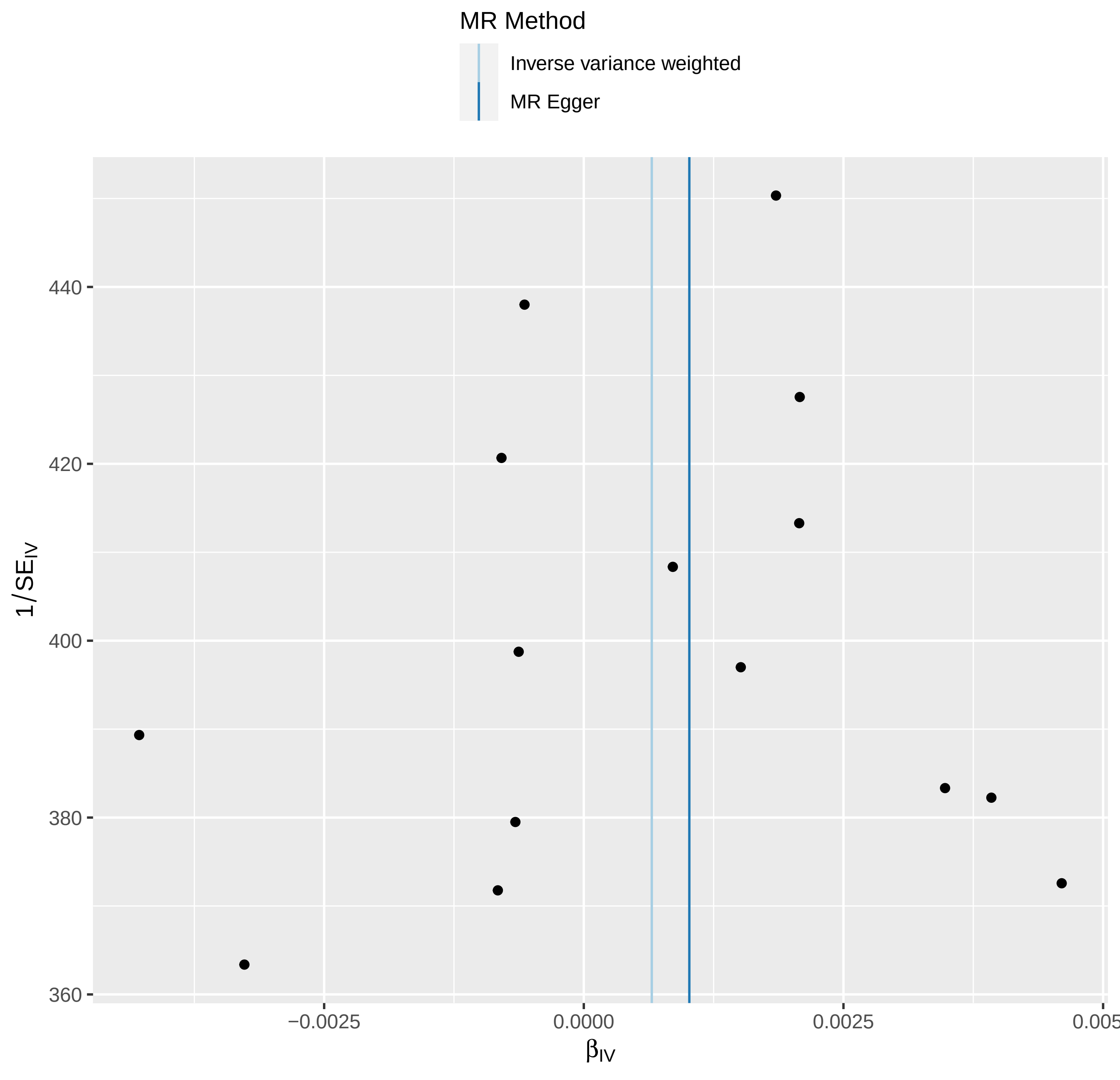

Supplement: Supplementary Figure 4 — Funnel plots of all the mendelian randomization analyses in this study. [file DataSheet4.pdf]
